# Spatiotemporal dynamics of the tumor microenvironment in hepatocellular carcinoma during combination immunotherapy

**DOI:** 10.1097/HC9.0000000000001014

**Published:** 2026-08-07

**Authors:** Hitoshi Iwasaki, Shinji Itoh, Katsuya Toshida, Junya Mita, Takuma Ishikawa, Norifumi Iseda, Kyohei Yugawa, Shohei Yoshiya, Takashi Motomura, Takeo Toshima, Shinichi Aishima, Yoshinao Oda, Tomoharu Yoshizumi

**Affiliations:** 1 Department of Surgery and Science, Graduate School of Medical Sciences, Kyushu University, Fukuoka, Japan; 2 Department of Scientific Pathology, Graduate School of Medical Sciences, Kyushu University, Fukuoka, Japan; 3 Department of Anatomic Pathology, Graduate School of Medical Sciences, Kyushu University, Fukuoka, Japan

**Keywords:** atezolizumab, bevacizumab, poliovirus receptor, single-cell gene expression analysis, TIGIT

## Abstract

**Background:**

Atezolizumab plus bevacizumab (ATZ/BEV) is a standard first-line therapy for advanced hepatocellular carcinoma (HCC); however, many patients do not achieve meaningful tumor regression. The temporal and spatial immune remodeling associated with ATZ/BEV remains poorly understood.

**Methods:**

We performed single-cell RNA sequencing of paired hepatectomy specimens obtained before and after ATZ/BEV from one patient and of tumor center and margin samples from another patient after ATZ/BEV. Cell composition, subclusters, and cell–cell communication were analyzed. In addition, candidate molecules identified by transcriptomic analysis were further assessed using serum-based assays and immunohistochemistry.

**Results:**

These single-cell analyses suggested that ATZ/BEV was associated with a shift toward an immune-active tumor microenvironment, with increased CD8^+^ T cells together with reduced endothelial cells. CD8^+^ T cells showed increased effector and exhaustion signatures, indicating coexistence of activation and dysfunction. CellChat analysis demonstrated selective activation of the TIGIT–PVR/NECTIN2 axis after treatment. Spatial analysis showed that the tumor margin was enriched for CD8^+^ T cells and exhibited stronger effector and exhaustion activity than the tumor center. Immunoregulatory signaling was also more prominent at the margin. Serum TIGIT levels were significantly higher after ATZ/BEV than in upfront resection cases (*p*=0.0325). Immunohistochemistry showed greater margin-to-center differences in TIGIT (*p*=0.0019) and PVR (*p*=0.0008) in the tumor after ATZ/BEV.

**Conclusions:**

Our exploratory findings suggest that ATZ/BEV may remodel the HCC microenvironment toward a state characterized by concurrent CD8^+^ T-cell activation and inhibitory signaling through the TIGIT–PVR/NECTIN2 axis, particularly at the tumor margin.

## Graphical abstract

**Figure:**
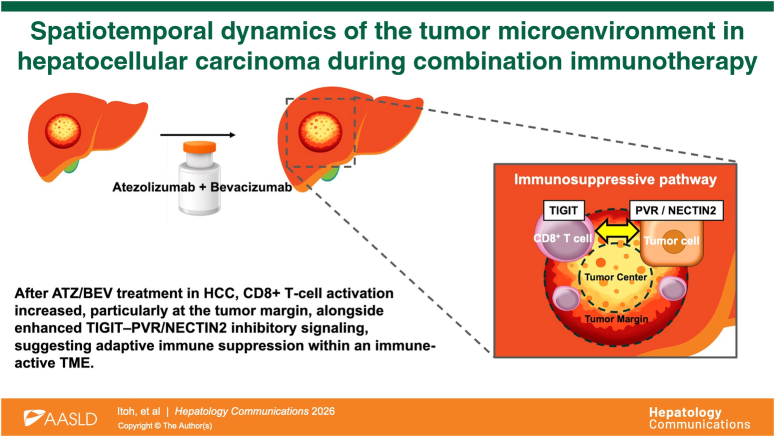

## INTRODUCTION

Hepatocellular carcinoma (HCC) is a highly lethal malignancy, ranking as the third leading cause of cancer-related deaths worldwide, with ~800,000 deaths annually.^1^ This poor prognosis is primarily attributable to the aggressive nature of advanced-stage HCC and the historical lack of effective systemic therapies. In fact, most HCC patients are diagnosed at an unresectable or advanced stage, for which systemic pharmacotherapy is the mainstay of treatment.^2^ Since the approval of the multikinase inhibitor sorafenib in 2007, it remained the standard first-line therapy for nearly a decade, although its survival benefit was modest.^3^ In 2018, lenvatinib was introduced as a first-line alternative after demonstrating non-inferiority to sorafenib.^4^ However, the advent of immune checkpoint inhibitors (ICIs) has fundamentally reshaped the therapeutic landscape of HCC. Notably, the combination of atezolizumab plus bevacizumab (ATZ/BEV) demonstrated superior overall survival and objective response rates compared with sorafenib in the IMbrave150 trial, establishing a new standard of care for advanced HCC.^5^ Furthermore, conversion surgery strategies, in which systemic pharmacotherapies including molecular targeted agents and immunotherapies are employed to downsize or downstage initially unresectable HCC, thereby permitting subsequent curative resection, have garnered increasing attention, and, with appropriate patient selection, have been associated with further improvements in prognosis.^6^^,^^7^

Although ATZ/BEV has been shown to prolong survival in advanced HCC, the objective response rate remains limited to 27%–30%, and ~65%–70% of patients fail to achieve meaningful tumor shrinkage.^5^ A principal contributor to this suboptimal efficacy is the immunosuppressive tumor microenvironment (TME) that is characteristic of HCC.^8^^–^^10^ Arising against a backdrop of chronic inflammation and cirrhosis, HCC lesions are heavily infiltrated by regulatory T cells (Tregs), myeloid‑derived suppressor cells (MDSCs), and tumor‑associated macrophages (TAMs), all of which act to inhibit the infiltration and activation of effector populations such as CD8^+^ T cells.^11^^,^^12^ Based on the inflammatory milieu of the TME, tumors can be classified as “immune‑hot” or “immune‑cold,” with ICIs demonstrating high response rates in immune‑hot tumors but predominantly encountering primary resistance in immune‑cold lesions.^13^^,^^14^ Moreover, it has been reported that the immune cell composition of HCC undergoes temporal remodeling in response to molecular targeted therapies and chemotherapy; for example, sorafenib may foster an immunosuppressive TME by enhancing or modulating MDSC function and Treg activity. In terms of spatial heterogeneity, our department has previously demonstrated that higher densities of CD8^+^ T-cell infiltration within the tumor center correlate with improved prognosis.^10^ Collectively, these observations suggest that the temporal and spatial dynamics of the TME represent critical determinants of ATZ/BEV therapeutic efficacy.

Single-cell RNA sequencing (scRNA-seq) has emerged as a powerful tool to dissect cell–cell interactions in the TME at high resolution. Unlike bulk RNA-seq and flow cytometry, which average signals across heterogeneous populations, scRNA-seq enables comprehensive profiling of individual cells and can uncover rare subsets such as suppressive macrophages and regulatory T cells often missed by conventional methods.^15^ In this exploratory study, we applied scRNA-seq to paired HCC specimens from one patient before and after treatment, as well as tumor margin and center samples from another patient, to characterize temporal and spatial TME dynamics following ATZ/BEV. Although limited by the very small number of cases, this dataset may provide hypothesis-generating insights into molecular networks associated with primary resistance.

## METHODS

### Patients and sample collection

To investigate temporal and spatial changes associated with ATZ/BEV, we analyzed samples from 2 patients (cohorts 1 and 2, Supplemental Table S1, https://links.lww.com/HC9/C466). The therapeutic response to ATZ/BEV for HCC was assessed according to the Response Evaluation Criteria in Solid Tumors (RECIST) version 1.1. For temporal changes, paired hepatectomy specimens were collected before and after ATZ/BEV in one patient (cohort 1): a salvage hepatectomy was performed for a residual/recurrent lesion after ATZ/BEV, with the overall response classified as stable disease (SD) according to RECIST v1.1 (Supplemental Figure S1A, https://links.lww.com/HC9/C462). For spatial changes, tumor margin and center samples were obtained from a single HCC specimen resected after ATZ/BEV in another patient (cohort 2, Supplemental Figure S1B, https://links.lww.com/HC9/C462) with SD. This retrospective study was approved by the Ethics Committee of Kyushu University Hospital, Kyushu University (Approval No. 2021-466), and was conducted in accordance with the Declarations of Helsinki and Istanbul. The requirement for written informed consent was waived by the Ethics Committee because of the retrospective nature of the study. Information regarding the study was made available to the participants, who were provided with the opportunity to opt out.

### Preparation of single-cell suspensions, library construction, and sequencing

Cryopreserved specimens were processed at a later time point, and single-cell suspensions were prepared by mechanical dissociation. The resulting cell suspensions were filtered through a sterile 30-µm cell strainer, after which cell number and viability were assessed. Gene expression libraries were then generated using the 10x Genomics Chromium platform. Library preparation was performed using the Chromium Fixed RNA Kit, Human Transcriptome. The libraries were sequenced on the NovaSeq X Plus system with 150-bp paired-end reads.

### Single-cell RNA-seq data analysis

Secondary analysis was performed using count matrices generated by Cell Ranger (v8.0.1). Ambient RNA contamination was first corrected using CellBender (v0.2.1), followed by sample-specific cell-level quality control (QC) and doublet detection. Cells failing the predefined QC criteria and barcodes classified as doublets were excluded before normalization, dimensionality reduction, integration, clustering, and UMAP visualization using Seurat (v5.4.0). SCTransform was used for normalization, and Harmony (v1.2.4) was applied to correct for batch effects across samples. After the initial cell-level QC, residual clusters showing low-quality profiles or mixed lineage-marker expression were further assessed based on QC metrics and canonical lineage-marker expression. Clusters considered to be low-quality, contamination-associated, or doublet-associated were excluded as an additional cluster-level QC step. The remaining high-quality cells were then reprocessed using SCTransform normalization, principal component analysis, Harmony integration, neighbor graph construction, clustering, and UMAP visualization. Major cell populations were identified based on SingleR (v2.8.0) annotation and known marker genes, including CellMarker 2.0.^16^ Each major cell population was then extracted and subjected to lineage-specific subclustering analysis. During this process, residual clusters showing low-quality profiles or mixed lineage-marker expression were reassessed using the same cluster-level QC strategy and were excluded when considered low-quality, contamination-associated, or doublet-associated. The remaining lineage-specific high-quality cells were then used for cell-state annotation, gene FeaturePlot generation, differential expression analysis, and downstream biological interpretation. All analyses were conducted in R 4.4.2 with Bioconductor 3.20.

### Downstream analysis

For downstream analyses, we used the QC-processed dataset after ambient RNA correction, cell-level QC, doublet removal, and cluster-level exclusion of residual low-quality or mixed-profile clusters at the major cell population level. For lineage-specific analyses, each major cell population was extracted from this QC-processed dataset and subjected to subclustering. Because subclustering can reveal residual low-quality, contamination-associated, or doublet-associated clusters that are not evident in the global analysis, each lineage-specific subcluster was reassessed based on QC metrics and canonical lineage-marker expression. When such clusters were identified, they were excluded where applicable, and the remaining lineage-specific cells were reprocessed by SCTransform normalization, principal component analysis (PCA), clustering, and UMAP visualization before downstream analysis. Gene signature scoring, differential expression analysis, Gene Ontology biological process (GO-BP) analysis, and gene FeaturePlots were generated from the corresponding lineage-specific QC-filtered datasets. Cell–cell communication analysis was performed using CellChat (v2.2.0.9001) on the final annotated high-quality cell populations within each cohort to infer intercellular signaling networks. Comparisons were made between before and after treatment in cohort 1 and between the tumor center and tumor margin in Cohort 2. Further details regarding sample collection, single-cell preparation, library construction, sequencing, data processing, downstream analyses, and in silico analyses are provided in the Supplemental Methods, https://links.lww.com/HC9/C465.

### Serum TIGIT levels by ELISA

Serum TIGIT concentrations were quantified using a sandwich ELISA with the Human TIGIT ELISA Kit (Cat.# ELH-TIGIT; RayBiotech, Norcross, GA, USA). Serum samples were obtained from 2 independent patient groups: patients who underwent upfront resection without ATZ/BEV treatment (n=8) and patients who underwent resection after ATZ/BEV treatment (n=8); these were not paired longitudinal samples from the same individuals. Samples were thawed at room temperature and diluted 1:2 according to the manufacturer’s protocol. Following incubation at room temperature, absorbance was measured at 450 nm, and TIGIT concentrations were calculated by applying a four-parameter logistic regression to the standard curve (1.563–100 ng/mL).

### Immunohistochemistry

Formalin-fixed, paraffin-embedded tissue sections (4 µm) were deparaffinized, rehydrated, and subjected to heat-induced epitope retrieval in 10 mM citrate buffer (pH 6.0, 105 °C). Sections were incubated overnight at 4 °C with anti-TIGIT (E5Y1W) XP rabbit monoclonal antibody (mAb) (#99567; Cell Signaling Technology, Danvers, MA, USA; 1:200) and anti-PVR/CD155 (D8A5G) rabbit mAb (#81254; Cell Signaling Technology, Danvers, MA, USA; 1:200). A total of 8 ATZ/BEV cases were initially identified. Three were excluded (2 with complete tumor necrosis and 1 with tumor thrombus), leaving 5 cases for analysis. In addition, 31 upfront hepatectomy cases were included. The cohort included 5 ATZ/BEV cases (3 excluded: 2 with complete necrosis, 1 with tumor thrombus) and 31 upfront hepatectomy cases. The “tumor margin” was defined as the area within 1000 µm of the tumor–normal interface, and the “tumor center” was defined as the region inside this boundary. Staining intensity for TIGIT and PVR was graded as follows: 0=negative; 1=weak; 2=moderate; and 3=strong. The proportion of positively stained cells was recorded as a percentage (0%–100%). H-scores (0–300) were calculated as intensity × percentage of positive cells.^17^^–^^19^ Counts were obtained in five random high-power fields (×400) by 2 blinded observers (Hitoshi Iwasaki and Shinji Itoh).

### Statistical analysis

All statistical analyses were conducted using JMP Pro 15 (SAS Institute Inc., Cary, NC, USA). The distribution of continuous variables was assessed with the Shapiro–Wilk test. Because the serum TIGIT analysis compared independent groups rather than paired longitudinal samples, between-group comparisons of non-normally distributed data were performed using the two-tailed Mann–Whitney *U* test.

## RESULTS

### Single-cell transcriptomic profiling revealed marked remodeling of the tumor microenvironment after ATZ/BEV treatment

**FIGURE 1:**
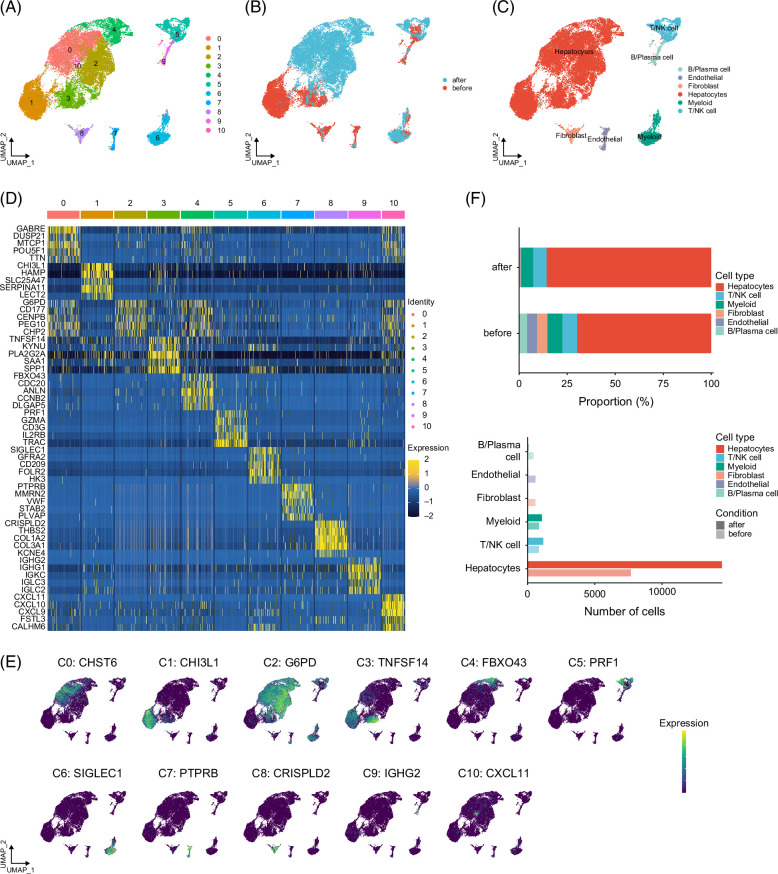
QC-filtered single-cell landscape of the HCC tumor microenvironment before and after ATZ/BEV. (A) UMAP visualization of high-quality cells after ambient RNA correction, cell-level QC, doublet removal, cluster-level exclusion of residual low-quality or mixed-profile clusters, and reprocessing by SCTransform normalization, PCA, Harmony integration, clustering, and UMAP visualization. (B) UMAP visualization after batch-effect correction with Harmony, with cells colored by treatment status (before vs. after treatment). (C) Manual annotation based on cluster-specific genes identified by FindAllMarkers and reference annotation by SingleR. (D) Heatmap showing the expression of 5 representative marker genes across clusters 0–10. (E) Feature plots showing canonical marker genes used for cell-type assignment. Color scales are shown individually for each gene and should not be compared directly across markers. (F) Bar plots showing the proportions (top) and absolute numbers (bottom) of the major annotated clusters. Abbreviations: ATZ, atezolizumab; BEV, bevacizumab; HCC, hepatocellular carcinoma; PCA, principal component analysis; QC, quality control; UMAP, Uniform Manifold Approximation and Projection.

scRNA-seq was performed on HCC specimens obtained from a primary hepatectomy (before ATZ/BEV) and a recurrent hepatectomy (after ATZ/BEV, Supplemental Figure S1A, https://links.lww.com/HC9/C462) in cohort 1. After ambient RNA removal using CellBender (Supplemental Figure S2, https://links.lww.com/HC9/C462), quality control (Supplemental Figure S3, https://links.lww.com/HC9/C462), and integration, 27,850 cells (before: 10,995 cells, after: 16,855 cells) were segregated into 11 clusters and visualized by UMAP (Figure 1A). Compared with the pre-integration UMAP (Supplemental Figure S4F, https://links.lww.com/HC9/C462), cells from both groups showed substantially greater overlap within the same clusters in the integrated visualization (Figure 1B). Cell-type identities of each cluster were assigned based on significantly differentially expressed genes identified by FindAllMarkers (Figure 1C and Supplemental Tables S2, https://links.lww.com/HC9/C466, S5A, S5B, https://links.lww.com/HC9/C467) and known canonical markers for each major lineage on UMAPs and feature plots (Supplemental Figure S5, https://links.lww.com/HC9/C462), together with annotation results generated by SingleR (Supplemental Figures S6A, B, https://links.lww.com/HC9/C462). The top-expressed genes in each cluster were visualized by heatmap analysis (Figure 1D) and FeaturePlot (Figure 1E).

Cell-type composition differed markedly between time points (Figure 1F). The proportions of endothelial cells, fibroblasts, and B/plasma cells were higher in the before sample, whereas these populations were nearly absent in the after sample.

### ATZ/BEV remodels the TME toward immune-active and immunoregulatory states

**FIGURE 2:**
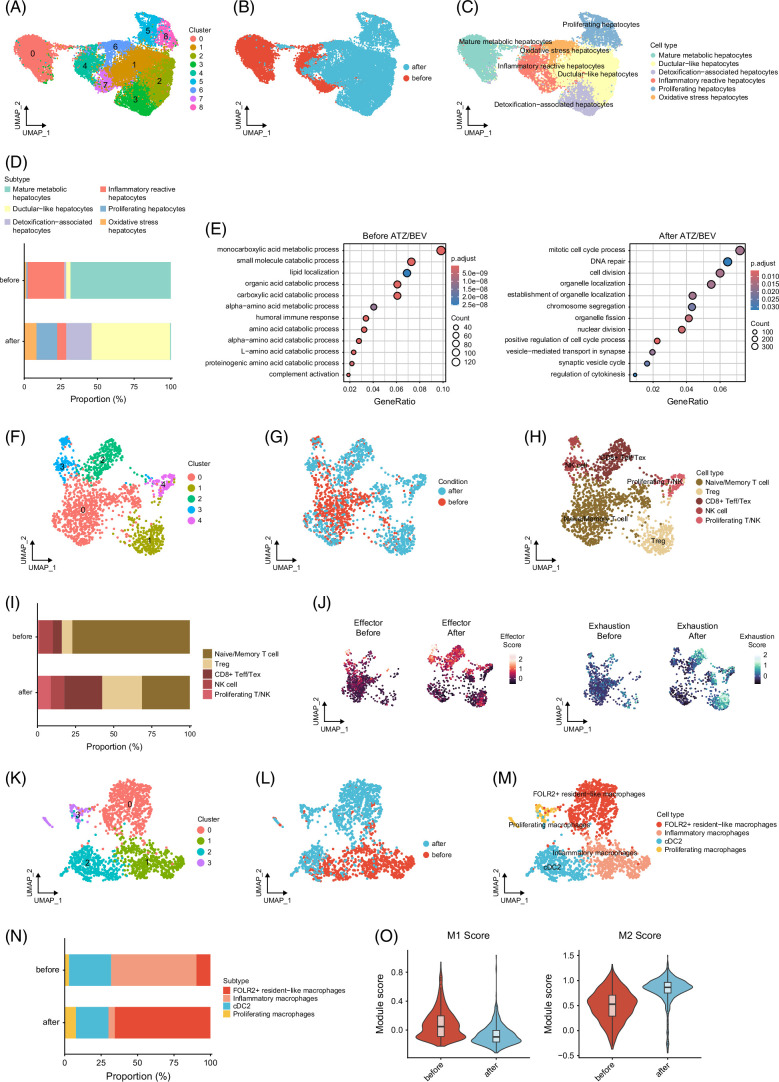
Subcluster analyses reveal treatment-associated remodeling of hepatocyte, T/NK-cell, and myeloid populations after ATZ/BEV. (A) Subcluster analysis of hepatocytes identified 9 clusters. (B) Following batch-effect correction with Harmony, cells were displayed on the UMAP according to the before and after treatment groups. (C) Cluster-specific genes were identified using FindAllMarkers, followed by manual annotation. (D) Bar plots show the cluster composition based on manual annotation. (E) After stratification by treatment status, differential expression and Gene Ontology biological process (GO-BP) over-representation analyses were performed. (F–J) T/NK-cell subclustering identified 5 clusters. UMAPs show the distribution of cells before and after treatment. Cluster-specific genes identified by FindAllMarkers were used for manual annotation. Bar plots show cluster composition, and AddModuleScore values for effector and exhaustion signatures were projected onto UMAPs split by treatment status. Color scales are shown individually for each gene and should not be compared directly across markers. (K–O) Myeloid subclustering identified 4 clusters. UMAPs show the distribution of cells before and after treatment. Cluster-specific genes identified by FindAllMarkers were used for manual annotation. Bar plots show cluster composition, and AddModuleScore values for M1 and M2 signatures were compared between before and after treatment using violin plots. Abbreviations: ATZ, atezolizumab; BEV, bevacizumab; GO-BP, Gene Ontology biological process; NK, natural killer; T, T lymphocyte; UMAP, Uniform Manifold Approximation and Projection.

Lineage-specific subclustering was performed for major cell populations, and residual low-quality, contamination-associated, or doublet-associated clusters were excluded where applicable. The remaining cells were reprocessed before cell-state annotation and downstream analyses. Hepatocyte clusters were further subclustered and visualized by UMAP (Figure 2A). The distribution of hepatocyte subclusters before and after ATZ/BEV treatment was shown (Figure 2B). Cluster-specific marker genes were identified using FindAllMarkers and used for annotation (Figure 2C and Supplemental Figure S7, https://links.lww.com/HC9/C462, Supplemental Tables S5C, S5D, https://links.lww.com/HC9/C467). Before treatment, hepatocytes were mainly composed of mature metabolic hepatocytes, whereas after treatment, ductular-like hepatocytes, proliferating hepatocytes, oxidative stress hepatocytes, and detoxification-associated hepatocytes were markedly increased (Figure 2D). GO-BP analysis was then performed for hepatocytes from the before and after treatment samples. Before treatment, enriched pathways were primarily related to amino acid metabolism, consistent with the mature metabolic hepatocyte phenotype (Figure 2E). In contrast, after treatment, pathways related to cell differentiation were predominantly enriched, reflecting the expansion of proliferating hepatocytes. Together, these results indicate that the hepatocyte compartment shifted from a predominantly metabolic state before treatment to a more diverse state after treatment, characterized by increased representation of proliferative, ductular-like, oxidative stress, and detoxification-associated phenotypes.

Subcluster analysis was then performed for T/NK cells and visualized by UMAP (Figures 2F, G). Cluster-specific marker genes were identified using FindAllMarkers and used for annotation (Figure 2H and Supplemental Figure S8, https://links.lww.com/HC9/C463, Supplemental Tables S5E-S5F, https://links.lww.com/HC9/C468). Before treatment, naive/memory T cells were predominant, whereas after ATZ/BEV treatment, CD8^+^ Teff/Tex cells, Treg cells, and proliferating T/NK cells were increased (Figure 2I). The CD8^+^ Teff/Tex cluster expressed *CD8A, CCL5,* and *NKG7*, together with *PDCD1, TO*X, and *LAG3*, indicating a tumor-reactive CD8^+^ T-cell population with features of both effector and exhaustion programs (Supplemental Table S5F, https://links.lww.com/HC9/C468). AddModuleScore analysis showed significant increases in both effector and exhaustion scores, particularly CD8^+^ Teff/Tex cells after treatment, suggesting that CD8^+^ T-cell activation was enhanced after ATZ/BEV treatment, along with increased exhaustion associated with persistent antigen stimulation (Figure 2J). Together, these findings indicate that the T-cell compartment after ATZ/BEV treatment was shifted toward a more immune-active state, accompanied by increased exhaustion.

Subcluster analysis was then performed for myeloid cells and visualized by UMAP (Figures 2K, L). Cluster-specific marker genes were identified using FindAllMarkers and used for annotation (Figure 2M and Supplemental Figure S9, https://links.lww.com/HC9/C463, Supplemental Tables S5G, S5H, https://links.lww.com/HC9/C468). Inflammatory macrophages, which were predominant before treatment, were markedly decreased after ATZ/BEV treatment, whereas FOLR2+ resident-like macrophages were substantially increased (Figure 2N). The FOLR2+ resident-like macrophage cluster expressed tissue-resident macrophage-associated genes, including *FOLR2, F13A1, CD209*, and *DAB2*, together with *SPP1* and *TREM2*, suggesting that this cluster represented a macrophage state with both resident-like and immunoregulatory features rather than a purely tissue-resident phenotype. AddModuleScore-based M1/M2 analysis showed a shift toward higher M2 scores in macrophages after treatment, suggesting polarization toward a reparative and immunoregulatory state (Figure 2O). Together, these findings indicate that the myeloid compartment after ATZ/BEV treatment was characterized by a decrease in inflammatory macrophages and an expansion of FOLR2+ resident-like macrophages with immunoregulatory features.

### ATZ/BEV amplifies immunoregulatory cell–cell communication via TIGIT/PVR and TIGIT/NECTIN2 signaling axes

**FIGURE 3:**
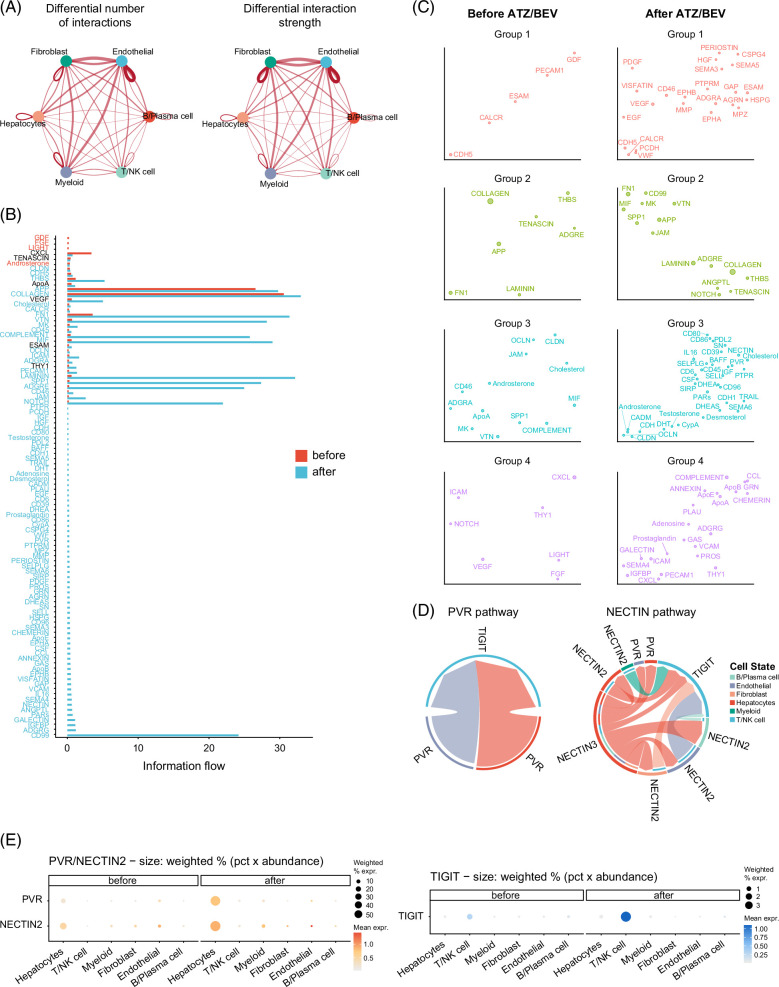
CellChat-based intercellular communication analysis before and after ATZ/BEV. (A) Differences in the number and strength of cell–cell interactions between before and after treatment. Nodes represent cell types, and edges represent ligand–receptor interactions. Red edges indicate interactions increased after treatment, whereas blue edges indicate decreased interactions; edge thickness reflects the magnitude of the difference. (B) Comparison of cumulative signaling strength across ligand–receptor pathways before and after treatment. (C) Functional embedding of CellChat-inferred signaling pathways. Each dot represents a pathway, and pathways with similar communication patterns are positioned closer together. Functionally related pathways are grouped into distinct clusters. (D) Chord plots showing ligand–receptor interactions (gene pairs) in the PVR and NECTIN pathways after treatment. (E) Dot plots showing the expression of *PVR, NECTIN2,* and *TIGIT*. Dot color indicates the mean normalized expression level, and dot size indicates the proportion of expressing cells weighted by cell-type composition. Abbreviations: ATZ, atezolizumab; BEV, bevacizumab; PVR, poliovirus receptor.

To characterize differences in cell–cell communication between hepatectomy specimens obtained before and after ATZ/BEV treatment, CellChat analysis was performed on the scRNA-seq data. The sample after treatment showed increases in both the total number and overall strength of inferred intercellular interactions compared with the pretreatment sample (Figure 3A and Supplemental Figure S10A, https://links.lww.com/HC9/C463). The signaling strength of each pathway in each sample is summarized in bar plots (Figure 3B and Supplemental Figure S10B, https://links.lww.com/HC9/C463).

Pathway functional embedding showed that, before ATZ/BEV treatment, intercellular communication was mainly composed of relatively limited programs related to extracellular matrix signaling (group 2), angiogenic and growth factor signaling (group 4), and vascular endothelial cell adhesion (group 1) (Figure 3C). After treatment, these basal communication programs were retained, while immune-related pathways prominently emerged as a distinct cluster (group 3), encompassing immune checkpoint molecules, T-cell regulatory signals, and cell adhesion mediators. Notably, PVR and NECTIN signaling, involving ligands for the immune checkpoint receptor TIGIT, were detected specifically within this group in the post-treatment sample. These pathways were further visualized using chord plots (Figure 3D). PVR and NECTIN signaling were mainly observed as signals from hepatocytes and endothelial cells to T/NK cells, corresponding to the *PVR–TIGIT* and *NECTIN2–TIGIT* axes. Finally, dot plot analysis confirmed cluster-specific expression of *PVR, NECTIN2*, and *TIGIT*, all of which were upregulated after ATZ/BEV treatment (Figure 3E and Supplemental Figure S11, https://links.lww.com/HC9/C463).

### scRNA-seq revealed the spatial characteristics of HCC after ATZ/BEV treatment

**FIGURE 4:**
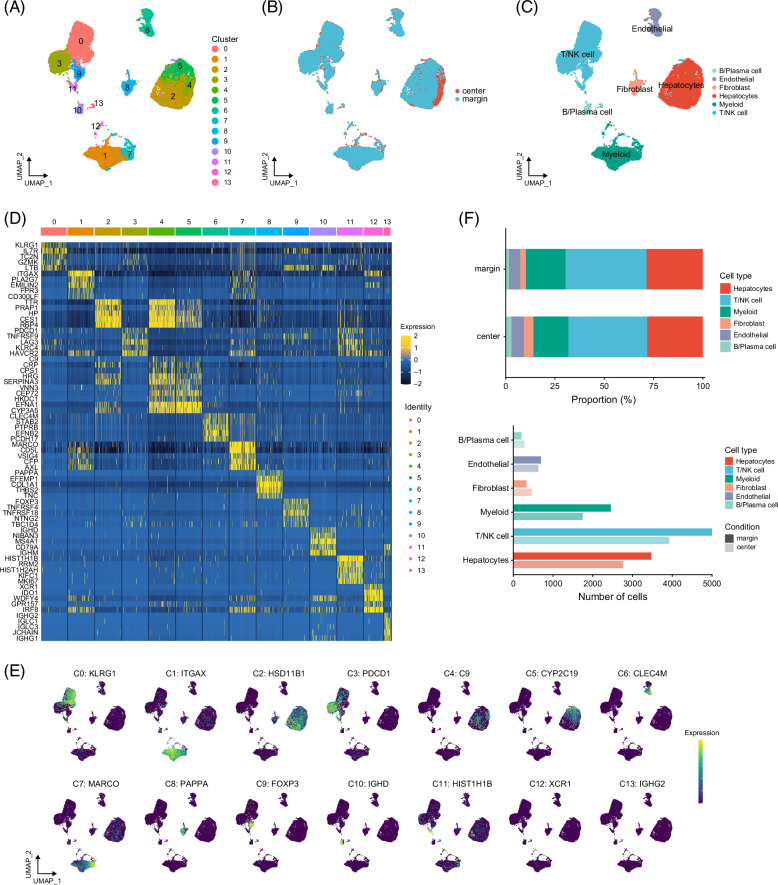
QC-filtered single-cell landscape of the HCC tumor microenvironment in the tumor margin and center after ATZ/BEV. (A) UMAP visualization of high-quality cells after ambient RNA correction, cell-level QC, doublet removal, cluster-level exclusion of residual low-quality or mixed-profile clusters, and reprocessing by SCTransform normalization, PCA, Harmony integration, clustering, and UMAP visualization, colored by 14 clusters (clusters 0–13). (B) UMAP visualization after batch-effect correction with Harmony, with cells colored by tumor region (tumor margin vs. tumor center). (C) Manual annotation based on cluster-specific genes identified by FindAllMarkers and reference annotation by SingleR. (D) Heatmap showing the expression of 5 representative marker genes across clusters 0–13. (E) Feature plots showing canonical marker genes used for cell-type assignment. Color scales are shown individually for each gene and should not be compared directly across markers. (F) Bar plots showing the proportions (top) and absolute numbers (bottom) of the major annotated clusters. Abbreviations: ATZ, atezolizumab; BEV, bevacizumab; HCC, hepatocellular carcinoma; PCA, principal component analysis; QC, quality control; UMAP, Uniform Manifold Approximation and Projection.

scRNA-seq was performed on tumor center and tumor margin regions of HCC specimens resected after ATZ/BEV (Supplemental Figure S1B, https://links.lww.com/HC9/C462) in cohort 2, yielding 21,975 cells (tumor margin; 12,166 cells, tumor center; 9809 cells) grouped into 14 clusters (Figure 4A) after ambient RNA removal using CellBender (Supplemental Figure S12, https://links.lww.com/HC9/C463), quality control (Supplemental Figure S13, https://links.lww.com/HC9/C463), and integration. Cells from both groups showed substantially greater overlap within the same clusters in the integrated visualization (Figure 4B and Supplemental Figure S14F, https://links.lww.com/HC9/C463). Cell-type identities of each cluster were assigned based on significantly differentially expressed genes identified by FindAllMarkers (Figure 4C and Supplemental Tables S3, https://links.lww.com/HC9/C466, S5I, S5J, https://links.lww.com/HC9/C468) and known canonical markers for each major lineage on UMAPs and feature plots (Supplemental Figure S15, https://links.lww.com/HC9/C463), together with annotation results generated by SingleR (Supplemental Figure S16, https://links.lww.com/HC9/C464). Cluster specificity was validated by heatmaps of top marker genes (Figure 4D) and feature plots of representative markers (Figure 4E).

Upon examining the cluster proportions using a bar plot, no extreme differences in clustering were observed between the tumor margin and center (Figure 4F).

### Spatial subclustering analysis identified region-specific phenotypes after ATZ/BEV treatment

**FIGURE 5:**
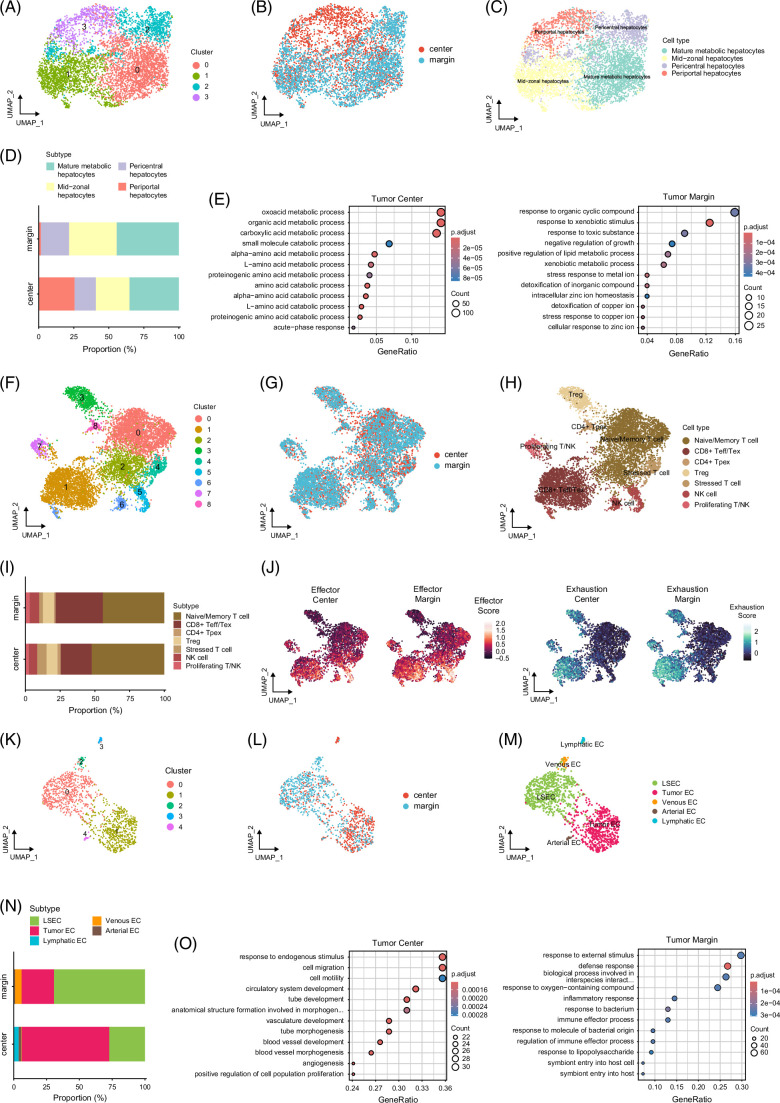
Subcluster analyses reveal region-specific phenotypes in hepatocyte, T/NK-cell, and endothelial populations after ATZ/BEV. (A) Subcluster analysis of hepatocytes identified 4 clusters. (B) Following batch-effect correction with Harmony, cells were visualized on the UMAP according to tumor margin and tumor center. (C) Cluster-specific genes were identified using FindAllMarkers, followed by manual annotation. (D) Bar plots show the cluster composition based on manual annotation. (E) After stratification by tumor region, differential expression and GO-BP over-representation analyses were performed. (F–J) T/NK-cell subclustering identified 9 clusters. UMAPs show the distribution of cells in the tumor margin and tumor center. Cluster-specific genes identified by FindAllMarkers were used for manual annotation, and doublet/contamination clusters were excluded. Bar plots show cluster composition, and AddModuleScore values for effector and exhaustion signatures were projected onto UMAPs split by tumor region. Color scales are shown individually for each gene and should not be compared directly across markers. (K–O) Endothelial subclustering identified 5 clusters. UMAPs show the distribution of cells in the tumor margin and tumor center. Cluster-specific genes identified by FindAllMarkers were used for manual annotation. Bar plots show cluster composition, and GO-BP over-representation analyses were performed. Abbreviations: ATZ, atezolizumab; BEV, bevacizumab; GO-BP, Gene Ontology biological process; NK, natural killer; PCA, principal component analysis; T, T lymphocyte; UMAP, Uniform Manifold Approximation and Projection.

Lineage-specific subclustering was performed for major cell populations, and residual low-quality, contamination-associated, or doublet-associated clusters were excluded where applicable. The remaining cells were reprocessed before cell-state annotation and downstream analyses. Subclustering analysis of hepatocytes after ATZ/BEV treatment identified 4 subclusters, which were visualized by UMAP (Figure 5A). The distribution of hepatocyte subclusters in the tumor center and tumor margin was shown (Figure 5B). Cluster-specific marker genes were identified using FindAllMarkers and used for annotation (Figure 5C and Supplemental Figures S17, https://links.lww.com/HC9/C464, Supplemental Tables S5K, S5L, https://links.lww.com/HC9/C468). Periportal hepatocytes were enriched in the tumor center but largely absent in the margin (Figure 5D). GO-BP analysis showed predominant enrichment of amino acid metabolic pathways in the tumor center (Figure 5E). In contrast, hepatocytes in the tumor margin showed expansion of pericentral and mid-zonal hepatocytes (Figure 5D), and other xenobiotic metabolism-related genes, together with increased metal stress response, heat shock response, and integrated stress response (Figure 5E). These findings suggest that, after ATZ/BEV treatment, hepatocytes in the tumor center retain an amino acid metabolism-oriented state, whereas those in the tumor margin exhibit cytoprotective stress-response programs.

Subclustering analysis was then performed for T/NK cells, which were classified into 9 subclusters (Figure 5F). The distribution of T/NK cells in the tumor center and tumor margin was shown (Figure 5G). Cluster-specific marker genes were identified using FindAllMarkers and used for annotation (Figure 5H and Supplemental Figure S18, https://links.lww.com/HC9/C464, Supplemental Tables S5M, S5N, https://links.lww.com/HC9/C468). Whereas naïve/memory T cells accounted for more than half of T/NK cells in the tumor center, CD8^+^ Teff/Tex cells were markedly increased in the tumor margin (Figure 5I). Effector and exhaustion scores were calculated using AddModuleScore and visualized on UMAP (Figure 5J). CD8^+^ Teff/Tex cells in the tumor margin showed stronger effector and exhaustion activity than those in the tumor center.

Subclustering analysis of endothelial cells identified 5 subclusters (Figure 5K). The distribution of endothelial cells in the tumor center and tumor margin is shown in Figure 5L. Cluster-specific marker genes were identified using FindAllMarkers and used for annotation (Figure 5M and Supplemental Figure S19, https://links.lww.com/HC9/C464, Supplemental Tables S5O, S5P, https://links.lww.com/HC9/C468). Endothelial cells in the tumor margin were composed predominantly of liver sinusoidal endothelial cells (LSECs), whereas tumor ECs were the dominant endothelial population in the tumor center (Figure 5N). This difference was also reflected in GO-BP analysis, which showed significant enrichment in the tumor center for pathways related to vascular formation and remodeling, including vasculature development, blood vessel morphogenesis, and angiogenesis (Figure 5O). These findings suggest that endothelial cells in the tumor center are primarily involved in the maintenance and remodeling of tumor vasculature.

### CellChat analysis revealed enhanced immunoregulatory signaling in the tumor margin after ATZ/BEV treatment

**FIGURE 6:**
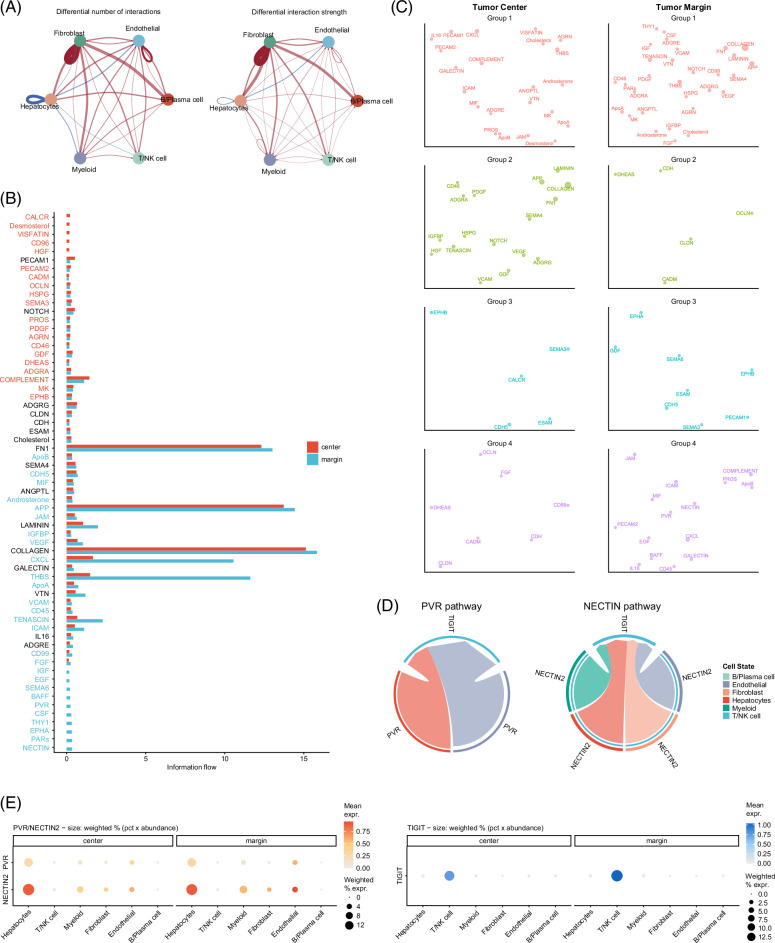
CellChat analysis comparing tumor margin versus center in HCC. (A) Differences in the number and strength of cell–cell interactions between the tumor margin and tumor center. Nodes represent cell types, and edges represent ligand–receptor interactions. Red edges indicate interactions stronger in the tumor margin, whereas blue edges indicate interactions stronger in the tumor center; edge thickness reflects the magnitude of the difference. (B) Comparison of cumulative signaling strength across ligand–receptor pathways in the tumor margin and tumor center. (C) Functional embedding of CellChat-inferred signaling pathways. Each dot represents a pathway, and pathways with similar communication patterns are positioned closer together. Functionally related pathways are grouped into distinct clusters. (D) Chord plots showing ligand–receptor interactions (gene pairs) in the PVR and NECTIN pathways in the tumor margin. (E) Dot plots showing the expression of *PVR, NECTIN2*, and *TIGIT*. Dot color indicates the mean normalized expression level, and dot size indicates the proportion of expressing cells weighted by cell-type composition. Abbreviations: HCC, hepatocellular carcinoma; PVR, poliovirus receptor.

CellChat analysis showed that both the number and strength of intercellular signaling interactions were higher in the tumor margin than in the tumor center (Figure 6A, Supplemental Figure S20A, https://links.lww.com/HC9/C464). Bar plots summarize the signaling pathways detected in the tumor center and tumor margin (Figure 6B, Supplemental Figure S20B, https://links.lww.com/HC9/C464). Pathway functional embedding identified distinct signaling groups, with center group 1 and margin group 4 both showing enrichment of immune-related pathways, although their compositions differed (Figure 6C). Center group 1 was mainly characterized by pathways involved in immune cell adhesion, including ICAM and PECAM, whereas margin group 4 included these pathways together with additional immunoregulatory pathways, including PVR and NECTIN. These findings indicate that immunoregulatory signaling was more prominent in the tumor margin, consistent with the increased abundance of CD8^+^ Teff/Tex cells in this region.

Chord plot analysis showed that PVR signaling was transmitted mainly from hepatocyte and endothelial cell clusters to T/NK cells, whereas NECTIN signaling was transmitted mainly from hepatocyte, endothelial cell, myeloid cell, and fibroblast clusters to T/NK cells (Figure 6D). Dot plot analysis showed that expression levels of *PVR* and *NECTIN* were largely similar between regions, whereas expression of the receptor *TIGIT* was markedly increased in T/NK-cell clusters in the tumor margin (Figure 6E and Supplemental Figure S21, https://links.lww.com/HC9/C464).

### Clinical validation of spatiotemporal TIGIT–PVR axis activation in HCC with ATZ/BEV

**FIGURE 7:**
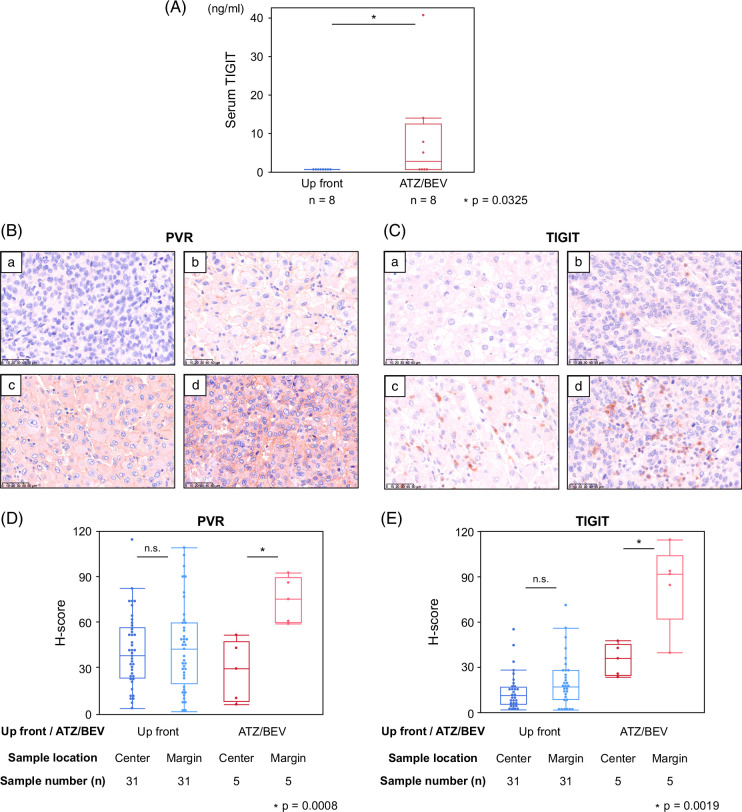
Comparative assessment of serum TIGIT concentrations and immunohistochemistry (IHC) H-scores of TIGIT and PVR. (A) Serum TIGIT concentrations measured by ELISA in 2 independent groups: patients who underwent upfront resection (n=8) and patients who underwent resection after ATZ/BEV treatment (n=8). These were not longitudinal paired samples from the same individuals. (B, C) Representative immunohistochemical staining intensities for PVR and TIGIT used for H-score assessment (a, negative; b, weak; c, moderate; d, strong). (D, E) Immunohistochemical H-scores for TIGIT and PVR in the tumor margin and tumor center of upfront resection cases (n=31) and post-ATZ/BEV resection cases (n=5). Abbreviations: ATZ, atezolizumab; BEV, bevacizumab; ELISA, enzyme-linked immunosorbent assay; IHC, immunohistochemistry; PVR, poliovirus receptor.

To assess treatment-associated differences in circulating TIGIT levels, serum TIGIT concentrations were measured by ELISA in upfront resection cases and post-ATZ/BEV resection cases. The ATZ/BEV-treated group showed significantly higher levels compared with the upfront resection group (*p*=0.0325) (Figure 7A), with a similar increase observed in scRNA-seq cases (Supplemental Figure S22, https://links.lww.com/HC9/C464).

Spatial expression of TIGIT and its ligand PVR was examined by IHC in tumor samples from 5 post-ATZ/BEV hepatectomy patients and 31 upfront hepatectomy patients (PVR, Figure 7B; TIGIT, Figure 7C; Supplemental Table S4, https://links.lww.com/HC9/C466). H-score analysis revealed significantly greater margin-to-center differences in the post-ATZ/BEV group (PVR, *p*=0.0008; TIGIT, *p*=0.0019) (Figures 7D, E).

In silico analysis using TIMER2.0 showed that high PVR expression correlated with reduced CD8^+^ T-cell infiltration across multiple cancers, and with increased Treg infiltration in LIHC (Supplemental Figures S23A, C, D, https://links.lww.com/HC9/C464). By contrast, NECTIN2 expression showed variable associations with CD8^+^ T cells and Tregs depending on cancer type, without a consistent trend (Supplemental Figures S23B, E, F, https://links.lww.com/HC9/C464).^20^

Using RNA expression data from the LIHC cohort available in the Human Protein Atlas, survival analysis demonstrated that patients with high PVR (*p*=0.014) or NECTIN2 (*p*=0.017) expression had significantly shorter overall survival compared with those with low expression (Supplemental Figures S23G, H, https://links.lww.com/HC9/C464).^21^

Collectively, these results indicate that TIGIT–PVR axis activation is enriched at the tumor margin following ATZ/BEV in HCC and is associated with an immunosuppressive TME and unfavorable clinical features.

## DISCUSSION

In this study, we used scRNA-seq to characterize the TME of HCC after ATZ/BEV treatment from both temporal and spatial perspectives. Our analysis showed that, after ATZ/BEV, the TME was remodeled toward an immune-active state centered on CD8^+^ T cells, while immunosuppressive networks were concurrently reinforced. In particular, CellChat analysis identified activation of the TIGIT–PVR/NECTIN2 axis, suggesting that an adaptive inhibitory program persists even after immune checkpoint-based therapy.

In the temporal analysis, endothelial cells were markedly reduced after treatment. Bevacizumab-mediated inhibition of VEGF has been reported to normalize abnormal tumor vasculature and relieve endothelial barriers that restrict lymphocyte infiltration, thereby promoting T-cell entry into tumors.^22^^,^^23^ Our findings are consistent with this concept. ATZ/BEV also induced a substantial change in T-cell composition. Whereas Naïve/Memory T cells predominated before treatment, CD8^+^ Teff/Tex cells, Treg cells, and proliferating T/NK cells increased after treatment. Together with the increase in effector activity in CD8^+^ Teff/Tex cells, these findings suggest that ATZ/BEV shifts the TME toward a more immune-active state, in line with previous reports.^24^

However, this immune activation was accompanied by a clear immunosuppressive counterpart. In CD8^+^ Teff/Tex cells, exhaustion activity was also increased after treatment, indicating that the expanded CD8^+^ T-cell population was not composed solely of functional antitumor effectors, but rather represented a state in which activation and dysfunction coexisted. Thus, the TME after ATZ/BEV appears to be not simply immune-hot, but rather immune-active under persistent inhibitory constraint.

This suppressive aspect was characterized by the enhanced TIGIT–PVR/NECTIN2 signaling identified by CellChat analysis. PVR (CD155) is a ligand for multiple receptors, including the activating receptor DNAM-1 and the inhibitory receptors TIGIT and CD96, and therefore plays a central role in regulating T-cell and NK-cell responses.^25^^–^^28^ Because TIGIT binds PVR with higher affinity than DNAM-1, PVR can favor inhibitory signaling and attenuate cytotoxic function and cytokine production.^28^^,^^29^ NECTIN2 can similarly engage TIGIT and contribute to immune suppression.^29^ The selective increase in TIGIT–PVR/NECTIN2 signaling after treatment suggests that this axis may represent a candidate adaptive resistance mechanism associated with constrained CD8^+^ T-cell responses after ATZ/BEV. The increase in serum TIGIT levels and the immunohistochemical findings further support this interpretation.

The spatial analysis also highlighted this duality. In the tumor center, naïve/memory T cells remained relatively enriched, whereas the tumor margin showed a higher proportion of CD8^+^ Teff/Tex cells together with stronger effector and exhaustion activity. Moreover, the PVR/NECTIN–TIGIT signaling observed in the before/after comparison was more prominent at the tumor margin, and IHC similarly showed greater margin-to-center differences in TIGIT and PVR expression in tumors resected after ATZ/BEV. These findings suggest that the tumor margin may represent a region where antitumor immune responses are more evident after ATZ/BEV, while inhibitory programs are also more prominent.

Previous studies have shown that immunologically active, or “hot,” HCC can exhibit both sensitivity to immune checkpoint blockade and coexisting checkpoint-mediated resistance.^30^ Our findings extend this concept at single-cell resolution by showing that, after ATZ/BEV, CD8^+^ T cells display both effector and exhaustion features, with the TIGIT–PVR/NECTIN2 axis emerging as a candidate mechanism underlying this balance. Therefore, the presence of infiltrating CD8^+^ T cells alone may not indicate effective antitumor immunity, and inhibitory programs must be evaluated in parallel.

This study has several important limitations. First, the single-cell analyses were based on 4 specimens from only 2 patients: the temporal comparison (before vs. after ATZ/BEV) was derived from one patient, and the spatial comparison (tumor margin vs. center) was derived from another. Therefore, these analyses should be considered exploratory and hypothesis-generating rather than definitive. Second, because statistical comparisons in single-cell datasets are performed at the cellular level, the possibility of pseudoreplication should be acknowledged, and the results should not be overinterpreted as directly generalizable at the patient level. Third, the temporal and spatial analyses were performed in different patients, and interpatient differences may have influenced the findings. Fourth, our spatial analyses demonstrate association rather than direct causality, and further spatially resolved or functional studies will be required to determine whether TIGIT–PVR/NECTIN2 signaling directly limits T-cell penetration into the tumor center. Nevertheless, resected HCC specimens obtained after ATZ/BEV are extremely rare, and the opportunity to perform both temporal and spatial single-cell analyses is itself valuable. In addition, the scRNA-seq findings were supported by ELISA and immunohistochemical analyses, which strengthen the validity of our observations.

Taken together, these exploratory findings suggest that, in these cases, the HCC microenvironment after ATZ/BEV was characterized by both CD8^+^ T-cell activation and concurrent reinforcement of inhibitory networks mediated by the TIGIT–PVR/NECTIN2 axis. The tumor margin may represent a site where this balance between immune activation and immune resistance is particularly evident. These observations warrant validation in larger, patient-level cohorts.

## CONCLUSION

Our exploratory single-cell analyses suggest that ATZ/BEV may remodel the HCC microenvironment toward a state marked by both CD8^+^ T-cell activation and persistent inhibitory signaling through the TIGIT–PVR/NECTIN2 axis. These findings are hypothesis-generating and should be validated in larger studies.

## Supplementary Material
