# Supplementary material for: Spatiotemporal dynamics of the tumor microenvironment in hepatocellular carcinoma during combination immunotherapy
Source: Hepatol Commun. 2026 Aug 7;10(9):e1014. doi: 10.1097/HC9.0000000000001014 (PMC13456720; doi:10.1097/HC9.0000000000001014)
Supplement: Supplementary file 2 [file hc9-10-e1014-s002.pdf]

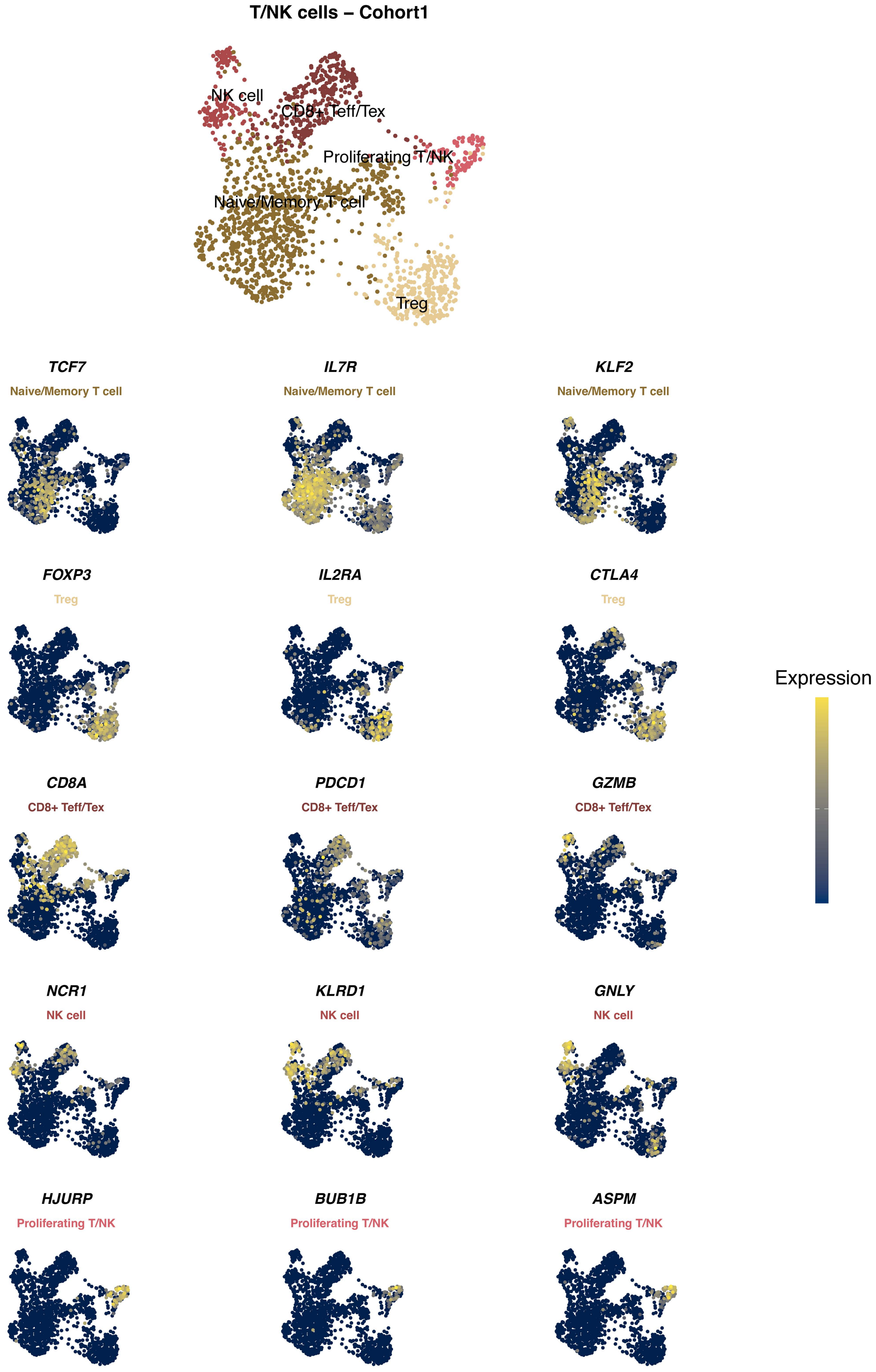


Supplementary Figure. 8 T/NK-cell subcluster annotation and marker gene expression before and after treatment.

UMAP embedding of T/NK cells, colored by annotated cell type. Five populations were identified: Naive/Memory T cells, CD8+ effector/exhausted T cells (CD8+ Teff/Tex), regulatory T cells (Treg), NK cells, and Proliferating T/NK cells. Feature plots showing normalized expression of representative marker genes for each subpopulation. Color scale indicates log-normalized gene expression (dark blue = low; yellow = high). Color scales are shown individually for each gene and should not be compared directly across markers. Marker genes used for annotation: TCF7, IL7R, and KLF2 for Naive/Memory T cells; FOXP3, IL2RA, and CTLA4 for Tregs; CD8A, PDCD1, and GZMB for CD8+ Teff/Tex; NCR1, KLRD1, and GNLY for NK cells; and HJURP, BUB1B, and ASPM for Proliferating T/NK cells.

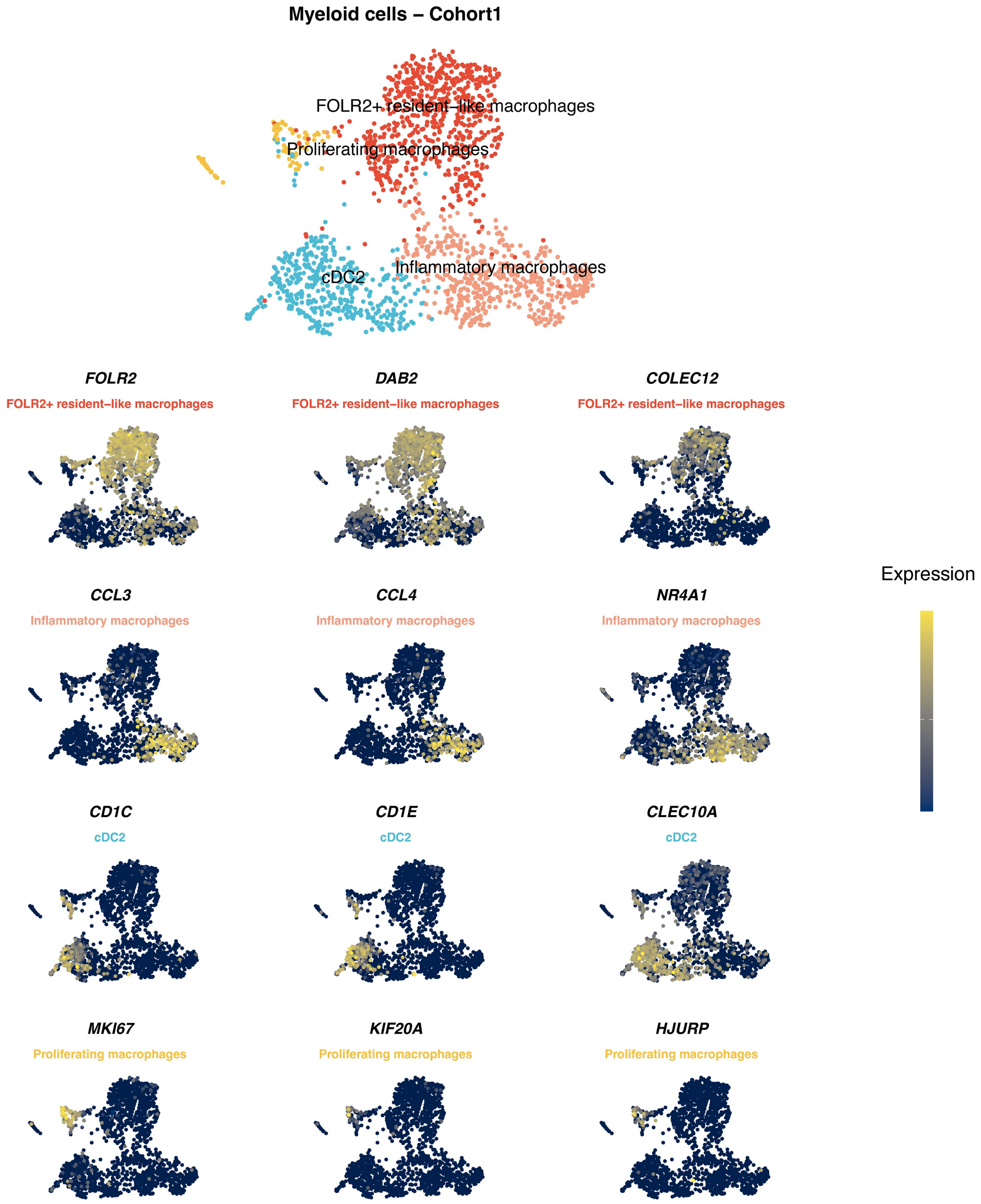


Supplementary Figure. 9 Myeloid cell subcluster annotation and marker gene expression before and after treatment.

UMAP embedding of myeloid cells, colored by annotated cell type. Four populations were identified: FOLR2+ resident-like macrophages, inflammatory macrophages, conventional type 2 dendritic cells (cDC2), and proliferating macrophages. Feature plots showing normalized expression of representative marker genes for each subpopulation. Color scale indicates log-normalized gene expression (dark blue = low; yellow = high). Color scales are shown individually for each gene and should not be compared directly across markers. Marker genes used for annotation: FOLR2, DAB2, and COLEC12 for FOLR2+ resident-like macrophages; CCL3, CCL4, and NR4A1 for inflammatory macrophages; CD1C, CD1E, and CLEC10A for cDC2; and MKI67, KIF20A, and HJURP for proliferating macrophages.

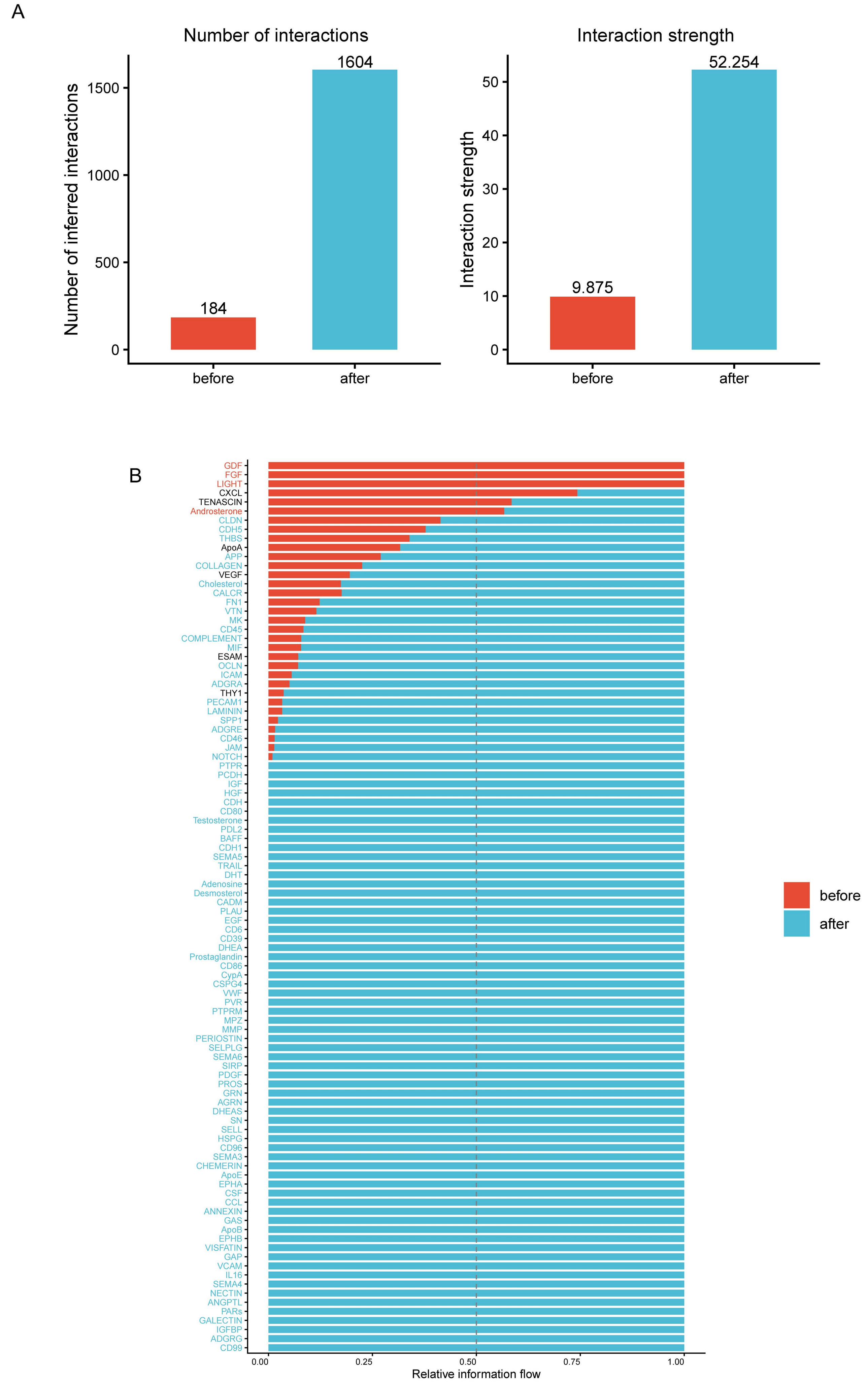


Supplementary Figure. 10 Global comparison of CellChat-inferred intercellular communication before and after treatment.

(A) Bar plots comparing the number of inferred cell-cell interactions (left) and the total interaction strength (right) between the pre-treatment (Before; red) and post-treatment (After; cyan) condition. The number of interactions increased from 184 (Before) to 1,604 (After), and the total interaction strength increased from 9.875 (Before) to 52.254 (After), indicating a marked increase in intercellular communication following ATZ/BEV treatment. Interaction strengths were computed using the CellChat framework. (B) Horizontal bar plot showing the relative information flow for each CellChat signaling pathway in the pre-treatment (Before; red) and post-treatment (After; cyan) conditions. Pathways are ranked in descending order of total information flow (Before + After combined). The x-axis represents the relative information flow, normalized to a maximum of 1.0 across all pathways. Pathway names highlighted in red indicate pathways with significantly higher relative information flow in the Before condition; names in cyan indicate pathways significantly enriched after treatment; and names in black (e.g., TENASCIN, VEGF, ESAM) indicate pathways showing no statistically significant difference between conditions. The dashed vertical line at 0.5 serves as a reference.

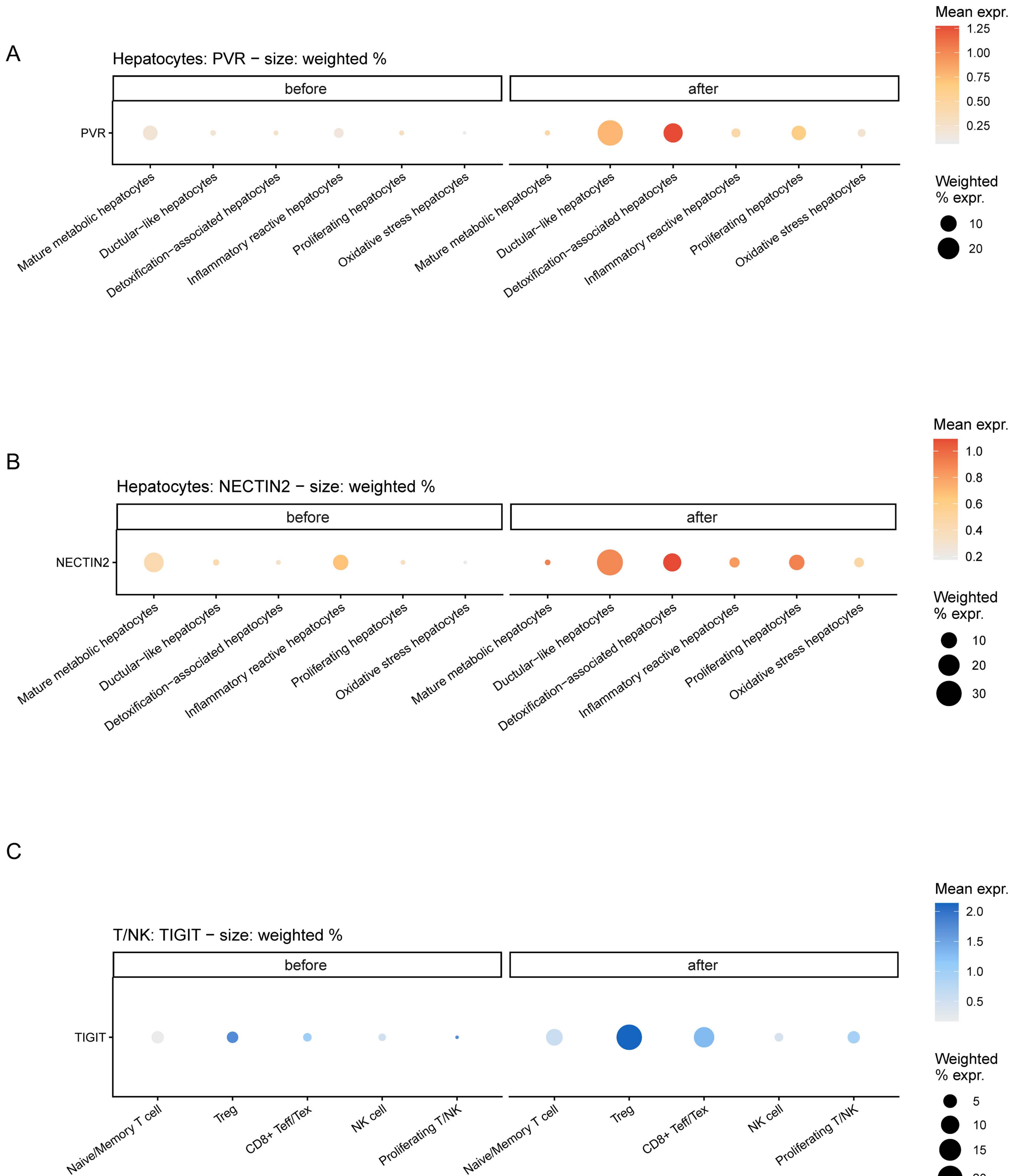


Supplementary Figure. 11 Expression of NECTIN2/PVR–TIGIT axis genes across hepatocyte and T/NK-cell subtypes before and after treatment.
Dot plots showing the expression of the immune checkpoint-related ligands PVR and NECTIN2 in hepatocyte subtypes (A, B), and the cognate receptor TIGIT in T/NK cell subtypes (C), stratified by treatment status. Dot size represents the weighted percentage of expressing cells (% expressing cells × cell type abundance [%]), which reflects the proportional contribution of each cell subtype to total ligand or receptor expression within the sample. Dot color indicates mean log-normalized gene expression (red scale: PVR and NECTIN2; blue scale: TIGIT). Six hepatocyte subtypes were examined for NECTIN2 and PVR: Mature metabolic, Ductular-like, Detoxification-associated, Inflammatory reactive, Proliferating, and Oxidative stress hepatocytes. Five T/NK cell subtypes were examined for TIGIT: Naive/Memory T cells, Tregs, CD8+ Teff/Tex, NK cells, and Proliferating T/NK cells.

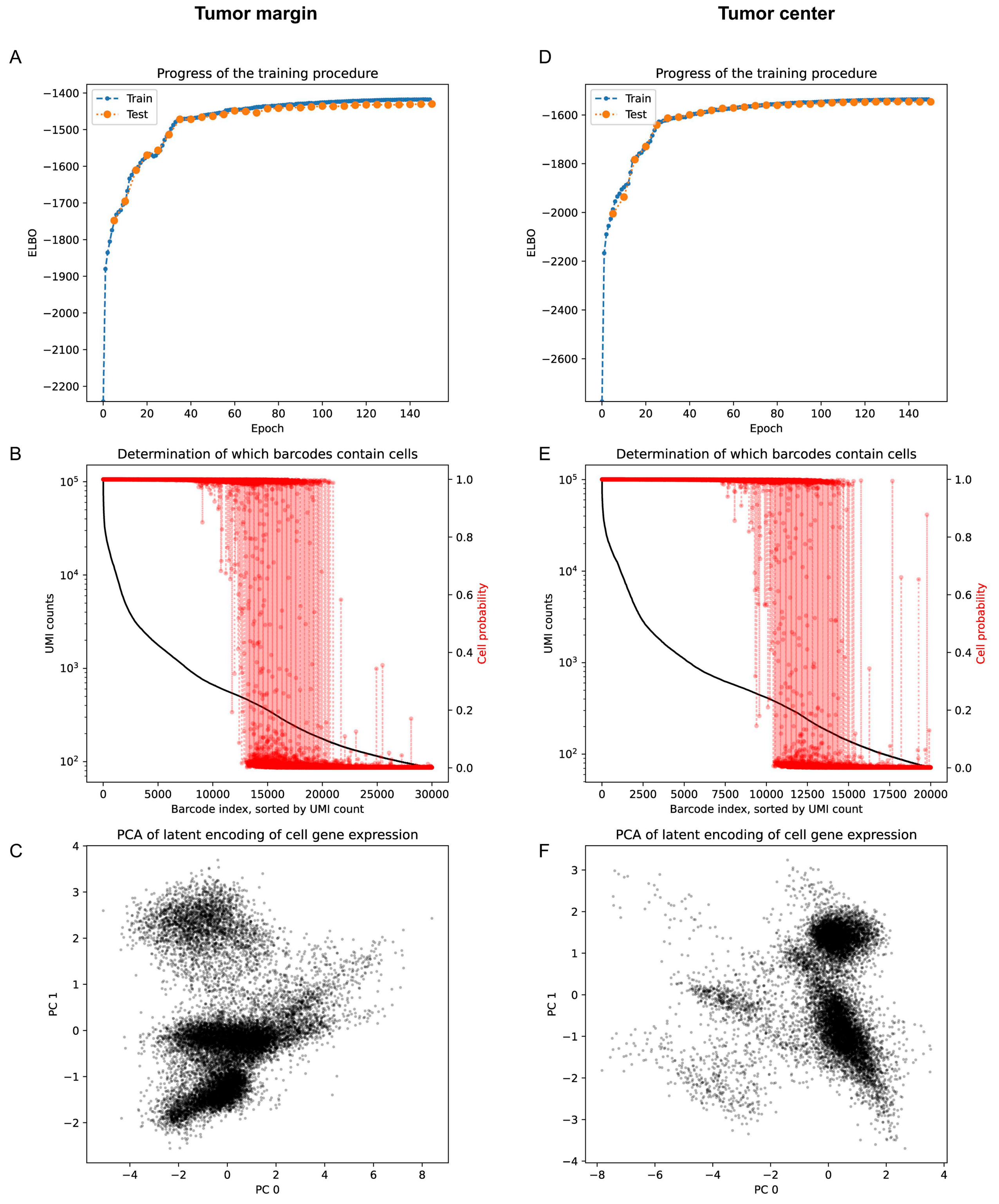


Supplementary Figure. 12 CellBender ambient RNA removal quality control for margin and center samples. Each panel set contains three plots: (A, D) Training curve showing the ELBO over 150 epochs, with convergence of train (blue) and test (orange) curves indicating stable model fitting. (B, E) Barcode rank plot showing UMI counts per barcode (black line) and CellBender-estimated cell probability (red dots) as a function of barcode index sorted by decreasing UMI count; high-probability barcodes (~1.0) are classified as cell-containing droplets, while low-probability barcodes represent empty droplets used to estimate the ambient RNA profile. The center sample contained approximately 10,000 cell barcodes and the margin sample approximately 12,500 cell barcodes. (C, F) PCA of the CellBender latent representation of decontaminated cell gene expression after ambient RNA correction.

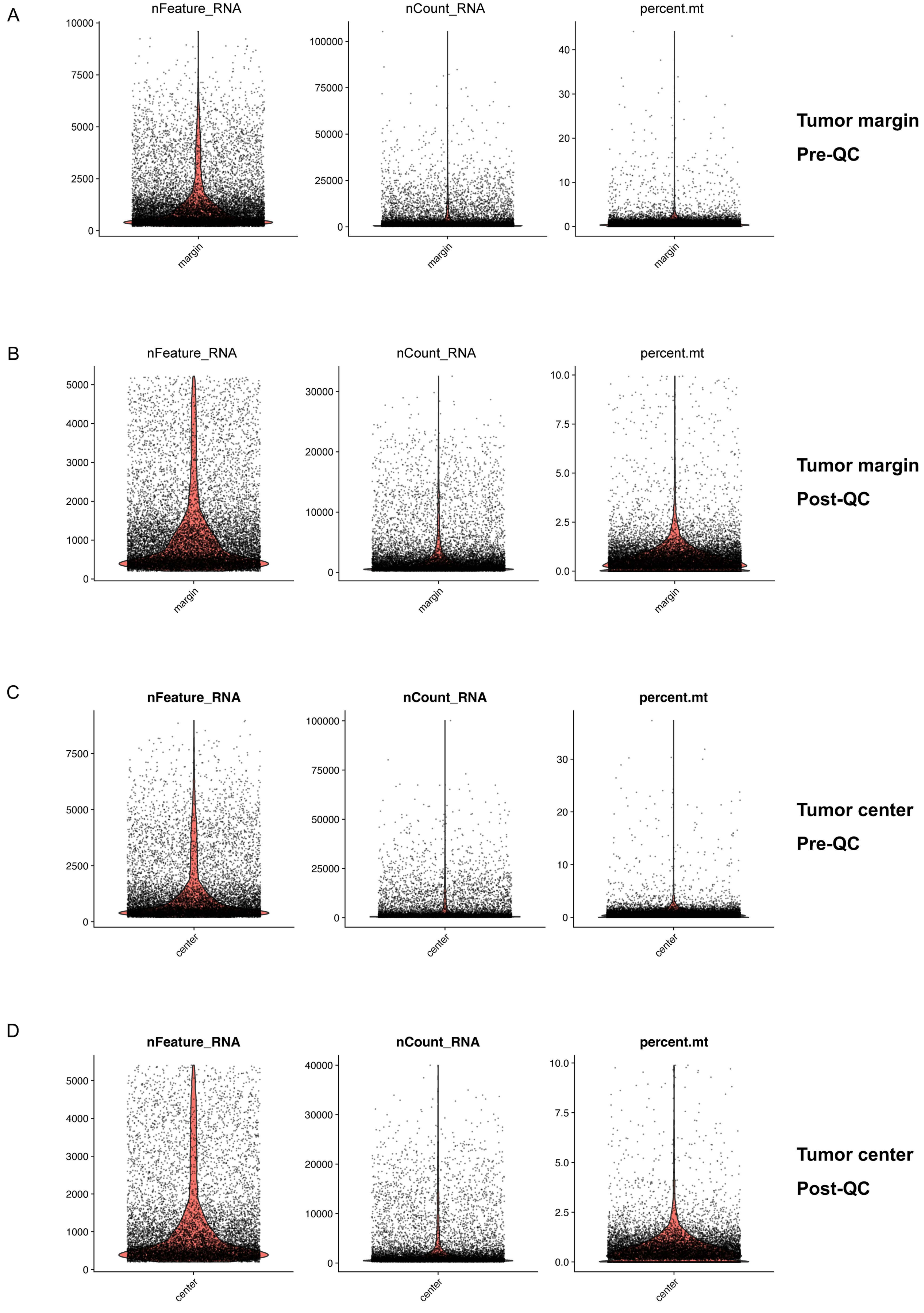


Supplementary Figure. 13 Quality control metrics of single-cell RNA-sequencing samples from margin and center regions.

Violin plots show the quality control (QC) results for the margin and center tumor samples. Samples from the margin region are shown before QC (A) and after QC (B), whereas samples from the center region are shown before QC (C) and after QC (D). The quality control metrics include the number of detected genes per cell (nFeature_RNA), total RNA counts per cell (nCount_RNA), and the percentage of mitochondrial transcripts (percent.mt).

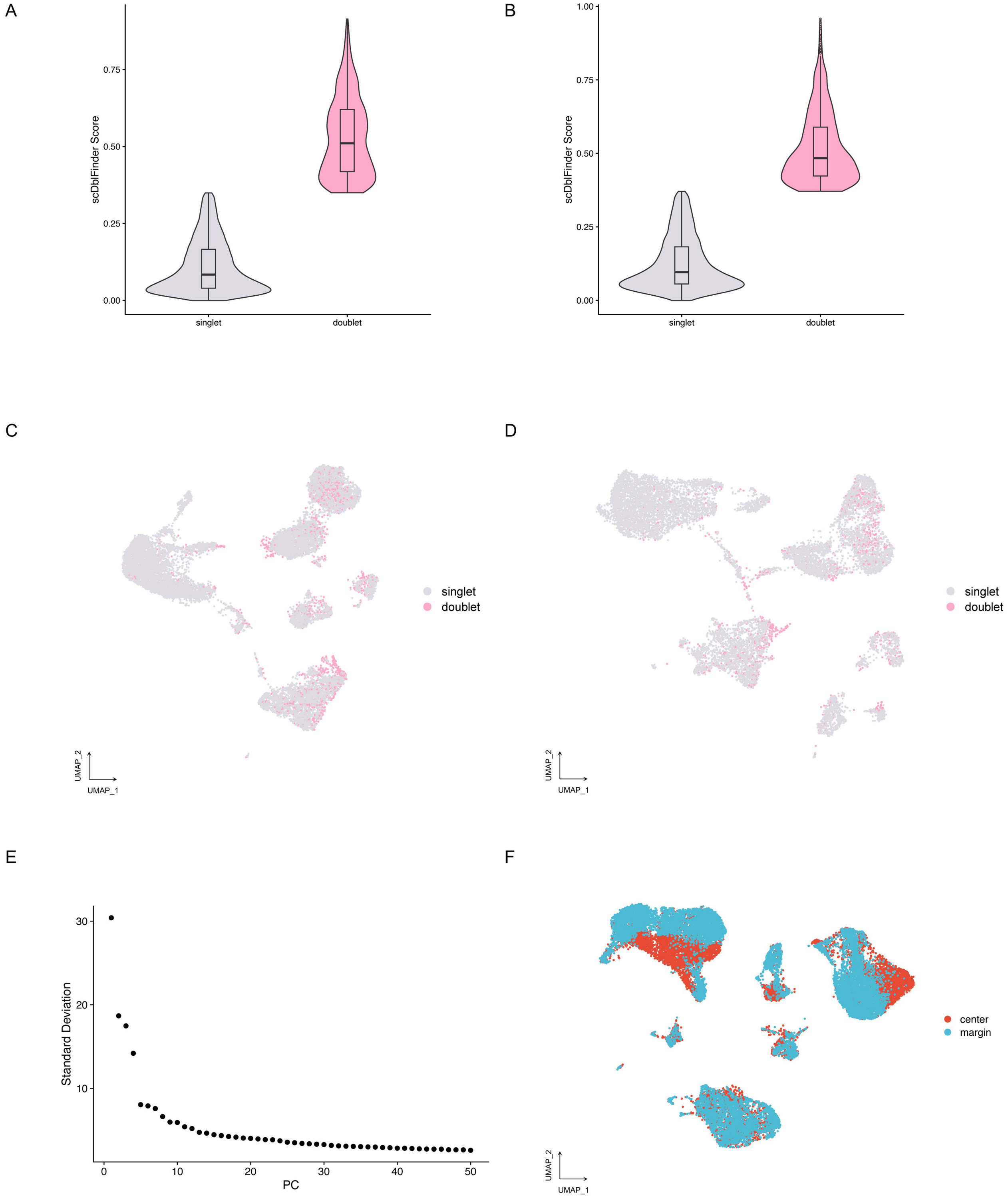


Supplementary Figure. 14 Doublet detection and batch-effect assessment in scRNA-seq data before downstream integration.

Violin plots of scDblFinder scores for the margin (A) and center (B) samples. Barcodes classified as singlets (grey) show consistently low scores (median ~0.12), whereas barcodes classified as doublets (pink) show markedly higher scores (median ~0.48–0.51), demonstrating clear separation between the two populations. The overlaid box plots indicate the median and interquartile range. Barcodes classified as doublets by scDblFinder were excluded before downstream integration and clustering. Residual mixed-profile clusters identified during subsequent lineage-specific subclustering were evaluated separately and excluded where applicable before downstream analyses. (C, D) UMAP visualization of doublet distribution prior to removal for the margin (C) and center (D) samples. Each dot represents a single barcode, colored by scDblFinder classification: singlets (grey) and doublets (pink). UMAP embedding was computed from the top 30 principal components of normalized gene expression before Harmony batch correction.

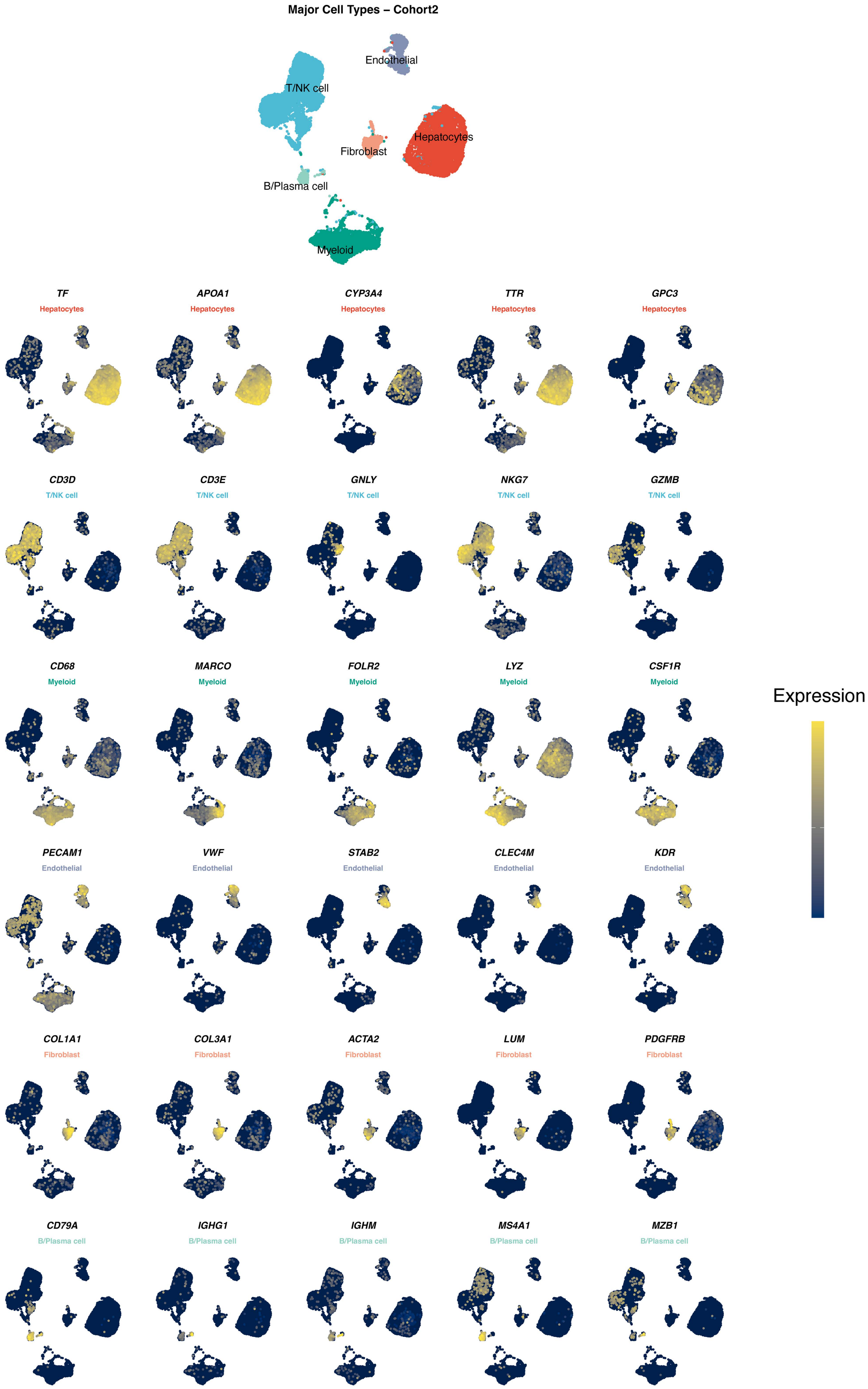


Supplementary Figure. 15 Expression of canonical cell-type marker genes across intratumoral regions. UMAP visualization of QC-filtered high-quality single cells from tumor margin and tumor center samples (n = 21,975 cells), colored by annotated major cell type. Feature plots show normalized expression of canonical marker genes for each cell population: Hepatocytes (TF, APOA1, CYP3A4, TTR, GPC3); T/NK cells (CD3D, CD3E, GNLY, NKG7, GZMB); Myeloid cells (CD68, MARCO, FOLR2, LYZ, CSF1R); Endothelial cells (PECAM1, VWF, STAB2, CLEC4M, KDR); Fibroblasts (COL1A1, COL3A1, ACTA2, LUM, PDGFRB); B/Plasma cells (CD79A, IGHG1, IGHM, MS4A1, MZB1). Color scale represents normalized expression level (cividis palette; low to high). Color scales are shown individually for each gene and should not be compared directly across markers. Cell type labels in feature plot subtitles are color-coded to match the UMAP.
