# Supplementary material for: Spatiotemporal dynamics of the tumor microenvironment in hepatocellular carcinoma during combination immunotherapy
Source: Hepatol Commun. 2026 Aug 7;10(9):e1014. doi: 10.1097/HC9.0000000000001014 (PMC13456720; doi:10.1097/HC9.0000000000001014)
Supplement: Supplementary file 3 [file hc9-10-e1014-s003.pdf]

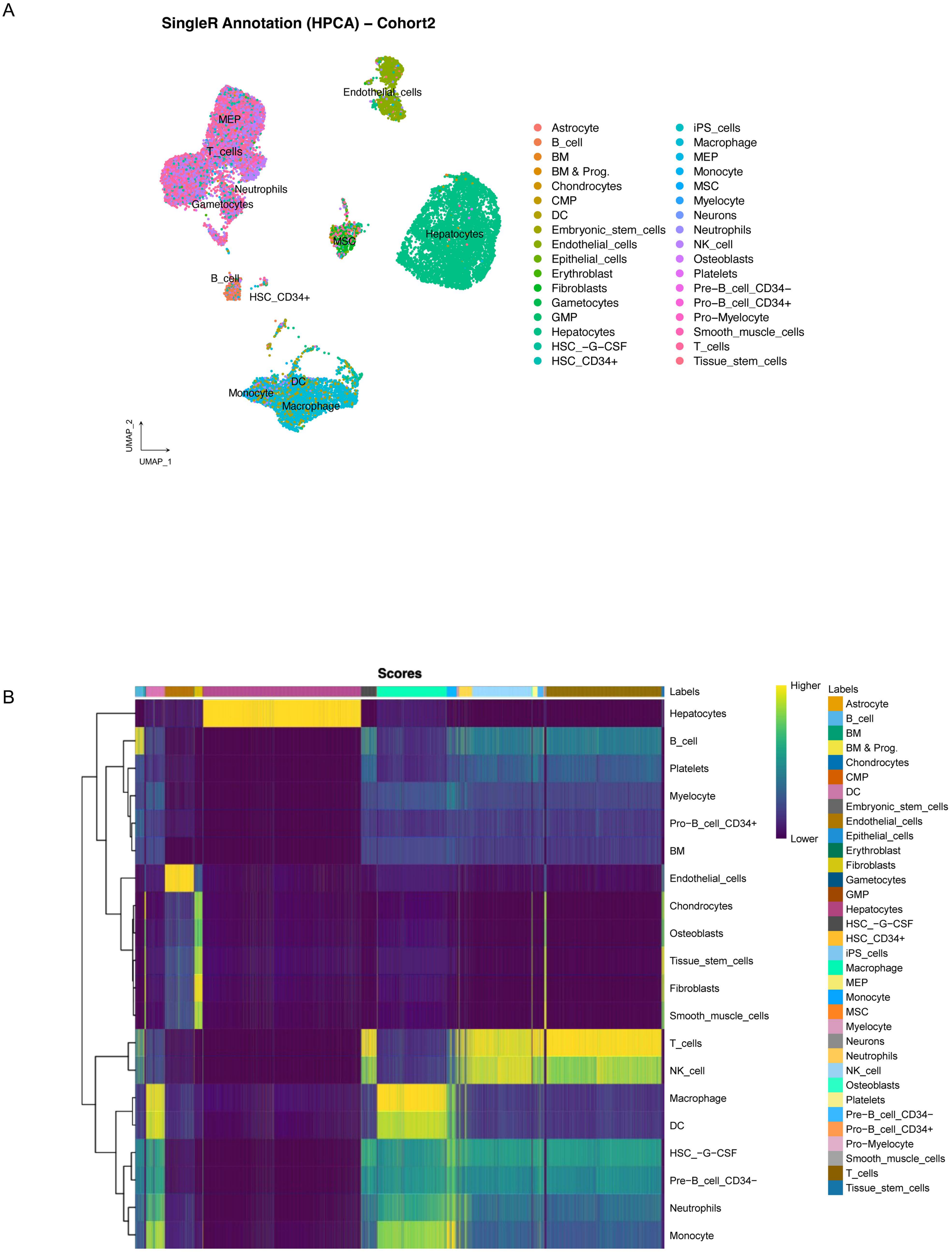

Supplementary Figure. 16 SingleR-based cell type annotation and reference score validation of integrated scRNA-seq data from margin and center regions

(A) UMAP of SingleR-assigned cell type labels using the Human Primary Cell Atlas (HPCA, v2.8.0) as reference. The dominant cluster corresponds to Hepatocytes, consistent with the liver tissue origin, with additional immune and stromal populations including T cells, Macrophages, Monocytes, DC, B cells, Fibroblasts, and Endothelial cells. These labels were used as one line of evidence to guide manual cell type annotation. (B) Heatmap of per-cell SingleR correlation scores against all HPCA reference cell types. Columns represent individual cells grouped by their final SingleR label (top color bar); rows represent reference cell types. Color indicates score magnitude (yellow: higher; purple: lower). Diagonal enrichment confirms transcriptionally distinct classification of each cell population.

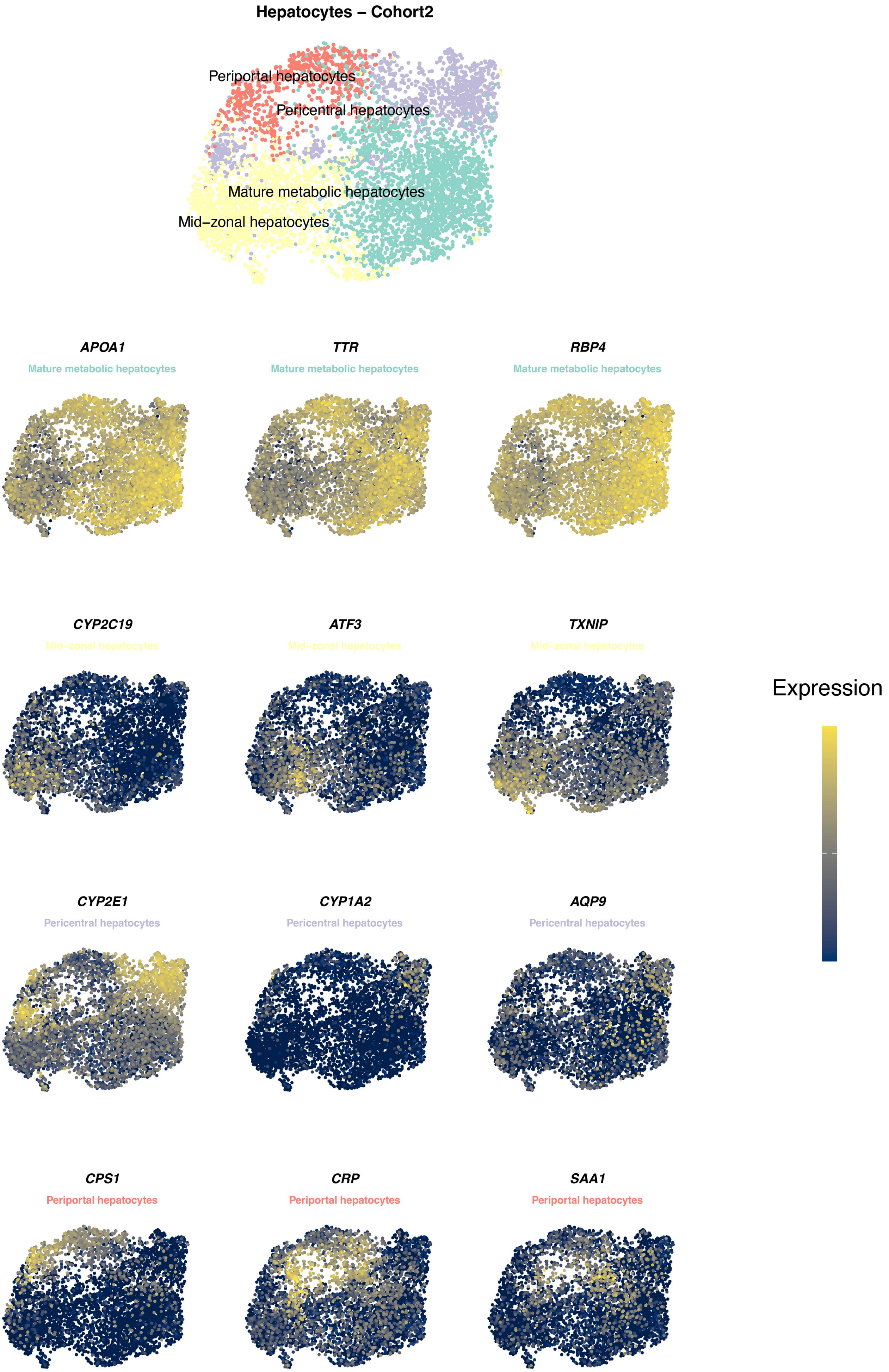

Supplementary Figure. 17 Hepatocyte subcluster annotation and marker gene expression in margin and center regions.

UMAP of hepatocyte subclusters: mature metabolic, mid-zonal, periportal, and pericentral. Feature plots showing expression of subcluster marker genes. Color scale indicates normalized log-transformed expression (dark blue: low; yellow: high). Color scales are shown individually for each gene and should not be compared directly across markers. Mature metabolic hepatocytes: APOA1, TTR, RBP4; mid-zonal hepatocytes: CYP2C19, ATF3, TXNIP; pericentral hepatocytes: CYP2E1, CYP1A2, AQP9, periportal hepatocytes: CPS1, CRP, SAA1.

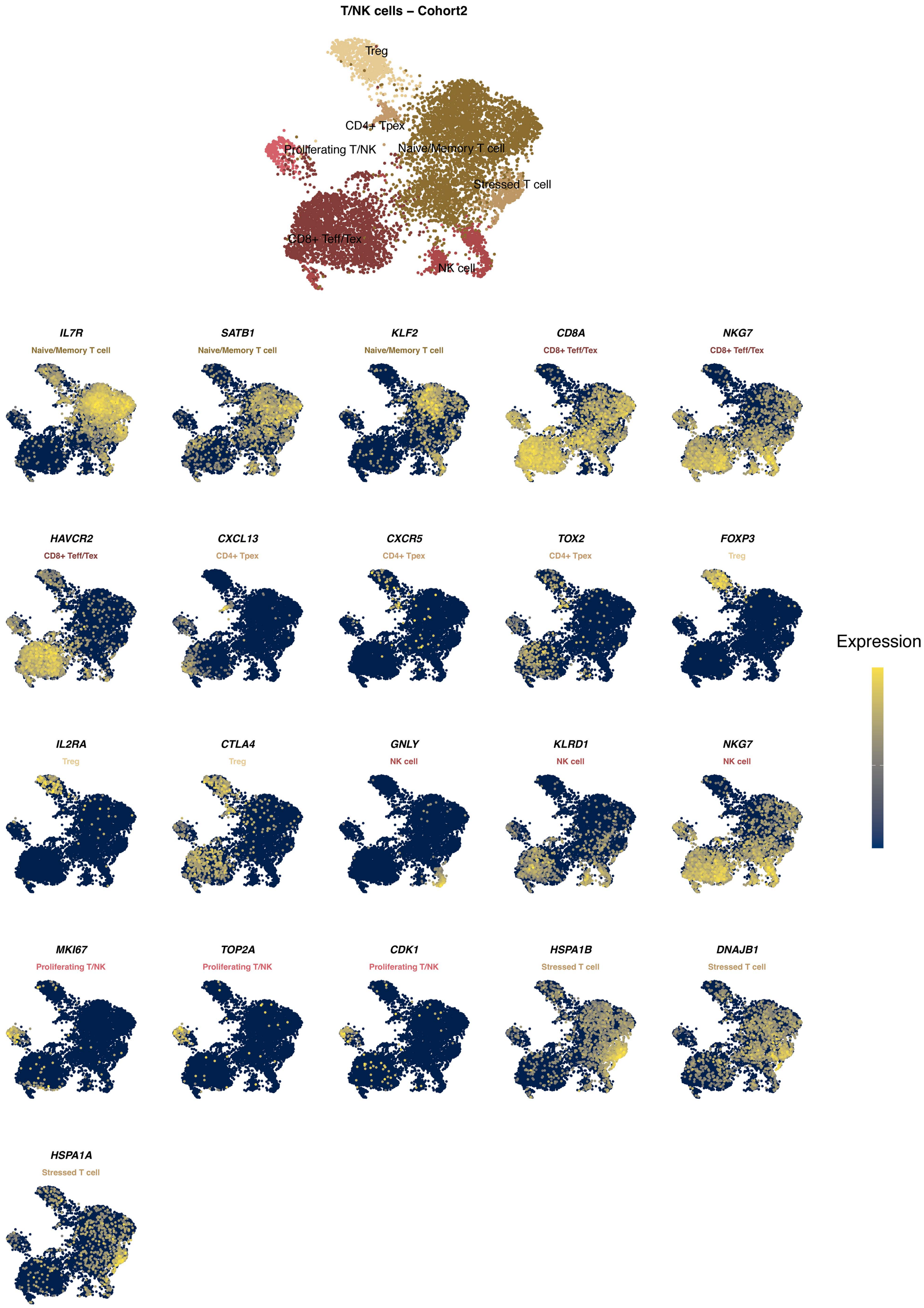

Supplementary Figure. 18 T/NK-cell subcluster annotation and marker gene expression in margin and center regions.
UMAP of T/NK cell subclusters: naive/memory T cells, CD8+ effector/exhausted T cells (Teff/Tex), CD4+ precursor exhausted T cells (Tpex), regulatory T cells (Treg), NK cells, stressed T cells, and Proliferating T/NK cells. Feature plots showing expression of subcluster marker genes. Color scale indicates normalized log-transformed expression (dark blue: low; yellow: high). Color scales are shown individually for each gene and should not be compared directly across markers. Naive/memory T cells: IL7R, SATB1, KLF2; CD8+ Teff/Tex: CD8A, NKG7, HAVCR2; CD4+ Tpex: CXCL13, CXCR5, TOX2; Treg: FOXP3, IL2RA, CTLA4; NK cells: GNLY, KLRD1, NKG7; Proliferating T/NK cells: MKI67, TOP2A, CDK1; Stressed T cells: HSPA1B, DNAJB1, HSPA1A.

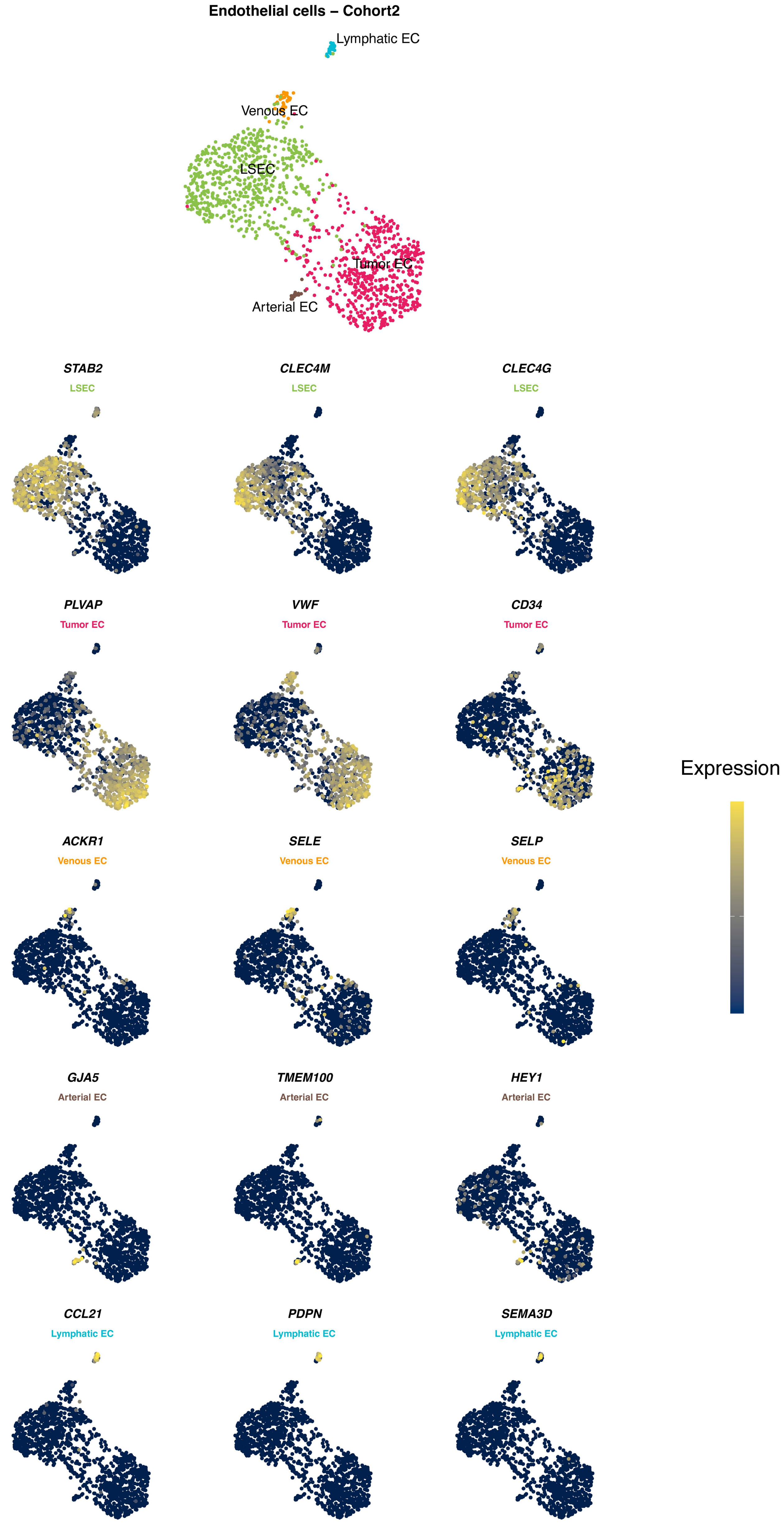

Supplementary Figure. 19 Endothelial cell subcluster annotation and marker gene expression in margin and center regions

UMAP of endothelial cell subclusters: liver sinusoidal endothelial cell (LSEC), Tumor EC, Venous EC, Arterial EC, and Lymphatic EC. Feature plots showing expression of subcluster marker genes. Color scale indicates normalized log-transformed expression (dark blue: low; yellow: high). Color scales are shown individually for each gene and should not be compared directly across markers. LSEC: STAB2, CLEC4M, CLEC4G; Tumor EC: PLVAP, VWF, CD34; Venous EC: ACKR1, SELE, SELP; Arterial EC: GJA5, TMEM100, HEY1; Lymphatic EC: CCL21, PDPN, SEMA3D.

A

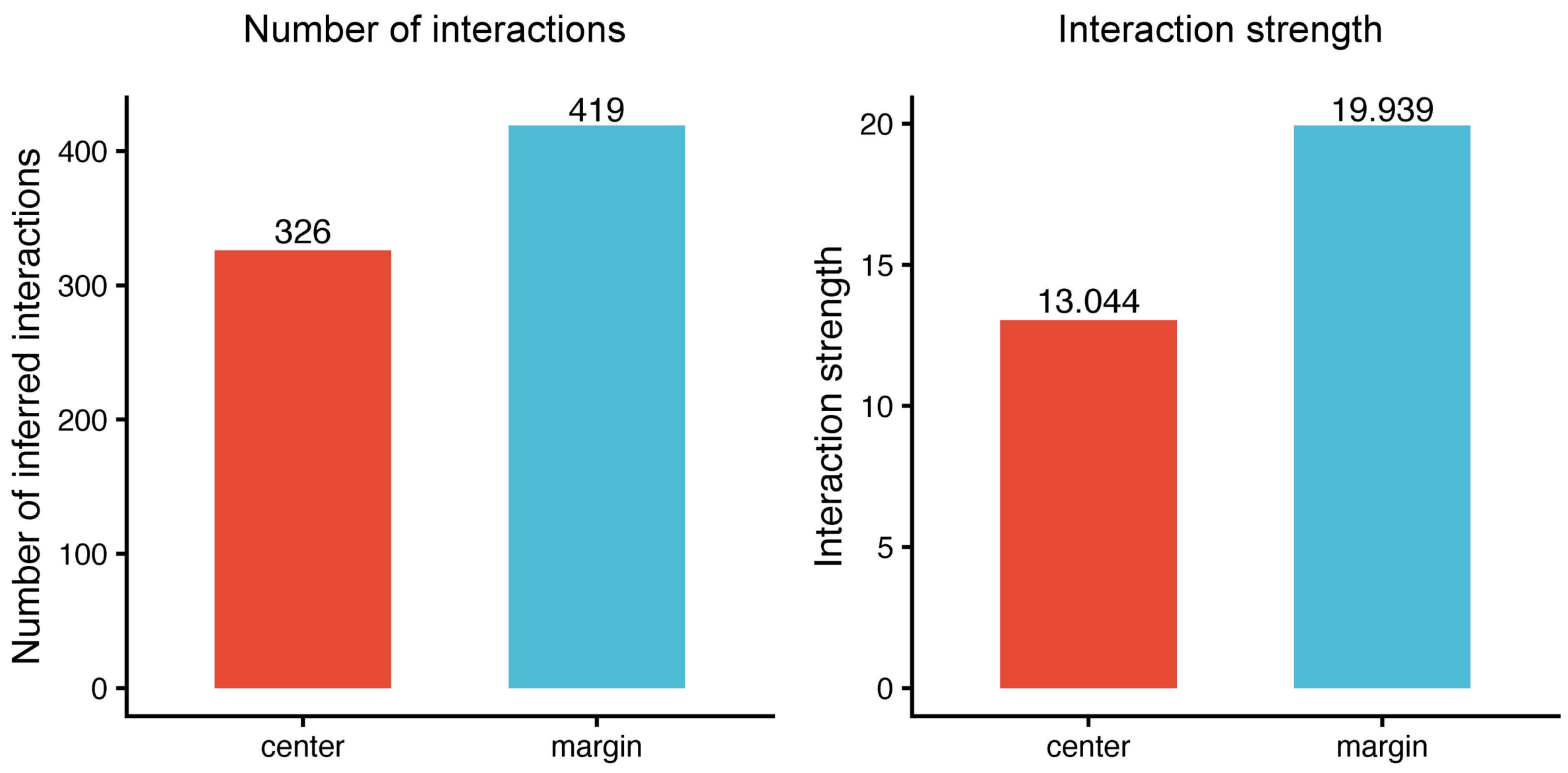

B

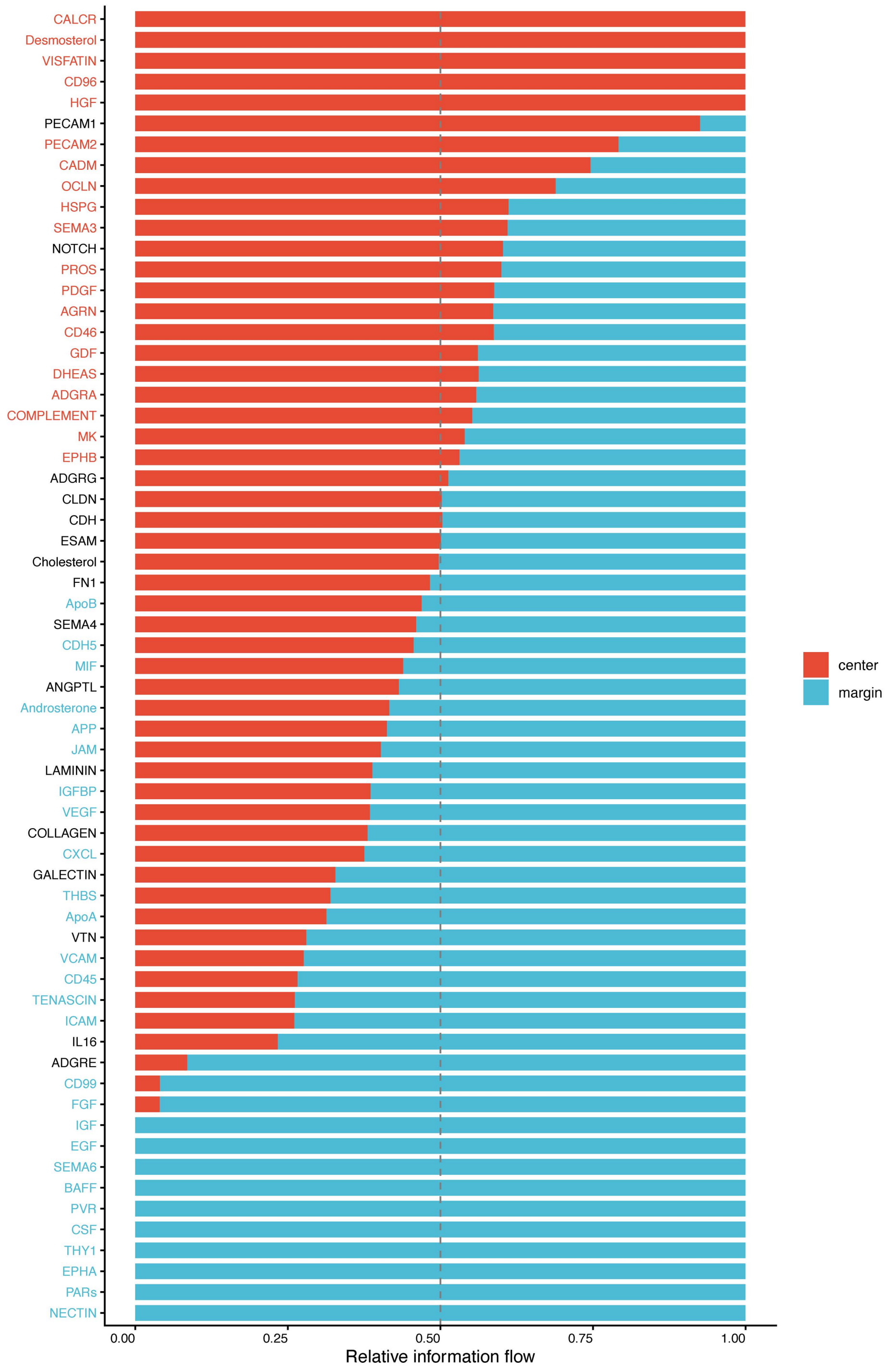

Supplementary Figure. 20 Global comparison of CellChat-inferred intercellular communication between margin and center regions.
(A) Bar plots comparing the number of inferred cell-cell interactions (left) and the total interaction strength (right) between the tumor center (Center; red) and tumor margin (Margin; cyan) conditions. The number of interactions increased from 326 (Center) to 419 (Margin), and the total interaction strength increased from 13.044 (Center) to 19.939 (Margin), indicating greater intercellular communication at the tumor margin. Interaction strengths were computed using the CellChat framework. (B) Horizontal bar plot showing the relative information flow for each CellChat signaling pathway in the tumor center (Center; red) and tumor margin (Margin; cyan) conditions. Pathways are ranked in descending order of total information flow (Center + Margin combined). The x-axis represents the relative information flow, normalized to a maximum of 1.0 across all pathways. Pathway names highlighted in red indicate pathways with significantly higher relative information flow in the Center condition; names in cyan indicate pathways significantly enriched at the tumor margin; and names in black indicate pathways showing no statistically significant difference between conditions. The dashed vertical line at 0.5 serves as a reference.

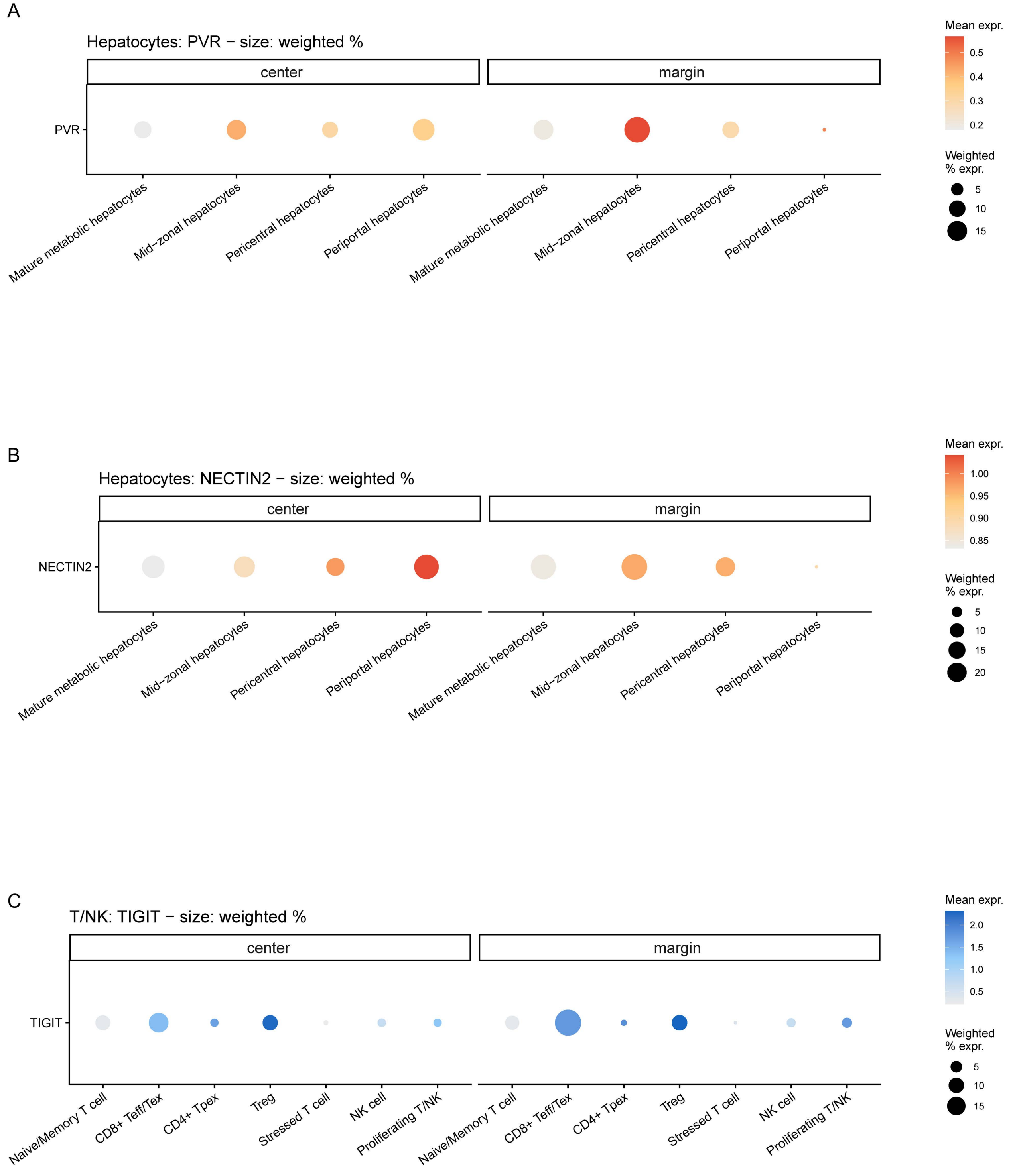

Supplementary Figure. 21 Expression of NECTIN2/PVR signaling axis genes across hepatocyte and T/NK-cell subtypes in margin and center regions.
Dot plots showing the expression of the immune checkpoint-related ligands PVR and NECTIN2 in hepatocyte subtypes (A, B), and the cognate receptors TIGIT, CD96, PVRIG, and CD226 in T/NK cell subtypes (C), stratified by tumor location (center vs. margin). Dot size represents the weighted percentage of expressing cells (% expressing cells × cell type abundance [%]), which reflects the proportional contribution of each cell subtype to total ligand or receptor expression within the sample. Dot color indicates mean log-normalized gene expression (red scale: NECTIN2 and PVR; blue scale: receptors). Five hepatocyte subtypes were examined for NECTIN2 and PVR: Mature metabolic, Mid-zonal, Periportal, Pericentral, and Proliferating hepatocytes. Seven T/NK cell subtypes were examined for receptors: CD8+ Teff/Tex, CD4+ Tpex, Naive/Memory T cells, NK cells, Tregs, Proliferating T/NK cells, and Stressed T cells.

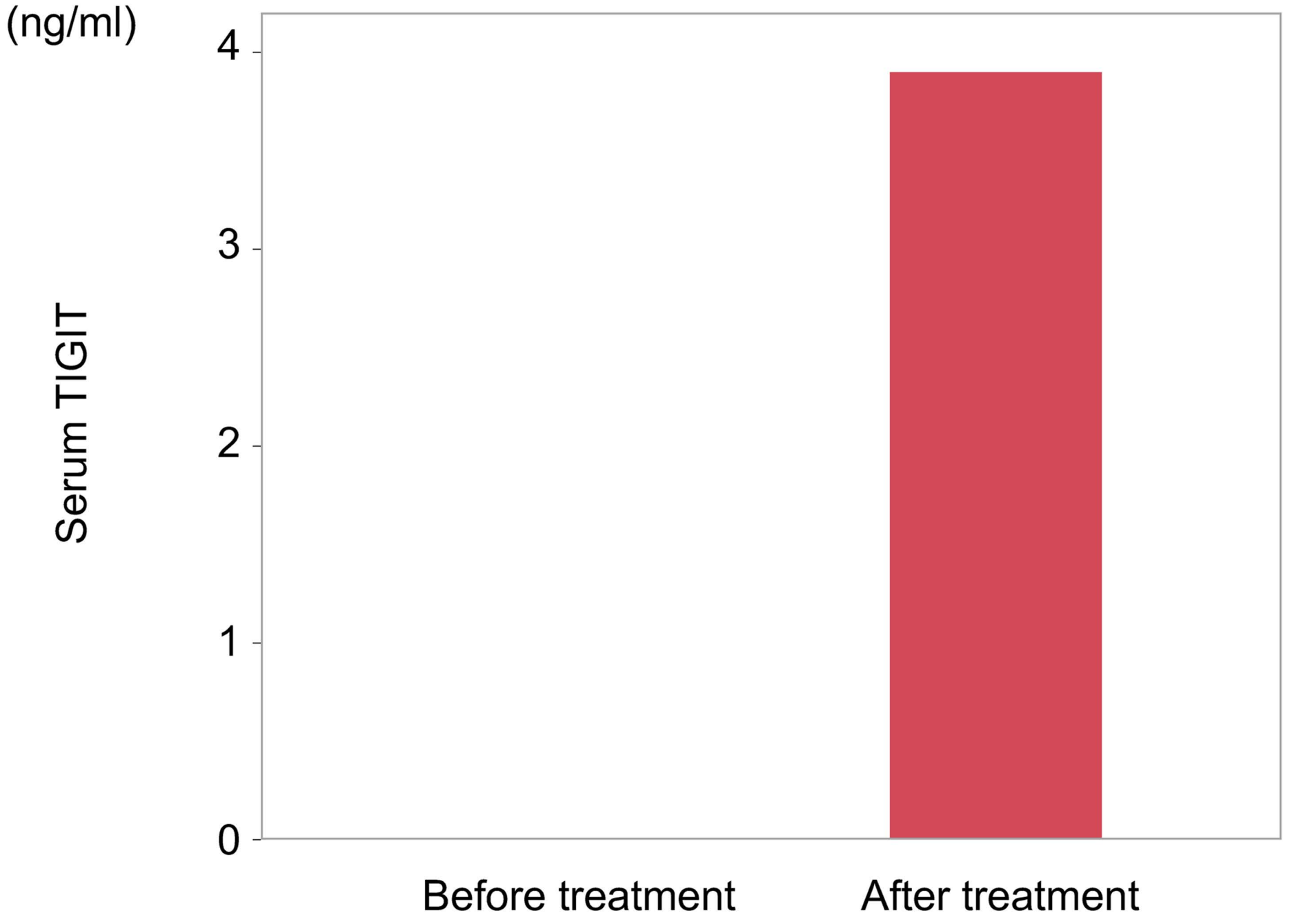

Supplementary Figure. 22 Increase in Serum TIGIT Levels in the samples after treatment.
Serum TIGIT levels were measured in the Pre- and Post-treatment samples that had been submitted for single-cell RNA sequencing. The Post-treatment samples showed an increased TIGIT concentration, indicating a treatment-associated rise in circulating TIGIT.

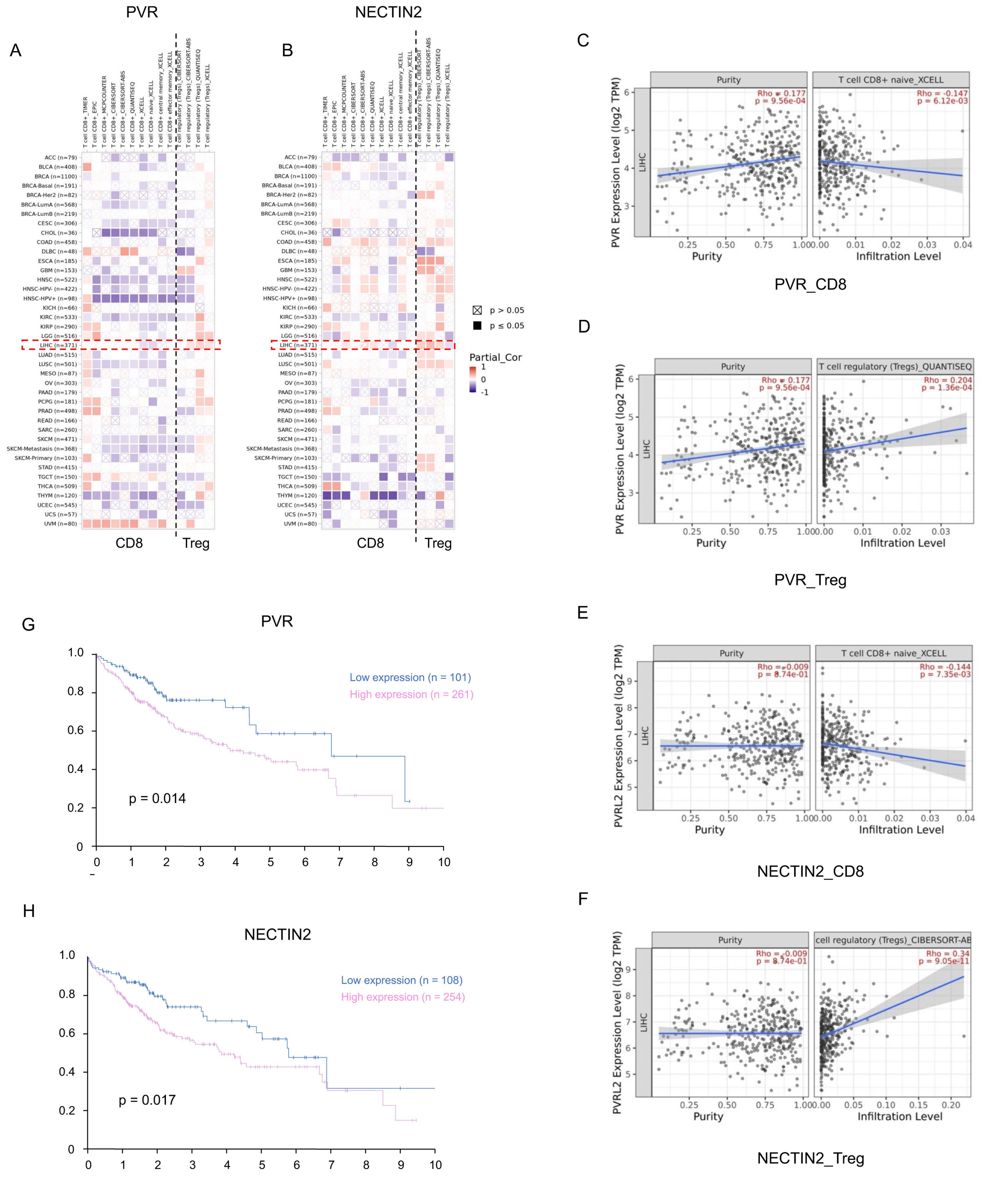

Supplementary Figure. 23 In silico analysis of the PVR/NECTIN expression.
(A, B) Heatmaps showing the partial correlation between PVR (A) or NECTIN2 (B) expression and the infiltration levels of CD8$^+$ T cells and regulatory T cells (Tregs) across multiple TCGA cancer types, analyzed using TIMER2.0. Immune cell infiltration estimates were derived from multiple algorithms, including TIMER, EPIC, CIBERSORT, quanTIseq, xCell, and others. Color intensity represents the strength and direction of partial correlations (red, positive; blue, negative). Filled squares denote statistically significant correlations ($p \leq 0.05$), whereas squares with cross marks indicate non-significant results ($p > 0.05$). The red dashed box highlights liver hepatocellular carcinoma (LIHC).
(C-F) Scatter plots showing the association between PVR (C, D) or NECTIN2 (E, F) expression levels and tumor purity (left) or immune cell infiltration (right) in LIHC (TCGA-LIHC). Immune infiltration levels of CD8$^+$ T cells (XCELL) and regulatory T cells (Tregs; QUANTISEQ or CIBERSORT-ABS) were obtained from the TIMER2.0 platform. Partial correlation coefficients (Rho) and p-values are indicated in each panel. Blue regression lines with 95% confidence intervals illustrate the direction and magnitude of the correlation. These analyses demonstrate that PVR expression negatively correlates with CD8$^+$ T-cell infiltration and positively correlates with Treg infiltration, whereas NECTIN2 shows weaker or algorithm-dependent associations.
(G, H) Overall survival analysis of PVR and NECTIN2 expression in hepatocellular carcinoma based on the Human Protein Atlas (HPA). (G) Kaplan–Meier overall survival curve comparing high versus low PVR expression groups in LIHC (HPA dataset). (H) Kaplan–Meier overall survival curve comparing high versus low NECTIN2 expression groups in LIHC (HPA dataset). Expression groups were stratified according to HPA-defined cutoffs. High expression of PVR and NECTIN2 was associated with significantly poorer overall survival (PVR: $p = 0.014$; NECTIN2: $p = 0.017$).
