# Supplementary material for: Spatiotemporal dynamics of the tumor microenvironment in hepatocellular carcinoma during combination immunotherapy
Source: Hepatol Commun. 2026 Aug 7;10(9):e1014. doi: 10.1097/HC9.0000000000001014 (PMC13456720; doi:10.1097/HC9.0000000000001014)
Supplement: Supplementary file 4 [file hc9-10-e1014-s006.pdf]

SupTable5A_C1_Total_SeuratCluster

p_val	avg_log2FC	pct.1	pct.2	p_val_adj	cluster	gene
0	3.77704677544956	0.422	0.027	0	0	CHST6
0	3.62529102502018	0.926	0.103	0	0	GABRE
0	3.30706344437103	0.33	0.03	0	0	DUSP21
0	3.24876177427279	0.703	0.079	0	0	MTCP1
0	3.02737580983097	0.295	0.028	0	0	PCDHGA8
0	2.89239299339411	0.529	0.057	0	0	BTNL3
0	2.88657442139444	0.287	0.027	0	0	PCDHGB6
0	2.85965789293423	0.467	0.054	0	0	POU5F1
0	2.76780017035934	0.297	0.037	0	0	ARHGEF38
0	2.76291564322612	0.31	0.034	0	0	C6orf141
0	2.75221574451162	0.35	0.038	0	0	XKR9
0	2.73482623973183	0.408	0.037	0	0	TTN
0	2.73173500114941	0.251	0.027	0	0	MPP3
0	2.68764069098072	0.913	0.211	0	0	SULT1C2
0	2.65874314822447	0.465	0.046	0	0	CCDC141
0	2.65619087694499	0.381	0.041	0	0	PCDHGA5
0	2.6394674242157	0.886	0.167	0	0	SLC17A4
0	2.63758210954481	0.674	0.095	0	0	PCSK5
0	2.63514613048052	0.415	0.049	0	0	KCNMB3
0	2.57590754622759	0.257	0.024	0	0	PCDHGA6
0	2.56669257444827	0.698	0.096	0	0	ZMAT1
0	2.56086673048525	0.279	0.027	0	0	PCDHGB7
0	2.5606369184194	0.952	0.401	0	0	SMARCA1
0	2.55392582612609	0.423	0.052	0	0	DNM1
0	2.52922185614877	0.655	0.094	0	0	WDYHV1
0	2.52790753510874	0.456	0.054	0	0	UGT1A3
0	2.50986910925277	0.348	0.045	0	0	CES4A
0	2.47428553770649	0.545	0.072	0	0	CACNA1H
0	2.4529766044617	0.491	0.066	0	0	ANKS1B
0	2.44190804427109	0.271	0.033	0	0	PHOSPHO2
0	2.43348161350685	0.926	0.186	0	0	CEP70
0	2.42259523288903	0.9	0.238	0	0	CTPS1
0	2.42203709534972	0.317	0.041	0	0	ALPK2
0	2.42164926115054	0.687	0.098	0	0	ZNF75D
0	2.40390197614541	0.578	0.085	0	0	PTEN
0	2.36803511094696	0.547	0.082	0	0	SHOX2
0	2.35324736659543	0.267	0.036	0	0	FSBP
0	2.32199739296461	0.842	0.159	0	0	BTNL8
0	2.3201033406791	0.543	0.08	0	0	LAMA3
0	2.31792064787753	0.259	0.037	0	0	SLC5A1
0	2.31338736252682	0.254	0.036	0	0	TTLL6
0	2.31086870802438	0.309	0.035	0	0	CEACAM19
0	2.29888121839637	0.461	0.067	0	0	ZNF436
0	2.27763382983156	0.808	0.13	0	0	CCDC14
0	2.27024279322527	0.445	0.058	0	0	NOL3
0	2.21575532195784	0.38	0.055	0	0	DEPRECATED-ENSG00000125675
0	2.20397144591705	0.879	0.199	0	0	NR1I2
0	2.2022564038673	0.344	0.043	0	0	PCDHGA3
0	2.19657359954669	0.563	0.078	0	0	FRAS1
0	2.19095055031334	0.524	0.079	0	0	CCDC198
0	2.18540737762975	0.369	0.054	0	0	FSD1L
0	2.18317707916241	0.306	0.038	0	0	PPP1R3E
0	2.17505236249718	0.28	0.038	0	0	ADCY10
0	2.17385909246299	0.615	0.103	0	0	TMC5
0	2.16961790160116	0.672	0.11	0	0	MGAM2
0	2.16854116478205	0.322	0.06	0	0	MUC1
0	2.16782915178495	0.699	0.115	0	0	TBC1D8B
0	2.16483098871964	0.257	0.034	0	0	CUX2

0	2.16015269096007	0.345	0.043	0	0	SPATA6L
0	2.15164856697788	0.514	0.07	0	0	ART4
0	2.15111857033768	0.36	0.059	0	0	B3GALT5
0	2.13212798995433	0.322	0.047	0	0	HCN3
0	2.12262158917387	0.534	0.074	0	0	MFSD3
0	2.10753707294284	0.836	0.177	0	0	TBC1D8
0	2.10536280880942	0.251	0.028	0	0	C14orf180
0	2.10196015468605	0.813	0.175	0	0	SLC15A1
0	2.10043592216925	0.5	0.079	0	0	FZD6
0	2.10004013601066	0.612	0.098	0	0	USP54
0	2.09325581034967	0.713	0.112	0	0	RBM12B
0	2.08398718973499	0.324	0.052	0	0	ATP2C2
0	2.07447598728742	0.749	0.133	0	0	CLMN
0	2.05339632640318	0.586	0.097	0	0	DEPRECATED-ENSG00000183747
0	2.04920813461161	0.321	0.046	0	0	RPA4
0	2.03504225636458	0.734	0.13	0	0	KCNJ3
0	2.03212807210816	0.619	0.12	0	0	GRIA3
0	2.02974504769861	0.304	0.045	0	0	C3orf85
0	2.02737406855799	0.828	0.207	0	0	NECAB3
0	2.02070935690633	0.413	0.056	0	0	MROH6
0	2.01935345887976	0.511	0.075	0	0	SLC10A5
0	2.01326127210991	0.326	0.05	0	0	MYO7B
0	2.00514190996145	0.315	0.043	0	0	ZNF552
0	2.0043795660456	0.448	0.07	0	0	PIK3C2G
0	2.00103778345165	0.495	0.069	0	0	VIPR1
0	1.99580419482674	0.736	0.155	0	0	KCNK5
0	1.99256040932141	0.401	0.065	0	0	POLE2
0	1.98575837174706	0.513	0.084	0	0	GREB1L
0	1.98124550282966	0.877	0.218	0	0	SMOC1
0	1.96683950477821	0.851	0.188	0	0	MLPH
0	1.96561312436877	0.264	0.038	0	0	ZNF782
0	1.96304884700277	0.252	0.038	0	0	FHOD3
0	1.95792101958449	0.93	0.269	0	0	PTPRK
0	1.9482753342911	0.837	0.177	0	0	PVR
0	1.94732193772042	0.96	0.418	0	0	MUC3A
0	1.93009500530793	0.411	0.061	0	0	PTPRD
0	1.91866323769942	0.272	0.04	0	0	PPP1R9A
0	1.91211382599051	0.406	0.062	0	0	THSD4
0	1.91126279308487	0.86	0.245	0	0	SLC6A19
0	1.89881867511709	0.462	0.073	0	0	ZNF251
0	1.89066410485611	0.411	0.063	0	0	CHST5
0	1.88582389625545	0.552	0.111	0	0	HAO2
0	1.88274287591311	0.257	0.038	0	0	ANKRD29
0	1.88272471333032	0.58	0.095	0	0	GLI4
0	1.87549802437617	0.637	0.114	0	0	MLH3
0	1.87004985367185	0.674	0.133	0	0	AKR1C8P
0	1.86673842289047	0.781	0.165	0	0	SH3RF2
0	1.86064096317327	0.73	0.142	0	0	NIPAL2
0	1.8540981684324	0.603	0.102	0	0	BCL2L2
0	1.84827890048344	0.509	0.09	0	0	HSD11B2
0	1.8459390189192	0.925	0.275	0	0	ERO1B
0	1.84403416240118	0.434	0.077	0	0	TSPYL4
0	1.84394112967504	0.487	0.072	0	0	GOLGA8B
0	1.84087631068478	0.322	0.047	0	0	LIG4
0	1.83946136538103	0.902	0.245	0	0	OCRL
0	1.83571813089803	0.581	0.105	0	0	CCL28
0	1.83209870033644	0.506	0.099	0	0	PLGLB2
0	1.81939899402294	0.435	0.072	0	0	ZSWIM5
0	1.81592488087068	0.304	0.046	0	0	MIPOL1
0	1.81527768441479	0.639	0.127	0	0	MS4A8

0	1.80933293916132	0.664	0.12	0	0	EXOC3L4
0	1.80589067690881	0.532	0.092	0	0	UGGT2
0	1.78320101959119	0.516	0.089	0	0	MAP3K21
0	1.78144431274177	0.685	0.123	0	0	ABCA5
0	1.77623981535294	0.72	0.146	0	0	WDFY2
0	1.77250612441028	0.582	0.108	0	0	PRELID2
0	1.77130739903979	0.425	0.07	0	0	RAVER2
0	1.77074966900181	0.613	0.119	0	0	EPPK1
0	1.75949500957854	0.478	0.08	0	0	CES3
0	1.75546035216706	0.84	0.204	0	0	ARHGEF26
0	1.74669454457477	0.498	0.091	0	0	TIGD2
0	1.74543458019112	0.292	0.049	0	0	CCDC7
0	1.74492023009519	0.704	0.135	0	0	BIVM
0	1.73882339673512	0.525	0.091	0	0	DPH6
0	1.7358363396899	0.541	0.086	0	0	FBXL8
0	1.73199293775568	0.643	0.139	0	0	SI
0	1.72744922834078	0.297	0.053	0	0	WDR5B
0	1.72698447361945	0.532	0.093	0	0	DNAH14
0	1.72640960534693	0.739	0.153	0	0	CYP4F12
0	1.7260924106579	0.932	0.292	0	0	ZBTB16
0	1.72590428215141	0.571	0.103	0	0	SLC2A13
0	1.71670961184409	0.348	0.058	0	0	NRXN3
0	1.71352880332436	0.64	0.135	0	0	ST3GAL6
0	1.71169479359071	0.9	0.249	0	0	HNF4G
0	1.71093126774743	0.436	0.067	0	0	NPIPB15
0	1.71011053148635	0.573	0.103	0	0	SPIRE2
0	1.70952425809011	0.758	0.17	0	0	GPT
0	1.70525057183123	0.835	0.202	0	0	ATAD2
0	1.70418198113103	0.771	0.169	0	0	DIAPH2
0	1.70073510960556	0.645	0.125	0	0	CRACR2B
0	1.69942320617262	0.456	0.076	0	0	SLC45A3
0	1.69783717905438	0.347	0.057	0	0	TMEM171
0	1.69651873480528	0.455	0.082	0	0	ACYP1
0	1.6850408945544	0.707	0.143	0	0	BDH2
0	1.68268333362128	0.495	0.088	0	0	NR3C2
0	1.68102859414077	0.964	0.404	0	0	NOTCH2NLC
0	1.67828458393174	0.707	0.127	0	0	PIEZO2
0	1.67676199271333	0.553	0.115	0	0	POF1B
0	1.67369850105412	0.338	0.05	0	0	DEPRECATED-ENSG00000186204
0	1.67188503371045	0.697	0.149	0	0	INTS6
0	1.67176768395468	0.593	0.122	0	0	ASB9
0	1.66875575795701	0.398	0.071	0	0	FZD8
0	1.66438439422396	0.919	0.268	0	0	F2RL1
0	1.6621508002679	0.555	0.087	0	0	DGKE
0	1.65769405056526	0.725	0.162	0	0	LRRC31
0	1.65762273748885	0.255	0.042	0	0	ARSJ
0	1.65634365284963	0.35	0.063	0	0	RADX
0	1.65351911162504	0.258	0.041	0	0	ZNF234
0	1.64630519509453	0.436	0.074	0	0	MTERF2
0	1.64355720248968	0.957	0.432	0	0	SELENBP1
0	1.63845703327264	0.658	0.129	0	0	NT5DC3
0	1.63801065969052	0.368	0.06	0	0	B4GALNT2
0	1.63789204763047	0.417	0.072	0	0	DCUN1D2
0	1.63743471285071	0.273	0.052	0	0	TTLL7
0	1.63664786727644	0.509	0.092	0	0	USP53
0	1.63617045931563	0.694	0.171	0	0	B3GNT3
0	1.63530018697971	0.867	0.234	0	0	IL6R
0	1.6281828599593	0.915	0.287	0	0	C2orf72
0	1.62745164295136	0.937	0.315	0	0	ALG13
0	1.61849256490294	0.329	0.05	0	0	AP002495.1

0	1.61751141175784	0.912	0.271	0	0	NOTCH2NLB
0	1.61367948351387	0.299	0.051	0	0	ADGRG2
0	1.61269412378168	0.671	0.144	0	0	PDX1
0	1.60795397801743	0.501	0.091	0	0	ERMARD
0	1.60756093544581	0.264	0.045	0	0	JRK
0	1.60732123398793	0.412	0.071	0	0	FRY
0	1.60658283854014	0.404	0.074	0	0	PTGR2
0	1.60576737709056	0.353	0.058	0	0	CLRN3
0	1.60560117562979	0.286	0.046	0	0	SYDE2
0	1.60304880930602	0.599	0.12	0	0	UGT1A9
0	1.60148047796215	0.597	0.123	0	0	FAIM
0	1.60113778537091	0.73	0.158	0	0	CDS1
0	1.5995250438164	0.696	0.194	0	0	FXYD3
0	1.59048512394775	0.888	0.261	0	0	DEGS1
0	1.59020432812874	0.644	0.129	0	0	MOCOS
0	1.5894593781385	0.626	0.115	0	0	TARBP1
0	1.58741941293508	0.633	0.124	0	0	GSAP
0	1.57844447286598	0.656	0.136	0	0	RBM41
0	1.57792471998632	0.792	0.191	0	0	NEB
0	1.57687175988453	0.578	0.101	0	0	SAMD12
0	1.57669981738775	0.314	0.054	0	0	TCAF2
0	1.57476149867436	0.381	0.14	0	0	AGR2
0	1.57349608265101	0.75	0.191	0	0	DUSP9
0	1.57149434455436	0.471	0.089	0	0	ZNF75A
0	1.57006005888503	0.266	0.047	0	0	POP1
0	1.56926693733712	0.303	0.054	0	0	PPM1L
0	1.56645323001644	0.491	0.087	0	0	FANCL
0	1.56063011576305	0.548	0.11	0	0	L3HYPDH
0	1.55894296991828	0.345	0.056	0	0	SLC22A5
0	1.55823425912492	0.796	0.184	0	0	TAZ
0	1.55768555464038	0.402	0.072	0	0	GTF2IRD1
0	1.55684796472464	0.638	0.124	0	0	GTF2H2C
0	1.55627790796415	0.755	0.174	0	0	IMPA1
0	1.55584507214374	0.292	0.046	0	0	SRCIN1
0	1.55413321431301	0.431	0.084	0	0	BRCC3
0	1.55387540407136	0.494	0.091	0	0	MTERF3
0	1.55379494279689	0.289	0.052	0	0	PTPRM
0	1.55256639175567	0.722	0.154	0	0	PRKAG2
0	1.55076041612015	0.317	0.051	0	0	WDR19
0	1.55050885445874	0.781	0.192	0	0	OPLAH
0	1.54934755329986	0.451	0.082	0	0	TMEM39A
0	1.5486535540827	0.39	0.074	0	0	RWDD3
0	1.54701087364776	0.897	0.278	0	0	LIPG
0	1.54664767569847	0.391	0.072	0	0	ENDOV
0	1.54646342048954	0.494	0.092	0	0	ATPSCKMT
0	1.54563251621696	0.634	0.133	0	0	CCT8
0	1.54358597686174	0.492	0.096	0	0	SLC26A6
0	1.54319418774313	0.442	0.082	0	0	LRIG3
0	1.54299703659237	0.843	0.257	0	0	CLDN7
0	1.54197769283787	0.269	0.045	0	0	IGSF3
0	1.54152656832464	0.327	0.052	0	0	ANKRD26
0	1.54134815230904	0.496	0.087	0	0	SLC25A29
0	1.54069137619487	0.642	0.13	0	0	DLG5
0	1.53807396189857	0.631	0.151	0	0	RHOBTB3
0	1.53486308153479	0.745	0.253	0	0	MUC5B
0	1.5308352451298	0.406	0.078	0	0	MMP28
0	1.52884888957769	0.724	0.156	0	0	TRMT2B
0	1.52693873947029	0.451	0.08	0	0	ZMYM5
0	1.52191086197049	0.749	0.176	0	0	KCNK1
0	1.52178807972054	0.439	0.072	0	0	TMEM8B

0	1.51615225188169	0.279	0.051	0	0	MECOM
0	1.51462651774876	0.926	0.323	0	0	ELL2
0	1.514383816799	0.574	0.112	0	0	STXBP5
0	1.51404227989878	0.31	0.054	0	0	STXBP4
0	1.51281023840692	0.666	0.153	0	0	KLB
0	1.51258515131015	0.52	0.093	0	0	ZNF337
0	1.5103910758957	0.445	0.083	0	0	CSPP1
0	1.50931239066603	0.597	0.121	0	0	ACACA
0	1.50815104488564	0.663	0.133	0	0	DEPRECATED-ENSG00000134250
0	1.50743061821462	0.467	0.082	0	0	NBEAL1
0	1.50682285662229	0.375	0.069	0	0	ZNF700
0	1.50523673862289	0.769	0.198	0	0	SYTL4
0	1.50377176305951	0.268	0.049	0	0	ZNF619
0	1.49890720071043	0.661	0.14	0	0	PDLIM5
0	1.49868309623174	0.316	0.05	0	0	ZNF248
0	1.49818720692476	0.681	0.134	0	0	ARL17A
0	1.49593498351267	0.913	0.288	0	0	ABCD1
0	1.49484104632361	0.336	0.058	0	0	ZNF839
0	1.49353781375852	0.394	0.073	0	0	PHYHIPL
0	1.49218893775946	0.3	0.058	0	0	KRT15
0	1.49154030896298	0.665	0.144	0	0	ACACB
0	1.48879592256174	0.913	0.31	0	0	PGM2
0	1.48853603975839	0.386	0.073	0	0	PSD3
0	1.48636068936699	0.637	0.129	0	0	ADCY6
0	1.48561689862769	0.291	0.056	0	0	SPAG1
0	1.48314377490607	0.337	0.062	0	0	GPR180
0	1.48300895480813	0.817	0.202	0	0	THOC2
0	1.48204870805888	0.26	0.049	0	0	ZXDB
0	1.47644115426498	0.693	0.153	0	0	PKP2
0	1.47526923050612	0.253	0.044	0	0	WNT5B
0	1.4751424196564	0.478	0.089	0	0	HSF4
0	1.46234164954835	0.616	0.13	0	0	RAB3IP
0	1.46054998602801	0.575	0.116	0	0	CLDN14
0	1.45905077590796	0.761	0.175	0	0	SAMD5
0	1.45882925951513	0.697	0.144	0	0	NOTCH2
0	1.45815069676919	0.584	0.121	0	0	OTOP3
0	1.45724532093026	0.395	0.075	0	0	ACTR3C
0	1.45692913424963	0.736	0.157	0	0	RBM26
0	1.45522469533691	0.415	0.079	0	0	NVL
0	1.4551766582093	0.594	0.146	0	0	ZNF189
0	1.45384402001713	0.4	0.076	0	0	ARMCX5
0	1.44949327661573	0.91	0.356	0	0	ACSM2A
0	1.44948882017715	0.911	0.289	0	0	TMEM45B
0	1.44864882379874	0.568	0.125	0	0	CCNE2
0	1.44854786087035	0.698	0.152	0	0	TRIO
0	1.44767467059985	0.761	0.19	0	0	NBPF26
0	1.44714470383968	0.951	0.467	0	0	ERRFI1
0	1.44684336903145	0.819	0.237	0	0	SLC35D2
0	1.44367319835811	0.912	0.301	0	0	NFIB
0	1.44289619497501	0.521	0.107	0	0	GTF2H2
0	1.43783662037681	0.778	0.192	0	0	ACMSD
0	1.43704236226586	0.819	0.239	0	0	TM7SF3
0	1.4366978895405	0.281	0.052	0	0	ZNF57
0	1.43558360832185	0.966	0.549	0	0	FMO5
0	1.43551846313143	0.275	0.041	0	0	GOLGA8A
0	1.42861560642185	0.292	0.051	0	0	NAALADL2
0	1.4262005875701	0.574	0.123	0	0	APPL2
0	1.42591846503989	0.803	0.214	0	0	GPRC5C
0	1.42424589234756	0.337	0.063	0	0	C1orf116
0	1.42219636383478	0.584	0.115	0	0	DCAF16

0	1.42073606091834	0.308	0.059	0	0	CPLANE1
0	1.42045810372264	0.379	0.076	0	0	TINAG
0	1.41869697055281	0.336	0.058	0	0	TSPOAP1
0	1.41751848275466	0.472	0.092	0	0	FAM221A
0	1.4142626842949	0.679	0.156	0	0	RAB33B
0	1.41412244262146	0.956	0.457	0	0	ATP1B1
0	1.41252010164141	0.914	0.303	0	0	PUM1
0	1.40772676449369	0.417	0.085	0	0	TNFRSF11A
0	1.40501402744896	0.515	0.103	0	0	C8orf76
0	1.40397113612652	0.29	0.052	0	0	MFSD8
0	1.40320860874684	0.304	0.055	0	0	AASDH
0	1.40092883389561	0.838	0.224	0	0	TMEM63A
0	1.39888676865909	0.358	0.071	0	0	FAM200B
0	1.3986165781442	0.465	0.089	0	0	ADCK5
0	1.39706060710079	0.428	0.081	0	0	RBMX2
0	1.39568867213547	0.252	0.047	0	0	EFNA5
0	1.3938119262782	0.828	0.216	0	0	XIAP
0	1.39263080309088	0.514	0.103	0	0	ENPP4
0	1.39229098433899	0.271	0.044	0	0	ZNF587B
0	1.39061360416012	0.579	0.125	0	0	CXADR
0	1.39004837657641	0.896	0.308	0	0	CDH17
0	1.38945615197253	0.94	0.359	0	0	MUC13
0	1.38598039780704	0.792	0.181	0	0	VPS13B
0	1.38332172996647	0.907	0.318	0	0	TMEM37
0	1.38188221435548	0.462	0.088	0	0	ZNF608
0	1.38065429627147	0.852	0.279	0	0	SEC16B
0	1.38034481604831	0.828	0.241	0	0	RAP2C
0	1.37889059627581	0.499	0.106	0	0	TBCE.1
0	1.3784168549219	0.318	0.056	0	0	ZFP41
0	1.37737486446455	0.317	0.062	0	0	CDC7
0	1.37439982400449	0.326	0.06	0	0	LRRC8E
0	1.3743240842306	0.424	0.089	0	0	SPHK1
0	1.3738222586565	0.9	0.315	0	0	C6
0	1.37172815412056	0.594	0.126	0	0	LRRC14
0	1.36796720499294	0.503	0.096	0	0	BAIAP2L2
0	1.36720734172243	0.78	0.196	0	0	INTS8
0	1.3646215021549	0.359	0.069	0	0	TP53I3
0	1.3628082485671	0.364	0.066	0	0	GCNT2
0	1.36214510485066	0.946	0.417	0	0	HGD
0	1.3614826665411	0.483	0.096	0	0	PAQR7
0	1.35963953035022	0.444	0.093	0	0	KNTC1
0	1.3589033963176	0.402	0.074	0	0	ANKRD36B
0	1.35769869229203	0.754	0.191	0	0	DEPRECATED-ENSG00000057294
0	1.35768721518021	0.351	0.063	0	0	ZNF606
0	1.35758738957763	0.399	0.076	0	0	GRAMD2B
0	1.35665810752794	0.288	0.051	0	0	PACRG
0	1.35642340061653	0.962	0.419	0	0	FKBP5
0	1.354953281187	0.949	0.507	0	0	SGK1
0	1.35445588365336	0.606	0.14	0	0	INAVA
0	1.3543244448343	0.612	0.134	0	0	PHF6
0	1.35432304753455	0.501	0.098	0	0	UVSSA
0	1.3541804301823	0.783	0.216	0	0	MATN2
0	1.35320141311686	0.433	0.088	0	0	UPF3B
0	1.35238628873383	0.395	0.076	0	0	C6orf132
0	1.35218143197845	0.471	0.109	0	0	ITGA3
0	1.35135635694522	0.542	0.108	0	0	ZNF91
0	1.34770717496759	0.819	0.213	0	0	PWWP3A
0	1.34454901155818	0.795	0.221	0	0	EHF
0	1.34423861932752	0.893	0.312	0	0	ZKSCAN1
0	1.34108522512262	0.372	0.071	0	0	DPY19L3

0	1.33903258395574	0.33	0.06	0	0	OTUD3
0	1.33865649384211	0.93	0.346	0	0	SERINC5
0	1.33835331658064	0.49	0.103	0	0	ZNF7
0	1.33804377579336	0.609	0.135	0	0	THOP1
0	1.33441573375287	0.799	0.208	0	0	HGSNAT
0	1.33335257525456	0.907	0.328	0	0	ATP1A1
0	1.3330812558128	0.424	0.076	0	0	CASZ1
0	1.33235123779663	0.567	0.12	0	0	DMXL1
0	1.33132147154368	0.709	0.173	0	0	TMEM68
0	1.32433097196712	0.878	0.305	0	0	ATRN
0	1.32318973150576	0.933	0.497	0	0	SERPINA7
0	1.32238122879262	0.362	0.073	0	0	PRIM1
0	1.32004152790271	0.323	0.055	0	0	RAPGEFL1
0	1.31968432553002	0.929	0.322	0	0	IVNS1ABP
0	1.31858242221636	0.327	0.065	0	0	FPGT
0	1.31838255374171	0.354	0.061	0	0	FBLIM1
0	1.31372643179352	0.333	0.068	0	0	ARV1
0	1.31292471498843	0.468	0.097	0	0	ALG6
0	1.31010755834485	0.654	0.151	0	0	LNX2
0	1.31007359851471	0.555	0.119	0	0	ANAPC4
0	1.30842264920836	0.337	0.064	0	0	RBL1
0	1.30794658925225	0.878	0.304	0	0	CLDN4
0	1.30781016050896	0.424	0.07	0	0	TMEM44
0	1.30664962936411	0.315	0.058	0	0	DEPRECATED-ENSG00000185619
0	1.30429311435197	0.422	0.089	0	0	SUSD4
0	1.30422312724501	0.3	0.053	0	0	ADAMTS10
0	1.30390092591833	0.266	0.048	0	0	TEFM
0	1.30306843111733	0.51	0.12	0	0	RNF125
0	1.29819107875623	0.368	0.108	0	0	DUOXA2
0	1.29756520898797	0.563	0.129	0	0	DONSON
0	1.29720847946729	0.615	0.137	0	0	MBOAT1
0	1.29719346075814	0.261	0.051	0	0	HELQ
0	1.29631699469736	0.462	0.091	0	0	DGKQ
0	1.29611940575965	0.38	0.074	0	0	FBXO4
0	1.29571754582533	0.403	0.081	0	0	ZFP62
0	1.2923717217691	0.279	0.055	0	0	TRMT61A
0	1.29233832468432	0.433	0.085	0	0	FAM76B
0	1.29118409520354	0.76	0.196	0	0	MCTS1
0	1.29004161348264	0.52	0.106	0	0	ADGRA3
0	1.28967409791098	0.945	0.427	0	0	F5
0	1.28953287745705	0.392	0.069	0	0	THBS3
0	1.28919816137934	0.651	0.134	0	0	ZBTB1
0	1.28841602456182	0.391	0.075	0	0	AC253572.1
0	1.28812614870478	0.466	0.094	0	0	AHI1
0	1.28543752107415	0.341	0.056	0	0	EPB41L4A
0	1.28535401849605	0.786	0.227	0	0	TMPRSS2
0	1.28522938872364	0.363	0.072	0	0	ZNF449
0	1.28347208107198	0.845	0.246	0	0	CYP4V2
0	1.28231593149162	0.815	0.269	0	0	SLC6A8
0	1.28210456689227	0.681	0.163	0	0	UGT2B11
0	1.28198349061694	0.461	0.096	0	0	PLEKHA5
0	1.28115289392717	0.253	0.057	0	0	PPP1R1B
0	1.28057995990843	0.356	0.071	0	0	CDHR2
0	1.27887332881798	0.89	0.287	0	0	PAG1
0	1.27784525640789	0.608	0.145	0	0	PLGLB1
0	1.27754325408692	0.329	0.064	0	0	ZSCAN26
0	1.27686026841054	0.956	0.467	0	0	TDO2
0	1.27529067335092	0.471	0.097	0	0	ANO8
0	1.27437280036311	0.476	0.097	0	0	PLPP2
0	1.27385522330669	0.251	0.05	0	0	OBI1

0	1.27329509321035	0.818	0.264	0	0	KLF5
0	1.27302440881575	0.357	0.069	0	0	FAN1
0	1.27112015148994	0.336	0.065	0	0	MCM9
0	1.270063241008	0.593	0.131	0	0	NFS1
0	1.26923029690133	0.868	0.286	0	0	ZHX1
0	1.26862098598762	0.51	0.125	0	0	PRR15L
0	1.26371932303939	0.335	0.064	0	0	NAIP
0	1.26188180149165	0.464	0.106	0	0	IPMK
0	1.26163678908903	0.476	0.095	0	0	D2HGDH
0	1.26074487975595	0.908	0.317	0	0	KCTD3
0	1.25768760525882	0.296	0.054	0	0	LPAR1
0	1.25657437365101	0.271	0.059	0	0	XRCC3
0	1.25471102862156	0.327	0.065	0	0	L2HGDH
0	1.24992272818476	0.335	0.062	0	0	PARD3B
0	1.24932066503774	0.899	0.337	0	0	OCLN
0	1.24625324233165	0.296	0.055	0	0	SMYD4
0	1.24624469403334	0.293	0.055	0	0	FUT6
0	1.2413821582784	0.368	0.072	0	0	CTPS2
0	1.24022748376716	0.375	0.079	0	0	KANSL1L
0	1.2387348640227	0.83	0.255	0	0	CPD
0	1.23855203389713	0.532	0.119	0	0	C1RL
0	1.23792799222957	0.688	0.171	0	0	FAM214A
0	1.23759176362004	0.829	0.252	0	0	CYP4F11
0	1.23746689127216	0.768	0.201	0	0	ACVR1B
0	1.23512288714744	0.855	0.263	0	0	FRK
0	1.2346924553457	0.398	0.078	0	0	METTL15
0	1.23420224582702	0.374	0.079	0	0	MSTO1
0	1.23310361270872	0.267	0.054	0	0	RWDD4
0	1.23199102198899	0.448	0.093	0	0	GEMIN7
0	1.23197807430553	0.708	0.179	0	0	TMEM164
0	1.23192616912761	0.495	0.107	0	0	UPF3A
0	1.23037504203219	0.763	0.205	0	0	SIPA1L2
0	1.22564368593532	0.948	0.386	0	0	TSPAN3
0	1.22530632500059	0.292	0.057	0	0	MFSD4B
0	1.2244563157441	0.355	0.065	0	0	ZNF346
0	1.22427636213652	0.468	0.134	0	0	DUOX2
0	1.22412782222192	0.345	0.073	0	0	SPATA33
0	1.2237054930237	0.903	0.346	0	0	SLC37A4
0	1.22260722735006	0.711	0.183	0	0	KIAA0232
0	1.22242143361986	0.709	0.174	0	0	SGK3
0	1.2215424331215	0.602	0.136	0	0	SLC46A1
0	1.22122374637229	0.861	0.256	0	0	N4BP2L2
0	1.22042867395044	0.575	0.136	0	0	APOOL
0	1.21928732702013	0.742	0.193	0	0	GPAT4
0	1.21867824404481	0.328	0.065	0	0	NFXL1
0	1.21831936111748	0.935	0.347	0	0	ANKRD10
0	1.21780615227234	0.631	0.153	0	0	SSX2IP
0	1.21656831691173	0.364	0.07	0	0	MFSD11
0	1.21611182748051	0.354	0.067	0	0	ZNF224
0	1.21531698623329	0.402	0.08	0	0	GK5
0	1.21528237740128	0.947	0.388	0	0	RNF128
0	1.2151344943225	0.268	0.054	0	0	ELAC1
0	1.21385664203289	0.348	0.068	0	0	ZNF26
0	1.21332611525293	0.696	0.169	0	0	STK39
0	1.21181645830198	0.339	0.057	0	0	RALGPS1
0	1.20922560001527	0.846	0.385	0	0	AKR1C2
0	1.20915913109608	0.952	0.364	0	0	ARGLU1
0	1.20911078896599	0.5	0.109	0	0	CBR4
0	1.20829683316884	0.313	0.064	0	0	FAHD2A
0	1.20720351015089	0.594	0.141	0	0	SH3RF1

0	1.20682576019162	0.274	0.048	0	0	MBD5
0	1.20663442460658	0.312	0.061	0	0	DHRS11
0	1.205837131711	0.348	0.074	0	0	AMMECR1
0	1.20541571515641	0.51	0.106	0	0	NSMAF
0	1.20490806250257	0.666	0.16	0	0	GALK2
0	1.20461545770609	0.936	0.347	0	0	ERICH5
0	1.20458565602392	0.43	0.084	0	0	PIBF1
0	1.20356263926785	0.76	0.202	0	0	DEPRECATED-ENSG00000213759
0	1.20154844446293	0.744	0.245	0	0	CLDN3
0	1.20009640144004	0.66	0.163	0	0	RAPH1
0	1.19897615881104	0.738	0.199	0	0	CYP4F2
0	1.19800524923929	0.653	0.156	0	0	ZDHHC14
0	1.19626321223484	0.306	0.064	0	0	ZNF33B
0	1.19453686879718	0.599	0.145	0	0	DCUN1D4
0	1.19371224848667	0.802	0.231	0	0	PARM1
0	1.19362181286866	0.399	0.087	0	0	XPO4
0	1.19353149384305	0.417	0.085	0	0	GUCY1A1
0	1.19301472998826	0.547	0.122	0	0	RCOR3
0	1.19271375109267	0.46	0.093	0	0	RAB28
0	1.19215493324513	0.626	0.147	0	0	RALGAPA2
0	1.19187808461495	0.572	0.122	0	0	PDE8A
0	1.1911805801842	0.297	0.057	0	0	VMAC
0	1.19082703786383	0.363	0.072	0	0	NIPAL1
0	1.18957327133686	0.657	0.15	0	0	IDUA
0	1.18831533180792	0.413	0.087	0	0	DNAAF2
0	1.18677240321167	0.415	0.091	0	0	HIBCH
0	1.18572931939541	0.433	0.092	0	0	CPSF1
0	1.18566092571357	0.505	0.114	0	0	TMLHE
0	1.18510398204641	0.411	0.087	0	0	RNF170
0	1.18478400838774	0.322	0.07	0	0	SLC1A1
0	1.18311594907388	0.889	0.319	0	0	ABCC3
0	1.18300468458021	0.87	0.276	0	0	IRF3
0	1.18275218212242	0.887	0.303	0	0	ZBTB7B
0	1.18258939040199	0.548	0.105	0	0	PRPF39
0	1.18095783316163	0.72	0.19	0	0	LYPLAL1
0	1.18019413475691	0.487	0.101	0	0	GALNS
0	1.18009356746334	0.436	0.123	0	0	ETNK2
0	1.18006624464942	0.252	0.047	0	0	ZNF696
0	1.17966746220883	0.566	0.117	0	0	TTLL3
0	1.17941184709899	0.49	0.106	0	0	TBC1D1
0	1.17586557471346	0.549	0.126	0	0	CEP170B
0	1.17583810533375	0.298	0.065	0	0	TMEM187
0	1.17496356222409	0.444	0.088	0	0	TMEM168
0	1.17472050798113	0.417	0.09	0	0	NSD2
0	1.1747123737758	0.392	0.088	0	0	SRGAP1
0	1.1734215102106	0.43	0.091	0	0	KCNT2
0	1.17142886380385	0.337	0.067	0	0	PRMT3
0	1.17110352697636	0.284	0.05	0	0	NINL
0	1.16990868001233	0.269	0.057	0	0	THAP5
0	1.16660766126579	0.413	0.078	0	0	KIF12
0	1.16642110768581	0.425	0.093	0	0	VSIG10
0	1.16637439584298	0.693	0.188	0	0	C1GALT1C1
0	1.16465003774186	0.53	0.128	0	0	MISP
0	1.16455847209335	0.514	0.099	0	0	KIFC2
0	1.16290476964383	0.685	0.182	0	0	TM4SF20
0	1.16129156382674	0.696	0.177	0	0	SLC51A
0	1.16077467486335	0.486	0.109	0	0	TRMT11
0	1.1604345691901	0.313	0.057	0	0	NEO1
0	1.16031400552991	0.541	0.131	0	0	ASRGL1
0	1.15918862494756	0.284	0.058	0	0	ATG10

0	1.15901961297466	0.378	0.077	0	0	ABHD13
0	1.15844487698655	0.374	0.078	0	0	PAQR9
0	1.1581808176773	0.463	0.102	0	0	MID2
0	1.15758336061378	0.356	0.072	0	0	GLCE
0	1.15692510478289	0.62	0.211	0	0	ACSM1
0	1.15573397996179	0.536	0.126	0	0	ELMOD2
0	1.15535345927974	0.297	0.062	0	0	SLC44A3
0	1.1547308945682	0.269	0.056	0	0	B3GLCT
0	1.15414828750123	0.327	0.066	0	0	SLC2A5
0	1.15400067432613	0.454	0.097	0	0	URGCP
0	1.15396223284457	0.319	0.065	0	0	ZNF133
0	1.15389480862396	0.355	0.068	0	0	CNTFR
0	1.15243559652377	0.286	0.055	0	0	MTCL1
0	1.15230683242843	0.352	0.076	0	0	TGDS
0	1.15207798683096	0.665	0.162	0	0	KDM6A
0	1.15183843088004	0.32	0.067	0	0	EID3
0	1.15125544753131	0.836	0.262	0	0	MEP1A
0	1.15091921865223	0.539	0.126	0	0	ADNP
0	1.15084110602229	0.28	0.056	0	0	B4GALT6
0	1.15055292420229	0.665	0.171	0	0	GATAD1
0	1.15024476575061	0.309	0.069	0	0	ITGB3BP
0	1.14894812762337	0.854	0.278	0	0	WWP1
0	1.14853443690746	0.4	0.086	0	0	DMAC2L
0	1.1478759185365	0.642	0.166	0	0	FTCD
0	1.14764258149637	0.707	0.18	0	0	INTS3
0	1.14703858973413	0.369	0.075	0	0	STOX2
0	1.14694453456219	0.271	0.057	0	0	TUBGCP5
0	1.14679864441081	0.51	0.111	0	0	ATG2B
0	1.14389229169239	0.664	0.17	0	0	SMYD2
0	1.14292191489051	0.387	0.083	0	0	TBCE
0	1.14280635365836	0.687	0.177	0	0	IL10RB
0	1.14212296090533	0.438	0.097	0	0	PLCXD1
0	1.14136246225867	0.366	0.076	0	0	ZNF44
0	1.1412721054691	0.49	0.106	0	0	SEMA6A
0	1.14050848338535	0.61	0.153	0	0	FBXO42
0	1.13929635127338	0.457	0.103	0	0	CLN5
0	1.13830742430897	0.667	0.177	0	0	PKDCC
0	1.1382385732573	0.77	0.213	0	0	LONRF3
0	1.13822492293846	0.726	0.172	0	0	COL27A1
0	1.13786818388849	0.421	0.087	0	0	VEZT
0	1.13738849229336	0.455	0.098	0	0	PLXNA2
0	1.13738564702063	0.365	0.079	0	0	CDKL1
0	1.13692423026987	0.607	0.136	0	0	TASOR2
0	1.13601066811124	0.701	0.184	0	0	STK3
0	1.13514338389453	0.303	0.063	0	0	CDC14B
0	1.13486172728479	0.411	0.089	0	0	RIC8B
0	1.13483394461885	0.578	0.134	0	0	C19orf25
0	1.13481211577718	0.733	0.193	0	0	SHROOM1
0	1.1334948132948	0.365	0.078	0	0	RNF217
0	1.13279760775917	0.365	0.072	0	0	MTMR11
0	1.1322311464546	0.315	0.07	0	0	FLVCR2
0	1.13208250805545	0.716	0.183	0	0	ATP9A
0	1.12944347493074	0.758	0.216	0	0	FAT1
0	1.12907179443521	0.376	0.079	0	0	KLHL42
0	1.12893542553162	0.33	0.064	0	0	SMYD3
0	1.12864162339605	0.741	0.207	0	0	NEDD4L
0	1.12647066820383	0.659	0.175	0	0	UBXN2B
0	1.12471534733248	0.615	0.153	0	0	SCML1
0	1.124324412465	0.79	0.254	0	0	CHPT1
0	1.12416467955641	0.477	0.109	0	0	DEPRECATED-ENSG00000134597

0	1.12245746027966	0.91	0.356	0	0	PCSK6
0	1.1207464103052	0.677	0.17	0	0	SOS 2
0	1.11846268205441	0.441	0.105	0	0	SLC7A1
0	1.11834693518392	0.831	0.301	0	0	BHMT
0	1.11695987816478	0.49	0.105	0	0	ZNF580
0	1.11660458956845	0.255	0.053	0	0	TFB1M
0	1.11631567636138	0.812	0.251	0	0	PLEKHA6
0	1.11628567229097	0.318	0.062	0	0	TMEM260
0	1.11546062089484	0.391	0.086	0	0	FNBP1L
0	1.11524512387115	0.574	0.138	0	0	AKAP8
0	1.11450342928865	0.553	0.129	0	0	SUSD3
0	1.11292992553789	0.393	0.081	0	0	SEC22A
0	1.11253511025687	0.354	0.072	0	0	CMTM4
0	1.1125053227289	0.341	0.071	0	0	SCYL3
0	1.11191100267483	0.276	0.058	0	0	CYB5RL
0	1.11170339874687	0.436	0.094	0	0	TRIP11
0	1.11033984324924	0.564	0.13	0	0	DPY19L4
0	1.11030224846343	0.356	0.082	0	0	HAL
0	1.10985505749018	0.256	0.05	0	0	PLEKHG6
0	1.10969616271463	0.428	0.097	0	0	MYO5C
0	1.10929693495563	0.328	0.069	0	0	C15orf40
0	1.1091911705475	0.655	0.142	0	0	LRBA
0	1.10901917294123	0.587	0.139	0	0	PCF11
0	1.10745495393878	0.465	0.1	0	0	TMEM50B
0	1.10687624598287	0.302	0.062	0	0	MOSMO
0	1.10561317620354	0.443	0.099	0	0	COG2
0	1.10557738034246	0.756	0.201	0	0	DLG1
0	1.10507659685973	0.433	0.092	0	0	SLC25A16
0	1.10492165640606	0.716	0.192	0	0	REPS1
0	1.10453657551628	0.465	0.103	0	0	CCDC125
0	1.10449531452171	0.775	0.22	0	0	PRKDC
0	1.10340892946068	0.863	0.325	0	0	VNN 1.00
0	1.10212405026261	0.477	0.117	0	0	HOXA13
0	1.10200758475766	0.729	0.192	0	0	HECTD1
0	1.10163470162948	0.257	0.051	0	0	CYP2R1
0	1.1016305522989	0.786	0.234	0	0	RNF103
0	1.10121448827526	0.899	0.345	0	0	TOR1AIP2
0	1.10053120063388	0.545	0.132	0	0	IGF2BP3
0	1.10040253799795	0.328	0.061	0	0	VCPKMT
0	1.09927988023574	0.949	0.409	0	0	NDRG1
0	1.09909604275085	0.675	0.187	0	0	PCDH1
0	1.09750006900349	0.256	0.056	0	0	C15orf41
0	1.09727718694484	0.303	0.058	0	0	LINS1
0	1.096549219204	0.565	0.136	0	0	TERF1
0	1.09645024867362	0.329	0.065	0	0	ZNF862
0	1.09642282354632	0.648	0.169	0	0	MAP3K5
0	1.09615438940746	0.295	0.062	0	0	ANKRA2
0	1.09470835368446	0.547	0.124	0	0	ATG12
0	1.09226797887362	0.304	0.058	0	0	CEP95
0	1.09171742757621	0.349	0.07	0	0	MAML3
0	1.09104719493568	0.484	0.108	0	0	PPP1R16A
0	1.08988356437971	0.396	0.082	0	0	SLC10A7
0	1.08675594181395	0.277	0.055	0	0	GSTA4
0	1.08517431417764	0.271	0.059	0	0	DRGX
0	1.08467948501317	0.544	0.126	0	0	SLC25A32
0	1.08461953323072	0.5	0.118	0	0	UTP23
0	1.08406199351954	0.714	0.186	0	0	NECTIN3
0	1.08283146911711	0.728	0.227	0	0	LSS
0	1.08269438904978	0.394	0.087	0	0	OMA1
0	1.08192142596212	0.403	0.09	0	0	FARSB

0	1.08136541766239	0.54	0.125	0	0	UGT1A4
0	1.08091996761964	0.399	0.084	0	0	KLHL23
0	1.07998904666828	0.932	0.494	0	0	PIGR
0	1.07962405354542	0.35	0.08	0	0	CLGN
0	1.07907395102898	0.509	0.12	0	0	PLD1
0	1.0789755955826	0.305	0.067	0	0	FAM184A
0	1.07872600063759	0.286	0.056	0	0	FAM171A1
0	1.07816830796361	0.271	0.058	0	0	C9orf85
0	1.07782790177229	0.664	0.171	0	0	COBLL1
0	1.07755654424889	0.498	0.125	0	0	UGT2B15
0	1.07622603570259	0.304	0.062	0	0	GALNT4
0	1.07578595260139	0.382	0.09	0	0	SSTR1
0	1.07367702980734	0.421	0.094	0	0	IFT88
0	1.07302060810382	0.773	0.228	0	0	PCCA
0	1.07264683791404	0.276	0.055	0	0	FAM120C
0	1.07143526280853	0.308	0.063	0	0	C9orf152
0	1.07104692892628	0.388	0.079	0	0	PEX6
0	1.07076444896463	0.255	0.054	0	0	STRADB
0	1.07024580743311	0.424	0.088	0	0	TEP1
0	1.07013720161899	0.451	0.096	0	0	VIL1
0	1.06947622437834	0.506	0.119	0	0	ELF2
0	1.06874336251953	0.563	0.13	0	0	SPIDR
0	1.06807610304604	0.426	0.096	0	0	POLB
0	1.06519331000823	0.429	0.093	0	0	GPAT3
0	1.06513542432823	0.309	0.069	0	0	FANCC
0	1.06494377915806	0.515	0.121	0	0	SIPA1L1
0	1.06348655684716	0.882	0.322	0	0	MIA2
0	1.06342729484607	0.97	0.584	0	0	ACSL1
0	1.06300921346704	0.475	0.125	0	0	CKB
0	1.06188311591809	0.269	0.069	0	0	MELTF
0	1.06059853826434	0.412	0.093	0	0	ARL4A
0	1.06026725588818	0.327	0.068	0	0	ATXN7L1
0	1.05927504610814	0.442	0.103	0	0	SLC30A5
0	1.05922931321913	0.935	0.446	0	0	PROX1
0	1.05682371315672	0.252	0.05	0	0	NAPB
0	1.05529451663671	0.946	0.405	0	0	MBNL3
0	1.05344090031929	0.369	0.071	0	0	MRI1
0	1.05230474056259	0.714	0.213	0	0	CNKSR3
0	1.05195213775142	0.971	0.533	0	0	ASPH
0	1.05178251514493	0.399	0.093	0	0	MSH2
0	1.05136240443221	0.452	0.108	0	0	PPIP5K2
0	1.05040865297761	0.308	0.061	0	0	LRRC75B
0	1.05011023271131	0.354	0.076	0	0	BTBD3
0	1.04930340930837	0.314	0.067	0	0	TTC21B
0	1.04905763148537	0.544	0.121	0	0	DTX4
0	1.04860748322235	0.756	0.254	0	0	HPGD
0	1.0464837368326	0.527	0.121	0	0	WDR20
0	1.0456643212131	0.409	0.095	0	0	NCEH1
0	1.04445092096719	0.273	0.068	0	0	FAM111B
0	1.04264991668348	0.501	0.116	0	0	TMEM43
0	1.04175326798688	0.532	0.125	0	0	WDR44
0	1.04143518918449	0.943	0.45	0	0	HSD17B11
0	1.04138334590255	0.334	0.065	0	0	ZNF692
0	1.0413611673819	0.578	0.146	0	0	LRRC58
0	1.04112094302713	0.718	0.188	0	0	ZMYM2
0	1.04053545011957	0.253	0.053	0	0	SPIN3
0	1.04048083138065	0.422	0.097	0	0	WFS1
0	1.03987426899502	0.772	0.233	0	0	EDEM3
0	1.03863830244212	0.676	0.173	0	0	CDC16
0	1.03758214458961	0.523	0.129	0	0	EPS8L3

0	1.03748540046712	0.895	0.345	0	0	IMMP2L
0	1.0370420509569	0.8	0.263	0	0	DGAT2
0	1.03454701637958	0.28	0.058	0	0	GUF1
0	1.03356054197282	0.393	0.086	0	0	GANC
0	1.03321237705123	0.571	0.138	0	0	MTMR1
0	1.03288906312662	0.253	0.058	0	0	AHCTF1
0	1.03206721839949	0.596	0.147	0	0	ZFAND1
0	1.03098994157943	0.335	0.077	0	0	CNTLN
0	1.03096936864473	0.876	0.283	0	0	IKBKB
0	1.03092357455869	0.271	0.06	0	0	KPTN
0	1.03058527702363	0.576	0.138	0	0	STRN3
0	1.02918487163572	0.284	0.06	0	0	SETD4
0	1.02815968215698	0.616	0.146	0	0	TMEM175
0	1.02782230015843	0.273	0.053	0	0	ME3
0	1.02739742784474	0.309	0.068	0	0	RAD51B
0	1.02706318858151	0.579	0.144	0	0	HELLS
0	1.02676369027291	0.706	0.187	0	0	TRAPPC9
0	1.02658693899343	0.42	0.101	0	0	ACSF2
0	1.02638399166526	0.571	0.145	0	0	DNASE1L1
0	1.02618696867536	0.506	0.126	0	0	FAM136A
0	1.02554502784056	0.491	0.117	0	0	ABCB10
0	1.02550077062	0.861	0.317	0	0	ENTPD5
0	1.0252966075304	0.431	0.097	0	0	HHAT
0	1.02471777954968	0.392	0.084	0	0	TMTC4
0	1.02456034139205	0.632	0.167	0	0	NREP
0	1.02262872175813	0.446	0.099	0	0	PLEKHH3
0	1.02180798854343	0.579	0.144	0	0	ARHGAP5
0	1.02080709410558	0.401	0.092	0	0	PDP1
0	1.02073564202802	0.473	0.108	0	0	CYP2C19
0	1.01715508737618	0.549	0.13	0	0	TJP3
0	1.01540337053522	0.315	0.073	0	0	TONSL
0	1.01510446154646	0.275	0.062	0	0	NDUFAF5
0	1.01480097599267	0.447	0.109	0	0	BAMBI
0	1.01476992793301	0.555	0.126	0	0	HIP1R
0	1.01275715715938	0.333	0.073	0	0	MBTPS2
0	1.01268037954351	0.773	0.241	0	0	STBD1
0	1.01034290277845	0.724	0.21	0	0	ZBTB33
0	1.01010184004546	0.354	0.08	0	0	PPP1R14D
0	1.00969417996292	0.54	0.131	0	0	SLC37A1
0	1.00955233808423	0.868	0.328	0	0	PNKD
0	1.0095289363086	0.507	0.12	0	0	AASS
0	1.00951232015726	0.56	0.168	0	0	FADS2
0	1.00939791096156	0.507	0.118	0	0	KLHL22
0	1.00908185601328	0.336	0.079	0	0	MOB3B
0	1.00899211049317	0.782	0.268	0	0	ONECUT1
0	1.00828371598314	0.474	0.112	0	0	LCLAT1
0	1.00705285210379	0.288	0.061	0	0	MSH5
0	1.00681359848004	0.756	0.243	0	0	ACSL3
0	1.00657386293335	0.618	0.166	0	0	ENOSF1
0	1.00646198154763	0.255	0.053	0	0	NAPEPLD
0	1.00469522237637	0.641	0.179	0	0	TIMM8A
0	1.00425320733183	0.709	0.199	0	0	PARP4
0	1.0041717261377	0.585	0.152	0	0	HOOK3
0	1.00407327580301	0.544	0.112	0	0	ZFYVE28
0	1.0040605051377	0.464	0.11	0	0	SNX24
0	1.00341976732569	0.282	0.06	0	0	ANKMY1
0	1.0030681240982	0.267	0.053	0	0	JRKL
0	1.00029673771876	0.279	0.06	0	0	PUS7L
0	0.999619648783475	0.333	0.08	0	0	RMI1
0	0.998725292447373	0.49	0.095	0	0	LAMB1

0	0.997254897603158	0.681	0.181	0	0	CEP57
0	0.996342697409754	0.735	0.202	0	0	SYBU
0	0.994807816961047	0.365	0.08	0	0	IPP
0	0.994442546425521	0.541	0.124	0	0	ABCG5
0	0.994019915943578	0.804	0.252	0	0	KLHDC2
0	0.993861437916541	0.713	0.186	0	0	MICAL3
0	0.993070784631813	0.609	0.171	0	0	TSPAN1
0	0.992358422116815	0.403	0.097	0	0	PIGM
0	0.99175032263757	0.615	0.142	0	0	FRYL
0	0.991066275644894	0.395	0.089	0	0	PM20D2
0	0.990828694138296	0.618	0.161	0	0	TMEM129
0	0.990508925618372	0.327	0.075	0	0	ZFP64
0	0.990468958147593	0.637	0.176	0	0	ACSS2
0	0.989127378938779	0.271	0.063	0	0	FBF1
0	0.98869638134011	0.701	0.192	0	0	BPTF
0	0.98830216241833	0.672	0.179	0	0	TUBGCP3
0	0.987031426640144	0.383	0.083	0	0	P4HTM
0	0.987005855856801	0.431	0.1	0	0	ETAA1
0	0.986982707038423	0.292	0.066	0	0	ARHGEF37
0	0.985396777076372	0.778	0.24	0	0	PLEKHF2
0	0.985337116455693	0.344	0.076	0	0	FGD6
0	0.984575201008306	0.349	0.068	0	0	TRIM52
0	0.984205489551428	0.462	0.103	0	0	THNSL2
0	0.983656563169027	0.439	0.101	0	0	CAMSAP3
0	0.982736714034253	0.833	0.257	0	0	AMOTL1
0	0.982567838445015	0.738	0.212	0	0	MAVS
0	0.982234065057002	0.745	0.211	0	0	MROH1
0	0.981849096097082	0.517	0.129	0	0	ZNF623
0	0.981596004178698	0.267	0.059	0	0	JKAMP
0	0.980526076880451	0.477	0.116	0	0	FGF13
0	0.97979722898526	0.611	0.172	0	0	PARP9
0	0.979787232537823	0.395	0.087	0	0	RECQL5
0	0.979602790953838	0.681	0.174	0	0	RNF157
0	0.979321077581446	0.484	0.109	0	0	MAP4K5
0	0.978833818262205	0.394	0.095	0	0	PIGP
0	0.978832029660456	0.458	0.105	0	0	TRIM56
0	0.978804047665353	0.545	0.131	0	0	CORO2A
0	0.977910218409829	0.255	0.053	0	0	DLG3
0	0.977719779279867	0.724	0.209	0	0	PEX2
0	0.97708174759195	0.786	0.243	0	0	PANK3
0	0.976416039716728	0.888	0.374	0	0	ADA
0	0.975726665040431	0.728	0.198	0	0	GRB10
0	0.975448284149458	0.89	0.358	0	0	TSPAN8
0	0.975442644469191	0.676	0.175	0	0	PPP4R3A
0	0.974293014332328	0.909	0.434	0	0	VEGFA
0	0.974276002383686	0.278	0.062	0	0	TCTEX1D2
0	0.974245762842211	0.754	0.243	0	0	ACSM2B
0	0.974137969601561	0.595	0.155	0	0	CYP2C18
0	0.973870311618033	0.279	0.056	0	0	USP30
0	0.97325335684066	0.367	0.084	0	0	HLCS
0	0.97290492155293	0.626	0.169	0	0	IL17RB
0	0.97249210877925	0.806	0.257	0	0	PDZD8
0	0.971736131526844	0.379	0.085	0	0	CEP83
0	0.971686240804033	0.26	0.053	0	0	CAPS
0	0.971234153856709	0.644	0.172	0	0	CXorf56
0	0.971103254757385	0.414	0.091	0	0	ABHD18
0	0.970894913189838	0.611	0.155	0	0	SEC24B
0	0.970713443504032	0.501	0.123	0	0	ATP6V0A1
0	0.970515672389384	0.323	0.075	0	0	CEP44
0	0.970072004225155	0.48	0.121	0	0	GCH1

0	0.969879660517709	0.798	0.246	0	0	NHSL1
0	0.968524533210714	0.457	0.11	0	0	OTUD6B
0	0.96753828023578	0.289	0.063	0	0	PPCDC
0	0.966293284874328	0.858	0.358	0	0	TFRC
0	0.965698731870085	0.592	0.145	0	0	UQCC1
0	0.964977034846875	0.313	0.077	0	0	POLD3
0	0.962854874222907	0.341	0.078	0	0	NSRP1
0	0.962474559018634	0.348	0.081	0	0	DIP2C
0	0.961724973177448	0.575	0.143	0	0	GBA2
0	0.960889638358049	0.284	0.066	0	0	LIAS
0	0.960189061238383	0.375	0.087	0	0	SLC23A1
0	0.959358492268383	0.41	0.1	0	0	SLC19A2
0	0.957475109669152	0.81	0.272	0	0	GRTP1
0	0.957439118445363	0.624	0.166	0	0	FBXO9
0	0.957365501831759	0.706	0.217	0	0	ANXA13
0	0.956762497743812	0.343	0.077	0	0	USP40
0	0.955836032679808	0.56	0.142	0	0	SMC6
0	0.955473295753315	0.307	0.073	0	0	SIMC1
0	0.955451034904862	0.54	0.137	0	0	TLE1
0	0.954956881093081	0.454	0.111	0	0	RMDN1
0	0.954921377906862	0.937	0.442	0	0	DNM2
0	0.954803982087039	0.432	0.105	0	0	NUP155
0	0.954773711803291	0.26	0.05	0	0	RALGAPA1
0	0.954728565844788	0.365	0.083	0	0	SCMH1
0	0.954216518354533	0.43	0.104	0	0	MAFG
0	0.954021418215924	0.26	0.055	0	0	RNF43
0	0.951936470028079	0.253	0.061	0	0	CHAF1B
0	0.951630713177597	0.667	0.159	0	0	MICALL2
0	0.951603310517962	0.979	0.538	0	0	CYP3A5
0	0.949765820161712	0.452	0.101	0	0	ABCC4
0	0.949366180410278	0.38	0.085	0	0	MNAT1
0	0.949131721715752	0.429	0.093	0	0	ZNF507
0	0.94875695642497	0.504	0.128	0	0	DDR1
0	0.948297506096838	0.281	0.064	0	0	DEPRECATED-ENSG00000050130
0	0.947584879482786	0.499	0.117	0	0	MED23
0	0.946970698633061	0.731	0.206	0	0	PATJ
0	0.94680720011114	0.848	0.308	0	0	NUDT16
0	0.946307700124469	0.595	0.146	0	0	KHDC4
0	0.946060037440887	0.601	0.152	0	0	KIF13A
0	0.945591917251544	0.374	0.085	0	0	GTPBP3
0	0.944813187250669	0.799	0.25	0	0	LLGL2
0	0.943997036373306	0.582	0.146	0	0	EPB41L5
0	0.943527136253212	0.676	0.175	0	0	TTC37
0	0.943214481369647	0.814	0.265	0	0	MARC2
0	0.942965477530891	0.454	0.106	0	0	MYNN
0	0.942914628610669	0.843	0.304	0	0	PPP1R15B
0	0.942170094093425	0.309	0.066	0	0	TMEM161B
0	0.941930909512162	0.485	0.117	0	0	PHLPP2
0	0.941632408018719	0.554	0.134	0	0	CLCC1
0	0.941490650047062	0.636	0.166	0	0	ARRB1
0	0.940479162314302	0.518	0.121	0	0	TMEM131
0	0.939587348262642	0.515	0.129	0	0	ZBTB41
0	0.938519972160513	0.834	0.273	0	0	STAU2
0	0.93847591583829	0.968	0.543	0	0	UGT2B4
0	0.938367186837194	0.37	0.085	0	0	PIAS1
0	0.938119873927602	0.806	0.28	0	0	KLF15
0	0.93768494935617	0.36	0.092	0	0	SLC35E3
0	0.937662580575902	0.307	0.068	0	0	TUBGCP4
0	0.937567886563324	0.298	0.064	0	0	ZNF791
0	0.937171025384155	0.408	0.104	0	0	FZD5

0	0.937131952147364	0.418	0.09	0	0	CPLX1
0	0.935620082434133	0.367	0.088	0	0	KLHL8
0	0.935301489617089	0.365	0.081	0	0	ATP11C
0	0.934993475824931	0.484	0.115	0	0	ARFGEF1
0	0.934047012316011	0.376	0.085	0	0	ZFX
0	0.933724739604077	0.423	0.098	0	0	SMC5
0	0.933544241268425	0.397	0.088	0	0	VPS13A
0	0.933501953507894	0.519	0.131	0	0	MANEAL
0	0.933473748460072	0.549	0.133	0	0	SHC2
0	0.932967514203778	0.737	0.216	0	0	FAM199X
0	0.932939088944735	0.692	0.197	0	0	ZDHHC9
0	0.932774851504765	0.297	0.064	0	0	TSNARE1
0	0.932544322147723	0.253	0.06	0	0	KIAA1328
0	0.931723880075868	0.332	0.081	0	0	POLA2
0	0.931693962319597	0.378	0.091	0	0	TBPL1
0	0.931662553142403	0.654	0.182	0	0	NUDT9
0	0.931294318635919	0.635	0.166	0	0	CPM
0	0.931239850615275	0.28	0.06	0	0	WNT4
0	0.929860219667154	0.446	0.105	0	0	CHM
0	0.929817649365924	0.314	0.068	0	0	XYLT2
0	0.929700132804024	0.661	0.193	0	0	SLC25A33
0	0.929662963893257	0.642	0.182	0	0	FGD4
0	0.928948590395257	0.457	0.102	0	0	ERMP1
0	0.928598835904012	0.591	0.149	0	0	NCKIPSD
0	0.928147901425415	0.686	0.182	0	0	HELZ
0	0.927822525803493	0.401	0.088	0	0	VPS8
0	0.927780021604625	0.339	0.082	0	0	TAF9
0	0.927329336641909	0.339	0.078	0	0	MYORG
0	0.926929196823592	0.605	0.158	0	0	ABCB6
0	0.926236088203353	0.339	0.085	0	0	LRRCC1
0	0.925855565616153	0.32	0.074	0	0	C1orf131
0	0.925387468620471	0.341	0.069	0	0	AP3M2
0	0.92538478920826	0.562	0.151	0	0	MARVELD2
0	0.925195623720396	0.511	0.128	0	0	SGCE
0	0.92516321218079	0.569	0.147	0	0	TAF2
0	0.924753302046814	0.5	0.121	0	0	MED16
0	0.924074048051065	0.433	0.099	0	0	ANKRD9
0	0.923976181793888	0.628	0.17	0	0	KIF1B
0	0.923873209165142	0.565	0.141	0	0	PTPRH
0	0.923487121469612	0.258	0.054	0	0	KIAA1211
0	0.923410929031038	0.273	0.059	0	0	IQCB1
0	0.923312273967036	0.386	0.086	0	0	USP2
0	0.922972377505382	0.381	0.091	0	0	ZHX3
0	0.922390184752238	0.54	0.142	0	0	EPB41L4B
0	0.922151518018621	0.509	0.131	0	0	EIF2S3
0	0.922075230733891	0.955	0.468	0	0	ACSL4
0	0.921795882060325	0.528	0.132	0	0	NPAS2
0	0.921247956559987	0.281	0.066	0	0	PAXIP1
0	0.921186238220802	0.575	0.148	0	0	NSMCE2
0	0.920970344941005	0.34	0.086	0	0	FANCA
0	0.920939147537404	0.352	0.082	0	0	ANO10
0	0.920702294516599	0.285	0.066	0	0	ADSSL1
0	0.920432476901881	0.586	0.151	0	0	FBXL6
0	0.919947658003952	0.748	0.225	0	0	FAM3A
0	0.918744317070694	0.268	0.06	0	0	SYT1
0	0.918718074983949	0.512	0.133	0	0	FAM8A1
0	0.91816124171233	0.801	0.29	0	0	ABHD2
0	0.918111818725976	0.394	0.095	0	0	PSMG4
0	0.916889687434137	0.551	0.147	0	0	ABHD3
0	0.916812908312296	0.482	0.116	0	0	SLC35D1

0	0.915365220499355	0.398	0.105	0	0	DSN1
0	0.914983093690451	0.66	0.179	0	0	FBXL17
0	0.913440065041612	0.43	0.104	0	0	NAGLU
0	0.91323357660081	0.345	0.081	0	0	SOCS5
0	0.912814596017768	0.94	0.489	0	0	ANPEP
0	0.912597173499799	0.259	0.059	0	0	SMUG1
0	0.912563369732971	0.456	0.112	0	0	CASP6
0	0.912083323554489	0.737	0.21	0	0	FAM122B
0	0.911511643191256	0.566	0.144	0	0	SPPL2B
0	0.910839663926287	0.825	0.298	0	0	PYGB
0	0.909846700827519	0.523	0.131	0	0	ATP8B1
0	0.90973715232181	0.346	0.079	0	0	C1GALT1
0	0.909328128426225	0.355	0.083	0	0	KYAT1
0	0.909037959493573	0.529	0.126	0	0	TTC14
0	0.908837261037515	0.545	0.145	0	0	COL5A3
0	0.908751859281513	0.275	0.059	0	0	DCBLD1
0	0.908121446597515	0.623	0.165	0	0	DAZAP1
0	0.907933912861499	0.484	0.116	0	0	CYHR1
0	0.907867139315852	0.62	0.175	0	0	PRKAB1
0	0.907417321022735	0.369	0.077	0	0	TTC13
0	0.907070847881746	0.454	0.109	0	0	CEP250
0	0.906825933362728	0.486	0.123	0	0	BCAT2
0	0.90592662264773	0.961	0.537	0	0	LAMP2
0	0.905429329443824	0.277	0.057	0	0	IL7
0	0.905307199374392	0.285	0.069	0	0	METTL27
0	0.904311001030588	0.45	0.109	0	0	RBM24
0	0.904009824273598	0.435	0.099	0	0	ACE
0	0.903910204906665	0.623	0.15	0	0	BCAR1
0	0.903655515878675	0.378	0.106	0	0	SLC4A4
0	0.903313094333872	0.499	0.122	0	0	MTMR4
0	0.903057554111844	0.705	0.216	0	0	SLC25A15
0	0.901524490797996	0.407	0.089	0	0	MYSM1
0	0.899090591970372	0.459	0.108	0	0	XPR1
0	0.898730321669528	0.41	0.095	0	0	PEX11A
0	0.89856181314542	0.605	0.144	0	0	TUT4
0	0.898157628292604	0.758	0.246	0	0	PBLD
0	0.897149184896896	0.57	0.152	0	0	RNF138
0	0.896989497732735	0.605	0.176	0	0	DHTKD1
0	0.895994433464864	0.423	0.099	0	0	RBBP6
0	0.895355787512477	0.383	0.087	0	0	TRAPPC8
0	0.895264809912684	0.261	0.05	0	0	PIGL
0	0.893159245652706	0.326	0.076	0	0	ZNF398
0	0.893009780971659	0.628	0.175	0	0	MYO19
0	0.892951883325687	0.432	0.094	0	0	TBCK
0	0.892074376945894	0.395	0.096	0	0	MBIP
0	0.89147933022352	0.643	0.174	0	0	LMBR1
0	0.890981489254952	0.314	0.07	0	0	SMARCAD1
0	0.890351291518476	0.407	0.091	0	0	EDARADD
0	0.890259271196605	0.36	0.085	0	0	DEPRECATED-ENSG00000232119
0	0.890045773047673	0.398	0.089	0	0	ASAP2
0	0.889698670961326	0.631	0.166	0	0	FOXP4
0	0.889457541667733	0.726	0.21	0	0	LRP5
0	0.888665123482778	0.593	0.165	0	0	NLK
0	0.887826734671451	0.695	0.222	0	0	NSDHL
0	0.887204241393194	0.694	0.216	0	0	C11orf54
0	0.885946018458647	0.466	0.111	0	0	POLK
0	0.885312645792933	0.598	0.166	0	0	HACD2
0	0.885285139885521	0.386	0.083	0	0	SHPRH
0	0.885152783149439	0.349	0.074	0	0	ABCG8
0	0.883924099163382	0.433	0.105	0	0	NDUFAF6

0	0.883686257654991	0.692	0.2	0	0	RTL8C
0	0.882634423843155	0.555	0.149	0	0	ZBTB10
0	0.882466847450498	0.811	0.227	0	0	NKTR
0	0.882332592416428	0.55	0.134	0	0	MON2
0	0.882039331658778	0.572	0.15	0	0	PHKA2
0	0.881269088202495	0.521	0.134	0	0	WWC2
0	0.881104534458037	0.345	0.086	0	0	SEPSECS
0	0.880078682233155	0.533	0.119	0	0	MTERF4
0	0.8792945983834	0.653	0.187	0	0	PLOD2
0	0.879072103733508	0.333	0.076	0	0	UBN2
0	0.878736435890335	0.468	0.117	0	0	FKBP3
0	0.878613580358493	0.563	0.15	0	0	MMGT1
0	0.878398031903561	0.429	0.104	0	0	SLC25A46
0	0.877883750514853	0.337	0.081	0	0	SEPTIN10
0	0.876277721505828	0.518	0.139	0	0	BTD
0	0.875754331012673	0.592	0.162	0	0	MALL
0	0.874015110679708	0.352	0.085	0	0	SQLE
0	0.873868395865199	0.851	0.304	0	0	SLC38A10
0	0.87302077307858	0.581	0.156	0	0	TOB2
0	0.872679555043792	0.889	0.436	0	0	BAAT
0	0.872273731417521	0.8	0.257	0	0	CD99L2
0	0.871077101924434	0.564	0.154	0	0	TRIM24
0	0.87083259792822	0.261	0.059	0	0	PPM1H
0	0.869662421892257	0.657	0.186	0	0	PARP10
0	0.869613348012339	0.862	0.307	0	0	CMC4
0	0.86959443468569	0.887	0.367	0	0	MAOA
0	0.869225086124911	0.541	0.15	0	0	PRKAB2
0	0.869006706751199	0.833	0.29	0	0	ABCA1
0	0.868572505564332	0.411	0.108	0	0	TOPBP1
0	0.866980483116831	0.895	0.389	0	0	DECR1
0	0.866705713985943	0.355	0.088	0	0	GUCY1B1
0	0.866003779063999	0.451	0.111	0	0	ZC3H14
0	0.865909417108174	0.395	0.104	0	0	GAS2L3
0	0.86582325393783	0.482	0.128	0	0	NDUFB4
0	0.865380182322962	0.286	0.06	0	0	TMEM184A
0	0.865261150975578	0.342	0.081	0	0	ZNF561
0	0.864844449711113	0.621	0.161	0	0	SH3GLB2
0	0.864376300729507	0.693	0.201	0	0	CUL4B
0	0.863562436542158	0.659	0.17	0	0	HECTD4
0	0.860690587671746	0.342	0.086	0	0	PIGC
0	0.86055978150693	0.552	0.151	0	0	LMBRD2
0	0.860523099132015	0.849	0.308	0	0	LAPTM4B
0	0.86026416920631	0.277	0.061	0	0	TBC1D2
0	0.860154049781445	0.562	0.148	0	0	MSH6
0	0.859025386685457	0.259	0.056	0	0	SZT2
0	0.858917659715837	0.51	0.11	0	0	DMXL2
0	0.85818436010551	0.711	0.21	0	0	PRPF3
0	0.857234425375315	0.896	0.387	0	0	CAPN2
0	0.856924592238402	0.323	0.076	0	0	PET117
0	0.856841860286874	0.317	0.07	0	0	ABCC6
0	0.856775480422573	0.402	0.095	0	0	FBXL12
0	0.856557612875714	0.304	0.068	0	0	ZNF397
0	0.85590876779445	0.416	0.103	0	0	SLC20A2
0	0.85507366021128	0.351	0.087	0	0	SLC28A1
0	0.854029935932774	0.536	0.138	0	0	PGAP3
0	0.853745006770135	0.309	0.07	0	0	MED20
0	0.853366065699284	0.509	0.134	0	0	EBAG9
0	0.853290614733415	0.625	0.17	0	0	SNX5
0	0.853171383403716	0.438	0.106	0	0	ZNF121
0	0.853077211816503	0.674	0.194	0	0	FNTA

0	0.852346601671906	0.375	0.083	0	0	ZNF266
0	0.852033337756235	0.64	0.173	0	0	PARP12
0	0.852011609620123	0.624	0.164	0	0	GOSR2
0	0.851876086999022	0.421	0.098	0	0	FLVCR1
0	0.851663509565297	0.783	0.247	0	0	POGZ
0	0.851443476288399	0.302	0.079	0	0	RFC4
0	0.851430763360879	0.418	0.132	0	0	S100P
0	0.851064657578941	0.493	0.129	0	0	HSPA4L
0	0.850946331117576	0.29	0.067	0	0	TBP
0	0.850928131546065	0.526	0.134	0	0	MTM1
0	0.849587278722861	0.71	0.202	0	0	UBR5
0	0.848554335996625	0.375	0.085	0	0	CALML4
0	0.847616587883406	0.67	0.189	0	0	SLC66A2
0	0.84719465116654	0.76	0.247	0	0	RREB1
0	0.847165132366714	0.351	0.072	0	0	LRIG2
0	0.846941338463235	0.676	0.194	0	0	VPS45
0	0.846728855145478	0.68	0.202	0	0	IPO9
0	0.846593705430454	0.426	0.109	0	0	PDS5B
0	0.846406125498686	0.327	0.079	0	0	SLC49A4
0	0.846078530038183	0.63	0.177	0	0	EEF2K
0	0.84584474043218	0.663	0.201	0	0	CYP4F3
0	0.845835801988396	0.778	0.268	0	0	GSTA2
0	0.844925341224669	0.521	0.13	0	0	VIRMA
0	0.844841107905554	0.422	0.099	0	0	LTN1
0	0.844592363602102	0.779	0.23	0	0	RNF19A
0	0.844288786917198	0.701	0.224	0	0	NDFIP2
0	0.843936996630205	0.439	0.109	0	0	RNF139
0	0.843505996474994	0.467	0.108	0	0	LRP6
0	0.843205900526882	0.438	0.104	0	0	PAN2
0	0.842713365117577	0.636	0.175	0	0	CARS2
0	0.842356841649591	0.365	0.075	0	0	GRAMD1B
0	0.841930294877375	0.477	0.126	0	0	MOAP1
0	0.841875481610929	0.343	0.084	0	0	TRAPPC11
0	0.841446068293528	0.432	0.103	0	0	RPTOR
0	0.840751874617673	0.583	0.161	0	0	ZMYND8
0	0.840289040318224	0.333	0.076	0	0	RAPGEF4
0	0.839969266810775	0.431	0.108	0	0	SMPD4
0	0.838053832008033	0.612	0.173	0	0	CANT1
0	0.837365233313793	0.495	0.124	0	0	NF1
0	0.83673013098753	0.299	0.063	0	0	ZDHHC17
0	0.836230614705013	0.551	0.143	0	0	TMEM185A
0	0.835525480734007	0.477	0.121	0	0	MYRF
0	0.834947862229964	0.487	0.12	0	0	NCOA6
0	0.834848857280563	0.324	0.077	0	0	MEST
0	0.833140146632447	0.678	0.203	0	0	LGR4
0	0.832866866887643	0.916	0.464	0	0	CES2
0	0.83229448204631	0.455	0.118	0	0	TMEM87A
0	0.832171895305259	0.426	0.098	0	0	USPL1
0	0.831797650056673	0.603	0.177	0	0	ABRACL
0	0.83081350671462	0.457	0.121	0	0	SLC25A43
0	0.830690959716874	0.354	0.084	0	0	ALKBH6
0	0.830076984293928	0.54	0.146	0	0	ODR4
0	0.829441183428278	0.339	0.077	0	0	MFSD6
0	0.829150080552424	0.663	0.209	0	0	GMDS
0	0.827426674716828	0.629	0.167	0	0	SIPA1L3
0	0.827071539888979	0.333	0.081	0	0	C4orf19
0	0.826821089333637	0.423	0.102	0	0	FRRS1
0	0.826190303992219	0.542	0.144	0	0	HMGXB4
0	0.825090690488574	0.597	0.168	0	0	SLC33A1
0	0.825075307788744	0.328	0.074	0	0	ZFYVE9

0	0.824331257295536	0.46	0.115	0	0	BORCS8
0	0.824134671651474	0.962	0.531	0	0	TSC22D3
0	0.823861254431986	0.306	0.079	0	0	DEPRECATED-ENSG00000147804
0	0.823271546260043	0.299	0.069	0	0	SNX7
0	0.822792856614322	0.737	0.243	0	0	SLC44A1
0	0.821894273490785	0.751	0.251	0	0	LACTB2
0	0.821607793673986	0.269	0.063	0	0	NUDCD1
0	0.821421345745024	0.356	0.083	0	0	CISD1
0	0.821342077651721	0.618	0.176	0	0	EGFR
0	0.820816327589403	0.286	0.065	0	0	FAM222A
0	0.820337170042335	0.717	0.228	0	0	TMEM64
0	0.82030974001143	0.309	0.078	0	0	PARD6B
0	0.819661783040745	0.365	0.092	0	0	PFN2
0	0.817881211913083	0.284	0.072	0	0	TMEM184C
0	0.817837262461416	0.868	0.329	0	0	TSPAN6
0	0.817760583778817	0.682	0.212	0	0	TMEM38B
0	0.816883113558188	0.582	0.157	0	0	FAM13A
0	0.816132993059601	0.462	0.112	0	0	ERCC6L2
0	0.8158800078077	0.437	0.11	0	0	PAWR
0	0.815869181728803	0.663	0.206	0	0	CTSO
0	0.81524692644916	0.626	0.174	0	0	PARP14
0	0.815002013546858	0.709	0.22	0	0	DST
0	0.81476965421525	0.348	0.085	0	0	TMCC3
0	0.814669846360659	0.306	0.07	0	0	PLAAT2
0	0.814377620134281	0.296	0.072	0	0	TTI1
0	0.81391400495583	0.44	0.112	0	0	ZCCHC2
0	0.81381383300298	0.408	0.088	0	0	DEPRECATED-ENSG00000135976
0	0.81337975034682	0.631	0.172	0	0	SHROOM3
0	0.813120508387716	0.305	0.072	0	0	ZNF263
0	0.812204766113205	0.293	0.068	0	0	PEX1
0	0.812064895320599	0.415	0.103	0	0	C2CD5
0	0.812025501282256	0.498	0.128	0	0	MORC4
0	0.81190714186339	0.444	0.105	0	0	PIGG
0	0.811771211015261	0.894	0.372	0	0	ATP6AP1
0	0.811499711063192	0.58	0.149	0	0	UBE2G2
0	0.81144308657962	0.347	0.082	0	0	ESCO1
0	0.811213761465004	0.33	0.075	0	0	USP37
0	0.811207858864957	0.672	0.186	0	0	FBXL5
0	0.810552054313605	0.25	0.059	0	0	NUDT12
0	0.810344832642716	0.551	0.15	0	0	ZFHX3
0	0.809823327787637	0.35	0.081	0	0	SLC12A8
0	0.80885265074546	0.366	0.104	0	0	CDC42EP5
0	0.808023558386075	0.827	0.279	0	0	STAG2
0	0.807411890957815	0.399	0.098	0	0	CADPS2
0	0.807039996854096	0.376	0.091	0	0	MINDY1
0	0.806767785429603	0.311	0.071	0	0	B4GAT1
0	0.806655473901168	0.327	0.074	0	0	TM2D1
0	0.806261310681326	0.444	0.105	0	0	EPM2AIP1
0	0.805869605362823	0.363	0.093	0	0	SPRY2
0	0.805619882021435	0.287	0.071	0	0	ST3GAL3
0	0.805121778180724	0.29	0.073	0	0	NHLRC3
0	0.805097866436328	0.435	0.104	0	0	KRIT1
0	0.805059229418124	0.361	0.09	0	0	PIGN
0	0.803776236813667	0.284	0.066	0	0	PTCD1
0	0.803342417235558	0.264	0.062	0	0	TRIM2
0	0.802655652442176	0.582	0.169	0	0	TRIQK
0	0.802522174086593	0.481	0.12	0	0	NEK9
0	0.802221115784271	0.581	0.165	0	0	ARHGEF40
0	0.801991587479879	0.423	0.107	0	0	CLCN3
0	0.801767559768736	0.545	0.156	0	0	CERS6

0	0.801081007086962	0.626	0.136	0	0	LYST
0	0.800183054737343	0.331	0.084	0	0	DDX60
0	0.800105484269347	0.381	0.092	0	0	COG1
0	0.800041799764422	0.406	0.095	0	0	TEAD3
0	0.7994210912797	0.331	0.081	0	0	ATR
0	0.799182585720979	0.673	0.191	0	0	TTC3
0	0.798828632272214	0.449	0.112	0	0	PCGF3
0	0.79787178528678	0.251	0.053	0	0	SLC7A9
0	0.797582022043077	0.416	0.109	0	0	CNOT10
0	0.797544046199697	0.324	0.076	0	0	EDC3
0	0.796969026243741	0.259	0.061	0	0	SFXN2
0	0.795618444646216	0.352	0.08	0	0	THOC1
0	0.795469057727858	0.62	0.189	0	0	GDA
0	0.795449609822381	0.371	0.096	0	0	POLA1
0	0.795153539937188	0.746	0.268	0	0	NBL1
0	0.794752177550608	0.484	0.12	0	0	PLCB1
0	0.794509951076641	0.281	0.066	0	0	PFKFB1
0	0.794048721465077	0.413	0.102	0	0	NSUN6
0	0.793391882112281	0.601	0.18	0	0	MARC1
0	0.79281463891289	0.31	0.07	0	0	FKBP9
0	0.79238090517132	0.25	0.06	0	0	B3GALNT2
0	0.792319976305419	0.395	0.096	0	0	RAB3GAP2
0	0.791660030542511	0.633	0.179	0	0	MAST4
0	0.791544207091824	0.613	0.17	0	0	TCEAL1
0	0.791485574334956	0.423	0.108	0	0	MCEE
0	0.790729772925462	0.505	0.132	0	0	ACBD6
0	0.790703953091008	0.494	0.126	0	0	SGMS1
0	0.790204344895841	0.417	0.101	0	0	NIPA1
0	0.789838320827114	0.841	0.326	0	0	INF2
0	0.789286917025095	0.585	0.167	0	0	TFDP1
0	0.78729629981553	0.713	0.222	0	0	FARP1
0	0.787188202702499	0.565	0.158	0	0	AQP3
0	0.786833647889278	0.335	0.077	0	0	EXD3
0	0.786671416755283	0.635	0.178	0	0	ASB13
0	0.786554527661318	0.406	0.095	0	0	IRAK4
0	0.786525003945105	0.558	0.152	0	0	KBTBD2
0	0.785849792313317	0.394	0.097	0	0	RAD50
0	0.785704554348413	0.748	0.219	0	0	DDX39B
0	0.785189012063909	0.574	0.158	0	0	PLA2G12A
0	0.784462706366683	0.524	0.137	0	0	RBCK1
0	0.784033667389402	0.309	0.076	0	0	UBE2K
0	0.783696769204261	0.342	0.083	0	0	TDRD7
0	0.783690521449693	0.283	0.059	0	0	DEPRECATED-ENSG00000197302
0	0.783253178131775	0.472	0.124	0	0	SLC30A9
0	0.782916893846309	0.951	0.435	0	0	MPP1
0	0.782808469353503	0.255	0.06	0	0	CXorf40B
0	0.781916104745868	0.541	0.144	0	0	SEMA4B
0	0.779760428598245	0.466	0.127	0	0	TOR1B
0	0.77947346545176	0.733	0.225	0	0	DIDO1
0	0.779228711844345	0.942	0.412	0	0	CCNL2
0	0.77916363926333	0.721	0.249	0	0	FDPS
0	0.779096608998098	0.554	0.145	0	0	RSBN1L
0	0.779021510988153	0.634	0.185	0	0	N4BP2L1
0	0.778622288829616	0.587	0.159	0	0	CAMK2D
0	0.777802329914044	0.974	0.644	0	0	PGRMC1
0	0.777340325599202	0.669	0.195	0	0	SCRIB
0	0.776839182385278	0.654	0.196	0	0	ILVBL
0	0.776666076044746	0.344	0.087	0	0	SOCS7
0	0.77654322274993	0.28	0.06	0	0	SENP7
0	0.775803236434564	0.605	0.161	0	0	PCMTD2

0	0.775139285656992	0.253	0.064	0	0	SLC30A6
0	0.775078833170189	0.516	0.139	0	0	TSNAX
0	0.774048625313785	0.269	0.064	0	0	GKAP1
0	0.77366755293433	0.763	0.225	0	0	KAT6A
0	0.77323030125403	0.756	0.265	0	0	BIRC3
0	0.773154175601543	0.574	0.159	0	0	NR5A2
0	0.773003954484393	0.275	0.068	0	0	ARL17B
0	0.772913714018374	0.651	0.195	0	0	ADAM15
0	0.772807301081629	0.455	0.118	0	0	CAMSAP2
0	0.772491495453277	0.407	0.098	0	0	SLC37A3
0	0.770875090351598	0.478	0.124	0	0	NUP133
0	0.770664471332003	0.387	0.093	0	0	FAM120B
0	0.77005546824292	0.348	0.085	0	0	SOCS4
0	0.769730132026878	0.388	0.12	0	0	TFF3
0	0.769684992958192	0.544	0.146	0	0	IL17RC
0	0.769489552259911	0.251	0.061	0	0	FAM76A
0	0.769133760166963	0.522	0.14	0	0	PHF14
0	0.768853740878346	0.32	0.077	0	0	WDR5
0	0.768314610266242	0.594	0.169	0	0	DNAJC22
0	0.76817393360797	0.485	0.123	0	0	LETMD1
0	0.767837274562768	0.695	0.205	0	0	MAN2A1
0	0.767082113205428	0.708	0.217	0	0	SPECC1L
0	0.766186003409769	0.563	0.155	0	0	RFC1
0	0.765679245054243	0.764	0.286	0	0	MSMO1
0	0.765333057087724	0.315	0.07	0	0	CASD1
0	0.765211696173876	0.465	0.121	0	0	FAF2
0	0.765024564812942	0.631	0.192	0	0	SGK2
0	0.764297838830863	0.84	0.405	0	0	GOLM1
0	0.763928546412576	0.354	0.092	0	0	XRCC5
0	0.763491173543933	0.442	0.112	0	0	RABGAP1
0	0.763191982986415	0.985	0.809	0	0	SELENOP
0	0.763015568977046	0.349	0.084	0	0	CHML
0	0.762675828895829	0.342	0.085	0	0	ABHD15
0	0.76261014162742	0.678	0.188	0	0	KMT2B
0	0.762108102825364	0.289	0.071	0	0	CLK2
0	0.761842330611616	0.606	0.19	0	0	TM7SF2
0	0.761728216867489	0.662	0.193	0	0	ENTPD6
0	0.76162794229878	0.709	0.216	0	0	NCOA2
0	0.761622754593012	0.431	0.11	0	0	BPNT1
0	0.760863710564089	0.274	0.06	0	0	MINDY3
0	0.760350390615051	0.435	0.115	0	0	INTS7
0	0.759207808486897	0.684	0.21	0	0	AR
0	0.758923402843732	0.37	0.087	0	0	PLXNA3
0	0.758415736642965	0.41	0.104	0	0	LARP1B
0	0.758373818777196	0.786	0.28	0	0	PDZK1
0	0.758001866236784	0.259	0.058	0	0	C2CD2
0	0.757483627386985	0.351	0.091	0	0	SLC36A1
0	0.75707301694784	0.615	0.178	0	0	PI4KB
0	0.756888965980746	0.338	0.09	0	0	SKP2
0	0.754657845171002	0.329	0.084	0	0	TMEM192
0	0.754615255654807	0.33	0.083	0	0	ZBTB37
0	0.753736622507781	0.25	0.061	0	0	TMEM30B
0	0.753696291977178	0.313	0.078	0	0	SPAG16
0	0.753625269873163	0.443	0.112	0	0	MCM3AP
0	0.753240922726296	0.627	0.172	0	0	PTK2
0	0.752546615773288	0.849	0.315	0	0	GPAA1
0	0.752537418050059	0.329	0.079	0	0	GPATCH2
0	0.752515420427019	0.709	0.229	0	0	CRY2
0	0.752347784982553	0.29	0.072	0	0	FBXL18
0	0.751485567220757	0.389	0.134	0	0	TFF1

0	0.75099137299754	0.409	0.095	0	0	DAPK2
0	0.750716074064218	0.895	0.392	0	0	SCCPDH
0	0.75069083697891	0.684	0.215	0	0	CCNG1
0	0.750409102217496	0.501	0.146	0	0	GSTM4
0	0.750108124083014	0.361	0.089	0	0	SDHAF4
0	0.750094767085897	0.269	0.065	0	0	PRLR
0	0.750049494537774	0.55	0.145	0	0	ZBED6
0	0.749777471525361	0.307	0.067	0	0	VCL
0	0.749185656351204	0.469	0.111	0	0	MAP3K4
0	0.749141748263038	0.678	0.188	0	0	SRSF5
0	0.748991036996048	0.658	0.205	0	0	SLC25A5
0	0.748592579914357	0.303	0.077	0	0	RASSF8
0	0.747757118058107	0.395	0.102	0	0	SLC35B4
0	0.747746105359035	0.437	0.106	0	0	APBB2
0	0.747453766752071	0.329	0.081	0	0	UMPS
0	0.747330394040846	0.334	0.087	0	0	RAP1GDS1
0	0.746799759167775	0.371	0.091	0	0	CAP2
0	0.74671594752766	0.324	0.079	0	0	ZNF654
0	0.746401016227571	0.736	0.238	0	0	SLC47A1
0	0.745862912708228	0.289	0.069	0	0	GABPA
0	0.745514289768912	0.414	0.114	0	0	LRRC45
0	0.745505773284205	0.514	0.137	0	0	TEAD1
0	0.745320683173123	0.655	0.209	0	0	IVD
0	0.745077178784747	0.581	0.165	0	0	GNPAT
0	0.745006063848334	0.339	0.088	0	0	C17orf75
0	0.744819004486494	0.295	0.071	0	0	DHRS4L2
0	0.744573459245954	0.747	0.242	0	0	UGT1A1
0	0.744279941814516	0.292	0.07	0	0	CYP20A1
0	0.74343122772859	0.264	0.065	0	0	PFKFB2
0	0.742951501440176	0.535	0.209	0	0	CLDN15
0	0.742784414198506	0.282	0.061	0	0	WDR27
0	0.742729738497448	0.368	0.092	0	0	TMEM251
0	0.741959232380821	0.83	0.326	0	0	PON2
0	0.741589366247659	0.767	0.26	0	0	VMA21
0	0.741583430241653	0.768	0.273	0	0	DNAJC3
0	0.741433154158968	0.862	0.318	0	0	MORF4L2
0	0.740861435441446	0.421	0.116	0	0	TMEM220
0	0.740759188594397	0.419	0.107	0	0	IRS1
0	0.74029752288812	0.495	0.144	0	0	GSKIP
0	0.740254655919426	0.375	0.089	0	0	PPP1R13B
0	0.740110726279506	0.503	0.132	0	0	MCM6
0	0.740033542771571	0.618	0.186	0	0	CHKA
0	0.739822477781442	0.529	0.148	0	0	GRAMD4
0	0.739752346544918	0.687	0.206	0	0	LRRFIP2
0	0.739194996249818	0.302	0.067	0	0	NR2C1
0	0.739070657308226	0.492	0.126	0	0	CDK13
0	0.73904284180917	0.721	0.21	0	0	BIRC6
0	0.73872650045044	0.372	0.094	0	0	GABPB2
0	0.738669119768026	0.482	0.126	0	0	FBXO21
0	0.738420801401704	0.661	0.179	0	0	EDNRB
0	0.738361355264508	0.711	0.234	0	0	SLC25A44
0	0.738251041792154	0.31	0.071	0	0	DDX31
0	0.738236551657065	0.857	0.327	0	0	IDH3G
0	0.738188323885753	0.38	0.1	0	0	NUP54
0	0.737408494543503	0.314	0.078	0	0	SLC35B3
0	0.736859408776583	0.251	0.049	0	0	HID1
0	0.736590099988945	0.93	0.456	0	0	UPB1
0	0.736456159190871	0.38	0.103	0	0	PLA2G4C
0	0.736406964711528	0.388	0.097	0	0	C6orf136
0	0.73625166042507	0.352	0.095	0	0	FBXO17

0	0.735107120516388	0.429	0.109	0	0	CUL5
0	0.734939719670743	0.39	0.101	0	0	FBXO38
0	0.73490827022749	0.511	0.153	0	0	ITPKC
0	0.734508164168942	0.313	0.077	0	0	SPATA5L1
0	0.734374872427458	0.396	0.105	0	0	PCSK9
0	0.734186553141179	0.361	0.087	0	0	KCTD13
0	0.734170196472993	0.438	0.108	0	0	EFNB1
0	0.733481657649858	0.635	0.177	0	0	PCID2
0	0.733030963609284	0.599	0.169	0	0	RBMX
0	0.732971830439863	0.495	0.126	0	0	DIS3
0	0.732804900799032	0.647	0.198	0	0	SORBS2
0	0.732794450564509	0.829	0.341	0	0	PCK2
0	0.73273788865642	0.311	0.08	0	0	EPDR1
0	0.732684070551697	0.57	0.185	0	0	TLCD4
0	0.732303940354144	0.341	0.087	0	0	NOL9
0	0.732204560208236	0.42	0.107	0	0	INPP1
0	0.73197919968391	0.834	0.284	0	0	MDM4
0	0.731845818337982	0.759	0.228	0	0	PILRB
0	0.731686957141524	0.593	0.17	0	0	SH3BP4
0	0.731599214273204	0.31	0.076	0	0	LRRC28
0	0.731458450566207	0.457	0.119	0	0	RHOT1
0	0.731432280538328	0.274	0.066	0	0	KNOP1
0	0.73135333011604	0.402	0.104	0	0	TMEM98
0	0.731294957619442	0.253	0.057	0	0	ZFP90
0	0.730720103853014	0.628	0.172	0	0	TPP2
0	0.730644476263843	0.734	0.241	0	0	PPARA
0	0.730095404898395	0.715	0.229	0	0	DGAT1
0	0.729871319925763	0.357	0.088	0	0	ANKRD46
0	0.728572109222254	0.344	0.09	0	0	CEP192
0	0.728395977315357	0.814	0.314	0	0	IL13RA1
0	0.727536729269617	0.431	0.115	0	0	SLC9A6
0	0.727131581913423	0.334	0.083	0	0	SLC16A13
0	0.726590421911744	0.275	0.067	0	0	MPV17L
0	0.726382614377236	0.365	0.089	0	0	NAA30
0	0.726288363057143	0.61	0.183	0	0	PLS1
0	0.726235994658145	0.611	0.178	0	0	ST5
0	0.726204781372743	0.576	0.149	0	0	PAN3
0	0.725664959974877	0.974	0.594	0	0	UGDH
0	0.725265298871675	0.577	0.162	0	0	NCKAP1
0	0.725239601209023	0.261	0.062	0	0	ALS2
0	0.724784650688771	0.54	0.153	0	0	SRP19
0	0.724219289629055	0.469	0.125	0	0	OSGEP
0	0.724015254813621	0.458	0.12	0	0	WBP1
0	0.723804313304768	0.488	0.133	0	0	SPATS2
0	0.723295836700654	0.345	0.079	0	0	NT5C3A
0	0.722786288847719	0.33	0.08	0	0	BCHE
0	0.722545800072988	0.29	0.068	0	0	B4GALT4
0	0.72170569205801	0.314	0.081	0	0	UHRF1BP1L
0	0.721654978852011	0.255	0.063	0	0	TXNDC16
0	0.721564049589712	0.298	0.07	0	0	SNAPC3
0	0.721478981477373	0.679	0.203	0	0	USP12
0	0.721181643450863	0.346	0.084	0	0	DEPRECATED-ENSG00000249915
0	0.72097741372768	0.35	0.085	0	0	ZNF618
0	0.720967118884645	0.459	0.118	0	0	NAE1
0	0.720773348796567	0.475	0.121	0	0	GNL3L
0	0.720721477147152	0.99	0.746	0	0	AGT
0	0.720472185986985	0.457	0.123	0	0	TRMT1L
0	0.718825931034023	0.309	0.075	0	0	HCFC2
0	0.718749406393644	0.464	0.123	0	0	PIK3CB
0	0.718739356847365	0.729	0.266	0	0	MCM3

0	0.718438112530752	0.54	0.149	0	0	GET1	
0	0.717971711058687	0.56	0.175	0	0	ME1	
0	0.717875371833129	0.406	0.099	0	0	CUL2	
0	0.717702238093946	0.281	0.07	0	0	ZNF236	
0	0.716978843273703	0.364	0.093	0	0	FAM110B	
0	0.716969867999876	0.366	0.095	0	0	PLXNC1	
0	0.716774270696378	0.762	0.275	0	0	HACD3	
0	0.716389061545891	0.641	0.184	0	0	MOGS	
0	0.716159191883715	0.97	0.555	0	0	ANXA4	
0	0.715685225877679	0.68	0.21	0	0	KLF3	
0	0.715545505004061	0.413	0.1	0	0	MRNIP	
0	0.715294673974848	0.306	0.081	0	0	PIGQ	
0	0.715178550151224	0.511	0.137	0	0	PMM2	
0	0.714771405205608	0.375	0.095	0	0	SUCO	
0	0.71436883217804	0.391	0.092	0	0	DPH7	
0	0.714158900699808	0.256	0.058	0	0	WDR75	
0	0.713988656657794	0.514	0.136	0	0	SCAF8	
0	0.713739093325872	0.495	0.134	0	0	DEPRECATED-ENSG00000146416	
0	0.71373549463932	0.668	0.19	0	0	ANKRD17	
0	0.713317263471022	0.45	0.114	0	0	CDC42BPA	
0	0.712324331222891	0.427	0.114	0	0	GART	
0	0.712181992929991	0.339	0.084	0	0	ATG4A	
0	0.71206122530896	0.371	0.084	0	0	ENGASE	
0	0.711986398901175	0.276	0.066	0	0	VAMP4	
0	0.7113819573917	0.476	0.127	0	0	MTFR1L	
0	0.710458708310472	0.643	0.194	0	0	APEX1	
0	0.709737115714756	0.474	0.137	0	0	PGAP2	
0	0.709485183293714	0.309	0.075	0	0	SIX5	
0	0.708446162786575	0.256	0.06	0	0	HNF1B	
0	0.707581872591662	0.306	0.073	0	0	DEPDC5	
0	0.706962744503357	0.438	0.114	0	0	FAM193A	
0	0.706784634560777	0.74	0.244	0	0	PA2G4	
0	0.706716746623399	0.515	0.12	0	0	ZBTB25	
0	0.706320819033524	0.6	0.176	0	0	VAMP7	
0	0.7057192399676	0.63	0.206	0	0	SLC25A30	
0	0.705662331258957	0.409	0.105	0	0	SMAD1	
0	0.705424939870675	0.548	0.146	0	0	BTBD7	
0	0.704837282795045	0.594	0.186	0	0	GFPT1	
0	0.704745585210797	0.711	0.204	0	0	FAM193B	
0	0.704499426713571	0.641	0.196	0	0	TMCO3	
0	0.703648401080775	0.53	0.144	0	0	DHX16	
0	0.703636797561994	0.429	0.111	0	0	VPS54	
0	0.703550034842417	0.63	0.191	0	0	FOXO3	
0	0.703509705166828	0.376	0.09	0	0	TRNT1	
0	0.703409061455223	0.936	0.507	0	0	LYZ	
0	0.703188329205386	0.609	0.188	0	0	DPP4	
0	0.701094882766367	0.642	0.201	0	0	NUP58	
0	0.700747029036662	0.303	0.073	0	0	DEPRECATED-ENSG00000072803	
0	0.700736997556029	0.287	0.071	0	0	HOOK2	
0	0.700550643861547	0.253	0.061	0	0	CHMP4C	
0	0.700530962483336	0.697	0.212	0	0	SEL1L3	
0	0.700254380440692	0.566	0.162	0	0	GINM1	
0	0.700162639069377	0.4	0.107	0	0	TMEM65	
0	0.699923583245185	0.549	0.149	0	0	ARHGAP12	
0	0.699511846307971	0.304	0.077	0	0	RPE	
0	0.699115269937341	0.522	0.142	0	0	BDP1	
0	0.698928610614615	0.412	0.11	0	0	VEZF1	
0	0.698676727348835	0.751	0.26	0	0	NIT1	
0	0.697834116596845	0.435	0.12	0	0	CCHCR1	
0	0.697786404047031	0.569	0.154	0	0	METTL17	

0	0.697721110810725	0.531	0.152	0	0	SLC22A23
0	0.697670066108241	0.256	0.06	0	0	CYP7A1
0	0.697557170243783	0.499	0.131	0	0	UBR3
0	0.69741416157847	0.485	0.136	0	0	COQ4
0	0.69661181949177	0.303	0.074	0	0	COMTD1
0	0.696186013385753	0.467	0.12	0	0	KAT2A
0	0.696048529577973	0.683	0.206	0	0	PHF3
0	0.69583034893067	0.625	0.195	0	0	TENT5A
0	0.695661457030145	0.526	0.14	0	0	LEMD2
0	0.695523082584967	0.305	0.077	0	0	WDFY3
0	0.694937809564039	0.615	0.184	0	0	CAPN5
0	0.694700169419142	0.442	0.118	0	0	MTX2
0	0.694053923372393	0.561	0.161	0	0	QSOX1
0	0.693715596622932	0.381	0.09	0	0	ABL2
0	0.693683317235221	0.448	0.117	0	0	CHD9
0	0.693255149100096	0.731	0.257	0	0	SLC25A4
0	0.693016933866818	0.457	0.124	0	0	CLYBL
0	0.692980521452491	0.254	0.061	0	0	FGFRL1
0	0.692956565690127	0.382	0.104	0	0	RANBP10
0	0.692922020886359	0.331	0.1	0	0	FAXDC2
0	0.692524838223245	0.359	0.092	0	0	THEM6
0	0.692418296292837	0.732	0.237	0	0	ARHGAP35
0	0.692263507101045	0.654	0.205	0	0	PACSIN2
0	0.692200235203714	0.365	0.094	0	0	C19orf33
0	0.692101435783103	0.617	0.185	0	0	BAIAP2L1
0	0.692053326664529	0.399	0.12	0	0	IL1RAP
0	0.691634166259873	0.93	0.487	0	0	IDH1
0	0.691524082222035	0.397	0.103	0	0	PARD3
0	0.691433552586729	0.291	0.075	0	0	UBXN2A
0	0.691369843051289	0.626	0.18	0	0	FAM83H
0	0.691034973730279	0.356	0.092	0	0	NOM1
0	0.690587688307659	0.305	0.081	0	0	AP5S1
0	0.690460137537252	0.253	0.059	0	0	TMEM116
0	0.689976781325864	0.494	0.141	0	0	COX11
0	0.689617318329099	0.367	0.097	0	0	BOP 1.00
0	0.689320482698177	0.439	0.126	0	0	SRD5A3
0	0.688975253611268	0.486	0.133	0	0	CSTF3
0	0.687910295236218	0.301	0.073	0	0	FAM133B
0	0.687620633266854	0.495	0.135	0	0	GTF2H1
0	0.687429556993087	0.333	0.088	0	0	MKKS
0	0.687253589177624	0.81	0.329	0	0	GCLC
0	0.687124517349432	0.613	0.186	0	0	DEPRECATED-ENSG00000133142
0	0.685360977160876	0.291	0.069	0	0	PKD1
0	0.685018439883421	0.397	0.102	0	0	USF3
0	0.684056072056129	0.423	0.115	0	0	PGM3
0	0.683369076935921	0.289	0.07	0	0	LIN52
0	0.683285207820875	0.31	0.072	0	0	ORC2
0	0.683212558347903	0.308	0.077	0	0	EXT2
0	0.683051035197417	0.27	0.067	0	0	TBCCD1
0	0.683006032506792	0.34	0.088	0	0	TIGD5
0	0.682707263074322	0.277	0.068	0	0	PTRH2
0	0.682575205527625	0.651	0.212	0	0	TM9SF1
0	0.682517490583774	0.615	0.177	0	0	MPHOSPH8
0	0.682474480394156	0.276	0.074	0	0	PIGV
0	0.682377767970966	0.47	0.129	0	0	PPA2
0	0.682363652073626	0.357	0.093	0	0	MBTD1
0	0.681437522675038	0.356	0.091	0	0	UCHL3
0	0.681422231427928	0.849	0.326	0	0	AFF1
0	0.681258897554753	0.494	0.126	0	0	CBFA2T2
0	0.680832609158955	0.767	0.241	0	0	STX16

0	0.679875313490355	0.512	0.151	0	0	DHFR
0	0.678951652040487	0.349	0.085	0	0	PPWD1
0	0.678680146126029	0.426	0.133	0	0	CD177
0	0.678271115405985	0.408	0.114	0	0	PIGX
0	0.678067305588272	0.282	0.062	0	0	NTN4
0	0.67789063000313	0.785	0.28	0	0	TCEAL4
0	0.677492641460868	0.431	0.114	0	0	FBXO28
0	0.676956706884682	0.484	0.134	0	0	SLC16A2
0	0.676702152326906	0.885	0.416	0	0	HPN
0	0.676513581224247	0.329	0.087	0	0	RBFA
0	0.676398806965605	0.862	0.363	0	0	TPD52
0	0.675293045492874	0.549	0.163	0	0	ZFYVE21
0	0.675273065040956	0.305	0.075	0	0	MEIS2
3.25928669083067E-177	0.674937572337511	0.296	0.128	5.46191263649404E-173	0	LCN2
0	0.674765752738297	0.704	0.224	0	0	ADAM9
0	0.67470881949883	0.726	0.239	0	0	AGFG2
0	0.6744379689087	0.709	0.244	0	0	SLC50A1
0	0.673589311466612	0.686	0.212	0	0	ECPAS
0	0.673265322984515	0.768	0.256	0	0	CHTOP
0	0.673114686949835	0.316	0.077	0	0	PCNX4
0	0.672994658717718	0.288	0.072	0	0	ZNF621
0	0.672541236446513	0.354	0.091	0	0	PLEKHA7
0	0.672498598232292	0.261	0.069	0	0	DIS3L
0	0.671940764112547	0.9	0.436	0	0	TM9SF2
0	0.671754876319262	0.29	0.072	0	0	CENPC
0	0.671455355926741	0.671	0.192	0	0	PHF20L1
0	0.67099357706669	0.763	0.246	0	0	ASH1L
0	0.670870580610672	0.323	0.083	0	0	CMTR2
0	0.670137410748325	0.418	0.107	0	0	CCNB1IP1
0	0.670103330923733	0.37	0.087	0	0	SLC35E2B
0	0.669571166857789	0.677	0.221	0	0	SPPL2A
0	0.669393970359883	0.499	0.127	0	0	FAM172A
0	0.669315441712339	0.706	0.231	0	0	KANK1
0	0.668475015826036	0.326	0.075	0	0	BRAF
0	0.668080209310991	0.296	0.072	0	0	SCAMP5
0	0.667458460838663	0.783	0.287	0	0	PDIA5
0	0.666787585138485	0.908	0.412	0	0	BCAP31
0	0.666476560685101	0.608	0.175	0	0	ZNF410
0	0.666350259782759	0.55	0.155	0	0	ESRRA
0	0.666079108765795	0.364	0.094	0	0	PSKH1
0	0.665893287578	0.478	0.136	0	0	E2F1
0	0.665574856383736	0.715	0.219	0	0	U2SURP
0	0.665475370653531	0.453	0.134	0	0	C19orf48
0	0.665351409279649	0.265	0.057	0	0	TRMT13
0	0.665169702822946	0.264	0.062	0	0	CAPRIN2
0	0.665017002953813	0.648	0.211	0	0	RHPN2
0	0.664972910186451	0.378	0.098	0	0	PRR14L
0	0.664688036083676	0.711	0.24	0	0	AKAP1
0	0.664617091771167	0.453	0.129	0	0	ADH5
0	0.664582084427849	0.318	0.079	0	0	PPIL4
0	0.664063252043226	0.446	0.113	0	0	PWWP2A
0	0.663353896539308	0.353	0.094	0	0	NOL11
0	0.663342807980831	0.415	0.111	0	0	TGS1
0	0.663184553483504	0.722	0.219	0	0	TCIM
0	0.662383311262973	0.645	0.197	0	0	RBM10
0	0.66221163306838	0.356	0.09	0	0	PDXDC1
0	0.662062766185031	0.492	0.143	0	0	GMPS
0	0.661963793309005	0.4	0.106	0	0	ITSN1
0	0.661903002016516	0.517	0.137	0	0	ZNF800
0	0.661499478908278	0.911	0.469	0	0	GOLIM4

	0	0.661194881758805	0.461	0.112	0	0	YTHDC2
	0	0.660644896020247	0.799	0.304	0	0	NAPRT
	0	0.66061123359522	0.675	0.215	0	0	CPNE3
	0	0.660391304466745	0.365	0.093	0	0	MRS2
	0	0.660303681841152	0.259	0.062	0	0	C6orf47
	0	0.660008771856488	0.264	0.067	0	0	GORAB
	0	0.659894775128615	0.261	0.069	0	0	ZBED3
	0	0.659781402561573	0.455	0.118	0	0	DOK4
	0	0.659755963141104	0.575	0.167	0	0	MAPK1IP1L
	0	0.65972312249043	0.74	0.272	0	0	MCFD2
	0	0.659372011555591	0.457	0.119	0	0	MGAT5
	0	0.658701497493968	0.345	0.088	0	0	LATS2
	0	0.658624807962072	0.287	0.069	0	0	UIMC1
	0	0.658502968501707	0.434	0.117	0	0	EXOSC2
	0	0.658302111401706	0.402	0.101	0	0	CYTH3
	0	0.657826820398307	0.26	0.069	0	0	ARMC10
	0	0.657697360057376	0.258	0.064	0	0	ZC3H11A
	0	0.657246065517734	0.359	0.088	0	0	MLLT10
	0	0.657168534452763	0.254	0.062	0	0	TMEM47
	0	0.656984837811968	0.336	0.091	0	0	ING2
	0	0.656530450311904	0.744	0.226	0	0	SUGP2
	0	0.656217007945846	0.929	0.501	0	0	TM4SF4
	0	0.654741693470487	0.42	0.112	0	0	GON7
	0	0.654111015551938	0.314	0.083	0	0	GDAP2
	0	0.654003269352861	0.291	0.078	0	0	DSG2
	0	0.653987850397783	0.492	0.141	0	0	CASP3
	0	0.653425757479577	0.956	0.546	0	0	LGALS4
	0	0.653000953767466	0.581	0.171	0	0	SEC23A
	0	0.652854242417226	0.545	0.182	0	0	TTPA
	0	0.652515617099089	0.367	0.087	0	0	RBM12
	0	0.65217634250844	0.515	0.138	0	0	TTC17
	0	0.651872997023711	0.272	0.065	0	0	NSUN3
	0	0.651533337151713	0.554	0.159	0	0	PSEN1
	0	0.651169408896803	0.324	0.082	0	0	COX19
	0	0.651126886928881	0.484	0.128	0	0	RALGAPB
	0	0.65102158928082	0.48	0.138	0	0	C6orf62
	0	0.650204180906327	0.691	0.218	0	0	RNF115
	0	0.649679343491276	0.397	0.102	0	0	NPHP3
	0	0.649411729407638	0.297	0.071	0	0	XPA
	0	0.649381077578277	0.68	0.22	0	0	NDUFV3
	0	0.64878410625829	0.627	0.208	0	0	EMP2
	0	0.648609804063375	0.776	0.279	0	0	FAM114A1
	0	0.648523830867224	0.31	0.08	0	0	ATP5F1C
	0	0.648363873301885	0.843	0.347	0	0	TMEM205
	0	0.64738868195518	0.271	0.072	0	0	KIAA1958
	0	0.647095854582385	0.6	0.177	0	0	DEPRECATED-ENSG00000174827
	0	0.64705276653282	0.253	0.074	0	0	ERI2
	0	0.647024251849384	0.547	0.137	0	0	DMTF1
	0	0.646965067075571	0.287	0.076	0	0	DGCR8
	0	0.646887549565611	0.275	0.073	0	0	BBS2
	0	0.645692489010648	0.853	0.326	0	0	UBE2A
	0	0.645531226790769	0.355	0.096	0	0	TCP11L1
	0	0.645387086323586	0.324	0.085	0	0	WDR76
	0	0.644029716564044	0.549	0.163	0	0	SLC35A3
	0	0.643644786708753	0.778	0.286	0	0	RAB4A
	0	0.64358035734679	0.251	0.054	0	0	PPM1K
	0	0.643470082319449	0.291	0.073	0	0	MAGIX
	0	0.642704056250865	0.578	0.168	0	0	NKAP
	0	0.641921676559613	0.608	0.188	0	0	CMTM8
	0	0.641641819520817	0.421	0.111	0	0	MED6

0	0.641380486169831	0.331	0.09	0	0	CMPK2
0	0.641327355975558	0.81	0.338	0	0	DHRS7
0	0.640822048137888	0.25	0.066	0	0	SULT1B1
0	0.640699871226808	0.498	0.131	0	0	ITGA6
0	0.640301241461546	0.469	0.133	0	0	TM2D2
0	0.639079322730193	0.488	0.125	0	0	TAPT1
0	0.638235912332917	0.327	0.085	0	0	DDX55
0	0.637883613695867	0.296	0.075	0	0	CROT
0	0.637325473219685	0.51	0.15	0	0	AS3MT
0	0.637219291576204	0.499	0.116	0	0	ATG16L2
0	0.637078074876316	0.739	0.245	0	0	PI4K2B
0	0.636000645953338	0.356	0.085	0	0	ZNF445
0	0.635982413407436	0.396	0.108	0	0	THUMPD3
0	0.635577743032879	0.588	0.164	0	0	KLHDC4
0	0.635511443362832	0.473	0.126	0	0	CDKAL1
0	0.634821241431208	0.447	0.128	0	0	DEPRECATED-ENSG00000078668
0	0.63457795570603	0.342	0.083	0	0	NPRL3
0	0.634367731468736	0.35	0.076	0	0	TANC1
0	0.633864136167361	0.35	0.086	0	0	FGFR3
0	0.633681999426066	0.853	0.345	0	0	MIDN
0	0.6336682589951	0.701	0.227	0	0	SLC35F5
0	0.633204964463209	0.304	0.076	0	0	PUS7
0	0.633083365526468	0.889	0.374	0	0	GDI1
0	0.633043130303365	0.284	0.074	0	0	COG8
0	0.632783146997458	0.544	0.16	0	0	RBBP7
0	0.632057809798482	0.318	0.085	0	0	DESI2
0	0.631904856822384	0.699	0.235	0	0	OCIAD1
0	0.631873994366194	0.658	0.207	0	0	ADHFE1
0	0.631867254933353	0.485	0.139	0	0	FAAH
0	0.631572801147806	0.264	0.07	0	0	ACTR6
0	0.631547253100126	0.454	0.111	0	0	ATAD2B
0	0.631511134110236	0.844	0.343	0	0	AFDN
0	0.63148974026222	0.454	0.126	0	0	POFUT1
0	0.631068550210436	0.52	0.163	0	0	FUCA2
0	0.630988909334931	0.385	0.098	0	0	MMP15
0	0.630795387774519	0.255	0.062	0	0	AGTR1
0	0.630771455064066	0.286	0.074	0	0	RABL3
0	0.630638655902771	0.515	0.152	0	0	LRP11
0	0.63050687206829	0.609	0.173	0	0	PDE4DIP
0	0.630246149097864	0.268	0.057	0	0	SCFD2
0	0.629438544635357	0.265	0.073	0	0	PKN3
0	0.629009912385626	0.341	0.09	0	0	ENOX2
0	0.628551007533217	0.252	0.066	0	0	DSTYK
0	0.628057905334573	0.345	0.09	0	0	KHDRBS3
0	0.627975533632471	0.279	0.07	0	0	SRSF10
0	0.62763975900873	0.438	0.127	0	0	TCAIM
0	0.626843356167838	0.435	0.119	0	0	PBX3
0	0.625680573084408	0.493	0.131	0	0	UBA6
0	0.625618022411536	0.769	0.277	0	0	PYCR2
0	0.625333903519051	0.854	0.363	0	0	SPTSSA
0	0.625014527588323	0.447	0.125	0	0	FNIP1
0	0.624531974445031	0.353	0.096	0	0	PMS1
0	0.624080299720849	0.381	0.095	0	0	PKD2
0	0.624076123890985	0.457	0.119	0	0	RASSF7
0	0.62389979356095	0.435	0.114	0	0	ZNF687
0	0.623897713207793	0.732	0.239	0	0	ABCC2
0	0.623880121482177	0.256	0.064	0	0	POLG2
0	0.623742354883015	0.473	0.122	0	0	SCAP
0	0.623578084338104	0.339	0.089	0	0	PIGF
0	0.623364599909148	0.348	0.085	0	0	PIGH

0	0.623195397981161	0.258	0.066	0	0	C7orf25
0	0.62198614007762	0.506	0.141	0	0	MAPK9
0	0.621724305075037	0.501	0.14	0	0	PSMC6
0	0.621341035924249	0.442	0.124	0	0	TOR1A
0	0.620807231613031	0.604	0.181	0	0	SRPK1
0	0.620750876377278	0.555	0.158	0	0	WDR33
0	0.620705094445511	0.553	0.152	0	0	SORBS1
0	0.620588918760497	0.565	0.17	0	0	SLC10A3
0	0.620515418024949	0.64	0.223	0	0	RDH10
0	0.620393863137513	0.636	0.224	0	0	C15orf48
0	0.620339218442399	0.585	0.171	0	0	MTIF3
0	0.620067828471713	0.317	0.079	0	0	C5orf51
0	0.619918055543867	0.681	0.202	0	0	SENP6
0	0.619541464367998	0.369	0.092	0	0	PIK3R4
0	0.619535233464615	0.427	0.104	0	0	DEPRECATED-ENSG00000122642
0	0.619496404104283	0.514	0.145	0	0	PRKCA
0	0.619041667692331	0.746	0.246	0	0	LIMD1
0	0.618065049066929	0.621	0.177	0	0	PCED1A
0	0.617954949293614	0.739	0.266	0	0	ABCD3
0	0.617867224256512	0.253	0.06	0	0	CPT1B
0	0.617690970020516	0.888	0.398	0	0	SSR4
0	0.617355356381786	0.745	0.254	0	0	MDM2
0	0.617152907165411	0.641	0.203	0	0	C5orf24
0	0.616882797096749	0.359	0.098	0	0	AGAP1
0	0.616388274357906	0.489	0.13	0	0	ZNF444
0	0.616120892519409	0.823	0.333	0	0	AIFM1
0	0.615775290621698	0.351	0.095	0	0	IPO5
0	0.615719970477061	0.313	0.085	0	0	STAM2
0	0.61542276852769	0.281	0.074	0	0	ZBED6CL
0	0.614901144168786	0.271	0.068	0	0	ZBTB48
0	0.614452138157517	0.395	0.106	0	0	VPS37A
0	0.614319244128908	0.576	0.165	0	0	ERCC5
0	0.61396923027502	0.696	0.226	0	0	MTUS1
0	0.613757198291154	0.511	0.145	0	0	NBN
0	0.613592811755386	0.709	0.251	0	0	SPSB1
0	0.613108145380386	0.535	0.161	0	0	BRI3BP
0	0.612924204177304	0.373	0.103	0	0	EXOC8
0	0.612889080134755	0.372	0.097	0	0	COQ8B
0	0.612807188653996	0.379	0.103	0	0	LIG3
0	0.612248994696022	0.459	0.114	0	0	TRADD
0	0.611722271228804	0.303	0.078	0	0	UBE3B
0	0.611621312621393	0.455	0.142	0	0	SLC1A5
0	0.611572171332734	0.389	0.108	0	0	OGFOD2
0	0.611131808302678	0.552	0.162	0	0	GOLGB1
0	0.610991606120808	0.762	0.227	0	0	OGT
0	0.610966869380062	0.341	0.097	0	0	SDCBP2
0	0.610744060508299	0.474	0.134	0	0	WWP2
0	0.610651379090599	0.772	0.3	0	0	PRG4
0	0.610358895794041	0.593	0.184	0	0	IGSF8
0	0.610348663251565	0.272	0.064	0	0	DEPRECATED-ENSG00000122912
0	0.609517848889003	0.435	0.127	0	0	EZH2
0	0.6095104003152	0.724	0.249	0	0	POLR2H
0	0.609367915659296	0.264	0.064	0	0	MIOS
0	0.608542124707296	0.314	0.084	0	0	HOXA10
0	0.607977732731456	0.346	0.091	0	0	SYNJ2BP
0	0.607913632537386	0.288	0.071	0	0	CLASP2
0	0.607655766918153	0.283	0.07	0	0	ACOX3
0	0.607580193269086	0.83	0.3	0	0	SETD5
0	0.606674218104016	0.597	0.175	0	0	SUN1
0	0.606650875274141	0.436	0.119	0	0	PER2

**0**	0.606616191850781	0.565	0.167	0	0	DYM
**0**	0.60658527619458	0.359	0.095	0	0	MTPAP
**0**	0.606473517222634	0.316	0.079	0	0	POLM
**0**	0.606458794888146	0.381	0.105	0	0	PUS1
**0**	0.606340893406297	0.33	0.08	0	0	EXOC6B
**0**	0.605558289920724	0.399	0.108	0	0	NSMCE4A
**0**	0.605431744995344	0.567	0.163	0	0	WWOX
**0**	0.605068479104199	0.616	0.184	0	0	SLC23A2
**0**	0.604943865732874	0.294	0.072	0	0	RRNAD1
**0**	0.604584325436628	0.703	0.233	0	0	LIMK2
**0**	0.604467594356869	0.258	0.067	0	0	UFSP2
**0**	0.604061975798893	0.343	0.089	0	0	AK3
**0**	0.60387015597688	0.473	0.133	0	0	VCPIP1
**0**	0.603746334322175	0.256	0.07	0	0	ETNPPL
**0**	0.603555156171233	0.385	0.108	0	0	PXMP4
**0**	0.602950080874925	0.289	0.067	0	0	MIGA2
**0**	0.602724656050772	0.4	0.108	0	0	SEPTIN7
**0**	0.60255769359636	0.541	0.157	0	0	GSK3B
**0**	0.601970713275581	0.293	0.078	0	0	RAD51D
**0**	0.601936151203538	0.387	0.106	0	0	GPD2
**0**	0.601680742345838	0.677	0.23	0	0	POGK
**0**	0.601614233501596	0.317	0.08	0	0	COX18
**0**	0.601578089348562	0.636	0.217	0	0	MCM4
**0**	0.601443620791492	0.395	0.102	0	0	PDS5A
**0**	0.601343940639526	0.304	0.083	0	0	KIFAP3
**0**	0.601200200187882	0.592	0.189	0	0	TDP2
**0**	0.601038872442756	0.255	0.064	0	0	NEPRO
**0**	0.60102753552652	0.275	0.07	0	0	ERCC6
**0**	0.600949598932304	0.261	0.069	0	0	CPSF4
**0**	0.600924304454857	0.764	0.297	0	0	CITED2
**0**	0.600878770038028	0.466	0.135	0	0	ELOVL6
**0**	0.600741430766052	0.783	0.309	0	0	CMBL
**0**	0.600293071044022	0.519	0.155	0	0	MTA1
**0**	0.600162893893843	0.715	0.236	0	0	HNRNPL
**0**	0.599770414114344	0.515	0.145	0	0	PPIC
**0**	0.59971195488467	0.711	0.246	0	0	DCAF6
**0**	0.598849942971326	0.372	0.093	0	0	PIAS3
**0**	0.598558630430971	0.517	0.152	0	0	UNC50
**0**	0.598417234043197	0.421	0.121	0	0	AK6
**0**	0.59815210097428	0.7	0.219	0	0	ANKHD1
**0**	0.59771412082437	0.448	0.122	0	0	SNX19
**0**	0.597564677173807	0.324	0.083	0	0	NUDT16L1
**0**	0.597075506630049	0.31	0.083	0	0	CLTC
**0**	0.596844440685522	0.369	0.096	0	0	FAM98C
**0**	0.596695035215525	0.538	0.132	0	0	DOCK9
**0**	0.59652439035197	0.561	0.175	0	0	ZNF664
**0**	0.596494054528913	0.387	0.106	0	0	SHLD2
**0**	0.595786469667723	0.601	0.187	0	0	TOR1AIP1
**0**	0.595566197551524	0.387	0.093	0	0	GYS1
**0**	0.595435398990599	0.742	0.244	0	0	DHX15
**0**	0.595401578410431	0.49	0.144	0	0	GORASP1
**0**	0.595190730171189	0.407	0.111	0	0	MDC 1.00
**0**	0.594956962750093	0.575	0.167	0	0	AP3B1
**0**	0.59479210743425	0.321	0.081	0	0	EXOC1
**0**	0.594565934010551	0.511	0.144	0	0	COMMD3
**0**	0.59432328926743	0.349	0.093	0	0	JAGN1
**0**	0.593916932083686	0.794	0.298	0	0	ECHDC2
**0**	0.593401655928894	0.531	0.158	0	0	CAND1
**0**	0.593284361666463	0.517	0.145	0	0	MTR
**0**	0.593236220340533	0.819	0.344	0	0	TP53INP1

0	0.592753062203559	0.653	0.208	0	0	COG3
0	0.592225459043366	0.883	0.39	0	0	RBM47
0	0.592119828289547	0.308	0.082	0	0	ACOT8
0	0.591856452238565	0.762	0.286	0	0	CPQ
0	0.591554568676832	0.274	0.074	0	0	REPS2
0	0.59138508111409	0.789	0.284	0	0	DPYD
0	0.591172639702661	0.429	0.122	0	0	PPP1CC
0	0.590783460079527	0.407	0.111	0	0	SETDB1
0	0.5894833008841	0.317	0.074	0	0	OSBPL10
0	0.589114285552043	0.835	0.351	0	0	TPM1
0	0.588880958388395	0.518	0.142	0	0	RELCH
0	0.588253584951193	0.523	0.155	0	0	ACTR10
0	0.587576319216512	0.75	0.241	0	0	MARCH6
0	0.586842324565765	0.295	0.076	0	0	FASTKD2
0	0.586824382391327	0.72	0.243	0	0	KIAA1522
0	0.586611322038588	0.387	0.106	0	0	FHL1
0	0.586398687739236	0.26	0.068	0	0	LTV1
0	0.586241678262386	0.301	0.083	0	0	PTPN18
0	0.586186657378216	0.502	0.14	0	0	ZNF33A
0	0.586164051738199	0.36	0.093	0	0	KLC4
0	0.586036877321844	0.468	0.135	0	0	ZYG11B
0	0.586005889804625	0.43	0.123	0	0	LYSMD3
0	0.585413966360491	0.549	0.154	0	0	ZNF326
0	0.585330626875404	0.893	0.427	0	0	ALDH3A2
0	0.585327405233978	0.261	0.07	0	0	PIGU
0	0.585320431066028	0.352	0.086	0	0	CLIC5
0	0.585297025061411	0.85	0.384	0	0	CDHR5
0	0.585289412405238	0.543	0.17	0	0	LIME1
0	0.585232279889854	0.963	0.546	0	0	AKR1C3
0	0.584790186564623	0.493	0.144	0	0	RO60
0	0.584158096232196	0.555	0.166	0	0	AIMP1
0	0.584115500097989	0.535	0.162	0	0	AKR7A2
0	0.583338017688951	0.71	0.245	0	0	EMC2
0	0.582826569586446	0.558	0.17	0	0	NTPCR
0	0.582795671378977	0.302	0.072	0	0	IRGQ
0	0.582630898729577	0.457	0.127	0	0	SFPQ
0	0.582402551609717	0.28	0.068	0	0	RNPC3
0	0.581952346788377	0.359	0.086	0	0	CYB561
0	0.581936103365446	0.291	0.074	0	0	SATB2
0	0.581828210780036	0.441	0.127	0	0	PLEKHG3
0	0.581817450092446	0.946	0.543	0	0	KHK
0	0.581164574726482	0.548	0.153	0	0	IP6K2
0	0.581134666395158	0.396	0.112	0	0	TMX1
0	0.580460526671619	0.363	0.095	0	0	TEF
0	0.579538306801142	0.63	0.203	0	0	SLC35F6
0	0.578889137280711	0.564	0.167	0	0	TMEM214
0	0.578647609650563	0.596	0.175	0	0	NUP210
0	0.578477357401574	0.533	0.166	0	0	GPC6
0	0.578391970583253	0.483	0.138	0	0	CENPV
0	0.57797812320781	0.428	0.121	0	0	MPHOSPH9
0	0.577881173862159	0.323	0.084	0	0	NAA40
0	0.577823611406951	0.516	0.148	0	0	HUS1
0	0.577570835020802	0.518	0.146	0	0	BRF1
0	0.577412798691954	0.321	0.086	0	0	FBXW11
0	0.576507275545229	0.306	0.074	0	0	ADCY9
0	0.576408164397682	0.551	0.162	0	0	PEX13
0	0.576063826820431	0.871	0.387	0	0	SMIM14
0	0.575897944613517	0.333	0.088	0	0	MPP6
0	0.5758676847229	0.692	0.226	0	0	SRP54
0	0.575087489439204	0.316	0.085	0	0	LCORL

0	0.574680517570091	0.286	0.075	0	0	CUEDC1
0	0.57467447239831	0.251	0.064	0	0	SMU1
2.86873489630836E-280	0.574481942035486	0.256	0.08	4.80742593923355E-276	0	DTL
0	0.57392655985841	0.475	0.128	0	0	TULP4
0	0.573773480202925	0.468	0.132	0	0	EEPD1
0	0.573477569553781	0.356	0.086	0	0	ANKRD36C
0	0.573403626281236	0.32	0.086	0	0	B4GALT7
0	0.57327163355006	0.432	0.127	0	0	NXT2
0	0.57322672292227	0.279	0.076	0	0	PRKRA
0	0.573215727253759	0.728	0.244	0	0	NR2F6
0	0.572720072221055	0.273	0.068	0	0	SSH3
0	0.572689350678127	0.941	0.529	0	0	SERPINA6
0	0.572573976832158	0.902	0.439	0	0	CRYL1
0	0.572473640615239	0.681	0.233	0	0	SEL1L
0	0.572470855660862	0.773	0.295	0	0	A1CF
0	0.572227656350634	0.33	0.086	0	0	CHD6
0	0.572192436719043	0.461	0.13	0	0	NUP42
0	0.572144603078851	0.263	0.062	0	0	RAB2B
0	0.571673481739026	0.873	0.375	0	0	FAM50A
0	0.571655556065607	0.665	0.215	0	0	ZBTB20
0	0.571265922835555	0.57	0.171	0	0	DOT1L
0	0.569088816113894	0.365	0.102	0	0	SNRPE
0	0.568977334325573	0.527	0.151	0	0	DICER1
0	0.568561934994293	0.27	0.074	0	0	POT1
0	0.568288555258423	0.659	0.222	0	0	UBL3
0	0.568201305580692	0.322	0.082	0	0	EED
0	0.568173055200834	0.363	0.102	0	0	ADRB2
0	0.567359546891711	0.465	0.138	0	0	RNF6
0	0.567072587528885	0.281	0.07	0	0	COG5
0	0.567054558590487	0.608	0.176	0	0	LRCH3
0	0.56678972777775	0.284	0.079	0	0	ZNF562
0	0.566136892554082	0.932	0.51	0	0	HSD17B4
0	0.566128311524659	0.526	0.15	0	0	COP 1
0	0.565906195753943	0.378	0.105	0	0	PHLPP1
0	0.565878850921256	0.503	0.141	0	0	POLR2B
0	0.565818070861879	0.359	0.095	0	0	PLA2G12B
0	0.565376913978728	0.449	0.132	0	0	FNIP2
0	0.565102360977981	0.286	0.076	0	0	THAP12
0	0.564951024655977	0.923	0.525	0	0	AKR1B10
0	0.56464919760252	0.411	0.106	0	0	SMPDL3A
0	0.563884440654602	0.35	0.085	0	0	SYNJ2
0	0.563812497901237	0.36	0.097	0	0	USP16
0	0.56377207679504	0.502	0.15	0	0	ZXDC
0	0.563645131743545	0.385	0.097	0	0	TBC1D22B
0	0.563573396946293	0.527	0.155	0	0	LMF2
0	0.563501486675239	0.386	0.105	0	0	RSPRY1
0	0.563020811801003	0.588	0.186	0	0	TEX2
0	0.562517864915693	0.551	0.16	0	0	ATP11A
0	0.561836004958856	0.255	0.074	0	0	PRIM2
0	0.56140337159726	0.442	0.13	0	0	ATP6V1H
0	0.561338866596392	0.775	0.274	0	0	FMR1
0	0.561269122539134	0.606	0.172	0	0	KDM4C
0	0.5612608081941	0.263	0.068	0	0	TJAP1
0	0.560691293254561	0.427	0.122	0	0	GOLPH3L
0	0.560677322730946	0.325	0.086	0	0	JMJD4
0	0.560632318169323	0.643	0.21	0	0	TOM1L1
0	0.560603700858684	0.462	0.135	0	0	ELF4
0	0.560307031993833	0.451	0.119	0	0	TELO2
0	0.560128871765393	0.519	0.159	0	0	EBPL
0	0.559176143096781	0.823	0.332	0	0	PEG10

0	0.559165443848159	0.25	0.07	0	0	CREB3L4	
0	0.559151015243617	0.26	0.067	0	0	TRIM32	
0	0.558495708252914	0.687	0.226	0	0	UBE2D3	
0	0.558354104645725	0.354	0.097	0	0	TTLL12	
0	0.558259634533917	0.285	0.069	0	0	HELZ2	
0	0.558245539119172	0.424	0.109	0	0	PKN2	
0	0.558232534691238	0.412	0.117	0	0	RNF14	
0	0.557899188133318	0.396	0.109	0	0	NDRG3	
0	0.557724191610966	0.762	0.276	0	0	VBP1	
0	0.557526409680831	0.368	0.101	0	0	GTPBP10	
0	0.557329491915026	0.26	0.071	0	0	TRAPPC13	
0	0.557300792936569	0.507	0.159	0	0	HLTF	
0	0.557001957586182	0.579	0.163	0	0	TUBGCP6	
0	0.556882680149426	0.332	0.092	0	0	ZNF282	
0	0.556581402734312	0.683	0.197	0	0	ERN 1.00	
0	0.556579126665174	0.531	0.169	0	0	CHAMP1	
0	0.556420354207802	0.632	0.2	0	0	VPS52	
0	0.556140211258713	0.415	0.112	0	0	TMEM181	
0	0.556070784877913	0.345	0.091	0	0	ACYP2	
0	0.555839097841174	0.734	0.252	0	0	STT3B	
0	0.555781168524319	0.44	0.126	0	0	RFNG	
0	0.555305966030096	0.289	0.077	0	0	C4orf33	
0	0.554837808046768	0.291	0.076	0	0	RPAP1	
0	0.554591786108439	0.355	0.099	0	0	GLRX2	
0	0.5544929318372	0.362	0.097	0	0	ADNP2	
0	0.554061248591425	0.289	0.075	0	0	SPRYD7	
0	0.553864698532989	0.462	0.13	0	0	ESRP2	
0	0.55378587854673	0.411	0.111	0	0	SMG1	
0	0.553765978349342	0.352	0.102	0	0	ARMC1	
0	0.553406659379204	0.704	0.225	0	0	SPG7	
0	0.552902413006992	0.379	0.11	0	0	KLHL12	
0	0.552779438559448	0.402	0.116	0	0	OSTC	
0	0.55253012600622	0.528	0.154	0	0	SAMD4B	
0	0.552318467508438	0.811	0.376	0	0	AOX1	
0	0.552157206287773	0.375	0.103	0	0	ALG2	
0	0.552109744685113	0.821	0.362	0	0	HABP2	
0	0.552085084203007	0.358	0.097	0	0	TMEM128	
0	0.551502919732973	0.325	0.088	0	0	CLOCK	
0	0.551375580728506	0.392	0.108	0	0	NUDT19	
0	0.551062473206353	0.33	0.091	0	0	ZNF512B	
0	0.550740638153837	0.699	0.241	0	0	PPM1B	
0	0.550005188263639	0.28	0.071	0	0	RIOK2	
0	0.549994833751309	0.371	0.101	0	0	TRAPPC6B	
0	0.549719564570043	0.764	0.305	0	0	STT3A	
0	0.549517734272217	0.385	0.103	0	0	RBFOX2	
0	0.548959242462921	0.636	0.207	0	0	ENAH	
0	0.548283568303531	0.366	0.096	0	0	MIGA1	
0	0.548131031402098	0.583	0.176	0	0	SEC24A	
0	0.547840965303969	0.375	0.1	0	0	YEATS2	
0	0.546470849860794	0.575	0.178	0	0	USO1	
0	0.546255103691145	0.316	0.081	0	0	ANGEL1	
0	0.545854533407314	0.701	0.256	0	0	CRYZ	
0	0.54518766159144	0.462	0.136	0	0	NCAPG2	
0	0.544940246521163	0.279	0.068	0	0	RUSC2	
0	0.544832836285279	0.289	0.084	0	0	SPIN4	
0	0.544641749968812	0.538	0.161	0	0	GNA11	
0	0.54449462110504	0.67	0.218	0	0	SRSF3	
0	0.544464407983494	0.416	0.122	0	0	NFYB	
0	0.544392870575676	0.522	0.147	0	0	ATXN2L	
0	0.54389855870914	0.574	0.167	0	0	DCAF5	

0	0.543722766059633	0.814	0.321	0	0	EGLN1
0	0.543679003785229	0.549	0.158	0	0	USP48
0	0.543535223663625	0.377	0.101	0	0	TMEM170A
0	0.542524150682459	0.334	0.096	0	0	SLC25A40
0	0.542300852106486	0.534	0.156	0	0	GOPC
0	0.542117015384468	0.519	0.153	0	0	MAP3K2
0	0.542102217250678	0.526	0.157	0	0	TMEM161A
0	0.541396941158492	0.335	0.085	0	0	BCAS3
0	0.541189686497121	0.592	0.175	0	0	DENND4C
0	0.541180958334148	0.344	0.094	0	0	FRS2
0	0.54116626760449	0.696	0.234	0	0	NUMB
0	0.541165956000852	0.565	0.181	0	0	PPP1R10
0	0.540693815716846	0.354	0.089	0	0	SLC2A1
0	0.540486166059931	0.361	0.094	0	0	ACVR1
0	0.53988112048246	0.61	0.203	0	0	TMEM9
0	0.539502359719065	0.813	0.319	0	0	SEC31A
0	0.5394444628365	0.42	0.128	0	0	ANKRD40
0	0.539422537960564	0.611	0.193	0	0	ITPRID2
0	0.539067445283129	0.269	0.071	0	0	LDAH
0	0.539032276022476	0.538	0.167	0	0	NUP85
0	0.538485368872424	0.647	0.204	0	0	SLC17A9
0	0.538116108014306	0.323	0.09	0	0	RNF31
0	0.537820362518775	0.398	0.115	0	0	CCNQ
0	0.537573982130435	0.624	0.184	0	0	TNRC6A
0	0.537083141093282	0.439	0.134	0	0	TTLL5
0	0.536766443438251	0.393	0.114	0	0	CCT4
0	0.53648014592683	0.535	0.157	0	0	DEPRECATED-ENSG00000085733
0	0.535721976214582	0.41	0.119	0	0	TBC1D16
0	0.535693801601154	0.457	0.124	0	0	RAB3GAP1
0	0.535605841940556	0.356	0.104	0	0	COX20
0	0.535200616700401	0.367	0.105	0	0	UEVLD
0	0.53471273108283	0.474	0.152	0	0	CPT2
0	0.534380739311922	0.592	0.192	0	0	PDCD6
0	0.534368803732629	0.76	0.302	0	0	SLC22A18
0	0.534119619124823	0.453	0.138	0	0	PNPLA3
0	0.534055889064952	0.428	0.124	0	0	SELENOI
0	0.533920707050111	0.327	0.096	0	0	WDR41
0	0.533756579893748	0.513	0.143	0	0	PIP4P1
0	0.533665606576925	0.294	0.078	0	0	TMEM209
0	0.533612381387211	0.261	0.069	0	0	FUNDC1
0	0.533457377388321	0.264	0.069	0	0	MRTFB
0	0.533166040497227	0.533	0.165	0	0	PLEKHJ1
0	0.532979720073584	0.469	0.136	0	0	CCS
0	0.532906658309105	0.543	0.167	0	0	YIPF4
0	0.532763030933941	0.732	0.276	0	0	CYSTM1
0	0.532281126762899	0.501	0.141	0	0	ZC3H3
0	0.531207298293282	0.719	0.253	0	0	F11
0	0.531151468360293	0.632	0.206	0	0	CD2AP
0	0.531104713164808	0.32	0.081	0	0	MPI
0	0.530832838240017	0.484	0.136	0	0	WASHC1
0	0.530799930927469	0.442	0.127	0	0	EPS15L1
0	0.53079336068648	0.335	0.093	0	0	ITPKA
0	0.530477445920223	0.307	0.092	0	0	SLC2A8
0	0.530307810004427	0.368	0.105	0	0	CPOX
0	0.529902392346849	0.285	0.082	0	0	ASF1A
0	0.529743588474645	0.506	0.156	0	0	ATP6V1C1
0	0.529679620214668	0.723	0.263	0	0	RAMP1
0	0.529588417926723	0.29	0.08	0	0	SDCCAG8
0	0.529557318201801	0.616	0.2	0	0	FAM20C
0	0.529506570278377	0.303	0.081	0	0	BACE1

0	0.529392713068887	0.482	0.138	0	0	TM9SF4
0	0.52936134013503	0.539	0.165	0	0	KDM4B
0	0.529217235786155	0.54	0.166	0	0	ASPSCR1
0	0.529103597389771	0.63	0.213	0	0	TMEM41B
0	0.528887057236746	0.457	0.13	0	0	DGCR2
0	0.528782641146625	0.337	0.097	0	0	COMMD2
0	0.528519392435626	0.703	0.241	0	0	SETD7
0	0.52843456768303	0.259	0.065	0	0	XRRA1
0	0.528318529353998	0.338	0.092	0	0	PIGO
0	0.528309555903688	0.627	0.203	0	0	SEPTIN8
0	0.52783638521613	0.426	0.124	0	0	GHR
0	0.527581961120132	0.705	0.26	0	0	AK4
0	0.527358687645051	0.289	0.08	0	0	ARHGAP21
0	0.527066426196364	0.819	0.338	0	0	PLS3
0	0.527007217006361	0.441	0.126	0	0	ARHGEF11
0	0.526420415649088	0.624	0.204	0	0	FAM20B
0	0.526336842117518	0.581	0.19	0	0	SKP1
0	0.525850614473948	0.407	0.11	0	0	METTL14
0	0.525446504022594	0.455	0.132	0	0	SEC24D
0	0.525172454226304	0.52	0.163	0	0	TIMMDC1
0	0.524454122085565	0.949	0.546	0	0	IFI27
0	0.524235260493838	0.766	0.287	0	0	IKBKG
0	0.524169634381481	0.62	0.19	0	0	NIBAN2
0	0.524115997237208	0.357	0.093	0	0	FARP2
0	0.523860027216447	0.56	0.152	0	0	NEMF
0	0.52362591997412	0.869	0.422	0	0	TRAM1
0	0.523382086149984	0.61	0.191	0	0	RB1CC1
0	0.523305956359277	0.809	0.335	0	0	PGRMC2
0	0.52326341721609	0.303	0.089	0	0	COQ2
0	0.523225380561489	0.887	0.377	0	0	MAT2A
0	0.522905338115741	0.821	0.355	0	0	TMBIM1
0	0.522510789851246	0.318	0.093	0	0	COQ6
0	0.522417120939561	0.353	0.101	0	0	IFI27L1
0	0.522304969013175	0.37	0.106	0	0	ANAPC7
0	0.522221434424451	0.391	0.114	0	0	HOMER2
0	0.521955599884583	0.829	0.296	0	0	LUC7L3
0	0.521913827791267	0.334	0.089	0	0	COG7
0	0.521887933678492	0.776	0.33	0	0	SEC61A1
0	0.521426357698258	0.441	0.121	0	0	CFAP97
0	0.521083714553049	0.835	0.301	0	0	NR1H4
0	0.521080889258367	0.381	0.106	0	0	TIMM9
0	0.520862770524921	0.334	0.095	0	0	KRCC1
0	0.520836448185771	0.602	0.192	0	0	NCLN
0	0.520655500455323	0.252	0.067	0	0	PPP1R14B
0	0.520644934468518	0.314	0.082	0	0	TBC1D24
0	0.52058822836081	0.504	0.155	0	0	ABHD10
0	0.520505727116174	0.432	0.13	0	0	MLIP
0	0.520476014245587	0.527	0.169	0	0	CADM1
0	0.520294748291512	0.409	0.12	0	0	MYO1D
0	0.518919337457902	0.511	0.162	0	0	ELOVL2
0	0.518656185821323	0.443	0.119	0	0	ANKRD27
0	0.518501361867371	0.383	0.11	0	0	RUSC1
0	0.518484364155195	0.593	0.201	0	0	ONECUT2
0	0.518432671544017	0.63	0.202	0	0	AGRN
0	0.518416909162808	0.572	0.182	0	0	CRAT
0	0.518413823859197	0.394	0.115	0	0	CNIH4
0	0.518361230536108	0.67	0.215	0	0	PCMTD1
0	0.518348752413214	0.392	0.109	0	0	FAM98B
0	0.51790034318019	0.291	0.081	0	0	PRUNE1
0	0.517724628189477	0.451	0.118	0	0	ZFYVE16

0	0.517547768581747	0.526	0.15	0	0	RSAD1
0	0.517339729510322	0.369	0.107	0	0	GFOD2
0	0.51723401081619	0.588	0.201	0	0	DNAJB9
0	0.516466512921172	0.405	0.115	0	0	CPSF2
0	0.516389307475403	0.436	0.124	0	0	MICU2
0	0.516387805618054	0.747	0.313	0	0	ETHE1
0	0.515535307618547	0.362	0.099	0	0	GOLGA1
0	0.514992512646498	0.45	0.126	0	0	PPARGC1A
0	0.51482630239423	0.293	0.079	0	0	HIRA
0	0.514748350952655	0.841	0.341	0	0	TBC1D9B
0	0.51444575603019	0.472	0.136	0	0	SLC39A11
0	0.514411769493883	0.657	0.229	0	0	STAP2
0	0.513798751731948	0.55	0.166	0	0	UBA2
0	0.513485527652343	0.407	0.111	0	0	CMTR1
0	0.513141650566384	0.402	0.109	0	0	STARD4
0	0.51268053804826	0.791	0.316	0	0	CD46
0	0.512676018511067	0.377	0.097	0	0	PAXBP1
0	0.51239778263998	0.263	0.066	0	0	FAM219B
0	0.512232384764578	0.485	0.146	0	0	TOMM6
0	0.511917351563189	0.489	0.142	0	0	RARS
0	0.511707806638592	0.858	0.448	0	0	SCD
0	0.511707015029584	0.479	0.132	0	0	ZNF219
0	0.511644184310682	0.642	0.221	0	0	MAPK6
0	0.511569744235021	0.275	0.075	0	0	DEPRECATED-ENSG00000116005
0	0.511493761364306	0.484	0.125	0	0	EPC1
0	0.511399488844248	0.732	0.287	0	0	ACSL5
0	0.511267591824699	0.261	0.063	0	0	TOP3B
0	0.511151348439936	0.535	0.171	0	0	HAUS4
0	0.51100121551953	0.432	0.112	0	0	SULF2
0	0.510892399316261	0.477	0.142	0	0	RIPK1
0	0.510393353932742	0.292	0.081	0	0	TFDP2
0	0.510374221650234	0.541	0.162	0	0	RPRD2
0	0.509824533288562	0.637	0.219	0	0	PSMD1
0	0.509770143258854	0.432	0.12	0	0	NAA15
0	0.509722461423976	0.336	0.093	0	0	COA8
0	0.509483405249865	0.721	0.252	0	0	SAFB2
0	0.509068000505746	0.449	0.129	0	0	DEPRECATED-ENSG00000188313
0	0.50850910390166	0.488	0.146	0	0	GATD1
0	0.508097799359449	0.342	0.095	0	0	XPO5
0	0.507777145151228	0.3	0.08	0	0	CDK8
0	0.507756452869908	0.307	0.082	0	0	TAF5L
0	0.506645589962426	0.359	0.104	0	0	DCAF10
0	0.506591126130093	0.511	0.17	0	0	PYGL
0	0.506499171929231	0.402	0.114	0	0	YTHDF2
0	0.506276694937815	0.319	0.089	0	0	SSR1
0	0.506021639371092	0.513	0.145	0	0	REV1
0	0.505974488685586	0.304	0.082	0	0	PSMG1
0	0.505765808838912	0.571	0.155	0	0	HERC3
0	0.505730947472447	0.843	0.331	0	0	SLC39A14
0	0.505646089678137	0.533	0.175	0	0	HIGD1A
0	0.505620573700301	0.607	0.224	0	0	SLC6A1
0	0.505359346782452	0.727	0.253	0	0	COPS5
0	0.504721460496748	0.698	0.25	0	0	SFT2D2
0	0.504717096315161	0.504	0.13	0	0	RASGEF1B
0	0.504389067486957	0.611	0.197	0	0	DKC1
0	0.504037630011868	0.888	0.434	0	0	NDUFA1
0	0.503790146203488	0.268	0.07	0	0	NBPF12
0	0.503575082693264	0.424	0.118	0	0	NUP214
0	0.503514991976568	0.442	0.123	0	0	EXOSC9
0	0.503454935214254	0.413	0.113	0	0	CXorf40A

0	0.503301455890089	0.322	0.094	0	0	SALL1
0	0.50271121179387	0.356	0.102	0	0	ZDHHC6
0	0.502592618739715	0.46	0.142	0	0	PSPH
0	0.502552814063849	0.361	0.099	0	0	CHD8
0	0.502509824714608	0.591	0.2	0	0	VDAC1
0	0.501939210130535	0.385	0.086	0	0	PIK3C2B
0	0.501876945139051	0.498	0.144	0	0	POMGNT1
0	0.50154150810574	0.596	0.199	0	0	HPRT1
0	0.501479609887547	0.542	0.158	0	0	TENT2
0	0.501356555157169	0.662	0.204	0	0	CREBZF
0	0.500819988254722	0.514	0.148	0	0	EIF2D
0	0.500435027874536	0.872	0.437	0	0	TMCO1
0	0.500430346495595	0.835	0.386	0	0	LSR
0	0.50033319159549	0.684	0.231	0	0	TRIM27
0	0.499915186262119	0.5	0.15	0	0	MED14
0	0.499655400226693	0.289	0.073	0	0	MICA
0	0.498993261128424	0.796	0.348	0	0	MGST2
0	0.498916939894355	0.818	0.319	0	0	HNRNPU
0	0.498752445490455	0.52	0.158	0	0	SCFD1
0	0.498449568646785	0.436	0.119	0	0	FBH1
0	0.497662019231235	0.615	0.198	0	0	AGFG1
0	0.497594994675117	0.968	0.592	0	0	S100A10
0	0.497346318691647	0.406	0.118	0	0	MOSPD2
0	0.49713500608153	0.574	0.176	0	0	CUL4A
0	0.497062632837909	0.525	0.157	0	0	YAP1
0	0.496980672081445	0.318	0.088	0	0	WDR36
0	0.496905318548224	0.483	0.15	0	0	DCUN1D1
0	0.496702720848882	0.4	0.117	0	0	BAG2
0	0.496547545535231	0.647	0.223	0	0	CRLS1
0	0.496516428406951	0.475	0.131	0	0	UQCRC2
0	0.495930967779482	0.393	0.115	0	0	SMIM20
0	0.495045702543169	0.705	0.255	0	0	ACBD3
0	0.494769585586274	0.475	0.127	0	0	DYRK2
0	0.494520796682166	0.276	0.069	0	0	MAP4K3
0	0.494355226515788	0.883	0.433	0	0	RARRES2
0	0.494146147902318	0.438	0.127	0	0	EEF1AKNMT
0	0.493909923657444	0.374	0.101	0	0	UPF2
0	0.493829969733513	0.808	0.312	0	0	ITPR2
0	0.493040665319168	0.391	0.109	0	0	SMO
0	0.492634292523829	0.346	0.098	0	0	DYNC1LI1
0	0.492425962613884	0.52	0.16	0	0	EXOSC4
0	0.492277586431178	0.373	0.107	0	0	UBE2W
0	0.49188372928716	0.384	0.109	0	0	SOS 1
0	0.491503194691181	0.672	0.256	0	0	RETSAT
0	0.49135033795928	0.944	0.598	0	0	HMGCS2
0	0.491219486770028	0.462	0.15	0	0	FADS1
0	0.490779491743509	0.293	0.075	0	0	CARD10
0	0.490208025174305	0.516	0.157	0	0	LARS
0	0.489755787916634	0.349	0.098	0	0	ERGIC2
0	0.489582644627634	0.665	0.205	0	0	ZNF638
0	0.489539686207796	0.519	0.15	0	0	MAN2A2
0	0.489440332218602	0.324	0.091	0	0	CAD
0	0.489339733596037	0.279	0.076	0	0	NFKBIL1
0	0.488940807931396	0.559	0.162	0	0	MYLK
0	0.488031509840475	0.649	0.23	0	0	NDUFS1
0	0.487775771363118	0.678	0.243	0	0	NUCB2
0	0.487733990810514	0.447	0.122	0	0	OTUD4
0	0.487646965863262	0.299	0.084	0	0	PSMA1
0	0.487352680112174	0.345	0.092	0	0	PRPF18
0	0.487198307674882	0.458	0.14	0	0	BTF3L4

0	0.487152477774672	0.274	0.071	0	0	ZNF12
0	0.486999020911818	0.342	0.087	0	0	TMEM94
0	0.486901822965717	0.272	0.079	0	0	HFE
0	0.486546384568752	0.291	0.078	0	0	TBC1D7
0	0.486515292140796	0.406	0.12	0	0	NRIP1
0	0.48642711494062	0.267	0.071	0	0	HAUS3
0	0.486257422348083	0.33	0.09	0	0	AP5M1
0	0.48623490163927	0.622	0.187	0	0	IDS
0	0.486095068455125	0.68	0.242	0	0	DTX3L
0	0.486043446651081	0.268	0.076	0	0	GTF2H3
0	0.486015610696134	0.73	0.252	0	0	ADD1
0	0.485933177719297	0.836	0.34	0	0	HBS1L
0	0.485783320811008	0.317	0.094	0	0	DDX59
0	0.485480897600508	0.268	0.072	0	0	ACP6
0	0.485359538098713	0.374	0.108	0	0	HSPA13
0	0.485221157336066	0.455	0.136	0	0	ACBD4
0	0.485004649944127	0.851	0.371	0	0	HNRNPH2
0	0.48484211781034	0.702	0.249	0	0	PTPRF
0	0.484549664023013	0.608	0.195	0	0	ERBB3
0	0.484517350033902	0.4	0.107	0	0	CLK4
0	0.48443250632833	0.57	0.185	0	0	CHP2
0	0.484393492698993	0.331	0.11	0	0	UHRF1
0	0.484216754704189	0.407	0.117	0	0	ITPRIPL2
0	0.484154933884413	0.286	0.074	0	0	ZMYND19
0	0.483791829082786	0.319	0.059	0	0	ZNF117
0	0.483645226189026	0.337	0.096	0	0	CDC42EP4
0	0.483286777705002	0.516	0.179	0	0	MCM2
0	0.483266554907423	0.402	0.123	0	0	ICK
0	0.482721988062991	0.392	0.11	0	0	ACAP3
0	0.482685273969617	0.371	0.111	0	0	LAMTOR3
0	0.482408173286492	0.415	0.116	0	0	STX17
0	0.482274316417999	0.519	0.166	0	0	MAL2
0	0.482239406697045	0.372	0.102	0	0	FAM126A
0	0.482223822576959	0.695	0.25	0	0	IMPAD1
0	0.482107270604098	0.281	0.074	0	0	PRMT7
0	0.481892167832864	0.589	0.183	0	0	KMT5B
0	0.480867707374094	0.642	0.221	0	0	TRAPPC4
0	0.480759625140524	0.425	0.133	0	0	TEX261
0	0.480546042800601	0.509	0.141	0	0	KIAA1217
0	0.480533068201013	0.782	0.282	0	0	IL1R1
0	0.480528068900806	0.503	0.157	0	0	LANCL1
0	0.480130993529733	0.461	0.141	0	0	SNX27
0	0.480007917556208	0.324	0.088	0	0	GTF2IRD2B
0	0.48000671851085	0.5	0.15	0	0	HDDC2
0	0.479950830690278	0.754	0.278	0	0	HNRNPD
0	0.479673807396211	0.385	0.116	0	0	CBX3
0	0.479174527414521	0.346	0.095	0	0	SESTD1
0	0.478826846842379	0.448	0.12	0	0	KIAA2026
0	0.478101175505944	0.781	0.305	0	0	GSR
0	0.477913434396635	0.335	0.094	0	0	MANBA
0	0.477691568293443	0.467	0.14	0	0	CC2D1A
0	0.477446529976328	0.257	0.062	0	0	SLC16A7
0	0.477391667825827	0.368	0.105	0	0	GALNT11
0	0.477160949065553	0.271	0.071	0	0	CNEP1R1
0	0.477156042707577	0.515	0.154	0	0	LARP1
0	0.476854433283792	0.831	0.351	0	0	NDUFV2
0	0.47651852565252	0.651	0.219	0	0	HSD3B7
0	0.476507598772533	0.783	0.315	0	0	RER1
0	0.476260899938595	0.308	0.091	0	0	CEP85
0	0.475789332471456	0.855	0.386	0	0	SDC1

0	0.475633555209352	0.637	0.217	0	0	MPV17
0	0.475612309018757	0.405	0.113	0	0	DHRSX
0	0.475509690022672	0.436	0.132	0	0	SYPL1
0	0.475420055932951	0.535	0.162	0	0	ZNF652
0	0.475358336743818	0.288	0.077	0	0	ZNF280D
0	0.475353781592873	0.254	0.069	0	0	SPRED2
0	0.47505720476477	0.9	0.464	0	0	MPC2
0	0.474539779251556	0.276	0.073	0	0	NICN1
0	0.474475665145033	0.796	0.317	0	0	STOM
0	0.474306124202217	0.37	0.112	0	0	SOX9
0	0.474305731338929	0.364	0.104	0	0	PATZ1
0	0.47426328637487	0.598	0.192	0	0	PSPC1
0	0.474162896489106	0.388	0.112	0	0	RPRD1B
0	0.474083233594162	0.424	0.124	0	0	GPD1L
0	0.473825510303456	0.478	0.146	0	0	DDX1
0	0.473579470025552	0.917	0.431	0	0	VMP1
0	0.473358360629344	0.636	0.19	0	0	TRPC4AP
0	0.473086421978872	0.31	0.086	0	0	ZMYM4
0	0.473066772978363	0.294	0.083	0	0	SOWAHB
0	0.472684588853268	0.266	0.071	0	0	MTX3
0	0.472622307872773	0.621	0.204	0	0	RABGGTB
0	0.472588238991295	0.43	0.125	0	0	C5orf15
0	0.472571830043359	0.578	0.157	0	0	CNST
0	0.471308135524359	0.328	0.088	0	0	TPCN1
0	0.47130411864092	0.466	0.141	0	0	ANKH
0	0.471298878023148	0.327	0.089	0	0	ERCC2
0	0.471121582197519	0.287	0.072	0	0	RNF121
0	0.470322347600473	0.394	0.117	0	0	ST7
0	0.470298131527356	0.255	0.07	0	0	VARS2
0	0.469589065455453	0.48	0.137	0	0	SELENOO
0	0.469293987314038	0.801	0.364	0	0	RRBP1
0	0.469206469006015	0.761	0.265	0	0	LBR
0	0.469201410608305	0.408	0.117	0	0	UBQLN4
0	0.469110458127653	0.552	0.165	0	0	DOCK5
0	0.468912179648791	0.709	0.271	0	0	MGAT2
0	0.468872910824062	0.401	0.114	0	0	UNG
0	0.468169660544465	0.449	0.136	0	0	ATL3
0	0.468150130794171	0.462	0.137	0	0	P2RX4
0	0.467996529776696	0.392	0.11	0	0	XYLB
0	0.467923373979906	0.286	0.074	0	0	ARHGEF16
0	0.467627502423062	0.395	0.115	0	0	SESN2
0	0.467172997176821	0.442	0.128	0	0	TNKS
0	0.46708054361766	0.453	0.135	0	0	LARP7
0	0.46698654358823	0.376	0.108	0	0	RDH14
0	0.46689189671703	0.523	0.153	0	0	PCGF2
0	0.466840411779692	0.635	0.205	0	0	UBE3C
0	0.466777118323256	0.333	0.103	0	0	PRRG4
0	0.466761292427496	0.313	0.085	0	0	NT5C
0	0.46676114728268	0.578	0.185	0	0	CDKN2A
0	0.466659175789434	0.56	0.191	0	0	EMC3
0	0.466181292190021	0.576	0.187	0	0	NET1
0	0.465544091899307	0.746	0.272	0	0	ERBIN
0	0.464503416673264	0.278	0.073	0	0	APC
0	0.464237978401338	0.577	0.18	0	0	HDHD3
0	0.464058664795124	0.425	0.126	0	0	CASP2
0	0.463969642312425	0.578	0.197	0	0	OCIAD2
0	0.463942148804566	0.3	0.091	0	0	GAR1
0	0.463734779833946	0.368	0.094	0	0	NOP9
0	0.463497747598492	0.39	0.115	0	0	QSER1
0	0.463074340493091	0.525	0.161	0	0	DEXI

0	0.462908128357258	0.453	0.136	0	0	WASHC5
0	0.462866325199985	0.533	0.166	0	0	FLNB
0	0.462369358216875	0.719	0.304	0	0	PLOD3
0	0.462094303197085	0.577	0.182	0	0	R3HDM2
0	0.461649882162635	0.851	0.358	0	0	CDK5RAP3
0	0.461308599769508	0.342	0.091	0	0	ITFG2
0	0.461138946327609	0.42	0.119	0	0	ASCC3
0	0.460739987959477	0.319	0.09	0	0	TMEM18
0	0.460614721701985	0.898	0.43	0	0	CIRBP
0	0.460293462239962	0.468	0.139	0	0	LARP4B
0	0.460202002566829	0.373	0.105	0	0	ZNF704
0	0.459791746273367	0.479	0.158	0	0	BCAP29
0	0.459650406555286	0.354	0.086	0	0	OFD1
0	0.45962790721882	0.28	0.082	0	0	COPG2
0	0.459487109117389	0.495	0.16	0	0	POMP
0	0.45928175372259	0.873	0.417	0	0	TMEM14A
0	0.458877450485054	0.423	0.121	0	0	TFCP2
0	0.458778636170265	0.551	0.168	0	0	NSD1
0	0.458570340652488	0.283	0.086	0	0	SEPHS1
0	0.458236010166509	0.426	0.132	0	0	GTPBP2
0	0.458216297487584	0.257	0.073	0	0	ALDH16A1
0	0.458035805077639	0.571	0.17	0	0	RNF152
0	0.457915993556997	0.347	0.091	0	0	MALT1
0	0.457686661079915	0.392	0.118	0	0	PSMC1
0	0.457681992461487	0.326	0.093	0	0	CCDC61
0	0.457087342425081	0.344	0.092	0	0	TTC33
0	0.456999682076082	0.29	0.081	0	0	SDHAF3
0	0.456924022376018	0.273	0.077	0	0	AIFM2
0	0.456510378897208	0.977	0.679	0	0	ALDH1A1
0	0.456389190999944	0.365	0.099	0	0	HEATR3
0	0.456247148732772	0.277	0.075	0	0	DEPRECATED-ENSG00000168924
0	0.455215252950122	0.771	0.331	0	0	GJB1
0	0.455098884504045	0.356	0.098	0	0	DENR
0	0.455001885504782	0.439	0.106	0	0	SLC25A37
0	0.45495886241253	0.816	0.349	0	0	DAP
0	0.454023163619032	0.927	0.516	0	0	HSD17B6
0	0.453887736593503	0.312	0.083	0	0	ZDHHC16
0	0.453844195423044	0.448	0.141	0	0	SLCO1B1
0	0.453816636476601	0.658	0.226	0	0	VDAC3
0	0.453773937183249	0.4	0.117	0	0	NHLRC2
0	0.453428677859827	0.395	0.115	0	0	SMARCE1
0	0.453408369200609	0.647	0.252	0	0	FDFT1
0	0.453146015488091	0.428	0.129	0	0	REPIN1
0	0.452883649624535	0.442	0.131	0	0	ITPR3
0	0.452735071335878	0.304	0.085	0	0	GGA1
0	0.45272496359851	0.654	0.226	0	0	UHMK1
0	0.452657961588491	0.372	0.11	0	0	CSTF2
0	0.452501654422114	0.276	0.073	0	0	DUS3L
0	0.452426714925624	0.41	0.116	0	0	TRPT1
0	0.452394753982185	0.486	0.143	0	0	S100PBP
0	0.45184469306174	0.347	0.099	0	0	METTL5
0	0.451412449178891	0.389	0.113	0	0	COPS4
0	0.451198158473851	0.935	0.566	0	0	METTL7A
0	0.450904236794954	0.282	0.083	0	0	GSTM1
0	0.450888883602171	0.547	0.171	0	0	NUP43
0	0.450770847872758	0.292	0.084	0	0	MGA
0	0.450756237988996	0.548	0.168	0	0	UBA1
0	0.450569457508683	0.696	0.278	0	0	CYP51A1
0	0.450553698905428	0.268	0.073	0	0	YBEY
0	0.450536016464988	0.426	0.132	0	0	GMFB

0	0.450249482358955	0.657	0.214	0	0	XRN1
0	0.449862213394893	0.353	0.103	0	0	DENND10
0	0.449284526863931	0.303	0.086	0	0	ZNF362
0	0.448613878510974	0.379	0.103	0	0	COBL
0	0.448562204852565	0.282	0.068	0	0	SLC35E2A
0	0.448449828967539	0.397	0.117	0	0	LAD1
0	0.448364723581115	0.645	0.229	0	0	TFG
0	0.447908328217673	0.371	0.102	0	0	SCRN2
0	0.447551727472669	0.256	0.074	0	0	G6PC3
0	0.447389160408318	0.269	0.072	0	0	E2F3
0	0.447233241882075	0.641	0.212	0	0	SPATA13
0	0.447148215266655	0.324	0.095	0	0	COG4
0	0.447125986316842	0.32	0.081	0	0	TAF1
0	0.447076085984602	0.714	0.254	0	0	BIN1
0	0.446804577527727	0.288	0.081	0	0	BLZF1
0	0.446676733476159	0.468	0.135	0	0	ATG4B
0	0.445791597005025	0.477	0.14	0	0	TAF8
0	0.444807075352196	0.509	0.148	0	0	PTAR1
0	0.444777133274486	0.661	0.237	0	0	DIO1
0	0.444408720185458	0.345	0.096	0	0	RIF1
0	0.444322756258871	0.499	0.151	0	0	SDR39U1
0	0.444122266109043	0.462	0.144	0	0	UBR7
0	0.44399988198573	0.37	0.106	0	0	MPP5
0	0.443272969292843	0.485	0.156	0	0	SCAMP4
0	0.443258973805209	0.568	0.176	0	0	PLPP1
0	0.442970305338243	0.383	0.102	0	0	MFSD10
0	0.442378133506455	0.275	0.077	0	0	TMEM101
0	0.441737179253987	0.277	0.072	0	0	KIZ
0	0.441584660119879	0.635	0.201	0	0	MSI2
0	0.441567795551268	0.353	0.109	0	0	PER3
0	0.44133672064512	0.591	0.195	0	0	ERAP1
0	0.44107324149848	0.624	0.205	0	0	TMUB2
0	0.440833246685856	0.491	0.149	0	0	NMRAL1
0	0.440621891467187	0.328	0.088	0	0	ARID2
0	0.440601573645158	0.303	0.078	0	0	PPARG
0	0.440471603426069	0.529	0.162	0	0	FASTK
0	0.440264694047293	0.356	0.094	0	0	THADA
0	0.44002080920468	0.421	0.122	0	0	MAPKAPK5
0	0.439852765638049	0.344	0.1	0	0	SAV1
0	0.439827223101614	0.453	0.138	0	0	CYREN
0	0.43981222396476	0.38	0.112	0	0	LYPLA1
0	0.439160846632048	0.598	0.21	0	0	NEU1
0	0.439113462944371	0.571	0.186	0	0	DNAJC1
0	0.439023405268835	0.407	0.118	0	0	ZNF768
0	0.438278797968806	0.286	0.074	0	0	GCFC2
0	0.43815937600138	0.615	0.216	0	0	SCAMP3
0	0.437764010352835	0.381	0.11	0	0	ARFRP1
0	0.437239934667463	0.403	0.12	0	0	RAB15
0	0.436751843297868	0.456	0.149	0	0	RASL11A
0	0.436451087830882	0.395	0.13	0	0	WEE1
0	0.436420368815848	0.55	0.167	0	0	KIAA0319L
0	0.435951624239519	0.477	0.137	0	0	CNNM3
0	0.435850923580062	0.517	0.134	0	0	DIP2A
0	0.435490411692869	0.443	0.141	0	0	MVK
0	0.43534217355954	0.671	0.223	0	0	TAF7
0	0.435253634993626	0.497	0.151	0	0	ATL2
0	0.434493996956597	0.495	0.154	0	0	AP1G1
0	0.434303051775489	0.277	0.074	0	0	TAPBPL
0	0.434261835369076	0.526	0.17	0	0	SUPT16H
0	0.434159887048045	0.677	0.228	0	0	YTHDF3

0	0.433799886866549	0.436	0.128	0	0	VAPB
0	0.433436929522051	0.371	0.092	0	0	CHD7
0	0.433435951811815	0.256	0.072	0	0	PPP1R13L
0	0.433430503015306	0.453	0.144	0	0	ZCCHC9
0	0.432854977860593	0.265	0.072	0	0	BIRC2
0	0.432831038881482	0.583	0.193	0	0	SYVN1
0	0.432774452086305	0.402	0.112	0	0	STRBP
0	0.432387684077004	0.262	0.075	0	0	OXLD1
0	0.431769965883346	0.272	0.075	0	0	ZSWIM9
0	0.431618440009838	0.466	0.129	0	0	NR2F1
0	0.431550637173497	0.322	0.091	0	0	SOX13
0	0.431421911352872	0.398	0.11	0	0	RBM4
0	0.431343244479105	0.576	0.194	0	0	FPGS
0	0.431181095956594	0.527	0.155	0	0	AUTS2
0	0.431177368575267	0.284	0.072	0	0	SEMA4G
0	0.431119222973857	0.646	0.227	0	0	ATP6V1A
0	0.430942641350523	0.349	0.097	0	0	LMAN2L
0	0.430932928372108	0.686	0.246	0	0	EPHX2
0	0.430931294575293	0.296	0.084	0	0	IFFO1
0	0.430836374138681	0.675	0.207	0	0	INSR
0	0.430815040065905	0.718	0.271	0	0	SCARB1
0	0.43075552087447	0.265	0.076	0	0	SLC9A8
0	0.430299097082113	0.518	0.153	0	0	PURA
0	0.430250441858741	0.877	0.392	0	0	WNK1
0	0.430177203656122	0.695	0.256	0	0	AIG1
2.64597475284918E-288	0.430154940222233	0.323	0.113	4.43412449082466E-284	0	CENPM
0	0.430074319938248	0.322	0.092	0	0	DCAKD
0	0.429817828182104	0.594	0.182	0	0	TRIM25
0	0.429793505081623	0.484	0.15	0	0	API5
0	0.429575197715291	0.714	0.284	0	0	PERP
0	0.429280121393244	0.313	0.089	0	0	DHX29
0	0.429179071593194	0.369	0.106	0	0	ZNF275
0	0.428759600620509	0.302	0.084	0	0	IL15RA
0	0.428721511099467	0.362	0.108	0	0	GNPDA1
0	0.428626157043327	0.29	0.082	0	0	MTG2
0	0.428185959240361	0.482	0.133	0	0	SLC5A3
0	0.428126191965559	0.255	0.071	0	0	L3MBTL4
0	0.427857939641248	0.647	0.235	0	0	SMIM19
0	0.427794964056405	0.686	0.284	0	0	CPEB4
0	0.427794355314081	0.611	0.139	0	0	RAPGEF5
0	0.427518946056606	0.334	0.085	0	0	DNAJB14
0	0.427492100154726	0.712	0.28	0	0	CAPN1
0	0.427452080710462	0.568	0.17	0	0	MORC3
0	0.427437711220883	0.267	0.074	0	0	EIF4EBP3
0	0.427169561268983	0.837	0.369	0	0	OAF
0	0.427110939245218	0.354	0.103	0	0	STXBP6
0	0.427015946052703	0.647	0.213	0	0	TMEM245
0	0.426413474755311	0.648	0.221	0	0	PAXX
0	0.426332067248377	0.252	0.071	0	0	RNF214
0	0.426242162743461	0.794	0.313	0	0	SREBF2
0	0.426130090378267	0.307	0.086	0	0	PCBP2
0	0.42579248398266	0.479	0.134	0	0	LAMC1
0	0.425769556420277	0.443	0.136	0	0	PSMA3
0	0.425233355914653	0.267	0.072	0	0	BRMS1L
0	0.425204610579687	0.316	0.093	0	0	SNRPG
0	0.42500770709025	0.627	0.207	0	0	RIOK3
0	0.424878663737478	0.491	0.144	0	0	NELFA
0	0.424568158736463	0.716	0.276	0	0	SRP9
0	0.424528089850048	0.453	0.144	0	0	RNF19B
0	0.424460413175571	0.543	0.179	0	0	EPRS

0	0.424065809628283	0.336	0.096	0	0	CLCN5
0	0.423742094686992	0.764	0.327	0	0	OTC
0	0.423554704241361	0.702	0.272	0	0	MLEC
0	0.423479610720921	0.712	0.232	0	0	KMT2C
0	0.423406200897661	0.29	0.078	0	0	BLOC1S4
0	0.422327830994806	0.706	0.266	0	0	DCAF11
0	0.422291054784872	0.294	0.088	0	0	POLH
0	0.422050431663297	0.463	0.132	0	0	SNX13
0	0.422015603227595	0.622	0.22	0	0	AHCYL1
0	0.421612507861447	0.365	0.105	0	0	TMEM138
0	0.421350665567386	0.5	0.143	0	0	MID1
0	0.421235376536171	0.456	0.144	0	0	NIT2
0	0.420955933736414	0.276	0.081	0	0	PKNOX1
0	0.420894066680727	0.319	0.09	0	0	SPOUT1
0	0.420772678418495	0.388	0.117	0	0	NUP160
0	0.420739905967451	0.546	0.168	0	0	SENP2
0	0.420696178051962	0.277	0.081	0	0	DNAAF5
0	0.420636704369065	0.545	0.168	0	0	GPR107
0	0.419909541127013	0.475	0.147	0	0	RTKN
0	0.419831241462384	0.462	0.144	0	0	HPS3
0	0.41947770114236	0.588	0.203	0	0	TP53BP2
0	0.419451600079957	0.26	0.071	0	0	TLK2
0	0.419449358147014	0.251	0.07	0	0	FBXO30
0	0.419184493408948	0.554	0.187	0	0	NDUFB1
0	0.419109590201975	0.322	0.088	0	0	INPP5E
0	0.418939330072063	0.489	0.152	0	0	MFF
0	0.418899047196948	0.523	0.172	0	0	SKAP2
0	0.418687364447775	0.498	0.16	0	0	MMAB
0	0.41831821045764	0.448	0.133	0	0	ZNF384
0	0.418291535608446	0.42	0.12	0	0	KHNYN
0	0.41815087917127	0.323	0.09	0	0	POM121
0	0.418073617787593	0.304	0.089	0	0	TRNAU1AP
0	0.417854849305792	0.425	0.131	0	0	PPCS
0	0.417832140803249	0.606	0.202	0	0	DHX9
0	0.417308452191299	0.423	0.125	0	0	RCOR1
0	0.416848374726052	0.31	0.089	0	0	RMND1
0	0.4166160570419	0.557	0.165	0	0	GPATCH2L
0	0.416610867835537	0.401	0.115	0	0	SLC17A5
0	0.41605207156047	0.534	0.178	0	0	PAM16
3.40769346792558E-299	0.416008512220928	0.25	0.073	5.71061271354969E-295	0	CDCA7L
0	0.415619393670783	0.426	0.131	0	0	GMPPA
0	0.415560686485409	0.677	0.236	0	0	EIF4A2
0	0.415519032882276	0.411	0.12	0	0	AAAS
0	0.414816516186761	0.507	0.136	0	0	SIN3B
0	0.414493874515347	0.528	0.169	0	0	AGGF1
0	0.414171922198928	0.29	0.086	0	0	BCS1L
0	0.413531459690175	0.753	0.287	0	0	NPLOC4
0	0.413420392367504	0.847	0.438	0	0	RNASE4
0	0.413352216915087	0.296	0.083	0	0	MAPK8
0	0.413055914890655	0.475	0.135	0	0	CHIC2
0	0.412874081646129	0.532	0.18	0	0	COQ8A
0	0.412704145908927	0.396	0.108	0	0	ELL
0	0.412466010864092	0.673	0.23	0	0	UPK3BL1
0	0.412245946021963	0.267	0.07	0	0	EVI5L
0	0.412209515578649	0.676	0.241	0	0	ETNK1
0	0.411923876789174	0.319	0.089	0	0	RAI14
0	0.411876313914078	0.276	0.079	0	0	ZNF318
0	0.410999563512959	0.323	0.096	0	0	ARFIP1
0	0.41028568476504	0.523	0.161	0	0	CCNY
0	0.410241565442686	0.451	0.129	0	0	EIF4G3

0	0.409677861528371	0.382	0.113	0	0	USP38
0	0.409095949396387	0.85	0.392	0	0	MTSS1
0	0.408628994309635	0.4	0.125	0	0	ACTL6A
0	0.408527100376049	0.418	0.131	0	0	COX7B
0	0.408347379472858	0.579	0.2	0	0	PSMB5
0	0.408243171156126	0.492	0.16	0	0	TECR
0	0.407934135159842	0.485	0.155	0	0	EDEM2
0	0.407852895470133	0.28	0.081	0	0	MTX1
0	0.407624203430261	0.808	0.339	0	0	CTNND1
0	0.406945934719206	0.332	0.108	0	0	TEX30
0	0.406899293443308	0.513	0.164	0	0	UBA5
0	0.406721318214184	0.325	0.091	0	0	DVL2
0	0.406493549170916	0.469	0.132	0	0	ARHGAP17
0	0.406421963495772	0.286	0.082	0	0	HEATR1
0	0.406091984546862	0.65	0.252	0	0	KDELR2
0	0.40564366211058	0.269	0.072	0	0	APTX
0	0.40555743143337	0.744	0.24	0	0	RABGAP1L
0	0.405188704393883	0.363	0.099	0	0	FBXO3
0	0.405071434783738	0.308	0.077	0	0	LTB4R
0	0.405058827027224	0.318	0.086	0	0	KLHL20
0	0.404767495950165	0.461	0.139	0	0	NUP153
0	0.404700754960633	0.344	0.092	0	0	CD58
0	0.404670903547165	0.501	0.15	0	0	PIP5K1A
0	0.404667565803297	0.587	0.188	0	0	CDC42BPB
0	0.404607745881293	0.637	0.233	0	0	PAPSS2
0	0.404222821275278	0.702	0.27	0	0	EIF2AK1
0	0.40403198720872	0.336	0.095	0	0	RGP1
0	0.403950884150791	0.422	0.132	0	0	USP1
0	0.403709683893311	0.849	0.359	0	0	CTSH
0	0.403387667288649	0.555	0.174	0	0	RBM14
0	0.402714056943574	0.434	0.13	0	0	FBXO46
0	0.402482798917804	0.396	0.12	0	0	MTMR10
0	0.402427054059293	0.717	0.284	0	0	CCL15
0	0.402381177840216	0.277	0.083	0	0	VTA1
0	0.402268176978335	0.637	0.224	0	0	PSMD10
3.15650040943335E-293	0.401593628830244	0.253	0.076	5.28966338612841E-289	0	LRIF1
2.03437901213847E-296	0.401566205301172	0.962	0.616	3.40921234854165E-292	0	MGST1
0	0.400724101548888	0.257	0.071	0	0	SBF2
0	0.400505517235131	0.602	0.189	0	0	CPSF6
0	0.400181215269674	0.419	0.12	0	0	CDK14
0	0.400064505342906	0.66	0.256	0	0	HADH
0	0.400063143954697	0.529	0.172	0	0	SLC39A9
0	0.399909096418507	0.486	0.149	0	0	MED13
0	0.399885237739374	0.533	0.176	0	0	SLC39A1
0	0.399793767378573	0.397	0.12	0	0	P4HA2
0	0.399792115024169	0.516	0.16	0	0	ZNF146
0	0.39973384236933	0.459	0.156	0	0	NSL1
0	0.399701460089566	0.495	0.144	0	0	SETX
0	0.399520978810264	0.277	0.083	0	0	WRAP73
0	0.398997570870133	0.799	0.348	0	0	RAB2A
0	0.398828813413671	0.326	0.09	0	0	ARFIP2
0	0.398438053048474	0.367	0.103	0	0	HDAC8
0	0.398330299700566	0.48	0.15	0	0	CTDSPL
0	0.398322635545789	0.609	0.212	0	0	PSMD12
0	0.398291599992992	0.539	0.189	0	0	DHCR7
0	0.398075940855723	0.518	0.169	0	0	SARDH
0	0.397219294355811	0.922	0.511	0	0	GSTA1
0	0.397194757264841	0.317	0.088	0	0	MYCBP2
2.43462203700317E-252	0.397001596806743	0.99	0.869	4.07993960960991E-248	0	ALB
0	0.39695745733852	0.501	0.155	0	0	TMBIM4

0	0.396814432593291	0.604	0.191	0	0	STX7
0	0.396695618522831	0.395	0.123	0	0	LNPK
0	0.396527771661223	0.672	0.227	0	0	PHF10
0	0.396491522357556	0.717	0.275	0	0	ERO1A
0	0.396392667806753	0.39	0.106	0	0	RIN2
0	0.395515416542	0.486	0.143	0	0	BOK
0	0.395467904039609	0.325	0.091	0	0	DNMBP
0	0.39517152875752	0.416	0.12	0	0	BRPF3
0	0.394809118970224	0.421	0.124	0	0	PHKB
0	0.394702422936375	0.316	0.092	0	0	PARN
0	0.394525150873514	0.306	0.093	0	0	AGBL5
0	0.39438372200985	0.42	0.119	0	0	TMEM165
0	0.394320992077937	0.448	0.141	0	0	MCCC2
0	0.394086577464976	0.489	0.16	0	0	AFG3L2
0	0.394033583193604	0.608	0.196	0	0	AGO2
0	0.393966925738091	0.3	0.088	0	0	DDX52
0	0.393631058754114	0.623	0.214	0	0	RAB11FIP1
0	0.39358696236429	0.731	0.3	0	0	DSTN
0	0.393089778563776	0.479	0.15	0	0	HDHD5
0	0.392926924260745	0.356	0.106	0	0	UCHL5
0	0.392299536510916	0.492	0.155	0	0	MPPE1
0	0.392246549707941	0.321	0.091	0	0	TBC1D13
0	0.392180659625194	0.431	0.131	0	0	RNF113A
0	0.392131280058824	0.968	0.636	0	0	MT-CYB
0	0.392097744711662	0.455	0.138	0	0	C2orf68
0	0.391979222206928	0.739	0.313	0	0	TMEM33
0	0.391332132908101	0.255	0.072	0	0	GIPC2
0	0.391090708551369	0.817	0.363	0	0	MTDH
0	0.390719904191341	0.282	0.08	0	0	USP21
0	0.390189599219269	0.341	0.096	0	0	MTREX
0	0.389913088864417	0.308	0.087	0	0	TMEM30A
0	0.388728824318243	0.865	0.438	0	0	CYC1
0	0.388666936883862	0.499	0.155	0	0	SCAF4
0	0.388561361332841	0.28	0.076	0	0	RTL10
0	0.388402151158261	0.494	0.159	0	0	HGH1
0	0.388044073942398	0.413	0.124	0	0	LMTK2
0	0.387757890827275	0.562	0.184	0	0	ARHGAP1
0	0.387420762780671	0.453	0.145	0	0	CPT1A
0	0.387308345542531	0.448	0.139	0	0	SFXN5
0	0.386854155147177	0.505	0.168	0	0	TMEM123
0	0.386563606001212	0.352	0.094	0	0	WDR37
0	0.386507755542366	0.338	0.101	0	0	GNE
0	0.386415136181487	0.462	0.129	0	0	ZNF655
0	0.386236217131801	0.288	0.086	0	0	RWDD1
0	0.386221817541965	0.296	0.088	0	0	CYCS
0	0.386152110662212	0.416	0.129	0	0	FGFR4
0	0.386041568010105	0.583	0.203	0	0	DPP9
0	0.385879860523898	0.692	0.254	0	0	FAM91A1
0	0.385502377327001	0.327	0.088	0	0	TRMU
0	0.385272056608038	0.342	0.105	0	0	MGME1
0	0.385245816033871	0.65	0.215	0	0	MKLN1
0	0.38518259273341	0.692	0.263	0	0	IARS2
0	0.384987695637947	0.428	0.128	0	0	UBAC2
0	0.384795223005608	0.354	0.105	0	0	GMPR2
0	0.384617701091027	0.492	0.149	0	0	LSM 7.00
0	0.384376436401252	0.832	0.371	0	0	PLXNB2
0	0.384276576720209	0.261	0.07	0	0	SNX21
0	0.384237658350325	0.341	0.096	0	0	TRMT2A
0	0.384083045678771	0.42	0.127	0	0	MVB12A
0	0.383359362895151	0.323	0.095	0	0	CNOT6

0	0.383351120209097	0.409	0.129	0	0	SMAP1
0	0.383293916565939	0.656	0.228	0	0	PRPS1
0	0.383211107577276	0.396	0.116	0	0	MAGEH1
0	0.382527660729304	0.861	0.396	0	0	TMEM106C
0	0.382129667053544	0.284	0.076	0	0	DPP8
0	0.381936967563055	0.487	0.163	0	0	ATPAF1
0	0.381895877372032	0.451	0.143	0	0	TNPO2
0	0.381826038949056	0.383	0.117	0	0	C12orf75
2.03769111215283E-293	0.381735342740657	0.988	0.778	3.41476276574571E-289	0	EPHX1
0	0.381229180278842	0.403	0.127	0	0	ZMPSTE24
0	0.381218720623494	0.372	0.101	0	0	ZNF407
0	0.381119699691892	0.634	0.213	0	0	PRKAA1
0	0.38094482939033	0.613	0.223	0	0	CISD2
0	0.380848969232394	0.515	0.165	0	0	SF3B3
0	0.380739071086087	0.431	0.132	0	0	COMMD6
0	0.38047255828402	0.272	0.077	0	0	RAP1GAP
0	0.380228885460576	0.489	0.144	0	0	DEPRECATED-ENSG00000197408
0	0.380069099419726	0.366	0.109	0	0	MTRR
0	0.379764387005841	0.323	0.097	0	0	DIPK2A
0	0.379642307674337	0.257	0.073	0	0	PTGFRN
0	0.379382804209258	0.411	0.116	0	0	VPS13D
0	0.379157479512224	0.514	0.171	0	0	CLN3
0	0.379063011168521	0.42	0.126	0	0	NUDT22
0	0.379026871775857	0.264	0.071	0	0	ADAT2
0	0.378908022267408	0.397	0.111	0	0	IFI44
0	0.378829682426642	0.737	0.28	0	0	USP9X
0	0.37865578403611	0.562	0.182	0	0	ASAP1
0	0.377685703685461	0.427	0.13	0	0	CASC4
0	0.377252435793912	0.25	0.071	0	0	ING5
0	0.377179572080616	0.486	0.155	0	0	CNOT11
0	0.376977853824735	0.503	0.145	0	0	CASK
0	0.376791962463871	0.742	0.315	0	0	RTN3
0	0.37623958072431	0.551	0.175	0	0	TCERG1
0	0.376091191578711	0.523	0.177	0	0	POLR2K
0	0.375934041455655	0.28	0.075	0	0	WDR45
0	0.375807972626771	0.358	0.111	0	0	TICAM1
0	0.375760012389617	0.812	0.364	0	0	COX6C
0	0.375588780015581	0.383	0.106	0	0	TP53I13
0	0.375386498989491	0.754	0.313	0	0	SAR1B
0	0.375247374912436	0.343	0.095	0	0	TSPAN7
0	0.375196979366402	0.293	0.079	0	0	CSNK1G3
0	0.375073201290511	0.502	0.15	0	0	KPNA1
0	0.3750327054831	0.33	0.091	0	0	CLEC16A
0	0.374831421624451	0.829	0.377	0	0	CPB2
0	0.3745698748385	0.845	0.366	0	0	G6PD
0	0.374565985411097	0.314	0.093	0	0	GRK3
0	0.374480548595502	0.406	0.121	0	0	MLH1
0	0.374286147939922	0.747	0.292	0	0	ZNF706
0	0.374145162617388	0.627	0.212	0	0	PYGO2
0	0.373660077496402	0.318	0.094	0	0	ZNF32
0	0.373395238944856	0.301	0.08	0	0	USP28
0	0.373313582922407	0.385	0.105	0	0	ANKRD13A
0	0.373162958846581	0.384	0.112	0	0	IREB2
0	0.372671529660187	0.282	0.077	0	0	VDR
0	0.37177109152015	0.285	0.076	0	0	CAMKMT
0	0.371657588416738	0.692	0.256	0	0	LMO7
0	0.371647855756935	0.491	0.163	0	0	DERA
0	0.371514552369979	0.342	0.097	0	0	EIF2AK4
0	0.371304364603375	0.328	0.092	0	0	DVL1
0	0.371098353164268	0.838	0.346	0	0	PICALM

0	0.370589968788384	0.564	0.193	0	0	GTF2I
0	0.370216591655248	0.58	0.186	0	0	NPEPPS
0	0.369869728355745	0.753	0.314	0	0	CLINT1
0	0.369848996002615	0.365	0.114	0	0	UQCRFS1
0	0.369679032423528	0.536	0.169	0	0	MYO6
0	0.369616413508517	0.495	0.165	0	0	PID1
0	0.369326096263468	0.541	0.171	0	0	UBE4A
0	0.368627635082764	0.337	0.094	0	0	GBP3
0	0.36796209507725	0.43	0.121	0	0	ARFGAP1
0	0.367747983346547	0.563	0.17	0	0	PHYKPL
0	0.367702262391591	0.756	0.284	0	0	LPP
0	0.367650327611496	0.378	0.12	0	0	CCT5
0	0.367454101497468	0.263	0.074	0	0	TARDBP
0	0.367453684172493	0.344	0.099	0	0	SUDS3
0	0.367076397827397	0.367	0.111	0	0	ALG5
0	0.366833006890235	0.675	0.235	0	0	SMARCA4
0	0.366809953316499	0.58	0.192	0	0	EWSR1
0	0.366721300682001	0.279	0.077	0	0	HARS2
0	0.366053312797848	0.362	0.115	0	0	CENPU
0	0.365875004147326	0.272	0.082	0	0	DEPRECATED-ENSG00000099810
0	0.365730495653457	0.316	0.088	0	0	SPTLC1
0	0.365635152793079	0.404	0.135	0	0	PCNA
0	0.365634707678735	0.435	0.126	0	0	CBWD1
0	0.365545700753712	0.361	0.11	0	0	DDX19A
0	0.365523669327186	0.46	0.142	0	0	ARFGEF2
0	0.365445660649543	0.363	0.108	0	0	BTBD1
0	0.365313685806655	0.693	0.226	0	0	CD47
0	0.365254846419482	0.28	0.072	0	0	DEPRECATED-ENSG00000197714
0	0.364979779534446	0.608	0.222	0	0	IRF6
0	0.364977219005724	0.714	0.264	0	0	PDCD6IP
0	0.364629840848472	0.481	0.15	0	0	PTGES2
0	0.364274183297201	0.868	0.408	0	0	SLC12A7
0	0.364209873536095	0.45	0.141	0	0	CUL3
0	0.364075871634829	0.844	0.422	0	0	ACAA2
0	0.364029754174627	0.287	0.087	0	0	LRRC8D
0	0.36363342191714	0.38	0.121	0	0	GGCT
0	0.363509065333019	0.773	0.325	0	0	NECTIN2
0	0.363391688903542	0.471	0.152	0	0	COMMD5
0	0.362829686301656	0.343	0.108	0	0	XPO1
0	0.362158245387718	0.303	0.086	0	0	CHPF2
0	0.361912900378788	0.781	0.329	0	0	ENY2
0	0.361524283905477	0.474	0.157	0	0	FOXA1
0	0.361488586363732	0.373	0.112	0	0	SEC22C
0	0.361307341377564	0.338	0.094	0	0	TSC2
0	0.361252874048242	0.371	0.116	0	0	ITGB1BP1
0	0.361219242080169	0.299	0.081	0	0	NACC2
0	0.361182734602427	0.752	0.313	0	0	SHMT1
0	0.361056803276679	0.43	0.135	0	0	ATF6
0	0.36091961195507	0.603	0.214	0	0	LRRC8A
0	0.360692286332565	0.541	0.172	0	0	VHL
0	0.359857801757631	0.37	0.113	0	0	RBX1
0	0.359574099500473	0.49	0.16	0	0	GSS
0	0.35953067499869	0.699	0.269	0	0	DERL1
0	0.359385941741505	0.799	0.364	0	0	GUSB
0	0.359346934196454	0.736	0.313	0	0	PRDX4
0	0.359233108647173	0.706	0.235	0	0	UBR2
0	0.359067934455495	0.637	0.238	0	0	ALAD
0	0.359004597638511	0.43	0.129	0	0	DLST
0	0.358957333992223	0.631	0.231	0	0	SLC39A7
0	0.358793634368704	0.332	0.093	0	0	TACC2

0	0.358716290672146	0.539	0.186	0	0	GALNT2
0	0.358557653482254	0.77	0.326	0	0	SCARB2
0	0.358330774434085	0.385	0.109	0	0	TBK1
0	0.35786490244585	0.531	0.177	0	0	CAMK1
0	0.357840979562305	0.447	0.137	0	0	SOX5
0	0.357742417803153	0.295	0.081	0	0	ZNF131
0	0.357723154543685	0.558	0.18	0	0	INTS1
0	0.357440393488108	0.284	0.079	0	0	MTF2
0	0.357383941348675	0.533	0.157	0	0	AEBP2
0	0.357135123095212	0.403	0.115	0	0	SLC35E1
0	0.357107241233239	0.355	0.103	0	0	RPUSD3
0	0.356945593030312	0.565	0.192	0	0	GIPC1
0	0.356783248897463	0.825	0.365	0	0	LPGAT1
0	0.35671404321616	0.371	0.113	0	0	KLHDC10
0	0.356540596219534	0.586	0.213	0	0	CETN2
0	0.356332077791466	0.327	0.097	0	0	MAIP1
0	0.355896116794636	0.426	0.137	0	0	PGK 1.00
0	0.355165431285902	0.571	0.193	0	0	SELENOS
0	0.354947029705335	0.489	0.145	0	0	PPP1R12B
0	0.354394952072114	0.394	0.117	0	0	MPZL1
0	0.353035700038755	0.272	0.069	0	0	TXLNG
0	0.352949773483286	0.324	0.094	0	0	NCBP1
1.39019810881215E-278	0.351706729397859	0.262	0.083	2.32969399074741E-274	0	TMEM60
0	0.351089938663856	0.345	0.1	0	0	SLC41A3
0	0.350927898410205	0.622	0.224	0	0	PIR
0	0.350793772911511	0.454	0.145	0	0	OXR1
0	0.350763962701946	0.384	0.12	0	0	TMEM230
0	0.350584285133644	0.496	0.165	0	0	BROX
0	0.350524681326245	0.334	0.097	0	0	CDYL
0	0.350462673801095	0.95	0.535	0	0	SPINK1
0	0.350023955017815	0.501	0.149	0	0	DYRK1A
0	0.349531091449256	0.811	0.379	0	0	CD151
0	0.349446958982406	0.378	0.114	0	0	PPP5C
0	0.349358215974542	0.46	0.155	0	0	ODC1
0	0.349130323706268	0.312	0.091	0	0	DDX18
0	0.34889774317058	0.617	0.214	0	0	NUS1
0	0.348849100569217	0.385	0.117	0	0	STRN
0	0.348843216875644	0.417	0.13	0	0	CEBPZOS
0	0.34876564743843	0.371	0.107	0	0	USP33
0	0.348362208992968	0.659	0.235	0	0	ATP2A2
0	0.34806878670386	0.286	0.082	0	0	RNF8
0	0.347846186435199	0.372	0.108	0	0	NT5DC1
0	0.347555724583461	0.332	0.101	0	0	BCL10
0	0.347500135608605	0.404	0.117	0	0	KIAA1109
0	0.347309288433903	0.313	0.085	0	0	SVIL
0	0.347158265047116	0.395	0.111	0	0	UNK
0	0.346918018866571	0.535	0.171	0	0	TNRC18
0	0.346327687522068	0.417	0.133	0	0	YIPF5
0	0.346225466932057	0.296	0.084	0	0	PACS2
0	0.346180368735161	0.401	0.115	0	0	MFN1
0	0.345857536508204	0.305	0.091	0	0	CCNYL1
0	0.345266292563556	0.362	0.113	0	0	PDHX
0	0.344877668767605	0.273	0.077	0	0	METTL21A
0	0.344601041253192	0.417	0.128	0	0	ME2
0	0.344596196103339	0.569	0.177	0	0	NME3
0	0.344567433720658	0.345	0.104	0	0	TIPARP
0	0.344358278573266	0.774	0.332	0	0	HM13
0	0.343618785090286	0.676	0.252	0	0	AMFR
0	0.343497931172168	0.313	0.09	0	0	SNRNP48
0	0.343115996555088	0.506	0.153	0	0	STK25

0	0.343035282013206	0.344	0.101	0	0	EXOC6
0	0.342847997904949	0.48	0.144	0	0	DNMT3A
0	0.342699345385094	0.283	0.074	0	0	ABCB1
0	0.342484453600342	0.42	0.137	0	0	GMNN
0	0.341377975019253	0.361	0.119	0	0	CLN6
0	0.341343445487939	0.82	0.399	0	0	MGST3
0	0.341228495837427	0.524	0.178	0	0	MTFR1
0	0.341201607001076	0.327	0.095	0	0	USP6NL
0	0.341130925062719	0.286	0.086	0	0	SLC52A2
0	0.340399874616716	0.503	0.16	0	0	AZIN1
0	0.340186503942507	0.608	0.213	0	0	SIL1
0	0.339739128188489	0.585	0.206	0	0	MBOAT7
2.76889995252572E-290	0.339172357230371	0.932	0.566	4.64012254044259E-286	0	BST2
0	0.338876976134911	0.346	0.104	0	0	APEX2
0	0.338285495694888	0.582	0.184	0	0	RORA
0	0.338232453722067	0.276	0.077	0	0	GALC
0	0.338208653473182	0.512	0.161	0	0	KDM3B
0	0.338168245647319	0.412	0.13	0	0	LEPROT
1.04198922843242E-294	0.33814010180261	0.258	0.077	1.74616554900705E-290	0	DEPRECATED-ENSG00000022267
0	0.337695985262542	0.287	0.086	0	0	DOCK7
0	0.337489995831932	0.364	0.114	0	0	CDC23
0	0.337395341293036	0.296	0.086	0	0	RBBP5
0	0.33723014631208	0.477	0.159	0	0	UQCC2
1.1960644559093E-291	0.336805386037764	0.287	0.093	2.00436481521281E-287	0	SLC1A4
0	0.336742864685261	0.284	0.08	0	0	GLTP
0	0.336741289326408	0.328	0.091	0	0	HERC2
0	0.336636909546563	0.679	0.267	0	0	SLC25A13
6.92251270932307E-307	0.336521108309825	0.283	0.088	1.16007467982836E-302	0	RNASEH1
0	0.336403337041153	0.406	0.125	0	0	NMI
0	0.336289651250704	0.588	0.209	0	0	DNMT1
0	0.335914876444198	0.506	0.157	0	0	TRRAP
0	0.33537789673609	0.331	0.097	0	0	MTMR6
0	0.334944917360815	0.778	0.333	0	0	RTN4
0	0.334794050476593	0.357	0.102	0	0	HDAC10
0	0.334730328373545	0.744	0.325	0	0	ALDH9A1
0	0.333203380174986	0.322	0.088	0	0	ELP1
0	0.332803500867257	0.686	0.252	0	0	MAGED2
0	0.33257061686951	0.43	0.132	0	0	SLC25A23
0	0.332569590498479	0.409	0.126	0	0	TM2D3
0	0.332414253678656	0.514	0.17	0	0	ABHD16A
0	0.332342785714439	0.385	0.12	0	0	GTF3A
0	0.332275320590481	0.484	0.157	0	0	TMEM189
0	0.331468014724912	0.42	0.136	0	0	DNAJC13
0	0.331453455346818	0.609	0.21	0	0	PLSCR1
1.74507840315776E-294	0.33132289000664	0.258	0.078	2.92440238801178E-290	0	WASHC3
0	0.331276417539598	0.346	0.104	0	0	CTBS
0	0.33110744660302	0.258	0.074	0	0	ARHGEF5
0	0.331013763411359	0.604	0.202	0	0	BACH1
1.35452422174603E-301	0.330988468078765	0.254	0.074	2.269911690802E-297	0	RRP15
0	0.330884373844652	0.251	0.068	0	0	MAP3K10
0	0.33052586812809	0.434	0.136	0	0	SNX14
4.72291035658235E-304	0.330496318912197	0.254	0.074	7.9146531755607E-300	0	ZNF414
0	0.330321181670275	0.348	0.1	0	0	CYP2D6
0	0.330244170476698	0.705	0.288	0	0	ATP6V0D1
0	0.330155560108308	0.334	0.093	0	0	PAM
0	0.329990004816263	0.318	0.093	0	0	DROSHA
0	0.329816831421768	0.501	0.171	0	0	MEGF9
0	0.329158384706209	0.412	0.131	0	0	FAHD1
3.39713597663818E-295	0.32895245415167	0.254	0.076	5.69292046965027E-291	0	SGCB
0	0.328273963356426	0.498	0.159	0	0	SUZ12

0	0.328190947366545	0.605	0.221	0	0	ACO1
0	0.328160895060416	0.337	0.101	0	0	ZNF639
0	0.328061892231813	0.325	0.083	0	0	ATP6V0A2
0	0.327555981328822	0.414	0.134	0	0	EHMT2
0	0.327179242046743	0.388	0.127	0	0	CENPX
0	0.326925580377523	0.431	0.14	0	0	CHCHD7
0	0.32691356766527	0.524	0.179	0	0	UBE2E3
0	0.326893149044159	0.324	0.099	0	0	YIPF2
0	0.326881856435811	0.532	0.188	0	0	LSM 6.00
0	0.326800426808769	0.356	0.111	0	0	LDLRAD4
0	0.326564567932567	0.321	0.097	0	0	AARS2
0	0.326562414833004	0.681	0.277	0	0	FH
0	0.326369472534072	0.471	0.152	0	0	ADIPOR2
0	0.326216009633762	0.548	0.203	0	0	NDUFA5
0	0.325873463628061	0.638	0.256	0	0	TMEM147
0	0.325734866728439	0.405	0.119	0	0	COPS2
0	0.325413005761541	0.257	0.073	0	0	CEP104
0	0.325076288256428	0.425	0.14	0	0	C8orf33
0	0.325059482325935	0.274	0.083	0	0	AP3S1
0	0.324867721660658	0.613	0.212	0	0	LGALS8
0	0.324660261679317	0.522	0.164	0	0	EIF2B5
0	0.324521797348502	0.488	0.182	0	0	SLC27A3
1.40183629188513E-293	0.324507311727133	0.252	0.075	2.3491972579411E-289	0	TOMM40
0	0.323972322572039	0.575	0.18	0	0	NIPBL
0	0.32368540895269	0.805	0.373	0	0	PRDX1
0	0.323082993152334	0.403	0.125	0	0	TBL2
0	0.322751378034864	0.555	0.175	0	0	ZRANB2
0	0.322681637347348	0.813	0.365	0	0	PSMB1
0	0.322478734208812	0.668	0.243	0	0	NAA10
0	0.322391026769319	0.942	0.481	0	0	NQO1
0	0.32107671466345	0.298	0.087	0	0	DCBLD2
0	0.320967022562309	0.338	0.103	0	0	SDF2
0	0.320547891228418	0.469	0.138	0	0	XAF1
0	0.320494420535237	0.38	0.117	0	0	ZADH2
0	0.320392770329017	0.528	0.187	0	0	PROS1
0	0.319716720182334	0.853	0.418	0	0	NFE2L1
0	0.319435629425521	0.471	0.152	0	0	PLRG1
0	0.319401912324091	0.349	0.107	0	0	DPAGT1
0	0.319391668061354	0.317	0.091	0	0	HEATR5B
0	0.31889675146757	0.531	0.184	0	0	WASL
0	0.318341136144318	0.251	0.068	0	0	ABHD4
0	0.318309340882885	0.443	0.149	0	0	ATRAID
0	0.31781627244576	0.439	0.143	0	0	HAT1
0	0.317589983103406	0.308	0.087	0	0	FLAD1
0	0.317582254924153	0.454	0.143	0	0	UGGT1
0	0.317504658714836	0.323	0.09	0	0	EPHB4
0	0.317271581820423	0.724	0.283	0	0	DCAF8
0	0.317178931094032	0.35	0.109	0	0	MED1
0	0.317144007178712	0.597	0.224	0	0	ELOVL1
2.52513847890848E-282	0.316706131117162	0.972	0.647	4.23162706295483E-278	0	PTGR1
0	0.316490571703843	0.294	0.084	0	0	DENND1B
0	0.316461322797507	0.283	0.082	0	0	THRB
0	0.316453847072237	0.728	0.357	0	0	SLPI
0	0.316441389443506	0.537	0.164	0	0	BRWD1
0	0.315967438242461	0.374	0.115	0	0	AP3S2
0	0.315438118539925	0.573	0.189	0	0	MSL1
0	0.31542362163879	0.264	0.077	0	0	ELP4
0	0.315133498655174	0.352	0.111	0	0	ZNF281
0	0.314936695733856	0.424	0.128	0	0	C1orf122
0	0.313835982061192	0.447	0.145	0	0	AGMO

0	0.3138238586265	0.668	0.225	0	0	SRRM2
0	0.313449530747373	0.649	0.248	0	0	C19orf12
0	0.313378401998149	0.398	0.116	0	0	SLC35C2
0	0.313083683872013	0.83	0.384	0	0	PUF60
0	0.31289448079934	0.663	0.247	0	0	WDR83OS
0	0.312160078031092	0.668	0.232	0	0	INTS11
0	0.311878123909245	0.896	0.362	0	0	LENG8
0	0.311544402910384	0.289	0.082	0	0	DENND5B
5.959367656719E-261	0.31143198846376	0.927	0.571	9.9867083191297E-257	0	GSTK1
0	0.310766243248232	0.569	0.203	0	0	ZFAND5
0	0.310725730972527	0.311	0.09	0	0	PDK1
0	0.310215740516137	0.528	0.168	0	0	AKAP9
0	0.310064542795216	0.361	0.112	0	0	ABHD11
0	0.309905871884168	0.415	0.122	0	0	TNS2
0	0.309829184614911	0.574	0.212	0	0	DNASE2
0	0.309801342908621	0.292	0.086	0	0	FBXO11
0	0.309367166954105	0.413	0.126	0	0	CAMK1D
0	0.309294475755772	0.405	0.124	0	0	PTPRJ
0	0.308265680339191	0.329	0.108	0	0	HMGB3
0	0.308265257619492	0.328	0.103	0	0	EEF1AKMT2
0	0.308203486576746	0.316	0.099	0	0	CHAF1A
0	0.308144445629929	0.507	0.18	0	0	DEPRECATED-ENSG00000001630
5.33015296057379E-236	0.308114472996219	0.926	0.561	8.93227033132955E-232	0	CERS2
0	0.306865502170628	0.431	0.139	0	0	ATXN10
0	0.306207428128881	0.335	0.1	0	0	STYX
0	0.305830329677216	0.58	0.19	0	0	EGLN2
0	0.305738187450755	0.432	0.147	0	0	PSMG3
0	0.305551040302367	0.275	0.082	0	0	HMGN5
0	0.305358738788931	0.541	0.19	0	0	KLKB1
0	0.30521614527619	0.418	0.124	0	0	GTF3C3
0	0.305057702691438	0.484	0.167	0	0	HECTD3
0	0.304804426621643	0.345	0.106	0	0	RAB22A
0	0.304536637932685	0.408	0.136	0	0	GLO1
7.19245137778842E-298	0.304020575330018	0.799	0.415	1.20531100188978E-293	0	ELF3
0	0.303687528971262	0.338	0.108	0	0	FDX1
0	0.303679216163402	0.435	0.141	0	0	UBTD2
0	0.30367358497298	0.324	0.102	0	0	ENDOG
0	0.30352089454138	0.478	0.153	0	0	TNPO3
0	0.303167627593111	0.332	0.095	0	0	AGO3
0	0.302820212563165	0.364	0.108	0	0	MAN1B1
0	0.302801492657898	0.51	0.166	0	0	UBE3A
0	0.302771592443165	0.32	0.098	0	0	ST14
0	0.302708115430336	0.527	0.175	0	0	BMI1
0	0.302286242838392	0.272	0.078	0	0	RABGGTA
0	0.30215285458064	0.421	0.135	0	0	EPS8
0	0.30210759075688	0.829	0.407	0	0	ACOX1
0	0.302020277633882	0.642	0.227	0	0	HGS
0	0.301654005929262	0.501	0.158	0	0	GIGYF1
0	0.301617469791847	0.311	0.091	0	0	ANKS4B
0	0.301560019183753	0.336	0.102	0	0	REST
0	0.301415053878489	0.309	0.092	0	0	C3orf38
0	0.301157991346404	0.322	0.094	0	0	ANAPC2
0	0.300680467359151	0.414	0.126	0	0	ILKAP
0	0.300335966697106	0.593	0.207	0	0	NUDCD3
0	0.300300501796705	0.334	0.102	0	0	GFM2
2.55908287651999E-272	0.300243261659464	0.272	0.09	4.28851108447221E-268	0	TIMELESS
0	0.300159825645622	0.71	0.297	0	0	LONP2
0	0.299971582079523	0.286	0.079	0	0	TWSG1
0	0.299924699872955	0.489	0.165	0	0	TMEM14C
0	0.299879677724231	0.338	0.097	0	0	FER

**0**	0.299463494210072	0.415	0.136	0	0	DEPRECATED-ENSG00000099797
**0**	0.29939603235842	0.537	0.181	0	0	ATP2C1
**0**	0.299389346749691	0.398	0.123	0	0	WAC
**0**	0.298548982431274	0.367	0.119	0	0	ADCK2
**0**	0.298500380456212	0.33	0.092	0	0	PDCL3
**0**	0.298187461496721	0.447	0.14	0	0	BCOR
**0**	0.297863251261774	0.412	0.133	0	0	EIF4E
**0**	0.29738393347057	0.604	0.211	0	0	MAPK14
**0**	0.29692869811676	0.469	0.16	0	0	TMEM19
**0**	0.2967062710077	0.408	0.125	0	0	TAF11
**0**	0.296677288325761	0.453	0.148	0	0	ATIC
**0**	0.296570726990702	0.295	0.084	0	0	UMAD1
**0**	0.296563390134255	0.403	0.118	0	0	BMPR2
**0**	0.296438570276173	0.431	0.14	0	0	PSMA4
**0**	0.296409437135921	0.489	0.158	0	0	SPATA20
**0**	0.296391314764785	0.563	0.194	0	0	PRCP
**0**	0.295826967776375	0.444	0.13	0	0	STIM2
**1.01568608837356E-299**	0.295751972913785	0.281	0.089	1.7020867468964E-295	0	C1orf35
**0**	0.295740978614513	0.789	0.374	0	0	TOB1
**0**	0.29508288172515	0.282	0.08	0	0	MIER3
**0**	0.295018496502998	0.347	0.109	0	0	KPNA3
**0**	0.294424700398345	0.472	0.146	0	0	TSPAN4
**0**	0.294325713293508	0.609	0.203	0	0	ZFR
**0**	0.294222856389117	0.369	0.111	0	0	CCDC130
**0**	0.293950017394841	0.322	0.094	0	0	ZKSCAN8
**0**	0.293234969462135	0.336	0.099	0	0	MFHAS1
**0**	0.293038122123549	0.481	0.171	0	0	DNAJA1
**0**	0.29291427758815	0.549	0.186	0	0	LRPPRC
**0**	0.292850559270674	0.515	0.179	0	0	TOMM70
**0**	0.292227401433489	0.43	0.132	0	0	LHFPL2
**0**	0.292159937022121	0.464	0.147	0	0	PIP4K2B
**0**	0.29212239239299	0.663	0.241	0	0	ITCH
**0**	0.292097609697274	0.507	0.166	0	0	MAEA
**0**	0.292022214766479	0.439	0.147	0	0	SLC7A2
**0**	0.292021232187202	0.321	0.101	0	0	CPEB2
**0**	0.291843102475701	0.573	0.211	0	0	MOCS2
**0**	0.291749839893422	0.392	0.115	0	0	CEBPZ
**0**	0.29126290943784	0.631	0.229	0	0	UPF1
**0**	0.2912180539205	0.461	0.156	0	0	RSRC1
**0**	0.291051678181661	0.509	0.175	0	0	PHACTR4
**0**	0.290890461852538	0.394	0.128	0	0	GANAB
**0**	0.290860921433454	0.489	0.181	0	0	DEPRECATED-ENSG00000132646
**0**	0.2907928513713	0.293	0.088	0	0	PDE6D
**0**	0.290695175727214	0.277	0.078	0	0	KLHL7
**0**	0.290397438990587	0.31	0.097	0	0	BRIX1
**0**	0.290009384721345	0.421	0.131	0	0	ROCK2
**0**	0.289679123812209	0.489	0.157	0	0	RC3H1
**2.55316278533667E-278**	0.289628853533474	0.252	0.077	4.2785901956672E-274	0	C17orf58
**0**	0.289457890956392	0.558	0.192	0	0	CLIP1
**0**	0.28937473308016	0.526	0.178	0	0	SLC35B2
**0**	0.28896029702393	0.458	0.137	0	0	OSBPL2
**0**	0.288660378431834	0.393	0.121	0	0	PRSS3
**1.2323085377183E-291**	0.288546096870867	0.281	0.091	2.06510264750833E-287	0	ZC3H13
**6.26956963178313E-301**	0.288503742855718	0.269	0.083	1.05065447889422E-296	0	ATP5MD
**0**	0.288221282111482	0.503	0.164	0	0	RCC1L
**0**	0.288128220248479	0.491	0.163	0	0	IFT27
**0**	0.287897380935267	0.402	0.123	0	0	AKAP8L
**2.14816314044166E-305**	0.287770236278956	0.257	0.075	3.59989179075214E-301	0	CD320
**0**	0.287078766591233	0.601	0.205	0	0	COPB1
**0**	0.286940979054151	0.307	0.093	0	0	RNF146

**0**	0.286902596949569	0.484	0.149	0	0	GOLGA3
**0**	0.286841053150205	0.773	0.345	0	0	HADHA
**0**	0.286519049741429	0.47	0.144	0	0	SKIL
**0**	0.28532707158603	0.541	0.175	0	0	USP24
**0**	0.285113026376073	0.386	0.115	0	0	PREPL
**0**	0.284539303451364	0.335	0.104	0	0	NUP188
**0**	0.284454400639217	0.546	0.19	0	0	SLC43A1
**3.61998118608698E-298**	0.28424574479368	0.268	0.082	6.06636447164456E-294	0	POLR3C
**0**	0.283892056460959	0.401	0.128	0	0	PPP2CA
**0**	0.283750520688938	0.409	0.135	0	0	MESD
**0**	0.283597121183615	0.323	0.09	0	0	MIB2
**0**	0.283557802641724	0.447	0.141	0	0	DNAJC11
**0**	0.28354364242964	0.346	0.104	0	0	TAF4
**0**	0.283026344893187	0.354	0.109	0	0	YIPF6
**0**	0.283005920996097	0.328	0.101	0	0	PAK4
**0**	0.282741964074252	0.253	0.061	0	0	BTBD9
**0**	0.282703546455457	0.351	0.109	0	0	ARPP19
**0**	0.282013521358271	0.774	0.313	0	0	HTATSF1
**0**	0.28166990177123	0.272	0.072	0	0	LMF1
**0**	0.281463121032024	0.573	0.194	0	0	MMP14
**0**	0.281059277182649	0.443	0.144	0	0	CHMP1A
**0**	0.280805344853329	0.358	0.111	0	0	UBL4A
**0**	0.280106600377561	0.269	0.079	0	0	DEPRECATED-ENSG00000164164
**0**	0.279821337573364	0.604	0.208	0	0	SMARCA5
**0**	0.279712460271115	0.488	0.16	0	0	USP32
**0**	0.279502326483436	0.575	0.198	0	0	RAF1
**1.37627258737366E-287**	0.279204119373178	0.257	0.079	2.30635760192078E-283	0	DTNBP1
**0**	0.278942082934949	0.746	0.318	0	0	CIAO2A
**0**	0.278851191327048	0.328	0.096	0	0	PPID
**0**	0.278532555453244	0.496	0.17	0	0	AHCY
**0**	0.278305872869274	0.42	0.13	0	0	GATD3B
**0**	0.278297018892274	0.365	0.105	0	0	TIMM10B
**2.74032396245682E-251**	0.278192788839608	0.943	0.556	4.59223489628514E-247	0	CFH
**0**	0.27818811163401	0.518	0.18	0	0	METTL23
**0**	0.278137119529588	0.609	0.196	0	0	MECP2
**0**	0.278109342549611	0.524	0.193	0	0	DEPRECATED-ENSG00000067064
**0**	0.277304432310737	0.49	0.18	0	0	AP1S1
**0**	0.277114326228993	0.328	0.1	0	0	CCDC93
**0**	0.276031736042491	0.618	0.236	0	0	CCT6A
**0**	0.275527749588467	0.316	0.1	0	0	CHRAC1
**0**	0.275343463508986	0.499	0.15	0	0	PIKFYVE
**0**	0.275284596291766	0.628	0.224	0	0	OTULIN
**0**	0.275266824526417	0.491	0.163	0	0	PALLD
**0**	0.275260584530029	0.472	0.149	0	0	SOAT1
**0**	0.275087417499139	0.747	0.298	0	0	ZNRF2
**0**	0.275050193893561	0.583	0.205	0	0	PPP4R3B
**0**	0.275015604759856	0.255	0.074	0	0	TAB1
**0**	0.274613559474638	0.432	0.138	0	0	MAN1A2
**0**	0.274437509710557	0.786	0.333	0	0	SHC1
**0**	0.273868270343681	0.468	0.15	0	0	WIZ
**0**	0.273696169363326	0.523	0.182	0	0	GORASP2
**0**	0.273619479619702	0.434	0.14	0	0	VARS
**0**	0.273506887673689	0.466	0.148	0	0	SGPP1
**0**	0.273299511535141	0.62	0.242	0	0	COX16
**0**	0.273056935798145	0.466	0.145	0	0	WDR13
**0**	0.273012668185719	0.483	0.169	0	0	KDELR1
**0**	0.272911021166817	0.336	0.108	0	0	PHAX
**0**	0.272607676827119	0.48	0.163	0	0	VPS26C
**0**	0.272263864590712	0.657	0.234	0	0	NRDC
**0**	0.272157151347742	0.41	0.129	0	0	PHKG2

0	0.272125221801747	0.586	0.213	0	0	NNT
0	0.271856320336527	0.318	0.101	0	0	SEC61G
0	0.27158824806888	0.366	0.121	0	0	C9orf64
0	0.271476911504953	0.628	0.216	0	0	FZR1
0	0.27125141130269	0.607	0.21	0	0	ANAPC16
0	0.271135806922318	0.451	0.14	0	0	DEAF1
6.02468815088942E-296	0.271060326050295	0.855	0.451	1.00961724032605E-291	0	DEPRECATED-ENSG00000198074
0	0.270919943597846	0.48	0.134	0	0	NOTCH1
0	0.270486122569697	0.319	0.096	0	0	SBNO1
0	0.270364259217013	0.541	0.196	0	0	CDH1
0	0.270131073646288	0.406	0.132	0	0	HSPB11
0	0.270122285134068	0.418	0.144	0	0	GBE1
4.0310195171535E-276	0.26942660200638	0.26	0.082	6.75518250684584E-272	0	XPOT
0	0.269294200956543	0.307	0.09	0	0	MED22
0	0.269244809547356	0.531	0.177	0	0	ITFG1
4.599223808544E-262	0.268946781432749	0.861	0.467	7.70737925835803E-258	0	APLP2
0	0.268749901939782	0.42	0.131	0	0	PCYOX1
0	0.268334031064956	0.702	0.256	0	0	NUFIP2
0	0.268267598498183	0.485	0.155	0	0	TYK2
0	0.267965815903301	0.817	0.314	0	0	SYNE2
0	0.267943704313542	0.27	0.08	0	0	RAE1
0	0.267607178145807	0.381	0.119	0	0	MIB1
0	0.267385349907288	0.491	0.167	0	0	EIF2S1
0	0.26734322955482	0.459	0.153	0	0	SMAD5
0	0.267234116449909	0.478	0.155	0	0	QTRT1
0	0.266862235928063	0.5	0.168	0	0	VAV2
0	0.266693834547297	0.466	0.145	0	0	HTT
0	0.266349512534057	0.391	0.127	0	0	RAB4B
0	0.266054910705656	0.388	0.122	0	0	RAPGEF2
0	0.265708096382054	0.336	0.102	0	0	PRR12
0	0.265579887399878	0.472	0.147	0	0	PRPF4B
0	0.265171341474592	0.404	0.119	0	0	ABCD4
0	0.264854578185538	0.537	0.187	0	0	USP14
0	0.264840599687608	0.26	0.069	0	0	RCHY1
0	0.264616524408821	0.743	0.313	0	0	LAGE3
0	0.264588072219382	0.571	0.194	0	0	RAB20
0	0.264177750600105	0.778	0.345	0	0	P4HB
0	0.264025061814931	0.623	0.224	0	0	AGPAT3
0	0.263611859330092	0.292	0.087	0	0	NFRKB
0	0.26352010860198	0.719	0.296	0	0	SLBP
0	0.263464402187861	0.328	0.098	0	0	DPY19L1
0	0.263098951399827	0.671	0.289	0	0	SERPINB1
0	0.26278617767506	0.61	0.227	0	0	MLX
0	0.262655766106517	0.466	0.152	0	0	DELE1
0	0.262498673516809	0.652	0.219	0	0	TIA1
0	0.262196542054412	0.463	0.152	0	0	UBE2J2
0	0.262120431046781	0.582	0.183	0	0	KMT2D
0	0.261776688425114	0.582	0.215	0	0	TJP1
0	0.261474300358955	0.423	0.138	0	0	CITED4
0	0.261324143204776	0.75	0.321	0	0	IRF2BP2
0	0.261312271348412	0.575	0.173	0	0	TAF1D
0	0.261263282811472	0.506	0.168	0	0	PNP
0	0.261207475567719	0.335	0.113	0	0	ICMT
0	0.260852420458824	0.376	0.101	0	0	C7orf26
0	0.260716220039417	0.394	0.14	0	0	ZNF680
0	0.260686133206288	0.525	0.176	0	0	TBC1D14
0	0.26051074396733	0.274	0.08	0	0	HEXD
0	0.260171112014573	0.548	0.204	0	0	HMGCR
0	0.259953891563103	0.344	0.092	0	0	ZZEF1
1.70226356163997E-307	0.25962515363438	0.282	0.088	2.85265327659627E-303	0	ALG8

0	0.259563956730303	0.37	0.122	0	0	NAXD
0	0.25951380721114	0.519	0.177	0	0	TTC1
0	0.259197569912852	0.493	0.171	0	0	NDUFA7
0	0.259051224092121	0.583	0.19	0	0	MED28
0	0.25867367694151	0.322	0.1	0	0	GTF2H5
0	0.258568850656734	0.277	0.084	0	0	POLR2D
0	0.258529264288636	0.446	0.138	0	0	ARL8B
0	0.258093854016638	0.462	0.159	0	0	ARL 1.00
0	0.257927602946312	0.349	0.111	0	0	DHX8
0	0.257714397340596	0.692	0.258	0	0	SMG5
0	0.257452308693274	0.359	0.115	0	0	HSD17B12
0	0.257386210922293	0.379	0.112	0	0	UBE2O
0	0.257157733490585	0.623	0.217	0	0	ECE1
0	0.257065187200828	0.302	0.086	0	0	ZNF76
0	0.256911043329091	0.331	0.097	0	0	AASDHPPT
0	0.256788869944531	0.725	0.283	0	0	PTOV1
0	0.256649170812825	0.466	0.149	0	0	KAT7
0	0.256479804361497	0.63	0.235	0	0	LMBRD1
0	0.256426675105319	0.404	0.134	0	0	FN3KRP
0	0.256309311779865	0.264	0.075	0	0	BFAR
0	0.256258334578318	0.615	0.233	0	0	GOLGA7
0	0.256159275845739	0.451	0.142	0	0	EAPP
0	0.255565139420744	0.283	0.081	0	0	BICRA
0	0.25544455744033	0.345	0.109	0	0	OSER1
0	0.255258939519926	0.509	0.156	0	0	ARID4B
0	0.254681537807594	0.433	0.14	0	0	DARS
0	0.254655646898029	0.718	0.277	0	0	TMPO
0	0.254643730783096	0.271	0.079	0	0	C5orf22
0	0.254192693361551	0.591	0.21	0	0	NFIA
0	0.254148523175831	0.561	0.2	0	0	CLPTM1L
0	0.254109638215984	0.486	0.15	0	0	FAM111A
7.68380681693928E-293	0.253875931137145	0.252	0.075	1.28765234638269E-288	0	IRAK2
0	0.25387190211	0.271	0.08	0	0	TUT1
0	0.253793858295381	0.414	0.135	0	0	CHMP5
0	0.253306831269513	0.475	0.157	0	0	ALDH5A1
0	0.253278735208754	0.616	0.229	0	0	NAA60
0	0.253150244439801	0.736	0.299	0	0	DSP
0	0.253097111735744	0.501	0.168	0	0	VPS35L
0	0.253027937504481	0.311	0.102	0	0	ZNF22
0	0.252627284980665	0.718	0.24	0	0	EHBP1L1
0	0.252558029903817	0.53	0.184	0	0	SNRPA
0	0.252399408096624	0.363	0.105	0	0	TRAF3
0	0.252283000755382	0.37	0.121	0	0	HSPA4
0	0.252130320921093	0.411	0.127	0	0	BOD1L1
0	0.252063083648633	0.318	0.103	0	0	AGA
0	0.251557292570082	0.373	0.112	0	0	SLC25A28
0	0.251428184283021	0.314	0.094	0	0	SIKE1
0	0.251371821308239	0.381	0.12	0	0	ERI3
0	0.251041511248315	0.344	0.105	0	0	SFT2D1
0	0.250814258288993	0.308	0.096	0	0	LRRC57
0	0.250763762495089	0.389	0.123	0	0	AVL9
0	0.250559978666098	0.423	0.134	0	0	TASOR
0	0.250394508305298	0.417	0.139	0	0	GALE
0	0.250376837224378	0.633	0.242	0	0	CALU
0	0.250180804735559	0.439	0.148	0	0	NELFB
0	5.79530040073846	0.538	0.02	0	1	CHI3L1
0	5.48388114216919	0.7	0.036	0	1	HAMP
0	5.47973471471227	0.376	0.009	0	1	SLC25A47
0	5.42130631924137	0.623	0.024	0	1	SERPINA11
0	5.3861254850573	0.264	0.006	0	1	LECT2

0	5.2312584860078	0.741	0.036	0	1	ADH4
0	5.04592311910205	0.319	0.01	0	1	F9
0	5.0093704041388	0.413	0.039	0	1	HSD17B13
0	4.87761822062975	0.848	0.07	0	1	IGFBP1
0	4.72750794994065	0.404	0.017	0	1	HSD11B1
0	4.61947744759227	0.339	0.018	0	1	ANGPTL8
0	4.59551017257579	0.909	0.153	0	1	CYP 20.00
0	4.57033108070795	0.866	0.077	0	1	AHSG
0	4.54020985595326	0.998	0.365	0	1	APOA2
0	4.519716628013	0.372	0.066	0	1	DEPRECATED-ENSG00000160868
0	4.42357802077534	0.254	0.011	0	1	EVA1A
0	4.35018749304429	0.338	0.017	0	1	GCGR
0	4.34090517271107	0.288	0.033	0	1	SLC10A1
0	4.31731122894119	0.301	0.023	0	1	CYP8B1
0	4.29792964088494	0.467	0.032	0	1	AFM
0	4.27642627414218	0.328	0.021	0	1	HJV
0	4.22429406821571	0.433	0.024	0	1	NR1I3
0	4.15969763228382	0.393	0.046	0	1	ABCB4
0	4.14598507324597	0.251	0.023	0	1	DEPRECATED-ENSG00000255974
0	4.14274796851427	0.54	0.035	0	1	APOC4
0	4.13799375083005	0.294	0.024	0	1	SERPINA4
0	4.12004414700609	0.756	0.076	0	1	APCS
0	4.05858161401287	0.972	0.172	0	1	HP
0	4.04970400336147	0.26	0.054	0	1	CYP3A4
0	4.0183905456637	0.738	0.199	0	1	ALAS1
0	3.96113646976633	0.805	0.088	0	1	HPR
0	3.95953828832092	0.401	0.027	0	1	CDO1
0	3.94526767973777	0.508	0.113	0	1	CYP2C9
0	3.89592357186651	0.636	0.14	0	1	ADH1B
0	3.89184146230097	0.596	0.156	0	1	G0S2
0	3.86989889002846	0.402	0.057	0	1	SLC22A7
0	3.81501789684658	0.314	0.048	0	1	CFHR5
0	3.77507113754219	0.829	0.237	0	1	AZGP1
0	3.74978774906629	0.308	0.029	0	1	ANGPTL3
0	3.72823806565873	0.908	0.105	0	1	HRG
0	3.63142173865117	0.589	0.057	0	1	SAA4
0	3.60830751470141	0.302	0.027	0	1	HGFAC
0	3.57925712966151	0.472	0.05	0	1	HPD
0	3.52707182458222	0.523	0.086	0	1	APOM
0	3.38772023551596	0.372	0.037	0	1	FGF21
0	3.37682980109079	0.865	0.291	0	1	SERPINC1
0	3.35604964572329	0.931	0.311	0	1	ITIH3
0	3.30330996001504	0.44	0.046	0	1	ID1
0	3.29343945343964	0.462	0.044	0	1	C9
0	3.28334572622629	0.853	0.144	0	1	HPX
0	3.2607205070499	0.964	0.519	0	1	FGB
0	3.1909655346156	0.85	0.403	0	1	PCK1
0	3.19004431926088	0.304	0.041	0	1	CHRD
0	3.16972087965217	0.618	0.146	0	1	MST1
0	3.16363179349643	0.446	0.085	0	1	RDH16
0	3.14796648170468	0.886	0.346	0	1	SERPING1
0	3.05219792173996	0.631	0.234	0	1	G6PC
0	3.04500522868838	0.434	0.047	0	1	MASP2
0	3.03676778212658	0.759	0.114	0	1	COL18A1
0	3.02407973193643	0.813	0.122	0	1	SERPINA3
0	3.01761238254829	0.466	0.17	0	1	ADH6
0	2.99996021640011	0.826	0.403	0	1	LEAP2
0	2.96553774108969	0.398	0.071	0	1	EGR1
0	2.95044115550234	0.3	0.097	0	1	CIDEB
0	2.9424621108394	0.628	0.161	0	1	CFHR2

0	2.91066578174331	0.448	0.133	0	1	MT2A
0	2.90883478983052	0.757	0.383	0	1	CYP2C8
0	2.90777880274286	0.984	0.669	0	1	FGA
0	2.90505670172939	0.468	0.12	0	1	TFR2
0	2.90203646113635	0.88	0.201	0	1	ITIH2
0	2.88481821209937	0.309	0.039	0	1	ALDH8A1
0	2.87037731895404	0.614	0.171	0	1	TAT
0	2.81049566426713	0.663	0.188	0	1	ITIH4
0	2.80521230069597	0.56	0.091	0	1	SLC38A3
0	2.80414547258871	0.392	0.05	0	1	MBL2
0	2.7995660693934	0.628	0.199	0	1	C5
6.8417174132259E-202	2.76977639601488	0.264	0.122	1.1465350041084E-197	1	DEFB1
0	2.75102730641524	0.963	0.255	0	1	APOE
0	2.71084088459153	0.329	0.104	0	1	FXYD1
0	2.70446469174371	0.972	0.648	0	1	FGG
0	2.68725747169156	0.57	0.278	0	1	PON3
0	2.68274280349564	0.514	0.153	0	1	PROC
0	2.6707320991773	0.872	0.394	0	1	C1R
0	2.66977644327906	0.893	0.382	0	1	CP
0	2.66410817934543	0.433	0.051	0	1	SLC38A4
0	2.64168217274197	0.538	0.141	0	1	IGFBP2
0	2.64032468431976	0.356	0.136	0	1	SERPINE1
0	2.63549386605732	0.302	0.069	0	1	RND1
0	2.6310768549865	0.982	0.729	0	1	RBP4
0	2.62286126619159	0.349	0.136	0	1	SLC27A5
0	2.60721113570489	0.978	0.724	0	1	APOA1
0	2.60603881348541	0.299	0.054	0	1	CPED1
0	2.56791766813226	0.399	0.175	0	1	ACADSB
0	2.54946419077642	0.995	0.92	0	1	SERPINA1
0	2.53919099053942	0.304	0.062	0	1	GCKR
0	2.53441759383577	0.434	0.119	0	1	TM4SF5
0	2.51426116443385	0.326	0.088	0	1	IL1RN
0	2.49874795721567	0.499	0.202	0	1	GADD45A
0	2.46889164204325	0.756	0.29	0	1	APOC1
0	2.45544458583821	0.398	0.202	0	1	SLC30A10
0	2.45298785650581	0.637	0.326	0	1	SERPINA10
0	2.4197364214643	0.354	0.142	0	1	RORC
0	2.3777004973789	0.782	0.399	0	1	F2
0	2.36777207780098	0.85	0.524	0	1	PLG
0	2.34239031452539	0.966	0.656	0	1	APOB
0	2.33016236793167	0.934	0.612	0	1	KNG1
0	2.32075332052412	0.65	0.259	0	1	NNMT
0	2.30946485740576	0.801	0.399	0	1	FGL1
0	2.30073907199961	0.693	0.408	0	1	POR
5.02352327776756E-165	2.29963640742915	0.258	0.13	8.41842030888287E-161	1	CYP2J2
0	2.24552491931686	0.406	0.185	0	1	F7
0	2.22039153344916	0.805	0.31	0	1	ITIH1
0	2.21534853808708	0.467	0.191	0	1	AQP9
3.55499540668958E-238	2.20956808248754	0.377	0.215	5.9574613025304E-234	1	MTTP
0	2.19217913925586	0.429	0.22	0	1	CXCL2
0	2.19058788077581	0.985	0.716	0	1	ORM1
2.11676561352448E-208	2.16657527473055	0.27	0.121	3.54727581514433E-204	1	ACSM5
0	2.14956857921295	0.552	0.313	0	1	PPP2R1B
0	2.14952526481368	0.303	0.06	0	1	MASP1
1.7734785772827E-193	2.14581949074139	0.278	0.134	2.97199539981034E-189	1	RBP5
0	2.14244566817143	0.696	0.46	0	1	INSIG1
0	2.13360616365756	0.919	0.62	0	1	ORM2
7.20356151942271E-91	2.12668746897621	0.27	0.18	1.20717283942486E-86	1	PPARGC1A
0	2.12460012993409	0.618	0.382	0	1	DCXR
3.92290722692959E-192	2.12431912359562	0.33	0.184	6.5740079308886E-188	1	DCPS

0	2.08563419963477	0.943	0.755	0	1	FABP1
1.02327492317647E-228	2.08242850567475	0.513	0.386	1.71480411625913E-224	1	HMGCS1
0	2.08001415254865	0.696	0.242	0	1	LBP
0	2.06980948394073	0.444	0.253	0	1	RGN
0	2.06300635720832	0.739	0.376	0	1	BHLHE40
0	2.05828809645967	0.654	0.393	0	1	C8A
0	2.05481443580245	0.985	0.807	0	1	APOC3
0	2.04777549244623	0.457	0.159	0	1	SLC9A3R2
2.74719956724414E-179	2.03972656982324	0.371	0.236	4.60375703478773E-175	1	SEC14L2
1.39591719037854E-142	2.03508537177657	0.285	0.164	2.33927802763636E-138	1	PEMT
1.14152268104999E-248	2.02810242483295	0.361	0.185	1.91296370890357E-244	1	GBP1
0	2.01697706004423	0.793	0.548	0	1	ALDH2
6.41973758804866E-219	1.99907104523714	0.383	0.225	1.07581962500519E-214	1	SLC25A25
0	1.99352947537882	0.579	0.329	0	1	ASGR1
0	1.98623921216507	0.921	0.643	0	1	TTR
5.5985327679279E-121	1.98157155519044	0.277	0.167	9.38202121249357E-117	1	DEPRECATED-ENSG00000187758
0	1.97637846017752	0.952	0.635	0	1	CFB
0	1.97017388421037	0.341	0.143	0	1	HES1
9.88801835595245E-164	1.96360931929925	0.308	0.173	1.65703411609051E-159	1	PPP1R3C
0	1.96057621748442	0.516	0.2	0	1	ATF3
5.20290442926924E-185	1.95254776106362	0.305	0.16	8.71902724256939E-181	1	KLF10
0	1.94507412286159	0.867	0.499	0	1	C1S
0	1.91421514166381	0.492	0.301	0	1	PON1
3.0394984396034E-161	1.91365389914849	0.278	0.145	5.09359148508738E-157	1	TRIP6
0	1.90937741253516	0.48	0.289	0	1	CYP27A1
0	1.90666042701079	0.806	0.536	0	1	MLXIPL
0	1.90328218986754	0.632	0.427	0	1	ACAT1
1.09465104875897E-268	1.89684980766679	0.429	0.25	1.83441622751027E-264	1	ORMDL3
5.35605572522069E-109	1.89203236834761	0.316	0.217	8.97567818432484E-105	1	ENPP1
0	1.88550980597281	0.726	0.45	0	1	SLC2A2
0	1.88034442021204	0.508	0.33	0	1	SLC30A1
0	1.87160553368458	0.569	0.366	0	1	ADH1A
0	1.85705191806136	0.535	0.319	0	1	C8B
6.94469345353842E-303	1.83875699753196	0.258	0.082	1.16379172894397E-298	1	SLC25A18
2.72306850258636E-73	1.83790828491475	0.25	0.17	4.56331819663422E-69	1	ACAD11
0	1.82323793756446	0.355	0.059	0	1	CPS1
0	1.81687799288141	0.585	0.364	0	1	GLYCTK
9.05760612674837E-263	1.81577760468095	0.487	0.325	1.51787363472049E-258	1	F11
3.44180792928346E-79	1.80948883853092	0.276	0.193	5.76778172789322E-75	1	SLCO1B1
1.0406726852974E-117	1.80779448165402	0.321	0.213	1.74395928602139E-113	1	HLF
9.69312492437938E-253	1.80337261560734	0.383	0.203	1.6243738748275E-248	1	REEP6
0	1.77275050729234	0.974	0.74	0	1	APOC2
0	1.76662922586678	0.654	0.356	0	1	SERPINF2
0	1.75693002003662	0.496	0.094	0	1	ARG1
1.58036748163953E-255	1.75241687128995	0.495	0.347	2.64837982573153E-251	1	EHHADH
1.58445544737346E-169	1.74040178584711	0.538	0.449	2.65523043870844E-165	1	FASN
0	1.73231792670301	0.564	0.392	0	1	DEPRECATED-ENSG00000147872
7.62081139930638E-283	1.72789367452234	0.523	0.369	1.27709557429576E-278	1	MPC1
0	1.72761408577001	0.875	0.649	0	1	C4BPA
5.35078755905884E-278	1.72681881299181	0.507	0.349	8.9668497914708E-274	1	FUOM
0	1.71645653873522	0.476	0.235	0	1	DEPRECATED-ENSG00000115738
1.83801885256524E-54	1.70685820204343	0.262	0.2	3.08015199312884E-50	1	AGMO
0	1.69729564328101	0.957	0.779	0	1	AMBP
0	1.693780163629	0.817	0.599	0	1	PRAP1
5.11147533849502E-101	1.69257034472757	0.273	0.173	8.56581037224995E-97	1	ACY1
0	1.68742123482119	0.903	0.628	0	1	AGXT
0	1.6753918836069	0.563	0.378	0	1	PCBD1
0	1.67454125344249	0.743	0.477	0	1	LRG1
8.82600869144031E-117	1.6670151413201	0.28	0.168	1.47906253651157E-112	1	PDK4
0	1.66291507770042	0.621	0.422	0	1	BHMT2

4.12441882316562E-265	1.66093002780622	0.483	0.311	6.91170106386094E-261	1	F10
2.2855076753024E-99	1.64384755490279	0.363	0.273	3.83005376227176E-95	1	LDLR
0	1.64341014138221	0.788	0.562	0	1	JUN
0	1.63258981881459	0.59	0.396	0	1	MAT1A
5.51233472956226E-244	1.63116208608941	0.499	0.352	9.23757053980044E-240	1	ABAT
0	1.61948072331852	0.604	0.308	0	1	PLPP3
2.89778301201753E-81	1.61875551796649	0.456	0.419	4.85610477153898E-77	1	CYP2B6
1.96243284423257E-83	1.61082498829929	0.331	0.253	3.28864496036494E-79	1	KLKB1
0	1.60703746513946	0.769	0.548	0	1	EFNA1
1.6192216503756E-182	1.591996845332	0.37	0.222	2.71349164169942E-178	1	NFIL3
0	1.5850415843285	0.479	0.122	0	1	CRP
0	1.57519969659021	0.625	0.456	0	1	ANG
2.7278049398636E-190	1.5743686787427	0.478	0.351	4.57125551822342E-186	1	ARHGEF10L
0	1.57079925799897	0.551	0.129	0	1	CD14
0	1.56290766561402	0.594	0.428	0	1	CFI
1.06374715665137E-95	1.56200413676052	0.355	0.267	1.78262748511636E-91	1	CGN
0	1.5583475479178	0.604	0.375	0	1	SERPINF1
0	1.55816324138305	0.856	0.583	0	1	CYP3A5
1.78633086853306E-94	1.55455200236431	0.358	0.272	2.9935332694877E-90	1	RAB17
1.27152719065066E-40	1.55124052144802	0.311	0.273	2.13082526609237E-36	1	HMGCR
0	1.55037643218161	0.698	0.531	0	1	ECHS1
1.86690255498513E-213	1.54946484427957	0.389	0.222	3.12855530164409E-209	1	ID2
4.97976171670879E-45	1.54320905045274	0.283	0.231	8.34508468486059E-41	1	CEBPA
3.47254380128286E-57	1.53815923413324	0.31	0.247	5.81928890218982E-53	1	EBNA1BP2
3.0643658165557E-206	1.53518802672408	0.396	0.232	5.13526423538404E-202	1	CREB3L3
4.82189681422194E-115	1.52703607560653	0.286	0.171	8.08053468127313E-111	1	PLIN4
9.41258055646425E-294	1.5262087105681	0.406	0.199	1.57736024965228E-289	1	MTSS2
0	1.52421133129243	0.74	0.564	0	1	PTMS
3.966012629644E-84	1.52092348391073	0.369	0.292	6.64624396475741E-80	1	ADHFE1
5.18414737258148E-172	1.51566252531232	0.341	0.194	8.68759416697205E-168	1	RGS3
0	1.5064837770047	0.673	0.506	0	1	DDT
4.08494147323455E-307	1.50618976344957	0.574	0.408	6.84554492084646E-303	1	BDH1
4.19447107298971E-61	1.50224870167881	0.264	0.191	7.02909462411616E-57	1	F13B
0	1.49832231369434	0.675	0.485	0	1	SERPIND1
7.55737449765881E-117	1.48412481047864	0.401	0.307	1.26646481831766E-112	1	ECI2
4.8608984986309E-186	1.46092962592512	0.493	0.378	8.14589370400566E-182	1	HAO1
0	1.45701045117989	0.681	0.516	0	1	FMO3
4.06846621765106E-104	1.45695458459659	0.389	0.304	6.81793568753965E-100	1	ALDH6A1
3.21243877719143E-78	1.45099372860502	0.356	0.286	5.38340490281739E-74	1	ACADM
1.50859371699622E-126	1.45061745616762	0.348	0.228	2.52810135094226E-122	1	PLIN5
5.97030721565445E-153	1.4313735137488	0.29	0.154	1.00050408319937E-148	1	DUSP10
0	1.41827667441824	0.838	0.662	0	1	CYB5A
1.92591057514471E-80	1.40564256351077	0.332	0.245	3.2274409418275E-76	1	OSGIN1
4.89842311951206E-191	1.40083808648921	0.541	0.443	8.2087774636783E-187	1	ACAA1
5.82556581078862E-150	1.39702314341715	0.449	0.342	9.76248318571958E-146	1	GRHPR
1.47954751834653E-235	1.39305170653074	0.498	0.332	2.47942573124511E-231	1	DPYS
0	1.38851073964602	0.789	0.444	0	1	PAH
7.6304941691856E-69	1.38755980285202	0.308	0.23	1.27871821287212E-64	1	PIPOX
0	1.38170240679126	0.64	0.483	0	1	ETS2
9.35279710611565E-244	1.36496947701791	0.542	0.395	1.56734173904286E-239	1	CCNL1
0	1.35373201411646	0.706	0.544	0	1	ADH1C
7.85782770389393E-116	1.35074833009453	0.291	0.173	1.31681476661854E-111	1	SERPINH1
0	1.34428639770593	0.966	0.772	0	1	TF
1.39338291543143E-103	1.32082524109445	0.421	0.338	2.33503108967998E-99	1	UGT1A1
3.44613977062559E-33	1.31540746779221	0.326	0.302	5.77504102761436E-29	1	UAP1
1.8092405097747E-131	1.30456256746377	0.354	0.227	3.03192524628045E-127	1	PABPC1L
0	1.28090686526408	0.855	0.68	0	1	APOH
1.07086597890515E-175	1.2794849044083	0.511	0.384	1.79455720744926E-171	1	ECHDC2
3.98666852405786E-74	1.26592136528852	0.374	0.307	6.68085911261616E-70	1	ZNHIT1
1.34620490487971E-77	1.26225629744055	0.373	0.294	2.25597017959742E-73	1	SLC6A1

8.38502392224952E-98	1.26033015913909	0.342	0.242	1.40516230889057E-93	1	NFKBIZ
0	1.25711494635567	0.82	0.631	0	1	GLUL
6.83354051132488E-46	1.25330544734453	0.353	0.318	1.14516471888782E-41	1	PECR
6.17880771301339E-38	1.24852920199135	0.316	0.278	1.03544459654678E-33	1	GOT2
1.97695259407159E-83	1.24096083871745	0.438	0.384	3.31297715714517E-79	1	MTHFD1
1.84066066510693E-39	1.23875406679301	0.341	0.308	3.08457914258619E-35	1	MTHFS
0	1.23840434014246	0.664	0.53	0	1	TMEM176A
4.77435431189728E-102	1.23224450015411	0.497	0.433	8.00086295587746E-98	1	SLC39A14
7.9165020220783E-37	1.22123752087838	0.282	0.234	1.32664740885988E-32	1	NCOA7
7.51448420386897E-12	1.21868879367741	0.25	0.242	1.25927726288436E-07	1	UGP2
4.49206937752136E-54	1.21591324923426	0.383	0.343	7.5278098628503E-50	1	ETFA
2.57682664214347E-133	1.21250615193724	0.352	0.226	4.31824608690403E-129	1	ARRDC3
0	1.21204537778543	0.74	0.569	0	1	C4BPB
2.82223520362449E-194	1.20419137423794	0.569	0.475	4.72950175423392E-190	1	NFE2L2
2.87244110675097E-48	1.20261571996311	0.368	0.331	4.81363680669328E-44	1	NADK2
3.53094729318591E-18	1.17606117653144	0.295	0.285	5.91716147392095E-14	1	ERBB3
6.02408114953686E-35	1.16697297627773	0.312	0.273	1.00951551903939E-30	1	DEPRECATED-ENSG00000100197
3.03916804007954E-80	1.16638210010158	0.417	0.353	5.0930378015653E-76	1	HSPE1
0	1.16069083749731	0.873	0.725	0	1	TMEM176B
1.3660700995715E-187	1.15227804146688	0.289	0.135	2.28926027286192E-183	1	TMEM140
1.81726826667632E-20	1.13604229001191	0.294	0.277	3.04537816129618E-16	1	DEPRECATED-ENSG00000109181
3.39846053211353E-26	1.13047517159373	0.317	0.29	5.69514015971585E-22	1	COL27A1
9.08365474230422E-55	1.12391261314892	0.389	0.344	1.52223886171534E-50	1	SERINC2
7.82095399765888E-59	1.11600802855857	0.438	0.405	1.31063547092768E-54	1	ASL
0	1.11106517101791	0.584	0.286	0	1	A2M
7.43628593940393E-32	1.10856460744499	0.325	0.292	1.24617279772531E-27	1	PKLR
3.45206930975869E-208	1.09923040219792	0.662	0.538	5.78497774929361E-204	1	UPB1
9.17951984619405E-27	1.0941410248331	0.281	0.247	1.5383039358252E-22	1	MGMT
7.26940039121487E-16	1.091979319312	0.268	0.253	1.21820611755979E-11	1	SH3BGRL2
3.16070521087285E-156	1.08833913565944	0.529	0.419	5.29670979238072E-152	1	GAMT
3.93318211953002E-278	1.0664620937674	0.656	0.501	6.5912265959084E-274	1	ALDH1L1
1.54787494879607E-14	1.06066614257599	0.302	0.296	2.59392883919245E-10	1	ACAT2
2.56252454846384E-14	1.06045923672212	0.26	0.243	4.29427863831571E-10	1	SARDH
6.1175966027678E-239	1.05824230354282	0.684	0.572	1.02518683869183E-234	1	SULT2A1
1.50124419638021E-66	1.05685910830709	0.442	0.398	2.51578502429396E-62	1	FAH
2.11960499306696E-91	1.05592234380995	0.428	0.349	3.55203404738162E-87	1	IFITM2
1.74943493065328E-13	1.04718892535086	0.269	0.258	2.93170305678876E-09	1	CHDH
4.12742939653028E-119	1.03753934309967	0.485	0.39	6.91674618270545E-115	1	KRT18
2.79987055937045E-113	1.03504608255895	0.523	0.458	4.69202308339301E-109	1	CAT
4.05390314647387E-14	1.0299694043388	0.307	0.302	6.79353089286091E-10	1	SLC17A9
1.64153130194606E-33	1.0261921046373	0.272	0.225	2.75087815580122E-29	1	AHR
3.44052238271409E-27	1.01886492952136	0.361	0.347	5.76562740895227E-23	1	HEBP2
5.92234747688841E-88	1.00117984353772	0.425	0.342	9.9246699017696E-84	1	LRP1
2.97159316197754E-06	1.00034458491794	0.25	0.256	0.0497979582084196	1	HIBADH
2.93831145559748E-27	0.997178803206455	0.28	0.243	4.92402233729027E-23	1	BCL6
2.04337872861861E-19	0.997045379579134	0.285	0.265	3.42429407341906E-15	1	ZFAND2A
1.5965935072164E-09	0.994073875606799	0.282	0.286	2.67557139939325E-05	1	GLUD1
1.21821893688367E-58	0.991202837289773	0.355	0.286	2.04149129442966E-54	1	BCL3
5.41854854944945E-51	0.986100367461631	0.388	0.339	9.08040365916738E-47	1	TCEA3
3.47635849526972E-130	0.980396042945254	0.564	0.5	5.825681566373E-126	1	HSPD1
8.39033465664628E-61	0.979689147366666	0.393	0.338	1.40605228176078E-56	1	GADD45B
3.13663377183488E-79	0.97803616441583	0.364	0.266	5.25637087484089E-75	1	SERPINA5
6.2846201846824E-151	0.971073250012193	0.591	0.511	1.05317665054908E-146	1	SDC4
0	0.963349994396054	0.265	0.07	0	1	MAFB
2.17578655604123E-24	0.958138810654593	0.349	0.338	3.64618311061389E-20	1	PRXL2A
7.50802861237121E-20	0.955918022100903	0.345	0.341	1.25819543486117E-15	1	NMD3
1.70307610798497E-303	0.949615238764554	0.723	0.593	2.85401494176121E-299	1	ASGR2
9.17876529905083E-26	0.945271524495029	0.348	0.329	1.53817748881494E-21	1	TNS3
5.78570168694172E-30	0.934696511207428	0.344	0.317	9.69567888697694E-26	1	SAT2
0	0.913857343996587	0.756	0.632	0	1	RHOB

5.6318936131608E-46	0.913166136490337	0.304	0.245	9.43792731693487E-42	1	IFRD1
0	0.912630816064618	0.963	0.791	0	1	CLU
6.23342616997573E-23	0.904294428468717	0.397	0.391	1.04459755756453E-18	1	MSMO1
8.22912063583077E-08	0.902511146219173	0.273	0.278	0.00137903603615252	1	HMGCL
1.01233647536605E-33	0.900649925004983	0.257	0.204	1.69647346541842E-29	1	TGM2
6.92882781953084E-35	0.89784329773792	0.438	0.428	1.16113296599698E-30	1	EXT1
2.01701576204454E-10	0.896678330156868	0.339	0.357	3.38011501403424E-06	1	AIG1
3.8467088820178E-21	0.890543077753313	0.358	0.346	6.44631474448543E-17	1	ABCC2
1.52028448573838E-42	0.883269206985628	0.442	0.423	2.54769274120037E-38	1	TNFRSF1A
3.84794160196934E-17	0.87853805416414	0.39	0.401	6.44838053658022E-13	1	SLC43A3
3.46436697651139E-71	0.866562164828242	0.544	0.49	5.80558617923779E-67	1	ELF3
0	0.866399598463886	0.92	0.782	0	1	ALDOB
2.3843051633681E-200	0.859677292428223	0.685	0.596	3.99561859277226E-196	1	GATM
5.07398159985607E-15	0.856752386079577	0.375	0.38	8.50297836503879E-11	1	CYP4V2
1.43501340767245E-14	0.856702782299562	0.383	0.399	2.40479546857749E-10	1	MYO18A
3.09519615500726E-48	0.855431183608121	0.45	0.427	5.18692971656117E-44	1	MPST
1.27297354434665E-08	0.854754652449927	0.266	0.261	0.000213324906561611	1	TTPA
4.45468606046695E-116	0.848692538185869	0.569	0.493	7.46516290013051E-112	1	AADAC
7.35610255848034E-16	0.83765235548154	0.347	0.353	1.23273566675014E-11	1	APMAP
3.2840344154789E-87	0.831121281254054	0.563	0.518	5.50338487345954E-83	1	FBP1
1.57969696457271E-15	0.821184844464276	0.319	0.31	2.64725617323095E-11	1	SHFL
4.65797616147316E-225	0.816701946347075	0.75	0.68	7.80583645139672E-221	1	SCP2
5.3363769069124E-88	0.792221414415765	0.573	0.507	8.9427004206038E-84	1	HPN
1.62844637264914E-40	0.79131127503481	0.496	0.489	2.72895043128543E-36	1	F11R
1.6317802039786E-36	0.790869411739835	0.31	0.259	2.73453726582734E-32	1	AKIRIN2
1.7458863208077E-52	0.788417724204034	0.5	0.472	2.92575629640954E-48	1	CPB2
4.69378702952418E-12	0.784547957148383	0.285	0.274	7.86584830407662E-08	1	COMT
3.37132460453881E-28	0.782777647427215	0.438	0.41	5.64966577228614E-24	1	LIPG
1.40413224846005E-47	0.782478285863173	0.484	0.471	2.35304482196935E-43	1	HAGH
3.2050408021977E-26	0.780175496179209	0.423	0.421	5.37100737632291E-22	1	MYO1B
1.74138909263479E-183	0.780151860598344	0.399	0.219	2.91821984143738E-179	1	DEPRECATED-ENSG00000175899
2.24249681362511E-30	0.775108385613874	0.408	0.393	3.75797616027296E-26	1	FNDC3B
2.20688355298355E-296	0.773648797308747	0.772	0.611	3.69829545808983E-292	1	CFH
1.30345510685793E-77	0.766485483092467	0.552	0.507	2.18433006807251E-73	1	H1F0
2.88651195454365E-58	0.766070560081285	0.527	0.468	4.83721673342426E-54	1	PCSK6
2.01362548163363E-09	0.76524834522841	0.301	0.302	3.37443358212163E-05	1	DUSP23
5.9675529464138E-10	0.756018701916243	0.346	0.358	1.00004252276002E-05	1	C8G
2.96962861271062E-15	0.755935584398987	0.182	0.262	4.97650362918045E-11	1	TKFC
6.75309948943669E-37	0.736533045625391	0.434	0.413	1.1316844124398E-32	1	CEBPB
2.03091676512021E-64	0.727954615140348	0.562	0.551	3.40341031498844E-60	1	PEBP1
1.12812324156088E-142	0.724263073130691	0.678	0.591	1.89050892820772E-138	1	HSD17B6
5.65018995408014E-28	0.722505003314112	0.445	0.436	9.46858832504749E-24	1	PON2
2.09186127135405E-79	0.703471612061296	0.583	0.539	3.50554111853512E-75	1	CLDN1
4.7842801518826E-07	0.701394165097661	0.363	0.394	0.00801749667852485	1	LONP2
1.82093778751215E-10	0.692559391048298	0.295	0.278	3.05152754431286E-06	1	CLDN15
1.29557188263624E-29	0.68288815457351	0.493	0.507	2.1711193609218E-25	1	DHRS3
2.74209756888358E-43	0.676361173453622	0.511	0.496	4.5952071059351E-39	1	IDH2
5.88875969757403E-297	0.675040076490857	0.801	0.724	9.86838350119456E-293	1	IFITM3
0	0.653770706509276	0.963	0.788	0	1	C3
8.88703393992245E-08	0.644754189033003	0.273	0.271	0.0014892891476522	1	IER2
6.74316716438497E-60	0.642517744414829	0.497	0.463	1.13001995340763E-55	1	CALD1
3.0950044855392E-11	0.640003390484578	0.372	0.391	5.1866085168666E-07	1	CNBP
5.62889977220097E-69	0.629184969824921	0.605	0.575	9.43291023825439E-65	1	C2
2.78177768239068E-25	0.613110294863855	0.429	0.417	4.6617030401503E-21	1	NR1H4
1.61533886507011E-22	0.612993051599132	0.533	0.559	2.7069848700845E-18	1	ACADVL
4.14212100389561E-08	0.611510667571684	0.198	0.266	0.000694136637832826	1	RALGPS2
0	0.599102398894496	0.906	0.772	0	1	GC
6.32251114005779E-296	0.584703518339383	0.876	0.833	1.05952641685088E-291	1	DEPRECATED-ENSG00000204389
1.53378817251949E-12	0.569154097632159	0.437	0.469	2.57032221950816E-08	1	STAU1
8.2942081331475E-56	0.56269962748741	0.628	0.562	1.38994339895286E-51	1	ERRFI1

1.10150900553452E-147	0.555551717018117	0.892	0.828	1.84590879147475E-143	1	HSPB1
4.23588860276348E-31	0.544327978426849	0.553	0.536	7.09850212051104E-27	1	VMP1
1.22031134104723E-47	0.543681591519833	0.59	0.585	2.04499774532696E-43	1	BRI3
4.67864531742369E-14	0.541094520759425	0.396	0.406	7.84047382293862E-10	1	GPX3
1.89353543998527E-13	0.536527858257598	0.24	0.331	3.17318669032732E-09	1	SHROOM1
2.25907684286073E-12	0.531255597843073	0.488	0.516	3.785760973266E-08	1	SLC12A7
5.42042409893438E-83	0.530556221735743	0.381	0.275	9.08354670499423E-79	1	DEPP1
2.64210751809038E-09	0.521703204577096	0.47	0.515	4.42764377881586E-05	1	PNPLA2
2.92469314426593E-09	0.518271162504327	0.501	0.538	4.90120077116085E-05	1	ALDH3A2
2.6841073968961E-294	0.503743082313211	0.658	0.447	4.49802717571849E-290	1	TXNIP
7.87415533501115E-19	0.501607328077616	0.182	0.269	1.31955095104117E-14	1	CYP2C18
1.64622363075296E-204	0.499267516839755	0.98	0.876	2.75874156041581E-200	1	ALB
5.66751224651679E-14	0.488355961351741	0.181	0.256	9.49761702271284E-10	1	RHEB
6.11863453824092E-09	0.486090480710859	0.196	0.261	0.000102536077591841	1	CADM1
1.69620385087896E-36	0.484236165898184	0.555	0.56	2.84249841330296E-32	1	OS9
3.63085352929834E-86	0.468902232424092	0.693	0.642	6.08458434439816E-82	1	NUPR1
4.00782863717018E-64	0.46578248870531	0.601	0.513	6.71631923016978E-60	1	CCNL2
2.45705052377952E-19	0.45591901495537	0.168	0.251	4.11752526774972E-15	1	MMAB
1.00430492096789E-30	0.454815949569884	0.549	0.524	1.68301418655799E-26	1	RNASE4
8.1383486622559E-18	0.434464558957627	0.19	0.276	1.36382446882084E-13	1	RNF152
1.33711987507237E-07	0.433031725611026	0.238	0.312	0.00224074548664627	1	PNN
7.75364051009468E-09	0.432907160795234	0.262	0.353	0.000129935507668167	1	ITCH
4.75445429830262E-74	0.43266251113945	0.793	0.779	7.96751451309553E-70	1	IL32
1.16236522454576E-22	0.430054971105303	0.177	0.269	1.94789164329379E-18	1	TCAF1
2.24828088250539E-25	0.426544997923221	0.192	0.294	3.76766910290253E-21	1	EGFR
1.75026803498699E-39	0.422671228535339	0.496	0.47	2.93309917303119E-35	1	ITM2B
1.07105849759007E-57	0.418567543073888	0.69	0.675	1.79487983026145E-53	1	ST6GAL1
8.13037631089555E-16	0.40352168714771	0.246	0.349	1.36248846217988E-11	1	DST
6.62242188467618E-08	0.403091150058043	0.285	0.371	0.00110978545943403	1	ERG28
1.7759630559166E-13	0.401624923933209	0.216	0.301	2.97615888910505E-09	1	N4BP2L1
9.91338885463779E-29	0.396947636236527	0.192	0.301	1.6612857042602E-24	1	COBLL1
2.38544613703704E-28	0.395086357273553	0.192	0.301	3.99753063644668E-24	1	SLC23A2
3.09499878099754E-09	0.391446754921173	0.247	0.331	5.18659895719568E-05	1	FAM3C
1.1799580256541E-06	0.387936126786267	0.679	0.732	0.0197737365939115	1	HSP90AA1
3.03071997785915E-13	0.387451825410851	0.23	0.317	5.07888053889636E-09	1	SLC25A30
5.56888681087218E-18	0.383404406432339	0.601	0.605	9.3323405176596E-14	1	HSD17B4
1.34787647202923E-14	0.379139099580412	0.195	0.274	2.25877139182659E-10	1	NDUFB6
1.31200245178568E-24	0.378670607171013	0.185	0.282	2.19865370870245E-20	1	DNAJC22
1.36296300127223E-06	0.376891461837487	0.27	0.35	0.0228405339753201	1	RMND5A
1.12473352323153E-24	0.375910252001876	0.229	0.339	1.8848284382314E-20	1	IDI1
7.46330985648614E-68	0.374481653724652	0.76	0.761	1.25070146574995E-63	1	HSPA1A
1.30880279325396E-09	0.372609264767446	0.259	0.348	2.19329172093499E-05	1	LARP4
3.5523508475726E-20	0.371796966645018	0.169	0.254	5.95302955036216E-16	1	CUL1
1.27104605129823E-11	0.370647916227003	0.275	0.373	2.13001897276557E-07	1	CRYZ
1.08615428069051E-06	0.36229699739992	0.239	0.307	0.0182017734358116	1	DEPRECATED-ENSG00000184500
4.05823506071088E-09	0.360546448820437	0.277	0.371	6.80079031473929E-05	1	CELF1
1.1297847871465E-14	0.359122823042308	0.232	0.324	1.8932933463001E-10	1	COG3
3.05224042265053E-08	0.356159653634617	0.297	0.388	0.000511494450027776	1	SCARB1
2.05477921610378E-09	0.346344661933495	0.217	0.289	3.44339901034672E-05	1	POLR3GL
4.19172697295926E-16	0.343171667482499	0.244	0.341	7.02449606128513E-12	1	C11orf54
2.42368995913997E-16	0.337357537173648	0.257	0.36	4.06161963352677E-12	1	SLC25A44
9.02877872948149E-104	0.333794836398148	0.876	0.841	1.51304273948651E-99	1	MT-CO3
5.15828794639614E-09	0.33255462297258	0.253	0.338	8.64425894057065E-05	1	SND1
6.54662841214023E-14	0.329741062033051	0.22	0.305	1.09708398930646E-09	1	CHP1
2.02756545281277E-06	0.328138672274368	0.38	0.488	0.0339779418582363	1	RXRA
3.3607086572808E-22	0.327369994968719	0.222	0.327	5.63187556787116E-18	1	ACO1
1.94815122335192E-08	0.326782855555462	0.258	0.34	0.000326471182009314	1	SNRNP70
2.79598593598361E-131	0.324444489208677	0.536	0.402	4.68551323152133E-127	1	JUNB
8.45156247523772E-10	0.321727552054455	0.258	0.343	1.41631283960034E-05	1	OSBP
9.61214870875814E-14	0.321512782916418	0.201	0.281	1.61080388061369E-09	1	FKBP2

4.0260194147768E-18	0.321129676498955	0.174	0.254	6.74680333528295E-14	1	NSUN2
2.49573040413336E-12	0.319575878271171	0.553	0.585	4.18234501124668E-08	1	ETFB
1.79272426322303E-26	0.317398886879968	0.164	0.257	3.00424732030916E-22	1	EBPL
9.79527266028295E-09	0.312862952664948	0.547	0.572	0.000164149179241022	1	DHCR24
1.93290751257405E-20	0.312481640957625	0.194	0.284	3.23916640957159E-16	1	DHCR7
1.09625049655868E-26	0.309719027836647	0.669	0.624	1.83709658213303E-22	1	KHK
7.10732426501367E-14	0.297921797094008	0.252	0.347	1.19104540033099E-09	1	NDUFB5
1.93314246141178E-21	0.289922776966267	0.214	0.316	3.23956013683386E-17	1	LTBR
1.0617001314847E-09	0.286499510447751	0.25	0.332	1.77919708034206E-05	1	TOMM7
3.44391846954282E-23	0.286369051020986	0.176	0.269	5.77131857125986E-19	1	DOCK5
1.20756620113366E-261	0.283229501339112	0.663	0.462	2.02363943985979E-257	1	PLA2G2A
1.68104617712135E-31	0.283065797040582	0.168	0.27	2.81709718361995E-27	1	NR5A2
6.81303528345236E-10	0.282895097439084	0.216	0.288	1.14172845280095E-05	1	STAT2
3.55324828040346E-51	0.282510463521413	0.728	0.662	5.95453346830012E-47	1	HMGCS2
2.40180415719033E-16	0.278171779377838	0.193	0.277	4.02494340661956E-12	1	PITPNB
2.52783790952866E-72	0.276738610795097	0.255	0.156	4.23615076878812E-68	1	FOS
7.70140607097308E-45	0.276104571517373	0.15	0.266	1.29060162937367E-40	1	PHKA2
1.70566320855815E-16	0.275290147025358	0.186	0.265	2.85835040490174E-12	1	SURF1
9.0270568483407E-24	0.274290776585622	0.272	0.394	1.51275418664494E-19	1	ABCD3
1.24691757542627E-18	0.257793935041146	0.229	0.325	2.08958447289934E-14	1	QDPR
3.0089111806034E-22	0.257044596254905	0.22	0.323	5.04233335645518E-18	1	TMEM97
1.15581506170464E-16	0.253445531730059	0.209	0.296	1.93691488040464E-12	1	MSRB2
6.7410132446815E-11	0.253158681829561	0.224	0.296	1.12965899954373E-06	1	HSD17B2
0	1.9837947302061	0.752	0.406	0	2	G6PD
1.40957804324574E-148	1.97531473318136	0.303	0.173	2.36217088487121E-144	2	CD177
3.52043470130994E-141	1.94279454713415	0.33	0.21	5.8995444724552E-137	2	CENPB
0	1.93055994515203	0.739	0.37	0	2	PEG10
2.15307101415767E-214	1.89910416883458	0.395	0.241	3.60811640552543E-210	2	CHP2
5.9902567143315E-137	1.87912284292064	0.291	0.167	1.00384722018767E-132	2	TFF1
3.84251156067445E-119	1.82561269306174	0.307	0.196	6.43928087337824E-115	2	MISP
9.12658622537786E-151	1.78426661972406	0.359	0.234	1.52943331964882E-146	2	MALL
2.61021391437911E-96	1.76904069247158	0.259	0.16	4.37419647771651E-92	2	TFF3
3.5423276109425E-61	1.76007076175788	0.255	0.184	5.93623261041744E-57	2	SLC25A23
0	1.72583889768866	0.768	0.501	0	2	STARD10
0	1.71343456117992	0.603	0.362	0	2	LAGE3
2.05311155438144E-82	1.69492490963018	0.261	0.172	3.44060434283241E-78	2	SFN
0	1.69039443723890	0.905	0.506	0	2	NQO1
7.37908083247087E-191	1.68816274931865	0.453	0.315	1.23658636590547E-186	2	SRXN1
0	1.68641313375078	0.975	0.604	0	2	S100A10
6.17523665817447E-114	1.659481986614	0.287	0.174	1.03484615917688E-109	2	S100P
0	1.64067197040862	0.808	0.412	0	2	ADA
0	1.62938732323162	0.591	0.395	0	2	IRAK1
1.68251953505625E-100	1.62874652429358	0.339	0.246	2.81956623684726E-96	2	PIK3R2
0	1.62367323015282	0.575	0.327	0	2	NBL1
3.99266942834727E-89	1.61145734112543	0.287	0.192	6.69091542802435E-85	2	PRR15L
0	1.60033774690457	0.884	0.522	0	2	PIGR
1.96317149998611E-66	1.59910396356862	0.305	0.234	3.28988279967672E-62	2	LRP3
0	1.59705640843344	0.965	0.572	0	2	ANXA4
3.10715639045441E-93	1.58014081138186	0.344	0.257	5.20697267912349E-89	2	SHROOM3
0	1.56925327781253	0.932	0.567	0	2	LGALS4
2.65245455460584E-134	1.56408302386295	0.366	0.245	4.44498334260847E-130	2	TSPAN1
7.31480286804594E-52	1.56205446850319	0.265	0.199	1.22581466462714E-47	2	E2F1
0	1.56040660252413	0.629	0.333	0	2	SLC6A8
0	1.56022306411111	0.856	0.57	0	2	GPX2
1.49524309620065E-113	1.54445972796019	0.337	0.228	2.50572838061305E-109	2	DEPRECATED-ENSG00000132646
6.36495643238212E-77	1.54159930665487	0.285	0.201	1.0666393989386E-72	2	RASL11A
4.28024872966008E-178	1.54061846506005	0.443	0.311	7.17284082116435E-174	2	GCHFR
2.00483313906722E-75	1.53015383600202	0.338	0.265	3.35969937444885E-71	2	ASB13
1.75449160596065E-203	1.52794645201739	0.448	0.297	2.94017703326886E-199	2	ANXA13
0	1.52199230473403	0.844	0.545	0	2	GSTA1

0	1.50951652916629	0.575	0.377	0	2	HTATSF1
0	1.5027282314183	0.572	0.37	0	2	ETHE1
1.81496383651192E-55	1.49612216667722	0.278	0.213	3.04151639722668E-51	2	KANK2
4.98176165857791E-46	1.48750179585583	0.271	0.213	8.34843618744487E-42	2	OTOP3
6.3081784620907E-105	1.48568616376512	0.338	0.234	1.05712454667716E-100	2	MCM2
0	1.48075549898403	0.604	0.411	0	2	CBR1
9.06222204390018E-108	1.46442596293869	0.363	0.267	1.51864717011679E-103	2	NIBAN2
5.38117215938703E-166	1.46360016343654	0.435	0.306	9.01776830470079E-162	2	IMPA2
3.43552895340361E-74	1.4579265389225	0.312	0.234	5.75725942011377E-70	2	AP1S1
6.15941403877622E-305	1.44498420318979	0.559	0.38	1.03219460461812E-300	2	CMBL
5.40129752213461E-32	1.43686060681689	0.252	0.209	9.05149438759318E-28	2	MANEAL
0	1.43483552934079	0.75	0.449	0	2	SSR4
1.90536170477252E-99	1.43325907022797	0.369	0.278	3.19300514485779E-95	2	CETN2
1.3305018264112E-228	1.43267459595062	0.499	0.348	2.22965496069988E-224	2	CYSTM1
2.43126810551015E-180	1.42997494354882	0.434	0.287	4.0743190912139E-176	2	C15orf48
0	1.42557400747613	0.601	0.306	0	2	MUC5B
0	1.42439084573325	0.624	0.432	0	2	PRDX1
0	1.42280649777939	0.909	0.575	0	2	AKR1C3
2.36719710054584E-56	1.42131040755925	0.302	0.238	3.96694890109472E-52	2	C7orf50
4.16959564715435E-62	1.41359581142425	0.301	0.229	6.98740838550126E-58	2	INAVA
0	1.41059558201018	0.853	0.478	0	2	MPP1
1.08652397000838E-121	1.40598898798093	0.403	0.301	1.82079686894004E-117	2	SYBU
0	1.39736931482308	0.708	0.475	0	2	S100A16
0	1.39584632711918	0.749	0.484	0	2	NDUFA1
7.71531188689215E-72	1.39331148254079	0.288	0.201	1.29293196600539E-67	2	TCF19
0	1.39329371795066	0.708	0.429	0	2	MAOA
2.28574755551934E-32	1.39293481041647	0.257	0.214	3.83045575353931E-28	2	CORO2A
1.94419021015702E-25	1.38945750863753	0.25	0.217	3.25807395418113E-21	2	CTDSPL
0	1.38255279331374	0.572	0.346	0	2	MEP1A
0	1.37734515848193	0.75	0.491	0	2	DEPRECATED-ENSG00000198074
7.13814971778118E-221	1.37372036384	0.501	0.337	1.19621112970577E-216	2	MCM3
0	1.3727309754746	0.695	0.377	0	2	CDH17
0	1.36847471806032	0.822	0.459	0	2	NDRG1
0	1.36457270882786	0.589	0.42	0	2	DAP
1.24273694056927E-120	1.3623490817928	0.399	0.289	2.08257856500598E-116	2	MCM4
2.13951560350292E-96	1.36227307420155	0.382	0.296	3.58540024835019E-92	2	PIR
1.71790099632135E-51	1.360089727861	0.328	0.275	2.87885848963532E-47	2	WSB2
0	1.35812436306986	0.967	0.692	0	2	ALDH1A1
1.14366788214428E-66	1.35760055573847	0.33	0.257	1.91655863689738E-62	2	C1orf115
3.84108860340442E-137	1.35478856283904	0.429	0.32	6.43689628158512E-133	2	FAM3A
0	1.34652453297606	0.994	0.874	0	2	FTL
5.90549485681727E-42	1.34553611479164	0.275	0.222	9.89642828105439E-38	2	PRELID1
0	1.34342194896064	0.586	0.34	0	2	KLF5
3.79696968484059E-203	1.33987163811477	0.503	0.357	6.36296179785586E-199	2	AMOTL1
6.77085885827054E-107	1.3288599413864	0.395	0.301	1.13466052746898E-102	2	TXNDC17
8.67135013005499E-95	1.32581337508796	0.37	0.275	1.45314485479461E-90	2	TM4SF20
1.57288318791634E-46	1.32509988723746	0.282	0.225	2.6358376463102E-42	2	PALLD
0	1.32471730060613	0.71	0.491	0	2	BEX3
4.77483827086741E-175	1.32142267088072	0.48	0.357	8.0016739743196E-171	2	DEPRECATED-ENSG00000159228
3.99459515037955E-41	1.3176725565624	0.267	0.213	6.69414255300605E-37	2	PYCARD
1.5212438679242E-40	1.31556974413069	0.268	0.215	2.54930047386737E-36	2	DEPRECATED-ENSG00000170421
0	1.31144993886533	0.615	0.434	0	2	SAP18
7.3107160853665E-44	1.30749871392189	0.317	0.272	1.22512980158572E-39	2	DEPRECATED-ENSG00000133142
1.96115977034047E-189	1.30499000740096	0.479	0.329	3.28651154313656E-185	2	PARM1
2.19900792891736E-35	1.30223852680415	0.29	0.248	3.68509748727971E-31	2	NTPCR
7.00934324750284E-33	1.30107944994155	0.282	0.241	1.17462574141653E-28	2	KIF1C
8.07737909398284E-276	1.30105552182857	0.574	0.396	1.35360718856964E-271	2	CMC4
2.05334318922109E-162	1.29650954250047	0.463	0.343	3.44099251649671E-158	2	TLE5
6.99326091846982E-175	1.29542381717365	0.481	0.356	1.17193066471717E-170	2	CCL15
0	1.29275952168537	0.684	0.426	0	2	TSPAN8

0	1.29165098205275	0.928	0.619	0	2	UGDH
0	1.28697792487162	0.895	0.563	0	2	SPINK1
0	1.28500942000473	0.875	0.578	0	2	IFI27
3.26582789103085E-45	1.28050058592408	0.31	0.259	5.4728743797895E-41	2	CRAT
1.92133439670544E-24	1.27984125359987	0.259	0.228	3.21977218199898E-20	2	HGH1
8.12680830419315E-49	1.27809080752945	0.316	0.261	1.36189053561669E-44	2	TMEM141
0	1.27738328873437	0.672	0.458	0	2	RBM47
2.1433738339067E-163	1.27493347723045	0.317	0.165	3.59186587086085E-159	2	AGR2
3.25842427751655E-247	1.26657769811713	0.551	0.383	5.46046740426224E-243	2	PYGB
1.16715537664613E-84	1.26247953824324	0.408	0.338	1.95591898018358E-80	2	NR2F6
1.07451219303949E-105	1.26060483774111	0.402	0.31	1.80066753309558E-101	2	GALK1
4.33846998823558E-90	1.2597277855524	0.383	0.3	7.27040800628519E-86	2	PSMD10
5.08112609534335E-179	1.2501208669327	0.493	0.356	8.51495111057639E-175	2	DGAT2
0	1.24859910780491	0.628	0.438	0	2	TPD52
8.11581665444728E-77	1.24676251841882	0.358	0.283	1.36004855495228E-72	2	RASSF3
0	1.24342078301594	0.824	0.564	0	2	AKR1B10
7.85463116461819E-49	1.24244658301528	0.318	0.264	1.31627909056672E-44	2	GIPC1
1.11347454443703E-55	1.24174853322221	0.349	0.295	1.86596064156758E-51	2	SLC25A5
0	1.24161019348225	0.707	0.502	0	2	CRYL1
7.47488631905432E-253	1.23806562030714	0.58	0.406	1.25264144934712E-248	2	SLPI
1.14173587665691E-96	1.23512868848998	0.415	0.333	1.91332098210165E-92	2	KIAA1522
9.42744834417136E-26	1.23165647606311	0.256	0.221	1.57985179351624E-21	2	COA6
0	1.22929801730203	0.734	0.52	0	2	KRT8
7.92444807935535E-304	1.22543842886091	0.634	0.474	1.32797900913837E-299	2	TSTA3
1.24627821719614E-117	1.22463505395151	0.455	0.364	2.0885130363773E-113	2	GSTA2
8.51636688617857E-155	1.22238159792685	0.477	0.362	1.4271727627858E-150	2	VBP1
8.56255274156439E-120	1.2199809243637	0.479	0.39	1.43491258843136E-115	2	SLC2A4RG
0	1.21893477016161	0.685	0.428	0	2	ERICH5
8.04034482271794E-49	1.21871655179938	0.33	0.277	1.34740098539107E-44	2	DNAJC4
3.93986293367461E-79	1.21734292419123	0.389	0.312	6.60242230425191E-75	2	NSDHL
9.68413232324152E-59	1.21700906448206	0.359	0.303	1.62286689472881E-54	2	KEAP1
5.74000675072755E-144	1.21691903978829	0.475	0.372	9.61910331286923E-140	2	ITPK1
2.6789098273184E-119	1.21220917675606	0.441	0.344	4.48931708862017E-115	2	SLC25A4
1.11248928493363E-37	1.20913461611438	0.294	0.249	1.86430954369178E-33	2	SLC10A3
4.45976838969683E-33	1.20755680742568	0.296	0.257	7.47367986745395E-29	2	LSM 6.00
6.88712873307772E-238	1.20691962270431	0.57	0.432	1.15414503308916E-233	2	ATP5F1D
8.44288240335782E-43	1.20669352862343	0.301	0.25	1.4148582331547E-38	2	NFIX
6.14433903022218E-80	1.20358792086535	0.387	0.311	1.02966833468463E-75	2	DEPRECATED-ENSG00000213759
4.25867519366567E-65	1.20345588510201	0.359	0.295	7.13668788954493E-61	2	SLC35A4
0	1.20143006057109	0.702	0.5	0	2	SCD
2.56815249135457E-81	1.19812154887355	0.361	0.27	4.303709945012E-77	2	B3GNT3
0	1.19441453020916	0.711	0.477	0	2	BCAP31
1.04912226123749E-42	1.19423224018995	0.323	0.278	1.75811908538179E-38	2	VDAC1
0	1.19382771038524	0.764	0.422	0	2	MUC13
6.51412254111628E-61	1.19268814974985	0.326	0.259	1.09163665544027E-56	2	FKBP8
0	1.1905534574408	0.673	0.482	0	2	TMEM14A
5.47799957558579E-17	1.18775760135999	0.26	0.241	9.18003168876667E-13	2	AKR7A2
5.63961161746702E-246	1.18292383403742	0.579	0.416	9.45086114855124E-242	2	PNKD
0	1.18202684894422	0.646	0.464	0	2	TMEM106C
1.0394182047167E-24	1.18147048671241	0.295	0.269	1.74185702746424E-20	2	NET1
0	1.18084502284535	0.922	0.671	0	2	PTGR1
5.79601608130531E-121	1.17915609978399	0.412	0.292	9.71296374905144E-117	2	DUSP9
1.36050790417001E-84	1.17751042043072	0.398	0.322	2.2799391458081E-80	2	TMEM64
8.7220620534718E-46	1.17537819991347	0.336	0.289	1.4616431589208E-41	2	DNPH1
2.49475865405521E-146	1.17026862522539	0.469	0.36	4.18071655246572E-142	2	ATP6V0D1
2.89317745062808E-46	1.16461540253112	0.327	0.276	4.84838677176253E-42	2	HEBP1
5.72736253118134E-130	1.1640653941688	0.47	0.371	9.59791412975368E-126	2	FAM114A1
8.9726850204229E-234	1.16339402105931	0.575	0.43	1.50364255572247E-229	2	ENO1
2.4429673663443E-45	1.16244422886287	0.336	0.287	4.09392471251978E-41	2	TMEM164
1.03827110288765E-230	1.16045341352689	0.6	0.472	1.73993471421913E-226	2	MGAT4B

2.78747737595726E-137	1.15789221577845	0.481	0.386	4.67125458662918E-133	2	UBE2Q1
5.5342133074558E-232	1.15553941599474	0.558	0.397	9.27423466063443E-228	2	LAPTM4B
8.74493031475135E-209	1.15132933604265	0.556	0.425	1.46547542214603E-204	2	NDUFB9
6.86483977421652E-92	1.15078354209529	0.422	0.348	1.1504098493632E-87	2	RAB5IF
1.08329171870464E-80	1.14781938753982	0.4	0.329	1.81538026220523E-76	2	AGPAT1
9.43804327499199E-19	1.14755709565235	0.263	0.24	1.58162729202316E-14	2	GNA11
9.42285624163631E-83	1.14596144411834	0.412	0.343	1.57908224897341E-78	2	PMVK
1.27688516438012E-139	1.14437307179113	0.486	0.388	2.1398041584682E-135	2	SLC25A1
2.02282674046565E-276	1.14219247128788	0.622	0.475	3.38985305167233E-272	2	BLVRB
9.19945325777697E-106	1.14103794465678	0.461	0.38	1.54164437693826E-101	2	DSP
2.40538653110726E-60	1.13764403456248	0.373	0.318	4.03094674882955E-56	2	TIMM13
3.73070528219361E-40	1.13684605005225	0.285	0.233	6.25191591190005E-36	2	PNP
5.43924640633709E-42	1.13435415715651	0.323	0.276	9.1150891277397E-38	2	FAM162A
1.01938457235333E-26	1.13411216535976	0.296	0.268	1.70828466634971E-22	2	BTBD2
2.54640989640175E-21	1.13296107246668	0.262	0.233	4.26727370439005E-17	2	JPT2
4.0618346745744E-89	1.13021781328784	0.418	0.337	6.80682254765178E-85	2	SPSB1
1.08595058989218E-16	1.12886412201459	0.258	0.239	1.81983599854131E-12	2	AHCY
1.0276829121124E-18	1.12882097486466	0.261	0.238	1.72219102411796E-14	2	NDUFA7
1.34764754420379E-21	1.12719280370683	0.263	0.235	2.25838775457671E-17	2	CCT3
2.16212801784704E-38	1.1253267088539	0.315	0.271	3.62329413230807E-34	2	SPR
1.46598995668697E-62	1.12303128463869	0.385	0.328	2.45670596941602E-58	2	UQCR10
1.51783234772413E-63	1.12208371319029	0.372	0.307	2.5435834483161E-59	2	TUBB2A
4.99844520848198E-82	1.11985429867676	0.424	0.36	8.3763944803741E-78	2	MAP2K2
9.19278019598801E-37	1.11921098833788	0.328	0.29	1.54052610524367E-32	2	EPN1
4.37343705460124E-114	1.11477800867558	0.449	0.354	7.32900581610075E-110	2	VMA21
0	1.11023581497151	0.623	0.386	0	2	CLDN4
2.57268435092842E-120	1.10976393711108	0.466	0.374	4.31130443528584E-116	2	TCEAL4
0	1.10949194161993	0.714	0.525	0	2	MPC2
1.99293964190869E-54	1.10844277986023	0.355	0.296	3.33976825191059E-50	2	IRF6
2.14858789919296E-35	1.10643366370598	0.292	0.244	3.60060360146756E-31	2	LIME1
0	1.1056221524524	0.671	0.51	0	2	PRDX2
0	1.10505588964226	0.741	0.523	0	2	CES2
3.08109462943905E-60	1.10407540260074	0.39	0.341	5.16329838001397E-56	2	COA3
6.50261862150695E-63	1.10117055322431	0.363	0.302	1.08970882859213E-58	2	CLTB
1.04981773789984E-39	1.10093022286871	0.319	0.274	1.75928456517255E-35	2	NDUFS6
2.25714344417776E-277	1.09798918989499	0.652	0.466	3.78252098375309E-273	2	GOLM1
3.43653111445836E-70	1.09779512016587	0.394	0.333	5.75893884160932E-66	2	UQCR11
2.43319429649621E-306	1.09696809897954	0.641	0.413	4.07754700206835E-302	2	ATP1A1
2.45287105086253E-23	1.09637258256701	0.271	0.241	4.11052130703543E-19	2	ARPC1A
8.02986121903643E-185	1.09529329170353	0.554	0.442	1.34564414308613E-180	2	COX6C
2.07905609735149E-112	1.09481110347485	0.449	0.359	3.48408220794162E-108	2	CAPN1
1.84570427445723E-50	1.09472958710283	0.35	0.298	3.09303122313543E-46	2	LSM 4.00
8.29736389639291E-167	1.09357913394251	0.508	0.376	1.39047224175752E-162	2	CITED2
6.57293434735278E-134	1.09233478082383	0.459	0.332	1.10149233792938E-129	2	CLDN3
9.91926227330027E-36	1.09206959492394	0.314	0.274	1.66226997175966E-31	2	MEA1
4.43598912468692E-171	1.0912956395219	0.535	0.42	7.43383057515035E-167	2	AIFM1
4.64131483890305E-179	1.08921484786423	0.55	0.426	7.77791540703373E-175	2	PCK2
0	1.089143799815	0.805	0.554	0	2	LYZ
2.02875717843379E-31	1.08516846508626	0.287	0.242	3.39979127961934E-27	2	SI
5.77444611686518E-66	1.08503424808956	0.408	0.353	9.67681680264268E-62	2	SHARPIN
1.51072148563216E-33	1.08273677766252	0.325	0.291	2.53166706562238E-29	2	FAM20B
4.94712171687135E-92	1.08035018489095	0.437	0.349	8.29038657313301E-88	2	HPGD
5.28124021710555E-41	1.0802819766117	0.347	0.308	8.85030235582549E-37	2	HSD3B7
0	1.07768434477952	0.986	0.774	0	2	CES1
2.81373442845728E-50	1.07736558947237	0.359	0.306	4.71525615520872E-46	2	DEPRECATED-ENSG00000057294
1.47748318396408E-32	1.07603105007322	0.317	0.277	2.47596631968701E-28	2	CDS1
3.3268562008397E-115	1.07490887056573	0.44	0.326	5.57514562136717E-111	2	EHF
2.66765716037493E-29	1.0748273304698	0.313	0.283	4.47045986935631E-25	2	DKC1
0	1.0744303928053	0.731	0.46	0	2	RNF128
5.98914246100603E-41	1.07416205956123	0.351	0.312	1.00366049361539E-36	2	CISD3

8.51952185683454E-53	1.07406698584399	0.364	0.313	1.42770147276833E-48	2	PRPS1
8.10742081907764E-103	1.07375909038462	0.469	0.396	1.35864158086103E-98	2	SLC25A39
1.60641258608667E-35	1.07330827958538	0.316	0.276	2.69202621176403E-31	2	TMEM238
8.37593868253416E-26	1.07234548317225	0.29	0.258	1.40363980441907E-21	2	POLR2I
6.38004879584925E-28	1.07146656893493	0.292	0.254	1.06916857720842E-23	2	PDX1
2.97973665161109E-262	1.07066001006693	0.616	0.454	4.99344268076987E-258	2	FAM50A
2.44758605277493E-114	1.0693397255821	0.469	0.383	4.10166470724023E-110	2	CHCHD2
6.94015697258348E-66	1.06871330180638	0.387	0.326	1.16303150546554E-61	2	NAA10
2.92790318153755E-134	1.0683466276761	0.487	0.387	4.90658015162063E-130	2	LASP1
3.01650381357869E-22	1.06800340388721	0.286	0.259	5.05505709079516E-18	2	SH3BP4
5.50414638417514E-102	1.06463722733123	0.451	0.365	9.2238485106007E-98	2	PGD
2.83118758404752E-217	1.06460321935792	0.569	0.393	4.74450415334684E-213	2	TMEM45B
1.06000227005292E-22	1.06393602911128	0.261	0.229	1.77635180415468E-18	2	PCGF2
4.41924107050344E-234	1.06359587634028	0.558	0.396	7.40576418594966E-230	2	EFHD2
8.20442123148621E-90	1.06245659866851	0.436	0.366	1.37489690997246E-85	2	CHMP2A
1.69750721158088E-91	1.06217728583901	0.421	0.344	2.84468258516723E-87	2	TALDO1
8.0590366970989E-19	1.06180374460596	0.293	0.274	1.35053336969983E-14	2	UGT2B11
1.28143358508358E-87	1.0603789567245	0.432	0.36	2.14742640188307E-83	2	RNPEP
3.365567711529E-170	1.05982411714225	0.543	0.419	5.6400183709803E-166	2	DHRS7
6.86973330218061E-50	1.05915824828625	0.348	0.294	1.15122990677943E-45	2	RNPEPL1
2.51286674104507E-45	1.05612693641144	0.35	0.303	4.21106208464332E-41	2	ISOC2
3.06032971307687E-27	1.0555025711295	0.31	0.28	5.12850053317422E-23	2	NACC1
0	1.05467828938636	0.727	0.459	0	2	TSPAN3
1.23130200593893E-18	1.05361098052503	0.264	0.235	2.06341590155246E-14	2	MS4A8
8.36832756082695E-254	1.0534034312278	0.626	0.463	1.40236433264338E-249	2	SMIM14
1.95323962521569E-172	1.05295656107676	0.537	0.409	3.27323896393646E-168	2	GPAA1
1.54954427626052E-74	1.05120109086784	0.403	0.336	2.59672629815738E-70	2	RNF187
4.41161799905577E-25	1.05081004098874	0.3	0.273	7.39298944281766E-21	2	PLS1
0	1.05007648434336	0.877	0.58	0	2	UGT2B4
3.52670265358439E-96	1.05001970607656	0.434	0.349	5.91004830687673E-92	2	RAMP1
6.00015371888436E-62	1.04850862438272	0.368	0.304	1.00550576021064E-57	2	PKN1
1.71153665583285E-64	1.04779335003241	0.409	0.355	2.86819312784468E-60	2	TMEM219
4.9404996120103E-209	1.04765538605114	0.587	0.445	8.27928924980686E-205	2	SPTSSA
1.17566679426545E-72	1.04448142087323	0.418	0.358	1.97018241383003E-68	2	AKT1
4.22241198218482E-296	1.04204350628305	0.633	0.451	7.07591799974532E-292	2	CALM3
2.55056251618295E-74	1.04049908276238	0.409	0.342	4.27423266461938E-70	2	LAMTOR1
7.13620158787708E-14	1.03961674242634	0.287	0.277	1.19588466209644E-09	2	FAM83H
1.79919779956447E-157	1.03920344942957	0.546	0.448	3.01509567251013E-153	2	POLR2L
8.38336334010815E-163	1.03915335680907	0.534	0.424	1.40488402853532E-158	2	PLS3
0	1.03901734415732	0.934	0.666	0	2	PGRMC1
4.99565767765883E-207	1.03863068877752	0.588	0.46	8.37172313622066E-203	2	NDUFC2
3.54701122998194E-34	1.03603534127592	0.258	0.204	5.94408141920373E-30	2	TYMS
0	1.03557860659738	0.888	0.647	0	2	MGST1
2.8786818055194E-46	1.0312117495	0.331	0.278	4.82409496968942E-42	2	VAT1
5.90449704118285E-11	1.03064176133385	0.272	0.267	9.89475614161421E-07	2	TCEAL1
1.50589450747028E-20	1.02948097016462	0.296	0.276	2.5235780156187E-16	2	CAPN5
2.30081298231411E-141	1.02792885669803	0.539	0.446	3.85570239576198E-137	2	CAMK2N1
5.87906915322533E-102	1.02663123539618	0.475	0.398	9.85214408697501E-98	2	CIAO2A
0	1.02644495690804	0.972	0.788	0	2	MT-CO2
1.62394054808183E-31	1.02563804438794	0.302	0.263	2.72139957047553E-27	2	MZT2B
6.11163362077601E-196	1.02552900113499	0.588	0.469	1.02418756216964E-191	2	MGST3
2.79631921297952E-28	1.02475585344064	0.315	0.286	4.68607173711108E-24	2	PEPD
9.20764969079515E-32	1.0236697135447	0.311	0.276	1.54301793518345E-27	2	SNRPB
9.39946190342664E-11	1.02343923828205	0.257	0.25	1.57516182577624E-06	2	FAM13A
0	1.02322305205767	0.984	0.786	0	2	EPHX1
2.04577136363506E-82	1.02175737966526	0.443	0.373	3.42830365117964E-78	2	PYCR2
1.08610817505699E-52	1.01843189671554	0.386	0.341	1.8201000797605E-48	2	PSMB4
7.97043409195802E-21	1.01650675603553	0.274	0.248	1.33568534513033E-16	2	GSK3A
1.38789938230064E-23	1.01596690813942	0.298	0.272	2.32584178485941E-19	2	GFPT1
5.23468788854415E-286	1.01596135470332	0.678	0.533	8.7722899636223E-282	2	SLC9A3R1

5.36840322332565E-28	1.01515415951588	0.317	0.29	8.99637012164913E-24	2	NAXE
7.45210527452207E-44	1.01484149188716	0.346	0.299	1.24882380190441E-39	2	CSTB
7.23318765689139E-151	1.01476803486962	0.53	0.424	1.21213758754186E-146	2	IDH3G
7.57533913764842E-88	1.00669361185208	0.441	0.363	1.26947533268712E-83	2	CD99L2
3.6591882087122E-14	1.00568826215797	0.251	0.233	6.13206760015991E-10	2	POLR2F
3.61303216196893E-57	1.00489540295801	0.381	0.317	6.05471929702754E-53	2	SIPA1L2
3.70164872696571E-177	1.00094766112519	0.56	0.422	6.20322293664914E-173	2	TSPAN6
3.27878241161902E-33	0.998339227600742	0.333	0.298	5.49458356539116E-29	2	PYGO2
3.97798425577502E-15	0.997710523372858	0.273	0.258	6.66630601582778E-11	2	ME1
1.22515689640858E-65	0.997606409056062	0.407	0.347	2.0531179270015E-61	2	AK4
3.09862817481181E-16	0.997183753287668	0.255	0.234	5.19268109534963E-12	2	KHSRP
5.81900470285654E-35	0.994946629066439	0.303	0.26	9.75148808104699E-31	2	CLIC1
2.20952690263469E-39	0.993156806650373	0.351	0.312	3.70272518343522E-35	2	UBXN6
7.49109514169249E-217	0.992862743815444	0.589	0.409	1.25535772384483E-212	2	PGM2
4.05950567645622E-54	0.992758547356682	0.381	0.329	6.80291961260533E-50	2	SMIM15
0	0.991503980369979	0.784	0.526	0	2	ACSL4
7.01421203570606E-24	0.990744284871057	0.308	0.282	1.17544165294362E-19	2	HPRT1
5.11104766734848E-40	0.988784137846093	0.348	0.305	8.56509368094258E-36	2	GCLM
1.13041889189559E-38	0.988041703259514	0.355	0.317	1.89435597903864E-34	2	STAP2
2.10467713397841E-117	0.987792106822282	0.494	0.407	3.52701794112103E-113	2	OTC
3.26299926274544E-50	0.987381998858744	0.38	0.331	5.46813416450881E-46	2	TMEM147
3.29000271565162E-60	0.986269178209016	0.411	0.361	5.51338655088899E-56	2	NENF
2.77558647667122E-155	0.985740476869085	0.544	0.426	4.65132781760563E-151	2	MDK
2.1555329019132E-168	0.985624690730477	0.572	0.466	3.61224203702613E-164	2	NDUFA2
1.37344636389327E-143	0.985197444690307	0.536	0.445	2.30162141661234E-139	2	UQCRQ
4.13339601039164E-13	0.983485780250581	0.277	0.268	6.92674503421431E-09	2	SIPA1L3
0	0.981237030566002	0.749	0.572	0	2	MT-ND2
4.18864841121795E-63	0.980873039364777	0.415	0.358	7.01933700751904E-59	2	NHSL1
2.55214066344056E-133	0.980407607948736	0.535	0.439	4.27687732379369E-129	2	TMEM205
1.94002921116994E-119	0.978608754469356	0.457	0.354	3.25110095207858E-115	2	CDKN1A
8.72793064948362E-52	0.978337581736771	0.37	0.319	1.46262661824046E-47	2	MAF 1.00
1.01425591168885E-252	0.978124006360206	0.647	0.506	1.69969005680817E-248	2	CYC1
7.97845054532154E-43	0.977039850262053	0.363	0.322	1.33702874238498E-38	2	RAD23A
2.5969194703577E-110	0.973843417067456	0.496	0.415	4.35191764842543E-106	2	EGLN1
1.26927292467867E-149	0.972985546296827	0.531	0.422	2.12704756717651E-145	2	UBE2A
3.29608999776493E-26	0.971054354502482	0.343	0.323	5.52358761825447E-22	2	SMIM19
1.75227461192064E-84	0.969338210118398	0.459	0.395	2.93646179465661E-80	2	PRDX4
5.00862818315585E-78	0.968131626848751	0.441	0.381	8.39345910933258E-74	2	ZNF706
2.86065233170621E-203	0.96812622771609	0.614	0.481	4.79388117747327E-199	2	ANXA2
4.00377221246466E-61	0.966253082820546	0.4	0.346	6.70952147364828E-57	2	BIN1
1.37763170667176E-229	0.965833646875592	0.555	0.34	2.30863521404054E-225	2	SLC6A19
1.61000418202359E-35	0.959694467473304	0.316	0.271	2.69804500823513E-31	2	MCM5
1.66111663808148E-47	0.959497169517015	0.361	0.314	2.78369926209695E-43	2	PAPSS2
3.17088260779701E-54	0.956040303183037	0.397	0.35	5.31376507414624E-50	2	MMP24OS
1.5238567742969E-40	0.95523703109776	0.352	0.312	2.55367918236675E-36	2	SNX17
9.58407337373514E-64	0.95460920624153	0.426	0.376	1.60609901597053E-59	2	COX7C
8.82667592698437E-68	0.952924751314	0.433	0.377	1.47917435184404E-63	2	SIVA1
2.34607218167186E-103	0.952180890886657	0.488	0.407	3.93154776204571E-99	2	MAOB
3.81679318203673E-29	0.952032889060284	0.328	0.298	6.39618201445716E-25	2	COPS6
9.12177645711552E-14	0.951575940865801	0.256	0.238	1.52862729868342E-09	2	POLD2
3.33225796311711E-73	0.949892831083623	0.41	0.325	5.58419789459165E-69	2	MATN2
1.95002766564413E-205	0.948989679592946	0.591	0.45	3.26785636208644E-201	2	HNRNPH2
2.47361372759242E-181	0.946473429553757	0.617	0.504	4.14528188469938E-177	2	TXNRD1
2.11900056896522E-28	0.945246980657145	0.325	0.295	3.55102115347191E-24	2	SSRP1
8.04769824853891E-21	0.945016127746518	0.316	0.3	1.34863327249015E-16	2	ATP5PD
8.81230756084858E-53	0.944658292563032	0.403	0.358	1.476766501047E-48	2	FH
6.2378240240691E-09	0.944237786299454	0.258	0.255	0.000104533345499535	2	SLC39A1
2.21212713178236E-66	0.942883770539806	0.428	0.378	3.70708264744089E-62	2	ATP5ME
0	0.942831544417109	0.74	0.474	0	2	MBNL3
3.89356806745168E-120	0.942333240659196	0.523	0.446	6.52484136743553E-116	2	NDUFB7

2.5493142164365E-47	0.942011613986936	0.36	0.313	4.2721407639043E-43	2	CAPNS1
1.23595156816722E-56	0.941284679644442	0.406	0.359	2.07120763793462E-52	2	ANAPC11
0	0.941126329730708	0.745	0.611	0	2	GPX4
6.11083513744747E-47	0.940787940134979	0.387	0.347	1.02405375233345E-42	2	ROMO1
1.16564328214234E-21	0.939941371180166	0.322	0.306	1.95338501221413E-17	2	CISD2
5.06952142251386E-20	0.938517437742378	0.317	0.298	8.49550399984873E-16	2	ATP9A
6.66867315519462E-30	0.938080994918083	0.332	0.3	1.11753624734751E-25	2	QDPR
8.70003028686396E-78	0.937932802831498	0.446	0.384	1.45795107547266E-73	2	DSTN
1.62806181740412E-31	0.936642706265282	0.351	0.318	2.72830599360582E-27	2	SLC25A15
3.1829422884673E-81	0.934806978420703	0.464	0.39	5.3339746870135E-77	2	PRG4
8.40536439142883E-10	0.933708500612237	0.275	0.271	1.40857096471564E-05	2	CDKN2A
1.99768673486736E-39	0.933229566401891	0.375	0.339	3.34772343029073E-35	2	HADH
7.89283961007305E-80	0.93315806906755	0.457	0.393	1.32268206185604E-75	2	SF3B5
4.90205129060529E-52	0.932106700399777	0.406	0.366	8.21485755279634E-48	2	SRP9
6.70831351822282E-163	0.931268949699987	0.551	0.397	1.12417917938378E-158	2	ABCD1
5.28792265362298E-20	0.930027440896655	0.321	0.307	8.86150078294138E-16	2	TM9SF1
5.81298378287082E-204	0.929803712468663	0.601	0.469	9.74139822333493E-200	2	EMD
3.8973246437765E-30	0.929462850234014	0.332	0.296	6.53113663804065E-26	2	KCNK1
3.00928052851244E-61	0.928617494334453	0.393	0.334	5.04295230968115E-57	2	PPP1CA
1.94695677311707E-14	0.928044806920588	0.265	0.249	3.26271016038958E-10	2	NME1
1.53843969986553E-36	0.927288354904283	0.347	0.311	2.57811724903466E-32	2	HIGD2A
8.72221323864794E-15	0.92593442733028	0.292	0.28	1.46166849453262E-10	2	ACADS
1.75244510238985E-170	0.925619124941113	0.601	0.496	2.93674750258491E-166	2	ACAA2
1.85863937820271E-09	0.923520818977216	0.252	0.247	3.1147078699921E-05	2	PSMD8
8.58970618006103E-33	0.923001886407786	0.364	0.338	1.43946296165463E-28	2	SETD7
1.08674065293762E-54	0.921162657068741	0.417	0.377	1.82115998619286E-50	2	NDUFS7
3.27406010677283E-43	0.919342000433128	0.382	0.341	5.4866699269299E-39	2	PHYH
0	0.918080083537167	0.805	0.521	0	2	TDO2
3.42645017900612E-17	0.917304165261931	0.322	0.313	5.74204520997845E-13	2	NAA60
2.41430729379656E-18	0.9166699645038	0.281	0.259	4.04589616294427E-14	2	PLAAT3
9.66694374964824E-25	0.916600594280418	0.335	0.315	1.61998643356605E-20	2	UBL3
3.17014318308058E-263	0.915147791164795	0.709	0.556	5.31252594620643E-259	2	IDH1
7.86106764745072E-15	0.914986153547238	0.276	0.26	1.31735771635979E-10	2	SLC25A11
1.19163615096215E-17	0.912640860212414	0.297	0.281	1.99694386178237E-13	2	NME2
1.16046158245468E-31	0.911881589911538	0.354	0.324	1.94470151987755E-27	2	NDFIP2
2.7362358984692E-12	0.911679402153626	0.288	0.282	4.585384118654 69E-08	2	IFI35
5.81165829017253E-18	0.909600657938401	0.307	0.295	9.73917696267112E-14	2	DUSP3
1.17226161608266E-12	0.909055259013515	0.289	0.28	1.96447601623132E-08	2	OCIAD2
0	0.907958372450842	0.774	0.597	0	2	CFL1
8.89095335621258E-24	0.90699909781333	0.322	0.298	1.4899459634341E-19	2	GUCD1
1.98602058497537E-15	0.906763104542558	0.293	0.279	3.32817329630172E-11	2	CMTM8
1.9891121999271E-28	0.905853433405194	0.353	0.332	3.33335422463783E-24	2	TXNL4A
1.53487829864474E-60	0.904901489571566	0.425	0.369	2.57214905286886E-56	2	PERP
1.22669400083481E-293	0.904594023042129	0.724	0.574	2.05569380659898E-289	2	MT-ND5
1.91779007143828E-49	0.902772206024515	0.377	0.312	3.21383260171628E-45	2	NEB
0	0.902405641131937	0.743	0.58	0	2	H3F3A
0	0.900603709788771	0.81	0.589	0	2	LAMP2
2.32465090605761E-08	0.900370065522483	0.272	0.272	0.000389564998837134	2	PYURF
4.5865183271634E-62	0.899883837377053	0.433	0.385	7.68608741266043E-58	2	DYNLL2
1.26262487568845E-14	0.899727759032682	0.254	0.235	2.1159067666787E-10	2	ALKBH5
2.84013945584806E-12	0.897880853277898	0.273	0.263	4.75950570011018E-08	2	PREB
1.58687827359802E-26	0.893781177248684	0.334	0.31	2.65929061089556E-22	2	JTB
1.37505349479958E-137	0.892237304959718	0.561	0.475	2.30431464658513E-133	2	PRKACA
2.9762857661132E-72	0.891770919358601	0.442	0.378	4.9876596868525E-68	2	SLBP
4.87259048266385E-286	0.891288086448458	0.714	0.569	8.16548713084807E-282	2	PSME1
4.58147777297072E-224	0.890274304062009	0.637	0.467	7.67764045194433E-220	2	CAPN2
5.56276304687746E-21	0.889896364265161	0.305	0.284	9.32207831395724E-17	2	PPIA
7.66136540760315E-85	0.889615323990818	0.461	0.379	1.28389161500614E-80	2	FRK
1.69797663054446E-47	0.889494910571391	0.404	0.36	2.84546923746641E-43	2	MGAT2
1.37973777444929E-10	0.889245972335121	0.294	0.293	2.31216456242212E-06	2	TMEM9

0	0.888439120505113	0.788	0.614	0	2	BST2
0	0.887049770033542	0.842	0.66	0	2	MT-ND3
3.09933816984586E-13	0.886482845668853	0.312	0.308	5.19387090502769E-09	2	RTL8C
2.58448112729374E-06	0.886138045197233	0.265	0.27	0.0433107347311885	2	FOXP4
1.34567960760461E-23	0.88609340836453	0.334	0.316	2.25508988642381E-19	2	ATP6V1A
2.12428188046073E-10	0.88563651862693	0.258	0.249	3.5598715752761E-06	2	SGTA
1.63801733101412E-63	0.87658674605514	0.453	0.41	2.74498944331347E-59	2	ALDH9A1
3.23110011059574E-96	0.87579177318384	0.5	0.433	5.41467756533635E-92	2	CUTA
8.67520018593209E-09	0.874083057651541	0.254	0.248	0.00014537900471585	2	AGMAT
2.20778255575614E-15	0.873226790081525	0.266	0.246	3.69980200693614E-11	2	CLPP
1.29041954675877E-16	0.872227363909911	0.251	0.226	2.16248507645834E-12	2	CALM1
1.56542941479654E-23	0.871167532571427	0.329	0.307	2.62334661331604E-19	2	GUK1
7.69130194317358E-07	0.86944719240729	0.258	0.26	0.0128890837963703	2	PSMB2
0	0.865452282967002	0.771	0.636	0	2	ECH1
4.7624654983015E-07	0.865077817817321	0.275	0.279	0.00798093968205365	2	TDP2
0	0.864933301165183	0.834	0.637	0	2	HMGCS2
9.10601140792826E-13	0.864558505024637	0.312	0.311	1.52598539174062E-08	2	MLX
3.35735379872391E-52	0.862982529301042	0.384	0.328	5.62625349590153E-48	2	MYH14
2.08176192186401E-10	0.861605908754659	0.275	0.268	3.4886166286597E-06	2	DDAH1
3.98005205715971E-55	0.861543980777486	0.428	0.386	6.66977123738825E-51	2	RNF5
2.50529800651992E-27	0.859875060651873	0.356	0.335	4.19837839932609E-23	2	SRI
5.12141029091446E-17	0.858551018853914	0.323	0.313	8.58245936551445E-13	2	PNPO
1.61604443798128E-270	0.858012581877481	0.747	0.551	2.70816726916903E-266	2	ANPEP
8.17219215015809E-08	0.856814212663341	0.259	0.254	0.00136949596052349	2	FADS2
1.27395756321714E-48	0.85574260859866	0.417	0.375	2.13489808443928E-44	2	HACD3
2.89077302500229E-141	0.855059745226447	0.559	0.472	4.84435743529884E-137	2	ARF1
4.9813425197181E-45	0.85478266678722	0.421	0.389	8.3477337945436E-41	2	RAB4A
0	0.854711709586804	0.776	0.519	0	2	ATP1B1
0	0.854605629929161	0.764	0.498	0	2	SELENBP1
1.04145689910644E-09	0.854397966479097	0.295	0.294	1.74527347152258E-05	2	TMCO3
1.3671516342076E-06	0.854046516162762	0.265	0.269	0.022910727086051	2	NDUFB1
2.22329370712933E-12	0.851435771953622	0.291	0.28	3.72579559440734E-08	2	TM7SF2
1.36396540284181E-09	0.850864746085665	0.255	0.247	2.28573322208231E-05	2	MIEN1
3.82319490246538E-29	0.850342489005386	0.36	0.336	6.40691001755149E-25	2	PSMC3
2.93383131349499E-41	0.849874907506441	0.269	0.21	4.91651451515491E-37	2	MARCKSL1
8.76963201942193E-87	0.847650770935107	0.395	0.284	1.46961493381473E-82	2	FXYD3
8.60882252413559E-13	0.846264251096884	0.31	0.306	1.44266647859464E-08	2	NQO2
3.73684664652286E-56	0.846018682818037	0.443	0.403	6.26220761024301E-52	2	ATOX1
1.47140736482465E-28	0.845976767212225	0.377	0.359	2.46578446197314E-24	2	LACTB2
0	0.844155634311207	0.791	0.595	0	2	KHK
5.28058126215069E-203	0.843789086739718	0.652	0.54	8.84919807911212E-199	2	MT-ND1
0	0.843026039030452	0.901	0.562	0	2	TSC22D3
7.82867320148527E-90	0.842639001904017	0.518	0.447	1.3119290551049E-85	2	GUSB
0	0.842259661487706	0.881	0.669	0	2	MT-CYB
1.8201353866533E-18	0.840233800832398	0.311	0.294	3.05018288095361E-14	2	NDUFB11
1.37438334693715E-13	0.839684070657683	0.311	0.306	2.30319161279727E-09	2	ZDHHC9
1.68994240711715E-30	0.839274510767483	0.381	0.36	2.83200548584692E-26	2	DCAF11
5.73132177742664E-22	0.8358739281551	0.338	0.323	9.60454903461156E-18	2	NEDD8
3.18873438635382E-09	0.835799664558565	0.282	0.28	5.34368108465173E-05	2	STOML2
4.03896634482068E-101	0.834670048036274	0.508	0.423	6.76849980065049E-97	2	MORF4L2
3.2708895749217E-22	0.834533311818592	0.321	0.299	5.48135674965379E-18	2	ACTR1B
4.69393265552826E-12	0.834155161574643	0.307	0.302	7.86609234413426E-08	2	ENAH
1.76666686420961E-06	0.831750173880292	0.251	0.251	0.0296058033104246	2	GLRX5
9.57748773202277E-198	0.831649399105933	0.648	0.534	1.60499539413238E-193	2	SOD1
2.24124016576716E-07	0.830531411724519	0.267	0.267	0.00375587026979261	2	NDUFA11
0	0.83013142514375	0.768	0.592	0	2	LMNA
2.56462057186334E-97	0.829667024235223	0.492	0.419	4.29779115432858E-93	2	STK24
2.04319216381435E-22	0.829019602347673	0.366	0.35	3.42398142812008E-18	2	LMO7
3.21966306061864E-41	0.82872269309796	0.416	0.386	5.39551135698471E-37	2	PGM1
4.06418151777165E-09	0.827287719266365	0.261	0.255	6.81075538748174E-05	2	PRDX5

2.14664622019603E-106	0.826343182166104	0.539	0.47	3.59734973580451E-102	2	PUF60
6.69627723319112E-117	0.824394782171687	0.535	0.439	1.12216213873817E-112	2	MIDN
1.53753982819068E-18	0.822206949649358	0.329	0.312	2.57660924408193E-14	2	RDH10
1.21193007262833E-133	0.820782168155573	0.562	0.463	2.03095241571056E-129	2	CNN3
2.27219498466964E-125	0.820669637788671	0.541	0.45	3.80774435530939E-121	2	PSMB8
6.13629299294211E-42	0.820648228618301	0.402	0.37	1.02831997975724E-37	2	NDUFB10
1.92680571510881E-30	0.819960419757584	0.361	0.335	3.22894101737934E-26	2	DYNLRB1
2.59815301758158E-11	0.819613378438394	0.294	0.285	4.35398482686321E-07	2	LRRC31
3.6082161010543E-22	0.818822014204277	0.366	0.356	6.0466485421468E-18	2	IARS2
3.77271575512573E-07	0.81651430844289	0.253	0.25	0.00632231706243969	2	CCDC124
6.49094576773114E-28	0.815349122107979	0.358	0.335	1.08775269175638E-23	2	POLR2E
1.56705076962398E-26	0.814330143740701	0.346	0.321	2.62606367973586E-22	2	NDUFA13
2.54794230783437E-31	0.813670258252277	0.351	0.32	4.26984171946883E-27	2	HNRNPAB
8.35003508447334E-14	0.813013253606454	0.283	0.269	1.39929887945604E-09	2	ABRACL
5.44307511448177E-14	0.812976755666526	0.327	0.326	9.12150527684856E-10	2	COX16
1.08800725808748E-19	0.811647665717372	0.319	0.302	1.82328256310301E-15	2	CORO1B
3.211870079489E-148	0.81132431603559	0.602	0.508	5.38245187920766E-144	2	HNRNPF
6.99932386685027E-39	0.809434186641466	0.414	0.387	1.17294669360677E-34	2	COX6A1
0	0.809330726878999	0.994	0.846	0	2	VTN
4.50614763432254E-141	0.8066504265511	0.588	0.484	7.55140220559771E-137	2	SCCPDH
3.1670305794699E-146	0.805426250331392	0.578	0.443	5.30730984507566E-142	2	TFRC
3.01640589479443E-25	0.804367472370139	0.326	0.3	5.05489299849651E-21	2	RAB11FIP1
2.84996064189793E-13	0.803483239614588	0.286	0.275	4.77596404369255E-09	2	RAB20
3.855402858188E-08	0.801727681134935	0.299	0.306	0.000646088410975145	2	PACSIN2
2.32530260621863E-09	0.801633154409796	0.295	0.296	3.89674210750118E-05	2	NEU1
1.55024926720064E-84	0.801054558859437	0.475	0.374	2.59790772197483E-80	2	HNF4G
5.12880030489622E-29	0.798317775209244	0.36	0.337	8.59484355094509E-25	2	SUB1
2.89974937596193E-34	0.794834461718621	0.412	0.393	4.859400004237E-30	2	HTATIP2
3.88321223035892E-27	0.793184863130224	0.319	0.28	6.50748705563547E-23	2	TUBB
1.57053311466176E-08	0.791799917513921	0.297	0.3	0.000263189939355018	2	ISOC1
8.07356664364171E-34	0.789432300798609	0.411	0.392	1.35296829814148E-29	2	POLDIP2
3.87534619936878E-135	0.788132310051819	0.548	0.381	6.49430516090221E-131	2	F2RL1
6.38762431044172E-51	0.78736859875219	0.44	0.402	1.07043808194382E-46	2	RTN3
7.69809404071903E-51	0.785229231693884	0.389	0.34	1.2900465993437E-46	2	G3BP2
3.31855918447953E-09	0.785019298486953	0.318	0.326	5.56124148135079E-05	2	ECI1
7.61202327510068E-71	0.784821998532109	0.486	0.439	1.27562286044137E-66	2	RAB2A
4.3611790679532E-13	0.784377097412223	0.304	0.296	7.30846388207597E-09	2	SEPTIN8
2.14135212226201E-20	0.784055179176156	0.353	0.335	3.58847788648668E-16	2	LSS
4.14647049499179E-66	0.783947696472677	0.486	0.443	6.94865525550725E-62	2	NDUFA3
3.65659804035423E-08	0.782196340681196	0.28	0.279	0.000612772699602563	2	MSRB2
8.4584088113691E-11	0.781979672462071	0.279	0.272	1.41746014860923E-06	2	PPM1G
2.24294695353041E-22	0.77854367458795	0.308	0.281	3.75873050472627E-18	2	EDNRB
1.9574777029403E-20	0.776350454071031	0.325	0.306	3.28034113458735E-16	2	RABAC1
4.0580518129601E-42	0.775414975812001	0.454	0.421	6.80048322815853E-38	2	NUDT16
2.32039346748488E-33	0.774460197070035	0.386	0.357	3.88851537281117E-29	2	COX8A
1.53196803449904E-40	0.7734119324674	0.39	0.351	2.56727203221349E-36	2	FLOT2
2.64263922266349E-127	0.77307163116338	0.54	0.448	4.42853480933947E-123	2	YWHAZ
6.50701039527391E-89	0.772643474388117	0.55	0.473	1.09044480204E-84	2	CDHR5
1.71059414194014E-51	0.772397830230233	0.456	0.423	2.86661366306328E-47	2	ENY2
2.42895277293712E-11	0.770071922166354	0.296	0.292	4.07043905688803E-07	2	APRT
1.46550867413777E-14	0.768224365900102	0.34	0.339	2.45589943612007E-10	2	MICOS10
3.07188917824247E-07	0.767154222094309	0.28	0.283	0.00514787188489873	2	ATP5F1A
1.43288509906913E-99	0.763946676436613	0.528	0.454	2.40122884902005E-95	2	CALM2
1.24756627383661E-61	0.761740135374349	0.484	0.439	2.09067156169539E-57	2	MGST2
9.52055845874117E-39	0.761355448424447	0.427	0.398	1.59545518651584E-34	2	SLC22A18
8.49589407205441E-07	0.76112783619206	0.267	0.268	0.0142374192859488	2	COX6B1
1.70661931302709E-13	0.760956663590618	0.332	0.331	2.8599526447708E-09	2	FAM199X
1.40891594513354E-184	0.757556063438106	0.693	0.528	2.36106134085479E-180	2	HSD17B11
3.23621404968394E-09	0.755163342754033	0.322	0.327	5.42324750446034E-05	2	ALAD
1.40127073590421E-29	0.752838584579891	0.398	0.374	2.34824949922827E-25	2	PDZD8

2.04518867302953E-24	0.752267666762677	0.376	0.363	3.42732717826289E-20	2	DBI
0	0.75128757062005	0.883	0.688	0	2	DEPRECATED-ENSG00000106538
2.5342468234859E-116	0.750804013875462	0.589	0.516	4.24689082679767E-112	2	ADI1
6.39915817134256E-72	0.75004381431644	0.499	0.448	1.07237092635359E-67	2	SEPHS2
1.81597065800898E-38	0.749404413790738	0.427	0.403	3.04320362869146E-34	2	ATP5IF1
3.10015810448029E-147	0.748325380710829	0.536	0.418	5.19524495148807E-143	2	EZR
1.83589730801141E-17	0.747329169671249	0.349	0.337	3.07659670876552E-13	2	FDFT1
5.75616908692405E-09	0.746698698048909	0.313	0.317	9.64618815586732E-05	2	TMEM38B
8.34206540870961E-132	0.746474776868905	0.617	0.513	1.39796332119156E-127	2	RARRES2
3.51152571107118E-15	0.743529725713117	0.329	0.321	5.88461478661309E-11	2	GOT1
4.68023272684683E-83	0.742942612505968	0.511	0.446	7.84313400364992E-79	2	TMBIM1
1.41699756748353E-07	0.742567931016477	0.284	0.282	0.0023746045235889	2	HSD17B2
3.37100038048014E-43	0.742107153129159	0.398	0.356	5.64912243760862E-39	2	RHOC
1.81149669553038E-37	0.742097931739251	0.43	0.406	3.03570616236982E-33	2	GSR
5.51566802717775E-23	0.741311318650343	0.399	0.39	9.24315647994447E-19	2	CPQ
1.56722848927443E-30	0.741181452930406	0.398	0.38	2.62636150232608E-26	2	EDF1
1.63384011110287E-92	0.741034249375503	0.522	0.45	2.7379892581862E-88	2	MIF
3.49809858621504E-10	0.740419911128465	0.322	0.327	5.86211361077916E-06	2	SIAH2
1.7472697163541E-45	0.739120824256123	0.433	0.402	2.9280745906662E-41	2	COX4I1
1.58872005558742E-17	0.737932680283157	0.292	0.267	2.66237706915339E-13	2	ARPC1B
5.46338505573339E-66	0.737222561285096	0.481	0.432	9.15554067639801E-62	2	P4HB
7.93722546887703E-85	0.73603483962649	0.519	0.431	1.33012024407441E-80	2	KCTD3
1.80942529814525E-10	0.735764043097532	0.308	0.308	3.03223491463181E-06	2	RBM42
6.4531972750988E-08	0.735548253984336	0.281	0.281	0.00108142679936106	2	DPP4
1.59026940271586E-07	0.734139703183328	0.28	0.282	0.00266497346507123	2	SMS
1.05277117581362E-08	0.732510551842209	0.308	0.313	0.000176423393642847	2	COPE
6.99982664598211E-31	0.731339790816918	0.408	0.388	1.17303094933368E-26	2	LAMTOR2
2.6514107780302E-23	0.731045257004453	0.364	0.349	4.44323418182301E-19	2	ATP5MG
4.16318448691402E-38	0.730428008819302	0.435	0.412	6.97666456317051E-34	2	EIF6
2.49068183385142E-65	0.72730334901675	0.486	0.434	4.17388461716821E-61	2	BSG
2.2672129624785E-19	0.725902576619682	0.365	0.357	3.79939548252147E-15	2	PTPA
3.28254692934959E-11	0.725689070228506	0.327	0.323	5.50089214420405E-07	2	CNKSR3
2.03377629940241E-07	0.723888179197703	0.315	0.324	0.00340820232253856	2	ACMSD
2.18197399197666E-39	0.723339983923365	0.373	0.33	3.65655201575448E-35	2	VASP
3.6734357564494E-15	0.720809759928067	0.352	0.348	6.15594364065791E-11	2	RETSAT
0	0.72013722164459	0.964	0.813	0	2	MT-ATP6
2.74031553872141E-12	0.720119798408578	0.26	0.244	4.59222077978934E-08	2	YWHAH
4.90380032070218E-299	0.718535772991679	0.838	0.581	8.21778857743272E-295	2	ASPH
8.53103261805767E-19	0.718276552582784	0.355	0.347	1.4296304461341E-14	2	CRTAP
4.58570694166848E-31	0.714914642320356	0.413	0.395	7.68472769284804E-27	2	UQCRC1
2.69858791798948E-48	0.714061107104522	0.475	0.444	4.52229363296677E-44	2	NFIC
3.0788737558984E-23	0.713944577224327	0.385	0.378	5.15957664013455E-19	2	VPS28
1.18447960598703E-07	0.713081095802044	0.288	0.292	0.00198495092371306	2	AP2A1
1.32130935674724E-40	0.711571225227657	0.445	0.424	2.21425022003702E-36	2	ARL6IP4
8.18930507064421E-14	0.709834898445006	0.355	0.358	1.37236374373856E-09	2	SNRPC
9.96199985589432E-08	0.709181864406155	0.313	0.324	0.00166943193585077	2	DUS1L
1.84731469235819E-85	0.708382599524518	0.579	0.523	3.09572996145385E-81	2	TMCO1
7.22608862363042E-185	0.708347837050235	0.719	0.567	1.21094793154799E-180	2	TM4SF4
5.18968537934904E-107	0.707441534304381	0.567	0.469	8.69687475871312E-103	2	ATP6AP1
5.48855847270526E-20	0.706280795574776	0.369	0.361	9.19772628855948E-16	2	ATP6V0E1
3.76345724703399E-55	0.705391248866473	0.433	0.387	6.30680165457956E-51	2	HNRNPUL1
3.94838960627381E-34	0.705234209283101	0.415	0.395	6.61671130219365E-30	2	SERF2
9.33686128477081E-17	0.70278696218597	0.307	0.279	1.56467121410189E-12	2	KCNK5
2.36252034329408E-18	0.702176629454725	0.344	0.333	3.95911159129222E-14	2	PSMB10
1.28727077881374E-08	0.70045897814526	0.314	0.316	0.000215720837113607	2	GRB10
1.79467691325128E-31	0.699322154027075	0.38	0.356	3.0075195712265E-27	2	MTCH1
1.30132259290819E-13	0.698690502081373	0.326	0.321	2.18075640119554E-09	2	ATP5F1E
5.68406844920139E-69	0.698054572463104	0.502	0.43	9.52536190717169E-65	2	VNN 1.00
1.06342380884478E-07	0.695802101451618	0.322	0.331	0.00178208561886208	2	POGK
3.86124128247214E-14	0.695119219511644	0.317	0.309	6.4706681411668E-10	2	DEPRECATED-ENSG00000179010

3.28223836871832E-10	0.694128713609689	0.345	0.354	5.50037505829816E-06	2	SLC44A1
3.30637749033715E-16	0.693986525958015	0.368	0.369	5.540827398307E-12	2	ELAVL1
1.44252052913743E-43	0.693875445270207	0.46	0.421	2.4173759027285E-39	2	GJB1
1.1790605226065E-38	0.693021943247648	0.447	0.417	1.97586962378398E-34	2	EBP
2.14651590078456E-14	0.692819755507749	0.363	0.367	3.59713134653476E-10	2	EIF2AK1
3.84965817248836E-45	0.691447158202599	0.448	0.417	6.45125716545599E-41	2	RAB5C
2.98014444517484E-17	0.689843697534088	0.376	0.376	4.99412606122399E-13	2	MCFD2
5.47732591866083E-09	0.687029189217553	0.32	0.326	9.17890277449182E-05	2	CTDSP1
7.08496355761401E-18	0.686473972242982	0.364	0.36	1.18729819298496E-13	2	RTF2
4.13740230047788E-102	0.683438851604554	0.587	0.506	6.93345877514082E-98	2	TRAM1
5.4547955460823E-44	0.683283547978302	0.457	0.433	9.14114637612473E-40	2	ATP5MC2
1.10359672341374E-179	0.683001747977157	0.684	0.521	1.84940738909675E-175	2	DNM2
2.46731926501756E-14	0.682614198489755	0.295	0.28	4.13473362431643E-10	2	TSPO
0	0.680901554268142	0.945	0.804	0	2	MT-ND4
8.27823616005343E-31	0.679695687642157	0.435	0.421	1.38726681570175E-26	2	ACBD5
7.31995641574293E-16	0.679379813107845	0.357	0.342	1.2266782961502E-11	2	GPRC5C
3.31659247323214E-14	0.678877364975263	0.343	0.34	5.55794566664243E-10	2	KDELR2
1.07978711575196E-56	0.678806931440816	0.476	0.427	1.80950724857713E-52	2	SDC2
1.80473760017053E-19	0.67804011385631	0.37	0.363	3.02437927036577E-15	2	ATP5MC3
8.7377340456046E-11	0.677126498301595	0.341	0.348	1.46426947136242E-06	2	SDHB
5.65336861495133E-38	0.676361703222523	0.426	0.378	9.47391512493544E-34	2	CLDN7
2.19568062750529E-227	0.676100531490398	0.898	0.806	3.67952159557336E-223	2	SQSTM1
1.06618204692563E-28	0.672520189997657	0.421	0.403	1.78670787423798E-24	2	STT3A
3.30337067837088E-48	0.672497817656818	0.478	0.452	5.53578858281392E-44	2	AKR1A1
2.87354792885348E-83	0.67225377720244	0.558	0.504	4.81549161917266E-79	2	COPG1
4.14438632543715E-15	0.672094537749252	0.359	0.353	6.94516260416757E-11	2	ERG28
6.85456677901111E-69	0.670299650182594	0.539	0.492	1.14868830082668E-64	2	ACOX1
1.10693416157993E-11	0.669410114410094	0.352	0.354	1.85500026797564E-07	2	FDPS
8.69557892690257E-50	0.669113669003867	0.477	0.432	1.45720511657033E-45	2	INF2
1.66645470851828E-74	0.666488895706928	0.542	0.472	2.79264480053493E-70	2	LSR
5.8642721797777E-11	0.664060529075917	0.305	0.297	9.82734731887147E-07	2	PKLR
0	0.662783870676818	0.886	0.754	0	2	OAZ1
2.06050260021187E-113	0.662292836667766	0.636	0.55	3.45299025743505E-109	2	GOLIM4
3.05676847026858E-13	0.661765530909	0.374	0.383	5.12253260247609E-09	2	SPATS2L
2.94766911014047E-10	0.66167931235649	0.333	0.337	4.9397038947734E-06	2	PXMP2
1.28560464693425E-09	0.659486984849579	0.302	0.301	2.15441626733242E-05	2	CDK16
2.75693742132027E-149	0.657820196249195	0.729	0.633	4.62007573064851E-145	2	NUPR1
7.1798021007903E-62	0.65656325215137	0.518	0.477	1.20319123605044E-57	2	SUMF2
2.51015657799711E-27	0.656418365349525	0.411	0.387	4.20652039340756E-23	2	GRTP1
1.71752021457402E-33	0.65631930764012	0.417	0.39	2.87822037558314E-29	2	S100A13
6.46757300928353E-18	0.656100663602309	0.384	0.383	1.08383588489573E-13	2	DEPRECATED-ENSG00000163463
2.14478800171634E-137	0.656073779236293	0.663	0.529	3.59423573327624E-133	2	PROX1
5.49413142981285E-14	0.654662143063366	0.348	0.345	9.20706545008038E-10	2	HSD17B10
4.41645861528181E-15	0.65076058700924	0.348	0.346	7.40110134748925E-11	2	AMFR
9.12009502641969E-07	0.650288697388501	0.283	0.288	0.0152834552452741	2	MECP2
0	0.650112561338606	0.987	0.879	0	2	MT-CO1
2.91571266554384E-77	0.649080533303509	0.539	0.488	4.88615128491837E-73	2	NUCKS1
1.17822715123138E-43	0.647393825338656	0.342	0.284	1.97447306003354E-39	2	MT-ND6
8.74767617088734E-08	0.647011477871572	0.273	0.27	0.0014659355727173	2	DEPRECATED-ENSG00000163931
1.01325279021203E-132	0.646145714055306	0.643	0.563	1.69800902583732E-128	2	ETFB
1.26231782230575E-259	0.645952751055871	0.793	0.667	2.11539220661997E-255	2	EEF2
2.62823805419552E-20	0.641282271971517	0.395	0.393	4.40440133122086E-16	2	SEC13
3.98163389785746E-20	0.640106277935537	0.391	0.388	6.67242208602954E-16	2	KRTCAP2
7.61530273057929E-15	0.63639539683597	0.359	0.355	1.27617243159048E-10	2	SMC1A
1.21643287373592E-39	0.634484802256714	0.454	0.428	2.03849820980665E-35	2	GSDMD
6.22546454259408E-94	0.63067936916478	0.585	0.513	1.04326334804792E-89	2	FBP1
0	0.629506106842138	0.958	0.773	0	2	ALDOB
1.15562269552879E-21	0.62807102884775	0.389	0.384	1.93659251316715E-17	2	ILRUN
2.97917364438652E-13	0.624852436448135	0.347	0.348	4.99249919326294E-09	2	RAB8A
1.08801717437842E-06	0.624569214092153	0.321	0.335	0.0182329918082336	2	AP3D1

7.31493117541333E-49	0.622652653167521	0.5	0.455	1.22583616637577E-44	2	HABP2
6.53082088744841E-22	0.620931290990243	0.385	0.371	1.0944349643186E-17	2	SERPINB1
7.80843001840862E-44	0.618983175074421	0.474	0.449	1.30853670248492E-39	2	MAPKAPK2
2.07193245203415E-85	0.616017092539593	0.6	0.544	3.47214440311883E-81	2	APLP2
2.80948601613918E-12	0.613821770013291	0.354	0.357	4.70813666584604E-08	2	PSMD3
6.64605393478738E-64	0.613552085335355	0.497	0.409	1.11374571839167E-59	2	C2orf72
1.83178947834986E-18	0.612305644698017	0.398	0.391	3.0697128078187E-14	2	PDZK1
1.48737092193338E-11	0.611934736329096	0.354	0.359	2.49253619097596E-07	2	C4orf3
4.59021880564992E-49	0.611552113391907	0.485	0.451	7.69228867450814E-45	2	UBB
8.28299433492455E-10	0.610471030610412	0.299	0.289	1.38806419064666E-05	2	MCM7
5.27740027922218E-32	0.60924960457077	0.434	0.416	8.84386738792053E-28	2	CTNNA1
3.98708519550286E-27	0.608248744654675	0.401	0.389	6.68155737062369E-23	2	EIF4H
2.41061875889511E-131	0.606534584526468	0.709	0.636	4.03971491615642E-127	2	GSTK1
9.40063930787413E-10	0.605196968476275	0.321	0.323	1.57535913521355E-05	2	EIF1B
2.31991122816051E-08	0.605109887118401	0.316	0.319	0.000388770723615138	2	IDI1
6.3606514643326E-42	0.604072718663581	0.482	0.458	1.06591797239286E-37	2	MYDGF
2.38495441068696E-06	0.601240286977785	0.33	0.348	0.039967066014292	2	HEXB
9.12003680160422E-12	0.600869872280904	0.337	0.339	1.52833576721284E-07	2	ENSA
7.12984951461689E-10	0.599748000405955	0.359	0.37	1.1948201816595E-05	2	MLEC
5.11873639351946E-79	0.596087178719849	0.547	0.474	8.57797844825991E-75	2	GDI1
2.37860985989106E-09	0.595280583205873	0.331	0.334	3.98607440320543E-05	2	PIK3AP1
2.99917686836247E-11	0.593373892044927	0.342	0.345	5.02602059600182E-07	2	HIPK2
5.51911885254145E-39	0.592565014001894	0.484	0.455	9.24893937308896E-35	2	RRBP1
6.77247858796432E-115	0.591877511300626	0.714	0.598	1.13493196177106E-110	2	SERPINA6
6.297470966751E-36	0.590495056382605	0.478	0.471	1.05533018460813E-31	2	NAPA
2.00744879323384E-25	0.5888519521087	0.435	0.41	3.36408268770127E-21	2	ZHX1
1.67185589519139E-222	0.588025905655316	0.95	0.765	2.80169610916174E-218	2	AGT
1.37672055206186E-28	0.587296771920068	0.463	0.46	2.30710830114527E-24	2	HINT1
3.08778219924069E-56	0.587207946476913	0.541	0.492	5.17450540948754E-52	2	DECR1
4.38261428742584E-13	0.584980537633122	0.364	0.366	7.34438502286822E-09	2	SNU13
6.50914627474337E-163	0.58462836497327	0.714	0.497	1.09080273272149E-158	2	MUC3A
4.87289529883189E-93	0.58455600920138	0.585	0.516	8.16599794178248E-89	2	ACTN4
4.63074752167544E-23	0.584283483820755	0.416	0.41	7.7602066968237E-19	2	MLF 2.00
1.59696135854595E-50	0.584201429541801	0.51	0.464	2.67618784465131E-46	2	OAF
5.33293525046781E-23	0.582155515237178	0.421	0.416	8.93693289273395E-19	2	ADIPOR1
1.50124999608164E-12	0.582128362582689	0.394	0.408	2.51579474343361E-08	2	DDRGK1
3.590773396118E-117	0.579558237010879	0.578	0.475	6.01741805721454E-113	2	NPC2
1.86864971940972E-15	0.57830144776265	0.364	0.348	3.13148319978681E-11	2	TMPRSS2
7.35240904322274E-169	0.576466080294684	0.733	0.494	1.23211670746327E-164	2	FKBP5
3.33462810173473E-08	0.575701620387269	0.373	0.393	0.000558816977288706	2	CLPTM1
1.09747803501552E-59	0.574882662074957	0.54	0.5	1.839153691079E-55	2	CYB5R3
4.34807482501079E-59	0.574344076157668	0.508	0.464	7.28650379175309E-55	2	SDF4
0	0.57426965858851	0.962	0.821	0	2	MT-CO3
1.14328720150342E-15	0.569045781553281	0.344	0.335	1.91592069227942E-11	2	TNIP1
6.363880686177E-48	0.566292533566186	0.51	0.484	1.06645912538954E-43	2	EIF1
2.33604921353955E-73	0.565684642607129	0.59	0.514	3.91475127204958E-69	2	RNASE4
2.49553902948849E-31	0.565626970159213	0.425	0.409	4.18202430561681E-27	2	SEPTIN9
9.89931606618185E-17	0.564344117323528	0.355	0.346	1.65892738637075E-12	2	PIM3
6.30090843291309E-65	0.561804439306621	0.587	0.528	1.05590623518758E-60	2	TM9SF2
7.0609050004091E-11	0.55618893534002	0.367	0.371	1.18326645996856E-06	2	CYP51A1
1.29640954144639E-199	0.555706308556569	0.845	0.724	2.17252310955586E-195	2	MT-ND4L
5.16151285538532E-09	0.552870935328332	0.356	0.36	8.64966324305472E-05	2	STBD1
4.9488752838835E-37	0.552760616208267	0.475	0.461	8.29332520073197E-33	2	CAST
0	0.550632749882245	0.956	0.767	0	2	FN1
3.92504978471811E-22	0.550254897974124	0.444	0.446	6.57759842923061E-18	2	LAMTOR4
1.80935831352212E-95	0.548264914814785	0.588	0.463	3.03212266180036E-91	2	AKR1C2
1.66547875023452E-06	0.54647710366341	0.355	0.377	0.0279100928964301	2	PIGT
2.03077253842914E-178	0.544656166090689	0.919	0.769	3.40316861989955E-174	2	GC
3.5627545169463E-10	0.540109493075467	0.34	0.344	5.97046401949861E-06	2	TMEM109
1.34582240154553E-21	0.538431096537521	0.442	0.44	2.25532918051E-17	2	PGRMC2

2.66163088697207E-42	0.53813449682894	0.525	0.506	4.4603610403878E-38	2	PRDX6
1.27218572192269E-22	0.53660692259982	0.458	0.466	2.13192883279805E-18	2	PSMB1
6.65901517685278E-18	0.534933584170182	0.299	0.264	1.11591776333699E-13	2	LMNB1
4.07141022254701E-20	0.527814192647838	0.417	0.409	6.82286925094428E-16	2	HNF4A
2.01040559315445E-08	0.525915434414567	0.38	0.402	0.000336903769300823	2	TAF10
5.21223775331166E-26	0.525792650017756	0.455	0.428	8.7346680270332E-22	2	ZBTB7B
3.51163514997049E-72	0.524990065692951	0.588	0.545	5.88479818432055E-68	2	PEBP1
3.27343562750663E-13	0.518542577408484	0.36	0.361	5.48562342457562E-09	2	SYAP1
1.11854918544144E-78	0.517873171657889	0.576	0.514	1.87446472496277E-74	2	APP
1.16153612311281E-15	0.513619025413251	0.427	0.431	1.94650223511245E-11	2	RIDA
1.16916673943449E-07	0.512314556569916	0.353	0.367	0.00195928962194431	2	NMT1
9.54727062779363E-28	0.511846186380491	0.453	0.448	1.59993161180566E-23	2	RACK1
6.76578096010412E-22	0.508840925292594	0.456	0.46	1.13380957329425E-17	2	ERGIC3
1.35028950758806E-47	0.506989041466506	0.482	0.446	2.26281515681608E-43	2	HNRNPA0
8.78946786963295E-261	0.503888324448545	0.971	0.819	1.47293902559309E-256	2	SELENOP
2.18933962380582E-07	0.501387632995839	0.366	0.382	0.00366889534157379	2	SERP1
3.02168411568729E-40	0.501296834077923	0.522	0.483	5.06373824106877E-36	2	SDC1
7.06034022162346E-11	0.500764234276221	0.186	0.254	1.18317181433966E-06	2	ARHGAP5
1.06180477898439E-14	0.500032746161678	0.405	0.413	1.77937244862204E-10	2	CLINT1
2.32493603731726E-07	0.499608278632775	0.337	0.347	0.00389612781133626	2	PSMB9
9.39422220736762E-07	0.499393697903408	0.196	0.253	0.0157428375751067	2	TIMMDC1
1.22641500755203E-06	0.497761907739159	0.366	0.388	0.020552262696557	2	CANX
1.76873302223851E-95	0.497172574422201	0.697	0.628	2.96404279866729E-91	2	CERS2
2.30020011511659E-09	0.497080087997756	0.37	0.371	3.85467535291238E-05	2	RAP2C
2.05603782662139E-46	0.494685994355293	0.522	0.49	3.44550818985213E-42	2	CD63
1.60840058240716E-21	0.493545946626747	0.447	0.448	2.69535769599792E-17	2	SSR2
9.91769223960472E-15	0.493198362635247	0.405	0.414	1.66200686551296E-10	2	PDXK
2.19128575775945E-25	0.490812463878591	0.451	0.449	3.67215667285329E-21	2	EEF1G
1.00413214200663E-29	0.486038484377095	0.5	0.462	1.6827246435747E-25	2	SLC37A4
3.7106823245967E-46	0.466236804832981	0.594	0.584	6.21836143955916E-42	2	BRI3
1.41579535480844E-27	0.465951511970407	0.48	0.473	2.37258985558798E-23	2	UBA52
5.59561134678417E-07	0.465256614189485	0.344	0.357	0.00937712549494091	2	GNB2
2.97960117180928E-15	0.463390640143406	0.442	0.43	4.99321564371799E-11	2	ATRN
3.87996525461678E-08	0.463389344727445	0.393	0.41	0.000650204577368681	2	FAH
6.09458803332775E-07	0.463059180876628	0.366	0.385	0.0102133106262506	2	ADRM1
5.47931327242394E-97	0.462807429033528	0.648	0.558	9.18223318192804E-93	2	ADH1C
1.93059291045786E-06	0.462175460903938	0.195	0.25	0.0323528759934528	2	MSH6
2.74107116362076E-06	0.458657522346833	0.209	0.269	0.0459348705599567	2	GNPAT
8.32032067994632E-23	0.458314145417536	0.421	0.413	1.3943193395454E-18	2	CDC37
1.34673267459129E-285	0.455575152633292	0.96	0.812	2.25685461608009E-281	2	HSPB1
1.77285036528188E-28	0.454671641192821	0.434	0.38	2.97094264213937E-24	2	OCRL
1.52172563681452E-06	0.45301083571243	0.39	0.419	0.0255010782217378	2	BLOC1S1
3.30141433835056E-12	0.452842448010988	0.188	0.256	5.53251014820787E-08	2	MOCOS
1.34758484968384E-09	0.452473701423528	0.442	0.473	2.25828269110017E-05	2	RXRA
1.78936544992394E-229	0.446725274226808	0.814	0.555	2.99861862098254E-225	2	SGK1
8.33181190077904E-07	0.44573860949511	0.397	0.424	0.0139624503833255	2	RER1
7.13999095594385E-09	0.443345805309798	0.371	0.382	0.000119651968439707	2	DAZAP2
7.13608157598916E-16	0.441923164259545	0.427	0.435	1.19586455050426E-11	2	TRIR
3.1152251814336E-07	0.441642286002374	0.196	0.251	0.00522049435904642	2	CRACR2B
8.47230029842063E-84	0.440560315019775	0.618	0.546	1.41978808400933E-79	2	ACTG1
9.95821596522841E-11	0.438184235053551	0.435	0.439	1.66879783145298E-06	2	ENTPD5
1.66720845279469E-06	0.432791430037646	0.21	0.268	0.0279390792519334	2	ARHGEF40
1.17941466392294E-188	0.431245067425171	0.995	0.872	1.97646309380207E-184	2	ALB
3.63072699453316E-17	0.428040590961699	0.47	0.476	6.08437229743867E-13	2	CD151
5.58070749862545E-07	0.427626082047049	0.222	0.286	0.00935214962619653	2	PRKAB1
2.75025193870798E-06	0.427530201495106	0.36	0.374	0.0460887219888684	2	VAMP8
1.3884333402253E-11	0.425015252653322	0.192	0.261	2.32673659154955E-07	2	FBXL6
9.1489195447011E-99	0.424957420654513	0.699	0.608	1.53317593730101E-94	2	IGFBP4
5.27970504746006E-07	0.424812016551603	0.221	0.283	0.00884772971853357	2	DHTKD1
1.76262654402959E-20	0.419927070220027	0.465	0.457	2.95380956248478E-16	2	TPM1

2.84308262650268E-06	0.418886193606296	0.234	0.299	0.0476443786549319	2	NUDT9
1.02391283424657E-11	0.416960605139641	0.41	0.423	1.7158731276304E-07	2	PHPT1
2.0564477387266E-06	0.414072740434337	0.216	0.276	0.0344619512055804	2	RBMX
1.56726375028467E-10	0.411961836211642	0.416	0.435	2.62642059272705E-06	2	RTN4
1.31685877001057E-07	0.411641619484012	0.415	0.441	0.00220679192678371	2	EIF4EBP2
7.76519584894623E-09	0.409975006048375	0.193	0.254	0.000130129152036641	2	LRRC58
2.90694042011508E-07	0.407460667321008	0.403	0.428	0.00487145075602884	2	DEPRECATED-ENSG00000089248
7.27378277049179E-07	0.404641753406497	0.342	0.355	0.0121894051667901	2	SDCBP
1.81841787627432E-25	0.404531896751126	0.538	0.543	3.04730467706051E-21	2	GOLPH3
3.85177635621007E-11	0.397343162349029	0.454	0.479	6.45480681773684E-07	2	NDFIP1
2.91188419478208E-07	0.393882278241814	0.213	0.275	0.00487973553361581	2	MTIF3
7.7204479935155E-09	0.393591890779344	0.198	0.261	0.000129379267475333	2	AGGF1
2.56930370833462E-150	0.392963142219479	0.964	0.791	4.30563915442716E-146	2	CLU
4.27162367977449E-09	0.392869263071856	0.217	0.286	7.1583869625661E-05	2	PTK2
4.52369932679785E-08	0.392324293700532	0.191	0.25	0.000758081533184783	2	RCC1L
1.28390857076137E-10	0.389999203222834	0.187	0.251	2.15157398288191E-06	2	TMEM161A
1.37229420941269E-06	0.386366980325613	0.231	0.294	0.0229969063613378	2	TRMT2B
8.28648766618487E-116	0.382838121316222	0.718	0.565	1.38864960309926E-111	2	SERPINA7
7.51478296443101E-12	0.382302222231133	0.455	0.48	1.25932732917935E-07	2	CMPK1
5.29347348331465E-83	0.379391763917166	0.749	0.681	8.87080286333869E-79	2	SCP2
8.94665767064578E-84	0.379161629676842	0.74	0.666	1.49928089244682E-79	2	LAMP1
5.54014818839023E-07	0.378444414224767	0.213	0.273	0.00928418033410434	2	WASL
8.49800829637135E-66	0.377965985633836	0.585	0.531	1.42409623030591E-61	2	CTSD
1.49802554871677E-108	0.375961641921524	0.709	0.615	2.51039121453957E-104	2	NFKBIA
4.96517275357997E-07	0.364555215984631	0.204	0.262	0.00832063650044932	2	TCAF1
1.61424124070556E-06	0.363741694887436	0.218	0.279	0.0270514547117437	2	SMIM7
2.05933903719377E-06	0.358499456226056	0.218	0.28	0.0345104035852933	2	ARHGAP1
8.11444549667791E-34	0.358437995261558	0.542	0.509	1.35981877633328E-29	2	H1F0
1.70980873820409E-09	0.357435907064675	0.188	0.25	2.86529748348242E-05	2	BROX
1.5118935342864E-10	0.351172779115365	0.198	0.267	2.53363118475715E-06	2	TOMM70
3.68094526077081E-35	0.350803808246563	0.683	0.639	6.16852806799972E-31	2	METTL7A
6.35058487555738E-22	0.349843142919718	0.527	0.518	1.06423101344591E-17	2	CREG1
2.07679206953305E-14	0.349347807159368	0.189	0.265	3.48028815012349E-10	2	PLA2G12A
3.52284882828489E-20	0.345224326298097	0.173	0.256	5.90359006643981E-16	2	LMBRD2
1.10298591471954E-07	0.345015519117455	0.224	0.291	0.00184838379588701	2	LMBR1
6.71647208024093E-105	0.339937317757257	0.797	0.639	1.12554639120678E-100	2	ACSL1
8.462862072496E-09	0.337689740316138	0.205	0.27	0.000141820642610888	2	MAPK1IP1L
5.94962266318086E-07	0.337052491158274	0.212	0.271	0.00997037765895848	2	COA4
2.94592682487822E-42	0.336861179450995	0.662	0.646	4.93678417313092E-38	2	NUCB1
3.85258268129904E-11	0.334434584530119	0.403	0.404	6.45615805732092E-07	2	PLAAT4
2.68522491926803E-09	0.331885498032877	0.191	0.253	4.49989991970936E-05	2	C6orf120
2.41585086713526E-08	0.328632829007903	0.231	0.3	0.000404848288314526	2	DNAJB9
2.75604703990275E-11	0.328626184750458	0.186	0.253	4.61858362946903E-07	2	TAF15
3.86890418543702E-57	0.326117349062072	0.675	0.647	6.48350963395536E-53	2	HSP90AB1
9.5404320299609E-16	0.326012406116621	0.511	0.507	1.59878559958085E-11	2	AADAC
1.2174228483698E-28	0.326004101800782	0.573	0.562	2.04015720929811E-24	2	XBP1
1.49394526804221E-08	0.325450028672616	0.23	0.296	0.000250355348018514	2	CYP4F12
2.31342973432563E-44	0.323364515992683	0.709	0.67	3.8768455487829E-40	2	ST6GAL1
1.77210471259964E-12	0.320354731978221	0.202	0.274	2.96969307737448E-08	2	ACACB
9.527466986487E-08	0.318404639597983	0.461	0.47	0.00159661291759549	2	TOR1AIP2
2.81877104968284E-105	0.31773816161396	0.656	0.566	4.72369652505851E-101	2	PFN1
4.10347921605998E-44	0.31768786436528	0.602	0.518	6.87661047027331E-40	2	HGD
3.84277266635383E-20	0.316198792699542	0.178	0.261	6.43971843427574E-16	2	RFC1
9.58798738080078E-11	0.314020076468861	0.425	0.439	1.60675492527459E-06	2	EPAS1
1.02425736469302E-06	0.311710288127998	0.21	0.269	0.0171645049175257	2	PRPF6
9.02570994646853E-24	0.311471111021303	0.514	0.467	1.5125284728292E-19	2	SERINC5
6.98409978458673E-09	0.308522033209902	0.204	0.267	0.000117039544190104	2	SUCLG1
9.26709604401004E-09	0.30785634601468	0.249	0.323	0.00015529799550552	2	LGR4
7.42500704773975E-09	0.30778515059891	0.208	0.274	0.000124428268106023	2	SEC23A
1.50321853731892E-08	0.305243393159929	0.196	0.256	0.000251909362483905	2	DHPS

8.51203055711002E-20	0.304325736730003	0.171	0.251	1.4264460807605E-15	2	GET1
8.58724969390545E-15	0.301911433933049	0.182	0.255	1.43905130370467E-10	2	CCNY
3.23079980485086E-08	0.301408253935033	0.2	0.262	0.000541417431296906	2	SKAP2
7.19992321213467E-12	0.300479429780909	0.476	0.472	1.20656313188953E-07	2	AOX1
2.5233687181824E-09	0.300047352735451	0.199	0.263	4.22866129793007E-05	2	TXLNA
3.34877362550901E-14	0.298980410034689	0.209	0.289	5.61187484162801E-10	2	CLIP1
4.37991460876746E-14	0.297420988294681	0.186	0.259	7.3398609013725E-10	2	ZNF652
4.47152418397538E-07	0.293941546144943	0.478	0.504	0.00749338022750594	2	IDH2
8.80680814856463E-07	0.293438232907323	0.21	0.268	0.0147584490953646	2	BUD23
2.23871491702234E-64	0.293224302507346	0.79	0.76	3.75163845794604E-60	2	FTH1
3.63941706344454E-16	0.29303201586854	0.178	0.254	6.09893511492036E-12	2	PCMT1
5.07426820121424E-08	0.291745738216781	0.217	0.281	0.000850345865159483	2	PSMC2
8.51686913070226E-16	0.288597754074632	0.181	0.255	1.42725692892308E-11	2	EXOC3L4
6.29814534857797E-26	0.287759257137803	0.591	0.562	1.0554431975147E-21	2	DHCR24
7.43741638629135E-08	0.284254383280059	0.193	0.25	0.0012463622380147	2	HCFC1
4.40094406091151E-07	0.282290857213892	0.241	0.309	0.00737510205727551	2	VPS52
5.93101699844239E-15	0.278912878234302	0.179	0.251	9.93919828598975E-11	2	SAMD4B
8.34316107325228E-13	0.278623321710325	0.21	0.285	1.39814693265562E-08	2	NREP
5.96542195948798E-13	0.277219977560997	0.19	0.261	9.99685411970997E-09	2	NCKIPSD
2.83006094799448E-10	0.274378923760014	0.221	0.293	4.74261613664915E-06	2	CHID1
6.83549380938686E-14	0.272025607434908	0.215	0.295	1.14549205257705E-09	2	SMYD2
3.94491333736774E-14	0.270023164870196	0.195	0.269	6.61088577076086E-10	2	METTL23
2.35214921241031E-09	0.269819426328703	0.252	0.331	3.9417316501572E-05	2	SSR3
7.14109859387656E-08	0.266141093320678	0.466	0.46	0.00119670530236183	2	OCLN
7.56203269322462E-07	0.263390099400311	0.23	0.292	0.0126724543873058	2	DEPRECATED-ENSG00000109181
5.20543221652702E-10	0.263311281536991	0.191	0.254	8.72326330845599E-06	2	MAEA
7.19475691907571E-07	0.262733350367568	0.267	0.343	0.0120569736449871	2	KTN1
1.1809594113142E-07	0.262521427907449	0.261	0.336	0.00197905178148034	2	TFG
1.77103028294957E-22	0.259582720755944	0.416	0.391	2.9678925481669E-18	2	FOSL2
2.20724804521704E-09	0.259471095469914	0.228	0.301	3.69890627417471E-05	2	IDH3B
1.70282154752626E-11	0.259247062355956	0.194	0.261	2.85358834934451E-07	2	RTCB
7.84412282826834E-11	0.259115407463661	0.195	0.262	1.31451810356121E-06	2	SUPT16H
1.9013681268743E-18	0.251622573809871	0.175	0.254	3.18631270701595E-14	2	SCFD1
0	3.86149161738018	0.368	0.085	0	3	TNFSF14
0	3.57081561220442	0.326	0.063	0	3	KYNU
0	3.45212224513383	0.82	0.462	0	3	PLA2G2A
0	3.38677551766865	0.335	0.078	0	3	SAA1
0	3.17814948151247	0.655	0.254	0	3	SPP1
0	3.1647213104768	0.502	0.153	0	3	CRP
0	3.11412149636799	0.331	0.078	0	3	SELENOM
0	2.89063419065465	0.389	0.083	0	3	CPS1
0	2.79305843215716	0.269	0.043	0	3	SDS
0	2.7799574230662	0.47	0.135	0	3	ARG1
2.26596775997571E-184	2.5260184902348	0.43	0.245	3.79730877221673E-180	3	NRBP2
6.74952212154339E-280	2.38062712216973	0.305	0.093	1.13108491712824E-275	3	SLC25A18
0	2.24380030725854	0.368	0.121	0	3	ICAM1
0	2.22658556635689	0.888	0.686	0	3	SOD2
3.1070931274269E-133	2.20974125661927	0.269	0.122	5.206866662942E-129	3	RGS14
1.22496905638749E-239	2.17706313729589	0.458	0.229	2.05280314469416E-235	3	DEPRECATED-ENSG00000175899
0	2.14168059474502	0.635	0.292	0	3	LBP
1.35708498230819E-239	2.13116093867056	0.465	0.239	2.27420301335207E-235	3	CREB3L3
1.85448382504098E-183	2.11285332303311	0.405	0.214	3.10774399400367E-179	3	ROBO1
2.67658113280465E-286	2.0977448838103	0.562	0.317	4.48541466235403E-282	3	A2M
8.78890950625865E-268	2.0207845672574	0.318	0.101	1.47284545505882E-263	3	SLC38A4
9.60113769168812E-62	1.99003762782232	0.263	0.164	1.6089586543731E-57	3	CD24
3.75168403791536E-195	1.9873764420567	0.424	0.217	6.28707211073856E-191	3	MTSS2
0	1.97991474411716	0.99	0.802	0	3	C3
0	1.90977698439243	0.715	0.368	0	3	ITIH1
5.2456937568202E-157	1.88453028378899	0.468	0.301	8.79073359767929E-153	3	UBD
1.93162838307068E-267	1.88132003346547	0.308	0.093	3.23702284434984E-263	3	MBL2

8.48603058530407E-92	1.8514056138741	0.311	0.179	1.42208900548526E-87	3	PLIN4
3.21238794238979E-160	1.80326189738765	0.257	0.096	5.38331971385681E-156	3	RND1
1.93547413683454E-255	1.7859240018778	0.689	0.512	3.24346755850731E-251	3	ALDH1L1
0	1.77045978172405	0.576	0.216	0	3	SERPINA3
0	1.722869359795	0.909	0.67	0	3	CFB
0	1.72106245477744	0.967	0.744	0	3	ORM1
1.50368817773967E-245	1.713802768625	0.395	0.156	2.51988064825614E-241	3	SLC38A3
0	1.70551137421131	0.734	0.35	0	3	APOE
1.47589997632825E-158	1.70339698563358	0.453	0.271	2.47331318033088E-154	3	TYMP
4.21465169154466E-227	1.69241052503887	0.303	0.1	7.06291330469055E-223	3	MASP2
5.48433241086572E-248	1.68345651968811	0.68	0.49	9.19064425412877E-244	3	PAH
0	1.67608525127511	0.675	0.291	0	3	ITIH2
8.29882293999373E-290	1.66129610720658	0.627	0.387	1.39071674828415E-285	3	SERPINF2
8.76374603873228E-55	1.64546201897626	0.337	0.241	1.46862856117076E-50	3	PLIN5
0	1.5810133135914	0.691	0.45	0	3	FGL1
8.69738791067308E-136	1.54893436688559	0.331	0.162	1.45750826607059E-131	3	TM4SF5
4.63028194889849E-189	1.543859436907	0.291	0.105	7.7594264899641E-185	3	C9
1.87342855626582E-63	1.48770934738277	0.337	0.232	3.13949157459025E-59	3	NCOA7
7.36888033224645E-77	1.48501024914279	0.4	0.287	1.23487696607786E-72	3	BCL3
0	1.46545756942519	0.959	0.764	0	3	APOC2
9.85427476574539E-46	1.46347442578065	0.272	0.182	1.65137936524361E-41	3	ALDH4A1
1.13169194233015E-248	1.42796539459675	0.563	0.306	1.89648935695687E-244	3	NNMT
9.68924735056083E-98	1.42713011193379	0.475	0.351	1.62372407100698E-93	3	DPYS
5.35462075675073E-24	1.41441177296172	0.312	0.265	8.97327346416287E-20	3	IL17RB
0	1.41276643174988	0.907	0.767	0	3	IL32
2.23022628760473E-26	1.39703197018073	0.307	0.254	3.737413212768E-22	3	CHDH
1.75134964535642E-81	1.39400467774178	0.495	0.398	2.9349117356883E-77	3	KRT18
7.1823730913896E-149	1.39112109714536	0.391	0.197	1.20362208265507E-144	3	IGFBP2
2.78906442439644E-204	1.37259944622852	0.49	0.254	4.67391416240355E-200	3	ITIH4
1.09678369832523E-199	1.36597481582855	0.663	0.512	1.83799012165342E-195	3	LRG1
0	1.36353119872241	0.961	0.784	0	3	FN1
0	1.36108917824197	0.841	0.727	0	3	IFITM3
9.57961073596304E-227	1.35984324427362	0.596	0.354	1.60535116713269E-222	3	APOC1
9.93874973209938E-146	1.35057570551633	0.456	0.276	1.66553568010521E-141	3	DEPP1
2.08794194611326E-27	1.34318644886283	0.304	0.245	3.49897311329659E-23	3	PABPC1L
1.65524516347312E-46	1.33600715849812	0.319	0.228	2.77385984494825E-42	3	REEP6
4.1655707434031E-43	1.30545451299495	0.28	0.193	6.98066345179491E-39	3	ISG15
1.42864299347477E-291	1.30244992084944	0.759	0.628	2.39411992846502E-287	3	CFH
2.90908366129648E-25	1.2884953092309	0.317	0.265	4.87504239960064E-21	3	DEPRECATED-ENSG00000163931
3.82924966940565E-267	1.28481942063483	0.698	0.454	6.41705659599E-263	3	CP
0	1.27983780483054	0.608	0.241	0	3	HPX
0	1.27737012937828	0.85	0.657	0	3	ORM2
7.31965028542856E-39	1.25846482967987	0.372	0.304	1.22662699483212E-34	3	SHFL
1.54936455985325E-99	1.25136434045932	0.412	0.265	2.59642512940208E-95	3	C5
2.21142247553636E-77	1.23539465443834	0.507	0.422	3.70590178450383E-73	3	MPST
1.6856809663646E-74	1.22762499214805	0.29	0.167	2.82486416343379E-70	3	DUSP10
2.63385111614927E-49	1.21918594356984	0.409	0.335	4.41380770044295E-45	3	TFPI
2.49078879720099E-16	1.21522707152335	0.284	0.247	4.17406386634943E-12	3	H6PD
0	1.20057037933942	0.905	0.813	0	3	SQSTM1
1.47082302079579E-26	1.19337672104246	0.379	0.334	2.46480521824959E-22	3	MYH14
1.96826441709534E-27	1.1701203150104	0.28	0.215	3.29841751016837E-23	3	RGS3
6.36746292759613E-70	1.16597584402601	0.506	0.43	1.06705943740656E-65	3	SNTB1
1.81583625755339E-21	1.16437902649655	0.252	0.198	3.04297840040797E-17	3	MGLL
3.63708926085578E-123	1.13607870636891	0.414	0.231	6.09503418334212E-119	3	CFHR2
4.78127024202875E-44	1.12667537468415	0.413	0.341	8.01245267159177E-40	3	TCEA3
5.61315784852348E-49	1.12019848309457	0.421	0.345	9.40652992255564E-45	3	LGALS3BP
3.58107334783251E-25	1.10887621555703	0.315	0.255	6.00116271629773E-21	3	OSGIN1
9.15262826784385E-19	1.10721236211655	0.375	0.345	1.53379744512527E-14	3	ABCC2
5.23423700129472E-14	1.10511626510182	0.308	0.28	8.77153436676969E-10	3	GLRX
1.21775413618356E-31	1.08157537096849	0.448	0.416	2.04071238141641E-27	3	NR1H4

1.52381993237425E-11	1.07845547523453	0.319	0.301	2.55361744267276E-07	3	SLC17A9
1.94463607549933E-20	1.07806879524966	0.253	0.199	3.25882113532178E-16	3	F13B
2.02758459972194E-10	1.06787691724981	0.259	0.235	3.39782627221403E-06	3	ABCA2
0	1.06693405785145	0.852	0.575	0	3	FGB
4.41487039018868E-27	1.05324166031874	0.314	0.252	7.3984397998782E-23	3	EBNA1BP2
1.52185507449189E-19	1.04955031256112	0.428	0.404	2.55032473383351E-15	3	GLYCTK
4.2117883976114E-10	1.04315216740017	0.29	0.27	7.05811499671718E-06	3	CRELD2
4.68537240650532E-66	1.03574802114306	0.47	0.366	7.85174707882161E-62	3	ASGR1
1.89444303905036E-11	1.03438302475366	0.298	0.274	3.1747076448406E-07	3	MANF
0	1.03429239197428	0.9	0.687	0	3	FGG
9.56891540642726E-120	1.03036158861959	0.31	0.141	1.60355884380908E-115	3	SAA4
1.49653121397466E-28	1.02777621960933	0.289	0.22	2.50788700837873E-24	3	F7
1.23373433723707E-39	1.0274299488304	0.406	0.336	2.06749200234189E-35	3	F10
1.44610431085458E-09	1.02496764425012	0.345	0.337	2.4233816041301E-05	3	LIMK2
6.72895047452565E-34	1.01367181346947	0.479	0.446	1.12763752052101E-29	3	TP53INP1
1.88758306062889E-16	1.00024748165371	0.415	0.403	3.1632116930019E-12	3	EPS8L2
3.64027265451159E-23	0.997028042981784	0.304	0.245	6.10036891443052E-19	3	NFIL3
1.73547940402998E-40	0.992127207682102	0.434	0.374	2.90831638527345E-36	3	ABAT
1.22486209036421E-261	0.991238402423047	0.765	0.546	2.05262389103234E-257	3	C1S
0	0.988934281753929	0.957	0.756	0	3	RBP4
4.29555078388458E-64	0.986553256243146	0.294	0.174	7.19848400363377E-60	3	TFR2
7.8772729593571E-55	0.986238310584738	0.322	0.21	1.32007340252906E-50	3	PROC
5.4749505541229E-09	0.986052382650645	0.252	0.226	9.17492213859915E-05	3	TSKU
5.83183350467393E-15	0.984435387498857	0.345	0.319	9.77298658713257E-11	3	FGGY
3.55203853406059E-62	0.98335705586303	0.575	0.521	5.95250617537874E-58	3	SDC4
2.73131089646617E-13	0.980250114246493	0.327	0.299	4.57713080029801E-09	3	DUSP23
2.10409648800529E-30	0.972607463606972	0.474	0.436	3.52604489459927E-26	3	BDH1
1.06758780579118E-178	0.972315320080325	0.724	0.605	1.78906364494485E-174	3	ASGR2
2.06030923087306E-248	0.970878591811061	0.685	0.4	3.45266620909708E-244	3	ITIH3
1.2257586651824E-158	0.964464399579756	0.296	0.112	2.05412637111267E-154	3	HPD
8.28592350376297E-41	0.962324625248457	0.498	0.463	1.3885550607606E-36	3	LPGAT1
2.08992948601547E-10	0.959420824265854	0.306	0.282	3.50230383266473E-06	3	ASS1
1.98879970165519E-06	0.957231390174162	0.279	0.268	0.0333283054003376	3	SLC43A1
0	0.956728693945437	0.587	0.22	0	3	HRG
1.66593259976983E-146	0.94905058212675	0.732	0.63	2.79176985069428E-142	3	PRAP1
3.14487820975541E-250	0.944255464224802	0.968	0.888	5.27018690390812E-246	3	FTL
9.33220924989564E-14	0.942594429912891	0.312	0.279	1.56389162609751E-09	3	IRF1
7.83179010678983E-47	0.935181873636743	0.496	0.433	1.31245138609584E-42	3	GAMT
9.92329326437134E-25	0.931773003550988	0.372	0.32	1.66294548524335E-20	3	CYP27A1
2.41276169036093E-20	0.930522703013235	0.422	0.407	4.04330604070684E-16	3	SEC11C
7.94112469086528E-93	0.919309971351251	0.64	0.559	1.3307736756952E-88	3	DHCR24
0	0.911175963457178	0.949	0.797	0	3	AMBP
0	0.908072651404304	0.933	0.705	0	3	FGA
0	0.90762252952503	0.966	0.797	0	3	CES1
1.33246125529666E-83	0.906686227691232	0.614	0.558	2.23293857162614E-79	3	XBP1
6.50511629981286E-20	0.904583535382586	0.391	0.359	1.09012738952264E-15	3	GRHPR
3.66659734542687E-33	0.877547806784537	0.411	0.352	6.14448383146636E-29	3	LRP1
6.85459874491065E-215	0.875184829426294	0.848	0.678	1.14869365767213E-210	3	TTR
0	0.872410252057406	0.989	0.861	0	3	VTN
2.51075787672624E-13	0.870113358923349	0.42	0.407	4.20752804981783E-09	3	ECHDC2
6.84152400769922E-08	0.862363261745152	0.371	0.376	0.00114650259321024	3	ARHGEF10L
1.63004546904072E-28	0.84913894834809	0.471	0.429	2.73163019701844E-24	3	MAT1A
1.89918930076213E-17	0.841270916927291	0.466	0.464	3.18266143021718E-13	3	MIF
7.18920178510547E-11	0.839119131154047	0.303	0.273	1.20476643514797E-06	3	COMT
2.89770461422809E-20	0.836228491614638	0.396	0.361	4.85597339252343E-16	3	IFITM2
5.62788083112005E-15	0.829809355606532	0.433	0.43	9.43120269679098E-11	3	EXT1
8.22273626803994E-07	0.828497593942305	0.316	0.303	0.0137796614379813	3	SAMD5
1.21303192390451E-14	0.82037240463617	0.423	0.409	2.03279889807917E-10	3	HNF4A
1.90432486257246E-17	0.819069689679792	0.259	0.209	3.19126760469893E-13	3	TGM2
7.96967034798058E-14	0.810547590666931	0.322	0.28	1.33555735691459E-09	3	SERPINA5

1.75778081101209E-133	0.808219886985805	0.629	0.454	2.94568908309407E-129	3	F2
9.07771617721147E-79	0.807888585074208	0.633	0.575	1.5212436769771E-74	3	C2
2.78830222152541E-33	0.80555146487116	0.292	0.207	4.67263686283228E-29	3	SLC9A3R2
2.42383664535571E-49	0.8042688367249	0.336	0.225	4.0618654502871E-45	3	MST1
2.74199325593636E-196	0.797238998535058	0.676	0.423	4.59503229829815E-192	3	SERPING1
7.70551009904743E-15	0.794688114626049	0.422	0.417	1.29128938239837E-10	3	CALR
1.61904447446803E-27	0.793198563265876	0.455	0.413	2.71319473031352E-23	3	CEBPB
2.80440355830902E-10	0.790523243063291	0.35	0.335	4.69961948301426E-06	3	TNIP1
1.60589078893284E-14	0.787700532410139	0.407	0.395	2.69115178409366E-10	3	FNDC3B
1.52936270849352E-20	0.78600964087522	0.331	0.279	2.56290602689343E-16	3	ORMDL3
1.64876444714019E-42	0.784403763154984	0.592	0.577	2.76299946051753E-38	3	ETFB
1.22128785873444E-22	0.783554877290048	0.336	0.274	2.04663419366718E-18	3	DEPRECATED-ENSG00000115738
9.42375971149291E-11	0.778737924718749	0.395	0.38	1.57923365245198E-06	3	ONECUT1
8.98866218835378E-08	0.768990823695048	0.36	0.355	0.00150632000952433	3	C8G
9.06301882292941E-09	0.768272335912926	0.343	0.333	0.000151878069434651	3	MAP3K13
8.78907847715782E-21	0.764236550582503	0.307	0.248	1.47287377120211E-16	3	ID2
2.03807078112396E-197	0.763212036350508	0.469	0.197	3.41539901500752E-193	3	HPR
1.73002104887431E-07	0.761545719194995	0.351	0.349	0.00289916927370357	3	PTPRF
6.03537085913734E-45	0.757221405971843	0.26	0.16	1.01140744857423E-40	3	APOM
1.91137323843094E-07	0.746216619408671	0.368	0.371	0.00320307927296256	3	SCARB1
5.02729626024368E-11	0.744057508343209	0.412	0.403	8.42474307291637E-07	3	SLC22A18
1.02609824593179E-153	0.737512907206312	0.832	0.705	1.71953544053249E-149	3	PTGR1
4.25061298818946E-11	0.72596900195208	0.425	0.43	7.1231772456079E-07	3	RIDA
1.11309338049624E-10	0.725155297956942	0.362	0.349	1.86532188703559E-06	3	CD59
4.45519923148241E-238	0.725062055989977	0.961	0.807	7.46602287211822E-234	3	CLU
6.69226773623456E-53	0.721524554406257	0.609	0.579	1.12149022723819E-48	3	GRINA
1.92238394353136E-244	0.715860395857123	0.87	0.697	3.22153101256985E-240	3	APOB
3.11487975465761E-27	0.709189070150232	0.538	0.538	5.21991549285522E-23	3	DDT
4.3843039252843E-08	0.708749065486895	0.429	0.443	0.000734721651799143	3	P4HB
5.46142863115742E-09	0.701427022292759	0.415	0.414	9.1522621000936E-05	3	PCBD1
1.04435289857638E-27	0.699823411558121	0.506	0.489	1.7501265874343E-23	3	F11R
7.77165894048422E-14	0.699810129447183	0.384	0.358	1.30237460524634E-09	3	C8B
0	0.690184820777684	0.722	0.278	0	3	HP
7.66178973014997E-11	0.685115587284109	0.507	0.499	1.28396272297853E-06	3	ELF3
1.37269782813593E-20	0.674182978446546	0.493	0.485	2.30036702039019E-16	3	CTSA
2.57266836686919E-66	0.66616885841896	0.38	0.242	4.31127764919939E-62	3	TAT
1.09253831059096E-79	0.652398496281959	0.769	0.765	1.83087570088833E-75	3	FTH1
7.79340527046331E-33	0.650366915717668	0.472	0.413	1.30601885522424E-28	3	SERPINF1
1.00487428493213E-08	0.647823648628032	0.58	0.633	0.000168396832668926	3	SPINK1
5.30555946732728E-13	0.643694642192402	0.457	0.464	8.89105655534705E-09	3	NDUFS2
9.49999449039068E-09	0.63965591781147	0.407	0.404	0.000159200907669967	3	PRG4
6.92963285799673E-38	0.62965578857755	0.606	0.609	1.16126787434309E-33	3	LMAN2
1.61403677734096E-27	0.629560285487058	0.603	0.589	2.70480283146798E-23	3	EFNA1
1.03008305963796E-13	0.627559702275422	0.471	0.459	1.72621319134129E-09	3	BHMT2
3.25150852708191E-58	0.627389156999826	0.626	0.531	5.44887798968387E-54	3	DEPRECATED-ENSG00000198074
7.46848881080126E-16	0.623812465983465	0.508	0.508	1.25156935491407E-11	3	AADAC
5.12497753290293E-138	0.620845177750466	0.606	0.377	8.58843734963873E-134	3	SERPINC1
1.29272301772295E-115	0.618206594548076	0.42	0.216	2.16634523310011E-111	3	COL18A1
3.30844390442181E-70	0.617928528026161	0.712	0.676	5.54429029503007E-66	3	LAMP1
2.58149573890473E-28	0.612953655056934	0.569	0.545	4.32607055925655E-24	3	CLDN1
2.67418981224254E-07	0.610740383526015	0.414	0.422	0.00448140728735605	3	MYO1B
3.36448437021864E-27	0.609500391936575	0.557	0.559	5.63820290761239E-23	3	OS9
6.81144457015917E-19	0.609120386736998	0.495	0.47	1.14146188106727E-14	3	AOX1
1.66738561466694E-09	0.603517443803756	0.287	0.258	2.79420481305887E-05	3	NFKBIZ
1.01372101774741E-06	0.600874572920892	0.399	0.408	0.0169879368154111	3	NUDC
2.60128406247656E-134	0.598144190698466	0.88	0.78	4.35923183189822E-130	3	FABP1
1.2321336992701E-18	0.598004929906255	0.536	0.525	2.06480965323683E-14	3	TXNRD1
2.21397990990749E-294	0.594042933131686	0.981	0.929	3.71018753302297E-290	3	SERPINA1
3.30280391460826E-55	0.593084531789124	0.694	0.647	5.53483880010052E-51	3	NUPR1
9.72645829933088E-30	0.577554031996933	0.574	0.554	1.62995988180187E-25	3	TMEM176A

1.25676270782906E-94	0.566746599797238	0.76	0.685	2.10608294577994E-90	3	C4BPA
4.98585679552736E-139	0.566647971771308	0.91	0.796	8.35529881794475E-135	3	ALDOB
9.18049608800632E-72	0.565026409017601	0.698	0.616	1.5384675344281E-67	3	GPX2
3.9514274299795E-152	0.557899691712584	0.94	0.793	6.62180208715965E-148	3	TF
3.67873930621735E-44	0.528098773008708	0.684	0.677	6.16483132935904E-40	3	ST6GAL1
1.97638141918948E-08	0.524961800634377	0.489	0.512	0.000331201998227773	3	PRDX6
5.36685366052181E-119	0.506945655934895	0.391	0.184	8.99377336430245E-115	3	APCS
4.19587191148964E-07	0.497505868294286	0.49	0.523	0.00703144214927435	3	CREG1
8.9053870740872E-57	0.492197208301535	0.691	0.57	1.49236476587553E-52	3	NQO1
1.00391886208711E-06	0.474085201477438	0.504	0.533	0.0168236722908558	3	ADI1
5.29360682519991E-32	0.470767668728352	0.657	0.622	8.87102631767002E-28	3	LMNA
1.80389880994592E-79	0.468990689564065	0.815	0.747	3.02297362570737E-75	3	TMEM176B
5.36159004691307E-60	0.462614574099426	0.764	0.671	8.98495260061693E-56	3	AGXT
2.77118505850235E-11	0.461504946945094	0.563	0.561	4.64395192103825E-07	3	KRT8
6.44390210757233E-54	0.460674411918045	0.749	0.71	1.07986911518697E-49	3	APOH
6.21234742521257E-108	0.453899167803776	0.781	0.661	1.04106518151712E-103	3	KNG1
1.07783530461348E-06	0.44888483023153	0.521	0.544	0.0180623640347126	3	PRDX2
1.16581157612139E-09	0.447696523521215	0.222	0.301	1.95366703926422E-05	3	SLC51A
7.63101874965424E-15	0.442472042456636	0.528	0.521	1.27880612206706E-10	3	SERPIND1
1.78214358862269E-07	0.433250598166683	0.201	0.264	0.0029865162258139	3	ABCB6
1.26179916453038E-24	0.430226240879233	0.649	0.641	2.11452303992001E-20	3	CERS2
2.62806715047356E-11	0.42734063799224	0.469	0.476	4.40411493076359E-07	3	PNRC1
3.335726439226E-28	0.420075079420362	0.687	0.692	5.59001036685494E-24	3	EEF2
1.05976654791792E-16	0.417954157011216	0.608	0.614	1.77595678100085E-12	3	GATM
1.22374192692832E-56	0.416062672453362	0.657	0.578	2.05074672114648E-52	3	PLG
8.40348689913508E-50	0.406153949913354	0.699	0.603	1.40825633455706E-45	3	AKR1B10
1.83612133749498E-07	0.401970423234139	0.209	0.277	0.00307697213737408	3	RHOT2
5.92556938694737E-107	0.397633352420566	0.647	0.466	9.9300691786464E-103	3	C1R
1.17706285926E-07	0.39526639430917	0.492	0.477	0.00197252193954791	3	ACSM2A
3.22112433932464E-18	0.387428920114805	0.605	0.602	5.39796016784023E-14	3	C4BPB
1.1831244701177E-07	0.380614441199836	0.49	0.474	0.00198267998702324	3	TSPAN8
4.23114479692421E-27	0.379245277787293	0.614	0.593	7.0905524506856E-23	3	ALDH2
8.26303656964472E-12	0.374850316656119	0.582	0.606	1.38471966834106E-07	3	TAPBP
4.89944407868412E-15	0.374544202398174	0.623	0.656	8.21048838705885E-11	3	HSP90AB1
3.0503238705372E-09	0.367922222852952	0.527	0.519	5.11173274224624E-05	3	HPN
1.14879720291502E-06	0.364370305895727	0.208	0.27	0.01925154352645	3	SEC16A
2.67247514084354E-13	0.357493558601785	0.619	0.653	4.47853384102561E-09	3	NUCB1
2.87193789140051E-06	0.355478572480751	0.198	0.253	0.0481279351840897	3	PFDN6
6.34722076036758E-08	0.347462154187242	0.54	0.536	0.0010636672550224	3	BAAT
1.15813083111759E-09	0.344083723916784	0.184	0.252	1.94079564678686E-05	3	TMEM120A
5.50519701171632E-08	0.342165466688472	0.225	0.293	0.00092256091522342	3	ANXA7
2.44685092044445E-27	0.341879356994807	0.713	0.62	4.1004327724808E-23	3	ASPH
9.59268097268514E-09	0.333058956945224	0.219	0.287	0.000160754147740258	3	SPR
3.72343269352646E-27	0.329408526265376	0.705	0.695	6.23972850781164E-23	3	CYB5A
2.16340868506759E-06	0.324896994620281	0.265	0.34	0.0362544027443626	3	AP3D1
4.83183150611748E-10	0.320516279816255	0.19	0.257	8.09718323795168E-06	3	SURF1
1.9396825414757E-09	0.314812189813864	0.265	0.354	3.25052000300497E-05	3	AKAP1
3.26634655740028E-07	0.303593322468499	0.192	0.254	0.00547374356089138	3	ATP11A
5.74913299912625E-07	0.30234602497248	0.251	0.319	0.00963439707993577	3	GHITM
1.194181888619E-10	0.299516149476704	0.251	0.339	2.00121000894772E-06	3	MROH1
8.09906757981201E-10	0.295702482919286	0.251	0.331	1.3572417450249E-05	3	OPLAH
1.40947907637524E-08	0.294624360001228	0.228	0.301	0.000236200503618962	3	TMEM9
2.05318272939992E-10	0.288593271345497	0.184	0.251	3.44072361792839E-06	3	MDH2
1.44137232424247E-10	0.287509103095139	0.198	0.272	2.41545174096554E-06	3	NME3
4.36484986982173E-07	0.285528324226652	0.261	0.337	0.00731461541184725	3	INTS11
6.09851429369894E-07	0.281409219728926	0.253	0.322	0.0102198902533807	3	ZBTB20
5.5255966779174E-13	0.280034999463059	0.214	0.299	9.25979491285398E-09	3	HDGFL2
1.21476741553386E-09	0.279638872249405	0.227	0.303	2.03570723495163E-05	3	TMCO3
2.00847745642472E-25	0.276437663714411	0.549	0.482	3.36580652147654E-21	3	PCK1
1.25886936507451E-12	0.273035414300699	0.177	0.252	2.10961328199186E-08	3	NOP56

9.81742348809591E-12	0.272346483839896	0.259	0.35	1.64520382813511E-07	3	PRXL2A
2.32081883448893E-10	0.270217631046966	0.2	0.273	3.88922820283655E-06	3	LRRC47
1.03101087933411E-07	0.26956739635138	0.253	0.329	0.0017277680315881	3	GNPTG
4.10359848004915E-09	0.269189707460441	0.191	0.256	6.87681033286636E-05	3	AGMAT
8.10552888438842E-10	0.268425543070663	0.217	0.289	1.35832453044581E-05	3	GPR108
3.93957955313613E-07	0.2683752887962	0.234	0.301	0.00660194741514552	3	DCTN2
7.31430943785688E-08	0.264259432906055	0.218	0.287	0.00122573197559606	3	SYVN1
3.41592778148501E-10	0.251879091006139	0.215	0.291	5.72441177621257E-06	3	IFI35
2.46370830749928E-07	0.251034201294081	0.259	0.332	0.0041286823817073	3	STAP2
7.41886672905484E-11	0.250105548212818	0.188	0.259	1.24325368645501E-06	3	PRPSAP1
0	5.40574959222839	0.398	0.015	0	4	FBXO43
0	5.2504479100683	0.347	0.012	0	4	DEPRECATED-ENSG00000117399
0	5.16929132183729	0.423	0.018	0	4	CDC20
0	5.08817453655265	0.341	0.009	0	4	CDC25C
0	5.00406653239773	0.642	0.037	0	4	ANLN
0	4.97956398452332	0.438	0.023	0	4	CCNB2
0	4.9570501544681	0.455	0.021	0	4	DLGAP5
0	4.90390224425177	0.427	0.017	0	4	CDCA3
0	4.89942790941246	0.512	0.027	0	4	NEK2
0	4.89535656407834	0.469	0.042	0	4	CCNB1
0	4.8847640654115	0.491	0.03	0	4	ASPM
0	4.81609336682647	0.472	0.019	0	4	HJURP
0	4.80366687483699	0.443	0.019	0	4	KIF14
0	4.78154044035102	0.53	0.025	0	4	BUB1
0	4.75812930959978	0.541	0.033	0	4	IQGAP3
0	4.75126919317586	0.646	0.074	0	4	CENPI
0	4.72637459841498	0.396	0.017	0	4	KIF20A
0	4.71458596159352	0.357	0.017	0	4	CENPA
0	4.68036237530814	0.295	0.01	0	4	BUB1B
0	4.67915223491072	0.392	0.021	0	4	NEIL3
0	4.60376450500926	0.468	0.024	0	4	CDCA8
0	4.5877617634621	0.436	0.023	0	4	SKA1
0	4.55881283723628	0.442	0.03	0	4	AURKA
0	4.54634767419565	0.475	0.027	0	4	PLK1
0	4.52782550624861	0.478	0.03	0	4	NUF2
0	4.52635986178074	0.461	0.021	0	4	CIT
0	4.52484965906636	0.748	0.089	0	4	TOP2A
0	4.52367218963806	0.625	0.058	0	4	HMMR
0	4.52056871620503	0.73	0.095	0	4	CENPF
0	4.51604950387941	0.344	0.015	0	4	TROAP
0	4.50713474334137	0.396	0.018	0	4	GTSE1
0	4.49947790781452	0.637	0.05	0	4	SPC24
0	4.47164435503584	0.79	0.129	0	4	HIST1H1B
0	4.46415051274008	0.652	0.044	0	4	KIFC1
0	4.45759305144508	0.446	0.022	0	4	KIF2C
0	4.45694148727767	0.709	0.066	0	4	TPX2
0	4.43608778089852	0.286	0.012	0	4	TTK
0	4.42924543523669	0.38	0.017	0	4	KIF18B
0	4.40775275174544	0.369	0.016	0	4	CKAP2L
0	4.40154780661469	0.594	0.044	0	4	BIRC5
0	4.36646463749504	0.398	0.016	0	4	AURKB
0	4.35992900326976	0.509	0.047	0	4	UBE2C
0	4.3523477946811	0.367	0.018	0	4	OIP5
0	4.34989206403681	0.466	0.024	0	4	ESPL1
0	4.34586508978709	0.448	0.029	0	4	ARHGAP11A
0	4.34169579171716	0.404	0.019	0	4	KIF4A
0	4.3369052059655	0.406	0.026	0	4	PBK
0	4.31955515268539	0.415	0.022	0	4	SPC25
0	4.29588138124766	0.314	0.013	0	4	POLQ
0	4.26980907475597	0.582	0.048	0	4	NCAPG

0	4.26842818355988	0.457	0.033	0	4	KIF23
0	4.25345748560589	0.349	0.017	0	4	FAM72C
0	4.24706175312299	0.664	0.074	0	4	PTTG1
0	4.20054966557679	0.343	0.02	0	4	SGO2
0	4.18848818825968	0.271	0.013	0	4	DEPRECATED-ENSG00000090889
0	4.17964721056791	0.554	0.042	0	4	FOXM1
0	4.167813970556	0.358	0.017	0	4	WDR62
0	4.15240770106155	0.381	0.019	0	4	KNL1
0	4.1319826174204	0.274	0.013	0	4	PARPBP
0	4.1208933900654	0.427	0.035	0	4	DEPDC1
0	4.09914057434809	0.559	0.045	0	4	PRC1
0	4.08179936683514	0.49	0.047	0	4	CCNA2
0	4.07172038975979	0.336	0.021	0	4	PRR11
0	4.06149036510542	0.322	0.021	0	4	ARHGEF39
0	4.05846168887751	0.308	0.016	0	4	CDCA5
0	4.0478580996654	0.645	0.076	0	4	CDK1
0	4.03268783048151	0.412	0.03	0	4	SKA3
0	3.99111985490935	0.413	0.027	0	4	DEPDC1B
0	3.9695651700002	0.384	0.028	0	4	CENPE
0	3.94073881832865	0.471	0.05	0	4	MAD2L1
0	3.94010672220426	0.637	0.046	0	4	TACC3
0	3.9254575170376	0.5	0.051	0	4	HIST1H2AH
0	3.91448202860687	0.271	0.012	0	4	DIAPH3
0	3.90926067188963	0.491	0.06	0	4	CDKN3
0	3.88901158597416	0.287	0.015	0	4	CDCA2
0	3.87694701083322	0.619	0.075	0	4	ECT2
0	3.87039673729627	0.372	0.035	0	4	GPSM2
0	3.85433428615304	0.269	0.013	0	4	KIF15
0	3.84660051296276	0.509	0.033	0	4	NDC80
0	3.84070349228731	0.654	0.066	0	4	MKI67
0	3.83336514597092	0.27	0.017	0	4	RACGAP1
0	3.79958954679682	0.306	0.016	0	4	PSRC1
0	3.73811958574525	0.735	0.121	0	4	NUSAP1
0	3.68271305971042	0.277	0.021	0	4	AC091057.6
0	3.66144814173529	0.424	0.039	0	4	MND1
0	3.65333254776262	0.33	0.02	0	4	SHCBP1
0	3.64537177015078	0.512	0.065	0	4	KNSTRN
0	3.61101779669188	0.257	0.017	0	4	PLK4
0	3.58324688469259	0.577	0.066	0	4	SPAG5
0	3.5827834406518	0.356	0.02	0	4	CEP55
0	3.54221943691493	0.549	0.072	0	4	CENPW
0	3.52741821372727	0.625	0.074	0	4	RRM2
0	3.52098999608792	0.463	0.047	0	4	CENPL
0	3.51574137299993	0.474	0.058	0	4	MELK
0	3.50311251434597	0.26	0.013	0	4	NCAPH
0	3.49083397793124	0.255	0.02	0	4	RAD54L
0	3.47168931128597	0.289	0.018	0	4	PKMYT1
0	3.46630654573274	0.396	0.036	0	4	KIF11
0	3.43790654014114	0.466	0.049	0	4	FAM83D
0	3.40896318539485	0.318	0.029	0	4	EXO1
0	3.40707134521449	0.344	0.032	0	4	BRCA2
0	3.39607080373962	0.838	0.281	0	4	HIST1H1C
0	3.37939158848119	0.431	0.044	0	4	DEPRECATED-ENSG00000188486
0	3.37250675200429	0.289	0.022	0	4	CIP2A
0	3.32886059348865	0.358	0.031	0	4	STIL
0	3.31808444231466	0.332	0.032	0	4	GINS4
0	3.31633556466633	0.572	0.097	0	4	HIST1H1E
0	3.303221839126	0.273	0.024	0	4	RAD51AP1
0	3.30204484992752	0.38	0.036	0	4	CDKN2D
0	3.28833996943869	0.469	0.062	0	4	UBE2S

0	3.26150580782405	0.657	0.113	0	4	NEURL1B
0	3.23661947885272	0.292	0.03	0	4	LIN9
0	3.22673606636039	0.652	0.125	0	4	UBE2T
0	3.19971818531751	0.582	0.082	0	4	NCAPD2
0	3.15882025855983	0.421	0.042	0	4	MXD3
0	3.15787797451585	0.336	0.039	0	4	CCNF
0	3.15363953204881	0.402	0.056	0	4	RECQL4
0	3.13179775248499	0.362	0.058	0	4	RAD54B
0	3.10114598642727	0.551	0.078	0	4	INCENP
0	3.09911269610836	0.265	0.028	0	4	E2F8
0	3.08912346968227	0.593	0.122	0	4	HIST1H4C
0	3.06121561143223	0.315	0.042	0	4	CDC45
0	3.03050784888876	0.485	0.094	0	4	KPNA2
0	3.03013076240804	0.364	0.039	0	4	CKAP2
0	2.97719308176785	0.448	0.059	0	4	H2AFX
0	2.95650669146935	0.619	0.104	0	4	KIF22
0	2.89986732179276	0.357	0.049	0	4	SUV39H1
0	2.89483470763347	0.33	0.038	0	4	ORC6
0	2.87408445708744	0.7	0.18	0	4	CDKN2C
0	2.8664499847639	0.336	0.037	0	4	CENPO
0	2.86633338841584	0.449	0.086	0	4	CENPJ
0	2.86010180156055	0.333	0.049	0	4	C21orf58
0	2.85714179924311	0.258	0.025	0	4	MYBL2
0	2.85481013619161	0.579	0.136	0	4	CENPU
0	2.83218239904899	0.286	0.023	0	4	POC1A
0	2.81762376359213	0.78	0.208	0	4	HMGB2
0	2.80741140844233	0.374	0.061	0	4	ARHGAP33
0	2.7795355261012	0.575	0.104	0	4	CKS2
0	2.77167307768224	0.659	0.163	0	4	SMC4
0	2.73336627430109	0.278	0.041	0	4	ATAD5
0	2.72539874435694	0.427	0.066	0	4	MIS18A
0	2.70498845998833	0.302	0.045	0	4	DSCC1
0	2.64493999454899	0.301	0.053	0	4	ZGRF1
0	2.64116085287056	0.653	0.158	0	4	TK1
0	2.63674109053906	0.326	0.049	0	4	FBXO5
0	2.63469552629615	0.281	0.047	0	4	BORA
0	2.63399275591587	0.529	0.105	0	4	STMN1
0	2.62124004188912	0.28	0.033	0	4	TEDC1
0	2.61360379045573	0.469	0.077	0	4	ASF1B
0	2.60749933342427	0.259	0.038	0	4	CENPK
0	2.59960204906357	0.756	0.24	0	4	H2AFZ
0	2.56820015318588	0.558	0.128	0	4	CENPM
0	2.55343661559542	0.78	0.229	0	4	LMNB1
0	2.54888373948571	0.44	0.091	0	4	FANCI
0	2.54145596083275	0.308	0.058	0	4	ATP8B3
0	2.54082260642422	0.313	0.055	0	4	BRCA1
0	2.51807980331727	0.398	0.082	0	4	GINS1
0	2.51705063267648	0.54	0.132	0	4	TUBB4B
0	2.51503480489117	0.334	0.052	0	4	GEN1
0	2.50922704980262	0.28	0.028	0	4	KIF20B
0	2.50810530830294	0.514	0.128	0	4	HMGB3
0	2.48878943736283	0.252	0.036	0	4	CDCA4
0	2.4632733247325	0.572	0.126	0	4	ZWINT
0	2.45000682988517	0.468	0.083	0	4	SMC2
0	2.44656741767041	0.414	0.089	0	4	TUBA1B
0	2.44254331538839	0.683	0.187	0	4	LMNB2
0	2.43437597288206	0.554	0.124	0	4	DTYMK
0	2.42692097907358	0.351	0.077	0	4	NUP62CL
0	2.41838598475473	0.531	0.138	0	4	MZT1
0	2.40174958882493	0.619	0.175	0	4	NCAPG2

0	2.39843965965947	0.508	0.141	0	4	GAS2L3
0	2.373982167726	0.713	0.223	0	4	CKS1B
0	2.34010435941929	0.4	0.038	0	4	MYBL1
0	2.3122017714927	0.591	0.149	0	4	CDC25B
0	2.29230793302812	0.336	0.05	0	4	DBF4
0	2.28736916087915	0.672	0.21	0	4	RAD21
0	2.27158486856897	0.478	0.12	0	4	EMC9
0	2.26745092342527	0.648	0.218	0	4	TUBA1C
0	2.24361628026192	0.555	0.16	0	4	CKAP5
0	2.23683610968599	0.479	0.116	0	4	PCLAF
0	2.2178940016498	0.427	0.112	0	4	RNF26
0	2.20587965708438	0.383	0.072	0	4	TBC1D31
0	2.20019776094907	0.503	0.133	0	4	SOGA1
0	2.17638594761668	0.294	0.051	0	4	RFC3
0	2.15226509319062	0.298	0.06	0	4	CENPH
0	2.14114874315272	0.387	0.087	0	4	MCM8
0	2.14088912407554	0.369	0.084	0	4	CENPP
1.16119940736659E-279	2.13907504309024	0.262	0.054	1.94593796686493E-275	4	BRIP1
0	2.13667815244196	0.275	0.049	0	4	CCDC34
0	2.12871168516836	0.297	0.057	0	4	NRM
0	2.12725798444117	0.545	0.19	0	4	ARL6IP1
0	2.12434056925873	0.631	0.18	0	4	TYMS
0	2.11512982909493	0.337	0.071	0	4	LRR1
0	2.10547042923758	0.351	0.064	0	4	VRK1
0	2.095013634261	0.501	0.124	0	4	CENPN
0	2.08526391268199	0.252	0.032	0	4	CHTF18
0	2.06796953958072	0.706	0.254	0	4	TUBB
0	2.06050927364776	0.4	0.082	0	4	NDC1
0	2.05044568528634	0.343	0.075	0	4	C9orf40
0	2.04871081849373	0.573	0.129	0	4	PHF19
1.69174238359856E-261	2.028230512072	0.352	0.1	2.83502188643446E-257	4	DTL
0	2.0260748556642	0.322	0.06	0	4	G2E3
0	2.0126667294452	0.418	0.12	0	4	FANCA
2.19675108536002E-302	2.00886086439705	0.401	0.114	3.68131546884633E-298	4	ALDH3A1
0	2.00585696512014	0.548	0.167	0	4	EZH2
0	1.98811330696547	0.586	0.188	0	4	TCF19
0	1.96283070437614	0.329	0.068	0	4	BARD1
0	1.95484766057825	0.338	0.079	0	4	MASTL
0	1.94607010281434	0.365	0.093	0	4	MELTF
3.30062415960742E-297	1.94049163651412	0.389	0.109	5.53118596667012E-293	4	SEPHS1
3.70425185332033E-264	1.92074463562303	0.275	0.062	6.20758525579421E-260	4	CHEK1
0	1.91523658956984	0.369	0.072	0	4	C1orf112
1.04147960421049E-283	1.91259746025336	0.359	0.098	1.74531152073594E-279	4	DEPRECATED-ENSG00000022267
1.10596330960672E-236	1.90097742791311	0.263	0.062	1.85337331423894E-232	4	STMND1
0	1.88693214791553	0.389	0.086	0	4	NAV2
0	1.83848720352374	0.406	0.091	0	4	BCL2L12
0	1.83385935106462	0.419	0.11	0	4	POLH
0	1.82486553330186	0.553	0.205	0	4	DHFR
1.26337081618428E-195	1.82227426876769	0.31	0.097	2.11715681376162E-191	4	FAM111B
4.93541941352452E-294	1.79360742460611	0.361	0.095	8.27077585318439E-290	4	PKN3
1.28291920759695E-190	1.79012183998474	0.273	0.078	2.14991600809097E-186	4	CDC6
2.54118654536446E-219	1.78937644809315	0.346	0.109	4.25852041272177E-215	4	TONSL
0	1.7767190125335	0.425	0.12	0	4	WDR34
0	1.77637601145022	0.322	0.073	0	4	RAD18
0	1.77411895577473	0.368	0.095	0	4	DARS2
0	1.76499428727297	0.259	0.041	0	4	CHEK2
5.9065691135827E-203	1.76329276687235	0.358	0.121	9.89822852054188E-199	4	POLE2
3.32848334081636E-299	1.7612555850068	0.402	0.115	5.57787238254006E-295	4	ZNF367
1.18285142153485E-235	1.75447807437918	0.325	0.092	1.9822224122081E-231	4	JPT1
0	1.75172799192221	0.471	0.144	0	4	FHL1

5.88447864159163E-289	1.75127131321253	0.386	0.108	9.86120930757926E-285	4	SPIN4
2.21728813972915E-224	1.72385596680222	0.303	0.084	3.7157314645581E-220	4	WDR90
1.40157551203023E-275	1.72233421328637	0.425	0.135	2.34876024306026E-271	4	FEN1
7.01298515788232E-255	1.72186240505241	0.257	0.056	1.17523605275792E-250	4	TNFAIP8L1
9.8595287406522E-306	1.71182939613994	0.323	0.074	1.65225982635849E-301	4	NUDT1
4.68263458270461E-267	1.71023341221611	0.256	0.053	7.84715903369638E-263	4	GSTCD
0	1.7079820981113	0.622	0.221	0	4	GGH
0	1.68763558225698	0.432	0.104	0	4	MIS18BP1
0	1.68087153290281	0.521	0.147	0	4	RANGAP1
2.8387273444423E-257	1.67879576663592	0.392	0.121	4.75713928381641E-253	4	LRRCC1
0	1.67759187732962	0.391	0.092	0	4	TDP1
0	1.67756644997495	0.562	0.212	0	4	RRM1
1.54545529380031E-234	1.67083774572201	0.271	0.066	2.58987398135056E-230	4	FANCG
8.64706394784707E-184	1.66198978843564	0.251	0.069	1.44907497638021E-179	4	RFC5
1.12727846303303E-307	1.66088276774386	0.357	0.089	1.88909324835074E-303	4	RFWD3
3.48717669215212E-229	1.64866370559762	0.337	0.099	5.84381070070852E-225	4	HRCT1
0	1.6449171156658	0.706	0.349	0	4	LBR
4.42805141180972E-294	1.62518310586666	0.438	0.135	7.42052855591072E-290	4	TEX30
1.26241354524403E-279	1.62511509773857	0.394	0.115	2.11555261911995E-275	4	COQ2
3.66563765194364E-187	1.6249844972043	0.266	0.075	6.14287557712716E-183	4	TP73
1.34335947473309E-200	1.62471210834819	0.341	0.111	2.25120180775771E-196	4	RFC4
7.61055335764283E-206	1.62242362451654	0.306	0.09	1.27537653167379E-201	4	XRCC3
4.74270704558529E-260	1.62022535829241	0.517	0.205	7.94782846699182E-256	4	TMEM19
2.81099423383615E-188	1.61619533822395	0.335	0.112	4.71066413706262E-184	4	POLD3
2.34396390682514E-253	1.61504321818974	0.335	0.092	3.92801471505757E-249	4	PARP2
8.42362903844002E-249	1.60436131974238	0.378	0.116	1.41163175426178E-244	4	DEPRECATED-ENSG00000120802
9.24560295557918E-172	1.59170461878735	0.476	0.221	1.54937814329596E-167	4	HELLS
1.746822184664E-225	1.58879109848148	0.37	0.119	2.92732461705993E-221	4	CDT1
0	1.58516750823966	0.354	0.087	0	4	CEP78
1.86788319349008E-274	1.58407641584093	0.514	0.201	3.13019865565067E-270	4	FAM111A
2.92843559328555E-298	1.58025646668205	0.452	0.143	4.90747236722792E-294	4	LIG1
0	1.57751665054454	0.373	0.093	0	4	SCLT1
4.70820464337745E-281	1.57437389931411	0.515	0.187	7.89000934137193E-277	4	TUBG1
5.15933342241848E-195	1.57047051738317	0.336	0.109	8.64601094928889E-191	4	RADX
0	1.56574554505674	0.361	0.09	0	4	CEP72
0	1.56208827478471	0.44	0.126	0	4	NDE1
5.33213485502926E-172	1.55350626517045	0.306	0.101	8.93559159005803E-168	4	ARHGEF37
7.19393626431626E-214	1.54936957492782	0.362	0.119	1.20555983917412E-209	4	POLA2
0	1.54450383144952	0.858	0.456	0	4	CALM3
3.719618014929E-288	1.54214144554887	0.662	0.317	6.23333586941802E-284	4	ATAD2
0	1.54093134468868	0.38	0.083	0	4	NEMP1
2.897566649086E-278	1.53922906273857	0.4	0.118	4.85574219053831E-274	4	CEP85
4.49410203343964E-183	1.53420375755254	0.311	0.099	7.53121618763815E-179	4	CNTNAP2
2.86927961045061E-221	1.53371657629857	0.314	0.089	4.80833877119313E-217	4	LIN54
0	1.52876446194158	0.272	0.047	0	4	CEP135
2.13416357350995E-268	1.52452225824935	0.316	0.078	3.57643131648798E-264	4	RCCD1
2.89426273003848E-195	1.51629167778387	0.276	0.078	4.85020548299848E-191	4	RHNO1
7.51455302794807E-217	1.5122702270561	0.38	0.128	1.25928879642354E-212	4	CHAF1A
2.44908191358275E-204	1.5049430350719	0.366	0.122	4.10417147078197E-200	4	AGBL5
0	1.50436850122183	0.552	0.198	0	4	ANP32E
2.13584999013507E-222	1.48667913037521	0.419	0.15	3.57925741346835E-218	4	DSN1
1.83192543818435E-229	1.48033572062364	0.332	0.096	3.06994064930933E-225	4	PRIM2
8.64281782722934E-186	1.46670580778581	0.277	0.08	1.44836341148709E-181	4	PAFAH1B3
0	1.46412658327319	0.574	0.21	0	4	GLA
8.79448711984975E-153	1.46382865913932	0.276	0.09	1.47378015154442E-148	4	ALG1L
5.97458393667384E-210	1.46165795770147	0.271	0.072	1.0012207761078E-205	4	RCC1
2.43538281358922E-277	1.45690753195583	0.314	0.075	4.08121451901281E-273	4	TTF2
0	1.45593138912654	0.351	0.081	0	4	FBLN1
3.84441468828608E-219	1.45428765710518	0.296	0.081	6.44247013462981E-215	4	VANGL1
0	1.45112805237153	0.671	0.276	0	4	FXYD3

1.4968179878312E-195	1.44839896390976	0.387	0.141	2.50836758400753E-191	4	UHRF1
1.37430377931298E-180	1.44811784899435	0.273	0.08	2.30305827337269E-176	4	TMEM201
2.65927951600354E-166	1.43845000337074	0.305	0.101	4.45642061291874E-162	4	TMEM187
0	1.43438873913559	0.735	0.346	0	4	TMPO
3.9364136819004E-190	1.43135703006119	0.394	0.146	6.59664204812869E-186	4	ACYP1
9.54266654293514E-272	1.43099399616246	0.576	0.239	1.59916005926507E-267	4	RBMX
5.19541064860264E-272	1.42841051868225	0.5	0.18	8.7064691649283E-268	4	DCK
6.26790936743253E-245	1.42798156257786	0.468	0.168	1.05037625179434E-240	4	CCHCR1
1.97986653857946E-176	1.42467145453517	0.307	0.099	3.31786034535145E-172	4	ZDHHC12
3.07637362227224E-154	1.41945382081465	0.447	0.205	5.15538691620383E-150	4	CCNE2
3.91736907959123E-295	1.41868602162412	0.425	0.127	6.56472710357898E-291	4	NCAPD3
3.07781930411582E-279	1.40748431205514	0.387	0.111	5.1578095898373E-275	4	KMT5A
7.62676495782E-216	1.39744789042043	0.266	0.068	1.27809327163148E-211	4	CEP97
4.05666912705884E-228	1.39625302549879	0.413	0.142	6.7981661231252E-224	4	NSD2
2.29094612181069E-245	1.39272786461153	0.445	0.155	3.83916751093035E-241	4	RUVBL2
0	1.39139722446143	0.627	0.259	0	4	RNF157
7.11251755006957E-255	1.39037455898782	0.442	0.151	1.19191569104066E-250	4	BRD8
5.09407662945455E-157	1.38228180871195	0.27	0.086	8.53665361563993E-153	4	PSAT1
9.04294911943378E-224	1.38089680652113	0.48	0.188	1.51541741343471E-219	4	MORC4
1.0778861980623E-214	1.37698857689181	0.422	0.15	1.8063216907128E-210	4	MYO5C
2.24358791353159E-195	1.37280248353246	0.275	0.077	3.75980462549624E-191	4	FAM122C
5.61859633503683E-202	1.3668072411673	0.271	0.073	9.41564373825472E-198	4	IFT122
9.05994349711962E-151	1.36654125874672	0.326	0.12	1.5182653312473E-146	4	HOXA10
8.40796346223375E-148	1.36372347916274	0.269	0.088	1.40900651700113E-143	4	TTLL7
3.96490652625806E-146	1.36124678838314	0.282	0.097	6.64439035670325E-142	4	CAMK2N2
1.59800746906541E-245	1.36088535159875	0.563	0.235	2.67794091665982E-241	4	NFIX
2.35283857117495E-190	1.36052812325433	0.402	0.152	3.94288687757498E-186	4	KNTC1
6.00167589220375E-162	1.35278516535575	0.344	0.126	1.0057608460155E-157	4	CDKL1
1.1242799966943E-259	1.35102300150329	0.637	0.29	1.88406841846031E-255	4	PSMD10
2.56443003768562E-217	1.34146765255446	0.388	0.132	4.29747185715356E-213	4	POLD1
1.92237834835228E-176	1.34045675542272	0.358	0.13	3.22152163616876E-172	4	ANKRD36B
1.06717404833977E-194	1.3338929081103	0.261	0.07	1.78837027020779E-190	4	HAUS7
9.17516213391505E-173	1.33378738000477	0.342	0.12	1.53757367040148E-168	4	RMI1
5.71663210432995E-177	1.33139243680937	0.294	0.092	9.57993208043613E-173	4	KATNA1
7.53333282863659E-186	1.32986164507628	0.272	0.078	1.26243591542292E-181	4	DNA2
1.58357264359198E-279	1.32944543467194	0.65	0.301	2.65375103613144E-275	4	FAM122B
2.28718043631153E-269	1.32742845492396	0.514	0.189	3.83285697517087E-265	4	SAE1
3.00211037464443E-301	1.32267637912417	0.642	0.274	5.03093656582914E-297	4	CDK16
7.57081165420958E-161	1.32246598524185	0.27	0.083	1.26871661701244E-156	4	CBX1
4.98949644237164E-242	1.3220308705481	0.461	0.167	8.36139813812639E-238	4	WEE1
4.06109417565134E-195	1.31935979839472	0.509	0.225	6.80558161955652E-191	4	NT5DC3
3.99804457403972E-176	1.30948456198906	0.416	0.164	6.69992309717576E-172	4	LAMA3
1.18120485044407E-210	1.30930831809079	0.48	0.191	1.97946308837417E-206	4	PSPH
0	1.30107392565492	0.891	0.495	0	4	MBNL3
0	1.29962763862148	0.83	0.471	0	4	NUCKS1
1.25676872006539E-220	1.29934639761521	0.467	0.178	2.10609302108558E-216	4	GMNN
1.16485656943031E-286	1.29903796927974	0.79	0.442	1.95206663905132E-282	4	CALM2
1.63930487665239E-195	1.29352599113902	0.257	0.068	2.74714711229407E-191	4	ODF2
1.55326469602963E-205	1.29170802586893	0.397	0.139	2.60296097760646E-201	4	FDXR
1.28847769665569E-151	1.29021890364204	0.336	0.124	2.15923092405561E-147	4	CLGN
8.36102237081233E-183	1.28639389521658	0.381	0.142	1.40114012890073E-178	4	ADRB2
1.20242866154676E-166	1.28345536056157	0.399	0.157	2.01502995102006E-162	4	C12orf75
5.1794686113097E-204	1.28294809700052	0.252	0.064	8.67975349883279E-200	4	PROSER3
0	1.28277297174012	0.485	0.156	0	4	DDX39A
1.82862563299819E-198	1.28161972674559	0.282	0.079	3.06441083577836E-194	4	AJUBA
5.94223690625739E-158	1.27772863450781	0.288	0.095	9.95800060750614E-154	4	FBF1
5.05065690312967E-194	1.27700378846739	0.296	0.088	8.4638908382647E-190	4	KIAA1328
2.27086642543199E-162	1.276075303964	0.307	0.104	3.80551795573894E-158	4	CDC7
3.2574721568506E-160	1.27594623185929	0.385	0.152	5.45887184045024E-156	4	PXMP4
7.04980919499469E-120	1.27555431334771	0.262	0.096	1.18140702489721E-115	4	HCN3

3.67734923768487E-218	1.2749462058644	0.492	0.195	6.1625018525123E-214	4	ILF2
3.38237620763535E-165	1.27401536113176	0.314	0.106	5.66818604875532E-161	4	ITGB3BP
6.90530721656833E-170	1.27343747451016	0.358	0.13	1.15719138335252E-165	4	GUCY1B1
5.01679655951671E-138	1.2675093366949	0.301	0.11	8.4071476744381E-134	4	HMGN5
3.58171635036692E-226	1.26585659968326	0.535	0.222	6.00224025994489E-222	4	RBBP7
6.65720470803484E-203	1.26390538360171	0.625	0.342	1.11561436497248E-198	4	MDM2
9.68627017147274E-134	1.26126213263591	0.493	0.263	1.6232251553354E-129	4	CCDC14
5.48118076457543E-188	1.25541822434639	0.281	0.082	9.18536272527551E-184	4	ZMYM1
6.30864787009402E-148	1.24981015338406	0.322	0.118	1.05720321007036E-143	4	SPATA33
2.25766396232591E-213	1.24879514820977	0.614	0.295	3.78339326806577E-209	4	TUBB2A
8.54536954513616E-154	1.2480123069282	0.307	0.107	1.43203302837392E-149	4	FANCC
6.14347318093598E-195	1.24265995704318	0.59	0.287	1.02952323566125E-190	4	MCM4
1.1558962639191E-290	1.24112251122133	0.765	0.386	1.93705095907564E-286	4	HTATSF1
1.07962343839105E-125	1.23763873344777	0.259	0.091	1.80923295805573E-121	4	CHAF1B
3.07368875650665E-166	1.23010356318659	0.338	0.12	5.15088761815385E-162	4	SLC1A4
3.84103715209183E-174	1.22849072016083	0.402	0.157	6.43681005947549E-170	4	TOPBP1
2.74835404513179E-158	1.22377538887901	0.395	0.159	4.60569170883185E-154	4	CNIH4
2.00056663921317E-159	1.22266496552304	0.348	0.129	3.35254957399343E-155	4	SKP2
2.48083119651823E-226	1.22014173514983	0.451	0.162	4.15737691912525E-222	4	CBX5
6.8911390246015E-220	1.21838557194037	0.484	0.189	1.15481707774272E-215	4	TNPO2
1.49943803326031E-260	1.2162735733062	0.686	0.332	2.51275825613763E-256	4	FLOT2
5.62088996801721E-221	1.21618187409994	0.584	0.259	9.41948740840324E-217	4	TMEM238
1.11979212769625E-268	1.21493755995868	0.435	0.14	1.87654764759338E-264	4	SAP30
1.49700728009465E-175	1.21367232266556	0.496	0.224	2.50868479998262E-171	4	DLG5
7.81447326855188E-284	1.21094733185583	0.777	0.412	1.30954943034392E-279	4	MORF4L2
7.48323897584146E-207	1.21007163937315	0.558	0.249	1.25404118757151E-202	4	CDKN2A
2.02946307560927E-210	1.20940017495389	0.456	0.176	3.40097422210602E-206	4	USP1
2.88573703565176E-254	1.20509641304638	0.472	0.168	4.83591812434521E-250	4	CDK5RAP2
3.85981865706892E-187	1.20446615456776	0.386	0.142	6.4682841055161E-183	4	CDK2
0	1.19821740023754	0.706	0.312	0	4	H1FX
9.99126231712566E-198	1.197956962237	0.39	0.139	1.67433573910392E-193	4	MGME1
6.53856348986839E-184	1.19686549549734	0.483	0.207	1.09573246963214E-179	4	FOXA1
1.45636152978934E-256	1.19684330046959	0.72	0.357	2.44057065162097E-252	4	VBP1
1.2130374526366E-152	1.19658829482866	0.32	0.115	2.03280816312841E-148	4	TIMELESS
1.88204537677285E-122	1.19638412318445	0.25	0.087	3.15393164239594E-118	4	DEPRECATED-ENSG00000165948
4.86312917131748E-177	1.19537498671261	0.612	0.329	8.14963186529384E-173	4	CEP70
6.55882848490205E-219	1.19505741483931	0.449	0.168	1.09912847749988E-214	4	MPHOSPH9
0	1.19410770005577	0.665	0.298	0	4	ADGRE5
1.00188967481006E-126	1.19097350481284	0.314	0.124	1.67896671704669E-122	4	WDR76
5.34508188665815E-127	1.18730593429503	0.276	0.099	8.95728822566172E-123	4	KRT15
1.37122193553401E-186	1.18660281089508	0.456	0.185	2.2978937195679E-182	4	C19orf48
1.67767959166808E-169	1.18566456553188	0.36	0.133	2.81145545971736E-165	4	TSPAN7
9.40840998133412E-204	1.18556883735498	0.49	0.202	1.57666134467197E-199	4	MEPCE
8.40228329939237E-218	1.18033265654828	0.347	0.106	1.40805463531217E-213	4	NUP107
4.34661616161129E-143	1.18009216814363	0.305	0.109	7.2840593636282E-139	4	PKNOX1
7.19279069532405E-244	1.17928180526817	0.6	0.264	1.2053678647224E-239	4	DEK
4.27766655949629E-156	1.17790332162388	0.418	0.177	7.16851362040388E-152	4	PCNA
5.43211949566785E-230	1.17687895369972	0.371	0.115	9.10314585084019E-226	4	THOC3
1.1973005818203E-198	1.17311565872978	0.528	0.229	2.00643631501446E-194	4	HAUS4
9.97016015574515E-255	1.17251286467606	0.714	0.361	1.67079943889977E-250	4	SIVA1
2.94240900859095E-152	1.17232105072595	0.349	0.132	4.93088901659671E-148	4	SLC25A40
4.16052339573168E-173	1.17007814790657	0.476	0.208	6.97220510656716E-169	4	FAIM
1.87196720774445E-113	1.16948167049978	0.273	0.104	3.13704264673814E-109	4	CETN3
1.40047786942229E-210	1.16831692508427	0.603	0.284	2.34692081357787E-206	4	LSM 4.00
4.42117113521603E-203	1.16587747794168	0.572	0.264	7.40899858839503E-199	4	HPRT1
4.6512046818927E-154	1.1614720666121	0.358	0.137	7.79448880591578E-150	4	PRRG4
3.81637850249261E-145	1.16074814374458	0.383	0.155	6.39548709447712E-141	4	FZD6
3.13967463301276E-132	1.15847457468815	0.258	0.087	5.26146675000279E-128	4	SMAGP
1.89824879997896E-221	1.1536756117024	0.655	0.32	3.18108533900474E-217	4	HADH
1.16585209919007E-236	1.1507716900563	0.641	0.289	1.95373494782273E-232	4	C15orf48

2.12960936184755E-134	1.14949999943596	0.341	0.136	3.56879936858412E-130	4	PAK4
1.83511585584439E-120	1.14897871380978	0.295	0.113	3.07528715122403E-116	4	RASSF8
8.72558518956281E-163	1.14816583374473	0.304	0.101	1.46223356606694E-158	4	XRCC1
1.01862485307745E-283	1.14581156070113	0.826	0.473	1.7070115287872E-279	4	TMEM106C
5.86431388067802E-159	1.14334384223437	0.428	0.18	9.82741720124023E-155	4	DEPRECATED-ENSG00000099797
4.56986367500985E-186	1.14292391110632	0.554	0.267	7.65817754658151E-182	4	TUBGCP3
1.77136366590176E-141	1.14090641528749	0.259	0.084	2.96845123131817E-137	4	EFCAB2
2.16169617717327E-161	1.13997281473805	0.404	0.161	3.62257045370696E-157	4	SLC7A1
2.9800502387768E-127	1.13453502866449	0.275	0.099	4.99396819014215E-123	4	CDCA7L
3.14818318398817E-152	1.13254902473709	0.458	0.207	5.27572537972738E-148	4	DONSON
9.49924272492191E-164	1.13241684432787	0.388	0.15	1.59188309584241E-159	4	CSTF2
4.95810105438172E-114	1.13229712536994	0.251	0.092	8.30878574693288E-110	4	B3GLCT
3.63632463282372E-194	1.13078460518902	0.37	0.127	6.093752819686E-190	4	PDE3B
8.87033695846531E-135	1.12959460048143	0.415	0.185	1.48649106749962E-130	4	CKB
1.18702696194255E-198	1.12922066987753	0.522	0.228	1.98921978282332E-194	4	NCAPH2
5.87581265209115E-140	1.12734741795335	0.336	0.13	9.84668684237436E-136	4	ING2
9.62469558553954E-197	1.12642523372197	0.359	0.119	1.61290648622472E-192	4	KATNAL1
5.32354908967477E-223	1.12606286391735	0.592	0.272	8.92120356447699E-219	4	NNT
5.9796647185762E-136	1.12519868929463	0.277	0.096	1.002072213539E-131	4	CREB3L4
1.21530063227216E-224	1.12497088926935	0.372	0.117	2.03660079956168E-220	4	DCAF15
6.60277986561219E-137	1.12484900443628	0.408	0.178	1.10649384987929E-132	4	NXT2
7.01715177115551E-150	1.12386721894232	0.267	0.086	1.17593429381024E-145	4	ANP32B
2.26206465219747E-158	1.12382892664448	0.493	0.229	3.79076794415251E-154	4	DEPRECATED-ENSG00000132646
1.07343750508608E-171	1.12015179649194	0.293	0.093	1.79886657102325E-167	4	FAM102B
3.0565379336204E-221	1.11715434136399	0.655	0.32	5.12214626916106E-217	4	KIF5B
2.71485244604076E-143	1.1160866422046	0.359	0.141	4.54954972907511E-139	4	ARMC1
4.44551696542201E-231	1.1137173642948	0.697	0.352	7.44979733065421E-227	4	CD99L2
1.18547478005617E-172	1.1134800346324	0.506	0.229	1.98661863641813E-168	4	SLC27A3
5.23816515361822E-189	1.1133773101299	0.259	0.07	8.77811716443342E-185	4	KIAA0586
6.84355094167794E-121	1.11249578967566	0.325	0.132	1.14684226680639E-116	4	KHDRBS3
1.90949998714549E-193	1.1099791181974	0.267	0.073	3.19994007845841E-189	4	METTL4
1.5390804965585E-165	1.10996401371476	0.423	0.173	2.57919109613273E-161	4	SEMA6A
1.50543359775349E-146	1.10972585655827	0.262	0.085	2.5228056231153E-142	4	DEPRECATED-ENSG00000102007
6.78196169894175E-148	1.10947895860139	0.265	0.085	1.13652114150866E-143	4	MAZ
2.70217042339654E-156	1.10268159133245	0.296	0.099	4.52829719552793E-152	4	ERI2
2.21863790235319E-146	1.10257931761998	0.312	0.113	3.71799339676348E-142	4	OSBPL10
3.04613041653264E-212	1.09731415187025	0.522	0.219	5.1047053520254E-208	4	RHEB
4.0101604293705E-262	1.09684609066988	0.75	0.36	6.72022684753908E-258	4	MEP1A
3.77539148123785E-212	1.09595283377252	0.321	0.093	6.3268010442584E-208	4	SPATA5
1.00609305902345E-163	1.09487178928479	0.381	0.147	1.68601074831149E-159	4	MED30
3.07536938337558E-157	1.0946118397588	0.45	0.194	5.1537040126608E-153	4	PRELID2
2.12591785333591E-187	1.09367653175265	0.484	0.203	3.56261313862033E-183	4	PPP2R5D
1.32497185208132E-202	1.09295027953173	0.634	0.316	2.22038782971788E-198	4	NUCB2
9.55343639347465E-220	1.08827281145851	0.678	0.361	1.60096487081848E-215	4	FMR1
1.09350824471973E-199	1.08814260916313	0.393	0.136	1.83250111650132E-195	4	TPGS2
1.44728025831815E-164	1.08569140429789	0.47	0.202	2.42535225688956E-160	4	ASRGL1
9.68533821289864E-120	1.08325314597184	0.398	0.18	1.62306897771755E-115	4	FGF13
2.76983776984428E-142	1.08208275709099	0.384	0.158	4.64169413470504E-138	4	C9orf64
2.88946417324678E-168	1.08123235664736	0.385	0.147	4.84216406152695E-164	4	ZNF704
3.6099489908888E-183	1.08079045332088	0.636	0.33	6.04955251893144E-179	4	LMO7
4.12728762561286E-180	1.07811597785938	0.462	0.19	6.91650860300203E-176	4	HSPA4L
4.07075040253856E-151	1.07781560839038	0.404	0.168	6.82176352457412E-147	4	CEP250
5.1979241178867E-105	1.07727588283988	0.254	0.096	8.71068123675452E-101	4	SPAG1
2.888410224652E-218	1.07539173625781	0.686	0.347	4.84039785447181E-214	4	VMA21
3.98569699269785E-128	1.07121348450227	0.346	0.141	6.67923102036306E-124	4	IFI27L1
2.7844950762928E-159	1.06944537182172	0.407	0.167	4.66625684885147E-155	4	SLC9A6
1.62049904435415E-156	1.06943371234286	0.301	0.102	2.71563229852868E-152	4	AACS
4.86811163169545E-246	1.06782761177958	0.771	0.435	8.15798147239523E-242	4	GUSB
9.54110928381749E-181	1.06688707419324	0.558	0.27	1.59889909378213E-176	4	MCM7
3.06046222994712E-184	1.06540707074203	0.482	0.203	5.12872260494538E-180	4	RSRC1

4.08710664713285E-122	1.0630669381747	0.312	0.123	6.84917331926523E-118	4	PRIM1
3.28154031227014E-106	1.06123438419808	0.325	0.143	5.4992052553023E-102	4	POLA1
1.38932444911569E-148	1.05738383912916	0.478	0.223	2.32822991182807E-144	4	MMGT1
1.47181606056947E-154	1.05644956074125	0.45	0.196	2.46646935430231E-150	4	UBR7
2.33259240375305E-86	1.05643088835398	0.274	0.119	3.90895835020935E-82	4	KCNMB3
2.70030160358904E-182	1.05606038303088	0.565	0.267	4.52516542729452E-178	4	DKC1
1.86471601390841E-142	1.05593059978318	0.325	0.121	3.12489109610771E-138	4	DESI2
4.84399352891056E-110	1.05580878327	0.252	0.093	8.11756435574832E-106	4	PRADC1
1.69114008356002E-133	1.05512985916303	0.361	0.148	2.83401255202989E-129	4	ARL4A
1.16121150574703E-190	1.05334298763882	0.557	0.247	1.94595824133088E-186	4	CHP2
9.50522668791109E-144	1.05215627418571	0.414	0.177	1.59288588836014E-139	4	REPIN1
3.17850550178549E-115	1.05120642515366	0.342	0.145	5.32653951989212E-111	4	ANKS1B
3.59648243364977E-115	1.0507482042316	0.333	0.14	6.02698526231029E-111	4	SSTR1
5.42848566190689E-178	1.04776442334106	0.358	0.126	9.09705627222356E-174	4	PLP2
1.74649385398028E-123	1.04651634658245	0.329	0.133	2.92677440050016E-119	4	CHRAC1
4.67414367172008E-164	1.04594789011681	0.531	0.249	7.83292996506851E-160	4	VAMP7
3.13738681531712E-151	1.04581876750791	0.407	0.17	5.25763282510843E-147	4	AK6
1.10446374234116E-194	1.04420632316222	0.488	0.201	1.85086033941532E-190	4	PGP
6.07362791501385E-163	1.04365894026371	0.339	0.121	1.01781856599802E-158	4	CDC27
1.44956970783959E-189	1.04349391660651	0.586	0.285	2.42918891639759E-185	4	FZR1
1.91163653494619E-120	1.04188099968068	0.348	0.146	3.20352050526282E-116	4	BRCC3
8.41600930973838E-222	1.04083626812033	0.42	0.145	1.41035484012596E-217	4	TCOF1
3.14148183536214E-164	1.03673393242525	0.476	0.207	5.26449525969987E-160	4	KIF16B
6.84832684507662E-182	1.03641748034275	0.613	0.306	1.14764261269794E-177	4	SIPA1L2
3.58632881148206E-234	1.03600010814283	0.786	0.452	6.00996982228164E-230	4	HNRNPH2
7.29214543074991E-185	1.03481495810666	0.448	0.18	1.22201773128507E-180	4	ITPR3
3.86617013211677E-157	1.03418306539541	0.307	0.105	6.47892790740129E-153	4	ZW10
2.25721456271876E-149	1.03215889987928	0.457	0.205	3.7826401642041E-145	4	IGF2BP3
2.87776516189107E-151	1.03046052021293	0.293	0.099	4.82255885829705E-147	4	ALDH16A1
1.31043351474765E-132	1.02734878734041	0.354	0.144	2.19602448401412E-128	4	COX20
1.29125532096786E-192	1.02670463929916	0.625	0.307	2.16388566687793E-188	4	IMPA2
1.35325462146363E-135	1.02667037926311	0.349	0.139	2.26778409464875E-131	4	PLXNC1
3.18370184243761E-122	1.02629345759145	0.426	0.202	5.33524754755694E-118	4	FADS1
2.13046785510908E-236	1.02536362000502	0.739	0.359	3.57023803159179E-232	4	KLF5
4.35917685210427E-135	1.02484234523757	0.385	0.164	7.30510856875633E-131	4	LRRC45
6.02056717874886E-240	1.02455721977645	0.762	0.407	1.00892664781473E-235	4	GPAA1
1.81252018190534E-123	1.02334237112147	0.285	0.106	3.03742132083697E-119	4	FAM184A
2.40702292941685E-126	1.02106952649918	0.342	0.139	4.03368902511675E-122	4	SAV1
1.2256516363664E-149	1.02072988628698	0.484	0.225	2.05394701222281E-145	4	BRI3BP
1.40445230351101E-108	1.02032341151106	0.282	0.111	2.35358117022375E-104	4	CCDC85C
3.03007147925982E-203	1.01896579153454	0.403	0.141	5.07779378494361E-199	4	OAS3
4.32428726050117E-142	1.01721444428837	0.302	0.108	7.24664059114785E-138	4	ZNF318
4.94458241455341E-166	1.0157364591472	0.559	0.273	8.2861312103086E-162	4	TMCO3
6.27692281891311E-137	1.01522339310507	0.322	0.121	1.05188672599346E-132	4	DBNDD2
3.09646429684103E-159	1.01451704860401	0.567	0.282	5.18905486864619E-155	4	NECTIN3
7.20625706926953E-190	1.01308147123727	0.651	0.323	1.20762455966819E-185	4	EHF
1.96707535425364E-121	1.01178322855719	0.372	0.162	3.29642487865825E-117	4	CCDC198
7.55230818752321E-105	1.01150405991966	0.296	0.121	1.26561580606514E-100	4	AMMECR1
1.02117980668865E-159	1.0113712725556	0.425	0.176	1.71129312004885E-155	4	EIF4E
4.60236756660692E-139	1.00746686152404	0.409	0.176	7.71264756811988E-135	4	DEPRECATED-ENSG00000171234
1.12100173376683E-142	1.00231265567834	0.387	0.16	1.87857470544645E-138	4	NUP155
5.05364258180828E-174	1.00144437547509	0.508	0.223	8.46889423859431E-170	4	GNA11
2.65705025439102E-156	0.994228000554934	0.448	0.192	4.45268481630846E-152	4	DDR1
6.74462523937972E-161	0.992835273272167	0.484	0.216	1.13026429761525E-156	4	HLTF
2.35696791461341E-181	0.990386134453821	0.614	0.301	3.94980683130914E-177	4	NEB
4.47683057730621E-172	0.988621192701277	0.575	0.279	7.50227268144974E-168	4	EMP2
3.5810303990723E-127	0.987811448855456	0.409	0.185	6.00109074276535E-123	4	DBP
2.65865631449479E-207	0.984199862294509	0.56	0.247	4.45537625183037E-203	4	TRIOBP
1.15923746247173E-122	0.983642454003853	0.741	0.497	1.94265013961012E-118	4	H1F0
3.1260164011673E-173	0.983394957554408	0.571	0.277	5.23857828507616E-169	4	NUP58

6.43507372581215E-137	0.983021953811765	0.422	0.189	1.0783896549716E-132	4	CLDN12
4.19222719781869E-152	0.98281598711952	0.487	0.225	7.02533433810456E-148	4	YWHAQ
1.06702866470054E-188	0.982023726784679	0.534	0.247	1.78812663630517E-184	4	CYTH2
1.70138463659124E-170	0.981684671285284	0.615	0.314	2.85118037399961E-166	4	GCHFR
2.729517120813E-160	0.978744309918016	0.369	0.14	4.57412479105843E-156	4	JADE1
6.66285170813543E-110	0.978193953032831	0.287	0.113	1.11656068924934E-105	4	PPIL1
3.97745316073741E-297	0.977589668021019	0.951	0.613	6.66541600676376E-293	4	AKR1C3
9.13559888668354E-251	0.976827723192837	0.805	0.469	1.53094366143043E-246	4	EMD
5.76160221276147E-136	0.97533617395466	0.414	0.182	9.65529298814567E-132	4	PLIN3
4.37399543035241E-173	0.974998645920316	0.498	0.22	7.32994154218457E-169	4	PRRC2A
1.01222664403888E-157	0.97380814451133	0.346	0.13	1.69628941008036E-153	4	ANKRD36C
9.60567198830688E-111	0.972415943429562	0.333	0.143	1.60971851180047E-106	4	PRPS2
2.07065120026897E-205	0.971678918537234	0.379	0.127	3.46999728141074E-201	4	FGFR1OP
1.02383971150383E-180	0.971067577752165	0.539	0.245	1.71575058853813E-176	4	SF3A2
2.92376310242761E-95	0.970657199071199	0.262	0.105	4.89964220704819E-91	4	METTL27
1.14518308216753E-129	0.969328464906693	0.325	0.127	1.91909780909635E-125	4	ALYREF
7.21459127772087E-172	0.967637093772794	0.381	0.142	1.20902120632046E-167	4	SRGAP2B
7.93319652080628E-141	0.965512572148632	0.388	0.16	1.32944507295672E-136	4	CCT5
1.45936919136957E-168	0.961353443147139	0.431	0.175	2.44561089089712E-164	4	AGR2
1.47144369345878E-145	0.960052155680656	0.315	0.113	2.46584534149822E-141	4	SRGAP2
3.08861914444397E-212	0.958263833741483	0.884	0.605	5.17590796225921E-208	4	LMNA
5.29704491060196E-156	0.958226221350378	0.581	0.298	8.87678786118676E-152	4	CCNG1
5.18074322519898E-112	0.952806586057552	0.286	0.112	8.68188949678844E-108	4	MEX3D
4.96368050388191E-75	0.952754331924128	0.254	0.113	8.3181357884053E-71	4	GPR180
2.08755857421607E-186	0.952252397138842	0.642	0.332	3.49833065867129E-182	4	SMC1A
1.73058140159457E-130	0.951198470023859	0.334	0.132	2.90010831279218E-126	4	TLNRD1
9.06341653782749E-113	0.950071633530247	0.265	0.099	1.51884734340913E-108	4	MSH5
2.69032507210109E-144	0.949924151375245	0.435	0.189	4.50844675582701E-140	4	COMMD4
6.9137913954966E-177	0.949815090286602	0.694	0.388	1.15861316205732E-172	4	GSR
1.56487259483543E-160	0.948145387956806	0.352	0.13	2.62241349442521E-156	4	CEP192
9.3052537754618E-142	0.946051324581169	0.5	0.241	1.55937442769189E-137	4	TFDP1
1.10926279116672E-146	0.945907401603479	0.534	0.261	1.85890258543719E-142	4	TIMM8A
4.20940747087582E-182	0.944399597311367	0.514	0.225	7.05412503969369E-178	4	YWHAH
2.40236399502119E-133	0.944193372839748	0.443	0.203	4.02588158285651E-129	4	DCTN3
7.13087570191045E-135	0.943838490376934	0.415	0.179	1.19499215012615E-130	4	HOXA13
2.10056022197746E-103	0.94315014334368	0.323	0.139	3.52011881998983E-99	4	PFN2
7.22496281411708E-120	0.942088165093013	0.461	0.228	1.21075926838974E-115	4	ZNF189
1.24299418501337E-135	0.941844608008547	0.346	0.137	2.08300965524541E-131	4	BCL7C
3.73472244584614E-85	0.94103991052786	0.258	0.109	6.25864787474897E-81	4	SNX7
1.4914987318583E-207	0.94102375996865	0.75	0.418	2.49945357484815E-203	4	UBE2A
2.21391859467002E-107	0.940087382702788	0.351	0.156	3.71008478094802E-103	4	HHAT
1.81316253302079E-164	0.938715694095819	0.492	0.218	3.03849777283624E-160	4	JPT2
7.82256030715592E-167	0.937761945463253	0.488	0.214	1.31090465627319E-162	4	PAF1
3.72318394801118E-106	0.937092413797423	0.362	0.164	6.23931166007714E-102	4	PIGX
1.45207122141007E-133	0.936406369203861	0.395	0.169	2.433380952839E-129	4	ACTL6A
3.59983536903668E-123	0.936280585206616	0.376	0.163	6.03260411143167E-119	4	TFF3
1.39787261818096E-142	0.936239440497034	0.317	0.115	2.34255493354765E-138	4	TMEM160
3.58618846311126E-211	0.935416685950133	0.815	0.481	6.00973462648186E-207	4	ANXA2
1.94515406195131E-116	0.934907708242054	0.494	0.258	3.259689177018E-112	4	MYO19
1.04869626096649E-118	0.934878054591917	0.301	0.118	1.75740519412764E-114	4	MAPRE1
1.34718383115645E-102	0.934837643515433	0.252	0.095	2.25761066425198E-98	4	GTF3C5
2.36192989566962E-119	0.934200297004611	0.39	0.174	3.95812211916315E-115	4	PPP1CC
4.58648076145703E-150	0.934043702726503	0.391	0.158	7.68602446004969E-146	4	CTCF
6.16976062426452E-141	0.93392192021629	0.355	0.143	1.03392848541425E-136	4	DEPRECATED-ENSG00000135976
5.32886183406121E-147	0.933171369652318	0.489	0.23	8.93010666151977E-143	4	SUPT16H
4.80873143771471E-146	0.931417848998757	0.412	0.173	8.05847214332231E-142	4	TFF1
2.92494228367634E-157	0.931051161433799	0.495	0.229	4.90161827898481E-153	4	PHACTR4
3.5842002048665E-123	0.92910807435724	0.324	0.13	6.00640270331528E-119	4	KANSL1L
1.79530045863418E-188	0.928098419316072	0.402	0.148	3.00856450857916E-184	4	PCNT
2.3497684905559E-152	0.927148970297883	0.479	0.223	3.93774203647357E-148	4	KBTBD2

4.36155464985996E-81	0.926256107681171	0.251	0.107	7.30909328223533E-77	4	C9orf152
3.45164335423048E-125	0.926037665196778	0.358	0.149	5.78426393301944E-121	4	UPF3B
1.294095699484E-135	0.92369300859827	0.576	0.305	2.16864557319528E-131	4	SYTL4
2.24601505514509E-165	0.923650735920541	0.641	0.34	3.76387202941215E-161	4	LACTB2
2.39318926844329E-131	0.923175291887583	0.37	0.155	4.01050657605727E-127	4	FZD5
9.06396884167445E-118	0.923137206910102	0.351	0.149	1.5189398984878E-113	4	MZT2A
1.42399696623073E-122	0.92235736223053	0.256	0.091	2.38633411600945E-118	4	TUBE1
1.43477942280301E-172	0.921333269353626	0.538	0.251	2.40440335673329E-168	4	NUP62
6.66465130775197E-92	0.921005832139509	0.269	0.111	1.11686226615307E-87	4	UBXN2A
1.17738938060683E-98	0.919165358388196	0.281	0.115	1.97306912402092E-94	4	PRUNE1
2.23133950971341E-127	0.91855953344319	0.396	0.174	3.73927875037774E-123	4	DEPRECATED-ENSG00000134597
4.52544338463562E-130	0.917171627358334	0.362	0.15	7.58373802397237E-126	4	KDM1A
3.23590613073714E-160	0.916282753118145	0.66	0.359	5.4227314938893E-156	4	PGD
2.63071012082037E-168	0.916043243544024	0.716	0.414	4.40854402047078E-164	4	SDC2
2.17997353085655E-111	0.915958715948036	0.273	0.105	3.65319964300941E-107	4	B4GALT4
3.43370694253986E-134	0.915465694027958	0.405	0.176	5.7542060943083E-130	4	HSPB11
3.6708400957857E-132	0.91305611346574	0.469	0.222	6.15159383251768E-128	4	PHF6
3.50261810240408E-184	0.911888716525979	0.357	0.122	5.86968741600876E-180	4	CCDC88A
1.19775508841855E-142	0.910997302453562	0.505	0.239	2.00719797717181E-138	4	SSX2IP
1.39879985969309E-119	0.910472220895799	0.356	0.151	2.34410880487368E-115	4	SLC4A4
1.09803037009516E-127	0.907584466822721	0.469	0.224	1.84007929420547E-123	4	FUCA2
7.15323076541005E-173	0.907571865416028	0.423	0.168	1.19873841166742E-168	4	HP1BP3
2.01897847754958E-92	0.907327014003388	0.26	0.106	3.38340413267759E-88	4	POT1
8.68267844418636E-132	0.906827632745821	0.566	0.299	1.45504325367675E-127	4	TXNDC17
4.23240726099902E-154	0.906816066773713	0.381	0.148	7.09266808798216E-150	4	DUOXA2
8.37614473755484E-128	0.905371856538095	0.44	0.205	1.40367433511944E-123	4	AAMDC
1.4924780992892E-160	0.904983770665178	0.58	0.294	2.50109479878884E-156	4	TMX4
1.29222566851673E-96	0.90353523766474	0.311	0.136	2.16551177530034E-92	4	GTPBP3
1.43823470788489E-139	0.902936731854423	0.524	0.253	2.4101937234735E-135	4	SHROOM3
9.69176554657247E-134	0.902788159013028	0.38	0.159	1.62414607029461E-129	4	CBX3
7.86530718257894E-130	0.902774305284964	0.459	0.214	1.31806817765658E-125	4	LRP11
3.21216407053104E-104	0.902498255757798	0.284	0.114	5.38294454939591E-100	4	ASF1A
4.5928322312652E-162	0.901332916450163	0.329	0.114	7.69666825315422E-158	4	RCC2
3.12166009940942E-159	0.900704381771444	0.48	0.214	5.23127799459031E-155	4	SUZ12
4.24618695462011E-102	0.900367595763246	0.311	0.132	7.11576009855238E-98	4	ENOX2
2.75789424707855E-141	0.899924846148646	0.537	0.273	4.62167917925424E-137	4	DNMT1
2.83847919437978E-134	0.899004635988248	0.501	0.243	4.75672343394163E-130	4	HACD2
1.44983170882923E-115	0.898728232016392	0.25	0.089	2.42962797765602E-111	4	TPRKB
4.60763257854113E-159	0.898532211247453	0.518	0.247	7.72147067511922E-155	4	DAZAP1
3.0240530114918E-147	0.897714359128887	0.493	0.23	5.06770803665796E-143	4	CHAMP1
1.67987687462902E-146	0.897486240253898	0.554	0.28	2.81513766650332E-142	4	SSRP1
1.43575049546959E-178	0.897234711064464	0.609	0.303	2.40603068030794E-174	4	HNRNPAB
5.47128610541637E-137	0.896652858537497	0.418	0.184	9.16878125545675E-133	4	TTLL5
5.87220453504697E-136	0.895424396541592	0.317	0.118	9.8406403598317E-132	4	DEPRECATED-ENSG00000140365
2.2534854635628E-134	0.894948638904763	0.307	0.113	3.77639093983854E-130	4	DEPRECATED-ENSG00000093000
2.16737573134701E-97	0.892352291125617	0.364	0.171	3.63208825059131E-93	4	IPMK
9.61217632673486E-123	0.891368704050365	0.35	0.146	1.61080850883423E-118	4	UCHL5
2.02883912757599E-126	0.890336470907506	0.358	0.149	3.39992860991185E-122	4	FOPNL
1.05412405949531E-117	0.889987740420584	0.32	0.129	1.76650109890224E-113	4	DDX59
2.10528872767497E-131	0.889503126494229	0.377	0.16	3.52804284983771E-127	4	MDC 1.00
7.52849775604488E-194	0.888940584369513	0.747	0.404	1.261625653958E-189	4	CMC4
2.91046611830998E-133	0.888536309380788	0.52	0.257	4.87735912106387E-129	4	CXorf56
2.98113391408026E-171	0.888266315713453	0.757	0.449	4.9957842132157E-167	4	SPTSSA
3.30657651631278E-249	0.888054544364928	0.91	0.52	5.54116092603696E-245	4	MPP1
4.52010960316188E-111	0.887726420242195	0.358	0.158	7.57479967297868E-107	4	CLN6
1.2058666398327E-147	0.88748513283115	0.557	0.276	2.02079131503165E-143	4	FAM20B
3.12186935431733E-110	0.886846907519116	0.368	0.163	5.23162866396498E-106	4	CCNQ
2.91115129439365E-103	0.886641469330483	0.281	0.112	4.87850733914488E-99	4	DSG2
5.54229811917216E-188	0.88584455016875	0.798	0.47	9.28778318810871E-184	4	SMIM14
2.29374536413249E-93	0.885570961082975	0.3	0.13	3.84385848121323E-89	4	MPP6

1.58647380404159E-172	0.884622048773379	0.677	0.378	2.65861280081289E-168	4	STAG2
1.71584527211351E-126	0.883608757777642	0.466	0.222	2.87541350700783E-122	4	THOP1
8.2449307777074E-139	0.883272708534712	0.524	0.257	1.38168549972821E-134	4	GFPT1
4.2627991853364E-131	0.88317632510362	0.294	0.108	7.14359887478673E-127	4	RAD51B
1.33776600918213E-98	0.882997329053956	0.327	0.144	2.24182827818742E-94	4	RBMX2
4.15602064441911E-125	0.88269128706912	0.332	0.132	6.96465939591754E-121	4	KPNB1
3.69874348717852E-144	0.882541258461377	0.566	0.284	6.19835433581377E-140	4	SLC25A5
2.92131016952102E-139	0.882124563861078	0.377	0.155	4.89553158208332E-135	4	SNHG32
4.60600186447269E-101	0.881971924929013	0.358	0.165	7.71873792448333E-97	4	ALG6
3.31607263649592E-130	0.879169549703622	0.308	0.116	5.55707452423987E-126	4	CEP44
3.13714983535002E-120	0.879047436365962	0.36	0.153	5.25723569407956E-116	4	GPD2
2.35655275010056E-138	0.875138871046766	0.447	0.202	3.94911109861851E-134	4	TEAD1
1.87969749357925E-171	0.874919157526406	0.741	0.425	3.14999705974011E-167	4	MDK
2.18198688580704E-109	0.873596371885682	0.374	0.17	3.65657362323544E-105	4	CENPX
4.85617272570967E-151	0.871650761133853	0.646	0.351	8.13797425374426E-147	4	SRP9
6.98185961572726E-120	0.870312192169099	0.381	0.167	1.17002003440357E-115	4	LNPK
1.38298989542642E-128	0.870015593003537	0.487	0.241	2.3176144667556E-124	4	DOT1L
3.4032902450508E-96	0.869901324154779	0.273	0.112	5.70323379265612E-92	4	RBL1
1.17079896538983E-130	0.868145823066028	0.526	0.268	1.96202490620028E-126	4	PPIA
2.09145767023865E-206	0.867801459547776	0.828	0.497	3.50486476378593E-202	4	BCAP31
6.76153824821181E-206	0.867030752054733	0.465	0.179	1.13309857963533E-201	4	BUB3
2.41516244928691E-173	0.866628138283576	0.633	0.332	4.047329232515E-169	4	PI4K2B
7.19792055825351E-117	0.866188672934592	0.387	0.174	1.20622752715212E-112	4	UGGT2
1.43632633401661E-115	0.866138062474295	0.359	0.155	2.40699567054503E-111	4	SEC22C
3.29203067019333E-244	0.865061119655741	0.862	0.457	5.51678499710998E-240	4	ADA
1.03392177304345E-214	0.864482350547068	0.616	0.297	1.73264610726621E-210	4	NID1
3.85647258255543E-121	0.863689723416333	0.311	0.122	6.46267675384639E-117	4	TRIM28
2.6908545546342E-150	0.863416204577837	0.625	0.34	4.50933406265599E-146	4	PCYT2
4.95368165921113E-151	0.862952704858804	0.59	0.301	8.30137972450602E-147	4	PRPS1
6.83691443965795E-129	0.862445769523379	0.391	0.168	1.14573012179788E-124	4	MBD3
2.12992803247982E-116	0.862143989579466	0.424	0.199	3.56933339682969E-112	4	EPS8L3
1.44366875338506E-148	0.861301785994372	0.529	0.255	2.41930009692269E-144	4	CRKL
3.28607562981841E-114	0.861239680262547	0.435	0.21	5.50680554044969E-110	4	DPY19L4
2.75840957950409E-110	0.859729384481586	0.385	0.175	4.62254277333295E-106	4	ACOT7
3.67221270615449E-192	0.85880091494909	0.694	0.372	6.1538940529737E-188	4	HNRNPUL1
3.12835829488807E-109	0.858654831465885	0.292	0.117	5.24250283057343E-105	4	C12orf65
1.0571565431789E-100	0.858326103962954	0.366	0.169	1.7715829350592E-96	4	MAP3K21
1.69630533174531E-140	0.857989091618946	0.482	0.23	2.84266847493878E-136	4	NUP85
1.59369347429896E-144	0.857197286553573	0.598	0.318	2.6707115242302E-140	4	THOC2
3.56746142214759E-109	0.856812664572053	0.385	0.176	5.97835185123493E-105	4	LCLAT1
2.11359695219392E-163	0.855785424371819	0.532	0.25	3.54196577248658E-159	4	CMC2
7.21322059219886E-115	0.855207778144637	0.336	0.141	1.20879150684069E-110	4	SRGAP1
3.03845849899613E-144	0.854398586533845	0.361	0.142	5.09184875261771E-140	4	BTG3
3.85000212946284E-147	0.854168233641079	0.523	0.249	6.45183356855383E-143	4	MZT2B
1.40843183251548E-131	0.854104920993888	0.482	0.228	2.36025006492945E-127	4	FBXL6
6.12637207396977E-101	0.853454011684379	0.341	0.151	1.02665743215585E-96	4	PHLPP1
1.41202404059683E-143	0.853337050183717	0.507	0.239	2.36626988723216E-139	4	NKAP
4.92310412296738E-105	0.852924718424793	0.254	0.095	8.25013788926874E-101	4	VKORC1L1
5.7942507913766E-173	0.852543460025919	0.666	0.376	9.7100054761889E-169	4	ZNRF2
1.87526629462458E-144	0.851957171602232	0.496	0.236	3.14257125653187E-140	4	SCML1
3.54959203779616E-161	0.85059728047081	0.344	0.124	5.94840633693881E-157	4	UBALD2
7.7234888268841E-126	0.849528817040646	0.318	0.123	1.29430225760924E-121	4	TTL
3.0850919683981E-132	0.849461089505093	0.563	0.292	5.16999712064153E-128	4	PIR
3.00911795062884E-100	0.848708651621899	0.299	0.126	5.04267986166382E-96	4	ARL4D
3.97293530268604E-121	0.848466364730332	0.364	0.155	6.65784498024126E-117	4	RUSC1
2.07908774626245E-146	0.848280488152581	0.414	0.175	3.48413524518662E-142	4	RAVER1
2.42272335673779E-88	0.847140433387242	0.324	0.152	4.05999980122118E-84	4	RFC2
3.15698787152651E-183	0.846514463612358	0.381	0.136	5.29048027510412E-179	4	DCP2
3.20522063778192E-95	0.8461753710345	0.273	0.112	5.37130874479495E-91	4	FSD1L
1.67181277522919E-128	0.845323203261386	0.32	0.124	2.80162384872907E-124	4	MAST2

1.26659085744984E-141	0.845241031701224	0.47	0.218	2.12255295891444E-137	4	TXNDC12
5.42509357704376E-125	0.845145509783096	0.407	0.182	9.09137181640993E-121	4	DEPRECATED-ENSG00000078668
9.6208758697069E-89	0.843377256312491	0.282	0.122	1.61226637824548E-84	4	FADD
2.28056859406267E-109	0.841709771611389	0.253	0.093	3.82177684993022E-105	4	ALMS1
1.16324711638592E-134	0.841678730662343	0.529	0.273	1.94936951763953E-130	4	CEP57
1.36520137140301E-167	0.840962113076871	0.589	0.296	2.28780445819716E-163	4	SRSF3
4.80226347561266E-119	0.840326972975035	0.328	0.133	8.0476331324317E-115	4	GLE1
1.15214451889199E-113	0.840242732520017	0.285	0.11	1.93076378475919E-109	4	SMG9
3.40807631783904E-202	0.838950036946697	0.815	0.456	5.71125429343466E-198	4	MAOA
5.40640487206265E-136	0.838116517738327	0.387	0.162	9.06005328460259E-132	4	PDS5B
1.47123893029493E-131	0.837473204332706	0.38	0.159	2.46550219938825E-127	4	QSER1
4.60862981352383E-123	0.837123906594702	0.542	0.289	7.72314184150323E-119	4	RBM42
3.66008940257411E-158	0.835712201900463	0.663	0.351	6.1335778208337E-154	4	NBL1
3.24996653702787E-82	0.835708208388218	0.381	0.198	5.44629392275131E-78	4	E2F1
1.21575163054285E-115	0.835233692915366	0.475	0.24	2.03735658246372E-111	4	ABCB6
3.10525696903166E-122	0.833347128910256	0.471	0.232	5.20378962870326E-118	4	DEPRECATED-ENSG00000049860
1.35231355261591E-133	0.832524786165303	0.454	0.211	2.26620705147375E-129	4	DEPRECATED-ENSG00000265241
5.06672045462667E-144	0.832188576561578	0.523	0.255	8.49081013786337E-140	4	SRPK1
6.77013938149011E-167	0.83159071415166	0.522	0.243	1.13453995755011E-162	4	MED25
5.59632017189499E-127	0.830585418192268	0.575	0.301	9.37831334406163E-123	4	MCTS1
2.21471091642327E-143	0.829991467124003	0.59	0.298	3.71141255374212E-139	4	SYBU
1.29534998596467E-141	0.829414652022138	0.634	0.346	2.17074750647959E-137	4	ANAPC11
8.36595068652252E-87	0.829236305181333	0.288	0.126	1.40196601604744E-82	4	BTBD3
7.84125944783678E-113	0.828434863948666	0.345	0.148	1.31403825826849E-108	4	ICMT
2.84433857378051E-112	0.828094074921624	0.272	0.103	4.76654258194137E-108	4	SGCB
7.92491684301105E-159	0.828002973219228	0.493	0.219	1.32805756455179E-154	4	TAF15
8.44541797452004E-78	0.827721519388735	0.257	0.111	1.41528314417007E-73	4	NRXN3
1.58879065420707E-95	0.826730727864802	0.309	0.133	2.66249537832022E-91	4	ITPKA
1.38099252076097E-96	0.82672920772669 1	0.32	0.142	2.31426726629124E-92	4	IRF2BP1
5.19123601351823E-150	0.826688309897135	0.608	0.315	8.69947331145385E-146	4	NAA10
1.04222247245933E-121	0.823842350471089	0.687	0.419	1.74655641934735E-117	4	SLPI
6.90058906579672E-139	0.823560567753227	0.557	0.287	1.15640071564621E-134	4	IPO9
3.64770780623109E-100	0.823167925538623	0.257	0.1	6.11282874168206E-96	4	MED21
9.90991493637814E-99	0.822923925233147	0.382	0.181	1.66070354503825E-94	4	GLO1
5.21773622334263E-118	0.822353804443017	0.447	0.216	8.74388236307758E-114	4	TECR
1.00601471586236E-123	0.822122090333304	0.43	0.198	1.68587946084213E-119	4	TMEM250
3.56627611550643E-108	0.821951539305373	0.486	0.251	5.97636551436567E-104	4	CRAT
1.60527685154735E-122	0.821335869397318	0.448	0.212	2.69012294782304E-118	4	UQCC2
1.66388324777536E-143	0.821252105481651	0.556	0.281	2.78833554662195E-139	4	HDGF
4.84923478805856E-116	0.821218624671854	0.354	0.152	8.12634765782853E-112	4	HSPA13
2.18587031029811E-109	0.819899169408601	0.38	0.173	3.66308146599757E-105	4	CASP6
2.75040355907551E-103	0.819546241382421	0.415	0.203	4.60912628429874E-99	4	COX11
6.42587789895734E-93	0.818612275962526	0.31	0.136	1.07684861830727E-88	4	RAVER2
1.12525259495722E-131	0.818074015385992	0.592	0.324	1.8856982986293E-127	4	HEXB
3.94465195895183E-78	0.817892854801428	0.301	0.142	6.61044775281147E-74	4	PIK3C2G
5.53801198519476E-93	0.817113699704114	0.306	0.134	9.28060048478938E-89	4	VRK3
1.61871914082349E-188	0.816792520142035	0.842	0.536	2.712649536192E-184	4	HSD17B11
1.67817777619996E-88	0.816407268859723	0.337	0.158	2.81229031735589E-84	4	ZNF608
1.86410971910941E-135	0.816292139798795	0.626	0.348	3.12387506728355E-131	4	MCM3
2.0030749866366E-60	0.816194455604269	0.274	0.136	3.35675306260561E-56	4	DEPRECATED-ENSG00000232119
5.67161548438253E-124	0.816163190634826	0.413	0.186	9.50449322872824E-120	4	NUP133
3.53826683629141E-170	0.816035023257347	0.73	0.387	5.92942756425714E-166	4	F2RL1
4.66793911898322E-84	0.815394112408563	0.317	0.147	7.82253237559208E-80	4	DNAAF2
9.38580303326425E-101	0.81515659659393	0.369	0.172	1.57287287231442E-96	4	GET3
3.14636359238314E-119	0.814431750614826	0.396	0.178	5.27267610811567E-115	4	CA2
1.02435516168623E-99	0.814296630918516	0.384	0.182	1.71661437995378E-95	4	GMFB
2.42455094275285E-83	0.813098596873664	0.302	0.138	4.06306246986522E-79	4	COMMD2
8.50197169705144E-111	0.813013514899798	0.5	0.264	1.42476041699188E-106	4	CLDN15
2.29948270313942E-118	0.811734858551555	0.451	0.221	3.85347311392104E-114	4	PCF11
1.73779611154684E-113	0.811027697850938	0.254	0.092	2.9121987237302E-109	4	FNTB

1.69268925604135E-158	0.810732351844481	0.309	0.105	2.83660865527409E-154	4	CENPC
4.94593133819823E-89	0.810579089667992	0.255	0.104	8.28839173655259E-85	4	RIOX1
4.31288923537743E-129	0.809949607596086	0.591	0.319	7.2275397806455E-125	4	SMIM15
1.9845983266805E-143	0.80991335464054	0.555	0.279	3.32578987585118E-139	4	SCRIB
4.53812423837032E-140	0.808084905533563	0.412	0.176	7.60498859866099E-136	4	FN3KRP
9.45834662361879E-116	0.807753009915549	0.355	0.154	1.58502972718604E-111	4	LSM 2.00
2.1471712779326E-137	0.807307929972741	0.579	0.301	3.59822962755945E-133	4	VDAC3
3.47792364948864E-94	0.806120710462065	0.302	0.13	5.82830445181306E-90	4	MKKS
8.28923603174784E-140	0.806063676267762	0.263	0.086	1.3891101742003E-135	4	POU2F1
7.08599129659514E-107	0.805782762762743	0.393	0.183	1.18747042148341E-102	4	ATP6V1H
1.50789132277769E-129	0.804841331453553	0.478	0.227	2.52692427871085E-125	4	SAP30L
6.93710982687634E-79	0.804245652198552	0.298	0.137	1.16252086478794E-74	4	CDC42EP4
7.75643933539238E-100	0.803450890738204	0.346	0.156	1.29982410382505E-95	4	SLC39A3
5.73096679542674E-243	0.803309039237553	0.969	0.651	9.60395415577612E-239	4	S100A10
9.80691297106961E-82	0.803282849903479	0.319	0.151	1.64344247569185E-77	4	AKR1C1
4.11552675495272E-135	0.803153205757918	0.67	0.388	6.89679973594977E-131	4	RTN3
1.49364507538265E-127	0.802766019530225	0.397	0.172	2.50305041732624E-123	4	SFN
1.22889046133219E-88	0.802430428238324	0.32	0.146	2.05937463510048E-84	4	STXBP6
1.442289370828E-121	0.802185675900957	0.587	0.322	2.41698852763356E-117	4	SRXN1
8.28758409136428E-103	0.798166172481021	0.391	0.184	1.38883334203083E-98	4	SYPL1
1.40736261514668E-113	0.797444533065761	0.502	0.254	2.35845827046281E-109	4	PKP2
4.20861072845661E-86	0.797322327397061	0.373	0.184	7.05278985874759E-82	4	TEX261
1.47660735039183E-147	0.796519842367505	0.368	0.143	2.47449859778663E-143	4	XPO1
1.92827488015288E-93	0.796136434228687	0.408	0.205	3.23140304416019E-89	4	ERH
1.70830469757868E-83	0.795998439442559	0.269	0.115	2.86277701220236E-79	4	ARV1
2.26108613128363E-156	0.795265786752773	0.404	0.163	3.7891281388051E-152	4	NUP205
6.59035260690384E-105	0.795199614160922	0.312	0.129	1.10441128986495E-100	4	COQ6
8.29201133650558E-118	0.794389529817233	0.494	0.25	1.38957525977161E-113	4	CLIC1
1.17552733887856E-100	0.794386342366692	0.258	0.1	1.96994871449269E-96	4	FOXRED1
3.68631049697206E-91	0.794215210354766	0.291	0.125	6.17751913082578E-87	4	DDA1
7.92876703804478E-136	0.794196981182603	0.455	0.214	1.32870278023554E-131	4	TGIF1
4.12083437365521E-79	0.793927082652032	0.321	0.153	6.9056942433714E-75	4	UBL4A
3.12569586000592E-127	0.793387496975166	0.577	0.32	5.23804112219791E-123	4	HGSNAT
5.0593425096911E-120	0.792544384072194	0.451	0.22	8.47844617774034E-116	4	LMF2
1.26716651178137E-148	0.792240857829259	0.734	0.425	2.12351764044321E-144	4	TSPAN6
3.65908293107912E-130	0.79118949674989	0.525	0.263	6.1318911759024E-126	4	SNRPB
5.53367759526829E-114	0.791134223432172	0.457	0.222	9.27333691415O6E-110	4	ELOVL2
1.59568049714588E-99	0.787133078895655	0.366	0.17	2.67404137711706E-95	4	ICK
2.78836002295858E-147	0.787041223935791	0.5	0.233	4.67273372647398E-143	4	H2AFY
1.34036122239308E-170	0.785875271184586	0.808	0.495	2.24617733648632E-166	4	TMEM14A
2.08270753734121E-97	0.785187995046936	0.261	0.104	3.4902012910764E-93	4	DEPRECATED-ENSG00000176101
5.37427518298002E-165	0.785018669042964	0.302	0.098	9.00621035163792E-161	4	CCNG2
4.71932734544263E-97	0.784469988393899	0.293	0.124	7.90864876549276E-93	4	ZNF700
1.44423130635199E-127	0.784420522018277	0.505	0.251	2.42024282318467E-123	4	CANT1
3.32196589985099E-113	0.784076059241443	0.411	0.191	5.56695045497029E-109	4	CYREN
3.60724620617402E-105	0.783974841581405	0.477	0.243	6.04502319230643E-101	4	TRIQK
4.88291241638594E-96	0.783741875379101	0.301	0.129	8.18278462737955E-92	4	UBE2M
2.69696789765708E-103	0.783062232391281	0.337	0.148	4.51957880289374E-99	4	LRIG3
2.05859113136605E-81	0.782415191138684	0.315	0.146	3.44978701794322E-77	4	TNFRSF11A
7.80308388810342E-116	0.782320895476538	0.509	0.26	1.30764079796837E-111	4	TDP2
1.12365486906501E-110	0.782191440095883	0.337	0.143	1.88302082957915E-106	4	CYB561A3
1.07040114942479E-76	0.782161460150639	0.257	0.113	1.79377824620606E-72	4	L2HGDH
1.07419651741346E-121	0.781742220201244	0.393	0.174	1.80013852388147E-117	4	RCOR1
4.31199746154126E-137	0.781588963886699	0.416	0.18	7.22604534605084E-133	4	GTPBP2
2.13278417385722E-117	0.780536239309169	0.351	0.148	3.57411971854992E-113	4	CPOX
1.62224312920459E-102	0.780403798600753	0.321	0.138	2.71855503592105E-98	4	SLC35E3
2.4415725916401E-143	0.780260930932601	0.644	0.343	4.09158734907048E-139	4	RAMP1
5.94957953332899E-109	0.780011200745299	0.488	0.246	9.97030538195273E-105	4	KCNJ3
1.01903204686587E-107	0.779968415299987	0.252	0.094	1.70769390413783E-103	4	TXNDC16
8.73387017789544E-93	0.778070630400473	0.292	0.125	1.46362196441172E-88	4	GAR1

5.78797456748992E-112	0.777936823774616	0.455	0.229	9.6994877801996E-108	4	RBM12B
2.01483324490024E-107	0.777828625032358	0.492	0.256	3.37645755180382E-103	4	PRKAB1
2.72645625400623E-150	0.777622460844678	0.376	0.147	4.56899539046364E-146	4	KRI1
2.69664850422079E-118	0.777471186670238	0.448	0.213	4.5190435633732E-114	4	ELOC
2.69306889040175E-171	0.777342283424749	0.756	0.442	4.51304484653525E-167	4	YWHAZ
2.6564859803178E-119	0.776959489615282	0.312	0.123	4.45173920581658E-115	4	HAUS2
2.67681081606976E-81	0.776195445451757	0.271	0.118	4.4857995655697E-77	4	SOWAHB
1.46863550905158E-137	0.775362512109903	0.543	0.278	2.46113938606863E-133	4	DCAF7
1.53810053186944E-126	0.774993480069057	0.53	0.271	2.5775488713068E-122	4	HDGFL2
8.1862401679412E-144	0.774912467680449	0.533	0.261	1.37185012734359E-139	4	HNRNPUL2
7.79551353833375E-121	0.774288366323446	0.427	0.196	1.30637215875397E-116	4	PIN1
3.92270193622374E-161	0.774086380377638	0.301	0.1	6.57366390472374E-157	4	HAUS3
3.66781177714705E-123	0.774071029348068	0.413	0.185	6.14651897614302E-119	4	RFX5
1.41675817632584E-107	0.774023366499467	0.539	0.294	2.37420335188683E-103	4	ISOC2
1.45729636769261E-103	0.773908321628113	0.458	0.235	2.44213725297927E-99	4	RBM41
8.28702512887183E-68	0.773322335106522	0.289	0.14	1.38873967109634E-63	4	THEM6
3.80596572244637E-166	0.771730677728189	0.821	0.51	6.37803735767563E-162	4	BEX3
6.32324655283276E-93	0.771716795942467	0.345	0.159	1.05964965732371E-88	4	ETAA1
1.73666661101058E-118	0.770128528464317	0.45	0.215	2.91030590673153E-114	4	KLHDC3
4.00758140726743E-87	0.768156787330489	0.297	0.133	6.71590492229876E-83	4	KLHL42
3.25839711056909E-107	0.767426381408599	0.479	0.244	5.46042187789168E-103	4	PDX1
1.7028306607771E-97	0.767105049242886	0.423	0.212	2.85360362133026E-93	4	COA6
1.62121689594685E-108	0.766444369135268	0.489	0.254	2.71683527422774E-104	4	RAB33B
2.55009553189304E-93	0.766236145272924	0.297	0.128	4.27345009234635E-89	4	CLOCK
1.36375270840654E-104	0.765726018706695	0.376	0.174	2.28537678874769E-100	4	DUT
6.58728689734077E-67	0.765367251787027	0.274	0.129	1.10389753825637E-62	4	CHST5
7.00866703622363E-152	0.764816573093378	0.763	0.451	1.17451242193036E-147	4	TPD52
2.37067880639944E-111	0.764708385265753	0.553	0.297	3.97278354376418E-107	4	TMEM38B
2.67720001496752E-81	0.763384334933442	0.34	0.165	4.48645178508258E-77	4	GGCT
2.10207754196997E-154	0.762911285629067	0.738	0.424	3.52266154483328E-150	4	KCTD3
6.04413491618094E-89	0.76281101695569	0.313	0.14	1.0128761292536E-84	4	NVL
4.61905843761795E-115	0.761572207213532	0.339	0.141	7.74061812976016E-111	4	AGAP1
1.28446311570994E-136	0.761471441052627	0.441	0.196	2.15250328930672E-132	4	PSMG2
1.04051558747758E-109	0.761322692722866	0.396	0.182	1.74369602149493E-105	4	SF3B4
1.01154198812113E-105	0.7603175953769	0.481	0.25	1.69514206369339E-101	4	HMG20B
7.76650898257749E-94	0.759711278968447	0.271	0.111	1.30151157530034E-89	4	TMEM60
1.39727585843648E-128	0.759490814841539	0.662	0.393	2.34155488356786E-124	4	NOTCH2NLB
1.31323835748894E-121	0.759479405829703	0.601	0.332	2.20072483947997E-117	4	SLC44A1
3.88492233460804E-108	0.759304997648506	0.448	0.219	6.51035284833616E-104	4	PALLD
5.09062214446107E-142	0.758541596570089	0.481	0.23	8.53086458968786E-138	4	RBM15
4.98962839194639E-114	0.758499695765078	0.307	0.123	8.36161925922375E-110	4	LCORL
5.90962113752576E-111	0.758186816629605	0.452	0.222	9.90334310226567E-107	4	PCMT1
4.3503577795656E-129	0.756703393623173	0.318	0.122	7.29032956696603E-125	4	TRNAU1AP
9.10122686085345E-108	0.755108757555863	0.379	0.172	1.52518359734182E-103	4	NFYB
1.92775839913564E-75	0.754964970996287	0.263	0.117	3.23053752527151E-71	4	UBE2K
3.12894230235087E-72	0.754697058327785	0.256	0.115	5.2434815102796E-68	4	TBC1D7
1.13312240851202E-121	0.754339246754625	0.459	0.219	1.89888653218443E-117	4	RAP2A
3.19438025719644E-105	0.753875000512735	0.432	0.213	5.3531424350098E-101	4	ZNF75D
3.53464140498285E-90	0.752589534849148	0.298	0.13	5.92335206647026E-86	4	SNRPG
2.00114473394493E-106	0.752543162217298	0.372	0.17	3.35351834514491E-102	4	ZCCHC2
4.29340334838439E-95	0.752237689951385	0.448	0.228	7.19488533122256E-91	4	AKR7A2
3.83282101059417E-152	0.751294676292922	0.685	0.366	6.4230414495537E-148	4	OCRL
3.53113055285497E-208	0.750792515929656	0.962	0.655	5.91746858047435E-204	4	UGDH
5.96858326318013E-92	0.75009909183174	0.413	0.207	1.00021518324373E-87	4	TJP3
5.60252473251867E-120	0.749504461056906	0.632	0.359	9.38871094675479E-116	4	CYP4F11
5.82288201752328E-120	0.748910647104607	0.534	0.278	9.75798568496552E-116	4	SEPTIN8
9.05646974982519E-106	0.748356593248401	0.446	0.221	1.51768320067571E-101	4	CHMP3
8.48198235151742E-119	0.74792675482781	0.565	0.303	1.42141060246729E-114	4	GOT1
7.98245385845713E-121	0.747064688966189	0.45	0.214	1.33769961760025E-116	4	WRNIP1
1.03743689763951E-139	0.747031545925719	0.653	0.372	1.73853675306429E-135	4	IKBKG

1.30433595917799E-94	0.746904710559316	0.254	0.1	2.18580620039047E-90	4	GNAI1
1.96043975317253E-111	0.746882704946586	0.412	0.193	3.28530493836652E-107	4	HAT1
8.30416456101686E-129	0.74644066407904	0.567	0.3	1.3916118971352E-124	4	TAZ
2.37811881846718E-146	0.746088573225633	0.701	0.384	3.9852515159873E-142	4	LAGE3
2.64639523477961E-82	0.74608475960553	0.294	0.133	4.43482913444368E-78	4	POU5F1
2.50804047618911E-109	0.745998894075451	0.413	0.193	4.20297422999771E-105	4	ZNF219
1.16684413683464E-115	0.745747111729867	0.507	0.264	1.95539740450749E-111	4	SMS
1.02934460655894E-85	0.745647261018274	0.298	0.133	1.72497569167147E-81	4	HINT3
5.97987160976404E-86	0.745537055516447	0.29	0.128	1.00210688436426E-81	4	DIP2C
1.37122137791229E-123	0.744844321407775	0.591	0.316	2.29789278510541E-119	4	TMEM64
1.46627066122734E-133	0.743884456431816	0.519	0.263	2.45717637408478E-129	4	PSPC1
1.99844066560138E-93	0.743427149600521	0.367	0.175	3.34898686741479E-89	4	PBX3
5.97912547817725E-90	0.743377698826884	0.272	0.114	1.00198184763294E-85	4	UBE2D2
8.9494267302476E-84	0.743281154344981	0.272	0.118	1.49974493145489E-79	4	CDK8
2.52347248851387E-144	0.742484768079602	0.671	0.403	4.22883519625154E-140	4	STOM
4.26256415899453E-124	0.741506906770486	0.669	0.392	7.14320501764303E-120	4	CIAO2A
2.19112275749365E-102	0.7413700553483	0.512	0.276	3.67188351700787E-98	4	TMEM9
6.43129131898529E-112	0.740959821666007	0.475	0.24	1.07775579923556E-107	4	ARHGEF40
5.99914477813857E-86	0.740454263916376	0.25	0.103	1.00533668192046E-81	4	AARS
4.300241801117E-101	0.740025980744936	0.26	0.101	7.20634521031186E-97	4	PPP1R13L
6.52115279383198E-119	0.739860142865418	0.537	0.277	1.09281478519036E-114	4	SLC25A33
6.47285010247782E-136	0.739427673996789	0.657	0.364	1.08472022017323E-131	4	PYCR2
3.99572599056128E-81	0.739192022775983	0.316	0.148	6.69603761498259E-77	4	GMPR2
7.89659148975396E-103	0.738847784476272	0.517	0.278	1.32331080185297E-98	4	CETN2
2.48778611593546E-109	0.738693341918344	0.36	0.158	4.16903197308465E-105	4	PPP5C
1.52238819958956E-109	0.738143769312111	0.405	0.189	2.55121814487218E-105	4	ATL3
2.11614995289968E-97	0.73781396187599	0.391	0.187	3.54624409106928E-93	4	AKIP1
3.15904417311442E-123	0.737677218785303	0.426	0.194	5.29392622530514E-119	4	SPATS2
4.21102552685496E-109	0.737473615739936	0.38	0.173	7.05683657790355E-105	4	CDC42BPA
4.59527149695568E-83	0.737395910798482	0.256	0.109	7.70075597459833E-79	4	NIPSNAP3A
1.11289777036039E-84	0.737154341071137	0.358	0.173	1.86499408356995E-80	4	RMDN1
3.21529692215169E-120	0.736633316682473	0.556	0.294	5.38819458214179E-116	4	KEAP1
1.48222603160843E-83	0.735164946934592	0.391	0.197	2.48391438376941E-79	4	POF1B
7.51397219185717E-124	0.735068282580919	0.48	0.233	1.25919145991142E-119	4	PRPSAP1
3.47086428588375E-123	0.734182267717328	0.574	0.309	5.81647437028399E-119	4	MAF 1.00
2.04166096605839E-141	0.733421924805629	0.662	0.406	3.42141544692065E-137	4	SYNE2
3.86770717682593E-96	0.733070977979741	0.33	0.148	6.48150368692489E-92	4	LAS1L
1.26397086198472E-84	0.732883918747825	0.321	0.149	2.11816237051399E-80	4	C6orf136
2.74622108368141E-74	0.732875529764175	0.355	0.182	4.60211729203331E-70	4	SRD5A3
1.86622603822142E-153	0.732787954143776	0.741	0.406	3.12742159485145E-149	4	CLDN4
7.08829291892016E-76	0.731212145147367	0.281	0.129	1.18785612735264E-71	4	STOX2
2.68532233524572E-137	0.730819421933845	0.601	0.321	4.50006316940478E-133	4	HNRNPL
1.64594835671976E-77	0.730709027244481	0.348	0.172	2.75828025619098E-73	4	SHOX2
5.79571358914078E-93	0.730589861499354	0.36	0.17	9.71245683268212E-89	4	PPIP5K2
5.95623785479574E-111	0.730188167057124	0.277	0.106	9.98146339706667E-107	4	ST3GAL3
2.63042574758899E-91	0.729619581444766	0.422	0.217	4.40806746780963E-87	4	ABHD10
1.09836768374019E-79	0.72955858763556	0.284	0.128	1.84064456441181E-75	4	EIF4EBP1
4.55976828897525E-137	0.729499297536181	0.693	0.408	7.64125969866472E-133	4	ENY2
8.26204708283006E-128	0.728854636820694	0.571	0.302	1.38455385014066E-123	4	EIF1B
3.53671610273462E-114	0.7286949642309	0.47	0.233	5.92682884496267E-110	4	GLRX5
1.25715342364804E-115	0.728379948680343	0.557	0.323	2.10673770734939E-111	4	SUGP2
1.68449699360168E-134	0.728315339168863	0.719	0.419	2.82288006187769E-130	4	AIFM1
2.60022142251045E-116	0.728123424646926	0.614	0.348	4.35745105984301E-112	4	MLEC
6.77043480464751E-134	0.726835768447979	0.465	0.214	1.13458946456283E-129	4	SH3GL1
4.57338322959592E-181	0.725836353008288	0.909	0.608	7.66407561615684E-177	4	ASPH
6.07287096734691E-76	0.724905566485584	0.259	0.115	1.017691716708E-71	4	RAD51D
4.72432152231833E-91	0.724799187748544	0.438	0.227	7.91701800710106E-87	4	AHCY
1.36773732134567E-123	0.724554636285325	0.534	0.267	2.29205420311108E-119	4	LRRC31
1.45884133283805E-94	0.724254620208644	0.403	0.198	2.44472630557001E-90	4	C6orf62
3.17431519126173E-120	0.723965042648776	0.425	0.197	5.31951739751641E-116	4	OXR1

9.56276770870264E-109	0.723293986997776	0.335	0.144	1.60252861262439E-104	4	NIPA2
8.95137579205569E-106	0.722620243215981	0.401	0.191	1.50007155523269E-101	4	ATF5
2.34208355880448E-85	0.722141951674003	0.303	0.137	3.92486362784454E-81	4	P4HTM
4.66986899607671E-195	0.721434323319963	0.84	0.575	7.82576646362535E-191	4	HNRNPA2B1
6.15887174873807E-123	0.721057163884746	0.585	0.323	1.03210372765353E-118	4	PPM1B
1.27779962792553E-111	0.721056107809426	0.615	0.351	2.1413366164776E-107	4	NENF
1.34513228249685E-118	0.719312926671744	0.58	0.317	2.25417267900823E-114	4	FAT1
1.30723487260164E-133	0.719233449499593	0.469	0.221	2.19066419950583E-129	4	HCFC1
1.5644198271882E-93	0.718933291481616	0.444	0.227	2.62165474640199E-89	4	INAVA
7.57977902791049E-101	0.718412583933184	0.277	0.111	1.27021936949724E-96	4	DGCR8
1.17841816501607E-132	0.71838397315404	0.387	0.163	1.97479316093393E-128	4	SMPD4
1.66162785575774E-118	0.718328776433919	0.609	0.338	2.78455596067882E-114	4	TALDO1
5.16251489732381E-115	0.718253872757457	0.558	0.301	8.65134246493524E-111	4	CCT6A
6.25387709779331E-82	0.717079358492484	0.437	0.239	1.0480247240482E-77	4	MCM2
5.11336700631035E-123	0.716793531764182	0.582	0.311	8.56898042917489E-119	4	FAM199X
3.21463929367001E-119	0.716619212184071	0.259	0.092	5.3870925283322E-115	4	DEPRECATED-ENSG00000179051
4.05849660736429E-74	0.716462418272789	0.324	0.157	6.80122861462108E-70	4	TMEM98
7.09401789783209E-94	0.715872271893795	0.398	0.195	1.1888155193187E-89	4	RNF6
1.23510632158568E-95	0.714996407701955	0.284	0.118	2.06979117371328E-91	4	SSNA1
2.17253407353697E-92	0.714924958818072	0.349	0.163	3.64073260043326E-88	4	PLEKHA5
3.20689970260538E-78	0.714506692945825	0.271	0.12	5.37412252162609E-74	4	C1orf35
8.10478267971183E-112	0.713566712017615	0.604	0.349	1.35819948146611E-107	4	MGAT2
3.15137312603125E-101	0.713154566436282	0.447	0.226	5.28107108460317E-97	4	LRRC58
5.37725238137273E-90	0.711959108812449	0.328	0.15	9.01119954070442E-86	4	CSPP1
1.3341169544656E-132	0.710951006202515	0.775	0.501	2.23571319229345E-128	4	TRAM1
1.16477171063484E-118	0.710598122267135	0.652	0.391	1.95192443268186E-114	4	MLF 2.00
1.61989758610413E-85	0.7105652974587	0.309	0.14	2.7146243747933E-81	4	TMTC4
3.53827173223672E-95	0.710451951994954	0.305	0.132	5.9294357688823E-91	4	CCDC61
2.61866025067486E-95	0.710247944859349	0.402	0.197	4.38835084808093E-91	4	CCDC117
1.81825635104355E-110	0.710244031934219	0.439	0.212	3.04703399307878E-106	4	BCAP29
5.76077658905514E-241	0.709942485836915	0.928	0.564	9.6539094079386E-237	4	PIGR
2.10603466546406E-89	0.70957965381282	0.431	0.223	3.52929289238466E-85	4	PID1
4.47808699221951E-108	0.70863152750557	0.56	0.303	7.50437818156146E-104	4	ACMSD
4.0452607211558E-102	0.708617472319085	0.456	0.23	6.77904791651288E-98	4	PLEKHJ1
2.54688262727885E-106	0.70798819861989	0.361	0.161	4.2680659067939E-102	4	STRN
3.54808160298334E-80	0.707228677268651	0.29	0.132	5.94587515027949E-76	4	CMPK2
1.67103747352096E-76	0.706729635726069	0.25	0.109	2.80032459812642E-72	4	TSPOAP1
2.33229670243835E-127	0.705946597300024	0.628	0.342	3.90846281394618E-123	4	SHARPIN
5.58403694092674E-80	0.70553789102401	0.315	0.149	9.35772910560503E-76	4	MSH2
4.7950569620991E-118	0.705414599269614	0.551	0.295	8.03555645708568E-114	4	KLF3
1.86285446373003E-106	0.70521490962692	0.49	0.256	3.12177151031878E-102	4	PPM1G
1.26013902864852E-115	0.70517357524545	0.563	0.295	2.11174098420919E-111	4	DUSP9
2.48550168361438E-93	0.704184954403758	0.316	0.141	4.16520372140097E-89	4	TCP11L1
1.73540470574296E-116	0.703295289869727	0.627	0.358	2.90819120588406E-112	4	PDZD8
1.29479697571103E-104	0.702743289455738	0.401	0.189	2.16982077189654E-100	4	GBE1
8.50955289542469E-94	0.702450792785428	0.474	0.251	1.42603087421527E-89	4	NDUFB1
8.57265094511542E-94	0.70183408045544	0.378	0.181	1.43660484538244E-89	4	S100P
8.72744397435675E-104	0.701485955367324	0.395	0.185	1.4625450612227E-99	4	COPRS
5.00420354118532E-68	0.701411120708622	0.305	0.151	8.38604429431836E-64	4	CDC42EP5
1.02118733924562E-90	0.700961881690316	0.381	0.185	1.71130574310781E-86	4	BANF1
1.37070389333311E-133	0.700030212174671	0.788	0.512	2.29702558444763E-129	4	RARRES2
3.65567653746488E-122	0.699441288703564	0.529	0.272	6.12618274148365E-118	4	AP2A1
7.34890735994898E-82	0.699266891093211	0.383	0.192	1.23152989538025E-77	4	NDUFB4
9.82178844480083E-119	0.698712911319174	0.384	0.168	1.64593530757972E-114	4	MLH1
2.4603226931182E-125	0.698521815443294	0.433	0.197	4.12300876912749E-121	4	HPS3
1.73682381812425E-80	0.69850490414525	0.338	0.165	2.91056935441262E-76	4	NR3C2
2.46367241939711E-108	0.698254685789522	0.455	0.225	4.12862224042568E-104	4	NUDCD2
3.17638194573763E-155	0.697864451869112	0.725	0.408	5.32298086466712E-151	4	IRAK1
8.8758428658056E-140	0.697659341377158	0.542	0.265	1.4874137474517E-135	4	PKDCC
8.83142615435861E-109	0.69738215579413	0.548	0.298	1.47997039494742E-104	4	NBPF26

2.323775086792E-146	0.697276217495902	0.525	0.251	3.89418229044604E-142	4	FUS
3.0431816165711E-104	0.696571272679362	0.354	0.157	5.09976375304985E-100	4	TMEM65
1.28296753343496E-164	0.696087608735973	0.983	0.811	2.1499969925303E-160	4	MT-CO2
5.43989520174894E-101	0.694914016308161	0.394	0.186	9.11617637909088E-97	4	OSGEP
6.83583255665364E-127	0.694467964807553	0.581	0.31	1.14554881984402E-122	4	SEPTIN11
2.53516868202757E-98	0.694247985670191	0.412	0.204	4.24843567734179E-94	4	DCAF16
2.61397555282911E-103	0.693795349815978	0.526	0.282	4.38050023143103E-99	4	C1GALT1C1
1.93030431093109E-80	0.69317444894262	0.425	0.226	3.23480396425831E-76	4	ARHGAP5
4.94978847940039E-110	0.692816720490998	0.343	0.147	8.29485553377917E-106	4	OSER1
2.16535460828684E-87	0.692637347474192	0.333	0.154	3.62870125256708E-83	4	CPSF1
1.2769031994976E-64	0.692586390395689	0.256	0.119	2.13983438171807E-60	4	DEPRECATED-ENSG00000147804
1.01089708132267E-104	0.692287091618558	0.602	0.347	1.69406132888053E-100	4	FH
1.81231985427085E-108	0.692005373842802	0.427	0.205	3.03708561178708E-104	4	MTM1
1.31678416329819E-126	0.691678903263162	0.628	0.344	2.20666690085511E-122	4	HPGD
1.37971115298913E-97	0.691280944698076	0.318	0.138	2.31211995017919E-93	4	IPO5
4.23066779171411E-183	0.691036432839462	0.905	0.578	7.08975308535451E-179	4	LYZ
1.46736752796314E-84	0.690851824066962	0.268	0.114	2.45901450336063E-80	4	WRAP73
4.85713772863163E-127	0.690684967216389	0.75	0.464	8.13959140564089E-123	4	LSR
2.98737011921005E-100	0.690427055528702	0.251	0.095	5.0062348457722E-96	4	PPOX
2.22595324761439E-161	0.689668440558888	0.858	0.591	3.7302524523522E-157	4	H3F3A
2.87918715069877E-120	0.689249960102241	0.377	0.163	4.82494182714099E-116	4	APBB2
2.97353734272788E-90	0.688235018143461	0.376	0.182	4.98305387894338E-86	4	PSMD2
9.24926716939541E-71	0.687783119031679	0.282	0.133	1.54999219224728E-66	4	FZD8
4.93218542511701E-130	0.687782456285437	0.72	0.423	8.26535633541108E-126	4	PLS3
4.53801733264051E-72	0.687733616564498	0.304	0.147	7.60480944603897E-68	4	RPUSD3
8.9037731871142E-102	0.686895626139723	0.443	0.221	1.4920943106966E-97	4	KHSRP
2.32823571671495E-81	0.686660149069059	0.326	0.154	3.90165741407091E-77	4	TIMM9
2.13879210811115E-112	0.686586672626828	0.539	0.283	3.58418781477266E-108	4	ENAH
3.16110563507069E-82	0.685650237959341	0.383	0.195	5.29738082325146E-78	4	HAO2
3.32679988717251E-132	0.684878806536214	0.696	0.411	5.57505125092369E-128	4	STK24
2.84882420155509E-139	0.684623339787435	0.454	0.206	4.77405959696602E-135	4	NUP50
5.00055495632637E-99	0.683706171204785	0.267	0.106	8.37992999581173E-95	4	MFSD14B
9.52646066830351E-82	0.682958949339857	0.389	0.198	1.5964442787943E-77	4	FIBP
4.92746707170464E-86	0.682718174760733	0.407	0.207	8.25744931876263E-82	4	GSKIP
1.85117777854718E-111	0.6821299162233	0.493	0.254	3.10220372128936E-107	4	DNAJC1
9.79357795083056E-73	0.68187506078111	0.277	0.128	1.64120779300019E-68	4	DEPRECATED-ENSG00000174903
3.29184236751893E-96	0.681754144543081	0.407	0.201	5.51646943948822E-92	4	ELMOD2
2.43561940261522E-101	0.680879826459465	0.436	0.217	4.08161099490258E-97	4	TMEM185A
8.55508977074817E-175	0.6807906765513	0.778	0.41	1.43366194378198E-170	4	CDH17
3.46932790796845E-129	0.680432184829215	0.583	0.31	5.81389970817352E-125	4	ADAM9
4.16316665780684E-117	0.680159606171952	0.36	0.154	6.9766346851527E-113	4	VPS37A
9.9012036656847E-99	0.680072098402152	0.336	0.148	1.65924371029544E-94	4	NUP54
3.43884372029188E-107	0.679940273634056	0.573	0.32	5.76281430646514E-103	4	MICOS10
9.34151405397209E-116	0.6793501748704	0.36	0.155	1.56545092516464E-111	4	GTF2A1
1.03228695891442E-116	0.679054117644224	0.492	0.248	1.72990648574878E-112	4	NOTCH2
3.07517933045481E-133	0.678209502060444	0.747	0.469	5.15338552197618E-129	4	SDC1
3.6627402937584E-115	0.67773858182153	0.671	0.404	6.13802018428033E-111	4	ACBD5
6.66028465951018E-97	0.677589706546763	0.532	0.291	1.11613050324072E-92	4	CISD2
6.87295328921401E-81	0.676983917408601	0.354	0.175	1.15176951220648E-76	4	PPP2CA
3.97433802992047E-97	0.67664695470062	0.377	0.179	6.66019567054072E-93	4	RPA1
9.60508223421063E-147	0.67641490905938	0.777	0.448	1.60961968080902E-142	4	G6PD
6.00530470334181E-119	0.676104806554309	0.417	0.191	1.00636896218602E-114	4	HNRNPR
2.83381549457148E-101	0.675570417325582	0.359	0.162	4.74890800580289E-97	4	VEZF1
5.43284824025173E-109	0.675545201581379	0.535	0.285	9.10436708101386E-105	4	COPS6
8.356902692991E-120	0.675496243243588	0.467	0.226	1.40044975329143E-115	4	KHDRBS1
2.19511833693309E-115	0.675244179575845	0.26	0.093	3.67857930903247E-111	4	PIGK
2.40143075752908E-81	0.674930356272557	0.306	0.141	4.02431766346724E-77	4	CHCHD3
2.21726452430403E-111	0.674401292165857	0.516	0.267	3.7156918898287E-107	4	NACC1
4.88320971101645E-81	0.673863571848199	0.298	0.137	8.18328283372136E-77	4	DCUN1D2
4.03825628463623E-93	0.673774600347485	0.3	0.13	6.76730988179339E-89	4	CHML

4.54030507236663E-102	0.673646383572842	0.4	0.19	7.60864324027201E-98	4	LMO4
1.80198082868932E-128	0.673305555829215	0.59	0.319	3.01975947271756E-124	4	HMGN1
3.25124313558998E-82	0.672515073402863	0.328	0.154	5.44843324662168E-78	4	VSIG10
2.03770089228011E-113	0.672267188696396	0.595	0.34	3.414779155283E-109	4	PLEKHF2
3.39238180027507E-92	0.67209373665453	0.335	0.153	5.68495342090097E-88	4	IFT88
3.17670060161983E-130	0.672045392555659	0.629	0.347	5.32351486819452E-126	4	ELAVL1
1.03254995361321E-93	0.671997305679548	0.321	0.142	1.73034721226502E-89	4	ZHX3
1.75812781288837E-106	0.671823321434939	0.543	0.296	2.94627058883832E-102	4	TRAPPC4
3.01094377622469E-137	0.671754556861725	0.831	0.539	5.04573958019733E-133	4	MPC2
2.23623734868436E-92	0.671655014972444	0.46	0.24	3.74748654892525E-88	4	NTPCR
2.30311658226601E-106	0.671427606206654	0.631	0.363	3.85956276856139E-102	4	HACD3
1.79224343640194E-121	0.671270881930879	0.662	0.373	3.00344155072238E-117	4	FRK
1.09719574680174E-104	0.671239981719425	0.483	0.249	1.83868063249035E-100	4	ENOSF1
1.74292619260779E-130	0.671220093978548	0.754	0.463	2.92079571357214E-126	4	FAM50A
3.85596681527695E-78	0.67051885057569	0.296	0.135	6.46182918904112E-74	4	WDR41
5.72852945499529E-118	0.670359432979849	0.77	0.506	9.59986966068111E-114	4	TXNRD1
9.06484333343568E-101	0.669772116217941	0.535	0.292	1.51908644581715E-96	4	CUL4B
1.07070549057088E-107	0.669247797330991	0.557	0.297	1.79428826109867E-103	4	DEPRECATED-ENSG00000057294
1.44929305233564E-91	0.66863137352491	0.604	0.373	2.42872529710407E-87	4	SLBP
1.30827938314294E-80	0.668198301524592	0.347	0.169	2.19241459027094E-76	4	ZNF768
1.48080380327632E-116	0.668135808346873	0.336	0.14	2.48153101353045E-112	4	JADE2
4.24430971978311E-98	0.66781669763463	0.497	0.265	7.11261422841254E-94	4	TRMT2B
3.29076289154347E-117	0.667804372788923	0.378	0.166	5.51466045364855E-113	4	TAF6
4.59587195708567E-86	0.667551551860456	0.336	0.156	7.70176222568416E-82	4	UQCRFS1
2.09186556697856E-97	0.667340749141271	0.449	0.231	3.50554831714267E-93	4	CLN3
9.18154430015712E-184	0.665119087197204	0.933	0.602	1.53864319382033E-179	4	SPINK1
2.89712189354697E-94	0.664952736507227	0.253	0.1	4.85499686920601E-90	4	CNOT1
5.20997729534757E-86	0.664886151827704	0.396	0.197	8.73087995154345E-82	4	USP54
6.05711232530999E-110	0.664784567961346	0.318	0.132	1.01505088347545E-105	4	SCMH1
8.68067402251647E-131	0.664430005700015	0.766	0.465	1.45470735269331E-126	4	AKR1C2
8.96033676395643E-95	0.66441180113316	0.396	0.192	1.50157323490382E-90	4	ELF4
4.56402237047052E-94	0.664159921584898	0.439	0.224	7.6483886884345E-90	4	LMBRD2
2.87272174036084E-105	0.663942361090344	0.423	0.204	4.8141070924967E-101	4	TIPRL
2.80513525197566E-117	0.663444183592216	0.335	0.138	4.70084565526081E-113	4	EEF1AKMT2
1.92470638534154E-94	0.662944752149001	0.256	0.102	3.22542296055535E-90	4	ENKD1
1.56479234001868E-122	0.662776102911432	0.734	0.447	2.62227900340331E-118	4	SAP18
9.41171105806613E-95	0.661748069241477	0.495	0.26	1.57721453911072E-90	4	PLS1
4.92366435341926E-96	0.660442122566951	0.427	0.212	8.25107672346E-92	4	HAX1
8.98043393435446E-80	0.660190099825604	0.323	0.154	1.50494111871912E-75	4	RALY
6.53246679832429E-115	0.659892608212472	0.649	0.375	1.09471078606318E-110	4	DSP
3.19333518364839E-110	0.65983795612043	0.39	0.176	5.35139110075797E-106	4	CASP2
3.33691972826741E-85	0.659390249415385	0.309	0.14	5.59201008063052E-81	4	ZFP62
4.42239307785867E-128	0.659184691487726	0.767	0.509	7.41104631987556E-124	4	NOTCH2NLC
3.83896828136577E-77	0.659137116738742	0.262	0.115	6.43334304591275E-73	4	ARHGAP21
1.11133553027645E-118	0.657865382880638	0.532	0.277	1.86237608163727E-114	4	ADAM15
5.87340321362096E-84	0.657583593595163	0.321	0.15	9.842649105386E-80	4	ARPP19
8.78956484000617E-85	0.657277543328793	0.382	0.189	1.47295527588823E-80	4	HINT2
1.46730540769298E-94	0.657130718028034	0.457	0.238	2.45891040221189E-90	4	POLR2K
2.25857152337882E-88	0.656842898257416	0.29	0.126	3.78491415887823E-84	4	USP5
2.91161404774641E-131	0.655791424235579	0.349	0.139	4.87928282121343E-127	4	NUP188
1.00414197144043E-87	0.655437183727958	0.365	0.177	1.68274111573987E-83	4	GPD1L
7.39912573445313E-96	0.655273093603129	0.513	0.283	1.23994549057966E-91	4	KIAA0232
2.16300154367472E-104	0.655066103022358	0.535	0.293	3.62475798689009E-100	4	HECTD1
3.54166823629235E-118	0.654979145814468	0.704	0.405	5.93512763037872E-114	4	LAPTM4B
8.33671430041969E-98	0.654140693255683	0.475	0.247	1.39706658246433E-93	4	SH3BP4
1.37665700662565E-164	0.654022731185788	0.954	0.707	2.30700181170327E-160	4	DEPRECATED-ENSG00000106538
2.16377200928589E-83	0.653558954382392	0.368	0.183	3.6260491331613E-79	4	FAF2
6.45412203746057E-141	0.653449476612602	0.939	0.691	1.08158177103764E-136	4	MT-CYB
3.46320613964099E-111	0.653322044416385	0.607	0.347	5.80364084881038E-107	4	EIF2AK1
3.87061745556022E-101	0.653256270195016	0.564	0.306	6.48638073202781E-97	4	ANXA13

8.024337609104E-92	0.652868549410059	0.422	0.213	1.34471849653365E-87	4	SCAMP4
1.95657732145767E-89	0.65273815216106	0.423	0.213	3.27883227529876E-85	4	EPPK1
5.94079536114219E-102	0.65169416300746	0.626	0.369	9.95558486620209E-98	4	KRTCAP2
1.16360067368936E-119	0.649812062574343	0.709	0.423	1.94996200896862E-115	4	IDH3G
1.80305501888546E-117	0.648719465166756	0.647	0.372	3.02155960064825E-113	4	ZNF706
5.93244056638371E-133	0.648478674839881	0.502	0.248	9.94158390114582E-129	4	TPR
2.32614252068852E-66	0.648028614467936	0.279	0.133	3.89814963616982E-62	4	C17orf75
5.06846927729602E-131	0.647439340344973	0.615	0.34	8.49374081489267E-127	4	NONO
1.70832451331293E-110	0.647370098380734	0.567	0.309	2.86281021940981E-106	4	POGK
4.60871251603324E-100	0.647237853166552	0.441	0.223	7.7232804343685E-96	4	ZBTB10
1.62439839079175E-65	0.646918122096435	0.322	0.165	2.72216682328881E-61	4	SMIM20
2.74001398213077E-117	0.646806883614569	0.48	0.234	4.59171543125474E-113	4	TPM4
6.4019171932375E-141	0.645759676600297	0.831	0.486	1.07283328324274E-136	4	RNF128
4.69704897265886E-112	0.645467517942259	0.455	0.222	7.87131466838172E-108	4	KDM3B
2.9661644297217E-82	0.645006210669879	0.344	0.165	4.97069835132763E-78	4	MAGOH
3.60662087560455E-90	0.6445463985791	0.494	0.26	6.0439752633381E-86	4	UGT2B11
5.58536649318395E-94	0.644115280634051	0.53	0.298	9.35995716927766E-90	4	RDH10
3.62616499123843E-91	0.643893860254145	0.511	0.282	6.07672729231736E-87	4	ISOC1
5.89038306157395E-87	0.643368258255566	0.55	0.327	9.87110393458563E-83	4	PRKDC
1.51555193851931E-90	0.643332601594243	0.279	0.117	2.53976193857066E-86	4	PARD6B
2.51146283262617E-96	0.643287993944677	0.444	0.226	4.20870941491493E-92	4	ARPC5
2.17257193923641E-82	0.643216868560378	0.344	0.166	3.64079605577237E-78	4	PDLIM1
5.90394390452806E-67	0.642321811696485	0.282	0.135	9.89382919520813E-63	4	ARFIP1
1.24510620641116E-87	0.642320835923437	0.427	0.219	2.08654898070383E-83	4	ATPAF1
3.81636754223411E-76	0.641777579258343	0.268	0.119	6.39546872727592E-72	4	SLC52A2
3.46917162385802E-94	0.641637141849492	0.388	0.187	5.81363780726128E-90	4	YIF1B
1.2664301260482E-100	0.641401885267546	0.567	0.306	2.12228360523157E-96	4	DEPRECATED-ENSG00000213759
1.65291487939674E-75	0.640858342830235	0.408	0.218	2.76995475489306E-71	4	PRELID1
1.91451724800659E-84	0.640415035023691	0.342	0.164	3.20834800420944E-80	4	CNOT10
2.45640311332314E-85	0.63990544891548	0.457	0.244	4.11644033730691E-81	4	ACACB
9.48779937543264E-121	0.639812246874346	0.74	0.463	1.589965419335E-116	4	PUF60
4.58207443449949E-130	0.639381787561554	0.655	0.377	7.67864033733425E-126	4	HNRNPA3
2.61654570627612E-111	0.639270668576537	0.339	0.145	4.38480729457752E-107	4	POLE
5.12135363603318E-116	0.639140619559457	0.426	0.2	8.58236442326441E-112	4	CORO1C
6.65397408557646E-99	0.638639443581028	0.587	0.339	1.1150729772609E-94	4	SNRPC
4.50505746600308E-80	0.638513592922478	0.382	0.194	7.54957530152796E-76	4	TM2D2
3.78914773395853E-104	0.63838497192553	0.469	0.239	6.3498537725677E-100	4	PIEZO2
3.5293797867467E-88	0.638346782053122	0.438	0.226	5.91453464663011E-84	4	SH3RF1
5.56089607455209E-169	0.638258276676175	0.335	0.115	9.31894964173438E-165	4	CEP120
2.14516878042313E-133	0.63804789665487	0.748	0.467	3.59487384223308E-129	4	GDI1
1.81978610902632E-92	0.6378409323116	0.44	0.227	3.04959756150631E-88	4	MBOAT1
2.22869910962655E-91	0.637415166890843	0.442	0.226	3.73485396791217E-87	4	MARVELD2
2.91444353101829E-80	0.637400496560532	0.302	0.139	4.88402446928045E-76	4	NOL11
7.33098556255412E-109	0.636433844160011	0.399	0.185	1.22852656057282E-104	4	PLCB1
6.39565486892828E-99	0.636428422528504	0.404	0.194	1.071783842935E-94	4	PSMG3
1.8495586008171E-87	0.636380624375218	0.369	0.178	3.09949030324929E-83	4	MVB12A
6.55445614900775E-102	0.635379655201555	0.454	0.228	1.09839576145072E-97	4	MCUR1
1.52287496859592E-115	0.635055131859999	0.52	0.267	2.55203387237304E-111	4	STK39
2.82829621035806E-92	0.634984062114045	0.429	0.217	4.73965878931804E-88	4	CENPB
2.3033257474661E-103	0.634581981697963	0.442	0.217	3.85991328760369E-99	4	MTMR1
2.42761883571498E-82	0.634250380156124	0.378	0.189	4.06820364489117E-78	4	CCDC28A
3.620119188848E-126	0.633987898561096	0.693	0.412	6.06659573667147E-122	4	TRIR
1.15255029056797E-89	0.63363474615363	0.284	0.122	1.9314437769338E-85	4	SP2
2.3916620430068E-100	0.633453608380451	0.632	0.375	4.0079472516708E-96	4	RNF5
2.89590801286236E-102	0.633425087076016	0.367	0.167	4.85296264795475E-98	4	MAD2L2
3.1592286710746E-92	0.633286919501806	0.306	0.134	5.29423540698682E-88	4	ARPIN
9.69747013979443E-77	0.633075014606312	0.452	0.251	1.62510204602675E-72	4	PPP1R10
2.41096628780081E-80	0.632770782547493	0.484	0.269	4.0402973050966E-76	4	GABRE
5.23128026756317E-76	0.632730697079777	0.316	0.15	8.76657947238235E-72	4	INO80C
2.73187939666464E-104	0.63262181722052	0.395	0.184	4.5780834929306E-100	4	ZCCHC3

2.30689965049712E-85	0.632511064486486	0.36	0.175	3.86590243430308E-81	4	ARHGEF18
1.51674165794994E-114	0.63219332332333	0.672	0.388	2.54175567039251E-110	4	ETHE1
1.15370614066099E-131	0.630774683593772	0.82	0.511	1.93338075051969E-127	4	NDUFA1
1.33725027192108E-86	0.630764371716126	0.378	0.186	2.24096400568534E-82	4	ABCB10
9.76565152032751E-79	0.6300640847113	0.388	0.197	1.63652788177648E-74	4	SLC16A2
3.68655226576241E-90	0.629718018589267	0.409	0.205	6.17792428696465E-86	4	GMPS
1.78715544352601E-84	0.628771010941546	0.416	0.215	2.9949150922609E-80	4	LRRC14
1.78567987379182E-85	0.628573344994174	0.252	0.105	2.99244233250033E-81	4	CD320
3.42885023310166E-85	0.628457767953137	0.494	0.27	5.74606722063176E-81	4	VDAC1
1.17866370299326E-102	0.628274431268857	0.441	0.217	1.9752046334761E-98	4	TCP1
4.06478086017377E-112	0.628217864398337	0.51	0.26	6.81175976547921E-108	4	FAM83H
8.7330869056552E-118	0.628098335261984	0.436	0.206	1.4634907036497E-113	4	RANBP9
2.98751845306194E-65	0.627957735639841	0.29	0.142	5.0064834236412E-61	4	ZSWIM5
1.35459994472104E-76	0.627756723941323	0.342	0.168	2.27003858736352E-72	4	ATPSCKMT
1.75227806031812E-76	0.62768375327767	0.334	0.163	2.9364675734811E-72	4	PDZD11
1.15547929266768E-86	0.626942131696763	0.372	0.18	1.93635219865251E-82	4	FKBP3
6.2853934620601E-83	0.626707036522391	0.401	0.205	1.05330623637203E-78	4	WWC2
3.75386984563767E-136	0.626489213234993	0.839	0.515	6.2907350873196E-132	4	FKBP5
1.99598099559422E-117	0.626101212216775	0.537	0.28	3.34486495241679E-113	4	ZFP91
7.43691448582947E-97	0.626084047266905	0.693	0.438	1.2462781295353E-92	4	UBB
1.52533950541384E-84	0.626057160610539	0.381	0.19	2.55616394317251E-80	4	PSMA4
8.85096515003482E-102	0.625358090452806	0.437	0.215	1.48324473984284E-97	4	HMGXB4
2.11088690925847E-107	0.625310027181985	0.629	0.356	3.53742428253534E-103	4	CYSTM1
8.08753274572486E-93	0.624869405693299	0.312	0.138	1.35530873752857E-88	4	RIF1
2.95917772124788E-125	0.624755419027577	0.33	0.131	4.9589900252672E-121	4	DDAH2
5.85591879620463E-102	0.623371309319493	0.582	0.335	9.81334871867972E-98	4	PA2G4
2.88289284902231E-96	0.623366546694757	0.434	0.217	4.83115183639159E-92	4	GSS
1.35477667959899E-70	0.62325113777496	0.255	0.114	2.27033475967199E-66	4	SIMC1
1.46603548285782E-97	0.62313893684521	0.404	0.198	2.45678226217314E-93	4	RNF19B
2.44517565786547E-93	0.623030246952372	0.492	0.262	4.09762536745095E-89	4	ASB13
2.02655594963935E-61	0.622714556667603	0.26	0.124	3.39610246040562E-57	4	CYCS
4.31759577736641E-71	0.622428023829733	0.34	0.171	7.23542700371064E-67	4	BAMBI
4.43824306205428E-93	0.621354244870401	0.461	0.237	7.43760772339057E-89	4	NCKAP1
1.79738664955055E-155	0.621088179892244	0.93	0.602	3.01206054731682E-151	4	TSC22D3
1.35402927254566E-87	0.620962572387957	0.523	0.296	2.26908225493201E-83	4	GCLM
3.25241627046274E-102	0.620016637124045	0.556	0.307	5.45039918604146E-98	4	NEDD4L
6.64442588289416E-102	0.619830176614475	0.54	0.291	1.1134728894554E-97	4	TM9SF1
7.28469910307092E-86	0.619342680168637	0.424	0.22	1.22076987569263E-81	4	GET1
2.35600009345772E-64	0.619277686924516	0.311	0.157	3.94818495661645E-60	4	THOC7
2.66130904476282E-68	0.617921503216133	0.272	0.127	4.45982169721353E-64	4	UQCC3
9.00305277416421E-91	0.61774740739377	0.39	0.191	1.50873158389444E-86	4	TADA3
4.31431768343157E-88	0.617618625833461	0.401	0.199	7.22993357389463E-84	4	CENPV
3.10678383491476E-67	0.617229365518251	0.264	0.123	5.20634835055015E-63	4	PDE6D
7.24602419436787E-91	0.616476824979884	0.485	0.257	1.21428873449217E-86	4	NET1
2.05548566687124E-66	0.616038321942406	0.251	0.115	3.44458288054283E-62	4	COPG2
1.56263242327176E-68	0.616038014155451	0.384	0.204	2.61865941491881E-64	4	CLDN14
4.1077064197943E-103	0.615583985858882	0.529	0.286	6.88369441829129E-99	4	PYGO2
1.15568929959489E-111	0.615314461268562	0.729	0.448	1.93670412826112E-107	4	PRDX1
1.0480648299725E-82	0.614851061450284	0.325	0.152	1.75634704206791E-78	4	PIGM
2.26515885313017E-104	0.614295790395503	0.579	0.326	3.79595320607554E-100	4	PSMB9
3.65697116219299E-74	0.613705927831332	0.3	0.142	6.12835227360301E-70	4	HCFC1R1
5.99668816584324E-91	0.613032337660492	0.406	0.2	1.00492500283201E-86	4	FAAH
3.47031190255096E-93	0.612984462973612	0.573	0.323	5.81554868629489E-89	4	AGPAT1
5.10143255012339E-103	0.612866215050501	0.689	0.429	8.54898066749678E-99	4	RAB2A
2.77034770144746E-91	0.61283308538972	0.415	0.208	4.64254867808565E-87	4	CTDSPL
5.45585488685188E-79	0.61225969808414	0.341	0.166	9.14292161938637E-75	4	CPSF2
5.11901790144578E-160	0.612071789127107	0.736	0.417	8.57845019924283E-156	4	EZR
1.6550634305313E-75	0.61155206388046	0.413	0.221	2.77355529688436E-71	4	FAM32A
3.93427183889559E-84	0.611081937435215	0.309	0.141	6.59305274762122E-80	4	GATA4
1.3476212316906E-96	0.611041893097914	0.528	0.29	2.25834366006711E-92	4	RHPN2

5.09970512763261E-99	0.61097504120646	0.423	0.207	8.54608585288674E-95	4	NSL1
6.832582490352E-69	0.610837991576079	0.285	0.136	1.14500417373319E-64	4	FRY
9.13166934568851E-106	0.610569011847682	0.569	0.318	1.53028514895048E-101	4	LONRF3
6.80026204150421E-66	0.609833744207346	0.336	0.172	1.13958791291528E-61	4	RBM24
1.21827408708203E-100	0.60974422597248	0.455	0.23	2.04158371513206E-96	4	ARPC1A
5.92777053995485E-89	0.609714350177944	0.387	0.19	9.93375787085633E-85	4	LARP7
1.12523191983787E-77	0.609624532368334	0.288	0.131	1.8856636512643E-73	4	LRRC57
1.61880736417754E-82	0.60929093026558	0.37	0.184	2.71279738088872E-78	4	RNF113A
4.61414294215537E-102	0.609180423976111	0.372	0.172	7.73238074246398E-98	4	PWWP2A
5.04068546301191E-76	0.608998600434705	0.425	0.228	8.44718069891536E-72	4	ENOPH1
1.903884590287E-88	0.608953232162138	0.406	0.203	3.19052979640295E-84	4	ADNP
3.82562362911655E-83	0.608791943196478	0.396	0.2	6.41098007767352E-79	4	ZBTB41
3.32239003628066E-66	0.608442579336382	0.355	0.187	5.56766122279914E-62	4	ZCRB1
1.90545678035331E-80	0.608099009716007	0.386	0.196	3.19316447251607E-76	4	TSR2
2.37168511764963E-90	0.607644139009347	0.327	0.149	3.97446992015725E-86	4	EXOC8
3.93028401432874E-80	0.607440759271353	0.409	0.212	6.58636995121211E-76	4	PRKCA
1.11091996177013E-93	0.607364556249419	0.394	0.19	1.86167967193438E-89	4	ELF2
2.51709110190603E-116	0.60704590640859	0.492	0.249	4.21814126857413E-112	4	TBCD
2.9959328242378E-67	0.606643283426078	0.288	0.139	5.0205842268577E-63	4	KLHL8
3.90194193603227E-76	0.606159025053304	0.441	0.24	6.53887429640288E-72	4	SLC39A1
1.10465312907948E-78	0.605565890987194	0.421	0.224	1.85117771371139E-74	4	PSENEN
3.37118276503891E-86	0.605520072484815	0.468	0.254	5.6494280776522E-82	4	IL17RB
2.47117000187787E-64	0.605353886332124	0.348	0.184	4.14118668914694E-60	4	AIMP2
5.10953205088563E-88	0.60490315829399	0.455	0.239	8.56255381087413E-84	4	CAMK1
1.7856218666784E-79	0.604820045779333	0.465	0.254	2.99234512417966E-75	4	CLMN
9.79608336142292E-88	0.604742429160793	0.399	0.201	1.64162764970725E-83	4	ANAPC4
4.21159663157408E-101	0.604621084701413	0.445	0.221	7.05779363519184E-97	4	AP2S1
1.33010296643101E-114	0.603737687536924	0.486	0.245	2.22898655114509E-110	4	PTBP1
8.61868257319721E-112	0.603514254757156	0.511	0.264	1.44431882561639E-107	4	FLOT1
1.54708281714969E-106	0.603486348608337	0.427	0.205	2.59260138497945E-102	4	MAPK9
8.27902476639013E-77	0.603301691619919	0.418	0.221	1.38739897035166E-72	4	PGLS
3.46082694887857E-104	0.602522419983965	0.411	0.196	5.7996538009307E-100	4	ITGA6
2.98631834785405E-80	0.602464750416865	0.461	0.249	5.00447228733382E-76	4	LSM 6.00
3.75561772449367E-99	0.601953860007242	0.347	0.155	6.29366418270649E-95	4	CDC23
2.34035091561244E-144	0.60171735346923	0.876	0.552	3.92196006438332E-140	4	ACSL4
1.95306659356927E-120	0.601564161318501	0.579	0.31	3.27294899750339E-116	4	PMF1
6.60111435801539E-49	0.60139806678581	0.26	0.135	1.10621474411622E-44	4	PHYHIPL
8.9302857921103E-112	0.600932681781985	0.719	0.431	1.49653729304184E-107	4	DAP
3.96611604540691E-69	0.600511597235138	0.268	0.125	6.64641726889291E-65	4	BAD
1.24608717092446E-77	0.600249889591607	0.281	0.127	2.08819288103521E-73	4	SRSF1
2.26008648049665E-79	0.599878142095163	0.286	0.129	3.78745292401628E-75	4	CAPRIN1
2.85888268232218E-99	0.599476222156495	0.461	0.239	4.7909155990355E-95	4	ERCC5
1.30610617723426E-85	0.598926446678735	0.367	0.179	2.18877273180917E-81	4	SNRPD2
7.53223588926407E-99	0.598501806434304	0.641	0.388	1.26225209032287E-94	4	STT3A
4.98047523778756E-80	0.598085689469752	0.515	0.294	8.34628040348439E-76	4	BTNL8
3.41547601964104E-97	0.598012004470995	0.607	0.355	5.72365471371446E-93	4	RNPEP
1.11733914848129E-79	0.597618709664081	0.342	0.167	1.87243694502494E-75	4	ANO8
1.54023042549654E-84	0.597334168196148	0.441	0.24	2.5811181470471E-80	4	ARL17A
1.04589066252821E-85	0.597327061374087	0.385	0.192	1.75270357226477E-81	4	UBTD2
2.46739279283279E-84	0.596891895165737	0.345	0.164	4.13485684222919E-80	4	ERAL1
1.47372367600764E-95	0.596770514905116	0.627	0.373	2.46966613625361E-91	4	CPQ
8.79777803627387E-109	0.596705678686615	0.859	0.601	1.47433164331878E-104	4	SERPINA6
9.18096946859476E-94	0.596667710776658	0.499	0.267	1.53854686354711E-89	4	PSMB5
9.35127500899746E-62	0.596630093413438	0.267	0.13	1.5670866660078E-57	4	MAGEF1
1.73054087577884E-92	0.596494584870299	0.376	0.179	2.90004039963018E-88	4	TSN
6.35238902667884E-83	0.596111246273873	0.369	0.182	1.06453335309084E-78	4	VAPB
2.26896801419818E-82	0.595428895682368	0.284	0.125	3.80233659819331E-78	4	C4orf19
2.37235365491178E-95	0.595413422118352	0.453	0.234	3.97559025490117E-91	4	DEPRECATED-ENSG00000134250
4.69877225534706E-94	0.595360592858526	0.64	0.388	7.8742025455106E-90	4	PRDX4
6.62348092915381E-80	0.594837430884651	0.485	0.264	1.1099629341076E-75	4	DEPRECATED-ENSG00000133142

6.95516562484675E-92	0.594287042240194	0.254	0.101	1.16554665541182E-87	4	IFT80
2.6547209729199E-63	0.593824519835623	0.27	0.129	4.44878140641917E-59	4	SLC2A8
7.28078490048615E-94	0.593710238995466	0.542	0.301	1.22011393362347E-89	4	RAB13
2.1222806718257E-94	0.593622539872253	0.638	0.385	3.5565179498455E-90	4	UBE2Q1
4.04296959642806E-104	0.593497956106828	0.619	0.337	6.77520844969414E-100	4	PARM1
3.20990837425328E-63	0.59339956957471	0.375	0.204	5.37916445357365E-59	4	EBAG9
2.32140106352388E-51	0.593160661343603	0.254	0.128	3.89020390225332E-47	4	TGDS
8.2120513179764E-84	0.592763652109128	0.406	0.207	1.37617555986648E-79	4	DEPRECATED-ENSG00000117691
7.84221594854417E-106	0.592736983068556	0.854	0.583	1.31419854865703E-101	4	MT-ND5
6.38678566107805E-109	0.592231372375739	0.469	0.237	1.07029754108346E-104	4	NUP43
2.96646707217173E-104	0.591799746309726	0.584	0.332	4.97120551954538E-100	4	FAM91A1
2.92408313484294E-77	0.591560096092475	0.344	0.169	4.90017851736979E-73	4	RAB15
3.54734364544116E-65	0.591070660315477	0.295	0.145	5.94463848103029E-61	4	XPO4
4.41870597399983E-99	0.590727023236121	0.521	0.283	7.40486747122891E-95	4	FNTA
1.53787027556133E-107	0.590715852641045	0.616	0.358	2.57716300778568E-103	4	ERBIN
4.51162815999964E-95	0.590710381082439	0.523	0.288	7.5605864705274E-91	4	APH1A
1.09105683760131E-78	0.590467618713976	0.417	0.219	1.82839304845228E-74	4	HGH1
1.69766577479567E-83	0.590215544306347	0.439	0.232	2.84494830540258E-79	4	PSMD8
2.58736267547944E-66	0.59009274063841	0.297	0.146	4.33590237156845E-62	4	DNTTIP1
3.17812542746605E-107	0.589301851999482	0.527	0.283	5.32590259134761E-103	4	LIX1L
6.4253126225817E-69	0.589188482913172	0.33	0.166	1.07675388929224E-64	4	PSMC1
5.16488889110034E-42	0.589060833361562	0.264	0.146	8.65532080370594E-38	4	TEF
9.2035378209256E-87	0.588689453722584	0.366	0.177	1.54232886803071E-82	4	EIF2AK2
5.10443211351118E-77	0.588496054297023	0.344	0.168	8.55400733582203E-73	4	PLPP2
9.66132049605159E-76	0.587647048952133	0.284	0.13	1.61904408872832E-71	4	SNUPN
3.29949106488156E-87	0.587140612483781	0.499	0.271	5.52928712652851E-83	4	ONECUT2
1.20229111447108E-101	0.587018193423484	0.591	0.339	2.01479944963064E-97	4	COPS5
3.99707221858606E-85	0.586702962563133	0.511	0.286	6.69829362390652E-81	4	TMEM97
4.90904131395971E-70	0.585734517911528	0.396	0.216	8.22657143393369E-66	4	ZMAT1
3.4100345099853E-79	0.585441473743093	0.48	0.266	5.71453583183336E-75	4	MEA1
1.5949450197205E-93	0.584456648872708	0.615	0.36	2.67280886404762E-89	4	PERP
2.96750377835394E-77	0.583682144007776	0.345	0.169	4.97294283176554E-73	4	RNF139
6.38446776047771E-87	0.583390687995773	0.455	0.238	1.06990910730085E-82	4	MTFR1
3.31609339004814E-133	0.582291093225471	0.766	0.473	5.55710930304268E-129	4	NPC2
2.93960711666577E-85	0.581788520044809	0.516	0.294	4.9261936061085E-81	4	DEPRECATED-ENSG00000179010
9.30791956936185E-65	0.58151904720649	0.278	0.133	1.55982116143366E-60	4	TIGD5
2.99231254882028E-104	0.581359702135713	0.913	0.677	5.01451736931303E-100	4	MT-ND3
1.4286210038592E-77	0.581048155594736	0.438	0.238	2.39408307826725E-73	4	GBA
2.22318361399082E-83	0.580884980379147	0.255	0.107	3.72561110032581E-79	4	GIT1
2.96219931231105E-90	0.580815562539452	0.357	0.168	4.96405360757086E-86	4	UBQLN4
5.19579570561238E-89	0.580759907550195	0.514	0.279	8.70711444346522E-85	4	TMEM164
2.57094332855198E-49	0.58067910420394	0.268	0.14	4.3083868299874E-45	4	SDHAF4
6.9283434637567E-78	0.580599786084916	0.404	0.212	1.16105179765635E-73	4	AURKAIP1
1.60749730975235E-99	0.580286602434987	0.595	0.337	2.69384399168299E-95	4	RNF103
3.42957965278143E-92	0.579921856533432	0.432	0.222	5.74728958213112E-88	4	SPPL2B
2.07902595312755E-92	0.579805745341777	0.53	0.284	3.48403169225114E-88	4	KCNK1
1.96041147693899E-76	0.579351948255699	0.264	0.117	3.28525755305436E-72	4	TFDP2
6.03369135713652E-92	0.579221746205421	0.518	0.279	1.01112599762894E-87	4	ILVBL
1.27004295634904E-70	0.57877362953898	0.302	0.146	2.12833798624972E-66	4	ASAP2
2.62567748479086E-87	0.578215868460597	0.491	0.267	4.40011032901252E-83	4	ESD
3.7960077669738E-133	0.57793172897264	0.83	0.484	6.36134981589469E-129	4	TSPAN3
7.03316600845145E-79	0.577905306965927	0.479	0.266	1.17861795969629E-74	4	NDUFS6
1.09832466722075E-73	0.57728081129172	0.376	0.195	1.84057247732854E-69	4	CNP
2.80856838383578E-69	0.577172641983586	0.325	0.163	4.706598897632E-65	4	TMX1
2.65653060137719E-123	0.577035574276723	0.411	0.183	4.45181398178789E-119	4	MUS81
5.27757550760626E-105	0.576818478902121	0.485	0.25	8.84416103564656E-101	4	STK11
2.45447324485101E-89	0.576617520742053	0.37	0.178	4.11320626372132E-85	4	MAP4K5
1.61840493811372E-76	0.576478206091771	0.406	0.212	2.71212299529097E-72	4	ASB9
1.60122746221292E-109	0.576354245175481	0.394	0.181	2.6833369811764E-105	4	TAGLN2
1.43031651674234E-70	0.575630752463755	0.284	0.133	2.39692441875681E-66	4	POP4

6.0310363199217E-63	0.575381401720478	0.27	0.13	1.01068106649248E-58	4	MRM2
3.15933442684741E-78	0.574997664202904	0.419	0.221	5.29441263251088E-74	4	ACTR10
1.51830841967173E-102	0.574687381489355	0.513	0.273	2.54438124968589E-98	4	AGFG1
4.75465693184664E-71	0.574612808983942	0.318	0.155	7.96785408638861E-67	4	RMND5B
4.32518895887991E-72	0.574132916413922	0.401	0.213	7.24815165729095E-68	4	TMEM258
3.39761688713904E-71	0.573431091352034	0.292	0.138	5.6937263794676E-67	4	DGCR6L
5.2373056011677E-72	0.572470905486592	0.363	0.186	8.77667672643684E-68	4	PHLPP2
5.8511469854923E-54	0.572028829853089	0.256	0.129	9.80535211828799E-50	4	AP1AR
1.20839410404452E-88	0.572027097537578	0.301	0.132	2.02502683955781E-84	4	AC253572.1
3.91039385674006E-91	0.571783001419918	0.453	0.237	6.55303802512499E-87	4	GSK3A
6.52827244220206E-89	0.571638921801609	0.56	0.322	1.09400789586422E-84	4	DYNLRB1
8.94690840077651E-78	0.570881767685768	0.33	0.159	1.49932290980213E-73	4	OARD1
2.37898772775435E-70	0.570816336810031	0.417	0.228	3.98670763417075E-66	4	NDUFA7
2.37233787098323E-107	0.570607918410353	0.788	0.521	3.9755638041937E-103	4	PRDX2
5.94453737345249E-86	0.570601015741619	0.428	0.223	9.96185573043168E-82	4	BROX
1.67560721946777E-83	0.570579979371275	0.486	0.266	2.80798257838408E-79	4	SOS 2
6.83758308395028E-119	0.570000851809151	0.381	0.165	1.14584217320839E-114	4	PSIP1
2.36392547009594E-103	0.569661239995428	0.53	0.285	3.96146630278677E-99	4	ACTR1B
5.24181757243877E-142	0.569504571222239	0.87	0.572	8.78423788789289E-138	4	SERPINA7
9.33720599115359E-77	0.569376880655251	0.327	0.157	1.56472897999752E-72	4	KLHDC10
1.32553454932958E-99	0.568435220961902	0.652	0.386	2.22133079776651E-95	4	SLC25A1
2.17461641458101E-65	0.567838538228317	0.293	0.142	3.64422218755486E-61	4	FNBP1L
1.50779608075094E-83	0.567708030258406	0.372	0.184	2.52676467212243E-79	4	CD177
3.3276958985219E-77	0.567545962345498	0.255	0.11	5.576552786743E-73	4	PRKRA
5.15529399875067E-67	0.567018763356975	0.314	0.156	8.63924168310638E-63	4	GTF2A2
4.94700726943114E-74	0.566324158726071	0.328	0.162	8.29019478211271E-70	4	ADCK2
8.04957680815497E-94	0.565991668797993	0.66	0.404	1.34894808151061E-89	4	EBP
3.77924757446527E-82	0.565836592959546	0.568	0.332	6.3332630852889E-78	4	RNF187
6.44955040906144E-64	0.565779211314734	0.288	0.142	1.08081565755052E-59	4	SPRY2
2.30265417272379E-90	0.564791490625395	0.432	0.219	3.85878786265053E-86	4	PCGF2
1.6536099763648E-126	0.564720267772812	0.265	0.092	2.77111959839213E-122	4	EXOSC8
1.04770525417431E-132	0.564690990859632	0.478	0.225	1.75574446494531E-128	4	NFATC2IP
8.36455962542928E-104	0.564625585359097	0.463	0.234	1.40173290202944E-99	4	UBA2
1.01667889470486E-74	0.564224095064789	0.374	0.191	1.7037504917464E-70	4	PSMA3
1.75836861599157E-59	0.563391536229853	0.372	0.205	2.94667412667867E-55	4	RASL11A
3.32457198650903E-83	0.563084818349438	0.295	0.132	5.57131773499183E-79	4	SOCS7
6.76778905753911E-136	0.562704563901599	0.719	0.431	1.1341460902624E-131	4	HNRNPA0
4.57110699748804E-97	0.562644999467303	0.448	0.229	7.66026110639047E-93	4	ZNF652
3.29050894763736E-65	0.562640287777893	0.286	0.138	5.51423489445068E-61	4	PRDX3
8.24103981396234E-65	0.5622994250786	0.307	0.153	1.38103345202381E-60	4	ZNF275
1.03144613067587E-73	0.561798127808066	0.417	0.225	1.72849742578662E-69	4	KDELR1
1.12218362051028E-89	0.561535181048536	0.667	0.426	1.88055531125112E-85	4	BSG
1.03978175578054E-79	0.560909712101047	0.548	0.323	1.74246626633702E-75	4	KDELR2
9.90486461715646E-96	0.560602873296925	0.446	0.226	1.65985721254308E-91	4	NSMCE2
3.54967451151208E-138	0.559730264413345	0.751	0.416	5.94854454639195E-134	4	PEG10
3.62989313722898E-65	0.558884524736801	0.387	0.209	6.08297491936832E-61	4	CORO2A
6.71934318175455E-98	0.558707844778346	0.853	0.586	1.12602753039843E-93	4	MT-ND2
3.7670362576929E-102	0.558297951146304	0.421	0.204	6.31279936064176E-98	4	DR1
8.96011833754375E-98	0.55822379424696	0.402	0.194	1.50153663100558E-93	4	BABAM1
1.85603352299459E-64	0.557858380494302	0.262	0.124	3.11034097783433E-60	4	NXT1
4.58552796856218E-128	0.557763103914427	0.788	0.508	7.68442776971649E-124	4	ACTN4
1.35788492593349E-98	0.557406403876908	0.746	0.485	2.27554355887935E-94	4	TSTA3
2.10473334719583E-74	0.557342468777392	0.503	0.287	3.52711214323078E-70	4	ATP5PD
1.00097853562476E-93	0.556755162970335	0.443	0.227	1.67743982999997E-89	4	PNP
2.71691097670468E-121	0.556621371658345	0.464	0.22	4.55299941476169E-117	4	SRRT
7.35445445598505E-79	0.556107488126946	0.542	0.312	1.23245947773397E-74	4	NDFIP2
1.82538580243905E-71	0.556061916997283	0.305	0.146	3.05898152772736E-67	4	APEX2
1.6385653069434E-117	0.555910473840345	0.631	0.356	2.74590774137574E-113	4	MAPK1
1.76261299790469E-91	0.555851734983794	0.547	0.307	2.95378686188868E-87	4	NCOA2
7.95269397876897E-115	0.555808632293809	0.812	0.531	1.3327124569621E-110	4	DNM2

3.62952275955243E-102	0.555638670260952	0.426	0.206	6.08235424045796E-98	4	GATD1
4.26876716165871E-90	0.555578679621199	0.717	0.471	7.15360000950767E-86	4	EIF1
2.45043314624189E-89	0.555378519878003	0.391	0.191	4.10643586647215E-85	4	EXOC5
2.8099607045926E-152	0.555264882155206	0.958	0.622	4.70893214875628E-148	4	ANXA4
8.67109205710292E-88	0.554984934542107	0.63	0.377	1.45310160692931E-83	4	DSTN
3.53570882742987E-72	0.554561872521543	0.382	0.199	5.92514085300697E-68	4	CC2D1A
1.07559519227821E-64	0.554508950843796	0.259	0.121	1.80248242321983E-60	4	PSMG1
2.80253137191849E-89	0.55447715724169	0.343	0.159	4.696482073061E-85	4	ITGB1BP1
3.7750527221572E-82	0.554366761351406	0.544	0.309	6.32623335179104E-78	4	SMIM19
3.17154584000805E-70	0.55430513892339	0.409	0.223	5.31487651868549E-66	4	TMEM14C
6.4392908999032E-89	0.553508400313945	0.87	0.634	1.07909636900578E-84	4	NUPR1
1.4681333438513E-79	0.553397056546333	0.454	0.242	2.460297857626E-75	4	MALL
1.08558927793277E-72	0.55296467704118	0.44	0.243	1.81923051195974E-68	4	COQ8A
1.63845816685819E-70	0.551917878754474	0.31	0.15	2.74572819602096E-66	4	RPP21
5.91333543740514E-67	0.551907965563989	0.385	0.206	9.90956752600353E-63	4	PSMA2
2.0316233027028E-97	0.551586596791396	0.436	0.215	3.40459433066936E-93	4	TERF1
3.68655654665545E-73	0.551389402637477	0.336	0.167	6.1779314608852E-69	4	BAG2
3.56402092804481E-75	0.55121164617276	0.312	0.148	5.97258627121749E-71	4	RIC8B
2.52421850024026E-67	0.551122959507008	0.301	0.148	4.23008536270263E-63	4	GUCY1A1
5.35135959348458E-79	0.550347531304146	0.265	0.116	8.96780840676145E-75	4	POLR2D
1.09114470189956E-77	0.550116776053346	0.275	0.122	1.82854029144328E-73	4	DCBLD2
1.6769348848442E-64	0.55000440434141	0.274	0.131	2.8102074800219E-60	4	RAP1GDS1
1.04581484453253E-62	0.549887167083502	0.31	0.157	1.75257651646762E-58	4	SOX9
8.0974467028629E-71	0.549799485349488	0.41	0.22	1.35697011846577E-66	4	POMP
2.93321763308191E-66	0.549347485304394	0.31	0.154	4.91548610951866E-62	4	UCK2
1.00929075930833E-67	0.548097037961936	0.342	0.175	1.69136945444889E-63	4	CMAS
4.216713325769E-85	0.547930907426515	0.6	0.356	7.06636819132369E-81	4	IL6R
1.8355852321033E-85	0.547472900613198	0.292	0.129	3.07607373195871E-81	4	TNPO1
1.2162430643172E-87	0.54693113558672	0.508	0.281	2.03818012718276E-83	4	STK3
2.55312755145764E-82	0.546888804461832	0.584	0.341	4.27853115073271E-78	4	STBD1
5.34160627799853E-59	0.54675905281474	0.25	0.12	8.95146380066993E-55	4	RTRAF
4.06198584555939E-78	0.545930005378684	0.547	0.322	6.80707587998842E-74	4	UQCR10
7.32245286218035E-92	0.545454470871817	0.606	0.357	1.22709665064418E-87	4	PLEKHA6
8.77759414735638E-73	0.545097456923436	0.342	0.17	1.47094922721398E-68	4	DEPRECATED-ENSG00000109084
7.22247544034302E-78	0.545041704610307	0.396	0.204	1.21034243429268E-73	4	CASP3
6.66535707327299E-94	0.544723781854585	0.296	0.126	1.11698053833909E-89	4	ADAT1
4.85154654639212E-89	0.543647600670096	0.516	0.285	8.13022170244391E-85	4	IMPA1
1.05524415525309E-88	0.543025498655507	0.344	0.16	1.76837815537313E-84	4	BAG5
1.46256765862673E-80	0.543020191639073	0.293	0.133	2.45097088232667E-76	4	SALL1
3.63347678148305E-78	0.542531207051155	0.392	0.202	6.08898039040929E-74	4	PTDSS1
4.23839640319634E-68	0.541469349660063	0.37	0.196	7.10270469247642E-64	4	METAP2
4.79585137286814E-77	0.541076822703686	0.336	0.164	8.03688773065243E-73	4	SESN2
1.93390176739047E-92	0.54088935312757	0.678	0.432	3.24083258179296E-88	4	HBS1L
2.39871508623996E-71	0.540815452383185	0.355	0.182	4.01976674152093E-67	4	UBAC2
8.88715805670779E-82	0.540450090363756	0.62	0.382	1.48930994714309E-77	4	CHCHD2
9.01665069595457E-57	0.539218183511107	0.257	0.126	1.51101032362807E-52	4	MOB3B
4.22107770395402E-75	0.539170907100824	0.565	0.341	7.07368201628615E-71	4	IARS2
7.39665436047334E-73	0.538958679863537	0.386	0.201	1.23953133772812E-68	4	EIF2S3
8.64790561458432E-81	0.538496911967716	0.446	0.24	1.44921602289204E-76	4	SLC35B2
1.41314967895066E-98	0.53751496563521	0.314	0.134	2.36815623198552E-94	4	C1orf174
1.16380839064632E-88	0.537447238835835	0.392	0.194	1.95031010104511E-84	4	VCPIP1
2.06271995708915E-122	0.537264325900571	0.25	0.086	3.45670610409E-118	4	RGS12
2.33642487586977E-101	0.537197988159993	0.424	0.206	3.91538080698256E-97	4	MID1
1.38214331613089E-118	0.53693737826314	0.501	0.253	2.31619576917215E-114	4	MICALL2
3.35764552245052E-58	0.536477034182992	0.323	0.17	5.62674236652258E-54	4	MID2
3.02314059137411E-60	0.536395030991483	0.322	0.167	5.06617900302473E-56	4	GREB1L
1.35815912689017E-78	0.535902089933592	0.328	0.157	2.27600306484255E-74	4	FLVCR1
4.64743715012514E-84	0.53579504896281	0.527	0.289	7.78817517617971E-80	4	ZDHHC9
7.7027195913669E-70	0.53554740235925	0.441	0.246	1.29082174912127E-65	4	ME1
1.20162463892725E-75	0.535368477534225	0.369	0.187	2.01368256991429E-71	4	TOMM5

4.55242134998706E-76	0.535194320018733	0.451	0.247	7.62894769830831E-72	4	SLC25A11
8.80833452141641E-94	0.535001917202567	0.4	0.194	1.47610069909896E-89	4	CUL3
1.54232914224809E-93	0.534984491791652	0.574	0.327	2.58463517657935E-89	4	PPP1CA
3.49021693651021E-88	0.534852641874742	0.57	0.328	5.8489055422038E-84	4	AMFR
7.19096216816842E-90	0.534616770084282	0.463	0.241	1.20506144014166E-85	4	SLC10A3
1.17607518025744E-111	0.53441879113324	0.448	0.216	1.97086678707542E-107	4	RNF168
2.04014027047283E-72	0.534272603711689	0.265	0.121	3.41886706525838E-68	4	XRCC6
9.99793529958205E-60	0.53422861772172	0.255	0.122	1.67545399750396E-55	4	UBE2L3
6.59769036025799E-61	0.533951037533459	0.313	0.16	1.10564095057203E-56	4	FAM98B
4.16185748283435E-74	0.533375893066639	0.414	0.219	6.9744407697338E-70	4	APOOL
2.52456607682117E-77	0.533018730534363	0.522	0.305	4.23066783153691E-73	4	RPN1
1.65326928709465E-90	0.532773207341202	0.516	0.288	2.77054867131322E-86	4	ATF6B
8.35879596137542E-76	0.532707239661272	0.288	0.133	1.40076702720729E-71	4	UBN1
1.86888866156106E-86	0.532650985672707	0.496	0.276	3.13188361904403E-82	4	SGK3
2.5370351557683E-84	0.532539818581705	0.696	0.45	4.25156351403652E-80	4	TOR1AIP2
1.9078930373568E-64	0.532034985682997	0.29	0.142	3.19724715200253E-60	4	DYNC1LI1
2.24936602935412E-83	0.531674061639648	0.525	0.297	3.76948759199163E-79	4	JTB
6.07063000468316E-78	0.531480782067012	0.331	0.159	1.0173161761848E-73	4	NANS
3.56635491715004E-86	0.531458227401625	0.327	0.151	5.97649757016003E-82	4	ANAPC7
8.76331348560263E-110	0.531042430537843	0.433	0.209	1.46855607391729E-105	4	SLC29A1
7.53511311451624E-108	0.530939835364816	0.512	0.269	1.26273425573063E-103	4	MECP2
1.59898766672562E-103	0.530916120503984	0.703	0.404	2.67958353189879E-99	4	ABCD1
4.49359476070486E-145	0.530614912117901	0.977	0.726	7.5303660999892E-141	4	ALDH1A1
2.8399129014352E-90	0.530407650274254	0.351	0.163	4.7591260402251E-86	4	NUP160
4.48068126296857E-119	0.530331793642247	0.663	0.4	7.50872566048273E-115	4	ILF3
1.07169822385533E-82	0.530175989115749	0.499	0.278	1.79595188353676E-78	4	NFIA
2.26888816315115E-49	0.529746310184154	0.262	0.136	3.8022027838087E-45	4	GTF2IRD1
2.92166954745149E-85	0.529376071954117	0.347	0.165	4.89613382761921E-81	4	NAA38
6.23050721676911E-93	0.528792790726338	0.559	0.318	1.04410839938617E-88	4	KANK1
1.0446273628504E-90	0.528580074257557	0.286	0.123	1.75058653466471E-86	4	EED
1.05299413310891E-87	0.528274455850199	0.533	0.3	1.76460756826391E-83	4	GOLGA7
6.28246469245024E-87	0.528138719316193	0.579	0.333	1.05281543316081E-82	4	XIAP
2.80720499854947E-84	0.527403832115693	0.372	0.182	4.7043141365692E-80	4	CARM1
1.40078639336363E-72	0.527352552760511	0.263	0.118	2.34743783799877E-68	4	SPATA5L1
2.24259561534024E-60	0.527286942209319	0.281	0.139	3.75814173218717E-56	4	UCHL3
8.36203248990795E-76	0.526852738082348	0.487	0.276	1.40130940465877E-71	4	NUDT5
2.57337384945586E-93	0.526791173002337	0.385	0.185	4.31245989691813E-89	4	SFPQ
4.09064831006509E-65	0.526511465241443	0.279	0.134	6.85510843800707E-61	4	GPR137
4.23507148056853E-58	0.52650579019585	0.282	0.143	7.09713278713674E-54	4	FAM110B
9.58438287683717E-80	0.526489141400859	0.274	0.121	1.60615088250037E-75	4	TAF5L
3.30319449982296E-67	0.526440880298285	0.433	0.241	5.53549334280331E-63	4	PPP1R7
2.65024588238033E-70	0.526211726593916	0.283	0.133	4.44128204969297E-66	4	BRIX1
5.57570893031084E-99	0.526169610620593	0.356	0.162	9.3437730254149E-95	4	STRBP
2.70176069806969E-57	0.525955659232324	0.268	0.133	4.52761057782519E-53	4	NOL9
2.0485807711116E-89	0.525644529296839	0.345	0.162	3.43301165622882E-85	4	ZADH2
3.54364765923238E-100	0.52526880052598	0.77	0.477	5.93844474734162E-96	4	RBM47
7.07284565939324E-98	0.524949650338813	0.451	0.229	1.18526747560112E-93	4	ATF2
5.36247128743939E-121	0.524848921701254	0.504	0.251	8.98642938349094E-117	4	RNF4
8.2899274844136E-92	0.524480515671297	0.252	0.1	1.38922604783803E-87	4	SLF2
1.07910598627415E-93	0.524137411552683	0.428	0.216	1.80836581179822E-89	4	SEMA4B
9.36979903073675E-72	0.524000932908383	0.449	0.247	1.57019092157086E-67	4	TMEM129
1.38505366851196E-102	0.523338042895496	0.413	0.198	2.32107293769235E-98	4	YY1
1.52556648502407E-71	0.52278538642065	0.527	0.31	2.55654431560333E-67	4	COX16
3.19095430919611E-45	0.521978507020861	0.26	0.138	5.34740123135084E-41	4	ANKRD46
9.09665269047104E-74	0.521820180292212	0.496	0.287	1.52441705786914E-69	4	DIAPH2
1.1423190016803E-55	0.52165159408554	0.331	0.178	1.91429818301585E-51	4	MTX2
8.39796085878776E-69	0.521417827359051	0.276	0.13	1.40733028071565E-64	4	TMEM18
1.70940245033743E-90	0.521169826793612	0.25	0.1	2.86461662627547E-86	4	DEPRECATED-ENSG00000084463
1.03854485455566E-82	0.521079805538105	0.553	0.323	1.74039346726437E-78	4	PSMC3
1.73415324336749E-72	0.520515247857905	0.353	0.179	2.90609400523524E-68	4	NABP2

6.80671715676791E-82	0.520444917696501	0.292	0.131	1.14066966113117E-77	4	DCAKD
1.73622974495306E-77	0.520425292025265	0.389	0.201	2.90957380659234E-73	4	WDR44
1.17878018888152E-66	0.520280396038912	0.421	0.231	1.97539984052764E-62	4	PYGL
1.07976385367502E-53	0.520043277677387	0.261	0.13	1.8094682659886E-49	4	RAI14
6.39079641657731E-69	0.520027082371743	0.378	0.199	1.07096966349003E-64	4	TIMM50
1.39234198712361E-102	0.519948923729097	0.713	0.448	2.33328670202175E-98	4	PSMB8
1.44848296576028E-85	0.518888164884051	0.791	0.548	2.42736775402108E-81	4	GOLIM4
1.66382463288623E-69	0.518795211310571	0.323	0.161	2.78823731979075E-65	4	ANKRD9
2.74216030466334E-79	0.518644070379649	0.447	0.24	4.59531223855482E-75	4	UBE2E3
6.47846937760667E-67	0.518444471138669	0.461	0.265	1.08566189829933E-62	4	STOML2
3.28056010020359E-76	0.518256107832252	0.566	0.34	5.49756261592118E-72	4	ACSL3
1.36727342592837E-74	0.518107890207999	0.386	0.201	2.29127680717077E-70	4	ZNF623
3.18380202245017E-89	0.518081034293243	0.511	0.283	5.335415429222E-85	4	LRRC8A
3.26172860066104E-67	0.518056936323242	0.382	0.203	5.46600478898776E-63	4	VIRMA
9.94172322890419E-72	0.518004267237914	0.358	0.184	1.66603397869976E-67	4	TIMM10
4.35923024917251E-73	0.517849019491739	0.566	0.346	7.3051980515633E-69	4	COX8A
2.43930704968953E-78	0.517137797407795	0.58	0.341	4.08779075386971E-74	4	AK4
9.47330484322713E-56	0.516997464483746	0.274	0.137	1.587536425628E-51	4	ARMCX5
4.88608878205395E-81	0.516989954752457	0.414	0.216	8.18810758096601E-77	4	IRF2BPL
3.49929910255538E-76	0.516496297755656	0.439	0.235	5.86412543606231E-72	4	PLA2G12A
7.98608130024887E-75	0.516236196585633	0.487	0.277	1.33830750429571E-70	4	TMEM68
2.98788687246117E-62	0.515794502477001	0.349	0.184	5.00710082087043E-58	4	CHMP5
1.74591559669966E-99	0.515573620120055	0.78	0.513	2.9258053569493E-95	4	CYC1
8.19077477417209E-69	0.515484290180186	0.34	0.171	1.37261003665576E-64	4	TMEM220
5.03446838901544E-73	0.51512567851298	0.261	0.117	8.43676212631208E-69	4	SS18L2
2.06205137242201E-64	0.514646446521519	0.264	0.124	3.45558568990481E-60	4	MAD2L1BP
4.49401767700372E-67	0.514350819089814	0.362	0.192	7.53107482312283E-63	4	KLHL22
4.10030096572541E-76	0.514279867523346	0.292	0.137	6.87128435836264E-72	4	TMEM44
8.23257135906974E-78	0.514231677556183	0.506	0.285	1.37961430835291E-73	4	SH3RF2
7.3911219070303E-84	0.513733255311692	0.381	0.189	1.23860420918014E-79	4	NAA50
1.04764834263554E-69	0.513713868909864	0.436	0.243	1.75564909258864E-65	4	RHOBTB3
4.00744037327426E-78	0.51359679661076	0.451	0.253	6.71566857753301E-74	4	NUP210
5.84950647424455E-55	0.513235493475661	0.29	0.15	9.80260294953902E-51	4	PDP1
8.32810753852807E-94	0.513203824621502	0.579	0.329	1.39562426130653E-89	4	RAB8A
1.52422799348999E-72	0.513200245747349	0.393	0.208	2.55430127149053E-68	4	DPY30
1.32273443731599E-65	0.513151893972391	0.28	0.134	2.21663837005413E-61	4	PLCB3
2.55325444207065E-95	0.512805292789651	0.474	0.246	4.27874379402199E-91	4	CKAP4
5.03622805260947E-104	0.512532869777063	0.499	0.262	8.43971097056296E-100	4	CEP350
4.67601457819977E-112	0.512205352064894	0.279	0.106	7.83606523014718E-108	4	FAM13B
3.31600352716061E-71	0.511790493217012	0.296	0.141	5.55695871081575E-67	4	LMAN2L
4.72946078764273E-71	0.510682291879561	0.485	0.268	7.92563038793168E-67	4	CDS1
3.68100193458548E-97	0.510512688057913	0.557	0.314	6.16862304197834E-93	4	PUM2
1.69331321017397E-83	0.509744539367454	0.631	0.393	2.83765427760954E-79	4	SHMT1
1.18668398597539E-83	0.509577356077512	0.497	0.272	1.98864502369756E-79	4	RAB1B
1.52352485043875E-71	0.509179096100344	0.449	0.249	2.55312294436526E-67	4	GDI2
6.006314408201E-63	0.509149953194011	0.262	0.124	1.00653816852632E-58	4	FAM200B
7.8220357096609E-97	0.508661789368249	0.403	0.195	1.31081674422497E-92	4	NUP153
6.86923417648848E-56	0.508535996374752	0.263	0.131	1.15114626329594E-51	4	TDRD7
9.18445254480898E-65	0.507708136371501	0.364	0.192	1.53913055745909E-60	4	PPA2
1.75105923753709E-69	0.507493028814888	0.42	0.23	2.93442507026466E-65	4	MAL2
3.81286929127539E-129	0.507340202783161	0.855	0.502	6.3896063583193E-125	4	NDRG1
3.22348810709644E-87	0.507195437971177	0.362	0.172	5.40192136987222E-83	4	HYI
2.8297415787462E-77	0.506909663210242	0.37	0.185	4.74208093766289E-73	4	GTF2H2
1.81671714064362E-86	0.506820803899213	0.396	0.198	3.04445458429058E-82	4	DEPRECATED-ENSG00000205937
5.70139196657011E-82	0.506767705920312	0.324	0.152	9.55439265757819E-78	4	RECQL
1.38562072202875E-83	0.506552557206292	0.453	0.24	2.32202320597578E-79	4	SMARCD2
2.67831603210246E-77	0.506512055107788	0.363	0.181	4.48832200659731E-73	4	TOR1A
1.63086664595395E-90	0.506273853617089	0.408	0.202	2.73300632528964E-86	4	SP1
7.00855503076617E-121	0.506157458764913	0.916	0.615	1.1744936520558E-116	4	UGT2B4
1.52404391455426E-74	0.505683975407201	0.4	0.209	2.55399279201002E-70	4	PCSK5

1.26041115115911E-90	0.505652007207823	0.792	0.521	2.11219700711244E-86	4	CRYL1
9.11849032594863E-71	0.505353369511603	0.6	0.362	1.52807660882247E-66	4	CCL15
5.64251555803515E-67	0.505180256881319	0.351	0.181	9.45572757215531E-63	4	TBCE.1
3.9436051028138E-90	0.505063410740135	0.716	0.443	6.60869343129536E-86	4	COX6C
4.91143171002531E-93	0.504718864788916	0.983	0.831	8.23057725966041E-89	4	MT-ATP6
1.23031619533792E-94	0.503166472635994	0.456	0.236	2.06176388014729E-90	4	TNRC18
4.4414411903276E-75	0.502999810524484	0.583	0.356	7.442967146751E-71	4	CHPT1
4.2864636612186E-63	0.502653367693201	0.294	0.145	7.18325580347013E-59	4	BSCL2
1.23515593906936E-90	0.502072364534777	0.442	0.227	2.06987432269243E-86	4	SF3B3
4.94428538490983E-88	0.501720444915192	0.546	0.312	8.2856334480319E-84	4	RAD23A
5.43949962003255E-60	0.501590712486314	0.283	0.14	9.11551346325054E-56	4	AGA
1.47560029294427E-65	0.501579408426919	0.251	0.115	2.47281097091601E-61	4	TMEM209
1.64383496651093E-75	0.50125013635702	0.429	0.229	2.75473863687902E-71	4	TBC1D8B
8.91563442575145E-72	0.500321812454476	0.698	0.469	1.49408201706743E-67	4	NDUFA2
4.22611890670525E-85	0.499913178088021	0.583	0.343	7.08213006385666E-81	4	RREB1
3.27713479883732E-70	0.499862625122851	0.302	0.145	5.49182249589158E-66	4	PM20D2
3.65390599113809E-60	0.499521289559575	0.282	0.139	6.12321565994921E-56	4	XRCC5
2.99119929605288E-87	0.4994489074215	0.345	0.163	5.01265178032541E-83	4	ABCB8
4.89531796066651E-89	0.499304563897447	0.385	0.188	8.20357383848494E-85	4	CHERP
7.04441114228819E-81	0.499205637078086	0.534	0.308	1.18050241922466E-76	4	AP1M1
1.79563092850498E-71	0.499195205889954	0.289	0.136	3.00911830998864E-67	4	HLCS
4.47146848526407E-94	0.499161740298943	0.35	0.16	7.49328688760552E-90	4	SNRNP40
1.79448475011662E-139	0.498584494423127	0.99	0.811	3.00719754424543E-135	4	EPHX1
1.89540043041416E-60	0.498530570321357	0.28	0.138	3.17631204128805E-56	4	COPS8
1.71416611096891E-103	0.498377079202292	0.744	0.433	2.8725995687617E-99	4	ATP1A1
3.19513244155086E-76	0.498304507707228	0.364	0.182	5.35440294555094E-72	4	SLC25A43
9.16568381546433E-72	0.498153871049057	0.432	0.237	1.53598529379551E-67	4	GINM1
7.75514679412721E-80	0.497988772994963	0.293	0.134	1.29960749975984E-75	4	SBNO1
3.6284553523505E-77	0.497284974148844	0.506	0.286	6.08056547946897E-73	4	GUCD1
5.15085444277414E-73	0.497230978225734	0.341	0.169	8.63180187520091E-69	4	PAWR
2.16428140037657E-74	0.497100115605266	0.362	0.182	3.62690277075105E-70	4	EHMT2
6.85868045655528E-60	0.497075585700239	0.371	0.203	1.14937767090953E-55	4	COPS7A
8.14894436704565E-74	0.497053558589766	0.272	0.123	1.36560009702951E-69	4	CNTLN
3.43681468508397E-59	0.496818166005119	0.401	0.228	5.75941404926371E-55	4	POLD2
5.81107264183402E-59	0.496626654403799	0.386	0.216	9.73819553318544E-55	4	EIF3H
1.18482231125771E-76	0.496196645056626	0.299	0.139	1.98552522920566E-72	4	NAA30
4.64705250101063E-69	0.495901435141745	0.366	0.19	7.78753058119362E-65	4	C8orf33
1.59190271044028E-71	0.49583367214802	0.35	0.176	2.66771056215583E-67	4	SNX24
1.71792930693992E-59	0.494976416636332	0.404	0.227	2.87890593256991E-55	4	CCT3
3.37049519099697E-76	0.494911356759685	0.32	0.152	5.64827584107273E-72	4	CADPS2
7.60204605637576E-58	0.494864607639169	0.265	0.13	1.27395087812745E-53	4	ATG4A
4.08773394996089E-74	0.494700914676463	0.637	0.401	6.85022455334446E-70	4	RER1
3.11941005390729E-60	0.494656612722674	0.345	0.184	5.22750736833783E-56	4	CASC4
2.03444238374203E-75	0.494368875012981	0.316	0.15	3.40931854667489E-71	4	MED1
5.30078315138943E-89	0.494323267443983	0.764	0.507	8.88305240509841E-85	4	HNRNPF
7.3637886223332E-75	0.49423138598201	0.409	0.217	1.2340236973306E-70	4	FAF1
5.2126123668497E-55	0.494226803188318	0.275	0.14	8.73529580436673E-51	4	ZFX
9.83150750388366E-43	0.493830506881935	0.252	0.135	1.64756402750082E-38	4	TINAG
1.33605624852951E-55	0.493790258375941	0.264	0.132	2.23896306128576E-51	4	FBXO4
5.41419163278538E-73	0.492856569743105	0.304	0.145	9.07310233822173E-69	4	BOD1
1.05976656078054E-104	0.492682091940091	0.26	0.1	1.77595680255603E-100	4	BICDL1
1.37654143329673E-91	0.492590834870588	0.44	0.226	2.30680813391867E-87	4	MAEA
7.31450043314436E-68	0.492434110042195	0.52	0.31	1.22576398258633E-63	4	NEDD8
2.6357258850911E-96	0.491546008731172	0.406	0.197	4.41694943823567E-92	4	NELFB
2.28893996031925E-85	0.491305174700716	0.372	0.179	3.83580558550301E-81	4	MICU2
3.82666575652328E-107	0.49047882325357	0.298	0.12	6.41272647478172E-103	4	FBXO34
2.52393841563271E-86	0.490406243553802	0.34	0.159	4.22961599691729E-82	4	C2CD5
1.39999843030476E-65	0.490379981375148	0.495	0.292	2.34611736950471E-61	4	ELOVL1
8.30876034377709E-97	0.490181213822961	0.693	0.43	1.39238205841017E-92	4	NFIC
1.71379026435366E-88	0.489777257599145	0.698	0.442	2.87196972500387E-84	4	OCLN

4.7397736228153E-87	0.489548952147463	0.292	0.127	7.94291263711388E-83	4	CPSF3
9.03863814777093E-62	0.489404110230349	0.359	0.192	1.51469498080345E-57	4	ELP5
7.44487136928589E-49	0.48896924489319	0.298	0.161	1.24761154406493E-44	4	LYPLA1
2.0282229919387E-68	0.488899673675792	0.365	0.192	3.39889608989087E-64	4	C6orf89
4.62268287010034E-59	0.488609624104706	0.393	0.22	7.74669195371415E-55	4	AFG3L2
1.16479416795494E-63	0.48845310270951	0.286	0.14	1.95196206665889E-59	4	DYNC1H1
5.90036871858592E-45	0.488130470460199	0.298	0.167	9.88783789860629E-41	4	FANCL
1.40734553461691E-80	0.487993917119257	0.781	0.542	2.35842964691102E-76	4	ACTG1
6.51675563877384E-69	0.487756413598176	0.453	0.254	1.09207790994572E-64	4	DDAH1
3.86829239165395E-74	0.487698185791966	0.511	0.295	6.4824843899337E-70	4	PSMD1
2.46986664992318E-101	0.487630076611352	0.788	0.484	4.13900253194127E-97	4	SSR4
4.97291596737452E-54	0.48752840966931	0.271	0.137	8.33361257812622E-50	4	PTGR2
2.76625285588027E-59	0.487088594080758	0.449	0.264	4.63568653588415E-55	4	EEF2K
8.59057751926235E-55	0.486758370833048	0.27	0.136	1.43960898067798E-50	4	NDUFB3
1.18752437823269E-61	0.486656569496482	0.399	0.221	1.99005335304234E-57	4	LANCL1
3.55565780009924E-89	0.486383727829816	0.606	0.354	5.9585713414063E-85	4	MAP2K2
4.14219095347597E-75	0.486324795353789	0.417	0.221	6.94148359983503E-71	4	IFT27
3.81950150519719E-78	0.485743301348798	0.714	0.466	6.40072062240944E-74	4	NDUFC2
5.79965922545933E-67	0.485610538681599	0.352	0.182	9.71906893002474E-63	4	C8orf76
6.5586520295316E-78	0.485446207342491	0.336	0.162	1.0990989071089E-73	4	RALBP1
1.53299286885224E-77	0.48527055635044	0.547	0.319	2.56898944962259E-73	4	TXNL4A
1.00589640724237E-61	0.485185798031683	0.37	0.199	1.68568119925676E-57	4	ATP5PF
1.63009661584211E-68	0.485125249554479	0.301	0.147	2.73171590882821E-64	4	PKD2
4.99519117184045E-69	0.485103209627202	0.564	0.339	8.37094136577023E-65	4	CLDN3
4.67859638678973E-60	0.485064286715288	0.27	0.132	7.84039182498224E-56	4	TBC1D13
8.92685882802722E-82	0.484799205910192	0.652	0.396	1.4959630024008E-77	4	ZHX1
4.97365097320651E-83	0.484379914042668	0.469	0.255	8.33484430089947E-79	4	SNX5
2.29342093002507E-70	0.483848471196785	0.563	0.34	3.84331479453602E-66	4	PMVK
1.57369420458672E-93	0.48352723306487	0.479	0.257	2.63719674804643E-89	4	TPP2
6.83739778387175E-82	0.483313117363128	0.594	0.357	1.14581112062123E-77	4	EFR3A
1.14660790276622E-60	0.482302078113284	0.286	0.142	1.92148552345564E-56	4	PPP1R13B
9.26432357534425E-56	0.48229454380159	0.375	0.211	1.55251534475619E-51	4	PYCARD
3.97026153649126E-82	0.481841508203127	0.693	0.439	6.65336428285205E-78	4	ENO1
3.09912263288122E-71	0.481831416933171	0.41	0.221	5.19350970818235E-67	4	AKAP8
6.54018613654275E-92	0.48154441514786	0.729	0.462	1.09600439276183E-87	4	CNN3
4.07802460313061E-69	0.481522787322459	0.436	0.241	6.83395362992628E-65	4	TMEM214
1.563080114126E-65	0.480887264660378	0.252	0.115	2.61940965525234E-61	4	SNW1
1.70027402824365E-77	0.480129307088297	0.531	0.309	2.84931921653071E-73	4	INTS8
9.61670552445205E-64	0.48001304827438	0.343	0.179	1.61156751178767E-59	4	PPP1R16A
1.4106859493075E-51	0.479857997486981	0.288	0.152	2.36402751384951E-47	4	NRAS
5.03749181138144E-82	0.479752804348794	0.323	0.151	8.44182877751302E-78	4	DHX8
2.12509455843143E-92	0.479176002429994	0.651	0.39	3.56123346101939E-88	4	SLC25A39
8.20766638304884E-83	0.478560350531752	0.398	0.205	1.37544073247133E-78	4	S100PBP
5.38223571441741E-68	0.47815508809487	0.388	0.202	9.0195506102207E-64	4	TMC5
9.85322916620927E-88	0.478078258296182	0.421	0.213	1.65120414367335E-83	4	NUDT21
5.41972392461443E-82	0.477634812837652	0.297	0.133	9.08237335286886E-78	4	COG4
6.88497868636067E-70	0.477123355815308	0.43	0.235	1.15378472826032E-65	4	C7orf50
9.40596096237514E-74	0.476849012159157	0.279	0.128	1.57625093807483E-69	4	TAF9
5.11190748252541E-77	0.476742175818838	0.677	0.431	8.56653455921609E-73	4	NDUFB9
3.74395696101733E-57	0.47628184112022	0.268	0.133	6.27412307527284E-53	4	IPP
2.95977791507568E-86	0.475758131340268	0.584	0.334	4.95999583008382E-82	4	SPSB1
1.64978509219103E-56	0.475222833814869	0.391	0.224	2.76470985749372E-52	4	DERA
1.80016634104836E-80	0.474646328894094	0.337	0.161	3.01671875432885E-76	4	ZNF75A
2.89445811810822E-90	0.474612135882623	0.425	0.213	4.85053291432576E-86	4	ZC3H4
3.53509735026274E-65	0.4745555649286	0.266	0.126	5.9241161395703E-61	4	GPC1
1.08243641340398E-72	0.47375706449523	0.527	0.307	1.81394694158239E-68	4	PEX2
9.30486152846955E-57	0.473621190686186	0.473	0.283	1.55930869494093E-52	4	DNPH1
2.49101098733213E-44	0.472772988914981	0.289	0.16	4.17443621257119E-40	4	VPS72
6.14520222211218E-73	0.472699223736927	0.469	0.262	1.02981298838156E-68	4	EIF4E2
1.07094193485467E-45	0.472377343174209	0.422	0.268	1.79468449442946E-41	4	MCM5

1.09772537549886E-61	0.47236071233351	0.262	0.125	1.83956818426099E-57	4	TAF12
1.90558254077739E-70	0.471984411712148	0.271	0.124	3.19337522183475E-66	4	PCBP2
1.17855378032393E-66	0.471517418336375	0.339	0.173	1.97502042506685E-62	4	HNF1A
6.76442914295901E-71	0.471454806920374	0.28	0.131	1.13358303577707E-66	4	PPP1R37
6.34679153908185E-81	0.471295346276184	0.761	0.521	1.06359532611934E-76	4	TM9SF2
5.58040831250429E-102	0.471137724780983	0.88	0.611	9.35164825009469E-98	4	LAMP2
6.7407259541588E-77	0.470940246340642	0.677	0.423	1.12961085539793E-72	4	DHRS7
1.56960777759279E-62	0.470552142226538	0.359	0.192	2.63034871369E-58	4	CHCHD7
1.55363800374745E-64	0.470536504522752	0.452	0.258	2.60358656667997E-60	4	EMC3
7.25611181735926E-81	0.470521710894166	0.716	0.481	1.21597921835307E-76	4	IDH2
5.11994457913295E-76	0.470165880474151	0.42	0.223	8.580003125711E-72	4	RAB3IP
2.7739294998412E-82	0.470098228147555	0.25	0.105	4.64855105583389E-78	4	STK35
3.06714977252174E-62	0.469978466692926	0.312	0.16	5.13992958879192E-58	4	NIPA1
9.17146459764968E-96	0.469675760164016	0.459	0.234	1.53695403727413E-91	4	SGTA
6.78005643211684E-71	0.469018081542378	0.573	0.349	1.13620185689414E-66	4	DBI
7.49744153178288E-58	0.469015930443619	0.26	0.127	1.25642125189618E-53	4	TARS2
1.4831982168671E-85	0.468775147918343	0.695	0.437	2.48554357182588E-81	4	ELL2
5.26469010562349E-42	0.468712782912089	0.275	0.151	8.82256767900385E-38	4	EDARADD
2.23640000603021E-103	0.468658291376034	0.493	0.257	3.74775913010543E-99	4	FBXL3
4.28798907595664E-58	0.468579691187815	0.283	0.143	7.18581209348813E-54	4	NOSIP
2.7758549010813E-86	0.468519320868508	0.514	0.283	4.65177764323205E-82	4	CD2AP
8.53509729689722E-75	0.468224433737308	0.478	0.263	1.43031160501404E-70	4	CAPN5
5.92017121046892E-73	0.467865923291779	0.452	0.249	9.92102291450382E-69	4	PIK3R2
1.99357482193249E-73	0.46776138784069	0.495	0.281	3.34083268659447E-69	4	NDUFB11
1.94282171906057E-70	0.467639270578992	0.453	0.247	3.2557806368017E-66	4	LNX2
6.1377975409133E-134	0.467620368223996	0.347	0.136	1.02857211190625E-129	4	CRYBG1
1.90406474732203E-63	0.467473021331393	0.301	0.15	3.19083170356226E-59	4	CREB3
1.59186220298358E-76	0.467326546637621	0.618	0.373	2.66764267975989E-72	4	TCEAL4
1.18991892571524E-77	0.467096153294547	0.433	0.23	1.9940661357136E-73	4	TRIM24
1.34417475330847E-72	0.466918969703835	0.384	0.201	2.25256805159434E-68	4	STXBP5
3.19036534961914E-87	0.466879559866503	0.524	0.299	5.34641425289175E-83	4	PAXX
4.2585235942826E-50	0.466782275223409	0.26	0.133	7.13643383929879E-46	4	ENDOV
4.79132947608301E-95	0.465928909026438	0.761	0.479	8.02930993601991E-91	4	CAPN2
1.49239236621376E-71	0.465639684529961	0.724	0.483	2.50095112730102E-67	4	DECR1
2.51227868769117E-72	0.465569360420683	0.437	0.235	4.21007662483287E-68	4	TOB2
7.31581375881071E-59	0.465012360707236	0.375	0.205	1.2259840697015E-54	4	WDYHV1
3.59424521349289E-127	0.465000683782742	0.45	0.207	6.02323612877139E-123	4	CKLF
1.5117516373046E-62	0.464907045273971	0.311	0.158	2.53339339379504E-58	4	SMC5
1.5474032903902E-89	0.464843292978228	0.563	0.321	2.5931384340359E-85	4	ENSA
2.04967727353478E-67	0.464746583144667	0.448	0.25	3.43484917498959E-63	4	POLR2I
1.81583522048206E-71	0.464361834358234	0.383	0.2	3.04297666248383E-67	4	NSF
3.07313026806286E-71	0.464220015683105	0.436	0.236	5.14995170321974E-67	4	SI
2.88810666004532E-71	0.464005944963671	0.475	0.269	4.83988914090395E-67	4	ZFAND5
2.43695386772187E-81	0.463978673949933	0.441	0.232	4.08384729152831E-77	4	FLNB
5.07006924511533E-95	0.463729721648132	0.398	0.192	8.49642204096427E-91	4	DUOX2
3.36167773848239E-62	0.463529412557746	0.435	0.245	5.6334995541488E-58	4	PSMB2
1.11378199965664E-84	0.463301749776453	0.479	0.257	1.86647587502459E-80	4	MARC1
7.1880607616101E-91	0.463157346699884	0.75	0.481	1.20457522243062E-86	4	GOLM1
1.2580651629881E-76	0.463035840573132	0.277	0.124	2.10826560013546E-72	4	SAP130
2.39098436778388E-66	0.462536608177771	0.367	0.193	4.00681160353223E-62	4	KCTD5
5.27581462146009E-61	0.462281347022313	0.345	0.182	8.84121014264283E-57	4	COX7B
1.221879371358E-77	0.462197074598121	0.496	0.277	2.04762545052173E-73	4	RPN2
7.87890873806593E-79	0.461894179955246	0.59	0.352	1.32034752632509E-74	4	AKT1
1.44658527920412E-50	0.461799792057008	0.313	0.17	2.42418761089027E-46	4	MTERF3
9.93635856566666E-69	0.461635431531147	0.314	0.156	1.66513496843442E-64	4	RUVBL1
4.11626680510766E-49	0.461508716975362	0.283	0.151	6.89803991199941E-45	4	SNRPE
2.37248627933076E-56	0.461342341033101	0.25	0.121	3.97581250690249E-52	4	AP5S1
9.19689059096694E-70	0.461147247533063	0.25	0.112	1.54121492523424E-65	4	THAP12
1.76990044053662E-65	0.461120613571753	0.402	0.22	2.96599915825127E-61	4	VPS26C
4.31188635549286E-91	0.460887879294796	0.517	0.283	7.22585915453493E-87	4	DYNLT1

5.8244663322966E-66	0.460307041719575	0.268	0.126	9.76064067966265E-62	4	ZNF672
2.94290002038633E-52	0.460291130892475	0.28	0.146	4.9317118541634E-48	4	ATP6V1D
1.30499890229561E-73	0.459969151298279	0.425	0.228	2.18691716046698E-69	4	CFDP1
1.54371610865744E-49	0.459493470408308	0.271	0.141	2.58695945488813E-45	4	TBCE
6.10344182552734E-129	0.459429433843102	0.91	0.556	1.02281478112187E-124	4	NQO1
8.50165323948834E-79	0.459391347087545	0.318	0.15	1.42470704987346E-74	4	FAM76B
2.69372032289235E-49	0.459380983568167	0.253	0.129	4.51413651710299E-45	4	THSD4
8.14358672220008E-75	0.459316374670003	0.595	0.358	1.36470226290629E-70	4	CAPN1
1.08679876569269E-78	0.459203430711548	0.93	0.732	1.82125737154781E-74	4	MT-ND4L
1.18504133759837E-79	0.459142944168396	0.379	0.191	1.98589227354734E-75	4	KLHL2
1.24233432969187E-64	0.459059884139544	0.467	0.268	2.08190386969763E-60	4	ACADS
4.01479529213855E-57	0.458918065247204	0.345	0.187	6.72799395056579E-53	4	COMMD6
1.50908207446132E-64	0.458687680855119	0.427	0.239	2.52891974038228E-60	4	CLTA
1.08658208207017E-77	0.458685406032139	0.654	0.422	1.8208942531332E-73	4	HADHA
6.86209411798764E-77	0.458655791207773	0.66	0.414	1.14994973229237E-72	4	ZBTB7B
2.27640883193513E-46	0.458277710533807	0.271	0.144	3.8148059205569E-42	4	KLHL23
5.85528249798247E-61	0.45816927346946	0.382	0.207	9.81228241011903E-57	4	NPAS2
2.00638379993227E-71	0.457798865286049	0.266	0.12	3.3622979719265E-67	4	PIGQ
2.60733425383671E-71	0.457444230267667	0.319	0.156	4.36937074257956E-67	4	CYTH3
1.1375681139815E-55	0.457402320573356	0.25	0.122	1.90633664541019E-51	4	ZNF398
1.166899079486E-37	0.457377259600849	0.251	0.139	1.95548947740264E-33	4	C6orf132
2.65604135050988E-92	0.457222049385897	0.654	0.412	4.45099409518446E-88	4	HNRNPU
2.20083084714276E-103	0.4569848064152	0.25	0.093	3.68815233364183E-99	4	ZBTB5
6.73123745576915E-55	0.456484379607125	0.266	0.132	1.12802077283779E-50	4	SOX13
1.58241201362971E-79	0.45590128206375	0.676	0.435	2.65180605244066E-75	4	ALG13
2.65966266023646E-75	0.455772623760101	0.448	0.245	4.45706268602426E-71	4	RAC1
2.52007583229149E-109	0.455712513588093	0.531	0.278	4.22314307975408E-105	4	MSI2
1.26308322013059E-76	0.455477682092804	0.412	0.217	2.11667486029485E-72	4	SCAF4
2.47724286500581E-69	0.454205618096027	0.422	0.231	4.15136359317673E-65	4	RFC1
4.71272230822021E-74	0.454152010342639	0.638	0.407	7.89758004411543E-70	4	SCARB2
2.31769797896279E-50	0.453728945163011	0.34	0.19	3.88399827314584E-46	4	PGK 1.00
1.71266115611144E-54	0.452963594558825	0.281	0.145	2.87007756541155E-50	4	HSBP1
8.54925375737208E-90	0.452481224667598	0.421	0.213	1.43268394466021E-85	4	WDTC1
1.39477102533109E-84	0.452274277782327	0.977	0.819	2.33735728424984E-80	4	MT-ND4
8.66495031405591E-115	0.451735443115105	0.67	0.367	1.45207237362949E-110	4	SLC6A8
2.9471168853149E-75	0.45117305857411	0.271	0.121	4.9387784764107E-71	4	RBBP5
1.2497028397411E-80	0.451068175600439	0.624	0.365	2.09425201883814E-76	4	AMOTL1
1.18518128690478E-71	0.451026377775788	0.371	0.191	1.98612680059503E-67	4	EFTUD2
7.35271809021034E-74	0.450904069839706	0.68	0.446	1.23216849755745E-69	4	MTDH
1.52411598932366E-76	0.450568919530563	0.477	0.27	2.55411357490859E-72	4	RBM26
2.78139197829642E-87	0.450539787081143	0.452	0.236	4.66105667722913E-83	4	SENP2
8.7536405502291E-70	0.450384198607885	0.432	0.236	1.46693508340739E-65	4	FAM13A
2.10490319184182E-77	0.450290423863268	0.587	0.346	3.52739676888852E-73	4	DCAF11
5.60404757563398E-76	0.450275166488933	0.439	0.238	9.39126292724743E-72	4	BMI1
2.54576291398669E-72	0.449652280478033	0.344	0.173	4.2661894912589E-68	4	INTS7
8.91694009711849E-74	0.449178199286087	0.346	0.172	1.49430082147512E-69	4	ZNF687
9.37122578558583E-63	0.449165582512307	0.317	0.161	1.57043001714847E-58	4	RPRD1B
7.16898859663217E-64	0.449067632319691	0.413	0.228	1.20137910902362E-59	4	TIMMDC1
2.65688585071047E-63	0.448992836736674	0.346	0.181	4.45240930862061E-59	4	CEBPZOS
8.07434064861472E-73	0.448887364047594	0.528	0.306	1.35309800589485E-68	4	NDUFS1
9.42327420605419E-50	0.448585270206078	0.258	0.132	1.57915229145056E-45	4	ABHD15
3.82203043631741E-64	0.448550948412298	0.51	0.303	6.40495860518072E-60	4	UBL3
1.38925395255046E-75	0.448289929163348	0.329	0.159	2.32811177368407E-71	4	ISG20L2
7.3038626122438E-64	0.448240822153764	0.343	0.178	1.22398129655982E-59	4	DNAH14
9.5485804278546E-79	0.448096028584984	0.299	0.136	1.60015110809987E-74	4	PIAS4
2.85102542010296E-66	0.448034462273004	0.413	0.228	4.77774839900855E-62	4	CDK4
3.98048817170802E-55	0.447993565540925	0.418	0.243	6.6705020781483E-51	4	LAPTM4A
4.36999080066187E-61	0.447814764197541	0.276	0.134	7.32323058374917E-57	4	MRGBP
9.50812341408631E-73	0.447792739096158	0.554	0.326	1.59337132173258E-68	4	SETD7
2.20710236174835E-54	0.447539054232298	0.294	0.152	3.69866213781788E-50	4	MBIP

2.74408419268587E-74	0.447167156708321	0.444	0.245	4.59853629010298E-70	4	EPRS
1.76885865839588E-66	0.446911390586314	0.278	0.132	2.96425333973982E-62	4	DVL2
1.65995433719906E-41	0.446857831671959	0.277	0.153	2.78175147827818E-37	4	SUSD4
1.66016479161194E-96	0.446778170546087	0.336	0.15	2.78210415778329E-92	4	KIF2A
1.38813371222074E-69	0.446662585044473	0.304	0.147	2.32623447493952E-65	4	ZDHHC6
5.79891560313217E-76	0.446587399622714	0.5	0.284	9.71782276772888E-72	4	PSMD12
2.26656793239763E-84	0.445950317829732	0.427	0.222	3.79831454111194E-80	4	SAMD4B
2.20755637538931E-98	0.445859623340774	0.379	0.176	3.69942297387741E-94	4	PHKG2
7.86962798763789E-54	0.445686882387241	0.315	0.168	1.31879225816836E-49	4	TMEM230
7.13931657001024E-56	0.445449413812209	0.319	0.169	1.19640667080232E-51	4	DEPRECATED-ENSG00000147123
8.70614195820124E-91	0.445049465884396	0.512	0.281	1.45897526935536E-86	4	PHF20L1
5.56577614264636E-69	0.444566894434929	0.589	0.367	9.32712765984677E-65	4	DEPRECATED-ENSG00000163463
5.27428813468436E-62	0.444318973014148	0.583	0.367	8.83865205610405E-58	4	DNAJC3
4.12522562943758E-70	0.444061657943425	0.455	0.252	6.91305310981149E-66	4	SMIM7
1.06554408539311E-85	0.443731308563016	0.461	0.245	1.78563877830178E-81	4	SNRPA
5.39504405402889E-70	0.443211933893639	0.694	0.451	9.04101482574162E-66	4	SLC37A4
1.72069997132902E-61	0.443172410220532	0.502	0.298	2.88354901195318E-57	4	CLTB
8.19869101201323E-71	0.443124061407632	0.574	0.345	1.37393663979318E-66	4	RAB5IF
1.15794697372122E-60	0.442836003929156	0.266	0.128	1.94048753856202E-56	4	FBXW11
1.782954133469E-67	0.442784863192656	0.354	0.183	2.98787453686735E-63	4	TCAIM
1.82912359698113E-61	0.442603180631798	0.283	0.14	3.06524532382098E-57	4	NOM1
1.0835863239145E-62	0.442550208802333	0.334	0.172	1.81587396161593E-58	4	CDC123
1.93722162414279E-72	0.442308239265232	0.325	0.16	3.24639599773849E-68	4	ACAP3
8.10526762559677E-61	0.442297490760916	0.313	0.161	1.35828074869751E-56	4	SAMD1
1.97821976784332E-54	0.442272034192003	0.375	0.211	3.31510068695184E-50	4	DEPRECATED-ENSG00000181061
5.97747492250226E-42	0.442058045845483	0.264	0.144	1.00170524751293E-37	4	USP2
3.50304711787723E-83	0.441896781366214	0.471	0.256	5.87040636013867E-79	4	AATF
4.08452410194841E-53	0.441771542600867	0.318	0.17	6.84484549004515E-49	4	TIGD2
3.45978541322566E-60	0.441612069115687	0.421	0.237	5.79790839548356E-56	4	YIPF4
2.6748351197932E-76	0.441456521014564	0.324	0.154	4.48248869374944E-72	4	SLC37A3
3.63018934809914E-84	0.441422991437455	0.426	0.22	6.08347130954454E-80	4	BTBD7
4.04811230831524E-75	0.440813741725907	0.447	0.24	6.78382660627467E-71	4	PDLIM5
8.20125226191966E-73	0.440600487819556	0.648	0.396	1.3743658540525E-68	4	CMBL
5.48781924361594E-53	0.440541050449021	0.25	0.124	9.1964874884516E-49	4	SWI5
1.40839357923156E-75	0.440102996148944	0.403	0.211	2.36018596007624E-71	4	LEMD2
1.79155625212369E-68	0.439426229317873	0.388	0.204	3.00228996730888E-64	4	ATP8B1
2.49875369253688E-63	0.439005985069956	0.401	0.22	4.1874114379533E-59	4	ATP6V1C1
4.90585764537274E-76	0.438996775094653	0.605	0.363	8.22123624211564E-72	4	ATP6V0D1
2.66045848563066E-53	0.438798466682597	0.254	0.126	4.45839633021985E-49	4	FGD6
3.6758208174101E-71	0.438540621531145	0.416	0.226	6.15994052581584E-67	4	H2AFJ
1.56324789352122E-81	0.438358567161921	0.406	0.208	2.61969081996287E-77	4	PLRG1
7.04644100773267E-82	0.438306763507643	0.568	0.342	1.18084258407584E-77	4	STT3B
1.29457449336136E-64	0.438036718777885	0.631	0.418	2.16944793597497E-60	4	ZBTB16
8.21113758801279E-64	0.4378598462296	0.272	0.13	1.37602243699918E-59	4	MCRIP1
1.73257337575672E-82	0.437528691877628	0.479	0.259	2.90344646309311E-78	4	PMM1
8.08177703316531E-68	0.437409343123168	0.549	0.332	1.35434419521784E-63	4	CRTAP
2.2571363853278E-52	0.437395308674513	0.258	0.129	3.78250915453233E-48	4	PTPRD
1.22344319992915E-73	0.437349917531354	0.296	0.138	2.05024611444128E-69	4	PIGN
2.26865250130354E-60	0.436990815978163	0.271	0.132	3.80180786168446E-56	4	MMD
2.55858422888418E-59	0.436977289408177	0.321	0.167	4.28767545076411E-55	4	MAGEH1
3.11306408004807E-48	0.436598521905798	0.267	0.139	5.21687278534456E-44	4	PDXDC1
3.3770549171628E-70	0.436578847109628	0.407	0.219	5.65926863018142E-66	4	PRCC
1.28873187458959E-75	0.43649687403288	0.417	0.219	2.15965687543723E-71	4	KCMF1
4.13458412912737E-74	0.43609446092068	0.443	0.24	6.92873608359165E-70	4	SCAF1
4.44562326791499E-71	0.435589272180508	0.345	0.174	7.44997547237194E-67	4	EXOSC2
7.03868246861863E-60	0.43535471501661	0.319	0.165	1.17954240809111E-55	4	CLCN3
9.94802290911517E-56	0.4353433953581	0.453	0.267	1.66708967910952E-51	4	CMTM8
3.92803095737382E-72	0.434419085126667	0.343	0.171	6.58259427836705E-68	4	PAGR1
1.53813661927499E-102	0.434074695095815	0.344	0.151	2.57760934658102E-98	4	ARID1A
2.71427803654638E-91	0.434067180734319	0.391	0.19	4.54858713364443E-87	4	LSM 8.00

9.98853138636291E-103	0.433609592252375	0.379	0.175	1.6738780897267E-98	4	SNTB2
6.71736841596784E-52	0.433584875677916	0.344	0.189	1.12569659914789E-47	4	TMLHE
2.5338099609172E-71	0.433298708588662	0.363	0.185	4.24615873250504E-67	4	EPS8
4.8684619369419E-77	0.433203343056695	0.523	0.3	8.15856851392725E-73	4	LONP1
1.85415950253667E-73	0.433192858466568	0.423	0.226	3.10720049435095E-69	4	CAND1
8.12266379487857E-65	0.432791029842884	0.611	0.389	1.36119599874575E-60	4	SF3B5
7.18234227503764E-55	0.43278400198468	0.365	0.201	1.20361691845081E-50	4	SGCE
9.0328423639806E-65	0.43253859661191	0.3	0.148	1.51372372335587E-60	4	TOR3A
2.44131665760022E-83	0.432501145455468	0.533	0.302	4.09115845480645E-79	4	SEL1L3
1.80141489653907E-68	0.432483416342819	0.666	0.44	3.01881108362017E-64	4	AKR1A1
2.97750387964656E-68	0.432289300985212	0.575	0.345	4.9897010015117E-64	4	SLC25A4
6.12220309057639E-54	0.432117575228113	0.272	0.139	1.02595879391879E-49	4	YIPF2
8.18937375478564E-89	0.431570093033131	0.493	0.267	1.37237525382698E-84	4	RNPS1
1.16312138937504E-67	0.431345196477588	0.608	0.374	1.94915882431469E-63	4	GRTP1
5.85833951599677E-80	0.43119573015674	0.362	0.177	9.81740536090739E-76	4	BORCS8
1.54068995489223E-66	0.430613904555344	0.391	0.212	2.5818882264084E-62	4	SPIDR
3.34643823992568E-67	0.430156571731481	0.481	0.276	5.60796120246745E-63	4	GNB1
4.12220336761895E-71	0.430054927361811	0.443	0.245	6.90798840345583E-67	4	TMX2
7.00892811868864E-66	0.429620207725861	0.483	0.28	1.17455617412984E-61	4	LRRC59
1.50545422459845E-86	0.429218330820548	0.274	0.116	2.52284018958208E-82	4	INIP
7.38166112036928E-59	0.429019917499254	0.449	0.26	1.23701877055148E-54	4	GIPC1
1.65504762615463E-64	0.428611674832924	0.612	0.378	2.77352881190993E-60	4	RAB4A
5.66059687132442E-64	0.428244667363358	0.337	0.174	9.48602823696546E-60	4	SNRNP25
8.05151468053775E-75	0.427359377710272	0.569	0.334	1.34927283016452E-70	4	TMPRSS2
6.72025891358759E-75	0.427295680622813	0.271	0.121	1.12618098873901E-70	4	EIF1AD
5.46155348669227E-86	0.427133970581992	0.475	0.253	9.1524713329989E-82	4	SDHA
1.34337463904405E-61	0.426635185465976	0.285	0.14	2.25122722011001E-57	4	MNAT1
1.56550470017364E-59	0.426631122738907	0.41	0.229	2.62347277655099E-55	4	SNF8
4.85917141370608E-64	0.426391071639035	0.266	0.126	8.14299945508865E-60	4	ZNF449
8.56424597934122E-45	0.426311748827228	0.297	0.164	1.435196341218E-40	4	ANAPC13
3.54811527657785E-62	0.426062475385251	0.418	0.235	5.94593158048916E-58	4	AP1S1
4.29993468069477E-64	0.425970794593342	0.748	0.516	7.2058305379083E-60	4	TMCO1
5.72371720393327E-64	0.425795099264746	0.378	0.202	9.59180529035137E-60	4	CHMP2B
5.68943570823352E-63	0.425755769303585	0.426	0.24	9.53435635985773E-59	4	TOMM70
7.87944242332985E-73	0.425667230026646	0.347	0.174	1.32043696130162E-68	4	GANAB
1.46511382885283E-69	0.425655221967891	0.536	0.31	2.45523775439158E-65	4	NSDHL
5.71771183724263E-75	0.425440263717498	0.465	0.259	9.5817414968512E-71	4	EGFR
3.01530355168722E-63	0.425285020193127	0.734	0.498	5.05304569191744E-59	4	ACAA2
1.50391577623508E-61	0.424774109063117	0.36	0.192	2.52026205781475E-57	4	MED16
2.21311923797364E-43	0.424652195684072	0.288	0.159	3.70874521899622E-39	4	LAMTOR3
7.82552883208708E-67	0.424475555496818	0.365	0.19	1.31140212168115E-62	4	UTP23
6.29153222689645E-60	0.424302418114114	0.334	0.175	1.05433497058331E-55	4	MTCH2
3.17197487453586E-81	0.424251450551215	0.355	0.172	5.3155954947472E-77	4	RAB11FIP4
1.10880868861677E-50	0.4241952283389	0.331	0.184	1.85814160038398E-46	4	C19orf53
2.8703534963932E-72	0.424147967315014	0.332	0.164	4.81013838925572E-68	4	RETREG2
5.48363573639665E-74	0.423808978302302	0.437	0.238	9.1894767670535E-70	4	WWOX
2.79117192399201E-62	0.423720031744584	0.445	0.254	4.6774459102258E-58	4	TCEAL1
7.68606016730187E-57	0.423561910923748	0.99	0.892	1.28802996283645E-52	4	MT-CO1
9.94721868786304E-56	0.423274696157264	0.305	0.159	1.66695490771209E-51	4	RBX1
3.30373247753385E-58	0.423171567619115	0.364	0.198	5.53639488585123E-54	4	EAPP
2.58658830963284E-57	0.422781804259857	0.26	0.126	4.33460468928271E-53	4	STAM2
7.15876978127796E-59	0.422589399071081	0.298	0.15	1.19966663994656E-54	4	PSMG4
2.32645283531831E-84	0.422558488602236	0.468	0.248	3.89866966142642E-80	4	USP14
1.03603053710073E-59	0.421986240572546	0.622	0.402	1.7361799740734E-55	4	ALDH9A1
8.99814549606322E-54	0.421792189060704	0.324	0.176	1.50790922223028E-49	4	TBL2
2.72641799293633E-82	0.421544088020017	0.501	0.277	4.5689312725627E-78	4	HNRNPM
1.25052712647016E-66	0.421330621133942	0.346	0.177	2.09563335853869E-62	4	VTI1B
6.17584672212419E-66	0.421087957660331	0.71	0.475	1.03494839369357E-61	4	MGST3
2.47968651956078E-50	0.42089437438634	0.357	0.203	4.15545866947995E-46	4	ATRAID
1.18756601651929E-46	0.420638039063211	0.283	0.152	1.99012313048302E-42	4	BTNL3

4.84241198675523E-51	0.420406968003139	0.254	0.128	8.11491400740441E-47	4	DPY19L3
9.90508743445994E-71	0.420367708702389	0.369	0.189	1.6598945522668E-66	4	TOR1B
5.89154376619012E-78	0.420089861852262	0.448	0.242	9.87304904338141E-74	4	UBP1
2.94560296697291E-88	0.419817383227721	0.556	0.319	4.93624145205321E-84	4	TBL1XR1
3.02776698004144E-79	0.419276063933368	0.401	0.207	5.07393190515344E-75	4	POLR2B
6.49096568953857E-64	0.419264564840397	0.558	0.34	1.08775603025287E-59	4	PSMD3
1.7773308512658E-61	0.418878651874827	0.324	0.166	2.97845104055122E-57	4	SLC30A5
4.09725129062211E-66	0.418321874978103	0.359	0.187	6.86617371282453E-62	4	TSR3
1.42948344524008E-64	0.418192033028568	0.464	0.267	2.39552835753333E-60	4	CDIPT
2.5998753479719E-69	0.418080062977463	0.518	0.302	4.35687110813131E-65	4	HIGD2A
5.79717850805085E-110	0.417698503827231	0.447	0.215	9.71491174379161E-106	4	POLR2A
2.96618933469766E-65	0.41757132369347	0.648	0.418	4.97074008708634E-61	4	GCLC
2.78218187246778E-78	0.416940256830161	0.533	0.304	4.66238038188151E-74	4	POLR1D
1.54695521996841E-75	0.416836820264358	0.517	0.297	2.59238755762306E-71	4	LRRFIP2
1.22162421141437E-89	0.416503333325818	0.417	0.21	2.0471978534882E-85	4	CNOT8
6.44267711244199E-63	0.416468693266147	0.354	0.186	1.07966383050303E-58	4	PIK3CB
2.71146061985598E-67	0.416416528297856	0.349	0.181	4.54386570675465E-63	4	EXOSC9
4.76775151929197E-86	0.416198204014048	0.491	0.267	7.98979799602949E-82	4	RB1CC1
4.9847109609383E-57	0.416105581225718	0.381	0.211	8.3533786283404E-53	4	APPL2
1.199155889574E-57	0.416012140552331	0.309	0.16	2.00954543974811E-53	4	SLC10A5
1.50215356100983E-65	0.415675310478032	0.387	0.208	2.51730893754027E-61	4	SLC37A1
7.33857401376876E-72	0.415298444532729	0.422	0.227	1.22979823322737E-67	4	PSMD7
3.06385995142696E-61	0.415281232408287	0.487	0.291	5.13441650660129E-57	4	ATP5F1B
2.00529898060774E-64	0.415273357064713	0.294	0.144	3.36048003170246E-60	4	U2AF2
5.19473064640336E-79	0.415120486114879	0.295	0.134	8.70532961724275E-75	4	CNOT6
5.10464501441319E-79	0.41511112968291	0.429	0.229	8.55436411515363E-75	4	NOP56
2.77493529250252E-71	0.414943645294943	0.657	0.412	4.65023656317572E-67	4	EGLN1
1.4219545218943E-72	0.414878301853366	0.481	0.269	2.38291138779047E-68	4	GDA
2.76993801270561E-73	0.414762619703892	0.639	0.373	4.64186212169206E-69	4	HNF4G
1.71199873475989E-77	0.414518017274437	0.426	0.225	2.86896747971062E-73	4	TAF2
6.4099451998343E-56	0.414473537257413	0.358	0.196	1.07417861658823E-51	4	KIF3B
1.1482568536024E-75	0.414450464558207	0.336	0.164	1.9242488352669E-71	4	CCNK
9.21311314700019E-51	0.414381961710541	0.334	0.186	1.54393350117429E-46	4	NDUFS8
1.94278721516281E-54	0.414208480833004	0.3	0.157	3.25572281516983E-50	4	SLC35B4
3.66255979108513E-63	0.413801108315883	0.415	0.231	6.13771769790045E-59	4	CCT8
4.14164542628053E-70	0.413697205169266	0.542	0.323	6.94056940536092E-66	4	DTX3L
1.48095334077933E-70	0.413525078378798	0.468	0.262	2.481781608478E-66	4	CARS2
7.05758422970439E-69	0.413378295273439	0.506	0.298	1.18270996521386E-64	4	CMTM6
3.75618577051061E-123	0.413092233812177	0.83	0.46	6.29461611422169E-119	4	MUC13
1.33003135702755E-66	0.412873530698397	0.523	0.308	2.22886654810676E-62	4	ECI1
7.29964074566E-85	0.412649715562269	0.352	0.167	1.2232737961577E-80	4	LSM14A
1.8062811109727E-56	0.412603635439707	0.445	0.258	3.02696588576805E-52	4	PYURF
1.65682917945781E-96	0.41232129218042	0.451	0.229	2.7765143389354E-92	4	STRN4
3.9208278877499E-65	0.412280401758579	0.504	0.299	6.57052337429129E-61	4	MAPK6
5.5304742453321E-77	0.412258407436135	0.434	0.234	9.26796874032753E-73	4	ATF4
6.8341814327862E-89	0.412151952612945	0.555	0.314	1.14527212450631E-84	4	SMARCA4
8.81295520825694E-48	0.411215827738599	0.259	0.133	1.4768750337997E-43	4	PIGC
7.8131070156931E-66	0.410563640307675	0.291	0.14	1.30932047368985E-61	4	NUBP2
3.15787834005079E-65	0.41032891278676	0.451	0.258	5.29197252225711E-61	4	TRIO
3.27185999443062E-93	0.410228779726734	0.403	0.197	5.48298297866683E-89	4	ADSS
1.28414450921665E-72	0.410000588063302	0.365	0.186	2.15196936854527E-68	4	VCP
1.2474374176702E-66	0.409773522408556	0.403	0.219	2.09045562453172E-62	4	C19orf25
2.60874816966852E-64	0.409619727554907	0.398	0.217	4.37174018273051E-60	4	MLH3
8.0030195926008E-70	0.409585490998306	0.265	0.121	1.34114602332804E-65	4	PLAA
1.82939977662598E-52	0.409531490543199	0.301	0.158	3.06570814566982E-48	4	POLB
4.90323837775823E-64	0.408588468877067	0.504	0.293	8.21684687344724E-60	4	RTL8C
3.13057164939847E-45	0.408575926994141	0.424	0.26	5.24621197006196E-41	4	TMEM141
1.98014012316194E-66	0.408405564612212	0.486	0.281	3.31831881839479E-62	4	RBM10
1.09248181612896E-38	0.40811911381394	0.256	0.141	1.83078102746891E-34	4	JAGN1
1.09414550234201E-80	0.40792520861043	0.459	0.247	1.83356903282474E-76	4	SH3GLB2

7.67336728896399E-47	0.40790580274159	0.33	0.185	1.28590289028459E-42	4	CBR4
8.76051005936613E-56	0.407872185194297	0.481	0.291	1.46808627574858E-51	4	NDUFAB1
2.8517617467361E-57	0.407871449393016	0.328	0.173	4.77898233518035E-53	4	USP53
2.12971437796932E-62	0.407455146565619	0.608	0.392	3.56897535460099E-58	4	TMEM33
2.26184253937598E-80	0.407235416779735	0.628	0.399	3.79039572748626E-76	4	EIF5
7.04932852012678E-62	0.40701433235116	0.409	0.228	1.18132647340285E-57	4	VAV2
1.49272074194487E-88	0.406882056885395	0.392	0.192	2.50150141935121E-84	4	HDAC6
3.53898685192629E-68	0.406537651025213	0.365	0.189	5.93063416645808E-64	4	SLC16A1
3.07031007427474E-80	0.406261081861608	0.342	0.164	5.14522562246961E-76	4	MIB1
4.34893498915736E-39	0.405316886294483	0.293	0.167	7.2879452548299E-35	4	CACNA1H
4.87173399378477E-76	0.405298875583781	0.529	0.306	8.16405182678452E-72	4	TAF7
2.39029201920692E-62	0.405172289284327	0.393	0.215	4.00565136578695E-58	4	CNOT11
2.03762704468237E-62	0.405018603027134	0.717	0.469	3.41465540147872E-58	4	ATP6AP1
4.23692061471136E-64	0.404735842753751	0.392	0.212	7.1002315661333E-60	4	COMMD3
7.72521247602333E-54	0.404482631130403	0.273	0.139	1.29459110673199E-49	4	MLST8
8.52168695995999E-77	0.404445271073569	0.511	0.292	1.4280643007501E-72	4	USP12
6.14116750700492E-71	0.40433007433459	0.437	0.238	1.02913685082388E-66	4	TTC1
1.01291026628918E-73	0.404309830833978	0.478	0.269	1.69743502424741E-69	4	CDC16
3.60471770874273E-67	0.404278244245925	0.671	0.443	6.04078593631106E-63	4	HINT1
1.03411014676064E-71	0.40426878408837	0.414	0.222	1.73296178394148E-67	4	AZIN1
8.67355682754309E-61	0.404132418263338	0.298	0.149	1.45351465315967E-56	4	DEPRECATED-ENSG00000167797
7.93757681306684E-61	0.404105875238655	0.386	0.213	1.33017912233374E-56	4	NBN
7.07345365132645E-63	0.404030041708717	0.388	0.21	1.18536936288929E-58	4	ZMYND11
1.61246294394926E-67	0.403919397163664	0.468	0.264	2.70216540147017E-63	4	SMYD2
9.91738759619789E-65	0.403878192021244	0.255	0.118	1.66195581337084E-60	4	ZNF131
2.04506449442136E-62	0.403683963991619	0.427	0.238	3.42711907975131E-58	4	NME1
7.48828054518524E-65	0.4035097681409	0.499	0.293	1.25488605376214E-60	4	LGR4
4.8084877218314E-69	0.403179614597388	0.664	0.437	8.05806372424505E-65	4	AFDN
1.88256098678909E-67	0.403071586114216	0.717	0.473	3.15479570166115E-63	4	PRKACA
2.66772029179417E-52	0.402908529315998	0.278	0.144	4.47056566498866E-48	4	TMEM251
4.19618036884736E-96	0.402627591160274	0.567	0.324	7.0319590621144E-92	4	EIF4A3
1.09369126941077E-79	0.402572656309249	0.562	0.338	1.83280782927856E-75	4	DHX15
2.97528647306549E-72	0.402391113077872	0.342	0.171	4.98598507156314E-68	4	UFD1
9.11668589769121E-73	0.401874762152551	0.401	0.212	1.52777422273509E-68	4	TLE1
1.09350872199036E-96	0.40176514744288	0.4	0.191	1.83250191631144E-92	4	SMC3
6.50179393014486E-53	0.401639110108592	0.427	0.253	1.08957062681367E-48	4	STMP1
1.05357576322484E-73	0.401388055913682	0.513	0.293	1.76558226401219E-69	4	GMDS
2.54733019767919E-63	0.401167005121758	0.367	0.197	4.26881594527078E-59	4	MED29
1.68276427307162E-72	0.400974316264381	0.592	0.367	2.81997636881342E-68	4	HNRNPD
4.29276356518648E-71	0.400916008777928	0.382	0.199	7.1938131825395E-67	4	LARP4B
6.07797293416515E-60	0.400697487387067	0.369	0.2	1.0185467043074E-55	4	SNX27
9.9762900516056E-102	0.400619103552552	0.953	0.698	1.67182668684807E-97	4	PGRMC1
1.18000961571289E-67	0.400548960770688	0.587	0.351	1.97746011401165E-63	4	NHSL1
5.94239503795504E-64	0.400351246949406	0.46	0.264	9.95826560460506E-60	4	CAV2
2.98328934865992E-82	0.400099770036853	0.793	0.52	4.9993962904843E-78	4	DEPRECATED-ENSG00000198074
5.0466169631402E-64	0.399784039976975	0.517	0.302	8.45712070683035E-60	4	CYP4F2
3.17811104762637E-65	0.399747883063839	0.472	0.271	5.32587849361227E-61	4	FBXL17
9.29385618637877E-72	0.399674081687222	0.531	0.312	1.55746441971335E-67	4	KTN1
2.24126806654971E-80	0.399276528952666	0.5	0.277	3.755917025924E-76	4	UBE2Z
2.72534613013031E-64	0.399229980174611	0.3	0.147	4.56713504487238E-60	4	PHAX
4.65675128619782E-55	0.399020137630501	0.282	0.143	7.8037838054103E-51	4	TTLL12
2.89444782265651E-73	0.398916037066241	0.378	0.194	4.85051566120777E-69	4	C2orf68
3.49794138307638E-75	0.398819698769691	0.697	0.443	5.86185016975941E-71	4	UQCRQ
2.23986559133344E-62	0.398539168213908	0.422	0.234	3.75356675795658E-58	4	ZFYVE21
2.27510312487096E-47	0.398249374867778	0.365	0.212	3.81261781665876E-43	4	CPT2
2.33300907222067E-69	0.398182881531194	0.372	0.192	3.9096566032274E-65	4	WASHC5
1.2351794338545E-74	0.398120770781143	0.513	0.293	2.06991369525338E-70	4	AHCYL1
5.83026990469231E-59	0.398064861763471	0.401	0.227	9.77036630628338E-55	4	MSH6
6.13282494372682E-70	0.398004275542003	0.371	0.192	1.02773880406974E-65	4	SLC25A10
1.45659886382252E-72	0.397979966258618	0.452	0.251	2.44096837599378E-68	4	ASAP1

2.73088635562988E-68	0.397794621684339	0.362	0.188	4.57641935476456E-64	4	NCOA6
2.46264718607965E-48	0.397063263897552	0.365	0.21	4.12690415443228E-44	4	ATP5MF
6.89561929694729E-73	0.396513551176621	0.434	0.235	1.15556788178243E-68	4	CLPP
4.86383735222214E-54	0.396212241714191	0.432	0.258	8.15081863485386E-50	4	TLCD4
6.22288413251607E-59	0.396063517462284	0.347	0.185	1.04283092292704E-54	4	DEPRECATED-ENSG00000134049
1.11248776108484E-80	0.396031376474763	0.43	0.222	1.86430699002597E-76	4	ABHD12
1.63193966326648E-71	0.39588460435736	0.546	0.32	2.73480448770196E-67	4	DGAT1
1.05066446084084E-84	0.395829970641388	0.537	0.308	1.76070350347708E-80	4	PHF10
1.28036756678651E-62	0.395772486559005	0.405	0.225	2.14563996842083E-58	4	EIF2S1
1.30432939481352E-68	0.395283101628332	0.379	0.199	2.1857951998285E-64	4	MBD6
1.13896675559638E-68	0.395151541908669	0.367	0.192	1.90868048902842E-64	4	FBXO21
5.17463707141221E-66	0.395050528389402	0.32	0.161	8.67165680427258E-62	4	TIMM17B
3.28692907913888E-53	0.394781100098336	0.349	0.191	5.50823575082094E-49	4	BCAT2
5.18499596560115E-57	0.394685213953362	0.462	0.271	8.6890162391544E-53	4	HEBP1
2.47416240294066E-81	0.39464953939719	0.269	0.116	4.14620135484796E-77	4	TTC39B
1.11849738274182E-104	0.394533572225583	0.439	0.213	1.87437791399874E-100	4	MTA2
1.2622184311501E-69	0.394431972640423	0.42	0.228	2.11522564692133E-65	4	MLLT1
5.39696499011549E-59	0.394389265405877	0.378	0.209	9.04423393043554E-55	4	FBL
4.10098357004536E-59	0.394294790126636	0.326	0.17	6.87242826668202E-55	4	MANBAL
1.34386314209102E-60	0.394156882542057	0.379	0.207	2.25204585351613E-56	4	FAM168B
3.33866226112401E-58	0.393880112506634	0.306	0.158	5.59493021719162E-54	4	LEO1
4.05837987287949E-81	0.393879063099587	0.471	0.255	6.80103299097145E-77	4	VAPA
3.34439455483072E-53	0.393806137049415	0.263	0.133	5.60453639498532E-49	4	CAD
1.71094547661594E-64	0.393762539039525	0.323	0.163	2.86720242971299E-60	4	RAB28
4.61795594060013E-76	0.393544142744862	0.49	0.274	7.73877056525769E-72	4	MBOAT7
3.8853564790744E-51	0.393366211650622	0.312	0.168	6.51108038763288E-47	4	SNRPB2
4.35909709334222E-67	0.393317419472477	0.562	0.331	7.3049749090229E-63	4	KIAA1522
2.6541847208521E-50	0.3931473478698	0.356	0.2	4.44788275520395E-46	4	PPDPF
3.21051879415636E-61	0.393047108284381	0.685	0.468	5.38018739524723E-57	4	SUMF2
3.92980141191877E-63	0.392905576220764	0.427	0.241	6.58556120609348E-59	4	FBXO42
1.00311274097509E-59	0.392717356105863	0.32	0.165	1.68101633132606E-55	4	SMARCE1
4.32558552009385E-55	0.392077197645384	0.427	0.247	7.24881621457327E-51	4	ZNF664
3.68795747101615E-91	0.392013665778283	0.573	0.333	6.18027912992886E-87	4	BAX
8.31165195110656E-49	0.391943351814826	0.265	0.138	1.39286663396644E-44	4	UQCRH
1.0791253470395E-64	0.391382415278158	0.431	0.239	1.80839825656879E-60	4	CAMK2D
2.04789398284005E-57	0.391214096038173	0.291	0.148	3.43186073644335E-53	4	GLYR1
3.16837596009934E-63	0.391199633623089	0.374	0.199	5.30956443393447E-59	4	PGAP2
3.69880936622536E-55	0.391033592446679	0.309	0.162	6.19846473592045E-51	4	SMAD1
7.38297468419746E-79	0.390345357702665	0.385	0.196	1.23723889757781E-74	4	CHIC2
3.89159075091326E-71	0.39029149205065	0.285	0.133	6.52152778038044E-67	4	KIF21A
1.4614430320232E-57	0.390198431803266	0.539	0.334	2.44908623306448E-53	4	DCAF6
6.91258808921536E-66	0.389952887655428	0.56	0.333	1.15841151199071E-61	4	NR1I2
3.84055616717538E-98	0.389754952254395	0.607	0.355	6.43600402495251E-94	4	ARF6
6.27435835130297E-58	0.38963818640089	0.388	0.216	1.05145697251135E-53	4	AGTRAP
3.80360237308553E-78	0.389566845847937	0.571	0.323	6.37407685681674E-74	4	MATN2
3.61063087666717E-35	0.389273064170659	0.337	0.207	6.05069522311884E-31	4	MANEAL
1.22340388778045E-63	0.389069324842377	0.385	0.206	2.05018023514249E-59	4	CEP170B
3.14500666198242E-71	0.388565155120657	0.525	0.308	5.27040216415014E-67	4	DLG1
1.73048189248536E-69	0.388554693032218	0.429	0.234	2.89994155542697E-65	4	KIF1C
3.97042934013819E-75	0.388363747066402	0.594	0.353	6.65364548820358E-71	4	RAP2C
3.03067739188182E-60	0.388050089175592	0.339	0.178	5.07880917331555E-56	4	KIAA1191
2.60487235289598E-77	0.387988908765021	0.52	0.299	4.36524508898309E-73	4	CYB5B
6.32726174930464E-51	0.387838549716815	0.297	0.157	1.06032252394847E-46	4	RDH14
3.22212615748025E-64	0.387746287108927	0.448	0.255	5.39963901470541E-60	4	SIPA1L3
3.75595962351853E-46	0.387682281084573	0.306	0.17	6.29423713709236E-42	4	CXorf40A
1.12284652539804E-60	0.387670434316773	0.486	0.29	1.88166620726203E-56	4	SAMD5
4.48322454989823E-45	0.387371086735822	0.275	0.149	7.51298770071945E-41	4	PEF1
7.31848233153085E-76	0.387345910044227	0.347	0.171	1.22643126911794E-71	4	WAC
5.26989769619163E-77	0.387216880231255	0.566	0.335	8.83129455927793E-73	4	SDCBP
1.23810481922142E-49	0.386805599875323	0.312	0.169	2.07481605605125E-45	4	GON7

2.60099711460867E-87	0.386785693794756	0.604	0.355	4.35875096466121E-83	4	CDKN1A
8.65611836679782E-71	0.386675153114395	0.32	0.157	1.45059231590798E-66	4	MARCH8
2.95404720009649E-49	0.386593218367973	0.307	0.166	4.9503922979217E-45	4	MYO5B
2.41904949740385E-66	0.386583140907853	0.387	0.208	4.05384314774937E-62	4	RPRD1A
2.2052865259139E-89	0.386478078890402	0.46	0.242	3.69561916012652E-85	4	RBM14
3.27602365462205E-76	0.38608740776502	0.3	0.14	5.48996044041563E-72	4	H3F3B
7.01573900932739E-68	0.385918194956281	0.424	0.232	1.17569754318308E-63	4	DEPRECATED-ENSG00000137575
9.92534822210557E-76	0.3858942455178	0.418	0.221	1.66328985506045E-71	4	PAFAH1B2
1.40297353622724E-58	0.38582477354892	0.47	0.278	2.35110305200961E-54	4	DNASE2
1.76340398686836E-53	0.385770267665878	0.375	0.21	2.955112401194E-49	4	BTD
5.65165149158267E-71	0.385512935682528	0.409	0.221	9.47103756959424E-67	4	SMC6
2.32688672211757E-75	0.385438330384147	0.405	0.213	3.89939676892463E-71	4	RRP1B
1.14839294916779E-70	0.385406369548247	0.345	0.173	1.92447690421538E-66	4	OTUD7B
5.85065018813583E-59	0.38522191335593	0.372	0.203	9.80451958527802E-55	4	MAPK3
8.75765187956253E-66	0.385003518113426	0.427	0.235	1.46760730197709E-61	4	SKAP2
9.87294247590693E-67	0.384781649045762	0.305	0.149	1.65450770011248E-62	4	DPAGT1
7.57562277839896E-62	0.384227943469144	0.319	0.163	1.2695228652041E-57	4	COPS4
4.51166437422927E-66	0.38412073562523	0.35	0.18	7.56064715833342E-62	4	UPF3A
1.12633681031841E-87	0.384115081647276	0.417	0.209	1.8875152267316E-83	4	TNPO3
2.09741275732869E-66	0.384095171848045	0.391	0.209	3.51484429873142E-62	4	CNIH1
7.91040475714241E-70	0.384092201354135	0.319	0.156	1.32562562920193E-65	4	PARD3
3.14449025742215E-54	0.384088594919313	0.449	0.269	5.26953677338803E-50	4	HSD17B2
3.29845168334084E-78	0.384061659528853	0.346	0.167	5.52754533094258E-74	4	NAGA
2.19415652875094E-65	0.383951209478135	0.554	0.332	3.67696751088083E-61	4	EMC2
3.20441140079159E-72	0.383929403109708	0.637	0.386	5.36995262544654E-68	4	PRG4
3.40049336252333E-74	0.383384099376825	0.251	0.109	5.6985467769166E-70	4	TTC9
1.58425630431432E-52	0.383261379093768	0.395	0.227	2.65489671476993E-48	4	DNASE1L1
1.76531575283371E-63	0.383107593000621	0.526	0.317	2.95831613859873E-59	4	SLC35F5
3.82134917549134E-52	0.382146982821361	0.358	0.199	6.40381694828839E-48	4	CPTP
1.93005250607309E-57	0.381932025739973	0.349	0.186	3.23438198967729E-53	4	ADGRA3
9.98676848198804E-57	0.381602487011853	0.466	0.276	1.67358266221156E-52	4	IL10RB
1.2818107965518E-66	0.381542764030563	0.459	0.258	2.14805853286151E-62	4	BTBD2
3.54596752174603E-50	0.381282075916225	0.342	0.189	5.94233237294199E-46	4	ADH5
9.10059060679488E-52	0.380850650252389	0.458	0.276	1.52507697388669E-47	4	C1orf43
3.30547118012135E-69	0.380798880411448	0.342	0.173	5.53930860364735E-65	4	CLIC4
1.72710598801068E-46	0.380791544035397	0.443	0.272	2.89428421470829E-42	4	FAM162A
5.95935489733356E-57	0.380762941328823	0.281	0.142	9.98668693695158E-53	4	PMS1
3.40524343954399E-54	0.380368308579838	0.469	0.281	5.70650695598782E-50	4	NAXE
3.10954829926335E-54	0.380299358372191	0.363	0.201	5.21098103990552E-50	4	COQ4
2.828672989865E-61	0.380003919721697	0.257	0.122	4.74029019641577E-57	4	PPIL4
3.09149690695035E-59	0.379983627315588	0.506	0.302	5.1807305166674E-55	4	CRLS1
1.43362932947582E-61	0.37978535186048	0.512	0.307	2.40247603033558E-57	4	PAPSS2
1.26627626825623E-71	0.379475503179023	0.591	0.369	2.12202577034379E-67	4	FAM107B
3.58091701991563E-48	0.379398818882832	0.328	0.182	6.00090074197462E-44	4	VPS25
1.0778107502602E-84	0.379130523596781	0.296	0.131	1.80619525528605E-80	4	CCDC82
2.01411094059931E-53	0.378960646558922	0.572	0.363	3.37524711425632E-49	4	CHMP2A
3.61333645351634E-67	0.378715519296948	0.492	0.279	6.05522922880269E-63	4	PCDH1
1.76381115685901E-43	0.378607331299571	0.285	0.158	2.95579473666433E-39	4	NUDT2
3.33664102010543E-55	0.378598042678228	0.282	0.144	5.59154302149268E-51	4	SLC41A3
2.04882368643181E-60	0.378200126407494	0.399	0.221	3.43341873372242E-56	4	NHP2
3.49809602955286E-92	0.378007991396513	0.991	0.801	5.86210932632469E-88	4	CES1
4.17271899983123E-77	0.377723023979958	0.423	0.225	6.99264249991718E-73	4	SET
1.91450430976327E-65	0.377564903053499	0.7	0.458	3.20832632230128E-61	4	SERINC5
8.0174887154477E-64	0.376566420993198	0.462	0.265	1.34357075893473E-59	4	CLPTM1L
6.95559882167444E-57	0.376475297018588	0.342	0.184	1.1656192505362E-52	4	MESD
6.14524717337514E-64	0.376416219335351	0.469	0.271	1.02982052131421E-59	4	DDB1
3.47627210226788E-56	0.376230130899166	0.384	0.214	5.82553678898051E-52	4	TMEM70
4.34596570243421E-63	0.376127147357223	0.656	0.419	7.28296932413924E-59	4	NFIB
2.04961909862414E-63	0.376065686623259	0.591	0.382	3.43475168547433E-59	4	STAU2
3.60081164438628E-58	0.375829732981956	0.335	0.177	6.03424015366252E-54	4	ZNF7

2.32747572595255E-62	0.375786018638479	0.45	0.257	3.90038382155129E-58	4	ZNF410
6.30371944679492E-56	0.375760310990284	0.373	0.208	1.05637730489389E-51	4	PSMC6
3.35960583447978E-57	0.375545510707493	0.452	0.267	5.63002745742122E-53	4	YWHAE
3.24065876350728E-52	0.37478602227038	0.261	0.132	5.43069595588549E-48	4	TRAPPC11
4.74282560837113E-122	0.373497924320753	0.403	0.178	7.94802715450834E-118	4	SLC44A2
1.671009327659E-53	0.373433324452871	0.77	0.544	2.80027743129095E-49	4	MT-ND1
7.96829772992635E-66	0.373203889669778	0.368	0.195	1.33532733358106E-61	4	SIPA1L1
4.8157789100261E-56	0.373170587940798	0.496	0.3	8.07028229742173E-52	4	PNPO
7.17858119045982E-65	0.373007710309047	0.643	0.416	1.20298663589726E-60	4	GSDMD
4.29940838702998E-55	0.372936543715594	0.283	0.145	7.20494857498484E-51	4	CIZ1
3.70348602160099E-78	0.37280173885604	0.491	0.272	6.20630187499894E-74	4	PRPF19
4.54314631329215E-60	0.372617429790005	0.437	0.252	7.61340459181499E-56	4	RRP36
4.11502601318278E-47	0.372459686426111	0.313	0.172	6.8959605928917E-43	4	HSD11B2
3.06073866868444E-77	0.372267100044117	0.319	0.151	5.12918586098139E-73	4	APAF1
2.0758717290224E-61	0.372033488853527	0.313	0.158	3.47874584349574E-57	4	KLHL12
5.02312093215493E-61	0.371870282003919	0.258	0.122	8.41774605810523E-57	4	UHRF1BP1L
2.68179614340946E-72	0.371814641134061	0.279	0.128	4.49415397712557E-68	4	SNRNP48
1.54087704258273E-66	0.371511088032872	0.342	0.175	2.58220174796014E-62	4	EMC8
3.33047469160479E-57	0.371148160487638	0.578	0.368	5.58120948819131E-53	4	CANX
1.53572830912048E-71	0.370879421471847	0.556	0.333	2.57357350042411E-67	4	SFT2D2
4.82140128632854E-75	0.370693274617649	0.313	0.149	8.07970427562936E-71	4	FAM126A
1.30378540244801E-64	0.37069058404536	0.541	0.323	2.18488357742238E-60	4	WDR83OS
1.73873029487593E-88	0.370436082731107	0.491	0.265	2.91376422815309E-84	4	PEA15
1.32016207434982E-66	0.370420354924548	0.358	0.186	2.21232760419543E-62	4	AP2M1
1.15373704891858E-46	0.370119820675716	0.354	0.205	1.93343254657775E-42	4	SNX12
9.94006206359477E-52	0.370058847544618	0.375	0.214	1.66575560061721E-47	4	TIMM17A
2.42653252825921E-48	0.369536718447397	0.474	0.294	4.06638321085678E-44	4	CSTB
2.99908380774291E-83	0.369468451043394	0.338	0.161	5.02586464501557E-79	4	UNK
9.03288837677603E-75	0.368879542105914	0.418	0.221	1.51373143418013E-70	4	SZRD1
2.45488279118057E-70	0.368871605523228	0.768	0.518	4.11389258146041E-66	4	BAAT
1.13885513691088E-57	0.368844164170351	0.487	0.286	1.90849343843526E-53	4	ATP9A
2.19925867084387E-51	0.36849150030499	0.333	0.183	3.68551768060015E-47	4	RTF1
5.34874107295043E-73	0.368212753787067	0.568	0.341	8.96342029005033E-69	4	ADD1
2.23689803281676E-64	0.368120359011455	0.728	0.502	3.74859372339432E-60	4	CREG1
8.00558939619023E-63	0.367897016032475	0.41	0.23	1.34157667101356E-58	4	WIPF2
1.31716586705408E-69	0.367359570795912	0.448	0.248	2.20730656000923E-65	4	MTIF3
2.16407604193318E-56	0.366958823430036	0.399	0.225	3.62655863107162E-52	4	DCTD
1.2791441894743E-44	0.366573185797288	0.384	0.223	2.14358983272103E-40	4	MGAM2
7.73409515529898E-70	0.366453553102183	0.275	0.128	1.296079666125E-65	4	CCNYL1
1.46729417445031E-50	0.366099144512729	0.254	0.129	2.45889157754383E-46	4	SEPTIN10
5.965259059941E-88	0.365767497847382	0.496	0.27	9.99658113264914E-84	4	AGO2
1.41590230071935E-64	0.365162443860618	0.526	0.315	2.37276907554548E-60	4	FARP1
5.16640699118197E-39	0.365140716748994	0.296	0.17	8.65786483582274E-35	4	MPND
1.51959529680911E-54	0.36505433594306	0.361	0.201	2.5465377983927E-50	4	TIMM23
1.30907469894425E-60	0.364949186154981	0.421	0.239	2.19374738049077E-56	4	UBA1
4.07005533973057E-61	0.364877840841834	0.502	0.299	6.82059873832049E-57	4	NAA60
9.98216668963559E-63	0.36460470092605	0.408	0.227	1.67281149384913E-58	4	CCNY
3.79795144292E-51	0.364392608571165	0.527	0.326	6.36460702804533E-47	4	TMEM147
1.02285562099196E-66	0.36438694443768	0.434	0.238	1.71410144965833E-62	4	RALGAPA2
1.99193041084418E-70	0.364272204138899	0.519	0.302	3.33807698249268E-66	4	UPF1
2.21680782379235E-80	0.36424724237387	0.453	0.241	3.71492655111122E-76	4	AP3B1
2.43870813937899E-83	0.364045946083536	0.448	0.235	4.08678709997131E-79	4	TXLNA
1.46241125893426E-70	0.363885458971885	0.567	0.346	2.45070878772204E-66	4	HNRNPC
9.93946129017654E-66	0.363860903979538	0.346	0.179	1.66565492300778E-61	4	GTF2F1
2.22604538061905E-67	0.363591842134734	0.413	0.225	3.7304068488414E-63	4	ALKBH5
1.42095587984019E-61	0.363483872533609	0.344	0.189	2.38123786343619E-57	4	ATG16L2
3.38776196549456E-58	0.363013713945872	0.287	0.143	5.67721150177578E-54	4	PRR12
1.34240770262421E-60	0.362370098840627	0.379	0.205	2.24960682805766E-56	4	SLC25A32
1.51004466201816E-57	0.362098951084861	0.316	0.165	2.53053284461003E-53	4	RBM22
2.62528626784759E-52	0.361866728794288	0.318	0.17	4.39945472765899E-48	4	BPNT1

5.5631640809012E-66	0.361705217438292	0.31	0.154	9.32275036677424E-62	4	FOXK2
1.56958390324855E-69	0.361609716423652	0.318	0.156	2.63030870506391E-65	4	MAP1S
2.9581364051194E-55	0.361565435031728	0.433	0.252	4.95724498769909E-51	4	PREB
9.29924126064861E-58	0.36146317102699	0.269	0.132	1.55836685045949E-53	4	DNMBP
7.68158771393762E-77	0.361308506612161	0.708	0.45	1.28728046910167E-72	4	TFRC
1.01681624191388E-55	0.361301851905339	0.269	0.133	1.70398065819929E-51	4	KYAT1
4.12273660840003E-54	0.361232707734058	0.276	0.14	6.90888200835677E-50	4	PAPSS1
5.41326017250216E-56	0.361072155180666	0.298	0.153	9.07154139707912E-52	4	LIG3
9.3000904853668E-64	0.361033592003675	0.345	0.178	1.55850916353777E-59	4	LYSMD3
1.09772141499667E-68	0.360830060240033	0.256	0.116	1.83956154725141E-64	4	DEPRECATED-ENSG00000188529
4.71977459040925E-58	0.36060636708452	0.445	0.254	7.90939825860783E-54	4	TSPAN1
2.29143248981507E-49	0.360558641136623	0.339	0.19	3.8399825664321E-45	4	PIH1D1
1.04290649882299E-104	0.360481721252254	0.568	0.322	1.74770271072756E-100	4	ASXL1
1.62170995106149E-63	0.360447276260961	0.506	0.3	2.71766153598884E-59	4	GPAT4
3.22492890919133E-68	0.360169681031854	0.318	0.158	5.40433586602282E-64	4	PCIF1
4.67018182357485E-43	0.360141639280565	0.352	0.207	7.82629069994673E-39	4	GADD45GIP1
1.96476423218828E-70	0.359879218714027	0.448	0.249	3.29255190030112E-66	4	CUL4A
4.02574911158137E-78	0.359789200159253	0.547	0.317	6.74635036118806E-74	4	DVL3
1.95122114613138E-52	0.359570187672872	0.295	0.155	3.26985639668696E-48	4	GJB2
4.63943725931629E-43	0.35929771535903	0.253	0.135	7.77476895916224E-39	4	LAMTOR5
1.53848436220368E-57	0.359204260783726	0.575	0.371	2.57819209418093E-53	4	ACSL5
4.55157987984524E-68	0.359068619367756	0.403	0.216	7.62753756264465E-64	4	IMMT
6.6171129534603E-51	0.358880621825199	0.258	0.131	1.10889578874088E-46	4	UBL7
1.84776285181793E-80	0.358804191386784	0.984	0.837	3.09648098707649E-76	4	SELENOP
8.04255491681453E-59	0.358641666003717	0.298	0.152	1.34777135295978E-54	4	TK2
1.92124269475678E-51	0.357967846081067	0.337	0.185	3.21961850787341E-47	4	TRMT1L
6.01656599256328E-49	0.35769398147469	0.354	0.199	1.00825612903375E-44	4	PRR15L
1.07001063176043E-52	0.357493464189727	0.325	0.175	1.79312381670412E-48	4	OTUD6B
1.3681539773866E-65	0.357390530726117	0.266	0.125	2.29275243530446E-61	4	NFYA
3.08592531800193E-76	0.357258862423083	0.493	0.281	5.17139364790764E-72	4	KMT2B
2.11479150359621E-42	0.357195584673586	0.323	0.186	3.54396760172652E-38	4	NDUFC1
4.94912863183702E-50	0.356884335792183	0.277	0.145	8.29374976123248E-46	4	PSMD11
5.54411048692065E-78	0.356250650334345	0.875	0.604	9.29082035398162E-74	4	GPX2
2.0560247125143E-63	0.356169145345784	0.609	0.372	3.44548621323146E-59	4	FAM114A1
1.47326627383317E-62	0.355560421861359	0.395	0.218	2.46889962168963E-58	4	CALM1
4.23248784133238E-68	0.355496716002011	0.578	0.348	7.0928031245048E-64	4	TLE5
2.9544193152575E-61	0.355402840479944	0.419	0.235	4.95101588850852E-57	4	SLC35A3
2.11251982820154E-88	0.355352958497374	0.339	0.156	3.54016072810015E-84	4	CCM2
5.91901315573464E-63	0.35532302913939	0.521	0.308	9.91908224638011E-59	4	ZBTB33
1.04735954632814E-73	0.355259213141948	0.454	0.249	1.75516512773669E-69	4	DNAJC8
2.54173275315257E-56	0.355095642383027	0.487	0.293	4.25943574773307E-52	4	SLC35A4
8.28948781505708E-80	0.35486013485765	0.374	0.185	1.38915236804727E-75	4	DNAJC13
4.63570359400509E-55	0.354799977219732	0.29	0.148	7.76851208283373E-51	4	RAB22A
2.65793390807599E-45	0.354621973593479	0.3	0.166	4.45416564315375E-41	4	DFFA
3.40912205270117E-65	0.354617281216474	0.254	0.117	5.71300673591662E-61	4	PPARG
1.26382155964701E-64	0.354544001234893	0.436	0.244	2.11791216965647E-60	4	DPM1
5.33393177170213E-63	0.35453963406132	0.305	0.152	8.93860286301842E-59	4	TEAD3
1.37492651308935E-54	0.354354719542109	0.277	0.14	2.30410185063513E-50	4	SEC22A
6.68492654514058E-68	0.354277317104524	0.922	0.674	1.12025999043466E-63	4	MGST1
1.31534911196236E-48	0.354276676846642	0.277	0.146	2.20426204182653E-44	4	GABPB2
3.18961752490106E-43	0.354265376751721	0.975	0.838	5.3451610482292E-39	4	MT-CO3
2.87968293137845E-64	0.354179082070285	0.323	0.164	4.82577265640401E-60	4	CCT4
2.55121947010811E-74	0.354118923372681	0.582	0.357	4.27533358800716E-70	4	BIRC3
3.26915097156708E-40	0.3540293017936	0.378	0.23	5.47844319815212E-36	4	ADCY6
2.6137258134724E-65	0.354027346883118	0.268	0.125	4.38008171821705E-61	4	TSG101
4.62371399925755E-47	0.353846366065688	0.26	0.135	7.7484199199558E-43	4	ADPRHL2
1.16303564832837E-51	0.353654499361423	0.347	0.193	1.94901513946869E-47	4	REEP3
1.86317172553812E-45	0.353530285387854	0.521	0.336	3.12230317765678E-41	4	PSMB4
7.65133887616015E-52	0.353502915021397	0.354	0.196	1.28221136886692E-47	4	ELOVL6
6.48299216264938E-55	0.353397914698668	0.3	0.155	1.08641982661678E-50	4	TICAM1

1.8641728634225E-62	0.353392164392197	0.431	0.244	3.12398088452343E-58	4	MAPK1IP1L
2.92001680717561E-71	0.353101636019946	0.295	0.138	4.89336416546489E-67	4	XPO5
4.08769148734483E-66	0.352803371710156	0.876	0.653	6.85015339449247E-62	4	ACSL1
1.00092779953182E-50	0.352258220677483	0.359	0.202	1.67735480645543E-46	4	C14orf119
6.26946037815312E-81	0.352215046184751	0.334	0.158	1.0506361701709E-76	4	RBBP6
2.56015535161715E-44	0.352190052958452	0.339	0.196	4.29030833824002E-40	4	ZNF358
1.01271046358852E-44	0.351508994427897	0.271	0.145	1.69710019488164E-40	4	TBPL1
4.90936793223191E-59	0.351469581400661	0.326	0.17	8.22711878083424E-55	4	MOSPD2
9.50208646772523E-85	0.351157277260515	0.435	0.225	1.59235965026139E-80	4	SORBS1
4.49301784052147E-66	0.351020770767379	0.255	0.117	7.52939929714588E-62	4	PPME1
9.23382785082808E-54	0.350967767488508	0.36	0.2	1.54740487124177E-49	4	SRP68
5.88183224006373E-77	0.3504917944052	0.365	0.182	9.8567744678988E-73	4	DLST
2.16415285612833E-76	0.350435735217884	0.531	0.306	3.62668735629986E-72	4	RNF115
8.58887701742752E-52	0.350318485001231	0.348	0.193	1.4393240105805E-47	4	TULP4
5.88755227407317E-46	0.35001235370128	0.304	0.168	9.86636010089182E-42	4	SLC35E1
8.31566873442961E-49	0.349886222074591	0.367	0.21	1.39353976651571E-44	4	TOMM6
2.5621375186639E-40	0.349847907856661	0.399	0.245	4.29363005377697E-36	4	PRDX5
4.09113113796326E-55	0.349732834879398	0.464	0.278	6.85591756099883E-51	4	EMC7
1.88479581674984E-56	0.349682941895153	0.577	0.363	3.15854082970938E-52	4	VPS28
1.56604396591623E-66	0.349639998538048	0.284	0.135	2.62437647808243E-62	4	COA8
4.68035235121584E-85	0.349600136010516	0.294	0.13	7.8433344701675E-81	4	C1orf21
6.77012458214221E-68	0.349389463098871	0.307	0.15	1.13453747747539E-63	4	PSMD9
1.71598069114377E-49	0.349083625410136	0.338	0.188	2.87564044221873E-45	4	IER3IP1
6.63234928331903E-54	0.34873325088733	0.363	0.201	1.1114490928986E-49	4	CPT1A
1.94121700226317E-38	0.348540551458096	0.314	0.184	3.25309145239262E-34	4	FAHD1
9.46136603020282E-62	0.348538239901163	0.564	0.347	1.58553571934139E-57	4	PANK3
1.06161692393126E-83	0.348504636726776	0.454	0.242	1.779057641124E-79	4	TCERG1
6.47008063578919E-42	0.348490931259905	0.363	0.214	1.08425611294555E-37	4	DEPRECATED-ENSG00000170421
3.59822042586029E-81	0.348190699304591	0.67	0.407	6.02989778965667E-77	4	EFHD2
2.52225811631049E-59	0.348119474908537	0.383	0.21	4.22680015131313E-55	4	ODC1
1.30957945521468E-50	0.347652300290996	0.289	0.153	2.19459325104875E-46	4	PER3
8.77711712093056E-86	0.347627347698016	0.377	0.182	1.47086928712554E-81	4	PLEKHG3
2.72957790133447E-57	0.347613193254746	0.361	0.196	4.57422664705631E-53	4	ZYG11B
1.98444634685058E-53	0.347567948964897	0.251	0.125	3.3255351880522E-49	4	STARD3NL
1.38000967161313E-51	0.347475592377898	0.445	0.267	2.31262020768928E-47	4	PDCD6
3.04547131395665E-52	0.346387971944483	0.516	0.335	5.10360082792856E-48	4	PCCA
3.88780852754908E-66	0.346315630846084	0.489	0.284	6.51518953046675E-62	4	DCTN1
3.41731800366229E-64	0.3461004986835	0.403	0.22	5.72674151053727E-60	4	SLAIN2
5.1701200565149E-87	0.345902203267896	0.431	0.22	8.66408719070767E-83	4	ENPP1
1.52034775613762E-60	0.345726410965385	0.29	0.144	2.54779876973543E-56	4	TCF3
4.93305608968724E-77	0.345638561356172	0.41	0.214	8.26681539509789E-73	4	BRF1
8.84012081554994E-84	0.345480096659054	0.639	0.395	1.48142744626986E-79	4	SPTAN1
1.25688011912402E-79	0.345310016000886	0.39	0.199	2.10627970362803E-75	4	HMGN4
1.19342165741581E-79	0.345145082625254	0.979	0.829	1.99993601349742E-75	4	HSPB1
6.83563627782759E-59	0.344993533246536	0.298	0.151	1.14551592743835E-54	4	KPNA3
8.57283823098698E-56	0.34494302014401	0.335	0.18	1.4366362307488E-51	4	SMAP1
3.04160107403259E-48	0.344866222842859	0.38	0.222	5.09711507986381E-44	4	UBL5
1.51297749792852E-67	0.344836829352347	0.542	0.327	2.53544769102861E-63	4	TMEM109
3.00563458059766E-51	0.34408677411156	0.251	0.126	5.03684243016556E-47	4	SLC27A4
4.93158050957454E-66	0.344055325318889	0.383	0.201	8.26434261794501E-62	4	ACBD6
7.37723882923308E-63	0.34399026036091	0.404	0.22	1.23627768300288E-58	4	LARP1
7.01688782242681E-66	0.343859961705352	0.276	0.13	1.17589006128229E-61	4	EPHB4
1.67870071297444E-55	0.343393641153906	0.539	0.338	2.81316665480256E-51	4	EDEM3
5.51196495609369E-71	0.343373448499642	0.43	0.232	9.23695087342181E-67	4	NCKIPSD
5.73992457040327E-58	0.343172294895513	0.621	0.4	9.6189655950818E-54	4	ADIPOR1
8.42131908784565E-56	0.342967650977094	0.507	0.304	1.41124465274117E-51	4	ATP6V1A
2.45847212713846E-81	0.342941848444265	0.354	0.171	4.11990759065864E-77	4	ADD3
9.11350507106894E-75	0.342665677927924	0.324	0.155	1.52724117980973E-70	4	ZBTB44
9.17905457082528E-50	0.342623379500944	0.341	0.19	1.5382259649789E-45	4	SLC30A9
1.2835868076292E-68	0.34259097759579	0.656	0.4	2.15103477222502E-64	4	ERO1B

3.03671568754286E-59	0.342563853849907	0.293	0.147	5.08892814918432E-55	4	TAF4
2.29473205153008E-75	0.342075620144842	0.41	0.215	3.84551197195411E-71	4	CTNNB1
7.17362051582999E-62	0.341539016117631	0.703	0.472	1.20215532604279E-57	4	ARF1
3.18111917166263E-55	0.341381570772726	0.35	0.19	5.33091950787224E-51	4	DGCR2
1.01384461792348E-68	0.341160567225965	0.571	0.348	1.69900081071617E-64	4	DERL1
2.40112973270878E-76	0.340848530301219	0.253	0.109	4.02381320607337E-72	4	ZCCHC8
1.1895299283712E-52	0.340488735086738	0.259	0.13	1.99341425396446E-48	4	NUBP1
1.09658337351227E-72	0.340347953536025	0.51	0.298	1.83765441733186E-68	4	DDX46
1.51124649540251E-51	0.340229985956014	0.305	0.163	2.53254687699552E-47	4	PTPRA
1.09414051719324E-65	0.339572408378273	0.312	0.155	1.83356067871242E-61	4	PDS5A
2.6083499023276E-57	0.339337838155108	0.552	0.345	4.37107276632059E-53	4	CCDC47
1.04790630055565E-64	0.339279075135137	0.567	0.348	1.75608137847116E-60	4	NMT1
2.30932319231183E-64	0.339123520204212	0.47	0.272	3.86996380567617E-60	4	HMGN3
2.46520090073702E-62	0.339080060897439	0.457	0.262	4.13118366945509E-58	4	MANF
6.1964047762307E-52	0.338668121603203	0.357	0.2	1.03839351240074E-47	4	PDK2
5.6142083860009E-83	0.338123170571203	0.415	0.211	9.40829041326031E-79	4	BDP1
1.42443101273193E-47	0.338081428675966	0.452	0.274	2.38706149113617E-43	4	DNAJC4
7.5287027538997E-46	0.337841696219783	0.297	0.163	1.26166000749851E-41	4	NDRG3
5.26430311659428E-51	0.337699321404833	0.376	0.216	8.8219191627887E-47	4	IRF9
3.6586682278799E-67	0.337183698907337	0.484	0.278	6.13119621628113E-63	4	DCTN2
1.27739534067621E-76	0.337085250938245	0.811	0.531	2.14065911190519E-72	4	STARD10
5.03518346623315E-54	0.337062204068366	0.308	0.163	8.43796045271352E-50	4	SMARCB1
4.4948807105716E-66	0.337048612763313	0.454	0.255	7.5325210947759E-62	4	PDE4DIP
4.15092488293029E-68	0.336996329494068	0.563	0.342	6.95611991881457E-64	4	PTPA
1.22382894047555E-63	0.336608048907816	0.557	0.337	2.05089253844893E-59	4	FDPS
2.01022965810798E-94	0.336492775753483	0.354	0.162	3.36874286105734E-90	4	TUBA4A
1.33383207441248E-60	0.335670204598296	0.365	0.196	2.23523579030044E-56	4	USP10
1.32908809653115E-62	0.335624126036612	0.461	0.267	2.2272858321669E-58	4	RAF1
4.57220471101034E-74	0.335600110519491	0.499	0.287	7.66210065471113E-70	4	MICAL3
1.34011447574688E-52	0.335348638452584	0.589	0.376	2.24576383845662E-48	4	ITPK1
1.24733722071161E-51	0.335174938165942	0.251	0.125	2.09028771446851E-47	4	DDX52
3.20169254002471E-68	0.335109926574894	0.382	0.2	5.36539635857341E-64	4	ACAD9
1.03639265274949E-57	0.33490220266441	0.72	0.486	1.73678680747759E-53	4	BLVRB
8.2174564936194E-66	0.334835736176827	0.407	0.223	1.37708135920074E-61	4	ABHD3
2.13523494785506E-46	0.334452370341186	0.435	0.264	3.5782267256155E-42	4	SKP1
3.78874852806312E-72	0.334164762892165	0.339	0.168	6.34918478332817E-68	4	SMARCC2
1.1327455985684E-57	0.333711203893062	0.469	0.278	1.89825507408093E-53	4	NAA20
2.78670304446589E-56	0.333663486291978	0.271	0.134	4.66995696191593E-52	4	PSME2
9.5748819358582E-67	0.333465692585172	0.38	0.202	1.60455871481112E-62	4	TAF8
3.98927886375084E-46	0.333428813480064	0.485	0.301	6.68523351987367E-42	4	HSD3B7
3.20741682991834E-74	0.333178237907686	0.472	0.262	5.37498912357716E-70	4	EWSR1
1.16554226441214E-57	0.332911277046759	0.34	0.181	1.95321572670186E-53	4	CSNK2B
1.92336099209688E-52	0.332861119789542	0.425	0.249	3.22316835055595E-48	4	PROS1
5.43909432769659E-72	0.332323620894365	0.339	0.169	9.11483427435395E-68	4	GMPPB
8.99615842314416E-52	0.331902826567954	0.275	0.141	1.5075762285505E-47	4	DCAF12
8.06762393029489E-45	0.331676838625658	0.302	0.167	1.35197241823882E-40	4	GRPEL1
1.12564254772168E-56	0.331540600201642	0.271	0.135	1.88635178147199E-52	4	ZNF445
6.28827636286042E-74	0.331486784611315	0.404	0.214	1.05378935288815E-69	4	KPNA1
2.46651690365168E-67	0.331470080265717	0.432	0.237	4.13338902713949E-63	4	KIF13A
2.75092522584494E-61	0.331132394929749	0.271	0.131	4.61000049347094E-57	4	PIP4K2C
6.45617156670035E-41	0.331004914898683	0.25	0.135	1.08192523114765E-36	4	ZNF32
1.25890303512163E-42	0.330874926211451	0.27	0.147	2.10966970625683E-38	4	GLRX2
4.12527822454924E-48	0.330711600685684	0.328	0.182	6.91314124869962E-44	4	DOK4
2.01015906216865E-58	0.330587403759298	0.542	0.337	3.36862455638223E-54	4	NARS
1.87525846916359E-44	0.330532401307515	0.333	0.191	3.14255814262434E-40	4	ALKBH7
1.15402701358737E-68	0.330489678847423	0.336	0.167	1.93391846936971E-64	4	MED6
2.06429667530025E-58	0.330466189797387	0.595	0.37	3.45934836846816E-54	4	CLDN7
3.39530887846216E-60	0.330376732627284	0.26	0.124	5.68985861852689E-56	4	GPKOW
3.30116658876972E-56	0.33025795671778	0.351	0.19	5.5320949694603E-52	4	ATG3
6.90047009063896E-67	0.329941410930795	0.429	0.237	1.15638077778928E-62	4	ARFGAP3

1.04971120503625E-52	0.3297051654637	0.312	0.166	1.75910603739974E-48	4	POP7
7.28449269945322E-58	0.329537090757937	0.304	0.156	1.22073528657437E-53	4	MBP
1.96727412335906E-56	0.329529778784577	0.564	0.357	3.29675797592512E-52	4	PIGT
3.18921377807257E-65	0.328718578562274	0.547	0.331	5.34448444929401E-61	4	ARHGAP35
4.42011828539717E-98	0.328675803244736	0.505	0.272	7.40723422266858E-94	4	NUMA1
2.85701137601294E-61	0.328646001712743	0.273	0.133	4.78777966392249E-57	4	SYNJ2
4.35989777571589E-53	0.327704105596806	0.569	0.361	7.30631669254468E-49	4	NDUFB10
3.02456010202312E-49	0.327475176273421	0.64	0.433	5.06855781897035E-45	4	SSR2
3.26612455248361E-74	0.327201373414048	0.311	0.147	5.47337152505204E-70	4	UBAC1
5.68402583963802E-99	0.326417802928521	0.361	0.164	9.52529050206539E-95	4	C1orf198
7.39256424467084E-50	0.32640426713408	0.382	0.219	1.23884591612194E-45	4	PSMD4
3.90905216813001E-64	0.326117601942886	0.375	0.201	6.55078962335227E-60	4	DAXX
2.320340852336E-71	0.326007870486496	0.312	0.15	3.88842720034467E-67	4	TMEM170A
2.28589725984545E-61	0.325978646626287	0.26	0.123	3.830706628049E-57	4	ZNF654
9.25931680713778E-61	0.325703969445783	0.344	0.182	1.55167631054015E-56	4	YME1L1
3.38580919429129E-45	0.32529301480049	0.394	0.235	5.67393904779335E-41	4	LRP3
1.01334915188896E-50	0.325007654113673	0.532	0.334	1.69817050873553E-46	4	IMPAD1
7.07450350958883E-54	0.324514270919132	0.36	0.201	1.1855452981369E-49	4	DDX23
1.88722242139522E-56	0.324391858066044	0.271	0.134	3.16260733377411E-52	4	GNPNAT1
2.47349526413676E-76	0.324318064196065	0.867	0.593	4.14508336364039E-72	4	AKR1B10
8.14457663756408E-57	0.324272682007952	0.469	0.277	1.36486815292299E-52	4	MOCS2
7.93976085625075E-52	0.323979264933777	0.405	0.234	1.3305451242905E-47	4	GPC6
4.17977382727653E-88	0.323803120769282	0.925	0.614	7.00446497975001E-84	4	LGALS4
2.06914609194536E-56	0.323764241444719	0.661	0.448	3.46747502088203E-52	4	MIF
4.35052829542534E-45	0.323740569299987	0.405	0.243	7.29061531747379E-41	4	FADS2
6.51053191970141E-68	0.323502243321416	0.498	0.294	1.09103493910356E-63	4	TMEM245
6.18094065325328E-49	0.323364355800735	0.262	0.135	1.03580203467219E-44	4	RWDD3
3.54410038724293E-53	0.323349966808614	0.527	0.331	5.9392034289417E-49	4	UBE2R2
3.44047139206001E-87	0.323248189534059	0.448	0.231	5.76554195881416E-83	4	BAZ1B
1.1530545369725E-79	0.323220700208694	0.469	0.254	1.93228879305851E-75	4	HDAC1
3.2277804726446E-50	0.323121690470336	0.344	0.191	5.40911451605782E-46	4	GLI4
4.33980685283009E-50	0.322594494675805	0.268	0.138	7.27264832397266E-46	4	LRRC42
4.5606365636625E-52	0.322229951833367	0.432	0.254	7.64271475338562E-48	4	NDUFA11
5.4223583570235E-48	0.322074806180212	0.301	0.163	9.08678813469998E-44	4	USP38
1.62438417350954E-54	0.321039630200956	0.416	0.241	2.72214299796729E-50	4	CWC15
7.87053476051318E-63	0.320878353485663	0.426	0.241	1.3189442151668E-58	4	SUMO3
8.34166283032194E-47	0.320779240285602	0.572	0.377	1.39789585710535E-42	4	LAMTOR2
9.79502505421681E-65	0.3207681101567	0.434	0.243	1.64145029858565E-60	4	TMUB1
5.57788806621122E-77	0.320630085857757	0.41	0.212	9.34742482135677E-73	4	MTR
1.12019841468876E-51	0.320556977464441	0.261	0.133	1.87722850333542E-47	4	DEPRECATED-ENSG00000249915
6.90755365398797E-65	0.320442515247187	0.466	0.268	1.1575678413353E-60	4	ERAP1
1.63710193276092E-50	0.320215981032021	0.578	0.372	2.74345541892075E-46	4	ATP5ME
3.22407136549963E-71	0.320183628308751	0.557	0.338	5.40289879430427E-67	4	SP3
2.58246868182533E-49	0.320095292568819	0.291	0.154	4.32770101700289E-45	4	NCEH1
6.64202374196035E-54	0.320011168532055	0.439	0.257	1.11307033867772E-49	4	NREP
7.45791981343512E-48	0.31978617406378	0.392	0.228	1.24979820233546E-43	4	CRACR2B
4.73601926608014E-86	0.319739843966385	0.448	0.234	7.9366210860971E-82	4	HIPK1
7.70846758667656E-44	0.319480485055728	0.268	0.144	1.29178499817526E-39	4	CASZ1
1.63532215892909E-67	0.31935106192821	0.344	0.175	2.74047287393337E-63	4	LSG1
2.93007035677256E-77	0.31927576970742	0.505	0.285	4.91021190387945E-73	4	UBE2I
8.65881795583067E-53	0.319158181779444	0.25	0.124	1.4510447130381E-48	4	ZNF76
1.32483932733103E-60	0.319105113436135	0.446	0.257	2.22016574474135E-56	4	FARSA
2.31137247131353E-55	0.31898043500381	0.536	0.332	3.87339798742722E-51	4	AGFG2
2.14706149804744E-66	0.31891807944555	0.356	0.184	3.5980456584279E-62	4	ARFGEF1
7.74531407989269E-51	0.318766652474503	0.262	0.134	1.29795973350842E-46	4	AP5M1
1.6356302486461E-51	0.318568521843433	0.321	0.174	2.74098917068114E-47	4	FBXO28
2.29380579909614E-49	0.318411143906906	0.294	0.157	3.84395975812532E-45	4	CUL2
4.21850967587166E-64	0.317886436939874	0.511	0.3	7.06937851482572E-60	4	GRB10
8.49053658892833E-53	0.317858427240944	0.632	0.43	1.42284412157261E-48	4	LAMTOR4
4.54446915433715E-57	0.317443865515493	0.273	0.135	7.6156214088382E-53	4	ZNF282

2.30812410802077E-49	0.317159570965628	0.321	0.175	3.8679543802212E-45	4	SUCLG2
6.03819448423024E-80	0.316991680007481	0.436	0.232	1.0118806316673E-75	4	FRYL
1.3908578194038E-55	0.316587468321626	0.414	0.237	2.33079953375689E-51	4	CCDC124
3.64359910118725E-56	0.316579800396229	0.255	0.124	6.10594337376959E-52	4	ZDHHC16
1.48205110873837E-62	0.31633196239039	0.26	0.122	2.48362124802375E-58	4	MAP3K20
2.18097029705101E-47	0.315968485656846	0.288	0.156	3.65487002379809E-43	4	NT5DC1
6.22261711736832E-61	0.315658972789629	0.407	0.225	1.04278617652858E-56	4	RCC1L
2.33531065071931E-50	0.315597334354236	0.401	0.232	3.91351358847542E-46	4	MEGF9
5.30066045428595E-52	0.314886469811728	0.478	0.293	8.8828467892924E-48	4	NQO2
8.3129612979816E-62	0.314718515290947	0.497	0.299	1.39308605431576E-57	4	MAN2A1
3.36383471023182E-73	0.314711058904559	0.374	0.192	5.63711420740649E-69	4	TAPT1
2.70814136722464E-54	0.314566520419782	0.337	0.181	4.53830330319506E-50	4	ANKRD40
1.84545151631106E-47	0.314459194870887	0.479	0.296	3.09260765103407E-43	4	RABAC1
2.13133101583585E-83	0.314159772620319	0.54	0.309	3.57168451633771E-79	4	SRSF2
3.52253610254348E-53	0.314077750889661	0.561	0.355	5.90306600064236E-49	4	ABCD3
3.71878139411713E-54	0.313868714950531	0.329	0.176	6.23193386026149E-50	4	ALDH7A1
2.767237210893E-54	0.313563546326487	0.775	0.543	4.63733611801449E-50	4	SLC9A3R1
2.32153687095403E-46	0.313544964619781	0.501	0.315	3.89043148834476E-42	4	TIMM13
8.1817700842648E-60	0.313260669901449	0.464	0.271	1.3711010307211E-55	4	ITPRID2
2.49659324224885E-39	0.312964364150482	0.257	0.141	4.18379095536062E-35	4	GTF2H5
4.35116361212934E-51	0.31287620554574	0.498	0.303	7.29167998120635E-47	4	CPNE3
1.46182441591321E-76	0.312564562906507	0.351	0.173	2.44972535618736E-72	4	FAM193A
1.47749654060576E-57	0.312496388771169	0.29	0.146	2.47598870274714E-53	4	POLE3
3.45294816714248E-80	0.312485579219979	0.512	0.292	5.78645053849736E-76	4	CGGBP1
5.158271191836E-50	0.312251947599266	0.438	0.262	8.64423086327876E-46	4	POLR3GL
6.88104541472271E-57	0.312116607418763	0.51	0.314	1.15312559059923E-52	4	CARHSP1
2.96218327119737E-52	0.312052998811911	0.59	0.378	4.96402672587255E-48	4	RAB10
1.96571881274547E-50	0.311743504334141	0.522	0.325	3.29415158639885E-46	4	FDFT1
1.408836126475E-40	0.311459800960763	0.265	0.146	2.3609275807468E-36	4	TMEM128
1.00825473543868E-48	0.31120672634158	0.294	0.157	1.68963328564814E-44	4	EIF3K
5.8291205782786E-54	0.31115302177819	0.355	0.195	9.76844026507928E-50	4	SLC25A38
2.50454185537136E-45	0.310835605882642	0.373	0.219	4.19711124123133E-41	4	TXN2
1.26562961705148E-55	0.310564924424623	0.388	0.218	2.12094211225487E-51	4	CDC5L
1.23375426305499E-54	0.310405140779418	0.546	0.345	2.06752539402755E-50	4	SYAP1
3.56510359464503E-43	0.310357073508998	0.496	0.318	5.97440060390615E-39	4	RDH11
8.16271702267701E-43	0.310297064620184	0.367	0.214	1.36790811866021E-38	4	CXADR
7.72129472869487E-74	0.310025531151373	0.383	0.197	1.29393457063469E-69	4	CSTF3
4.51209375994786E-54	0.309579913874328	0.358	0.198	7.56136672292062E-50	4	FAM136A
1.47111636361214E-44	0.309327827407986	0.26	0.137	2.46529680214122E-40	4	SLIRP
1.28670375331991E-49	0.308973365079605	0.254	0.129	2.15625814981351E-45	4	MED22
2.04635347548598E-61	0.30894343973791	0.323	0.165	3.42927915421941E-57	4	AHI1
3.27042093911834E-45	0.308676732044203	0.264	0.14	5.48057140977452E-41	4	BLVRA
4.42818815168966E-62	0.308441086956088	0.33	0.17	7.42075770460153E-58	4	P4HA2
9.08092361828145E-60	0.307952648745395	0.321	0.165	1.52178117995161E-55	4	HSPA4
1.11413303192078E-59	0.307735070038902	0.49	0.292	1.86706413489284E-55	4	PAK2
6.46205062156177E-75	0.30766564214648	0.477	0.266	1.08291044316132E-70	4	RBM23
7.13767453153249E-41	0.307566595007695	0.316	0.181	1.19613149799421E-36	4	GHR
2.33764821112199E-51	0.307535014366742	0.353	0.197	3.91743087219822E-47	4	ANO6
2.64047105343909E-56	0.307463797902301	0.283	0.143	4.42490139135322E-52	4	SUDS3
3.39385428596023E-49	0.307282521762214	0.518	0.323	5.68742101241215E-45	4	OCIAD1
1.98751971944279E-51	0.306996558799825	0.571	0.366	3.33068554584222E-47	4	SPATS2L
1.13346851631464E-48	0.306917170547541	0.61	0.409	1.89946653964007E-44	4	TMED5
1.08563488592095E-61	0.306911740433059	0.302	0.152	1.81930694182633E-57	4	TMEM138
4.0135919165673E-56	0.306587215399875	0.513	0.313	6.72597733378347E-52	4	MAVS
5.51352821190185E-53	0.306547480293935	0.509	0.309	9.23957057750512E-49	4	STAP2
4.23433821642652E-44	0.306533351463509	0.397	0.236	7.09590398308755E-40	4	HSD17B8
3.02631619718571E-55	0.306456832668246	0.317	0.167	5.07150068324381E-51	4	MPZL1
1.3394437936865E-44	0.306049512489372	0.531	0.34	2.24463990945983E-40	4	ROMO1
1.37778045585592E-46	0.305502049075927	0.258	0.134	2.30888448792335E-42	4	TMEM14B
1.32820882903515E-54	0.305441509882307	0.438	0.257	2.22581235569711E-50	4	INTS6

1.95066486103116E-70	0.305100176788204	0.369	0.191	3.26892417411602E-66	4	BRD7
1.89442438241881E-74	0.304809749405224	0.473	0.26	3.17467638005745E-70	4	CDC42BPB
9.43152156834122E-48	0.304757459330234	0.268	0.139	1.58053438442262E-43	4	SLC36A1
9.77978143127674E-56	0.304638267357705	0.365	0.202	1.63889577225336E-51	4	MCRS1
3.17214042328228E-57	0.30459594692015	0.404	0.23	5.31587292133645E-53	4	YWHAB
3.49420678025528E-70	0.304516412272253	0.64	0.414	5.8555917223518E-66	4	LUC7L2
3.8814605551527E-48	0.303899911240642	0.396	0.231	6.50455159832489E-44	4	MOCOS
3.78819893750995E-55	0.303620802842735	0.278	0.14	6.34826377947918E-51	4	CEP83
7.77885711927671E-69	0.303571316534986	0.481	0.275	1.30358087604839E-64	4	TENT5A
1.48454617298493E-47	0.303255970202839	0.267	0.139	2.48780247668814E-43	4	NGDN
2.43039139735148E-51	0.303085848127013	0.272	0.139	4.07284990368161E-47	4	FRS2
2.75211997066233E-69	0.302925689010939	0.496	0.285	4.61200264683593E-65	4	SETD3
9.61851095850883E-46	0.302850552512588	0.32	0.18	1.61187006642691E-41	4	GOLPH3L
2.32280432806795E-53	0.302844496333754	0.406	0.233	3.89255549297627E-49	4	ABHD16A
1.07418695720051E-64	0.302717252699349	0.456	0.258	1.80012250287662E-60	4	DYNC1I2
1.88573183245778E-49	0.302623625526167	0.286	0.151	3.16010940483275E-45	4	POLR2G
1.12892109541286E-40	0.302460085085001	0.481	0.306	1.89184597169287E-36	4	CISD3
2.47591366254091E-78	0.302350069285686	0.406	0.209	4.14913611568605E-74	4	CASK
3.047820963274E-59	0.3019347682193	0.332	0.174	5.10753837025456E-55	4	SF1
2.58238403146374E-37	0.301753317579583	0.256	0.143	4.32755915992694E-33	4	ENDOG
2.00693549839549E-48	0.30138438560663	0.319	0.176	3.36322250821116E-44	4	ELOA
1.84051880862029E-48	0.301037595308411	0.274	0.143	3.08434141948588E-44	4	GNE
2.04926073745268E-54	0.300858441590624	0.554	0.35	3.4341511438232E-50	4	HNRNPK
6.91059479559962E-58	0.300527037421396	0.259	0.126	1.15807747584659E-53	4	ZDHHC5
4.03386753688295E-60	0.300221561511365	0.593	0.377	6.75995521830845E-56	4	CEBPG
1.14472314465495E-92	0.300026672240101	0.384	0.185	1.91832704581277E-88	4	NUP98
6.63334204166088E-59	0.299872602636237	0.253	0.121	1.11161545934153E-54	4	WDR5
1.1252755231691E-49	0.299840862559603	0.616	0.409	1.88573672172678E-45	4	IL13RA1
8.36260258495087E-55	0.299149224746603	0.328	0.175	1.40140494118607E-50	4	ZC3H14
1.50995204533814E-65	0.299019833432026	0.514	0.3	2.53037763757766E-61	4	PKN1
2.95873281350197E-55	0.298930053292937	0.279	0.141	4.9582444488666E-51	4	SH3BP5L
1.37639114214318E-43	0.298440950922553	0.456	0.285	2.30655627600355E-39	4	TMBIM6
4.71065242716216E-65	0.298137837289558	0.426	0.234	7.89411133743835E-61	4	AGGF1
5.31101931462542E-41	0.298060748909564	0.709	0.487	8.90020616744928E-37	4	SCCPDH
3.20907681720851E-65	0.297938433914823	0.604	0.367	5.37777093027802E-61	4	CTPS1
1.81070067956189E-46	0.297668946808331	0.518	0.331	3.03437219880981E-42	4	HSD17B10
3.60761157062109E-48	0.297616844884195	0.375	0.216	6.04563547004682E-44	4	HDDC2
8.76667362242844E-56	0.297518417578621	0.531	0.33	1.46911916564656E-51	4	VKORC1
3.27861901616694E-58	0.29745415415416	0.357	0.191	5.49430974729255E-54	4	ATXN10
1.30093074235212E-46	0.297256860903261	0.53	0.336	2.18009973803368E-42	4	COA3
3.96202437944921E-53	0.297181731993124	0.343	0.187	6.63956045508099E-49	4	POFUT1
1.94964831096389E-49	0.297174768671224	0.328	0.181	3.26722063951329E-45	4	RCAN1
9.6405016163255E-62	0.297166550984962	0.346	0.182	1.61555526086383E-57	4	SHKBP1
2.18055201628255E-38	0.297004772025575	0.356	0.215	3.6541690688863E-34	4	NMRAL1
4.17676655114347E-67	0.296917218967718	0.568	0.344	6.99942538640623E-63	4	MTCH1
1.35144620374645E-61	0.296745050155422	0.389	0.212	2.26475354823831E-57	4	IPO8
1.76258653933923E-50	0.296069570693953	0.499	0.308	2.95374252262468E-46	4	SPPL2A
2.17674481255192E-52	0.295634766422138	0.632	0.413	3.64778895687451E-48	4	RIDA
8.62792641155957E-49	0.295514223445838	0.49	0.301	1.44586790804915E-44	4	SSR3
7.06596618421933E-58	0.295340148611725	0.469	0.275	1.18411461315147E-53	4	PPP4R3B
1.11513252012621E-68	0.295245580810638	0.556	0.337	1.8687390772275E-64	4	HUWE1
2.07603397672925E-47	0.295197894694681	0.332	0.186	3.47901773820287E-43	4	RRAGA
2.79925083548698E-67	0.295163397948843	0.375	0.195	4.69098455010909E-63	4	STX10
9.64039152438684E-63	0.294996071246255	0.397	0.216	1.61553681165675E-58	4	TMEM189
4.34491579854008E-51	0.294966952503439	0.394	0.226	7.28120989519347E-47	4	YAP1
9.52447610138364E-48	0.2949501774401	0.442	0.267	1.59611170506987E-43	4	NDUFA5
1.14548919242692E-59	0.29486738163933	0.331	0.172	1.91961078866904E-55	4	NRIP1
5.13043419330579E-35	0.294722395734676	0.294	0.172	8.59758162114184E-31	4	PRSS3
1.31431583100595E-68	0.294704738369518	0.556	0.332	2.20253046959977E-64	4	G3BP2
3.0618731325283E-56	0.294656815759582	0.454	0.267	5.13108699549092E-52	4	GPR108

3.79291321316506E-51	0.294499240848573	0.76	0.539	6.35616396262201E-47	4	SOD1
5.6679055276701E-47	0.294437029031455	0.38	0.221	9.49827608326956E-43	4	DEPRECATED-ENSG00000213246
4.26170737343888E-53	0.294236570913021	0.644	0.415	7.14176921640888E-49	4	ATRN
1.51268861753414E-69	0.29381620823596	0.283	0.132	2.53496358526372E-65	4	SOCS4
2.09444344972851E-50	0.293550971671269	0.479	0.292	3.50986833305504E-46	4	ACO1
4.97204411353307E-49	0.293544140264247	0.498	0.309	8.33215152545871E-45	4	TBC1D8
2.47144991263221E-49	0.293358052186848	0.291	0.154	4.14165576358906E-45	4	BZW1
5.08068090609505E-50	0.293261704373946	0.278	0.144	8.51420506243409E-46	4	METTL5
8.52303096171325E-68	0.29322540321818	0.297	0.144	1.42828952856391E-63	4	GSPT1
1.23108784365108E-42	0.293138598987619	0.494	0.312	2.06305700839048E-38	4	NDUFA13
5.53684400106242E-52	0.293085288891991	0.457	0.276	9.2786431769804E-48	4	SGK2
4.28220129205242E-35	0.29290970759569	0.26	0.148	7.17611292522144E-31	4	TSPYL4
3.79420044829481E-66	0.292862774115416	0.465	0.264	6.35832111125244E-62	4	PCID2
9.43777226288993E-53	0.292709279249207	0.389	0.221	1.58158187581509E-48	4	TRAF7
6.20472303320027E-55	0.292578486500676	0.397	0.225	1.0397874859037E-50	4	PET100
2.53023826484573E-70	0.291766491223073	0.303	0.146	4.24017328422848E-66	4	TBC1D10B
1.69289709806399E-51	0.291528791674886	0.354	0.197	2.83695695693563E-47	4	GOSR1
3.1099910086751E-60	0.29148147370243	0.323	0.167	5.21172293233773E-56	4	ST7
1.16530101880175E-48	0.29138041416016	0.514	0.325	1.95281144730796E-44	4	SRI
1.25414285935914E-64	0.291367118052363	0.328	0.166	2.10169260371404E-60	4	TGS1
1.37752690411633E-55	0.291057065742207	0.462	0.273	2.30845958591815E-51	4	DPP9
2.34341439889055E-45	0.291016402058137	0.609	0.4	3.92709384966078E-41	4	PTPRK
1.06656871135772E-59	0.290717680912046	0.293	0.147	1.78735584649327E-55	4	DEPRECATED-ENSG00000107438
2.84306219193766E-46	0.290702343172132	0.315	0.175	4.76440362124913E-42	4	RFXANK
2.48966254862881E-57	0.290556889720511	0.369	0.203	4.17217649899216E-53	4	SOCS6
8.20687421052878E-59	0.290545440832832	0.548	0.34	1.37530798020041E-54	4	BAG6
6.30531503242922E-65	0.290026149726995	0.372	0.197	1.05664469313449E-60	4	CNPPD1
1.08905491668242E-49	0.289664114540179	0.329	0.182	1.8250382293764E-45	4	ATP6V1E1
4.10250472505259E-47	0.28916015399651	0.438	0.266	6.87497741824313E-43	4	COX7A2
2.4245897873604E-55	0.288806088271455	0.532	0.326	4.06312756565855E-51	4	SUB1
4.27377303322198E-48	0.288805554947824	0.405	0.238	7.1619888490734E-44	4	GLB1
5.99861392271028E-44	0.288721998202685	0.581	0.382	1.00524772116779E-39	4	HTATIP2
1.33993514478251E-61	0.288635377911732	0.309	0.157	2.24546331562653E-57	4	ABL1
2.27838589997145E-46	0.288286435309442	0.492	0.308	3.81811909117216E-42	4	CAPNS1
2.76113250395985E-57	0.288200103123434	0.365	0.198	4.62710585013592E-53	4	CHMP1A
4.42712986967998E-58	0.288132940976045	0.289	0.145	7.41898423560971E-54	4	ADNP2
1.1098444587857E-69	0.288068914955367	0.307	0.148	1.85987734403308E-65	4	RELL1
7.33056077735612E-37	0.287948567722449	0.267	0.15	1.22845537506934E-32	4	RNF170
2.06427756278658E-47	0.287882017344421	0.359	0.206	3.45931633971776E-43	4	VAMP3
4.51347355999933E-44	0.287809890647084	0.635	0.433	7.56367899184687E-40	4	CBR1
9.07257855444633E-50	0.287550077415867	0.269	0.139	1.52038271415412E-45	4	PRPF18
2.3977704748948E-77	0.286837455238786	0.438	0.234	4.0181837618287E-73	4	POLDIP3
2.11561199345991E-83	0.286749337853962	0.542	0.312	3.54534257864011E-79	4	CD47
4.35493006847482E-52	0.286325995706373	0.564	0.365	7.2979918087501E-48	4	DAZAP2
1.05934364693796E-55	0.286179262730724	0.425	0.247	1.77524808353863E-51	4	ATP2C1
1.14092155088073E-41	0.286118571041443	0.288	0.16	1.91195633496594E-37	4	DAD1
1.47527138124494E-49	0.286061775999399	0.618	0.407	2.47225978069026E-45	4	RAB5C
7.1878752729931E-44	0.285911661849069	0.306	0.171	1.20454413824818E-39	4	TMEM50B
7.40257439324261E-50	0.285824599043387	0.34	0.188	1.2405234168196E-45	4	MYRF
6.54043464187268E-43	0.285684471930604	0.467	0.293	1.09604603728502E-38	4	QDPR
3.84475542319901E-57	0.285532398663078	0.36	0.194	6.4430411381969E-53	4	SLC2A13
2.10090830564151E-67	0.28549783081263	0.383	0.203	3.52070213859405E-63	4	NIN
3.10096050452686E-55	0.285375704820711	0.658	0.407	5.19658961348611E-51	4	C2orf72
4.17504186634148E-59	0.285284655161493	0.343	0.181	6.99653515961506E-55	4	ZDHHC3
8.23381307951047E-58	0.285016875317324	0.479	0.281	1.37982239586436E-53	4	VPS52
2.6589328059015E-76	0.284989390126815	0.463	0.253	4.45583959612973E-72	4	SUN1
2.75234596156551E-43	0.284916684692321	0.632	0.431	4.61238136239149E-39	4	CUTA
4.51069615081861E-51	0.284889668841101	0.371	0.212	7.55902460954182E-47	4	KIAA2013
2.0030514310992E-66	0.284766693238175	0.444	0.247	3.35671358823604E-62	4	CDC34
4.93488427837121E-47	0.284725979761825	0.27	0.142	8.26987907369448E-43	4	GK5

1.0920758043227E-54	0.284581120975764	0.585	0.375	1.83010063288397E-50	4	EIF4H
1.53095535054897E-46	0.284040714246172	0.358	0.204	2.56557497644997E-42	4	NDUFB2
1.02735195161205E-44	0.283983852301301	0.416	0.251	1.72163640051147E-40	4	PLAAT3
4.07191512354492E-49	0.283651344334889	0.283	0.149	6.82371536403657E-45	4	SAP30BP
2.63583242770058E-54	0.283634107617154	0.297	0.154	4.41712798234063E-50	4	MPP5
1.06561872964909E-71	0.28343547339875	0.574	0.347	1.78576386714594E-67	4	HSF1
1.88659405439377E-45	0.283402540094321	0.643	0.429	3.16155431635307E-41	4	C6
3.37068326746914E-41	0.283240347428623	0.281	0.157	5.64859101962479E-37	4	BRMS1
2.46409285783325E-58	0.283221479284431	0.366	0.2	4.12932681115696E-54	4	CNNM3
7.85500611169439E-41	0.282899290787537	0.492	0.314	1.31634192419775E-36	4	GALK1
4.70393817662082E-47	0.28276686384148	0.428	0.257	7.88285959638116E-43	4	ARRB1
2.94004889838349E-60	0.282691068314544	0.281	0.139	4.92693394391105E-56	4	CDYL
4.85769712225514E-59	0.282633988519112	0.491	0.291	8.14052883747516E-55	4	TMEM41B
4.99662511809776E-81	0.282405901428801	0.391	0.196	8.37334437290823E-77	4	TRAF2
2.23926712763441E-55	0.282076774193654	0.404	0.229	3.75256385248974E-51	4	RNF167
1.32179623677882E-51	0.281707814828783	0.341	0.188	2.21506613359394E-47	4	PBDC1
1.30270950169865E-54	0.281691102133232	0.466	0.278	2.18308058294659E-50	4	DHX9
1.86739205379813E-66	0.281667144985201	0.422	0.23	3.1293756037549E-62	4	DEPRECATED-ENSG00000073578
7.44334419869217E-74	0.281585566580771	0.363	0.183	1.24735562081683E-69	4	FNIP1
3.19505681078978E-49	0.281521226392983	0.332	0.183	5.35427620352152E-45	4	RHOT1
4.2409456506981E-51	0.281445045820402	0.604	0.398	7.10697672143988E-47	4	BLOC1S1
7.17160143401421E-58	0.281326230547894	0.542	0.339	1.2018169683121E-53	4	GNB2
9.57781873200022E-48	0.2810922680332	0.649	0.431	1.6050508631086E-43	4	MGST2
6.5702913655857E-56	0.280729745057795	0.265	0.131	1.10104942704485E-51	4	ANKRD52
1.63058146976981E-33	0.28064546635139	0.266	0.154	2.73252842704025E-29	4	PYM1
7.07409045004305E-53	0.280578158321257	0.405	0.233	1.18547607761821E-48	4	DCUN1D4
8.08384969610969E-72	0.280394367236699	0.553	0.333	1.35469153207406E-67	4	SNRNP200
2.56894194867256E-50	0.280182712722926	0.354	0.197	4.30503291758548E-46	4	PQBP1
1.52800165287829E-76	0.280021166805611	0.405	0.213	2.56062516989344E-72	4	DYRK1A
1.72481070525193E-67	0.279967788783437	0.346	0.176	2.89043777986119E-63	4	ERLIN1
2.78419476223339E-56	0.279737199007615	0.325	0.172	4.66575358255071E-52	4	TRIM56
2.81571545900961E-94	0.279600750077209	0.489	0.255	4.7185759662083E-90	4	NAP1L4
1.85705531757296E-56	0.279449383115478	0.343	0.184	3.11205330118876E-52	4	FAM168A
6.43754741114114E-106	0.279310651764321	0.37	0.163	1.07880419515903E-101	4	TENT4A
1.11822408881297E-55	0.279210140118364	0.453	0.265	1.87391992803278E-51	4	DNAJB2
3.60677929391946E-57	0.279201015860419	0.405	0.229	6.04424074075023E-53	4	VPS35L
3.2894000890295E-54	0.279154040706641	0.979	0.786	5.51237666919563E-50	4	AGT
8.42004302086729E-54	0.279138374264328	0.402	0.229	1.41103080943694E-49	4	CERS6
3.51087055392922E-68	0.278712560031053	0.39	0.208	5.88351687427459E-64	4	ZC3H3
1.74920865745137E-55	0.27866213557355	0.844	0.614	2.931323868157E-51	4	CFL1
2.47736931156062E-47	0.278204933477245	0.303	0.165	4.15157549231328E-43	4	ARF5
1.65197079717135E-34	0.278077673238898	0.276	0.16	2.76837266189976E-30	4	UROD
3.74744866620653E-65	0.277180760538073	0.381	0.203	6.2799744748289E-61	4	RCOR3
5.09989660684436E-65	0.276995701091888	0.603	0.376	8.54640673374978E-61	4	ACTN1
1.24961656121521E-28	0.27697470739907	0.276	0.17	2.09410743328445E-24	4	UNG
4.23604371730834E-45	0.276861360113908	0.669	0.458	7.09876206146532E-41	4	NDFIP1
3.22595071213538E-62	0.276110030439343	0.311	0.157	5.40604820339646E-58	4	AK1
2.0591459612237E-53	0.276076023033699	0.316	0.168	3.45071680181867E-49	4	PDCD5
1.20351365862027E-42	0.275960749700682	0.399	0.242	2.01684818911585E-38	4	PSMC4
3.70186450440819E-74	0.275805677120731	0.549	0.328	6.20358453648725E-70	4	KAT6A
1.67933289572884E-47	0.275537642381712	0.476	0.291	2.8142260666624E-43	4	PACSIN2
7.63400574446924E-50	0.275359298425454	0.843	0.625	1.27930668265815E-45	4	FMO5
9.94406440147322E-67	0.275032473570911	0.316	0.155	1.66642631239888E-62	4	ZNF507
4.33442125123135E-61	0.275014067069064	0.326	0.167	7.2636231328135E-57	4	GTF3A
1.55568319736779E-57	0.274952962835182	0.252	0.12	2.60701390214895E-53	4	AQR
2.10377797643721E-52	0.274928216681425	0.32	0.172	3.52551113291348E-48	4	GART
8.49564213513246E-51	0.274843327960952	0.297	0.157	1.4236997090055E-46	4	NBEAL1
1.53712186827507E-77	0.274741646142045	0.633	0.361	2.57590882685536E-73	4	SLC6A19
2.84355239106638E-53	0.27464949797966	0.313	0.166	4.76522509694903E-49	4	ERI3
5.66221448092826E-56	0.274354659732602	0.318	0.167	9.48873902713957E-52	4	DHRSX

4.65960314775885E-44	0.274269446233142	0.397	0.238	7.80856295501428E-40	4	CAPZA2
1.99750288259466E-54	0.274149667639393	0.445	0.259	3.34741533065214E-50	4	PTK2
3.65800968876665E-49	0.273903663436884	0.806	0.58	6.13009263643516E-45	4	PSME1
1.26696635674169E-41	0.273900610120299	0.313	0.178	2.12318222062772E-37	4	ZMPSTE24
7.06799538297905E-86	0.273660889866105	0.497	0.28	1.18445466627963E-81	4	SUN2
1.15223903294125E-68	0.273631671334178	0.276	0.129	1.93092217140295E-64	4	INPP5E
4.47680105654703E-67	0.273475639685908	0.585	0.349	7.50222321056151E-63	4	SULT1C2
1.56250236700575E-46	0.273364303965813	0.593	0.393	2.61844146662823E-42	4	ATP5IF1
1.40739667543194E-67	0.273259977527394	0.388	0.205	2.35851534868884E-63	4	SGPP1
2.16219844785621E-64	0.27323954851976	0.477	0.273	3.62341215891743E-60	4	TP53BP2
6.24561249915695E-55	0.273166620768349	0.292	0.149	1.04663974260872E-50	4	PRR14L
1.36364840145369E-41	0.273099888606998	0.403	0.246	2.2852019911561E-37	4	EI24
1.30025276348323E-55	0.273063038738219	0.537	0.333	2.1789635810452E-51	4	MAGED2
3.96906079430783E-38	0.272987117506706	0.299	0.173	6.65135207910106E-34	4	RNF14
1.40949516762587E-49	0.272753895355721	0.453	0.273	2.36203200190743E-45	4	NUDT9
3.09158098848132E-52	0.272740174019849	0.352	0.194	5.18087142049699E-48	4	TAF13
6.65068112170984E-42	0.272461062093026	0.745	0.526	1.11452114237614E-37	4	F5
1.32217775012151E-47	0.272459870632848	0.576	0.374	2.21570547365362E-43	4	CLPTM1
1.21890978247195E-38	0.272319591597113	0.291	0.167	2.04264901346649E-34	4	CCNB1IP1
7.58201871807869E-42	0.272155770452467	0.251	0.134	1.27059469677563E-37	4	PSD3
2.42933150068535E-37	0.272064150211449	0.253	0.139	4.07107372884852E-33	4	SYNJ2BP
3.7977676882294E-56	0.271622915348909	0.428	0.243	6.36429909193482E-52	4	GNPAT
1.15286082485343E-43	0.271537521179511	0.335	0.192	1.93196417028938E-39	4	LIMA1
2.79951442136801E-47	0.271510259171114	0.271	0.143	4.69142626732851E-43	4	BCL10
2.61843350631241E-59	0.271054403092391	0.321	0.166	4.38797086987834E-55	4	ADSL
9.9710091573096E-52	0.270614483857997	0.564	0.348	1.67094171458194E-47	4	SMOC1
1.69959721564923E-67	0.27044622677258	0.286	0.136	2.84818501398499E-63	4	MTOR
9.89988957651654E-57	0.270216529202235	0.682	0.406	1.65902349523264E-52	4	TMEM45B
2.71381946633676E-48	0.26993597701662	0.539	0.339	4.54781866168714E-44	4	CRYZ
3.01247784663709E-47	0.269554074873982	0.523	0.331	5.04831037539444E-43	4	ARHGEF26
1.57021651102072E-71	0.26954822294937	0.506	0.288	2.63136882916853E-67	4	FAM177A1
1.84241968688647E-50	0.269253343242787	0.356	0.2	3.08752691128435E-46	4	WDR20
2.96032179076185E-58	0.269235783509402	0.342	0.181	4.96090725695871E-54	4	CAMSAP2
1.116762658355E-50	0.269181390396692	0.381	0.217	1.87147086287131E-46	4	AP1G1
1.5460118283505E-36	0.268772051713052	0.378	0.236	2.59080662194976E-32	4	ST3GAL6
5.13612837061011E-29	0.268334823521299	0.281	0.171	8.60712392346842E-25	4	OSTC
4.27811357302578E-53	0.267836394067186	0.501	0.306	7.16926272567659E-49	4	UHMK1
5.94961803365546E-52	0.267444648211486	0.36	0.202	9.97036990079982E-48	4	BUD31
5.28872144398932E-45	0.267125534754987	0.66	0.449	8.86283939583731E-41	4	PSMB1
2.09040415024258E-59	0.266691743729701	0.327	0.169	3.50309927497651E-55	4	CHM
3.79953687136238E-66	0.266638511957364	0.286	0.138	6.36726388902908E-62	4	DPY19L1
9.41798849810625E-59	0.266629252407682	0.356	0.19	1.57826651251265E-54	4	FNIP2
4.84409916216302E-41	0.266618004588996	0.64	0.464	8.1177413759528E-37	4	ANKRD10
5.37260501713316E-62	0.266381089259945	0.475	0.274	9.00341148771174E-58	4	PPM1A
5.27897793174271E-60	0.266046039869304	0.3	0.151	8.84651121801444E-56	4	KDM4A
1.01305134430709E-40	0.265890367807647	0.295	0.166	1.69767144278983E-36	4	COG2
2.34997538304597E-56	0.265724620234245	0.271	0.135	3.93808874690844E-52	4	NCBP1
2.78078615900624E-61	0.265677676312244	0.549	0.339	4.66004144526266E-57	4	AAMP
6.94756764147877E-75	0.26558096780894	0.446	0.24	1.16427338535901E-70	4	TNFSF10
1.0702410913737E-48	0.265541710765368	0.37	0.213	1.79351002092405E-44	4	DEPRECATED-ENSG00000196821
5.76946480616269E-42	0.265103054275303	0.35	0.207	9.66846912216743E-38	4	COX17
4.57750733984539E-50	0.264713642003914	0.519	0.324	7.67098680011291E-46	4	UFC1
5.07377995394736E-35	0.26447655826584	0.384	0.239	8.50264044682499E-31	4	ALG3
5.85913635876394E-76	0.264087519005392	0.394	0.202	9.81874071001662E-72	4	RANBP3
2.0072622925262E-52	0.264035518408505	0.468	0.283	3.3637701498154E-48	4	MAT2B
7.04791927841348E-28	0.263994239152723	0.32	0.206	1.18109031267653E-23	4	MCM6
1.41603618605563E-40	0.26387787114156	0.567	0.378	2.37299344059202E-36	4	COX6A1
7.14817048066432E-52	0.263849363444571	0.506	0.311	1.19789040914973E-47	4	SPECC1L
1.89990929921072E-45	0.263829664100947	0.465	0.284	3.18386800361733E-41	4	EPN1
2.61044435147663E-41	0.263636409269285	0.379	0.225	4.37458264420454E-37	4	NDUFA9

8.75529160287706E-49	0.263581170111052	0.32	0.176	1.46721176681014E-44	4	AMACR
1.21730526312827E-53	0.263539286144582	0.361	0.199	2.03996015995035E-49	4	RCN1
1.16727969680472E-59	0.263456309054125	0.321	0.165	1.95612731590535E-55	4	CMTR1
1.48751797128294E-55	0.263328880288186	0.392	0.221	2.49278261627595E-51	4	PURA
1.20023206406345E-62	0.26332156348788	0.255	0.119	2.01134889295753E-58	4	USP28
1.80975730862954E-44	0.263319937518291	0.567	0.371	3.03279129780138E-40	4	HADHB
1.15464851678062E-44	0.263282023192811	0.637	0.424	1.93495998442097E-40	4	PGRMC2
4.95208377996259E-49	0.262890706636275	0.562	0.352	8.2987019984613E-45	4	TM7SF3
9.42478081660588E-46	0.262824309045587	0.329	0.185	1.57940476924681E-41	4	YIPF5
1.67326085241459E-49	0.262536620694931	0.366	0.209	2.80405053647637E-45	4	GORASP1
7.13239405703428E-72	0.262519816024624	0.334	0.164	1.1952465960778E-67	4	MTMR12
1.30808431513143E-39	0.262346750052596	0.722	0.527	2.19208769529725E-35	4	GOLPH3
1.60301550793459E-42	0.262344944656841	0.757	0.538	2.68633338819678E-38	4	APLP2
1.17948981406585E-55	0.262340026854325	0.653	0.439	1.97658903041155E-51	4	PICALM
8.7146110766752E-54	0.262324760305396	0.396	0.224	1.46039452422923E-49	4	HOOK1
1.33994406783492E-45	0.261990878393545	0.274	0.147	2.24547826887775E-41	4	FAM98C
6.37488982841139E-40	0.261976224902719	0.339	0.201	1.06830403744518E-35	4	SLC1A5
4.59508251119226E-47	0.261821469767986	0.37	0.212	7.70043927225599E-43	4	DCUN1D1
4.39738240901788E-58	0.261274699239035	0.323	0.169	7.36913344103217E-54	4	ERMP1
2.91943967116093E-60	0.26127314729908	0.281	0.137	4.89239700093149E-56	4	GTF2H4
6.74440514364849E-57	0.261215749957364	0.27	0.133	1.13022741397261E-52	4	ATE1
1.70516333579049E-54	0.261047796821513	0.285	0.145	2.85751271811771E-50	4	PLEKHA3
6.46700236468726E-48	0.260649024752334	0.604	0.384	1.08374025627429E-43	4	DEGS1
7.89987110260479E-36	0.260598334822255	0.838	0.648	1.32386039937451E-31	4	ECH1
6.33958397036084E-37	0.260493619154061	0.291	0.167	1.06238748175307E-32	4	CAMSAP3
2.02368316302233E-52	0.260010202492638	0.269	0.136	3.39128824459281E-48	4	OGG1
1.34509067255941E-44	0.25971774671527	0.313	0.176	2.25410294907505E-40	4	CPPED1
5.40446014024432E-45	0.25939996428225	0.253	0.132	9.05679430302142E-41	4	NSA2
2.7258995132036E-52	0.25934943074126	0.503	0.307	4.5680624042266E-48	4	FGGY
1.05802142364804E-58	0.259308927766235	0.483	0.287	1.77303230174938E-54	4	VPS45
1.29030346770133E-44	0.2589484660079	0.6	0.39	2.16229055117388E-40	4	WWP1
4.44325796569414E-49	0.258756391998821	0.426	0.254	7.44601169891024E-45	4	SRA1
1.351821475157E-59	0.258167333147611	0.278	0.136	2.2653824280681E-55	4	DIPK2A
2.07342625331806E-49	0.25790172659801	0.544	0.341	3.4746477153104E-45	4	ACSM2B
1.27801253250021E-45	0.257878297693324	0.274	0.146	2.14169340196385E-41	4	DMAC2L
4.74687041156156E-62	0.257876425362502	0.27	0.129	7.95480543569485E-58	4	PATL1
3.43335416793971E-57	0.257425496845063	0.344	0.184	5.75361491463337E-53	4	EEF1AKNMT
2.08773468512403E-48	0.257299499454994	0.34	0.189	3.49862578533086E-44	4	GCH1
2.25360810844095E-55	0.2572540617746	0.395	0.223	3.77659646812535E-51	4	RMDN3
1.70848957774933E-82	0.257229309282114	0.451	0.238	2.86308683439232E-78	4	TRA2B
1.63110905085473E-39	0.256540506543007	0.273	0.152	2.73341254742235E-35	4	VEZT
1.58555654300289E-54	0.256516643369525	0.433	0.25	2.65707565476423E-50	4	VPS4A
6.34102129874648E-41	0.256511758486892	0.306	0.174	1.06262834924393E-36	4	GTF3C6
4.64670375304892E-55	0.256448894021134	0.286	0.146	7.78694614935939E-51	4	BCL7B
4.38817742506314E-50	0.256224666966792	0.439	0.26	7.3537077289208E-46	4	RAPH1
8.19611400392215E-37	0.256186762538936	0.408	0.256	1.37350478477727E-32	4	COX6B1
8.0000601315626E-97	0.2561267896298	0.298	0.124	1.34065007684726E-92	4	PNRC2
1.62202904155301E-35	0.256064971894719	0.548	0.362	2.71819626783453E-31	4	MCFD2
2.99545691059457E-45	0.255992167407029	0.612	0.408	5.01978669077437E-41	4	DEPRECATED-ENSG00000089248
9.89463401112894E-60	0.255866935862248	0.619	0.39	1.65814276758499E-55	4	SLC2A4RG
2.32448123178261E-40	0.255684501373803	0.278	0.154	3.8953656482213E-36	4	YIPF6
1.91062798104312E-49	0.255436523945358	0.539	0.351	3.20183037063205E-45	4	HSP90B1
8.66998082189661E-49	0.255386674581337	0.273	0.143	1.45291538613343E-44	4	STYX
1.37415641714938E-62	0.255359799159725	0.385	0.207	2.30281132385893E-58	4	PAPOLA
2.67135150874633E-52	0.255312763413433	0.397	0.227	4.4766508583571E-48	4	GOPC
8.3428091314486E-57	0.255221195468791	0.407	0.23	1.39808795424816E-52	4	AFTPH
1.9530753817563E-45	0.255071167168482	0.393	0.233	3.27296372474721E-41	4	GNAI3
7.68472095670135E-88	0.254880100908107	0.304	0.135	1.28780553792401E-83	4	MALT1
1.89211056515423E-59	0.254803505203042	0.891	0.615	3.17079888508546E-55	4	IFI27
9.49579616416324E-57	0.254766733490552	0.39	0.22	1.59130552119048E-52	4	AIP

8.74142686470254E-43	0.254734026326861	0.365	0.215	1.46488831398685E-38	4	ARL 1.00
7.8090620897637E-56	0.254391777928892	0.28	0.141	1.3086426250026E-51	4	SESTD1
8.73402637140626E-52	0.254240892084153	0.303	0.16	1.46364813932026E-47	4	OGFOD2
3.2399506404559E-44	0.254195735282094	0.616	0.413	5.429509283276E-40	4	ARL6IP4
1.54131131238561E-53	0.253992582796266	0.487	0.3	2.58292949729581E-49	4	UXT
4.20506425769994E-32	0.253742463433809	0.252	0.147	7.04684668305356E-28	4	PIAS3
2.38610332919929E-44	0.253535305559058	0.256	0.135	3.99863195907217E-40	4	MSTO1
1.16334187332491E-35	0.253497837792032	0.427	0.27	1.94952831131788E-31	4	OCIAD2
8.46129378048044E-38	0.252910559797439	0.498	0.326	1.41794361173291E-33	4	POLR2E
6.40409477171607E-50	0.252581210968188	0.396	0.229	1.07319820184418E-45	4	SLC4A2
1.25999818435753E-41	0.252519987861238	0.254	0.136	2.11150495734635E-37	4	URM1
9.23672002255252E-47	0.252218049156158	0.434	0.264	1.54788954137935E-42	4	NDUFAF3
4.1145171638266E-77	0.252128590592221	0.458	0.247	6.89510786314061E-73	4	RUFY1
2.02631374490854E-52	0.252067726858768	0.3	0.158	3.39569657371772E-48	4	SLC19A2
7.94776072612106E-72	0.251660675308713	0.307	0.147	1.33188574248337E-67	4	ZDHHC20
4.72593488339225E-35	0.251365428968185	0.261	0.147	7.91972167758873E-31	4	COG1
3.54405127055516E-38	0.251304563460825	0.552	0.367	5.93912111919634E-34	4	DEPRECATED-ENSG00000159228
5.14112687316835E-97	0.251271463239148	0.39	0.183	8.61550041405552E-93	4	CENPT
1.16142123360628E-47	0.250993266655384	0.457	0.275	1.9463097032774E-43	4	FOXO3
1.64422276437311E-54	0.250914607082996	0.318	0.168	2.75538850853645E-50	4	TMEM8A
6.0100660288554E-51	0.250655639524198	0.264	0.135	1.00716686511559E-46	4	EEFSEC
1.48583336350771E-49	0.250602355851528	0.445	0.267	2.48995955056622E-45	4	IFI6
2.62028518310315E-103	0.250564276599161	0.748	0.487	4.39107390984426E-99	4	BTG1
4.02795277122732E-56	0.25041308129458	0.4	0.225	6.75004325402275E-52	4	GTF2H2C
5.03235777635665E-31	0.250321766385648	0.252	0.146	8.43322516161847E-27	4	C19orf33
3.19281275908762E-53	0.250104127767465	0.418	0.244	5.35051562167903E-49	4	TMEM183A
0	8.47154417996964	0.387	0.004	0	5	PRF1
0	8.14247473025893	0.311	0.008	0	5	GZMA
0	7.87114292909888	0.315	0.004	0	5	CD3G
0	7.81575460151806	0.466	0.006	0	5	IL2RB
0	7.65153598288216	0.676	0.013	0	5	TRAC
0	7.63604702227465	0.296	0.004	0	5	CXCR6
0	7.56694400378697	0.315	0.004	0	5	TIGIT
0	7.4010698071399	0.252	0.005	0	5	KLRK1
0	7.38723636546461	0.311	0.01	0	5	NKG7
0	7.36022866412259	0.492	0.007	0	5	CD3E
0	7.35584325522862	0.262	0.006	0	5	CTSW
0	7.33466580496358	0.313	0.003	0	5	CD247
0	7.29292195877843	0.339	0.004	0	5	LCK
0	7.2814629129559	0.396	0.006	0	5	CD7
0	7.27267219722516	0.509	0.011	0	5	CD2
0	7.27010386582963	0.397	0.008	0	5	TRBC1
0	7.15889246211546	0.454	0.008	0	5	CD96
0	7.14565311650026	0.37	0.01	0	5	CD3D
0	7.12992028555145	0.653	0.02	0	5	TRBC2
0	7.12617508231672	0.254	0.003	0	5	ICOS
0	6.91724365146118	0.286	0.005	0	5	CD27
0	6.85537147291011	0.57	0.012	0	5	SPOCK2
0	6.82806406253831	0.339	0.006	0	5	ZAP70
0	6.821200932323	0.259	0.004	0	5	SLAMF1
0	6.79171737054161	0.298	0.005	0	5	PYHIN1
0	6.78772305018445	0.392	0.005	0	5	RASGRP1
0	6.77252177209929	0.447	0.007	0	5	ITK
0	6.74250983268037	0.36	0.005	0	5	CD5
0	6.68258930175718	0.57	0.019	0	5	IL7R
0	6.48595671302038	0.37	0.019	0	5	CCL5
0	6.42182795254782	0.351	0.005	0	5	CD6
0	6.38588770915166	0.481	0.012	0	5	IKZF3
0	6.31641876868048	0.364	0.01	0	5	SLAMF6
0	6.31057881130696	0.28	0.008	0	5	IPCEF1

0	6.22628977487779	0.361	0.024	0	5	BCL11B
0	6.17593326127112	0.35	0.01	0	5	CD69
0	6.09933984491295	0.325	0.013	0	5	CCR5
0	6.09672922192305	0.412	0.016	0	5	ITM2A
0	6.06394598239684	0.291	0.013	0	5	CD28
0	6.04506347112668	0.272	0.006	0	5	SKAP1
0	6.03364271085169	0.388	0.013	0	5	CST7
0	5.88964964930167	0.392	0.011	0	5	MYO1G
0	5.79382129850417	0.271	0.017	0	5	PTPN22
0	5.76252441726072	0.365	0.015	0	5	PTPN7
0	5.70646976899166	0.361	0.013	0	5	CCND2
0	5.69746096863672	0.264	0.009	0	5	RHOH
0	5.69667270555386	0.438	0.02	0	5	LTB
0	5.60653465194599	0.329	0.008	0	5	TRAF3IP3
0	5.60489001684607	0.381	0.022	0	5	STAT4
0	5.58904371704068	0.443	0.026	0	5	SEPTIN1
0	5.55137096977226	0.261	0.012	0	5	PSTPIP1
0	5.54932890622796	0.513	0.017	0	5	ACAP1
0	5.51244020348745	0.337	0.012	0	5	TBC1D10C
0	5.47183458598555	0.284	0.011	0	5	CYFIP2
0	5.46873170298024	0.444	0.019	0	5	SPN
0	5.46630393096019	0.287	0.018	0	5	LAT
0	5.4654152937128	0.388	0.014	0	5	FYN
0	5.44528579432281	0.375	0.018	0	5	DGKA
0	5.44305110382852	0.421	0.034	0	5	S100A4
0	5.36971406302759	0.363	0.017	0	5	RASAL3
0	5.28776244105452	0.301	0.016	0	5	SEMA4D
0	5.24636280877073	0.291	0.014	0	5	SYTL1
0	5.23605112902645	0.52	0.043	0	5	ITGAL
0	5.20780539046643	0.587	0.037	0	5	IKZF1
0	5.18729874788212	0.46	0.034	0	5	PTPRC
0	5.16791722154873	0.294	0.028	0	5	EMB
0	5.15799753147597	0.588	0.035	0	5	CORO1A
0	5.14524325495265	0.521	0.041	0	5	RAC2
0	5.13031201062294	0.317	0.013	0	5	CCDC88C
0	5.05413348097254	0.326	0.021	0	5	GBP5
0	5.05294538185135	0.307	0.026	0	5	PRKCH
0	5.03126055798677	0.672	0.076	0	5	ETS1
0	4.9779891243258	0.271	0.01	0	5	FCMR
0	4.961456173261	0.347	0.018	0	5	DUSP2
0	4.95004973039874	0.255	0.02	0	5	DENND1C
0	4.91142250307721	0.743	0.13	0	5	IL2RG
0	4.90138372166223	0.678	0.049	0	5	CYTIP
0	4.88281873241844	0.275	0.016	0	5	GNG2
0	4.87269320256603	0.278	0.012	0	5	ATP2A3
0	4.86440524773228	0.317	0.022	0	5	CRIP1
0	4.79628848844994	0.324	0.031	0	5	CD52
0	4.74014823267725	0.376	0.026	0	5	DEF6
0	4.72193873108765	0.524	0.048	0	5	SLA
0	4.67309002842423	0.372	0.037	0	5	SELPLG
0	4.65900429471181	0.404	0.024	0	5	TMC8
0	4.64272621290741	0.462	0.042	0	5	PRDM1
0	4.63703008342663	0.935	0.103	0	5	CXCR4
0	4.60712896823035	0.371	0.023	0	5	JAK3
0	4.60035831643547	0.385	0.03	0	5	ARHGAP9
0	4.60008079464354	0.365	0.027	0	5	SASH3
0	4.58766095387002	0.262	0.023	0	5	FGD3
0	4.56243579211068	0.32	0.037	0	5	SLFN5
0	4.5613420588767	0.394	0.046	0	5	FYB1
0	4.52503821401648	0.402	0.055	0	5	TRAF1

0	4.4583822381979	0.624	0.144	0	5	AKNA
0	4.45088180633833	0.308	0.026	0	5	ARHGAP15
0	4.4435597518962	0.405	0.034	0	5	FMNL1
0	4.41163218178924	0.692	0.079	0	5	WIPF1
0	4.36395289316438	0.549	0.128	0	5	STK17B
0	4.36128728538958	0.343	0.03	0	5	ARHGAP30
0	4.24573082717262	0.327	0.033	0	5	MDFIC
0	4.24396388244587	0.449	0.047	0	5	ITGA4
0	4.22892500813781	0.454	0.112	0	5	CLEC2D
0	4.18417513014695	0.646	0.063	0	5	CELF2
0	4.14948156133587	0.339	0.034	0	5	DOCK8
0	4.14641862929278	0.294	0.029	0	5	LBH
0	4.14025144277379	0.539	0.059	0	5	CD53
0	4.1304116527464	0.362	0.06	0	5	BCL2
0	4.12380234901331	0.326	0.028	0	5	TAGAP
0	4.09659192767896	0.433	0.062	0	5	PIK3IP1
0	4.08611709603441	0.644	0.09	0	5	LCP1
1.24121525958843E-269	4.07462716720339	0.359	0.116	2.08002853201829E-265	5	PARP8
0	4.04011053648089	0.7	0.281	0	5	ZFP36L2
0	3.9955695699743	0.561	0.085	0	5	ARHGDIB
0	3.97775565347021	0.342	0.046	0	5	GPSM3
0	3.94800755699107	0.373	0.045	0	5	IL10RA
0	3.93795310222113	0.275	0.041	0	5	LIMD2
0	3.9320880919902	0.322	0.079	0	5	NBEAL2
0	3.93081605233269	0.407	0.109	0	5	NLRC5
4.32077519548834E-278	3.92121580904866	0.353	0.108	7.24075507259936E-274	5	STK17A
0	3.88939442924427	0.325	0.05	0	5	ARHGAP45
0	3.85208687505953	0.576	0.136	0	5	CD44
0	3.84048851331906	0.312	0.037	0	5	S100A6
0	3.83305413847294	0.296	0.033	0	5	IL16
4.91389999963454E-296	3.82296007767755	0.321	0.084	8.23471361938756E-292	5	CHD3
0	3.77365318742437	0.303	0.07	0	5	LPXN
0	3.77032075929717	0.389	0.045	0	5	DOCK2
0	3.76148119414102	0.314	0.069	0	5	ISG20
0	3.74513055445422	0.469	0.074	0	5	FXYD5
0	3.74472693201726	0.508	0.077	0	5	RGS1
0	3.74451740027374	0.331	0.076	0	5	CCDC88B
0	3.74173217181469	0.394	0.037	0	5	ITPKB
0	3.73532132575294	0.269	0.047	0	5	GIMAP4
4.09944455900509E-178	3.73070556097546	0.261	0.083	6.86984919198073E-174	5	OXNAD1
0	3.71623679970963	0.254	0.044	0	5	SEPTIN6
0	3.71566010032663	0.261	0.038	0	5	LDHB
0	3.67287529289312	0.385	0.048	0	5	UCP2
0	3.67104374751058	0.487	0.103	0	5	TRIM22
0	3.60954606832974	0.333	0.035	0	5	CD37
0	3.58891011542207	0.419	0.052	0	5	CHST11
6.82476566420816E-205	3.57044828259052	0.288	0.09	1.143694230008E-200	5	DENND2D
0	3.56663217601513	0.262	0.035	0	5	INPP5D
0	3.54623521565086	0.412	0.056	0	5	LCP2
0	3.52991566642057	0.411	0.106	0	5	TNFRSF1B
3.03513057514933E-202	3.52763551390197	0.255	0.072	5.08627181783525E-198	5	ICAM3
6.83208929667637E-282	3.5256578134357	0.503	0.228	1.14492152433703E-277	5	STK4
0	3.52449073419868	0.259	0.035	0	5	ST8SIA4
4.25516937464501E-274	3.52441011662282	0.333	0.096	7.13081283803011E-270	5	ANKRD44
0	3.5237539404786	0.825	0.134	0	5	SRGN
2.53735181806489E-179	3.51522296319019	0.258	0.08	4.25209417671314E-175	5	CASP8
0	3.51515987636122	0.384	0.107	0	5	STK10
0	3.51208492597479	0.572	0.067	0	5	LSP1
2.66032742849295E-231	3.49102249295065	0.302	0.09	4.45817670466849E-227	5	PIM2
3.86950233419123E-280	3.44451046849181	0.29	0.07	6.48451201163766E-276	5	RASSF5

0	3.43230570245661	0.311	0.048	0	5	RCSD1
3.56318861208026E-203	3.42738642850093	0.469	0.237	5.97119147612411E-199	5	TNFAIP3
0	3.42308201119459	0.512	0.17	0	5	ARHGEF1
3.92385574038799E-307	3.42216853371401	0.289	0.064	6.57559744974219E-303	5	SOCS1
4.91237285605175E-265	3.3764155571475	0.271	0.064	8.23215443217153E-261	5	KCNAB2
2.71687601620429E-166	3.36620392530096	0.29	0.106	4.55294082795515E-162	5	TRAF5
4.00921064824718E-154	3.36484756374484	0.318	0.133	6.71863520433263E-150	5	GPRIN3
1.59471506185253E-240	3.36013974143841	0.396	0.152	2.67242350065246E-236	5	CBLB
1.30384997598582E-233	3.35803339064679	0.449	0.205	2.18499178975703E-229	5	RESF1
5.60723062351123E-250	3.33183023963649	0.364	0.122	9.39659707888012E-246	5	ARID5B
6.80900100315513E-279	3.31230401580823	0.297	0.074	1.14105238810874E-274	5	PTK2B
0	3.30451470879907	0.271	0.044	0	5	NCKAP1L
0	3.28901464079871	0.44	0.085	0	5	IFI16
2.37541478439407E-148	3.28251364289702	0.459	0.278	3.98072009568758E-144	5	CNOT6L
1.76131175455354E-128	3.27644850385849	0.361	0.185	2.95160623828082E-124	5	LEPROTL1
2.43737325961144E-151	3.26644639038062	0.408	0.216	4.08455010845686E-147	5	SMCHD1
0	3.22942366690746	0.262	0.044	0	5	MYO1F
1.06691667021591E-171	3.22732890137531	0.366	0.162	1.78793895594782E-167	5	KAT2B
0	3.22691238701581	0.39	0.112	0	5	ARHGAP4
1.15965136303895E-121	3.22258991675405	0.305	0.141	1.94334375418068E-117	5	CCSER2
1.13555230800725E-88	3.20765751621379	0.29	0.151	1.90295855775856E-84	5	PBXIP1
0	3.18913572805321	0.554	0.076	0	5	ITGB2
1.0102457194953E-135	3.17401713907073	0.289	0.12	1.69296977673022E-131	5	PHTF2
0	3.14745060754222	0.296	0.053	0	5	TMEM173
0	3.14140541130706	0.441	0.1	0	5	ANXA6
0	3.14099343306596	0.344	0.047	0	5	DOK2
1.1836045506523E-139	3.13727698639333	0.265	0.099	1.98348450598313E-135	5	SH3KBP1
0	3.10986994457203	0.329	0.052	0	5	CMTM3
1.01547705293968E-150	3.06009697257777	0.256	0.088	1.70173644531632E-146	5	PREX1
4.06865111961589E-133	3.05755214344291	0.296	0.127	6.81824554625231E-129	5	DOCK10
1.48010096464398E-275	3.04905112838923	0.304	0.076	2.48035319655038E-271	5	NLRP1
7.0561249594674E-141	3.02406905542029	0.289	0.116	1.18246542070755E-136	5	MOB3A
1.21473998246449E-136	3.01469263037291	0.252	0.091	2.035661262614E-132	5	MICAL1
3.54817354179901E-183	3.00970756426684	0.339	0.13	5.94602922134678E-179	5	PIM1
6.88189686940677E-114	3.00634994586397	0.394	0.235	1.15326827737519E-109	5	SLC38A1
1.07907580341788E-305	2.97253833269076	0.259	0.05	1.80831523136769E-301	5	LPAR6
9.95623590967083E-53	2.96965074118019	0.321	0.223	1.66846601374264E-48	5	PPP2R5C
0	2.94880763243418	0.688	0.151	0	5	LAPTM5
5.71303897718496E-105	2.93236605867888	0.365	0.208	9.57391071796655E-101	5	HECA
5.71571521873154E-54	2.92248117535753	0.266	0.162	9.57839556355032E-50	5	PITPNC1
3.46017766889592E-67	2.90493647016798	0.279	0.162	5.79856573753577E-63	5	PDE7A
0	2.9030985228763	0.427	0.07	0	5	HCLS1
7.82040932162523E-80	2.88290577027927	0.284	0.153	1.31054419411796E-75	5	PCSK7
3.09926658061024E-92	2.88025846717178	0.272	0.132	5.19375093578664E-88	5	INPP4A
1.38330553867494E-68	2.87301272295463	0.286	0.165	2.31814342171147E-64	5	HNRNPLL
6.51770733929238E-103	2.87163113551021	0.277	0.129	1.09223739591862E-98	5	FOXO1
9.88810797473071E-137	2.85503888101236	0.32	0.14	1.65704913440537E-132	5	SIPA1
3.78441189738287E-174	2.82581919800054	0.26	0.081	6.34191745763421E-170	5	FERMT3
8.38186960859868E-227	2.82399838075924	0.256	0.063	1.40463370900897E-222	5	SERPINB9
4.45467264135956E-188	2.82192069010241	0.366	0.149	7.46514041239036E-184	5	FLNA
1.45359072165677E-167	2.78668563459274	0.26	0.083	2.43592733135241E-163	5	ABCC1
7.28943145105451E-270	2.78119540541293	0.57	0.306	1.22156292256771E-265	5	ADGRE5
4.048347650844E-125	2.7657753832127	0.336	0.164	6.78422099328437E-121	5	PLCG1
1.00730134983183E-91	2.76425532317012	0.264	0.126	1.68803560204817E-87	5	PIP4K2A
0	2.76097125618978	0.339	0.073	0	5	CD4
0	2.75994761029095	0.66	0.408	0	5	EVL
6.07955498236364E-214	2.72931027537109	0.323	0.106	1.0188118239445E-209	5	TSPAN14
2.23856789019943E-81	2.71897851123754	0.294	0.158	3.7513920703962E-77	5	FNBP1
1.42876392321513E-95	2.68704427775236	0.274	0.13	2.39432258252392E-91	5	PKM
0	2.67456920120286	0.787	0.464	0	5	TXNIP

0	2.67367820060449	0.47	0.081	0	5	CYBA
3.49076614163857E-93	2.66886612417101	0.424	0.293	5.84982590015791E-89	5	RBL2
4.36417921223809E-73	2.6657027768279	0.349	0.225	7.31349152386859E-69	5	ELF1
4.49347703104561E-84	2.64812007508084	0.341	0.205	7.53016880862624E-80	5	AAK1
3.03356635633527E-91	2.63839877740024	0.322	0.178	5.08365049994664E-87	5	CCND3
1.58331400448513E-38	2.63221181780149	0.3	0.219	2.65331760871618E-34	5	DUSP16
2.95128320859629E-221	2.63156849864405	0.412	0.166	4.94576040096567E-217	5	ARL4C
4.15227306623306E-211	2.62535643003782	0.548	0.316	6.95837920439336E-207	5	SMAP2
3.33352641231748E-59	2.62343594649308	0.28	0.17	5.58632356176163E-55	5	LNPEP
6.32929554802746E-217	2.59933025120618	0.524	0.284	1.06066334793844E-212	5	OGA
7.16404817104085E-108	2.59875008861336	0.435	0.286	1.20055119250303E-103	5	SUN2
0	2.59520269722196	0.6	0.157	0	5	VIM
1.60376443407715E-86	2.594547754389	0.358	0.22	2.68758843862648E-82	5	SYNRG
2.47596581669433E-40	2.58408335924635	0.318	0.235	4.14922351561636E-36	5	SARAF
1.61526456234063E-114	2.57481500749312	0.361	0.196	2.70686035357043E-110	5	ARL6IP5
2.26330750896423E-83	2.56784885694107	0.355	0.221	3.79285072352225E-79	5	CBFB
3.10169625194446E-245	2.56672761737204	0.582	0.344	5.19782257900852E-241	5	MSN
7.75639227966425E-39	2.55600270526149	0.313	0.239	1.29981621822613E-34	5	LYST
2.60915092286811E-45	2.54510458890555	0.303	0.21	4.37241511654238E-41	5	ATM
1.79324581035178E-63	2.52723759155486	0.316	0.201	3.00512132898752E-59	5	RAB27A
1.45691689250262E-121	2.50853418972196	0.427	0.257	2.44150132845589E-117	5	ZC3HAV1
2.11137796974249E-129	2.50600412667214	0.542	0.396	3.53824720169447E-125	5	CDKN1B
1.28991927833749E-26	2.49362107116854	0.27	0.206	2.16164672663797E-22	5	BRD1
8.66450340710649E-285	2.48921832160088	0.691	0.492	1.45199748096291E-280	5	BTG1
1.04748370981226E-106	2.46161833259943	0.354	0.194	1.75537320090338E-102	5	CSK
9.24964394413568E-51	2.45898280873537	0.366	0.275	1.55005533215826E-46	5	CDC42SE2
3.98645434067262E-48	2.45382809393977	0.259	0.161	6.68050018409917E-44	5	CYLD
2.2189027196037E-134	2.44270459830967	0.316	0.137	3.71843717751188E-130	5	PPP1R18
1.55619233120594E-75	2.43447002536944	0.443	0.338	2.60786710863491E-71	5	PDCD4
2.0499174936923E-43	2.43217441298779	0.295	0.204	3.43525173592955E-39	5	NFATC2
2.53679106015704E-72	2.43174819054854	0.409	0.292	4.25115445861117E-68	5	CYTH1
3.93377480915079E-38	2.41425464399954	0.267	0.184	6.59221982517489E-34	5	GNPTAB
8.13449213990732E-47	2.37172834952454	0.282	0.185	1.36317819280567E-42	5	WDR82
1.4498261063265E-198	2.35383193128647	0.6	0.412	2.42961858898195E-194	5	SYNE2
1.18664194304919E-09	2.34884550284696	0.253	0.233	1.98857456816183E-05	5	CLDND1
5.68900491940997E-107	2.33975714421731	0.475	0.333	9.53363444394722E-103	5	CDC42SE1
1.10759022120766E-46	2.31767631177189	0.45	0.395	1.85609969269979E-42	5	EML4
1.78042828381577E-39	2.31571559460444	0.27	0.183	2.98364171801847E-35	5	FGFR1OP2
1.00049697179664E-88	2.28841898886864	0.42	0.283	1.6766328253368E-84	5	GRK2
5.13558715179E-25	2.28077918681049	0.259	0.198	8.60621694896969E-21	5	PCNX1
4.8251777203714E-126	2.27686922019496	0.47	0.302	8.0860328237984E-122	5	MACF1
2.02147638820346E-52	2.27250226762962	0.259	0.153	3.38759013135135E-48	5	PACS1
1.58086713333568E-32	2.26496337231466	0.271	0.197	2.64921714204393E-28	5	KMT2A
1.07799780357168E-72	2.245309459021	0.357	0.23	1.80650871922542E-68	5	CNN2
6.57657223228138E-108	2.2236311257012	0.476	0.335	1.10210197468571E-103	5	OGT
9.3547287328502E-86	2.22250211342404	0.44	0.314	1.56766544105104E-81	5	JAK1
5.49252490632749E-101	2.21765632394438	0.446	0.299	9.20437323802361E-97	5	AKAP13
2.11744163991028E-25	2.19486653097134	0.267	0.205	3.54840870016165E-21	5	TNRC6B
3.79885632731952E-195	2.18599757310063	0.618	0.448	6.36612343332205E-191	5	RNF213
3.52308499152215E-42	2.18064768167782	0.37	0.291	5.90398582879282E-38	5	KMT2E
6.77353170371186E-51	2.17816425560893	0.303	0.201	1.13510844290803E-46	5	APBB1IP
3.36190980157646E-19	2.17168185945549	0.31	0.27	5.63388844548184E-15	5	RORA
8.41318007977294E-76	2.16180804783644	0.415	0.293	1.40988071776835E-71	5	MLLT6
1.63009094967316E-92	2.16144474764759	0.427	0.29	2.73170641346228E-88	5	SH3BGRL3
7.85789112799554E-22	2.12668166169551	0.258	0.2	1.31682539522949E-17	5	TERF2IP
3.59186664554265E-39	2.11635998254241	0.301	0.219	6.01925012460038E-35	5	CKLF
4.04388406542833E-86	2.11440468995224	0.467	0.359	6.7767409168448E-82	5	MBNL1
7.63452028422366E-21	2.10939629021591	0.283	0.232	1.2793929092302E-16	5	CEMIP2
9.08544214312408E-40	2.09823976274314	0.327	0.251	1.52253839434473E-35	5	LRBA
1.33945497518968E-106	2.09570848976094	0.489	0.352	2.24465864742287E-102	5	TAP1

7.15429678435469E-17	2.09417416035564	0.258	0.216	1.19891705512216E-12	5	DIP2A
0	2.09189686999041	0.832	0.707	0	5	TMSB4X
5.13386771888455E-45	2.06051469185161	0.341	0.253	8.60333552330674E-41	5	TSC22D4
4.22148553177339E-16	2.05078975528136	0.26	0.221	7.07436545414584E-12	5	CASK
4.47515762245622E-78	2.03132077817729	0.338	0.199	7.49946914371214E-74	5	LY6E
1.1641771349536E-28	2.01940338132968	0.277	0.208	1.95092804275524E-24	5	ATP2B4
1.11670620848137E-53	2.00586806613871	0.355	0.255	1.87137626417308E-49	5	MYH9
4.50063938186587E-32	1.99077381275562	0.33	0.263	7.54217147613082E-28	5	NUP210
9.77518201622731E-123	1.99029132586071	0.521	0.368	1.63812500227937E-118	5	TMSB10
5.61943870233557E-59	1.984808085274	0.374	0.269	9.41705537737395E-55	5	SP100
3.75888980535832E-65	1.981650753115	0.457	0.366	6.29914753581947E-61	5	DIAPH1
1.96771706957611E-10	1.96906422985694	0.269	0.248	3.29750026519564E-06	5	HIPK1
1.02754928917535E-14	1.96837543592223	0.426	0.435	1.72196709880005E-10	5	PIK3R1
6.78592415701141E-35	1.94484665986447	0.399	0.336	1.13718517023197E-30	5	RNF149
6.24839044800741E-30	1.94442648377815	0.294	0.225	1.04710527127708E-25	5	CD99
1.32763215942002E-38	1.92332557004866	0.406	0.347	2.22484597275607E-34	5	DDX17
1.84487142530679E-36	1.90667868195199	0.319	0.237	3.09163553452913E-32	5	LRCH4
8.25890963244592E-48	1.90269904188129	0.396	0.316	1.38402807620529E-43	5	TGFBR2
7.02235435896287E-25	1.88576415888365	0.253	0.185	1.176806143475E-20	5	MAP7D1
7.01811807427365E-29	1.87712376546383	0.312	0.248	1.17609622688678E-24	5	VPS13C
4.94638211295813E-44	1.87530217695369	0.37	0.289	8.28914714489524E-40	5	ESYT1
2.87802789909417E-28	1.87058270086865	0.306	0.24	4.822999153302E-24	5	GBP2
2.28510998642835E-31	1.86907745294352	0.394	0.349	3.82938731525663E-27	5	RABGAP1L
3.47464773084587E-20	1.86686580765197	0.266	0.212	5.82281466735151E-16	5	KLC1
2.88240664927392E-10	1.86428272309297	0.253	0.228	4.83033706285324E-06	5	RNF44
2.88561044735377E-43	1.83194139789134	0.345	0.26	4.83570598767544E-39	5	TLN1
4.97094608605448E-35	1.82530254402112	0.399	0.346	8.3303114510101E-31	5	SNRNP200
1.42252122775733E-17	1.82064309589771	0.274	0.228	2.38386107347573E-13	5	PPP6R1
5.48061670324601E-20	1.81883535437874	0.335	0.301	9.18441747129966E-16	5	ATP11B
1.15961081355391E-66	1.81078130138087	0.444	0.338	1.94327580135364E-62	5	CTSC
7.41280637589944E-16	1.80586610944982	0.302	0.271	1.24223809247323E-11	5	NAP1L4
3.19812749556436E-29	1.79749879483007	0.319	0.255	5.35942205706675E-25	5	STIM1
2.09848598746678E-13	1.79340655051037	0.276	0.245	3.51664281779684E-09	5	TAB2
8.11411927138888E-14	1.76553890224435	0.28	0.246	1.35976410749935E-09	5	TIAL1
2.42958421995362E-25	1.75760280690312	0.365	0.326	4.07149723579828E-21	5	FUBP1
1.05114733738264E-30	1.75470725233805	0.348	0.281	1.76151270798582E-26	5	FAM214A
2.73533365781758E-16	1.75381349580507	0.288	0.248	4.5838721437707E-12	5	LTBP3
4.87150069406416E-09	1.75281415992492	0.254	0.233	8.16366086311272E-05	5	USP34
9.86137703996498E-21	1.74939418044093	0.326	0.284	1.65256956435733E-16	5	MED15
9.38863536137364E-125	1.73202390836123	0.628	0.579	1.57334751385899E-120	5	PFN1
1.03244444433903E-18	1.72063555120687	0.275	0.225	1.73017039982334E-14	5	FLII
8.03316237426396E-39	1.71330709462302	0.287	0.193	1.34619735067915E-34	5	LGALS1
1.69144547037506E-25	1.70752538455446	0.378	0.341	2.83452431925452E-21	5	DDX6
6.88225288989383E-07	1.70263265728862	0.287	0.279	0.0115332793928841	5	NOP53
1.13673493122226E-12	1.7023449166201	0.254	0.22	1.90494039774226E-08	5	HERC4
1.69818837307293E-16	1.70019859824809	0.331	0.305	2.84582407559562E-12	5	NCOR1
3.96305553042362E-07	1.69966910844563	0.25	0.239	0.0066412884578839	5	MSL2
1.431895967381E-12	1.69316116907671	0.275	0.247	2.39957126213708E-08	5	RBM15
6.63077632774382E-33	1.69082552911015	0.455	0.429	1.11118549700331E-28	5	MAF
9.44498949046812E-15	1.68612653116414	0.362	0.351	1.58279133881265E-10	5	RNF19A
3.87387783738616E-08	1.68510283174582	0.269	0.258	0.000649184447989173	5	TAP2
2.22797963043498E-08	1.67690073194544	0.324	0.33	0.000373364826468293	5	CD47
2.99028706148447E-10	1.67517345068327	0.287	0.266	5.01112305763568E-06	5	PNISR
9.18084532816133E-09	1.67102117501364	0.303	0.297	0.000153852606009328	5	PAFAH1B1
1.72622362004335E-18	1.66740018221576	0.296	0.256	2.89280554246864E-14	5	ADAM10
1.66487150066631E-91	1.64488864773362	0.557	0.468	2.7899916608166E-87	5	CFLAR
5.63685873792823E-09	1.63938285131945	0.253	0.231	9.44624787302013E-05	5	AIP
1.15542639320256E-19	1.6393099477752	0.273	0.22	1.93626354972885E-15	5	PTPN1
1.00707232176894E-33	1.63778872829127	0.427	0.385	1.68765179682038E-29	5	SMARCA2
1.88135312468629E-13	1.63238314050065	0.312	0.29	3.15277156634928E-09	5	KDM5C

1.28337229144656E-15	1.63210290746096	0.275	0.234	2.15067528600614E-11	5	SHISA5
1.69555755236138E-13	1.60502133721762	0.326	0.306	2.84141534624721E-09	5	MARCH7
3.10423760671675E-63	1.59901264380476	0.545	0.495	5.20208138133593E-59	5	DDX5
2.65817449975589E-58	1.59669335742921	0.534	0.471	4.45456882669091E-54	5	PNRC1
1.91048298531567E-08	1.5917424707887	0.283	0.271	0.000320158738679201	5	RAP1A
1.51147250861099E-13	1.58737598499308	0.335	0.321	2.53292562993029E-09	5	TBC1D2B
0	1.58498278330161	0.925	0.89	0	5	B2M
2.49315497194845E-110	1.58013120428713	0.607	0.47	4.17802910199121E-106	5	LENG8
1.85411871851959E-14	1.5741261975364	0.317	0.288	3.10713214849513E-10	5	NUMA1
2.29821200413764E-29	1.57234186320137	0.412	0.369	3.85134367653385E-25	5	CLK1
1.2739997859272E-22	1.56960628785832	0.388	0.354	2.13496884125679E-18	5	CHD2
1.3034013153304E-14	1.56875450825611	0.4	0.399	2.18423992423068E-10	5	TUT7
8.01188936834545E-40	1.56834257073532	0.527	0.519	1.34263242034733E-35	5	AHNAK
1.92900341776131E-146	1.54104533034086	0.69	0.618	3.23262392748441E-142	5	MCL1
1.12557580326764E-28	1.53610421911683	0.407	0.372	1.88623993111591E-24	5	ARF6
9.22989245767239E-46	1.52198912551425	0.389	0.3	1.54674537805674E-41	5	SAMHD1
5.67323024343184E-63	1.5033289009118	0.546	0.496	9.50719924194309E-59	5	WNK1
3.48572056947073E-25	1.50166236009375	0.431	0.411	5.84137053031906E-21	5	SEPTIN9
1.24185763377626E-10	1.50104325294527	0.259	0.23	2.08110502268226E-06	5	POLR2A
1.45220425990813E-16	1.49259086869868	0.318	0.283	2.43360389875404E-12	5	MAP4
1.88409206076953E-11	1.48712355207253	0.275	0.245	3.15736147543758E-07	5	M6PR
1.39937752649135E-10	1.46871496586322	0.347	0.341	2.3450768588942E-06	5	H1FX
1.80274609965428E-12	1.46080944599276	0.255	0.395	3.02104191380064E-08	5	PER1
2.52088681157786E-23	1.45783685993799	0.437	0.412	4.22450211884218E-19	5	LUC7L3
4.70698272797343E-22	1.45054144639459	0.463	0.463	7.88796165553788E-18	5	RSRP1
7.50557683458187E-09	1.44356986742692	0.287	0.267	0.000125778456593923	5	AKIRIN2
2.91484343050661E-07	1.43654233566255	0.263	0.246	0.00488469462084297	5	GNA13
6.09806897151449E-11	1.42731624018551	0.35	0.347	1.0219143982464E-06	5	STAT1
8.2683286810742E-07	1.42013901036506	0.347	0.358	0.0138560652037441	5	STX16
3.47389438400885E-11	1.41767743308393	0.344	0.338	5.82155220872203E-07	5	CCNI
6.29195431285538E-11	1.29594560492658	0.348	0.339	1.0544057037483E-06	5	APOL6
2.78542149043814E-20	1.23800674969074	0.453	0.44	4.66780933367624E-16	5	EZR
1.36162807028442E-10	1.23306550261084	0.4	0.414	2.28181632018264E-06	5	SPTAN1
4.46584356013307E-09	1.22594615312827	0.376	0.387	7.483860638071E-05	5	CAPZB
6.10782974874466E-13	1.19289417112557	0.418	0.414	1.02355010929463E-08	5	IKBKB
2.07176761020628E-163	1.18769620378592	0.778	0.849	3.47186816118369E-159	5	ACTB
5.11911473981834E-09	1.14871391179608	0.347	0.346	8.57861248098758E-05	5	EHBP1L1
3.45685173003538E-43	1.12614353073456	0.602	0.603	5.79299212919329E-39	5	TAPBP
1.78874716510395E-06	1.124118927725	0.495	0.552	0.0299758249928121	5	JUND
2.2640699186047E-06	1.09717448581152	0.184	0.258	0.0379412836959775	5	PPIG
1.31476360921508E-30	1.09259111047234	0.485	0.441	2.20328085632263E-26	5	DDIT4
2.57874712457011E-08	1.08132287877435	0.203	0.294	0.000432146443135459	5	MECP2
1.30145339404965E-11	1.0481394500109	0.164	0.259	2.1809755977484E-07	5	KIAA0319L
1.20387195521445E-20	1.04610629799914	0.517	0.563	2.01744862254837E-16	5	ACTG1
1.15530849659701E-06	1.04508733927532	0.182	0.26	0.0193606597859728	5	VHL
4.69911114608185E-15	1.03211729445871	0.153	0.254	7.87477045860396E-11	5	BRWD1
5.9370612156345E-08	1.02748935226749	0.187	0.275	0.00099493271851603	5	KDM4C
2.33943161855844E-07	0.995165310196962	0.195	0.28	0.00392041950638023	5	KDM6A
9.59936282476772E-07	0.986745522732117	0.197	0.28	0.0160866122217457	5	MPHOSPH8
2.28250526406635E-08	0.986619393848445	0.181	0.265	0.000382502232152238	5	RBM14
1.83896070906668E-08	0.981025260193409	0.178	0.262	0.000308173035625394	5	ERCC5
5.4909914631546E-20	0.976474937993614	0.529	0.492	9.20180349395448E-16	5	ARGLU1
3.76489985514682E-07	0.96317904269029	0.235	0.333	0.00630921917725504	5	PHF10
4.04750443421777E-13	0.954285489710656	0.463	0.456	6.78280793086213E-09	5	IVNS1ABP
2.29924242304711E-07	0.945718135476673	0.182	0.263	0.00385307045254236	5	DCAF5
6.7775676576019E-07	0.935905788206224	0.204	0.288	0.0113578478806093	5	GGNBP2
3.7720117149625E-14	0.934346332863366	0.194	0.306	6.32113723193416E-10	5	PHF20L1
1.62140622076933E-10	0.925601800267641	0.2	0.299	2.71715254476524E-06	5	B4GALT1
4.35998188065042E-07	0.924919757967172	0.184	0.264	0.00730645763559398	5	MORC3
1.14902792221805E-10	0.919662395886724	0.191	0.292	1.925540992053E-06	5	CDC73

6.9602097005619E-10	0.91015572052762	0.186	0.28	1.16639194162016E-05	5	TPP2
1.14327857805762E-08	0.907877585120601	0.188	0.278	0.000191590624110896	5	LRCH3
2.88156405205421E-06	0.902084875434432	0.233	0.324	0.0482892503843244	5	SUPT5H
3.65308265236907E-10	0.900973908896793	0.165	0.251	6.12183590884008E-06	5	GRIPAP1
9.95236195680916E-13	0.896337918084288	0.168	0.265	1.66781681672208E-08	5	KCTD20
3.15905201957021E-07	0.892355480377047	0.226	0.323	0.00529393937439575	5	PCMTD1
2.3330677613073E-06	0.877579651921411	0.214	0.299	0.0390975495439878	5	ZFR
6.74438928624973E-12	0.875497873078334	0.183	0.284	1.13022475658973E-07	5	PARP12
3.19460433004792E-10	0.866166150989356	0.164	0.252	5.3535179362943E-06	5	METTL17
1.5438877150716E-12	0.862778435313277	0.244	0.37	2.58724703291698E-08	5	CSNK1D
1.88272283288962E-08	0.861838419139889	0.185	0.27	0.000315506692335643	5	PRPF40A
3.3400380099513E-10	0.861196594240996	0.213	0.317	5.5972356970764E-06	5	TMEM245
5.82410264870592E-08	0.858766255869786	0.179	0.26	0.000976003121870138	5	NUP43
2.44259215541309E-06	0.857924862383225	0.254	0.345	0.0409329593404126	5	PFKFB3
1.33128231432547E-14	0.85725033274947	0.235	0.365	2.23096290234663E-10	5	SAFB2
1.72144690192241E-15	0.849156051650408	0.168	0.274	2.88480071824158E-11	5	DEPRECATED-ENSG00000161547
6.24216544514384E-07	0.838479835651393	0.194	0.274	0.010460620852972	5	STK11
1.63115972736217E-07	0.833111070161413	0.251	0.362	0.00273349747111352	5	MARCH6
9.90622176259822E-08	0.832372498529617	0.211	0.303	0.00166008464297621	5	SMARCA5
4.7557592215786E-11	0.831534051883435	0.178	0.274	7.96970130352141E-07	5	TBCD
6.76337975208652E-08	0.83137908539606	0.2	0.292	0.00113340717885466	5	MOGS
3.01341971363639E-08	0.823267596985379	0.217	0.312	0.000504988875611186	5	ZMYM2
2.38896685918194E-17	0.822047294955646	0.153	0.259	4.0034306626171E-13	5	STX5
9.80183415859444E-11	0.821485613768282	0.205	0.307	1.64259136829726E-06	5	RAB21
1.9817534034179E-12	0.819817471608533	0.21	0.322	3.32102235344772E-08	5	MAN2A1
4.91126711321667E-09	0.813919747438073	0.539	0.62	8.2303014283285E-05	5	HSPA8
3.65325289343939E-14	0.810362941436181	0.168	0.272	6.12212119882573E-10	5	NME3
1.67255068575806E-06	0.805934349637741	0.224	0.314	0.0280286043919337	5	PAK2
7.76131144686127E-13	0.805137271697219	0.165	0.262	1.30064057226501E-08	5	DNAJC5
1.00109201924825E-11	0.799300898472636	0.162	0.253	1.67763000585621E-07	5	RPRD2
5.97251013698763E-08	0.798737719457884	0.2	0.286	0.00100087324875639	5	CEP350
4.14547267894588E-16	0.798201863365305	0.19	0.309	6.94698311537751E-12	5	LGALS8
4.95341654440653E-26	0.793928251383281	0.211	0.372	8.30093544511646E-22	5	UBE2B
2.62022849559701E-16	0.792548620883747	0.154	0.256	4.39097891292148E-12	5	PLEKHB2
3.24209202588661E-07	0.791787541945557	0.215	0.306	0.00543309781698078	5	HIPK3
1.72561851047771E-08	0.789157968574134	0.23	0.332	0.000289179149985855	5	PPP3R1
1.48927472289695E-07	0.789024445426722	0.193	0.276	0.00249572658063071	5	HDAC1
2.90447769770602E-08	0.788951682005975	0.221	0.319	0.000486732372581575	5	PHF3
3.41697625072859E-12	0.77849860751817	0.22	0.335	5.72616880097097E-08	5	SRSF2
1.13719207247202E-08	0.776159607923041	0.19	0.278	0.000190570647504862	5	CPSF7
5.48623837600639E-15	0.773889840129559	0.175	0.283	9.1938382705115E-11	5	FBXL3
8.05816924841437E-15	0.773392805581508	0.179	0.29	1.35038800264928E-10	5	RBM23
8.62339222365082E-09	0.773367444474902	0.173	0.257	0.00014451080688394	5	UBA2
2.98780218229923E-13	0.772769843266832	0.161	0.258	5.00695889709706E-09	5	GPR107
2.8926152257608E-15	0.769931436097967	0.179	0.291	4.84744459532995E-11	5	ARSA
2.66897029156557E-06	0.769133179438065	0.192	0.266	0.0447266041460559	5	OGFR
1.35810986169857E-11	0.768572556181317	0.219	0.331	2.27592050623447E-07	5	RNF115
3.36194268616122E-07	0.767192156159358	0.194	0.275	0.00563394355346898	5	SUPT6H
1.36126807059296E-18	0.76546308430999	0.203	0.337	2.28121303269968E-14	5	BSDC1
1.35202835237095E-06	0.764708765087119	0.207	0.29	0.0226572911290325	5	TNFRSF14
1.6236749582028E-10	0.760901346822694	0.171	0.26	2.72095449495625E-06	5	UBE4A
2.67164624736598E-13	0.759102898375856	0.192	0.301	4.47714478133591E-09	5	COPB1
1.33533226198974E-11	0.756643333109323	0.162	0.252	2.23774980464241E-07	5	ATF2
1.12540580381651E-13	0.75292229221271	0.175	0.277	1.88595504603571E-09	5	SDHA
2.78667646562963E-06	0.751729601639973	0.222	0.308	0.0466991242110213	5	GPBP1L1
6.2363679120369E-07	0.750693257377575	0.304	0.416	0.0104509053469914	5	IRF3
5.3534359577393E-08	0.749831790516223	0.189	0.273	0.000897128797797952	5	DAZAP1
5.24330821549937E-08	0.742104078979662	0.185	0.27	0.000878673590753384	5	DNAJC8
6.77236750210857E-21	0.740697499024205	0.172	0.296	1.13491334600335E-16	5	RNF157
5.1788002881005E-07	0.738037534415004	0.197	0.278	0.00867863352279881	5	TXNDC15

1.36522908027568E-16	0.737068146165493	0.165	0.274	2.28785089272598E-12	5	KLHL21
5.43080901412237E-07	0.73688752303384	0.22	0.308	0.00910094974586626	5	UBE2I
1.74242956969292E-11	0.736844917373144	0.246	0.37	2.9199634728914E-07	5	TM9SF3
1.85985148447895E-09	0.735168637805576	0.205	0.301	3.11673911768982E-05	5	UBE2Z
1.16752917706273E-10	0.733298966243831	0.244	0.364	1.95654539492172E-06	5	DHX15
3.64620829492167E-08	0.733154013796414	0.176	0.256	0.000611031586062974	5	CLPP
2.83008381489912E-12	0.731103720282364	0.168	0.263	4.74265445700794E-08	5	SEC24B
2.75430682349305E-06	0.726921855156825	0.222	0.309	0.0461566737480966	5	TRAPPC9
4.30476578087454E-16	0.723223132777682	0.209	0.334	7.21392649558955E-12	5	STAT6
2.03085949912647E-07	0.722120017760035	0.181	0.261	0.00340331434863614	5	DYM
1.07116313112946E-11	0.721982517857383	0.17	0.262	1.79505517514675E-07	5	DEPRECATED-ENSG00000062485
4.3792709429493E-08	0.71612966262991	0.233	0.335	0.000733878224619444	5	NRDC
4.69790998579119E-09	0.715660544486104	0.171	0.254	7.87275755418888E-05	5	ZSWIM8
2.94376834071551E-07	0.715203295335751	0.18	0.257	0.00493316698537106	5	IWS1
4.56936150176482E-19	0.708115562271686	0.182	0.308	7.65733600465749E-15	5	TXNDC11
6.81420843672245E-07	0.706025031953514	0.319	0.44	0.0114192504982595	5	PRKAR1A
3.0528826580941E-21	0.705937457211854	0.143	0.259	5.11602075843409E-17	5	TMEM175
7.39276180620671E-17	0.703582835567034	0.23	0.367	1.23887902348412E-12	5	FBXO7
9.00812354050891E-18	0.70303393418607	0.183	0.307	1.50958134291848E-13	5	TTC3
2.66529318016205E-12	0.70281090261881	0.18	0.279	4.46649831131557E-08	5	AATF
2.61420156797533E-12	0.702183014626164	0.193	0.296	4.38087898761307E-08	5	SERBP1
3.5083560103091E-20	0.701032039913795	0.151	0.265	5.879303002076E-16	5	TMEM183A
9.52326483863523E-15	0.700798064860047	0.202	0.324	1.59590872165849E-10	5	UBR5
1.53921727288395E-21	0.696883684043941	0.222	0.371	2.57942030589892E-17	5	SYAP1
4.03136053710451E-09	0.696665558056649	0.201	0.296	6.75575398807974E-05	5	PPP4R3B
9.97366492389129E-11	0.69525617578074	0.163	0.25	1.6713867679457E-06	5	KHDRBS1
8.98257429854461E-14	0.693346324591352	0.172	0.274	1.50529980095011E-09	5	TPR
1.12101653299001E-06	0.692228678120535	0.233	0.322	0.0187859950598466	5	TMX4
3.62527257909454E-14	0.690973805868047	0.174	0.279	6.07523178804663E-10	5	ZNF410
3.68619397902852E-16	0.690683523065288	0.174	0.287	6.17732387005599E-12	5	PCID2
6.39992628540083E-24	0.69046700846646	0.139	0.261	1.07249964690747E-19	5	SENP2
4.5382594246241E-09	0.688837008470389	0.194	0.284	7.60521514378506E-05	5	EWSR1
4.75152917898692E-11	0.688082387743822	0.219	0.327	7.96261259814628E-07	5	TOP 1.00
6.80377375178971E-20	0.688046888734419	0.194	0.326	1.14017640532492E-15	5	LPIN2
4.58786871947542E-11	0.687979696430796	0.233	0.345	7.68835040009691E-07	5	TNIP1
2.94533820455641E-20	0.687667713953821	0.198	0.332	4.93579776319564E-16	5	PATJ
1.38642609289576E-09	0.686445421407881	0.17	0.251	2.32337284647472E-05	5	CIC
4.31422681130298E-14	0.685987063056938	0.156	0.253	7.22978129038154E-10	5	KHDC4
3.42398924913111E-12	0.68516350973251	0.169	0.265	5.73792118369391E-08	5	TCERG1
4.16351887682468E-11	0.68465819252946	0.226	0.336	6.97722493378279E-07	5	UBE2D3
6.18543689691176E-09	0.677494281940163	0.248	0.356	0.000103655551518447	5	TMEM248
3.42950694146054E-09	0.677292334492778	0.257	0.372	5.74716773249958E-05	5	PDCD6IP
1.13715042737218E-13	0.676264161558058	0.218	0.338	1.90563668619031E-09	5	U2SURP
5.90565482407101E-13	0.676145242044706	0.623	0.794	9.8966963541782E-09	5	IL32
5.56692641372677E-12	0.671646808417408	0.182	0.282	9.32905528412331E-08	5	SRPK1
1.26016954150573E-23	0.671423444233057	0.158	0.286	2.1117921176553E-19	5	EGLN2
9.86155301469152E-08	0.670879056929834	0.21	0.298	0.001652599054202	5	GNB1
5.58742732273487E-07	0.668057850371203	0.257	0.359	0.00936341070743909	5	APMAP
2.2616729829345E-18	0.66653462370557	0.181	0.305	3.79011158480163E-14	5	RABGGTB
1.99818409295643E-09	0.666276688038687	0.233	0.339	3.34855690297639E-05	5	SPG7
2.59679468418087E-09	0.664518253324633	0.308	0.443	4.3517085317503E-05	5	SNX1
4.51386736113043E-12	0.664394666147707	0.189	0.289	7.56433892378237E-08	5	KPNA4
7.2561796877702E-08	0.660975592637618	0.21	0.3	0.00121599059207653	5	DNMT1
1.07102213869537E-13	0.655984100798832	0.198	0.305	1.79481890002569E-09	5	RASSF3
1.20163062985377E-15	0.655091037122249	0.203	0.323	2.01369260950894E-11	5	DDX46
1.26962530942038E-08	0.653263189543463	0.194	0.278	0.000212763809352668	5	IER2
6.64575993177811E-08	0.644466727187381	0.267	0.379	0.00111369644936738	5	NBR1
1.50579863518052E-12	0.640994650939586	0.191	0.299	2.52341735283551E-08	5	AGFG1
8.57724816211983E-07	0.640793045766004	0.235	0.327	0.0143737524700804	5	SNX17
2.01589659487231E-47	0.639118887748297	0.139	0.317	3.37823951368701E-43	5	FAM177A1

1.99940285002148E-06	0.638593087065371	0.547	0.638	0.03350599296066	5	CFL1
2.83561193101705E-12	0.637808151040367	0.2	0.307	4.75191847399837E-08	5	DCTN1
3.65685478695883E-20	0.634891670643095	0.141	0.251	6.1281572519856E-16	5	ZFAND2B
2.68207771267277E-11	0.633938628087571	0.199	0.3	4.49462583089703E-07	5	APRT
1.87682229939475E-12	0.633015521264484	0.195	0.3	3.14517880932572E-08	5	DHX9
2.0713043675327E-11	0.62755589983183	0.183	0.278	3.47109185911129E-07	5	DNAJC7
2.74999032936099E-11	0.627319652732702	0.167	0.258	4.60843379394314E-07	5	SMAD2
1.52936496672235E-17	0.626781529503504	0.164	0.276	2.56290981123331E-13	5	CYTH2
1.19753225239246E-08	0.626029803345252	0.185	0.27	0.000200682454855928	5	EXOC3
4.91580088756893E-13	0.620681176389725	0.18	0.281	8.23789912738801E-09	5	PMPCB
5.89081215454836E-10	0.620036157943091	0.229	0.335	9.87182300859214E-06	5	ZMIZ2
1.2053981542481E-28	0.61875407028557	0.19	0.345	2.02000622688896E-24	5	RAB18
1.80857769357523E-07	0.617887703330318	0.214	0.305	0.00303081449889336	5	UBE2V1
7.1876752262737E-08	0.615585835533785	0.275	0.392	0.00120451061441895	5	HNRNPD
2.23498587202989E-11	0.613535296056491	0.18	0.274	3.74538932434768E-07	5	SF3A2
4.3191547142492E-08	0.612559928026633	0.18	0.263	0.000723803947013881	5	AP3B1
6.29912768103826E-12	0.61190359826768	0.169	0.263	1.05560781678839E-07	5	PPP6C
2.923413622824E-06	0.610810653673474	0.278	0.385	0.0489905654912847	5	ERBIN
8.94519064025418E-10	0.609709902473494	0.173	0.259	1.4990350474938E-05	5	UBA1
6.56084795625842E-12	0.606272700872876	0.184	0.281	1.09946690050979E-07	5	ZBTB7A
1.27006026375729E-15	0.605764057002508	0.206	0.328	2.12836699000447E-11	5	HGS
1.68827782033613E-09	0.605565295470371	0.333	0.473	2.82921597131929E-05	5	SERINC1
1.04371361470435E-17	0.603469826498988	0.166	0.28	1.74905527552155E-13	5	FUS
1.02460307918482E-10	0.602912456358308	0.182	0.274	1.71702984009792E-06	5	CHURC1
3.91473027197844E-07	0.59957043937714	0.213	0.301	0.00656030498978147	5	SEC11A
1.91715017400306E-17	0.596932905178372	0.2	0.327	3.21276026159433E-13	5	SRSF3
2.35792063677445E-17	0.596826860222137	0.147	0.251	3.95140340310663E-13	5	EIF2B5
1.02030985910984E-19	0.596380227992581	0.205	0.341	1.70983526189627E-15	5	TRIM27
2.38150718465239E-16	0.596312595614705	0.172	0.284	3.99092974004048E-12	5	PCED1A
4.77318116329767E-14	0.590662571146546	0.168	0.27	7.99889699345423E-10	5	PTBP1
1.88010594430073E-20	0.589280515649478	0.152	0.266	3.15068154145917E-16	5	MAPK1IP1L
5.25948856981694E-11	0.586470010946571	0.167	0.255	8.81385094529923E-07	5	CIAO1
1.68966232174137E-13	0.585284671676923	0.201	0.311	2.83153611877419E-09	5	MICAL3
3.36158084799454E-18	0.584500931562686	0.162	0.275	5.63333718506925E-14	5	ZC3H15
1.43766271095925E-10	0.582642621027635	0.213	0.315	2.40923517102551E-06	5	FZR1
3.42460970493495E-19	0.582347090604222	0.231	0.374	5.73896094353E-15	5	TMED4
1.74364194390835E-14	0.58155103606135	0.255	0.39	2.92199516960161E-10	5	TMEM259
1.8056439750182E-21	0.580989913717249	0.139	0.253	3.0258981733355E-17	5	DHPS
3.49264545692855E-18	0.578653904166561	0.165	0.281	5.85297525672086E-14	5	KMT5B
4.73558360930086E-10	0.577823751463008	0.17	0.255	7.93589101246638E-06	5	ATF4
2.8247292667355E-13	0.577558611464321	0.186	0.294	4.73368130519536E-09	5	RBM26
4.639331262866E-12	0.576353619250479	0.215	0.326	7.77459133031085E-08	5	UPF1
1.0641647185629E-16	0.574908068665638	0.225	0.359	1.7833272353677E-12	5	DNAJA2
5.91344670378796E-15	0.573032176533173	0.256	0.391	9.90975398620786E-11	5	DCAF8
2.11586159435644E-23	0.570757133809955	0.164	0.293	3.54576085982252E-19	5	MOV10
2.1518776134736E-11	0.568451902793372	0.175	0.267	3.60611650465905E-07	5	PITPNB
3.02565816477802E-12	0.567770442870838	0.193	0.299	5.07039795253501E-08	5	PARP10
1.14907232536001E-18	0.567487411205085	0.158	0.271	1.9256154028383E-14	5	RUFY1
3.94726201480557E-21	0.559750844334927	0.141	0.253	6.61482168441117E-17	5	RNF138
1.66404621028775E-07	0.559213831672584	0.227	0.317	0.00278860863920021	5	EIF4A1
3.06978562656363E-26	0.558567100146709	0.186	0.332	5.14434675299534E-22	5	DUS1L
2.81897454533424E-07	0.558334666434276	0.277	0.386	0.00472403754307112	5	SRPRA
1.10339732961999E-29	0.55777107140056	0.121	0.25	1.84907324497717E-25	5	IP6K2
2.11997039557448E-12	0.553952439335873	0.213	0.323	3.55264638890371E-08	5	CYB5B
3.33822432412915E-12	0.552059343466256	0.69	0.786	5.59419632237564E-08	5	OAZ1
2.77452358538626E-10	0.551698184851542	0.216	0.321	4.64954662439029E-06	5	CMTM6
1.79340146119672E-21	0.551610456734836	0.167	0.291	3.00538216867347E-17	5	TNRC6A
1.21049539826156E-16	0.55062616836421	0.715	0.62	2.02854818840673E-12	5	TSC22D3
3.48136099524174E-15	0.549669209104034	0.215	0.338	5.83406475582611E-11	5	INTS11
2.76388189289721E-08	0.548912091473573	0.194	0.279	0.000463171327611714	5	DYNC1I2

2.39442151380043E-09	0.548396319886129	0.253	0.364	4.01257157282676E-05	5	BIN1
5.61688235720586E-22	0.545207883379004	0.194	0.331	9.41277145420558E-18	5	PRPF3
6.66731686590011E-23	0.543944912677624	0.215	0.364	1.11730896038754E-18	5	C11orf58
4.65288880376714E-11	0.541557814302468	0.23	0.34	7.79731105735298E-07	5	SMARCA4
3.35550170084368E-18	0.541150886055474	0.158	0.269	5.62314975027383E-14	5	SNRPA
9.45669743581093E-21	0.541122825662466	0.15	0.266	1.5847533562932E-16	5	TAX1BP1
2.0028180479474E-17	0.539126690163471	0.194	0.321	3.35632248475025E-13	5	PARP4
4.39188544659248E-09	0.537966084447985	0.3	0.427	7.35992163139968E-05	5	SETD5
9.44810623767268E-13	0.537900752250458	0.17	0.267	1.58331364330919E-08	5	ATP2C1
2.01326593455366E-18	0.537699754438613	0.161	0.269	3.37383105312503E-14	5	NFKBIZ
3.0945246685325E-16	0.537113908960954	0.224	0.352	5.18580443952677E-12	5	PPM1B
1.15001716917403E-20	0.536352288015896	0.184	0.316	1.92719877210184E-16	5	PRKAA1
9.31916222545984E-12	0.535603133993756	0.245	0.363	1.56170520574256E-07	5	GNB2
2.36491513720857E-19	0.535486149354731	0.166	0.284	3.96312478693412E-15	5	MAP3K5
2.93993472179606E-17	0.535068339107984	0.153	0.258	4.92674260678584E-13	5	ARHGEF7
2.21671168229813E-26	0.533494122987	0.168	0.308	3.71476543719521E-22	5	DNAJC21
3.2610326526645E-10	0.532636344536711	0.255	0.374	5.46483851933516E-06	5	HNRNPK
6.34343709135807E-14	0.531331546533	0.165	0.263	1.06303318776979E-09	5	SMARCD2
2.69774022888503E-21	0.530596652809956	0.187	0.321	4.52087307556554E-17	5	MKLN1
5.49149146484183E-14	0.530590645603036	0.23	0.354	9.20264139678193E-10	5	UBE2R2
3.28683418167414E-22	0.527082960227189	0.178	0.31	5.50807672164953E-18	5	SETD3
1.57548241843269E-21	0.526896268484323	0.272	0.435	2.6401934368095E-17	5	CCNL1
4.09511218386028E-10	0.526629673699583	0.319	0.459	6.86258899771305E-06	5	EEF1G
8.94183158698575E-10	0.525286620371551	0.2	0.293	1.49847213734707E-05	5	DDB1
5.1352528852711E-11	0.524265646979028	0.331	0.476	8.60565678513731E-07	5	DEPRECATED-ENSG00000132002
1.36593352069785E-11	0.522433587900934	0.166	0.255	2.28903139398546E-07	5	EXOC7
9.42439069814182E-26	0.521859224674933	0.151	0.281	1.57933939319461E-21	5	CMC2
2.50048197021912E-11	0.521806705264057	0.205	0.306	4.1903076856932E-07	5	PPP4C
2.63747436363095E-08	0.520786394430338	0.308	0.441	0.000441987953857274	5	LUC7L2
1.45413507660573E-13	0.518990420977175	0.155	0.25	2.43683956137588E-09	5	WIPF2
3.21104226926158E-18	0.518224790655722	0.171	0.29	5.38106463482856E-14	5	DPP4
1.97370796060434E-10	0.514837145481385	0.169	0.257	3.30753980038075E-06	5	TXLNA
4.41207597880064E-25	0.51409626262882	0.159	0.295	7.39375692527412E-21	5	NUB1
4.39528373994905E-10	0.513764906760394	0.192	0.287	7.36561649140662E-06	5	MAST4
4.40350673182648E-14	0.512629577808562	0.195	0.306	7.37939658119482E-10	5	MAPK14
1.05323923744147E-18	0.512125595464874	0.178	0.298	1.76501831410441E-14	5	PPM1A
3.3696997748082E-21	0.511829024740273	0.168	0.294	5.64694288262358E-17	5	CDC16
5.99091471738596E-11	0.510988509451079	0.209	0.311	1.00395748833954E-06	5	ACTR1B
4.43117956180683E-10	0.500969005483604	0.253	0.369	7.42577070967589E-06	5	NONO
6.72421495455589E-12	0.500106475082931	0.254	0.379	1.12684394208448E-07	5	SURF4
1.57962220824425E-22	0.499908533990617	0.176	0.306	2.64713089657571E-18	5	NELFCD
8.13729530100777E-10	0.499525893024728	0.228	0.332	1.36364794654288E-05	5	CTDSP1
1.2320230436135E-06	0.498826149136153	0.331	0.457	0.0206462421648751	5	DRAP1
3.13896565523646E-22	0.497706386019336	0.158	0.283	5.26027864504526E-18	5	NPEPPS
1.60515780404948E-17	0.496459391172834	0.249	0.398	2.68992344802612E-13	5	RAB14
1.289466956113E-18	0.49520475071462	0.145	0.253	2.16088872505416E-14	5	ZNF652
8.01690327036365E-25	0.494982460379959	0.19	0.334	1.34347265004754E-20	5	SNRNP70
6.7891398409509E-08	0.492452812053181	0.318	0.442	0.00113772405454655	5	STK24
3.39118981203535E-09	0.489637134339427	0.286	0.406	5.68295588700883E-05	5	STAU2
1.87227454625694E-12	0.487056945959797	0.181	0.28	3.13755768461738E-08	5	NUP62
4.34933556543259E-09	0.485694750791107	0.268	0.382	7.28861654055193E-05	5	ERO1A
1.02814921026736E-13	0.484359497538533	0.203	0.312	1.72297244656604E-09	5	PYGO2
6.3931198036218E-16	0.481658035103315	0.233	0.368	1.07135901669094E-11	5	IQGAP2
2.38290247766506E-22	0.476757525327383	0.138	0.253	3.9932679720711E-18	5	UBE2G2
5.23485778513631E-25	0.470394942549323	0.135	0.256	8.77257467633142E-21	5	SPAG9
3.58227113573292E-21	0.467907022890693	0.218	0.363	6.00316996926123E-17	5	CELF1
4.9282092760551E-15	0.467517174278021	0.162	0.263	8.25869310481314E-11	5	URI1
5.68775852395245E-09	0.465930185341821	0.263	0.371	9.53154573443951E-05	5	MDM2
2.68341021342295E-21	0.465877155023208	0.244	0.399	4.49685883565417E-17	5	CNBP
1.23372354133738E-17	0.463762681466677	0.155	0.262	2.06747391057319E-13	5	RBM17

6.83350865919173E-25	0.463030054483594	0.196	0.341	1.14515938110735E-20	5	RNF7
1.72419540563135E-18	0.461756442093077	0.246	0.392	2.88940666075702E-14	5	USP9X
2.76059676966855E-24	0.460524168752924	0.236	0.396	4.62620806661055E-20	5	ILRUN
1.46452487046987E-24	0.458859829395056	0.197	0.345	2.45425077793342E-20	5	EIF4A2
6.00004290806168E-18	0.457235288983879	0.161	0.273	1.00548719053298E-13	5	USP14
2.98645401264625E-14	0.452514689477907	0.19	0.297	5.00469963439258E-10	5	DEK
1.96158471404917E-14	0.452480235086058	0.191	0.302	3.2872236638036E-10	5	HNRNPM
2.85217968631502E-23	0.449708697265493	0.193	0.332	4.77968271832672E-19	5	SND1
6.88156533828267E-22	0.449232438748784	0.157	0.28	1.15321271938941E-17	5	SMAD4
8.71038477004132E-18	0.448495965666157	0.275	0.424	1.45968627976352E-13	5	SELENOF
5.49380791251554E-14	0.44720152160879	0.161	0.257	9.20652329979354E-10	5	CNST
2.87698576623046E-21	0.445837353663286	0.14	0.252	4.821252747049E-17	5	ABCF1
5.69447409890202E-12	0.44415482931071	0.203	0.307	9.54279969494001E-08	5	ZFP91
2.97488891056277E-16	0.442344568388765	0.186	0.3	4.98531883632109E-12	5	MCM7
5.30618101731902E-13	0.440108840292891	0.283	0.424	8.89209814882321E-09	5	UBXN4
1.88841872767329E-22	0.439537961833002	0.159	0.284	3.1646121038349E-18	5	PI4KB
3.22116414429639E-22	0.436648620987222	0.159	0.283	5.39802687301188E-18	5	ARCN1
9.53178173325476E-16	0.435511800343696	0.171	0.279	1.59733598285883E-11	5	DNAJC1
1.16217401322049E-14	0.434381608263458	0.249	0.385	1.94757121135489E-10	5	TMPO
6.63494531569558E-09	0.434380977113572	0.246	0.351	0.000111188413600427	5	PRKDC
1.65114885421057E-27	0.432924129520625	0.162	0.304	2.76699524988608E-23	5	FBXL5
3.01214028908416E-26	0.432758545776575	0.141	0.269	5.04774469644724E-22	5	PCMTD2
2.78404828820667E-27	0.431207921494494	0.167	0.311	4.66550812137673E-23	5	RIOK3
3.79991861697598E-28	0.430221343282537	0.135	0.264	6.36790361832834E-24	5	LIN7C
4.07193246093693E-15	0.429784469466894	0.167	0.27	6.82374441803811E-11	5	PSMD13
1.10764036507249E-13	0.428666379017182	0.185	0.289	1.85618372378848E-09	5	RNASEK
5.60522785190221E-10	0.428425604808901	0.194	0.286	9.39324083421773E-06	5	MCM5
1.02950007426741E-15	0.424405396038733	0.157	0.261	1.72523622445733E-11	5	TBC1D14
1.04820993708908E-11	0.424229150716321	0.184	0.277	1.75659021257388E-07	5	LMNB1
2.17511009154431E-13	0.421824501155642	0.166	0.264	3.64504949140996E-09	5	UBP1
1.60611130257897E-23	0.420162830883218	0.161	0.291	2.69152132086184E-19	5	RAF1
3.3315558267614E-16	0.418973197896528	0.165	0.272	5.58302125448675E-12	5	INTS1
2.28237558184881E-12	0.417955845716331	0.225	0.337	3.82480500006223E-08	5	NCL
7.52222014071532E-19	0.415450815547529	0.259	0.407	1.26057365118107E-14	5	DPYD
6.75661888333914E-20	0.413120630264702	0.252	0.403	1.13227419246997E-15	5	RAB11B
1.32299708456424E-21	0.412723017228515	0.144	0.258	2.21707851431275E-17	5	NCAPH2
8.50096827333114E-09	0.412287721158616	0.206	0.301	0.000142459226324483	5	ITGB1
5.19432214135381E-29	0.410256938156274	0.207	0.368	8.70464504448072E-25	5	NDUFA10
4.8829373867566E-30	0.408870619425476	0.137	0.273	8.18282647272671E-26	5	SH3GLB2
2.1106832463996E-16	0.406454730905945	0.155	0.255	3.53708298431644E-12	5	NAGK
1.58362754535186E-14	0.404934677184956	0.169	0.27	2.65384304050065E-10	5	SEC16A
4.61404754946151E-28	0.40406772478097	0.189	0.341	7.7322208833876E-24	5	SRP54
5.52852606141427E-19	0.403571378302383	0.256	0.408	9.26470397371804E-15	5	ZNRF2
1.73366698420201E-20	0.402797102467519	0.156	0.273	2.90527913212573E-16	5	CHMP4B
2.87179001379371E-06	0.401940393109132	0.19	0.264	0.0481254570511549	5	FKBP1A
7.68256290093941E-11	0.400765573778648	0.229	0.332	1.28744389093943E-06	5	WSB1
1.97997420318097E-26	0.400136238379261	0.257	0.433	3.31804076969067E-22	5	SREBF2
1.54971978413733E-11	0.399651468887464	0.183	0.281	2.59702041425735E-07	5	CLIP1
4.734435240963E-20	0.398584238104221	0.21	0.345	7.93396657680579E-16	5	PPP6R2
1.04087590888547E-25	0.395571151410306	0.132	0.253	1.74429984811026E-21	5	DEPRECATED-ENSG00000073578
1.09376305083762E-22	0.395469450191983	0.144	0.264	1.83292812059368E-18	5	METTL23
7.28716246707478E-12	0.394191049350475	0.164	0.258	1.22118268623239E-07	5	YIPF4
2.60634057555811E-17	0.392508599310055	0.152	0.256	4.36770553652028E-13	5	GOLGB1
4.28246572211878E-23	0.390525608436221	0.147	0.267	7.17655605712666E-19	5	DPM1
6.80779034222348E-23	0.389944355151239	0.286	0.461	1.14084950554981E-18	5	AHSA1
4.15987148667914E-29	0.389062235252849	0.124	0.251	6.97111263737691E-25	5	STUB1
1.23014624142274E-17	0.387478171818158	0.158	0.268	2.06147907137624E-13	5	UBQLN2
1.00488284798319E-31	0.387295171842154	0.158	0.309	1.68398267665022E-27	5	TMUB2
2.23729044543326E-35	0.382395512664666	0.113	0.252	3.74925132845705E-31	5	ZNF326
1.33362811283103E-14	0.378522364632792	0.221	0.344	2.23489399148224E-10	5	ITCH

4.16463441626719E-17	0.377073627772034	0.278	0.429	6.97909435478056E-13	5	LRPAP1
2.67722337544531E-19	0.375899580779192	0.142	0.25	4.48649093257126E-15	5	RBM3
1.18576479728396E-16	0.375524319731641	0.205	0.323	1.98710464728846E-12	5	HERPUD1
5.480527904549E-21	0.374244862385545	0.148	0.264	9.18426866244321E-17	5	ITFG1
9.35325964734798E-24	0.374189906133772	0.164	0.295	1.56741925170257E-19	5	FBXL17
1.99030449593527E-20	0.372054333933564	0.248	0.4	3.33535227428833E-16	5	LPP
1.9976934027924E-25	0.371883594392726	0.146	0.273	3.3477346043995E-21	5	MED25
4.22131434249311E-19	0.369695594407072	0.154	0.265	7.07407857514995E-15	5	PRPF6
2.50470996119291E-28	0.36843352688382	0.184	0.335	4.19739295296708E-24	5	DLG1
2.04187082632971E-20	0.366228032512822	0.153	0.268	3.42176713076332E-16	5	EPRS
3.54778397412855E-13	0.365542767922515	0.173	0.272	5.94537638384462E-09	5	ATP6AP2
1.97897107728364E-22	0.364811272211439	0.219	0.366	3.31635973131192E-18	5	BAG6
2.16685462163921E-15	0.364666042753369	0.209	0.329	3.63121497494299E-11	5	UHMK1
7.21680721173168E-21	0.362828778825473	0.232	0.376	1.209392552542E-16	5	SNU13
1.88346847161576E-16	0.360471687856506	0.324	0.484	3.15631646473369E-12	5	SDF4
4.48282639244373E-23	0.357994414373661	0.138	0.255	7.51232046845721E-19	5	SRP72
2.42449945463407E-23	0.355162334141224	0.273	0.435	4.06297618607577E-19	5	PPP1R15B
2.10333015189383E-20	0.353337008590586	0.292	0.457	3.52476066854368E-16	5	LAMTOR4
3.76627315861191E-15	0.352928077786727	0.266	0.4	6.31152055920184E-11	5	SLBP
4.56460345581513E-07	0.350120944835535	0.276	0.379	0.007649362471255	5	VAMP8
4.18218463662926E-22	0.341395213899	0.144	0.261	7.00850501406332E-18	5	GSK3A
5.91107103433457E-19	0.339541201293289	0.264	0.411	9.90577283933788E-15	5	STAG2
6.2763893618409E-29	0.338460813973737	0.154	0.295	1.0517973292573E-24	5	UBE2H
5.11712782876533E-20	0.336286600888155	0.16	0.279	8.57528281544493E-16	5	INTS6
8.63228956749422E-28	0.335771182722411	0.141	0.272	1.44659908572068E-23	5	CDC34
3.58888102193851E-26	0.335223550807463	0.137	0.262	6.01424681656456E-22	5	TTC1
7.73194996699643E-22	0.333512819107108	0.154	0.274	1.29572017546926E-17	5	RRP36
1.33042594578824E-34	0.331844008431113	0.121	0.262	2.22952779995193E-30	5	TNRC18
1.44543948716606E-20	0.32816240434358	0.181	0.307	2.42226749259288E-16	5	PNN
8.69346571625643E-27	0.326793600543594	0.157	0.294	1.45685098473025E-22	5	RB1CC1
1.44789568568666E-22	0.326609067855073	0.302	0.477	2.42638359007371E-18	5	CAST
6.79978504580426E-33	0.322864876361749	0.161	0.315	1.13950797797588E-28	5	NUS1
7.61436497255063E-34	0.317719791791907	0.206	0.378	1.27601528210003E-29	5	POGZ
4.32872336595594E-18	0.316397966628555	0.171	0.289	7.25407461666896E-14	5	CLPTM1L
2.45777306895148E-25	0.316355512331221	0.254	0.419	4.11873610894889E-21	5	CALCOCO2
3.7590809082454E-33	0.316141429169823	0.209	0.382	6.29946778603764E-29	5	CHTOP
5.48312909777748E-26	0.315012283667711	0.239	0.403	9.18862774205551E-22	5	EIF4H
3.88973622979483E-19	0.312994331944707	0.147	0.254	6.51841997389018E-15	5	PLEKHJ1
9.39568242888441E-17	0.312729770949221	0.182	0.296	1.57452846143245E-12	5	DPP9
1.51228103155369E-19	0.310689484592355	0.144	0.251	2.53428055267768E-15	5	PNP
2.12960812486053E-29	0.31024465740019	0.127	0.254	3.56879729564128E-25	5	STRN4
6.51577436457569E-11	0.304672895860599	0.244	0.357	1.09191346801559E-06	5	RNF187
2.60064246784738E-18	0.304265906362439	0.167	0.282	4.35815664761864E-14	5	BTBD2
3.86119072284366E-26	0.302017925716367	0.214	0.37	6.47058341334141E-22	5	POLR2J3
2.01657991204957E-15	0.301199448793224	0.154	0.252	3.37938461661267E-11	5	YWHAQ
3.12390387979034E-23	0.300087618896347	0.148	0.267	5.23503812175266E-19	5	GABARAP
1.02212547088072E-16	0.299896907853471	0.186	0.302	1.71287786410191E-12	5	DCTN2
1.06474493379595E-24	0.29845850923858	0.177	0.312	1.78429956005525E-20	5	YBX1
1.31888863070444E-15	0.295133937093243	0.216	0.338	2.2101935673345E-11	5	MKNK2
5.78420739051564E-36	0.293666153192786	0.169	0.334	9.6931747450261E-32	5	LMBRD1
1.44411830916957E-35	0.292633290907135	0.183	0.351	2.42005346250637E-31	5	MIA3
7.54432712404688E-30	0.289465640394767	0.139	0.273	1.26427833944778E-25	5	VPS4A
2.45201048804532E-18	0.289041433666049	0.165	0.276	4.10907917586635E-14	5	HMCES
2.54035739141524E-31	0.288243421869027	0.208	0.376	4.25713091653365E-27	5	HSF1
3.23189338760933E-18	0.285588231371923	0.195	0.319	5.41600693895571E-14	5	PSMC5
1.07340107245977E-22	0.285537682199496	0.137	0.254	1.79880551722808E-18	5	RFC1
5.1216674341257E-20	0.285401212206652	0.22	0.359	8.58289028610784E-16	5	SFT2D2
1.51043284445666E-23	0.284626733009061	0.196	0.339	2.53118336074047E-19	5	KTN1
4.90733106672763E-28	0.282564409585101	0.18	0.328	8.22370540162216E-24	5	GOLGA7
1.45670474710682E-22	0.281644650159848	0.138	0.255	2.4411458152016E-18	5	PSEN1

8.47477892596569E-20	0.280756146087039	0.256	0.405	1.42020345241333E-15	5	DYNLL2
1.76746899802113E-40	0.27825507951227	0.127	0.283	2.96192454688381E-36	5	VAPA
1.52894321014038E-58	0.275955998309693	0.135	0.332	2.56220303155324E-54	5	FURIN
2.09479520121569E-21	0.274914008252332	0.167	0.29	3.51045779819725E-17	5	GPR108
4.17375523054449E-17	0.273468511635855	0.467	0.646	6.99437901534645E-13	5	NFKBIA
4.93231356641551E-28	0.272692510762625	0.135	0.263	8.26557107459912E-24	5	TRA2B
3.02064757885384E-23	0.271584239872973	0.245	0.402	5.06200121264326E-19	5	NPLOC4
4.19735076198425E-28	0.2705977915781	0.162	0.303	7.0339204069332E-24	5	NUDCD3
1.6824562154623E-30	0.270432622280851	0.141	0.28	2.81946012587172E-26	5	R3HDM2
1.22684435143982E-23	0.269095703049974	0.187	0.327	2.05594576414284E-19	5	ECPAS
2.81206952465036E-19	0.268049159896026	0.148	0.256	4.71246610940907E-15	5	AQP3
4.46715828564507E-12	0.265505551874454	0.162	0.252	7.486063855084E-08	5	GNAI3
1.20975897487697E-16	0.265204343757181	0.222	0.348	2.02731409009883E-12	5	SRI
1.12143498461425E-13	0.264699129923457	0.247	0.371	1.87930074721656E-09	5	ATP6V0E1
3.76431499862009E-36	0.263597135317421	0.129	0.275	6.30823907468754E-32	5	CUL4A
9.28984650900147E-28	0.26301142648209	0.147	0.279	1.55679247797847E-23	5	AP2A2
1.38036235362379E-27	0.25796399187867	0.149	0.281	2.31321123220274E-23	5	FKBP8
4.27639158836835E-31	0.257085883294794	0.121	0.254	7.16637702378768E-27	5	UQCC1
1.88116598613212E-17	0.253727924247529	0.207	0.334	3.1524579595602E-13	5	AP1M1
4.16044139885291E-26	0.25171388765755	0.18	0.324	6.9720676961977E-22	5	UXT
1.68384776426027E-13	0.250472056023474	0.167	0.26	2.82179208334736E-09	5	LIME1
2.67614056465638E-09	0.250429594923424	0.258	0.368	4.48467635825115E-05	5	EID1
5.05984348656361E-29	0.250125664862215	0.128	0.258	8.4792857147833E-25	5	PEX13
5.6152833786623E-17	0.250095960979083	0.177	0.289	9.41009188596229E-13	5	DPYSL2
0	8.12623299052739	0.424	0.003	0	6	SIGLEC1
0	8.10420799557858	0.275	0.002	0	6	GFRA2
0	8.0504404566431	0.456	0.007	0	6	CD209
0	7.88210128060507	0.505	0.011	0	6	FOLR2
0	7.84263602734468	0.286	0.001	0	6	HK3
0	7.82834484460686	0.743	0.029	0	6	CD163
0	7.79985951110671	0.33	0.005	0	6	CD163L1
0	7.79636696219106	0.346	0.003	0	6	PTAFR
0	7.77344103742099	0.755	0.017	0	6	CSF1R
0	7.6577221662965	0.557	0.025	0	6	F13A1
0	7.60949687559794	0.774	0.033	0	6	C1QC
0	7.60056314459793	0.294	0.002	0	6	IGSF21
0	7.56438033276735	0.394	0.004	0	6	SLC1A3
0	7.55814182966959	0.489	0.009	0	6	MSR1
0	7.51951130660664	0.495	0.005	0	6	FPR3
0	7.51904298932151	0.295	0.006	0	6	MMP9
0	7.4426508856436	0.347	0.002	0	6	LILRB5
0	7.40708829564295	0.422	0.004	0	6	LRRC25
0	7.3593895742969	0.525	0.016	0	6	GPNMB
0	7.35633739813341	0.481	0.007	0	6	SLAMF8
0	7.35148098934733	0.36	0.008	0	6	GPR34
0	7.28257459888104	0.337	0.003	0	6	LILRB2
0	7.17838549505698	0.799	0.056	0	6	C1QB
0	7.11028436190323	0.356	0.003	0	6	TFEC
0	7.1065264184797	0.392	0.008	0	6	DEPRECATED-ENSG00000110079
0	7.10323278999543	0.56	0.008	0	6	EMILIN2
0	7.09460434584356	0.341	0.003	0	6	CMKLR1
0	7.06829639968224	0.79	0.052	0	6	C1QA
0	7.04890487573311	0.509	0.022	0	6	FMN1
0	7.04398606528881	0.612	0.017	0	6	MS4A7
0	7.00480195118528	0.481	0.013	0	6	MS4A4A
0	6.97223863782582	0.826	0.04	0	6	FGL2
0	6.95140596964399	0.373	0.006	0	6	VSIG4
0	6.86163985084855	0.601	0.009	0	6	CSF2RA
0	6.78805310422335	0.258	0.005	0	6	P2RY13
0	6.78422921242007	0.311	0.005	0	6	IL18

0	6.77314161245239	0.34	0.003	0	6	CD86
0	6.70382627065809	0.463	0.012	0	6	IGSF6
0	6.65918124138635	0.326	0.006	0	6	SLC37A2
0	6.62499442203666	0.699	0.019	0	6	MPEG1
0	6.5112028325414	0.287	0.005	0	6	DEPRECATED-ENSG00000079215
0	6.50454552765255	0.458	0.006	0	6	HCK
0	6.45231191922222	0.407	0.015	0	6	C5AR1
0	6.44136554893467	0.254	0.004	0	6	PILRA
0	6.42418340664259	0.472	0.024	0	6	TLR2
0	6.38156619803904	0.303	0.012	0	6	PLA2G7
0	6.37410935005153	0.312	0.004	0	6	MS4A4E
0	6.32187831923483	0.261	0.002	0	6	SIGLEC10
0	6.30691651776663	0.73	0.021	0	6	SPI1
0	6.29348833489895	0.506	0.024	0	6	CPVL
0	6.24233957646558	0.33	0.011	0	6	CLEC10A
0	6.23495342472551	0.366	0.006	0	6	TM6SF1
0	6.21517254111847	0.548	0.014	0	6	LAIR1
0	6.17862339740609	0.656	0.032	0	6	CYBB
0	6.1613906912629	0.278	0.004	0	6	PHACTR1
0	6.08885206542596	0.465	0.013	0	6	C3AR1
0	6.02235411659567	0.477	0.01	0	6	CLEC7A
0	6.02205667813166	0.629	0.038	0	6	FCER1G
0	5.93076018842503	0.603	0.02	0	6	FCGR2A
0	5.92965771218164	0.261	0.006	0	6	MNDA
0	5.91881957891147	0.602	0.044	0	6	MRC1
0	5.85123840162179	0.855	0.087	0	6	MS4A6A
0	5.78136398240326	0.467	0.029	0	6	ADAP2
0	5.75918273903426	0.614	0.015	0	6	ITGAX
0	5.75235230494836	0.434	0.011	0	6	RNASE6
0	5.71266905985991	0.341	0.008	0	6	ZNF385A
0	5.69375902023318	0.445	0.009	0	6	AXL
0	5.67323895471826	0.359	0.005	0	6	EPB41L3
0	5.63437326933529	0.551	0.009	0	6	FGD2
0	5.61274724470339	0.26	0.006	0	6	CCR1
0	5.6061380616389	0.35	0.012	0	6	PLAU
0	5.59169126421851	0.281	0.007	0	6	PLBD1
0	5.57469085366291	0.588	0.029	0	6	TYROBP
0	5.56859091779806	0.341	0.006	0	6	MARCH1
0	5.52477317632349	0.594	0.036	0	6	AIF1
0	5.4918527899967	0.31	0.017	0	6	KCNMA1
0	5.48046869061464	0.48	0.009	0	6	CD300A
0	5.46533397336239	0.503	0.022	0	6	FCGR3A
0	5.45173724080467	0.293	0.004	0	6	TRPM2
0	5.42803613998476	0.427	0.014	0	6	SLC8A1
0	5.3829969473142	0.411	0.023	0	6	PLAUR
0	5.36986183168758	0.533	0.074	0	6	RNASE1
0	5.35971039356676	0.292	0.006	0	6	IL4I1
0	5.34333362585975	0.369	0.01	0	6	TBXAS1
0	5.32021224145007	0.315	0.008	0	6	TLR4
0	5.30902909080555	0.41	0.023	0	6	SLC7A7
0	5.30183148512204	0.26	0.005	0	6	LY86
0	5.24714447810304	0.314	0.005	0	6	ALOX5
0	5.21540121886972	0.631	0.08	0	6	RGS2
0	5.21094338144602	0.34	0.008	0	6	RAB31
0	5.19855720752357	0.514	0.053	0	6	TNFSF13
0	5.16584710132456	0.441	0.013	0	6	PLEK
0	5.15895403857712	0.256	0.006	0	6	CD72
0	5.12805274583644	0.4	0.011	0	6	OGFRL1
0	5.12355452732822	0.331	0.018	0	6	DNAJA4
0	5.05245314690677	0.743	0.275	0	6	LGMN

0	5.02531308749926	0.464	0.018	0	6	PLXDC2
0	5.01166937425489	0.456	0.016	0	6	GPR183
0	5.0030011959729	0.255	0.011	0	6	LST1
0	4.96919822967128	0.591	0.079	0	6	DAB2
0	4.88154611175637	0.518	0.059	0	6	SLC15A3
0	4.86063436616369	0.32	0.034	0	6	NR4A2
0	4.8582786205801	0.428	0.065	0	6	CD83
0	4.85452590594541	0.252	0.01	0	6	TMEM86A
0	4.79789114008662	0.354	0.009	0	6	RENBP
0	4.77574039876444	0.424	0.02	0	6	NCF2
0	4.75741569664943	0.348	0.014	0	6	NFKBID
0	4.71508485254793	0.556	0.021	0	6	ADA2
0	4.71198211172539	0.578	0.019	0	6	THEMIS2
0	4.69047168142255	0.296	0.008	0	6	FGR
0	4.69035182884702	0.275	0.006	0	6	RAB3IL1
0	4.68596160766637	0.308	0.008	0	6	GAS7
0	4.67023171608878	0.375	0.017	0	6	DEPRECATED-ENSG00000119900
0	4.65799446612751	0.322	0.009	0	6	BASP1
0	4.64432766977595	0.428	0.031	0	6	MERTK
0	4.63189715843827	0.285	0.009	0	6	NRROS
0	4.62498155356537	0.658	0.071	0	6	HSPA6
0	4.61916885890009	0.314	0.006	0	6	WDFY4
0	4.59666592143731	0.495	0.023	0	6	PLTP
0	4.59260345685237	0.316	0.011	0	6	NCF4
0	4.56707213172129	0.257	0.012	0	6	AMPD3
0	4.55601137194787	0.279	0.036	0	6	KLF4
0	4.54671671095697	0.26	0.006	0	6	LRRK1
0	4.51645723847543	0.313	0.009	0	6	IRAK3
0	4.500976307615	0.322	0.009	0	6	CSF2RB
0	4.48743121751309	0.335	0.012	0	6	FMNL2
0	4.46464163269952	0.438	0.047	0	6	RGL1
0	4.44807647395533	0.392	0.03	0	6	OTULINL
0	4.41688276680032	0.625	0.069	0	6	MAFB
0	4.40882300617776	0.417	0.015	0	6	AOAH
0	4.38843099776388	0.574	0.105	0	6	CIITA
0	4.38807868023752	0.438	0.046	0	6	SYK
0	4.34387274089092	0.487	0.023	0	6	JAML
0	4.29402020221283	0.399	0.018	0	6	CYTH4
0	4.29149429559243	0.536	0.029	0	6	KLHL6
0	4.27266282362053	0.522	0.109	0	6	SDC3
0	4.23721183656261	0.586	0.031	0	6	STAB1
0	4.22489692500089	0.562	0.024	0	6	ZEB2
0	4.21225466448998	0.469	0.046	0	6	CAPG
0	4.2029753833213	0.376	0.016	0	6	TRPS1
0	4.19463269602633	0.435	0.058	0	6	RNF144B
0	4.18845543351033	0.399	0.066	0	6	ENPP2
0	4.18258773693428	0.261	0.037	0	6	PTPRE
0	4.15504354509204	0.282	0.008	0	6	PALD1
0	4.08618787719081	0.569	0.192	0	6	HMOX1
0	4.04091103662936	0.289	0.012	0	6	GNB4
0	4.03827795453783	0.988	0.83	0	6	CD74
0	4.03216205075264	0.395	0.035	0	6	TMEM106A
0	4.02479834345731	0.282	0.012	0	6	GIMAP8
0	4.01334956840511	0.302	0.016	0	6	RGS10
0	4.00352801548918	0.275	0.014	0	6	LAT2
0	4.00015312415298	0.477	0.025	0	6	HAVCR2
0	3.97737608647668	0.303	0.045	0	6	RTN1
0	3.95306272064561	0.67	0.05	0	6	CD4
0	3.95196872264275	0.661	0.139	0	6	FOS
0	3.93553695849253	0.472	0.026	0	6	PLCB2

0	3.92471419532404	0.418	0.032	0	6	AKR1B1
0	3.89779098695538	0.27	0.015	0	6	IL2RA
0	3.891403762434	0.316	0.019	0	6	DEPRECATED-ENSG00000163191
0	3.89124941520719	0.556	0.174	0	6	SLCO2B1
0	3.88634466617651	0.532	0.034	0	6	DOK2
0	3.87049180986831	0.309	0.016	0	6	APOBR
0	3.86885835847628	0.373	0.031	0	6	SPINT2
0	3.85448897278586	0.553	0.154	0	6	IER5
0	3.84467829929295	0.377	0.059	0	6	PDGFC
0	3.83331309206453	0.268	0.03	0	6	P2RX7
0	3.83168962082814	0.418	0.021	0	6	CD84
0	3.82558442851124	0.431	0.023	0	6	PARVG
0	3.81656338057999	0.662	0.07	0	6	ITGB2
0	3.81522135798265	0.573	0.057	0	6	GSN
0	3.81489330326278	0.894	0.562	0	6	CTSB
0	3.80963831022288	0.291	0.014	0	6	ABI3
0	3.77762103548661	0.307	0.035	0	6	ETV5
0	3.76755978843176	0.279	0.054	0	6	NR4A1
0	3.75710631472055	0.335	0.031	0	6	VCAM1
0	3.75675217093071	0.393	0.024	0	6	ACP5
0	3.74846147067678	0.537	0.041	0	6	RIN3
0	3.74340714793279	0.423	0.059	0	6	IL18BP
0	3.73147867434855	0.698	0.208	0	6	KCTD12
0	3.72743179598995	0.347	0.029	0	6	DSE
0	3.71109498183643	0.517	0.118	0	6	ATP6V1B2
0	3.70663486755403	0.567	0.068	0	6	TGFBI
0	3.6978354591497	0.665	0.053	0	6	HCLS1
0	3.69316932479099	0.262	0.011	0	6	TMEM273
0	3.69258921544867	0.282	0.014	0	6	VASH1
0	3.69096878647932	0.582	0.04	0	6	TIMP2
0	3.68862679432819	0.305	0.028	0	6	RELT
0	3.68346058609863	0.32	0.044	0	6	SLC43A2
0	3.65660357055493	0.855	0.453	0	6	PSAP
0	3.65584414896558	0.735	0.332	0	6	CD68
0	3.65172423726861	0.487	0.028	0	6	MYO1F
0	3.62203770957012	0.496	0.022	0	6	CD93
0	3.6023013183778	0.344	0.063	0	6	SLC31A2
0	3.59313697510874	0.533	0.048	0	6	PLEKHO1
0	3.57034200782204	0.354	0.033	0	6	MAP3K8
0	3.55793891034956	0.252	0.024	0	6	ZNF331
0	3.53482226352941	0.32	0.015	0	6	PMP22
0	3.51688541876488	0.298	0.017	0	6	ENTPD1
0	3.46460600797005	0.335	0.029	0	6	S100A11
0	3.44852560852595	0.536	0.036	0	6	METRNL
0	3.44251649870974	0.273	0.021	0	6	MYO5A
0	3.43711405975768	0.856	0.48	0	6	CTSZ
0	3.43649485672402	0.668	0.068	0	6	CYBA
0	3.4314234428028	0.4	0.028	0	6	PLEKHO2
0	3.41946206080671	0.822	0.143	0	6	LAPTM5
0	3.41838907746619	0.362	0.018	0	6	PIK3R5
0	3.41516741505383	0.463	0.031	0	6	NCKAP1L
0	3.41022436528424	0.382	0.031	0	6	SLC16A3
0	3.39978383207989	0.364	0.025	0	6	FAR1
0	3.39304408378349	0.374	0.023	0	6	ADCY7
0	3.37922605914246	0.469	0.112	0	6	PPT1
0	3.37893069484941	0.344	0.065	0	6	NPL
0	3.36722269857512	0.722	0.3	0	6	MFSD1
0	3.35065071292523	0.617	0.332	0	6	PLD3
0	3.32249430563768	0.372	0.031	0	6	GSTP1
0	3.32102639143167	0.29	0.016	0	6	TNFSF12

0	3.31388785194323	0.432	0.036	0	6	ANXA1
0	3.30698262123895	0.447	0.139	0	6	MCOLN1
0	3.29491152987926	0.397	0.082	0	6	ARHGAP18
0	3.27332540333715	0.877	0.507	0	6	SAT1
0	3.24543417878109	0.418	0.075	0	6	NABP1
0	3.22812275716999	0.548	0.229	0	6	LIPA
0	3.21452961167919	0.38	0.033	0	6	COTL1
0	3.20855265335311	0.277	0.027	0	6	RILPL2
0	3.19070507147635	0.331	0.023	0	6	WAS
0	3.17330087702134	0.657	0.177	0	6	CD14
0	3.16851937028835	0.604	0.072	0	6	RGS1
0	3.1648968958079	0.296	0.015	0	6	NRP2
8.04011901108184E-213	3.16219236024693	0.269	0.071	1.3473631438771E-208	6	FAM49A
0	3.16097826358935	0.62	0.187	0	6	IRF8
0	3.14383743146319	0.424	0.03	0	6	DENND3
0	3.11880271222269	0.836	0.468	0	6	IFI30
0	3.11828821214262	0.509	0.05	0	6	LCP2
2.90590941172022E-272	3.10544662828024	0.301	0.074	4.86972299216075E-268	6	TNFRSF21
0	3.09485997585556	0.265	0.014	0	6	RASSF2
1.50442691728767E-272	3.07786907984171	0.315	0.08	2.52111862799068E-268	6	CALHM6
3.7041047480518E-126	3.07728684856648	0.451	0.248	6.2073387367852E-122	6	CHMP1B
0	3.06837970749863	0.433	0.037	0	6	LPAR6
1.77209855528915E-281	3.0554744918021	0.258	0.052	2.96968275895356E-277	6	PLA2G15
0	3.04440242403654	0.47	0.121	0	6	DAPK1
0	3.04169420864841	0.498	0.181	0	6	TNFAIP2
2.92843193891895E-257	3.04131872884191	0.317	0.086	4.90746624324037E-253	6	STARD13
0	3.03811034281375	0.379	0.092	0	6	RAP2B
0	3.03045780226071	0.431	0.069	0	6	FERMT3
3.73634788445632E-279	3.03008604001427	0.631	0.393	6.2613717847719E-275	6	SLC40A1
0	3.02885341031896	0.477	0.042	0	6	UCP2
0	3.01850453292088	0.418	0.042	0	6	RCSD1
0	3.01515135204875	0.277	0.013	0	6	PDGFB
0	3.00559570473249	0.845	0.135	0	6	SRGN
0	2.98186658527426	0.463	0.153	0	6	TTYH3
0	2.98023954925255	0.372	0.052	0	6	PTGER4
0	2.9686980735653	0.387	0.042	0	6	SH3BGRL
0	2.96785937181782	0.316	0.025	0	6	GLIPR1
0	2.96498931138404	0.672	0.322	0	6	CTSC
0	2.96162293917313	0.472	0.038	0	6	IL10RA
5.94355763795644E-221	2.95688873929516	0.436	0.182	9.9602138896874E-217	6	DMXL2
0	2.9440641113865	0.305	0.044	0	6	PLEKHA2
0	2.92739019372269	0.374	0.096	0	6	ITGAM
0	2.92708964979919	0.534	0.158	0	6	ARL4C
3.23595589412138E-290	2.91343533862872	0.398	0.125	5.42281488736862E-286	6	ASAH1
0	2.91016900383339	0.308	0.019	0	6	GIMAP5
1.38039056015261E-235	2.90918796403985	0.404	0.148	2.31325850070374E-231	6	FCHO2
0	2.90378822665864	0.315	0.067	0	6	CLN8
3.45419105710981E-172	2.90080065600616	0.25	0.074	5.78853337350462E-168	6	LPCAT2
0	2.89915993324687	0.417	0.08	0	6	MICAL1
0	2.89901334747995	0.464	0.041	0	6	DOCK2
0	2.88975551592714	0.532	0.072	0	6	LSP1
0	2.87940082434528	0.321	0.031	0	6	ST8SIA4
7.33518940838599E-191	2.87594921702604	0.272	0.08	1.22923104105732E-186	6	BMP2K
0	2.86739379076003	0.551	0.181	0	6	CXCL16
0	2.8656046442059	0.654	0.281	0	6	SAMHD1
0	2.8511857603557	0.753	0.406	0	6	FCGRT
0	2.83341484144189	0.392	0.056	0	6	KCNAB2
0	2.82053218501224	0.324	0.069	0	6	SH3PXD2B
0	2.8101419599439	0.531	0.103	0	6	ARHGAP4
0	2.80785742552518	0.457	0.044	0	6	CMTM3

0	2.79969735112726	0.466	0.155	0	6	SIRPA
0	2.79464794366374	0.338	0.077	0	6	VSIR
0	2.79155416169462	0.37	0.031	0	6	DOCK11
0	2.78561294656802	0.578	0.223	0	6	GNA13
0	2.78452424875541	0.317	0.063	0	6	RHBDF2
8.98089442549297E-173	2.76404906274697	0.289	0.095	1.50501828782411E-168	6	ABCC5
0	2.76279947663365	0.59	0.245	0	6	MAN2B1
0	2.75758884887708	0.466	0.05	0	6	CHST11
6.17269803706054E-273	2.7565745578554	0.419	0.143	1.0344207370506E-268	6	GM2A
1.03428775012545E-241	2.75506030353074	0.61	0.318	1.73325941166023E-237	6	PFKFB3
0	2.75149066761175	0.408	0.096	0	6	REL
0	2.74234446200569	0.536	0.179	0	6	ARRB2
1.47387282157759E-248	2.73161197940791	0.347	0.105	2.46991607439973E-244	6	DEPRECATED-ENSG00000196743
0	2.72900260619007	0.399	0.033	0	6	PECAM1
0	2.72236260008876	0.431	0.094	0	6	LGALS3
1.26762401037964E-302	2.71760540855384	0.296	0.063	2.1242843165942E-298	6	CASP1
1.74649765031001E-303	2.71098857591311	0.413	0.127	2.92678076238951E-299	6	RNASET2
6.87530470125165E-261	2.70865153159197	0.416	0.147	1.15216356183575E-256	6	UNC93B1
7.36962742817793E-152	2.70714151354095	0.252	0.08	1.23500216441406E-147	6	AVPI1
8.67684097765452E-303	2.70631432409774	0.318	0.074	1.45406501103535E-298	6	WDR81
0	2.70503668084588	0.334	0.034	0	6	SFMBT2
1.45186645087585E-241	2.7043117120613	0.301	0.08	2.43303779837776E-237	6	PI4K2A
2.2569322492998E-138	2.69605254943968	0.368	0.166	3.7821670633766E-134	6	PPP1R15A
0	2.69013848427261	0.729	0.452	0	6	GRN
0	2.68684393506117	0.328	0.069	0	6	FKBP15
3.37129757696212E-290	2.68630588179872	0.401	0.121	5.64962047947312E-286	6	PRNP
0	2.67657898187004	0.456	0.064	0	6	SH3BP5
0	2.67264302644919	0.503	0.123	0	6	PLXND1
3.87212858033342E-154	2.66557004586291	0.293	0.102	6.48891307492274E-150	6	IER3
0	2.6596103458241	0.556	0.06	0	6	CD53
0	2.65767673559152	0.542	0.227	0	6	NAGK
2.50652555895916E-229	2.63323561858062	0.273	0.069	4.20043553170377E-225	6	CTBP2
0	2.62624239719059	0.556	0.244	0	6	MARCKS
1.72310964296965E-241	2.61580055799703	0.367	0.118	2.88758713968854E-237	6	FRMD4A
0	2.60887024477153	0.633	0.093	0	6	LCP1
2.51859797476315E-233	2.6054078535724	0.347	0.108	4.22066648610808E-229	6	JAK2
1.14336594930031E-144	2.60103518666188	0.274	0.097	1.91605265783746E-140	6	PAK1
3.14556603342924E-284	2.59365259676509	0.453	0.164	5.27133955882073E-280	6	DEPRECATED-ENSG00000026297
5.87883810130823E-254	2.58220075594081	0.326	0.089	9.85175689017233E-250	6	FRMD4B
8.66622101619645E-287	2.57687563011905	0.26	0.051	1.4522853178942E-282	6	RASA4
0	2.56891216295253	0.368	0.07	0	6	IGFLR1
3.33855532780861E-280	2.5672602321686	0.528	0.224	5.59475101834167E-276	6	HEXA
3.41631395188772E-293	2.56573631965422	0.329	0.079	5.72505892057345E-289	6	RCN3
0	2.55341407144583	0.358	0.065	0	6	ARHGEF2
0	2.54797387468919	0.634	0.066	0	6	CELF2
2.33292967139586E-233	2.54136790866603	0.396	0.141	3.90952354332518E-229	6	PRKCD
0	2.5390909952848	0.283	0.027	0	6	SAMSN1
3.70570294902154E-235	2.53382591129132	0.447	0.166	6.2100170019703E-231	6	P4HA1
2.40559294376257E-227	2.52978429729152	0.385	0.136	4.03129265515732E-223	6	ABR
1.08117925166297E-97	2.52809712688425	0.264	0.114	1.8118401899368E-93	6	NAIP
1.42948277376262E-254	2.52778009532814	0.419	0.148	2.3955272322714E-250	6	LGALS9
2.18002238959141E-204	2.52700472001242	0.358	0.126	3.65328152047728E-200	6	GRK3
1.89179219372724E-295	2.52156267498726	0.499	0.184	3.17026535582481E-291	6	ARAP1
0	2.5127575650076	0.331	0.03	0	6	INPP5D
6.75779468604305E-256	2.50368779819865	0.501	0.218	1.13247123348709E-251	6	RASSF4
9.38754742892665E-211	2.4919824443596	0.268	0.071	1.57316519813953E-206	6	CTTNBP2NL
0	2.48677595334898	0.386	0.109	0	6	SESN1
1.64982453889679E-151	2.48449987708603	0.272	0.093	2.76477596228324E-147	6	DRAM1
1.4655674158833E-215	2.4831312304932	0.299	0.084	2.45599787553724E-211	6	FES
0	2.47508766193948	0.254	0.023	0	6	EVI2A

8.04619101307182E-272	2.47508280147592	0.397	0.124	1.34838068997058E-267	6	EPB41L2
5.98181781584372E-201	2.46989469177907	0.396	0.152	1.00243302957909E-196	6	MKNK1
5.57407556227049E-51	2.46783580434413	0.293	0.178	9.34103582725289E-47	6	APBB3
0	2.46160834167365	0.258	0.022	0	6	PRKCB
2.68352987611639E-161	2.45617123074772	0.352	0.142	4.49705936639584E-157	6	ATG7
1.26699168470444E-137	2.44934934958763	0.314	0.127	2.1232246652277E-133	6	CCDC88A
0	2.44500311155914	0.265	0.033	0	6	TAGAP
2.75310195447397E-266	2.44171770254166	0.505	0.216	4.61364825530748E-262	6	AP1B1
0	2.43618113722958	0.826	0.609	0	6	CST3
0	2.43228849371805	0.784	0.401	0	6	JUNB
3.9880075065015E-202	2.43189486470221	0.353	0.123	6.68310297939522E-198	6	JDP2
0	2.42545835180418	0.364	0.084	0	6	DOCK4
0	2.42486965946349	0.419	0.105	0	6	SEC14L1
0	2.42479348620663	0.326	0.071	0	6	ADAMTSL4
0	2.41491993895429	0.325	0.036	0	6	CLEC2B
1.51913438355319E-172	2.39578127110257	0.34	0.128	2.54576539995844E-168	6	NAAA
1.31944678020168E-227	2.39524019926234	0.39	0.138	2.21112891426198E-223	6	ADPGK
2.5265028558901E-295	2.39047421757349	0.443	0.148	4.23391348590063E-291	6	RHOG
2.91224328876885E-157	2.39000558139553	0.336	0.132	4.88033730331883E-153	6	GPR137B
2.02064362275795E-160	2.38523422743647	0.493	0.265	3.38619458301777E-156	6	NAMPT
3.77689976058879E-113	2.3810730579852	0.268	0.105	6.3293286187947E-109	6	ACOT9
1.6037207980735E-269	2.36798407364635	0.261	0.054	2.68751531341158E-265	6	SLC6A6
5.8120601491486E-234	2.36566791526757	0.277	0.069	9.73985039794322E-230	6	MVB12B
0	2.35985381201204	0.254	0.018	0	6	DEPRECATED-ENSG00000196329
0	2.3551313309017	0.321	0.033	0	6	ARHGAP30
0	2.35218471812025	0.427	0.077	0	6	CD81
0	2.34185314607867	0.472	0.102	0	6	TNFRSF1B
0	2.33802338720184	0.454	0.048	0	6	ITGA4
1.43903399567689E-244	2.3318914131515	0.43	0.162	2.41153316995534E-240	6	NAGA
1.82231059937214E-204	2.32803001202016	0.483	0.239	3.05382810242784E-200	6	IFNGR1
0	2.32410038486249	0.787	0.478	0	6	LITAF
0	2.31969678272019	0.34	0.035	0	6	DOCK8
5.51140402185323E-247	2.30938006355922	0.347	0.102	9.23601085982164E-243	6	GAS6
1.63085501742713E-148	2.3069418142651	0.309	0.117	2.73298683820438E-144	6	CEP170
0	2.30082607265286	0.488	0.052	0	6	SLA
0	2.29962762323762	0.36	0.068	0	6	CNTRL
2.83859927183292E-294	2.29029367654352	0.256	0.048	4.75692465973761E-290	6	GMIP
9.53854398993158E-245	2.28254533279595	0.577	0.295	1.59846920183273E-240	6	HERPUD1
0	2.27698746695629	0.593	0.268	0	6	TYMP
0	2.27267288624007	0.294	0.034	0	6	IL16
1.64280938229388E-282	2.26949118867148	0.528	0.227	2.75301996284809E-278	6	M6PR
7.45390133374608E-263	2.26486255425119	0.581	0.303	1.24912478550917E-258	6	MGAT1
0	2.26202099607407	0.528	0.089	0	6	ARHGDIB
0	2.24296183006128	0.389	0.037	0	6	SELPLG
1.7877494237847E-163	2.24193007538931	0.348	0.134	2.99591048437841E-159	6	ETV6
0	2.22251992560064	0.303	0.032	0	6	EVI2B
5.51931244287963E-163	2.21337593089151	0.388	0.167	9.24926379177769E-159	6	PTPN6
5.08682440287624E-140	2.2091889264178	0.32	0.128	8.52450033434E-136	6	RHOQ
0	2.20675841236213	0.377	0.049	0	6	FYB1
8.35334062144535E-268	2.20317926098732	0.265	0.055	1.39985282134181E-263	6	EMP3
1.04314186060668E-163	2.19486023590189	0.313	0.111	1.74809713000468E-159	6	SH2B3
1.68176186030599E-133	2.19258070988298	0.305	0.121	2.81829652550077E-129	6	GCA
5.9306076604959E-204	2.19162645211927	0.534	0.291	9.93851231745902E-200	6	CTSL
0	2.1887523284002	0.42	0.041	0	6	GPSM3
1.39529962673502E-126	2.18694647215692	0.441	0.258	2.33824311448255E-122	6	CPM
2.80816844820227E-228	2.18019375277904	0.507	0.235	4.70592868549736E-224	6	COLGALT1
0	2.17976706701681	0.742	0.463	0	6	TPP1
6.54629688612793E-135	2.1731224670057	0.288	0.107	1.09702843217732E-130	6	RALGDS
9.97291733927662E-232	2.1645461557584	0.367	0.12	1.67126148771598E-227	6	SWAP70
1.6799971726698E-262	2.15863039085937	0.348	0.098	2.81533926196005E-258	6	IQGAP1

5.76431304878218E-215	2.14959727289157	0.416	0.158	9.65983580714918E-211	6	SH3TC1
0	2.14948158171678	0.681	0.357	0	6	TMSB10
1.51296743495706E-122	2.13289068901324	0.258	0.095	2.53543082750103E-118	6	GPR155
0	2.12816522462792	0.662	0.154	0	6	VIM
1.34050031867957E-136	2.12749742510516	0.277	0.101	2.24641043404322E-132	6	SNX29
0	2.12692626030275	0.923	0.701	0	6	TMSB4X
3.44778410918562E-131	2.12661520005109	0.334	0.142	5.77779661017325E-127	6	PABPC4
2.65611286280828E-149	2.12287474377703	0.357	0.149	4.45111393549412E-145	6	MEF2A
6.87159186690442E-180	2.11815115700884	0.378	0.15	1.15154136505584E-175	6	PLBD2
1.56101378364334E-166	2.11599846037783	0.292	0.098	2.61594689862952E-162	6	TCN2
0	2.10674128915617	0.345	0.057	0	6	SERPINB9
8.62597599827911E-141	2.1028367568237	0.272	0.095	1.44554105779161E-136	6	USP4
3.90851889663832E-145	2.10219888428481	0.481	0.284	6.5498959669865E-141	6	NRP1
8.20500452925414E-180	2.09660863415732	0.255	0.069	1.37499465901241E-175	6	SOCS3
8.19248375539702E-175	2.09398102577595	0.329	0.118	1.37289642772943E-170	6	ALCAM
1.6744489301254E-209	2.08878543697808	0.404	0.153	2.80604151710415E-205	6	FCHSD2
9.04823339583653E-162	2.08037736844963	0.472	0.254	1.51630295247429E-157	6	AP2A2
7.14561224497334E-95	2.06988306942395	0.294	0.136	1.19746170001263E-90	6	MANBA
2.08811407029802E-281	2.06703381221588	0.332	0.083	3.49926155900543E-277	6	PREX1
1.05014317134341E-227	2.06287368642763	0.598	0.304	1.75982992653729E-223	6	WSB1
7.95036110996513E-245	2.06137890921308	0.412	0.143	1.33232151480796E-240	6	NECAP2
6.84404543805826E-219	2.06074559644116	0.326	0.098	1.1469251345098E-214	6	CCNG2
1.34314975485939E-160	2.05972728533056	0.282	0.092	2.25085035919337E-156	6	CD40
3.9697907862757E-90	2.05356653652989	0.288	0.132	6.65257539964081E-86	6	CACUL1
2.74501869439024E-132	2.05339229603434	0.309	0.123	4.60010232805917E-128	6	ZMIZ1
0	2.04784996735063	0.296	0.04	0	6	LIMD2
6.38889872176925E-30	2.03810811212608	0.295	0.207	1.07065164779409E-25	6	RASGEF1B
2.08018043909136E-123	2.03261983665583	0.414	0.217	3.48596637982931E-119	6	NINJ1
1.2088418432384E-148	2.03034372332217	0.265	0.087	2.02577716089891E-144	6	OSBPL3
9.58138670474266E-145	2.02951923632638	0.338	0.136	1.60564878398077E-140	6	PLEKHM2
0	2.02817583893335	0.332	0.041	0	6	FMNL1
2.07070985462719E-92	2.02776748457501	0.285	0.131	3.47009557438424E-88	6	USP36
0	2.02086379625768	0.359	0.041	0	6	GIMAP4
1.67192464190075E-153	2.0039656150007	0.458	0.23	2.80181131489727E-149	6	MYO9B
4.46936815645213E-212	2.00261581099602	0.625	0.417	7.48976715658248E-208	6	MAF
1.10206395633402E-36	2.00150308071034	0.334	0.231	1.84683877802455E-32	6	GLA
1.04268488359974E-304	2.00142315236832	0.822	0.618	1.74733132793644E-300	6	DUSP1
1.53669346766419E-276	2.00023386507412	0.698	0.48	2.57519091311165E-272	6	NPC2
3.9138237137555E-116	1.99735914523997	0.311	0.135	6.55878577951147E-112	6	ARPIN
1.3225477495605E-47	1.99695607086388	0.373	0.253	2.21632551871348E-43	6	ATF3
0	1.99676454054782	0.412	0.065	0	6	PIK3IP1
8.12004895373798E-115	1.98340245394414	0.377	0.189	1.36075780366741E-110	6	DRAM2
6.31291372486587E-246	1.97704180177425	0.667	0.46	1.05791808201302E-241	6	ITM2B
5.7386703259513E-276	1.97408677607249	0.677	0.442	9.61686373222918E-272	6	CTSS
0	1.97231849540454	0.519	0.102	0	6	TRIM22
1.86462242630542E-173	1.96749391770151	0.532	0.31	3.12473426200262E-169	6	GNS
4.29831667630293E-125	1.96336855414073	0.439	0.237	7.20311908614845E-121	6	RUNX1
2.13110831437018E-251	1.96237338607622	0.568	0.275	3.57131131322155E-247	6	ZYX
0	1.9585472547478	0.259	0.031	0	6	ARHGAP15
3.82766801796605E-226	1.95773907732309	0.606	0.327	6.4144060645075E-222	6	EHBP1L1
1.31206395956801E-125	1.95513772318319	0.297	0.118	2.19875678344407E-121	6	SNX8
3.32933092803998E-167	1.95318533713632	0.355	0.139	5.5792927692094E-163	6	KIAA0930
9.99422841453316E-213	1.95105500076033	0.364	0.124	1.67483279770747E-208	6	PKM
7.65147422880692E-170	1.94938871963582	0.496	0.264	1.28223405126346E-165	6	GAA
4.38000506932084E-47	1.94524756346646	0.311	0.193	7.34001249516786E-43	6	ATG16L2
0	1.94303125484415	0.354	0.049	0	6	ARHGAP45
1.01289610396018E-167	1.94112574006801	0.444	0.21	1.69741129101648E-163	6	IFNGR2
2.64087043594992E-162	1.94100691404745	0.433	0.207	4.42557067656487E-158	6	CYFIP1
1.22648706638136E-173	1.93235375062118	0.458	0.218	2.05534702584188E-169	6	CNPY3
2.21886373502649E-228	1.92930906064932	0.67	0.453	3.7183718471574E-224	6	CTSH

2.73901094172144E-231	1.92288464754899	0.607	0.321	4.59003453613679E-227	6	RNF149
8.2565625964743E-131	1.91889784944575	0.32	0.131	1.38363475991716E-126	6	TGFBR1
4.06840553618379E-104	1.91698389450148	0.361	0.186	6.8178339975368E-100	6	LHFPL2
9.5023061053413E-176	1.91663389933422	0.377	0.148	1.59239645713309E-171	6	CORO7
2.12381875215451E-140	1.91455676963055	0.292	0.107	3.55909546486052E-136	6	EPSTI1
3.89272702326939E-195	1.91335924148601	0.276	0.076	6.52343194559484E-191	6	PTK2B
8.0547122876449E-237	1.90902676512369	0.449	0.171	1.34980868516353E-232	6	MAP7D1
9.04081077032884E-128	1.90868234670828	0.359	0.163	1.51505906889171E-123	6	ANKS1A
8.98792282723858E-144	1.90288895540965	0.415	0.204	1.50619610738864E-139	6	SCPEP1
0	1.90237491062985	0.478	0.083	0	6	IFI16
2.77505457982507E-150	1.90213162100638	0.336	0.133	4.65043646487085E-146	6	MYO1E
2.48995963123853E-77	1.89741087412503	0.615	0.473	4.17267435002952E-73	6	HSPH1
0	1.89223971501083	0.299	0.056	0	6	PRDM1
0	1.89121013533159	0.452	0.076	0	6	FXYD5
6.45604763019676E-188	1.89098386079192	0.512	0.264	1.08190446186837E-183	6	DPYSL2
1.87870073407864E-193	1.88811022682288	0.396	0.151	3.14832669016899E-189	6	FNBP1
5.97452461543767E-103	1.88700054319465	0.377	0.207	1.00121083505504E-98	6	TSPAN4
0	1.8839693655493	0.6	0.088	0	6	WIPF1
8.42238112369666E-83	1.8766414739153	0.304	0.152	1.41142262870909E-78	6	LPCAT1
3.04372562271495E-109	1.87173557219087	0.253	0.097	5.10067539854572E-105	6	FEZ2
1.30806695410261E-105	1.86827815289795	0.305	0.134	2.19205860168515E-101	6	DDAH2
6.02604785836363E-66	1.86375169735887	0.251	0.125	1.00984510010458E-61	6	SLC49A4
4.29976841163391E-87	1.85582530290284	0.471	0.33	7.20555190421611E-83	6	FUCA1
0	1.85363653536267	0.308	0.037	0	6	ARHGAP9
1.48085722567514E-137	1.84228086235697	0.315	0.125	2.48162053878639E-133	6	SLC11A2
2.94224636244121E-86	1.84058275138252	0.261	0.115	4.93061645417899E-82	6	CYB5R4
0	1.83882917633876	0.5	0.143	0	6	CD44
1.33816058789926E-110	1.83655788117906	0.269	0.106	2.24248951320159E-106	6	GNG10
1.71039590325047E-108	1.83584516062716	0.282	0.116	2.86628145466713E-104	6	HPS1
4.12024880905323E-224	1.8305298509689	0.284	0.072	6.9047129542114E-220	6	PLCG2
4.8106871888072E-301	1.83037387458574	0.336	0.078	8.06174959100311E-297	6	ABCC1
3.89560996063387E-254	1.82772980100178	0.477	0.179	6.52826317203024E-250	6	LGALS1
1.48668547286751E-44	1.81739070000824	0.262	0.156	2.49138751543138E-40	6	APAF1
3.73372170666954E-124	1.81357851543952	0.447	0.242	6.25697083603682E-120	6	CLCN7
4.85498463943715E-220	1.80214647438216	0.441	0.172	8.13598325876878E-216	6	PPP1R9B
4.49523974552881E-188	1.80070791366675	0.464	0.211	7.53312276555719E-184	6	GNAI2
3.45105758828071E-68	1.79862196465429	0.287	0.15	5.78328230644082E-64	6	BTG2
4.38899728675762E-55	1.79814768447629	0.267	0.147	7.35508165314843E-51	6	SRGAP1
3.39259022647557E-144	1.79052270401474	0.501	0.294	5.68530270152776E-140	6	RNF130
1.15083925817538E-59	1.78825815323457	0.254	0.132	1.9285764288503E-55	6	UVRAG
3.37385584094463E-18	1.78415106402506	0.359	0.293	5.65390761825501E-14	6	SPP1
1.64557236731535E-140	1.7839198771341	0.384	0.176	2.75765017314706E-136	6	RBPJ
1.2620469214316E-179	1.78129637835336	0.671	0.422	2.11493823093508E-175	6	KLF6
1.16932465278001E-76	1.76882338438949	0.49	0.332	1.95955425312874E-72	6	EIF4A3
2.36417266941175E-118	1.76280770166761	0.253	0.093	3.96188055940021E-114	6	AMDHD2
3.1401604806248E-275	1.76261276892786	0.422	0.133	5.26228093343104E-271	6	SIPA1
2.72363067835666E-57	1.75981592815055	0.391	0.26	4.56426029079009E-53	6	ZC3HAV1
7.36391202494418E-65	1.75117567497018	0.271	0.14	1.23404437714015E-60	6	SLC12A9
5.98883354231683E-86	1.7488464297016	0.328	0.168	1.00360872502145E-81	6	PNPLA6
2.12290570521073E-117	1.73555379005651	0.33	0.146	3.55756538079213E-113	6	CSGALNACT2
2.74794840259537E-92	1.73503409916154	0.31	0.149	4.60501193306933E-88	6	SLC38A7
6.49127934669908E-54	1.73337744774278	0.255	0.139	1.08780859291983E-49	6	ST14
5.56474267553463E-87	1.72036446868081	0.591	0.538	9.32539577566093E-83	6	CTSD
2.64388590174074E-108	1.71991005597742	0.266	0.105	4.43062399413713E-104	6	RIPOR1
5.76873727375102E-113	1.71915093648933	0.439	0.252	9.66724992335196E-109	6	ATP6AP2
1.1454092059145E-172	1.7172026368837	0.587	0.354	1.91947674727152E-168	6	QKI
3.56520064064461E-132	1.7161456505759	0.412	0.203	5.97456323359224E-128	6	OSTF1
4.48140593702928E-116	1.71439105706172	0.49	0.301	7.50994006927367E-112	6	LAP3
8.2866398195436E-117	1.71392157834304	0.355	0.165	1.38867510095912E-112	6	BNIP3L
2.3308458208191E-92	1.70595882844683	0.339	0.173	3.90603142652864E-88	6	VOPP1

1.76183938624037E-108	1.69647175689569	0.297	0.128	2.95249044346162E-104	6	DOCK10
5.44608220366221E-142	1.69640763596079	0.355	0.15	9.12654455689714E-138	6	ABCG1
9.7194404161683E-180	1.69563327766669	0.374	0.131	1.62878382494148E-175	6	ICAM1
1.34092658140931E-185	1.69367087405056	0.371	0.134	2.24712476512572E-181	6	PPP1R18
8.90998880653383E-224	1.6896722713953	0.581	0.315	1.49313592419894E-219	6	SMAP2
3.86074477219302E-69	1.68729208760834	0.296	0.155	6.46983608924106E-65	6	GPCPD1
6.28025275881592E-161	1.68663607356419	0.512	0.284	1.05244475732237E-156	6	SH3BGRL3
5.42198049493946E-303	1.67965266526187	0.385	0.102	9.08615491341954E-299	6	TSPAN14
1.21386813759159E-119	1.67116993817868	0.336	0.149	2.03420022497598E-115	6	SAP30
1.00844703817025E-82	1.66189965795788	0.628	0.48	1.68995554656571E-78	6	MAT2A
7.9469218312106E-80	1.65863802009406	0.263	0.122	1.33174516047427E-75	6	MTMR14
0	1.65853301049934	0.765	0.118	0	6	CXCR4
1.13124043463881E-151	1.65135410572027	0.56	0.358	1.89573272036772E-147	6	ANXA5
6.7175223906798E-153	1.6507171775056	0.398	0.173	1.12572240223012E-148	6	TWF2
7.31585563036922E-84	1.64734936591367	0.302	0.149	1.22599108653727E-79	6	SRGAP2B
2.60319706355234E-111	1.64606605827559	0.271	0.107	4.36243763910101E-107	6	SLC4A7
7.49410194982384E-169	1.6460092767515	0.258	0.074	1.25586160475148E-164	6	LPXN
4.29662982206874E-258	1.64542539838828	0.799	0.61	7.2002922558228E-254	6	MCL1
1.67246763084277E-90	1.64276681647541	0.37	0.201	2.80272125576631E-86	6	CREM
2.76751728785792E-78	1.64271594122145	0.296	0.147	4.63780547099231E-74	6	SNAP23
6.15425807306567E-115	1.64062657247449	0.576	0.398	1.03133056788435E-110	6	ABCA1
1.45931816223508E-26	1.63495359942396	0.27	0.186	2.44552537627355E-22	6	ZFYVE16
1.30928876282036E-184	1.63362155371826	0.333	0.111	2.19410610873436E-180	6	STK10
0	1.63203455146279	0.294	0.039	0	6	CD37
4.13553335946638E-72	1.62832543716523	0.357	0.206	6.93032680379377E-68	6	LYN
1.13958036373606E-111	1.62227637693027	0.346	0.16	1.90970877354889E-107	6	HPS5
1.89892664623049E-41	1.62179205777491	0.357	0.243	3.18222127375306E-37	6	DOCK5
3.8500356821605E-57	1.61731336676776	0.301	0.17	6.45188979616456E-53	6	DENND4B
0	1.61709535708163	0.255	0.037	0	6	SASH3
4.16498757173791E-136	1.61697096549293	0.429	0.214	6.97968617271839E-132	6	FLII
3.19844025603124E-69	1.60999757834927	0.274	0.138	5.35994618105715E-65	6	GIT2
6.03710872897617E-89	1.599895986646	0.36	0.192	1.01169868080183E-84	6	CREBL2
1.50997294010324E-40	1.59681143092351	0.268	0.164	2.53041265302501E-36	6	ITSN1
6.70962988355267E-113	1.59525878666156	0.429	0.235	1.12439977588576E-108	6	PLEKHB2
4.75278507874653E-115	1.58877685068875	0.289	0.116	7.96471723496343E-111	6	MOB3A
1.5024675156053E-105	1.58698596804343	0.461	0.265	2.51783506265135E-101	6	SLC3A2
1.08923791033981E-269	1.57791520524814	0.272	0.056	1.82534489014745E-265	6	TMEM173
3.30648036696569E-112	1.57728074698476	0.422	0.231	5.5409997989611E-108	6	SUSD6
3.05310007234067E-94	1.57623100302099	0.655	0.518	5.11638510122849E-90	6	ZFP36
4.69477024355853E-81	1.57602115559615	0.429	0.271	7.86749597415539E-77	6	PEA15
6.18238386522636E-82	1.57545677809724	0.415	0.253	1.03604388813463E-77	6	FAM49B
7.49332885876582E-89	1.5730831710321	0.365	0.195	1.25573205015198E-84	6	OAZ2
1.56151241927507E-83	1.57208287497653	0.262	0.118	2.61678251222116E-79	6	SRGAP2
2.61687442378359E-102	1.57200206262527	0.409	0.221	4.38535815937654E-98	6	AHR
5.80698707763781E-69	1.57004298892863	0.382	0.236	9.73134894470544E-65	6	DEPRECATED-ENSG00000137575
3.30451332170168E-107	1.56857894305082	0.432	0.24	5.53770342450768E-103	6	VPS13C
2.13430008302324E-10	1.56579223649648	0.268	0.222	3.57666007913035E-06	6	SELENOK
6.35793027945578E-96	1.56275099722752	0.455	0.271	1.0654619562312E-91	6	TNFRSF14
3.59314313571129E-118	1.55814822701762	0.385	0.19	6.02138926682498E-114	6	VEGFB
4.71644050964704E-137	1.55725846791934	0.876	0.756	7.90381100606652E-133	6	HSPA1B
2.01710270395743E-94	1.54410982960814	0.505	0.341	3.38026071129187E-90	6	SDCBP
3.3063017444324E-58	1.53411680982091	0.347	0.213	5.54070046331982E-54	6	MED13L
3.25033606713315E-73	1.53106250799895	0.322	0.172	5.44691318130174E-69	6	RAB35
1.6615698837805E-10	1.52187922340218	0.306	0.267	2.78445881123936E-06	6	PDE4DIP
0	1.5096036882671	0.857	0.654	0	6	GLUL
2.24216365682538E-114	1.50857695170371	0.493	0.297	3.75741785610796E-110	6	EIF4A1
2.08337958238048E-35	1.50827141831692	0.33	0.228	3.4913275041532E-31	6	RUFY3
6.38318501293969E-140	1.50496685405921	0.481	0.256	1.06969414446843E-135	6	ARPC1B
6.95620800656406E-98	1.5025748391241	0.423	0.239	1.16572133774001E-93	6	SERPINB6
1.551102596532E-65	1.50012762478665	0.302	0.163	2.59933773126833E-61	6	ARMH3

2.69318799276577E-30	1.49904880950597	0.259	0.17	4.51324443827688E-26	6	SPIN1
8.9698516216683E-25	1.49821847709721	0.337	0.256	1.50316773475917E-20	6	DDX3X
2.61841419424733E-36	1.49455593318732	0.297	0.188	4.38793850671968E-32	6	SERPINH1
5.98153827670883E-222	1.48786235627091	0.699	0.472	1.00238618441087E-217	6	TXNIP
2.35474128123135E-62	1.48111937051872	0.414	0.273	3.9460754390875E-58	6	STX7
9.41911237555714E-90	1.47195174979271	0.323	0.161	1.57845485189586E-85	6	MAP3K3
4.44205525382003E-81	1.46815446942173	0.316	0.153	7.4439961943516E-77	6	TMEM140
5.79353457755752E-129	1.46553262212519	0.497	0.278	9.70880524507089E-125	6	GRK2
5.22588656689691E-78	1.46225244795605	0.429	0.271	8.75754070880585E-74	6	CHKA
1.28763515124551E-54	1.45720933882133	0.259	0.141	2.15781898645723E-50	6	BLVRA
5.2125213658547E-63	1.45678819112473	0.36	0.214	8.7351433048993E-59	6	PTPN1
1.4826018811382E-74	1.4535151350678	0.397	0.242	2.4845442324114E-70	6	TBC1D14
9.47091306313587E-50	1.44855757824297	0.27	0.149	1.58713561112031E-45	6	ODF3B
2.28328989469861E-61	1.44589663119175	0.25	0.123	3.82633720553593E-57	6	NT5C2
2.84772027705745E-107	1.44494341407338	0.503	0.307	4.77220964029288E-103	6	NCOR2
1.83459085691958E-43	1.4381712626816	0.382	0.27	3.07440735802584E-39	6	RAB20
5.13825972498986E-53	1.43417825978302	0.496	0.363	8.61069564713801E-49	6	CLK1
1.3033863310122E-24	1.42394514896969	0.279	0.197	2.18421481351024E-20	6	FNIP2
7.07338195283102E-68	1.42281267464008	0.309	0.167	1.18535734765542E-63	6	WASHC4
3.56136915759099E-114	1.41587342774632	0.548	0.386	5.96814243429098E-110	6	RNH1
2.97317696734419E-65	1.41340302139711	0.341	0.194	4.9824499618754E-61	6	JMJD1C
2.91305390090602E-56	1.40509057545183	0.284	0.159	4.88169572713831E-52	6	SPTLC2
2.81173318166983E-81	1.40154937561653	0.345	0.181	4.71190246584231E-77	6	BNIP2
1.38637768078834E-55	1.39912707254586	0.342	0.212	2.32329171746511E-51	6	RNF13
6.11491215226212E-25	1.39872214402394	0.378	0.299	1.02473697847609E-20	6	UGCG
5.6844504533495E-39	1.39554624569229	0.297	0.188	9.52600206972309E-35	6	TRIM38
6.08982118890185E-65	1.38932895187039	0.473	0.332	1.02053223483617E-60	6	GNAQ
3.93622787325229E-87	1.38735315341665	0.429	0.267	6.59633066999618E-83	6	MMP14
2.25732643071675E-127	1.38161382314714	0.577	0.37	3.78282763259514E-123	6	PER1
3.55741320445422E-192	1.38155720306671	0.285	0.078	5.96151304802439E-188	6	NLRP1
5.08229264968087E-88	1.37627838543229	0.376	0.205	8.5169060223352E-84	6	ATM
9.08001402837917E-47	1.36620102740315	0.267	0.153	1.52162875087578E-42	6	RIC1
1.02851379528833E-97	1.36578055111568	0.445	0.259	1.72358341814418E-93	6	GGA2
3.89877658873149E-62	1.36413556411734	0.314	0.177	6.53356980739624E-58	6	PTPRJ
4.6909519169349E-91	1.36274660601219	0.549	0.435	7.8610972223995E-87	6	NCOA4
4.81214652773921E-28	1.36214769807025	0.277	0.188	8.06419515118537E-24	6	NXF1
7.89214498801136E-91	1.35859788045205	0.511	0.347	1.32256565709094E-86	6	LRP1
1.73438813624085E-87	1.35242478501603	0.467	0.288	2.90648763871242E-83	6	CYTH1
5.05738328739424E-74	1.35061116939875	0.319	0.168	8.47516291301527E-70	6	RAPGEF1
3.54598545834828E-44	1.34952244554859	0.274	0.164	5.94236243110005E-40	6	ACP2
2.52292921684936E-59	1.34922140356036	0.378	0.244	4.22792478159615E-55	6	ATP6V0C
1.33229130213599E-47	1.34894589956043	0.371	0.244	2.23265376411949E-43	6	H2AFY
3.64170357244003E-50	1.34836541037117	0.749	0.658	6.102766846695E-46	6	DNAJB1
9.12858195110154E-58	1.3456155860825	0.267	0.142	1.5297677633656E-53	6	PAPSS1
2.56788623892376E-129	1.34408556227111	0.278	0.101	4.30326375918843E-125	6	ANKRD44
5.07460766027336E-43	1.33982906589851	0.381	0.264	8.5040275170861E-39	6	DEDD2
6.6942237817831E-126	1.33876395772084	0.36	0.161	1.12181802135121E-121	6	ITM2C
9.3574039227408E-77	1.33510657916058	0.281	0.136	1.5681137493729E-72	6	GPRIN3
3.4340865586554E-39	1.33379124805474	0.256	0.151	5.75484225499472E-35	6	RAB12
7.52069739006603E-41	1.32637510566784	0.321	0.21	1.26031846862727E-36	6	ITSN2
3.49370221565997E-45	1.32482148054563	0.468	0.348	5.85474617300297E-41	6	CHD2
1.0412022511645E-66	1.3217712599703	0.351	0.201	1.74484673250147E-62	6	SLC1A5
8.80644208821968E-167	1.31782352606991	0.49	0.237	1.47578356514385E-162	6	DEPRECATED-ENSG00000175899
9.89147061986661E-113	1.3174508296096	0.387	0.194	1.65761264647725E-108	6	ARL6IP5
2.17054074046034E-61	1.31504082395428	0.282	0.15	3.63739217286343E-57	6	CREBRF
3.26309237618268E-75	1.30605304302631	0.261	0.121	5.46829020400693E-71	6	RAB8B
1.2103667591162E-58	1.30274028854581	0.323	0.186	2.02833261492694E-54	6	MRTFA
1.41621566288528E-21	1.29910336939341	0.277	0.2	2.37329420786316E-17	6	MYD88
6.80990264119986E-61	1.29654373485952	0.906	0.836	1.14120348461227E-56	6	DEPRECATED-ENSG00000204389
1.47074527002171E-79	1.29645757988334	0.323	0.167	2.46467492350238E-75	6	PSME4

3.28228625298003E-59	1.29537237272135	0.356	0.22	5.50045530274393E-55	6	SNX2
9.05728170338845E-55	1.29478238373921	0.358	0.229	1.51781926785384E-50	6	ABHD12
8.59587065649016E-44	1.29139156691212	0.299	0.184	1.44049600461462E-39	6	OSBPL8
2.33456792045945E-89	1.28982873970386	0.521	0.36	3.91226892110595E-85	6	VAMP8
2.53227340923131E-42	1.28652616323258	0.305	0.191	4.24358377918983E-38	6	INPPL1
6.95475954702777E-44	1.28565620293753	0.362	0.244	1.16547860489091E-39	6	GPATCH2L
0	1.28516563053028	0.301	0.048	0	6	PTPRC
4.94460238413054E-69	1.28339277307254	0.488	0.332	8.28616467532596E-65	6	TACC1
5.3560950936586E-122	1.28329010013084	0.264	0.089	8.97574415795309E-118	6	CPED1
8.05611439366351E-79	1.2811901422874	0.452	0.291	1.35004365009013E-74	6	GRB2
1.61949737779444E-110	1.28001985362545	0.529	0.329	2.71395370570793E-106	6	CDC42SE1
9.87268102835013E-74	1.27725007248117	0.321	0.17	1.65446388673091E-69	6	ARPC2
1.08390839094028E-53	1.27623430109012	0.308	0.181	1.81641368153772E-49	6	PDK4
1.00755629399231E-31	1.27607694065859	0.392	0.291	1.68846283747232E-27	6	PLSCR1
3.37181180963764E-27	1.27347748417687	0.25	0.163	5.65048223059075E-23	6	GAS2L3
1.35287924773433E-60	1.26694628546099	0.402	0.263	2.26715504335319E-56	6	SNX6
4.72945090140628E-97	1.26202850860114	0.449	0.255	7.92561382057665E-93	6	AKIRIN2
6.29947099283259E-47	1.26193069948352	0.288	0.17	1.05566534897889E-42	6	CEPT1
4.90685219706057E-22	1.2608913491769	0.415	0.343	8.2229029118341E-18	6	GADD45B
2.45192089396349E-99	1.25793706344732	0.636	0.463	4.10892903410401E-95	6	CFLAR
1.89955688672137E-67	1.24706758831855	0.349	0.201	3.18327743076767E-63	6	PRMT2
6.5155329936498E-96	1.24094696519971	0.42	0.23	1.09187301907583E-91	6	LRCH4
3.66425154887269E-72	1.23875341590422	0.313	0.165	6.14055274560085E-68	6	WASF2
1.54203830227319E-63	1.2385311945324	0.37	0.222	2.58414778694941E-59	6	POLR2A
4.57524734172964E-19	1.23847595612928	0.514	0.462	7.66719949527053E-15	6	DEPRECATED-ENSG00000132002
4.39596406839645E-41	1.23762270730624	0.266	0.157	7.36675658581878E-37	6	LRRFIP1
3.15302776250391E-38	1.23151335835358	0.26	0.158	5.28384392440406E-34	6	DIP2B
9.41625899660664E-28	1.22660961473752	0.327	0.237	1.57797668265134E-23	6	TENT2
1.11450771448271E-18	1.22458274653612	0.266	0.195	1.86769202793012E-14	6	UTRN
5.92534464744443E-82	1.22428902206073	0.499	0.349	9.92969256018738E-78	6	SCAMP2
1.18338885511149E-83	1.2239640849042	0.519	0.376	1.98312304339584E-79	6	CAPZB
4.38943645610745E-57	1.22345047467003	0.299	0.168	7.35581761314486E-53	6	USP15
1.78498640171932E-45	1.22292668640918	0.261	0.15	2.99128021200124E-41	6	ANKFY1
0	1.21327337785821	0.359	0.075	0	6	CYTIP
9.73786759303014E-30	1.21170306643476	0.7	0.653	1.63187185123999E-25	6	RHOB
5.39031581487403E-79	1.21116729081911	0.359	0.198	9.03309124256589E-75	6	APBB1IP
6.89455585238558E-62	1.20891553403019	0.286	0.154	1.15538966974278E-57	6	ACTR2
9.13449276290655E-23	1.20499248036774	0.341	0.259	1.53075829720788E-18	6	STX4
8.8725669179695E-65	1.20407432357096	0.466	0.323	1.48686476411333E-60	6	TNS3
1.64981581231496E-139	1.20129159727194	0.403	0.179	2.76476133827741E-135	6	ARHGEF1
2.46965931191651E-62	1.20126711026891	0.297	0.16	4.13865507490969E-58	6	EHD4
2.86785748403939E-30	1.19811940268625	0.427	0.324	4.80595557175321E-26	6	ADAM9
2.88315745987488E-297	1.19580156463436	0.298	0.059	4.83159527125833E-293	6	CORO1A
3.23080310214823E-60	1.19315842331775	0.316	0.18	5.41417983858E-56	6	GNPTAB
1.20230202611407E-29	1.1883842763739	0.263	0.173	2.01481773536195E-25	6	MAP2K1
1.01031112405802E-24	1.18574484726975	0.317	0.233	1.69307938169643E-20	6	ZFHX3
6.44135852925964E-45	1.17627185025747	0.356	0.241	1.07944286233333E-40	6	MGAT4A
7.92583707763829E-98	1.17486102764257	0.491	0.309	1.32821177747062E-93	6	TGFBR2
1.48477105928652E-42	1.17173057288191	0.288	0.176	2.48817934115235E-38	6	TRIM14
2.68388605865998E-88	1.17060430312978	0.513	0.342	4.49765625710239E-84	6	DPP7
2.74788100741463E-26	1.16890169619663	0.26	0.176	4.60489899222544E-22	6	ITPRIPL2
2.87643012405806E-21	1.16743474777993	0.257	0.174	4.82032160189649E-17	6	DUSP10
6.09785971225747E-83	1.16724049932471	0.622	0.465	1.02187933058011E-78	6	PNRC1
2.95318210581449E-18	1.16592349234469	0.354	0.283	4.94894257292392E-14	6	PPP1CB
4.56903557441663E-43	1.16571568363977	0.447	0.34	7.65678981560738E-39	6	STAT1
8.88640889000174E-79	1.16428443590414	0.577	0.45	1.48918440178649E-74	6	SYNGR2
2.51566969462665E-60	1.16297384521579	0.341	0.201	4.21575927425534E-56	6	NFATC2
1.6537615580732E-31	1.16047238738874	0.334	0.233	2.77137361901906E-27	6	DENND1A
1.69460738331491E-68	1.159882552584	0.564	0.48	2.83982305295912E-64	6	CTSA
1.77975604477064E-41	1.15618867901567	0.351	0.234	2.98251517982663E-37	6	DEPRECATED-ENSG00000124831

3.3333068570624E-27	1.14965717652581	0.298	0.209	5.58595563106517E-23	6	ABI1
1.62227712177126E-42	1.14830712833955	0.336	0.22	2.71861200066427E-38	6	ROCK1
6.37954994604797E-27	1.14648158365022	0.334	0.242	1.06908497995872E-22	6	AKAP9
8.26975044515831E-69	1.1440734836152	0.461	0.308	1.38584477959963E-64	6	MAX
2.43697571790163E-34	1.14311862351317	0.352	0.252	4.08388390805955E-30	6	TPD52L2
9.9546036618733E-60	1.14023699847204	0.354	0.209	1.66819248165673E-55	6	UBE2L6
1.6938226370318E-195	1.13285487573155	0.854	0.843	2.83850797513789E-191	6	ACTB
7.75219292879E-40	1.13168554401571	0.458	0.35	1.29911249100663E-35	6	ARRDC2
3.2384245974802E-47	1.13121423664425	0.332	0.208	5.42695194045732E-43	6	NIN
2.98143813788187E-57	1.12709965739668	0.374	0.229	4.99629403146244E-53	6	CNN2
2.13903371679751E-21	1.1237822968914	0.287	0.209	3.58459270260927E-17	6	CERT1
2.19050883840684E-48	1.11806436379906	0.419	0.293	3.67085471140218E-44	6	STXBP2
2.41098049775116E-49	1.11625310178923	0.453	0.324	4.0403211181314E-45	6	PIK3AP1
4.7373756459898E-25	1.11331621016196	0.268	0.183	7.93889410754971E-21	6	FGFR1OP2
3.19122182468295E-59	1.10937759697727	0.366	0.224	5.34784953380369E-55	6	ELF1
3.43620050810794E-36	1.1080803253633	0.316	0.212	5.75838481148729E-32	6	SGPP1
1.88400637041294E-49	1.10703879395453	0.426	0.296	3.157217875538E-45	6	CORO1B
2.09788292356385E-48	1.10619553160176	0.398	0.265	3.51563220330829E-44	6	STAT2
5.15222937419259E-41	1.10546222017248	0.323	0.209	8.63410598527194E-37	6	CORO1C
2.62968793327872E-17	1.09866714262904	0.284	0.216	4.40683103858849E-13	6	SOAT1
1.15206937867792E-23	1.09605543584475	0.261	0.18	1.93063786478846E-19	6	RAB4B
9.7157038339367E-86	1.0926379418882	0.48	0.297	1.62815764849111E-81	6	AKAP13
2.8018386747953E-29	1.09202348204992	0.25	0.159	4.69532125122196E-25	6	LRMDA
9.25288650967109E-37	1.08934702545006	0.263	0.161	1.55059872129068E-32	6	FOXN2
1.27578077881921E-24	1.08872869370668	0.306	0.221	2.13795342914523E-20	6	PRPF38B
1.83757255657418E-20	1.08528182967632	0.311	0.231	3.07940409030701E-16	6	GSAP
4.5527343505095E-50	1.08487218963825	0.324	0.195	7.62947222458382E-46	6	DCK
1.20864267104326E-129	1.08032844385706	0.344	0.145	2.0254433881343E-125	6	STK17B
1.43180515026823E-17	1.08001250483728	0.312	0.242	2.39941907081949E-13	6	IFNAR2
1.92483076842238E-29	1.07842747818353	0.3	0.207	3.22563140172222E-25	6	STX6
1.55249578854682E-23	1.0753556243536	0.403	0.316	2.60167244244675E-19	6	TBC1D2B
8.42176330374287E-24	1.07533933058983	0.277	0.194	1.41131909444123E-19	6	SNX10
3.80429621631133E-54	1.07274053295122	0.33	0.196	6.37523959929452E-50	6	CSK
2.32926614887762E-23	1.07226673347555	0.275	0.193	3.90338421228911E-19	6	TUBGCP2
1.03818047201905E-28	1.07197067678223	0.324	0.231	1.73978283500953E-24	6	TSPAN33
2.51766476298032E-132	1.06844231048426	0.859	0.759	4.21910260980243E-128	6	FTH1
1.556752262164E-10	1.06837237950203	0.331	0.283	2.60880544093443E-06	6	SLC20A1
5.79208169254129E-54	1.06764914824522	0.508	0.388	9.70637050036068E-50	6	DPYD
2.15486222564157E-76	1.06320300425367	0.568	0.416	3.61111811773014E-72	6	EFHD2
6.79971645351014E-43	1.06214176015014	0.433	0.313	1.13949648327923E-38	6	MINK1
1.56090642067818E-22	1.05316081799764	0.396	0.312	2.6157669797725E-18	6	MYO15B
7.64555102676746E-33	1.05215084986599	0.371	0.273	1.28124144106569E-28	6	CHID1
1.21576450633318E-17	1.05052219906692	0.348	0.279	2.03737815971315E-13	6	FGD4
8.02852442498412E-32	1.04628820703167	0.258	0.163	1.34542012313884E-27	6	PITPNA
1.74354197292371E-35	1.04592437981967	0.254	0.153	2.92182763822556E-31	6	SORBS3
2.38675618279781E-38	1.04535069987152	0.293	0.187	3.99972601113257E-34	6	MGAT5
2.67365557832324E-29	1.04452673216446	0.293	0.199	4.48051201815408E-25	6	FOXN3
2.06583414839356E-51	1.04437670376855	0.312	0.179	3.46192486587792E-47	6	IRS2
2.84112496128147E-42	1.04423278637302	0.271	0.158	4.76115721011549E-38	6	ST3GAL1
2.14425713117303E-25	1.04415139620116	0.266	0.18	3.59334610041976E-21	6	CPPED1
1.33817852295632E-89	1.0416577750206	0.774	0.59	2.24251956877021E-85	6	LYZ
7.37623568974355E-63	1.03808046451875	0.55	0.448	1.23610957688722E-58	6	PICALM
2.35178223609802E-114	1.03456331950435	0.521	0.311	3.94111667125305E-110	6	ADGRE5
4.14158743391562E-22	1.02881436762615	0.343	0.261	6.94047222175579E-18	6	ASAP1
6.38251389816464E-34	1.02862527442359	0.335	0.23	1.06958167905443E-29	6	ELMO1
2.33157326811191E-23	1.02836446598457	0.267	0.186	3.90725048270194E-19	6	RCAN1
2.16020270281575E-24	1.0261516870855	0.33	0.244	3.62006768937864E-20	6	SKAP2
2.63305526822622E-15	1.01748945339563	0.285	0.221	4.4124740184935E-11	6	RELCH
0	1.01687075994777	0.322	0.059	0	6	IKZF1
4.90600101964056E-20	1.01496178748019	0.316	0.242	8.22147650871366E-16	6	YWHAH

6.67417447596402E-36	1.00727165924592	0.382	0.274	1.11845815868205E-31	6	RNASEK
2.77006957246261E-48	1.00715708443214	0.439	0.319	4.64208258953284E-44	6	CD164
1.53273758361373E-44	1.0054949427676	0.538	0.413	2.56856164261989E-40	6	ITPR2
4.66255017768704E-49	1.00458271585037	0.377	0.253	7.81350158776795E-45	6	ATP6V0B
2.01018195964344E-18	1.00441945295463	0.256	0.185	3.36866292797048E-14	6	CAMK1D
5.83028924803019E-105	1.00253459668357	0.538	0.348	9.77039872184898E-101	6	MSN
2.34245883508744E-20	0.999226048802676	0.261	0.188	3.92549251583953E-16	6	PTPN12
1.97098030114407E-56	0.997494680258618	0.577	0.49	3.30296878865724E-52	6	CD63
7.39634627463661E-178	0.99288454786998	0.557	0.293	1.2394797087036E-173	6	ZFP36L2
3.13740328964161E-41	0.988894249004221	0.829	0.756	5.25766043278141E-37	6	HSPA1A
1.63774668459605E-17	0.984672593118258	0.297	0.228	2.74453589404606E-13	6	USP32
4.36599704168695E-19	0.980087517714004	0.27	0.196	7.31653784245899E-15	6	STARD3
2.80614603178978E-19	0.978414835037161	0.282	0.207	4.70253952007332E-15	6	RBM6
6.10583062736397E-30	0.977996242670336	0.382	0.285	1.02321509653365E-25	6	BACH1
6.99925891446796E-25	0.974927998244649	0.276	0.189	1.17293580888654E-20	6	KLHL24
1.34685604570844E-13	0.968724778878271	0.302	0.243	2.25706136139821E-09	6	HERC3
7.59674780350617E-80	0.966462815052974	0.693	0.679	1.27306299691156E-75	6	LAMP1
3.97954239247249E-13	0.964905670248391	0.26	0.202	6.6689171413054E-09	6	ARHGAP17
2.14189355337979E-17	0.964810259625474	0.25	0.183	3.58938521675385E-13	6	KIDINS220
5.98043316898474E-62	0.963762538807858	0.404	0.256	1.00220099045846E-57	6	TLN1
1.19677216261059E-12	0.963008243983336	0.251	0.197	2.00555079010282E-08	6	ATF6
2.77290541844107E-10	0.962692277266368	0.252	0.203	4.64683490022355E-06	6	ATP6V0A1
5.81155943087327E-13	0.962671923454977	0.285	0.228	9.73901129425742E-09	6	TGIF1
2.17117761217241E-34	0.959102674030338	0.374	0.268	3.63845944247852E-30	6	CMIP
3.16787067656526E-32	0.958182501133916	0.309	0.211	5.30871767978806E-28	6	SNX9
1.70117377320513E-22	0.957847151004548	0.314	0.231	2.85082700913715E-18	6	RAP2A
2.01531544804574E-15	0.956530408257274	0.321	0.254	3.37726562783505E-11	6	ADAM10
5.40941973050422E-37	0.956269447879046	0.326	0.217	9.06510558437897E-33	6	TMEM127
2.52958903876104E-09	0.949948174473706	0.301	0.255	4.23908531115574E-05	6	MORC3
0	0.949808461852685	0.447	0.054	0	6	ENG
6.30350643238274E-25	0.94004454515772	0.261	0.178	1.0563416079387E-20	6	NCOA3
1.09718717832752E-13	0.931830541010644	0.269	0.209	1.83866627344125E-09	6	TAOK3
3.77328483277444E-55	0.924861820366794	0.527	0.4	6.32327072276341E-51	6	DBNL
2.67648013529738E-48	0.923281291492872	0.649	0.531	4.48524541073135E-44	6	VMP1
7.74810042379845E-41	0.9200237809357	0.321	0.207	1.29842666902015E-36	6	AAK1
7.2643186990427E-51	0.917641723134383	0.407	0.267	1.21735452758558E-46	6	COMT
1.64218838621842E-37	0.916821297357882	0.364	0.251	2.75197929762483E-33	6	GABARAP
3.70292942493086E-58	0.912880372652846	0.479	0.343	6.20536913029913E-54	6	LGALS3BP
9.78770746518559E-48	0.909784790344778	0.522	0.398	1.6402240170158E-43	6	CDKN1B
1.6516047587587E-13	0.908939750150337	0.274	0.213	2.76775925472782E-09	6	VAMP3
1.78050496477388E-28	0.908805333392104	0.403	0.308	2.98377021996806E-24	6	TCIRG1
7.58870593095595E-47	0.906717381977343	0.473	0.35	1.2717153399096E-42	6	RHOA
5.35858639761715E-102	0.905288910232448	0.528	0.329	8.97991908512682E-98	6	A2M
2.42667286118079E-25	0.903278827599166	0.266	0.18	4.06661838076676E-21	6	TMEM165
1.03588268799803E-30	0.898921220317392	0.293	0.195	1.73593220854711E-26	6	RFX5
2.62809845919826E-30	0.898371709447732	0.351	0.253	4.40416739792444E-26	6	ARPC4
1.38939418307514E-31	0.895612979587086	0.383	0.28	2.32834677199733E-27	6	MED15
2.22677066847316E-34	0.888822044849635	0.554	0.517	3.73162228622731E-30	6	CREG1
4.18272938460252E-22	0.887831408976186	0.408	0.333	7.00941790271691E-18	6	VASP
9.41715984634114E-22	0.886899474923936	0.306	0.223	1.57812764704985E-17	6	SCAMP4
1.22425101092253E-37	0.885416000047536	0.251	0.147	2.05159984410398E-33	6	EPS15
3.5291147634954E-264	0.885301061259336	0.905	0.892	5.91409052066656E-260	6	B2M
1.03213342630541E-19	0.884006106966262	0.784	0.718	1.72964919580261E-15	6	HSP90AA1
7.04986592823791E-08	0.882572747539402	0.256	0.216	0.00118141653225411	6	DNMT3A
1.03363900081984E-31	0.881392086762687	0.254	0.156	1.73217223757388E-27	6	PPFIBP2
2.97796583311795E-19	0.880962305564172	0.4	0.322	4.99047514313906E-15	6	ZMIZ2
2.52223187224538E-17	0.879906825648904	0.262	0.194	4.22675617150881E-13	6	DNAJC13
1.31142678118939E-22	0.878724940996146	0.334	0.253	2.19768899991717E-18	6	TAP2
1.18391973390225E-26	0.876704882349048	0.254	0.167	1.98401269007339E-22	6	CLSTN1
1.93861801867559E-10	0.876198900116695	0.295	0.244	3.24873607569656E-06	6	SPAG9

8.37203316181827E-37	0.875043471213954	0.35	0.24	1.40298531725751E-32	6	STK4
2.15509216977743E-18	0.873928807127104	0.295	0.222	3.61150345811301E-14	6	PIKFYVE
5.20051436637236E-19	0.873682762539435	0.387	0.308	8.7150219751668E-15	6	CMTM6
2.93632249708314E-35	0.873663638471878	0.318	0.212	4.92068924061192E-31	6	HECA
2.64699392511938E-19	0.871987967555555	0.331	0.259	4.43583241971506E-15	6	NADK
5.18550455618973E-40	0.866320433501811	0.518	0.423	8.68986853526275E-36	6	CNDP2
7.26617403511427E-27	0.862673841633029	0.336	0.244	1.21766544480445E-22	6	GLB1
4.9596054665238E-15	0.862076982023655	0.282	0.217	8.31130684080059E-11	6	ELMSAN1
5.81593528556363E-33	0.858296914677306	0.292	0.188	9.74634435154753E-29	6	UBXN1
7.65325543213336E-24	0.854660671943581	0.262	0.18	1.28253254531691E-19	6	PPP1R12A
8.79691452773303E-16	0.854021618182563	0.325	0.257	1.4741869365575E-11	6	SBNO2
4.04773772764867E-12	0.852516838076001	0.329	0.277	6.78319888399364E-08	6	YPEL5
2.72455235094872E-40	0.850880130694682	0.673	0.613	4.56580482971986E-36	6	ZFP36L1
1.15791097701817E-43	0.847053533846828	0.476	0.362	1.94042721528704E-39	6	ANXA11
1.49495597833391E-24	0.844594151851074	0.27	0.187	2.50524722849196E-20	6	ORMDL1
2.96067779289664E-17	0.832332108525855	0.334	0.26	4.96150384533619E-13	6	IRF2
3.86790641131826E-23	0.832045692347677	0.33	0.247	6.48183756408713E-19	6	GBA
2.23685151919153E-14	0.828957935172151	0.257	0.196	3.74851577586116E-10	6	CD9
2.15891893526928E-08	0.821545567517283	0.305	0.261	0.000361791635172427	6	EXOC3
6.32916580860455E-27	0.820637784774906	0.369	0.277	1.06064160620595E-22	6	TSPO
9.84065401665733E-26	0.820131094113785	0.301	0.209	1.64909680011143E-21	6	KLC1
2.08294462051437E-23	0.818442043881226	0.343	0.254	3.49059859505798E-19	6	OGFR
1.79738404560379E-23	0.81455247614736	0.388	0.301	3.01205618362284E-19	6	MARCH7
4.19902244681473E-09	0.813120807596765	0.256	0.208	7.03672181637213E-05	6	MEF2D
6.26935028590538E-25	0.806598093025052	0.413	0.318	1.05061772091202E-20	6	STAT6
6.79564028755007E-37	0.805860570535427	0.295	0.185	1.13881339938764E-32	6	CTDSP2
1.97357951761923E-08	0.802847352044383	0.397	0.352	0.000330732455562631	6	NUFIP2
1.79022007758885E-17	0.799006345532254	0.323	0.245	3.0000508060234E-13	6	ARRDC3
2.21202475615526E-239	0.798110851483181	0.275	0.061	3.70691108636499E-235	6	RAC2
2.83162979428352E-61	0.797380543359862	0.647	0.578	4.74524520926032E-57	6	PFN1
7.76265387907864E-72	0.795787735213692	0.734	0.688	1.300865537056E-67	6	CEBPD
7.84231955712001E-17	0.793014271823956	0.285	0.217	1.31421591138217E-12	6	SETX
1.11190559913681E-16	0.791265796600737	0.33	0.263	1.86333140303347E-12	6	DENND4C
1.66058127348328E-10	0.790609616790806	0.386	0.335	2.78280209810329E-06	6	KMT2C
3.00770735183836E-15	0.790484139341167	0.361	0.293	5.04031598021072E-11	6	RASSF3
1.63114410632727E-38	0.785252014325998	0.565	0.466	2.73347129338324E-34	6	PLXNB2
1.14324823600579E-23	0.785209489224684	0.376	0.286	1.9158553938985E-19	6	B4GALT1
5.55944547219503E-33	0.782158300614659	0.511	0.421	9.31651872230442E-29	6	TNFRSF1A
2.79473170509735E-14	0.780930428486579	0.262	0.2	4.68341139140214E-10	6	TMEM9B
3.91859222445927E-17	0.779553080311654	0.345	0.284	6.56677684974885E-13	6	VAT1
1.40342860308649E-30	0.77784366930385	0.419	0.317	2.35186565305234E-26	6	CYBC1
6.32041373863479E-10	0.777065477179191	0.29	0.238	1.05917493432042E-05	6	CERS6
1.02601601806421E-12	0.772373378424772	0.394	0.335	1.71939764307201E-08	6	HNRNPH3
6.45947365810535E-18	0.771609885463486	0.479	0.413	1.08247859562529E-13	6	CEBPB
1.50695939430578E-35	0.770477200153312	0.296	0.188	2.52536255297763E-31	6	ACTR1A
1.30002999688226E-06	0.770003402256971	0.25	0.213	0.021785902687753	6	BTAF1
1.96596400583282E-19	0.762736120268637	0.322	0.245	3.29456248097464E-15	6	BCL2L1
3.47922974720396E-29	0.762353378477923	0.666	0.611	5.83049321036439E-25	6	HSPA8
3.99080701700559E-23	0.752425488917406	0.373	0.287	6.68779439909797E-19	6	SPG21
1.3418119943086E-21	0.751275097369054	0.332	0.25	2.24860854006236E-17	6	DNAJC5
2.19403007802109E-06	0.748705471202973	0.329	0.294	0.0367675560474774	6	HNRNPDL
8.39026767863107E-120	0.745312927641179	0.836	0.775	1.406041057585E-115	6	OAZ1
4.3178352409017E-17	0.74428566994192	0.26	0.189	7.23582829670306E-13	6	SHKBP1
3.15028336624218E-22	0.744205124316188	0.334	0.253	5.27924486514864E-18	6	MXD4
8.31553791522969E-17	0.74408686342487	0.344	0.275	1.39351784383419E-12	6	PTBP3
2.2422037836689E-26	0.743170916835743	0.313	0.217	3.75748510067234E-22	6	PYCARD
1.05718106200941E-13	0.742478846774834	0.464	0.409	1.77162402371537E-09	6	PDXK
7.71399259281119E-21	0.737190918052946	0.258	0.18	1.2927108787033E-16	6	RSU1
4.51099372517687E-17	0.735820919465488	0.408	0.335	7.5595232846514E-13	6	KIAA0100
1.51876525826628E-27	0.735248204490283	0.381	0.292	2.54514681980263E-23	6	DCAF7

5.8238046381872E-11	0.725590427608433	0.33	0.281	9.7595318126741E-07	6	ZFAND5
2.56711197325106E-10	0.7207230807956	0.325	0.273	4.30196624477413E-06	6	SELENOS
6.51534557323563E-15	0.720572007791865	0.367	0.303	1.09184161116283E-10	6	CSTB
3.79480501279886E-07	0.720215223360135	0.321	0.284	0.00635933424044834	6	C16orf72
1.61061140456178E-24	0.716943142625021	0.301	0.213	2.69906259176463E-20	6	TBC1D15
2.45402956083009E-12	0.716553089460877	0.292	0.236	4.11246273803906E-08	6	ITGB5
2.08125592018135E-17	0.714843144492318	0.366	0.293	3.4877686710399E-13	6	TMUB2
2.10649473196151E-07	0.712034355241521	0.413	0.375	0.0035300638718211	6	UBC
7.99890818951643E-43	0.711066245216684	0.622	0.554	1.34045703439916E-38	6	OS9
7.04089565961834E-11	0.708081940471307	0.34	0.285	1.17991329463884E-06	6	TAOK1
8.40509395439966E-14	0.703498511003108	0.294	0.231	1.40852564487829E-09	6	ITGAV
3.9595568864659E-08	0.697623272545384	0.222	0.296	0.000663542543033955	6	TENT5A
5.1679531914271E-07	0.693815476421637	0.318	0.28	0.00866045595819354	6	RBM25
3.1885979825091E-13	0.690380207959256	0.391	0.331	5.34345249908874E-09	6	RAB7A
4.45004755319588E-14	0.689530706324877	0.336	0.271	7.45738968964565E-10	6	EP300
1.39330913691188E-06	0.689163544284924	0.314	0.277	0.0233490745163693	6	CEP350
2.26053801095285E-15	0.687227195301302	0.361	0.295	3.78820959875479E-11	6	HIPK3
3.74563207037816E-14	0.686829778344302	0.444	0.39	6.27693022353973E-10	6	RAB10
3.27070039584843E-09	0.683359800914365	0.313	0.264	5.48103972336281E-05	6	PNISR
1.25426424593627E-23	0.68289288136121	0.35	0.266	2.10189602333999E-19	6	RAP1A
1.55840933152931E-06	0.682768612785497	0.307	0.267	0.0261158235777681	6	ARRB1
3.32447209407192E-40	0.680705067414879	0.577	0.515	5.57115033524573E-36	6	AHNAK
3.70092627796229E-17	0.678309112666532	0.305	0.234	6.2020122566092E-13	6	QARS
5.17230014770802E-17	0.675953067612606	0.428	0.363	8.6677405875291E-13	6	MBNL1
1.64786460259709E-16	0.674639673901576	0.457	0.395	2.7614915010322E-12	6	TUT7
1.3912424067871E-12	0.674467317825737	0.255	0.197	2.33144402529383E-08	6	OAS1
2.50541509121889E-86	0.674139761764419	0.34	0.167	4.19857460986462E-82	6	AKNA
8.94910549146646E-16	0.673691776706859	0.547	0.489	1.49969109825995E-11	6	NFE2L2
2.51983972077952E-12	0.667420946490203	0.645	0.602	4.22274740408233E-08	6	JUN
8.38719730773189E-21	0.663842734043113	0.439	0.358	1.40552652482971E-16	6	USF2
1.8773647264333E-09	0.662715909933053	0.253	0.205	3.14608780855692E-05	6	SPG11
4.7871985984206E-07	0.66194975541965	0.254	0.213	0.00802238741123324	6	PFDN5
7.25364734762651E-11	0.659564387337254	0.253	0.199	1.21556622251525E-06	6	ZNF655
1.9197598993743E-15	0.658978355707163	0.421	0.358	3.21713363937146E-11	6	TAP1
4.40209425193394E-36	0.65623152009422	0.621	0.579	7.37702954739089E-32	6	GRINA
8.91370390401055E-10	0.656065411647015	0.348	0.299	1.49375850023409E-05	6	YBX1
3.00980885412742E-16	0.653912669119763	0.376	0.305	5.04383767774674E-12	6	MPV17
3.03875577739889E-14	0.653126902438763	0.426	0.362	5.09234693176506E-10	6	MBD2
1.91161286802286E-09	0.651230420304332	0.265	0.214	3.20348084423271E-05	6	TFE3
4.38077106548536E-12	0.650150795391471	0.309	0.251	7.34129615154036E-08	6	PCM1
7.56116528694428E-07	0.649492028430047	0.258	0.221	0.0126710007878612	6	PBX2
4.56925654030715E-11	0.648253897622702	0.432	0.384	7.65716011024672E-07	6	RERE
1.49784837513827E-08	0.646280183333197	0.317	0.271	0.000251009430705671	6	PARP14
4.37852460307978E-27	0.63948354112722	0.525	0.438	7.33753152984109E-23	6	DDIT4
8.27466745210275E-25	0.637517358664043	0.518	0.407	1.38666877162338E-20	6	IKBKB
6.72984463623086E-10	0.633230873248564	0.32	0.269	1.12778736413957E-05	6	NUP62
7.13914701301746E-25	0.632869255740208	0.538	0.457	1.19637825644147E-20	6	RSRP1
2.66681337664665E-11	0.630544626121045	0.292	0.237	4.46904585658446E-07	6	ARL8A
1.30668519625637E-16	0.627951033609072	0.283	0.209	2.18974305188643E-12	6	HBP1
1.50539314872399E-08	0.627563212496633	0.254	0.207	0.000252273783863166	6	AUP1
4.96577497398547E-20	0.618547258429311	0.365	0.284	8.32164570140485E-16	6	NUMA1
4.46225775700943E-19	0.614025428768362	0.285	0.208	7.47785154919641E-15	6	QRICH1
5.42073622183915E-11	0.613247991571424	0.286	0.23	9.08406976055805E-07	6	TRIM8
1.07493181675802E-07	0.612888437424727	0.311	0.269	0.00180137073852309	6	ISCU
2.41080418995113E-15	0.612521351967909	0.484	0.43	4.04002566152011E-11	6	SNX1
5.33125515411407E-18	0.61240564520557	0.436	0.37	8.93411738726435E-14	6	PTTG1IP
5.29327608268723E-09	0.602827209869072	0.259	0.213	8.87047205936727E-05	6	HTT
1.41944514279967E-10	0.598329071636091	0.332	0.281	2.37870617030369E-06	6	CYB5R1
1.95616019184456E-11	0.594513753840179	0.267	0.211	3.27813324949311E-07	6	LCOR
6.20293930029196E-07	0.590465321045205	0.356	0.318	0.0103948856794293	6	GNPTG

6.64432802759232E-28	0.588751897889577	0.546	0.454	1.11345649086392E-23	6	RNF213
1.43559061097568E-08	0.585478710888906	0.289	0.241	0.000240576274587304	6	GBP2
2.46037909311449E-17	0.584579160379287	0.452	0.383	4.12310328424126E-13	6	SMARCA2
2.42312167791144E-14	0.581119251415096	0.436	0.376	4.060667307844E-10	6	NEK6
4.53750318513835E-07	0.571179664945234	0.264	0.222	0.00760394783765484	6	FAM111A
3.90100881263582E-19	0.571136042038577	0.361	0.286	6.53731056821511E-15	6	PHC2
1.23583324949163E-11	0.568340750516713	0.292	0.234	2.07100935949808E-07	6	AP2S1
1.71037658598286E-14	0.56447032877591	0.541	0.45	2.86624908279008E-10	6	IVNS1ABP
6.5506207406562E-07	0.56382274813968	0.321	0.282	0.0109775302371917	6	IST1
1.11324612125687E-07	0.561922833833489	0.267	0.224	0.00186557785000226	6	KIF16B
2.02636988028278E-10	0.558974610083901	0.386	0.335	3.39579064537788E-06	6	CCNI
2.21620798136171E-08	0.558934211374218	0.251	0.204	0.000371392133516595	6	APOL2
1.00232821445109E-12	0.554594977872639	0.316	0.25	1.67970162177714E-08	6	SUMO3
2.57619680531286E-10	0.553791641104921	0.59	0.545	4.31719060634329E-06	6	JUND
2.43240914280008E-08	0.55272338753324	0.262	0.219	0.000407623124150437	6	DELE1
3.04514180355946E-19	0.550633432565594	0.303	0.226	5.10304863440494E-15	6	CBFB
2.3219455277288E-09	0.549040630109467	0.203	0.279	3.89111631536792E-05	6	TRIM25
6.69999451166153E-08	0.544565998180999	0.25	0.205	0.00112278508026424	6	TMOD3
9.95544569781709E-09	0.54363981786306	0.416	0.373	0.000166833359004019	6	TCF25
4.05523278524697E-10	0.540720183337978	0.292	0.239	6.79575910151687E-06	6	ARPC5
6.82964732514121E-12	0.539915459582146	0.663	0.63	1.14451229874716E-07	6	NFKBIA
1.89040585463063E-09	0.536692501424026	0.296	0.245	3.16794213119001E-05	6	TIAL1
5.30644033456682E-08	0.532374832524105	0.338	0.293	0.000889253271266707	6	KMT2E
2.33102374121626E-14	0.528514929275166	0.516	0.447	3.90632958553021E-10	6	TBC1D9B
2.79108893990935E-09	0.524035028287845	0.45	0.415	4.67730684550009E-05	6	LRPAP1
2.39548005401524E-19	0.523952758366136	0.428	0.349	4.01434547451874E-15	6	LAMTOR1
1.44063230422452E-06	0.521925659551923	0.297	0.26	0.0241421161541945	6	RUFY1
2.44235071120983E-33	0.517761671196755	0.344	0.234	4.09289132184543E-29	6	SARAF
5.8971906854832E-10	0.515699025940944	0.478	0.428	9.88251215073274E-06	6	RTN4
2.37548914478713E-06	0.51504971056844	0.335	0.294	0.0398084470883428	6	PCBP1
1.32567521217968E-12	0.513482373485872	0.289	0.227	2.22156652057071E-08	6	PPP6R1
5.055899007803E-12	0.513255076178209	0.479	0.427	8.47267555727626E-08	6	PRKAR1A
1.50048525287521E-06	0.510998344759095	0.259	0.22	0.0251451318676828	6	HERC4
4.36179001702261E-11	0.510581390743392	0.532	0.496	7.30948771052649E-07	6	DDX5
1.2546337343263E-22	0.504716769932551	0.404	0.317	2.10251521198401E-18	6	JAK1
1.015879611201E-13	0.503739061299422	0.47	0.42	1.70241105245064E-09	6	RAB5C
1.26247004546063E-12	0.501357236345269	0.392	0.33	2.11564730218292E-08	6	TRIP12
8.66393825555779E-15	0.49906331051050	0.485	0.421	1.45190277286637E-10	6	DEPRECATED-ENSG00000147872
1.23419402341351E-07	0.498663226656538	0.268	0.226	0.00206826234443636	6	EDEM2
1.8672218285067E-08	0.496112872510877	0.29	0.244	0.000312909034021153	6	TAB2
8.86662242527223E-09	0.482891369132089	0.292	0.245	0.000148586858602712	6	RTCB
6.43008308659775E-09	0.482806146812507	0.486	0.439	0.000107755332365205	6	AFF1
2.12821336744258E-08	0.480320868070577	0.397	0.35	0.000356645996116027	6	NARS
2.06716259446103E-28	0.475287687414092	0.41	0.307	3.4641510757978E-24	6	MACF1
3.32454624908798E-18	0.475082359382428	0.551	0.483	5.57127460422163E-14	6	ATP6AP1
6.6170348809922E-16	0.469863367455432	0.328	0.254	1.10888270535667E-11	6	TSC22D4
1.42103707478618E-06	0.451135166958929	0.29	0.251	0.0238137392992668	6	CLTA
3.17895351877249E-08	0.448778593326476	0.327	0.279	0.000532729030675893	6	ARSA
1.9318651772213E-08	0.446782654391912	0.34	0.294	0.000323741966398745	6	PAFAH1B1
7.39062359672955E-14	0.438799936770809	0.251	0.188	1.23852070233994E-09	6	WDR82
5.61219866595531E-15	0.437294426801297	0.613	0.584	9.40492252440791E-11	6	BRI3
5.4713452761691E-07	0.427233675818269	0.319	0.278	0.00916888041380418	6	FOXP1
7.99169151471544E-07	0.41472205207945	0.311	0.27	0.0133924766403601	6	DYNC1I2
1.21498711172316E-94	0.396421732855008	0.286	0.113	2.03607540182568E-90	6	ANXA6
2.18344708525866E-07	0.393995859448938	0.383	0.34	0.00365902062547646	6	EIF3A
1.58405965658212E-07	0.389282086048469	0.391	0.356	0.00265456717250031	6	IQGAP2
5.65972024539762E-08	0.386124125255613	0.292	0.245	0.000948455918723733	6	ZSWIM8
3.8426573583789E-09	0.380826751923984	0.406	0.359	6.43952520117135E-05	6	ATP6V0E1
2.30027886767439E-120	0.361390087645989	0.372	0.159	3.85480732644873E-116	6	IL2RG
1.9299180973933E-10	0.356049787534346	0.179	0.255	3.2341567476117E-06	6	BCL6

4.62207575109333E-09	0.348847763483145	0.562	0.559	7.7456745436822E-05	6	ACTG1
5.66866954695404E-07	0.342791786983484	0.29	0.251	0.00949955642678559	6	TNFAIP3
1.494482940127E-08	0.334265564847902	0.4	0.351	0.000250445451106482	6	GNB2
8.57367857963958E-09	0.333164317672571	0.71	0.597	0.0001436777056376	6	SGK1
5.87224509920703E-07	0.323691463410086	0.326	0.283	0.00984070833725113	6	MAP4
4.53660242915073E-08	0.316519834789086	0.574	0.553	0.000760243835077079	6	TGOLN2
9.54373671765142E-126	0.306664963118987	0.329	0.124	1.59933939914402E-121	6	TIMP1
3.44123368743426E-07	0.299709376712758	0.391	0.349	0.00576681941340233	6	APMAP
1.2320379602508E-09	0.295800163303874	0.303	0.246	2.06464921378829E-05	6	QSOX1
8.52729701290449E-12	0.284204003821296	0.285	0.226	1.42900443342253E-07	6	CD99
1.51260888957542E-08	0.27957579125854	0.364	0.312	0.000253482997715048	6	PKN1
4.2575535324454E-15	0.277812823619809	0.323	0.245	7.134808209672E-11	6	CSRP1
1.15044197764307E-12	0.274543387900539	0.365	0.297	1.92791066613426E-08	6	OGA
0	9.03150575001849	0.543	0.003	0	7	PTPRB
0	8.85581398877488	0.498	0.003	0	7	MMRN2
0	8.77991402197202	0.316	0.003	0	7	VWF
0	8.75923979738053	0.377	0.002	0	7	TEK
0	8.73969722135205	0.269	0.001	0	7	STAB2
0	8.7258443852362	0.423	0.007	0	7	PLVAP
0	8.65520322522352	0.379	0.003	0	7	EFNB2
0	8.64577347125472	0.601	0.006	0	7	FLT1
0	8.41687392013619	0.396	0.002	0	7	FLT4
0	8.39791158056699	0.288	0.003	0	7	ADGRL4
0	8.37366531930636	0.254	0.001	0	7	ROBO4
0	8.37101091242545	0.64	0.004	0	7	RAMP3
0	8.26705028868396	0.427	0.004	0	7	SEMA3F
0	8.13379562454235	0.761	0.007	0	7	CDH5
0	8.09648842730082	0.538	0.006	0	7	AFAP1L1
0	8.09091699406023	0.255	0.002	0	7	KDR
0	8.06122782528316	0.427	0.003	0	7	PDE2A
0	8.05105969254054	0.385	0.006	0	7	RASIP1
0	8.04186036586054	0.62	0.012	0	7	FCN3
0	8.01820648576819	0.435	0.003	0	7	PCDH17
0	7.97818007002278	0.433	0.004	0	7	OIT3
0	7.95472352871805	0.316	0.002	0	7	TIE1
0	7.89241384372644	0.291	0.003	0	7	CLEC14A
0	7.75185451843482	0.31	0.003	0	7	ECSCR
0	7.74704371438543	0.612	0.019	0	7	DNASE1L3
0	7.72066319769634	0.761	0.033	0	7	EGFL7
0	7.66708648659699	0.454	0.012	0	7	FCN2
0	7.58772079911423	0.452	0.022	0	7	PODXL
0	7.39727239916353	0.46	0.011	0	7	ESAM
0	7.37752131226863	0.463	0.005	0	7	LDB2
0	7.27309173810652	0.43	0.009	0	7	CRHBP
0	7.25804385749858	0.308	0.004	0	7	APLNR
0	7.24929498447839	0.347	0.004	0	7	RAMP2
0	7.23357297183208	0.418	0.006	0	7	HECW2
0	7.09091847427231	0.279	0.005	0	7	PCDH12
0	7.03769588538401	0.332	0.014	0	7	AQP1
0	6.9848085012035	0.315	0.006	0	7	IL33
0	6.94128620592224	0.338	0.005	0	7	CALCRL
0	6.91012064403959	0.282	0.007	0	7	TMEM255B
0	6.90086839117553	0.28	0.004	0	7	DCHS1
0	6.88660010380417	0.341	0.006	0	7	STC2
0	6.79510159793789	0.332	0.005	0	7	CAVIN2
0	6.37627788472086	0.266	0.006	0	7	ARHGAP23
0	6.35102826189869	0.762	0.035	0	7	NPDC1
0	6.33201494412923	0.283	0.017	0	7	SHANK3
0	6.30314385841306	0.531	0.008	0	7	ADAMTS4
0	6.14711668674011	0.291	0.008	0	7	GNG11

0	6.06268750265134	0.448	0.016	0	7	NAV1
6.18341303660886E-197	6.03560285869612	0.266	0.036	1.03621635667491E-192	7	NOSTRIN
0	5.93134663675786	0.69	0.112	0	7	HYAL2
0	5.90145287992711	0.737	0.127	0	7	HSPG2
0	5.88651557994921	0.418	0.01	0	7	MCAM
0	5.87138396491194	0.402	0.014	0	7	SMAD6
0	5.86547763328812	0.405	0.02	0	7	DYSF
0	5.8367756475709	0.427	0.057	0	7	AKAP12
0	5.6761534584001	0.311	0.013	0	7	FGFR1
0	5.66464615935611	0.357	0.015	0	7	FSCN1
0	5.65364945895967	0.36	0.026	0	7	ALPL
0	5.64203034573809	0.261	0.012	0	7	ZNF160
0	5.52654471775022	0.423	0.028	0	7	LIMS2
0	5.49756028395948	0.853	0.063	0	7	ENG
0	5.42771801680002	0.305	0.012	0	7	LIFR
0	5.41993983667844	0.487	0.038	0	7	ITPRIP
0	5.38894989173613	0.377	0.014	0	7	FXYD6
0	5.35469052699749	0.382	0.026	0	7	NRP2
0	5.32963189328767	0.294	0.02	0	7	LMO2
3.42779561622863E-245	5.30220188311644	0.293	0.035	5.74429989367593E-241	7	PLK2
2.23217379786256E-273	5.28503450248857	0.322	0.039	3.74067685045808E-269	7	ADAMTS9
0	5.26061505325783	0.471	0.048	0	7	PECAM1
1.03963618725259E-300	5.22942226226504	0.502	0.093	1.74222232259789E-296	7	PPFIBP1
1.58193313409947E-252	5.19864058955032	0.515	0.118	2.6510035461239E-248	7	MCF2L
0	5.17768035774698	0.313	0.02	0	7	THBD
0	5.17673235396091	0.585	0.042	0	7	CD93
0	5.07373883307282	0.712	0.029	0	7	COL4A1
1.96959569666917E-217	5.0689310309293	0.282	0.037	3.3006484684782E-213	7	SPNS2
3.15958686975717E-256	5.03822315019242	0.469	0.093	5.29483567633907E-252	7	ADM
0	4.99842734089917	0.263	0.01	0	7	PXDN
1.78731448993617E-166	4.98404987131226	0.283	0.048	2.99518162223504E-162	7	SPRY1
3.73947516150266E-227	4.90100166446673	0.34	0.052	6.26661247564616E-223	7	ARHGAP29
0	4.87820168312721	0.687	0.027	0	7	COL4A2
0	4.87136198781139	0.351	0.019	0	7	S1PR1
0	4.83595478183764	0.257	0.02	0	7	GASK1B
0	4.80478213445146	0.304	0.013	0	7	TINAGL1
0	4.7463439292363	0.804	0.038	0	7	SPARC
0	4.7388902771324	0.393	0.028	0	7	GRASP
2.29817681228909E-290	4.69360245351169	0.336	0.039	3.85128470203405E-286	7	SMTN
0	4.5774980888256	0.718	0.054	0	7	STAB1
2.36265936854618E-162	4.55330163048969	0.371	0.085	3.95934456980968E-158	7	FZD4
0	4.5415095568864	0.36	0.027	0	7	TCF4
2.1563464071925E-123	4.53099167851059	0.268	0.055	3.61360530917319E-119	7	DLL1
3.38743811187704E-208	4.41514457363682	0.635	0.234	5.67666878788355E-204	7	RAPGEF5
3.05505624842552E-133	4.38302546590656	0.29	0.06	5.11966326111149E-129	7	SHE
7.7570207973719E-159	4.35893115020394	0.257	0.04	1.29992154522358E-154	7	SCARF1
2.32758109624141E-153	4.35110466650447	0.379	0.092	3.90056040108136E-149	7	WWTR1
4.24018845955445E-188	4.33082252044129	0.254	0.034	7.10570782052135E-184	7	ANGPTL4
0	4.31677517777046	0.285	0.012	0	7	TNXB
8.37788478933959E-107	4.29025298762983	0.338	0.098	1.40396593299753E-102	7	GDF15
1.61940925691714E-73	4.23680415960438	0.257	0.076	2.71380603274175E-69	7	MAN1C1
2.39557730305135E-69	4.22719301162586	0.271	0.088	4.01450844445345E-65	7	LAMA4
1.22539144217993E-141	4.14912849256746	0.255	0.044	2.05351097880513E-137	7	DLC1
0	4.13503328000255	0.476	0.039	0	7	SLC2A3
1.03308901165956E-77	4.09995780849206	0.311	0.106	1.73125056573909E-73	7	DOCK6
2.56139050462741E-278	4.09447954218618	0.357	0.046	4.29237820765462E-274	7	PLEKHO2
7.16856978484777E-230	4.09046245732576	0.252	0.027	1.20130892454479E-225	7	VASH1
3.65698189947679E-159	4.07897145118849	0.537	0.192	6.12837026714321E-155	7	CD9
0	4.05406699448451	0.499	0.043	0	7	DEPRECATED-ENSG00000059804
0	4.05235362200325	0.255	0.017	0	7	ICAM2

1.22825880679906E-283	4.05234705707761	0.343	0.041	2.05831610843386E-279	7	LAMB2
0	4.04097585306721	0.501	0.081	0	7	SH3BP5
0	3.99668951604607	0.535	0.071	0	7	SOCS3
2.05592842626213E-101	3.98525926532906	0.285	0.073	3.44532485673008E-97	7	CAVIN1
0	3.98171425749281	0.391	0.049	0	7	DENND3
6.92859240815147E-97	3.97168954353933	0.274	0.07	1.16109351575802E-92	7	KALRN
2.08670402138503E-138	3.96831315845238	0.338	0.079	3.49689859903704E-134	7	SMAD7
1.28030680971912E-50	3.94657912101104	0.318	0.145	2.1455381517273E-46	7	TMEM44
2.0520314901379E-279	3.84905448978523	0.318	0.036	3.43879437117309E-275	7	DUSP5
3.27080232115479E-301	3.82050120844449	0.316	0.033	5.48121052979119E-297	7	CRIM1
6.3023321308963E-174	3.79917834671427	0.493	0.146	1.0561448184956E-169	7	ITGA5
2.57051811483902E-263	3.7877585996679	0.48	0.092	4.30767425684723E-259	7	TIMP3
6.7456377625096E-101	3.75676583509508	0.288	0.073	1.13043397624136E-96	7	CHSY1
5.52204514899817E-79	3.7444308476065	0.526	0.302	9.25384326069113E-75	7	CTSL
2.18610520289544E-198	3.69203367236064	0.313	0.048	3.66347509901217E-194	7	SFMBT2
9.30655658733208E-242	3.67360221444088	0.784	0.355	1.55959275290511E-237	7	PLPP3
3.75541089644681E-64	3.65043723290689	0.308	0.117	6.29331758026557E-60	7	EMP1
1.19158064045124E-106	3.64517325330298	0.427	0.158	1.99685083726819E-102	7	ABCG1
5.23425982037747E-138	3.63683681964272	0.532	0.211	8.77157260698857E-134	7	PXN
0	3.61926144396051	0.288	0.025	0	7	ITGA9
7.94089302441272E-64	3.56652298715602	0.313	0.121	1.33073485303108E-59	7	NEDD9
0	3.52073276375613	0.715	0.044	0	7	IGFBP7
3.66782137506724E-186	3.51859029998611	0.368	0.071	6.14653506033769E-182	7	HDAC7
6.40048907640722E-94	3.50735335841869	0.285	0.076	1.07259395942432E-89	7	TM4SF1
8.95258144997055E-191	3.50248386067596	0.407	0.085	1.50027359938606E-186	7	GSN
1.77784507074657E-88	3.4823499785287	0.335	0.109	2.9793127695571E-84	7	TSPAN9
1.44674128571733E-48	3.47918223056608	0.269	0.108	2.42444904660511E-44	7	CHST12
6.50857573437876E-226	3.47170466906733	0.335	0.048	1.09070712156719E-221	7	PLTP
9.36227776708103E-117	3.45605326186652	0.599	0.325	1.56893050820744E-112	7	TCIM
9.23092364758134E-46	3.43642607476963	0.294	0.131	1.54691818486168E-41	7	WARS
5.26801973488959E-91	3.43595432908103	0.357	0.121	8.82814747172797E-87	7	SEC14L1
6.05206120535812E-101	3.39932817662282	0.31	0.084	1.01420441679391E-96	7	TNS1
1.08095151714678E-34	3.39013135148944	0.269	0.131	1.81145855243458E-30	7	KIAA0355
3.84519976520411E-302	3.37631763245485	0.408	0.055	6.44378576652905E-298	7	ITPKB
2.07291874662233E-87	3.34562496774635	0.532	0.306	3.4737972355897E-83	7	INSR
3.10615439404945E-36	3.33217042935161	0.308	0.162	5.20529353354807E-32	7	ARHGAP26
5.29857509083581E-51	3.31716583009833	0.297	0.124	8.87935213722265E-47	7	FAM219A
1.49523437944182E-62	3.30131316974367	0.343	0.143	2.5057137730686E-58	7	PGS1
3.09800608029434E-113	3.29253352186391	0.499	0.208	5.19163858935726E-109	7	TGM2
2.52970843772849E-54	3.29088810474876	0.319	0.137	4.2392853999454E-50	7	HMBOX1
4.81773964731445E-118	3.2716927434913	0.426	0.142	8.07356810096956E-114	7	PLXND1
2.78979131075161E-29	3.20622185972564	0.26	0.133	4.67513227855755E-25	7	PTPRG
2.2065236955229E-79	3.19913946509997	0.463	0.23	3.69769240895727E-75	7	CEMIP2
1.09223324214914E-112	3.19811579193726	0.318	0.08	1.83036446719353E-108	7	PALM2-AKAP2
4.08588962057877E-88	3.17654159408255	0.347	0.116	6.8471338261659E-84	7	TSPAN14
8.00424487808507E-38	3.1707526079119	0.391	0.247	1.3413513566695E-33	7	CNST
0	3.16545771174675	0.638	0.093	0	7	RNASE1
3.45027775454728E-307	3.15309483780787	0.26	0.021	5.78197546107034E-303	7	C7
6.50602436885707E-91	3.04961040586068	0.319	0.095	1.09027956373307E-86	7	ID3
3.27227112037643E-44	3.04855252096584	0.329	0.163	5.48367194352682E-40	7	TMEM184B
8.51121510903825E-52	3.04520295002735	0.426	0.246	1.42630942797263E-47	7	ARHGEF7
4.93048896231182E-19	3.03836963508579	0.252	0.153	8.26251340304215E-15	7	PRSS23
1.45527995306547E-47	3.03644326309692	0.343	0.17	2.43875814534711E-43	7	SNRK
9.79444198098026E-49	3.01462064956356	0.391	0.217	1.64135258717267E-44	7	MARCKSL1
1.12446616940774E-43	3.00341851651299	0.332	0.165	1.8843804066935E-39	7	TNFRSF10B
8.08758073417816E-120	2.98975065588179	0.399	0.116	1.35531677943358E-115	7	ID1
8.98107012757125E-29	2.97470689634287	0.282	0.154	1.50504773197839E-24	7	MXRA7
1.4855444447349E-46	2.91180061613762	0.46	0.301	2.48947538048674E-42	7	UGCG
5.03969363185627E-157	2.91044394837114	0.729	0.438	8.44551858826474E-153	7	DDIT4
1.40619672510317E-19	2.90487643470225	0.344	0.261	2.35650447192789E-15	7	PLPP1

1.59870552701178E-37	2.88611278833382	0.41	0.272	2.67911072216634E-33	7	PRCP
4.3917632569402E-37	2.87615460113756	0.266	0.121	7.35971686598039E-33	7	LAMA5
4.96461594530563E-29	2.87432653109724	0.333	0.208	8.31970340114317E-25	7	NOTCH1
5.52773737842841E-18	2.86125467629534	0.274	0.178	9.26338229877032E-14	7	SLC25A37
1.54803456331203E-14	2.85433873482331	0.255	0.174	2.5941963211983E-10	7	NR2F2
5.89647321775982E-18	2.8535866999729	0.286	0.194	9.8813098183219E-14	7	PELO
2.71785068263497E-26	2.83343721464427	0.335	0.216	4.55457417395969E-22	7	SIN3B
4.90294669468007E-164	2.82595787885008	0.332	0.063	8.21635807094486E-160	7	NR4A1
6.95646119915651E-121	2.82090647943262	0.279	0.058	1.16576376775465E-116	7	GIMAP4
0	2.7983728909601	0.538	0.029	0	7	COL6A2
1.26727052002686E-39	2.78957315946647	0.346	0.191	2.12369193746102E-35	7	SLC44A2
2.35549092127983E-176	2.78518197512888	0.366	0.07	3.94733168588073E-172	7	TIMP2
3.81063003673307E-79	2.75507114221848	0.455	0.211	6.38585381555728E-75	7	SLC9A3R2
1.03213588769308E-41	2.74509675030511	0.335	0.172	1.72965332059607E-37	7	PLCG1
8.99349380050282E-32	2.71117510611271	0.401	0.283	1.50712969108826E-27	7	SELENON
8.78187710325132E-116	2.69654750169237	0.257	0.051	1.47166696496286E-111	7	CLEC2B
2.96551767105458E-24	2.66002077904638	0.305	0.191	4.96961451315327E-20	7	JUP
4.66835362756643E-13	2.65510671534558	0.286	0.221	7.82322700907582E-09	7	DOCK9
1.17289537118231E-263	2.63868941450592	0.573	0.128	1.96553806302732E-259	7	TIMP1
1.5004521779887E-16	2.6364773463226	0.275	0.185	2.51445775987346E-12	7	DAPK3
1.80075355104388E-15	2.6282979564245	0.279	0.193	3.01770280083933E-11	7	MED10
8.1766973821413E-98	2.62773540249497	0.465	0.182	1.37025094729924E-93	7	VIM
8.4931057440741E-100	2.61496553335885	0.29	0.072	1.42327466059194E-95	7	SERPINB9
7.79375600613969E-54	2.6054857715016	0.482	0.317	1.30607763150889E-49	7	NID1
2.77333690337985E-14	2.5990324933577	0.261	0.181	4.64755798268396E-10	7	SULF2
2.06639723568171E-18	2.59262255960769	0.28	0.184	3.46286848755541E-14	7	CLIC4
9.34639103513712E-42	2.58737406996031	0.365	0.2	1.56626820966828E-37	7	JMJD1C
4.34687594279008E-64	2.57113433513197	0.52	0.32	7.28449470492761E-60	7	WSB1
1.21208173750793E-86	2.55242486838917	0.266	0.068	2.0312065757158E-82	7	CMTM3
1.4967179943448E-13	2.52821263998228	0.268	0.188	2.50820001492301E-09	7	SNTB2
5.63158899434509E-51	2.52817822120545	0.43	0.246	9.4374168367235E-47	7	ARRDC3
1.81820527308066E-66	2.52644575341601	0.257	0.077	3.04694839662857E-62	7	PLEKHO1
4.43771131370411E-164	2.48922487394105	0.448	0.112	7.43671661950535E-160	7	ETS1
5.44724294972841E-124	2.48547229325234	0.715	0.521	9.12848973515487E-120	7	APP
1.03547145772532E-99	2.44246760851476	0.473	0.189	1.73524306885609E-95	7	SERPINH1
2.01537209655152E-63	2.43506079594548	0.509	0.31	3.37736055940103E-59	7	MACF1
4.68414677732861E-85	2.41416714765805	0.618	0.419	7.84969316944729E-81	7	STOM
5.94079602499634E-24	2.38965540758238	0.402	0.32	9.95558597868887E-20	7	TGFBR2
4.86033675707617E-71	2.37712969536771	0.321	0.109	8.14495233750824E-67	7	DAB2
5.40309634792843E-13	2.3751374255605	0.26	0.185	9.05450885985847E-09	7	TNS2
2.36483371094616E-18	2.35542152793966	0.308	0.209	3.96298833280357E-14	7	XAF1
1.75076342582064E-40	2.35371539502923	0.448	0.292	2.93392934899023E-36	7	DEPP1
1.78835708135272E-106	2.34185445273054	0.365	0.104	2.99692879693089E-102	7	IFI16
3.89179484903942E-27	2.33310969416384	0.421	0.315	6.52186980802026E-23	7	MYO15B
3.72387540982753E-13	2.30058038408911	0.336	0.285	6.24047041178897E-09	7	EDNRB
3.05800511066396E-26	2.29718620419741	0.397	0.296	5.12460496445067E-22	7	NRP1
9.77736310268415E-22	2.29106026792399	0.387	0.305	1.63849050874781E-17	7	ECE1
1.53068662051209E-11	2.28292657952792	0.372	0.344	2.56512463865416E-07	7	SLC38A2
4.65765075507733E-94	2.26664123409244	0.335	0.097	7.80529113535858E-90	7	FXYD5
4.33353273085635E-43	2.26161319695528	0.527	0.434	7.26213415036906E-39	7	EPAS1
3.44921458982412E-29	2.26151237958228	0.274	0.14	5.78019380962726E-25	7	KLF2
1.25961572960321E-25	2.25434851812314	0.305	0.178	2.11086403966906E-21	7	HES1
1.11639592312468E-07	2.253436298821	0.275	0.234	0.00187085628797234	7	PCGF2
5.05226103462447E-08	2.2489788591401	0.302	0.267	0.000846657904182369	7	DENND4C
4.67934867089796E-09	2.24398100779877	0.299	0.254	7.8416525026908E-05	7	BCAR1
1.84769763993692E-12	2.23180676703756	0.318	0.258	3.09637170500628E-08	7	FKBP1A
1.80813705044177E-16	2.19980069426336	0.261	0.167	3.03007606913032E-12	7	CBLB
1.99846699107846E-25	2.19767257270971	0.493	0.423	3.34903098364929E-21	7	DEPRECATED-ENSG00000147872
1.26073653298561E-97	2.16643860436649	0.304	0.077	2.11274228197729E-93	7	MRC1
1.34902931094296E-10	2.13296177111295	0.362	0.327	2.26070331927821E-06	7	BSDC1

5.41642870635428E-53	2.11869367227388	0.573	0.428	9.0768512261085E-49	7	MAF
7.38841682227129E-14	2.11803615957584	0.368	0.317	1.23815089107622E-09	7	UBR4
4.23044225569075E-20	2.10489094333965	0.415	0.349	7.08937513208656E-16	7	CD59
1.07169281731987E-11	2.10314994037115	0.308	0.246	1.79594282326464E-07	7	SPAG9
4.96292299294084E-10	2.09907739793675	0.329	0.295	8.31686635157026E-06	7	ADAM15
5.56049487393935E-116	2.08745702933121	0.732	0.489	9.31827730974757E-112	7	ARGLU1
5.24659341654307E-21	1.9543323429332	0.427	0.362	8.79224124744288E-17	7	RHOC
1.26564067598452E-06	1.94625286704234	0.3	0.277	0.0212096064481486	7	MMP14
1.29961922858645E-25	1.93752316538465	0.423	0.321	2.17790190326517E-21	7	JAK1
2.339886722996E-120	1.93640957104423	0.784	0.629	3.9211821703967E-116	7	DUSP1
8.30339905476674E-11	1.91959194622751	0.302	0.24	1.39148361359781E-06	7	SARAF
1.6476981754813E-07	1.89848310946805	0.304	0.27	0.00276121260247157	7	TRIOBP
5.03737046767308E-13	1.86488417267786	0.274	0.195	8.44162542972655E-09	7	LEPROTL1
2.99654893609397E-27	1.84464176843935	0.474	0.394	5.02161670710627E-23	7	FOSL2
6.00328483578961E-15	1.84271812803563	0.335	0.257	1.00603047278162E-10	7	LUC7L
1.83928207122446E-19	1.83841485749961	0.388	0.298	3.08226889495795E-15	7	CYTH1
1.00973943176089E-12	1.8219495365613	0.33	0.264	1.69212133974489E-08	7	MARCKS
2.75188322245638E-06	1.81563455459637	0.293	0.268	0.0461160590419241	7	CYTH2
1.13061793215476E-06	1.79953034991934	0.268	0.228	0.0189468953070495	7	GNAI2
2.6382335458084E-53	1.79732988135547	0.335	0.135	4.42115177606571E-49	7	MS4A6A
3.55030781169431E-07	1.78917668692662	0.308	0.281	0.00594960583083733	7	PEA15
2.86217541108978E-09	1.77679566355284	0.338	0.3	4.79643355390425E-05	7	ATN1
5.7665293322696E-15	1.74485212880925	0.255	0.162	9.6635498550174E-11	7	TMEM140
1.57456102196212E-60	1.73313318502625	0.628	0.442	2.63864936060411E-56	7	BHLHE40
2.21059290199537E-08	1.73124533026644	0.275	0.221	0.000370451158516385	7	RGS3
2.73735525608577E-34	1.72432257702721	0.527	0.427	4.58725993814853E-30	7	FCGRT
5.28300451623318E-09	1.71174692343503	0.43	0.457	8.85325896830356E-05	7	IVNS1ABP
6.86796987318567E-14	1.70837630429596	0.25	0.162	1.15093439134845E-09	7	FLNA
8.33829621242477E-13	1.69876472149316	0.266	0.184	1.39733167927814E-08	7	P4HA1
2.10867577048476E-12	1.68681751270299	0.413	0.384	3.53371885617837E-08	7	PER1
1.5261211170116E-11	1.68393127663379	0.255	0.178	2.55747376788804E-07	7	PPP1R15A
1.56350334796695E-24	1.66534803344208	0.377	0.251	2.62011891052301E-20	7	DEPRECATED-ENSG00000175899
2.17882397670477E-54	1.63889796622816	0.592	0.424	3.65127322016185E-50	7	JUNB
2.60695287584717E-29	1.63549279348677	0.36	0.207	4.36873162934469E-25	7	CD14
2.42464437997995E-10	1.62693790210569	0.326	0.27	4.06321905197041E-06	7	IER2
3.13442228083821E-24	1.60650408180755	0.448	0.359	5.25266485822868E-20	7	MSN
7.79605964476861E-37	1.55358792374493	0.498	0.339	1.30646367527032E-32	7	A2M
4.84523014163515E-17	1.49013924418664	0.438	0.378	8.11963667135218E-13	7	TMSB10
3.59985386007328E-08	1.45835313177696	0.138	0.265	0.00060326350987108	7	IGFBP3
8.18001670960853E-07	1.44830112685845	0.322	0.294	0.013708072001962	7	ZYX
4.38134598225983E-35	1.44054493392835	0.576	0.478	7.34225959707103E-31	7	LENG8
6.10753797314809E-26	1.38442315151886	0.258	0.128	1.02350121354016E-21	7	TRIM22
1.52706158166614E-12	1.35992025885357	0.457	0.439	2.55904979855612E-08	7	KLF6
4.34170414963757E-25	1.27760811229219	0.629	0.656	7.27582781396264E-21	7	RHOB
8.62456480073263E-37	1.25564177596113	0.263	0.106	1.44530456930677E-32	7	CYBA
2.81955854430516E-06	1.17841014805861	0.437	0.476	0.0472501620854659	7	ITM2B
1.00854248731789E-07	1.1201335071903	0.426	0.425	0.00169011550024731	7	SYNE2
1.98131603486952E-06	1.05948919428504	0.146	0.254	0.0332028941123433	7	KIF13A
1.41477158996477E-07	1.02875992260417	0.153	0.28	0.00237087423046296	7	RAB20
5.77632328852309E-09	1.01083271798804	0.531	0.627	9.679962566907E-05	7	IGFBP4
2.45862764904801E-19	0.97976159390709	0.567	0.529	4.12016821427465E-15	7	CCNL2
1.96891627848108E-06	0.955439953428747	0.171	0.292	0.0329950989947859	7	TAOK1
3.39460782816086E-08	0.937339753326098	0.142	0.269	0.000568868379843196	7	SEC24A
2.83024203809358E-06	0.932774743041705	0.156	0.27	0.0474291960743722	7	ZC3H15
1.24245815210453E-06	0.901472129062254	0.155	0.271	0.0208211137129678	7	NIPBL
1.23743177015147E-32	0.901344045484173	0.429	0.233	2.07368816041983E-28	7	COL18A1
1.7437087526072E-69	0.867228940332882	0.457	0.177	2.92210712761914E-65	7	SRGN
9.93165880709469E-07	0.862375179959525	0.152	0.266	0.0166434738289293	7	RUFY1
6.42000231394198E-08	0.862362239918812	0.142	0.265	0.0010758639877704	7	STK38
2.67497118586149E-09	0.860135069224719	0.141	0.274	4.48271671326669E-05	7	SNX5

5.52195413230754E-07	0.857442062544201	0.203	0.351	0.00925369073492098	7	AGFG2
2.4724948982097E-06	0.857357410900779	0.203	0.34	0.0414340695041982	7	NUMB
7.7828646218488E-07	0.849019992948176	0.538	0.607	0.0130425245332942	7	JUN
1.85376235147458E-08	0.842535093219373	0.164	0.301	0.00031065349486011	7	UBE2V1
7.42855556800452E-10	0.841838462442893	0.139	0.277	1.2448773420862E-05	7	TRIM25
3.934130769287E-07	0.83725750474178	0.141	0.252	0.00659281634317116	7	ATF4
1.43855497096505E-06	0.826745157736759	0.153	0.266	0.0241073042034324	7	SEC16A
1.21303164640526E-07	0.815006611429526	0.167	0.299	0.00203279843304594	7	ANKRD17
6.53915644155302E-07	0.811514787474961	0.174	0.297	0.0109583183647546	7	ITGB1
8.0724273472441E-09	0.795623126194392	0.131	0.255	0.000135277737485117	7	KIAA0319L
8.11135347621847E-08	0.784166463715399	0.155	0.278	0.00135930061554469	7	WDR26
1.58394236644E-06	0.771998977204808	0.163	0.278	0.0265437061768014	7	PCED1A
1.55355154416713E-06	0.770552172442097	0.163	0.281	0.0260344167771528	7	CAV2
8.61776725230537E-09	0.745716289783477	0.136	0.261	0.000144416543614133	7	MORC3
1.96990971448507E-07	0.743532694042911	0.18	0.314	0.00330117469953408	7	MKLN1
3.8785575522532E-13	0.742070779240334	0.626	0.718	6.49968674606591E-09	7	TMSB4X
1.69054581038048E-07	0.739455168441854	0.16	0.283	0.00283301666903561	7	CPSF6
4.84039954002323E-07	0.739056581728193	0.175	0.304	0.00811154154917094	7	CDK16
2.19575065976101E-06	0.735480064845889	0.194	0.327	0.036796389556275	7	SRRM2
4.4814886477638E-07	0.727978798446302	0.196	0.334	0.00751007867592258	7	PDIA6
2.97787683980174E-09	0.721680426779143	0.138	0.267	4.99032600813976E-05	7	MED25
2.83843862306408E-07	0.72129284968483	0.142	0.256	0.00475665544453078	7	NCKAP1
6.86760689683487E-15	0.715466287114143	0.102	0.255	1.15087356377159E-10	7	FAM13A
1.35307495735015E-10	0.709052261391627	0.189	0.358	2.26748301352738E-06	7	MARCH6
4.31786829209412E-08	0.707473381846187	0.138	0.257	0.000723588368389132	7	ANAPC5
3.05639344241087E-07	0.706847378587163	0.156	0.274	0.00512190413079213	7	SYMPK
3.71096333981262E-11	0.703789724161015	0.119	0.255	6.21883236485799E-07	7	ARFGAP3
4.44916301843711E-12	0.702765044825365	0.124	0.27	7.45590738629691E-08	7	SRA1
5.29454032071753E-08	0.697701053460649	0.136	0.253	0.000887259066945843	7	SLC27A3
1.43947893315256E-10	0.696189563842267	0.122	0.253	2.41227879617706E-06	7	SKAP2
1.61681037559834E-06	0.693573774126308	0.216	0.359	0.027094508274277	7	SAFB2
1.32850582190132E-08	0.682404024002371	0.163	0.296	0.000222631005634223	7	COPB1
1.55867338323576E-08	0.679607480107516	0.163	0.298	0.000261202485562648	7	PCDH1
1.32772875278264E-09	0.676671113710138	0.167	0.318	2.22500784391315E-05	7	UBR5
8.82869277921518E-09	0.676191690162357	0.164	0.304	0.000147951233594088	7	LGALS8
4.65541113399793E-09	0.669665856694427	0.15	0.283	7.80153797835373E-05	7	SLC23A2
1.35954308207174E-09	0.669192062118028	0.13	0.256	2.27832229693583E-05	7	CLTA
4.82107560970571E-07	0.665467971687973	0.155	0.273	0.00807915850674483	7	ARRB1
5.72893265409164E-16	0.656997190799758	0.133	0.314	9.60054534172677E-12	7	CUL4B
1.11613460991656E-06	0.652841905014591	0.196	0.332	0.0187041837929817	7	UPK3BL1
3.78355811678213E-08	0.650847369102555	0.133	0.25	0.00063404866921035	7	FRYL
2.9973035917282E-08	0.650218763370756	0.191	0.337	0.000502288135901812	7	EIF3B
5.36241027619329E-10	0.647023272102562	0.135	0.266	8.98632714084471E-06	7	PTBP1
3.48021772615236E-09	0.644090233229591	0.138	0.265	5.83214886548612E-05	7	PSMD13
2.04936322722271E-09	0.63292257916134	0.197	0.362	3.43432289617981E-05	7	POLR2J3
1.52918029064418E-13	0.631864694760513	0.116	0.267	2.56260033106152E-09	7	DCTN4
1.7696906347659E-07	0.630945105544014	0.163	0.286	0.00296564756574069	7	RBM25
9.14473974558931E-07	0.63033036235366	0.211	0.357	0.0153247548656586	7	RSRC2
1.35376337155925E-07	0.629502895997055	0.224	0.385	0.00226863665805899	7	PTOV1
1.18040505336694E-07	0.627840967681518	0.161	0.288	0.00197812278843232	7	NIBAN2
5.15006617222794E-07	0.626449209849082	0.199	0.337	0.00863048089141959	7	DVL3
1.11591360840995E-07	0.62362969761526	0.155	0.28	0.00187004802497339	7	MED28
1.48474305797717E-06	0.621701589560747	0.171	0.289	0.0248813241655815	7	C16orf72
6.49342404421574E-11	0.615114837184343	0.144	0.289	1.08816800132967E-06	7	ITPRID2
5.30829383570971E-10	0.61128456450117	0.177	0.335	8.89563880988234E-06	7	TRIM27
5.04196320527768E-07	0.60947187623528	0.153	0.271	0.00844932193940434	7	STK11
2.31525253576447E-08	0.607455777096538	0.249	0.433	0.00038799001994341	7	SEC31A
2.81982366325E-10	0.607316753734266	0.191	0.359	4.72546049487436E-06	7	NUFIP2
2.12249621804511E-06	0.598114539566809	0.288	0.467	0.0355687916219999	7	NDUFS2
1.35238569907473E-07	0.596984585259648	0.247	0.42	0.00226632795450944	7	IRF2BP2

5.68270950822874E-10	0.589079599512999	0.138	0.269	9.52308459388972E-06	7	ZDHHC14
5.73984998109833E-12	0.588264760438354	0.114	0.255	9.61884059832458E-08	7	PI4KA
4.59666203919212E-10	0.583582864417285	0.21	0.386	7.70308624527815E-06	7	PPP2R5A
3.22754404181192E-07	0.582758124216936	0.186	0.318	0.00540871830526842	7	TIA1
2.03037855044901E-09	0.58184050750785	0.144	0.282	3.40250837484246E-05	7	HECTD4
9.7036491866614E-08	0.578876280062394	0.147	0.265	0.00162613753070072	7	CDC34
1.30969874454629E-12	0.57648484685363	0.11	0.25	2.19479315611067E-08	7	SRP72
1.20096545978593E-09	0.57260670899373	0.141	0.274	2.01257791750926E-05	7	VAPA
1.223651363493E-12	0.569874351828924	0.114	0.259	2.05059495494158E-08	7	PDLIM5
5.18843483821063E-10	0.557344872818133	0.171	0.321	8.69477910187338E-06	7	LONP1
3.15134158148402E-11	0.555733460238038	0.149	0.298	5.28101822225093E-07	7	DEPRECATED-ENSG00000184500
3.15007175680695E-10	0.550644960083988	0.171	0.325	5.27889025005709E-06	7	IGF2R
1.44090304859407E-10	0.544093239459324	0.121	0.251	2.41466532883394E-06	7	RTCB
2.75284093251364E-06	0.543594884089053	0.171	0.283	0.0461321083470635	7	IFI6
2.80757330118389E-08	0.542649951944226	0.164	0.297	0.000470493133812396	7	UBE2Z
3.65797786401605E-08	0.541969550034808	0.18	0.319	0.00061300393045181	7	PCMTD1
1.59217385815067E-12	0.536616705055303	0.199	0.391	2.6681649514889E-08	7	UBAP2L
6.00924382042342E-08	0.534590435091332	0.156	0.284	0.00100702907942656	7	YWHAE
6.99082077518994E-12	0.534214121881964	0.147	0.301	1.17152174550633E-07	7	SLC35F6
9.99737941182177E-10	0.53048511239424	0.196	0.361	1.67536084183309E-05	7	LIMD1
8.63271666033649E-08	0.527896162910932	0.196	0.342	0.00144667065793919	7	KMT2C
5.29596562723873E-11	0.527205505030782	0.167	0.328	8.87497919812666E-07	7	LRP5
2.14750330391387E-07	0.526951819333732	0.192	0.332	0.00359878603669887	7	FUBP1
5.57514634383267E-07	0.526705220135492	0.221	0.37	0.00934283024299479	7	MBD2
7.11076476222891E-08	0.525256421233274	0.152	0.273	0.00119162195885432	7	DEDD
2.00347853569684E-08	0.519663795160133	0.156	0.287	0.000335742933012076	7	IST1
2.17288109118757E-06	0.518538387025518	0.221	0.361	0.0364131413261213	7	STX16
5.88484257757499E-12	0.51588109645286	0.131	0.278	9.86181919150017E-08	7	PARP14
7.53825508255387E-09	0.514346925771845	0.191	0.341	0.000126326078673438	7	BRD4
6.65386467860193E-09	0.513061849041529	0.153	0.285	0.000111505464284011	7	RNASEK
1.69477556267077E-08	0.511856385941495	0.207	0.362	0.000284010488792368	7	CPNE1
1.24194954026477E-07	0.511222697257487	0.239	0.4	0.0020812590395757	7	MYO18A
2.3457240594703E-09	0.50446165109803	0.13	0.254	3.93096437886032E-05	7	SMAD2
5.36491930601445E-09	0.504212551712398	0.185	0.334	8.99053177301901E-05	7	ADAM9
4.54358481818872E-12	0.503836773482338	0.178	0.352	7.61413943832066E-08	7	TMEM248
6.82025504770139E-12	0.497883682373457	0.116	0.254	1.1429383408938E-07	7	NR5A2
2.2057740385174E-06	0.497609321742043	0.147	0.251	0.0369643613374745	7	KIF1C
1.58476549935837E-08	0.496929914249633	0.205	0.36	0.000265575002382475	7	FBXO7
8.64894164984608E-07	0.495628529676381	0.263	0.434	0.0144938964168121	7	EGLN1
3.78397676477782E-07	0.494461375054789	0.222	0.373	0.00634118826241467	7	TRA2A
1.24082881983104E-07	0.49408843922869	0.235	0.405	0.00207938093627286	7	SERINC3
1.541191863894E-06	0.494041204993202	0.146	0.251	0.0258272932551357	7	TIAL1
6.97490183591592E-09	0.491908244888475	0.149	0.277	0.000116885404966279	7	STAT2
4.69598103846686E-11	0.491395296083093	0.188	0.359	7.86952502426276E-07	7	DHX15
4.57688656224505E-12	0.490312697454014	0.119	0.259	7.66994650101025E-08	7	DYM
2.2776831705726E-12	0.484592240933528	0.138	0.29	3.81694145724557E-08	7	HMGN3
7.25096629217908E-08	0.483287133845954	0.139	0.257	0.00121511693124337	7	SUMO3
6.92692285640175E-10	0.4812455403468	0.194	0.357	1.1608137322758E-05	7	C11orf58
2.64215448118753E-12	0.480827756261982	0.185	0.362	4.42772247957407E-08	7	C4orf3
2.63732352813412E-09	0.478586214067124	0.135	0.263	4.41962676844717E-05	7	ZRANB2
1.62791845417905E-11	0.47765769459811	0.167	0.33	2.72806574551326E-07	7	SRSF2
1.86255530772733E-06	0.477622201819659	0.327	0.537	0.0312127018468946	7	SAT1
4.04449921827417E-08	0.47740773664628	0.152	0.275	0.000677777178998385	7	FUS
3.11242570548872E-09	0.473226640661609	0.202	0.364	5.215802997258E-05	7	UBE2B
1.5947395688692E-12	0.470262443712988	0.146	0.305	2.67246456951101E-08	7	SETD3
6.23922641793591E-09	0.46646307854453	0.2	0.362	0.00010455695631177	7	ADD1
1.08293024561502E-13	0.464586953826743	0.147	0.316	1.81477450560165E-09	7	XRN1
3.59481095613611E-10	0.45980350722834	0.142	0.278	6.02418420029289E-06	7	GTF2I
2.6518870822881E-13	0.459507851836232	0.153	0.323	4.4440323724984E-09	7	HGS
3.67389965098603E-10	0.459268398952102	0.139	0.276	6.1567210351224E-06	7	SNX6

7.9093296157205E-12	0.456623442583726	0.16	0.321	1.32544545700244E-07	7	ECPAS
2.47326328007588E-16	0.449311813311781	0.13	0.304	4.14469460475116E-12	7	C5orf24
1.04390733542707E-10	0.449201723903725	0.124	0.257	1.74937991270868E-06	7	CWC15
7.92865626327242E-11	0.448845600260004	0.13	0.267	1.32868421659919E-06	7	PRPF40A
1.18385415813941E-08	0.446881498714907	0.164	0.298	0.000198390279821003	7	ESYT1
1.74416746820031E-10	0.445984499784431	0.182	0.343	2.92287584321008E-06	7	TMED9
1.13364808710637E-09	0.445926013801152	0.172	0.321	1.89976746437286E-05	7	KXD1
1.95490724546055E-11	0.444725667314822	0.133	0.276	3.27603356194279E-07	7	TXNDC15
6.70450937048571E-07	0.441706990070403	0.207	0.344	0.0112354168030599	7	SUGP2
2.46800845899027E-10	0.437133361804779	0.243	0.436	4.1358885755759E-06	7	RTN4
2.1921784740206E-14	0.430290204108235	0.131	0.296	3.67365268676373E-10	7	DHX9
4.2360799996336E-11	0.427727035543505	0.121	0.254	7.09882286338598E-07	7	GPR107
7.892878645232E-13	0.426819226214297	0.153	0.316	1.32268860336798E-08	7	PARP4
1.13549616378106E-13	0.423180216762403	0.114	0.267	1.9028644712643E-09	7	SH3GLB2
1.58217188013217E-07	0.422951065326976	0.152	0.269	0.00265140363672549	7	DDX24
4.96105567719038E-15	0.422400515009047	0.106	0.264	8.31373710383564E-11	7	KDSR
1.58397032740702E-12	0.420580298686057	0.122	0.269	2.65441747466869E-08	7	IRF2
1.98310744248914E-10	0.416677987211729	0.21	0.384	3.3232914521233E-06	7	FAM120A
2.29946129315361E-11	0.416447683426625	0.128	0.266	3.85343723506683E-07	7	MGRN1
1.37943821077681E-11	0.415493896719122	0.155	0.31	2.31166255361979E-07	7	TMEM263
2.53159804540671E-15	0.413453669711378	0.147	0.329	4.24245200449257E-11	7	CTDSP1
2.77319435979698E-06	0.413071797018847	0.177	0.293	0.0464731910814778	7	CNOT6L
2.34594921264373E-08	0.412070123771859	0.221	0.382	0.000393134169054836	7	OPTN
2.54683528124703E-10	0.411970175730432	0.125	0.256	4.26798656431378E-06	7	UBA1
4.21919998508029E-07	0.410658069322437	0.141	0.25	0.00707053533499755	7	YWHAH
6.71164305861002E-09	0.410170697330482	0.169	0.307	0.000112473714376187	7	ACTR1B
3.01861122754487E-08	0.409226912392669	0.258	0.437	0.000505858869511969	7	GSDMD
4.14265693233344E-08	0.406266595970379	0.186	0.327	0.000694226448720437	7	SNRNP70
9.65663169282446E-13	0.402842130773169	0.127	0.277	1.61825833908352E-08	7	ZBTB7A
1.58579406325694E-14	0.399041216756923	0.131	0.298	2.65747369120599E-10	7	HELZ
1.38226486997867E-12	0.398777199594255	0.117	0.26	2.31639946911025E-08	7	AP3B1
6.80711621084463E-16	0.396899053691608	0.1	0.259	1.14073653461334E-11	7	TMEM214
2.59887986358226E-11	0.39611171934368	0.153	0.306	4.35520287539115E-07	7	MICAL3
9.16291938512117E-12	0.393878695876756	0.167	0.333	1.53552203055861E-07	7	CD47
4.14693323588656E-10	0.392660846672369	0.144	0.281	6.9494307166987E-06	7	KANSL1
6.86173787383498E-11	0.388687278647242	0.138	0.28	1.14989003289727E-06	7	SELENOS
8.73839174421797E-12	0.387376570996923	0.114	0.25	1.46437968849605E-07	7	TUT4
2.40759603950436E-11	0.386338185273143	0.164	0.323	4.03464944300141E-07	7	SLC39A7
1.74263521899228E-15	0.383673630328225	0.136	0.311	2.92030809998727E-11	7	SLC35A4
2.34326763326836E-10	0.379083362205786	0.183	0.343	3.92684789983112E-06	7	UBR2
1.18746367733498E-12	0.375113791024168	0.133	0.285	1.98995163047796E-08	7	TNRC6A
5.32004458515127E-13	0.371887087486149	0.131	0.284	8.9153307157965E-09	7	CLPTM1L
3.65204140058774E-09	0.37145382996178	0.197	0.356	6.12009097910493E-05	7	RABGAP1L
1.71124000322498E-08	0.370566160866501	0.313	0.533	0.000286769599740443	7	ZFP36
5.2457054025679E-14	0.369612545536715	0.117	0.272	8.7907531136233E-10	7	ARNT
2.8937929787398E-14	0.369601850932257	0.158	0.339	4.84941827377215E-10	7	MAP3K13
5.18049574234287E-11	0.360161190101466	0.169	0.327	8.68147476501818E-07	7	RNF10
1.90923767111338E-15	0.358823599996268	0.113	0.274	3.19950048925181E-11	7	CANT1
9.65821006983398E-12	0.358762539154353	0.169	0.336	1.61852284350278E-07	7	RAB1A
6.90438496253214E-13	0.357422303058593	0.11	0.253	1.15703683202114E-08	7	POLDIP3
5.17631279709711E-11	0.356967150978448	0.144	0.293	8.67446498537534E-07	7	RAB1B
5.28258476110041E-13	0.354315450520362	0.161	0.329	8.85255554265207E-09	7	ANKHD1
2.22455647546024E-10	0.35369530580125	0.146	0.288	3.72791174157627E-06	7	UBE2H
7.38123828958535E-14	0.351372378388366	0.113	0.263	1.23694791256871E-09	7	DOT1L
2.31407940202282E-08	0.350653906687952	0.213	0.369	0.000387793426190984	7	POGZ
1.27295819210723E-09	0.350481500742597	0.133	0.261	2.13322333833329E-05	7	TAP2
5.23789656454355E-14	0.349869010859467	0.133	0.297	8.77766706286208E-10	7	FBXL5
7.47263617356421E-11	0.347997755401578	0.13	0.264	1.25226436996589E-06	7	RAC1
5.03272363543831E-15	0.346646518059477	0.102	0.255	8.43383826826752E-11	7	UBA2
4.58294774812031E-07	0.34571432069079	0.219	0.363	0.00768010383630002	7	PPP2R1B

6.53020049428986E-17	0.344614517946559	0.139	0.332	1.09433099883309E-12	7	ACVR1B
3.97160722289191E-14	0.343081450595507	0.138	0.305	6.65561938412227E-10	7	PRRC2C
2.0028792464691E-11	0.342441424137498	0.147	0.293	3.35642504123292E-07	7	NRBP1
1.25065741115111E-10	0.342381207523527	0.124	0.255	2.09585168960703E-06	7	IWS1
2.25799494614672E-12	0.34192214046364	0.246	0.455	3.78394793075267E-08	7	NFIC
2.96958273058241E-11	0.340867746724819	0.119	0.252	4.97642673991E-07	7	CIAO1
9.17311917346954E-12	0.34081088620224	0.156	0.314	1.53723131109003E-07	7	MPV17
8.52745477983483E-13	0.340587780452761	0.225	0.428	1.42903087200472E-08	7	COPA
9.60898188367409E-12	0.339851315033389	0.192	0.368	1.6102731840661E-07	7	PDCD6IP
2.60974672836611E-13	0.339576894947179	0.208	0.409	4.37341356739593E-09	7	SREBF1
3.39467307252856E-11	0.338108985425827	0.208	0.393	5.68879313494336E-07	7	LPP
5.52480424332704E-10	0.333867511770424	0.172	0.325	9.25846695096746E-06	7	SAT2
1.38916626984817E-14	0.332326269927393	0.13	0.295	2.32796483501156E-10	7	AGFG1
4.64265564856637E-12	0.329677557355416	0.163	0.327	7.78016233586752E-08	7	GPBP1
1.50037728418829E-11	0.329615166720214	0.293	0.187	2.51433225284273E-07	7	LAPTM5
7.88537529025968E-14	0.329558013693366	0.119	0.273	1.32143119114172E-09	7	SUPT6H
4.40042269464432E-11	0.328244720223241	0.156	0.309	7.37422835168495E-07	7	ATF6B
4.79972694412602E-12	0.327075979288216	0.144	0.295	8.04338241296638E-08	7	THRAP3
1.00903992612935E-15	0.326779441451947	0.11	0.275	1.69094910820756E-11	7	DNAJC1
3.87552454196417E-11	0.324320598518203	0.164	0.318	6.49460402742355E-07	7	LAP3
1.73178178088845E-11	0.321167201576703	0.188	0.359	2.90211990841286E-07	7	BAG6
1.44744208469543E-15	0.313100465636777	0.155	0.342	2.4256234455326E-11	7	DIDO1
4.39002121216239E-13	0.311650590162075	0.146	0.305	7.35679754734173E-09	7	GPBP1L1
1.62071914785236E-17	0.309555803719511	0.142	0.333	2.71600114797098E-13	7	MAVS
5.56927945938616E-12	0.308298232025435	0.13	0.275	9.33299851803933E-08	7	CPSF7
4.45992213975224E-13	0.30465179684562	0.147	0.31	7.47393752179681E-09	7	COG3
1.16193991450807E-14	0.304183118640457	0.18	0.376	1.94717890873263E-10	7	FIS1
3.27166810199292E-13	0.303479747760485	0.139	0.3	5.48266140531973E-09	7	LTBR
3.70177164028704E-15	0.303025755970554	0.097	0.25	6.20342891479303E-11	7	ST3GAL6
1.91319763115296E-12	0.30265063083235	0.127	0.275	3.20613659028613E-08	7	KIF1B
4.93292073742162E-12	0.30240041704982	0.144	0.296	8.26658857177115E-08	7	SPG21
4.1294028930973E-15	0.300647744681819	0.156	0.339	6.92005336825245E-11	7	ITCH
7.75343316433655E-13	0.300171048997186	0.15	0.313	1.29932032967952E-08	7	RABAC1
1.20853663166953E-12	0.299998272534853	0.172	0.342	2.0252656873518E-08	7	CCNI
6.29992412490287E-09	0.297101377541577	0.244	0.424	0.000105574128485122	7	ILF3
4.74469882983646E-11	0.294217407014521	0.175	0.338	7.95116629903993E-07	7	TRIP12
1.88013802720529E-14	0.293068514441614	0.125	0.285	3.15073530599062E-10	7	HNRNPUL2
5.10857231830546E-13	0.290793863575964	0.119	0.267	8.56094549101628E-09	7	EXOC3
4.20910207348046E-15	0.290015765427689	0.122	0.288	7.05361325473855E-11	7	NSFL1C
5.17296402822931E-15	0.28930418898634	0.111	0.269	8.66885311850668E-11	7	LRRC47
1.0321537801106E-12	0.289069431545947	0.205	0.394	1.72968330470935E-08	7	SELENOH
1.6663364261863E-15	0.28837619810643	0.106	0.265	2.792446583003E-11	7	SNRPA
1.79401825404872E-13	0.287353813316026	0.185	0.369	3.00641579013484E-09	7	EMC4
4.6056248552069E-17	0.287054419544963	0.18	0.389	7.71810613235573E-13	7	HNRNPD
9.9938877167858E-17	0.286680948604701	0.108	0.275	1.67477570357896E-12	7	ZNF410
1.1291433384831E-12	0.281265014457475	0.18	0.358	1.89221840662998E-08	7	GNB2
1.34277695061894E-16	0.279902825111309	0.102	0.265	2.25022561384722E-12	7	WASL
7.64267393721614E-12	0.278200487928769	0.144	0.297	1.28075929839868E-07	7	DCTN2
6.36725749922178E-14	0.277728835927055	0.111	0.262	1.06702501171959E-09	7	RBM14
2.43315230667514E-14	0.275205891833793	0.146	0.318	4.0774766355262E-10	7	DDX46
6.86170111102973E-14	0.274679800663435	0.166	0.341	1.14988387218636E-09	7	ETNK1
1.07953508972311E-06	0.274632645952559	0.177	0.296	0.0180908490335799	7	GRK2
1.63355889807963E-11	0.273787124509318	0.178	0.348	2.73751800140184E-07	7	KAT6A
1.38500394802163E-13	0.272402116222881	0.124	0.278	2.32098961609464E-09	7	ARCN1
1.21545162940783E-12	0.27191506268934	0.135	0.285	2.03685384056164E-08	7	KPNA4
2.60236255123448E-14	0.27155027271245	0.133	0.296	4.36103916335874E-10	7	NUDCD3
1.3581352343738E-14	0.269178199185238	0.122	0.283	2.27596302576361E-10	7	CEP350
2.99155185156426E-09	0.268912832809265	0.172	0.315	5.01324259285139E-05	7	SHFL
6.01724331864416E-13	0.266081335687415	0.147	0.311	1.00836963533839E-08	7	ELOVL1
1.66023782215277E-09	0.26583772697237	0.142	0.275	2.78222654236362E-05	7	SNX3

6.82415841134092E-12	0.265269116678986	0.131	0.276	1.14359246657251E-07	7	SPEN
1.00839770615471E-19	0.264357867045716	0.11	0.299	1.68987287597406E-15	7	NFIA
4.90232863697807E-18	0.263271304833438	0.142	0.338	8.21532232984785E-14	7	RAB18
7.30960245483707E-17	0.262355363886945	0.117	0.29	1.2249431793816E-12	7	TTC37
1.15520562712797E-14	0.261763062709195	0.15	0.326	1.93589358994105E-10	7	SND1
7.76329808888942E-15	0.261533854964918	0.138	0.309	1.30097349373609E-10	7	ZMYM2
3.06045696590037E-18	0.259805494829606	0.105	0.277	5.12871378345584E-14	7	TPP2
5.36984062886332E-14	0.258006865447991	0.11	0.259	8.99877892584915E-10	7	SCAF1
4.06426189159777E-14	0.257955410185034	0.172	0.356	6.81089007793955E-10	7	SDCBP
4.94413296917096E-13	0.257673547263987	0.11	0.252	8.28537802973669E-09	7	HOOK3
2.205777991317E-14	0.25606920990578	0.133	0.298	3.69644275784903E-10	7	SEC11A
2.77897951487766E-11	0.254837158900776	0.122	0.259	4.65701387103199E-07	7	SH3BGRL2
1.3355616870214E-07	0.25185555687905	0.815	0.894	0.00223813427511047	7	B2M
3.13559670471214E-10	0.25069977454248	0.327	0.559	5.2546329577566E-06	7	ACADVL
5.18350044841536E-20	0.250277202518182	0.122	0.318	8.68651005145445E-16	7	LRRFIP2
1.0297518901821E-15	0.250093473289636	0.13	0.3	1.72565821756715E-11	7	SMARCA5
0	9.66090580899585	0.608	0.002	0	8	CRISPLD2
0	9.42085672070574	0.746	0.005	0	8	THBS2
0	9.39063076609149	0.333	0.001	0	8	MXRA8
0	9.37819596373013	0.768	0.006	0	8	COL1A2
0	9.36721874171988	0.847	0.01	0	8	COL3A1
0	9.32925473883773	0.331	0.001	0	8	KCNE4
0	9.32370329522255	0.256	0.001	0	8	HEYL
0	9.30478811766426	0.854	0.012	0	8	COL1A1
0	9.29829773354603	0.653	0.005	0	8	COL6A3
0	9.00221133660854	0.395	0.001	0	8	LAMC3
0	8.78492837218496	0.383	0.002	0	8	NFASC
0	8.76080662214482	0.424	0.002	0	8	PODN
0	8.71743627899546	0.334	0.004	0	8	SVEP1
0	8.5742244982201	0.251	0.005	0	8	PRKG1
0	8.5545380277594	0.399	0.002	0	8	PRELP
0	8.53747351031487	0.294	0.001	0	8	LOXL1
0	8.40190465572317	0.632	0.007	0	8	EFEMP1
0	8.36783208215505	0.744	0.008	0	8	AEBP1
0	8.29128905911517	0.37	0.008	0	8	COL14A1
0	8.24413322302035	0.32	0.001	0	8	HAND2
0	8.19776976379543	0.339	0.003	0	8	CCDC80
0	8.19442164469479	0.764	0.013	0	8	DCN
0	8.1923698766252	0.302	0.002	0	8	ID4
0	8.16362896357381	0.268	0.001	0	8	SMOC2
0	8.14815077157653	0.476	0.006	0	8	LUM
0	8.06359392654129	0.439	0.002	0	8	MFAP4
0	7.96390759146917	0.564	0.007	0	8	GGT5
0	7.95664207930596	0.392	0.003	0	8	FBLN5
0	7.91435023399753	0.622	0.007	0	8	EMILIN1
0	7.87968265002242	0.831	0.015	0	8	BGN
0	7.84050097658009	0.394	0.003	0	8	ELN
0	7.80014587826865	0.354	0.003	0	8	DKK 3.00
0	7.7831388961276	0.701	0.012	0	8	COLEC11
0	7.73959039203556	0.605	0.009	0	8	COL5A1
0	7.63324584541407	0.27	0.004	0	8	ITGBL1
0	7.58055205022792	0.899	0.088	0	8	COL6A1
0	7.5477410251643	0.347	0.004	0	8	ANGPTL2
0	7.54038144328288	0.285	0.002	0	8	PTPRS
0	7.53934137141135	0.564	0.008	0	8	C11orf96
0	7.485269789616	0.902	0.021	0	8	COL6A2
0	7.42648741191736	0.413	0.009	0	8	COL5A2
0	7.38271283318156	0.537	0.012	0	8	FBN1
0	7.30573202899793	0.469	0.008	0	8	THY1
0	7.2788085189049	0.492	0.006	0	8	ADAMTS2

0	7.203196044645	0.458	0.027	0	8	ACTA2
0	7.10336079629164	0.556	0.046	0	8	PDGFRB
0	7.0006906185002	0.404	0.007	0	8	ADAMTS1
0	6.98359699837496	0.778	0.041	0	8	TAGLN
0	6.96600234254014	0.384	0.006	0	8	CCL21
0	6.95554051441188	0.265	0.008	0	8	SOD3
0	6.93031877368581	0.564	0.014	0	8	MYL9
0	6.92372164499955	0.686	0.024	0	8	CXCL12
0	6.90799973334792	0.519	0.024	0	8	ADAMTSL2
0	6.85568592896633	0.265	0.009	0	8	COLEC10
0	6.78851557921065	0.574	0.014	0	8	C7
0	6.76401707479794	0.489	0.039	0	8	GEM
0	6.73541369404909	0.272	0.01	0	8	HGF
0	6.68403043104154	0.551	0.027	0	8	LTBP4
0	6.61858986120222	0.503	0.011	0	8	LRRC32
0	6.59080854701691	0.957	0.039	0	8	IGFBP7
0	6.5161642213515	0.302	0.003	0	8	FBLN2
0	6.47896216140747	0.426	0.011	0	8	C1QTNF1
0	6.38165038972504	0.322	0.005	0	8	ADGRA2
0	6.37036631936471	0.323	0.007	0	8	EHD2
0	6.30723427499454	0.352	0.023	0	8	LOXL2
0	6.30091217396888	0.405	0.009	0	8	PTGDS
0	6.29444311173747	0.272	0.004	0	8	FKBP10
0	6.26955930629356	0.58	0.044	0	8	THBS1
8.94377251629436E-266	6.1679297776317	0.334	0.043	1.49879739828061E-261	8	COL16A1
0	6.14701153983018	0.407	0.009	0	8	TNXB
0	6.10817957301731	0.309	0.025	0	8	IFITM10
0	6.06151991759895	0.357	0.04	0	8	CCN1
0	5.95381455536347	0.383	0.014	0	8	MMP2
1.74129683961671E-165	5.93452704235568	0.273	0.044	2.91806524382968E-161	8	ADAMTS13
0	5.92922702739123	0.397	0.02	0	8	MEGF6
0	5.8671813181317	0.32	0.01	0	8	PHLDB1
4.59061487792278E-291	5.84904246219355	0.395	0.056	7.69295241242299E-287	8	LTBP2
0	5.67267712824267	0.375	0.009	0	8	INMT
0	5.59105413233194	0.876	0.037	0	8	SPARC
1.21631851793224E-186	5.58717521393567	0.307	0.05	2.03830657235085E-182	8	CCN2
0	5.55508032959692	0.759	0.026	0	8	COL4A2
0	5.51077902108327	0.625	0.116	0	8	FSTL1
0	5.43802716483218	0.785	0.028	0	8	COL4A1
0	5.41625844963932	0.704	0.125	0	8	TIMP1
0	5.37155133720299	0.375	0.023	0	8	ITGA9
0	5.30613070585951	0.315	0.013	0	8	TINAGL1
0	5.30565387351743	0.281	0.012	0	8	MRC2
0	5.26831307162551	0.423	0.03	0	8	CRIM1
0	5.23496353966371	0.268	0.018	0	8	TPM2
0	5.2174778340191	0.318	0.008	0	8	PXDN
2.74744306114191E-119	5.20826204019049	0.262	0.054	4.60416508186161E-115	8	FGFR2
4.50806452565314E-149	4.96001842297746	0.254	0.041	7.55461453208953E-145	8	ARVCF
4.13118917468568E-179	4.94579191411308	0.309	0.052	6.92304681893826E-175	8	JAG1
5.79989755245841E-122	4.91163981274797	0.277	0.059	9.71946831840981E-118	8	LMCD1
3.75600626130205E-135	4.75236849530679	0.352	0.087	6.29431529268998E-131	8	MYH10
0	4.73417284852512	0.54	0.091	0	8	TIMP3
0	4.72536668394742	0.317	0.012	0	8	LIFR
0	4.65070564778169	0.28	0.02	0	8	OSMR
0	4.62427959707588	0.293	0.016	0	8	FXYD6
4.46249829028506E-118	4.58317088002613	0.297	0.069	7.47825463485971E-114	8	KALRN
7.62789808496006E-161	4.5101947652249	0.273	0.045	1.27828316107761E-156	8	CYBRD1
3.2313743310184E-110	4.47456505557071	0.54	0.255	5.41513710392063E-106	8	IGFBP3
3.63911079083037E-103	4.47360081696195	0.333	0.096	6.09842186327354E-99	8	FBLN1
1.31653983557092E-252	4.4734807911909	0.301	0.035	2.20625745644975E-248	8	GRK5

6.29859190464965E-242	4.45101522965769	0.257	0.026	1.05551803138119E-237	8	RBP1
0	4.29062782194843	0.265	0.017	0	8	SMAD6
8.26279466250696E-148	4.26822189619896	0.262	0.044	1.38467912954292E-143	8	SLCO3A1
5.71835390262686E-83	4.25135410652107	0.288	0.085	9.5828174700221E-79	8	CCDC3
1.11505182825495E-286	4.11354050089232	0.362	0.045	1.86860385378964E-282	8	PCOLCE
8.59478580514006E-209	4.0682133745536	0.26	0.031	1.44031420522537E-204	8	PMP22
0	4.03291393644051	0.674	0.067	0	8	ENG
0	4.02845522406132	0.365	0.041	0	8	LAMB2
0	3.93195056967912	0.548	0.066	0	8	TIMP2
8.41533604263051E-121	3.91569608298939	0.309	0.072	1.41024201402402E-116	8	CAVIN1
1.43993418725242E-83	3.86306418770061	0.27	0.074	2.4130417109976E-79	8	CHSY1
1.87810909070474E-188	3.85446646678466	0.423	0.093	3.14733521420301E-184	8	ID3
1.81544752153733E-52	3.83973322027504	0.267	0.101	3.04232695659225E-48	8	GATA6
1.30664511344043E-98	3.83550052093421	0.408	0.15	2.18967588110348E-94	8	PRSS23
7.00124691332384E-149	3.7864983946396	0.376	0.09	1.17326895773481E-144	8	RCN3
9.1072473151382E-126	3.7406410297366	0.339	0.084	1.52619250507086E-121	8	TNS1
1.24970428422365E-54	3.73327578715305	0.262	0.094	2.09425443950199E-50	8	UACA
1.12150769737781E-79	3.71572737313151	0.408	0.174	1.87942259926573E-75	8	C1orf198
3.9928013809875E-85	3.62884883360063	0.395	0.156	6.69113655425885E-81	8	ITGA1
3.09129293810623E-119	3.60856955051325	0.363	0.1	5.18038870567842E-115	8	MASP1
4.59398156618054E-78	3.50314988576164	0.402	0.17	7.69859430860535E-74	8	NR2F2
8.6140912411402E-36	3.43191736962054	0.277	0.134	1.44354941019027E-31	8	TANC1
2.20386959036169E-75	3.4278431620178	0.376	0.152	3.69324465952812E-71	8	MXRA7
3.0607815454104E-79	3.38196759516677	0.497	0.266	5.12925771379874E-75	8	MICALL2
3.57313908894004E-114	3.33383643683376	0.461	0.169	5.98786648524572E-110	8	ITM2C
1.91707167267183E-49	3.31118494377832	0.355	0.178	3.21262870906345E-45	8	LAMB1
2.75370973130143E-107	3.30012768744887	0.342	0.095	4.61466676771493E-103	8	CD81
2.86223231829719E-72	3.26435618937949	0.268	0.08	4.79652891900243E-68	8	SMAD7
1.40767442213646E-122	3.26419756922327	0.6	0.289	2.35898079661628E-118	8	DEPP1
0	3.21134351316356	0.728	0.176	0	8	VIM
9.98595828025305E-39	3.16908942839371	0.395	0.247	1.67344688860481E-34	8	QSOX1
2.3109570466167E-84	3.1601242208017	0.281	0.078	3.87270181872027E-80	8	SLC12A4
3.52478599794262E-65	3.15786013429613	0.27	0.087	5.90683637535225E-61	8	PHLDA1
1.13969897653037E-103	3.13878930799978	0.278	0.064	1.90990754486959E-99	8	NR4A1
5.21440834800839E-41	3.08647063571051	0.256	0.106	8.73830550959246E-37	8	LZTS2
1.69582897306012E-175	3.06655728023766	0.402	0.085	2.84187019305415E-171	8	GSN
2.83617410369278E-107	3.06462868651145	0.342	0.094	4.75286056296837E-103	8	IER5L
4.66097573817886E-79	2.99391334890596	0.254	0.066	7.81086314204014E-75	8	TMEM173
2.50749969696423E-73	2.91195321734808	0.526	0.317	4.20206799217266E-69	8	NID1
2.124227885067E-64	2.86093497663249	0.371	0.16	3.55978108979527E-60	8	FLNA
8.80475715083613E-59	2.85509969336305	0.447	0.246	1.47550120333712E-54	8	CSRP1
1.40780501548462E-146	2.84262811875744	0.732	0.464	2.35919964494912E-142	8	CALD1
2.42529092056174E-32	2.75029505774267	0.371	0.235	4.06430252467737E-28	8	MPRIP
2.52644429022321E-137	2.74884853457634	0.526	0.188	4.23381534155606E-133	8	SERPINH1
9.90887751133473E-63	2.67545991331468	0.26	0.082	1.66052969334947E-58	8	PALM2-AKAP2
3.41703864466708E-21	2.65561041277167	0.342	0.248	5.72627336073309E-17	8	MYLK
3.46666848270354E-60	2.64605006321416	0.559	0.4	5.80944304331459E-56	8	GPX3
3.74415722316466E-29	2.60529995011973	0.355	0.224	6.27445867457933E-25	8	RBPMS
4.71984288648634E-39	2.60088280665629	0.262	0.111	7.9095127091738E-35	8	IER3
1.36344966603206E-69	2.59235513795125	0.424	0.194	2.28486895033653E-65	8	LGALS1
1.59621598476394E-18	2.57753484874015	0.33	0.238	2.67493874726741E-14	8	ITGB5
2.01087020036346E-71	2.56901978628803	0.548	0.348	3.36981628176908E-67	8	LGALS3BP
1.53714070888812E-30	2.55401474098837	0.278	0.142	2.57594039995471E-26	8	SPON2
4.565935269967E-189	2.52523389634519	0.828	0.478	7.65159432541069E-185	8	C1R
1.98933606898288E-36	2.49916768154756	0.434	0.295	3.33372938440151E-32	8	NRP1
1.02423529649226E-45	2.49398300346231	0.264	0.102	1.71641350986173E-41	8	SELENOM
1.35385909568208E-08	2.48852403350616	0.254	0.203	0.000226879707254403	8	NR2F1
5.26658320795255E-19	2.46301560967493	0.325	0.233	8.82574013988688E-15	8	ITGAV
6.27095208113286E-53	2.41126545948057	0.254	0.087	1.05088614975624E-48	8	SH3BP5
1.35734534621358E-24	2.4049744499639	0.302	0.175	2.27463933118472E-20	8	SERPINE1

1.42555026093331E-10	2.38690074096994	0.259	0.195	2.38893712727204E-06	8	PELO
5.14516659316012E-35	2.37850675232921	0.415	0.278	8.62227017681772E-31	8	DPYSL2
9.07897013383582E-34	2.35008327508757	0.281	0.138	1.52145381502821E-29	8	HSPG2
9.11744018946434E-09	2.3318358356715	0.272	0.22	0.000152790062695043	8	PPP1R12B
4.14087188230342E-85	2.31639117956377	0.264	0.066	6.93927310036407E-81	8	METRNL
3.62779332121212E-33	2.25579621172501	0.293	0.146	6.07945604768727E-29	8	PLXND1
3.52003533487248E-08	2.21443015909233	0.257	0.21	0.00058988752141793	8	LAMC1
7.66488706618419E-54	2.19936168864013	0.574	0.456	1.28448177455115E-49	8	TPM1
1.01925404439382E-26	2.1572062461056	0.371	0.248	1.70806592759517E-22	8	LTBP3
3.82974076895334E-35	2.1548349353369	0.514	0.434	6.41787958061201E-31	8	EPAS1
1.42125699444949E-13	2.1421124752274	0.339	0.276	2.38174247129845E-09	8	MMP14
5.45410744919265E-08	2.14170627205389	0.317	0.285	0.000913999326335704	8	PKDCC
5.8694451136786E-08	2.0754107762495	0.315	0.286	0.00098360161215026	8	EDNRB
1.15173131433634E-11	2.05127328513101	0.376	0.344	1.93007133656485E-07	8	SLC38A2
3.80093023092463E-21	2.0000113897092	0.447	0.392	6.3695988809835E-17	8	ACTN1
7.64027385527875E-28	1.99962001613898	0.457	0.356	1.28035709266761E-23	8	LRP1
5.40365993187698E-77	1.97752371714145	0.524	0.231	9.05545331383944E-73	8	COL18A1
1.08642955233722E-108	1.96679722093091	0.767	0.565	1.82063864380672E-104	8	C1S
1.24972108344468E-28	1.96117906382725	0.495	0.417	2.09428259163659E-24	8	SERPINF1
1.01944951511703E-23	1.95337511753578	0.379	0.263	1.70839349743311E-19	8	MARCKS
1.85238878798582E-31	1.87084153474276	0.264	0.122	3.10423313090664E-27	8	ANXA6
1.07283232872385E-25	1.74719118650441	0.278	0.145	1.79785241647543E-21	8	ICAM1
1.05094293448407E-06	1.73602117210049	0.323	0.301	0.017611701696084	8	ATN1
8.74881453070549E-63	1.71531804234954	0.686	0.624	1.46612633905563E-58	8	IGFBP4
3.1976642737447E-18	1.64635257815007	0.465	0.416	5.35864578994137E-14	8	CEBPB
9.58172661406772E-61	1.62288855239304	0.621	0.423	1.60570574598547E-56	8	JUNB
8.26355692549454E-40	1.5167316397212	0.26	0.1	1.38480686957438E-35	8	LSP1
1.28473080775614E-06	1.37269223638845	0.342	0.324	0.0215295188763774	8	WSB1
1.05015967733561E-08	1.33626379526806	0.449	0.473	0.000175985758727902	8	PLXNB2
2.85731332950953E-13	1.29010317142679	0.508	0.526	4.78828567759207E-09	8	APP
4.67386979556143E-25	1.26432204714162	0.54	0.444	7.83247100340185E-21	8	BHLHE40
9.71612417441957E-10	1.21378659159008	0.267	0.192	1.62822808914923E-05	8	MT2A
4.27580331056772E-28	1.18295942300845	0.664	0.656	7.16539118784938E-24	8	RHOB
6.61401266764019E-17	0.975180909839322	0.445	0.332	1.10837624284314E-12	8	NNMT
2.26531377254972E-06	0.916286346615034	0.14	0.25	0.0379621282003882	8	GPC6
5.13275915571623E-07	0.841967216808548	0.174	0.308	0.00860147779314925	8	SAMD5
7.12614172139864E-10	0.814549462568698	0.117	0.25	1.19419882967198E-05	8	NCKIPSD
9.25555009816722E-08	0.807796298986122	0.135	0.253	0.00155104508545086	8	BCL2L1
8.49636848721724E-08	0.779543558778498	0.158	0.288	0.00142382143108787	8	NIBAN2
9.08950539473704E-08	0.767321882713417	0.185	0.334	0.00152321931405003	8	MTUS1
2.22766381460649E-12	0.759783995365535	0.621	0.693	3.73311902051755E-08	8	EEF2
2.09989561194937E-06	0.737609146368573	0.17	0.293	0.0351900506650475	8	PLOD2
9.66873122481872E-09	0.724270845400265	0.175	0.326	0.000162028597865512	8	PAPSS2
1.4420987571355E-06	0.719873212266618	0.148	0.262	0.0241666909720767	8	NFIX
2.16416217881502E-06	0.710653941188426	0.158	0.275	0.0362670297925822	8	TXNDC15
1.22355046555659E-08	0.687601955073664	0.13	0.254	0.000205042587017974	8	GPR107
1.11217129377451E-08	0.684789209836664	0.129	0.252	0.000186377665410732	8	ATF4
5.41987887727941E-08	0.677100735796427	0.159	0.291	0.000908263302254484	8	VAT1
4.87972759710613E-08	0.673071012926431	0.146	0.273	0.000817744750723045	8	TRIOBP
4.95165455768799E-08	0.657661429004259	0.166	0.304	0.000829798270777353	8	LRRC8A
6.47027482671193E-09	0.654911505987499	0.129	0.257	0.000108428865546039	8	NCKAP1
9.95002650178318E-13	0.647628105827755	0.641	0.718	1.66742544116883E-08	8	TMSB4X
8.28138763900494E-11	0.632577351091339	0.135	0.284	1.38779494054445E-06	8	CPSF6
1.89816782273259E-08	0.630759729976312	0.159	0.297	0.000318094963733527	8	FBXL5
1.18625774950611E-09	0.626890032819085	0.137	0.276	1.98793073662234E-05	8	DYNC1I2
1.25734865642126E-06	0.62667591823567	0.156	0.274	0.0210706487843075	8	ISCU
2.40742520218297E-08	0.625900743838869	0.233	0.409	0.000403436315381823	8	SREBF1
2.09908738396534E-06	0.621159509743462	0.191	0.325	0.0351765063804911	8	TBC1D2B
3.78760125335465E-09	0.614569368187368	0.14	0.276	6.34726218037172E-05	8	TRIO
2.47379946002865E-07	0.613366620563025	0.24	0.411	0.004145593135116	8	CALCOCO2

1.13118460958094E-13	0.607838559382461	0.156	0.34	1.89563916873575E-09	8	KANK1
2.06628754546178E-10	0.604751557732585	0.148	0.298	3.46268466868484E-06	8	NFIA
3.86213919840982E-08	0.602481496110031	0.164	0.303	0.000647217286869517	8	RAB21
1.01237832600682E-06	0.59787069751423	0.212	0.358	0.0169654359872223	8	AAMP
2.77075867753309E-10	0.589567235215588	0.137	0.28	4.64323739180995E-06	8	EGLN2
1.7485941322282E-09	0.584304380180643	0.183	0.343	2.93029404678801E-05	8	TMED9
1.15494081817715E-06	0.578891393538797	0.159	0.279	0.0193544982310127	8	SELENOS
2.89117334916682E-09	0.57191838565451	0.146	0.285	4.84502829853376E-05	8	TNRC6A
2.14342687922572E-08	0.571003758602063	0.132	0.254	0.000359195476420646	8	RUNX1
3.75184700774035E-08	0.562136840554062	0.177	0.321	0.000628734521557127	8	UBR4
6.91416101603599E-16	0.557695945533269	0.096	0.261	1.15867510306731E-11	8	ARHGEF40
1.04981073190222E-11	0.554556975037594	0.113	0.255	1.75927282452174E-07	8	GINM1
1.70360615531749E-09	0.554389084555045	0.13	0.263	2.85490319508105E-05	8	KDSR
9.07284514595748E-09	0.552998519526777	0.175	0.323	0.000152042738955956	8	UBXN6
7.58033223553524E-10	0.548003990736609	0.135	0.271	1.270312076031E-05	8	ERF
1.56622712000885E-16	0.545286736129946	0.116	0.292	2.62468340771083E-12	8	TOMM20
4.70102140579751E-08	0.542020039861846	0.235	0.403	0.000787797167183547	8	KDM2A
4.5342314967592E-12	0.539752754062043	0.125	0.276	7.59846514226907E-08	8	PTK2
2.47453579159261E-06	0.535940352112507	0.264	0.435	0.041468270795509	8	PRKAR1A
6.70959978554204E-12	0.534452193781437	0.113	0.254	1.12439473206114E-07	8	TCAF1
1.79712374400185E-07	0.533723473388705	0.156	0.281	0.0030116199701983	8	CAV2
2.81527239541071E-08	0.529746144455392	0.191	0.344	0.000471783348022927	8	ASXL1
1.23112997773782E-08	0.528307848376076	0.129	0.25	0.000206312761669304	8	NR3C1
2.67554608253919E-07	0.522437493262985	0.206	0.353	0.00448368012511918	8	CD59
5.94086304559125E-09	0.519587460399714	0.159	0.301	9.95569829180181E-05	8	PLSCR1
1.40373022149829E-08	0.519244252902812	0.238	0.42	0.000235237110518683	8	IRF2BP2
1.73088005313597E-10	0.515679483115007	0.183	0.351	2.90060879304525E-06	8	PIM3
3.13728529114143E-11	0.51275463441387	0.135	0.283	5.25746269089481E-07	8	GAA
4.60778392647576E-07	0.50698944715919	0.183	0.318	0.00772172430398807	8	ZBTB20
1.97086323392399E-11	0.496689976336541	0.122	0.267	3.30277260740982E-07	8	MED25
1.02126629408525E-09	0.486389261122564	0.188	0.352	1.71143805562807E-05	8	PWWP3A
2.22097300375234E-16	0.483222654345012	0.178	0.391	3.72190655968818E-12	8	UBAP2L
6.69050356077319E-07	0.477066014966192	0.182	0.312	0.0112119458671437	8	RABAC1
3.57977961524475E-09	0.474310871593296	0.183	0.341	5.99899467922715E-05	8	BRD4
3.82092736407929E-12	0.47425061320366	0.138	0.295	6.40311007672408E-08	8	AP2A1
2.37988410132223E-08	0.473671726817552	0.186	0.336	0.000398820977699579	8	RAB1A
2.62615114849421E-09	0.471739939041541	0.141	0.277	4.40090409464659E-05	8	RAPH1
7.94747611135205E-14	0.471125592519248	0.132	0.302	1.33183804674038E-09	8	TTC3
2.19923334354993E-08	0.467654098405341	0.182	0.328	0.000368547523712098	8	TMEM50A
2.60215217977603E-07	0.460857502744853	0.151	0.271	0.00436068662286867	8	CYTH2
1.24119646495942E-12	0.458990651212263	0.122	0.275	2.079997035979E-08	8	LRCH3
1.01917769842651E-12	0.458453891075199	0.145	0.312	1.70793798702314E-08	8	SENP6
4.1565161341154E-12	0.458087649877547	0.124	0.271	6.96548973755059E-08	8	RAB6A
1.43050534661661E-11	0.45755496730011	0.125	0.272	2.39724085986011E-07	8	SUN1
1.16301840881965E-09	0.456971099167214	0.235	0.422	1.94898624949997E-05	8	LRPAP1
1.29165615628433E-07	0.453802405418545	0.257	0.438	0.00216455738670127	8	SHC1
2.1021734699109E-11	0.453717788313577	0.154	0.319	3.52282230087668E-07	8	TIA1
2.04203365996744E-09	0.451156162679832	0.132	0.263	3.42204000737344E-05	8	PITPNB
2.38208932731815E-12	0.447779353225579	0.169	0.347	3.99190529471975E-08	8	C16orf58
2.82197503166849E-11	0.447466670937637	0.185	0.362	4.72906575807005E-07	8	CPNE1
1.40514514206649E-07	0.447118790834543	0.156	0.279	0.00235474222907502	8	MANF
7.00830866794291E-07	0.443997199914147	0.204	0.342	0.0117445236657387	8	APOL6
4.0942108889547E-07	0.44167376418813	0.297	0.494	0.00686107860771028	8	MAT2A
3.54099194667298E-08	0.438561824604615	0.204	0.358	0.000593399430423457	8	NDST1
1.25661132815127E-10	0.435688228391359	0.133	0.278	2.10582926371591E-06	8	SIK2
1.24274113005684E-08	0.43522460177681	0.198	0.362	0.000208258558574925	8	ADD1
9.55216371799515E-11	0.431493239634227	0.129	0.268	1.60075159586163E-06	8	IRF2
8.47399133625808E-08	0.431454539232052	0.161	0.291	0.00142007146813013	8	PPP1CB
6.15871822726414E-08	0.428705045343098	0.14	0.26	0.00103207800052492	8	KCTD20
1.68021685069975E-15	0.427205135677309	0.148	0.337	2.81570739840264E-11	8	SLC35F5

1.37155257785415E-08	0.426014974628133	0.135	0.258	0.000229844780996798	8	FADS2
4.70794008049892E-15	0.42428643809814	0.188	0.401	7.8895659869001E-11	8	LMAN1
2.50452354045353E-11	0.422788232946522	0.143	0.297	4.19708054909202E-07	8	DCTN2
3.29810198817959E-07	0.422136476571109	0.186	0.322	0.00552695931179136	8	MAX
1.4684859185389E-09	0.420563319835818	0.13	0.263	2.46088870228748E-05	8	STIM1
9.46081240441546E-09	0.419758221063349	0.138	0.267	0.000158544294273194	8	NME3
3.00502895911216E-10	0.418554291049535	0.174	0.339	5.03582752968016E-06	8	DYNC1LI2
4.90327147478837E-08	0.413467066940376	0.138	0.259	0.000821690233745036	8	CLCN7
2.89550355164623E-08	0.412953125415483	0.175	0.315	0.000485228485184876	8	ARHGDIA
5.03375167631038E-12	0.412332099749813	0.132	0.284	8.43556105916093E-08	8	NEK7
4.82895295115685E-13	0.410978810466054	0.143	0.309	8.09235935554865E-09	8	CREBZF
2.33361087722486E-12	0.40864119351836	0.116	0.263	3.91066510805342E-08	8	PCMTD2
8.77666223128193E-10	0.405222843883688	0.161	0.311	1.47079305671823E-05	8	MARCH7
1.16396581215776E-10	0.398784822908119	0.162	0.319	1.95057390801398E-06	8	TOMM7
4.5796059886002E-16	0.398018833559301	0.151	0.346	7.67450371569621E-12	8	MATN2
8.0941499447863E-10	0.396286441889421	0.137	0.274	1.35641764774729E-05	8	DNAJC7
1.06079826654882E-15	0.388225319634733	0.096	0.255	1.77768573508252E-11	8	UBA2
1.86005839043252E-07	0.387325849315437	0.145	0.261	0.00311708585068681	8	MXD4
3.08373316145947E-11	0.378853196854399	0.121	0.262	5.16772003197379E-07	8	TPD52L2
7.15215657184576E-09	0.377245873325001	0.18	0.331	0.000119855839830991	8	BSDC1
7.97361249233924E-15	0.375958134552694	0.166	0.365	1.33621798146621E-10	8	EID1
1.93971431964184E-10	0.375093745177977	0.24	0.429	3.25057325685579E-06	8	SCARB2
2.44288422697913E-11	0.373893897556019	0.164	0.327	4.09378538757162E-07	8	SRRM2
2.03534613794792E-11	0.372081011074953	0.116	0.254	3.41083305797313E-07	8	TXLNA
1.19881477717103E-09	0.371826260974323	0.153	0.295	2.00897380358321E-05	8	KDM5C
5.32861109083708E-12	0.370655124773815	0.241	0.455	8.92968646602479E-08	8	NFIC
1.92408805289485E-11	0.370500495063449	0.124	0.267	3.22438675904119E-07	8	GALNT2
3.9369276260469E-07	0.370025517851544	0.206	0.348	0.00659750331572939	8	VKORC1
2.73828301213606E-13	0.366088662022954	0.141	0.311	4.5888146717376E-09	8	ZNF638
2.09743162727678E-12	0.36451075731479	0.166	0.344	3.51487592099042E-08	8	UBR2
1.46792057584909E-09	0.364363770405929	0.132	0.262	2.4599413010079E-05	8	TSC22D4
4.5829386505059E-13	0.363678787759458	0.129	0.288	7.68008859051778E-09	8	IST1
4.28792814952423E-17	0.36194391574666	0.133	0.325	7.1857099929727E-13	8	GNPTG
2.33719972742298E-12	0.360369946225523	0.137	0.296	3.91667930321542E-08	8	THRAP3
8.33413302570807E-17	0.357545961027219	0.113	0.291	1.39663401244816E-12	8	HMGN3
8.49795580622622E-07	0.355740192168466	0.211	0.349	0.0142408743400739	8	EHBP1L1
3.29734966237802E-16	0.355393973860901	0.178	0.387	5.52569856421308E-12	8	YIPF3
3.21157963618989E-10	0.353999631946659	0.146	0.292	5.38196515432702E-06	8	TAOK1
1.91636498407887E-11	0.352282524753512	0.116	0.254	3.21144444031937E-07	8	SMAD2
3.67263579362513E-07	0.350986054338792	0.342	0.544	0.00615460306295699	8	VEGFA
1.75010149496212E-07	0.34705031613337	0.198	0.342	0.00293282008525752	8	HNRNPH3
1.86999723365202E-08	0.345257969677768	0.193	0.344	0.000313374136415406	8	ESYT2
9.37345439060778E-14	0.340696194317623	0.158	0.338	1.57080348677805E-09	8	RAB18
3.51857203608876E-11	0.340054908507917	0.198	0.377	5.89642301807754E-07	8	CPEB4
1.81077643455531E-11	0.33813502017555	0.148	0.304	3.03449914902779E-07	8	PLEC
8.67946218654563E-11	0.33554705933306	0.178	0.343	1.45450427322132E-06	8	SEC22B
2.38075909495564E-08	0.327425147838792	0.153	0.284	0.000398967609132667	8	YPEL5
2.51979314961593E-09	0.324808633538449	0.196	0.358	4.22266936012637E-05	8	DPP7
8.04538201331172E-17	0.323915777364337	0.092	0.257	1.34824511779078E-12	8	CAMK2D
6.12528223339798E-14	0.320901614611742	0.22	0.438	1.02647479667283E-09	8	PDIA3
5.87529267399024E-13	0.315045395326941	0.158	0.333	9.84581546307284E-09	8	ATP2A2
1.45431433427832E-13	0.314564260371216	0.117	0.275	2.4371399613836E-09	8	FUS
5.73884660384038E-17	0.314342785653984	0.159	0.363	9.61715913871571E-13	8	C4orf3
3.86954332168052E-13	0.312587915269381	0.124	0.281	6.48458069847221E-09	8	MED28
3.45576978294819E-11	0.312030021918342	0.132	0.275	5.79117900226458E-07	8	CRELD2
2.30573771736655E-13	0.311211689171163	0.127	0.285	3.86395526676286E-09	8	CDIPT
5.00133972011289E-12	0.309869048196176	0.122	0.269	8.38124510296518E-08	8	RC3H2
3.60697136365964E-09	0.307112370132026	0.127	0.251	6.04456261122083E-05	8	KDM4B
1.39749923842136E-13	0.306593525437731	0.153	0.328	2.34192922374652E-09	8	SNRNP70
1.16466694995437E-06	0.306027193645919	0.15	0.258	0.0195174887473354	8	SLC25A25

2.4247512602896E-12	0.30454486786325	0.138	0.297	4.06339816199332E-08	8	UBE2Z
4.85232198108604E-08	0.303411115237714	0.133	0.251	0.000813152117590399	8	NAGK
2.45219085503144E-15	0.302902591112828	0.093	0.25	4.10938143486169E-11	8	ABHD16A
1.19676662754353E-09	0.302749628458192	0.125	0.253	2.00554151443744E-05	8	MYO6
9.60907519092179E-13	0.301058463445648	0.117	0.266	1.61028882049467E-08	8	SEC16A
2.68164210183231E-10	0.292080886106956	0.153	0.302	4.49389583425059E-06	8	TXNDC11
5.89248103734131E-14	0.2874585248007	0.124	0.286	9.87461972237657E-10	8	RBM25
3.42907302079574E-12	0.284763991343457	0.114	0.26	5.74644056824949E-08	8	DCAF5
1.53741424249793E-11	0.283149751269483	0.241	0.448	2.57639878757803E-07	8	CTNND1
1.49941270646674E-10	0.278082341428242	0.14	0.283	2.51271581349696E-06	8	YWHAG
2.98338017238499E-14	0.277160497857386	0.167	0.353	4.99954849288277E-10	8	HDLBP
3.41210491966541E-15	0.275302175085001	0.1	0.258	5.7180054243753E-11	8	LIN7C
1.34320307632235E-14	0.275066821309453	0.119	0.282	2.25093971530099E-10	8	HECTD4
7.52468320144583E-16	0.271017572551956	0.169	0.369	1.26098641089829E-11	8	HSF1
7.00936709120665E-08	0.27000572206483	0.318	0.531	0.00117462973714441	8	SDC4
6.60763274019853E-10	0.26940320780363	0.156	0.3	1.10730709460247E-05	8	SUN2
4.75089769916686E-10	0.268721592492618	0.172	0.327	7.96155436426383E-06	8	RNF10
2.45424198780718E-12	0.26851084704557	0.153	0.319	4.11281872316728E-08	8	FAM3C
3.63236690085871E-10	0.267815412810821	0.191	0.357	6.08712045245903E-06	8	RSRC2
8.87840663516589E-13	0.267305430942235	0.161	0.333	1.4878433839211E-08	8	INTS11
3.85062591933575E-13	0.266906320236459	0.117	0.271	6.45287891562286E-09	8	NIPBL
2.27657642433723E-16	0.266183928396202	0.117	0.296	3.81508677190434E-12	8	EMC7
2.05816484309047E-13	0.266122294353853	0.121	0.278	3.44907264405101E-09	8	ARCN1
1.38355987558328E-14	0.265555229378452	0.122	0.293	2.31856963950247E-10	8	FOXO3
8.90449354648186E-14	0.265305370498713	0.114	0.271	1.49221502851943E-09	8	ZC3H15
1.59913663798006E-10	0.263965470984767	0.174	0.335	2.67983317792699E-06	8	ADAM9
1.12421403336229E-15	0.262405672681542	0.162	0.359	1.88395787710852E-11	8	NUFIP2
1.78516086418953E-13	0.261701033015744	0.217	0.424	2.99157257620882E-09	8	CTNNA1
3.80760891110046E-12	0.261462373158852	0.169	0.339	6.38079101322215E-08	8	RAB7A
3.58390742551529E-09	0.261458262119483	0.135	0.265	6.00591206367853E-05	8	TAF1D
1.10259929246088E-13	0.259754282019911	0.22	0.433	1.84773589430595E-09	8	SEC31A
6.04416870234204E-08	0.259701567066927	0.166	0.3	0.00101288179113848	8	KMT2E
1.75030150510921E-11	0.255669021178415	0.122	0.265	2.93315526226202E-07	8	CDC34
1.65342542847799E-14	0.254162829245035	0.111	0.272	2.77081033304342E-10	8	TCEAL1
1.75050177192417E-10	0.25152620212701	0.215	0.4	2.93349086939053E-06	8	REEP5
6.08056335522846E-07	0.250563164288443	0.26	0.427	0.0101898080706919	8	STOM
3.15256994134995E-11	0.250466677657191	0.169	0.332	5.28307670771425E-07	8	FUBP1
0	9.98232697117648	0.251	0.004	0	9	IGHG2
0	9.01690409885309	0.685	0.042	0	9	IGHG1
0	8.939442305507	0.544	0.026	0	9	DEPRECATED-ENSG00000211592
0	8.81597233364067	0.535	0.025	0	9	IGKC
0	8.52356992485858	0.302	0.009	0	9	IGLC3
0	8.17709004837187	0.469	0.044	0	9	IGLC2
0	8.11289196385923	0.278	0.009	0	9	IGHM
0	7.75455352762374	0.27	0.003	0	9	CD79A
0	7.65393239184759	0.25	0.004	0	9	MZB1
1.89031762938871E-107	4.27215771814637	0.265	0.052	3.16779428332959E-103	9	CD37
1.18072964352969E-81	4.19250071311646	0.332	0.101	1.97866673662705E-77	9	PIM2
8.51299366239147E-12	3.05256323716887	0.291	0.215	1.42660747794356E-07	9	IRF8
1.4553072697795E-17	3.00869127856239	0.263	0.158	2.43880392269649E-13	9	BTG2
4.48211129423374E-23	2.95595412761751	0.263	0.137	7.5111221068769E-19	9	ARID5B
5.90161235654315E-275	2.727527906151	0.698	0.152	9.88992198709502E-271	9	CXCR4
9.92693641098355E-148	2.58969679012101	0.413	0.088	1.66355600375262E-143	9	CYTIP
5.75935014860672E-26	2.47692369772821	0.311	0.172	9.65151897903514E-22	9	ITM2C
6.37024695242233E-15	2.34294462732303	0.405	0.332	1.06752598428693E-10	9	SMAP2
2.24630398601063E-64	2.28397484822065	0.266	0.073	3.76435621975662E-60	9	IKZF1
3.93660333435852E-87	2.2661428206644	0.368	0.104	6.596959867718E-83	9	CYBA
5.60002126207193E-70	2.26496221436902	0.454	0.184	9.38451563098015E-66	9	LAPTM5
3.83512771137145E-08	2.22604508030682	0.257	0.194	0.000642690701871627	9	ARHGEF1
3.63210744338068E-08	2.1218138199622	0.302	0.247	0.000608668565361735	9	STK4

1.35122877176446E-54	2.09240487926779	0.332	0.119	2.26438917572289E-50	9	WIPF1
4.38985819989076E-37	1.97804915248596	0.266	0.102	7.35652437137694E-33	9	CELF2
5.55946912503443E-54	1.94457893500265	0.302	0.1	9.3165583597327E-50	9	LSP1
8.13043161492287E-25	1.73831326966515	0.568	0.486	1.36249773002877E-20	9	TXNIP
6.29663212589068E-28	1.60267087726054	0.28	0.127	1.05518961165676E-23	9	LCP1
2.99737371181372E-25	1.56695599402375	0.323	0.171	5.02299886625743E-21	9	IL2RG
9.35708231630718E-45	1.55842097001404	0.407	0.184	1.56805985456676E-40	9	VIM
1.27253323458332E-08	1.52423361542861	0.143	0.285	0.000213251119451473	9	STX7
1.92447277328121E-08	1.46098782689411	0.246	0.444	0.000322503147346466	9	EZR
1.0260248699034E-07	1.3103122816455	0.139	0.268	0.00171941247698411	9	RC3H2
9.57928173343642E-07	1.27596325526657	0.167	0.3	0.0160529603288928	9	PAFAH1B1
1.73340758595454E-08	1.26884291761882	0.135	0.271	0.000290484443254262	9	KDM4C
3.24531525152702E-57	1.25982223139322	0.863	0.84	5.43849929850898E-53	9	CD74
3.25419277163842E-09	1.24097379775353	0.126	0.265	5.45337624671166E-05	9	STK38
1.99568900325271E-07	1.21328125350129	0.137	0.261	0.0033443756316509	9	MORC3
8.26305086672198E-09	1.17294344630633	0.169	0.328	0.000138472206424527	9	STAT6
1.24152738371292E-09	1.17291494808852	0.152	0.306	2.08055158962611E-05	9	MICAL3
4.30047519136653E-07	1.17278109266349	0.137	0.255	0.00720673632569202	9	PPIG
1.46055445817534E-08	1.16563624386031	0.176	0.333	0.000244759716101023	9	CD47
3.43512847679891E-08	1.14983161188559	0.128	0.255	0.000575658830141961	9	RALGPS2
5.11005841274318E-08	1.14824025450624	0.171	0.32	0.000856343588807502	9	MYO15B
1.35229732316239E-08	1.13081491661172	0.126	0.259	0.000226617985415554	9	CLCN7
2.26468635447809E-07	1.12546694599899	0.163	0.299	0.00379516139283439	9	HNRNPDL
6.14581427244931E-07	1.11048424256526	0.169	0.304	0.0102991555577705	9	MLLT6
1.7330966587213E-06	1.10138859982466	0.158	0.283	0.0290432338068515	9	YPEL5
3.96565452139575E-08	1.09926140823377	0.184	0.343	0.0006645643846955	9	UBR2
7.75045479565195E-07	1.05644033056813	0.201	0.355	0.0129882121465535	9	RABGAP1L
1.82279038963558E-07	1.04947003039584	0.131	0.25	0.00305463213495131	9	TAB2
2.65081639149456E-12	1.04632257364532	0.118	0.278	4.44223810886659E-08	9	CMIP
1.47206703379669E-12	1.04175727693634	0.116	0.277	2.46688993523649E-08	9	PMPCB
7.32183082237337E-11	1.04001416664217	0.176	0.355	1.22699240921333E-06	9	BAX
1.58695227752812E-10	1.03896834692987	0.124	0.275	2.65941462668162E-06	9	TSPYL1
2.93075355498201E-09	1.02201667522005	0.124	0.262	4.91135680743885E-05	9	ZRANB2
1.105719252041E-09	1.01491346889678	0.12	0.258	1.85296432257031E-05	9	USP24
2.62821377341468E-06	0.998438322100493	0.188	0.326	0.0440436064148832	9	SRRM2
6.33895785534136E-10	0.992688337012517	0.206	0.393	1.06228255739811E-05	9	RBM39
2.07311851500848E-08	0.988550565809926	0.154	0.295	0.000347413200745121	9	KDM5C
1.87758696907841E-06	0.987672611776256	0.156	0.277	0.031464602427816	9	YTHDC1
1.38034210541804E-07	0.986097791484868	0.174	0.319	0.00231317730025956	9	PCMTD1
3.16244558307365E-10	0.965001723354285	0.135	0.285	5.29962630811482E-06	9	GGNBP2
2.65962609525412E-09	0.960005081188419	0.169	0.331	4.45700141042685E-05	9	ZMIZ2
3.97276591423579E-12	0.959342562556846	0.141	0.311	6.65756111907634E-08	9	SENP6
4.39701299538798E-09	0.955636292304833	0.169	0.326	7.36851437767118E-05	9	GPBP1
1.01464820681213E-10	0.954883730493621	0.146	0.31	1.70034746497577E-06	9	ZNF638
8.68228210104579E-11	0.949325318048676	0.64	0.718	1.45497683449325E-06	9	TMSB4X
2.01187761713735E-09	0.94590291804117	0.165	0.327	3.37150451079878E-05	9	CYBC1
7.40929383751352E-10	0.943048308718369	0.15	0.304	1.24164946129052E-05	9	UBE2I
4.25095349754371E-08	0.937676952215585	0.163	0.31	0.000712374787118375	9	MARCH7
1.75597216282641E-09	0.934287326070879	0.128	0.269	2.94265815046449E-05	9	DEPRECATED-ENSG00000161547
7.72137614645485E-09	0.924979013009543	0.126	0.253	0.00012939482146229	9	STX5
5.0313301842863E-33	0.923731742665917	0.385	0.18	8.43150312282699E-29	9	SRGN
1.25681261739146E-08	0.921071882925704	0.216	0.39	0.000210616658422461	9	RERE
7.54501555250403E-14	0.920252295456607	0.109	0.275	1.26439370628862E-09	9	KMT2D
4.67429940306046E-10	0.916713425456417	0.144	0.3	7.83319093964871E-06	9	MSI2
5.98550426749586E-12	0.916675293986408	0.186	0.378	1.00305080514696E-07	9	ELOVL5
2.92065517273878E-11	0.915870428487623	0.169	0.34	4.89443393847565E-07	9	ETNK1
4.80792653799452E-09	0.91404974037133	0.133	0.271	8.05712329237122E-05	9	NUP210
3.0924321624065E-10	0.912208954989725	0.159	0.322	5.18229781776081E-06	9	MAX
4.87502821529204E-11	0.903267001643613	0.12	0.271	8.1695722831864E-07	9	NIPBL
1.97233011001467E-11	0.901957501674553	0.161	0.34	3.30523079836258E-07	9	DDX39B

1.62707117416627E-09	0.901395441498976	0.133	0.274	2.72664587366783E-05	9	MARK3	
9.49601378610371E-11	0.893692894576832	0.173	0.344	1.59134199027526E-06	9	ASXL1	
1.29980041206382E-10	0.890887988230871	0.109	0.25	2.17820553053655E-06	9	HERC3	
1.07777059471096E-10	0.889024829739779	0.212	0.413	1.80612796261662E-06	9	DBNL	
5.10020064011573E-12	0.881106409936912	0.124	0.284	8.54691623270594E-08	9	NEK7	
3.26293627254858E-12	0.879480798781837	0.131	0.295	5.46802860553691E-08	9	CCDC50	
5.42036684877773E-08	0.877318118528896	0.191	0.347	0.000908345076518172	9	DDX6	
1.10314702618385E-11	0.874979514031885	0.135	0.3	1.8486537864789E-07	9	SRSF5	
1.88887231345469E-08	0.873066417818293	0.131	0.262	0.000316537222288738	9	TSC22D4	
2.00719810292972E-08	0.872217111456554	0.129	0.259	0.000336366258088963	9	NDUFB8	
4.54648151618589E-11	0.870620789166736	0.159	0.33	7.61899372482432E-07	9	SRSF2	
1.75373609483473E-11	0.866068160960622	0.109	0.253	2.93891094772404E-07	9	TMEM175	
1.25899207955297E-11	0.862708140423561	0.152	0.322	2.10981892691487E-07	9		TOP 1.00
3.1078040682282E-15	0.854467413598616	0.098	0.266	5.20805805753682E-11	9	RUFY1	
6.03284533566058E-09	0.847011841182858	0.139	0.275	0.000101098422135	9	DEDD2	
5.4900125232329E-09	0.839139113096742	0.195	0.361	9.2001629864337E-05	9	RHOA	
1.57986385878355E-07	0.830917125948794	0.139	0.262	0.00264753585454947	9	ANKRD11	
2.84986769163039E-09	0.824175822892571	0.137	0.279	4.7758082776342E-05	9	CASC3	
4.52097961900108E-11	0.822146228646256	0.152	0.319	7.57625764552201E-07	9	FAM193B	
3.59576027121539E-13	0.821771618531712	0.116	0.278	6.02577506250275E-09	9	PARP14	
2.3319031179532E-09	0.820357085006555	0.143	0.287	3.90780324506598E-05	9	IST1	
5.81123600093335E-14	0.815353685195871	0.188	0.403	9.7384692903641E-10	9	EML4	
4.51925814582353E-12	0.812575233802796	0.199	0.404	7.57337280077107E-08	9	KDM2A	
6.62382081183437E-13	0.811190615418342	0.118	0.284	1.1100198916472E-08	9	CPSF6	
1.24369913647341E-14	0.808635974017196	0.158	0.36	2.08419101290214E-10	9	SAFB2	
4.61540931872051E-12	0.808247017066082	0.114	0.268	7.73450293631184E-08	9	GAK	
2.45258814894005E-13	0.804819382998034	0.098	0.255	4.11004721999374E-09	9	IWS1	
1.49904678440308E-11	0.804427900602653	0.12	0.274	2.51210260130267E-07	9	SYMPK	
3.75836867730716E-14	0.803127132447287	0.143	0.333	6.29827422943133E-10	9	INTS11	
3.5787716991879E-10	0.799830696238315	0.171	0.341	5.99730561349908E-06	9	BRD4	
1.3559627193653E-08	0.796762019194728	0.148	0.286	0.000227232232511236	9	TNFRSF14	
1.62885591525263E-11	0.789967485408018	0.107	0.252	2.72963674278036E-07	9	POLDIP3	
1.68739273770068E-08	0.789521236236055	0.24	0.43	0.00028277327498388	9	EVL	
1.51435021723365E-11	0.789501066454368	0.107	0.252	2.53774809404016E-07	9	RBM15	
4.70942205189696E-13	0.785735415187859	0.128	0.295	7.89204947456892E-09	9	B4GALT1	
4.96673294700718E-14	0.784430679081892	0.133	0.316	8.32325107259464E-10	9	XRN1	
1.08397229148592E-13	0.783887906794663	0.129	0.305	1.8165207660721E-09	9	STXBP2	
7.0821420430403E-12	0.775397770941527	0.124	0.279	1.18682536357269E-07	9	EP300	
1.4807146573704E-07	0.775385705821717	0.131	0.25	0.00248138162282131	9	GNA13	
2.60961237392504E-14	0.770849108407747	0.105	0.271	4.37318841622357E-10	9	STK11	
8.92763829945156E-14	0.76753566924619	0.109	0.275	1.49609362622209E-09	9	CPSF7	
2.08784842192411E-13	0.766561684976615	0.131	0.305	3.49881638546043E-09	9	PRRC2C	
3.07151774405322E-15	0.766217812857456	0.109	0.286	5.14724943548438E-11	9	NCLN	
1.2700700123276E-13	0.765899551111324	0.165	0.361	2.1283833266586E-09	9	NKTR	
1.06974747579675E-06	0.765368705474832	0.24	0.399	0.0179268281994019	9	FNDC3B	
5.2480508101438E-11	0.76427993047744	0.137	0.294	8.79468354763898E-07	9	BACH1	
5.37205295147875E-14	0.762413807368854	0.122	0.295	9.00248633608809E-10	9	THRAP3	
4.3147383386904E-11	0.759891105620151	0.111	0.255	7.23063850797738E-07	9	GPATCH2L	
1.74669014550243E-14	0.757980612400356	0.129	0.311	2.92710334583297E-10	9	FZR1	
2.37422452590499E-12	0.755167489776982	0.116	0.275	3.97872546051158E-08	9	RAP1A	
2.22787938163946E-13	0.753422453259729	0.101	0.26	3.7334802677514E-09	9	SKI	
8.9324073711869E-11	0.752201135165126	0.152	0.313	1.4968928272635E-06	9	EIF4A1	
9.36561466563029E-12	0.750383316426232	0.189	0.378	1.56948970566632E-07	9	BIRC3	
9.24447092176791E-12	0.748979175555079	0.167	0.35	1.54918843706987E-07	9	PILRB	
5.10080943228882E-12	0.741964117500616	0.109	0.261	8.54793644662961E-08	9	KLHDC4	
8.53108811494855E-16	0.741172772822856	0.109	0.289	1.42963974630308E-11	9	RBM26	
2.48053682058925E-08	0.740041962738284	0.156	0.299	0.000415688360394346	9	SUN2	
2.56316702318893E-15	0.739495984332391	0.098	0.267	4.29535529746001E-11	9	DNAJC8	
8.00936827416362E-14	0.727638376594846	0.135	0.314	1.34220993538434E-09	9	CDK9	
1.17054696104665E-10	0.72691956807744	0.116	0.261	1.96160259732197E-06	9	TCERG1	

3.11006515979733E-12	0.724558981664254	0.15	0.324	5.21184719478837E-08	9	MINK1
7.80468576344193E-15	0.723132859713798	0.12	0.301	1.3079092402376E-10	9	PNN
5.30146008430262E-09	0.722726501039348	0.171	0.325	8.88418680927433E-05	9	TGFBR2
4.49940535343583E-10	0.715915947109405	0.139	0.288	7.54010349128777E-06	9	MOGS
3.43616426465302E-13	0.715513217876747	0.169	0.362	5.75832407470554E-09	9	CPNE1
9.61623498981469E-11	0.71385286352652	0.124	0.269	1.61148865959315E-06	9	DDX24
1.93198740444891E-09	0.711926812311682	0.118	0.25	3.23762449237549E-05	9	M6PR
1.7406467519783E-15	0.692442711655832	0.18	0.393	2.91697582696524E-11	9	SELENOH
5.52178700620795E-11	0.691155094596743	0.15	0.309	9.25341066500329E-07	9	CREBZF
2.67930305134302E-07	0.690785331036357	0.182	0.324	0.00448997605344063	9	SND1
2.58744491094759E-09	0.68759814044638	0.233	0.428	4.33604018176597E-05	9	RBM33
3.26147554189742E-18	0.681837026920154	0.118	0.328	5.4655807131117E-14	9	TRAF4
2.27450208482639E-09	0.68108407874022	0.141	0.285	3.81161059375206E-05	9	RBM25
1.59356517269393E-13	0.68067817318163	0.152	0.339	2.67049651640049E-09	9	SAFB
1.33059876480059E-13	0.679875886202217	0.184	0.389	2.22981741005284E-09	9	PER1
1.6676093433885E-11	0.678490631772457	0.216	0.416	2.79457973765044E-07	9	SEPTIN9
1.51280418389571E-09	0.677618149532141	0.188	0.356	2.53515725137242E-05	9	RSRC2
1.36917493582854E-11	0.677337748397274	0.15	0.318	2.29446335746147E-07	9	TIA1
5.31656018400892E-11	0.675933720800858	0.111	0.253	8.90949155636214E-07	9	CNST
6.24159284008046E-13	0.671582639225752	0.176	0.371	1.04596612814068E-08	9	MBNL1
4.11685039782756E-12	0.670250407641424	0.137	0.306	6.89901789667942E-08	9	RBL2
1.74511959273183E-13	0.667900087957159	0.116	0.285	2.92447141349999E-09	9	RBM23
9.61943052998265E-08	0.666412288845684	0.334	0.552	0.00161202416821449	9	JUND
1.7074158974681E-12	0.665670100622545	0.101	0.251	2.86128756097705E-08	9	KDM4B
4.86730155828545E-14	0.665484915798642	0.152	0.342	8.15662395137476E-10	9	KMT2C
1.34864107089469E-11	0.66342407545093	0.109	0.258	2.26005270660532E-07	9	PCM1
1.84701882416897E-06	0.660460026601811	0.308	0.503	0.0309523414554237	9	LITAF
2.47207619941823E-08	0.660077853025845	0.144	0.279	0.000414270529498507	9	SELENOS
1.94595192040255E-13	0.655060120234123	0.118	0.285	3.26102622821059E-09	9	HNRNPUL2
2.62912588692011E-14	0.647284643485701	0.118	0.292	4.40588916130072E-10	9	TRPC4AP
6.06274147550113E-14	0.642258101857493	0.101	0.261	1.01599421646448E-09	9	KCTD20
4.28466142532217E-14	0.641434806462562	0.154	0.346	7.1802356165549E-10	9	TACC1
3.9934765802163E-21	0.641171716039543	0.098	0.309	6.69226805312647E-17	9	RAB11FIP1
2.27179520136647E-13	0.636965855839857	0.133	0.309	3.80707439844993E-09	9	TMEM263
7.89006225139149E-17	0.636782441250325	0.116	0.312	1.32221663208819E-12	9	CGGBP1
8.15129050684247E-16	0.63517751318556	0.161	0.37	1.36599326313666E-11	9	POGZ
2.00440012395E-10	0.629557866590319	0.161	0.326	3.35897372771541E-06	9	TAF7
1.63460826445178E-14	0.628890180712481	0.109	0.28	2.73927652956829E-10	9	MED28
6.97449513147762E-12	0.62689981730896	0.189	0.384	1.16878589413302E-07	9	TMEM259
7.12395681657673E-15	0.625822238566573	0.126	0.31	1.19383268332193E-10	9	NCOR1
4.37474875766283E-12	0.623688782861713	0.152	0.327	7.33120396809138E-08	9	SNRNP70
4.02505556561187E-14	0.622028610985703	0.139	0.321	6.74518811685237E-10	9	SUPT5H
5.35804644281971E-10	0.620220271437392	0.191	0.366	8.97901422887728E-06	9	TAP1
1.19475998744327E-15	0.617693715503342	0.114	0.296	2.00217878695743E-11	9	ZFR
1.61073077579824E-13	0.616130421666912	0.169	0.363	2.69926263408269E-09	9	PLEKHF2
3.60774439821677E-10	0.615552164021373	0.158	0.317	6.04585806253167E-06	9	MACF1
1.3253012722273E-08	0.614701367915302	0.137	0.269	0.000222093987199851	9	TLN1
2.78509539774481E-14	0.612402645200259	0.158	0.353	4.66726286754076E-10	9	DNAJA2
2.91702235803106E-08	0.608254629712298	0.161	0.302	0.000488834606758845	9	SH3BGRL3
7.68181398398233E-11	0.607141968478338	0.223	0.421	1.28731838743576E-06	9	SETD5
1.16688098191401E-16	0.603512039646373	0.133	0.336	1.9554591494915E-12	9	RAB1A
1.26995810341969E-15	0.603507490560172	0.116	0.3	2.12819578971071E-11	9	ZNF644
9.00914676873656E-11	0.603090249197983	0.163	0.332	1.50975281550487E-06	9	FUBP1
6.81354710223875E-13	0.59580250484589	0.114	0.276	1.14181422339317E-08	9	SPEN
1.72499054091923E-07	0.595165626006232	0.148	0.271	0.00289073914847245	9	AKIRIN2
4.25801147952635E-12	0.591863622408932	0.135	0.296	7.13557563739026E-08	9	MBNL2
5.35040648104288E-17	0.59042182711469	0.096	0.277	8.96621118093166E-13	9	ZBTB7A
4.39339628974779E-12	0.588930063704414	0.144	0.317	7.36245350235935E-08	9	TCIRG1
2.8625575018462E-13	0.578731851356793	0.114	0.278	4.79707386159386E-09	9	FBXL3
8.15233093658676E-13	0.574715233044875	0.126	0.29	1.36616761835321E-08	9	MED15

**4.51881885308737E-13**	0.573107127735849	0.148	0.326	7.57263663400381E-09	9	LMBRD1
**2.29796262212784E-10**	0.570565790776251	0.116	0.256	3.85092576216184E-06	9	TRA2B
**1.2502035742851E-16**	0.569904910385423	0.105	0.291	2.09509114978698E-12	9	AGO2
**9.21340306462296E-15**	0.569361200677957	0.111	0.282	1.54398208556952E-10	9	KANSL1
**2.39366260462694E-11**	0.566285997094018	0.12	0.27	4.01129979283382E-07	9	ZC3H15
**6.29891679049875E-14**	0.565574171413981	0.111	0.275	1.05557247575178E-09	9	LRCH3
**3.81105581979326E-17**	0.564207338516926	0.099	0.283	6.38656734280954E-13	9	DNAJB2
**4.71161917249353E-15**	0.564136611885691	0.171	0.378	7.89573140926466E-11	9	ARF6
**5.46343450011559E-16**	0.55966852976691	0.141	0.343	9.1556235352937E-12	9	ENSA
**5.85637764005017E-13**	0.556475547875371	0.101	0.254	9.81411764919608E-09	9	SMAD2
**6.08074904247725E-14**	0.553296926151649	0.094	0.251	1.01901192453834E-09	9	BAZ1B
**8.07896153452109E-18**	0.55021303896645	0.079	0.257	1.35387237395504E-13	9	COLGALT1
**1.4310039428456E-15**	0.549775387391607	0.137	0.333	2.39807640742066E-11	9	U2SURP
**2.99652349828752E-13**	0.548915367775394	0.214	0.428	5.02157407843022E-09	9	CCNL1
**1.22996936870537E-10**	0.548643817157042	0.133	0.285	2.06118266807647E-06	9	ARSA
**1.14631618883805E-15**	0.544252721402764	0.116	0.302	1.92099666925481E-11	9	TMUB2
**2.49104864226727E-14**	0.543758103999736	0.203	0.417	4.17449931471148E-10	9	SELENOF
**1.48722909695604E-13**	0.542993668064685	0.103	0.263	2.49229852067893E-09	9	OGFR
**2.23134455076942E-07**	0.541358894438563	0.379	0.607	0.0037392871981794	9	TAPBP
**1.65391256780287E-16**	0.537431910659503	0.141	0.344	2.77162668112405E-12	9	KIAA0100
**3.26366398757428E-13**	0.536266287222601	0.131	0.305	5.46924811037699E-09	9	GPBP1L1
**1.60137941159896E-14**	0.535155279888014	0.152	0.348	2.68359161795754E-10	9	KAT6A
**1.51867341357478E-12**	0.534908223312807	0.101	0.251	2.54499290646861E-08	9	NR3C1
**1.2131658722123E-19**	0.534739012546806	0.133	0.356	2.03302336865337E-15	9	RNF19A
**1.45131563470731E-17**	0.531104128993087	0.092	0.277	2.43211474064251E-13	9	KMT5B
**4.28602042051973E-11**	0.520561819725541	0.113	0.254	7.18251302070696E-07	9	LTBP3
**2.61530847688349E-12**	0.519225562547232	0.111	0.263	4.38273394556136E-08	9	STIM1
**3.46924548985405E-15**	0.517552019862974	0.094	0.259	5.81376159189742E-11	9	LRBA
**1.09904744801922E-11**	0.515592865441587	0.143	0.306	1.84178371339062E-07	9	SLC17A9
**6.223502226224E-14**	0.514947448496156	0.103	0.266	1.04293450307062E-09	9	MTIF3
**4.07275470952476E-15**	0.512347849307001	0.111	0.286	6.8251223422216E-11	9	PSPC1
**1.98585303134747E-16**	0.512236920323507	0.216	0.459	3.32789250993209E-12	9	MAPKAPK2
**5.79764274774906E-11**	0.510702046626719	0.165	0.335	9.71568971667787E-07	9	SEL1L
**2.41455938457266E-08**	0.510572996723836	0.18	0.327	0.000404631861666687	9	WSB1
**1.09722245869344E-14**	0.510437964614282	0.111	0.283	1.83872539627846E-10	9	CEP350
**2.63536936815082E-07**	0.509955215712652	0.135	0.253	0.00441635198714715	9	ARRDC3
**3.39305676538582E-08**	0.509313977528604	0.18	0.328	0.000568608452743356	9	ADGRE5
**3.88891442399854E-19**	0.504472425382781	0.086	0.277	6.51704279173676E-15	9	KDM6A
**2.83537624618861E-13**	0.503639727154091	0.128	0.297	4.75152351336287E-09	9	COPB1
**7.26974508988659E-15**	0.502945254650403	0.221	0.457	1.2182638821632E-10	9	HNRNPA0
**2.40972582483532E-18**	0.50249579371393	0.129	0.342	4.03821853725903E-14	9	DIDO1
**1.33866594753672E-13**	0.497395340857499	0.167	0.361	2.24333639488203E-09	9	STX16
**2.67981707199414E-14**	0.496761397404956	0.15	0.34	4.49083744924778E-10	9	TNIP1
**6.63504003245918E-16**	0.496466680874822	0.098	0.273	1.11190000863951E-11	9	RHOT2
**2.62584481821641E-18**	0.492119387700586	0.103	0.3	4.40039074636706E-14	9	ANKRD17
**1.67331453320588E-14**	0.491271298034808	0.092	0.253	2.80414049474641E-10	9	NCAPH2
**2.07965343208695E-11**	0.487586571634083	0.141	0.301	3.48508322149131E-07	9	ANAPC16
**4.14154978844336E-17**	0.485935977492284	0.113	0.301	6.94040913547339E-13	9	PCBP1
**3.25534181121079E-19**	0.485400385653452	0.141	0.365	5.45530180722703E-15	9	ASH1L
**3.78845648126232E-14**	0.485374096491625	0.109	0.275	6.34869537129939E-10	9	FUS
**8.39865369409926E-17**	0.484875031706933	0.094	0.274	1.40744638605715E-12	9	DEDD
**2.03407326967009E-10**	0.484344701143529	0.131	0.278	3.40869998531314E-06	9	WDR26
**6.72503995694624E-20**	0.483979171888505	0.077	0.268	1.12698219598505E-15	9	CUL4A
**1.41869724174597E-17**	0.483208259340121	0.242	0.505	2.3774528377179E-13	9	WNK1
**3.61746599560301E-19**	0.481519401374357	0.167	0.405	6.06214951543152E-15	9	PJA2
**8.5441210005919E-16**	0.480221176928946	0.111	0.29	1.43182379727919E-11	9	SLC20A1
**2.66709455020436E-13**	0.478735270148893	0.118	0.284	4.46951704723246E-09	9	ATXN7L3B
**6.46939054613804E-16**	0.4782315076012	0.118	0.304	1.08414046772181E-11	9	LIX1L
**3.52022299742476E-19**	0.472424104229202	0.139	0.361	5.89918969908442E-15	9	LIMD1
**5.79778079854784E-14**	0.466353426714204	0.113	0.283	9.71592106220647E-10	9	ZNF217

3.51455121830852E-16	0.466279390980024	0.084	0.255	5.88968493164141E-12	9	PI4KA
1.76997157312385E-16	0.465504542071455	0.159	0.367	2.96611836224095E-12	9	TMED4
9.24423898189973E-16	0.460835129166242	0.308	0.601	1.54914956858676E-11	9	HNRNPA2B1
8.40926606436313E-16	0.459835802867307	0.178	0.392	1.40922480706597E-11	9	SMARCA2
1.01560652473192E-15	0.457503825173608	0.107	0.289	1.70195341414575E-11	9	NUB1
1.25053709627061E-16	0.45631452301227	0.118	0.309	2.09565006593029E-12	9	ZMYM2
1.81760964114226E-13	0.45374678468561	0.113	0.274	3.04595023662621E-09	9	DNAJC7
8.49551087258007E-13	0.452162615111943	0.291	0.539	1.42367771202697E-08	9	CIRBP
3.86873156897096E-15	0.451305053632762	0.124	0.307	6.48322036328154E-11	9	NUS1
7.68367006274105E-14	0.451261834838668	0.099	0.259	1.28762942911414E-09	9	IFNGR1
1.53428158073672E-15	0.449159326841715	0.135	0.327	2.5711490729986E-11	9	RNF10
1.45647435677995E-15	0.448525086725564	0.113	0.292	2.44075972709185E-11	9	PPP1CB
1.12207575660039E-16	0.448127787265157	0.171	0.384	1.88037455291094E-12	9	EMC10
1.95237217543909E-15	0.44439910680318	0.092	0.259	3.27178529160083E-11	9	DYM
1.02416326658249E-12	0.438724076372353	0.154	0.331	1.71629280213893E-08	9	NRDC
1.53388803097494E-17	0.438178055889459	0.111	0.305	2.57048956230781E-13	9	AFF4
2.46603406108214E-14	0.437936145735493	0.203	0.419	4.13257987956145E-10	9	IKBKB
2.00013367533047E-15	0.436960698292127	0.099	0.269	3.35182401311879E-11	9	GOSR2
4.12896155321049E-13	0.435087697693761	0.255	0.487	6.91931377087015E-09	9	TPP1
1.85191511573363E-19	0.432379975231902	0.084	0.278	3.10343935094642E-15	9	NPEPPS
2.03810268028804E-12	0.424393695575037	0.118	0.273	3.41545247162669E-08	9	RNF4
3.00152660967969E-16	0.422765299076159	0.086	0.257	5.02995829250122E-12	9	NUP43
3.10592587482424E-18	0.421881351568012	0.152	0.374	5.20491058103046E-14	9	CHTOP
9.04012255802508E-18	0.416985897618933	0.139	0.349	1.51494373827384E-13	9	OXA1L
1.91646038867118E-18	0.413134113635628	0.099	0.293	3.21160431933516E-14	9	RAB1B
1.81042883442437E-13	0.412383736838334	0.124	0.293	3.03391664072836E-09	9	NRBP1
5.07627688609481E-17	0.411754853740322	0.086	0.262	8.50682480571768E-13	9	RBM14
3.5061781345443E-16	0.41015275720654	0.094	0.266	5.87565331786933E-12	9	PTBP1
1.18443077177724E-14	0.409373429499551	0.135	0.32	1.9848690873443E-10	9	TOMM7
7.99202194222736E-21	0.407664719502932	0.191	0.45	1.33930303707846E-16	9	LAMTOR4
5.05173537062452E-21	0.405617089092959	0.129	0.355	8.46569813409258E-17	9	FAM91A1
2.53102523235801E-14	0.403328790717257	0.171	0.367	4.24149208438556E-10	9	MDM2
1.19075919515589E-13	0.403259675733934	0.098	0.257	1.99547425924225E-09	9	VHL
4.21213239167235E-15	0.402585153744526	0.118	0.296	7.05869146196452E-11	9	SPG21
5.37397485618552E-16	0.402004657323423	0.231	0.473	9.0057070639957E-12	9	CTSH
1.37668875380169E-12	0.401520208550176	0.137	0.303	2.30705501362087E-08	9	RAB21
1.19078760348413E-13	0.400994071611224	0.118	0.288	1.9955218659187E-09	9	UBE2H
3.12423914795778E-17	0.397843127334861	0.111	0.3	5.23559996414765E-13	9	MAT2B
4.58522211097738E-11	0.396355247982063	0.173	0.347	7.6839152135759E-07	9	EIF3A
8.66437561945975E-15	0.390949975797405	0.099	0.267	1.45197606630907E-10	9	MED25
2.7209967814011E-12	0.390326724918713	0.165	0.344	4.55984640627197E-08	9	SUGP2
4.47578956108292E-16	0.387638273668196	0.098	0.272	7.50052814646275E-12	9	HMCES
5.29198536864729E-16	0.386490686559734	0.156	0.359	8.86830908077913E-12	9	NUFIP2
9.84432008823868E-18	0.383651610351209	0.09	0.27	1.64971116038704E-13	9	TPR
1.13477252096249E-13	0.381358327915165	0.103	0.264	1.90165179062895E-09	9	PITPNB
2.27858348049075E-17	0.380495906732815	0.212	0.454	3.81845019660639E-13	9	EEF1G
2.10460548199105E-16	0.375972320599129	0.096	0.273	3.5268978667206E-12	9	SDHA
2.33590101122843E-16	0.374711261215935	0.126	0.318	3.9145029146166E-12	9	DDX46
4.93946874617075E-20	0.373819266940594	0.098	0.302	8.27756172483293E-16	9	DNAJC21
3.10336944907138E-16	0.371182981594674	0.09	0.26	5.20062652275381E-12	9	PRPF6
7.40673894064165E-17	0.371030474717333	0.092	0.273	1.24122131167273E-12	9	SIPA1L3
5.68761863227518E-15	0.37014143302924	0.096	0.26	9.53131130396674E-11	9	AP3B1
4.22791501465913E-19	0.369859110872771	0.092	0.286	7.08513998156578E-15	9	KPNA4
1.01133016494695E-16	0.366466214730958	0.212	0.453	1.6947870904181E-12	9	RACK1
3.38624248320085E-25	0.364105243940198	0.096	0.334	5.67466515334799E-21	9	MROH1
8.81531604609248E-19	0.363407761761162	0.088	0.277	1.47727066300418E-14	9	TPP2
4.09803036441485E-19	0.362571235171926	0.073	0.256	6.8674792846864E-15	9	UBA1
7.85116685185519E-13	0.361603083690668	0.111	0.268	1.31569854103389E-08	9	IRF2
1.21672931388342E-15	0.361182994064195	0.128	0.315	2.03899498420584E-11	9	MKLN1
3.33034437467126E-10	0.360363430329949	0.129	0.274	5.58099110307409E-06	9	IER2

5.22565613968781E-17	0.359958580925089	0.184	0.412	8.75715455888883E-13	9	IRF3
2.87293976419527E-16	0.358118097449874	0.143	0.344	4.81447245683844E-12	9	SEC22B
1.32447456075148E-20	0.355103280896635	0.146	0.383	2.21955446890733E-16	9	OPTN
7.01272673643378E-21	0.353812756291079	0.116	0.336	1.17519274649157E-16	9	TRIM27
3.73337393339755E-18	0.351366094741531	0.124	0.328	6.25638803758762E-14	9	PPP3R1
7.98180398046235E-21	0.351043381748806	0.131	0.363	1.33759071104588E-16	9	POLR2J3
2.70567015259368E-20	0.350931517252491	0.139	0.365	4.53416204171649E-16	9	TMEM63A
1.08628796083449E-16	0.350689232131259	0.092	0.266	1.82040136476643E-12	9	MGRN1
4.72678967065885E-18	0.350395516730166	0.139	0.348	7.9211541300901E-14	9	HIPK2
8.7140256210235E-19	0.349521724350388	0.094	0.286	1.46029641357112E-14	9	TNRC6A
3.7127388444256E-14	0.349127446314353	0.124	0.298	6.22180775548842E-10	9	ESYT1
1.20557994853016E-15	0.34750480729519	0.208	0.444	2.02031087774685E-11	9	KLF6
2.20440377919724E-17	0.346774517184645	0.131	0.332	3.69413985317874E-13	9	UPK3BL1
2.44897280495827E-18	0.346697751973736	0.128	0.335	4.10398862654907E-14	9	SPG7
8.059143820149E-18	0.34538442311332	0.146	0.365	1.35055132138057E-13	9	CSNK1D
1.50984453019882E-16	0.342457028628595	0.083	0.252	2.53019746370718E-12	9	EXOC7
3.358393157707E-21	0.34234433266464	0.113	0.33	5.62799525368539E-17	9	ANKHD1
2.69838590374038E-13	0.337129828695015	0.101	0.254	4.52195509748814E-09	9	RUNX1
8.13252370794824E-11	0.336316956593908	0.255	0.464	1.36284832297797E-06	9	RNF213
1.98457138092953E-14	0.335200470620542	0.116	0.289	3.32574472016171E-10	9	MAP4
3.80060891203043E-15	0.331719395786153	0.12	0.298	6.36906041478059E-11	9	SEC11A
4.72063091634534E-18	0.33166687463187	0.079	0.259	7.91083328961153E-14	9	DNAJC5
6.16070957670393E-20	0.330586754197711	0.114	0.325	1.03241171086404E-15	9	PRPF3
3.98376651056429E-13	0.329728954319159	0.105	0.263	6.67599591840363E-09	9	EPRS
3.09958716781267E-17	0.32944441696962	0.163	0.385	5.19428817582048E-13	9	DCAF8
4.15277120958847E-22	0.329180025064203	0.114	0.339	6.95921399302837E-18	9	PPP6R2
9.24208334556248E-18	0.327325757991672	0.109	0.302	1.54878832704936E-13	9	PPP4C
4.58863384917126E-16	0.324143724963738	0.131	0.319	7.6896326044412E-12	9	PKN1
3.745989064407E-17	0.323490020907327	0.114	0.309	6.27752847413326E-13	9	ATF6B
2.20284128883667E-15	0.323370333199288	0.098	0.267	3.6915214318325E-11	9	EXOC3
3.58379648437931E-17	0.321507826175536	0.128	0.326	6.00572614852286E-13	9	RNF114
4.86901524359023E-16	0.314873329153461	0.255	0.524	8.1594957452085E-12	9	AHNAK
1.75090474072089E-22	0.313786865193442	0.158	0.412	2.93416616450007E-18	9	CALCOCO2
3.07082478627998E-17	0.306891033623118	0.111	0.295	5.14608817684799E-13	9	GNB1
2.39776450727449E-21	0.304086019917451	0.066	0.26	4.01817376129059E-17	9	URI1
4.41859342385134E-17	0.303736972866525	0.083	0.256	7.40467885969008E-13	9	WWOX
2.11505368790177E-17	0.303096787157107	0.098	0.283	3.54440697018579E-13	9	PTBP3
2.19777726738436E-18	0.301269149002945	0.133	0.345	3.68303514468271E-14	9	ESYT2
3.41244549572707E-18	0.294649868846345	0.107	0.301	5.71857616173943E-14	9	KMT2B
8.32162956524916E-25	0.294629612495412	0.184	0.467	1.39453868254445E-20	9	BRD2
6.13365096003559E-15	0.289493445024158	0.096	0.262	1.02787722788276E-10	9	ADAM10
1.41924225779758E-16	0.286904535614393	0.143	0.342	2.37836617561718E-12	9	CCNI
1.36914161633335E-17	0.283521840446708	0.148	0.362	2.29440752065143E-13	9	ADD1
3.82035473091606E-19	0.283471407558044	0.105	0.303	6.40215045806913E-15	9	ZFP91
1.9106230522435E-19	0.282689642904265	0.092	0.287	3.20182211094966E-15	9	IDS
1.95193444648125E-16	0.282021079847785	0.109	0.292	3.27105174541328E-12	9	TAOK1
1.31419015526994E-17	0.280936864461727	0.12	0.316	2.20231986220136E-13	9	ARHGDIA
2.1820295544135E-09	0.279105613175569	0.323	0.534	3.65664512728615E-05	9	CCNL2
1.63897524690669E-13	0.277968315688453	0.116	0.283	2.74659471876623E-09	9	CLPTM1L
4.03398777881297E-20	0.275972722355365	0.131	0.353	6.76015671973478E-16	9	G3BP2
8.67424650934765E-16	0.275432677482053	0.099	0.272	1.45363023003648E-11	9	CYTH2
1.56808828904764E-11	0.275324891816568	0.124	0.275	2.62780235478603E-07	9	TXNDC15
2.14648228583692E-22	0.275180907312597	0.174	0.439	3.59707501460552E-18	9	SNX1
1.0642911241356E-20	0.273062295768627	0.173	0.418	1.78353906582644E-16	9	UBXN4
4.57736934801825E-11	0.27261683331108	0.113	0.253	7.67075555340898E-07	9	ARHGEF7
1.22188584566179E-15	0.272308505740013	0.135	0.327	2.04763630016003E-11	9	BIRC6
1.15537427131688E-15	0.27020535348942	0.18	0.392	1.93617620387283E-11	9	CNBP
9.33715014024565E-18	0.270117799846265	0.107	0.302	1.56471962050237E-13	9	UBE2V1
3.95140542436478E-18	0.269315793207079	0.131	0.338	6.6217652101505E-14	9	TRIP12
5.34157640998539E-12	0.264999597576355	0.118	0.275	8.95141374785352E-08	9	CRELD2

9.14857158063657E-20	0.264684425450293	0.113	0.319	1.53311762548308E-15	9	ZBTB20
1.65543612053057E-19	0.263460524054234	0.12	0.333	2.77417985078513E-15	9	MKNK2
2.42120454007138E-16	0.262883952252062	0.174	0.39	4.05745456825162E-12	9	CAPZB
5.67485919243577E-16	0.262866552899827	0.09	0.258	9.50992903468386E-12	9	ZMYND8
1.72682565832397E-15	0.261625208881421	0.096	0.267	2.89381443821931E-11	9	NADK
2.22917926353925E-16	0.261545789390685	0.148	0.352	3.73565860983907E-12	9	RAB8A
4.99046133472707E-16	0.260924119948096	0.084	0.252	8.36301510473562E-12	9	CIAO1
4.76859675310498E-18	0.25899628847839	0.084	0.268	7.99121443885332E-14	9	PRPF40A
3.00569960309953E-13	0.257803782560446	0.098	0.252	5.03695139487419E-09	9	ZBTB1
3.74269868516927E-19	0.256480519310135	0.086	0.278	6.27201445660666E-15	9	MPHOSPH8
1.15735382126051E-13	0.252737472381174	0.212	0.43	1.93949353366836E-09	9	SYNE2
4.56990608029379E-16	0.2521045299802	0.084	0.251	7.65824860935633E-12	9	FRYL
4.17197094244932E-16	0.250903381853179	0.159	0.366	6.99138890535657E-12	9	HNRNPC
0	6.33661561180607	0.536	0.017	0	10	CXCL11
4.84566623881254E-243	6.21141957226409	0.764	0.069	8.12036748300206E-239	10	CXCL10
1.35674685175104E-232	5.50746953828983	0.814	0.083	2.27363637416439E-228	10	CXCL9
1.20331265413523E-85	4.47175309765676	0.407	0.049	2.01651134579982E-81	10	FSTL3
1.38980781718448E-69	2.88406329259292	0.3	0.032	2.32903994003775E-65	10	CX3CL1
4.43904528381864E-70	2.577808866023	0.529	0.094	7.43895208662328E-66	10	CALHM6
5.9213192392727E-46	2.55723033471065	0.464	0.104	9.92294678117319E-42	10	DRGX
4.39116152985307E-61	2.37857293526336	0.871	0.317	7.35870849172777E-57	10	UBD
2.31920946643881E-25	2.17573806874657	0.421	0.128	3.88653122385816E-21	10	MX1
4.46721432502494E-44	2.16206997758582	0.836	0.372	7.48615776587679E-40	10	BIRC3
2.20641461059912E-30	2.12959283156485	0.421	0.114	3.697509604442E-26	10	CCL20
2.9457496692505E-34	2.08672152522892	0.457	0.122	4.93648729572998E-30	10	PHLDA2
2.26364922347169E-17	2.06178183955163	0.279	0.08	3.79342336869386E-13	10	CCL25
1.60493157951895E-15	2.04881497491017	0.471	0.201	2.68954434095785E-11	10	ISG15
4.44622461288436E-24	2.01902637742119	0.321	0.084	7.4509832062716E-20	10	CTSE
6.80680228672761E-42	1.97232823889864	0.814	0.36	1.14068392720981E-37	10	TAP1
1.97961197662882E-44	1.94920171387831	0.371	0.068	3.31743375043458E-40	10	AKR1B15
9.33276493385743E-27	1.9078026975133	0.493	0.164	1.56398474761583E-22	10	DUOXA2
1.15659922428058E-12	1.87105965090117	0.25	0.084	1.9382289800494E-08	10	TTLL6
1.08245119481166E-14	1.83836068058388	0.336	0.125	1.81397171226538E-10	10	SLC1A1
3.98651350270176E-16	1.81762262812819	0.35	0.127	6.68059932782761E-12	10	B4GALNT2
8.53447329913164E-28	1.80475255322146	0.493	0.162	1.43020703546848E-23	10	SPHK1
3.86914866512691E-23	1.78491085967701	0.35	0.1	6.48391933301968E-19	10	OAS2
3.44443910487875E-20	1.76217976438271	0.314	0.09	5.77219105195581E-16	10	DHX58
5.57220147268526E-22	1.71597741172161	0.45	0.163	9.33789522792596E-18	10	NCEH1
2.93610583234044E-26	1.70158119482741	0.557	0.206	4.9203261538361E-22	10	DUOX2
4.99684845119166E-19	1.67865827646244	0.386	0.134	8.37371863450698E-15	10	WARS
1.09705056285896E-34	1.67548854503006	0.843	0.401	1.83843733323905E-30	10	PLAAT4
3.16488860670159E-41	1.67079725021152	0.457	0.105	5.30372032711052E-37	10	GBP4
7.8810312390017E-19	1.65089265176349	0.279	0.077	1.32070321503191E-14	10	HERC6
4.42653313361994E-20	1.64909063142574	0.464	0.173	7.4179842253203E-16	10	IFI44
1.06982604152506E-25	1.64521147949101	0.679	0.312	1.7928144803877E-21	10	LAP3
1.33875325579579E-30	1.63415051219243	0.729	0.344	2.24348270606258E-26	10	PSMB9
2.0483364475679E-14	1.57903365195383	0.279	0.091	3.43260221883429E-10	10	CASP10
3.7761321602931E-32	1.57802117852966	0.371	0.086	6.32804227421918E-28	10	DEPRECATED-ENSG00000227471
2.33240500710103E-22	1.56143384315743	0.421	0.137	3.90864431089991E-18	10	DDX60
3.73374757680347E-19	1.5598521044467	0.607	0.279	6.25701418920726E-15	10	IFI6
3.1081649993743E-27	1.53994156775531	0.464	0.142	5.20866290595146E-23	10	TINAG
1.8985282461411E-19	1.53588797286942	0.257	0.065	3.18155363488326E-15	10	PPP4R4
5.11839225651976E-17	1.52051343795751	0.557	0.253	8.5774017434758E-13	10	FADS2
3.53013954809681E-28	1.51722082834784	0.664	0.281	5.91580785470064E-24	10	IFI35
3.70935106006677E-11	1.49785260800147	0.314	0.127	6.21613050645989E-07	10	PRUNE1
2.25707803279992E-32	1.49664988629776	0.564	0.18	3.7824113673661E-28	10	PAQR7
1.55383464164338E-29	1.48751520931518	0.743	0.313	2.60391609246598E-25	10	C15orf48
5.50399947387627E-22	1.48489388057002	0.429	0.142	9.22360231832185E-18	10	TMCC3
9.1804015436177E-13	1.46359370963251	0.293	0.106	1.53845169067945E-08	10	XKR9
2.6424166093982E-14	1.45462204732336	0.457	0.2	4.4281617540295E-10	10	OAS1

1.3083893845622E-38	1.44747093275489	0.964	0.634	2.19259893064933E-34	10	IFI27
3.66350892733768E-28	1.42639768030851	0.636	0.256	6.13930826043248E-24	10	TAP2
3.88613910786014E-17	1.41095498777122	0.3	0.093	6.51239191695202E-13	10	HID1
1.4010276641538E-13	1.40860857248793	0.407	0.166	2.34784215958894E-09	10	IFIH1
5.03910378124577E-13	1.38853970989601	0.3	0.107	8.44453011661166E-09	10	SPAG1
1.46967823678515E-36	1.38688298349509	0.771	0.291	2.46288678920455E-32	10	TM4SF20
2.78278731894008E-33	1.3787695338935	0.971	0.781	4.66339498907978E-29	10	IL32
6.53095979833716E-15	1.36852773143385	0.393	0.153	1.09445824300534E-10	10	C19orf33
1.40915292166871E-15	1.36309134216135	0.293	0.093	2.36145846613243E-11	10	FBXO6
2.09733530376861E-27	1.35902151868983	0.557	0.198	3.51471450205544E-23	10	DEPRECATED-ENSG00000188313
2.57519346901052E-24	1.35718536705099	0.643	0.273	4.31550921536782E-20	10	PARP14
2.53871423079544E-27	1.34516278695203	0.729	0.322	4.254377307967E-23	10	STAP2
3.03359652592618E-37	1.34466483451759	0.571	0.162	5.08370105814709E-33	10	PLA2G4C
6.14407668206875E-16	1.33043974788489	0.25	0.071	1.02962437038108E-11	10	SYT7
7.58928774111442E-16	1.32924514484952	0.3	0.097	1.27181283965596E-11	10	BMP2
4.62162537707623E-16	1.32452232823402	0.464	0.196	7.74491980690435E-12	10	HOXA13
3.48182808803884E-17	1.31400374152712	0.464	0.183	5.8348475099355E-13	10	BAMBI
4.41789150559944E-29	1.30550807921562	0.75	0.345	7.40350258508353E-25	10	STAT1
8.01086601546759E-23	1.30190785413352	0.514	0.188	1.34246092687206E-18	10	ITGA3
1.17272489019773E-18	1.29862144870317	0.407	0.142	1.96525237099335E-14	10	CMPK2
8.57925147607292E-21	1.2977646980066	0.4	0.131	1.4377109623603E-16	10	IL15RA
3.03033472374558E-09	1.29054764063841	0.329	0.148	5.07823493005284E-05	10	SEC61G
9.39002128862868E-12	1.2812153760075	0.257	0.089	1.57357976754839E-07	10	COX7A1
1.96118587134697E-08	1.27979960191807	0.25	0.103	0.000328655528320326	10	CHMP4C
1.31480531614863E-13	1.27571565888181	0.307	0.108	2.20335074880188E-09	10	DEPRECATED-ENSG00000109536
3.07486911935589E-10	1.27192423022043	0.257	0.096	5.15286567021661E-06	10	SLC7A9
2.25234788848449E-12	1.2546695947033	0.293	0.106	3.7744845915223E-08	10	FAM171A1
1.21471337991633E-15	1.25447806651588	0.45	0.184	2.03561668206379E-11	10	FRAS1
2.49320354111895E-13	1.25360115515525	0.257	0.082	4.17811049420714E-09	10	TLCD2
3.56422451477236E-08	1.25150205872199	0.279	0.125	0.000597292744185553	10	B3GALT5
4.86963420397648E-11	1.24694396892734	0.329	0.138	8.16053299902378E-07	10	PTPRD
6.73766964512527E-16	1.24327553361277	0.371	0.133	1.12909867913009E-11	10	AMMECR1
3.76297575678652E-20	1.23008794808447	0.536	0.212	6.30599477322286E-16	10	ANKH
2.18652042994893E-13	1.22820686603214	0.35	0.134	3.66417093650842E-09	10	NT5C
7.95184690600087E-22	1.22675334181801	0.471	0.165	1.33257050450763E-17	10	LGALS9
7.82393387229579E-16	1.21948887436061	0.429	0.168	1.31113483831933E-11	10	ART4
1.65647854623625E-14	1.21828592577528	0.393	0.157	2.7759267477827E-10	10	TNFRSF11A
9.43081930569377E-22	1.21779548451917	0.493	0.183	1.58041669924816E-17	10	APOL3
4.30558149305124E-28	1.21475280362848	0.821	0.391	7.21529346605527E-24	10	HNF4G
3.9687810531702E-14	1.21453011766144	0.343	0.125	6.65088328890263E-10	10	COPG2
2.08920016256805E-12	1.21210032260045	0.4	0.17	3.50108163243154E-08	10	ZNF608
2.73157986950273E-22	1.20678498589821	0.593	0.239	4.57758154531268E-18	10	EXOC3L4
6.20872439095824E-21	1.2047929571955	0.45	0.159	1.04045803343678E-16	10	OAS3
5.27944940001712E-13	1.20418251974036	0.55	0.282	8.8473013045487E-09	10	IRF1
1.73081067386188E-19	1.1996953684283	0.543	0.222	2.90049252725774E-15	10	PYCARD
2.24766545209053E-13	1.19848418801783	0.257	0.084	3.76663776461331E-09	10	TMEM41A
1.87124379908559E-19	1.19735517586481	0.657	0.301	3.13583035850763E-15	10	KCNK1
5.9607877638809E-08	1.18781783228935	0.271	0.116	0.000998908813471161	10	TRANK1
2.34471007323305E-22	1.18467495781667	0.736	0.356	3.92926514072394E-18	10	PARM1
2.61432914031601E-24	1.18218876987749	0.564	0.206	4.38109277334157E-20	10	APOL2
2.22709157980365E-27	1.17636688930649	0.693	0.296	3.73216006943496E-23	10	PLSCR1
2.56933923226411E-15	1.17324905523764	0.529	0.231	4.3056986854282E-11	10	ITPKC
1.34599040699603E-14	1.1731783794688	0.286	0.092	2.25561072404394E-10	10	WDR1
5.55064872514518E-11	1.16354748125319	0.286	0.109	9.3017771335983E-07	10	PCDHGA3
8.67199524289634E-17	1.16229584474551	0.464	0.18	1.45325296280457E-12	10	CLN5
2.03275675947978E-17	1.16021344229148	0.629	0.308	3.40649377753622E-13	10	TM9SF1
5.98321908662422E-34	1.15813111127271	0.757	0.288	1.00266785453649E-29	10	TYMP
1.20775818407808E-24	1.15806344251683	0.943	0.592	2.02396116487805E-20	10	SERPINA7
1.1401259054283E-12	1.15665863845265	0.271	0.094	1.91062299231675E-08	10	IGSF3
9.97176945478094E-19	1.15489729619182	0.507	0.195	1.67106912523219E-14	10	FGF13

2.53278851456598E-13	1.15397710804721	0.357	0.14	4.24444699270967E-09	10	PPP1R14D
2.25863018883494E-19	1.15221685448336	0.629	0.267	3.78501247044959E-15	10	TSPAN1
1.10862860082607E-16	1.15084594056355	0.55	0.244	1.85783980926433E-12	10	ZNF189
1.93225017433762E-17	1.14994678200825	0.786	0.442	3.23806484215498E-13	10	VNN 1.00
2.79369679486907E-11	1.14600341579275	0.371	0.16	4.68167708884159E-07	10	BRCC3
1.3493519433729E-09	1.14584152310036	0.314	0.134	2.2612439867043E-05	10	CDHR2
1.75754762837898E-13	1.14372025048938	0.407	0.173	2.94529831563749E-09	10	TRIM47
3.15624907446468E-17	1.14324206261518	0.493	0.205	5.28924219898791E-13	10	RNF125
1.29208107891122E-17	1.14187490086699	0.55	0.235	2.16526947203942E-13	10	ABHD3
1.80900410001528E-10	1.14058124465127	0.35	0.152	3.0315290708056E-06	10	PFN2
1.65803605816829E-26	1.12798591952452	0.964	0.573	2.77853682627842E-22	10	TDO2
1.07679902210944E-18	1.12643030643361	0.464	0.182	1.804499801251E-14	10	SULF2
4.52964102201131E-10	1.12329021512529	0.314	0.13	7.59077242468656E-06	10	ATP5F1C
4.41743282004703E-12	1.12263490489237	0.286	0.104	7.40273391983481E-08	10	GSTA4
6.51101964007383E-27	1.12119712023971	0.936	0.486	1.09111667128357E-22	10	MUC13
2.1235208195612E-16	1.1189841265547	0.429	0.168	3.55859618942066E-12	10	SOX9
3.49885298753429E-28	1.11568100528558	0.857	0.456	5.86337783650996E-24	10	CTSS
3.69594095385866E-17	1.1134965841648	0.564	0.256	6.19365785047634E-13	10	MALL
1.14688338725615E-11	1.11136606161602	0.421	0.188	1.92194718036386E-07	10	SFN
4.77357969929442E-20	1.11048591095854	0.529	0.207	7.99956486007759E-16	10	CNP
4.60815456776556E-09	1.10924571987187	0.25	0.097	7.72234542466153E-05	10	ALG14
7.18014992557652E-16	1.10917061745976	0.493	0.209	1.20324952452811E-11	10	RCN1
1.38870245747913E-18	1.10850052965555	0.293	0.082	2.32718757824353E-14	10	CELSR1
1.99465279466697E-15	1.10143624626757	0.407	0.158	3.34263915330291E-11	10	STXBP6
4.18981215395638E-07	1.10050525011185	0.286	0.139	0.0070212872076001	10	CLGN
2.68811639095193E-12	1.09998397491254	0.264	0.091	4.50474544795725E-08	10	EMC6
1.39331717510522E-15	1.09973574587273	0.771	0.457	2.33492092204133E-11	10	TPM1
6.47669189941688E-26	1.09826184075081	0.864	0.442	1.08536402850428E-21	10	PGM2
2.29693134097809E-12	1.09490823767972	0.414	0.18	3.84919754121108E-08	10	TIGD2
1.10391539925281E-14	1.09464932370225	0.307	0.102	1.84994142606786E-10	10	MIPOL1
3.54779183352424E-20	1.08967164545264	0.7	0.325	5.94538955461993E-16	10	NSDHL
2.94517707096767E-18	1.08524855606126	0.357	0.115	4.93552773552762E-14	10	HELZ2
1.94064177281541E-20	1.08313586653733	0.357	0.108	3.25212748288406E-16	10	RELB
1.03702419673494E-14	1.0815399710716	0.436	0.181	1.73784514888842E-10	10	MID2
8.55333423557768E-24	1.08093346157527	0.879	0.546	1.43336775119811E-19	10	CLDN1
2.742829202098E-07	1.07970180869462	0.279	0.131	0.00459643317687583	10	PSMG1
1.47718110532864E-10	1.07687996941547	0.364	0.161	2.47546009630974E-06	10	CDC42EP5
2.23583181655155E-18	1.07033336932718	0.814	0.43	3.74680695817708E-14	10	CLDN4
3.62625146503926E-15	1.06894521201457	0.307	0.101	6.0768722051128E-11	10	CSRP2
1.24296509084776E-18	1.06746961879171	0.707	0.366	2.08296089924267E-14	10	TM7SF3
1.15675114464691E-13	1.06667967987738	0.286	0.095	1.9384835681993E-09	10	ZXDB
1.25663658727567E-15	1.06480162908284	0.393	0.145	2.10587159295656E-11	10	CYB561
5.69656113211264E-19	1.06427627811727	0.686	0.322	9.54629714519435E-15	10	CNKSR3
6.72080183979982E-13	1.06263374279121	0.329	0.123	1.12627197231365E-08	10	SLC2A5
3.96681158006449E-13	1.06027554869686	0.329	0.122	6.64758284587207E-09	10	GPR180
6.82818359120545E-10	1.06003094718165	0.436	0.21	1.14426700621421E-05	10	DDR1
1.695254400209E-09	1.05921150027136	0.3	0.126	2.84090732387024E-05	10	DEPRECATED-ENSG00000125675
2.17834292194861E-20	1.0581457854754	0.636	0.283	3.65046706860148E-16	10	NUB1
2.09909155217453E-08	1.05407748945436	0.321	0.15	0.000351765762313408	10	MMP28
6.51076970543265E-13	1.05314487137667	0.443	0.202	1.0910747872364E-08	10	TNFAIP2
2.22877771100386E-11	1.05144588050524	0.279	0.104	3.73498568810027E-07	10	SF3B6
1.30983348367622E-17	1.05055853151282	0.6	0.27	2.19501895194461E-13	10	CDKN2A
1.3721354175084E-14	1.05003984689718	0.786	0.484	2.29942453266057E-10	10	LSR
1.01198749260993E-28	1.04907689832763	0.957	0.646	1.69588864011572E-24	10	BST2
3.81063822703514E-18	1.04832966919288	0.521	0.217	6.38586754086549E-14	10	UBE2L6
3.74749521086773E-15	1.04828834806182	0.4	0.152	6.28005247437214E-11	10	ZSWIM5
2.09887901767664E-12	1.04441702084312	0.457	0.211	3.51730145782252E-08	10	USP54
1.52763327791024E-09	1.03736348827153	0.257	0.099	2.56000784712198E-05	10	IPO11
9.06727667279849E-10	1.03618900495059	0.257	0.098	1.51949422482757E-05	10	H2AFY2
1.17997782206407E-18	1.03618390437815	0.721	0.349	1.97740683421497E-14	10	TMPRSS2

1.03373860115981E-12	1.03516791183944	0.364	0.144	1.73233914782361E-08	10	POU5F1
6.00975512123936E-27	1.0328427239291	0.929	0.601	1.00711476321729E-22	10	TAPBP
1.45301697392403E-22	1.0291692825927	0.843	0.466	2.43496584490189E-18	10	PSMB8
1.33826460370622E-18	1.02843073740886	0.643	0.294	2.24266382289088E-14	10	CETN2
4.5859370592018E-22	1.02706169788563	0.836	0.425	7.68511332381038E-18	10	TMEM45B
8.30780257062524E-15	1.02659218909185	0.364	0.135	1.39222155478538E-10	10	MOB3B
9.06267571462811E-22	1.02275506209565	0.35	0.099	1.51872319625738E-17	10	SRD5A1
7.10769344237327E-13	1.02166700167281	0.286	0.101	1.19110726707291E-08	10	C2CD2
1.36544910866084E-09	1.02105748158456	0.321	0.139	2.28821961629383E-05	10	CHST5
1.0344224936573E-24	1.01867800870972	0.443	0.135	1.7334852148709E-20	10	CIITA
3.64527145959989E-11	1.01757841355762	0.257	0.093	6.10874591199749E-07	10	ZNF697
1.67823215665813E-11	1.01597599394647	0.321	0.127	2.81238144812769E-07	10	OSBPL10
2.13891230040288E-10	1.01496691165488	0.293	0.117	3.58438923301515E-06	10	PAIP2
5.33824196968074E-12	1.01319188063039	0.293	0.108	8.94582589279099E-08	10	CHPF
4.1317067493809E-18	1.01283847831005	0.407	0.142	6.9239141706125E-14	10	MMD
5.54695021405602E-12	1.01245650480374	0.436	0.198	9.29557916871508E-08	10	PHLPP2
3.2779401592991E-12	1.01157565523645	0.321	0.121	5.49317211895344E-08	10	PARVA
4.30575220657364E-19	1.00764842161569	0.65	0.283	7.21557954777611E-15	10	CDS1
1.00915167054258E-12	1.00750056173201	0.364	0.146	1.69113636949526E-08	10	WDR41
1.20577042693636E-16	1.00688350625641	0.679	0.343	2.02063008145996E-12	10	NECAB3
1.51116652551862E-13	1.00278215330445	0.257	0.081	2.5324128634641E-09	10	EFNA2
8.66714347638358E-24	1.00142415180947	0.571	0.212	1.45243990377236E-19	10	APOL1
7.34340039753715E-16	1.00116201259577	0.443	0.175	1.23060703861928E-11	10	INPP1
1.92044162360668E-14	1.0003589647212	0.379	0.147	3.21827607284007E-10	10	GBP3
5.83820747346431E-22	0.997611798169021	0.664	0.287	9.78366808403149E-18	10	TP53BP2
9.88720992043666E-16	0.994884727188873	0.571	0.262	1.65689863846678E-11	10	CKAP4
1.02380988200386E-18	0.989693883463957	0.679	0.303	1.71570060026207E-14	10	PYGO2
3.05282471057758E-18	0.988627885446787	0.679	0.319	5.11592364998591E-14	10	SYBU
3.74733997227548E-21	0.986644594624736	0.9	0.536	6.27979232553925E-17	10	MUC3A
1.02584867667935E-12	0.986270607231038	0.464	0.211	1.71911721237925E-08	10	E2F1
7.74471278902345E-22	0.986200664505128	0.771	0.378	1.29785896918455E-17	10	CCL15
3.90734910075092E-08	0.98586497334661	0.264	0.114	0.00065479356230384	10	DEPRECATED-ENSG00000186204
1.14916390941178E-15	0.985539514339988	0.371	0.134	1.92576887939225E-11	10	MSANTD3
5.3967859513442E-17	0.984960548970445	0.514	0.216	9.0439338972626E-13	10	MANEAL
1.36634688643353E-13	0.9843915772124	0.4	0.163	2.28972411228532E-09	10	AKR1C1
3.4523497165626E-15	0.981845908965586	0.486	0.206	5.7854476550156E-11	10	ELF4
8.47119002366919E-21	0.981699530605235	0.65	0.292	1.41960202416648E-16	10	UBE2Z
1.37510498961456E-14	0.97992584336483	0.479	0.208	2.30440094159607E-10	10	LY6E
1.22005642119756E-12	0.979226960802319	0.479	0.225	2.04457055064286E-08	10	CXADR
9.59636222215173E-18	0.975374495716788	0.414	0.146	1.60815838118819E-13	10	GUCY1B1
1.100056753076E-15	0.975242027060958	0.393	0.143	1.84347510680475E-11	10	PHYHIPL
5.78224381403978E-10	0.971797155407953	0.443	0.218	9.68988418356786E-06	10	GBP1
1.3310245131196E-09	0.970512060067067	0.264	0.103	2.23053087908583E-05	10	PACRG
4.46442270182354E-13	0.967014592700704	0.357	0.137	7.48147956371588E-09	10	THSD4
3.36485108143417E-13	0.965804479253907	0.421	0.173	5.63881744226737E-09	10	ANAPC13
4.4330769472494E-22	0.965455815020729	0.843	0.411	7.42895034820054E-18	10	F2RL1
6.98361226612051E-17	0.963938617885459	0.471	0.18	1.17031374355648E-12	10	PLPP2
3.4314412402897E-13	0.962461204198563	0.479	0.216	5.75040923047748E-09	10	MTCP1
6.41286997604291E-10	0.961634673847919	0.293	0.12	1.07466875058527E-05	10	TMEM171
1.76122008252635E-08	0.960297336079326	0.257	0.107	0.000295145261429767	10	GUF1
8.44417002266441E-17	0.958696906267337	0.586	0.268	1.4150740123981E-12	10	PARP9
2.23853201427943E-16	0.958355397635683	0.657	0.308	3.75133194952947E-12	10	GMDS
1.17806787559216E-15	0.956866026446117	0.593	0.271	1.97420614591735E-11	10	NIPAL2
3.13864351629088E-16	0.956807169364199	0.55	0.238	5.25973880460026E-12	10	LMBRD2
1.83634637387016E-15	0.9547843983974	0.7	0.367	3.07734925333161E-11	10	MGAT2
1.30222972781785E-23	0.953003929431997	0.907	0.595	2.18227657787716E-19	10	PSME1
1.05887233399027E-19	0.951675548763736	0.836	0.455	1.77445825730089E-15	10	TMEM205
2.53742534661924E-13	0.94931669826984	0.314	0.111	4.25221739586453E-09	10	TCAF2
6.39878035693773E-20	0.944380314618477	0.786	0.398	1.07230761221562E-15	10	DEGS1
9.12589933977704E-13	0.943794172553134	0.407	0.171	1.52931821135984E-08	10	FZD6

1.18863990404249E-10	0.943559647180323	0.264	0.097	1.9919227511944E-06	10	NCOA5
6.40028922019272E-08	0.942873648948581	0.25	0.106	0.0010725604675199	10	ADGRG2
1.24577467045321E-09	0.93833527236971	0.293	0.119	2.08766919274549E-05	10	SNX7
4.56808823071562E-16	0.936259859853409	0.45	0.177	7.65520225703324E-12	10	CEACAM1
1.05368793864683E-10	0.935868154994643	0.25	0.089	1.76577024758436E-06	10	TMEM104
9.89388218147539E-13	0.935413119202926	0.3	0.108	1.65801677597165E-08	10	SCO2
5.70602568342251E-07	0.935099668298188	0.314	0.156	0.00956215784027945	10	PRPS2
1.03150511946742E-14	0.935004082395898	0.543	0.247	1.7285962792035E-10	10	CADM1
2.69338833240423E-09	0.933954917370451	0.371	0.175	4.51358016744301E-05	10	C12orf75
5.12045193715981E-15	0.9337897290637	0.6	0.289	8.58085335629241E-11	10	PARP10
9.35832789491668E-11	0.930840676934398	0.336	0.139	1.56826858863014E-06	10	AGBL5
1.28034371022713E-19	0.930384508552783	0.9	0.553	2.14559998959862E-15	10	PROX1
1.34757527097812E-12	0.929870259305223	0.25	0.079	2.25826663910513E-08	10	SOCS1
1.97710030209787E-24	0.929124122887279	0.943	0.63	3.3132246862556E-20	10	LAMP2
1.23964758037657E-10	0.926653896801154	0.264	0.099	2.07740141519505E-06	10	ARNTL2
3.19851522953732E-13	0.925616581673092	0.264	0.087	5.36007182165864E-09	10	SNX33
3.99532148424404E-12	0.923021737511397	0.507	0.237	6.69535974329616E-08	10	PSENEN
2.12543269952653E-13	0.922811140066419	0.436	0.185	3.56180011786656E-09	10	BAIAP2L2
1.39940964162747E-18	0.922686959935225	0.671	0.305	2.34513067743931E-14	10	CTSO
1.8048231675046E-12	0.922566679138946	0.657	0.355	3.0245226641042E-08	10	CLDN3
8.87040321907435E-12	0.922195589145836	0.286	0.103	1.48650217145248E-07	10	RAB32
2.43972028604865E-16	0.921183703572227	0.486	0.201	4.08848325536034E-12	10	RFX5
1.54354245844148E-12	0.920539436616889	0.557	0.285	2.58666845185623E-08	10	VDAC1
1.00100299158756E-08	0.918530173996988	0.271	0.112	0.000167748081330243	10	NDUFA12
3.8462023089762E-11	0.917962825971209	0.257	0.092	6.44546582938231E-07	10	SGMS2
2.01997710149253E-10	0.917812759108724	0.336	0.143	3.38507762668118E-06	10	ATP11C
8.12443941582943E-19	0.913553073787553	0.686	0.315	1.3614935573047E-14	10	DEPRECATED-ENSG00000057294
1.12407062162229E-23	0.913392794781142	0.986	0.674	1.88371754771464E-19	10	S100A10
5.79163517093826E-14	0.91192946502848	0.436	0.178	9.70562221945833E-10	10	SLC7A1
2.20615868981469E-20	0.910689658821624	0.936	0.636	3.69708073239146E-16	10	LGALS4
1.72634135896625E-15	0.910684375087726	0.521	0.224	2.89300284935564E-11	10	SHC2
1.31585005197891E-15	0.909391561229793	0.536	0.241	2.20510151710626E-11	10	MBOAT1
8.16750058921881E-14	0.90886926508044	0.8	0.525	1.36870974874129E-09	10	SDC4
6.28405071488552E-09	0.908842151199097	0.314	0.137	0.000105308121880051	10	NT5C3A
9.38989464640643E-12	0.907982038480997	0.321	0.124	1.57355854484479E-07	10	DSG2
2.44289125169134E-13	0.905293171082998	0.486	0.216	4.09379715958434E-09	10	MISP
2.41335054953932E-13	0.904057451743725	0.493	0.22	4.04429285091799E-09	10	HIP1R
1.94101702857221E-13	0.902413756676169	0.471	0.204	3.25275633648131E-09	10	IRF7
7.32964218962627E-21	0.901910985894849	0.879	0.518	1.22830143813757E-16	10	S100A16
3.99042526516294E-08	0.901222442568068	0.336	0.161	0.000668715465936006	10	LRIG3
9.69791365277461E-16	0.900633028032697	0.407	0.153	1.62517636993197E-11	10	PSMD11
1.94346022892653E-17	0.899317281204914	0.764	0.411	3.25685065163508E-13	10	CIAO2A
8.71154333911122E-13	0.897940141204012	0.371	0.149	1.45988043276826E-08	10	UCHL3
2.55400108405663E-16	0.897892738829741	0.593	0.275	4.27999501666211E-12	10	PARP12
5.69731554583923E-21	0.897632694445746	0.843	0.438	9.54756139171739E-17	10	INF2
7.77782868348985E-15	0.895851235796684	0.543	0.243	1.30340853077923E-10	10	TBC1D8B
4.20953948206363E-09	0.89531820796344	0.264	0.107	7.05434626404224E-05	10	NKIRAS2
6.5162606446262E-21	0.893611510423727	0.464	0.16	1.09199495882646E-16	10	CREB3
5.14215692200654E-16	0.893090020837449	0.636	0.295	8.61722656989856E-12	10	FAM20B
1.38381456789793E-15	0.891942584297242	0.486	0.206	2.31899645288336E-11	10	SGMS1
2.55464321136959E-14	0.891494710308114	0.4	0.158	4.28107109361315E-10	10	DNAAF2
4.80349228559661E-14	0.891296504684207	0.636	0.323	8.0496923722028E-10	10	NEB
1.28952635636124E-11	0.891179169532234	0.307	0.116	2.16098826799017E-07	10	PCNX3
3.54290449754445E-07	0.890850180159917	0.257	0.116	0.00593719935698499	10	PCDHGA5
4.56271927913489E-17	0.890273373849388	0.629	0.281	7.64620496797424E-13	10	CYP4F12
1.53380882042024E-08	0.890256406723967	0.3	0.132	0.000257035682126025	10	BMS1
1.10099130300472E-10	0.889648307594673	0.486	0.242	1.84504122557531E-06	10	INAVA
5.8819924321334E-11	0.888771419970457	0.457	0.217	9.85704291776914E-07	10	ADNP
1.19096101219921E-10	0.887562586147657	0.421	0.195	1.99581246424343E-06	10	SLC25A43
1.24509135748381E-14	0.886031656537064	0.507	0.222	2.08652409687137E-10	10	PCSK5

1.10749036925102E-15	0.883080488371111	0.586	0.276	1.85593236079086E-11	10	MMP14
6.31369006364136E-16	0.880315361220812	0.521	0.224	1.05804818086502E-11	10	DEPRECATED-ENSG00000170421
5.50294479876104E-21	0.879810138989394	0.886	0.454	9.22183489376374E-17	10	ATP1A1
3.73509980283581E-18	0.877836788036958	0.793	0.435	6.25928024959226E-14	10	NFIB
1.76290195641218E-09	0.876222149031025	0.293	0.122	2.95427109855552E-05	10	XYLT2
3.95593486728862E-11	0.875788198867437	0.35	0.147	6.62935565060228E-07	10	ARMCX5
9.35218422690318E-10	0.875282600292555	0.35	0.157	1.56723903274444E-05	10	DEPRECATED-ENSG00000107438
2.27434238766976E-13	0.874492843405485	0.471	0.203	3.81134297325699E-09	10	DARS
2.09335374729798E-11	0.874478465060591	0.429	0.19	3.50804220972196E-07	10	SEMA6A
1.86563648453825E-13	0.872999310226294	0.379	0.151	3.1264336207892E-09	10	CSF1
1.55661739526497E-10	0.87296271605863	0.343	0.145	2.60857943098504E-06	10	DEPRECATED-ENSG00000232119
5.65851716861482E-12	0.872955229607916	0.421	0.19	9.48254307116472E-08	10	EIF2AK2
1.0008181251747E-21	0.872107568036496	0.879	0.505	1.67717101416777E-17	10	SSR4
1.18653901576842E-10	0.871745980903165	0.45	0.217	1.98840208262472E-06	10	CLDN14
3.36842850790541E-11	0.870956256587399	0.493	0.234	5.64481249354788E-07	10	MGAM2
4.15440433984213E-09	0.870544349189734	0.293	0.125	6.96195079270744E-05	10	PPIL1
3.22411284483752E-12	0.86780416480868	0.414	0.176	5.40296830537872E-08	10	MYO5B
4.66567263006673E-09	0.86731441309561	0.25	0.099	7.81873419346582E-05	10	KIAA1211
6.3036330938905E-10	0.867158910904514	0.314	0.131	1.05636283387417E-05	10	CNTFR
1.92951930141752E-10	0.864524790834793	0.314	0.126	3.23348844531548E-06	10	MAP3K14
2.51576311055678E-11	0.864439983510857	0.507	0.251	4.21591582067104E-07	10	TPM4
4.12519251406766E-17	0.863236921353695	0.771	0.393	6.91299761507459E-13	10	FRK
7.65984507710543E-07	0.860562138146877	0.329	0.164	0.0128363683802133	10	ACYP1
8.04068053255458E-13	0.859182519677196	0.514	0.239	1.3474572436455E-08	10	MS4A8
2.74910538605417E-09	0.858844730647292	0.264	0.105	4.60695080594958E-05	10	B4GALT6
8.40980617651725E-17	0.858169765869999	0.614	0.285	1.40931531906076E-12	10	EDNRB
1.92063072504115E-09	0.858049211005403	0.429	0.206	3.21859296902396E-05	10	SAMD12
5.25704285898773E-13	0.856042316875957	0.464	0.199	8.80975242309163E-09	10	TOMM5
2.26288656831688E-07	0.853044436441445	0.3	0.143	0.00379214531118542	10	NOL3
6.42548500476902E-19	0.851041668786633	0.7	0.333	1.07678277709919E-14	10	PSMB10
2.81533512580121E-08	0.850033240309181	0.321	0.148	0.000471793860381767	10	CDC42EP4
1.19706832203091E-08	0.84716912033947	0.271	0.113	0.00020060470940594	10	ENKD1
3.5145244277016E-08	0.846573426980898	0.321	0.148	0.000588964003594235	10	RAVER2
2.64794853193052E-10	0.845591521036575	0.343	0.147	4.43743214980917E-06	10	SYNJ2BP
1.2955252055103E-19	0.845108707139086	0.9	0.51	2.17104113939415E-15	10	RNF128
1.42622751346232E-15	0.843417468207827	0.536	0.237	2.39007206706015E-11	10	RAB3IP
1.49299943976744E-11	0.841991893935916	0.329	0.13	2.50196846116227E-07	10	RWDD1
9.80571966531201E-20	0.841320943024777	0.336	0.095	1.64324250151299E-15	10	GBP7
1.95697800591557E-12	0.839199213830395	0.414	0.177	3.27950374231332E-08	10	PSMC1
2.34415804476691E-11	0.837488846616274	0.371	0.16	3.92834005142039E-07	10	PDP1
4.88331729706975E-12	0.837365591872604	0.414	0.181	8.18346312642948E-08	10	P4HA2
3.19125385372739E-15	0.836432349307712	0.729	0.382	5.34790320807636E-11	10	VBP1
5.05122788504588E-10	0.836175077661854	0.421	0.2	8.46484768975989E-06	10	ADH5
1.83333118068076E-18	0.834892380464562	0.614	0.269	3.07229639258482E-14	10	IL17RB
5.03629321618848E-10	0.833451713487971	0.364	0.163	8.43982017168866E-06	10	GOLGA8B
5.81220162459374E-11	0.83219063851453	0.443	0.208	9.7400874824942E-07	10	VCPIP1
1.91625936427763E-08	0.830837839209494	0.293	0.129	0.000321126744265644	10	SPAG16
1.065437319288E-23	0.829676980814773	0.993	0.716	1.78545985966282E-19	10	PGRMC1
9.62082006481156E-15	0.829473494338607	0.793	0.456	1.61225702646112E-10	10	ENO1
1.91887476055475E-13	0.829189594525288	0.293	0.1	3.21565032373765E-09	10	PACSIN3
1.59619825656047E-11	0.828612018577895	0.536	0.273	2.67490903834404E-07	10	GIPC1
7.07277751273865E-10	0.828443790471523	0.4	0.19	1.18525605558474E-05	10	ACOT7
1.7993181427758E-10	0.82805728278715	0.414	0.194	3.01529734366369E-06	10	EIF4E
4.00813123905732E-19	0.826731237165506	0.764	0.37	6.71682633041225E-15	10	VMA21
8.2461131113515E-12	0.824300940630315	0.264	0.092	1.38188363520028E-07	10	MDP1
4.95336466673373E-12	0.823908666172276	0.386	0.165	8.30084850851239E-08	10	TICAM1
2.39196448619747E-09	0.823789590739426	0.336	0.146	4.00845408596971E-05	10	FRY
9.86016205681272E-20	0.823228949451402	0.793	0.431	1.65236595748068E-15	10	GSDMD
1.27247707481417E-09	0.82283288024834	0.486	0.248	2.13241708197359E-05	10	GOLGB1
4.66028823978493E-14	0.821608139617362	0.607	0.295	7.80971103223158E-10	10	TMEM164

2.89384369654677E-14	0.821326427899494	0.721	0.39	4.84950326667308E-10	10	CPQ
8.14035782512591E-10	0.821149492356265	0.321	0.134	1.3641611643346E-05	10	PSMB3
1.26264464474967E-09	0.82027069263166	0.357	0.16	2.11593989567149E-05	10	SNRPE
8.1612861945662E-07	0.818334830696679	0.264	0.121	0.013676683404854	10	RABGGTA
3.85717937137579E-11	0.817216676106614	0.364	0.151	6.46386119055154E-07	10	THEM6
1.09834196718566E-07	0.815538181870366	0.25	0.106	0.00184060146860973	10	VKORC1L1
2.07631411693937E-11	0.814931532633069	0.521	0.258	3.47948719716699E-07	10	SSX2IP
5.27725780762046E-07	0.814869765123141	0.279	0.129	0.00884362863401037	10	DEPRECATED-ENSG00000147804
2.06247609520629E-11	0.814541227321765	0.457	0.217	3.4562974403467E-07	10	SEC61B
9.68320549877502E-10	0.814382768447602	0.393	0.186	1.62271157748472E-05	10	PDGFA
8.56561724075345E-12	0.811342597003756	0.4	0.172	1.43542613720546E-07	10	NDRG3
1.6660301657501E-14	0.811017738696458	0.529	0.232	2.79193335176402E-10	10	HGH1
5.43473129443929E-08	0.809203221987526	0.3	0.138	0.000910752270322137	10	CCDC141
8.78177615063498E-12	0.806600741775668	0.479	0.225	1.47165004732341E-07	10	UGT1A9
2.37800920624058E-07	0.805775296468997	0.307	0.145	0.00398506782781796	10	ING2
1.5417920259283E-14	0.805374443274285	0.579	0.275	2.58373507705065E-10	10	CXorf56
4.9474429692081E-12	0.804658003562543	0.321	0.12	8.29092492779894E-08	10	AIFM2
4.50089799968135E-12	0.804313709152605	0.471	0.215	7.542604867866E-08	10	EBAG9
7.44458896687512E-09	0.802665395202896	0.414	0.204	0.000124756421906893	10	ACO2
1.71420102082318E-10	0.801736057831969	0.293	0.113	2.87265807069548E-06	10	FAM222A
1.15171075092207E-15	0.801374239807162	0.343	0.118	1.9300368763952E-11	10	EPSTI1
1.23343068506198E-08	0.799223699184607	0.357	0.168	0.000206698314202687	10	WFS1
2.00231192789295E-11	0.795853845246886	0.693	0.402	3.355474328763E-07	10	ABHD2
1.26250550391827E-14	0.794572065659402	0.593	0.281	2.11570672346624E-10	10	ERAP1
6.19493364842023E-10	0.79449884742671	0.407	0.193	1.03814698080226E-05	10	PTEN
2.41797512582081E-14	0.794485545110851	0.843	0.538	4.05204271585052E-10	10	SCD
9.54661712664558E-08	0.794446327821771	0.35	0.172	0.00159982209808327	10	AP3S2
3.13560984953125E-16	0.794402457434516	0.579	0.249	5.25465498584446E-12	10	SI
5.84695198162758E-14	0.793624203161662	0.486	0.222	9.7983221308115E-10	10	KIAA1217
1.40117145659525E-13	0.792503571975249	0.536	0.254	2.34808312696232E-09	10	SLC39A1
1.19587372364398E-10	0.792499828573448	0.471	0.228	2.00404518608258E-06	10	ZNF75D
2.33488805096589E-12	0.789816098483389	0.307	0.112	3.91280539580864E-08	10	PAIP1
1.0781077904088E-09	0.787374690872874	0.379	0.178	1.80669303516707E-05	10	MPZL1
1.6018580995672E-17	0.786520033247075	0.857	0.52	2.68439380325472E-13	10	TRAM1
5.21483831896801E-08	0.786424534900699	0.414	0.212	0.000873902605492659	10	PRELID2
3.02757987615514E-07	0.786289528697856	0.35	0.182	0.00507361835646079	10	THNSL2
6.07811159550239E-13	0.783526952596026	0.564	0.262	1.01856994117429E-08	10	SH3BP4
2.50644428627832E-09	0.782120437704108	0.457	0.23	4.20029933494521E-05	10	GRIA3
5.24086295473988E-13	0.781972659438212	0.479	0.21	8.78263813955309E-09	10	SLC16A2
1.92266648703349E-11	0.779437132221378	0.493	0.233	3.22200449897073E-07	10	IL17RC
6.60477421771757E-12	0.779232957296533	0.579	0.283	1.10682806340511E-07	10	KCNK5
4.24626195886703E-16	0.778610755203266	0.836	0.474	7.11588579066938E-12	10	TSPAN8
3.39652201215199E-09	0.778440159728112	0.321	0.141	5.69189158796431E-05	10	ANO10
1.60303369749531E-10	0.778396719861692	0.436	0.21	2.68636387026263E-06	10	XAF1
4.25852497723149E-07	0.777368906644412	0.257	0.116	0.00713643615684453	10	CETN3
2.51810119150466E-14	0.776424983930615	0.671	0.35	4.2198339767235E-10	10	XIAP
6.69333092540839E-19	0.775734902529973	0.821	0.413	1.12166839647994E-14	10	HTATSF1
1.3969287814476E-08	0.774643452901746	0.279	0.119	0.000234097325194988	10	TSPOAP1
9.99863182722907E-18	0.774551257712772	0.921	0.603	1.67557072160705E-13	10	SGK1
9.93694808376919E-14	0.774352572428282	0.357	0.135	1.66523375987804E-09	10	GPC1
5.83136648184302E-15	0.773327126648177	0.743	0.385	9.77220395027254E-11	10	KLF5
8.50047812100766E-16	0.773215974423566	0.521	0.227	1.42451012351846E-11	10	IRF9
1.16423462339843E-10	0.7716769224462	0.436	0.206	1.95102438189109E-06	10	DIS3
1.17512659139047E-13	0.771395305820331	0.436	0.18	1.96927714185214E-09	10	CHM
1.62082965794289E-13	0.77112089651826	0.65	0.33	2.7161863407807E-09	10	FAM199X
9.60551173132191E-14	0.771019946369343	0.636	0.32	1.60969165593493E-09	10	MCTS1
1.3648147868604E-06	0.770212129932078	0.286	0.14	0.0228715661982065	10	LRRCC1
4.54782327490654E-14	0.769681885997581	0.514	0.231	7.62124224408838E-10	10	PRELID1
5.54985180049594E-16	0.769174061710042	0.514	0.216	9.3004416472711E-12	10	ATP8B1
1.15184825631179E-06	0.769152926910416	0.25	0.115	0.019302673079273	10	SGCB

4.63813744480291E-15	0.769098455554667	0.371	0.135	7.77259073000071E-11	10	PML
9.12975108554058E-14	0.768626903176397	0.464	0.198	1.52996368691489E-09	10	MYRF
6.4712171762803E-10	0.768567280485654	0.307	0.126	1.08444657440105E-05	10	RADX
1.00440179297148E-08	0.768038457031444	0.371	0.18	0.000168317652466161	10	ITPRIPL2
5.29458553801254E-07	0.767635836681539	0.414	0.222	0.00887266644460141	10	TJP3
1.90638932650083E-15	0.766709407958382	0.743	0.38	3.1947272333501E-11	10	SLC6A19
1.72355559110923E-08	0.76518741990969	0.279	0.119	0.000288833445958084	10	TTN
7.61908639993914E-11	0.764592726763185	0.357	0.15	1.2768064989018E-06	10	DYNC1H1
2.53597650251138E-18	0.763756570171053	0.764	0.405	4.24978942290857E-14	10	CALCOCO2
1.56288000185509E-13	0.76341362619908	0.621	0.321	2.61907430710876E-09	10	SPPL2A
4.45850504766154E-11	0.76330255551558	0.529	0.265	7.47156275887121E-07	10	STMP1
5.08005828949667E-10	0.762969235744192	0.336	0.142	8.51316168153852E-06	10	FAM53B
1.081253815442E-13	0.762469508107704	0.743	0.414	1.8119651439177E-09	10	PYGB
1.21990566828927E-11	0.762061327929214	0.35	0.145	2.04431791891916E-07	10	MXD1
8.67993682329339E-10	0.76205332926954	0.664	0.364	1.45458381284751E-05	10	DBI
8.63499067622718E-10	0.762011395349949	0.357	0.156	1.44705173752215E-05	10	IFI27L1
1.18397083799831E-13	0.761684120398233	0.471	0.206	1.98409833031756E-09	10	TM2D2
6.19703562261051E-18	0.761575965142198	0.907	0.523	1.03849922963707E-13	10	MBNL3
2.92619216765002E-11	0.761283471743944	0.429	0.195	4.90371283454791E-07	10	CASC4
1.27697782711545E-16	0.760903529014803	0.857	0.475	2.13995944268007E-12	10	ERICH5
3.9469090623997E-11	0.760729921978108	0.464	0.218	6.61423020676941E-07	10	BOK
7.4359037450677E-13	0.760534242087215	0.864	0.56	1.24610874959845E-08	10	KRT8
1.05470689847356E-10	0.758324129253985	0.479	0.238	1.76747782046199E-06	10	RCC1L
1.07798833028963E-14	0.75787913575413	0.657	0.322	1.80649284389936E-10	10	TBC1D8
4.42638054642183E-11	0.757419709655646	0.421	0.184	7.4177285196937E-07	10	IPMK
1.62907722498404E-10	0.756030871493329	0.393	0.175	2.73000761362826E-06	10	CCT4
3.64639950433275E-21	0.755710600403376	0.95	0.707	6.11063628936083E-17	10	SOD2
4.92401391356256E-12	0.754937900480892	0.493	0.227	8.25166251634814E-08	10	FAIM
1.31781590339207E-18	0.753972081887343	0.943	0.623	2.20839589090443E-14	10	GPX2
1.25011444715121E-13	0.753884932588161	0.614	0.293	2.094941790536E-09	10	NAXE
3.08125328497834E-15	0.753746135440197	0.671	0.338	5.1635642549667E-11	10	DTX3L
1.61583285922224E-11	0.752564387834968	0.536	0.26	2.70781270548462E-07	10	PDX1
2.1744136259071E-16	0.751595840577405	0.821	0.448	3.64388235429512E-12	10	NDUFB9
1.03910960224879E-12	0.75118292385002	0.321	0.119	1.74133987144853E-08	10	ATMIN
2.43416730124325E-17	0.751030352017495	0.936	0.548	4.07917756342344E-13	10	MPP1
9.40463563135247E-14	0.750928332084262	0.607	0.298	1.57602883910205E-09	10	ISOC1
3.00371126569339E-11	0.750457352435268	0.45	0.207	5.03361933904898E-07	10	ELOVL6
1.11188380602146E-10	0.749829516857814	0.671	0.394	1.86329488213075E-06	10	RAB4A
1.09113295047156E-11	0.749269877009445	0.407	0.176	1.82852059840024E-07	10	PLEKHA5
4.64354880349626E-15	0.748849294769741	0.436	0.173	7.78165908489904E-11	10	COPS4
1.80600079050592E-12	0.748706782125511	0.286	0.1	3.02649612472981E-08	10	LRP12
9.6074438483713E-10	0.748679889356513	0.743	0.46	1.61001544011006E-05	10	OCLN
4.66553917943642E-11	0.748665202306676	0.343	0.142	7.81851055689955E-07	10	CISD1
5.32687381839076E-13	0.748303287465773	0.607	0.307	8.92677514485924E-09	10	RTL8C
1.99254741975226E-12	0.746303496660521	0.293	0.104	3.33911096602084E-08	10	DRAM1
6.40926673710368E-13	0.746301022318011	0.693	0.375	1.07406491980384E-08	10	CYSTM1
5.22224598379744E-13	0.74542200847784	0.736	0.429	8.75143981964775E-09	10	EGLN1
5.30162862225711E-08	0.744614708647426	0.264	0.112	0.000888446924517846	10	JOSD2
8.24364062487062E-11	0.744478271888117	0.286	0.107	1.38146929591582E-06	10	SMYD4
7.11321292461709E-12	0.744140849736533	0.429	0.186	1.19203222190733E-07	10	NMI
2.44655726642514E-16	0.743604071462636	0.343	0.114	4.09994066707524E-12	10	ITGAM
7.43798211066513E-10	0.743393217879751	0.279	0.11	1.24645704210526E-05	10	STXBP4
5.24729508929544E-13	0.743239512813847	0.771	0.425	8.7934171106413E-09	10	ABCD1
1.94523800335403E-15	0.742202560766408	0.693	0.355	3.25982984602069E-11	10	RNF103
1.17280804144525E-13	0.740634060982483	0.45	0.191	1.96539171585394E-09	10	MVB12A
1.27898635511597E-11	0.740148080543233	0.329	0.128	2.14332533390334E-07	10	CCDC167
3.70478866858883E-13	0.739918517715857	0.257	0.083	6.20848485082116E-09	10	GPR157
2.55198946324622E-07	0.739752721239229	0.3	0.143	0.00427662394250802	10	PAQR9
8.98473347864626E-10	0.739713170276192	0.364	0.162	1.50566163635154E-05	10	MED1
4.02636111005122E-09	0.739379442772965	0.293	0.122	6.74737594822384E-05	10	ACLY

1.39653840606601E-12	0.738635480167372	0.9	0.631	2.34031906088542E-08	10	NFKBIA
2.01541841543528E-17	0.73860944209788	0.336	0.105	3.37743818058645E-13	10	RFTN1
4.24788583692991E-09	0.738344333369167	0.393	0.189	7.11860708552714E-05	10	GOLPH3L
4.2167057972551E-11	0.737794423393266	0.457	0.212	7.0663555750401E-07	10	NSF
8.54802584722267E-12	0.737305124606136	0.293	0.107	1.43247817147758E-07	10	NDUFA6
7.21941533131155E-10	0.735507760314881	0.329	0.141	1.20982962122119E-05	10	NSA2
1.06593233457427E-12	0.735377540917192	0.614	0.306	1.78628940627957E-08	10	LYPLAL1
1.18942580325358E-15	0.734601617317297	0.529	0.223	1.99323976109234E-11	10	DESI1
1.96314933880584E-15	0.734535169500922	0.55	0.239	3.28984566197083E-11	10	ARHGAP5
5.06848784001644E-10	0.733989844317927	0.329	0.138	8.49377192229955E-06	10	TAF9
2.32491853574336E-06	0.730977856186559	0.314	0.159	0.0389609848219873	10	PRR14L
1.13100861357052E-15	0.730853384546906	0.514	0.211	1.89534423462148E-11	10	TIMM50
5.18528439095617E-14	0.730686851880649	0.721	0.377	8.68949958236435E-10	10	PDZD8
2.35662599122889E-12	0.730611966270455	0.679	0.372	3.94923383610137E-08	10	SERPINB1
5.83264622933307E-08	0.730587623928727	0.5	0.286	0.000977434855111636	10	DNAJB9
1.37577825891632E-10	0.730295536470746	0.329	0.136	2.30552920629196E-06	10	MTMR11
6.23402869960606E-16	0.729895587529708	0.664	0.314	1.04469852947998E-11	10	PSMD10
2.50659425386555E-06	0.729822717973407	0.329	0.175	0.0420055065062789	10	COG2
3.31158747252672E-09	0.729580096462296	0.279	0.113	5.54955828646027E-05	10	MELTF
4.1278176892339E-10	0.729306168625063	0.443	0.213	6.91739688361816E-06	10	STXBP5
2.3976851168089E-08	0.729233042198291	0.457	0.244	0.000401804071874835	10	MCUR1
3.11307704402253E-14	0.729014846572572	0.671	0.347	5.21689451037296E-10	10	KIAA1522
1.1419971891285E-06	0.728690848128384	0.293	0.142	0.0191375888954154	10	PSD3
2.19068491275651E-10	0.728651086208926	0.45	0.225	3.67114977679736E-06	10	IFNGR2
1.76330065406996E-09	0.728622028794149	0.536	0.288	2.95493923609044E-05	10	PPP4R3B
1.39421930970712E-15	0.728591785980977	0.607	0.284	2.3364327192072E-11	10	ANXA7
5.39734605306846E-13	0.728491687103192	0.529	0.251	9.04487251573213E-09	10	TTC1
6.95352366060271E-12	0.728486009018873	0.421	0.182	1.1652714950438E-07	10	MYNN
6.91949277750876E-13	0.726975464356559	0.757	0.414	1.15956859965492E-08	10	PTPRK
5.12049761928128E-11	0.726860578638586	0.571	0.294	8.58092991039157E-07	10	NEU1
4.5826681625045E-16	0.725255459295341	0.814	0.455	7.67963530672505E-12	10	ADAR
1.23014690231581E-11	0.725061949327899	0.464	0.214	2.06148017890083E-07	10	WDR44
6.80438606172251E-07	0.724476469946716	0.386	0.203	0.0114027901622346	10	NR2F1
1.1665142758399E-14	0.724089283265496	0.629	0.314	1.95484462345251E-10	10	HSD3B7
7.41583817291187E-10	0.722420243309087	0.421	0.204	1.24274616101657E-05	10	MYD88
8.99760944577733E-15	0.721528708523032	0.907	0.567	1.50781939092337E-10	10	ATP1B1
4.0416276561313E-12	0.721148029164921	0.521	0.254	6.77295962614483E-08	10	PDLIM5
1.11155189978025E-15	0.720082943195461	0.907	0.508	1.86273867365174E-11	10	TSPAN3
8.78254082802018E-10	0.719251663656551	0.429	0.203	1.47177819195962E-05	10	PSMA4
6.25453498520938E-11	0.719124937338623	0.479	0.232	1.04813497282139E-06	10	APOOL
9.68718615097979E-09	0.718922520630787	0.314	0.14	0.000162337865518119	10	TDRD7
1.57288277700902E-10	0.718783805286349	0.45	0.214	2.63583695771172E-06	10	EIF2S3
1.32893000638559E-07	0.718721401346439	0.336	0.16	0.00222702090470097	10	EDARADD
7.51368697616868E-15	0.718326606111603	0.729	0.376	1.25914366346635E-10	10	CD99L2
4.16573494633358E-12	0.717273398097809	0.614	0.313	6.98093862306581E-08	10	PNPO
3.02693270012693E-16	0.716832181148124	0.457	0.178	5.07253381887271E-12	10	CYP7B1
2.8776360488042E-08	0.716007868356872	0.3	0.133	0.000482234249058607	10	CMTM4
3.32856754671043E-13	0.715720398323485	0.536	0.251	5.57801349477734E-09	10	DEPRECATED-ENSG00000001630
2.18089018859311E-09	0.714048830276936	0.357	0.161	3.65473577804433E-05	10	RPP21
4.70396429355348E-11	0.713294209984966	0.479	0.233	7.88290336313691E-07	10	POMP
1.06143047381573E-14	0.713100135893353	0.786	0.426	1.7787451880204E-10	10	LAPTM4B
3.17633780964637E-14	0.712964405270508	0.757	0.406	5.32290690140539E-10	10	LAGE3
2.06797118763096E-08	0.712952591130475	0.286	0.125	0.000346550611623196	10	MTX1
1.88477487744425E-14	0.712650717308181	0.564	0.252	3.15850573962108E-10	10	NCKAP1
7.77695203408978E-14	0.711943506656822	0.707	0.367	1.30326162187277E-09	10	NHSL1
6.20433400465316E-12	0.711660112009617	0.421	0.181	1.03972229249978E-07	10	RNF139
8.37629749494376E-09	0.711533015505347	0.343	0.158	0.000140369993420268	10	GUCY1A1
6.67811597899034E-14	0.7109064882096	0.536	0.245	1.1191186757592E-09	10	ABHD16A
3.26608838108895E-13	0.710877734664926	0.464	0.203	5.47331090902886E-09	10	PSMA3
1.56490557555464E-14	0.709496073692873	0.65	0.329	2.62246876351446E-10	10	ADAM9

1.86214504436503E-15	0.709361973925174	0.871	0.531	3.12058266534692E-11	10	BEX3
3.39223614241882E-08	0.709328505026043	0.314	0.144	0.000568470932746546	10	GPR137
5.07656779433177E-15	0.709185139811857	0.636	0.305	8.50731230974118E-11	10	ZDHHC9
2.36694937041306E-09	0.709022997376441	0.4	0.197	3.9665337549382E-05	10	LHFPL2
9.65149865780414E-11	0.707832796730786	0.557	0.276	1.61739814507482E-06	10	UGT2B11
3.37174034771246E-07	0.707596322946238	0.307	0.146	0.00565036247469654	10	C6orf132
2.14172521459689E-12	0.706745497823037	0.5	0.234	3.58910311462147E-08	10	PTPRH
2.84676064972257E-16	0.706386817652985	0.764	0.383	4.77060149680508E-12	10	CTPS1
8.76721477066812E-15	0.706168510471358	0.421	0.163	1.46920985126856E-10	10	ARL4A
2.40850313016802E-08	0.705446824471077	0.25	0.103	0.000403616954553557	10	ATP5MPL
6.5855094972298E-11	0.705356598109028	0.4	0.178	1.10359968154577E-06	10	OSTC
6.72284919471644E-10	0.704505952555233	0.443	0.209	1.12661506805058E-05	10	OST4
1.17179371495267E-14	0.703621134506514	0.779	0.457	1.96369190751769E-10	10	SYNGR2
3.59885007391585E-13	0.703571502763643	0.779	0.44	6.03095295386818E-09	10	AIFM1
3.70176475631406E-11	0.702866807294231	0.279	0.101	6.20341737863111E-07	10	CYP21A2
2.39443197600528E-08	0.701892569784655	0.329	0.15	0.000401258910538965	10	SDCBP2
1.41478077994039E-14	0.701095877657848	0.593	0.276	2.37088963102411E-10	10	RAB20
1.65751893056951E-13	0.700121691902881	0.907	0.538	2.77767022384839E-09	10	FKBP5
2.40071369115733E-08	0.700056026611582	0.357	0.169	0.000402311600364146	10	SEC22C
1.1547904344938E-14	0.699801074803198	0.614	0.299	1.93519781012471E-10	10	PSMD12
3.16379986421356E-12	0.699286242443475	0.686	0.382	5.30189581244909E-08	10	HACD3
5.61389121068486E-16	0.698850349602922	0.657	0.308	9.40775889086568E-12	10	CISD2
2.35907810850283E-10	0.696999109283437	0.35	0.148	3.95334309422904E-06	10	PRDX3
2.73210210225443E-06	0.696425024662819	0.386	0.21	0.0457845670295797	10	CCL28
3.42044638141039E-12	0.69641512554899	0.364	0.147	5.73198404596753E-08	10	LRRC42
4.97224356083935E-11	0.696009241424399	0.379	0.163	8.33248575925459E-07	10	DAPK2
7.99817562182847E-12	0.695959560214026	0.564	0.271	1.34033427070602E-07	10	PKP2
2.00763674342958E-08	0.695825187640297	0.493	0.261	0.000336439765463929	10	TMEM129
1.86365264426351E-07	0.695752139406277	0.386	0.202	0.00312310910125679	10	DGCR2
4.99794805475653E-09	0.695426794669205	0.35	0.158	8.375561350161E-05	10	RNF170
2.41238739944458E-13	0.69462280793733	0.536	0.251	4.04267880398923E-09	10	UBA1
5.18889651776048E-13	0.69426021467757	0.507	0.22	8.69555278446301E-09	10	GSKIP
3.15838440158767E-17	0.694026840606601	0.957	0.646	5.29282058018063E-13	10	ANXA4
3.71108139713042E-08	0.693879550700348	0.307	0.138	0.000621903020531116	10	SLC16A13
6.24384640199495E-16	0.693799442609533	0.807	0.446	1.04634378004631E-11	10	SSR2
3.70075339866939E-13	0.693206075731352	0.607	0.312	6.20172254549016E-09	10	LRRFIP2
7.45407235862803E-15	0.693157243677331	0.693	0.347	1.24915344585889E-10	10	EMC2
3.35863123051785E-07	0.693039747431574	0.286	0.131	0.00562839421610181	10	XRCC6
5.163013789519E-10	0.692892761150504	0.307	0.125	8.65217850847595E-06	10	TP53INP2
4.46683174214574E-14	0.692785745629897	0.779	0.412	7.48551663348783E-10	10	NOTCH2NLB
1.46656162095379E-17	0.691567332619696	0.743	0.394	2.45766396439436E-13	10	FOSL2
8.72706581538842E-08	0.691235715040906	0.414	0.212	0.00146248168934279	10	PSPH
1.61200231046304E-10	0.690908967040645	0.493	0.246	2.70139347187396E-06	10	ATP11A
1.05965440654368E-09	0.689988524937378	0.471	0.233	1.7757688544859E-05	10	MAP3K2
8.76031973415347E-15	0.689729099289219	0.786	0.389	1.46805438104944E-10	10	GRTP1
5.38462240781795E-12	0.689678821685334	0.307	0.115	9.02355023102133E-08	10	SRSF10
1.38016873217387E-18	0.689539579011081	0.75	0.376	2.31288676137698E-14	10	OPTN
6.81299652189427E-16	0.689067909061753	0.393	0.143	1.14172195713904E-11	10	PSME2
2.62537516554718E-09	0.688932524325091	0.429	0.209	4.39960370242396E-05	10	HSPA4L
1.96185468026285E-12	0.688741981080014	0.307	0.111	3.28767607318448E-08	10	PLEKHF1
7.89731498517075E-10	0.68776479886663	0.35	0.156	1.32343204521491E-05	10	SCAND1
4.98441972841436E-11	0.687734729419475	0.629	0.342	8.35289058087678E-07	10	PRKDC
7.42986125708317E-07	0.687615420246256	0.257	0.116	0.01245096149462	10	TMEM187
8.17497168440124E-13	0.686445295026346	0.829	0.486	1.36996175487196E-08	10	ATP6AP1
1.74366337133496E-12	0.68601902119192	0.586	0.296	2.92203107768312E-08	10	SLC35F6
2.47273552885909E-17	0.685883347397382	0.8	0.439	4.14381019926206E-13	10	EZR
6.35155706081914E-09	0.685718735625413	0.286	0.119	0.000106439393225207	10	ZNF839
8.69832659559044E-12	0.685416288519193	0.479	0.234	1.45766557088905E-07	10	ITGAV
8.513672585194E-10	0.684893866692831	0.414	0.192	1.42672125182681E-05	10	TBCE.1
5.08021762832966E-12	0.68438938036356	0.779	0.441	8.51342870155484E-08	10	UBE2A

**2.5609999608315E-11**	0.682394284443849	0.521	0.263	4.29172373436142E-07	10	POLR2I
**7.13698048201003E-11**	0.681755160927376	0.471	0.225	1.19601518917524E-06	10	NBN
**1.28134673513538E-16**	0.680714757687142	0.821	0.443	2.14728085873986E-12	10	IDH3G
**1.25971322006742E-12**	0.680539205108414	0.593	0.289	2.11102741418898E-08	10	AP2A1
**1.44791053553741E-08**	0.679685338202314	0.293	0.125	0.000242640847545359	10	ARHGAP21
**6.71068938752278E-09**	0.679613544757235	0.364	0.169	0.000112457732756107	10	PXMP4
**1.82949588371844E-10**	0.679453821385483	0.386	0.171	3.06586920193536E-06	10	GEMIN7
**5.68686936688611E-11**	0.679328562058625	0.479	0.23	9.53005568502775E-07	10	IRF2BPL
**3.78721216114812E-15**	0.679092676629421	0.464	0.19	6.34661013965201E-11	10	TBC1D1
**9.77633079115045E-10**	0.679005459425661	0.386	0.176	1.63831751398099E-05	10	SMARCE1
**3.22945975936527E-13**	0.678746324956253	0.8	0.467	5.41192866474432E-09	10	SAP18
**2.30263988078664E-12**	0.678667342751639	0.621	0.308	3.85876391222225E-08	10	CUL4B
**1.36647261099807E-14**	0.678240665076583	0.936	0.527	2.28993480151057E-10	10	NDRG1
**7.19026907456223E-08**	0.678046234098526	0.393	0.198	0.00120494529151514	10	VCP
**2.13626308037654E-15**	0.677199187326397	0.564	0.242	3.57994967009501E-11	10	TMEM123
**1.77173868090172E-14**	0.677072898083315	0.579	0.261	2.9690796814551E-10	10	LNX2
**2.06023416615781E-11**	0.677071277389218	0.586	0.299	3.45254041564725E-07	10	FNTA
**3.35020604136859E-14**	0.676901560184714	0.357	0.131	5.61427528412549E-10	10	PSMA1
**6.05254376254567E-09**	0.676641271578312	0.286	0.118	0.00010142852837274	10	TMEM184C
**3.98337357722229E-13**	0.67623201062433	0.786	0.461	6.67533744070911E-09	10	MYDGF
**9.82348321295629E-08**	0.67611843808416	0.443	0.237	0.00164621931682722	10	DCTD
**1.79269781062151E-10**	0.676110622576251	0.429	0.2	3.00420299103953E-06	10	CITED4
**3.60154465827397E-08**	0.6756416011143	0.379	0.184	0.000603546853833552	10	MBD3
**2.22560631868744E-07**	0.674921058855712	0.307	0.144	0.00372967106885642	10	FEM1B
**1.28788574101183E-11**	0.674830424268924	0.75	0.459	2.15823892478762E-07	10	RRBP1
**6.12829607923837E-08**	0.674614616635014	0.314	0.144	0.00102697985695877	10	KLHL42
**1.2988853042544E-11**	0.67444135877084	0.407	0.176	2.17667199286952E-07	10	CNIH4
**2.83341893849543E-14**	0.673074614258572	0.843	0.491	4.74824345713064E-10	10	MGST3
**9.91846996249624E-10**	0.672684631174282	0.257	0.098	1.66213719631512E-05	10	RAB24
**1.92450527235919E-13**	0.672262828793038	0.629	0.32	3.22508593541952E-09	10	TFG
**1.73081289549977E-11**	0.671525340054972	0.514	0.252	2.90049625027851E-07	10	SLC35B1
**9.63209223766765E-13**	0.671479395792789	0.721	0.385	1.61414601718834E-08	10	CLDN7
**4.24134971701627E-08**	0.670847006095217	0.257	0.108	0.000710765385577587	10	RAB11A
**2.76313554436462E-12**	0.670641200663384	0.507	0.238	4.63046254524623E-08	10	SLC46A1
**6.69085586450474E-10**	0.670415176476412	0.479	0.237	1.1212536257737E-05	10	PLOD1
**1.73478301684494E-09**	0.669595825115431	0.486	0.241	2.90714937962876E-05	10	MMGT1
**2.4663736854652E-10**	0.669136836029747	0.414	0.191	4.13314902210258E-06	10	LCLAT1
**1.79035477357852E-09**	0.669061780233366	0.514	0.271	3.00027652956289E-05	10	SNX6
**7.98854388740787E-12**	0.667726181201501	0.607	0.311	1.33872018465181E-07	10	PARP4
**2.95539750103308E-06**	0.667327910525942	0.264	0.126	0.0495265513223123	10	SIX5
**3.86901881240408E-10**	0.667319988402105	0.507	0.25	6.48370172582675E-06	10	RBM41
**3.74175682528992E-13**	0.666993053976587	0.357	0.135	6.27043608782085E-09	10	NUDT16L1
**1.95739440299006E-13**	0.666962060760649	0.736	0.38	3.28020154053075E-09	10	DGAT2
**2.20172242847128E-07**	0.666401119778984	0.25	0.108	0.00368964644563217	10	USP31
**8.30355447938597E-09**	0.666251875915392	0.286	0.12	0.00013915096596555	10	VCL
**2.29023405770052E-15**	0.666073685654626	0.85	0.471	3.83797423389453E-11	10	G6PD
**3.9677930589973E-13**	0.6651355233277	0.621	0.315	6.64922760826767E-09	10	TMEM38B
**2.02242556264009E-09**	0.664947255305676	0.4	0.194	3.38918075787227E-05	10	FKBP3
**4.98937713377881E-12**	0.664783606654262	0.279	0.096	8.36119820078653E-08	10	CBX1
**1.96046977312684E-08**	0.664520023626336	0.393	0.189	0.000328535524580596	10	MTX2
**9.40082790163564E-09**	0.664478975462466	0.393	0.189	0.00015753907397561	10	PPP1CC
**1.97536843697547E-15**	0.664042040184826	0.836	0.439	3.3103224266835E-11	10	PEG10
**7.14080226732839E-14**	0.663791389289141	0.557	0.262	1.19665564395889E-09	10	PLAAT3
**1.77435163908829E-13**	0.663199907230461	0.8	0.447	2.97345847678416E-09	10	TSPAN6
**7.5185160898948E-09**	0.66260302442343	0.55	0.298	0.000125995292634457	10	NUP58
**7.99433569477378E-11**	0.662581355087696	0.507	0.25	1.33969077573019E-06	10	GINM1
**4.29455401750403E-11**	0.66195963000955	0.536	0.263	7.19681362253325E-07	10	KCNJ3
**1.85926545612637E-14**	0.661415008910967	0.814	0.447	3.11575705137658E-10	10	MDK
**1.44097242223791E-13**	0.66134364685497	0.693	0.361	2.41478158518628E-09	10	RAB5IF
**2.9273683236785E-08**	0.661105371977681	0.3	0.133	0.000490568383682044	10	UBQLN1

2.50589090243195E-09	0.660668455510288	0.314	0.135	4.19937197429546E-05	10	COX19
2.23502149065908E-09	0.660188351412307	0.371	0.168	3.74544901404649E-05	10	HSD17B12
3.04079220772317E-14	0.659950629415963	0.936	0.636	5.09575958170249E-10	10	UGT2B4
2.20782413482269E-07	0.659608433967311	0.279	0.125	0.00369987168513586	10	DEPRECATED-ENSG00000188529
2.02344003138325E-11	0.659073991501093	0.5	0.247	3.39088080459205E-07	10	FLNB
1.59888889763959E-18	0.65876343652652	0.993	0.874	2.67941801466442E-14	10	VTN
2.73073693725836E-11	0.658712445606763	0.257	0.089	4.57616895945755E-07	10	SPATA2
9.66373678079432E-09	0.658575375360783	0.25	0.099	0.000161944900972551	10	POU2F1
2.01682529400996E-13	0.658531926827745	0.807	0.455	3.37979582770189E-09	10	ELL2
3.67570625976866E-10	0.658429724039115	0.521	0.267	6.15974855012031E-06	10	NDUFB1
6.03101901647566E-11	0.658152112544411	0.807	0.513	1.01067816678099E-06	10	COPG1
1.23564597435004E-13	0.657678400673281	0.721	0.394	2.0706955238158E-09	10	DSTN
4.59518678537272E-10	0.657647583277806	0.686	0.4	7.7006140149276E-06	10	CITED2
2.63141236379883E-14	0.657518889250396	0.529	0.245	4.40972083925408E-10	10	IFNAR2
2.58094398210666E-12	0.657172866552947	0.529	0.24	4.32514592521434E-08	10	PSMD7
6.93269222462531E-12	0.656715220227037	0.514	0.242	1.16178056300271E-07	10	WIPF2
8.79800702929804E-13	0.656332131705266	0.486	0.218	1.47437001796977E-08	10	FADS1
8.94378463715407E-09	0.656029608864353	0.307	0.132	0.000149879942949428	10	TP53I3
3.74168682940689E-10	0.655631259985512	0.343	0.146	6.27031878872006E-06	10	ITPKA
4.33533219851276E-10	0.655586509672439	0.443	0.209	7.26514969826768E-06	10	RNF6
4.8030618760726E-07	0.655198314717357	0.307	0.147	0.00804897109192246	10	ZNF22
2.84049851852101E-13	0.655111458646256	0.821	0.47	4.76010741733752E-09	10	SPTSSA
5.08413909897081E-12	0.655022502254656	0.643	0.324	8.52000030205529E-08	10	DEPRECATED-ENSG00000213759
7.2621426932749E-14	0.654499474339843	0.636	0.318	1.21698987253901E-09	10	SLC39A7
5.72597044219461E-11	0.654086826354952	0.507	0.244	9.59558126702973E-07	10	RBM12B
1.20050749882245E-08	0.653959267152066	0.429	0.215	0.000201181046652666	10	TMC5
7.69989171837917E-12	0.653910896152533	0.657	0.352	1.29034785416598E-07	10	FDPS
7.66726806021103E-09	0.652827779995269	0.379	0.178	0.000128488078153016	10	PDCD5
1.14445456571404E-10	0.651774658926974	0.536	0.271	1.91787696122359E-06	10	SNX3
1.98161473633065E-10	0.651486787714909	0.35	0.148	3.3207899751429E-06	10	FRS2
4.8277369309952E-10	0.650890597095471	0.371	0.167	8.09032154896175E-06	10	RDH14
4.78776922547572E-11	0.650605821895903	0.436	0.199	8.0233436680522E-07	10	ABCB10
4.04758853356707E-12	0.650341170144663	0.907	0.563	6.78294886455169E-08	10	CES2
3.71606306706263E-11	0.64974221580689	0.636	0.337	6.22737848778355E-07	10	LSS
1.95728844968828E-07	0.649486611577505	0.514	0.286	0.00328002398398761	10	HPRT1
2.55408731323442E-09	0.649142462543061	0.35	0.155	4.28013951951824E-05	10	ATP6V1D
1.54752995267937E-06	0.648870801402183	0.307	0.15	0.0259335069470009	10	AGA
1.99084027103042E-11	0.648845784289026	0.493	0.235	3.33625012619278E-07	10	PALLD
4.95750181693246E-13	0.648808772306019	0.836	0.537	8.30778154481541E-09	10	TM9SF2
1.25046164889819E-12	0.648493275604535	0.843	0.558	2.09552363122358E-08	10	ACTG1
6.93427259714236E-08	0.648474454320991	0.421	0.22	0.00116204540182912	10	ERH
3.30945603067891E-10	0.648273723577729	0.636	0.355	5.54598641621171E-06	10	PSMD3
5.17237101075272E-08	0.648204268184033	0.364	0.181	0.000866785933981941	10	EFNB1
2.3950554112586E-08	0.64810994589798	0.257	0.106	0.000401363385818717	10	HSBP1L1
8.41609665216733E-13	0.647810810108696	0.529	0.239	1.4103694769702E-08	10	PHF6
1.5701485634419E-10	0.647607458101169	0.336	0.14	2.63125496261593E-06	10	RBFA
2.95238193550476E-13	0.646187455317601	0.8	0.447	4.94760164751887E-09	10	RAB2A
7.30566975985187E-09	0.645205391601628	0.579	0.317	0.000122428413835598	10	CYP4F2
7.58937455549088E-09	0.644942198228477	0.329	0.146	0.000127182738800916	10	PAM
4.90380314368424E-07	0.644918196926662	0.286	0.133	0.00821779330818604	10	PSMB6
1.02511345630883E-10	0.644863166445077	0.564	0.292	1.71788513008234E-06	10	TMEM9
1.16747074696566E-10	0.64485596095108	0.286	0.108	1.95644747776506E-06	10	XDH
3.1357054379169E-09	0.644523964135983	0.414	0.197	5.25481517286114E-05	10	ADGRA3
2.56321896011199E-11	0.644462631498256	0.564	0.294	4.29544233335568E-07	10	LRRC59
1.4474175437303E-10	0.644442341318223	0.421	0.196	2.42558231978323E-06	10	TAGLN2
2.28431492313538E-06	0.64404268035799	0.257	0.12	0.0382805494819028	10	ABCB1
9.16964800491337E-09	0.643775401714157	0.386	0.188	0.000153664961266338	10	SNX24
3.63246257411086E-15	0.6437561305718	0.943	0.637	6.08728078169498E-11	10	AKR1C3
1.46131494507628E-08	0.643657864347087	0.436	0.222	0.000244887158495882	10	CPT2
5.94085125548289E-10	0.643466836947538	0.529	0.27	9.95567853393823E-06	10	DHTKD1

2.62492921273498E-13	0.643265382978754	0.75	0.404	4.39885637470129E-09	10	WWP1
1.39336850426245E-08	0.642503959170615	0.314	0.137	0.000233500693944302	10	WDR76
1.82511002559042E-10	0.642446221975748	0.35	0.147	3.05851938088442E-06	10	CHRAC1
6.98658058442334E-10	0.64235820721012	0.686	0.423	1.17081117433766E-05	10	NECTIN2
3.7457623798456E-11	0.642034812473669	0.693	0.378	6.27714859614526E-07	10	PERP
4.50969229470895E-12	0.64172458678102	0.45	0.199	7.55734234747326E-08	10	SNX10
6.51050579404155E-14	0.64155534755068	0.786	0.44	1.09103056096548E-09	10	P4HB
1.23491721735969E-08	0.64112353952326	0.379	0.182	0.000206947427285137	10	PGM3
6.64686391312589E-10	0.640313516575142	0.357	0.156	1.11388145456164E-05	10	SUCO
3.64831857920498E-09	0.640166510362107	0.479	0.249	6.11385227503171E-05	10	QSOX1
9.95258723560236E-07	0.640058505025713	0.35	0.182	0.0166785456894224	10	HSD11B2
1.48720047954648E-09	0.640038385515586	0.5	0.259	2.49225056362399E-05	10	UBQLN2
2.28561124663185E-11	0.63887190535078	0.786	0.467	3.83022732710565E-07	10	TOR1AIP2
3.52793545041354E-10	0.638511357840228	0.486	0.241	5.91211422780301E-06	10	TIMMDC1
1.59301713090549E-11	0.638470312855736	0.607	0.316	2.66957810797141E-07	10	SRPRB
4.24064183381969E-08	0.638290331470288	0.357	0.169	0.000710646758511503	10	SLC25A28
1.5836964685313E-08	0.637885827233264	0.429	0.212	0.000265395854196476	10	SGCE
2.05732181070001E-11	0.637693038038462	0.55	0.272	3.44765989037108E-07	10	NET1
1.81112558857949E-07	0.637634982910847	0.329	0.156	0.00303508426134152	10	SMPD1
2.20277005901394E-11	0.637387964808802	0.514	0.25	3.69140206489556E-07	10	FAM13A
3.74816316633262E-12	0.637127173145014	0.593	0.303	6.2811718341402E-08	10	SAMD5
5.85921588405636E-10	0.635964047354875	0.336	0.143	9.81887397850166E-06	10	C17orf75
2.22526123558708E-13	0.635685658586342	0.614	0.306	3.72909277859682E-09	10	CSTB
3.44446610143215E-10	0.63567040471618	0.35	0.15	5.77223629278E-06	10	NAA30
3.23217916900994E-11	0.635468862937909	0.443	0.199	5.41648585142686E-07	10	TMLHE
2.75333807358866E-09	0.635293440365653	0.471	0.241	4.61404394371987E-05	10	MAEA
1.19820127599436E-12	0.634899925770406	0.793	0.48	2.00794569831136E-08	10	CNN3
2.95299390569818E-12	0.634883369239744	0.65	0.339	4.948627187169E-08	10	FAM3A
8.19630727454219E-11	0.63445640657982	0.457	0.213	1.37353717306778E-06	10	ATRAID
5.49478089294111E-09	0.6342006423662	0.379	0.177	9.20815382039072E-05	10	SLC30A5
4.13970925591552E-11	0.633914587432125	0.429	0.194	6.93732477106323E-07	10	TMEM63B
4.6554416569755E-08	0.633895066268572	0.279	0.122	0.000780158912875955	10	FKBP9
9.43960329923173E-17	0.633856344739298	0.693	0.335	1.58188872088525E-12	10	C19orf12
1.35829382198552E-08	0.633784008668725	0.45	0.237	0.000227622878688334	10	PID1
2.59025822348876E-13	0.633531886164762	0.671	0.338	4.34075473092247E-09	10	MICOS10
2.05625167290309E-10	0.633467820484533	0.564	0.289	3.445866553451E-06	10	IL10RB
1.86003260529669E-11	0.633270514833343	0.293	0.108	3.11704263995619E-07	10	SAMM50
1.35870959397685E-06	0.632498189171149	0.35	0.181	0.0227692553758641	10	IL1RAP
1.18873473350572E-10	0.632348643816149	0.521	0.262	1.99208166640888E-06	10	PXDC1
2.53657156330705E-13	0.632105372897976	0.729	0.391	4.25078662578995E-09	10	ZNF706
2.15142644620063E-14	0.632025383680083	0.486	0.21	3.60536043854301E-10	10	UBA6
3.39149834841922E-10	0.631884839505662	0.307	0.123	5.68347293228093E-06	10	CASP7
3.15879248974572E-11	0.631818385446015	0.486	0.229	5.29350445431588E-07	10	MLH3
3.5746612603201E-09	0.631565085550221	0.486	0.244	5.99041734004442E-05	10	PYGL
4.10046722767009E-11	0.631514941396269	0.443	0.203	6.87156298012954E-07	10	KLHL22
5.81328369260873E-07	0.631141772141461	0.379	0.2	0.0097419008120737	10	SLC30A9
7.12715657955657E-10	0.631120681846504	0.414	0.194	1.19436889960209E-05	10	COX7B
9.14175348069588E-10	0.630866483550855	0.55	0.281	1.53197504829502E-05	10	MEA1
3.9328597431305E-07	0.630518596630397	0.293	0.137	0.00659068635753809	10	SEPTIN10
8.40652157590402E-16	0.6303157187028	0.564	0.243	1.40876488569E-11	10	GBP2
4.09668392950672E-12	0.630249123715187	0.764	0.417	6.86522292906736E-08	10	ERO1B
3.59860849796729E-12	0.62959014067044	0.721	0.429	6.03054812089359E-08	10	HM13
3.18453177332597E-08	0.629531752294448	0.257	0.107	0.000533663834573966	10	PRDM4
5.33072406419249E-11	0.62952102585872	0.45	0.211	8.93322738677377E-07	10	LARP4B
3.45199234978261E-10	0.629486710809301	0.6	0.316	5.78484877976569E-06	10	CPNE3
3.14200876764975E-13	0.62934809067453	0.593	0.295	5.26537829282744E-09	10	SMARCA5
4.53012942975066E-11	0.62842195963433	0.443	0.205	7.59159089837616E-07	10	PLD1
3.62657950369346E-11	0.627151712242431	0.664	0.372	6.07742193228949E-07	10	CHPT1
4.37057229385148E-08	0.626425817232456	0.371	0.179	0.000732420505003631	10	DEPRECATED-ENSG00000147123
2.40370856841392E-09	0.625778810381779	0.707	0.457	4.02813481894804E-05	10	TMBIM1

3.78256171465599E-10	0.625479299566989	0.464	0.226	6.33881692142051E-06	10	COA6
4.11499731702425E-11	0.625002520266597	0.286	0.106	6.89591250386924E-07	10	MTCL1
1.01463072200045E-13	0.624872189619873	0.486	0.211	1.70031816392836E-09	10	UGGT1
4.40389405320085E-10	0.624841859035364	0.429	0.209	7.38004565435398E-06	10	LAMC1
1.72745385982654E-07	0.624696641210741	0.257	0.109	0.00289486717829731	10	FBXO30
3.15825994347373E-11	0.623851059551485	0.564	0.288	5.29261201327327E-07	10	C1orf43
8.51876385452513E-07	0.623698028075451	0.364	0.188	0.0142757444674132	10	WEE1
5.61293392688944E-07	0.62368780510926	0.307	0.148	0.00940615467468133	10	KRCC1
6.16196115407187E-11	0.623594922660724	0.529	0.264	1.03262145019936E-06	10	PREB
6.26285016870068E-11	0.623584895924225	0.557	0.283	1.04952843127086E-06	10	NME2
6.62896339042223E-12	0.623361173502549	0.779	0.428	1.11088168496696E-07	10	CMC4
2.19389480537576E-11	0.623315354498646	0.664	0.347	3.6765289148487E-07	10	EHF
2.0627096489631E-10	0.623315333054993	0.357	0.152	3.45668882973236E-06	10	CASZ1
5.55044500095479E-08	0.623282187225692	0.25	0.104	0.000930143573260004	10	CCDC25
6.63479955931753E-09	0.622735394113074	0.393	0.189	0.000111185971015043	10	PLGLB2
2.35166663520513E-14	0.621826618266979	0.886	0.533	3.94092294727676E-10	10	NDUFA1
1.12875213560813E-07	0.621605608606924	0.293	0.135	0.0018915628288521	10	TMEM30A
2.92972416026865E-13	0.621583905177647	0.557	0.256	4.90963174777821E-09	10	SLC10A3
6.6173771194574E-14	0.62155216782514	0.6	0.299	1.10894005767867E-09	10	CD2AP
4.68970495568623E-10	0.621303477400724	0.414	0.194	7.85900756473899E-06	10	ROCK2
2.8202614794544E-10	0.62118793372588	0.336	0.14	4.72619418726968E-06	10	TMEM18
8.7103727190273E-13	0.62071632095479	0.579	0.278	1.4596842602546E-08	10	ASB13
1.08243929340114E-07	0.620554212167823	0.307	0.143	0.00181395176788163	10	NOL9
6.61628311341716E-09	0.620472890596532	0.307	0.133	0.000110875672414645	10	ADGRV1
1.45877707821208E-07	0.62006536211213	0.343	0.169	0.0024446186276678	10	KLHL12
6.30158646242709E-13	0.619857164313837	0.586	0.283	1.05601985937353E-08	10	ZFAND5
4.34751349433534E-12	0.619627931587643	0.829	0.505	7.28556311380716E-08	10	ANXA2
1.22173010923323E-08	0.619294235730402	0.393	0.191	0.000204737531705305	10	FAM234A
9.326855325287E-14	0.61927331435301	0.971	0.692	1.5629944154116E-09	10	MGST1
9.78541002069273E-10	0.61905932403872	0.686	0.406	1.63983901126769E-05	10	TMEM33
1.3014271631777E-10	0.618673208021487	0.464	0.229	2.18093164005318E-06	10	AP1G1
7.60028683832921E-12	0.616978644263232	0.6	0.297	1.27365606836721E-07	10	STK3
3.7129910961746E-12	0.616854275679217	0.321	0.12	6.2222304789694E-08	10	AIDA
1.12968995886088E-10	0.616612509691957	0.5	0.244	1.89313443305907E-06	10	DCUN1D4
1.1295353623963E-07	0.616461307172862	0.514	0.295	0.00189287536030372	10	RBM10
1.84064196584054E-08	0.616308527898743	0.371	0.174	0.000308454780635558	10	ATP5PB
1.46163569640434E-13	0.61615739415642	0.679	0.337	2.4494091000344E-09	10	THOC2
4.89529355481865E-11	0.615915180442886	0.457	0.213	8.20353293916509E-07	10	C6orf62
1.47514182479776E-11	0.615366966736595	0.629	0.326	2.47204266999609E-07	10	GALK1
3.96291041805692E-09	0.615118025315593	0.307	0.13	6.64104527857978E-05	10	LANCL2
7.42404475619161E-14	0.614996776727	0.693	0.372	1.24412142024259E-09	10	ERO1A
1.08085620979632E-11	0.614885137140293	0.45	0.203	1.81129883637667E-07	10	REEP3
4.92669267517514E-13	0.614814872395904	0.9	0.558	8.2561515850585E-09	10	HSD17B11
7.20461309029412E-11	0.614191353889511	0.479	0.223	1.20734906167149E-06	10	OTOP3
1.13734519224792E-07	0.61253166186933	0.257	0.112	0.00190596307316907	10	PPP1R13L
1.27557492506291E-08	0.612365084489573	0.336	0.152	0.000213760845942043	10	FAM110B
1.34617827234931E-08	0.611863951345829	0.329	0.15	0.000225592554880298	10	SLC23A1
1.53835427157021E-09	0.611547059073645	0.457	0.227	2.57797408829736E-05	10	CNOT11
3.36660068557802E-13	0.61071748495819	0.679	0.351	5.64174942889165E-09	10	SPSB1
1.30436626344565E-08	0.610262390510566	0.371	0.18	0.000218585698428223	10	BPNT1
1.239945270353E-07	0.609855460949935	0.35	0.169	0.00207790028405756	10	TMEM98
5.19913228803376E-07	0.6092898158452	0.386	0.202	0.00871270588828698	10	UTP23
4.80842459927851E-09	0.608224676760354	0.464	0.235	8.05795794347092E-05	10	DERA
1.32690064996181E-08	0.607836819796765	0.4	0.196	0.0002223620109206	10	FAF2
4.38849193553973E-12	0.607801590426279	0.5	0.233	7.35423478557747E-08	10	ATP6V1C1
5.96145697581215E-08	0.60752903473552	0.536	0.3	0.0009990209600066	10	EMP2
9.41295102140861E-08	0.60718873107927	0.4	0.208	0.00157742233216765	10	GOSR1
1.52546647230861E-09	0.607150726392997	0.35	0.156	2.55637671429478E-05	10	DNTTIP1
1.04533830731176E-12	0.607031212605755	0.571	0.277	1.75177793539305E-08	10	LMBR1
1.73090116264479E-07	0.606746520049663	0.314	0.15	0.00290064416836014	10	NOL11

9.59401494467405E-15	0.606397471046488	0.829	0.436	1.60776502442848E-10	10	CDH17
1.19675101947916E-13	0.60633783263894	0.707	0.367	2.00551535844318E-09	10	ELAVL1
1.04974176917798E-10	0.605992320637271	0.543	0.269	1.75915725678846E-06	10	INTS6
6.45998786736085E-10	0.605334870639446	0.35	0.153	1.08256476681233E-05	10	PRRG4
1.35064485080726E-11	0.605044895401541	0.579	0.285	2.2634106409828E-07	10	NUDT9
4.60585386876229E-07	0.604090314864704	0.279	0.128	0.00771848991327185	10	LACTB
2.270172261063E-13	0.603557207871408	0.843	0.502	3.80435467508938E-09	10	SCCPDH
1.98319683418733E-12	0.603346409117448	0.464	0.209	3.32344125473112E-08	10	DEPRECATED-ENSG00000176485
4.52808199859274E-12	0.602927528976283	0.679	0.37	7.58815981324172E-08	10	RAP2C
1.94824837970898E-13	0.602677621268029	0.921	0.574	3.26487463471631E-09	10	ACSL4
2.98185038106107E-10	0.602634797193365	0.486	0.237	4.99698486858214E-06	10	YAP1
3.95642941747331E-13	0.601489334428814	0.457	0.194	6.63018441780177E-09	10	NXT2
1.28692112977705E-12	0.600890974823986	0.814	0.451	2.15662242928038E-08	10	ALG13
8.70144928386275E-10	0.600812105941296	0.364	0.161	1.45818887098972E-05	10	NUP54
4.35904250425747E-07	0.600193457926779	0.364	0.193	0.00730488342863467	10	OTUD4
2.26272111577991E-13	0.60016089508898	0.693	0.361	3.79186804582397E-09	10	ATP6V0E1
1.41043987865075E-11	0.599634265171512	0.693	0.391	2.36361514864292E-07	10	MSMO1
4.2099707574711E-14	0.598894535870061	0.714	0.348	7.05506899537007E-10	10	NR1I2
2.29595876874713E-11	0.598266390511728	0.507	0.239	3.84756770466643E-07	10	THOP1
8.41705571148561E-09	0.598024748758376	0.471	0.244	0.000141053019613076	10	ZNF652
2.44041315517586E-07	0.597643730633244	0.393	0.2	0.00408964436544371	10	GCH1
5.56397954078881E-12	0.596736996812073	0.464	0.206	9.32411691445389E-08	10	CYREN
5.28001074810387E-12	0.596221886496897	0.629	0.322	8.84824201167247E-08	10	ZBTB33
7.80587759481172E-09	0.595402133756198	0.5	0.26	0.000130810896733855	10	BIVM
2.98845754235999E-11	0.595351525670307	0.436	0.195	5.00805714948687E-07	10	EHMT2
5.97619952422854E-09	0.59519086572978	0.293	0.123	0.000100149151627022	10	C4orf33
4.49372446733832E-09	0.595125637290454	0.414	0.202	7.53058346236556E-05	10	C8orf33
1.4208432320456E-07	0.594752864517897	0.271	0.12	0.00238104908826201	10	TMEM101
3.73540107105106E-11	0.593649284329138	0.621	0.325	6.25978511486736E-07	10	NEDD8
2.97068484587824E-10	0.593365169234655	0.479	0.231	4.97827366472276E-06	10	HMGXB4
1.62170476893292E-11	0.593268343236422	0.386	0.163	2.7176528517778E-07	10	BZW1
9.46158878193105E-10	0.592602795206766	0.521	0.266	1.585573048076E-05	10	KLB
8.58400454010329E-09	0.592564863649693	0.579	0.332	0.000143850748083051	10	PIK3AP1
2.19718917566498E-13	0.592544203361736	0.707	0.369	3.68204962057937E-09	10	CYP51A1
2.04517143054473E-09	0.592343502204849	0.479	0.246	3.42729828330686E-05	10	GPC6
1.41567264828957E-11	0.590713797129718	0.743	0.443	2.37238422400367E-07	10	PLS3
4.04584757148233E-11	0.590556399811796	0.543	0.275	6.78003136029009E-07	10	SKP1
9.67810312898269E-10	0.589997275253879	0.514	0.268	1.62185652235492E-05	10	TMEM179B
1.20651924150375E-10	0.589534728717215	0.429	0.198	2.02188494491199E-06	10	GTF2H2
6.59927782135848E-09	0.589389825539562	0.443	0.22	0.000110590697730325	10	SMAD5
3.67012937596946E-10	0.58921249243622	0.379	0.169	6.15040280824962E-06	10	JCAD
8.78591557151662E-09	0.589084421861052	0.536	0.291	0.000147234373147475	10	SLC51A
7.84788027826635E-09	0.588481786090145	0.371	0.171	0.000131514777703188	10	SMO
2.14588078242339E-07	0.588186936619366	0.371	0.188	0.00359606701518512	10	ZNF7
3.01749101454012E-12	0.587964750561483	0.671	0.347	5.05671144216633E-08	10	RETSAT
5.87394857594698E-14	0.587050400568293	0.971	0.708	9.84356302357194E-10	10	MT-CYB
1.07524238516714E-07	0.586760688755283	0.293	0.13	0.00180189118906309	10	LRRC8D
3.62166994744694E-11	0.586515040306066	0.564	0.28	6.06919449793158E-07	10	OCIAD2
1.00318645651386E-11	0.586187610607107	0.743	0.43	1.68113986382593E-07	10	RTN4
7.59738473086048E-11	0.585632054996797	0.757	0.446	1.2731697331976E-06	10	MGST2
1.19354235528407E-10	0.585613604664728	0.614	0.34	2.00013827898504E-06	10	SUB1
8.10344390776269E-09	0.584458560563668	0.393	0.188	0.000135797513006287	10	PBX3
1.64982604813054E-10	0.584453519427211	0.493	0.245	2.76477849145716E-06	10	DEPRECATED-ENSG00000137575
1.19574960842119E-11	0.584032739180049	0.493	0.227	2.00383719379222E-07	10	EIF3H
2.65009158774135E-10	0.58390165839479	0.571	0.291	4.44102348273696E-06	10	TMEM68
2.13516780287595E-11	0.583872580438452	0.793	0.473	3.57811420405951E-07	10	TPD52
4.2924059109329E-11	0.58365434884266	0.529	0.264	7.19321382554135E-07	10	RRP36
1.89769986950339E-09	0.583242211589724	0.586	0.318	3.18016544131377E-05	10	ATP6V1A
2.36705072260739E-09	0.583007310850231	0.786	0.474	3.96670360094547E-05	10	SERINC5
4.59489509590966E-11	0.582339001644885	0.393	0.17	7.70012520172541E-07	10	HHAT

5.32982871073943E-07	0.581778045642212	0.264	0.12	0.00893172695345713	10	SNAPC3
5.66485010234366E-08	0.581498523602546	0.321	0.15	0.00094931558015075	10	KLHL8
2.79932392137535E-11	0.581465801887828	0.7	0.383	4.69110702744081E-07	10	AMOTL1
7.06592815100254E-10	0.580705459510771	0.664	0.378	1.18410823954501E-05	10	CYP4V2
8.12461681116528E-07	0.580419591927192	0.35	0.178	0.0136152328521508	10	STX12
8.09690878919465E-10	0.580194264625606	0.45	0.219	1.35687997489324E-05	10	PSMC6
7.58538258188727E-09	0.579265387986758	0.5	0.259	0.000127115841307267	10	TRIQK
1.01985638363563E-11	0.579005131437196	0.25	0.084	1.70907532769659E-07	10	IL18BP
1.79772867410705E-12	0.578918083490957	0.6	0.299	3.01263371206859E-08	10	KIAA0232
5.38393491403231E-12	0.578769003719885	0.793	0.459	9.02239812893535E-08	10	GUSB
6.15131795675252E-09	0.578169675842677	0.436	0.217	0.000103083786319259	10	ATG12
3.35252008818639E-11	0.578078946512191	0.55	0.279	5.61815316378274E-07	10	MAST4
2.93041474776096E-12	0.577269541606167	0.593	0.301	4.91078903429781E-08	10	ENAH
3.67765276297797E-11	0.577255189106973	0.393	0.168	6.16301050019848E-07	10	EHD4
1.66126155781974E-08	0.57657399472643	0.293	0.126	0.000278394211859433	10	THRB
3.61785788922479E-11	0.576411920693327	0.671	0.372	6.0628062507629E-07	10	SRP9
1.11535985543761E-09	0.576146604532949	0.45	0.219	1.86912004574235E-05	10	GMPS
2.95476523439676E-09	0.576005211918042	0.336	0.146	4.9515955798021E-05	10	KPNB1
1.92297087771366E-10	0.575877609528929	0.757	0.432	3.22251459687255E-06	10	GPAA1
1.46838271881836E-07	0.575844931569925	0.586	0.353	0.00246071576019581	10	ERG28
7.91778387205499E-13	0.575784693341292	0.664	0.348	1.32686222127898E-08	10	PSMB4
8.22892136612243E-10	0.575643811038157	0.486	0.241	1.3790026425348E-05	10	ARPC5
2.87029926254022E-10	0.57561538631027	0.514	0.26	4.8100475041649E-06	10	ZNF664
2.46161748050827E-09	0.575590984916634	0.486	0.247	4.12517857383576E-05	10	PSMD8
1.01837247385212E-06	0.574986948753816	0.286	0.137	0.0170658859168139	10	GAR1
1.73410140601594E-07	0.574830907230223	0.393	0.208	0.00290600713620151	10	ZYG11B
6.26515811839855E-09	0.574666690966488	0.421	0.206	0.000104991519748123	10	SLC2A13
8.30631009831821E-12	0.573770292389577	0.35	0.138	1.39197144627617E-07	10	DEPRECATED-ENSG00000116489
7.4911272016182E-10	0.573754117880932	0.679	0.378	1.25536309644718E-05	10	CHMP2A
1.16238597423798E-07	0.573171059324063	0.35	0.17	0.00194792641562801	10	KNTC1
3.70909444542496E-14	0.572718800763202	0.607	0.279	6.21570047164315E-10	10	DPP4
7.05734602653889E-07	0.571954642518003	0.314	0.158	0.0118267004712739	10	PKD2
1.23377310036126E-08	0.571536653417148	0.357	0.168	0.00020675569615854	10	LAMTOR3
4.46125145591866E-08	0.57149102177391	0.607	0.358	0.000747616518982849	10	STBD1
5.81358100986758E-09	0.571211087982259	0.307	0.132	9.74239905633608E-05	10	RBBP5
6.11579046921337E-10	0.570915288174982	0.607	0.325	1.02488416683078E-05	10	SMIM19
2.68323226464853E-08	0.570461705889356	0.421	0.213	0.0004496560629098	10	CPT1A
1.10390450689264E-10	0.569781358537602	0.614	0.318	1.84992317265068E-06	10	UBXN6
3.81677526646837E-08	0.569729327030292	0.364	0.177	0.00063961519915477	10	SLC25A29
2.2891628052752E-08	0.568766622591125	0.436	0.222	0.000383617902908018	10	CCNE2
5.19675958471149E-12	0.56856934692225	0.607	0.318	8.70872971205951E-08	10	TOP 1.00
9.90651675244649E-12	0.567800526237922	0.786	0.448	1.66013407737498E-07	10	PCK2
2.13590190074037E-08	0.567229252180352	0.379	0.183	0.000357934440526071	10	MAP3K21
5.02346916088373E-07	0.567108282496575	0.329	0.163	0.00841832961980895	10	TMEM39A
2.01475253968054E-20	0.567092196297803	0.993	0.892	3.37632230599666E-16	10	B2M
9.80645608929918E-11	0.567037274415053	0.6	0.315	1.64336591144476E-06	10	SLC15A1
8.14117675201843E-07	0.566889886061355	0.314	0.153	0.0136429840010325	10	ZNF436
4.18523306632925E-11	0.566657010029433	0.729	0.422	7.01361357255456E-07	10	COPA
2.06692923896479E-11	0.566095905746553	0.686	0.361	3.4637600186572E-07	10	SLC25A4
4.12328381939494E-11	0.566027303411802	0.593	0.302	6.90979902454203E-07	10	NECTIN3
3.66820903025836E-10	0.565699440278945	0.521	0.262	6.14718469290696E-06	10	VPS4A
1.53210966948115E-11	0.565413207573007	0.636	0.335	2.56750938411651E-07	10	LONRF3
3.74942851511617E-11	0.564645914434948	0.807	0.446	6.28329230563168E-07	10	KCTD3
2.42367411018176E-11	0.564596109585734	0.486	0.237	4.06159307384259E-07	10	NFKB1
1.12996618261999E-12	0.564339672532596	0.507	0.232	1.89359732883458E-08	10	CENPB
4.05710507721713E-08	0.563268640692029	0.429	0.219	0.000679889668840046	10	SIAE
9.45135537257918E-09	0.562704502406734	0.321	0.14	0.000158385813333682	10	ARID2
3.05264193297901E-13	0.56203148417273	0.864	0.52	5.11561735128623E-09	10	BCAP31
4.01601338135804E-10	0.561149130478142	0.693	0.39	6.73003522447981E-06	10	TCEAL4
8.35025894400134E-11	0.561078277732835	0.329	0.133	1.39933639383574E-06	10	RIPK2

6.63038123337099E-07	0.560941614147118	0.429	0.233	0.0111111928708831	10	MTMR1
7.17247562797426E-10	0.559948163270051	0.479	0.235	1.20196346573593E-05	10	PGLS
8.10895400972431E-10	0.559883793298329	0.536	0.281	1.3588985129496E-05	10	SNRPB
4.23220490186524E-13	0.559323882139565	0.807	0.452	7.09232897454577E-09	10	AFDN
4.2226589092154E-10	0.559289743011597	0.471	0.231	7.07633180006316E-06	10	WRNIP1
5.70760272851348E-07	0.559105800890363	0.45	0.244	0.0095648006524429	10	GNAI3
1.74870909806663E-10	0.558807154080981	0.643	0.351	2.93048670654006E-06	10	SDCBP
4.35967194806199E-10	0.558218118347459	0.436	0.206	7.30593825056228E-06	10	ATP6V0A1
1.99796208376335E-06	0.557785966669703	0.35	0.18	0.0334818485997062	10	TRPT1
1.12826801836786E-13	0.557591162534727	0.979	0.847	1.89075154518086E-09	10	SELENOP
4.28165966806972E-09	0.55705362435928	0.643	0.367	7.17520527175123E-05	10	ANAPC11
1.00478774678706E-10	0.557015368326216	0.721	0.459	1.68382330606576E-06	10	RNF213
8.69607274576464E-11	0.55696760991606	0.75	0.413	1.45728787073524E-06	10	ZHX1
1.8899073715095E-10	0.556722919676029	0.521	0.255	3.16710677317562E-06	10	NTPCR
3.71972509117202E-07	0.556370446634182	0.371	0.19	0.00623351530778607	10	DNAH14
7.30379510040974E-10	0.555862946786546	0.436	0.204	1.22396998292666E-05	10	ATL3
1.02584305964115E-09	0.555736907171398	0.557	0.292	1.71910779934664E-05	10	SGK3
9.36723699340853E-10	0.554231871062871	0.586	0.321	1.5697615753554E-05	10	CAPNS1
7.61707952826634E-09	0.554180774504827	0.357	0.164	0.000127647018734687	10	UPF3B
3.09664637487478E-10	0.55415054879133	0.436	0.21	5.18935999501516E-06	10	TMEM208
2.5804077822365E-09	0.554115749287021	0.329	0.142	4.32424736147193E-05	10	TMEM14B
4.0366278334738E-12	0.553956722892217	0.479	0.213	6.76458092333539E-08	10	TM9SF4
2.77263095862966E-08	0.553181073625171	0.436	0.222	0.000464637496047159	10	BTD
1.21696796764883E-08	0.553152161101282	0.664	0.381	0.000203939492018591	10	CPD
2.45696733776455E-13	0.55264547296352	0.464	0.195	4.11738586462584E-09	10	CARM1
5.52065370138097E-13	0.552146781724472	0.693	0.372	9.25151147277423E-09	10	ELOVL5
5.30372746826703E-12	0.551969072365156	0.679	0.38	8.88798649132189E-08	10	SPATS2L
2.98219617642154E-11	0.551731126246548	0.321	0.125	4.99756435244721E-07	10	UBE2D2
6.35252723555423E-07	0.551301242413759	0.3	0.141	0.0106455651413418	10	ARFIP2
8.61417594971513E-09	0.551142888301648	0.371	0.176	0.000144356360565326	10	VEZF1
9.28744667747407E-07	0.551101926530325	0.357	0.183	0.015563903142111	10	TRIM56
2.69863457721034E-11	0.550539061981961	0.379	0.16	4.52237182448908E-07	10	UTP6
9.78820450158006E-15	0.550393983508201	0.514	0.22	1.64030731037479E-10	10	POLR2B
3.62276032520289E-12	0.550109217256686	0.814	0.487	6.071021752975E-08	10	ARF1
2.86645877249933E-11	0.54993728473365	0.729	0.405	4.80361161095437E-07	10	STT3A
6.8131700834269E-07	0.549647545820034	0.314	0.154	0.0114175104258068	10	TEF
1.16269716187239E-10	0.549317503541112	0.636	0.339	1.94844790386575E-06	10	NUCB2
8.12172328945885E-12	0.54847985836702	0.814	0.484	1.36103838884751E-07	10	FAM50A
1.22534374128795E-11	0.548203160648328	0.921	0.601	2.05343104165035E-07	10	GSTA1
2.675223551348E-08	0.54720248799967	0.286	0.122	0.000448313962734898	10	PARD3B
4.63749653822441E-08	0.5467878146866	0.371	0.178	0.000777151669875647	10	GGCT
1.88076505119881E-09	0.546378254523829	0.557	0.29	3.15178607279897E-05	10	MOCS2
9.24320635348621E-08	0.546200241742892	0.464	0.241	0.00154897652071722	10	NDUFA7
2.12283181654083E-06	0.546175932651529	0.343	0.179	0.0355744155815912	10	NAGA
3.14086859853572E-11	0.545952040221691	0.286	0.105	5.26346759742616E-07	10	ABTB2
2.53189169641785E-11	0.545725313980592	0.614	0.327	4.24294410485704E-07	10	DST
1.26552278669031E-07	0.545720364942924	0.443	0.241	0.00212076308593562	10	MARVELD2
1.2404779331401E-07	0.545713675221345	0.386	0.197	0.00207879292035618	10	SQOR
2.05458145647517E-11	0.545492873129506	0.664	0.374	3.4430676047611E-07	10	EFR3A
7.9966104194744E-07	0.545480081772821	0.307	0.149	0.0134007197409552	10	CPEB2
5.7444772574669E-10	0.545326296160383	0.536	0.283	9.62659498806302E-06	10	PSMB5
5.43544206823612E-08	0.545243775745781	0.757	0.489	0.000910871381795008	10	F11R
2.8707187154044E-07	0.544901424734763	0.264	0.118	0.0048107504232747	10	ELP4
1.61226153920146E-07	0.544813215739184	0.529	0.31	0.00270182788739381	10	MLX
2.94982187415948E-10	0.544417230888844	0.457	0.218	4.94331149671646E-06	10	DCAF16
9.79426360618499E-08	0.544337324094214	0.486	0.273	0.00164132269512448	10	RAPH1
3.58639427617391E-12	0.544133685246104	0.629	0.323	6.01007952801223E-08	10	DLG1
3.5277517715606E-11	0.54388612063126	0.75	0.428	5.91180641878125E-07	10	SEC61A1
6.59596089782256E-10	0.54350290104244	0.614	0.341	1.10535112725711E-05	10	HNRNPL
1.00414211696745E-07	0.542635557703019	0.257	0.11	0.00168274135961406	10	ARMC10

1.85956946169321E-07	0.542211552594644	0.25	0.108	0.00311626650390549	10	ABHD4
5.27523187706944E-08	0.542055352375542	0.543	0.296	0.000884023357959297	10	DNPH1
1.29809147034237E-08	0.54161950233941	0.45	0.223	0.000217534168599974	10	ATP13A1
1.38994615631942E-08	0.541460532479475	0.55	0.3	0.000232927176876008	10	ATP9A
9.50589741298679E-08	0.541220172723573	0.264	0.114	0.00159299828846833	10	RUSC2
2.93015355399929E-11	0.54116288998569	0.436	0.195	4.91035132579201E-07	10	VAPB
3.55837862501228E-10	0.540457294779459	0.4	0.18	5.96313089979558E-06	10	TMEM50B
6.91298803208663E-08	0.53926905509193	0.307	0.139	0.00115847853441708	10	RAI14
1.65593359303349E-09	0.539154885705144	0.457	0.227	2.77501351520552E-05	10	ALDH5A1
9.36055707197573E-08	0.539107443302409	0.364	0.18	0.00156864215412169	10	MTERF3
4.05211766625937E-10	0.538812585234608	0.864	0.547	6.79053878511746E-06	10	SELENBP1
3.76798748635989E-11	0.538441225349598	0.557	0.277	6.31439342964191E-07	10	PLS1
1.17471560766056E-09	0.538258411741815	0.421	0.196	1.96858841531757E-05	10	PLEKHG3
9.45158697396924E-07	0.537958193068011	0.286	0.135	0.0158389694509776	10	BCHE
2.16750483328042E-09	0.537878541183434	0.671	0.395	3.63230459961133E-05	10	PLOD3
1.3459842310452E-10	0.537600884518014	0.514	0.253	2.25560037438554E-06	10	POLR2K
8.68199024308938E-10	0.537411373750535	0.821	0.498	1.45492792493692E-05	10	RBM47
1.11852139093639E-09	0.537259923129521	0.4	0.185	1.8744181469312E-05	10	GET3
2.94323678078435E-06	0.537116340744481	0.4	0.216	0.0493227619723842	10	RASL11A
4.41950279834589E-07	0.536856870157382	0.307	0.146	0.00740620278946804	10	KHDRBS3
7.19364440302938E-09	0.53680080692862	0.536	0.297	0.000120551092905966	10	MAPK14
2.15480086545903E-10	0.536571491240633	0.957	0.744	3.61101529033623E-06	10	ALDH1A1
1.17895340790207E-07	0.536501688999197	0.579	0.339	0.00197569012096228	10	FDFT1
2.502608557554E-11	0.536419752934058	0.821	0.476	4.19387142074899E-07	10	HNRNPH2
7.3602617421766E-09	0.535991977316907	0.679	0.395	0.000123343266275395	10	HTATIP2
1.80482019367397E-08	0.535956223447836	0.564	0.318	0.000302451768055884	10	CCNG1
4.24282501969893E-10	0.535509605203627	0.621	0.35	7.11012616801147E-06	10	FAM91A1
2.50436310625071E-07	0.534995346470589	0.386	0.198	0.00419681169345495	10	IER3IP1
7.72997433491892E-11	0.53387955457228	0.593	0.31	1.29538909904571E-06	10	COPE
1.49338326692828E-13	0.533572211627791	0.364	0.137	2.50261167871841E-09	10	DDA1
3.99911380389525E-10	0.533567435450379	0.593	0.326	6.70171491256766E-06	10	YTHDF3
1.67046362881651E-10	0.533491962379083	0.464	0.223	2.79936294917071E-06	10	API5
8.52736567741076E-09	0.533426058395411	0.45	0.227	0.00014290159402205	10	EPPK1
1.36130671436874E-08	0.53341463382629	0.471	0.251	0.000228127779193914	10	ARFGAP3
1.47512498197305E-09	0.532202789652863	0.7	0.409	2.47201444479043E-05	10	ATOX1
6.19185215310191E-07	0.531310961620723	0.286	0.133	0.0103763058381682	10	PDE6D
4.57769100296587E-12	0.531296547060577	0.657	0.339	7.6712945827702E-08	10	TMEM147
1.6220593901733E-08	0.53096544298437	0.364	0.172	0.000271824712605242	10	SMAD1
5.18360647258436E-11	0.529720699028274	0.693	0.375	8.68668772675687E-07	10	CAPN1
1.38809251486668E-06	0.529661514568748	0.414	0.231	0.0232616543641358	10	PSMD4
4.05995751717519E-10	0.529516136783884	0.443	0.209	6.80367680728218E-06	10	EAPP
3.2756893142521E-07	0.529508944802262	0.279	0.129	0.00548940015282367	10	RTRAF
2.28225176654432E-09	0.529399560817551	0.529	0.271	3.82459751037498E-05	10	GALK2
7.7799020959993E-12	0.529266695876553	0.679	0.357	1.30375599324756E-07	10	COPS5
1.2077366952899E-07	0.529089073267919	0.293	0.133	0.00202392515396682	10	CLTC
2.88969622748944E-09	0.528076670737211	0.743	0.437	4.8425529380268E-05	10	MORF4L2
8.01245154745653E-09	0.527891112437813	0.379	0.177	0.000134272663032277	10	CCNQ
2.06953267905319E-07	0.52785000202776	0.514	0.286	0.00346812286355734	10	B3GNT3
5.60872048518848E-08	0.527685088808304	0.636	0.382	0.000939909378907886	10	DNAJC3
4.29079182507402E-08	0.527451558584641	0.379	0.186	0.000719050894045905	10	PWWP2A
3.41651733647254E-09	0.527343870548899	0.507	0.271	5.72539975246067E-05	10	KIF1B
8.50368389403385E-11	0.526836066308247	0.614	0.337	1.42504734696219E-06	10	MYH14
1.9332761809894E-08	0.526775151650336	0.593	0.321	0.000323978422410204	10	OPLAH
1.15206083289518E-12	0.525623214999849	0.657	0.35	1.93062354376573E-08	10	RNF19A
6.48643993709842E-10	0.525505197533531	0.507	0.255	1.08699760465895E-05	10	LAPTM4A
9.89929862601597E-09	0.525272063075731	0.593	0.319	0.000165892446374776	10	TAZ
5.15877733131135E-09	0.525231293119096	0.464	0.235	8.64507905181157E-05	10	RSBN1L
6.37750486105448E-08	0.524289396918266	0.279	0.119	0.00106874226461551	10	NIPSNAP3A
1.33730869969186E-09	0.523684086088614	0.436	0.209	2.24106191894361E-05	10	DHX36
2.28705355232279E-10	0.523657540053168	0.514	0.254	3.83264434298253E-06	10	UBE2E3

1.10218335311881E-07	0.523124154543688	0.321	0.149	0.0018470388631565	10	JAGN1
1.79141254713048E-06	0.523014437626454	0.386	0.208	0.0300204914648125	10	USP10
7.25386053667842E-08	0.521777346748955	0.45	0.233	0.00121560194873657	10	SUZ12
2.73376333438916E-12	0.521493218971443	0.743	0.416	4.58124059576936E-08	10	CALR
6.97200477674372E-10	0.521458468819464	0.779	0.468	1.16836856048671E-05	10	SLC37A4
9.68334938878855E-07	0.521321717657179	0.314	0.155	0.0162273569057319	10	COG1
6.66244965239943E-09	0.521120066040617	0.314	0.135	0.00011164933127491	10	CMTR2
2.96067039410754E-10	0.520971204102044	0.379	0.165	4.96149144644542E-06	10	KCNT2
1.59256924008233E-08	0.520686251307504	0.521	0.282	0.000266882753252997	10	TMEM238
6.4043171575881E-09	0.52043801922246	0.521	0.272	0.000107323546926861	10	WDFY2
1.30091591215661E-11	0.520370810308528	0.721	0.384	2.18007488559205E-07	10	ACSL5
3.62260263643324E-09	0.519855023719962	0.607	0.339	6.07075749813483E-05	10	KDELR2
8.64735219752407E-07	0.519806942548559	0.35	0.181	0.0144912328126108	10	MET
6.51192002884878E-07	0.519543846333048	0.5	0.28	0.0109126755843448	10	CMTM8
1.97546820849712E-08	0.51877514222529	0.457	0.239	0.000331048962379948	10	MSH6
4.49730896586021E-09	0.518507955603146	0.864	0.605	7.53659036498853E-05	10	MT-ND2
1.35968812539025E-09	0.518054337663234	0.471	0.24	2.27856536052899E-05	10	TASOR2
8.97578494653118E-12	0.517607734099646	0.35	0.138	1.5041620413397E-07	10	JMJD4
1.19634670640366E-06	0.516238473251388	0.407	0.221	0.0200483781059125	10	TSNAX
5.08716272314558E-13	0.515761057496036	0.586	0.287	8.52506729144736E-09	10	SEC23B
6.4958412242831E-11	0.515409504879746	0.543	0.27	1.08857307236536E-06	10	ABRACL
1.10859422288322E-07	0.515121304615948	0.357	0.176	0.0018577821987077	10	CLCN3
1.62413951839683E-11	0.514491194202673	0.9	0.559	2.7217330049294E-07	10	MPC2
8.3834023401974E-08	0.51346581436505	0.729	0.461	0.00140489056417028	10	UQCRQ
2.10384690128118E-11	0.513094943963854	0.564	0.281	3.525626637167E-07	10	STX7
2.04595109478142E-10	0.512973457388088	0.507	0.243	3.4286048446347E-06	10	GPS1
1.59264669336936E-09	0.512614175733814	0.529	0.269	2.66895732874837E-05	10	NREP
8.03188188569956E-08	0.512588109433335	0.379	0.186	0.00134598276640553	10	PCGF3
2.42786590352081E-10	0.512533583459408	0.5	0.244	4.06861768112017E-06	10	RFC1
1.94303998187159E-09	0.512502865308054	0.464	0.228	3.25614640162041E-05	10	ZMAT1
2.14044343755463E-07	0.512487807605911	0.364	0.183	0.00358695511265405	10	NRIP1
3.26110390746015E-11	0.512316869756776	0.636	0.33	5.46495792812171E-07	10	RDH11
1.50633369183576E-11	0.512197619113627	0.786	0.451	2.52431400077837E-07	10	DAP
1.43285011330852E-10	0.512194790914403	0.664	0.362	2.40117021988242E-06	10	PANK3
1.48038792364464E-08	0.511897377497202	0.479	0.254	0.000248083408244368	10	TMEM214
5.16118633349933E-07	0.511375216333496	0.264	0.12	0.00864911605767818	10	PRKRA
6.9225848607063E-09	0.511134196012776	0.564	0.318	0.000116008677095716	10	TXNDC17
7.13116117536254E-12	0.510983676551584	0.493	0.222	1.19503998976725E-07	10	ZMYND11
1.97929197247794E-11	0.510261129643748	0.493	0.234	3.31689748747854E-07	10	PCGF2
5.37856486637391E-10	0.509312425228955	0.671	0.379	9.01339900306939E-06	10	CYP4F11
4.43324544031961E-08	0.509114709471958	0.393	0.194	0.000742923270888761	10	PCNA
1.47668758639101E-08	0.508850130796074	0.321	0.142	0.000247463305727406	10	ENDOV
4.01426197169057E-08	0.508627204413383	0.336	0.156	0.000672710021215906	10	FAM98C
8.47926295398021E-10	0.508514802662208	0.536	0.281	1.420954885828E-05	10	UBXN2B
3.23626292664588E-07	0.508347790877478	0.307	0.146	0.00542332941247317	10	ARFIP1
6.70811874145341E-11	0.507896385024616	0.636	0.346	1.12414653869276E-06	10	SLC50A1
1.38382269625139E-09	0.507862141440433	0.493	0.243	2.31901007437807E-05	10	CCT8
2.53596570688411E-09	0.507490149981446	0.579	0.306	4.2497713315964E-05	10	ELOVL1
2.07227802848313E-07	0.507475316861805	0.371	0.186	0.00347272352013203	10	RMDN1
1.12289492383207E-09	0.506987515069737	0.679	0.385	1.88174731335779E-05	10	PYCR2
6.35632730391375E-08	0.505952162230554	0.45	0.225	0.00106519332958987	10	ASB9
1.14420133356796E-08	0.505725703626657	0.25	0.099	0.000191745259479319	10	GPR89A
8.23348319804922E-10	0.50527160882141	0.743	0.431	1.37976711432909E-05	10	ATRN
7.28698758430254E-10	0.50510650342413	0.436	0.207	1.22115337937742E-05	10	KIF3B
9.1683385935749E-08	0.505085867710666	0.55	0.313	0.00153643018151128	10	TRAPPC4
3.72728028027502E-07	0.504878474457469	0.321	0.155	0.00624617629368487	10	TPGS2
3.92650619289181E-08	0.504697632948618	0.643	0.37	0.00065800390780481	10	VAMP8
2.1114990701092E-08	0.504685950155104	0.6	0.335	0.0003538450141689	10	FAT1
2.06355868566489E-11	0.504401726718554	0.571	0.281	3.45811164543723E-07	10	SOS 2
5.82274536771287E-08	0.503867210767388	0.307	0.138	0.000975775668721323	10	SCLY

5.05451337557821E-15	0.5036501458743	0.364	0.129	8.47035351479396E-11	10	NLRC5
1.81552889693758E-07	0.502385291568838	0.657	0.388	0.003042463325488	10	MEP1A
1.89960250287902E-06	0.502170552540161	0.371	0.197	0.0318335387432466	10	SF3B4
1.93094455590273E-08	0.501999646506796	0.4	0.198	0.000323587688678179	10	TADA2B
1.06997992508652E-09	0.501846180550066	0.436	0.208	1.79307235845999E-05	10	DNAJC11
2.41062211891074E-10	0.50160798565905	0.45	0.211	4.03972054687062E-06	10	CNNM3
6.62592637273553E-07	0.501141393457464	0.329	0.162	0.0111037274154302	10	MBIP
4.45928481707634E-07	0.501070075938522	0.257	0.115	0.00747286949645654	10	FXR2
1.01904591108295E-13	0.501038206726732	0.971	0.802	1.7077171377928E-09	10	FN1
3.54554008804597E-10	0.500924052530708	0.5	0.244	5.94161607954743E-06	10	AKR7A2
5.03243719895172E-10	0.500756419090996	0.821	0.481	8.43335825800329E-06	10	MAOA
6.30285259139995E-09	0.500722758047471	0.314	0.137	0.00010562320372668	10	SRSF1
6.60267820853026E-10	0.50064221699244	0.821	0.502	1.1064768141855E-05	10	BLVRB
5.82942478003053E-13	0.500461934710935	0.529	0.241	9.76895004637516E-09	10	RNF167
4.67299936687027E-10	0.500338082397917	0.493	0.247	7.8310123390012E-06	10	RTCB
2.64465333261127E-06	0.500089661711838	0.364	0.193	0.0443191005478997	10	FAHD1
2.06934331854177E-06	0.500011402072004	0.279	0.133	0.034678055332123	10	DCBLD2
1.64559922930892E-09	0.499514374402413	0.621	0.324	2.75769518847589E-05	10	ANXA13
1.76932526200274E-08	0.49942964321378	0.357	0.166	0.000296503527406419	10	IFT88
1.72484084466141E-11	0.49937614610902	0.557	0.272	2.89048828748359E-07	10	SHROOM3
2.79481588409628E-07	0.499164634659707	0.386	0.197	0.00468355245856855	10	TEX261
3.32471306324997E-07	0.499134527022854	0.364	0.18	0.00557155415139429	10	ATPSCKMT
1.41375538866926E-06	0.498125449480192	0.429	0.233	0.0236917128033194	10	NFS1
6.233463965253E-13	0.497601460377192	0.743	0.435	1.0446038912971E-08	10	IL6ST
1.03867457731819E-07	0.496493251524199	0.429	0.222	0.00174061085666982	10	DEPRECATED-ENSG00000181061
5.15844753516025E-07	0.496355609356585	0.286	0.13	0.00864452637942155	10	ZFP64
2.00256062347908E-10	0.495942920097645	0.471	0.219	3.35589109282624E-06	10	NPAS2
6.59411764378777E-12	0.495790037789942	0.757	0.422	1.10504223474595E-07	10	RAB5C
3.5463242527501E-11	0.49570894847832	0.55	0.268	5.94293018275862E-07	10	FOXP4
1.06195702678264E-06	0.495269941557211	0.336	0.167	0.0177962758548234	10	CSTF2
8.77647784850999E-11	0.495070551776623	0.536	0.264	1.4707621578533E-06	10	IRF2
1.11267771268431E-07	0.49503655997293	0.271	0.118	0.00186462531091637	10	LRRC8E
2.68574209554384E-08	0.494766693541058	0.45	0.231	0.000450076660371237	10	FAF1
1.9349439497665E-11	0.49464012281695	0.593	0.296	3.24257907101869E-07	10	TTC3
1.63199253919267E-09	0.494107787440764	0.657	0.367	2.73489309717908E-05	10	MCM3
1.49639806574653E-07	0.493692471400595	0.55	0.308	0.00250766387857803	10	TMEM245
7.26549111263479E-10	0.493555620514722	0.357	0.154	1.21755100065534E-05	10	TMEM128
2.13558411273274E-08	0.493493434718599	0.314	0.141	0.000357881185611752	10	RNF217
1.72077999835042E-07	0.493436139478091	0.343	0.169	0.00288368312123563	10	FLVCR1
7.01046674351578E-11	0.492989735074635	0.607	0.308	1.17481401687837E-06	10	RABAC1
2.02570334263972E-08	0.492834162919989	0.471	0.237	0.000339467366159564	10	JPT2
8.29272624307209E-09	0.492663654045019	0.314	0.137	0.000138969506381402	10	FBXW11
4.58774169678166E-10	0.492571453456008	0.321	0.133	7.6881375354667E-06	10	GDAP2
5.77744922760033E-09	0.492436353229242	0.314	0.135	9.68184941561263E-05	10	FGD6
7.5270967704112E-09	0.491314417859131	0.607	0.342	0.000126139087678551	10	SETD7
3.60458470783233E-07	0.490896940044047	0.35	0.176	0.00604056305338542	10	RETREG2
5.33893399452356E-10	0.490591232143841	0.621	0.328	8.94698558802258E-06	10	NDFIP2
1.49738964943994E-07	0.490445013331061	0.371	0.184	0.00250932557453145	10	SLC9A6
2.17223990719847E-07	0.490232106301786	0.393	0.201	0.0036402396364832	10	FNIP2
2.38892094949967E-08	0.489716523345325	0.443	0.227	0.000400335372717155	10	HAX1
1.21978145489545E-09	0.489415093661347	0.636	0.352	2.0441097621138E-05	10	EDEM3
1.61345607955236E-09	0.489077757872306	0.75	0.441	2.70382969811384E-05	10	DHRS7
4.26191151044043E-09	0.489053652888188	0.321	0.137	7.14211130919607E-05	10	NFU1
9.28408816310181E-07	0.488945439860954	0.364	0.189	0.015558274943726	10	SNTB2
1.81417646627136E-11	0.488830076779233	0.464	0.211	3.04019692217754E-07	10	SNX27
9.14133025636228E-09	0.488426313394044	0.657	0.384	0.000153190412436119	10	COX7C
1.63749526756629E-08	0.488034148290357	0.493	0.257	0.000274411456938759	10	DPM1
2.91158704084561E-10	0.488029301013232	0.657	0.349	4.87923756304907E-06	10	CEP70
1.57024074083001E-09	0.487973429600794	0.279	0.11	2.63140943348293E-05	10	DIS3L
6.4622979831777E-08	0.487809268129206	0.307	0.139	0.00108295189602092	10	MRM2

1.04764586321488E-10	0.487757222090722	0.886	0.516	1.7556449375755E-06	10	TMEM14A
5.15809158830794E-11	0.487477117254495	0.436	0.192	8.64392988368645E-07	10	GSDMB
1.74121327089786E-07	0.487308783757628	0.286	0.127	0.00291792519937064	10	MED18
1.82091952512074E-09	0.48730763278023	0.757	0.447	3.05149694019734E-05	10	TMEM37
1.45086603406986E-08	0.48669386812982	0.371	0.173	0.000243136129989428	10	DAG1
1.06300136654883E-11	0.486158422544292	0.479	0.221	1.78137769006253E-07	10	POMGNT1
4.67176078394027E-09	0.48563200780159	0.45	0.226	7.8289367217271E-05	10	MFF
6.10367511667749E-08	0.48523282303855	0.321	0.148	0.00102285387605281	10	DCUN1D2
2.42907699404744E-08	0.485067012580602	0.507	0.267	0.00040706472266247	10	BDH2
7.19086433954286E-09	0.485046298139267	0.55	0.299	0.000120504504602059	10	C5orf24
6.52420635854451E-09	0.484984423024488	0.779	0.486	0.000109332650156489	10	AKR1C2
1.69213778872397E-09	0.484739629611411	0.386	0.179	2.83568450634363E-05	10	CXorf40A
2.18746648281462E-06	0.484707430084691	0.321	0.166	0.0366575633190073	10	CUL2
1.66564778493966E-06	0.484439876734093	0.321	0.161	0.0279129255800189	10	OSER1
6.0242648648176E-10	0.484246196027291	0.579	0.311	1.00954630604613E-05	10	XRN1
1.71405636770491E-11	0.483987355916364	0.507	0.235	2.87241566099989E-07	10	POLR2F
2.13666425243776E-09	0.483882806610873	0.371	0.168	3.5806219542352E-05	10	LZIC
1.24311950651103E-09	0.48384629463404	0.45	0.218	2.08321966901118E-05	10	RARS
4.57166511212486E-09	0.483682370742332	0.8	0.514	7.66119639489884E-05	10	ACAA2
1.50660453495697E-09	0.483641441645232	0.721	0.423	2.52476787968089E-05	10	IL13RA1
3.40505421946035E-09	0.483121008827113	0.364	0.164	5.70618986097165E-05	10	GJB2
2.76396766431507E-08	0.482895923557139	0.443	0.222	0.000463185701185919	10	CORO2A
3.24185097301977E-07	0.482714459917925	0.307	0.144	0.00543269386058654	10	PDCL3
1.32108623984296E-08	0.482671001001838	0.4	0.196	0.000221387632072884	10	PSMD2
6.67831379379958E-09	0.482460788927875	0.407	0.193	0.000111915182556493	10	C8orf76
4.10720971147687E-14	0.482285423918122	0.643	0.315	6.88286203449294E-10	10	LPIN2
2.76599562442586E-09	0.481766911033392	0.786	0.49	4.63525546741286E-05	10	PRKACA
2.01736596771345E-09	0.481550001996998	0.443	0.211	3.3807018886942E-05	10	ATP5PF
1.68224763447416E-06	0.481511766490556	0.457	0.261	0.0281911058585179	10	HACD2
9.9880377410824E-10	0.481068323072868	0.5	0.25	1.67379536465059E-05	10	KIF13A
1.03243623089999E-10	0.480917954977067	0.829	0.498	1.7301566357422E-06	10	CAPN2
1.28384620514676E-11	0.480904263893931	0.614	0.301	2.15146947058494E-07	10	DIAPH2
1.67909759809112E-11	0.480805527735459	0.943	0.581	2.81383175488109E-07	10	NQO1
3.43265146848242E-10	0.480467367734231	0.586	0.312	5.75243733088284E-06	10	CLTB
1.80818705420165E-06	0.480418978461261	0.264	0.124	0.0303015986543113	10	AP3S1
2.58336874917309E-10	0.480405285189438	0.971	0.677	4.32920934986427E-06	10	UGDH
2.1897990282853E-08	0.480341684883585	0.329	0.148	0.000366966521160051	10	GTF2H5
6.13327114995044E-09	0.48025049839402	0.364	0.166	0.00010278135793087	10	SLC35B4
1.07105963619733E-08	0.479680653418572	0.621	0.352	0.000179488173833949	10	LMO7
9.19027051042307E-10	0.478988492227836	0.279	0.109	1.5401055321367E-05	10	OSGIN2
2.97492262401255E-08	0.478964818791791	0.329	0.149	0.000498537533332023	10	SEC22A
3.25477968673123E-10	0.478828975499756	0.479	0.233	5.4543597990242E-06	10	KCMF1
5.60449882911227E-11	0.478760917097651	0.7	0.398	9.39201913782635E-07	10	TMED7
7.14437997919667E-09	0.4781802386584	0.479	0.247	0.000119725519691378	10	ABCA5
5.03868375236178E-08	0.478070000657	0.421	0.212	0.000844382623220788	10	BCL2L2
2.33639751386145E-08	0.47786713677673	0.35	0.163	0.000391533495372902	10	MZT2A
4.46671493584485E-07	0.477737103208657	0.407	0.216	0.0074853208894888	10	GALNT1
3.81646911023957E-08	0.477265546875091	0.271	0.114	0.000639563893493947	10	DEPRECATED-ENSG00000185619
2.59820529433536E-07	0.477247226390593	0.579	0.348	0.00435407243224719	10	IMPAD1
3.36357951097925E-07	0.477033168751091	0.407	0.211	0.00563668654449904	10	FIBP
1.43528319145916E-07	0.476877678602776	0.5	0.279	0.00240524757224726	10	DEPRECATED-ENSG00000133142
2.09757340484892E-09	0.476723991859106	0.407	0.19	3.51511351184582E-05	10	LYSMD3
4.60670272257165E-10	0.476418086374049	0.429	0.201	7.71991242248557E-06	10	PRKD2
4.07264393461797E-09	0.475995656236258	0.536	0.285	6.8249367056328E-05	10	RBM26
7.5586147635213E-07	0.475953578135841	0.343	0.173	0.012666726620709	10	ZNF75A
1.88067592793316E-07	0.475835166043861	0.336	0.16	0.00315163672003039	10	MSH2
5.9275414376112E-07	0.475613453150966	0.343	0.169	0.00993337394114884	10	DSN1
3.72958638752248E-08	0.475384152970535	0.707	0.417	0.000625004086821017	10	ALDH9A1
8.72048509517884E-07	0.474845211521779	0.414	0.222	0.0146137889225007	10	CTDSPL
1.75580259609132E-07	0.474813697764023	0.429	0.224	0.00294237399052984	10	DEPRECATED-ENSG00000196821

1.57978996690213E-09	0.474418901365184	0.679	0.374	2.64741202653459E-05	10	MCFD2
9.68481016811963E-13	0.474101126657353	0.879	0.521	1.62298048797349E-08	10	SMARCA1
2.02784808128311E-08	0.474001204802319	0.586	0.322	0.000339826781461423	10	PVR
1.23094851696111E-07	0.473964566026428	0.436	0.231	0.00206282352472343	10	SEMA4B
5.83012869839779E-08	0.473880295304638	0.257	0.107	0.000977012967277502	10	ATG10
1.17199799273641E-06	0.47377014399491	0.657	0.398	0.0196403423622768	10	CHCHD2
3.80634220379816E-11	0.473676956365785	0.807	0.494	6.37866826512496E-07	10	CD63
2.84162854630948E-09	0.4735135496418	0.714	0.415	4.76200111790542E-05	10	EIF6
6.18829835211791E-09	0.473197263368651	0.4	0.19	0.000103703503784792	10	SMAP1
5.28158536379315E-07	0.472347077971795	0.393	0.202	0.00885088075264456	10	PRPF39
2.869768448488E-09	0.471765510188646	0.536	0.272	4.80915796597619E-05	10	KDM6A
4.94476830651099E-10	0.47112318525104	0.579	0.31	8.28644272805111E-06	10	PSMD1
2.20788639716329E-09	0.470835848538929	0.421	0.202	3.69997602436625E-05	10	ORMDL2
1.49496810668023E-08	0.470565799053223	0.486	0.254	0.000250526755317474	10	SLC35B2
5.94782013458626E-09	0.470134584149453	0.357	0.162	9.96735698153966E-05	10	FAM76B
2.01946367410771E-09	0.469791650704059	0.479	0.235	3.38421722506971E-05	10	ACTR10
2.96827133242264E-08	0.46960283873794	0.721	0.413	0.000497422909887385	10	CMBL
1.32776292001153E-09	0.469212131363598	0.371	0.167	2.22506510135533E-05	10	PARD3
6.38362238909717E-09	0.469181248344075	0.707	0.428	0.00010697674399649	10	ENY2
9.49165854137181E-11	0.468993822052389	0.314	0.124	1.59061213836309E-06	10	ORC2
6.40638706035597E-10	0.468952007075501	0.7	0.399	1.07358234357445E-05	10	STAG2
2.38700261498246E-08	0.468337848715793	0.307	0.135	0.000400013898218761	10	TSG101
3.44186731970475E-10	0.467810793122889	0.621	0.338	5.76788125436122E-06	10	UQCR10
9.48873061133737E-07	0.467525417489307	0.321	0.158	0.0159012147584792	10	PHAX
5.56896956339271E-10	0.467253208735792	0.45	0.217	9.3324791943335E-06	10	TSPAN4
1.18454337699948E-07	0.466538110285315	0.443	0.238	0.00198505779117573	10	EIF2S1
5.52537457311747E-10	0.465759570310228	0.65	0.371	9.25942270963026E-06	10	MAP2K2
2.19263990505743E-10	0.465520080509706	0.714	0.412	3.67442595289524E-06	10	UBXN4
1.44714207605618E-06	0.465471931982953	0.364	0.19	0.0242512069105495	10	GHR
5.83150144706389E-10	0.465156976065644	0.486	0.243	9.77243012498967E-06	10	RNF138
7.83958103352114E-11	0.464840507810203	0.793	0.462	1.31375698959747E-06	10	HABP2
9.98540202690245E-10	0.464632547326229	0.686	0.391	1.67335367166831E-05	10	COX6A1
9.54312845820527E-10	0.464458189744781	0.35	0.152	1.59923746702604E-05	10	PIK3R4
1.54598908115777E-06	0.464275037063501	0.35	0.181	0.0259076850220418	10	STX17
1.1534561670565E-07	0.463645435571184	0.336	0.162	0.00193296184475328	10	ALG2
1.63423324884364E-10	0.463046701884044	0.671	0.373	2.73864807841217E-06	10	PPIB
9.10160045621011E-09	0.462092637492654	0.386	0.186	0.000152524620445169	10	OTUD6B
2.54423595067921E-09	0.461878938056325	0.493	0.243	4.26363060614822E-05	10	MAL2
1.96650792740294E-07	0.461174340441776	0.479	0.259	0.00329547398474184	10	TFDP1
4.7272238191568E-07	0.461003652728805	0.507	0.284	0.00792188167614297	10	WSB2
6.78865757637978E-09	0.460929081095487	0.293	0.122	0.000113764323664972	10	DLAT
4.46276677705748E-07	0.460821642448838	0.336	0.166	0.00747870456499293	10	VSIG10
7.80539067159708E-11	0.460655729986612	0.736	0.422	1.30802736874624E-06	10	PPP1R15B
7.71131199319608E-09	0.460327712010152	0.364	0.167	0.00012922616638198	10	GPAT3
1.10325767141511E-07	0.460205880548116	0.507	0.279	0.00184883920575744	10	GATAD1
1.43397213257764E-08	0.460094865302213	0.607	0.336	0.000240305049977361	10	NAA10
1.6231335088066E-08	0.459655290889087	0.693	0.396	0.00027200471340581	10	PDIA5
7.17008978767034E-10	0.459528507377528	0.443	0.209	1.2015636466178E-05	10	WWP2
3.20247426023915E-12	0.459290655284197	0.643	0.329	5.36670636530877E-08	10	TCIM
1.03299067584223E-11	0.459211586239052	0.371	0.154	1.7310857745764E-07	10	JADE2
8.0744854459932E-12	0.459122848530407	0.343	0.136	1.35312227103954E-07	10	TANC1
9.01114482245873E-07	0.458157367328423	0.286	0.134	0.0151008764934763	10	ABCG8
2.740305567345E-06	0.458038309950117	0.536	0.319	0.0459220406975675	10	CCT6A
4.41617752949252E-07	0.457774754949501	0.293	0.139	0.00740063030392357	10	NUBP1
5.29176341472584E-11	0.457271771618247	0.621	0.328	8.86793713039756E-07	10	SRP54
2.39625428691569E-09	0.457030555967701	0.629	0.356	4.01564293401332E-05	10	ARRDC2
3.08048817131556E-08	0.457022491081328	0.514	0.278	0.000516228207749061	10	MSRB2
1.20971836492924E-08	0.457008954551873	0.443	0.219	0.000202724603594843	10	CEP170B
5.17412761325631E-07	0.456730614494398	0.329	0.162	0.00867080305429492	10	ZMYM5
4.82595955864028E-09	0.4560582279565	0.314	0.135	8.08734302836937E-05	10	STRIP1

2.92512911506271E-09	0.455923109975166	0.55	0.302	4.9019313710221E-05	10	NUS1
1.56766608810556E-08	0.455791997974701	0.371	0.173	0.000262709483044729	10	TNKS1BP1
9.68205549466163E-09	0.455694165987454	0.507	0.271	0.00016225188597954	10	CDH1
1.99776307987521E-06	0.455586019771253	0.407	0.225	0.0334785136925488	10	LEMD2
1.08853378604914E-06	0.455586019113425	0.371	0.193	0.0182416491866114	10	TSN
3.90792598848566E-09	0.455270854045585	0.3	0.127	6.54890237150427E-05	10	TTC39B
1.56186448416814E-09	0.455199185324704	0.557	0.299	2.61737250256897E-05	10	DYNLT1
5.72002783640808E-10	0.454823674560298	0.421	0.195	9.58562264825267E-06	10	GLO1
1.71532399762532E-09	0.454127975975039	0.571	0.312	2.87453995522051E-05	10	GCLM
2.7981847652015E-08	0.453922338146681	0.393	0.197	0.000468919802952468	10	EPS8
2.60911374657387E-07	0.453856034597402	0.371	0.184	0.0043723528165085	10	SHOX2
1.58811023967794E-10	0.453601395086625	0.493	0.235	2.6613551396523E-06	10	NDUFA9
2.23876769280346E-09	0.4535683811983	0.75	0.463	3.75172689960003E-05	10	PSMB1
2.00628626437844E-08	0.452778805023122	0.4	0.194	0.00033621345218454	10	MAP3K11
6.89839437568004E-10	0.452693387228719	0.621	0.338	1.15603292947646E-05	10	DYNLRB1
1.48230719994165E-08	0.451894643328618	0.321	0.143	0.000248405040566222	10	KIF21A
1.5221538392721E-07	0.451643252038906	0.407	0.212	0.00255082540385219	10	CC2D1A
3.3321154749774E-10	0.45134498336663	0.729	0.412	5.58395911296712E-06	10	SELENOF
6.80732251440977E-10	0.451327468051116	0.779	0.462	1.14077110696479E-05	10	MTDH
4.48486682116291E-10	0.451044391102734	0.329	0.135	7.51573981890481E-06	10	PITPNM2
1.21227101885573E-09	0.451028190000554	0.479	0.235	2.03152377339844E-05	10	RMDN3
2.06033373051667E-08	0.450998991268387	0.664	0.379	0.000345270726559983	10	ATP6V0D1
3.52845962431196E-10	0.450914120485122	0.714	0.404	5.91299263842199E-06	10	SF3B5
8.88923351231063E-08	0.450773423146631	0.407	0.209	0.00148965775199302	10	CUL3
1.82252292528062E-09	0.450682116056964	0.271	0.108	3.05418391818526E-05	10	MAP3K10
1.3698417889495E-08	0.450578142880903	0.493	0.255	0.000229558086992157	10	SCML1
2.95139853179773E-09	0.450433879278532	0.879	0.602	4.94595365958663E-05	10	MT-ND5
6.98425878835305E-07	0.450299597345882	0.579	0.359	0.011704220877522	10	CCDC47
8.6771921497424E-09	0.448928811953944	0.443	0.219	0.000145412386045383	10	TEAD1
3.25816045176664E-13	0.448737877102418	0.5	0.222	5.46002528507054E-09	10	MED13
2.11526825184562E-08	0.448273320972288	0.693	0.418	0.000354476653644289	10	CTNNA1
1.68779283320057E-07	0.448185993545802	0.5	0.278	0.00282840322987752	10	TOR1AIP1
6.18897164296715E-10	0.448095622367856	0.779	0.488	1.03714786792844E-05	10	EIF1
1.87057204473883E-06	0.447810237460688	0.307	0.154	0.0313470463257333	10	TBPL1
6.24082866187065E-09	0.44778125101313	0.407	0.197	0.000104583806715628	10	CD177
2.96551419227017E-09	0.447700321454513	0.421	0.2	4.96960868340636E-05	10	PGK 1.00
1.43484187751258E-07	0.447446859288073	0.264	0.115	0.00240450801833559	10	E2F3
3.76021890115158E-08	0.447201810447079	0.871	0.587	0.000630137483454981	10	ANPEP
4.84369349463364E-10	0.446865879083576	0.65	0.362	8.11706155830705E-06	10	RHOC
8.31351319681617E-08	0.446163309316505	0.314	0.144	0.00139317854152245	10	PLCB3
1.45328746784712E-09	0.446149147952177	0.414	0.194	2.4354191386182E-05	10	UBAC2
2.38188499787322E-08	0.445882261135482	0.9	0.629	0.000399156287943594	10	ASPH
3.77432186757107E-08	0.445712856802845	0.364	0.172	0.00063250085856756	10	SUPT4H1
7.12059357173211E-08	0.445144015896401	0.343	0.162	0.00119326907075087	10	UEVLD
6.09632940061539E-10	0.444761899416251	0.814	0.498	1.02162288095513E-05	10	TMEM106C
9.72204286453588E-09	0.444198780826531	0.579	0.304	0.000162921994323892	10	SLC25A5
5.060102674571E-07	0.44413616225629	0.393	0.207	0.00847972006204607	10	UGT2B15
5.03811966262308E-07	0.444110375881922	0.343	0.173	0.00844288093062376	10	MON1B
3.19475266698281E-13	0.443914574395205	0.5	0.22	5.35376651932979E-09	10	MARCKSL1
1.48818593915291E-09	0.443268363842345	0.421	0.197	2.49390199683245E-05	10	STAT5B
2.57957368228518E-07	0.442660927156315	0.507	0.284	0.00432284957677351	10	HEBP1
7.07409747509725E-08	0.442350532290125	0.293	0.129	0.0011854772548768	10	PGAM5
5.58138581156918E-07	0.44197843867556	0.5	0.288	0.00935328634302764	10	DKC1
2.64567968334598E-09	0.441528751493904	0.764	0.476	4.43363001335119E-05	10	ANKRD10
2.88863951849374E-10	0.441329140504936	0.657	0.343	4.8407821050918E-06	10	GPRC5C
8.56050902883819E-08	0.440823966371405	0.85	0.565	0.0014345701030527	10	GOLIM4
8.18191177630443E-09	0.440664727202938	0.264	0.106	0.00013711247754731	10	RPP38
7.13101660387227E-12	0.440652691943759	0.657	0.342	1.19501576247691E-07	10	TMEM109
2.35527477844037E-07	0.440554441309138	0.507	0.281	0.00394696947371037	10	TM7SF2
6.55988381413911E-10	0.440262905019698	0.75	0.441	1.09930532957343E-05	10	AFF1

2.07622613329234E-06	0.440229397829791	0.271	0.129	0.034793397541713	10	LYSMD2
2.71267317225069E-11	0.440050099607413	0.6	0.295	4.5458977020577E-07	10	NDUFB11
3.98844682751068E-08	0.439882175648681	0.35	0.164	0.00066838391935424	10	PIGM
5.63937697216496E-11	0.43988025532085	0.471	0.217	9.45046792995404E-07	10	SLC25A32
3.29078901648142E-07	0.439752376255582	0.257	0.114	0.00551470423381956	10	GALNT10
3.46604342067733E-09	0.439637504043391	0.329	0.144	5.80839556437108E-05	10	CCDC61
1.9652408539436E-08	0.439565244028702	0.407	0.204	0.000329335062303868	10	TULP4
1.17895018126785E-07	0.439038202984723	0.279	0.125	0.00197568471376867	10	FEM1A
2.96700718010385E-09	0.43897983266117	0.471	0.232	4.97211063241803E-05	10	UBL5
1.10077870148188E-08	0.438941548553072	0.457	0.235	0.000184468494794333	10	AZIN1
8.95552721615494E-09	0.438841676370686	0.814	0.532	0.000150076725088324	10	TMCO1
7.67457464500905E-07	0.438649390605653	0.3	0.144	0.0128610521901062	10	MSTO1
1.77907050180965E-08	0.43860672032552	0.829	0.535	0.000298136634693262	10	BAAT
5.00066569882473E-09	0.438218220605027	0.571	0.299	8.38011557809048E-05	10	C1GALT1C1
1.7610151145249E-10	0.438116725600924	0.657	0.353	2.95110912892082E-06	10	SMG5
5.07102970314681E-07	0.438062518957859	0.786	0.554	0.00849803157653342	10	APLP2
1.39412744880644E-06	0.43698371757616	0.286	0.137	0.0233627877870984	10	CTPS2
7.58015828917981E-09	0.436855832121811	0.271	0.11	0.000127028292610075	10	GORAB
5.35599133349419E-10	0.436591983275456	0.543	0.279	8.97557027666956E-06	10	PDCD6
1.19852316467965E-07	0.436579047340093	0.25	0.106	0.00200848511937015	10	OLA1
2.48158404521967E-09	0.436556977134692	0.336	0.145	4.15863854297912E-05	10	TACC2
1.69174211436865E-08	0.4365400155361	0.564	0.32	0.000283502143525898	10	TMED10
4.47602544950851E-08	0.436347568751767	0.486	0.255	0.000750092344828636	10	LRBA
2.15186864824332E-09	0.436084288808857	0.579	0.314	3.60610148072615E-05	10	TMX4
2.1756109699426E-08	0.435827018328025	0.421	0.209	0.000364588886342981	10	C1RL
2.07538463338285E-08	0.435671465926806	0.357	0.167	0.000347792956862298	10	CYTH3
9.15139279796723E-13	0.435267935399847	0.643	0.328	1.53359040508335E-08	10	CD47
5.23860989973075E-07	0.434886581507475	0.371	0.193	0.00877886246996879	10	RPA1
2.85334172283718E-09	0.434720041752066	0.364	0.164	4.78163005913054E-05	10	CADPS2
1.43219119221846E-09	0.434313883361855	0.807	0.513	2.4000659999197E-05	10	NFE2L1
1.25999515104553E-07	0.434165078444402	0.464	0.246	0.0021114998741221	10	YWHAH
3.26676340764982E-07	0.4339921638605	0.293	0.136	0.00547444211853958	10	LCORL
2.26755505650242E-07	0.433805709684829	0.543	0.314	0.00379996876368676	10	CYB5B
1.36665268531538E-06	0.433703598212188	0.35	0.178	0.0229023657005152	10	TMEM181
1.06770631914911E-07	0.43362577332948	0.386	0.194	0.00178926224963007	10	NSMAF
2.13421034790455E-17	0.433268602733349	0.85	0.491	3.57650970101844E-13	10	IFI30
2.59668975400104E-08	0.433064498898834	0.421	0.211	0.000435153268975495	10	SRP68
3.50127648123779E-07	0.43301363283869	0.664	0.418	0.00586743912725829	10	RER1
3.4469317535291E-10	0.43290750543	0.514	0.248	5.77636823256406E-06	10	DEPRECATED-ENSG00000132646
1.17944660430049E-08	0.432633060277548	0.321	0.141	0.000197651661948677	10	TSPAN31
5.84105584634818E-08	0.432371192101592	0.393	0.196	0.000978844138731028	10	TRMT1L
1.40487510808574E-07	0.432156974904572	0.357	0.172	0.00235428970613009	10	USP38
4.03161069496238E-09	0.431884097251183	0.857	0.581	6.75617320261796E-05	10	GRINA
8.40736221852181E-11	0.431854959420565	0.771	0.466	1.40890576057989E-06	10	LPGAT1
1.03778240867686E-09	0.431677564284067	0.714	0.389	1.73911576046068E-05	10	OCRL
5.04071638264353E-09	0.43139776549603	0.771	0.446	8.44723251403403E-05	10	PNKD
2.87879180048151E-07	0.431029421286985	0.371	0.191	0.00482427929924692	10	CFAP97
6.69292487494404E-07	0.430928717236905	0.35	0.174	0.0112160035054312	10	RAB28
1.4367799471151E-07	0.430812934884276	0.557	0.315	0.00240775583537548	10	NBPF26
1.73658965706027E-07	0.430802463238772	0.429	0.221	0.00291017694730161	10	ITPA
2.64793545209941E-08	0.430613646610881	0.729	0.435	0.00044374102306282	10	SDC2
5.99804708992978E-10	0.430307637428842	0.55	0.279	1.00515273133043E-05	10	PRKAG2
1.20551787364807E-10	0.429867273336896	0.329	0.134	2.02020685265943E-06	10	DDX52
6.54481029765301E-07	0.429746825803021	0.429	0.232	0.0109677930968069	10	TECR
1.73962873963638E-07	0.429684293105723	0.357	0.178	0.00291526984188264	10	CPSF2
1.42333249154059E-06	0.429451325030701	0.55	0.325	0.0238522058932372	10	NEDD4L
6.62954703628499E-10	0.428966433974282	0.671	0.382	1.11097949234064E-05	10	CANX
8.6885705430016E-08	0.428808945186216	0.471	0.25	0.00145603065159621	10	GLB1
1.37564089732126E-07	0.428742656780953	0.529	0.291	0.00230529901573097	10	CEP57
1.0436679109871E-08	0.427812614549503	0.421	0.208	0.000174897868523219	10	PQBP1

6.09621921673839E-08	0.427078572608531	0.343	0.162	0.00102160441634102	10	CPOX
4.95991911676385E-09	0.426684296406884	0.714	0.413	8.31183245587287E-05	10	CDC37
3.29804487170392E-09	0.426634514851103	0.457	0.229	5.52686359600142E-05	10	LRP10
5.10249793842877E-08	0.426577235813059	0.414	0.212	0.000855076604521894	10	RNF19B
2.12009293197839E-06	0.426414165106371	0.307	0.155	0.0355285173540938	10	MKRN1
1.45674486840076E-09	0.42615789981206	0.486	0.244	2.441213050466E-05	10	TRIM24
1.41700111424124E-09	0.426106065193505	0.514	0.263	2.37461046724546E-05	10	STX4
1.05877663375375E-11	0.426066282762787	0.514	0.238	1.77429788284454E-07	10	KDELR1
8.48268502582646E-08	0.425750673411458	0.464	0.243	0.001421528356628	10	AQP9
1.31792679001717E-07	0.424880066181421	0.443	0.232	0.00220858171471078	10	AFG3L2
2.57023054040117E-11	0.424695581173082	0.543	0.26	4.30719233960428E-07	10	SEC23A
7.19101617052517E-12	0.424639712165025	0.514	0.239	1.20507048985661E-07	10	TAF15
1.31026447507488E-09	0.424213080501568	0.443	0.209	2.19574120733048E-05	10	BTF3L4
5.4114950107239E-07	0.423948694793666	0.414	0.22	0.00906858333897111	10	MTM1
3.18431002451639E-07	0.423307893250357	0.414	0.216	0.00533626673908457	10	FAM8A1
4.03338818204629E-09	0.42322305450199	0.543	0.284	6.75915191547318E-05	10	MOGS
2.14817077796543E-10	0.423082355458673	0.471	0.228	3.59990458971447E-06	10	CTNNB1
7.65426255526642E-08	0.422451736600887	0.386	0.193	0.00128270131901155	10	ABHD14B
5.51393198360945E-08	0.422442755748634	0.607	0.348	0.000924024721813272	10	RNF187
5.71286388364128E-09	0.421539679230945	0.279	0.114	9.57361729620605E-05	10	BRMS1L
9.82114180084849E-08	0.421348825325106	0.436	0.227	0.00164582694298619	10	SDR39U1
3.16226749513856E-09	0.4210783044121	0.371	0.167	5.29932786835319E-05	10	GTF2A2
1.76528453042724E-08	0.420880165429156	0.279	0.119	0.000295826381608996	10	TAPBPL
2.07893218606122E-06	0.420629095721345	0.336	0.169	0.0348387455740138	10	SMC5
3.11546872081036E-07	0.420604423981683	0.421	0.226	0.005220902482334	10	TMEM258
2.61466767412681E-07	0.420396906072086	0.643	0.38	0.00438166008830171	10	GSTA2
8.15585532788983E-08	0.420157433026016	0.386	0.192	0.00136675823584778	10	UPF3A
1.84761280141237E-08	0.420108110711183	0.636	0.36	0.000309622953260685	10	MTCH1
1.36668619298799E-07	0.419348240678704	0.393	0.195	0.00229029272220928	10	NDUFC1
1.96184091867141E-09	0.419266066388265	0.314	0.133	3.28765301150955E-05	10	TMEM35B
4.40042662827405E-10	0.41923664074716	0.3	0.119	7.37423494366165E-06	10	ZBED6CL
7.58540368166474E-11	0.419146455256081	0.464	0.215	1.27116194897338E-06	10	WBP2
1.42305830573504E-07	0.41900281826417	0.614	0.369	0.00238476110875078	10	SURF4
6.22080223533408E-11	0.418828393367394	0.579	0.295	1.04248203859729E-06	10	RABGGTB
7.78878172926907E-07	0.418639826446666	0.264	0.122	0.0130524404219091	10	IRGQ
8.93138866491867E-07	0.418625981438904	0.343	0.176	0.0149672211246707	10	GRPEL1
4.83254816171944E-08	0.418585124190844	0.543	0.306	0.000809838420940943	10	PAK2
9.45935636334357E-08	0.418457252289049	0.25	0.104	0.00158519893936912	10	AGTR1
6.15611804051354E-07	0.418266587880324	0.407	0.214	0.0103164226122926	10	TRAPPC10
6.60453454007567E-10	0.418000070872418	0.514	0.266	1.10678789822588E-05	10	DENND4C
4.58564402165025E-07	0.417654944087555	0.443	0.237	0.0076846222514815	10	BROX
1.68877696829046E-06	0.417461171248173	0.257	0.117	0.0283005244346115	10	VAMP5
7.42171717344724E-12	0.417268230915028	0.629	0.312	1.24373136392629E-07	10	KLF3
9.7988211331571E-09	0.416824584179044	0.321	0.14	0.000164208644549447	10	UBALD2
3.96567634643579E-08	0.416388290676423	0.707	0.453	0.000664568042135709	10	STAT3
4.03377903230958E-08	0.416339904527219	0.629	0.356	0.00067598069023444	10	ACSL3
8.83100798447406E-09	0.41632406183246	0.7	0.423	0.000147990031803816	10	SCARB2
9.05666246442298E-07	0.416184123454153	0.293	0.142	0.01517715495788	10	VWA1
3.4103069171096E-08	0.415677240245849	0.471	0.251	0.000571499233169226	10	SENP2
1.38310173142437E-06	0.415615258552632	0.614	0.365	0.0231780188152096	10	SLC35D2
4.42987695185423E-08	0.415439575860215	0.643	0.364	0.000742358779591732	10	RAMP1
1.41883473271693E-07	0.415425972981084	0.264	0.115	0.00237768324508703	10	TSNARE1
5.20069012201844E-08	0.415313863676111	0.371	0.183	0.00087153165064785	10	WAC
4.08518965878816E-09	0.41469708617525	0.714	0.405	6.84596083019719E-05	10	PRDX4
1.08885530536044E-08	0.4146191971247	0.536	0.287	0.000182470372072302	10	VAT1
9.16471108575768E-08	0.414567037507357	0.55	0.309	0.00153582228375127	10	MCM4
3.96721945658012E-08	0.41451870859372	0.3	0.131	0.000664826636533696	10	MAP3K20
1.0949985213479E-08	0.414134439054242	0.45	0.228	0.000183499852207481	10	AGTRAP
4.88378112254724E-08	0.413922960656458	0.671	0.408	0.000818424040516467	10	RTN3
6.58777653940234E-08	0.413601691805065	0.571	0.322	0.00110397959247304	10	PEX2

7.28906487498113E-09	0.413468976445344	0.514	0.269	0.000122150149174934	10	MZT2B
1.00303567421089E-07	0.413138842519148	0.679	0.431	0.0016808871828426	10	STK24
5.58470312284399E-07	0.413043698598664	0.414	0.222	0.00935884549326196	10	APPL2
1.16477492051891E-07	0.412212112089431	0.429	0.225	0.0019519298118056	10	TLE1
2.8905709421989E-08	0.412147212489843	0.379	0.178	0.000484401878493691	10	WHRN
9.40662606850694E-07	0.411885135430129	0.3	0.145	0.0157636239656039	10	HINT3
5.3626160551995E-08	0.411715941000319	0.421	0.214	0.000898667198530332	10	MCM6
6.2923456341283E-09	0.411584474193814	0.979	0.823	0.000105447128136722	10	MT-CO2
1.78256617719324E-07	0.411506631622354	0.471	0.248	0.00298722439974043	10	CHAMP1
4.88566253831893E-07	0.411165422138801	0.471	0.265	0.00818739328171486	10	DHCR7
7.51356545891117E-11	0.410371542105861	0.593	0.303	1.25912329960433E-06	10	RAB11FIP1
6.78332345801632E-11	0.410308289254179	0.686	0.364	1.13674934509438E-06	10	HNRNPK
3.12996492705686E-09	0.410041170523177	0.979	0.842	5.24519522476188E-05	10	MT-ATP6
4.49596795211467E-09	0.409950493561191	0.543	0.286	7.53434309415377E-05	10	DPP9
1.25859374469693E-06	0.409804671131411	0.936	0.718	0.0210915139736312	10	PTGR1
4.96850333558965E-07	0.409007160622521	0.336	0.167	0.00832621788978114	10	UBAP1
6.29523257960055E-11	0.408697527007733	0.579	0.288	1.05495507568946E-06	10	PPM1A
4.67936019099041E-08	0.408309758256187	0.514	0.281	0.000784167180806173	10	ATP5F1A
5.92346859050712E-10	0.407992594643321	0.286	0.111	9.92654866397182E-06	10	DEPRECATED-ENSG00000050130
7.61918002038765E-08	0.407572556995873	0.829	0.54	0.00127682218781656	10	CRYL1
2.85607336841849E-08	0.40753937378261	0.436	0.226	0.00047862077507957	10	PPIC
6.08125789299697E-07	0.406892463499254	0.55	0.321	0.0101909719770843	10	NDUFV3
7.7586225844145E-11	0.406132141696362	0.45	0.212	1.30018997269618E-06	10	SYNPO
5.69787726304436E-08	0.406062238875547	0.75	0.468	0.000954850271740974	10	PRDX1
1.04575948252074E-06	0.405439265521766	0.764	0.5	0.0175248374080825	10	DECR1
8.62831535865386E-07	0.405122276663264	0.321	0.156	0.0144593308780321	10	BOP 1.00
2.09785578806584E-07	0.404596282626603	0.443	0.228	0.00351558672964074	10	SCAMP4
2.20463510704097E-09	0.404570033787854	0.764	0.459	3.69452751237926E-05	10	NDUFB7
7.58185237935194E-09	0.40412377549283	0.593	0.327	0.00012705668217318	10	UBE2D3
5.33367652638364E-08	0.403790630094529	0.35	0.165	0.00089381751229137	10	UBE2W
3.82628137535942E-09	0.40367637144678	0.671	0.388	6.41208232882731E-05	10	CLPTM1
1.75315113658795E-10	0.40366405321361	0.586	0.297	2.93793067469408E-06	10	DNAJC21
1.30788327577923E-08	0.403522478650114	0.393	0.187	0.000219175079355083	10	TOX4
1.49070436179333E-07	0.403018541231394	0.514	0.294	0.00249812236949326	10	DLD
3.35487100051307E-07	0.402373727924194	0.364	0.182	0.0056220928226598	10	DEPRECATED-ENSG00000109084
1.64231291883721E-08	0.402359794860048	0.7	0.424	0.00027521879893874	10	SLC38A10
1.52623718135039E-10	0.402271866482388	0.407	0.181	2.55766826850698E-06	10	PAWR
1.49685050361912E-07	0.402207528342279	0.643	0.363	0.00250842207396493	10	SMOC1
1.46561299175446E-09	0.401934682453806	0.636	0.335	2.45607425158212E-05	10	DGAT1
1.13499262742004E-08	0.401840116202025	0.586	0.318	0.00019020206450305	10	TUBB2A
2.559106725212E-09	0.401713439424095	0.571	0.306	4.28855105011026E-05	10	IRF6
1.9023310444358E-08	0.401564660864208	0.493	0.265	0.000318792636426551	10	ZDHHC14
1.46351816341562E-07	0.401561158771375	0.657	0.39	0.0024525637382519	10	ITPK1
3.79244427654171E-08	0.401391465846943	0.493	0.256	0.000635537811862859	10	ARHGEF40
4.62217135336639E-09	0.400303225051272	0.543	0.278	7.74583475397139E-05	10	SMYD2
2.6946811659687E-06	0.400275407428132	0.457	0.255	0.0451574669793035	10	PRDX5
4.48844491944723E-08	0.400255263864772	0.386	0.186	0.000752173599600967	10	AK6
2.11377859059407E-08	0.400142071225657	0.75	0.464	0.000354227016211754	10	YWHAZ
1.23988624763906E-08	0.400087861407387	0.679	0.394	0.000207780137379353	10	POLDIP2
1.64619117192098E-10	0.399805352217898	0.507	0.246	2.75868716590518E-06	10	NCKIPSD
1.96294256577978E-08	0.399617560789241	0.436	0.227	0.000328949915173376	10	HMOX2
1.33978368278724E-07	0.398530916733737	0.407	0.204	0.00224520949561485	10	ELF2
1.99219970370795E-06	0.398347937129284	0.3	0.149	0.0333852826347378	10	CDYL
9.71461298027534E-09	0.398310887826074	0.471	0.236	0.000162797484323454	10	TMEM14C
1.62850119593196E-06	0.398043703839512	0.321	0.162	0.0272904230414277	10	RAB3GAP2
7.3696644419824E-08	0.397986553161459	0.329	0.155	0.00123500836718741	10	PRSS23
9.23466944313739E-07	0.397779483377656	0.464	0.266	0.0154754590528096	10	DEPRECATED-ENSG00000067064
5.37126894700354E-08	0.397778940491944	0.829	0.58	0.000900117250138853	10	C2
2.31271431156545E-07	0.397565807772333	0.264	0.117	0.00387564664332138	10	TRAFD1
2.99089178888352E-08	0.397547632784178	0.529	0.292	0.0005012136459811	10	RPN2

**1.28908047571776E-06**	0.397307348083617	0.35	0.179	0.0216024106120783	10	PHF11
**2.01435116235893E-08**	0.397234656565005	0.471	0.239	0.00033756496778811	10	HELLS
**1.25505976253703E-06**	0.396358961082486	0.75	0.485	0.0210322915005955	10	NDUFA2
**5.78210443743459E-10**	0.39610217232336	0.721	0.405	9.68965061625288E-06	10	LASP1
**4.0822276718887E-07**	0.395923051485848	0.407	0.21	0.00684099713255109	10	CSTF3
**3.18652150202315E-08**	0.395901488728657	0.436	0.22	0.000533997273309039	10	TOMM6
**6.27099629135878E-07**	0.395590473073343	0.25	0.111	0.010508935585059	10	SLC39A6
**4.17070484166508E-08**	0.395212690982969	0.521	0.283	0.000698926717366233	10	GDA
**2.59144118671016E-11**	0.394846776858889	0.579	0.284	4.34273714068888E-07	10	SELENON
**5.15355655708578E-10**	0.393271805210438	0.729	0.448	8.63633007836435E-06	10	TP53INP1
**1.88498941206006E-06**	0.392371453552436	0.4	0.217	0.0315886525673025	10	WDYHV1
**1.0195212325008E-07**	0.392075472154771	0.536	0.296	0.00170851368142484	10	DUSP3
**1.51100978577964E-07**	0.392051038226587	0.629	0.371	0.00253215019900953	10	LLGL2
**5.69315465053022E-07**	0.391802415972111	0.314	0.153	0.00954058856335855	10	TCF12
**5.90585958386661E-07**	0.391270459163574	0.314	0.153	0.00989703949064367	10	CAP2
**2.30496974616865E-06**	0.391253928092356	0.45	0.249	0.0386266830062943	10	TOB2
**1.13664746378211E-09**	0.391248431270378	0.629	0.325	1.90479381980606E-05	10	SLC17A4
**4.56702322950714E-08**	0.391158135821812	0.657	0.373	0.000765341752800807	10	IL6R
**2.17080325942383E-13**	0.391002856517357	0.443	0.181	3.63783210214246E-09	10	ENPEP
**1.96337075987742E-12**	0.390413309722529	0.579	0.282	3.29021671940258E-08	10	IDS
**1.20022276881306E-07**	0.390342298799152	0.75	0.509	0.00201133331597692	10	SLC12A7
**2.61290114274313E-08**	0.390156966510894	0.893	0.612	0.000437869973500893	10	GATM
**2.90249917375642E-08**	0.390131274321758	0.771	0.465	0.000486400811538101	10	IMMP2L
**4.69730940545753E-08**	0.38939134404427	0.379	0.183	0.000787175110166573	10	LNPK
**7.52213080012317E-10**	0.389369999310362	0.443	0.209	1.26055867948464E-05	10	PSMG3
**1.32043084932175E-08**	0.388636109242424	0.8	0.493	0.000221277801729339	10	SMIM14
**1.39438979541099E-09**	0.387728110537555	0.4	0.181	2.33671841914973E-05	10	MOSPD2
**4.23759946140401E-12**	0.387523874884344	0.407	0.173	7.10136917742084E-08	10	TCF7L2
**5.21908970788088E-09**	0.387274196269415	0.521	0.278	8.74615053246677E-05	10	DNAJB2
**9.41942019686464E-08**	0.387189464461097	0.45	0.231	0.00157850643659058	10	ABHD10
**4.82534665677805E-09**	0.387062328132198	0.536	0.279	8.08631592742866E-05	10	COBLL1
**5.03668601988908E-07**	0.387058738555562	0.321	0.156	0.00844047843213012	10	JADE1
**1.83628981668511E-09**	0.386698827393389	0.557	0.291	3.07725447480091E-05	10	EMC7
**1.36031069685939E-07**	0.386280080400148	0.843	0.559	0.00227960866579697	10	SLC9A3R1
**6.78323041851213E-07**	0.385808506217137	0.421	0.221	0.0113673375353426	10	MID1
**2.11444698693583E-09**	0.385391105619574	0.421	0.199	3.54339026070706E-05	10	PLIN3
**2.60399661636247E-06**	0.384966056583036	0.35	0.183	0.0436377752970023	10	UFD1
**4.22458011475854E-07**	0.384939318595527	0.721	0.432	0.00707955135631236	10	ZBTB7B
**8.98331682613295E-07**	0.384395589626302	0.45	0.248	0.0150542423372336	10	AQP3
**6.86009142871057E-08**	0.383868746229463	0.557	0.307	0.00114961412162332	10	RHPN2
**6.38050659910424E-07**	0.383668458758787	0.293	0.139	0.0106924529587789	10	SOCS5
**3.01210772850874E-09**	0.383659290791137	0.65	0.362	5.04769013143494E-05	10	SHARPIN
**1.26675162630862E-11**	0.38365850848193	0.307	0.113	2.12282237536798E-07	10	IRAK2
**1.74653940749541E-09**	0.383439101165454	0.407	0.189	2.92685073908081E-05	10	KIAA1191
**1.21969722764777E-08**	0.383396113386723	0.671	0.394	0.000204396861409213	10	DNAJB11
**1.85931439855313E-08**	0.383366156479534	0.814	0.486	0.00031158390690953 4	10	GDI1
**2.20768238864389E-06**	0.383256865338778	0.371	0.196	0.0369963414688943	10	DEPRECATED-ENSG00000134049
**1.47943391303696E-06**	0.382946047342235	0.443	0.245	0.0247923535146734	10	PSEN1
**1.79799820404568E-07**	0.382280829433634	0.407	0.208	0.00301308539033975	10	SPG11
**3.2757840946477E-09**	0.38222567998232	0.436	0.205	5.48955898581061E-05	10	SART1
**1.70083454638514E-06**	0.382000858181792	0.729	0.5	0.0285025853283223	10	ACOX1
**2.05535712089757E-07**	0.381865197550434	0.693	0.422	0.00344436746320014	10	ACBD5
**3.87062183195422E-08**	0.381734717785509	0.657	0.382	0.000648638806598888	10	DEPRECATED-ENSG00000163463
**2.49676355651628E-08**	0.381604989191849	0.486	0.252	0.000418407636800999	10	GSK3A
**2.62268821306132E-09**	0.38149058211185	0.55	0.289	4.39510090744817E-05	10	PLOD2
**2.11793502397462E-07**	0.38129633076816	0.679	0.408	0.00354923551317667	10	ETHE1
**5.77448880653227E-08**	0.381211070909918	0.371	0.18	0.000967688834198677	10	PRSS3
**5.64594210574152E-07**	0.38115928330418	0.521	0.296	0.00946146978080164	10	AGRN
**1.65899029050163E-07**	0.380789817422511	0.336	0.161	0.00278013592882262	10	VEZT
**2.09305195196327E-09**	0.380627763947188	0.679	0.384	3.50753646110004E-05	10	FMR1

2.41327615132222E-07	0.38060767636507	0.271	0.12	0.00404416817438577	10	RABL3
1.15619855220335E-07	0.38040090902351	0.357	0.169	0.00193755753378237	10	ARFRP1
3.27332091871328E-07	0.380076363761226	0.343	0.167	0.00548543119557972	10	RSPRY1
1.44680551656081E-06	0.379921029123718	0.829	0.551	0.024245566846526	10	DNM2
3.63728723415131E-07	0.379634528667498	0.386	0.198	0.00609536594699077	10	ATP6V1H
1.06591398497347E-07	0.379498572611846	0.65	0.382	0.00178625865601854	10	HNRNPD
3.33801195560291E-09	0.379386794362414	0.471	0.237	5.59384043519936E-05	10	RASSF4
4.97442983927047E-09	0.378959739823817	0.536	0.291	8.33614952464945E-05	10	MBNL2
6.73192407204134E-09	0.378795969810461	0.664	0.365	0.000112813583599269	10	EIF2AK1
2.03233371558143E-07	0.378686840541147	0.486	0.272	0.00340578484057135	10	PTK2
7.68437635784416E-09	0.378655175407495	0.471	0.243	0.000128774779004752	10	KLHL5
9.2322698678894E-09	0.378571242111609	0.514	0.267	0.000154714378446091	10	TCEAL1
4.3358260168223E-07	0.377782140692952	0.421	0.223	0.0072659772389908	10	SMIM26
3.08085623378044E-07	0.377489622810828	0.536	0.314	0.00516289887656926	10	SSR3
3.46182068603682E-07	0.377485182634828	0.443	0.242	0.0058013191056605	10	SNF8
5.71801058552038E-08	0.377451926274739	0.357	0.168	0.000958224213921506	10	VPS72
2.96943232757899E-09	0.37725366869363	0.943	0.625	4.97617469455688E-05	10	SPINK1
5.00022996920333E-08	0.377128048481763	0.4	0.201	0.000837938538239095	10	ATG3
1.51215859878356E-09	0.376655841310879	0.364	0.161	2.53407537984149E-05	10	PPP1R3B
5.52492467635428E-07	0.376483292427502	0.829	0.531	0.00925866877263451	10	RARRES2
1.75453716153751E-07	0.376415521846785	0.314	0.145	0.00294025337530457	10	ACTR3C
1.36230408672359E-06	0.376108459938094	0.257	0.119	0.022829491885314	10	TRAPPC1
3.37055029823297E-08	0.376101179188639	0.736	0.472	0.000564836818977881	10	PLXNB2
5.20423331223161E-09	0.376033096776025	0.571	0.305	8.72125418463774E-05	10	APH1A
9.88499642614067E-07	0.375989182824445	0.393	0.205	0.0165652770109265	10	NDUFB4
3.62381246018736E-09	0.375942763733064	0.486	0.25	6.07278492078197E-05	10	CCDC124
2.52190573938873E-08	0.375779221660265	0.271	0.114	0.000422620963806764	10	HAUS3
2.22581348872466E-08	0.375712615006265	0.743	0.462	0.000373001824440478	10	STAU1
2.59778684683677E-07	0.375330946506473	0.271	0.122	0.00435337119792905	10	SPRYD7
3.22028919994961E-09	0.374860112089432	0.364	0.161	5.39656064127555E-05	10	ICMT
9.26862035416453E-08	0.374580527261072	0.814	0.527	0.00155323539895089	10	NOTCH2NLC
3.30711930355555E-07	0.374510547883244	0.521	0.287	0.00554207052889839	10	ONECUT2
1.20568477040229E-06	0.374448209290333	0.45	0.25	0.0202048653824016	10	SLC39A9
7.4617434256012E-09	0.374411944495358	0.636	0.354	0.000125043896326225	10	ACBD3
6.63721740109876E-07	0.374053949711895	0.307	0.147	0.0111226489207613	10	ANKRD46
1.22645900067029E-09	0.373973340570118	0.493	0.248	2.05529999332326E-05	10	EXOC7
3.73144511146072E-08	0.373950477990078	0.85	0.56	0.000625315571778588	10	MT-ND1
4.38110106212838E-07	0.373536960376726	0.429	0.227	0.00734184915991474	10	FOXA1
1.44431500935692E-10	0.373437369677134	0.557	0.28	2.42038309268032E-06	10	RNASEK
7.06517560038124E-08	0.37335995653916	0.35	0.166	0.00118398212711189	10	NUDT7
8.27017062428614E-07	0.37327420809096	0.721	0.425	0.0138591519321787	10	C2orf72
3.61489313000723E-10	0.372804739668108	0.5	0.243	6.05783790726612E-06	10	GNA11
1.22653066364508E-06	0.372720022081264	0.357	0.181	0.0205542008613643	10	ERMARD
1.14431362644466E-08	0.372153739777156	0.343	0.157	0.000191764077519596	10	ANKFY1
2.75671370014832E-07	0.371885065289408	0.479	0.262	0.00461970081870856	10	SLC33A1
2.47336802341269E-11	0.371367826040774	0.443	0.195	4.14487013363499E-07	10	PELO
7.44017123352092E-09	0.371288245498815	0.543	0.286	0.000124682389531343	10	DNAJC4
9.80232063548861E-07	0.371150145489657	0.357	0.182	0.0164267289209518	10	LAMA3
4.25238927932018E-08	0.370391823768846	0.321	0.148	0.000712615395428476	10	ETV6
1.4014915574701E-07	0.370373937794643	0.471	0.254	0.00234861955200839	10	PPP1R7
8.98033885144414E-13	0.370253448446255	0.707	0.368	1.50492518472501E-08	10	QKI
6.30508596895908E-12	0.369677207861918	0.364	0.144	1.05660630667816E-07	10	SLC28A1
8.30311034206023E-08	0.369364263417576	0.786	0.486	0.00139143523112245	10	CDHR5
2.19062777464764E-07	0.369267818063939	0.307	0.142	0.00367105402475451	10	ALG9
2.78589617833508E-09	0.369086492286675	0.507	0.265	4.66860481565393E-05	10	SRA1
6.65789704048637E-08	0.368986341958025	0.386	0.192	0.00111573038604471	10	C1orf122
2.0006031348443E-07	0.368888778403807	0.671	0.415	0.00335261073337207	10	ADIPOR1
5.52856071453667E-07	0.368624614024719	0.436	0.238	0.00926476204542056	10	SCFD1
5.56747979222153E-07	0.36862064162817	0.25	0.112	0.00932998263580484	10	SBF2
1.00512905592959E-06	0.368541368178232	0.279	0.13	0.016843952719268	10	DEPRECATED-ENSG00000128228

6.83556831465006E-07	0.368371188814277	0.493	0.273	0.0114550453816906	10	SNX4
1.04530165239253E-08	0.368314388040363	0.493	0.252	0.000175171650907941	10	BMI1
8.06727137382031E-10	0.368093713966839	0.429	0.205	1.35191333682481E-05	10	TAX1BP3
1.99536125367038E-06	0.367970261070992	0.479	0.269	0.0334382638890083	10	CANT1
2.1085634728281E-08	0.367875630214653	0.579	0.324	0.000353353066776533	10	TMEM50A
8.99629369416316E-09	0.367610502609669	0.557	0.298	0.000150759889726786	10	ZFP91
6.61787441385162E-07	0.36696654092963	0.35	0.174	0.0110902339427325	10	TMX1
3.43986018920583E-09	0.366618473310122	0.743	0.419	5.76451770507114E-05	10	PAG1
3.82779060769111E-08	0.366426316770963	0.486	0.256	0.000641461150036877	10	HHEX
1.08174470724276E-07	0.366362095423899	0.479	0.263	0.00181278778039742	10	FAM49B
3.49334177287486E-07	0.366086248137406	0.314	0.149	0.0058541421429837	10	ZFX
1.84541517051964E-06	0.365996476177418	0.386	0.209	0.0309254674275681	10	TSR2
5.13856050256918E-08	0.365870917169763	0.586	0.339	0.000861119969020543	10	PSMC3
8.44964110897963E-09	0.365820451320185	0.621	0.348	0.000141599085704281	10	PTPRF
7.95976146929348E-09	0.365493865739941	0.686	0.394	0.00013338968270242	10	REEP5
1.31049905009079E-07	0.365399589217912	0.879	0.625	0.00219613430814215	10	LMNA
8.14033093795656E-08	0.364715779770201	0.521	0.286	0.00136415665858276	10	MECP2
8.89022939913604E-09	0.36468591645009	0.707	0.409	0.000148982464270722	10	KLF9
2.08479137395606E-08	0.36438207119094	0.307	0.135	0.000349369338447556	10	STAM2
2.40355509204362E-08	0.364119942756461	0.621	0.348	0.00040278776232467	10	DCAF6
3.31952404063733E-07	0.363435807745753	0.436	0.232	0.00556285838730004	10	PRCC
2.70012390303043E-07	0.363066246880136	0.379	0.187	0.0045248676366984	10	DPH6
3.49843029518917E-08	0.362997267063152	0.75	0.457	0.000586266948867801	10	ATP5F1D
2.35240463748116E-07	0.362897063195423	0.386	0.195	0.00394215969149092	10	CBR4
4.45376026992466E-07	0.362843071025453	0.386	0.199	0.00746361146033975	10	HARS
1.09302886214982E-06	0.362668561142732	0.264	0.123	0.0183169776719068	10	TSEN54
3.00633050248933E-07	0.362604439148425	0.557	0.32	0.00503800865607162	10	RPN1
9.79645038676186E-10	0.36234359298969	0.557	0.287	1.64168915581355E-05	10	TUBB
4.40352369630518E-09	0.362319528067595	0.714	0.427	7.37942501026822E-05	10	SEC31A
1.23499970574314E-06	0.361979556968718	0.493	0.278	0.0206961250688436	10	FAM83H
2.19399188968393E-08	0.361785227434144	0.457	0.237	0.000367669160873233	10	PET100
4.46378690509241E-08	0.361346353281759	0.486	0.252	0.000748041409555387	10	MCM2
1.81050969045945E-07	0.361330907080952	0.421	0.219	0.00303405213927195	10	HDHD2
1.23668531549982E-06	0.361012852765116	0.336	0.167	0.020724372517146	10	DEPRECATED-ENSG00000178950
1.41943565355925E-07	0.360909707456405	0.743	0.471	0.00237869026823459	10	NAPA
7.5020027740892E-09	0.360904198372478	0.586	0.313	0.000125718562488187	10	JTB
5.77324781799297E-11	0.360457838175414	0.357	0.145	9.67480869339262E-07	10	ENOX2
1.16555725426626E-10	0.360444428698379	0.7	0.391	1.9532408466994E-06	10	IKBKG
2.41780965213512E-10	0.360304446015719	0.571	0.296	4.05176541504804E-06	10	MSI2
6.87139673674829E-09	0.359995480207578	0.693	0.397	0.000115150866514428	10	SERF2
7.69959494858917E-10	0.359394973294942	0.714	0.388	1.29029812148457E-05	10	SLC6A8
6.93185777610974E-10	0.359379942450673	0.543	0.274	1.16164072612047E-05	10	PRCP
2.14946482042954E-07	0.358776666616036	0.571	0.335	0.00360207314607582	10	TXNL4A
1.99524410451556E-09	0.358376099181585	0.664	0.366	3.34363007034718E-05	10	SULT1C2
7.55415625976678E-08	0.35819487147622	0.307	0.141	0.00126592550601172	10	PPP1R37
2.82233806172185E-07	0.357372180916157	0.45	0.241	0.00472967412383347	10	PCYT1A
1.56746562002615E-08	0.357261940649389	0.543	0.284	0.000262675888603982	10	NIBAN2
3.18543869399876E-07	0.357175263344655	0.4	0.208	0.00533815816340313	10	TMEM131
7.4834509745127E-07	0.356904016747679	0.307	0.148	0.0125407671430884	10	TBC1D23
1.92466262776844E-09	0.356078184912666	0.571	0.296	3.22534963161435E-05	10	ILVBL
3.83909778977095E-11	0.355905886513969	0.621	0.316	6.43356007609815E-07	10	GOLGA7
1.21439520526931E-08	0.35539965105711	0.757	0.45	0.000203508348490903	10	NDUFA3
3.86247647088123E-08	0.355112223584824	0.514	0.269	0.000647273806990277	10	CHP2
2.27772817840562E-07	0.355109942332343	0.671	0.403	0.00381701688137214	10	PRG4
1.31008462339592E-08	0.354883842188839	0.464	0.233	0.000219543981188688	10	LARP1
3.07520239617714E-10	0.354502267402686	0.343	0.144	5.15342417551365E-06	10	TINF2
1.43315848123358E-07	0.354289178496763	0.307	0.142	0.00240168698285124	10	DEPRECATED-ENSG00000249915
2.56439434040271E-07	0.354270310610514	0.764	0.506	0.00429741203564686	10	CYB5R3
6.88872444903647E-08	0.353775470150439	0.386	0.196	0.00115441244316953	10	GMPPA
9.04363237221589E-07	0.353728127980902	0.479	0.269	0.0151553191293594	10	VAMP7

2.05066981511678E-06	0.353723767314495	0.871	0.584	0.034365124761727	10	IDH1
2.47947363005496E-07	0.353317914844377	0.486	0.263	0.00415510190924611	10	RBMX
1.16182717608759E-09	0.352944085942339	0.714	0.392	1.94698998168758E-05	10	RAB10
8.64844948879102E-10	0.352710901518623	0.429	0.202	1.4493071653316E-05	10	GOLT1B
2.32032653334084E-08	0.352443746511241	0.45	0.23	0.000388840320457258	10	SC5D
8.83441127560752E-07	0.352259643231043	0.571	0.344	0.0148047064156631	10	PPP1CA
3.00837846769326E-08	0.351725360921895	0.35	0.163	0.000504144063616037	10	NLN
1.22113285578447E-06	0.35151265137488	0.407	0.212	0.0204637443972361	10	DDX23
5.55512429647516E-09	0.350965599926762	0.536	0.276	9.30927729603308E-05	10	GFPT1
1.62710550658846E-09	0.350797761478989	0.5	0.252	2.72670340794094E-05	10	CAMK2D
4.62237767081543E-09	0.350152530116465	0.414	0.198	7.7461805007525E-05	10	ZCRB1
1.66455084447214E-08	0.349950465500679	0.279	0.118	0.000278945430516642	10	PJA1
1.66430710979204E-07	0.34974071378122	0.621	0.361	0.0027890458545895	10	COX8A
1.39223785119783E-06	0.349589674782575	0.35	0.179	0.0233311219103732	10	ANO8
4.16984229987023E-08	0.349485684730573	0.293	0.128	0.000698782172612253	10	CDK8
1.04611252907362E-07	0.348677568388607	0.421	0.216	0.00175307537622157	10	VIRMA
1.37456407313979E-07	0.348146883727685	0.486	0.266	0.00230349447376767	10	SMIM7
1.0846562112553E-06	0.348095096817996	0.393	0.202	0.0181766687882163	10	BCAT2
1.43598848392601E-07	0.347972205813173	0.657	0.396	0.00240642950136321	10	HNRNPA3
1.20891854518609E-08	0.347849115540401	0.443	0.222	0.000202590569802284	10	ADIPOR2
2.43336993199513E-07	0.34782816295774	0.471	0.253	0.00407784133203743	10	TOMM70
2.67842577597087E-10	0.347638861384691	0.671	0.359	4.48850591537199E-06	10	TMEM63A
2.80990395643867E-10	0.347373423500588	0.414	0.185	4.70883705019993E-06	10	TMEM167A
1.54635805925998E-08	0.347311869430546	0.579	0.319	0.000259138683570788	10	UHMK1
4.60673946157087E-07	0.347021841633348	0.6	0.343	0.00771997398970047	10	HEXB
7.36712149873263E-08	0.347010507297814	0.493	0.268	0.00123458222075761	10	SIPA1L3
1.48457430509267E-08	0.346986013154493	0.55	0.294	0.00024878496204743	10	COL27A1
2.08359655993272E-08	0.346676117942311	0.379	0.181	0.000349169111513526	10	VPS54
9.69314970595215E-11	0.346551467398679	0.571	0.295	1.62437802772346E-06	10	TJP1
2.28361960230406E-08	0.346394027528394	0.379	0.178	0.000382688972954114	10	PDLIM1
1.61236288495101E-07	0.346274713733791	0.421	0.218	0.00270199772260091	10	CASP3
1.55421916026708E-07	0.34617918557566	0.621	0.365	0.00260456046877557	10	FH
8.29848243655428E-09	0.346025884624733	0.443	0.22	0.000139065968671777	10	SLC37A1
7.66475481406159E-08	0.345387269566067	0.793	0.532	0.00128445961174044	10	CYC1
1.09063532952274E-07	0.345223085288901	0.979	0.814	0.0018276866852142	10	CES1
2.70126739058286E-06	0.345192999380685	0.386	0.203	0.0452678389313876	10	HINT2
1.05857469201123E-08	0.344553072792968	0.471	0.241	0.000177395946887243	10	VPS35L
1.30853093269664E-06	0.343698159356118	0.793	0.525	0.0219283613701303	10	TXNRD1
9.78872358217355E-07	0.343635261913411	0.371	0.192	0.0164039429790064	10	SNRPD2
7.86922761178176E-10	0.343629259709591	0.271	0.104	1.31872516318239E-05	10	CBWD3
7.76428569434196E-10	0.343586257919277	0.536	0.273	1.30113899665783E-05	10	ARCN1
4.51552650202568E-11	0.343572650150163	0.25	0.087	7.56711931209464E-07	10	BTN3A3
2.32585361505298E-07	0.343301734223677	0.521	0.284	0.00389766548810578	10	FAM162A
2.27798066330996E-07	0.343263335610814	0.65	0.407	0.00381743999557483	10	SEC11C
1.01746854919988E-07	0.34299215568491	0.75	0.462	0.00170507379474916	10	COX6C
1.57258878818065E-12	0.342332828250414	0.471	0.202	2.63534429123314E-08	10	TMEM9B
1.64182820700782E-07	0.342285838028277	0.371	0.185	0.0027513757093037	10	XPR1
3.76413812997822E-08	0.342214844458223	0.793	0.483	0.00063079426782175	10	NDUFC2
9.09417063956096E-08	0.342146528571153	0.35	0.167	0.00152400111577763	10	ALG5
2.87852976922933E-06	0.341600620001747	0.55	0.321	0.0482384018727451	10	ACMSD
9.20745335380206E-07	0.340532261379451	0.314	0.153	0.0154298503303015	10	TTLL12
3.43023928253481E-09	0.340414370522016	0.536	0.279	5.74839498967184E-05	10	ZMAT2
9.39730491956577E-07	0.340399545896941	0.371	0.192	0.0157480035842083	10	ATP6V1E1
4.19920821104819E-09	0.340054198351532	0.421	0.201	7.03703312007455E-05	10	YIF1B
5.26099146261165E-07	0.339460587027602	0.329	0.162	0.00881636949304461	10	TK2
3.21184315883946E-08	0.33938383134488	0.679	0.397	0.000538240676558317	10	SLC43A3
1.39454900061678E-08	0.33932517622779	0.593	0.331	0.00023369852152336	10	SLC35F5
3.29598835670494E-09	0.339309862264377	0.5	0.262	5.52341728816615E-05	10	MGRN1
7.37025777133177E-07	0.337919355110203	0.407	0.218	0.0123510779731978	10	PSMA2
2.61087741467239E-07	0.337755597570395	0.586	0.338	0.00437530837150799	10	SMIM15

**2.92034452509805E-07**	0.337632967570925	0.629	0.366	0.00489391335515932	10	MLEC
**1.26093279817667E-08**	0.337476457607206	0.521	0.274	0.000211307118318447	10	EP300
**2.25615911214417E-06**	0.337394633435942	0.4	0.22	0.0378087144013121	10	RNF13
**2.28987312813153E-06**	0.337310901133498	0.457	0.256	0.0383736938812282	10	GNPAT
**2.68721608981926E-08**	0.336808020534584	0.664	0.395	0.000450323672331912	10	LMAN1
**2.97860304887166E-09**	0.3367341422631	0.571	0.316	4.99154298929912E-05	10	MYO15B
**4.70417457217132E-08**	0.336313721774999	0.314	0.143	0.000788325574804469	10	CRIPT
**1.69987935341002E-06**	0.335445093955829	0.271	0.126	0.0284865782044452	10	ARV1
**9.58594342488525E-07**	0.334799125619533	0.329	0.162	0.0160641239914227	10	HIBCH
**2.2468777865755E-07**	0.334717513615592	0.279	0.125	0.00376531779474322	10	ADCY9
**1.69327782372097E-07**	0.334500431141124	0.671	0.42	0.00283759497699161	10	MYO1B
**3.3636872521101E-07**	0.334464345332933	0.436	0.232	0.00563686709708611	10	SH3GL1
**4.91956178569401E-09**	0.333987240265121	0.414	0.196	8.24420164046602E-05	10	GMFB
**1.27263255967667E-07**	0.333652669089554	0.743	0.456	0.00213267764350616	10	MIDN
**1.67388039338486E-09**	0.333216583691297	0.25	0.095	2.80508876323434E-05	10	EIF2AK3
**7.07088620001634E-09**	0.333020936172307	0.379	0.175	0.000118493910939874	10	PDZD11
**3.81343781065051E-07**	0.332808012085752	0.757	0.482	0.00639055908308813	10	PUF60
**2.95478853366132E-08**	0.332680760479562	0.336	0.154	0.000495163462470963	10	USP16
**5.31882653968653E-08**	0.332504901493572	0.429	0.213	0.000891328951520668	10	HPS3
**5.21623052948645E-07**	0.332314997335637	0.357	0.177	0.0087413591213134	10	YTHDF2
**2.88138293460612E-06**	0.332292653105776	0.443	0.254	0.0482862152181294	10	FBXO42
**3.79722400961731E-08**	0.332079242447426	0.571	0.314	0.000636338799531669	10	ZBTB20
**3.87748062463969E-08**	0.332010819363453	0.8	0.528	0.00064978820307712	10	ACTN4
**9.77356944885322E-09**	0.33180257087007	0.35	0.158	0.000163785476823882	10	FDX1
**4.3562811896761E-09**	0.331599274458932	0.421	0.201	7.30025601765921E-05	10	AKIP1
**3.82760342072856E-08**	0.330935371745487	0.4	0.198	0.000641429781245692	10	TACO1
**1.18485165823876E-08**	0.330799550299617	0.621	0.345	0.000198557440887652	10	AMFR
**1.66135453735754E-07**	0.329982944244402	0.393	0.196	0.00278409793370376	10	RRAGA
**3.51539177627612E-08**	0.329925215328965	0.786	0.493	0.000589109353868352	10	EMD
**1.58825026735886E-06**	0.329594044503069	0.664	0.403	0.0266158979803997	10	SLC22A18
**5.17475445081838E-07**	0.329485019867032	0.464	0.249	0.00867185350868144	10	ALG3
**3.63675278667303E-07**	0.329436046569106	0.621	0.373	0.00609447031990666	10	CDKN1A
**2.06832439142491E-08**	0.329323847107306	0.407	0.198	0.000346609801514986	10	GMNN
**5.03715129440459E-10**	0.329180478601123	0.507	0.251	8.44125813916321E-06	10	NME1
**4.64182202661232E-07**	0.328959815777957	0.657	0.407	0.00777876535219693	10	IRF3
**4.97292702973165E-07**	0.328581123007894	0.414	0.215	0.0083336311164243	10	SNX12
**2.54313398774295E-06**	0.328448812336072	0.879	0.648	0.0426178393665963	10	NUCB1
**4.13261903685263E-08**	0.328427986532469	0.321	0.144	0.000692544298195764	10	ADPRHL2
**5.70580375854065E-08**	0.327991707077088	0.421	0.211	0.000956178593856243	10	PRRC1
**1.99320744356534E-08**	0.32781356162177	0.543	0.296	0.00033402170339268	10	SEPTIN8
**7.19741584758956E-08**	0.327809186767463	0.521	0.285	0.00120614294773906	10	FBXL17
**2.08119523598794E-09**	0.327775769447861	0.471	0.237	3.48766697646858E-05	10	KHSRP
**7.27155034246953E-07**	0.327646966789612	0.729	0.458	0.0121856640639104	10	ERGIC3
**2.58337146648099E-08**	0.327156856997836	0.414	0.205	0.000432921390352884	10	NF1
**1.69088579180237E-06**	0.327047068941069	0.343	0.175	0.028335864099024	10	FYTTD1
**1.27778534779603E-09**	0.326924384399117	0.557	0.299	2.14131268583659E-05	10	PLEC
**2.24556443014823E-07**	0.326512235162109	0.479	0.267	0.00376311687204241	10	CLIC1
**5.30189299888896E-07**	0.326422447803678	0.729	0.471	0.00888491228753812	10	SDF4
**2.71605397875657E-07**	0.326258114934691	0.757	0.459	0.00455156325760026	10	HINT1
**7.57402773068068E-07**	0.326030192150074	0.293	0.138	0.0126925556710747	10	DEPRECATED-ENSG00000174903
**2.52524471169809E-08**	0.325899508079521	0.421	0.218	0.000423180508786366	10	CHD4
**4.12146492647754E-07**	0.325852892624755	0.607	0.35	0.00690675092379106	10	NR2F6
**4.05767824123974E-08**	0.325736743712899	0.55	0.31	0.000679985719666955	10	GUK1
**1.29777758879366E-06**	0.325594790393076	0.379	0.2	0.0217481568330042	10	NCOA6
**2.7516273025725E-08**	0.32556197792803	0.664	0.392	0.0004611177033651	10	SEC13
**2.38708991554003E-08**	0.325398312236961	0.529	0.284	0.000400028528046198	10	STK39
**2.25179178384582E-07**	0.325202529301059	0.279	0.125	0.00377355267136883	10	TBC1D7
**4.11910705518721E-07**	0.324827989740198	0.829	0.54	0.00690279960308273	10	PRDX2
**1.49437651034405E-07**	0.324664020982433	0.479	0.253	0.00250427615603455	10	CWC15
**3.41954680152425E-08**	0.324611297659685	0.4	0.196	0.000573047652999434	10	CHMP5

2.82435368409268E-08	0.324411197522185	0.3	0.131	0.000473305190380251	10	KIFAP3
8.32350076650869E-07	0.323063587239216	0.457	0.248	0.0139485225845153	10	SUPT16H
4.93298239904156E-07	0.322917467872188	0.929	0.693	0.00826669190431385	10	MT-ND3
2.55689747648334E-07	0.322854740791718	0.571	0.328	0.00428484879109079	10	CALU
2.27807930638715E-08	0.322310026017632	0.536	0.29	0.000381760530164358	10	AGFG1
1.16008637662679E-07	0.321939913323781	0.493	0.269	0.00194407274995118	10	PDE4DIP
2.74378374151633E-09	0.321166021335295	0.593	0.317	4.59803279403306E-05	10	IDI1
7.97474428666353E-08	0.321071810613798	0.343	0.162	0.00133640764755907	10	BID
6.38953809354114E-08	0.320806745504732	0.321	0.146	0.00107075879371562	10	SLIRP
3.59542822716227E-08	0.320766116482944	0.45	0.23	0.000602521862307853	10	CALM1
1.11295635869135E-07	0.320739556998863	0.371	0.184	0.00186509226589496	10	STXBP3
4.41677285908312E-08	0.320499550827625	0.457	0.236	0.000740162795725149	10	CHMP3
2.91046149558045E-08	0.320469514251698	0.507	0.279	0.000487735137429372	10	CLPTM1L
1.557544763937E-07	0.320363627168613	0.821	0.486	0.00261013351540562	10	ADA
1.53754756474752E-08	0.320279023482779	0.664	0.382	0.000257662220900389	10	EDF1
1.38818830953321E-06	0.32023613088673	0.307	0.15	0.0232632596911576	10	TBCE
1.31993504116833E-06	0.320202821711662	0.8	0.525	0.0221194714198988	10	HNRNPF
3.98168931503801E-07	0.320188738488246	0.336	0.165	0.00667251495414069	10	UBL4A
3.18178927961559E-08	0.319849951608068	0.457	0.234	0.000533204247477981	10	SMC6
7.92540992449614E-08	0.319761787127926	0.564	0.323	0.00132814019514706	10	AP1M1
1.80478368594654E-07	0.319736934715667	0.379	0.188	0.00302445650090921	10	ARHGEF18
2.96090941639426E-06	0.319002867833408	0.379	0.206	0.049618919999935	10	L3HYPDH
5.04369063509626E-07	0.318761316391476	0.707	0.465	0.00845221676629432	10	TOB1
1.85639265766342E-06	0.31850014712333	0.507	0.291	0.0311094281571236	10	DNASE2
1.50313904732793E-07	0.317353820774184	0.443	0.233	0.00251896041551215	10	TMEM185A
4.94100715231322E-08	0.316751086825073	0.636	0.354	0.00082801397858465	10	SMC1A
5.42706887924579E-07	0.316373312340851	0.436	0.232	0.0090946820278401	10	VPS26C
4.12465732388525E-08	0.316240495838282	0.5	0.271	0.000691210074336691	10	DNAJC1
4.30235836224915E-07	0.316145527708124	0.343	0.171	0.00720989214345712	10	ACP2
2.02335606818719E-06	0.315942193624506	0.25	0.115	0.0339074009906809	10	RLIM
1.04674721274893E-06	0.315349960233055	0.879	0.606	0.0175413897912466	10	HSD17B6
1.47103734674399E-07	0.314856225361379	0.607	0.359	0.00246516438567358	10	SYAP1
1.23779220475536E-06	0.314411982791445	0.379	0.192	0.0207429217672903	10	ANKRD40
1.26976977281971E-07	0.313806940802799	0.421	0.216	0.00212788018529126	10	UBTF
2.25671191480815E-07	0.313600397058771	0.35	0.172	0.00378179782683549	10	TIMM17B
3.77448649557549E-08	0.313290219608697	0.464	0.243	0.00063252844692854	10	YWHAQ
1.73881983979651E-07	0.312872048436547	0.25	0.107	0.002913914287531	10	MINDY3
3.19419065552972E-07	0.312722180836307	0.321	0.153	0.00535282470053671	10	CCNC
4.10955762302897E-07	0.312715387164137	0.429	0.23	0.00688679666467195	10	ELOC
2.53868388389826E-08	0.312442011502039	0.664	0.394	0.000425432645263671	10	S100A13
1.81305973486018E-07	0.312228255404149	0.379	0.188	0.00303832550367869	10	CDC42BPA
3.42272746392565E-08	0.311363139986597	0.486	0.258	0.00057358066840466	10	FKBP1A
9.35161595189984E-07	0.310540858897228	0.907	0.63	0.0156714380121938	10	CFL1
5.45482872157284E-12	0.310499061759943	0.379	0.147	9.14120197161176E-08	10	ICAM1
2.19435350923289E-06	0.310456516274208	0.45	0.246	0.0367729761077248	10	PLGLB1
2.01186369830608E-10	0.310438275246402	0.507	0.238	3.37148118562132E-06	10	DNAJA1
1.29346566738052E-09	0.310420494850543	0.65	0.356	2.16758976539628E-05	10	ADD1
6.49273293087686E-07	0.310249078859182	0.35	0.177	0.0108805218455634	10	NDUFAF6
6.18982652763263E-07	0.310214657686635	0.771	0.467	0.0103729112950068	10	CTSH
1.83008912251851E-06	0.310196919481958	0.557	0.333	0.0306686335151652	10	EIF3B
1.62596671655499E-07	0.309962827827301	0.529	0.297	0.00272479502360284	10	UBE2V1
4.72411609469275E-08	0.309872197314375	0.436	0.216	0.000791667375148611	10	ELMOD2
9.96419841741559E-08	0.309805050955297	0.286	0.126	0.0016698003707905	10	ASF1A
1.77876042126469E-07	0.30963388532126	0.314	0.148	0.00298084671395537	10	CLCN5
1.71679312536186E-09	0.309495587760168	0.629	0.336	2.8770019194814E-05	10	ETNK1
8.41461817366777E-08	0.309016045896111	0.564	0.313	0.00141012171354324	10	NAA60
9.8113900126272E-08	0.30893935731584	0.314	0.144	0.00164419273831607	10	CLIP2
1.94420139543863E-06	0.308742417205838	0.264	0.124	0.0325809269847606	10	SMARCAD1
9.7376454383873E-08	0.308061086175971	0.407	0.209	0.00163183462256494	10	NUP153
1.50926403403231E-09	0.307785490188081	0.7	0.388	2.52922466823135E-05	10	SLBP

2.79418937514503E-08	0.307754180442223	0.636	0.341	0.000468250255486805	10	ATAD2
5.44949095572945E-07	0.307223875730271	0.764	0.493	0.00913225694361141	10	MTSS1
1.86699543312402E-06	0.306828322093102	0.379	0.197	0.0312871094682923	10	SLC25A23
3.73674320965602E-09	0.306628725810937	0.507	0.255	6.26203427074156E-05	10	NDUFS4
6.1522728744964E-07	0.306559177115769	0.45	0.241	0.0103099788830811	10	TRMT112
5.4419080821532E-09	0.306411981866887	0.364	0.166	9.11954956407234E-05	10	SLC37A3
5.83494980923317E-07	0.306406451279099	0.986	0.83	0.00977820889031294	10	MT-ND4
1.22989003452578E-08	0.306200335026308	0.586	0.317	0.00020610497198583	10	UPF1
5.28096695447108E-08	0.306164336736721	0.579	0.335	0.000884984442230263	10	TNIP1
1.57287891924606E-06	0.305524585245149	0.671	0.422	0.0263583049287255	10	EBP
1.35306389955914E-07	0.304989564075905	0.386	0.192	0.00226746448288121	10	FGFR4
2.10201186953613E-08	0.304588519068433	0.293	0.127	0.000352255149096864	10	SS18L2
8.5524518572978E-08	0.304289274186947	0.464	0.249	0.00143321988224597	10	MYLK
2.76222368794481E-07	0.303690885331631	0.514	0.288	0.00462893445625791	10	GGCX
2.05266696804347E-07	0.30368131392235	0.414	0.217	0.00343985930504725	10	SP1
5.80749682839108E-07	0.302933390658369	0.486	0.27	0.00973220318501777	10	VAPA
9.61620329137299E-08	0.3028825441684	0.293	0.129	0.00161148334756828	10	SNX8
5.88771743577818E-11	0.302806931091617	0.464	0.216	9.86663687887708E-07	10	SNX9
1.9261086422287E-06	0.302034143988871	0.343	0.176	0.0322777286264685	10	SMIM20
2.4488024802804E-06	0.30176457278876	0.257	0.119	0.0410370319645389	10	TMEM161B
1.9735796624755E-09	0.301245063650929	0.657	0.347	3.30732479837644E-05	10	CRTAP
4.78328685416649E-07	0.300922346071617	0.3	0.141	0.0080158321102122	10	ERCC2
9.71231900645864E-07	0.300903606565764	0.686	0.428	0.0162759041910234	10	CNDP2
9.76306751310769E-07	0.300814187204924	0.421	0.229	0.0163609485384659	10	EIF2D
4.26942069567748E-07	0.300389923636336	0.436	0.233	0.00715469520181633	10	TRRAP
9.02564738273907E-09	0.300058687960645	0.829	0.484	0.000151251798839941	10	CALM3
7.66964343554564E-12	0.299929783844267	0.293	0.105	1.28527884692874E-07	10	SELENOM
1.49116145981079E-06	0.299905925424124	0.264	0.122	0.0249888837435092	10	RAE1
8.56972428701113E-09	0.299526460985145	0.571	0.304	0.000143611439601733	10	CORO1B
6.43611602268693E-09	0.299360770148647	0.257	0.101	0.000107856432308188	10	NHEJ1
3.50132991144409E-09	0.298362927463752	0.471	0.231	5.86752866559801E-05	10	ATP5MC1
8.48925487369077E-07	0.298272979007338	0.421	0.227	0.014226293317331	10	HDDC2
2.60405039142346E-07	0.298007371788756	0.429	0.228	0.00436386764594744	10	EDEM2
1.14035904030393E-07	0.29772970466734	0.479	0.25	0.00191101367974133	10	DEPRECATED-ENSG00000134250
6.61653737165557E-09	0.297678309545404	0.55	0.3	0.000110879933274204	10	PRRC2C
1.83041559696244E-07	0.297478146823751	0.743	0.455	0.00306741045738966	10	AKR1A1
1.68545960585593E-06	0.297423736140633	0.55	0.318	0.0282449320749337	10	CISD3
1.50003654955618E-08	0.29712560039385	0.429	0.212	0.000251376124974625	10	TIMM23
3.3724955403365E-07	0.296709892644457	0.564	0.31	0.0056516280264959	10	GHITM
2.52487722999137E-09	0.296396158708896	0.371	0.166	4.23118926201954E-05	10	HSPA13
2.62556468187631E-07	0.295949417981826	0.479	0.262	0.00439992129388831	10	MTIF3
1.57907443899625E-06	0.295378204139409	0.45	0.246	0.0264621294486992	10	CLN3
1.00939273625571E-06	0.295079799535241	0.371	0.193	0.0169154034741731	10	RHOT1
3.25076678611423E-09	0.294709525965252	0.679	0.389	5.44763498017023E-05	10	NPLOC4
1.22034884225884E-18	0.293621674737736	0.293	0.08	2.04506058985736E-14	10	RHBDF2
8.58082966657668E-08	0.293617566989888	0.364	0.173	0.00143797543552492	10	SMPDL3A
1.2956931020904E-06	0.293603356871942	0.479	0.272	0.021713225004831	10	TRIO
1.54046619693582E-06	0.293558196365048	0.457	0.249	0.0258151325282505	10	ZGPAT
1.4040433064571E-06	0.293458508865367	0.386	0.203	0.0235289577296081	10	ZNF148
1.72749072561095E-09	0.292819878075465	0.536	0.265	2.89492895797884E-05	10	NOTCH2
2.47575162354072E-07	0.292052310879574	0.486	0.262	0.00414886457072953	10	SH3GLB2
5.4917850514294E-08	0.291289667452022	0.679	0.396	0.000920313338918538	10	ZNRF2
1.67830026622795E-07	0.291231871119512	0.479	0.254	0.0028124955861448	10	DYM
2.56216955368911E-08	0.291207664381895	0.736	0.442	0.000429368373807221	10	CTNND1
6.83117331851979E-08	0.290370273424145	0.529	0.281	0.00114476802471755	10	TRMT2B
6.72431446572252E-09	0.290196259393563	0.364	0.164	0.000112686061816578	10	DIP2B
1.02673228765691E-07	0.290114838659654	0.629	0.36	0.00172059796765545	10	RTF2
4.27331116124527E-09	0.290096349943191	0.579	0.307	7.16121484401482E-05	10	REPS1
3.96280145904768E-09	0.289843060009637	0.521	0.27	6.6408626850721E-05	10	ZNF410
1.51758811820469E-08	0.289761521505034	0.571	0.32	0.000254317416848742	10	FGGY

4.54270903080744E-08	0.289101990357891	0.486	0.26	0.000761267179382711	10	ATP6V0B
3.77723764191314E-08	0.289089904802801	0.407	0.203	0.000632989484031804	10	INSIG2
3.9822602603986E-09	0.288156117925503	0.479	0.239	6.67347174437597E-05	10	CCT3
1.04734552196865E-07	0.288022880662742	0.536	0.3	0.00175514162571507	10	SIL1
8.58394794510837E-11	0.287689572682563	0.543	0.271	1.43849799664126E-06	10	RORA
1.11125415339638E-06	0.287225224687025	0.507	0.288	0.0186223971026165	10	RAB1B
9.83257632129477E-28	0.286973843324875	1	0.84	1.64774313992258E-23	10	CD74
2.89579815974578E-07	0.286716267886563	0.514	0.289	0.00485277855610198	10	TENT5A
1.97828717351902E-07	0.28638876697379	0.457	0.242	0.00331521364538318	10	EPB41L5
1.41089439433094E-07	0.285412088952217	0.55	0.316	0.00236437682601978	10	ECPAS
1.69716854684682E-09	0.285358005858189	0.621	0.338	2.8441150508059E-05	10	SLC25A3
6.07816119629389E-07	0.285353262528021	0.55	0.314	0.0101857825327493	10	UBR5
1.77296072983398E-06	0.285249112788716	0.571	0.336	0.0297112759105578	10	TBL1XR1
2.46093727107304E-06	0.285218592257154	0.286	0.14	0.041240386788642	10	DCTN6
1.53980784439138E-12	0.285109060551627	0.993	0.821	2.58040998563107E-08	10	C3
1.4050722382262E-06	0.284511760343479	0.586	0.346	0.0235462005681947	10	ARHGAP35
1.63732710538774E-07	0.28444267276661	0.3	0.138	0.00274383276320878	10	WTAP
2.39581367877067E-06	0.283994598195306	0.543	0.322	0.0401490456288389	10	TAF7
1.23645935385509E-06	0.283971936589278	0.543	0.322	0.0207205858519035	10	PRPS1
2.24080402009199E-07	0.283852151029287	0.514	0.288	0.00375513937687015	10	TAOK1
1.79066606449877E-06	0.283816221093046	0.25	0.114	0.0300079819088704	10	LRIF1
2.74618623394474E-07	0.283709041001203	0.407	0.214	0.00460205889084459	10	TAF8
1.2084983015633E-07	0.283443087226357	0.514	0.281	0.00202520145375977	10	ARSA
1.95181896509409E-06	0.2832450108524	0.379	0.197	0.0327085822170467	10	DEPRECATED-ENSG00000099797
4.51897587760794E-08	0.28301454716283	0.421	0.213	0.000757289977569538	10	GNG12
3.67602146447756E-07	0.282960590822509	0.407	0.212	0.0061602767701715	10	ECHDC1
6.05036661631083E-07	0.282260443955895	0.343	0.17	0.0101392043756137	10	LATS1
1.37097013133817E-06	0.281104663837925	0.45	0.246	0.0229747174609651	10	FBXL6
7.58679116538037E-08	0.281072298293339	0.493	0.266	0.00127139446349444	10	COX6B1
2.43520721565772E-08	0.28100594690075	0.65	0.358	0.00040809202519992	10	PLEKHF2
3.84437759780867E-08	0.280711489484776	0.379	0.18	0.000644240797840777	10	VPS13D
5.90665652819734E-08	0.280388477561949	0.3	0.131	0.00098983750099531	10	MAPRE1
1.97460285123792E-06	0.27967721848733	0.371	0.196	0.0330903945810451	10	METTL26
2.14797278929996E-06	0.279564728927601	0.357	0.185	0.0359957280030888	10	RFXANK
1.79913893736098E-06	0.279503876263726	0.464	0.256	0.0301499703122953	10	ABCB6
1.81733535901344E-06	0.278870666184577	0.557	0.324	0.0304549059463473	10	INTS8
9.95925847039224E-07	0.27850015775581	0.714	0.442	0.0166897253446833	10	BSG
1.28052082694186E-07	0.278441541085449	0.557	0.306	0.00214589680178916	10	RBM42
1.14290306239511E-07	0.27766835081046	0.436	0.226	0.00191527695196173	10	CLCC1
9.24141057368666E-07	0.277430619636292	0.407	0.219	0.0154867558393841	10	RTKN
3.625678366285E-08	0.277365327800699	0.521	0.283	0.00060759118062204	10	MOV10
3.23570214124674E-07	0.277176781683216	0.679	0.432	0.00542238964830128	10	PDIA3
1.47480252859261E-06	0.276984972011665	0.3	0.144	0.024714740774155	10	TAOK2
1.4570044951463E-09	0.276836262499724	0.393	0.174	2.44164813296618E-05	10	BCKDK
3.46323885980674E-07	0.276364055528159	0.714	0.422	0.00580369568126414	10	OTC
7.16675062363586E-07	0.275561652415332	0.35	0.175	0.012010040695089	10	STRN
7.2715520787358E-07	0.274451956498901	0.614	0.363	0.0121856669735454	10	NMT1
1.42156560683404E-07	0.274194610814822	0.464	0.246	0.00238225964393248	10	LRP3
1.10115695262459E-07	0.273538451096017	0.686	0.41	0.00184531882120829	10	CLINT1
1.78167630120417E-08	0.272853002360112	0.643	0.356	0.000298573314555794	10	IARS2
1.61361072558395E-08	0.272613808803316	0.393	0.191	0.000270408885393358	10	TNIP2
1.90404394710554E-06	0.272434585952187	0.65	0.386	0.0319079684655947	10	SIVA1
7.72616403876457E-11	0.270868378739418	0.343	0.138	1.29475056961617E-06	10	DOCK10
3.80610626109161E-07	0.270552561983322	0.257	0.114	0.00637827287233732	10	RNF145
2.5403983670576E-06	0.270256490935851	0.407	0.217	0.0425719958351513	10	TCF19
1.29536692535104E-08	0.270084967731269	0.371	0.176	0.000217077589350328	10	CMTR1
2.6344074028427E-07	0.270073349034305	0.257	0.111	0.0044147399256838	10	SNX21
5.54882904095297E-08	0.269988547983048	0.364	0.175	0.000929872770682899	10	ZADH2
5.51875861227605E-09	0.269647534059524	0.607	0.327	9.2483356824522E-05	10	KTN1
1.42445575840653E-08	0.269274832905776	0.45	0.228	0.000238710295993767	10	RETREG3

3.13905968515647E-07	0.26921203532179	0.271	0.121	0.00526043622038521	10	RTL10
1.62794334738432E-06	0.268925634891631	0.721	0.467	0.0272810746154664	10	CALM2
3.10024779641218E-07	0.268589783973624	0.521	0.289	0.00519539525722753	10	PCCB
4.13750090583899E-07	0.268193173035884	0.386	0.194	0.00693362401800498	10	MESD
4.2357410385618E-08	0.267651582826733	0.507	0.273	0.000709825483242186	10	TRIM25
2.50294797568573E-06	0.267643442969395	0.321	0.158	0.0419444021765415	10	RAB22A
5.46027766916318E-08	0.267494021814918	0.514	0.278	0.000915033331798366	10	SPR
1.01609021563419E-07	0.267195956343042	0.293	0.129	0.00170276398335977	10	MAPK8
4.87017778110008E-08	0.26706552121019	0.429	0.215	0.000816144392556751	10	TMEM250
2.24454440498636E-06	0.266942669252017	0.371	0.195	0.0376140751387614	10	EEF1AKNMT
3.85318710351069E-07	0.266900471205618	0.321	0.151	0.00645717094806322	10	IFT20
1.15649774448286E-06	0.266470233229223	0.264	0.12	0.0193805892020438	10	TUBGCP4
1.70692364021066E-08	0.266131821940425	0.457	0.233	0.000286046263626502	10	ROBO1
1.55172519009764E-06	0.266080933035465	0.336	0.168	0.0260038107356563	10	AK1
1.68146032014629E-08	0.266024858172581	0.321	0.141	0.000281779120450116	10	UBE2M
1.34647484526677E-06	0.265915381416224	0.471	0.266	0.0225642254569806	10	NDUFA11
1.43137776663678E-06	0.265537633241346	0.614	0.364	0.0239870286132992	10	TMEM219
1.15108471106039E-07	0.265354500781419	0.579	0.328	0.001928987758795	10	NDUFB5
8.66605667922352E-07	0.265277154729137	0.507	0.293	0.0145225777830428	10	HELZ
7.014693891224E-08	0.265237801652298	0.329	0.153	0.00117552240229132	10	PHF5A
1.73246709524316E-08	0.265041170992761	0.471	0.238	0.000290326835820848	10	ZBTB10
2.27421492226519E-08	0.264898509003715	0.5	0.255	0.000381112936673201	10	PIEZO2
5.89730550064226E-07	0.264826275259668	0.364	0.188	0.00988270455797629	10	TM2D3
1.976088497862E-08	0.264625084716156	0.464	0.236	0.000331152910471715	10	SAMD4B
2.90074701702956E-06	0.264377017841761	0.593	0.347	0.0486107185113814	10	PHYH
1.21307209620833E-09	0.264289807184123	0.614	0.33	2.03286621882592E-05	10	TRIM27
1.40081473002502E-09	0.264269988120188	0.379	0.168	2.34748532457593E-05	10	DAD1
5.63683030090055E-08	0.264204807307911	0.629	0.357	0.000944620021824915	10	C4orf3
3.96193064907215E-09	0.26394506751928	0.407	0.192	6.63940338171512E-05	10	LEPROT
4.81808681882307E-07	0.263908953431118	0.464	0.25	0.00807414989098369	10	NR5A2
2.35943699032415E-06	0.263822524901403	0.336	0.173	0.0395394450838521	10	ETAA1
4.95045235153673E-07	0.263536713817255	0.6	0.348	0.00829596805070526	10	SFT2D2
8.87909695286985E-08	0.263118814815547	0.464	0.24	0.00148795906736193	10	CAND1
1.14731013575612E-08	0.262103542686663	0.664	0.378	0.00019226623255001	10	VPS28
3.6018760091611E-07	0.261569867747646	0.65	0.386	0.00603602381615218	10	ATP5ME
7.44422304185629E-07	0.261318822932868	0.45	0.245	0.0124750289735428	10	TMED2
9.19757780080653E-08	0.260834142780638	0.4	0.198	0.00154133008785916	10	COMMD6
9.50215042293001E-10	0.260796883132775	0.336	0.143	1.59237036787461E-05	10	ST3GAL2
3.86170029810819E-07	0.260319642467757	0.314	0.15	0.00647143735956971	10	CEP83
1.52153550924729E-08	0.260282910410209	0.521	0.272	0.000254978920639662	10	CLIP1
7.32636765947808E-10	0.259546333885751	0.543	0.275	1.22775269237534E-05	10	CDC42BPB
7.13848714510718E-07	0.259326921149839	0.464	0.259	0.0119626767577706	10	TRAP1
1.0456487893213E-07	0.259247722028581	0.55	0.304	0.00175229824114464	10	BPTF
7.02670294038788E-08	0.257441498433272	0.4	0.194	0.0011775348787502	10	PCYOX1
9.24323824201087E-08	0.257213847770337	0.6	0.321	0.00154898186459618	10	PAPSS2
1.50772339439094E-08	0.257213393764849	0.421	0.208	0.000252664286432033	10	METAP2
1.03179205290776E-07	0.256995163318264	0.557	0.305	0.00172907712226282	10	TMEM263
4.69489958234702E-07	0.25694351177388	0.593	0.353	0.00786771272009714	10	SP3
2.0822301849567E-07	0.256091340187849	0.636	0.381	0.00348940134395044	10	YIPF3
4.11984760736963E-07	0.255997142101827	0.693	0.409	0.00690404062043003	10	GSR
1.3237996432023E-06	0.255257360553478	0.707	0.439	0.0221842344207842	10	PGRMC2
5.95368469537033E-07	0.255192879402998	0.493	0.272	0.00997718481250161	10	PPM1G
1.38210071602352E-06	0.254964089638951	0.343	0.172	0.0231612437991221	10	FRRS1
5.38060114526113E-07	0.254382478877464	0.479	0.264	0.00901681139922861	10	LRRC47
4.57401286031404E-08	0.254021553722784	0.4	0.198	0.000766513075131426	10	GNL3L
7.2284434804788E-08	0.253893814997503	0.343	0.16	0.00121134255845864	10	RNF181
1.18415231158475E-07	0.253594764280203	0.279	0.123	0.00198440244375372	10	ZNF280D
9.63463777317361E-07	0.253447908025152	0.357	0.179	0.0161457259802843	10	ALG6
8.58221582328652E-07	0.253380944517215	0.679	0.415	0.0143820772766636	10	EIF5
1.38045094362431E-07	0.252391785875841	0.621	0.353	0.00231335969132561	10	CRYZ

**1.7765023607077E-07**	0.251886834872638	0.357	0.175	0.00297706265607396	10	ZC3H7B
**4.11043495026887E-07**	0.251845423228784	0.464	0.249	0.00688826688966057	10	BCL2L1
**2.39831782415119E-08**	0.251539109848039	0.607	0.323	0.000401910100971256	10	SLC25A15
**4.57203556976356E-07**	0.251323614890856	0.507	0.283	0.00766181720780977	10	RHOU

SupTable5B_C1_Total_MajorCelltype

p_val	avg_log2FC	pct.1	pct.2	p_val_adj	cluster	gene
0	3.2774425402296	0.929	0.192	0	Hepatocytes	RBP4
0	3.42461535923875	0.865	0.138	0	Hepatocytes	APOB
0	3.15939253979598	0.971	0.254	0	Hepatocytes	CLU
0	3.36439691543955	0.947	0.233	0	Hepatocytes	AGT
0	3.28346313343497	0.92	0.207	0	Hepatocytes	APOA1
0	3.17082699178675	0.959	0.251	0	Hepatocytes	AMBP
0	3.42681907177751	0.826	0.121	0	Hepatocytes	AGXT
0	3.34890244440259	0.953	0.252	0	Hepatocytes	TF
0	3.05827554332587	0.966	0.267	0	Hepatocytes	C3
0	3.07147743159946	0.956	0.267	0	Hepatocytes	CES1
0	3.16214066539014	0.909	0.222	0	Hepatocytes	ORM1
0	3.00634704550323	0.947	0.271	0	Hepatocytes	ALDOB
0	3.07014900728491	0.864	0.189	0	Hepatocytes	DEPRECATED-ENSG00000106538
0	3.24187433420069	0.831	0.159	0	Hepatocytes	C4BPA
0	3.22790507928821	0.812	0.142	0	Hepatocytes	KNG1
0	3.61717319046018	0.773	0.106	0	Hepatocytes	CYP3A5
0	3.22641013651135	0.815	0.148	0	Hepatocytes	ORM2
0	3.33521622479769	0.831	0.173	0	Hepatocytes	CFB
0	3.44618015813581	0.775	0.124	0	Hepatocytes	FMO5
0	3.41515607638083	0.863	0.212	0	Hepatocytes	FGA
0	3.17220123753472	0.808	0.16	0	Hepatocytes	HMGCS2
0	3.47979098931358	0.735	0.088	0	Hepatocytes	C4BPB
0	3.11850201631264	0.773	0.13	0	Hepatocytes	PRAP1
0	2.99620317666653	0.922	0.282	0	Hepatocytes	FABP1
0	3.64277093727041	0.719	0.079	0	Hepatocytes	MLXIPL
0	2.82452973458442	0.956	0.317	0	Hepatocytes	EPHX1
0	3.33158068523791	0.764	0.125	0	Hepatocytes	KHK
0	3.08791314301283	0.973	0.334	0	Hepatocytes	APOC3
0	2.95654233093198	0.845	0.208	0	Hepatocytes	APOH
0	3.07166174056849	0.748	0.115	0	Hepatocytes	ASGR2
0	2.8834053001895	0.915	0.282	0	Hepatocytes	APOC2
0	3.02994279421293	0.823	0.191	0	Hepatocytes	MGST1
0	2.8711867495717	0.826	0.195	0	Hepatocytes	TTR
0	1.98578112816278	0.93	0.311	0	Hepatocytes	FN1
0	3.3170381524392	0.714	0.095	0	Hepatocytes	PLG
0	2.90195111098107	0.924	0.31	0	Hepatocytes	GC
0	2.75544243026834	0.822	0.211	0	Hepatocytes	CYB5A
0	2.96200846426573	0.716	0.107	0	Hepatocytes	EFNA1
0	3.20708756428036	0.835	0.227	0	Hepatocytes	FGG
0	3.19100406035201	0.745	0.137	0	Hepatocytes	SERPINA6
0	3.23964607048871	0.732	0.127	0	Hepatocytes	HSD17B6
0	3.544178816183	0.685	0.082	0	Hepatocytes	UPB1
0	3.13787663943066	0.72	0.117	0	Hepatocytes	TM4SF4
0	3.20629223714501	0.759	0.167	0	Hepatocytes	UGT2B4
0	2.82244102847717	0.839	0.254	0	Hepatocytes	PTGR1
0	3.09974673015252	0.713	0.133	0	Hepatocytes	SULT2A1
0	3.0373716863038	0.685	0.113	0	Hepatocytes	DHCR24
0	3.47796657330566	0.652	0.08	0	Hepatocytes	HGD
0	2.96669314251857	0.835	0.265	0	Hepatocytes	PGRMC1
0	3.19887282099157	0.664	0.095	0	Hepatocytes	FMO3
0	3.10995168720788	0.647	0.082	0	Hepatocytes	ALDH1L1
0	3.26864965700643	0.644	0.08	0	Hepatocytes	LRG1
0	3.12724137664513	0.753	0.189	0	Hepatocytes	LGALS4
0	3.27465789947654	0.67	0.107	0	Hepatocytes	PROX1
0	2.8595906315464	0.663	0.103	0	Hepatocytes	CLDN1
0	3.56875126798241	0.635	0.076	0	Hepatocytes	HPN
0	1.58646684477218	0.869	0.31	0	Hepatocytes	TMEM176B
0	2.97380487388461	0.676	0.118	0	Hepatocytes	KRT8
0	2.98503498470133	0.739	0.183	0	Hepatocytes	GPX2
0	3.11370964701785	0.679	0.124	0	Hepatocytes	CES2
0	3.06595497458889	0.656	0.103	0	Hepatocytes	F5

0	3.49044910158946	0.624	0.072	0	Hepatocytes	PAH
0	3.27323524407354	0.635	0.085	0	Hepatocytes	SERPIND1
0	2.14712315577535	0.764	0.216	0	Hepatocytes	NUPR1
0	2.82719922482209	0.987	0.442	0	Hepatocytes	VTN
0	3.28398474698929	0.706	0.161	0	Hepatocytes	SERPINA7
0	2.86409433173073	0.687	0.143	0	Hepatocytes	ADH1C
0	2.99350877605031	0.714	0.171	0	Hepatocytes	GSTA1
0	2.84997397330913	0.789	0.248	0	Hepatocytes	UGDH
0	2.57354097915968	0.856	0.315	0	Hepatocytes	ALDH1A1
0	3.35638183953982	0.61	0.073	0	Hepatocytes	ELF3
0	3.17015531864648	0.659	0.124	0	Hepatocytes	SELENBP1
0	2.99997046449775	0.723	0.191	0	Hepatocytes	AKR1B10
0	3.15408080334416	0.638	0.107	0	Hepatocytes	RNASE4
0	3.25401369162642	0.612	0.082	0	Hepatocytes	SLC2A2
0	3.07721156698271	0.645	0.116	0	Hepatocytes	BAAT
0	3.74416574748993	0.587	0.059	0	Hepatocytes	ACSM2A
0	3.38440877278118	0.587	0.061	0	Hepatocytes	PCSK6
0	2.39571339755169	0.749	0.226	0	Hepatocytes	CERS2
0	3.54327063560609	0.597	0.074	0	Hepatocytes	PCK1
0	3.48638209585058	0.595	0.073	0	Hepatocytes	CDHR5
0	2.90737430934764	0.746	0.225	0	Hepatocytes	AKR1C3
0	3.09820767929789	0.682	0.162	0	Hepatocytes	TDO2
0	3.04866449235309	0.597	0.078	0	Hepatocytes	SDC1
0	2.05305740419966	0.749	0.231	0	Hepatocytes	CFH
0	2.26208675860609	0.776	0.258	0	Hepatocytes	ACSL1
0	3.1195853528763	0.658	0.142	0	Hepatocytes	STARD10
0	2.59208253142987	0.68	0.167	0	Hepatocytes	ERRFI1
0	3.36902831026495	0.583	0.071	0	Hepatocytes	CPB2
0	3.34070143827476	0.585	0.075	0	Hepatocytes	CP
0	2.32451409425739	0.718	0.208	0	Hepatocytes	GATM
0	2.80452655842347	0.612	0.103	0	Hepatocytes	AADAC
0	3.33221981544991	0.577	0.068	0	Hepatocytes	AOX1
0	3.34987197055215	0.564	0.057	0	Hepatocytes	BHMT2
0	3.13482884817375	0.592	0.089	0	Hepatocytes	ANG
0	3.20435609601555	0.708	0.206	0	Hepatocytes	FGB
0	2.96284397648026	0.644	0.142	0	Hepatocytes	DEPRECATED-ENSG00000198074
0	3.30473654003228	0.576	0.075	0	Hepatocytes	F2
0	3.20804703583291	0.615	0.115	0	Hepatocytes	RNF128
0	3.54795074028389	0.556	0.06	0	Hepatocytes	CYP2C8
0	3.20415024638344	0.693	0.198	0	Hepatocytes	PIGR
0	2.02385962865787	0.697	0.202	0	Hepatocytes	ALDH2
0	1.91498857346918	0.795	0.301	0	Hepatocytes	SCP2
0	2.73976179766478	0.634	0.14	0	Hepatocytes	RARRES2
0	2.87812511076332	0.587	0.093	0	Hepatocytes	LSR
0	1.20024934589555	0.671	0.178	0	Hepatocytes	C1S
0	3.14205648367381	0.565	0.072	0	Hepatocytes	HABP2
0	2.67945238874899	0.628	0.136	0	Hepatocytes	FBP1
0	3.26422879497886	0.583	0.093	0	Hepatocytes	MAOA
0	3.08930358246821	0.576	0.088	0	Hepatocytes	FGL1
0	3.15949911049704	0.561	0.077	0	Hepatocytes	OCLN
0	2.02264072522687	0.688	0.205	0	Hepatocytes	ANPEP
0	3.13487709332224	0.544	0.062	0	Hepatocytes	SLC39A14
0	2.82522651021553	0.682	0.201	0	Hepatocytes	NQO1
0	3.1841108215886	0.542	0.062	0	Hepatocytes	C8A
0	1.84377199545505	0.746	0.266	0	Hepatocytes	METTL7A
0	3.34798781104645	0.543	0.068	0	Hepatocytes	C6
0	3.35699861953474	0.531	0.056	0	Hepatocytes	MAT1A
0	2.16994038973597	0.676	0.202	0	Hepatocytes	ETFB
0	3.09034054194853	0.598	0.124	0	Hepatocytes	PLA2G2A
0	2.85542766372069	0.557	0.085	0	Hepatocytes	CFI
0	2.40509503987218	0.727	0.255	0	Hepatocytes	ASPH
0	2.7024975075292	0.744	0.273	0	Hepatocytes	ANXA4
0	3.15123009490674	0.635	0.164	0	Hepatocytes	MUC3A

0	1.30397899719131	0.836	0.365	0	Hepatocytes	IFITM3
0	2.93760615571555	0.581	0.111	0	Hepatocytes	LEAP2
0	2.21837022108021	0.732	0.262	0	Hepatocytes	IFI27
0	1.55877547851443	0.677	0.208	0	Hepatocytes	C2
0	3.18575735616117	0.584	0.115	0	Hepatocytes	AKR1C2
0	3.31289199314901	0.584	0.117	0	Hepatocytes	MUC13
0	3.4975427598313	0.522	0.056	0	Hepatocytes	CYP2B6
0	2.20767009496449	0.652	0.186	0	Hepatocytes	SOD1
0	2.5876589745356	0.559	0.096	0	Hepatocytes	CAMK2N1
0	2.48337764103655	0.656	0.193	0	Hepatocytes	MPC2
0	2.67885378321447	0.722	0.259	0	Hepatocytes	SPINK1
0	2.91263447713258	0.544	0.082	0	Hepatocytes	TSPAN6
0	2.30129409143175	0.568	0.106	0	Hepatocytes	OAF
0	2.31258258282415	0.596	0.135	0	Hepatocytes	GOLM1
0	1.81852107370232	0.698	0.24	0	Hepatocytes	HSD17B4
0	3.665699590682	0.524	0.067	0	Hepatocytes	ITIH3
0	3.13658983349749	0.534	0.077	0	Hepatocytes	BDH1
0	2.06096504687657	0.65	0.193	0	Hepatocytes	TMEM176A
0	3.56470982177477	0.52	0.064	0	Hepatocytes	C2orf72
0	3.06573798496947	0.522	0.067	0	Hepatocytes	GJB1
0	2.56009658521458	0.612	0.157	0	Hepatocytes	TMEM14A
0	1.76011567528289	0.613	0.159	0	Hepatocytes	S100A16
0	2.76124697555179	0.618	0.165	0	Hepatocytes	MBNL3
0	3.24938194494193	0.524	0.073	0	Hepatocytes	CLDN4
0	1.85912060852325	0.619	0.168	0	Hepatocytes	SDC4
0	2.39154558763565	0.661	0.211	0	Hepatocytes	ATP1B1
0	3.35724909925255	0.508	0.058	0	Hepatocytes	LIPG
0	3.39444929470482	0.503	0.053	0	Hepatocytes	HNF4A
0	2.99519867292173	0.568	0.118	0	Hepatocytes	TSPAN8
0	2.90865843675905	0.53	0.083	0	Hepatocytes	ENTPD5
0	3.41176123353237	0.51	0.064	0	Hepatocytes	BHMT
0	2.81235395712184	0.558	0.113	0	Hepatocytes	FASN
0	2.755843392553	0.987	0.543	0	Hepatocytes	ALB
0	1.49116961447383	0.931	0.488	0	Hepatocytes	HSPB1
0	3.32061943528409	0.495	0.052	0	Hepatocytes	EPS8L2
0	1.82188493637412	0.676	0.234	0	Hepatocytes	IDH1
0	3.35002498243493	0.518	0.076	0	Hepatocytes	TMEM45B
0	2.73270606641927	0.527	0.087	0	Hepatocytes	SDC2
0	3.33534971616241	0.496	0.056	0	Hepatocytes	ITIH1
0	2.87567497859154	0.513	0.073	0	Hepatocytes	MAOB
0	2.05496775529559	0.653	0.217	0	Hepatocytes	ECHS1
0	3.24618264407048	0.496	0.06	0	Hepatocytes	SEC16B
0	2.38529750609483	0.584	0.149	0	Hepatocytes	SMIM14
0	1.03247086036459	0.797	0.363	0	Hepatocytes	SOD2
0	2.23221331334468	0.631	0.197	0	Hepatocytes	CRYL1
0	2.30750997235253	0.605	0.172	0	Hepatocytes	ACAA2
0	2.10989533030842	0.627	0.194	0	Hepatocytes	DDT
0	2.2361711608911	0.51	0.078	0	Hepatocytes	MYO1B
0	1.48460279052872	0.766	0.334	0	Hepatocytes	ST6GAL1
0	1.98896695379755	0.642	0.211	0	Hepatocytes	PEBP1
0	3.22173668740981	0.48	0.049	0	Hepatocytes	GRTP1
0	2.95486334012244	0.566	0.135	0	Hepatocytes	ERICH5
0	3.45314333655546	0.474	0.044	0	Hepatocytes	SERPINA10
0	2.33956647275501	0.592	0.162	0	Hepatocytes	SCCPDH
0	3.12534704801253	0.488	0.06	0	Hepatocytes	HAO1
0	2.68880280265081	0.496	0.069	0	Hepatocytes	KRT18
0	2.062399918048	0.618	0.191	0	Hepatocytes	ALDH3A2
0	2.98822371033324	0.484	0.058	0	Hepatocytes	KLF15
0	3.15688182051132	0.501	0.075	0	Hepatocytes	SERPINF2
0	3.14809105298143	0.611	0.185	0	Hepatocytes	SMARCA1
0	3.04184057439947	0.488	0.063	0	Hepatocytes	A1CF
0	1.59053853320328	0.749	0.324	0	Hepatocytes	ECH1
0	3.47134626198754	0.469	0.045	0	Hepatocytes	ONECUT1

0	2.62993081305498	0.527	0.104	0	Hepatocytes	GAMT
0	2.95415240179631	0.492	0.069	0	Hepatocytes	ADH1A
0	1.94115409808306	0.577	0.154	0	Hepatocytes	F11R
0	3.09741795788138	0.526	0.104	0	Hepatocytes	SLPI
0	3.54220622693511	0.488	0.066	0	Hepatocytes	SERPINC1
0	3.04126197632348	0.491	0.069	0	Hepatocytes	PRG4
0	2.55726462379475	0.549	0.128	0	Hepatocytes	POR
0	2.4971629033858	0.502	0.081	0	Hepatocytes	PTPRK
0	1.31859926622886	0.506	0.085	0	Hepatocytes	NR1H4
0	1.58294585028257	0.647	0.227	0	Hepatocytes	SLC9A3R1
0	2.70945746148273	0.518	0.098	0	Hepatocytes	MPST
0	1.92704296613168	0.602	0.182	0	Hepatocytes	H1F0
0	2.18835471475749	0.591	0.171	0	Hepatocytes	TSTA3
0	0.577271755087251	0.868	0.449	0	Hepatocytes	IL32
0	0.56179923798757	0.711	0.292	0	Hepatocytes	IGFBP4
0	3.14017837245591	0.499	0.081	0	Hepatocytes	F2RL1
0	2.01599287422508	0.552	0.135	0	Hepatocytes	IMMP2L
0	3.32067206897909	0.466	0.049	0	Hepatocytes	CYP4F11
0	2.85688470732985	0.472	0.056	0	Hepatocytes	MARC2
0	1.25157846781394	0.933	0.518	0	Hepatocytes	SELENOP
0	2.9929160695329	0.481	0.066	0	Hepatocytes	DSP
0	1.67723052718084	0.522	0.108	0	Hepatocytes	NFIB
0	2.92599336570552	0.477	0.063	0	Hepatocytes	PDZK1
0	2.07300493467633	0.586	0.172	0	Hepatocytes	ACOX1
0	3.00727145939492	0.526	0.113	0	Hepatocytes	PEG10
0	3.29629776193241	0.462	0.049	0	Hepatocytes	ASGR1
0	1.84174256991251	0.614	0.201	0	Hepatocytes	ADI1
0	2.06173825240236	0.644	0.232	0	Hepatocytes	HSD17B11
0	2.36006015379705	0.533	0.121	0	Hepatocytes	MDK
0	2.54837529047306	0.514	0.104	0	Hepatocytes	RIDA
0	2.25450350526333	0.53	0.121	0	Hepatocytes	ABCC3
0	2.68308930880684	0.508	0.099	0	Hepatocytes	OTC
0	1.80803396442934	0.594	0.185	0	Hepatocytes	PRDX6
0	3.2896743522114	0.459	0.051	0	Hepatocytes	PLEKHA6
0	3.20247325468895	0.522	0.114	0	Hepatocytes	CDH17
0	3.47243759020802	0.448	0.04	0	Hepatocytes	DPYS
0	0.726990693035351	0.569	0.163	0	Hepatocytes	C1R
0	2.27642127606068	0.552	0.149	0	Hepatocytes	SLC37A4
0	2.63257368091311	0.531	0.128	0	Hepatocytes	CBR1
0	1.94133802140396	0.714	0.311	0	Hepatocytes	LAMP2
0	1.85177695649357	0.616	0.214	0	Hepatocytes	BEX3
0	3.04816616751848	0.477	0.075	0	Hepatocytes	FRK
0	2.42977254563612	0.532	0.131	0	Hepatocytes	PCK2
0	2.79495533381454	0.497	0.097	0	Hepatocytes	CMBL
0	1.33118302262533	0.905	0.505	0	Hepatocytes	SQSTM1
0	2.07329875763664	0.621	0.222	0	Hepatocytes	SCD
0	2.17992388126484	0.548	0.149	0	Hepatocytes	ACAT1
0	2.64264959295876	0.526	0.128	0	Hepatocytes	VNN 1.00
0	3.24902895754079	0.464	0.067	0	Hepatocytes	DGAT2
0	2.11158234341422	0.539	0.142	0	Hepatocytes	TMEM205
0	3.15548854161938	0.475	0.078	0	Hepatocytes	HNF4G
0	3.4174311214451	0.442	0.046	0	Hepatocytes	C8B
0	3.49516395739808	0.43	0.034	0	Hepatocytes	ABCC2
0	3.04085076237625	0.457	0.062	0	Hepatocytes	EHHADH
0	3.40651488737747	0.463	0.068	0	Hepatocytes	SLC6A19
0	3.22322293852726	0.435	0.041	0	Hepatocytes	UGT1A1
0	3.50000924491974	0.437	0.044	0	Hepatocytes	F11
0	3.4642536276371	0.445	0.052	0	Hepatocytes	SMOC1
0	2.09593095917126	0.657	0.264	0	Hepatocytes	ACSL4
0	2.78312850661115	0.46	0.068	0	Hepatocytes	PERP
0	3.37895800317999	0.436	0.045	0	Hepatocytes	C8G
0	1.29948090583598	0.644	0.253	0	Hepatocytes	XBP1
0	2.00156433401023	0.579	0.188	0	Hepatocytes	TMEM106C

0	2.31460220618426	0.528	0.138	0	Hepatocytes	KCTD3
0	2.4577100014729	0.491	0.102	0	Hepatocytes	ASL
0	2.16914612525979	0.554	0.165	0	Hepatocytes	TPD52
0	2.9366097870781	0.472	0.083	0	Hepatocytes	MSMO1
0	1.52040057869837	0.646	0.258	0	Hepatocytes	GOLIM4
0	1.68480683647715	0.613	0.225	0	Hepatocytes	CYC1
0	2.05501496913043	0.537	0.149	0	Hepatocytes	SEPHS2
0	3.19961503455816	0.469	0.081	0	Hepatocytes	MEP1A
0	3.09989528405484	0.467	0.08	0	Hepatocytes	KLF5
0	3.14472473738108	0.431	0.046	0	Hepatocytes	SERINC2
0	3.65796376665511	0.429	0.044	0	Hepatocytes	NR1I2
0	2.60211061806014	0.486	0.101	0	Hepatocytes	FAH
0	3.46014078122175	0.436	0.051	0	Hepatocytes	ACSM2B
0	2.429639482348	0.487	0.103	0	Hepatocytes	ECHDC2
0	1.92755757211572	0.547	0.163	0	Hepatocytes	TOR1AIP2
0	2.03186296101324	0.541	0.157	0	Hepatocytes	UQCRQ
0	3.2319466658788	0.435	0.051	0	Hepatocytes	CLDN3
0	2.82425807034357	0.469	0.085	0	Hepatocytes	OCRL
0	3.09353821042696	0.438	0.055	0	Hepatocytes	STBD1
0	3.44742354611333	0.43	0.047	0	Hepatocytes	AZGP1
0	3.48778265487629	0.43	0.047	0	Hepatocytes	TMPRSS2
0	1.90212992134264	0.607	0.225	0	Hepatocytes	NDRG1
0	3.30053168053097	0.422	0.041	0	Hepatocytes	F10
0	1.82676344895651	0.552	0.171	0	Hepatocytes	HAGH
0	0.851775317215762	0.56	0.18	0	Hepatocytes	CNN3
0	3.03300020992769	0.423	0.043	0	Hepatocytes	ARHGEF26
0	1.84261981952252	0.604	0.224	0	Hepatocytes	TXNRD1
0	2.06693208146784	0.55	0.171	0	Hepatocytes	SPTSSA
0	1.89033159022085	0.612	0.234	0	Hepatocytes	NDUFA1
0	1.58134934598959	0.553	0.176	0	Hepatocytes	SERINC5
0	2.28662544353625	0.525	0.148	0	Hepatocytes	PNKD
0	1.61926612389055	0.619	0.242	0	Hepatocytes	PRDX2
0	1.87465264313085	0.522	0.145	0	Hepatocytes	PLS3
0	0.896136508417698	0.703	0.326	0	Hepatocytes	LMNA
0	3.35093142991133	0.427	0.052	0	Hepatocytes	CEP70
0	3.14703541898522	0.426	0.051	0	Hepatocytes	SLC47A1
0	2.65828070614826	0.457	0.082	0	Hepatocytes	CCL15
0	2.71615040958807	0.426	0.052	0	Hepatocytes	PTPRF
0	1.45851019421553	0.974	0.6	0	Hepatocytes	FTL
0	1.25928751520508	0.674	0.301	0	Hepatocytes	PTMS
0	1.80699999876488	0.515	0.142	0	Hepatocytes	SNTB1
0	2.22060673969629	0.49	0.117	0	Hepatocytes	NAPRT
0	2.21077661664894	0.533	0.161	0	Hepatocytes	ATP1A1
0	2.67994646703363	0.466	0.094	0	Hepatocytes	SLC6A8
0	3.41579321530079	0.411	0.04	0	Hepatocytes	MLPH
0	2.35987422133496	0.504	0.133	0	Hepatocytes	DCXR
0	1.78771347728992	0.522	0.152	0	Hepatocytes	MIA2
0	1.99578842518518	0.473	0.104	0	Hepatocytes	PDIA5
0	1.38651902771187	0.579	0.211	0	Hepatocytes	BLVRB
0	2.59986561415794	0.486	0.119	0	Hepatocytes	HMGCS1
0	3.43224905431381	0.41	0.043	0	Hepatocytes	PON3
0	3.44311034642474	0.438	0.071	0	Hepatocytes	MUC5B
0	3.23125849208806	0.423	0.056	0	Hepatocytes	EHF
0	1.78108068162121	0.542	0.176	0	Hepatocytes	LPGAT1
0	1.03307126879031	0.569	0.203	0	Hepatocytes	MTSS1
0	2.84625930650875	0.434	0.069	0	Hepatocytes	PBLD
0	3.0746459567469	0.414	0.049	0	Hepatocytes	MYH14
0	2.56894366914879	0.563	0.198	0	Hepatocytes	ADA
0	3.56052963889284	0.401	0.037	0	Hepatocytes	SLC17A4
0	2.70736004448891	0.46	0.097	0	Hepatocytes	CTPS1
0	2.42310361987321	0.455	0.092	0	Hepatocytes	ABAT
0	2.36341548429953	0.481	0.119	0	Hepatocytes	GLYCTK
0	3.33933169700619	0.412	0.05	0	Hepatocytes	PON1

0	0.909105322704917	0.711	0.349	0	Hepatocytes	GPX4
0	1.79307681879296	0.581	0.219	0	Hepatocytes	SSR4
0	1.73895956691931	0.513	0.152	0	Hepatocytes	MORF4L2
0	2.23620461740518	0.501	0.141	0	Hepatocytes	ABCD1
0	1.14809093063934	0.578	0.219	0	Hepatocytes	DHRS3
0	1.50771344934634	0.571	0.212	0	Hepatocytes	MGAT4B
0	0.430353146056234	0.613	0.254	0	Hepatocytes	VEGFA
0	2.23508081384819	0.478	0.119	0	Hepatocytes	SLC22A18
0	2.69299389287134	0.418	0.059	0	Hepatocytes	GPRC5C
0	1.75135626178656	0.446	0.087	0	Hepatocytes	LLGL2
0	3.04514191290027	0.441	0.083	0	Hepatocytes	SULT1C2
0	2.0588576056503	0.478	0.12	0	Hepatocytes	HSDL2
0	2.52776512916353	0.422	0.065	0	Hepatocytes	TCEA3
0	2.44117529652233	0.436	0.079	0	Hepatocytes	SLC25A4
0	1.34571293372688	0.579	0.222	0	Hepatocytes	INSIG1
0	1.86189712369838	0.507	0.15	0	Hepatocytes	GPAA1
0	2.0481283981222	0.458	0.101	0	Hepatocytes	AMOTL1
0	2.44380967583498	0.426	0.069	0	Hepatocytes	LMO7
0	2.70108889552838	0.422	0.067	0	Hepatocytes	KIAA1522
0	2.5244427017336	0.439	0.084	0	Hepatocytes	HPGD
0	1.98881603697962	0.496	0.141	0	Hepatocytes	EBP
0	3.19143029992441	0.402	0.048	0	Hepatocytes	LBP
0	2.76047544159415	0.443	0.089	0	Hepatocytes	CYP51A1
0	2.09549248330965	0.479	0.125	0	Hepatocytes	SLC25A1
0	1.69623433785403	0.557	0.204	0	Hepatocytes	NDUFC2
0	1.01362313660765	0.92	0.568	0	Hepatocytes	MT-CO3
0	3.42136354375616	0.391	0.039	0	Hepatocytes	CYP4F2
0	2.34775323276774	0.545	0.193	0	Hepatocytes	G6PD
0	3.09268668801918	0.417	0.065	0	Hepatocytes	NECAB3
0	2.21133494521607	0.502	0.15	0	Hepatocytes	CMC4
0	2.55487178411808	0.459	0.108	0	Hepatocytes	CLDN7
0	2.03113006885413	0.747	0.396	0	Hepatocytes	S100A10
0	1.4456612595147	0.564	0.213	0	Hepatocytes	MGST3
0	2.25659464510341	0.451	0.1	0	Hepatocytes	CYP4V2
0	2.27208306357237	0.486	0.135	0	Hepatocytes	PCBD1
0	2.11534431388949	0.487	0.137	0	Hepatocytes	ZHX1
0	3.694152623826	0.383	0.033	0	Hepatocytes	G6PC
0	1.99626148047676	0.409	0.059	0	Hepatocytes	LONRF3
0	3.48076831727135	0.387	0.038	0	Hepatocytes	AR
0	2.06504744443176	0.437	0.088	0	Hepatocytes	RAMP1
0	1.47287141516203	0.528	0.18	0	Hepatocytes	ELL2
0	1.6815665021053	0.542	0.194	0	Hepatocytes	CAT
0	2.33886760612939	0.442	0.094	0	Hepatocytes	ABCD3
0	1.51072286040453	0.509	0.161	0	Hepatocytes	PON2
0	1.55884498611862	0.573	0.225	0	Hepatocytes	DECR1
0	1.85525988677209	0.513	0.165	0	Hepatocytes	AIFM1
0	2.2779635469692	0.48	0.132	0	Hepatocytes	LAGE3
0	2.91835126796306	0.402	0.055	0	Hepatocytes	DIO1
0	1.43965976728378	0.52	0.173	0	Hepatocytes	TMEM37
0	1.15354839978578	0.781	0.434	0	Hepatocytes	MT-CYB
0	2.51683132802979	0.452	0.105	0	Hepatocytes	DEPRECATED-ENSG00000159228
0	1.73488414767807	0.499	0.152	0	Hepatocytes	LAPTM4B
0	1.69827763691881	0.621	0.274	0	Hepatocytes	MPP1
0	3.46979528206172	0.387	0.041	0	Hepatocytes	SLC15A1
0	2.61995504347873	0.43	0.084	0	Hepatocytes	AK4
0	1.38797107871083	0.57	0.224	0	Hepatocytes	RBM47
0	3.26995766946777	0.38	0.035	0	Hepatocytes	SLC6A1
0	2.46245178723254	0.432	0.087	0	Hepatocytes	PCYT2
0	0.857455270071961	0.565	0.22	0	Hepatocytes	EMD
0	3.26114817607679	0.395	0.051	0	Hepatocytes	NEB
0	1.84154909683154	0.46	0.116	0	Hepatocytes	FAM114A1
0	3.53957886702676	0.384	0.04	0	Hepatocytes	SHROOM1
0	1.94493683408941	0.514	0.171	0	Hepatocytes	PGM2

0	2.38299639774799	0.407	0.064	0	Hepatocytes	FAT1
0	1.69009434764864	0.512	0.169	0	Hepatocytes	ZKSCAN1
0	3.68195391174147	0.381	0.039	0	Hepatocytes	BTNL8
0	1.95538308902247	0.423	0.081	0	Hepatocytes	SPSB1
0	1.97730490630404	0.406	0.065	0	Hepatocytes	KANK1
0	3.41814160228792	0.378	0.037	0	Hepatocytes	RHPN2
0	1.62317043039084	0.489	0.148	0	Hepatocytes	ERO1B
0	3.52671187558384	0.373	0.032	0	Hepatocytes	CYP4F3
0	1.66142480982998	0.52	0.18	0	Hepatocytes	NDUFB9
0	3.20501980016779	0.402	0.062	0	Hepatocytes	ITIH2
0	1.97041369146761	0.463	0.123	0	Hepatocytes	CEBPG
0	2.4373728023434	0.428	0.088	0	Hepatocytes	PARM1
0	2.44445977214413	0.421	0.081	0	Hepatocytes	PPARA
0	2.28384994845452	0.413	0.074	0	Hepatocytes	EPHX2
0	1.05396575932632	0.536	0.199	0	Hepatocytes	RXRA
0	1.38449611432409	0.603	0.266	0	Hepatocytes	TMCO1
0	0.817708027209195	0.499	0.162	0	Hepatocytes	EXT1
0	1.71116129407202	0.48	0.143	0	Hepatocytes	SHMT1
0	3.04238050402003	0.391	0.055	0	Hepatocytes	ACMSD
0	2.56424391630028	0.394	0.058	0	Hepatocytes	SYTL4
0	3.40893224391615	0.384	0.048	0	Hepatocytes	DUSP9
0	2.62197560164065	0.408	0.072	0	Hepatocytes	NADK2
0	1.81471819970582	0.501	0.165	0	Hepatocytes	ATRN
0	1.58339880463409	0.591	0.255	0	Hepatocytes	BCAP31
0	2.84864715632393	0.395	0.06	0	Hepatocytes	CYP27A1
0	1.66099745861883	0.521	0.186	0	Hepatocytes	DAP
0	1.77011656109102	0.41	0.075	0	Hepatocytes	MATN2
0	2.23704704766135	0.42	0.085	0	Hepatocytes	NR2F6
0	2.95742980242535	0.389	0.055	0	Hepatocytes	SYBU
0	2.44034333758976	0.422	0.088	0	Hepatocytes	AIG1
0	3.63923315774561	0.369	0.035	0	Hepatocytes	GPT
0	2.56080353059786	0.417	0.083	0	Hepatocytes	PHYH
0	1.19373226409851	0.464	0.131	0	Hepatocytes	S100A13
0	3.44868135417506	0.37	0.037	0	Hepatocytes	SH3RF2
0	2.77171932706337	0.392	0.06	0	Hepatocytes	STAP2
0	1.70594726016998	0.537	0.206	0	Hepatocytes	PRDX1
0	0.945589906806472	0.91	0.579	0	Hepatocytes	MT-ATP6
0	2.83265447117388	0.393	0.062	0	Hepatocytes	SLC25A15
0	1.84857352666756	0.578	0.247	0	Hepatocytes	TSPAN3
0	3.40651083890318	0.358	0.027	0	Hepatocytes	RGN
0	2.98246630532383	0.375	0.045	0	Hepatocytes	IRF6
0	1.71470114931956	0.459	0.13	0	Hepatocytes	PGM1
0	1.47388649211221	0.515	0.187	0	Hepatocytes	MGST2
0	2.84439989614573	0.39	0.062	0	Hepatocytes	OPLAH
0	3.29468176607752	0.37	0.043	0	Hepatocytes	KCNK1
0	3.38308652743644	0.369	0.042	0	Hepatocytes	ACSM1
0	2.55825775604589	0.447	0.12	0	Hepatocytes	APOC1
0	1.93121326886811	0.442	0.116	0	Hepatocytes	IL6R
0	1.29817157802301	0.528	0.202	0	Hepatocytes	RRBP1
0	1.29154791242966	0.491	0.165	0	Hepatocytes	ACBD5
0	0.959275758827102	0.486	0.16	0	Hepatocytes	SERPINF1
0	1.1880202855493	0.507	0.182	0	Hepatocytes	PGRMC2
0	1.62479922869744	0.391	0.066	0	Hepatocytes	LRP5
0	2.44782364084379	0.403	0.078	0	Hepatocytes	PXMP2
0	1.69544469525821	0.463	0.138	0	Hepatocytes	DSTN
0	2.28992651450152	0.421	0.096	0	Hepatocytes	CRYZ
0	3.58620236416268	0.356	0.031	0	Hepatocytes	RAB17
0	2.7667784387472	0.388	0.065	0	Hepatocytes	FGGY
0	3.08972696630564	0.365	0.042	0	Hepatocytes	PKLR
0	2.21717690584921	0.429	0.106	0	Hepatocytes	LACTB2
0	1.37960305906968	0.508	0.185	0	Hepatocytes	P4HB
0	1.09789033563982	0.564	0.241	0	Hepatocytes	NUCKS1
0	2.84490281862656	0.392	0.07	0	Hepatocytes	ANXA13

0	3.30031082790631	0.353	0.031	0	Hepatocytes	ERBB3
0	3.0846041661371	0.356	0.034	0	Hepatocytes	SGK2
0	1.49676077790588	0.529	0.208	0	Hepatocytes	COX6C
0	1.06864410732895	0.627	0.306	0	Hepatocytes	MT-ND1
0	3.1236229715048	0.35	0.029	0	Hepatocytes	CGN
0	1.85843066967218	0.443	0.123	0	Hepatocytes	CYSTM1
0	1.25779612092816	0.552	0.233	0	Hepatocytes	NDUFA2
0	1.54016240688573	0.461	0.143	0	Hepatocytes	RAB4A
0	2.13448390231993	0.389	0.071	0	Hepatocytes	CNKSR3
0	1.14686138694335	0.519	0.201	0	Hepatocytes	AFDN
0	1.87299911816984	0.459	0.142	0	Hepatocytes	MTHFD1
0	1.91907158594449	0.37	0.053	0	Hepatocytes	SAMD5
0	2.76161591667301	0.374	0.057	0	Hepatocytes	LGR4
0	2.54641479139275	0.385	0.068	0	Hepatocytes	ALDH6A1
0	1.8629859557279	0.443	0.126	0	Hepatocytes	CD99L2
0	1.91408671529438	0.48	0.163	0	Hepatocytes	HTATSF1
0	2.09290595638686	0.406	0.09	0	Hepatocytes	FAM3A
0	1.99067196406731	0.431	0.115	0	Hepatocytes	FH
0	2.60031021690114	0.372	0.056	0	Hepatocytes	ADHFE1
0	1.84203341744986	0.448	0.132	0	Hepatocytes	HACD3
0	2.47300143191063	0.372	0.056	0	Hepatocytes	ZDHHC9
0	0.701639381076938	0.571	0.255	0	Hepatocytes	PNPLA2
0	2.53450765493258	0.387	0.072	0	Hepatocytes	GOT1
0	1.19918447504605	0.408	0.093	0	Hepatocytes	TFPI
0	0.689564559005322	0.715	0.4	0	Hepatocytes	GSTK1
0	3.48524991221992	0.348	0.033	0	Hepatocytes	CYP4F12
0	1.13438885580208	0.548	0.234	0	Hepatocytes	PUF60
0	0.580100717414431	0.523	0.209	0	Hepatocytes	TPM1
0	2.7268866475148	0.384	0.07	0	Hepatocytes	TUBB2A
0	1.28198041315726	0.525	0.212	0	Hepatocytes	NDUFB7
0	3.12777785461467	0.357	0.044	0	Hepatocytes	SLC51A
0	3.10019238978812	0.353	0.04	0	Hepatocytes	ONECUT2
0	2.45923038371367	0.366	0.053	0	Hepatocytes	ATP9A
0	2.69490003710295	0.377	0.064	0	Hepatocytes	PIR
0	1.31712641204635	0.475	0.162	0	Hepatocytes	SAR1B
0	1.17533829369296	0.497	0.185	0	Hepatocytes	ZBTB7B
0	2.8179526811124	0.349	0.037	0	Hepatocytes	GDA
0	2.61451328969416	0.386	0.074	0	Hepatocytes	C11orf54
0	2.59740485163034	0.37	0.058	0	Hepatocytes	TOM1L1
0	1.92413777350672	0.448	0.136	0	Hepatocytes	VBP1
0	1.01546893274993	0.539	0.228	0	Hepatocytes	CD151
0	1.26755646398327	0.533	0.222	0	Hepatocytes	SAP18
0	2.45998829468651	0.4	0.089	0	Hepatocytes	GCHFR
0	1.25100316454751	0.522	0.212	0	Hepatocytes	ATP5F1D
0	1.88641471679241	0.437	0.127	0	Hepatocytes	CHPT1
0	1.44816101337881	0.455	0.146	0	Hepatocytes	TCEAL4
0	2.27795755295103	0.367	0.058	0	Hepatocytes	NECTIN3
0	1.95777896756156	0.418	0.109	0	Hepatocytes	ERG28
0	3.49344639556574	0.389	0.08	0	Hepatocytes	HP
0	3.52145868469426	0.335	0.026	0	Hepatocytes	FTCD
0	0.930830535891052	0.578	0.269	0	Hepatocytes	COPG1
0	1.51244442348567	0.476	0.167	0	Hepatocytes	CIAO2A
0	1.62241428026459	0.47	0.161	0	Hepatocytes	PRDX4
0	3.25420697612563	0.344	0.035	0	Hepatocytes	DEPRECATED-ENSG00000100197
0	2.1406337686189	0.366	0.058	0	Hepatocytes	ENAH
0	2.13135429845973	0.361	0.053	0	Hepatocytes	SORBS2
0	3.33341726289889	0.343	0.036	0	Hepatocytes	ITIH4
0	1.78904473612144	0.439	0.132	0	Hepatocytes	MCFD2
0	2.62134175697082	0.379	0.072	0	Hepatocytes	DEPRECATED-ENSG00000057294
0	0.741916634307296	0.512	0.206	0	Hepatocytes	TP53INP1
0	1.27121176592665	0.379	0.073	0	Hepatocytes	GRB10
0	2.87300011248796	0.345	0.039	0	Hepatocytes	HSD17B2
0	3.48750028520831	0.35	0.044	0	Hepatocytes	B3GNT3

0	2.69077729480587	0.388	0.082	0	Hepatocytes	DEPRECATED-ENSG00000213759
0	3.49538016014959	0.336	0.031	0	Hepatocytes	MARC1
0	2.04989561588949	0.394	0.089	0	Hepatocytes	IMPA2
0	3.12905979826324	0.347	0.042	0	Hepatocytes	CDS1
0	2.61902113989825	0.39	0.085	0	Hepatocytes	NSDHL
0	1.02127215676075	0.524	0.22	0	Hepatocytes	ACAA1
0	3.19827080634254	0.343	0.039	0	Hepatocytes	DEPRECATED-ENSG00000109181
0	2.03084543170004	0.444	0.14	0	Hepatocytes	GSTA2
0	2.80693602243298	0.343	0.04	0	Hepatocytes	BAIAP2L1
0	2.24415067339891	0.405	0.102	0	Hepatocytes	ATAD2
0	1.0608725697777	0.386	0.083	0	Hepatocytes	TRAF4
0	1.35930688541245	0.454	0.152	0	Hepatocytes	CPQ
0	3.29034194662865	0.343	0.041	0	Hepatocytes	HPX
0	1.59127487602869	0.357	0.055	0	Hepatocytes	COL27A1
0	1.93579268701216	0.388	0.087	0	Hepatocytes	NEDD4L
0	0.952202985877351	0.547	0.246	0	Hepatocytes	FAM50A
0	1.2624729961722	0.498	0.197	0	Hepatocytes	GCLC
0	1.44429453057255	0.431	0.13	0	Hepatocytes	NHSL1
0	3.56781432072396	0.36	0.059	0	Hepatocytes	CYP 20.00
0	3.23165191008625	0.346	0.045	0	Hepatocytes	SERPINA5
0	1.58080594736487	0.385	0.084	0	Hepatocytes	PVR
0	2.8438233078215	0.548	0.248	0	Hepatocytes	APOA2
0	1.05361195084338	0.522	0.222	0	Hepatocytes	HINT1
0	1.18509946293322	0.522	0.222	0	Hepatocytes	GUSB
0	1.85408181943118	0.441	0.141	0	Hepatocytes	FUOM
0	1.92708617279062	0.377	0.077	0	Hepatocytes	RDH10
0	2.97657688683468	0.347	0.047	0	Hepatocytes	GABRE
0	2.09273009626487	0.368	0.069	0	Hepatocytes	UAP1
0	2.31766510715216	0.376	0.077	0	Hepatocytes	MTHFS
0	1.23131235758877	0.47	0.171	0	Hepatocytes	SEC11C
0	2.46917517161095	0.403	0.105	0	Hepatocytes	SRXN1
0	1.66213498638543	0.425	0.127	0	Hepatocytes	SLC30A1
0	2.82418394726008	0.335	0.038	0	Hepatocytes	EGFR
0	1.5045677346744	0.458	0.161	0	Hepatocytes	HTATIP2
0	1.62499887187415	0.432	0.135	0	Hepatocytes	NENF
0	1.35898478976667	0.476	0.179	0	Hepatocytes	PYGB
0	3.18255854664752	0.34	0.044	0	Hepatocytes	FAM83H
0	0.946341723027068	0.538	0.242	0	Hepatocytes	HNRNPH2
0	3.10078998375008	0.332	0.036	0	Hepatocytes	DEPRECATED-ENSG00000174827
0	1.25148064884418	0.472	0.176	0	Hepatocytes	GSR
0	1.08642287611627	0.49	0.194	0	Hepatocytes	ENY2
0	1.23504820304079	0.431	0.135	0	Hepatocytes	SCARB1
0	1.8273975470598	0.411	0.115	0	Hepatocytes	COA3
0	1.80958607995609	0.435	0.14	0	Hepatocytes	NBL1
0	3.02830316686225	0.366	0.071	0	Hepatocytes	FXYD3
0	2.50984059984283	0.331	0.037	0	Hepatocytes	TLCD4
0	2.26619372899177	0.387	0.093	0	Hepatocytes	ALAD
0	1.42709991145612	0.456	0.162	0	Hepatocytes	POLDIP2
0	3.3021746922278	0.324	0.03	0	Hepatocytes	CREB3L3
0	1.74388152892525	0.356	0.062	0	Hepatocytes	PCDH1
0	3.289458295524	0.323	0.029	0	Hepatocytes	TTPA
0	1.84723643919757	0.385	0.092	0	Hepatocytes	PECR
0	2.97314705021362	0.338	0.045	0	Hepatocytes	PLS1
0	2.95862397479333	0.334	0.041	0	Hepatocytes	SHROOM3
0	2.98706947724712	0.328	0.035	0	Hepatocytes	KLKB1
0	1.36774867703498	0.439	0.146	0	Hepatocytes	PDZD8
0	1.09587305036168	0.505	0.212	0	Hepatocytes	IDH3G
0	0.932165156686456	0.884	0.591	0	Hepatocytes	MT-CO2
0	2.52920311637503	0.357	0.064	0	Hepatocytes	SLC25A33
0	3.57106054797363	0.324	0.033	0	Hepatocytes	KCNJ3
0	3.09457164513985	0.33	0.039	0	Hepatocytes	IL17RB
0	2.44621155691716	0.369	0.078	0	Hepatocytes	HAAO
0	3.33353369754732	0.32	0.029	0	Hepatocytes	CHDH

0	2.9362697076886	0.347	0.056	0	Hepatocytes	LRRC31
0	2.33644525684528	0.34	0.05	0	Hepatocytes	COBLL1
0	2.45257138575303	0.339	0.049	0	Hepatocytes	CAPN5
0	2.16616389604887	0.373	0.083	0	Hepatocytes	GCLM
0	1.36685307759304	0.458	0.169	0	Hepatocytes	MPC1
0	1.08052818935562	0.508	0.219	0	Hepatocytes	RAB2A
0	1.53440056247485	0.404	0.115	0	Hepatocytes	AKAP1
0	1.57993518434243	0.409	0.12	0	Hepatocytes	HEBP2
0	4.06141607762151	0.312	0.023	0	Hepatocytes	AKR1C8P
0	1.28731746347029	0.419	0.13	0	Hepatocytes	PPP2R1B
0	2.26835944457708	0.359	0.07	0	Hepatocytes	SLC25A30
0	2.84060115629629	0.341	0.053	0	Hepatocytes	TM7SF2
0	1.58358198367538	0.431	0.143	0	Hepatocytes	VMA21
0	1.20049386078019	0.416	0.128	0	Hepatocytes	RNF103
0	1.21454368268475	0.408	0.12	0	Hepatocytes	PCCA
0	2.51544994658659	0.343	0.056	0	Hepatocytes	ASS1
0	1.72171225815029	0.413	0.126	0	Hepatocytes	FDPS
0	3.44611293664127	0.31	0.023	0	Hepatocytes	PLIN5
0	1.94940676420513	0.381	0.095	0	Hepatocytes	PEX19
0	1.32826743479268	0.427	0.141	0	Hepatocytes	NIT1
0	1.5442641510388	0.432	0.146	0	Hepatocytes	SRP9
0	1.22179244205821	0.394	0.108	0	Hepatocytes	CRY2
0	2.79755983046982	0.322	0.036	0	Hepatocytes	DNAJC22
0	2.2460304085722	0.374	0.088	0	Hepatocytes	HSD3B7
0	1.64939224838013	0.407	0.121	0	Hepatocytes	RETSAT
0	1.81534897408748	0.388	0.103	0	Hepatocytes	SIPA1L2
0	1.56109923966894	0.388	0.103	0	Hepatocytes	NDFIP2
0	2.13961700123415	0.376	0.091	0	Hepatocytes	CRLS1
0	1.43729812530386	0.399	0.115	0	Hepatocytes	KDELR2
0	2.4776992170997	0.34	0.056	0	Hepatocytes	CLDN15
0	3.30989512778423	0.32	0.036	0	Hepatocytes	PDX1
0	2.77625871935226	0.331	0.047	0	Hepatocytes	CDH1
0	1.80610116782958	0.361	0.077	0	Hepatocytes	SLC17A9
0	3.35492197090919	0.327	0.044	0	Hepatocytes	TSPAN1
0	3.05235984677739	0.32	0.037	0	Hepatocytes	OSGIN1
0	1.56526606815436	0.421	0.138	0	Hepatocytes	GRHPR
0	2.42888690573005	0.361	0.078	0	Hepatocytes	TMEM97
0	2.87484828671149	0.329	0.047	0	Hepatocytes	C1orf115
0	1.48190076160131	0.424	0.142	0	Hepatocytes	SLC35D2
0	3.45152665936843	0.315	0.033	0	Hepatocytes	TAT
0	2.07153982083047	0.377	0.095	0	Hepatocytes	CISD3
0	2.06915788211388	0.385	0.103	0	Hepatocytes	GALK1
0	0.706633639265521	0.889	0.607	0	Hepatocytes	MT-ND4
0	1.36023272500701	0.417	0.135	0	Hepatocytes	SLC25A13
0	3.39451690872573	0.31	0.028	0	Hepatocytes	CYP2C18
0	1.64005325112833	0.41	0.129	0	Hepatocytes	SLC44A1
0	2.42978820361576	0.327	0.047	0	Hepatocytes	DDAH1
0	2.03954536199781	0.339	0.059	0	Hepatocytes	CMTM8
0	2.94453910151909	0.325	0.045	0	Hepatocytes	KLB
0	2.23327259865137	0.342	0.062	0	Hepatocytes	KCNK5
0	1.1683062122752	0.429	0.149	0	Hepatocytes	RAP2C
0	1.90967604674444	0.384	0.105	0	Hepatocytes	SMIM19
0	2.77643250651453	0.32	0.041	0	Hepatocytes	SEC14L2
0	1.83417324183073	0.374	0.096	0	Hepatocytes	TMEM38B
0	0.942738447262199	0.384	0.106	0	Hepatocytes	ACVR1B
0	1.6256885072518	0.395	0.117	0	Hepatocytes	LSS
0	1.75250048069587	0.363	0.086	0	Hepatocytes	ACO1
0	2.15223736881618	0.357	0.08	0	Hepatocytes	ISOC1
0	1.32627967939161	0.354	0.077	0	Hepatocytes	TJP1
0	0.991358694256664	0.343	0.068	0	Hepatocytes	PKDCC
0	1.83476814845997	0.359	0.084	0	Hepatocytes	IMPA1
0	3.11319146261524	0.307	0.033	0	Hepatocytes	CFHR2
0	1.70714150590776	0.402	0.128	0	Hepatocytes	HADH

0	2.55309233048487	0.329	0.055	0	Hepatocytes	PKP2
0	2.50855310561748	0.326	0.052	0	Hepatocytes	CLMN
0	2.05727726995767	0.372	0.098	0	Hepatocytes	C15orf48
0	2.28419024893402	0.338	0.064	0	Hepatocytes	TIMM8A
0	3.42952196407742	0.296	0.022	0	Hepatocytes	SLC30A10
0	1.65538803643678	0.39	0.117	0	Hepatocytes	NNMT
0	2.98062298093319	0.337	0.065	0	Hepatocytes	C5
0	2.67570411723389	0.349	0.077	0	Hepatocytes	TM4SF20
0	1.94911933752912	0.375	0.104	0	Hepatocytes	UBD
0	1.88143180004337	0.506	0.235	0	Hepatocytes	SERPING1
0	3.43437675338604	0.311	0.04	0	Hepatocytes	SERPINA3
0	2.89795636978713	0.326	0.055	0	Hepatocytes	CHP2
0	1.97971819709798	0.368	0.097	0	Hepatocytes	ISOC2
0	0.84782394997056	0.955	0.684	0	Hepatocytes	MT-CO1
0	2.22149434278992	0.335	0.065	0	Hepatocytes	SPR
0	1.95496326810524	0.99	0.72	0	Hepatocytes	SERPINA1
0	1.22047862925149	0.35	0.08	0	Hepatocytes	NFIA
0	3.06841174225348	0.302	0.033	0	Hepatocytes	MTTP
0	3.4909718738014	0.298	0.03	0	Hepatocytes	INAVA
0	2.72240382049956	0.312	0.046	0	Hepatocytes	MALL
0	2.61521533503147	0.358	0.092	0	Hepatocytes	ALAS1
0	2.42497396533685	0.334	0.068	0	Hepatocytes	ASB13
0	1.39938337855297	0.349	0.083	0	Hepatocytes	DEPRECATED-ENSG00000184500
0	3.12408329586527	0.302	0.037	0	Hepatocytes	PLGLB1
0	2.32815303022674	0.314	0.049	0	Hepatocytes	ACACB
0	2.90800605093469	0.295	0.03	0	Hepatocytes	EXOC3L4
0	1.74099005320715	0.351	0.087	0	Hepatocytes	TMEM164
0	2.1573989792229	0.317	0.054	0	Hepatocytes	LNX2
0	1.95014409387067	0.343	0.081	0	Hepatocytes	GGCX
0	3.18213365219279	0.298	0.036	0	Hepatocytes	AQP9
0	2.83588424027043	0.298	0.036	0	Hepatocytes	TBC1D8B
0	2.93444044596135	0.304	0.043	0	Hepatocytes	SI
0	3.21179922784108	0.314	0.053	0	Hepatocytes	HRG
0	1.9875995940704	0.304	0.044	0	Hepatocytes	NR5A2
0	2.38969619459813	0.32	0.06	0	Hepatocytes	DHCR7
0	2.13454172141634	0.337	0.077	0	Hepatocytes	TMEM238
0	3.3675439566895	0.294	0.034	0	Hepatocytes	MS4A8
0	2.41248087746584	0.296	0.037	0	Hepatocytes	EPB41L5
0	1.19938172327391	0.312	0.053	0	Hepatocytes	RNF152
0	3.14732020110057	0.288	0.029	0	Hepatocytes	MGAM2
0	2.69008560350896	0.288	0.029	0	Hepatocytes	PTPRH
0	3.55112534221687	0.28	0.022	0	Hepatocytes	F7
0	2.89895438368029	0.298	0.04	0	Hepatocytes	PIPOX
0	2.61253187230513	0.312	0.055	0	Hepatocytes	SSX2IP
0	3.08926940716345	0.29	0.033	0	Hepatocytes	MST1
0	3.04602196609749	0.288	0.031	0	Hepatocytes	ADH1B
0	2.05461870160901	0.322	0.065	0	Hepatocytes	SLC43A1
0	2.2564998352387	0.321	0.065	0	Hepatocytes	CRAT
0	2.86038717590765	0.296	0.04	0	Hepatocytes	MOCOS
0	3.43313270762826	0.28	0.025	0	Hepatocytes	ADH6
0	3.24804919606753	0.283	0.028	0	Hepatocytes	EPB41L4B
0	1.91331327116416	0.308	0.055	0	Hepatocytes	SH3BGRL2
0	2.98815195946284	0.29	0.037	0	Hepatocytes	REEP6
0	3.2680473639817	0.283	0.03	0	Hepatocytes	GRIA3
0	1.6322863641259	0.316	0.064	0	Hepatocytes	SH3BP4
0	1.89285545484684	0.309	0.058	0	Hepatocytes	ABCB6
0	3.26037301483381	0.278	0.027	0	Hepatocytes	UGT1A9
0	2.9763576285795	0.291	0.04	0	Hepatocytes	ELOVL2
0	2.75544480454027	0.296	0.045	0	Hepatocytes	MAL2
0	3.49804207026607	0.274	0.024	0	Hepatocytes	PROC
0	2.67668929139375	0.303	0.053	0	Hepatocytes	GGH
0	3.12315448603993	0.28	0.03	0	Hepatocytes	EPPK1
0	2.07579260053587	0.3	0.05	0	Hepatocytes	CADM1

0	2.47598870629045	0.304	0.054	0	Hepatocytes	DEPRECATED-ENSG00000001630
0	3.6122067167513	0.268	0.019	0	Hepatocytes	ABCG5
0	3.3703314696953	0.279	0.03	0	Hepatocytes	FOXA1
0	2.38378049859199	0.3	0.051	0	Hepatocytes	AGMAT
0	2.70222806229275	0.293	0.044	0	Hepatocytes	MARVELD2
0	2.31188942941218	0.312	0.063	0	Hepatocytes	TRIQK
0	2.32840281131431	0.288	0.039	0	Hepatocytes	HOOK1
0	2.08491905372029	0.295	0.047	0	Hepatocytes	DLG5
0	3.61981407315017	0.28	0.033	0	Hepatocytes	AHSG
0	2.81396780178181	0.273	0.027	0	Hepatocytes	TJP3
0	3.37148794489354	0.276	0.031	0	Hepatocytes	HPR
0	2.26693621931322	0.286	0.041	0	Hepatocytes	ENPP1
0	3.50094657080508	0.274	0.03	0	Hepatocytes	OTOP3
0	2.58602129034623	0.298	0.054	0	Hepatocytes	HSD17B8
0	3.56783000363684	0.266	0.024	0	Hepatocytes	EPS8L3
0	2.87058517279603	0.28	0.038	0	Hepatocytes	AS3MT
0	3.20890246202205	0.267	0.026	0	Hepatocytes	UGT1A4
0	3.39668782587496	0.27	0.029	0	Hepatocytes	DEPRECATED-ENSG00000197408
0	3.23885282740667	0.261	0.021	0	Hepatocytes	AGMO
0	2.34348076118571	0.272	0.033	0	Hepatocytes	PCSK5
0	3.48935765642226	0.258	0.019	0	Hepatocytes	SLCO1B1
0	2.78466936905298	0.275	0.037	0	Hepatocytes	CXADR
0	2.72210023658664	0.274	0.037	0	Hepatocytes	DEPRECATED-ENSG00000170421
0	3.17559804293104	0.265	0.03	0	Hepatocytes	TMC5
0	3.40963873479002	0.265	0.03	0	Hepatocytes	MISP
0	3.12064757432629	0.266	0.031	0	Hepatocytes	CLDN14
0	3.16274996480604	0.265	0.03	0	Hepatocytes	MTCP1
0	3.66607072379301	0.254	0.019	0	Hepatocytes	DEPRECATED-ENSG00000183747
0	3.28833414265263	0.251	0.019	0	Hepatocytes	ESRP2
0	3.20239952578147	0.252	0.023	0	Hepatocytes	F13B
0	3.4413295302677	0.256	0.028	0	Hepatocytes	HAO2
0	3.25130713085884	0.254	0.027	0	Hepatocytes	SPIRE2
6.40465149906801E-308	3.33994969516861	0.257	0.029	1.07329149821382E-303	Hepatocytes	PRR15L
2.24066606783399E-307	3.1103127446245	0.244	0.02	3.7549081964762E-303	Hepatocytes	PPARGC1A
2.86380587196332E-307	2.20422385973674	0.305	0.059	4.79916588023613E-303	Hepatocytes	MTFR1
7.56685156515466E-306	2.60531569861154	0.263	0.032	1.26805298528862E-301	Hepatocytes	FAAH
3.78749528858552E-305	1.31789825582506	0.399	0.125	6.34708460461161E-301	Hepatocytes	MID1IP1
1.10494595191785E-304	1.75100636631627	0.298	0.052	1.85166842622394E-300	Hepatocytes	GPC6
3.66858338911683E-304	1.3813677211129	0.39	0.118	6.14781204348199E-300	Hepatocytes	MAP3K13
1.96109128979122E-303	0.575500476708426	0.529	0.222	3.28639678343212E-299	Hepatocytes	TOB1
3.01486871152404E-303	1.38325736813404	0.392	0.122	5.05231698677199E-299	Hepatocytes	TMEM64
1.98489549147923E-302	2.02221643134682	0.348	0.093	3.32628786462089E-298	Hepatocytes	CETN2
2.12263617172672E-302	1.10461864408751	0.467	0.177	3.55711369657963E-298	Hepatocytes	ATP5IF1
3.10914863284844E-302	1.7779471779585	0.283	0.044	5.21031127892742E-298	Hepatocytes	COL5A3
1.09975494824463E-301	2.0319570901553	0.347	0.092	1.84296934226835E-297	Hepatocytes	NAXE
1.16688661367784E-301	1.93074690116228	0.346	0.091	1.95546858720132E-297	Hepatocytes	TMEM9
4.34692013314861E-301	1.94416207598756	0.35	0.094	7.28456875913043E-297	Hepatocytes	ACAT2
9.16345808565143E-301	1.75913373429762	0.32	0.069	1.53561230599347E-296	Hepatocytes	HDHD3
9.57296627494E-301	2.53972095361659	0.257	0.03	1.60423768835445E-296	Hepatocytes	CCL28
1.0145683895329E-300	1.9291998033239	0.342	0.088	1.70021370717924E-296	Hepatocytes	PCCB
1.23090944577316E-300	1.21758678660583	0.461	0.182	2.06275804922666E-296	Hepatocytes	ABHD2
2.54703996375892E-300	3.24790568739855	0.254	0.029	4.2683295712672E-296	Hepatocytes	DUOX2
6.43506038455699E-300	2.77976155667879	0.267	0.037	1.07838741924406E-295	Hepatocytes	RTKN
1.27697799551308E-299	3.21564437114539	0.253	0.029	2.13995972488081E-295	Hepatocytes	APCS
1.68400043428997E-299	2.56369714222106	0.284	0.049	2.82204792778314E-295	Hepatocytes	BPHL
2.39783041050647E-299	1.23833118020359	0.476	0.194	4.01828420192674E-295	Hepatocytes	ALDH9A1
4.52915275196753E-299	1.71379701319187	0.369	0.111	7.58995418174719E-295	Hepatocytes	PSMD10
5.17552696231997E-299	1.68880739374534	0.29	0.048	8.67314808345581E-295	Hepatocytes	CRACR2B
6.34080046008871E-299	2.75667195111478	0.271	0.04	1.06259134110167E-294	Hepatocytes	IGF2BP3
9.06095867304644E-299	2.08460825582386	0.329	0.079	1.51843545442912E-294	Hepatocytes	UGT2B11
1.18747158608379E-298	2.47205080403993	0.258	0.031	1.98996488395921E-294	Hepatocytes	SLC16A2
1.32656546437909E-298	1.47605296999815	0.418	0.146	2.22305840520648E-294	Hepatocytes	RAB5IF
2.86284204243467E-298	1.0445442357718	0.467	0.177	4.79755069471203E-294	Hepatocytes	SLC2A4RG

3.0627876663596E-298	1.79026455046803	0.359	0.101	5.13261957128542E-294	Hepatocytes	QDPR
5.03930316328689E-298	0.646143745091967	0.379	0.103	8.44486424103617E-294	Hepatocytes	PATJ
1.072776635941E-297	3.11589141544646	0.254	0.03	1.79775908650993E-293	Hepatocytes	UGT2B15
1.75682073983219E-297	2.83840477251626	0.259	0.033	2.94408019581078E-293	Hepatocytes	USP54
3.26527902345124E-297	1.34595490630238	0.378	0.113	5.47195458749959E-293	Hepatocytes	TBC1D8
1.40075210418333E-296	0.786094775138087	0.511	0.214	2.34738037619042E-292	Hepatocytes	NFIC
2.0924337019144E-296	3.3764468042624	0.242	0.023	3.50650039766816E-292	Hepatocytes	HOXA13
2.74632592951745E-296	0.638821411505745	0.387	0.111	4.60229299268534E-292	Hepatocytes	MTUS1
3.78811065662163E-296	1.28597986717468	0.464	0.195	6.34811583836653E-292	Hepatocytes	ETHE1
4.25066744766633E-296	0.995349069114673	0.49	0.202	7.12326850879924E-292	Hepatocytes	EGLN1
3.53587930283768E-295	1.5440863769708	0.378	0.115	5.92542653569538E-291	Hepatocytes	ECI1
3.69330151846624E-295	1.56540216982036	0.32	0.072	6.18923468464573E-291	Hepatocytes	FOXP4
3.70898754643495E-295	2.89741201775606	0.254	0.031	6.21552133031568E-291	Hepatocytes	PNPLA3
6.54609980375391E-295	0.981569615246162	0.528	0.233	1.09699540511308E-290	Hepatocytes	POLR2L
7.23775009038312E-295	1.17382362511024	0.452	0.172	1.2129021601464E-290	Hepatocytes	RNF5
8.61486192793229E-295	0.939465106342317	0.45	0.171	1.44367856188289E-290	Hepatocytes	ZNF706
1.95996979690754E-294	1.65050813211667	0.352	0.098	3.28451738565765E-290	Hepatocytes	C1GALT1C1
7.99629891432817E-294	2.67018701676221	0.256	0.032	1.34001977206311E-289	Hepatocytes	HSPA4L
1.44535481001816E-293	1.05005095938713	0.492	0.203	2.42212559062843E-289	Hepatocytes	NDUFA4
2.62536680973168E-293	1.31961689695889	0.343	0.087	4.39958969974834E-289	Hepatocytes	PLOD2
4.40216238983657E-293	2.6119575857027	0.274	0.045	7.37714373288813E-289	Hepatocytes	TST
6.47866343582565E-293	1.0220223908774	0.44	0.161	1.08569441857566E-288	Hepatocytes	CPD
1.02577178946762E-292	2.26316448115656	0.267	0.038	1.71898836478984E-288	Hepatocytes	NPAS2
1.23089025500384E-292	1.39894679756355	0.302	0.06	2.06272588933543E-288	Hepatocytes	RALGPS2
3.71113060190424E-292	1.38558515819222	0.395	0.128	6.21911266267112E-288	Hepatocytes	PRXL2A
1.27079911137668E-291	0.941338842856844	0.5	0.223	2.12960515084504E-287	Hepatocytes	UBE2A
1.8222247701097E-291	3.03280644999729	0.238	0.022	3.05368426974983E-287	Hepatocytes	PLIN4
3.20283756201771E-291	1.76181239460784	0.367	0.111	5.36731518642928E-287	Hepatocytes	PNPO
5.05259150508471E-291	1.15756289556741	0.456	0.176	8.46713284422095E-287	Hepatocytes	SLC43A3
5.15328329398689E-291	1.10468205136012	0.399	0.13	8.63587214406323E-287	Hepatocytes	SETD7
1.36744027707634E-290	0.862216432616464	0.45	0.17	2.29155641632453E-286	Hepatocytes	IKBKG
3.79586721813108E-290	1.14661826367711	0.44	0.162	6.36111428414407E-286	Hepatocytes	DEPRECATED-ENSG00000163463
4.47907388464517E-290	1.9495105595898	0.331	0.084	7.50603201588837E-286	Hepatocytes	DEPRECATED-ENSG00000133142
2.76099879087817E-289	2.96697128362717	0.232	0.019	4.62688177375364E-285	Hepatocytes	CYP2C9
6.44012353084998E-289	1.67927943147657	0.31	0.066	1.07923590129984E-284	Hepatocytes	NFIX
1.82056366400918E-288	1.19416172571261	0.486	0.203	3.05090058814658E-284	Hepatocytes	NUDT16
2.67522230793921E-288	3.20664420297202	0.265	0.043	4.48313754364452E-284	Hepatocytes	IGFBP1
3.51415385160425E-288	2.94654397439519	0.257	0.036	5.8890190245184E-284	Hepatocytes	POF1B
1.41323831482935E-287	2.42211048306729	0.289	0.054	2.36830476799103E-283	Hepatocytes	G0S2
1.56495623104602E-287	1.5481240044838	0.394	0.131	2.62255365198693E-283	Hepatocytes	FDFT1
4.92391209740227E-287	3.11330095405865	0.237	0.023	8.25149189282673E-283	Hepatocytes	FGFR4
5.70982759270998E-287	2.4480473686157	0.275	0.048	9.56852907986338E-283	Hepatocytes	FAIM
1.95281895343737E-286	1.86487223592191	0.322	0.077	3.27253400217035E-282	Hepatocytes	CDKN2A
7.61869831161614E-286	1.23468395964499	0.372	0.113	1.27674146306063E-281	Hepatocytes	CCNG1
8.45822302961442E-286	1.46899355372449	0.374	0.117	1.41742901530278E-281	Hepatocytes	MCTS1
9.59096162921734E-286	1.15025418141597	0.397	0.129	1.60725334982424E-281	Hepatocytes	NMD3
9.78226910392246E-286	2.16624901694261	0.266	0.04	1.63931265643533E-281	Hepatocytes	BOK
1.05577665867608E-284	2.06635743593891	0.261	0.037	1.76927052460938E-280	Hepatocytes	DTX4
1.28149750719318E-283	1.17945929315866	0.376	0.117	2.14753352255433E-279	Hepatocytes	PRPS1
1.58997130328499E-283	2.27593023104785	0.275	0.047	2.66447391004498E-279	Hepatocytes	ALDH5A1
2.20209399900084E-283	3.0113533648498	0.241	0.027	3.6902691235256E-279	Hepatocytes	MLIP
4.09350963515442E-283	0.797079903742906	0.359	0.096	6.85990344659178E-279	Hepatocytes	CTTN
6.52193823236045E-283	1.18516978577938	0.377	0.118	1.09294640897896E-278	Hepatocytes	ZBTB33
1.10467606475782E-282	3.44046009440715	0.232	0.022	1.85121614932116E-278	Hepatocytes	CYP2C19
1.37656525171943E-282	0.802750002762156	0.442	0.166	2.30684804883143E-278	Hepatocytes	FMR1
1.61766918552216E-282	0.639166354159378	0.664	0.378	2.71089002109803E-278	Hepatocytes	SGK1
1.65351226164637E-282	3.37566363956525	0.224	0.017	2.77095584806698E-278	Hepatocytes	SLC38A3
3.26079915191751E-282	1.41038337624602	0.305	0.066	5.46444721878336E-278	Hepatocytes	SCML1
7.43816816129099E-282	0.55921324176418	0.589	0.251	1.24648822046914E-277	Hepatocytes	CREG1
2.25969606396804E-281	0.860972462969485	0.419	0.149	3.78679866399764E-277	Hepatocytes	PANK3
1.09651955169521E-280	4.10135842278845	0.221	0.016	1.83754746473083E-276	Hepatocytes	GREB1L
2.76579432826918E-280	0.851806697890142	0.482	0.202	4.63491813531349E-276	Hepatocytes	IL13RA1
6.10413045818296E-280	0.697088626432776	0.605	0.3	1.0229301821823E-275	Hepatocytes	GOLPH3

**2.77975032743929E-279**	1.05931995239827	0.462	0.189	4.65830559872276E-275	Hepatocytes	WWP1
**3.30503269503714E-279**	1.34268851442298	0.318	0.076	5.53857379034324E-275	Hepatocytes	BDH2
**9.01376817191635E-279**	0.700998345176936	0.339	0.086	1.51052727024974E-274	Hepatocytes	RNF157
**1.01302615941083E-278**	1.09823521897879	0.432	0.156	1.69762923794066E-274	Hepatocytes	ARHGEF10L
**1.32877640960243E-278**	1.52688012839622	0.412	0.152	2.22676350721175E-274	Hepatocytes	TALDO1
**1.49667454062626E-278**	3.44982284468899	0.227	0.02	2.50812719518148E-274	Hepatocytes	SHOX2
**1.68722665953593E-278**	1.68369814505795	0.275	0.048	2.82745443605032E-274	Hepatocytes	TSKU
**2.11063977992657E-278**	1.81767409240661	0.266	0.042	3.53701014320095E-274	Hepatocytes	CEP170B
**2.22975522335199E-277**	1.76294923612351	0.35	0.102	3.73662380329326E-273	Hepatocytes	ACADM
**2.2372090938787E-277**	1.66058184549379	0.341	0.092	3.74911499952193E-273	Hepatocytes	LDLR
**2.61661910551236E-277**	0.865646336357915	0.431	0.158	4.38493029701762E-273	Hepatocytes	KLHDC2
**3.44365674473462E-277**	1.35885780016857	0.289	0.057	5.77087997282628E-273	Hepatocytes	ADCY6
**3.61353255313722E-277**	1.39249392935184	0.358	0.109	6.05555785254735E-273	Hepatocytes	CTSO
**6.23766657386939E-277**	1.48156916356507	0.286	0.054	1.04530816444903E-272	Hepatocytes	YAP1
**6.60162057050011E-277**	2.24769870263868	0.302	0.07	1.10629957520441E-272	Hepatocytes	HIBADH
**8.77694182302494E-277**	1.35492032683115	0.391	0.128	1.47083991070252E-272	Hepatocytes	C19orf12
**2.68059878355456E-276**	1.11534257208912	0.392	0.134	4.49214744148074E-272	Hepatocytes	THOC2
**3.43023514896973E-276**	1.66969350480033	0.332	0.09	5.74838806264347E-272	Hepatocytes	ACADS
**3.56158627563543E-276**	3.06958251164221	0.228	0.022	5.96850628070986E-272	Hepatocytes	HNF1A
**5.06214045413817E-276**	2.10277419273201	0.281	0.053	8.48313497304475E-272	Hepatocytes	HLF
**9.3291980654016E-276**	1.32375567646737	0.358	0.109	1.5633870118E-271	Hepatocytes	NQO2
**1.31174154011413E-275**	1.1817992696224	0.448	0.176	2.19821647292327E-271	Hepatocytes	COX6A1
**2.64257111333321E-275**	1.06677416203361	0.474	0.196	4.4284206717238E-271	Hepatocytes	EIF6
**3.44548792872223E-275**	1.31547815160934	0.408	0.145	5.77394867095272E-271	Hepatocytes	ROMO1
**7.44641261683171E-275**	0.692973201318266	0.411	0.14	1.24786982632866E-270	Hepatocytes	PI4K2B
**1.11292836510936E-274**	1.76576378992507	0.361	0.113	1.86504535425027E-270	Hepatocytes	MCM4
**1.29226936318404E-274**	0.779417932432505	0.403	0.132	2.16558499882382E-270	Hepatocytes	AGFG2
**2.87059779747909E-274**	2.00245194450669	0.33	0.09	4.81054778901546E-270	Hepatocytes	HMGCR
**4.97009461462808E-274**	2.99751507580316	0.23	0.023	8.32888455519373E-270	Hepatocytes	TFR2
**5.54336343460779E-274**	3.28107890279172	0.232	0.025	9.28956844371574E-270	Hepatocytes	PLGLB2
**1.05840653203978E-273**	0.856393183275951	0.468	0.192	1.77367766639226E-269	Hepatocytes	MLF 2.00
**3.7304410012098E-273**	0.610726222290425	0.305	0.065	6.25147302982739E-269	Hepatocytes	PIEZO2
**6.47873252919612E-273**	3.01347650365729	0.23	0.024	1.08570599724269E-268	Hepatocytes	DEPRECATED-ENSG00000187758
**7.92199934933721E-273**	1.84455726864744	0.32	0.083	1.32756865096193E-268	Hepatocytes	DHTKD1
**1.2160781776474E-272**	1.64437686239761	0.263	0.042	2.03790381010151E-268	Hepatocytes	ATP8B1
**2.00647305778677E-272**	1.42694492561653	0.422	0.164	3.36244755023907E-268	Hepatocytes	MCM3
**2.19244250960536E-272**	0.962455884012029	0.443	0.173	3.67409515759666E-268	Hepatocytes	SIVA1
**2.72322049623869E-272**	1.76761450767664	0.356	0.11	4.56357290759679E-268	Hepatocytes	SLC25A5
**2.90670724377949E-272**	1.22511844127115	0.455	0.186	4.87105999912567E-268	Hepatocytes	CHCHD2
**3.48592011171779E-272**	1.23032396002688	0.288	0.056	5.84170492321668E-268	Hepatocytes	DEPRECATED-ENSG00000085733
**3.56153306680369E-272**	1.25068528086244	0.369	0.114	5.96841711334962E-268	Hepatocytes	MALSU1
**4.06563554524604E-272**	2.05521384301773	0.25	0.035	6.81319204672332E-268	Hepatocytes	SOX5
**7.9581114911809E-272**	1.4858029815483	0.311	0.075	1.3336203236921E-267	Hepatocytes	PROS1
**2.09542940659581E-271**	1.88095736927512	0.298	0.067	3.51152059957326E-267	Hepatocytes	H6PD
**5.03205156009188E-271**	0.697440210487612	0.41	0.139	8.43271200440198E-267	Hepatocytes	NDST1
**1.24945507250886E-270**	0.778011662419689	0.754	0.463	2.09383681051034E-266	Hepatocytes	MT-ND3
**1.28885225105047E-270**	1.94723987064066	0.285	0.06	2.15985860231037E-266	Hepatocytes	EBPL
**1.57832231997145E-270**	1.56313891815153	0.321	0.082	2.64495254380815E-266	Hepatocytes	RAB33B
**2.09028663110467E-270**	1.32007445564612	0.404	0.142	3.50290233640521E-266	Hepatocytes	ETFA
**6.98882825466096E-270**	0.956118557747261	0.522	0.239	1.17118783891608E-265	Hepatocytes	MIF
**2.07137027670657E-269**	1.15061186290649	0.422	0.159	3.47120230970487E-265	Hepatocytes	ANAPC11
**2.0723126958134E-269**	0.899565962050289	0.577	0.305	3.4727816156441E-265	Hepatocytes	TRAM1
**2.2222240903967E-269**	2.46972330953313	0.262	0.044	3.72400313068679E-265	Hepatocytes	MANEAL
**2.7608418107386E-269**	1.10748968318933	0.401	0.14	4.62661870643575E-265	Hepatocytes	SLC50A1
**3.28630529373501E-269**	1.02547181922357	0.313	0.071	5.50719041124113E-265	Hepatocytes	CKAP4
**3.37854319853904E-269**	2.38766132550607	0.258	0.042	5.66176269211173E-265	Hepatocytes	DEPRECATED-ENSG00000146416
**3.97281488322904E-269**	3.55054431284697	0.235	0.03	6.65764318131523E-265	Hepatocytes	ETNK2
**4.91989627979349E-269**	0.847544332308702	0.522	0.236	8.24476218567793E-265	Hepatocytes	STAU1
**7.44971555827475E-269**	1.53564487479704	0.266	0.044	1.24842333325568E-264	Hepatocytes	TEAD1
**1.80023786228997E-268**	2.57707749482215	0.268	0.051	3.01683860962554E-264	Hepatocytes	CCNE2
**1.97507128011567E-268**	1.00845505919006	0.383	0.13	3.30982445121784E-264	Hepatocytes	FAM199X
**2.30449595656449E-268**	3.25288926903946	0.219	0.019	3.86187432401077E-264	Hepatocytes	CCDC198
**4.13477910236185E-268**	2.15954754481488	0.259	0.043	6.92906281973799E-264	Hepatocytes	CENPV

9.22665152386414E-268	1.70568100128489	0.288	0.062	1.54620226236915E-263	Hepatocytes	SH3RF1
1.71740818103595E-267	2.9997363275381	0.221	0.02	2.87803262978005E-263	Hepatocytes	TM4SF5
1.92065639705513E-267	1.54727731451228	0.31	0.075	3.21863599018498E-263	Hepatocytes	BIVM
1.94903618780969E-267	1.20076160438699	0.402	0.145	3.26619484353148E-263	Hepatocytes	DCAF6
4.12920822048636E-267	1.26680521116208	0.391	0.137	6.91972713589105E-263	Hepatocytes	SMIM15
7.41939727144732E-267	2.30640125255546	0.242	0.032	1.24334259474914E-262	Hepatocytes	PPP1R3C
1.51195200580985E-266	1.13755328252654	0.295	0.065	2.53372917133615E-262	Hepatocytes	ABCA5
1.77551232335307E-266	0.696566744929667	0.535	0.244	2.97540355147507E-262	Hepatocytes	CMPK1
3.3310267282326E-266	0.262166399196084	0.462	0.178	5.5821345911722E-262	Hepatocytes	GPX3
1.42195796652182E-265	3.24263988727542	0.212	0.016	2.38291716029726E-261	Hepatocytes	SLC10A5
1.63456490991889E-265	3.04278402473856	0.224	0.023	2.73920387604207E-261	Hepatocytes	LAMA3
2.78012685174825E-265	1.64057759779291	0.335	0.097	4.65893657815971E-261	Hepatocytes	VDAC1
3.04663950943703E-265	1.42115436552222	0.321	0.086	5.10555848991458E-261	Hepatocytes	NIPAL2
3.14547043813246E-265	2.36143921733341	0.241	0.032	5.27117936022237E-261	Hepatocytes	ADGRA3
3.37430164099308E-265	1.30309591741927	0.331	0.092	5.65465468997621E-261	Hepatocytes	UBXN2B
3.88238858940618E-265	2.95624330353034	0.224	0.023	6.50610679812687E-261	Hepatocytes	HSD11B2
5.03560095579972E-265	2.35002756235115	0.243	0.034	8.43866008172918E-261	Hepatocytes	MYRF
7.3536433037143E-265	1.43179962670861	0.272	0.049	1.23232354483644E-260	Hepatocytes	NT5E
1.41652109504292E-264	1.2952347398899	0.377	0.124	2.37380605107292E-260	Hepatocytes	ECI2
2.5715832998831E-264	2.26926590307275	0.271	0.054	4.30945929394409E-260	Hepatocytes	ASB9
3.14809014139052E-264	2.1521001681558	0.263	0.047	5.27556945894223E-260	Hepatocytes	WDYHV1
6.44125192476406E-264	1.87235940270751	0.293	0.067	1.07942499755196E-259	Hepatocytes	NT5DC3
7.06540058590647E-264	1.76672999352974	0.256	0.041	1.18401983018621E-259	Hepatocytes	SLC7A2
9.51510402111941E-264	2.28722919160814	0.227	0.024	1.59454113185919E-259	Hepatocytes	FRAS1
2.16788432776355E-263	0.920762657138726	0.504	0.224	3.63294055646617E-259	Hepatocytes	CUTA
2.48576793666828E-263	1.30736176466797	0.446	0.183	4.16564990826871E-259	Hepatocytes	LAMTOR2
2.63693826709855E-263	0.825753666393991	0.251	0.037	4.41898114800376E-259	Hepatocytes	SAMD12
2.72564860131495E-263	0.927246809578069	0.404	0.148	4.5676419260836E-259	Hepatocytes	XIAP
2.76361400828835E-263	1.53714729581027	0.325	0.09	4.63126435508962E-259	Hepatocytes	GFPT1
3.73373434213947E-263	1.0947695669096	0.328	0.086	6.25699201055733E-259	Hepatocytes	CAV2
4.90285429411706E-263	0.900511097833008	0.467	0.209	8.21620322608138E-259	Hepatocytes	NOTCH2NLB
5.37040885166804E-263	1.28604570975874	0.41	0.152	8.9997311536253E-259	Hepatocytes	PMVK
5.99777351129941E-263	1.62593755948398	0.272	0.051	1.00510688502356E-258	Hepatocytes	TLE1
9.7668658966406E-263	1.40255347038693	0.347	0.103	1.63673138695903E-258	Hepatocytes	SIGMAR1
3.26621347007479E-262	3.1056330051232	0.24	0.035	5.47352053315134E-258	Hepatocytes	CD177
4.57605715469628E-262	3.16749651727768	0.217	0.02	7.66855657984003E-258	Hepatocytes	CAMSAP3
2.1075206032979E-261	2.83275304181394	0.233	0.03	3.53178302700661E-257	Hepatocytes	GHR
3.19213469324097E-261	1.7412218255186	0.313	0.08	5.34937931893321E-257	Hepatocytes	NRBP2
5.73438279563451E-261	1.14154656757904	0.44	0.176	9.60967868892432E-257	Hepatocytes	HADHB
2.22313150513119E-260	1.20147975275381	0.301	0.07	3.72552377629885E-256	Hepatocytes	NCKAP1
5.81754383334385E-260	1.29017569008153	0.347	0.105	9.74903995591763E-256	Hepatocytes	FAM20B
1.54832059508173E-259	2.04502621548369	0.249	0.04	2.59467565323796E-255	Hepatocytes	CLDN12
1.57906505048194E-259	2.35173317032963	0.286	0.066	2.64619721159764E-255	Hepatocytes	CEBPA
4.79370709936059E-259	1.6538056006499	0.351	0.111	8.03329435710848E-255	Hepatocytes	DUSP23
5.44386836181861E-259	0.794323963049736	0.348	0.1	9.12283460073562E-255	Hepatocytes	AGRN
6.37311473295432E-259	1.19322854283452	0.418	0.162	1.06800656694849E-254	Hepatocytes	DBI
8.05402148833708E-259	2.69237319286096	0.226	0.026	1.34969292101553E-254	Hepatocytes	RBM24
9.77923879843769E-259	1.36254752527965	0.307	0.077	1.63880483784219E-254	Hepatocytes	CXCL2
2.59694710191968E-258	2.85276775621724	0.236	0.033	4.351963953397E-254	Hepatocytes	DEPRECATED-ENSG00000171234
3.13947429091059E-258	2.37198066319613	0.262	0.049	5.26113101670796E-254	Hepatocytes	ACADSB
3.25174052711827E-258	1.62163719349909	0.324	0.09	5.44926677534479E-254	Hepatocytes	PMM1
3.94117407888492E-258	1.32516247525983	0.402	0.149	6.60461952139536E-254	Hepatocytes	PSMB4
7.34872077967939E-258	0.980297767424896	0.383	0.131	1.23149862825867E-253	Hepatocytes	FARP1
2.02526865843783E-257	2.39423229027484	0.232	0.03	3.39394521781012E-253	Hepatocytes	DNAH14
3.52192109191913E-257	1.05588840652828	0.443	0.177	5.90203536583808E-253	Hepatocytes	LONP2
8.22134426783614E-257	2.35248872841561	0.261	0.049	1.37773287240398E-252	Hepatocytes	RASL11A
2.30755526014491E-256	1.83539549332766	0.283	0.061	3.86700110495085E-252	Hepatocytes	PRKAB2
2.31988333239446E-256	3.08877826222458	0.209	0.017	3.88766048842664E-252	Hepatocytes	PCSK9
3.08534600365076E-256	2.67989957728944	0.227	0.028	5.17042283291794E-252	Hepatocytes	BAIAP2L2
3.52760699016503E-256	1.54818220918979	0.321	0.089	5.91156379411855E-252	Hepatocytes	NET1
6.34089531097713E-256	4.01094033615616	0.2	0.013	1.06260723621355E-251	Hepatocytes	BTNL3
1.02901716601264E-255	0.986559982797699	0.495	0.238	1.72442696680398E-251	Hepatocytes	DHRS7
2.85787670462971E-255	1.04538057824936	0.463	0.204	4.78922978161846E-251	Hepatocytes	RTN3

5.43578811949945E-255	1.96684747120099	0.257	0.046	9.10929373065719E-251	Hepatocytes	SGCE
1.14456345761154E-254	1.83043174882974	0.273	0.058	1.91805944226542E-250	Hepatocytes	ZNF75D
1.27946611167481E-254	1.4878493203398	0.297	0.071	2.14412930994465E-250	Hepatocytes	PLA2G12A
2.18525883720239E-254	1.25997027457343	0.397	0.145	3.66205675938377E-250	Hepatocytes	HSD17B10
8.23960417449078E-254	1.94719136416946	0.249	0.041	1.38079286756116E-249	Hepatocytes	AASS
9.82582605150245E-254	1.24838280444084	0.346	0.109	1.64661192971078E-249	Hepatocytes	ILVBL
2.54406661455512E-253	0.916841525183179	0.44	0.176	4.26334683267147E-249	Hepatocytes	ACSL5
4.84781520069504E-253	0.978056167583486	0.417	0.159	8.12396871332475E-249	Hepatocytes	DCAF11
6.79989385223016E-253	2.02407075839627	0.266	0.054	1.13952621175673E-248	Hepatocytes	ASRGL1
7.18168693269862E-253	1.03658487992603	0.348	0.108	1.20350709618163E-248	Hepatocytes	STK3
9.28182232812076E-253	1.86456671021306	0.264	0.051	1.55544778574648E-248	Hepatocytes	SIAE
1.17445267295915E-252	2.31637718896291	0.259	0.049	1.96814778934495E-248	Hepatocytes	FN3K
2.55691325563952E-252	0.80821476757913	0.298	0.068	4.2848752338007E-248	Hepatocytes	MYO6
1.10067184303735E-251	2.55493823117221	0.216	0.023	1.84450587456199E-247	Hepatocytes	CACNA1H
1.17822764221934E-251	1.10792067965375	0.331	0.095	1.97447388283117E-247	Hepatocytes	TRMT2B
1.30008583752858E-251	1.17565782521458	0.419	0.168	2.1786838465304E-247	Hepatocytes	TM7SF3
1.5769733474532E-251	1.65571615474624	0.266	0.053	2.64269193566208E-247	Hepatocytes	CORO2A
1.76163665754957E-251	0.884678449148631	0.401	0.147	2.95215071072156E-247	Hepatocytes	EMC2
3.31783540380768E-251	0.815719220026767	0.665	0.378	5.5600285697009E-247	Hepatocytes	MT-ND2
3.72337905436257E-251	1.2164299629554	0.351	0.116	6.23963861930079E-247	Hepatocytes	DIAPH2
4.53307611361444E-251	0.758748567907044	0.481	0.205	7.59652895119509E-247	Hepatocytes	SCARB2
5.85829517685785E-251	1.46019747230212	0.343	0.108	9.81733105737838E-247	Hepatocytes	TMCO3
7.41027707822864E-251	1.31491164740001	0.31	0.082	1.24181423276956E-246	Hepatocytes	PLAAT3
2.07352685393359E-250	1.69262464408198	0.302	0.078	3.47481630182191E-246	Hepatocytes	NTPCR
2.2584261705977E-250	2.52586116972559	0.248	0.043	3.78467057668762E-246	Hepatocytes	SLC25A10
3.43272623859381E-250	0.851852592241074	0.513	0.242	5.7525626306355E-246	Hepatocytes	UBB
5.35176376060149E-250	1.53305550674142	0.368	0.13	8.96848571001597E-246	Hepatocytes	TXNDC17
5.42654375169224E-250	1.31084323299445	0.359	0.12	9.09380201908586E-246	Hepatocytes	TM9SF1
9.14085611457687E-250	1.80324223952081	0.278	0.062	1.53182466768079E-245	Hepatocytes	APOOL
1.28557900558272E-249	1.06027429683576	0.366	0.125	2.15437329755552E-245	Hepatocytes	NBPF26
1.46015286345817E-249	0.7719457267844	0.392	0.139	2.4469241685832E-245	Hepatocytes	SLC25A44
1.79607165126169E-249	2.86301711949885	0.222	0.028	3.00985687318434E-245	Hepatocytes	MET
2.31175693160889E-249	1.2980833737322	0.326	0.096	3.87404226599018E-245	Hepatocytes	ACSS2
3.10659095282446E-249	1.59063724550115	0.317	0.09	5.20602511874323E-245	Hepatocytes	VAMP7
5.43953062328213E-249	3.05444170836492	0.236	0.037	9.11556541849619E-245	Hepatocytes	S100P
5.74502571236227E-249	0.745793822529716	0.364	0.117	9.62751408877669E-245	Hepatocytes	LRRFIP2
1.54766750794606E-248	0.607979261231069	0.617	0.312	2.59358120981601E-244	Hepatocytes	APLP2
3.32203808214081E-248	1.65966142845977	0.279	0.062	5.56707141805157E-244	Hepatocytes	IL17RC
9.75142829942582E-248	1.12339225468713	0.429	0.174	1.63414435441778E-243	Hepatocytes	NDUFB10
1.57711627479085E-247	3.29728985268552	0.214	0.024	2.6429314532945E-243	Hepatocytes	VIL1
2.37464771144532E-247	1.73638559979357	0.249	0.043	3.97943463484007E-243	Hepatocytes	EEPD1
3.76423781029168E-247	0.719636705026736	0.411	0.16	6.3081097224868E-243	Hepatocytes	PLEKHF2
1.00619570785836E-246	0.492973089466294	0.662	0.369	1.68618276722904E-242	Hepatocytes	LYZ
2.20573644342963E-246	3.07342568035497	0.202	0.017	3.69637313189937E-242	Hepatocytes	COBL
2.29119640487366E-246	2.4108810366397	0.26	0.053	3.83958693528728E-242	Hepatocytes	IGFBP2
2.54868520191789E-246	0.511418674722941	0.569	0.283	4.271086661374E-242	Hepatocytes	SLC12A7
6.57209389368853E-246	0.924331807396661	0.441	0.184	1.10135149470432E-241	Hepatocytes	KRTCAP2
1.27106781583267E-245	3.36526591885526	0.203	0.018	2.13005544577239E-241	Hepatocytes	GJB2
7.8658318659913E-245	1.3717849939892	0.289	0.069	1.31815610410282E-240	Hepatocytes	PHKA2
1.41102030608732E-244	2.21733061928374	0.232	0.035	2.36458782894113E-240	Hepatocytes	PPP1R16A
1.91219822853235E-244	4.00835705765581	0.197	0.015	3.20446179137451E-240	Hepatocytes	ANKS1B
2.79286223250904E-244	1.63893155533117	0.263	0.054	4.68027852923865E-240	Hepatocytes	WWC2
3.2451768824347E-244	1.67099763354076	0.286	0.068	5.43826741958407E-240	Hepatocytes	TSTD1
1.22387976408547E-243	1.38297950912415	0.303	0.082	2.05097770865443E-239	Hepatocytes	ARHGEF40
4.35809757265954E-243	2.8244567871059	0.232	0.037	7.30329991226286E-239	Hepatocytes	ALDH4A1
5.31219061982186E-243	1.0770128639779	0.327	0.097	8.90216904069747E-239	Hepatocytes	PRKAG2
8.53196049429547E-243	0.882083420677258	0.372	0.131	1.42978593963403E-238	Hepatocytes	PAPSS2
8.62344779445645E-243	1.94687586663372	0.289	0.072	1.44511738139501E-238	Hepatocytes	NIPSNAP1
2.42865345466019E-242	2.19167289720547	0.254	0.05	4.06993745931954E-238	Hepatocytes	MAP7
2.54014573957701E-242	3.41080721043279	0.206	0.021	4.25677623038315E-238	Hepatocytes	CES3
3.73404702587169E-242	2.99794922878923	0.21	0.022	6.25751600595578E-238	Hepatocytes	ARG1
3.77075574817729E-242	1.10604057154667	0.358	0.119	6.3190324827955E-238	Hepatocytes	RTL8C
4.9469151380911E-242	1.14979417144954	0.336	0.106	8.29004038841307E-238	Hepatocytes	DKC1

4.96473401573111E-242	2.2985588617328	0.222	0.03	8.31990126356219E-238	Hepatocytes	RORC
7.29777653165248E-242	2.47966557354667	0.224	0.031	1.22296139117432E-237	Hepatocytes	MAP3K21
7.67140618261683E-242	1.8514769456707	0.255	0.051	1.28557424808293E-237	Hepatocytes	PRELID2
1.21531179139989E-241	1.48064256043305	0.286	0.069	2.03661950002794E-237	Hepatocytes	VAV2
2.77303455698378E-241	0.720823866002113	0.4	0.147	4.64705131059342E-237	Hepatocytes	PWWP3A
3.57909296511262E-241	0.922255374239736	0.458	0.199	5.99784399093572E-237	Hepatocytes	DDRGK1
6.41099758935589E-241	0.928691645731248	0.44	0.185	1.07435497602426E-236	Hepatocytes	ATP5ME
7.93942783265243E-241	3.62767843605052	0.192	0.013	1.33048931619589E-236	Hepatocytes	RDH16
9.17337506239074E-241	3.23310169386392	0.21	0.024	1.53727419295544E-236	Hepatocytes	ADH4
1.82310391747064E-240	0.645409286292643	0.575	0.277	3.0551575448973E-236	Hepatocytes	ETS2
1.94122655816833E-240	0.915468238454933	0.349	0.113	3.25310746617848E-236	Hepatocytes	KIAA0232
2.86294525750279E-240	1.01459667046431	0.451	0.193	4.79772366252318E-236	Hepatocytes	UQCRC1
3.19608665519042E-240	1.58976591418188	0.325	0.101	5.3560020167681E-236	Hepatocytes	MSRB2
3.37217848717595E-240	1.2007965541455	0.369	0.13	5.65109670880946E-236	Hepatocytes	ZNHIT1
3.94411829223837E-240	2.68712717063	0.237	0.04	6.60955343413306E-236	Hepatocytes	FGF13
4.14879339748205E-240	2.10503229143269	0.224	0.031	6.95254797550042E-236	Hepatocytes	BAMBI
5.30066674932386E-240	2.73995930052693	0.233	0.038	8.88285733851692E-236	Hepatocytes	SORD
6.18620350091888E-240	2.90566569137532	0.209	0.023	1.03668398268399E-235	Hepatocytes	APOM
1.25609733723996E-239	1.134367182776	0.323	0.097	2.10496791774672E-235	Hepatocytes	CXorf56
1.27157171033005E-239	0.971067260548782	0.464	0.203	2.1308998721711E-235	Hepatocytes	ATOX1
2.04203657889049E-239	1.85624592586179	0.289	0.074	3.42204489890468E-235	Hepatocytes	AKR7A2
2.22264337590383E-239	3.32094970116584	0.2	0.018	3.72470576933963E-235	Hepatocytes	SUSD4
5.67488254737495E-239	1.27148492419349	0.396	0.154	9.50996817289095E-235	Hepatocytes	SDHB
7.91953724101408E-239	2.44166037198892	0.231	0.037	1.32715605084914E-234	Hepatocytes	METTL7B
1.36474494625807E-238	1.86652144674748	0.278	0.067	2.28703958093927E-234	Hepatocytes	MMAB
1.7421065930685E-238	1.00169552058236	0.269	0.057	2.9194222286642E-234	Hepatocytes	SHC2
1.99532325257883E-238	1.67186163969634	0.318	0.095	3.3437627066716E-234	Hepatocytes	TMEM141
3.94988146571214E-238	1.77111993160436	0.293	0.079	6.6192113602404E-234	Hepatocytes	AP1S1
1.88196940448302E-237	0.848261825550304	0.404	0.156	3.15380432803265E-233	Hepatocytes	EDEM3
3.75334525522836E-237	1.94137017349065	0.267	0.06	6.28985597871169E-233	Hepatocytes	GSTM4
4.03871137916708E-237	1.00579333757436	0.371	0.131	6.7680725292082E-233	Hepatocytes	TFG
5.24190332048426E-237	1.14983007517374	0.367	0.13	8.78438158446753E-233	Hepatocytes	IDI1
6.11574184852589E-237	2.11001579995661	0.22	0.03	1.02487601897597E-232	Hepatocytes	PLPP2
6.4700032884964E-237	1.1817495864729	0.329	0.099	1.08424315108623E-232	Hepatocytes	OCIAD2
7.09423374284675E-237	0.563974468718323	0.436	0.174	1.18885169062626E-232	Hepatocytes	PPP2R5A
2.7349886022047E-236	1.03271973968655	0.317	0.093	4.58329389957464E-232	Hepatocytes	NREP
2.79957227784462E-236	1.04726313432483	0.407	0.166	4.69152322321201E-232	Hepatocytes	ACSL3
2.99072035714351E-236	0.698361298323338	0.506	0.239	5.01184917450109E-232	Hepatocytes	NDUFA3
8.99301274975023E-236	0.436686135889038	0.49	0.215	1.50704907660314E-231	Hepatocytes	SHC1
1.05122748487983E-235	1.15897335049336	0.357	0.125	1.76164701916162E-231	Hepatocytes	CISD2
1.43701659690503E-235	0.313880528591613	0.302	0.075	2.40815241309346E-231	Hepatocytes	BCAR1
1.90099218019651E-235	0.797421604135813	0.514	0.246	3.18568269557332E-231	Hepatocytes	ERGIC3
2.9966371423982E-235	2.99515930910215	0.211	0.026	5.0217645232309E-231	Hepatocytes	FRRS1
6.38905499356154E-235	2.72688930225326	0.229	0.037	1.07067783582104E-230	Hepatocytes	SFN
8.45669096567126E-235	1.13861798107756	0.368	0.13	1.41717227202719E-230	Hepatocytes	RAB13
8.98114077153625E-235	1.06554181193568	0.39	0.147	1.50505957049404E-230	Hepatocytes	NUCB2
1.08927794956177E-234	0.999172928768691	0.415	0.167	1.82541198787562E-230	Hepatocytes	SHARPIN
1.29924115514151E-234	2.15642941864139	0.26	0.057	2.17726832778615E-230	Hepatocytes	ECHDC3
1.35653988436124E-234	2.15114833685228	0.254	0.053	2.27328953821256E-230	Hepatocytes	PSPH
1.42901266077877E-234	1.0929885393352	0.38	0.14	2.39473941693307E-230	Hepatocytes	SEM1
1.78451437849463E-234	1.84837735486199	0.291	0.078	2.99048919548131E-230	Hepatocytes	LRP3
1.99877973682033E-234	1.12215397694993	0.413	0.168	3.3495550829635E-230	Hepatocytes	COX8A
3.61163602583703E-234	2.94672883695621	0.215	0.029	6.0523796520977E-230	Hepatocytes	SLC27A5
5.12949482262119E-234	3.72568864667768	0.189	0.014	8.5960074237486E-230	Hepatocytes	PLA2G12B
8.77355411201804E-234	1.59905818907955	0.282	0.071	1.47027219809198E-229	Hepatocytes	MTSS2
1.96823466081808E-233	0.628457563373595	0.669	0.375	3.29836764459894E-229	Hepatocytes	LMAN2
2.17988168014139E-233	2.60635196853332	0.206	0.024	3.65304571958094E-229	Hepatocytes	SOX9
4.18931237023694E-233	3.50822767646789	0.19	0.014	7.02044967004306E-229	Hepatocytes	PIK3C2G
8.72889480185597E-233	2.26734569595344	0.217	0.03	1.46278819089502E-228	Hepatocytes	LAD1
1.29370804290527E-232	0.976602637375561	0.435	0.184	2.16799593830065E-228	Hepatocytes	EDF1
1.42580987442072E-232	2.86105357605996	0.201	0.021	2.38937218755424E-228	Hepatocytes	AKR1C1
5.30784009386388E-232	2.77982247869005	0.21	0.027	8.89487842929709E-228	Hepatocytes	XYLB
5.42216659963278E-232	1.9898094608838	0.303	0.089	9.08646678766461E-228	Hepatocytes	EBNA1BP2

1.01968985824506E-231	1.25041263606459	0.348	0.12	1.70879626444708E-227	Hepatocytes	EMP2
1.58274114212146E-231	1.66619924072865	0.275	0.067	2.65235760596715E-227	Hepatocytes	LRP11
4.82646889134502E-231	0.872665254298401	0.484	0.233	8.08819656811599E-227	Hepatocytes	IRAK1
5.68876417378204E-231	1.43070454462073	0.288	0.075	9.53323100242394E-227	Hepatocytes	TMEM120A
5.69545641340043E-231	1.0581264441634	0.333	0.108	9.54444585757645E-227	Hepatocytes	NUDT9
1.58528608695381E-230	0.733492959013288	0.406	0.156	2.6566224245172E-226	Hepatocytes	POLR2H
1.99716867817456E-230	2.72564581524907	0.219	0.033	3.34685527088493E-226	Hepatocytes	TIGD2
2.21286481245401E-230	1.50505391180387	0.321	0.098	3.70831885271043E-226	Hepatocytes	IGSF8
2.98203124764648E-230	3.48621203924816	0.184	0.012	4.99728796480596E-226	Hepatocytes	MOGAT3
3.67601630888048E-230	0.718224900805381	0.593	0.331	6.16026813042191E-226	Hepatocytes	TM9SF2
4.29508222947697E-230	1.45025271444433	0.298	0.083	7.1976988001575E-226	Hepatocytes	POLR2K
5.54612865651254E-230	3.1726444887633	0.19	0.016	9.29420240258372E-226	Hepatocytes	CYP2J2
1.10688293893789E-229	3.01431473945297	0.195	0.018	1.85491442907212E-225	Hepatocytes	SAA4
2.25096099319638E-229	2.90078115709392	0.22	0.033	3.7721604323985E-225	Hepatocytes	PRSS3
4.98106398750182E-229	0.774636561703258	0.298	0.079	8.34726703025555E-225	Hepatocytes	RALGAPA2
9.71446673318549E-229	2.60347158935455	0.199	0.02	1.62795033514722E-224	Hepatocytes	PLXNB1
1.32223185141743E-228	1.43425193052893	0.251	0.052	2.21579613660533E-224	Hepatocytes	MORC4
3.56877955369566E-228	1.77707169560997	0.267	0.063	5.98056077608318E-224	Hepatocytes	MMUT
5.2046623633563E-228	0.829282593440223	0.266	0.057	8.72197318851249E-224	Hepatocytes	KIAA1217
2.84116458922328E-227	1.07260303310436	0.377	0.141	4.76122361862037E-223	Hepatocytes	TIMM13
5.83154975654499E-227	1.17001974382492	0.295	0.081	9.7725110820181E-223	Hepatocytes	RBM41
6.05977923418976E-227	1.45979870190069	0.331	0.108	1.01549780406552E-222	Hepatocytes	GOT2
6.22934368192835E-227	2.21073749179247	0.204	0.024	1.04391341421755E-222	Hepatocytes	KCNT2
7.2664996848439E-227	0.744700707051501	0.449	0.19	1.21772001718614E-222	Hepatocytes	MYO18A
2.35330225532723E-226	0.744059105189272	0.66	0.381	3.94366391947737E-222	Hepatocytes	MT-ND5
2.81694613918971E-226	1.81172422067789	0.206	0.025	4.72063834005412E-222	Hepatocytes	ART4
4.77670190354041E-226	2.18460189402195	0.253	0.055	8.00479704995302E-222	Hepatocytes	E2F1
8.70347789866653E-226	0.666534295483336	0.264	0.058	1.45852882625854E-221	Hepatocytes	HIP1R
9.22352975512359E-226	0.878970870225626	0.436	0.192	1.54567911636361E-221	Hepatocytes	PYCR2
1.09702850978159E-225	0.776843970346201	0.436	0.187	1.83840037669199E-221	Hepatocytes	NDUFS7
4.37196947049362E-225	1.49828089110226	0.301	0.089	7.3265464386532E-221	Hepatocytes	RHOBTB3
5.83110436104159E-225	2.29879907151676	0.208	0.027	9.77176468823349E-221	Hepatocytes	MYO5C
8.63261725890393E-225	3.1738044056432	0.187	0.016	1.44665400024712E-220	Hepatocytes	KIF12
9.32497939377041E-225	2.70963811678198	0.221	0.036	1.56268004680805E-220	Hepatocytes	DEPRECATED-ENSG00000109084
1.16504063008691E-224	2.30462184036383	0.21	0.028	1.95237508789965E-220	Hepatocytes	FZD6
6.98679561914913E-224	3.32228109473752	0.202	0.025	1.17084720985701E-219	Hepatocytes	DUOXA2
1.06792561382275E-223	1.80689950716223	0.242	0.049	1.78962974364416E-219	Hepatocytes	CKB
1.72361008893774E-223	1.02825370185844	0.429	0.187	2.88842578704187E-219	Hepatocytes	CHMP2A
3.56577126860335E-223	2.401324932011	0.221	0.036	5.97551949192549E-219	Hepatocytes	THNSL2
5.98241781347971E-223	1.417266556714	0.285	0.078	1.00253357718293E-218	Hepatocytes	MMGT1
7.24941089102298E-223	1.26244917380154	0.33	0.11	1.21485627711763E-218	Hepatocytes	FAM162A
1.50192753299958E-222	1.22146199207045	0.387	0.153	2.5169301598007E-218	Hepatocytes	UQCR10
3.72921161049605E-222	0.98124080018345	0.281	0.073	6.24941281686929E-218	Hepatocytes	RAB3IP
5.15061206618558E-222	1.54349480789839	0.25	0.054	8.63139570051379E-218	Hepatocytes	DEPRECATED-ENSG00000176485
7.18148847419201E-222	0.605546702413184	0.703	0.43	1.2034738385051E-217	Hepatocytes	BST2
7.8438302599584E-222	1.6728579678879	0.252	0.056	1.31446907496383E-217	Hepatocytes	DDR1
1.04684588364726E-221	0.585663654743445	0.337	0.11	1.75430433181608E-217	Hepatocytes	TENT5A
2.45236699536029E-221	2.49386335665143	0.225	0.04	4.10967661082477E-217	Hepatocytes	AMACR
3.56456023510798E-221	4.01658880351048	0.177	0.012	5.97349004199396E-217	Hepatocytes	UGT1A3
5.2551900275486E-221	1.86303557656919	0.241	0.049	8.80664744816595E-217	Hepatocytes	CITED4
5.56351102404173E-221	1.21624856998849	0.264	0.063	9.32333177408913E-217	Hepatocytes	ODC1
6.4197392075724E-221	3.22432690437755	0.19	0.019	1.07581989640498E-216	Hepatocytes	CYP2D6
7.78024546450781E-221	0.754302364279917	0.386	0.149	1.30381353494222E-216	Hepatocytes	NAA10
2.05958258380424E-220	1.50714889510771	0.267	0.067	3.45144849393915E-216	Hepatocytes	DONSON
5.05148026622162E-220	1.13087920509708	0.288	0.079	8.46527063013419E-216	Hepatocytes	GNA11
5.8983327697224E-220	1.36611136207133	0.307	0.096	9.8844260555008E-216	Hepatocytes	PIK3R2
6.94950635693676E-220	3.08115333604471	0.229	0.044	1.16459827529546E-215	Hepatocytes	CRP
3.76604084079347E-219	0.801303415861086	0.447	0.201	6.3111312410017E-215	Hepatocytes	PLOD3
4.53814800727848E-219	3.53530177321893	0.179	0.013	7.60502843059727E-215	Hepatocytes	SLC28A1
5.79185896294042E-219	1.49953631647467	0.294	0.085	9.70599725009556E-215	Hepatocytes	GLRX5
1.93707248168722E-218	0.764359677605209	0.429	0.184	3.24614606481145E-214	Hepatocytes	VPS28
2.96140504483273E-218	2.37123313546381	0.213	0.033	4.96272257413069E-214	Hepatocytes	HOMER2
5.45060018863536E-218	1.90421843454308	0.237	0.048	9.13411579611513E-214	Hepatocytes	CLYBL

6.01297412518398E-218	2.43469029627258	0.208	0.03	1.00765420389833E-213	Hepatocytes	FZD5
3.2482897317312E-217	0.537303189871749	0.578	0.335	5.44348393243514E-213	Hepatocytes	NOTCH2NLC
3.2543997693037E-217	1.32947250383631	0.33	0.112	5.45372313339915E-213	Hepatocytes	GLUD1
3.72881800858442E-217	0.727155113874735	0.338	0.114	6.24875321878576E-213	Hepatocytes	CEP57
4.12025432882529E-217	3.15161080539091	0.177	0.013	6.90472220424542E-213	Hepatocytes	HAL
6.70793152261592E-217	1.90344148180503	0.246	0.054	1.12411516455998E-212	Hepatocytes	ACBD4
8.87064810493117E-217	1.2707195738237	0.227	0.04	1.48654320942437E-212	Hepatocytes	CDC42BPA
1.3749082556363E-216	1.03000736941674	0.392	0.159	2.30407125479532E-212	Hepatocytes	UQCR11
2.36368908907205E-216	0.569874603113258	0.552	0.299	3.96107017546694E-212	Hepatocytes	CAPN2
2.43266529384534E-216	1.33996051829136	0.27	0.069	4.07666049942602E-212	Hepatocytes	ETFDH
5.43307161669866E-216	0.448083969655031	0.478	0.218	9.10474141526361E-212	Hepatocytes	NECTIN2
1.54541042741926E-215	1.80965389423843	0.2	0.025	2.58979879426919E-211	Hepatocytes	TEAD3
1.54556070276037E-215	0.497321166618077	0.408	0.167	2.59005062568583E-211	Hepatocytes	COPS5
3.00687493887849E-215	2.90394206847795	0.193	0.023	5.03892102257258E-211	Hepatocytes	GNMT
4.24730464654731E-215	1.31550660215966	0.255	0.058	7.11763312668398E-211	Hepatocytes	GNG12
5.72168426030383E-215	2.34377187305569	0.202	0.027	9.58839848341717E-211	Hepatocytes	SLC4A4
6.76033972894896E-215	0.597503309519228	0.375	0.142	1.13289773177727E-210	Hepatocytes	FAM122B
7.94575931518541E-215	2.47537183843616	0.216	0.037	1.33155034603877E-210	Hepatocytes	TFF3
8.48488133390643E-215	0.857693088623044	0.273	0.07	1.42189641393604E-210	Hepatocytes	SEMA4B
1.0505446026947E-214	1.25411472989611	0.288	0.082	1.76050264519577E-210	Hepatocytes	DCUN1D4
2.45364329932246E-214	1.63244693613045	0.219	0.038	4.11181544100458E-210	Hepatocytes	MID2
2.61587984518899E-214	0.737829547423722	0.436	0.19	4.3836914445677E-210	Hepatocytes	COX7C
3.70375084414918E-214	1.42624810897568	0.301	0.092	6.2067456646252E-210	Hepatocytes	COQ8A
7.27825842507229E-214	1.59176809916477	0.264	0.067	1.21969054687361E-209	Hepatocytes	DEPRECATED-ENSG00000181061
1.18153263543295E-213	0.971208747915991	0.354	0.13	1.98001239045853E-209	Hepatocytes	IVD
1.24810673015452E-213	1.10115599316291	0.23	0.043	2.09157725839295E-209	Hepatocytes	SEMA6A
3.47602511466759E-213	1.36902406406739	0.257	0.061	5.82512288715994E-209	Hepatocytes	FAM8A1
7.11486219986387E-213	0.71887770583178	0.456	0.216	1.19230860745319E-208	Hepatocytes	STT3A
7.93432649210441E-213	0.456243066533577	0.485	0.235	1.32963443354686E-208	Hepatocytes	ZBTB16
9.28183143008948E-213	1.27556865002435	0.323	0.108	1.55544931105439E-208	Hepatocytes	COX7A2
1.35076268061087E-212	1.13377029299063	0.226	0.041	2.2636081001677E-208	Hepatocytes	LRP6
1.40056982586313E-212	1.57134772920572	0.249	0.057	2.34707491418143E-208	Hepatocytes	ZNF219
1.54608856797472E-212	2.04557828066187	0.213	0.035	2.59093522221203E-208	Hepatocytes	MFSD3
4.97936495779957E-212	0.855313694354778	0.302	0.091	8.34441979628052E-208	Hepatocytes	NKAP
6.58281432476297E-212	1.83429978293693	0.246	0.056	1.10314802454378E-207	Hepatocytes	TTC38
1.11257100189018E-211	0.902965698402866	0.346	0.123	1.86444648496756E-207	Hepatocytes	SCRIB
1.69938152724028E-211	1.0472245441878	0.298	0.09	2.84782356334926E-207	Hepatocytes	PDLIM5
2.02346984735928E-211	1.31516790314578	0.219	0.038	3.39093077020468E-207	Hepatocytes	PAWR
2.17040229473371E-211	0.85680434675352	0.338	0.117	3.63716016551476E-207	Hepatocytes	TMEM68
2.27432980408809E-211	0.78973275681609	0.432	0.194	3.81132188569081E-207	Hepatocytes	DNAJC3
3.14227165866581E-211	1.468865270377	0.231	0.045	5.26581884559216E-207	Hepatocytes	DOK4
5.63662854646695E-211	3.18191682995007	0.185	0.02	9.44586211816932E-207	Hepatocytes	SLC23A1
7.78149670335687E-211	2.04458606921116	0.247	0.058	1.30402321754854E-206	Hepatocytes	ALDOC
1.05725365445945E-210	0.992332210096343	0.215	0.035	1.77174567414314E-206	Hepatocytes	NR3C2
1.76323371370518E-210	0.971848224393035	0.312	0.101	2.95482705742714E-206	Hepatocytes	TCEAL1
1.76714819275639E-210	0.746782192678527	0.313	0.1	2.96138694142117E-206	Hepatocytes	SIPA1L3
2.97194416428628E-210	1.75114821536697	0.248	0.056	4.98038403051095E-206	Hepatocytes	ELOVL6
1.15089283547367E-209	1.15488930178582	0.351	0.129	1.92866621368678E-205	Hepatocytes	NDUFAB1
1.27139090038308E-209	0.601210127176014	0.318	0.1	2.13059687086196E-205	Hepatocytes	PTK2
1.87227465495284E-209	2.97055419358821	0.189	0.022	3.13755786676997E-205	Hepatocytes	SSTR1
2.28745904209673E-209	3.05794907242524	0.185	0.02	3.8333238627457E-205	Hepatocytes	GPAM
2.78552900621811E-209	0.57160501330579	0.376	0.145	4.66798950862031E-205	Hepatocytes	DST
4.55937205725227E-209	1.68243593000943	0.204	0.03	7.64059569354335E-205	Hepatocytes	PARD3
4.56352316827642E-209	1.10456474232122	0.324	0.112	7.64755212539763E-205	Hepatocytes	CCDC14
6.42301421540961E-209	0.994412318769952	0.372	0.146	1.07636872221834E-204	Hepatocytes	COX16
7.01854337541115E-209	0.646858667931536	0.291	0.081	1.1761674988514E-204	Hepatocytes	FLNB
7.766697396701E-209	1.64071129799741	0.213	0.035	1.30154314973915E-204	Hepatocytes	IRS1
8.5277189689291E-209	1.56453929755078	0.207	0.032	1.42907514481314E-204	Hepatocytes	ZNF608
2.0821666613906E-208	2.82071005051213	0.196	0.027	3.48929489115836E-204	Hepatocytes	RBP5
4.63741019445677E-208	0.537279120738341	0.366	0.141	7.77137200387065E-204	Hepatocytes	TAZ
5.09803010231773E-208	1.02674126458525	0.268	0.07	8.54327884546406E-204	Hepatocytes	PPIC
6.5641551048756E-208	1.61356041118423	0.239	0.054	1.10002111247505E-203	Hepatocytes	GALE
7.69699312503742E-208	2.39085574926027	0.2	0.029	1.28986210789377E-203	Hepatocytes	PEX11A

1.29871870165742E-207	0.652785620002099	0.451	0.204	2.17639280023751E-203	Hepatocytes	TMED7
1.75228810142404E-207	1.03395232054196	0.279	0.078	2.93648440036641E-203	Hepatocytes	EHBP1
1.76091872520735E-207	0.675190732005738	0.51	0.253	2.95094759970248E-203	Hepatocytes	AKR1A1
1.78699951700955E-207	0.538795846107725	0.385	0.152	2.9946537906046E-203	Hepatocytes	DGAT1
2.08078825979396E-207	0.912362027354205	0.277	0.076	3.48698496576272E-203	Hepatocytes	PALLD
2.3242977559256E-207	0.738548481504269	0.326	0.108	3.89505817938012E-203	Hepatocytes	DPP4
2.55277933439765E-207	1.64427610594949	0.247	0.058	4.27794760858358E-203	Hepatocytes	MACROD1
3.98669341188908E-207	2.04371679644651	0.22	0.042	6.68090081964373E-203	Hepatocytes	TMEM220
4.23317546255929E-207	1.07494960652868	0.333	0.118	7.09395544015686E-203	Hepatocytes	CHP1
5.70551619653159E-207	0.837537198103881	0.37	0.143	9.56130404214764E-203	Hepatocytes	PEX2
6.09791945698929E-207	1.53434394596249	0.32	0.111	1.02188934260226E-202	Hepatocytes	HMGCL
7.18508434031652E-207	0.817204172874174	0.415	0.178	1.20407643375024E-202	Hepatocytes	EIF2AK1
1.13166985611705E-206	1.36416921094871	0.307	0.1	1.89645234488095E-202	Hepatocytes	POLR2I
1.26190342998213E-206	1.64554157792899	0.282	0.083	2.11469776796406E-202	Hepatocytes	POLD2
1.54538682961242E-206	0.37170023178182	0.45	0.201	2.5897592490645E-202	Hepatocytes	PDHA1
2.02546229513015E-206	0.932548525567047	0.36	0.137	3.3942697141791E-202	Hepatocytes	JTB
2.18568172503307E-206	0.596121317017021	0.361	0.138	3.66276543481043E-202	Hepatocytes	GPAT4
3.23265315308059E-206	1.37146286211756	0.281	0.081	5.41728015393245E-202	Hepatocytes	EXOSC4
9.80348903069437E-206	3.05268813853679	0.187	0.022	1.64286869176376E-201	Hepatocytes	ZSWIM5
2.24575664105565E-205	2.11213844501492	0.243	0.059	3.76343897908107E-201	Hepatocytes	DEPRECATED-ENSG00000245848
2.50700494997837E-205	0.410838728105049	0.36	0.137	4.20123889517375E-201	Hepatocytes	KLF3
3.25077077030536E-205	1.66616444634556	0.213	0.038	5.44764165687772E-201	Hepatocytes	PLEKHA5
3.38818860905165E-205	0.756246207777982	0.438	0.199	5.67792647104876E-201	Hepatocytes	CLPTM1
4.16260876063635E-205	2.66999976550562	0.187	0.023	6.97569976107439E-201	Hepatocytes	GAS2
4.70927145815046E-205	2.06801224927037	0.211	0.036	7.89179710956853E-201	Hepatocytes	CD24
5.25309152448997E-205	0.746765075511214	0.289	0.084	8.8031307767403E-201	Hepatocytes	ZFAND1
5.79692767104257E-205	0.485628847528818	0.563	0.295	9.71449139113314E-201	Hepatocytes	CYB5R3
6.07074543006929E-205	1.13936918170254	0.297	0.091	1.01733551917101E-200	Hepatocytes	HIGD1A
7.49826233613155E-205	2.01963881198295	0.197	0.028	1.25655880228893E-200	Hepatocytes	LRIG3
8.81605834477447E-205	2.87152065716473	0.178	0.017	1.47739505741731E-200	Hepatocytes	SALL1
9.06673262127054E-205	0.350884742195663	0.431	0.186	1.51940305267252E-200	Hepatocytes	PTOV1
1.67233291671613E-204	3.26117756357792	0.23	0.054	2.80249550183289E-200	Hepatocytes	AGR2
1.95573562992893E-204	2.28863276081586	0.187	0.022	3.2774217686349E-200	Hepatocytes	USP2
2.16921969544606E-204	3.23598879278987	0.171	0.014	3.63517836562851E-200	Hepatocytes	SERPINA11
2.64352190617542E-204	2.02427815438837	0.188	0.023	4.43001401036877E-200	Hepatocytes	KLHL23
3.1976510404945E-204	1.4563324732854	0.279	0.082	5.35862361366067E-200	Hepatocytes	PID1
3.9179195523537E-204	0.537140444638609	0.517	0.26	6.56564958583433E-200	Hepatocytes	PSMB1
4.82511909623498E-204	1.09918659552657	0.281	0.082	8.08593458147058E-200	Hepatocytes	THOP1
7.24063990402239E-204	2.71216419968932	0.2	0.03	1.21338643511607E-199	Hepatocytes	LCN2
7.63138943257796E-204	1.12777106148421	0.311	0.102	1.27886824111142E-199	Hepatocytes	NDUFB1
8.660869938035E-204	0.444864102492576	0.462	0.216	1.4513885842159E-199	Hepatocytes	CLINT1
1.03466706090551E-203	1.49812502635492	0.283	0.085	1.73389506066545E-199	Hepatocytes	AHCY
1.80322789325152E-203	2.19428766625207	0.207	0.035	3.02184930351089E-199	Hepatocytes	SMO
2.65699586781063E-203	0.614248334288213	0.486	0.234	4.45259367527706E-199	Hepatocytes	COPB2
3.43339804786359E-203	0.855820606315557	0.441	0.203	5.7536884486098E-199	Hepatocytes	ITPK1
4.0742530398215E-203	1.45847575197007	0.291	0.091	6.82763324413287E-199	Hepatocytes	CHAMP1
1.33697492335939E-202	0.732529584466231	0.355	0.135	2.24050257656567E-198	Hepatocytes	CUL4B
1.47463118210458E-202	1.02843675484203	0.266	0.07	2.47118693497085E-198	Hepatocytes	KANK2
1.818382154582E-202	2.63763859263707	0.178	0.018	3.04724481464852E-198	Hepatocytes	FGFR3
1.97968484927798E-202	2.44190143758323	0.187	0.023	3.31755587042004E-198	Hepatocytes	FAM110B
2.36409776100109E-202	0.871676479424556	0.363	0.141	3.96175502788563E-198	Hepatocytes	CPNE3
5.82157453079407E-202	2.31820107142234	0.193	0.027	9.7557945987047E-198	Hepatocytes	STXBP6
9.41022950994877E-202	1.57460295039345	0.261	0.07	1.57696626127721E-197	Hepatocytes	DEPRECATED-ENSG00000117691
1.01586758594504E-201	0.723885927983085	0.491	0.241	1.7023909005267E-197	Hepatocytes	HADHA
1.25048849059316E-201	2.69504215370403	0.19	0.026	2.09556861253602E-197	Hepatocytes	AKR1C4
1.35513343053249E-201	1.48093123523173	0.291	0.091	2.27093260288635E-197	Hepatocytes	ALG3
2.32984663438889E-201	1.12537236085368	0.33	0.12	3.9043569899089E-197	Hepatocytes	HPRT1
2.54447299952308E-201	1.44256578803559	0.276	0.08	4.26402785260078E-197	Hepatocytes	HLTF
2.60990242895133E-201	0.764991520644736	0.475	0.227	4.37367449043663E-197	Hepatocytes	TMED5
2.78137620030102E-201	1.08237408070177	0.319	0.109	4.66103023646444E-197	Hepatocytes	MYO19
3.39028487270179E-201	0.574489438143869	0.395	0.161	5.68143938967365E-197	Hepatocytes	ARHGAP35
4.23516093245917E-201	0.758829089922071	0.333	0.119	7.09728269061508E-197	Hepatocytes	TP53BP2
5.95452077409748E-201	1.34102226999009	0.262	0.07	9.97858591323256E-197	Hepatocytes	BTD

7.16228388749425E-201	3.38802170149069	0.172	0.016	1.20025553386629E-196	Hepatocytes	CHST5
1.05983128829084E-200	0.578161823339916	0.385	0.161	1.77606527291778E-196	Hepatocytes	HGSNAT
1.13537378824535E-200	1.80100365235988	0.261	0.072	1.90265939434155E-196	Hepatocytes	GSKIP
4.48538238477143E-200	2.22277023369436	0.187	0.024	7.51660380039996E-196	Hepatocytes	CAP2
1.05051219566589E-199	1.07325921356249	0.27	0.075	1.7604483374969E-195	Hepatocytes	MLH3
1.54029049432389E-199	1.11461373087198	0.323	0.114	2.58121881038798E-195	Hepatocytes	NDUFA5
1.54576293614146E-199	1.12146426577997	0.298	0.097	2.59038952838586E-195	Hepatocytes	HHEX
1.91338324080005E-199	1.40468847388073	0.301	0.099	3.20644763493272E-195	Hepatocytes	COA4
1.98783200507077E-199	0.669421958158013	0.288	0.083	3.3312088740976E-195	Hepatocytes	SARDH
2.03671349113939E-199	1.41363765182834	0.249	0.061	3.41312446845138E-195	Hepatocytes	C1RL
2.73143248363872E-199	1.09734478709492	0.317	0.109	4.57733455608176E-195	Hepatocytes	SNX4
3.31553310917754E-199	2.58370412868613	0.184	0.023	5.55617038435973E-195	Hepatocytes	UGT2B10
4.08221965108649E-199	0.552674175896126	0.34	0.125	6.84098369129074E-195	Hepatocytes	SLC66A2
5.57413796787766E-199	3.36513810284692	0.164	0.011	9.34114040656938E-195	Hepatocytes	APOC4
6.95777575469847E-199	0.457212946485795	0.647	0.35	1.16598406097237E-194	Hepatocytes	BRI3
1.6108645626672E-198	0.556048639059711	0.525	0.267	2.69948683411769E-194	Hepatocytes	NAPA
1.93711795255964E-198	1.09477963122263	0.286	0.087	3.24622226489944E-194	Hepatocytes	RBBP7
2.1923383170444E-198	3.43506996828282	0.177	0.02	3.673920551703E-194	Hepatocytes	PHYHIPL
2.62778155820763E-198	2.08011563288607	0.178	0.019	4.40363633524435E-194	Hepatocytes	POU5F1
2.88938525125413E-198	0.996037643631214	0.266	0.073	4.84203180405167E-194	Hepatocytes	DPY19L4
3.29261035648489E-198	3.46168184741045	0.168	0.015	5.51775643539738E-194	Hepatocytes	C4orf19
3.46002561249949E-198	0.696819412788648	0.293	0.091	5.79831092142665E-194	Hepatocytes	KIF13A
4.00056848158489E-198	0.645001629851117	0.469	0.226	6.70415266143996E-194	Hepatocytes	RER1
5.43789525481536E-198	0.724903197279928	0.376	0.148	9.11282486801957E-194	Hepatocytes	CARHSP1
8.85031259871892E-198	1.60193535254199	0.206	0.036	1.48313538529332E-193	Hepatocytes	HHAT
1.04890781562407E-197	1.76286349298833	0.235	0.054	1.75775971742282E-193	Hepatocytes	SLC25A23
1.17876063376671E-197	1.06879833233398	0.276	0.079	1.97536707006626E-193	Hepatocytes	GET1
1.4663030289681E-197	0.960464153828849	0.303	0.099	2.45723061594475E-193	Hepatocytes	WASL
2.2324847450448E-197	3.69543112369042	0.156	0.008	3.74119793574608E-193	Hepatocytes	ACOT12
5.11655260094357E-197	0.56026446960011	0.355	0.135	8.57431884866124E-193	Hepatocytes	GMDS
6.77884642576885E-197	0.74996593152469	0.297	0.095	1.13599908403034E-192	Hepatocytes	TMEM214
1.37885112256522E-196	0.668678003144905	0.351	0.132	2.31067871119479E-192	Hepatocytes	TMEM41B
3.93522880059366E-196	0.936308839448911	0.274	0.079	6.59465642403485E-192	Hepatocytes	NFS1
5.07639946786579E-196	2.11567459124085	0.223	0.048	8.50703022824949E-192	Hepatocytes	ALDH7A1
6.76609099486501E-196	0.78774342381518	0.431	0.198	1.13386152891948E-191	Hepatocytes	CANX
9.17312912308387E-196	0.51783257328075	0.372	0.147	1.5372329784464E-191	Hepatocytes	NCOA2
1.16670601922199E-195	1.03344972433347	0.3	0.099	1.95516594701221E-191	Hepatocytes	NDUFB6
2.56342582221831E-195	0.526204795295275	0.579	0.32	4.29578899287345E-191	Hepatocytes	HNRNPF
3.27143155743666E-195	2.75135244156157	0.169	0.016	5.48226500395235E-191	Hepatocytes	THSD4
5.0454447649984E-195	0.861689704122741	0.316	0.11	8.45515633718432E-191	Hepatocytes	RAPH1
5.22827706201304E-195	3.35251484559855	0.166	0.015	8.76154670052146E-191	Hepatocytes	ABCG8
1.13290068941668E-194	1.88525364798111	0.231	0.054	1.89851497532448E-190	Hepatocytes	NXT2
1.66172528813549E-194	1.18828090870906	0.236	0.056	2.78471923785746E-190	Hepatocytes	PHLPP2
2.42085117399025E-194	0.931756978167935	0.276	0.078	4.05686239737285E-190	Hepatocytes	KIAA1671
2.82283624228871E-194	1.46278581699781	0.194	0.03	4.73050897482742E-190	Hepatocytes	SLC45A3
3.38142823146326E-194	1.87058051414313	0.204	0.036	5.66659743028613E-190	Hepatocytes	TMEM98
3.89472157114326E-194	0.699856007131763	0.582	0.374	6.52677440892188E-190	Hepatocytes	FKBP5
4.41446014674207E-194	1.08268774403106	0.329	0.122	7.39775231391035E-190	Hepatocytes	DNAJB9
6.62335663347628E-194	1.66187590118038	0.218	0.044	1.10994210463795E-189	Hepatocytes	RAB15
7.47640986876337E-194	1.95167011982797	0.228	0.053	1.25289676580737E-189	Hepatocytes	ACY1
8.58049512386368E-194	0.721948968218339	0.352	0.137	1.43791937285708E-189	Hepatocytes	LYPLAL1
8.76602104278608E-194	2.88368248441144	0.226	0.054	1.46900980635009E-189	Hepatocytes	TFF1
9.28668927988984E-194	1.50078719694999	0.286	0.091	1.55626338952394E-189	Hepatocytes	TKFC
1.00536149532826E-193	1.6056494972584	0.229	0.051	1.6847847938711E-189	Hepatocytes	TRMT11
1.01686787476967E-193	0.770971480018003	0.453	0.216	1.70406718453902E-189	Hepatocytes	UBE2Q1
1.04267745233877E-193	3.20020234192025	0.168	0.016	1.74731887462931E-189	Hepatocytes	NIPAL1
1.08111596508269E-193	0.937786822491188	0.374	0.154	1.81173413428557E-189	Hepatocytes	NDUFB5
1.35087910797216E-193	0.511819326503078	0.446	0.203	2.26380320913974E-189	Hepatocytes	LMAN1
2.22093436544209E-193	0.625910886816314	0.459	0.22	3.72184180960786E-189	Hepatocytes	SLC25A39
2.30809125535353E-193	1.50902770797058	0.226	0.05	3.86789932572145E-189	Hepatocytes	UGGT2
4.3320269259989E-193	2.19823182844564	0.201	0.035	7.25961072258895E-189	Hepatocytes	NUDT7
6.52192107161172E-193	0.267114994423323	0.475	0.227	1.09294353318069E-188	Hepatocytes	PPP1R15B
9.67520107683013E-193	1.37103366653781	0.261	0.074	1.62137019645519E-188	Hepatocytes	CNIH1

1.21067165552325E-192	1.48523240595804	0.245	0.063	2.02884356032586E-188	Hepatocytes	SFXN5
3.01319730933131E-192	2.77823900934005	0.179	0.022	5.04951605097741E-188	Hepatocytes	ITPKA
3.65426590206834E-192	1.64563719749832	0.226	0.051	6.12381879868613E-188	Hepatocytes	CCHCR1
5.43298110214691E-192	0.395691238509893	0.609	0.34	9.1045897309778E-188	Hepatocytes	ACADVL
6.38819409955707E-192	0.435855195648894	0.651	0.384	1.07053356720377E-187	Hepatocytes	PSME1
7.50481491244968E-192	2.9390289032916	0.197	0.035	1.25765688302832E-187	Hepatocytes	HAMP
1.20994637972431E-191	3.00817933401584	0.175	0.021	2.027628143142E-187	Hepatocytes	TINAG
1.3799964281604E-191	2.78992718741987	0.167	0.016	2.31259801431119E-187	Hepatocytes	TRIM55
1.80095466313451E-191	1.13661879761853	0.328	0.123	3.01803982448081E-187	Hepatocytes	HEBP1
3.13450500415308E-191	1.05385626491684	0.288	0.09	5.25280348595973E-187	Hepatocytes	FBXL6
3.31570121465612E-191	1.41743005857925	0.25	0.066	5.55645209552073E-187	Hepatocytes	DHRS1
4.95410272331905E-191	2.79248994479773	0.17	0.018	8.30208534373806E-187	Hepatocytes	PTPRD
5.46787549815123E-191	1.10366364084094	0.217	0.045	9.16306575980183E-187	Hepatocytes	ENPEP
5.69405218574637E-191	0.550705228337736	0.419	0.186	9.54209265287377E-187	Hepatocytes	EIF4G1
6.17242450376192E-191	0.843508434757049	0.294	0.094	1.03437489834042E-186	Hepatocytes	LIME1
6.5632356912018E-191	0.846015847685116	0.42	0.196	1.0998670371316E-186	Hepatocytes	RNPEP
8.82057991981126E-191	0.572282064338057	0.518	0.265	1.47815278296197E-186	Hepatocytes	MAN1A1
9.55632706748006E-191	1.08263874153121	0.321	0.115	1.60144928996831E-186	Hepatocytes	TDP2
1.59967455607722E-190	0.703929205949825	0.27	0.079	2.68073462107421E-186	Hepatocytes	SC5D
1.76282443738986E-190	0.889316744394793	0.371	0.152	2.95414119217793E-186	Hepatocytes	SIAH2
2.42861380234622E-190	2.45170035937146	0.183	0.026	4.0698710099718E-186	Hepatocytes	MMP28
4.7060390302834E-190	0.285897716018473	0.538	0.294	7.88638020694891E-186	Hepatocytes	GDI1
4.84398844042007E-190	3.8791685531742	0.157	0.012	8.11755582845595E-186	Hepatocytes	GOLT1A
4.90215889058464E-190	2.6709171260057	0.175	0.021	8.21503786884175E-186	Hepatocytes	PAQR9
4.93677018697963E-190	1.85434244996647	0.242	0.063	8.27303947934046E-186	Hepatocytes	C19orf48
5.70578017629423E-190	1.09933422524891	0.268	0.08	9.56174641943386E-186	Hepatocytes	ZMAT1
7.93362639039397E-190	0.8061386081953	0.404	0.178	1.32951711050222E-185	Hepatocytes	IARS2
8.47279172310782E-190	1.16585270843991	0.324	0.121	1.41987043695841E-185	Hepatocytes	NDUFS6
1.05121373761925E-189	0.575428526338733	0.514	0.269	1.76162398150233E-185	Hepatocytes	MTDH
1.14895877244059E-189	1.16363074912879	0.215	0.043	1.92542511085593E-185	Hepatocytes	CYP7B1
1.24361622434906E-189	0.977302796319291	0.332	0.126	2.08405206876416E-185	Hepatocytes	C1orf43
1.75170889028766E-189	0.956060591527991	0.339	0.128	2.93551375834406E-185	Hepatocytes	LRRC59
2.18625725157604E-189	0.53006167281589	0.384	0.158	3.66372990219113E-185	Hepatocytes	LIMK2
2.29859121184048E-189	2.21128641122582	0.172	0.019	3.85197915280228E-185	Hepatocytes	SLC12A8
4.06927546783164E-189	0.821926906991625	0.334	0.125	6.81929182899226E-185	Hepatocytes	MOCS2
9.69139090339565E-189	3.09605931872275	0.176	0.022	1.62408328759104E-184	Hepatocytes	SQLE
1.35079633501222E-188	0.507951039031055	0.341	0.128	2.26366449821348E-184	Hepatocytes	SEPTIN8
1.35552196913389E-188	0.810296397326236	0.411	0.187	2.27158371587457E-184	Hepatocytes	ATP5MC3
1.84781347692138E-188	1.38670753370417	0.28	0.089	3.09656582462486E-184	Hepatocytes	CCT3
3.0433058900858E-188	0.553149972369033	0.49	0.248	5.09997201060578E-184	Hepatocytes	INF2
5.54845616675606E-188	1.23435700975673	0.297	0.101	9.29810284424981E-184	Hepatocytes	PRDX5
6.95405430228447E-188	1.25961258396218	0.281	0.09	1.16536041997683E-183	Hepatocytes	NDUFA7
8.74194660019339E-188	2.09604926206152	0.211	0.044	1.46497541126041E-183	Hepatocytes	MYO5B
1.19250506825113E-187	0.320232207406861	0.291	0.091	1.99839999337525E-183	Hepatocytes	MYLK
1.50468026831342E-187	1.96321590494817	0.22	0.05	2.52154319363964E-183	Hepatocytes	IPMK
1.87387419057596E-187	2.97941128165121	0.17	0.02	3.14023836856719E-183	Hepatocytes	ANKS4B
3.18875859772655E-187	1.02737349207694	0.345	0.137	5.34372165807015E-183	Hepatocytes	GUCD1
3.31890117612809E-187	0.642408901158374	0.303	0.104	5.56181459095546E-183	Hepatocytes	SEC23A
3.34948791189403E-187	3.52427249403673	0.157	0.012	5.61307184275201E-183	Hepatocytes	DEPRECATED-ENSG00000125675
4.23704448403323E-187	0.653848499470072	0.513	0.267	7.1004391463429E-183	Hepatocytes	MYDGF
5.43584615467551E-187	0.879516441758336	0.197	0.034	9.10939098600521E-183	Hepatocytes	VIPR1
6.7838128485759E-187	1.31238295742861	0.283	0.091	1.13683135716435E-182	Hepatocytes	MDH2
8.14693041982997E-187	0.379305332114734	0.644	0.342	1.36526259975511E-182	Hepatocytes	GRINA
8.38288931101517E-187	1.76184726547029	0.184	0.027	1.40480459073992E-182	Hepatocytes	FBXO17
9.62633163170517E-187	3.22278650417825	0.166	0.017	1.61318065484115E-182	Hepatocytes	BCHE
9.83909491114618E-187	1.74504787349703	0.249	0.07	1.64883552520988E-182	Hepatocytes	DCPS
1.61564661641763E-186	2.30348208039469	0.181	0.026	2.70750059979266E-182	Hepatocytes	DEFB1
2.55353103407208E-186	3.31778832760201	0.153	0.01	4.27920730689799E-182	Hepatocytes	SLC22A7
3.27622640366855E-186	1.00318933999769	0.285	0.092	5.49030020726775E-182	Hepatocytes	TRIM24
4.45058027699739E-186	0.839048838460397	0.403	0.18	7.45828242819223E-182	Hepatocytes	SNRPC
8.93484681572077E-186	0.88463516594832	0.269	0.08	1.49730162937849E-181	Hepatocytes	LRRC14
1.18301508797978E-185	0.988950252131375	0.286	0.095	1.98249668443651E-181	Hepatocytes	ST3GAL6
1.19174334779663E-185	0.351968845836789	0.369	0.148	1.99712350223759E-181	Hepatocytes	DLG1

1.36955227047179E-185	0.300510065848958	0.36	0.14	2.29509569485662E-181	Hepatocytes	ZBTB20
1.41395394919873E-185	2.75566499436414	0.173	0.022	2.36950402806724E-181	Hepatocytes	CGNL1
2.41696691452584E-185	3.37659616315887	0.155	0.012	4.0503531553624E-181	Hepatocytes	GPD1
2.48143513137995E-185	2.33663793592823	0.178	0.024	4.15838899316652E-181	Hepatocytes	ACTR3C
2.99045606032012E-185	1.7855349996419	0.196	0.034	5.01140626588446E-181	Hepatocytes	CPLX1
3.01679181294079E-185	0.988293094875278	0.365	0.153	5.05553972012618E-181	Hepatocytes	RPN1
5.40355108969025E-185	3.4392800576483	0.159	0.014	9.05527091610292E-181	Hepatocytes	SOWAHB
5.4225267215756E-185	0.528129976329625	0.498	0.28	9.08707028001638E-181	Hepatocytes	ALG13
6.1675710592284E-185	3.46150558360772	0.154	0.011	1.03356155810549E-180	Hepatocytes	ABCC6
8.84286389223865E-185	0.608312717526016	0.536	0.294	1.48188713106135E-180	Hepatocytes	CALM3
2.15707975467968E-184	3.58584143385029	0.151	0.009	3.6148342528922E-180	Hepatocytes	MASP2
2.19590203295566E-184	0.838699067129259	0.28	0.089	3.67989262682709E-180	Hepatocytes	PHF6
2.79764576844076E-184	1.02084463028087	0.346	0.139	4.68829477875303E-180	Hepatocytes	AGPAT2
4.21593156930357E-184	1.07836477277365	0.273	0.083	7.06505812383893E-180	Hepatocytes	ROBO1
4.72211051788821E-184	0.759279489339071	0.389	0.171	7.91331280587706E-180	Hepatocytes	HEXB
4.744241292107E-184	3.47456930879466	0.154	0.012	7.9503995573129E-180	Hepatocytes	C9
5.06152636662373E-184	1.85555679942699	0.185	0.028	8.48210588518804E-180	Hepatocytes	PFN2
5.46095608756661E-184	0.67654201980647	0.426	0.198	9.15147021154412E-180	Hepatocytes	SERP1
6.72097995713744E-184	1.10332007965724	0.278	0.088	1.12630182121709E-179	Hepatocytes	TMEM161A
1.01818484690978E-183	0.588845351644577	0.373	0.158	1.70627416645141E-179	Hepatocytes	POGK
1.05643922888455E-183	0.264737303793935	0.508	0.263	1.77038085976473E-179	Hepatocytes	NDUFV2
1.46715880334294E-183	0.409906046016708	0.342	0.127	2.4586647226421E-179	Hepatocytes	NDRG2
1.36665078943189E-182	0.398144283655418	0.423	0.192	2.29023339292996E-178	Hepatocytes	TMPO
1.54190895054646E-182	3.31826698414227	0.164	0.018	2.58393101932575E-178	Hepatocytes	CDHR2
1.63986503240449E-182	1.2414075752338	0.221	0.05	2.74808582130345E-178	Hepatocytes	OTUD7B
2.49357174379377E-182	1.44319059144897	0.239	0.064	4.17872752824959E-178	Hepatocytes	BCAT2
2.55837928820363E-182	2.09996372103107	0.209	0.045	4.28733201117165E-178	Hepatocytes	MSRB1
2.65465415150611E-182	1.2014158423049	0.293	0.1	4.44866942709395E-178	Hepatocytes	MCM2
2.75916263343757E-182	0.791069505483803	0.358	0.147	4.62380474111467E-178	Hepatocytes	NAA60
2.95950942485852E-182	1.32976156480946	0.245	0.067	4.9595458941779E-178	Hepatocytes	MCCC2
3.54545174060908E-182	0.562098644205499	0.544	0.29	5.9414680269127E-178	Hepatocytes	PRKACA
6.15082021552123E-182	0.690525117745436	0.327	0.123	1.03075445171705E-177	Hepatocytes	NIBAN2
7.4535968586427E-182	1.1232660406339	0.3	0.108	1.24907376157134E-177	Hepatocytes	ME1
9.5483266857358E-182	0.574814895311833	0.406	0.186	1.60010858599561E-177	Hepatocytes	RREB1
1.05263388631669E-181	1.09874137050149	0.254	0.072	1.76400386668951E-177	Hepatocytes	EBAG9
1.10048055136288E-181	1.27989678252332	0.288	0.098	1.84418530797391E-177	Hepatocytes	DEPRECATED-ENSG00000132646
1.47079427854061E-181	2.92870989215488	0.161	0.016	2.46475705197836E-177	Hepatocytes	HPD
1.50273848270734E-181	0.631829913851175	0.443	0.212	2.51828914932096E-177	Hepatocytes	DNAJB11
1.53540072612339E-181	1.78860816951615	0.2	0.039	2.57302453683758E-177	Hepatocytes	VSIG10
1.89885796245206E-181	1.62831832654631	0.212	0.046	3.18210617347717E-177	Hepatocytes	NDUFAF6
2.18366440414276E-181	0.555125184823978	0.455	0.224	3.65938480846244E-177	Hepatocytes	TMEM33
2.30042069030705E-181	3.17209265240775	0.181	0.029	3.85504499281655E-177	Hepatocytes	ACSM5
2.57392236326208E-181	0.507526187347542	0.343	0.134	4.31337909635459E-177	Hepatocytes	CD2AP
2.99517601706403E-181	2.9575240111124	0.171	0.022	5.0193159693959E-177	Hepatocytes	CLGN
5.6240462603694E-181	2.97998109560363	0.164	0.018	9.42477672312705E-177	Hepatocytes	ADGRV1
6.79242804619256E-181	0.540605402607813	0.355	0.142	1.13827509198095E-176	Hepatocytes	SHFL
1.25074677194178E-180	0.464874150444018	0.401	0.178	2.09600144042004E-176	Hepatocytes	ACBD3
1.40730880530291E-180	2.6208141664931	0.17	0.022	2.35836809592661E-176	Hepatocytes	RHOD
1.56873345677378E-180	2.85053827864434	0.171	0.023	2.6288835268615E-176	Hepatocytes	PPP1R14D
2.52048722658189E-180	3.04049860934385	0.154	0.013	4.22383249430594E-176	Hepatocytes	DSG2
2.75444154412066E-180	0.856877576772171	0.308	0.11	4.6158931396374E-176	Hepatocytes	NDUFA11
3.15213778194282E-180	1.32333011065647	0.186	0.03	5.28235249497978E-176	Hepatocytes	TMEM8B
3.73072896840115E-180	1.60766760543055	0.26	0.08	6.25195560524665E-176	Hepatocytes	CPT2
4.10416088781565E-180	0.640535524537157	0.461	0.228	6.87775281580147E-176	Hepatocytes	BLOC1S1
7.8048208160603E-180	0.342968189170289	0.47	0.229	1.30793187235539E-175	Hepatocytes	PHPT1
1.07422943461253E-179	1.74674948030882	0.229	0.058	1.80019368652367E-175	Hepatocytes	FAHD1
1.08254539435109E-179	2.98988024780178	0.153	0.012	1.81412957185355E-175	Hepatocytes	SLC38A4
1.69882881363471E-179	3.0009905222163	0.166	0.02	2.84689732588905E-175	Hepatocytes	ALDH3A1
4.21801752705514E-179	0.715855211987024	0.284	0.093	7.06855377183901E-175	Hepatocytes	CCT8
6.27855181631945E-179	2.63066112377154	0.178	0.027	1.05215971337881E-174	Hepatocytes	C6orf132
1.09837426534487E-178	0.822727832796952	0.384	0.169	1.84065559386493E-174	Hepatocytes	PSMC3
1.13209403935172E-178	1.47498853873567	0.268	0.086	1.89716319114562E-174	Hepatocytes	DHFR
1.18400709973585E-178	1.73109344151516	0.254	0.078	1.98415909773735E-174	Hepatocytes	TCF19

2.23150714935494E-178	0.574463339719313	0.404	0.185	3.73955968088901E-174	Hepatocytes	MMP24OS
2.47410900503135E-178	3.12806546836805	0.157	0.016	4.14611187063153E-174	Hepatocytes	B4GALNT2
3.27350742986727E-178	1.17689050007789	0.3	0.108	5.48574375097158E-174	Hepatocytes	CKS1B
3.89215172401553E-178	0.666626884997375	0.335	0.129	6.52246785910522E-174	Hepatocytes	FAM20C
3.98417271741354E-178	3.07835842205274	0.155	0.014	6.67667663984162E-174	Hepatocytes	SLC1A1
4.07139980364106E-178	3.70813612978599	0.142	0.007	6.82285179094168E-174	Hepatocytes	PFKFB1
5.78935262969107E-178	0.69668201713977	0.453	0.223	9.70179713683629E-174	Hepatocytes	SF3B5
7.30589255442733E-178	0.471374922355886	0.369	0.156	1.22432147427093E-173	Hepatocytes	INTS8
9.54709630819062E-178	0.56021242782482	0.365	0.153	1.59990239932658E-173	Hepatocytes	VDAC3
1.24317747631781E-177	0.997571861502374	0.291	0.1	2.08331681481339E-173	Hepatocytes	PRPSAP1
1.56138285038282E-177	0.913964045782931	0.239	0.063	2.61656538067152E-173	Hepatocytes	GLI4
1.58455900863768E-177	1.12287102579679	0.321	0.123	2.65540398667502E-173	Hepatocytes	STOML2
1.80167354493734E-177	1.13609648552747	0.187	0.031	3.01924452660599E-173	Hepatocytes	HYAL1
1.80483841698705E-177	1.62499409938669	0.213	0.048	3.0245482191869E-173	Hepatocytes	BAG2
4.55054430756752E-177	0.700591209302351	0.361	0.153	7.62580215062165E-173	Hepatocytes	UBL3
4.87865459889254E-177	1.54407059209028	0.236	0.065	8.17564937682412E-173	Hepatocytes	COPRS
7.55343153066762E-177	0.295888675256921	0.274	0.085	1.26580405590928E-172	Hepatocytes	PCGF2
8.39333918940075E-177	1.6515398492441	0.235	0.063	1.40655578135978E-172	Hepatocytes	GMNN
1.02095894631573E-176	1.40522322010908	0.244	0.069	1.7109230022359E-172	Hepatocytes	MVK
1.44351441073617E-176	1.21865099299443	0.26	0.081	2.41904144951168E-172	Hepatocytes	CTDSPL
1.45126407096655E-176	0.750693668603631	0.365	0.155	2.43202833012574E-172	Hepatocytes	NDUFV3
1.67379085361073E-176	1.8032747895828	0.219	0.053	2.80493871248085E-172	Hepatocytes	CENPX
1.70453333950527E-176	0.632795779050023	0.477	0.246	2.85645697034294E-172	Hepatocytes	SEC61A1
2.62621279337398E-176	1.39892626908904	0.27	0.087	4.40100739913611E-172	Hepatocytes	ATP5MC1
5.0391652423866E-176	0.957731620773595	0.345	0.142	8.44463311319147E-172	Hepatocytes	ATP5PD
6.05917441285291E-176	1.71015016861336	0.241	0.068	1.01539644810589E-171	Hepatocytes	GBE1
6.75124107220424E-176	0.75003521241889	0.356	0.148	1.13137297887999E-171	Hepatocytes	CLTB
6.78178345999345E-176	1.2453991858059	0.301	0.11	1.1364912722257E-171	Hepatocytes	HACD2
8.26270982097527E-176	1.26190783070577	0.215	0.05	1.38466491179903E-171	Hepatocytes	MTERF3
8.97997324274577E-176	1.88752610062848	0.17	0.023	1.50486391601934E-171	Hepatocytes	STOX2
1.08980262561382E-175	0.262896742180834	0.361	0.149	1.82629124000365E-171	Hepatocytes	FURIN
1.20987778941806E-175	0.96731148338922	0.317	0.12	2.02751319950679E-171	Hepatocytes	SKP1
2.09020460099291E-175	2.20797227759634	0.166	0.022	3.50276487034392E-171	Hepatocytes	MTMR11
2.7540313895269E-175	1.65802959379955	0.223	0.056	4.61520580256917E-171	Hepatocytes	WEE1
4.97253332506011E-175	0.908083455486193	0.307	0.113	8.33297134613573E-171	Hepatocytes	COX6B1
4.99535594452021E-175	3.38543283111814	0.154	0.015	8.37121749182697E-171	Hepatocytes	B3GALT5
5.98820030019797E-175	0.931154926279855	0.264	0.082	1.00350260630718E-170	Hepatocytes	ACACA
8.83275934018211E-175	1.63788336423662	0.193	0.037	1.48019381022772E-170	Hepatocytes	EDARADD
1.11989698642379E-174	0.456874610366333	0.39	0.175	1.87672336984899E-170	Hepatocytes	AMFR
2.06386676702844E-174	0.84840874839048	0.338	0.137	3.45862792818626E-170	Hepatocytes	NDUFB11
2.08831130963521E-174	1.90005322156437	0.177	0.028	3.49959209268668E-170	Hepatocytes	KHDRBS3
2.15461545466452E-174	0.911143085829122	0.305	0.113	3.61070457892681E-170	Hepatocytes	PREB
2.2100242228701E-174	2.34563790824558	0.198	0.041	3.70355859268571E-170	Hepatocytes	UBL4A
2.65824909646504E-174	3.42492243146946	0.143	0.009	4.45469383585612E-170	Hepatocytes	AFM
2.833139201673E-174	0.867942537898342	0.236	0.063	4.74777467416361E-170	Hepatocytes	GCH1
4.75435767887331E-174	0.695160023310858	0.258	0.079	7.96735259825589E-170	Hepatocytes	SLC37A1
6.44344079787456E-174	1.18132786770706	0.25	0.073	1.07979180890782E-169	Hepatocytes	CPT1A
7.16242460385256E-174	3.94822674545755	0.142	0.009	1.20027911511361E-169	Hepatocytes	CHST6
7.33702854027187E-174	1.03946231268219	0.29	0.101	1.22953924277876E-169	Hepatocytes	TCAF1
7.55107063029494E-174	0.70630119645669	0.289	0.099	1.26540841622483E-169	Hepatocytes	TOB2
7.70712426239872E-174	3.15858757653005	0.149	0.012	1.29155988389278E-169	Hepatocytes	TMEM171
8.91321247693832E-174	0.638604211930835	0.37	0.159	1.49367614688532E-169	Hepatocytes	OSBP
1.082033630997E-173	2.05274160859571	0.174	0.027	1.81327195882478E-169	Hepatocytes	NOL3
1.17991276585035E-173	1.77304815177752	0.177	0.028	1.97729781301201E-169	Hepatocytes	SH3D19
1.30531082237255E-173	1.19572882378098	0.274	0.091	2.18743987613191E-169	Hepatocytes	POLR2F
1.6235354061717E-173	0.365357933423988	0.539	0.297	2.72072063366254E-169	Hepatocytes	ARF1
1.81652466495448E-173	0.835747262369118	0.221	0.054	3.04413203353071E-169	Hepatocytes	FBXL8
1.88017253923107E-173	1.37930261924886	0.217	0.053	3.15079314124343E-169	Hepatocytes	CCDC125
1.9343814192163E-173	1.31604527673913	0.194	0.038	3.24163638232267E-169	Hepatocytes	MMP15
2.17779866254166E-173	1.12790773562505	0.199	0.04	3.64955499868732E-169	Hepatocytes	RBFOX2
2.26617597608196E-173	2.04761874899531	0.202	0.044	3.79765770071814E-169	Hepatocytes	PXMP4
2.32126501351697E-173	1.0077930804827	0.282	0.097	3.88997590965173E-169	Hepatocytes	BRI3BP
2.78253113291968E-173	0.50946580389649	0.319	0.12	4.66296567254681E-169	Hepatocytes	LMBR1

**3.72024328362303E-173**	0.566820281957184	0.308	0.112	6.23438369469548E-169	Hepatocytes	FBXO9
**5.74821497628347E-173**	1.3651321883599	0.229	0.059	9.63285865725584E-169	Hepatocytes	RASSF7
**7.9139556759226E-173**	0.778809026594729	0.324	0.127	1.32622069217111E-168	Hepatocytes	RHOU
**1.19872156999543E-172**	1.52741921922709	0.239	0.068	2.00881760699835E-168	Hepatocytes	HINT2
**1.60582828023597E-172**	0.547737108699353	0.319	0.116	2.69104703201943E-168	Hepatocytes	SPTBN1
**2.15683013919599E-172**	1.04071762453426	0.325	0.127	3.61441594726464E-168	Hepatocytes	ORMDL3
**3.13587502087564E-172**	1.09461039859434	0.225	0.058	5.2550993599834E-168	Hepatocytes	DEPRECATED-ENSG00000134597
**3.1715802290972E-172**	0.829183691076452	0.346	0.142	5.31493414792108E-168	Hepatocytes	SCAMP3
**3.76034346915837E-172**	1.29093486816391	0.271	0.089	6.30158358561559E-168	Hepatocytes	HGH1
**4.71280319140905E-172**	0.432651508534369	0.292	0.101	7.89771558816328E-168	Hepatocytes	BMI1
**5.1416364384618E-172**	1.34910799624045	0.191	0.037	8.61635434357427E-168	Hepatocytes	DNAAF2
**9.8620071967262E-172**	0.612005156365089	0.379	0.169	1.65267516602738E-167	Hepatocytes	TXNL4A
**1.01890071476466E-171**	0.993393577783105	0.426	0.213	1.70747381780261E-167	Hepatocytes	PGD
**1.05467600066838E-171**	0.260540465057734	0.477	0.24	1.76742604192007E-167	Hepatocytes	SEC31A
**1.54305459351548E-171**	0.420377456402476	0.441	0.213	2.58585088781324E-167	Hepatocytes	DYNLL2
**1.56305659830553E-171**	0.981492631151078	0.278	0.094	2.61937024744041E-167	Hepatocytes	LMBRD2
**2.40129999296694E-171**	1.53144703660082	0.205	0.046	4.024098528214E-167	Hepatocytes	SLC20A2
**2.45036235124795E-171**	0.823228596220869	0.306	0.114	4.10631722822132E-167	Hepatocytes	ENOSF1
**2.6621389676483E-171**	1.59857970433319	0.179	0.03	4.46121248198502E-167	Hepatocytes	RAVER2
**5.44792824527502E-171**	2.67248221650854	0.165	0.022	9.12963815343188E-167	Hepatocytes	HOXA10
**5.51331797231773E-171**	0.941284023162762	0.333	0.135	9.23921825801005E-167	Hepatocytes	NAA20
**1.25423318292802E-170**	0.87787897330875	0.322	0.127	2.10184396795078E-166	Hepatocytes	MEA1
**1.37308809007063E-170**	2.05875843457556	0.168	0.024	2.30102102134036E-166	Hepatocytes	ARL4D
**2.46726850087434E-170**	2.35031951316463	0.166	0.023	4.13464855376521E-166	Hepatocytes	MYORG
**2.61654184905842E-170**	1.26523554367599	0.259	0.083	4.3848008306521E-166	Hepatocytes	AAMDC
**2.82730728328839E-170**	0.598447494934032	0.288	0.098	4.73800154533468E-166	Hepatocytes	AQP3
**3.47208954442982E-170**	1.10553737945676	0.283	0.099	5.81852765855549E-166	Hepatocytes	LPCAT3
**4.51458184147041E-170**	1.02234583848536	0.338	0.14	7.56553624993611E-166	Hepatocytes	DNPH1
**5.58145667845763E-170**	0.522179054021237	0.318	0.121	9.3534051017593E-166	Hepatocytes	CARS2
**6.64715858773677E-170**	0.56594135049931	0.393	0.177	1.11393083613293E-165	Hepatocytes	IMPAD1
**7.37049376485906E-170**	1.19875430185777	0.223	0.058	1.23514734511508E-165	Hepatocytes	MAPRE3
**8.45829580831986E-170**	0.839263342288466	0.383	0.176	1.41744121155824E-165	Hepatocytes	TMEM147
**1.47315103230655E-169**	0.417020978273683	0.237	0.063	2.46870649993932E-165	Hepatocytes	PLCB1
**1.54592150662263E-169**	1.97587298465181	0.172	0.027	2.59065526079821E-165	Hepatocytes	SOX13
**1.74857045715759E-169**	3.46457393755441	0.143	0.01	2.9302543721047E-165	Hepatocytes	CPS1
**2.27530512676323E-169**	3.14116934735501	0.152	0.016	3.81295633142982E-165	Hepatocytes	C1orf116
**2.65822697420067E-169**	0.845799216887203	0.296	0.106	4.45465676336548E-165	Hepatocytes	PAM16
**2.9980419290652E-169**	0.681794840754574	0.443	0.237	5.02411866472747E-165	Hepatocytes	DEGS1
**3.22430592046972E-169**	0.994396551292381	0.251	0.076	5.40329186152316E-165	Hepatocytes	ZBTB41
**3.71759125819753E-169**	0.770427370921392	0.369	0.16	6.22993943048742E-165	Hepatocytes	NDUFA13
**3.86393510278277E-169**	1.09705349480872	0.274	0.092	6.47518244524336E-165	Hepatocytes	SDHC
**4.49656884648134E-169**	0.63685886411902	0.288	0.1	7.53535007293343E-165	Hepatocytes	ASPSCR1
**5.32097598773406E-169**	2.52406647528495	0.163	0.022	8.91689156024474E-165	Hepatocytes	HKDC1
**5.85812558156416E-169**	0.357394075327591	0.4	0.18	9.81704684958522E-165	Hepatocytes	SMG5
**7.00540317743049E-169**	1.2495873715875	0.238	0.068	1.1739654644738E-164	Hepatocytes	C8orf33
**9.98895051662469E-169**	0.635907424492493	0.271	0.088	1.67394832757597E-164	Hepatocytes	ODR4
**1.07424972217831E-168**	1.48405994727256	0.233	0.066	1.80022768442642E-164	Hepatocytes	ACOT13
**1.10042084178538E-168**	1.05874398318358	0.283	0.099	1.84408524666393E-164	Hepatocytes	MCUR1
**1.27507546372961E-168**	1.15283418553133	0.223	0.058	2.13677146211808E-164	Hepatocytes	ITGA3
**1.4330701194175E-168**	0.369830045118558	0.351	0.146	2.40153890611984E-164	Hepatocytes	REPS1
**1.87320408983531E-168**	1.01628376666563	0.266	0.087	3.13911541374601E-164	Hepatocytes	UQCC2
**2.41163392127055E-168**	2.96649265922012	0.151	0.016	4.04141612526519E-164	Hepatocytes	CLRN3
**5.27128475899717E-168**	0.778541162620293	0.342	0.142	8.83361899912746E-164	Hepatocytes	HDGF
**5.56282998579651E-168**	2.34865062693668	0.17	0.027	9.32219049019779E-164	Hepatocytes	PANK1
**6.13972126116332E-168**	0.52417798070551	0.439	0.218	1.02889448894575E-163	Hepatocytes	SEC13
**6.39457659192604E-168**	0.672777182821465	0.409	0.192	1.07160314527497E-163	Hepatocytes	GSTO1
**1.40457760254866E-167**	1.48512868288618	0.235	0.067	2.35379114635105E-163	Hepatocytes	ADH5
**1.47482456043901E-167**	1.20487363720052	0.227	0.062	2.47151099838369E-163	Hepatocytes	TOP1MT
**2.2015452002809E-167**	0.60831929634621	0.342	0.14	3.68934944663073E-163	Hepatocytes	SIL1
**2.28404806212134E-167**	0.661824471285523	0.342	0.14	3.82760774250294E-163	Hepatocytes	TMEM106B
**2.30188267358771E-167**	3.62269818817609	0.146	0.013	3.85749498439828E-163	Hepatocytes	LRRC8E
**2.68107710806033E-167**	0.553447453396145	0.416	0.197	4.4929490176875E-163	Hepatocytes	FIS1
**2.72085058453096E-167**	0.390590285466399	0.277	0.094	4.55960140955698E-163	Hepatocytes	ZBTB10

2.80037310943675E-167	0.827185113958621	0.294	0.106	4.6928652567941E-163	Hepatocytes	UBE2E3
3.10992253301663E-167	1.23108553333318	0.22	0.057	5.21160818082926E-163	Hepatocytes	HYI
4.76289142999684E-167	3.03130878577259	0.143	0.011	7.98165345838871E-163	Hepatocytes	MBL2
6.01454212724424E-167	1.03801513881369	0.248	0.075	1.00791696968359E-162	Hepatocytes	ZCCHC9
6.92239706437178E-167	0.486170281097416	0.49	0.263	1.16005530004742E-162	Hepatocytes	BSG
8.17565490843112E-167	1.15643585926474	0.269	0.09	1.37007624955489E-162	Hepatocytes	CNPY2
9.03092741457523E-167	1.22080148100564	0.294	0.109	1.51340281613452E-162	Hepatocytes	SLC25A25
1.16170813768439E-166	0.509330928342324	0.319	0.124	1.9467904971315E-162	Hepatocytes	SMYD2
1.46151163590169E-166	1.85689483048133	0.182	0.034	2.44920119944404E-162	Hepatocytes	THEM6
2.28795986945761E-166	1.55924641723398	0.22	0.059	3.83416314923707E-162	Hepatocytes	PEMT
3.14836355650259E-166	2.60083759469164	0.149	0.015	5.27602764798705E-162	Hepatocytes	MAGIX
3.25744179187204E-166	0.663693748774937	0.318	0.125	5.45882095481917E-162	Hepatocytes	FPGS
4.26337116035897E-166	1.401609263473	0.233	0.067	7.14455739052956E-162	Hepatocytes	TACO1
4.71887467339529E-166	1.33864637810942	0.268	0.091	7.90789017767582E-162	Hepatocytes	PSMD4
6.52705282545006E-166	1.19887728589574	0.283	0.102	1.09380351248892E-161	Hepatocytes	PYGL
6.8831514300003E-166	3.0541700185876	0.143	0.012	1.15347851663945E-161	Hepatocytes	SLC25A18
1.15544683861857E-165	2.26617769540898	0.174	0.03	1.93629781215699E-161	Hepatocytes	GNPNAT1
1.31512105734408E-165	3.59203225504534	0.141	0.011	2.20387986789721E-161	Hepatocytes	DEPRECATED-ENSG00000186204
1.83599776086717E-165	0.643224186478515	0.371	0.163	3.07676504766121E-161	Hepatocytes	DAP3
1.97875580834366E-165	0.556473775160996	0.296	0.108	3.31599898362231E-161	Hepatocytes	GNPAT
2.05499619260984E-165	1.96391819806715	0.173	0.029	3.44376261957557E-161	Hepatocytes	FZD8
2.24679079832507E-165	1.36139720535496	0.227	0.063	3.76517201983315E-161	Hepatocytes	TBCE.1
3.5913045777331E-165	0.812199628167697	0.355	0.153	6.01830821136512E-161	Hepatocytes	KEAP1
3.60240799560029E-165	3.35158640167943	0.136	0.008	6.03691531902697E-161	Hepatocytes	TMEM184A
3.97868940081693E-165	2.10983247855363	0.188	0.039	6.6674876976889E-161	Hepatocytes	TEX30
7.82432869405384E-165	0.91567603333325	0.302	0.115	1.31120100254954E-160	Hepatocytes	PXDC1
9.03098141487421E-165	1.07810688492938	0.29	0.107	1.51341186550462E-160	Hepatocytes	NME1
1.13011738843475E-164	3.02356716470784	0.146	0.014	1.89385071953895E-160	Hepatocytes	SEMA4G
1.44822391778994E-164	1.38055885512545	0.176	0.03	2.42693364143238E-160	Hepatocytes	TACC2
1.51361969749101E-164	2.74033040058834	0.148	0.015	2.53652388905543E-160	Hepatocytes	ARHGEF16
2.43895700856601E-164	1.79146252432138	0.161	0.022	4.08720415495493E-160	Hepatocytes	CNTFR
3.31698209949082E-164	2.14971141003505	0.154	0.019	5.55859860232672E-160	Hepatocytes	RADX
3.94822266905818E-164	1.1970933222784	0.247	0.076	6.6164315488077E-160	Hepatocytes	PGAP2
4.22185233016992E-164	0.5926869920824	0.314	0.12	7.07498013489875E-160	Hepatocytes	ST5
6.32276476721948E-164	1.48499092976616	0.246	0.077	1.05956891969064E-159	Hepatocytes	PDK2
7.4128560329928E-164	0.901593676830578	0.348	0.152	1.24224641400893E-159	Hepatocytes	LSM 4.00
9.13596404099878E-164	1.52208982443725	0.217	0.058	1.53100485399058E-159	Hepatocytes	ICK
1.2009934264242E-163	0.973454680901629	0.263	0.088	2.01262478400167E-159	Hepatocytes	PRKCA
1.23945785632964E-163	0.878133650937779	0.288	0.104	2.07708347563722E-159	Hepatocytes	DNAJA3
1.33387161043035E-163	0.374665041155866	0.398	0.183	2.23530204475918E-159	Hepatocytes	PA2G4
1.88728279753598E-163	3.5976626140805	0.138	0.011	3.16270851211079E-159	Hepatocytes	APOA5
2.10783044009554E-163	3.00914699318898	0.153	0.018	3.5323022515121E-159	Hepatocytes	DEPRECATED-ENSG00000160868
2.2163395147604E-163	0.546011892173073	0.515	0.285	3.71414175883547E-159	Hepatocytes	CALM2
2.52942881901246E-163	2.72340616700316	0.152	0.018	4.23881681490109E-159	Hepatocytes	RASD1
2.61783907441596E-163	0.533173140046628	0.341	0.138	4.38697472090626E-159	Hepatocytes	BCL3
5.55300025624192E-163	0.791775849152967	0.298	0.109	9.30571782941022E-159	Hepatocytes	GADD45A
5.58686219896178E-163	3.18528504114589	0.137	0.01	9.36246367302015E-159	Hepatocytes	PRODH2
5.81104904013583E-163	0.954525754056547	0.292	0.108	9.73815598145963E-159	Hepatocytes	TOMM70
6.17595771358965E-163	0.37304612628311	0.512	0.273	1.03496699364335E-158	Hepatocytes	NDUFS2
8.80318548810448E-163	1.29266429066733	0.247	0.078	1.47523782409655E-158	Hepatocytes	TUBG1
1.18243279933577E-162	0.411615624186513	0.505	0.278	1.98152088512688E-158	Hepatocytes	TMBIM1
1.46579607659937E-162	1.58817558817181	0.214	0.057	2.45638106516522E-158	Hepatocytes	PAQR7
1.48922464711277E-162	3.46292531726875	0.137	0.01	2.49564266363159E-158	Hepatocytes	HSD17B13
1.63541229189877E-162	0.841901050576502	0.298	0.112	2.74062391876395E-158	Hepatocytes	TRAP1
1.7535847355388E-162	2.01418563908286	0.187	0.04	2.93865729981592E-158	Hepatocytes	HMGB3
1.82591091184878E-162	1.79062326173707	0.163	0.024	3.05986150607618E-158	Hepatocytes	TPD52L1
2.14400522947113E-162	1.46702539763971	0.185	0.037	3.59292396354773E-158	Hepatocytes	MTERF2
2.51879866500421E-162	0.373712337838225	0.468	0.239	4.22100280281405E-158	Hepatocytes	CD46
2.6276840677108E-162	0.740739340496562	0.208	0.051	4.40347296066976E-158	Hepatocytes	PLEKHH3
2.91797734570237E-162	1.80024290020075	0.173	0.03	4.88994643592803E-158	Hepatocytes	KYAT1
3.01436083240498E-162	1.32652391129609	0.222	0.06	5.05146588294427E-158	Hepatocytes	ZNF7
3.03235955783901E-162	0.956895993064841	0.276	0.097	5.08162814702661E-158	Hepatocytes	SLC46A1
3.17312628326238E-162	2.43911288551385	0.157	0.021	5.3175250254911E-158	Hepatocytes	PARD6B

3.34073938790343E-162	0.78928876229739	0.344	0.147	5.59841106624857E-158	Hepatocytes	COPS6
6.78812939007861E-162	1.75994379521681	0.177	0.033	1.13755472318937E-157	Hepatocytes	FXYD1
8.88688668207144E-162	0.8503891683108	0.211	0.052	1.48926447018153E-157	Hepatocytes	CEACAM1
9.7307406549938E-162	0.510977978738522	0.377	0.169	1.63067751896386E-157	Hepatocytes	RMND5A
1.05659774553747E-161	1.03273611578314	0.263	0.089	1.7706465019717E-157	Hepatocytes	TMEM70
1.38066411613043E-161	0.410809645538808	0.368	0.163	2.31371692581138E-157	Hepatocytes	SPECC1L
1.47817571004965E-161	0.789642787951973	0.212	0.054	2.47712685490121E-157	Hepatocytes	ANO8
1.59415931749307E-161	1.08585236338053	0.271	0.094	2.67149218425489E-157	Hepatocytes	ATPAF1
1.79768981968636E-161	0.74015026713232	0.268	0.09	3.01256859983041E-157	Hepatocytes	TERF1
3.28911957715601E-161	0.751058426312104	0.359	0.158	5.51190658739805E-157	Hepatocytes	SRPRB
5.53101348007206E-161	0.930625008789082	0.277	0.099	9.26887238990476E-157	Hepatocytes	ARHGAP5
5.54795967393704E-161	2.14236648948833	0.176	0.033	9.2972708215837E-157	Hepatocytes	PTGR2
5.66575555654868E-161	0.444904959909911	0.381	0.171	9.49467316166427E-157	Hepatocytes	OCIAD1
8.21500780444513E-161	1.46682653211725	0.23	0.067	1.37667100786892E-156	Hepatocytes	GLO1
8.25927984367307E-161	0.256066439772978	0.35	0.143	1.38409011620273E-156	Hepatocytes	SPATA13
8.9002819755749E-161	1.31816674882318	0.208	0.053	1.49150925346684E-156	Hepatocytes	FAM221A
8.90327569950484E-161	0.941524871496521	0.279	0.099	1.49201094172302E-156	Hepatocytes	RHEB
8.91274962710047E-161	2.05938142253733	0.163	0.025	1.4935985825095E-156	Hepatocytes	CMTM4
9.67313392401138E-161	1.42607123867625	0.184	0.037	1.62102378298583E-156	Hepatocytes	FNBP1L
1.05593202297729E-160	0.650228884180188	0.411	0.201	1.76953088410535E-156	Hepatocytes	MGAT2
1.10608555479762E-160	1.12306803512973	0.303	0.118	1.85357817272985E-156	Hepatocytes	LSM 6.00
1.18576490152243E-160	1.05507606042717	0.305	0.119	1.98710482197129E-156	Hepatocytes	DEPRECATED-ENSG00000067064
2.18431849471835E-160	1.39584798106772	0.187	0.038	3.66048093344902E-156	Hepatocytes	TSHZ2
2.46806939214808E-160	0.95840048178431	0.298	0.115	4.13599068736176E-156	Hepatocytes	TFDP1
2.54321526411614E-160	0.29310458287508	0.365	0.16	4.26192013960583E-156	Hepatocytes	EIF1B
2.58997214969267E-160	0.300249414438074	0.301	0.113	4.34027532845498E-156	Hepatocytes	IDUA
2.84830633198371E-160	0.522846823615398	0.417	0.206	4.77319175113831E-156	Hepatocytes	CPEB4
7.33933647869214E-160	0.809953225199063	0.196	0.044	1.22992600709923E-155	Hepatocytes	CADPS2
7.35343206422581E-160	1.02712435890583	0.195	0.044	1.23228814532296E-155	Hepatocytes	ARL4A
1.11698245981436E-159	0.787519098606843	0.231	0.065	1.8718392061569E-155	Hepatocytes	SLC35D1
1.17493077362296E-159	1.02031009343569	0.203	0.049	1.96894899043736E-155	Hepatocytes	LARP1B
1.32142217274741E-159	0.70394576000139	0.407	0.196	2.2144392770901E-155	Hepatocytes	SPCS1
1.3947485605969E-159	3.08755815120172	0.136	0.01	2.33731963784829E-155	Hepatocytes	SPATA6L
2.00980539080819E-159	0.747077575230005	0.292	0.108	3.36803187391636E-155	Hepatocytes	SLC35B1
2.22132926972693E-159	1.95446196990561	0.193	0.045	3.72250359020838E-155	Hepatocytes	CDC42EP5
2.67481914201495E-159	3.19723326644684	0.132	0.009	4.48246191818865E-155	Hepatocytes	XKR9
2.82277975977314E-159	0.447163220348142	0.48	0.252	4.73041432142782E-155	Hepatocytes	GSDMD
2.8878102488433E-159	0.713294838310355	0.296	0.112	4.8393924150116E-155	Hepatocytes	EI24
5.00695330527364E-159	1.689221425815	0.177	0.034	8.39065234897757E-155	Hepatocytes	ARMCX5
6.32205407111596E-159	1.79340842983465	0.183	0.038	1.05944982123761E-154	Hepatocytes	GNE
6.70289144634901E-159	0.551975173507194	0.312	0.122	1.12327054857917E-154	Hepatocytes	GALK2
7.19023035349208E-159	1.06162767360805	0.229	0.066	1.2049388026382E-154	Hepatocytes	PCYOX1
1.34661539722924E-158	1.72376515878157	0.179	0.035	2.25665808267675E-154	Hepatocytes	CDC42EP4
1.45931912689216E-158	3.42129308743604	0.138	0.012	2.44552699284588E-154	Hepatocytes	KRT15
1.46484052917E-158	1.43603672795327	0.228	0.068	2.45477975878308E-154	Hepatocytes	SRD5A3
1.51118586417714E-158	2.91561631125181	0.148	0.018	2.53244527118804E-154	Hepatocytes	AL135905.2
1.78275886585436E-158	2.69403793628771	0.147	0.017	2.98754730739874E-154	Hepatocytes	RAP1GAP
4.66584915146558E-158	1.01660963956938	0.225	0.064	7.81903000802602E-154	Hepatocytes	LCLAT1
4.91641102409271E-158	0.543865736709043	0.426	0.209	8.23892159417457E-154	Hepatocytes	SPATS2L
5.14578847537191E-158	1.16735754474283	0.211	0.055	8.62331232702825E-154	Hepatocytes	ST7
6.19212932453989E-158	0.818027121110723	0.264	0.09	1.0376770322064E-153	Hepatocytes	HAX1
6.51687252969196E-158	1.88136306791892	0.182	0.038	1.09209749852578E-153	Hepatocytes	KLC4
7.03977233130754E-158	0.504450551523094	0.424	0.213	1.17972504728052E-153	Hepatocytes	ATP6V0D1
8.47297516547385E-158	0.281718933000674	0.391	0.177	1.41990117823011E-153	Hepatocytes	TSC22D2
1.10597957408396E-157	0.305835958988971	0.319	0.127	1.8534005702499E-153	Hepatocytes	SLC23A2
1.15046903806827E-157	1.23942000827258	0.226	0.065	1.92795601399481E-153	Hepatocytes	GSDMB
1.48032443615844E-157	3.11173031240402	0.149	0.019	2.48072769011432E-153	Hepatocytes	PLAAT2
1.60260265345567E-157	0.626047006175017	0.272	0.094	2.68564152666101E-153	Hepatocytes	IFT27
3.18424876579168E-157	1.00712752519312	0.221	0.061	5.3361640817137E-153	Hepatocytes	CASP6
3.33637774645049E-157	1.33356621156943	0.19	0.042	5.59110182750173E-153	Hepatocytes	ZNF251
3.75590465019681E-157	3.48785314338899	0.135	0.011	6.29414501279981E-153	Hepatocytes	NGEF
3.82681690656382E-157	3.05257385381238	0.143	0.015	6.41297977201966E-153	Hepatocytes	ARHGEF37
4.56826720660925E-157	2.070209101637	0.149	0.018	7.65550218483579E-153	Hepatocytes	NRXN3

4.76733062209068E-157	0.411426793714296	0.248	0.076	7.98909265649957E-153	Hepatocytes	SYNPO
1.11991703349107E-156	1.0120818225342	0.228	0.066	1.87675696472433E-152	Hepatocytes	TMEM63B
1.19542431502634E-156	0.628033378116186	0.364	0.164	2.00329206712113E-152	Hepatocytes	NDUFS1
1.23235804668646E-156	1.82799053719516	0.177	0.035	2.06518561463716E-152	Hepatocytes	ANKRD46
1.53349460152248E-156	1.77895725494026	0.184	0.039	2.56983025323137E-152	Hepatocytes	TEF
1.5369768828819E-156	0.475616599200155	0.463	0.238	2.5756658603335E-152	Hepatocytes	ADIPOR1
1.66107411362984E-156	2.13707125833681	0.163	0.027	2.78362799962089E-152	Hepatocytes	DNM1
2.03673172467195E-156	0.361676777435491	0.372	0.167	3.41315502420525E-152	Hepatocytes	PMF1
2.18364805870033E-156	0.512029418365689	0.295	0.112	3.65935741677001E-152	Hepatocytes	SLC10A3
2.44359858309585E-156	0.999947274443765	0.248	0.08	4.09498250555203E-152	Hepatocytes	NIT2
3.35209644567205E-156	1.32913070312334	0.231	0.07	5.61744322365722E-152	Hepatocytes	DEPRECATED-ENSG00000099797
3.83177084331885E-156	0.941155701375252	0.249	0.081	6.42128157923373E-152	Hepatocytes	TYMS
4.52705834312473E-156	0.68848624340268	0.336	0.144	7.58644437140842E-152	Hepatocytes	DUSP3
4.58631143962589E-156	0.695243001102424	0.272	0.096	7.68574071052507E-152	Hepatocytes	SLC22A23
5.01052265202367E-156	1.13461088875732	0.234	0.072	8.39663386026126E-152	Hepatocytes	TMLHE
5.59819675530985E-156	0.507072412761376	0.548	0.308	9.38145812254824E-152	Hepatocytes	IDH2
5.84573740606877E-156	0.902511319882051	0.272	0.099	9.79628674509005E-152	Hepatocytes	TMEM14C
6.70798690362286E-156	0.71942734579432	0.269	0.094	1.12412444530912E-151	Hepatocytes	C19orf25
6.83329698607716E-156	0.354099101105205	0.341	0.142	1.14512390892681E-151	Hepatocytes	LRRC8A
8.65264083788107E-156	2.86669648804438	0.134	0.011	1.45000955161211E-151	Hepatocytes	GRB14
9.88053330592686E-156	0.550013382071959	0.355	0.158	1.65577977140722E-151	Hepatocytes	MAPK6
1.0006138981122E-155	1.18987980347286	0.246	0.08	1.67682877045642E-151	Hepatocytes	ATP5PF
1.0939030560777E-155	1.02829599972381	0.297	0.116	1.83316274137501E-151	Hepatocytes	C18orf32
1.17473162542622E-155	1.26758226189204	0.204	0.051	1.96861525788925E-151	Hepatocytes	SMAD1
1.26630679110549E-155	1.79204607572393	0.183	0.04	2.12207692053458E-151	Hepatocytes	PRRG4
1.57527051597973E-155	0.674866831578187	0.384	0.18	2.63983833067884E-151	Hepatocytes	AGPAT1
1.61565004189525E-155	1.25757964648688	0.213	0.057	2.70750634020806E-151	Hepatocytes	ATPSCKMT
2.8847184286058E-155	3.35434193145355	0.127	0.007	4.8342111426576E-151	Hepatocytes	NR1I3
3.21559665181246E-155	1.35719843046027	0.212	0.057	5.38869686910732E-151	Hepatocytes	GON7
3.23872222975636E-155	0.453826317479474	0.34	0.145	5.4274507126257E-151	Hepatocytes	C5orf24
6.72165322273406E-155	0.664011075043392	0.183	0.038	1.12641464706577E-150	Hepatocytes	CASZ1
1.07930321588384E-154	0.743244052596082	0.3	0.118	1.80869632917813E-150	Hepatocytes	TMEM129
1.83832862179362E-154	3.4484671517857	0.146	0.019	3.08067110440175E-150	Hepatocytes	CHI3L1
2.63694373906688E-154	0.481108163766755	0.348	0.153	4.41899031792828E-150	Hepatocytes	IPO9
2.76576851562653E-154	1.23989253669609	0.193	0.045	4.63487487848694E-150	Hepatocytes	PPP1R3B
2.79419922805691E-154	0.450437782372876	0.244	0.077	4.68251906637776E-150	Hepatocytes	ZNF444
3.63579004960868E-154	0.962769806490868	0.284	0.106	6.09285696513422E-150	Hepatocytes	PFDN6
4.92735011804115E-154	2.86813849846971	0.143	0.017	8.25725332781336E-150	Hepatocytes	HRCT1
6.80617503229339E-154	1.0142950154642	0.29	0.112	1.14057881191173E-149	Hepatocytes	MGMT
7.22501666288794E-154	0.476769480903433	0.211	0.055	1.21076829236676E-149	Hepatocytes	APBB2
7.62699049499093E-154	1.03043980005614	0.176	0.034	1.27813106715058E-149	Hepatocytes	FRY
1.08279957693805E-153	2.97354884989283	0.144	0.017	1.81455553103279E-149	Hepatocytes	C9orf152
1.08696810767354E-153	0.476393585662431	0.285	0.106	1.82154115483932E-149	Hepatocytes	HSPA9
1.19167673937392E-153	1.25130203388306	0.219	0.063	1.99701187984281E-149	Hepatocytes	RMDN1
1.23074069948571E-153	0.403177505038145	0.34	0.146	2.06247526419815E-149	Hepatocytes	PSMD12
1.53857405163048E-153	1.88111954126894	0.195	0.048	2.57834239572236E-149	Hepatocytes	UBE2T
1.62715866234801E-153	0.370508469275485	0.336	0.142	2.7267924863628E-149	Hepatocytes	VPS52
3.50658557245851E-153	2.70257284071411	0.131	0.01	5.87633610232596E-149	Hepatocytes	ADGRG2
4.02444794416513E-153	1.28469531439557	0.213	0.059	6.74416986483192E-149	Hepatocytes	CLN5
4.64462485196495E-153	1.24241585764604	0.245	0.08	7.78346232692287E-149	Hepatocytes	CPTP
5.36425697203402E-153	1.68237635537828	0.191	0.045	8.98942183373461E-149	Hepatocytes	BRCC3
6.4541043307554E-153	0.755618839071845	0.31	0.126	1.08157880374799E-148	Hepatocytes	PYURF
8.85859301231221E-153	2.76725317017152	0.139	0.015	1.48452301700328E-148	Hepatocytes	RND1
1.21187061492522E-152	1.10855503711586	0.262	0.093	2.03085277649168E-148	Hepatocytes	COA6
1.41913707030854E-152	0.637924377929224	0.41	0.204	2.37818990242306E-148	Hepatocytes	MLEC
1.58995434054501E-152	1.06029098620814	0.211	0.057	2.66444548388533E-148	Hepatocytes	ALG6
1.62660383891215E-152	1.16779385996143	0.212	0.059	2.72586271324897E-148	Hepatocytes	LRRC45
2.25084876918977E-152	0.834082866551447	0.31	0.127	3.77197236740821E-148	Hepatocytes	EMC3
2.67416259897243E-152	1.09621312380485	0.197	0.049	4.481361683358E-148	Hepatocytes	IFT88
2.91789604703059E-152	2.92390025748051	0.138	0.014	4.88981019561386E-148	Hepatocytes	ATP2C2
3.09808308826997E-152	3.29559933617035	0.133	0.011	5.19176763932282E-148	Hepatocytes	FUT6
3.37337055458676E-152	0.519507850030873	0.366	0.169	5.65309437537649E-148	Hepatocytes	HNRNPAB
4.84939881276331E-152	0.45232541054189	0.403	0.195	8.12662253042875E-148	Hepatocytes	CCDC47

4.97003514182401E-152	2.29902138938609	0.135	0.012	8.32878489066868E-148	Hepatocytes	PCDHGA3
6.3406437189597E-152	0.476150262498647	0.313	0.126	1.06256507442327E-147	Hepatocytes	TEX2
6.55498350087035E-152	0.80571791314205	0.218	0.061	1.09848413507585E-147	Hepatocytes	CEP250
8.05833596529623E-152	0.57875319854146	0.361	0.164	1.35041594106434E-147	Hepatocytes	CCT6A
1.23787047736686E-151	1.06591262270424	0.224	0.066	2.07442334597138E-147	Hepatocytes	SELENOI
1.36277205680814E-151	1.97305313481577	0.158	0.026	2.28373341279907E-147	Hepatocytes	KCNMB3
1.44327356345293E-151	0.731633812441625	0.222	0.064	2.41863783763442E-147	Hepatocytes	PER2
1.44851750998806E-151	0.486091759215492	0.288	0.11	2.427425643238E-147	Hepatocytes	FAM13A
1.49777772669172E-151	1.16043015535383	0.203	0.052	2.50997591438998E-147	Hepatocytes	ADCK5
1.88798995887775E-151	0.743417414628501	0.322	0.136	3.16389357308733E-147	Hepatocytes	PSMB5
2.21514244968273E-151	0.498279546959728	0.371	0.172	3.71213571717831E-147	Hepatocytes	RAD23A
4.092675616489E-151	0.852895672376352	0.24	0.077	6.85850579811226E-147	Hepatocytes	PTS
6.2144913364705E-151	0.374377094372219	0.523	0.287	1.04142445816573E-146	Hepatocytes	NDFIP1
6.34555648645783E-151	3.15584858450115	0.137	0.014	1.0633883560006E-146	Hepatocytes	CTH
7.23849089269741E-151	0.394573435879005	0.347	0.15	1.21302630379823E-146	Hepatocytes	COG3
7.75178906539409E-151	2.68629079645428	0.133	0.012	1.29904481157874E-146	Hepatocytes	GCKR
8.89591054874063E-151	1.93072104574721	0.159	0.027	1.49077668975796E-146	Hepatocytes	MEST
1.00694343831035E-150	0.485403870592422	0.463	0.241	1.68743581392048E-146	Hepatocytes	CALR
1.01571212494941E-150	1.08601103850579	0.195	0.048	1.70213037899023E-146	Hepatocytes	ACYP1
1.04065326999193E-150	3.55688375834797	0.136	0.014	1.74392674985248E-146	Hepatocytes	CAMK2N2
1.48914103136951E-150	1.31746809947896	0.229	0.071	2.49550254036903E-146	Hepatocytes	AIMP2
1.68041666675345E-150	0.30701014550568	0.291	0.111	2.81604225014543E-146	Hepatocytes	CAMK2D
1.78630560891324E-150	0.89547464153209	0.188	0.044	2.99349093941681E-146	Hepatocytes	RBMX2
2.19405759219919E-150	2.05670661400075	0.167	0.032	3.6768017130074E-146	Hepatocytes	POLE2
2.23484044793951E-150	0.847255940320038	0.258	0.09	3.74514562265703E-146	Hepatocytes	APPL2
3.89276404372995E-150	0.408934423916217	0.799	0.546	6.52349398448265E-146	Hepatocytes	MT-ND4L
4.41038304673529E-150	3.16283332991983	0.124	0.008	7.39091990971899E-146	Hepatocytes	SRCIN1
5.77222439324586E-150	1.03215654500237	0.282	0.109	9.6730936382014E-146	Hepatocytes	TMED2
5.95020353162044E-150	2.55477266231576	0.156	0.026	9.97135107828954E-146	Hepatocytes	DEPRECATED-ENSG00000147804
7.3538884580585E-150	1.73020889864709	0.18	0.04	1.23236462780144E-145	Hepatocytes	PAK4
7.56968643325136E-150	3.48744691317679	0.127	0.009	1.26852805248426E-145	Hepatocytes	TMEM30B
1.02960906975462E-149	0.634492736839661	0.285	0.108	1.72541887909479E-145	Hepatocytes	MIEN1
1.12831482794918E-149	1.49987804006859	0.207	0.058	1.89082998867724E-145	Hepatocytes	POP7
1.35554219031193E-149	1.36596939698977	0.199	0.051	2.27161760252474E-145	Hepatocytes	CSTF2
1.36731983686286E-149	1.1101000641228	0.252	0.087	2.29135458261479E-145	Hepatocytes	SEC61B
1.61161944509627E-149	2.95322843845633	0.138	0.015	2.70075186609233E-145	Hepatocytes	DEPRECATED-ENSG00000108448
2.09841406789142E-149	2.54376727643884	0.137	0.015	3.51652229497244E-145	Hepatocytes	ETNPPL
3.06374141810694E-149	0.701194548521865	0.195	0.048	5.1342178684636E-145	Hepatocytes	ZNF704
3.19250634946268E-149	0.86173477616572	0.237	0.077	5.35000214042956E-145	Hepatocytes	PSMA3
6.64055672707041E-149	0.993674126754569	0.278	0.106	1.11282449632246E-144	Hepatocytes	NCOA7
6.84111860330144E-149	0.688418409368861	0.249	0.083	1.14643465554126E-144	Hepatocytes	ZNF623
7.40982914615448E-149	0.625524339966411	0.182	0.04	1.24173916831257E-144	Hepatocytes	PPP1R13B
7.41397142215842E-149	1.09979446655264	0.267	0.098	1.24243333092531E-144	Hepatocytes	PRELID1
1.27642793003551E-148	2.36418273029702	0.148	0.021	2.1390379251535E-144	Hepatocytes	SATB2
1.3340769819061E-148	0.915697855449322	0.274	0.102	2.23564620627824E-144	Hepatocytes	JPT2
1.94004460523937E-148	2.08029652794847	0.137	0.015	3.25112674946014E-144	Hepatocytes	MYO7B
2.72905181874286E-148	0.614982136478109	0.257	0.088	4.57334503784928E-144	Hepatocytes	POMGNT1
2.81563769858945E-148	0.57699819661582	0.415	0.21	4.71844565529621E-144	Hepatocytes	PIGT
3.01024410250719E-148	3.47155662513018	0.126	0.009	5.04456706698155E-144	Hepatocytes	SLC22A1
3.67058460449267E-148	0.35419672593072	0.363	0.163	6.15116568020881E-144	Hepatocytes	SAT2
3.74615509543759E-148	0.515446547563928	0.35	0.157	6.27780670893432E-144	Hepatocytes	DEPRECATED-ENSG00000179010
4.19319352652598E-148	0.526445863086973	0.345	0.154	7.02695371175224E-144	Hepatocytes	GID8
4.86696992402053E-148	0.38457399284262	0.464	0.245	8.15606819867361E-144	Hepatocytes	CTNNA1
4.93531010150334E-148	0.822784160479783	0.286	0.112	8.2705926680993E-144	Hepatocytes	SLC27A3
5.37410958678925E-148	0.312560520682207	0.318	0.131	9.00593284554142E-144	Hepatocytes	SYVN1
5.47944453619841E-148	0.347108849111082	0.355	0.16	9.1824531537613E-144	Hepatocytes	TRAPPC4
9.21835897015119E-148	1.35442356928649	0.219	0.066	1.54481259621794E-143	Hepatocytes	MTCH2
9.22483284054361E-148	2.48555407210143	0.134	0.013	1.5458974874183E-143	Hepatocytes	XDH
1.46535406837379E-147	1.30955498248663	0.228	0.072	2.45564034778079E-143	Hepatocytes	NDUFC1
1.54897141989925E-147	0.645332474347139	0.258	0.089	2.59576630546716E-143	Hepatocytes	SUSD3
1.87445173574808E-147	2.72444948201675	0.139	0.017	3.14120621876663E-143	Hepatocytes	RAPGEFL1
2.05792524379987E-147	1.99357471644553	0.183	0.043	3.44867112355983E-143	Hepatocytes	C19orf33
3.42536408583543E-147	0.825307961313159	0.283	0.11	5.74022513504302E-143	Hepatocytes	PSMD8

4.96024795397591E-147	0.814987502906181	0.215	0.062	8.31238352127284E-143	Hepatocytes	PGM3
5.02805022254469E-147	1.22864483883044	0.245	0.083	8.4260065629404E-143	Hepatocytes	TMEM208
5.25172039117081E-147	0.369198928165449	0.344	0.151	8.80083303152405E-143	Hepatocytes	PYGO2
5.26886494559224E-147	2.28798515682479	0.146	0.021	8.82956387582347E-143	Hepatocytes	SLC22A5
5.8042937423627E-147	3.14915267404161	0.126	0.009	9.72683545345141E-143	Hepatocytes	PTPN3
6.01920456723628E-147	3.32912059756893	0.122	0.008	1.00869830137746E-142	Hepatocytes	CDO1
7.6139861329516E-147	1.17470910296249	0.228	0.072	1.27595179616003E-142	Hepatocytes	SLC25A43
7.66832840700232E-147	1.26079338971999	0.209	0.058	1.28505847444545E-142	Hepatocytes	UNG
1.18586110950729E-146	0.361377937449937	0.415	0.208	1.98726604731232E-142	Hepatocytes	MAP2K2
1.22281499750844E-146	0.583014514227196	0.209	0.057	2.04919337282464E-142	Hepatocytes	PLXNA2
1.69236711881112E-146	2.15185376229359	0.153	0.025	2.83606881770368E-142	Hepatocytes	THRB
2.13675613307437E-146	1.05629832379031	0.268	0.099	3.58077592780603E-142	Hepatocytes	UBL5
3.29666688006485E-146	1.05786987536195	0.269	0.101	5.52455435761267E-142	Hepatocytes	POMP
3.53986864514533E-146	3.57574350964299	0.133	0.014	5.93211187553454E-142	Hepatocytes	DEPDC7
4.63799684552138E-146	3.43539173230227	0.125	0.01	7.77235511372474E-142	Hepatocytes	FGF21
5.08140335598007E-146	0.366151951997212	0.351	0.158	8.5154157439514E-142	Hepatocytes	HECTD1
6.03319488755706E-146	0.391947540400229	0.282	0.108	1.01104279925681E-141	Hepatocytes	RBM12B
6.03502601596151E-146	0.446590800098707	0.306	0.124	1.01134965975483E-141	Hepatocytes	HMCES
6.77106109560168E-146	0.269428756220424	0.381	0.18	1.13469441840093E-141	Hepatocytes	WDR83OS
7.93260295348213E-146	0.851834769999231	0.274	0.104	1.32934560294454E-141	Hepatocytes	KDELR1
8.29436144297792E-146	0.94088360220081	0.246	0.084	1.38996909061424E-141	Hepatocytes	CC2D1A
1.01230574670595E-145	0.734085093380273	0.243	0.081	1.69642197032983E-141	Hepatocytes	ERGIC1
1.21803239462768E-145	2.95416092797207	0.127	0.01	2.04117868691706E-141	Hepatocytes	TOX3
1.28062431970622E-145	1.00280052801482	0.261	0.095	2.14607023496368E-141	Hepatocytes	AURKAIP1
1.42670420529144E-145	0.523081922511197	0.211	0.059	2.39087090722739E-141	Hepatocytes	PDLIM1
1.62157757129743E-145	0.782147275643345	0.253	0.088	2.71743969398023E-141	Hepatocytes	COX11
1.77059529055311E-145	0.79090927427053	0.321	0.137	2.9671635879089E-141	Hepatocytes	IFI35
2.19103266177414E-145	1.38626935125487	0.227	0.072	3.67173253460111E-141	Hepatocytes	TK1
2.25137579839832E-145	3.27122118540577	0.133	0.015	3.77285556295591E-141	Hepatocytes	UGT2B7
2.61012737123086E-145	0.781135798691271	0.278	0.107	4.37405144870867E-141	Hepatocytes	NUDCD2
2.88083443935575E-145	3.30264854770291	0.138	0.018	4.82770235347236E-141	Hepatocytes	ABCB4
3.60351228294302E-145	0.877730024766592	0.277	0.107	6.03876588375592E-141	Hepatocytes	FUCA2
3.82558881415792E-145	2.64155620980182	0.132	0.014	6.41092173476584E-141	Hepatocytes	SPAG1
4.12872370861224E-145	0.992587278877809	0.215	0.063	6.91891519089238E-141	Hepatocytes	SLC26A6
8.55267587791894E-145	1.37655985371092	0.207	0.059	1.43325742362166E-140	Hepatocytes	NAXD
1.0197016836849E-144	0.361941557009017	0.357	0.163	1.70881608151915E-140	Hepatocytes	GOLGA7
1.04641007265764E-144	0.360900814439775	0.401	0.196	1.75357399975967E-140	Hepatocytes	PFDN2
1.47573501600603E-144	0.284069742855382	0.194	0.049	2.4730367398229E-140	Hepatocytes	DAPK2
2.2214163609418E-144	0.868545699035884	0.265	0.098	3.72264953766627E-140	Hepatocytes	ELOC
2.49932599109911E-144	0.524205164126998	0.277	0.104	4.18837049588389E-140	Hepatocytes	DEXI
2.55455934220071E-144	0.762535266747454	0.252	0.089	4.28093054565994E-140	Hepatocytes	C16orf70
2.82194490529521E-144	0.724030027847597	0.33	0.144	4.72901527229372E-140	Hepatocytes	NUDT5
2.83956450984285E-144	2.99432067545087	0.125	0.01	4.75854220559465E-140	Hepatocytes	CYP21A2
2.88934458604268E-144	3.44441798150873	0.127	0.011	4.84196365729032E-140	Hepatocytes	C3orf85
3.03092351070828E-144	0.33152582051912	0.349	0.156	5.07922161924493E-140	Hepatocytes	AHCYL1
3.19308456566872E-144	0.651327544728012	0.372	0.176	5.35097111514764E-140	Hepatocytes	RDH11
3.27040747937945E-144	1.41037404933199	0.198	0.053	5.48054885394408E-140	Hepatocytes	DEPRECATED-ENSG00000114021
6.38623389487672E-144	0.300399299861737	0.277	0.104	1.07020507610344E-139	Hepatocytes	UBA5
7.16706703033222E-144	0.908239218947892	0.258	0.094	1.20105709294307E-139	Hepatocytes	SMIM26
7.23775066774864E-144	0.817013550738701	0.219	0.066	1.21290225690132E-139	Hepatocytes	DPH6
8.63775860816379E-144	0.896958643522037	0.231	0.074	1.44751558755609E-139	Hepatocytes	POFUT1
9.41913593182956E-144	0.837803229784768	0.267	0.101	1.578458799456E-139	Hepatocytes	TECR
1.14984911618448E-143	0.96309235462685	0.21	0.06	1.92691714890196E-139	Hepatocytes	SLC7A1
1.1541161474003E-143	0.527563909619326	0.334	0.147	1.93406783981341E-139	Hepatocytes	NNT
1.31274134840301E-143	1.96090620532874	0.166	0.034	2.19989195165377E-139	Hepatocytes	SLC16A13
1.36509844266687E-143	2.42712032403292	0.15	0.024	2.28763197022113E-139	Hepatocytes	ACOT2
1.97666455728115E-143	0.830416992395438	0.321	0.14	3.31249446509176E-139	Hepatocytes	ESD
2.08040529380094E-143	0.570233257354474	0.279	0.106	3.48634319135161E-139	Hepatocytes	FASTK
2.53646875225165E-143	0.662010299588944	0.355	0.164	4.25061433502332E-139	Hepatocytes	SSR3
2.56233690231776E-143	2.72336408056512	0.127	0.012	4.2939641809041E-139	Hepatocytes	HNF1B
3.02149454549757E-143	0.81743978540397	0.219	0.065	5.06342055934483E-139	Hepatocytes	FBXO31
3.46839014104662E-143	1.86771625356687	0.169	0.036	5.81232819836593E-139	Hepatocytes	LRRCC1
4.06254953511919E-143	0.448584711452949	0.337	0.152	6.80802051095273E-139	Hepatocytes	NUP58

4.4719819176588E-143	1.60074004201797	0.17	0.036	7.49414729761261E-139	Hepatocytes	CDKL1
5.7701034997301E-143	0.663890104132723	0.283	0.11	9.6695394448477E-139	Hepatocytes	CLN3
7.17142776641667E-143	0.532013793101931	0.38	0.184	1.2017878650961E-138	Hepatocytes	MICOS10
7.45018894334312E-143	0.768565968578665	0.203	0.057	1.24850266312544E-138	Hepatocytes	ANKRD9
7.96947791653079E-143	1.19831789549404	0.209	0.061	1.33552510925223E-138	Hepatocytes	PIGX
1.08069760511055E-142	1.09105745891298	0.234	0.078	1.81103304664426E-138	Hepatocytes	ALKBH7
1.32335963519177E-142	0.668291513689669	0.236	0.077	2.21768607665436E-138	Hepatocytes	UTP23
1.93073320267025E-142	1.5608136714108	0.142	0.02	3.2355227010348E-138	Hepatocytes	PCDHGA5
2.28846823447713E-142	0.541349867847894	0.315	0.135	3.83501506733677E-138	Hepatocytes	EEF2K
3.04919038687571E-142	0.484005411674971	0.452	0.24	5.10983325032631E-138	Hepatocytes	COX4I1
3.05167604241166E-142	1.06149791768973	0.269	0.102	5.11399871187347E-138	Hepatocytes	NHP2
3.31617485976772E-142	0.430476604187302	0.272	0.102	5.55724582999874E-138	Hepatocytes	SPPL2B
3.46396930138695E-142	0.8548324650536	0.302	0.127	5.80491975526425E-138	Hepatocytes	STMP1
4.38763566115542E-142	0.40700040225866	0.364	0.17	7.35279984096425E-138	Hepatocytes	UQCRB
4.58760003335152E-142	0.42694414357548	0.443	0.24	7.68790013589047E-138	Hepatocytes	CITED2
5.61560363751131E-142	0.812511196144271	0.26	0.095	9.41062857574145E-138	Hepatocytes	ARL 1.00
6.47983329230698E-142	0.352662465545191	0.339	0.151	1.0858904631248E-137	Hepatocytes	FNTA
7.12486714793898E-142	1.57556353414321	0.133	0.015	1.19398523665161E-137	Hepatocytes	WNT4
1.00397511685385E-141	2.58135763916527	0.14	0.019	1.68246150082368E-137	Hepatocytes	CNTNAP2
1.23358354981867E-141	0.467532379664188	0.379	0.181	2.06723931278613E-137	Hepatocytes	UFC1
1.26762574434222E-141	0.720131401802197	0.297	0.121	2.12428722236869E-137	Hepatocytes	ZNF664
1.36085320602757E-141	2.43864554270772	0.137	0.017	2.28051780266101E-137	Hepatocytes	SYT17
1.67804792585103E-141	0.485701108293997	0.266	0.099	2.81207271414116E-137	Hepatocytes	HMGXB4
1.82803687504867E-141	3.20124253223957	0.135	0.017	3.06342419520656E-137	Hepatocytes	NAGS
2.05838507152114E-141	0.70386356378095	0.336	0.152	3.44944170285512E-137	Hepatocytes	TMBIM6
3.95445588363283E-141	0.770783227533044	0.261	0.097	6.6268771697919E-137	Hepatocytes	TOLLIP
4.34354422424308E-141	1.01736281791828	0.208	0.06	7.27891141098656E-137	Hepatocytes	SLC30A5
5.70233118987398E-141	1.65431837124189	0.174	0.039	9.55596660799082E-137	Hepatocytes	DEPRECATED-ENSG00000232119
5.74185739810974E-141	0.475832983269954	0.238	0.078	9.62220462775231E-137	Hepatocytes	FBXO21
1.15784774129774E-140	2.99326668188763	0.124	0.011	1.94032124486676E-136	Hepatocytes	RNF43
1.30774985501463E-140	0.731920622175151	0.248	0.089	2.19152720703352E-136	Hepatocytes	SOCS6
1.54679242377251E-140	0.327131898448035	0.536	0.307	2.59211474375798E-136	Hepatocytes	EIF1
1.62973424661866E-140	2.30553723823427	0.14	0.019	2.73110865048356E-136	Hepatocytes	ARHGEF5
1.73341147026964E-140	3.23065286979474	0.13	0.014	2.90485094187787E-136	Hepatocytes	DRGX
1.96711176031644E-140	3.34358388174961	0.127	0.013	3.29648588793829E-136	Hepatocytes	CYP7A1
2.04466113750019E-140	0.301928729418669	0.562	0.327	3.42644313422282E-136	Hepatocytes	NFE2L1
2.16416052324361E-140	0.486790286832536	0.281	0.108	3.62670020485164E-136	Hepatocytes	RFC1
2.43754726252865E-140	0.935939805151871	0.295	0.122	4.08484170254551E-136	Hepatocytes	PSMB2
2.8599918931894E-140	1.07765976798567	0.227	0.074	4.79277441460679E-136	Hepatocytes	TCAIM
3.06251609190319E-140	0.647958255152453	0.25	0.087	5.13216446681137E-136	Hepatocytes	ELMOD2
3.1242061708231E-140	1.05981340564059	0.205	0.059	5.23554470106534E-136	Hepatocytes	HSF4
3.29527603044488E-140	0.44618026698662	0.278	0.107	5.52222357181953E-136	Hepatocytes	CFDP1
3.77981828293348E-140	3.26556300286401	0.125	0.012	6.33421947853992E-136	Hepatocytes	PITX1
3.88863487143738E-140	2.75604947380465	0.143	0.022	6.51657431755477E-136	Hepatocytes	MUC1
3.8908244524084E-140	0.533696648831435	0.35	0.162	6.520243617346E-136	Hepatocytes	PSMD1
4.50412125349177E-140	0.425577615019431	0.534	0.31	7.54800639660151E-136	Hepatocytes	ATP6AP1
4.8669238405872E-140	0.314276355920527	0.362	0.167	8.15599097205603E-136	Hepatocytes	POLR1D
5.10001960240262E-140	0.361869090506445	0.332	0.147	8.54661284970631E-136	Hepatocytes	APEX1
5.77023636919627E-140	0.803919711325664	0.27	0.106	9.66976210749911E-136	Hepatocytes	DERA
5.95040088720817E-140	3.77069167263615	0.118	0.008	9.97168180678345E-136	Hepatocytes	C6orf141
1.18588945869461E-139	2.03965924811106	0.138	0.019	1.98731355488043E-135	Hepatocytes	MELTF
1.2084192703622E-139	1.18865618067784	0.207	0.061	2.02506901327297E-135	Hepatocytes	CNIH4
1.23611045679422E-139	1.34355225444294	0.217	0.068	2.07147390349576E-135	Hepatocytes	SNRNP25
1.2402427645369E-139	0.951722751222121	0.285	0.117	2.07839882481093E-135	Hepatocytes	TUBA1C
1.85411633596857E-139	0.809159759136717	0.184	0.045	3.10712815581613E-135	Hepatocytes	AGAP1
2.2773857924906E-139	3.83071546280572	0.119	0.009	3.81644311105574E-135	Hepatocytes	DUSP21
2.46717727605143E-139	0.745247516356806	0.255	0.092	4.13449567920699E-135	Hepatocytes	SMAD5
2.52037413083816E-139	0.353588174340125	0.264	0.097	4.22364296845859E-135	Hepatocytes	EIF2D
2.92290660631879E-139	1.05733993061487	0.226	0.075	4.89820689086903E-135	Hepatocytes	PCNA
2.95838294355664E-139	0.334697907647483	0.39	0.19	4.95765813681222E-135	Hepatocytes	HDLBP
3.03592091998503E-139	2.12626911707299	0.158	0.031	5.08759627771091E-135	Hepatocytes	TRIB3
3.12312071411641E-139	0.392695463171319	0.38	0.184	5.23372569271628E-135	Hepatocytes	DYNLRB1
3.6292002615067E-139	0.432612848018207	0.319	0.14	6.08181379823293E-135	Hepatocytes	SOS 2

4.66220305235695E-139	0.360936651292095	0.482	0.26	7.81291987513977E-135	Hepatocytes	EIF4EBP2
5.27540734605571E-139	0.378427616352737	0.512	0.303	8.84052763052016E-135	Hepatocytes	TFRC
6.12350626067191E-139	1.10074994842161	0.233	0.079	1.0261771791634E-134	Hepatocytes	SLC41A2
7.51656244681832E-139	0.537709741897131	0.212	0.063	1.25962553483781E-134	Hepatocytes	P4HA2
1.2399811300857E-138	0.853985964826118	0.203	0.058	2.07796037779762E-134	Hepatocytes	DGKQ
1.54521702236135E-138	0.885161617330264	0.177	0.041	2.58947468607315E-134	Hepatocytes	NPIPB15
1.84162328162099E-138	1.6731087509857	0.164	0.034	3.08619229534046E-134	Hepatocytes	TGDS
2.11280019842931E-138	1.25476823558916	0.205	0.061	3.54063057252784E-134	Hepatocytes	C9orf64
2.25480888740709E-138	1.075309935734	0.279	0.115	3.77860873351681E-134	Hepatocytes	ZNF189
2.37743564103068E-138	0.537094021622044	0.277	0.109	3.98410664723921E-134	Hepatocytes	ESRRA
2.58892526306038E-138	0.939861579782971	0.285	0.116	4.33852095583659E-134	Hepatocytes	C7orf50
2.70790378681523E-138	0.986430974877108	0.285	0.116	4.53790516594497E-134	Hepatocytes	SURF1
2.76524416420927E-138	1.61480291966388	0.187	0.049	4.6339961703819E-134	Hepatocytes	FDX1
3.01269458629645E-138	2.43939587964514	0.15	0.027	5.04867358771558E-134	Hepatocytes	HMGN5
3.37876216912383E-138	0.621059678795841	0.311	0.134	5.66212964301772E-134	Hepatocytes	GIPC1
3.95096758358404E-138	2.61214965504344	0.125	0.012	6.62103147657013E-134	Hepatocytes	ALPK2
5.38333949473313E-138	0.424726418218256	0.219	0.067	9.02140032527378E-134	Hepatocytes	PDGFA
5.48512353933239E-138	2.54661202635061	0.16	0.034	9.19197002721322E-134	Hepatocytes	STEAP3
9.40326066739897E-138	0.659055406806387	0.185	0.047	1.57579842264272E-133	Hepatocytes	PM20D2
1.13829308219596E-137	1.63856021057469	0.15	0.026	1.90755154714399E-133	Hepatocytes	CCDC85C
1.19665082453096E-137	0.547948894510153	0.301	0.128	2.00534745174899E-133	Hepatocytes	FKBP2
1.73135827373957E-137	0.477852859901457	0.3	0.126	2.90141019513278E-133	Hepatocytes	RBMX
2.01261991999509E-137	2.50987461028361	0.132	0.017	3.37274846192777E-133	Hepatocytes	HCN3
2.45900543047382E-137	0.888710739578278	0.243	0.085	4.12080130038803E-133	Hepatocytes	CCS
2.76754435758982E-137	1.05159618826375	0.206	0.06	4.63785083444902E-133	Hepatocytes	URGCP
2.78725512682671E-137	0.657768425415214	0.24	0.083	4.6708821415362E-133	Hepatocytes	L3HYPDH
3.0497614252137E-137	2.9148673195881	0.134	0.018	5.11079019637311E-133	Hepatocytes	PRLR
3.34716347291287E-137	0.773630859212093	0.234	0.079	5.60917654790739E-133	Hepatocytes	TOR1B
3.65492696422075E-137	1.18314783215737	0.159	0.031	6.12492660664113E-133	Hepatocytes	RAPGEF4
4.1508356463308E-137	0.867392470866233	0.19	0.051	6.95597037612115E-133	Hepatocytes	C6orf136
4.37820761856502E-137	1.68585426638285	0.17	0.038	7.33700032719126E-133	Hepatocytes	WDR34
4.95315538478516E-137	1.05180744006489	0.239	0.083	8.30049779382297E-133	Hepatocytes	YIF1A
5.96127307396364E-137	0.651835177193632	0.267	0.103	9.98990141734827E-133	Hepatocytes	GSS
6.58470466622749E-137	0.282738863085378	0.272	0.104	1.1034648079664E-132	Hepatocytes	GTF2H2C
7.46493204410712E-137	1.78157412065589	0.175	0.042	1.25097331195147E-132	Hepatocytes	LRRC42
7.6168911590108E-137	2.5381784695725	0.14	0.022	1.27643862042703E-132	Hepatocytes	CDC14B
8.17385666784574E-137	3.29016753971604	0.119	0.01	1.36977490039759E-132	Hepatocytes	CBLC
1.75534108445121E-136	0.401072824867005	0.262	0.098	2.94160058932334E-132	Hepatocytes	HDDC2
1.76103938441367E-136	2.00745799778069	0.13	0.015	2.95114980040042E-132	Hepatocytes	ABTB2
1.81369546129985E-136	1.95724940847386	0.152	0.028	3.03939085404629E-132	Hepatocytes	TONSL
1.87292536868685E-136	1.77876108035806	0.14	0.021	3.13864833284543E-132	Hepatocytes	LRRC75B
2.75432550418061E-136	0.565159802953911	0.349	0.165	4.61569867990586E-132	Hepatocytes	MLX
2.84515229849252E-136	0.896609572780607	0.242	0.086	4.76790622181376E-132	Hepatocytes	FAM136A
3.48488008424405E-136	0.928012900480199	0.238	0.083	5.83996204517617E-132	Hepatocytes	NDUFB4
4.37248363187169E-136	0.385432336011339	0.392	0.194	7.32740807029059E-132	Hepatocytes	ATP5MG
4.57225857973052E-136	1.60976910900019	0.161	0.034	7.6621909279124E-132	Hepatocytes	MROH6
4.89275789128633E-136	0.775499844415875	0.246	0.088	8.19928367421763E-132	Hepatocytes	COQ4
5.54093296591009E-136	0.300895208769641	0.381	0.186	9.28549546427214E-132	Hepatocytes	SUB1
5.60859527743254E-136	0.401781117087622	0.397	0.198	9.39888396592146E-132	Hepatocytes	PSMD3
6.15144715890651E-136	1.14500362354374	0.226	0.075	1.03085951488955E-131	Hepatocytes	COX7B
8.90338405015324E-136	0.554400773386455	0.286	0.115	1.49202909912468E-131	Hepatocytes	SLC39A9
9.6517982563231E-136	1.14070471573793	0.205	0.061	1.61744835179462E-131	Hepatocytes	C12orf75
1.34357603824821E-135	0.371560841534875	0.48	0.262	2.25156472489636E-131	Hepatocytes	PSMF1
1.57724957708176E-135	0.782672298224502	0.226	0.075	2.64315484127361E-131	Hepatocytes	REPIN1
2.09510497197533E-135	2.66409461451564	0.141	0.023	3.51097691203626E-131	Hepatocytes	DECR2
2.32904256674481E-135	0.460519470115736	0.334	0.151	3.90300953335095E-131	Hepatocytes	LTBR
4.2832689579124E-135	2.70438554678597	0.136	0.02	7.1779021196696E-131	Hepatocytes	GIPC2
4.36998546515658E-135	0.469547966302057	0.299	0.125	7.3232216425094E-131	Hepatocytes	SLC33A1
4.41087473932838E-135	0.354730122813704	0.274	0.107	7.3917438881665E-131	Hepatocytes	MSH6
5.51727931671586E-135	0.618963381160562	0.229	0.076	9.24585667895243E-131	Hepatocytes	CYHR1
5.89337792302218E-135	3.79431137047022	0.117	0.009	9.87612272340056E-131	Hepatocytes	ARHGEF38
6.14295180163812E-135	0.339987688613618	0.474	0.26	1.02943586291852E-130	Hepatocytes	HM13
7.25725742103314E-135	1.13540789758369	0.251	0.095	1.21617119861673E-130	Hepatocytes	FADS1

1.07035906669008E-134	0.293132954752777	0.251	0.091	1.79370772395923E-130	Hepatocytes	SLC25A32
1.16199389066795E-134	1.99625004405718	0.138	0.021	1.94726936198135E-130	Hepatocytes	FAM222A
1.2362006000407E-134	0.515836262030504	0.259	0.098	2.07162496554821E-130	Hepatocytes	COMMD3
1.41797966434509E-134	1.48227831676598	0.196	0.057	2.3762503215095E-130	Hepatocytes	ABHD11
1.99658971597374E-134	0.877121815093093	0.175	0.042	3.34588504602879E-130	Hepatocytes	GUCY1B1
2.08985866717148E-134	0.471107660712901	0.245	0.089	3.50218515444597E-130	Hepatocytes	BCL2L2
2.36773891211844E-134	2.85643374894771	0.14	0.022	3.96785686892809E-130	Hepatocytes	CCL20
2.4284046862619E-134	3.11693975752956	0.124	0.014	4.06952057323769E-130	Hepatocytes	NR0B2
2.71441539892142E-134	0.524912520030215	0.204	0.059	4.54881732551251E-130	Hepatocytes	TNKS1BP1
2.78532809251623E-134	0.880570745089167	0.258	0.098	4.6676528174387E-130	Hepatocytes	DESI1
3.39587055733245E-134	2.717690764438	0.131	0.017	5.69079987997772E-130	Hepatocytes	FOXA2
3.93684918667575E-134	0.814990940354435	0.24	0.084	6.59737186703121E-130	Hepatocytes	KIF3B
9.58771269680711E-134	0.955646606017875	0.228	0.078	1.60670889373094E-129	Hepatocytes	GMFB
1.14972417364856E-133	0.309118117576424	0.334	0.151	1.92670777020025E-129	Hepatocytes	RBM10
1.17351908411528E-133	2.09852976760638	0.148	0.027	1.96658328116039E-129	Hepatocytes	L2HGDH
1.19006222659605E-133	0.468749658045173	0.275	0.108	1.99430627932966E-129	Hepatocytes	HELLS
1.31193153586064E-133	2.23156975577609	0.152	0.03	2.19853486779526E-129	Hepatocytes	GSTM1
1.31999250693923E-133	0.765040732739473	0.179	0.044	2.21204344312876E-129	Hepatocytes	SDCBP2
2.77243349047221E-133	1.33566394123923	0.19	0.053	4.64604404333333E-129	Hepatocytes	PIGP
3.47340102095092E-133	1.7979733409285	0.152	0.03	5.82072543090955E-129	Hepatocytes	SIX5
3.61327255523055E-133	1.58503359096526	0.176	0.045	6.05512214805536E-129	Hepatocytes	GTF2H5
3.84951477255488E-133	0.606532254780331	0.309	0.135	6.45101685584747E-129	Hepatocytes	PRKAB1
4.06958719149028E-133	0.925898052471755	0.242	0.087	6.81981421549942E-129	Hepatocytes	OST4
5.4466078836709E-133	0.416936002691267	0.268	0.105	9.1274254914557E-129	Hepatocytes	TCP1
5.5783822953341E-133	0.769738541593656	0.244	0.088	9.34825305052089E-129	Hepatocytes	CCDC117
5.74774882988616E-133	0.6228402697397	0.256	0.095	9.63207748912323E-129	Hepatocytes	ADIPOR2
5.89855351837967E-133	0.581013147221739	0.253	0.094	9.88479598610064E-129	Hepatocytes	GMPS
5.90861303094289E-133	1.09492180765006	0.172	0.041	9.90165371725409E-129	Hepatocytes	GTF2IRD1
5.97849265179231E-133	0.25433150866463	0.292	0.121	1.00187579858736E-128	Hepatocytes	TMUB1
7.44907029297947E-133	0.482905775007247	0.281	0.114	1.2483151996975E-128	Hepatocytes	ARPC1A
8.39741652546902E-133	0.602260106202803	0.27	0.107	1.4072390613381E-128	Hepatocytes	ELOB
8.82874234513181E-133	0.468760633526897	0.226	0.074	1.47952064219719E-128	Hepatocytes	C8orf76
1.07825573678039E-132	0.751209527021947	0.265	0.104	1.80694096369657E-128	Hepatocytes	ABHD10
1.58920972958279E-132	0.781460727655206	0.197	0.057	2.66319766483484E-128	Hepatocytes	SLC19A2
1.65922317239064E-132	0.968639869416354	0.208	0.064	2.78052619229223E-128	Hepatocytes	CCNQ
2.22496618799046E-132	2.58647738627202	0.12	0.012	3.72859833783441E-128	Hepatocytes	NUP62CL
2.81386510947387E-132	1.07059104090784	0.23	0.08	4.71547515045631E-128	Hepatocytes	ZCRB1
3.99237550656599E-132	0.844171995623232	0.247	0.092	6.69042287390329E-128	Hepatocytes	UBR7
7.70774848869516E-132	2.82286028429014	0.119	0.012	1.29166449173553E-127	Hepatocytes	SLC7A9
9.4708107066409E-132	0.88624754944058	0.217	0.071	1.58711845821888E-127	Hepatocytes	AK6
1.32535485116519E-131	0.709836665510929	0.277	0.114	2.22102965958262E-127	Hepatocytes	TMEM123
1.45762883341293E-131	2.18791504598513	0.144	0.026	2.44269439903339E-127	Hepatocytes	SNX7
1.49774419748352E-131	0.454333266312965	0.283	0.116	2.50991972614289E-127	Hepatocytes	AGGF1
1.50468639389724E-131	0.890000177796942	0.193	0.055	2.521553458893E-127	Hepatocytes	ZNF275
1.81302944956949E-131	0.495360650012458	0.364	0.178	3.03827475158854E-127	Hepatocytes	NEDD8
2.50241265383629E-131	0.254480944010281	0.216	0.068	4.19354312529886E-127	Hepatocytes	RAB11FIP4
2.81160522204371E-131	0.30946303844875	0.261	0.1	4.71168803110085E-127	Hepatocytes	ATL2
3.977673967805E-131	0.414759011671244	0.286	0.118	6.66578603524761E-127	Hepatocytes	GINM1
4.00427237074613E-131	0.74490787270366	0.247	0.092	6.71035963889636E-127	Hepatocytes	GOLGA5
4.2234234173497E-131	0.331164491798828	0.472	0.259	7.07761296279463E-127	Hepatocytes	ARL6IP4
4.34875284730329E-131	2.18807776514807	0.122	0.013	7.28764002151085E-127	Hepatocytes	SYDE2
4.56725528212112E-131	0.768586286649311	0.227	0.078	7.65380640177857E-127	Hepatocytes	GMPPA
4.75412024342543E-131	3.00445180370529	0.118	0.011	7.96695470393233E-127	Hepatocytes	MARVELD3
5.79783630849168E-131	0.257414483473784	0.228	0.077	9.71601408577036E-127	Hepatocytes	PLEKHG3
6.36250321925552E-131	0.801903254637611	0.239	0.087	1.06622828948284E-126	Hepatocytes	NCAPG2
6.52552336159055E-131	0.584832179831758	0.197	0.057	1.09354720493535E-126	Hepatocytes	GPAT3
7.89708815570905E-131	1.12676157974307	0.192	0.055	1.32339403313372E-126	Hepatocytes	SPHK1
8.02971751308809E-131	1.36638179994485	0.198	0.06	1.3456200608433E-126	Hepatocytes	DSN1
8.35113572582559E-131	1.12893817290187	0.156	0.032	1.39948332493385E-126	Hepatocytes	ZFYVE9
1.06659435251821E-130	0.668256730128018	0.283	0.12	1.78739881595002E-126	Hepatocytes	DEPRECATED-ENSG00000049860
1.76883811884557E-130	0.426507999044108	0.218	0.07	2.9642189195614E-126	Hepatocytes	LMTK2
2.042022176563E-130	0.831179629849441	0.227	0.077	3.42202076348427E-126	Hepatocytes	CBR4
2.74981737962451E-130	0.429133522635809	0.275	0.11	4.60814396477476E-126	Hepatocytes	SLC4A2

4.17387107693917E-130	0.491649878064274	0.244	0.089	6.99457315073467E-126	Hepatocytes	PRRC1
4.51717439652327E-130	1.5915322552952	0.172	0.043	7.56988085369369E-126	Hepatocytes	ING2
4.65696035370477E-130	0.792910540312087	0.314	0.144	7.80413416073845E-126	Hepatocytes	H2AFZ
4.79183198908209E-130	0.951751263877861	0.172	0.042	8.03015204730377E-126	Hepatocytes	AK3
6.10347265526823E-130	0.968784849067857	0.288	0.127	1.02281994756985E-125	Hepatocytes	FADS2
6.31683406522543E-130	0.403391662209488	0.591	0.402	1.05857505265048E-125	Hepatocytes	DNM2
8.10676086570615E-130	0.644626517290389	0.324	0.149	1.35853098587504E-125	Hepatocytes	PPIA
8.6061751857211E-130	0.917024149238937	0.272	0.111	1.44222283762314E-125	Hepatocytes	PSENEN
1.00853368393628E-129	0.905335879838273	0.245	0.091	1.69010074754042E-125	Hepatocytes	ACAD9
1.04835271008013E-129	0.309173517428808	0.492	0.278	1.75682947155228E-125	Hepatocytes	SSR2
1.12154470485085E-129	1.74039901873308	0.127	0.016	1.87948461638905E-125	Hepatocytes	FGFRL1
1.21802102335076E-129	1.67503141799631	0.152	0.031	2.0411596309312E-125	Hepatocytes	RASSF8
1.29127460827673E-129	0.437497289157559	0.23	0.079	2.16391798855014E-125	Hepatocytes	GTF2H2
1.38462327034001E-129	0.31871140755002	0.197	0.058	2.32035167643578E-125	Hepatocytes	FHL1
2.26436911239719E-129	1.68807255464093	0.126	0.016	3.79462975855521E-125	Hepatocytes	MIPOL1
2.42810818896613E-129	0.494799309258249	0.416	0.223	4.06902370306945E-125	Hepatocytes	CAPN1
2.95307105628178E-129	2.77274090459858	0.121	0.014	4.94875647611701E-125	Hepatocytes	CFHR5
3.32288984129009E-129	0.325019500874302	0.254	0.096	5.56849879603394E-125	Hepatocytes	MTM1
3.81500396948424E-129	2.59370244241345	0.13	0.019	6.39318365206169E-125	Hepatocytes	SULT1B1
3.95917827823918E-129	3.24549153068647	0.122	0.015	6.63479095867322E-125	Hepatocytes	PPP1R1B
4.49189998342557E-129	1.46143805910097	0.165	0.039	7.52752599222457E-125	Hepatocytes	SCLY
5.11732451331052E-129	2.71537092885366	0.119	0.012	8.57561241940578E-125	Hepatocytes	GBP7
6.93384090041344E-129	1.32866764984365	0.152	0.031	1.16197305809128E-124	Hepatocytes	LIN7A
8.80542849309626E-129	0.752838066266878	0.256	0.099	1.47561370687307E-124	Hepatocytes	DCUN1D1
1.06452392597664E-128	2.23592733438969	0.138	0.023	1.78392919515166E-124	Hepatocytes	SLC44A3
1.09884354641235E-128	3.09698699702112	0.115	0.01	1.84144201507782E-124	Hepatocytes	CYP3A4
1.33799597928186E-128	0.712434297895973	0.23	0.081	2.24221366208053E-124	Hepatocytes	SYPL1
1.66551551226348E-128	0.505638554501392	0.189	0.053	2.79107089545114E-124	Hepatocytes	PIBF1
1.94589085814891E-128	0.70011045749531	0.196	0.058	3.26092390008594E-124	Hepatocytes	SLC25A16
2.12229017125092E-128	0.619965479296121	0.224	0.078	3.55653386898229E-124	Hepatocytes	CA2
2.95402252051893E-128	2.52306830319702	0.123	0.015	4.95035093988563E-124	Hepatocytes	TTLL7
3.11202660563585E-128	0.897744058147189	0.236	0.086	5.21513418572456E-124	Hepatocytes	PPA2
3.27543458200943E-128	1.1027037538479	0.16	0.035	5.48897327253141E-124	Hepatocytes	AMMECR1
3.47962563681177E-128	0.413571653275648	0.267	0.105	5.83115664216916E-124	Hepatocytes	MTMR1
5.74603065795836E-128	2.94821797680554	0.123	0.015	9.62919817660662E-124	Hepatocytes	C11orf52
5.87568264481272E-128	0.313178038219796	0.305	0.135	9.84646897617716E-124	Hepatocytes	CANT1
6.12153775199756E-128	0.340131612079868	0.423	0.225	1.02584729647975E-123	Hepatocytes	YIPF3
8.75711314266919E-128	3.02013345530486	0.112	0.009	1.4675170204485E-123	Hepatocytes	OGDHL
1.31347782907917E-127	0.264851657131705	0.269	0.106	2.20112614597087E-123	Hepatocytes	STRN3
1.33474182767548E-127	0.951173416276607	0.251	0.097	2.23676035481856E-123	Hepatocytes	PSMA2
1.45167192683602E-127	0.626168529175976	0.151	0.03	2.43271181499181E-123	Hepatocytes	MEIS2
1.68182533624386E-127	0.528636334125947	0.212	0.068	2.81840289847746E-123	Hepatocytes	ERMARD
1.73152212288036E-127	0.603758030555426	0.244	0.092	2.9016847735229E-123	Hepatocytes	TIMM50
1.77828175648253E-127	0.622262615965799	0.256	0.1	2.98004456751343E-123	Hepatocytes	RSRC1
3.9307830920812E-127	0.40978099831191	0.316	0.142	6.58720630570968E-123	Hepatocytes	GATAD1
4.34116363640221E-127	0.509519706863128	0.247	0.092	7.27492202188282E-123	Hepatocytes	WDR44
5.81272912442906E-127	1.11019547135734	0.203	0.064	9.74097146671822E-123	Hepatocytes	ANAPC13
6.8772146112763E-127	0.68924902185885	0.21	0.068	1.15248362455768E-122	Hepatocytes	BPNT1
6.94090175162459E-127	1.96139717350899	0.14	0.025	1.16315631553725E-122	Hepatocytes	DHRS11
7.00867744614951E-127	1.99420905605074	0.14	0.025	1.17451416642573E-122	Hepatocytes	TMEM187
7.00986953262236E-127	2.98407935598092	0.123	0.016	1.17471393627685E-122	Hepatocytes	TUFT1
8.09200040480753E-127	2.96639183994938	0.118	0.013	1.35605742783765E-122	Hepatocytes	PPL
8.13562867453604E-127	2.75041737921712	0.126	0.017	1.36336865327875E-122	Hepatocytes	PACRG
8.23024537891075E-127	1.65224943753649	0.148	0.03	1.37922452059786E-122	Hepatocytes	SLC2A5
1.01136025780456E-126	3.05555894137529	0.112	0.009	1.69483752002887E-122	Hepatocytes	ALDH8A1
1.01537278536133E-126	0.622813672102528	0.267	0.106	1.70156171370852E-122	Hepatocytes	LANCL1
1.13243174800498E-126	0.359730015040488	0.398	0.208	1.89772912330675E-122	Hepatocytes	FLOT2
1.15997097655807E-126	0.962352903787551	0.194	0.057	1.94387936251602E-122	Hepatocytes	PHLPP1
1.25464233289741E-126	2.22358157918162	0.129	0.019	2.10252962146948E-122	Hepatocytes	SYT1
1.57654237392872E-126	0.539683421448898	0.305	0.135	2.64196971022975E-122	Hepatocytes	MZT2B
2.50899742956234E-126	0.617142174563103	0.289	0.125	4.20457789246057E-122	Hepatocytes	SLC39A1
4.11349025139579E-126	0.709695301856695	0.191	0.056	6.89338696328907E-122	Hepatocytes	CSPP1
5.09970414413054E-126	1.75885259341329	0.148	0.03	8.54608420473397E-122	Hepatocytes	PHLDA2

6.99017887166863E-126	1.76556735441287	0.192	0.06	1.17141417531423E-121	Hepatocytes	QPRT
8.46743233315852E-126	0.663856679427467	0.231	0.083	1.4189723103907E-121	Hepatocytes	ABCB10
8.47140093894904E-126	1.24672966738828	0.166	0.041	1.41963736934908E-121	Hepatocytes	AGBL5
1.00020606973986E-125	0.636398657441309	0.216	0.072	1.67614533167006E-121	Hepatocytes	ZC3H14
2.15963964735623E-125	2.83914050076392	0.116	0.012	3.61912412103956E-121	Hepatocytes	AJUBA
2.27996022800491E-125	1.23812195838726	0.157	0.035	3.82075735009063E-121	Hepatocytes	GCNT2
2.46216866862776E-125	0.8046850757513	0.205	0.065	4.1261022548864E-121	Hepatocytes	CCNB1IP1
2.64255540748113E-125	0.278487965916261	0.355	0.172	4.42839435185687E-121	Hepatocytes	LONP1
2.71087238065796E-125	0.535876417187961	0.332	0.156	4.54287993550661E-121	Hepatocytes	GPI
2.85299807939558E-125	2.9644280591815	0.117	0.013	4.78105418145112E-121	Hepatocytes	PLEKHG6
2.89994823998565E-125	0.296358613435174	0.481	0.268	4.85973326056794E-121	Hepatocytes	ATP5MC2
2.92932716363445E-125	3.16105537567579	0.113	0.011	4.90896646081861E-121	Hepatocytes	CYP17A1
3.16520988719824E-125	2.5826196472655	0.126	0.018	5.30425872896681E-121	Hepatocytes	CHMP4C
3.99113675616151E-125	0.759264890190822	0.258	0.102	6.68834697597546E-121	Hepatocytes	TIMM17A
4.28434434166052E-125	1.20896235476932	0.169	0.043	7.1797042477547E-121	Hepatocytes	CISD1
6.01564971392258E-125	0.271045322440661	0.413	0.216	1.00810257905915E-120	Hepatocytes	HSPA5
6.04164112292787E-125	2.39001283446722	0.133	0.022	1.01245821938025E-120	Hepatocytes	SH2D4A
6.19028015862754E-125	2.69401895614959	0.119	0.014	1.0373671489828E-120	Hepatocytes	LDHD
6.44227008729769E-125	0.418723693846541	0.377	0.191	1.07959562122935E-120	Hepatocytes	POLR2E
7.15323583346319E-125	0.44097085163298	0.229	0.081	1.19873926097176E-120	Hepatocytes	EPS8
7.33046696137073E-125	1.2594739289266	0.188	0.056	1.22843965338651E-120	Hepatocytes	SNRPE
7.72110170559648E-125	0.670571202913691	0.168	0.042	1.29390222382386E-120	Hepatocytes	MPP6
1.18130466131608E-124	0.72794177787923	0.277	0.116	1.97963035143348E-120	Hepatocytes	MEGF9
1.65351106649167E-124	2.66373450463899	0.128	0.019	2.77095384522673E-120	Hepatocytes	SAA1
1.75065040822447E-124	2.06410214640968	0.138	0.025	2.93373995410257E-120	Hepatocytes	DEPRECATED-ENSG00000198736
1.91566438322447E-124	0.27196969275537	0.255	0.098	3.21027037340757E-120	Hepatocytes	RBCK1
2.63836778404011E-124	3.58618855428316	0.107	0.008	4.42137673249442E-120	Hepatocytes	DMGDH
3.0112815650928E-124	0.532455344159638	0.334	0.158	5.04630564678252E-120	Hepatocytes	EPN1
3.024397894608E-124	0.652602602601096	0.198	0.061	5.06828599178409E-120	Hepatocytes	FLVCR1
5.21270191150012E-124	0.602620821171175	0.175	0.047	8.73544586329191E-120	Hepatocytes	DCUN1D2
5.6953532642069E-124	0.718963120041602	0.197	0.061	9.54427300015792E-120	Hepatocytes	POLB
7.4222430638244E-124	1.15676364187853	0.181	0.051	1.24381949263569E-119	Hepatocytes	SAV1
9.05956719915716E-124	2.81101243537902	0.113	0.011	1.51820227123476E-119	Hepatocytes	EFNA5
1.19970077608082E-123	0.253918083030449	0.274	0.112	2.01045856055624E-119	Hepatocytes	TAF2
1.26814272122362E-123	0.720049201609517	0.231	0.084	2.12515357222654E-119	Hepatocytes	COQ9
1.28974777934284E-123	0.271994995163173	0.409	0.214	2.16135932862273E-119	Hepatocytes	ELAVL1
1.36470257378202E-123	0.781847539851715	0.179	0.049	2.2869685731439E-119	Hepatocytes	TMTC4
1.5462328154445E-123	0.42091136921729	0.259	0.103	2.5911769521219E-119	Hepatocytes	PGAP3
1.73310467247375E-123	0.406663376854627	0.21	0.069	2.90433681013151E-119	Hepatocytes	CHM
2.8406444480415E-123	0.876049737472697	0.227	0.082	4.76035196602795E-119	Hepatocytes	DEPRECATED-ENSG00000134049
3.13957797333023E-123	2.48783364645801	0.116	0.013	5.26130476770679E-119	Hepatocytes	IGSF3
3.18364190417161E-123	1.1396897659136	0.205	0.068	5.33514710301079E-119	Hepatocytes	SMIM20
5.76302380453102E-123	0.742701478231299	0.276	0.116	9.65767529163308E-119	Hepatocytes	UGP2
6.84306188696794E-123	0.569527492780596	0.288	0.125	1.14676031101809E-118	Hepatocytes	SUCLG1
7.03876700294997E-123	1.06627511540523	0.265	0.111	1.17955657435436E-118	Hepatocytes	CENPB
7.83467344924622E-123	1.08583592115915	0.221	0.079	1.31293457662468E-118	Hepatocytes	NABP2
8.17738178143948E-123	1.09798179323716	0.228	0.084	1.37036563893363E-118	Hepatocytes	SF3B4
8.68557339272932E-123	0.538490829101021	0.222	0.078	1.45552838915358E-118	Hepatocytes	CAMSAP2
9.53303914502453E-123	0.501122675662004	0.192	0.059	1.59754669992321E-118	Hepatocytes	MED30
9.71377559416172E-123	0.277095535772629	0.22	0.075	1.62783451406962E-118	Hepatocytes	GPHN
9.84357538299218E-123	0.855182622997032	0.168	0.044	1.64958636268183E-118	Hepatocytes	DNMBP
1.40127852611106E-122	0.481943514025961	0.298	0.132	2.34826255405691E-118	Hepatocytes	GALNT2
1.85939966336607E-122	1.15435744265191	0.229	0.085	3.11598195586886E-118	Hepatocytes	BANF1
1.97664995162769E-122	0.564485618805185	0.186	0.055	3.31246998893768E-118	Hepatocytes	GUCY1A1
3.30120762712428E-122	0.536673022698323	0.228	0.083	5.53216374153487E-118	Hepatocytes	RNF113A
3.41512479096692E-122	0.878409936691134	0.168	0.044	5.72306612470236E-118	Hepatocytes	PSD3
6.59464934989205E-122	0.289881252697961	0.305	0.137	1.10513133805491E-117	Hepatocytes	INTS6
7.33328098706052E-122	2.9430781958906	0.117	0.015	1.2289112278116E-117	Hepatocytes	BBOX1
7.518635518437E-122	0.639858199840651	0.217	0.075	1.25997294017967E-117	Hepatocytes	NFYB
8.81648383168923E-122	2.79090619539781	0.112	0.011	1.47746636051448E-117	Hepatocytes	HSD11B1
1.03715943816621E-121	0.671695628320212	0.192	0.059	1.73807178647893E-117	Hepatocytes	UPF3B
1.11518258995105E-121	2.20727602126732	0.122	0.017	1.86882298423997E-117	Hepatocytes	PACSIN3
1.43150928693347E-121	0.396482272576682	0.3	0.134	2.39892326304311E-117	Hepatocytes	USO1

1.6209027851913E-121	0.957560225682564	0.147	0.031	2.71630888742358E-117	Hepatocytes	CARD10
1.89137105481816E-121	0.557486585348058	0.309	0.141	3.16955961366427E-117	Hepatocytes	POLR3GL
2.14098758028346E-121	1.58888868607149	0.126	0.019	3.58786698703902E-117	Hepatocytes	GSTA4
2.37438103144816E-121	0.94694048132694	0.223	0.08	3.97898773250082E-117	Hepatocytes	VPS25
2.70077493651048E-121	1.05000288811994	0.185	0.055	4.52595863860427E-117	Hepatocytes	RNF170
3.56331491466752E-121	0.468930192356691	0.331	0.159	5.97140313399983E-117	Hepatocytes	NEU1
3.65344245786383E-121	4.02912839547621	0.104	0.007	6.1224388708882E-117	Hepatocytes	SHANK2
3.88087154832729E-121	2.05682217530558	0.136	0.025	6.50356454068686E-117	Hepatocytes	MPV17L
4.19493875359498E-121	1.57240337307932	0.127	0.019	7.02987836327446E-117	Hepatocytes	NAALADL2
4.84067545653388E-121	0.897208060138467	0.208	0.07	8.11200393005948E-117	Hepatocytes	DEPRECATED-ENSG00000147123
6.12625000960187E-121	3.13202863758727	0.106	0.009	1.02663697660908E-116	Hepatocytes	SCN8A
6.14117081719056E-121	0.867738071643401	0.199	0.065	1.02913740554479E-116	Hepatocytes	ACSF2
1.22716674132048E-120	0.660514104193492	0.201	0.065	2.05648602510486E-116	Hepatocytes	RPRD1B
1.30390595350668E-120	1.32386722458644	0.177	0.051	2.18508559688649E-116	Hepatocytes	ENDOG
1.80559559708679E-120	0.286414344682503	0.324	0.151	3.02581710159805E-116	Hepatocytes	TOMM20
2.02945073758008E-120	0.581604415323485	0.205	0.068	3.4009535460367E-116	Hepatocytes	SESN2
5.82690795206563E-120	2.339828246525	0.122	0.017	9.76473234607158E-116	Hepatocytes	CRB3
7.40880081422359E-120	1.18959805061564	0.165	0.043	1.24156684044759E-115	Hepatocytes	GTF2IRD2B
9.54282167993116E-120	1.07517213708401	0.176	0.05	1.59918605712286E-115	Hepatocytes	JAGN1
1.15155916496438E-119	0.282878236525301	0.323	0.153	1.92978284864731E-115	Hepatocytes	TUBGCP3
1.37956153384721E-119	1.19130284146731	0.161	0.04	2.31186921842115E-115	Hepatocytes	UMPS
1.92764257633243E-119	1.79619761699084	0.147	0.032	3.23034342941789E-115	Hepatocytes	CROT
1.92770396259045E-119	1.01006828378552	0.197	0.064	3.23044630050908E-115	Hepatocytes	UQCRFS1
2.05990833811831E-119	0.551768869038471	0.311	0.143	3.45199439301867E-115	Hepatocytes	MANF
2.18334634509219E-119	0.446305038509742	0.341	0.166	3.65885180510549E-115	Hepatocytes	PACSIN2
4.57444643686723E-119	1.88571445678607	0.144	0.031	7.6658573389021E-115	Hepatocytes	AIFM2
5.60719032952655E-119	0.500288714418198	0.195	0.062	9.3965295542206E-115	Hepatocytes	CPSF1
6.27209930917075E-119	0.386751151503404	0.224	0.081	1.05107840223083E-114	Hepatocytes	FKBP3
6.71182299778197E-119	1.20392741159216	0.175	0.05	1.1247672979683E-114	Hepatocytes	SDHAF4
8.40187882954767E-119	0.369786147609473	0.222	0.079	1.4079868542556E-114	Hepatocytes	UPF3A
9.84992126826786E-119	3.52655503885924	0.107	0.01	1.65064980613633E-114	Hepatocytes	DEPRECATED-ENSG00000227471
1.58709835692812E-118	1.13454833127404	0.185	0.057	2.65965942654014E-114	Hepatocytes	TNFRSF11A
1.65221179321301E-118	3.38093969894918	0.102	0.007	2.76877652306636E-114	Hepatocytes	SLC10A1
1.82198652177512E-118	0.726630319107257	0.201	0.066	3.05328501319075E-114	Hepatocytes	ETAA1
1.83115405628388E-118	2.31095212985447	0.115	0.014	3.06864796752053E-114	Hepatocytes	MUC20
2.29225151697323E-118	3.65483935831771	0.103	0.008	3.84135509214374E-114	Hepatocytes	SLC44A4
2.43879409851066E-118	0.803521227654712	0.18	0.053	4.08693115028417E-114	Hepatocytes	ZNF436
2.54091637022388E-118	0.250254729879587	0.377	0.194	4.25806765322119E-114	Hepatocytes	DTX3L
2.58200829027237E-118	0.787119616324177	0.303	0.143	4.32692949283844E-114	Hepatocytes	DEPRECATED-ENSG00000163931
3.18191545378458E-118	0.668896404258595	0.24	0.093	5.33225391745221E-114	Hepatocytes	PSMG3
3.21079038103052E-118	3.32970579803234	0.11	0.012	5.38064252053095E-114	Hepatocytes	CNGA1
3.2552098911849E-118	0.796051822501616	0.17	0.046	5.45508073564766E-114	Hepatocytes	DVL1
4.33828388137035E-118	0.749860922054022	0.19	0.059	7.27009612840044E-114	Hepatocytes	HIBCH
4.58563884376666E-118	1.45591996820294	0.183	0.056	7.68461357438416E-114	Hepatocytes	FEN1
4.95416780279954E-118	0.558072849964137	0.281	0.123	8.30219440393148E-114	Hepatocytes	ZFYVE21
5.2577157846651E-118	3.8735169561007	0.105	0.009	8.81088011194178E-114	Hepatocytes	CTSE
5.92925375792762E-118	0.865326497102659	0.187	0.058	9.9362434475351E-114	Hepatocytes	RIC8B
6.63004386468483E-118	0.796589968115332	0.177	0.051	1.11106275084388E-113	Hepatocytes	FMC1
7.03896324925798E-118	1.7004128586108	0.127	0.021	1.17958946131065E-113	Hepatocytes	B4GALT6
7.68028689610233E-118	0.420638962377322	0.29	0.128	1.28706247804883E-113	Hepatocytes	NDUFS4
9.66884339064577E-118	1.36457636853719	0.173	0.05	1.62030477540442E-113	Hepatocytes	SYNJ2BP
9.77839921841658E-118	0.463798833385468	0.325	0.155	1.63866414102225E-113	Hepatocytes	HDGFL2
1.590010629988E-117	0.803313288711337	0.17	0.047	2.66453981373388E-113	Hepatocytes	KANSL1L
2.2601622916353E-117	0.464962294356575	0.15	0.034	3.78757996832243E-113	Hepatocytes	FBLIM1
2.56752847930735E-117	0.30705183978583	0.304	0.139	4.30266422562326E-113	Hepatocytes	DNPEP
2.7749927197989E-117	2.78860996636384	0.104	0.009	4.65033279983899E-113	Hepatocytes	ACY3
3.25889487744395E-117	0.316879954566216	0.2	0.066	5.46125603562057E-113	Hepatocytes	PLCXD1
3.74947363754699E-117	0.78246612212003	0.169	0.045	6.28336792180125E-113	Hepatocytes	IPP
4.09273209873576E-117	0.923038748389247	0.171	0.048	6.85860045106139E-113	Hepatocytes	ENOX2
4.82607405154342E-117	0.893520772894633	0.142	0.029	8.08753489557646E-113	Hepatocytes	TPST1
4.88953294946766E-117	0.514942863453254	0.328	0.158	8.1938793167179E-113	Hepatocytes	RPN2
6.50658546232663E-117	0.622138549480283	0.207	0.071	1.0903735917767E-112	Hepatocytes	PDCD5
6.53527348145163E-117	0.521862451206013	0.264	0.111	1.09518113002166E-112	Hepatocytes	NDUFS3

7.31698735761265E-117	0.615862204955555	0.169	0.046	1.22618074138873E-112	Hepatocytes	KIF21A
8.77295209562857E-117	2.53424956150622	0.112	0.013	1.47017131218544E-112	Hepatocytes	PPP1R9A
1.41434080302138E-116	0.251952140614107	0.354	0.176	2.37015231770323E-112	Hepatocytes	KXD1
1.4811245614802E-116	1.07629763644188	0.128	0.022	2.48206854012852E-112	Hepatocytes	MTCL1
2.01629675296165E-116	0.427903947572304	0.246	0.097	3.37891009861314E-112	Hepatocytes	ACBD6
2.39281064528475E-116	1.14514485972028	0.147	0.034	4.00987207936818E-112	Hepatocytes	FSD1L
3.14282534310637E-116	1.14681271559119	0.134	0.026	5.26674670997766E-112	Hepatocytes	TCAF2
3.24761417862395E-116	0.904403853498224	0.21	0.075	5.44235184053801E-112	Hepatocytes	ZNF768
3.33701577204257E-116	2.55973795530684	0.107	0.011	5.59217103078894E-112	Hepatocytes	RAB26
3.69708393836104E-116	0.56989668435825	0.228	0.086	6.19557326390543E-112	Hepatocytes	PIK3CB
4.70479853696085E-116	0.59275237030922	0.2	0.066	7.88430138823899E-112	Hepatocytes	CXXC5
5.15662216758428E-116	0.420840581838525	0.228	0.085	8.64146742843774E-112	Hepatocytes	DEPRECATED-ENSG00000078668
7.70940538976609E-116	1.13659097546127	0.193	0.063	1.291942155217E-111	Hepatocytes	TICAM1
8.54866101296911E-116	1.10661624887191	0.198	0.067	1.43258461255336E-111	Hepatocytes	LYPLA1
9.20961170668522E-116	0.346116969889078	0.234	0.09	1.54334672980631E-111	Hepatocytes	MTFR1L
9.51850420432226E-116	1.44916110508356	0.196	0.066	1.59511093456032E-111	Hepatocytes	CENPU
9.95002499249073E-116	3.31999741234728	0.106	0.01	1.6674251882416E-111	Hepatocytes	SLC5A1
1.05934465238309E-115	0.427468823546661	0.499	0.298	1.77524976846358E-111	Hepatocytes	ENO1
1.06290249025081E-115	0.673876261396472	0.146	0.033	1.78121199316231E-111	Hepatocytes	PARD3B
1.30790831891512E-115	1.23237902032202	0.17	0.049	2.19179276083795E-111	Hepatocytes	HINT3
2.02913473069424E-115	0.420296812967083	0.223	0.083	3.4004239816974E-111	Hepatocytes	ENPP4
2.34858630932355E-115	0.592971562753074	0.288	0.13	3.9357609371644E-111	Hepatocytes	LAPTM4A
2.38983064449034E-115	0.506270469282309	0.177	0.052	4.00487819403692E-111	Hepatocytes	GK5
2.98219573861116E-115	0.508637896629519	0.221	0.081	4.99756361876458E-111	Hepatocytes	MVB12A
3.29383574867076E-115	0.842213156010952	0.19	0.06	5.51980994762245E-111	Hepatocytes	YIPF6
3.35839330853339E-115	0.401132546590471	0.166	0.044	5.62799550644026E-111	Hepatocytes	THBS3
3.55200830696711E-115	1.11730371522107	0.183	0.056	5.95245552081549E-111	Hepatocytes	IFI27L1
4.39108534712607E-115	1.5841623408153	0.12	0.018	7.35858082471387E-111	Hepatocytes	KIAA1211
4.54062641967156E-115	0.87672361727493	0.208	0.074	7.6091817540856E-111	Hepatocytes	DEPRECATED-ENSG00000148834
4.5899472271002E-115	1.99465666070335	0.135	0.027	7.69183356317452E-111	Hepatocytes	FAM111B
5.54860016828108E-115	0.352954031306998	0.246	0.098	9.29834416200544E-111	Hepatocytes	CHMP2B
6.01298489502129E-115	0.42573165290336	0.306	0.141	1.00765600870767E-110	Hepatocytes	CRELD2
6.40001034145856E-115	0.39978104269642	0.317	0.151	1.07251373302163E-110	Hepatocytes	SNRPB
6.60393067592025E-115	1.444385431342	0.128	0.023	1.10668670267071E-110	Hepatocytes	FAM171A1
7.81936113440127E-115	1.27152529499215	0.123	0.02	1.31036853890297E-110	Hepatocytes	NINL
9.2832234436504E-115	0.342716077977816	0.184	0.056	1.55568258468693E-110	Hepatocytes	ASAP2
9.72521422334631E-115	0.75549182337968	0.137	0.027	1.62975139954837E-110	Hepatocytes	NEO1
1.0433733046122E-114	1.21373373992192	0.166	0.046	1.74848498386913E-110	Hepatocytes	FAM210B
1.0866784463291E-114	2.10314191203875	0.116	0.016	1.82105574035831E-110	Hepatocytes	ZXDB
1.2175464480083E-114	0.668209867069985	0.218	0.08	2.04036433757232E-110	Hepatocytes	PPP1CC
1.26891883011637E-114	0.8642864192576	0.166	0.045	2.12645417550901E-110	Hepatocytes	RBFA
1.33495953772533E-114	2.82931618787312	0.107	0.012	2.23712519332011E-110	Hepatocytes	GAL3ST1
1.77178028910765E-114	0.376297286317005	0.21	0.074	2.96914940848661E-110	Hepatocytes	RNF139
2.03928118355166E-114	3.15984894803798	0.104	0.01	3.41742740739587E-110	Hepatocytes	TTLL6
2.15080330257829E-114	0.911258669805198	0.251	0.105	3.60431617446069E-110	Hepatocytes	ATP5MF
2.17876815501162E-114	2.25994261333598	0.112	0.014	3.65117967416848E-110	Hepatocytes	ADCY10
2.31946514145034E-114	0.397621351282768	0.233	0.09	3.88695968404247E-110	Hepatocytes	MOAP1
2.46085987468458E-114	0.257521505481169	0.206	0.071	4.12390897799641E-110	Hepatocytes	SLC25A29
2.63603930493138E-114	2.50032564322775	0.107	0.011	4.41747466720401E-110	Hepatocytes	PCDHGA8
3.86334403360875E-114	2.7723964696956	0.113	0.015	6.47419193152154E-110	Hepatocytes	DEPRECATED-ENSG00000178445
4.93659408276129E-114	1.08878533186302	0.207	0.074	8.27274436389137E-110	Hepatocytes	MPND
5.14234173880181E-114	1.08152295607506	0.185	0.059	8.61753628588407E-110	Hepatocytes	UHRF1
5.91443747174117E-114	0.523004655389172	0.264	0.114	9.91141431514385E-110	Hepatocytes	GRAMD4
6.13232444828989E-114	0.501266528971288	0.204	0.069	1.02765493104442E-109	Hepatocytes	FANCL
6.42237332645028E-114	0.339961926071092	0.269	0.115	1.07626132204654E-109	Hepatocytes	NDUFAF8
6.79222948053214E-114	2.08571038521968	0.107	0.012	1.13824181634758E-109	Hepatocytes	FSBP
7.18718116540177E-114	0.297126927502538	0.309	0.144	1.20442781969803E-109	Hepatocytes	GTF2I
9.10223101356187E-114	0.703254862636107	0.229	0.089	1.5253518732527E-109	Hepatocytes	TOMM5
1.03766101443084E-113	0.271384847943861	0.295	0.134	1.73891232798321E-109	Hepatocytes	SMDT1
1.99682076547946E-113	1.93069487596186	0.128	0.023	3.34627223879049E-109	Hepatocytes	XRCC3
2.17457968815496E-113	0.727672326799909	0.206	0.073	3.64416064141008E-109	Hepatocytes	PSMC1
2.36720799053766E-113	1.63424966258665	0.109	0.013	3.96696715054301E-109	Hepatocytes	ARSJ
2.55245737853795E-113	0.744124397540386	0.264	0.114	4.27740807495389E-109	Hepatocytes	AFG3L2

2.72277651613218E-113	0.40639155787564	0.302	0.14	4.56282888573432E-109	Hepatocytes	COX14
3.24254409093778E-113	0.795506304042152	0.18	0.055	5.43385538759354E-109	Hepatocytes	MINDY1
4.39581010085473E-113	0.419334352025057	0.335	0.167	7.36649856701235E-109	Hepatocytes	SSRP1
7.02989648608435E-113	1.28107942914817	0.169	0.049	1.17807005313801E-108	Hepatocytes	TIGD5
7.40641059288303E-113	1.91492397940972	0.128	0.024	1.24116628715534E-108	Hepatocytes	HSBP1L1
7.43318616351022E-113	0.65783402222456	0.189	0.061	1.24565333728104E-108	Hepatocytes	INO80C
1.00130781427416E-112	1.14785321179414	0.126	0.023	1.67799163516063E-108	Hepatocytes	AGTR1
1.13978653440849E-112	0.554729182632529	0.342	0.174	1.91005427436174E-108	Hepatocytes	RBM42
1.20305997888457E-112	0.28034885094921	0.236	0.094	2.01608791261477E-108	Hepatocytes	SLC2A13
1.73570997302146E-112	1.49252860283536	0.155	0.04	2.90870277278936E-108	Hepatocytes	NTHL1
2.0109083075029E-112	0.874925349285676	0.176	0.053	3.36988014171337E-108	Hepatocytes	TBCE
2.04730850864494E-112	0.273323235548518	0.25	0.102	3.43087959878719E-108	Hepatocytes	PMM2
2.11080927806977E-112	0.395501616199001	0.252	0.105	3.53729418818933E-108	Hepatocytes	DPY30
2.24307460149257E-112	2.52852333103346	0.103	0.01	3.75894441718125E-108	Hepatocytes	RAC3
2.38800743130705E-112	0.655059941752794	0.217	0.081	4.00182285338435E-108	Hepatocytes	SNX24
2.48940041752166E-112	0.593612457114282	0.226	0.087	4.17173721968279E-108	Hepatocytes	NDUFS8
3.14704222362997E-112	0.630644697463413	0.257	0.109	5.2738133583591E-108	Hepatocytes	TMEM258
3.24967642060366E-112	0.364688405973384	0.21	0.075	5.44580774564761E-108	Hepatocytes	UBQLN4
3.35538002374768E-112	1.23060215914407	0.158	0.042	5.62294584379636E-108	Hepatocytes	SWI5
3.36301417161895E-112	1.41221067174012	0.153	0.04	5.63573914879904E-108	Hepatocytes	SEPHS1
4.02730070518032E-112	1.03927057335296	0.142	0.032	6.74895052174118E-108	Hepatocytes	FAM184A
4.30232715600199E-112	1.5051109939475	0.128	0.024	7.20983984802814E-108	Hepatocytes	TRIM2
5.05306332748795E-112	0.973452749887278	0.222	0.084	8.46792352420431E-108	Hepatocytes	ABHD14B
5.31841266471327E-112	0.61213042142892	0.252	0.105	8.9125959435265E-108	Hepatocytes	TOMM6
5.34100427209171E-112	2.29440994201967	0.131	0.026	8.95045495917129E-108	Hepatocytes	TADA1
5.38485561446998E-112	0.650263851110471	0.318	0.155	9.0239410387288E-108	Hepatocytes	NME2
5.82513032335874E-112	0.455545258484554	0.251	0.104	9.76175339588458E-108	Hepatocytes	GORASP1
6.31784815913765E-112	0.264183580625482	0.439	0.245	1.05874499450829E-107	Hepatocytes	SERF2
7.41596624873495E-112	0.333386485122724	0.265	0.113	1.242767623963E-107	Hepatocytes	SRF
7.48666370727324E-112	0.382685029878119	0.321	0.157	1.25461510406485E-107	Hepatocytes	DNAJC4
7.98509270142825E-112	1.22483928005971	0.181	0.057	1.33814183490535E-107	Hepatocytes	GLRX2
9.61391637148911E-112	0.349486356165107	0.235	0.093	1.61110010553414E-107	Hepatocytes	PLD1
9.63318443373401E-112	0.765504588260529	0.167	0.048	1.61432904740514E-107	Hepatocytes	ENDOV
1.17941190787675E-111	2.61244187121836	0.102	0.01	1.97645847521986E-107	Hepatocytes	ANGPTL3
1.1913271506341E-111	0.677603394711096	0.251	0.104	1.99642603903262E-107	Hepatocytes	ERH
1.26269268417307E-111	0.34899559058279	0.403	0.217	2.11602040013723E-107	Hepatocytes	EMC4
1.70303480687413E-111	2.21776047611756	0.125	0.023	2.85394572935966E-107	Hepatocytes	ALG1L
1.75606182007887E-111	0.391672626259218	0.367	0.191	2.94280839808818E-107	Hepatocytes	PDIA6
1.76321366895521E-111	0.444816162296963	0.253	0.103	2.95479346643514E-107	Hepatocytes	RGS3
2.16931345088496E-111	0.668597763946128	0.168	0.049	3.63533548099302E-107	Hepatocytes	ZNF618
2.7502284632246E-111	0.416238200328554	0.205	0.072	4.60883285867178E-107	Hepatocytes	MCEE
2.91633949954076E-111	0.795710341972151	0.175	0.053	4.88720173333041E-107	Hepatocytes	UCHL3
3.10972430162656E-111	0.324072779822733	0.206	0.071	5.21127598466578E-107	Hepatocytes	SERPINE1
3.2357060845243E-111	1.7465216075889	0.113	0.016	5.42239625644583E-107	Hepatocytes	PTPRU
3.23753164670705E-111	0.623652098134658	0.243	0.1	5.42545553355168E-107	Hepatocytes	GALT
3.95158939149591E-111	0.788617291499605	0.181	0.057	6.62207350226884E-107	Hepatocytes	ZHX3
4.21609500149029E-111	1.22748232863475	0.204	0.074	7.06533200349743E-107	Hepatocytes	SLC5A6
5.51208821130757E-111	0.667796887103769	0.228	0.089	9.23715742450922E-107	Hepatocytes	IER3IP1
6.74185590826384E-111	2.02924455655971	0.124	0.022	1.12980021310686E-106	Hepatocytes	ZNF57
7.73095186359766E-111	1.1430681482903	0.154	0.04	1.2955529133017E-106	Hepatocytes	MBTPS2
9.15783991780201E-111	0.661589323794263	0.199	0.069	1.53467081342526E-106	Hepatocytes	OARD1
1.14900145806671E-110	0.796568177755705	0.184	0.059	1.9254966434282E-106	Hepatocytes	COQ8B
1.21460068825567E-110	0.477244501704886	0.221	0.084	2.03542783337885E-106	Hepatocytes	RNF185
1.37896492553711E-110	0.281527709221243	0.318	0.153	2.3108694222151E-106	Hepatocytes	FGD4
1.55915882745023E-110	1.24513737070057	0.17	0.051	2.6128383630411E-106	Hepatocytes	SKP2
1.59213468152159E-110	1.61016417507391	0.128	0.025	2.66809929929389E-106	Hepatocytes	CACFD1
1.65171555541268E-110	0.927539913836776	0.187	0.061	2.76794492776057E-106	Hepatocytes	MSH2
1.65984333149469E-110	0.391307951587282	0.324	0.158	2.78156545491881E-106	Hepatocytes	COX7A2L
1.68193981502724E-110	0.33890907642546	0.274	0.121	2.81859474202264E-106	Hepatocytes	LRRC58
2.06578166824395E-110	0.925504211940906	0.192	0.065	3.46183691964322E-106	Hepatocytes	UCK2
2.75126138460997E-110	0.339816199705605	0.187	0.06	4.61056382832939E-106	Hepatocytes	VEZT
3.51644505367966E-110	2.84992999832941	0.101	0.01	5.89285862095638E-106	Hepatocytes	EFNA2
3.70696588184214E-110	0.5865664441415	0.195	0.067	6.21213342479106E-106	Hepatocytes	WFS1

4.71668194841969E-110	0.638601382402245	0.206	0.074	7.90421560916172E-106	Hepatocytes	IFRD2
6.98145316058242E-110	0.96136896303994	0.153	0.04	1.1699519206504E-105	Hepatocytes	SPAG16
7.2645437848633E-110	1.2758486045858	0.122	0.021	1.21739224746739E-105	Hepatocytes	MECOM
8.09196828374685E-110	0.251220395504019	0.25	0.103	1.3560520449903E-105	Hepatocytes	PSMC6
8.75890392804388E-110	0.418173451908253	0.246	0.102	1.46781712026159E-105	Hepatocytes	VIRMA
8.83552841608736E-110	0.747491303099927	0.165	0.047	1.48065785196792E-105	Hepatocytes	ATG4A
8.89413169318026E-110	0.315992520868228	0.474	0.274	1.49047858914315E-105	Hepatocytes	PDIA3
1.13069022962193E-109	1.21408110615965	0.137	0.03	1.89481068680043E-105	Hepatocytes	ADSSL1
1.30355697133723E-109	1.68207815589766	0.138	0.031	2.18450077256693E-105	Hepatocytes	C17orf58
1.3614843324396E-109	0.51581374674401	0.165	0.046	2.28157544430228E-105	Hepatocytes	RAI14
1.4033009972008E-109	1.30697112166525	0.149	0.038	2.3516518111091E-105	Hepatocytes	PHB
1.81625667616351E-109	0.61466052318716	0.318	0.157	3.04368293791481E-105	Hepatocytes	WSB2
2.14457429496735E-109	0.746697512789268	0.214	0.079	3.59387760350629E-105	Hepatocytes	ACTL6A
2.22713186608277E-109	0.77409717686396	0.148	0.037	3.73222758118151E-105	Hepatocytes	ADCY9
2.38679741857437E-109	0.350953714497145	0.207	0.074	3.99979511404693E-105	Hepatocytes	CXorf40A
3.85954768069077E-109	0.805878353651423	0.233	0.094	6.4678300033016E-105	Hepatocytes	CLUH
3.98860217138849E-109	1.34769876877286	0.234	0.099	6.68409951881283E-105	Hepatocytes	ATF5
4.72276258549005E-109	2.64669149205421	0.115	0.018	7.91440554076423E-105	Hepatocytes	PC
1.19780484253709E-108	0.915221730762572	0.14	0.032	2.00728135512366E-104	Hepatocytes	DEPRECATED-ENSG00000022267
1.22455980939292E-108	0.845120655368001	0.185	0.061	2.05211732858066E-104	Hepatocytes	CENPM
1.48832168193138E-108	0.43537176776201	0.224	0.087	2.4941294745806E-104	Hepatocytes	EZH2
1.83446031543625E-108	0.75025997644452	0.173	0.053	3.07418859660807E-104	Hepatocytes	PDXDC1
1.87694855754088E-108	2.98602108180441	0.102	0.011	3.14539039272701E-104	Hepatocytes	CUX2
2.05501818907978E-108	1.69870559320373	0.16	0.046	3.4437994812599E-104	Hepatocytes	ZNF367
2.27757466997897E-108	1.95163262752151	0.112	0.017	3.81675963195075E-104	Hepatocytes	RTN4RL2
2.317868202691E-108	0.976935802447495	0.188	0.063	3.88428353406957E-104	Hepatocytes	RPP21
2.9240048133528E-108	1.26754141440588	0.176	0.056	4.90004726621662E-104	Hepatocytes	DCTPP1
2.99155639284462E-108	0.478571898199812	0.221	0.085	5.01325020312901E-104	Hepatocytes	C1orf122
4.22166604280302E-108	0.965024850389245	0.194	0.067	7.0746679545293E-104	Hepatocytes	MPLKIP
4.84355528931752E-108	2.19416976710851	0.12	0.021	8.1168299538383E-104	Hepatocytes	PSAT1
4.8746703666713E-108	0.315398168608779	0.27	0.119	8.16897260046776E-104	Hepatocytes	GBA2
6.70603729187952E-108	0.425784252462564	0.223	0.088	1.12379772937317E-103	Hepatocytes	ARHGEF11
7.52240979923796E-108	0.364168605790078	0.245	0.102	1.2606054341563E-103	Hepatocytes	NDUFB2
1.00346229333623E-107	1.01850025588654	0.182	0.059	1.68160211117286E-103	Hepatocytes	BOP 1.00
1.03614464858981E-107	1.60068742083709	0.143	0.035	1.73637120210681E-103	Hepatocytes	DHRS4L2
1.11689824156327E-107	1.40036830297165	0.118	0.02	1.87169807321173E-103	Hepatocytes	BMP2
1.32118840241052E-107	0.462940088710408	0.246	0.103	2.21404752475956E-103	Hepatocytes	ILF2
1.50579511789921E-107	1.03786265615912	0.158	0.044	2.5234114585755E-103	Hepatocytes	FAM200B
1.56147845671952E-107	0.734837558150733	0.225	0.089	2.61672559777057E-103	Hepatocytes	PSMD2
2.00514635940335E-107	0.413100032808858	0.193	0.066	3.36022426908814E-103	Hepatocytes	TIMM9
2.15304908081701E-107	0.458557794717728	0.267	0.119	3.60807964963315E-103	Hepatocytes	ACTR10
2.99276492466769E-107	0.422914099236447	0.277	0.126	5.01527546075811E-103	Hepatocytes	SAP30L
3.42957036400244E-107	2.15179539407988	0.111	0.016	5.74727401599529E-103	Hepatocytes	CHRD
3.58464527157969E-107	0.761917459324258	0.166	0.049	6.00714854611324E-103	Hepatocytes	FBXO4
3.60630991458984E-107	0.694063514565185	0.178	0.057	6.04345415486966E-103	Hepatocytes	NVL
4.03407155085142E-107	2.54877217937156	0.12	0.022	6.76029710491681E-103	Hepatocytes	LEPR
4.39569851293914E-107	0.54984305945093	0.245	0.103	7.36631156798341E-103	Hepatocytes	NDUFA8
4.64622800563706E-107	0.318533240843629	0.197	0.068	7.78614889184659E-103	Hepatocytes	NSUN6
4.94757653586103E-107	0.681553468359949	0.176	0.056	8.29114875879591E-103	Hepatocytes	IPO5
5.3046196532704E-107	2.08946029250005	0.122	0.023	8.88948161495053E-103	Hepatocytes	EEF1AKMT4
6.62155588059435E-107	1.00616689227479	0.205	0.076	1.10964033447E-102	Hepatocytes	GGCT
8.37118622343971E-107	0.696195474580507	0.173	0.054	1.40284338732403E-102	Hepatocytes	GTPBP3
9.03697588692383E-107	0.504300998772134	0.16	0.045	1.5144164191307E-102	Hepatocytes	GPC1
1.07552108481852E-106	0.673286232014933	0.21	0.079	1.80235823393888E-102	Hepatocytes	TBC1D16
1.17111940824307E-106	1.56978129323029	0.106	0.013	1.96256190433374E-102	Hepatocytes	ANKRD29
1.47969496586179E-106	1.54278819273974	0.14	0.034	2.47967282379119E-102	Hepatocytes	REPS2
1.53651877684096E-106	1.5712338705979	0.141	0.035	2.57489816623009E-102	Hepatocytes	DEPRECATED-ENSG00000116005
1.9291271654683E-106	0.385628470840485	0.224	0.088	3.23283130389178E-102	Hepatocytes	VAPB
2.01301865335904E-106	0.618328767474926	0.179	0.058	3.37341665929908E-102	Hepatocytes	CDC37L1
3.00954618592417E-106	0.462906611095638	0.292	0.137	5.04339749837172E-102	Hepatocytes	FAM174C
3.14110962474672E-106	2.16575426347465	0.12	0.022	5.26387150915056E-102	Hepatocytes	FXYD2
3.14855641504701E-106	0.822213245858237	0.141	0.034	5.27635084033578E-102	Hepatocytes	HOOK2
3.42988484374037E-106	1.93295238749437	0.12	0.023	5.74780102114011E-102	Hepatocytes	VNN 2.00

3.53950823673827E-106	0.642207386588115	0.215	0.082	5.93150790312599E-102	Hepatocytes	CMAS
3.67209495590854E-106	1.29030389119996	0.11	0.016	6.15369672711154E-102	Hepatocytes	BEND7
4.98639923237604E-106	0.594879559672299	0.145	0.036	8.35620783361576E-102	Hepatocytes	PARVA
5.7645099890556E-106	1.26560174035778	0.167	0.051	9.66016583965937E-102	Hepatocytes	TMEM14B
5.82810633475339E-106	0.261191733676301	0.347	0.178	9.76674059577973E-102	Hepatocytes	AGPAT3
6.31485140221722E-106	0.582702457014725	0.217	0.084	1.05824279798356E-101	Hepatocytes	GOLPH3L
6.9687604962357E-106	0.498722828641255	0.216	0.084	1.16782488395918E-101	Hepatocytes	PBX3
7.22522146368441E-106	0.865008765665026	0.162	0.047	1.21080261288423E-101	Hepatocytes	SEPTIN10
7.45573834700793E-106	0.253317093906531	0.292	0.137	1.24943263219159E-101	Hepatocytes	UBQLN2
1.12699746738616E-105	0.309036038212513	0.342	0.174	1.88862235584572E-101	Hepatocytes	SLC35A4
1.38211254459353E-105	0.390788547487245	0.208	0.077	2.31614420222983E-101	Hepatocytes	TRPT1
1.56204302536364E-105	0.625521325836814	0.169	0.052	2.61767170190439E-101	Hepatocytes	CCDC61
1.60995768763325E-105	0.532619730054164	0.18	0.059	2.69796709293579E-101	Hepatocytes	TBPL1
1.66966260084463E-105	0.493416960117782	0.217	0.083	2.79802058649543E-101	Hepatocytes	MTX2
1.80236819506785E-105	0.620697376613855	0.134	0.03	3.02040862129471E-101	Hepatocytes	PPP1R13L
1.85959727096944E-105	0.944763493864337	0.145	0.037	3.11631310669058E-101	Hepatocytes	EID3
1.94118073637671E-105	0.282315840854935	0.303	0.145	3.25303067802009E-101	Hepatocytes	LMNB1
2.80470189132934E-105	0.812045251064771	0.183	0.061	4.7001194294897E-101	Hepatocytes	ARMC1
3.02482872105554E-105	3.19641543685707	0.1	0.011	5.06900797074487E-101	Hepatocytes	LYPD6
3.14132578046191E-105	1.08301260334144	0.147	0.038	5.26423374289807E-101	Hepatocytes	B4GAT1
3.32298869534228E-105	1.91542327646864	0.12	0.022	5.56866445565459E-101	Hepatocytes	DEPRECATED-ENSG00000100522
3.69184324464133E-105	1.08462461029757	0.156	0.044	6.18679090936994E-101	Hepatocytes	TP53I3
4.05403062834882E-105	0.469990740822929	0.169	0.051	6.79374452698696E-101	Hepatocytes	SPON2
8.1751731370159E-105	1.03449619125486	0.136	0.031	1.36999551430112E-100	Hepatocytes	CDH2
1.06071820376569E-104	0.564743118087134	0.246	0.106	1.77755156587054E-100	Hepatocytes	RAD23B
1.08166129699467E-104	1.34537804968488	0.137	0.033	1.81264800150367E-100	Hepatocytes	KIAA1958
1.34256677313524E-104	1.17611752754005	0.152	0.041	2.24987339842003E-100	Hepatocytes	C1orf131
1.69547082951241E-104	2.26629770092106	0.106	0.015	2.84127001609689E-100	Hepatocytes	GLDC
2.54030500824321E-104	0.489084310285658	0.324	0.166	4.25704313281398E-100	Hepatocytes	PEPD
4.49165879001219E-104	0.309499797526667	0.213	0.081	7.52712180030243E-100	Hepatocytes	INTS7
4.75569425367845E-104	2.63558049346917	0.107	0.016	7.96959243031435E-100	Hepatocytes	FMO1
5.14269548594518E-104	2.94906511612418	0.1	0.012	8.61812909534694E-100	Hepatocytes	LRRC26
5.29831663141718E-104	0.392423787863283	0.19	0.066	8.87891901092891E-100	Hepatocytes	LIG3
5.45324383845937E-104	0.302240141580892	0.269	0.121	9.13854602449021E-100	Hepatocytes	TMEM59
6.89868035872212E-104	0.54517870214643	0.234	0.097	1.15608085451465E-99	Hepatocytes	UBTD2
1.10117968766922E-103	0.267469667654722	0.326	0.161	1.84535692059609E-99	Hepatocytes	EMC7
1.10144734388569E-103	1.49179793577349	0.118	0.021	1.84580545888363E-99	Hepatocytes	WASF3
1.10684484722332E-103	1.81586701053878	0.112	0.019	1.85485059497683E-99	Hepatocytes	TMEM54
1.12461725297612E-103	0.503451386261811	0.241	0.101	1.88463359253738E-99	Hepatocytes	FEM1C
1.36885047903201E-103	1.50442271993252	0.161	0.049	2.29391963276184E-99	Hepatocytes	UQCC3
1.5957106890404E-103	0.254508096351348	0.27	0.122	2.6740919726939E-99	Hepatocytes	DNAJA1
1.63904579188211E-103	2.8540990237724	0.1	0.012	2.74671293803604E-99	Hepatocytes	DEPRECATED-ENSG00000187017
1.74784188514758E-103	0.99106479001914	0.166	0.051	2.92903343113031E-99	Hepatocytes	MKKS
1.81711872328173E-103	0.560232273463381	0.32	0.163	3.04512755647552E-99	Hepatocytes	TUBB
2.53438798006611E-103	0.61892151147319	0.161	0.048	4.24712737699478E-99	Hepatocytes	BTBD3
2.94404993905706E-103	0.457705074465706	0.187	0.064	4.93363888787182E-99	Hepatocytes	SCRN2
3.03947617320164E-103	1.24518560026791	0.159	0.047	5.0935541710513E-99	Hepatocytes	RMI1
3.1933644821952E-103	0.483156684981936	0.181	0.06	5.35144019926271E-99	Hepatocytes	DMAC2L
3.99820403911104E-103	2.5744213394392	0.101	0.013	6.70019032874228E-99	Hepatocytes	GUCY2C
4.42520558390762E-103	0.677770387952599	0.174	0.056	7.4157595175124E-99	Hepatocytes	CALML4
4.59050930413806E-103	0.317024947730504	0.375	0.201	7.69277549187455E-99	Hepatocytes	TUFM
4.79531500475175E-103	0.56223534233444	0.157	0.045	8.03598888496298E-99	Hepatocytes	SPATA33
6.00080648525319E-103	1.19988440346899	0.103	0.014	1.00561515079873E-98	Hepatocytes	PCDHGB6
6.18586484230904E-103	0.255410601119239	0.272	0.126	1.03662723027415E-98	Hepatocytes	STUB1
6.24408492274361E-103	0.863361057782352	0.128	0.027	1.04638375135337E-98	Hepatocytes	RPA4
6.63771868479582E-103	0.264722043980508	0.265	0.119	1.11234889719808E-98	Hepatocytes	SMC6
7.15448078020184E-103	0.533507724405795	0.285	0.135	1.19894788914623E-98	Hepatocytes	PSMC4
7.50981450719354E-103	0.917432708308201	0.151	0.042	1.25849471511549E-98	Hepatocytes	LRRC28
8.56806670313649E-103	1.0201484582263	0.17	0.055	1.43583661811161E-98	Hepatocytes	EMC9
8.7117469139626E-103	0.372262747195137	0.381	0.207	1.45991454784185E-98	Hepatocytes	VKORC1
8.90850662392504E-103	0.632227254141942	0.281	0.133	1.49288754003736E-98	Hepatocytes	CCDC124
1.00788499392068E-102	0.538361324536014	0.229	0.093	1.68901367281227E-98	Hepatocytes	SLC30A9
1.31930504748852E-102	1.04177326536636	0.147	0.039	2.21089139858127E-98	Hepatocytes	SIMC1

1.52295999047275E-102	1.12261865772371	0.193	0.07	2.55217635203423E-98	Hepatocytes	MZT1
1.57957059095438E-102	0.569587704938679	0.197	0.071	2.64704439632136E-98	Hepatocytes	RUSC1
2.15044160807332E-102	0.473465831760448	0.261	0.117	3.60371004680927E-98	Hepatocytes	SPCS2
2.17657502643257E-102	0.383652606860502	0.268	0.123	3.6475044292957E-98	Hepatocytes	RRM1
2.22425636326557E-102	0.328511049289032	0.232	0.095	3.72740881356045E-98	Hepatocytes	SEC24D
2.40415485816785E-102	0.651321215964601	0.198	0.073	4.02888271131769E-98	Hepatocytes	MTLN
2.86653078914695E-102	1.42638440505407	0.103	0.014	4.80373229645247E-98	Hepatocytes	FHOD3
2.91453495310603E-102	0.283250344373061	0.289	0.137	4.88417767441509E-98	Hepatocytes	GORASP2
3.41105804536626E-102	0.692672198605536	0.158	0.047	5.71625107242479E-98	Hepatocytes	FGD6
6.5413546142021E-102	0.666082573115904	0.23	0.095	1.09620020624799E-97	Hepatocytes	SLC16A1
1.100622087076E-101	0.298747753324364	0.205	0.077	1.84442249352196E-97	Hepatocytes	MPZL1
1.31199489760159E-101	0.358738684754338	0.205	0.077	2.19864104940074E-97	Hepatocytes	GTF3A
1.31435262817379E-101	1.138135527902	0.126	0.027	2.20259213429365E-97	Hepatocytes	USP30
1.34289575057121E-101	1.06535132430464	0.173	0.057	2.25042469880724E-97	Hepatocytes	SEC61G
1.42237681845193E-101	0.940260888571756	0.122	0.025	2.38361907236175E-97	Hepatocytes	MOCS1
1.57867152426554E-101	0.271950681465585	0.217	0.085	2.64553774036419E-97	Hepatocytes	KIAA1191
1.67328930077159E-101	0.504850993161437	0.164	0.05	2.80409821023302E-97	Hepatocytes	IFT43
1.82433510722834E-101	0.660388819005643	0.158	0.047	3.05722077269324E-97	Hepatocytes	BAD
2.034911415779E-101	0.482371445581448	0.168	0.052	3.41010455056245E-97	Hepatocytes	SOCS7
2.19328650227919E-101	0.852720220252528	0.206	0.079	3.67550952051947E-97	Hepatocytes	OSTC
2.95000576471159E-101	0.438507810026907	0.279	0.131	4.94361966050368E-97	Hepatocytes	KIF1C
3.98971769158767E-101	0.85279966236937	0.167	0.053	6.68596890756261E-97	Hepatocytes	C17orf75
4.56097751614286E-101	0.272086756907791	0.169	0.054	7.6432861215522E-97	Hepatocytes	SCMH1
4.96710614067452E-101	0.686670753663488	0.193	0.07	8.32387647054237E-97	Hepatocytes	PDHX
5.03141958682767E-101	1.85083455244766	0.124	0.026	8.43165294360581E-97	Hepatocytes	CHAF1B
5.6496561555535E-101	2.03602025595905	0.116	0.022	9.46769378547655E-97	Hepatocytes	EEF1AKMT1
6.53763342296349E-101	0.250426677134374	0.34	0.176	1.09557660902022E-96	Hepatocytes	ATP5F1B
7.1524981907428E-101	1.48899026585376	0.181	0.064	1.19861564680468E-96	Hepatocytes	PCTP
1.32702402639449E-100	1.67171221863522	0.136	0.034	2.22382686343189E-96	Hepatocytes	PDXP
1.4953153510466E-100	0.55488741516095	0.258	0.116	2.50584946528389E-96	Hepatocytes	PARK7
1.85180760772298E-100	0.562707310114116	0.223	0.091	3.10325918902217E-96	Hepatocytes	PPCS
2.13408720570495E-100	1.02253237454198	0.117	0.023	3.57630333932036E-96	Hepatocytes	PPP1R3E
2.44050828967066E-100	1.62574910035843	0.114	0.021	4.0898037918301E-96	Hepatocytes	ZDHHC23
3.5549607539836E-100	0.479341429126972	0.21	0.083	5.95740323152571E-96	Hepatocytes	KLF11
3.96681680089137E-100	0.75965092467802	0.227	0.095	6.64759159493376E-96	Hepatocytes	GFM1
4.21340734184152E-100	1.39341479748999	0.138	0.035	7.06082802345802E-96	Hepatocytes	METTL27
4.90583439448255E-100	0.473009521153978	0.201	0.076	8.22119727827386E-96	Hepatocytes	COG2
5.12344548146268E-100	0.457365178814977	0.273	0.127	8.58586993783516E-96	Hepatocytes	ENOPH1
5.71285897028848E-100	0.444855862664429	0.192	0.07	9.57360906240944E-96	Hepatocytes	USF3
5.73760585481131E-100	1.90942344259651	0.115	0.022	9.61507989149279E-96	Hepatocytes	ABHD17C
5.8196159929974E-100	1.52954719142508	0.153	0.045	9.75251248106504E-96	Hepatocytes	ATP5F1C
6.83143138383088E-100	0.679639348379306	0.185	0.066	1.14481127130238E-95	Hepatocytes	ZCCHC14
6.84737001600615E-100	0.657269070161981	0.267	0.126	1.14748226728231E-95	Hepatocytes	COL18A1
7.87169959867726E-100	0.556311748763479	0.263	0.121	1.31913941874633E-95	Hepatocytes	PRCC
8.15076868275669E-100	0.342332316154293	0.171	0.056	1.36590581585637E-95	Hepatocytes	PEX6
8.58845662510051E-100	1.75739493000409	0.13	0.031	1.43925356123434E-95	Hepatocytes	CREB3L4
1.4472594378519E-99	2.1269670276129	0.102	0.015	2.42531736595222E-95	Hepatocytes	ELOVL7
1.68609877637336E-99	0.333937487916847	0.239	0.102	2.82556432944647E-95	Hepatocytes	RCN1
1.74999790418031E-99	0.331533285426212	0.549	0.341	2.93264648782536E-95	Hepatocytes	ANXA2
1.89052684542059E-99	2.51590124312105	0.136	0.036	3.16814488755582E-95	Hepatocytes	TNFSF14
2.8363724650979E-99	0.585119444877728	0.25	0.111	4.75319297701106E-95	Hepatocytes	DCTN3
2.89906706108008E-99	0.80400504737889	0.153	0.044	4.85825658095801E-95	Hepatocytes	SLC25A42
2.932564444551E-99	0.462371949073258	0.281	0.134	4.91439149617857E-95	Hepatocytes	NFIL3
3.06708799037833E-99	1.25372070141291	0.15	0.044	5.13982605427601E-95	Hepatocytes	RFC4
3.39335887239666E-99	0.434377977662368	0.266	0.123	5.68659079836233E-95	Hepatocytes	NDUFA9
3.4635632347574E-99	0.416305750503408	0.202	0.078	5.80423926880646E-95	Hepatocytes	MAFG
3.4849678041648E-99	1.20722172321883	0.115	0.022	5.84010904621938E-95	Hepatocytes	MAGI3
4.17518488951236E-99	0.476127634363955	0.261	0.12	6.99677483784481E-95	Hepatocytes	ITPKC
4.94337590445174E-99	1.08873263238354	0.161	0.05	8.28410934068022E-95	Hepatocytes	RMND1
6.24202118179671E-99	0.410984955608901	0.213	0.085	1.04603790964549E-94	Hepatocytes	TBL2
6.66741606144044E-99	0.431534591121412	0.13	0.031	1.11732558357619E-94	Hepatocytes	GKAP1
6.81971921676416E-99	0.367821974207889	0.223	0.092	1.14284854634534E-94	Hepatocytes	YIPF5
7.23397956658971E-99	0.266730021380001	0.188	0.067	1.2122702957691E-94	Hepatocytes	FBXL12

7.28904231171258E-99	1.09637456069153	0.184	0.067	1.22149771059679E-94	Hepatocytes	ZWINT
7.47110913777647E-99	0.781782035575318	0.115	0.022	1.25200846930858E-94	Hepatocytes	CEACAM19
8.27073726253855E-99	1.01005844655685	0.137	0.035	1.38601015045621E-94	Hepatocytes	ACP6
1.10179090515883E-98	1.05676495770871	0.154	0.046	1.84638119886517E-94	Hepatocytes	MAP3K20
1.12983481539141E-98	1.07531000558074	0.109	0.018	1.89337718363292E-94	Hepatocytes	TTC22
1.60358954262522E-98	0.578757019879672	0.259	0.119	2.68729535553135E-94	Hepatocytes	TXN2
2.11665737202032E-98	0.392334288786764	0.181	0.063	3.54709442403166E-94	Hepatocytes	FAM98C
2.44679595902708E-98	0.711710213173665	0.244	0.109	4.10034066813759E-94	Hepatocytes	NME4
2.63467478484865E-98	2.00045084001949	0.121	0.026	4.41518800444937E-94	Hepatocytes	NUDT12
3.22498118977047E-98	0.408215798287691	0.235	0.1	5.40442347781735E-94	Hepatocytes	CYREN
3.24713746729243E-98	0.615523896747667	0.194	0.073	5.44155296768866E-94	Hepatocytes	DAD1
3.43181043837344E-98	1.22377636599521	0.144	0.04	5.75102793262621E-94	Hepatocytes	UBXN2A
3.57267604908513E-98	1.49763088080553	0.112	0.02	5.98709052305685E-94	Hepatocytes	ABCA6
5.40627553897779E-98	1.25689788555464	0.114	0.022	9.05983654821898E-94	Hepatocytes	IFT81
5.87635115906701E-98	0.31229955740469	0.238	0.103	9.84758927236449E-94	Hepatocytes	TSR2
6.24115996690536E-98	0.538850492605867	0.211	0.084	1.045893587254E-93	Hepatocytes	MBD3
6.95313651967146E-98	2.0696704300574	0.115	0.022	1.16520661796654E-93	Hepatocytes	NUDT8
8.08512390931361E-98	0.497737550124802	0.157	0.048	1.35490506472277E-93	Hepatocytes	GLCE
9.24908444367275E-98	0.476225822691567	0.165	0.053	1.54996157107068E-93	Hepatocytes	RNF217
1.0595897960536E-97	0.38724608974234	0.186	0.066	1.77566058022662E-93	Hepatocytes	PSMG4
1.06628695425431E-97	1.79654410392383	0.112	0.021	1.78688367793938E-93	Hepatocytes	TRIM16
1.13237426781529E-97	0.369812296882986	0.183	0.064	1.89763279800487E-93	Hepatocytes	FDXR
1.21643102689098E-97	0.536901084082622	0.315	0.161	2.0384951148639E-93	Hepatocytes	GLRX
1.25751309320129E-97	0.442189320445525	0.272	0.128	2.10734044158673E-93	Hepatocytes	SNF8
2.2841913033E-97	0.875545127100473	0.142	0.038	3.82784778607014E-93	Hepatocytes	ZBED6CL
2.52751078901769E-97	0.403945516030239	0.183	0.065	4.23560258023585E-93	Hepatocytes	RPUSD3
2.79856542783098E-97	0.473304830999419	0.156	0.048	4.68983594395915E-93	Hepatocytes	DCBLD2
3.46239854338581E-97	0.584857075247626	0.233	0.1	5.80228747900595E-93	Hepatocytes	TMEM134
3.58988099185971E-97	1.13406159351085	0.173	0.059	6.0159225661585E-93	Hepatocytes	PRDX3
3.77659945021967E-97	2.7578253665146	0.106	0.018	6.32882535867813E-93	Hepatocytes	TPRN
3.80628651080593E-97	1.1008297186064	0.171	0.058	6.37857493480858E-93	Hepatocytes	COQ5
4.22298954854596E-97	0.251381545658511	0.354	0.188	7.07688588545332E-93	Hepatocytes	ATP6V1A
4.37996859241305E-97	0.744432719097392	0.247	0.112	7.3399513671658E-93	Hepatocytes	CDKN2C
5.26382828868204E-97	0.7213490617637	0.192	0.072	8.82112344617337E-93	Hepatocytes	SLC35B4
5.46494853646454E-97	0.812225539510094	0.13	0.032	9.15816075740727E-93	Hepatocytes	WDR19
5.46752628299848E-97	0.585903713876433	0.187	0.068	9.16248054504885E-93	Hepatocytes	MBIP
5.46857703831226E-97	1.14824234404699	0.161	0.051	9.16424140080368E-93	Hepatocytes	TMEM192
6.56557540063313E-97	1.26675191254674	0.146	0.042	1.1002591256381E-92	Hepatocytes	NR2C2AP
6.58902635365335E-97	0.347830958028696	0.223	0.093	1.10418903634523E-92	Hepatocytes	TRMT1L
6.72055394351538E-97	0.838666331541695	0.179	0.063	1.12623042985431E-92	Hepatocytes	IRF2BP1
7.1093735134157E-97	0.725465435165779	0.217	0.09	1.1913888133782E-92	Hepatocytes	ACOT7
7.75139958323518E-97	0.724006376752995	0.151	0.045	1.29897954215855E-92	Hepatocytes	SPIN4
7.85307496893704E-97	0.872435539240667	0.173	0.059	1.31601830329447E-92	Hepatocytes	DGCR6L
9.57781343922366E-97	0.598298081164631	0.192	0.072	1.6050499761451E-92	Hepatocytes	RMND5B
1.06767845465143E-96	0.373719073429369	0.281	0.136	1.78921555430487E-92	Hepatocytes	HAUS4
1.42096347676148E-96	0.320531059557386	0.286	0.137	2.38125059435688E-92	Hepatocytes	TNFSF10
1.67483323810368E-96	0.867422493652299	0.14	0.038	2.80668554041415E-92	Hepatocytes	KLHL25
1.878781871314E-96	0.551913984679585	0.203	0.079	3.148462659948E-92	Hepatocytes	RUVBL2
3.53663340035683E-96	0.460951652199672	0.196	0.074	5.92669025231797E-92	Hepatocytes	NUDT19
3.54598938190996E-96	1.04631001805088	0.147	0.042	5.94236900620471E-92	Hepatocytes	GLTP
3.77462879655086E-96	0.638092825142125	0.177	0.062	6.32552293725993E-92	Hepatocytes	GFM2
3.84270987505489E-96	1.60829391350996	0.414	0.302	6.43961320861699E-92	Hepatocytes	APOE
4.48703642426434E-96	0.295047567095089	0.353	0.19	7.51937563978218E-92	Hepatocytes	UBXN6
4.65411972781077E-96	0.423834776942058	0.258	0.119	7.7993738398653E-92	Hepatocytes	BCAP29
4.74308316529924E-96	0.331276182521038	0.234	0.101	7.94845876840847E-92	Hepatocytes	HDAC6
5.70920220575711E-96	0.294772239184279	0.2	0.078	9.56748105640777E-92	Hepatocytes	RRP1
8.66142080574665E-96	0.990602566369593	0.128	0.031	1.45148089862702E-91	Hepatocytes	LPAR1
8.89683239353291E-96	0.633045450517212	0.144	0.04	1.49093117250825E-91	Hepatocytes	FANCC
1.03842354955356E-95	0.402787086957867	0.213	0.086	1.74019018434185E-91	Hepatocytes	OGFOD3
1.04374147111678E-95	0.359620277357811	0.238	0.105	1.74910195729749E-91	Hepatocytes	RNF6
1.19769149901702E-95	0.532125036371113	0.221	0.092	2.00709141405271E-91	Hepatocytes	PTEN
1.19839225216844E-95	0.917919227120179	0.166	0.056	2.00826573618387E-91	Hepatocytes	TMCC3
1.2240442922011E-95	1.00977904198442	0.131	0.033	2.0512534248706E-91	Hepatocytes	GORAB

1.40828129596113E-95	0.79853055116057	0.145	0.041	2.35999779577166E-91	Hepatocytes	GPR180
1.49134866633961E-95	0.417432251892293	0.231	0.099	2.49920209505191E-91	Hepatocytes	MGLL
1.52018840529489E-95	0.369812150937763	0.299	0.149	2.54753172959317E-91	Hepatocytes	PSMC2
1.74166967135352E-95	0.696389627735094	0.167	0.056	2.91869003525422E-91	Hepatocytes	RWDD3
1.95068890886348E-95	0.545190237604534	0.189	0.07	3.26896447347342E-91	Hepatocytes	PIGM
2.566536685151E-95	0.714156435461318	0.126	0.03	4.30100217697605E-91	Hepatocytes	KPTN
3.80085392932645E-95	0.972972234976677	0.13	0.033	6.36947101476527E-91	Hepatocytes	PARP2
3.88641011733137E-95	1.17414440934997	0.153	0.047	6.51284607462391E-91	Hepatocytes	PET117
4.14687567786208E-95	0.848996123610554	0.174	0.061	6.94933426096128E-91	Hepatocytes	KLHL8
4.20200977150876E-95	0.882408819778762	0.165	0.055	7.04172797509438E-91	Hepatocytes	PIGC
4.28237240012503E-95	0.481941083392938	0.233	0.102	7.17639966812952E-91	Hepatocytes	ZNF358
4.33798857380664E-95	0.506028531890125	0.166	0.055	7.26960125198517E-91	Hepatocytes	ZNF32
6.23966557141789E-95	0.401786456670429	0.203	0.08	1.04564315645821E-90	Hepatocytes	NUP155
6.5713884865612E-95	0.276718108247832	0.4	0.228	1.10123328257793E-90	Hepatocytes	TMEM219
6.70603646435859E-95	0.335354149904969	0.251	0.114	1.12379759069721E-90	Hepatocytes	HDHD5
7.79994935439554E-95	0.699776293605386	0.204	0.081	1.3071155128096E-90	Hepatocytes	TMEM230
8.1329819985324E-95	0.894081782359091	0.149	0.044	1.36292512331406E-90	Hepatocytes	PRUNE1
8.82540736245188E-95	0.876861194351123	0.109	0.02	1.47896176579969E-90	Hepatocytes	CREB3L1
1.08897246734425E-94	0.289677217757686	0.243	0.108	1.8249000607755E-90	Hepatocytes	ATIC
1.11318016254347E-94	1.89243200716271	0.109	0.02	1.86546731639035E-90	Hepatocytes	LMNTD2
1.39250985257872E-94	0.93975946459075	0.147	0.043	2.33356801095142E-90	Hepatocytes	MTX1
1.53004473703914E-94	1.27867684998231	0.127	0.031	2.5640489703302E-90	Hepatocytes	RBBP9
1.5348714625651E-94	0.465511951073693	0.314	0.162	2.5721375969666E-90	Hepatocytes	ATP5F1A
1.67272414975868E-94	0.441882961777203	0.231	0.101	2.8031511301656E-90	Hepatocytes	CHCHD7
1.9892251115904E-94	0.405491790472362	0.149	0.044	3.3335434420032E-90	Hepatocytes	DENND5B
2.74930985895385E-94	0.863168569688216	0.16	0.052	4.60729346163487E-90	Hepatocytes	CDT1
3.23850085065474E-94	1.96776792185695	0.11	0.021	5.42707972552722E-90	Hepatocytes	PDSS1
3.42338186538083E-94	0.402676215173647	0.183	0.067	5.73690333000519E-90	Hepatocytes	FARSB
3.44014963222711E-94	0.340910092834359	0.235	0.103	5.7650027536862E-90	Hepatocytes	ELF4
3.49086875350798E-94	0.450938180511147	0.144	0.041	5.84999785712867E-90	Hepatocytes	MED20
3.56007474456329E-94	0.284529352176478	0.227	0.096	5.96597325693916E-90	Hepatocytes	SCAP
3.56391101165779E-94	0.446121145316989	0.24	0.106	5.97240207333613E-90	Hepatocytes	TIMM23
3.92187196924339E-94	0.5852071801239	0.152	0.047	6.57227304605807E-90	Hepatocytes	WDR5
4.38136193342452E-94	0.739838045570578	0.162	0.053	7.34228632803281E-90	Hepatocytes	DIP2C
4.71800602830584E-94	1.52506139359943	0.117	0.025	7.90643450223493E-90	Hepatocytes	STRADB
6.03858203426651E-94	0.357713405669688	0.182	0.066	1.01194557730238E-89	Hepatocytes	ARL 16.00
6.54805594479031E-94	0.387822512257937	0.344	0.186	1.09732321522796E-89	Hepatocytes	COPE
7.82137051950241E-94	0.27862188572388	0.338	0.18	1.31070527165821E-89	Hepatocytes	APH1A
9.49096914841408E-94	0.335059863906392	0.238	0.106	1.59049660989123E-89	Hepatocytes	SLC39A11
9.56716542788878E-94	0.551484595633616	0.221	0.094	1.6032655824056E-89	Hepatocytes	C19orf53
1.39293733656742E-93	2.27129783903734	0.137	0.039	2.33428438861969E-89	Hepatocytes	CENPI
1.5661321589858E-93	0.532288509060845	0.223	0.096	2.6245242720284E-89	Hepatocytes	TIMM10
3.13863221589156E-93	0.583413317722234	0.192	0.074	5.25971986739107E-89	Hepatocytes	ZNF277
3.49721596809947E-93	0.27592946723149	0.25	0.114	5.86063451934109E-89	Hepatocytes	TSNAX
5.89417678251631E-93	0.385794484711808	0.225	0.096	9.87746145214083E-89	Hepatocytes	COMMD6
6.31935830425368E-93	0.7301542857735	0.148	0.044	1.05899806462683E-88	Hepatocytes	ARV1
6.44148467251506E-93	0.575084604282528	0.186	0.07	1.07946400142007E-88	Hepatocytes	NUP54
7.27796483163174E-93	1.47276345234882	0.117	0.026	1.21964134648485E-88	Hepatocytes	NAPEPLD
8.75901944344199E-93	1.58224316262643	0.139	0.04	1.46783647833201E-88	Hepatocytes	DTL
9.40406847071714E-93	2.06722992131739	0.111	0.023	1.57593379432278E-88	Hepatocytes	CDKN3
9.79464035220017E-93	1.80717691632035	0.113	0.024	1.6413858302217E-88	Hepatocytes	ALKBH2
1.15938606897421E-92	0.606267526694725	0.143	0.041	1.94289917438698E-88	Hepatocytes	ITGB3BP
1.30582525978761E-92	0.316967827390352	0.233	0.102	2.18830197035207E-88	Hepatocytes	TAF13
1.33326601092775E-92	0.703390807923363	0.15	0.046	2.23428718111273E-88	Hepatocytes	MTG2
1.5056873104049E-92	1.09690524216142	0.146	0.044	2.52323079477653E-88	Hepatocytes	MEX3D
1.61753056313903E-92	0.724840780643119	0.202	0.082	2.71065771770839E-88	Hepatocytes	GRPEL1
1.92738771896931E-92	0.620900398233906	0.178	0.065	3.22991633944876E-88	Hepatocytes	PSMD11
1.97412205595413E-92	0.901398490969958	0.137	0.038	3.30823374136793E-88	Hepatocytes	EIF4EBP3
2.09555009128262E-92	0.606099281653452	0.194	0.075	3.51172284297141E-88	Hepatocytes	KLHDC10
2.33374078804951E-92	1.01190975759353	0.169	0.059	3.91088281261338E-88	Hepatocytes	NDUFB3
2.80274311342815E-92	0.272097041733715	0.276	0.134	4.69683690948289E-88	Hepatocytes	PLEKHJ1
2.90982892570108E-92	0.417689853893636	0.165	0.056	4.87629131368987E-88	Hepatocytes	DCAKD
4.08405657749444E-92	0.752436896696008	0.153	0.048	6.84406201256519E-88	Hepatocytes	SCYL3

4.11745091602141E-92	0.589479948673647	0.249	0.115	6.90002424506867E-88	Hepatocytes	SNAP29
4.19146135969689E-92	0.372255222183142	0.224	0.095	7.02405094658005E-88	Hepatocytes	FAF2
5.3979123294745E-92	0.686882244653734	0.177	0.065	9.04582148173337E-88	Hepatocytes	TTLL12
8.19044091347526E-92	0.625636853017877	0.18	0.066	1.37255408828018E-87	Hepatocytes	BSCL2
1.08372801910773E-91	0.435107520930954	0.135	0.037	1.81611141442073E-87	Hepatocytes	BRMS1L
1.63104008195698E-91	0.422815023158793	0.273	0.132	2.73329696934351E-87	Hepatocytes	GPS1
1.64365656076083E-91	0.375943686588796	0.234	0.104	2.754439664523E-87	Hepatocytes	TM2D2
2.90065925252299E-91	0.368315833989684	0.219	0.094	4.86092477537803E-87	Hepatocytes	SNRPD2
3.02919342638365E-91	0.848651976369813	0.144	0.043	5.07632234393373E-87	Hepatocytes	FPGT
3.09051580804393E-91	0.285682829904184	0.195	0.076	5.17908639112001E-87	Hepatocytes	OGFOD2
3.28520938424746E-91	0.667489938095867	0.2	0.081	5.50535388612189E-87	Hepatocytes	PDZD11
3.37557651753126E-91	0.466950945013817	0.25	0.116	5.65679112807888E-87	Hepatocytes	NSL1
3.85549234596285E-91	0.700309874419547	0.214	0.092	6.46103407336454E-87	Hepatocytes	ERLIN1
4.22702904144904E-91	0.394435774059689	0.202	0.082	7.0836552676603E-87	Hepatocytes	ERI3
4.93158153258003E-91	2.0153214571551	0.1	0.017	8.26434433229761E-87	Hepatocytes	ZBED8
5.40539651477104E-91	1.14620927215115	0.11	0.022	9.05836347945331E-87	Hepatocytes	RAB23
6.68802115331905E-91	0.992138592702503	0.139	0.041	1.12077858487321E-86	Hepatocytes	FAHD2A
6.91478596059113E-91	0.743433037801887	0.233	0.105	1.15877983127586E-86	Hepatocytes	SDF2L1
8.62425889639139E-91	1.02589797789647	0.152	0.049	1.44525330585727E-86	Hepatocytes	AP5S1
9.78783752078674E-91	0.644762090235803	0.155	0.05	1.64024581173344E-86	Hepatocytes	TBC1D24
1.1715377778283E-90	1.03603142972394	0.141	0.041	1.96326300808467E-86	Hepatocytes	RABL3
1.22062002410146E-90	0.36013865478721	0.262	0.124	2.04551503638923E-86	Hepatocytes	TRAF7
1.30575583685542E-90	0.402694251118195	0.184	0.07	2.18818563140231E-86	Hepatocytes	GTPBP10
1.37981929795154E-90	0.610293284650742	0.186	0.072	2.31230117950718E-86	Hepatocytes	PER3
1.54837776368575E-90	0.281092176756624	0.17	0.059	2.59477145638457E-86	Hepatocytes	HLCS
1.70707723882104E-90	0.25053509756705	0.253	0.118	2.86072003681631E-86	Hepatocytes	NMRAL1
1.71398279114137E-90	0.742026158316485	0.111	0.023	2.8722923613947E-86	Hepatocytes	MYRIP
1.73793801147717E-90	0.781043166046826	0.172	0.062	2.91243651963344E-86	Hepatocytes	PLEKHA7
1.9246894137411E-90	0.507975932557767	0.136	0.038	3.22539451954733E-86	Hepatocytes	PKN3
2.15195922947205E-90	0.441702622771384	0.188	0.072	3.60625327674926E-86	Hepatocytes	FOPNL
2.75539628208895E-90	1.77135782443863	0.103	0.019	4.61749308952466E-86	Hepatocytes	PHOSPHO2
2.76211410730375E-90	0.29338728510425	0.199	0.079	4.62875082101963E-86	Hepatocytes	COPS4
2.82800117255614E-90	1.16160937781521	0.115	0.026	4.73916436496958E-86	Hepatocytes	FUNDC2
3.01037379365631E-90	1.78248550476885	0.105	0.02	5.04478440340924E-86	Hepatocytes	CIART
3.18285420434496E-90	1.11761435618677	0.142	0.043	5.33382707564129E-86	Hepatocytes	SCAMP5
3.85167387326563E-90	0.289140811742577	0.173	0.062	6.45463507681855E-86	Hepatocytes	NOM1
3.86786719558133E-90	0.84731737728658	0.123	0.03	6.4817718463552E-86	Hepatocytes	B3GLCT
4.75571595980815E-90	0.417246461124305	0.2	0.081	7.9696288054465E-86	Hepatocytes	DFFA
5.64480960831168E-90	0.71675877334725	0.119	0.028	9.45957194160871E-86	Hepatocytes	SMAGP
5.8228650331836E-90	0.286811745452734	0.271	0.131	9.75795722260908E-86	Hepatocytes	TIMMDC1
6.74961068552511E-90	0.502568365278683	0.124	0.03	1.1310997586803E-85	Hepatocytes	TMEM47
7.55049893395116E-90	0.96140631434915	0.143	0.043	1.26531261135154E-85	Hepatocytes	SPRYD7
7.73184191129604E-90	0.908553866746975	0.176	0.066	1.29570206749499E-85	Hepatocytes	MICOS13
9.9783165742308E-90	0.312937623578978	0.294	0.149	1.6721662915096E-85	Hepatocytes	GDI2
1.02044667311941E-89	0.900707054248632	0.168	0.059	1.7100645348135E-85	Hepatocytes	COX5B
1.26155653263342E-89	0.477322367742275	0.164	0.057	2.11411643738709E-85	Hepatocytes	FANCA
1.33568639776795E-89	0.376467301725512	0.167	0.058	2.23834326537953E-85	Hepatocytes	KLHL42
1.3583988423009E-89	0.64320072264811	0.174	0.064	2.27640477992786E-85	Hepatocytes	FAXDC2
1.46392131867883E-89	0.63016462744367	0.203	0.083	2.45323934584198E-85	Hepatocytes	MAGOH
1.74426335023748E-89	1.7303798887616	0.12	0.03	2.92303652232797E-85	Hepatocytes	DHODH
1.75443493257175E-89	0.388318120656313	0.251	0.119	2.94008206000375E-85	Hepatocytes	LMNB2
1.90014140993912E-89	0.531289282304088	0.177	0.066	3.18425697477597E-85	Hepatocytes	BLNK
2.15149672625932E-89	1.17765656331719	0.12	0.029	3.60547821386536E-85	Hepatocytes	CSRP2
2.21853364238064E-89	0.733274939418248	0.175	0.065	3.71781867790147E-85	Hepatocytes	CENPN
2.60753920647805E-89	0.285377789361641	0.222	0.096	4.36971420221592E-85	Hepatocytes	RFNG
2.99221299656627E-89	1.22839793339596	0.11	0.023	5.01435053964576E-85	Hepatocytes	ZNF696
3.07496153102929E-89	0.332760608159936	0.242	0.111	5.15302053369889E-85	Hepatocytes	EIF2S3
3.3400580460313E-89	0.296418694163647	0.244	0.112	5.59726927353925E-85	Hepatocytes	MFN2
3.44359146683322E-89	2.191907659618	0.115	0.027	5.77077058011911E-85	Hepatocytes	GSTZ1
3.45278555351601E-89	0.613308085623602	0.196	0.078	5.78617803058213E-85	Hepatocytes	THUMPD3
3.72416188277552E-89	0.296038055171251	0.357	0.196	6.24095048315522E-85	Hepatocytes	HYOU1
3.9722525900182E-89	0.487955724284565	0.223	0.099	6.6567008903525E-85	Hepatocytes	DDX49
4.16534348232145E-89	0.377823229974578	0.189	0.073	6.98028260767429E-85	Hepatocytes	ZNF281

**4.35364301100651E-89**	0.538954245445198	0.159	0.053	7.29583495784471E-85	Hepatocytes	PRIM1
**5.56199824421389E-89**	0.339934743427873	0.17	0.061	9.32079665765364E-85	Hepatocytes	MAIP1
**5.94907736017614E-89**	0.385783176749938	0.315	0.165	9.96946384018317E-85	Hepatocytes	CYB5R1
**6.06775069185974E-89**	0.790245740125322	0.183	0.071	1.01683366094185E-84	Hepatocytes	DEPRECATED-ENSG00000167797
**6.57427628392379E-89**	0.411070332907006	0.192	0.075	1.10171721965995E-84	Hepatocytes	RDH14
**6.92151623094941E-89**	0.539743509249585	0.156	0.052	1.1599076899825E-84	Hepatocytes	MSANTD3
**9.00692116300176E-89**	0.544286620629147	0.164	0.057	1.50937984849583E-84	Hepatocytes	ANO10
**9.7926555750806E-89**	0.347167818088484	0.285	0.142	1.64105322127201E-84	Hepatocytes	CAMK1
**1.10915063082488E-88**	0.440826209437232	0.243	0.112	1.85871462713633E-84	Hepatocytes	TMEM250
**1.61583412858339E-88**	2.1214055260392	0.102	0.019	2.70781483268005E-84	Hepatocytes	DEPRECATED-ENSG00000221926
**1.88166821854264E-88**	0.302029611019103	0.188	0.074	3.15329960063376E-84	Hepatocytes	MPP5
**2.16602200297381E-88**	0.571130608975142	0.176	0.066	3.62981967258351E-84	Hepatocytes	TMEM251
**2.45974199518033E-88**	1.81414613384407	0.112	0.025	4.1220356355232E-84	Hepatocytes	PAICS
**3.15554614530069E-88**	0.26927298629167	0.22	0.095	5.2880642302949E-84	Hepatocytes	TOR1A
**4.15200831988871E-88**	0.897990525691667	0.136	0.04	6.9579355424695E-84	Hepatocytes	YBEY
**4.19947757554396E-88**	0.303754204717243	0.245	0.114	7.03748452109657E-84	Hepatocytes	GADD45GIP1
**4.56053603650094E-88**	1.28836169472696	0.124	0.032	7.64254628996827E-84	Hepatocytes	SCOC
**4.61654961132593E-88**	0.531017699149267	0.255	0.123	7.73641383865999E-84	Hepatocytes	TMEM19
**5.34572917409842E-88**	0.392186326974081	0.141	0.042	8.95837294995413E-84	Hepatocytes	RRNAD1
**5.57208361014339E-88**	0.631188581320178	0.228	0.103	9.33769771387828E-84	Hepatocytes	NSMCE1
**6.63523089863633E-88**	0.579410732152487	0.164	0.058	1.11193199399348E-83	Hepatocytes	TBC1D13
**6.81316796107613E-88**	0.504680130574105	0.177	0.066	1.14175068691714E-83	Hepatocytes	METTL5
**7.29446952047894E-88**	0.363553770310466	0.167	0.059	1.22240720224186E-83	Hepatocytes	MSTO1
**7.35128619660697E-88**	0.626758470215364	0.238	0.111	1.2319285408274E-83	Hepatocytes	PPDPF
**9.33292892682199E-88**	0.920918048341343	0.131	0.037	1.56401222955683E-83	Hepatocytes	GNAI1
**1.13201661519858E-87**	0.602881290219398	0.173	0.064	1.89703344374978E-83	Hepatocytes	COMMD2
**1.25831103546007E-87**	0.566615218561252	0.181	0.07	2.10867763322399E-83	Hepatocytes	PHAX
**1.44806067294046E-87**	0.671827752307143	0.157	0.054	2.42666007571362E-83	Hepatocytes	FLAD1
**1.5925041888758E-87**	0.579740142434077	0.279	0.144	2.66871851971807E-83	Hepatocytes	PABPC1L
**1.67440645518685E-87**	0.889225405896381	0.18	0.07	2.80597033760212E-83	Hepatocytes	PRPS2
**1.7544503713091E-87**	0.25840902414316	0.246	0.115	2.94010793223978E-83	Hepatocytes	DDX1
**1.77666869976753E-87**	1.27554965594586	0.131	0.037	2.97734140707042E-83	Hepatocytes	ZBED3
**1.8344072147602E-87**	0.42135731364371	0.23	0.103	3.07409961049514E-83	Hepatocytes	ATXN10
**1.95781244805338E-87**	0.874070655516999	0.118	0.029	3.28090210044785E-83	Hepatocytes	TCFL5
**2.43284249049056E-87**	2.06842355305277	0.106	0.022	4.07695744556408E-83	Hepatocytes	MCRIP2
**2.87622761424627E-87**	0.38094288524604	0.237	0.109	4.8199822359539E-83	Hepatocytes	CHMP1A
**2.96568610828983E-87**	0.596026368712823	0.213	0.093	4.96989678027209E-83	Hepatocytes	KLF10
**3.425911230886E-87**	1.03085021112947	0.125	0.033	5.74114204071876E-83	Hepatocytes	CYB5RL
**3.42801253794557E-87**	0.474282519412034	0.236	0.109	5.74466341108919E-83	Hepatocytes	NUTF2
**4.53575827261023E-87**	1.01411228757546	0.179	0.069	7.60102371324022E-83	Hepatocytes	DTYMK
**5.26703595575679E-87**	0.68610654657158	0.223	0.1	8.82649885465722E-83	Hepatocytes	TEX261
**7.94344868864247E-87**	0.552522071868288	0.154	0.052	1.33116313124271E-82	Hepatocytes	THAP7
**8.04615010278819E-87**	0.409744800127936	0.26	0.126	1.34837383422525E-82	Hepatocytes	PINK1
**9.21319250727745E-87**	0.506830740936355	0.235	0.108	1.54394680036956E-82	Hepatocytes	METAP2
**1.02629486606578E-86**	0.430506016768769	0.343	0.19	1.71986493655303E-82	Hepatocytes	GHITM
**1.11287678713016E-86**	0.850227332585918	0.112	0.025	1.86495891987273E-82	Hepatocytes	BCL7A
**1.45752730819974E-86**	0.252895632832059	0.214	0.092	2.44252426308112E-82	Hepatocytes	FAM160A2
**1.60631135597042E-86**	1.00117316569106	0.101	0.019	2.69185657033523E-82	Hepatocytes	GPRASP2
**1.84047496176992E-86**	0.897019278264019	0.152	0.051	3.08426794093403E-82	Hepatocytes	C1orf35
**1.94964392037928E-86**	0.299128576183204	0.189	0.075	3.2672132817716E-82	Hepatocytes	RANBP10
**2.16930212200799E-86**	0.30809592559756	0.234	0.108	3.63531649606099E-82	Hepatocytes	ANO6
**2.49123209885097E-86**	0.296841206892317	0.247	0.116	4.17480675125446E-82	Hepatocytes	HDHD2
**3.01339480995462E-86**	0.32293650140392	0.172	0.063	5.04984702252196E-82	Hepatocytes	METTL15
**3.46872698520921E-86**	0.666334988913053	0.157	0.054	5.81289268181359E-82	Hepatocytes	BORCS7
**4.372097337435E-86**	1.36138302473068	0.107	0.022	7.32676071807356E-82	Hepatocytes	TP73
**4.87226538803706E-86**	0.322260620312385	0.324	0.176	8.16494233727251E-82	Hepatocytes	POLD4
**4.94156633165183E-86**	1.02900544590283	0.133	0.038	8.28107685858214E-82	Hepatocytes	PAXIP1
**5.54362248802117E-86**	0.326737364100055	0.262	0.127	9.29000256542587E-82	Hepatocytes	ATP6V1C1
**5.93409260592383E-86**	0.761658783664363	0.115	0.028	9.94435238900716E-82	Hepatocytes	EFCAB2
**8.27290481880596E-86**	0.901950564584107	0.118	0.03	1.3863733895355E-81	Hepatocytes	TSPAN12
**1.05098279429367E-85**	1.58204348503898	0.103	0.021	1.76123696667734E-81	Hepatocytes	TMEM177
**1.31462952575219E-85**	0.300042292215068	0.221	0.098	2.20305615925552E-81	Hepatocytes	EEF1AKNMT
**1.32135487716144E-85**	0.628015386397332	0.122	0.032	2.21432650314715E-81	Hepatocytes	PPM1H

1.384976016861E-85	1.31301861215163	0.164	0.06	2.32094280905566E-81	Hepatocytes	CENPF
1.64366629216023E-85	1.26256808299222	0.119	0.031	2.75445597240212E-81	Hepatocytes	CHCHD4
1.86343287482917E-85	0.402358565360764	0.206	0.088	3.12274081163872E-81	Hepatocytes	RNF14
2.03692432633289E-85	0.289909332391822	0.179	0.068	3.41347778606866E-81	Hepatocytes	TSPYL4
2.6390196097325E-85	0.731921628975809	0.15	0.049	4.42246906198972E-81	Hepatocytes	CCDC167
2.95198845088093E-85	0.394320344778884	0.111	0.025	4.94694224598626E-81	Hepatocytes	ADAMTSL3
3.1145535922784E-85	1.04212930891987	0.123	0.033	5.21936890994015E-81	Hepatocytes	SPRYD4
3.45455197277339E-85	0.455603016830495	0.148	0.048	5.78913819597364E-81	Hepatocytes	TFDP2
3.46259378224908E-85	0.343526871939406	0.162	0.058	5.802614660293E-81	Hepatocytes	ESCO1
3.74350012384609E-85	1.38978573021129	0.13	0.038	6.27335750754128E-81	Hepatocytes	RITA1
3.80114977710258E-85	0.587934387479699	0.128	0.036	6.3699667964685E-81	Hepatocytes	PPM1L
4.10890347491545E-85	0.355784563478762	0.197	0.082	6.88570044326332E-81	Hepatocytes	SHB
4.46366126634487E-85	0.494264404981311	0.192	0.078	7.48020355014074E-81	Hepatocytes	SHLD2
5.30344019663938E-85	0.253251050648478	0.219	0.097	8.88750508152826E-81	Hepatocytes	GATD3B
5.85333597435515E-85	0.495454940959074	0.112	0.026	9.80902042582436E-81	Hepatocytes	LARGE1
6.02494838741048E-85	0.495864797597146	0.17	0.063	1.00966085076225E-80	Hepatocytes	GRAMD2B
6.45974326297175E-85	0.355309634698296	0.243	0.115	1.08252377600881E-80	Hepatocytes	WBP2
7.23077022146323E-85	0.595377138830501	0.164	0.059	1.21173247371281E-80	Hepatocytes	ERCC2
1.19174085614877E-84	0.273895657658298	0.26	0.128	1.99711932673411E-80	Hepatocytes	VPS26C
1.30643887129225E-84	0.405961390974069	0.233	0.108	2.18933026051156E-80	Hepatocytes	COMMD4
1.48735686288256E-84	0.73690416652557	0.115	0.028	2.49251263081859E-80	Hepatocytes	SPIN3
1.89682752705645E-84	1.04034749885383	0.119	0.03	3.1787035698412E-80	Hepatocytes	WDR90
2.2505640159499E-84	0.760030024609473	0.169	0.063	3.77149517792884E-80	Hepatocytes	CHRAC1
2.25986655951174E-84	0.260649975858146	0.224	0.102	3.78708438042977E-80	Hepatocytes	ATP6V1H
2.65173853812536E-84	0.887019308620653	0.185	0.075	4.44378344219049E-80	Hepatocytes	TUBB4B
3.72696177995711E-84	0.832723035836762	0.133	0.039	6.24564255085213E-80	Hepatocytes	ENKD1
4.20600438802728E-84	0.989929070312476	0.142	0.046	7.04842215345611E-80	Hepatocytes	HFE
5.10905882794557E-84	0.633093135893184	0.146	0.048	8.56176078387119E-80	Hepatocytes	TP53INP2
7.93848365556527E-84	0.984773608846453	0.117	0.03	1.33033109099963E-79	Hepatocytes	DEPRECATED-ENSG00000165948
8.65715088347157E-84	0.859480227390542	0.166	0.062	1.45076534505217E-79	Hepatocytes	LAMTOR5
9.21164516015605E-84	0.342351657711952	0.26	0.128	1.54368749593895E-79	Hepatocytes	DEPRECATED-ENSG00000213246
1.60361741246355E-83	0.380842008387376	0.169	0.063	2.68734205980641E-79	Hepatocytes	CHAF1A
2.17043904733293E-83	0.670596840272	0.228	0.105	3.63722175552052E-79	Hepatocytes	BCKDHA
2.257949085353E-83	0.913554429006118	0.119	0.031	3.78387107723455E-79	Hepatocytes	LRP12
2.93645135499412E-83	0.425794209236554	0.201	0.085	4.92090518069915E-79	Hepatocytes	CCT5
2.95999103084859E-83	1.34689492137187	0.12	0.032	4.96035296949606E-79	Hepatocytes	UNC13B
5.39174558816605E-83	0.469611587064782	0.221	0.099	9.03548725664866E-79	Hepatocytes	CASC4
5.58576195557649E-83	0.952903705453809	0.123	0.034	9.36061988515509E-79	Hepatocytes	APOO
7.37160609234192E-83	0.803599724748109	0.122	0.033	1.23533374895466E-78	Hepatocytes	THAP5
8.09214568409215E-83	0.333821192258371	0.222	0.1	1.35608177374016E-78	Hepatocytes	CHMP5
8.78237543254906E-83	1.38245508438672	0.104	0.023	1.47175047498657E-78	Hepatocytes	MAP10
9.58752192852746E-83	0.99132189136626	0.127	0.036	1.60667692478263E-78	Hepatocytes	USP31
1.01429601493272E-82	1.00931173073352	0.123	0.034	1.69975726182425E-78	Hepatocytes	TRMT61A
1.12148996747731E-82	1.18544431763243	0.114	0.029	1.87939288749848E-78	Hepatocytes	SOWAHC
1.20289798885955E-82	0.873832161301387	0.114	0.028	2.01581644973084E-78	Hepatocytes	BCAR3
1.72561023400997E-82	0.362359069577331	0.23	0.107	2.8917776301539E-78	Hepatocytes	ELP5
2.21131015714369E-82	1.19488259424483	0.101	0.021	3.70571356134139E-78	Hepatocytes	POGLUT2
2.89931479913802E-82	1.53513306388258	0.124	0.035	4.85867174039549E-78	Hepatocytes	GINS1
3.04870828145846E-82	1.1268834376519	0.127	0.037	5.10902533806808E-78	Hepatocytes	C7orf25
3.13676940684943E-82	1.80688021528857	0.11	0.027	5.25659817199828E-78	Hepatocytes	CDC6
3.85191449770171E-82	0.866202871833455	0.137	0.043	6.45503831524853E-78	Hepatocytes	DEPRECATED-ENSG00000185201
3.98403382288023E-82	1.42133036400843	0.111	0.027	6.6764438803827E-78	Hepatocytes	CDKN2AIPNL
5.62956002556355E-82	0.399754944603266	0.159	0.057	9.43401669083941E-78	Hepatocytes	WDR76
7.63755177670927E-82	1.55382505225936	0.118	0.031	1.27990092674094E-77	Hepatocytes	SRD5A1
7.70291969299785E-82	0.661760574079155	0.19	0.079	1.29085528215258E-77	Hepatocytes	NUDT2
8.14330966750701E-82	0.651461380131996	0.148	0.05	1.36465583408083E-77	Hepatocytes	SDHAF3
8.3991522367939E-82	0.387011145909418	0.264	0.133	1.40752993184192E-77	Hepatocytes	HECTD3
9.00645888689301E-82	0.260148620475736	0.218	0.099	1.50930238026553E-77	Hepatocytes	CEBPZOS
9.28976351227338E-82	0.658454349347775	0.168	0.064	1.55677856938677E-77	Hepatocytes	SLIRP
1.24336472746985E-81	1.20570389974318	0.156	0.058	2.08363061029397E-77	Hepatocytes	CIDEB
1.31591994511977E-81	0.734016143433585	0.117	0.03	2.20521864403171E-77	Hepatocytes	SNTA1
1.40408133307926E-81	0.770877816705235	0.184	0.076	2.35295949797422E-77	Hepatocytes	C11orf24
1.63479435105238E-81	0.400112193621047	0.163	0.061	2.73958837349358E-77	Hepatocytes	UBE2M

3.40255458669356E-81	0.396994021505988	0.176	0.069	5.70200097638107E-77	Hepatocytes	SLC41A3
3.73009712052186E-81	0.711310913311734	0.17	0.066	6.25089675457053E-77	Hepatocytes	YIPF2
3.74293068893434E-81	1.13847031225055	0.123	0.035	6.27240324851617E-77	Hepatocytes	CCDC25
3.9555617205355E-81	0.433202349240237	0.154	0.054	6.62873033127339E-77	Hepatocytes	FAN1
5.06349264011977E-81	0.268891750619449	0.129	0.038	8.48540096631271E-77	Hepatocytes	STXBP4
6.45250391509561E-81	0.796210900062415	0.205	0.092	1.08131060609172E-76	Hepatocytes	IL1RAP
8.27913963489415E-81	0.669738986758897	0.187	0.078	1.38741822001556E-76	Hepatocytes	NLN
8.47262085513705E-81	0.937991066380383	0.155	0.056	1.41984180290387E-76	Hepatocytes	TOMM40L
1.11833335894192E-80	0.599985892688434	0.2	0.088	1.87410304291487E-76	Hepatocytes	TIMM8B
1.18342253719874E-80	0.592377661526234	0.187	0.078	1.98317948783765E-76	Hepatocytes	RFC2
1.43781173436334E-80	0.850075845115253	0.133	0.041	2.40948490444608E-76	Hepatocytes	PTCD1
1.89199703261927E-80	1.73882984611261	0.108	0.026	3.17060862726337E-76	Hepatocytes	DEPRECATED-ENSG00000101444
1.8969309138923E-80	0.866258967031824	0.129	0.039	3.17887682550072E-76	Hepatocytes	CEP72
1.95672299878076E-80	1.18077782278588	0.133	0.042	3.27907640135679E-76	Hepatocytes	METRN
2.64690274220617E-80	0.324456057824147	0.21	0.094	4.43567961538911E-76	Hepatocytes	SUCLG2
3.46091072345578E-80	1.4982657430617	0.11	0.027	5.79979419036719E-76	Hepatocytes	GINS2
3.53911402195106E-80	0.49622660695332	0.228	0.108	5.93084727798558E-76	Hepatocytes	CCDC28A
4.0767107116304E-80	1.20594104734854	0.118	0.032	6.83175181055022E-76	Hepatocytes	C15orf41
5.77335486746506E-80	0.518249781025611	0.21	0.094	9.67498808689794E-76	Hepatocytes	RFXANK
8.27558377206776E-80	0.29761274461676	0.157	0.057	1.38682232852311E-75	Hepatocytes	NUDT16L1
1.0549128754127E-79	0.887349414452995	0.152	0.055	1.76782299661661E-75	Hepatocytes	EPDR1
1.06382317442418E-79	1.28393041732804	0.101	0.022	1.78275487570005E-75	Hepatocytes	OXER1
1.13421743337185E-79	0.251652475272115	0.239	0.114	1.90072157484455E-75	Hepatocytes	DNAJC15
1.4852863924523E-79	0.257788785255934	0.208	0.093	2.48904293647157E-75	Hepatocytes	SLC9A6
1.53981422960109E-79	1.2161172600904	0.13	0.04	2.58042068596551E-75	Hepatocytes	RPP25L
1.80422709570521E-79	0.294974310528241	0.264	0.135	3.0235237669828E-75	Hepatocytes	CHMP3
2.21834761272549E-79	1.53826592893429	0.115	0.031	3.71750692940537E-75	Hepatocytes	RABEPK
2.39170532985721E-79	1.04465575475322	0.132	0.042	4.00801979177472E-75	Hepatocytes	ERI2
2.55896214267994E-79	0.883442816620925	0.128	0.039	4.28830875870304E-75	Hepatocytes	TCTEX1D2
2.78967631344509E-79	0.302168425097466	0.181	0.074	4.67493956607129E-75	Hepatocytes	ECSIT
3.55588294145309E-79	0.631356143917328	0.177	0.072	5.95894863328708E-75	Hepatocytes	HSBP1
4.82836973381811E-79	0.283033682702739	0.133	0.041	8.09138199993238E-75	Hepatocytes	ZFP41
5.8980855460661E-79	0.31752319689742	0.266	0.136	9.88401175809757E-75	Hepatocytes	DNASE1L1
6.82221543220874E-79	0.38122951989979	0.186	0.077	1.14326686212954E-74	Hepatocytes	MZT2A
6.91376430677673E-79	1.08856489907614	0.143	0.05	1.15860862252965E-74	Hepatocytes	ATP5MD
7.95267286218581E-79	1.03001009669712	0.136	0.044	1.3327089182451E-74	Hepatocytes	CETN3
8.92371805029398E-79	1.22847038033925	0.109	0.027	1.49543667086826E-74	Hepatocytes	SGMS2
9.29155258144322E-79	0.609071146861701	0.177	0.072	1.55707838159825E-74	Hepatocytes	ATP6V1D
1.4478684885165E-78	0.568328702312831	0.161	0.06	2.42633801305595E-74	Hepatocytes	MAGEF1
1.65894587822504E-78	0.418602550039482	0.165	0.063	2.78006150272951E-74	Hepatocytes	BRIX1
1.94754024735007E-78	0.606384932500731	0.175	0.071	3.26368794650924E-74	Hepatocytes	CCNC
1.99657540524456E-78	0.681338411411538	0.155	0.057	3.34586106410884E-74	Hepatocytes	DEPRECATED-ENSG00000120802
2.06487731302277E-78	0.601901529127379	0.158	0.059	3.46032140116356E-74	Hepatocytes	GAR1
2.47659343606082E-78	0.51206747820754	0.116	0.031	4.15027528015072E-74	Hepatocytes	F8
2.53937748385084E-78	0.68050754882663	0.129	0.04	4.25548878743723E-74	Hepatocytes	ABLIM3
3.84405917916844E-78	1.17862271672041	0.158	0.061	6.44187437245047E-74	Hepatocytes	TOP2A
4.38505344609969E-78	0.612299013915683	0.154	0.057	7.34847256497385E-74	Hepatocytes	CNTLN
5.38913993012747E-78	0.335793715319872	0.198	0.086	9.03112069490762E-74	Hepatocytes	TOPBP1
7.92949723007635E-78	0.665448276832856	0.151	0.054	1.32882514581619E-73	Hepatocytes	ZFP64
9.62031661273568E-78	0.815195000881448	0.127	0.039	1.61217265796225E-73	Hepatocytes	CMSS1
9.7500926632994E-78	0.458576465971069	0.182	0.076	1.63392052851571E-73	Hepatocytes	GMPR2
2.83116547869357E-77	0.278802521194063	0.338	0.191	4.74446710919468E-73	Hepatocytes	CSTB
3.24725737015755E-77	0.481420682500497	0.239	0.118	5.44175390091002E-73	Hepatocytes	ATRAID
3.40806167328239E-77	0.257693311658556	0.153	0.055	5.71122975208664E-73	Hepatocytes	RBBP5
4.14706476110789E-77	0.838731829070376	0.185	0.08	6.9496511266646E-73	Hepatocytes	PLPBP
6.04329636477218E-77	0.498248589456916	0.13	0.041	1.01273560480852E-72	Hepatocytes	L3MBTL4
6.26152831292675E-77	1.24450582540384	0.126	0.039	1.04930691468027E-72	Hepatocytes	CENPW
6.53939937266284E-77	1.16432174329568	0.163	0.065	1.09587254687084E-72	Hepatocytes	PCLAF
7.71530722259926E-77	0.254613100370684	0.177	0.072	1.29293118436318E-72	Hepatocytes	COG1
8.41344631183876E-77	0.958081540162196	0.15	0.055	1.40992533293794E-72	Hepatocytes	GFER
8.69246989959968E-77	0.460420859993848	0.19	0.081	1.45668410577491E-72	Hepatocytes	ALG5
9.04890744192003E-77	0.49435944895498	0.19	0.082	1.51641590911696E-72	Hepatocytes	NUSAP1
9.12036931270925E-77	0.524283975090612	0.13	0.041	1.52839148942382E-72	Hepatocytes	SMIM13

1.22072877032246E-76	0.34144675127153	0.182	0.076	2.04569727330637E-72	Hepatocytes	COX20
1.43445013972899E-76	0.8670870805786	0.106	0.026	2.40385154415784E-72	Hepatocytes	HACL1
1.58257132503447E-76	1.149956239457	0.109	0.028	2.65207302649276E-72	Hepatocytes	OCEL1
1.98295656183082E-76	0.298323102555908	0.201	0.09	3.32303860631608E-72	Hepatocytes	ERAL1
2.34142181833215E-76	0.618516356967193	0.191	0.084	3.92375468316102E-72	Hepatocytes	VPS72
3.26147801325587E-76	0.998558091423297	0.127	0.04	5.46558485461419E-72	Hepatocytes	NDUFAF5
3.27613534211792E-76	0.297092685705427	0.261	0.134	5.4901476063212E-72	Hepatocytes	FAM32A
3.27961431535769E-76	0.909014811325062	0.119	0.035	5.49597766967641E-72	Hepatocytes	ELAC1
3.55100604885557E-76	0.440238338548211	0.13	0.042	5.95077593667216E-72	Hepatocytes	SNX21
3.63033091826758E-76	0.655665969359216	0.13	0.041	6.08370855283281E-72	Hepatocytes	VARS2
3.85016132933945E-76	0.638391927828933	0.116	0.033	6.45210035570706E-72	Hepatocytes	PEX5
4.14437145609577E-76	0.675715696336407	0.191	0.084	6.94513768612528E-72	Hepatocytes	HSD17B12
4.16099693367884E-76	0.390571225902147	0.139	0.047	6.972998661459E-72	Hepatocytes	FBXL18
4.37735644793221E-76	1.06169216869725	0.112	0.03	7.3355739354448E-72	Hepatocytes	POP1
4.89055110928165E-76	0.715372312894718	0.117	0.033	8.19558554893419E-72	Hepatocytes	DEPRECATED-ENSG00000104442
4.99849420208828E-76	0.361145821698408	0.161	0.062	8.37647658385955E-72	Hepatocytes	CLOCK
5.18474275085448E-76	0.584358865666817	0.164	0.064	8.68859190188194E-72	Hepatocytes	COQ6
7.70021909860302E-76	0.364667763058725	0.225	0.108	1.29040271654389E-71	Hepatocytes	PGK 1.00
1.03613960278277E-75	0.755465918941274	0.156	0.06	1.73636274634336E-71	Hepatocytes	STMN1
1.10497741291264E-75	0.338542510160638	0.238	0.117	1.85172114855901E-71	Hepatocytes	ANKH
1.19374219970759E-75	0.865507481839988	0.145	0.052	2.00047317826999E-71	Hepatocytes	PPIL1
1.31606909501683E-75	0.362604559090276	0.159	0.06	2.20546858942921E-71	Hepatocytes	AP1AR
1.62707443012088E-75	0.796731643530757	0.143	0.051	2.72665132999657E-71	Hepatocytes	TWF1
1.66610798984813E-75	0.391744741116563	0.212	0.099	2.79206376938749E-71	Hepatocytes	ZMPSTE24
1.69112326019103E-75	0.523461885570985	0.195	0.087	2.83398435942813E-71	Hepatocytes	ERLIN2
1.82187120939351E-75	0.376388979559145	0.175	0.072	3.05309177270164E-71	Hepatocytes	CHCHD3
2.61463813590242E-75	0.460300375838815	0.1	0.023	4.38161058814527E-71	Hepatocytes	PRRG1
2.78457277600678E-75	0.334471023135763	0.113	0.03	4.66638705803216E-71	Hepatocytes	NUAK1
3.71825314396082E-75	0.30583084413571	0.164	0.065	6.23104861864954E-71	Hepatocytes	DDX59
3.87701134245867E-75	0.610604828623694	0.165	0.065	6.49709560769223E-71	Hepatocytes	POP4
5.02020736194641E-75	0.614445850972336	0.126	0.039	8.41286349714979E-71	Hepatocytes	CHPF
5.26198838563105E-75	0.350525474634639	0.184	0.078	8.81804013664051E-71	Hepatocytes	COASY
5.49100447615962E-75	0.442756866078399	0.115	0.032	9.20182530114829E-71	Hepatocytes	DLG3
7.44698843979254E-75	1.11090660929038	0.115	0.033	1.24796632274043E-70	Hepatocytes	ALG14
1.20479103590581E-74	0.338014217714381	0.164	0.065	2.01898881797095E-70	Hepatocytes	ZNF512B
1.23791561246134E-74	0.261836561786077	0.195	0.087	2.07449898336272E-70	Hepatocytes	NDRG3
1.64241653046398E-74	0.408763970962611	0.151	0.056	2.75236162175154E-70	Hepatocytes	PSMG1
1.88705966598652E-74	0.29690136714304	0.139	0.048	3.16233458826021E-70	Hepatocytes	ZNF839
2.22209751605189E-74	0.403019734559035	0.158	0.061	3.72379101739975E-70	Hepatocytes	DDA1
2.4041204607429E-74	0.368340069010176	0.213	0.1	4.02882506811296E-70	Hepatocytes	VTI1B
3.71800373538665E-74	0.506914960754264	0.163	0.065	6.23063065976094E-70	Hepatocytes	SEPSECS
4.42091442393316E-74	0.472635875675312	0.171	0.07	7.40856839162719E-70	Hepatocytes	AGA
4.74199623851251E-74	0.614112178194816	0.15	0.057	7.94663729649927E-70	Hepatocytes	LANCL2
6.37468886418368E-74	0.284285931511237	0.314	0.176	1.0682703598599E-69	Hepatocytes	NACC1
6.9824450088129E-74	0.332085005928088	0.12	0.037	1.17011813457687E-69	Hepatocytes	CXorf40B
9.31769542416565E-74	0.302980850104916	0.175	0.073	1.56145939918168E-69	Hepatocytes	PSKH1
9.41845708258337E-74	0.42441043256121	0.152	0.058	1.57834503789932E-69	Hepatocytes	DEPRECATED-ENSG00000140365
1.0398811609845E-73	0.74778988256208	0.108	0.029	1.74263284957782E-69	Hepatocytes	GPC3
1.40172477832E-73	0.807393326759756	0.14	0.05	2.34901038350866E-69	Hepatocytes	SHMT2
1.52431458172853E-73	1.72617388899185	0.102	0.026	2.55444637606067E-69	Hepatocytes	ALDH1B1
1.98950418999898E-73	0.459365358400528	0.182	0.078	3.33401112160029E-69	Hepatocytes	DCAF10
2.00206804918075E-73	0.685353588941189	0.107	0.029	3.3550656368171E-69	Hepatocytes	ZNF554
2.89206886817349E-73	0.483660634492169	0.15	0.056	4.84652900928513E-69	Hepatocytes	SLC35B3
3.77727455066861E-73	0.343667552499322	0.132	0.044	6.32995669201045E-69	Hepatocytes	NBPF12
3.78272682889411E-73	0.436813423396843	0.144	0.052	6.33909361986075E-69	Hepatocytes	COMTD1
4.13313777681577E-73	1.1679801509722	0.121	0.038	6.92631228638787E-69	Hepatocytes	BCKDHB
4.67291248173616E-73	0.331262154652588	0.128	0.042	7.83086673689346E-69	Hepatocytes	DEPRECATED-ENSG00000122912
5.62334839460917E-73	0.545162105681732	0.182	0.079	9.42360723968605E-69	Hepatocytes	RNF181
5.786437012315E-73	0.510771617120528	0.132	0.045	9.69691114523748E-69	Hepatocytes	PTRH2
6.73369677817342E-73	0.304443800082068	0.208	0.097	1.1284329060863E-68	Hepatocytes	ARF4
7.51125750083064E-73	0.831528956561708	0.111	0.031	1.2587365319892E-68	Hepatocytes	SETD6
7.55089418139825E-73	0.524783078376367	0.14	0.05	1.26537884691872E-68	Hepatocytes	TUBGCP4
9.79491407128067E-73	0.738018057285913	0.1	0.025	1.64143170006522E-68	Hepatocytes	MANSC1

1.15676721109579E-72	0.688864324942676	0.139	0.05	1.93851049235432E-68	Hepatocytes	IQCE
1.20574520110001E-72	0.46009429011839	0.132	0.045	2.0205878080034E-68	Hepatocytes	LIAS
1.22758642658799E-72	0.446283480862812	0.236	0.119	2.05718933367615E-68	Hepatocytes	FIBP
1.96509566639547E-72	1.44866552143703	0.104	0.027	3.29310731774552E-68	Hepatocytes	DEPRECATED-ENSG00000172301
2.10978853936629E-72	0.684967006054173	0.132	0.045	3.53558363427002E-68	Hepatocytes	BCL2L12
2.40914403439685E-72	0.70020688784746	0.164	0.066	4.03724357284224E-68	Hepatocytes	MOSPD3
2.4380980672229E-72	1.09867528425228	0.103	0.027	4.08576474105214E-68	Hepatocytes	PUS3
3.53977761313252E-72	0.656300236604058	0.104	0.027	5.93195932408747E-68	Hepatocytes	ZNF782
3.56802629952907E-72	0.603934562887651	0.156	0.061	5.97929847275082E-68	Hepatocytes	SLC1A4
5.66372936700466E-72	0.577201623552162	0.11	0.031	9.49127767322641E-68	Hepatocytes	ZNF697
7.14646024667465E-72	0.352435583063868	0.163	0.065	1.19760380813774E-67	Hepatocytes	SNRPG
8.1011863793677E-72	1.11755968279392	0.117	0.035	1.35759681345444E-67	Hepatocytes	ARNTL2
1.2273794698284E-71	0.389590395987823	0.226	0.111	2.05684251553843E-67	Hepatocytes	AKIP1
1.30488724306919E-71	0.480075142712862	0.117	0.035	2.18673004193535E-67	Hepatocytes	PRKCZ
1.45310929974153E-71	0.648532278327157	0.13	0.044	2.43512056450686E-67	Hepatocytes	PIGU
1.77904732078089E-71	0.391561241331609	0.148	0.056	2.98132750016462E-67	Hepatocytes	DYNLT3
2.43126371653915E-71	0.364671158507134	0.208	0.098	4.07431173617631E-67	Hepatocytes	ACAD11
2.71720346883828E-71	0.532461508149144	0.125	0.041	4.55348957307919E-67	Hepatocytes	ZNF576
3.0155399774953E-71	0.495076343683708	0.15	0.058	5.05344189428663E-67	Hepatocytes	BCS1L
5.51583315133735E-71	0.575710202408064	0.156	0.062	9.24343319501112E-67	Hepatocytes	SLC25A20
7.34007199755093E-71	0.689906278284536	0.162	0.067	1.23004926534958E-66	Hepatocytes	ZMAT5
7.88583039664169E-71	0.453229042739164	0.171	0.072	1.32150745786921E-66	Hepatocytes	TSFM
1.02031609346468E-70	0.50155912278956	0.169	0.071	1.70984570942811E-66	Hepatocytes	MLST8
1.67536409271931E-70	0.659590815123076	0.164	0.068	2.80757514657902E-66	Hepatocytes	GPR137
1.77669014185376E-70	0.931746809897634	0.109	0.031	2.97737733971852E-66	Hepatocytes	FITM2
2.20086243871838E-70	1.10188136612778	0.119	0.038	3.68820527480427E-66	Hepatocytes	TRUB2
3.48318569256847E-70	0.798609774905106	0.129	0.044	5.83712258360625E-66	Hepatocytes	FUNDC1
3.98003578956765E-70	0.775140786202549	0.135	0.048	6.66974397615746E-66	Hepatocytes	ST13
4.0680110576148E-70	0.875754218318818	0.144	0.055	6.81717293035087E-66	Hepatocytes	RIOX2
4.14649855022576E-70	0.745360778536203	0.116	0.036	6.94870227046832E-66	Hepatocytes	RBIS
4.33198782328301E-70	0.406032167139304	0.182	0.081	7.25954519425767E-66	Hepatocytes	CTNNBIP1
4.36998017708683E-70	0.766643582340371	0.11	0.032	7.32321278076211E-66	Hepatocytes	DEPRECATED-ENSG00000132570
4.98971610563539E-70	0.417744510624411	0.196	0.091	8.36176624982379E-66	Hepatocytes	TOMM22
5.67882859788398E-70	0.582019895414518	0.149	0.058	9.51658096433397E-66	Hepatocytes	TIMELESS
7.61639842674422E-70	0.432319559888223	0.194	0.09	1.2763560483538E-65	Hepatocytes	TRAPPC6A
9.76181861562495E-70	0.748008541407641	0.131	0.046	1.63588556360643E-65	Hepatocytes	PAIP1
1.00895006885611E-69	0.766631547758644	0.167	0.07	1.69079852538907E-65	Hepatocytes	UQCRH
1.04317633781412E-69	0.474115497836036	0.155	0.062	1.7481549069089E-65	Hepatocytes	MOB3B
1.96169196634029E-69	0.806566060875402	0.11	0.032	3.28740339719305E-65	Hepatocytes	DOLPP1
2.75777562673313E-69	0.930781546361018	0.109	0.032	4.62148039527937E-65	Hepatocytes	TTPAL
2.81292579839649E-69	0.85967721677709	0.118	0.037	4.71390105295284E-65	Hepatocytes	RWDD4
3.04308872335227E-69	0.501337566574039	0.148	0.057	5.09960808259373E-65	Hepatocytes	POLD3
3.35125632676591E-69	0.551078784136064	0.138	0.051	5.61603535239431E-65	Hepatocytes	CFAP20
3.53330778543881E-69	0.511177612106651	0.14	0.052	5.92111718683835E-65	Hepatocytes	POLE4
3.88776075370926E-69	0.497594904683878	0.106	0.03	6.51510947106599E-65	Hepatocytes	SPATA2
1.24336667565762E-68	0.276412396933796	0.154	0.061	2.08363387506704E-64	Hepatocytes	MAD2L1BP
1.26466517696667E-68	1.17449683332202	0.113	0.035	2.11932590356075E-64	Hepatocytes	TIMM21
1.28158533804368E-68	0.856367511237055	0.12	0.039	2.1476807094936E-64	Hepatocytes	HSD17B7
2.46179096662575E-68	0.531918538699953	0.135	0.049	4.12546930187144E-64	Hepatocytes	CRADD
2.59408567902111E-68	0.627797484294236	0.141	0.053	4.34716878090357E-64	Hepatocytes	TAB3
2.68786752969864E-68	0.798221492843563	0.123	0.041	4.50432840626899E-64	Hepatocytes	OLA1
2.74939222398661E-68	0.697809958311633	0.121	0.04	4.60743148895677E-64	Hepatocytes	COQ7
2.96708125817642E-68	0.363572015005371	0.128	0.044	4.97223477245204E-64	Hepatocytes	AASDH
3.18914852251132E-68	0.315011380455572	0.145	0.056	5.34437509402447E-64	Hepatocytes	C15orf40
3.61062060337657E-68	0.917098890555069	0.124	0.042	6.05067800713845E-64	Hepatocytes	SIRT5
4.0122870714875E-68	0.74383662693759	0.132	0.047	6.72379067439876E-64	Hepatocytes	TOMM40
4.12836912024584E-68	0.353159003744664	0.151	0.06	6.91832097170798E-64	Hepatocytes	ACOT8
4.95604908686666E-68	0.959703604750225	0.114	0.036	8.30534705977114E-64	Hepatocytes	FAM189B
8.53981277364556E-68	0.661359687704627	0.124	0.042	1.43110182460752E-63	Hepatocytes	VKORC1L1
1.00084981111929E-67	0.666683684561137	0.122	0.041	1.67722411347371E-63	Hepatocytes	JKAMP
6.10741041191892E-67	0.423635369781012	0.19	0.088	1.02347983682937E-62	Hepatocytes	LAMTOR3
8.38260927604732E-67	1.00215489435594	0.108	0.033	1.40475766248001E-62	Hepatocytes	ZNF593
8.57314614246907E-67	0.578430316090666	0.149	0.06	1.43668783055497E-62	Hepatocytes	RWDD1

9.83777025022686E-67	0.253484911986487	0.186	0.086	1.64861353853302E-62	Hepatocytes	ACP1
1.01947309351227E-66	0.28739076719557	0.142	0.055	1.70843301010786E-62	Hepatocytes	PKNOX1
1.1201233499586E-66	0.617667278833695	0.14	0.054	1.87710270986062E-62	Hepatocytes	DLAT
1.70811653067639E-66	0.371975991574919	0.172	0.076	2.86246168210749E-62	Hepatocytes	DEPRECATED-ENSG00000100412
2.37918148576407E-66	0.306364557677013	0.153	0.062	3.98703233384343E-62	Hepatocytes	MPI
2.6251422205358E-66	0.637508147730286	0.125	0.044	4.3992133331739E-62	Hepatocytes	SLC48A1
3.0310088808888E-66	0.422886720917871	0.131	0.048	5.07936468259346E-62	Hepatocytes	AARS
3.24796476379428E-66	0.736448572534509	0.137	0.052	5.44293935116646E-62	Hepatocytes	PIGV
3.63386151972169E-66	0.311467888848766	0.125	0.044	6.08962513474961E-62	Hepatocytes	ATG10
4.50926515399457E-66	0.462715470101032	0.135	0.05	7.55662654506409E-62	Hepatocytes	POT1
7.4383724818993E-66	1.1014175536027	0.101	0.028	1.24652246051669E-61	Hepatocytes	DPM3
8.10953403617447E-66	0.300663951639355	0.168	0.073	1.35899571378212E-61	Hepatocytes	SLC25A40
9.00326834253363E-66	1.1296535630553	0.103	0.03	1.50876770884179E-61	Hepatocytes	TNFRSF12A
1.0106831146132E-65	0.725156744839712	0.111	0.035	1.6937027634688E-61	Hepatocytes	TEN1
1.55121458325141E-65	0.38478502120515	0.125	0.044	2.59952539861271E-61	Hepatocytes	LIG4
1.82067284378494E-65	0.402917167032083	0.115	0.037	3.0510835516148E-61	Hepatocytes	C11orf71
1.993945294604E-65	0.335734401347346	0.103	0.03	3.34145352469738E-61	Hepatocytes	ZNF823
2.05573503164056E-65	0.634794995793764	0.129	0.047	3.44500076602324E-61	Hepatocytes	DEPRECATED-ENSG00000050130
2.20339245779364E-65	0.286738836939711	0.163	0.07	3.69244508077059E-61	Hepatocytes	CMPK2
3.02779504127253E-65	1.10078290239917	0.119	0.041	5.0739789301645E-61	Hepatocytes	DEPRECATED-ENSG00000111912
3.13152069392828E-65	0.313001861770542	0.189	0.089	5.24780237888502E-61	Hepatocytes	THOC7
3.38421680598748E-65	0.82921199826495	0.154	0.064	5.67127052347383E-61	Hepatocytes	PSMB3
3.53893869588395E-65	0.855879569587712	0.126	0.046	5.93055346656233E-61	Hepatocytes	EARS2
4.74412359071523E-65	0.879528391861343	0.1	0.028	7.95020231332058E-61	Hepatocytes	DEPRECATED-ENSG00000071894
6.73868373791634E-65	0.635092287915429	0.111	0.035	1.12926862080002E-60	Hepatocytes	C9orf40
1.06351415099403E-64	0.840061488192793	0.121	0.042	1.7822370142358E-60	Hepatocytes	PRADC1
3.31864158507059E-64	0.726026002118554	0.132	0.049	5.5613795682613E-60	Hepatocytes	LRIF1
3.62028859888611E-64	0.961323702411012	0.14	0.056	6.06687963401334E-60	Hepatocytes	GADD45G
6.39162482528378E-64	0.371414604301798	0.216	0.11	1.07110848822106E-59	Hepatocytes	IMP4
6.65660769576539E-64	0.835372217977675	0.1	0.029	1.11551431765636E-59	Hepatocytes	NECTIN1
6.99499788565083E-64	0.355184027155099	0.183	0.086	1.17222174567737E-59	Hepatocytes	NOP16
7.54280332441574E-64	0.664521489173725	0.128	0.047	1.26402298110559E-59	Hepatocytes	ARMC10
1.23881655014851E-63	0.439976220245854	0.153	0.064	2.07600877473887E-59	Hepatocytes	CYCS
1.42166200320997E-63	0.461889867618604	0.117	0.04	2.38242118497927E-59	Hepatocytes	AHCTF1
1.89757352827339E-63	0.787234797817967	0.152	0.065	3.17995371868054E-59	Hepatocytes	EGR1
2.34306199448897E-63	0.379843043817769	0.11	0.035	3.92650329036462E-59	Hepatocytes	MSH3
2.40648278017823E-63	0.88944747446963	0.104	0.032	4.03278384302268E-59	Hepatocytes	PPP2CB
3.55922831772067E-63	0.376991260314754	0.131	0.049	5.9645548148363E-59	Hepatocytes	RRP15
4.38796242618931E-63	0.683744970948143	0.142	0.057	7.35334743380805E-59	Hepatocytes	ATP6V0E2
8.79160231399226E-63	0.472978871071667	0.133	0.051	1.47329671577882E-58	Hepatocytes	COA5
1.03303509754392E-62	0.374472262547014	0.131	0.05	1.73116021646409E-58	Hepatocytes	FLYWCH2
1.25118821063652E-62	0.5253834937699	0.143	0.058	2.09674120338468E-58	Hepatocytes	VTA1
1.28527411281066E-62	0.641993826184348	0.138	0.055	2.1538623582481E-58	Hepatocytes	TMEM101
1.29170728769957E-62	0.682435158171096	0.123	0.044	2.16464307272694E-58	Hepatocytes	WDR5B
1.59048360757096E-62	0.25129943302287	0.207	0.104	2.66533242956741E-58	Hepatocytes	MED8
2.20187016167877E-62	0.8296650765667	0.122	0.045	3.68989401694128E-58	Hepatocytes	RPP38
3.65880291461961E-62	0.779125049976679	0.101	0.03	6.13142192431955E-58	Hepatocytes	TUSC1
4.83225645194014E-62	0.431834554701729	0.113	0.038	8.09789536216128E-58	Hepatocytes	TMEM120B
5.03047522087476E-62	0.328604708838152	0.148	0.062	8.43007037514193E-58	Hepatocytes	SNAPC4
5.89308660665229E-62	0.505194327750287	0.119	0.042	9.87563453542791E-58	Hepatocytes	OPA3
7.50452073663621E-62	0.462960901846863	0.114	0.039	1.2576075850455E-57	Hepatocytes	EID2
8.25494275501641E-62	0.855602850287214	0.115	0.039	1.38336330688565E-57	Hepatocytes	UTP25
1.55659924866873E-61	0.328498726855677	0.173	0.08	2.60854902091905E-57	Hepatocytes	HCFC1R1
1.59682311525983E-61	0.331618033330374	0.223	0.118	2.67595617655243E-57	Hepatocytes	DBP
1.96737421755476E-61	0.287405271861492	0.113	0.038	3.29692571377827E-57	Hepatocytes	ZNF619
2.4776859385207E-61	0.313926373836795	0.126	0.047	4.15210609577299E-57	Hepatocytes	MCM8
2.72969413520941E-61	0.343940888788513	0.103	0.031	4.57442143178393E-57	Hepatocytes	MYB
3.5348644970283E-61	0.861521615304309	0.117	0.041	5.92372592412002E-57	Hepatocytes	SLC25A22
5.30399867373225E-61	0.686107117197084	0.121	0.044	8.8884409774405E-57	Hepatocytes	CFL2
8.28566074845205E-61	0.504025270533751	0.132	0.051	1.38851102822559E-56	Hepatocytes	DARS2
1.11628809569684E-60	0.926011045730801	0.103	0.033	1.87067559076876E-56	Hepatocytes	RSPH3
1.17046937052495E-60	0.336576816593287	0.105	0.034	1.9614725711257E-56	Hepatocytes	PDP2
1.2599542402155E-60	0.383733592210168	0.155	0.067	2.11143131575313E-56	Hepatocytes	TARS2

1.4158358780559E-60	0.823251818153936	0.124	0.047	2.37265776444608E-56	Hepatocytes	NDUFA6
1.81195072000243E-60	0.356049188110148	0.136	0.055	3.03646701658007E-56	Hepatocytes	DEPRECATED-ENSG00000168924
2.02721187522634E-60	0.489396262180208	0.149	0.063	3.3972016605043E-56	Hepatocytes	ALG8
2.7199261517621E-60	0.340730827136716	0.136	0.055	4.55805224512293E-56	Hepatocytes	CLK2
3.02856834989153E-60	0.343499843139472	0.13	0.051	5.07527484074823E-56	Hepatocytes	EVI5L
3.71793389443046E-60	0.428319538630265	0.12	0.044	6.23051362028656E-56	Hepatocytes	SF3B6
4.92802328232245E-60	0.252913685044628	0.141	0.058	8.25838141651596E-56	Hepatocytes	MPHOSPH10
2.11297948955638E-59	0.559957159010499	0.145	0.062	3.54093102859859E-55	Hepatocytes	IMPACT
2.20648083972558E-59	0.505391434334831	0.151	0.066	3.69762059121213E-55	Hepatocytes	RRS1
2.21782187788408E-59	0.796256834364296	0.106	0.035	3.71662590295814E-55	Hepatocytes	LGALSL
2.84910599445313E-59	0.290374554398785	0.176	0.084	4.77453182550456E-55	Hepatocytes	HIST1H4C
3.31894350864095E-59	0.355807584873834	0.142	0.059	5.5618855317805E-55	Hepatocytes	AP3S1
3.87098873138404E-59	0.608391617668764	0.119	0.044	6.48700291605337E-55	Hepatocytes	SMUG1
5.02475182691594E-59	0.663904492209072	0.147	0.063	8.42047911154574E-55	Hepatocytes	PGAM5
5.2473367404779E-59	0.759853609285961	0.116	0.042	8.79348690969287E-55	Hepatocytes	RFK
6.25762194938768E-59	1.35552848024686	0.114	0.041	1.04865228627839E-54	Hepatocytes	KNSTRN
7.03290852991633E-59	0.343762373109461	0.142	0.06	1.17857481144338E-54	Hepatocytes	YIPF1
1.12410594496944E-58	0.47867792725155	0.136	0.055	1.88377674257979E-54	Hepatocytes	TMEM184C
1.15167934623945E-58	0.565769328981779	0.13	0.052	1.92998424842808E-54	Hepatocytes	CENPJ
1.22282515751092E-58	0.438539997329138	0.143	0.06	2.0492103989568E-54	Hepatocytes	COPG2
1.47117245752588E-58	0.747519030066997	0.126	0.049	2.46539080432187E-54	Hepatocytes	TRAPPC2L
2.22393889981139E-58	0.286578022833455	0.186	0.091	3.72687680830393E-54	Hepatocytes	PUS1
3.0218305606257E-58	0.255728975448704	0.159	0.071	5.06398365349656E-54	Hepatocytes	TDRD7
4.46829062977609E-58	0.267297110556935	0.153	0.068	7.48796143737877E-54	Hepatocytes	TAF12
5.44327062584953E-58	0.62500401081715	0.137	0.057	9.12183291479865E-54	Hepatocytes	COG8
6.02349982951519E-58	0.956274067653853	0.12	0.046	1.00941810143016E-53	Hepatocytes	SPAG5
6.07045431818519E-58	0.630360574040175	0.188	0.095	1.01728673464147E-53	Hepatocytes	UROD
6.38850316853106E-58	0.521717061986283	0.145	0.063	1.07058536098244E-53	Hepatocytes	GPATCH4
6.81322203950224E-58	1.070542888074	0.112	0.041	1.14175974937978E-53	Hepatocytes	GCDH
8.26780269527224E-58	0.592588177085566	0.128	0.051	1.38551837567372E-53	Hepatocytes	SHPK
8.68526859957613E-58	0.328307125594645	0.203	0.104	1.45547731191697E-53	Hepatocytes	MAD2L2
9.01118573451517E-58	0.596985037451113	0.108	0.037	1.51009450539005E-53	Hepatocytes	IBA57
9.81896150014415E-58	0.454575142594109	0.103	0.034	1.64546156819416E-53	Hepatocytes	DEPRECATED-ENSG00000130021
1.51953100761438E-57	0.578121433837552	0.108	0.038	2.54643006256018E-53	Hepatocytes	DOLK
1.58700935642082E-57	0.565401060998956	0.148	0.065	2.65951027949001E-53	Hepatocytes	DEPRECATED-ENSG00000128228
1.63693198022054E-57	0.475949105570843	0.148	0.065	2.74317061245359E-53	Hepatocytes	SMIM10L1
2.24489394666409E-57	0.40992635736931	0.157	0.072	3.76199327581967E-53	Hepatocytes	SMCO4
2.50710665225324E-57	0.733712521585567	0.131	0.054	4.20140932784598E-53	Hepatocytes	RRM2
3.00456154201729E-57	0.939274799691524	0.131	0.055	5.03504423211257E-53	Hepatocytes	ECT2
3.06618132699319E-57	0.730879748718943	0.131	0.053	5.13830666777518E-53	Hepatocytes	ZDHHC12
4.05206796436463E-57	0.341147949721804	0.141	0.06	6.79045549468225E-53	Hepatocytes	DNAAF5
4.98260655940682E-57	0.772934703499742	0.151	0.068	8.34985207225395E-53	Hepatocytes	SLC35C1
5.17738286404044E-57	0.622917987667083	0.124	0.049	8.67625820355897E-53	Hepatocytes	STYXL1
5.49121173961468E-57	0.555702970317856	0.123	0.048	9.20217263324627E-53	Hepatocytes	C1D
5.82677105109047E-57	0.413336004868721	0.176	0.085	9.7645029274174E-53	Hepatocytes	SCAND1
6.20098856226594E-57	0.27877342243657	0.109	0.038	1.03916166326453E-52	Hepatocytes	OBI1
6.42283343750118E-57	0.841977869901676	0.105	0.036	1.07633842745645E-52	Hepatocytes	PDIA4
8.18056004217989E-57	0.449348315504897	0.112	0.04	1.37089825186851E-52	Hepatocytes	PHB2
9.01761005467014E-57	0.621234903920524	0.104	0.036	1.51117109296162E-52	Hepatocytes	INTS12
1.23659592438456E-56	0.951666441235718	0.121	0.048	2.07228745008364E-52	Hepatocytes	ASF1B
1.62707757474451E-56	0.680573692269827	0.109	0.039	2.72665659975684E-52	Hepatocytes	DEPRECATED-ENSG00000056050
2.75804045054996E-56	0.552544420212141	0.105	0.037	4.62192418703162E-52	Hepatocytes	LRR1
3.81702035798537E-56	0.389959604590889	0.136	0.057	6.39656271591188E-52	Hepatocytes	ATMIN
4.92146223029351E-56	0.825813487218598	0.101	0.034	8.24738640552586E-52	Hepatocytes	LBHD1
6.04895361745991E-56	0.375943848576748	0.129	0.052	1.01368364721393E-51	Hepatocytes	APIP
9.18745124445819E-56	0.263630428652338	0.148	0.066	1.5396330795463E-51	Hepatocytes	VWA8
1.26233764716927E-55	0.466230088375374	0.129	0.052	2.11542542912627E-51	Hepatocytes	NDUFA12
1.89673658207786E-55	0.374150835186144	0.112	0.041	3.17855116424608E-51	Hepatocytes	C19orf47
3.66733944041938E-55	0.297262823805744	0.147	0.065	6.1457274342548E-51	Hepatocytes	BACE1
4.87069471180636E-55	0.715402569547556	0.131	0.055	8.1623101980451E-51	Hepatocytes	CHCHD5
4.87937069741292E-55	0.291081328103781	0.15	0.068	8.17684941472458E-51	Hepatocytes	PDE6D
7.44955835327609E-55	0.510758766640746	0.164	0.078	1.24839698884201E-50	Hepatocytes	DEPRECATED-ENSG00000105701
7.7013222009704E-55	0.469891380501258	0.134	0.057	1.29058757443862E-50	Hepatocytes	GTF2H3

1.10967010360291E-54	1.38321986956969	0.115	0.045	1.85958515961775E-50	Hepatocytes	HMMR
1.5973009684531E-54	0.270840783309709	0.102	0.035	2.67675696293371E-50	Hepatocytes	MEGF8
1.60915751488345E-54	0.444801008417668	0.189	0.098	2.69662616344169E-50	Hepatocytes	SHTN1
1.6808188692387E-54	0.666432994024686	0.153	0.071	2.81671626107021E-50	Hepatocytes	THYN1
2.12810949901956E-54	0.452530180966889	0.172	0.085	3.56628589845697E-50	Hepatocytes	SNAPIN
3.10125954959655E-54	0.75363531645993	0.104	0.037	5.1970907532139E-50	Hepatocytes	WNT5B
3.40211190530036E-54	0.69964774019772	0.119	0.047	5.70125913090234E-50	Hepatocytes	ATP5MPL
4.48277986886512E-54	0.42269463947016	0.165	0.079	7.51224250424417E-50	Hepatocytes	BZW2
5.22413690588874E-54	0.534800595059873	0.103	0.036	8.75460862688836E-50	Hepatocytes	PGBD2
1.03761840806063E-53	0.411719290462575	0.107	0.039	1.738840928228E-49	Hepatocytes	NELFE
1.07185602829807E-53	0.26608079234457	0.124	0.05	1.7962163322219E-49	Hepatocytes	PDIK1L
1.40368424284407E-53	0.457765212852925	0.17	0.083	2.3522940541581E-49	Hepatocytes	MTIF2
2.17147471072609E-53	0.537648065491584	0.129	0.054	3.63895732023478E-49	Hepatocytes	PTGFRN
2.8654450254449E-53	0.254276569465303	0.107	0.039	4.80191277364056E-49	Hepatocytes	PSMB7
3.0111127125667E-53	0.286711236810864	0.143	0.064	5.04602268371928E-49	Hepatocytes	PDRG1
6.3670360988311E-53	0.312381027091924	0.123	0.051	1.06698790944212E-48	Hepatocytes	NAV2
7.71224622252926E-53	0.402589519574206	0.111	0.042	1.29241822197145E-48	Hepatocytes	METTL2A
1.30432056154333E-52	0.342524225206687	0.128	0.054	2.18578039703431E-48	Hepatocytes	CDCA7L
1.40338331290874E-52	0.374214647964081	0.238	0.137	2.35178975577247E-48	Hepatocytes	ARL6IP1
1.45130995945604E-52	0.576589174999629	0.116	0.046	2.43210523005643E-48	Hepatocytes	MLYCD
2.76064676405483E-52	0.382220346775267	0.113	0.044	4.62629184720308E-48	Hepatocytes	DPM2
2.87224554400645E-52	1.06897695748223	0.107	0.04	4.81330908264601E-48	Hepatocytes	MIS18A
3.24230561416204E-52	1.36835385132835	0.108	0.041	5.43345574821275E-48	Hepatocytes	SPC24
4.19772018485961E-52	0.44673410708303	0.13	0.056	7.03453948578773E-48	Hepatocytes	PICK1
5.07504931614151E-52	0.753669155654598	0.116	0.046	8.50476764398995E-48	Hepatocytes	MDH1
6.42380564839298E-52	0.278636567924144	0.127	0.053	1.0765013505577E-47	Hepatocytes	TMEM203
6.61213509035545E-52	0.452929860972729	0.127	0.054	1.10806159844177E-47	Hepatocytes	KNOP1
1.00597256540963E-51	0.499985648618213	0.171	0.087	1.68580882511347E-47	Hepatocytes	CHCHD10
1.12686949772634E-51	0.466865995570413	0.15	0.07	1.88840790428981E-47	Hepatocytes	CLTC
3.27305117806905E-51	0.555607951877331	0.106	0.039	5.48497916420812E-47	Hepatocytes	CINP
8.58262170297508E-51	1.00950618322181	0.13	0.058	1.43827574498456E-46	Hepatocytes	TPX2
8.94378747087249E-51	0.566505889435924	0.12	0.049	1.49879990436881E-46	Hepatocytes	GPN3
5.01540746258977E-50	0.376776198088957	0.137	0.062	8.40481982580794E-46	Hepatocytes	AIDA
5.5178233776202E-50	0.468924129301642	0.117	0.048	9.24676841621594E-46	Hepatocytes	FBXO8
6.64746517732352E-50	0.719318731115933	0.134	0.06	1.11398221441588E-45	Hepatocytes	CDK1
6.66554573264101E-50	0.573162839118913	0.144	0.067	1.11701215387598E-45	Hepatocytes	MED18
7.69759990347927E-50	0.632129083800047	0.1	0.037	1.28996379182506E-45	Hepatocytes	STS
8.6781138036291E-50	0.597085359402145	0.117	0.048	1.45427831121216E-45	Hepatocytes	HSPBP1
1.41321021242161E-49	0.28285763987851	0.106	0.041	2.36825767397613E-45	Hepatocytes	MDP1
1.52974410194688E-49	0.330096686843705	0.165	0.082	2.56354516604257E-45	Hepatocytes	KRCC1
2.002598407279E-49	0.902983431941932	0.103	0.039	3.35595441091816E-45	Hepatocytes	MELK
2.34481148970957E-49	0.328406699110706	0.112	0.045	3.9294350944553E-45	Hepatocytes	SIRT3
5.74470613767881E-49	0.7276000504834	0.116	0.048	9.62697854552215E-45	Hepatocytes	LHPP
5.80637496502217E-49	0.287447010079936	0.157	0.077	9.73032316638415E-45	Hepatocytes	MRM2
9.1092812915352E-49	0.569778583694981	0.115	0.048	1.52653335883547E-44	Hepatocytes	GATD3A
1.52077406870918E-48	0.252461651773682	0.15	0.072	2.54851318434284E-44	Hepatocytes	FADD
1.56536405303005E-48	0.325754617893494	0.127	0.056	2.62323708006776E-44	Hepatocytes	FOXRED1
1.83869440949182E-48	0.726326116297414	0.106	0.042	3.08128409142639E-44	Hepatocytes	TMCC1
1.94254211792355E-48	0.376766186595804	0.11	0.044	3.25531208121629E-44	Hepatocytes	MRFAP1
2.64282175163626E-48	0.925832697208078	0.101	0.039	4.42884069139204E-44	Hepatocytes	NCAPG
3.06652320120375E-48	0.27816298347025	0.132	0.06	5.13887958057724E-44	Hepatocytes	CAAP1
3.95594258315235E-48	0.638526320576155	0.113	0.047	6.62936858084671E-44	Hepatocytes	DBT
6.85707405074959E-48	0.270047409261938	0.18	0.095	1.14910846942462E-43	Hepatocytes	PYM1
8.00446776576633E-48	0.265432203799573	0.118	0.05	1.34138870818712E-43	Hepatocytes	ZNF322
9.04405484181962E-48	0.258160688788416	0.134	0.061	1.51560271039213E-43	Hepatocytes	ARMC6
1.80789673268214E-47	0.353752889444557	0.108	0.044	3.02967334462874E-43	Hepatocytes	TMEM201
2.54805556210752E-47	0.755301590405169	0.106	0.042	4.27003151097978E-43	Hepatocytes	CDK5
3.12720735792465E-47	0.251649955474496	0.186	0.101	5.24057409041013E-43	Hepatocytes	GAS2L3
4.52990734992152E-47	0.730588772156905	0.109	0.045	7.59121873699848E-43	Hepatocytes	SFXN4
5.82327308231068E-47	0.469029192401899	0.101	0.039	9.75864103133624E-43	Hepatocytes	NT5DC2
8.99343856762309E-47	0.741971257531585	0.103	0.041	1.50712043516228E-42	Hepatocytes	CDK18
1.1325019219432E-46	0.517422433144016	0.11	0.045	1.89784672079242E-42	Hepatocytes	HMBS
7.12082316861141E-46	0.496858535600053	0.108	0.044	1.1933075465959E-41	Hepatocytes	MYCBP

4.67756216501573E-45	0.722326157067219	0.105	0.044	7.83865867613336E-41	Hepatocytes	UBE2S
4.85243121745278E-45	0.326305954596847	0.127	0.058	8.13170423420737E-41	Hepatocytes	JOSD2
5.02619270581881E-45	0.60485473224488	0.101	0.04	8.42289373641116E-41	Hepatocytes	NMNAT1
6.80033940450366E-45	0.474670096239385	0.13	0.061	1.13960087740672E-40	Hepatocytes	TPMT
6.22781306265869E-44	0.468853085977426	0.151	0.077	1.04365691304034E-39	Hepatocytes	RNF26
2.04987071016266E-43	0.367901891950541	0.145	0.073	3.43517333609059E-39	Hepatocytes	TMEM160
2.85832845546873E-43	0.299364974118896	0.125	0.058	4.7899868256745E-39	Hepatocytes	BRK1
1.3022927761331E-42	0.64765096012004	0.104	0.044	2.18238223424386E-38	Hepatocytes	MYCL
1.58688163987341E-42	0.302983382665205	0.145	0.073	2.65929625209986E-38	Hepatocytes	CHCHD1
4.01907879984328E-42	0.412112357142485	0.109	0.048	6.73517225277737E-38	Hepatocytes	DEPRECATED-ENSG00000180817
4.14162259250989E-42	0.492131899694506	0.101	0.042	6.94053114052808E-38	Hepatocytes	GEMIN6
1.89497703419382E-41	0.366995381728268	0.105	0.045	3.17560251390201E-37	Hepatocytes	RYK
2.42144437801428E-41	0.516567271859138	0.108	0.047	4.05785648867632E-37	Hepatocytes	PAFAH1B3
7.00817662316015E-41	0.535077726146696	0.155	0.083	1.17443023850918E-36	Hepatocytes	EIF4EBP1
1.03585951223547E-40	0.279596051516483	0.119	0.056	1.7358933706042E-36	Hepatocytes	POP5
1.29131263099119E-39	0.29639830472198	0.113	0.052	2.16398170701504E-35	Hepatocytes	POLR1B
1.76929825421061E-39	0.497729481934107	0.122	0.06	2.96499001440614E-35	Hepatocytes	HDDC3
3.31609113428286E-39	0.399057730481129	0.113	0.053	5.55710552283121E-35	Hepatocytes	IPO13
1.56533400855248E-38	0.423074784092056	0.152	0.082	2.62318673153225E-34	Hepatocytes	RGS14
2.29821702422558E-38	0.739046171368696	0.104	0.047	3.85135208919722E-34	Hepatocytes	POLR3H
6.651293446723E-38	0.393529074870045	0.114	0.054	1.11462375580184E-33	Hepatocytes	PLGRKT
1.21640845989514E-37	0.52545155953823	0.104	0.047	2.03845729709227E-33	Hepatocytes	EMC6
3.27812166331353E-36	0.650648359146104	0.209	0.133	5.49347628338081E-32	Hepatocytes	MT2A
4.82433742642199E-36	0.300936061857831	0.121	0.06	8.08462465919798E-32	Hepatocytes	DEPRECATED-ENSG00000109536
6.26338922685558E-36	0.423321117523968	0.105	0.049	1.04961876663646E-31	Hepatocytes	ABHD6
9.73701783183434E-36	0.252272387271459	0.137	0.073	1.6317294482588E-31	Hepatocytes	FLVCR2
2.7616082629248E-35	0.263937493611827	0.111	0.054	4.62790312700939E-31	Hepatocytes	H2AFY2
8.99716579509972E-35	0.563284920893305	0.101	0.047	1.50774504394281E-30	Hepatocytes	KIFC1
1.41710384554692E-34	0.425647752566974	0.105	0.051	2.37478262436753E-30	Hepatocytes	EIF3M
3.64847470411179E-33	0.37975430388151	0.1	0.048	6.11411390915053E-29	Hepatocytes	HOMEZ
7.64121662473622E-33	0.29803147204204	0.163	0.096	1.2805150819733E-28	Hepatocytes	SLC31A1
7.181390087183E-30	0.404201924398847	0.104	0.053	1.20345735081013E-25	Hepatocytes	FBXO6
2.84612676628617E-29	0.660314141823073	0.13	0.075	4.76953923494236E-25	Hepatocytes	PTTG1
2.03842340231633E-28	0.446950684043679	0.103	0.054	3.41598993760171E-24	Hepatocytes	GNG5
2.97457094227378E-28	0.421188706333905	0.104	0.055	4.98478598506241E-24	Hepatocytes	C1orf112
0	6.59080854701691	0.957	0.039	0	Fibroblast	IGFBP7
0	7.485269789616	0.902	0.021	0	Fibroblast	COL6A2
0	9.30478811766426	0.854	0.012	0	Fibroblast	COL1A1
0	5.59105413233194	0.876	0.037	0	Fibroblast	SPARC
0	9.36721874171988	0.847	0.01	0	Fibroblast	COL3A1
0	7.87968265002242	0.831	0.015	0	Fibroblast	BGN
0	7.58055205022792	0.899	0.088	0	Fibroblast	COL6A1
0	9.37819596373013	0.768	0.006	0	Fibroblast	COL1A2
0	5.43802716483218	0.785	0.028	0	Fibroblast	COL4A1
0	8.19442164469479	0.764	0.013	0	Fibroblast	DCN
0	9.42085672070574	0.746	0.005	0	Fibroblast	THBS2
0	6.98359699837496	0.778	0.041	0	Fibroblast	TAGLN
0	8.36783208215505	0.744	0.008	0	Fibroblast	AEBP1
0	5.55508032959692	0.759	0.026	0	Fibroblast	COL4A2
0	7.7831388961276	0.701	0.012	0	Fibroblast	COLEC11
0	6.92372164499955	0.686	0.024	0	Fibroblast	CXCL12
0	9.29829773354603	0.653	0.005	0	Fibroblast	COL6A3
0	8.40190465572317	0.632	0.007	0	Fibroblast	EFEMP1
0	7.91435023399753	0.622	0.007	0	Fibroblast	EMILIN1
0	4.03291393644051	0.674	0.067	0	Fibroblast	ENG
0	9.66090580899585	0.608	0.002	0	Fibroblast	CRISPLD2
0	7.73959039203556	0.605	0.009	0	Fibroblast	COL5A1
0	5.41625844963932	0.704	0.125	0	Fibroblast	TIMP1
0	6.78851557921065	0.574	0.014	0	Fibroblast	C7
0	7.96390759146917	0.564	0.007	0	Fibroblast	GGT5
0	7.53934137141135	0.564	0.008	0	Fibroblast	C11orf96
0	3.21134351316356	0.728	0.176	0	Fibroblast	VIM
0	6.93031877368581	0.564	0.014	0	Fibroblast	MYL9

0	6.26955930629356	0.58	0.044	0	Fibroblast	THBS1
0	7.38271283318156	0.537	0.012	0	Fibroblast	FBN1
0	6.68403043104154	0.551	0.027	0	Fibroblast	LTBP4
0	7.10336079629164	0.556	0.046	0	Fibroblast	PDGFRB
0	5.51077902108327	0.625	0.116	0	Fibroblast	FSTL1
0	6.90799973334792	0.519	0.024	0	Fibroblast	ADAMTSL2
0	6.61858986120222	0.503	0.011	0	Fibroblast	LRRC32
0	7.2788085189049	0.492	0.006	0	Fibroblast	ADAMTS2
0	3.93195056967912	0.548	0.066	0	Fibroblast	TIMP2
0	8.14815077157653	0.476	0.006	0	Fibroblast	LUM
0	7.30573202899793	0.469	0.008	0	Fibroblast	THY1
0	6.76401707479794	0.489	0.039	0	Fibroblast	GEM
0	4.73417284852512	0.54	0.091	0	Fibroblast	TIMP3
0	8.06359392654129	0.439	0.002	0	Fibroblast	MFAP4
0	7.203196044645	0.458	0.027	0	Fibroblast	ACTA2
0	8.76080662214482	0.424	0.002	0	Fibroblast	PODN
0	6.47896216140747	0.426	0.011	0	Fibroblast	C1QTNF1
0	7.42648741191736	0.413	0.009	0	Fibroblast	COL5A2
0	6.14701153983018	0.407	0.009	0	Fibroblast	TNXB
0	8.5545380277594	0.399	0.002	0	Fibroblast	PRELP
0	7.0006906185002	0.404	0.007	0	Fibroblast	ADAMTS1
0	6.30091217396888	0.405	0.009	0	Fibroblast	PTGDS
0	9.00221133660854	0.395	0.001	0	Fibroblast	LAMC3
0	5.26831307162551	0.423	0.03	0	Fibroblast	CRIM1
0	7.84050097658009	0.394	0.003	0	Fibroblast	ELN
0	7.95664207930596	0.392	0.003	0	Fibroblast	FBLN5
0	8.78492837218496	0.383	0.002	0	Fibroblast	NFASC
0	6.96600234254014	0.384	0.006	0	Fibroblast	CCL21
0	5.92922702739123	0.397	0.02	0	Fibroblast	MEGF6
0	5.95381455536347	0.383	0.014	0	Fibroblast	MMP2
0	5.67267712824267	0.375	0.009	0	Fibroblast	INMT
0	8.29128905911517	0.37	0.008	0	Fibroblast	COL14A1
0	5.37155133720299	0.375	0.023	0	Fibroblast	ITGA9
0	7.80014587826865	0.354	0.003	0	Fibroblast	DKK 3.00
0	7.5477410251643	0.347	0.004	0	Fibroblast	ANGPTL2
0	8.19776976379543	0.339	0.003	0	Fibroblast	CCDC80
0	9.39063076609149	0.333	0.001	0	Fibroblast	MXRA8
0	9.32925473883773	0.331	0.001	0	Fibroblast	KCNE4
0	8.71743627899546	0.334	0.004	0	Fibroblast	SVEP1
0	6.30723427499454	0.352	0.023	0	Fibroblast	LOXL2
0	4.02845522406132	0.365	0.041	0	Fibroblast	LAMB2
0	8.24413322302035	0.32	0.001	0	Fibroblast	HAND2
0	6.06151991759895	0.357	0.04	0	Fibroblast	CCN1
0	6.38165038972504	0.322	0.005	0	Fibroblast	ADGRA2
0	6.37036631936471	0.323	0.007	0	Fibroblast	EHD2
0	5.2174778340191	0.318	0.008	0	Fibroblast	PXDN
0	5.8671813181317	0.32	0.01	0	Fibroblast	PHLDB1
0	4.72536668394742	0.317	0.012	0	Fibroblast	LIFR
0	5.30613070585951	0.315	0.013	0	Fibroblast	TINAGL1
0	8.1923698766252	0.302	0.002	0	Fibroblast	ID4
0	6.5161642213515	0.302	0.003	0	Fibroblast	FBLN2
0	8.53747351031487	0.294	0.001	0	Fibroblast	LOXL1
0	6.10817957301731	0.309	0.025	0	Fibroblast	IFITM10
0	7.54038144328288	0.285	0.002	0	Fibroblast	PTPRS
0	4.62427959707588	0.293	0.016	0	Fibroblast	FXYD6
0	5.30565387351743	0.281	0.012	0	Fibroblast	MRC2
0	6.29444311173747	0.272	0.004	0	Fibroblast	FKBP10
0	8.16362896357381	0.268	0.001	0	Fibroblast	SMOC2
0	7.63324584541407	0.27	0.004	0	Fibroblast	ITGBL1
0	6.73541369404909	0.272	0.01	0	Fibroblast	HGF
0	4.65070564778169	0.28	0.02	0	Fibroblast	OSMR
0	6.95554051441188	0.265	0.008	0	Fibroblast	SOD3
0	6.85568592896633	0.265	0.009	0	Fibroblast	COLEC10

0	9.32370329522255	0.256	0.001	0	Fibroblast	HEYL
0	5.23496353966371	0.268	0.018	0	Fibroblast	TPM2
0	4.29062782194843	0.265	0.017	0	Fibroblast	SMAD6
0	8.5742244982201	0.251	0.005	0	Fibroblast	PRKG1
0	7.77698166000113	0.249	0.003	0	Fibroblast	ARHGEF17
0	8.41469710139842	0.244	0.001	0	Fibroblast	ASPN
0	6.97601390355742	0.243	0.004	0	Fibroblast	DPYSL3
0	6.89606226115808	0.243	0.004	0	Fibroblast	LHFPL6
0	9.43550664028824	0.24	0.001	0	Fibroblast	NOTCH3
0	7.22435500291394	0.244	0.008	0	Fibroblast	FHL2
0	8.71200323069058	0.236	0.001	0	Fibroblast	ISLR
0	7.85590672052217	0.236	0.003	0	Fibroblast	PTGIR
0	6.10377632269623	0.243	0.011	0	Fibroblast	HMCN1
0	5.73460563498104	0.236	0.007	0	Fibroblast	VCAN
0	9.10647537507203	0.222	0.001	0	Fibroblast	LAMA2
0	5.41955418486568	0.227	0.006	0	Fibroblast	PTGIS
0	4.08606011449384	0.235	0.015	0	Fibroblast	ADAMTS4
0	6.39253129409202	0.223	0.004	0	Fibroblast	MGP
0	7.40993075770059	0.219	0.002	0	Fibroblast	KIRREL1
0	9.46117775366508	0.217	0	0	Fibroblast	FAM180A
0	5.34917473400398	0.22	0.007	0	Fibroblast	TMEM204
0	8.30969232823064	0.209	0.001	0	Fibroblast	CD248
0	6.17911064487586	0.217	0.011	0	Fibroblast	MFGE8
0	6.21419928727039	0.207	0.006	0	Fibroblast	CLEC11A
0	5.06777900407559	0.207	0.007	0	Fibroblast	GJA4
0	7.51729148840219	0.199	0.002	0	Fibroblast	PDGFRA
0	6.46586648068145	0.201	0.004	0	Fibroblast	PGF
0	5.94641777908129	0.206	0.009	0	Fibroblast	ECM1
0	7.6969188254364	0.182	0.001	0	Fibroblast	STEAP4
0	6.72452230547637	0.186	0.007	0	Fibroblast	OLFML3
0	8.70461325300587	0.175	0	0	Fibroblast	PTH1R
0	6.04952313508114	0.177	0.004	0	Fibroblast	TGFB1I1
0	5.95262684222715	0.175	0.005	0	Fibroblast	PBX1
0	5.23637070093112	0.17	0.006	0	Fibroblast	HSPB8
0	6.65151752107117	0.164	0.002	0	Fibroblast	KAZN
0	5.46390451374971	0.167	0.005	0	Fibroblast	PPP1R14A
0	7.62407764622309	0.162	0.002	0	Fibroblast	DENND2A
0	6.96545020551793	0.159	0.002	0	Fibroblast	SYNPO2
0	7.59031141673261	0.158	0.002	0	Fibroblast	FMOD
0	9.22221101255579	0.156	0	0	Fibroblast	PAPPA
0	10.6331000661362	0.154	0	0	Fibroblast	NGFR
0	7.72746161892373	0.154	0.001	0	Fibroblast	MRVI1
0	7.33710398447385	0.156	0.003	0	Fibroblast	CYGB
0	8.30154279637273	0.151	0.001	0	Fibroblast	PLA2G5
0	5.79039121412304	0.151	0.003	0	Fibroblast	NID2
0	5.85093986939871	0.151	0.004	0	Fibroblast	RBMS3
0	7.55436934403148	0.151	0.004	0	Fibroblast	TBX2
0	10.569746825186	0.146	0	0	Fibroblast	ALDH1A3
0	7.37515878329791	0.15	0.004	0	Fibroblast	CCDC102B
0	6.57763768456197	0.151	0.005	0	Fibroblast	HSPB6
0	7.55498244753171	0.145	0.001	0	Fibroblast	DPT
0	5.99739127557262	0.148	0.004	0	Fibroblast	CRYAB
0	6.7588402881829	0.146	0.003	0	Fibroblast	SAMD11
0	5.79711500727812	0.146	0.003	0	Fibroblast	MAP1B
0	8.61674199826459	0.143	0.001	0	Fibroblast	NFATC4
0	8.63906935384208	0.141	0	0	Fibroblast	ITGA11
0	8.23002479603732	0.141	0.001	0	Fibroblast	SSC5D
0	8.42763924984034	0.14	0.001	0	Fibroblast	ADAMTS12
0	7.10882309127601	0.141	0.002	0	Fibroblast	LOX
0	6.29643650741902	0.141	0.002	0	Fibroblast	CPXM2
0	7.10560247310444	0.141	0.002	0	Fibroblast	CDH11
0	8.6242230680065	0.137	0.001	0	Fibroblast	FRZB
0	6.62158478007077	0.138	0.003	0	Fibroblast	CTHRC1

0	7.74980697080413	0.135	0.001	0	Fibroblast	ADAM33
0	8.53935515466988	0.132	0	0	Fibroblast	FOXF1
0	7.70465992543381	0.129	0.001	0	Fibroblast	MXRA5
0	8.62718444720604	0.127	0	0	Fibroblast	SPON1
0	9.58618142020266	0.127	0.001	0	Fibroblast	SLIT3
0	6.8356475687803	0.127	0.001	0	Fibroblast	MOXD1
0	5.54021869048468	0.129	0.004	0	Fibroblast	DCLK2
0	6.69097666258526	0.127	0.002	0	Fibroblast	CERCAM
0	6.91988924972465	0.124	0.001	0	Fibroblast	CACNA1C
0	9.34688882801555	0.122	0.001	0	Fibroblast	TNC
0	8.03673712713083	0.122	0.002	0	Fibroblast	CEMIP
0	6.81265070305213	0.121	0.002	0	Fibroblast	ABI3BP
0	8.58060061115459	0.119	0.001	0	Fibroblast	PAMR1
0	7.07366551610768	0.117	0.001	0	Fibroblast	COL8A1
0	6.34144769496322	0.119	0.003	0	Fibroblast	DAAM2
0	7.40019131182742	0.119	0.003	0	Fibroblast	GALNT16
0	7.56366193327895	0.113	0.001	0	Fibroblast	CXCL14
0	7.28333589298319	0.113	0.001	0	Fibroblast	GDF2
0	7.04910092592721	0.111	0.002	0	Fibroblast	KIAA1755
0	9.61469927827825	0.108	0.001	0	Fibroblast	PCDH18
0	8.7712918622947	0.106	0.001	0	Fibroblast	EPHA3
0	7.33473316776426	0.106	0.001	0	Fibroblast	P3H3
0	7.54551017298359	0.106	0.002	0	Fibroblast	GAS1
0	7.66402875447636	0.105	0.002	0	Fibroblast	SULF1
0	8.66290859441575	0.103	0.001	0	Fibroblast	LMOD1
0	7.670966588679	0.103	0.001	0	Fibroblast	GLI2
0	7.93621135551911	0.1	0	0	Fibroblast	TCF21
0	6.94758720105239	0.101	0.001	0	Fibroblast	IGFBP6
1.18063280344938E-306	7.47618675126164	0.108	0.002	1.97850445202048E-302	Fibroblast	SHISA3
8.75295457121573E-300	5.30720239548994	0.122	0.004	1.46682012704433E-295	Fibroblast	RIPOR3
1.47235800470562E-296	6.8564975200607	0.121	0.004	2.46737754428568E-292	Fibroblast	SUSD2
2.67364712103267E-291	5.11096336319067	0.156	0.007	4.48049784542655E-287	Fibroblast	HOXB3
4.59061487792278E-291	5.84904246219355	0.395	0.056	7.69295241242299E-287	Fibroblast	LTBP2
1.11505182825495E-286	4.11354050089232	0.362	0.045	1.86860385378964E-282	Fibroblast	PCOLCE
3.54597093778336E-280	4.36931642804575	0.143	0.006	5.94233809753736E-276	Fibroblast	PEAR1
1.65321727807469E-271	6.47851112245056	0.133	0.005	2.77046151459757E-267	Fibroblast	DEPRECATED-ENSG00000107796
8.94377251629436E-266	6.1679297776317	0.334	0.043	1.49879739828061E-261	Fibroblast	COL16A1
5.34392023034494E-264	5.17944324563442	0.117	0.004	8.95534152201204E-260	Fibroblast	TBXA2R
7.83335272819389E-259	5.01587215256382	0.207	0.015	1.31271325019073E-254	Fibroblast	SEPTIN4
5.60928676994721E-257	6.11492634138139	0.151	0.008	9.40004276907754E-253	Fibroblast	CCL19
1.31653983557092E-252	4.4734807911909	0.301	0.035	2.20625745644975E-248	Fibroblast	GRK5
2.431837011593E-248	6.80245921023387	0.122	0.005	4.07527246402756E-244	Fibroblast	MMP23B
6.29859190464965E-242	4.45101522965769	0.257	0.026	1.05551803138119E-237	Fibroblast	RBP1
9.5516789325471E-242	5.45192876156047	0.209	0.017	1.60067035551624E-237	Fibroblast	PDLIM3
4.32267722372972E-240	4.86144695232022	0.217	0.019	7.24394249152627E-236	Fibroblast	MYOF
1.55288507070471E-237	5.66811513023089	0.117	0.005	2.60232480148695E-233	Fibroblast	SRPX
2.82548953012773E-229	5.17563120617888	0.214	0.019	4.73495535458806E-225	Fibroblast	EFEMP2
2.97242747770307E-218	5.90223413797932	0.227	0.023	4.98119396713481E-214	Fibroblast	COL4A4
2.04105755904064E-217	4.58947208346877	0.148	0.009	3.42040425744031E-213	Fibroblast	MYO10
1.07410046174086E-209	4.23994032175287	0.114	0.005	1.79997755378533E-205	Fibroblast	JAM3
8.59478580514006E-209	4.0682133745536	0.26	0.031	1.44031420522537E-204	Fibroblast	PMP22
1.22658303436804E-208	5.48044659511962	0.156	0.011	2.05550784899396E-204	Fibroblast	SCARF2
1.79895686357658E-208	5.46707229800375	0.185	0.015	3.01469191198163E-204	Fibroblast	SRPX2
2.79816305027472E-206	5.07524117704647	0.124	0.006	4.68916163965038E-202	Fibroblast	TUSC3
4.91830765422494E-202	5.19091486967007	0.137	0.008	8.24209996695015E-198	Fibroblast	HOXB4
2.96093466006987E-200	4.8207179315622	0.172	0.014	4.96193430334508E-196	Fibroblast	CPE
4.565935269967E-189	2.52523389634519	0.828	0.478	7.65159432541069E-185	Fibroblast	C1R
5.54745172999044E-189	4.48648950479123	0.125	0.007	9.29641960911798E-185	Fibroblast	COL15A1
1.87810909070474E-188	3.85446646678466	0.423	0.093	3.14733521420301E-184	Fibroblast	ID3
1.21631851793224E-186	5.58717521393567	0.307	0.05	2.03830657235085E-182	Fibroblast	CCN2
7.51902708455271E-180	2.41677655475003	0.117	0.007	1.26003855882934E-175	Fibroblast	IGFBP5
4.13118917468568E-179	4.94579191411308	0.309	0.052	6.92304681893826E-175	Fibroblast	JAG1
1.73207129408433E-176	5.38896626491619	0.133	0.009	2.90260507462653E-172	Fibroblast	TGFB3

1.69582897306012E-175	3.06655728023766	0.402	0.085	2.84187019305415E-171	Fibroblast	GSN
3.66083549510166E-169	3.97221258468816	0.121	0.008	6.13482812269136E-165	Fibroblast	HIC1
1.97895755523547E-167	3.97559301225883	0.108	0.006	3.3163370710636E-163	Fibroblast	NES
5.64018307543989E-166	6.85963956342125	0.119	0.008	9.45181879782217E-162	Fibroblast	ADRA2A
1.74129683961671E-165	5.93452704235568	0.273	0.044	2.91806524382968E-161	Fibroblast	ADAMTS13
1.87182413740099E-164	4.29878877015735	0.169	0.016	3.13680288945658E-160	Fibroblast	AFAP1
9.34639534382425E-162	4.65109392889347	0.138	0.011	1.56626893171807E-157	Fibroblast	NKD2
7.62789808496006E-161	4.5101947652249	0.273	0.045	1.27828316107761E-156	Fibroblast	CYBRD1
2.7914679349046E-159	5.30064387187814	0.109	0.007	4.67794196531313E-155	Fibroblast	SEPTIN5
5.17434806494314E-157	4.69999638357289	0.188	0.021	8.67117248723172E-153	Fibroblast	HES4
2.11591787720744E-153	3.57015513302179	0.166	0.017	3.54585517862424E-149	Fibroblast	FGFR1
2.47155145893272E-153	4.47292279929078	0.117	0.008	4.14182593487945E-149	Fibroblast	PTGS2
2.70103855636286E-152	5.28316304687125	0.106	0.007	4.52640041275288E-148	Fibroblast	EPHB3
4.50806452565314E-149	4.96001842297746	0.254	0.041	7.55461453208953E-145	Fibroblast	ARVCF
7.00124691332384E-149	3.7864983946396	0.376	0.09	1.17326895773481E-144	Fibroblast	RCN3
8.26279466250696E-148	4.26822189619896	0.262	0.044	1.38467912954292E-143	Fibroblast	SLCO3A1
9.15826750908668E-147	5.60092664899963	0.243	0.039	1.53474246917275E-142	Fibroblast	ANK3
1.40780501548462E-146	2.84262811875744	0.732	0.464	2.35919964494912E-142	Fibroblast	CALD1
1.65282094453874E-142	5.15717263417444	0.108	0.007	2.76979733885802E-138	Fibroblast	LXN
2.52644429022321E-137	2.74884853457634	0.526	0.188	4.23381534155606E-133	Fibroblast	SERPINH1
3.75600626130205E-135	4.75236849530679	0.352	0.087	6.29431529268998E-131	Fibroblast	MYH10
3.21959200947919E-133	4.57521957507825	0.108	0.008	5.39539228948522E-129	Fibroblast	CCL2
5.06676375770656E-132	4.47208691621114	0.111	0.009	8.49088270516465E-128	Fibroblast	BACE2
1.62091713931054E-126	3.86376891857644	0.24	0.042	2.7163329420566E-122	Fibroblast	SMTN
9.1072473151382E-126	3.7406410297366	0.339	0.084	1.52619250507086E-121	Fibroblast	TNS1
1.40767442213646E-122	3.26419756922327	0.6	0.289	2.35898079661628E-118	Fibroblast	DEPP1
5.79989755245841E-122	4.91163981274797	0.277	0.059	9.71946831840981E-118	Fibroblast	LMCD1
8.41533604263051E-121	3.91569608298939	0.309	0.072	1.41024201402402E-116	Fibroblast	CAVIN1
3.18652274821202E-120	3.81565658906248	0.177	0.024	5.3399748214537E-116	Fibroblast	CHST15
3.7571585484847E-120	5.15563388462069	0.172	0.023	6.29624629555067E-116	Fibroblast	ISYNA1
3.96595944592367E-120	4.02304360580247	0.146	0.017	6.64615483947889E-116	Fibroblast	MCAM
2.74744306114191E-119	5.20826204019049	0.262	0.054	4.60416508186161E-115	Fibroblast	FGFR2
3.09129293810623E-119	3.60856955051325	0.363	0.1	5.18038870567842E-115	Fibroblast	MASP1
3.97626250466042E-118	3.57709155517255	0.249	0.048	6.66342070530993E-114	Fibroblast	VCAM1
4.46249829028506E-118	4.58317088002613	0.297	0.069	7.47825463485971E-114	Fibroblast	KALRN
8.4661486296752E-118	4.12634643912082	0.138	0.015	1.41875718736097E-113	Fibroblast	RNF122
4.39615377315202E-115	4.86673627509719	0.143	0.017	7.36707449304816E-111	Fibroblast	ANTXR1
3.57313908894004E-114	3.33383643683376	0.461	0.169	5.98786648524572E-110	Fibroblast	ITM2C
1.24026696353251E-113	3.57234776106957	0.116	0.011	2.07843937748778E-109	Fibroblast	TEAD2
9.86422764577825E-113	3.62338073087816	0.164	0.022	1.65304726887952E-108	Fibroblast	RGS16
6.64994261772349E-111	3.0459886102865	0.196	0.031	1.1143973838781E-106	Fibroblast	TCF4
1.94266640104442E-110	3.30679202418935	0.108	0.01	3.25552035487023E-106	Fibroblast	ARHGAP23
3.2313743310184E-110	4.47456505557071	0.54	0.255	5.41513710392063E-106	Fibroblast	IGFBP3
1.84928362545604E-109	5.02298417194158	0.246	0.051	3.09902949953924E-105	Fibroblast	SERPINE2
1.08642955233722E-108	1.96679722093091	0.767	0.565	1.82063864380672E-104	Fibroblast	C1S
2.75370973130143E-107	3.30012768744887	0.342	0.095	4.61466676771493E-103	Fibroblast	CD81
2.83617410369278E-107	3.06462868651145	0.342	0.094	4.75286056296837E-103	Fibroblast	IER5L
4.71154198529439E-107	3.96794550735749	0.235	0.047	7.89560205895634E-103	Fibroblast	FSTL3
1.13969897653037E-103	3.13878930799978	0.278	0.064	1.90990754486959E-99	Fibroblast	NR4A1
2.6592197681444E-103	3.18139950541476	0.212	0.039	4.45632048745638E-99	Fibroblast	TRIB2
3.63911079083037E-103	4.47360081696195	0.333	0.096	6.09842186327354E-99	Fibroblast	FBLN1
8.3689109611288E-101	3.74480570187197	0.153	0.021	1.40246209886596E-96	Fibroblast	S1PR3
2.97750168498709E-99	3.76384404525463	0.138	0.018	4.98969732370136E-95	Fibroblast	DLG4
4.28001594811327E-99	3.53577064006776	0.117	0.013	7.17245072584821E-95	Fibroblast	OBSCN
1.30664511344043E-98	3.83550052093421	0.408	0.15	2.18967588110348E-94	Fibroblast	PRSS23
1.58594901698624E-98	1.98259642893223	0.246	0.051	2.65773336266553E-94	Fibroblast	PLTP
1.91618037128262E-97	1.82746697745573	0.235	0.047	3.21113506619541E-93	Fibroblast	NPDC1
2.28206344698343E-94	3.22217889932467	0.219	0.044	3.82428192445484E-90	Fibroblast	LBH
1.66856185308432E-92	2.76431790105804	0.177	0.03	2.7961759533987E-88	Fibroblast	ALPL
5.84408041535092E-92	4.4946618882883	0.101	0.01	9.79350996004508E-88	Fibroblast	ABCC9
4.56802748243718E-91	4.58677534732508	0.105	0.011	7.65510045506823E-87	Fibroblast	GLIS2
1.40569725057535E-90	3.76999557565412	0.101	0.011	2.35566745251418E-86	Fibroblast	HOXB2
3.56048200417017E-86	3.92144787182027	0.105	0.012	5.96665574258838E-82	Fibroblast	PLXDC1

3.09033530121691E-85	3.92407149626688	0.238	0.057	5.1787838977793E-81	Fibroblast	PDLIM7
3.9928013809875E-85	3.62884883360063	0.395	0.156	6.69113655425885E-81	Fibroblast	ITGA1
4.14087188230342E-85	2.31639117956377	0.264	0.066	6.93927310036407E-81	Fibroblast	METRNL
2.3109570466167E-84	3.1601242208017	0.281	0.078	3.87270181872027E-80	Fibroblast	SLC12A4
1.43993418725242E-83	3.86306418770061	0.27	0.074	2.4130417109976E-79	Fibroblast	CHSY1
5.02813001128231E-83	3.17358098507969	0.145	0.023	8.42614027290689E-79	Fibroblast	SPRY4
5.71835390262686E-83	4.25135410652107	0.288	0.085	9.5828174700221E-79	Fibroblast	CCDC3
1.62914509231101E-82	2.34237771591391	0.241	0.057	2.73012134569479E-78	Fibroblast	ZEB2
1.12150769737781E-79	3.71572737313151	0.408	0.174	1.87942259926573E-75	Fibroblast	C1orf198
3.0607815454104E-79	3.38196759516677	0.497	0.266	5.12925771379874E-75	Fibroblast	MICALL2
4.66097573817886E-79	2.99391334890596	0.254	0.066	7.81086314204014E-75	Fibroblast	TMEM173
8.96687891045768E-79	6.74924214649469	0.117	0.016	1.5026695678145E-74	Fibroblast	RGS5
3.7049148037784E-78	3.16275933729537	0.178	0.035	6.20869622817184E-74	Fibroblast	AXL
4.59398156618054E-78	3.50314988576164	0.402	0.17	7.69859430860535E-74	Fibroblast	NR2F2
5.40365993187698E-77	1.97752371714145	0.524	0.231	9.05545331383944E-73	Fibroblast	COL18A1
2.20386959036169E-75	3.4278431620178	0.376	0.152	3.69324465952812E-71	Fibroblast	MXRA7
8.14463261746312E-75	4.72874636725678	0.174	0.036	1.36487753403447E-70	Fibroblast	LTBP1
2.15556949130071E-74	3.01263568699185	0.166	0.032	3.61230335352173E-70	Fibroblast	ZNF83
2.50749969696423E-73	2.91195321734808	0.526	0.317	4.20206799217266E-69	Fibroblast	NID1
5.78845357141736E-73	3.14316275665217	0.203	0.047	9.7002904949812E-69	Fibroblast	DSE
2.86223231829719E-72	3.26435618937949	0.268	0.08	4.79652891900243E-68	Fibroblast	SMAD7
2.01087020036346E-71	2.56901978628803	0.548	0.348	3.36981628176908E-67	Fibroblast	LGALS3BP
1.36344966603206E-69	2.59235513795125	0.424	0.194	2.28486895033653E-65	Fibroblast	LGALS1
1.76779083558547E-68	4.19276601330416	0.151	0.029	2.96246388227413E-64	Fibroblast	VASN
7.8102598127238E-67	3.9359795166417	0.195	0.048	1.30884333941625E-62	Fibroblast	F2R
3.36402010185193E-66	3.98251707173531	0.182	0.042	5.63742488668347E-62	Fibroblast	ADAMTS9
7.62357216044806E-66	4.43735915065478	0.103	0.015	1.27755822264789E-61	Fibroblast	MARVELD1
3.52478599794262E-65	3.15786013429613	0.27	0.087	5.90683637535225E-61	Fibroblast	PHLDA1
5.57905520797707E-65	2.50001073231786	0.211	0.054	9.34938071752798E-61	Fibroblast	S100A6
1.95035003180847E-64	2.99240512057776	0.146	0.029	3.26839658330464E-60	Fibroblast	PDGFB
2.07789321245269E-64	2.23353703395132	0.124	0.021	3.48213344542821E-60	Fibroblast	NR4A3
2.124227885067E-64	2.86093497663249	0.371	0.16	3.55978108979527E-60	Fibroblast	FLNA
1.19392170959354E-63	4.13843602797727	0.125	0.022	2.00077400093685E-59	Fibroblast	PFKM
8.74881453070549E-63	1.71531804234954	0.686	0.624	1.46612633905563E-58	Fibroblast	IGFBP4
9.90887751133473E-63	2.67545991331468	0.26	0.082	1.66052969334947E-58	Fibroblast	PALM2-AKAP2
1.35385749492431E-62	4.17957245678062	0.119	0.02	2.26879438999417E-58	Fibroblast	EVA1B
3.50677990461791E-62	3.34260622808069	0.153	0.032	5.8766617641587E-58	Fibroblast	TRIP10
6.77148086239511E-62	3.31668472825303	0.195	0.05	1.13476476292017E-57	Fibroblast	SPRY1
9.58172661406772E-61	1.62288855239304	0.621	0.423	1.60570574598547E-56	Fibroblast	JUNB
3.46666848270354E-60	2.64605006321416	0.559	0.4	5.80944304331459E-56	Fibroblast	GPX3
3.70729709432681E-60	4.19093923861761	0.166	0.039	6.21268847067287E-56	Fibroblast	ZNF703
8.80475715083613E-59	2.85509969336305	0.447	0.246	1.47550120333712E-54	Fibroblast	CSRP1
9.12548836812149E-58	2.92516482593233	0.186	0.048	1.5292493407298E-53	Fibroblast	MAGED1
1.1251711607966E-55	3.18556735664717	0.138	0.029	1.88556183126294E-51	Fibroblast	GNB4
2.89824612648956E-55	3.74105218273443	0.246	0.084	4.8568808587712E-51	Fibroblast	ST3GAL4
4.58107370370165E-55	4.00554375107663	0.103	0.017	7.67696331266323E-51	Fibroblast	OLFML2B
9.36032260411466E-55	2.21542974083883	0.244	0.078	1.56860286199754E-50	Fibroblast	SOCS3
1.24970428422365E-54	3.73327578715305	0.262	0.094	2.09425443950199E-50	Fibroblast	UACA
1.80285295228196E-54	3.10805373557666	0.177	0.046	3.02122097743412E-50	Fibroblast	DLC1
2.20111417866241E-54	2.99891780961816	0.219	0.067	3.68862714060246E-50	Fibroblast	MYADM
2.52264855939467E-54	4.15882806298995	0.132	0.028	4.22745445583359E-50	Fibroblast	NLGN2
2.88456143607485E-54	3.19365605651482	0.109	0.019	4.83394805457423E-50	Fibroblast	SYNGAP1
3.01631512521582E-54	2.95369817566719	0.158	0.037	5.05474088683667E-50	Fibroblast	PHLDB2
7.66488706618419E-54	2.19936168864013	0.574	0.456	1.28448177455115E-49	Fibroblast	TPM1
6.27095208113286E-53	2.41126545948057	0.254	0.087	1.05088614975624E-48	Fibroblast	SH3BP5
1.81544752153733E-52	3.83973322027504	0.267	0.101	3.04232695659225E-48	Fibroblast	GATA6
4.04139249331962E-51	2.70846917630624	0.143	0.033	6.77256554030503E-47	Fibroblast	SEMA4C
6.88385533577564E-51	3.27403146536109	0.135	0.03	1.15359647716928E-46	Fibroblast	EPHA2
4.88417728146346E-50	3.10237159460714	0.129	0.028	8.18490428827647E-46	Fibroblast	UAP1L1
1.91707167267183E-49	3.31118494377832	0.355	0.178	3.21262870906345E-45	Fibroblast	LAMB1
1.12273403470139E-48	3.43442861412398	0.106	0.02	1.88147769535258E-44	Fibroblast	FHOD1
3.4885034462561E-48	2.63936376966858	0.101	0.018	5.84603407523598E-44	Fibroblast	ENO2
3.80956361157715E-47	2.89072635593842	0.13	0.029	6.38406670028099E-43	Fibroblast	DOCK1

4.23845617448281E-47	4.14475231949995	0.117	0.025	7.1028048571983E-43	Fibroblast	FILIP1L
1.70620692722898E-46	2.56942940143252	0.167	0.046	2.85926156865032E-42	Fibroblast	PLXDC2
9.5903720688284E-46	3.64621955247049	0.222	0.079	1.60715455129426E-41	Fibroblast	SYTL2
1.02423529649226E-45	2.49398300346231	0.264	0.102	1.71641350986173E-41	Fibroblast	SELENOM
2.14978819226071E-42	2.32999554921985	0.161	0.045	3.6026150525905E-38	Fibroblast	ITPRIP
5.21440834800839E-41	3.08647063571051	0.256	0.106	8.73830550959246E-37	Fibroblast	LZTS2
5.71758255013441E-40	3.11330090474695	0.127	0.032	9.58152483751524E-36	Fibroblast	RND3
8.26355692549454E-40	1.5167316397212	0.26	0.1	1.38480686957438E-35	Fibroblast	LSP1
1.60045518999006E-39	2.82290894834999	0.108	0.024	2.68204280738534E-35	Fibroblast	HAPLN3
4.71984288648634E-39	2.60088280665629	0.262	0.111	7.9095127091738E-35	Fibroblast	IER3
9.98595828025305E-39	3.16908942839371	0.395	0.247	1.67344688860481E-34	Fibroblast	QSOX1
3.38916926584398E-38	2.15756013877814	0.133	0.035	5.67956985570135E-34	Fibroblast	LIMS2
9.12726803738396E-38	4.75058602535527	0.125	0.033	1.5295475777048E-33	Fibroblast	F3
9.00907572127026E-37	1.30943872868841	0.167	0.051	1.50974090937047E-32	Fibroblast	DEPRECATED-ENSG00000059804
1.66105540436762E-36	4.10530540652379	0.185	0.066	2.78359664663926E-32	Fibroblast	ADRA1B
1.98933606898288E-36	2.49916768154756	0.434	0.295	3.33372938440151E-32	Fibroblast	NRP1
3.07299788035037E-36	2.96322129889166	0.238	0.1	5.14972984789115E-32	Fibroblast	CTIF
4.05765339732938E-36	3.77342661361534	0.154	0.049	6.79981556324457E-32	Fibroblast	AMOTL2
8.6140912411402E-36	3.43191736962054	0.277	0.134	1.44354941019027E-31	Fibroblast	TANC1
1.15951732052457E-35	2.72760852172197	0.185	0.065	1.94311912573507E-31	Fibroblast	PDLIM2
1.89710800667358E-35	4.64452658150639	0.15	0.047	3.17917359758359E-31	Fibroblast	ADCY5
2.32844129110297E-35	2.44530488907463	0.109	0.027	3.90200191563036E-31	Fibroblast	YBX3
3.43278198763277E-35	3.03685433156179	0.1	0.023	5.75265605487499E-31	Fibroblast	CALHM2
3.82974076895334E-35	2.1548349353369	0.514	0.434	6.41787958061201E-31	Fibroblast	EPAS1
5.14516659316012E-35	2.37850675232921	0.415	0.278	8.62227017681772E-31	Fibroblast	DPYSL2
1.2984471659164E-34	3.15258671846586	0.103	0.025	2.1759377606427E-30	Fibroblast	RELN
6.84961669205213E-34	3.10422971279991	0.207	0.082	1.1478587652541E-29	Fibroblast	BMP1
9.07897013383582E-34	2.35008327508757	0.281	0.138	1.52145381502821E-29	Fibroblast	HSPG2
9.88674504530356E-34	1.91689221184953	0.103	0.025	1.65682073469197E-29	Fibroblast	NAV1
3.62779332121212E-33	2.25579621172501	0.293	0.146	6.07945604768727E-29	Fibroblast	PLXND1
2.42529092056174E-32	2.75029505774267	0.371	0.235	4.06430252467737E-28	Fibroblast	MPRIP
3.08126693158407E-32	3.11066498688592	0.101	0.025	5.16358712394858E-28	Fibroblast	LFNG
5.09892865626621E-32	2.34984627342856	0.158	0.052	8.54478464217092E-28	Fibroblast	GSTP1
1.25705836334446E-31	4.11837989228234	0.133	0.041	2.10657840529265E-27	Fibroblast	OLFML1
1.85238878798582E-31	1.87084153474276	0.264	0.122	3.10423313090664E-27	Fibroblast	ANXA6
8.00715593320759E-31	3.40680411401415	0.209	0.089	1.34183919128693E-26	Fibroblast	LAMA4
8.4669915412406E-31	3.33395781823309	0.235	0.108	1.4188984424811E-26	Fibroblast	TRAM2
1.1848352749664E-30	1.6804423839887	0.148	0.047	1.98554695378869E-26	Fibroblast	SLC2A3
1.53714070888812E-30	2.55401474098837	0.278	0.142	2.57594039995471E-26	Fibroblast	SPON2
6.8752662699305E-30	2.07177706748945	0.193	0.076	1.15215712151495E-25	Fibroblast	HDAC7
1.92382287739005E-29	2.04624458636438	0.117	0.034	3.22394237793025E-25	Fibroblast	RASA3
3.74415722316466E-29	2.60529995011973	0.355	0.224	6.27445867457933E-25	Fibroblast	RBPMS
1.24972108344468E-28	1.96117906382725	0.495	0.417	2.09428259163659E-24	Fibroblast	SERPINF1
2.58103866648561E-28	1.00495988782702	0.222	0.089	4.32530459729659E-24	Fibroblast	CD4
4.27580331056772E-28	1.18295942300845	0.664	0.656	7.16539118784938E-24	Fibroblast	RHOB
4.46469275392434E-28	3.50283141796583	0.154	0.056	7.4819321170264E-24	Fibroblast	DMD
7.64027385527875E-28	1.99962001613898	0.457	0.356	1.28035709266761E-23	Fibroblast	LRP1
1.77438692925864E-27	1.97648348585976	0.113	0.033	2.97351761605163E-23	Fibroblast	PLAU
4.44161000290046E-27	3.27921421245129	0.167	0.065	7.44325004286059E-23	Fibroblast	INHBA
1.01925404439382E-26	2.1572062461056	0.371	0.248	1.70806592759517E-22	Fibroblast	LTBP3
1.44491234799795E-26	1.90710795314012	0.162	0.061	2.42138411277497E-22	Fibroblast	ANXA1
1.07283232872385E-25	1.74719118650441	0.278	0.145	1.79785241647543E-21	Fibroblast	ICAM1
2.62363297065487E-25	3.90348075881541	0.133	0.048	4.39668413222344E-21	Fibroblast	TNFRSF11B
2.65713713025076E-25	3.32498665687236	0.195	0.087	4.45283040287423E-21	Fibroblast	ADCY3
4.04176065679165E-25	2.25033487143095	0.1	0.029	6.77318250865145E-21	Fibroblast	BASP1
4.67386979556143E-25	1.26432204714162	0.54	0.444	7.83247100340185E-21	Fibroblast	BHLHE40
1.35734534621358E-24	2.4049744499639	0.302	0.175	2.27463933118472E-20	Fibroblast	SERPINE1
1.79195418054525E-24	2.5751597779008	0.14	0.051	3.00295681575772E-20	Fibroblast	RBMS1
7.97858832980223E-24	2.33196944295608	0.215	0.104	1.33705183230826E-19	Fibroblast	RFTN1
1.01944951511703E-23	1.95337511753578	0.379	0.263	1.70839349743311E-19	Fibroblast	MARCKS
3.87978142197098E-23	0.992396906516173	0.233	0.107	6.50173770693898E-19	Fibroblast	CYBA
1.34940342294393E-22	2.80728875354095	0.235	0.125	2.26133025616945E-18	Fibroblast	HTRA1
2.26252958186339E-22	1.66639154230968	0.175	0.075	3.79154707328668E-18	Fibroblast	SERPINB9

3.22713438156315E-22	1.63146937618722	0.169	0.07	5.40803179662353E-18	Fibroblast	CMTM3
9.97104486965698E-22	2.68655519106211	0.103	0.033	1.67094769925712E-17	Fibroblast	GYPC
3.41703864466708E-21	2.65561041277167	0.342	0.248	5.72627336073309E-17	Fibroblast	MYLK
3.80093023092463E-21	2.0000113897092	0.447	0.392	6.3695988809835E-17	Fibroblast	ACTN1
9.9928316860023E-21	1.85297410390612	0.204	0.099	1.67459873394027E-16	Fibroblast	CPED1
2.23303566319832E-20	1.88770086496074	0.204	0.1	3.74212116438774E-16	Fibroblast	TGFBI
2.56538946576931E-20	2.17312608209056	0.109	0.038	4.29907966673621E-16	Fibroblast	MSC
9.66586011007095E-20	3.42448095721767	0.174	0.083	1.61980483724569E-15	Fibroblast	PAPLN
5.26658320795255E-19	2.46301560967493	0.325	0.233	8.82574013988688E-15	Fibroblast	ITGAV
8.94576841690163E-19	2.81462819301715	0.133	0.055	1.49913187130437E-14	Fibroblast	KSR1
1.14355841933651E-18	0.257648266389814	0.055	0.21	1.91637519912413E-14	Fibroblast	SLC2A13
1.59621598476394E-18	2.57753484874015	0.33	0.238	2.67493874726741E-14	Fibroblast	ITGB5
2.62178996885718E-18	2.54958933214443	0.117	0.045	4.39359562981087E-14	Fibroblast	PLSCR3
3.1976642737447E-18	1.64635257815007	0.465	0.416	5.35864578994137E-14	Fibroblast	CEBPB
1.46873661281395E-17	3.48813405117919	0.141	0.064	2.46130881575362E-13	Fibroblast	SEMA5A
1.75336230412377E-17	0.277713824842934	0.072	0.229	2.93828454925061E-13	Fibroblast	LSM 7.00
1.84255593296333E-17	3.08673970608618	0.114	0.045	3.08775523245995E-13	Fibroblast	UBE2E2
3.67261947172087E-17	0.333362521106135	0.076	0.235	6.15457571070983E-13	Fibroblast	TMBIM4
4.28792814952423E-17	0.36194391574666	0.133	0.325	7.1857099929727E-13	Fibroblast	GNPTG
5.73884660384038E-17	0.314342785653984	0.159	0.363	9.61715913871571E-13	Fibroblast	C4orf3
6.57932618495716E-17	0.470046165407148	0.048	0.189	1.10256348207512E-12	Fibroblast	FRAS1
6.61401266764019E-17	0.975180909839322	0.445	0.332	1.10837624284314E-12	Fibroblast	NNMT
8.04538201331172E-17	0.323915777364337	0.092	0.257	1.34824511779078E-12	Fibroblast	CAMK2D
8.19893769214871E-17	3.2025533395714	0.207	0.119	1.37397797845028E-12	Fibroblast	VCL
8.33413302570807E-17	0.357545961027219	0.113	0.291	1.39663401244816E-12	Fibroblast	HMGN3
1.37082448045755E-16	2.54767472566847	0.244	0.152	2.29722766435077E-12	Fibroblast	NEURL1B
1.56622712000885E-16	0.545286736129946	0.116	0.292	2.62468340771083E-12	Fibroblast	TOMM20
2.22097300375234E-16	0.483222654345012	0.178	0.391	3.72190655968818E-12	Fibroblast	UBAP2L
2.27657642433723E-16	0.266183928396202	0.117	0.296	3.81508677190434E-12	Fibroblast	EMC7
2.32156318501619E-16	0.280485727152768	0.05	0.185	3.89047558545014E-12	Fibroblast	DMAP1
2.74523778892494E-16	0.281535857976398	0.087	0.245	4.60046948668042E-12	Fibroblast	UBA5
3.29734966237802E-16	0.355393973860901	0.178	0.387	5.52569856421308E-12	Fibroblast	YIPF3
3.7917036724359E-16	1.70541839659613	0.233	0.137	6.35413701426808E-12	Fibroblast	ARID5B
4.5796059886002E-16	0.398018833559301	0.151	0.346	7.67450371569621E-12	Fibroblast	MATN2
6.91416101603599E-16	0.557695945533269	0.096	0.261	1.15867510306731E-11	Fibroblast	ARHGEF40
7.52468320144583E-16	0.271017572551956	0.169	0.369	1.26098641089829E-11	Fibroblast	HSF1
9.5717937697115E-16	1.1523482083914	0.14	0.061	1.60404119992825E-11	Fibroblast	ITPKB
1.03222076076845E-15	2.50980089554478	0.105	0.041	1.72979555089577E-11	Fibroblast	SOX4
1.06079826654882E-15	0.388225319634733	0.096	0.255	1.77768573508252E-11	Fibroblast	UBA2
1.12421403336229E-15	0.262405672681542	0.162	0.359	1.88395787710852E-11	Fibroblast	NUFIP2
1.68021685069975E-15	0.427205135677309	0.148	0.337	2.81570739840264E-11	Fibroblast	SLC35F5
2.33135399823895E-15	0.251192482836926	0.092	0.245	3.90688303024882E-11	Fibroblast	VPS35L
2.45219085503144E-15	0.302902591112828	0.093	0.25	4.10938143486169E-11	Fibroblast	ABHD16A
3.05064038404925E-15	3.36143266690732	0.1	0.039	5.11226315558973E-11	Fibroblast	RHOBTB1
3.41210491966541E-15	0.275302175085001	0.1	0.258	5.7180054243753E-11	Fibroblast	LIN7C
3.44036058726895E-15	0.403467158036163	0.077	0.223	5.76535627214531E-11	Fibroblast	TBC1D17
4.56080935739374E-15	0.507319706180916	0.079	0.225	7.64300432112042E-11	Fibroblast	PDE8A
4.70794008049892E-15	0.42428643809814	0.188	0.401	7.8895659869001E-11	Fibroblast	LMAN1
7.50239639681186E-15	0.29620787929881	0.082	0.23	1.25725158817773E-10	Fibroblast	TLE1
7.67262000169482E-15	0.255735009035565	0.088	0.238	1.28577765988402E-10	Fibroblast	TIMM44
7.85479687788085E-15	0.268720145830207	0.09	0.242	1.31630686079527E-10	Fibroblast	ZBTB10
7.97361249233924E-15	0.375958134552694	0.166	0.365	1.33621798146621E-10	Fibroblast	EID1
1.16074065300011E-14	0.497329051937794	0.035	0.152	1.94516918629758E-10	Fibroblast	PLEKHA7
1.1874453302734E-14	0.31320322318149	0.095	0.247	1.98992088447216E-10	Fibroblast	UQCC1
1.34320307632235E-14	0.275066821309453	0.119	0.282	2.25093971530099E-10	Fibroblast	HECTD4
1.34444473179813E-14	0.342572932482853	0.093	0.243	2.25302048154731E-10	Fibroblast	ABCF3
1.36972905512448E-14	0.292238101759896	0.088	0.238	2.29539195057761E-10	Fibroblast	MAP3K2
1.38355987558328E-14	0.265555229378452	0.122	0.293	2.31856963950247E-10	Fibroblast	FOXO3
1.65342542847799E-14	0.254162829245035	0.111	0.272	2.77081033304342E-10	Fibroblast	TCEAL1
2.43612958459655E-14	0.315087174367904	0.058	0.186	4.0824659578669E-10	Fibroblast	TBC1D16
2.81869705551427E-14	0.25068726364168	0.074	0.212	4.72357252563081E-10	Fibroblast	ZYG11B
2.98338017238499E-14	0.277160497857386	0.167	0.353	4.99954849288277E-10	Fibroblast	HDLBP
3.17175690741493E-14	0.253136983337506	0.077	0.215	5.31523022544593E-10	Fibroblast	PRRC1

3.50118919701507E-14	2.75792765648248	0.153	0.078	5.86729285635786E-10	Fibroblast	MAN1C1
3.71477550716338E-14	2.23172829159632	0.227	0.141	6.2252207949044E-10	Fibroblast	EPB41L2
4.45843282328877E-14	0.273129131771062	0.043	0.16	7.47144172526732E-10	Fibroblast	CHD8
4.50838342307272E-14	0.298998217570987	0.082	0.22	7.55514894038526E-10	Fibroblast	RAD23B
4.80761176411922E-14	1.82893301516151	0.103	0.042	8.05659579431099E-10	Fibroblast	CRIP1
5.89248103734131E-14	0.2874585248007	0.124	0.286	9.87461972237657E-10	Fibroblast	RBM25
6.12528223339798E-14	0.320901614611742	0.22	0.438	1.02647479667283E-09	Fibroblast	PDIA3
7.28386929918585E-14	0.334218846116719	0.045	0.163	1.22063081715756E-09	Fibroblast	PDP1
7.94747611135205E-14	0.471125592519248	0.132	0.302	1.33183804674038E-09	Fibroblast	TTC3
8.90449354648186E-14	0.265305370498713	0.114	0.271	1.49221502851943E-09	Fibroblast	ZC3H15
9.18327078071515E-14	0.378636389618571	0.076	0.212	1.53893251743224E-09	Fibroblast	ATG4B
9.37345439060778E-14	0.340696194317623	0.158	0.338	1.57080348677805E-09	Fibroblast	RAB18
9.96181739743749E-14	0.515691226303606	0.032	0.14	1.66940135946258E-09	Fibroblast	DPY19L3
1.10259929246088E-13	0.259754282019911	0.22	0.433	1.84773589430595E-09	Fibroblast	SEC31A
1.13118460958094E-13	0.607838559382461	0.156	0.34	1.89563916873575E-09	Fibroblast	KANK1
1.16200619669739E-13	2.62373248049684	0.156	0.082	1.94728998442548E-09	Fibroblast	PEAK1
1.27885068440233E-13	0.28473385459942	0.082	0.218	2.14309797692142E-09	Fibroblast	TM9SF4
1.30509079989712E-13	0.555195969318109	0.066	0.196	2.1870711624676E-09	Fibroblast	CAMSAP2
1.39749923842136E-13	0.306593525437731	0.153	0.328	2.34192922374652E-09	Fibroblast	SNRNP70
1.41621673837018E-13	0.295699510707049	0.058	0.182	2.37329601016075E-09	Fibroblast	SLC17A5
1.42125699444949E-13	2.1421124752274	0.339	0.276	2.38174247129845E-09	Fibroblast	MMP14
1.45431433427832E-13	0.314564260371216	0.117	0.275	2.4371399613836E-09	Fibroblast	FUS
1.51369127606902E-13	0.426747072555777	0.1	0.246	2.53664384043647E-09	Fibroblast	NEMF
1.54664268159932E-13	0.603689756689815	0.095	0.241	2.59186380582414E-09	Fibroblast	EHBP1
1.64396266929236E-13	1.49388942926777	0.154	0.078	2.75495264120014E-09	Fibroblast	TRAF1
1.78516086418953E-13	0.261701033015744	0.217	0.424	2.99157257620882E-09	Fibroblast	CTNNA1
2.05816484309047E-13	0.266122294353853	0.121	0.278	3.44907264405101E-09	Fibroblast	ARCN1
2.22342371124251E-13	0.328671381528007	0.08	0.213	3.7260134553002E-09	Fibroblast	CNPPD1
2.30573771736655E-13	0.311211689171163	0.127	0.285	3.86395526676286E-09	Fibroblast	CDIPT
2.71365553266282E-13	0.52262243935853	0.092	0.235	4.54754394163635E-09	Fibroblast	SEMA4B
2.73828301213606E-13	0.366088662022954	0.141	0.311	4.5888146717376E-09	Fibroblast	ZNF638
2.85731332950953E-13	1.29010317142679	0.508	0.526	4.78828567759207E-09	Fibroblast	APP
2.9072762768E-13	0.300301220412747	0.074	0.206	4.87201358466144E-09	Fibroblast	PRPF39
3.04855766321139E-13	0.312014144878054	0.103	0.248	5.10877293200964E-09	Fibroblast	USP48
3.14047459992034E-13	2.52874332417952	0.129	0.062	5.2628073345465E-09	Fibroblast	CRIP2
3.71791695082095E-13	0.285476690513424	0.061	0.184	6.23048522618575E-09	Fibroblast	TMEM50B
3.85062591933575E-13	0.266906320236459	0.117	0.271	6.45287891562286E-09	Fibroblast	NIPBL
3.86954332168052E-13	0.312587915269381	0.124	0.281	6.48458069847221E-09	Fibroblast	MED28
4.5829386505059E-13	0.363678787759458	0.129	0.288	7.68008859051778E-09	Fibroblast	IST1
4.78117116058842E-13	0.926827103841996	0.111	0.049	8.01228663091407E-09	Fibroblast	EGFL7
4.82895295115685E-13	0.410978810466054	0.143	0.309	8.09235935554865E-09	Fibroblast	CREBZF
4.88279098813248E-13	0.251201603694565	0.056	0.174	8.18258113791241E-09	Fibroblast	PMPCA
5.34193505370464E-13	2.6822766557712	0.199	0.122	8.95201476299824E-09	Fibroblast	MICAL2
5.42503267212542E-13	2.61872690854784	0.161	0.088	9.09126975194778E-09	Fibroblast	ADGRL2
5.54394133258255E-13	0.325505045199128	0.071	0.197	9.29053688514184E-09	Fibroblast	EIF3L
5.87529267399024E-13	0.315045395326941	0.158	0.333	9.84581546307284E-09	Fibroblast	ATP2A2
6.11848702881935E-13	1.11819058769432	0.195	0.108	1.02533605628955E-08	Fibroblast	IFI16
6.21816676554433E-13	0.415815362552998	0.066	0.19	1.04204038656992E-08	Fibroblast	PEX11B
6.47680866073992E-13	0.374209632041946	0.045	0.156	1.0853835953668E-08	Fibroblast	KLHL23
7.77051185509019E-13	3.44136822800652	0.103	0.046	1.30218237667601E-08	Fibroblast	CTSK
8.59627243316079E-13	0.300501101811951	0.039	0.144	1.44056333434908E-08	Fibroblast	TRAPPC11
8.84759066214057E-13	0.437920128226537	0.076	0.203	1.48267924316152E-08	Fibroblast	NEK9
8.87840663516589E-13	0.267305430942235	0.161	0.333	1.4878433839211E-08	Fibroblast	INTS11
9.17413518457029E-13	0.39253537015766	0.056	0.173	1.53740157423029E-08	Fibroblast	KLHDC10
9.60907519092179E-13	0.301058463445648	0.117	0.266	1.61028882049467E-08	Fibroblast	SEC16A
9.95002650178318E-13	0.647628105827755	0.641	0.718	1.66742544116883E-08	Fibroblast	TMSB4X
1.00323809846721E-12	0.353455424010385	0.056	0.174	1.68122640541136E-08	Fibroblast	RBBP6
1.01917769842651E-12	0.458453891075199	0.145	0.312	1.70793798702314E-08	Fibroblast	SENP6
1.065291009188E-12	0.272849783523154	0.061	0.179	1.78521467319726E-08	Fibroblast	ABCB8
1.06614592126474E-12	0.47050381324499	0.101	0.244	1.78664733485545E-08	Fibroblast	SCAF11
1.21042267109749E-12	0.345258306741669	0.068	0.189	2.02842631222518E-08	Fibroblast	TBL2
1.24119646495942E-12	0.458990651212263	0.122	0.275	2.079997035979E-08	Fibroblast	LRCH3
1.32395158615236E-12	0.498523980624124	0.059	0.177	2.21867806807412E-08	Fibroblast	TRIM47

1.54695758106876E-12	0.301864150218323	0.048	0.158	2.59239151435502E-08	Fibroblast	CIZ1
1.64468988262212E-12	0.55790171042503	0.071	0.195	2.75617130529815E-08	Fibroblast	TELO2
1.87237451414367E-12	0.361781468333958	0.093	0.23	3.13772521080196E-08	Fibroblast	MTR
1.93245088466644E-12	0.413594761689824	0.092	0.226	3.23840119252403E-08	Fibroblast	PRRC2B
1.98630366312169E-12	0.319129776440448	0.063	0.18	3.32864767865932E-08	Fibroblast	ERAL1
2.09743162727678E-12	0.36451075731479	0.166	0.344	3.51487592099042E-08	Fibroblast	UBR2
2.15610523615569E-12	0.277395123482868	0.087	0.218	3.61320115474971E-08	Fibroblast	COPS7A
2.19598959818922E-12	0.422218891684897	0.092	0.225	3.68003936864549E-08	Fibroblast	TTC17
2.22766381460649E-12	0.759783995365535	0.621	0.693	3.73311902051755E-08	Fibroblast	EEF2
2.33361087722486E-12	0.40864119351836	0.116	0.263	3.91066510805342E-08	Fibroblast	PCMTD2
2.33545821619774E-12	0.36958231041676	0.096	0.232	3.91376087870418E-08	Fibroblast	AGTRAP
2.33719972742298E-12	0.360369946225523	0.137	0.296	3.91667930321542E-08	Fibroblast	THRAP3
2.3417403894345E-12	0.345083176988796	0.04	0.145	3.92428854461434E-08	Fibroblast	MPP6
2.38208932731815E-12	0.447779353225579	0.169	0.347	3.99190529471975E-08	Fibroblast	C16orf58
2.4247512602896E-12	0.30454486786325	0.138	0.297	4.06339816199332E-08	Fibroblast	UBE2Z
2.45424198780718E-12	0.26851084704557	0.153	0.319	4.11281872316728E-08	Fibroblast	FAM3C
2.70943604149014E-12	0.275064864207821	0.064	0.183	4.54047291832917E-08	Fibroblast	MED6
2.74998212305632E-12	0.340061707179048	0.09	0.223	4.60842004181778E-08	Fibroblast	DR1
3.32540016800261E-12	0.262418209792513	0.072	0.195	5.57270560153878E-08	Fibroblast	ZFYVE16
3.42907302079574E-12	0.284763991343457	0.114	0.26	5.74644056824949E-08	Fibroblast	DCAF5
3.80760891110046E-12	0.261462373158852	0.169	0.339	6.38079101322215E-08	Fibroblast	RAB7A
3.82092736407929E-12	0.47425061320366	0.138	0.295	6.40311007672408E-08	Fibroblast	AP2A1
3.89729121007381E-12	0.503455447616509	0.042	0.145	6.53108060984168E-08	Fibroblast	DEPRECATED-ENSG00000249915
4.1565161341154E-12	0.458087649877547	0.124	0.271	6.96548973755059E-08	Fibroblast	RAB6A
4.1985567093314E-12	0.501476564508073	0.042	0.145	7.03594133349755E-08	Fibroblast	ENDOV
4.22134947772737E-12	0.434732057543288	0.092	0.224	7.07413745477553E-08	Fibroblast	SMAD5
4.29581842256508E-12	0.44021901462135	0.108	0.247	7.19893251253456E-08	Fibroblast	SUSD6
4.5342314967592E-12	0.539752754062043	0.125	0.276	7.59846514226907E-08	Fibroblast	PTK2
5.00133972011289E-12	0.309869048196176	0.122	0.269	8.38124510296518E-08	Fibroblast	RC3H2
5.03375167631038E-12	0.412332099749813	0.132	0.284	8.43556105916093E-08	Fibroblast	NEK7
5.08473343906258E-12	0.483092039100093	0.074	0.197	8.52099629718108E-08	Fibroblast	YME1L1
5.32861109083708E-12	0.370655124773815	0.241	0.455	8.92968646602479E-08	Fibroblast	NFIC
6.04234753364496E-12	0.424315350660199	0.077	0.199	1.01257659968822E-07	Fibroblast	YIPF5
6.11421820900731E-12	0.623263487110792	0.072	0.193	1.02462068746544E-07	Fibroblast	ERCC6L2
6.41205265579525E-12	0.380332337469472	0.096	0.23	1.07453178405817E-07	Fibroblast	QTRT1
6.5271685808054E-12	0.548010012057422	0.101	0.236	1.09382291077137E-07	Fibroblast	SON
6.61340219320142E-12	0.488404935572661	0.093	0.225	1.10827393953669E-07	Fibroblast	DDX42
6.642426142681E-12	0.37878899698869	0.058	0.17	1.11313777299048E-07	Fibroblast	DDI2
6.70959978554204E-12	0.534452193781437	0.113	0.254	1.12439473206114E-07	Fibroblast	TCAF1
6.94349727575245E-12	0.33566185009344	0.101	0.237	1.1635912734706E-07	Fibroblast	SRF
7.69558398766502E-12	0.293981718731326	0.077	0.199	1.2896259646529E-07	Fibroblast	GMPPA
8.3397914147805E-12	2.54074684444306	0.113	0.054	1.39758224528892E-07	Fibroblast	SPINT2
8.6872265670621E-12	0.351814028150654	0.1	0.232	1.45580542810827E-07	Fibroblast	DDX56
8.71643642157183E-12	0.261735006721597	0.088	0.215	1.46070041552701E-07	Fibroblast	UBR3
9.0910904718113E-12	0.271401984164733	0.061	0.173	1.52348494126614E-07	Fibroblast	TBK1
9.18844435009356E-12	0.268013994020619	0.066	0.18	1.53979950418868E-07	Fibroblast	KRIT1
9.64972708181565E-12	0.417711047218507	0.106	0.242	1.61710126437067E-07	Fibroblast	ZNF146
1.04981073190222E-11	0.554556975037594	0.113	0.255	1.75927282452174E-07	Fibroblast	GINM1
1.15173131433634E-11	2.05127328513101	0.376	0.344	1.93007133656485E-07	Fibroblast	SLC38A2
1.22801373032412E-11	0.348568885708937	0.095	0.224	2.05790540927717E-07	Fibroblast	SOAT1
1.3004216230106E-11	0.72565519538635	0.034	0.129	2.17924655584116E-07	Fibroblast	ZNF606
1.43050534661661E-11	0.45755496730011	0.125	0.272	2.39724085986011E-07	Fibroblast	SUN1
1.43704104718012E-11	0.293991340375163	0.04	0.139	2.40819338686445E-07	Fibroblast	COPS7B
1.49896186846436E-11	0.492564776697457	0.072	0.189	2.51196029917257E-07	Fibroblast	FAM193A
1.53741424249793E-11	0.283149751269483	0.241	0.448	2.57639878757803E-07	Fibroblast	CTNND1
1.75030150510921E-11	0.255669021178415	0.122	0.265	2.93315526226202E-07	Fibroblast	CDC34
1.81077643455531E-11	0.33813502017555	0.148	0.304	3.03449914902779E-07	Fibroblast	PLEC
1.91636498407887E-11	0.352282524753512	0.116	0.254	3.21144444031937E-07	Fibroblast	SMAD2
1.92408805289485E-11	0.370500495063449	0.124	0.267	3.22438675904119E-07	Fibroblast	GALNT2
1.97086323392399E-11	0.496689976336541	0.122	0.267	3.30277260740982E-07	Fibroblast	MED25
1.99391343477725E-11	0.294558108603793	0.101	0.232	3.34140013399971E-07	Fibroblast	JMJD6
2.03534613794792E-11	0.372081011074953	0.116	0.254	3.41083305797313E-07	Fibroblast	TXLNA
2.04494513054708E-11	0.449666612289422	0.055	0.16	3.4269190497708E-07	Fibroblast	SFT2D1

2.09935390445724E-11	0.404192117050913	0.053	0.158	3.51809727308944E-07	Fibroblast	GABPB2
2.1021734699109E-11	0.453717788313577	0.154	0.319	3.52282230087668E-07	Fibroblast	TIA1
2.12391636055108E-11	0.445477155846124	0.109	0.246	3.5592590370115E-07	Fibroblast	RNF40
2.16653848612387E-11	0.314916082900111	0.095	0.224	3.63068519504638E-07	Fibroblast	UBE2J2
2.44288422697913E-11	0.373893897556019	0.164	0.327	4.09378538757162E-07	Fibroblast	SRRM2
2.46974936976096E-11	0.259152522928558	0.074	0.19	4.13880599384542E-07	Fibroblast	SGSM3
2.50452354045353E-11	0.422788232946522	0.143	0.297	4.19708054909202E-07	Fibroblast	DCTN2
2.64088060449307E-11	0.348926379621952	0.08	0.2	4.42558771700948E-07	Fibroblast	PLEKHG3
2.64384725078127E-11	0.444451194650016	0.058	0.166	4.43055922285925E-07	Fibroblast	EMG1
2.82197503166849E-11	0.447466670937637	0.185	0.362	4.72906575807005E-07	Fibroblast	CPNE1
2.93865736930478E-11	0.401095151067073	0.064	0.175	4.92460201948095E-07	Fibroblast	EIF3G
3.08373316145947E-11	0.378853196854399	0.121	0.262	5.16772003197379E-07	Fibroblast	TPD52L2
3.13728529114143E-11	0.51275463441387	0.135	0.283	5.25746269089481E-07	Fibroblast	GAA
3.15256994134995E-11	0.250466677657191	0.169	0.332	5.28307670771425E-07	Fibroblast	FUBP1
3.20063651255646E-11	0.490058655881595	0.04	0.137	5.36362666774211E-07	Fibroblast	TPD52L1
3.20371698772111E-11	2.37802253022743	0.161	0.093	5.36878892802304E-07	Fibroblast	MTHFD1L
3.21032365486887E-11	0.273643513576784	0.093	0.219	5.37986038082924E-07	Fibroblast	MBTPS1
3.22968136728487E-11	0.253808645657591	0.058	0.165	5.41230003529598E-07	Fibroblast	PPP1R3B
3.35996023740505E-11	1.98815168675393	0.232	0.159	5.63062136584338E-07	Fibroblast	PACS1
3.41541345463287E-11	0.430866949813093	0.043	0.141	5.72354986727376E-07	Fibroblast	JMJD4
3.43166998278436E-11	0.33810497222983	0.08	0.198	5.75079255715003E-07	Fibroblast	MESD
3.45576978294819E-11	0.312030021918342	0.132	0.275	5.79117900226458E-07	Fibroblast	CRELD2
3.50152258505742E-11	0.260718626301135	0.05	0.15	5.86785154803922E-07	Fibroblast	SLC2A1
3.51857203608876E-11	0.340054908507917	0.198	0.377	5.89642301807754E-07	Fibroblast	CPEB4
3.54505573442397E-11	0.545191394008744	0.058	0.165	5.94080439974768E-07	Fibroblast	GPATCH8
3.65300166429873E-11	0.488685286119049	0.063	0.171	6.1217001890318E-07	Fibroblast	PARD3
4.27587263288663E-11	0.296219929040055	0.021	0.103	7.16550735819142E-07	Fibroblast	ELAC1
4.76156945329369E-11	0.609122951525054	0.051	0.153	7.97943808982957E-07	Fibroblast	NUBP2
4.8586086827121E-11	0.722359864339723	0.068	0.179	8.14205643048895E-07	Fibroblast	AHI1
4.87471338216572E-11	0.646256731191062	0.056	0.162	8.16904468583332E-07	Fibroblast	SLC45A3
4.87807942842199E-11	0.283264172166612	0.048	0.148	8.17468550614957E-07	Fibroblast	SCMH1
4.89734721524954E-11	0.371245001498538	0.069	0.181	8.20697446331519E-07	Fibroblast	SEH1L
5.26933154788721E-11	0.441304057213642	0.045	0.143	8.83034580794938E-07	Fibroblast	SLC12A8
6.65614375243482E-11	0.359571883352209	0.105	0.234	1.11543657003303E-06	Fibroblast	STK25
6.84062474502734E-11	0.275650869146809	0.042	0.136	1.14635189477168E-06	Fibroblast	THAP7
7.0708878719783E-11	0.330561307039378	0.063	0.168	1.18493938958612E-06	Fibroblast	UCK2
7.14138409315819E-11	0.571547689275499	0.109	0.242	1.19675314633145E-06	Fibroblast	ZFHX3
7.25449754177099E-11	0.38936537437444	0.079	0.195	1.21570869804998E-06	Fibroblast	C5orf15
7.56937885229663E-11	0.321744928978127	0.023	0.105	1.26847650806787E-06	Fibroblast	MIPOL1
7.93842410681428E-11	0.4099770796527	0.105	0.235	1.33032111181994E-06	Fibroblast	SPATA20
8.28138763900494E-11	0.632577351091339	0.135	0.284	1.38779494054445E-06	Fibroblast	CPSF6
8.44854793729148E-11	0.27340086331722	0.042	0.136	1.41580766333131E-06	Fibroblast	PSMB6
8.55431978196788E-11	0.521423722249748	0.045	0.142	1.43353290906218E-06	Fibroblast	MAGEF1
8.67946218654563E-11	0.33554705933306	0.178	0.343	1.45450427322132E-06	Fibroblast	SEC22B
9.23486411398699E-11	0.320160968612835	0.061	0.166	1.54757852822194E-06	Fibroblast	EIF2B1
9.44953854258804E-11	0.330521856138232	0.082	0.197	1.5835536689669E-06	Fibroblast	PPP6R3
9.55216371799515E-11	0.431493239634227	0.129	0.268	1.60075159586163E-06	Fibroblast	IRF2
1.02657498044845E-10	0.523642072780242	0.072	0.184	1.72033435223551E-06	Fibroblast	MANBAL
1.10704736144773E-10	0.490213385352291	0.079	0.193	1.85518996831411E-06	Fibroblast	CAMK1D
1.16232078047234E-10	0.284289521488997	0.058	0.16	1.94781716391554E-06	Fibroblast	TPGS1
1.16396581215776E-10	0.398784822908119	0.162	0.319	1.95057390801398E-06	Fibroblast	TOMM7
1.19200152105959E-10	0.723651747568471	0.085	0.202	1.99755614899167E-06	Fibroblast	VCP
1.2267156658244E-10	0.255929981500234	0.068	0.175	2.05573011278853E-06	Fibroblast	SACM1L
1.24780017631063E-10	0.434356916756168	0.039	0.129	2.09106353546135E-06	Fibroblast	BAZ1A
1.25386548344903E-10	0.585169152939435	0.037	0.127	2.10122777716388E-06	Fibroblast	BABAM2
1.25661132815127E-10	0.435688228391359	0.133	0.278	2.10582926371591E-06	Fibroblast	SIK2
1.39414588138924E-10	0.386345874189148	0.09	0.209	2.33630966803208E-06	Fibroblast	UBTD2
1.40581352779031E-10	0.448586016749543	0.066	0.172	2.35586230987099E-06	Fibroblast	TMEM98
1.42555026093331E-10	2.38690074096994	0.259	0.195	2.38893712727204E-06	Fibroblast	PELO
1.44877807777259E-10	0.297404297097095	0.071	0.18	2.42786230273131E-06	Fibroblast	MCEE
1.49941270646674E-10	0.278082341428242	0.14	0.283	2.51271581349696E-06	Fibroblast	YWHAG
1.56292230844706E-10	0.589723218602054	0.09	0.21	2.61914520449558E-06	Fibroblast	L3HYPDH
1.57034517871434E-10	0.361828748183422	0.1	0.223	2.63158445048949E-06	Fibroblast	WIZ

1.59607276510942E-10	0.512583176372455	0.051	0.149	2.67469873977036E-06	Fibroblast	SH3D19
1.59913663798006E-10	0.263965470984767	0.174	0.335	2.67983317792699E-06	Fibroblast	ADAM9
1.61093475171866E-10	0.384014334691537	0.045	0.139	2.69960445693013E-06	Fibroblast	ATP6V0A2
1.67120508844145E-10	0.37179501612292	0.074	0.184	2.80060548721018E-06	Fibroblast	VPS13D
1.73088005313597E-10	0.515679483115007	0.183	0.351	2.90060879304525E-06	Fibroblast	PIM3
1.74616938016846E-10	0.287057153574754	0.045	0.139	2.92623064728631E-06	Fibroblast	KATNAL1
1.75050177192417E-10	0.25152620212701	0.215	0.4	2.93349086939053E-06	Fibroblast	REEP5
1.7711648486235E-10	0.640824687015168	0.035	0.124	2.96811805332326E-06	Fibroblast	XPA
1.83270582570758E-10	0.484794404914726	0.079	0.191	3.07124842272076E-06	Fibroblast	TAF11
1.89770982819085E-10	0.293983822452061	0.024	0.105	3.18018213008222E-06	Fibroblast	KMT5C
1.93971431964184E-10	0.375093745177977	0.24	0.429	3.25057325685579E-06	Fibroblast	SCARB2
1.95889046188276E-10	0.258307213541147	0.042	0.133	3.28270863602313E-06	Fibroblast	POLM
2.06628754546178E-10	0.604751557732585	0.148	0.298	3.46268466868484E-06	Fibroblast	NFIA
2.08231308000494E-10	0.413215079913518	0.085	0.2	3.48954025947227E-06	Fibroblast	RAB3GAP1
2.2211391432077E-10	0.319070738582257	0.063	0.165	3.72218497618747E-06	Fibroblast	TK2
2.2980491866627E-10	1.89683089788495	0.148	0.083	3.85107082700936E-06	Fibroblast	ARHGEF2
2.36431200524585E-10	0.288604458284935	0.032	0.118	3.962114058391E-06	Fibroblast	LRRC75B
2.414876824424E-10	1.0019806938802	0.103	0.048	4.04685058236974E-06	Fibroblast	PLAUR
2.56403866860289E-10	0.39125735107796	0.047	0.14	4.29681600084472E-06	Fibroblast	SEPTIN10
2.68164210183231E-10	0.292080886106956	0.153	0.302	4.49389583425059E-06	Fibroblast	TXNDC11
2.71051804389338E-10	0.380179824307834	0.079	0.19	4.54228613795652E-06	Fibroblast	KIDINS220
2.77075867753309E-10	0.589567235215588	0.137	0.28	4.64323739180995E-06	Fibroblast	EGLN2
2.81533531980316E-10	0.452594585018781	0.069	0.175	4.71793892892613E-06	Fibroblast	CNOT2
2.9146076476588E-10	0.404358821645966	0.109	0.236	4.88429949594662E-06	Fibroblast	TRAF7
2.93494879525829E-10	0.286732733802104	0.098	0.218	4.91838719109385E-06	Fibroblast	BTAF1
2.94605654982919E-10	0.256889177493418	0.071	0.177	4.93700156620376E-06	Fibroblast	ACAP2
3.00502895911216E-10	0.418554291049535	0.174	0.339	5.03582752968016E-06	Fibroblast	DYNC1LI2
3.13572050848226E-10	0.579830200138563	0.047	0.139	5.25484042811458E-06	Fibroblast	UBLCP1
3.19697804312198E-10	0.256870347351895	0.035	0.121	5.35749580466381E-06	Fibroblast	DEPRECATED-ENSG00000116005
3.21157963618989E-10	0.353999631946659	0.146	0.292	5.38196515432702E-06	Fibroblast	TAOK1
3.21486706148661E-10	0.277276437442823	0.055	0.151	5.38747422163926E-06	Fibroblast	COPS8
3.22600044216977E-10	0.312979084311302	0.076	0.184	5.4061315409881E-06	Fibroblast	EFNB1
3.24695380907151E-10	0.286024664361409	0.048	0.142	5.44124519324204E-06	Fibroblast	DNAJB14
3.37207651166707E-10	0.375510082623339	0.096	0.217	5.65092581825168E-06	Fibroblast	GNG12
3.38269623924761E-10	0.637169017154108	0.095	0.213	5.66872235773114E-06	Fibroblast	BTF3L4
3.38355302572672E-10	0.319412615726449	0.058	0.156	5.67015816051283E-06	Fibroblast	ZNF436
3.44998294464421E-10	0.619566563801136	0.056	0.155	5.78148141863476E-06	Fibroblast	LMAN2L
3.46649814982195E-10	0.277992968297211	0.066	0.168	5.80915759947162E-06	Fibroblast	ZBED1
3.47963624155326E-10	0.309612633050453	0.042	0.132	5.83117441359496E-06	Fibroblast	TTC9C
3.63236690085871E-10	0.267815412810821	0.191	0.357	6.08712045245903E-06	Fibroblast	RSRC2
3.64959383399025E-10	0.422690582255381	0.077	0.187	6.11598934700086E-06	Fibroblast	SPCS3
4.06216456903996E-10	0.372695740071239	0.061	0.161	6.80737538479716E-06	Fibroblast	MFSD14A
4.1885680179032E-10	0.275329264630534	0.034	0.118	7.01920228440219E-06	Fibroblast	YBEY
4.37070246195673E-10	0.341911364374751	0.077	0.184	7.32442318574709E-06	Fibroblast	GMPPB
4.46697788809394E-10	0.316251677199714	0.08	0.19	7.48576154486783E-06	Fibroblast	TRPM7
4.60130202319006E-10	0.607218947106383	0.085	0.199	7.7108619304619E-06	Fibroblast	RFNG
4.64393931772364E-10	0.54129301891954	0.043	0.134	7.78231350864127E-06	Fibroblast	MEST
4.73863084457842E-10	0.301666782926341	0.043	0.133	7.94099756934452E-06	Fibroblast	BRAF
4.75089769916686E-10	0.268721592492618	0.172	0.327	7.96155436426383E-06	Fibroblast	RNF10
4.80849072937013E-10	0.420944136378706	0.116	0.243	8.05806876427847E-06	Fibroblast	DEPRECATED-ENSG00000085733
4.88304900226966E-10	0.495511993431929	0.106	0.229	8.18301351800349E-06	Fibroblast	ARL 1.00
4.90229412342226E-10	0.332300449273128	0.077	0.186	8.21526449203102E-06	Fibroblast	ALG12
5.01455422273965E-10	0.28829533167116	0.098	0.216	8.4033899664671E-06	Fibroblast	QRICH1
5.18992304006083E-10	0.547811967461216	0.106	0.231	8.69727303053394E-06	Fibroblast	PIP5K1A
5.44175128625012E-10	0.41181205954819	0.061	0.161	9.11928680549794E-06	Fibroblast	PAXBP1
5.98810612618222E-10	0.541482011234042	0.096	0.214	1.00348682462562E-05	Fibroblast	SLC1A5
5.99713163246048E-10	0.626812172481165	0.087	0.2	1.00499931896773E-05	Fibroblast	KAT2A
6.04729512903474E-10	0.407294048356943	0.087	0.198	1.01340571772364E-05	Fibroblast	FAM168A
6.34233711386722E-10	0.487535461739221	0.069	0.173	1.06284885354187E-05	Fibroblast	RUSC1
6.53624153540472E-10	0.601610304465494	0.039	0.125	1.09534335650312E-05	Fibroblast	MED20
6.60763274019853E-10	0.26940320780363	0.156	0.3	1.10730709460247E-05	Fibroblast	SUN2
7.12614172139864E-10	0.814549462568698	0.117	0.25	1.19419882967198E-05	Fibroblast	NCKIPSD
7.17639318326194E-10	2.76312524378331	0.127	0.07	1.20261996965104E-05	Fibroblast	ZNF532

7.58033223553524E-10	0.548003990736609	0.135	0.271	1.270312076031E-05	Fibroblast	ERF
7.65788234627245E-10	0.348226908323501	0.077	0.181	1.28330792358834E-05	Fibroblast	DENND4B
7.93222269331773E-10	0.677610753906188	0.113	0.238	1.32928187894619E-05	Fibroblast	PGPEP1
8.08883837254078E-10	0.609289579443769	0.039	0.124	1.35552753447038E-05	Fibroblast	NRXN3
8.0941499447863E-10	0.396286441889421	0.137	0.274	1.35641764774729E-05	Fibroblast	DNAJC7
8.11810247294695E-10	0.594238084604288	0.061	0.158	1.36043161241645E-05	Fibroblast	BSCL2
8.77666223128193E-10	0.405222843883688	0.161	0.311	1.47079305671823E-05	Fibroblast	MARCH7
8.91270532097715E-10	0.578802246638251	0.053	0.147	1.49359115768935E-05	Fibroblast	GIGYF2
9.18293253656825E-10	0.427037875637165	0.069	0.171	1.53887583447811E-05	Fibroblast	TBCK
9.3214374521373E-10	0.357381635951326	0.093	0.208	1.56208648822917E-05	Fibroblast	PHRF1
9.48055217875509E-10	1.61790815692796	0.127	0.068	1.58875093411578E-05	Fibroblast	EMP3
9.71612417441957E-10	1.21378659159008	0.267	0.192	1.62822808914923E-05	Fibroblast	MT2A
1.02126629408525E-09	0.486389261122564	0.188	0.352	1.71143805562807E-05	Fibroblast	PWWP3A
1.03037626514519E-09	2.43580544049296	0.127	0.07	1.72670454513031E-05	Fibroblast	NCKAP5L
1.03608960931039E-09	0.331439327248642	0.053	0.145	1.73627896728236E-05	Fibroblast	MORF4L1
1.04312476509216E-09	0.93139249317301	0.087	0.199	1.74806848134143E-05	Fibroblast	FNIP1
1.04482354811376E-09	0.323465994685996	0.063	0.16	1.75091530192904E-05	Fibroblast	ANKFY1
1.04808866900485E-09	0.495534111035572	0.082	0.19	1.75638699151833E-05	Fibroblast	R3HDM4
1.04863688785887E-09	0.499108282297727	0.119	0.245	1.75730569667389E-05	Fibroblast	NAB1
1.10666394111044E-09	0.349216849553987	0.085	0.195	1.85454743251287E-05	Fibroblast	TMEM87A
1.10929031694803E-09	0.648554627852878	0.066	0.165	1.8589487131415E-05	Fibroblast	TMEM138
1.13429038167355E-09	0.467802777432602	0.09	0.203	1.90084382160853E-05	Fibroblast	DEPRECATED-ENSG00000188313
1.16301840881965E-09	0.456971099167214	0.235	0.422	1.94898624949997E-05	Fibroblast	LRPAP1
1.17244840121218E-09	0.564423200156662	0.034	0.116	1.96478903075138E-05	Fibroblast	ZFP41
1.18625774950611E-09	0.626890032819085	0.137	0.276	1.98793073662234E-05	Fibroblast	DYNC1I2
1.19676662754353E-09	0.302749628458192	0.125	0.253	2.00554151443744E-05	Fibroblast	MYO6
1.19881477717103E-09	0.371826260974323	0.153	0.295	2.00897380358321E-05	Fibroblast	KDM5C
1.22636647140427E-09	0.362135730535802	0.111	0.233	2.05514493277927E-05	Fibroblast	LRP10
1.23097237185696E-09	0.384967193363332	0.047	0.136	2.0628635007579E-05	Fibroblast	ZDHHC16
1.26352379738289E-09	3.11114183108253	0.154	0.094	2.11741317965425E-05	Fibroblast	PDE3A
1.26562060789914E-09	0.40078473099793	0.088	0.199	2.12092701471739E-05	Fibroblast	EPS15L1
1.33621448251058E-09	0.887465394600109	0.098	0.216	2.23922822979122E-05	Fibroblast	BCL2L2
1.36157857941188E-09	0.254560675470455	0.037	0.119	2.28173338337843E-05	Fibroblast	RIC8A
1.38646504982841E-09	0.461585229024288	0.055	0.147	2.32343813050245E-05	Fibroblast	PLCB3
1.41486310425576E-09	0.867431220235663	0.069	0.171	2.3710275901118E-05	Fibroblast	WFS1
1.43758012654861E-09	0.677740410836423	0.084	0.193	2.40909677607015E-05	Fibroblast	CHD9
1.46792057584909E-09	0.364363770405929	0.132	0.262	2.4599413010079E-05	Fibroblast	TSC22D4
1.4684859185389E-09	0.420563319835818	0.13	0.263	2.46088870228748E-05	Fibroblast	STIM1
1.5046013647584E-09	0.453824244610871	0.035	0.117	2.52141096706213E-05	Fibroblast	COX15
1.56474008229872E-09	0.600859664447845	0.111	0.234	2.62219142991619E-05	Fibroblast	ERLEC1
1.60342597163565E-09	0.387356760299575	0.056	0.15	2.68702124326702E-05	Fibroblast	ENGASE
1.69485986202915E-09	0.396285107262314	0.061	0.156	2.84024615678846E-05	Fibroblast	MRS2
1.70360615531749E-09	0.554389084555045	0.13	0.263	2.85490319508105E-05	Fibroblast	KDSR
1.73543776845341E-09	2.73189454433077	0.111	0.058	2.90824661237423E-05	Fibroblast	PLXNA1
1.7485941322282E-09	0.584304380180643	0.183	0.343	2.93029404678801E-05	Fibroblast	TMED9
1.75083245888969E-09	0.252770761194107	0.026	0.101	2.93404503460735E-05	Fibroblast	SYDE2
1.84963747218375E-09	0.262830214323676	0.029	0.106	3.09962247588552E-05	Fibroblast	GSTA4
1.87001399438634E-09	0.554858878237407	0.061	0.157	3.13376945179263E-05	Fibroblast	ABL2
1.92226371797113E-09	0.433631110332402	0.05	0.138	3.22132953857602E-05	Fibroblast	STRIP1
1.97450136254515E-09	0.451216337518582	0.096	0.21	3.30886938335316E-05	Fibroblast	APOL2
1.99350487216673E-09	0.491902801403826	0.074	0.177	3.34071546477701E-05	Fibroblast	UNK
2.00558089275153E-09	0.57294666572938	0.077	0.181	3.36095246007302E-05	Fibroblast	MAGEH1
2.04203365996744E-09	0.451156162679832	0.132	0.263	3.42204000737344E-05	Fibroblast	PITPNB
2.0580248708139E-09	0.451503614012504	0.074	0.175	3.44883807850993E-05	Fibroblast	ITGB1BP1
2.07092466548748E-09	0.333197899575487	0.105	0.22	3.47045555442392E-05	Fibroblast	PIP4K2B
2.11136993427034E-09	0.330769925334144	0.069	0.168	3.53823373585023E-05	Fibroblast	DDX19A
2.20685446089554E-09	0.338836387349115	0.056	0.148	3.69824670556875E-05	Fibroblast	COG4
2.35152685964053E-09	0.359865552092645	0.035	0.115	3.9406887113856E-05	Fibroblast	ANKRD26
2.51395961458268E-09	0.556960768740067	0.058	0.15	4.21289352211765E-05	Fibroblast	ZNF22
2.51979314961593E-09	0.324808633538449	0.196	0.358	4.22266936012637E-05	Fibroblast	DPP7
2.54143846644133E-09	0.361675115524897	0.066	0.163	4.25894258206238E-05	Fibroblast	CDK2
2.55101674210639E-09	0.537066759843737	0.096	0.209	4.27499385642189E-05	Fibroblast	TAF13
2.62244657059636E-09	0.584657383503892	0.069	0.166	4.39469596300538E-05	Fibroblast	SUGT1

2.62615114849421E-09	0.471739939041541	0.141	0.277	4.40090409464659E-05	Fibroblast	RAPH1
2.69112521051022E-09	0.465469425982279	0.117	0.241	4.50978762777303E-05	Fibroblast	AUTS2
2.72403404656012E-09	1.43677036527259	0.105	0.052	4.56493625522546E-05	Fibroblast	PLEKHO2
2.79069075334032E-09	0.437466318418142	0.059	0.153	4.67663956444771E-05	Fibroblast	SUPT20H
2.8366873719653E-09	0.869626395023452	0.034	0.113	4.75372069793944E-05	Fibroblast	EXOSC3
2.89117334916682E-09	0.57191838565451	0.146	0.285	4.84502829853376E-05	Fibroblast	TNRC6A
2.9095568112021E-09	0.678497606777174	0.058	0.151	4.87583530421249E-05	Fibroblast	TBC1D23
2.91744846636944E-09	0.320125938001885	0.101	0.215	4.8890601399419E-05	Fibroblast	ADSS
2.98561588296106E-09	0.480252695074296	0.058	0.15	5.00329509666614E-05	Fibroblast	KCTD13
2.98755579477588E-09	0.300801513100538	0.076	0.176	5.00654600088542E-05	Fibroblast	SMARCB1
3.06417925921643E-09	0.253315250684789	0.055	0.144	5.13495160259489E-05	Fibroblast	PRMT1
3.14556407125018E-09	1.90285280512372	0.162	0.1	5.27133627060105E-05	Fibroblast	ADM
3.21018893433461E-09	0.322718903235081	0.056	0.146	5.37963461615794E-05	Fibroblast	EXOSC6
3.43457693267858E-09	0.532410039787704	0.1	0.214	5.75566402378277E-05	Fibroblast	TNPO2
3.46980826898221E-09	0.633109741388568	0.058	0.149	5.81470469716038E-05	Fibroblast	ACYP2
3.57977961524475E-09	0.474310871593296	0.183	0.341	5.99899467922715E-05	Fibroblast	BRD4
3.58390742551529E-09	0.261458262119483	0.135	0.265	6.00591206367853E-05	Fibroblast	TAF1D
3.60697136365964E-09	0.307112370132026	0.127	0.251	6.04456261122083E-05	Fibroblast	KDM4B
3.66087520421859E-09	0.515063425624719	0.045	0.129	6.13489466722951E-05	Fibroblast	EIF2B3
3.67900469844614E-09	0.754562001814651	0.111	0.232	6.16527607365604E-05	Fibroblast	SCAMP4
3.78760125335465E-09	0.614569368187368	0.14	0.276	6.34726218037172E-05	Fibroblast	TRIO
3.81229305604222E-09	0.31519065365947	0.055	0.143	6.38864070331554E-05	Fibroblast	TPCN1
3.89072219184398E-09	0.313359925556699	0.045	0.129	6.52007224909214E-05	Fibroblast	RNF8
3.94017241102545E-09	0.558577434221259	0.103	0.217	6.60294092639645E-05	Fibroblast	STXBP5
4.07571615456357E-09	0.445001176477303	0.117	0.238	6.83008513181763E-05	Fibroblast	USP34
4.08961044942073E-09	0.487376573963569	0.055	0.144	6.85336919113926E-05	Fibroblast	POM121
4.09959027857283E-09	0.602613558120076	0.04	0.122	6.87009338883234E-05	Fibroblast	VCPKMT
4.17187069601192E-09	1.67586563758289	0.203	0.133	6.99122091237677E-05	Fibroblast	EGR1
4.56057384554396E-09	0.295121355122951	0.113	0.229	7.64260965036257E-05	Fibroblast	IFNGR2
4.57968002618061E-09	0.341769089869578	0.034	0.111	7.67462778787346E-05	Fibroblast	DEPRECATED-ENSG00000197302
4.62038947454082E-09	0.574640584443407	0.029	0.103	7.7428486814355E-05	Fibroblast	CSRP2
5.16054624868342E-09	0.289923546438009	0.063	0.155	8.64804340354367E-05	Fibroblast	SPRY2
5.64073504770789E-09	0.352585016120313	0.077	0.175	9.45274379294889E-05	Fibroblast	CFAP298
5.84674713318135E-09	0.990761187024652	0.085	0.191	9.79797884578531E-05	Fibroblast	CDC42BPA
5.8945597666655E-09	0.335206659035181	0.072	0.169	9.87810325697805E-05	Fibroblast	CHD1
5.92239561100527E-09	0.761368373957409	0.117	0.238	9.92475056492264E-05	Fibroblast	PCGF2
5.94086304559125E-09	0.519587460399714	0.159	0.301	9.95569829180181E-05	Fibroblast	PLSCR1
5.9713683094605E-09	0.769594471421105	0.039	0.119	0.000100068190129939	Fibroblast	PCDHGA5
5.97363169139677E-09	0.724307141901481	0.095	0.206	0.000100106119884427	Fibroblast	SEC24D
6.08137579229087E-09	0.69236255011527	0.051	0.138	0.00010191169552721	Fibroblast	COX19
6.14163606625008E-09	0.579414003443094	0.087	0.192	0.000102921537198219	Fibroblast	ARFGAP1
6.22892974852713E-09	2.05488811077285	0.175	0.113	0.000104384404725818	Fibroblast	CDH2
6.25228445688694E-09	0.349213470431979	0.048	0.132	0.00010477578292851	Fibroblast	CHCHD1
6.47027482671193E-09	0.654911505987499	0.129	0.257	0.000108428865546039	Fibroblast	NCKAP1
6.52316636100795E-09	0.262377078097387	0.064	0.155	0.000109315221877771	Fibroblast	ABR
6.54547448755367E-09	0.371551339558354	0.034	0.11	0.000109689061462424	Fibroblast	SMYD4
6.66593127823216E-09	0.506240537600618	0.074	0.172	0.000111707676360615	Fibroblast	ARFRP1
6.68936291189679E-09	0.507640022594151	0.095	0.203	0.000112100343677566	Fibroblast	EIF4G3
6.8018923136647E-09	0.357611973799461	0.059	0.148	0.000113986111392393	Fibroblast	MMADHC
7.05853551234479E-09	0.316817182649731	0.068	0.161	0.000118286938115874	Fibroblast	ZC3H7A
7.15215657184576E-09	0.377245873325001	0.18	0.331	0.000119855839830991	Fibroblast	BSDC1
7.279372371908E-09	0.394944928590501	0.04	0.118	0.000121987722208434	Fibroblast	FTSJ3
7.30358827014083E-09	0.82206661780115	0.059	0.15	0.00012239353223102	Fibroblast	COMMD1
7.37386312170714E-09	0.6523100548418	0.045	0.127	0.000123571198193568	Fibroblast	B3GAT3
7.40966100188005E-09	0.583948162145868	0.037	0.114	0.000124171099069506	Fibroblast	FOXRED1
7.54085537753555E-09	0.539904564873912	0.074	0.172	0.000126369654416741	Fibroblast	PCIF1
7.63240331581905E-09	0.528906885638789	0.037	0.114	0.000127903814766496	Fibroblast	MRTFB
7.68962568397039E-09	0.408161061158502	0.032	0.106	0.000128862747211976	Fibroblast	AAR2
7.7906345514072E-09	0.424685439058561	0.032	0.107	0.000130555453812482	Fibroblast	TRMT61A
8.69785906967373E-09	0.682551449052313	0.051	0.138	0.000145758722289592	Fibroblast	USP40
8.72266989700606E-09	0.503250357082104	0.037	0.114	0.000146174502134028	Fibroblast	CEP95
8.7540046358675E-09	0.374176604023072	0.048	0.132	0.000146699609687867	Fibroblast	CEP44
9.07284514595748E-09	0.552998519526777	0.175	0.323	0.000152042738955956	Fibroblast	UBXN6

9.11744018946434E-09	2.3318358356715	0.272	0.22	0.000152790062695043	Fibroblast	PPP1R12B
9.23222822181292E-09	0.290177473766967	0.039	0.116	0.000154713680541141	Fibroblast	WDR70
9.25559520720655E-09	0.442927826284669	0.061	0.151	0.000155105264482367	Fibroblast	DPY19L1
9.29999170154646E-09	0.62905568830086	0.066	0.158	0.000155849260934515	Fibroblast	BRAT1
9.39252961451552E-09	0.411181414701193	0.034	0.108	0.000157400011280051	Fibroblast	RAB2B
9.46027491423606E-09	0.897790225710711	0.079	0.179	0.000158535287012768	Fibroblast	NAGLU
9.46081240441546E-09	0.419758221063349	0.138	0.267	0.000158544294273194	Fibroblast	NME3
9.66873122481872E-09	0.724270845400265	0.175	0.326	0.000162028597865512	Fibroblast	PAPSS2
9.7834567470301E-09	1.67726020449373	0.105	0.054	0.00016395116816673	Fibroblast	MAP3K8
9.91336422920469E-09	0.353731215579813	0.084	0.184	0.000166128157753012	Fibroblast	ZER1
1.02779471502054E-08	0.531760204957564	0.085	0.187	0.000172237838343143	Fibroblast	AAAS
1.05015967733561E-08	1.33626379526806	0.449	0.473	0.000175985758727902	Fibroblast	PLXNB2
1.06149722808399E-08	1.03982008876045	0.122	0.244	0.000177885705482316	Fibroblast	ADCY6
1.07730488255718E-08	0.30444926664063	0.064	0.155	0.000180534752218932	Fibroblast	PFN2
1.08384626574322E-08	0.298629232650956	0.082	0.181	0.000181630957213249	Fibroblast	TENT4A
1.09613125833051E-08	0.363643492240759	0.061	0.151	0.000183689676271027	Fibroblast	AASDHPPT
1.11217129377451E-08	0.684789209836664	0.129	0.252	0.000186377665410732	Fibroblast	ATF4
1.12176972096162E-08	0.605127610228017	0.096	0.205	0.000187986169838748	Fibroblast	YIF1B
1.12762797612582E-08	0.487868134136621	0.064	0.154	0.000188967896239166	Fibroblast	IFT20
1.14878695095314E-08	0.76989602630946	0.117	0.237	0.000192513717240727	Fibroblast	RSAD1
1.1672822494122E-08	0.353059495745005	0.08	0.179	0.000195613159356496	Fibroblast	WASHC4
1.17093420642036E-08	0.395252187232339	0.108	0.218	0.000196225154311925	Fibroblast	NME4
1.21104833046507E-08	0.467598027229826	0.093	0.197	0.000202947479219337	Fibroblast	OTUD5
1.21239143928852E-08	0.457394169634429	0.095	0.199	0.000203172557395971	Fibroblast	CLASRP
1.22355046555659E-08	0.687601955073664	0.13	0.254	0.000205042587017974	Fibroblast	GPR107
1.23112997773782E-08	0.528307848376076	0.129	0.25	0.000206312761669304	Fibroblast	NR3C1
1.23387046302064E-08	0.32643139000434	0.031	0.103	0.000206772012192998	Fibroblast	SF3A3
1.24274113005684E-08	0.43522460177681	0.198	0.362	0.000208258558574925	Fibroblast	ADD1
1.24517487601319E-08	0.446980750001351	0.042	0.121	0.00020866640572229	Fibroblast	HOOK2
1.25721861214649E-08	0.607784738389887	0.066	0.158	0.000210684695023509	Fibroblast	TSPYL4
1.28182383340145E-08	0.35734169941205	0.071	0.164	0.000214808038001415	Fibroblast	PSMD9
1.28186092119049E-08	2.91453386075535	0.127	0.074	0.000214814253173103	Fibroblast	HSPA2
1.2901771267194E-08	0.356791261965172	0.031	0.103	0.000216207882895637	Fibroblast	ZFP90
1.315308784299E-08	0.417241968307855	0.039	0.115	0.000220419446072826	Fibroblast	RPP30
1.35385909568208E-08	2.48852403350616	0.254	0.203	0.000226879707254403	Fibroblast	NR2F1
1.37155257785415E-08	0.426014974628133	0.135	0.258	0.000229844780996798	Fibroblast	FADS2
1.38125866182913E-08	0.53474659843537	0.045	0.125	0.000231471326549325	Fibroblast	SMG9
1.38508422842352E-08	0.556585988832226	0.061	0.15	0.000232112414999214	Fibroblast	GTF2H4
1.40373022149829E-08	0.519244252902812	0.238	0.42	0.000235237110518683	Fibroblast	IRF2BP2
1.40997028345712E-08	0.683576632167762	0.074	0.17	0.000236282820101745	Fibroblast	NPHP3
1.42041380073395E-08	0.816602457437916	0.105	0.216	0.000238032944726996	Fibroblast	BLCAP
1.45949628656895E-08	0.261369264991084	0.043	0.122	0.000244582387703225	Fibroblast	ZCCHC8
1.46582292826227E-08	0.467132791819616	0.045	0.124	0.000245642606318191	Fibroblast	KLHL7
1.46900618433954E-08	0.306077283907062	0.084	0.183	0.00024617605637162	Fibroblast	KIAA1109
1.47890849660743E-08	0.607634820160266	0.074	0.171	0.000247835485861474	Fibroblast	SLC19A2
1.51039966441478E-08	0.464669528614302	0.111	0.225	0.000253112775762629	Fibroblast	HERC4
1.52445361609964E-08	0.431711429116708	0.08	0.178	0.000255467936985978	Fibroblast	RBM5
1.5419414624154E-08	0.393337638580431	0.056	0.142	0.000258398550271572	Fibroblast	DIP2C
1.57213806906213E-08	0.619778324471246	0.061	0.15	0.000263458897613432	Fibroblast	MED12
1.62138359918277E-08	0.283837089767134	0.072	0.166	0.000271711463551048	Fibroblast	THAP11
1.65047846795817E-08	0.457986742377236	0.043	0.122	0.00027658718166043	Fibroblast	ACD
1.66227955302152E-08	0.901800852564008	0.092	0.198	0.000278564807495346	Fibroblast	PCYOX1
1.66680266541812E-08	0.413540233620672	0.059	0.146	0.000279322790670769	Fibroblast	XPO6
1.73074878142668E-08	0.561699611452299	0.039	0.115	0.000290038880791483	Fibroblast	PPP1R13L
1.75437485990348E-08	0.435590185684261	0.127	0.247	0.000293998139022626	Fibroblast	HEXA
1.75745774973489E-08	0.426840354904264	0.056	0.141	0.000294514769700573	Fibroblast	EXOSC10
1.78077056764774E-08	0.468279306193973	0.098	0.205	0.000298421531726408	Fibroblast	DDOST
1.78621050716862E-08	0.523673448960456	0.093	0.197	0.000299333156791317	Fibroblast	SNX19
1.79621552370804E-08	0.525821082595053	0.039	0.114	0.000301009797462993	Fibroblast	KNOP1
1.84614401280566E-08	0.289720140673539	0.035	0.109	0.000309376813665973	Fibroblast	FAM210A
1.86999723365202E-08	0.345257969677768	0.193	0.344	0.000313374136415406	Fibroblast	ESYT2
1.87141991487313E-08	0.395193780221565	0.101	0.208	0.00031361254933444	Fibroblast	PPP1R12C
1.88241149127789E-08	0.707302640792457	0.066	0.157	0.000315454517708349	Fibroblast	PIAS3

**1.89816782273259E-08**	0.630759729976312	0.159	0.297	0.000318094963733527	Fibroblast	FBXL5
**1.96079022662702E-08**	0.383653190672346	0.08	0.176	0.000328589226178156	Fibroblast	VPS39
**1.97146042534469E-08**	0.321188413814287	0.031	0.101	0.000330377338079263	Fibroblast	PPM1D
**1.97510007843862E-08**	0.539135649291637	0.048	0.129	0.000330987271144744	Fibroblast	HCFC2
**2.10137572183704E-08**	0.456492111390358	0.034	0.106	0.000352148543465451	Fibroblast	NAALADL2
**2.12585640128981E-08**	0.438347273983675	0.047	0.126	0.000356251015728146	Fibroblast	UFL1
**2.14342687922572E-08**	0.571003758602063	0.132	0.254	0.000359195476420646	Fibroblast	RUNX1
**2.19923334354993E-08**	0.467654098405341	0.182	0.328	0.000368547523712098	Fibroblast	TMEM50A
**2.22434754140777E-08**	2.39370610812568	0.127	0.074	0.000372756160989114	Fibroblast	PKIG
**2.24844316891879E-08**	0.43116561229438	0.09	0.191	0.000376794106247411	Fibroblast	TRAPPC12
**2.24850542241697E-08**	0.39789261401091	0.051	0.132	0.000376804538688637	Fibroblast	PES 1.00
**2.26293206410549E-08**	0.658776542537588	0.071	0.163	0.000379222155302799	Fibroblast	TRAPPC6B
**2.29282230076094E-08**	0.436030775965626	0.031	0.1	0.000384231161161518	Fibroblast	PEX5
**2.32592754923572E-08**	0.6124312962311	0.114	0.227	0.000389778938700922	Fibroblast	TMEM127
**2.34241511344414E-08**	0.374111725481758	0.051	0.133	0.00039254192471097	Fibroblast	EPDR1
**2.35357402899659E-08**	0.357764666782438	0.034	0.105	0.000394411935779248	Fibroblast	FNTB
**2.37988410132223E-08**	0.473671726817552	0.186	0.336	0.000398820977699579	Fibroblast	RAB1A
**2.38075909495564E-08**	0.327425147838792	0.153	0.284	0.000398967609132667	Fibroblast	YPEL5
**2.40742520218297E-08**	0.625900743838869	0.233	0.409	0.000403436315381823	Fibroblast	SREBF1
**2.44531996919385E-08**	0.554405735367101	0.071	0.161	0.000409786720437505	Fibroblast	DEPRECATED-ENSG00000196313
**2.4531449909548E-08**	0.385163428996513	0.037	0.111	0.000411098037584205	Fibroblast	COMMD9
**2.46976944303122E-08**	0.873776372923591	0.09	0.192	0.000413883963263172	Fibroblast	FBH1
**2.48473219608485E-08**	0.336249180952348	0.048	0.128	0.0004163914214199	Fibroblast	IDH3A
**2.54922200483401E-08**	0.43032824409899	0.045	0.123	0.000427198623570083	Fibroblast	GALC
**2.65920971868901E-08**	0.926166401583774	0.076	0.171	0.000445630364657904	Fibroblast	ART4
**2.67448055974577E-08**	0.480193418116863	0.079	0.174	0.000448189452202197	Fibroblast	UBE2O
**2.71102330163173E-08**	2.7204895854461	0.105	0.057	0.000454313284887445	Fibroblast	DMPK
**2.81527239541071E-08**	0.529746144455392	0.191	0.344	0.000471783348022927	Fibroblast	ASXL1
**2.84199213995186E-08**	0.5620724432484	0.106	0.215	0.000476261042813132	Fibroblast	BET1L
**2.84827961650225E-08**	0.397924744974844	0.071	0.162	0.000477314698133448	Fibroblast	AKAP11
**2.8538352289608E-08**	0.569125220065053	0.059	0.144	0.000478245707669252	Fibroblast	CCDC6
**2.87350068950972E-08**	0.572853774861775	0.108	0.216	0.000481541245548039	Fibroblast	PURB
**2.87449068375614E-08**	0.532717029867453	0.127	0.244	0.000481707148783854	Fibroblast	RBM3
**2.89550355164623E-08**	0.412953125415483	0.175	0.315	0.000485228485184876	Fibroblast	ARHGDIA
**2.90663335211927E-08**	0.772700858379221	0.072	0.165	0.000487093617148147	Fibroblast	CSNK1A1
**2.95130255008124E-08**	0.677953014817858	0.059	0.146	0.000494579281342614	Fibroblast	RWDD3
**2.97630303351745E-08**	0.259766555415396	0.042	0.116	0.000498768862356854	Fibroblast	ADSSL1
**2.98900414958754E-08**	0.586238409264681	0.066	0.154	0.000500897315387879	Fibroblast	FBXO17
**3.05210533492533E-08**	0.439703868370114	0.087	0.186	0.000511471812026786	Fibroblast	VPS26A
**3.21209141032127E-08**	0.517204540915446	0.119	0.232	0.000538282278541639	Fibroblast	DEPRECATED-ENSG00000166136
**3.2916820891495E-08**	0.260657329305238	0.088	0.187	0.000551620084499674	Fibroblast	ADD3
**3.29505460349469E-08**	0.297188663936149	0.032	0.102	0.000552185250453641	Fibroblast	EXD2
**3.42878285819042E-08**	0.412163427152372	0.082	0.176	0.000574595431375551	Fibroblast	TTYH3
**3.43234596560453E-08**	0.62290018946575	0.048	0.128	0.000575192536916007	Fibroblast	BICRA
**3.52003533487248E-08**	2.21443015909233	0.257	0.21	0.00058988752141793	Fibroblast	LAMC1
**3.54099194667298E-08**	0.438561824604615	0.204	0.358	0.000593399430423457	Fibroblast	NDST1
**3.58955872575383E-08**	0.619840921523776	0.055	0.137	0.000601538251261827	Fibroblast	GPKOW
**3.59781916883605E-08**	1.4590836530857	0.117	0.065	0.000602922536313544	Fibroblast	SH3BGRL
**3.60527962886535E-08**	0.413586087386255	0.058	0.141	0.000604172760205255	Fibroblast	DHX29
**3.61895987690119E-08**	0.28924009253471	0.059	0.143	0.000606465296171102	Fibroblast	PIAS2
**3.75184700774035E-08**	0.562136840554062	0.177	0.321	0.000628734521557127	Fibroblast	UBR4
**3.86213919840982E-08**	0.602481496110031	0.164	0.303	0.000647217286869517	Fibroblast	RAB21
**4.01071530900433E-08**	0.519917114715279	0.047	0.124	0.000672115671482946	Fibroblast	CUEDC1
**4.27811717157604E-08**	0.609975296430192	0.072	0.163	0.000716926875612713	Fibroblast	TCTA
**4.31626554918975E-08**	0.754985444597143	0.051	0.131	0.000723319780733219	Fibroblast	AP3M2
**4.51133848817972E-08**	0.283938592985189	0.056	0.138	0.000756010103849158	Fibroblast	NAT9
**4.68169920919242E-08**	0.466699474343466	0.066	0.152	0.000784559153476466	Fibroblast	TRMT2A
**4.70102140579751E-08**	0.542020039861846	0.235	0.403	0.000787797167183547	Fibroblast	KDM2A
**4.75972165386937E-08**	0.404222452460643	0.072	0.163	0.000797634154755429	Fibroblast	RIC1
**4.79247967014377E-08**	0.70771553928897	0.042	0.116	0.000803123743122692	Fibroblast	FLYWCH2
**4.830011249071E-08**	0.64412434564591	0.048	0.126	0.000809413285119317	Fibroblast	FASTKD2
**4.85232198108604E-08**	0.303411115237714	0.133	0.251	0.000813152117590399	Fibroblast	NAGK
**4.87972759710613E-08**	0.673071012926431	0.146	0.273	0.000817744750723045	Fibroblast	TRIOBP

4.90327147478837E-08	0.413467066940376	0.138	0.259	0.000821690233745036	Fibroblast	CLCN7
4.90357847242146E-08	0.538891071435113	0.09	0.189	0.000821741680408389	Fibroblast	GALNS
4.95165455768799E-08	0.657661429004259	0.166	0.304	0.000829798270777353	Fibroblast	LRRC8A
4.99863829365836E-08	0.393414807831401	0.109	0.217	0.000837671805251268	Fibroblast	CERT1
4.99977001694229E-08	0.56939012364288	0.04	0.114	0.000837861459439189	Fibroblast	SBF2
5.15245748083074E-08	0.411451796850103	0.074	0.165	0.000863448824637616	Fibroblast	PCSK7
5.17897340396495E-08	0.382128940504905	0.034	0.103	0.000867892363036446	Fibroblast	GPN2
5.19924272790969E-08	0.546637256734164	0.042	0.116	0.000871289096343106	Fibroblast	TXLNG
5.21911030181435E-08	0.440206940516278	0.047	0.123	0.000874618504378049	Fibroblast	IQCE
5.25124718892655E-08	0.475652807161797	0.058	0.14	0.000880004003920311	Fibroblast	MED22
5.2676726101528E-08	0.550402981565568	0.085	0.182	0.000882756576009407	Fibroblast	PDLIM1
5.29094563681546E-08	0.771247144171565	0.066	0.152	0.000886656669817536	Fibroblast	SHPRH
5.3143470360242E-08	0.609926010517265	0.05	0.128	0.000890578276296936	Fibroblast	DEPRECATED-ENSG00000188529
5.37653843827034E-08	0.485282809718641	0.058	0.14	0.000901000311485343	Fibroblast	VPS11
5.41987887727941E-08	0.677100735796427	0.159	0.291	0.000908263302254484	Fibroblast	VAT1
5.45410744919265E-08	2.14170627205389	0.317	0.285	0.000913999326335704	Fibroblast	PKDCC
5.47815980666026E-08	0.729037741992425	0.066	0.151	0.000918030020400127	Fibroblast	SLC12A9
5.4846686434157E-08	0.859432092800562	0.08	0.174	0.000919120771263602	Fibroblast	CLK4
5.49751183241242E-08	0.301529107938793	0.068	0.153	0.000921273032875674	Fibroblast	SRSF8
5.51250120452932E-08	0.44732619581434	0.13	0.247	0.000923784951855024	Fibroblast	CREBBP
5.61874767231543E-08	0.314433124895183	0.043	0.118	0.00094158973492662	Fibroblast	SLC35E2A
5.68816352426885E-08	0.442468852111362	0.058	0.14	0.000953222443396974	Fibroblast	ZBTB37
5.73796407594726E-08	0.484703203384704	0.043	0.117	0.000961568019847241	Fibroblast	ERCC6
5.77174363854453E-08	0.487005332037799	0.108	0.213	0.000967228798947292	Fibroblast	RCN1
5.77207955121106E-08	2.20404078941553	0.235	0.181	0.000967285091191949	Fibroblast	CLSTN3
5.83278521299655E-08	0.28758945543607	0.04	0.113	0.000977458145993962	Fibroblast	L3MBTL4
5.8694451136786E-08	2.0754107762495	0.315	0.286	0.00098360161215026	Fibroblast	EDNRB
5.87918034808431E-08	0.586640019065173	0.034	0.103	0.000985233042731968	Fibroblast	NINL
5.97847924602535E-08	0.720253888300692	0.117	0.231	0.00100187355204893	Fibroblast	DMTF1
5.97870201646178E-08	0.671177216899108	0.117	0.229	0.00100191088391867	Fibroblast	SRRM1
6.04416870234204E-08	0.259701567066927	0.166	0.3	0.00101288179113848	Fibroblast	KMT2E
6.04505134579222E-08	0.461076425613853	0.056	0.137	0.00101302970452786	Fibroblast	FBRSL1
6.12343410137332E-08	0.39199887494761	0.055	0.134	0.00102616508670814	Fibroblast	NT5C2
6.14897847820955E-08	0.433633494941216	0.045	0.121	0.00103044581337836	Fibroblast	SDHAF2
6.15871822726414E-08	0.428705045343098	0.14	0.26	0.00103207800052492	Fibroblast	KCTD20
6.32316157658915E-08	0.461002252351014	0.055	0.136	0.00105963541700481	Fibroblast	LRIG2
6.35607955920635E-08	0.971439770561577	0.056	0.137	0.0010651518125318	Fibroblast	BAD
6.49132566703117E-08	0.72752661218766	0.039	0.11	0.00108781635528108	Fibroblast	SENP7
6.59269473494398E-08	0.657600340179457	0.08	0.174	0.00110480378368191	Fibroblast	ZNF330
6.6153653436041E-08	0.441295275772049	0.045	0.12	0.00110860292428118	Fibroblast	DUS3L
6.89969866130255E-08	2.64470849964231	0.178	0.124	0.00115625150166108	Fibroblast	TWSG1
7.00936709120665E-08	0.27000572206483	0.318	0.531	0.00117462973714441	Fibroblast	SDC4
7.04355570708698E-08	0.676908822244009	0.032	0.1	0.00118035906539364	Fibroblast	TRMT61B
7.14734314309608E-08	0.402509911819264	0.061	0.144	0.00119775176392004	Fibroblast	MIB2
7.19331785812619E-08	0.607948444460282	0.064	0.149	0.00120545620666479	Fibroblast	GLMP
7.67076142649492E-08	0.589563320803232	0.084	0.176	0.00128546619985202	Fibroblast	TCF7L2
7.90764129164387E-08	0.545899306787469	0.053	0.131	0.00132516252765368	Fibroblast	NACC2
7.90877368799832E-08	0.628984501519596	0.072	0.161	0.00132535229463476	Fibroblast	GYS1
7.96249409996461E-08	0.441771418135139	0.074	0.163	0.00133435476127207	Fibroblast	YEATS2
8.35077838537965E-08	0.316334620315101	0.037	0.106	0.00139942344182192	Fibroblast	TADA2A
8.42469009902604E-08	0.66178198523073	0.04	0.112	0.00141180956679478	Fibroblast	FBXO30
8.47399133625808E-08	0.431454539232052	0.161	0.291	0.00142007146813013	Fibroblast	PPP1CB
8.49636848721724E-08	0.779543558778498	0.158	0.288	0.00142382143108787	Fibroblast	NIBAN2
8.50021829134008E-08	0.364152045336094	0.069	0.155	0.00142446658126277	Fibroblast	MAN2B2
8.51037224070719E-08	0.409737096205696	0.047	0.122	0.00142616818009771	Fibroblast	ZNF621
8.63549733989808E-08	0.367523033763553	0.074	0.162	0.00144713664422012	Fibroblast	MCOLN1
8.65330175325872E-08	0.737097322751548	0.095	0.195	0.0014501203078111	Fibroblast	FAM234A
8.81739865105453E-08	0.72799075650073	0.127	0.244	0.00147761966594372	Fibroblast	CDK4
8.89529301984357E-08	0.820896112165792	0.084	0.179	0.00149067320426538	Fibroblast	IRS1
9.02801056927628E-08	0.360853628080204	0.047	0.121	0.00151291401119932	Fibroblast	FAM13B
9.08950539473704E-08	0.767321882713417	0.185	0.334	0.00152321931405003	Fibroblast	MTUS1
9.22542708835627E-08	0.528789175285023	0.066	0.15	0.00154599707146674	Fibroblast	XPNPEP1
9.25555009816722E-08	0.807796298986122	0.135	0.253	0.00155104508545086	Fibroblast	BCL2L1

9.36592930498078E-08	0.440145356103901	0.034	0.101	0.00156954243292868	Fibroblast	PRKCI
9.5052296490801E-08	0.491441372931227	0.13	0.245	0.00159288638459284	Fibroblast	DEPRECATED-ENSG00000124831
9.60228693039756E-08	0.470614523868353	0.077	0.166	0.00160915124379602	Fibroblast	SS18
1.0107786632919E-07	0.568602214388158	0.077	0.167	0.00169386288394457	Fibroblast	CAMLG
1.04981094042611E-07	0.385600277847626	0.074	0.161	0.00175927317396608	Fibroblast	CREBRF
1.05058764726991E-07	0.280257239115275	0.098	0.197	0.00176057477929491	Fibroblast	KLHL24
1.09493784770203E-07	0.367531042160062	0.043	0.116	0.00183489684517907	Fibroblast	G6PC3
1.09943277802028E-07	0.661316473631688	0.103	0.205	0.00184242944940638	Fibroblast	KMT2A
1.10788389496436E-07	0.455697457686643	0.043	0.116	0.00185659183118128	Fibroblast	CEP104
1.10999227967131E-07	0.509944824633083	0.063	0.144	0.00186012506227319	Fibroblast	SIKE1
1.11243800965165E-07	0.33838370598212	0.077	0.165	0.00186422361657423	Fibroblast	GRAMD1A
1.14314710906844E-07	0.421890171485416	0.058	0.137	0.00191568592537689	Fibroblast	ZMYM4
1.17730352440852E-07	0.74118537506699	0.108	0.213	0.0019729252462038	Fibroblast	CHIC2
1.22047487286436E-07	0.344070601847043	0.039	0.107	0.00204527179194609	Fibroblast	PIGK
1.23323176254727E-07	0.326342219943909	0.034	0.1	0.00206664978767672	Fibroblast	STX8
1.23553273484674E-07	2.00645518849697	0.177	0.121	0.00207050575705617	Fibroblast	LDB1
1.26522938652068E-07	0.454283291644327	0.109	0.211	0.00212027140593136	Fibroblast	SLK
1.29165615628433E-07	0.453802405418545	0.257	0.438	0.00216455738670127	Fibroblast	SHC1
1.37689678864768E-07	0.37896684887878	0.04	0.11	0.00230740363841578	Fibroblast	XRRA1
1.40514514206649E-07	0.447118790834543	0.156	0.279	0.00235474222907502	Fibroblast	MANF
1.45441315642874E-07	0.623370135331051	0.055	0.132	0.00243730556754329	Fibroblast	PCNX4
1.47435016639153E-07	0.509872817815413	0.048	0.122	0.00247071600883892	Fibroblast	PPP4R1
1.47767638857727E-07	0.525646574653189	0.064	0.146	0.00247629009197779	Fibroblast	ZKSCAN8
1.48545950158973E-07	0.32839585040348	0.047	0.119	0.00248933303276407	Fibroblast	PPP1R8
1.49853384941842E-07	0.263978650594541	0.053	0.128	0.00251124302485538	Fibroblast	SDCCAG8
1.5193500339017E-07	0.680619907951671	0.037	0.105	0.00254612678681247	Fibroblast	FAM120C
1.57189459636033E-07	0.287555584545516	0.035	0.102	0.00263418096458065	Fibroblast	NOL12
1.60820415997204E-07	0.674525372286187	0.061	0.141	0.00269502853128115	Fibroblast	ZNF622
1.61273584117046E-07	0.702670439978893	0.048	0.122	0.00270262272263346	Fibroblast	ZNF397
1.66602726240768E-07	0.537555076958531	0.039	0.106	0.0027919284863428	Fibroblast	ZBTB5
1.67668957512284E-07	0.362491896654026	0.063	0.142	0.00280979638999086	Fibroblast	PPP3CA
1.70897627831479E-07	0.633165632979161	0.047	0.118	0.00286390244719992	Fibroblast	BFAR
1.75010149496212E-07	0.34705031613337	0.198	0.342	0.00293282008525752	Fibroblast	HNRNPH3
1.78351508685574E-07	0.308135243763313	0.074	0.159	0.00298881458255284	Fibroblast	WDR6
1.79712374400185E-07	0.533723473388705	0.156	0.281	0.0030116199701983	Fibroblast	CAV2
1.86005839043252E-07	0.387325849315437	0.145	0.261	0.00311708585068681	Fibroblast	MXD4
1.87971766401523E-07	0.525270943706056	0.076	0.16	0.00315003086135673	Fibroblast	MTPN
1.92720149525295E-07	0.546025526787449	0.114	0.219	0.00322960426574489	Fibroblast	WDR13
1.95294547531233E-07	0.587823265798822	0.048	0.121	0.0032727460275284	Fibroblast	CLASP2
2.05735658630082E-07	0.648589040871808	0.103	0.202	0.00344771816732291	Fibroblast	PLIN3
2.22309890333198E-07	0.399868317728862	0.037	0.103	0.00372546914220374	Fibroblast	B3GALNT2
2.30753181081046E-07	0.856258584047669	0.09	0.184	0.00386696180855617	Fibroblast	BRD3
2.43620860562412E-07	0.718711041152614	0.069	0.151	0.00408259838130489	Fibroblast	KAT8
2.45342608702638E-07	0.503372266632669	0.072	0.156	0.00411145143663881	Fibroblast	HCFC1R1
2.45949452021837E-07	0.884723850807345	0.124	0.234	0.00412162091698195	Fibroblast	IRF2BPL
2.47379946002865E-07	0.613366620563025	0.24	0.411	0.004145593135116	Fibroblast	CALCOCO2
2.48330121860457E-07	0.320430568571171	0.047	0.117	0.00416151618213753	Fibroblast	FXR2
2.49823530694092E-07	0.293081484755515	0.04	0.108	0.0041865427273716	Fibroblast	TOP3B
2.52168003784501E-07	0.548653152604157	0.066	0.147	0.00422583140742067	Fibroblast	SPPL3
2.54240730550411E-07	0.412406915739552	0.039	0.105	0.00426056616256379	Fibroblast	FAM76A
2.56014745331365E-07	0.345336002520266	0.053	0.126	0.00429029510226302	Fibroblast	DNAAF5
2.60215217977603E-07	0.460857502744853	0.151	0.271	0.00436068662286867	Fibroblast	CYTH2
2.67554608253919E-07	0.522437493262985	0.206	0.353	0.00448368012511918	Fibroblast	CD59
2.76553958863293E-07	0.719378842946769	0.061	0.139	0.00463449124263107	Fibroblast	RB1
2.77185900415459E-07	0.659205122181795	0.085	0.175	0.00464508131916225	Fibroblast	SECISBP2L
2.8032462051213E-07	0.571694521431272	0.035	0.1	0.00469767999054227	Fibroblast	MAZ
2.83679216254785E-07	0.407194225365406	0.103	0.199	0.00475389630599768	Fibroblast	GLRX3
2.89318756832569E-07	0.706933637432962	0.069	0.151	0.00484840372700019	Fibroblast	CDYL
2.9109837933928E-07	0.427365849558324	0.051	0.124	0.00487822664096766	Fibroblast	GTF3C2
2.94987377210774E-07	0.632298097389003	0.042	0.11	0.00494339846729815	Fibroblast	ZNF335
2.98514203721675E-07	0.506001801561726	0.077	0.163	0.00500250102596782	Fibroblast	RXRB
3.136451943756E-07	0.599733199849766	0.058	0.134	0.0052560661673463	Fibroblast	IL15RA
3.17770892338605E-07	0.856751049289044	0.043	0.112	0.00532520461381035	Fibroblast	NIPSNAP2

3.23522671831143E-07	0.460100503278939	0.058	0.134	0.0054215929345463	Fibroblast	MAP3K20
3.25373377412426E-07	0.539388521115451	0.068	0.148	0.00545260705867743	Fibroblast	ITFG2
3.27071605799106E-07	0.603757898819274	0.042	0.11	0.00548106596998142	Fibroblast	VPS35
3.29028490459073E-07	0.879002979251736	0.127	0.236	0.00551385944311315	Fibroblast	TMEM185A
3.29810198817959E-07	0.422136476571109	0.186	0.322	0.00552695931179136	Fibroblast	MAX
3.39642376933121E-07	0.283394217818959	0.043	0.111	0.00569172695264524	Fibroblast	JARID2
3.56714063583053E-07	0.710092710695585	0.048	0.119	0.0059778142775248	Fibroblast	KIZ
3.603464815082E-07	0.902379608891722	0.072	0.155	0.00603868633711442	Fibroblast	ACVR1
3.67263579362513E-07	0.350986054338792	0.342	0.544	0.00615460306295699	Fibroblast	VEGFA
3.68602755160571E-07	0.535693743281489	0.108	0.208	0.00617704497098085	Fibroblast	SOX5
3.71414708183389E-07	0.364795942569651	0.095	0.188	0.00622416767973723	Fibroblast	APBB3
3.74108076058122E-07	0.371058440457719	0.047	0.117	0.006269303138582	Fibroblast	GALNT10
3.79096603364567E-07	0.609814426704581	0.132	0.243	0.00635290087918341	Fibroblast	IP6K2
3.82468395606735E-07	0.41173299305572	0.055	0.128	0.00640940537357766	Fibroblast	MXI1
3.84259036501455E-07	0.478983327404916	0.068	0.147	0.00643941293369139	Fibroblast	ADPRHL2
3.86961200581478E-07	0.426899031005527	0.05	0.121	0.00648469579934441	Fibroblast	TSHZ1
3.91301494507866E-07	0.654046796626306	0.085	0.174	0.00655743044496282	Fibroblast	ELL
3.9369276260469E-07	0.370025517851544	0.206	0.348	0.00659750331572939	Fibroblast	VKORC1
4.0167429093136E-07	0.707570743021294	0.117	0.22	0.00673125776742773	Fibroblast	TFE3
4.0942108889547E-07	0.44167376418813	0.297	0.494	0.00686107860771028	Fibroblast	MAT2A
4.18501285017721E-07	0.914234922967213	0.111	0.213	0.00701324453432697	Fibroblast	TRAF2
4.22658215459411E-07	0.480238893052736	0.058	0.133	0.00708290637466881	Fibroblast	KDM3A
4.23537601964889E-07	0.954355692166046	0.05	0.121	0.00709764313372761	Fibroblast	EPB41L4A
4.42760574862503E-07	0.584252047489957	0.072	0.153	0.00741978171354582	Fibroblast	FARP2
4.42840240904009E-07	0.7314705051326	0.076	0.16	0.00742111675706939	Fibroblast	ASAP2
4.46665434203901E-07	0.329344630083601	0.055	0.128	0.00748521934638897	Fibroblast	CCNH
4.59570486808771E-07	0.451101640885833	0.098	0.189	0.00770148221794139	Fibroblast	SETD1B
4.60021383612421E-07	0.349431954829778	0.048	0.118	0.00770903834657694	Fibroblast	RGL3
4.60778392647576E-07	0.50698944715919	0.183	0.318	0.00772172430398807	Fibroblast	ZBTB20
4.76575024751647E-07	0.635437479161774	0.121	0.225	0.0079864442647881	Fibroblast	CYFIP1
4.8521492026927E-07	0.334639740133219	0.066	0.144	0.00813123163387243	Fibroblast	TSPAN31
4.99073179659095E-07	0.59887247470504	0.1	0.194	0.00836346834472711	Fibroblast	TNIP2
4.99620204668232E-07	0.411651994672926	0.051	0.122	0.00837263538983023	Fibroblast	NIPSNAP3A
5.00567101600099E-07	0.717995247286702	0.087	0.175	0.00838850348861447	Fibroblast	RABL6
5.13275915571623E-07	0.841967216808548	0.174	0.308	0.00860147779314925	Fibroblast	SAMD5
5.13368590268335E-07	0.310226130501547	0.092	0.181	0.00860303083571676	Fibroblast	PNPLA6
5.29001844621584E-07	0.830644980589767	0.042	0.108	0.00886501291216851	Fibroblast	ALS2
5.34728828555225E-07	0.33553973303326	0.05	0.119	0.00896098570892846	Fibroblast	MFSD14B
5.45762808470205E-07	0.91265362351467	0.058	0.134	0.0091458931443437	Fibroblast	CNTFR
5.51172375273458E-07	0.614542149805328	0.037	0.1	0.00923654666483261	Fibroblast	SDHAF1
5.61043685388882E-07	0.500609423272146	0.037	0.1	0.00940197007974688	Fibroblast	ZNF276
5.82523700911403E-07	0.378124073141095	0.039	0.102	0.00976193217987329	Fibroblast	POLG
5.99053186598723E-07	0.345949476110448	0.106	0.202	0.0100389333010214	Fibroblast	CD9
6.03439512367892E-07	0.357931919432752	0.087	0.172	0.0101124393482611	Fibroblast	PITPNA
6.08056335522846E-07	0.250563164288443	0.26	0.427	0.0101898080706919	Fibroblast	STOM
6.22923936383437E-07	0.443731435865787	0.066	0.143	0.0104389593259136	Fibroblast	DENND6A
6.36426367987644E-07	0.533558170247828	0.039	0.102	0.0106652330747369	Fibroblast	RNASEH2C
6.46549895220756E-07	0.447478494685114	0.059	0.133	0.0108348831441094	Fibroblast	FBXO11
6.58976989050667E-07	0.381945440692884	0.059	0.133	0.0110431363825111	Fibroblast	SHOC2
6.69050356077319E-07	0.477066014966192	0.182	0.312	0.0112119458671437	Fibroblast	RABAC1
6.98288859750187E-07	0.767082854787872	0.058	0.131	0.0117019247116936	Fibroblast	ANKZF1
7.00830866794291E-07	0.443997199914147	0.204	0.342	0.0117445236657387	Fibroblast	APOL6
7.04946286407198E-07	0.469559377284354	0.048	0.117	0.0118134898676118	Fibroblast	RNPC3
7.08745384254153E-07	2.22285262952883	0.114	0.068	0.0118771551493311	Fibroblast	PPP2R5B
7.09334054031918E-07	0.60328909337787	0.072	0.152	0.0118870200774669	Fibroblast	SLC35E2B
7.11826894149164E-07	0.62807394646591	0.087	0.173	0.0119287950921517	Fibroblast	ATXN2
7.3680428843987E-07	0.379701971434038	0.066	0.142	0.0123473662656753	Fibroblast	INPP5E
7.46022033985988E-07	0.563239411585578	0.055	0.126	0.0125018372455372	Fibroblast	SETDB2
7.60308230517711E-07	0.446968291804164	0.08	0.163	0.0127412453270158	Fibroblast	RBM15B
7.72858108615479E-07	0.353696891977397	0.042	0.106	0.0129515561841782	Fibroblast	ARL 3.00
7.84199467358517E-07	0.478069879241532	0.039	0.102	0.013141614673994	Fibroblast	TSPAN12
7.9090620702472E-07	0.563227506439089	0.055	0.126	0.0132540062173203	Fibroblast	CASP7
8.15755186819973E-07	0.585525647754503	0.076	0.157	0.0136704254207291	Fibroblast	PABPC4

8.18179172063475E-07	0.540076556255751	0.082	0.164	0.0137110465654397	Fibroblast	ATRX
8.3683580441684E-07	0.494891967385529	0.059	0.133	0.0140236944104174	Fibroblast	PACS2
8.49795580622622E-07	0.355740192168466	0.211	0.349	0.0142408743400739	Fibroblast	EHBP1L1
8.5137387882465E-07	0.901054040277922	0.077	0.16	0.0142673234613435	Fibroblast	SH2B1
9.09607662978474E-07	0.696594171255156	0.043	0.108	0.0152432052161933	Fibroblast	CAPRIN2
9.28923940190156E-07	0.570240994168518	0.039	0.101	0.0155669073897066	Fibroblast	ZNF207
9.35705069987881E-07	0.531433603977901	0.039	0.101	0.0156805455628569	Fibroblast	TRIM23
9.51100885294408E-07	0.276625930467256	0.04	0.103	0.0159385486357637	Fibroblast	PPM1F
9.53130353396398E-07	0.554780755928154	0.127	0.231	0.0159725584622168	Fibroblast	CRK
9.72745564518455E-07	0.310964903499174	0.058	0.129	0.0163012701702003	Fibroblast	DEPRECATED-ENSG00000272391
9.98406125853371E-07	0.720575262547714	0.063	0.136	0.0167312898570508	Fibroblast	STARD3NL
1.01237832600682E-06	0.59787069751423	0.212	0.358	0.0169654359872223	Fibroblast	AAMP
1.01613951841174E-06	0.59305674956612	0.055	0.125	0.0170284660495439	Fibroblast	NDUFS5
1.01754865894781E-06	0.66448304357285	0.088	0.175	0.0170520804266473	Fibroblast	TOM1
1.04256208813883E-06	0.332532304351117	0.103	0.194	0.0174712554730305	Fibroblast	WDR82
1.04967151856354E-06	0.541244069749938	0.047	0.113	0.0175903953080878	Fibroblast	SLC39A6
1.05094293448407E-06	1.73602117210049	0.323	0.301	0.017611701696084	Fibroblast	ATN1
1.14528228664677E-06	0.511398930018802	0.069	0.145	0.0191926405596266	Fibroblast	NSD3
1.15494081817715E-06	0.578891393538797	0.159	0.279	0.0193544982310127	Fibroblast	SELENOS
1.16466694995437E-06	0.306027193645919	0.15	0.258	0.0195174887473354	Fibroblast	SLC25A25
1.1803332522306E-06	0.460057810160712	0.068	0.142	0.0197800246408804	Fibroblast	RNF146
1.19404799613181E-06	1.97056660595462	0.109	0.065	0.0200098563191769	Fibroblast	HK1
1.20139888250305E-06	0.470196620669978	0.079	0.159	0.0201330424729862	Fibroblast	EHD1
1.22497821989059E-06	0.648819980103023	0.1	0.189	0.0205281850089266	Fibroblast	HP1BP3
1.25734865642126E-06	0.62667591823567	0.156	0.274	0.0210706487843075	Fibroblast	ISCU
1.26593150426826E-06	0.46622272211703	0.059	0.131	0.0212144801485275	Fibroblast	WASHC2A
1.27559371026759E-06	0.39525742585093	0.056	0.126	0.0213763993966643	Fibroblast	MIER3
1.28473080775614E-06	1.37269223638845	0.342	0.324	0.0215295188763774	Fibroblast	WSB1
1.29227237726925E-06	0.759456715401739	0.103	0.194	0.0216559004982781	Fibroblast	MAP4K5
1.30065365371701E-06	1.10642321593152	0.072	0.151	0.0217963539289896	Fibroblast	P4HTM
1.36088543354719E-06	0.305214295002962	0.077	0.155	0.0228057180953838	Fibroblast	TMEM11
1.42742341488699E-06	0.615799923639157	0.051	0.119	0.0239207615866762	Fibroblast	BBS2
1.4420987571355E-06	0.719873212266618	0.148	0.262	0.0241666909720767	Fibroblast	NFIX
1.46724890395186E-06	0.41344736439805	0.04	0.102	0.0245881571324253	Fibroblast	SNX30
1.47181404771462E-06	0.81536925637964	0.111	0.207	0.0246646598116015	Fibroblast	DYRK2
1.48938720618422E-06	0.478487411865474	0.042	0.103	0.0249591508012352	Fibroblast	PCGF1
1.5651138267647E-06	0.440558882090933	0.114	0.208	0.0262281775089228	Fibroblast	MAP4K4
1.56595763633761E-06	0.631391500454677	0.119	0.218	0.0262423180697457	Fibroblast	RNF11
1.61479658446095E-06	0.476210858903519	0.1	0.189	0.0270607611623967	Fibroblast	FBRS
1.64081285709973E-06	0.342316627380077	0.051	0.117	0.0274967418592773	Fibroblast	ZFYVE1
1.68238323544882E-06	0.295136596207817	0.08	0.161	0.0281933782596513	Fibroblast	DEPRECATED-ENSG00000135976
1.68464056838293E-06	0.997984287576626	0.048	0.113	0.0282312066449612	Fibroblast	POLR1A
1.69706096188273E-06	0.590921387109629	0.114	0.208	0.0284393475992307	Fibroblast	UBE4B
1.73580862036199E-06	0.542835094549155	0.059	0.13	0.0290886808600262	Fibroblast	EMC1
1.76899535959724E-06	0.33879722822049	0.043	0.106	0.0296448242361306	Fibroblast	CNOT4
1.81227490284548E-06	1.00742604608907	0.069	0.144	0.0303701028218846	Fibroblast	PSD3
1.83490104050242E-06	0.980902800587776	0.047	0.111	0.0307492716367395	Fibroblast	UFSP2
1.84600329683663E-06	2.28996897995488	0.244	0.206	0.0309353232483882	Fibroblast	DEPTOR
1.92706151792411E-06	0.716903205236996	0.096	0.183	0.0322936969173722	Fibroblast	ITPRIPL2
1.97165878367922E-06	0.620360864288571	0.042	0.103	0.0330410578968963	Fibroblast	MLYCD
2.06098672469028E-06	0.59560178467093	0.093	0.178	0.0345380155323598	Fibroblast	ZC3H7B
2.08242297709866E-06	0.764979105170895	0.069	0.143	0.0348972442502194	Fibroblast	PPP1R37
2.09908738396534E-06	0.621159509743462	0.191	0.325	0.0351765063804911	Fibroblast	TBC1D2B
2.09989561194937E-06	0.737609146368573	0.17	0.293	0.0351900506650475	Fibroblast	PLOD2
2.10135195037293E-06	0.628627433147166	0.09	0.174	0.0352144559843496	Fibroblast	MAP3K3
2.16416217881502E-06	0.710653941188426	0.158	0.275	0.0362670297925822	Fibroblast	TXNDC15
2.23667564949383E-06	0.275564533169156	0.114	0.207	0.0374822105342175	Fibroblast	TERF2IP
2.26531377254972E-06	0.916286346615034	0.14	0.25	0.0379621282003882	Fibroblast	GPC6
2.27482777533462E-06	0.676979400848477	0.085	0.166	0.0381215638590576	Fibroblast	MAN1B1
2.27601340711341E-06	0.744011868880454	0.085	0.168	0.0381414326764066	Fibroblast	KCNT2
2.28364074584922E-06	0.280867859463629	0.053	0.12	0.0382692516189413	Fibroblast	RUBCN
2.3211586967102E-06	0.838651026179373	0.137	0.243	0.0388979774394696	Fibroblast	AEBP2
2.34633886459337E-06	0.608070783386373	0.058	0.127	0.0393199466928557	Fibroblast	MEIS2

**2.34714485184509E-06**	0.942782820163959	0.05	0.115	0.03933345342722	Fibroblast	ENKD1
**2.35099176273006E-06**	0.443491326424856	0.074	0.149	0.0393979199598303	Fibroblast	DHX30
**2.36194331454474E-06**	0.580366609352041	0.064	0.136	0.0395814460651407	Fibroblast	RCL1
**2.36637083511265E-06**	0.288727427337348	0.042	0.103	0.0396556424548178	Fibroblast	TOGARAM1
**2.36794038593679E-06**	0.517517985479796	0.1	0.187	0.0396819449875286	Fibroblast	ARF4
**2.43753385081755E-06**	0.872364057621859	0.114	0.209	0.0408481922720006	Fibroblast	MAML1
**2.47453579159261E-06**	0.535940352112507	0.264	0.435	0.041468270795509	Fibroblast	PRKAR1A
**2.55206992839798E-06**	0.592420879290659	0.056	0.124	0.0427675878600934	Fibroblast	WDR43
**2.55554987743693E-06**	0.565672443954945	0.047	0.109	0.042825904846088	Fibroblast	RRAGC
**2.57662057473983E-06**	0.590827477366692	0.063	0.133	0.04317900759149	Fibroblast	IFFO1
**2.61482895335262E-06**	0.444807542470915	0.061	0.13	0.0438193036002832	Fibroblast	MAP3K7
**2.62831869134646E-06**	0.788280786554881	0.05	0.114	0.0440453646295839	Fibroblast	FTO
**2.64843224876925E-06**	0.418876432820409	0.058	0.126	0.0443824276248751	Fibroblast	HMG20A
**2.64958927708718E-06**	0.490107693933621	0.074	0.15	0.044401817105427	Fibroblast	ORAI1
**2.65726218852197E-06**	0.48357906158302	0.135	0.238	0.0445303997552512	Fibroblast	PAFAH1B2
**2.75508663258187E-06**	0.616862758392053	0.051	0.117	0.046169741788807	Fibroblast	RLIM
**2.75569432820483E-06**	0.830337361440958	0.137	0.241	0.0461799255520566	Fibroblast	YAP1
**2.76768017140868E-06**	0.314588293618984	0.074	0.148	0.0463807843124666	Fibroblast	UBA3
**2.82179612957448E-06**	1.10424872747705	0.066	0.137	0.0472876595394092	Fibroblast	SVIL
**2.85141962395135E-06**	0.734727258761158	0.066	0.137	0.0477840900581768	Fibroblast	ELAC2
**2.88263922392657E-06**	0.674778961851076	0.113	0.205	0.0483072681145615	Fibroblast	VEGFB
**2.94184667001828E-06**	0.503669726340897	0.053	0.118	0.0492994664961663	Fibroblast	UBXN7
**0**	6.97223863782582	0.826	0.04	0	Myeloid	FGL2
**0**	5.85123840162179	0.855	0.087	0	Myeloid	MS4A6A
**0**	7.17838549505698	0.799	0.056	0	Myeloid	C1QB
**0**	7.60949687559794	0.774	0.033	0	Myeloid	C1QC
**0**	7.06829639968224	0.79	0.052	0	Myeloid	C1QA
**0**	7.77344103742099	0.755	0.017	0	Myeloid	CSF1R
**0**	7.82834484460686	0.743	0.029	0	Myeloid	CD163
**0**	3.00559570473249	0.845	0.135	0	Myeloid	SRGN
**0**	6.30691651776663	0.73	0.021	0	Myeloid	SPI1
**0**	6.62499442203666	0.699	0.019	0	Myeloid	MPEG1
**0**	3.41946206080671	0.822	0.143	0	Myeloid	LAPTM5
**0**	1.65853301049934	0.765	0.118	0	Myeloid	CXCR4
**0**	6.17862339740609	0.656	0.032	0	Myeloid	CYBB
**0**	3.95306272064561	0.67	0.05	0	Myeloid	CD4
**0**	3.6978354591497	0.665	0.053	0	Myeloid	HCLS1
**0**	3.43649485672402	0.668	0.068	0	Myeloid	CYBA
**0**	5.75918273903426	0.614	0.015	0	Myeloid	ITGAX
**0**	7.04398606528881	0.612	0.017	0	Myeloid	MS4A7
**0**	3.81656338057999	0.662	0.07	0	Myeloid	ITGB2
**0**	6.86163985084855	0.601	0.009	0	Myeloid	CSF2RA
**0**	6.02205667813166	0.629	0.038	0	Myeloid	FCER1G
**0**	4.62498155356537	0.658	0.071	0	Myeloid	HSPA6
**0**	5.93076018842503	0.603	0.02	0	Myeloid	FCGR2A
**0**	2.54797387468919	0.634	0.066	0	Myeloid	CELF2
**0**	4.71198211172539	0.578	0.019	0	Myeloid	THEMIS2
**0**	5.57469085366291	0.588	0.029	0	Myeloid	TYROBP
**0**	5.52477317632349	0.594	0.036	0	Myeloid	AIF1
**0**	5.91881957891147	0.602	0.044	0	Myeloid	MRC1
**0**	4.41688276680032	0.625	0.069	0	Myeloid	MAFB
**0**	4.23721183656261	0.586	0.031	0	Myeloid	STAB1
**0**	7.10323278999543	0.56	0.008	0	Myeloid	EMILIN2
**0**	5.21540121886972	0.631	0.08	0	Myeloid	RGS2
**0**	5.63437326933529	0.551	0.009	0	Myeloid	FGD2
**0**	3.69096878647932	0.582	0.04	0	Myeloid	TIMP2
**0**	2.60887024477153	0.633	0.093	0	Myeloid	LCP1
**0**	4.22489692500089	0.562	0.024	0	Myeloid	ZEB2
**0**	4.71508485254793	0.556	0.021	0	Myeloid	ADA2
**0**	6.21517254111847	0.548	0.014	0	Myeloid	LAIR1
**0**	3.16851937028835	0.604	0.072	0	Myeloid	RGS1
**0**	7.6577221662965	0.557	0.025	0	Myeloid	F13A1
**0**	3.95196872264275	0.661	0.139	0	Myeloid	FOS

0	3.81522135798265	0.573	0.057	0	Myeloid	GSN
0	1.8839693655493	0.6	0.088	0	Myeloid	WIPF1
0	4.96919822967128	0.591	0.079	0	Myeloid	DAB2
0	7.3593895742969	0.525	0.016	0	Myeloid	GPNMB
0	2.12816522462792	0.662	0.154	0	Myeloid	VIM
0	4.29149429559243	0.536	0.029	0	Myeloid	KLHL6
0	3.44852560852595	0.536	0.036	0	Myeloid	METRNL
0	3.70663486755403	0.567	0.068	0	Myeloid	TGFBI
0	3.88634466617651	0.532	0.034	0	Myeloid	DOK2
0	2.6596103458241	0.556	0.06	0	Myeloid	CD53
0	3.74846147067678	0.537	0.041	0	Myeloid	RIN3
0	7.88210128060507	0.505	0.011	0	Myeloid	FOLR2
0	3.73147867434855	0.698	0.208	0	Myeloid	KCTD12
0	7.51951130660664	0.495	0.005	0	Myeloid	FPR3
0	7.04890487573311	0.509	0.022	0	Myeloid	FMN1
0	3.59313697510874	0.533	0.048	0	Myeloid	PLEKHO1
0	6.29348833489895	0.506	0.024	0	Myeloid	CPVL
0	5.46533397336239	0.503	0.022	0	Myeloid	FCGR3A
0	3.17330087702134	0.657	0.177	0	Myeloid	CD14
0	7.55814182966959	0.489	0.009	0	Myeloid	MSR1
0	7.35633739813341	0.481	0.007	0	Myeloid	SLAMF8
0	3.62203770957012	0.496	0.022	0	Myeloid	CD93
0	4.59666592143731	0.495	0.023	0	Myeloid	PLTP
0	5.48046869061464	0.48	0.009	0	Myeloid	CD300A
0	4.38843099776388	0.574	0.105	0	Myeloid	CIITA
0	7.00480195118528	0.481	0.013	0	Myeloid	MS4A4A
0	5.05245314690677	0.743	0.275	0	Myeloid	LGMN
0	6.02235411659567	0.477	0.01	0	Myeloid	CLEC7A
0	4.34387274089092	0.487	0.023	0	Myeloid	JAML
0	5.19855720752357	0.514	0.053	0	Myeloid	TNFSF13
0	2.88975551592714	0.532	0.072	0	Myeloid	LSP1
0	3.11828821214262	0.509	0.05	0	Myeloid	LCP2
0	4.88154611175637	0.518	0.059	0	Myeloid	SLC15A3
0	5.36986183168758	0.533	0.074	0	Myeloid	RNASE1
0	3.65172423726861	0.487	0.028	0	Myeloid	MYO1F
0	6.08885206542596	0.465	0.013	0	Myeloid	C3AR1
0	6.50454552765255	0.458	0.006	0	Myeloid	HCK
0	4.00015312415298	0.477	0.025	0	Myeloid	HAVCR2
0	6.70382627065809	0.463	0.012	0	Myeloid	IGSF6
0	8.0504404566431	0.456	0.007	0	Myeloid	CD209
0	6.42418340664259	0.472	0.024	0	Myeloid	TLR2
0	5.02531308749926	0.464	0.018	0	Myeloid	PLXDC2
0	3.93553695849253	0.472	0.026	0	Myeloid	PLCB2
0	5.01166937425489	0.456	0.016	0	Myeloid	GPR183
0	2.26202099607407	0.528	0.089	0	Myeloid	ARHGDIB
0	5.78136398240326	0.467	0.029	0	Myeloid	ADAP2
0	2.30082607265286	0.488	0.052	0	Myeloid	SLA
0	5.69375902023318	0.445	0.009	0	Myeloid	AXL
0	3.02885341031896	0.477	0.042	0	Myeloid	UCP2
0	2.96162293917313	0.472	0.038	0	Myeloid	IL10RA
0	3.16097826358935	0.62	0.187	0	Myeloid	IRF8
0	3.41516741505383	0.463	0.031	0	Myeloid	NCKAP1L
0	2.8101419599439	0.531	0.103	0	Myeloid	ARHGAP4
0	5.16584710132456	0.441	0.013	0	Myeloid	PLEK
0	2.89901334747995	0.464	0.041	0	Myeloid	DOCK2
0	4.21225466448998	0.469	0.046	0	Myeloid	CAPG
0	5.75235230494836	0.434	0.011	0	Myeloid	RNASE6
0	3.36722269857512	0.722	0.3	0	Myeloid	MFSD1
0	8.12623299052739	0.424	0.003	0	Myeloid	SIGLEC1
0	7.40708829564295	0.422	0.004	0	Myeloid	LRRC25
0	1.97231849540454	0.519	0.102	0	Myeloid	TRIM22
0	2.75758884887708	0.466	0.05	0	Myeloid	CHST11
0	4.27266282362053	0.522	0.109	0	Myeloid	SDC3

0	2.80785742552518	0.457	0.044	0	Myeloid	CMTM3
0	5.42803613998476	0.427	0.014	0	Myeloid	SLC8A1
0	3.82558442851124	0.431	0.023	0	Myeloid	PARVG
0	2.33802338720184	0.454	0.048	0	Myeloid	ITGA4
0	4.77574039876444	0.424	0.02	0	Myeloid	NCF2
0	3.65584414896558	0.735	0.332	0	Myeloid	CD68
0	4.40882300617776	0.417	0.015	0	Myeloid	AOAH
0	3.65660357055493	0.855	0.453	0	Myeloid	PSAP
0	3.85448897278586	0.553	0.154	0	Myeloid	IER5
0	3.71109498183643	0.517	0.118	0	Myeloid	ATP6V1B2
0	4.64432766977595	0.428	0.031	0	Myeloid	MERTK
0	3.83168962082814	0.418	0.021	0	Myeloid	CD84
0	3.31388785194323	0.432	0.036	0	Myeloid	ANXA1
0	3.06837970749863	0.433	0.037	0	Myeloid	LPAR6
0	1.90237491062985	0.478	0.083	0	Myeloid	IFI16
0	3.14383743146319	0.424	0.03	0	Myeloid	DENND3
0	0.949808461852685	0.447	0.054	0	Myeloid	ENG
0	2.67657898187004	0.456	0.064	0	Myeloid	SH3BP5
0	4.38807868023752	0.438	0.046	0	Myeloid	SYK
0	6.45231191922222	0.407	0.015	0	Myeloid	C5AR1
0	4.46464163269952	0.438	0.047	0	Myeloid	RGL1
0	7.56438033276735	0.394	0.004	0	Myeloid	SLC1A3
0	5.12805274583644	0.4	0.011	0	Myeloid	OGFRL1
0	5.3829969473142	0.411	0.023	0	Myeloid	PLAUR
0	5.30902909080555	0.41	0.023	0	Myeloid	SLC7A7
0	3.92471419532404	0.418	0.032	0	Myeloid	AKR1B1
0	7.1065264184797	0.392	0.008	0	Myeloid	DEPRECATED-ENSG00000110079
0	2.43228849371805	0.784	0.401	0	Myeloid	JUNB
0	3.89124941520719	0.556	0.174	0	Myeloid	SLCO2B1
0	4.29402020221283	0.399	0.018	0	Myeloid	CYTH4
0	2.67264302644919	0.503	0.123	0	Myeloid	PLXND1
0	2.1887523284002	0.42	0.041	0	Myeloid	GPSM3
0	4.19463269602633	0.435	0.058	0	Myeloid	RNF144B
0	4.08618787719081	0.569	0.192	0	Myeloid	HMOX1
0	3.01850453292088	0.418	0.042	0	Myeloid	RCSD1
0	2.92708964979919	0.534	0.158	0	Myeloid	ARL4C
0	1.89121013533159	0.452	0.076	0	Myeloid	FXYD5
0	3.43711405975768	0.856	0.48	0	Myeloid	CTSZ
0	2.8656046442059	0.654	0.281	0	Myeloid	SAMHD1
0	3.4314234428028	0.4	0.028	0	Myeloid	PLEKHO2
0	2.86739379076003	0.551	0.181	0	Myeloid	CXCL16
0	2.34185314607867	0.472	0.102	0	Myeloid	TNFRSF1B
0	3.27332540333715	0.877	0.507	0	Myeloid	SAT1
0	3.75675217093071	0.393	0.024	0	Myeloid	ACP5
0	3.11880271222269	0.836	0.468	0	Myeloid	IFI30
0	6.95140596964399	0.373	0.006	0	Myeloid	VSIG4
0	2.72900260619007	0.399	0.033	0	Myeloid	PECAM1
0	3.74340714793279	0.423	0.059	0	Myeloid	IL18BP
0	4.8582786205801	0.428	0.065	0	Myeloid	CD83
0	4.44807647395533	0.392	0.03	0	Myeloid	OTULINL
0	3.03045780226071	0.431	0.069	0	Myeloid	FERMT3
0	4.2029753833213	0.376	0.016	0	Myeloid	TRPS1
0	6.23495342472551	0.366	0.006	0	Myeloid	TM6SF1
0	4.03216205075264	0.395	0.035	0	Myeloid	TMEM106A
0	5.34333362585975	0.369	0.01	0	Myeloid	TBXAS1
0	4.67023171608878	0.375	0.017	0	Myeloid	DEPRECATED-ENSG00000119900
0	2.74234446200569	0.536	0.179	0	Myeloid	ARRB2
0	3.37922605914246	0.469	0.112	0	Myeloid	PPT1
0	1.83882917633876	0.5	0.143	0	Myeloid	CD44
0	2.78561294656802	0.578	0.223	0	Myeloid	GNA13
0	5.67323895471826	0.359	0.005	0	Myeloid	EPB41L3
0	7.11028436190323	0.356	0.003	0	Myeloid	TFEC
0	2.24296183006128	0.389	0.037	0	Myeloid	SELPLG

0	7.35148098934733	0.36	0.008	0	Myeloid	GPR34
0	3.39304408378349	0.374	0.023	0	Myeloid	ADCY7
0	3.41022436528424	0.382	0.031	0	Myeloid	SLC16A3
0	2.96498931138404	0.672	0.322	0	Myeloid	CTSC
0	2.35218471812025	0.427	0.077	0	Myeloid	CD81
0	3.04440242403654	0.47	0.121	0	Myeloid	DAPK1
0	3.21452961167919	0.38	0.033	0	Myeloid	COTL1
0	2.8511857603557	0.753	0.406	0	Myeloid	FCGRT
0	1.99676454054782	0.412	0.065	0	Myeloid	PIK3IP1
0	2.9686980735653	0.387	0.042	0	Myeloid	SH3BGRL
0	2.76279947663365	0.59	0.245	0	Myeloid	MAN2B1
0	7.4426508856436	0.347	0.002	0	Myeloid	LILRB5
0	4.79789114008662	0.354	0.009	0	Myeloid	RENBP
0	3.41838907746619	0.362	0.018	0	Myeloid	PIK3R5
0	7.79636696219106	0.346	0.003	0	Myeloid	PTAFR
0	3.24543417878109	0.418	0.075	0	Myeloid	NABP1
0	3.86885835847628	0.373	0.031	0	Myeloid	SPINT2
0	3.32249430563768	0.372	0.031	0	Myeloid	GSTP1
0	3.39978383207989	0.364	0.025	0	Myeloid	FAR1
0	2.79155416169462	0.37	0.031	0	Myeloid	DOCK11
0	7.09460434584356	0.341	0.003	0	Myeloid	CMKLR1
0	5.6061380616389	0.35	0.012	0	Myeloid	PLAU
0	6.77314161245239	0.34	0.003	0	Myeloid	CD86
0	2.89915993324687	0.417	0.08	0	Myeloid	MICAL1
0	2.72236260008876	0.431	0.094	0	Myeloid	LGALS3
0	2.83341484144189	0.392	0.056	0	Myeloid	KCNAB2
0	5.56859091779806	0.341	0.006	0	Myeloid	MARCH1
0	7.28257459888104	0.337	0.003	0	Myeloid	LILRB2
0	4.75741569664943	0.348	0.014	0	Myeloid	NFKBID
0	4.18845543351033	0.399	0.066	0	Myeloid	ENPP2
0	5.71266905985991	0.341	0.008	0	Myeloid	ZNF385A
0	5.21094338144602	0.34	0.008	0	Myeloid	RAB31
0	3.81489330326278	0.894	0.562	0	Myeloid	CTSB
0	2.20675841236213	0.377	0.049	0	Myeloid	FYB1
0	7.79985951110671	0.33	0.005	0	Myeloid	CD163L1
0	2.27698746695629	0.593	0.268	0	Myeloid	TYMP
0	2.14948158171678	0.681	0.357	0	Myeloid	TMSB10
0	4.48743121751309	0.335	0.012	0	Myeloid	FMNL2
0	3.57034200782204	0.354	0.033	0	Myeloid	MAP3K8
0	2.98023954925255	0.372	0.052	0	Myeloid	PTGER4
0	6.65918124138635	0.326	0.006	0	Myeloid	SLC37A2
0	3.22812275716999	0.548	0.229	0	Myeloid	LIPA
0	6.24233957646558	0.33	0.011	0	Myeloid	CLEC10A
0	3.84467829929295	0.377	0.059	0	Myeloid	PDGFC
0	2.02086379625768	0.359	0.041	0	Myeloid	GIMAP4
0	3.72743179598995	0.347	0.029	0	Myeloid	DSE
0	3.04169420864841	0.498	0.181	0	Myeloid	TNFAIP2
0	2.65767673559152	0.542	0.227	0	Myeloid	NAGK
0	3.29491152987926	0.397	0.082	0	Myeloid	ARHGAP18
0	2.42486965946349	0.419	0.105	0	Myeloid	SEC14L1
0	4.65799446612751	0.322	0.009	0	Myeloid	BASP1
0	5.12355452732822	0.331	0.018	0	Myeloid	DNAJA4
0	4.500976307615	0.322	0.009	0	Myeloid	CSF2RB
0	2.62624239719059	0.556	0.244	0	Myeloid	MARCKS
0	2.75149066761175	0.408	0.096	0	Myeloid	REL
0	2.79969735112726	0.466	0.155	0	Myeloid	SIRPA
0	2.98186658527426	0.463	0.153	0	Myeloid	TTYH3
0	2.32410038486249	0.787	0.478	0	Myeloid	LITAF
0	5.24714447810304	0.314	0.005	0	Myeloid	ALOX5
0	4.61916885890009	0.314	0.006	0	Myeloid	WDFY4
0	6.37410935005153	0.312	0.004	0	Myeloid	MS4A4E
0	3.30698262123895	0.447	0.139	0	Myeloid	MCOLN1
0	3.19070507147635	0.331	0.023	0	Myeloid	WAS

0	5.32021224145007	0.315	0.008	0	Myeloid	TLR4
0	3.46460600797005	0.335	0.029	0	Myeloid	S100A11
0	6.78422921242007	0.311	0.005	0	Myeloid	IL18
0	2.31969678272019	0.34	0.035	0	Myeloid	DOCK8
0	3.53482226352941	0.32	0.015	0	Myeloid	PMP22
0	1.94303125484415	0.354	0.049	0	Myeloid	ARHGAP45
0	4.59260345685237	0.316	0.011	0	Myeloid	NCF4
0	3.75710631472055	0.335	0.031	0	Myeloid	VCAM1
0	4.51645723847543	0.313	0.009	0	Myeloid	IRAK3
0	2.5127575650076	0.331	0.03	0	Myeloid	INPP5D
0	2.70503668084588	0.334	0.034	0	Myeloid	SFMBT2
0	4.68596160766637	0.308	0.008	0	Myeloid	GAS7
0	2.56891216295253	0.368	0.07	0	Myeloid	IGFLR1
0	3.891403762434	0.316	0.019	0	Myeloid	DEPRECATED-ENSG00000163191
0	2.55341407144583	0.358	0.065	0	Myeloid	ARHGEF2
0	5.4918527899967	0.31	0.017	0	Myeloid	KCNMA1
0	3.87049180986831	0.309	0.016	0	Myeloid	APOBR
0	7.60056314459793	0.294	0.002	0	Myeloid	IGSF21
0	2.29962762323762	0.36	0.068	0	Myeloid	CNTRL
0	2.02817583893335	0.332	0.041	0	Myeloid	FMNL1
0	6.38156619803904	0.303	0.012	0	Myeloid	PLA2G7
0	2.96785937181782	0.316	0.025	0	Myeloid	GLIPR1
0	2.87940082434528	0.321	0.031	0	Myeloid	ST8SIA4
0	2.41491993895429	0.325	0.036	0	Myeloid	CLEC2B
0	2.91016900383339	0.308	0.019	0	Myeloid	GIMAP5
0	7.51904298932151	0.295	0.006	0	Myeloid	MMP9
0	5.45173724080467	0.293	0.004	0	Myeloid	TRPM2
0	2.3551313309017	0.321	0.033	0	Myeloid	ARHGAP30
0	4.69047168142255	0.296	0.008	0	Myeloid	FGR
0	2.10674128915617	0.345	0.057	0	Myeloid	SERPINB9
0	3.03811034281375	0.379	0.092	0	Myeloid	RAP2B
0	4.86063436616369	0.32	0.034	0	Myeloid	NR4A2
0	4.01334956840511	0.302	0.016	0	Myeloid	RGS10
0	5.35971039356676	0.292	0.006	0	Myeloid	IL4I1
0	7.84263602734468	0.286	0.001	0	Myeloid	HK3
0	3.35065071292523	0.617	0.332	0	Myeloid	PLD3
0	1.21327337785821	0.359	0.075	0	Myeloid	CYTIP
0	6.5112028325414	0.287	0.005	0	Myeloid	DEPRECATED-ENSG00000079215
0	3.1648968958079	0.296	0.015	0	Myeloid	NRP2
0	3.6023013183778	0.344	0.063	0	Myeloid	SLC31A2
0	3.51688541876488	0.298	0.017	0	Myeloid	ENTPD1
0	2.42545835180418	0.364	0.084	0	Myeloid	DOCK4
0	3.37893069484941	0.344	0.065	0	Myeloid	NPL
0	2.17976706701681	0.742	0.463	0	Myeloid	TPP1
0	2.92739019372269	0.374	0.096	0	Myeloid	ITGAM
0	2.48677595334898	0.386	0.109	0	Myeloid	SESN1
0	4.04091103662936	0.289	0.012	0	Myeloid	GNB4
0	3.68862679432819	0.305	0.028	0	Myeloid	RELT
0	2.69013848427261	0.729	0.452	0	Myeloid	GRN
0	3.80963831022288	0.291	0.014	0	Myeloid	ABI3
0	3.68346058609863	0.32	0.044	0	Myeloid	SLC43A2
0	4.63189715843827	0.285	0.009	0	Myeloid	NRROS
0	6.1613906912629	0.278	0.004	0	Myeloid	PHACTR1
0	5.59169126421851	0.281	0.007	0	Myeloid	PLBD1
0	4.15504354509204	0.282	0.008	0	Myeloid	PALD1
0	3.32102639143167	0.29	0.016	0	Myeloid	TNFSF12
0	8.10420799557858	0.275	0.002	0	Myeloid	GFRA2
0	3.77762103548661	0.307	0.035	0	Myeloid	ETV5
0	1.85363653536267	0.308	0.037	0	Myeloid	ARHGAP9
0	2.22251992560064	0.303	0.032	0	Myeloid	EVI2B
0	4.02479834345731	0.282	0.012	0	Myeloid	GIMAP8
0	4.69035182884702	0.275	0.006	0	Myeloid	RAB3IL1
0	3.69258921544867	0.282	0.014	0	Myeloid	VASH1

0	3.01515135204875	0.277	0.013	0	Myeloid	PDGFB
0	1.01687075994777	0.322	0.059	0	Myeloid	IKZF1
0	4.00352801548918	0.275	0.014	0	Myeloid	LAT2
0	2.9440641113865	0.305	0.044	0	Myeloid	PLEKHA2
0	2.79464794366374	0.338	0.077	0	Myeloid	VSIR
0	2.27267288624007	0.294	0.034	0	Myeloid	IL16
0	2.68684393506117	0.328	0.069	0	Myeloid	FKBP15
0	6.32187831923483	0.261	0.002	0	Myeloid	SIGLEC10
0	3.97737608647668	0.303	0.045	0	Myeloid	RTN1
0	2.04784996735063	0.296	0.04	0	Myeloid	LIMD2
0	2.5390909952848	0.283	0.027	0	Myeloid	SAMSN1
0	2.42479348620663	0.326	0.071	0	Myeloid	ADAMTSL4
0	5.92965771218164	0.261	0.006	0	Myeloid	MNDA
0	2.82053218501224	0.324	0.069	0	Myeloid	SH3PXD2B
0	5.30183148512204	0.26	0.005	0	Myeloid	LY86
0	3.89779098695538	0.27	0.015	0	Myeloid	IL2RA
0	1.63203455146279	0.294	0.039	0	Myeloid	CD37
0	5.61274724470339	0.26	0.006	0	Myeloid	CCR1
0	4.54671671095697	0.26	0.006	0	Myeloid	LRRK1
0	2.78452424875541	0.317	0.063	0	Myeloid	RHBDF2
0	1.28516563053028	0.301	0.048	0	Myeloid	PTPRC
0	6.78805310422335	0.258	0.005	0	Myeloid	P2RY13
0	3.44251649870974	0.273	0.021	0	Myeloid	MYO5A
0	3.69316932479099	0.262	0.011	0	Myeloid	TMEM273
0	3.09485997585556	0.265	0.014	0	Myeloid	RASSF2
0	6.44136554893467	0.254	0.004	0	Myeloid	PILRA
0	5.15895403857712	0.256	0.006	0	Myeloid	CD72
0	3.20855265335311	0.277	0.027	0	Myeloid	RILPL2
0	2.90378822665864	0.315	0.067	0	Myeloid	CLN8
0	7.17140312667977	0.249	0.002	0	Myeloid	CD300LF
0	4.56707213172129	0.257	0.012	0	Myeloid	AMPD3
0	5.0030011959729	0.255	0.011	0	Myeloid	LST1
0	6.52771523117161	0.246	0.002	0	Myeloid	ADGRE2
0	4.55601137194787	0.279	0.036	0	Myeloid	KLF4
0	1.89223971501083	0.299	0.056	0	Myeloid	PRDM1
0	4.85452590594541	0.252	0.01	0	Myeloid	TMEM86A
0	6.71889124059123	0.248	0.006	0	Myeloid	COLEC12
0	3.83331309206453	0.268	0.03	0	Myeloid	P2RX7
0	7.60181306317132	0.238	0.001	0	Myeloid	ADORA3
0	2.46160834167365	0.258	0.022	0	Myeloid	PRKCB
0	2.35985381201204	0.254	0.018	0	Myeloid	DEPRECATED-ENSG00000196329
0	3.80148407219151	0.245	0.01	0	Myeloid	GASK1B
0	3.43307511592073	0.245	0.01	0	Myeloid	PTGS1
0	4.2694557117411	0.242	0.008	0	Myeloid	FCGR2B
0	5.6201682063193	0.237	0.004	0	Myeloid	EBI3
0	2.44500311155914	0.265	0.033	0	Myeloid	TAGAP
0	2.47508766193948	0.254	0.023	0	Myeloid	EVI2A
0	2.57521647353522	0.249	0.019	0	Myeloid	TRPV2
0	4.37147874351329	0.238	0.009	0	Myeloid	ATP8B4
0	1.9585472547478	0.259	0.031	0	Myeloid	ARHGAP15
0	3.55793891034956	0.252	0.024	0	Myeloid	ZNF331
0	5.27486966020779	0.233	0.006	0	Myeloid	BCAT1
0	3.21635748556567	0.244	0.018	0	Myeloid	DISC1
0	5.12822876594242	0.232	0.006	0	Myeloid	SCIMP
0	3.76755978843176	0.279	0.054	0	Myeloid	NR4A1
0	3.57431658532137	0.249	0.024	0	Myeloid	STX11
0	3.64228676417858	0.235	0.01	0	Myeloid	MEF2C
0	4.18258773693428	0.261	0.037	0	Myeloid	PTPRE
0	4.78215759124199	0.237	0.013	0	Myeloid	C1orf162
0	3.35485995213249	0.236	0.012	0	Myeloid	THBD
0	6.34511618230933	0.226	0.003	0	Myeloid	FPR1
0	2.12692626030275	0.923	0.701	0	Myeloid	TMSB4X
0	6.19494787659459	0.224	0.003	0	Myeloid	MILR1

0	3.80994716740654	0.238	0.017	0	Myeloid	SLC9A9
0	6.83300297046045	0.223	0.003	0	Myeloid	ANKRD22
0	5.70308200412745	0.223	0.003	0	Myeloid	P2RY6
0	3.25637875072303	0.231	0.011	0	Myeloid	DEPRECATED-ENSG00000118263
0	2.49236673958763	0.241	0.023	0	Myeloid	FLI1
0	1.61709535708163	0.255	0.037	0	Myeloid	SASH3
0	3.60213466751808	0.226	0.009	0	Myeloid	KLF7
0	5.61400910024837	0.225	0.008	0	Myeloid	NR4A3
0	2.43618113722958	0.826	0.609	0	Myeloid	CST3
0	2.96067005879491	0.249	0.033	0	Myeloid	SUSD1
0	3.48797746381944	0.227	0.011	0	Myeloid	BEX4
0	4.82065032513431	0.219	0.004	0	Myeloid	LILRB1
0	2.06411604116063	0.246	0.032	0	Myeloid	GIMAP6
0	1.85925367689654	0.239	0.025	0	Myeloid	FGD3
0	5.30638327728028	0.215	0.002	0	Myeloid	CSF3R
0	2.2571055000641	0.237	0.024	0	Myeloid	HCST
0	2.53328064428169	0.232	0.021	0	Myeloid	TCF4
0	3.93647329569087	0.22	0.009	0	Myeloid	DEPRECATED-ENSG00000072694
0	4.68095665798341	0.215	0.005	0	Myeloid	OLFML2B
0	2.93728657689986	0.222	0.012	0	Myeloid	LMO2
0	4.87191330060154	0.219	0.009	0	Myeloid	RNF135
0	2.99622080328107	0.225	0.016	0	Myeloid	UAP1L1
0	2.36950785848593	0.244	0.035	0	Myeloid	CYBRD1
0	3.26507091924615	0.248	0.039	0	Myeloid	GAB2
0	3.52074843608104	0.22	0.012	0	Myeloid	VAV1
0	3.0239698623977	0.23	0.023	0	Myeloid	SAMD9L
0	6.22544626347151	0.211	0.004	0	Myeloid	CFD
0	5.95002166820186	0.209	0.002	0	Myeloid	CD33
0	3.69236339363865	0.219	0.013	0	Myeloid	LY96
0	3.28295415418484	0.22	0.014	0	Myeloid	YBX3
0	2.64163031328023	0.235	0.029	0	Myeloid	FMNL3
0	7.35975805366484	0.209	0.004	0	Myeloid	TREM2
0	2.63150458347937	0.243	0.038	0	Myeloid	RGS19
0	1.51620307296533	0.242	0.038	0	Myeloid	DEF6
0	3.7478662148176	0.243	0.039	0	Myeloid	SPRED1
0	1.5096036882671	0.857	0.654	0	Myeloid	GLUL
0	3.12869645048435	0.217	0.015	0	Myeloid	SLFN11
0	2.21656539708547	0.225	0.025	0	Myeloid	PIK3CD
0	3.1618557043485	0.222	0.022	0	Myeloid	GRASP
0	3.51020859649925	0.219	0.019	0	Myeloid	HOMER3
0	2.46000346036154	0.217	0.018	0	Myeloid	PPM1M
0	3.04801353763077	0.223	0.024	0	Myeloid	IGF1
0	6.45367466300832	0.201	0.002	0	Myeloid	SIGLEC9
0	5.06232407140686	0.204	0.005	0	Myeloid	MCTP1
0	6.28880561124579	0.199	0.004	0	Myeloid	NFAM1
0	3.75552877780929	0.205	0.01	0	Myeloid	ARHGAP31
0	3.53803465344194	0.205	0.01	0	Myeloid	PARVB
0	6.02641208581458	0.196	0.003	0	Myeloid	CR1
0	5.76075526169421	0.193	0.002	0	Myeloid	LOXL3
0	2.06964687110255	0.214	0.023	0	Myeloid	BIN2
0	2.51504934508057	0.212	0.021	0	Myeloid	ALOX5AP
0	4.25140781988695	0.195	0.006	0	Myeloid	GNA15
0	2.9174224436167	0.199	0.014	0	Myeloid	SLC25A24
0	2.15365112091956	0.201	0.016	0	Myeloid	ZC3H12D
0	1.29164846786947	0.211	0.026	0	Myeloid	CXCL12
0	4.49372235137072	0.186	0.003	0	Myeloid	BTK
0	2.3156074741009	0.197	0.015	0	Myeloid	SNX20
0	3.98643416841564	0.199	0.018	0	Myeloid	ALDH3B1
0	5.06632743377184	0.19	0.009	0	Myeloid	CCL4L2
0	3.14894378190587	0.203	0.023	0	Myeloid	GAB3
0	1.84442034028643	0.202	0.023	0	Myeloid	RASA3
0	1.34584179145335	0.203	0.024	0	Myeloid	DENND1C
0	2.27631957868361	0.205	0.026	0	Myeloid	SLC2A9

0	4.85162044708369	0.185	0.007	0	Myeloid	SEMA4A
0	2.50641076950386	0.192	0.014	0	Myeloid	GIMAP1
0	4.16617284553512	0.185	0.007	0	Myeloid	ADAM28
0	3.51699031954644	0.197	0.021	0	Myeloid	DSC2
0	4.5936154597523	0.179	0.003	0	Myeloid	DPEP2
0	2.2361545318979	0.198	0.023	0	Myeloid	GYPC
0	6.34444098942656	0.179	0.006	0	Myeloid	S100B
0	3.11766007040769	0.183	0.011	0	Myeloid	MYOF
0	3.291939865361	0.177	0.005	0	Myeloid	MARVELD1
0	1.95010478616076	0.19	0.019	0	Myeloid	ITGA9
0	5.09739654656076	0.176	0.005	0	Myeloid	PDCD1LG2
0	3.61811284033242	0.173	0.005	0	Myeloid	HVCN1
0	3.65797179600599	0.181	0.014	0	Myeloid	SECTM1
0	5.04428935341376	0.169	0.003	0	Myeloid	SLC7A8
0	3.74727035527333	0.177	0.011	0	Myeloid	C1orf54
0	2.29007496183917	0.181	0.015	0	Myeloid	GPR132
0	3.42757857315565	0.173	0.007	0	Myeloid	TANC2
0	3.45365890289911	0.173	0.008	0	Myeloid	PAQR8
0	4.54423663014423	0.167	0.003	0	Myeloid	KCNQ1
0	2.22374236522105	0.181	0.017	0	Myeloid	DYSF
0	3.40519673172249	0.174	0.011	0	Myeloid	PLAGL1
0	4.51833451638526	0.179	0.016	0	Myeloid	TTYH2
0	4.34085186899831	0.166	0.004	0	Myeloid	TNFAIP8L2
0	2.66508159493252	0.173	0.012	0	Myeloid	SLC25A36
0	5.43646006276938	0.162	0.002	0	Myeloid	GBGT1
0	4.2811035789216	0.171	0.012	0	Myeloid	HRH2
0	4.50371036873874	0.163	0.004	0	Myeloid	DOK3
0	4.03827795453783	0.988	0.83	0	Myeloid	CD74
0	2.87500834226809	0.167	0.009	0	Myeloid	KCNK6
0	5.65120822352327	0.164	0.007	0	Myeloid	CDCP1
0	4.0768281110548	0.162	0.006	0	Myeloid	SPIRE1
0	4.14182599731553	0.16	0.005	0	Myeloid	CD80
0	2.77432116370849	0.169	0.014	0	Myeloid	SLC49A3
0	6.86327251160698	0.16	0.005	0	Myeloid	CCL13
0	5.98039763067014	0.155	0.001	0	Myeloid	GPR141
0	6.23143217588243	0.156	0.002	0	Myeloid	KCNJ5
0	3.23473070293745	0.168	0.015	0	Myeloid	MTHFD2
0	3.67583058375414	0.16	0.007	0	Myeloid	SCRN1
0	6.20638864872803	0.154	0.001	0	Myeloid	SIGLEC7
0	4.09285478749741	0.158	0.007	0	Myeloid	DOK1
0	2.42724044834271	0.164	0.014	0	Myeloid	PIK3CG
0	3.77913543933991	0.165	0.016	0	Myeloid	MITF
0	5.70016008029475	0.152	0.003	0	Myeloid	CXorf21
0	4.05084418158428	0.155	0.008	0	Myeloid	HDAC9
0	3.13969750862882	0.158	0.012	0	Myeloid	GCNT1
0	7.49640524658622	0.147	0.001	0	Myeloid	RAB39A
0	2.63560623461479	0.154	0.009	0	Myeloid	SPART
0	5.08466236721837	0.146	0.002	0	Myeloid	NLRP3
0	2.97458104503763	0.152	0.008	0	Myeloid	CD200R1
0	6.06163837178695	0.144	0.002	0	Myeloid	FCGR1A
0	3.52842579591928	0.15	0.008	0	Myeloid	DAPP1
0	4.56390570138564	0.143	0.003	0	Myeloid	KCNE3
0	3.24316000105686	0.147	0.007	0	Myeloid	CTAG2
0	5.947443600789	0.141	0.002	0	Myeloid	SIRPB2
0	8.03215002289451	0.139	0.001	0	Myeloid	OSCAR
0	6.004606927219	0.138	0.002	0	Myeloid	DPEP3
0	3.05134617511054	0.144	0.009	0	Myeloid	SCN9A
0	3.18271282772366	0.145	0.01	0	Myeloid	PDE4A
0	6.62764387253622	0.136	0.002	0	Myeloid	PTPRO
0	4.92383552281932	0.135	0.003	0	Myeloid	HPGDS
0	5.9857744629133	0.134	0.002	0	Myeloid	TMEM236
0	3.43805443633463	0.14	0.008	0	Myeloid	LRMP
0	6.24932217721397	0.135	0.003	0	Myeloid	MMP19

0	7.98311321013754	0.129	0.001	0	Myeloid	LILRB4
0	3.95556419284618	0.129	0.002	0	Myeloid	B3GNT7
0	6.67585510807712	0.128	0.001	0	Myeloid	CARD9
0	4.14038478676427	0.131	0.004	0	Myeloid	DEPRECATED-ENSG00000176788
0	4.72066681097183	0.131	0.005	0	Myeloid	ACOT11
0	4.6793607559801	0.135	0.011	0	Myeloid	FABP3
0	2.11669886367366	0.134	0.01	0	Myeloid	TGFB1
0	3.15230482079383	0.132	0.008	0	Myeloid	RPP25
0	5.54664784011446	0.124	0.001	0	Myeloid	GAL3ST4
0	4.8247410957228	0.126	0.003	0	Myeloid	PIK3R6
0	4.6706921671388	0.13	0.008	0	Myeloid	CCL3
0	4.96378391936713	0.126	0.007	0	Myeloid	CCRL2
0	5.43388395643992	0.121	0.002	0	Myeloid	PLD4
0	5.94542772394945	0.12	0.003	0	Myeloid	DHRS9
0	4.64567185794721	0.122	0.007	0	Myeloid	BEST1
0	4.51072947763787	0.117	0.003	0	Myeloid	CREB5
0	5.06398522696145	0.117	0.003	0	Myeloid	CEP295NL
0	5.76113040357702	0.116	0.002	0	Myeloid	TLR7
0	6.78431511549218	0.113	0.002	0	Myeloid	CD1C
0	6.86382125017747	0.112	0.004	0	Myeloid	CD5L
0	3.30844751220259	0.111	0.006	0	Myeloid	ARHGAP10
0	6.39359440492635	0.11	0.005	0	Myeloid	MMP12
0	7.55728256877575	0.105	0.001	0	Myeloid	CD1E
0	5.12933993749214	0.105	0.002	0	Myeloid	NCKAP5
0	5.62337814006978	0.104	0.001	0	Myeloid	SIGLEC5
0	3.68757648626666	0.106	0.004	0	Myeloid	SPECC1
0	5.09482366987662	0.104	0.003	0	Myeloid	RGS18
0	6.61075537642077	0.101	0.001	0	Myeloid	TLR8
0	5.48558255840699	0.102	0.002	0	Myeloid	CD180
0	6.7391726525838	0.1	0.001	0	Myeloid	NLRC4
0	5.93986382296358	0.102	0.003	0	Myeloid	HRH1
1.19258583486918E-307	2.99900854076928	0.128	0.01	1.99853534207377E-303	Myeloid	MRC2
1.04268488359974E-304	2.00142315236832	0.822	0.618	1.74733132793644E-300	Myeloid	DUSP1
1.12399881551532E-304	1.20452822864381	0.209	0.029	1.88359721504057E-300	Myeloid	GIMAP7
8.36122577117961E-304	5.03942426233874	0.106	0.006	1.40117421473428E-299	Myeloid	FOSB
1.74649765031001E-303	2.71098857591311	0.413	0.127	2.92678076238951E-299	Myeloid	RNASET2
5.42198049493946E-303	1.67965266526187	0.385	0.102	9.08615491341954E-299	Myeloid	TSPAN14
8.67684097765452E-303	2.70631432409774	0.318	0.074	1.45406501103535E-298	Myeloid	WDR81
1.26762401037964E-302	2.71760540855384	0.296	0.063	2.1242843165942E-298	Myeloid	CASP1
5.8364371009118E-302	2.36897666170118	0.166	0.018	9.780701293708E-298	Myeloid	ARHGAP25
4.8106871888072E-301	1.83037387458574	0.336	0.078	8.06174959100311E-297	Myeloid	ABCC1
7.57864977748361E-300	2.67628667155171	0.205	0.03	1.2700301297107E-295	Myeloid	HSD17B14
2.88315745987488E-297	1.19580156463436	0.298	0.059	4.83159527125833E-293	Myeloid	CORO1A
1.89179219372724E-295	2.52156267498726	0.499	0.184	3.1702653582481E-291	Myeloid	ARAP1
2.5265028558901E-295	2.39047421757349	0.443	0.148	4.23391348590063E-291	Myeloid	RHOG
2.83859927183292E-294	2.29029367654352	0.256	0.048	4.75692465973761E-290	Myeloid	GMIP
3.41631395188772E-293	2.56573631965422	0.329	0.079	5.72505892057345E-289	Myeloid	RCN3
3.72968184069516E-293	2.26959966886667	0.213	0.033	6.25020082863695E-289	Myeloid	SCARF1
8.21740253923408E-292	4.50104180255707	0.107	0.007	1.37707231752485E-287	Myeloid	CFP
4.11669613405153E-291	2.88669926519421	0.108	0.007	6.89875938144356E-287	Myeloid	PLXDC1
3.23595589412138E-290	2.91343533862872	0.398	0.125	5.42281488736862E-286	Myeloid	ASAH1
3.37129757696212E-290	2.68630588179872	0.401	0.121	5.64962047947312E-286	Myeloid	PRNP
8.66622101619645E-287	2.57687563011905	0.26	0.051	1.4522853178942E-282	Myeloid	RASA4
3.14556603342924E-284	2.59365259676509	0.453	0.164	5.27133955882073E-280	Myeloid	DEPRECATED-ENSG00000026297
4.95231439489828E-284	2.07759401117661	0.148	0.015	8.29908846297054E-280	Myeloid	IL12RB1
1.02669824016638E-282	3.61396630927141	0.129	0.011	1.72054091087082E-278	Myeloid	LPAR5
1.20442892828825E-282	2.43615592773492	0.144	0.015	2.01838199802545E-278	Myeloid	FAM78A
1.59931246976515E-282	2.73633854071976	0.155	0.017	2.68012783683243E-278	Myeloid	CGAS
1.64280938229388E-282	2.26949118867148	0.528	0.227	2.75301996284809E-278	Myeloid	M6PR
1.77209855528915E-281	3.0554744918021	0.258	0.052	2.96968275895356E-277	Myeloid	PLA2G15
2.08811407029802E-281	2.06703381221588	0.332	0.083	3.49926155900543E-277	Myeloid	PREX1
1.95837812744568E-280	2.08065895353994	0.152	0.016	3.28185006597347E-276	Myeloid	SYT11
3.33855532780861E-280	2.5672602321686	0.528	0.224	5.59475101834167E-276	Myeloid	HEXA

3.73634788445632E-279	3.03008604001427	0.631	0.393	6.2613717847719E-275	Myeloid	SLC40A1
1.25734097907757E-278	1.89971904532609	0.148	0.016	2.10705201273819E-274	Myeloid	CALHM2
1.36180258926526E-277	3.25357214864441	0.133	0.013	2.28210877909072E-273	Myeloid	CLEC4A
1.53669346766419E-276	2.00023386507412	0.698	0.48	2.57519091311165E-272	Myeloid	NPC2
5.7386703259513E-276	1.97408677607249	0.677	0.442	9.61686373222918E-272	Myeloid	CTSS
3.04321271795736E-275	1.54670706400766	0.228	0.039	5.09981587275294E-271	Myeloid	TMC8
3.1401604806248E-275	1.76261276892786	0.422	0.133	5.26228093343104E-271	Myeloid	SIPA1
6.66687356777825E-275	3.8698090338981	0.21	0.035	1.11723467248828E-270	Myeloid	TNFSF13B
6.17269803706054E-273	2.7565745578554	0.419	0.143	1.0344207370506E-268	Myeloid	GM2A
7.8645296880195E-273	3.44201001055384	0.102	0.007	1.31793788511831E-268	Myeloid	ADPRH
1.50442691728767E-272	3.07786907984171	0.315	0.08	2.52111862799068E-268	Myeloid	CALHM6
2.90590941172022E-272	3.10544662828024	0.301	0.074	4.86972299216075E-268	Myeloid	TNFRSF21
8.04619101307182E-272	2.47508280147592	0.397	0.124	1.34838068997058E-267	Myeloid	EPB41L2
9.65373491077381E-271	2.45716617666795	0.175	0.024	1.61777289634748E-266	Myeloid	TSPAN15
1.08923791033981E-269	1.57791520524814	0.272	0.056	1.82534489014745E-265	Myeloid	TMEM173
1.6037207980735E-269	2.36798407364635	0.261	0.054	2.68751531341158E-265	Myeloid	SLC6A6
8.35334062144535E-268	2.20317926098732	0.265	0.055	1.39985282134181E-263	Myeloid	EMP3
2.75310195447397E-266	2.44171770254166	0.505	0.216	4.61364825530748E-262	Myeloid	AP1B1
3.27276732806099E-265	3.24837833248663	0.107	0.008	5.48450348836461E-261	Myeloid	MX2
6.50758792919754E-265	2.4230670948824	0.111	0.009	1.09054158517492E-260	Myeloid	CSGALNACT1
7.60725722791497E-265	4.45516027235335	0.126	0.012	1.27482416625399E-260	Myeloid	HBEGF
3.5291147634954E-264	0.885301061259336	0.905	0.892	5.9140905206656E-260	Myeloid	B2M
2.13836194843949E-263	2.9542452937038	0.161	0.02	3.5834669531949E-259	Myeloid	ZNF141
3.5447433426981E-263	2.73972433410261	0.152	0.018	5.94028089369347E-259	Myeloid	APH1B
7.45390133374608E-263	2.26486255425119	0.581	0.303	1.24912478550917E-258	Myeloid	MGAT1
1.6799971726698E-262	2.15863039085937	0.348	0.098	2.81533926196005E-258	Myeloid	IQGAP1
5.99926729850199E-262	3.48466379660641	0.203	0.034	1.00535721388296E-257	Myeloid	CLIC2
6.87530470125165E-261	2.70865153159197	0.416	0.147	1.15216356183575E-256	Myeloid	UNC93B1
2.05984568945557E-260	2.28804685368085	0.21	0.036	3.45188940638964E-256	Myeloid	SESN3
1.05600287754516E-259	2.79397589955552	0.15	0.018	1.76964962219017E-255	Myeloid	FILIP1L
4.29662982206874E-258	1.64542539838828	0.799	0.61	7.2002922558228E-254	Myeloid	MCL1
2.92843193891895E-257	3.04131872884191	0.317	0.086	4.90746624324037E-253	Myeloid	STARD13
5.19903270247101E-256	2.06389353147479	0.135	0.014	8.71253900280091E-252	Myeloid	FSCN1
6.75779468604305E-256	2.50368779819865	0.501	0.218	1.13247123348709E-251	Myeloid	RASSF4
1.3406593699687E-255	1.90959020398416	0.215	0.037	2.24667697219354E-251	Myeloid	SLC2A3
1.42948277376262E-254	2.52778009532814	0.419	0.148	2.3955272322714E-250	Myeloid	LGALS9
3.89560996063387E-254	1.82772980100178	0.477	0.179	6.52826317203024E-250	Myeloid	LGALS1
5.87883810130823E-254	2.58220075594081	0.326	0.089	9.85175689017233E-250	Myeloid	FRMD4B
2.13110831437018E-251	1.96237338607622	0.568	0.275	3.57131131322155E-247	Myeloid	ZYX
2.16999131591771E-251	4.42444460650945	0.125	0.013	3.6364714472149E-247	Myeloid	ARHGAP22
2.01778145909344E-250	1.71848129453847	0.224	0.041	3.38139816914879E-246	Myeloid	DEPRECATED-ENSG00000059804
3.85876584828672E-250	2.18867137910478	0.226	0.043	6.46651980855888E-246	Myeloid	ARHGEF6
6.48659788725739E-250	1.83650946709926	0.181	0.027	1.08702407394659E-245	Myeloid	TMC6
1.47387282157759E-248	2.73161197940791	0.347	0.105	2.46991607439973E-244	Myeloid	DEPRECATED-ENSG00000196743
3.30603044791608E-248	3.2285951714872	0.158	0.021	5.54024582461776E-244	Myeloid	FAM174B
6.86389912993843E-248	2.78315632393622	0.119	0.011	1.15025221619508E-243	Myeloid	LY75
5.51140402185323E-247	2.30938006355922	0.347	0.102	9.23601085982164E-243	Myeloid	GAS6
5.16124611152121E-246	2.24868497513699	0.132	0.014	8.64921623368724E-242	Myeloid	EVA1B
6.31291372486587E-246	1.97704180177425	0.667	0.46	1.05791808201302E-241	Myeloid	ITM2B
7.95036110996513E-245	2.06137890921308	0.412	0.143	1.33232151480796E-240	Myeloid	NECAP2
9.53854398993158E-245	2.28254533279595	0.577	0.295	1.59846920183273E-240	Myeloid	HERPUD1
1.43903399567689E-244	2.3318914131515	0.43	0.162	2.41153316995534E-240	Myeloid	NAGA
1.03428775012545E-241	2.75506030353074	0.61	0.318	1.73325941166023E-237	Myeloid	PFKFB3
1.38297600706865E-241	1.84565510369062	0.218	0.041	2.31759119264565E-237	Myeloid	RBMS1
1.45186645087585E-241	2.7043117120613	0.301	0.08	2.43303779837776E-237	Myeloid	PI4K2A
1.72310964296965E-241	2.61580055799703	0.367	0.118	2.88758713968854E-237	Myeloid	FRMD4A
2.21202475615526E-239	0.798110851483181	0.275	0.061	3.70691108636499E-235	Myeloid	RAC2
6.7827521687159E-239	3.74682949658652	0.171	0.027	1.13665360843341E-234	Myeloid	TLR1
5.59483623344364E-238	3.29891855739262	0.109	0.01	9.37582656000485E-234	Myeloid	ARMCX1
8.0547122876449E-237	1.90902676512369	0.449	0.171	1.34980868516353E-232	Myeloid	MAP7D1
3.55893162311771E-236	1.92714423084111	0.106	0.009	5.96405761402066E-232	Myeloid	HGF
5.46768927256384E-236	2.93306888327586	0.12	0.012	9.16275368296247E-232	Myeloid	ANTXR1
1.38039056015261E-235	2.90918796403985	0.404	0.148	2.31325850070374E-231	Myeloid	FCHO2

3.70570294902154E-235	2.53382591129132	0.447	0.166	6.2100170019703E-231	Myeloid	P4HA1
5.8120601491486E-234	2.36566791526757	0.277	0.069	9.73985039794322E-230	Myeloid	MVB12B
2.33292967139586E-233	2.54136790866603	0.396	0.141	3.90952354332518E-229	Myeloid	PRKCD
2.51859797476315E-233	2.6054078535724	0.347	0.108	4.22066648610808E-229	Myeloid	JAK2
9.26965017891653E-232	1.89131133102251	0.141	0.017	1.55340797698283E-227	Myeloid	HAPLN3
9.97291733927662E-232	2.1645461557584	0.367	0.12	1.67126148771598E-227	Myeloid	SWAP70
2.73901094172144E-231	1.92288464754899	0.607	0.321	4.59003453613679E-227	Myeloid	RNF149
1.17735198554064E-229	2.60631714867659	0.245	0.055	1.97300645736901E-225	Myeloid	STARD8
2.50652555895916E-229	2.63323561858062	0.273	0.069	4.20043553170377E-225	Myeloid	CTBP2
2.21886373502649E-228	1.92930906064932	0.67	0.453	3.7183718471574E-224	Myeloid	CTSH
2.80816844820227E-228	2.18019375277904	0.507	0.235	4.70592868549736E-224	Myeloid	COLGALT1
1.05014317134341E-227	2.06287368642763	0.598	0.304	1.75982992653729E-223	Myeloid	WSB1
1.31944678020168E-227	2.39524019926234	0.39	0.138	2.21112891426198E-223	Myeloid	ADPGK
2.40559294376257E-227	2.52978429729152	0.385	0.136	4.03129265515732E-223	Myeloid	ABR
3.82766801796605E-226	1.95773907732309	0.606	0.327	6.4144060645075E-222	Myeloid	EHBP1L1
4.12024880905323E-224	1.8305298509689	0.284	0.072	6.9047129542114E-220	Myeloid	PLCG2
8.90998880653383E-224	1.6896722713953	0.581	0.315	1.49313592419894E-219	Myeloid	SMAP2
2.1937145485987E-222	3.02567301989899	0.215	0.045	3.6762268405417E-218	Myeloid	LRRK2
5.98153827670883E-222	1.48786235627091	0.699	0.472	1.00238618441087E-217	Myeloid	TXNIP
1.41117005892038E-221	2.93350646276901	0.188	0.034	2.36483878473878E-217	Myeloid	RCBTB2
1.68975494957629E-221	1.52169276747991	0.223	0.045	2.83169134449995E-217	Myeloid	S100A6
5.94355763795644E-221	2.95688873929516	0.436	0.182	9.9602138896874E-217	Myeloid	DMXL2
4.85498463943715E-220	1.80214647438216	0.441	0.172	8.13598325876878E-216	Myeloid	PPP1R9B
6.84404543805826E-219	2.06074559644116	0.326	0.098	1.1469251345098E-214	Myeloid	CCNG2
1.4655674158833E-215	2.4831312304932	0.299	0.084	2.45599787553724E-211	Myeloid	FES
5.76431304878218E-215	2.14959727289157	0.416	0.158	9.65983580714918E-211	Myeloid	SH3TC1
8.04011901108184E-213	3.16219236024693	0.269	0.071	1.3473631438771E-208	Myeloid	FAM49A
9.99422841453316E-213	1.95105500076033	0.364	0.124	1.67483279770747E-208	Myeloid	PKM
4.46936815645213E-212	2.00261581099602	0.625	0.417	7.48976715658248E-208	Myeloid	MAF
4.83092573125084E-212	2.92736199398526	0.118	0.013	8.09566534043016E-208	Myeloid	SLAMF7
8.45075297096238E-212	2.59273884105168	0.138	0.019	1.41617718287388E-207	Myeloid	LFNG
8.69146020439626E-211	2.73418431892532	0.225	0.051	1.45651490105272E-206	Myeloid	C20orf194
9.38754742892665E-211	2.4919824443596	0.268	0.071	1.57316519813953E-206	Myeloid	CTTNBP2NL
1.6744489301254E-209	2.08878543697808	0.404	0.153	2.80604151710415E-205	Myeloid	FCHSD2
5.47244844436361E-208	1.99458678285681	0.122	0.015	9.17072910306455E-204	Myeloid	FHOD1
1.42643877876399E-206	2.13632077423935	0.181	0.033	2.39042610545269E-202	Myeloid	LRRC8C
4.64020998300868E-206	3.766129680607	0.209	0.046	7.77606388952595E-202	Myeloid	OTUD1
1.82231059937214E-204	2.32803001202016	0.483	0.239	3.05382810242784E-200	Myeloid	IFNGR1
2.18002238959141E-204	2.52700472001242	0.358	0.126	3.65328152047728E-200	Myeloid	GRK3
2.82172678571922E-204	2.89931077859422	0.187	0.037	4.72864974750826E-200	Myeloid	UBE2E2
5.9306076604959E-204	2.19162645211927	0.534	0.291	9.93851231745902E-200	Myeloid	CTSL
3.9880075065015E-202	2.43189486470221	0.353	0.123	6.68310297939522E-198	Myeloid	JDP2
4.88147838252552E-202	1.7522066655808	0.181	0.033	8.18038147343627E-198	Myeloid	GBP5
5.98181781584372E-201	2.46989469177907	0.396	0.152	1.00243302957909E-196	Myeloid	MKNK1
1.24327163038651E-200	2.89008702755133	0.246	0.063	2.08347459820172E-196	Myeloid	TBC1D12
7.54293493526197E-199	1.86441516788583	0.114	0.013	1.2640450364512E-194	Myeloid	GMFG
2.83581293631886E-197	2.58654620206677	0.104	0.011	4.75225531868314E-193	Myeloid	MCOLN2
1.6938226370318E-195	1.13285487573155	0.854	0.843	2.83850797513789E-191	Myeloid	ACTB
3.89272702326939E-195	1.91335924148601	0.276	0.076	6.52343194559484E-191	Myeloid	PTK2B
1.87870073407864E-193	1.88811022682288	0.396	0.151	3.14832669016899E-189	Myeloid	FNBP1
5.46009946735508E-193	1.15565236574298	0.117	0.014	9.15003468739364E-189	Myeloid	DEPRECATED-ENSG00000183943
9.15460940934694E-193	1.66381958796653	0.149	0.024	1.53412944481836E-188	Myeloid	UNC13D
3.36180046816585E-192	2.60264724101211	0.236	0.06	5.63370522455233E-188	Myeloid	MOB1B
3.55741320445422E-192	1.38155720306671	0.285	0.078	5.96151304802439E-188	Myeloid	NLRP1
2.67081206167924E-191	2.99297202291538	0.143	0.023	4.47574685296207E-187	Myeloid	TLR6
7.33518940838599E-191	2.87594921702604	0.272	0.08	1.22923104105732E-186	Myeloid	BMP2K
1.11713988479807E-189	1.56048346119235	0.185	0.037	1.87210301894461E-185	Myeloid	ARID5A
4.49523974552881E-188	1.80070791366675	0.464	0.211	7.53312276555719E-184	Myeloid	GNAI2
6.45604763019676E-188	1.89098386079192	0.512	0.264	1.08190446186837E-183	Myeloid	DPYSL2
7.20928343659466E-187	1.41911072997486	0.155	0.026	1.20813171830453E-182	Myeloid	ZNF83
1.34092658140931E-185	1.69367087405056	0.371	0.134	2.24712476512572E-181	Myeloid	PPP1R18
5.11781967630264E-185	2.95073624585412	0.111	0.014	8.57644221354797E-181	Myeloid	DLG4
1.30928876282036E-184	1.63362155371826	0.333	0.111	2.19410610873436E-180	Myeloid	STK10

1.58527754550723E-184	1.76501504851302	0.101	0.011	2.65660811076102E-180	Myeloid	GPR65
3.45958989478168E-183	2.84440743172765	0.229	0.06	5.79758074567514E-179	Myeloid	TMEM51
5.15541196231058E-183	1.02431778089956	0.222	0.051	8.63943936644008E-179	Myeloid	ITPKB
8.954039945015E-182	0.605973452834795	0.135	0.02	1.50051801398561E-177	Myeloid	MEGF6
8.21875313922184E-181	2.03199573495168	0.106	0.013	1.3772986510708E-176	Myeloid	VAV3
1.54985549371629E-180	1.2728470315131	0.2	0.043	2.59724783636976E-176	Myeloid	LDHB
4.60223994266201E-180	2.05290030640641	0.228	0.058	7.71243369591299E-176	Myeloid	STAT5A
6.87159186690442E-180	2.11815115700884	0.378	0.15	1.15154136505584E-175	Myeloid	PLBD2
7.63482597319859E-180	2.89669344726988	0.226	0.059	1.27944413658862E-175	Myeloid	AP1S2
8.20500452925414E-180	2.09660863415732	0.255	0.069	1.37499465901241E-175	Myeloid	SOCS3
9.7194404161683E-180	1.69563327766669	0.374	0.131	1.62878382494148E-175	Myeloid	ICAM1
1.2620469214316E-179	1.78129637835336	0.671	0.422	2.11493823093508E-175	Myeloid	KLF6
2.94070483664845E-179	2.67020356638536	0.205	0.049	4.92803316525548E-175	Myeloid	PACC1
2.49448326516798E-178	1.19942999542433	0.121	0.017	4.1802550557685E-174	Myeloid	NFATC1
7.39634627463661E-178	0.99288454786998	0.557	0.293	1.2394797087036E-173	Myeloid	ZFP36L2
1.13793924779207E-177	1.85932382833272	0.128	0.019	1.90695859144995E-173	Myeloid	NAV1
3.76096283895599E-177	3.32823967745609	0.134	0.022	6.30262152552246E-173	Myeloid	STAC3
9.5023061053413E-176	1.91663389933422	0.377	0.148	1.59239645713309E-171	Myeloid	CORO7
8.19248375539702E-175	2.09398102577595	0.329	0.118	1.37289642772943E-170	Myeloid	ALCAM
1.85198216511917E-174	1.86376975577603	0.219	0.054	3.1035517123067E-170	Myeloid	HK1
1.22648706638136E-173	1.93235375062118	0.458	0.218	2.05534702584188E-169	Myeloid	CNPY3
1.86462242630542E-173	1.96749391770151	0.532	0.31	3.12473426200262E-169	Myeloid	GNS
1.90873351336174E-173	3.05874119559522	0.218	0.057	3.19865562169161E-169	Myeloid	STON2
8.98089442549297E-173	2.76404906274697	0.289	0.095	1.50501828782411E-168	Myeloid	ABCC5
1.1454092059145E-172	1.7172026368837	0.587	0.354	1.91947674727152E-168	Myeloid	QKI
1.51913438355319E-172	2.39578127110257	0.34	0.128	2.54576539995844E-168	Myeloid	NAAA
3.45419105710981E-172	2.90080065600616	0.25	0.074	5.78853337350462E-168	Myeloid	LPCAT2
1.2324458316961E-170	2.5971044710551	0.224	0.06	2.06533272475632E-166	Myeloid	CCSAP
3.28330262344633E-170	1.83439872400969	0.121	0.018	5.50215853637136E-166	Myeloid	DEPRECATED-ENSG00000141524
7.65147422880692E-170	1.94938871963582	0.496	0.264	1.28223405126346E-165	Myeloid	GAA
2.35912401109227E-169	0.759131306187489	0.248	0.065	3.95342001778843E-165	Myeloid	ITGAL
7.49410194982384E-169	1.6460092767515	0.258	0.074	1.25586160475148E-164	Myeloid	LPXN
1.01289610396018E-167	1.94112574006801	0.444	0.21	1.69741129101648E-163	Myeloid	IFNGR2
3.32933092803998E-167	1.95318533713632	0.355	0.139	5.5792927692094E-163	Myeloid	KIAA0930
8.80644208821968E-167	1.31782352606991	0.49	0.237	1.47578356514385E-162	Myeloid	DEPRECATED-ENSG00000175899
1.56101378364334E-166	2.11599846037783	0.292	0.098	2.61594689862952E-162	Myeloid	TCN2
3.88782150977273E-166	1.27192585177572	0.185	0.04	6.51521128607715E-162	Myeloid	SPN
1.77061092748238E-164	1.02222404517415	0.196	0.044	2.96718979227497E-160	Myeloid	MDFIC
1.04314186060668E-163	2.19486023590189	0.313	0.111	1.74809713000468E-159	Myeloid	SH2B3
1.16491184362097E-163	3.05687987306129	0.171	0.038	1.95215926754002E-159	Myeloid	EAF2
1.7877494237847E-163	2.24193007538931	0.348	0.134	2.99591048437841E-159	Myeloid	ETV6
4.43645369281792E-163	1.91778710924889	0.12	0.018	7.43460909842427E-159	Myeloid	IL21R
5.51931244287963E-163	2.21337593089151	0.388	0.167	9.24926379177769E-159	Myeloid	PTPN6
2.64087043594992E-162	1.94100691404745	0.433	0.207	4.42557067656487E-158	Myeloid	CYFIP1
9.04823339583653E-162	2.08037736844963	0.472	0.254	1.51630295247429E-157	Myeloid	AP2A2
2.36439856088638E-161	3.63383935196144	0.109	0.016	3.96225910833339E-157	Myeloid	SRGAP3
2.68352987611639E-161	2.45617123074772	0.352	0.142	4.49705936639584E-157	Myeloid	ATG7
6.28025275881592E-161	1.68663607356419	0.512	0.284	1.05244475732237E-156	Myeloid	SH3BGRL3
1.34314975485939E-160	2.05972728533056	0.282	0.092	2.25085035919337E-156	Myeloid	CD40
2.02064362275795E-160	2.38523422743647	0.493	0.265	3.38619458301777E-156	Myeloid	NAMPT
8.15744973498892E-160	2.22437876981348	0.221	0.06	1.36702542658944E-155	Myeloid	NCKAP5L
3.8627875003866E-158	1.35204630196266	0.157	0.031	6.47325929314787E-154	Myeloid	ZNF264
2.91224328876885E-157	2.39000558139553	0.336	0.132	4.88033730331883E-153	Myeloid	GPR137B
1.54758888072859E-156	3.39815555202646	0.127	0.022	2.59344944632497E-152	Myeloid	TGFA
3.87212858033342E-154	2.66557004586291	0.293	0.102	6.48891307492274E-150	Myeloid	IER3
1.67192464190075E-153	2.0039656150007	0.458	0.23	2.80181131489727E-149	Myeloid	MYO9B
6.7175223906798E-153	1.6507171775056	0.398	0.173	1.12572240223012E-148	Myeloid	TWF2
7.36962742817793E-152	2.70714151354095	0.252	0.08	1.23500216441406E-147	Myeloid	AVPI1
1.13124043463881E-151	1.65135410572027	0.56	0.358	1.89573272036772E-147	Myeloid	ANXA5
1.64982453889679E-151	2.48449987708603	0.272	0.093	2.76477596228324E-147	Myeloid	DRAM1
8.88020086002288E-151	1.9659067506847	0.119	0.019	1.48814406012263E-146	Myeloid	ICA1
2.77505457982507E-150	1.90213162100638	0.336	0.133	4.65043646487085E-146	Myeloid	MYO1E
2.4998552851265E-149	2.73418210046617	0.103	0.015	4.18925748681499E-145	Myeloid	TEC

2.57862850983608E-149	2.25000069852103	0.105	0.015	4.32126565678331E-145	Myeloid	HAGHL
2.65611286280828E-149	2.12287474377703	0.357	0.149	4.45111393549412E-145	Myeloid	MEF2A
1.2088418432384E-148	2.03034372332217	0.265	0.087	2.02577716089891E-144	Myeloid	OSBPL3
1.63085501742713E-148	2.3069418142651	0.309	0.117	2.73298683820438E-144	Myeloid	CEP170
3.90851889663832E-145	2.10219888428481	0.481	0.284	6.5498959669865E-141	Myeloid	NRP1
9.58138670474266E-145	2.02951923632638	0.338	0.136	1.60564878398077E-140	Myeloid	PLEKHM2
1.14336594930031E-144	2.60103518666188	0.274	0.097	1.91605265783746E-140	Myeloid	PAK1
2.60610503381254E-144	3.43164436756365	0.114	0.019	4.36731081566306E-140	Myeloid	BMF
3.39259022647557E-144	1.79052270401474	0.501	0.294	5.68530270152776E-140	Myeloid	RNF130
8.98792282723858E-144	1.90288895540965	0.415	0.204	1.50619610738864E-139	Myeloid	SCPEP1
1.10069403921843E-143	1.06671136922531	0.118	0.02	1.84454307092224E-139	Myeloid	APOBEC3C
1.84804750962068E-143	3.82432278801883	0.117	0.02	3.09695801662233E-139	Myeloid	CYP2S1
5.44608220366221E-142	1.69640763596079	0.355	0.15	9.12654455689714E-138	Myeloid	ABCG1
8.62597599827911E-141	2.1028367568237	0.272	0.095	1.44554105779161E-136	Myeloid	USP4
1.64557236731535E-140	1.7839198771341	0.384	0.176	2.75765017314706E-136	Myeloid	RBPJ
2.12381875215451E-140	1.91455676963055	0.292	0.107	3.55909546486052E-136	Myeloid	EPSTI1
2.40810583935356E-140	2.7345918358575	0.12	0.022	4.03550376558869E-136	Myeloid	NFE2L3
5.08682440287624E-140	2.2091889264178	0.32	0.128	8.52450033434E-136	Myeloid	RHOQ
6.38318501293969E-140	1.50496685405921	0.481	0.256	1.06969414446843E-135	Myeloid	ARPC1B
9.82112060707001E-140	3.60579515350205	0.118	0.021	1.64582339133279E-135	Myeloid	SLC15A2
1.30579802499313E-139	0.671570247831047	0.167	0.038	2.18825633028349E-135	Myeloid	EMB
1.64981581231496E-139	1.20129159727194	0.403	0.179	2.76476133827741E-135	Myeloid	ARHGEF1
2.2569322492998E-138	2.69605254943968	0.368	0.166	3.7821670633766E-134	Myeloid	PPP1R15A
2.52552937343901E-138	0.847993657306761	0.143	0.029	4.2322821240091E-134	Myeloid	SEMA4D
4.87263699121324E-138	1.92989356634562	0.112	0.019	8.16556506987514E-134	Myeloid	ARMCX3
8.70232228125936E-138	0.747051882784302	0.168	0.039	1.45833516789344E-133	Myeloid	ITPRIP
1.26699168470444E-137	2.44934934958763	0.314	0.127	2.1232246652277E-133	Myeloid	CCDC88A
1.48085722567514E-137	1.84228086235697	0.315	0.125	2.48162053878639E-133	Myeloid	SLC11A2
4.71644050964704E-137	1.55725846791934	0.876	0.756	7.90381100606652E-133	Myeloid	HSPA1B
1.34050031867957E-136	2.12749742510516	0.277	0.101	2.24641043404322E-132	Myeloid	SNX29
1.93611929756872E-136	2.88898308239001	0.1	0.015	3.24454871886565E-132	Myeloid	KMO
2.37113797744955E-136	2.69551144096224	0.15	0.034	3.97355302260996E-132	Myeloid	TMEM144
4.16498757173791E-136	1.61697096549293	0.429	0.214	6.97968617271839E-132	Myeloid	FLII
1.02701637796428E-135	2.27774794377633	0.199	0.056	1.72107404619253E-131	Myeloid	PAPOLG
2.70371144005087E-135	3.20131790330556	0.104	0.017	4.53087963123725E-131	Myeloid	CCL4
6.54629688612793E-135	2.1731224670057	0.288	0.107	1.09702843217732E-130	Myeloid	RALGDS
7.11690649310244E-135	0.720527510007465	0.133	0.026	1.19265119011411E-130	Myeloid	CD28
1.02960709538703E-133	2.6464082486121	0.102	0.016	1.72541557044959E-129	Myeloid	ZMYND15
1.68176186030599E-133	2.19258070988298	0.305	0.121	2.81829652550077E-129	Myeloid	GCA
2.51766476298032E-132	1.06844231048426	0.859	0.759	4.21910260980243E-128	Myeloid	FTH1
2.74501869439024E-132	2.05339229603434	0.309	0.123	4.60010232805917E-128	Myeloid	ZMIZ1
3.56520064064461E-132	1.7161456505759	0.412	0.203	5.97456323359224E-128	Myeloid	OSTF1
9.36410403229208E-132	1.71668716869079	0.142	0.031	1.56923655373151E-127	Myeloid	AP1G2
9.72749151781281E-132	2.55718229334103	0.217	0.068	1.63013302855507E-127	Myeloid	ARHGAP27
3.44778410918562E-131	2.12661520005109	0.334	0.142	5.77779661017325E-127	Myeloid	PABPC4
8.2565625964743E-131	1.91889784944575	0.32	0.131	1.38363475991716E-126	Myeloid	TGFBR1
1.63453879781628E-130	2.5455818889324	0.133	0.028	2.73916011738051E-126	Myeloid	SLC8B1
2.41240518302654E-130	1.65288616780199	0.123	0.024	4.04270860571588E-126	Myeloid	PDE4B
4.87649146287502E-130	1.61000399596913	0.115	0.021	8.17202439348596E-126	Myeloid	KCTD17
6.29117868067316E-130	0.943635468254364	0.193	0.052	1.05427572330721E-125	Myeloid	S100A4
6.63698752864541E-130	1.5513359922898	0.175	0.044	1.1122263700504E-125	Myeloid	SATB1
1.20864267104326E-129	1.08032844385706	0.344	0.145	2.0254433881343E-125	Myeloid	STK17B
2.56788623892376E-129	1.34408556227111	0.278	0.101	4.30326375918843E-125	Myeloid	ANKRD44
5.79353457755752E-129	1.46553262212519	0.497	0.278	9.70880524507089E-125	Myeloid	GRK2
9.04081077032884E-128	1.90868234670828	0.359	0.163	1.51505906889171E-123	Myeloid	ANKS1A
2.25732643071675E-127	1.38161382314714	0.577	0.37	3.78282763259514E-123	Myeloid	PER1
5.98048451530132E-127	1.93203553873783	0.112	0.021	1.00220959507419E-122	Myeloid	STK32C
1.39529962673502E-126	2.18694647215692	0.441	0.258	2.33824311448255E-122	Myeloid	CPM
2.35619536514342E-126	1.90936096467174	0.123	0.024	3.94851219290734E-122	Myeloid	GLIPR2
3.7041047480518E-126	3.07728684856648	0.451	0.248	6.2073387367852E-122	Myeloid	CHMP1B
6.6942237817831E-126	1.33876395772084	0.36	0.161	1.12181802135121E-121	Myeloid	ITM2C
9.54373671765142E-126	0.306664963118987	0.329	0.124	1.59933399914402E-121	Myeloid	TIMP1
1.31206395956801E-125	1.95513772318319	0.297	0.118	2.19875678344407E-121	Myeloid	SNX8

1.56247154905877E-125	2.44157937035552	0.188	0.054	2.61838982191268E-121	Myeloid	SLC29A3
2.21461169069919E-125	1.60134189470249	0.244	0.08	3.7112462712737E-121	Myeloid	KYNU
4.29831667630293E-125	1.96336855414073	0.439	0.237	7.20311908614845E-121	Myeloid	RUNX1
6.79375706706187E-125	2.15967402669796	0.223	0.072	1.13849780929823E-120	Myeloid	KIAA0513
3.73372170666954E-124	1.81357851543952	0.447	0.242	6.25697083603682E-120	Myeloid	CLCN7
8.25274176738699E-124	1.51055500560456	0.15	0.035	1.38299446537871E-119	Myeloid	MFNG
2.08018043909136E-123	2.03261983665583	0.414	0.217	3.48596637982931E-119	Myeloid	NINJ1
1.51296743495706E-122	2.13289068901324	0.258	0.095	2.53543082750103E-118	Myeloid	GPR155
4.85383625554116E-122	2.01776652467256	0.108	0.02	8.13405879703587E-118	Myeloid	TLE4
5.3560950936586E-122	1.28329010013084	0.264	0.089	8.97574415795309E-118	Myeloid	CPED1
2.79772649010203E-121	2.95662562389005	0.118	0.024	4.68843005211298E-117	Myeloid	SPINT1
3.55233086014412E-121	2.04422686874186	0.177	0.049	5.95299605542951E-117	Myeloid	TTC7B
2.30027886767439E-120	0.361390087645989	0.372	0.159	3.85480732644873E-116	Myeloid	IL2RG
2.42452223650157E-120	1.89065871640347	0.235	0.08	4.06301436392933E-116	Myeloid	ANKRD13D
4.14465190830432E-120	1.65095651588159	0.145	0.034	6.94560766793638E-116	Myeloid	DUSP2
5.4501871537541E-120	1.64106619724478	0.122	0.025	9.13342363226111E-116	Myeloid	ZDHHC8
8.39026767863107E-120	0.745312927641179	0.836	0.775	1.406041057585E-115	Myeloid	OAZ1
1.21386813759159E-119	1.67116993817868	0.336	0.149	2.03420022497598E-115	Myeloid	SAP30
2.3427318402952E-119	1.15444615111044	0.13	0.028	3.92595001796669E-115	Myeloid	GNG2
1.58814542052132E-118	1.11474455638433	0.16	0.04	2.66141409570963E-114	Myeloid	JAK3
2.36417266941175E-118	1.76280770166761	0.253	0.093	3.96188055940021E-114	Myeloid	AMDHD2
2.84157899947115E-118	2.16277842351968	0.19	0.057	4.76191808731375E-114	Myeloid	MFSD12
3.59314313571129E-118	1.55814822701762	0.385	0.19	6.02138926682498E-114	Myeloid	VEGFB
4.02989477248438E-118	1.80212336043361	0.133	0.03	6.75329765972933E-114	Myeloid	SAP25
7.27824888369677E-118	2.44815637531743	0.205	0.066	1.2196889479299E-113	Myeloid	HSPBAP1
2.12290570521073E-117	1.73555379005651	0.33	0.146	3.55756538079213E-113	Myeloid	CSGALNACT2
8.2866398195436E-117	1.71392157834304	0.355	0.165	1.38867510095912E-112	Myeloid	BNIP3L
3.63704620472722E-116	2.12140933340143	0.249	0.093	6.09496202988188E-112	Myeloid	CMTM7
3.9138237137555E-116	1.99735914523997	0.311	0.135	6.55878577951147E-112	Myeloid	ARPIN
4.48140593702928E-116	1.71439105706172	0.49	0.301	7.50994006927367E-112	Myeloid	LAP3
4.75278507874653E-115	1.58877685068875	0.289	0.116	7.96471723496343E-111	Myeloid	MOB3A
6.15425807306567E-115	1.64062657247449	0.576	0.398	1.03133056788435E-110	Myeloid	ABCA1
8.12004895373798E-115	1.98340245394414	0.377	0.189	1.36075780366741E-110	Myeloid	DRAM2
2.24216365682538E-114	1.50857695170371	0.493	0.297	3.75741785610796E-110	Myeloid	EIF4A1
2.35178223609802E-114	1.03456331950435	0.521	0.311	3.94111667125305E-110	Myeloid	ADGRE5
3.56136915759099E-114	1.41587342774632	0.548	0.386	5.96814243429098E-110	Myeloid	RNH1
9.50640751651755E-114	1.6986740121607	0.191	0.058	1.59308377161801E-109	Myeloid	SFXN3
3.77689976058879E-113	2.3810730579852	0.268	0.105	6.3293286187947E-109	Myeloid	ACOT9
5.76873727375102E-113	1.71915093648933	0.439	0.252	9.66724992335196E-109	Myeloid	ATP6AP2
6.25859283346803E-113	1.85929689968358	0.237	0.085	1.04881498703257E-108	Myeloid	SH3BP1
6.50882139726593E-113	1.94100944920054	0.183	0.055	1.09074828975382E-108	Myeloid	CHMP4A
6.70962988355267E-113	1.59525878666156	0.429	0.235	1.12439977588576E-108	Myeloid	PLEKHB2
9.89147061986661E-113	1.3174508296096	0.387	0.194	1.65761264647725E-108	Myeloid	ARL6IP5
3.30648036696569E-112	1.57728074698476	0.422	0.231	5.5409997989611E-108	Myeloid	SUSD6
1.13958036373606E-111	1.62227637693027	0.346	0.16	1.90970877354889E-107	Myeloid	HPS5
2.60319706355234E-111	1.64606605827559	0.271	0.107	4.36243763910101E-107	Myeloid	SLC4A7
1.10931153717583E-110	1.92766019146672	0.109	0.022	1.85898427399926E-106	Myeloid	CHST15
1.33816058789926E-110	1.83655788117906	0.269	0.106	2.24248951320159E-106	Myeloid	GNG10
1.61949737779444E-110	1.28001985362545	0.529	0.329	2.71395370570793E-106	Myeloid	CDC42SE1
2.11859243028564E-110	1.89611012492901	0.247	0.092	3.55033719467268E-106	Myeloid	BAZ2B
2.6468450590339E-110	2.08060805531817	0.248	0.093	4.43558294992901E-106	Myeloid	SH3BP2
2.66381184227426E-110	1.23468965020632	0.227	0.079	4.4640158852832E-106	Myeloid	RASSF1
5.35624639830928E-110	1.97439850242118	0.153	0.041	8.97599771428669E-106	Myeloid	TRPM4
1.3457970620952E-109	2.32503254691029	0.234	0.086	2.25528671665913E-105	Myeloid	SGPL1
3.04372562271495E-109	1.87173557219087	0.253	0.097	5.10067539854572E-105	Myeloid	FEZ2
4.78560923511874E-109	1.20413624592126	0.119	0.025	8.01972395621199E-105	Myeloid	DOCK1
9.03624889398999E-109	2.56449644204661	0.182	0.056	1.51429458965484E-104	Myeloid	ZNF267
1.71039590325047E-108	1.83584516062716	0.282	0.116	2.86628145466713E-104	Myeloid	HPS1
1.76183938624037E-108	1.69647175689569	0.297	0.128	2.95249044346162E-104	Myeloid	DOCK10
2.64388590174074E-108	1.71991005597742	0.266	0.105	4.43062399413713E-104	Myeloid	RIPOR1
2.15271627779193E-107	2.05763948640777	0.169	0.049	3.60752193832371E-103	Myeloid	UBE2D1
2.40090372774395E-107	0.703842457533665	0.156	0.04	4.02343446695331E-103	Myeloid	LAMB2
2.84772027705745E-107	1.44494341407338	0.503	0.307	4.77220964029288E-103	Myeloid	NCOR2

3.30451332170168E-107	1.56857894305082	0.432	0.24	5.53770342450768E-103	Myeloid	VPS13C
4.10014870250256E-107	2.30135148079049	0.157	0.044	6.8710291956538E-103	Myeloid	CTNS
1.30806695410261E-105	1.86827815289795	0.305	0.134	2.19205860168515E-101	Myeloid	DDAH2
1.5024675156053E-105	1.58698596804343	0.461	0.265	2.51783506265135E-101	Myeloid	SLC3A2
5.30148610277148E-105	2.59806922003909	0.119	0.027	8.88423041102445E-101	Myeloid	TMEM268
5.83028924803019E-105	1.00253459668357	0.538	0.348	9.77039872184898E-101	Myeloid	MSN
7.62966011446499E-105	3.16062290939008	0.13	0.032	1.27857844198204E-100	Myeloid	LGALS2
1.75939044293386E-104	0.944270264603231	0.145	0.038	2.94838650426856E-100	Myeloid	NIBAN1
3.32176847348194E-104	2.32508736818306	0.141	0.037	5.56661960786103E-100	Myeloid	TIGAR
4.06840553618379E-104	1.91698389450148	0.361	0.186	6.8178339975368E-100	Myeloid	LHFPL2
3.65361794059981E-103	1.74195543352121	0.211	0.074	6.12273294485717E-99	Myeloid	PEAK1
5.97452461543767E-103	1.88700054319465	0.377	0.207	1.00121083505504E-98	Myeloid	TSPAN4
2.61687442378359E-102	1.57200206262527	0.409	0.221	4.38535815937654E-98	Myeloid	AHR
4.13712536781018E-102	1.24321849277864	0.198	0.064	6.9329946913763E-98	Myeloid	ADGRG6
5.35858639761715E-102	0.905288910232448	0.528	0.329	8.97991908512682E-98	Myeloid	A2M
5.97365171217592E-102	1.9409695921533	0.211	0.075	1.00106455392644E-97	Myeloid	NAGPA
1.25510073288845E-101	1.85751796628124	0.239	0.092	2.10329780817447E-97	Myeloid	PSTPIP2
1.56265419091317E-101	1.35313932566241	0.221	0.075	2.6186958931323E-97	Myeloid	FAM20A
1.60976499413345E-101	1.6643562466336	0.229	0.086	2.69764417716883E-97	Myeloid	CD276
7.08996362362021E-101	2.59682031994827	0.215	0.078	1.18813610404627E-96	Myeloid	UBXN11
1.00432973552617E-99	4.30752836463313	0.104	0.022	1.68305577079475E-95	Myeloid	FCGBP
1.29081919985918E-99	2.04197842821164	0.166	0.05	2.16315481512402E-95	Myeloid	CD55
1.48623974682373E-99	1.81994273524439	0.191	0.064	2.4906405677272E-95	Myeloid	CBL
2.45192089396349E-99	1.25793706344732	0.636	0.463	4.10892903410401E-95	Myeloid	CFLAR
4.84059439681321E-99	2.63412991337138	0.206	0.074	8.11186809017959E-95	Myeloid	C9orf72
4.87968089646344E-99	2.03275751763392	0.241	0.095	8.17736924629343E-95	Myeloid	DEPRECATED-ENSG00000068305
6.95620800656406E-98	1.5025748391241	0.423	0.239	1.16572133774001E-93	Myeloid	SERPINB6
7.92583707763829E-98	1.17486102764257	0.491	0.309	1.32821177747062E-93	Myeloid	TGFBR2
1.02851379528833E-97	1.36578055111568	0.445	0.259	1.72358341814418E-93	Myeloid	GGA2
1.08117925166297E-97	2.52809712688425	0.264	0.114	1.8118401899368E-93	Myeloid	NAIP
1.11427205858178E-97	3.06634312114684	0.136	0.037	1.86729711577135E-93	Myeloid	S100A9
1.12648479244452E-97	2.3354913191489	0.108	0.024	1.88776321517853E-93	Myeloid	CPNE2
4.72945090140628E-97	1.26202850860114	0.449	0.255	7.92561382057665E-93	Myeloid	AKIRIN2
2.14417467323562E-96	1.43811341024648	0.188	0.062	3.59320791740825E-92	Myeloid	MYADM
6.35793027945578E-96	1.56275099722752	0.455	0.271	1.0654619562312E-91	Myeloid	TNFRSF14
6.5155329936498E-96	1.24094696519971	0.42	0.23	1.09187301907583E-91	Myeloid	LRCH4
1.17845636206727E-95	2.10760381681534	0.131	0.034	1.97485717155233E-91	Myeloid	RHOBTB1
1.50039897847441E-95	1.95363632815418	0.23	0.09	2.51436860812742E-91	Myeloid	TET3
2.37223825471566E-95	0.994233986179935	0.108	0.024	3.97539686725251E-91	Myeloid	PSTPIP1
5.74924707059777E-95	2.55186535261954	0.227	0.089	9.63458824090774E-91	Myeloid	MLKL
7.14561224497334E-95	2.06988306942395	0.294	0.136	1.19746170001263E-90	Myeloid	MANBA
1.21498711172316E-94	0.396421732855008	0.286	0.113	2.03607540182568E-90	Myeloid	ANXA6
2.01710270395743E-94	1.54410982960814	0.505	0.341	3.38026071129187E-90	Myeloid	SDCBP
3.05310007234067E-94	1.57623100302099	0.655	0.518	5.11638510122849E-90	Myeloid	ZFP36
1.96778689115351E-93	1.68075801063954	0.227	0.089	3.29761727219505E-89	Myeloid	LIMK1
2.30398452555206E-93	1.64814047016546	0.149	0.043	3.86101726792014E-89	Myeloid	RNF166
1.90655883176199E-92	1.27717251412402	0.218	0.08	3.19501129026674E-88	Myeloid	CD302
2.07070985462719E-92	2.02776748457501	0.285	0.131	3.47009557438424E-88	Myeloid	USP36
2.3308458208191E-92	1.70595882844683	0.339	0.173	3.90603142652864E-88	Myeloid	VOPP1
2.74794840259537E-92	1.73503409916154	0.31	0.149	4.60501193306933E-88	Myeloid	SLC38A7
6.0165739839107E-92	0.568458225277556	0.214	0.075	1.00825746822376E-87	Myeloid	DTNA
7.32209478309274E-92	2.88727761316316	0.13	0.035	1.22703664375068E-87	Myeloid	TLR5
1.23038699089993E-91	2.33320513176873	0.197	0.072	2.06188251935011E-87	Myeloid	ZSWIM6
4.6909519169349E-91	1.36274660601219	0.549	0.435	7.8610972223995E-87	Myeloid	NCOA4
7.1866412604989E-91	1.24081220563901	0.105	0.023	1.2043373424344E-86	Myeloid	MAP4K1
7.89214498801136E-91	1.35859788045205	0.511	0.347	1.32256565709094E-86	Myeloid	LRP1
9.33725720946825E-91	1.90637054834572	0.182	0.062	1.56473756316269E-86	Myeloid	ARSB
1.67246763084277E-90	1.64276681647541	0.37	0.201	2.80272125576631E-86	Myeloid	CREM
2.64664003665362E-90	2.45129242982282	0.134	0.038	4.43523937342414E-86	Myeloid	HK2
3.9697907862757E-90	2.05356653652989	0.288	0.132	6.65257539964081E-86	Myeloid	CACUL1
7.63028234194702E-90	3.00886352411118	0.116	0.029	1.27868271486348E-85	Myeloid	EDA
9.41911237555714E-90	1.47195174979271	0.323	0.161	1.57845485189586E-85	Myeloid	MAP3K3
1.33817852295632E-89	1.0416577750206	0.774	0.59	2.24251956877021E-85	Myeloid	LYZ

2.33456792045945E-89	1.28982873970386	0.521	0.36	3.91226892110595E-85	Myeloid	VAMP8
6.03710872897617E-89	1.599895986646	0.36	0.192	1.01169868080183E-84	Myeloid	CREBL2
7.49332885876582E-89	1.5730831710321	0.365	0.195	1.25573205015198E-84	Myeloid	OAZ2
2.68388605865998E-88	1.17060430312978	0.513	0.342	4.49765625710239E-84	Myeloid	DPP7
5.08229264968087E-88	1.37627838543229	0.376	0.205	8.5169060223352E-84	Myeloid	ATM
1.3808978390174E-87	3.08311402427384	0.119	0.032	2.31410859862537E-83	Myeloid	SUCNR1
1.73438813624085E-87	1.35242478501603	0.467	0.288	2.90648763871242E-83	Myeloid	CYTH1
3.93622787325229E-87	1.38735315341665	0.429	0.267	6.59633066999618E-83	Myeloid	MMP14
4.29976841163391E-87	1.85582530290284	0.471	0.33	7.20555190421611E-83	Myeloid	FUCA1
5.56474267553463E-87	1.72036446868081	0.591	0.538	9.32539577566093E-83	Myeloid	CTSD
1.01311823794779E-86	1.80079252912947	0.247	0.107	1.6977835431529E-82	Myeloid	DAGLB
2.50541509121889E-86	0.674139761764419	0.34	0.167	4.19857460986462E-82	Myeloid	AKNA
2.94224636244121E-86	1.84058275138252	0.261	0.115	4.93061645417899E-82	Myeloid	CYB5R4
5.98883354231683E-86	1.7488464297016	0.328	0.168	1.00360872502145E-81	Myeloid	PNPLA6
9.7157038339367E-86	1.0926379418882	0.48	0.297	1.62815764849111E-81	Myeloid	AKAP13
2.48848337256629E-84	1.71814871810636	0.155	0.049	4.1702004357466E-80	Myeloid	SLC46A3
3.55006992553606E-84	2.4476676328724	0.109	0.027	5.94920718121332E-80	Myeloid	ZBTB46
7.31585563036922E-84	1.64734936591367	0.302	0.149	1.22599108653727E-79	Myeloid	SRGAP2B
1.18338885511149E-83	1.2239640849042	0.519	0.376	1.98312304339584E-79	Myeloid	CAPZB
1.56151241927507E-83	1.57208287497653	0.262	0.118	2.61678251222116E-79	Myeloid	SRGAP2
6.09785971225747E-83	1.16724049932471	0.622	0.465	1.02187933058011E-78	Myeloid	PNRC1
8.42238112369666E-83	1.8766414739153	0.304	0.152	1.41142262870909E-78	Myeloid	LPCAT1
1.00844703817025E-82	1.66189965795788	0.628	0.48	1.68995554656571E-78	Myeloid	MAT2A
2.14540854528228E-82	1.64101609107583	0.212	0.084	3.59527564018404E-78	Myeloid	BICD2
5.92534464744443E-82	1.22428902206073	0.499	0.349	9.92969256018738E-78	Myeloid	SCAMP2
6.18238386522636E-82	1.57545677809724	0.415	0.253	1.03604388813463E-77	Myeloid	FAM49B
1.2852656011852E-81	1.1251180778736	0.216	0.086	2.15384809446615E-77	Myeloid	CCDC88B
2.81173318166983E-81	1.40154937561653	0.345	0.181	4.71190246584231E-77	Myeloid	BNIP2
4.44205525382003E-81	1.46815446942173	0.316	0.153	7.4439961943516E-77	Myeloid	TMEM140
4.69477024355853E-81	1.57602115559615	0.429	0.271	7.86749597415539E-77	Myeloid	PEA15
2.21291009054712E-80	0.96408672522834	0.16	0.052	3.70839472973887E-76	Myeloid	SEPTIN6
7.59674780350617E-80	0.966462815052974	0.693	0.679	1.27306299691156E-75	Myeloid	LAMP1
7.9469218312106E-80	1.65863802009406	0.263	0.122	1.33174516047427E-75	Myeloid	MTMR14
1.47074527002171E-79	1.29645757988334	0.323	0.167	2.46467492350238E-75	Myeloid	PSME4
2.3879124416727E-79	2.67409192357855	0.223	0.096	4.00166366975511E-75	Myeloid	GZF1
4.02238637237545E-79	0.923354248419298	0.113	0.029	6.74071508282678E-75	Myeloid	CCR5
5.39031581487403E-79	1.21116729081911	0.359	0.198	9.03309124256589E-75	Myeloid	APBB1IP
8.05611439366351E-79	1.2811901422874	0.452	0.291	1.35004365009013E-74	Myeloid	GRB2
8.88640889000174E-79	1.16428443590414	0.577	0.45	1.48918440178649E-74	Myeloid	SYNGR2
2.76751728785792E-78	1.64271594122145	0.296	0.147	4.63780547099231E-74	Myeloid	SNAP23
5.22588656689691E-78	1.46225244795605	0.429	0.271	8.75754070880585E-74	Myeloid	CHKA
1.72081680809387E-77	1.73419331457331	0.158	0.054	2.88374480700037E-73	Myeloid	GHDC
2.48995963123853E-77	1.89741087412503	0.615	0.473	4.17267435002952E-73	Myeloid	HSPH1
9.3574039227408E-77	1.33510657916058	0.281	0.136	1.5681137493729E-72	Myeloid	GPRIN3
1.16932465278001E-76	1.76882338438949	0.49	0.332	1.95955425312874E-72	Myeloid	EIF4A3
1.25576710262124E-76	1.80185778972349	0.116	0.032	2.10441451057268E-72	Myeloid	PLK3
2.15486222564157E-76	1.06320300425367	0.568	0.416	3.61111811773014E-72	Myeloid	EFHD2
2.43373169970047E-75	2.13010270338716	0.122	0.036	4.07844758235805E-71	Myeloid	FUT4
3.26309237618268E-75	1.30605304302631	0.261	0.121	5.46829020400693E-71	Myeloid	RAB8B
1.2528802895268E-74	2.09009537193186	0.188	0.075	2.09957678918901E-70	Myeloid	ACER3
1.4826018811382E-74	1.4535151350678	0.397	0.242	2.4845442324114E-70	Myeloid	TBC1D14
1.89109942551791E-74	2.76328163376408	0.193	0.079	3.16910441728292E-70	Myeloid	CXCL9
2.40196261049664E-74	2.30352430966423	0.113	0.032	4.02520894267026E-70	Myeloid	ARID3A
2.44931873681944E-74	1.7279758730434	0.237	0.106	4.10456833916201E-70	Myeloid	TBC1D22A
2.59540350137641E-74	1.98610057157763	0.18	0.068	4.34937718760658E-70	Myeloid	ST3GAL5
5.05738328739424E-74	1.35061116939875	0.319	0.168	8.47516291301527E-70	Myeloid	RAPGEF1
9.87268102835013E-74	1.27725007248117	0.321	0.17	1.65446388673091E-69	Myeloid	ARPC2
3.25033606713315E-73	1.53106250799895	0.322	0.172	5.44691318130174E-69	Myeloid	RAB35
4.13234156472317E-73	1.27788640154521	0.221	0.091	6.92497799416308E-69	Myeloid	IER5L
4.52204472993761E-73	1.83197332993435	0.214	0.091	7.57804255842945E-69	Myeloid	NFKBIE
8.4358279913988E-73	2.30258938687344	0.165	0.06	1.41367605479861E-68	Myeloid	SDS
9.7922572750091E-73	1.87695118090687	0.171	0.064	1.64098647414603E-68	Myeloid	BTN2A2
3.66425154887269E-72	1.23875341590422	0.313	0.165	6.14055274560085E-68	Myeloid	WASF2

3.7507551294624E-72	1.85788103912345	0.1	0.026	6.2855154459531E-68	Myeloid	ADAM8
4.13553335946638E-72	1.62832543716523	0.357	0.206	6.93032680379377E-68	Myeloid	LYN
7.76265387907864E-72	0.795787735213692	0.734	0.688	1.300865537056E-67	Myeloid	CEBPD
1.37666321366413E-71	1.94243873086513	0.128	0.04	2.30701221345836E-67	Myeloid	SNX18
3.07382875136801E-71	1.44254371115471	0.245	0.112	5.15112222154252E-67	Myeloid	MAPKAPK3
9.90823713582386E-71	1.51898175177456	0.17	0.064	1.66042237922136E-66	Myeloid	FUT11
1.12652922907207E-70	1.85091971006735	0.119	0.036	1.88783768207897E-66	Myeloid	CPEB3
1.36021823099464E-70	1.20510790103286	0.247	0.113	2.27945371150081E-66	Myeloid	LDB1
2.2051307334265E-70	2.26515928798407	0.204	0.089	3.69535808307612E-66	Myeloid	SLC18B1
3.12189996017558E-69	1.50967640043507	0.226	0.101	5.23167995326223E-65	Myeloid	JARID2
3.19844025603124E-69	1.60999757834927	0.274	0.138	5.35994618105715E-65	Myeloid	GIT2
3.86074477219302E-69	1.68729208760834	0.296	0.155	6.46983608924106E-65	Myeloid	GPCPD1
4.94460238413054E-69	1.28339277307254	0.488	0.332	8.28616467532596E-65	Myeloid	TACC1
5.80698707763781E-69	1.57004298892863	0.382	0.236	9.73134894470544E-65	Myeloid	DEPRECATED-ENSG00000137575
8.26975044515831E-69	1.1440734836152	0.461	0.308	1.38584477959963E-64	Myeloid	MAX
1.37929640377342E-68	2.23833451266375	0.132	0.044	2.31142491344349E-64	Myeloid	SLC27A1
1.69460738331491E-68	1.159882552584	0.564	0.48	2.83982305295912E-64	Myeloid	CTSA
1.77427325482365E-68	0.66770363919089	0.123	0.037	2.97332712043347E-64	Myeloid	CRIP1
2.85507844234585E-68	1.58777274872667	0.11	0.032	4.78454045368317E-64	Myeloid	IL27RA
3.45105758828071E-68	1.79862196465429	0.287	0.15	5.78328230644082E-64	Myeloid	BTG2
7.07338195283102E-68	1.42281267464008	0.309	0.167	1.18535734765542E-63	Myeloid	WASHC4
1.89955688672137E-67	1.24706758831855	0.349	0.201	3.18327743076767E-63	Myeloid	PRMT2
1.0412022511645E-66	1.3217712599703	0.351	0.201	1.74484673250147E-62	Myeloid	SLC1A5
2.06941154067426E-66	1.9857592251966	0.22	0.1	3.46791985986192E-62	Myeloid	KDM6B
6.02604785836363E-66	1.86375169735887	0.251	0.125	1.00984510010458E-61	Myeloid	SLC49A4
8.47169314207143E-66	1.72191935877158	0.207	0.091	1.41968633674833E-61	Myeloid	UPP1
1.551102596532E-65	1.50012762478665	0.302	0.163	2.59933773126833E-61	Myeloid	ARMH3
2.33809119170334E-65	1.22385218001607	0.178	0.071	3.91817321905646E-61	Myeloid	CHSY1
2.97317696734419E-65	1.41340302139711	0.341	0.194	4.9824499618754E-61	Myeloid	JMJD1C
6.08982118890185E-65	1.38932895187039	0.473	0.332	1.02053223483617E-60	Myeloid	GNAQ
7.14471626458334E-65	1.80558842107506	0.239	0.116	1.19731155161888E-60	Myeloid	SETDB2
7.36391202494418E-65	1.75117567497018	0.271	0.14	1.23404437714015E-60	Myeloid	SLC12A9
8.8725669179695E-65	1.20407432357096	0.466	0.323	1.48686476411333E-60	Myeloid	TNS3
2.19851935261973E-64	1.45161078553174	0.202	0.088	3.68427873112015E-60	Myeloid	PHACTR2
3.23665139136045E-64	1.55387154338632	0.246	0.12	5.42398040164184E-60	Myeloid	WASHC2A
3.84115854018958E-64	1.94713067137445	0.169	0.068	6.43701348164969E-60	Myeloid	SRGAP2C
5.11227361391345E-64	2.46712876426959	0.112	0.035	8.56714812219617E-60	Myeloid	IRF5
6.68934196363452E-64	1.18354014387899	0.213	0.096	1.12099992626587E-59	Myeloid	GIMAP2
7.46574054884874E-64	0.339074423771783	0.119	0.036	1.25110880117607E-59	Myeloid	RASAL3
1.39392528078568E-63	1.10654061106843	0.133	0.045	2.33593998554064E-59	Myeloid	ACSS1
1.54203830227319E-63	1.2385311945324	0.37	0.222	2.58414778694941E-59	Myeloid	POLR2A
1.90434009048535E-63	1.80021399589483	0.182	0.076	3.19129312363535E-59	Myeloid	PWWP2B
1.94043625766442E-63	2.06784574182379	0.127	0.043	3.25178308059404E-59	Myeloid	FAM117B
2.96050960399485E-63	1.65169901688027	0.173	0.07	4.96122199437457E-59	Myeloid	SSH2
5.2125213658547E-63	1.45678819112473	0.36	0.214	8.7351433048993E-59	Myeloid	PTPN1
7.33203894707636E-63	1.73093136989367	0.169	0.068	1.22870308675106E-58	Myeloid	AKAP10
7.37623568974355E-63	1.03808046451875	0.55	0.448	1.23610957688722E-58	Myeloid	PICALM
1.78435913389422E-62	2.17450940428799	0.223	0.107	2.99022903657993E-58	Myeloid	ATP1B3
2.35474128123135E-62	1.48111937051872	0.414	0.273	3.9460754390875E-58	Myeloid	STX7
2.46965931191651E-62	1.20126711026891	0.297	0.16	4.13865507490969E-58	Myeloid	EHD4
3.89877658873149E-62	1.36413556411734	0.314	0.177	6.53356980739624E-58	Myeloid	PTPRJ
5.98043316898474E-62	0.963762538807858	0.404	0.256	1.00220099045846E-57	Myeloid	TLN1
6.89455585238558E-62	1.20891553403019	0.286	0.154	1.15538966974278E-57	Myeloid	ACTR2
7.21702605423366E-62	1.79595585353124	0.158	0.062	1.20942922616848E-57	Myeloid	DEPRECATED-ENSG00000168214
2.17054074046034E-61	1.31504082395428	0.282	0.15	3.63739217286343E-57	Myeloid	CREBRF
2.28328989469861E-61	1.44589663119175	0.25	0.123	3.82633720553593E-57	Myeloid	NT5C2
2.83162979428352E-61	0.797380543359862	0.647	0.578	4.74524520926032E-57	Myeloid	PFN1
3.01064242171144E-61	1.64891112270642	0.194	0.085	5.04523457030403E-57	Myeloid	ERICH1
6.80990264119986E-61	1.29654373485952	0.906	0.836	1.14120348461227E-56	Myeloid	DEPRECATED-ENSG00000204389
1.35287924773433E-60	1.26694628546099	0.402	0.263	2.26715504335319E-56	Myeloid	SNX6
2.51566969462665E-60	1.16297384521579	0.341	0.201	4.21575927425534E-56	Myeloid	NFATC2
3.23080310214823E-60	1.19315842331775	0.316	0.18	5.41417983858E-56	Myeloid	GNPTAB
9.9546036618733E-60	1.14023699847204	0.354	0.209	1.66819248165673E-55	Myeloid	UBE2L6

1.15083925817538E-59	1.78825815323457	0.254	0.132	1.9285764288503E-55	Myeloid	UVRAG
1.88119067856009E-59	1.85495612969683	0.143	0.054	3.15249933913099E-55	Myeloid	FIG4
2.52292921684936E-59	1.34922140356036	0.378	0.244	4.22792478159615E-55	Myeloid	ATP6V0C
2.77371381645196E-59	1.67395770134874	0.155	0.061	4.64818961361019E-55	Myeloid	P2RY11
3.19122182468295E-59	1.10937759697727	0.366	0.224	5.34784953380369E-55	Myeloid	ELF1
3.28228625298003E-59	1.29537237272135	0.356	0.22	5.50045530274393E-55	Myeloid	SNX2
3.68051300928406E-59	2.01053479152618	0.174	0.074	6.16780370095822E-55	Myeloid	SLC38A6
4.42585965779513E-59	2.27552951653149	0.111	0.036	7.41685561453307E-55	Myeloid	ZNF703
1.15279709791821E-58	1.76960441244491	0.242	0.123	1.93185737669134E-54	Myeloid	IFFO1
1.2103667591162E-58	1.30274028854581	0.323	0.186	2.02833261492694E-54	Myeloid	MRTFA
2.45430955972549E-58	1.74611253661703	0.16	0.066	4.11293196018797E-54	Myeloid	SORT1
3.3063017444324E-58	1.53411680982091	0.347	0.213	5.54070046331982E-54	Myeloid	MED13L
3.70292942493086E-58	0.912880372652846	0.479	0.343	6.20536913029913E-54	Myeloid	LGALS3BP
5.51476567014527E-58	2.31506368127559	0.139	0.052	9.24164431002944E-54	Myeloid	ZKSCAN4
6.44189998700626E-58	1.49189217931328	0.164	0.067	1.07953359982251E-53	Myeloid	TNFAIP8
9.12858195110154E-58	1.3456155860825	0.267	0.142	1.5297677633656E-53	Myeloid	PAPSS1
1.9900966046543E-57	1.86639033780629	0.112	0.037	3.33500389007967E-53	Myeloid	SERPINB8
2.72363067835666E-57	1.75981592815055	0.391	0.26	4.56426029079009E-53	Myeloid	ZC3HAV1
2.98143813788187E-57	1.12709965739668	0.374	0.229	4.99629403146244E-53	Myeloid	CNN2
3.38185902152303E-57	2.13244440416501	0.14	0.053	5.66731934826829E-53	Myeloid	PLXNA1
3.8500356821605E-57	1.61731336676776	0.301	0.17	6.45188979616456E-53	Myeloid	DENND4B
4.38943645610745E-57	1.22345047467003	0.299	0.168	7.35581761314486E-53	Myeloid	USP15
1.97098030114407E-56	0.997494680258618	0.577	0.49	3.30296878865724E-52	Myeloid	CD63
2.91305390090602E-56	1.40509057545183	0.284	0.159	4.88169572713831E-52	Myeloid	SPTLC2
3.46154252568376E-56	1.31000595822121	0.182	0.079	5.80085296454084E-52	Myeloid	STX3
4.03580152743497E-56	1.03005845340778	0.13	0.046	6.76319619967552E-52	Myeloid	CD52
4.80468964491576E-56	1.10484424667182	0.104	0.033	8.05169890694984E-52	Myeloid	LPAR2
1.38637768078834E-55	1.39912707254586	0.342	0.212	2.32329171746511E-51	Myeloid	RNF13
3.77328483277444E-55	0.924861820366794	0.527	0.4	6.32327072276341E-51	Myeloid	DBNL
4.38899728675762E-55	1.79814768447629	0.267	0.147	7.35508165314843E-51	Myeloid	SRGAP1
7.65636577590929E-55	1.17857357549845	0.169	0.071	1.28305377672688E-50	Myeloid	ACSF3
9.05728170338845E-55	1.29478238373921	0.358	0.229	1.51781926785384E-50	Myeloid	ABHD12
1.28763515124551E-54	1.45720933882133	0.259	0.141	2.15781898645723E-50	Myeloid	BLVRA
1.49257650997962E-54	1.82444265603554	0.14	0.054	2.50125971542384E-50	Myeloid	SMCR8
1.87521135933113E-54	1.36322481725615	0.14	0.054	3.1424791959671E-50	Myeloid	AGO1
1.95611483698252E-54	1.6682674922017	0.239	0.126	3.2780572438153E-50	Myeloid	IL17RA
3.80429621631133E-54	1.07274053295122	0.33	0.196	6.37523959929452E-50	Myeloid	CSK
5.55916436348539E-54	1.29713842060651	0.212	0.101	9.31604764032882E-50	Myeloid	NPEPL1
5.79208169254129E-54	1.06764914824522	0.508	0.388	9.70637050036068E-50	Myeloid	DPYD
6.49127934669908E-54	1.73337744774278	0.255	0.139	1.08780859291983E-49	Myeloid	ST14
6.55378206499428E-54	2.0480918173627	0.187	0.087	1.09828279845174E-49	Myeloid	HPSE
8.20864571852112E-54	1.43476807210241	0.173	0.076	1.37560484950977E-49	Myeloid	ADAM17
1.08390839094028E-53	1.27623430109012	0.308	0.181	1.81641368153772E-49	Myeloid	PDK4
1.72104802504155E-53	1.1621188491122	0.192	0.088	2.88413228036462E-49	Myeloid	LIMS1
2.02258063949223E-53	2.582106506657	0.236	0.127	3.38944063566108E-49	Myeloid	RIPK2
3.36349522526744E-53	1.84726568880677	0.114	0.04	5.63654529850317E-49	Myeloid	TMOD2
5.13825972498986E-53	1.43417825978302	0.496	0.363	8.61069564713801E-49	Myeloid	CLK1
9.3108291866219E-53	1.39227800681197	0.151	0.062	1.5603087550941E-48	Myeloid	GLS
2.16276156401835E-52	1.76572126560961	0.231	0.12	3.62435582898194E-48	Myeloid	WDFY3
2.17317187405299E-52	1.63512040001543	0.109	0.037	3.641801426538E-48	Myeloid	SUFU
2.75201005524623E-52	1.56989939634026	0.2	0.096	4.61181845058163E-48	Myeloid	RP2
3.11064848508805E-52	1.82231457135502	0.196	0.093	5.21282473131055E-48	Myeloid	SNX30
3.15872966084381E-52	1.63388434130184	0.233	0.121	5.29339916564205E-48	Myeloid	MIS18BP1
6.78730726475441E-52	1.87348342139216	0.102	0.034	1.13741695142754E-47	Myeloid	WDR91
2.06583414839356E-51	1.04437670376855	0.312	0.179	3.46192486587792E-47	Myeloid	IRS2
2.37746776127669E-51	1.30098861852147	0.217	0.108	3.98416047434747E-47	Myeloid	INPP5B
5.57407556227049E-51	2.46783580434413	0.293	0.178	9.34103582725289E-47	Myeloid	APBB3
7.2643186990427E-51	0.917641723134383	0.407	0.267	1.21735452758558E-46	Myeloid	COMT
1.09447715986136E-50	0.636690329403926	0.106	0.035	1.83412482449567E-46	Myeloid	CST7
1.38370225412694E-50	1.64320768174934	0.241	0.129	2.31880823746593E-46	Myeloid	DBNDD2
2.48751660107141E-50	1.79520627164553	0.156	0.067	4.16858032007546E-46	Myeloid	PLD2
3.64170357244003E-50	1.34836541037117	0.749	0.658	6.102766846695E-46	Myeloid	DNAJB1
4.5527343505095E-50	1.08487218963825	0.324	0.195	7.62947222458382E-46	Myeloid	DCK

7.26709557816523E-50	1.46826097982489	0.18	0.083	1.21781987698893E-45	Myeloid	B3GNT2
9.47091306313587E-50	1.44855757824297	0.27	0.149	1.58713561112031E-45	Myeloid	ODF3B
1.00731713800785E-49	1.85646726768011	0.136	0.055	1.68806205987356E-45	Myeloid	TMPPE
1.88400637041294E-49	1.10703879395453	0.426	0.296	3.157217875538E-45	Myeloid	CORO1B
2.284401454562E-49	1.05405070481287	0.188	0.088	3.828199957555E-45	Myeloid	TP53
2.41098049775116E-49	1.11625310178923	0.453	0.324	4.0403211181314E-45	Myeloid	PIK3AP1
2.74241227418765E-49	1.46154671215749	0.199	0.097	4.59573448908366E-45	Myeloid	RAB32
4.66255017768704E-49	1.00458271585037	0.377	0.253	7.81350158776795E-45	Myeloid	ATP6V0B
7.28326261205571E-49	1.70801092261555	0.12	0.045	1.2205291485283E-44	Myeloid	TPK1
2.09788292356385E-48	1.10619553160176	0.398	0.265	3.51563220330829E-44	Myeloid	STAT2
2.19050883840684E-48	1.11806436379906	0.419	0.293	3.67085471140218E-44	Myeloid	STXBP2
2.67648013529738E-48	0.923281291492872	0.649	0.531	4.48524541073135E-44	Myeloid	VMP1
2.77006957246261E-48	1.00715708443214	0.439	0.319	4.64208258953284E-44	Myeloid	CD164
4.31986657006364E-48	0.728668798718219	0.15	0.062	7.23923239811264E-44	Myeloid	PDLIM2
5.17573393789804E-48	1.76794378809764	0.161	0.072	8.67349493312953E-44	Myeloid	ATG4C
6.00476718455701E-48	1.91424196954625	0.114	0.042	1.00627888478806E-43	Myeloid	AFF3
9.78770746518559E-48	0.909784790344778	0.522	0.398	1.6402240170158E-43	Myeloid	CDKN1B
1.3225477495605E-47	1.99695607086388	0.373	0.253	2.21632551871348E-43	Myeloid	ATF3
1.33229130213599E-47	1.34894589956043	0.371	0.244	2.23265376411949E-43	Myeloid	H2AFY
1.60995102194118E-47	1.72220328173265	0.249	0.142	2.69795592256903E-43	Myeloid	SLC36A1
2.1332925865905E-47	1.48694725136331	0.12	0.046	3.57497171660836E-43	Myeloid	PCYOX1L
3.2384245974802E-47	1.13121423664425	0.332	0.208	5.42695194045732E-43	Myeloid	NIN
3.24248789812516E-47	1.5961634533133	0.198	0.098	5.43376121967814E-43	Myeloid	DGLUCY
3.32813031839493E-47	1.86977636535035	0.16	0.072	5.57728078756623E-43	Myeloid	FOXRED2
4.38000506932084E-47	1.94524756346646	0.311	0.193	7.34001249516786E-43	Myeloid	ATG16L2
6.29947099283259E-47	1.26193069948352	0.288	0.17	1.05566534897889E-42	Myeloid	CEPT1
7.58870593095595E-47	0.906717381977343	0.473	0.35	1.2717153399096E-42	Myeloid	RHOA
7.95212433129763E-47	1.90104909984395	0.107	0.039	1.33261699543886E-42	Myeloid	REEP4
9.08001402837917E-47	1.36620102740315	0.267	0.153	1.52162875087578E-42	Myeloid	RIC1
9.45237806288608E-47	1.36292386676072	0.118	0.045	1.58402951577845E-42	Myeloid	MAPK13
1.51193458403925E-46	1.44036736170093	0.211	0.109	2.53369997593297E-42	Myeloid	WDR11
1.51770161303281E-46	1.3956702579953	0.248	0.137	2.54336436312039E-42	Myeloid	FEM1B
3.3372216503359E-46	1.12219509958284	0.225	0.119	5.59251604163291E-42	Myeloid	PIP5K1C
3.61600975466865E-46	1.23520769925757	0.174	0.081	6.05970914687372E-42	Myeloid	TBC1D10A
4.76236988887235E-46	1.04643713389353	0.194	0.094	7.98077945977228E-42	Myeloid	OAS2
7.32201647426549E-46	1.19569905503221	0.245	0.135	1.22702352075741E-41	Myeloid	INPP4A
1.11676472569766E-45	1.99494589472	0.238	0.133	1.87147432732415E-41	Myeloid	CKS2
1.14554023562927E-45	2.03587951739848	0.153	0.069	1.91969632686753E-41	Myeloid	SLC45A4
1.63148576606219E-45	1.68703364813407	0.212	0.111	2.73404384676701E-41	Myeloid	TBC1D9
1.78498640171932E-45	1.22292668640918	0.261	0.15	2.99128021200124E-41	Myeloid	ANKFY1
2.01677131856572E-45	0.604155236097195	0.166	0.074	3.37970537565244E-41	Myeloid	SOCS1
3.49370221565997E-45	1.32482148054563	0.468	0.348	5.85474617300297E-41	Myeloid	CHD2
5.64566808037936E-45	1.68451346893152	0.231	0.128	9.46101056909973E-41	Myeloid	WARS
6.44135852925964E-45	1.17627185025747	0.356	0.241	1.07944286233333E-40	Myeloid	MGAT4A
7.49489635525703E-45	1.73549515010645	0.151	0.067	1.25599473121397E-40	Myeloid	C2CD2L
1.16269984798512E-44	1.53786083322565	0.129	0.053	1.94845240525347E-40	Myeloid	ZNF589
1.48668547286751E-44	1.81739070000824	0.262	0.156	2.49138751543138E-40	Myeloid	APAF1
1.53273758361373E-44	1.0054949427676	0.538	0.413	2.56856164261989E-40	Myeloid	ITPR2
3.54598545834828E-44	1.34952244554859	0.274	0.164	5.94236243110005E-40	Myeloid	ACP2
4.15832771515502E-44	2.4335886777459	0.159	0.075	6.96852558505678E-40	Myeloid	B3GNT5
6.95475954702777E-44	1.28565620293753	0.362	0.244	1.16547860489091E-39	Myeloid	GPATCH2L
8.59587065649016E-44	1.29139156691212	0.299	0.184	1.44049600461462E-39	Myeloid	OSBPL8
1.15791097701817E-43	0.847053533846828	0.476	0.362	1.94042721528704E-39	Myeloid	ANXA11
1.83459085691958E-43	1.4381712626816	0.382	0.27	3.07440735802584E-39	Myeloid	RAB20
4.56903557441663E-43	1.16571568363977	0.447	0.34	7.65678981560738E-39	Myeloid	STAT1
5.07460766027336E-43	1.33982906589851	0.381	0.264	8.5040275170861E-39	Myeloid	DEDD2
6.79971645351014E-43	1.06214176015014	0.433	0.313	1.13949648327923E-38	Myeloid	MINK1
7.99890818951643E-43	0.711066245216684	0.622	0.554	1.34045703439916E-38	Myeloid	OS9
1.21160045451353E-42	1.74819046913646	0.227	0.126	2.03040004167377E-38	Myeloid	TESK1
1.48477105928652E-42	1.17173057288191	0.288	0.176	2.48817934115235E-38	Myeloid	TRIM14
1.62227712177126E-42	1.14830712833955	0.336	0.22	2.71861200066427E-38	Myeloid	ROCK1
2.36238037010386E-42	1.12416294834478	0.226	0.124	3.95887702422005E-38	Myeloid	ZBTB4
2.53227340923131E-42	1.28652616323258	0.305	0.191	4.24358377918983E-38	Myeloid	INPPL1

**2.76971183207364E-42**	0.9273614033106	0.235	0.129	4.641483088189E-38	Myeloid	PML
**2.84112496128147E-42**	1.04423278637302	0.271	0.158	4.76115721011549E-38	Myeloid	ST3GAL1
**3.10049339361853E-42**	2.41030085178191	0.108	0.042	5.19580682902593E-38	Myeloid	FZD1
**8.72254978645667E-42**	1.0480960537491	0.113	0.044	1.46172489321441E-37	Myeloid	SLCO3A1
**1.43633714325393E-41**	0.89310566475882	0.203	0.105	2.40701378466493E-37	Myeloid	SH3KBP1
**1.77975604477064E-41**	1.15618867901567	0.351	0.234	2.98251517982663E-37	Myeloid	DEPRECATED-ENSG00000124831
**1.85787135945311E-41**	0.76964166016698	0.199	0.099	3.11342082417153E-37	Myeloid	RFTN1
**1.89892664623049E-41**	1.62179205777491	0.357	0.243	3.18222127375306E-37	Myeloid	DOCK5
**2.85223308520309E-41**	1.44016917577308	0.205	0.11	4.77977220418334E-37	Myeloid	SGSH
**3.13740328964161E-41**	0.988894249004221	0.829	0.756	5.25766043278141E-37	Myeloid	HSPA1A
**3.38185042959925E-41**	1.56233675194861	0.146	0.067	5.66730494992243E-37	Myeloid	SENP3
**4.39596406839645E-41**	1.23762270730624	0.266	0.157	7.36675658581878E-37	Myeloid	LRRFIP1
**5.15222937419259E-41**	1.10546222017248	0.323	0.209	8.63410598527194E-37	Myeloid	CORO1C
**5.18588788519019E-41**	0.434513317405604	0.237	0.13	8.69051091800172E-37	Myeloid	CLEC2D
**6.76813225502972E-41**	1.52838628718224	0.167	0.08	1.13420360329788E-36	Myeloid	RASSF5
**7.52069739006603E-41**	1.32637510566784	0.321	0.21	1.26031846862727E-36	Myeloid	ITSN2
**7.74810042379845E-41**	0.9200237809357	0.321	0.207	1.29842666902015E-36	Myeloid	AAK1
**1.17428225430648E-40**	1.4115382071459	0.212	0.114	1.9678622017668E-36	Myeloid	KDM2B
**1.50997294010324E-40**	1.59681143092351	0.268	0.164	2.53041265302501E-36	Myeloid	ITSN1
**2.56853308956838E-40**	2.2306561960915	0.128	0.055	4.30434775149869E-36	Myeloid	LACC1
**2.72455235094872E-40**	0.850880130694682	0.673	0.613	4.56580482971986E-36	Myeloid	ZFP36L1
**3.32447209407192E-40**	0.680705067414879	0.577	0.515	5.57115033524573E-36	Myeloid	AHNAK
**5.08616248341492E-40**	1.31339960657401	0.228	0.128	8.52339108970673E-36	Myeloid	TNK2
**5.18550455618973E-40**	0.866320433501811	0.518	0.423	8.68986853526275E-36	Myeloid	CNDP2
**7.75219292879E-40**	1.13168554401571	0.458	0.35	1.29911249100663E-35	Myeloid	ARRDC2
**1.08381214260825E-39**	1.2846799494747	0.213	0.118	1.8162523885829E-35	Myeloid	ZNF562
**2.69030179642445E-39**	0.920005908319561	0.161	0.076	4.5084077504481E-35	Myeloid	BCL2
**3.4340865586554E-39**	1.33379124805474	0.256	0.151	5.75484225499472E-35	Myeloid	RAB12
**5.6844504533495E-39**	1.39554624569229	0.297	0.188	9.52600206972309E-35	Myeloid	TRIM38
**5.90632208002374E-39**	1.87746353202396	0.14	0.065	9.89781454170377E-35	Myeloid	SASH1
**1.10884294481895E-38**	1.54013816912024	0.135	0.061	1.85819900692759E-34	Myeloid	ANKRD37
**1.33572195782717E-38**	1.25019516324562	0.223	0.125	2.23840285692678E-34	Myeloid	DUSP22
**1.63114410632727E-38**	0.785252014325998	0.565	0.466	2.73347129338324E-34	Myeloid	PLXNB2
**2.38675618279781E-38**	1.04535069987152	0.293	0.187	3.99972601113257E-34	Myeloid	MGAT5
**2.43585276511016E-38**	1.30164196243959	0.214	0.12	4.08200206377161E-34	Myeloid	VAC14
**3.15302776250391E-38**	1.23151335835358	0.26	0.158	5.28384392440406E-34	Myeloid	DIP2B
**3.66477508464335E-38**	1.39257360728184	0.186	0.097	6.14143008684532E-34	Myeloid	MAP3K1
**4.59649219119801E-38**	1.44503864892227	0.24	0.141	7.70280161400963E-34	Myeloid	SPNS1
**6.31207291973777E-38**	0.911514883330505	0.169	0.084	1.05777717988965E-33	Myeloid	ADCY3
**8.90308719904794E-38**	1.16310118440793	0.135	0.061	1.49197935281645E-33	Myeloid	CAP1
**9.42978275727504E-38**	1.48090516424188	0.147	0.07	1.58024299446415E-33	Myeloid	TFEB
**1.22425101092253E-37**	0.885416000047536	0.251	0.147	2.05159984410398E-33	Myeloid	EPS15
**1.31321572240323E-37**	1.60020272582406	0.177	0.091	2.20068690760333E-33	Myeloid	AGTPBP1
**1.64218838621842E-37**	0.916821297357882	0.364	0.251	2.75197929762483E-33	Myeloid	GABARAP
**1.8030909856986E-37**	1.29695095132594	0.227	0.131	3.02161987383371E-33	Myeloid	TTL
**2.74975058038973E-37**	1.0564330026237	0.194	0.103	4.60803202261711E-33	Myeloid	LZTS2
**5.40941973050422E-37**	0.956269447879046	0.326	0.217	9.06510558437897E-33	Myeloid	TMEM127
**6.79564028755007E-37**	0.805860570535427	0.295	0.185	1.13881339938764E-32	Myeloid	CTDSP2
**8.29263126115862E-37**	1.1974619403209	0.151	0.072	1.38967914674496E-32	Myeloid	SDSL
**8.37203316181827E-37**	0.875043471213954	0.35	0.24	1.40298531725751E-32	Myeloid	STK4
**9.25288650967109E-37**	1.08934702545006	0.263	0.161	1.55059872129068E-32	Myeloid	FOXN2
**1.09368685091043E-36**	1.61673101235996	0.15	0.073	1.8328004247557E-32	Myeloid	ZDHHC7
**1.10206395633402E-36**	2.00150308071034	0.334	0.231	1.84683877802455E-32	Myeloid	GLA
**2.2473266098636E-36**	1.17757353613295	0.246	0.148	3.76606993280943E-32	Myeloid	TANGO2
**2.61841419424733E-36**	1.49455593318732	0.297	0.188	4.38793850671968E-32	Myeloid	SERPINH1
**3.24689322217101E-36**	1.0317632822921	0.176	0.091	5.44114366171418E-32	Myeloid	DEPRECATED-ENSG00000150867
**3.43620050810794E-36**	1.1080803253633	0.316	0.212	5.75838481148729E-32	Myeloid	SGPP1
**4.40209425193394E-36**	0.65623152009422	0.621	0.579	7.37702954739089E-32	Myeloid	GRINA
**6.67417447596402E-36**	1.00727165924592	0.382	0.274	1.11845815868205E-31	Myeloid	RNASEK
**1.27426865628636E-35**	1.97882205363425	0.177	0.095	2.13541941420468E-31	Myeloid	ID3
**1.50695939430578E-35**	0.770477200153312	0.296	0.188	2.52536255297763E-31	Myeloid	ACTR1A
**1.74354197292371E-35**	1.04592437981967	0.254	0.153	2.92182763822556E-31	Myeloid	SORBS3
**1.82358206711625E-35**	1.25807253964428	0.185	0.099	3.0559588280734E-31	Myeloid	SLC66A3

2.08337958238048E-35	1.50827141831692	0.33	0.228	3.4913275041532E-31	Myeloid	RUFY3
2.16305001516087E-35	1.80616075823743	0.142	0.069	3.62483921540659E-31	Myeloid	PPARGC1B
2.93632249708314E-35	0.873663638471878	0.318	0.212	4.92068924061192E-31	Myeloid	HECA
3.87170781326369E-35	1.06076716479148	0.197	0.108	6.48820795346728E-31	Myeloid	CTDNEP1
4.08318980252932E-35	1.43727644749363	0.156	0.079	6.84260947107863E-31	Myeloid	CARD8
5.66573454451644E-35	0.802820034314143	0.224	0.129	9.49463794970064E-31	Myeloid	PIP4K2A
7.25766750984546E-35	1.83990427372537	0.245	0.152	1.2162399212999E-30	Myeloid	HEATR3
2.08936947565074E-34	2.02075979629551	0.224	0.138	3.50136536729552E-30	Myeloid	KLF2
2.17117761217241E-34	0.959102674030338	0.374	0.268	3.63845944247852E-30	Myeloid	CMIP
2.22677066847316E-34	0.888822044849635	0.554	0.517	3.73162228622731E-30	Myeloid	CREG1
2.41813734078759E-34	1.21440379524456	0.195	0.108	4.05231455569184E-30	Myeloid	RNF145
2.43697571790163E-34	1.14311862351317	0.352	0.252	4.08388390805955E-30	Myeloid	TPD52L2
2.45981177642088E-34	0.930220879670542	0.162	0.082	4.12215257492612E-30	Myeloid	FRAT2
3.84283500181235E-34	1.05761624298986	0.166	0.085	6.43982289603714E-30	Myeloid	SLC15A4
3.92955707985648E-34	1.14663562953055	0.244	0.147	6.58515175442348E-30	Myeloid	TCF12
4.25590433071875E-34	1.49427834499565	0.122	0.055	7.13204447741848E-30	Myeloid	NEK3
6.33278144765328E-34	1.04001444419203	0.229	0.137	1.06124751499774E-29	Myeloid	PRKACB
6.38251389816464E-34	1.02862527442359	0.335	0.23	1.06958167905443E-29	Myeloid	ELMO1
9.53981343666728E-34	1.32880380396593	0.223	0.132	1.5986819357167E-29	Myeloid	VPS11
1.55197068600829E-33	1.21584182004987	0.211	0.122	2.60079247561269E-29	Myeloid	VPS53
1.9807746051613E-33	1.30901428902347	0.201	0.115	3.31938208332931E-29	Myeloid	NHLRC3
2.44235071120983E-33	0.517761671196755	0.344	0.234	4.09289132184543E-29	Myeloid	SARAF
5.23003520303503E-33	1.51680775893662	0.144	0.072	8.7644929932461E-29	Myeloid	ZMYM6
5.55944547219503E-33	0.782158300614659	0.511	0.421	9.31651872230442E-29	Myeloid	TNFRSF1A
5.81593528556363E-33	0.858296914677306	0.292	0.188	9.74634435154753E-29	Myeloid	UBXN1
7.64555102676746E-33	1.05215084986599	0.371	0.273	1.28124144106569E-28	Myeloid	CHID1
8.24737477367077E-33	0.906227956019331	0.153	0.076	1.38209506457175E-28	Myeloid	DUSP6
2.30241890230094E-32	1.424708115877	0.206	0.12	3.85839359647591E-28	Myeloid	EVI5
2.31374093278077E-32	1.13688043613475	0.246	0.153	3.87736705515402E-28	Myeloid	APPL1
3.16787067656526E-32	0.958182501133916	0.309	0.211	5.30871767978806E-28	Myeloid	SNX9
7.08008444951036E-32	1.12712943659965	0.221	0.13	1.18648055204895E-27	Myeloid	ZNF609
8.02852442498412E-32	1.04628820703167	0.258	0.163	1.34542012313884E-27	Myeloid	PITPNA
1.00755629399231E-31	1.27607694065859	0.392	0.291	1.68846283747232E-27	Myeloid	PLSCR1
1.03363900081984E-31	0.881392086762687	0.254	0.156	1.73217223757388E-27	Myeloid	PPFIBP2
1.04052541031193E-31	1.02938202353024	0.184	0.102	1.74371248260073E-27	Myeloid	GBP4
1.38939418307514E-31	0.895612979587086	0.383	0.28	2.32834677199733E-27	Myeloid	MED15
1.6537615580732E-31	1.16047238738874	0.334	0.233	2.77137361901906E-27	Myeloid	DENND1A
2.6648816369342E-31	0.933770423063029	0.198	0.112	4.46580864717433E-27	Myeloid	PBRM1
2.94323166235747E-31	2.18713440577967	0.224	0.14	4.93226761977865E-27	Myeloid	MXD1
3.3152984764975E-31	0.707738774098598	0.215	0.127	5.55577718691452E-27	Myeloid	PARP8
4.21063753591203E-31	0.744574479290823	0.218	0.128	7.05618638268138E-27	Myeloid	TMEM35B
6.01626292963928E-31	1.14824981524035	0.129	0.062	1.00820534174895E-26	Myeloid	MAD1L1
1.03588268799803E-30	0.898921220317392	0.293	0.195	1.73593220854711E-26	Myeloid	RFX5
1.08046535860968E-30	1.2951626622027	0.249	0.163	1.8106438479581E-26	Myeloid	WLS
1.26143711142594E-30	0.957861567114435	0.11	0.049	2.11391631132758E-26	Myeloid	CEP85L
1.37211660161407E-30	1.17450625460967	0.227	0.139	2.29939300098485E-26	Myeloid	SPPL3
1.40342860308649E-30	0.77784366930385	0.419	0.317	2.35186565305234E-26	Myeloid	CYBC1
1.49519056404114E-30	1.36592061826991	0.12	0.056	2.50564034722015E-26	Myeloid	SLC39A10
1.7393446414431E-30	0.967711219643008	0.173	0.095	2.91479375013035E-26	Myeloid	ARPC3
1.92265206945428E-30	1.42255054150939	0.235	0.149	3.22198033799148E-26	Myeloid	PLXNC1
2.28636806009829E-30	1.27063100509717	0.189	0.108	3.83149559511272E-26	Myeloid	MIF4GD
2.51295991855672E-30	1.24321832988227	0.14	0.071	4.21121823151735E-26	Myeloid	CRLF3
2.62809845919826E-30	0.898371709447732	0.351	0.253	4.40416739792444E-26	Myeloid	ARPC4
2.69318799276577E-30	1.49904880950597	0.259	0.17	4.51324443827688E-26	Myeloid	SPIN1
2.86785748403939E-30	1.19811940268625	0.427	0.324	4.80595557175321E-26	Myeloid	ADAM9
3.27944594852527E-30	0.919769260504341	0.238	0.149	5.49569552053865E-26	Myeloid	DCP2
5.99217921827364E-30	0.691687918205456	0.14	0.069	1.0041693933983E-25	Myeloid	AHDC1
6.10583062736397E-30	0.977996242670336	0.382	0.285	1.02321509653365E-25	Myeloid	BACH1
6.38889872176925E-30	2.03810811212608	0.295	0.207	1.07065164779409E-25	Myeloid	RASGEF1B
6.61893953989182E-30	1.43642597283817	0.116	0.055	1.10920188809507E-25	Myeloid	SNX11
9.73786759303014E-30	1.21170306643476	0.7	0.653	1.63187185123999E-25	Myeloid	RHOB
1.02169081293185E-29	0.782946633291923	0.22	0.133	1.71214946431119E-25	Myeloid	NPC1
1.04777564667633E-29	1.12236092324268	0.125	0.06	1.75586242870019E-25	Myeloid	TRIM65

1.0559270848707E-29	1.15064355768381	0.15	0.078	1.76952260882631E-25	Myeloid	CCDC115
1.20230202611407E-29	1.1883842763739	0.263	0.173	2.01481773536195E-25	Myeloid	MAP2K1
1.36104553758544E-29	1.47666155684692	0.18	0.103	2.28084011188569E-25	Myeloid	AGO4
1.92483076842238E-29	1.07842747818353	0.3	0.207	3.22563140172222E-25	Myeloid	STX6
2.10351782093711E-29	1.60169036611647	0.246	0.161	3.52507516432641E-25	Myeloid	STK38L
2.67365557832324E-29	1.04452673216446	0.293	0.199	4.48051201815408E-25	Myeloid	FOXN3
2.8018386747953E-29	1.09202348204992	0.25	0.159	4.69532125122196E-25	Myeloid	LRMDA
3.47922974720396E-29	0.762353378477923	0.666	0.611	5.83049321036439E-25	Myeloid	HSPA8
7.59488801152626E-29	1.47300155583648	0.121	0.058	1.27275133297157E-24	Myeloid	INTS9
8.33817585066518E-29	1.32225065670125	0.207	0.125	1.39731150905447E-24	Myeloid	DOCK7
8.41821664152635E-29	1.07452044313796	0.242	0.153	1.41072474478699E-24	Myeloid	UBAC1
8.77671751267544E-29	1.84049846954409	0.188	0.11	1.47080232077415E-24	Myeloid	CNEP1R1
8.89794717323763E-29	1.58572379987592	0.113	0.053	1.49111798729116E-24	Myeloid	RASAL2
1.03818047201905E-28	1.07197067678223	0.324	0.231	1.73978283500953E-24	Myeloid	TSPAN33
1.14499722701294E-28	1.70968298253253	0.186	0.108	1.91878635302828E-24	Myeloid	SCLT1
1.18740131944047E-28	1.13821930426398	0.184	0.106	1.98984713111834E-24	Myeloid	RNF111
1.78050496477388E-28	0.908805333392104	0.403	0.308	2.98377021996806E-24	Myeloid	TCIRG1
2.06716259446103E-28	0.475287687414092	0.41	0.307	3.4641510757978E-24	Myeloid	MACF1
2.41366254405392E-28	1.40589575475512	0.229	0.144	4.04481569132555E-24	Myeloid	FER
2.92428025193947E-28	1.43654563171043	0.167	0.093	4.90050884620016E-24	Myeloid	SCN1B
4.69203092704329E-28	1.04203622143546	0.131	0.066	7.86290542753914E-24	Myeloid	SLC12A6
4.81214652773921E-28	1.36214769807025	0.277	0.188	8.06419515118537E-24	Myeloid	NXF1
6.64432802759232E-28	0.588751897889577	0.546	0.454	1.11345649086392E-23	Myeloid	RNF213
8.79962074360145E-28	1.33933555017389	0.163	0.09	1.47464044421273E-23	Myeloid	TDRKH
9.41625899660664E-28	1.22660961473752	0.327	0.237	1.57797668265134E-23	Myeloid	TENT2
1.1918643989038E-27	0.807748373737305	0.244	0.155	1.99732635968298E-23	Myeloid	PACS1
1.24421979137133E-27	1.62937124767945	0.13	0.067	2.08506352638008E-23	Myeloid	ZNF532
1.26329752833037E-27	1.30341108267782	0.248	0.168	2.11703399797604E-23	Myeloid	ACE
1.42736035333498E-27	1.21076412101062	0.204	0.123	2.39197048011876E-23	Myeloid	LACTB
1.48861469908052E-27	0.93654395915218	0.2	0.12	2.49462051271914E-23	Myeloid	ANTXR2
1.51876525826628E-27	0.735248204490283	0.381	0.292	2.54514681980263E-23	Myeloid	DCAF7
1.84509577732726E-27	0.892208753734459	0.112	0.053	3.09201150364502E-23	Myeloid	KSR1
2.5049414790449E-27	1.14025537947708	0.248	0.161	4.19778093058344E-23	Myeloid	WIPI2
2.76496915878749E-27	1.08874908959821	0.159	0.088	4.63353531629607E-23	Myeloid	ARF3
3.06391895003382E-27	0.825163574644731	0.201	0.121	5.13451537646668E-23	Myeloid	SLC25A6
3.3333068570624E-27	1.14965717652581	0.298	0.209	5.58595563106517E-23	Myeloid	ABI1
3.37181180963764E-27	1.27347748417687	0.25	0.163	5.65048223059075E-23	Myeloid	GAS2L3
4.37852460307978E-27	0.63948354112722	0.525	0.438	7.33753152984109E-23	Myeloid	DDIT4
4.69336454035913E-27	1.04800487653734	0.22	0.137	7.86514029673383E-23	Myeloid	FAM53B
5.01407725698227E-27	1.19219623516564	0.248	0.164	8.40259067725089E-23	Myeloid	ZNF106
6.04301544339058E-27	1.03292845329608	0.248	0.162	1.01268852800339E-22	Myeloid	ATP6V1F
6.32916580860455E-27	0.820637784774906	0.369	0.277	1.06064160620595E-22	Myeloid	TSPO
6.37954994604797E-27	1.14648158365022	0.334	0.242	1.06908497995872E-22	Myeloid	AKAP9
6.91407282165889E-27	1.21855446251571	0.167	0.095	1.1586603234536E-22	Myeloid	AGAP3
7.18712478928263E-27	0.860436461009109	0.22	0.135	1.20441837218798E-22	Myeloid	MCRIP1
7.26617403511427E-27	0.862673841633029	0.336	0.244	1.21766544480445E-22	Myeloid	GLB1
8.13804748171502E-27	0.946287141601733	0.247	0.161	1.3637739969858E-22	Myeloid	IFIH1
1.18391973390225E-26	0.876704882349048	0.254	0.167	1.98401269007339E-22	Myeloid	CLSTN1
1.45441147027009E-26	1.21018468026978	0.198	0.12	2.43730274187861E-22	Myeloid	MADD
1.45931816223508E-26	1.63495359942396	0.27	0.186	2.44552537627355E-22	Myeloid	ZFYVE16
2.2422037836689E-26	0.743170916835743	0.313	0.217	3.75748510067234E-22	Myeloid	PYCARD
2.27475861272509E-26	0.855646708380451	0.203	0.123	3.8120404832047E-22	Myeloid	SP110
2.74788100741463E-26	1.16890169619663	0.26	0.176	4.60489899222544E-22	Myeloid	ITPRIPL2
3.12757615902424E-26	0.901595408385538	0.207	0.126	5.24119212729282E-22	Myeloid	SP140L
3.56194558088084E-26	1.19094411921485	0.245	0.16	5.96910840444011E-22	Myeloid	ZBED1
3.6778127731516E-26	0.94810197391221	0.202	0.123	6.16327864524745E-22	Myeloid	MAP3K7
9.84065401665733E-26	0.820131094113785	0.301	0.209	1.64909680011143E-21	Myeloid	KLC1
1.26303875468237E-25	0.818659600460545	0.152	0.081	2.11660034509671E-21	Myeloid	PALM2-AKAP2
1.42948718492398E-25	0.991527204218166	0.2	0.121	2.3955346244956E-21	Myeloid	LRCH1
1.45329472642781E-25	1.29654706291913	0.207	0.13	2.43543130254772E-21	Myeloid	TES
1.62893290743711E-25	1.53948304420724	0.206	0.129	2.72976576628311E-21	Myeloid	SRC
2.14425713117303E-25	1.04415139620116	0.266	0.18	3.59334610041976E-21	Myeloid	CPPED1
2.33550056483072E-25	1.16349740862448	0.177	0.105	3.91383184654332E-21	Myeloid	CHFR

2.42667286118079E-25	0.903278827599166	0.266	0.18	4.06661838076676E-21	Myeloid	TMEM165
3.33556053907181E-25	0.764738709282957	0.219	0.137	5.58973235137653E-21	Myeloid	YPEL3
3.88377635989955E-25	1.43804595036043	0.104	0.05	6.50843242391967E-21	Myeloid	GTDC1
3.892954052375E-25	1.05479501515741	0.187	0.113	6.52381240097002E-21	Myeloid	EPG5
4.52736869333396E-25	1.44934562556536	0.196	0.121	7.58696445628904E-21	Myeloid	SDCCAG8
4.7373756459898E-25	1.11331621016196	0.268	0.183	7.93889410754971E-21	Myeloid	FGFR1OP2
5.67356539945715E-25	2.60472781403716	0.129	0.07	9.5077608964103E-21	Myeloid	SNAPC1
6.11491215226212E-25	1.39872214402394	0.378	0.299	1.02473697847609E-20	Myeloid	UGCG
6.26935028590538E-25	0.806598093025052	0.413	0.318	1.05061772091202E-20	Myeloid	STAT6
6.30350643238274E-25	0.94004454515772	0.261	0.178	1.0563416079387E-20	Myeloid	NCOA3
6.72231019697733E-25	0.753068131916114	0.203	0.123	1.12652474280946E-20	Myeloid	MAPRE2
6.99925891446796E-25	0.974927998244649	0.276	0.189	1.17293580888654E-20	Myeloid	KLHL24
7.11147233492297E-25	1.26735217952173	0.208	0.13	1.19174053388639E-20	Myeloid	LMBR1L
7.13914701301746E-25	0.632869255740208	0.538	0.457	1.19637825644147E-20	Myeloid	RSRP1
7.18162478706415E-25	0.98296539778208	0.162	0.092	1.20349668181621E-20	Myeloid	NUP93
8.27466745210275E-25	0.637517358664043	0.518	0.407	1.38666877162338E-20	Myeloid	IKBKB
8.9698516216683E-25	1.49821847709721	0.337	0.256	1.50316773475917E-20	Myeloid	DDX3X
1.01031112405802E-24	1.18574484726975	0.317	0.233	1.69307938169643E-20	Myeloid	ZFHX3
1.05006908270973E-24	0.954616559328128	0.178	0.106	1.75970576880496E-20	Myeloid	TRAF6
1.15256852252865E-24	1.04737997962081	0.133	0.071	1.93147433005351E-20	Myeloid	ABTB1
1.27578077881921E-24	1.08872869370668	0.306	0.221	2.13795342914523E-20	Myeloid	PRPF38B
1.3033863310122E-24	1.42394514896969	0.279	0.197	2.18421481351024E-20	Myeloid	FNIP2
1.47036279526734E-24	1.55956810669464	0.137	0.076	2.46403397230902E-20	Myeloid	ALPK1
1.49495597833391E-24	0.844594151851074	0.27	0.187	2.50524722849196E-20	Myeloid	ORMDL1
1.61061140456178E-24	0.716943142625021	0.301	0.213	2.69906259176463E-20	Myeloid	TBC1D15
2.14637446470256E-24	1.25073486973606	0.154	0.087	3.59689432794855E-20	Myeloid	STRADA
2.16020270281575E-24	1.0261516870855	0.33	0.244	3.62006768937864E-20	Myeloid	SKAP2
2.17853047038783E-24	0.933208522530715	0.166	0.097	3.65078136227593E-20	Myeloid	MOB1A
2.99853438399221E-24	1.0213198461386	0.239	0.16	5.02494392069415E-20	Myeloid	ARHGAP26
7.65325543213336E-24	0.854660671943581	0.262	0.18	1.28253254531691E-19	Myeloid	PPP1R12A
8.42176330374287E-24	1.07533933058983	0.277	0.194	1.41131909444123E-19	Myeloid	SNX10
8.74922222434802E-24	0.810872186818106	0.224	0.144	1.46619466035624E-19	Myeloid	SLC31A1
1.14324823600579E-23	0.785209489224684	0.376	0.286	1.9158553938985E-19	Myeloid	B4GALT1
1.15206937867792E-23	1.09605543584475	0.261	0.18	1.93063786478846E-19	Myeloid	RAB4B
1.1916714817621E-23	0.972975372371888	0.137	0.076	1.99700306913693E-19	Myeloid	MAN1C1
1.25426424593627E-23	0.68289288136121	0.35	0.266	2.10189602333999E-19	Myeloid	RAP1A
1.3324854313877E-23	1.27004272172942	0.106	0.053	2.23297908591951E-19	Myeloid	EOGT
1.46392380600898E-23	0.874970950906494	0.213	0.136	2.45324351410985E-19	Myeloid	GDE1
1.55249578854682E-23	1.0753556243536	0.403	0.316	2.60167244244675E-19	Myeloid	TBC1D2B
1.79738404560379E-23	0.81455247614736	0.388	0.301	3.01205618362284E-19	Myeloid	MARCH7
1.98115299670484E-23	0.8237417915307	0.22	0.142	3.32001619187797E-19	Myeloid	GLMP
2.08294462051437E-23	0.818442043881226	0.343	0.254	3.49059859505798E-19	Myeloid	OGFR
2.32926614887762E-23	1.07226673347555	0.275	0.193	3.90338421228911E-19	Myeloid	TUBGCP2
2.33157326811191E-23	1.02836446598457	0.267	0.186	3.90725048270194E-19	Myeloid	RCAN1
2.42665423243561E-23	1.45630534856796	0.12	0.063	4.0665871627156E-19	Myeloid	ZNF641
2.73422628070541E-23	0.261583930352064	0.179	0.103	4.58201640120612E-19	Myeloid	MYC
3.55922374607557E-23	1.20134042506346	0.218	0.143	5.96454715367344E-19	Myeloid	PGS1
3.67377975015471E-23	0.962812157882356	0.195	0.121	6.15652010530926E-19	Myeloid	PPP1R21
3.85176558646289E-23	0.97070452399871	0.212	0.136	6.4547887697945E-19	Myeloid	PIAS2
3.86790641131826E-23	0.832045692347677	0.33	0.247	6.48183756408713E-19	Myeloid	GBA
3.99080701700559E-23	0.752425488917406	0.373	0.287	6.68779439909797E-19	Myeloid	SPG21
4.38742368666635E-23	1.43593660735447	0.171	0.104	7.35244461411547E-19	Myeloid	VPS35
4.66863983669706E-23	1.74295541203503	0.171	0.104	7.82370663833693E-19	Myeloid	SCO2
4.92113517888933E-23	1.10549050347922	0.174	0.105	8.24683833278274E-19	Myeloid	COMMD9
6.01100968791003E-23	0.740007613833191	0.147	0.083	1.00732500349996E-18	Myeloid	BTN3A3
7.81945952032911E-23	0.971262675035488	0.237	0.16	1.31038502641675E-18	Myeloid	ARID1A
8.38278304818555E-23	1.43241191788606	0.233	0.159	1.40478678321493E-18	Myeloid	NCEH1
8.66892046944497E-23	0.677027472106851	0.232	0.155	1.45273769226959E-18	Myeloid	RELL1
9.13449276290655E-23	1.20499248036774	0.341	0.259	1.53075829720788E-18	Myeloid	STX4
9.26815481986659E-23	0.767866244986277	0.238	0.159	1.55315738471324E-18	Myeloid	DNAJB12
1.15231213905784E-22	1.08130448260291	0.17	0.103	1.93104468263313E-18	Myeloid	RALB
1.18101245194435E-22	1.57875322780479	0.116	0.061	1.97914066696834E-18	Myeloid	TET2
1.2546337343263E-22	0.504716769932551	0.404	0.317	2.10251521198401E-18	Myeloid	JAK1

1.31142678118939E-22	0.878724940996146	0.334	0.253	2.19768899991717E-18	Myeloid	TAP2
1.56090642067818E-22	1.05316081799764	0.396	0.312	2.6157669797725E-18	Myeloid	MYO15B
1.70117377320513E-22	0.957847151004548	0.314	0.231	2.85082700913715E-18	Myeloid	RAP2A
2.0895446637414E-22	1.75615015547998	0.153	0.09	3.50165894749784E-18	Myeloid	SLC26A11
2.90265861882365E-22	1.23669686110317	0.246	0.169	4.86427531342468E-18	Myeloid	ACAP2
3.15028336624218E-22	0.744205124316188	0.334	0.253	5.27924486514864E-18	Myeloid	MXD4
3.26652918943329E-22	0.881702404657189	0.185	0.113	5.4740496156523E-18	Myeloid	VAMP5
3.48035222903638E-22	1.13674608882665	0.151	0.086	5.83237426541916E-18	Myeloid	NAB2
4.14158743391562E-22	1.02881436762615	0.343	0.261	6.94047222175579E-18	Myeloid	ASAP1
4.18272938460252E-22	0.887831408976186	0.408	0.333	7.00941790271691E-18	Myeloid	VASP
4.69494082641334E-22	0.90352648499866	0.221	0.147	7.86778183690347E-18	Myeloid	CRYBG1
4.90685219706057E-22	1.2608913491769	0.415	0.343	8.2229029118341E-18	Myeloid	GADD45B
5.02435928453547E-22	0.924080476212182	0.185	0.114	8.41982128902455E-18	Myeloid	PPP3CB
6.53126482027514E-22	0.939667556725215	0.138	0.077	1.09450935858171E-17	Myeloid	SMIM29
7.17460628140502E-22	1.59638456235863	0.146	0.085	1.20232052063785E-17	Myeloid	IQSEC2
8.56720002874295E-22	2.45949291703748	0.137	0.079	1.43569138081674E-17	Myeloid	PRMT9
9.41715984634114E-22	0.886899474923936	0.306	0.223	1.57812764704985E-17	Myeloid	SCAMP4
9.58828103565548E-22	0.775239118126131	0.242	0.164	1.60680413595514E-17	Myeloid	CAB39
1.3418119943086E-21	0.751275097369054	0.332	0.25	2.24860854006236E-17	Myeloid	DNAJC5
1.41621566288528E-21	1.29910336939341	0.277	0.2	2.37329420786316E-17	Myeloid	MYD88
1.6798692279775E-21	1.8751477600081	0.11	0.058	2.8151248522447E-17	Myeloid	NTAN1
2.13903371679751E-21	1.1237822968914	0.287	0.209	3.58459270260927E-17	Myeloid	CERT1
2.14259446538982E-21	0.811449119454541	0.237	0.16	3.59055980510026E-17	Myeloid	STK40
2.29844247289965E-21	0.95599295919006	0.189	0.118	3.85172989608524E-17	Myeloid	INTS10
2.77748658479981E-21	0.753982527211785	0.212	0.139	4.65451201880753E-17	Myeloid	XPO6
2.86151506069932E-21	0.963637423288476	0.134	0.076	4.79532693871992E-17	Myeloid	TFPT
2.87643012405806E-21	1.16743474777993	0.257	0.174	4.82032160189649E-17	Myeloid	DUSP10
3.22501288799026E-21	1.12997228545991	0.177	0.111	5.40447659769408E-17	Myeloid	RRAGD
3.27813051786191E-21	1.84848646640084	0.163	0.1	5.493491121833E-17	Myeloid	C15orf39
3.69389813798528E-21	0.986274599028186	0.242	0.165	6.19023449963573E-17	Myeloid	SHTN1
4.97202855157424E-21	1.46029634961313	0.181	0.114	8.3321254467281E-17	Myeloid	MTHFR
6.73921133848957E-21	1.03345454029603	0.158	0.095	1.12935703610408E-16	Myeloid	TGFBRAP1
7.38762505846095E-21	1.30924173548013	0.146	0.084	1.23801820729689E-16	Myeloid	GABARAPL1
7.71399259281119E-21	0.737190918052946	0.258	0.18	1.2927108787033E-16	Myeloid	RSU1
7.92902998986166E-21	1.01820747829018	0.206	0.136	1.32874684570102E-16	Myeloid	HEATR5B
8.04111371587652E-21	1.13855494178002	0.23	0.158	1.34752983650659E-16	Myeloid	TK2
8.38719730773189E-21	0.663842734043113	0.439	0.358	1.40552652482971E-16	Myeloid	USF2
1.12158579139387E-20	1.07311557903131	0.206	0.136	1.87955346921784E-16	Myeloid	HMBOX1
1.83757255657418E-20	1.08528182967632	0.311	0.231	3.07940409030701E-16	Myeloid	GSAP
1.9119366520494E-20	0.470522835446729	0.229	0.154	3.20402344150439E-16	Myeloid	PIEZO1
2.16224859918218E-20	0.969929962407906	0.238	0.163	3.62349620250949E-16	Myeloid	UBA7
2.34245883508744E-20	0.999226048802676	0.261	0.188	3.92549251583953E-16	Myeloid	PTPN12
2.40119071065929E-20	0.636001726765014	0.127	0.07	4.02391539292283E-16	Myeloid	SLC7A6
3.55126938858492E-20	0.720850274379638	0.139	0.078	5.95121724139061E-16	Myeloid	SLC12A4
4.49435012410491E-20	0.665206925177654	0.136	0.077	7.53163193797502E-16	Myeloid	PISD
4.90600101964056E-20	1.01496178748019	0.316	0.242	8.22147650871366E-16	Myeloid	YWHAH
4.96577497398547E-20	0.618547258429311	0.365	0.284	8.32164570140485E-16	Myeloid	NUMA1
5.24292780002577E-20	1.14980062597651	0.197	0.13	8.78609840728318E-16	Myeloid	EXOC1
6.3634745993973E-20	1.92472733603637	0.132	0.077	1.066391073367E-15	Myeloid	TICAM2
6.51041274296424E-20	0.854818777115034	0.184	0.117	1.09101496746595E-15	Myeloid	ATXN7
7.68236282271305E-20	1.18380248128555	0.166	0.104	1.28741036183025E-15	Myeloid	MIOS
8.26057129466825E-20	1.58748685602343	0.152	0.092	1.3843065375605E-15	Myeloid	BLOC1S6
1.03213342630541E-19	0.884006106966262	0.784	0.718	1.72964919580261E-15	Myeloid	HSP90AA1
1.12381539246039E-19	1.01017492132627	0.217	0.147	1.88328983468512E-15	Myeloid	RABEP1
1.40091803772149E-19	1.15165899781406	0.162	0.1	2.34765844761368E-15	Myeloid	PHIP
1.79957442039467E-19	0.92255740796619	0.197	0.129	3.01572681369739E-15	Myeloid	CDK2AP1
1.96596400583282E-19	0.762736120268637	0.322	0.245	3.29456248097464E-15	Myeloid	BCL2L1
2.39548005401524E-19	0.523952758366136	0.428	0.349	4.01434547451874E-15	Myeloid	LAMTOR1
2.58882306341648E-19	1.2791651579869	0.125	0.071	4.33834968967333E-15	Myeloid	ZFYVE26
2.64699392511938E-19	0.871987967555555	0.331	0.259	4.43583241971506E-15	Myeloid	NADK
2.6528624698499E-19	0.615773420627616	0.196	0.128	4.44566692697446E-15	Myeloid	PHTF2
2.80614603178978E-19	0.978414835037161	0.282	0.207	4.70253952007332E-15	Myeloid	RBM6
2.94742506566329E-19	1.01130960448057	0.2	0.133	4.93929492503855E-15	Myeloid	RB1

2.97796583311795E-19	0.880962305564172	0.4	0.322	4.99047514313906E-15	Myeloid	ZMIZ2
3.04514180355946E-19	0.550633432565594	0.303	0.226	5.10304863440494E-15	Myeloid	CBFB
3.11345693333777E-19	1.26991340911589	0.21	0.144	5.21753112888744E-15	Myeloid	CD58
3.18518970142146E-19	1.28759505062758	0.177	0.114	5.33774090164209E-15	Myeloid	RUBCN
3.90100881263582E-19	0.571136042038577	0.361	0.286	6.53731056821511E-15	Myeloid	PHC2
4.22283261459806E-19	1.01623338216324	0.22	0.153	7.07662289554342E-15	Myeloid	CYB561A3
4.36599704168695E-19	0.980087517714004	0.27	0.196	7.31653784245899E-15	Myeloid	STARD3
4.46225775700943E-19	0.614025428768362	0.285	0.208	7.47785154919641E-15	Myeloid	QRICH1
4.57524734172964E-19	1.23847595612928	0.514	0.462	7.66719949527053E-15	Myeloid	DEPRECATED-ENSG00000132002
4.69720005813229E-19	1.51337252610551	0.242	0.174	7.8715678574181E-15	Myeloid	TSEN34
5.20051436637236E-19	0.873682762539435	0.387	0.308	8.7150219751668E-15	Myeloid	CMTM6
7.71052911726131E-19	1.00631853846391	0.21	0.142	1.29213046947065E-14	Myeloid	UBA3
1.10786405693792E-18	1.4853499749368	0.155	0.096	1.85655858661657E-14	Myeloid	RIT1
1.11450771448271E-18	1.22458274653612	0.266	0.195	1.86769202793012E-14	Myeloid	UTRN
1.16359287728898E-18	1.05677228185817	0.195	0.129	1.94994894376088E-14	Myeloid	GGA1
2.01018195964344E-18	1.00441945295463	0.256	0.185	3.36866292797048E-14	Myeloid	CAMK1D
2.15509216977743E-18	0.873928807127104	0.295	0.222	3.61150345811301E-14	Myeloid	PIKFYVE
2.33328404566449E-18	0.539919502840082	0.205	0.135	3.91011740372456E-14	Myeloid	STIMATE
2.40825180058075E-18	0.997675408658732	0.241	0.171	4.03574836741322E-14	Myeloid	RBM5
2.47912906639894E-18	1.40035273007423	0.108	0.059	4.15452448947134E-14	Myeloid	CLCN6
2.56592865284901E-18	1.08320474327048	0.101	0.054	4.29998323644437E-14	Myeloid	UNC119
2.95318210581449E-18	1.16592349234469	0.354	0.283	4.94894257292392E-14	Myeloid	PPP1CB
3.16185227445522E-18	0.653345547991749	0.228	0.159	5.29863204153205E-14	Myeloid	N4BP1
3.32454624908798E-18	0.475082359382428	0.551	0.483	5.57127460422163E-14	Myeloid	ATP6AP1
3.37385584094463E-18	1.78415106402506	0.359	0.293	5.65390761825501E-14	Myeloid	SPP1
3.69001009073109E-18	1.34777966958774	0.154	0.098	6.18371891004716E-14	Myeloid	PDK3
5.33125515411407E-18	0.61240564520557	0.436	0.37	8.93411738726435E-14	Myeloid	PTTG1IP
6.45947365810535E-18	0.771609885463486	0.479	0.413	1.08247859562529E-13	Myeloid	CEBPB
7.4788324620824E-18	0.299925208734616	0.103	0.054	1.25330274399577E-13	Myeloid	SLFN5
7.77538708869817E-18	1.15314084152743	0.168	0.108	1.30299936832404E-13	Myeloid	KDM7A
8.29899232949029E-18	0.607705381051722	0.211	0.143	1.39074513457598E-13	Myeloid	ORAI1
1.19811456465256E-17	1.13505186302791	0.187	0.126	2.00780038744476E-13	Myeloid	RCC2
1.21576450633318E-17	1.05052219906692	0.348	0.279	2.03737815971315E-13	Myeloid	FGD4
1.43180515026823E-17	1.08001250483728	0.312	0.242	2.39941907081949E-13	Myeloid	IFNAR2
1.56169739560398E-17	1.17258243776205	0.168	0.108	2.61709249555315E-13	Myeloid	VTI1A
1.63774668459605E-17	0.984672593118258	0.297	0.228	2.74453589404606E-13	Myeloid	USP32
1.79022007758885E-17	0.799006345532254	0.323	0.245	3.0000508060234E-13	Myeloid	ARRDC3
2.02424442660059E-17	1.11946532178507	0.128	0.076	3.39222881009727E-13	Myeloid	NOD1
2.08125592018135E-17	0.714843144492318	0.366	0.293	3.4877686710399E-13	Myeloid	TMUB2
2.14189355337979E-17	0.964810259625474	0.25	0.183	3.58938521675385E-13	Myeloid	KIDINS220
2.46037909311449E-17	0.584579160379287	0.452	0.383	4.12310328424126E-13	Myeloid	SMARCA2
2.47705351754558E-17	1.01194314662943	0.154	0.097	4.15104628470288E-13	Myeloid	PPM1F
2.52223187224538E-17	0.879906825648904	0.262	0.194	4.22675617150881E-13	Myeloid	DNAJC13
2.62968793327872E-17	1.09866714262904	0.284	0.216	4.40683103858849E-13	Myeloid	SOAT1
2.96067779289664E-17	0.832332108525855	0.334	0.26	4.96150384533619E-13	Myeloid	IRF2
3.2342219758081E-17	0.992420460372523	0.173	0.114	5.41990918705922E-13	Myeloid	HLX
3.70092627796229E-17	0.678309112666532	0.305	0.234	6.2020122566092E-13	Myeloid	QARS
3.72703540699834E-17	0.859874086047685	0.143	0.088	6.24576593504781E-13	Myeloid	DNAJB6
3.91859222445927E-17	0.779553080311654	0.345	0.284	6.56677684974885E-13	Myeloid	VAT1
4.3178352409017E-17	0.74428566994192	0.26	0.189	7.23582829670306E-13	Myeloid	SHKBP1
4.51099372517687E-17	0.735820919465488	0.408	0.335	7.5595232846514E-13	Myeloid	KIAA0100
4.82765051764135E-17	1.15699648535127	0.224	0.159	8.09017673746337E-13	Myeloid	SERTAD2
5.11699647832665E-17	0.933102137898395	0.104	0.058	8.5750626983798E-13	Myeloid	SIDT2
5.17230014770802E-17	0.675953067612606	0.428	0.363	8.6677405875291E-13	Myeloid	MBNL1
7.84231955712001E-17	0.793014271823956	0.285	0.217	1.31421591138217E-12	Myeloid	SETX
8.31553791522969E-17	0.74408686342487	0.344	0.275	1.39351784383419E-12	Myeloid	PTBP3
1.03290120430766E-16	0.819504916752825	0.146	0.09	1.73093583817877E-12	Myeloid	MTHFD1L
1.11190559913681E-16	0.791265796600737	0.33	0.263	1.86333140303347E-12	Myeloid	DENND4C
1.30668519625637E-16	0.627951033609072	0.283	0.209	2.18974305188643E-12	Myeloid	HBP1
1.64786460259709E-16	0.674639673901576	0.457	0.395	2.7614915010322E-12	Myeloid	TUT7
1.69167259372072E-16	1.61177299932448	0.105	0.059	2.83490493255718E-12	Myeloid	TCEAL9
1.72640956854123E-16	0.559592606416666	0.17	0.111	2.8931171549614E-12	Myeloid	BAIAP2
2.21164599304575E-16	0.775110745855424	0.223	0.16	3.70627635514606E-12	Myeloid	GNPDA1

2.89631305059562E-16	0.770949100044747	0.198	0.135	4.85364141018813E-12	Myeloid	OSTM1
2.99702934700642E-16	0.520700428735952	0.151	0.094	5.02242177971336E-12	Myeloid	CCDC85B
3.00980885412742E-16	0.653912669119763	0.376	0.305	5.04383767774674E-12	Myeloid	MPV17
3.38562237025582E-16	1.42915607388388	0.196	0.137	5.67362596807471E-12	Myeloid	TPCN1
3.55866692047571E-16	0.78712604929746	0.227	0.164	5.9636140253332E-12	Myeloid	MAP1S
3.68638061362691E-16	1.11752788393627	0.156	0.101	6.17763663231597E-12	Myeloid	DEPRECATED-ENSG00000179051
6.13835359333919E-16	1.01522642713271	0.242	0.177	1.02866529517178E-11	Myeloid	NCK1
6.29969956760306E-16	1.06639242294455	0.16	0.105	1.05570365353892E-11	Myeloid	SUMF1
6.6170348809922E-16	0.469863367455432	0.328	0.254	1.10888270535667E-11	Myeloid	TSC22D4
7.83847216938613E-16	1.03394691051982	0.24	0.176	1.31357116614573E-11	Myeloid	MTMR10
8.09810661196678E-16	0.854769563575021	0.167	0.11	1.35708070603339E-11	Myeloid	TUBA1B
8.79691452773303E-16	0.854021618182563	0.325	0.257	1.4741869365575E-11	Myeloid	SBNO2
8.94910549146646E-16	0.673691776706859	0.547	0.489	1.49969109825995E-11	Myeloid	NFE2L2
1.19381001681834E-15	0.916092355957421	0.173	0.117	2.00058682618418E-11	Myeloid	GALC
1.65381919489588E-15	1.06883424201337	0.153	0.1	2.77147020680651E-11	Myeloid	TMX3
1.71851527157696E-15	0.966470289360571	0.204	0.144	2.87988789210866E-11	Myeloid	SF3B1
1.9197598993743E-15	0.658978355707163	0.421	0.358	3.21713363937146E-11	Myeloid	TAP1
2.01531544804574E-15	0.956530408257274	0.321	0.254	3.37726562783505E-11	Myeloid	ADAM10
2.26053801095285E-15	0.687227195301302	0.361	0.295	3.78820959875479E-11	Myeloid	HIPK3
2.26371227275144E-15	1.01310468183786	0.17	0.115	3.79352902667686E-11	Myeloid	DTNBP1
2.41080418995113E-15	0.612521351967909	0.484	0.43	4.04002566152011E-11	Myeloid	SNX1
2.63305526822622E-15	1.01748945339563	0.285	0.221	4.4124740184935E-11	Myeloid	RELCH
2.63840688887407E-15	0.802401440364694	0.248	0.185	4.42144226437517E-11	Myeloid	PHKB
2.72759379286566E-15	0.901657802951334	0.234	0.17	4.57090167808427E-11	Myeloid	VPS39
2.99619142117596E-15	0.303220220095697	0.187	0.126	5.02101758360668E-11	Myeloid	NLRC5
2.99682619841565E-15	1.03537020602737	0.203	0.145	5.02208134330494E-11	Myeloid	MTMR6
3.00770735183836E-15	0.790484139341167	0.361	0.293	5.04031598021072E-11	Myeloid	RASSF3
3.26345656498746E-15	0.52741835957367	0.156	0.101	5.46890051160599E-11	Myeloid	DENND2D
3.82845844947995E-15	0.676756923277522	0.114	0.068	6.41573066963851E-11	Myeloid	BORCS6
4.2575535324454E-15	0.277812823619809	0.323	0.245	7.134808209672E-11	Myeloid	CSRP1
4.9596054665238E-15	0.862076982023655	0.282	0.217	8.31130684080059E-11	Myeloid	ELMSAN1
5.09972005349722E-15	0.727673604614483	0.174	0.115	8.54611086565065E-11	Myeloid	MIIP
5.3101421110353E-15	0.736410973490775	0.212	0.149	8.89873614967295E-11	Myeloid	DDX21
5.61219866595531E-15	0.437294426801297	0.613	0.584	9.40492252440791E-11	Myeloid	BRI3
6.51534557323563E-15	0.720572007791865	0.367	0.303	1.09184161116283E-10	Myeloid	CSTB
8.07394568622806E-15	0.885253590687653	0.144	0.092	1.3530318180981E-10	Myeloid	HERC1
8.66393825555779E-15	0.4990633105105	0.485	0.421	1.45190277286637E-10	Myeloid	DEPRECATED-ENSG00000147872
1.05097967204226E-14	1.08867815388564	0.24	0.179	1.76123173440841E-10	Myeloid	VPS26A
1.05782106465741E-14	1.0294437653246	0.111	0.066	1.77269654015288E-10	Myeloid	ACCS
1.14246399186348E-14	0.580290800618337	0.058	0.118	1.91454115756482E-10	Myeloid	IRAK2
1.38928299634525E-14	1.089955742499	0.117	0.071	2.32816044527537E-10	Myeloid	SIRT1
1.71037658598286E-14	0.56447032877591	0.541	0.45	2.86624908279008E-10	Myeloid	IVNS1ABP
1.9416831902903E-14	0.974345842537252	0.236	0.175	3.25387269028849E-10	Myeloid	DDX27
2.09548686547684E-14	0.91699082581874	0.101	0.058	3.51161688916609E-10	Myeloid	RABEP2
2.23264095051373E-14	0.943381995592309	0.133	0.084	3.7414597048709E-10	Myeloid	MAP1LC3B
2.23685151919153E-14	0.828957935172151	0.257	0.196	3.74851577586116E-10	Myeloid	CD9
2.33102374121626E-14	0.528514929275166	0.516	0.447	3.90632958553021E-10	Myeloid	TBC1D9B
2.42312167791144E-14	0.581119251415096	0.436	0.376	4.060667307844E-10	Myeloid	NEK6
2.46896198862663E-14	1.13740761774391	0.118	0.072	4.13748650054051E-10	Myeloid	TATDN3
2.49468920090921E-14	1.49067212535903	0.157	0.107	4.18060016288366E-10	Myeloid	ATP2B1
2.50766728848965E-14	1.21629771107292	0.157	0.106	4.20234884205095E-10	Myeloid	FAM53C
2.6169924524173E-14	1.13381996360943	0.112	0.068	4.38555595176091E-10	Myeloid	SAMD8
2.79473170509735E-14	0.780930428486579	0.262	0.2	4.68341139140214E-10	Myeloid	TMEM9B
3.00222861368037E-14	0.805170542636637	0.191	0.136	5.03113471080556E-10	Myeloid	SLC2A8
3.03875577739889E-14	0.653126902438763	0.426	0.362	5.09234693176506E-10	Myeloid	MBD2
3.23301558583002E-14	0.702272825701765	0.229	0.168	5.41788751873395E-10	Myeloid	CLN6
3.40707844411177E-14	1.03016275226923	0.207	0.15	5.7095820566425E-10	Myeloid	RGL2
3.43084298713739E-14	0.765167542885885	0.147	0.095	5.74940667784484E-10	Myeloid	ENTPD4
3.74563207037816E-14	0.686829778344302	0.444	0.39	6.27693022353973E-10	Myeloid	RAB10
4.45004755319588E-14	0.689530706324877	0.336	0.271	7.45738968964565E-10	Myeloid	EP300
5.26828794062474E-14	1.01372804725838	0.16	0.108	8.82859693089894E-10	Myeloid	PTPN9
6.36046367972825E-14	0.744752320491058	0.213	0.154	1.06588650344886E-09	Myeloid	TRABD
6.49894707880041E-14	1.13360531080137	0.173	0.121	1.08909355146537E-09	Myeloid	FLVCR2

7.39062359672955E-14	0.438799936770809	0.251	0.188	1.23852070233994E-09	Myeloid	WDR82
8.40509395439966E-14	0.703498511003108	0.294	0.231	1.40852564487829E-09	Myeloid	ITGAV
1.015879611201E-13	0.503739061299422	0.47	0.42	1.70241105245064E-09	Myeloid	RAB5C
1.05718106200941E-13	0.742478846774834	0.464	0.409	1.77162402371537E-09	Myeloid	PDXK
1.09718717832752E-13	0.931830541010644	0.269	0.209	1.83866627344125E-09	Myeloid	TAOK3
1.14574101380872E-13	0.687815612686451	0.185	0.129	1.92003279094065E-09	Myeloid	PTPN23
1.31451272588193E-13	0.27081816612358	0.248	0.184	2.20286042603293E-09	Myeloid	CCND3
1.34685604570844E-13	0.968724778878271	0.302	0.243	2.25706136139821E-09	Myeloid	HERC3
1.36835179240732E-13	0.711778943162427	0.123	0.078	2.29308393371618E-09	Myeloid	DEPRECATED-ENSG00000175826
1.6516047587587E-13	0.908939750150337	0.274	0.213	2.76775925472782E-09	Myeloid	VAMP3
1.72110608749843E-13	1.06541254341952	0.19	0.135	2.88422958142988E-09	Myeloid	ZMYM3
1.72709487457564E-13	1.38632340353551	0.14	0.092	2.89426559081386E-09	Myeloid	CLPB
1.84313507504683E-13	0.993918083872593	0.227	0.171	3.08872575876347E-09	Myeloid	VPS41
1.89390614582955E-13	1.06323929454357	0.167	0.116	3.17380791918115E-09	Myeloid	FAM13B
3.04754242355173E-13	0.786392653766153	0.199	0.143	5.10707159338799E-09	Myeloid	YPEL2
3.1885979825091E-13	0.690380207959256	0.391	0.331	5.34345249908874E-09	Myeloid	RAB7A
3.42409820334606E-13	0.705252748683901	0.196	0.141	5.73810376916733E-09	Myeloid	ADPRHL2
3.77255786953619E-13	0.707128850055841	0.238	0.178	6.32205247776874E-09	Myeloid	NFKB2
3.97954239247249E-13	0.964905670248391	0.26	0.202	6.6689171413054E-09	Myeloid	ARHGAP17
4.61784327957103E-13	1.19713817420413	0.156	0.107	7.73858176790514E-09	Myeloid	WBP1L
4.83263329980215E-13	0.697131847163412	0.147	0.098	8.09852688380844E-09	Myeloid	CHD3
4.84863238876235E-13	1.88737687627663	0.206	0.156	8.12533815708795E-09	Myeloid	NR1H3
5.11206614574808E-13	0.745206859444124	0.17	0.117	8.56680044704463E-09	Myeloid	AP5Z1
5.66640834830046E-13	0.851710928304261	0.209	0.154	9.49576711008191E-09	Myeloid	PITHD1
5.81155943087327E-13	0.962671923454977	0.285	0.228	9.73901129425742E-09	Myeloid	TGIF1
6.02098378088584E-13	0.714693598418266	0.233	0.175	1.00899646200085E-08	Myeloid	STX12
6.97162694269256E-13	0.437510650991819	0.168	0.116	1.16830524305642E-08	Myeloid	PRDM2
7.04096228936613E-13	0.804302008137061	0.173	0.12	1.17992446045198E-08	Myeloid	DEPRECATED-ENSG00000111328
7.69283764521955E-13	1.50144686804808	0.141	0.095	1.28916573258589E-08	Myeloid	RGS12
7.98797663656701E-13	1.1713072226376	0.156	0.107	1.3386251247559E-08	Myeloid	NIPSNAP2
8.55411092176531E-13	0.84885187591596	0.231	0.176	1.43349790826943E-08	Myeloid	NHLRC2
9.03561012494558E-13	1.11151146949286	0.14	0.093	1.51418754473838E-08	Myeloid	MPHOSPH6
1.00232821445109E-12	0.554594977872639	0.316	0.25	1.67970162177714E-08	Myeloid	SUMO3
1.02601601806421E-12	0.772373378424772	0.394	0.335	1.71939764307201E-08	Myeloid	HNRNPH3
1.15044197764307E-12	0.274543387900539	0.365	0.297	1.92791066613426E-08	Myeloid	OGA
1.19677216261059E-12	0.963008243983336	0.251	0.197	2.00555079010282E-08	Myeloid	ATF6
1.26247004546063E-12	0.501357236345269	0.392	0.33	2.11564730218292E-08	Myeloid	TRIP12
1.31113629629665E-12	1.02288709116318	0.169	0.118	2.19720220533393E-08	Myeloid	FLCN
1.32567521217968E-12	0.513482373485872	0.289	0.227	2.22156652057071E-08	Myeloid	PPP6R1
1.3912424067871E-12	0.674467317825737	0.255	0.197	2.33144402529383E-08	Myeloid	OAS1
1.73209739505426E-12	0.828290857082456	0.191	0.137	2.90264881463193E-08	Myeloid	UBL7
2.28792157544086E-12	0.710670214570035	0.201	0.146	3.83409897612379E-08	Myeloid	NDE1
2.45402956083009E-12	0.716553089460877	0.292	0.236	4.11246273803906E-08	Myeloid	ITGB5
2.45583767258401E-12	1.1020948123125	0.137	0.092	4.11549277171628E-08	Myeloid	ZNF518A
2.51983972077952E-12	0.667420946490203	0.645	0.602	4.22274740408233E-08	Myeloid	JUN
2.55049734634754E-12	1.06463860860553	0.118	0.076	4.2741234530092E-08	Myeloid	ZDHHC21
2.67054955826606E-12	0.744201494442687	0.208	0.154	4.47530694974226E-08	Myeloid	SFT2D1
2.69966892015105E-12	0.557964826843295	0.219	0.162	4.52410517638913E-08	Myeloid	MARK2
2.97715604626991E-12	1.24729093466724	0.128	0.085	4.98911810233912E-08	Myeloid	ARL14EP
3.31976447150785E-12	0.734880675922774	0.147	0.101	5.56326130135286E-08	Myeloid	PXK
4.04773772764867E-12	0.852516838076001	0.329	0.277	6.78319888399364E-08	Myeloid	YPEL5
4.38077106548536E-12	0.650150795391471	0.309	0.251	7.34129615154036E-08	Myeloid	PCM1
5.055899007803E-12	0.513255076178209	0.479	0.427	8.47267555727626E-08	Myeloid	PRKAR1A
5.62554305483843E-12	0.972377755002415	0.225	0.172	9.42728505129824E-08	Myeloid	DNM1L
5.7191092045302E-12	1.36040365734939	0.132	0.088	9.5840832049517E-08	Myeloid	RAB9A
6.43068220829557E-12	1.07346273768748	0.157	0.11	1.07765372446617E-07	Myeloid	ALDH16A1
6.82964732514121E-12	0.539915459582146	0.663	0.63	1.14451229874716E-07	Myeloid	NFKBIA
7.0340642448501E-12	0.530473379246535	0.155	0.106	1.17876848615198E-07	Myeloid	SLC25A45
8.46608253278154E-12	1.08903587489744	0.127	0.085	1.41874611084353E-07	Myeloid	SLC9A7
8.52729701290449E-12	0.284204003821296	0.285	0.226	1.42900443342253E-07	Myeloid	CD99
1.09382641197216E-11	1.18776176571947	0.1	0.062	1.83303430118295E-07	Myeloid	ATP10D
1.23583324949163E-11	0.568340750516713	0.292	0.234	2.07100935949808E-07	Myeloid	AP2S1
1.43992446228748E-11	0.779108216237206	0.194	0.143	2.41302541390136E-07	Myeloid	SMNDC1

1.62380607802474E-11	0.769768629252087	0.206	0.154	2.72117422555386E-07	Myeloid	PARL
1.73564553098107E-11	0.764514525176195	0.213	0.161	2.90859478081807E-07	Myeloid	BTBD1
1.95616019184456E-11	0.594513753840179	0.267	0.211	3.27813324949311E-07	Myeloid	LCOR
2.66681337664665E-11	0.630544626121045	0.292	0.237	4.46904585658446E-07	Myeloid	ARL8A
2.70098348259984E-11	1.01281351642461	0.115	0.075	4.5263081201408E-07	Myeloid	PHETA1
2.79936633743804E-11	0.867975016625846	0.177	0.129	4.69117810827867E-07	Myeloid	WAPL
3.31172719806144E-11	0.810315038321784	0.176	0.128	5.54979243851136E-07	Myeloid	FBXO11
3.61513214405221E-11	0.831569273887522	0.156	0.111	6.05823844700269E-07	Myeloid	G6PC3
3.72071882040178E-11	0.53503188217167	0.241	0.188	6.2351805992293E-07	Myeloid	GTF2F1
3.83970730165038E-11	0.829574069222669	0.217	0.167	6.43458149610571E-07	Myeloid	ACTR3
4.27156802169832E-11	1.17557127233853	0.213	0.166	7.15829369076205E-07	Myeloid	RIN2
4.36179001702261E-11	0.510581390743392	0.532	0.496	7.30948771052649E-07	Myeloid	DDX5
4.56925654030715E-11	0.648253897622702	0.432	0.384	7.65716011024672E-07	Myeloid	RERE
4.80889868520315E-11	0.84019737245614	0.168	0.121	8.05875241666343E-07	Myeloid	ARL 2.00
5.42073622183915E-11	0.613247991571424	0.286	0.23	9.08406976055805E-07	Myeloid	TRIM8
5.8238046381872E-11	0.725590427608433	0.33	0.281	9.7595318126741E-07	Myeloid	ZFAND5
5.85290999807434E-11	0.603567314747331	0.146	0.101	9.80830657477299E-07	Myeloid	CAMTA2
7.04089565961834E-11	0.708081940471307	0.34	0.285	1.17991329463884E-06	Myeloid	TAOK1
7.25364734762651E-11	0.659564387337254	0.253	0.199	1.21556622251525E-06	Myeloid	ZNF655
7.59309066878806E-11	0.749414576076831	0.16	0.115	1.2724501342755E-06	Myeloid	TRAFD1
7.75376089050773E-11	0.74020300628839	0.221	0.171	1.29937525003129E-06	Myeloid	ADCK2
8.24107814919766E-11	0.967803594981628	0.195	0.147	1.38103987624254E-06	Myeloid	TATDN2
9.23085058299956E-11	0.74535382964326	0.205	0.155	1.54690594069907E-06	Myeloid	CAPZA1
1.05183834462253E-10	0.355215735698599	0.209	0.157	1.76267069791843E-06	Myeloid	RBM15B
1.07331918591839E-10	0.762788929485948	0.192	0.144	1.79866829176204E-06	Myeloid	USP3
1.09722756010525E-10	1.2236370018603	0.147	0.105	1.83873394522438E-06	Myeloid	DDHD1
1.13863879429987E-10	0.850647311423639	0.127	0.085	1.90813089148773E-06	Myeloid	MAPK7
1.18566763244973E-10	0.756921445935713	0.179	0.132	1.98694181845926E-06	Myeloid	RASA1
1.22994118532752E-10	1.01322290635094	0.2	0.152	2.06113543837185E-06	Myeloid	SIAH1
1.3872320730877E-10	0.756337913787963	0.239	0.188	2.32472350808037E-06	Myeloid	CASP4
1.41944514279967E-10	0.598329071636091	0.332	0.281	2.37870617030369E-06	Myeloid	CYB5R1
1.556752262164E-10	1.06837237950203	0.331	0.283	2.60880544093443E-06	Myeloid	SLC20A1
1.66058127348328E-10	0.790609616790806	0.386	0.335	2.78280209810329E-06	Myeloid	KMT2C
1.6615698837805E-10	1.52187922340218	0.306	0.267	2.78445881123936E-06	Myeloid	PDE4DIP
1.85001556714833E-10	0.696532956863166	0.18	0.134	3.10025608742718E-06	Myeloid	ZNFX1
1.9299180973933E-10	0.356049787534346	0.179	0.255	3.2341567476117E-06	Myeloid	BCL6
1.93861801867559E-10	0.876198900116695	0.295	0.244	3.24873607569656E-06	Myeloid	SPAG9
1.98435551935046E-10	0.90074640563421	0.237	0.189	3.3253829793275E-06	Myeloid	ME2
2.02636988028278E-10	0.558974610083901	0.386	0.335	3.39579064537788E-06	Myeloid	CCNI
2.05127729766743E-10	0.51695476101274	0.202	0.153	3.43753049543108E-06	Myeloid	RNF38
2.13430008302324E-10	1.56579223649648	0.268	0.222	3.57666007913035E-06	Myeloid	SELENOK
2.29654143726705E-10	0.866721168202035	0.173	0.128	3.84854414057213E-06	Myeloid	PTPN18
2.56711197325106E-10	0.7207230807956	0.325	0.273	4.30196624477413E-06	Myeloid	SELENOS
2.57619680531286E-10	0.553791641104921	0.59	0.545	4.31719060634329E-06	Myeloid	JUND
2.63114640168575E-10	0.966551577778652	0.156	0.113	4.40927513994497E-06	Myeloid	PRKD3
2.68359689216117E-10	0.801636107521532	0.185	0.137	4.49717167188368E-06	Myeloid	PPP3CA
2.77290541844107E-10	0.962692277266368	0.252	0.203	4.64683490022355E-06	Myeloid	ATP6V0A1
2.80719708619969E-10	0.965201770236275	0.109	0.072	4.70430087705343E-06	Myeloid	SLC9A1
2.82168179336728E-10	0.590168472788542	0.172	0.126	4.72857434932488E-06	Myeloid	EIF3D
2.87326426791496E-10	1.4637700960921	0.157	0.115	4.81501626017188E-06	Myeloid	MYLIP
2.95187560999828E-10	1.00969663232551	0.15	0.107	4.94675314723512E-06	Myeloid	PHF20
3.32863307295821E-10	0.633899514200315	0.11	0.172	5.57812330366338E-06	Myeloid	GPAT3
3.34073807905237E-10	0.517182468677889	0.211	0.161	5.59840887287596E-06	Myeloid	CDV3
3.5461490435945E-10	0.738092350886318	0.128	0.088	5.94263656725566E-06	Myeloid	WDR77
3.74046991932451E-10	0.690340467473482	0.178	0.132	6.26827949080401E-06	Myeloid	ELAC2
4.05523278524697E-10	0.540720183337978	0.292	0.239	6.79575910151687E-06	Myeloid	ARPC5
4.72029808490494E-10	1.08196764267297	0.154	0.112	7.9102755306837E-06	Myeloid	WDR70
5.31491359142185E-10	0.826405050194878	0.141	0.1	8.90673219650473E-06	Myeloid	TMEM243
5.36657324020343E-10	1.20436007322939	0.174	0.129	8.99330343593291E-06	Myeloid	ZNF516
5.68404318765294E-10	0.894223316400255	0.127	0.087	9.52531957386879E-06	Myeloid	PHF21A
5.8971906854832E-10	0.515699025940944	0.478	0.428	9.88251215073274E-06	Myeloid	RTN4
6.1525071060647E-10	0.505960965744479	0.238	0.19	1.03103714083432E-05	Myeloid	SPOP
6.32041373863479E-10	0.777065477179191	0.29	0.238	1.05917493432042E-05	Myeloid	CERS6

6.72984463623086E-10	0.633230873248564	0.32	0.269	1.12778736413957E-05	Myeloid	NUP62
7.06684577515197E-10	1.16602054246084	0.102	0.067	1.18426201499997E-05	Myeloid	MIER2
7.57174009162825E-10	0.936173064036531	0.138	0.097	1.26887220455506E-05	Myeloid	SLC35A1
7.58627626092728E-10	0.865949270473536	0.18	0.134	1.27130817580619E-05	Myeloid	PNRC2
7.83770507777103E-10	0.612100992725476	0.11	0.073	1.31344261693287E-05	Myeloid	ASCC1
8.91370390401055E-10	0.656065411647015	0.348	0.299	1.49375850023409E-05	Myeloid	YBX1
9.77729312506215E-10	1.29021794003297	0.198	0.154	1.63847878189791E-05	Myeloid	VPS8
1.06905452856621E-09	1.10087480696613	0.113	0.076	1.79152157897125E-05	Myeloid	BLOC1S2
1.2320379602508E-09	0.295800163303874	0.303	0.246	2.06464921378829E-05	Myeloid	QSOX1
1.2360321960142E-09	0.689887840651179	0.151	0.109	2.0713427540806E-05	Myeloid	SYNE3
1.24898120051041E-09	1.52236473363989	0.11	0.075	2.09304269581534E-05	Myeloid	NKRF
1.26805927219014E-09	1.15165257349087	0.163	0.121	2.12501372833623E-05	Myeloid	DENND5A
1.41708990700581E-09	1.35225974248735	0.106	0.071	2.37475926616034E-05	Myeloid	HECTD2
1.46458427294229E-09	0.737144270222139	0.141	0.099	2.4543503245967E-05	Myeloid	TCEAL8
1.49733827035848E-09	0.329039664776954	0.119	0.178	2.50923947346673E-05	Myeloid	TCF7L2
1.4996007342402E-09	0.57723513403959	0.216	0.169	2.51303091043974E-05	Myeloid	MLXIP
1.49961717689748E-09	1.71147817710037	0.144	0.105	2.5130584650448E-05	Myeloid	FAM102B
1.73227878742778E-09	1.11174783259543	0.189	0.145	2.90295279197147E-05	Myeloid	PIAS1
1.86943407522409E-09	1.15258129313616	0.149	0.109	3.13279762326053E-05	Myeloid	SAMD4A
1.8773647264333E-09	0.662715909933053	0.253	0.205	3.14608780855692E-05	Myeloid	SPG11
1.89040585463063E-09	0.536692501424026	0.296	0.245	3.16794213119001E-05	Myeloid	TIAL1
1.91161286802286E-09	0.651230420304332	0.265	0.214	3.20348084423271E-05	Myeloid	TFE3
2.06230457148097E-09	0.592775235535392	0.109	0.072	3.45601000088781E-05	Myeloid	WWC3
2.06988329997182E-09	0.726294757769105	0.171	0.129	3.46871043409277E-05	Myeloid	MAPRE1
2.24039089773441E-09	0.465108367608374	0.07	0.117	3.75444706642332E-05	Myeloid	ARL5B
2.31418761896499E-09	0.937741875713668	0.167	0.125	3.87811561186153E-05	Myeloid	GGA3
2.3219455277288E-09	0.549040630109467	0.203	0.279	3.89111631536792E-05	Myeloid	TRIM25
2.43948083833394E-09	0.545658025838324	0.202	0.154	4.08808198888002E-05	Myeloid	C17orf49
2.46452101598191E-09	1.34435895681503	0.102	0.069	4.13004431858248E-05	Myeloid	OSBPL1A
2.52958903876104E-09	0.949948174473706	0.301	0.255	4.23908531115574E-05	Myeloid	MORC3
2.5643336753676E-09	1.25836172434494	0.191	0.149	4.29731037318102E-05	Myeloid	EIF2AK4
2.79108893990935E-09	0.524035028287845	0.45	0.415	4.67730684550009E-05	Myeloid	LRPAP1
2.83873166540996E-09	0.906979904652982	0.178	0.135	4.75714652489401E-05	Myeloid	STX2
3.04368318864498E-09	0.482568109412094	0.202	0.155	5.10060428753126E-05	Myeloid	MTPN
3.14158610581028E-09	0.6726826774489	0.164	0.121	5.26466999611687E-05	Myeloid	KCTD9
3.27070039584843E-09	0.683359800914365	0.313	0.264	5.48103972336281E-05	Myeloid	PNISR
3.4056363740589E-09	0.74374133794704	0.192	0.149	5.70716543564791E-05	Myeloid	THADA
3.8426573583789E-09	0.380826751923984	0.406	0.359	6.43952520117135E-05	Myeloid	ATP6V0E1
4.19442684840053E-09	0.799232940605497	0.114	0.078	7.0290205125496E-05	Myeloid	NOL8
4.19902244681473E-09	0.813120807596765	0.256	0.208	7.03672181637213E-05	Myeloid	MEF2D
4.37030844454025E-09	0.7656764335764	0.14	0.1	7.32376289136054E-05	Myeloid	TECPR1
4.62207575109333E-09	0.348847763483145	0.562	0.559	7.7456745436822E-05	Myeloid	ACTG1
4.87994882463521E-09	0.943186643814917	0.178	0.136	8.17781824032368E-05	Myeloid	CCNYL1
5.03368546577266E-09	0.546640395833342	0.071	0.119	8.43545010354182E-05	Myeloid	TSPYL2
5.04961300000658E-09	0.689494724620543	0.193	0.15	8.46214146541103E-05	Myeloid	TBC1D5
5.25575628014859E-09	1.02471820227917	0.107	0.073	8.807596374273E-05	Myeloid	DDX60L
5.29327608268723E-09	0.602827209869072	0.259	0.213	8.87047205936727E-05	Myeloid	HTT
5.41941288426421E-09	0.72398490467369	0.203	0.158	9.08185211144996E-05	Myeloid	YEATS2
5.65887000910345E-09	0.685748913851159	0.171	0.128	9.48313436125557E-05	Myeloid	SHOC2
5.67622169057863E-09	0.680338203463439	0.246	0.199	9.51221230907167E-05	Myeloid	ATG3
6.22237920350219E-09	1.8563281451514	0.207	0.171	0.00010427463069229	Myeloid	SMPDL3A
6.43008308659775E-09	0.482806146812507	0.486	0.439	0.000107755332365205	Myeloid	AFF1
6.53309766168018E-09	0.714144212669969	0.14	0.101	0.000109481650614437	Myeloid	ZNF394
6.69097327281814E-09	0.922432466886139	0.187	0.144	0.000112127330105886	Myeloid	TBRG1
7.13678840537498E-09	0.727298443300533	0.123	0.086	0.000119598300097274	Myeloid	MARCH2
7.81267950866104E-09	0.619351120962334	0.232	0.186	0.000130924883206142	Myeloid	TRAPPC12
8.57367857963958E-09	0.333164317672571	0.71	0.597	0.0001436777056376	Myeloid	SGK1
8.66866386896574E-09	0.729177821420837	0.19	0.146	0.000145269469116128	Myeloid	YJU2
8.86662242527223E-09	0.482891369132089	0.292	0.245	0.000148586858602712	Myeloid	RTCB
9.95544569781709E-09	0.54363981786306	0.416	0.373	0.000166833359004019	Myeloid	TCF25
1.18106278623294E-08	0.663341990262087	0.144	0.105	0.000197922501716916	Myeloid	RRAGC
1.19670091905315E-08	0.287243159181148	0.073	0.119	0.000200543140014926	Myeloid	TSNARE1
1.28364697857505E-08	0.675934997299838	0.166	0.125	0.000215113560669607	Myeloid	PIK3C2A

1.3991412373943E-08	1.00707417453485	0.131	0.094	0.000234468088562537	Myeloid	FAM118B
1.43559061097568E-08	0.585478710888906	0.289	0.241	0.000240576274587304	Myeloid	GBP2
1.494482940127E-08	0.334265564847902	0.4	0.351	0.000250445451106482	Myeloid	GNB2
1.49784837513827E-08	0.646280183333197	0.317	0.271	0.000251009430705671	Myeloid	PARP14
1.50539314872399E-08	0.627563212496633	0.254	0.207	0.000252273783863166	Myeloid	AUP1
1.51260888957542E-08	0.27957579125854	0.364	0.312	0.000253482997715048	Myeloid	PKN1
1.59945751875355E-08	1.02759454433211	0.192	0.152	0.00026803709099272	Myeloid	GANC
1.63933563055545E-08	1.34117290918138	0.169	0.131	0.000274719864968483	Myeloid	PPARD
1.65749312911551E-08	1.79086217479872	0.177	0.14	0.000277762698577177	Myeloid	CHML
1.8672218285067E-08	0.496112872510877	0.29	0.244	0.000312909034021153	Myeloid	TAB2
1.87888768888193E-08	1.52677935095511	0.114	0.081	0.000314863998902835	Myeloid	TFCP2L1
1.90286903708456E-08	0.684436382018961	0.19	0.147	0.00031888279323463	Myeloid	CREB3L2
1.91697610794202E-08	1.26227681600863	0.13	0.094	0.000321246856168923	Myeloid	KDM1B
1.9318651772213E-08	0.446782654391912	0.34	0.294	0.000323741966398745	Myeloid	PAFAH1B1
1.95555440391438E-08	1.03503098876367	0.101	0.068	0.000327711807007971	Myeloid	GPATCH3
1.97357951761923E-08	0.802847352044383	0.397	0.352	0.000330732455562631	Myeloid	NUFIP2
2.07276692205599E-08	0.879326225545658	0.142	0.106	0.000347354280798143	Myeloid	TMEM87B
2.08764859666131E-08	0.73401483453348	0.159	0.12	0.000349848151828502	Myeloid	MPDU1
2.12821336744258E-08	0.480320868070577	0.397	0.35	0.000356645996116027	Myeloid	NARS
2.15891893526928E-08	0.821545567517283	0.305	0.261	0.000361791635172427	Myeloid	EXOC3
2.21620798136171E-08	0.558934211374218	0.251	0.204	0.000371392133516595	Myeloid	APOL2
2.42656016368724E-08	0.513559410897191	0.206	0.162	0.000406642952230708	Myeloid	RECQL
2.43240914280008E-08	0.55272338753324	0.262	0.219	0.000407623124150437	Myeloid	DELE1
3.08613885406889E-08	0.835226957655825	0.211	0.171	0.000517175149164864	Myeloid	TRIM47
3.08839097248179E-08	0.972823681223485	0.149	0.111	0.000517552559168499	Myeloid	RPP30
3.17895351877249E-08	0.448778593326476	0.327	0.279	0.000532729030675893	Myeloid	ARSA
3.49429466749508E-08	0.674970977833433	0.123	0.088	0.000585573900378826	Myeloid	MCUB
3.6651191913139E-08	0.784089691300524	0.201	0.16	0.000614200674080383	Myeloid	WDFY1
3.9595568864659E-08	0.697623272545384	0.222	0.296	0.000663542543033955	Myeloid	TENT5A
4.15288547540276E-08	0.762434360360989	0.189	0.149	0.000695940547967995	Myeloid	DCAF12
4.47534560300968E-08	0.555482154910246	0.159	0.12	0.00074997841615236	Myeloid	CTDP1
4.53660242915073E-08	0.316519834789086	0.574	0.553	0.000760243835077079	Myeloid	TGOLN2
4.91104046614325E-08	0.51890081044486	0.124	0.089	0.000822992161316286	Myeloid	UBE2E1
5.17096540158448E-08	0.846253593687425	0.139	0.102	0.000866550381997527	Myeloid	NEMP1
5.30644033456682E-08	0.532374832524105	0.338	0.293	0.000889253271266707	Myeloid	KMT2E
5.3784726286436E-08	0.872476577189471	0.101	0.069	0.000901324443108095	Myeloid	ERMAP
5.65972024539762E-08	0.386124125255613	0.292	0.245	0.000948455918723733	Myeloid	ZSWIM8
6.69999451166153E-08	0.544565998180999	0.25	0.205	0.00112278508026424	Myeloid	TMOD3
6.89073255951083E-08	0.277986843651887	0.154	0.115	0.00115474896232282	Myeloid	SPRYD3
7.0184567319304E-08	1.12633260222548	0.186	0.146	0.0011761529791369	Myeloid	DNAJB4
7.04986592823791E-08	0.882572747539402	0.256	0.216	0.00118141653225411	Myeloid	DNMT3A
7.63899093807369E-08	0.868454561193512	0.15	0.113	0.00128014210140239	Myeloid	MARF1
7.69020184480202E-08	1.0384178636807	0.181	0.143	0.00128872402515192	Myeloid	HNMT
9.3051224457405E-08	1.10769743448224	0.101	0.07	0.00155935241945719	Myeloid	SGSM2
9.94093187831572E-08	0.960855198560586	0.118	0.085	0.00166590136416815	Myeloid	UTP18
1.02737894671806E-07	0.878646555931239	0.105	0.072	0.00172168163891012	Myeloid	PLIN2
1.07493181675802E-07	0.612888437424727	0.311	0.269	0.00180137073852309	Myeloid	ISCU
1.09455801903423E-07	0.915926643124465	0.216	0.176	0.00183426032829756	Myeloid	GTPBP1
1.11324612125687E-07	0.561922833833489	0.267	0.224	0.00186557785000226	Myeloid	KIF16B
1.1547300697058E-07	1.12700798367561	0.166	0.13	0.00193509665081298	Myeloid	PELI1
1.17236611120799E-07	0.947232061897564	0.135	0.101	0.00196465112916235	Myeloid	SLC35A5
1.23419402341351E-07	0.498663226656538	0.268	0.226	0.00206826234443636	Myeloid	EDEM2
1.24447710753051E-07	0.825440224641607	0.165	0.126	0.00208549473679963	Myeloid	TMF1
1.58405965658212E-07	0.389282086048469	0.391	0.356	0.00265456717250031	Myeloid	IQGAP2
1.66696865062275E-07	0.261135118143619	0.113	0.079	0.0027935060647136	Myeloid	BCAM
1.78107325308637E-07	0.38742219158368	0.213	0.171	0.00298472255752214	Myeloid	BAK1
1.809398842826E-07	0.436147706099778	0.14	0.104	0.00303219058080781	Myeloid	MMS19
1.8583533459305E-07	0.463023582353313	0.213	0.172	0.00311422853711033	Myeloid	BCKDK
1.99802266243011E-07	0.565696532448042	0.24	0.2	0.00334828637770038	Myeloid	YIF1B
2.06335160670275E-07	0.419020914123236	0.142	0.106	0.00345776462251248	Myeloid	PHF23
2.10649473196151E-07	0.712034355241521	0.413	0.375	0.0035300638718211	Myeloid	UBC
2.18344708525866E-07	0.393995859448938	0.383	0.34	0.00365902062547646	Myeloid	EIF3A
2.27668417187319E-07	0.998101364616021	0.124	0.091	0.00381526733522509	Myeloid	TTF2

2.36569857886535E-07	0.396768634180372	0.163	0.125	0.00396443767846255	Myeloid	SF3A1
2.43911020261809E-07	0.58646327113948	0.202	0.16	0.00408746087754739	Myeloid	RYBP
2.58798628291387E-07	0.697390207654536	0.156	0.12	0.00433694741290706	Myeloid	DPP8
2.5977513992331E-07	0.519589296156357	0.238	0.197	0.00435331179483483	Myeloid	PLIN3
2.86597753183439E-07	0.821847128689973	0.168	0.132	0.00480280514784807	Myeloid	STARD3NL
2.97853463276153E-07	2.07535957722106	0.228	0.2	0.00499142833758178	Myeloid	BAG3
3.03625443478151E-07	0.509913935772218	0.179	0.14	0.00508815518180685	Myeloid	SPAG7
3.06238299655636E-07	0.729582154747507	0.158	0.122	0.00513194142562915	Myeloid	HMG20A
3.44123368743426E-07	0.299709376712758	0.391	0.349	0.00576681941340233	Myeloid	APMAP
3.49760320745679E-07	0.639350454184943	0.2	0.16	0.00586128345505609	Myeloid	ATRX
3.61770502019346E-07	0.674931228736437	0.142	0.107	0.00606255007284021	Myeloid	FAM219B
3.69261329953396E-07	0.493897183541546	0.209	0.171	0.00618808136735902	Myeloid	BAG5
3.79480501279886E-07	0.720215223360135	0.321	0.284	0.00635933424044834	Myeloid	C16orf72
4.01574612679573E-07	1.35964671897552	0.181	0.147	0.00672958735928429	Myeloid	CLCN5
4.23712953720574E-07	0.516117407666785	0.114	0.083	0.00710058167844937	Myeloid	CHST14
4.41774278779231E-07	0.779675056955932	0.241	0.202	0.00740325336378236	Myeloid	FNBP4
4.48154830987756E-07	0.433903175783194	0.086	0.128	0.00751017865769281	Myeloid	TBC1D7
4.53750318513835E-07	0.571179664945234	0.264	0.222	0.00760394783765484	Myeloid	FAM111A
4.7871985984206E-07	0.66194975541965	0.254	0.213	0.00802238741123324	Myeloid	PFDN5
5.1679531914271E-07	0.693815476421637	0.318	0.28	0.00866045595819354	Myeloid	RBM25
5.22794249919092E-07	0.992069997655822	0.108	0.077	0.00876098604014414	Myeloid	R3HCC1L
5.25146453551993E-07	0.879283442764294	0.192	0.155	0.0088004042686243	Myeloid	MTMR3
5.4713452761691E-07	0.427233675818269	0.319	0.278	0.00916888041380418	Myeloid	FOXP1
5.66866954695404E-07	0.342791786983484	0.29	0.251	0.00949955642678559	Myeloid	TNFAIP3
5.69490452290253E-07	0.548325088888762	0.188	0.15	0.00954352099948006	Myeloid	MAN2B2
5.87224509920703E-07	0.323691463410086	0.326	0.283	0.00984070833725113	Myeloid	MAP4
6.07840145280588E-07	0.950040744210215	0.106	0.076	0.0101861851546121	Myeloid	MSANTD4
6.20293930029196E-07	0.590465321045205	0.356	0.318	0.0103948856794293	Myeloid	GNPTG
6.24962102328654E-07	0.700971540210041	0.173	0.137	0.0104731149108236	Myeloid	NUBP1
6.5506207406562E-07	0.56382274813968	0.321	0.282	0.0109775302371917	Myeloid	IST1
7.56116528694428E-07	0.649492028430047	0.258	0.221	0.0126710007878612	Myeloid	PBX2
7.78821309619211E-07	1.08048232452024	0.129	0.097	0.0130514875065987	Myeloid	PPM1D
7.8335663811992E-07	0.60471154875794	0.169	0.134	0.0131274905416136	Myeloid	NAA40
7.99169151471544E-07	0.41472205207945	0.311	0.27	0.0133924766403601	Myeloid	DYNC1I2
8.4045156728394E-07	0.874365723193803	0.156	0.122	0.0140842873645443	Myeloid	EZH1
8.99007565621291E-07	0.995049055767116	0.133	0.101	0.0150655687846816	Myeloid	CXorf38
9.69103917243441E-07	0.619624556514418	0.202	0.165	0.0162402434451656	Myeloid	CYLD
1.03478805286184E-06	0.807090010817307	0.181	0.146	0.0173409781898588	Myeloid	ZZEF1
1.30002999688226E-06	0.770003402256971	0.25	0.213	0.021785902687753	Myeloid	BTAF1
1.34856747836416E-06	0.499499878387706	0.167	0.13	0.0225992938024266	Myeloid	SRPK2
1.38281946123987E-06	0.593135265466517	0.148	0.113	0.0231732885314578	Myeloid	SEC23IP
1.39330913691188E-06	0.689163544284924	0.314	0.277	0.0233490745163693	Myeloid	CEP350
1.40887570680848E-06	0.631635901216004	0.156	0.121	0.0236099390946965	Myeloid	DNAJC10
1.42103707478618E-06	0.451135166958929	0.29	0.251	0.0238137392992668	Myeloid	CLTA
1.42881626929376E-06	0.738680288832801	0.166	0.132	0.0239441030408249	Myeloid	ZNF362
1.44063230422452E-06	0.521925659551923	0.297	0.26	0.0241421161541945	Myeloid	RUFY1
1.45323604074914E-06	1.08050873568582	0.102	0.075	0.0243533295708741	Myeloid	BTN2A1
1.49275250544594E-06	0.392245188015255	0.164	0.128	0.0250155464862631	Myeloid	KDM3A
1.50048525287521E-06	0.510998344759095	0.259	0.22	0.0251451318676828	Myeloid	HERC4
1.53010770420643E-06	0.371726906868317	0.099	0.143	0.0256415449070914	Myeloid	SNRNP48
1.55840933152931E-06	0.682768612785497	0.307	0.267	0.0261158235777681	Myeloid	ARRB1
1.56003454115458E-06	0.829530299719389	0.241	0.207	0.0261430588406684	Myeloid	P2RX4
1.57056127951618E-06	0.882251818459279	0.15	0.117	0.0263194659221322	Myeloid	CREB1
1.64955772904699E-06	0.65955516380376	0.207	0.17	0.0276432884233694	Myeloid	NANS
1.72905758014979E-06	0.428183956247066	0.179	0.144	0.0289755469281502	Myeloid	TNKS2
1.73587073692353E-06	0.260208777485313	0.141	0.108	0.0290897218093645	Myeloid	CCDC97
1.85835776093168E-06	0.514511652639406	0.193	0.157	0.0311423593576931	Myeloid	AKAP11
1.92412128186573E-06	0.399304681294766	0.192	0.157	0.0322442244415059	Myeloid	PRPF8
2.03927786567686E-06	0.319400815057445	0.207	0.17	0.0341742184730128	Myeloid	SACM1L
2.19403007802109E-06	0.748705471202973	0.329	0.294	0.0367675560474774	Myeloid	HNRNPDL
2.23973733223788E-06	0.973812583388362	0.145	0.112	0.0375335182136424	Myeloid	EAF1
2.27955501858921E-06	0.89820628471723	0.222	0.187	0.038200783001518	Myeloid	AKAP17A
2.30483063376263E-06	1.02857928489134	0.114	0.086	0.0386243517605942	Myeloid	DOP1B

2.33895492928848E-06	0.850927343099458	0.21	0.176	0.0391962067050163	Myeloid	ARRDC1
2.36992975358171E-06	0.34257968991008	0.184	0.24	0.0397152828105223	Myeloid	AZIN1
2.37548914478713E-06	0.51504971056844	0.335	0.294	0.0398084470883428	Myeloid	PCBP1
2.46645375410961E-06	0.657598102732208	0.175	0.14	0.0413328320113689	Myeloid	CCDC6
2.59761641754467E-06	0.696790103872892	0.249	0.213	0.0435308559252136	Myeloid	GTF2H1
2.6305866874079E-06	0.810757890501445	0.148	0.116	0.0440833717075816	Myeloid	OSBPL11
2.63112437949875E-06	0.635619381956613	0.141	0.109	0.0440923823516401	Myeloid	SLF2
2.64062784727389E-06	0.906128265726995	0.189	0.155	0.0442516414646159	Myeloid	ARIH1
2.65597221324963E-06	0.719710636590211	0.188	0.153	0.0445087823496372	Myeloid	PGGT1B
2.83120495874622E-06	0.38768098451982	0.246	0.207	0.0474453326986692	Myeloid	CNPPD1
2.85569513398566E-06	0.423209256044915	0.185	0.149	0.0478557390553316	Myeloid	CDS2
0	5.49756028395948	0.853	0.063	0	Endothelial	ENG
0	4.7463439292363	0.804	0.038	0	Endothelial	SPARC
0	8.13379562454235	0.761	0.007	0	Endothelial	CDH5
0	7.72066319769634	0.761	0.033	0	Endothelial	EGFL7
0	6.35102826189869	0.762	0.035	0	Endothelial	NPDC1
0	5.07373883307282	0.712	0.029	0	Endothelial	COL4A1
0	3.52073276375613	0.715	0.044	0	Endothelial	IGFBP7
0	4.5774980888256	0.718	0.054	0	Endothelial	STAB1
0	4.87820168312721	0.687	0.027	0	Endothelial	COL4A2
0	8.37101091242545	0.64	0.004	0	Endothelial	RAMP3
0	5.90145287992711	0.737	0.127	0	Endothelial	HSPG2
0	8.04186036586054	0.62	0.012	0	Endothelial	FCN3
0	8.64577347125472	0.601	0.006	0	Endothelial	FLT1
0	7.74704371438543	0.612	0.019	0	Endothelial	DNASE1L3
0	5.93134663675786	0.69	0.112	0	Endothelial	HYAL2
0	3.16545771174675	0.638	0.093	0	Endothelial	RNASE1
0	5.17673235396091	0.585	0.042	0	Endothelial	CD93
0	9.03150575001849	0.543	0.003	0	Endothelial	PTPRB
0	8.09648842730082	0.538	0.006	0	Endothelial	AFAP1L1
0	6.30314385841306	0.531	0.008	0	Endothelial	ADAMTS4
0	2.7983728909601	0.538	0.029	0	Endothelial	COL6A2
0	8.85581398877488	0.498	0.003	0	Endothelial	MMRN2
0	3.99668951604607	0.535	0.071	0	Endothelial	SOCS3
0	7.37752131226863	0.463	0.005	0	Endothelial	LDB2
0	4.05406699448451	0.499	0.043	0	Endothelial	DEPRECATED-ENSG00000059804
0	5.41993983667844	0.487	0.038	0	Endothelial	ITPRIP
0	7.39727239916353	0.46	0.011	0	Endothelial	ESAM
0	7.66708648659699	0.454	0.012	0	Endothelial	FCN2
0	4.13503328000255	0.476	0.039	0	Endothelial	SLC2A3
0	6.06268750265134	0.448	0.016	0	Endothelial	NAV1
0	8.01820648576819	0.435	0.003	0	Endothelial	PCDH17
0	7.58772079911423	0.452	0.022	0	Endothelial	PODXL
0	7.97818007002278	0.433	0.004	0	Endothelial	OIT3
0	8.06122782528316	0.427	0.003	0	Endothelial	PDE2A
0	8.26705028868396	0.427	0.004	0	Endothelial	SEMA3F
0	5.26061505325783	0.471	0.048	0	Endothelial	PECAM1
0	7.27309173810652	0.43	0.009	0	Endothelial	CRHBP
0	4.04097585306721	0.501	0.081	0	Endothelial	SH3BP5
0	8.7258443852362	0.423	0.007	0	Endothelial	PLVAP
0	7.23357297183208	0.418	0.006	0	Endothelial	HECW2
0	5.88651557994921	0.418	0.01	0	Endothelial	MCAM
0	5.52654471775022	0.423	0.028	0	Endothelial	LIMS2
0	8.41687392013619	0.396	0.002	0	Endothelial	FLT4
0	5.87138396491194	0.402	0.014	0	Endothelial	SMAD6
0	5.86547763328812	0.405	0.02	0	Endothelial	DYSF
0	8.05105969254054	0.385	0.006	0	Endothelial	RASIP1
0	8.65520322522352	0.379	0.003	0	Endothelial	EFNB2
0	8.75923979738053	0.377	0.002	0	Endothelial	TEK
0	5.8367756475709	0.427	0.057	0	Endothelial	AKAP12
0	4.7388902771324	0.393	0.028	0	Endothelial	GRASP
0	5.38894989173613	0.377	0.014	0	Endothelial	FXYD6
0	5.35469052699749	0.382	0.026	0	Endothelial	NRP2

**0**	7.24929498447839	0.347	0.004	0	Endothelial	RAMP2	
**0**	3.98171425749281	0.391	0.049	0	Endothelial	DENND3	
**0**	5.66464615935611	0.357	0.015	0	Endothelial	FSCN1	
**0**	6.88660010380417	0.341	0.006	0	Endothelial	STC2	
**0**	5.65364945895967	0.36	0.026	0	Endothelial	ALPL	
**0**	6.94128620592224	0.338	0.005	0	Endothelial	CALCRL	
**0**	4.5415095568864	0.36	0.027	0	Endothelial	TCF4	
**0**	4.87136198781139	0.351	0.019	0	Endothelial	S1PR1	
**0**	6.79510159793789	0.332	0.005	0	Endothelial	CAVIN2	
**0**	7.03769588538401	0.332	0.014	0	Endothelial	AQP1	
**0**	7.95472352871805	0.316	0.002	0	Endothelial	TIE1	
**0**	8.77991402197202	0.316	0.003	0	Endothelial	VWF	
**0**	6.9848085012035	0.315	0.006	0	Endothelial	IL33	
**0**	7.75185451843482	0.31	0.003	0	Endothelial	ECSCR	
**0**	7.25804385749858	0.308	0.004	0	Endothelial	APLNR	
**0**	5.6761534584001	0.311	0.013	0	Endothelial	FGFR1	
**0**	5.42771801680002	0.305	0.012	0	Endothelial	LIFR	
**0**	5.17768035774698	0.313	0.02	0	Endothelial	THBD	
**0**	4.80478213445146	0.304	0.013	0	Endothelial	TINAGL1	
**0**	7.89241384372644	0.291	0.003	0	Endothelial	CLEC14A	
**0**	8.39791158056699	0.288	0.003	0	Endothelial	ADGRL4	
**0**	6.14711668674011	0.291	0.008	0	Endothelial	GNG11	
**0**	6.90086839117553	0.28	0.004	0	Endothelial	DCHS1	
**0**	6.91012064403959	0.282	0.007	0	Endothelial	TMEM255B	
**0**	7.09091847427231	0.279	0.005	0	Endothelial	PCDH12	
**0**	5.32963189328767	0.294	0.02	0	Endothelial	LMO2	
**0**	4.31677517777046	0.285	0.012	0	Endothelial	TNXB	
**0**	8.73969722135205	0.269	0.001	0	Endothelial	STAB2	
**0**	6.33201494412923	0.283	0.017	0	Endothelial	SHANK3	
**0**	3.61926144396051	0.288	0.025	0	Endothelial	ITGA9	
**0**	6.37627788472086	0.266	0.006	0	Endothelial	ARHGAP23	
**0**	4.99842734089917	0.263	0.01	0	Endothelial	PXDN	
**0**	8.09091699406023	0.255	0.002	0	Endothelial	KDR	
**0**	8.37366531930636	0.254	0.001	0	Endothelial	ROBO4	
**0**	5.64203034573809	0.261	0.012	0	Endothelial	ZNF160	
**0**	8.1555998956559	0.247	0.001	0	Endothelial	CLDN5	
**0**	6.1412066881525	0.243	0.005	0	Endothelial	COL15A1	
**0**	8.04586259812539	0.239	0.001	0	Endothelial	ADCY4	
**0**	4.05235362200325	0.255	0.017	0	Endothelial	ICAM2	
**0**	8.71610195199238	0.239	0.001	0	Endothelial	NOVA2	
**0**	4.83595478183764	0.257	0.02	0	Endothelial	GASK1B	
**0**	9.27278828691981	0.238	0.003	0	Endothelial	STC1	
**0**	8.21689139404141	0.235	0.001	0	Endothelial	EXOC3L2	
**0**	8.06427357721709	0.233	0.003	0	Endothelial	NPR 1.00	
**0**	7.74820958851485	0.232	0.002	0	Endothelial	ERG	
**0**	6.53437436675924	0.235	0.007	0	Endothelial	ADGRF5	
**0**	6.2418403004639	0.228	0.004	0	Endothelial	PEAR1	
**0**	8.33712850501437	0.222	0.001	0	Endothelial	EMCN	
**0**	7.07620263916143	0.218	0.003	0	Endothelial	CYYR1	
**0**	8.01470181063363	0.219	0.005	0	Endothelial	DLL4	
**0**	5.28477996158985	0.218	0.006	0	Endothelial	GJA4	
**0**	6.4412461501572	0.214	0.002	0	Endothelial	GPR4	
**0**	6.59112606382236	0.213	0.005	0	Endothelial	FGD5	
**0**	7.17237092950205	0.21	0.002	0	Endothelial	TEAD4	
**0**	4.44118269255681	0.216	0.013	0	Endothelial	INMT	
**0**	6.66721344037533	0.208	0.007	0	Endothelial	FAM167B	
**0**	4.21974450516684	0.214	0.013	0	Endothelial	PHLDB1	
**0**	7.40338590333164	0.205	0.004	0	Endothelial	CLEC4G	
**0**	7.9197775308186	0.2	0.001	0	Endothelial	SOX18	
**0**	9.19050692529475	0.199	0.001	0	Endothelial	CD34	
**0**	5.17754282515972	0.205	0.008	0	Endothelial	MYO10	
**0**	5.03653326056436	0.207	0.011	0	Endothelial	SH2D3C	
**0**	6.10388030736037	0.199	0.004	0	Endothelial	NES	

0	7.83448248204035	0.194	0.001	0	Endothelial	PGM5
0	7.125953532372	0.194	0.002	0	Endothelial	KANK3
0	7.37304063037364	0.191	0.005	0	Endothelial	IGFBP5
0	5.2801051308947	0.191	0.006	0	Endothelial	HIC1
0	7.62622134827532	0.183	0.001	0	Endothelial	MYCT1
0	8.50729096688902	0.183	0.001	0	Endothelial	PREX2
0	5.19871870026489	0.191	0.009	0	Endothelial	TEAD2
0	7.06776133544368	0.182	0.003	0	Endothelial	BCL6B
0	7.15260806472305	0.178	0.002	0	Endothelial	GJA1
0	5.32200337605798	0.182	0.007	0	Endothelial	IL3RA
0	5.69016401537636	0.177	0.004	0	Endothelial	JAM3
0	7.20299677023484	0.174	0.002	0	Endothelial	TSPAN18
0	6.91057838711282	0.174	0.002	0	Endothelial	RHOJ
0	8.6015142451623	0.166	0.001	0	Endothelial	SLCO2A1
0	7.89441309549128	0.166	0.001	0	Endothelial	SEMA6B
0	5.65113257696245	0.169	0.005	0	Endothelial	EHD3
0	4.47863266676911	0.171	0.008	0	Endothelial	TMEM204
0	7.50234063978588	0.161	0.002	0	Endothelial	PDE10A
0	8.19543735843291	0.16	0.001	0	Endothelial	ARHGEF15
0	6.18522909348692	0.16	0.005	0	Endothelial	EGLN3
0	5.83186223434057	0.156	0.004	0	Endothelial	LTK
0	6.43382348515818	0.147	0.005	0	Endothelial	CLEC1A
0	7.41456468538661	0.142	0.001	0	Endothelial	BMPER
0	7.78753118208956	0.142	0.001	0	Endothelial	NOS3
0	6.14438727694353	0.139	0.003	0	Endothelial	CDH13
0	6.10893980784509	0.133	0.003	0	Endothelial	TDRP
0	10.0138858950001	0.13	0	0	Endothelial	DIPK2B
0	7.95166236994027	0.13	0.001	0	Endothelial	NXF3
0	5.57857007124966	0.13	0.003	0	Endothelial	OLFML2A
0	7.33761332684	0.128	0.001	0	Endothelial	ADAMTS7
0	9.62003962429632	0.127	0	0	Endothelial	EXOC3L1
0	8.06591352473253	0.127	0.001	0	Endothelial	JAM2
0	5.39787688102989	0.127	0.003	0	Endothelial	A4GALT
0	6.56188027411203	0.125	0.003	0	Endothelial	SLCO4A1
0	4.8717792425589	0.125	0.004	0	Endothelial	TBXA2R
0	6.26035759439155	0.122	0.002	0	Endothelial	PRICKLE2
0	5.99684455590322	0.119	0.003	0	Endothelial	VEGFC
0	6.46947254411404	0.116	0.002	0	Endothelial	ST6GALNAC3
0	9.02880286926297	0.113	0	0	Endothelial	CCM2L
0	7.13265896958407	0.114	0.001	0	Endothelial	DEPRECATED-ENSG00000165682
0	6.84050737435943	0.114	0.002	0	Endothelial	TM4SF18
0	6.21526430467025	0.114	0.002	0	Endothelial	OLFM1
0	6.5486202411578	0.106	0.002	0	Endothelial	TFPI2
2.97345192562511E-307	4.67488093465949	0.164	0.007	4.98291073696256E-303	Endothelial	PTGIS
3.45027775454728E-307	3.15309483780787	0.26	0.021	5.78197546107034E-303	Endothelial	C7
4.15243309309131E-305	4.03338797736154	0.236	0.017	6.95864737740242E-301	Endothelial	MMP2
3.84519976520411E-302	3.37631763245485	0.408	0.055	6.44378576652905E-298	Endothelial	ITPKB
3.27080232115479E-301	3.82050120844449	0.316	0.033	5.48121052979119E-297	Endothelial	CRIM1
5.19623561179165E-301	6.41152641581319	0.144	0.006	8.70785163824046E-297	Endothelial	FUT1
1.03963618725259E-300	5.22942226226504	0.502	0.093	1.74222232259789E-296	Endothelial	PPFIBP1
5.81871656915538E-300	6.25089772925094	0.182	0.01	9.75100522659058E-296	Endothelial	HSPA12B
9.53499197515283E-299	3.55085297908413	0.196	0.012	1.59787395519611E-294	Endothelial	ADAMTS1
2.29817681228909E-290	4.69360245351169	0.336	0.039	3.85128470203405E-286	Endothelial	SMTN
1.22825880679906E-283	4.05234705707761	0.343	0.041	2.05831610843386E-279	Endothelial	LAMB2
1.14603954968276E-280	5.14343549993167	0.124	0.004	1.92053307735837E-276	Endothelial	DBN1
2.0520314901379E-279	3.84905448978523	0.318	0.036	3.43879437117309E-275	Endothelial	DUSP5
2.56139050462741E-278	4.09447954218618	0.357	0.046	4.29237820765462E-274	Endothelial	PLEKHO2
4.14785222786147E-278	4.69444933788209	0.158	0.008	6.95097076345026E-274	Endothelial	GABBR1
1.39792959027961E-275	5.11928054643295	0.116	0.004	2.34265040739057E-271	Endothelial	RIPOR3
1.64012744464063E-274	2.99077453129681	0.202	0.014	2.74852557172877E-270	Endothelial	THY1
2.23217379786256E-273	5.28503450248857	0.322	0.039	3.74067685045808E-269	Endothelial	ADAMTS9
2.68285219019629E-269	5.98618908731496	0.11	0.003	4.49592370033094E-265	Endothelial	RAPGEF3
1.27540727225173E-268	4.85189662867474	0.238	0.02	2.13732750683945E-264	Endothelial	SPRY4

1.01568836611711E-267	4.65631822816646	0.121	0.004	1.70209056393906E-263	Endothelial	RASGRF2
1.17289537118231E-263	2.63868941450592	0.573	0.128	1.96553806302732E-259	Endothelial	TIMP1
2.57051811483902E-263	3.7877585996679	0.48	0.092	4.30767425684723E-259	Endothelial	TIMP3
3.15958686975717E-256	5.03822315019242	0.469	0.093	5.29483567633907E-252	Endothelial	ADM
3.84994819231866E-253	4.40080125454045	0.205	0.016	6.45174318068761E-249	Endothelial	RGCC
1.58193313409947E-252	5.19864058955032	0.515	0.118	2.6510035461239E-248	Endothelial	MCF2L
3.42779561622863E-245	5.30220188311644	0.293	0.035	5.74429989367593E-241	Endothelial	PLK2
7.34115193672285E-245	5.85755631474114	0.194	0.015	1.23023024155602E-240	Endothelial	CD36
4.45348335779291E-242	3.60913872888614	0.155	0.009	7.46314741098936E-238	Endothelial	ADGRA2
9.30655658733208E-242	3.67360221444088	0.784	0.355	1.55959275290511E-237	Endothelial	PLPP3
2.52670855981041E-237	3.56361464005575	0.208	0.017	4.23425820453029E-233	Endothelial	LRRC32
1.69489988434587E-235	4.03656494225016	0.139	0.007	2.8403132261868E-231	Endothelial	FKBP10
1.01209475703424E-232	4.56644655399777	0.11	0.004	1.69606839383798E-228	Endothelial	MAP1B
7.16856978484777E-230	4.09046245732576	0.252	0.027	1.20130892454479E-225	Endothelial	VASH1
1.48823906693856E-229	5.74633413981192	0.147	0.009	2.49399102837564E-225	Endothelial	C5AR2
1.23367578380493E-227	4.86688777727654	0.141	0.008	2.0673938785003E-223	Endothelial	BACE2
3.73947516150266E-227	4.90100166446673	0.34	0.052	6.26661247564616E-223	Endothelial	ARHGAP29
6.50857573437876E-226	3.47170466906733	0.335	0.048	1.09070712156719E-221	Endothelial	PLTP
8.56115876795633E-226	5.4743440500545	0.124	0.006	1.43467898633412E-221	Endothelial	GNA14
6.04881964978335E-224	4.50185041138552	0.227	0.022	1.01366119691069E-219	Endothelial	PALD1
2.49323035590801E-223	4.61341692050953	0.103	0.004	4.17815543043064E-219	Endothelial	CD200
3.85761257191078E-221	4.90504341270963	0.117	0.005	6.46458714800808E-217	Endothelial	PMEPA1
3.54819959428001E-220	3.84959416158405	0.149	0.009	5.94607288009445E-216	Endothelial	VCAN
1.96959569666917E-217	5.0689310309293	0.282	0.037	3.3006484684782E-213	Endothelial	SPNS2
2.46260047439824E-215	5.1495639945201	0.11	0.005	4.12682587499658E-211	Endothelial	HDGFL3
1.7709588223189E-209	5.64138841114893	0.202	0.019	2.96777279444202E-205	Endothelial	LIMCH1
3.38743811187704E-208	4.41514457363682	0.635	0.234	5.67666878788355E-204	Endothelial	RAPGEF5
3.55473203687034E-208	3.80671764720073	0.156	0.011	5.95701994738731E-204	Endothelial	EHD2
3.49730006003885E-203	4.64598178872748	0.235	0.026	5.86077544061311E-199	Endothelial	PDGFB
1.78148522249963E-198	5.05552791302969	0.208	0.021	2.98541293586488E-194	Endothelial	PTGS1
2.18610520289544E-198	3.69203367236064	0.313	0.048	3.66347509901217E-194	Endothelial	SFMBT2
6.18341303660886E-197	6.03560285869612	0.266	0.036	1.03621635667491E-192	Endothelial	NOSTRIN
4.23401915135461E-196	3.78738900776573	0.221	0.024	7.09536929384005E-192	Endothelial	YBX3
6.21540111440985E-196	5.13213690727005	0.113	0.006	1.0415769187528E-191	Endothelial	LDLRAD3
3.49945296180474E-195	3.94884472844754	0.147	0.01	5.86438327339238E-191	Endothelial	TBKBP1
1.02879398094956E-191	4.99508511443572	0.102	0.005	1.72405295327528E-187	Endothelial	SH3RF3
6.69318315671704E-191	5.08494990244401	0.177	0.016	1.12164363340264E-186	Endothelial	RBMS2
8.95258144997055E-191	3.50248386067596	0.407	0.085	1.50027359938606E-186	Endothelial	GSN
4.24018845955445E-188	4.33082252044129	0.254	0.034	7.10570782052135E-184	Endothelial	ANGPTL4
3.66782137506724E-186	3.51859029998611	0.368	0.071	6.14653506033769E-182	Endothelial	HDAC7
3.079519321274E-184	4.61221000261264	0.205	0.022	5.16065847859097E-180	Endothelial	DEPRECATED-ENSG00000118263
3.72489838119135E-181	4.7924605843018	0.196	0.021	6.24218470720046E-177	Endothelial	RASGRP3
2.35549092127983E-176	2.78518197512888	0.366	0.07	3.94733168588073E-172	Endothelial	TIMP2
6.3023321308963E-174	3.79917834671427	0.493	0.146	1.0561448184956E-169	Endothelial	ITGA5
5.30264933304921E-170	4.25221629415522	0.142	0.011	8.88617975232387E-166	Endothelial	PLEKHG5
3.01675111485183E-167	5.74735918277466	0.136	0.011	5.0554715182687E-163	Endothelial	SPARCL1
1.78731448993617E-166	4.98404987131226	0.283	0.048	2.99518162223504E-162	Endothelial	SPRY1
4.43771131370411E-164	2.48922487394105	0.448	0.112	7.43671661950535E-160	Endothelial	ETS1
4.90294669468007E-164	2.82595787885008	0.332	0.063	8.21635807094486E-160	Endothelial	NR4A1
5.7468755549486E-164	4.15647655003284	0.131	0.01	9.63061405498287E-160	Endothelial	ZSCAN18
2.36265936854618E-162	4.55330163048969	0.371	0.085	3.95934456980968E-158	Endothelial	FZD4
9.52120774897663E-162	5.18738906439209	0.105	0.006	1.5955639945735E-157	Endothelial	UNC5B
3.65698189947679E-159	4.07897145118849	0.537	0.192	6.12837026714321E-155	Endothelial	CD9
7.7570207973719E-159	4.35893115020394	0.257	0.04	1.29992154522358E-154	Endothelial	SCARF1
5.03969363185627E-157	2.91044394837114	0.729	0.438	8.44551858826474E-153	Endothelial	DDIT4
2.46938868738703E-154	4.38064212718832	0.164	0.017	4.13820156232318E-150	Endothelial	ENO2
2.32758109624141E-153	4.35110466650447	0.379	0.092	3.90056040108136E-149	Endothelial	WWTR1
4.63872753512209E-150	5.0274281937961	0.106	0.007	7.7735796033576E-146	Endothelial	DEPRECATED-ENSG00000176014
2.54630395928845E-149	3.97655203432827	0.113	0.008	4.26709617497558E-145	Endothelial	RIMKLB
2.73551962954587E-144	5.58814701448289	0.1	0.006	4.58418379519298E-140	Endothelial	P3H2
5.54954242432138E-144	5.1013626420106	0.192	0.025	9.29992319467777E-140	Endothelial	DIPK1B
1.22539144217993E-141	4.14912849256746	0.255	0.044	2.05351097880513E-137	Endothelial	DLC1
2.08670402138503E-138	3.96831315845238	0.338	0.079	3.49689859903704E-134	Endothelial	SMAD7

5.23425982037747E-138	3.63683681964272	0.532	0.211	8.77157260698857E-134	Endothelial	PXN
6.13194115650687E-138	3.58237190039497	0.155	0.017	1.02759069900742E-133	Endothelial	HBEGF
1.15850868742998E-137	4.30602197824067	0.133	0.012	1.94142885839517E-133	Endothelial	ALPK3
9.69707502094877E-135	5.24476504688547	0.196	0.028	1.62503583201059E-130	Endothelial	ITGB3
3.05505624842552E-133	4.38302546590656	0.29	0.06	5.11966326111149E-129	Endothelial	SHE
9.98273515819893E-132	4.55519486573498	0.197	0.028	1.67290675781098E-127	Endothelial	EPHA2
1.3724880081186E-131	5.31064589120089	0.18	0.024	2.30001540400515E-127	Endothelial	APOLD1
1.17408754032134E-127	2.55539625786565	0.113	0.009	1.9675359000705E-123	Endothelial	ANGPTL2
6.24746301445459E-127	3.39735478602237	0.189	0.027	1.0469498519623E-122	Endothelial	LOXL2
2.45169708911342E-126	3.31550553093579	0.1	0.007	4.10855398193627E-122	Endothelial	LHFPL6
5.44724294972841E-124	2.48547229325234	0.715	0.521	9.12848973515487E-120	Endothelial	APP
8.00647960951808E-124	4.57829056773418	0.141	0.015	1.34172585296304E-119	Endothelial	JAG2
2.1563464071925E-123	4.53099167851059	0.268	0.055	3.61360530917319E-119	Endothelial	DLL1
2.59334076461891E-123	4.23920415564257	0.103	0.008	4.34592045334837E-119	Endothelial	HOXB3
1.85471977111123E-121	4.22632946128693	0.163	0.021	3.10813939242821E-117	Endothelial	FCGR2B
6.95646119915651E-121	2.82090647943262	0.279	0.058	1.16576376775465E-116	Endothelial	GIMAP4
2.01858194991676E-120	5.66123689728361	0.13	0.013	3.38273963167051E-116	Endothelial	FOXC1
2.339886722996E-120	1.93640957104423	0.784	0.629	3.9211821703967E-116	Endothelial	DUSP1
8.08758073417816E-120	2.98975065588179	0.399	0.116	1.35531677943358E-115	Endothelial	ID1
1.37754273950243E-119	3.36069318266368	0.2	0.031	2.30848612285818E-115	Endothelial	SEMA4C
2.11504684111905E-119	4.23219055104404	0.16	0.02	3.54439549634731E-115	Endothelial	DEPRECATED-ENSG00000072694
2.62735329031228E-118	3.69480369498135	0.153	0.019	4.40291864390532E-114	Endothelial	FHOD1
4.81773964731445E-118	3.2716927434913	0.426	0.142	8.07356810096956E-114	Endothelial	PLXND1
1.00202644012768E-117	4.97101994179758	0.1	0.008	1.67919590836597E-113	Endothelial	PROCR
3.05001579698446E-117	3.7598540635221	0.196	0.031	5.11121647258655E-113	Endothelial	FMNL2
9.36227776708103E-117	3.45605326186652	0.599	0.325	1.56893050820744E-112	Endothelial	TCIM
5.56049487393935E-116	2.08745702933121	0.732	0.489	9.31827730974757E-112	Endothelial	ARGLU1
8.78187710325132E-116	2.69654750169237	0.257	0.051	1.47166696496286E-111	Endothelial	CLEC2B
1.39602009971131E-115	2.54278943881416	0.161	0.021	2.33945048309621E-111	Endothelial	FBN1
1.28715018052723E-113	4.80265511751009	0.2	0.034	2.15700627252754E-109	Endothelial	CCL14
3.09800608029434E-113	3.29253352186391	0.499	0.208	5.19163858935726E-109	Endothelial	TGM2
1.09223324214914E-112	3.19811579193726	0.318	0.08	1.83036446719353E-108	Endothelial	PALM2-AKAP2
8.03483786569203E-112	3.70043008636675	0.141	0.017	1.34647812953267E-107	Endothelial	AFAP1
7.1503615842673E-111	4.70561968336528	0.208	0.037	1.19825759429151E-106	Endothelial	PLEKHG1
1.74190279269015E-110	3.93773576363843	0.191	0.031	2.91908069999015E-106	Endothelial	TRIP10
1.06278317200779E-108	3.66392504473037	0.16	0.022	1.78101203965065E-104	Endothelial	MEF2C
1.52803602813535E-108	4.01130773059637	0.102	0.009	2.56068277594922E-104	Endothelial	HOXB4
8.37788478933959E-107	4.29025298762983	0.338	0.098	1.40396593299753E-102	Endothelial	GDF15
1.19158064045124E-106	3.64517325330298	0.427	0.158	1.99685083726819E-102	Endothelial	ABCG1
1.78835708135272E-106	2.34185445273054	0.365	0.104	2.99692879693089E-102	Endothelial	IFI16
2.97773795173313E-106	1.88754506268116	0.149	0.019	4.99009325951438E-102	Endothelial	COL5A1
7.78414285728414E-104	3.49923331930901	0.185	0.03	1.30446666002368E-99	Endothelial	DEPRECATED-ENSG00000196329
1.24543554997221E-101	4.9951069943801	0.196	0.035	2.08710089464342E-97	Endothelial	ARAP3
2.05592842626213E-101	3.98525926532906	0.285	0.073	3.44532485673008E-97	Endothelial	CAVIN1
6.05206120535812E-101	3.39932817662282	0.31	0.084	1.01420441679391E-96	Endothelial	TNS1
6.7456377625096E-101	3.75676583509508	0.288	0.073	1.13043397624136E-96	Endothelial	CHSY1
2.81358504495123E-100	4.23516495933753	0.116	0.013	4.71500581832928E-96	Endothelial	CD109
8.4931057440741E-100	2.61496553335885	0.29	0.072	1.42327466059194E-95	Endothelial	SERPINB9
8.81813951229178E-100	4.26495073553788	0.166	0.026	1.47774381946986E-95	Endothelial	ELK3
1.03547145772532E-99	2.44246760851476	0.473	0.189	1.73524306885609E-95	Endothelial	SERPINH1
3.09742708667454E-98	4.42946952529835	0.146	0.02	5.19066831184919E-94	Endothelial	MAPK12
5.25470252905652E-98	4.13144461223927	0.155	0.023	8.80583049819292E-94	Endothelial	ICA1
6.2353516546757E-98	2.1479293198928	0.205	0.038	1.04492023029055E-93	Endothelial	FYN
8.1766973821413E-98	2.62773540249497	0.465	0.182	1.37025094729924E-93	Endothelial	VIM
1.26073653298561E-97	2.16643860436649	0.304	0.077	2.11274228197729E-93	Endothelial	MRC1
2.08173973161114E-97	3.70583416224373	0.214	0.043	3.48857944223394E-93	Endothelial	PLSCR3
4.15694620299944E-97	3.91635432735283	0.11	0.012	6.96621044698646E-93	Endothelial	TOX2
4.82912821605054E-97	4.93862849551185	0.1	0.01	8.09265306445749E-93	Endothelial	DNM3
6.92859240815147E-97	3.97168954353933	0.274	0.07	1.16109351575802E-92	Endothelial	KALRN
7.48458978997148E-96	4.1325169116182	0.122	0.015	1.25426755700342E-91	Endothelial	CPE
1.00795642503635E-95	3.23776484920041	0.205	0.04	1.68913337707591E-91	Endothelial	FMNL3
1.19787335678023E-95	3.82243692998834	0.144	0.02	2.00739617129232E-91	Endothelial	ARHGAP31
3.00541371709148E-94	3.66424017005521	0.106	0.011	5.0364723071019E-90	Endothelial	CFP

4.58104498043137E-94	4.05370888866966	0.2	0.039	7.67691517820689E-90	Endothelial	SOX4
4.65765075507733E-94	2.26664123409244	0.335	0.097	7.80529113535858E-90	Endothelial	FXYD5
6.40048907640722E-94	3.50735335841869	0.285	0.076	1.07259395942432E-89	Endothelial	TM4SF1
2.40927615436845E-93	3.42490265891333	0.183	0.033	4.03746497949065E-89	Endothelial	ENTPD1
1.5160136200159E-91	3.19996484697769	0.152	0.023	2.54053562442265E-87	Endothelial	OSMR
5.26801973488959E-91	3.43595432908103	0.357	0.121	8.82814747172797E-87	Endothelial	SEC14L1
6.50602436885707E-91	3.04961040586068	0.319	0.095	1.09027956373307E-86	Endothelial	ID3
5.16731219166221E-90	1.10087016880316	0.149	0.022	8.65938177078753E-86	Endothelial	AEBP1
4.36112742162901E-89	3.59149108552286	0.213	0.046	7.30837733316589E-85	Endothelial	ACP5
5.27477106683157E-89	3.57208485276339	0.178	0.033	8.83946135379634E-85	Endothelial	MAFF
1.0849955096966E-88	3.28556821852264	0.142	0.021	1.81823547514957E-84	Endothelial	NFATC1
1.77784507074657E-88	3.4823499785287	0.335	0.109	2.9793127695571E-84	Endothelial	TSPAN9
4.08588962057877E-88	3.17654159408255	0.347	0.116	6.8471338261659E-84	Endothelial	TSPAN14
2.07291874662233E-87	3.34562496774635	0.532	0.306	3.4737972355897E-83	Endothelial	INSR
1.21208173750793E-86	2.55242486838917	0.266	0.068	2.0312065757158E-82	Endothelial	CMTM3
2.14824317621015E-86	2.89961844580118	0.247	0.06	3.60002591469296E-82	Endothelial	LPAR6
3.31270576317194E-86	3.03914771130341	0.128	0.018	5.55143231792354E-82	Endothelial	C1QTNF1
9.01311076256551E-86	1.62606441616313	0.13	0.018	1.51041710159073E-81	Endothelial	C11orf96
4.68414677732861E-85	2.41416714765805	0.618	0.419	7.84969316944729E-81	Endothelial	STOM
1.34989854644703E-84	3.15461860446273	0.183	0.035	2.26215998413593E-80	Endothelial	GIMAP5
3.79837069223851E-84	3.72035097987957	0.169	0.031	6.36530960605329E-80	Endothelial	TSPAN15
2.45668284583131E-81	3.66613067449883	0.155	0.027	4.11690911304411E-77	Endothelial	UAP1L1
4.88318676914881E-80	4.49694594684018	0.106	0.013	8.18324438773958E-76	Endothelial	NXPE3
2.2065236955229E-79	3.19913946509997	0.463	0.23	3.69769240895727E-75	Endothelial	CEMIP2
3.81063003673307E-79	2.75507114221848	0.455	0.211	6.38585381555728E-75	Endothelial	SLC9A3R2
5.52204514899817E-79	3.7444308476065	0.526	0.302	9.25384326069113E-75	Endothelial	CTSL
1.03308901165956E-77	4.09995780849206	0.311	0.106	1.73125056573909E-73	Endothelial	DOCK6
2.8351923018813E-77	3.75916955560186	0.219	0.054	4.75121525949269E-73	Endothelial	JAG1
3.57415427892148E-77	3.84687884656934	0.23	0.059	5.98956774061662E-73	Endothelial	CRIP2
8.97953675770557E-75	3.38511340192887	0.183	0.039	1.5047907698563E-70	Endothelial	TRIB2
2.34886751274915E-74	3.50026553642974	0.128	0.02	3.93623217786503E-70	Endothelial	SLC25A36
1.02528301127652E-73	3.2447854869342	0.153	0.029	1.7181692702972E-69	Endothelial	GNB4
1.61940925691714E-73	4.23680415960438	0.257	0.076	2.71380603274175E-69	Endothelial	MAN1C1
4.98626431247552E-73	3.51979633550377	0.153	0.029	8.35598173484648E-69	Endothelial	RASSF2
1.89343521932826E-72	3.03867410314886	0.211	0.052	3.1730187405503E-68	Endothelial	SLC16A3
4.20945295419528E-72	4.55958583998476	0.171	0.036	7.05420126064046E-68	Endothelial	CDC42EP2
4.86033675707617E-71	2.37712969536771	0.321	0.109	8.14495233750824E-67	Endothelial	DAB2
3.56101510174122E-70	3.16488564671283	0.155	0.03	5.96754910749793E-66	Endothelial	ABI3
1.7437087526072E-69	0.867228940332882	0.457	0.177	2.92210712761914E-65	Endothelial	SRGN
2.39557730305135E-69	4.22719301162586	0.271	0.088	4.01450844445345E-65	Endothelial	LAMA4
1.12407036013598E-68	4.49127500668182	0.124	0.021	1.88371710951588E-64	Endothelial	INHBB
6.06157573347306E-68	3.28747171303235	0.138	0.025	1.01579886141542E-63	Endothelial	CHST15
7.66547819314378E-68	3.193335266713	0.172	0.038	1.28458083560703E-63	Endothelial	GRK5
8.33454536375579E-68	3.52554025161571	0.177	0.04	1.39670311205819E-63	Endothelial	MFNG
1.80199551406675E-67	3.93467480073232	0.224	0.062	3.01978408247306E-63	Endothelial	RASA4
1.81820527308066E-66	2.52644575341601	0.257	0.077	3.04694839662857E-62	Endothelial	PLEKHO1
8.0850375705902E-66	3.01923997115317	0.16	0.033	1.35489059607951E-61	Endothelial	PMP22
1.08007153003735E-65	3.30017343413396	0.152	0.031	1.8099838700366E-61	Endothelial	DISC1
4.69207148696233E-65	3.8326120176226	0.225	0.065	7.86297339785147E-61	Endothelial	STARD8
6.64804817704642E-65	3.44057084095107	0.155	0.032	1.11407991350944E-60	Endothelial	PALMD
3.75541089644681E-64	3.65043723290689	0.308	0.117	6.29331758026557E-60	Endothelial	EMP1
4.34687594279008E-64	2.57113433513197	0.52	0.32	7.28449470492761E-60	Endothelial	WSB1
7.58652715713504E-64	3.12343719238939	0.146	0.029	1.27135022099269E-59	Endothelial	DOCK1
7.94089302441272E-64	3.56652298715602	0.313	0.121	1.33073485303108E-59	Endothelial	NEDD9
1.14851158629873E-63	2.78142951874131	0.232	0.067	1.92467571631941E-59	Endothelial	TMEM173
2.01537209655152E-63	2.43506079594548	0.509	0.31	3.37736055940103E-59	Endothelial	MACF1
1.49523437944182E-62	3.30131316974367	0.343	0.143	2.5057137730686E-58	Endothelial	PGS1
3.62605647155853E-62	2.60946959059858	0.103	0.016	6.07654543503779E-58	Endothelial	PTGDS
7.01702936761764E-62	3.31034799026857	0.233	0.071	1.17591378142536E-57	Endothelial	ADGRG1
6.40314850174622E-61	3.21362278760667	0.106	0.017	1.07303962592263E-56	Endothelial	SPART
1.57456102196212E-60	1.73313318502625	0.628	0.442	2.63864936060411E-56	Endothelial	BHLHE40
1.23048178547051E-59	2.35383231835604	0.1	0.015	2.06204137609149E-55	Endothelial	ADAMTS2
1.339231997654E-58	4.2443885370472	0.175	0.044	2.24428498166858E-54	Endothelial	FAM43A

1.32521782523485E-57	3.63429513001469	0.182	0.047	2.22080003152857E-53	Endothelial	HEG1
2.34207788657321E-57	2.86864315396955	0.199	0.055	3.92485412231939E-53	Endothelial	CD55
3.20762856373994E-57	3.55279988681694	0.108	0.018	5.3753439471154E-53	Endothelial	DLG4
4.1138733336731E-57	3.52846321274867	0.243	0.081	6.89402893256938E-53	Endothelial	CTTNBP2NL
1.12249941587331E-56	3.42526901817014	0.241	0.08	1.88108452112049E-52	Endothelial	PEAK1
1.36950098192074E-56	2.79616767128109	0.243	0.079	2.29500974550278E-52	Endothelial	SLC12A4
4.55614927574782E-56	3.0360062641866	0.158	0.037	7.63519495629819E-52	Endothelial	PHLDB2
1.93512423481047E-54	3.56161152126051	0.116	0.022	3.24288119269539E-50	Endothelial	KLF7
2.17882397670477E-54	1.63889796622816	0.592	0.424	3.65127322016185E-50	Endothelial	JUNB
2.52970843772849E-54	3.29088810474876	0.319	0.137	4.2392853999454E-50	Endothelial	HMBOX1
3.96639471053261E-54	2.81371944798316	0.144	0.032	6.64688425591055E-50	Endothelial	ZNF83
7.79375600613969E-54	2.6054857715016	0.482	0.317	1.30607763150889E-49	Endothelial	NID1
1.31786891256954E-53	1.91944333045404	0.221	0.067	2.20848472368404E-49	Endothelial	METRNL
2.6382335458084E-53	1.79732988135547	0.335	0.135	4.42115177606571E-49	Endothelial	MS4A6A
5.41642870635428E-53	2.11869367227388	0.573	0.428	9.0768512261085E-49	Endothelial	MAF
7.9081374215931E-53	2.7354385929488	0.172	0.045	1.32524566911057E-48	Endothelial	FAR1
8.87440650360679E-53	1.62325453943874	0.102	0.017	1.48717304187443E-48	Endothelial	GGT5
8.51121510903825E-52	3.04520295002735	0.426	0.246	1.42630942797263E-47	Endothelial	ARHGEF7
5.29857509083581E-51	3.31716583009833	0.297	0.124	8.87935213722265E-47	Endothelial	FAM219A
5.63158899434509E-51	2.52817822120545	0.43	0.246	9.4374168367235E-47	Endothelial	ARRDC3
8.92417355252105E-51	3.33530628015136	0.111	0.021	1.49551300393148E-46	Endothelial	PARVB
1.28030680971912E-50	3.94657912101104	0.318	0.145	2.1455381517273E-46	Endothelial	TMEM44
1.78057510199917E-50	1.79179343336245	0.11	0.021	2.98388775593021E-46	Endothelial	PPP1R16B
1.94648761314607E-50	3.53493791198391	0.135	0.031	3.26192394211019E-46	Endothelial	ZBTB46
3.82390109806784E-49	2.94473546137137	0.116	0.024	6.40809346014209E-45	Endothelial	BEX4
9.79444198098026E-49	3.01462064956356	0.391	0.217	1.64135258717267E-44	Endothelial	MARCKSL1
1.44674128571733E-48	3.47918223056608	0.269	0.108	2.42444904660511E-44	Endothelial	CHST12
2.66146507888508E-48	3.08276391681745	0.128	0.029	4.46008317919562E-44	Endothelial	GIMAP8
4.2193881282633E-48	2.12036056451279	0.199	0.061	7.07085062534364E-44	Endothelial	KLHL6
1.45527995306547E-47	3.03644326309692	0.343	0.17	2.43875814534711E-43	Endothelial	SNRK
7.33498911731801E-47	0.852712162777285	0.136	0.031	1.22919747628015E-42	Endothelial	BGN
1.4855444447349E-46	2.91180061613762	0.46	0.301	2.48947538048674E-42	Endothelial	UGCG
9.23092364758134E-46	3.43642607476963	0.294	0.131	1.54691818486168E-41	Endothelial	WARS
1.6660244778395E-45	1.92373821219028	0.244	0.088	2.79192381996343E-41	Endothelial	NLRP1
1.57080625539289E-44	2.69670782377757	0.16	0.044	2.63235712278741E-40	Endothelial	GIMAP6
3.27227112037643E-44	3.04855252096584	0.329	0.163	5.48367194352682E-40	Endothelial	TMEM184B
1.12446616940774E-43	3.00341851651299	0.332	0.165	1.8843804066935E-39	Endothelial	TNFRSF10B
3.50143356398071E-43	3.96923089528101	0.146	0.04	5.86770236651887E-39	Endothelial	HSD17B14
4.33353273085635E-43	2.26161319695528	0.527	0.434	7.26213415036906E-39	Endothelial	EPAS1
8.02371447868101E-43	3.74345238420374	0.135	0.034	1.34461407233736E-38	Endothelial	GPRC5B
1.37414702261362E-42	3.53388550570105	0.244	0.098	2.3027955804959E-38	Endothelial	PPM1F
9.34639103513712E-42	2.58737406996031	0.365	0.2	1.56626820966828E-37	Endothelial	JMJD1C
1.03213588769308E-41	2.74509675030511	0.335	0.172	1.72965332059607E-37	Endothelial	PLCG1
1.3231924598591E-41	2.51763858730139	0.102	0.021	2.21740592423188E-37	Endothelial	MYOF
1.75076342582064E-40	2.35371539502923	0.448	0.292	2.93392934899023E-36	Endothelial	DEPP1
2.09858800597992E-40	3.89966938110026	0.116	0.028	3.51681378042115E-36	Endothelial	CPNE2
9.17547384178895E-40	2.95237577158917	0.211	0.078	1.53762590640699E-35	Endothelial	BCAM
1.26727052002686E-39	2.78957315946647	0.346	0.191	2.12369193746102E-35	Endothelial	SLC44A2
2.13030587463905E-39	2.83131539623798	0.213	0.079	3.56996658472012E-35	Endothelial	ACKR3
3.57614068411892E-39	2.6055396213778	0.152	0.044	5.99289655844648E-35	Endothelial	ARID5A
8.57945895756689E-39	3.29812063270368	0.102	0.023	1.43774573210906E-34	Endothelial	PFKM
3.10855571067804E-38	3.39763840565436	0.233	0.096	5.20931765995426E-34	Endothelial	UPP1
3.55385419674505E-38	3.77699047122536	0.146	0.043	5.95554886290535E-34	Endothelial	GRPEL2
5.35847504001151E-38	2.09300314166822	0.141	0.039	8.97973247205129E-34	Endothelial	GIMAP7
8.00424487808507E-38	3.1707526079119	0.391	0.247	1.3413513566695E-33	Endothelial	CNST
1.59870552701178E-37	2.88611278833382	0.41	0.272	2.67911072216634E-33	Endothelial	PRCP
1.91513761670159E-37	3.38474799504694	0.114	0.028	3.20938761806852E-33	Endothelial	MAPK11
3.67849820492176E-37	2.37950045478176	0.169	0.054	6.16442729180788E-33	Endothelial	COTL1
4.3917632569402E-37	2.87615460113756	0.266	0.121	7.35971686598039E-33	Endothelial	LAMA5
7.79605964476861E-37	1.55358792374493	0.498	0.339	1.30646367527032E-32	Endothelial	A2M
8.62456480073263E-37	1.25564177596113	0.263	0.106	1.44530456930677E-32	Endothelial	CYBA
1.14629429404852E-36	3.54618198012094	0.153	0.048	1.92095997796651E-32	Endothelial	CAV1
1.4719017167169E-36	2.42817980549188	0.228	0.093	2.46661289687418E-32	Endothelial	ABCC1

3.10615439404945E-36	3.33217042935161	0.308	0.162	5.20529353354807E-32	Endothelial	ARHGAP26
4.38134598225983E-35	1.44054493392835	0.576	0.478	7.34225959707103E-31	Endothelial	LENG8
4.80328252585028E-35	2.58859877132167	0.236	0.1	8.04934085681989E-31	Endothelial	DOCK4
5.1081831641941E-35	2.46561744405065	0.21	0.082	8.56029334655647E-31	Endothelial	ARHGEF2
9.07037565391922E-35	4.64585201341681	0.247	0.114	1.52001355208378E-30	Endothelial	ZNF117
9.95000518226387E-35	3.10310591772493	0.11	0.028	1.66742186844378E-30	Endothelial	IRAK3
1.08095151714678E-34	3.39013135148944	0.269	0.131	1.81145855243458E-30	Endothelial	KIAA0355
2.73735525608577E-34	1.72432257702721	0.527	0.427	4.58725993814853E-30	Endothelial	FCGRT
3.71959173842244E-34	1.68789606298316	0.142	0.042	6.23329183524832E-30	Endothelial	ITM2A
1.23743177015147E-32	0.901344045484173	0.429	0.233	2.07368816041983E-28	Endothelial	COL18A1
1.6490050115921E-32	2.23576841729799	0.141	0.044	2.76340259842604E-28	Endothelial	PRKCH
3.12115253784347E-32	3.30240824596691	0.238	0.109	5.23042742291809E-28	Endothelial	TCN2
5.14248827794234E-32	3.13071695394178	0.113	0.031	8.61778185617577E-28	Endothelial	RENBP
8.99349380050282E-32	2.71117510611271	0.401	0.283	1.50712969108826E-27	Endothelial	SELENON
1.11802582830456E-30	3.42877803233096	0.141	0.047	1.87358768307278E-26	Endothelial	TRPM4
1.78365647037836E-30	3.5879122633556	0.11	0.031	2.98905151306005E-26	Endothelial	HOMER3
1.79422064711379E-30	2.79852935208329	0.149	0.051	3.00675496043329E-26	Endothelial	KLF4
1.84330363602734E-30	2.96944570647822	0.232	0.107	3.08900823325461E-26	Endothelial	LZTS2
2.48204179190171E-30	2.51789779666601	0.116	0.033	4.15940563486888E-26	Endothelial	GYPC
4.1203587957752E-30	3.78538845769236	0.197	0.083	6.90489726996008E-26	Endothelial	MAP3K6
4.24698818021249E-30	1.53292759865243	0.116	0.033	7.11710279240009E-26	Endothelial	ADAMTSL2
8.56707544984662E-30	3.17671632528828	0.177	0.069	1.4356705038853E-25	Endothelial	FUT11
2.1568731275158E-29	2.63723621287547	0.139	0.046	3.61448798709098E-25	Endothelial	MPDZ
2.60695287584717E-29	1.63549279348677	0.36	0.207	4.36873162934469E-25	Endothelial	CD14
2.78979131075161E-29	3.20622185972564	0.26	0.133	4.67513227855755E-25	Endothelial	PTPRG
3.44921458982412E-29	2.26151237958228	0.274	0.14	5.78019380962726E-25	Endothelial	KLF2
4.96461594530563E-29	2.87432653109724	0.333	0.208	8.31970340114317E-25	Endothelial	NOTCH1
5.72189252922785E-29	2.9958455509687	0.153	0.055	9.58874750048003E-25	Endothelial	KSR1
6.22109805240419E-29	2.88609555583114	0.142	0.049	1.04253161162189E-24	Endothelial	F2R
8.98107012757125E-29	2.97470689634287	0.282	0.154	1.50504773197839E-24	Endothelial	MXRA7
1.02367128677233E-28	3.19088506164442	0.174	0.069	1.71546834237307E-24	Endothelial	SYNE1
4.28558530206714E-28	1.80252307865247	0.141	0.047	7.18178384920411E-24	Endothelial	PLAUR
1.00167072702623E-27	1.96055302276182	0.138	0.046	1.67859980435056E-23	Endothelial	TNFRSF25
1.01398804261223E-27	2.03262779205588	0.103	0.029	1.69924116180958E-23	Endothelial	PDE4B
1.85957662074605E-27	3.7556897297679	0.169	0.068	3.11627850104623E-23	Endothelial	PIK3R3
1.872028186071E-27	0.70209281230012	0.227	0.103	3.13714483421779E-23	Endothelial	COL6A1
2.86964974460753E-27	3.23710864375062	0.225	0.108	4.8089590420133E-23	Endothelial	TRAF6
2.99654893609397E-27	1.84464176843935	0.474	0.394	5.02161670710627E-23	Endothelial	FOSL2
3.68852865937198E-27	2.02683581292255	0.218	0.098	6.18123632737556E-23	Endothelial	CD81
3.89179484903942E-27	2.33310969416384	0.421	0.315	6.52186980802026E-23	Endothelial	MYO15B
5.80677367505437E-27	2.81820372888484	0.156	0.059	9.73099132465612E-23	Endothelial	PDLIM7
1.18580279155268E-26	2.73597961488981	0.208	0.095	1.98716831808397E-22	Endothelial	CCDC85B
2.19340877756051E-26	3.56179962324063	0.111	0.034	3.6757144294359E-22	Endothelial	RPGR
2.71785068263497E-26	2.83343721464427	0.335	0.216	4.55457417395969E-22	Endothelial	SIN3B
3.0008907868791E-26	3.65726060814029	0.141	0.051	5.028892780652E-22	Endothelial	PTPN14
3.05800511066396E-26	2.29718620419741	0.397	0.296	5.12460496445067E-22	Endothelial	NRP1
5.3656883356516E-26	3.0449045303345	0.114	0.036	8.99182051288494E-22	Endothelial	SLC2A9
6.10753797314809E-26	1.38442315151886	0.258	0.128	1.02350121354016E-21	Endothelial	TRIM22
7.08033488422175E-26	4.47746494983377	0.147	0.056	1.18652251989788E-21	Endothelial	ERV3-1
8.18028680227654E-26	2.3094097747987	0.138	0.049	1.3708524623255E-21	Endothelial	DSE
1.25961572960321E-25	2.25434851812314	0.305	0.178	2.11086403966906E-21	Endothelial	HES1
1.29961922858645E-25	1.93752316538465	0.423	0.321	2.17790190326517E-21	Endothelial	JAK1
1.40770130403002E-25	2.54228394793738	0.1	0.029	2.3590258452935E-21	Endothelial	PRKAR1B
1.99846699107846E-25	2.19767257270971	0.493	0.423	3.34903098364929E-21	Endothelial	DEPRECATED-ENSG00000147872
4.02987210188592E-25	2.11392303882438	0.211	0.097	6.75325966834042E-21	Endothelial	IER5L
4.34170414963757E-25	1.27760811229219	0.629	0.656	7.27582781396264E-21	Endothelial	RHOB
4.66302106888774E-25	2.10388866706163	0.144	0.053	7.81429070724207E-21	Endothelial	GSTP1
5.83968473339511E-25	2.14278555820355	0.133	0.047	9.78614367622353E-21	Endothelial	PLXDC2
1.56350334796695E-24	1.66534803344208	0.377	0.251	2.62011891052301E-20	Endothelial	DEPRECATED-ENSG00000175899
1.98175455116264E-24	2.35262094416574	0.139	0.051	3.32102427683836E-20	Endothelial	RBMS1
2.96551767105458E-24	2.66002077904638	0.305	0.191	4.96961451315327E-20	Endothelial	JUP
3.13442228083821E-24	1.60650408180755	0.448	0.359	5.25266485822868E-20	Endothelial	MSN
5.94079602499634E-24	2.38965540758238	0.402	0.32	9.95558597868887E-20	Endothelial	TGFBR2

1.03126045054166E-23	2.71121906948812	0.189	0.087	1.72818626301772E-19	Endothelial	RASSF1
1.05978631737594E-23	4.08961218784442	0.121	0.042	1.77598991065861E-19	Endothelial	FRMD8
1.12029610997765E-23	3.59941720552257	0.142	0.056	1.87739222110055E-19	Endothelial	TIAM1
1.45296395646106E-23	3.30301230081535	0.227	0.119	2.43487699823744E-19	Endothelial	TP53I11
1.53586876110499E-23	3.17477255042096	0.144	0.056	2.57380886985974E-19	Endothelial	TTC7B
2.24854201052431E-23	2.7126100548108	0.224	0.114	3.76810670123663E-19	Endothelial	RIPOR1
3.96860520981463E-23	3.59563911406707	0.133	0.05	6.65058861060735E-19	Endothelial	GDPD5
1.52106536198758E-22	3.76553255745955	0.141	0.056	2.54900133361879E-18	Endothelial	CASKIN2
2.21004727585423E-22	3.13747564814218	0.247	0.139	3.70359722487652E-18	Endothelial	EPHB4
9.77736310268415E-22	2.29106026792399	0.387	0.305	1.63849050874781E-17	Endothelial	ECE1
2.1363310450943E-21	3.27167223564312	0.13	0.05	3.58006356536903E-17	Endothelial	TGFBR3
2.55452902484726E-21	2.99459500091468	0.183	0.087	4.28087973983904E-17	Endothelial	ADGRL2
5.24659341654307E-21	1.9543323429332	0.427	0.362	8.79224124744288E-17	Endothelial	RHOC
1.40639739462668E-20	3.08392433691629	0.121	0.046	2.35684075391539E-16	Endothelial	NRARP
2.39618382674341E-20	2.56913527708128	0.108	0.038	4.01552485685661E-16	Endothelial	ZNF264
2.86214434872372E-20	2.95090578664315	0.152	0.066	4.7963814995912E-16	Endothelial	SLC6A6
3.67205696819746E-20	1.26677858862663	0.125	0.047	6.1536330673053E-16	Endothelial	JAK3
4.16624129090432E-20	3.34643920938926	0.197	0.102	6.98178715529746E-16	Endothelial	MPZL2
4.23044225569075E-20	2.10489094333965	0.415	0.349	7.08937513208656E-16	Endothelial	CD59
5.18350044841536E-20	0.250277202518182	0.122	0.318	8.68651005145445E-16	Endothelial	LRRFIP2
7.04594549127639E-20	4.08329027380825	0.153	0.068	1.1807595454281E-15	Endothelial	ADAMTSL1
1.00839770615471E-19	0.264357867045716	0.11	0.299	1.68987287597406E-15	Endothelial	NFIA
1.05413274605505E-19	0.873122207252359	0.108	0.037	1.76651565583906E-15	Endothelial	DNAJA4
1.08910949582257E-19	2.88186301435596	0.194	0.099	1.82512969309947E-15	Endothelial	CDK17
1.40619672510317E-19	2.90487643470225	0.344	0.261	2.35650447192789E-15	Endothelial	PLPP1
1.83928207122446E-19	1.83841485749961	0.388	0.298	3.08226889495795E-15	Endothelial	CYTH1
2.45862764904801E-19	0.97976159390709	0.567	0.529	4.12016821427465E-15	Endothelial	CCNL2
4.93048896231182E-19	3.03836963508579	0.252	0.153	8.26251340304215E-15	Endothelial	PRSS23
6.29749225541344E-19	2.96291799294679	0.122	0.048	1.05533375216219E-14	Endothelial	DUSP7
1.48695384623975E-18	3.44698239481503	0.144	0.064	2.49183725552857E-14	Endothelial	ANKRD37
2.06639723568171E-18	2.59262255960769	0.28	0.184	3.46286848755541E-14	Endothelial	CLIC4
2.36483371094616E-18	2.35542152793966	0.308	0.209	3.96298833280357E-14	Endothelial	XAF1
3.06045696590037E-18	0.259805494829606	0.105	0.277	5.12871378345584E-14	Endothelial	TPP2
4.90232863697807E-18	0.263271304833438	0.142	0.338	8.21532232984785E-14	Endothelial	RAB18
5.52773737842841E-18	2.86125467629534	0.274	0.178	9.26338229877032E-14	Endothelial	SLC25A37
5.89647321775982E-18	2.8535866999729	0.286	0.194	9.8813098183219E-14	Endothelial	PELO
9.10216706082239E-18	2.31208709524712	0.175	0.087	1.52534115605262E-13	Endothelial	GABARAPL1
1.17623270275507E-17	2.61378235436773	0.219	0.125	1.97113076327694E-13	Endothelial	HTRA1
1.26767812288828E-17	2.26882617637154	0.133	0.057	2.12437499833619E-13	Endothelial	AKR1B1
1.62071914785236E-17	0.309555803719511	0.142	0.333	2.71600114797098E-13	Endothelial	MAVS
2.18964609348378E-17	2.9998300980699	0.192	0.104	3.66940892346012E-13	Endothelial	ARL 15.00
2.63395758398554E-17	2.69411933607612	0.2	0.109	4.41398611924296E-13	Endothelial	NOL4L
3.9849516869209E-17	2.94025400265511	0.232	0.14	6.67798203694204E-13	Endothelial	VWA1
4.6056248552069E-17	0.287054419544963	0.18	0.389	7.71810613235573E-13	Endothelial	HNRNPD
4.84523014163515E-17	1.49013924418664	0.438	0.378	8.11963667135218E-13	Endothelial	TMSB10
5.11737151161545E-17	2.62725634603929	0.227	0.135	8.57569117916516E-13	Endothelial	SWAP70
6.52094104063039E-17	2.7570414393193	0.18	0.094	1.09277929958884E-12	Endothelial	HERC1
6.53020049428986E-17	0.344614517946559	0.139	0.332	1.09433099883309E-12	Endothelial	ACVR1B
7.30960245483707E-17	0.262355363886945	0.117	0.29	1.2249431793816E-12	Endothelial	TTC37
7.77030652914754E-17	1.83995281956797	0.125	0.052	1.30214796815454E-12	Endothelial	LDHB
9.9938877167858E-17	0.286680948604701	0.108	0.275	1.67477570357896E-12	Endothelial	ZNF410
1.34277695061894E-16	0.279902825111309	0.102	0.265	2.25022561384722E-12	Endothelial	WASL
1.5004521779887E-16	2.6364773463226	0.275	0.185	2.51445775987346E-12	Endothelial	DAPK3
1.55544986163003E-16	1.35984735397517	0.131	0.055	2.6066228781196E-12	Endothelial	S100A6
1.80813705044177E-16	2.19980069426336	0.261	0.167	3.03007606913032E-12	Endothelial	CBLB
2.47326328007588E-16	0.449311813311781	0.13	0.304	4.14469460475116E-12	Endothelial	C5orf24
3.67219145406375E-16	0.338852238984914	0.078	0.225	6.15385843872004E-12	Endothelial	ZC3H3
4.8687383004011E-16	0.310166394421131	0.085	0.235	8.15903164381216E-12	Endothelial	HMGXB4
5.19838743592918E-16	0.599316448333134	0.083	0.234	8.71145766513012E-12	Endothelial	SC5D
5.57702688324401E-16	0.340944009769265	0.088	0.239	9.34598165094031E-12	Endothelial	IFT27
5.58567699568435E-16	2.00404352266826	0.214	0.125	9.36047750936783E-12	Endothelial	FSTL1
5.72893265409164E-16	0.656997190799758	0.133	0.314	9.60054534172677E-12	Endothelial	CUL4B
6.73525093885677E-16	2.80936975170648	0.135	0.062	1.12869335233362E-11	Endothelial	IL4R

6.80711621084463E-16	0.396899053691608	0.1	0.259	1.14073653461334E-11	Endothelial	TMEM214
7.96587735241381E-16	2.76163803360794	0.211	0.124	1.33492172671751E-11	Endothelial	PIP5K1C
1.00903992612935E-15	0.326779441451947	0.11	0.275	1.69094910820756E-11	Endothelial	DNAJC1
1.0297518901821E-15	0.250093473289636	0.13	0.3	1.72565821756715E-11	Endothelial	SMARCA5
1.0906245370855E-15	1.25703401490361	0.135	0.06	1.82766859924788E-11	Endothelial	ZEB2
1.44744208469543E-15	0.313100465636777	0.155	0.342	2.4256234455326E-11	Endothelial	DIDO1
1.60154945518121E-15	1.4021374305726	0.128	0.056	2.68387657699267E-11	Endothelial	TAGLN
1.63672143310577E-15	3.06897475136817	0.11	0.046	2.74281777759866E-11	Endothelial	MCC
1.6663364261863E-15	0.28837619810643	0.106	0.265	2.792446583003E-11	Endothelial	SNRPA
1.74263521899228E-15	0.383673630328225	0.136	0.311	2.92030809998727E-11	Endothelial	SLC35A4
1.80075355104388E-15	2.6282979564245	0.279	0.193	3.01770280083933E-11	Endothelial	MED10
1.90923767111338E-15	0.358823599996268	0.113	0.274	3.19950048925181E-11	Endothelial	CANT1
2.07659670402389E-15	0.275868485226286	0.092	0.242	3.47996075660324E-11	Endothelial	ZNF146
2.53159804540671E-15	0.413453669711378	0.147	0.329	4.24245200449257E-11	Endothelial	CTDSP1
2.89532706572284E-15	1.83685974217771	0.113	0.047	4.85198909673833E-11	Endothelial	LBH
3.70177164028704E-15	0.303025755970554	0.097	0.25	6.20342891479303E-11	Endothelial	ST3GAL6
3.90870190615466E-15	0.258039031333376	0.092	0.24	6.55020265433398E-11	Endothelial	NDUFA9
4.1294028930973E-15	0.300647744681819	0.156	0.339	6.92005336825245E-11	Endothelial	ITCH
4.20910207348046E-15	0.290015765427689	0.122	0.288	7.05361325473855E-11	Endothelial	NSFL1C
4.68234565392752E-15	2.36375007029629	0.144	0.069	7.84667484685174E-11	Endothelial	NCKAP5L
4.96105567719038E-15	0.422400515009047	0.106	0.264	8.31373710383564E-11	Endothelial	KDSR
4.97377783013491E-15	3.19164712837914	0.117	0.051	8.33505688774008E-11	Endothelial	TCF7L1
5.03272363543831E-15	0.346646518059477	0.102	0.255	8.43383826826752E-11	Endothelial	UBA2
5.17296402822931E-15	0.28930418898634	0.111	0.269	8.66885311850668E-11	Endothelial	LRRC47
5.7665293322696E-15	1.74485212880925	0.255	0.162	9.6635498550174E-11	Endothelial	TMEM140
6.00328483578961E-15	1.84271812803563	0.335	0.257	1.00603047278162E-10	Endothelial	LUC7L
6.86760689683487E-15	0.715466287114143	0.102	0.255	1.15087356377159E-10	Endothelial	FAM13A
7.02803939067877E-15	0.307596346155789	0.091	0.237	1.17775884108995E-10	Endothelial	PURA
7.29727781726316E-15	2.29688204800548	0.189	0.106	1.22287781661696E-10	Endothelial	KDM6B
7.76329808888942E-15	0.261533854964918	0.138	0.309	1.30097349373609E-10	Endothelial	ZMYM2
1.15520562712797E-14	0.261763062709195	0.15	0.326	1.93589358994105E-10	Endothelial	SND1
1.16193991450807E-14	0.304183118640457	0.18	0.376	1.94717890873263E-10	Endothelial	FIS1
1.29433575706239E-14	2.49075349431583	0.239	0.155	2.16904786168515E-10	Endothelial	SSH1
1.3581352343738E-14	0.269178199185238	0.122	0.283	2.27596302576361E-10	Endothelial	CEP350
1.38916626984817E-14	0.332326269927393	0.13	0.295	2.32796483501156E-10	Endothelial	AGFG1
1.54803456331203E-14	2.85433873482331	0.255	0.174	2.5941963211983E-10	Endothelial	NR2F2
1.58579406325694E-14	0.399041216756923	0.131	0.298	2.65747369120599E-10	Endothelial	HELZ
1.88013802720529E-14	0.293068514441614	0.125	0.285	3.15073530599062E-10	Endothelial	HNRNPUL2
1.89719668340099E-14	0.408277907606351	0.099	0.245	3.17932220204338E-10	Endothelial	UBA5
2.1921784740206E-14	0.430290204108235	0.131	0.296	3.67365268676373E-10	Endothelial	DHX9
2.205777991317E-14	0.25606920990578	0.133	0.298	3.69644275784903E-10	Endothelial	SEC11A
2.30032403636327E-14	0.283821993602189	0.064	0.19	3.85488302013757E-10	Endothelial	GANAB
2.43315230667514E-14	0.275205891833793	0.146	0.318	4.0774766355262E-10	Endothelial	DDX46
2.60236255123448E-14	0.27155027271245	0.133	0.296	4.36103916335874E-10	Endothelial	NUDCD3
2.77333690337985E-14	2.5990324933577	0.261	0.181	4.64755798268396E-10	Endothelial	SULF2
2.8937929787398E-14	0.369601850932257	0.158	0.339	4.84941827377215E-10	Endothelial	MAP3K13
2.9034871953342E-14	0.293876006445008	0.078	0.211	4.86566384194106E-10	Endothelial	DIS3
3.71716106230581E-14	0.303681051944944	0.053	0.174	6.22921850821208E-10	Endothelial	USPL1
3.81108454127518E-14	0.289456128076624	0.088	0.226	6.38661547426894E-10	Endothelial	TSNAX
3.97160722289191E-14	0.343081450595507	0.138	0.305	6.65561938412227E-10	Endothelial	PRRC2C
4.06426189159777E-14	0.257955410185034	0.172	0.356	6.81089007793955E-10	Endothelial	SDCBP
4.11438057869294E-14	0.559435832349787	0.077	0.212	6.89487897377362E-10	Endothelial	P2RX4
4.3062893048773E-14	2.93356977393059	0.155	0.081	7.21647961711338E-10	Endothelial	CTBP2
4.70702259410072E-14	3.0924344995786	0.227	0.148	7.88802846319399E-10	Endothelial	TSPAN7
4.80353533228124E-14	0.311023058695203	0.1	0.247	8.0497645098369E-10	Endothelial	UQCC1
5.1809087599412E-14	3.74144399274176	0.136	0.067	8.68216689990946E-10	Endothelial	RPH3AL
5.23789656454355E-14	0.349869010859467	0.133	0.297	8.77766706286208E-10	Endothelial	FBXL5
5.2457054025679E-14	0.369612545536715	0.117	0.272	8.7907531136233E-10	Endothelial	ARNT
5.36984062886332E-14	0.258006865447991	0.11	0.259	8.99877892584915E-10	Endothelial	SCAF1
6.04065378494494E-14	2.2266537154181	0.188	0.107	1.01229276128107E-09	Endothelial	MYC
6.36725749922178E-14	0.277728835927055	0.111	0.262	1.06702501171959E-09	Endothelial	RBM14
6.80743677645035E-14	2.65416642074153	0.178	0.101	1.14079025499755E-09	Endothelial	ZEB1
6.86170111102973E-14	0.274679800663435	0.166	0.341	1.14988387218636E-09	Endothelial	ETNK1

6.86796987318567E-14	1.70837630429596	0.25	0.162	1.15093439134845E-09	Endothelial	FLNA
6.93901911331023E-14	2.59630534117242	0.203	0.122	1.16284082300853E-09	Endothelial	DENND5A
7.38123828958535E-14	0.351372378388366	0.113	0.263	1.23694791256871E-09	Endothelial	DOT1L
7.38841682227129E-14	2.11803615957584	0.368	0.317	1.23815089107622E-09	Endothelial	UBR4
7.88537529025968E-14	0.329558013693366	0.119	0.273	1.32143119114172E-09	Endothelial	SUPT6H
8.06540819821223E-14	2.84026793084558	0.167	0.092	1.35160110585641E-09	Endothelial	MTHFD1L
9.42094315881433E-14	0.263377887741926	0.085	0.217	1.57876165455411E-09	Endothelial	OXR1
1.08293024561502E-13	0.464586953826743	0.147	0.316	1.81477450560165E-09	Endothelial	XRN1
1.13549616378106E-13	0.423180216762403	0.114	0.267	1.9028644712643E-09	Endothelial	SH3GLB2
1.1421447296876E-13	0.277413412784677	0.097	0.238	1.91400613801047E-09	Endothelial	DICER1
1.16233741907272E-13	2.48306087741123	0.135	0.066	1.94784504688207E-09	Endothelial	SFXN3
1.2595875425969E-13	0.860317547043435	0.077	0.21	2.11081680388389E-09	Endothelial	SAMD12
1.38500394802163E-13	0.272402116222881	0.124	0.278	2.32098961609464E-09	Endothelial	ARCN1
1.4967179943448E-13	2.52821263998228	0.268	0.188	2.50820001492301E-09	Endothelial	SNTB2
1.52918029064418E-13	0.631864694760513	0.116	0.267	2.56260033106152E-09	Endothelial	DCTN4
1.79401825404872E-13	0.287353813316026	0.185	0.369	3.00641579013484E-09	Endothelial	EMC4
1.79901602703655E-13	2.19623533522375	0.111	0.049	3.01479105810785E-09	Endothelial	S100A11
1.95352202141389E-13	2.24163841432005	0.172	0.095	3.2737122034854E-09	Endothelial	RCN3
1.98168909146303E-13	0.390390783617198	0.064	0.186	3.32091457947374E-09	Endothelial	TBC1D16
2.13122097999168E-13	0.489179700326128	0.078	0.209	3.57150011827006E-09	Endothelial	WASHC5
2.55112886192104E-13	2.20191527376929	0.127	0.06	4.27518174680728E-09	Endothelial	TCEAL9
2.60974672836611E-13	0.339576894947179	0.208	0.409	4.37341356739593E-09	Endothelial	SREBF1
2.6518870822881E-13	0.459507851836232	0.153	0.323	4.4440323724984E-09	Endothelial	HGS
2.78760708976117E-13	0.30510173171233	0.095	0.232	4.67147196102176E-09	Endothelial	BRF1
3.01285858422834E-13	0.322624839658846	0.075	0.202	5.04894841544985E-09	Endothelial	YY1AP1
3.27166810199292E-13	0.303479747760485	0.139	0.3	5.48266140531973E-09	Endothelial	LTBR
3.48505856187799E-13	0.337458320817959	0.089	0.219	5.84026113799513E-09	Endothelial	SNX12
3.72387540982753E-13	2.30058038408911	0.336	0.285	6.24047041178897E-09	Endothelial	EDNRB
3.75751443565586E-13	0.347516614041603	0.095	0.233	6.29684269127209E-09	Endothelial	HUS1
3.8785575522532E-13	0.742070779240334	0.626	0.718	6.49968674606591E-09	Endothelial	TMSB4X
4.01928956173667E-13	2.34590404335132	0.142	0.072	6.7355254475583E-09	Endothelial	RGL1
4.39002121216239E-13	0.311650590162075	0.146	0.305	7.35679754734173E-09	Endothelial	GPBP1L1
4.45992213975224E-13	0.30465179684562	0.147	0.31	7.47393752179681E-09	Endothelial	COG3
4.48295407333346E-13	0.343955180283495	0.077	0.204	7.5125344360922E-09	Endothelial	OAS1
4.66835362756643E-13	2.65510671534558	0.286	0.221	7.82322700907582E-09	Endothelial	DOCK9
4.94413296917096E-13	0.257673547263987	0.11	0.252	8.28537802973669E-09	Endothelial	HOOK3
5.03737046767308E-13	1.86488417267786	0.274	0.195	8.44162542972655E-09	Endothelial	LEPROTL1
5.10857231830546E-13	0.290793863575964	0.119	0.267	8.56094549101628E-09	Endothelial	EXOC3
5.28258476110041E-13	0.354315450520362	0.161	0.329	8.85255554265207E-09	Endothelial	ANKHD1
5.32004458515127E-13	0.371887087486149	0.131	0.284	8.9153307157965E-09	Endothelial	CLPTM1L
5.40309634792843E-13	2.3751374255605	0.26	0.185	9.05450885985847E-09	Endothelial	TNS2
5.55026510686743E-13	1.71485539960623	0.133	0.064	9.30113426608843E-09	Endothelial	SH3BGRL
6.01242643386221E-13	0.466291876198018	0.085	0.216	1.00756242178663E-08	Endothelial	WASHC1
6.01724331864416E-13	0.266081335687415	0.147	0.311	1.00836963533839E-08	Endothelial	ELOVL1
6.90438496253214E-13	0.357422303058593	0.11	0.253	1.15703683202114E-08	Endothelial	POLDIP3
7.09333411753936E-13	1.77311033228769	0.105	0.046	1.18870093141725E-08	Endothelial	RELT
7.75343316433655E-13	0.300171048997186	0.15	0.313	1.29932032967952E-08	Endothelial	RABAC1
7.892878645232E-13	0.426819226214297	0.153	0.316	1.32268860336798E-08	Endothelial	PARP4
8.33829621242477E-13	1.69876472149316	0.266	0.184	1.39733167927814E-08	Endothelial	P4HA1
8.52745477983483E-13	0.340587780452761	0.225	0.428	1.42903087200472E-08	Endothelial	COPA
8.682966571635E-13	2.2759292004575	0.221	0.141	1.45509153807459E-08	Endothelial	EPB41L2
8.7519708059494E-13	1.45617105683537	0.1	0.043	1.466655267661E-08	Endothelial	RILPL2
8.7973941917819E-13	0.402948968776808	0.064	0.183	1.47426731865881E-08	Endothelial	CXorf40A
8.83833167764461E-13	0.341310766422497	0.067	0.184	1.48112762253968E-08	Endothelial	TMEM50B
8.91069960651799E-13	0.369161027953575	0.097	0.229	1.49325504006028E-08	Endothelial	IPO8
9.65663169282446E-13	0.402842130773169	0.127	0.277	1.61825833908352E-08	Endothelial	ZBTB7A
1.00973943176089E-12	1.8219495365613	0.33	0.264	1.69212133974489E-08	Endothelial	MARCKS
1.0200363043519E-12	0.367750096193311	0.088	0.219	1.70937683883291E-08	Endothelial	NDUFA8
1.0321537801106E-12	0.289069431545947	0.205	0.394	1.72968330470935E-08	Endothelial	SELENOH
1.04031695017179E-12	0.447217562141033	0.106	0.245	1.74336314509789E-08	Endothelial	ZNF326
1.06489719222289E-12	0.284390539918423	0.061	0.175	1.78455471472712E-08	Endothelial	AP3S2
1.12884828961288E-12	2.16134710682289	0.106	0.048	1.89172396373326E-08	Endothelial	SLCO3A1
1.1291433384831E-12	0.281265014457475	0.18	0.358	1.89221840662998E-08	Endothelial	GNB2

1.1836222227786E-12	0.307736998736192	0.053	0.164	1.98351412093238E-08	Endothelial	VEZT
1.18746367733498E-12	0.375113791024168	0.133	0.285	1.98995163047796E-08	Endothelial	TNRC6A
1.20853663166953E-12	0.299998272534853	0.172	0.342	2.0252656873518E-08	Endothelial	CCNI
1.21545162940783E-12	0.27191506268934	0.135	0.285	2.03685384056164E-08	Endothelial	KPNA4
1.223651363493E-12	0.569874351828924	0.114	0.259	2.05059495494158E-08	Endothelial	PDLIM5
1.24259743043796E-12	0.361989581003831	0.072	0.192	2.08234477392794E-08	Endothelial	NAA15
1.30969874454629E-12	0.57648484685363	0.11	0.25	2.19479315611067E-08	Endothelial	SRP72
1.38226486997867E-12	0.398777199594255	0.117	0.26	2.31639946911025E-08	Endothelial	AP3B1
1.44790173182399E-12	0.421911539154645	0.095	0.228	2.42639372219065E-08	Endothelial	PHF14
1.52706158166614E-12	1.35992025885357	0.457	0.439	2.55904979855612E-08	Endothelial	KLF6
1.56516216464168E-12	0.346390995978383	0.089	0.215	2.62289875550652E-08	Endothelial	NELFB
1.58397032740702E-12	0.420580298686057	0.122	0.269	2.65441747466869E-08	Endothelial	IRF2
1.59217385815067E-12	0.536616705055303	0.199	0.391	2.6681649514889E-08	Endothelial	UBAP2L
1.5947395688692E-12	0.470262443712988	0.146	0.305	2.67246456951101E-08	Endothelial	SETD3
1.84769763993692E-12	2.23180676703756	0.318	0.258	3.09637170500628E-08	Endothelial	FKBP1A
1.88627657177463E-12	0.284404361243186	0.089	0.216	3.16102227897993E-08	Endothelial	DEPRECATED-ENSG00000205937
1.91319763115296E-12	0.30265063083235	0.127	0.275	3.20613659028613E-08	Endothelial	KIF1B
2.01371138833769E-12	1.95661678851235	0.11	0.05	3.37457754457629E-08	Endothelial	MAGED1
2.10867577048476E-12	1.68681751270299	0.413	0.384	3.53371885617837E-08	Endothelial	PER1
2.20303941723854E-12	0.268156360780035	0.08	0.202	3.69185345540835E-08	Endothelial	DNAJC13
2.25799494614672E-12	0.34192214046364	0.246	0.455	3.78394793075267E-08	Endothelial	NFIC
2.2776831705726E-12	0.484592240933528	0.138	0.29	3.81694145724557E-08	Endothelial	HMGN3
2.3809809804128E-12	1.4841558498938	0.219	0.138	3.99004792697578E-08	Endothelial	ARID5B
2.41484795664957E-12	0.379018192809177	0.033	0.128	4.04680220575335E-08	Endothelial	TBC1D7
2.64215448118753E-12	0.480827756261982	0.185	0.362	4.42772247957407E-08	Endothelial	C4orf3
3.0267576126978E-12	2.08147517109859	0.153	0.083	5.07224040735898E-08	Endothelial	IL18BP
3.03439854582175E-12	0.460708500243287	0.066	0.181	5.08504508308808E-08	Endothelial	CEPT1
3.15087941358805E-12	0.472805711088982	0.077	0.196	5.28024372129086E-08	Endothelial	UPF3A
3.22205334586165E-12	0.354459821318491	0.097	0.227	5.39951696699496E-08	Endothelial	ATP13A1
3.46262111879491E-12	0.344016590773465	0.086	0.211	5.80266047087652E-08	Endothelial	ANO6
4.13130197929328E-12	0.294144905959793	0.072	0.19	6.92323585689968E-08	Endothelial	UVSSA
4.20248882958062E-12	0.297802810770335	0.066	0.179	7.0425307806112E-08	Endothelial	HSPA4
4.20637448616065E-12	0.315701768328276	0.074	0.19	7.04904236390802E-08	Endothelial	BCLAF1
4.44916301843711E-12	0.702765044825365	0.124	0.27	7.45590738629691E-08	Endothelial	SRA1
4.5389801865007E-12	0.529697681613733	0.042	0.142	7.60642299653787E-08	Endothelial	STOX2
4.54358481818872E-12	0.503836773482338	0.178	0.352	7.61413943832066E-08	Endothelial	TMEM248
4.57423436329998E-12	0.274662300069538	0.058	0.166	7.66550194601811E-08	Endothelial	KIF2A
4.57688656224505E-12	0.490312697454014	0.119	0.259	7.66994650101025E-08	Endothelial	DYM
4.64265564856637E-12	0.329677557355416	0.163	0.327	7.78016233586752E-08	Endothelial	GPBP1
4.67413980984999E-12	0.400843353941857	0.102	0.236	7.83292349334661E-08	Endothelial	USP32
4.74970704977108E-12	0.453546634751132	0.095	0.222	7.95955907400637E-08	Endothelial	RARS
4.79972694412602E-12	0.327075979288216	0.144	0.295	8.04338241296638E-08	Endothelial	THRAP3
4.93292073742162E-12	0.30240041704982	0.144	0.296	8.26658857177115E-08	Endothelial	SPG21
5.03925050123667E-12	0.434384289328394	0.111	0.248	8.4447759899724E-08	Endothelial	GNAI3
5.05831284653663E-12	0.31131242360296	0.095	0.222	8.47672066822609E-08	Endothelial	KAT7
5.06671240780143E-12	0.372327478495361	0.058	0.165	8.49079665299363E-08	Endothelial	HIBCH
5.14962267296353E-12	2.63983887061309	0.139	0.073	8.62973767535229E-08	Endothelial	WWC3
5.23943549981004E-12	0.457510413612802	0.086	0.21	8.78024601058166E-08	Endothelial	RALGAPB
5.56927945938616E-12	0.308298232025435	0.13	0.275	9.33299851803933E-08	Endothelial	CPSF7
5.64535619494526E-12	0.35537620075707	0.081	0.202	9.46048791148926E-08	Endothelial	ATG2B
5.73984998109833E-12	0.588264760438354	0.114	0.255	9.61884059832458E-08	Endothelial	PI4KA
5.7790871206891E-12	2.96448774392544	0.116	0.056	9.68459419685079E-08	Endothelial	RASAL2
5.88484257757499E-12	0.51588109645286	0.131	0.278	9.86181919150017E-08	Endothelial	PARP14
6.11663445648384E-12	0.321708798947545	0.078	0.196	1.02502560221756E-07	Endothelial	CSNK2B
6.57520938859774E-12	0.409110609965855	0.108	0.242	1.10187358934121E-07	Endothelial	SUMO2
6.82025504770139E-12	0.497883682373457	0.116	0.254	1.1429383408938E-07	Endothelial	NR5A2
6.82415841134092E-12	0.265269116678986	0.131	0.276	1.14359246657251E-07	Endothelial	SPEN
6.98833777916244E-12	0.40095414645979	0.067	0.182	1.17110564503204E-07	Endothelial	ABCC4
6.99082077518994E-12	0.534214121881964	0.147	0.301	1.17152174550633E-07	Endothelial	SLC35F6
7.0291744038248E-12	2.82119513651357	0.131	0.068	1.17794904659296E-07	Endothelial	PPP2R5B
7.16394309094425E-12	0.354974120267218	0.086	0.211	1.20053358318044E-07	Endothelial	CBFA2T2
7.33935065789588E-12	0.45568049900634	0.1	0.231	1.22992838325019E-07	Endothelial	TYK2
7.64267393721614E-12	0.278200487928769	0.144	0.297	1.28075929839868E-07	Endothelial	DCTN2

7.83561461497855E-12	0.443547279103771	0.085	0.206	1.3130922971781E-07	Endothelial	CDKAL1
7.9093296157205E-12	0.456623442583726	0.16	0.321	1.32544545700244E-07	Endothelial	ECPAS
7.96542289607549E-12	0.303691111581054	0.092	0.216	1.33484556892433E-07	Endothelial	CDK12
8.2256920409606E-12	0.350993565323546	0.061	0.17	1.37846147222418E-07	Endothelial	CUL2
8.68059605649644E-12	0.485144919018504	0.099	0.227	1.45469428714767E-07	Endothelial	KIAA2013
8.73839174421797E-12	0.387376570996923	0.114	0.25	1.46437968849605E-07	Endothelial	TUT4
8.90227641797519E-12	0.321440900779827	0.11	0.242	1.49184348212428E-07	Endothelial	HCFC1
9.16291938512117E-12	0.393878695876756	0.167	0.333	1.53552203055861E-07	Endothelial	CD47
9.17311917346954E-12	0.34081088620224	0.156	0.314	1.53723131109003E-07	Endothelial	MPV17
9.31809278812552E-12	0.47019084593074	0.113	0.249	1.56152598943407E-07	Endothelial	KHDC4
9.50272590590479E-12	2.6036371941718	0.239	0.168	1.59246680731152E-07	Endothelial	JCAD
9.53957879971877E-12	0.418619620807359	0.089	0.211	1.59864261525687E-07	Endothelial	TM2D2
9.60898188367409E-12	0.339851315033389	0.192	0.368	1.6102731840661E-07	Endothelial	PDCD6IP
9.65821006983398E-12	0.358762539154353	0.169	0.336	1.61852284350278E-07	Endothelial	RAB1A
1.02330136545497E-11	0.617068885982692	0.105	0.238	1.71484842822945E-07	Endothelial	BTBD7
1.06015567998231E-11	0.273022431553646	0.063	0.17	1.77660888851435E-07	Endothelial	MBD4
1.07169281731987E-11	2.10314994037115	0.308	0.246	1.79594282326464E-07	Endothelial	SPAG9
1.11722177467428E-11	0.391513713595578	0.058	0.164	1.87224024999916E-07	Endothelial	PSMG4
1.13410371849403E-11	2.82204631024157	0.13	0.067	1.9005310114523E-07	Endothelial	STXBP1
1.13684234000324E-11	0.286501590238806	0.111	0.242	1.90512039337742E-07	Endothelial	ABCF3
1.16696530946921E-11	0.345013804207043	0.08	0.197	1.95560046560851E-07	Endothelial	GATD3B
1.24209779835837E-11	0.253826624742911	0.039	0.134	2.08150749048896E-07	Endothelial	ZNF398
1.30959213519576E-11	0.384767963455419	0.07	0.185	2.19461450016106E-07	Endothelial	STX17
1.37943821077681E-11	0.415493896719122	0.155	0.31	2.31166255361979E-07	Endothelial	TMEM263
1.45545978319186E-11	0.414460858477078	0.099	0.225	2.43905950467292E-07	Endothelial	TTC17
1.46454225778013E-11	0.401351093164867	0.092	0.214	2.45427991558794E-07	Endothelial	TNPO2
1.50037728418829E-11	0.329615166720214	0.293	0.187	2.51433225284273E-07	Endothelial	LAPTM5
1.5261211170116E-11	1.68393127663379	0.255	0.178	2.55747376788804E-07	Endothelial	PPP1R15A
1.53068662051209E-11	2.28292657952792	0.372	0.344	2.56512463865416E-07	Endothelial	SLC38A2
1.62620172478644E-11	0.630975269186793	0.094	0.218	2.72518885039711E-07	Endothelial	TAF8
1.62791845417905E-11	0.47765769459811	0.167	0.33	2.72806574551326E-07	Endothelial	SRSF2
1.63355889807963E-11	0.273787124509318	0.178	0.348	2.73751800140184E-07	Endothelial	KAT6A
1.65714303289414E-11	0.270746838965333	0.086	0.205	2.777040294524E-07	Endothelial	STIP1
1.73178178088845E-11	0.321167201576703	0.188	0.359	2.90211990841286E-07	Endothelial	BAG6
1.75458119326341E-11	0.325796895679335	0.05	0.15	2.94032716367083E-07	Endothelial	MAIP1
1.94437119155654E-11	0.379496656432654	0.081	0.198	3.25837724281045E-07	Endothelial	RNF126
1.95490724546055E-11	0.444725667314822	0.133	0.276	3.27603356194279E-07	Endothelial	TXNDC15
2.0012582637772E-11	3.16366414550814	0.149	0.083	3.35370859843784E-07	Endothelial	RILPL1
2.0028792464691E-11	0.342441424137498	0.147	0.293	3.35642504123292E-07	Endothelial	NRBP1
2.09252846322952E-11	2.3262797541982	0.177	0.106	3.50665919868003E-07	Endothelial	SH3PXD2A
2.18559072089956E-11	0.365157818343875	0.069	0.179	3.66261293008348E-07	Endothelial	HDAC3
2.29946129315361E-11	0.416447683426625	0.128	0.266	3.85343723506683E-07	Endothelial	MGRN1
2.40178651210927E-11	0.447173211982208	0.102	0.229	4.02491383699272E-07	Endothelial	SPIDR
2.40759603950436E-11	0.386338185273143	0.164	0.323	4.03464944300141E-07	Endothelial	SLC39A7
2.52577815224055E-11	0.772685078246171	0.078	0.195	4.23269902752471E-07	Endothelial	ME2
2.59887986358226E-11	0.39611171934368	0.153	0.306	4.35520287539115E-07	Endothelial	MICAL3
2.60892521587513E-11	0.337592995041975	0.064	0.173	4.37203687676355E-07	Endothelial	NUDT19
2.63609132500159E-11	0.589657056803236	0.063	0.17	4.41756184243766E-07	Endothelial	NBEAL1
2.69398134142172E-11	0.437037823352113	0.102	0.228	4.51457393195451E-07	Endothelial	FAM111A
2.77897951487766E-11	0.254837158900776	0.122	0.259	4.65701387103199E-07	Endothelial	SH3BGRL2
2.80776969437879E-11	0.409588176911108	0.095	0.216	4.70526045383997E-07	Endothelial	DDX23
2.83263318965573E-11	0.368909208676318	0.058	0.161	4.74692669922507E-07	Endothelial	PEF1
2.86939676625638E-11	0.40085527731793	0.075	0.186	4.80853510089244E-07	Endothelial	PAGR1
2.96958273058241E-11	0.340867746724819	0.119	0.252	4.97642673991E-07	Endothelial	CIAO1
3.04332934207455E-11	0.52235963289254	0.091	0.21	5.10001131144852E-07	Endothelial	CDK13
3.06241758417229E-11	0.334949202151303	0.064	0.168	5.13199938755592E-07	Endothelial	MTRR
3.1102580721522E-11	0.369235820830017	0.08	0.196	5.21217047731266E-07	Endothelial	RNF185
3.15134158148402E-11	0.555733460238038	0.149	0.298	5.28101822225093E-07	Endothelial	DEPRECATED-ENSG00000184500
3.19725193722892E-11	0.263524341076667	0.089	0.209	5.35795479640822E-07	Endothelial	PLD1
3.24560984228998E-11	0.367031372184092	0.077	0.189	5.43899297370955E-07	Endothelial	KHNYN
3.27746613640358E-11	0.502677864832414	0.103	0.229	5.49237775138512E-07	Endothelial	BDP1
3.32299042737438E-11	2.32213704030457	0.175	0.106	5.56866735819399E-07	Endothelial	GBP4
3.39467307252856E-11	0.338108985425827	0.208	0.393	5.68879313494336E-07	Endothelial	LPP

3.62994171889971E-11	0.400792106078342	0.064	0.17	6.08305633253214E-07	Endothelial	SLC25A16
3.71096333981262E-11	0.703789724161015	0.119	0.255	6.21883236485799E-07	Endothelial	ARFGAP3
3.87552454196417E-11	0.324320598518203	0.164	0.318	6.49460402742355E-07	Endothelial	LAP3
3.95948548730387E-11	0.322171485303506	0.092	0.212	6.63530577962382E-07	Endothelial	DNAJC11
3.99017885137742E-11	0.510539025630404	0.094	0.216	6.68674171913829E-07	Endothelial	BET1L
4.19798556064485E-11	0.334783605679086	0.097	0.217	7.03498420252864E-07	Endothelial	MVP
4.20951093871995E-11	0.433274569204683	0.089	0.208	7.0542984311069E-07	Endothelial	PHRF1
4.2360799996336E-11	0.427727035543505	0.121	0.254	7.09882286338598E-07	Endothelial	GPR107
4.25087624783592E-11	0.352963988661962	0.044	0.139	7.12361841612343E-07	Endothelial	LEMD3
4.40042269464432E-11	0.328244720223241	0.156	0.309	7.37422835168495E-07	Endothelial	ATF6B
4.42476461402336E-11	0.642357857870983	0.088	0.205	7.41502054018035E-07	Endothelial	SNX14
4.42965233233627E-11	2.37367054773435	0.144	0.08	7.42321137852912E-07	Endothelial	DUSP6
4.48130478061929E-11	0.434833817358466	0.114	0.245	7.5097705513618E-07	Endothelial	CDK4
4.48651933318271E-11	0.284618204917354	0.086	0.2	7.51850909854759E-07	Endothelial	DDX49
4.53466959628167E-11	0.309493671901776	0.094	0.212	7.59919930944883E-07	Endothelial	PHC3
4.53607961602534E-11	0.40386558299294	0.092	0.209	7.60156222053526E-07	Endothelial	ZNF358
4.54091465272809E-11	0.651392295036586	0.088	0.205	7.60966477504173E-07	Endothelial	YIF1B
4.69598103846686E-11	0.491395296083093	0.188	0.359	7.86952502426276E-07	Endothelial	DHX15
4.74469882983646E-11	0.294217407014521	0.175	0.338	7.95116629903993E-07	Endothelial	TRIP12
4.84810367606088E-11	0.543844436252219	0.067	0.173	8.12445214034282E-07	Endothelial	TRIP11
4.93092808599037E-11	0.30218226960158	0.072	0.179	8.26324928650266E-07	Endothelial	CMTR1
4.93639272548315E-11	0.283681031337136	0.049	0.146	8.27240692936466E-07	Endothelial	ZBTB21
5.17631279709711E-11	0.356967150978448	0.144	0.293	8.67446498537534E-07	Endothelial	RAB1B
5.18049574234287E-11	0.360161190101466	0.169	0.327	8.68147476501818E-07	Endothelial	RNF10
5.29596562723873E-11	0.527205505030782	0.167	0.328	8.87497919812666E-07	Endothelial	LRP5
5.30079864008831E-11	0.374224973987534	0.094	0.214	8.88307836105999E-07	Endothelial	PPP2R5E
5.50530999002141E-11	0.561125756679828	0.105	0.23	9.22579848127788E-07	Endothelial	QTRT1
5.690181746794E-11	0.293976033351561	0.078	0.188	9.53560657127738E-07	Endothelial	XPR1
5.8057636009976E-11	0.522033076483702	0.094	0.212	9.72929864255178E-07	Endothelial	NOP14
6.05449342655231E-11	0.288280083243229	0.056	0.157	1.01461200842164E-06	Endothelial	VPS4B
6.06595496106333E-11	0.401226446618431	0.095	0.214	1.01653273237499E-06	Endothelial	FIP1L1
6.24944501128454E-11	0.406279666127304	0.072	0.18	1.04728199499106E-06	Endothelial	NUP160
6.33505897147278E-11	0.318950837840297	0.044	0.136	1.06162918243941E-06	Endothelial	IL17RA
6.46932531173588E-11	0.278151552766844	0.07	0.177	1.0841295357407E-06	Endothelial	RBM4
6.49342404421574E-11	0.615114837184343	0.144	0.289	1.08816800132967E-06	Endothelial	ITPRID2
6.86173787383498E-11	0.388687278647242	0.138	0.28	1.14989003289727E-06	Endothelial	SELENOS
6.87736992589295E-11	0.4073280318376	0.094	0.211	1.15250965218114E-06	Endothelial	BCOR
7.16339013278287E-11	0.436691606151357	0.07	0.177	1.20044091845175E-06	Endothelial	VPS41
7.39311589299526E-11	0.328928481445175	0.083	0.195	1.23893836134815E-06	Endothelial	C5orf15
7.47263617356421E-11	0.347997755401578	0.13	0.264	1.25226436996589E-06	Endothelial	RAC1
7.92865626327242E-11	0.448845600260004	0.13	0.267	1.32868421659919E-06	Endothelial	PRPF40A
8.29883066056085E-11	0.265440319080019	0.045	0.14	1.39071804209679E-06	Endothelial	DPY19L3
8.30339905476674E-11	1.91959194622751	0.302	0.24	1.39148361359781E-06	Endothelial	SARAF
8.47516216632099E-11	0.424339635076578	0.088	0.201	1.42026767583207E-06	Endothelial	TUBGCP2
8.60745913802932E-11	0.480789202641494	0.1	0.22	1.44243800235095E-06	Endothelial	GALNT1
8.61887991097779E-11	0.270837924980479	0.114	0.241	1.44435189548166E-06	Endothelial	LRRC41
8.80884632358211E-11	0.632324402811833	0.094	0.212	1.47618646690589E-06	Endothelial	FAM172A
8.9890189637348E-11	0.623757566623923	0.094	0.214	1.50637979794268E-06	Endothelial	MTERF4
9.38042210500395E-11	0.488590299735326	0.108	0.233	1.57197113635656E-06	Endothelial	BCL2L13
9.93986387627127E-11	0.258602217978681	0.075	0.182	1.66572238838554E-06	Endothelial	SSU72
1.01600247125052E-10	2.79817987202123	0.182	0.115	1.70261694132162E-06	Endothelial	PRKD3
1.04358116391593E-10	0.826885185293933	0.088	0.202	1.74883331449032E-06	Endothelial	GNL3L
1.04390733542707E-10	0.449201723903725	0.124	0.257	1.74937991270868E-06	Endothelial	CWC15
1.04985150413205E-10	0.261228956628859	0.08	0.192	1.75934115062448E-06	Endothelial	INTS4
1.0623151415539E-10	0.34296475235587	0.07	0.177	1.78022771421603E-06	Endothelial	ARMC8
1.06976296073124E-10	0.482454446346054	0.091	0.209	1.79270876959341E-06	Endothelial	TAF13
1.13882765911436E-10	0.58488521013303	0.102	0.224	1.90844739114384E-06	Endothelial	NELFA
1.17268429789232E-10	0.46563576312186	0.092	0.208	1.96518434640795E-06	Endothelial	TAPT1
1.18719342711757E-10	0.390589520532458	0.074	0.179	1.98949874516362E-06	Endothelial	CLCN3
1.21263585560609E-10	0.28792286324816	0.099	0.217	2.03213516682468E-06	Endothelial	NUDT3
1.25065741115111E-10	0.342381207523527	0.124	0.255	2.09585168960703E-06	Endothelial	IWS1
1.25940287513472E-10	0.467907945580498	0.092	0.208	2.11050733815077E-06	Endothelial	VARS
1.31534150329111E-10	0.344758520847154	0.069	0.171	2.20424929121525E-06	Endothelial	KRR1

1.34266764130237E-10	0.785787136649461	0.074	0.181	2.25004243329451E-06	Endothelial	SLC17A5
1.34829836975475E-10	0.28083486539875	0.086	0.2	2.25947840803501E-06	Endothelial	AMPD2
1.34902931094296E-10	2.13296177111295	0.362	0.327	2.26070331927821E-06	Endothelial	BSDC1
1.35307495735015E-10	0.709052261391627	0.189	0.358	2.26748301352738E-06	Endothelial	MARCH6
1.36438998951202E-10	0.322254900862744	0.113	0.236	2.28644474442424E-06	Endothelial	RNF168
1.43947893315256E-10	0.696189563842267	0.122	0.253	2.41227879617706E-06	Endothelial	SKAP2
1.44090304859407E-10	0.544093239459324	0.121	0.251	2.41466532883394E-06	Endothelial	RTCB
1.48274358595558E-10	0.250304434524547	0.063	0.163	2.48478170134436E-06	Endothelial	UTP6
1.54053010837308E-10	0.328228944328662	0.055	0.15	2.58162035561161E-06	Endothelial	PEX6
1.58662965328671E-10	0.755054820103583	0.064	0.165	2.65887397297787E-06	Endothelial	ABHD18
1.59397684814945E-10	0.316738487881431	0.095	0.213	2.67118640212885E-06	Endothelial	TRAF2
1.59694107319277E-10	0.778651698057517	0.099	0.216	2.67615385045644E-06	Endothelial	BCL2L2
1.61600631349778E-10	0.301379327451321	0.052	0.147	2.70810338015958E-06	Endothelial	DVL1
1.7165255401791E-10	0.401431781679958	0.053	0.148	2.87655350023213E-06	Endothelial	NCBP1
1.74416746820031E-10	0.445984499784431	0.182	0.343	2.92287584321008E-06	Endothelial	TMED9
1.8000368930469E-10	0.353604828624087	0.077	0.183	3.01650182536799E-06	Endothelial	SMARCC2
1.81084479667923E-10	0.487470814004578	0.111	0.235	3.03461371027505E-06	Endothelial	SPATA20
1.83082225336388E-10	0.429449066047524	0.088	0.2	3.06809193218719E-06	Endothelial	FBXO46
1.88623879669999E-10	0.527145125553247	0.097	0.215	3.16095897550985E-06	Endothelial	UBR3
1.89500925789916E-10	0.741436781340826	0.055	0.151	3.17565651438741E-06	Endothelial	RAVER2
1.89981890396942E-10	0.352387665443428	0.059	0.16	3.18371651927195E-06	Endothelial	VPS8
1.95537103400743E-10	1.37157677291512	0.105	0.05	3.27681077878965E-06	Endothelial	ST8SIA4
1.9567043049229E-10	0.715206052413459	0.111	0.238	3.27904507418979E-06	Endothelial	ZBED6
1.97970240446519E-10	0.51363726960976	0.103	0.224	3.31758528940277E-06	Endothelial	UBE2J2
1.98310744248914E-10	0.416677987211729	0.21	0.384	3.3232914521233E-06	Endothelial	FAM120A
1.99058677068945E-10	0.567093943589146	0.11	0.233	3.33582531032137E-06	Endothelial	USF1
2.01227059460999E-10	0.452895410730245	0.083	0.194	3.37216306244742E-06	Endothelial	RCAN1
2.11452636792654E-10	0.587655682976992	0.047	0.138	3.54352328737129E-06	Endothelial	COX19
2.21057930036718E-10	0.666981462774234	0.056	0.153	3.70448879155532E-06	Endothelial	NAA30
2.22455647546024E-10	0.35369530580125	0.146	0.288	3.72791174157627E-06	Endothelial	UBE2H
2.25311632435444E-10	0.567581070523807	0.069	0.172	3.77577233635317E-06	Endothelial	PCIF1
2.3094182942666E-10	0.289452355853679	0.102	0.221	3.87012317753196E-06	Endothelial	DNMT3A
2.3126885834953E-10	0.278681310850177	0.081	0.189	3.87560352822142E-06	Endothelial	TMEM167A
2.34326763326836E-10	0.379083362205786	0.183	0.343	3.92684789983112E-06	Endothelial	UBR2
2.38282757557911E-10	0.338084399045949	0.05	0.143	3.99314245115547E-06	Endothelial	THBS3
2.41847994766251E-10	0.426121718724994	0.106	0.227	4.05288869629283E-06	Endothelial	MEPCE
2.42464437997995E-10	1.62693790210569	0.326	0.27	4.06321905197041E-06	Endothelial	IER2
2.43712418899405E-10	0.490695793477292	0.105	0.224	4.08413271591622E-06	Endothelial	PPP1R12B
2.46800845899027E-10	0.437133361804779	0.243	0.436	4.1358885755759E-06	Endothelial	RTN4
2.54683528124703E-10	0.411970175730432	0.125	0.256	4.26798656431378E-06	Endothelial	UBA1
2.62112213484725E-10	0.280421660957075	0.049	0.139	4.39247647357703E-06	Endothelial	TRNAU1AP
2.68205750769007E-10	0.250316591505623	0.05	0.143	4.49459197138703E-06	Endothelial	RNF146
2.68517122074025E-10	0.402919445714246	0.075	0.18	4.49980993171652E-06	Endothelial	PAN2
2.6887167222731E-10	0.41177055770651	0.055	0.149	4.50575148318527E-06	Endothelial	CEP192
2.81982366325E-10	0.607316753734266	0.191	0.359	4.72546049487436E-06	Endothelial	NUFIP2
3.06141827952148E-10	0.694118244235366	0.058	0.155	5.1303247528221E-06	Endothelial	PMS1
3.08790366458193E-10	0.400454320312183	0.067	0.169	5.1747089611064E-06	Endothelial	PDS5A
3.13559670471214E-10	0.25069977454248	0.327	0.559	5.2546329577566E-06	Endothelial	ACADVL
3.14624590952834E-10	1.61444030323695	0.113	0.056	5.27247889518759E-06	Endothelial	SLFN5
3.15007175680695E-10	0.550644960083988	0.171	0.325	5.27889025005709E-06	Endothelial	IGF2R
3.15156971515938E-10	0.71444399783895	0.058	0.156	5.28140052866409E-06	Endothelial	TRNT1
3.16945997596381E-10	2.18764501956935	0.203	0.135	5.31138102772015E-06	Endothelial	PML
3.28748734689698E-10	0.35210426715835	0.047	0.137	5.50917129592996E-06	Endothelial	GPATCH2
3.30915957504244E-10	0.457512021591968	0.056	0.151	5.54548961585612E-06	Endothelial	DPY19L1
3.51718736117627E-10	0.30574198131089	0.088	0.197	5.8941025798592E-06	Endothelial	TMEM167B
3.56627389589684E-10	2.26929290252811	0.122	0.064	5.97636179474392E-06	Endothelial	HK1
3.59481095613611E-10	0.45980350722834	0.142	0.278	6.02418420029289E-06	Endothelial	GTF2I
3.65148719320787E-10	0.45959849368965	0.075	0.179	6.11916223837775E-06	Endothelial	STRN
3.67389965098603E-10	0.459268398952102	0.139	0.276	6.1567210351224E-06	Endothelial	SNX6
3.71554162118643E-10	0.47447491140353	0.085	0.193	6.22650464878422E-06	Endothelial	CHD9
3.83076322858595E-10	0.255065645131322	0.038	0.12	6.41959301846434E-06	Endothelial	PAIP2
3.83282051367416E-10	0.848375969116026	0.07	0.173	6.42304061681517E-06	Endothelial	ZNF608
3.9163300125847E-10	0.28689999269115	0.081	0.187	6.56298583508943E-06	Endothelial	MYO1D

4.02601659309507E-10	0.484328759526745	0.117	0.244	6.74679860670872E-06	Endothelial	SCAF11
4.14693323588656E-10	0.392660846672369	0.144	0.281	6.9494307166987E-06	Endothelial	KANSL1
4.50038932567865E-10	2.37888646696266	0.166	0.101	7.54175243197229E-06	Endothelial	BAZ2B
4.5179372885329E-10	0.546089250735497	0.055	0.149	7.57115930812344E-06	Endothelial	AARS2
4.59666203919212E-10	0.583582864417285	0.21	0.386	7.70308624527815E-06	Endothelial	PPP2R5A
4.63749590198191E-10	0.51235403801045	0.081	0.187	7.77151563254128E-06	Endothelial	ARF4
4.72760314310064E-10	0.356703176625347	0.059	0.154	7.92251734720806E-06	Endothelial	THEM6
4.88188545284216E-10	0.514420323749371	0.102	0.219	8.18106364187289E-06	Endothelial	MBTPS1
4.96292299294084E-10	2.09907739793675	0.329	0.295	8.31686635157026E-06	Endothelial	ADAM15
5.18843483821063E-10	0.557344872818133	0.171	0.321	8.69477910187338E-06	Endothelial	LONP1
5.24439415938973E-10	0.284549938818091	0.058	0.15	8.78855573230531E-06	Endothelial	PIAS1
5.30829383570971E-10	0.61128456450117	0.177	0.335	8.89563880988234E-06	Endothelial	TRIM27
5.36241027619329E-10	0.647023272102562	0.135	0.266	8.98632714084471E-06	Endothelial	PTBP1
5.44280080157767E-10	0.390496179211643	0.059	0.155	9.12104558328386E-06	Endothelial	ZNF639
5.50409819082528E-10	0.534036509149092	0.086	0.194	9.223767748185E-06	Endothelial	SRSF9
5.52480424332704E-10	0.333867511770424	0.172	0.325	9.25846695096746E-06	Endothelial	SAT2
5.68270950822874E-10	0.589079599512999	0.138	0.269	9.52308459388972E-06	Endothelial	ZDHHC14
5.6878683370758E-10	0.488678797675406	0.089	0.199	9.53172975927162E-06	Endothelial	METTL26
5.8048460350659E-10	0.59058669315691	0.075	0.177	9.72776098556343E-06	Endothelial	SNRNP40
5.86655411112704E-10	0.284990503033219	0.125	0.249	9.83117137942669E-06	Endothelial	IFNAR2
6.42657323068936E-10	0.389267879224808	0.081	0.188	1.07696514199892E-05	Endothelial	STXBP3
6.52225356028748E-10	0.352134993982486	0.074	0.175	1.09299925163298E-05	Endothelial	FAAP20
6.66854948983017E-10	0.350647647072851	0.114	0.235	1.11751552350574E-05	Endothelial	TRIM44
6.92692285640175E-10	0.4812455403468	0.194	0.357	1.1608137322758E-05	Endothelial	C11orf58
6.94942730812249E-10	0.326374554044144	0.034	0.116	1.16458502829517E-05	Endothelial	ANKRA2
7.29196899074843E-10	0.370644171284165	0.042	0.127	1.22198816346962E-05	Endothelial	ATXN7L1
7.42120227906813E-10	0.331057143434585	0.05	0.139	1.24364507792624E-05	Endothelial	NAA40
7.42855556800452E-10	0.841838462442893	0.139	0.277	1.2448773420862E-05	Endothelial	TRIM25
7.52086619967604E-10	0.531051101107167	0.045	0.131	1.26034675774171E-05	Endothelial	SPIN4
7.5813379783598E-10	1.24943881596032	0.136	0.074	1.27048061841354E-05	Endothelial	RIN3
8.33429141137281E-10	0.474143550380327	0.074	0.174	1.39666055471786E-05	Endothelial	ZNF330
8.34962831359158E-10	0.412800999905524	0.1	0.214	1.39923071279168E-05	Endothelial	SIRT2
8.3847906767488E-10	0.464349251677659	0.095	0.205	1.40512322160956E-05	Endothelial	CHERP
8.48238485086884E-10	0.566165519817197	0.066	0.163	1.4214780533086E-05	Endothelial	TRAPPC6B
8.51737790974385E-10	0.498577357880005	0.117	0.239	1.42734219011487E-05	Endothelial	LARS
8.75769795046773E-10	0.492785447980383	0.124	0.249	1.46761502253938E-05	Endothelial	ATF2
8.80701213783127E-10	0.33900114710117	0.042	0.127	1.47587909405776E-05	Endothelial	MIER3
9.26101526345042E-10	3.04617318362962	0.106	0.054	1.55196093784902E-05	Endothelial	SOCS2
9.41814125623189E-10	0.290245228695043	0.063	0.158	1.57829211171934E-05	Endothelial	PGGT1B
9.60962112702011E-10	0.880223327384694	0.067	0.166	1.61038030846603E-05	Endothelial	ARL4A
9.63850873651795E-10	0.358069394192558	0.089	0.196	1.61522129406568E-05	Endothelial	RAVER1
9.65599981179554E-10	0.331911945771853	0.063	0.156	1.6181524484607E-05	Endothelial	ESF1
9.73676583989253E-10	0.613605941425735	0.058	0.152	1.63168721944919E-05	Endothelial	RBM12
9.86242401381898E-10	0.313672258528043	0.05	0.138	1.65274501623578E-05	Endothelial	NSRP1
9.90167998586122E-10	0.256381629112816	0.066	0.163	1.65932353203062E-05	Endothelial	PLCL2
9.96049138505445E-10	0.347505795138627	0.067	0.164	1.66917914630742E-05	Endothelial	CREB3
9.99737941182177E-10	0.53048511239424	0.196	0.361	1.67536084183309E-05	Endothelial	LIMD1
1.01357209333534E-09	0.349359748116526	0.072	0.171	1.69854411401136E-05	Endothelial	CCDC130
1.02318872285052E-09	0.386964091788143	0.069	0.167	1.71465966175291E-05	Endothelial	ZNF704
1.02351736887459E-09	0.471461559830909	0.085	0.191	1.71521040676005E-05	Endothelial	FAM160A2
1.09025988630799E-09	0.462530672329634	0.074	0.173	1.82705751747493E-05	Endothelial	LATS1
1.12232185412498E-09	0.536029156761788	0.074	0.173	1.88078696314265E-05	Endothelial	ITSN1
1.13364808710637E-09	0.445926013801152	0.172	0.321	1.89976746437286E-05	Endothelial	KXD1
1.17971965080216E-09	0.477507414372626	0.053	0.143	1.97697419081426E-05	Endothelial	ATG4A
1.19966355720251E-09	0.329171558400214	0.044	0.129	2.01039618915997E-05	Endothelial	RNF8
1.20096545978593E-09	0.57260670899373	0.141	0.274	2.01257791750926E-05	Endothelial	VAPA
1.2101100226458E-09	0.30005680458081	0.077	0.176	2.02790237594983E-05	Endothelial	PTPRA
1.22878639707523E-09	0.379212794044296	0.083	0.187	2.05920024421867E-05	Endothelial	SLC30A7
1.27295819210723E-09	0.350481500742597	0.133	0.261	2.13322333833329E-05	Endothelial	TAP2
1.27559984406714E-09	0.416905531865278	0.041	0.123	2.13765021868771E-05	Endothelial	ZNF133
1.29699755603042E-09	0.398230860004338	0.122	0.244	2.17350850439577E-05	Endothelial	ARL8A
1.29776109527622E-09	0.389085001012877	0.078	0.179	2.17478804346389E-05	Endothelial	CCNK
1.32772875278264E-09	0.676671113710138	0.167	0.318	2.22500784391315E-05	Endothelial	UBR5

1.35954308207174E-09	0.669192062118028	0.13	0.256	2.27832229693583E-05	Endothelial	CLTA
1.49448059730674E-09	0.380426158722713	0.077	0.176	2.50445058496664E-05	Endothelial	ACE
1.52431388716494E-09	0.620746088343262	0.069	0.167	2.554445212111E-05	Endothelial	MPP5
1.57318646234204E-09	0.568239444947342	0.083	0.187	2.6363458735928E-05	Endothelial	MBD3
1.57896701986272E-09	0.295775281764582	0.039	0.119	2.64603293188595E-05	Endothelial	RIOK2
1.60015340576694E-09	0.431961903178796	0.066	0.159	2.68153707738424E-05	Endothelial	DNTTIP1
1.62834641416583E-09	2.33573467828335	0.158	0.097	2.72878292085909E-05	Endothelial	MLKL
1.63806001992915E-09	2.56227972059899	0.163	0.101	2.74506098139727E-05	Endothelial	TECPR1
1.66023782215277E-09	0.26583772697237	0.142	0.275	2.78222654236362E-05	Endothelial	SNX3
1.66811265261607E-09	0.418943574148389	0.078	0.179	2.795423183254E-05	Endothelial	CEBPZ
1.6689242874041E-09	1.43931585426197	0.102	0.05	2.7967833208318E-05	Endothelial	INPP5D
1.75729139593054E-09	0.453218394238167	0.056	0.146	2.94486892130039E-05	Endothelial	NOL9
1.79206074269127E-09	0.406497411948789	0.074	0.171	3.00313539260203E-05	Endothelial	LEO1
1.81151101018683E-09	0.502428561733718	0.103	0.215	3.03573015087109E-05	Endothelial	MBD6
1.81465754573067E-09	2.16727388244393	0.144	0.084	3.04100311513545E-05	Endothelial	BMP1
1.82888894634639E-09	0.287693890446576	0.094	0.2	3.06485209628728E-05	Endothelial	GTPBP6
1.83593645888789E-09	0.351938916291703	0.05	0.136	3.07666231780432E-05	Endothelial	GGA1
2.03037855044901E-09	0.58184050750785	0.144	0.282	3.40250837484246E-05	Endothelial	HECTD4
2.04936322722271E-09	0.63292257916134	0.197	0.362	3.43432289617981E-05	Endothelial	POLR2J3
2.07288408780361E-09	0.404932211969658	0.097	0.206	3.47373915434128E-05	Endothelial	ZNF384
2.08834335919172E-09	0.695938728175043	0.113	0.231	3.49964580133349E-05	Endothelial	PIP5K1A
2.12190821484558E-09	0.264012035820175	0.075	0.172	3.55589378643823E-05	Endothelial	CAMTA1
2.14566238724864E-09	0.4927289724695	0.072	0.17	3.59570102855127E-05	Endothelial	MED17
2.2137491624208E-09	0.440053732844307	0.078	0.177	3.70980084638478E-05	Endothelial	DAG1
2.24964511785638E-09	0.40046775855544	0.055	0.143	3.76995528850372E-05	Endothelial	BCAS3
2.2683919329139E-09	2.49356478257333	0.199	0.137	3.80137120117712E-05	Endothelial	CSNK1E
2.33348946316272E-09	0.668511477309786	0.085	0.188	3.91046164236808E-05	Endothelial	MCM3AP
2.3457240594703E-09	0.50446165109803	0.13	0.254	3.93096437886032E-05	Endothelial	SMAD2
2.38530740658283E-09	0.683192394567741	0.058	0.146	3.99729815195151E-05	Endothelial	ZNF618
2.43746936891571E-09	0.314839535805517	0.044	0.126	4.08471116842895E-05	Endothelial	ZNF263
2.45941633972284E-09	0.585561563652834	0.113	0.23	4.12148990210754E-05	Endothelial	PIKFYVE
2.46909201321593E-09	0.30863363367724	0.119	0.237	4.13770439574726E-05	Endothelial	CLDND1
2.51248396195287E-09	0.445053675367034	0.058	0.146	4.21042062344062E-05	Endothelial	PIGF
2.63732352813412E-09	0.478586214067124	0.135	0.263	4.41962676844717E-05	Endothelial	ZRANB2
2.64110568010597E-09	0.492860387432866	0.064	0.157	4.42596489872158E-05	Endothelial	SAV1
2.67497118586149E-09	0.860135069224719	0.141	0.274	4.48271671326669E-05	Endothelial	SNX5
2.68587326790991E-09	0.25199730935615	0.049	0.132	4.50098642236342E-05	Endothelial	PCNX4
2.7228742344491E-09	0.710249222359883	0.119	0.241	4.5629926420898E-05	Endothelial	AUTS2
2.80802206762834E-09	0.260942735460794	0.066	0.159	4.70568338093157E-05	Endothelial	ODF3B
2.80898288635098E-09	0.338945947458636	0.061	0.15	4.70729352094697E-05	Endothelial	GTF2H4
2.82205327637233E-09	0.678555169834816	0.102	0.214	4.72919688054475E-05	Endothelial	UBA6
2.86217541108978E-09	1.77679566355284	0.338	0.3	4.79643355390425E-05	Endothelial	ATN1
2.87401015063664E-09	0.30128511431202	0.044	0.125	4.81626621043687E-05	Endothelial	POLR3C
2.88843326260946E-09	0.333635582539985	0.047	0.131	4.84043646148094E-05	Endothelial	L3MBTL2
2.97143489682774E-09	0.696523339447127	0.088	0.191	4.97953060010393E-05	Endothelial	TM2D3
2.97787683980174E-09	0.721680426779143	0.138	0.267	4.99032600813976E-05	Endothelial	MED25
2.99155185156426E-09	0.268912832809265	0.172	0.315	5.01324259285139E-05	Endothelial	SHFL
3.11242570548872E-09	0.473226640661609	0.202	0.364	5.215802997258E-05	Endothelial	UBE2B
3.13187114942037E-09	0.342435839640346	0.103	0.215	5.24838967219865E-05	Endothelial	FEM1C
3.19182308477274E-09	0.409751419732685	0.125	0.246	5.34885712546215E-05	Endothelial	LRCH4
3.21887555777996E-09	2.27320600912854	0.224	0.162	5.39419165972766E-05	Endothelial	MEF2A
3.28529936354236E-09	0.530297445869289	0.099	0.207	5.50550467342429E-05	Endothelial	EFTUD2
3.34511614875677E-09	2.52765454229946	0.125	0.07	5.60574564208659E-05	Endothelial	TNFAIP8L1
3.40399472765324E-09	0.691352094518759	0.102	0.212	5.7044143646013E-05	Endothelial	ZNF444
3.47761935142153E-09	0.516550675796379	0.094	0.199	5.8277945091122E-05	Endothelial	VAPB
3.48021772615236E-09	0.644090233229591	0.138	0.265	5.83214886548612E-05	Endothelial	PSMD13
3.54185311738999E-09	2.29411755511864	0.156	0.097	5.93543745412214E-05	Endothelial	UACA
3.57118938807449E-09	0.546970123631591	0.049	0.131	5.98459917653523E-05	Endothelial	CDK8
3.58201685715694E-09	0.459722536299748	0.041	0.119	6.0027438492236E-05	Endothelial	DEPRECATED-ENSG00000022267
3.65204140058774E-09	0.37145382996178	0.197	0.356	6.12009097910493E-05	Endothelial	RABGAP1L
3.68781261813528E-09	0.556595251237494	0.122	0.245	6.1800363854711E-05	Endothelial	VPS35L
3.70833323928265E-09	0.363421458233463	0.085	0.184	6.21442484238987E-05	Endothelial	GMPPB
3.84314356623814E-09	0.292023502441238	0.031	0.105	6.44033998830187E-05	Endothelial	CXorf40B

3.88393506762744E-09	0.436523455677161	0.058	0.146	6.50869838633007E-05	Endothelial	CMPK2
3.89184674045886E-09	0.404126387595632	0.111	0.223	6.52195676766096E-05	Endothelial	STRAP
3.98360542923593E-09	0.47667883617312	0.078	0.177	6.67572597831356E-05	Endothelial	UNK
4.05727738374896E-09	0.540697060524969	0.08	0.179	6.79918543968651E-05	Endothelial	MAFG
4.1327282707413E-09	0.566948217338609	0.088	0.19	6.92562603610827E-05	Endothelial	EMC8
4.32252214592758E-09	0.367694638424247	0.036	0.111	7.24368261214544E-05	Endothelial	PSMA5
4.39092451388531E-09	0.258806716847923	0.063	0.152	7.358311300369E-05	Endothelial	TFIP11
4.49755390078247E-09	3.18658685303931	0.167	0.11	7.53700082693126E-05	Endothelial	NTN4
4.554368289747E-09	0.276657381651401	0.053	0.138	7.63221037995802E-05	Endothelial	USP40
4.56165662936145E-09	0.445197606969759	0.081	0.18	7.64442417948392E-05	Endothelial	YTHDF2
4.65541113399793E-09	0.669665856694427	0.15	0.283	7.80153797835373E-05	Endothelial	SLC23A2
4.67934867089796E-09	2.24398100779877	0.299	0.254	7.8416525026908E-05	Endothelial	BCAR1
4.81101370377749E-09	0.282428725672874	0.08	0.175	8.06229676479032E-05	Endothelial	RABL6
4.86212506645749E-09	0.558231187224083	0.045	0.127	8.14794918636946E-05	Endothelial	ORC2
4.90709099579691E-09	0.254567169928566	0.044	0.122	8.22330309075646E-05	Endothelial	ZMYND19
5.01994275992594E-09	0.416735192792852	0.058	0.145	8.41242007708389E-05	Endothelial	SYNJ2
5.09813820180021E-09	0.649770055924553	0.127	0.247	8.5434599985768E-05	Endothelial	GNA11
5.22127339735423E-09	0.428007937651557	0.102	0.21	8.74980995928621E-05	Endothelial	APOL2
5.25160623933039E-09	0.319219503808486	0.083	0.181	8.80064173586987E-05	Endothelial	MPZL1
5.28300451623318E-09	1.71174692343503	0.43	0.457	8.85325896830356E-05	Endothelial	IVNS1ABP
5.29369577639947E-09	2.39127493128449	0.235	0.177	8.87117538209023E-05	Endothelial	BNIP3L
5.29489867958403E-09	0.382800088836301	0.044	0.123	8.87319120724691E-05	Endothelial	XPA
5.36491930601445E-09	0.504212551712398	0.185	0.334	8.99053177301901E-05	Endothelial	ADAM9
5.549437131215E-09	0.37054109915975	0.119	0.234	9.2997467444901E-05	Endothelial	CALM1
5.65794903495963E-09	0.331079755541862	0.049	0.131	9.48159099278534E-05	Endothelial	MX1
5.72547593195811E-09	0.253502583577635	0.075	0.168	9.5947525667754E-05	Endothelial	ZBED1
5.73700214994154E-09	0.538113958769351	0.127	0.246	9.61406820287203E-05	Endothelial	WIPF2
5.77632328852309E-09	1.01083271798804	0.531	0.627	9.679962566907E-05	Endothelial	IGFBP4
5.96193432099302E-09	0.406093344473382	0.092	0.194	9.99100953512011E-05	Endothelial	MED4
6.1029079740387E-09	0.68155597037348	0.086	0.188	0.000102272531828941	Endothelial	CEP250
6.19960570262697E-09	0.539486100188803	0.122	0.24	0.000103892992364623	Endothelial	CUL1
6.23922641793591E-09	0.46646307854453	0.2	0.362	0.00010455695631177	Endothelial	ADD1
6.28939720396476E-09	0.648681426058728	0.103	0.214	0.000105397718344042	Endothelial	PPP1R35
6.29992412490287E-09	0.297101377541577	0.244	0.424	0.000105574128485122	Endothelial	ILF3
6.32598540085331E-09	0.602251280104564	0.125	0.244	0.0001060108633475	Endothelial	RBM3
6.33580469798186E-09	2.3666714900406	0.188	0.126	0.00010617541512878	Endothelial	PIK3C2A
6.38223037735453E-09	2.32032913174438	0.128	0.073	0.000106953416663707	Endothelial	AHDC1
6.65386467860193E-09	0.513061849041529	0.153	0.285	0.000111505464284011	Endothelial	RNASEK
6.69828748365408E-09	0.617472705028787	0.113	0.226	0.000112249901651075	Endothelial	ZMYND11
6.71164305861002E-09	0.410170697330482	0.169	0.307	0.000112473714376187	Endothelial	ACTR1B
6.96210818182103E-09	0.610928051909976	0.074	0.167	0.000116671008910957	Endothelial	MFSD10
6.97490183591592E-09	0.491908244888475	0.149	0.277	0.000116885404966279	Endothelial	STAT2
7.07431115726879E-09	0.443932598426844	0.05	0.132	0.00011855130637351	Endothelial	MGA
7.11086118408885E-09	0.449253494794447	0.07	0.163	0.000119163811722961	Endothelial	OAS3
7.25445101344418E-09	0.424712545757277	0.058	0.143	0.000121570090083298	Endothelial	FAM210B
7.33666383201895E-09	0.806513195840692	0.116	0.228	0.000122947812496974	Endothelial	LEMD2
7.44157435219547E-09	0.642550194372076	0.117	0.232	0.000124705902994092	Endothelial	SNX2
7.44481880883126E-09	0.482365017947039	0.08	0.175	0.000124760273598394	Endothelial	ARL2BP
7.45561329452573E-09	0.644042420411731	0.091	0.192	0.000124941167589662	Endothelial	PHKB
7.46803173037793E-09	0.33592048381233	0.077	0.172	0.000125149275737673	Endothelial	RBM27
7.51554996507499E-09	0.507144342882144	0.044	0.122	0.000125945586314727	Endothelial	NIPSNAP3A
7.53825508255387E-09	0.514346925771845	0.191	0.341	0.000126326078673438	Endothelial	BRD4
7.93786071475371E-09	0.450765325558505	0.085	0.182	0.000133022669857843	Endothelial	B4GALT5
7.99483869265846E-09	0.600390092614326	0.088	0.188	0.00013397750681157	Endothelial	SF1
8.0724273472441E-09	0.795623126194392	0.131	0.255	0.000135277737485117	Endothelial	KIAA0319L
8.08106945550743E-09	0.384042046142805	0.072	0.165	0.000135422561935394	Endothelial	TEP1
8.17033000864257E-09	0.362061994930775	0.069	0.16	0.000136918390284832	Endothelial	SLU7
8.18346878003804E-09	2.1106920111568	0.169	0.108	0.000137138569815878	Endothelial	SIGIRR
8.18782797594857E-09	0.374371974148235	0.11	0.22	0.000137211621220946	Endothelial	VAMP3
8.19560998721148E-09	0.42786368839394	0.085	0.184	0.00013734203216569	Endothelial	ITPRIPL2
8.34650134813606E-09	0.830737671519097	0.075	0.17	0.000139870669592064	Endothelial	RSPRY1
8.42314932908673E-09	3.01684635999817	0.149	0.092	0.000141155136456835	Endothelial	HID1
8.61776725230537E-09	0.745716289783477	0.136	0.261	0.000144416543614133	Endothelial	MORC3

8.63986544801863E-09	0.456834045685544	0.059	0.145	0.000144786865177896	Endothelial	SNRPG
8.68424223377788E-09	1.5770595471944	0.216	0.152	0.00014553053135365	Endothelial	SIPA1
8.82869277921518E-09	0.676191690162357	0.164	0.304	0.000147951233594088	Endothelial	LGALS8
8.86096176878469E-09	0.25631383049246	0.036	0.11	0.000148491997321294	Endothelial	SMYD4
8.92098800619558E-09	0.506288668401106	0.07	0.161	0.000149497917007826	Endothelial	PRPF8
8.96128453791304E-09	0.26847579041125	0.058	0.141	0.000150173206286347	Endothelial	LSM 1.00
9.12101291173566E-09	0.451210985896303	0.074	0.165	0.000152849934374866	Endothelial	EXOC8
9.19365656202344E-09	0.402309013955787	0.13	0.248	0.000154067296666389	Endothelial	MYO9B
9.3525088333815E-09	0.328944840787703	0.047	0.126	0.000156729343029807	Endothelial	UFL1
9.36674251032061E-09	0.526361746741889	0.088	0.187	0.000156967870987953	Endothelial	CDC123
9.39184923756225E-09	0.288551320025731	0.077	0.169	0.000157388609523068	Endothelial	BRMS1
9.64624515208153E-09	0.264337618647194	0.074	0.164	0.000161651776258582	Endothelial	RNF141
9.74581239077794E-09	0.613908613312886	0.041	0.117	0.000163320324044657	Endothelial	BRMS1L
9.84626770203453E-09	0.270353733080493	0.056	0.14	0.000165003754150695	Endothelial	SEPTIN10
9.96819455733745E-09	0.720174500628544	0.189	0.118	0.000167047004391861	Endothelial	ARHGDIB
1.00090870522504E-08	0.515379709017528	0.033	0.105	0.000167732280821611	Endothelial	KMT5C
1.00416921307885E-08	0.58772229382836	0.088	0.186	0.000168278676727753	Endothelial	TRIM56
1.0099905030268E-08	0.426353215699229	0.041	0.118	0.000169254208497231	Endothelial	SLC35E2A
1.03707133356779E-08	0.37083307234134	0.05	0.13	0.000173792414079291	Endothelial	CARNMT1
1.0741255917378E-08	0.522377741643305	0.116	0.229	0.000180001966663421	Endothelial	SRRM1
1.10426748764936E-08	0.346241430513539	0.049	0.128	0.000185053145580279	Endothelial	HIRA
1.13300702311954E-08	0.5688165617353	0.119	0.231	0.000189869316934372	Endothelial	TXNL1
1.18385415813941E-08	0.446881498714907	0.164	0.298	0.000198390279821003	Endothelial	ESYT1
1.22909438778953E-08	0.69634824096768	0.108	0.218	0.00020597163750577	Endothelial	ABI1
1.24542342000243E-08	0.276675145822825	0.058	0.14	0.000208708056724008	Endothelial	USP5
1.25553188028165E-08	2.76245356800273	0.233	0.181	0.000210402032497598	Endothelial	LAMB1
1.28798750528272E-08	0.532191859240375	0.095	0.197	0.000215840946135278	Endothelial	SNX19
1.32850582190132E-08	0.682404024002371	0.163	0.296	0.000222631005634223	Endothelial	COPB1
1.34640895872278E-08	0.368847922655855	0.061	0.145	0.000225631213302764	Endothelial	DVL2
1.35752809307659E-08	0.256210302919249	0.097	0.197	0.000227494557837775	Endothelial	IMP4
1.36906136294288E-08	0.427296067012004	0.056	0.14	0.000229427303201968	Endothelial	MED22
1.37131121075075E-08	0.558060072050224	0.095	0.197	0.00022980433269761	Endothelial	SYF2
1.37964037719869E-08	2.70437877911361	0.113	0.062	0.000231200134410956	Endothelial	CDYL2
1.38374154751304E-08	0.258573520514353	0.044	0.121	0.000231887408532236	Endothelial	CLK2
1.45841902604545E-08	0.498183856810816	0.061	0.146	0.000244401860384696	Endothelial	UBN1
1.4774606667364E-08	0.416999641053242	0.083	0.177	0.000247592858531686	Endothelial	BCKDK
1.4936858386467E-08	0.271811341456933	0.042	0.118	0.000250311872840415	Endothelial	LMF1
1.51933606108865E-08	0.430993164405354	0.041	0.116	0.000254610337117236	Endothelial	CEP104
1.52864582030357E-08	0.41077272293252	0.072	0.162	0.000256170466566473	Endothelial	MOB4
1.52899439704962E-08	0.651995422374029	0.128	0.246	0.000256228881057575	Endothelial	PAN3
1.55757394910887E-08	0.367292249883974	0.066	0.151	0.000261018242391665	Endothelial	CDYL
1.55867338323576E-08	0.679607480107516	0.163	0.298	0.000261202485562648	Endothelial	PCDH1
1.57539729253139E-08	0.270595160516921	0.033	0.103	0.00026400507828241	Endothelial	SLX4IP
1.58476549935837E-08	0.496929914249633	0.205	0.36	0.000265575002382475	Endothelial	FBXO7
1.59200536546059E-08	0.414406578077371	0.042	0.118	0.000266788259143886	Endothelial	SRSF10
1.59881521321659E-08	2.32754942225567	0.138	0.083	0.000267929453430836	Endothelial	CLN8
1.66772391523617E-08	0.438555877470534	0.058	0.14	0.000279477173715277	Endothelial	TPRG1L
1.67301356322595E-08	2.19393952203108	0.219	0.162	0.000280363612925405	Endothelial	GRAMD1A
1.69477556267077E-08	0.511856385941495	0.207	0.362	0.000284010488792368	Endothelial	CPNE1
1.70463924407389E-08	0.434207435111198	0.08	0.172	0.000285663444521902	Endothelial	SOS 1
1.70968976008556E-08	0.354548664838996	0.063	0.147	0.000286509809995138	Endothelial	ZNF282
1.71124000322498E-08	0.370566160866501	0.313	0.533	0.000286769599740443	Endothelial	ZFP36
1.73730430451777E-08	0.930446956376669	0.11	0.219	0.000291137455351087	Endothelial	HTT
1.73911556050195E-08	0.95085238624822	0.069	0.158	0.000291440985628917	Endothelial	SIAH1
1.78643025897621E-08	0.563685634403558	0.113	0.221	0.000299369982799233	Endothelial	TSPAN4
1.78738362808883E-08	2.53469243327494	0.16	0.103	0.000299529748395126	Endothelial	PTPRM
1.79844830352933E-08	0.314098407121605	0.044	0.119	0.000301383966705446	Endothelial	COG5
1.80488320772749E-08	0.497613057698405	0.061	0.145	0.000302462327950972	Endothelial	ENDOV
1.82650746295236E-08	0.55506901410483	0.127	0.241	0.000306086120641556	Endothelial	MCMBP
1.85376235147458E-08	0.842535093219373	0.164	0.301	0.00031065349486011	Endothelial	UBE2V1
1.85585745344403E-08	2.78816062031023	0.131	0.078	0.000311004592048151	Endothelial	NOD1
1.96303899556187E-08	0.606983149320813	0.095	0.195	0.000328966074876259	Endothelial	DOK4
1.97671420779524E-08	0.367584550849219	0.099	0.199	0.000331257766942326	Endothelial	TAGLN2

1.98695167208746E-08	0.402953430933917	0.097	0.197	0.000332973361208417	Endothelial	SHKBP1
2.00347853569684E-08	0.519663795160133	0.156	0.287	0.000335742933012076	Endothelial	IST1
2.04280488504043E-08	0.569262002721659	0.081	0.175	0.000342333242635076	Endothelial	SEPTIN7
2.06707636180706E-08	0.421509097703385	0.07	0.16	0.000346400656711627	Endothelial	PITHD1
2.09455848682248E-08	0.591376200140994	0.086	0.184	0.000351006111221712	Endothelial	EXOSC7
2.10213489331034E-08	0.815161322780024	0.085	0.179	0.000352275765420947	Endothelial	KRIT1
2.16455621444873E-08	0.265037064865457	0.049	0.126	0.000362736330417319	Endothelial	DDX31
2.21059290199537E-08	1.73124533026644	0.275	0.221	0.000370451158516385	Endothelial	RGS3
2.23544285647582E-08	0.879908252947077	0.078	0.171	0.000374615513888217	Endothelial	FHL1
2.25161811515402E-08	0.300199579354015	0.056	0.138	0.00037732616373751	Endothelial	NFYA
2.26279332260963E-08	0.591531819058398	0.103	0.208	0.000379198905002922	Endothelial	EXOC5
2.29822473806671E-08	0.344515315211412	0.058	0.141	0.000385136501605219	Endothelial	DDX55
2.31407940202282E-08	0.350653906687952	0.213	0.369	0.000387793426190984	Endothelial	POGZ
2.31525253576447E-08	0.607455777096538	0.249	0.433	0.00038799001994341	Endothelial	SEC31A
2.34594921264373E-08	0.412070123771859	0.221	0.382	0.000393134169054836	Endothelial	OPTN
2.35275400461027E-08	0.662745321415902	0.111	0.221	0.000394274516092589	Endothelial	GCN1
2.39271930667288E-08	0.754791107612995	0.078	0.171	0.00040097190141224	Endothelial	WFS1
2.39634543331584E-08	0.43820225997365	0.105	0.209	0.000401579567715068	Endothelial	SMC3
2.46384021159166E-08	0.382506462398668	0.078	0.168	0.000412890342658531	Endothelial	TCOF1
2.48089913540292E-08	0.590797253486534	0.085	0.178	0.000415749077110822	Endothelial	DNM1L
2.53920971431701E-08	0.66718736244536	0.097	0.198	0.000425520763925245	Endothelial	DLST
2.56448377752263E-08	0.455393258591597	0.088	0.183	0.000429756191437242	Endothelial	EIF4G2
2.63121774150131E-08	0.436932253334701	0.072	0.159	0.00044093946912079	Endothelial	CHD8
2.65598209177896E-08	0.614721997728549	0.056	0.137	0.000445089478940317	Endothelial	DDX52
2.67037768122595E-08	0.375710537137934	0.034	0.104	0.000447501891819844	Endothelial	SLC25A17
2.6909551403114E-08	0.517355457606082	0.089	0.185	0.000450950262413385	Endothelial	COPS2
2.70956987739115E-08	0.852917963211592	0.058	0.141	0.000454069720053209	Endothelial	DDX60
2.77783158599158E-08	0.441887285113355	0.053	0.131	0.000465509017180469	Endothelial	USP28
2.80757330118389E-08	0.542649951944226	0.164	0.297	0.000470493133812396	Endothelial	UBE2Z
2.8487455216831E-08	0.757187286586419	0.119	0.231	0.000477392774523654	Endothelial	WDTC1
2.8544515489094E-08	0.395650326667666	0.13	0.245	0.000478348990566237	Endothelial	ZFAND2B
2.87009719759101E-08	0.92395066415822	0.067	0.154	0.000480970888372301	Endothelial	TMTC4
2.90438510113325E-08	1.12492150254072	0.135	0.078	0.000486716855247911	Endothelial	CHST11
2.91481440911171E-08	2.36347161629206	0.192	0.135	0.000488464598678941	Endothelial	PIP4K2A
2.9303845933655E-08	0.38430788390666	0.066	0.15	0.00049107385015619	Endothelial	TSC2
2.9709758983863E-08	0.488479217837164	0.085	0.179	0.000497876141051576	Endothelial	USP15
2.9973035917282E-08	0.650218763370756	0.191	0.337	0.000502288135901812	Endothelial	EIF3B
3.01861122754487E-08	0.409226912392669	0.258	0.437	0.000505858869511969	Endothelial	GSDMD
3.07838187787521E-08	0.382707348469166	0.047	0.123	0.000515875235094328	Endothelial	NFKBIL1
3.17435887414676E-08	0.468884320677878	0.127	0.238	0.000531959060129514	Endothelial	KIAA1671
3.2523725059967E-08	0.499726451177379	0.049	0.126	0.000545032584554927	Endothelial	ZNF513
3.30495188939449E-08	0.842706306276178	0.102	0.207	0.000553843837624728	Endothelial	INSIG2
3.32673002397063E-08	0.581152781287844	0.083	0.175	0.000557493417416999	Endothelial	GNL2
3.38076167074394E-08	0.636186546998542	0.05	0.126	0.00056654804078327	Endothelial	DNAAF5
3.39460782816086E-08	0.937339753326098	0.142	0.269	0.000568868379843196	Endothelial	SEC24A
3.40981343205775E-08	0.412275017604006	0.052	0.129	0.000571416534944239	Endothelial	EIF2B3
3.46141296639883E-08	0.377729706616859	0.067	0.152	0.000580063584909117	Endothelial	SHPRH
3.4774341194614E-08	0.479239606911758	0.059	0.142	0.00058274840973934	Endothelial	SNRNP48
3.57255351834677E-08	0.607607571335253	0.125	0.237	0.000598688518604551	Endothelial	TRRAP
3.59704026564685E-08	0.271239917391302	0.045	0.12	0.000602792007717099	Endothelial	SLC9A8
3.59985386007328E-08	1.45835313177696	0.138	0.265	0.00060326350987108	Endothelial	IGFBP3
3.60480367579399E-08	0.93379819964686	0.103	0.206	0.000604092999989557	Endothelial	SMIM12
3.62646112146512E-08	0.390000992236857	0.066	0.15	0.000607722354735125	Endothelial	ATG13
3.64484445382997E-08	0.398984236133764	0.092	0.188	0.000610803033572826	Endothelial	ASCC3
3.65797786401605E-08	0.541969550034808	0.18	0.319	0.00061300393045181	Endothelial	PCMTD1
3.65919806781538E-08	1.4037305600524	0.103	0.055	0.000613208412204501	Endothelial	THBS1
3.67666258517527E-08	0.432913590711642	0.13	0.243	0.000616135116023672	Endothelial	AEBP2
3.78355811678213E-08	0.650847369102555	0.133	0.25	0.00063404866921035	Endothelial	FRYL
3.79657952010799E-08	2.23813029597825	0.244	0.188	0.000636230795979698	Endothelial	PDK4
3.8973697628586E-08	0.766058716329239	0.13	0.245	0.000653121224859845	Endothelial	RNF40
3.98075018622329E-08	0.683971135884932	0.055	0.133	0.000667094116207299	Endothelial	USP37
4.04449921827417E-08	0.47740773664628	0.152	0.275	0.000677777178998385	Endothelial	FUS
4.04998641109924E-08	2.85669241983637	0.119	0.069	0.00067869672277201	Endothelial	SASH1

4.10646753271542E-08	0.333905459556938	0.066	0.147	0.00068816182913245	Endothelial	SBNO1
4.14265693233344E-08	0.406266595970379	0.186	0.327	0.000694226448720437	Endothelial	SNRNP70
4.17791482248807E-08	0.560802970254765	0.058	0.137	0.000700134965952552	Endothelial	STAM2
4.25636786867797E-08	0.254119734085056	0.049	0.123	0.000713282127433054	Endothelial	USP47
4.29869486549899E-08	0.706273354950722	0.045	0.12	0.00072037528556032	Endothelial	ST3GAL3
4.31786829209412E-08	0.707473381846187	0.138	0.257	0.000723588368389132	Endothelial	ANAPC5
4.42071685655288E-08	0.958532649593952	0.058	0.139	0.000740823730821132	Endothelial	CTPS2
4.4536147226493E-08	0.496954418672198	0.055	0.133	0.000746336755221569	Endothelial	MAPRE1
4.45712863565464E-08	0.369287953559303	0.113	0.215	0.000746925616763005	Endothelial	QRICH1
4.46686992094474E-08	0.250177976577518	0.044	0.117	0.00074855806135192	Endothelial	CNEP1R1
4.53154271575401E-08	0.340486040014021	0.055	0.132	0.000759395928306057	Endothelial	TTC9C
4.64174618839142E-08	0.499860229602515	0.077	0.164	0.000777863826250634	Endothelial	NRAS
4.74197146547841E-08	0.441243011142674	0.055	0.133	0.000794659578184872	Endothelial	PIGQ
4.8690111944887E-08	0.307473309519946	0.052	0.128	0.000815948895972417	Endothelial	WRAP73
4.92899488118705E-08	1.16324846046337	0.106	0.209	0.000826000962189326	Endothelial	SLC2A13
4.95162627488569E-08	0.266997983168245	0.061	0.141	0.000829793531145344	Endothelial	PPTC7
4.97214968390942E-08	0.52496048431961	0.05	0.126	0.000833232844029541	Endothelial	SMARCAD1
4.98334091130509E-08	0.560231072386426	0.075	0.163	0.000835108269916508	Endothelial	RIC1
4.99470446765697E-08	0.396880289001126	0.083	0.174	0.000837012574689956	Endothelial	KANSL3
5.0156137310752E-08	0.372064974810663	0.056	0.134	0.000840516549053582	Endothelial	KIFAP3
5.05226103462447E-08	2.2489788591401	0.302	0.267	0.000846657904182369	Endothelial	DENND4C
5.05679722356401E-08	0.2543965348253	0.041	0.112	0.000847418078724857	Endothelial	SRRD
5.06385245010557E-08	0.572909160209728	0.117	0.222	0.000848600393588692	Endothelial	TEX264
5.08768269529877E-08	0.319946885046735	0.075	0.163	0.000852593866078168	Endothelial	PITPNM1
5.10497053203317E-08	0.298195335096983	0.039	0.108	0.000855490961758119	Endothelial	TRIM2
5.11211182997862E-08	0.469134612189736	0.055	0.132	0.000856687700467817	Endothelial	CZIB
5.18771731058967E-08	0.548938565541096	0.061	0.142	0.000869357666908617	Endothelial	DNAJB14
5.29454032071753E-08	0.697701053460649	0.136	0.253	0.000887259066945843	Endothelial	SLC27A3
5.29730464734905E-08	0.383816739366138	0.121	0.228	0.000887722312802754	Endothelial	MTG1
5.35088345953487E-08	0.830103208834272	0.058	0.138	0.000896701050148854	Endothelial	ZNF449
5.38324275208489E-08	0.845539919001238	0.069	0.154	0.000902123820394386	Endothelial	SLC10A7
5.40677495754894E-08	0.358039272431712	0.039	0.109	0.000906067347386052	Endothelial	GTF3C5
5.41633218856714E-08	2.14611286872579	0.228	0.174	0.000907668948160081	Endothelial	SH3TC1
5.55175557907667E-08	0.35073786645456	0.07	0.154	0.000930363199941668	Endothelial	BCL10
5.68555210163809E-08	0.412529731994331	0.088	0.181	0.00095278482119251	Endothelial	TMEM8A
5.7396559859068E-08	0.578818756839976	0.067	0.151	0.000961851550118262	Endothelial	ANP32A
5.82419381936931E-08	0.772320231179452	0.061	0.141	0.000976018400249909	Endothelial	ZNF561
5.82812313015388E-08	0.538335109929141	0.106	0.207	0.000976676874151188	Endothelial	CSNK1G2
5.84498479921147E-08	0.47594970181715	0.128	0.237	0.000979502552651858	Endothelial	CNPY3
5.99391295358394E-08	0.500060496929894	0.05	0.125	0.0010044599327616	Endothelial	SMYD3
6.00924382042342E-08	0.534590435091332	0.156	0.284	0.00100702907942656	Endothelial	YWHAE
6.02831142760684E-08	2.48246155491169	0.139	0.086	0.00101022442903835	Endothelial	ST3GAL4
6.10014929222434E-08	0.30503691295967	0.047	0.121	0.00102226301839095	Endothelial	NR2C1
6.10036160145193E-08	0.623598664624195	0.044	0.116	0.00102229859717131	Endothelial	ADSSL1
6.1386287033331E-08	0.485911028217051	0.041	0.111	0.00102871139810456	Endothelial	TRIM32
6.17659800474971E-08	0.469133228925055	0.099	0.195	0.00103507429363596	Endothelial	OSBPL8
6.24268751056269E-08	0.491948344090615	0.108	0.209	0.0010461495730201	Endothelial	FNDC3A
6.29997704918984E-08	0.253580239273951	0.058	0.135	0.00105575015390323	Endothelial	HS2ST1
6.38962276660507E-08	0.362474726705827	0.072	0.157	0.00107077298322768	Endothelial	NOP9
6.42000231394198E-08	0.862362239918812	0.142	0.265	0.0010758639877704	Endothelial	STK38
6.62389255684878E-08	0.253421232303373	0.095	0.189	0.00111003191467672	Endothelial	R3HDM4
6.82758702820971E-08	0.792742589723085	0.103	0.204	0.00114416703418738	Endothelial	TPRA1
6.83796266822417E-08	0.710630252940293	0.11	0.212	0.00114590578394101	Endothelial	SRCAP
6.84154070515789E-08	0.40003140710033	0.063	0.141	0.00114650539137036	Endothelial	ELP1
6.96322542376704E-08	0.608446165598063	0.102	0.2	0.00116689731651488	Endothelial	TNKS
6.96746836324147E-08	0.422996178151476	0.061	0.141	0.00116760834831201	Endothelial	WDR36
7.04130241286122E-08	0.577840228145877	0.089	0.182	0.00117998145834728	Endothelial	NHLRC2
7.0647109075725E-08	0.463163422739421	0.052	0.127	0.001183904253891	Endothelial	TMEM135
7.07128140827316E-08	0.372408732558375	0.07	0.153	0.00118500533839842	Endothelial	DYNC1H1
7.11076476222891E-08	0.525256421233274	0.152	0.273	0.00119162195885432	Endothelial	DEDD
7.12360601545107E-08	0.572843320383975	0.133	0.246	0.00119377389606929	Endothelial	KHDRBS1
7.25096629217908E-08	0.483287133845954	0.139	0.257	0.00121511693124337	Endothelial	SUMO3
7.39909997935952E-08	0.624464031134488	0.088	0.18	0.00123994117454107	Endothelial	MED24

7.44200429855402E-08	0.436432060172237	0.078	0.164	0.00124713108035168	Endothelial	PPP1R3B
7.63642223024369E-08	0.997023078473835	0.117	0.225	0.00127971163734424	Endothelial	HDHD5
7.94866314638248E-08	0.598692311934774	0.106	0.205	0.00133203697007078	Endothelial	UFM1
8.09852975872971E-08	2.73365649606117	0.205	0.151	0.00135715161696792	Endothelial	PIK3C2B
8.11135347621847E-08	0.784166463715399	0.155	0.278	0.00135930061554469	Endothelial	WDR26
8.11756131924022E-08	0.286881762260309	0.106	0.206	0.00136034092587828	Endothelial	ZNF592
8.28292151977427E-08	0.59440286601726	0.105	0.205	0.00138805198828377	Endothelial	DRAM2
8.4393170447241E-08	0.538946074723641	0.099	0.195	0.00141426075035486	Endothelial	ORMDL1
8.49414857570911E-08	0.356816733850202	0.042	0.113	0.00142344941831733	Endothelial	TJAP1
8.57063064420176E-08	0.77787739128243	0.133	0.248	0.00143626628335533	Endothelial	GRIPAP1
8.63271666033649E-08	0.527896162910932	0.196	0.342	0.00144667065793919	Endothelial	KMT2C
8.76496471791503E-08	0.778216495055932	0.131	0.245	0.0014688327874282	Endothelial	GSK3B
8.78939443551276E-08	0.386926092473352	0.041	0.11	0.00147292671950323	Endothelial	ZNF335
8.88625887774989E-08	0.337559234066846	0.056	0.133	0.00148915926273333	Endothelial	RNASEH1
9.27210373686883E-08	0.262677844985935	0.088	0.177	0.00155381914422448	Endothelial	PREPL
9.39386135550059E-08	0.280274673107183	0.05	0.123	0.00157422328595479	Endothelial	SCAMP5
9.7036491866614E-08	0.578876280062394	0.147	0.265	0.00162613753070072	Endothelial	CDC34
9.77181472376265E-08	0.720490822681886	0.102	0.201	0.00163756071140814	Endothelial	DEPRECATED-ENSG00000163374
9.88719940452286E-08	0.527097280375733	0.064	0.144	0.00165689687620994	Endothelial	POM121
1.00854248731789E-07	1.1201335071903	0.426	0.425	0.00169011550024731	Endothelial	SYNE2
1.01243044628741E-07	0.561844989179651	0.108	0.207	0.00169663094188844	Endothelial	EPC1
1.01297504467778E-07	0.76380851012996	0.113	0.217	0.00169754357987103	Endothelial	YY1
1.0161440545857E-07	0.623350152432058	0.092	0.186	0.00170285420667471	Endothelial	NRIP1
1.04032571433391E-07	0.873645717881102	0.063	0.142	0.00174337783208077	Endothelial	DIP2C
1.06118445895943E-07	0.662279183319171	0.086	0.176	0.00177833291632422	Endothelial	SMARCB1
1.1037027739605E-07	0.31591038003586	0.072	0.154	0.001849585108603	Endothelial	IFT20
1.11591360840995E-07	0.62362969761526	0.155	0.28	0.00187004802497339	Endothelial	MED28
1.11639592312468E-07	2.253436298821	0.275	0.234	0.00187085628797234	Endothelial	PCGF2
1.12578583045728E-07	0.424366481636269	0.1	0.197	0.0018865918946803	Endothelial	OTUD5
1.13577291685127E-07	0.706604945349644	0.128	0.239	0.00190332825405936	Endothelial	MTA1
1.15231641148403E-07	0.504074629566854	0.113	0.213	0.00193105184236493	Endothelial	PRMT2
1.17287863872811E-07	0.521325719830744	0.1	0.195	0.00196551002278057	Endothelial	KIAA2026
1.18040505336694E-07	0.627840967681518	0.161	0.288	0.00197812278843232	Endothelial	NIBAN2
1.18995698702958E-07	0.855633960798826	0.108	0.208	0.00199412991886416	Endothelial	NF1
1.19715781909715E-07	0.361129540490886	0.058	0.133	0.00200619707324301	Endothelial	BCS1L
1.21303164640526E-07	0.815006611429526	0.167	0.299	0.00203279843304594	Endothelial	ANKRD17
1.22274018727762E-07	0.536221877734899	0.058	0.135	0.00204906800583984	Endothelial	FBXO34
1.2323960798894E-07	2.96735520404336	0.171	0.118	0.00206524935067866	Endothelial	EPB41L4A
1.24082881983104E-07	0.49408843922869	0.235	0.405	0.00207938093627286	Endothelial	SERINC3
1.24194954026477E-07	0.511222697257487	0.239	0.4	0.0020812590395757	Endothelial	MYO18A
1.24875658596476E-07	0.512081447491473	0.114	0.216	0.00209266628675974	Endothelial	USP25
1.25380289243768E-07	0.250634804500112	0.091	0.181	0.00210112288714707	Endothelial	DDX27
1.30156509657086E-07	0.699393005332282	0.088	0.178	0.00218116278883344	Endothelial	RBM22
1.31412466447732E-07	0.532178917102755	0.106	0.205	0.00220221011273109	Endothelial	VEGFB
1.31723487935824E-07	0.59158164200948	0.128	0.237	0.00220742221082854	Endothelial	ARID4B
1.32417218359764E-07	0.407963973978956	0.072	0.153	0.00221904774527292	Endothelial	ARID4A
1.32680815140807E-07	0.475938122018539	0.119	0.222	0.00222346510012965	Endothelial	KPNA6
1.3355616870214E-07	0.25185555687905	0.815	0.894	0.00223813427511047	Endothelial	B2M
1.34533824439228E-07	0.359433173128169	0.045	0.115	0.00225451782995259	Endothelial	PPCDC
1.34635067882094E-07	0.660513977041626	0.108	0.207	0.00225621446756813	Endothelial	REEP3
1.35238569907473E-07	0.596984585259648	0.247	0.42	0.00226632795450944	Endothelial	IRF2BP2
1.35376337155925E-07	0.629502895997055	0.224	0.385	0.00226863665805899	Endothelial	PTOV1
1.36384049746331E-07	0.391102863514471	0.127	0.233	0.00228552390564902	Endothelial	NINJ1
1.39365761847791E-07	0.614411991897841	0.063	0.142	0.00233549143704528	Endothelial	PATL1
1.41477158996477E-07	1.02875992260417	0.153	0.28	0.00237087423046296	Endothelial	RAB20
1.41567957101151E-07	0.638579404033511	0.131	0.241	0.00237239582510108	Endothelial	ALKBH5
1.43763694109708E-07	0.565898149813828	0.069	0.15	0.00240919198589049	Endothelial	CD58
1.44729217714923E-07	0.604414747399311	0.124	0.231	0.00242537223046669	Endothelial	DMTF1
1.46876843036573E-07	0.529368535892657	0.097	0.192	0.0024613621356069	Endothelial	GTF3C3
1.48722961857885E-07	0.64232328663072	0.067	0.147	0.00249229939481444	Endothelial	ADPRHL2
1.49967431182179E-07	0.311360435757729	0.058	0.134	0.00251315421175096	Endothelial	MTMR14
1.503247045998E-07	0.522184644092877	0.053	0.127	0.00251914139968345	Endothelial	DERL2
1.51825078521491E-07	0.613570084009327	0.078	0.164	0.00254428466586314	Endothelial	TIMM10B

1.52921547559106E-07	0.37068170712583	0.117	0.219	0.0025626592939955	Endothelial	ATM
1.53718455654942E-07	0.573903823409146	0.041	0.108	0.00257601387986551	Endothelial	ALS2
1.58217188013217E-07	0.422951065326976	0.152	0.269	0.00265140363672549	Endothelial	DDX24
1.5830559029821E-07	0.780471140835796	0.099	0.195	0.00265288508221741	Endothelial	CCDC159
1.60400448835429E-07	0.410095358852704	0.078	0.162	0.00268799072158411	Endothelial	CDK2
1.6476981754813E-07	1.89848310946805	0.304	0.27	0.00276121260247157	Endothelial	TRIOBP
1.68242232759725E-07	2.07221896655419	0.124	0.074	0.00281940333658748	Endothelial	PKIG
1.69054581038048E-07	0.739455168441854	0.16	0.283	0.00283301666903561	Endothelial	CPSF6
1.69571753438167E-07	0.614138809003572	0.055	0.128	0.0028416834441168	Endothelial	VAC14
1.7696906347659E-07	0.630945105544014	0.163	0.286	0.00296564756574069	Endothelial	RBM25
1.82846496207674E-07	0.584953991072901	0.103	0.199	0.00306414158344819	Endothelial	RRAGA
1.85747358607549E-07	0.259747375065601	0.036	0.1	0.0031127542355453	Endothelial	INAFM1
1.96990971448507E-07	0.743532694042911	0.18	0.314	0.00330117469953408	Endothelial	MKLN1
1.98678177468653E-07	0.738125240779175	0.099	0.192	0.00332944889801969	Endothelial	POLK
1.99849852165221E-07	0.364758283776452	0.039	0.105	0.00334908382258478	Endothelial	LCMT1
2.02130169202735E-07	0.475361289103754	0.07	0.15	0.00338729737549944	Endothelial	RGP1
2.02685886586595E-07	2.34938475761575	0.21	0.159	0.00339661008741817	Endothelial	RELL1
2.02821670252255E-07	0.33968032199757	0.116	0.216	0.0033988855500873	Endothelial	PURB
2.04056215632454E-07	0.490101249786394	0.047	0.116	0.00341957406156866	Endothelial	TDP1
2.04701037038338E-07	0.624919266950966	0.056	0.13	0.00343037997868847	Endothelial	EMC1
2.12307351451115E-07	0.498899169948657	0.081	0.167	0.00355784659561778	Endothelial	DIP2B
2.13178207646278E-07	0.714006067891829	0.105	0.201	0.00357244040373633	Endothelial	AP2M1
2.13202646973237E-07	0.755743871269086	0.15	0.091	0.0035728499579775	Endothelial	CD4
2.14750330391387E-07	0.526951819333732	0.192	0.332	0.00359878603669887	Endothelial	FUBP1
2.18003267630613E-07	0.526308451545844	0.083	0.167	0.00365329875895381	Endothelial	RANBP10
2.24534422994916E-07	0.271146696427016	0.056	0.13	0.0037627478605488	Endothelial	SF3A1
2.33896132343329E-07	0.418114085413674	0.097	0.189	0.00391963138580951	Endothelial	ATAD2B
2.40012093164458E-07	0.292339197475568	0.042	0.109	0.00402212265724999	Endothelial	KANSL2
2.40240127170889E-07	0.510321309198312	0.086	0.171	0.00402594405112976	Endothelial	ZNF106
2.41284593291196E-07	0.267484879169333	0.047	0.115	0.00404344721437386	Endothelial	ARL5B
2.4192286778243E-07	0.57497137577374	0.091	0.18	0.00405414341829797	Endothelial	ARRDC1
2.43655347591231E-07	0.306361952555803	0.059	0.134	0.00408317631493384	Endothelial	CDIP1
2.45882271273875E-07	0.428701074028921	0.038	0.102	0.00412049510200759	Endothelial	WDR90
2.48352561249682E-07	0.564600624122291	0.058	0.132	0.00416189222142216	Endothelial	AQR
2.49316181349103E-07	0.739874540744505	0.124	0.23	0.00417804056704827	Endothelial	MTR
2.5570123130512E-07	0.76718460804595	0.072	0.152	0.00428504123421119	Endothelial	CCDC93
2.56565727683209E-07	0.379656926725683	0.055	0.127	0.00429952846451522	Endothelial	MEX3D
2.66382970359623E-07	0.313388898820343	0.049	0.118	0.00446404581728656	Endothelial	MIGA2
2.6741405952099E-07	0.388469728090676	0.083	0.167	0.00448132480945275	Endothelial	CHKB
2.69265968848125E-07	0.446678729014751	0.045	0.113	0.00451235910595687	Endothelial	VARS2
2.73007485908361E-07	0.495915207041011	0.127	0.229	0.00457505944885231	Endothelial	XRN2
2.7599892918949E-07	0.304624563374996	0.039	0.104	0.00462519005535747	Endothelial	ASH2L
2.76437268715101E-07	0.564272529844805	0.049	0.117	0.00463253574912766	Endothelial	ZDHHC17
2.77437355501651E-07	0.527774463218316	0.113	0.211	0.00464929520349667	Endothelial	DEAF1
2.80761035209248E-07	1.06271870426942	0.089	0.18	0.00470499342803657	Endothelial	SLC25A29
2.81973599193647E-07	0.341678523113896	0.042	0.108	0.00472531357528714	Endothelial	SLC30A6
2.83547952182944E-07	2.33352954022315	0.146	0.095	0.00475169658268177	Endothelial	CD276
2.83843862306408E-07	0.721292849684831	0.142	0.256	0.00475665544453078	Endothelial	NCKAP1
2.84463295523465E-07	0.439783856905652	0.067	0.145	0.00476703590638223	Endothelial	SOCS4
2.86070716697279E-07	0.39631732106403	0.083	0.167	0.004793973070413	Endothelial	STK40
2.86550287988426E-07	1.04601169162133	0.113	0.214	0.00480200972611005	Endothelial	SPATS2
2.90077739069147E-07	0.36516081304658	0.041	0.105	0.00486112275132076	Endothelial	SLC35A5
2.91026633634314E-07	0.528900736580567	0.125	0.229	0.00487702432644384	Endothelial	IFNGR2
2.96474732711479E-07	0.553595416585456	0.081	0.165	0.00496832357077896	Endothelial	GPATCH8
2.99040977737669E-07	0.746328418608147	0.066	0.145	0.00501132870492786	Endothelial	ALG9
2.99690893274451E-07	2.72538139030211	0.192	0.142	0.00502221998949325	Endothelial	C1orf21
3.01742057208914E-07	0.876271254161255	0.064	0.142	0.00505659339470699	Endothelial	THOC1
3.02252821280153E-07	0.49907097542741	0.047	0.115	0.00506515277901281	Endothelial	ADAT2
3.02274885394831E-07	0.328914503569314	0.075	0.157	0.00506552252944658	Endothelial	PABPC4
3.02393684339824E-07	0.391276472047297	0.067	0.144	0.00506751336216676	Endothelial	TSPAN31
3.04881327860097E-07	0.654475344763098	0.075	0.157	0.00510920129227951	Endothelial	TCF3
3.05639344241087E-07	0.706847378587163	0.156	0.274	0.00512190413079213	Endothelial	SYMPK
3.07819881116483E-07	0.579802685758666	0.088	0.174	0.00515844556775002	Endothelial	UBE2O

3.0836898913288E-07	0.763141245145557	0.125	0.23	0.00516764751988881	Endothelial	KPNA1
3.08383695125477E-07	0.441718293503289	0.064	0.14	0.00516789396291274	Endothelial	ZBTB37
3.20701225840579E-07	0.250209974557048	0.069	0.146	0.00537431114263642	Endothelial	ZNF512B
3.22754404181192E-07	0.582758124216936	0.186	0.318	0.00540871830526842	Endothelial	TIA1
3.24468531040095E-07	0.487697208409644	0.083	0.167	0.00543744364316991	Endothelial	RECQL
3.27855478301036E-07	0.481197537902601	0.044	0.11	0.00549420210536876	Endothelial	UROS
3.42623544118832E-07	0.570206448165077	0.138	0.247	0.00574168535234338	Endothelial	CREBBP
3.46033099940131E-07	0.678514234326282	0.103	0.195	0.00579882268879672	Endothelial	LEPROT
3.55030781169431E-07	1.78917668692662	0.308	0.281	0.00594960583083733	Endothelial	PEA15
3.5608193860822E-07	0.439812216395198	0.072	0.151	0.00596722112719655	Endothelial	CLCN5
3.62559513176528E-07	1.91141721267635	0.177	0.123	0.00607577232181226	Endothelial	SH2B3
3.63164270006152E-07	0.467784074703238	0.111	0.208	0.0060859068367631	Endothelial	ELP2
3.68292354150607E-07	0.332231900123702	0.078	0.159	0.00617184327085587	Endothelial	SMPD1
3.77702150475757E-07	0.263503171260481	0.059	0.132	0.00632953263767274	Endothelial	MAPK8
3.78397676477782E-07	0.494461375054789	0.222	0.373	0.00634118826241467	Endothelial	TRA2A
3.80333487434053E-07	0.399140827723848	0.044	0.11	0.00637362858241985	Endothelial	CCT2
3.80497819987556E-07	0.817090281727381	0.056	0.129	0.00637638246735146	Endothelial	ZNF606
3.83804855611919E-07	0.553327146148938	0.053	0.124	0.00643180177034454	Endothelial	KLHL7
3.91049709852456E-07	0.474791596914568	0.094	0.182	0.00655321103770745	Endothelial	SART3
3.934130769287E-07	0.83725750474178	0.141	0.252	0.00659281634317116	Endothelial	ATF4
3.95510891807E-07	0.823076987662548	0.08	0.162	0.00662797152490171	Endothelial	SRGAP2B
3.95984654714836E-07	0.885932423340681	0.122	0.223	0.00663591084371121	Endothelial	GORASP1
4.07982580782607E-07	1.01120031063997	0.061	0.135	0.00683697208875493	Endothelial	ZNF654
4.21919998508029E-07	0.410658069322437	0.141	0.25	0.00707053533499755	Endothelial	YWHAH
4.24706510357865E-07	0.297357588596392	0.077	0.157	0.0071172317005771	Endothelial	DCP2
4.32583924316797E-07	2.02925345654366	0.161	0.11	0.00724924140370089	Endothelial	PDE4D
4.33325992649407E-07	0.686934023556474	0.055	0.126	0.00726167698481877	Endothelial	FASTKD2
4.34527377658801E-07	0.334079844727968	0.07	0.147	0.00728180979480619	Endothelial	FEM1B
4.36085405038855E-07	0.27118334368421	0.074	0.151	0.00730791921764114	Endothelial	SLC12A9
4.4577860768968E-07	0.650930583237557	0.066	0.141	0.00747035790766366	Endothelial	RTCA
4.4814886477638E-07	0.727978798446302	0.196	0.334	0.00751007867592258	Endothelial	PDIA6
4.56652650423412E-07	0.771392568226827	0.128	0.232	0.00765258511579554	Endothelial	DDX56
4.58294774812031E-07	0.34571432069079	0.219	0.363	0.00768010383630002	Endothelial	PPP2R1B
4.63166524042113E-07	0.555991465984958	0.094	0.181	0.00776174460989773	Endothelial	IFRD2
4.70768533089841E-07	0.532145473602782	0.059	0.132	0.00788913907751956	Endothelial	WDR18
4.77236582898044E-07	0.369207394428782	0.045	0.111	0.00799753065620542	Endothelial	DEPRECATED-ENSG00000197302
4.82107560970571E-07	0.665467971687973	0.155	0.273	0.00807915850674483	Endothelial	ARRB1
4.84039954002323E-07	0.739056581728193	0.175	0.304	0.00811154154917094	Endothelial	CDK16
4.88994320122049E-07	0.285136962999664	0.077	0.156	0.00819456681660529	Endothelial	NSMCE3
4.92662146983689E-07	0.876366869965748	0.106	0.202	0.00825603225915265	Endothelial	VCP
4.93102332152927E-07	1.43986343072236	0.041	0.105	0.00826340888221876	Endothelial	PPM1H
5.04196320527768E-07	0.60947187623528	0.153	0.271	0.00844932193940434	Endothelial	STK11
5.07566489084508E-07	0.69164315310806	0.066	0.142	0.00850579922407818	Endothelial	U2AF1L4
5.0942321246764E-07	0.381875926446548	0.041	0.104	0.00853691419453271	Endothelial	CPT1B
5.15006617222794E-07	0.626449209849082	0.199	0.337	0.00863048089141959	Endothelial	DVL3
5.16821768726079E-07	0.775335177994201	0.059	0.132	0.00866089920031162	Endothelial	BRAF
5.19391091423524E-07	1.04280074141707	0.074	0.153	0.00870395591007541	Endothelial	FARP2
5.20095476301557E-07	0.484309259304859	0.072	0.15	0.0087157599918615	Endothelial	ZBTB11
5.26642696358362E-07	0.579196248933118	0.069	0.145	0.00882547830557343	Endothelial	COG7
5.52195413230754E-07	0.857442062544201	0.203	0.351	0.00925369073492098	Endothelial	AGFG2
5.5355406375963E-07	0.564489562978831	0.042	0.105	0.00927645900048388	Endothelial	FNTB
5.57514634383267E-07	0.526705220135492	0.221	0.37	0.00934283024299479	Endothelial	MBD2
5.59622219278493E-07	0.789046751853959	0.141	0.249	0.00937814915066899	Endothelial	BRWD1
5.61172652015605E-07	0.513864970715192	0.053	0.122	0.00940413130247751	Endothelial	WDR45
5.79395270974392E-07	0.512192911846196	0.045	0.111	0.00970950595098886	Endothelial	UFSP2
5.82880517587929E-07	0.303039130598722	0.045	0.11	0.00976791171373852	Endothelial	SAMM50
5.8727294375726E-07	0.767714858743688	0.075	0.155	0.00984151999148416	Endothelial	TBC1D5
6.01527339458938E-07	0.647349509093923	0.095	0.182	0.0100803951546529	Endothelial	PHF11
6.05084515806316E-07	0.435408849415603	0.075	0.152	0.0101400063158822	Endothelial	SLC35E2B
6.0864848152223E-07	0.425140425597373	0.085	0.167	0.0101997312533495	Endothelial	MAN1B1
6.13976154480131E-07	0.275830533550729	0.045	0.11	0.010289012396778	Endothelial	TMEM87B
6.27614499538167E-07	0.486604508394683	0.099	0.186	0.0105175637832606	Endothelial	SIN3A
6.37559891155656E-07	0.701921645742743	0.074	0.152	0.0106842286559865	Endothelial	NMRK1

6.43360592982673E-07	0.770346597934487	0.125	0.227	0.0107814368172036	Endothelial	CNOT9
6.50145406283339E-07	0.94702070574981	0.183	0.124	0.0108951367184962	Endothelial	ANXA6
6.53915644155302E-07	0.811514787474961	0.174	0.297	0.0109583183647546	Endothelial	ITGB1
6.56557245849425E-07	0.71251285083108	0.08	0.161	0.0110025863259447	Endothelial	PAXBP1
6.58696955274119E-07	0.415771393737343	0.064	0.138	0.0110384435764837	Endothelial	NAT9
6.70450937048571E-07	0.441706990070403	0.207	0.344	0.0112354168030599	Endothelial	SUGP2
6.77206887699147E-07	0.613664436541635	0.106	0.198	0.0113486330240623	Endothelial	ULK1
6.82006157162796E-07	1.14435339786481	0.061	0.134	0.0114290591817341	Endothelial	MAML3
6.88392461773402E-07	0.557545552776675	0.078	0.158	0.0115360808743987	Endothelial	AGAP1
6.89020152945183E-07	0.572711082416753	0.097	0.185	0.0115465997230554	Endothelial	PLPP5
6.97052811341122E-07	0.417269838258171	0.042	0.106	0.0116812110124545	Endothelial	CBWD3
7.21199773106017E-07	0.555536630026162	0.085	0.167	0.0120858657977106	Endothelial	CAMLG
7.4080164648371E-07	0.610624296753061	0.113	0.205	0.012414353991774	Endothelial	TRAK1
7.54526211602112E-07	1.02093096456597	0.103	0.196	0.0126443502540282	Endothelial	CBWD1
7.63022704075526E-07	0.692456122209117	0.085	0.167	0.0127867344748977	Endothelial	TRIM41
7.6718896073136E-07	0.632885428764364	0.056	0.125	0.0128565526039361	Endothelial	ALG1
7.7828646218488E-07	0.849019992948176	0.538	0.607	0.0130425245332942	Endothelial	JUN
7.82258895394093E-07	0.709968354038011	0.055	0.123	0.0131090945690142	Endothelial	RAD51B
7.82348061724284E-07	0.59564726140802	0.05	0.116	0.0131105888183756	Endothelial	FLYWCH2
7.84899674741044E-07	0.781209034470018	0.113	0.209	0.0131533487493104	Endothelial	MAML1
8.18001670960853E-07	1.44830112685845	0.322	0.294	0.013708072001962	Endothelial	ZYX
8.25348484603217E-07	0.666455298400056	0.099	0.186	0.0138311899049807	Endothelial	KLF11
8.26258603431406E-07	0.498994072010258	0.045	0.11	0.0138464416763035	Endothelial	PEX14
8.34094162815515E-07	0.595214800923049	0.063	0.134	0.0139777499804624	Endothelial	EIF1AD
8.36787609360201E-07	2.10625441948288	0.185	0.135	0.0140228867576582	Endothelial	ZMIZ1
8.48373695472704E-07	0.428357286269403	0.052	0.119	0.0142170463887316	Endothelial	ACOX3
8.54092985789642E-07	0.633144686174128	0.113	0.207	0.0143128902558628	Endothelial	EDC4
8.5569296839584E-07	0.352268855006782	0.102	0.19	0.0143397027643775	Endothelial	KLF10
8.64894164984608E-07	0.495628529676381	0.263	0.434	0.0144938964168121	Endothelial	EGLN1
8.69802101568134E-07	2.25008262530421	0.133	0.085	0.0145761436180788	Endothelial	STX3
8.69959202875007E-07	0.578507173690878	0.053	0.122	0.0145787763217794	Endothelial	ROGDI
8.71551390382394E-07	0.329530250149341	0.059	0.129	0.0146054582000282	Endothelial	GPATCH4
8.72789011075573E-07	0.38919142313938	0.064	0.136	0.0146261982476045	Endothelial	ZNF362
8.74385781201572E-07	0.561852898410474	0.086	0.169	0.0146529569213759	Endothelial	DDI2
8.90359315454787E-07	0.50976234939137	0.056	0.125	0.0149206414083913	Endothelial	FAM133B
8.93932530792567E-07	0.938811760922525	0.108	0.202	0.0149805213510218	Endothelial	DEPRECATED-ENSG00000188313
8.96714007578287E-07	0.314831841002118	0.097	0.182	0.0150271333389969	Endothelial	FKBP11
9.03596478702487E-07	2.42086105735209	0.146	0.097	0.0151424697900963	Endothelial	TNFAIP1
9.14473974558931E-07	0.63033036235366	0.211	0.357	0.0153247548656586	Endothelial	RSRC2
9.24090072706973E-07	2.65608902972158	0.175	0.127	0.0154859014384234	Endothelial	PPARG
9.34124925102197E-07	0.596203242462275	0.131	0.234	0.0156540654948626	Endothelial	IRF2BPL
9.60157415166042E-07	0.630976805650677	0.097	0.184	0.0160903179633525	Endothelial	KDM5A
9.68170086761438E-07	0.579678101727954	0.088	0.169	0.0162245943139482	Endothelial	WIPI2
9.93165880709469E-07	0.862375179959525	0.152	0.266	0.0166434738289293	Endothelial	RUFY1
1.00686766025573E-06	2.53297863472674	0.105	0.061	0.0168730882505655	Endothelial	MAGI1
1.02153392084188E-06	0.775354004740651	0.114	0.208	0.0171188654454683	Endothelial	SART1
1.05119444821842E-06	0.888469890099683	0.092	0.178	0.0176159165632443	Endothelial	STRBP
1.05250042871222E-06	0.752652809113867	0.088	0.169	0.0176378021843593	Endothelial	PCNT
1.06791572676971E-06	0.720514769834626	0.095	0.181	0.0178961317492068	Endothelial	MAP2K1
1.07237705167213E-06	0.785385578870496	0.131	0.234	0.0179708946319215	Endothelial	WRNIP1
1.07868585705007E-06	0.361931330116984	0.064	0.136	0.018076617592445	Endothelial	ZNF76
1.07953508972311E-06	0.274632645952559	0.177	0.296	0.0180908490335799	Endothelial	GRK2
1.09930931262761E-06	0.405389168353564	0.11	0.2	0.0184222254610134	Endothelial	GSE1
1.1022793155383E-06	0.482191742728644	0.05	0.115	0.0184719967697908	Endothelial	TAB1
1.10414096882985E-06	0.745167458328502	0.089	0.173	0.0185031943556506	Endothelial	OXSR1
1.10484126892698E-06	0.723267592521205	0.117	0.212	0.0185149299846784	Endothelial	DHX36
1.10780545657976E-06	0.316587440834335	0.061	0.13	0.0185646038413637	Endothelial	TMEM126B
1.11013718607355E-06	0.927555913298962	0.08	0.16	0.0186036789642206	Endothelial	FAM120B
1.11613460991656E-06	0.652841905014591	0.196	0.332	0.0187041837929817	Endothelial	UPK3BL1
1.12797072203548E-06	0.449263446158301	0.074	0.15	0.0189025333598705	Endothelial	SF3B1
1.13061793215476E-06	1.79953034991934	0.268	0.228	0.0189468953070495	Endothelial	GNAI2
1.15405582341148E-06	0.437290636983852	0.077	0.153	0.0193396674887296	Endothelial	JMJD8
1.1760270193805E-06	0.612612395850667	0.072	0.147	0.0197078607907785	Endothelial	URM1

1.19418859759213E-06	0.497631839619962	0.066	0.137	0.020012212518449	Endothelial	SLC27A4
1.21649860759911E-06	0.756549960536134	0.055	0.122	0.020386083666146	Endothelial	ZCCHC8
1.23122477777567E-06	0.884207133133384	0.105	0.195	0.0206328648259646	Endothelial	CAMSAP2
1.24245815210453E-06	0.901472129062254	0.155	0.271	0.0208211137129678	Endothelial	NIPBL
1.25545514403988E-06	0.549162310034858	0.086	0.167	0.0210389173038204	Endothelial	USP33
1.26564067598452E-06	1.94625286704234	0.3	0.277	0.0212096064481486	Endothelial	MMP14
1.26569319806677E-06	0.796007474810786	0.125	0.224	0.0212104866132029	Endothelial	DDX42
1.26754965681656E-06	0.604072939071094	0.056	0.124	0.0212415971489318	Endothelial	HARS2
1.27262390680875E-06	2.67433356214676	0.124	0.078	0.021326631430301	Endothelial	RAI1
1.29842325803642E-06	0.969075298170288	0.045	0.108	0.0217589769581743	Endothelial	CC2D1B
1.32381950059268E-06	0.613926665269937	0.113	0.204	0.0221845671909322	Endothelial	MSRA
1.37519117100217E-06	0.589799923941409	0.113	0.205	0.0230454536436544	Endothelial	KMT2A
1.39853792266338E-06	0.80384302715809	0.114	0.209	0.023436698507993	Endothelial	TBC1D20
1.43855497096505E-06	0.826745157736759	0.153	0.266	0.0241073042034324	Endothelial	SEC16A
1.44848877400621E-06	0.706823464200167	0.058	0.127	0.0242737748747961	Endothelial	USP21
1.47102178384203E-06	0.504292023287706	0.058	0.126	0.0246513830536247	Endothelial	MTF2
1.47583286620114E-06	1.00749469233703	0.127	0.229	0.0247320071717987	Endothelial	REV1
1.48432340635881E-06	0.754151771313799	0.089	0.172	0.024874291643761	Endothelial	ARFRP1
1.48474305797717E-06	0.621701589560747	0.171	0.289	0.0248813241655815	Endothelial	C16orf72
1.50752867640936E-06	0.682589088435738	0.095	0.18	0.025263165559268	Endothelial	NAA38
1.53265087559124E-06	0.378406584035224	0.044	0.105	0.0256841633731581	Endothelial	LRSAM1
1.541191863894E-06	0.494041204993202	0.146	0.251	0.0258272932551357	Endothelial	TIAL1
1.55355154416713E-06	0.770552172442097	0.163	0.281	0.0260344167771528	Endothelial	CAV2
1.55835125718094E-06	0.271812706696118	0.078	0.153	0.0261148503678382	Endothelial	MAP2K3
1.5627057929261E-06	0.628504248426141	0.111	0.2	0.0261878236778556	Endothelial	RAB3GAP1
1.58394236644E-06	0.771998977204808	0.163	0.278	0.0265437061768014	Endothelial	PCED1A
1.60951028434743E-06	1.0600267583154	0.106	0.198	0.0269721733450943	Endothelial	FNIP1
1.61138851502408E-06	0.573067930534065	0.044	0.105	0.0270036487347735	Endothelial	ZNF395
1.61231278063757E-06	0.368859010091731	0.042	0.103	0.0270191375779243	Endothelial	GMEB2
1.61681037559834E-06	0.693573774126308	0.216	0.359	0.027094508274277	Endothelial	SAFB2
1.64652838345304E-06	2.51535272637965	0.205	0.16	0.027592522649906	Endothelial	ITGA1
1.75637511162802E-06	0.440824871177475	0.072	0.144	0.0294333341206623	Endothelial	AMBRA1
1.76214945402776E-06	0.831455913163587	0.106	0.196	0.0295301005505972	Endothelial	OTUD4
1.78287572008191E-06	0.630662284729608	0.056	0.124	0.0298774313171327	Endothelial	TTC21B
1.79380410080701E-06	0.628717917753367	0.052	0.116	0.0300605691213239	Endothelial	MAP4K3
1.86255530772733E-06	0.477622201819659	0.327	0.537	0.0312127018468946	Endothelial	SAT1
1.8908276332017E-06	0.511621419264659	0.053	0.118	0.0316864894771941	Endothelial	HELZ2
1.90500700343596E-06	0.726400808820668	0.103	0.189	0.0319241073635798	Endothelial	HP1BP3
1.9050106143238E-06	0.30407662082676	0.063	0.13	0.0319241678748382	Endothelial	EXT2
1.90682498709014E-06	0.646192691348926	0.08	0.156	0.0319545731336566	Endothelial	ZNF428
1.9095603765669E-06	0.707943513147945	0.064	0.134	0.0320004127905081	Endothelial	NFRKB
1.93430565100369E-06	0.486353563724174	0.049	0.111	0.0324150940995199	Endothelial	GTF2B
1.93688707111034E-06	0.818819819058098	0.081	0.159	0.032458353537667	Endothelial	TBC1D10B
1.94157962530346E-06	0.293216329990487	0.089	0.17	0.0325369913608354	Endothelial	FNBP1
1.94347863772013E-06	0.694079001923688	0.044	0.105	0.032568815010914	Endothelial	SIRT6
1.95394615619877E-06	0.393333230221299	0.081	0.158	0.032744229685579	Endothelial	PRR14
1.96891627848108E-06	0.955439953428747	0.171	0.292	0.0329950989947859	Endothelial	TAOK1
1.98131603486952E-06	1.05948919428504	0.146	0.254	0.0332028941123433	Endothelial	KIF13A
1.98995880953585E-06	0.324522368591993	0.08	0.156	0.0333477297302018	Endothelial	PIGS
1.99719580155904E-06	0.341685190275657	0.053	0.118	0.0334690072425264	Endothelial	FBXW8
2.00007172882689E-06	0.787559779429409	0.099	0.184	0.033517202031681	Endothelial	P4HA2
2.02033997238376E-06	0.387821340690539	0.042	0.101	0.033856857257207	Endothelial	TRIM23
2.03103336630905E-06	0.572744504325626	0.072	0.143	0.034036057152607	Endothelial	CUEDC2
2.03975284455673E-06	0.505635497553438	0.114	0.204	0.0341821781690817	Endothelial	CERK
2.04631803782186E-06	0.700389451577698	0.094	0.175	0.0342921976778187	Endothelial	PPP5C
2.06843255492424E-06	0.288057636482113	0.061	0.128	0.0346627927554205	Endothelial	SLC25A6
2.06863797796379E-06	0.384885504881241	0.059	0.126	0.0346662352347173	Endothelial	RBM28
2.1035981137791E-06	0.988215292566554	0.092	0.176	0.0352520971907102	Endothelial	BAG5
2.11434710338007E-06	0.529750125358539	0.052	0.115	0.0354322287584432	Endothelial	ALDH16A1
2.12249621804511E-06	0.598114539566809	0.288	0.467	0.0355687916219999	Endothelial	NDUFS2
2.13467172140328E-06	0.338103617822884	0.05	0.113	0.0357728287072761	Endothelial	ABCB7
2.14581468827814E-06	1.97041602267265	0.108	0.064	0.0359595625461651	Endothelial	RFX3
2.15842025049231E-06	0.781156661247663	0.103	0.19	0.0361708065577501	Endothelial	TRPM7

2.17288109118757E-06	0.518538387025518	0.221	0.361	0.0364131413261213	Endothelial	STX16
2.19250118256396E-06	0.427919532694922	0.047	0.108	0.0367419348174069	Endothelial	POLG2
2.19575065976101E-06	0.735480064845889	0.194	0.327	0.036796389556275	Endothelial	SRRM2
2.2057740385174E-06	0.497609321742043	0.147	0.251	0.0369643613374745	Endothelial	KIF1C
2.21866008899092E-06	0.262006401900656	0.064	0.133	0.0371803057713099	Endothelial	KMT5A
2.2219220739683E-06	0.689073538867191	0.081	0.158	0.0372349701155607	Endothelial	BCL7B
2.22436435545224E-06	0.645587129452284	0.075	0.148	0.0372758978686686	Endothelial	PIGO
2.23185743844575E-06	0.250403707372948	0.066	0.134	0.0374014669534739	Endothelial	WAPL
2.24361650424925E-06	0.830842646713403	0.07	0.142	0.0375985253782089	Endothelial	DCTN6
2.31329165441746E-06	0.689843265661628	0.092	0.174	0.0387661415447278	Endothelial	CLK4
2.31636784999235E-06	0.420050440052665	0.055	0.12	0.0388176924301717	Endothelial	TPST1
2.32247671824274E-06	2.51120669849148	0.124	0.079	0.0389200648443119	Endothelial	ZSWIM6
2.34008831352474E-06	0.895725919180625	0.066	0.136	0.0392151999580476	Endothelial	TAF1
2.37195111609532E-06	0.803777533878527	0.067	0.139	0.0397491568035254	Endothelial	CDC27
2.37705409054681E-06	0.358170763385211	0.069	0.139	0.0398346724493834	Endothelial	UBLCP1
2.38720846745999E-06	0.777907763361817	0.094	0.174	0.0400048394976945	Endothelial	CFAP298
2.45673829479083E-06	0.851844382850805	0.125	0.22	0.0411700203441047	Endothelial	ATP8B1
2.45785914981621E-06	0.763153421162377	0.061	0.128	0.0411888036326201	Endothelial	CSNK1G3
2.45951330037363E-06	0.306831260184781	0.058	0.124	0.0412165238876612	Endothelial	WDR43
2.4724948982097E-06	0.857357410900779	0.203	0.34	0.0414340695041982	Endothelial	NUMB
2.50811291921216E-06	2.29074114515579	0.183	0.135	0.0420309563001574	Endothelial	PITPNM2
2.55476777834538E-06	0.60106323601094	0.127	0.223	0.0428127984295119	Endothelial	ZC3H18
2.56660670677031E-06	0.453684955743346	0.072	0.143	0.0430111951920568	Endothelial	LRRC40
2.63743793179678E-06	0.574915464316161	0.08	0.155	0.0441981848610505	Endothelial	E4F1
2.64401539591572E-06	0.272659884047409	0.075	0.148	0.0443084100047557	Endothelial	ANKRD36C
2.65144053520074E-06	0.339084235922766	0.059	0.126	0.0444328404888939	Endothelial	JAK2
2.65476156337698E-06	0.511791176030624	0.103	0.187	0.0444884942790715	Endothelial	AKAP8L
2.65895682914881E-06	0.515467448102578	0.088	0.165	0.0445587985428758	Endothelial	SRM
2.69296971639664E-06	0.459883386694989	0.097	0.178	0.0451287865073748	Endothelial	FYTTD1
2.72890729356028E-06	0.603110123250658	0.141	0.241	0.0457310284254831	Endothelial	QARS
2.75188322245638E-06	1.81563455459637	0.293	0.268	0.0461160590419241	Endothelial	CYTH2
2.75284093251364E-06	0.543594884089053	0.171	0.283	0.0461321083470635	Endothelial	IFI6
2.77319435979698E-06	0.413071797018847	0.177	0.293	0.0464731910814778	Endothelial	CNOT6L
2.81955854430516E-06	1.17841014805861	0.437	0.476	0.0472501620854659	Endothelial	ITM2B
2.83024203809358E-06	0.932774743041705	0.156	0.27	0.0474291960743722	Endothelial	ZC3H15
2.8306438453519E-06	0.315118870181704	0.044	0.103	0.0474359295604072	Endothelial	UNC13B
2.85879728132655E-06	0.561734663635757	0.094	0.172	0.0479077248404703	Endothelial	CCM2
2.87654292576918E-06	0.851265290988906	0.091	0.171	0.04820510635004	Endothelial	TBCK
2.89782425737492E-06	0.279280925584872	0.135	0.232	0.0485617389050889	Endothelial	SMCHD1
2.9020777338155E-06	0.413751683520555	0.07	0.14	0.0486330186632802	Endothelial	BAG1
0	9.01690409885309	0.685	0.042	0	B/Plasma cell	IGHG1
0	8.939442305507	0.544	0.026	0	B/Plasma cell	DEPRECATED-ENSG00000211592
0	8.81597233364067	0.535	0.025	0	B/Plasma cell	IGKC
0	8.17709004837187	0.469	0.044	0	B/Plasma cell	IGLC2
0	8.52356992485858	0.302	0.009	0	B/Plasma cell	IGLC3
0	8.11289196385923	0.278	0.009	0	B/Plasma cell	IGHM
0	7.75455352762374	0.27	0.003	0	B/Plasma cell	CD79A
0	9.98232697117648	0.251	0.004	0	B/Plasma cell	IGHG2
0	7.65393239184759	0.25	0.004	0	B/Plasma cell	MZB1
0	7.46062811179765	0.24	0.004	0	B/Plasma cell	POU2AF1
0	7.43625869183704	0.225	0.004	0	B/Plasma cell	MS4A1
0	9.30461856087144	0.167	0	0	B/Plasma cell	FCRL5
0	8.48005288460732	0.146	0.003	0	B/Plasma cell	JCHAIN
0	8.06501028704745	0.116	0.001	0	B/Plasma cell	FCRL1
0	7.83232904598727	0.111	0.001	0	B/Plasma cell	NIBAN3
0	7.76675270669576	0.103	0.001	0	B/Plasma cell	IGHD
0	7.95586421235319	0.103	0.002	0	B/Plasma cell	CD22
2.99527748955938E-290	8.19181687160131	0.116	0.003	5.01948601700362E-286	B/Plasma cell	IGHG3
5.90161235654315E-275	2.727527906151	0.698	0.152	9.88992198709502E-271	B/Plasma cell	CXCR4
1.93380785647396E-240	6.70344769081289	0.118	0.004	3.24067520587907E-236	B/Plasma cell	BANK1
6.28723642153514E-184	6.8818387741507	0.191	0.017	1.05361507952086E-179	B/Plasma cell	TNFRSF13C
8.59407602435008E-183	7.64080687712676	0.105	0.005	1.44019526016059E-178	B/Plasma cell	IGHA1
1.01527815157737E-172	5.06356427010145	0.189	0.017	1.70140312641335E-168	B/Plasma cell	LY9

7.23579966527765E-162	5.1982700961127	0.143	0.01	1.21257530790723E-157	B/Plasma cell	CD79B
9.92693641098355E-148	2.58969679012101	0.413	0.088	1.66355600375262E-143	B/Plasma cell	CYTIP
1.22681512122128E-108	3.79402179921734	0.154	0.018	2.05589678014262E-104	B/Plasma cell	SLAMF7
1.89031762938871E-107	4.27215771814637	0.265	0.052	3.16779428332959E-103	B/Plasma cell	CD37
2.24188137895596E-103	5.13756576394633	0.114	0.01	3.7569448148544E-99	B/Plasma cell	P2RX5
4.97432791701664E-93	2.98703466371211	0.246	0.049	8.33597872333649E-89	B/Plasma cell	ACAP1
9.43262634879615E-93	2.71048977599332	0.238	0.046	1.58071952353126E-88	B/Plasma cell	LTB
2.9393846535138E-90	3.82057602591223	0.171	0.026	4.92582080235843E-86	B/Plasma cell	FCMR
3.93660333435852E-87	2.2661428206644	0.368	0.104	6.596959867718E-83	B/Plasma cell	CYBA
1.18072964352969E-81	4.19250071311646	0.332	0.101	1.97866673662705E-77	B/Plasma cell	PIM2
6.25126520368296E-81	4.51389403101878	0.137	0.019	1.04758702283319E-76	B/Plasma cell	SELL
9.06038492144029E-81	3.78489807736865	0.137	0.019	1.51833930513496E-76	B/Plasma cell	RASGRP2
3.69010564042174E-79	4.25260827423562	0.156	0.025	6.18387903221874E-75	B/Plasma cell	WDFY4
2.69136003145211E-72	3.17789836419321	0.173	0.031	4.51018114070744E-68	B/Plasma cell	SYTL1
2.39344970622063E-70	5.03252760983522	0.161	0.029	4.01094301768454E-66	B/Plasma cell	DERL3
5.60002126207193E-70	2.26496221436902	0.454	0.184	9.38451563098015E-66	B/Plasma cell	LAPTM5
3.50598712221544E-69	3.09294647083473	0.242	0.06	5.87533321940863E-65	B/Plasma cell	KLHL6
2.24630398601063E-64	2.28397484822065	0.266	0.073	3.76435621975662E-60	B/Plasma cell	IKZF1
1.0732659157753E-61	3.8573027907238	0.107	0.015	1.79857902165625E-57	B/Plasma cell	CD38
2.69127193057741E-58	3.0898057626283	0.161	0.033	4.51003350126162E-54	B/Plasma cell	CCDC88C
3.24531525152702E-57	1.25982223139322	0.863	0.84	5.43849929850898E-53	B/Plasma cell	CD74
1.35122877176446E-54	2.09240487926779	0.332	0.119	2.26438917572289E-50	B/Plasma cell	WIPF1
5.55946912503443E-54	1.94457893500265	0.302	0.1	9.3165583597327E-50	B/Plasma cell	LSP1
1.37265250600359E-53	4.43298478532367	0.158	0.035	2.30029106956082E-49	B/Plasma cell	HSH2D
1.31694450751647E-52	3.26452812915858	0.143	0.029	2.2069356056961E-48	B/Plasma cell	ATP2A3
3.79827759829142E-52	3.26356483215604	0.111	0.018	6.36515359921677E-48	B/Plasma cell	CARMIL2
9.04219214047278E-52	2.62036465455344	0.188	0.046	1.51529055890043E-47	B/Plasma cell	TAGAP
8.44318735824101E-51	2.84269358854889	0.189	0.048	1.41490933749403E-46	B/Plasma cell	ADAM19
3.55516894208228E-49	2.21411647000787	0.178	0.044	5.95775211314149E-45	B/Plasma cell	IKZF3
2.93296579730166E-47	3.79374058430597	0.118	0.022	4.91506408311812E-43	B/Plasma cell	PARP15
4.43859726123437E-47	3.12793655867455	0.186	0.05	7.43820129037656E-43	B/Plasma cell	IL16
1.20489524618971E-46	3.36273584396857	0.131	0.027	2.01916345356471E-42	B/Plasma cell	SP140
1.40922572475635E-45	3.02759754107192	0.246	0.083	2.36158046954669E-41	B/Plasma cell	ISG20
9.35708231630718E-45	1.55842097001404	0.407	0.184	1.56805985456676E-40	B/Plasma cell	VIM
3.40973878702222E-44	2.21137277337145	0.229	0.072	5.71404025929184E-40	B/Plasma cell	CORO1A
4.43694803013956E-42	3.05797455153762	0.199	0.061	7.43543750890787E-38	B/Plasma cell	TENT5C
3.59469271226334E-40	1.62176332604047	0.193	0.057	6.02398604721091E-36	B/Plasma cell	IL7R
4.38985819989076E-37	1.97804915248596	0.266	0.102	7.35652437137694E-33	B/Plasma cell	CELF2
1.17739401459415E-36	2.26204135700445	0.214	0.073	1.97307688965687E-32	B/Plasma cell	RAC2
1.04931061187365E-34	2.51224338451074	0.156	0.045	1.75843472337786E-30	B/Plasma cell	ARHGAP15
3.25534369780486E-34	2.75028785736026	0.197	0.068	5.45530496878139E-30	B/Plasma cell	ARHGAP45
3.67621091460011E-34	2.72702892372629	0.113	0.026	6.16059425068687E-30	B/Plasma cell	RHOH
6.44649684589956E-34	2.32283606082303	0.182	0.059	1.08030394143585E-29	B/Plasma cell	FMNL1
1.10487977192256E-33	2.55630856861	0.163	0.049	1.85155752178783E-29	B/Plasma cell	DEF6
5.0313301842863E-33	0.923731742665917	0.385	0.18	8.43150312282699E-29	B/Plasma cell	SRGN
2.13130800857786E-32	2.77648937669363	0.161	0.049	3.57164596077478E-28	B/Plasma cell	TMC8
4.78167844029395E-32	1.89969110018794	0.105	0.024	8.0131367302446E-28	B/Plasma cell	CD27
9.85668879434434E-32	2.56738031154183	0.154	0.046	1.65178390815622E-27	B/Plasma cell	JAK3
2.24076701317254E-31	3.08532322392682	0.114	0.029	3.75507736067453E-27	B/Plasma cell	BASP1
4.36966967616384E-31	2.72797701067063	0.126	0.034	7.32269244331536E-27	B/Plasma cell	TBC1D10C
7.69882429800831E-30	1.12228476253934	0.188	0.064	1.29016897586023E-25	B/Plasma cell	TRBC2
1.32424646797737E-29	2.44095076608383	0.189	0.068	2.21917223103648E-25	B/Plasma cell	DOCK2
1.32832150543657E-29	1.98279585991346	0.201	0.074	2.2260011788106E-25	B/Plasma cell	ITGA4
5.34055935401547E-29	2.87004480512376	0.165	0.056	8.94970936545912E-25	B/Plasma cell	LIMD2
2.07441391254228E-28	2.50210083369335	0.165	0.056	3.47630283463835E-24	B/Plasma cell	THEMIS2
2.09395774425535E-28	2.49401406884789	0.163	0.055	3.50905438782312E-24	B/Plasma cell	DENND3
6.29663212589068E-28	1.60267087726054	0.28	0.127	1.05518961165676E-23	B/Plasma cell	LCP1
7.67164797570805E-28	2.5711571609164	0.161	0.054	1.28561476776916E-23	B/Plasma cell	SEPTIN1
1.98200987436683E-27	3.03048841112614	0.141	0.044	3.32145214746393E-23	B/Plasma cell	FGD2
2.04994893598374E-27	2.71309948277918	0.152	0.05	3.43530442692156E-23	B/Plasma cell	SASH3
2.6241967067993E-27	2.24843904799609	0.137	0.042	4.39762884125427E-23	B/Plasma cell	DGKA
1.88791065841058E-26	2.83062663610803	0.111	0.03	3.16376068136445E-22	B/Plasma cell	TRAF3IP3
5.72113286358991E-26	2.22750333033473	0.126	0.037	9.58747445280397E-22	B/Plasma cell	MYO1G

5.75935014860672E-26	2.47692369772821	0.311	0.172	9.65151897903514E-22	B/Plasma cell	ITM2C
1.09194445337587E-25	2.21586161264249	0.169	0.061	1.82988051496728E-21	B/Plasma cell	ITPKB
2.99737371181372E-25	1.56695599402375	0.323	0.171	5.02299886625743E-21	B/Plasma cell	IL2RG
3.38624248320085E-25	0.364105243940198	0.096	0.334	5.67466515334799E-21	B/Plasma cell	MROH1
8.13043161492287E-25	1.73831326966515	0.568	0.486	1.36249773002877E-20	B/Plasma cell	TXNIP
8.32162956524916E-25	0.294629612495412	0.184	0.467	1.39453868254445E-20	B/Plasma cell	BRD2
1.7701671046484E-23	1.99730381338526	0.225	0.1	2.96644603396979E-19	B/Plasma cell	FXYD5
1.80147308892692E-23	2.65882275764682	0.169	0.065	3.01890860242374E-19	B/Plasma cell	GPSM3
2.080469528048E-23	2.33567357182805	0.103	0.029	3.48645083510285E-19	B/Plasma cell	PDE4B
4.48211129423374E-23	2.95595412761751	0.263	0.137	7.5111221068769E-19	B/Plasma cell	ARID5B
9.05368413255517E-23	2.51713238769413	0.118	0.037	1.5172163869336E-18	B/Plasma cell	PIK3CD
1.75090474072089E-22	0.313786865193442	0.158	0.412	2.93416616450007E-18	B/Plasma cell	CALCOCO2
2.14648228583692E-22	0.275180907312597	0.174	0.439	3.59707501460552E-18	B/Plasma cell	SNX1
2.68806268342051E-22	1.69216715023372	0.248	0.117	4.50465544487609E-18	B/Plasma cell	ARHGDIB
4.15277120958847E-22	0.329180025064203	0.114	0.339	6.95921399302837E-18	B/Plasma cell	PPP6R2
2.39776450727449E-21	0.304086019917451	0.066	0.26	4.01817376129059E-17	B/Plasma cell	URI1
3.358393157707E-21	0.34234433266464	0.113	0.33	5.62799525368539E-17	B/Plasma cell	ANKHD1
3.9934765802163E-21	0.641171716039543	0.098	0.309	6.69226805312647E-17	B/Plasma cell	RAB11FIP1
5.05173537062452E-21	0.405617089092959	0.129	0.355	8.46569813409258E-17	B/Plasma cell	FAM91A1
6.39663991477004E-21	2.82882205684808	0.184	0.08	1.07194891691716E-16	B/Plasma cell	BCL2
7.01272673643378E-21	0.353812756291079	0.116	0.336	1.17519274649157E-16	B/Plasma cell	TRIM27
7.98180398046235E-21	0.351043381748806	0.131	0.363	1.33759071104588E-16	B/Plasma cell	POLR2J3
7.99202194222736E-21	0.407664719502932	0.191	0.45	1.33930303707846E-16	B/Plasma cell	LAMTOR4
1.0642911241356E-20	0.273062295768627	0.173	0.418	1.78353906582644E-16	B/Plasma cell	UBXN4
1.32447456075148E-20	0.355103280896635	0.146	0.383	2.21955446890733E-16	B/Plasma cell	OPTN
1.84590930874061E-20	2.64898430448917	0.107	0.034	3.09337481958752E-16	B/Plasma cell	TCF4
2.70567015259368E-20	0.350931517252491	0.139	0.365	4.53416204171649E-16	B/Plasma cell	TMEM63A
3.42198996660227E-20	2.46971726819678	0.137	0.05	5.73457078603208E-16	B/Plasma cell	CD52
4.03398777881297E-20	0.275972722355365	0.131	0.353	6.76015671973478E-16	B/Plasma cell	G3BP2
4.93946874617075E-20	0.373819266940594	0.098	0.302	8.27756172483293E-16	B/Plasma cell	DNAJC21
5.83276867284267E-20	2.83152685014294	0.133	0.049	9.77455374194974E-16	B/Plasma cell	EVI2B
6.16070957670393E-20	0.330586754197711	0.114	0.325	1.03241171086404E-15	B/Plasma cell	PRPF3
6.72503995694624E-20	0.483979171888505	0.077	0.268	1.12698219598505E-15	B/Plasma cell	CUL4A
9.14857158063657E-20	0.264684425450293	0.113	0.319	1.53311762548308E-15	B/Plasma cell	ZBTB20
9.88594716898461E-20	2.7265744396442	0.133	0.049	1.65668702657844E-15	B/Plasma cell	INPP5D
1.2131658722123E-19	0.534739012546806	0.133	0.356	2.03302336865337E-15	B/Plasma cell	RNF19A
1.65543612053057E-19	0.263460524054234	0.12	0.333	2.77417985078513E-15	B/Plasma cell	MKNK2
1.85191511573363E-19	0.432379975231902	0.084	0.278	3.10343935094642E-15	B/Plasma cell	NPEPPS
1.9106230522435E-19	0.282689642904265	0.092	0.287	3.20182211094966E-15	B/Plasma cell	IDS
2.01695437139192E-19	2.44524292684253	0.244	0.129	3.38001213557857E-15	B/Plasma cell	TRIM22
2.33365894969885E-19	0.264241387734737	0.062	0.238	3.91074566790534E-15	B/Plasma cell	MAP3K2
3.06273487222476E-19	0.282173342302678	0.045	0.21	5.13253109887425E-15	B/Plasma cell	ATP6V0A1
3.25534181121079E-19	0.485400385653452	0.141	0.365	5.45530180722703E-15	B/Plasma cell	ASH1L
3.52022299742476E-19	0.472424104229202	0.139	0.361	5.89918969908442E-15	B/Plasma cell	LIMD1
3.61746599560301E-19	0.481519401374357	0.167	0.405	6.06214951543152E-15	B/Plasma cell	PJA2
3.74269868516927E-19	0.256480519310135	0.086	0.278	6.27201445660666E-15	B/Plasma cell	MPHOSPH8
3.82035473091606E-19	0.283471407558044	0.105	0.303	6.40215045806913E-15	B/Plasma cell	ZFP91
3.88891442399854E-19	0.504472425382781	0.086	0.277	6.51704279173676E-15	B/Plasma cell	KDM6A
4.09803036441485E-19	0.362571235171926	0.073	0.256	6.8674792846864E-15	B/Plasma cell	UBA1
4.22791501465913E-19	0.369859110872771	0.092	0.286	7.08513998156578E-15	B/Plasma cell	KPNA4
4.23232515836106E-19	0.298507224440202	0.066	0.243	7.09253050038147E-15	B/Plasma cell	TAF15
6.84057394946885E-19	0.259441951197175	0.069	0.247	1.14634338245199E-14	B/Plasma cell	TENT2
8.7140256210235E-19	0.349521724350388	0.094	0.286	1.46029641357112E-14	B/Plasma cell	TNRC6A
8.81531604609248E-19	0.363407761761162	0.088	0.277	1.47727066300418E-14	B/Plasma cell	TPP2
9.69776959478425E-19	1.69288230812483	0.146	0.056	1.62515222869394E-14	B/Plasma cell	ADA2
1.91646038867118E-18	0.413134113635628	0.099	0.293	3.21160431933516E-14	B/Plasma cell	RAB1B
2.19777726738436E-18	0.301269149002945	0.133	0.345	3.68303514468271E-14	B/Plasma cell	ESYT2
2.30066404307818E-18	2.36849194558686	0.139	0.054	3.85545280339042E-14	B/Plasma cell	ARHGAP9
2.40972582483532E-18	0.50249579371393	0.129	0.342	4.03821853725903E-14	B/Plasma cell	DIDO1
2.44897280495827E-18	0.346697751973736	0.128	0.335	4.10398862654907E-14	B/Plasma cell	SPG7
2.62584481821641E-18	0.492119387700586	0.103	0.3	4.40039074636706E-14	B/Plasma cell	ANKRD17
3.10592587482424E-18	0.421881351568012	0.152	0.374	5.20491058103046E-14	B/Plasma cell	CHTOP
3.26147554189742E-18	0.681837026920154	0.118	0.328	5.4655807131117E-14	B/Plasma cell	TRAF4

3.41244549572707E-18	0.294649868846345	0.107	0.301	5.71857616173943E-14	B/Plasma cell	KMT2B
3.73337393339755E-18	0.351366094741531	0.124	0.328	6.25638803758762E-14	B/Plasma cell	PPP3R1
3.95140542436478E-18	0.269315793207079	0.131	0.338	6.6217652101505E-14	B/Plasma cell	TRIP12
4.18198333466638E-18	1.79351399464856	0.103	0.034	7.00816767223391E-14	B/Plasma cell	CD69
4.64995817573816E-18	3.25334088541453	0.107	0.037	7.79239991090201E-14	B/Plasma cell	PRKCB
4.72063091634534E-18	0.33166687463187	0.079	0.259	7.91083328961153E-14	B/Plasma cell	DNAJC5
4.72678967065885E-18	0.350395516730166	0.139	0.348	7.9211541300901E-14	B/Plasma cell	HIPK2
4.76859675310498E-18	0.25899628847839	0.084	0.268	7.99121443885332E-14	B/Plasma cell	PRPF40A
5.77044186544409E-18	1.77810521802505	0.197	0.092	9.67010647811121E-14	B/Plasma cell	CD53
8.059143820149E-18	0.34538442311332	0.146	0.365	1.35055132138057E-13	B/Plasma cell	CSNK1D
8.07896153452109E-18	0.55021303896645	0.079	0.257	1.35387237395504E-13	B/Plasma cell	COLGALT1
9.04012255802508E-18	0.416985897618933	0.139	0.349	1.51494373827384E-13	B/Plasma cell	OXA1L
9.18142942412744E-18	0.250972182074808	0.058	0.222	1.53862394289528E-13	B/Plasma cell	DIP2A
9.24208334556248E-18	0.327325757991672	0.109	0.302	1.54878832704936E-13	B/Plasma cell	PPP4C
9.33715014024565E-18	0.270117799846265	0.107	0.302	1.56471962050237E-13	B/Plasma cell	UBE2V1
9.84432008823868E-18	0.383651610351209	0.09	0.27	1.64971116038704E-13	B/Plasma cell	TPR
1.23972696512394E-17	0.266522135549011	0.058	0.225	2.0775344481547E-13	B/Plasma cell	PPP1R12B
1.31419015526994E-17	0.280936864461727	0.12	0.316	2.20231986220136E-13	B/Plasma cell	ARHGDIA
1.32417075026793E-17	0.256101629247204	0.047	0.203	2.219045343299E-13	B/Plasma cell	ATF6
1.36914161633335E-17	0.283521840446708	0.148	0.362	2.29440752065143E-13	B/Plasma cell	ADD1
1.41869724174597E-17	0.483208259340121	0.242	0.505	2.3774528377179E-13	B/Plasma cell	WNK1
1.42280475187927E-17	1.90063222119726	0.218	0.108	2.38433620319929E-13	B/Plasma cell	IFI16
1.45131563470731E-17	0.531104128993087	0.092	0.277	2.43211474064251E-13	B/Plasma cell	KMT5B
1.4553072697795E-17	3.00869127856239	0.263	0.158	2.43880392269649E-13	B/Plasma cell	BTG2
1.4656364408635E-17	2.1371168069075	0.163	0.071	2.45611354759905E-13	B/Plasma cell	UCP2
1.50650896276909E-17	0.260244542551378	0.058	0.22	2.52460771980844E-13	B/Plasma cell	RCOR3
1.53388803097494E-17	0.438178055889459	0.111	0.305	2.57048956230781E-13	B/Plasma cell	AFF4
1.74349799431477E-17	0.500984026940774	0.06	0.223	2.9217539388727E-13	B/Plasma cell	PAPOLA
1.95446357821989E-17	0.30870659091377	0.073	0.242	3.27529006438089E-13	B/Plasma cell	MCMBP
2.11505368790177E-17	0.303096787157107	0.098	0.283	3.54440697018579E-13	B/Plasma cell	PTBP3
2.20440377919724E-17	0.346774517184645	0.131	0.332	3.69413985317874E-13	B/Plasma cell	UPK3BL1
2.22597928602791E-17	0.271802528175971	0.064	0.229	3.73029608752557E-13	B/Plasma cell	NBN
2.27858348049075E-17	0.380495906732815	0.212	0.454	3.81845019660639E-13	B/Plasma cell	EEF1G
3.07082478627998E-17	0.306891033623118	0.111	0.295	5.14608817684799E-13	B/Plasma cell	GNB1
3.09958716781267E-17	0.32944441696962	0.163	0.385	5.19428817582048E-13	B/Plasma cell	DCAF8
3.12423914795778E-17	0.397843127334861	0.111	0.3	5.23559996414765E-13	B/Plasma cell	MAT2B
3.47638285190767E-17	0.298779421140649	0.062	0.223	5.82572238322687E-13	B/Plasma cell	FXR1
3.58379648437931E-17	0.321507826175536	0.128	0.326	6.00572614852286E-13	B/Plasma cell	RNF114
3.745989064407E-17	0.323490020907327	0.114	0.309	6.27752847413326E-13	B/Plasma cell	ATF6B
3.81105581979326E-17	0.564207338516926	0.099	0.283	6.38656734280954E-13	B/Plasma cell	DNAJB2
4.14154978844336E-17	0.485935977492284	0.113	0.301	6.94040913547339E-13	B/Plasma cell	PCBP1
4.211673116543E-17	1.51669135029173	0.199	0.093	7.05792180870276E-13	B/Plasma cell	HCLS1
4.41859342385134E-17	0.303736972866525	0.083	0.256	7.40467885969008E-13	B/Plasma cell	WWOX
4.88406564244705E-17	0.266998715103447	0.068	0.233	8.18471720361276E-13	B/Plasma cell	DDX56
5.07627688609481E-17	0.411754853740322	0.086	0.262	8.50682480571768E-13	B/Plasma cell	RBM14
5.22565613968781E-17	0.359958580925089	0.184	0.412	8.75715455888883E-13	B/Plasma cell	IRF3
5.26616694307297E-17	0.631881002464651	0.073	0.242	8.82504256320168E-13	B/Plasma cell	SRRT
5.27742078826247E-17	0.304753193286162	0.054	0.211	8.84390175697025E-13	B/Plasma cell	ZFYVE28
5.35040648104288E-17	0.59042182711469	0.096	0.277	8.96621118093166E-13	B/Plasma cell	ZBTB7A
5.68010008281649E-17	0.326769718031816	0.069	0.235	9.51871171878387E-13	B/Plasma cell	SEMA4B
6.41896469070719E-17	1.62232611401973	0.152	0.064	1.07569010286871E-12	B/Plasma cell	MPEG1
7.40673894064165E-17	0.371030474717333	0.092	0.273	1.24122131167273E-12	B/Plasma cell	SIPA1L3
7.89006225139149E-17	0.636782441250325	0.116	0.312	1.32221663208819E-12	B/Plasma cell	CGGBP1
8.39865369409926E-17	0.484875031706933	0.094	0.274	1.40744638605715E-12	B/Plasma cell	DEDD
1.01133016494695E-16	0.366466214730958	0.212	0.453	1.6947870904181E-12	B/Plasma cell	RACK1
1.08628796083449E-16	0.350689232131259	0.092	0.266	1.82040136476643E-12	B/Plasma cell	MGRN1
1.12207575660039E-16	0.448127787265157	0.171	0.384	1.88037455291094E-12	B/Plasma cell	EMC10
1.16688098191401E-16	0.603512039646373	0.133	0.336	1.9554591494915E-12	B/Plasma cell	RAB1A
1.2502035742851E-16	0.569904910385423	0.105	0.291	2.09509114978698E-12	B/Plasma cell	AGO2
1.25053709627061E-16	0.45631452301227	0.118	0.309	2.09565006593029E-12	B/Plasma cell	ZMYM2
1.36461896268525E-16	0.415775961597451	0.079	0.247	2.28682845766794E-12	B/Plasma cell	ABCF1
1.41924225779758E-16	0.286904535614393	0.143	0.342	2.37836617561718E-12	B/Plasma cell	CCNI
1.50984453019882E-16	0.342457028628595	0.083	0.252	2.53019746370718E-12	B/Plasma cell	EXOC7

1.64631010733275E-16	0.304853654316378	0.038	0.179	2.75888647786822E-12	B/Plasma cell	CCNK
1.65391256780287E-16	0.537431910659503	0.141	0.344	2.77162668112405E-12	B/Plasma cell	KIAA0100
1.76997157312385E-16	0.465504542071455	0.159	0.367	2.96611836224095E-12	B/Plasma cell	TMED4
1.79572116941976E-16	0.289985448928237	0.049	0.198	3.00926953571364E-12	B/Plasma cell	WBP1
1.95193444648125E-16	0.282021079847785	0.109	0.292	3.27105174541328E-12	B/Plasma cell	TAOK1
1.98585303134747E-16	0.512236920323507	0.216	0.459	3.32789250993209E-12	B/Plasma cell	MAPKAPK2
2.10460548199105E-16	0.375972320599129	0.096	0.273	3.5268978667206E-12	B/Plasma cell	SDHA
2.22917926353925E-16	0.261545789390685	0.148	0.352	3.73565860983907E-12	B/Plasma cell	RAB8A
2.33590101122843E-16	0.374711261215935	0.126	0.318	3.9145029146166E-12	B/Plasma cell	DDX46
2.42120454007138E-16	0.262883952252062	0.174	0.39	4.05745456825162E-12	B/Plasma cell	CAPZB
2.47754816238981E-16	2.71933794566411	0.176	0.085	4.15187521053284E-12	B/Plasma cell	PLCG2
2.87293976419527E-16	0.358118097449874	0.143	0.344	4.81447245683844E-12	B/Plasma cell	SEC22B
3.00152660967969E-16	0.422765299076159	0.086	0.257	5.02995829250122E-12	B/Plasma cell	NUP43
3.08080790900458E-16	0.519103619439162	0.079	0.246	5.16281789390987E-12	B/Plasma cell	EIF2B5
3.09859216881138E-16	2.20165008124314	0.111	0.041	5.19262075649411E-12	B/Plasma cell	RASAL3
3.10336944907138E-16	0.371182981594674	0.09	0.26	5.20062652275381E-12	B/Plasma cell	PRPF6
3.40679721295536E-16	0.398715159224991	0.064	0.221	5.7091107694706E-12	B/Plasma cell	ATG12
3.5061781345443E-16	0.41015275720654	0.094	0.266	5.87565331786933E-12	B/Plasma cell	PTBP1
3.51455121830852E-16	0.466279390980024	0.084	0.255	5.88968493164141E-12	B/Plasma cell	PI4KA
3.93349781915067E-16	0.460687113069493	0.039	0.182	6.5917556453327E-12	B/Plasma cell	SMPD4
4.17197094244932E-16	0.250903381853179	0.159	0.366	6.99138890535657E-12	B/Plasma cell	HNRNPC
4.22245520801501E-16	0.632198524971765	0.045	0.192	7.07599043759156E-12	B/Plasma cell	GTF3C3
4.47578956108292E-16	0.387638273668196	0.098	0.272	7.50052814646275E-12	B/Plasma cell	HMCES
4.56990608029379E-16	0.2521045299802	0.084	0.251	7.65824860935633E-12	B/Plasma cell	FRYL
4.58863384917126E-16	0.324143724963738	0.131	0.319	7.6896326044412E-12	B/Plasma cell	PKN1
4.83473886325347E-16	0.299014185513237	0.081	0.245	8.10205538704017E-12	B/Plasma cell	VPS35L
4.86901524359023E-16	0.314873329153461	0.255	0.524	8.1594957452085E-12	B/Plasma cell	AHNAK
4.99046133472707E-16	0.260924119948096	0.084	0.252	8.36301510473562E-12	B/Plasma cell	CIAO1
5.29198536864729E-16	0.386490686559734	0.156	0.359	8.86830908077913E-12	B/Plasma cell	NUFIP2
5.37397485618552E-16	0.402004657323423	0.231	0.473	9.0057070639957E-12	B/Plasma cell	CTSH
5.46343450011559E-16	0.55966852976691	0.141	0.343	9.1556235352937E-12	B/Plasma cell	ENSA
5.47758278744402E-16	1.53868138163508	0.129	0.051	9.17933323519869E-12	B/Plasma cell	SPOCK2
5.67485919243577E-16	0.262866552899827	0.09	0.258	9.50992903468386E-12	B/Plasma cell	ZMYND8
6.46939054613804E-16	0.4782315076012	0.118	0.304	1.08414046772181E-11	B/Plasma cell	LIX1L
6.63504003245918E-16	0.496466680874822	0.098	0.273	1.11190000863951E-11	B/Plasma cell	RHOT2
6.77657077644366E-16	0.2780918433145	0.062	0.215	1.13561773071643E-11	B/Plasma cell	NELFB
8.15129050684247E-16	0.63517751318556	0.161	0.37	1.36599326313666E-11	B/Plasma cell	POGZ
8.26821133234414E-16	0.488194512452382	0.077	0.235	1.38558685507423E-11	B/Plasma cell	MAN2A2
8.40926606436313E-16	0.459835802867307	0.178	0.392	1.40922480706597E-11	B/Plasma cell	SMARCA2
8.53108811494855E-16	0.741172772822856	0.109	0.289	1.42963974630308E-11	B/Plasma cell	RBM26
8.5441210005919E-16	0.480221176928946	0.111	0.29	1.43182379727919E-11	B/Plasma cell	SLC20A1
8.67424650934765E-16	0.275432677482053	0.099	0.272	1.45363023003648E-11	B/Plasma cell	CYTH2
9.24423898189973E-16	0.460835129166242	0.308	0.601	1.54914956858676E-11	B/Plasma cell	HNRNPA2B1
1.01560652473192E-15	0.457503825173608	0.107	0.289	1.70195341414575E-11	B/Plasma cell	NUB1
1.14631618883805E-15	0.544252721402764	0.116	0.302	1.92099666925481E-11	B/Plasma cell	TMUB2
1.15537427131688E-15	0.27020535348942	0.18	0.392	1.93617620387283E-11	B/Plasma cell	CNBP
1.19475998744327E-15	0.617693715503342	0.114	0.296	2.00217878695743E-11	B/Plasma cell	ZFR
1.20557994853016E-15	0.34750480729519	0.208	0.444	2.02031087774685E-11	B/Plasma cell	KLF6
1.21672931388342E-15	0.361182994064195	0.128	0.315	2.03899498420584E-11	B/Plasma cell	MKLN1
1.22188584566179E-15	0.272308505740013	0.135	0.327	2.04763630016003E-11	B/Plasma cell	BIRC6
1.26995810341969E-15	0.603507490560172	0.116	0.3	2.12819578971071E-11	B/Plasma cell	ZNF644
1.30000593862238E-15	0.28870998669595	0.077	0.238	2.17854995194339E-11	B/Plasma cell	DICER1
1.4310039428456E-15	0.549775387391607	0.137	0.333	2.39807640742066E-11	B/Plasma cell	U2SURP
1.45647435677995E-15	0.448525086725564	0.113	0.292	2.44075972709185E-11	B/Plasma cell	PPP1CB
1.53428158073672E-15	0.449159326841715	0.135	0.327	2.5711490729986E-11	B/Plasma cell	RNF10
1.65278973052003E-15	2.80495794092895	0.176	0.088	2.76974503040546E-11	B/Plasma cell	CD83
1.65502246569605E-15	0.590499496860017	0.073	0.232	2.77348664801345E-11	B/Plasma cell	WDTC1
1.72682565832397E-15	0.261625208881421	0.096	0.267	2.89381443821931E-11	B/Plasma cell	NADK
1.7406467519783E-15	0.692442711655832	0.18	0.393	2.91697582696524E-11	B/Plasma cell	SELENOH
1.95237217543909E-15	0.44439910680318	0.092	0.259	3.27178529160083E-11	B/Plasma cell	DYM
2.00013367533047E-15	0.436960698292127	0.099	0.269	3.35182401311879E-11	B/Plasma cell	GOSR2
2.20284128883667E-15	0.323370333199288	0.098	0.267	3.6915214318325E-11	B/Plasma cell	EXOC3
2.23355217078612E-15	0.539222282079266	0.075	0.231	3.74298672780338E-11	B/Plasma cell	KPNA1

2.31370180785656E-15	0.350432110151727	0.084	0.245	3.87730148960602E-11	B/Plasma cell	RBM3
2.56316702318893E-15	0.739495984332391	0.098	0.267	4.29535529746001E-11	B/Plasma cell	DNAJC8
2.79451454387568E-15	0.440103234441372	0.083	0.243	4.68304747262686E-11	B/Plasma cell	SET
3.07151774405322E-15	0.766217812857456	0.109	0.286	5.14724943548438E-11	B/Plasma cell	NCLN
3.0801530516787E-15	0.536512978486958	0.066	0.215	5.16172048400316E-11	B/Plasma cell	LARP4B
3.1078040682282E-15	0.854467413598616	0.098	0.266	5.20805805753682E-11	B/Plasma cell	RUFY1
3.46924548985405E-15	0.517552019862974	0.094	0.259	5.81376159189742E-11	B/Plasma cell	LRBA
3.56865407486417E-15	0.273826587271104	0.083	0.24	5.98035049865737E-11	B/Plasma cell	TSPAN33
3.80060891203043E-15	0.331719395786153	0.12	0.298	6.36906041478059E-11	B/Plasma cell	SEC11A
3.86873156897096E-15	0.451305053632762	0.124	0.307	6.48322036328154E-11	B/Plasma cell	NUS1
4.07275470952476E-15	0.512347849307001	0.111	0.286	6.8251223422216E-11	B/Plasma cell	PSPC1
4.12480360342181E-15	0.384458736223238	0.066	0.215	6.91234587861427E-11	B/Plasma cell	UGGT1
4.21213239167235E-15	0.402585153744526	0.118	0.296	7.05869146196452E-11	B/Plasma cell	SPG21
4.35796232428206E-15	0.462404126845859	0.075	0.23	7.30307326303188E-11	B/Plasma cell	REV1
4.71161917249353E-15	0.564136611885691	0.171	0.378	7.89573140926466E-11	B/Plasma cell	ARF6
4.81492207920484E-15	1.93904094221707	0.111	0.042	8.06884642033147E-11	B/Plasma cell	CRIP1
4.84645595452088E-15	0.577849810579012	0.086	0.249	8.1216908885861E-11	B/Plasma cell	KHDC4
5.35603578517645E-15	0.593665285668276	0.083	0.242	8.9756447687987E-11	B/Plasma cell	MRFAP1L1
5.48605573362188E-15	0.371731558750065	0.079	0.235	9.19353219840354E-11	B/Plasma cell	GNL1
5.58278300495751E-15	0.473596100366397	0.088	0.248	9.3556277597078E-11	B/Plasma cell	STRN4
5.68761863227518E-15	0.37014143302924	0.096	0.26	9.53131130396674E-11	B/Plasma cell	AP3B1
5.76123401875333E-15	0.453562094693577	0.054	0.197	9.65467596862683E-11	B/Plasma cell	OTUD4
6.13365096003559E-15	0.289493445024158	0.096	0.262	1.02787722788276E-10	B/Plasma cell	ADAM10
6.37024695242233E-15	2.34294462732303	0.405	0.332	1.06752598428693E-10	B/Plasma cell	SMAP2
7.12395681657673E-15	0.625822238566573	0.126	0.31	1.19383268332193E-10	B/Plasma cell	NCOR1
7.26974508988659E-15	0.502945254650403	0.221	0.457	1.2182638821632E-10	B/Plasma cell	HNRNPA0
7.80468576344193E-15	0.723132859713798	0.12	0.301	1.3079092402376E-10	B/Plasma cell	PNN
8.48637842850222E-15	0.298079532652612	0.03	0.156	1.4221472970484E-10	B/Plasma cell	TMEM8B
8.66437561945975E-15	0.390949975797405	0.099	0.267	1.45197606630907E-10	B/Plasma cell	MED25
9.21340306462296E-15	0.569361200677957	0.111	0.282	1.54398208556952E-10	B/Plasma cell	KANSL1
9.29878083382141E-15	2.32454314515131	0.176	0.088	1.55828969213179E-10	B/Plasma cell	PTK2B
9.50365249709862E-15	0.645166667199887	0.073	0.225	1.59262208546379E-10	B/Plasma cell	INO80E
1.09722245869344E-14	0.510437964614282	0.111	0.283	1.83872539627846E-10	B/Plasma cell	CEP350
1.18443077177724E-14	0.409373429499551	0.135	0.32	1.9848690873443E-10	B/Plasma cell	TOMM7
1.24369913647341E-14	0.808635974017196	0.158	0.36	2.08419101290214E-10	B/Plasma cell	SAFB2
1.29548561179058E-14	2.75953892280643	0.218	0.124	2.17097478823865E-10	B/Plasma cell	STK17A
1.32109548747079E-14	0.272609402763242	0.073	0.225	2.21389181790355E-10	B/Plasma cell	TTC17
1.35698775104513E-14	0.364418187582549	0.068	0.216	2.27404007320144E-10	B/Plasma cell	PURB
1.44176351898295E-14	0.378834289053251	0.068	0.213	2.41610730511163E-10	B/Plasma cell	CNPPD1
1.53434146769629E-14	0.307823929814789	0.071	0.222	2.57124943156544E-10	B/Plasma cell	CHD4
1.58729237262516E-14	0.4409664157437	0.088	0.248	2.65998455804524E-10	B/Plasma cell	CREBBP
1.60137941159896E-14	0.535155279888014	0.152	0.348	2.68359161795754E-10	B/Plasma cell	KAT6A
1.63460826445178E-14	0.628890180712481	0.109	0.28	2.73927652956829E-10	B/Plasma cell	MED28
1.67331453320588E-14	0.491271298034808	0.092	0.253	2.80414049474641E-10	B/Plasma cell	NCAPH2
1.6872169203283E-14	0.267574948429138	0.043	0.175	2.82743811508617E-10	B/Plasma cell	C2CD5
1.72006671463095E-14	0.317539704912634	0.049	0.185	2.88248780037855E-10	B/Plasma cell	ABCD4
1.74669014550243E-14	0.757980612400356	0.129	0.311	2.92710334583297E-10	B/Plasma cell	FZR1
1.75517725142768E-14	0.328215124416607	0.077	0.23	2.9413260379425E-10	B/Plasma cell	MTR
1.92125031912587E-14	0.281603687881438	0.062	0.204	3.21963128479113E-10	B/Plasma cell	STARD3
1.97993031032434E-14	0.480184873308945	0.053	0.191	3.31796721404154E-10	B/Plasma cell	MAPKAPK5
1.98169205503824E-14	0.440932965664507	0.064	0.208	3.32091954583309E-10	B/Plasma cell	LSM 8.00
1.98457138092953E-14	0.335200470620542	0.116	0.289	3.32574472016171E-10	B/Plasma cell	MAP4
2.03946919153756E-14	0.629401007376337	0.073	0.224	3.41774247117864E-10	B/Plasma cell	NELFA
2.052431133022E-14	0.357854579436886	0.054	0.193	3.43946409271827E-10	B/Plasma cell	CHD9
2.17624767635203E-14	0.437373902485959	0.084	0.239	3.64695585603073E-10	B/Plasma cell	AP1B1
2.33475229752074E-14	0.534878572134041	0.053	0.191	3.91257790018525E-10	B/Plasma cell	TFCP2
2.46603406108214E-14	0.437936145735493	0.203	0.419	4.13257987956145E-10	B/Plasma cell	IKBKB
2.49104864226727E-14	0.543758103999736	0.203	0.417	4.17449931471148E-10	B/Plasma cell	SELENOF
2.53102523235801E-14	0.403328790717257	0.171	0.367	4.24149208438556E-10	B/Plasma cell	MDM2
2.56792837760268E-14	0.455406858669178	0.077	0.229	4.30333437518657E-10	B/Plasma cell	LEMD2
2.59859654124501E-14	0.259678332528897	0.056	0.193	4.35472808381838E-10	B/Plasma cell	EIF2AK2
2.60961237392504E-14	0.770849108407747	0.105	0.271	4.37318841622357E-10	B/Plasma cell	STK11
2.62912588692011E-14	0.647284643485701	0.118	0.292	4.40588916130072E-10	B/Plasma cell	TRPC4AP

2.67981707199414E-14	0.496761397404956	0.15	0.34	4.49083744924778E-10	B/Plasma cell	TNIP1
2.72559047411155E-14	0.578703752534071	0.045	0.177	4.56754451651613E-10	B/Plasma cell	VPS41
2.77255536793015E-14	0.470016567978125	0.088	0.243	4.64624828557734E-10	B/Plasma cell	AEBP2
2.78509539774481E-14	0.612402645200259	0.158	0.353	4.66726286754076E-10	B/Plasma cell	DNAJA2
2.85269545518653E-14	0.310833334374387	0.088	0.243	4.78054704380158E-10	B/Plasma cell	DENND1A
3.09047277504624E-14	0.503635570575891	0.084	0.239	5.1790142764225E-10	B/Plasma cell	MTA1
3.11005446206887E-14	0.303138504435289	0.068	0.211	5.21182926753502E-10	B/Plasma cell	TMOD3
3.19326079202997E-14	0.426165204956183	0.088	0.248	5.35126643528382E-10	B/Plasma cell	UBE2G2
3.22266792164228E-14	1.10105396309492	0.206	0.107	5.40054690308814E-10	B/Plasma cell	RGS1
3.58783350143051E-14	0.524981212279818	0.077	0.228	6.01249138169724E-10	B/Plasma cell	PIP4P1
3.6381258065782E-14	0.300989929360152	0.084	0.237	6.09677122666374E-10	B/Plasma cell	TIMM44
3.68298795132216E-14	0.494530594195643	0.073	0.221	6.17195120882567E-10	B/Plasma cell	SIN3B
3.7127388444256E-14	0.349127446314353	0.124	0.298	6.22180775548842E-10	B/Plasma cell	ESYT1
3.75836867730716E-14	0.803127132447287	0.143	0.333	6.29827422943133E-10	B/Plasma cell	INTS11
3.78845648126232E-14	0.485374096491625	0.109	0.275	6.34869537129939E-10	B/Plasma cell	FUS
3.81476561493074E-14	0.275442531689494	0.06	0.2	6.39278421750093E-10	B/Plasma cell	GTPBP6
3.91313297330433E-14	0.533488114451065	0.068	0.212	6.5576282366634E-10	B/Plasma cell	FAM172A
3.99804188836985E-14	0.372939499858549	0.066	0.207	6.6999185965302E-10	B/Plasma cell	EFTUD2
4.02505556561187E-14	0.622028610985703	0.139	0.321	6.74518811685237E-10	B/Plasma cell	SUPT5H
4.10325517819126E-14	0.609850887880085	0.088	0.244	6.87623502761291E-10	B/Plasma cell	ARL8A
4.10488835962961E-14	0.283365696167804	0.054	0.192	6.87897191306729E-10	B/Plasma cell	NAA15
4.16016229329707E-14	0.288712701802258	0.056	0.194	6.97159997110723E-10	B/Plasma cell	PHKG2
4.27648658584543E-14	0.48478444068867	0.054	0.188	7.16653622055976E-10	B/Plasma cell	NUP214
4.28466142532217E-14	0.641434806462562	0.154	0.346	7.1802356165549E-10	B/Plasma cell	TACC1
4.33591463837744E-14	0.500457558444457	0.083	0.237	7.26612575099292E-10	B/Plasma cell	RNF168
4.36755613812259E-14	0.35917872171286	0.075	0.22	7.31915057626583E-10	B/Plasma cell	SNX9
4.36899858047112E-14	0.288032171639846	0.062	0.201	7.3215678211535E-10	B/Plasma cell	SQOR
4.48519942421828E-14	0.695861188129279	0.083	0.237	7.516297195105E-10	B/Plasma cell	GIGYF1
4.56071693751988E-14	0.431173020934204	0.088	0.24	7.64284944389582E-10	B/Plasma cell	TXNDC12
4.60563997618812E-14	0.391134735878921	0.066	0.208	7.71813147209606E-10	B/Plasma cell	ACO2
4.68833230170515E-14	0.761520868682712	0.077	0.225	7.85670727119749E-10	B/Plasma cell	DDX42
4.76752292903845E-14	0.458184801519883	0.053	0.188	7.98941492448264E-10	B/Plasma cell	RAB4B
4.86730155828545E-14	0.665484915798642	0.152	0.342	8.15662395137476E-10	B/Plasma cell	KMT2C
4.96673294700718E-14	0.784430679081892	0.133	0.316	8.32325107259464E-10	B/Plasma cell	XRN1
5.21013314473784E-14	0.294135295021528	0.084	0.235	8.73114112395168E-10	B/Plasma cell	PHF12
5.30522387568095E-14	0.714670315423369	0.068	0.212	8.89049417086614E-10	B/Plasma cell	TMEM131
5.37205295147875E-14	0.762413807368854	0.122	0.295	9.00248633608809E-10	B/Plasma cell	THRAP3
5.49483299430845E-14	0.747688418860676	0.079	0.231	9.2082411318621E-10	B/Plasma cell	PTAR1
5.79778079854784E-14	0.466353426714204	0.113	0.283	9.71592106220647E-10	B/Plasma cell	ZNF217
5.81123600093335E-14	0.815353685195871	0.188	0.403	9.7384692903641E-10	B/Plasma cell	EML4
6.06274147550113E-14	0.642258101857493	0.101	0.261	1.01599421646448E-09	B/Plasma cell	KCTD20
6.08074904247725E-14	0.553296926151649	0.094	0.251	1.01901192453834E-09	B/Plasma cell	BAZ1B
6.09352248777634E-14	0.438497751153644	0.058	0.195	1.02115249850156E-09	B/Plasma cell	TELO2
6.20307029807119E-14	0.529182772332834	0.073	0.218	1.03951052055077E-09	B/Plasma cell	GTF2H1
6.223502226224E-14	0.514947448496156	0.103	0.266	1.04293450307062E-09	B/Plasma cell	MTIF3
6.29891679049875E-14	0.565574171413981	0.111	0.275	1.05557247575178E-09	B/Plasma cell	LRCH3
6.4840639191519E-14	0.489114395249492	0.051	0.183	1.08659943157148E-09	B/Plasma cell	MED6
6.50039534563134E-14	0.316134074211864	0.071	0.213	1.0893362520209E-09	B/Plasma cell	NUP153
6.6088334498027E-14	0.581767648101659	0.056	0.193	1.10750830951794E-09	B/Plasma cell	POLK
6.93829088190095E-14	2.44219595275322	0.118	0.049	1.16271878598896E-09	B/Plasma cell	PARVG
7.54501555250403E-14	0.920252295456607	0.109	0.275	1.26439370628862E-09	B/Plasma cell	KMT2D
7.61426408375263E-14	0.346175527439411	0.066	0.206	1.27599837515527E-09	B/Plasma cell	NUP42
7.68367006274105E-14	0.451261834838668	0.099	0.259	1.28762942911414E-09	B/Plasma cell	IFNGR1
8.00936827416362E-14	0.727638376594846	0.135	0.314	1.34220993538434E-09	B/Plasma cell	CDK9
8.92763829945156E-14	0.76753566924619	0.109	0.275	1.49609362622209E-09	B/Plasma cell	CPSF7
9.0225922007853E-14	0.440436819253169	0.064	0.202	1.5120060010076E-09	B/Plasma cell	TUBGCP2
9.16747854537569E-14	0.534057221304756	0.075	0.219	1.53628605463406E-09	B/Plasma cell	HTT
9.37492446088423E-14	0.272847577165019	0.09	0.241	1.57104984115498E-09	B/Plasma cell	PET100
9.95654033573986E-14	0.262975509323415	0.049	0.179	1.66851702946329E-09	B/Plasma cell	HDAC3
9.97339014594922E-14	0.362035066522261	0.092	0.245	1.67134072065817E-09	B/Plasma cell	DEPRECATED-ENSG00000124831
1.0257781377526E-13	0.441630615809158	0.086	0.237	1.7189990032458E-09	B/Plasma cell	TRRAP
1.05014982889847E-13	0.608432547563207	0.088	0.242	1.75984108326806E-09	B/Plasma cell	ZNF146
1.08397229148592E-13	0.783887906794663	0.129	0.305	1.8165207660721E-09	B/Plasma cell	STXBP2

1.13477252096249E-13	0.381358327915165	0.103	0.264	1.90165179062895E-09	B/Plasma cell	PITPNB	
1.15735382126051E-13	0.252737472381174	0.212	0.43	1.93949353366836E-09	B/Plasma cell	SYNE2	
1.18002120522949E-13	0.276236539664034	0.041	0.165	1.97747953572358E-09	B/Plasma cell	RAB3GAP2	
1.18475312761581E-13	0.474183403014768	0.045	0.171	1.98540929125858E-09	B/Plasma cell		LSM 2.00
1.19075919515589E-13	0.403259675733934	0.098	0.257	1.99547425924225E-09	B/Plasma cell	VHL	
1.19078760348413E-13	0.400994071611224	0.118	0.288	1.9955218659187E-09	B/Plasma cell	UBE2H	
1.23775239474381E-13	0.315870116193297	0.092	0.246	2.07422546311167E-09	B/Plasma cell	ZFAND2B	
1.25558799529068E-13	0.494005876437963	0.049	0.179	2.10411436250813E-09	B/Plasma cell	FYTTD1	
1.2700700123276E-13	0.765899551111324	0.165	0.361	2.12838332665886E-09	B/Plasma cell	NKTR	
1.28876644033388E-13	0.252464113920796	0.066	0.204	2.15971480071151E-09	B/Plasma cell	MED23	
1.29320741144744E-13	1.98825267390182	0.221	0.123	2.16715698010362E-09	B/Plasma cell	ANXA6	
1.31140871834277E-13	0.444059868831376	0.069	0.21	2.19765873019881E-09	B/Plasma cell	SLC25A38	
1.32152298676599E-13	0.303851914914039	0.073	0.216	2.21460822122245E-09	B/Plasma cell	DNAJC15	
1.33059876480059E-13	0.679875886202217	0.184	0.389	2.22981741005284E-09	B/Plasma cell	PER1	
1.33866594753672E-13	0.497395340857499	0.167	0.361	2.24333639488203E-09	B/Plasma cell	STX16	
1.35319412969086E-13	0.386516842856236	0.079	0.225	2.26768272253594E-09	B/Plasma cell	DELE1	
1.36689232778281E-13	0.288244906808497	0.051	0.179	2.29063816289844E-09	B/Plasma cell	RETREG2	
1.42394137742038E-13	0.57879953815215	0.081	0.228	2.38624096028107E-09	B/Plasma cell	CNOT8	
1.43380565310077E-13	0.355628761881464	0.051	0.179	2.40277151346626E-09	B/Plasma cell	AHI1	
1.46261060318395E-13	0.712646054920697	0.079	0.228	2.45104284881567E-09	B/Plasma cell		LSM 7.00
1.46790163299152E-13	0.464166732113697	0.077	0.222	2.45990955656719E-09	B/Plasma cell	DMXL1	
1.48722909695604E-13	0.542993668064685	0.103	0.263	2.49229852067893E-09	B/Plasma cell	OGFR	
1.50276192824902E-13	0.444716810856883	0.066	0.204	2.5183284393597E-09	B/Plasma cell	SLC41A2	
1.54973853625761E-13	0.275707078522349	0.041	0.165	2.59705183906051E-09	B/Plasma cell	RNF141	
1.59356517269393E-13	0.68067817318163	0.152	0.339	2.67049651640049E-09	B/Plasma cell	SAFB	
1.61073077579824E-13	0.616130421666912	0.169	0.363	2.69926263408269E-09	B/Plasma cell	PLEKHF2	
1.63897524690669E-13	0.277968315688453	0.116	0.283	2.74659471876623E-09	B/Plasma cell	CLPTM1L	
1.64923301467201E-13	0.408643665899729	0.064	0.2	2.76378468598736E-09	B/Plasma cell	GTPBP2	
1.71892869013752E-13	0.737545452555233	0.062	0.198	2.88058069893246E-09	B/Plasma cell	NSMAF	
1.74511959273183E-13	0.667900087957159	0.116	0.285	2.92447141349999E-09	B/Plasma cell	RBM23	
1.81042883442437E-13	0.412383736838334	0.124	0.293	3.03391664072836E-09	B/Plasma cell	NRBP1	
1.81760964114226E-13	0.45374678468561	0.113	0.274	3.04595023662621E-09	B/Plasma cell	DNAJC7	
1.86410329777539E-13	0.874267433370363	0.071	0.213	3.123864306412E-09	B/Plasma cell	DHX36	
1.94595192040255E-13	0.655060120234123	0.118	0.285	3.26102622821059E-09	B/Plasma cell	HNRNPUL2	
1.97634303297599E-13	0.368596768645115	0.011	0.114	3.31195565466117E-09	B/Plasma cell	MYO7B	
1.9806977728086E-13	0.279243176414883	0.053	0.183	3.31925332767266E-09	B/Plasma cell	ZNF121	
2.03200880620367E-13	0.32469831277705	0.066	0.203	3.4052403574361E-09	B/Plasma cell	SNX10	
2.08784842192411E-13	0.766561684976615	0.131	0.305	3.49881638546043E-09	B/Plasma cell	PRRC2C	
2.22353742111774E-13	0.293584114620555	0.039	0.161	3.7262040103091E-09	B/Plasma cell	PAXBP1	
2.22787938163946E-13	0.753422453259729	0.101	0.26	3.7334802677514E-09	B/Plasma cell	SKI	
2.27179520136647E-13	0.636965855839857	0.133	0.309	3.80707439844993E-09	B/Plasma cell	TMEM263	
2.32668337470739E-13	0.528507688781052	0.083	0.228	3.89905599933464E-09	B/Plasma cell	RELCH	
2.3701598681501E-13	0.755281040770403	0.081	0.227	3.97191390704594E-09	B/Plasma cell	API5	
2.43251459567081E-13	1.00160264794443	0.073	0.214	4.07640795942515E-09	B/Plasma cell	MTERF4	
2.45258814894005E-13	0.804819382998034	0.098	0.255	4.11004721999374E-09	B/Plasma cell	IWS1	
2.64697459396471E-13	0.549912161335742	0.054	0.184	4.43580002456606E-09	B/Plasma cell	PSIP1	
2.66709455020436E-13	0.478735270148893	0.118	0.284	4.46951704723246E-09	B/Plasma cell	ATXN7L3B	
2.69838590374038E-13	0.337129828695015	0.101	0.254	4.52195509748814E-09	B/Plasma cell	RUNX1	
2.83537624618861E-13	0.503639727154091	0.128	0.297	4.75152351336287E-09	B/Plasma cell	COPB1	
2.8625575018462E-13	0.578731851356793	0.114	0.278	4.79707386159386E-09	B/Plasma cell	FBXL3	
2.87732207877444E-13	0.311005967481857	0.064	0.198	4.8218163396102E-09	B/Plasma cell	PDHB	
2.90731915633622E-13	0.792952681497128	0.084	0.234	4.87208544218823E-09	B/Plasma cell	NR1H2	
2.93329274439994E-13	0.283438757679042	0.054	0.184	4.91561198106542E-09	B/Plasma cell	VPS13D	
2.99652349828752E-13	0.548915367775394	0.214	0.428	5.02157407843022E-09	B/Plasma cell	CCNL1	
3.00569960309953E-13	0.257803782560446	0.098	0.252	5.03695139487419E-09	B/Plasma cell	ZBTB1	
3.08722539674208E-13	0.561101074860717	0.081	0.226	5.17357231986037E-09	B/Plasma cell	RANBP9	
3.26366398757428E-13	0.536266287222601	0.131	0.305	5.46924811037699E-09	B/Plasma cell	GPBP1L1	
3.31631441100555E-13	0.464318967002997	0.081	0.224	5.5574796899631E-09	B/Plasma cell	ZNF800	
3.43616426465302E-13	0.715513217876747	0.169	0.362	5.75832407470554E-09	B/Plasma cell	CPNE1	
3.45100196030987E-13	1.86765451281844	0.139	0.064	5.78318908508728E-09	B/Plasma cell	PTPRC	
3.59576027121539E-13	0.821771618531712	0.116	0.278	6.02577506250275E-09	B/Plasma cell	PARP14	
3.69637193887388E-13	0.339011146548766	0.045	0.167	6.19438009516484E-09	B/Plasma cell	BZW1	
3.90307233123861E-13	0.860507619851928	0.088	0.237	6.54076861268966E-09	B/Plasma cell	CLDND1	

3.98376651056429E-13	0.329728954319159	0.105	0.263	6.67599591840363E-09	B/Plasma cell	EPRS
4.04705648008E-13	0.559733103633917	0.053	0.181	6.78205724931807E-09	B/Plasma cell	DENND4B
4.09143461974459E-13	0.720271730322407	0.073	0.216	6.85642613576799E-09	B/Plasma cell	ECHDC1
4.12337884346853E-13	0.327991044961346	0.051	0.178	6.90995826588456E-09	B/Plasma cell	RRP1
4.12896155321049E-13	0.435087697693761	0.255	0.487	6.91931377087015E-09	B/Plasma cell	TPP1
4.16337037746377E-13	0.313069872868498	0.058	0.188	6.97697607855379E-09	B/Plasma cell	MCM3AP
4.30621197816651E-13	0.508712373966828	0.079	0.221	7.21635003301144E-09	B/Plasma cell	DNMT3A
4.36287324719669E-13	0.411127555048039	0.068	0.202	7.31130298765221E-09	B/Plasma cell	ATG2B
4.3859780017581E-13	0.450799737468903	0.047	0.17	7.35002193534622E-09	B/Plasma cell	RSPRY1
4.46464228452978E-13	0.27924852685934	0.039	0.157	7.481847540415E-09	B/Plasma cell	NUP188
4.51881885308737E-13	0.573107127735849	0.148	0.326	7.57263663400381E-09	B/Plasma cell	LMBRD1
4.57045705524478E-13	0.889505194629279	0.094	0.246	7.65917193317921E-09	B/Plasma cell	TLK1
4.70804256634405E-13	0.318473687195114	0.045	0.167	7.88973773267935E-09	B/Plasma cell	ANAPC7
4.70942205189696E-13	0.785735415187859	0.128	0.295	7.89204947456892E-09	B/Plasma cell	B4GALT1
4.76495032797108E-13	0.452157138845717	0.098	0.249	7.98510375961394E-09	B/Plasma cell	WDR33
5.05195639691292E-13	0.469157709233258	0.081	0.223	8.46606852994667E-09	B/Plasma cell	ZC3H18
5.19335576110168E-13	0.400295855620229	0.041	0.162	8.7030255844542E-09	B/Plasma cell	MYSM1
5.21950962223609E-13	0.250182925923088	0.028	0.139	8.74685422494325E-09	B/Plasma cell	HAUS2
5.40386879571148E-13	0.550039377553704	0.06	0.19	9.0558033278533E-09	B/Plasma cell	UVSSA
5.4422527333894E-13	0.537282543224018	0.086	0.231	9.12012713061396E-09	B/Plasma cell	CRK
5.80739329033601E-13	0.919581251495544	0.081	0.223	9.73202967594509E-09	B/Plasma cell	ZNF33A
5.85637764005017E-13	0.556475547875371	0.101	0.254	9.81411764919608E-09	B/Plasma cell	SMAD2
6.01590171881714E-13	0.435111097366177	0.077	0.215	1.00814481003938E-08	B/Plasma cell	UBR3
6.22355655635437E-13	0.297911016451206	0.054	0.179	1.04294360771386E-08	B/Plasma cell	CMTR1
6.24159284008046E-13	0.671582639225752	0.176	0.371	1.04596612814068E-08	B/Plasma cell	MBNL1
6.53518167316024E-13	0.266323570572674	0.096	0.245	1.09516574478819E-08	B/Plasma cell	TRMT112
6.60326919498167E-13	0.673662057093303	0.081	0.223	1.10657585169503E-08	B/Plasma cell	KPNA6
6.62382081183437E-13	0.811190615418342	0.118	0.284	1.1100198916472E-08	B/Plasma cell	CPSF6
6.81354710223875E-13	0.59580250484589	0.114	0.276	1.14181422339317E-08	B/Plasma cell	SPEN
7.49967996382203E-13	0.483868564013157	0.086	0.23	1.2567963683373E-08	B/Plasma cell	PRPF38B
7.59279266106312E-13	0.49553536711911	0.073	0.21	1.27240019414096E-08	B/Plasma cell	CDK13
7.69215011986556E-13	0.462152174828464	0.09	0.237	1.28905051708707E-08	B/Plasma cell	ARID4B
7.71934048725034E-13	0.302780049878523	0.054	0.181	1.29360707885341E-08	B/Plasma cell	SEH1L
7.85116685185519E-13	0.361603083690668	0.111	0.268	1.31569854103389E-08	B/Plasma cell	IRF2
8.15233093658676E-13	0.574715233044875	0.126	0.29	1.36616761835321E-08	B/Plasma cell	MED15
8.39112395341786E-13	0.30048668037108	0.066	0.199	1.40618455211376E-08	B/Plasma cell	GTPBP4
8.39556264273044E-13	0.307320884823684	0.032	0.144	1.40692838766877E-08	B/Plasma cell	MIB2
8.49551087258007E-13	0.452162615111943	0.291	0.539	1.42367771202697E-08	B/Plasma cell	CIRBP
8.75878746974874E-13	0.296366344832141	0.068	0.2	1.46779760418049E-08	B/Plasma cell	CYHR1
9.20687375002163E-13	2.11152957469916	0.248	0.156	1.54288790302862E-08	B/Plasma cell	STK17B
9.57108614581753E-13	2.56893138245316	0.12	0.053	1.6039226163161E-08	B/Plasma cell	DOCK11
1.02416326658249E-12	0.438724076372353	0.154	0.331	1.71629280213893E-08	B/Plasma cell	NRDC
1.02893602898157E-12	1.29148237357246	0.128	0.056	1.72429099736731E-08	B/Plasma cell	S100A6
1.03773748439419E-12	0.57718677972609	0.092	0.24	1.73904047634778E-08	B/Plasma cell	ELMO1
1.05573100240516E-12	0.889409677505608	0.083	0.226	1.76919401383057E-08	B/Plasma cell	PTGES2
1.06382422770579E-12	0.379993522574294	0.045	0.166	1.78275664078936E-08	B/Plasma cell	EIF2B1
1.07211290248632E-12	0.572682175397688	0.075	0.211	1.79664680198657E-08	B/Plasma cell	SIPA1L1
1.08945750693372E-12	0.490029186394833	0.053	0.175	1.82571289011953E-08	B/Plasma cell	SEPTIN7
1.14415031969561E-12	1.30978424794066	0.15	0.071	1.91736710574591E-08	B/Plasma cell	PRDM1
1.1728328343583E-12	0.410692745631938	0.075	0.21	1.96543326381764E-08	B/Plasma cell	HNRNPR
1.2791940708429E-12	0.522527003717401	0.073	0.207	2.14367342391854E-08	B/Plasma cell	CSNK1G2
1.30707228036463E-12	0.264099588695124	0.054	0.179	2.19039172743504E-08	B/Plasma cell	ANKS1A
1.30821894984444E-12	0.624592168942174	0.069	0.203	2.19231331614931E-08	B/Plasma cell	SORL1
1.32918323790751E-12	0.682339716513232	0.054	0.179	2.2274452700854E-08	B/Plasma cell	SETDB1
1.37668875380169E-12	0.401520208550176	0.137	0.303	2.30705501362087E-08	B/Plasma cell	RAB21
1.45012781024184E-12	0.442455581254294	0.086	0.228	2.43012418440327E-08	B/Plasma cell	XAB2
1.47206703379669E-12	1.04175727693634	0.116	0.277	2.46688993523649E-08	B/Plasma cell	PMPCB
1.51867341357478E-12	0.534908223312807	0.101	0.251	2.54499290646861E-08	B/Plasma cell	NR3C1
1.61081346954079E-12	0.48838846368964	0.088	0.231	2.69940121225646E-08	B/Plasma cell	PIP5K1A
1.66662953126281E-12	0.334013553042602	0.094	0.238	2.79293776849021E-08	B/Plasma cell	RSBN1L
1.7074158974681E-12	0.665670100622545	0.101	0.251	2.86128756097705E-08	B/Plasma cell	KDM4B
1.71463851558096E-12	0.399728011732875	0.051	0.173	2.87339122441057E-08	B/Plasma cell	SLC39A3
1.73710807595216E-12	0.553132331313537	0.049	0.17	2.91104571368064E-08	B/Plasma cell	SPTLC2

1.77558515108687E-12	0.647966763414954	0.081	0.222	2.97552559619137E-08	B/Plasma cell	TBC1D15
1.80116925400133E-12	0.346920785809909	0.062	0.189	3.01839943585543E-08	B/Plasma cell	TMEM165
1.80168566622572E-12	0.358521252087318	0.083	0.222	3.01926483946106E-08	B/Plasma cell	SGPP1
1.81171533833297E-12	0.295718374415137	0.064	0.194	3.03607256397839E-08	B/Plasma cell	SS18L1
1.85881426990919E-12	0.654438086734254	0.079	0.216	3.11500095351383E-08	B/Plasma cell	OXR1
1.90876731906944E-12	0.725151298316422	0.056	0.182	3.19871227329656E-08	B/Plasma cell	PIGG
2.01565787270062E-12	0.274916091853534	0.051	0.173	3.3778394630717E-08	B/Plasma cell	OGFOD2
2.03810268028804E-12	0.424393695575037	0.118	0.273	3.41545247162669E-08	B/Plasma cell	RNF4
2.05769185568801E-12	0.502193190228398	0.051	0.173	3.44828001176196E-08	B/Plasma cell	CAMTA1
2.08230162462048E-12	0.286711273990375	0.045	0.162	3.489521062539E-08	B/Plasma cell	TOR3A
2.09203353633367E-12	0.258619454176603	0.034	0.144	3.50582980018797E-08	B/Plasma cell	FANCA
2.12390537442117E-12	0.300194308649572	0.069	0.2	3.559240626455E-08	B/Plasma cell	TNKS
2.17183297097042E-12	0.279342518883818	0.053	0.175	3.63955769275222E-08	B/Plasma cell	NANS
2.17944909925161E-12	0.465646453051721	0.09	0.233	3.65232080052585E-08	B/Plasma cell	RC3H1
2.20783798495283E-12	0.574499122977228	0.073	0.206	3.69989489518395E-08	B/Plasma cell	PRPF31
2.32094317955867E-12	0.395339907947073	0.09	0.232	3.88943658030442E-08	B/Plasma cell	RETREG3
2.37422452590499E-12	0.755167489776982	0.116	0.275	3.97872546051158E-08	B/Plasma cell	RAP1A
2.43098760653167E-12	0.83928408580583	0.088	0.233	4.07384903102578E-08	B/Plasma cell	ATXN2L
2.47744629108518E-12	0.362441406264792	0.043	0.158	4.15170449460055E-08	B/Plasma cell	MIGA1
2.52846549605523E-12	0.725729348606831	0.099	0.246	4.23720247828935E-08	B/Plasma cell	AFTPH
2.57375244476347E-12	0.509292781394138	0.062	0.188	4.31309434693463E-08	B/Plasma cell	SF1
2.60762575888538E-12	0.678676896918509	0.064	0.191	4.36985924674012E-08	B/Plasma cell	TRAPPC12
2.61530847688349E-12	0.519225562547232	0.111	0.263	4.38273394556136E-08	B/Plasma cell	STIM1
2.62714216809329E-12	0.278575950839123	0.079	0.213	4.40256484529074E-08	B/Plasma cell	PRMT2
2.64065853677532E-12	0.501700707525688	0.032	0.141	4.42521557592808E-08	B/Plasma cell	ZNF561
2.65081639149456E-12	1.04632257364532	0.118	0.278	4.44223810886659E-08	B/Plasma cell	CMIP
2.7209967814011E-12	0.390326724918713	0.165	0.344	4.55984640627197E-08	B/Plasma cell	SUGP2
2.8611230433544E-12	0.443173696900031	0.086	0.226	4.7946699960533E-08	B/Plasma cell	PRRC2B
2.86165894424117E-12	0.517307736635131	0.054	0.177	4.79556805875936E-08	B/Plasma cell	SNRNP40
2.97730466063063E-12	0.827831101554234	0.034	0.144	4.98936715028481E-08	B/Plasma cell	PPP1R37
3.00064108588808E-12	0.381932025570666	0.038	0.15	5.02847433173125E-08	B/Plasma cell	AGO3
3.1030026513981E-12	0.674331494136293	0.099	0.248	5.20001184321294E-08	B/Plasma cell	CIC
3.11006515979733E-12	0.724558981664254	0.15	0.324	5.21184719478837E-08	B/Plasma cell	MINK1
3.11976415440237E-12	0.355090590658982	0.036	0.146	5.22810076994748E-08	B/Plasma cell	INPP5A
3.12628041040228E-12	0.515919805200949	0.084	0.222	5.23902071175215E-08	B/Plasma cell	KAT7
3.22739823238231E-12	0.539052184069277	0.071	0.203	5.40847395782628E-08	B/Plasma cell	EPB41
3.26293627254858E-12	0.879480798781837	0.131	0.295	5.46802860553691E-08	B/Plasma cell	CCDC50
3.36246124572227E-12	0.373051983748854	0.056	0.18	5.63481255558139E-08	B/Plasma cell	PAN2
3.40706095221251E-12	0.643722047670416	0.09	0.229	5.70955274371773E-08	B/Plasma cell	QTRT1
3.42346343813814E-12	0.582015519306892	0.081	0.216	5.7370400296319E-08	B/Plasma cell	CDK12
3.4309423912569E-12	0.280962574489724	0.043	0.159	5.74957325926831E-08	B/Plasma cell	BCL7B
3.47961514222691E-12	0.5111569194891	0.086	0.225	5.83113905534386E-08	B/Plasma cell	HERC4
3.49229757136931E-12	0.559037338165634	0.086	0.224	5.85239227010069E-08	B/Plasma cell	RPRD1A
3.53771637810831E-12	0.962277355225612	0.075	0.208	5.92850510643391E-08	B/Plasma cell	TAPT1
3.58622656704986E-12	0.558949816725277	0.056	0.178	6.00979848106215E-08	B/Plasma cell	CTCF
3.66483957397149E-12	0.292670823671594	0.039	0.152	6.14153815806142E-08	B/Plasma cell	ZFX
3.69199850277362E-12	0.280718762564044	0.058	0.18	6.18705109094802E-08	B/Plasma cell	ADSL
3.85369178149521E-12	0.48650623799258	0.068	0.196	6.45801668742967E-08	B/Plasma cell	RPA1
3.94889323484569E-12	0.500263009653624	0.09	0.23	6.61755528295441E-08	B/Plasma cell	DYRK1A
3.9712538602129E-12	0.741712488666873	0.073	0.204	6.65502721894479E-08	B/Plasma cell	RFFL
3.97276591423579E-12	0.959342562556846	0.141	0.311	6.65756111907634E-08	B/Plasma cell	SENP6
4.04344627980109E-12	0.38273015181931	0.054	0.175	6.77600727569068E-08	B/Plasma cell	PLCXD1
4.10398933197689E-12	0.641271283329668	0.09	0.229	6.87746532252687E-08	B/Plasma cell	MON2
4.11685039782756E-12	0.670250407641424	0.137	0.306	6.89901789667942E-08	B/Plasma cell	RBL2
4.23494523883221E-12	0.26933008285266	0.054	0.174	7.09692123123502E-08	B/Plasma cell	SNHG32
4.25801147952635E-12	0.591863622408932	0.135	0.296	7.13557563739026E-08	B/Plasma cell	MBNL2
4.35608961396034E-12	0.377489134100181	0.066	0.192	7.29993497507473E-08	B/Plasma cell	NTMT1
4.37474875766283E-12	0.623688782861713	0.152	0.327	7.33120396809138E-08	B/Plasma cell	SNRNP70
4.39339628974779E-12	0.588930063704414	0.144	0.317	7.36245350235935E-08	B/Plasma cell	TCIRG1
4.51925814582353E-12	0.812575233802796	0.199	0.404	7.57337280077107E-08	B/Plasma cell	KDM2A
4.54654176562304E-12	0.250367482049265	0.036	0.145	7.61909469083109E-08	B/Plasma cell	DCAKD
4.58902573544334E-12	0.723475012587196	0.073	0.205	7.69028932745595E-08	B/Plasma cell	CHERP
4.61540931872051E-12	0.808247017066082	0.114	0.268	7.73450293631184E-08	B/Plasma cell	GAK

4.68168631779359E-12	0.724508696225752	0.054	0.175	7.84556993135851E-08	B/Plasma cell	TCF20
4.74124014518783E-12	0.338267926556985	0.039	0.151	7.94537023530577E-08	B/Plasma cell	PDXDC1
4.78356850775735E-12	0.970600553321249	0.058	0.182	8.01630410529977E-08	B/Plasma cell	SSU72
4.81773499893389E-12	0.528773419699639	0.039	0.153	8.07356031121341E-08	B/Plasma cell	MTREX
5.10020064011573E-12	0.881106409936912	0.124	0.284	8.54691623270594E-08	B/Plasma cell	NEK7
5.10080943228882E-12	0.741964117500616	0.109	0.261	8.54793644662961E-08	B/Plasma cell	KLHDC4
5.25600291065337E-12	0.576134424208297	0.064	0.189	8.80800967767292E-08	B/Plasma cell	KHNYN
5.34157640998539E-12	0.264999597576355	0.118	0.275	8.95141374785352E-08	B/Plasma cell	CRELD2
5.37693890208738E-12	0.339860058581023	0.068	0.194	9.01067421211803E-08	B/Plasma cell	NUDT22
5.5290551299084E-12	0.588831410212305	0.094	0.237	9.2655905867005E-08	B/Plasma cell	RUFY3
5.71203146543957E-12	0.350053124968129	0.038	0.147	9.57222232978364E-08	B/Plasma cell	ANAPC2
5.85004231517046E-12	0.409534622151977	0.041	0.153	9.80350091176266E-08	B/Plasma cell	NAA30
5.98550426749586E-12	0.916675293986408	0.186	0.378	1.00305080514696E-07	B/Plasma cell	ELOVL5
6.21384901085178E-12	0.415512077990236	0.053	0.171	1.04131681723854E-07	B/Plasma cell	TBCK
6.24778669643495E-12	0.270625188730229	0.047	0.162	1.04700409458857E-07	B/Plasma cell	RHBDD2
6.54078350277596E-12	0.697019327857322	0.086	0.223	1.09610449939519E-07	B/Plasma cell	PMM2
6.55684845270657E-12	0.785600140451104	0.077	0.212	1.09879666370457E-07	B/Plasma cell	ATG4B
6.74901975285381E-12	0.268958222870185	0.077	0.208	1.13100073018324E-07	B/Plasma cell	ISY1
6.89373380364948E-12	0.680893337973012	0.034	0.141	1.15525191081558E-07	B/Plasma cell	CCNYL1
6.89588327708728E-12	0.559481882712793	0.079	0.211	1.15561211957429E-07	B/Plasma cell	CBFA2T2
6.97449513147762E-12	0.62689981730896	0.189	0.384	1.16878589413302E-07	B/Plasma cell	TMEM259
7.0821420430403E-12	0.775397770941527	0.124	0.279	1.18682536357269E-07	B/Plasma cell	EP300
7.28188402329638E-12	0.405401007638866	0.039	0.15	1.22029812462401E-07	B/Plasma cell	PPID
7.34693102089873E-12	0.381365739859484	0.049	0.163	1.23119870048221E-07	B/Plasma cell	YEATS2
7.43513499672438E-12	0.444313936556844	0.051	0.167	1.24597992275107E-07	B/Plasma cell	LRRFIP1
7.69941870113278E-12	0.51449609590163	0.032	0.138	1.29026858593583E-07	B/Plasma cell	USP40
7.70566318667738E-12	0.320787033290694	0.077	0.207	1.2913150368234E-07	B/Plasma cell	EPC1
8.36720846646597E-12	0.425691747922536	0.041	0.152	1.40217679481037E-07	B/Plasma cell	TRMT2A
8.42974149296805E-12	0.476856165827283	0.103	0.249	1.41265607939159E-07	B/Plasma cell	IFNAR2
8.51299366239147E-12	3.05256323716887	0.291	0.215	1.42660747794356E-07	B/Plasma cell	IRF8
9.24447092176791E-12	0.748979175555079	0.167	0.35	1.54918843706987E-07	B/Plasma cell	PILRB
9.36561466563029E-12	0.750383316426232	0.189	0.378	1.56948970566632E-07	B/Plasma cell	BIRC3
9.40540575375069E-12	0.719633126075432	0.069	0.197	1.57615789621354E-07	B/Plasma cell	TMEM167B
9.41661581427185E-12	0.344318842345539	0.041	0.15	1.57803647815568E-07	B/Plasma cell	TTC33
9.53640043035275E-12	0.537701830977539	0.096	0.236	1.59810998411851E-07	B/Plasma cell	SON
9.58005855372507E-12	0.664295895999028	0.075	0.207	1.60542621243325E-07	B/Plasma cell	ZNF91
1.05626941080596E-11	0.323207948704802	0.066	0.189	1.77009627862863E-07	B/Plasma cell	PEX11B
1.05661552533537E-11	0.610209248885526	0.083	0.217	1.77067629735701E-07	B/Plasma cell	YY1
1.08426955945825E-11	0.334791211887842	0.066	0.187	1.81701892774014E-07	B/Plasma cell	STXBP3
1.09904744801922E-11	0.515592865441587	0.143	0.306	1.84178371339062E-07	B/Plasma cell	SLC17A9
1.10314702618385E-11	0.874979514031885	0.135	0.3	1.8486537864789E-07	B/Plasma cell	SRSF5
1.10424223872937E-11	0.30183113396488	0.06	0.181	1.85048914366267E-07	B/Plasma cell	SLC35C2
1.1135086989781E-11	0.82906483409179	0.079	0.209	1.86601787774749E-07	B/Plasma cell	ARHGAP17
1.18700336534679E-11	0.67163165170029	0.081	0.212	1.98918023964815E-07	B/Plasma cell	BABAM1
1.19650640234459E-11	0.298066517426276	0.047	0.159	2.00510542904906E-07	B/Plasma cell	ATG7
1.21121948499427E-11	0.394169649081812	0.079	0.209	2.02976161295339E-07	B/Plasma cell	SMC3
1.21984968053846E-11	2.36311703419823	0.118	0.054	2.04422409464636E-07	B/Plasma cell	GSTP1
1.25634867845329E-11	0.525500626134324	0.103	0.249	2.10538911535202E-07	B/Plasma cell	E2F4
1.25899207955297E-11	0.862708140423561	0.152	0.322	2.10981892691487E-07	B/Plasma cell	TOP 1.00
1.31339875943997E-11	0.505561616814513	0.062	0.184	2.20099364106949E-07	B/Plasma cell	MANBAL
1.34864107089469E-11	0.66342407545093	0.109	0.258	2.26005270660532E-07	B/Plasma cell	PCM1
1.36536660542115E-11	0.720151695340026	0.053	0.17	2.28808135736476E-07	B/Plasma cell	TP53I13
1.36917493582854E-11	0.677337748397274	0.15	0.318	2.29446335746147E-07	B/Plasma cell	TIA1
1.36958512682323E-11	0.300016222770109	0.051	0.165	2.29515075553037E-07	B/Plasma cell	TMEM138
1.40210283370695E-11	0.660003194660917	0.086	0.218	2.34964392872611E-07	B/Plasma cell	ABI1
1.43718724481565E-11	0.289337088243758	0.034	0.137	2.40843838486206E-07	B/Plasma cell	BAD
1.43749106039968E-11	0.410157272870667	0.083	0.215	2.40894751901778E-07	B/Plasma cell	ADSS
1.4500218459226E-11	0.271148670322482	0.075	0.202	2.4299466093971E-07	B/Plasma cell	ZCCHC3
1.45698532652601E-11	0.703719747206753	0.101	0.242	2.44161601019228E-07	B/Plasma cell	SUMO2
1.49555601353561E-11	0.338461282908199	0.079	0.208	2.50625276748298E-07	B/Plasma cell	EXOC5
1.49904678440308E-11	0.804427900602653	0.12	0.274	2.51210260130267E-07	B/Plasma cell	SYMPK
1.51435021723365E-11	0.789501066454368	0.107	0.252	2.53774809404016E-07	B/Plasma cell	RBM15
1.54568126019386E-11	0.774923803795775	0.069	0.196	2.59025265583287E-07	B/Plasma cell	RAVER1

1.55305782654925E-11	0.390729028870102	0.032	0.135	2.60261430573123E-07	B/Plasma cell	CDIP1
1.56808828904764E-11	0.275324891816568	0.124	0.275	2.62780235478603E-07	B/Plasma cell	TXNDC15
1.61519888541568E-11	0.450458498946763	0.051	0.165	2.7067502921796E-07	B/Plasma cell	ACTR2
1.62885591525263E-11	0.789967485408018	0.107	0.252	2.72963674278036E-07	B/Plasma cell	POLDIP3
1.63905296563134E-11	0.301704844504749	0.069	0.193	2.746724959805E-07	B/Plasma cell	ZNF580
1.66637814336085E-11	0.500241043623756	0.066	0.187	2.79251649264412E-07	B/Plasma cell	FBXO28
1.6676093433885E-11	0.678490631772457	0.216	0.416	2.79457973765044E-07	B/Plasma cell	SEPTIN9
1.68873066469744E-11	0.484786613528883	0.079	0.209	2.82997484789997E-07	B/Plasma cell	SART1
1.73899361153498E-11	0.425934240896054	0.062	0.182	2.91420549421031E-07	B/Plasma cell	SF3B2
1.75373609483473E-11	0.866068160960622	0.109	0.253	2.93891094772404E-07	B/Plasma cell	TMEM175
1.76882189477792E-11	0.420319342235503	0.051	0.164	2.96419173126884E-07	B/Plasma cell	CREB3
1.82538867241111E-11	0.765164158929173	0.088	0.223	3.05898633722654E-07	B/Plasma cell	SCAF8
1.86298811618697E-11	0.687973014125792	0.056	0.173	3.12199548510612E-07	B/Plasma cell	RBBP6
1.8724564592211E-11	0.574478891681454	0.039	0.146	3.13786253436272E-07	B/Plasma cell	SOCS4
1.88530272040948E-11	0.583222254736973	0.103	0.242	3.1593902988622E-07	B/Plasma cell	NCBP2
1.94178646501219E-11	0.508335693931445	0.06	0.178	3.25404575806744E-07	B/Plasma cell	ZC3H7B
1.97233011001467E-11	0.901957501674553	0.161	0.34	3.30523079836258E-07	B/Plasma cell	DDX39B
2.00653323470379E-11	0.622351491815132	0.105	0.248	3.36254839471662E-07	B/Plasma cell	GRIPAP1
2.07162789255586E-11	0.567557573577326	0.094	0.231	3.47163402234511E-07	B/Plasma cell	IRF9
2.07965343208695E-11	0.487586571634083	0.141	0.301	3.48508322149131E-07	B/Plasma cell	ANAPC16
2.32361557581863E-11	0.599627703083418	0.086	0.215	3.89391498195686E-07	B/Plasma cell	BET1L
2.33668115820238E-11	0.439486045500628	0.043	0.152	3.91581028491555E-07	B/Plasma cell	XPO5
2.35301123180743E-11	2.15909590396477	0.124	0.058	3.94317622226289E-07	B/Plasma cell	SEPTIN6
2.39366260462694E-11	0.566285997094018	0.12	0.27	4.01129979283382E-07	B/Plasma cell	ZC3H15
2.42906796553402E-11	0.362885853195452	0.034	0.135	4.07063209664191E-07	B/Plasma cell	PPARD
2.4604184218518E-11	0.45663734073575	0.073	0.199	4.12316919133925E-07	B/Plasma cell	ULK1
2.48666609686946E-11	0.851423160753311	0.043	0.154	4.16715504513384E-07	B/Plasma cell	FARP2
2.50835413900712E-11	0.381668860251094	0.032	0.135	4.20349986614813E-07	B/Plasma cell	ALCAM
2.51441643181602E-11	0.516439686725527	0.096	0.231	4.21365905643729E-07	B/Plasma cell	SDR39U1
2.61743132820284E-11	0.460840161894401	0.075	0.201	4.38629141980231E-07	B/Plasma cell	FBXW4
2.6175960748848E-11	0.354477965226973	0.053	0.165	4.38656750229194E-07	B/Plasma cell	EXOC8
2.61904279142534E-11	0.64644069558326	0.092	0.228	4.38899190987058E-07	B/Plasma cell	SRSF7
2.63075306603923E-11	0.256550281938166	0.064	0.181	4.40861598806855E-07	B/Plasma cell	MAGEH1
2.63233108370129E-11	0.309184057345977	0.069	0.192	4.41126043006662E-07	B/Plasma cell	ARFGAP1
2.75024182050523E-11	0.475888810705016	0.015	0.105	4.60885524280266E-07	B/Plasma cell	TRMT13
2.75202026246447E-11	1.15965348455734	0.049	0.162	4.61183555583797E-07	B/Plasma cell	HIF1A
2.88796835767684E-11	0.676412804429963	0.062	0.179	4.83965737379485E-07	B/Plasma cell	MIB1
2.88997326166246E-11	0.567844352924806	0.034	0.135	4.84301719189395E-07	B/Plasma cell	FAN1
2.92065517273878E-11	0.915870428487623	0.169	0.34	4.89443393847565E-07	B/Plasma cell	ETNK1
2.97529730052335E-11	0.670575398116725	0.062	0.181	4.98600321621704E-07	B/Plasma cell	GTF3A
3.07195781601358E-11	0.46896790959678	0.079	0.208	5.14798690807556E-07	B/Plasma cell	PPP1R12C
3.2062831383162E-11	0.57357112854122	0.075	0.202	5.37308928319029E-07	B/Plasma cell	NUP98
3.28119301572285E-11	0.505328206421428	0.049	0.16	5.49862325574836E-07	B/Plasma cell	NIPA2
3.32975445785596E-11	0.844457648205734	0.043	0.152	5.58000252047503E-07	B/Plasma cell	CDYL
3.48647691988592E-11	0.594757374984119	0.081	0.211	5.84263802234483E-07	B/Plasma cell	PHC3
3.48716835932774E-11	0.749454234037709	0.068	0.189	5.84379673656143E-07	B/Plasma cell	GALNS
3.50778560064892E-11	0.811106613855447	0.068	0.191	5.87834710956746E-07	B/Plasma cell	TAF11
3.56481970546365E-11	0.424751730481709	0.079	0.205	5.97392486241599E-07	B/Plasma cell	ZNF655
3.6161906160436E-11	0.384645308846292	0.079	0.203	6.06001223436587E-07	B/Plasma cell	PIH1D1
3.76170271201946E-11	0.315766999997905	0.043	0.151	6.3038614048022E-07	B/Plasma cell	NCAPD3
3.82719797820735E-11	0.70717162771345	0.058	0.175	6.41361837187988E-07	B/Plasma cell	NSMCE4A
3.85672610473575E-11	0.412732595316746	0.024	0.12	6.46310160631616E-07	B/Plasma cell	SSBP1
3.90124246134818E-11	0.698013732097935	0.06	0.177	6.53770211672729E-07	B/Plasma cell	RAB5A
3.97125371098287E-11	2.72555972017038	0.169	0.093	6.6550269688651E-07	B/Plasma cell	CCDC88B
3.98132682764277E-11	1.08964463017508	0.064	0.181	6.67190749776376E-07	B/Plasma cell	PKN2
3.98383407356707E-11	0.904384196538468	0.107	0.249	6.67610914048369E-07	B/Plasma cell	BRWD1
4.0360996118577E-11	0.613873254595439	0.053	0.163	6.76369572955113E-07	B/Plasma cell	RBM15B
4.12912829065149E-11	0.287677488232074	0.079	0.205	6.91959318947376E-07	B/Plasma cell	CDKAL1
4.1452105603183E-11	0.777866826886484	0.06	0.175	6.94654385698141E-07	B/Plasma cell	TIMM17B
4.25625089321694E-11	0.794641336194218	0.051	0.163	7.13262524685294E-07	B/Plasma cell	RXRB
4.26377904491852E-11	0.719557685090597	0.105	0.247	7.14524092347446E-07	B/Plasma cell	SUSD6
4.28602042051973E-11	0.520561819725541	0.113	0.254	7.18251302070696E-07	B/Plasma cell	LTBP3
4.3147383386904E-11	0.759891105620151	0.111	0.255	7.23063850797738E-07	B/Plasma cell	GPATCH2L

4.36915028192463E-11	0.413407600500491	0.045	0.151	7.32182204244929E-07	B/Plasma cell	COPS8
4.40147218187509E-11	0.357536688578271	0.06	0.175	7.37598708238628E-07	B/Plasma cell	CFAP298
4.44257802005158E-11	0.769362478710315	0.083	0.21	7.44487224600244E-07	B/Plasma cell	ARL6IP5
4.52097961900108E-11	0.822146228646256	0.152	0.319	7.57625764552201E-07	B/Plasma cell	FAM193B
4.53264761124422E-11	0.382711325135163	0.03	0.129	7.59581086692306E-07	B/Plasma cell	ZNF606
4.54374424625574E-11	0.254510820410693	0.053	0.164	7.61440660787536E-07	B/Plasma cell	LAS1L
4.54648151618589E-11	0.870620789166736	0.159	0.33	7.61899372482432E-07	B/Plasma cell	SRSF2
4.57736934801825E-11	0.27261683331108	0.113	0.253	7.67075555340898E-07	B/Plasma cell	ARHGEF7
4.58522211097738E-11	0.396355247982063	0.173	0.347	7.6839152135759E-07	B/Plasma cell	EIF3A
4.58828631118302E-11	0.831120369025197	0.069	0.192	7.6890502002805E-07	B/Plasma cell	YTHDC2
4.87502821529204E-11	0.903267001643613	0.12	0.271	8.1695722831864E-07	B/Plasma cell	NIPBL
4.93586515231387E-11	0.346390579972128	0.054	0.166	8.27152282224759E-07	B/Plasma cell	FOPNL
4.98751996071462E-11	0.484008797648959	0.039	0.144	8.35808595016556E-07	B/Plasma cell	ARFIP2
5.0554974173076E-11	0.772678483469312	0.041	0.147	8.47200257192407E-07	B/Plasma cell	PDCL3
5.19134386865578E-11	0.358963869494946	0.066	0.183	8.69965405509336E-07	B/Plasma cell	LSM14A
5.2480508101438E-11	0.76427993047744	0.137	0.294	8.79468354763898E-07	B/Plasma cell	BACH1
5.25976497208697E-11	0.586607617719353	0.071	0.193	8.81431414022335E-07	B/Plasma cell	CDK5RAP2
5.31656018400892E-11	0.675933720800858	0.111	0.253	8.90949155636214E-07	B/Plasma cell	CNST
5.33057053467693E-11	0.702454162617268	0.107	0.249	8.93297010201159E-07	B/Plasma cell	METTL17
5.33382744788423E-11	0.591443283117201	0.039	0.143	8.9384280371644E-07	B/Plasma cell	RNF146
5.5058902562793E-11	0.569123713760832	0.036	0.136	9.22677089147286E-07	B/Plasma cell	LRIG2
5.52089585786526E-11	0.844210179041595	0.06	0.176	9.2519172786106E-07	B/Plasma cell	UNK
5.52178700620795E-11	0.691155094596743	0.15	0.309	9.25341066500329E-07	B/Plasma cell	CREBZF
5.57758858384999E-11	0.300869706280674	0.064	0.179	9.34692294881581E-07	B/Plasma cell	IAH1
5.58979286769363E-11	0.320700413323958	0.058	0.171	9.36737488768098E-07	B/Plasma cell	MKNK1
5.59670542662209E-11	0.867877072751762	0.096	0.232	9.3789589539333E-07	B/Plasma cell	RNF44
5.59722369405156E-11	0.317775448929605	0.034	0.133	9.37982746649161E-07	B/Plasma cell	BRAF
5.59861376450139E-11	0.939082905430449	0.083	0.211	9.38215694655142E-07	B/Plasma cell	SLK
5.63654077714986E-11	0.467256261877999	0.075	0.197	9.44571503434774E-07	B/Plasma cell	RTF1
5.64961631718207E-11	0.443527357260632	0.075	0.198	9.46762702433371E-07	B/Plasma cell	CBWD2
5.67743642245359E-11	0.346073418228308	0.054	0.167	9.51424795674773E-07	B/Plasma cell	DIP2B
5.72056954072446E-11	0.798403732827376	0.101	0.238	9.58653043634606E-07	B/Plasma cell	AKT2
5.79764274774906E-11	0.510702046626719	0.165	0.335	9.71568971667787E-07	B/Plasma cell	SEL1L
5.83217216385456E-11	0.521976368244231	0.064	0.182	9.77355411218747E-07	B/Plasma cell	SLC25A37
5.97506009345294E-11	0.956376360467108	0.096	0.229	1.00130057046084E-06	B/Plasma cell	BDP1
6.08642653753845E-11	0.799372650869453	0.054	0.167	1.01996335916069E-06	B/Plasma cell	RECQL
6.26765537135728E-11	0.284686071008362	0.045	0.15	1.05033368713205E-06	B/Plasma cell	TLNRD1
6.58584724316685E-11	0.581016477285907	0.079	0.202	1.1036562810099E-06	B/Plasma cell	YY1AP1
6.87031976989911E-11	0.499485056279452	0.032	0.13	1.15132818703969E-06	B/Plasma cell	UBE3B
6.87268040113216E-11	0.567647881334201	0.068	0.187	1.15172378162173E-06	B/Plasma cell	HDAC5
7.20176718651052E-11	0.406352681599566	0.049	0.157	1.20687214511543E-06	B/Plasma cell	TANGO2
7.23500248336412E-11	0.567812193637277	0.092	0.222	1.21244171616216E-06	B/Plasma cell	GON4L
7.32183082237337E-11	1.04001416664217	0.176	0.355	1.22699240921333E-06	B/Plasma cell	BAX
7.38934143547851E-11	0.364935591552513	0.064	0.179	1.23830583775749E-06	B/Plasma cell	EIF4B
7.44155615756533E-11	0.253311233823959	0.053	0.163	1.2470559808848E-06	B/Plasma cell	ZNF407
7.61954145457747E-11	0.292729935497606	0.049	0.156	1.27688275695809E-06	B/Plasma cell	ESF1
7.68026864197726E-11	0.564302791814976	0.092	0.221	1.28705941902255E-06	B/Plasma cell	GCN1
7.68181398398233E-11	0.607141968478338	0.223	0.421	1.28731838743576E-06	B/Plasma cell	SETD5
7.73093721602731E-11	0.655075251172069	0.028	0.124	1.29555045866186E-06	B/Plasma cell	RAD51B
7.77129801144136E-11	0.428403964119038	0.062	0.174	1.30231412075734E-06	B/Plasma cell	MLXIP
7.78487238656986E-11	0.27286944504716	0.058	0.171	1.30458891454138E-06	B/Plasma cell	CCDC130
7.93988248140109E-11	0.476479512166	0.083	0.208	1.33056550623319E-06	B/Plasma cell	PHRF1
8.13252370794824E-11	0.336316956593908	0.255	0.464	1.36284832297797E-06	B/Plasma cell	RNF213
8.37379381540199E-11	0.457470666301134	0.098	0.23	1.40328036758506E-06	B/Plasma cell	ROCK1
8.68228210104579E-11	0.949325318048676	0.64	0.718	1.45497683449325E-06	B/Plasma cell	TMSB4X
8.68276251392842E-11	0.782042377671867	0.081	0.204	1.45505734208412E-06	B/Plasma cell	CERK
8.9324073711869E-11	0.752201135165126	0.152	0.313	1.4968928272635E-06	B/Plasma cell	EIF4A1
9.00914676873656E-11	0.603090249197983	0.163	0.332	1.50975281550487E-06	B/Plasma cell	FUBP1
9.08804876863552E-11	0.537509865225604	0.058	0.173	1.52297521264794E-06	B/Plasma cell	KNTC1
9.49601378610371E-11	0.893692894576832	0.173	0.344	1.59134199027526E-06	B/Plasma cell	ASXL1
9.61623498981469E-11	0.71385286352652	0.124	0.269	1.61148865959315E-06	B/Plasma cell	DDX24
9.78825874198928E-11	0.639117362410521	0.071	0.191	1.64031639998256E-06	B/Plasma cell	ARHGEF18
9.79681480509957E-11	0.562162423963169	0.036	0.134	1.64175022503859E-06	B/Plasma cell	MAML3

9.93134909758151E-11	0.450275237458857	0.094	0.224	1.66429548177271E-06	B/Plasma cell	POLR2B
9.9394108093441E-11	0.900149053631775	0.066	0.184	1.66564646342988E-06	B/Plasma cell	ATP6V1G1
1.01112055301552E-10	0.834354295759121	0.036	0.135	1.69443582274341E-06	B/Plasma cell	FBXO34
1.01464820681213E-10	0.954883730493621	0.146	0.31	1.70034746497577E-06	B/Plasma cell	ZNF638
1.03802608007112E-10	0.721554594745698	0.021	0.11	1.73952410498319E-06	B/Plasma cell	LIG4
1.03977909561827E-10	0.572732976086353	0.068	0.184	1.7424618084371E-06	B/Plasma cell	ZER1
1.04847050698783E-10	0.715946246093214	0.098	0.23	1.75702687561021E-06	B/Plasma cell	SEC63
1.06022306736055E-10	0.498769022371556	0.071	0.19	1.77672181628281E-06	B/Plasma cell	KIDINS220
1.07777059471096E-10	0.889024829739779	0.212	0.413	1.80612796261662E-06	B/Plasma cell	DBNL
1.08854668949531E-10	0.348172488551257	0.051	0.158	1.82418654225624E-06	B/Plasma cell	BRAT1
1.12754162467322E-10	0.293698144433498	0.06	0.172	1.88953425462738E-06	B/Plasma cell	SOS 1
1.14445436140931E-10	0.513942005031503	0.045	0.151	1.91787661884972E-06	B/Plasma cell	NGDN
1.15577587276648E-10	0.348224981502326	0.051	0.157	1.93684920758207E-06	B/Plasma cell	NOP9
1.17054696104665E-10	0.72691956807744	0.116	0.261	1.96160259732197E-06	B/Plasma cell	TCERG1
1.17203061889807E-10	0.640764454499777	0.086	0.214	1.96408891114938E-06	B/Plasma cell	PPP1R35
1.18568328471708E-10	0.44714973628648	0.045	0.148	1.98696804852888E-06	B/Plasma cell	YPEL2
1.18902241519675E-10	0.688999729199622	0.068	0.184	1.99256376338672E-06	B/Plasma cell	STX17
1.20386246874233E-10	0.818889759505412	0.066	0.182	2.0174327251184E-06	B/Plasma cell	PHF11
1.21745551551695E-10	0.356733063469217	0.064	0.179	2.0402119529033E-06	B/Plasma cell	SLC25A46
1.22996936870537E-10	0.548643817157042	0.133	0.285	2.06118266807647E-06	B/Plasma cell	ARSA
1.25873709000448E-10	0.90383381756099	0.053	0.162	2.10939161542951E-06	B/Plasma cell	ADRB2
1.26695942297275E-10	0.349327682983807	0.019	0.107	2.12317060101774E-06	B/Plasma cell	ALMS1
1.29980041206382E-10	0.890887988230871	0.109	0.25	2.17820553053655E-06	B/Plasma cell	HERC3
1.31452878020136E-10	0.687738968194391	0.056	0.166	2.20288732986144E-06	B/Plasma cell	IRAK4
1.33783699921658E-10	0.273704490572374	0.043	0.146	2.24194724328715E-06	B/Plasma cell	TGFBR1
1.3629184906079E-10	0.675178428654109	0.079	0.202	2.28397880656073E-06	B/Plasma cell	MVD
1.41212394089002E-10	0.274306130103627	0.043	0.145	2.36643730014349E-06	B/Plasma cell	CCDC92
1.45784609099237E-10	0.289555512382275	0.024	0.115	2.44305847928501E-06	B/Plasma cell	MIF4GD
1.4773098838647E-10	0.601785945644406	0.028	0.122	2.47567590338047E-06	B/Plasma cell	OTUD3
1.48437806989388E-10	0.517599852131013	0.041	0.144	2.48752076952817E-06	B/Plasma cell	CCDC6
1.51398363437799E-10	0.403393103456303	0.036	0.133	2.53713377449064E-06	B/Plasma cell	IFFO1
1.58279239296644E-10	0.727566459136696	0.028	0.122	2.65244349213316E-06	B/Plasma cell	RNF121
1.58656738759801E-10	0.354988074937393	0.043	0.145	2.65876962813675E-06	B/Plasma cell	AP5M1
1.58695227752812E-10	1.03896834692987	0.124	0.275	2.65941462668162E-06	B/Plasma cell	TSPYL1
1.59305707035513E-10	0.713615054029344	0.049	0.154	2.66964503850112E-06	B/Plasma cell	DEPRECATED-ENSG00000185658
1.59678356609257E-10	0.420423919512529	0.094	0.223	2.67588990005793E-06	B/Plasma cell	MARCKSL1
1.61884261581002E-10	0.966447073982398	0.101	0.235	2.71285645557443E-06	B/Plasma cell	TRIM44
1.65859790819408E-10	0.298890192733731	0.041	0.142	2.77947837455164E-06	B/Plasma cell	THOC1
1.72862699219486E-10	0.349431622834432	0.06	0.169	2.89683311352015E-06	B/Plasma cell	RALY
1.75867212123782E-10	0.512221688503052	0.066	0.182	2.94718274077034E-06	B/Plasma cell	TTC7A
1.84548775546371E-10	0.310819545669496	0.045	0.147	3.09266838060608E-06	B/Plasma cell	AC253572.1
1.9601619126268E-10	0.363113491950042	0.056	0.166	3.28483933317999E-06	B/Plasma cell	TMEM39A
1.96654999243913E-10	0.631329945645946	0.028	0.121	3.29554447732949E-06	B/Plasma cell	LIN52
1.97486538565669E-10	1.45151352818406	0.15	0.077	3.30947941328349E-06	B/Plasma cell	CHST11
1.97773447630735E-10	0.329728171245542	0.053	0.16	3.31428743539586E-06	B/Plasma cell	C17orf49
1.98115032880401E-10	0.91032516207472	0.043	0.145	3.32001172100975E-06	B/Plasma cell	CHML
2.00440012395E-10	0.629557866590319	0.161	0.326	3.35897372771541E-06	B/Plasma cell	TAF7
2.03407326967009E-10	0.484344701143529	0.131	0.278	3.40869998531314E-06	B/Plasma cell	WDR26
2.04422538891408E-10	0.630111782589408	0.084	0.206	3.42571290674222E-06	B/Plasma cell	DCK
2.06424130557049E-10	0.328198235114525	0.041	0.14	3.45925557987503E-06	B/Plasma cell	KLHL20
2.06620807332405E-10	0.482017151043381	0.099	0.23	3.46255148927644E-06	B/Plasma cell	TYK2
2.17617934143207E-10	0.48546604552494	0.019	0.105	3.64684134037186E-06	B/Plasma cell	KIAA1328
2.18464796060219E-10	0.34334802240714	0.047	0.151	3.66103305237715E-06	B/Plasma cell	TBC1D23
2.20556114853536E-10	0.791560759278635	0.054	0.162	3.69607937271556E-06	B/Plasma cell	ARFGAP2
2.23355173626594E-10	0.474181348489159	0.051	0.155	3.74298599963447E-06	B/Plasma cell	TBC1D5
2.23827149390197E-10	0.496975885658966	0.064	0.178	3.75089536948092E-06	B/Plasma cell	NISCH
2.2595954370409E-10	0.800226807994418	0.056	0.164	3.78663003339314E-06	B/Plasma cell	FAM126A
2.26202113061385E-10	0.707817084710866	0.051	0.156	3.79069501068269E-06	B/Plasma cell	PIGS
2.29796262212784E-10	0.570565790776251	0.116	0.256	3.85092576216184E-06	B/Plasma cell	TRA2B
2.33072355572042E-10	1.04912367504634	0.053	0.159	3.90582653467627E-06	B/Plasma cell	TBC1D10B
2.52225816810328E-10	0.261573802447723	0.047	0.149	4.22680023810749E-06	B/Plasma cell	UBA3
2.5596701564753E-10	0.519011767180174	0.047	0.149	4.28949524822131E-06	B/Plasma cell	MTOR
2.57527895141373E-10	0.816894868757239	0.111	0.243	4.31565246677913E-06	B/Plasma cell	IFNAR1

2.66763451879043E-10	0.499571851192278	0.039	0.138	4.470421926589E-06	B/Plasma cell	ZDHHC5
2.67110563013464E-10	0.572885227274879	0.043	0.145	4.47623881497962E-06	B/Plasma cell	NR1D2
2.70332326432801E-10	0.574052770616864	0.038	0.135	4.53022912636088E-06	B/Plasma cell	WDR59
2.75400494781265E-10	0.649182278940595	0.036	0.131	4.61516149154444E-06	B/Plasma cell	ZNF346
2.77789913831638E-10	0.380678161311073	0.056	0.163	4.65520337599059E-06	B/Plasma cell	PATZ1
2.82351499973942E-10	0.704225057370317	0.101	0.232	4.73164643656331E-06	B/Plasma cell	DEPRECATED-ENSG00000166136
2.90914698265125E-10	0.599736522236202	0.041	0.14	4.87514851352696E-06	B/Plasma cell	NT5C3A
2.94920502988111E-10	0.786814319743677	0.084	0.207	4.94227778907477E-06	B/Plasma cell	BRD7
2.9720729392819E-10	0.85213483251867	0.109	0.247	4.98059983164861E-06	B/Plasma cell	LYST
2.99448769128595E-10	0.353167055320135	0.06	0.168	5.01816247305699E-06	B/Plasma cell	UNC93B1
3.00959629937488E-10	0.672682646903112	0.101	0.233	5.04348147849242E-06	B/Plasma cell	USF1
3.0924321624065E-10	0.912208954989725	0.159	0.322	5.18229781776081E-06	B/Plasma cell	MAX
3.09781454376535E-10	0.649198056844494	0.09	0.213	5.19131761244197E-06	B/Plasma cell	NFATC2
3.12759001146606E-10	0.313250860091798	0.043	0.143	5.24121534121483E-06	B/Plasma cell	PIAS2
3.16244558307365E-10	0.965001723354285	0.135	0.285	5.29962630811482E-06	B/Plasma cell	GGNBP2
3.18413970528126E-10	0.524740058504375	0.039	0.138	5.33598131811033E-06	B/Plasma cell	TMEM30A
3.30368201157685E-10	1.26144641851675	0.096	0.224	5.53631031500049E-06	B/Plasma cell	HIP1R
3.33034437467126E-10	0.360363430329949	0.129	0.274	5.58099110307409E-06	B/Plasma cell	IER2
3.33195766898965E-10	0.809605936481685	0.024	0.113	5.58369466169286E-06	B/Plasma cell	MSH5
3.5787716991879E-10	0.799830696238315	0.171	0.341	5.99730561349908E-06	B/Plasma cell	BRD4
3.60774439821677E-10	0.615552164021373	0.158	0.317	6.04585806253167E-06	B/Plasma cell	MACF1
3.61674261510943E-10	0.372144456388104	0.086	0.205	6.06093727440038E-06	B/Plasma cell	VEGFB
3.68358149206897E-10	0.852113851588546	0.034	0.129	6.17294586440918E-06	B/Plasma cell	BLZF1
3.75249550010165E-10	0.343568363166839	0.049	0.152	6.28843195907034E-06	B/Plasma cell	CSAD
3.75508886271117E-10	0.542487370792102	0.101	0.23	6.29277791613139E-06	B/Plasma cell	SEPTIN2
3.75749786402639E-10	1.51570717706607	0.06	0.17	6.29681492053543E-06	B/Plasma cell	DEPRECATED-ENSG00000178950
3.81977199634449E-10	0.281384143968153	0.068	0.179	6.4011739114741E-06	B/Plasma cell	METTL3
3.82376480637715E-10	0.425144170392439	0.098	0.224	6.40786506252683E-06	B/Plasma cell	MED13L
3.89517958455094E-10	0.475899983076509	0.036	0.128	6.52754194779047E-06	B/Plasma cell	PRMT3
3.95429420970113E-10	0.580938621551098	0.062	0.172	6.62660623661715E-06	B/Plasma cell	RBM27
3.98148875216911E-10	0.336775296529945	0.096	0.221	6.67217885088499E-06	B/Plasma cell	UBE2L6
4.12204081272768E-10	0.873867226109408	0.038	0.133	6.90771599396904E-06	B/Plasma cell	C5orf51
4.1739348749334E-10	0.717716078913471	0.111	0.246	6.99468006341339E-06	B/Plasma cell	KLHL5
4.18785395290966E-10	0.46097808874688	0.019	0.102	7.01800565428601E-06	B/Plasma cell	EXD2
4.25725763633746E-10	0.378971318465749	0.043	0.141	7.13431234697432E-06	B/Plasma cell	RTCA
4.42248650849522E-10	0.743681650259373	0.075	0.189	7.41120289093629E-06	B/Plasma cell	HP1BP3
4.42771865109565E-10	0.474009200282015	0.083	0.2	7.41997091550608E-06	B/Plasma cell	STAT5B
4.47019046969451E-10	0.548695266113983	0.09	0.214	7.49114518911406E-06	B/Plasma cell	SPSB3
4.47064177966214E-10	1.05813715085363	0.03	0.122	7.49190149435782E-06	B/Plasma cell	TMEM161B
4.49940535343583E-10	0.715915947109405	0.139	0.288	7.54010349128777E-06	B/Plasma cell	MOGS
4.63768620334122E-10	0.355727490734394	0.043	0.141	7.77183453955921E-06	B/Plasma cell	AZI2
4.67429940306046E-10	0.916713425456417	0.144	0.3	7.83319093964871E-06	B/Plasma cell	MSI2
4.70697966481773E-10	0.69427003572511	0.03	0.121	7.88795652230155E-06	B/Plasma cell	TTN
4.75707809546137E-10	0.546503014812781	0.109	0.242	7.97191147237416E-06	B/Plasma cell	MSL2
4.7793386262357E-10	0.462857955209375	0.032	0.123	8.00921566984578E-06	B/Plasma cell	DEPRECATED-ENSG00000164164
4.84336465453119E-10	0.556223430544596	0.086	0.207	8.11651048806337E-06	B/Plasma cell	ZNF592
4.93199054517683E-10	0.614384107249118	0.056	0.161	8.26502975560734E-06	B/Plasma cell	PRKCD
5.13298892886584E-10	0.791686751920394	0.043	0.141	8.60186284699338E-06	B/Plasma cell	ZNF622
5.21186705578456E-10	0.390227162083413	0.071	0.184	8.73404681208376E-06	B/Plasma cell	CLSTN3
5.35804644281971E-10	0.620220271437392	0.191	0.366	8.97901422887728E-06	B/Plasma cell	TAP1
5.41007439494294E-10	0.598371509293153	0.071	0.183	9.06620267104539E-06	B/Plasma cell	CCDC9
5.4475948484987E-10	0.508315559032157	0.024	0.111	9.12907944711412E-06	B/Plasma cell	ZNF248
5.44823999360117E-10	0.432182305126569	0.041	0.136	9.13016058127683E-06	B/Plasma cell	THOC3
5.44964896333606E-10	0.403261605680494	0.064	0.173	9.13252173275856E-06	B/Plasma cell	LATS1
5.5019260408848E-10	0.265940667141558	0.019	0.101	9.22012765931475E-06	B/Plasma cell	GPR89A
5.62266272847593E-10	0.580875671025901	0.066	0.174	9.42245820037996E-06	B/Plasma cell	ELL
5.6516067332638E-10	0.872485127881333	0.071	0.186	9.47096256360347E-06	B/Plasma cell	TRIM14
6.09098400998665E-10	0.464637211015295	0.094	0.215	1.02072710039356E-05	B/Plasma cell	QRICH1
6.10041723099943E-10	0.30713433218747	0.038	0.131	1.02230791957088E-05	B/Plasma cell	LACTB
6.11681452142526E-10	0.442914136379583	0.032	0.123	1.02505577750044E-05	B/Plasma cell	GALC
6.20097369986436E-10	0.984552426517207	0.092	0.217	1.03915917262327E-05	B/Plasma cell	DLGAP4
6.33895785534136E-10	0.992688337012517	0.206	0.393	1.06228255739811E-05	B/Plasma cell	RBM39
6.48960546857903E-10	0.768024860965513	0.084	0.2	1.08752808442447E-05	B/Plasma cell	DDX54

6.4997080082513E-10	2.87464000035841	0.21	0.138	1.08922106802275E-05	B/Plasma cell	PDK1
6.52387616652757E-10	0.967774265692361	0.068	0.179	1.09327116798669E-05	B/Plasma cell	PHF2
6.57277799606545E-10	0.426818331328711	0.041	0.137	1.10146613658065E-05	B/Plasma cell	MFSD6
6.61278777977165E-10	1.04516220029046	0.06	0.167	1.10817097613413E-05	B/Plasma cell	MFSD10
6.67903044710444E-10	0.286909809774365	0.041	0.136	1.11927192232576E-05	B/Plasma cell	UBQLN1
6.72662699520631E-10	0.492530516276993	0.073	0.186	1.12724815185667E-05	B/Plasma cell	VPS26A
6.74079732274337E-10	0.885436067491729	0.038	0.132	1.12962281534533E-05	B/Plasma cell	ZNF26
6.82376517983915E-10	0.654747035867321	0.083	0.201	1.14352656883744E-05	B/Plasma cell	EXOC4
6.88120759975489E-10	0.283221039587992	0.047	0.145	1.15315276956692E-05	B/Plasma cell	TMEM94
7.06275562076377E-10	0.367913440281347	0.026	0.112	1.18357658692759E-05	B/Plasma cell	STXBP4
7.0894648929722E-10	0.92107487567268	0.045	0.143	1.18805252676428E-05	B/Plasma cell	TMEM18
7.11917335827244E-10	0.561169283467524	0.038	0.132	1.1930310713793E-05	B/Plasma cell	MAPK8
7.14697224411921E-10	0.898437383316803	0.113	0.247	1.1976896086695E-05	B/Plasma cell	NFATC2IP
7.40929383751352E-10	0.943048308718369	0.15	0.304	1.24164946129052E-05	B/Plasma cell	UBE2I
7.58264278661604E-10	0.394136002720683	0.075	0.187	1.27069927818112E-05	B/Plasma cell	ADD3
7.58682644530174E-10	0.315321631738782	0.045	0.142	1.27140037570367E-05	B/Plasma cell	DCTN6
7.7326640115801E-10	0.720504365329234	0.079	0.195	1.29583983506059E-05	B/Plasma cell	CTDSP2
7.79347222628152E-10	0.65227085174578	0.045	0.142	1.30603007568026E-05	B/Plasma cell	DNAJB14
7.81472904628129E-10	0.32677208927887	0.043	0.139	1.30959229357582E-05	B/Plasma cell	ZNF672
7.90703785810874E-10	0.526438795519573	0.073	0.186	1.32506140426186E-05	B/Plasma cell	VOPP1
7.94157844463749E-10	0.480694463056413	0.03	0.119	1.33084971575235E-05	B/Plasma cell	ZNF33B
8.14082502463984E-10	0.270846602467746	0.026	0.112	1.36423945762914E-05	B/Plasma cell	PARP2
8.26971062926062E-10	0.815290333024878	0.056	0.16	1.38583810725149E-05	B/Plasma cell	MTPN
8.48713264448436E-10	0.419874257289033	0.064	0.17	1.42227368856269E-05	B/Plasma cell	ZBTB44
8.6406171628203E-10	0.361262858857088	0.081	0.197	1.44799462414543E-05	B/Plasma cell	SHKBP1
8.86043709876483E-10	0.88668757582817	0.066	0.174	1.48483204901101E-05	B/Plasma cell	UBE2O
8.88483219830087E-10	0.573380586829304	0.086	0.206	1.48892017979126E-05	B/Plasma cell	GOLT1B
8.98761422536319E-10	0.279070965821586	0.024	0.108	1.50614439188636E-05	B/Plasma cell	POLG2
9.01395625178692E-10	0.525501833699071	0.073	0.184	1.51055878867445E-05	B/Plasma cell	DMAP1
9.11981802874178E-10	0.463254900061289	0.06	0.166	1.52829910525655E-05	B/Plasma cell	EMG1
9.13415478593076E-10	0.373859805318624	0.021	0.103	1.53070165902628E-05	B/Plasma cell	B3GALNT2
9.26408759856751E-10	0.336637053686551	0.047	0.145	1.55247579976794E-05	B/Plasma cell	PLP2
9.31822431710543E-10	0.468462469442542	0.053	0.153	1.56154803106053E-05	B/Plasma cell	SESTD1
9.35572857306849E-10	0.422675581614393	0.056	0.159	1.56783299427482E-05	B/Plasma cell	GOLGA1
9.57920329233003E-10	0.62941769509863	0.092	0.214	1.60528288772867E-05	B/Plasma cell	UBA6
9.79218606733877E-10	0.796317810275991	0.066	0.173	1.64097454116463E-05	B/Plasma cell	USPL1
9.91784910914656E-10	0.811283339792515	0.058	0.161	1.66203315371078E-05	B/Plasma cell	PWP1
1.02170533961802E-09	0.56781340218792	0.021	0.103	1.71217380813188E-05	B/Plasma cell	PDK3
1.05204566188045E-09	1.13906407865739	0.103	0.232	1.76301812017926E-05	B/Plasma cell	SNX2
1.07241783341709E-09	0.505495587734409	0.028	0.116	1.79715780524036E-05	B/Plasma cell	NBPF12
1.07500550657648E-09	0.255392610427568	0.049	0.147	1.80149422792087E-05	B/Plasma cell	SBNO1
1.105719252041E-09	1.01491346889678	0.12	0.258	1.85296432257031E-05	B/Plasma cell	USP24
1.11613004370492E-09	0.308794989692898	0.026	0.111	1.8704107272407E-05	B/Plasma cell	GKAP1
1.14079770431663E-09	0.726232986192117	0.062	0.165	1.91174879289381E-05	B/Plasma cell	ALG2
1.15073413324578E-09	0.932351954954089	0.034	0.123	1.92840026049328E-05	B/Plasma cell	USP47
1.15255371112988E-09	0.363565927224901	0.062	0.168	1.93144950911145E-05	B/Plasma cell	MTRR
1.16056014359018E-09	1.01239662025364	0.034	0.123	1.94486668862843E-05	B/Plasma cell	GCFC2
1.16346777868081E-09	0.920811682603859	0.081	0.198	1.94973930351331E-05	B/Plasma cell	MAST3
1.20174097670537E-09	0.479005783063278	0.096	0.218	2.01387752876286E-05	B/Plasma cell	LCOR
1.20874796054422E-09	0.655301093040934	0.051	0.149	2.02561983228001E-05	B/Plasma cell	ANKRD36B
1.22332082839037E-09	0.668947858742601	0.045	0.141	2.05004104421658E-05	B/Plasma cell	ZMYM3
1.24118016695633E-09	0.291035764087117	0.058	0.161	2.07996972378541E-05	B/Plasma cell	ZDHHC20
1.24152738371292E-09	1.17291494808852	0.152	0.306	2.08055158962611E-05	B/Plasma cell	MICAL3
1.24938776677211E-09	0.342490459658318	0.028	0.114	2.0937240195567E-05	B/Plasma cell	AP002495.1
1.2709804239299E-09	0.334930276026331	0.021	0.101	2.12990899442173E-05	B/Plasma cell	SAYSD1
1.27200398210541E-09	0.734683007478527	0.098	0.222	2.13162427321225E-05	B/Plasma cell	S100PBP
1.29547950516447E-09	1.99607721305297	0.114	0.055	2.17096455475462E-05	B/Plasma cell	DOCK8
1.30938953138395E-09	0.58834794781834	0.066	0.172	2.19427497669323E-05	B/Plasma cell	PITPNC1
1.31442853258278E-09	0.321539668249612	0.068	0.174	2.20271933490223E-05	B/Plasma cell	KANSL3
1.31650926391511E-09	0.846535135886191	0.034	0.123	2.20620622446895E-05	B/Plasma cell	RTL10
1.38121450886136E-09	0.810432358377269	0.084	0.201	2.31463927394987E-05	B/Plasma cell	KIF13B
1.39124362019907E-09	2.32052465641853	0.208	0.136	2.3314460587296E-05	B/Plasma cell	CLEC2D
1.40373351588307E-09	0.80890181698522	0.06	0.165	2.35237662591685E-05	B/Plasma cell	APAF1

1.43528673867862E-09	0.270405871765045	0.077	0.188	2.40525351667763E-05	B/Plasma cell	ZC3H12A
1.44506247531164E-09	0.328438648879816	0.028	0.114	2.42163569612725E-05	B/Plasma cell	CEP95
1.45228710648023E-09	0.644270374918851	0.086	0.205	2.43374273303957E-05	B/Plasma cell	DRAM2
1.45520237840241E-09	0.537640370970089	0.06	0.163	2.43862814572675E-05	B/Plasma cell	UTP6
1.47451682238067E-09	0.743756477010394	0.03	0.117	2.47099529094552E-05	B/Plasma cell	GALNT4
1.51280418389571E-09	0.677618149532141	0.188	0.356	2.53515725137242E-05	B/Plasma cell	RSRC2
1.52826801737321E-09	0.339788242285304	0.077	0.188	2.56107154351403E-05	B/Plasma cell	CLIC4
1.53090099633679E-09	1.11750457268687	0.06	0.164	2.56548388966119E-05	B/Plasma cell	TMEM170A
1.53882958621451E-09	0.526009866900024	0.073	0.183	2.57877062057827E-05	B/Plasma cell	MTMR10
1.55229157972905E-09	0.935893383437918	0.032	0.12	2.60133022930995E-05	B/Plasma cell	DTNBP1
1.55938742559849E-09	0.644401211045124	0.094	0.217	2.61322144781795E-05	B/Plasma cell	TAF8
1.58747374350685E-09	0.476736040786816	0.064	0.169	2.66028849936879E-05	B/Plasma cell	STK38L
1.59443271804866E-09	0.762244831669242	0.049	0.146	2.67195034890594E-05	B/Plasma cell	ST3GAL2
1.61694730321824E-09	0.566091804680066	0.075	0.186	2.70968029073313E-05	B/Plasma cell	WAC
1.62707117416627E-09	0.901395441498976	0.133	0.274	2.72664587366783E-05	B/Plasma cell	MARK3
1.65795621528629E-09	0.659003242964321	0.028	0.114	2.77840302557677E-05	B/Plasma cell	LINS1
1.67744623787732E-09	0.789671526083402	0.053	0.152	2.81106440543481E-05	B/Plasma cell	CEP83
1.69018409017675E-09	0.336675673616735	0.039	0.132	2.83241049831821E-05	B/Plasma cell	MGA
1.71385955347138E-09	0.831406761625381	0.039	0.133	2.87208583970734E-05	B/Plasma cell	SLC25A42
1.71990949911366E-09	0.744646512057928	0.047	0.143	2.88222433861467E-05	B/Plasma cell	NPRL3
1.72225600056145E-09	0.56446215578574	0.064	0.168	2.88615660574087E-05	B/Plasma cell	MARK2
1.73876059502011E-09	0.807639562162692	0.107	0.235	2.91381500513471E-05	B/Plasma cell	AIP
1.75597216282641E-09	0.934287326070879	0.128	0.269	2.94265815046449E-05	B/Plasma cell	DEPRECATED-ENSG00000161547
1.79718434592262E-09	0.873975190042256	0.036	0.125	3.01172152689713E-05	B/Plasma cell	SMYD3
1.79751508025617E-09	0.709270339699237	0.043	0.138	3.01227577149329E-05	B/Plasma cell	SAP130
1.85831962867054E-09	0.718104019301635	0.064	0.168	3.11417203372608E-05	B/Plasma cell	LIG1
1.86411889727402E-09	0.9097009161988	0.06	0.162	3.12389044805181E-05	B/Plasma cell	PRR14L
1.90247570477016E-09	0.281855683788203	0.028	0.113	3.18816878605384E-05	B/Plasma cell	CPSF4
1.91600717942922E-09	0.593368081952961	0.053	0.153	3.21084483128749E-05	B/Plasma cell	SUPT20H
1.93198740444891E-09	0.711926812311682	0.118	0.25	3.23762449237549E-05	B/Plasma cell	M6PR
1.94025297103256E-09	0.386405575279928	0.068	0.172	3.25147592885637E-05	B/Plasma cell	CCM2
1.94124882976969E-09	0.257482994235844	0.03	0.115	3.25314478892805E-05	B/Plasma cell	TRAPPC13
1.94608071566189E-09	0.533039523322417	0.038	0.128	3.26124206330619E-05	B/Plasma cell	ABLIM1
1.95484091679758E-09	0.522208233879399	0.096	0.217	3.27592240836939E-05	B/Plasma cell	AAK1
1.98560222413914E-09	0.641268458013913	0.038	0.127	3.32747220721237E-05	B/Plasma cell	BLOC1S4
1.99202021361192E-09	0.604482978711638	0.047	0.143	3.33822747397085E-05	B/Plasma cell	ANKRD52
2.01187761713735E-09	0.94590291804117	0.165	0.327	3.37150451079878E-05	B/Plasma cell	CYBC1
2.07686228469428E-09	0.586546738459758	0.079	0.193	3.48040581669068E-05	B/Plasma cell	TRADD
2.08076350258655E-09	0.353127394199679	0.077	0.187	3.48694347763453E-05	B/Plasma cell	AKAP8L
2.0844121113765E-09	0.702765322937362	0.06	0.164	3.49305781624474E-05	B/Plasma cell	PANK4
2.09866899955127E-09	0.775501105820051	0.084	0.2	3.51694950944802E-05	B/Plasma cell	RAB3GAP1
2.12201587609047E-09	0.458723227691874	0.047	0.141	3.55607420515241E-05	B/Plasma cell	EXOSC10
2.14879485820291E-09	0.340961300323005	0.062	0.165	3.60095042337644E-05	B/Plasma cell	MED1
2.15809758867311E-09	0.329170477581281	0.056	0.156	3.6165399390984E-05	B/Plasma cell	VPS4B
2.17742145823189E-09	0.554522895276526	0.038	0.127	3.648922879705E-05	B/Plasma cell	TWSG1
2.1820295544135E-09	0.279105613175569	0.323	0.534	3.65664512728615E-05	B/Plasma cell	CCNL2
2.18607409691488E-09	0.461154235519851	0.026	0.109	3.66342297160995E-05	B/Plasma cell	KATNA1
2.20487848909237E-09	1.11231529250221	0.073	0.183	3.694935372021E-05	B/Plasma cell	NUP205
2.21756015486324E-09	0.289734804602759	0.06	0.161	3.71618730751982E-05	B/Plasma cell	CSGALNACT2
2.23677361093417E-09	0.73678904915923	0.069	0.176	3.74838521720349E-05	B/Plasma cell	RBM4
2.25698334724172E-09	0.404910040746085	0.036	0.124	3.78225269330768E-05	B/Plasma cell	PELP1
2.27450208482639E-09	0.68108407874022	0.141	0.285	3.81161059375206E-05	B/Plasma cell	RBM25
2.31471796063767E-09	0.363124821856255	0.03	0.116	3.8790043584366E-05	B/Plasma cell	ARL5B
2.3319031179532E-09	0.820357085006555	0.143	0.287	3.90780324506598E-05	B/Plasma cell	IST1
2.37662100329603E-09	0.341419434399499	0.051	0.146	3.98274147732349E-05	B/Plasma cell	DDX59
2.3967832373155E-09	0.920841933907415	0.032	0.118	4.01652934909331E-05	B/Plasma cell	TBP
2.45017831196259E-09	0.751395369220365	0.056	0.157	4.10600881518692E-05	B/Plasma cell	JADE2
2.46883591545764E-09	0.940076147535974	0.105	0.231	4.13727522712391E-05	B/Plasma cell	GATAD2B
2.46910470925918E-09	0.734708180812614	0.073	0.181	4.13772567177653E-05	B/Plasma cell	CRTC2
2.49018289279061E-09	0.472824302391414	0.058	0.16	4.1730484917385E-05	B/Plasma cell	SFT2D1
2.54293934726818E-09	1.05283482128765	0.058	0.16	4.26145775815202E-05	B/Plasma cell	RELA
2.54521223730526E-09	0.897639221899599	0.047	0.141	4.26526666727616E-05	B/Plasma cell	RSF1
2.57671568794419E-09	0.786150326180076	0.058	0.159	4.31806014985687E-05	B/Plasma cell	VPS8

2.58744491094759E-09	0.68759814044638	0.233	0.428	4.33604018176597E-05	B/Plasma cell	RBM33
2.60490557947626E-09	0.277456501215763	0.028	0.112	4.36530077008632E-05	B/Plasma cell	FBXO30
2.6383230241761E-09	0.940159226169912	0.06	0.161	4.42130172391431E-05	B/Plasma cell	MFSD14A
2.6462581718052E-09	0.801402515465646	0.028	0.112	4.43459944431116E-05	B/Plasma cell	AXIN1
2.65266948862089E-09	0.779005951991408	0.077	0.187	4.44534352903088E-05	B/Plasma cell	SLC30A7
2.65962609525412E-09	0.960005081188419	0.169	0.331	4.45700141042685E-05	B/Plasma cell	ZMIZ2
2.7019913825435E-09	0.606796560307206	0.083	0.195	4.5279971588664E-05	B/Plasma cell	SPOP
2.74618264109793E-09	0.372696698653825	0.053	0.148	4.60205286995191E-05	B/Plasma cell	OGG1
2.84986769163039E-09	0.824175822892571	0.137	0.279	4.7758082776342E-05	B/Plasma cell	CASC3
2.88418208456103E-09	0.724548809024729	0.047	0.141	4.83331233730737E-05	B/Plasma cell	SNRNP48
2.9000156130983E-09	0.593404186925262	0.088	0.202	4.85984616443014E-05	B/Plasma cell	STIM2
2.93075355498201E-09	1.02201667522005	0.124	0.262	4.91135680743885E-05	B/Plasma cell	ZRANB2
2.99302697594251E-09	0.471928430844433	0.049	0.145	5.01571460628446E-05	B/Plasma cell	PRKACB
3.0120486976558E-09	0.700851703664748	0.099	0.219	5.0475912075316E-05	B/Plasma cell	NIN
3.08070536051962E-09	0.353768747939818	0.032	0.117	5.16264604315878E-05	B/Plasma cell	REXO4
3.08908297359462E-09	1.02013941149472	0.079	0.19	5.17668524714987E-05	B/Plasma cell	NCK2
3.14147184118699E-09	0.648466134040599	0.075	0.185	5.26447851146116E-05	B/Plasma cell	CHORDC1
3.14913132347013E-09	0.390719258756951	0.039	0.129	5.27731427187124E-05	B/Plasma cell	PPME1
3.184218275201E-09	0.880753337736459	0.088	0.204	5.33611298558183E-05	B/Plasma cell	PCNX1
3.19922145680889E-09	0.657740436390578	0.066	0.17	5.36125531732034E-05	B/Plasma cell	UCKL1
3.2113704891427E-09	0.759029592536442	0.105	0.229	5.38161466570534E-05	B/Plasma cell	IFNGR2
3.22831847736148E-09	0.77650443115061	0.058	0.158	5.41001610436236E-05	B/Plasma cell	RNF169
3.23654632409316E-09	0.917506551914612	0.079	0.192	5.42380432991532E-05	B/Plasma cell	CAMK1D
3.25379253888828E-09	0.5274199808331	0.038	0.126	5.45270553666898E-05	B/Plasma cell	DEPDC5
3.25419277163842E-09	1.24097379775353	0.126	0.265	5.45337624671166E-05	B/Plasma cell	STK38
3.28372275165592E-09	0.683355800358491	0.083	0.194	5.502862587225E-05	B/Plasma cell	GTF2F1
3.29662304719128E-09	0.488676372739988	0.075	0.183	5.52448090248315E-05	B/Plasma cell	NUDT4
3.45172009457037E-09	0.590844548684122	0.049	0.145	5.78439253448103E-05	B/Plasma cell	CLPX
3.46690586887346E-09	0.362624510828949	0.041	0.132	5.80984085505815E-05	B/Plasma cell	ZNF224
3.47412726442966E-09	0.897150328236215	0.081	0.193	5.82194246973122E-05	B/Plasma cell	SH3GLB1
3.54026398559657E-09	0.900122466876829	0.066	0.17	5.93277438706273E-05	B/Plasma cell	TBRG4
3.63811663498031E-09	0.572020626570621	0.045	0.137	6.0967558569E-05	B/Plasma cell	ELAC2
3.63838786816295E-09	0.395761064557652	0.026	0.108	6.09721038946747E-05	B/Plasma cell	FOXJ2
3.66326596953772E-09	0.5824466275294	0.092	0.209	6.13890111175131E-05	B/Plasma cell	MAML1
3.73531429545182E-09	0.709194433850174	0.098	0.219	6.25963969631816E-05	B/Plasma cell	SKIL
3.79074904292984E-09	0.727831410896264	0.041	0.132	6.35253724614182E-05	B/Plasma cell	TM2D1
3.79524393516394E-09	0.545101720902637	0.051	0.145	6.36006978654772E-05	B/Plasma cell	CYB561D2
3.98078842018208E-09	0.470947024619356	0.069	0.174	6.67100523454113E-05	B/Plasma cell	HPS5
4.05017274536073E-09	0.96179710734044	0.116	0.246	6.78727948667551E-05	B/Plasma cell	PAN3
4.08444897514714E-09	0.852862152792621	0.069	0.174	6.84471959255158E-05	B/Plasma cell	GEMIN7
4.09932499858359E-09	0.925437211347444	0.047	0.14	6.86964883262638E-05	B/Plasma cell	MED22
4.11825489423457E-09	1.41928791195079	0.088	0.205	6.9013715517583E-05	B/Plasma cell	PRPF39
4.1798937785514E-09	0.616350342247739	0.049	0.144	7.00466599409643E-05	B/Plasma cell	TTC13
4.18715953212061E-09	0.537660853855532	0.041	0.132	7.01684194392772E-05	B/Plasma cell	SCNM1
4.255627870577E-09	0.696214443738421	0.084	0.198	7.13158118551294E-05	B/Plasma cell	MED10
4.26097714844984E-09	0.830899648945435	0.066	0.17	7.14054550537224E-05	B/Plasma cell	UBAP1
4.31526964659968E-09	0.788234726290394	0.071	0.176	7.23152887377175E-05	B/Plasma cell	VPS39
4.31770281865085E-09	0.660855595718913	0.054	0.151	7.23560638349509E-05	B/Plasma cell	RBM12
4.38990134415917E-09	0.57980425755075	0.053	0.148	7.35659667254194E-05	B/Plasma cell	SIRT7
4.39701299538798E-09	0.955636292304833	0.169	0.326	7.36851437767118E-05	B/Plasma cell	GPBP1
4.42540459079735E-09	0.684760608403395	0.064	0.165	7.41609301325819E-05	B/Plasma cell	N4BP1
4.44309951744521E-09	0.489970945649411	0.041	0.131	7.44574617133468E-05	B/Plasma cell	WASHC2A
4.48920182668381E-09	0.98000945976255	0.113	0.241	7.52300442115674E-05	B/Plasma cell	NFX1
4.53446240004415E-09	1.04479995977332	0.092	0.21	7.59885208999398E-05	B/Plasma cell	ZBTB25
4.5638031178988E-09	0.308464107555123	0.058	0.156	7.6480212649748E-05	B/Plasma cell	PRR12
4.57245315559387E-09	0.840025582566573	0.069	0.174	7.6625169981442E-05	B/Plasma cell	TRIM33
4.58665978796775E-09	1.18644185605583	0.062	0.165	7.68632447267635E-05	B/Plasma cell	TEP1
4.59547372807115E-09	1.04370069463609	0.066	0.169	7.70109487350163E-05	B/Plasma cell	PCNT
4.61895419487839E-09	0.389278218520745	0.049	0.142	7.7404434397772E-05	B/Plasma cell	PATL1
4.65901787255413E-09	0.593132108823414	0.058	0.157	7.8075821508262E-05	B/Plasma cell	EXOC6
4.761695576648E-09	0.506393662845533	0.049	0.142	7.97649447346720E-05	B/Plasma cell	BCAS3
4.77909594295616E-09	0.702514841227768	0.054	0.151	8.00880898120594E-05	B/Plasma cell	ANP32A
4.80792653799452E-09	0.91404974037133	0.133	0.271	8.05712329237122E-05	B/Plasma cell	NUP210

4.82256444909826E-09	0.308997546044032	0.03	0.112	8.08165350379887E-05	B/Plasma cell	VMAC
4.88622351011957E-09	0.925809757198363	0.083	0.194	8.18833335825837E-05	B/Plasma cell	TNIP2
4.92208297722008E-09	0.524602850104361	0.036	0.122	8.24842665322541E-05	B/Plasma cell	PDPK1
4.93356549236289E-09	0.293496346908573	0.028	0.11	8.26766905210173E-05	B/Plasma cell	ZNF335
4.93769316206675E-09	0.777424567678808	0.068	0.171	8.27458620099146E-05	B/Plasma cell	TNFRSF10B
4.94971755501166E-09	0.641422647407345	0.043	0.133	8.29473667868854E-05	B/Plasma cell	KMT5A
5.00281696231937E-09	0.684245921438696	0.047	0.139	8.3837206654548E-05	B/Plasma cell	CDC27
5.0222436228426E-09	0.689821880239503	0.084	0.196	8.41627586315962E-05	B/Plasma cell	NXF1
5.03402225408955E-09	0.849842844983627	0.086	0.199	8.43601449340327E-05	B/Plasma cell	PHF8
5.07509695095301E-09	0.445092757001076	0.053	0.148	8.50484747040705E-05	B/Plasma cell	IGBP1
5.10184872042597E-09	0.614268507080433	0.068	0.168	8.54967808568985E-05	B/Plasma cell	ERCC1
5.13484998974462E-09	1.11449013822527	0.113	0.24	8.60498161281403E-05	B/Plasma cell	FAM102A
5.30146008430262E-09	0.722726501039348	0.171	0.325	8.88418680927433E-05	B/Plasma cell	TGFBR2
5.4900125232329E-09	0.839139113096742	0.195	0.361	9.2001629864337E-05	B/Plasma cell	RHOA
5.56463377086729E-09	1.0329065390024	0.098	0.215	9.3252132732194E-05	B/Plasma cell	CCNDBP1
5.68594591495827E-09	1.25898840288067	0.056	0.154	9.52850816428708E-05	B/Plasma cell	PIK3C2B
5.73816593086935E-09	0.67303882048001	0.09	0.202	9.61601846695086E-05	B/Plasma cell	OTUB1
5.74736030718999E-09	0.383767746852027	0.043	0.132	9.63142640278899E-05	B/Plasma cell	SNAPC4
5.77777363471842E-09	1.07244422331025	0.034	0.119	9.68239305706113E-05	B/Plasma cell	KRAS
5.78470389141055E-09	0.604897361387681	0.069	0.174	9.6940067812258E-05	B/Plasma cell	SIK3
5.92317872432561E-09	0.551016557423308	0.032	0.115	9.92606290622485E-05	B/Plasma cell	SCLT1
5.97635055677435E-09	0.585828825852407	0.075	0.182	0.000100151682630425	B/Plasma cell	PPHLN1
6.03284533566058E-09	0.847011841182858	0.139	0.275	0.000101098422135	B/Plasma cell	DEDD2
6.03442560087137E-09	0.618404000190907	0.069	0.172	0.000101124904219402	B/Plasma cell	EIF3J
6.14146164584078E-09	0.539954379444242	0.047	0.138	0.000102918614261	B/Plasma cell	DCAF15
6.15316750196028E-09	0.610924249440393	0.083	0.191	0.00010311478099785	B/Plasma cell	NARF
6.19998490240011E-09	0.354805565940827	0.075	0.182	0.000103899346994421	B/Plasma cell	EPM2AIP1
6.33924941564255E-09	1.12100445258076	0.064	0.166	0.000106233141707338	B/Plasma cell	KIF2A
6.42563309212058E-09	0.423581677915261	0.043	0.133	0.000107680759357757	B/Plasma cell	POLM
6.47875842285431E-09	0.6262388804833	0.069	0.172	0.000108571033650192	B/Plasma cell	PITPNA
6.60461499334033E-09	0.578530894106756	0.056	0.152	0.000110680138058397	B/Plasma cell	NMRK1
6.89334677324083E-09	0.326141009981288	0.032	0.114	0.00011551870522597	B/Plasma cell	TLK2
6.92794916697321E-09	0.608202351788374	0.051	0.144	0.000116098572140137	B/Plasma cell	SIKE1
6.94743784299822E-09	0.856332673875926	0.054	0.15	0.000116425163372964	B/Plasma cell	YAF2
7.01429060393414E-09	0.812001612929619	0.105	0.226	0.000117545481940728	B/Plasma cell	GOLGA3
7.11022771549099E-09	0.866380935114369	0.101	0.218	0.000119153196056198	B/Plasma cell	MBTPS1
7.1927803083774E-09	0.446107245192344	0.034	0.117	0.000120536612407788	B/Plasma cell	ERCC6
7.21295980773535E-09	0.574845186114678	0.047	0.138	0.000120874780458029	B/Plasma cell	COPS7B
7.2642658081663E-09	0.265140796880682	0.034	0.117	0.000121734566413251	B/Plasma cell	ZDHHC17
7.31123023876599E-09	1.2473373592904	0.045	0.135	0.00012252159634124	B/Plasma cell	POLH
7.43188215451374E-09	0.546655702523419	0.024	0.103	0.000124543481145341	B/Plasma cell	TMA16
7.56795154039659E-09	0.478947288245164	0.047	0.137	0.000126823731913966	B/Plasma cell	PCBP2
7.72137614645485E-09	0.924979013009543	0.126	0.253	0.00012939482146229	B/Plasma cell	STX5
7.8254472732667E-09	1.19502566292781	0.038	0.124	0.000131138845405403	B/Plasma cell	TTC21B
7.84952043066959E-09	0.523850885844781	0.039	0.126	0.000131542263377161	B/Plasma cell	DENND5A
7.97481958847169E-09	0.53569968977721	0.032	0.114	0.000133642026663609	B/Plasma cell	ACTR6
8.00538909324387E-09	0.595795451115879	0.101	0.218	0.000134154310424581	B/Plasma cell	PFDN5
8.11996893220601E-09	1.03665466870002	0.075	0.181	0.000136074439365908	B/Plasma cell	MFN1
8.14004842922166E-09	0.472813629879757	0.047	0.136	0.000136410931576897	B/Plasma cell	SP2
8.14859543614345E-09	0.589549479452016	0.056	0.151	0.000136554162318892	B/Plasma cell	MTMR6
8.17302550874801E-09	0.386619704978084	0.036	0.12	0.000136963561475599	B/Plasma cell	DEPRECATED-ENSG00000197714
8.19049498342699E-09	0.675631537543248	0.049	0.14	0.000137256314932269	B/Plasma cell	SRSF1
8.21881072857165E-09	0.874373109975824	0.036	0.12	0.000137730830189404	B/Plasma cell	APC
8.26305086672198E-09	1.17294344630633	0.169	0.328	0.000138472206424527	B/Plasma cell	STAT6
8.2702774157834E-09	0.531096135150007	0.045	0.135	0.000138593308933698	B/Plasma cell	SMURF1
8.3129817244278E-09	0.524383196791375	0.062	0.16	0.000139308947737961	B/Plasma cell	PHAX
8.3747337519493E-09	0.619763980791736	0.058	0.154	0.000140343788215166	B/Plasma cell	H3F3B
8.38084962686918E-09	0.360569210094037	0.056	0.15	0.000140446278047074	B/Plasma cell	XPNPEP1
8.46749612344189E-09	0.7640485904244	0.088	0.199	0.000141898300036639	B/Plasma cell	FBXO46
8.5434225113273E-09	0.662790347832602	0.073	0.177	0.000143170674444823	B/Plasma cell	WASF2
8.62236046991721E-09	0.945437589958894	0.051	0.145	0.000144493516754873	B/Plasma cell	ZBTB21
8.71199554214514E-09	1.18730520568562	0.054	0.15	0.000145995621295268	B/Plasma cell	COMMD1
8.74445967502121E-09	0.404630414942992	0.034	0.117	0.000146539655234005	B/Plasma cell	C16orf87

8.76902724214565E-09	0.531796250920523	0.051	0.141	0.000146951358523877	B/Plasma cell	DEPRECATED-ENSG00000116489
8.92304652411274E-09	0.45557777380691	0.066	0.164	0.000149532413651081	B/Plasma cell	TIMM10B
9.2508031922595E-09	0.542865054986232	0.028	0.107	0.000155024959895885	B/Plasma cell	TXNDC16
9.34058980666399E-09	1.12332430876164	0.06	0.159	0.000156529603980075	B/Plasma cell	CYB561A3
9.39546977815915E-09	0.477902111252375	0.041	0.128	0.000157449282542391	B/Plasma cell	HIRA
9.45806394454887E-09	0.394091550952211	0.032	0.113	0.00015849823558275	B/Plasma cell	NOL4L
9.51674776617357E-09	0.582144081255726	0.039	0.126	0.000159481659065537	B/Plasma cell	ZNF263
9.5309061782146E-09	0.545410184038467	0.049	0.139	0.00015971892573452	B/Plasma cell	MRI1
9.55153383846208E-09	0.985649155640465	0.047	0.137	0.000160064604064948	B/Plasma cell	SPOPL
9.72378598130946E-09	1.0345201145279	0.045	0.134	0.000162951205474784	B/Plasma cell	KLF16
9.7679155579499E-09	0.468732353353979	0.094	0.205	0.000163690728920124	B/Plasma cell	UFM1
9.80877359045567E-09	0.874989424960619	0.075	0.18	0.000164375427828856	B/Plasma cell	SMG1
9.81821240448872E-09	0.68297529059345	0.049	0.138	0.000164533603474422	B/Plasma cell	CMTR2
9.91863647874291E-09	0.467569542927344	0.036	0.12	0.000166216510110774	B/Plasma cell	SLC9A8
1.01825784131505E-08	0.881886412888634	0.12	0.248	0.000170639649047576	B/Plasma cell	MYO9B
1.02001554257511E-08	0.537124918206093	0.038	0.121	0.000170934204624737	B/Plasma cell	PRMT7
1.02461742910996E-08	0.253070182951319	0.036	0.119	0.000171705388770247	B/Plasma cell	TRAFD1
1.02669652969717E-08	0.83022028939791	0.058	0.154	0.000172053804446652	B/Plasma cell	BCL10
1.03164576195478E-08	0.990621808196629	0.071	0.174	0.000172883196788383	B/Plasma cell	EIF3G
1.04206649910905E-08	0.830104596439551	0.038	0.122	0.000174629503920694	B/Plasma cell	ZMYND19
1.08390362946838E-08	1.34994575376891	0.107	0.23	0.000181640570226311	B/Plasma cell	PIKFYVE
1.1038923986621E-08	0.84765515829769	0.092	0.203	0.000184990288167794	B/Plasma cell	ATG16L2
1.1101694493204E-08	0.432973886873901	0.036	0.119	0.000186042196317112	B/Plasma cell	BBS2
1.110356867076E-08	0.689301606178843	0.051	0.142	0.000186073603784596	B/Plasma cell	ZNF44
1.12789588028841E-08	0.825745214547342	0.081	0.188	0.000189012791618732	B/Plasma cell	ASCC3
1.14241927593121E-08	0.49057065654103	0.081	0.187	0.000191446622260553	B/Plasma cell	MYO1D
1.1488436338475E-08	0.491148659320597	0.024	0.101	0.000192523216160163	B/Plasma cell	INVS
1.17420831532188E-08	1.74266845996298	0.24	0.166	0.00019677382948164	B/Plasma cell	CD44
1.1760307548886E-08	0.478836752574841	0.062	0.158	0.000197079233904231	B/Plasma cell	GTF3C1
1.18415988691025E-08	1.07475558893048	0.109	0.231	0.00019844151384842	B/Plasma cell	DMTF1
1.18490175407944E-08	0.291493044126909	0.038	0.12	0.000198565835948633	B/Plasma cell	OSBPL11
1.18797949319176E-08	1.17491830350584	0.041	0.128	0.000199081603469075	B/Plasma cell	CSNK1G3
1.19782710546553E-08	0.496598614825409	0.047	0.136	0.000200731866333914	B/Plasma cell	TAF1
1.20004333879433E-08	0.764574522741832	0.086	0.193	0.000201103262715154	B/Plasma cell	KIFC2
1.22287212985907E-08	0.354686843357781	0.034	0.116	0.000204928911521783	B/Plasma cell	RPIA
1.24089109067121E-08	0.418909206622905	0.032	0.112	0.000207948528974681	B/Plasma cell	BAP1
1.25681261739146E-08	0.921071882925704	0.216	0.39	0.000210616658422461	B/Plasma cell	RERE
1.26326309925348E-08	0.396749667646533	0.032	0.112	0.000211697630172898	B/Plasma cell	TOM1L2
1.2666511740402E-08	0.875705490364379	0.073	0.175	0.000212265403745657	B/Plasma cell	USP38
1.26980184274719E-08	0.841712945375134	0.062	0.158	0.000212793392807574	B/Plasma cell	PGGT1B
1.27253323458332E-08	1.52423361542861	0.143	0.285	0.000213251119451473	B/Plasma cell	STX7
1.27567484251654E-08	0.293838343094012	0.032	0.112	0.000213777590108922	B/Plasma cell	ING5
1.28172349009963E-08	0.377092858579346	0.034	0.116	0.000214791222470897	B/Plasma cell	ZNF414
1.30164218866552E-08	1.01532306543091	0.064	0.164	0.000218129197976568	B/Plasma cell	OSER1
1.3253012722273E-08	0.614701367915302	0.137	0.269	0.000222093987199851	B/Plasma cell	TLN1
1.34867167038891E-08	0.687721596613472	0.064	0.161	0.000226010398523773	B/Plasma cell	PRPF8
1.35229732316239E-08	1.13081491661172	0.126	0.259	0.000226617985415554	B/Plasma cell	CLCN7
1.3559627193653E-08	0.796762019194728	0.148	0.286	0.000227232232511236	B/Plasma cell	TNFRSF14
1.35599696664162E-08	0.906328870197182	0.062	0.159	0.000227237971669802	B/Plasma cell	MTMR3
1.36157664477203E-08	0.44260669827308	0.026	0.104	0.000228173014130897	B/Plasma cell	CACTIN
1.38014912070569E-08	0.590573819502985	0.032	0.112	0.000231285389647859	B/Plasma cell	WDR27
1.38284618120409E-08	0.304137327453728	0.032	0.111	0.000231737363046181	B/Plasma cell	JPT1
1.41277796341652E-08	0.780894992204268	0.109	0.229	0.00023675333110934	B/Plasma cell	SRRM1
1.42073099554037E-08	0.296831105667794	0.049	0.137	0.000238086100232655	B/Plasma cell	RASA1
1.43315030026688E-08	0.950959405623193	0.101	0.221	0.000240167327318724	B/Plasma cell	PRPF4B
1.44380665610475E-08	0.319130389943647	0.034	0.115	0.000241953119430034	B/Plasma cell	RPP30
1.45078895819525E-08	0.638102302677429	0.034	0.115	0.00024312321361436	B/Plasma cell	AARS
1.45271600065977E-08	0.307359184033082	0.024	0.1	0.000243446147390564	B/Plasma cell	NOMO1
1.46055445817534E-08	1.16563624386031	0.176	0.333	0.000244759716101023	B/Plasma cell	CD47
1.48675525909115E-08	0.842937372920256	0.084	0.192	0.000249150446318495	B/Plasma cell	GNPTAB
1.52324546487185E-08	0.290310070991863	0.036	0.118	0.000255265475003225	B/Plasma cell	MIGA2
1.52824299152059E-08	0.906448463554791	0.068	0.167	0.00025610296051902	B/Plasma cell	KRI1
1.56212851407381E-08	0.722890877044303	0.06	0.155	0.000261781496388488	B/Plasma cell	EEF1AKMT2

1.58262282701481E-08	0.39225284915524	0.073	0.172	0.000265215933351142	B/Plasma cell	PCIF1
1.58880254962827E-08	0.64514091595541	0.077	0.18	0.000266251531266706	B/Plasma cell	LNPEP
1.62790412444632E-08	0.310398648946798	0.056	0.147	0.000272804173174715	B/Plasma cell	TINF2
1.66938040568977E-08	0.723453544404362	0.092	0.203	0.000279754768385492	B/Plasma cell	OAS1
1.66948528158918E-08	0.514875370077434	0.032	0.112	0.000279772343488714	B/Plasma cell	NIPSNAP2
1.68539976515622E-08	0.833864003929484	0.051	0.141	0.000282439292644879	B/Plasma cell	CCDC141
1.68739273770068E-08	0.789521236236055	0.24	0.43	0.00028277327498388	B/Plasma cell	EVL
1.68812521636542E-08	0.80300067494672	0.083	0.189	0.000282896023758517	B/Plasma cell	ATAD2B
1.72811445973026E-08	0.465651698353526	0.062	0.159	0.000289597421161597	B/Plasma cell	ODF3B
1.73340758595454E-08	1.26884291761882	0.135	0.271	0.000290484443254262	B/Plasma cell	KDM4C
1.79284064532362E-08	1.96809801443191	0.116	0.06	0.000300444235343332	B/Plasma cell	NCKAP1L
1.84015276996244E-08	0.619491355173312	0.043	0.129	0.000308372801190306	B/Plasma cell	HEATR1
1.88887231345469E-08	0.873066417818293	0.131	0.262	0.000316537222288738	B/Plasma cell	TSC22D4
1.89815966166373E-08	0.771582876561093	0.032	0.112	0.000318093596101608	B/Plasma cell	PHF20
1.91284216254054E-08	0.526522777409362	0.054	0.144	0.000320554089598544	B/Plasma cell	CACUL1
1.92447277328121E-08	1.46098782689411	0.246	0.444	0.000322503147346466	B/Plasma cell	EZR
1.93648949787628E-08	0.835464997613312	0.062	0.157	0.000324516910054107	B/Plasma cell	USP16
1.98578925599576E-08	0.398249343630047	0.083	0.188	0.000332778563519769	B/Plasma cell	PPP1R12A
1.99206309136178E-08	0.950906629259212	0.075	0.178	0.000333829932850407	B/Plasma cell	TUBA4A
2.00719810292972E-08	0.872217111456554	0.129	0.259	0.000336366258088963	B/Plasma cell	NDUFB8
2.07311851500848E-08	0.988550565809926	0.154	0.295	0.000347413200745121	B/Plasma cell	KDM5C
2.08509941784479E-08	0.570742621337243	0.032	0.112	0.000349420960442429	B/Plasma cell	ATG4D
2.08837851398905E-08	0.690957384315645	0.051	0.142	0.000349970471374285	B/Plasma cell	CHD6
2.09120001090245E-08	1.0927789950247	0.113	0.232	0.000350443297827033	B/Plasma cell	MTA2
2.17699324802683E-08	0.398423866641747	0.028	0.104	0.000364820528504336	B/Plasma cell	WDR75
2.1801137362444E-08	0.302895690978911	0.03	0.106	0.000365343459919836	B/Plasma cell	BAHD1
2.31732456258058E-08	0.624686955832211	0.043	0.128	0.000388337250197254	B/Plasma cell	DEPRECATED-ENSG00000188529
2.35975753387949E-08	0.606281892984566	0.049	0.136	0.000395448167527524	B/Plasma cell	KIAA0355
2.37023404258673E-08	0.3353737396248	0.039	0.121	0.000397203820856684	B/Plasma cell	CREB1
2.38805769971409E-08	0.650656353887543	0.073	0.174	0.000400190709318088	B/Plasma cell	SACM1L
2.3941518006036E-08	0.942066450724075	0.092	0.202	0.000401211958745151	B/Plasma cell	UTRN
2.41455938457266E-08	0.510572996723836	0.18	0.327	0.000404631861666687	B/Plasma cell	WSB1
2.43607806334755E-08	0.621228130417397	0.036	0.116	0.000408237961855783	B/Plasma cell	TXLNG
2.44515079819087E-08	0.848182002159741	0.056	0.147	0.000409758370760825	B/Plasma cell	URM1
2.45310724345518E-08	0.832902155555522	0.054	0.145	0.00041109171185822	B/Plasma cell	EXOSC6
2.47207619941823E-08	0.660077853025845	0.144	0.279	0.000414270529498507	B/Plasma cell	SELENOS
2.48053682058925E-08	0.740041962738284	0.156	0.299	0.000415688360394346	B/Plasma cell	SUN2
2.49819683178894E-08	0.74833826533476	0.028	0.103	0.00041864782507119	B/Plasma cell	TMEM116
2.51846051935168E-08	0.855909742755271	0.041	0.124	0.000422043613832955	B/Plasma cell	THAP12
2.52559971023238E-08	0.71068178323557	0.047	0.133	0.000423239999440743	B/Plasma cell	PRPF4
2.55782627560298E-08	0.711800420058468	0.045	0.13	0.000428640527265547	B/Plasma cell	CARNMT1
2.56593030107666E-08	0.697004512875333	0.032	0.11	0.000429998599854427	B/Plasma cell	SENP7
2.58144384248261E-08	0.663559290180464	0.039	0.121	0.000432598359123236	B/Plasma cell	PJA1
2.61011448356211E-08	0.874833571586808	0.114	0.235	0.000437402985155338	B/Plasma cell	POLR2A
2.61780186183424E-08	1.04806964969087	0.069	0.167	0.000438691236006181	B/Plasma cell	MAN1B1
2.63520492400636E-08	0.316213109583299	0.039	0.121	0.000441607641164986	B/Plasma cell	RALGDS
2.6509495186371E-08	0.352074026630516	0.026	0.101	0.000444246120333205	B/Plasma cell	ANP32B
2.65910414276427E-08	0.748996487994722	0.064	0.159	0.000445612672244437	B/Plasma cell	SLU7
2.76295351033556E-08	0.787391176285028	0.045	0.131	0.000463015749262032	B/Plasma cell	DHDDS
2.76465451210195E-08	0.408101335793126	0.026	0.1	0.000463300803138045	B/Plasma cell	EID2
2.7668588554491E-08	0.900476430233821	0.062	0.155	0.000463670206996161	B/Plasma cell	TMEM11
2.77315370235417E-08	0.482654002002072	0.06	0.152	0.000464725097440513	B/Plasma cell	PLXNA3
2.79689316335047E-08	0.599470685595304	0.086	0.19	0.000468703356314272	B/Plasma cell	VDAC2
2.89900351288314E-08	0.345879619867463	0.028	0.104	0.000485815008688957	B/Plasma cell	HCCS
2.91702235803106E-08	0.608254629712298	0.161	0.302	0.000488834606758845	B/Plasma cell	SH3BGRL3
3.00399971069787E-08	0.51536982846123	0.038	0.119	0.000503410271518749	B/Plasma cell	ACOX3
3.03752934441874E-08	0.550252852825371	0.079	0.179	0.000509029167537693	B/Plasma cell	MED24
3.07835529510044E-08	0.754914270447955	0.062	0.155	0.000515870780352931	B/Plasma cell	MAN2B2
3.11809361001413E-08	0.99614012448717	0.039	0.122	0.000522530127166168	B/Plasma cell	ZNF397
3.13262578528461E-08	0.612408303798278	0.026	0.1	0.000524965429097996	B/Plasma cell	TTC28
3.1562406536384E-08	0.524075656919873	0.039	0.122	0.000528922808736722	B/Plasma cell	CENPC
3.17978571530666E-08	0.27044649215881	0.032	0.108	0.000532868490171091	B/Plasma cell	ZC3H11A
3.2363760653525E-08	0.416462916134893	0.041	0.124	0.000542351901031771	B/Plasma cell	RAE1

3.24373581834412E-08	1.12564631827212	0.051	0.138	0.000543585248438107	B/Plasma cell	LCORL
3.29459770202469E-08	0.669865998110109	0.073	0.171	0.000552108682905297	B/Plasma cell	SECISBP2
3.39305676538582E-08	0.509313977528604	0.18	0.328	0.000568608452743356	B/Plasma cell	ADGRE5
3.43085972172789E-08	0.438393440877337	0.064	0.157	0.000574943472167159	B/Plasma cell	MKRN1
3.43512847679891E-08	1.14983161188559	0.128	0.255	0.000575658830141961	B/Plasma cell	RALGPS2
3.45147933573725E-08	0.386837562494873	0.068	0.163	0.000578398907082848	B/Plasma cell	PLCL2
3.48654120892687E-08	0.616463200287919	0.06	0.151	0.000584274575791965	B/Plasma cell	YJU2
3.51509597222009E-08	0.639219291720456	0.079	0.179	0.000589059783024642	B/Plasma cell	MAFK
3.51583213015802E-08	1.30354214676814	0.043	0.126	0.000589183148371881	B/Plasma cell	DEPRECATED-ENSG00000072803
3.52211726880412E-08	2.09071808925432	0.101	0.05	0.000590236411906195	B/Plasma cell	ST8SIA4
3.53012424875402E-08	0.947052474278964	0.077	0.178	0.000591578221606198	B/Plasma cell	STRBP
3.55240038012583E-08	0.284853418409035	0.039	0.12	0.000595311255701487	B/Plasma cell	CCDC107
3.62676538200186E-08	0.727510458436878	0.056	0.145	0.000607773342715872	B/Plasma cell	SPAG7
3.63210744338068E-08	2.1218138199622	0.302	0.247	0.000608668565361735	B/Plasma cell	STK4
3.63831852699966E-08	0.918471498921952	0.045	0.13	0.000609709418754603	B/Plasma cell	PIK3C2A
3.65278293191729E-08	1.18964196041783	0.099	0.212	0.000612133363730699	B/Plasma cell	SRCAP
3.75250488917733E-08	0.317178671370733	0.034	0.111	0.000628844769328337	B/Plasma cell	CHFR
3.83512771137145E-08	2.22604508030682	0.257	0.194	0.000642690701871627	B/Plasma cell	ARHGEF1
3.8373193768587E-08	0.503915610314851	0.038	0.116	0.000643057981173981	B/Plasma cell	TDP1
3.85839956315336E-08	0.838905096289769	0.062	0.155	0.00064659059879324	B/Plasma cell	TRAPPC8
3.86982392678567E-08	0.41124012186453	0.043	0.126	0.000648505093650743	B/Plasma cell	HMG20A
3.89242420621203E-08	0.898963447480453	0.047	0.132	0.000652292448477011	B/Plasma cell	AQR
3.90071603351427E-08	0.681651771735851	0.098	0.208	0.000653681992896321	B/Plasma cell	UBE4B
3.92361987728973E-08	1.02291388291513	0.075	0.176	0.000657520219036214	B/Plasma cell	BAK1
3.95956540501428E-08	1.40271611471326	0.056	0.148	0.000663543970572292	B/Plasma cell	USP6NL
3.96565452139575E-08	1.09926140823377	0.184	0.343	0.0006645643846955	B/Plasma cell	UBR2
4.00235638825814E-08	0.955649697807066	0.105	0.219	0.0006707148835443	B/Plasma cell	EEF1B2
4.01152778403833E-08	0.624082111117435	0.043	0.126	0.000672251826049144	B/Plasma cell	CASD1
4.03369252899059E-08	0.63427288167249	0.049	0.134	0.000675966194008242	B/Plasma cell	PPIL2
4.08069935844931E-08	0.299500379110581	0.064	0.156	0.000683843598488936	B/Plasma cell	ERGIC2
4.08210734614467E-08	1.09948083547232	0.094	0.203	0.000684079549066924	B/Plasma cell	ARID1B
4.09674961482356E-08	0.534517927714113	0.045	0.13	0.000686533300452132	B/Plasma cell	RAPGEF6
4.10124668003264E-08	1.01573112780382	0.06	0.152	0.000687286918639869	B/Plasma cell	USP8
4.10895988539631E-08	0.45866873757014	0.03	0.105	0.000688579497594714	B/Plasma cell	PAK1IP1
4.12064890752142E-08	0.465794550851833	0.036	0.113	0.00069053834392244	B/Plasma cell	POLR1A
4.23622953666684E-08	0.866101201232849	0.038	0.117	0.000709907345754628	B/Plasma cell	SLC35E2A
4.25095349754371E-08	0.937676952215585	0.163	0.31	0.000712374787118375	B/Plasma cell	MARCH7
4.27482957394648E-08	0.971989274783898	0.111	0.225	0.000716375940001951	B/Plasma cell	PBX2
4.35362190418074E-08	0.286185342713366	0.058	0.146	0.000729579958702608	B/Plasma cell	CARD19
4.36277251929761E-08	0.467769323259314	0.038	0.116	0.000731113418783893	B/Plasma cell	HAUS3
4.38973159846887E-08	1.04043603876951	0.105	0.217	0.000735631221271412	B/Plasma cell	TTC14
4.44505507607568E-08	0.829462144474498	0.043	0.125	0.000744902329648762	B/Plasma cell	PDSS2
4.45665492335274E-08	1.13882149414825	0.081	0.182	0.000746846232055452	B/Plasma cell	DDX39A
4.53498431137312E-08	0.99288876254628	0.028	0.102	0.000759972670899907	B/Plasma cell	COA1
4.5797997148985E-08	1.16839248955516	0.103	0.217	0.00076748283622269	B/Plasma cell	TRAPPC10
4.63761439288323E-08	0.806577193885963	0.045	0.128	0.000777171419959371	B/Plasma cell	POLR2D
4.65287521982855E-08	0.971770813896264	0.107	0.22	0.000779728829338868	B/Plasma cell	NFAT5
4.7073105603495E-08	0.480589194153906	0.047	0.131	0.000788851103703369	B/Plasma cell	EIF3D
4.70965802497455E-08	1.00328734126051	0.028	0.102	0.000789244491825236	B/Plasma cell	CWC25
4.73401640464475E-08	0.617232480691261	0.051	0.137	0.000793326469090368	B/Plasma cell	NFYA
4.86310810453962E-08	1.00833571484886	0.079	0.181	0.000814959656158749	B/Plasma cell	TENT4A
4.92384454877549E-08	0.527761910066156	0.034	0.11	0.000825137869483797	B/Plasma cell	VPS35
4.98814715835075E-08	0.519119494466327	0.116	0.231	0.000835913700796419	B/Plasma cell	GNAI2
5.02858024572546E-08	0.68491277951169	0.028	0.101	0.000842689477578672	B/Plasma cell	CRNKL1
5.06181158127087E-08	0.557439620906316	0.036	0.113	0.000848258384789372	B/Plasma cell	SYS1
5.10319824646352E-08	0.431820700833958	0.064	0.156	0.000855193962142356	B/Plasma cell	SDE2
5.10967649747311E-08	0.495144665423862	0.101	0.212	0.000856279587446543	B/Plasma cell	SLC5A3
5.11005841274318E-08	1.14824025450624	0.171	0.32	0.000856343588807502	B/Plasma cell	MYO15B
5.15721284687264E-08	0.880306651829932	0.036	0.113	0.000864245728878917	B/Plasma cell	INO80
5.17823002241902E-08	0.728889502826061	0.069	0.164	0.00086776778715698	B/Plasma cell	C7orf26
5.29990409664449E-08	0.750033648482148	0.096	0.203	0.000888157928515684	B/Plasma cell	BUB3
5.42036684877773E-08	0.877318118528896	0.191	0.347	0.000908345076518172	B/Plasma cell	DDX6
5.47351385830843E-08	0.53282156075967	0.032	0.107	0.000917251452375327	B/Plasma cell	DRAM1

5.47476902614213E-08	0.966153030041651	0.109	0.224	0.000917461793400898	B/Plasma cell	SETX
5.51183854474652E-08	0.34646514122894	0.049	0.132	0.000923673903328621	B/Plasma cell	SPTY2D1
5.5135065432562E-08	1.20824195319271	0.105	0.22	0.000923953426518875	B/Plasma cell	DENND11
5.55544409079249E-08	0.527500848180691	0.043	0.125	0.000930981320735006	B/Plasma cell	SMG9
5.56652334642346E-08	0.863451507426801	0.054	0.143	0.000932837982393643	B/Plasma cell	RNF31
5.70595106495854E-08	0.852380209532459	0.051	0.137	0.000956203279465751	B/Plasma cell	FBRSL1
5.82158656819688E-08	0.682239538012274	0.058	0.148	0.000975581477098433	B/Plasma cell	TNKS2
5.86645973996359E-08	0.856436699837081	0.081	0.181	0.000983101323223098	B/Plasma cell	PNPLA6
5.96229342630956E-08	0.602261664427169	0.039	0.118	0.000999161132380956	B/Plasma cell	ZNF236
5.98784152921326E-08	0.588051998753397	0.039	0.119	0.00100344248346556	B/Plasma cell	SPRYD3
6.02887801903708E-08	1.31273121172397	0.054	0.143	0.00101031937843023	B/Plasma cell	TRMU
6.06929314710866E-08	0.956523320306206	0.047	0.131	0.00101709214559247	B/Plasma cell	USP28
6.115829341674E-08	0.498587170227962	0.058	0.146	0.00102489068107773	B/Plasma cell	ZKSCAN8
6.14141014867745E-08	0.611132852236026	0.034	0.109	0.00102917751271537	B/Plasma cell	TSPAN17
6.19773937154902E-08	0.912862471483296	0.088	0.192	0.00103861716388418	B/Plasma cell	ILKAP
6.29012409859481E-08	0.697377403532768	0.032	0.107	0.00105409899644252	B/Plasma cell	PIGK
6.33220112024235E-08	0.959005045302585	0.075	0.173	0.00106115026373021	B/Plasma cell	OXSR1
6.44629175921014E-08	0.726406574549522	0.06	0.149	0.00108026957300844	B/Plasma cell	MALT1
6.53840945131468E-08	0.867000328462597	0.038	0.115	0.00109570665585131	B/Plasma cell	ALDH16A1
6.84053990232334E-08	0.634486854809551	0.045	0.128	0.00114633767683134	B/Plasma cell	AAGAB
7.10167611803575E-08	0.335802313168251	0.032	0.106	0.00119009888386043	B/Plasma cell	FAM174A
7.12482205706787E-08	0.643741816812118	0.064	0.156	0.00119397768032343	B/Plasma cell	CDR2
7.14167571233547E-08	0.925533181473777	0.064	0.155	0.00119680201587318	B/Plasma cell	TRNT1
7.14822976558993E-08	0.717401059296218	0.056	0.144	0.00119790034411756	B/Plasma cell	ALG9
7.29467244555765E-08	1.3204296376284	0.071	0.167	0.00122244120842655	B/Plasma cell	USP33
7.35324507414531E-08	0.686861847494117	0.051	0.135	0.00123225680952527	B/Plasma cell	ZNRD2
7.35616599370771E-08	0.667318834143796	0.045	0.125	0.00123274629722554	B/Plasma cell	ZNF280D
7.52512286631451E-08	0.729803016467054	0.03	0.103	0.00126106008993698	B/Plasma cell	ATG5
7.64338728156831E-08	0.880091299124487	0.083	0.182	0.00128087884064522	B/Plasma cell	CCNT2
7.68370506540583E-08	1.13979011398847	0.077	0.175	0.00128763529486071	B/Plasma cell	AKIRIN1
7.69503091379013E-08	1.06886407535071	0.056	0.144	0.00128953328053295	B/Plasma cell	FOXK1
8.19152698095037E-08	0.628833017945412	0.045	0.127	0.00137273609146766	B/Plasma cell	RNF123
8.19916035682784E-08	0.550155512269838	0.049	0.132	0.00137401529259721	B/Plasma cell	ATXN1L
8.266633555716E-08	0.686993362224868	0.054	0.141	0.00138532245126689	B/Plasma cell	CCDC88A
8.30475603482159E-08	0.516058506874957	0.036	0.111	0.0013917110163154	B/Plasma cell	SRSF11
8.36958379789967E-08	0.464211980701855	0.036	0.111	0.00140257485285203	B/Plasma cell	PLA2G6
8.49629530115701E-08	0.968136336260769	0.105	0.216	0.00142380916656789	B/Plasma cell	CERT1
8.66824978385546E-08	0.369838308768611	0.039	0.118	0.0014526252987785	B/Plasma cell	FAM98A
8.90067013150683E-08	1.07297927952998	0.045	0.126	0.00149157430063791	B/Plasma cell	MIER3
9.06449511445496E-08	1.01909920205461	0.077	0.174	0.00151902809128036	B/Plasma cell	CLK4
9.13837489581531E-08	1.40530963083953	0.124	0.067	0.00153140886504073	B/Plasma cell	IL10RA
9.32302267975803E-08	0.911340981224914	0.113	0.226	0.00156235214067385	B/Plasma cell	ATP13A1
9.36762241231981E-08	0.731172032541733	0.06	0.148	0.00156982616385655	B/Plasma cell	MMADHC
9.61943052998265E-08	0.666412288845684	0.334	0.552	0.00161202416821449	B/Plasma cell	JUND
9.73931989935181E-08	0.831473789741663	0.045	0.125	0.00163211522873338	B/Plasma cell	IRGQ
1.00136400658446E-07	0.686905695140918	0.032	0.104	0.00167808580223424	B/Plasma cell	PHTF1
1.00670596323149E-07	0.415661990305131	0.036	0.11	0.00168703785318332	B/Plasma cell	NUDCD1
1.01452009871245E-07	0.506455814568678	0.039	0.117	0.00170013278142232	B/Plasma cell	LDAH
1.01493812098221E-07	2.13318770750638	0.101	0.052	0.00170083330314199	B/Plasma cell	ARHGAP30
1.02104164114276E-07	0.610574653789718	0.041	0.12	0.00171106158222704	B/Plasma cell	ZSWIM9
1.0260248699034E-07	1.3103122816455	0.139	0.268	0.00171941247698411	B/Plasma cell	RC3H2
1.02796843893557E-07	0.836917040673252	0.105	0.214	0.00172266950996822	B/Plasma cell	SPATS2
1.05337046623306E-07	0.737254439814319	0.071	0.164	0.00176523822731336	B/Plasma cell	HDAC8
1.06362216192839E-07	0.790763347381967	0.032	0.105	0.0017824180189596	B/Plasma cell	NIPAL3
1.06446975818325E-07	0.34228744710677	0.038	0.113	0.00178383842076349	B/Plasma cell	SSBP3
1.0695852179596E-07	0.509214122725227	0.094	0.197	0.0017924109082567	B/Plasma cell	ACTR1A
1.07317655853517E-07	1.28047718242544	0.062	0.153	0.00179842927679323	B/Plasma cell	ARID4A
1.0738009185734E-07	0.894353561410233	0.06	0.148	0.00179947557934531	B/Plasma cell	AARS2
1.12365308701556E-07	1.17015659664343	0.068	0.16	0.00188301784322067	B/Plasma cell	CAPZA1
1.17330898158886E-07	0.695189795489415	0.088	0.189	0.00196623119134661	B/Plasma cell	VPS36
1.22113558289647E-07	0.507495315943813	0.03	0.102	0.0020463790098179	B/Plasma cell	JRKL
1.23138892802715E-07	0.264455247674393	0.045	0.124	0.0020635615655879	B/Plasma cell	STAM
1.26428159441649E-07	0.548709931755824	0.073	0.167	0.00211868309592316	B/Plasma cell	ST3GAL1

1.26669115281555E-07	0.297099057740521	0.043	0.122	0.0021227210338883	B/Plasma cell	ZCCHC8
1.27039323252168E-07	0.330988251451037	0.088	0.188	0.00212892497905984	B/Plasma cell	P4HA1
1.28055435153072E-07	1.25949463386768	0.073	0.167	0.00214595298229519	B/Plasma cell	CHKB
1.30620666418315E-07	0.343685345830779	0.043	0.12	0.00218894112783812	B/Plasma cell	MTHFR
1.33311118698671E-07	1.97639763517122	0.122	0.066	0.00223402772715232	B/Plasma cell	RCSD1
1.33456300163193E-07	1.83881628231915	0.133	0.076	0.00223646067813479	B/Plasma cell	SERPINB9
1.336011050188E-07	0.858692679049539	0.077	0.171	0.00223888731790505	B/Plasma cell	MARCH8
1.34249268005909E-07	0.928185395360655	0.06	0.146	0.00224974923324303	B/Plasma cell	XPO6
1.35182674642065E-07	0.858900687112722	0.128	0.247	0.00226539126165173	B/Plasma cell	USP48
1.36413932868384E-07	1.44965595265701	0.073	0.169	0.00228602468700839	B/Plasma cell	MBD4
1.38034210541804E-07	0.986097791484868	0.174	0.319	0.00231317730025956	B/Plasma cell	PCMTD1
1.39499582351777E-07	0.37696266423656	0.051	0.131	0.00233773400105108	B/Plasma cell	CEP170
1.39769914193179E-07	0.269151184060073	0.06	0.145	0.00234226422204929	B/Plasma cell	CRIPT
1.43048213353035E-07	0.513536619231423	0.03	0.101	0.00239720195937015	B/Plasma cell	PPP2R3C
1.4314648960144E-07	0.580309241693809	0.034	0.106	0.00239884887274094	B/Plasma cell	CBWD3
1.4807146573704E-07	0.775385705821717	0.131	0.25	0.00248138162282131	B/Plasma cell	GNA13
1.50575139247949E-07	0.43568737879956	0.03	0.101	0.00252333818351713	B/Plasma cell	BCLAF3
1.5577361123269E-07	1.05171470741459	0.045	0.124	0.00261045417703743	B/Plasma cell	HARS2
1.56776643582255E-07	1.24162371066904	0.062	0.15	0.00262726299315143	B/Plasma cell	ATG13
1.57241269260753E-07	1.08141596842872	0.083	0.182	0.0026350491902717	B/Plasma cell	KIAA1109
1.57986385878355E-07	0.830917125948794	0.139	0.262	0.00264753585454947	B/Plasma cell	ANKRD11
1.58120690683347E-07	0.645472085170616	0.084	0.183	0.00264978653447153	B/Plasma cell	HEXIM1
1.60566037921731E-07	0.838974775378964	0.036	0.11	0.00269076566349238	B/Plasma cell	CRAMP1
1.63147125235743E-07	1.0512886938481	0.096	0.197	0.00273401952470058	B/Plasma cell	KLHL24
1.65503881750732E-07	0.963202384487449	0.06	0.146	0.00277351405037876	B/Plasma cell	CLEC16A
1.7189522297537E-07	0.971638714785829	0.069	0.159	0.00288062014662125	B/Plasma cell	PITHD1
1.72499054091923E-07	0.595165626006232	0.148	0.271	0.00289073914847245	B/Plasma cell	AKIRIN2
1.75622923418189E-07	0.899248034379445	0.069	0.16	0.00294308895064201	B/Plasma cell	SH2B1
1.75718283534173E-07	0.92176715781545	0.051	0.132	0.00294468699546567	B/Plasma cell	DENND1B
1.79676755596061E-07	0.639609072495169	0.056	0.141	0.00301102307027878	B/Plasma cell	STIMATE
1.79943699151181E-07	1.09615682355982	0.053	0.135	0.0030154965103755	B/Plasma cell	GGA1
1.81359319716836E-07	0.704464697997122	0.084	0.182	0.00303921947981474	B/Plasma cell	SEC24C
1.82279038963558E-07	1.04947003039584	0.131	0.25	0.00305463213495131	B/Plasma cell	TAB2
1.83889785670568E-07	1.01973084939057	0.088	0.19	0.00308162502826739	B/Plasma cell	TRPM7
1.84870777716209E-07	0.745402838073245	0.045	0.122	0.00309806449296823	B/Plasma cell	STAMBP
1.88610966852846E-07	1.04229115424458	0.084	0.182	0.00316074258252	B/Plasma cell	SART3
1.93939456920551E-07	0.672752965652661	0.118	0.23	0.00325003741907459	B/Plasma cell	ATXN1
1.96566545753653E-07	1.18622878704217	0.105	0.212	0.00329406217373971	B/Plasma cell	ZNF24
1.96681625781355E-07	0.252455428540043	0.039	0.115	0.00329599068484395	B/Plasma cell	PPCDC
1.99568900325271E-07	1.21328125350129	0.137	0.261	0.0033443756316509	B/Plasma cell	MORC3
2.0196848137657E-07	0.450909812422046	0.03	0.1	0.00338458781090856	B/Plasma cell	THOC5
2.01988934268046E-07	0.673469155770569	0.034	0.106	0.00338493056046391	B/Plasma cell	MFSD8
2.04789986744665E-07	0.932783318218347	0.068	0.156	0.0034318705978671	B/Plasma cell	ZMAT3
2.08284707834792E-07	0.74267283410647	0.041	0.116	0.00349043513389544	B/Plasma cell	WDR70
2.09088595237308E-07	0.712947197842573	0.043	0.12	0.0035039066789868	B/Plasma cell	TMEM260
2.12249250341296E-07	1.14089284087735	0.069	0.161	0.00355687293721944	B/Plasma cell	APPL1
2.22888468420809E-07	0.487660516369388	0.045	0.122	0.00373516495379591	B/Plasma cell	ATAD1
2.23134455076942E-07	0.541358894438563	0.379	0.607	0.0037392871981794	B/Plasma cell	TAPBP
2.26468635447809E-07	1.12546694599899	0.163	0.299	0.00379516139283439	B/Plasma cell	HNRNPDL
2.29223869656844E-07	0.786107507501155	0.069	0.159	0.00384133360770939	B/Plasma cell	ANKFY1
2.29517784788628E-07	0.804982958705714	0.109	0.215	0.00384625903748783	B/Plasma cell	HMGN4
2.31941888793793E-07	0.371419524967413	0.043	0.118	0.00388688217240638	B/Plasma cell	EIF2A
2.343713409607E-07	0.429585461626325	0.032	0.102	0.00392759493181941	B/Plasma cell	TFAM
2.41951768974283E-07	1.81655764723866	0.137	0.08	0.00405462774447103	B/Plasma cell	PLEKHO1
2.43433309901415E-07	0.336048214572458	0.047	0.123	0.00407945540732792	B/Plasma cell	FLCN
2.43619839265103E-07	0.922517758576198	0.069	0.16	0.0040825812664046	B/Plasma cell	DPH7
2.50108596402758E-07	0.825368733923947	0.101	0.205	0.00419131985851743	B/Plasma cell	TRAK1
2.57445746233169E-07	0.443080260681649	0.045	0.121	0.00431427581537544	B/Plasma cell	TRAPPC1
2.6073816851596E-07	0.423857557404577	0.049	0.127	0.00436945022799046	B/Plasma cell	CCNH
2.61028022667872E-07	0.65525055175797	0.107	0.213	0.00437430760386821	B/Plasma cell	BRD1
2.63536936815082E-07	0.509955215712652	0.135	0.253	0.00441635198714715	B/Plasma cell	ARRDC3
2.64540308772542E-07	0.626552382307715	0.066	0.152	0.00443316649441026	B/Plasma cell	FER
2.66162802464185E-07	0.491985830198004	0.034	0.104	0.00446035624369481	B/Plasma cell	NDUFAF7

2.67774530589614E-07	1.18506546974536	0.036	0.108	0.00448736558362076	B/Plasma cell	NEPRO
2.67930305134302E-07	0.690785331036357	0.182	0.324	0.00448997605344063	B/Plasma cell	SND1
2.71835739365044E-07	1.878348454311	0.122	0.068	0.00455542332027941	B/Plasma cell	EMP3
2.7227188066886E-07	0.80320676190703	0.053	0.131	0.00456273217624876	B/Plasma cell	TTC9C
2.7963769218825E-07	0.778422243851328	0.038	0.11	0.00468616844569069	B/Plasma cell	LRRC8B
2.83810718083889E-07	0.670163193759714	0.051	0.128	0.00475610001364982	B/Plasma cell	TMEM199
2.84307871981414E-07	0.957235924061508	0.066	0.152	0.00476443131866454	B/Plasma cell	SLC35E2B
2.85218820851485E-07	0.984696266132758	0.049	0.128	0.00477969699982918	B/Plasma cell	PPP1R21
2.97651023327E-07	1.00859934733025	0.069	0.158	0.00498803584891387	B/Plasma cell	METTL9
2.97762816599886E-07	0.832178111606627	0.043	0.118	0.00498990928058088	B/Plasma cell	ZNF12
3.00714095030509E-07	1.00528771818352	0.064	0.15	0.00503936680452127	B/Plasma cell	ZBTB11
3.00987994702832E-07	1.1538281222105	0.054	0.137	0.00504395681523006	B/Plasma cell	CCNT1
3.02478355970401E-07	0.9628637170133	0.038	0.11	0.00506893228935198	B/Plasma cell	MAP3K10
3.03659238961734E-07	0.5242962660357	0.041	0.116	0.00508872152652073	B/Plasma cell	IRAK2
3.04724732178239E-07	0.799481561707363	0.066	0.151	0.00510657706184293	B/Plasma cell	CCDC12
3.08426285860493E-07	0.424174741484067	0.069	0.156	0.00516860769845015	B/Plasma cell	GBF1
3.10131787169453E-07	0.47940498941549	0.051	0.13	0.00519718848938569	B/Plasma cell	MAP3K7
3.13477491425396E-07	1.02472135055269	0.084	0.18	0.00525325580130679	B/Plasma cell	MCEE
3.17125991743269E-07	0.856948406432191	0.066	0.152	0.00531439736963371	B/Plasma cell	PAPSS1
3.19172015686193E-07	0.77300546844394	0.049	0.126	0.00534868463886922	B/Plasma cell	ZNF562
3.19549560676905E-07	1.01814427149719	0.049	0.126	0.00535501153782358	B/Plasma cell	MTF2
3.20849672281825E-07	0.562091338264441	0.039	0.113	0.00537679880809882	B/Plasma cell	RINT1
3.28456465464898E-07	0.957912648864682	0.094	0.194	0.00550427344826075	B/Plasma cell	CASP4
3.44846999116811E-07	0.609737674968335	0.032	0.1	0.00577894601119953	B/Plasma cell	ZNF84
3.44956342361933E-07	0.39497394112087	0.038	0.109	0.00578077838530128	B/Plasma cell	CDK11B
3.47784077510002E-07	0.624816095225688	0.049	0.125	0.00582816557091262	B/Plasma cell	SLC2A5
3.48067074088792E-07	0.921311976539206	0.041	0.115	0.00583290802757998	B/Plasma cell	CCAR2
3.51589075782645E-07	0.869101858732017	0.092	0.19	0.00589192973196557	B/Plasma cell	CCND3
3.62540687172974E-07	1.29785660880431	0.124	0.069	0.0060754568356447	B/Plasma cell	SPI1
3.65420615486068E-07	0.744237657174333	0.068	0.153	0.00612371867431552	B/Plasma cell	RNF220
3.92507678948005E-07	0.458683499341775	0.039	0.111	0.00657764368381066	B/Plasma cell	GAPVD1
3.97230471945888E-07	0.804573596690714	0.058	0.139	0.00665678824886919	B/Plasma cell	ZNFX1
4.02760218668879E-07	1.03523413712727	0.045	0.121	0.00674945574445307	B/Plasma cell	DNAJC2
4.05435800040389E-07	0.836940765804436	0.105	0.21	0.00679429313707683	B/Plasma cell	APOL2
4.08717610943328E-07	0.528963409980455	0.038	0.108	0.00684928972418829	B/Plasma cell	AP2B1
4.12072898073548E-07	0.719095594884399	0.039	0.111	0.00690551762591652	B/Plasma cell	SRRD
4.18127190383148E-07	0.806689323913545	0.056	0.136	0.00700697545644079	B/Plasma cell	SRC
4.20436572369638E-07	0.619961981074562	0.036	0.105	0.00704567607977039	B/Plasma cell	ZNF395
4.2088210564318E-07	0.554174756887865	0.034	0.103	0.00705314232636841	B/Plasma cell	TMCO4
4.30047519136653E-07	1.17278109266349	0.137	0.255	0.00720673632569202	B/Plasma cell	PPIG
4.33293438206704E-07	1.14406273244419	0.094	0.191	0.00726113143746794	B/Plasma cell	PHKB
4.38699144536656E-07	1.00586323902172	0.038	0.108	0.00735172026414528	B/Plasma cell	KLC2
4.40997815673953E-07	1.05906652993547	0.053	0.132	0.00739024139506411	B/Plasma cell	TRIM52
4.41016244447342E-07	1.36088360000524	0.043	0.118	0.00739055022444856	B/Plasma cell	SRSF10
4.44090046488315E-07	1.1790630745275	0.079	0.172	0.00744206099905119	B/Plasma cell	SLC25A28
4.49352767345382E-07	0.673628527868573	0.109	0.211	0.00753025367517392	B/Plasma cell	LY6E
4.54682883272936E-07	0.527377562657339	0.034	0.103	0.00761957575788786	B/Plasma cell	HABP4
4.54903891091099E-07	1.03776435293181	0.036	0.106	0.00762327940690464	B/Plasma cell	EXOSC8
4.5920557420195E-07	1.0195407106122	0.101	0.202	0.00769536701247628	B/Plasma cell	ITPR3
4.60231282890422E-07	0.607124692880059	0.066	0.15	0.00771255583867769	B/Plasma cell	ZZEF1
4.86582939179275E-07	0.8188778522	0.056	0.138	0.00815415689476629	B/Plasma cell	NAT9
4.90255728054735E-07	0.982096161089785	0.129	0.244	0.00821570549074125	B/Plasma cell	SARAF
4.90376703164653E-07	0.884743387060724	0.062	0.145	0.00821773279163325	B/Plasma cell	PPP4R2
4.9918992873219E-07	0.287241448772494	0.043	0.116	0.00836542482569404	B/Plasma cell	EIF2B4
5.00205457164045E-07	0.82538669557765	0.038	0.108	0.00838244305115506	B/Plasma cell	PPOX
5.13112189527548E-07	0.876549842967392	0.049	0.125	0.00859873407210265	B/Plasma cell	HPS4
5.41759527011196E-07	1.15066904175216	0.105	0.206	0.00907880615365363	B/Plasma cell	ARRB2
5.51222914697387E-07	0.949704180153582	0.069	0.154	0.00923739360449881	B/Plasma cell	PCGF5
5.57351176852421E-07	0.616445989157864	0.051	0.128	0.00934009102169288	B/Plasma cell	VAC14
5.61339811013954E-07	0.771563298852816	0.062	0.144	0.00940693255297183	B/Plasma cell	FAM53B
5.62766780868212E-07	1.03734380047881	0.098	0.196	0.00943084571378949	B/Plasma cell	CBWD1
5.68451261438711E-07	0.528290536260536	0.038	0.108	0.00952610623918992	B/Plasma cell	KARS
5.80825822067095E-07	0.941750997283519	0.103	0.204	0.00973347912620039	B/Plasma cell	PRKD2

5.81807731521673E-07	0.937837451720923	0.062	0.144	0.0097499339648402	B/Plasma cell	TSPAN31
5.91587361313074E-07	0.83410115556133	0.098	0.195	0.0099138210008845	B/Plasma cell	ORMDL1
5.97447407069242E-07	1.14170115282943	0.058	0.138	0.0100120236476664	B/Plasma cell	STRIP1
6.09654693355582E-07	0.588743767678388	0.034	0.101	0.0102165933512528	B/Plasma cell	TRIM23
6.14581427244931E-07	1.11048424256526	0.169	0.304	0.0102991555577705	B/Plasma cell	MLLT6
6.18201025763455E-07	0.638568129134906	0.039	0.109	0.010359812789744	B/Plasma cell	SMYD4
6.20815626174353E-07	0.390755026651263	0.058	0.139	0.0104036282634298	B/Plasma cell	RB1
6.25815483970346E-07	1.13538791208269	0.058	0.138	0.0104874158803751	B/Plasma cell	NAA40
6.29834625088899E-07	0.939552953242549	0.053	0.129	0.0105547686472398	B/Plasma cell	LTB4R
6.3807561256615E-07	0.400390731275929	0.088	0.181	0.0106928711153835	B/Plasma cell	PPP1R15A
6.39515297727048E-07	0.860539082010264	0.083	0.174	0.0107169973593099	B/Plasma cell	RMC1
6.58927828860658E-07	0.371969517934126	0.038	0.106	0.0110423125560469	B/Plasma cell	GZF1
6.60686838735778E-07	0.727570499831412	0.077	0.166	0.0110717900435342	B/Plasma cell	SS18
6.76758416799992E-07	0.952403738874309	0.043	0.115	0.0113411175487343	B/Plasma cell	ARL17B
6.78464518921323E-07	1.3280215295357	0.086	0.179	0.0113697084080835	B/Plasma cell	MTO1
6.8003500685714E-07	0.395580681287534	0.039	0.109	0.011396026644912	B/Plasma cell	MINDY3
6.80179852007288E-07	0.512149656303328	0.045	0.117	0.0113984539599381	B/Plasma cell	ZFYVE1
7.00996158905606E-07	0.992865557482835	0.084	0.176	0.0117472936309401	B/Plasma cell	SNRK
7.07853503086244E-07	1.71668175848699	0.058	0.139	0.0118622090047193	B/Plasma cell	ATP6V0A2
7.10707897320048E-07	0.952010565943478	0.038	0.107	0.0119100429432894	B/Plasma cell	SLC16A7
7.18856613147961E-07	0.758505050158345	0.098	0.194	0.0120465991231335	B/Plasma cell	OSBPL8
7.24776585556255E-07	0.586941952510894	0.047	0.12	0.0121458060207517	B/Plasma cell	BICRAL
7.32862043306379E-07	0.870382780485357	0.086	0.179	0.0122813021217283	B/Plasma cell	WASHC4
7.37854679084947E-07	1.07145814183333	0.047	0.121	0.0123649687121055	B/Plasma cell	ACD
7.43537091305852E-07	0.450944964388748	0.038	0.106	0.0124601945761035	B/Plasma cell	CNOT4
7.52444490685644E-07	0.698460545394597	0.047	0.121	0.01260946477491	B/Plasma cell	RNMT
7.56680271837995E-07	0.774556572672868	0.051	0.126	0.012680447995461	B/Plasma cell	NCOA1
7.72814621809677E-07	1.19584972128983	0.081	0.171	0.0129508274322866	B/Plasma cell	MRNIP
7.75045479565195E-07	1.05644033056813	0.201	0.355	0.0129882121465535	B/Plasma cell	RABGAP1L
7.80102740338312E-07	0.571307065448044	0.047	0.121	0.0130729617225894	B/Plasma cell	MIIP
7.99043772722675E-07	0.944549747755195	0.06	0.139	0.0133903755432866	B/Plasma cell	UBLCP1
8.16222065311491E-07	0.747889228357286	0.049	0.122	0.01367824937049	B/Plasma cell	USP7
8.18552756381575E-07	1.0826234836608	0.111	0.213	0.0137173070914424	B/Plasma cell	AUP1
8.304728848122E-07	1.069195150716	0.092	0.186	0.0139170646036829	B/Plasma cell	SIN3A
8.51179564379599E-07	0.657273397917446	0.092	0.186	0.0142640671398733	B/Plasma cell	SPCS3
8.62277762162592E-07	0.818114078564695	0.045	0.118	0.0144500507383207	B/Plasma cell	UBXN7
8.67900131167432E-07	0.639390869884047	0.062	0.142	0.0145442703981038	B/Plasma cell	DDX18
8.75303553648252E-07	0.77166592867678	0.039	0.109	0.0146683369520374	B/Plasma cell	RALB
9.14745610250085E-07	1.07887819519766	0.034	0.101	0.0153293069365709	B/Plasma cell	ZBTB22
9.15732677451416E-07	0.752702454944983	0.041	0.111	0.0153458482087308	B/Plasma cell	RELB
9.26269310802069E-07	0.639875135260976	0.049	0.123	0.0155224211104211	B/Plasma cell	USP22
9.29022540889174E-07	1.10988983582743	0.06	0.14	0.0155685597402208	B/Plasma cell	STX2
9.42148770281822E-07	0.458426787847961	0.036	0.102	0.0157885290923828	B/Plasma cell	COIL
9.42219262266067E-07	0.754863851570065	0.056	0.133	0.0157897103970547	B/Plasma cell	CLASP1
9.57928173343642E-07	1.27596325526657	0.167	0.3	0.0160529603288928	B/Plasma cell	PAFAH1B1
9.85076743307734E-07	0.754766937602783	0.099	0.197	0.016507916064351	B/Plasma cell	MRTFA
9.92822744253572E-07	0.950974818545949	0.039	0.108	0.0166377235482014	B/Plasma cell	AMD 1.00
9.96839786674506E-07	1.23250458086972	0.039	0.108	0.0167050411450914	B/Plasma cell	CNOT3
1.00894160723162E-06	0.327301079256647	0.041	0.111	0.0169078434539875	B/Plasma cell	ZFAND6
1.01770589377402E-06	0.867875659859317	0.043	0.114	0.0170547153678649	B/Plasma cell	RIOK1
1.01936710958898E-06	0.930909284422949	0.09	0.183	0.0170825540224921	B/Plasma cell	KDM5B
1.04526256288799E-06	0.731966703168274	0.058	0.136	0.017516510028877	B/Plasma cell	CHPF2
1.06536813760647E-06	0.871957564647612	0.105	0.204	0.0178534392500093	B/Plasma cell	RFX5
1.06974747579675E-06	0.765368705474832	0.24	0.399	0.0179268281994019	B/Plasma cell	FNDC3B
1.07357181893079E-06	0.682170847000774	0.038	0.106	0.0179909165416421	B/Plasma cell	ZBTB5
1.07887452053591E-06	0.517204207141606	0.038	0.105	0.0180797792151408	B/Plasma cell	SLC35A5
1.09213263091468E-06	0.909198600454814	0.039	0.107	0.0183019586288681	B/Plasma cell	AGPAT5
1.0938101445985E-06	1.07053872026273	0.041	0.111	0.0183300704031816	B/Plasma cell	FAM219B
1.11019937100315E-06	0.47016439889031	0.041	0.11	0.0186047210592708	B/Plasma cell	UROS
1.1251766015954E-06	0.556022885964831	0.077	0.161	0.0188557094895356	B/Plasma cell	PIEZO1
1.13477550837361E-06	0.821838195540551	0.045	0.117	0.019016567969325	B/Plasma cell	ITGAM
1.13736866882273E-06	1.31466252977104	0.094	0.19	0.0190600241521313	B/Plasma cell	BCLAF1
1.19857033684285E-06	0.514187698128241	0.051	0.124	0.0200856417048125	B/Plasma cell	WDR43

1.21770138709544E-06	0.712206263836611	0.038	0.105	0.0204062398449453	B/Plasma cell	MEN1
1.23807967148348E-06	0.422253870312709	0.047	0.118	0.0207477391347202	B/Plasma cell	ACOT9
1.25231267416076E-06	0.973677451914752	0.053	0.127	0.020986255793586	B/Plasma cell	TRMT1
1.26212899389297E-06	0.934526559857792	0.073	0.158	0.0211507576796584	B/Plasma cell	KLHL36
1.2868010986142E-06	1.199633527241	0.111	0.21	0.0215642128105767	B/Plasma cell	SPG11
1.29428523838857E-06	0.758509997042023	0.068	0.149	0.0216896320249157	B/Plasma cell	GIT2
1.32004764025924E-06	1.17120285519277	0.128	0.236	0.0221213583554644	B/Plasma cell	CNPY3
1.3523353483873E-06	0.685390738911433	0.071	0.155	0.0226624357682743	B/Plasma cell	CSTF2T
1.35701350167608E-06	0.700678978376095	0.056	0.132	0.0227408322610877	B/Plasma cell	KDM3A
1.37571240404073E-06	1.76100103261502	0.126	0.074	0.0230541884669146	B/Plasma cell	CYBB
1.3825383465105E-06	0.741656420807356	0.131	0.24	0.0231685776108229	B/Plasma cell	MPRIP
1.40918748651886E-06	0.741588452149056	0.088	0.178	0.023615163899083	B/Plasma cell	RBM5
1.41401637294499E-06	1.0567508292184	0.071	0.154	0.0236960863778122	B/Plasma cell	UBE2G1
1.45713394731655E-06	1.10470870662523	0.084	0.174	0.0244186506891307	B/Plasma cell	CNOT2
1.46728717055778E-06	0.688179760694501	0.056	0.131	0.0245887984042073	B/Plasma cell	SNX8
1.50367899105575E-06	0.861238753743734	0.053	0.126	0.0251986525321123	B/Plasma cell	ORC2
1.51521336512379E-06	1.12129917565524	0.062	0.139	0.0253919455727445	B/Plasma cell	PNRC2
1.54285380509913E-06	0.493416175729475	0.062	0.14	0.0258551440658513	B/Plasma cell	CSNK1E
1.55152816137392E-06	0.49081509166352	0.038	0.104	0.0260005089283041	B/Plasma cell	CPT1B
1.5524971057978E-06	0.806194468368428	0.038	0.103	0.0260167464989595	B/Plasma cell	SARS
1.56496589691495E-06	1.09107095105821	0.077	0.161	0.0262256985005007	B/Plasma cell	SMARCC1
1.59705879965416E-06	0.721208805339685	0.047	0.117	0.0267635113646044	B/Plasma cell	FXR2
1.68320648970195E-06	0.434588554094744	0.039	0.106	0.0282071743544253	B/Plasma cell	SLC66A3
1.69267953360189E-06	1.49171841733395	0.041	0.109	0.0283659236241006	B/Plasma cell	PRDM4
1.70689223251338E-06	0.558086952479766	0.069	0.149	0.0286041000324592	B/Plasma cell	ZNF266
1.71207808600468E-06	1.25287625273479	0.133	0.244	0.0286910045652665	B/Plasma cell	NEMF
1.7330966587213E-06	1.10138859982466	0.158	0.283	0.0290432338068515	B/Plasma cell	YPEL5
1.74204892324012E-06	0.595563387126609	0.111	0.06	0.0291932558556579	B/Plasma cell	TRAC
1.76261627452914E-06	0.591582345831363	0.047	0.116	0.0295379235285594	B/Plasma cell	ATP1B3
1.81103363442077E-06	0.761932270856483	0.064	0.142	0.0303493016456233	B/Plasma cell	ASXL2
1.83360444016208E-06	1.11196966548605	0.056	0.132	0.030727543208236	B/Plasma cell	TRIM5
1.84555991826716E-06	0.96463568763406	0.103	0.2	0.0309278931103211	B/Plasma cell	CENPT
1.84701882416897E-06	0.660460026601811	0.308	0.503	0.0309523414554237	B/Plasma cell	LITAF
1.87758696907841E-06	0.987672611776256	0.156	0.277	0.031464602427816	B/Plasma cell	YTHDC1
1.89048020907587E-06	0.543935456606746	0.045	0.114	0.0316806673436935	B/Plasma cell	DRG1
1.9027462184772E-06	0.27895657348394	0.051	0.122	0.0318862211292409	B/Plasma cell	AP5Z1
1.92320170495178E-06	1.15356913790187	0.047	0.118	0.0322290141715819	B/Plasma cell	MOB2
1.94510949005484E-06	1.29660285369379	0.135	0.249	0.032596144834339	B/Plasma cell	TUT4
1.9488048497368E-06	1.26739164188475	0.088	0.18	0.0326580716718893	B/Plasma cell	PSME4
1.95833304067671E-06	0.611117569606031	0.041	0.108	0.0328177450956603	B/Plasma cell	ATG14
2.02223065546573E-06	0.822738682777721	0.051	0.122	0.0338885413242947	B/Plasma cell	METTL21A
2.03485816334865E-06	0.624664201333893	0.081	0.163	0.0341001531013966	B/Plasma cell	NECAP2
2.03785780985446E-06	0.354727589360816	0.038	0.102	0.0341504211775411	B/Plasma cell	ARPC3
2.05091466796234E-06	1.44420241847996	0.062	0.141	0.0343692280057128	B/Plasma cell	PPTC7
2.12889150440774E-06	1.09992693710902	0.058	0.133	0.0356759638308649	B/Plasma cell	MTMR14
2.19577134362737E-06	1.59234094629861	0.146	0.091	0.0367967361765074	B/Plasma cell	NLRP1
2.21208885876236E-06	0.813086124536971	0.083	0.169	0.0370701850951397	B/Plasma cell	FOXN2
2.29873122174875E-06	0.344128194156557	0.041	0.107	0.0385221378140656	B/Plasma cell	ARL 15.00
2.32799102526202E-06	0.906543490730282	0.041	0.108	0.039012473601341	B/Plasma cell	IQCB1
2.33082456334038E-06	0.879500450027234	0.083	0.169	0.0390599580324582	B/Plasma cell	CHD1
2.40013116655518E-06	1.01149239504759	0.038	0.102	0.0402213980891318	B/Plasma cell	SZT2
2.44015184380276E-06	0.476113479195713	0.064	0.14	0.0408920645984467	B/Plasma cell	BAG1
2.54361571798604E-06	1.12002784617065	0.105	0.204	0.0426259122020101	B/Plasma cell	KMT2A
2.54644216194481E-06	0.450348984578845	0.043	0.109	0.0426732777498711	B/Plasma cell	ASMTL
2.57622893336618E-06	1.11495967690157	0.079	0.162	0.0431724444653504	B/Plasma cell	PITPNM1
2.60420352195692E-06	1.54235279794555	0.116	0.218	0.043641242620954	B/Plasma cell	LYN
2.62821377341468E-06	0.998438322100493	0.188	0.326	0.0440436064148832	B/Plasma cell	SRRM2
2.66922598577001E-06	0.893723359942652	0.054	0.128	0.0447308890695339	B/Plasma cell	ZNF830
2.68728287312635E-06	0.813193372652411	0.038	0.101	0.0450334863878514	B/Plasma cell	POU2F1
2.69769083820507E-06	1.09741925504143	0.088	0.177	0.0452079030666406	B/Plasma cell	NFATC3
2.70755472130413E-06	0.853226056182736	0.06	0.133	0.0453732020196147	B/Plasma cell	NCBP3
2.75540169201725E-06	0.783634550690978	0.039	0.104	0.0461750215548251	B/Plasma cell	CXorf38
2.78161881529321E-06	0.510940766432458	0.096	0.186	0.0466143681066836	B/Plasma cell	ARL4C

**2.79274448769432E-06**	0.797897960831266	0.045	0.112	0.0468008121247814	B/Plasma cell	STK16
**2.79600858776311E-06**	0.618938251680176	0.038	0.101	0.0468555119137342	B/Plasma cell	FAM192A
**2.80700425020579E-06**	1.33493808593728	0.045	0.114	0.0470397772249487	B/Plasma cell	CDCA7L
**2.82700043949931E-06**	1.04446525392157	0.079	0.161	0.0473748733651295	B/Plasma cell	AKAP11
**2.85632108571158E-06**	0.688019374278906	0.041	0.108	0.0478662287543547	B/Plasma cell	RAB2B
**2.88323803649465E-06**	1.18831702314669	0.096	0.186	0.0483173030155773	B/Plasma cell	HERPUD2
**2.91178323693389E-06**	0.872421814589094	0.09	0.178	0.0487956634845381	B/Plasma cell	NR2C2
**2.97095241645567E-06**	1.12859898473483	0.049	0.118	0.0497872205949642	B/Plasma cell	FTSJ3
**0**	4.63703008342663	0.935	0.103	0	T/NK cell	CXCR4
**0**	3.5237539404786	0.825	0.134	0	T/NK cell	SRGN
**0**	7.65153598288216	0.676	0.013	0	T/NK cell	TRAC
**0**	7.12992028555145	0.653	0.02	0	T/NK cell	TRBC2
**0**	4.90138372166223	0.678	0.049	0	T/NK cell	CYTIP
**0**	4.41163218178924	0.692	0.079	0	T/NK cell	WIPF1
**0**	4.91142250307721	0.743	0.13	0	T/NK cell	IL2RG
**0**	5.03126055798677	0.672	0.076	0	T/NK cell	ETS1
**0**	4.18417513014695	0.646	0.063	0	T/NK cell	CELF2
**0**	6.85537147291011	0.57	0.012	0	T/NK cell	SPOCK2
**0**	4.08611709603441	0.644	0.09	0	T/NK cell	LCP1
**0**	5.15799753147597	0.588	0.035	0	T/NK cell	CORO1A
**0**	6.68258930175718	0.57	0.019	0	T/NK cell	IL7R
**0**	5.20780539046643	0.587	0.037	0	T/NK cell	IKZF1
**0**	2.94880763243418	0.688	0.151	0	T/NK cell	LAPTM5
**0**	3.51208492597479	0.572	0.067	0	T/NK cell	LSP1
**0**	7.27267219722516	0.509	0.011	0	T/NK cell	CD2
**0**	5.54932890622796	0.513	0.017	0	T/NK cell	ACAP1
**0**	7.36022866412259	0.492	0.007	0	T/NK cell	CD3E
**0**	4.14025144277379	0.539	0.059	0	T/NK cell	CD53
**0**	5.14524325495265	0.521	0.041	0	T/NK cell	RAC2
**0**	4.4583822381979	0.624	0.144	0	T/NK cell	AKNA
**0**	3.18913572805321	0.554	0.076	0	T/NK cell	ITGB2
**0**	5.23605112902645	0.52	0.043	0	T/NK cell	ITGAL
**0**	4.72193873108765	0.524	0.048	0	T/NK cell	SLA
**0**	3.9955695699743	0.561	0.085	0	T/NK cell	ARHGDIB
**0**	6.38588770915166	0.481	0.012	0	T/NK cell	IKZF3
**0**	7.81575460151806	0.466	0.006	0	T/NK cell	IL2RB
**0**	7.15889246211546	0.454	0.008	0	T/NK cell	CD96
**0**	2.59520269722196	0.6	0.157	0	T/NK cell	VIM
**0**	6.77252177209929	0.447	0.007	0	T/NK cell	ITK
**0**	3.85208687505953	0.576	0.136	0	T/NK cell	CD44
**0**	3.74472693201726	0.508	0.077	0	T/NK cell	RGS1
**0**	5.18729874788212	0.46	0.034	0	T/NK cell	PTPRC
**0**	5.46873170298024	0.444	0.019	0	T/NK cell	SPN
**0**	4.36395289316438	0.549	0.128	0	T/NK cell	STK17B
**0**	4.64272621290741	0.462	0.042	0	T/NK cell	PRDM1
**0**	4.04011053648089	0.7	0.281	0	T/NK cell	ZFP36L2
**0**	5.69667270555386	0.438	0.02	0	T/NK cell	LTB
**0**	5.58904371704068	0.443	0.026	0	T/NK cell	SEPTIN1
**0**	4.24396388244587	0.449	0.047	0	T/NK cell	ITGA4
**0**	6.09672922192305	0.412	0.016	0	T/NK cell	ITM2A
**0**	3.74513055445422	0.469	0.074	0	T/NK cell	FXYD5
**0**	7.2814629129559	0.396	0.006	0	T/NK cell	CD7
**0**	7.27010386582963	0.397	0.008	0	T/NK cell	TRBC1
**0**	2.67367820060449	0.47	0.081	0	T/NK cell	CYBA
**0**	5.44305110382852	0.421	0.034	0	T/NK cell	S100A4
**0**	6.78772305018445	0.392	0.005	0	T/NK cell	RASGRP1
**0**	3.67104374751058	0.487	0.103	0	T/NK cell	TRIM22
**0**	8.47154417996964	0.387	0.004	0	T/NK cell	PRF1
**0**	5.88964964930167	0.392	0.011	0	T/NK cell	MYO1G
**0**	4.65900429471181	0.404	0.024	0	T/NK cell	TMC8
**0**	6.03364271085169	0.388	0.013	0	T/NK cell	CST7
**0**	5.4654152937128	0.388	0.014	0	T/NK cell	FYN
**0**	4.4435597518962	0.405	0.034	0	T/NK cell	FMNL1

0	4.09659192767896	0.433	0.062	0	T/NK cell	PIK3IP1
0	3.58891011542207	0.419	0.052	0	T/NK cell	CHST11
0	7.14565311650026	0.37	0.01	0	T/NK cell	CD3D
0	5.60489001684607	0.381	0.022	0	T/NK cell	STAT4
0	3.74173217181469	0.394	0.037	0	T/NK cell	ITPKB
0	2.9030985228763	0.427	0.07	0	T/NK cell	HCLS1
0	5.44528579432281	0.375	0.018	0	T/NK cell	DGKA
0	3.54623521565086	0.412	0.056	0	T/NK cell	LCP2
0	3.28901464079871	0.44	0.085	0	T/NK cell	IFI16
0	6.74250983268037	0.36	0.005	0	T/NK cell	CD5
0	4.60035831643547	0.385	0.03	0	T/NK cell	ARHGAP9
0	6.31641876868048	0.364	0.01	0	T/NK cell	SLAMF6
0	6.48595671302038	0.37	0.019	0	T/NK cell	CCL5
0	5.76252441726072	0.365	0.015	0	T/NK cell	PTPN7
0	4.74014823267725	0.376	0.026	0	T/NK cell	DEF6
0	4.5613420588767	0.394	0.046	0	T/NK cell	FYB1
0	5.70646976899166	0.361	0.013	0	T/NK cell	CCND2
0	4.60712896823035	0.371	0.023	0	T/NK cell	JAK3
0	4.52503821401648	0.402	0.055	0	T/NK cell	TRAF1
0	6.42182795254782	0.351	0.005	0	T/NK cell	CD6
0	5.36971406302759	0.363	0.017	0	T/NK cell	RASAL3
0	3.77032075929717	0.389	0.045	0	T/NK cell	DOCK2
0	4.22892500813781	0.454	0.112	0	T/NK cell	CLEC2D
0	3.42308201119459	0.512	0.17	0	T/NK cell	ARHGEF1
0	3.14140541130706	0.441	0.1	0	T/NK cell	ANXA6
0	6.17593326127112	0.35	0.01	0	T/NK cell	CD69
0	4.60008079464354	0.365	0.027	0	T/NK cell	SASH3
0	3.67287529289312	0.385	0.048	0	T/NK cell	UCP2
0	6.22628977487779	0.361	0.024	0	T/NK cell	BCL11B
0	7.29292195877843	0.339	0.004	0	T/NK cell	LCK
0	4.67309002842423	0.372	0.037	0	T/NK cell	SELPLG
0	6.82806406253831	0.339	0.006	0	T/NK cell	ZAP70
0	4.961456173261	0.347	0.018	0	T/NK cell	DUSP2
0	3.94800755699107	0.373	0.045	0	T/NK cell	IL10RA
0	5.51244020348745	0.337	0.012	0	T/NK cell	TBC1D10C
0	2.67456920120286	0.787	0.464	0	T/NK cell	TXNIP
0	5.60653465194599	0.329	0.008	0	T/NK cell	TRAF3IP3
0	4.36128728538958	0.343	0.03	0	T/NK cell	ARHGAP30
0	6.09933984491295	0.325	0.013	0	T/NK cell	CCR5
0	7.56694400378697	0.315	0.004	0	T/NK cell	TIGIT
0	7.87114292909888	0.315	0.004	0	T/NK cell	CD3G
0	7.33466580496358	0.313	0.003	0	T/NK cell	CD247
0	4.14948156133587	0.339	0.034	0	T/NK cell	DOCK8
0	3.52991566642057	0.411	0.106	0	T/NK cell	TNFRSF1B
0	5.05413348097254	0.326	0.021	0	T/NK cell	GBP5
0	5.13031201062294	0.317	0.013	0	T/NK cell	CCDC88C
0	8.14247473025893	0.311	0.008	0	T/NK cell	GZMA
0	4.1304116527464	0.362	0.06	0	T/NK cell	BCL2
0	7.38723636546461	0.311	0.01	0	T/NK cell	NKG7
0	3.60954606832974	0.333	0.035	0	T/NK cell	CD37
0	4.12380234901331	0.326	0.028	0	T/NK cell	TAGAP
0	3.93081605233269	0.407	0.109	0	T/NK cell	NLRC5
0	3.14099343306596	0.344	0.047	0	T/NK cell	DOK2
0	3.97775565347021	0.342	0.046	0	T/NK cell	GPSM3
0	4.86440524773228	0.317	0.022	0	T/NK cell	CRIP1
0	4.24573082717262	0.327	0.033	0	T/NK cell	MDFIC
0	4.79628848844994	0.324	0.031	0	T/NK cell	CD52
0	6.79171737054161	0.298	0.005	0	T/NK cell	PYHIN1
0	7.63604702227465	0.296	0.004	0	T/NK cell	CXCR6
0	5.28776244105452	0.301	0.016	0	T/NK cell	SEMA4D
0	4.56243579211068	0.32	0.037	0	T/NK cell	SLFN5
0	4.45088180633833	0.308	0.026	0	T/NK cell	ARHGAP15
0	6.91724365146118	0.286	0.005	0	T/NK cell	CD27

**0**	5.05294538185135	0.307	0.026	0	T/NK cell	PRKCH
**0**	3.22691238701581	0.39	0.112	0	T/NK cell	ARHGAP4
**0**	6.06394598239684	0.291	0.013	0	T/NK cell	CD28
**0**	3.51515987636122	0.384	0.107	0	T/NK cell	STK10
**0**	3.10986994457203	0.329	0.052	0	T/NK cell	CMTM3
**0**	5.24636280877073	0.291	0.014	0	T/NK cell	SYTL1
**0**	3.84048851331906	0.312	0.037	0	T/NK cell	S100A6
**0**	3.88939442924427	0.325	0.05	0	T/NK cell	ARHGAP45
**0**	5.47183458598555	0.284	0.011	0	T/NK cell	CYFIP2
**0**	6.31057881130696	0.28	0.008	0	T/NK cell	IPCEF1
**0**	5.46630393096019	0.287	0.018	0	T/NK cell	LAT
**0**	2.76097125618978	0.339	0.073	0	T/NK cell	CD4
**0**	4.87269320256603	0.278	0.012	0	T/NK cell	ATP2A3
**0**	6.04506347112668	0.272	0.006	0	T/NK cell	SKAP1
**0**	5.16791722154873	0.294	0.028	0	T/NK cell	EMB
**0**	4.14641862929278	0.294	0.029	0	T/NK cell	LBH
**0**	3.43230570245661	0.311	0.048	0	T/NK cell	RCSD1
**0**	3.83305413847294	0.296	0.033	0	T/NK cell	IL16
**0**	4.9779891243258	0.271	0.01	0	T/NK cell	FCMR
**0**	4.88281873241844	0.275	0.016	0	T/NK cell	GNG2
**0**	7.35584325522862	0.262	0.006	0	T/NK cell	CTSW
**0**	6.821200932323	0.259	0.004	0	T/NK cell	SLAMF1
**0**	5.69746096863672	0.264	0.009	0	T/NK cell	RHOH
**0**	3.74451740027374	0.331	0.076	0	T/NK cell	CCDC88B
**0**	5.79382129850417	0.271	0.017	0	T/NK cell	PTPN22
**0**	2.75994761029095	0.66	0.408	0	T/NK cell	EVL
**0**	7.12617508231672	0.254	0.003	0	T/NK cell	ICOS
**0**	5.55137096977226	0.261	0.012	0	T/NK cell	PSTPIP1
**0**	7.4010698071399	0.252	0.005	0	T/NK cell	KLRK1
**0**	3.76148119414102	0.314	0.069	0	T/NK cell	ISG20
**0**	7.29436460383406	0.248	0.004	0	T/NK cell	THEMIS
**0**	3.9320880919902	0.322	0.079	0	T/NK cell	NBEAL2
**0**	3.14745060754222	0.296	0.053	0	T/NK cell	TMEM173
**0**	4.58766095387002	0.262	0.023	0	T/NK cell	FGD3
**0**	4.95004973039874	0.255	0.02	0	T/NK cell	DENND1C
**0**	3.93795310222113	0.275	0.041	0	T/NK cell	LIMD2
**0**	3.77365318742437	0.303	0.07	0	T/NK cell	LPXN
**0**	7.778285358123	0.235	0.004	0	T/NK cell	CD8A
**0**	6.34081931147994	0.231	0.003	0	T/NK cell	SH2D2A
**0**	4.90344088660611	0.241	0.013	0	T/NK cell	SP140
**0**	3.30451470879907	0.271	0.044	0	T/NK cell	NCKAP1L
**0**	3.56663217601513	0.262	0.035	0	T/NK cell	INPP5D
**0**	5.51858527256319	0.234	0.008	0	T/NK cell	CARD11
**0**	6.38436640546386	0.228	0.003	0	T/NK cell	SIT 1.00
**0**	3.52449073419868	0.259	0.035	0	T/NK cell	ST8SIA4
**0**	4.56432646532238	0.247	0.023	0	T/NK cell	HCST
**0**	6.53463975744376	0.227	0.003	0	T/NK cell	GPR174
**0**	3.71566010032663	0.261	0.038	0	T/NK cell	LDHB
**0**	3.73532132575294	0.269	0.047	0	T/NK cell	GIMAP4
**0**	7.57942409716228	0.224	0.003	0	T/NK cell	TNFRSF18
**0**	4.62720396143128	0.247	0.026	0	T/NK cell	GIMAP7
**0**	4.14344322261586	0.243	0.023	0	T/NK cell	PIK3CD
**0**	4.60385429541855	0.249	0.029	0	T/NK cell	NIBAN1
**0**	6.37278928407314	0.224	0.004	0	T/NK cell	NLRC3
**0**	3.22942366690746	0.262	0.044	0	T/NK cell	MYO1F
**0**	4.88509407389366	0.231	0.013	0	T/NK cell	MAP4K1
**0**	8.01363574863834	0.22	0.002	0	T/NK cell	SIRPG
**0**	4.29434292907859	0.245	0.028	0	T/NK cell	SAMSN1
**0**	4.71609654584221	0.242	0.026	0	T/NK cell	PCED1B
**0**	7.15766080221827	0.215	0.002	0	T/NK cell	SLFN12L
**0**	4.5408494335185	0.231	0.021	0	T/NK cell	BIN2
**0**	3.71623679970963	0.254	0.044	0	T/NK cell	SEPTIN6
**0**	5.43718527526866	0.216	0.008	0	T/NK cell	PPP1R16B

0	3.9696220229569	0.23	0.023	0	T/NK cell	TMC6
0	5.26252403878641	0.225	0.02	0	T/NK cell	ITGB7
0	3.91666282510757	0.239	0.036	0	T/NK cell	EVI2B
0	5.52919151176171	0.208	0.005	0	T/NK cell	P2RY10
0	2.87640232942733	0.244	0.042	0	T/NK cell	PLCB2
0	6.26322397413183	0.204	0.004	0	T/NK cell	CCR4
0	7.471038813008	0.203	0.004	0	T/NK cell	GZMK
0	6.57918614081495	0.204	0.005	0	T/NK cell	SH2D1A
0	6.59389625058284	0.201	0.003	0	T/NK cell	GRAP2
0	5.81431624638954	0.218	0.021	0	T/NK cell	LAG3
0	5.48814197678943	0.203	0.006	0	T/NK cell	CARMIL2
0	3.41187365025795	0.238	0.042	0	T/NK cell	CLEC2B
0	6.8917272368739	0.198	0.003	0	T/NK cell	PDCD1
0	6.99184465001474	0.196	0.004	0	T/NK cell	TRAT1
0	7.316105461687	0.195	0.004	0	T/NK cell	CTLA4
0	5.87711334077698	0.197	0.007	0	T/NK cell	SYTL3
0	4.39284775601545	0.208	0.02	0	T/NK cell	SLC7A5
0	5.60001739872082	0.2	0.012	0	T/NK cell	APOBEC3G
0	5.49952686485161	0.193	0.008	0	T/NK cell	RUNX3
0	4.5612896269976	0.194	0.011	0	T/NK cell	PARP15
0	3.85763451339593	0.217	0.034	0	T/NK cell	GIMAP6
0	4.8505974166388	0.196	0.013	0	T/NK cell	APOBEC3C
0	5.73932595855273	0.188	0.008	0	T/NK cell	TC2N
0	7.92255982467925	0.18	0.003	0	T/NK cell	FOXP3
0	4.23469692569833	0.198	0.022	0	T/NK cell	ALOX5AP
0	5.54733807188452	0.186	0.01	0	T/NK cell	DUSP4
0	4.88524977620551	0.183	0.008	0	T/NK cell	RIPOR2
0	4.83434035905907	0.202	0.028	0	T/NK cell	TOX
0	4.27247895004287	0.185	0.013	0	T/NK cell	IL21R
0	6.82537645487991	0.172	0.002	0	T/NK cell	PRKCQ
0	5.34443465828691	0.177	0.008	0	T/NK cell	BATF
0	7.01304013227917	0.168	0.002	0	T/NK cell	SAMD3
0	4.00581271351123	0.177	0.011	0	T/NK cell	CCL4
0	6.52096920434575	0.167	0.003	0	T/NK cell	CXCR3
0	6.3799407469525	0.168	0.005	0	T/NK cell	SLA2
0	6.88818860394653	0.164	0.002	0	T/NK cell	TNFRSF9
0	4.18100208373014	0.179	0.017	0	T/NK cell	ARHGAP25
0	5.23458945412211	0.17	0.01	0	T/NK cell	UBASH3B
0	4.00427867673443	0.178	0.021	0	T/NK cell	UNC13D
0	4.20728654247943	0.178	0.022	0	T/NK cell	ATP8B2
0	4.12811556084704	0.171	0.016	0	T/NK cell	PFKP
0	4.44172895708448	0.169	0.014	0	T/NK cell	HIST1H1D
0	5.05985149018071	0.164	0.009	0	T/NK cell	AGAP2
0	6.79822590890333	0.156	0.002	0	T/NK cell	GATA3
0	7.368510403548	0.156	0.004	0	T/NK cell	KLRD1
0	6.56561411295353	0.153	0.003	0	T/NK cell	TESPA1
0	6.28634011505085	0.153	0.003	0	T/NK cell	PBX4
0	7.62034997981612	0.152	0.003	0	T/NK cell	KLRC4
0	4.76644123748197	0.163	0.014	0	T/NK cell	IL12RB1
0	5.37525654060224	0.165	0.017	0	T/NK cell	CDC14A
0	7.50863874298779	0.149	0.002	0	T/NK cell	LINC02694
0	7.38204820967059	0.145	0.002	0	T/NK cell	TBX21
0	7.90478971654455	0.14	0.002	0	T/NK cell	EOMES
0	3.27708617271493	0.149	0.011	0	T/NK cell	LY9
0	4.93530544996738	0.15	0.014	0	T/NK cell	RHOF
0	5.07903188488938	0.139	0.005	0	T/NK cell	RUNX2
0	6.71017104127644	0.136	0.003	0	T/NK cell	CD40LG
0	6.28170779344919	0.134	0.004	0	T/NK cell	KLRB1
0	5.08955323570389	0.134	0.004	0	T/NK cell	ZBP1
0	6.99254790396002	0.129	0.001	0	T/NK cell	ZNF831
0	5.39589039308461	0.136	0.009	0	T/NK cell	APOBEC3D
0	5.4247845285032	0.132	0.006	0	T/NK cell	C16orf54
0	2.09189686999041	0.832	0.707	0	T/NK cell	TMSB4X

0	4.2174940984471	0.13	0.009	0	T/NK cell	CD48
0	5.83544434896998	0.124	0.003	0	T/NK cell	KCNA3
0	6.63265678125293	0.125	0.004	0	T/NK cell	GPR171
0	7.76780082057715	0.122	0.001	0	T/NK cell	GZMM
0	5.75491434800298	0.128	0.007	0	T/NK cell	MATK
0	6.45121443177027	0.121	0.002	0	T/NK cell	IL12RB2
0	7.94569321361794	0.121	0.002	0	T/NK cell	GNLY
0	8.01366541425195	0.12	0.001	0	T/NK cell	GZMH
0	6.81621281620562	0.121	0.002	0	T/NK cell	PTGER2
0	5.33297972866999	0.125	0.009	0	T/NK cell	ITPRIPL1
0	6.27049452976124	0.117	0.004	0	T/NK cell	GFI1
0	5.85010409680359	0.116	0.004	0	T/NK cell	TNFRSF4
0	3.72484442733556	0.116	0.004	0	T/NK cell	P2RX5
0	5.36695595645927	0.115	0.003	0	T/NK cell	S1PR4
0	5.32072662133607	0.117	0.006	0	T/NK cell	SCML4
0	4.99694981317264	0.117	0.006	0	T/NK cell	CD226
0	4.16529327113454	0.116	0.007	0	T/NK cell	OBSCN
0	3.50390922476497	0.113	0.005	0	T/NK cell	CD79B
0	6.93103221125666	0.106	0.001	0	T/NK cell	KLRC3
0	7.1296870887227	0.106	0.001	0	T/NK cell	Z82206.1
0	6.50697805617445	0.105	0.001	0	T/NK cell	NTNG2
0	8.78564571730373	0.102	0.001	0	T/NK cell	FASLG
0	6.05655642838026	0.104	0.004	0	T/NK cell	TSPAN32
0	6.56308434284173	0.104	0.006	0	T/NK cell	IL18RAP
0	1.58498278330161	0.925	0.89	0	T/NK cell	B2M
3.92385574038799E-307	3.42216853371401	0.289	0.064	6.57559744974219E-303	T/NK cell	SOCS1
1.07907580341788E-305	2.97253833269076	0.259	0.05	1.80831523136769E-301	T/NK cell	LPAR6
1.07999008075037E-304	3.73929081506427	0.224	0.037	1.80984737732146E-300	T/NK cell	ADAM19
6.6499286322833E-304	3.48795776087731	0.206	0.031	1.11439504019804E-299	T/NK cell	WAS
1.37871139745864E-303	4.77200266269813	0.115	0.008	2.31044455986119E-299	T/NK cell	CRACR2A
2.45958590031625E-302	3.78336034452511	0.148	0.015	4.12177405174997E-298	T/NK cell	DEPRECATED-ENSG00000141524
4.91389999963454E-296	3.82296007767755	0.321	0.084	8.23471361938756E-292	T/NK cell	CHD3
7.63865956237532E-292	4.43472596866214	0.155	0.018	1.28008656946286E-287	T/NK cell	SNX20
1.66758425689236E-288	4.98456586545861	0.149	0.017	2.79453769770022E-284	T/NK cell	IKZF2
3.45359918822612E-286	5.0035950923425	0.118	0.009	5.78754151962933E-282	T/NK cell	MCTP2
4.98324961689134E-285	4.96377150957454	0.109	0.008	8.35092970798651E-281	T/NK cell	OXCT1
8.66450340710649E-285	2.48921832160088	0.691	0.492	1.45199748096291E-280	T/NK cell	BTG1
6.83208929667637E-282	3.5256578134357	0.503	0.228	1.14492152433703E-277	T/NK cell	STK4
7.42808713324962E-281	3.87905533052156	0.159	0.02	1.24479884178997E-276	T/NK cell	CCDC69
3.86950233419123E-280	3.44451046849181	0.29	0.07	6.48451201163766E-276	T/NK cell	RASSF5
1.97481000052679E-279	3.84718619600544	0.161	0.02	3.30938659888279E-275	T/NK cell	PDE4B
4.04327114135373E-279	2.96857121313451	0.225	0.04	6.77571377868057E-275	T/NK cell	DEPRECATED-ENSG00000059804
6.80900100315513E-279	3.31230401580823	0.297	0.074	1.14105238810874E-274	T/NK cell	PTK2B
4.32077519548834E-278	3.92121580904866	0.353	0.108	7.24075507259936E-274	T/NK cell	STK17A
1.41276623149808E-275	3.56089889906982	0.182	0.027	2.36751365074449E-271	T/NK cell	PRKCB
1.48010096464398E-275	3.04905112838923	0.304	0.076	2.48035319655038E-271	T/NK cell	NLRP1
2.86200608048277E-274	3.00117981820887	0.195	0.031	4.79614978967303E-270	T/NK cell	TRIB2
4.25516937464501E-274	3.52441011662282	0.333	0.096	7.13081283803011E-270	T/NK cell	ANKRD44
3.34342191993721E-271	4.77323209615556	0.105	0.008	5.60290645343078E-267	T/NK cell	PRR5L
6.63068855668925E-271	4.08622002795016	0.129	0.013	1.11117078832998E-266	T/NK cell	RASGRP2
7.28943145105451E-270	2.78119540541293	0.57	0.306	1.22156292256771E-265	T/NK cell	ADGRE5
8.40939945067146E-270	3.43006794641638	0.221	0.041	1.40924715994352E-265	T/NK cell	DOCK11
1.24121525958843E-269	4.07462716720339	0.359	0.116	2.08002853201829E-265	T/NK cell	PARP8
1.41876536965504E-268	3.73220854879451	0.129	0.013	2.37756700646791E-264	T/NK cell	SELL
1.38804778109331E-266	4.00486766387756	0.16	0.021	2.32609047155616E-262	T/NK cell	ADAM8
1.74175781603044E-265	3.90097603224231	0.179	0.027	2.91883774810381E-261	T/NK cell	AP1G2
4.91237285605175E-265	3.3764155571475	0.271	0.064	8.23215443217153E-261	T/NK cell	KCNAB2
4.00301588556517E-258	2.79582185281073	0.225	0.044	6.70825402103012E-254	T/NK cell	COTL1
6.86758986377414E-254	3.98949147744508	0.143	0.017	1.15087070937127E-249	T/NK cell	GPR132
5.18863794496394E-253	3.26017113510783	0.248	0.056	8.69511946817057E-249	T/NK cell	EMP3
1.34306988802745E-252	3.47966082425301	0.137	0.016	2.2507165183564E-248	T/NK cell	HS3ST3B1
4.44504350686714E-250	4.22156185737289	0.218	0.044	7.44900390880795E-246	T/NK cell	ARHGEF3
5.60723062351123E-250	3.33183023963649	0.364	0.122	9.39659707888012E-246	T/NK cell	ARID5B

3.0949166769106E-245	3.09636556950474	0.219	0.044	5.18646136716679E-241	T/NK cell	HAVCR2
3.10169625194446E-245	2.56672761737204	0.582	0.344	5.19782257900852E-241	T/NK cell	MSN
2.78884761754946E-244	4.58001692444766	0.171	0.027	4.67355083748939E-240	T/NK cell	LPAR2
1.77962139443125E-243	3.55682570779147	0.183	0.031	2.98228953278789E-239	T/NK cell	PIK3R5
2.05119711795905E-241	4.42662265463503	0.197	0.037	3.43739613027578E-237	T/NK cell	TNFRSF25
1.59471506185253E-240	3.36013974143841	0.396	0.152	2.67242350065246E-236	T/NK cell	CBLB
3.1588277523534E-234	3.74079430946371	0.136	0.017	5.29356354739383E-230	T/NK cell	SYT11
1.30384997598582E-233	3.35803339064679	0.449	0.205	2.18499178975703E-229	T/NK cell	RESF1
1.66565975496301E-233	3.53854247869744	0.136	0.017	2.79131261736701E-229	T/NK cell	HAPLN3
1.3128824593948E-232	5.47496291063313	0.114	0.012	2.2001284254538E-228	T/NK cell	AKAP5
2.66032742849295E-231	3.49102249295065	0.302	0.09	4.45817670466849E-227	T/NK cell	PIM2
8.38186960859868E-227	2.82399838075924	0.256	0.063	1.40463370900897E-222	T/NK cell	SERPINB9
2.51270431315531E-224	3.52872424413505	0.16	0.026	4.21078988798567E-220	T/NK cell	TRPV2
2.95128320859629E-221	2.63156849864405	0.412	0.166	4.94576040096567E-217	T/NK cell	ARL4C
5.75891340236487E-218	2.70644021438891	0.195	0.038	9.65078707968305E-214	T/NK cell	SLC2A3
6.32929554802746E-217	2.59933025120618	0.524	0.284	1.06066334793844E-212	T/NK cell	OGA
4.07442300941966E-215	3.88776444652481	0.199	0.042	6.82791807918546E-211	T/NK cell	CEP85L
6.07955498236364E-214	2.72931027537109	0.323	0.106	1.0188118239445E-209	T/NK cell	TSPAN14
9.5098811329233E-214	4.29145798869101	0.118	0.014	1.59366588025529E-209	T/NK cell	DEPRECATED-ENSG00000183943
6.34101865080141E-213	3.7983521180119	0.124	0.016	1.0626279055013E-208	T/NK cell	FAM78A
4.15227306623306E-211	2.62535643003782	0.548	0.316	6.95837920439336E-207	T/NK cell	SMAP2
1.52611803698464E-210	4.21157531669434	0.113	0.013	2.55746860637886E-206	T/NK cell	GMFG
1.74847033402215E-210	3.56899403925856	0.203	0.044	2.93008658575432E-206	T/NK cell	ARHGEF6
4.01070588811692E-208	3.16728786885403	0.184	0.036	6.72114092730633E-204	T/NK cell	ADCY7
3.6355506676954E-206	2.73574827840442	0.193	0.04	6.09245580892395E-202	T/NK cell	PARVG
6.82476566420816E-205	3.57044828259052	0.288	0.09	1.143694230008E-200	T/NK cell	DENND2D
2.96526050407208E-204	4.0151797850668	0.162	0.029	4.969183552724E-200	T/NK cell	EVI2A
3.56318861208026E-203	3.42738642850093	0.469	0.237	5.97119147612411E-199	T/NK cell	TNFAIP3
3.03513057514933E-202	3.52763551390197	0.255	0.072	5.08627181783525E-198	T/NK cell	ICAM3
1.52803106964425E-199	3.62372414779648	0.124	0.017	2.56067446650983E-195	T/NK cell	CALHM2
1.4498261063265E-198	2.35383193128647	0.6	0.412	2.42961858898195E-194	T/NK cell	SYNE2
3.52191731908163E-197	3.08642664498843	0.183	0.037	5.90202904331699E-193	T/NK cell	CD84
5.08124222217621E-197	3.90805524726795	0.121	0.016	8.5151457159229E-193	T/NK cell	PIK3CG
3.79885632731952E-195	2.18599757310063	0.618	0.448	6.36612343332205E-191	T/NK cell	RNF213
5.84768903280431E-192	2.92852775212064	0.154	0.027	9.79955728117347E-188	T/NK cell	APOBR
9.93588620857311E-192	3.36834627039823	0.116	0.015	1.66505581083268E-187	T/NK cell	ICAM2
1.38193445402261E-190	3.42365529755488	0.231	0.062	2.31584575805108E-186	T/NK cell	SLC7A6
4.15484366648885E-190	3.85202053705565	0.151	0.027	6.96268701630201E-186	T/NK cell	RASA3
4.45467264135956E-188	2.82192069010241	0.366	0.149	7.46514041239036E-184	T/NK cell	FLNA
1.56674430940346E-184	3.57134801081929	0.171	0.035	2.62555011369832E-180	T/NK cell	GLIPR1
3.54817354179901E-183	3.00970756426684	0.339	0.13	5.94602922134678E-179	T/NK cell	PIM1
1.10184883531355E-182	2.72228367743978	0.192	0.044	1.84647827821844E-178	T/NK cell	SFMBT2
2.16681432654917E-181	3.58053925663741	0.237	0.068	3.63114744843109E-177	T/NK cell	EIF4E3
2.53735181806489E-179	3.51522296319019	0.258	0.08	4.25209417671314E-175	T/NK cell	CASP8
4.09944455900509E-178	3.73070556097546	0.261	0.083	6.86984919198073E-174	T/NK cell	OXNAD1
1.1777567215651E-177	3.64999547940361	0.225	0.062	1.97368471399879E-173	T/NK cell	PTGER4
5.77339168569921E-177	3.79864204372469	0.135	0.023	9.67504978689474E-173	T/NK cell	GLIPR2
1.71010679150966E-176	3.0781110344432	0.106	0.014	2.86579696121188E-172	T/NK cell	ENO2
3.8043010503531E-175	5.22851494198392	0.108	0.015	6.37524770018172E-171	T/NK cell	CCR6
1.23923905742938E-174	3.75357278580475	0.137	0.024	2.07671681244016E-170	T/NK cell	PPM1M
1.37047214830807E-174	3.28762431176534	0.108	0.015	2.29663722613467E-170	T/NK cell	SYNGAP1
3.78441189738287E-174	2.82581919800054	0.26	0.081	6.34191745763421E-170	T/NK cell	FERMT3
6.67335493500355E-172	3.7267222088659	0.183	0.043	1.1183208200079E-167	T/NK cell	SATB1
1.06691667021591E-171	3.22732890137531	0.366	0.162	1.78793895594782E-167	T/NK cell	KAT2B
1.57896826223351E-170	3.2974596802601	0.17	0.037	2.64603501385091E-166	T/NK cell	ARID5A
1.37679665193026E-167	2.8702729218137	0.231	0.066	2.30723582930473E-163	T/NK cell	HDAC7
1.45359072165677E-167	2.78668563459274	0.26	0.083	2.43592733135241E-163	T/NK cell	ABCC1
2.71687601620429E-166	3.36620392530096	0.29	0.106	4.55294082795515E-162	T/NK cell	TRAF5
4.96207417022088E-166	3.00781652323242	0.15	0.03	8.31544389445614E-162	T/NK cell	FLI1
2.07176761020628E-163	1.18769620378592	0.778	0.849	3.47186816118369E-159	T/NK cell	ACTB
8.13605955518237E-160	4.12323160903955	0.177	0.043	1.36344086025746E-155	T/NK cell	ANO9
3.59415208192341E-156	2.54985947413585	0.183	0.045	6.02308005888725E-152	T/NK cell	JAML
8.55017860905365E-155	3.26641350728639	0.132	0.024	1.43283893130521E-150	T/NK cell	IL2RA

1.35869789523485E-154	2.62877459105756	0.139	0.027	2.27690593283456E-150	T/NK cell	ZNF83
4.00921064824718E-154	3.36484756374484	0.318	0.133	6.71863520433263E-150	T/NK cell	GPRIN3
1.25837824495327E-152	2.26898921433198	0.159	0.035	2.1087902628927E-148	T/NK cell	CYTH4
2.43737325961144E-151	3.26644639038062	0.408	0.216	4.08455010845686E-147	T/NK cell	SMCHD1
1.01547705293968E-150	3.06009697257777	0.256	0.088	1.70173644531632E-146	T/NK cell	PREX1
2.37541478439407E-148	3.28251364289702	0.459	0.278	3.98072009568758E-144	T/NK cell	CNOT6L
4.46092631226253E-147	3.37704447220819	0.102	0.016	7.47562031408954E-143	T/NK cell	PHC1
1.92900341776131E-146	1.54104533034086	0.69	0.618	3.23262392748441E-142	T/NK cell	MCL1
7.0561249594674E-141	3.02406905542029	0.289	0.116	1.18246542070755E-136	T/NK cell	MOB3A
7.9911501405467E-141	3.44337784286981	0.103	0.016	1.33915694055282E-136	T/NK cell	CDC42EP3
1.1836045506523E-139	3.13727698639333	0.265	0.099	1.98348450598313E-135	T/NK cell	SH3KBP1
7.10668837860232E-139	2.69322510039615	0.152	0.035	1.19093883848618E-134	T/NK cell	FMNL3
9.88810797473071E-137	2.85503888101236	0.32	0.14	1.65704913440537E-132	T/NK cell	SIPA1
1.21473998246449E-136	3.01469263037291	0.252	0.091	2.035661262614E-132	T/NK cell	MICAL1
1.0102457194953E-135	3.17401713907073	0.289	0.12	1.69296977673022E-131	T/NK cell	PHTF2
2.2189027196037E-134	2.44270459830967	0.316	0.137	3.71843717751188E-130	T/NK cell	PPP1R18
2.85756847002983E-134	3.02965072569198	0.106	0.018	4.788713242076E-130	T/NK cell	CDK6
2.93999588136263E-134	3.03498889615217	0.229	0.077	4.92684509798749E-130	T/NK cell	CNTRL
4.06865111961589E-133	3.05755214344291	0.296	0.127	6.81824554625231E-129	T/NK cell	DOCK10
8.3820984530325E-132	3.00432875382937	0.135	0.03	1.40467205875919E-127	T/NK cell	SAMD9L
9.91365732381674E-132	2.97090798355159	0.212	0.069	1.66133069432521E-127	T/NK cell	CASP1
1.85872172603456E-131	3.14904193231227	0.108	0.02	3.11484586848872E-127	T/NK cell	VAV1
5.23703194260107E-130	2.39658053248617	0.111	0.02	8.77621812941088E-126	T/NK cell	LFNG
2.11137796974249E-129	2.50600412667214	0.542	0.396	3.53824720169447E-125	T/NK cell	CDKN1B
1.76131175455354E-128	3.27644850385849	0.361	0.185	2.95160623828082E-124	T/NK cell	LEPROTL1
1.84474278817151E-128	3.2400643762356	0.146	0.035	3.09141996441782E-124	T/NK cell	MFNG
8.53032563327159E-127	2.87929453835816	0.187	0.056	1.42951196962365E-122	T/NK cell	HK1
4.8251777203714E-126	2.27686922019496	0.47	0.302	8.0860328237984E-122	T/NK cell	MACF1
4.048347650844E-125	2.7657753832127	0.336	0.164	6.78422099328437E-121	T/NK cell	PLCG1
9.38863536137364E-125	1.73202390836123	0.628	0.579	1.57334751385899E-120	T/NK cell	PFN1
1.58992605113541E-123	2.31072515167007	0.205	0.065	2.66439807649272E-119	T/NK cell	RIN3
8.1706118751744E-123	4.00847496458486	0.112	0.022	1.36923113804173E-118	T/NK cell	ZC3H12D
9.77518201622731E-123	1.99029132586071	0.521	0.368	1.63812500227937E-118	T/NK cell	TMSB10
1.15965136303895E-121	3.22258991675405	0.305	0.141	1.94334375418068E-117	T/NK cell	CCSER2
1.23622730572369E-121	3.65565079412488	0.181	0.055	2.07166971893175E-117	T/NK cell	PGGHG
1.45691689250262E-121	2.50853418972196	0.427	0.257	2.44150132845589E-117	T/NK cell	ZC3HAV1
1.55117210363635E-121	3.10247090667531	0.107	0.02	2.59945421127379E-117	T/NK cell	CGAS
2.47682162282497E-119	3.28357747780977	0.1	0.018	4.15065767553009E-115	T/NK cell	NFATC1
5.65344618480603E-116	1.37092333720817	0.16	0.043	9.47404511649794E-112	T/NK cell	NPDC1
9.71625023194178E-116	2.91061640381634	0.178	0.054	1.6282492138688E-111	T/NK cell	ANXA1
1.19060206684235E-115	2.58365226797326	0.124	0.028	1.99521094361441E-111	T/NK cell	GYPC
3.97478647039181E-115	3.86976546290534	0.153	0.042	6.66094716708259E-111	T/NK cell	TRERF1
1.61526456234063E-114	2.57481500749312	0.361	0.196	2.70686035357043E-110	T/NK cell	ARL6IP5
6.88189686940677E-114	3.00634994586397	0.394	0.235	1.15326827737519E-109	T/NK cell	SLC38A1
7.63208958267878E-114	3.13016038973912	0.174	0.053	1.27898557226531E-109	T/NK cell	GMIP
2.00930631843944E-113	2.5739258759985	0.135	0.033	3.36719552844082E-109	T/NK cell	TRPS1
2.17175375602367E-113	3.00452511014809	0.131	0.032	3.63942494434447E-109	T/NK cell	GIMAP5
1.10027937474834E-112	3.66399317660018	0.199	0.069	1.84384817620327E-108	T/NK cell	HELB
2.49315497194845E-110	1.58013120428713	0.607	0.47	4.17802910199121E-106	T/NK cell	LENG8
1.87941542844309E-109	3.68050402430912	0.171	0.053	3.14952437498494E-105	T/NK cell	ATP8A1
6.57657223228138E-108	2.2236311257012	0.476	0.335	1.10210197468571E-103	T/NK cell	OGT
7.16404817104085E-108	2.59875008861336	0.435	0.286	1.20055119250303E-103	T/NK cell	SUN2
4.31861031629628E-107	3.19345878723968	0.118	0.027	7.23712716804931E-103	T/NK cell	DEPRECATED-ENSG00000196329
5.31377536823333E-107	2.65686890195316	0.139	0.037	8.90482476208542E-103	T/NK cell	RILPL2
5.68900491940997E-107	2.33975714421731	0.475	0.333	9.53363444394722E-103	T/NK cell	CDC42SE1
1.04748370981226E-106	2.46161833259943	0.354	0.194	1.75537320090338E-102	T/NK cell	CSK
1.33945497518968E-106	2.09570848976094	0.489	0.352	2.24465864742287E-102	T/NK cell	TAP1
5.71303897718496E-105	2.93236605867888	0.365	0.208	9.57391071796655E-101	T/NK cell	HECA
1.10999957481105E-104	4.2513092296398	0.163	0.051	1.86013728746836E-100	T/NK cell	TCF7
2.71713188751136E-103	3.10430346661326	0.183	0.062	4.55336961709153E-99	T/NK cell	GLCCI1
6.07527056978434E-103	3.73001123158617	0.1	0.021	1.01809384208446E-98	T/NK cell	CLIP4
6.51770733929238E-103	2.87163113551021	0.277	0.129	1.09223739591862E-98	T/NK cell	FOXO1
2.00691970401181E-102	3.71895062275615	0.169	0.055	3.363196039983E-98	T/NK cell	IL18R1

4.45257836802608E-101	2.41350379646966	0.185	0.061	7.46163082913811E-97	T/NK cell	METRNL
5.49252490632749E-101	2.21765632394438	0.446	0.299	9.20437323802361E-97	T/NK cell	AKAP13
3.43033823165888E-100	3.56004310588302	0.121	0.031	5.74856080861396E-96	T/NK cell	IL27RA
2.64544830235511E-99	2.17711681051854	0.176	0.057	4.4332422650867E-95	T/NK cell	SH3BGRL
5.6573023932465E-97	4.0920499970086	0.102	0.023	9.48050735060248E-93	T/NK cell	TRIM59
4.20896514416435E-96	4.17219594416623	0.135	0.038	7.05338378859061E-92	T/NK cell	FLT3LG
1.42876392321513E-95	2.68704427775236	0.274	0.13	2.39432258252392E-91	T/NK cell	PKM
6.8322804449305E-94	2.67281861925009	0.24	0.105	1.14495355696145E-89	T/NK cell	IQGAP1
2.0930209982502E-93	1.31297681838055	0.125	0.032	3.50748458886769E-89	T/NK cell	DNAJA4
3.49076614163857E-93	2.66886612417101	0.424	0.293	5.84982590015791E-89	T/NK cell	RBL2
3.56679677901092E-93	3.2906262100051	0.141	0.043	5.9772380422665E-89	T/NK cell	MAPK13
1.63009094967316E-92	2.16144474764759	0.427	0.29	2.73170641346228E-88	T/NK cell	SH3BGRL3
3.09926658061024E-92	2.88025846717178	0.272	0.132	5.19375093578664E-88	T/NK cell	INPP4A
1.00730134983183E-91	2.76425532317012	0.264	0.126	1.68803560204817E-87	T/NK cell	PIP4K2A
1.66487150066631E-91	1.64488864773362	0.557	0.468	2.7899916608166E-87	T/NK cell	CFLAR
3.03356635633527E-91	2.63839877740024	0.322	0.178	5.08365049994664E-87	T/NK cell	CCND3
5.49739619949017E-91	3.01518511764465	0.212	0.087	9.21253655110562E-87	T/NK cell	SH3BP1
3.94745564339618E-90	3.1681283681701	0.178	0.065	6.61514616720332E-86	T/NK cell	ZNF101
7.73077656399945E-89	1.40619874801943	0.122	0.033	1.29552353659503E-84	T/NK cell	CRIM1
1.00049697179664E-88	2.28841898886864	0.42	0.283	1.6766328253368E-84	T/NK cell	GRK2
1.13555230800725E-88	3.20765751621379	0.29	0.151	1.90295855775856E-84	T/NK cell	PBXIP1
1.83075182543502E-88	2.26384397441077	0.138	0.041	3.06797390906401E-84	T/NK cell	FAR1
1.60376443407715E-86	2.594547754389	0.358	0.22	2.68758843862648E-82	T/NK cell	SYNRG
4.04388406542833E-86	2.11440468995224	0.467	0.359	6.7767409168448E-82	T/NK cell	MBNL1
7.20389324090112E-86	2.95051010197693	0.237	0.109	1.20722842931021E-81	T/NK cell	SLC4A7
9.3547287328502E-86	2.22250211342404	0.44	0.314	1.56766544105104E-81	T/NK cell	JAK1
1.57797136386671E-85	3.42902330613935	0.126	0.037	2.64436441156784E-81	T/NK cell	LRRC8C
3.04927711215031E-84	1.5048970799154	0.14	0.042	5.10997858454149E-80	T/NK cell	ACP5
4.49347703104561E-84	2.64812007508084	0.341	0.205	7.53016880862624E-80	T/NK cell	AAK1
2.26330750896423E-83	2.56784885694107	0.355	0.221	3.79285072352225E-79	T/NK cell	CBFB
7.3670408688642E-82	2.50461752791797	0.167	0.06	1.23456870880426E-77	T/NK cell	PDLIM2
2.23856789019943E-81	2.71897851123754	0.294	0.158	3.7513920703962E-77	T/NK cell	FNBP1
4.78418865789265E-81	3.02935677010629	0.246	0.12	8.01734335289649E-77	T/NK cell	ERAP2
6.83775729400196E-81	2.39963681991912	0.166	0.06	1.14587136732885E-76	T/NK cell	SLC6A6
2.55582022048785E-80	3.57792408697626	0.136	0.044	4.28304352549354E-76	T/NK cell	RNF166
4.17172450701726E-80	2.70299734711319	0.196	0.081	6.99097592885952E-76	T/NK cell	ADCY3
4.63785519777151E-80	2.9041561294413	0.205	0.088	7.7721177404255E-76	T/NK cell	DEPRECATED-ENSG00000150867
7.82040932162523E-80	2.88290577027927	0.284	0.153	1.31054419411796E-75	T/NK cell	PCSK7
9.9569531370366E-80	2.39551301229465	0.211	0.09	1.66858620670459E-75	T/NK cell	NABP1
2.66966037915242E-79	1.96862294399452	0.152	0.051	4.47381686338362E-75	T/NK cell	ADA2
3.62939807061424E-78	3.25250951451653	0.111	0.031	6.08214528673534E-74	T/NK cell	SAP25
4.47515762245622E-78	2.03132077817729	0.338	0.199	7.49946914371214E-74	T/NK cell	LY6E
1.75720617565812E-77	3.1216711562872	0.131	0.042	2.94472610916787E-73	T/NK cell	SESN3
6.32927042923116E-76	2.62848606018694	0.139	0.046	1.06065913853056E-71	T/NK cell	RBMS1
8.41318007977294E-76	2.16180804783644	0.415	0.293	1.40988071776835E-71	T/NK cell	MLLT6
1.55619233120594E-75	2.43447002536944	0.443	0.338	2.60786710863491E-71	T/NK cell	PDCD4
4.36417921223809E-73	2.6657027768279	0.349	0.225	7.31349152386859E-69	T/NK cell	ELF1
1.07799780357168E-72	2.245309459021	0.357	0.23	1.80650871922542E-68	T/NK cell	CNN2
1.11163547967401E-72	2.11200205530334	0.114	0.034	1.8628787368377E-68	T/NK cell	ZNF264
2.17922863657123E-72	2.79247333860234	0.111	0.033	3.65195134916607E-68	T/NK cell	MYO5A
2.53679106015704E-72	2.43174819054854	0.409	0.292	4.25115445861117E-68	T/NK cell	CYTH1
6.57701736561686E-72	2.51734795975331	0.233	0.113	1.10217657013007E-67	T/NK cell	LDB1
4.86722893697828E-70	2.73821612511345	0.24	0.122	8.1565022525882E-66	T/NK cell	RAB8B
1.38330553867494E-68	2.87301272295463	0.286	0.165	2.31814342171147E-64	T/NK cell	HNRNPLL
5.06237921936364E-68	2.45891998179703	0.227	0.111	8.48353509580958E-64	T/NK cell	CISH
6.61668029282205E-68	2.00070079544961	0.18	0.074	1.10882328347112E-63	T/NK cell	PLEKHO1
3.46017766889592E-67	2.90493647016798	0.279	0.162	5.79856573753577E-63	T/NK cell	PDE7A
5.84717816332445E-67	2.59920420294549	0.102	0.03	9.79870116609912E-63	T/NK cell	TNFSF12
1.15961081355391E-66	1.81078130138087	0.444	0.338	1.94327580135364E-62	T/NK cell	CTSC
1.66733282774933E-66	2.51264029239239	0.147	0.055	2.79411635274232E-62	T/NK cell	PLEKHA2
5.01021234023385E-66	2.86982619469726	0.182	0.08	8.39611383976388E-62	T/NK cell	BTN3A3
3.75888980535832E-65	1.981650753115	0.457	0.366	6.29914753581947E-61	T/NK cell	DIAPH1
1.79324581035178E-63	2.52723759155486	0.316	0.201	3.00512132898752E-59	T/NK cell	RAB27A

3.10423760671675E-63	1.59901264380476	0.545	0.495	5.20208138133593E-59	T/NK cell	DDX5
5.67323024343184E-63	1.5033289009118	0.546	0.496	9.50719924194309E-59	T/NK cell	WNK1
6.05856098107193E-62	2.75875130150563	0.155	0.064	1.01529364920803E-57	T/NK cell	SLC12A6
6.75377897027658E-61	2.65687741859494	0.21	0.104	1.13179827983895E-56	T/NK cell	SYNE3
1.59950667625719E-60	2.03188180947492	0.106	0.034	2.6804532880718E-56	T/NK cell	DEPRECATED-ENSG00000163191
2.72356856267368E-60	2.34488758132422	0.197	0.094	4.56415619732856E-56	T/NK cell	OAS2
3.33352641231748E-59	2.62343594649308	0.28	0.17	5.58632356176163E-55	T/NK cell	LNPEP
5.61943870233557E-59	1.984808085274	0.374	0.269	9.41705537737395E-55	T/NK cell	SP100
1.52894321014038E-58	0.275955998309693	0.135	0.332	2.56220303155324E-54	T/NK cell	FURIN
2.28698227937043E-58	3.27233940619412	0.108	0.036	3.83252490376897E-54	T/NK cell	SEC31B
2.65817449975589E-58	1.59669335742921	0.534	0.471	4.45456882669091E-54	T/NK cell	PNRC1
7.54344909380557E-58	3.16853026745814	0.207	0.106	1.26413119913994E-53	T/NK cell	ARAP2
4.61424675847983E-57	3.00922827911116	0.135	0.052	7.7325547178605E-53	T/NK cell	RBM38
8.32156841834252E-56	1.66302875053569	0.183	0.084	1.39452843554584E-51	T/NK cell	PHLDA1
9.55987863074501E-56	2.97811100735834	0.155	0.067	1.60204446094025E-51	T/NK cell	TNFAIP8
2.29462292197558E-55	3.13715843949412	0.211	0.111	3.84532909264668E-51	T/NK cell	TNIK
1.49170357157511E-54	2.29506144694951	0.108	0.037	2.49979684524557E-50	T/NK cell	DUSP5
5.71571521873154E-54	2.92248117535753	0.266	0.162	9.57839556355032E-50	T/NK cell	PITPNC1
1.11670620848137E-53	2.00586806613871	0.355	0.255	1.87137626417308E-49	T/NK cell	MYH9
9.95623590967083E-53	2.96965074118019	0.321	0.223	1.66846601374264E-48	T/NK cell	PPP2R5C
1.63527321847786E-52	3.01499945511324	0.171	0.081	2.7403908595252E-48	T/NK cell	SP4
2.02147638820346E-52	2.27250226762962	0.259	0.153	3.38759013135135E-48	T/NK cell	PACS1
1.08998606219127E-51	1.92410158083753	0.158	0.069	1.82659864302014E-47	T/NK cell	CAPG
6.77353170371186E-51	2.17816425560893	0.303	0.201	1.13510844290803E-46	T/NK cell	APBB1IP
9.24964394413568E-51	2.45898280873537	0.366	0.275	1.55005533215826E-46	T/NK cell	CDC42SE2
4.24030576266961E-50	2.18450288396357	0.168	0.078	7.10590439708173E-46	T/NK cell	ARHGEF2
1.09165363158226E-49	2.18430897518512	0.195	0.099	1.82939315580555E-45	T/NK cell	RFTN1
2.63122155984596E-49	2.75956915205528	0.136	0.058	4.40940108998986E-45	T/NK cell	CHMP4A
1.38063548743557E-48	3.68843923409894	0.128	0.053	2.31366894984453E-44	T/NK cell	RCAN3
3.61285146664309E-48	2.82618417555564	0.134	0.057	6.05441648780048E-44	T/NK cell	STN 1.00
3.98645434067262E-48	2.45382809393977	0.259	0.161	6.68050018409917E-44	T/NK cell	CYLD
8.25890963244592E-48	1.90269904188129	0.396	0.316	1.38402807620529E-43	T/NK cell	TGFBR2
1.13531358070022E-47	2.57586676532427	0.229	0.134	1.90255849853743E-43	T/NK cell	ASXL2
2.01589659487231E-47	0.639118887748297	0.139	0.317	3.37823951368701E-43	T/NK cell	FAM177A1
2.88367636084237E-47	2.93102121004308	0.14	0.062	4.83246484549965E-43	T/NK cell	GLS
4.70781330761001E-47	3.12693378970077	0.115	0.046	7.88935354089286E-43	T/NK cell	PCYOX1L
5.3255352503584E-47	3.06403501314338	0.162	0.079	8.9245319725506E-43	T/NK cell	PCNX2
8.13449213990732E-47	2.37172834952454	0.282	0.185	1.36317819280567E-42	T/NK cell	WDR82
1.10759022120766E-46	2.31767631177189	0.45	0.395	1.85609969269979E-42	T/NK cell	EML4
3.33982632417714E-46	2.46007411063248	0.23	0.136	5.59688095405605E-42	T/NK cell	FAM53B
4.07152567413088E-46	3.00519442643367	0.198	0.109	6.82306272470853E-42	T/NK cell	RASA2
9.22989245767239E-46	1.52198912551425	0.389	0.3	1.54674537805674E-41	T/NK cell	SAMHD1
2.60915092286811E-45	2.54510458890555	0.303	0.21	4.37241511654238E-41	T/NK cell	ATM
5.13386771888455E-45	2.06051469185161	0.341	0.253	8.60333552330674E-41	T/NK cell	TSC22D4
7.28152454926208E-45	2.47698861949526	0.131	0.056	1.22023788396534E-40	T/NK cell	EML2
1.60436775996888E-44	0.387592739569685	0.097	0.249	2.68859949215584E-40	T/NK cell	AUTS2
4.94638211295813E-44	1.87530217695369	0.37	0.289	8.28914714489524E-40	T/NK cell	ESYT1
2.0499174936923E-43	2.43217441298779	0.295	0.204	3.43525173592955E-39	T/NK cell	NFATC2
2.88561044735377E-43	1.83194139789134	0.345	0.26	4.83570598767544E-39	T/NK cell	TLN1
3.45685173003538E-43	1.12614353073456	0.602	0.603	5.79299212919329E-39	T/NK cell	TAPBP
2.74370475342089E-42	2.43682736132132	0.191	0.104	4.59790042578273E-38	T/NK cell	LPIN1
3.52308499152215E-42	2.18064768167782	0.37	0.291	5.90398582879282E-38	T/NK cell	KMT2E
6.51188618238564E-42	2.86138714850316	0.164	0.084	1.09126188644419E-37	T/NK cell	FAS
9.84642956757822E-41	2.72072710890483	0.204	0.119	1.65006466693476E-36	T/NK cell	BTN3A1
1.10003141766121E-40	2.65844497285877	0.183	0.101	1.84343264971665E-36	T/NK cell	RSBN1
1.76746899802113E-40	0.27825507951227	0.127	0.283	2.96192454688381E-36	T/NK cell	VAPA
2.47596581669433E-40	2.58408335924635	0.318	0.235	4.14922351561636E-36	T/NK cell	SARAF
3.76272921082905E-40	0.278238825861167	0.102	0.247	6.30558161150733E-36	T/NK cell	RMDN3
4.42311607527911E-40	3.32499609844068	0.129	0.059	7.41225791895273E-36	T/NK cell	TENT5C
8.01188936834545E-40	1.56834257073532	0.527	0.519	1.34263242034733E-35	T/NK cell	AHNAK
9.08544214312408E-40	2.09823976274314	0.327	0.251	1.52253839434473E-35	T/NK cell	LRBA
1.78042828381577E-39	2.31571559460444	0.27	0.183	2.98364171801847E-35	T/NK cell	FGFR1OP2
2.36391817038087E-39	2.5189190964953	0.237	0.151	3.96145406992426E-35	T/NK cell	EHD1

3.59186664554265E-39	2.11635998254241	0.301	0.219	6.01925012460038E-35	T/NK cell	CKLF
7.75639227966425E-39	2.55600270526149	0.313	0.239	1.29981621822613E-34	T/NK cell	LYST
8.03316237426396E-39	1.71330709462302	0.287	0.193	1.34619735067915E-34	T/NK cell	LGALS1
9.50897685338628E-39	2.52949345297417	0.186	0.103	1.59351434109047E-34	T/NK cell	SIGIRR
1.32763215942002E-38	1.92332557004866	0.406	0.347	2.22484597275607E-34	T/NK cell	DDX17
1.58331400448513E-38	2.63221181780149	0.3	0.219	2.65331760871618E-34	T/NK cell	DUSP16
3.4921933054167E-38	2.85346478394576	0.205	0.123	5.8522175412173E-34	T/NK cell	ATF7IP
3.93377480915079E-38	2.41425464399954	0.267	0.184	6.59221982517489E-34	T/NK cell	GNPTAB
1.16355620038584E-37	0.264591964154729	0.066	0.187	1.94988748060658E-33	T/NK cell	PAN2
1.65522947213579E-37	2.85481782840994	0.176	0.098	2.77383354940516E-33	T/NK cell	GIMAP2
1.84487142530679E-36	1.90667868195199	0.319	0.237	3.09163553452913E-32	T/NK cell	LRCH4
2.02817817834619E-36	2.52801986910735	0.16	0.085	3.39882099127255E-32	T/NK cell	ANKRD13D
3.76431499862009E-36	0.263597135317421	0.129	0.275	6.30823907468754E-32	T/NK cell	CUL4A
5.78420739051564E-36	0.293666153192786	0.169	0.334	9.6931747450261E-32	T/NK cell	LMBRD1
1.32736316637946E-35	2.45029580887831	0.163	0.088	2.2243951942187E-31	T/NK cell	KLF12
1.44411830916957E-35	0.292633290907135	0.183	0.351	2.42005346250637E-31	T/NK cell	MIA3
2.23729044543326E-35	0.382395512664666	0.113	0.252	3.74925132845705E-31	T/NK cell	ZNF326
4.97094608605448E-35	1.82530254402112	0.399	0.346	8.3303114510101E-31	T/NK cell	SNRNP200
6.39464999668734E-35	2.9268594468905	0.131	0.064	1.07161544644486E-30	T/NK cell	PPP3CC
6.78592415701141E-35	1.94484665986447	0.399	0.336	1.13718517023197E-30	T/NK cell	RNF149
7.3675637553177E-35	0.714918493547709	0.049	0.154	1.23465633411614E-30	T/NK cell	CYB561
8.45016232595542E-35	3.37595077314861	0.136	0.069	1.41607820258361E-30	T/NK cell	KIF21B
9.46528469699898E-35	2.03815543225696	0.195	0.116	1.58619240952309E-30	T/NK cell	MAPKAPK3
1.33042594578824E-34	0.331844008431113	0.121	0.262	2.22952779995193E-30	T/NK cell	TNRC18
4.86292724347904E-34	2.83456172623679	0.183	0.107	8.14929347462217E-30	T/NK cell	BICDL1
5.83021705941557E-34	2.3569972223832	0.156	0.084	9.7702777481686E-30	T/NK cell	RASSF1
7.61436497255063E-34	0.317719791791907	0.206	0.378	1.27601528210003E-29	T/NK cell	POGZ
8.12334210644683E-34	2.57124661193925	0.159	0.087	1.36130967019836E-29	T/NK cell	TBC1D4
1.00707232176894E-33	1.63778872829127	0.427	0.385	1.68765179682038E-29	T/NK cell	SMARCA2
2.27894712790862E-33	0.260392300064971	0.09	0.214	3.81905959694927E-29	T/NK cell	FBXO21
3.7590809082454E-33	0.316141429169823	0.209	0.382	6.29946778603764E-29	T/NK cell	CHTOP
6.63077632774382E-33	1.69082552911015	0.455	0.429	1.11118549700331E-28	T/NK cell	MAF
6.79978504580426E-33	0.322864876361749	0.161	0.315	1.13950797797588E-28	T/NK cell	NUS1
1.40631964599142E-32	2.31102072525143	0.219	0.143	2.35671046275242E-28	T/NK cell	CD58
1.58086713333568E-32	2.26496337231466	0.271	0.197	2.64921714204393E-28	T/NK cell	KMT2A
1.66196526153358E-32	2.11337522128714	0.2	0.122	2.78512138527797E-28	T/NK cell	SP110
2.12834575760233E-32	2.38641000825211	0.179	0.106	3.56668182058998E-28	T/NK cell	CHST12
4.06838434077948E-32	0.343426204555043	0.051	0.151	6.81779847827826E-28	T/NK cell	ZBTB21
4.50063938186587E-32	1.99077381275562	0.33	0.263	7.54217147613082E-28	T/NK cell	NUP210
9.47750899575059E-32	2.36547044432711	0.181	0.108	1.58824095750788E-27	T/NK cell	KAT6B
1.00488284798319E-31	0.387295171842154	0.158	0.309	1.68398267665022E-27	T/NK cell	TMUB2
1.41139908539214E-31	0.375743887936513	0.11	0.238	2.36522258730014E-27	T/NK cell	BRF1
1.73222059240359E-31	0.251612005695681	0.087	0.204	2.90285526874994E-27	T/NK cell	CBWD2
2.28510998642835E-31	1.86907745294352	0.394	0.349	3.82938731525663E-27	T/NK cell	RABGAP1L
2.54035739141524E-31	0.288243421869027	0.208	0.376	4.25713091653365E-27	T/NK cell	HSF1
3.62042916458371E-31	2.27122484378561	0.224	0.149	6.06711519400938E-27	T/NK cell	PSD4
4.27639158836835E-31	0.257085883294794	0.121	0.254	7.16637702378768E-27	T/NK cell	UQCC1
4.38014750281393E-31	1.54393161578193	0.115	0.053	7.34025118521559E-27	T/NK cell	THEMIS2
7.96143547602558E-31	0.451095495133717	0.114	0.241	1.33417735707237E-26	T/NK cell	TGIF1
9.87753120821168E-31	0.273295708464432	0.112	0.239	1.65527667987211E-26	T/NK cell	EIF2D
1.05114733738264E-30	1.75470725233805	0.348	0.281	1.76151270798582E-26	T/NK cell	FAM214A
1.31476360921508E-30	1.09259111047234	0.485	0.441	2.20328085632263E-26	T/NK cell	DDIT4
1.41739761058979E-30	0.397678828315522	0.056	0.156	2.37527491582637E-26	T/NK cell	PLEKHA1
1.5029359662158E-30	1.04003217943087	0.108	0.049	2.51862009218444E-26	T/NK cell	PLEKHO2
1.6824562154623E-30	0.270432622280851	0.141	0.28	2.81946012587172E-26	T/NK cell	R3HDM2
1.71822175784883E-30	0.507772423116636	0.103	0.226	2.87939602180306E-26	T/NK cell	RARA
1.73263938937547E-30	0.300876224138159	0.114	0.243	2.90355708871541E-26	T/NK cell	RSAD1
3.60068263175309E-30	2.20666587637822	0.187	0.114	6.03402395429183E-26	T/NK cell	EPSTI1
4.1186737717456E-30	2.34267221825692	0.16	0.092	6.90207350669128E-26	T/NK cell	DGKZ
4.18503353638004E-30	2.70521110765563	0.194	0.123	7.01327920026567E-26	T/NK cell	RAPGEF6
4.8829373867566E-30	0.408870619425476	0.137	0.273	8.18282647272671E-26	T/NK cell	SH3GLB2
4.9632151601104E-30	1.91207235609759	0.11	0.051	8.317355965313E-26	T/NK cell	SLC16A3
4.97854130564231E-30	2.92609917345944	0.122	0.061	8.34303951999538E-26	T/NK cell	DDB2

5.06823840089327E-30	0.499075382896542	0.09	0.205	8.49335391221693E-26	T/NK cell	PLEKHG3
6.24839044800741E-30	1.94442648377815	0.294	0.225	1.04710527127708E-25	T/NK cell	CD99
7.54432712404688E-30	0.289465640394767	0.139	0.273	1.26427833944778E-25	T/NK cell	VPS4A
1.10339732961999E-29	0.55777107140056	0.121	0.25	1.84907324497717E-25	T/NK cell	IP6K2
1.40956138809374E-29	0.634619165667	0.068	0.172	2.36214297416748E-25	T/NK cell	DAPK2
2.12960812486053E-29	0.31024465740019	0.127	0.254	3.56879729564128E-25	T/NK cell	STRN4
2.29821200413764E-29	1.57234186320137	0.412	0.369	3.85134367653385E-25	T/NK cell	CLK1
3.19812749556436E-29	1.79749879483007	0.319	0.255	5.35942205706675E-25	T/NK cell	STIM1
3.46892671320635E-29	2.86477246979119	0.166	0.099	5.81322738599121E-25	T/NK cell	PTPN4
4.15987148667914E-29	0.389062235252849	0.124	0.251	6.97111263737691E-25	T/NK cell	STUB1
4.9233044000913E-29	0.442364827801227	0.089	0.201	8.25047351367301E-25	T/NK cell	CCDC159
5.05984348656361E-29	0.250125664862215	0.128	0.258	8.4792857147833E-25	T/NK cell	PEX13
5.19432214135381E-29	0.410256938156274	0.207	0.368	8.70464504448072E-25	T/NK cell	NDUFA10
6.2763893618409E-29	0.338460813973737	0.154	0.295	1.0517973292573E-24	T/NK cell	UBE2H
7.01811807427365E-29	1.87712376546383	0.312	0.248	1.17609622688678E-24	T/NK cell	VPS13C
7.41440603308661E-29	0.354559598969	0.093	0.206	1.24250616302465E-24	T/NK cell	RNF113A
7.43742725870474E-29	0.284662540291741	0.123	0.248	1.24636406001374E-24	T/NK cell	ABCF3
7.7684902725846E-29	0.330062330339779	0.089	0.201	1.30184359987973E-24	T/NK cell	PTPN12
1.12557580326764E-28	1.53610421911683	0.407	0.372	1.88623993111591E-24	T/NK cell	ARF6
1.1641771349536E-28	2.01940338132968	0.277	0.208	1.95092804275524E-24	T/NK cell	ATP2B4
1.1975595523303E-28	0.351456918013979	0.099	0.215	2.00687029779511E-24	T/NK cell	ARHGAP17
1.2053981542481E-28	0.61875407028557	0.19	0.345	2.02000622688896E-24	T/NK cell	RAB18
2.01486340893666E-28	1.55680931983148	0.107	0.051	3.37650810069605E-24	T/NK cell	GSTP1
2.11642555481164E-28	0.31284008780049	0.082	0.19	3.54670594475334E-24	T/NK cell	MOSPD2
2.15393646706074E-28	0.317318763226878	0.082	0.19	3.60956673150039E-24	T/NK cell	RNF139
2.4894954361556E-28	0.349884114940928	0.092	0.204	4.17189645190955E-24	T/NK cell	DLST
2.50470996119291E-28	0.36843352688382	0.184	0.335	4.19739295296708E-24	T/NK cell	DLG1
2.73806507949706E-28	0.430315203321195	0.122	0.246	4.58844946022118E-24	T/NK cell	SAMD4B
2.87802789909417E-28	1.87058270086865	0.306	0.24	4.822999153302E-24	T/NK cell	GBP2
3.18097123442988E-28	2.77931419148869	0.1	0.047	5.3306715946576E-24	T/NK cell	ACSS1
3.46182792710078E-28	0.494574538514429	0.06	0.156	5.80133124023549E-24	T/NK cell	PLXND1
3.79991861697598E-28	0.430221343282537	0.135	0.264	6.36790361832834E-24	T/NK cell	LIN7C
4.19735076198425E-28	0.2705977915781	0.162	0.303	7.0339204069332E-24	T/NK cell	NUDCD3
4.61404754946151E-28	0.40406772478097	0.189	0.341	7.7322208833876E-24	T/NK cell	SRP54
4.68030824356066E-28	0.354858833518481	0.097	0.21	7.84326055455896E-24	T/NK cell	CRELD1
4.90733106672763E-28	0.282564409585101	0.18	0.328	8.22370540162216E-24	T/NK cell	GOLGA7
4.93231356641551E-28	0.272692510762625	0.135	0.263	8.26557107459912E-24	T/NK cell	TRA2B
5.61295600981232E-28	2.21430725473603	0.151	0.086	9.40619168124349E-24	T/NK cell	IGFLR1
8.63228956749422E-28	0.335771182722411	0.141	0.272	1.44659908572068E-23	T/NK cell	CDC34
9.28984650900147E-28	0.26301142648209	0.147	0.279	1.55679247797847E-23	T/NK cell	AP2A2
1.03043690305782E-27	2.06495445040833	0.245	0.175	1.7268061621443E-23	T/NK cell	ARPC2
1.26636167896226E-27	2.24737365390815	0.101	0.048	2.12216890160495E-23	T/NK cell	RGS19
1.38036235362379E-27	0.25796399187867	0.149	0.281	2.31321123220274E-23	T/NK cell	FKBP8
1.56964198678563E-27	0.348899103168736	0.114	0.234	2.63040604145535E-23	T/NK cell	LSM 7.00
1.65114885421057E-27	0.432924129520625	0.162	0.304	2.76699524988608E-23	T/NK cell	FBXL5
1.70937690570088E-27	0.35038988297627	0.116	0.237	2.86457381857353E-23	T/NK cell	CRK
2.14679891677954E-27	0.402812902925732	0.111	0.229	3.59760562473916E-23	T/NK cell	TBC1D17
2.70081061930636E-27	2.29669128403368	0.16	0.095	4.52601843583359E-23	T/NK cell	OSBPL3
2.78404828820667E-27	0.431207921494494	0.167	0.311	4.66550812137673E-23	T/NK cell	RIOK3
3.00298938425939E-27	0.284610861658092	0.09	0.199	5.03240961014189E-23	T/NK cell	LYSMD3
3.83335692204845E-27	0.443865320668201	0.111	0.228	6.4239395299688E-23	T/NK cell	PAPOLA
4.33754833418067E-27	0.301040240767257	0.058	0.15	7.26886349841997E-23	T/NK cell	PIGH
4.72593880156728E-27	0.285256351979948	0.065	0.16	7.91972824366646E-23	T/NK cell	CASZ1
4.85915709380947E-27	0.330586837173279	0.078	0.18	8.14297545780591E-23	T/NK cell	TIMM17B
5.49156065320709E-27	0.288720553435371	0.118	0.239	9.20275734264443E-23	T/NK cell	USF1
6.00271888287317E-27	0.275473419045376	0.101	0.214	1.00593563039189E-22	T/NK cell	MTMR4
7.34453697333777E-27	0.904563849369042	0.12	0.059	1.23079750599194E-22	T/NK cell	KLHL6
8.1680162284549E-27	3.2090677024989	0.118	0.061	1.36879615956447E-22	T/NK cell	MYBL1
8.62512186208689E-27	0.266141747749088	0.069	0.165	1.44539792164852E-22	T/NK cell	ARL 16.00
8.66326458509621E-27	2.75891834874007	0.102	0.05	1.45178987917042E-22	T/NK cell	C21orf91
8.69346571625643E-27	0.326793600543594	0.157	0.294	1.45685098473025E-22	T/NK cell	RB1CC1
9.16123425095189E-27	3.15119331018659	0.11	0.055	1.53523963577452E-22	T/NK cell	CTC1
1.17910618302284E-26	0.470959191765814	0.074	0.172	1.97594614150968E-22	T/NK cell	CAMLG

1.21350029241971E-26	0.359930881475512	0.093	0.201	2.03358379003695E-22	T/NK cell	CSNK2B
1.28991927833749E-26	2.49362107116854	0.27	0.206	2.16164672663797E-22	T/NK cell	BRD1
1.32897384683933E-26	2.38517200588275	0.154	0.091	2.22709437253335E-22	T/NK cell	PHACTR2
1.35147096834209E-26	0.341932783295714	0.111	0.227	2.26479504874767E-22	T/NK cell	SLC25A32
1.37796974293655E-26	0.345451609942945	0.127	0.247	2.30920169521307E-22	T/NK cell	SRRT
1.97997420318097E-26	0.400136238379261	0.257	0.433	3.31804076969067E-22	T/NK cell	SREBF2
2.21671168229813E-26	0.533494122987	0.168	0.308	3.71476543719521E-22	T/NK cell	DNAJC21
2.36606926457592E-26	2.14500289308298	0.249	0.182	3.96505887357633E-22	T/NK cell	CD82
2.56087372435864E-26	0.404090686317393	0.092	0.199	4.29151218728021E-22	T/NK cell	RCOR1
2.60890484853703E-26	0.634821306722973	0.097	0.205	4.37200274517835E-22	T/NK cell	AMPD2
3.01214028908416E-26	0.432758545776575	0.141	0.269	5.04774469644724E-22	T/NK cell	PCMTD2
3.06978562656363E-26	0.558567100146709	0.186	0.332	5.14434675299534E-22	T/NK cell	DUS1L
3.58888102193851E-26	0.335223550807463	0.137	0.262	6.01424681656456E-22	T/NK cell	TTC1
3.86119072284366E-26	0.302017925716367	0.214	0.37	6.47058341334141E-22	T/NK cell	POLR2J3
4.12533413180301E-26	0.296889396977892	0.086	0.19	6.91323493807549E-22	T/NK cell	STX17
4.16044139885291E-26	0.25171388765755	0.18	0.324	6.9720676961977E-22	T/NK cell	UXT
4.68235211764732E-26	0.282766479219035	0.083	0.186	7.84668567875337E-22	T/NK cell	MFN1
4.79451628529211E-26	0.278530358698846	0.102	0.213	8.03465039089252E-22	T/NK cell	MED16
4.95341654440653E-26	0.793928251383281	0.211	0.372	8.30093544511646E-22	T/NK cell	UBE2B
5.48312909777748E-26	0.315012283667711	0.239	0.403	9.18862774205551E-22	T/NK cell	EIF4H
5.59605537199586E-26	2.17543436030234	0.107	0.053	9.37786959239067E-22	T/NK cell	KSR1
7.24916281693076E-26	0.46294880697337	0.112	0.226	1.21481470486126E-21	T/NK cell	DENND11
7.27461942956423E-26	0.323730483847494	0.116	0.232	1.21908072400637E-21	T/NK cell	ZMYND11
9.42439069814182E-26	0.521859224674933	0.151	0.281	1.57933939319461E-21	T/NK cell	CMC2
9.61016016339748E-26	0.400489317816185	0.104	0.216	1.61047064018215E-21	T/NK cell	UQCRC2
1.04087590888547E-25	0.395571151410306	0.132	0.253	1.74429984811026E-21	T/NK cell	DEPRECATED-ENSG00000073578
1.17699522324122E-25	0.407177475113266	0.12	0.237	1.97240859510764E-21	T/NK cell	ARAF
1.42528378081082E-25	0.568240359682486	0.096	0.204	2.38849055988278E-21	T/NK cell	ULK1
1.69144547037506E-25	1.70752538455446	0.378	0.341	2.83452431925452E-21	T/NK cell	DDX6
1.9976934027924E-25	0.371883594392726	0.146	0.273	3.3477346043995E-21	T/NK cell	MED25
2.11744163991028E-25	2.19486653097134	0.267	0.205	3.54840870016165E-21	T/NK cell	TNRC6B
2.23425780837649E-25	0.34955945071853	0.076	0.173	3.74416923527732E-21	T/NK cell	MTRR
2.42958421995362E-25	1.75760280690312	0.365	0.326	4.07149723579828E-21	T/NK cell	FUBP1
2.45777306895148E-25	0.316355512331221	0.254	0.419	4.11873610894889E-21	T/NK cell	CALCOCO2
2.88563357385278E-25	1.79303698563126	0.249	0.185	4.83574474306248E-21	T/NK cell	SNTB2
2.90765940695178E-25	0.651587694014201	0.123	0.241	4.87265563416979E-21	T/NK cell	GNL1
3.13796087391776E-25	0.475534361394479	0.106	0.216	5.25859483251137E-21	T/NK cell	OSBPL2
3.20601948948089E-25	1.87424573540135	0.186	0.12	5.37264746047208E-21	T/NK cell	NEDD9
3.48572056947073E-25	1.50166236009375	0.431	0.411	5.84137053031906E-21	T/NK cell	SEPTIN9
3.67949544618771E-25	2.2290777394713	0.168	0.105	6.16609846872136E-21	T/NK cell	CABIN1
3.89645623769129E-25	2.04578784508904	0.191	0.124	6.52968136312307E-21	T/NK cell	MAPRE2
4.41207597880064E-25	0.51409626262882	0.159	0.295	7.39375692527412E-21	T/NK cell	NUB1
5.13558715179E-25	2.28077918681049	0.259	0.198	8.60621694896969E-21	T/NK cell	PCNX1
5.14912849824202E-25	0.441010947801737	0.095	0.199	8.62890953735397E-21	T/NK cell	KIFC2
5.23485778513631E-25	0.470394942549323	0.135	0.256	8.77257467633142E-21	T/NK cell	SPAG9
6.10141835371169E-25	0.587231199277455	0.125	0.244	1.022475687715E-20	T/NK cell	AKT2
6.71232254697134E-25	0.471140586815397	0.104	0.214	1.12485101242146E-20	T/NK cell	SNX13
6.83350865919173E-25	0.463030054483594	0.196	0.341	1.14515938110735E-20	T/NK cell	RNF7
7.02235435896287E-25	1.88576415888365	0.253	0.185	1.176806143475E-20	T/NK cell	MAP7D1
7.59471404833794E-25	0.439586546659744	0.088	0.189	1.27272218022047E-20	T/NK cell	ABCD4
8.01690327036365E-25	0.494982460379959	0.19	0.334	1.34347265004754E-20	T/NK cell	SNRNP70
9.44928816927328E-25	0.306704437997426	0.121	0.237	1.58351171140682E-20	T/NK cell	TARBP1
1.06474493379595E-24	0.29845850923858	0.177	0.312	1.78429956005525E-20	T/NK cell	YBX1
1.08621998634749E-24	0.288790787471744	0.107	0.216	1.82028745312112E-20	T/NK cell	STX10
1.25836028633043E-24	0.385075824361795	0.103	0.211	2.10876016783254E-20	T/NK cell	NUP42
1.29860597905271E-24	0.3612109138268	0.13	0.249	2.17620389969653E-20	T/NK cell	TAF2
1.3315334659843E-24	2.62142798221044	0.156	0.096	2.23138378229649E-20	T/NK cell	USP20
1.46452487046987E-24	0.458859829395056	0.197	0.345	2.45425077793342E-20	T/NK cell	EIF4A2
1.48699989287288E-24	0.422079487020197	0.131	0.248	2.49191442047637E-20	T/NK cell	TAF15
1.53250879876172E-24	0.473860963719592	0.09	0.192	2.56817824496489E-20	T/NK cell	HDAC5
1.78811401576803E-24	1.76652899801567	0.238	0.17	2.99652146762406E-20	T/NK cell	ITM2C
1.94287881429755E-24	0.296244760249329	0.063	0.151	3.25587631699983E-20	T/NK cell	MRGBP
2.12588599874906E-24	0.3377321284236	0.073	0.166	3.56255975670368E-20	T/NK cell	POLR2C

2.70928522925359E-24	0.326323692736622	0.089	0.19	4.54022018718317E-20	T/NK cell	DMAP1
2.76059676966855E-24	0.460524168752924	0.236	0.396	4.62620806661055E-20	T/NK cell	ILRUN
3.33051282460052E-24	0.414567129902528	0.113	0.226	5.58127339146555E-20	T/NK cell	ATG12
3.97516671445114E-24	0.352433747521418	0.047	0.128	6.66158438007722E-20	T/NK cell	FLCN
4.15713229170699E-24	2.80540561588561	0.126	0.071	6.96652229444257E-20	T/NK cell	ADGRG1
4.53712425391646E-24	0.384239455737495	0.124	0.238	7.60331282471321E-20	T/NK cell	HUS1
6.39992628540083E-24	0.69046700846646	0.139	0.261	1.07249964690747E-19	T/NK cell	SENP2
6.94472785318545E-24	0.298024037096835	0.125	0.239	1.16379749363682E-19	T/NK cell	ERP44
8.90773939738676E-24	0.389184986702718	0.122	0.235	1.49275896821407E-19	T/NK cell	SEC63
9.35325964734798E-24	0.374189906133772	0.164	0.295	1.56741925170257E-19	T/NK cell	FBXL17
1.0416193900831E-23	0.342568150618736	0.082	0.178	1.74554577390126E-19	T/NK cell	USPL1
1.06470281709836E-23	2.33852971276556	0.204	0.142	1.78422898089342E-19	T/NK cell	ANKRD36C
1.11950605684774E-23	0.360268357052068	0.069	0.158	1.87606825006545E-19	T/NK cell	SLC10A7
1.13955447032701E-23	0.478934893176477	0.1	0.204	1.909665381374E-19	T/NK cell	CLASRP
1.15910125227554E-23	0.377332238954996	0.083	0.179	1.94242187856335E-19	T/NK cell	TMEM65
1.22684435143982E-23	0.269095703049974	0.187	0.327	2.05594576414284E-19	T/NK cell	ECPAS
1.26016954150573E-23	0.671423444233057	0.158	0.286	2.1117921176553E-19	T/NK cell	EGLN2
1.27784840775853E-23	2.01483349451781	0.237	0.174	2.14141836172174E-19	T/NK cell	RAPGEF1
1.32724104853058E-23	0.26752415131741	0.129	0.242	2.22419054912755E-19	T/NK cell	RNF168
1.38905445705741E-23	0.558577094980656	0.08	0.176	2.3277774591368E-19	T/NK cell	MARCH8
1.45958133317783E-23	0.527189513559596	0.042	0.119	2.4459663981394E-19	T/NK cell	ANKRD26
1.51043284445666E-23	0.284626733009061	0.196	0.339	2.53118336074047E-19	T/NK cell	KTN1
1.60611130257897E-23	0.420162830883218	0.161	0.291	2.69152132086184E-19	T/NK cell	RAF1
1.7467822439071E-23	0.534582779845922	0.075	0.168	2.92725768433952E-19	T/NK cell	UTP6
2.11586159435644E-23	0.570757133809955	0.164	0.293	3.54576085982252E-19	T/NK cell	MOV10
2.42449945463407E-23	0.355162334141224	0.273	0.435	4.06297618607577E-19	T/NK cell	PPP1R15B
2.48897907210713E-23	0.264747555856261	0.09	0.188	4.17103112903712E-19	T/NK cell	CHMP6
2.51803052955851E-23	0.288745916500682	0.109	0.217	4.21971556143414E-19	T/NK cell	TMEM131
2.52088681157786E-23	1.45783685993799	0.437	0.412	4.22450211884218E-19	T/NK cell	LUC7L3
2.85217968631502E-23	0.449708697265493	0.193	0.332	4.77968271832672E-19	T/NK cell	SND1
2.9049739258323E-23	0.310008536939002	0.111	0.217	4.86815530490977E-19	T/NK cell	MED29
3.02064757885384E-23	0.271584239872973	0.245	0.402	5.06200121264326E-19	T/NK cell	NPLOC4
3.06902732208975E-23	0.394167097144776	0.131	0.247	5.14307598635801E-19	T/NK cell	SRP19
3.12390387979034E-23	0.300087618896347	0.148	0.267	5.23503812175266E-19	T/NK cell	GABARAP
3.48965490295322E-23	0.650429944098895	0.111	0.218	5.847963686369E-19	T/NK cell	EAPP
3.77464351307588E-23	0.577775847433997	0.065	0.151	6.32554759921257E-19	T/NK cell	ANAPC2
4.25588811899212E-23	0.488024358689223	0.071	0.16	7.132017309807E-19	T/NK cell	ZNF639
4.28246572211878E-23	0.390525608436221	0.147	0.267	7.17655605712666E-19	T/NK cell	DPM1
4.48282639244373E-23	0.357994414373661	0.138	0.255	7.51232046845721E-19	T/NK cell	SRP72
4.490798613502E-23	0.328389178661608	0.125	0.236	7.52568031650665E-19	T/NK cell	HDDC2
4.501650464642E-23	0.271255878811646	0.084	0.18	7.54386584864706E-19	T/NK cell	TOM1
4.60829891581644E-23	0.40533921682978	0.115	0.222	7.72258732312519E-19	T/NK cell	DLGAP4
4.74024378747089E-23	0.296424715798348	0.101	0.202	7.94370053904372E-19	T/NK cell	EIF4E
4.82535000056525E-23	1.85731374208574	0.216	0.154	8.08632153094725E-19	T/NK cell	ZBTB38
4.992110784755E-23	0.287908710333002	0.059	0.142	8.36577925309243E-19	T/NK cell	LCORL
5.19912076102452E-23	2.11296446503578	0.178	0.116	8.71268657132489E-19	T/NK cell	KDM2B
5.485465278093E-23	0.343518103530367	0.113	0.221	9.19254271302826E-19	T/NK cell	PITRM1
5.56139803816577E-23	0.301416866745148	0.056	0.138	9.31979083235819E-19	T/NK cell	C5orf51
6.33333678891276E-23	0.430785493070589	0.116	0.224	1.061340579086E-18	T/NK cell	TTLL3
6.66731686590011E-23	0.543944912677624	0.215	0.364	1.11730896038754E-18	T/NK cell	C11orf58
6.80779034222348E-23	0.389944355151239	0.286	0.461	1.14084950554981E-18	T/NK cell	AHSA1
7.22166547505187E-23	0.361523374439463	0.114	0.222	1.21020670030919E-18	T/NK cell	STXBP5
7.62643067130738E-23	0.342296999789639	0.093	0.191	1.27803725189769E-18	T/NK cell	UFD1
8.5359010654137E-23	0.436155314817954	0.094	0.194	1.43044630054203E-18	T/NK cell	OTUD6B
1.07340107245977E-22	0.285537682199496	0.137	0.254	1.79880551722808E-18	T/NK cell	RFC1
1.08214183880081E-22	0.422526338604581	0.058	0.14	1.81345329346239E-18	T/NK cell	ZNF76
1.08808026048634E-22	0.479876005038131	0.065	0.15	1.823404900523E-18	T/NK cell	SYNJ2
1.09376305083762E-22	0.395469450191983	0.144	0.264	1.83292812059368E-18	T/NK cell	METTL23
1.15667191946889E-22	0.306555133998172	0.052	0.13	1.93835080264597E-18	T/NK cell	ZNF692
1.19158744961359E-22	0.347282706201544	0.064	0.148	1.99686224806246E-18	T/NK cell	ASNSD1
1.21649348216999E-22	0.263295627296231	0.063	0.147	2.03859977742047E-18	T/NK cell	NPRL3
1.2739997859272E-22	1.56960628785832	0.388	0.354	2.13496884125679E-18	T/NK cell	CHD2
1.31749361279864E-22	0.316045743146453	0.099	0.2	2.20785579632796E-18	T/NK cell	TELO2

1.31942263427151E-22	0.354527807338754	0.064	0.148	2.2110884505122E-18	T/NK cell	MIB2
1.44789568568666E-22	0.326609067855073	0.302	0.477	2.42638359007371E-18	T/NK cell	CAST
1.45670474710682E-22	0.281644650159848	0.138	0.255	2.4411458152016E-18	T/NK cell	PSEN1
1.57962220824425E-22	0.499908533990617	0.176	0.306	2.64713089657571E-18	T/NK cell	NELFCD
1.8448028617117E-22	2.88667039398917	0.198	0.139	3.09152063565647E-18	T/NK cell	KLF2
1.88841872767329E-22	0.439537961833002	0.159	0.284	3.1646121038349E-18	T/NK cell	PI4KB
1.90174845303149E-22	0.316220458136217	0.07	0.157	3.18695005759018E-18	T/NK cell	MNAT1
1.97897107728364E-22	0.364811272211439	0.219	0.366	3.31635973131192E-18	T/NK cell	BAG6
2.38290247766506E-22	0.476757525327383	0.138	0.253	3.9932679720711E-18	T/NK cell	UBE2G2
2.45870280931634E-22	0.275316552553425	0.129	0.24	4.12029416785231E-18	T/NK cell	FAF1
2.48404092489102E-22	0.488207131094739	0.126	0.236	4.16275578193238E-18	T/NK cell	PIP5K1A
2.49212175913502E-22	0.725689588307871	0.098	0.199	4.17629764395847E-18	T/NK cell	CFAP97
2.53720172765668E-22	0.284066317792901	0.129	0.241	4.25184265520706E-18	T/NK cell	KLHDC3
2.63566534492801E-22	0.413468663308797	0.087	0.182	4.41684798503035E-18	T/NK cell	PREPL
2.64492532078997E-22	0.303083943944426	0.056	0.135	4.43236585257983E-18	T/NK cell	POFUT2
2.70912885744622E-22	0.256583061936504	0.129	0.242	4.53995813930837E-18	T/NK cell	MTMR1
2.83093061176989E-22	0.360189545153363	0.067	0.151	4.74407351920398E-18	T/NK cell	SENP5
2.86337643528021E-22	0.277056505893401	0.125	0.233	4.79844623024257E-18	T/NK cell	DEPRECATED-ENSG00000196821
3.13896565523646E-22	0.497706386019336	0.158	0.283	5.26027864504526E-18	T/NK cell	NPEPPS
3.22116414429639E-22	0.436648620987222	0.159	0.283	5.39802687301188E-18	T/NK cell	ARCN1
3.28683418167414E-22	0.527082960227189	0.178	0.31	5.50807672164953E-18	T/NK cell	SETD3
3.39369291468854E-22	0.301151549637094	0.115	0.221	5.68715058643506E-18	T/NK cell	BLCAP
4.18218463662926E-22	0.341395213899	0.144	0.261	7.00850501406332E-18	T/NK cell	GSK3A
4.30886025333701E-22	2.13329419110197	0.162	0.103	7.22078801254216E-18	T/NK cell	GBP4
4.58035443550647E-22	0.416291690247264	0.12	0.228	7.67575796302174E-18	T/NK cell	WIZ
4.70698272797343E-22	1.45054144639459	0.463	0.463	7.88796165553788E-18	T/NK cell	RSRP1
4.79616031449809E-22	0.259871412784971	0.091	0.186	8.0374054550359E-18	T/NK cell	GTF3A
4.93543412339842E-22	0.285496433138254	0.135	0.247	8.27080050399107E-18	T/NK cell	EIF2S1
4.95323409675989E-22	0.27141993054213	0.085	0.178	8.30062969935022E-18	T/NK cell	ZNF330
5.58580563477427E-22	0.341259723585845	0.047	0.122	9.36069308275472E-18	T/NK cell	DAGLB
5.61688235720586E-22	0.545207883379004	0.194	0.331	9.41277145420558E-18	T/NK cell	PRPF3
6.09613863697137E-22	0.625482320948799	0.103	0.203	1.02159091278366E-17	T/NK cell	RNF126
6.35375185132659E-22	0.369393882341912	0.076	0.164	1.06476173524531E-17	T/NK cell	GOLGA1
6.88156533828267E-22	0.449232438748784	0.157	0.28	1.15321271938941E-17	T/NK cell	SMAD4
7.73194996699643E-22	0.333512819107108	0.154	0.274	1.29572017546926E-17	T/NK cell	RRP36
7.85789112799554E-22	2.12668166169551	0.258	0.2	1.31682539522949E-17	T/NK cell	TERF2IP
8.00373249734938E-22	0.359056539923496	0.057	0.136	1.34126549190581E-17	T/NK cell	SCNM1
8.17312570028258E-22	0.788001199026718	0.118	0.224	1.36965240485335E-17	T/NK cell	SKIL
8.59572813650923E-22	2.46998526973886	0.14	0.085	1.44047212111622E-17	T/NK cell	N4BP2
1.07674975357825E-21	0.299245037264706	0.094	0.19	1.80441723704644E-17	T/NK cell	CIAO2B
1.13641212644384E-21	0.399432241056245	0.102	0.2	1.90439944149459E-17	T/NK cell	ZDHHC3
1.28590903181052E-21	0.318820536042429	0.1	0.199	2.15492635550807E-17	T/NK cell	ME2
1.32299708456424E-21	0.412723017228515	0.144	0.258	2.21707851431275E-17	T/NK cell	NCAPH2
1.3566540181584E-21	0.402269164550944	0.132	0.243	2.27348080362985E-17	T/NK cell	ZBED6
1.41115278322764E-21	0.545256975912218	0.099	0.197	2.36480983413288E-17	T/NK cell	ARFGAP1
1.4805865892314E-21	0.540467039854173	0.12	0.226	2.48116700623398E-17	T/NK cell	SIN3B
1.51830474354405E-21	0.476734317887256	0.083	0.173	2.54437508923112E-17	T/NK cell	TMEM184B
1.53921727288395E-21	0.696883684043941	0.222	0.371	2.57942030589892E-17	T/NK cell	SYAP1
1.57548241843269E-21	0.526896268484323	0.272	0.435	2.6401934368095E-17	T/NK cell	CCNL1
1.69150435406205E-21	0.511053486600643	0.124	0.231	2.83462299653719E-17	T/NK cell	PTGES2
1.77442445290614E-21	0.422328573289534	0.096	0.193	2.97358049818011E-17	T/NK cell	XPR1
1.79340146119672E-21	0.551610456734836	0.167	0.291	3.00538216867347E-17	T/NK cell	TNRC6A
1.8056439750182E-21	0.580989913717249	0.139	0.253	3.0258981733355E-17	T/NK cell	DHPS
1.97040419712267E-21	0.947720676873847	0.074	0.161	3.30200335353817E-17	T/NK cell	CHD7
1.99049891680381E-21	0.322636341029358	0.083	0.173	3.33567808477982E-17	T/NK cell	ZBED1
1.99953512446343E-21	0.594212840023109	0.064	0.146	3.35082096157581E-17	T/NK cell	DCTN6
2.08838398740265E-21	0.272412602708424	0.083	0.173	3.49971388608936E-17	T/NK cell	SLC37A3
2.09479520121569E-21	0.274914008252332	0.167	0.29	3.51045779819725E-17	T/NK cell	GPR108
2.12262798719403E-21	0.564040659228846	0.109	0.211	3.55709998093975E-17	T/NK cell	LETMD1
2.51678797887E-21	0.329004536847788	0.124	0.229	4.21763329499034E-17	T/NK cell	DPY30
2.51684871690463E-21	0.494456803594504	0.077	0.165	4.21773507978878E-17	T/NK cell	FAM120B
2.68341021342295E-21	0.465877155023208	0.244	0.399	4.49685883565417E-17	T/NK cell	CNBP
2.69774022888503E-21	0.530596652809956	0.187	0.321	4.52087307556554E-17	T/NK cell	MKLN1

2.84932117393295E-21	2.73301405756231	0.117	0.066	4.77489242327684E-17	T/NK cell	STAT5A
2.87698576623046E-21	0.445837353663286	0.14	0.252	4.821252747049E-17	T/NK cell	ABCF1
2.92505833081638E-21	0.288165314789546	0.101	0.198	4.90181275078209E-17	T/NK cell	BORCS8
2.97998057495484E-21	0.473591191886596	0.13	0.239	4.99385144750932E-17	T/NK cell	NR1H2
3.0528826580941E-21	0.705937457211854	0.143	0.259	5.11602075843409E-17	T/NK cell	TMEM175
3.08593685064155E-21	2.3643778611843	0.155	0.099	5.1714129743051E-17	T/NK cell	DGKH
3.15650179952159E-21	0.472317592785839	0.073	0.16	5.28966571563828E-17	T/NK cell	MTPAP
3.3696997748082E-21	0.511829024740273	0.168	0.294	5.64694288262358E-17	T/NK cell	CDC16
3.38947856617689E-21	0.313016002203515	0.088	0.179	5.68008818119924E-17	T/NK cell	HPS5
3.44013899376668E-21	0.806084096735948	0.048	0.121	5.7649849257542E-17	T/NK cell	LMF1
3.46182124440693E-21	0.610919580389929	0.13	0.241	5.80132004137713E-17	T/NK cell	COP 1
3.58227113573292E-21	0.467907022890693	0.218	0.363	6.00316996926123E-17	T/NK cell	CELF1
3.59012228993508E-21	0.339724315216418	0.041	0.111	6.0163269334732E-17	T/NK cell	TXNDC16
3.71937510367912E-21	0.362894197302505	0.05	0.125	6.23292879874547E-17	T/NK cell	ZNF839
3.82894625219312E-21	0.318482343230754	0.115	0.218	6.41654812942523E-17	T/NK cell	BTF3L4
3.94726201480557E-21	0.559750844334927	0.141	0.253	6.61482168441117E-17	T/NK cell	RNF138
4.41713922253044E-21	0.547597520382251	0.073	0.157	7.40224190911651E-17	T/NK cell	NOL11
4.79207484686557E-21	0.558506680176168	0.068	0.15	8.03055902837732E-17	T/NK cell	ATP11C
4.83232297830309E-21	1.42686669104054	0.103	0.053	8.09800684704032E-17	T/NK cell	DENND3
4.95488595701998E-21	0.540311429324297	0.073	0.157	8.30339788677408E-17	T/NK cell	NAA30
5.480527904549E-21	0.374244862385545	0.148	0.264	9.18426866244321E-17	T/NK cell	ITFG1
5.49943371576965E-21	0.463943375583029	0.066	0.148	9.21595102088679E-17	T/NK cell	DEPRECATED-ENSG00000249915
5.64338627554518E-21	0.323598144314767	0.068	0.15	9.45718672055862E-17	T/NK cell	NBPF15
5.88898880219275E-21	0.311600536577253	0.089	0.179	9.8687674347146E-17	T/NK cell	SUPT4H1
6.77236750210857E-21	0.740697499024205	0.172	0.296	1.13491334600335E-16	T/NK cell	RNF157
7.21680721173168E-21	0.362828778825473	0.232	0.376	1.209392552542E-16	T/NK cell	SNU13
7.25658974444012E-21	0.513270201776137	0.127	0.233	1.21605930937328E-16	T/NK cell	MTG1
7.63452028422366E-21	2.10939629021591	0.283	0.232	1.2793929092302E-16	T/NK cell	CEMIP2
8.84473146938836E-21	0.396219976984395	0.077	0.161	1.4822000996401E-16	T/NK cell	PABPC4
8.85031705670118E-21	0.688992116415241	0.126	0.233	1.48313613236198E-16	T/NK cell	PIP4P1
9.053121214336E-21	0.39681749191673	0.092	0.185	1.51712205309843E-16	T/NK cell	CEPT1
9.41412923892607E-21	0.520760468517312	0.134	0.244	1.57761977785923E-16	T/NK cell	LARS
9.45669743581093E-21	0.541122825662466	0.15	0.266	1.5847533562932E-16	T/NK cell	TAX1BP1
9.80923931004954E-21	0.364421665066344	0.098	0.191	1.6438323235781E-16	T/NK cell	MARCH5
9.86137703996498E-21	1.74939418044093	0.326	0.284	1.65256956435733E-16	T/NK cell	MED15
1.09966580113773E-20	0.308851853770726	0.132	0.242	1.84281994954662E-16	T/NK cell	TMEM185A
1.15001716917403E-20	0.536352288015896	0.184	0.316	1.92719877210184E-16	T/NK cell	PRKAA1
1.20387195521445E-20	1.04610629799914	0.517	0.563	2.01744862254837E-16	T/NK cell	ACTG1
1.21380424557755E-20	0.278902217703547	0.117	0.218	2.03409315473885E-16	T/NK cell	CHMP1A
1.24474826312883E-20	0.49743789792655	0.14	0.249	2.0859491393513E-16	T/NK cell	ARL8A
1.28437156608725E-20	0.385891238503501	0.065	0.145	2.15234987044902E-16	T/NK cell	WDR36
1.3547561710526E-20	0.482774330362042	0.096	0.191	2.27030039144995E-16	T/NK cell	MYNN
1.37258980222538E-20	0.447674094102066	0.1	0.195	2.3001859905693E-16	T/NK cell	MAPKAPK5
1.44543948716606E-20	0.32816240434358	0.181	0.307	2.42226749259288E-16	T/NK cell	PNN
1.46812564593456E-20	0.555430412342051	0.095	0.187	2.46028495745713E-16	T/NK cell	NUDT4
1.57106180737741E-20	0.485276032771085	0.114	0.215	2.63278537680306E-16	T/NK cell	DIS3
1.60704303532942E-20	2.75740178256033	0.117	0.067	2.69308271860504E-16	T/NK cell	SYNE1
1.62045914841617E-20	0.409986258403657	0.097	0.19	2.71556544091582E-16	T/NK cell	ENTR1
1.73366698420201E-20	0.402797102467519	0.156	0.273	2.90527913212573E-16	T/NK cell	CHMP4B
1.82870955661395E-20	0.480618823026578	0.083	0.171	3.06455147497366E-16	T/NK cell	BZW1
1.88010594430073E-20	0.589280515649478	0.152	0.266	3.15068154145917E-16	T/NK cell	MAPK1IP1L
1.99030449593527E-20	0.372054333933564	0.248	0.4	3.33535227428833E-16	T/NK cell	LPP
2.02903096601512E-20	2.66929600565435	0.135	0.083	3.40025009284814E-16	T/NK cell	GRK6
2.04187082632971E-20	0.366228032512822	0.153	0.268	3.42176713076332E-16	T/NK cell	EPRS
2.08012037284587E-20	0.342742477206004	0.116	0.217	3.48586572081512E-16	T/NK cell	TSR2
2.10333015189383E-20	0.353337008590586	0.292	0.457	3.52476066854368E-16	T/NK cell	LAMTOR4
2.32285187956768E-20	0.507956677318007	0.116	0.217	3.89263517977951E-16	T/NK cell	BABAM1
2.34241593807901E-20	0.256815053383847	0.111	0.209	3.9254206290328E-16	T/NK cell	TTLL5
2.42996373808716E-20	0.343920604485726	0.059	0.136	4.07213323228647E-16	T/NK cell	MAPK8
2.64476208215431E-20	0.580040649844149	0.052	0.125	4.43209229727419E-16	T/NK cell	TSHZ1
2.78542149043814E-20	1.23800674969074	0.453	0.44	4.66780933367624E-16	T/NK cell	EZR
2.94533820455641E-20	0.687667713953821	0.198	0.332	4.93579776319564E-16	T/NK cell	PATJ
3.47464773084587E-20	1.86686580765197	0.266	0.212	5.82281466735151E-16	T/NK cell	KLC1

3.5083560103091E-20	0.701032039913795	0.151	0.265	5.879303002076E-16	T/NK cell	TMEM183A
3.65685478695883E-20	0.634891670643095	0.141	0.251	6.1281572519856E-16	T/NK cell	ZFAND2B
4.0728482150847E-20	0.643730926236155	0.117	0.217	6.82527903883895E-16	T/NK cell	NOP14
4.07926445174749E-20	0.467714654009615	0.134	0.243	6.83603136823845E-16	T/NK cell	BTBD7
4.71792645475604E-20	2.37439231435064	0.171	0.115	7.90630115288017E-16	T/NK cell	PRDM2
4.734435240963E-20	0.398584238104221	0.21	0.345	7.93396657680579E-16	T/NK cell	PPP6R2
4.82668361340743E-20	2.34338626928213	0.201	0.144	8.08855639934818E-16	T/NK cell	ORAI1
4.99048701523309E-20	0.836963293362125	0.057	0.132	8.36305814012761E-16	T/NK cell	MXI1
5.11712782876533E-20	0.336286600888155	0.16	0.279	8.57528281544493E-16	T/NK cell	INTS6
5.1216674341257E-20	0.285401212206652	0.22	0.359	8.58289028610784E-16	T/NK cell	SFT2D2
5.22148943083465E-20	0.347364199194775	0.055	0.129	8.7501719881927E-16	T/NK cell	ZNF263
5.36955885892488E-20	0.52195205927605	0.11	0.208	8.99830673578632E-16	T/NK cell	NCOA6
5.48061670324601E-20	1.81883535437874	0.335	0.301	9.18441747129966E-16	T/NK cell	ATP11B
5.4909914631546E-20	0.976474937993614	0.529	0.492	9.20180349395448E-16	T/NK cell	ARGLU1
5.85923995470703E-20	0.525126733018116	0.088	0.177	9.81891431609805E-16	T/NK cell	CRCP
5.90416805874942E-20	0.442189001109924	0.082	0.169	9.89420483285228E-16	T/NK cell	RAB3GAP2
6.52481277561149E-20	0.281671321382201	0.076	0.159	1.09342812493697E-15	T/NK cell	SPRY2
6.75661888333914E-20	0.413120630264702	0.252	0.403	1.13227419246997E-15	T/NK cell	RAB11B
6.80377375178971E-20	0.688046888734419	0.194	0.326	1.14017640532492E-15	T/NK cell	LPIN2
6.89502536245993E-20	0.417093860157721	0.077	0.161	1.15546835024104E-15	T/NK cell	TBPL1
7.23411247581753E-20	0.378861772675555	0.128	0.234	1.2122925686975E-15	T/NK cell	NBN
7.51627312353094E-20	0.345831847670608	0.14	0.247	1.25957705004131E-15	T/NK cell	SUMO2
7.56776453338208E-20	0.377809712255528	0.114	0.213	1.26820598050417E-15	T/NK cell	NF1
7.63439397461622E-20	0.283318261474131	0.058	0.131	1.27937174226619E-15	T/NK cell	IDH3A
8.47477892596569E-20	0.280756146087039	0.256	0.405	1.42020345241333E-15	T/NK cell	DYNLL2
8.62301146397373E-20	0.384803363353377	0.067	0.146	1.44504426113272E-15	T/NK cell	PATL1
9.06410400287184E-20	0.334650421673863	0.069	0.149	1.51896254880126E-15	T/NK cell	LRRC57
9.11506085455708E-20	0.354552764068225	0.062	0.139	1.52750189800668E-15	T/NK cell	UBN2
9.24930665714301E-20	0.394756814286123	0.102	0.196	1.54999880960403E-15	T/NK cell	FBH1
9.2680783426073E-20	0.309954390580369	0.11	0.207	1.55314456865413E-15	T/NK cell	SCAP
1.02030985910984E-19	0.596380227992581	0.205	0.341	1.70983526189627E-15	T/NK cell	TRIM27
1.04107208439628E-19	0.447227941382643	0.077	0.159	1.74462859903129E-15	T/NK cell	NOSIP
1.05105577331257E-19	0.499849874061904	0.072	0.153	1.7613592649172E-15	T/NK cell	HLCS
1.06055081274739E-19	2.48417237639257	0.173	0.118	1.77727105200207E-15	T/NK cell	ATXN7
1.15542639320256E-19	1.6393099477752	0.273	0.22	1.93626354972885E-15	T/NK cell	PTPN1
1.22253070608192E-19	0.754236443304369	0.113	0.213	2.04871695725209E-15	T/NK cell	TAPT1
1.35075279506919E-19	2.17570493356139	0.228	0.173	2.26359153397695E-15	T/NK cell	USP15
1.39290330631233E-19	2.1098912410992	0.184	0.128	2.33422736071819E-15	T/NK cell	SP140L
1.4395103295901E-19	0.252393970527605	0.067	0.144	2.4123314103271E-15	T/NK cell	CCNYL1
1.46713305098082E-19	2.23798026557144	0.214	0.158	2.45862156683367E-15	T/NK cell	PHF19
1.49519892280717E-19	1.97264915914283	0.221	0.165	2.50565435484025E-15	T/NK cell	RHOG
1.51228103155369E-19	0.310689484592355	0.144	0.251	2.53428055267768E-15	T/NK cell	PNP
1.67099022911144E-19	0.38402487101696	0.068	0.146	2.80024542594496E-15	T/NK cell	TMEM18
1.75010215464513E-19	0.601319856135954	0.125	0.228	2.93282119075431E-15	T/NK cell	NELFA
1.85246736905567E-19	2.22903528616537	0.195	0.14	3.10436481706349E-15	T/NK cell	NSD3
1.91655767843296E-19	0.317618859208952	0.114	0.21	3.21176735751795E-15	T/NK cell	CHERP
1.92222689793509E-19	0.346612847849591	0.087	0.174	3.22126783555963E-15	T/NK cell	MAP1S
1.96255857527649E-19	0.575135560974459	0.115	0.214	3.28885566044834E-15	T/NK cell	ARL8B
2.14341805153545E-19	0.531155130577032	0.102	0.194	3.59193997076311E-15	T/NK cell	EXOSC2
2.31389422171963E-19	0.673562545099515	0.11	0.206	3.87762393675776E-15	T/NK cell	DEPRECATED-ENSG00000163374
2.32270952871476E-19	0.493360295810373	0.073	0.153	3.8923966282202E-15	T/NK cell	PPID
2.3381792264144E-19	0.472553203183553	0.135	0.24	3.91832074762526E-15	T/NK cell	WRNIP1
2.36491513720857E-19	0.535486149354731	0.166	0.284	3.96312478693412E-15	T/NK cell	MAP3K5
2.58610581655106E-19	0.655651566374512	0.096	0.187	4.33379612737627E-15	T/NK cell	MED6
2.67722337544531E-19	0.375899580779192	0.142	0.25	4.48649093257126E-15	T/NK cell	RBM3
2.76161716656801E-19	0.483384414963799	0.094	0.183	4.62791804773468E-15	T/NK cell	STRBP
2.78081420928916E-19	0.878710577948668	0.063	0.138	4.66008845192678E-15	T/NK cell	NFRKB
2.81206952465036E-19	0.268049159896026	0.148	0.256	4.71246610940907E-15	T/NK cell	AQP3
3.01173108416575E-19	0.391760913809651	0.11	0.204	5.04705895084497E-15	T/NK cell	GSE1
3.08695233233749E-19	0.337052604026172	0.042	0.108	5.17311471853117E-15	T/NK cell	MED9
3.19905541473581E-19	0.63104889520519	0.093	0.182	5.36097706401427E-15	T/NK cell	RBM22
3.20809772332465E-19	0.626724057497361	0.11	0.206	5.37613016474745E-15	T/NK cell	DNAJC13
3.36190980157646E-19	2.17168185945549	0.31	0.27	5.63388844548184E-15	T/NK cell	RORA

3.42460970493495E-19	0.582347090604222	0.231	0.374	5.73896094353E-15	T/NK cell	TMED4
3.52373580840151E-19	0.624483990556937	0.084	0.169	5.90507646771925E-15	T/NK cell	TMEM138
3.53188131841136E-19	0.646266215908976	0.114	0.212	5.91872671339376E-15	T/NK cell	ELF2
3.55525190619147E-19	1.73696304426899	0.232	0.178	5.95789114439567E-15	T/NK cell	PTPN6
3.65664161490079E-19	0.53312932706121	0.11	0.207	6.12780001825074E-15	T/NK cell	NEK9
3.88973622979483E-19	0.312994331944707	0.147	0.254	6.51841997389018E-15	T/NK cell	PLEKHJ1
4.22131434249311E-19	0.369695594407072	0.154	0.265	7.07407857514995E-15	T/NK cell	PRPF6
4.41620458786092E-19	0.958963828022521	0.082	0.167	7.40067564833734E-15	T/NK cell	TBC1D22B
4.56936150176482E-19	0.708115562271686	0.182	0.308	7.65733600465749E-15	T/NK cell	TXNDC11
4.6048887366508E-19	0.653472023819528	0.087	0.173	7.71687254487941E-15	T/NK cell	CUL2
4.68167987001083E-19	0.410419306399612	0.048	0.115	7.84555912616415E-15	T/NK cell	TIFA
4.79268813166757E-19	0.279601392036688	0.046	0.113	8.03158677104852E-15	T/NK cell	GUF1
4.81885476935256E-19	0.599424107831493	0.081	0.165	8.07543682248103E-15	T/NK cell	PAXBP1
5.30137188473584E-19	0.57813430706562	0.12	0.218	8.88403900444033E-15	T/NK cell	AUP1
5.32493562452655E-19	0.639996343526662	0.056	0.128	8.92352711958159E-15	T/NK cell	IRGQ
5.51635379960416E-19	0.885602936786063	0.094	0.182	9.24430569737665E-15	T/NK cell	SH3TC1
5.52852606141427E-19	0.403571378302383	0.256	0.408	9.26470397371804E-15	T/NK cell	ZNRF2
5.55041253316016E-19	0.622275355848829	0.107	0.202	9.3013813230698E-15	T/NK cell	NSMAF
5.57689148358881E-19	2.29204180519204	0.195	0.139	9.34575474819813E-15	T/NK cell	YPEL3
5.68292018101444E-19	0.359612669834425	0.092	0.178	9.523437639344E-15	T/NK cell	ISG20L2
5.87608805841813E-19	0.277798953361826	0.121	0.219	9.84714836829711E-15	T/NK cell	SRP68
5.91107103433457E-19	0.339541201293289	0.264	0.411	9.90577283933788E-15	T/NK cell	STAG2
5.92045214501729E-19	0.753098207746441	0.09	0.176	9.92149370461997E-15	T/NK cell	CAMTA1
6.00043621751445E-19	0.660126004716156	0.12	0.218	1.00555310133107E-14	T/NK cell	TRAF2
6.13992617866622E-19	0.669140674001588	0.057	0.13	1.02892882902089E-14	T/NK cell	DDX31
6.40319294836222E-19	0.320052794238601	0.113	0.208	1.07304707428654E-14	T/NK cell	BCCIP
6.43326359510953E-19	0.302045172836027	0.123	0.223	1.07808631326846E-14	T/NK cell	ATIC
7.16368497542196E-19	0.329371262195745	0.097	0.186	1.20049032818121E-14	T/NK cell	CUL5
7.36992695029504E-19	0.42845532879709	0.064	0.138	1.23505235833044E-14	T/NK cell	EIF1AD
7.52222014071532E-19	0.415450815547529	0.259	0.407	1.26057365118107E-14	T/NK cell	DPYD
7.7112128115466E-19	0.354218596643075	0.123	0.222	1.29224504295898E-14	T/NK cell	EIF2S3
7.83972484835504E-19	0.410395322734649	0.122	0.218	1.31378109008734E-14	T/NK cell	GNL3
8.33051044403483E-19	0.497890721836448	0.117	0.215	1.39602694021136E-14	T/NK cell	SGMS1
8.3651733131811E-19	0.406940943807724	0.08	0.162	1.40183574382289E-14	T/NK cell	PGGT1B
8.4907139913009E-19	0.601741885781014	0.071	0.15	1.4228738506622E-14	T/NK cell	ALKBH6
9.01352568950844E-19	0.49301065185131	0.069	0.147	1.51048663504782E-14	T/NK cell	TNPO1
9.63018692764967E-19	0.295574506713619	0.066	0.142	1.61382672533553E-14	T/NK cell	COX19
1.03244444433903E-18	1.72063555120687	0.275	0.225	1.73017039982334E-14	T/NK cell	FLII
1.05323923744147E-18	0.512125595464874	0.178	0.298	1.76501831410441E-14	T/NK cell	PPM1A
1.06520936752633E-18	0.442417174340319	0.112	0.204	1.78507785810062E-14	T/NK cell	GTPBP6
1.08624552713785E-18	0.410817070282006	0.069	0.145	1.8203302543776E-14	T/NK cell	TAF9
1.09007270143252E-18	0.272129871566257	0.08	0.161	1.82674383306061E-14	T/NK cell	PIAS3
1.12073001995106E-18	0.372407414244615	0.14	0.245	1.87811936743398E-14	T/NK cell	DCTD
1.14907232536001E-18	0.567487411205085	0.158	0.271	1.9256154028383E-14	T/NK cell	RUFY1
1.1664396868063E-18	2.63197578971835	0.188	0.135	1.95471962715E-14	T/NK cell	NPC1
1.17928870559693E-18	2.64408096808897	0.157	0.106	1.97625201283933E-14	T/NK cell	SFI1
1.20070848144758E-18	0.3440161596297	0.076	0.155	2.01214727320986E-14	T/NK cell	YJU2
1.25339339287671E-18	0.552785609395421	0.109	0.204	2.10043664778279E-14	T/NK cell	EPS15L1
1.289466956113E-18	0.49520475071462	0.145	0.253	2.16088872505416E-14	T/NK cell	ZNF652
1.34720366704233E-18	0.698830988383486	0.128	0.228	2.25764390522954E-14	T/NK cell	STRAP
1.36126807059296E-18	0.76546308430999	0.203	0.337	2.28121303269968E-14	T/NK cell	BSDC1
1.3871933506235E-18	0.2855881574675	0.082	0.164	2.32465861697485E-14	T/NK cell	TAF4
1.48247100913873E-18	0.267737468242862	0.05	0.117	2.48432491711468E-14	T/NK cell	AP002495.1
1.49542421935487E-18	0.692482695322609	0.049	0.117	2.50603190679489E-14	T/NK cell	CEP95
1.53168444164826E-18	0.329205908836154	0.052	0.121	2.56679678731416E-14	T/NK cell	FBXW8
1.55682972610607E-18	0.317515193265487	0.128	0.228	2.60893525500856E-14	T/NK cell	UBE2J2
1.59315452841473E-18	0.455035935729394	0.098	0.186	2.6698083587174E-14	T/NK cell	B4GALT5
1.60370332040107E-18	0.683131477490501	0.113	0.21	2.68748602432812E-14	T/NK cell	NUP133
1.70309740799063E-18	0.399560840674661	0.042	0.106	2.8540506363107E-14	T/NK cell	TPRKB
1.72419540563135E-18	0.461756442093077	0.246	0.392	2.88940666075702E-14	T/NK cell	USP9X
1.72622362004335E-18	1.66740018221576	0.296	0.256	2.89280554246864E-14	T/NK cell	ADAM10
1.81112478298435E-18	0.519103811114762	0.126	0.222	3.03508291132518E-14	T/NK cell	RNF11
1.8191262436563E-18	0.312317683662719	0.064	0.138	3.04849175911924E-14	T/NK cell	DEPRECATED-ENSG00000186591

1.94243678115859E-18	0.449557747496934	0.074	0.152	3.25513555786556E-14	T/NK cell	PIGO
1.9603642292477E-18	0.618341946349122	0.047	0.113	3.28517837537329E-14	T/NK cell	ABHD4
2.01326593455366E-18	0.537699754438613	0.161	0.269	3.37383105312503E-14	T/NK cell	NFKBIZ
2.19630104439756E-18	0.395411542437448	0.072	0.149	3.68056129020143E-14	T/NK cell	LSM14B
2.22309386816313E-18	0.422291368934663	0.1	0.188	3.72546070426777E-14	T/NK cell	ATP6V1G1
2.23478730532536E-18	0.359502715572431	0.117	0.212	3.74505656626424E-14	T/NK cell	ATL3
2.2616729829345E-18	0.66653462370557	0.181	0.305	3.79011158480163E-14	T/NK cell	RABGGTB
2.29620618942135E-18	0.404622516363957	0.123	0.22	3.8479823322323E-14	T/NK cell	STX6
2.39899549355034E-18	2.23810378463325	0.147	0.095	4.02023664809166E-14	T/NK cell	ENTPD4
2.45201048804532E-18	0.289041433666049	0.165	0.276	4.10907917586635E-14	T/NK cell	HMCES
2.55977158044631E-18	0.270272974138125	0.117	0.212	4.28966521451193E-14	T/NK cell	LARP7
2.58558480956491E-18	0.406537189122599	0.124	0.223	4.33292302386888E-14	T/NK cell	ANAPC4
2.60064246784738E-18	0.304265906362439	0.167	0.282	4.35815664761864E-14	T/NK cell	BTBD2
2.72286679076998E-18	0.451385197515711	0.141	0.246	4.56298016797233E-14	T/NK cell	ZNF146
2.73179551876608E-18	0.352184342187776	0.119	0.215	4.5779429303482E-14	T/NK cell	BCOR
2.9094739483047E-18	0.412148973691746	0.117	0.21	4.87569644256902E-14	T/NK cell	VEGFB
2.99115460284283E-18	0.279633028681462	0.077	0.154	5.01257688344401E-14	T/NK cell	THOC6
3.06466812281731E-18	0.514792629795944	0.117	0.213	5.13577084021725E-14	T/NK cell	KCTD5
3.21104226926158E-18	0.518224790655722	0.171	0.29	5.38106463482856E-14	T/NK cell	DPP4
3.23189338760933E-18	0.285588231371923	0.195	0.319	5.41600693895571E-14	T/NK cell	PSMC5
3.24377104392632E-18	0.319366199762485	0.045	0.11	5.43591151541173E-14	T/NK cell	C6orf47
3.35550170084368E-18	0.541150886055474	0.158	0.269	5.62314975027383E-14	T/NK cell	SNRPA
3.36158084799454E-18	0.584500931562686	0.162	0.275	5.63333718506925E-14	T/NK cell	ZC3H15
3.37118851899144E-18	0.326955622670942	0.063	0.136	5.64943772012585E-14	T/NK cell	WDR18
3.38491828014698E-18	0.40649557545601	0.085	0.169	5.67244605387032E-14	T/NK cell	RAD50
3.49264545692855E-18	0.578653904166561	0.165	0.281	5.85297525672086E-14	T/NK cell	KMT5B
3.51280641713939E-18	0.311448438345758	0.141	0.244	5.88676099384219E-14	T/NK cell	TXNDC12
3.69940251990134E-18	0.354839234957314	0.056	0.126	6.19945874285067E-14	T/NK cell	PRKRA
3.89116349827063E-18	2.96100560264558	0.119	0.072	6.52081179040192E-14	T/NK cell	CRLF3
3.98933646282791E-18	2.17267560321679	0.183	0.132	6.68533004440701E-14	T/NK cell	MFSD6
3.99396631894814E-18	0.707251628750818	0.106	0.197	6.69308875729329E-14	T/NK cell	NTMT1
4.16871003471507E-18	0.748837147349286	0.134	0.236	6.98592427617551E-14	T/NK cell	SDR39U1
4.24704814237972E-18	0.430505101381772	0.096	0.182	7.11720327699994E-14	T/NK cell	CCT4
4.28410136349795E-18	0.43401795918341	0.06	0.13	7.17929706494987E-14	T/NK cell	USP21
4.32872336595594E-18	0.316397966628555	0.171	0.289	7.25407461666896E-14	T/NK cell	CLPTM1L
4.38703679554179E-18	0.636460962358951	0.105	0.195	7.35179626196893E-14	T/NK cell	TAF11
4.46028794701751E-18	2.24717189024242	0.116	0.069	7.47455054161195E-14	T/NK cell	CBL
4.58641370300197E-18	0.525567852603096	0.082	0.162	7.6859120834907E-14	T/NK cell	FAM98C
6.00004290806168E-18	0.457235288983879	0.161	0.273	1.00548719053298E-13	T/NK cell	USP14
6.28993461891788E-18	0.416984506674659	0.049	0.115	1.05406724343826E-13	T/NK cell	DEPRECATED-ENSG00000122912
6.44743657325912E-18	0.568050932464132	0.061	0.132	1.08046142094676E-13	T/NK cell	BLZF1
6.51671010573167E-18	0.484840810468298	0.143	0.245	1.09207027951851E-13	T/NK cell	PET100
6.52424488956661E-18	0.351292496252808	0.128	0.225	1.09333295859357E-13	T/NK cell	SCYL1
6.62681779307616E-18	0.275420071275798	0.07	0.145	1.1105221257637E-13	T/NK cell	CEP85
7.16980940633846E-18	0.3244509940027	0.073	0.148	1.2015166603142E-13	T/NK cell	PRMT1
7.37515971646054E-18	0.379592041475872	0.099	0.186	1.23592926528446E-13	T/NK cell	SMPD4
7.68269338510884E-18	0.607827563428578	0.111	0.203	1.28746575747654E-13	T/NK cell	GTPBP4
7.69606848945636E-18	0.35059174237461	0.077	0.154	1.2897071574631E-13	T/NK cell	TSC2
7.88992253659359E-18	0.452192142234678	0.099	0.185	1.32219321868235E-13	T/NK cell	TENT4A
8.48862682603562E-18	0.488164030158003	0.123	0.219	1.42252408350705E-13	T/NK cell	LARP4B
8.50155850418425E-18	0.401335602082042	0.068	0.141	1.4246911741312E-13	T/NK cell	TSG101
8.71038477004132E-18	0.448495965666157	0.275	0.424	1.45968627976352E-13	T/NK cell	SELENOF
9.00812354050891E-18	0.70303393418607	0.183	0.307	1.50958134291848E-13	T/NK cell	TTC3
9.53008425473457E-18	0.256736125161062	0.057	0.125	1.59705151940842E-13	T/NK cell	FBXL18
1.04371361470435E-17	0.603469826498988	0.166	0.28	1.74905527552155E-13	T/NK cell	FUS
1.05289548312321E-17	2.11091617080743	0.12	0.072	1.76444225061787E-13	T/NK cell	ST3GAL5
1.06328683188059E-17	0.317190251819097	0.058	0.127	1.78185607286549E-13	T/NK cell	XPA
1.07605052497213E-17	0.312204988147915	0.136	0.235	1.8032454697483E-13	T/NK cell	ZXDC
1.09937273794142E-17	0.356696465544709	0.114	0.204	1.84232883424223E-13	T/NK cell	TAGLN2
1.10063508983351E-17	0.436815691802384	0.081	0.161	1.844444283543E-13	T/NK cell	U2AF2
1.12883109762548E-17	0.260064736683835	0.117	0.21	1.89169515340077E-13	T/NK cell	PRPF31
1.13248071134822E-17	2.45469081917302	0.138	0.089	1.89781117607734E-13	T/NK cell	MEX3C
1.17128121442207E-17	0.425620553566265	0.086	0.167	1.96283305912851E-13	T/NK cell	PATZ1

1.23014624142274E-17	0.387478171818158	0.158	0.268	2.06147907137624E-13	T/NK cell	UBQLN2
1.23372354133738E-17	0.463762681466677	0.155	0.262	2.06747391057319E-13	T/NK cell	RBM17
1.3071599580623E-17	0.474469538729669	0.093	0.176	2.1905386577208E-13	T/NK cell	SEC22C
1.33089067169435E-17	1.96677141949013	0.215	0.162	2.2303065876254E-13	T/NK cell	ST3GAL1
1.41434311489105E-17	2.90786034897252	0.115	0.069	2.37015619193442E-13	T/NK cell	LRIG1
1.42252122775733E-17	1.82064309589771	0.274	0.228	2.38386107347573E-13	T/NK cell	PPP6R1
1.43588254715984E-17	0.392333927514818	0.097	0.183	2.40625197253046E-13	T/NK cell	VEZF1
1.52936496672235E-17	0.626781529503504	0.164	0.276	2.56290981123331E-13	T/NK cell	CYTH2
1.58723355426766E-17	1.43318585064374	0.104	0.058	2.65988599024174E-13	T/NK cell	ZEB2
1.60515780404948E-17	0.496459391172834	0.249	0.398	2.68992344802612E-13	T/NK cell	RAB14
1.65004332492123E-17	0.300934664178486	0.074	0.148	2.765142603903E-13	T/NK cell	UBE2M
1.6942683419575E-17	0.253195594860331	0.138	0.238	2.83925488745238E-13	T/NK cell	ERLEC1
1.70356484433958E-17	0.48051343196098	0.121	0.216	2.85483396614427E-13	T/NK cell	ATG4B
1.72572572420092E-17	0.565798636512834	0.134	0.233	2.89197116861591E-13	T/NK cell	IPO8
1.88116598613212E-17	0.253727924247529	0.207	0.334	3.1524579595602E-13	T/NK cell	AP1M1
1.89527354133809E-17	0.31275820581959	0.114	0.204	3.17609940057438E-13	T/NK cell	GTPBP2
1.91306994433488E-17	0.320175982868674	0.058	0.126	3.20592261271639E-13	T/NK cell	MRRF
1.91715017400306E-17	0.596932905178372	0.2	0.327	3.21276026159433E-13	T/NK cell	SRSF3
1.93127207072022E-17	0.775486815878799	0.057	0.125	3.23642573611294E-13	T/NK cell	OTUD3
1.95186827807276E-17	0.470450373482202	0.05	0.115	3.27094086039434E-13	T/NK cell	WDR27
1.96568779216176E-17	0.267483745444968	0.075	0.149	3.29409960210468E-13	T/NK cell	KIF21A
1.97652369046328E-17	0.309961748108897	0.077	0.154	3.31225840047837E-13	T/NK cell	PIAS4
2.0028180479474E-17	0.539126690163471	0.194	0.321	3.35632248475025E-13	T/NK cell	PARP4
2.00941916487369E-17	0.26636072087688	0.05	0.115	3.36738463649533E-13	T/NK cell	STXBP4
2.12889212709077E-17	0.449729186356927	0.067	0.138	3.56759742657872E-13	T/NK cell	ZNRD2
2.20871514560001E-17	0.495724245699672	0.056	0.123	3.7013648409965E-13	T/NK cell	TMEM260
2.35792063677445E-17	0.596826860222137	0.147	0.251	3.95140340310663E-13	T/NK cell	EIF2B5
2.38896685918194E-17	0.822047294955646	0.153	0.259	4.0034306626171E-13	T/NK cell	STX5
2.4429164099703E-17	0.488634752747535	0.049	0.113	4.09383931982822E-13	T/NK cell	XRRA1
2.46010045574725E-17	0.298973900209718	0.091	0.173	4.12263634374123E-13	T/NK cell	MBD4
2.60634057555811E-17	0.392508599310055	0.152	0.256	4.36770553652028E-13	T/NK cell	GOLGB1
2.61084949896266E-17	0.455141973389517	0.053	0.118	4.37526159036162E-13	T/NK cell	ALDH16A1
2.75321563889597E-17	0.409946280340204	0.103	0.189	4.61383876766187E-13	T/NK cell	PLPP5
2.93993472179606E-17	0.535068339107984	0.153	0.258	4.92674260678584E-13	T/NK cell	ARHGEF7
3.07464948516223E-17	0.346060078704427	0.083	0.161	5.15249760723486E-13	T/NK cell	PLXNC1
3.07555764468106E-17	0.566592067127654	0.092	0.174	5.15401950095651E-13	T/NK cell	GPD2
3.10966961219073E-17	0.65791131399422	0.096	0.18	5.21118433610923E-13	T/NK cell	STARD4
3.115482491796E-17	0.361862518491496	0.042	0.102	5.22092555975174E-13	T/NK cell	TMEM126A
3.13754547274877E-17	0.326101921751177	0.114	0.205	5.25789870323239E-13	T/NK cell	COMMD6
3.62947855338144E-17	0.383949069100543	0.099	0.184	6.08228015975662E-13	T/NK cell	MED24
3.74446371134628E-17	0.251460676619516	0.061	0.13	6.2749722874741E-13	T/NK cell	FEM1A
3.7950168307524E-17	0.59579972209868	0.089	0.171	6.35968920497487E-13	T/NK cell	MAN1B1
3.80167333581608E-17	0.728845723401952	0.111	0.201	6.37084417616058E-13	T/NK cell	YME1L1
3.84702281549597E-17	0.735617838647472	0.104	0.192	6.44684083420815E-13	T/NK cell	BRPF3
4.07111103743552E-17	0.547875116567763	0.104	0.191	6.82236787653445E-13	T/NK cell	RABGAP1
4.16463441626719E-17	0.377073627772034	0.278	0.429	6.97909435478056E-13	T/NK cell	LRPAP1
4.17375523054449E-17	0.273468511635855	0.467	0.646	6.99437901534645E-13	T/NK cell	NFKBIA
4.29887786863881E-17	0.647020795462057	0.1	0.185	7.20405953226491E-13	T/NK cell	TGS1
4.61080948351271E-17	0.473192054464554	0.141	0.241	7.7267945324706E-13	T/NK cell	KCMF1
4.61743260755763E-17	0.354535577099241	0.117	0.209	7.73789356374507E-13	T/NK cell	DRAM2
4.62328409755619E-17	0.463860491962784	0.075	0.149	7.74769949068466E-13	T/NK cell	ATE1
4.91738845421361E-17	0.43294206346163	0.14	0.242	8.24055957157116E-13	T/NK cell	MAP3K2
4.98969512568526E-17	0.486508774586435	0.133	0.231	8.36173109162336E-13	T/NK cell	API5
5.05747082666427E-17	0.588113239907659	0.093	0.175	8.47530961132398E-13	T/NK cell	TNFRSF10B
5.0684101324782E-17	0.388551394806503	0.062	0.132	8.49364170000696E-13	T/NK cell	RPE
5.08253853803414E-17	0.476562990826063	0.093	0.176	8.51731808203761E-13	T/NK cell	TRIP11
5.6152833786623E-17	0.250095960979083	0.177	0.289	9.41009188596229E-13	T/NK cell	DPYSL2
5.78964223284837E-17	0.322693356949546	0.058	0.124	9.7022824538073E-13	T/NK cell	DNAJC2
5.79715039398547E-17	0.316800928117845	0.046	0.108	9.71486463024085E-13	T/NK cell	KIAA1328
5.99873137848115E-17	1.88173601035414	0.219	0.17	1.00526740440587E-12	T/NK cell	CLSTN1
6.35258490974544E-17	0.812617745161372	0.125	0.219	1.06456617917514E-12	T/NK cell	CNNM3
7.11433792427954E-17	0.649036502988539	0.055	0.12	1.19222074935076E-12	T/NK cell	APTX
7.15429678435469E-17	2.09417416035564	0.258	0.216	1.19891705512216E-12	T/NK cell	DIP2A

7.39276180620671E-17	0.703582835567034	0.23	0.367	1.23887902348412E-12	T/NK cell	FBXO7
7.41235719498017E-17	0.675260180188094	0.108	0.197	1.24216281873478E-12	T/NK cell	DGKE
7.49582415065753E-17	0.513273430280631	0.078	0.154	1.25615021116719E-12	T/NK cell	TBC1D23
7.53644854375452E-17	0.483748261632305	0.062	0.129	1.26295804696238E-12	T/NK cell	SH2B3
7.54444790427602E-17	0.76039362479421	0.125	0.22	1.26429857979857E-12	T/NK cell	WASHC1
7.69752501392693E-17	0.374088302987611	0.113	0.201	1.28995124183387E-12	T/NK cell	SYF2
7.71436723329157E-17	0.266821110955053	0.098	0.181	1.292773660955E-12	T/NK cell	CBX3
7.94931977432615E-17	0.709247500417324	0.092	0.174	1.33214700778158E-12	T/NK cell	CCDC130
8.05430314310362E-17	0.463275236787667	0.046	0.108	1.3497401207213E-12	T/NK cell	FAM120C
8.15228326332778E-17	0.535225671536617	0.07	0.142	1.36615962926847E-12	T/NK cell	CDC27
8.3114109054572E-17	0.512047495482499	0.114	0.203	1.39282623953652E-12	T/NK cell	MAPKAP1
8.44820275711697E-17	1.83345623076734	0.172	0.12	1.41574981803766E-12	T/NK cell	ST6GALNAC6
8.60540100132928E-17	2.38382587064228	0.177	0.127	1.44209309980276E-12	T/NK cell	ZBTB4
8.91393094705345E-17	0.319520124028301	0.054	0.118	1.49379654810722E-12	T/NK cell	ANKRA2
9.03262554939389E-17	0.432407722965618	0.108	0.194	1.51368738956743E-12	T/NK cell	LMTK2
9.39568242888441E-17	0.312729770949221	0.182	0.296	1.57452846143245E-12	T/NK cell	DPP9
9.8189531938531E-17	0.494251511674592	0.119	0.211	1.6454601762259E-12	T/NK cell	EFTUD2
1.01849289649678E-16	0.838893529354891	0.078	0.153	1.7067903959493E-12	T/NK cell	TTC33
1.02212547088072E-16	0.299896907853471	0.186	0.302	1.71287786410191E-12	T/NK cell	DCTN2
1.02294300117622E-16	0.306938663050184	0.123	0.216	1.71424788137111E-12	T/NK cell	HAT1
1.06058601924732E-16	0.894697687207315	0.07	0.142	1.77733005105466E-12	T/NK cell	MRI1
1.0641647185629E-16	0.574908068665638	0.225	0.359	1.7833272353677E-12	T/NK cell	DNAJA2
1.10579120647788E-16	0.531192027633659	0.057	0.124	1.85308490381563E-12	T/NK cell	LIN52
1.15681248665567E-16	0.718378718248345	0.096	0.179	1.93858636513757E-12	T/NK cell	NSMCE4A
1.18576479728396E-16	0.375524319731641	0.205	0.323	1.98710464728846E-12	T/NK cell	HERPUD1
1.1933317117984E-16	0.503942335014937	0.112	0.199	1.99978528263175E-12	T/NK cell	SNRPD2
1.20975897487697E-16	0.265204343757181	0.222	0.348	2.02731409009883E-12	T/NK cell	SRI
1.21049539826156E-16	0.55062616836421	0.715	0.62	2.02854818840673E-12	T/NK cell	TSC22D3
1.316783518896E-16	0.457343616383199	0.083	0.161	2.20666582096592E-12	T/NK cell	SDF2
1.32459148863031E-16	0.528623526046965	0.087	0.167	2.21975041664668E-12	T/NK cell	TRAPPC6B
1.36522908027568E-16	0.737068146165493	0.165	0.274	2.28785089272598E-12	T/NK cell	KLHL21
1.40607061031964E-16	0.725212452691641	0.101	0.185	2.35629312877366E-12	T/NK cell	TMEM181
1.40899633555839E-16	0.408713645302018	0.053	0.117	2.36119605912876E-12	T/NK cell	TBCCD1
1.45220425990813E-16	1.49259086869868	0.318	0.283	2.43360389875404E-12	T/NK cell	MAP4
1.46980293473702E-16	0.48949041898333	0.109	0.197	2.4630957580323E-12	T/NK cell	NUDT22
1.50989259200757E-16	0.340191792814753	0.077	0.15	2.53027800568628E-12	T/NK cell	PDCL3
1.52038740819046E-16	0.69754088771206	0.126	0.22	2.54786521864557E-12	T/NK cell	BET1L
1.52353381396184E-16	0.652697058333867	0.12	0.211	2.55313796543725E-12	T/NK cell	ZNF91
1.53604952942992E-16	0.294002826354632	0.059	0.125	2.57411180141866E-12	T/NK cell	PPP4R1
1.53629201338074E-16	0.768228898340384	0.095	0.177	2.57451815602345E-12	T/NK cell	SNHG32
1.55767525213346E-16	0.482987441285874	0.085	0.163	2.61035218752525E-12	T/NK cell	SUCO
1.63803936643137E-16	1.62349441936886	0.137	0.087	2.74502637026569E-12	T/NK cell	TACC3
1.67344768816312E-16	0.397762776573932	0.066	0.134	2.80436363582375E-12	T/NK cell	RTRAF
1.68564911730299E-16	0.348642076961835	0.087	0.165	2.82481079077634E-12	T/NK cell	PWP1
1.69818837307293E-16	1.70019859824809	0.331	0.305	2.84582407559562E-12	T/NK cell	NCOR1
1.86444524882909E-16	0.582933279883301	0.048	0.109	3.12443734798779E-12	T/NK cell	PNPLA8
1.88346847161576E-16	0.360471687856506	0.324	0.484	3.15631646473369E-12	T/NK cell	SDF4
1.89381000815054E-16	0.60133350304777	0.132	0.226	3.17364681165867E-12	T/NK cell	FXR1
1.91518550356191E-16	0.359133357804385	0.098	0.178	3.20946786686904E-12	T/NK cell	SAMD1
2.00734054960109E-16	0.378427563116476	0.147	0.246	3.36390129302151E-12	T/NK cell	RCC1L
2.1106832463996E-16	0.406454730905945	0.155	0.255	3.53708298431644E-12	T/NK cell	NAGK
2.2444299210107E-16	0.428809925642344	0.046	0.106	3.76121566162973E-12	T/NK cell	TMEM218
2.26738055403231E-16	0.779000498588486	0.085	0.163	3.79967633244734E-12	T/NK cell	TIPARP
2.34983071414317E-16	0.478660045118351	0.104	0.188	3.93784631076113E-12	T/NK cell	ZER1
2.38150718465239E-16	0.596312595614705	0.172	0.284	3.99092974004048E-12	T/NK cell	PCED1A
2.42210376131085E-16	0.511572121908718	0.097	0.18	4.05896148320472E-12	T/NK cell	ZNF75A
2.47443171175169E-16	0.488769412596603	0.092	0.172	4.14665266255349E-12	T/NK cell	KDM1A
2.62022849559701E-16	0.792548620883747	0.154	0.256	4.39097891292148E-12	T/NK cell	PLEKHB2
2.69626888093165E-16	0.387852500290679	0.109	0.195	4.51840739066526E-12	T/NK cell	ARHGEF18
2.73038562205293E-16	0.7044405305938	0.074	0.145	4.5755802254363E-12	T/NK cell	INPP5E
2.73533365781758E-16	1.75381349580507	0.288	0.248	4.5838721437707E-12	T/NK cell	LTBP3
2.79412927909647E-16	0.637762309408275	0.112	0.2	4.68240184590987E-12	T/NK cell	NAE1
2.92683345770536E-16	0.526691338437343	0.13	0.223	4.90478750842263E-12	T/NK cell	PPP1R11

2.97488891056277E-16	0.442344568388765	0.186	0.3	4.98531883632109E-12	T/NK cell	MCM7
3.08171807607641E-16	0.539399700659221	0.146	0.245	5.16434315188885E-12	T/NK cell	NFX1
3.0945246685325E-16	0.537113908960954	0.224	0.352	5.18580443952677E-12	T/NK cell	PPM1B
3.11410849482455E-16	0.808066030157782	0.119	0.21	5.21862301562698E-12	T/NK cell	NAA50
3.14565577266822E-16	0.655149829768674	0.067	0.136	5.27148994383741E-12	T/NK cell	POLM
3.16490729791658E-16	0.567835409924685	0.079	0.153	5.30375164984861E-12	T/NK cell	RNF20
3.19852642164775E-16	0.429165127641099	0.069	0.138	5.36009057739731E-12	T/NK cell	WDR59
3.3315558267614E-16	0.418973197896528	0.165	0.272	5.58302125448675E-12	T/NK cell	INTS1
3.5112047777489E-16	0.281523073758989	0.068	0.138	5.8840769665516E-12	T/NK cell	UHRF1BP1L
3.57248372946499E-16	0.605147596330938	0.135	0.231	5.98676823383742E-12	T/NK cell	ATP13A1
3.68619397902852E-16	0.690683523065288	0.174	0.287	6.17732387005599E-12	T/NK cell	PCID2
3.71995522533759E-16	0.453460663915375	0.047	0.108	6.23390096662073E-12	T/NK cell	TUBE1
3.77053863162313E-16	0.268233770800584	0.059	0.124	6.31866863887404E-12	T/NK cell	DEPRECATED-ENSG00000168924
3.82115111492508E-16	0.598655226185472	0.061	0.128	6.40348503839145E-12	T/NK cell	NFXL1
3.82216101642317E-16	0.534808209700242	0.071	0.141	6.40517743132195E-12	T/NK cell	NUDT16L1
3.99568072293647E-16	0.610102184344246	0.117	0.204	6.69596175549693E-12	T/NK cell	SQOR
4.11810136572804E-16	0.350558812498836	0.062	0.129	6.90111426868704E-12	T/NK cell	ZNF513
4.14547267894588E-16	0.798201863365305	0.19	0.309	6.94698311537751E-12	T/NK cell	LGALS8
4.2054103993262E-16	0.376405061143526	0.051	0.113	7.04742674719085E-12	T/NK cell	SUMF1
4.22148553177339E-16	2.05078975528136	0.26	0.221	7.07436545414584E-12	T/NK cell	CASK
4.30476578087454E-16	0.723223132777682	0.209	0.334	7.21392649558955E-12	T/NK cell	STAT6
4.44006696958E-16	0.910843944068909	0.107	0.194	7.44066422762216E-12	T/NK cell	ZNF337
4.46604789847531E-16	0.25816597873343	0.052	0.114	7.48420306826493E-12	T/NK cell	UFSP2
4.51707123629794E-16	0.504092202190131	0.118	0.209	7.56970797778809E-12	T/NK cell	DGCR2
4.5546387736173E-16	0.362040692411586	0.075	0.147	7.63266365682787E-12	T/NK cell	NSA2
4.59034828740268E-16	0.409713444832862	0.133	0.229	7.69250566002941E-12	T/NK cell	DELE1
4.81975020381188E-16	2.35232576483961	0.111	0.068	8.07693739154795E-12	T/NK cell	PNMA1
4.91031072821579E-16	0.697375432072723	0.067	0.136	8.22869871834403E-12	T/NK cell	UMAD1
4.9552213301551E-16	0.802746979480331	0.064	0.131	8.30395990507392E-12	T/NK cell	DEPRECATED-ENSG00000188529
4.97047301988809E-16	0.250503785209525	0.058	0.122	8.32951868672846E-12	T/NK cell	GABPA
5.181912244606E-16	2.03798578040929	0.231	0.187	8.68384853951074E-12	T/NK cell	RBPJ
5.25541208552302E-16	0.405732212912262	0.085	0.162	8.80701957291947E-12	T/NK cell	XPO4
5.3620766626853E-16	0.418247511872806	0.124	0.215	8.98576807132803E-12	T/NK cell	C2orf68
5.52084913609277E-16	0.622417518285141	0.073	0.144	9.25183898226426E-12	T/NK cell	JMJD4
5.6929864411086E-16	0.422628187728451	0.043	0.101	9.54030667800979E-12	T/NK cell	WDR60
5.74395573284687E-16	0.603198968284219	0.07	0.139	9.62572101710478E-12	T/NK cell	ZDHHC16
5.83699909277932E-16	0.656789133976978	0.137	0.236	9.78164307967959E-12	T/NK cell	DHX16
5.84544732904217E-16	0.446859290142003	0.085	0.161	9.79580063400887E-12	T/NK cell	COG1
6.03679683800995E-16	0.670413043094585	0.101	0.182	1.01164641411371E-11	T/NK cell	MDC 1.00
6.21991894719103E-16	0.579007188301511	0.085	0.161	1.04233401717027E-11	T/NK cell	DENR
6.22410822768326E-16	0.395598450353258	0.06	0.125	1.04303605679516E-11	T/NK cell	WDR45
6.23325183595029E-16	0.507210426117623	0.066	0.134	1.04456834266855E-11	T/NK cell	ZNF346
6.3931198036218E-16	0.481658035103315	0.233	0.368	1.07135901669094E-11	T/NK cell	IQGAP2
6.39649277759272E-16	0.628444787881556	0.084	0.159	1.07192425966899E-11	T/NK cell	E4F1
6.74757313544925E-16	1.77482200572376	0.238	0.191	1.13075830603859E-11	T/NK cell	SLC44A2
7.11206494075483E-16	0.831723778059894	0.078	0.15	1.19183984277169E-11	T/NK cell	ZNF445
7.24596863572778E-16	0.530258636026174	0.098	0.178	1.21427942397526E-11	T/NK cell	MLXIP
7.3226682804203E-16	0.762919591883409	0.09	0.168	1.22713275043283E-11	T/NK cell	TIMM10B
7.41280637589944E-16	1.80586610944982	0.302	0.271	1.24223809247323E-11	T/NK cell	NAP1L4
7.91775929128467E-16	0.82904521853267	0.138	0.234	1.32685810203349E-11	T/NK cell	MTR
8.2641344027924E-16	0.611208422226463	0.06	0.125	1.38490364321995E-11	T/NK cell	PDPK1
8.78487315435175E-16	0.284239576306158	0.139	0.232	1.47216904320627E-11	T/NK cell	SELENOK
9.00795812761197E-16	0.919311802517914	0.09	0.168	1.50955362302521E-11	T/NK cell	FAM76B
9.01226582828218E-16	0.393686582126994	0.083	0.155	1.51027550750353E-11	T/NK cell	CSAD
9.23912715316697E-16	0.570083455012856	0.056	0.12	1.54829292832772E-11	T/NK cell	ERCC6
9.53178173325476E-16	0.435511800343696	0.171	0.279	1.59733598285883E-11	T/NK cell	DNAJC1
9.77359440481621E-16	0.742884858068349	0.081	0.155	1.6378589503591E-11	T/NK cell	P4HTM
9.93807656845495E-16	0.587433698060421	0.064	0.13	1.66542287134168E-11	T/NK cell	MIER3
1.00233297835587E-15	0.693966140152115	0.083	0.159	1.67970960512877E-11	T/NK cell	PIK3R4
1.02416322892787E-15	1.89751213113308	0.164	0.115	1.71629273903733E-11	T/NK cell	PPP3CB
1.02950007426741E-15	0.424405396038733	0.157	0.261	1.72523622445733E-11	T/NK cell	TBC1D14
1.05611612451465E-15	0.28064036196304	0.098	0.177	1.76983940146164E-11	T/NK cell	ATXN2
1.11613730233982E-15	0.400444484672343	0.068	0.136	1.87042289126106E-11	T/NK cell	PIGQ

1.13475399123444E-15	0.356330932455523	0.092	0.17	1.90162073851068E-11	T/NK cell	FBXO38
1.16850645569834E-15	0.460477249230689	0.086	0.161	1.95818311845927E-11	T/NK cell	TMEM128
1.19085331012483E-15	0.675203561259052	0.089	0.166	1.99563197710718E-11	T/NK cell	RELL1
1.20163062985377E-15	0.655091037122249	0.203	0.323	2.01369260950894E-11	T/NK cell	DDX46
1.266643134523E-15	0.715206536444528	0.05	0.109	2.12264056483364E-11	T/NK cell	CBWD3
1.27006026375729E-15	0.605764057002508	0.206	0.328	2.12836699000447E-11	T/NK cell	HGS
1.28337229144656E-15	1.63210290746096	0.275	0.234	2.15067528600614E-11	T/NK cell	SHISA5
1.28454050363957E-15	0.395578820230945	0.063	0.129	2.1526329759992E-11	T/NK cell	DEPRECATED-ENSG00000072803
1.31888863070444E-15	0.295133937093243	0.216	0.338	2.2101935673345E-11	T/NK cell	MKNK2
1.36210121068216E-15	0.554424845123415	0.086	0.16	2.28260920886117E-11	T/NK cell	SDE2
1.44101259281398E-15	0.569779700564259	0.053	0.114	2.41484890303766E-11	T/NK cell	ZFAND6
1.4500205744079E-15	0.438255487651404	0.088	0.164	2.42994447859275E-11	T/NK cell	GLYR1
1.50795761152489E-15	0.28419072648255	0.074	0.143	2.52703536539341E-11	T/NK cell	SRSF1
1.53183886320397E-15	0.429706348733793	0.077	0.147	2.56705556695721E-11	T/NK cell	ERC1
1.53390400739293E-15	0.696893456989176	0.102	0.185	2.57051633558907E-11	T/NK cell	SLC35C2
1.54252100061679E-15	0.393436278352058	0.088	0.163	2.58495669283362E-11	T/NK cell	POLA1
1.64167911468593E-15	0.781095356916181	0.084	0.159	2.75112586039069E-11	T/NK cell	SUDS3
1.65132201289083E-15	0.6082789491018	0.073	0.142	2.76728542920246E-11	T/NK cell	ZNF672
1.68960690571086E-15	0.610672786653281	0.058	0.121	2.83144325259026E-11	T/NK cell	SRSF10
1.69390755070044E-15	0.678600258230936	0.098	0.177	2.8386502734638E-11	T/NK cell	UBE2O
1.71684666466777E-15	0.773168918543651	0.06	0.124	2.87709164065025E-11	T/NK cell	PJA1
1.72144690192241E-15	0.849156051650408	0.168	0.274	2.88480071824158E-11	T/NK cell	DEPRECATED-ENSG00000161547
1.80130565227736E-15	0.388065143749467	0.075	0.144	3.0186280120864E-11	T/NK cell	AZI2
1.81214081241459E-15	2.41995508076252	0.166	0.119	3.03678557344436E-11	T/NK cell	STAM
1.87778262332837E-15	0.379553596505052	0.064	0.129	3.14678812017369E-11	T/NK cell	ZNF562
1.97385565225743E-15	0.289886626366306	0.076	0.147	3.307787302053E-11	T/NK cell	LRRC40
2.01657991204957E-15	0.301199448793224	0.154	0.252	3.37938461661267E-11	T/NK cell	YWHAQ
2.03596976746629E-15	1.94099263593681	0.225	0.18	3.41187813632E-11	T/NK cell	NCOA3
2.08127451048506E-15	0.576623854610461	0.108	0.192	3.48779982467087E-11	T/NK cell	NUP214
2.09530112393594E-15	0.571401268527864	0.137	0.231	3.51130562349185E-11	T/NK cell	RAB5B
2.16685462163921E-15	0.364666042753369	0.209	0.329	3.63121497494299E-11	T/NK cell	UHMK1
2.17244662047118E-15	0.285394864106292	0.064	0.129	3.6405860465856E-11	T/NK cell	ARL 2.00
2.1765326365926E-15	0.34185312101621	0.084	0.156	3.64743339240189E-11	T/NK cell	POLD1
2.28501075465817E-15	2.65491456849712	0.101	0.061	3.82922102265616E-11	T/NK cell	AEN
2.37275141221618E-15	0.60979512695305	0.077	0.148	3.97625681659187E-11	T/NK cell	ALG9
2.58231684612809E-15	0.424587605225809	0.122	0.21	4.32744657074145E-11	T/NK cell	ZNF148
2.84552050507386E-15	0.436916868199552	0.072	0.14	4.76852326240277E-11	T/NK cell	FBRSL1
2.88342028699618E-15	0.367339881001322	0.082	0.154	4.8320357169482E-11	T/NK cell	COPS8
2.8926152257608E-15	0.769931436097967	0.179	0.291	4.84744459532995E-11	T/NK cell	ARSA
2.93638723566867E-15	0.572963777877136	0.093	0.169	4.92079772953356E-11	T/NK cell	SUGT1
2.96858870038498E-15	0.85796828594744	0.06	0.124	4.97476094410515E-11	T/NK cell	RALGPS1
3.0400882093706E-15	0.811258431482346	0.087	0.161	5.09457982126325E-11	T/NK cell	MKRN1
3.13639455399648E-15	0.551515895802831	0.081	0.152	5.2559699935873E-11	T/NK cell	VRK3
3.33028480218366E-15	0.42383345040104	0.063	0.128	5.58089127149938E-11	T/NK cell	SMG9
3.34795625736081E-15	0.615942969426633	0.087	0.161	5.61050509608525E-11	T/NK cell	MIGA1
3.48136099524174E-15	0.549669209104034	0.215	0.338	5.83406475582611E-11	T/NK cell	INTS11
3.53206332181855E-15	0.323058905092724	0.074	0.142	5.91903171470353E-11	T/NK cell	POLA2
3.67564925038527E-15	0.605374106657006	0.07	0.137	6.15965301379564E-11	T/NK cell	PLAA
3.76627315861191E-15	0.352928077786727	0.266	0.4	6.31152055920184E-11	T/NK cell	SLBP
4.07193246093693E-15	0.429784469466894	0.167	0.27	6.82374441803811E-11	T/NK cell	PSMD13
4.10610734823615E-15	0.556963131787983	0.11	0.194	6.88101469417414E-11	T/NK cell	PPP2CA
4.11561649278976E-15	0.750329087417105	0.124	0.212	6.89695011861707E-11	T/NK cell	PHRF1
4.15053332319328E-15	0.619790707350066	0.083	0.157	6.95546374300729E-11	T/NK cell	MBTD1
4.47437689964994E-15	0.627739738161812	0.078	0.149	7.49816080843337E-11	T/NK cell	ABHD13
4.52465976804355E-15	0.640231951860358	0.089	0.163	7.58242483928737E-11	T/NK cell	DPH7
4.59590498809248E-15	0.382540519565525	0.063	0.126	7.70181757904539E-11	T/NK cell	RABGGTA
4.69911114608185E-15	1.03211729445871	0.153	0.254	7.87477045860396E-11	T/NK cell	BRWD1
4.7022665241428E-15	0.524757590407451	0.12	0.206	7.8800582411585E-11	T/NK cell	SFPQ
4.74007920437631E-15	0.771913972335582	0.131	0.221	7.94342473069382E-11	T/NK cell	YY1
4.81942929363022E-15	0.443115107640508	0.055	0.116	8.07639961026553E-11	T/NK cell	RNF214
4.9282092760551E-15	0.467517174278021	0.162	0.263	8.25869310481314E-11	T/NK cell	URI1
4.96974662256991E-15	2.154584396745	0.203	0.158	8.32830139010266E-11	T/NK cell	PITPNM1
5.28156929744977E-15	0.399187974194142	0.064	0.128	8.85085382866632E-11	T/NK cell	RPAP1

5.3560947354409E-15	0.889008783991433	0.109	0.192	8.97574355765186E-11	T/NK cell	RAB11FIP4
5.36348779506609E-15	0.251868203581496	0.098	0.176	8.98813284697175E-11	T/NK cell	RBX1
5.45516905249697E-15	0.564820379260764	0.145	0.241	9.14177229817442E-11	T/NK cell	PURA
5.48623837600639E-15	0.773889840129559	0.175	0.283	9.1938382705115E-11	T/NK cell	FBXL3
5.5004287846964E-15	0.383005606472498	0.145	0.242	9.21761855739423E-11	T/NK cell	SMC6
5.52579599283513E-15	0.466873408339813	0.143	0.236	9.26012892479311E-11	T/NK cell	DEPRECATED-ENSG00000265241
5.56823378997714E-15	0.337864447885229	0.065	0.129	9.33124618524369E-11	T/NK cell	RBM28
5.59872040308681E-15	0.631157154020781	0.065	0.129	9.38233565149287E-11	T/NK cell	FASTKD2
5.60116654057675E-15	0.661501028123665	0.075	0.143	9.38643488869851E-11	T/NK cell	CSNK1E
5.61247256453811E-15	0.464927003958698	0.077	0.147	9.40538152365297E-11	T/NK cell	TSPAN31
5.62588073475586E-15	0.295037536857604	0.054	0.114	9.42785093530388E-11	T/NK cell	NIPSNAP2
5.66144933800695E-15	0.395586212294682	0.101	0.18	9.48745680063204E-11	T/NK cell	VPS41
5.73448104460411E-15	0.622855987492427	0.109	0.192	9.60984333454756E-11	T/NK cell	INTS7
5.91344670378796E-15	0.573032176533173	0.256	0.391	9.90975398620786E-11	T/NK cell	DCAF8
6.04982025359529E-15	0.452683304284927	0.143	0.238	1.0138288780975E-10	T/NK cell	SCAF4
6.09916585279566E-15	0.503346005167316	0.063	0.127	1.0220982136115E-10	T/NK cell	HARS2
6.18694882520772E-15	0.6363703327735	0.07	0.136	1.03680888412831E-10	T/NK cell	ZNF398
6.30155696685191E-15	0.259641282197661	0.053	0.113	1.05601491650504E-10	T/NK cell	VPS35
6.47967987120705E-15	0.817079975822522	0.119	0.206	1.08586475281688E-10	T/NK cell	YY1AP1
6.7665077480468E-15	0.568845266810303	0.05	0.108	1.13393136841768E-10	T/NK cell	RP2
6.83085571798583E-15	0.534899233973584	0.107	0.187	1.14471480122007E-10	T/NK cell	VPS13D
7.07551285577635E-15	0.720736827394829	0.099	0.178	1.185714444371E-10	T/NK cell	FAM98B
7.31400642524449E-15	0.341187350194719	0.073	0.14	1.22568119674247E-10	T/NK cell	EXOC6B
7.43705947680376E-15	0.570437386960383	0.121	0.206	1.24630242712277E-10	T/NK cell	MUS81
7.497971353113E-15	2.32222933036225	0.138	0.093	1.25651003935468E-10	T/NK cell	HERC1
7.65791307859501E-15	0.755350907908027	0.056	0.117	1.28331307371095E-10	T/NK cell	KDM7A
7.6640161995018E-15	2.12167172910636	0.197	0.152	1.28433583471251E-10	T/NK cell	DCP2
7.69193656717655E-15	0.696950934672257	0.103	0.182	1.28901472992745E-10	T/NK cell	RETREG2
7.82989317940897E-15	0.852876764836381	0.085	0.157	1.31213349900535E-10	T/NK cell	DEPRECATED-ENSG00000185658
7.83487616533201E-15	0.770291757330066	0.114	0.199	1.31296854778634E-10	T/NK cell	TMEM87A
7.94210399683711E-15	0.430922998627599	0.058	0.119	1.33093778778996E-10	T/NK cell	DEPRECATED-ENSG00000185619
8.05816924841437E-15	0.773392805581508	0.179	0.29	1.35038800264928E-10	T/NK cell	RBM23
8.13603160665718E-15	0.519926475183335	0.073	0.14	1.36343617664361E-10	T/NK cell	DDX52
8.47337817931301E-15	0.641668074752797	0.128	0.216	1.41996871528927E-10	T/NK cell	ZNF24
9.4381643028404E-15	0.564746664498746	0.054	0.113	1.58164757386999E-10	T/NK cell	MAP3K10
9.44498949046812E-15	1.68612653116414	0.362	0.351	1.58279133881265E-10	T/NK cell	RNF19A
9.51765248065731E-15	0.699145558561751	0.092	0.167	1.59496820270855E-10	T/NK cell	LAS1L
9.52326483863523E-15	0.700798064860047	0.202	0.324	1.59590872165849E-10	T/NK cell	UBR5
9.78544970933791E-15	0.368326236260718	0.046	0.102	1.63984566229085E-10	T/NK cell	TFB1M
1.02754928917535E-14	1.96837543592223	0.426	0.435	1.72196709880005E-10	T/NK cell	PIK3R1
1.10246000931724E-14	0.496011536247939	0.101	0.18	1.84750248361383E-10	T/NK cell	BAG5
1.11350130867393E-14	0.36859660715702	0.054	0.112	1.86600549307577E-10	T/NK cell	HIP1
1.16217401322049E-14	0.434381608263458	0.249	0.385	1.94757121135489E-10	T/NK cell	TMPO
1.16712280241026E-14	0.570132385975551	0.113	0.195	1.95586439227911E-10	T/NK cell	TRAPPC12
1.21232347680604E-14	0.667673852243043	0.052	0.111	2.03161168243156E-10	T/NK cell	PPOX
1.30004742924554E-14	0.886989754358363	0.098	0.176	2.17861948192968E-10	T/NK cell	LTN1
1.30081432306969E-14	0.296941591953636	0.075	0.143	2.17990464260019E-10	T/NK cell	KLHL20
1.3034013153304E-14	1.56875450825611	0.4	0.399	2.18423992423068E-10	T/NK cell	TUT7
1.33128231432547E-14	0.85725033274947	0.235	0.365	2.23096290234663E-10	T/NK cell	SAFB2
1.33362811283103E-14	0.378522364632792	0.221	0.344	2.23489399148224E-10	T/NK cell	ITCH
1.34439607482383E-14	0.617657498909383	0.086	0.158	2.25293894218978E-10	T/NK cell	DYNC1LI1
1.34443588993536E-14	0.525249684248118	0.121	0.204	2.25300566435367E-10	T/NK cell	CENPT
1.3535103239866E-14	0.829843654708894	0.052	0.111	2.26821260093674E-10	T/NK cell	RAB2B
1.37890411053547E-14	0.594843810416102	0.083	0.155	2.31076750843534E-10	T/NK cell	CDYL
1.39469126389341E-14	0.399759282347619	0.115	0.197	2.33722362003258E-10	T/NK cell	SMAP1
1.41467176829736E-14	0.689380591288451	0.066	0.13	2.37070694931272E-10	T/NK cell	ATXN7L1
1.42177538097051E-14	0.446616375579271	0.082	0.152	2.38261118343038E-10	T/NK cell	AARS2
1.43320028115197E-14	0.330557987296783	0.132	0.221	2.40175703115447E-10	T/NK cell	HPS3
1.44103580206816E-14	0.413868661119196	0.121	0.205	2.41488779710582E-10	T/NK cell	TUBGCP2
1.44314482765693E-14	2.27372033782235	0.136	0.092	2.41842210218749E-10	T/NK cell	VSIR
1.56354813719022E-14	1.03564257585242	0.115	0.2	2.62019396830336E-10	T/NK cell	MICU2
1.58362754535186E-14	0.404934677184956	0.169	0.27	2.65384304050065E-10	T/NK cell	SEC16A
1.64138423177496E-14	0.679474343448007	0.118	0.201	2.75063169560848E-10	T/NK cell	TMEM167B

1.64870184405606E-14	0.498661080904422	0.081	0.15	2.76289455026915E-10	T/NK cell	NCBP1
1.68633195027787E-14	0.339443479043652	0.091	0.164	2.82595508227566E-10	T/NK cell	FDXR
1.72947111972316E-14	0.29935758693649	0.048	0.104	2.89824770243207E-10	T/NK cell	SNX30
1.74364194390835E-14	0.58155103606135	0.255	0.39	2.92199516960161E-10	T/NK cell	TMEM259
1.76375596075561E-14	0.437431006504318	0.075	0.143	2.95570223903425E-10	T/NK cell	NFU1
1.77701332076131E-14	0.919956044974138	0.073	0.14	2.97791892293181E-10	T/NK cell	MFSD11
1.85411871851959E-14	1.5741261975364	0.317	0.288	3.10713214849513E-10	T/NK cell	NUMA1
1.90913305035753E-14	0.611118869416939	0.054	0.112	3.19932516578915E-10	T/NK cell	DBR1
1.93663387413629E-14	0.471109687541465	0.083	0.153	3.24541104627759E-10	T/NK cell	C1orf174
1.93831613704957E-14	0.486929519908979	0.116	0.199	3.24823018246767E-10	T/NK cell	HSPB11
1.96158471404917E-14	0.452480235086058	0.191	0.302	3.2872236638036E-10	T/NK cell	HNRNPM
2.01809289183416E-14	0.774194910303863	0.117	0.201	3.38192006813569E-10	T/NK cell	UBAC2
2.04671791314452E-14	0.359938429904593	0.073	0.139	3.42988987884758E-10	T/NK cell	GPKOW
2.05324844311524E-14	0.481401988463182	0.119	0.202	3.44083374097252E-10	T/NK cell	EZH2
2.1351195788415E-14	0.920372103383218	0.108	0.19	3.57803339022258E-10	T/NK cell	WAC
2.26539358521933E-14	0.41974307410011	0.134	0.223	3.79634657011055E-10	T/NK cell	UBTF
2.26689039655975E-14	2.1022692239732	0.204	0.16	3.79885492655483E-10	T/NK cell	N4BP1
2.42969234033805E-14	0.96802787186372	0.112	0.193	4.07167842393851E-10	T/NK cell	NMI
2.57639939834281E-14	0.849128668919315	0.086	0.158	4.31753011174288E-10	T/NK cell	PMS1
2.73394108689224E-14	0.675951224678054	0.058	0.118	4.58153847341402E-10	T/NK cell	ZBTB48
2.7717646087343E-14	0.899233676143217	0.071	0.137	4.64492313131693E-10	T/NK cell	PPIL4
2.86740154102883E-14	0.474688993672006	0.058	0.118	4.80519150245612E-10	T/NK cell	CPLANE1
2.90333933661702E-14	0.656842257013979	0.133	0.221	4.8654160603028E-10	T/NK cell	MCRS1
2.91475194962501E-14	0.656521194180653	0.083	0.153	4.88454131718159E-10	T/NK cell	PIAS1
2.93908351100857E-14	0.382372036013879	0.069	0.133	4.92531614774816E-10	T/NK cell	AC118549.1
2.95311895510188E-14	0.679514773970197	0.082	0.152	4.94883674495974E-10	T/NK cell	ACYP2
2.97927631876288E-14	0.315219800653745	0.064	0.126	4.99267125498283E-10	T/NK cell	NHLRC3
2.98645401264625E-14	0.452514689477907	0.19	0.297	5.00469963439258E-10	T/NK cell	DEK
3.01127333219263E-14	0.680937960716445	0.057	0.116	5.04629185008841E-10	T/NK cell	CPSF4
3.02175136465679E-14	0.911315850310016	0.108	0.187	5.06385093689184E-10	T/NK cell	CCDC9
3.04046964253074E-14	0.924605770910109	0.074	0.14	5.09521902695301E-10	T/NK cell	NFYA
3.24453002923314E-14	0.464822017875642	0.064	0.126	5.43718342298889E-10	T/NK cell	RTL10
3.36072293209678E-14	0.80337365324608	0.152	0.249	5.63189948960779E-10	T/NK cell	MAEA
3.43575536668931E-14	0.627494889946998	0.097	0.173	5.75763884349795E-10	T/NK cell	TBRG4
3.51052119728E-14	0.502493350805801	0.083	0.153	5.88293142240183E-10	T/NK cell	MLLT10
3.62088199834139E-14	0.572123642500045	0.046	0.101	6.06787405282051E-10	T/NK cell	CYP2R1
3.62527257909454E-14	0.690973805868047	0.174	0.279	6.07523178804663E-10	T/NK cell	ZNF410
3.65274013120801E-14	0.650017924154815	0.113	0.193	6.12126191187839E-10	T/NK cell	SGSM3
3.65325289343939E-14	0.810362941436181	0.168	0.272	6.12212119882573E-10	T/NK cell	NME3
3.67026830554389E-14	0.530224200975033	0.066	0.128	6.15063562643045E-10	T/NK cell	POLR3C
3.7720117149625E-14	0.934346332863366	0.194	0.306	6.32113723193416E-10	T/NK cell	PHF20L1
3.81472024366891E-14	0.309986667982186	0.048	0.104	6.39270818434035E-10	T/NK cell	SAYSD1
3.8746287453899E-14	2.00646702524033	0.117	0.075	6.49310285152439E-10	T/NK cell	ACSF3
3.88875688855214E-14	0.322866137595379	0.062	0.123	6.51677879383568E-10	T/NK cell	DTNBP1
4.03796018844561E-14	0.763637788707325	0.148	0.244	6.76681368379716E-10	T/NK cell	ABHD3
4.31422681130298E-14	0.685987063056938	0.156	0.253	7.22978129038154E-10	T/NK cell	KHDC4
4.40350673182648E-14	0.512629577808562	0.195	0.306	7.37939658119482E-10	T/NK cell	MAPK14
4.5940778800579E-14	1.65730360721528	0.183	0.137	7.69875571140103E-10	T/NK cell	PRNP
4.73829465507013E-14	0.528643882138712	0.148	0.241	7.94043418296652E-10	T/NK cell	SLAIN2
4.77318116329767E-14	0.590662571146546	0.168	0.27	7.99889699345423E-10	T/NK cell	PTBP1
4.8821759802379E-14	0.593233144696299	0.105	0.183	8.18155050768268E-10	T/NK cell	MIB1
4.90192759189514E-14	0.364007764847694	0.132	0.219	8.21465025849788E-10	T/NK cell	NELFB
4.96777545199801E-14	0.278346826854571	0.056	0.114	8.32499810245827E-10	T/NK cell	SRRD
4.97473773238597E-14	0.984004773453024	0.083	0.152	8.33666549193242E-10	T/NK cell	KCTD13
5.07324269735618E-14	0.491181435084835	0.141	0.23	8.50174011222949E-10	T/NK cell	PPP2R5D
5.3082079211766E-14	0.809967275563002	0.064	0.125	8.89549483430775E-10	T/NK cell	MICA
5.356750145402E-14	0.694108783463902	0.13	0.218	8.97684189366468E-10	T/NK cell	WDR20
5.46739993529778E-14	0.311184162120185	0.149	0.239	9.16226881157201E-10	T/NK cell	CSDE1
5.49149146484183E-14	0.530590645603036	0.23	0.354	9.20264139678193E-10	T/NK cell	UBE2R2
5.49380791251554E-14	0.44720152160879	0.161	0.257	9.20652329979354E-10	T/NK cell	CNST
5.67332937131322E-14	0.740388849325999	0.078	0.145	9.50736536044669E-10	T/NK cell	BCAS3
5.84244146774704E-14	1.01055052396882	0.092	0.166	9.79076341165049E-10	T/NK cell	RXRB
5.88346907408835E-14	0.752281026812886	0.071	0.135	9.85951747435725E-10	T/NK cell	C12orf65

6.06583891714803E-14	0.425829721196975	0.076	0.142	1.01651328573567E-09	T/NK cell	HAUS2
6.34343709135807E-14	0.531331546533	0.165	0.263	1.06303318776979E-09	T/NK cell	SMARCD2
6.40760028145723E-14	0.52302395853463	0.065	0.126	1.0737856551666E-09	T/NK cell	ETF1
6.87656001129113E-14	0.269546943949943	0.064	0.125	1.15237392669217E-09	T/NK cell	ZBED6CL
6.88902061510749E-14	0.740783848762788	0.102	0.177	1.15446207467971E-09	T/NK cell	ELL
7.13283456335752E-14	0.805471063698618	0.074	0.139	1.19532041612745E-09	T/NK cell	KIAA0355
7.19547646902268E-14	0.67450342033192	0.103	0.178	1.20581794667882E-09	T/NK cell	FAAP20
7.36067911756251E-14	0.543982397366756	0.151	0.244	1.23350260652113E-09	T/NK cell	CUL1
7.5547008523671E-14	0.526984643179373	0.15	0.243	1.26601676883968E-09	T/NK cell	PAFAH1B2
7.91363896575518E-14	0.310018865581798	0.059	0.118	1.32616761788125E-09	T/NK cell	TRAPPC13
8.11411927138888E-14	1.76553890224435	0.28	0.246	1.35976410749935E-09	T/NK cell	TIAL1
8.32050021205019E-14	1.00892679058883	0.084	0.153	1.39434942553537E-09	T/NK cell	PRPF18
8.37891864867326E-14	0.636915329632684	0.089	0.16	1.40413918714466E-09	T/NK cell	TCF3
8.44405895145627E-14	0.657242569128353	0.105	0.182	1.41505539908504E-09	T/NK cell	SPIN1
8.54683821497845E-14	0.813910992863942	0.116	0.197	1.43227914806609E-09	T/NK cell	ANKRD27
8.71743806535524E-14	0.495812722317378	0.088	0.16	1.46086827099223E-09	T/NK cell	OMA1
8.72769293524014E-14	0.488901194922722	0.059	0.118	1.46258678208754E-09	T/NK cell	SSH3
8.73889536382361E-14	0.257066418066707	0.055	0.111	1.46446408506956E-09	T/NK cell	GTF3C5
8.9208032870604E-14	0.502809393631007	0.117	0.199	1.49494821484558E-09	T/NK cell	GRSF1
8.98257429854461E-14	0.693346324591352	0.172	0.274	1.50529980095011E-09	T/NK cell	TPR
9.29388431185896E-14	0.528384006019879	0.06	0.119	1.55746913298132E-09	T/NK cell	MAP4K3
9.39166884221041E-14	0.645766224519627	0.065	0.127	1.57385586457762E-09	T/NK cell	XYLT2
9.62754871717485E-14	0.69363343830086	0.138	0.227	1.61338461402416E-09	T/NK cell	SOAT1
9.95973053483788E-14	0.339151700247991	0.124	0.206	1.66905164302813E-09	T/NK cell	SNX10
1.01274957845283E-13	0.645576336714625	0.14	0.228	1.69716574357125E-09	T/NK cell	MED13L
1.02511459014294E-13	0.377730974059758	0.155	0.247	1.71788703016154E-09	T/NK cell	HELLS
1.02814921026736E-13	0.484359497538533	0.203	0.312	1.72297244656604E-09	T/NK cell	PYGO2
1.07102213869537E-13	0.655984100798832	0.198	0.305	1.79481890002569E-09	T/NK cell	RASSF3
1.08355807681458E-13	0.523952149017223	0.073	0.136	1.81582662512588E-09	T/NK cell	PSMA1
1.09418060645148E-13	2.05927449022445	0.154	0.11	1.83362786029138E-09	T/NK cell	CCNG2
1.10764036507249E-13	0.428666379017182	0.185	0.289	1.85618372378848E-09	T/NK cell	RNASEK
1.12143498461425E-13	0.264699129923457	0.247	0.371	1.87930074721656E-09	T/NK cell	ATP6V0E1
1.12540580381651E-13	0.75292229221271	0.175	0.277	1.88595504603571E-09	T/NK cell	SDHA
1.13715042737218E-13	0.676264161558058	0.218	0.338	1.90563668619031E-09	T/NK cell	U2SURP
1.16692164872037E-13	0.297026386321442	0.089	0.159	1.9555272989256E-09	T/NK cell	ACVR1
1.19276978067964E-13	0.495079887261619	0.144	0.235	1.99884359846293E-09	T/NK cell	CNOT11
1.2513235706387E-13	0.572159645934282	0.066	0.127	2.09696803967634E-09	T/NK cell	KLHL7
1.38774423959073E-13	0.70734255393323	0.142	0.232	2.32558179670614E-09	T/NK cell	LEMD2
1.42668582726184E-13	0.442472675901427	0.133	0.22	2.39084010932539E-09	T/NK cell	C14orf119
1.45413507660573E-13	0.518990420977175	0.155	0.25	2.43683956137588E-09	T/NK cell	WIPF2
1.47658869316171E-13	0.554577878357724	0.081	0.147	2.4744673320004E-09	T/NK cell	NAAA
1.51147250861099E-13	1.58737598499308	0.335	0.321	2.53292562993029E-09	T/NK cell	TBC1D2B
1.53368797596972E-13	0.316240480078221	0.069	0.131	2.57015431013005E-09	T/NK cell	PIP5K1C
1.57523566675345E-13	0.313568565430808	0.068	0.128	2.63977993034543E-09	T/NK cell	KPNA2
1.58038050488537E-13	0.621840123330154	0.054	0.11	2.64840165008691E-09	T/NK cell	CBLL1
1.68384776426027E-13	0.250472056023474	0.167	0.26	2.82179208334736E-09	T/NK cell	LIME1
1.68966232174137E-13	0.585284671676923	0.201	0.311	2.83153611877419E-09	T/NK cell	MICAL3
1.69555755236138E-13	1.60502133721762	0.326	0.306	2.84141534624721E-09	T/NK cell	MARCH7
1.70333279376071E-13	0.657385784241654	0.122	0.206	2.8544450957842E-09	T/NK cell	HARS
1.77442321769822E-13	0.951340067069714	0.065	0.125	2.97357842821867E-09	T/NK cell	VCPKMT
1.83971839937796E-13	0.472384259950841	0.069	0.13	3.08300009367759E-09	T/NK cell	RAD51D
1.85520514870135E-13	0.934779493397987	0.058	0.116	3.10895278819373E-09	T/NK cell	RINT1
1.88135312468629E-13	1.63238314050065	0.312	0.29	3.15277156634928E-09	T/NK cell	KDM5C
1.89198662670581E-13	1.81626275494551	0.237	0.198	3.1705911890336E-09	T/NK cell	BUB3
1.95959962610395E-13	0.493646830140421	0.106	0.183	3.283897053425E-09	T/NK cell	SMARCE1
1.97636350066086E-13	0.601210378302364	0.108	0.183	3.31198995440747E-09	T/NK cell	BNIP3L
1.97768687150639E-13	0.378164403142192	0.054	0.109	3.31420765927041E-09	T/NK cell	ARL 3.00
2.03875017833261E-13	1.81725148896913	0.213	0.172	3.41653754884979E-09	T/NK cell	NFATC3
2.04679536249345E-13	0.772919872119497	0.139	0.228	3.43001966846652E-09	T/NK cell	TTC17
2.09848598746678E-13	1.79340655051037	0.276	0.245	3.51664281779684E-09	T/NK cell	TAB2
2.11807259551409E-13	0.276472155125878	0.063	0.123	3.5494660555625E-09	T/NK cell	ZSWIM9
2.13849361294964E-13	0.732788809446946	0.094	0.165	3.58368759658101E-09	T/NK cell	DEPRECATED-ENSG00000196313
2.17511009154431E-13	0.421824501155642	0.166	0.264	3.64504949140996E-09	T/NK cell	UBP1

2.22676226764347E-13	0.443137997042554	0.091	0.161	3.73160820811693E-09	T/NK cell	GABPB2
2.27696076367545E-13	0.669131665093222	0.112	0.19	3.81573084776733E-09	T/NK cell	MLH1
2.29917905891542E-13	0.612647878740939	0.152	0.245	3.85296426693046E-09	T/NK cell	BROX
2.49408410592217E-13	0.506450086970708	0.104	0.178	4.17958614470437E-09	T/NK cell	USP38
2.50896502380836E-13	0.693902903213724	0.102	0.175	4.20452358689805E-09	T/NK cell	ARFRP1
2.61721335326083E-13	0.640516072941537	0.102	0.176	4.3859261373945E-09	T/NK cell	OXSR1
2.63258136690762E-13	0.502091443066963	0.08	0.146	4.41167985466378E-09	T/NK cell	BECN1
2.65975906470322E-13	0.639200451504312	0.076	0.139	4.45722424062966E-09	T/NK cell	STARD3NL
2.66662563443244E-13	2.23561187541086	0.108	0.069	4.46873123818189E-09	T/NK cell	SBF1
2.67164624736598E-13	0.759102898375856	0.192	0.301	4.47714478133591E-09	T/NK cell	COPB1
2.67798171410168E-13	0.523638758187044	0.084	0.152	4.48776175649159E-09	T/NK cell	DEPRECATED-ENSG00000132463
2.68867125657373E-13	0.924782352763388	0.093	0.163	4.50567529176626E-09	T/NK cell	RELA
2.75543725765646E-13	0.387779103686798	0.109	0.185	4.6175617563807E-09	T/NK cell	PDS5B
2.75553450173897E-13	0.343431939289802	0.063	0.123	4.61772471801417E-09	T/NK cell	FCF1
2.76575972293502E-13	0.537286132217054	0.088	0.158	4.63486014369451E-09	T/NK cell	ZFP62
2.8247292667355E-13	0.577558611464321	0.186	0.294	4.73368130519536E-09	T/NK cell	RBM26
2.855792324117E-13	0.934412848063537	0.101	0.175	4.78573677675527E-09	T/NK cell	SECISBP2
2.98780218229923E-13	0.772769843266832	0.161	0.258	5.00695889709706E-09	T/NK cell	GPR107
3.03970430902483E-13	0.758906923989789	0.127	0.21	5.0939364810638E-09	T/NK cell	BRD7
3.07838226527421E-13	0.603836430222187	0.095	0.167	5.15875300014651E-09	T/NK cell	UCHL5
3.1199081411258E-13	0.315969272951331	0.051	0.104	5.22834206289862E-09	T/NK cell	COA1
3.17968083507969E-13	0.58262030948484	0.1	0.173	5.32850914342655E-09	T/NK cell	WIPI2
3.38163440817405E-13	0.777932327874792	0.065	0.124	5.66694294121807E-09	T/NK cell	ACD
3.42075793633838E-13	0.679749876301195	0.108	0.185	5.73250614971586E-09	T/NK cell	CPSF2
3.45560879945389E-13	0.754706775596669	0.108	0.184	5.79090922612483E-09	T/NK cell	SLC35E1
3.46695974117614E-13	0.57400574943576	0.068	0.13	5.80993113426297E-09	T/NK cell	UTP3
3.54778397412855E-13	0.365542767922515	0.173	0.272	5.94537638384462E-09	T/NK cell	ATP6AP2
3.58619610778754E-13	0.775591444921523	0.118	0.2	6.00974743743036E-09	T/NK cell	OTUD4
4.04750443421777E-13	0.954285489710656	0.463	0.456	6.78280793086213E-09	T/NK cell	IVNS1ABP
4.2121314277283E-13	0.50141014194849	0.054	0.108	7.05868984658708E-09	T/NK cell	AMDHD2
4.44689918481201E-13	0.486692589420271	0.132	0.215	7.45211365390797E-09	T/NK cell	USP10
4.57650142659278E-13	1.05421701348394	0.057	0.113	7.66930109068418E-09	T/NK cell	ANKMY1
4.64829889075006E-13	2.36859131476611	0.104	0.066	7.78961928111895E-09	T/NK cell	VAMP1
4.85698951543096E-13	0.603437203307863	0.087	0.155	8.1393430299592E-09	T/NK cell	MTREX
4.91580088756893E-13	0.620681176389725	0.18	0.281	8.23789912738801E-09	T/NK cell	PMPCB
5.30618101731902E-13	0.440108840292891	0.283	0.424	8.89209814882321E-09	T/NK cell	UBXN4
5.46892150505469E-13	0.279149838026694	0.073	0.135	9.16481865817065E-09	T/NK cell	SLC52A2
5.51790673531943E-13	0.397206288282849	0.125	0.205	9.2469081070483E-09	T/NK cell	VCP
5.5334387616259E-13	0.632985819352988	0.141	0.227	9.27293667673269E-09	T/NK cell	ELMSAN1
5.64639034998029E-13	0.366402480640206	0.113	0.19	9.46222094849698E-09	T/NK cell	ATF7
5.6608840584486E-13	0.859873614116959	0.143	0.231	9.48650950514816E-09	T/NK cell	XAB2
5.69580973134405E-13	0.879957105822398	0.089	0.158	9.54503794778636E-09	T/NK cell	TRNT1
5.72726220593688E-13	0.442130992559175	0.05	0.103	9.59774600470902E-09	T/NK cell	BCAS2
5.73412916047535E-13	0.833962270780465	0.088	0.155	9.60925364712459E-09	T/NK cell	CCDC93
5.86262515113589E-13	2.36336841141281	0.134	0.093	9.82458722827352E-09	T/NK cell	GFOD1
5.90565482407101E-13	0.676145242044706	0.623	0.794	9.8966963541782E-09	T/NK cell	IL32
5.92070212439184E-13	0.47050161251966	0.143	0.23	9.92191262005584E-09	T/NK cell	VPS26B
6.02574891497843E-13	0.579396492393041	0.08	0.145	1.00979500317209E-08	T/NK cell	UVRAG
6.10782974874466E-13	1.19289417112557	0.418	0.414	1.02355010929463E-08	T/NK cell	IKBKB
6.23928244166414E-13	0.363226001921268	0.11	0.185	1.04557895157408E-08	T/NK cell	TMEM8A
6.68099834415089E-13	0.647501635256541	0.147	0.238	1.11960170251281E-08	T/NK cell	TMBIM4
6.98283561586099E-13	0.521730885697361	0.048	0.1	1.17018359250599E-08	T/NK cell	MPHOSPH6
7.02958645703164E-13	0.417785945840578	0.083	0.149	1.17801809846936E-08	T/NK cell	ST3GAL2
7.61744526157328E-13	0.599129748049241	0.092	0.161	1.27653147693445E-08	T/NK cell	PLEKHA3
7.63413956695987E-13	0.290185633626576	0.097	0.167	1.27932910863113E-08	T/NK cell	KPNA3
7.76131144686127E-13	0.805137271697219	0.165	0.262	1.30064057226501E-08	T/NK cell	DNAJC5
7.88142307542369E-13	0.509477027243326	0.138	0.224	1.3207688789795E-08	T/NK cell	OPA1
8.38294686154908E-13	0.781337961082997	0.102	0.175	1.4048142350584E-08	T/NK cell	EIF3J
8.73116746265811E-13	0.852200827807611	0.052	0.105	1.46316904339225E-08	T/NK cell	HERC5
9.07827379889422E-13	1.07903203148664	0.115	0.192	1.52133712321869E-08	T/NK cell	PWWP2A
9.08325034302714E-13	0.301342133531687	0.104	0.176	1.52217109248449E-08	T/NK cell	KNTC1
9.10254320819885E-13	2.23048543891922	0.188	0.149	1.52540419082996E-08	T/NK cell	THADA
9.44810623767268E-13	0.537900752250458	0.17	0.267	1.58331364330919E-08	T/NK cell	ATP2C1

9.554261380134E-13	0.89889156607046	0.087	0.154	1.60110312208286E-08	T/NK cell	DPY19L1
9.59675686045006E-13	0.421651683688188	0.076	0.137	1.60822451467422E-08	T/NK cell	PTPN23
9.95236195680916E-13	0.896337918084288	0.168	0.265	1.66781681672208E-08	T/NK cell	KCTD20
1.02447645954582E-12	0.366704705544499	0.058	0.113	1.71681765090689E-08	T/NK cell	WDR55
1.03886024038992E-12	2.05048020825992	0.181	0.14	1.74092199084543E-08	T/NK cell	PRKACB
1.05013011613847E-12	0.431958836227739	0.133	0.213	1.75980804862485E-08	T/NK cell	ELF4
1.05403102062538E-12	0.546981658557023	0.079	0.142	1.76634518436401E-08	T/NK cell	SLC11A2
1.07949548520298E-12	0.528973426737696	0.074	0.136	1.80901853410315E-08	T/NK cell	USP37
1.07980215078609E-12	0.830710047813251	0.137	0.224	1.80953244428733E-08	T/NK cell	SNX9
1.12952180990145E-12	0.366254101005046	0.135	0.22	1.89285264903285E-08	T/NK cell	C6orf62
1.13673493122226E-12	1.7023449166201	0.254	0.22	1.90494039774226E-08	T/NK cell	HERC4
1.13896470047656E-12	0.533142474174241	0.101	0.173	1.90867704505863E-08	T/NK cell	RSPRY1
1.16769593054527E-12	0.516220810124819	0.131	0.214	1.95682484040777E-08	T/NK cell	SIPA1L1
1.18220290658024E-12	0.556994213635719	0.083	0.148	1.98113563084716E-08	T/NK cell	TMEM94
1.18640084784426E-12	0.666060215220393	0.11	0.185	1.98817054081742E-08	T/NK cell	SNRPB2
1.26873095399513E-12	0.736091586316602	0.076	0.137	2.12613933270504E-08	T/NK cell	BMS1
1.30256753768336E-12	0.483270430859652	0.07	0.129	2.18284267964977E-08	T/NK cell	COMTD1
1.34475024694749E-12	0.416453005813982	0.061	0.116	2.25353246383461E-08	T/NK cell	GRWD1
1.3492162876701E-12	1.31689263978128	0.228	0.186	2.26101665487755E-08	T/NK cell	TWF2
1.36340749074943E-12	0.450058013741492	0.049	0.1	2.2847982729979E-08	T/NK cell	ZMYM1
1.3839786278538E-12	0.568364586641233	0.053	0.106	2.31927138455739E-08	T/NK cell	CACTIN
1.39707110107799E-12	0.467738413030333	0.072	0.133	2.34121175118649E-08	T/NK cell	EMC1
1.4076385774547E-12	0.575006636938657	0.139	0.225	2.35892072809858E-08	T/NK cell	CASP3
1.41192480012941E-12	0.995151956495496	0.08	0.145	2.36610358005687E-08	T/NK cell	THOC1
1.431895967381E-12	1.69316116907671	0.275	0.247	2.39957126213708E-08	T/NK cell	RBM15
1.46053000133588E-12	0.416609216611441	0.069	0.128	2.44755617623867E-08	T/NK cell	HPS4
1.50346948353129E-12	0.666216122094016	0.122	0.202	2.51951416050173E-08	T/NK cell	ARFGEF1
1.50579863518052E-12	0.640994650939586	0.191	0.299	2.52341735283551E-08	T/NK cell	AGFG1
1.54305785369701E-12	0.63039883791806	0.154	0.244	2.58585635122545E-08	T/NK cell	LMF2
1.5438877150716E-12	0.862778435313277	0.244	0.37	2.58724703291698E-08	T/NK cell	CSNK1D
1.56817262381769E-12	0.909736256666168	0.062	0.119	2.62794368299369E-08	T/NK cell	EIF2B4
1.5771825422169E-12	0.620146961800589	0.066	0.125	2.64304250424708E-08	T/NK cell	ZNF397
1.65245953887397E-12	0.838142433583563	0.145	0.233	2.76919169524499E-08	T/NK cell	REV1
1.67109642215099E-12	0.6870622084783	0.155	0.245	2.80042338424062E-08	T/NK cell	NSUN2
1.68624532555128E-12	1.77027954623347	0.19	0.149	2.82580991655884E-08	T/NK cell	CSF1
1.74454121128532E-12	0.925393618178125	0.107	0.182	2.92350216187194E-08	T/NK cell	CEBPZ
1.78691411694345E-12	2.10614506431473	0.124	0.084	2.99451067717383E-08	T/NK cell	TBC1D10A
1.80274609965428E-12	1.46080944599276	0.255	0.395	3.02104191380064E-08	T/NK cell	PER1
1.84782237486159E-12	0.405465742810691	0.082	0.146	3.09658073579306E-08	T/NK cell	RNF31
1.87227454625694E-12	0.487056945959797	0.181	0.28	3.13755768461738E-08	T/NK cell	NUP62
1.87682229939475E-12	0.633015521264484	0.195	0.3	3.14517880932572E-08	T/NK cell	DHX9
1.88285887505232E-12	0.4583870897916	0.066	0.123	3.15529490281268E-08	T/NK cell	ARMC6
1.91736782711915E-12	0.682203531001777	0.055	0.108	3.21312500468628E-08	T/NK cell	C2orf49
1.9817534034179E-12	0.819817471608533	0.21	0.322	3.32102235344772E-08	T/NK cell	MAN2A1
1.98541470603001E-12	0.66756953089573	0.062	0.118	3.3271579643651E-08	T/NK cell	ZNF414
2.02295359295122E-12	0.655834478574494	0.106	0.178	3.39006563106766E-08	T/NK cell	BRD8
2.02483637939076E-12	0.632645534187575	0.126	0.205	3.39322080458303E-08	T/NK cell	OTUB1
2.03939341276552E-12	0.433834795893706	0.08	0.143	3.41761548111246E-08	T/NK cell	USP5
2.0414837122692E-12	0.372459942621968	0.071	0.129	3.42111840502073E-08	T/NK cell	RNF123
2.11407426296755E-12	0.475418723864208	0.06	0.115	3.54276564988102E-08	T/NK cell	EXOSC3
2.11997039557448E-12	0.553952439335873	0.213	0.323	3.55264638890371E-08	T/NK cell	CYB5B
2.17699197030139E-12	0.512914153196278	0.132	0.213	3.64820314383108E-08	T/NK cell	FNDC3A
2.28237558184881E-12	0.417955845716331	0.225	0.337	3.82480500006223E-08	T/NK cell	NCL
2.30200649956506E-12	0.512128033313049	0.132	0.212	3.85770249197112E-08	T/NK cell	EXOC5
2.34745348063723E-12	0.683825888921743	0.111	0.185	3.93386254285187E-08	T/NK cell	SSU72
2.38454354993378E-12	0.704057397863557	0.077	0.139	3.99601808097903E-08	T/NK cell	PPIE
2.42198509790183E-12	0.415733878583076	0.095	0.164	4.05876262706388E-08	T/NK cell	RAB22A
2.48241112666727E-12	0.731326896705837	0.094	0.161	4.16002456606901E-08	T/NK cell	BCL7B
2.61420156797533E-12	0.702183014626164	0.193	0.296	4.38087898761307E-08	T/NK cell	SERBP1
2.64973746113053E-12	0.775635965246357	0.148	0.236	4.44043003736255E-08	T/NK cell	CTNNB1
2.66529318016205E-12	0.70281090261881	0.18	0.279	4.46649831131557E-08	T/NK cell	AATF
2.74748893454585E-12	0.403325878370685	0.096	0.162	4.60424195651194E-08	T/NK cell	HIST1H4C
2.83008381489912E-12	0.731103720282364	0.168	0.263	4.74265445700794E-08	T/NK cell	SEC24B

2.83561193101705E-12	0.637808151040367	0.2	0.307	4.75191847399837E-08	T/NK cell	DCTN1
2.93004946723039E-12	0.78549108924777	0.051	0.102	4.91017689718469E-08	T/NK cell	CTU2
3.02313819813532E-12	0.561492126139443	0.052	0.103	5.06617499243517E-08	T/NK cell	PRKCI
3.02565816477802E-12	0.567770442870838	0.193	0.299	5.07039795253501E-08	T/NK cell	PARP10
3.09217782032755E-12	0.588523229855914	0.083	0.146	5.18187159130491E-08	T/NK cell	TRMU
3.14213005149786E-12	0.689009945087104	0.072	0.132	5.26558154030012E-08	T/NK cell	HCFC2
3.22189961245698E-12	0.648650641623577	0.072	0.131	5.39925937055541E-08	T/NK cell	POLR2D
3.25812167900049E-12	0.754821457139009	0.137	0.221	5.45996030966902E-08	T/NK cell	GTF2H1
3.26063360215467E-12	0.705189312021628	0.097	0.166	5.46416979049079E-08	T/NK cell	RIC1
3.27258662162973E-12	0.659740220109301	0.133	0.216	5.4842006605271E-08	T/NK cell	TERF2
3.33822432412915E-12	0.552059343466256	0.69	0.786	5.59419632237564E-08	T/NK cell	OAZ1
3.33910637109363E-12	0.766092748606293	0.099	0.169	5.59567445667871E-08	T/NK cell	EMG1
3.35946559888165E-12	0.608989760504254	0.054	0.106	5.62979245060587E-08	T/NK cell	SARS
3.41697625072859E-12	0.77849860751817	0.22	0.335	5.72616880097097E-08	T/NK cell	SRSF2
3.42398924913111E-12	0.68516350973251	0.169	0.265	5.73792118369391E-08	T/NK cell	TCERG1
3.4864363323612E-12	0.722944946188389	0.119	0.197	5.8425700057709E-08	T/NK cell	ZNF580
3.57375502861239E-12	0.836626106754722	0.148	0.236	5.98889867694864E-08	T/NK cell	DDX56
3.60508620974036E-12	1.64957990881561	0.228	0.188	6.04140347028289E-08	T/NK cell	PPP1R9B
3.64539231084859E-12	0.728899062392955	0.143	0.228	6.10894843452007E-08	T/NK cell	DDX42
3.65511012049654E-12	0.762661528017158	0.056	0.108	6.1252335399281E-08	T/NK cell	ZBTB5
3.66384498326618E-12	0.4340713145804	0.067	0.124	6.13987142295746E-08	T/NK cell	FAM184A
3.67604436711808E-12	0.569130609211651	0.1	0.169	6.16031515041648E-08	T/NK cell	ELOF1
3.71167652045068E-12	0.478658912056514	0.085	0.149	6.22002751297126E-08	T/NK cell	EEFSEC
3.8912177828002E-12	0.752488857672932	0.082	0.146	6.52090276041658E-08	T/NK cell	ABHD15
3.92790280249489E-12	0.584878012752017	0.085	0.148	6.58237951642093E-08	T/NK cell	CRIPT
4.0534654012593E-12	0.300590182910071	0.059	0.113	6.79279731943034E-08	T/NK cell	ZNF316
4.25440790038165E-12	0.60062468738503	0.147	0.234	7.12953675945958E-08	T/NK cell	SEPTIN2
4.35576138482496E-12	0.390710173181237	0.075	0.134	7.29938492868967E-08	T/NK cell	TTC9C
4.37023715992867E-12	0.552189848602042	0.135	0.217	7.32364343260846E-08	T/NK cell	NFYC
4.46715828564507E-12	0.265505551874454	0.162	0.252	7.486063855084E-08	T/NK cell	GNAI3
4.49862191787702E-12	0.566276297587522	0.107	0.177	7.53879060997831E-08	T/NK cell	CLK4
4.51386736113043E-12	0.664394666147707	0.189	0.289	7.56433892378237E-08	T/NK cell	KPNA4
4.51633308834174E-12	1.02821430952843	0.068	0.125	7.56847098944309E-08	T/NK cell	GALC
4.52489120132246E-12	0.638545085731247	0.142	0.228	7.58281267517618E-08	T/NK cell	RO60
4.59436906423881E-12	0.281434214674002	0.086	0.149	7.69924367785139E-08	T/NK cell	PSME2
4.639331262866E-12	0.576353619250479	0.215	0.326	7.77459133031085E-08	T/NK cell	UPF1
4.85622462847556E-12	0.267820093475886	0.059	0.112	8.13806123239935E-08	T/NK cell	PEX14
4.89036162878745E-12	0.733248823762393	0.098	0.167	8.19526801752201E-08	T/NK cell	TMEM170A
4.95636547055471E-12	0.709812038872611	0.11	0.185	8.30587725555558E-08	T/NK cell	PKN2
5.06005941377869E-12	0.492165712238689	0.138	0.219	8.47964756561033E-08	T/NK cell	SAE1
5.40450594964051E-12	0.905574995534655	0.114	0.188	9.05687107040756E-08	T/NK cell	COPS2
5.44782686265247E-12	0.803358506765371	0.093	0.159	9.12946825643301E-08	T/NK cell	EXOC6
5.56692641372677E-12	0.671646808417408	0.182	0.282	9.32905528412331E-08	T/NK cell	SRPK1
5.69447409890202E-12	0.44415482931071	0.203	0.307	9.54279969494001E-08	T/NK cell	ZFP91
5.81570729763123E-12	1.16161819803082	0.063	0.118	9.74596228937042E-08	T/NK cell	TXLNG
5.86629411902682E-12	0.924109109212053	0.135	0.218	9.83073568466514E-08	T/NK cell	UBA6
6.29912768103826E-12	0.61190359826768	0.169	0.263	1.05560781678839E-07	T/NK cell	PPP6C
6.3233754333911E-12	0.63592308679965	0.117	0.191	1.05967125512768E-07	T/NK cell	RSU1
6.52383795477391E-12	0.46586615071021	0.079	0.14	1.09326476446101E-07	T/NK cell	TMEM30A
6.56084795625842E-12	0.606272700872876	0.184	0.281	1.09946690050979E-07	T/NK cell	ZBTB7A
6.62601993293531E-12	0.712449698000208	0.158	0.249	1.1103884203613E-07	T/NK cell	RNF40
6.72421495455589E-12	0.500106475082931	0.254	0.379	1.12684394208448E-07	T/NK cell	SURF4
6.74438928624973E-12	0.875497873078334	0.183	0.284	1.13022475658973E-07	T/NK cell	PARP12
6.82368870140665E-12	0.690660137854555	0.147	0.234	1.14351375258173E-07	T/NK cell	MED14
6.85636938179976E-12	0.938222354978313	0.099	0.168	1.148990381002E-07	T/NK cell	ABHD18
6.91665743848237E-12	1.27215494419125	0.086	0.151	1.15909345354088E-07	T/NK cell	FGFR1OP
6.99794096006698E-12	0.605012111229657	0.079	0.139	1.17271494608803E-07	T/NK cell	UBE2J1
7.12790169882104E-12	0.770697940205868	0.118	0.194	1.19449376668843E-07	T/NK cell	NAA15
7.14378990111455E-12	0.947062100882788	0.082	0.144	1.19715631162878E-07	T/NK cell	ELP1
7.1456793751183E-12	0.511580890203583	0.05	0.1	1.19747294968232E-07	T/NK cell	TYW3
7.16124367972212E-12	0.332112179564755	0.105	0.174	1.20008121584783E-07	T/NK cell	LSM 2.00
7.28716246707478E-12	0.394191049350475	0.164	0.258	1.22118268623239E-07	T/NK cell	YIPF4
7.94395410818243E-12	0.468481074549592	0.076	0.136	1.33124782944921E-07	T/NK cell	LRRC8D

8.13482474212877E-12	0.447777206246139	0.093	0.157	1.36323393028594E-07	T/NK cell	ADK
8.51381019594633E-12	0.732391448932297	0.073	0.132	1.42674431263669E-07	T/NK cell	MAP3K14
8.51949317666071E-12	0.853916271797626	0.092	0.159	1.4276966665448E-07	T/NK cell	TCP11L1
8.58574780620376E-12	0.495428522436188	0.159	0.245	1.43879961736363E-07	T/NK cell	RRM1
8.69614896181206E-12	0.575506756357914	0.097	0.165	1.45730064302046E-07	T/NK cell	XPO1
8.8169614582163E-12	0.61907291302278	0.051	0.101	1.47754640116789E-07	T/NK cell	HSPA14.1
8.88220139678059E-12	0.646563063234897	0.156	0.243	1.48847931007249E-07	T/NK cell	HPCAL1
8.93904432632562E-12	0.401176034149246	0.059	0.111	1.49800504820565E-07	T/NK cell	RALB
8.98391165430966E-12	0.96874313090078	0.08	0.143	1.50552391502921E-07	T/NK cell	NT5C3A
8.99217497458253E-12	0.472112999891001	0.086	0.149	1.50690868224054E-07	T/NK cell	UBN1
9.01341460767273E-12	0.969487014651406	0.095	0.163	1.5104680199538E-07	T/NK cell	FBXO3
9.0230439369255E-12	0.706775139558311	0.075	0.135	1.51208170294997E-07	T/NK cell	ZNF26
9.31916222545984E-12	0.535603133993756	0.245	0.363	1.56170520574256E-07	T/NK cell	GNB2
9.756837692513E-12	0.633036427912823	0.092	0.157	1.63505086051133E-07	T/NK cell	LMAN2L
9.94431400649176E-12	0.612746875996548	0.078	0.139	1.66646814120789E-07	T/NK cell	GPATCH2
1.00109201924825E-11	0.799300898472636	0.162	0.253	1.67763000585621E-07	T/NK cell	RPRD2
1.02114485409156E-11	0.705210432881579	0.069	0.126	1.71123454648664E-07	T/NK cell	RAD51B
1.04820993708908E-11	0.424229150716321	0.184	0.277	1.75659021257388E-07	T/NK cell	LMNB1
1.04921095711813E-11	0.334334569607903	0.095	0.161	1.75826772193856E-07	T/NK cell	ATG7
1.05016935774892E-11	0.838263638164585	0.089	0.154	1.75987380971563E-07	T/NK cell	AASDHPPT
1.06811179917687E-11	0.65178226246202	0.06	0.114	1.7899417530606E-07	T/NK cell	TRIM32
1.07116313112946E-11	0.721982517857383	0.17	0.262	1.79505517514675E-07	T/NK cell	DEPRECATED-ENSG00000062485
1.08663885731988E-11	2.68079377981496	0.105	0.07	1.82098939709666E-07	T/NK cell	SLF1
1.10690905721111E-11	0.308288498746094	0.057	0.109	1.85495819807438E-07	T/NK cell	ALMS1
1.15323313803581E-11	0.942633152661511	0.066	0.121	1.9325880927204E-07	T/NK cell	KIZ
1.15907166791947E-11	0.972059850081657	0.071	0.128	1.94237230109944E-07	T/NK cell	TENT4B
1.17712365336476E-11	0.920406128883549	0.149	0.234	1.97262381830866E-07	T/NK cell	KPNA1
1.21577402066938E-11	0.79116905620211	0.056	0.108	2.03739410383774E-07	T/NK cell	MFSD8
1.21874856529073E-11	0.780159830335814	0.152	0.238	2.0423788457142E-07	T/NK cell	MAN2A2
1.23127418798265E-11	0.49183367975867	0.054	0.104	2.06336928422133E-07	T/NK cell	AGL
1.25507726925574E-11	0.872108320490105	0.068	0.124	2.10325848781877E-07	T/NK cell	TMEM161B
1.30145339404965E-11	1.0481394500109	0.164	0.259	2.1809755977484E-07	T/NK cell	KIAA0319L
1.30257004199067E-11	0.843214660320474	0.121	0.197	2.18284687636797E-07	T/NK cell	PHKG2
1.32336965833294E-11	1.04300365976769	0.087	0.15	2.21770287343434E-07	T/NK cell	PDE3B
1.33533226198974E-11	0.756643333109323	0.162	0.252	2.23774980464241E-07	T/NK cell	ATF2
1.35810986169857E-11	0.768572556181317	0.219	0.331	2.27592050623447E-07	T/NK cell	RNF115
1.3625824360664E-11	0.90340757803568	0.098	0.165	2.28341564636008E-07	T/NK cell	ADRB2
1.36593352069785E-11	0.522433587900934	0.166	0.255	2.28903139398546E-07	T/NK cell	EXOC7
1.37116283234627E-11	0.533796741115456	0.114	0.187	2.29779467444588E-07	T/NK cell	VPS54
1.37238703253755E-11	0.571913857128623	0.063	0.117	2.29984618912643E-07	T/NK cell	PPCDC
1.3771789598732E-11	0.423281515131584	0.1	0.167	2.30787650095551E-07	T/NK cell	PSMD9
1.39951645880763E-11	0.650848645292477	0.129	0.206	2.34530968166982E-07	T/NK cell	ZCCHC3
1.41535927473875E-11	2.06127020626546	0.171	0.133	2.37185907260719E-07	T/NK cell	TES
1.42921776295966E-11	0.583836378476772	0.084	0.146	2.39508312716779E-07	T/NK cell	TRAPPC11
1.45515987269472E-11	0.735913100879571	0.121	0.196	2.43855691466181E-07	T/NK cell	GPD1L
1.4750659065338E-11	0.364252107043361	0.07	0.126	2.47191544616934E-07	T/NK cell	EIF3I
1.47851577623607E-11	0.592971745355899	0.141	0.222	2.47769673781641E-07	T/NK cell	PTDSS1
1.51940282517635E-11	0.508517284011869	0.069	0.124	2.54621525443052E-07	T/NK cell	ZNF621
1.54971978413733E-11	0.399651468887464	0.183	0.281	2.59702041425735E-07	T/NK cell	CLIP1
1.5547410941181E-11	0.384672667993039	0.08	0.14	2.60543512552311E-07	T/NK cell	NT5C
1.68675749454788E-11	1.78523227866705	0.18	0.141	2.82666820936334E-07	T/NK cell	TGFBR1
1.69888406845608E-11	0.566561334567779	0.087	0.15	2.8469899219187E-07	T/NK cell	COG4
1.69948748780126E-11	0.48402458057458	0.057	0.109	2.84800113205736E-07	T/NK cell	TADA2A
1.74242956969292E-11	0.736844917373144	0.246	0.37	2.9199634728914E-07	T/NK cell	TM9SF3
1.75743385055449E-11	2.5740306973961	0.241	0.211	2.94510764675922E-07	T/NK cell	CREM
1.77098987482366E-11	0.864043146834454	0.151	0.237	2.96782483222949E-07	T/NK cell	STK25
1.78529599291228E-11	0.814850962722941	0.073	0.131	2.9917990249224E-07	T/NK cell	ABLIM1
1.79254272335907E-11	0.58793710890783	0.12	0.195	3.00394309580513E-07	T/NK cell	PHKB
1.81695821395352E-11	0.457165751553188	0.143	0.223	3.04485857494331E-07	T/NK cell	EEF1B2
1.82328566418774E-11	0.900665598406967	0.126	0.203	3.05546211604581E-07	T/NK cell	RAB3GAP1
1.83985975024479E-11	0.489513400021861	0.137	0.217	3.08323696946022E-07	T/NK cell	PPP2R5E
1.88409206076953E-11	1.48712355207253	0.275	0.245	3.15736147543758E-07	T/NK cell	M6PR
1.89838614924391E-11	0.646646802763963	0.086	0.147	3.18131550890294E-07	T/NK cell	CLPX

1.92352804257943E-11	0.462079557016447	0.069	0.124	3.22344829375461E-07	T/NK cell	STARD7
2.0713043675327E-11	0.62755589983183	0.183	0.278	3.47109185911129E-07	T/NK cell	DNAJC7
2.10420045835088E-11	0.820261568999972	0.093	0.157	3.5262191281044E-07	T/NK cell	H3F3B
2.12552320328793E-11	0.279615199545577	0.057	0.108	3.56195178406991E-07	T/NK cell	PPIL3
2.1518776134736E-11	0.568451902793372	0.175	0.267	3.60611650465905E-07	T/NK cell	PITPNB
2.15207616691349E-11	0.574819963702876	0.146	0.229	3.60644924051362E-07	T/NK cell	SUSD3
2.16648445173073E-11	0.860231213379521	0.128	0.204	3.63059464421036E-07	T/NK cell	TADA2B
2.23498587202989E-11	0.613535296056491	0.18	0.274	3.74538932434768E-07	T/NK cell	SF3A2
2.24095091879258E-11	0.573311207769723	0.063	0.116	3.7553855497126E-07	T/NK cell	ACTR6
2.36296083802842E-11	0.276746362618829	0.062	0.114	3.95984977236802E-07	T/NK cell	JPT1
2.36710740801305E-11	0.799737610104426	0.113	0.185	3.96679859434828E-07	T/NK cell	SF3B2
2.38368547370748E-11	0.856170745337909	0.059	0.111	3.99458011683899E-07	T/NK cell	IQCB1
2.43558818992713E-11	1.98749859031328	0.12	0.083	4.08155868867988E-07	T/NK cell	ERI1
2.47426705517998E-11	0.429993677247235	0.094	0.157	4.14637673107062E-07	T/NK cell	BCL7C
2.48311396489528E-11	0.47134938495519	0.097	0.163	4.1612023823715E-07	T/NK cell	MGME1
2.50048197021912E-11	0.521806705264057	0.205	0.306	4.1903076856932E-07	T/NK cell	PPP4C
2.52619004929697E-11	0.449097977752861	0.072	0.128	4.23338928461186E-07	T/NK cell	PDSS2
2.60167590226216E-11	2.03731771450916	0.165	0.126	4.35988847701093E-07	T/NK cell	TMF1
2.68207771267277E-11	0.633938628087571	0.199	0.3	4.49462583089703E-07	T/NK cell	APRT
2.74871605758874E-11	0.323586511982553	0.052	0.1	4.60629836930721E-07	T/NK cell	BLOC1S6
2.74999032936099E-11	0.627319652732702	0.167	0.258	4.60843379394314E-07	T/NK cell	SMAD2
2.75385914079674E-11	1.92217524015334	0.21	0.175	4.61491714814717E-07	T/NK cell	KLF13
2.79093190261876E-11	1.02373001849091	0.098	0.164	4.67704368240852E-07	T/NK cell	INO80B
2.8212138295555E-11	0.778307615610863	0.125	0.2	4.7277901355691E-07	T/NK cell	RTF1
2.83568338337421E-11	0.431422752205574	0.087	0.149	4.7520382138585E-07	T/NK cell	PIGF
2.94076166987297E-11	0.801199359678186	0.118	0.19	4.92812840637313E-07	T/NK cell	AKAP8L
3.05659971804513E-11	0.574343980509946	0.059	0.111	5.12224980750002E-07	T/NK cell	KATNA1
3.15918778448928E-11	0.799052962986526	0.106	0.174	5.29416688924713E-07	T/NK cell	KRR1
3.21262745596716E-11	0.671687033613596	0.065	0.119	5.38372109070977E-07	T/NK cell	MOSMO
3.22815421467828E-11	0.900582093900921	0.051	0.1	5.40974083295785E-07	T/NK cell	RALGAPA1
3.30286944526046E-11	1.64220671312488	0.156	0.117	5.53494861636747E-07	T/NK cell	RALGDS
3.31137951653764E-11	0.506512986249204	0.133	0.211	5.54920979381378E-07	T/NK cell	KLHL2
3.33893421971069E-11	0.708899358771186	0.099	0.164	5.59538596539117E-07	T/NK cell	POLE
3.38794354531184E-11	0.901899591428451	0.067	0.121	5.67751579323357E-07	T/NK cell	ZPR1
3.42640926925659E-11	0.419875999633261	0.106	0.173	5.74197665342019E-07	T/NK cell	RUVBL1
3.47389438400885E-11	1.41767743308393	0.344	0.338	5.82155220872203E-07	T/NK cell	CCNI
3.60761321814101E-11	0.674730476886075	0.061	0.113	6.04563823096071E-07	T/NK cell	FBF1
3.78437946680279E-11	0.892471951801644	0.076	0.134	6.34186311046811E-07	T/NK cell	PCNX4
3.88332184617928E-11	2.1257042510047	0.216	0.182	6.50767074982724E-07	T/NK cell	TRIM14
3.98388661832318E-11	0.728871207234756	0.132	0.21	6.67619719498598E-07	T/NK cell	TULP4
4.021630969298E-11	0.62325815097548	0.159	0.247	6.73944917834959E-07	T/NK cell	CAND1
4.09315572204828E-11	0.498763551758024	0.086	0.146	6.8593103590085E-07	T/NK cell	ALYREF
4.1029821912133E-11	0.701199161003457	0.112	0.182	6.87577755603524E-07	T/NK cell	HDAC3
4.12608755821167E-11	1.08433227818197	0.056	0.106	6.91449753005112E-07	T/NK cell	NR1D1
4.16351887682468E-11	0.68465819252946	0.226	0.336	6.97722493378279E-07	T/NK cell	UBE2D3
4.27454631127714E-11	0.331916629447616	0.063	0.115	7.16328470843823E-07	T/NK cell	MED21
4.28807005563722E-11	0.688409022473015	0.078	0.136	7.18594779923686E-07	T/NK cell	XRCC6
4.34429493222874E-11	0.440429616267435	0.064	0.115	7.28016944742892E-07	T/NK cell	TOMM34
4.48933019535856E-11	0.675226789264864	0.062	0.114	7.52321954138187E-07	T/NK cell	PRELID3B
4.57623904585092E-11	1.87404242639653	0.194	0.156	7.66886139303697E-07	T/NK cell	PIEZO1
4.58786871947542E-11	0.687979696430796	0.233	0.345	7.68835040009691E-07	T/NK cell	TNIP1
4.65288880376714E-11	0.541557814302468	0.23	0.34	7.79731105735298E-07	T/NK cell	SMARCA4
4.75152917898692E-11	0.688082387743822	0.219	0.327	7.96261259814628E-07	T/NK cell	TOP 1.00
4.7557592215786E-11	0.831534051883435	0.178	0.274	7.96970130352141E-07	T/NK cell	TBCD
5.1352528852711E-11	0.524265646979028	0.331	0.476	8.60565678513731E-07	T/NK cell	DEPRECATED-ENSG00000132002
5.14538772711794E-11	0.730783759286818	0.063	0.114	8.62264075310425E-07	T/NK cell	ING5
5.25948856981694E-11	0.586470010946571	0.167	0.255	8.81385094529923E-07	T/NK cell	CIAO1
5.29456515553403E-11	0.800086166059599	0.054	0.103	8.87263228764393E-07	T/NK cell	FAM192A
5.5777495677061E-11	0.67563222360358	0.092	0.154	9.34719272556189E-07	T/NK cell	KAT8
5.59229207857436E-11	1.04395339576855	0.092	0.155	9.3715630652749E-07	T/NK cell	RIF1
5.70797298665679E-11	0.662549874971886	0.155	0.24	9.56542113103944E-07	T/NK cell	SUZ12
5.7546907304282E-11	0.848468660546087	0.073	0.129	9.64371072605157E-07	T/NK cell	BLOC1S4
5.79402014800726E-11	2.88086102189482	0.159	0.123	9.70961896403056E-07	T/NK cell	VPS37B

5.8153614095456E-11	0.390613310925153	0.082	0.141	9.74538265011652E-07	T/NK cell	PRKAG1
5.87709628996281E-11	0.2526123712977	0.101	0.166	9.84883796271967E-07	T/NK cell	TCTA
5.91155931279438E-11	0.859249107137835	0.073	0.128	9.90659109638083E-07	T/NK cell	DEPDC5
5.99091471738596E-11	0.510988509451079	0.209	0.311	1.00395748833954E-06	T/NK cell	ACTR1B
6.09806897151449E-11	1.42731624018551	0.35	0.347	1.0219143982464E-06	T/NK cell	STAT1
6.29195431285538E-11	1.29594560492658	0.348	0.339	1.0544057037483E-06	T/NK cell	APOL6
6.51577436457569E-11	0.304672895860599	0.244	0.357	1.09191346801559E-06	T/NK cell	RNF187
6.53758531096621E-11	0.43176404088802	0.141	0.219	1.09556854641172E-06	T/NK cell	PIN1
6.56567646583299E-11	1.03390723787779	0.069	0.123	1.10027606214429E-06	T/NK cell	CLASP2
6.75776326356865E-11	0.533486393803913	0.078	0.136	1.13246596770883E-06	T/NK cell	EDC3
6.76201441813173E-11	0.687295980113126	0.083	0.143	1.13317837619052E-06	T/NK cell	SPTLC1
7.2880653486211E-11	0.619078299432813	0.118	0.19	1.22133399112192E-06	T/NK cell	CDC123
7.49417358946508E-11	0.764396634627042	0.136	0.213	1.25587361012256E-06	T/NK cell	TBC1D20
7.6188864837692E-11	0.700087736641131	0.052	0.1	1.27677299695004E-06	T/NK cell	ZCCHC4
7.68256290093941E-11	0.400765573778648	0.229	0.332	1.28744389093943E-06	T/NK cell	WSB1
7.95120913940177E-11	0.360379062799644	0.057	0.106	1.33246362758095E-06	T/NK cell	ASH2L
7.98712030658753E-11	0.742647389529261	0.107	0.175	1.33848162097794E-06	T/NK cell	SMC5
8.01551445511006E-11	1.0588257183122	0.117	0.189	1.34323991238734E-06	T/NK cell	ALG12
8.19294120251745E-11	0.996394899132574	0.061	0.112	1.37297308671787E-06	T/NK cell	AGO4
8.34135166032476E-11	0.766142350641384	0.099	0.162	1.39784371123722E-06	T/NK cell	TPGS1
8.60131042378179E-11	0.478351326112174	0.062	0.112	1.44140760081735E-06	T/NK cell	CCT2
8.64387676261729E-11	1.06064427105654	0.092	0.154	1.4485408678794E-06	T/NK cell	SHPRH
8.95758166626137E-11	0.668586328898678	0.092	0.154	1.50111153563208E-06	T/NK cell	ZFX
9.73942430576199E-11	1.03850650910102	0.132	0.209	1.63213272515959E-06	T/NK cell	GOLT1B
9.80183415859444E-11	0.821485613768282	0.205	0.307	1.64259136829726E-06	T/NK cell	RAB21
9.97366492389129E-11	0.69525617578074	0.163	0.25	1.6713867679457E-06	T/NK cell	KHDRBS1
9.98544378521738E-11	0.647568547957966	0.081	0.139	1.67336066952673E-06	T/NK cell	TTC31
1.02460307918482E-10	0.602912456358308	0.182	0.274	1.71702984009792E-06	T/NK cell	CHURC1
1.02976766619476E-10	0.70925120643643	0.143	0.222	1.72568465500917E-06	T/NK cell	RANBP3
1.03117685047946E-10	0.464826190647113	0.073	0.127	1.72804616603347E-06	T/NK cell	DGCR8
1.03237779623934E-10	0.351629169878678	0.053	0.1	1.73005871093789E-06	T/NK cell	MANEA
1.03380006017468E-10	0.917257929937831	0.088	0.149	1.73244214084073E-06	T/NK cell	ZKSCAN8
1.040020861107E-10	2.01099339683298	0.183	0.148	1.74286695904311E-06	T/NK cell	TATDN2
1.05807539880239E-10	0.857965974480043	0.054	0.102	1.77312275331304E-06	T/NK cell	MBD5
1.10759109370099E-10	0.639707691187145	0.126	0.2	1.85610115482413E-06	T/NK cell	SNX19
1.11343939403714E-10	0.467269810868775	0.083	0.14	1.86590173652744E-06	T/NK cell	TRIM28
1.12242935999059E-10	0.799363093647631	0.076	0.131	1.88096712147223E-06	T/NK cell	SS18L2
1.13030870307748E-10	0.7307005922546	0.074	0.129	1.89417132461724E-06	T/NK cell	TBL3
1.14552811615355E-10	0.784177020128571	0.071	0.125	1.91967601705013E-06	T/NK cell	C5orf22
1.14902792221805E-10	0.919662395886724	0.191	0.292	1.925540992053E-06	T/NK cell	CDC73
1.16380941136833E-10	0.364123442128226	0.094	0.156	1.95031181157105E-06	T/NK cell	CLNS1A
1.16752917706273E-10	0.733298966243831	0.244	0.364	1.95654539492172E-06	T/NK cell	DHX15
1.24185763377626E-10	1.50104325294527	0.259	0.23	2.08110502268226E-06	T/NK cell	POLR2A
1.29501422299416E-10	1.08565919980213	0.112	0.18	2.17018483489361E-06	T/NK cell	ARMC8
1.33225114197854E-10	0.643499460903014	0.134	0.21	2.23258646372764E-06	T/NK cell	C6orf89
1.36162807028442E-10	1.23306550261084	0.4	0.414	2.28181632018264E-06	T/NK cell	SPTAN1
1.38535577451913E-10	0.421138769125577	0.056	0.104	2.32157920693915E-06	T/NK cell	ZFP90
1.38795843324415E-10	0.370815298078426	0.072	0.126	2.32594074243055E-06	T/NK cell	XPOT
1.39937752649135E-10	1.46871496586322	0.347	0.341	2.3450768588942E-06	T/NK cell	H1FX
1.4305309380001E-10	0.676527685478414	0.088	0.147	2.39728374590057E-06	T/NK cell	DROSHA
1.43766271095925E-10	0.582642621027635	0.213	0.315	2.40923517102551E-06	T/NK cell	FZR1
1.53187017552899E-10	0.504186810625098	0.146	0.225	2.56710804015148E-06	T/NK cell	TEX264
1.54484371175838E-10	0.49162812897507	0.1	0.163	2.58884909216469E-06	T/NK cell	DENND10
1.58758226797983E-10	0.794205213033541	0.073	0.128	2.66047036468059E-06	T/NK cell	FAM133B
1.62140622076933E-10	0.925601800267641	0.2	0.299	2.71715254476524E-06	T/NK cell	B4GALT1
1.6236749582028E-10	0.760901346822694	0.171	0.26	2.72095449495625E-06	T/NK cell	UBE4A
1.63830804568596E-10	0.772768338914631	0.145	0.224	2.74547662296053E-06	T/NK cell	GCN1
1.66116413900281E-10	0.823297923999195	0.063	0.113	2.78377886414091E-06	T/NK cell	SETD4
1.67382933465323E-10	0.528664198605772	0.057	0.105	2.80500319901189E-06	T/NK cell	TMA16
1.70580043006828E-10	0.709036495598008	0.103	0.167	2.85858036070842E-06	T/NK cell	MED1
1.71086984504343E-10	0.908161957506064	0.123	0.195	2.86707568632377E-06	T/NK cell	POLK
1.73222083429966E-10	0.503256259578817	0.077	0.132	2.90285567411938E-06	T/NK cell	UBE3B
1.77535684416766E-10	0.779587982624153	0.076	0.131	2.97514299945617E-06	T/NK cell	CSNK1G3

1.85241237349703E-10	0.650266323384073	0.068	0.12	3.10427265550632E-06	T/NK cell	BFAR
1.85814274161301E-10	0.27460931731598	0.062	0.112	3.11387560639508E-06	T/NK cell	PUS7L
1.90931556720414E-10	0.395639712904193	0.079	0.135	3.19963102752071E-06	T/NK cell	SSNA1
1.93335307643972E-10	0.942518990758192	0.134	0.209	3.23991308549768E-06	T/NK cell	CREBL2
1.93497658954984E-10	0.681399244774795	0.079	0.136	3.24263376876762E-06	T/NK cell	MTMR14
1.95641601967228E-10	1.88838806543342	0.211	0.18	3.2785619657668E-06	T/NK cell	KDM5A
1.96771706957611E-10	1.96906422985694	0.269	0.248	3.29750026519564E-06	T/NK cell	HIPK1
1.97370796060434E-10	0.514837145481385	0.169	0.257	3.30753980038075E-06	T/NK cell	TXLNA
2.00158465905417E-10	1.99594003906755	0.196	0.161	3.35425557164298E-06	T/NK cell	CORO7
2.05220591118335E-10	0.844766616631136	0.136	0.211	3.43908666596106E-06	T/NK cell	ISY1
2.19995065402543E-10	0.489189768795698	0.09	0.149	3.68667730601582E-06	T/NK cell	SPPL3
2.20737567208257E-10	2.01625570770661	0.115	0.081	3.69912015127597E-06	T/NK cell	SUPV3L1
2.27761798700336E-10	0.711959696973494	0.131	0.206	3.81683222262023E-06	T/NK cell	ASCC2
2.3850491596406E-10	0.730638248137265	0.144	0.223	3.99686538172572E-06	T/NK cell	PIP4K2B
2.38978097818471E-10	0.808744533400384	0.088	0.147	4.00479496324193E-06	T/NK cell	COG7
2.43176379037884E-10	0.519808272166414	0.118	0.187	4.07514975991686E-06	T/NK cell	AVL9
2.54317963307511E-10	0.512211228434567	0.07	0.122	4.26186042910727E-06	T/NK cell	PAIP2
2.56274084242627E-10	0.643215479752006	0.058	0.106	4.29464110373795E-06	T/NK cell	ZBTB45
2.56920988488096E-10	0.680592362726017	0.138	0.215	4.30548192508351E-06	T/NK cell	WWP2
2.69610116206091E-10	1.02297572582416	0.121	0.192	4.51812632738167E-06	T/NK cell	KHNYN
2.77452358538626E-10	0.551698184851542	0.216	0.321	4.64954662439029E-06	T/NK cell	CMTM6
2.7837028215296E-10	0.72619573394311	0.144	0.222	4.66492918831931E-06	T/NK cell	RIPK1
2.80193118643324E-10	0.576795293328176	0.113	0.181	4.69547628222482E-06	T/NK cell	STRN
2.88240664927392E-10	1.86428272309297	0.253	0.228	4.83033706285324E-06	T/NK cell	RNF44
2.92295952394726E-10	0.307746341698665	0.145	0.221	4.89829557023082E-06	T/NK cell	TMEM250
2.99028706148447E-10	1.67517345068327	0.287	0.266	5.01112305763568E-06	T/NK cell	PNISR
3.11553680122175E-10	1.18863126347949	0.149	0.231	5.22101657148741E-06	T/NK cell	SRSF7
3.19460433004792E-10	0.866166150989356	0.164	0.252	5.3535179362943E-06	T/NK cell	METTL17
3.23634654767365E-10	1.01494443618623	0.098	0.161	5.4234695445915E-06	T/NK cell	VPS13A
3.25055488343894E-10	0.679028589234006	0.126	0.197	5.44727987366698E-06	T/NK cell	GTF2F1
3.2610326526645E-10	0.532636344536711	0.255	0.374	5.46483851933516E-06	T/NK cell	HNRNPK
3.32735878222339E-10	0.86738983086666	0.157	0.24	5.57598784724995E-06	T/NK cell	GIGYF1
3.3400380099513E-10	0.861196594240996	0.213	0.317	5.5972356970764E-06	T/NK cell	TMEM245
3.39418874571003E-10	0.6994467866303	0.082	0.139	5.68798150006086E-06	T/NK cell	THOC3
3.42130046821595E-10	1.11000101227801	0.085	0.142	5.73341532463629E-06	T/NK cell	STX2
3.46445357041896E-10	0.846411272384696	0.093	0.153	5.8057312933081E-06	T/NK cell	YAF2
3.5990790223731E-10	0.778430040052555	0.126	0.198	6.03133662569284E-06	T/NK cell	SPOP
3.65308265236907E-10	0.900973908896793	0.165	0.251	6.12183590884008E-06	T/NK cell	GRIPAP1
3.78902399248726E-10	0.619322461977848	0.075	0.128	6.34964640661016E-06	T/NK cell	CASP7
3.83954541786687E-10	0.774152911169023	0.139	0.215	6.43431021126131E-06	T/NK cell	CUL3
3.84469117762227E-10	0.485232295368235	0.065	0.114	6.4429334754594E-06	T/NK cell	BAP1
4.09511218386028E-10	0.526629673699583	0.319	0.459	6.86258899771305E-06	T/NK cell	EEF1G
4.39528373994905E-10	0.513764906760394	0.192	0.287	7.36561649140662E-06	T/NK cell	MAST4
4.43117956180683E-10	0.500969005483604	0.253	0.369	7.42577070967589E-06	T/NK cell	NONO
4.43523116173488E-10	0.673075785785001	0.089	0.147	7.43256038083531E-06	T/NK cell	SIKE1
4.5358494137562E-10	2.23700813659505	0.188	0.157	7.60117644757264E-06	T/NK cell	DEPRECATED-ENSG00000135976
4.55204760783846E-10	0.701055097620398	0.072	0.124	7.6283213812157E-06	T/NK cell	CSTF1
4.56742996370891E-10	0.70156116415386	0.062	0.111	7.65409913318339E-06	T/NK cell	MINDY3
4.58072184713137E-10	0.358226284713664	0.055	0.1	7.67637367142275E-06	T/NK cell	MED11
4.59303206214162E-10	0.86499731368604	0.133	0.206	7.69700312973693E-06	T/NK cell	ATG16L2
4.6388642419844E-10	0.900543504644047	0.124	0.194	7.77380869671745E-06	T/NK cell	TM2D3
4.66561897581264E-10	0.416501914802129	0.106	0.168	7.81864427966682E-06	T/NK cell	MZT2A
4.67288650847612E-10	1.02810041646933	0.133	0.207	7.83082321090427E-06	T/NK cell	MED23
4.70585223586491E-10	0.712780780537437	0.1	0.161	7.88606717686242E-06	T/NK cell	KLHL36
4.72558698776559E-10	1.40826767936343	0.097	0.16	7.91913867409758E-06	T/NK cell	NOP9
4.73058808009553E-10	0.712004981962399	0.14	0.215	7.9275195046241E-06	T/NK cell	PDCD10
4.73558360930086E-10	0.577823751463008	0.17	0.255	7.93589101246638E-06	T/NK cell	ATF4
4.74378405653673E-10	0.35491749164706	0.063	0.112	7.94963332194426E-06	T/NK cell	RRAGC
4.77671450062781E-10	0.650463469047751	0.063	0.112	8.00481816015209E-06	T/NK cell	FAM102B
4.86810943549298E-10	0.701623007550149	0.133	0.206	8.15797779199913E-06	T/NK cell	EIF4G3
4.92168851127991E-10	0.708343919181309	0.074	0.126	8.24776560720287E-06	T/NK cell	RAE1
4.94371365195292E-10	1.92965125623805	0.193	0.161	8.2846753379427E-06	T/NK cell	KIF2A
5.04976325622301E-10	0.599658413978367	0.083	0.137	8.46239326477853E-06	T/NK cell	PSMB6

5.09293787141877E-10	0.623960715410015	0.076	0.129	8.53474528492358E-06	T/NK cell	DERL2
5.45611983311788E-10	0.942685824452126	0.059	0.107	9.14336561633895E-06	T/NK cell	TRMT13
5.54809152657204E-10	0.929848672586806	0.147	0.225	9.29749178022943E-06	T/NK cell	TBC1D15
5.60522785190221E-10	0.428425604808901	0.194	0.286	9.39324083421773E-06	T/NK cell	MCM5
5.89081215454836E-10	0.620036157943091	0.229	0.335	9.87182300859214E-06	T/NK cell	ZMIZ2
5.9248648177093E-10	0.929522513003621	0.067	0.116	9.92888846151725E-06	T/NK cell	TLK2
5.94193976959721E-10	0.970025775310356	0.154	0.235	9.95750266589101E-06	T/NK cell	JMJD6
5.96389938314211E-10	0.696247390328641	0.067	0.117	9.99430258626955E-06	T/NK cell	RIOK1
6.10850705440558E-10	0.439551394282768	0.07	0.12	1.02366361217729E-05	T/NK cell	DEPRECATED-ENSG00000176101
6.1386751957488E-10	0.649375476309068	0.088	0.146	1.02871918930358E-05	T/NK cell	PIP4K2C
6.20493833634211E-10	0.592502563642489	0.089	0.146	1.03982356640421E-05	T/NK cell	POM121
6.5496961742734E-10	0.377066846387291	0.062	0.11	1.09759808488474E-05	T/NK cell	AMD 1.00
6.74804096930787E-10	0.602794595708853	0.065	0.115	1.13083670563661E-05	T/NK cell	NSUN3
6.80115114347313E-10	0.732776952784593	0.056	0.102	1.13973690862323E-05	T/NK cell	CAPS
6.9602097005619E-10	0.91015572052762	0.186	0.28	1.16639194162016E-05	T/NK cell	TPP2
7.06663109600962E-10	1.08129313473645	0.091	0.149	1.18422603906929E-05	T/NK cell	CLIC5
7.21227688109417E-10	0.543187012618537	0.07	0.12	1.20863335973376E-05	T/NK cell	FTSJ3
7.29739108999193E-10	0.78906875870004	0.128	0.2	1.22289679886085E-05	T/NK cell	WBP1
7.59070017006032E-10	0.619700163806613	0.095	0.154	1.27204953449871E-05	T/NK cell	SLC12A9
7.75873596649705E-10	0.655776635205875	0.063	0.112	1.30020897326558E-05	T/NK cell	MFSD4B
7.9141876758822E-10	0.607907673882282	0.137	0.21	1.32625957072434E-05	T/NK cell	IBTK
7.98010994305555E-10	0.574298559405493	0.065	0.113	1.33730682425725E-05	T/NK cell	MCM8
8.06959681086831E-10	0.693134557600869	0.063	0.111	1.35230303356531E-05	T/NK cell	CNOT3
8.13729530100777E-10	0.499525893024728	0.228	0.332	1.36364794654288E-05	T/NK cell	CTDSP1
8.2921171690363E-10	1.80421399151217	0.249	0.226	1.3895929951871E-05	T/NK cell	ATXN1
8.31017080417079E-10	0.709828006186504	0.122	0.191	1.39261842336294E-05	T/NK cell	SF1
8.63072397527517E-10	0.624014541051901	0.07	0.12	1.44633672377661E-05	T/NK cell	ZFYVE1
8.66858135209148E-10	1.13849787012981	0.137	0.214	1.45268086298349E-05	T/NK cell	CBFA2T2
8.67817622440385E-10	0.589753559032484	0.068	0.118	1.4542887716856E-05	T/NK cell	COQ10B
8.94183158698575E-10	0.525286620371551	0.2	0.293	1.49847213734707E-05	T/NK cell	DDB1
8.94519064025418E-10	0.609709902473494	0.173	0.259	1.4990350474938E-05	T/NK cell	UBA1
9.16549855519444E-10	2.13364333626914	0.134	0.1	1.53595424787948E-05	T/NK cell	ELMO2
9.19119027470638E-10	0.831177475984564	0.072	0.124	1.5402596662353E-05	T/NK cell	RNF121
9.34138995984761E-10	0.630445208335425	0.078	0.132	1.56543012947126E-05	T/NK cell	PIK3C2A
9.36118865109114E-10	0.685362171757527	0.058	0.104	1.56874799414985E-05	T/NK cell	RIT1
9.50388104893281E-10	0.870265878102498	0.077	0.13	1.59266038618016E-05	T/NK cell	BICRA
9.73352169187629E-10	0.697577278775672	0.077	0.129	1.63114356512463E-05	T/NK cell	TMEM209
1.05592659781322E-09	1.00021444387747	0.059	0.105	1.76952179261539E-05	T/NK cell	GMEB2
1.05830405576563E-09	1.05298039661512	0.1	0.161	1.77350593665204E-05	T/NK cell	PRKAR2A
1.09864944265452E-09	2.45643823512301	0.113	0.081	1.84111673600045E-05	T/NK cell	TNRC6C
1.12576067545253E-09	0.546509444064176	0.074	0.125	1.88654973992334E-05	T/NK cell	TTI1
1.15482148347143E-09	0.622199607450782	0.118	0.183	1.93524984200142E-05	T/NK cell	NAA38
1.16006533479815E-09	0.771511416663001	0.117	0.185	1.94403748805475E-05	T/NK cell	PIGG
1.174151822082E-09	1.04313151541555	0.101	0.162	1.96764362344502E-05	T/NK cell	NIPA2
1.18664194304919E-09	2.34884550284696	0.253	0.233	1.98857456816183E-05	T/NK cell	CLDND1
1.22921704873865E-09	1.16641299534366	0.071	0.121	2.05992193027622E-05	T/NK cell	SSBP1
1.26078108670154E-09	1.03089576716776	0.162	0.247	2.11281694509444E-05	T/NK cell	TASOR2
1.26396183984666E-09	2.11927157724307	0.134	0.1	2.11814725121504E-05	T/NK cell	TMEM243
1.29906366532727E-09	0.767550066959486	0.075	0.126	2.17697089035545E-05	T/NK cell	WDR43
1.29943561179005E-09	0.677533866145309	0.151	0.227	2.17759419823776E-05	T/NK cell	INO80E
1.31672300726285E-09	0.907398648523218	0.124	0.19	2.20656441557108E-05	T/NK cell	ZC3H12A
1.31695647119656E-09	0.622448060305091	0.076	0.127	2.2069556544312E-05	T/NK cell	HEXD
1.3438320123748E-09	0.767757608506516	0.126	0.195	2.25199368633769E-05	T/NK cell	ILKAP
1.37569809673515E-09	0.667542406776574	0.116	0.182	2.30539487050877E-05	T/NK cell	WASHC4
1.38642609289576E-09	0.686445421407881	0.17	0.251	2.32337284647472E-05	T/NK cell	CIC
1.3899047126824E-09	0.75884024909876	0.095	0.154	2.32920231751317E-05	T/NK cell	XPO5
1.40954508095852E-09	0.75497748371419	0.059	0.105	2.36211564667029E-05	T/NK cell	SLC38A9
1.44695127467082E-09	0.549146131924314	0.089	0.146	2.42480094609336E-05	T/NK cell	GDE1
1.44745187424027E-09	1.08102906214446	0.154	0.234	2.42563985085185E-05	T/NK cell	PTAR1
1.45035516943294E-09	1.67413177775068	0.214	0.183	2.43050519293572E-05	T/NK cell	ARPC5L
1.48563799270953E-09	0.980121108745744	0.059	0.105	2.48963214818263E-05	T/NK cell	PDK3
1.49314811205622E-09	0.817489849955985	0.088	0.145	2.50221760618381E-05	T/NK cell	C3orf38
1.54398594951044E-09	0.825189076931101	0.085	0.14	2.58741165418959E-05	T/NK cell	SAP130

1.54660369263328E-09	0.793201303144505	0.108	0.17	2.59179846811485E-05	T/NK cell	FOXK2
1.55558203652787E-09	0.708884486644771	0.088	0.144	2.60684437681341E-05	T/NK cell	DDX18
1.58291088685202E-09	0.859771296336205	0.117	0.182	2.65264206418661E-05	T/NK cell	MAP2K7
1.63070750323081E-09	0.776979820188954	0.133	0.203	2.73273963391419E-05	T/NK cell	DDX54
1.63834114692842E-09	0.829693270756793	0.094	0.151	2.74553209402264E-05	T/NK cell	GNB5
1.68827782033613E-09	0.605565295470371	0.333	0.473	2.82921597131929E-05	T/NK cell	SERINC1
1.78116011312706E-09	1.38870947781183	0.08	0.133	2.98486811757833E-05	T/NK cell	AP3M2
1.85985148447895E-09	0.735168637805576	0.205	0.301	3.11673911768982E-05	T/NK cell	UBE2Z
1.8627953464795E-09	0.98486267354653	0.117	0.182	3.12167244163035E-05	T/NK cell	MTO1
1.87284129483173E-09	1.09083301333534	0.114	0.179	3.13850744187901E-05	T/NK cell	UNK
1.89178317959783E-09	0.856189195577561	0.115	0.18	3.17025025237004E-05	T/NK cell	RALBP1
1.89516966454495E-09	0.351343097985623	0.092	0.149	3.17592532384443E-05	T/NK cell	URM1
1.94490839889018E-09	0.77571091069511	0.122	0.183	3.25927749486017E-05	T/NK cell	HIST1H1B
1.95701506759488E-09	0.933059968145578	0.073	0.124	3.27956585027549E-05	T/NK cell	PEX26
1.95757206011095E-09	0.589533607235648	0.066	0.113	3.28049925833393E-05	T/NK cell	FBXO30
1.98143279838284E-09	0.73313201096756	0.117	0.181	3.32048508352997E-05	T/NK cell	FYTTD1
1.99818409295643E-09	0.666276688038687	0.233	0.339	3.34855690297639E-05	T/NK cell	SPG7
2.02935845338714E-09	2.88082220726093	0.141	0.109	3.40079889618617E-05	T/NK cell	PDE4D
2.03416210788507E-09	1.02013828576859	0.11	0.173	3.4088488603938E-05	T/NK cell	AK1
2.0683660540948E-09	2.06350337584606	0.224	0.196	3.46616783345206E-05	T/NK cell	STAT5B
2.16600792963264E-09	0.873361856428488	0.082	0.134	3.62979608847838E-05	T/NK cell	TRIM5
2.18755546548417E-09	0.803237709533763	0.123	0.191	3.66590544905836E-05	T/NK cell	TMEM165
2.25635025060965E-09	0.765106283293372	0.136	0.208	3.78119174997164E-05	T/NK cell	ZNF384
2.281391550098E-09	0.503116357689881	0.085	0.14	3.82315595965423E-05	T/NK cell	CMTR2
2.39442151380043E-09	0.548396319886129	0.253	0.364	4.01257157282676E-05	T/NK cell	BIN1
2.41596772179059E-09	0.412029489320145	0.138	0.208	4.04867870817667E-05	T/NK cell	STIP1
2.43196385595729E-09	0.837895327281142	0.098	0.157	4.07548502981322E-05	T/NK cell	EEF1AKMT2
2.46911035054608E-09	0.888465909699213	0.093	0.15	4.13773512544512E-05	T/NK cell	GTF2E2
2.47614485158921E-09	0.758782839219821	0.071	0.12	4.14952354229319E-05	T/NK cell	ZNF236
2.49623743914708E-09	1.03374027247722	0.13	0.199	4.18319470052268E-05	T/NK cell	EXOSC9
2.54081109965156E-09	0.911592921104789	0.06	0.106	4.25789124079608E-05	T/NK cell	WDR75
2.54679607543376E-09	1.00167238199121	0.056	0.1	4.2679208632119E-05	T/NK cell	THAP6
2.55317366346897E-09	0.673943306151703	0.15	0.225	4.2786084252413E-05	T/NK cell	YKT6
2.55891293083596E-09	0.677066459633653	0.126	0.088	4.2882262894949E-05	T/NK cell	SH3BP5
2.59679468418087E-09	0.664518253324633	0.308	0.443	4.3517085317503E-05	T/NK cell	SNX1
2.6149468404104E-09	0.869494981242826	0.103	0.162	4.38212791515975E-05	T/NK cell	PITHD1
2.66023758581571E-09	1.08015608686784	0.095	0.152	4.45802614630997E-05	T/NK cell	XPNPEP1
2.67614056465638E-09	0.250429594923424	0.258	0.368	4.48467635825115E-05	T/NK cell	EID1
2.69587508285838E-09	0.761465241694361	0.154	0.23	4.51774746385407E-05	T/NK cell	ANP32E
2.71895218023625E-09	0.801367573206648	0.122	0.186	4.5564200636399E-05	T/NK cell	TAF6
2.79340910343565E-09	1.12549031772665	0.063	0.11	4.68119497553747E-05	T/NK cell	ATG14
2.82758063810086E-09	0.938970527862283	0.108	0.171	4.73845963332942E-05	T/NK cell	MBP
2.88581615627059E-09	0.836927593197731	0.08	0.132	4.83605071467826E-05	T/NK cell	TMEM126B
2.89687702250999E-09	0.786977094817626	0.093	0.15	4.85458651432223E-05	T/NK cell	C11orf68
2.91672257551396E-09	0.517492344606021	0.09	0.145	4.8878436920463E-05	T/NK cell	CAPRIN1
3.03416388027581E-09	0.485484323371063	0.093	0.149	5.0846518305662E-05	T/NK cell	FEM1B
3.08467671475978E-09	0.872969818222826	0.075	0.125	5.16930123859444E-05	T/NK cell	DEPRECATED-ENSG00000164164
3.17244536710432E-09	0.26752277353769	0.163	0.24	5.31638394619342E-05	T/NK cell	TRIM8
3.25926111569895E-09	0.797825592380996	0.064	0.11	5.46186977768831E-05	T/NK cell	ZC3H11A
3.30753719556071E-09	0.790251483926191	0.057	0.101	5.54277083232064E-05	T/NK cell	RNF41
3.33322137527487E-09	0.804699720597356	0.076	0.127	5.58581238068562E-05	T/NK cell	SMYD3
3.39118981203535E-09	0.489637134339427	0.286	0.406	5.68295588700883E-05	T/NK cell	STAU2
3.42950694146054E-09	0.677292334492778	0.257	0.372	5.74716773249958E-05	T/NK cell	PDCD6IP
3.5815815235364E-09	0.621478161393877	0.137	0.209	6.0020143171423E-05	T/NK cell	TMEM9B
3.63710210894742E-09	0.815983661189597	0.072	0.121	6.09505571417408E-05	T/NK cell	KRAS
3.84167903106947E-09	0.542697048702032	0.069	0.117	6.43788572026622E-05	T/NK cell	TAB1
3.91209902537739E-09	0.910253277073681	0.121	0.186	6.55589554672743E-05	T/NK cell	PSIP1
3.93933061586898E-09	0.714712972039596	0.071	0.119	6.60153024607324E-05	T/NK cell	FXR2
3.94480736637085E-09	0.855502441079326	0.071	0.119	6.61070818456427E-05	T/NK cell	HMGXB3
4.03136053710451E-09	0.696665558056649	0.201	0.296	6.75575398807974E-05	T/NK cell	PPP4R3B
4.11910541543903E-09	0.888384901158259	0.113	0.176	6.90279685519273E-05	T/NK cell	NIPA1
4.12179788953888E-09	0.915455328416249	0.106	0.167	6.90730890328925E-05	T/NK cell	EXOC8
4.27493279073114E-09	1.8306778918973	0.234	0.209	7.16393237070724E-05	T/NK cell	PRMT2

4.29777828444969E-09	0.786449340557828	0.086	0.141	7.20221684908078E-05	T/NK cell	ZNF700
4.32265159243571E-09	0.896059330676295	0.063	0.109	7.24389953860376E-05	T/NK cell	SMU1
4.34315676975016E-09	0.950670406492391	0.115	0.179	7.27826211474732E-05	T/NK cell	RBM4
4.34933556543259E-09	0.485694750791107	0.268	0.382	7.28861654055193E-05	T/NK cell	ERO1A
4.39188544659248E-09	0.537966084447985	0.3	0.427	7.35992163139968E-05	T/NK cell	SETD5
4.42046262270301E-09	0.563365255569147	0.098	0.154	7.40781126312571E-05	T/NK cell	PLEKHM2
4.43131987566576E-09	0.833409946295111	0.085	0.139	7.42600584764069E-05	T/NK cell	PCBP2
4.46584356013307E-09	1.22594615312827	0.376	0.387	7.483860638071E-05	T/NK cell	CAPZB
4.49819066251917E-09	0.854293714913817	0.091	0.147	7.53806791224962E-05	T/NK cell	SPAG7
4.53706983098226E-09	0.730701472041485	0.168	0.247	7.60322162276007E-05	T/NK cell	CBX4
4.5382594246241E-09	0.688837008470389	0.194	0.284	7.60521514378506E-05	T/NK cell	EWSR1
4.65171648336693E-09	0.841930369842929	0.088	0.142	7.79534648282629E-05	T/NK cell	WDR76
4.6834271197034E-09	0.883504250807326	0.081	0.134	7.84848716719895E-05	T/NK cell	CEP44
4.69790998579119E-09	0.715660544486104	0.171	0.254	7.87275755418888E-05	T/NK cell	ZSWIM8
4.77990117952626E-09	0.649904048139458	0.13	0.197	8.01015839665011E-05	T/NK cell	CASP2
4.82472594173785E-09	0.841936042334818	0.104	0.163	8.08527573316428E-05	T/NK cell	ZC3H7A
4.87150069406416E-09	1.75281415992492	0.254	0.233	8.16366086311272E-05	T/NK cell	USP34
4.88224433431067E-09	0.882812146422605	0.098	0.157	8.18166505543783E-05	T/NK cell	STYX
4.91126711321667E-09	0.813919747438073	0.539	0.62	8.2303014283285E-05	T/NK cell	HSPA8
4.92806476622826E-09	0.700132270875322	0.157	0.234	8.25845093524532E-05	T/NK cell	NUDT21
5.01901136812368E-09	0.864338128559866	0.06	0.105	8.41085925070166E-05	T/NK cell	CIAO3
5.05405316738042E-09	0.502646737328044	0.082	0.134	8.4695822978961E-05	T/NK cell	RAB29
5.11911473981834E-09	1.14871391179608	0.347	0.346	8.57861248098758E-05	T/NK cell	EHBP1L1
5.15858779991132E-09	0.862871225204992	0.106	0.167	8.6447614350914E-05	T/NK cell	NSD2
5.19211229811682E-09	0.913474852273105	0.097	0.154	8.70094178918416E-05	T/NK cell	NMRK1
5.23328746605758E-09	0.251338383295642	0.061	0.105	8.76994313561929E-05	T/NK cell	SLC25A17
5.45507085096954E-09	0.600125166621154	0.094	0.15	9.14160773205475E-05	T/NK cell	OGG1
5.4685923246193E-09	0.74898828027691	0.161	0.241	9.16426701759702E-05	T/NK cell	DICER1
5.56519255865015E-09	0.344881033162064	0.057	0.1	9.32614968978593E-05	T/NK cell	VANGL1
5.63685873792823E-09	1.63938285131945	0.253	0.231	9.44624787302013E-05	T/NK cell	AIP
5.68775852395245E-09	0.465930185341821	0.263	0.371	9.53154573443951E-05	T/NK cell	MDM2
6.04486596967695E-09	0.454613082708275	0.058	0.101	0.000101299863919846	T/NK cell	NCOA5
6.06592743864273E-09	1.07021544276981	0.062	0.107	0.000101652812016775	T/NK cell	SLC4A1AP
6.12941401277122E-09	0.994310788835664	0.116	0.182	0.00010271672002602	T/NK cell	KRIT1
6.15161634382992E-09	1.06640934189921	0.126	0.193	0.000103088786689902	T/NK cell	TOX4
6.18543689691176E-09	0.677494281940163	0.248	0.356	0.000103655551518447	T/NK cell	TMEM248
6.26125284954963E-09	1.66527596415581	0.24	0.217	0.000104926075252753	T/NK cell	NFAT5
6.27034866563494E-09	0.808929350255574	0.067	0.113	0.00010507850293871	T/NK cell	RELB
6.63494531569558E-09	0.434380977113572	0.246	0.351	0.000111188413600427	T/NK cell	PRKDC
6.73357114740786E-09	2.04458056251597	0.161	0.129	0.000112841185288261	T/NK cell	ZDHHC18
6.86434950563631E-09	0.66061643140671	0.141	0.211	0.000115032769015453	T/NK cell	IRF7
6.91247578846788E-09	0.862181861389013	0.165	0.245	0.000115839269263145	T/NK cell	KDM3B
6.98754072766606E-09	1.103941731499	0.133	0.202	0.000117097207514228	T/NK cell	LDLRAP1
6.99908582381616E-09	0.789397299756944	0.075	0.123	0.000117290680235511	T/NK cell	PRMT7
7.33448632631859E-09	0.815964022709773	0.059	0.103	0.000122911321856447	T/NK cell	PPM1D
7.47270546439842E-09	0.762809291999027	0.063	0.107	0.000125227598172389	T/NK cell	LRSAM1
7.50557683458187E-09	1.44356986742692	0.287	0.267	0.000125778456593923	T/NK cell	AKIRIN2
7.53388752865186E-09	0.791884705773533	0.119	0.183	0.000126252887205148	T/NK cell	MAGEH1
7.75301826650523E-09	0.771069972552078	0.097	0.154	0.000129925080110095	T/NK cell	TRMT2A
7.82388135851829E-09	1.01112377894487	0.067	0.113	0.000131112603806049	T/NK cell	ATG4D
7.87088237502172E-09	1.806989474443	0.211	0.184	0.000131900246840614	T/NK cell	PPP1R12A
7.99622181598407E-09	2.06056498320626	0.113	0.082	0.000134000685192261	T/NK cell	GABPB1
8.08197874561094E-09	0.67238562033136	0.09	0.143	0.000135437799818948	T/NK cell	LSM 1.00
8.12810820221878E-09	0.601908816502162	0.067	0.112	0.000136210837252782	T/NK cell	RAB11A
8.19999784625348E-09	0.476767640264663	0.076	0.124	0.000137415563907516	T/NK cell	STAMBP
8.34942339336288E-09	0.79324909162168	0.125	0.191	0.000139919637225975	T/NK cell	GALNS
8.49693437164598E-09	2.12318139916214	0.146	0.114	0.000142391626200043	T/NK cell	TRANK1
8.50096827333114E-09	0.412287721158616	0.206	0.301	0.000142459226324483	T/NK cell	ITGB1
8.62339222365082E-09	0.773367444474902	0.173	0.257	0.00014451080688394	T/NK cell	UBA2
8.82186728611471E-09	0.742857620209969	0.089	0.142	0.00014783685198071	T/NK cell	WTAP
8.99144230244422E-09	1.1268335517341	0.111	0.172	0.00015067859010436	T/NK cell	UBAP1
9.1539397409948E-09	0.851618541339885	0.154	0.232	0.000153401722179591	T/NK cell	MON2
9.18084532816133E-09	1.67102117501364	0.303	0.297	0.000153852606009328	T/NK cell	PAFAH1B1

9.36089502295932E-09	0.811631485369938	0.085	0.137	0.000156869878794752	T/NK cell	POLH
9.59795390400405E-09	0.964294329902162	0.083	0.134	0.0001608425115233	T/NK cell	LYSMD2
9.65151102783565E-09	0.920552796200583	0.121	0.185	0.00016174002180447	T/NK cell	ZNF121
1.02444444655583E-08	0.753556207487662	0.136	0.204	0.000171676400353826	T/NK cell	GNL3L
1.03260343870947E-08	0.953016298296368	0.08	0.131	0.000173043684258933	T/NK cell	ZRANB1
1.03886181361263E-08	0.784435620730122	0.088	0.141	0.000174092462725204	T/NK cell	PDCL
1.03927581841054E-08	0.778981467870057	0.073	0.121	0.000174161841649238	T/NK cell	RIC8A
1.06318136804353E-08	0.726812802332164	0.142	0.213	0.000178167933656734	T/NK cell	HNRNPR
1.08368668790367E-08	1.04855072793524	0.11	0.171	0.000181604215158898	T/NK cell	TMEM168
1.08682019523717E-08	2.28054854087825	0.101	0.071	0.000182129328317846	T/NK cell	KCTD7
1.09696151644878E-08	0.538018920427609	0.078	0.127	0.000183828810926487	T/NK cell	DNAJC10
1.13021571061013E-08	0.74268105172404	0.069	0.116	0.000189401548784046	T/NK cell	RABGEF1
1.13719207247202E-08	0.776159607923041	0.19	0.278	0.000190570647504862	T/NK cell	CPSF7
1.14327857805762E-08	0.907877585120601	0.188	0.278	0.000191590624110896	T/NK cell	LRCH3
1.17228242192242E-08	0.723728612167096	0.063	0.107	0.000196451088265759	T/NK cell	MEN1
1.17641885663961E-08	0.898169264793531	0.073	0.121	0.000197144271995666	T/NK cell	ZNF33B
1.17821664382021E-08	0.990418990120715	0.111	0.174	0.000197445545171391	T/NK cell	TBCK
1.18410845360367E-08	0.520989427311036	0.073	0.12	0.000198432894654902	T/NK cell	OXLD1
1.19045726257588E-08	1.19195477989046	0.137	0.205	0.000199496828062466	T/NK cell	SORL1
1.19753225239246E-08	0.626029803345252	0.185	0.27	0.000200682454855928	T/NK cell	EXOC3
1.24513451201422E-08	0.480160377361219	0.058	0.101	0.000208659641523344	T/NK cell	HELQ
1.26962530942038E-08	0.653263189543463	0.194	0.278	0.000212763809352668	T/NK cell	IER2
1.27458961488862E-08	1.90545603564148	0.152	0.12	0.000213595727663035	T/NK cell	ZNF292
1.34366651884956E-08	0.470417372065836	0.063	0.106	0.000225171635228809	T/NK cell	ZNF274
1.37642899961481E-08	0.871997677568566	0.147	0.218	0.00023066197175545	T/NK cell	DEPRECATED-ENSG00000205937
1.44017012673423E-08	0.633614573646879	0.072	0.119	0.000241343709838122	T/NK cell	C16orf87
1.45105635612471E-08	2.01331675959222	0.13	0.098	0.000243168024159379	T/NK cell	POLG
1.46027683009303E-08	0.881751295202752	0.087	0.14	0.00024471319118699	T/NK cell	STRIP1
1.49713441815073E-08	1.12837355226778	0.138	0.208	0.000250889785793699	T/NK cell	PRPF39
1.54086085533272E-08	1.76929593574792	0.166	0.134	0.000258217462136657	T/NK cell	ZNF609
1.54925525314215E-08	1.73141488214166	0.137	0.105	0.000259624195321562	T/NK cell	USP4
1.62035168675106E-08	1.98157895430807	0.138	0.106	0.000271538535665742	T/NK cell	NPEPL1
1.67314339651769E-08	0.942124791756687	0.081	0.131	0.000280385370388435	T/NK cell	DEPRECATED-ENSG00000093000
1.69076675736416E-08	0.852887738681344	0.114	0.174	0.000283338693199087	T/NK cell	RBM27
1.69473293361331E-08	0.838539235124071	0.089	0.142	0.000284003345014919	T/NK cell	C1GALT1
1.71581070718624E-08	1.87261069515044	0.186	0.157	0.00028753555831027	T/NK cell	CSGALNACT2
1.72561851047771E-08	0.789157968574134	0.23	0.332	0.000289179149985855	T/NK cell	PPP3R1
1.80932520931869E-08	0.793322605870693	0.079	0.127	0.000303206718577626	T/NK cell	LTA4H
1.82101125270831E-08	0.812677743823475	0.068	0.112	0.000305165065728859	T/NK cell	FAM53C
1.82783527046577E-08	2.12056202241431	0.182	0.153	0.000306308634624654	T/NK cell	TANK
1.83896070906668E-08	0.981025260193409	0.178	0.262	0.000308173035625394	T/NK cell	ERCC5
1.88272283288962E-08	0.861838419139889	0.185	0.27	0.000315506692335643	T/NK cell	PRPF40A
1.89591499944232E-08	0.759753989774359	0.11	0.168	0.000317717435606543	T/NK cell	DNAJB12
1.90323776776913E-08	0.865438645606241	0.105	0.162	0.000318944585122751	T/NK cell	SFT2D1
1.91048298531567E-08	1.5917424707887	0.283	0.271	0.000320158738679201	T/NK cell	RAP1A
1.92151913965271E-08	0.525291442093363	0.085	0.135	0.000322008177423	T/NK cell	VWA8
1.96937888277747E-08	0.610558957141146	0.125	0.189	0.000330028513175848	T/NK cell	VOPP1
2.11805421536831E-08	0.784586139165501	0.169	0.248	0.000354943525411421	T/NK cell	NAB1
2.16800675513313E-08	0.635967225481103	0.084	0.135	0.00036331457202521	T/NK cell	MAPRE1
2.22797963043498E-08	1.67690073194544	0.324	0.33	0.000373364826468293	T/NK cell	CD47
2.28250526406635E-08	0.986619393848445	0.181	0.265	0.000382502232152238	T/NK cell	RBM14
2.31910822393939E-08	0.874852273452733	0.083	0.134	0.000388636156167763	T/NK cell	TM2D1
2.33017498942782E-08	0.975548237796556	0.096	0.15	0.000390490724728314	T/NK cell	SIRT7
2.42270684473791E-08	1.08197566255272	0.138	0.206	0.000405997213041179	T/NK cell	STARD3
2.4386010591752E-08	0.746941420416506	0.109	0.167	0.000408660765496581	T/NK cell	CSNK1A1
2.46489188076929E-08	1.0202987561185	0.119	0.181	0.000413066581379318	T/NK cell	ABCB8
2.55559016907862E-08	0.694417138280422	0.13	0.196	0.000428265800534195	T/NK cell	EIF2AK2
2.56641728778717E-08	0.703174519415895	0.109	0.168	0.000430080209087373	T/NK cell	DHX8
2.57874712457011E-08	1.08132287877435	0.203	0.294	0.000432146443135459	T/NK cell	MECP2
2.63747436363095E-08	0.520786394430338	0.308	0.441	0.000441987953857274	T/NK cell	LUC7L2
2.63843607200798E-08	0.897913045080521	0.059	0.101	0.000442149116947097	T/NK cell	TRMT61B
2.69716537730317E-08	0.586737985655506	0.111	0.169	0.000451990973928465	T/NK cell	STK40
2.71559428259282E-08	0.883588366998959	0.141	0.21	0.000455079289876904	T/NK cell	LSM 8.00

2.76388189289721E-08	0.548912091473573	0.194	0.279	0.000463171327611714	T/NK cell	DYNC1I2
2.76712439080094E-08	0.819578919758481	0.076	0.124	0.000463714705410422	T/NK cell	USP19
2.78260670265006E-08	0.632437555654279	0.093	0.146	0.000466309231230097	T/NK cell	PARN
2.81260245043164E-08	1.78773791184167	0.195	0.168	0.000471335918643334	T/NK cell	CAB39
2.85502930635181E-08	0.821050544176795	0.1	0.155	0.000478445811158436	T/NK cell	DYNC1H1
2.87876217065715E-08	0.872277247478113	0.084	0.134	0.000482422964558725	T/NK cell	SELENOT
2.90447769770602E-08	0.788951682005975	0.221	0.319	0.000486732372581575	T/NK cell	PHF3
2.91275696301276E-08	0.591922474276937	0.167	0.244	0.000488119811861678	T/NK cell	QARS
2.92302932311336E-08	0.619435277954358	0.152	0.223	0.000489841253967337	T/NK cell	SP1
2.93296450996286E-08	0.476983694217878	0.127	0.189	0.000491506192579576	T/NK cell	CBX5
2.93545777258447E-08	0.555632509255184	0.167	0.239	0.000491924013529705	T/NK cell	AHR
2.97426171927323E-08	0.532326298615528	0.096	0.149	0.000498426778915807	T/NK cell	GIGYF2
3.00605358689118E-08	1.08291607235694	0.069	0.114	0.000503754460091224	T/NK cell	TOM1L2
3.01341971363639E-08	0.823267596985379	0.217	0.312	0.000504988875611186	T/NK cell	ZMYM2
3.12141358644669E-08	0.826343389315902	0.159	0.232	0.000523086488816736	T/NK cell	XRN2
3.1317155630625E-08	1.50858540396922	0.241	0.223	0.000524812894058014	T/NK cell	FAM111A
3.26899870968842E-08	0.894097726082763	0.066	0.109	0.000547818803769585	T/NK cell	FRMD4B
3.48832845667303E-08	0.842986884876975	0.168	0.245	0.000584574082769266	T/NK cell	MRFAP1L1
3.64620829492167E-08	0.733154013796414	0.176	0.256	0.000611031586062974	T/NK cell	CLPP
3.7193573559322E-08	1.87919668269616	0.181	0.152	0.000623289905707117	T/NK cell	EPS15
3.73219728223678E-08	1.04645733886348	0.073	0.119	0.00062544162055724	T/NK cell	CNEP1R1
3.87387783738616E-08	1.68510283174582	0.269	0.258	0.000649184447989173	T/NK cell	TAP2
3.90341409748858E-08	0.711124327556545	0.13	0.194	0.000654134134457136	T/NK cell	CKAP5
3.9411621450062E-08	2.39918280415952	0.142	0.112	0.000660459952260138	T/NK cell	ATG16L1
3.99021731393798E-08	0.856307368508904	0.077	0.124	0.000668680617469726	T/NK cell	ZCCHC8
4.10392215922416E-08	0.507962727658981	0.061	0.103	0.000687735275442785	T/NK cell	PRKCZ
4.11036152318948E-08	0.617190168807693	0.077	0.124	0.000688814384056093	T/NK cell	SNAPC3
4.1852933824043E-08	0.80258610357545	0.066	0.109	0.000701371465023312	T/NK cell	AGPAT5
4.3191547142492E-08	0.612559928026633	0.18	0.263	0.000723803947013881	T/NK cell	AP3B1
4.37461081300465E-08	0.797036460685807	0.134	0.198	0.00073309728004332	T/NK cell	EIF3L
4.3792709429493E-08	0.71612966262991	0.233	0.335	0.000733878224619444	T/NK cell	NRDC
4.59001657442068E-08	1.09591660570851	0.061	0.103	0.000769194977541417	T/NK cell	TRIM23
4.59614943661102E-08	0.309898592574116	0.09	0.14	0.000770222722587275	T/NK cell	PITPNM2
4.64199591375404E-08	0.301344710498994	0.105	0.16	0.000777905675226902	T/NK cell	TPGS2
4.89556085265328E-08	0.854174049070223	0.126	0.189	0.000820398087687637	T/NK cell	SPCS3
4.94929189097317E-08	0.719855805605858	0.072	0.117	0.000829402335089285	T/NK cell	RPP30
5.02833674035976E-08	0.58851475618226	0.07	0.114	0.000842648670949489	T/NK cell	VMAC
5.24330821549937E-08	0.742104078979662	0.185	0.27	0.000878673590753384	T/NK cell	DNAJC8
5.26372691944501E-08	1.70666627388497	0.216	0.191	0.000882095357160594	T/NK cell	OSBPL8
5.27673919713738E-08	0.609770333740717	0.146	0.215	0.000884275954656282	T/NK cell	CNPPD1
5.3534359577393E-08	0.749831790516223	0.189	0.273	0.000897128797797952	T/NK cell	DAZAP1
5.42697960750527E-08	0.476556460942596	0.12	0.181	0.000909453242625733	T/NK cell	INPP1
5.44059254403008E-08	0.944118145948949	0.075	0.121	0.000911734498528561	T/NK cell	AACS
5.49525568198043E-08	0.516136022490895	0.067	0.11	0.00092089494718628	T/NK cell	SPAST
5.79711292476146E-08	0.472577728308554	0.059	0.1	0.000971480183931525	T/NK cell	RFT1
5.82410264870592E-08	0.858766255869786	0.179	0.26	0.000976003121870138	T/NK cell	NUP43
5.9370612156345E-08	1.02748935226749	0.187	0.275	0.00099493271851603	T/NK cell	KDM4C
5.94974418921714E-08	0.658114161191501	0.114	0.17	0.000997058131229008	T/NK cell	LIG1
5.97251013698763E-08	0.798737719457884	0.2	0.286	0.00100087324875639	T/NK cell	CEP350
6.3879558582506E-08	1.41945146849286	0.129	0.195	0.00107049364272563	T/NK cell	MAP3K4
6.46126535440713E-08	0.607267090881244	0.166	0.24	0.00108277884809155	T/NK cell	MEAF6
6.64575993177811E-08	0.644466727187381	0.267	0.379	0.00111369644936738	T/NK cell	NBR1
6.73812373265912E-08	0.835907860779735	0.059	0.1	0.00112917477511902	T/NK cell	ATF1
6.76337975208652E-08	0.83137908539606	0.2	0.292	0.00113340717885466	T/NK cell	MOGS
6.7891398409509E-08	0.492452812053181	0.318	0.442	0.00113772405454655	T/NK cell	STK24
6.83843677480615E-08	1.12269524243494	0.063	0.105	0.00114598523472201	T/NK cell	TMEM116
7.1876752262737E-08	0.615585835533785	0.275	0.392	0.00120451061441895	T/NK cell	HNRNPD
7.21503926121845E-08	0.90061372001374	0.093	0.144	0.00120909627939499	T/NK cell	SPOUT1
7.2561796877702E-08	0.660975592637618	0.21	0.3	0.00121599059207653	T/NK cell	DNMT1
7.3196190225779E-08	0.85654145130279	0.07	0.114	0.0012266217558036	T/NK cell	NOA1
7.34150688883764E-08	0.977640568188004	0.127	0.191	0.00123028972443141	T/NK cell	FAM193A
7.37288652343303E-08	0.873826700752957	0.081	0.128	0.00123554832359691	T/NK cell	UFL1
7.81818535643951E-08	1.99120048729815	0.161	0.133	0.00131017150203213	T/NK cell	RASA1

8.26237268412966E-08	0.791665887215883	0.064	0.105	0.00138460841440645	T/NK cell	AHCYL2
8.35140448823672E-08	0.926739485666008	0.098	0.151	0.00139952836413871	T/NK cell	MTOR
8.36558564561534E-08	0.76238872264538	0.089	0.14	0.00140190484249222	T/NK cell	ATR
8.58541385605213E-08	0.729495151005318	0.099	0.152	0.00143874365399722	T/NK cell	GLE1
8.80672457977868E-08	0.708653910314474	0.121	0.18	0.00147583090507931	T/NK cell	ZC3H7B
8.92224714047531E-08	2.22546186067812	0.224	0.206	0.00149519017580085	T/NK cell	ZBTB25
8.92898036487048E-08	1.06155925495842	0.114	0.171	0.001496318529545	T/NK cell	IFIH1
9.18619542603057E-08	1.94875163613248	0.13	0.101	0.0015394226294942	T/NK cell	PHTF1
9.1937650418203E-08	0.919377857809827	0.067	0.11	0.00154069114570825	T/NK cell	DMAC1
9.33405828080435E-08	0.768916225759428	0.067	0.109	0.00156420148669719	T/NK cell	SLC30A6
9.52946392506236E-08	0.999446313848552	0.072	0.116	0.00159694756456195	T/NK cell	VAMP4
9.73527968964172E-08	1.99764047386292	0.142	0.112	0.00163143817039016	T/NK cell	RNF145
9.86155301469152E-08	0.670879056929834	0.21	0.298	0.001652599054202	T/NK cell	GNB1
9.90622176259822E-08	0.832372498529617	0.211	0.303	0.00166008464297621	T/NK cell	SMARCA5
1.01230570274028E-07	1.00391305750946	0.158	0.233	0.00169642189665215	T/NK cell	RRP1B
1.01743521622645E-07	0.879480849975504	0.137	0.202	0.00170501793535228	T/NK cell	FBXO46
1.03707657706285E-07	1.8086438677348	0.235	0.218	0.00173793292784193	T/NK cell	S100PBP
1.06361469806179E-07	0.932296526564883	0.067	0.11	0.00178240551101195	T/NK cell	CAPRIN2
1.07171281834175E-07	0.744697250729135	0.101	0.154	0.00179597634097711	T/NK cell	TFIP11
1.07686280207864E-07	0.99302793879311	0.121	0.182	0.00180460668372339	T/NK cell	YTHDF2
1.0780397272357E-07	0.938686743149507	0.062	0.103	0.00180657897490158	T/NK cell	ZBTB22
1.07821641976717E-07	0.739421394542994	0.103	0.155	0.00180687507624582	T/NK cell	RNF220
1.1455931499334E-07	1.59174662003174	0.245	0.229	0.00191978500065839	T/NK cell	MTA2
1.1845093144693E-07	0.902971954931218	0.123	0.183	0.00198500070918766	T/NK cell	ARRDC1
1.18824613128575E-07	0.959805766955233	0.111	0.169	0.00199126286680866	T/NK cell	BTBD1
1.18964106146577E-07	1.84116383168868	0.16	0.132	0.00199360049080433	T/NK cell	RNGTT
1.19283760962169E-07	0.682464437654576	0.17	0.248	0.00199895726620403	T/NK cell	GSK3B
1.19908632748736E-07	1.20158248406732	0.066	0.108	0.00200942886760332	T/NK cell	BAHD1
1.21589909275137E-07	0.840204969260374	0.083	0.131	0.00203760369963274	T/NK cell	TMEM185B
1.21927712863819E-07	1.29899057403489	0.249	0.227	0.00204326461217188	T/NK cell	GNAI2
1.21987368797878E-07	1.03339862390349	0.085	0.133	0.00204426432631484	T/NK cell	FAM241A
1.22085642963181E-07	0.54578300659719	0.075	0.119	0.00204591120477698	T/NK cell	RPAP3
1.23141014044757E-07	1.07848248511768	0.069	0.111	0.00206359711336203	T/NK cell	RUFY2
1.23169749882863E-07	0.386399386616351	0.061	0.101	0.00206407866853702	T/NK cell	ABCF2
1.27937656223126E-07	0.925988548570193	0.063	0.104	0.00214397924298714	T/NK cell	SZT2
1.32099435443377E-07	0.647807496343688	0.081	0.127	0.00221372233916011	T/NK cell	HMG20A
1.33840074673114E-07	1.90043617132052	0.137	0.108	0.00224289197137205	T/NK cell	JARID2
1.40125490870673E-07	0.630014801495679	0.081	0.128	0.00234822297601073	T/NK cell	SMARCAD1
1.40694188864954E-07	1.02264072751196	0.117	0.176	0.0023577532169989	T/NK cell	TRIM33
1.41842975976959E-07	1.27903185239363	0.078	0.124	0.00237700459142187	T/NK cell	ABCB1
1.48368017842757E-07	0.97971589448691	0.1	0.153	0.00248635124300892	T/NK cell	MTMR6
1.48927472289695E-07	0.789024445426722	0.193	0.276	0.00249572658063071	T/NK cell	HDAC1
1.50206182094195E-07	1.12353939981675	0.075	0.119	0.00251715519953452	T/NK cell	MIGA2
1.52619686974959E-07	0.883580462803002	0.093	0.144	0.00255760071432637	T/NK cell	ZNF44
1.57427763706571E-07	0.771876184249729	0.074	0.117	0.00263817446419473	T/NK cell	PRIM2
1.59078007624065E-07	0.581466080150896	0.062	0.101	0.00266582925176409	T/NK cell	THOC5
1.63115972736217E-07	0.833111070161413	0.251	0.362	0.00273349747111352	T/NK cell	MARCH6
1.64124993193326E-07	0.634868550221009	0.074	0.117	0.00275040663593376	T/NK cell	MFAP1
1.64147453585608E-07	0.762832218529693	0.085	0.132	0.00275078302718761	T/NK cell	WASHC2A
1.66404621028775E-07	0.559213831672584	0.227	0.317	0.00278860863920021	T/NK cell	EIF4A1
1.67205582329882E-07	0.801354337055249	0.138	0.202	0.00280203114868416	T/NK cell	TNKS
1.77167720234727E-07	2.22870334533926	0.153	0.126	0.00296897665569356	T/NK cell	TTC39B
1.78678313763879E-07	0.880093767353597	0.124	0.184	0.00299429118205509	T/NK cell	SLMAP
1.80727134815318E-07	1.49839001828628	0.105	0.16	0.0030286253252351	T/NK cell	GSPT1
1.80857769357523E-07	0.617887703330318	0.214	0.305	0.00303081449889336	T/NK cell	UBE2V1
1.83514320910475E-07	1.16270453944765	0.076	0.121	0.00307533298981775	T/NK cell	B4GALT4
1.84428811860356E-07	0.590876899240293	0.121	0.179	0.00309065802915584	T/NK cell	SNRNP40
1.87574572311237E-07	0.940297073378205	0.108	0.162	0.0031433746827917	T/NK cell	HDAC10
1.87865169936848E-07	0.802422702863347	0.077	0.122	0.00314824451780169	T/NK cell	CDC7
1.89387989533037E-07	2.11291568658522	0.199	0.176	0.00317376392859464	T/NK cell	BCL2L11
1.94656103374238E-07	0.700647802240949	0.097	0.149	0.00326204698034548	T/NK cell	ZNF282
1.95079537872094E-07	0.683952606461897	0.152	0.219	0.00326914289566055	T/NK cell	MVP
1.96687971518911E-07	1.78564506272052	0.209	0.187	0.00329609702671391	T/NK cell	ZFC3H1

1.99627322995775E-07	2.2882427694142	0.124	0.096	0.00334535467876319	T/NK cell	UHRF2
2.00473051109901E-07	0.954706012012579	0.106	0.16	0.00335952739049971	T/NK cell	ADNP2
2.03085949912647E-07	0.722120017760035	0.181	0.261	0.00340331434863614	T/NK cell	DYM
2.05968557737351E-07	1.00016465732668	0.082	0.129	0.00345162109056253	T/NK cell	SNW1
2.07206612828697E-07	0.909616606762549	0.122	0.18	0.00347236841778331	T/NK cell	RPTOR
2.0822941960524E-07	0.847970172041481	0.063	0.103	0.00348950861374461	T/NK cell	DEPRECATED-ENSG00000130305
2.13193674447428E-07	1.9223268496312	0.206	0.183	0.00357269959638999	T/NK cell	SIN3A
2.15275556965581E-07	0.82256126268556	0.074	0.117	0.00360758778362921	T/NK cell	CEP104
2.18411694434511E-07	0.988557441380925	0.064	0.104	0.00366014317533354	T/NK cell	TMCO4
2.26396431805371E-07	0.487503608416021	0.072	0.114	0.00379395140419441	T/NK cell	SLC39A6
2.29557252352129E-07	0.875753592248586	0.074	0.116	0.00384692043491697	T/NK cell	FTO
2.29924242304711E-07	0.945718135476673	0.182	0.263	0.00385307045254236	T/NK cell	DCAF5
2.33943161855844E-07	0.995165310196962	0.195	0.28	0.00392041950638023	T/NK cell	KDM6A
2.35411975867917E-07	0.82755716461996	0.063	0.103	0.00394503389159455	T/NK cell	TGFBRAP1
2.42255446164969E-07	1.92893118587325	0.208	0.187	0.00405971676683255	T/NK cell	NCK2
2.57512917691585E-07	2.01582264032695	0.114	0.087	0.00431540147467558	T/NK cell	FUT8
2.68003102267956E-07	0.834034532163762	0.133	0.193	0.0044911959878064	T/NK cell	NARF
2.68085231553441E-07	0.695356821346902	0.082	0.128	0.00449257231037257	T/NK cell	MCM9
2.81897454533424E-07	0.558334666434276	0.277	0.386	0.00472403754307112	T/NK cell	SRPRA
2.91484343050661E-07	1.43654233566255	0.263	0.246	0.00488469462084297	T/NK cell	GNA13
2.94376834071551E-07	0.715203295335751	0.18	0.257	0.00493316698537106	T/NK cell	IWS1
2.95681367986726E-07	1.04659990290187	0.085	0.132	0.00495502836472156	T/NK cell	ZNF131
3.00508701156336E-07	0.883960687146566	0.129	0.189	0.00503592481397787	T/NK cell	STXBP3
3.04795870438763E-07	1.03941683294338	0.155	0.226	0.00510776919681279	T/NK cell	ZNF33A
3.08758546528007E-07	0.890631654764821	0.106	0.159	0.00517417572271634	T/NK cell	USP16
3.0890767303073E-07	0.734454982920221	0.073	0.115	0.00517667478464897	T/NK cell	IFT80
3.11843915915915E-07	1.06560537945693	0.161	0.232	0.00522588034291891	T/NK cell	PIKFYVE
3.15905201957021E-07	0.892355480377047	0.226	0.323	0.00529393937439575	T/NK cell	PCMTD1
3.18872567319802E-07	0.862211003162284	0.106	0.158	0.00534366648314524	T/NK cell	TANGO2
3.22553696498701E-07	0.724797679334162	0.119	0.175	0.00540535484592523	T/NK cell	GTF2A1
3.24209202588661E-07	0.791787541945557	0.215	0.306	0.00543309781698078	T/NK cell	HIPK3
3.24367239086251E-07	1.90016658612494	0.199	0.175	0.00543574619260739	T/NK cell	TUBA4A
3.24671763813581E-07	1.70573766644907	0.242	0.226	0.005440849417988	T/NK cell	PRPF38B
3.32421732639798E-07	0.707848442153218	0.102	0.154	0.00557072339557774	T/NK cell	XRCC5
3.35274115086925E-07	0.556233144318149	0.093	0.142	0.00561852362062669	T/NK cell	DDX60
3.35426189012439E-07	1.29426519645709	0.063	0.103	0.00562107207547046	T/NK cell	JRKL
3.36194268616122E-07	0.767192156159358	0.194	0.275	0.00563394355346898	T/NK cell	SUPT6H
3.56402068745773E-07	1.23144951070205	0.107	0.161	0.00597258586804167	T/NK cell	JADE1
3.63960953297982E-07	0.879238982470203	0.077	0.122	0.00609925765536759	T/NK cell	GIT1
3.66383920996165E-07	0.420619131762068	0.066	0.105	0.00613986174805373	T/NK cell	PTPN11
3.69874058061956E-07	0.773929234688369	0.13	0.19	0.00619834946500226	T/NK cell	RAB4B
3.71057328406203E-07	0.757005233093782	0.164	0.233	0.00621817870943115	T/NK cell	IRF9
3.76489985514682E-07	0.96317904269029	0.235	0.333	0.00630921917725504	T/NK cell	PHF10
3.91473027197844E-07	0.59957043937714	0.213	0.301	0.00656030498978147	T/NK cell	SEC11A
3.9223247227795E-07	0.991316799989684	0.115	0.171	0.0065730317704339	T/NK cell	PDS5A
3.96305553042362E-07	1.69966910844563	0.25	0.239	0.0066412884578839	T/NK cell	MSL2
4.15796323776373E-07	0.757584991163557	0.089	0.135	0.00696791479384446	T/NK cell	NT5C2
4.32656438284163E-07	1.32276392144895	0.148	0.216	0.007250456592766	T/NK cell	MTERF4
4.35998188065042E-07	0.924919757967172	0.184	0.264	0.00730645763559398	T/NK cell	MORC3
4.37088315985689E-07	0.393202981592369	0.072	0.113	0.00732472599928817	T/NK cell	WBP1L
4.46934616455071E-07	0.9433521069704	0.114	0.168	0.00748973030255409	T/NK cell	SS18
4.54334125523595E-07	0.707086528619329	0.084	0.129	0.0076137312755244	T/NK cell	WRAP73
4.56460345581513E-07	0.350120944835535	0.276	0.379	0.007649362471255	T/NK cell	VAMP8
4.58727248473802E-07	0.748474202057329	0.064	0.102	0.00768735122992397	T/NK cell	POU2F1
4.6780647861548E-07	1.03198943052783	0.103	0.153	0.00783950096863822	T/NK cell	JAZF1
4.69612474250404E-07	2.06590141771892	0.141	0.114	0.00786976584348827	T/NK cell	VAMP2
5.10306720460205E-07	0.696709475548425	0.067	0.106	0.00855172002147211	T/NK cell	LCMT1
5.1788002881005E-07	0.738037534415004	0.197	0.278	0.00867863352279881	T/NK cell	TXNDC15
5.41674642801878E-07	0.972698182473253	0.067	0.107	0.00907738366407388	T/NK cell	SCFD2
5.43080901412237E-07	0.73688752303384	0.22	0.308	0.00910094974586626	T/NK cell	UBE2I
5.58742732273487E-07	0.668057850371203	0.257	0.359	0.00936341070743909	T/NK cell	APMAP
5.73491328876019E-07	0.856118082407846	0.154	0.22	0.00961056768930433	T/NK cell	PSMG2
5.87454255657338E-07	0.857330630604145	0.084	0.129	0.00984455841630566	T/NK cell	BABAM2

5.89964745797931E-07	1.14163873404597	0.098	0.147	0.00988662921008174	T/NK cell	CCDC82
5.96392664846395E-07	0.798430024684198	0.088	0.135	0.0099943482774959	T/NK cell	IFFO1
6.04727355525181E-07	0.731973228276939	0.084	0.129	0.010134021023891	T/NK cell	UBE2D2
6.12098736497845E-07	0.956079076842473	0.064	0.102	0.0102575506262309	T/NK cell	INVS
6.2363679120369E-07	0.750693257377575	0.304	0.416	0.0104509053469914	T/NK cell	IRF3
6.24216544514384E-07	0.838479835651393	0.194	0.274	0.010460620852972	T/NK cell	STK11
6.24481818717577E-07	1.04315551642274	0.113	0.166	0.0104650663180692	T/NK cell	SAP30
6.29529486246813E-07	1.08047886431255	0.078	0.122	0.0105496551305241	T/NK cell	ELP4
6.38160645305348E-07	0.715269908286801	0.09	0.137	0.010694296094027	T/NK cell	ZNF654
6.65191536666282E-07	0.740580574220221	0.122	0.179	0.0111472797714536	T/NK cell	TMX1
6.7775676576019E-07	0.935905788206224	0.204	0.288	0.0113578478806093	T/NK cell	GGNBP2
6.81420843672245E-07	0.706025031953514	0.319	0.44	0.0114192504982595	T/NK cell	PRKAR1A
6.88225288989383E-07	1.70263265728862	0.287	0.279	0.0115332793928841	T/NK cell	NOP53
7.04230730009695E-07	0.914121709458206	0.096	0.145	0.0118014985735025	T/NK cell	ANKRD52
7.11733391299104E-07	0.515730037776078	0.063	0.1	0.0119272281713904	T/NK cell	ANAPC15
7.1451456709169E-07	0.534852188385885	0.163	0.234	0.0119738351153225	T/NK cell	AGTRAP
7.27873919441176E-07	0.948169474081666	0.124	0.179	0.0121977111419952	T/NK cell	RANGAP1
7.2972303163992E-07	0.357375063179558	0.138	0.197	0.0122286985642218	T/NK cell	C9orf16
7.32105187192225E-07	0.77949213744335	0.065	0.103	0.0122686187269673	T/NK cell	FBXL4
7.48345212543903E-07	0.708991166092187	0.07	0.11	0.0125407690718107	T/NK cell	DSTYK
7.6490574997028E-07	0.745991917978055	0.109	0.161	0.012818290558002	T/NK cell	TBC1D10B
7.94507547388453E-07	1.49503698875531	0.22	0.2	0.0133143574791357	T/NK cell	PRKD2
8.10418638867498E-07	0.723490235942695	0.087	0.132	0.0135809955501415	T/NK cell	WDR46
8.14666138433986E-07	1.35732715850241	0.116	0.171	0.0136521751478767	T/NK cell	ANKRD13A
8.23883500563423E-07	1.72180198684762	0.229	0.212	0.0138066397024418	T/NK cell	QRICH1
8.2683286810742E-07	1.42013901036506	0.347	0.358	0.0138560652037441	T/NK cell	STX16
8.29882484449485E-07	1.23640263673398	0.074	0.114	0.0139071706744045	T/NK cell	RBMXL1
8.53372862132881E-07	0.911292926715527	0.127	0.184	0.0143008224236228	T/NK cell	TTC7A
8.57724816211983E-07	0.640793045766004	0.235	0.327	0.0143737524700804	T/NK cell	SNX17
8.69364270945766E-07	0.838821267050681	0.098	0.146	0.0145688064525092	T/NK cell	FOXK1
8.73338273800116E-07	1.03556341446909	0.13	0.189	0.0146354027923423	T/NK cell	SLC30A7
8.80182453389747E-07	0.59007302398093	0.148	0.21	0.0147500975539054	T/NK cell	TADA3
8.84897842853098E-07	1.06431363139044	0.112	0.166	0.0148291180505322	T/NK cell	ZMYM5
8.86559064908514E-07	0.942144124592621	0.11	0.162	0.0148569568097369	T/NK cell	SH2B1
8.93522054107722E-07	1.32800684514026	0.12	0.176	0.0149736425827372	T/NK cell	RMC1
9.04278169878724E-07	1.22726530961527	0.093	0.14	0.0151538935708277	T/NK cell	NAA40
9.04483023015775E-07	0.928526709111858	0.077	0.119	0.0151573264996984	T/NK cell	TARDBP
9.19193107752851E-07	0.539233298932589	0.09	0.135	0.0154038380997223	T/NK cell	UBE2L3
9.22940365559577E-07	1.35589302579599	0.077	0.119	0.0154666346460474	T/NK cell	SLC35E2A
9.23331481057261E-07	2.00812670354781	0.178	0.155	0.0154731889595576	T/NK cell	RNF38
9.46632846310667E-07	0.948430578100373	0.096	0.143	0.0158636732384742	T/NK cell	EXOSC10
9.52202704152939E-07	0.847458386571581	0.106	0.156	0.0159570129161949	T/NK cell	UBE2G1
9.59936282476772E-07	0.986745522732117	0.197	0.28	0.0160866122217457	T/NK cell	MPHOSPH8
9.65413995085274E-07	0.558315394710049	0.077	0.119	0.016178407729639	T/NK cell	SEC23IP
9.68339969726404E-07	0.813791754135189	0.077	0.118	0.0162274412126751	T/NK cell	MAPK8IP3
9.71078864450795E-07	0.453114898837584	0.12	0.171	0.0162733396104664	T/NK cell	NUSAP1
9.79382717814797E-07	0.855575403966389	0.099	0.146	0.0164124955851404	T/NK cell	PPP4R2
9.84850854520146E-07	0.709487111388086	0.071	0.111	0.0165041306200486	T/NK cell	ASMTL
1.00420165629166E-06	0.932147035430222	0.13	0.19	0.0168284113561357	T/NK cell	MCM3AP
1.01897663313662E-06	0.992065006375366	0.082	0.126	0.0170760104181036	T/NK cell	ACLY
1.07690028481404E-06	0.720623726236467	0.162	0.229	0.0180466949729137	T/NK cell	TMEM127
1.12101653299001E-06	0.692228678120535	0.233	0.322	0.0187859950598466	T/NK cell	TMX4
1.1281012717622E-06	0.879157519202588	0.063	0.101	0.018904721112191	T/NK cell	DLG3
1.13164998185978E-06	0.491464756836355	0.078	0.119	0.0189641903960061	T/NK cell	XRCC1
1.13497583738497E-06	0.956340449784603	0.148	0.211	0.0190199250828974	T/NK cell	RNF125
1.14048916293959E-06	1.59047357034348	0.183	0.159	0.0191123173925416	T/NK cell	ITPR1
1.15530849659701E-06	1.04508733927532	0.182	0.26	0.0193606597859728	T/NK cell	VHL
1.15717659699412E-06	0.92861391444713	0.175	0.249	0.0193919654124275	T/NK cell	TENT2
1.19318852837749E-06	1.23395693807107	0.065	0.103	0.01999545335855	T/NK cell	CWC25
1.21771205674843E-06	0.725754978855743	0.176	0.248	0.0204064186469901	T/NK cell	AFTPH
1.21774385162704E-06	0.845268477173665	0.072	0.111	0.020406951465566	T/NK cell	SERGEF
1.2320230436135E-06	0.498826149136153	0.331	0.457	0.0206462421648751	T/NK cell	DRAP1
1.23833168578931E-06	1.74047329614391	0.202	0.181	0.0207519623904572	T/NK cell	CLSTN3

1.25019714825E-06	0.409988821339761	0.111	0.161	0.0209508038103735	T/NK cell	SSH1
1.3147194793938E-06	0.668082556894214	0.073	0.113	0.0220320690356814	T/NK cell	GPALPP1
1.35202835237095E-06	0.764708765087119	0.207	0.29	0.0226572911290325	T/NK cell	TNFRSF14
1.35207603776176E-06	1.08601255600472	0.096	0.144	0.0226580902408116	T/NK cell	U2AF1L4
1.35797047180968E-06	0.820027509793819	0.07	0.109	0.0227568691665867	T/NK cell	PIK3CA
1.36761211481131E-06	0.95316688391819	0.11	0.162	0.022918443820008	T/NK cell	ZDHHC6
1.37408549741506E-06	2.13734857795398	0.114	0.089	0.0230269247656815	T/NK cell	ABHD17B
1.38926891573702E-06	0.675687513465019	0.114	0.165	0.023281368489921	T/NK cell	POLR2G
1.39247899359847E-06	1.8389114889253	0.169	0.146	0.0233351629747232	T/NK cell	ZZEF1
1.40844086782446E-06	2.15917023367222	0.173	0.15	0.0236026520630023	T/NK cell	CRYBG1
1.43238356273418E-06	1.69374378824423	0.116	0.09	0.0240038837442994	T/NK cell	ERICH1
1.47720730899828E-06	0.809493738580413	0.144	0.208	0.0247550400841932	T/NK cell	MAN1A2
1.48231055190747E-06	0.832119838183095	0.125	0.18	0.0248405602288653	T/NK cell	IAH1
1.49234089325912E-06	0.687329460540505	0.122	0.177	0.0250086486892363	T/NK cell	ITGB1BP1
1.49519100035501E-06	1.21233743947422	0.096	0.143	0.0250564107839492	T/NK cell	PPTC7
1.49603365825837E-06	0.743685236541273	0.075	0.114	0.0250705320450937	T/NK cell	ABCB7
1.51800642835417E-06	1.10080240638428	0.123	0.179	0.0254387517263592	T/NK cell	IFI44
1.54167981029339E-06	0.909234099929008	0.14	0.201	0.0258354702608966	T/NK cell	USP1
1.56221436855022E-06	1.34262204734583	0.147	0.21	0.0261795883881646	T/NK cell	ELP2
1.56821546428908E-06	0.803419312430901	0.09	0.135	0.0262801547505564	T/NK cell	DUSP22
1.62644427411845E-06	0.81953905392121	0.173	0.242	0.027255953145677	T/NK cell	ELMO1
1.67255068575806E-06	0.805934349637741	0.224	0.314	0.0280286043919337	T/NK cell	PAK2
1.68516035430904E-06	0.927084117799589	0.078	0.12	0.0282399172175109	T/NK cell	HELZ2
1.71060903071163E-06	1.92570693324221	0.137	0.112	0.0286663861366656	T/NK cell	ADAT2
1.74443091844294E-06	1.25624550677146	0.065	0.103	0.0292331733312667	T/NK cell	BCLAF3
1.76717929129541E-06	0.576036254623014	0.066	0.103	0.0296143905635285	T/NK cell	UBE2N
1.78874716510395E-06	1.124118927725	0.495	0.552	0.0299758249928121	T/NK cell	JUND
1.84711588228976E-06	0.90945964168529	0.089	0.134	0.0309539679554118	T/NK cell	AQR
1.9849498368883E-06	0.975041273275226	0.079	0.12	0.0332637893665742	T/NK cell	COG5
1.99940285002148E-06	0.638593087065371	0.547	0.638	0.03350599296066	T/NK cell	CFL1
2.02238113105514E-06	2.08554679556285	0.122	0.098	0.0338910629942221	T/NK cell	AP4B1
2.03634236132276E-06	1.05861894531069	0.152	0.216	0.0341250252910469	T/NK cell	FIP1L1
2.11711696947304E-06	0.899101358342788	0.094	0.14	0.0354786461744292	T/NK cell	COPS7B
2.13868622200015E-06	0.939586061043751	0.077	0.119	0.0358401037082785	T/NK cell	RIOX1
2.1633283009497E-06	1.05182623305651	0.174	0.246	0.036253055667315	T/NK cell	SCAF11
2.19868991717441E-06	1.76079262818005	0.203	0.185	0.0368456456320088	T/NK cell	PTPRJ
2.20680242911334E-06	0.915819340508021	0.176	0.248	0.0369815951070814	T/NK cell	KBTBD2
2.25088824437486E-06	1.55095192525745	0.071	0.109	0.0377203851992339	T/NK cell	FOXJ2
2.2555328917474E-06	0.489449364188526	0.095	0.141	0.0377982201999029	T/NK cell	TTL
2.2640699186047E-06	1.09717448581152	0.184	0.258	0.0379412836959775	T/NK cell	PPIG
2.3330677613073E-06	0.877579651921411	0.214	0.299	0.0390975495439878	T/NK cell	ZFR
2.3489530080949E-06	1.06111660306956	0.12	0.174	0.0393637545096543	T/NK cell	TBK1
2.35462642978778E-06	0.894067992133538	0.076	0.115	0.0394588297103837	T/NK cell	INO80
2.3765188248726E-06	1.7253877977144	0.153	0.128	0.039825702467215	T/NK cell	FAM89B
2.44259215541309E-06	0.857924862383225	0.254	0.345	0.0409329593404126	T/NK cell	PFKFB3
2.46045137850935E-06	0.956460432776275	0.135	0.194	0.0412322442010596	T/NK cell	YTHDC2
2.51026934565166E-06	0.761476384992483	0.076	0.115	0.0420670936944304	T/NK cell	CES4A
2.54174163802242E-06	0.932388790107975	0.083	0.125	0.0425945063699797	T/NK cell	THAP12
2.54537054621285E-06	0.799641012850373	0.066	0.102	0.042655319613435	T/NK cell	ANP32B
2.54987200567208E-06	1.72805370706526	0.125	0.1	0.0427307550710528	T/NK cell	CHMP7
2.66897029156557E-06	0.769133179438065	0.192	0.266	0.0447266041460559	T/NK cell	OGFR
2.67181825450843E-06	1.36047652985454	0.077	0.118	0.0447743303090522	T/NK cell	BIRC2
2.71073565835291E-06	0.935420283281864	0.081	0.122	0.0454265081626781	T/NK cell	CYP20A1
2.71693486910932E-06	1.81776547262922	0.176	0.155	0.045530394536534	T/NK cell	METTL9
2.75430682349305E-06	0.726921855156825	0.222	0.309	0.0461566737480966	T/NK cell	TRAPPC9
2.77711163813024E-06	1.02851758309646	0.114	0.166	0.0465388368317865	T/NK cell	UPF2
2.78667646562963E-06	0.751729601639973	0.222	0.308	0.0466991242110213	T/NK cell	GPBP1L1
2.79515042710517E-06	0.762642783218942	0.105	0.152	0.0468411308574285	T/NK cell	ETV6
2.87179001379371E-06	0.401940393109132	0.19	0.264	0.0481254570511549	T/NK cell	FKBP1A
2.88156405205421E-06	0.902084875434432	0.233	0.324	0.0482892503843244	T/NK cell	SUPT5H
2.923413622824E-06	0.610810653673474	0.278	0.385	0.0489905654912847	T/NK cell	ERBIN
2.93604715247458E-06	2.11775228598904	0.141	0.115	0.049202278181169	T/NK cell	REL
2.96019560907827E-06	0.851426382907614	0.164	0.229	0.0496069580169337	T/NK cell	NUP50

SupTable5C_C1_Hep_SeuratCluster

p_val	avg_log2FC	pct.1	pct.2	p_val_adj	cluster	gene
0	6.10695764369728	0.54	0.02	0	0	CHI3L1
0	5.99065984302081	0.707	0.034	0	0	HAMP
0	5.59749494229615	0.265	0.007	0	0	LECT2
0	5.51804604623758	0.622	0.027	0	0	SERPINA11
0	5.40316341820322	0.374	0.01	0	0	SLC25A47
0	5.18464703301105	0.321	0.011	0	0	F9
0	5.15454444128964	0.738	0.041	0	0	ADH4
0	4.9152896108564	0.414	0.05	0	0	HSD17B13
0	4.89492998968792	0.266	0.016	0	0	MAFB
0	4.8524806341748	0.851	0.078	0	0	IGFBP1
0	4.75563140156198	0.406	0.018	0	0	HSD11B1
0	4.531334939801	0.342	0.02	0	0	GCGR
0	4.51844080781512	0.44	0.04	0	0	ID1
0	4.51392106471295	0.373	0.082	0	0	DEPRECATED-ENSG00000160868
0	4.50420160411523	0.997	0.404	0	0	APOA2
0	4.451031741687	0.336	0.022	0	0	ANGPTL8
0	4.42006153320789	0.871	0.091	0	0	AHSG
0	4.40251726834359	0.912	0.186	0	0	CYP 20.00
0	4.32144270785511	0.252	0.014	0	0	EVA1A
0	4.25826783693719	0.329	0.024	0	0	HJV
0	4.21952463781051	0.302	0.029	0	0	CYP8B1
0	4.20653914589873	0.288	0.042	0	0	SLC10A1
0	4.08201247074454	0.464	0.041	0	0	AFM
0	4.06414812248031	0.251	0.03	0	0	DEPRECATED-ENSG00000255974
0	4.05719269443966	0.432	0.029	0	0	NR1I3
0	4.01849250128218	0.394	0.056	0	0	ABCB4
0	3.98385740840665	0.541	0.043	0	0	APOC4
0	3.98365234117729	0.741	0.237	0	0	ALAS1
0	3.93192662029806	0.755	0.092	0	0	APCS
0	3.92040792624057	0.292	0.031	0	0	SERPINA4
0	3.9053624562031	0.26	0.068	0	0	CYP3A4
0	3.89468850172383	0.551	0.076	0	0	CD14
0	3.84053768279081	0.597	0.192	0	0	G0S2
0	3.78560089764188	0.975	0.203	0	0	HP
0	3.78302433566495	0.298	0.031	0	0	CPED1
0	3.77951644023456	0.507	0.146	0	0	CYP2C9
0	3.7149022889219	0.316	0.06	0	0	CFHR5
0	3.7050061541184	0.805	0.107	0	0	HPR
0	3.70115394255033	0.633	0.177	0	0	ADH1B
0	3.68901201776324	0.401	0.034	0	0	CDO1
0	3.67758584225275	0.309	0.036	0	0	ANGPTL3
0	3.67617774053231	0.402	0.073	0	0	SLC22A7
0	3.55095926589769	0.302	0.033	0	0	HGFAC
0	3.51728601390597	0.829	0.305	0	0	AZGP1
0	3.47188747247864	0.91	0.124	0	0	HRG
0	3.41962121424923	0.758	0.11	0	0	COL18A1
0	3.40628591940817	0.59	0.07	0	0	SAA4
0	3.38247701412746	0.402	0.073	0	0	EGR1
0	3.31470398999986	0.47	0.062	0	0	HPD
0	3.29572614206587	0.523	0.109	0	0	APOM
0	3.26411378500151	0.45	0.133	0	0	MT2A
0	3.15148781367621	0.373	0.047	0	0	FGF21
3.98399153650677E-302	3.13344201668017	0.358	0.158	6.67637301687805E-298	0	SERPINE1
0	3.0939146638466	0.934	0.396	0	0	ITIH3
0	3.06991423321961	0.862	0.368	0	0	SERPINC1
0	3.06465556276818	0.885	0.385	0	0	SERPING1
0	3.02109622601667	0.304	0.05	0	0	CHRD
0	2.98838707604036	0.965	0.627	0	0	FGB

0	2.98726084569162	0.854	0.179	0	0	HPX
2.78522770681681E-291	2.98505233472912	0.298	0.112	4.6674845910836E-287	0	CIDEB
0	2.97208047222595	0.323	0.098	0	0	IL1RN
0	2.93806616334196	0.462	0.056	0	0	C9
0	2.9373623367603	0.618	0.187	0	0	MST1
2.18826019231498E-167	2.92142779640991	0.289	0.152	3.66708643028145E-163	0	SERPINH1
0	2.8839184474898	0.876	0.473	0	0	C1R
0	2.86928124906437	0.852	0.518	0	0	PCK1
0	2.830089247943	0.444	0.111	0	0	RDH16
0	2.77708174123718	0.52	0.21	0	0	ATF3
0	2.76644129611402	0.302	0.064	0	0	MASP1
0	2.74506458186924	0.825	0.506	0	0	LEAP2
0	2.72354002986523	0.816	0.15	0	0	SERPINA3
0	2.72158606332783	0.627	0.306	0	0	G6PC
0	2.6968097396088	0.434	0.06	0	0	MASP2
0	2.67987652868777	0.465	0.222	0	0	ADH6
0	2.67645109480356	0.739	0.378	0	0	BHLHE40
0	2.66627238784646	0.627	0.206	0	0	CFHR2
0	2.66293737629854	0.467	0.154	0	0	TFR2
0	2.66252819853178	0.5	0.235	0	0	GADD45A
0	2.64216466761143	0.964	0.24	0	0	APOE
6.32597227389845E-294	2.60486587299368	0.325	0.129	1.0601064336599E-289	0	FXYD1
0	2.59504430063025	0.983	0.826	0	0	FGA
0	2.57661445808761	0.875	0.25	0	0	ITIH2
0	2.57651699116216	0.752	0.496	0	0	CYP2C8
1.29981919567364E-112	2.55219916733115	0.265	0.156	2.17823700810988E-108	0	DEFB1
0	2.53881807367164	0.306	0.05	0	0	ALDH8A1
0	2.51137213676432	0.664	0.241	0	0	ITIH4
0	2.50584215367281	0.609	0.221	0	0	TAT
0	2.50381706260316	0.626	0.247	0	0	C5
2.70442869455831E-226	2.49388180480214	0.36	0.192	4.53208160634081E-222	0	GBP1
0	2.46632841067976	0.658	0.381	0	0	TXNIP
0	2.4584270002139	0.556	0.118	0	0	SLC38A3
9.02023548130102E-181	2.44437725490889	0.257	0.113	1.51161106195643E-176	0	FOS
0	2.42781305319805	0.539	0.172	0	0	IGFBP2
0	2.4034395293114	0.389	0.065	0	0	MBL2
0	2.39667758554408	0.97	0.793	0	0	FGG
0	2.38105808286699	0.994	0.989	0	0	SERPINA1
0	2.37125802866174	0.302	0.088	0	0	RND1
0	2.36743478724806	0.571	0.362	0	0	PON3
2.56046162246958E-244	2.36202504440607	0.339	0.16	4.29082158693452E-240	0	HES1
5.77672017049112E-261	2.36181888464501	0.399	0.22	9.68062766170902E-257	0	ACADSB
0	2.36146989223793	0.512	0.199	0	0	PROC
0	2.34557069480905	0.434	0.065	0	0	SLC38A4
0	2.34117733368075	0.892	0.491	0	0	CP
0	2.33294985107272	0.453	0.182	0	0	SLC9A3R2
1.30450943373454E-270	2.32711261701686	0.289	0.106	2.18609690905234E-266	0	TMEM140
0	2.29916552613109	0.603	0.355	0	0	PLPP3
0	2.29775181790857	0.981	0.912	0	0	RBP4
1.81163453160633E-228	2.29549458964463	0.347	0.175	3.03593714806589E-224	0	SLC27A5
0	2.2839484434308	0.977	0.902	0	0	APOA1
0	2.27998548255519	0.435	0.154	0	0	TM4SF5
0	2.26502753560668	0.304	0.08	0	0	GCKR
7.08298699186524E-105	2.26211454863947	0.279	0.176	1.18696696009678E-100	0	PDK4
1.9332020326238E-224	2.19020007078947	0.354	0.182	3.23965996627096E-220	0	RORC
0	2.17338194218731	0.75	0.35	0	0	APOC1
0	2.15111963343509	0.646	0.31	0	0	NNMT
0	2.1307044362875	0.636	0.425	0	0	SERPINA10
1.65389844728106E-128	2.11368342777724	0.278	0.157	2.77160301795359E-124	0	TRIP6
0	2.11109055554008	0.474	0.247	0	0	DEPRECATED-ENSG00000115738

2.86633039077084E-300	2.10275590779742	0.563	0.43	4.80339646885378E-296	0	DEPRECATED-ENSG00000147872
2.98572695787572E-165	2.09108918637473	0.394	0.268	5.00348123600814E-161	0	SLC30A10
4.21895095304764E-206	2.06586891177012	0.427	0.271	7.07011800711723E-202	0	CXCL2
0	2.06317243647587	0.552	0.38	0	0	PPP2R1B
5.71705274954309E-153	2.05921894206642	0.352	0.223	9.58063699768432E-149	0	ARRDC3
0	2.05788534031237	0.694	0.507	0	0	POR
0	2.04662733462859	0.78	0.51	0	0	F2
0	2.04347001042985	0.692	0.544	0	0	INSIG1
0	2.03371556201904	0.848	0.673	0	0	PLG
0	2.00637848323657	0.932	0.774	0	0	KNG1
0	2.00025989413021	0.965	0.835	0	0	APOB
2.64882317336623E-124	1.98006263087854	0.303	0.186	4.43889787392712E-120	0	KLF10
1.03625777263108E-79	1.97386611163013	0.258	0.171	1.73656077537516E-75	0	CYP2J2
7.68128786354072E-43	1.96620856211296	0.25	0.198	1.28723022017215E-38	0	ACAD11
0	1.96564767213367	0.8	0.507	0	0	FGL1
4.33282367218963E-107	1.93544743352834	0.329	0.226	7.26094590985539E-103	0	DCPS
1.60120890154564E-121	1.9280312475981	0.378	0.28	2.68330587721018E-117	0	MTTP
0	1.90769472051594	0.79	0.603	0	0	JUN
0	1.90644297752623	0.537	0.352	0	0	JUNB
2.11688749436868E-139	1.90469945661849	0.383	0.267	3.54748006306304E-135	0	SLC25A25
0	1.89686801715495	0.805	0.398	0	0	ITIH1
2.27121592532854E-208	1.88666749557057	0.404	0.243	3.80610364766558E-204	0	F7
3.62028349791064E-75	1.88019972493016	0.284	0.202	6.06687108579865E-71	0	PEMT
3.9346543155526E-306	1.87988757568988	0.461	0.246	6.59369370200306E-302	0	AQP9
6.77310690464403E-185	1.8742327996493	0.389	0.238	1.13503725508025E-180	0	ID2
0	1.86257665817808	0.984	0.885	0	0	ORM1
0	1.85243378162508	0.865	0.609	0	0	C1S
0	1.85097816020709	0.615	0.471	0	0	DCXR
1.37340573599669E-100	1.84883457750973	0.277	0.171	2.30155333238325E-96	0	RBP5
8.26665686285674E-116	1.81745824087051	0.27	0.154	1.38532635707753E-111	0	ACSM5
1.08730566258695E-172	1.79648556256959	0.425	0.294	1.82210682936321E-168	0	ORMDL3
0	1.79083598974403	0.915	0.783	0	0	ORM2
2.6354861488147E-108	1.7827082472339	0.512	0.482	4.41654768818368E-104	0	HMGCS1
0	1.77654633672447	0.79	0.669	0	0	ALDH2
0	1.76376413763975	0.94	0.918	0	0	FABP1
4.84688952895822E-26	1.73970629656755	0.267	0.239	8.12241747262819E-22	0	PPARGC1A
0	1.73574248528551	0.696	0.309	0	0	LBP
0	1.73066776511055	0.983	0.969	0	0	APOC3
5.36310376937428E-211	1.72818356946177	0.539	0.435	8.98748929671742E-207	0	CCNL1
8.47822299502911E-156	1.72695720523877	0.442	0.334	1.42078060950698E-151	0	RGN
0	1.72391481458044	0.653	0.51	0	0	C8A
1.06668570863827E-193	1.70601720541905	0.506	0.403	1.78755191053601E-189	0	SLC30A1
1.47871171090503E-77	1.70579033622493	0.369	0.306	2.47802508513464E-73	0	SEC14L2
7.8406836131825E-126	1.70092512695096	0.287	0.161	1.31394175989712E-121	0	DUSP10
3.72101828554825E-76	1.69555075738076	0.307	0.223	6.23568244292176E-72	0	PPP1R3C
6.97321802826781E-305	1.67885546708165	0.578	0.426	1.16857187717712E-300	0	ASGR1
0	1.65274214732432	0.917	0.797	0	0	TTR
0	1.64247483365702	0.628	0.526	0	0	ACAT1
2.17153639664084E-44	1.631062488912	0.273	0.218	3.63906069349072E-40	0	DEPRECATED-ENSG00000187758
6.54276356508436E-123	1.62950455839323	0.368	0.256	1.09643631823684E-118	0	NFIL3
0	1.62809226517377	0.953	0.794	0	0	CFB
2.15314517830047E-38	1.62770822473907	0.271	0.225	3.60824068979593E-34	0	AHR
4.94486141313395E-74	1.61497486974977	0.342	0.268	8.28659875612988E-70	0	NFKBIZ
7.2078708930531E-56	1.59101709519931	0.308	0.247	1.20789500425784E-51	0	AKIRIN2
5.11102965376394E-163	1.58430620176883	0.476	0.371	8.56506349377762E-159	0	CYP27A1
1.57862828713915E-176	1.57206074054467	0.522	0.442	2.6454652835878E-172	0	MPC1
1.83726785817037E-48	1.56913966330974	0.319	0.27	3.07889347672191E-44	0	HLF
8.88646550969496E-37	1.56096245131753	0.312	0.281	1.48919389011468E-32	0	ENPP1
0	1.55617453121116	0.808	0.696	0	0	MLXIPL
3.44044351014496E-161	1.55303457674225	0.488	0.39	5.76549523430093E-157	0	PON1

6.06707133979797E-213	1.55106541536597	0.564	0.469	1.01671981512334E-208	0	ADH1A
3.43161736981496E-255	1.54734990572722	0.581	0.453	5.75070438833591E-251	0	GLYCTK
8.92958507428278E-111	1.5462803781961	0.34	0.229	1.49641986674831E-106	0	RGS3
3.59868999176775E-180	1.52598728477082	0.259	0.107	6.0306846882044E-176	0	SLC25A18
0	1.51601341315804	0.722	0.58	0	0	SLC2A2
1.97464618287278E-208	1.5061822280326	0.531	0.415	3.3091120732582E-204	0	C8B
5.6178364837358E-159	1.50442532734358	0.504	0.423	9.41437037944446E-155	0	FUOM
3.84888625154345E-127	1.4780480812262	0.381	0.262	6.44996358033652E-123	0	REEP6
2.69854168232778E-63	1.47643695069814	0.54	0.569	4.5222161512449E-59	0	FASN
1.29662855919631E-40	1.47015016575756	0.364	0.338	2.17289013950117E-36	0	LDLR
1.26144186021654E-115	1.46821922740576	0.485	0.425	2.11392426935088E-111	0	F11
3.36757967289891E-293	1.46805834770749	0.602	0.451	5.64339001584399E-289	0	SERPINF1
7.33575710917373E-54	1.46645229074583	0.415	0.399	1.22932617635533E-49	0	HSPE1
2.86268844067907E-44	1.44968484202788	0.273	0.216	4.79729328888999E-40	0	ACY1
0	1.43839897718446	0.354	0.076	0	0	CPS1
0	1.43457580339459	0.972	0.896	0	0	APOC2
8.90816797728803E-105	1.43452651637999	0.476	0.423	1.49283078963393E-100	0	ARHGEF10L
8.80327927859351E-25	1.43289405589888	0.329	0.325	1.4752535415067E-20	0	RNF149
8.52403669559773E-19	1.42899133512833	0.272	0.256	1.42845806944827E-14	0	SLCO1B1
0	1.42293362292335	0.651	0.453	0	0	SERPINF2
5.93884890813623E-116	1.40961393110443	0.493	0.449	9.95232300025469E-112	0	EHHADH
6.72123507792255E-31	1.40551406964259	0.302	0.271	1.12634457435826E-26	0	IFRD1
1.44463406404614E-204	1.38991599441844	0.562	0.465	2.42091776452853E-200	0	PCBD1
0	1.38349734776688	0.873	0.818	0	0	C4BPA
0	1.38207506973283	0.493	0.12	0	0	ARG1
0	1.37890682306017	0.735	0.655	0	0	PTMS
0	1.37746438217207	0.818	0.703	0	0	GLUL
1.67356711359406E-176	1.36451958005768	0.405	0.244	2.80456376896092E-172	0	MTSS2
1.01857302574033E-09	1.3629795296196	0.259	0.264	1.70692467653564E-05	0	AGMO
0	1.35138591890647	0.953	0.961	0	0	AMBP
8.52795147773639E-157	1.35080116586815	0.569	0.532	1.42911410863906E-152	0	NFE2L2
6.01191534481483E-09	1.34838712611081	0.313	0.338	0.000100747677348407	0	HMGCR
0	1.34346124828694	0.815	0.76	0	0	PRAP1
0	1.34336775295729	0.903	0.803	0	0	AGXT
1.09934194812211E-53	1.33565199600083	0.4	0.373	1.84227723666304E-49	0	ECI2
0	1.3342136763913	0.584	0.286	0	0	A2M
3.77319585154641E-248	1.32857557750349	0.639	0.558	6.32312160802148E-244	0	ETS2
2.3533479643968E-17	1.327601952973	0.309	0.303	3.94374051873616E-13	0	EBNA1BP2
0	1.32348186787066	0.741	0.616	0	0	LRG1
5.09581763279499E-214	1.31040087015774	0.619	0.552	8.53957118903784E-210	0	BHMT2
2.13194242246494E-112	1.30941108868765	0.495	0.445	3.57270911156675E-108	0	ABAT
1.62591794726699E-71	1.30333718602641	0.424	0.378	2.72471329603002E-67	0	LRP1
1.49792236084507E-43	1.28542646355111	0.393	0.374	2.51021829230417E-39	0	GADD45B
1.57007825120306E-113	1.28415013047527	0.478	0.406	2.63113713336608E-109	0	F10
0	1.28351829834245	0.696	0.642	0	0	ECHS1
8.80646279578832E-10	1.27514156416595	0.282	0.29	1.47578703531821E-05	0	CEBPA
1.8126100948471E-173	1.27234882084699	0.586	0.516	3.03757199694478E-169	0	MAT1A
0	1.26923208885763	0.767	0.705	0	0	EFNA1
2.88548067921214E-20	1.26869265771654	0.329	0.331	4.8354885222237E-16	0	KLKB1
3.80653996320616E-11	1.2678013690527	0.454	0.546	6.37899967034089E-07	0	CYP2B6
3.69355986243336E-82	1.26572933402499	0.351	0.258	6.18966761746583E-78	0	PABPC1L
7.06454685071145E-103	1.25815400820633	0.536	0.525	1.18387676124223E-98	0	ACAA1
7.06805562970126E-278	1.24749339173826	0.673	0.617	1.18446476242534E-273	0	DDT
0	1.23341444588849	0.48	0.149	0	0	CRP
1.63233592023303E-166	1.2326591678174	0.593	0.55	2.7354685351265E-162	0	CFI
1.85912963441949E-179	1.23219765785924	0.62	0.584	3.11552944136019E-175	0	ANG
4.57180729136892E-25	1.22210840693056	0.354	0.351	7.66143465887603E-21	0	CGN
6.47790855825529E-07	1.21603126086974	0.274	0.289	0.0108556791619242	0	IER2
1.21664403922503E-16	1.21334809021266	0.348	0.363	2.03885208093331E-12	0	TNS3
0	1.20746844870361	0.756	0.703	0	0	RHOB

3.90162068246955E-27	1.20049977720094	0.356	0.352	6.53833593968248E-23	0	ACADM
9.66267869119778E-20	1.19961203903545	0.367	0.378	1.61927169507092E-15	0	ADHFE1
2.56713254720147E-72	1.19351319069521	0.449	0.415	4.30200072260022E-68	0	GRHPR
0	1.19066610524484	0.858	0.749	0	0	CYP3A5
3.60031640118998E-21	1.18075188680315	0.356	0.36	6.03341022511417E-17	0	RAB17
4.58984496499666E-40	1.17515491077098	0.283	0.226	7.69166219234141E-36	0	PLIN4
3.28091409817836E-13	1.16893995633724	0.254	0.239	5.4981558457273E-09	0	TGM2
3.98875263833662E-142	1.16607067591097	0.571	0.524	6.68435167132451E-138	0	BDH1
7.73349397845809E-87	1.16214386901984	0.395	0.304	1.29597892091001E-82	0	CREB3L3
2.90726587212632E-230	1.14820833856607	0.67	0.623	4.87199614850929E-226	0	SERPIND1
3.06335877531517E-30	1.14704612460148	0.385	0.387	5.13357663567317E-26	0	ALDH6A1
5.59578751016076E-60	1.13496761880149	0.427	0.393	9.3774207095274E-56	0	IFITM2
1.42976815123337E-08	1.13303517678227	0.278	0.285	0.000239600546783688	0	BCL6
7.63427038498406E-12	1.12410658885905	0.283	0.279	1.27935103111563E-07	0	NCOA7
1.97643586837547E-64	1.10936844896567	0.487	0.489	3.31211122822362E-60	0	HAO1
8.78322534772123E-205	1.09966583312637	0.679	0.664	1.47189290377112E-200	0	FMO3
3.8586855790183E-10	1.09619882863636	0.258	0.252	6.46638529331887E-06	0	F13B
0	1.09309344190935	0.833	0.818	0	0	CYB5A
1.38342182494201E-06	1.08914372792692	0.186	0.25	0.0231833829423781	0	ATF5
1.16818698886814E-38	1.07799722233447	0.345	0.302	1.95764775594524E-34	0	PLIN5
7.61685794894326E-26	1.06382527041561	0.371	0.371	1.27643305508391E-21	0	ZNHIT1
8.5715758222749E-19	1.04932998804796	0.33	0.319	1.43642467629683E-14	0	OSGIN1
1.17634455637244E-27	1.0478463156142	0.438	0.468	1.97131820756893E-23	0	TNFRSF1A
2.74037252399713E-204	1.04445558299134	0.396	0.212	4.59231627571439E-200	0	DEPRECATED-ENSG00000175899
4.45886595327447E-14	1.03889176952811	0.303	0.297	7.47216756449736E-10	0	PIPOX
2.40052076673438E-71	1.038514590757	0.495	0.482	4.02279270089348E-67	0	ITM2B
2.14992143939031E-14	1.02702250225959	0.381	0.415	3.60283834813028E-10	0	ETFA
6.28648240077874E-206	1.0264409212243	0.703	0.683	1.0534887207225E-201	0	ADH1C
3.41712772652443E-91	1.02446149285178	0.496	0.436	5.72642264410963E-87	0	DPYS
1.6788180309883E-07	1.01923072962157	0.313	0.339	0.00281336325633019	0	GOT2
1.04922203536316E-73	1.01877106797033	0.56	0.58	1.75828628686158E-69	0	HSPD1
3.31798933093977E-07	1.00833908534333	0.345	0.39	0.00556028652078887	0	APMAP
4.30807303250914E-26	0.99217072078781	0.437	0.47	7.21946878787882E-22	0	MTHFD1
0	0.992096429650732	0.784	0.576	0	0	PAH
2.89059063993499E-07	0.991941071255703	0.282	0.292	0.00484405179440306	0	COMT
0	0.984486668793443	0.965	0.949	0	0	TF
2.71170614198914E-24	0.979241094911594	0.422	0.444	4.54427715274541E-20	0	UGT1A1
5.88472117983782E-07	0.976813025998355	0.35	0.399	0.00986161575317221	0	PECR
1.55558071457042E-63	0.97664602939341	0.508	0.486	2.60684216147711E-59	0	ECHDC2
3.22318115300282E-171	0.960025756934714	0.663	0.648	5.40140697620213E-167	0	TMEM176A
1.12482808629485E-14	0.942125202791846	0.405	0.442	1.8849869070129E-10	0	FNDC3B
0	0.93411073640776	0.851	0.843	0	0	APOH
8.96698391736459E-25	0.927362110920917	0.357	0.34	1.50268716487196E-20	0	BCL3
0	0.921891274235433	0.869	0.869	0	0	TMEM176B
4.74231194962384E-08	0.921411804170111	0.341	0.372	0.000794716636517962	0	SAT2
6.88345466907237E-103	0.900080838947607	0.602	0.609	1.15352933344315E-98	0	CTSB
8.44561335277194E-17	0.894722100220454	0.493	0.564	1.41531588565752E-12	0	SLC39A14
1.02950155938935E-48	0.887862488794428	0.378	0.317	1.72523871322468E-44	0	DEPP1
3.54009781814697E-28	0.883670938162173	0.452	0.477	5.93249592365069E-24	0	PNRC1
2.76865960792582E-16	0.874608721204646	0.177	0.264	4.63971977096209E-12	0	FEM1C
1.05816046819948E-12	0.869520574258006	0.37	0.386	1.7732653126087E-08	0	SLC6A1
4.11354857402352E-20	0.843688382437726	0.178	0.275	6.89348470034862E-16	0	EEPD1
5.13358513149282E-174	0.833451708906237	0.736	0.738	8.60286196335567E-170	0	C4BPB
7.23794750490755E-07	0.824748665658698	0.435	0.513	0.0121293524287241	0	ASL
2.71796897723429E-11	0.820975370609476	0.2	0.283	4.55477241204923E-07	0	LUC7L
5.40264702504546E-14	0.776261878893035	0.199	0.289	9.05375588457118E-10	0	PNISR
2.57079893580654E-14	0.77105710752934	0.429	0.469	4.3081448566246E-10	0	CEBPB
2.30217721463528E-36	0.765612842051404	0.519	0.551	3.85798857628581E-32	0	CAT
1.09365233245161E-291	0.755108643538613	0.877	0.898	1.8327425787224E-287	0	DEPRECATED-ENSG00000204389
2.17309828681789E-17	0.740948537957094	0.214	0.321	3.64167810904941E-13	0	KMT2E

2.14891077678561E-46	0.736626930485787	0.525	0.53	3.60114467973732E-42	0	GAMT
7.37176283379162E-09	0.735254564467947	0.186	0.256	0.00012353600156868	0	RNF11
4.62584339305242E-10	0.732531882109149	0.441	0.504	7.75198835807724E-06	0	FAH
3.64209383736985E-54	0.728762638159308	0.661	0.697	6.1034208526644E-50	0	UPB1
2.2816917370276E-07	0.725672085149821	0.239	0.326	0.00382365901291084	0	PLGLB1
2.31635978526213E-31	0.716681188838313	0.147	0.251	3.88175572814227E-27	0	HERC4
3.91180091859333E-17	0.714281023931634	0.189	0.282	6.55539597937871E-13	0	MEAF6
4.94590641030434E-101	0.710975334005552	0.653	0.649	8.28834996238801E-97	0	ALDH1L1
4.56090159070513E-74	0.707776661749098	0.681	0.725	7.64315888570366E-70	0	SULT2A1
8.10937484293801E-10	0.707360315804434	0.192	0.266	1.35896903617955E-05	0	TTC38
5.34696303872641E-10	0.702157266281075	0.196	0.274	8.96044066029771E-06	0	C16orf70
1.11222011523154E-47	0.698554295848028	0.587	0.634	1.86385846910501E-43	0	SDC4
8.15026473341577E-32	0.687477060117949	0.166	0.278	1.36582136402581E-27	0	RUFY3
2.27155912510059E-10	0.684951705812087	0.299	0.423	3.80667878184357E-06	0	MAP3K13
6.95867743059307E-11	0.683075217673303	0.24	0.339	1.16613516381879E-06	0	SPG21
3.80309637123671E-09	0.675696496673518	0.288	0.401	6.37322889891847E-05	0	CLK1
9.48060358175008E-28	0.674021846366408	0.482	0.504	1.58875954822968E-23	0	KRT18
1.27439639152637E-07	0.661164462661602	0.32	0.443	0.00213563347291989	0	NDST1
3.11714115966888E-08	0.656578200623707	0.195	0.265	0.00052237051553731	0	RAD23B
1.20280581105177E-21	0.653800627772666	0.555	0.584	2.01566197816055E-17	0	VMP1
4.01267926213927E-10	0.652389961826538	0.196	0.27	6.72444790749299E-06	0	SOX5
2.72048969021468E-14	0.647702431415068	0.489	0.561	4.55899662286176E-10	0	CALD1
1.8007646372873E-06	0.641558808934972	0.257	0.344	0.0301772137916606	0	CCDC50
4.37617739637863E-13	0.641338385838149	0.233	0.334	7.33359808085131E-09	0	SIK2
1.32896061440392E-37	0.640425093356053	0.165	0.285	2.22707219761809E-33	0	MAN2A2
7.26301773199136E-15	0.628196795743403	0.258	0.378	1.21713651152711E-10	0	HNRNPH3
7.29916250667895E-38	0.628047211601341	0.176	0.303	1.22319365286926E-33	0	DEPRECATED-ENSG00000197408
1.8259171938648E-10	0.62536302678109	0.206	0.286	3.05987203347863E-06	0	CBX4
2.07300676205528E-13	0.624017572651259	0.244	0.353	3.47394473185224E-09	0	TXNDC11
6.34892687017659E-15	0.62256860374069	0.219	0.319	1.06395316490419E-10	0	RAB6A
1.53498572418427E-19	0.609201372058346	0.218	0.328	2.572329076588E-15	0	H6PD
1.16002474309236E-30	0.605079439087396	0.153	0.256	1.94396946447418E-26	0	LRCH4
2.6645441985426E-14	0.600022929766657	0.359	0.342	4.46524316791769E-10	0	SERPINA5
2.4236936893065E-17	0.597189356817061	0.51	0.563	4.06162588453984E-13	0	IDH2
3.89632606478186E-22	0.597088946459638	0.177	0.276	6.52946321936143E-18	0	TIAL1
6.37019489690238E-29	0.593769703338615	0.552	0.61	1.0675172608229E-24	0	OS9
1.89416109906726E-104	0.585594840262668	0.718	0.759	3.17423516981692E-100	0	ASGR2
1.70617556064072E-14	0.585567802394781	0.212	0.307	2.85920900452172E-10	0	STAT2
2.32546064510263E-17	0.583068193125413	0.213	0.316	3.89700694906298E-13	0	HOOK1
1.67546057015963E-07	0.581289055364247	0.325	0.45	0.00280773682347351	0	SLC25A13
1.02692232252457E-06	0.570694216681768	0.307	0.416	0.0172091642808667	0	TRAF4
2.25427485266339E-11	0.56629809080601	0.316	0.451	3.77771379809332E-07	0	RBM39
2.15938058948594E-16	0.565067199621407	0.291	0.427	3.61868999186053E-12	0	TSC22D2
8.85734183891702E-17	0.560322974541522	0.267	0.39	1.48431334536571E-12	0	CHD2
4.34186306788187E-25	0.553984548924659	0.227	0.354	7.27609412915644E-21	0	HDHD3
4.1072019834287E-20	0.551553258286076	0.251	0.378	6.88284908382982E-16	0	SND1
1.21817300641633E-37	0.551303668896021	0.155	0.271	2.04141432415249E-33	0	NOP58
1.09954333243048E-39	0.551049574770375	0.167	0.291	1.842614716487E-35	0	DEPRECATED-ENSG00000146416
0	0.550988123725184	0.959	0.975	0	0	CLU
1.87966850561849E-22	0.543221240115221	0.275	0.421	3.14994848171546E-18	0	CELF1
3.14542232390308E-32	0.542778831894064	0.156	0.262	5.27109873039678E-28	0	HDAC6
4.10048305441415E-11	0.542506920230076	0.184	0.257	6.87158950258723E-07	0	SELENOK
8.9305078798902E-07	0.540123930836534	0.202	0.263	0.01496574510512	0	ALDOC
9.32879282551491E-07	0.53832064256189	0.292	0.396	0.0156331910169979	0	MALSU1
4.83799253097188E-59	0.538166219714007	0.68	0.731	8.10750788340268E-55	0	GATM
2.58206857984951E-14	0.537882171615523	0.18	0.261	4.3270305261118E-10	0	DEPRECATED-ENSG00000166136
7.40318920732909E-17	0.535623598162128	0.276	0.407	1.24062644736421E-12	0	RSRC2
5.08971621571139E-81	0.534593717322209	0.746	0.812	8.52934643428915E-77	0	SCP2
1.74259808157391E-29	0.532817723619213	0.156	0.257	2.92024586510155E-25	0	SARAF
2.06426038522321E-12	0.530423666992602	0.3	0.424	3.45928755355705E-08	0	C19orf12

6.52066368527528E-47	0.528783306137619	0.6	0.549	1.09273282037843E-42	0	CCNL2
2.71455662799193E-12	0.527430659841867	0.288	0.405	4.54905399718888E-08	0	CYP4F3
1.16617531503317E-13	0.5211608236436	0.296	0.426	1.95427659293258E-09	0	UBE2B
4.3701567962621E-14	0.518752109749352	0.284	0.41	7.32350875917603E-10	0	MIA3
1.10516934207451E-26	0.516810937471484	0.172	0.278	1.85204278344846E-22	0	SEC63
3.74454520677621E-25	0.511483224417831	0.236	0.366	6.27510885751557E-21	0	PNN
7.44011035042901E-22	0.511263804141181	0.184	0.282	1.24681369252489E-17	0	SLC7A2
9.13431424100246E-23	0.510354489707707	0.171	0.266	1.53072838050719E-18	0	MLIP
2.22344517253676E-25	0.508418251142674	0.196	0.309	3.7260494201371E-21	0	EHBP1
1.16500314556616E-20	0.507198187690524	0.197	0.299	1.95231227133978E-16	0	NT5E
2.55847321689768E-60	0.505032412580633	0.76	0.823	4.28748941687714E-56	0	HSPA1A
1.39482211563707E-20	0.50440674110565	0.23	0.347	2.3374429013846E-16	0	UBE2H
3.78437100706834E-192	0.503680056435433	0.916	0.957	6.34184893364513E-188	0	ALDOB
4.39257321123932E-07	0.502919123707647	0.343	0.47	0.00736107418739484	0	PPP2R5A
2.56138840146412E-21	0.502287775373385	0.568	0.63	4.29237468317357E-17	0	AADAC
3.44741424679114E-55	0.502133284432505	0.125	0.25	5.77717679477259E-51	0	TNKS
2.15314012761491E-16	0.500866543278381	0.254	0.373	3.60823222585706E-12	0	TMEM106B
3.08407771977866E-39	0.495522144250257	0.168	0.293	5.16829744280508E-35	0	CUL1
6.88405256116032E-17	0.495228067059919	0.289	0.413	1.15362952819925E-12	0	PILRB
9.2714035903507E-07	0.483613546751896	0.262	0.344	0.0155370181367097	0	TTPA
1.03106902539064E-08	0.483485824707738	0.31	0.425	0.000172786547274963	0	CIB1
3.4644193872885E-14	0.483402346246839	0.646	0.723	5.80567400921808E-10	0	DNAJB1
2.05225691871911E-13	0.481808104749794	0.552	0.621	3.43917214438949E-09	0	H1F0
7.47643487681657E-09	0.481482277196512	0.557	0.653	0.000125290095665692	0	FBP1
3.31450927150853E-07	0.481421537227844	0.355	0.48	0.005554454637194	0	RBM33
3.86358119498523E-41	0.480033965484816	0.156	0.277	6.47458936655625E-37	0	FXR1
4.3716296030146E-20	0.477184280784869	0.236	0.348	7.32597688873187E-16	0	SPTBN1
2.44956921047835E-30	0.477043626916125	0.15	0.25	4.10498808291963E-26	0	AUP1
1.03588417786954E-53	0.476167724367929	0.144	0.276	1.73593470527377E-49	0	SELENOO
1.6272098926883E-13	0.474341098854423	0.221	0.312	2.72687833816706E-09	0	NIPSNAP1
3.93645485708826E-27	0.473863130095362	0.258	0.406	6.59671104950851E-23	0	ITCH
5.14728888465937E-18	0.472159775206636	0.588	0.667	8.62582671291218E-14	0	BRI3
1.19352188806187E-113	0.46663498172743	0.77	0.745	2.00010398001408E-109	0	CFH
1.13014472896962E-51	0.4665669669966	0.178	0.324	1.89389653680728E-47	0	TKFC
8.45462887071683E-37	0.4638269628271	0.173	0.294	1.41682670615473E-32	0	NSUN2
1.11403348952349E-30	0.462014397904301	0.192	0.314	1.86689732174347E-26	0	PITPNB
4.3682629155751E-23	0.461549124276375	0.283	0.426	7.32033499392074E-19	0	TRA2A
6.12961524332654E-11	0.460810224821197	0.293	0.412	1.02720092247666E-06	0	PEX19
3.07149459722807E-48	0.460679988815772	0.134	0.254	5.1472106460348E-44	0	LCOR
2.20193570892036E-19	0.457122641855438	0.209	0.314	3.69000386100875E-15	0	TNFSF10
1.45910845852457E-15	0.456987909295771	0.261	0.377	2.44517395479548E-11	0	TMEM263
2.42406386629853E-19	0.456291240165683	0.267	0.398	4.06224622714308E-15	0	RAB1A
1.86227636376174E-11	0.452579997408905	0.265	0.369	3.12080273039193E-07	0	PCCB
1.68984811926716E-46	0.450672929856751	0.142	0.263	2.83184747826791E-42	0	CYP2C19
1.28108944426849E-153	0.450270760847952	0.797	0.848	2.14684969070513E-149	0	IFITM3
1.5316687671759E-11	0.449309924589591	0.356	0.501	2.56677052003337E-07	0	CEBPG
3.95932216689028E-24	0.448883382881293	0.23	0.354	6.63503208727474E-20	0	SLC43A1
3.89171599195569E-24	0.448322788915166	0.255	0.389	6.52173765931934E-20	0	SNRNP70
2.27027301749391E-14	0.445667668460646	0.393	0.555	3.8045235227163E-10	0	HSPH1
1.65899193072839E-46	0.445640294356286	0.174	0.311	2.78013867751464E-42	0	DOCK5
7.26168511528167E-07	0.443090806268749	0.572	0.658	0.012169131916189	0	HPN
5.68146342006005E-08	0.44262245143921	0.56	0.67	0.000952099639933663	0	PEBP1
3.52026736929269E-28	0.440353270823846	0.184	0.295	5.89926405746068E-24	0	NDUFB8
1.3267074938147E-45	0.437249261898053	0.149	0.271	2.22329641813468E-41	0	HEXA
2.75189949709134E-12	0.436461526117393	0.356	0.503	4.61163317722567E-08	0	IL6ST
8.19582798489974E-14	0.432660263435486	0.255	0.358	1.3734568537095E-09	0	TLCD4
4.25353960633167E-39	0.432438814463112	0.214	0.358	7.12808167229061E-35	0	N4BP2L1
3.35735272983507E-43	0.4322775377121	0.146	0.264	5.62625170465761E-39	0	DUSP16
4.68185196525987E-18	0.431480232118505	0.233	0.344	7.84584752338249E-14	0	CTSL
1.39130722022157E-35	0.430279968744991	0.203	0.336	2.33155263964731E-31	0	B4GALT1

1.82770697039712E-39	0.42499261460107	0.161	0.28	3.0628713409915E-35	0	MTG1
1.49723952407436E-33	0.422673729249529	0.17	0.283	2.50907399444382E-29	0	PNPLA3
7.07809620705474E-13	0.419643080880114	0.214	0.297	1.18614736237823E-08	0	TSKU
5.47519852259962E-26	0.418234511935804	0.218	0.34	9.17533768417244E-22	0	WSB1
2.97357185016856E-42	0.418115287020937	0.14	0.254	4.98311170651246E-38	0	SELENOI
3.5337355958555E-57	0.41529545028039	0.154	0.295	5.92183411153464E-53	0	ZSWIM8
7.48520433881242E-27	0.414991213670946	0.328	0.492	1.25437054309819E-22	0	SREBF1
2.13244248542724E-09	0.410194667437139	0.296	0.403	3.57354711707898E-05	0	CARHSP1
2.74503157890231E-33	0.408366160073991	0.16	0.267	4.60012391992449E-29	0	TNFAIP3
7.27757022918911E-53	0.407802764512787	0.136	0.263	1.21957521900751E-48	0	FBXW7
7.98678838042927E-10	0.406652096365823	0.255	0.349	1.33842599679234E-05	0	POLD4
2.07218921648947E-18	0.405959656763257	0.317	0.464	3.47257468899306E-14	0	SLC47A1
4.57031265521451E-41	0.404135559265088	0.197	0.339	7.65892994760847E-37	0	RALGPS2
8.14532866426072E-18	0.403366868675897	0.241	0.353	1.36499417755681E-13	0	PMM1
2.52350377976177E-08	0.402888082581786	0.344	0.468	0.000422888763412478	0	C8G
1.66231909560255E-47	0.401777274467813	0.162	0.294	2.78571434041076E-43	0	NFX1
6.8606428658942E-44	0.399959635637799	0.174	0.308	1.14970653146655E-39	0	IWS1
1.341861593225E-44	0.398674364018063	0.149	0.272	2.24869165792646E-40	0	PTS
4.11157210841917E-14	0.39603687325893	0.282	0.4	6.89017253928885E-10	0	HAAO
1.48970608351091E-25	0.395292290275209	0.185	0.291	2.49644945474759E-21	0	SIAE
1.26546707947993E-41	0.394515051937235	0.193	0.332	2.12066973179247E-37	0	EPB41L5
3.10658799610621E-28	0.392837459696283	0.256	0.403	5.20602016387479E-24	0	LARP4
3.00624146536815E-31	0.392768878629945	0.194	0.315	5.03785944766394E-27	0	BPHL
1.73717447988912E-43	0.392559875616763	0.219	0.374	2.91115699339819E-39	0	TIA1
1.90779604071454E-51	0.391935840260882	0.147	0.278	3.19708460502943E-47	0	SRSF7
1.8914853602343E-27	0.391135701561293	0.179	0.284	3.16975116668064E-23	0	ARL8A
4.61033456051763E-45	0.386224595289497	0.137	0.252	7.72599865651544E-41	0	CRELD1
2.48683713910151E-29	0.385004507906771	0.24	0.379	4.16744167770631E-25	0	GGCX
5.55890766398026E-11	0.381306626782922	0.267	0.37	9.31561746329812E-07	0	ASS1
1.28100652342113E-07	0.381156414856574	0.223	0.292	0.00214671073194913	0	ROBO1
2.80533765696345E-29	0.379498915786146	0.21	0.333	4.70118484553936E-25	0	COA4
1.02278048571521E-08	0.378794888732005	0.601	0.704	0.000171397553796155	0	C2
1.64191365100287E-30	0.373942833561794	0.246	0.391	2.75151889635062E-26	0	FAM3C
2.02260545498304E-47	0.364115788138249	0.171	0.308	3.38948222146058E-43	0	PPIG
3.97821143604633E-45	0.363927016489565	0.223	0.383	6.66668672452644E-41	0	MKLN1
1.01560340181025E-27	0.358612594717343	0.236	0.365	1.70194818075362E-23	0	CDH1
9.99761139572108E-28	0.358145599850551	0.171	0.273	1.67539971769494E-23	0	MACROD1
2.85932804369388E-42	0.35628379460572	0.197	0.338	4.79166193562221E-38	0	PMPCB
1.59913977722265E-35	0.355193948512856	0.165	0.279	2.67983843866972E-31	0	CLDN12
8.97049152114567E-34	0.353936672516076	0.245	0.392	1.50327496911359E-29	0	TJP1
1.5768933285239E-31	0.353136051727823	0.249	0.394	2.64255783994036E-27	0	SGK2
2.21860682581974E-37	0.353122695029105	0.203	0.339	3.71794131870872E-33	0	DEPRECATED-ENSG00000001630
3.22426246522377E-44	0.351592337104742	0.152	0.274	5.403219039222E-40	0	ERGIC1
1.72254822374033E-61	0.351255209922294	0.192	0.358	2.88664631334404E-57	0	ZNF644
9.90948411540042E-18	0.350117444310807	0.673	0.752	1.6606313480588E-13	0	HSD17B6
2.85151884332044E-21	0.344152706174839	0.278	0.414	4.77857527763639E-17	0	SEM1
4.86838474341454E-54	0.342811877677727	0.14	0.269	8.15843915301409E-50	0	SKIL
1.92605622984858E-24	0.342380138763782	0.272	0.412	3.22768502998025E-20	0	TMEM248
9.39246760168848E-48	0.340127331193609	0.156	0.282	1.57398972069096E-43	0	C1RL
1.67746223252605E-68	0.339683729466128	0.189	0.364	2.81109120926715E-64	0	SLC23A2
7.19847672647841E-64	0.337597176746098	0.127	0.263	1.20632072982325E-59	0	SLC5A3
1.23044665719164E-44	0.333513458045404	0.272	0.451	2.06198250812174E-40	0	ELOVL5
6.77515513891224E-21	0.333278034535327	0.789	0.894	1.13538049817891E-16	0	IL32
3.34581824260856E-50	0.330366053649588	0.168	0.305	5.60692221096342E-46	0	SC5D
1.61393515469477E-43	0.328488780585118	0.18	0.314	2.70463253223749E-39	0	RHEB
3.83546861845885E-37	0.321217273679198	0.247	0.401	6.42747831081334E-33	0	SORBS2
2.27723230709741E-60	0.32011886255648	0.127	0.257	3.81618590023384E-56	0	KAT2A
5.29050386357105E-33	0.319384148756169	0.196	0.32	8.86582637457237E-29	0	TMEM120A
3.48132372501782E-27	0.314709879191108	0.247	0.38	5.83400229838486E-23	0	TOMM7
1.97939623248685E-63	0.314632518883328	0.189	0.357	3.31707220640147E-59	0	TNRC6A

8.04269127508478E-27	0.314454366571877	0.89	0.944	1.34779420387871E-22	0	HSPB1
2.7039888074638E-44	0.311468151200728	0.145	0.262	4.53134444354783E-40	0	GHR
2.54067532072031E-57	0.31078217338266	0.181	0.335	4.25766370246309E-53	0	RBM25
1.83593521816978E-44	0.307391633277889	0.159	0.281	3.07666023860892E-40	0	SLC29A1
1.19404608449099E-41	0.304901975690932	0.248	0.412	2.00098242839E-37	0	TOM1L1
4.10666771364447E-52	0.303312140946762	0.245	0.423	6.88195375452541E-48	0	DST
2.35891908026167E-61	0.301735550431656	0.144	0.283	3.95307659470251E-57	0	PPP1R12B
3.30108879170388E-178	0.301381517026251	0.964	0.967	5.53196459713737E-174	0	C3
1.10554799668377E-46	0.29921152986216	0.228	0.389	1.85267733284266E-42	0	COG3
7.23822700175598E-58	0.298526942891274	0.19	0.35	1.21298208095427E-53	0	UGCG
1.99074166378554E-35	0.297588575220284	0.231	0.373	3.33608488017181E-31	0	MARC1
7.81688148698007E-64	0.293463775061907	0.162	0.315	1.30995299958812E-59	0	PHYKPL
3.58696086171671E-61	0.293086353413503	0.187	0.349	6.01102901206486E-57	0	CHKA
2.08551735515683E-45	0.288632925958129	0.213	0.364	3.49490998377182E-41	0	GPBP1L1
3.43253662401465E-80	0.288466681328719	0.141	0.303	5.75224487452376E-76	0	ZBED6
4.83107376936077E-33	0.287945198027169	0.161	0.268	8.09591342269477E-29	0	NINJ1
1.939240536979E-37	0.285159085024378	0.321	0.498	3.24977929186941E-33	0	ITPR2
3.49590132332314E-29	0.284248209065008	0.164	0.265	5.85843143762492E-25	0	BUD31
1.19463688800634E-63	0.28421329350552	0.151	0.298	2.00197249692102E-59	0	PCM1
9.4691466086532E-44	0.281642409282122	0.204	0.351	1.5868395886781E-39	0	SEC11A
7.43977003356938E-18	0.279914862373911	0.27	0.394	1.24675666222556E-13	0	RAB7A
7.6677955579159E-23	0.279761959961392	0.366	0.541	1.28496917959555E-18	0	EPS8L2
2.73361790253063E-12	0.278458810695042	0.372	0.52	4.58099688106083E-08	0	EIF4EBP2
7.35559056762147E-73	0.273874402338125	0.12	0.263	1.23264986732201E-68	0	LRP6
4.54133334595739E-48	0.27042114252968	0.186	0.329	7.6103664211554E-44	0	STAG1
2.71880751635331E-51	0.267330464652706	0.136	0.259	4.55617763590488E-47	0	ZNF24
8.45552501339827E-31	0.26467217692421	0.266	0.416	1.41697688174528E-26	0	RMND5A
2.88385577641888E-72	0.263897366296569	0.146	0.301	4.83276551012277E-68	0	ATL2
2.21050981718023E-13	0.259657621651979	0.293	0.404	3.70437235163064E-09	0	UBD
7.22580412224198E-58	0.258085627394838	0.181	0.336	1.21090025480531E-53	0	CPSF7
1.35012953409655E-35	0.257649612180149	0.233	0.371	2.262547073239E-31	0	FTCD
9.82794719063392E-59	0.256967258957874	0.126	0.254	1.64696739020643E-54	0	BTAF1
3.94797408786666E-28	0.254098668114024	0.281	0.43	6.61601497644695E-24	0	IQGAP2
1.64246245572617E-25	0.254094737611354	0.217	0.331	2.75243858330591E-21	0	PXDC1
3.29095734073008E-27	0.252961434829136	0.317	0.483	5.51498631159547E-23	0	HADHB
5.14407321550909E-43	0.252384330292065	0.158	0.28	8.62043789455013E-39	0	LIPA
9.08903595317504E-44	0.252263942313904	0.285	0.467	1.52314064503307E-39	0	SRSF4
2.18972470280404E-60	1.5151621604864	0.313	0.225	3.66954065695901E-56	1	CD177
2.26141341934458E-58	1.49134676491092	0.297	0.211	3.78967660813765E-54	1	TFF1
0	1.37519015219678	0.721	0.478	0	1	PEG10
3.0406092090038E-31	1.3322998229456	0.262	0.207	5.09545291244857E-27	1	TFF3
0	1.31984095162389	0.913	0.643	0	1	PIGR
5.97242165863903E-75	1.31008630549722	0.399	0.311	1.00085842155473E-70	1	TSPAN1
2.31927481995304E-43	1.26850264335528	0.357	0.304	3.8866407432773E-39	1	MALL
2.36325134510294E-35	1.26509128391594	0.278	0.217	3.96033660412351E-31	1	KCTD12
1.00583021931643E-22	1.24988944086637	0.274	0.234	1.68557028153047E-18	1	MARCKSL1
2.40415541843746E-22	1.23426759383801	0.302	0.269	4.0288836502175E-18	1	OTOP3
8.14342950534608E-303	1.20230329139836	0.719	0.504	1.3646759165059E-298	1	G6PD
0	1.18834278177359	0.96	0.696	0	1	S100A10
0	1.18550216469359	0.95	0.693	0	1	ANXA4
6.81486035468681E-43	1.18319492878089	0.34	0.278	1.14203429823842E-38	1	DEPRECATED-ENSG00000132646
7.18354184597024E-48	1.18141895018789	0.373	0.317	1.20381794254769E-43	1	CHP2
1.03954484933097E-39	1.17162794147818	0.342	0.284	1.74206925850884E-35	1	MCM2
4.35750340051696E-255	1.16756273397384	0.631	0.393	7.30230419858632E-251	1	MUC5B
0	1.16754402146541	0.842	0.622	0	1	LYZ
0	1.15370653834749	0.752	0.54	0	1	SSR4
2.21116735468524E-205	1.14573278380123	0.615	0.437	3.70547425298152E-201	1	MEP1A
0	1.14374765733212	0.804	0.508	0	1	ADA
0	1.13869170641433	0.737	0.474	0	1	CDH17
9.23524297570916E-19	1.13315574039906	0.259	0.224	1.54764201786934E-14	1	SFN

4.15478134859366E-140	1.1271483818286	0.56	0.444	6.96258258397326E-136	1	ETHE1
1.46246946883941E-75	1.1004980062876	0.444	0.356	2.45080633588108E-71	1	C15orf48
5.15030720689187E-154	1.0878978323593	0.576	0.459	8.6308848173094E-150	1	HTATSF1
4.55894363190226E-75	1.08411160022287	0.455	0.379	7.6398777383418E-71	1	ANXA13
6.87684961860511E-19	1.08376511318359	0.286	0.255	1.15242245908584E-14	1	RASL11A
3.9582667249267E-103	1.0728126267009	0.511	0.41	6.63326337763217E-99	1	PARM1
0	1.05900581578836	0.895	0.679	0	1	SPINK1
2.27860712944103E-07	1.0586011297666	0.265	0.263	0.00381848982751727	1	MANEAL
1.09690642310551E-93	1.05819335240301	0.341	0.206	1.83819578384021E-89	1	AGR2
2.18230738945625E-140	1.0555349262182	0.578	0.477	3.65711072325079E-136	1	DHRS7
2.92950490135246E-46	1.04924538351497	0.423	0.382	4.90926431368645E-42	1	DEPRECATED-ENSG00000213759
5.53808856726792E-285	1.04880363145466	0.733	0.558	9.28072882102757E-281	1	BCAP31
2.16717265917811E-23	1.0451705433702	0.34	0.315	3.63174794225067E-19	1	RASSF3
0	1.04455276578435	0.838	0.57	0	1	MPP1
3.52069993402014E-149	1.04401263020439	0.574	0.458	5.89998894943094E-145	1	LAGE3
1.57060931408679E-30	1.03849486261944	0.378	0.343	2.63202708854664E-26	1	TM4SF20
0	1.03501284580226	0.877	0.634	0	1	NQO1
8.61930220724303E-32	1.0283056888927	0.343	0.297	1.44442266388979E-27	1	MT-ND6
0	1.02601072641048	0.919	0.713	0	1	LGALS4
5.62677573503581E-28	1.02105462571573	0.281	0.227	9.42935077677301E-24	1	S100P
1.09161364391672E-42	1.01814999850278	0.405	0.353	1.82932614447564E-38	1	MCM4
1.98283166485214E-86	1.01472498039879	0.496	0.407	3.32282930395922E-82	1	MCM3
4.98425001452085E-238	1.01174327114395	0.708	0.536	8.35260617433404E-234	1	TSPAN8
5.67674137616797E-22	1.01130233799105	0.328	0.3	9.51308319818228E-18	1	MCM5
1.67875788977531E-134	1.00686364116181	0.549	0.411	2.81326247168546E-130	1	NBL1
1.670767315303E-14	1.00468739873083	0.28	0.255	2.79987186698477E-10	1	PRR15L
3.33523369524173E-10	1.00286135750601	0.282	0.273	5.5891846264861E-06	1	PNP
1.84911273215548E-107	1.00144752766195	0.525	0.425	3.09874311654615E-103	1	EFHD2
1.94256559656575E-13	0.99969955938747	0.333	0.328	3.25535142672489E-09	1	EDNRB
7.14113165431586E-07	0.99905031479374	0.269	0.268	0.0119671084263025	1	QSOX1
1.46489554002633E-11	0.994052357784805	0.329	0.331	2.45487194597613E-07	1	UGT2B11
1.41522780506571E-23	0.98811356592567	0.367	0.346	2.37163875572911E-19	1	CETN2
2.34360438704639E-227	0.986985309696431	0.71	0.554	3.92741223181234E-223	1	MAOA
1.77448433661623E-193	0.985832987690141	0.716	0.598	2.97368085130148E-189	1	SCD
1.2589245169005E-135	0.984811155499935	0.605	0.523	2.10970570542186E-131	1	PRDX1
9.61861562694701E-14	0.981340012159244	0.286	0.263	1.61188760676378E-09	1	MISP
6.34261789298269E-218	0.977257552856486	0.688	0.497	1.06289590650604E-213	1	ATP1A1
2.2801297134507E-83	0.975114005591071	0.518	0.452	3.82104137380069E-79	1	DGAT2
1.61550873308035E-97	0.97330120346606	0.532	0.451	2.70726953489605E-93	1	EZR
3.8447565173739E-21	0.972097605514378	0.372	0.362	6.44304297181519E-17	1	TMEM109
4.4128773209221E-13	0.969281519197773	0.304	0.292	7.39509981440125E-09	1	AP1S1
1.48728011782791E-273	0.969035802023178	0.76	0.536	2.49238402145602E-269	1	TSPAN3
1.85230004766108E-296	0.967911132041281	0.822	0.688	3.10408441987044E-292	1	GSTA1
3.06645323389289E-08	0.966634382717167	0.251	0.24	0.00051387623293577	1	PYCARD
2.81829605926824E-68	0.966580540524285	0.498	0.448	4.72290053612171E-64	1	CCL15
2.22091669312571E-131	0.963879923267128	0.582	0.481	3.72181219434007E-127	1	LAPTM4B
0	0.955689173035316	0.868	0.698	0	1	IFI27
2.00068981899742E-60	0.951986686261771	0.465	0.417	3.35275599867588E-56	1	ATP6V0D1
1.64624441832712E-68	0.944106054726812	0.49	0.434	2.75877639623258E-64	1	CYSTM1
0	0.943490482884512	0.803	0.533	0	1	MUC13
1.18258488375132E-77	0.941842242077928	0.505	0.429	1.98177574819047E-73	1	CITED2
2.60121994768984E-25	0.937312992209935	0.389	0.374	4.35912438833864E-21	1	CD68
2.88476490193451E-168	0.936469751124	0.689	0.577	4.83428902266186E-164	1	GOLM1
1.30749437008611E-49	0.935430295935112	0.451	0.41	2.1910990653903E-45	1	CAPN1
1.33793813370535E-239	0.932104278270586	0.727	0.586	2.24211672446343E-235	1	NDUFA1
6.57041494125973E-07	0.926838759282773	0.295	0.302	0.011010701358563	1	PRCP
5.57216084904164E-32	0.926322496634676	0.389	0.345	9.33782715082399E-28	1	B3GNT3
4.42879898682521E-174	0.925512764790691	0.663	0.55	7.42178134212169E-170	1	RBM47
4.28671618001464E-26	0.92224810715751	0.393	0.377	7.18367897446853E-22	1	G3BP2
2.17536540346141E-12	0.920417387026844	0.333	0.328	3.64547734312064E-08	1	HEBP1

2.76757981336337E-24	0.919306468617455	0.396	0.381	4.63791025123433E-20	1	TMEM147
3.2704553632485E-132	0.917812599742918	0.601	0.518	5.48062909773184E-128	1	SAP18
7.59248536116845E-12	0.917326046523477	0.3	0.29	1.27234869682461E-07	1	CLIC1
6.14614273595727E-108	0.916505106069875	0.601	0.511	1.02997059969172E-103	1	SLPI
3.52302039873548E-252	0.912784998765072	0.774	0.577	5.90387758420092E-248	1	RNF128
2.65084372164997E-13	0.905744662293418	0.333	0.328	4.44228390874101E-09	1	NIBAN2
0	0.904642784180875	0.897	0.71	0	1	AKR1C3
3.86540832102799E-301	0.897334564186814	0.805	0.561	6.47765126437871E-297	1	NDRG1
2.73969373554091E-248	0.893268601832957	0.76	0.644	4.59117876201945E-244	1	CFL1
0	0.889270552318575	0.937	0.808	0	1	PGRMC1
1.77915907162726E-18	0.887989515212305	0.362	0.351	2.98151477223297E-14	1	VASP
1.97705803201326E-81	0.887983062190097	0.54	0.464	3.31315385004783E-77	1	PYGB
3.99904669669826E-24	0.887514431338078	0.386	0.366	6.70160245432694E-20	1	TXNDC17
8.87923056028184E-228	0.886458985553081	0.775	0.658	1.48798145729203E-223	1	CES2
6.55953910799691E-09	0.886058224167872	0.33	0.34	0.000109924756371812	1	VDAC1
1.90239155463221E-14	0.885789009938263	0.323	0.304	3.18802776725266E-10	1	SI
3.97622026518652E-129	0.884964755710127	0.614	0.491	6.66334992039957E-125	1	TFRC
2.65425682546183E-08	0.883403582161681	0.323	0.328	0.000444800358810894	1	DPP4
1.58463933706357E-120	0.881784024695138	0.582	0.443	2.65553860105112E-116	1	KLF5
4.05720447215325E-08	0.878343994899235	0.312	0.318	0.000679906325443441	1	VAT1
2.38050702670584E-101	0.869471027016735	0.588	0.522	3.98925367535365E-97	1	NPC2
6.39415035763993E-86	0.868203044789572	0.547	0.486	1.0715317169333E-81	1	CMBL
1.57851842818256E-32	0.860687862804032	0.428	0.404	2.64528118194833E-28	1	CDKN1A
0	0.858767620970355	0.895	0.726	0	1	UGT2B4
5.05776104804557E-09	0.857915147669622	0.346	0.361	8.47579596431477E-05	1	SLC25A5
3.04731016377382E-244	0.853369087952208	0.794	0.681	5.10668237245216E-240	1	BST2
1.61285154146002E-12	0.852472411779005	0.349	0.352	2.7028166131787E-08	1	CAPNS1
5.25489827544335E-281	0.852449466173742	0.846	0.682	8.80615852998797E-277	1	LAMP2
0	0.850338240590495	0.954	0.833	0	1	ALDH1A1
6.1074568798957E-12	0.849007781658346	0.333	0.328	1.02348762393292E-07	1	CTSC
1.12377504957869E-163	0.848363546188706	0.685	0.538	1.88322222808397E-159	1	ERICH5
1.19349807082838E-107	0.848293453288113	0.603	0.53	2.00006406709419E-103	1	TSPAN6
6.68032178801824E-57	0.843197030880213	0.488	0.426	1.1194883252361E-52	1	CLDN3
1.24384925600284E-09	0.842791043336437	0.337	0.345	2.08444258320957E-05	1	SNX17
5.12360863032776E-166	0.84250443004388	0.693	0.596	8.58614334270327E-162	1	BEX3
6.9560658818674E-128	0.842305598534491	0.628	0.539	1.16569752048334E-123	1	TPD52
9.31846838112593E-11	0.841057361641212	0.348	0.351	1.56158893130908E-06	1	CDS1
9.36160107742037E-23	0.838120857159978	0.396	0.385	1.56881710855411E-18	1	GALK1
6.80882936376875E-43	0.836347852435975	0.46	0.426	1.14102362478037E-38	1	VMA21
2.13425784515205E-34	0.830672125390603	0.464	0.437	3.57658929690581E-30	1	HPGD
5.20548370643052E-08	0.828333570769688	0.346	0.364	0.000872334959523626	1	TM9SF1
0	0.828010672826125	0.981	0.949	0	1	EPHX1
3.2695690101678E-84	0.827718729776537	0.578	0.521	5.47914374723919E-80	1	CBR1
1.74551860515088E-146	0.825650176224909	0.678	0.597	2.92514007851184E-142	1	TMEM14A
6.62028129738467E-89	0.817338037248179	0.556	0.488	1.10942673981572E-84	1	ENO1
8.29928410840941E-17	0.817145581525244	0.393	0.395	1.39079403088725E-12	1	TMEM64
2.37228155646343E-31	0.8141059432316	0.451	0.441	3.97546943232142E-27	1	ITPK1
0	0.812130093323504	0.963	0.863	0	1	MT-CO2
1.13233919696426E-21	0.804399846672768	0.419	0.412	1.89757402627271E-17	1	MGAT2
1.82085656230826E-30	0.80367440259636	0.439	0.407	3.05139142711618E-26	1	MATN2
2.55103889355706E-25	0.802780556262453	0.438	0.437	4.27503097782291E-21	1	SLC25A4
5.39914657822607E-10	0.800990298893759	0.267	0.247	9.04788983579125E-06	1	TYMS
0	0.80066622952431	0.915	0.759	0	1	UGDH
4.73181020286092E-85	0.800585477538136	0.598	0.559	7.92956753795432E-81	1	MGST3
6.70200031358105E-56	0.799140373886626	0.519	0.487	1.12312121254991E-51	1	GRN
1.81236650409878E-64	0.797756868493293	0.559	0.536	3.03716378756874E-60	1	TMEM205
6.7947907330507E-97	0.797310809600219	0.601	0.547	1.13867103104464E-92	1	NDUFC2
1.76248732956535E-133	0.796769006104989	0.646	0.499	2.95357626688561E-129	1	CLDN4
0	0.795985419395614	0.985	0.949	0	1	CES1
5.21229090592896E-66	0.79505364338731	0.558	0.517	8.73475710015575E-62	1	GUSB

6.64991554088711E-16	0.794835672481384	0.374	0.37	1.11439284634186E-11	1	PSMD10
8.69570606944239E-73	0.790124803337432	0.554	0.512	1.45722642311716E-68	1	NDUFB9
9.01850063499085E-25	0.787113855729815	0.426	0.421	1.51132033641177E-20	1	RNPEP
3.91999260962028E-13	0.785964263222424	0.387	0.392	6.56912361520167E-09	1	NSDHL
6.78436039001533E-164	0.785560381407672	0.711	0.639	1.13692311415877E-159	1	PSME1
8.24333011707408E-195	0.782035455412907	0.737	0.646	1.38141726101927E-190	1	MT-ND2
2.1535627846522E-32	0.781526870133559	0.463	0.447	3.60894051452015E-28	1	VBP1
1.00567730330636E-10	0.780975449971921	0.278	0.263	1.6853140248808E-06	1	CENPB
7.08876534382568E-92	0.779444086316265	0.592	0.501	1.18793529631831E-87	1	TMEM45B
2.79735621682809E-40	0.778279743562065	0.485	0.469	4.68780954816052E-36	1	PRDX4
1.17639479988011E-25	0.777386321851372	0.44	0.43	1.97140240563909E-21	1	SLBP
0	0.772704654785723	0.886	0.582	0	1	TSC22D3
3.05477660374826E-134	0.77230085994713	0.675	0.599	5.11919463256134E-130	1	S100A16
1.48716925595575E-14	0.772005323703944	0.388	0.391	2.49219823913065E-10	1	SYBU
6.96796079387816E-70	0.770602797174173	0.554	0.519	1.1676908698381E-65	1	IFI30
3.75280072902772E-112	0.765197558750782	0.637	0.534	6.28894346170465E-108	1	CAPN2
7.06589223003744E-178	0.763575589275892	0.725	0.642	1.18410221990967E-173	1	H3F3A
2.18869608586664E-12	0.762970542886215	0.381	0.393	3.66781690069531E-08	1	ATP6V0E1
7.54330409679655E-54	0.762865698106468	0.545	0.525	1.26410690054117E-49	1	SUMF2
5.0763696663889E-58	0.761650116726924	0.525	0.486	8.50698028693452E-54	1	BSG
2.093688431935E-91	0.761492427350872	0.595	0.522	3.50860307423667E-87	1	ATP6AP1
3.79639813390793E-09	0.759087200399942	0.363	0.382	6.36200399280291E-05	1	PRPS1
2.12694357535773E-53	0.757619927518043	0.534	0.509	3.56433204358449E-49	1	AIFM1
8.80272214006592E-17	0.757454603897776	0.379	0.377	1.47516017623225E-12	1	PPP1CA
1.42011437769835E-58	0.749633287029909	0.552	0.529	2.37982767414689E-54	1	CD63
7.8029989797609E-20	0.747389919728214	0.436	0.44	1.30762656902833E-15	1	PYCR2
1.76813257863428E-23	0.745059247007015	0.449	0.451	2.96303657527533E-19	1	HACD3
5.63701586832718E-164	0.743046649598517	0.719	0.644	9.44651119214268E-160	1	MT-ND5
1.13728858343835E-87	0.742912224383376	0.592	0.527	1.90586820812599E-83	1	HNRNPH2
1.5133014869455E-07	0.7407656567457	0.346	0.362	0.00253599063182326	1	PSMB10
1.40850665398106E-126	0.738701231701489	0.689	0.618	2.36037545074147E-122	1	CRYL1
4.20913731485865E-21	0.73860917628609	0.428	0.431	7.05367231224013E-17	1	CHMP2A
1.75341787096702E-11	0.738188995424962	0.276	0.251	2.93837766816654E-07	1	TCF19
1.8866235888288E-23	0.737765758822725	0.414	0.4	3.1616038101593E-19	1	GCHFR
5.28629778433448E-47	0.737318543454013	0.52	0.499	8.85877782698771E-43	1	PSMB8
3.85296258841556E-10	0.730793142942277	0.378	0.394	6.4567947056668E-06	1	NDFIP2
1.26910736292639E-22	0.728491175021823	0.455	0.459	2.12677011879204E-18	1	STT3A
1.02352743376672E-113	0.72795193137361	0.66	0.61	1.71522727350627E-109	1	PRDX2
1.23410582097226E-252	0.725990438213284	0.892	0.806	2.06811453478531E-248	1	MGST1
7.83501505138661E-80	0.723236548021317	0.598	0.537	1.31299182231137E-75	1	FAM50A
2.36742622970073E-52	0.721483561246674	0.539	0.521	3.96733287573249E-48	1	PLS3
5.34411377159343E-12	0.721149358096612	0.317	0.297	8.95566585843628E-08	1	IGFBP3
2.45547634276871E-81	0.721107486852095	0.597	0.54	4.1148872552118E-77	1	ANXA2
1.71825702158936E-25	0.720181963714014	0.452	0.455	2.87945511677945E-21	1	UBE2Q1
7.98346764449235E-66	0.720061910744914	0.554	0.491	1.33786950786403E-61	1	CMC4
1.88897713614608E-49	0.718973322435724	0.528	0.508	3.1655478847536E-45	1	SDF4
1.50092245801746E-56	0.710199015351721	0.554	0.537	2.51524585514566E-52	1	ARF1
5.60126332400767E-25	0.709922942947994	0.451	0.442	9.38659707837205E-21	1	CD99L2
4.08322698773861E-07	0.709509004899706	0.359	0.376	0.00684267178605235	1	KCNK1
3.07896544925047E-15	0.70920980266308	0.404	0.409	5.15973029985393E-11	1	FAM3A
1.08813811548743E-82	0.708861495780797	0.605	0.574	1.82350185393383E-78	1	BLVRB
1.13589544221128E-91	0.70832651051343	0.628	0.567	1.90353358205767E-87	1	TRAM1
6.27250444764247E-10	0.707931337220949	0.374	0.389	1.05114629533593E-05	1	AGPAT1
8.69829168927877E-201	0.705228007889348	0.82	0.719	1.45765972128934E-196	1	GPX2
4.1486304771371E-28	0.704773961231532	0.465	0.465	6.95227495358635E-24	1	RTN3
5.06517006961497E-238	0.703664261192806	0.838	0.732	8.48821200266077E-234	1	MT-ND3
2.24197610822225E-103	0.702413413341622	0.586	0.436	3.75710356215884E-99	1	SLC6A19
6.30378737987195E-29	0.699899135692519	0.496	0.493	1.05638868911894E-24	1	PRG4
1.1373152938424E-08	0.698787665296027	0.374	0.392	0.000190591296942109	1	UQCR10
2.09686692317448E-63	0.697547536240015	0.566	0.518	3.51392958985579E-59	1	PNKD

1.34813280007285E-57	0.695982889983441	0.548	0.516	2.25920094636209E-53	1	DAP
1.22108596962353E-25	0.694896275926438	0.456	0.457	2.04629586789512E-21	1	TCEAL4
2.7104070011726E-95	0.6918797703907	0.634	0.583	4.54210005256504E-91	1	PFN1
7.68929923107685E-15	0.690427610009142	0.411	0.422	1.28857276514386E-10	1	RAB5IF
1.34837566588261E-103	0.690383474318656	0.718	0.628	2.25960794088608E-99	1	DEPRECATED-ENSG00000198074
2.04486255927335E-75	0.689738004789795	0.615	0.589	3.42678067683028E-71	1	CTSD
4.7005149833586E-34	0.689246039375728	0.48	0.455	7.87712300911234E-30	1	AMOTL1
4.87557136052839E-136	0.689215815162441	0.764	0.67	8.17048248597347E-132	1	ANPEP
1.99482844622023E-22	0.687561452158323	0.441	0.438	3.34293351017586E-18	1	RAMP1
1.50813872092645E-09	0.685257857328561	0.379	0.392	2.52733886852855E-05	1	SMC1A
1.04235157611757E-11	0.684656333857151	0.379	0.387	1.74677277125782E-07	1	RNF187
8.32936806806101E-61	0.683521220121106	0.605	0.588	1.39583550084566E-56	1	APP
5.4498290672489E-96	0.680542100253025	0.605	0.521	9.1328235508957E-92	1	CALM3
4.07746081123357E-10	0.680329903691024	0.379	0.397	6.83300882746522E-06	1	UQCR11
1.56513791044185E-13	0.678648745724762	0.422	0.442	2.62285811031845E-09	1	COX7C
1.86663140460872E-54	0.67852388194245	0.561	0.528	3.12810090784329E-50	1	MDK
1.99348723585639E-24	0.676838875626275	0.447	0.422	3.34068590984814E-20	1	EHF
5.31095594072452E-09	0.676732798873934	0.397	0.422	8.90009996546614E-05	1	PIGT
3.12824856327871E-81	0.675245653026162	0.64	0.614	5.24231894234246E-77	1	APLP2
1.39102758925877E-81	0.674773050028094	0.629	0.57	2.33108403407985E-77	1	TMEM106C
1.44938933299996E-64	0.674303518156367	0.609	0.589	2.42888664424133E-60	1	TSTA3
5.01952613754086E-08	0.673942393069671	0.375	0.397	0.000841172190129098	1	CAPZB
4.39770168364254E-28	0.673935682615838	0.474	0.471	7.36966848144817E-24	1	STK24
2.12956843066484E-09	0.673715550762852	0.427	0.462	3.56873077610815E-05	1	CPQ
1.76049366672842E-23	0.672829119739377	0.455	0.457	2.95023528670349E-19	1	CHCHD2
3.13070689874311E-15	0.66947840270916	0.398	0.396	5.2464386209137E-11	1	IMPA2
1.22929315219562E-75	0.668817881118563	0.592	0.498	2.06004946444942E-71	1	PGM2
1.07542600408433E-27	0.666981764413908	0.447	0.437	1.80219889764453E-23	1	LASP1
5.32304237933235E-08	0.66598449187326	0.383	0.409	0.000892035441928515	1	PSMB4
1.47081836035144E-07	0.665784952299937	0.389	0.415	0.00246479740827695	1	RETSAT
1.64432225657158E-49	0.664992104654135	0.537	0.52	2.75555523756265E-45	1	ATP5F1D
3.61428252515352E-47	0.664210791757162	0.54	0.529	6.05681465565227E-43	1	COX6C
2.97872410758443E-57	0.66237881252839	0.578	0.545	4.99174585948998E-53	1	SPTSSA
9.81663299895462E-42	0.661985014792282	0.544	0.531	1.64507135796482E-37	1	PCK2
1.10696689311499E-31	0.661688353086742	0.512	0.517	1.85505511948209E-27	1	MGST2
4.71898776104266E-43	0.661019392916308	0.521	0.503	7.90807968995529E-39	1	YWHAZ
2.52911135046936E-17	0.660199371515111	0.4	0.394	4.23828480111655E-13	1	TLE5
7.22435409834954E-169	0.657695553181944	0.799	0.628	1.21065725980142E-164	1	ATP1B1
7.19040617032088E-40	0.65738840095844	0.528	0.505	1.20496826602237E-35	1	GPAA1
1.60355818134474E-52	0.657232334262605	0.556	0.544	2.68724280029752E-48	1	PRKACA
1.09733114832889E-156	0.656176268438174	0.778	0.629	1.83890753836955E-152	1	ACSL4
1.30797228104044E-16	0.653452767407921	0.45	0.465	2.19189994856757E-12	1	PERP
8.63875377884717E-73	0.652895967783194	0.592	0.478	1.44768235825921E-68	1	F2RL1
9.83293162959486E-08	0.652046944510292	0.389	0.412	0.00164780268248751	1	SERPINB1
4.14873384956064E-119	0.651578657855645	0.727	0.625	6.95244818509373E-115	1	HSD17B11
2.75305048112467E-179	0.65047832524991	0.809	0.652	4.61356199626872E-175	1	TDO2
2.51418435128468E-15	0.649481824800618	0.445	0.467	4.21327013588286E-11	1	FAM114A1
3.06329888056117E-38	0.649306138198744	0.52	0.503	5.1334762640444E-34	1	IDH3G
1.93288480224283E-15	0.648551110265414	0.409	0.415	3.23912835159854E-11	1	TALDO1
2.42001607664747E-77	0.648008359213308	0.636	0.585	4.05546294124583E-73	1	TM9SF2
5.37526436827166E-92	0.647373074970684	0.652	0.618	9.00786802834966E-88	1	MT-ND1
1.51352187737809E-21	0.64622789239676	0.456	0.466	2.5363599621102E-17	1	DSTN
3.10426037425012E-19	0.644689153497263	0.432	0.427	5.20211953516835E-15	1	PGD
1.98939067297282E-35	0.643722751452704	0.516	0.505	3.33382088976785E-31	1	TMBIM1
7.84743733747991E-113	0.641140083304297	0.702	0.647	1.31507354901488E-108	1	MPC2
4.22607911443326E-09	0.638069511768713	0.386	0.403	7.08206337996726E-05	1	FLOT2
2.93273069773159E-06	0.637349990868224	0.363	0.386	0.0491467010325859	1	LAMTOR1
2.02843480546775E-11	0.63717869091103	0.41	0.428	3.39925104700285E-07	1	HNRNPUL1
4.01980396709091E-14	0.634032869255435	0.454	0.483	6.73638748805095E-10	1	ALDH9A1
1.29976650958672E-58	0.631740671502474	0.616	0.603	2.17814871676543E-54	1	TMCO1

7.61869013890479E-07	0.631257575932508	0.379	0.407	0.0127674009347766	1	KDELR2
8.07364397630406E-21	0.629697417640767	0.468	0.479	1.35298125754903E-16	1	HNRNPA0
5.58970105155002E-11	0.626582986520056	0.42	0.447	9.36722102218753E-07	1	CDC37
3.19101793266152E-300	0.626478438029883	0.993	0.985	5.34750785155418E-296	1	VTN
4.88021391347823E-29	0.625955879278681	0.503	0.511	8.17826247620682E-25	1	OTC
2.01147739913343E-35	0.625347103857796	0.515	0.497	3.37083382546781E-31	1	UBE2A
7.83539265926995E-237	0.625079870654993	0.881	0.754	1.31305510184046E-232	1	MT-CYB
3.64562553346696E-35	0.62198492391989	0.529	0.514	6.10933926898393E-31	1	BTG1
1.52216094886073E-10	0.620772915780509	0.427	0.458	2.55083731810081E-06	1	ZNF706
7.23960358780962E-12	0.620161356850064	0.429	0.45	1.21321276924514E-07	1	GSTA2
3.2975980005569E-27	0.615542467897331	0.495	0.508	5.52611472933325E-23	1	CUTA
2.75409874497332E-44	0.611889854171416	0.566	0.561	4.61531867682629E-40	1	CNN3
4.16943945542161E-17	0.609202953010407	0.462	0.481	6.98714663939554E-13	1	CIAO2A
8.92769515380414E-53	0.608635920888811	0.592	0.57	1.4961031538745E-48	1	ACTN4
9.24732200039945E-17	0.607938349679577	0.47	0.49	1.54966622082694E-12	1	CTSS
1.72764169611715E-53	0.607327167179979	0.585	0.562	2.89518195435311E-49	1	EMD
1.01441396275592E-06	0.604619319249031	0.204	0.268	0.0169995491878636	1	UGT2B15
3.06218792224035E-38	0.604072280068704	0.591	0.598	5.13161452009037E-34	1	CDHR5
1.34078185699528E-97	0.600958840162455	0.701	0.671	2.24688223595269E-93	1	CST3
2.56525150014538E-15	0.600260901413413	0.443	0.456	4.29884846394362E-11	1	DEPRECATED-ENSG00000159228
1.99606871418476E-07	0.599093124866404	0.19	0.252	0.00334501195123083	1	RNF6
1.82682767512972E-08	0.598996451965479	0.419	0.451	0.000306139781798238	1	SIVA1
5.33183275170852E-60	0.598667223890153	0.606	0.578	8.93508532531313E-56	1	ACTG1
2.11205327989667E-09	0.59545831052032	0.415	0.447	3.53937888645083E-05	1	ATP5ME
2.69969821457406E-58	0.593096267062708	0.608	0.58	4.5241542679832E-54	1	SMIM14
8.84753208356982E-234	0.591905038701251	0.912	0.858	1.48266942656463E-229	1	ACTB
1.16574846493057E-38	0.591057421827735	0.554	0.553	1.95356127753066E-34	1	NDUFA2
4.61943951529773E-48	0.590024982929188	0.585	0.58	7.74125673973594E-44	1	HNRNPF
2.14134502358754E-126	0.589082318467712	0.742	0.59	3.58846599052799E-122	1	MBNL3
2.08870614105473E-12	0.589024264243125	0.445	0.477	3.50025375117952E-08	1	RTN4
1.87398704141223E-17	0.583188150537608	0.488	0.515	3.14042748399862E-13	1	RAB2A
2.71947631210241E-08	0.582348655777322	0.188	0.252	0.000455729840382122	1	FBXO21
9.51905125528219E-46	0.580895562931927	0.554	0.49	1.59520260936019E-41	1	ABCD1
5.00047988582793E-07	0.580781124476067	0.389	0.399	0.00837980419267045	1	NEB
5.52459870689146E-07	0.580340103345469	0.191	0.251	0.00925812251300872	1	NSF
1.46136679350995E-123	0.579496869892001	0.772	0.719	2.44895847256398E-119	1	LAMP1
6.51122439023578E-128	0.577763939914655	0.839	0.801	1.09115098331571E-123	1	HMGCS2
4.75057933468267E-09	0.577563012217166	0.431	0.466	7.96102084906123E-05	1	RAB5C
3.11310505923838E-106	0.576013582792152	0.761	0.709	5.21694145827167E-102	1	TM4SF4
2.59225022221129E-190	0.575993670003722	0.903	0.823	4.34409292238168E-186	1	PTGR1
9.98337470317427E-28	0.575294663323732	0.518	0.507	1.67301393275794E-23	1	MIDN
2.93416151965438E-06	0.57499834206601	0.405	0.442	0.0491706787463682	1	FH
1.85310618745604E-80	0.57449723670303	0.703	0.67	3.10543534893884E-76	1	IDH1
7.63426233224245E-24	0.570738663027425	0.514	0.532	1.27934968163719E-19	1	POLR2L
4.9134644875452E-09	0.57052661553772	0.186	0.253	8.23398378822824E-05	1	NDUFB4
3.61843626254892E-190	0.570468380139004	0.858	0.792	6.06377548877948E-186	1	OAZ1
1.33123605486143E-06	0.56860988497322	0.405	0.445	0.0223088538073679	1	NDUFS7
1.04584354708427E-161	0.567790565549551	0.973	0.941	1.75262461620383E-157	1	AGT
2.01807836912275E-08	0.567455315664517	0.195	0.261	0.000338189573097591	1	RETREG3
4.26205613643313E-17	0.566724036170527	0.464	0.485	7.14235367343463E-13	1	SLC25A1
3.04737517763055E-86	0.565339853043274	0.727	0.714	5.10679132267328E-82	1	GSTK1
1.78272882098374E-49	0.558878888450773	0.524	0.476	2.98749695820456E-45	1	IRAK1
1.4155894186992E-240	0.557768455156855	0.946	0.888	2.37224474785612E-236	1	B2M
2.21090726773196E-16	0.557340760345096	0.484	0.513	3.70503839926522E-12	1	NDUFA3
1.91870907384002E-87	0.553263292449073	0.79	0.708	3.21537266594111E-83	1	AKR1B10
1.42422257657759E-06	0.553119970025936	0.377	0.4	0.0238671219382872	1	RHOC
3.46117308369891E-271	0.552231606495459	0.94	0.875	5.80023385366263E-267	1	MT-ND4
1.05980986560302E-08	0.5502936005509	0.185	0.251	0.000177602937277753	1	PHLPP2
4.43109706988137E-26	0.548212293645435	0.467	0.431	7.4256324697072E-22	1	DDIT4
9.19617541564861E-07	0.546447741292407	0.207	0.271	0.0154109507615439	1	NFKB1

6.58791362761856E-08	0.54494510249956	0.209	0.279	0.00110400256571632	1	ATP8B1
1.64917182208206E-67	0.542735941084113	0.658	0.646	2.76368213944511E-63	1	SLC9A3R1
5.53013166485397E-65	0.542486496573753	0.669	0.643	9.26739464396228E-61	1	GOLIM4
4.73654217470105E-08	0.541293201855563	0.416	0.45	0.000793749737636401	1	KRTCAP2
3.29151990062278E-07	0.53756886867578	0.197	0.26	0.00551592904946366	1	CC2D1A
2.92500390447244E-20	0.534451921488612	0.504	0.531	4.90172154311492E-16	1	NDUFB7
5.42407712948551E-09	0.533226417763746	0.208	0.281	9.08966845359181E-05	1	MXD4
5.42033680553851E-09	0.533083220531918	0.427	0.459	9.08340041872144E-05	1	COX4I1
2.28325434800313E-07	0.532664183010996	0.201	0.265	0.00382627763638364	1	KIF16B
9.91927493331249E-23	0.532116716587277	0.5	0.512	1.66227209332451E-18	1	P4HB
2.93420044961342E-23	0.531613079828027	0.52	0.548	4.91713311346217E-19	1	UQCRQ
5.32097715566656E-12	0.531105911819247	0.396	0.385	8.91689351746602E-08	1	DUSP9
1.1057281861355E-275	0.529207851085922	0.961	0.897	1.85297929432587E-271	1	MT-ATP6
9.04602973880247E-10	0.529141705089291	0.185	0.252	1.51593366362852E-05	1	ANKH
5.77107844285012E-29	0.528204110294624	0.556	0.567	9.67117325452823E-25	1	CYB5R3
8.01814280620665E-27	0.527610228878737	0.536	0.526	1.34368037146411E-22	1	VNN 1.00
4.31061199169525E-07	0.52589988985419	0.242	0.319	0.0072237235756829	1	HACD2
1.86990406987068E-17	0.525813940783168	0.515	0.534	3.13358524028928E-13	1	RRBP1
9.10257715782567E-16	0.523335965685398	0.492	0.52	1.52540988010843E-11	1	MAOB
8.20515429768545E-25	0.522212064296358	0.514	0.519	1.37501975720613E-20	1	CALM2
3.86934384890704E-07	0.518583916387979	0.436	0.474	0.00648424642199842	1	CTNNA1
5.05076015463773E-07	0.517399745335695	0.237	0.313	0.0084640638671419	1	TMEM214
2.48566694225818E-32	0.515773591821598	0.588	0.611	4.16548066183626E-28	1	ACAA2
3.2150322053408E-07	0.512528535765425	0.192	0.253	0.00538775096971011	1	BCAT2
1.52895548256131E-11	0.511609049537427	0.46	0.488	2.56222359767624E-07	1	GSDMD
3.96124466637761E-23	0.51006435414342	0.533	0.554	6.63825381191559E-19	1	PUF60
3.54104715294476E-24	0.508675226091847	0.518	0.515	5.93408681890482E-20	1	MORF4L2
3.07298974024556E-36	0.508520366911656	0.594	0.594	5.1497162067035E-32	1	SCCPDH
9.82843368804612E-76	0.504415211089714	0.771	0.739	1.64704891744277E-71	1	SERPINA6
4.30794311752096E-09	0.504379213570295	0.226	0.304	7.21925107634162E-05	1	SLC39A9
3.42580224414669E-107	0.499415290267761	0.584	0.442	5.74095940074103E-103	1	SLC6A8
3.20270924394685E-07	0.499309911950761	0.249	0.327	0.00536710015100613	1	ABCB6
3.50199657679244E-10	0.497590437021497	0.206	0.283	5.86864586338877E-06	1	EIF2S1
6.95658987433108E-12	0.497559787060301	0.178	0.251	1.1657853311404E-07	1	CYREN
3.14688786072308E-08	0.497433840396987	0.193	0.257	0.000527355467699973	1	HOXA13
3.51992952823132E-08	0.495047538505626	0.212	0.284	0.000589869790341004	1	SF3B3
1.12106180027878E-09	0.494858203606454	0.195	0.266	1.87867536490719E-05	1	CPT1A
3.3316531367339E-149	0.494401743542627	0.888	0.858	5.58318432653866E-145	1	DEPRECATED-ENSG00000106538
1.20590990863601E-18	0.494341597271208	0.493	0.474	2.02086382489222E-14	1	HNF4G
1.2761555991747E-07	0.494119458492081	0.224	0.297	0.00213858155309696	1	PPP6C
1.673923078187E-53	0.492816466961165	0.624	0.613	2.80516029442578E-49	1	CYC1
5.00505262302265E-27	0.491315033218987	0.576	0.592	8.38746718566135E-23	1	LSR
6.59639935108916E-08	0.490799245739913	0.219	0.291	0.00110542460325552	1	FKBP1A
4.36359156717151E-24	0.490522228969901	0.543	0.541	7.31250674826602E-20	1	GDI1
2.0237769803104E-24	0.490287962084787	0.561	0.585	3.39144546360416E-20	1	COPG1
2.44896119409841E-12	0.490167902753278	0.468	0.484	4.10396916907011E-08	1	FRK
5.09780577700981E-07	0.488198703111318	0.21	0.276	0.00854290292111303	1	HDDC2
1.62901500415565E-99	0.487886148355594	0.748	0.694	2.72990334396403E-95	1	LMNA
1.44504817899421E-08	0.487758774701515	0.214	0.288	0.00024216117383585	1	ATPAF1
1.22321623442499E-07	0.486866564949577	0.43	0.46	0.0020498657656494	1	SF3B5
1.90866467758354E-14	0.486467516604808	0.493	0.52	3.1985402666945E-10	1	MYDGF
1.60549310599085E-07	0.486174082538635	0.228	0.301	0.00269048534701947	1	CAMK1
9.20179479416206E-10	0.486019886730194	0.46	0.494	1.54203677160568E-05	1	SYNGR2
5.29837795360354E-58	0.485894827650295	0.698	0.694	8.87902177464881E-54	1	TMSB4X
7.82073420445269E-09	0.483794564133122	0.236	0.321	0.000131059863798218	1	NKAP
2.21109360312618E-08	0.483599860161997	0.194	0.26	0.000370535066011885	1	KIAA2013
2.07530036646106E-82	0.483241108451569	0.749	0.75	3.47778835411544E-78	1	ECH1
6.01124011378248E-08	0.483112448872436	0.189	0.252	0.00100736361826767	1	PSMA3
2.02734567068199E-71	0.48257207004628	0.699	0.672	3.39742587492888E-67	1	KRT8
2.59493635860187E-132	0.482017540716443	0.843	0.786	4.34859434974501E-128	1	MT-ND4L

1.04038881774122E-06	0.481461759768086	0.227	0.297	0.0174348358077074	1	HAUS4
2.36468726926242E-11	0.4813846905662	0.188	0.262	3.96274292582997E-07	1	CHMP2B
1.23217296373675E-128	0.480965723805146	0.891	0.838	2.06487545263004E-124	1	CD74
8.22845564809474E-42	0.480078230877671	0.639	0.634	1.37892459750772E-37	1	RARRES2
9.18071739506339E-87	0.479886375965658	0.789	0.758	1.53850462106472E-82	1	KHK
1.11679916371525E-08	0.479400334266271	0.21	0.282	0.000187153203855402	1	UQCC2
1.7971952460869E-81	0.477749578411009	0.732	0.548	3.01173979339243E-77	1	FKBP5
7.27564854266584E-07	0.477523587547549	0.259	0.341	0.0121925318277994	1	NPTN
2.74772144482085E-11	0.47751706646379	0.206	0.285	4.60463159723078E-07	1	GLB1
1.0969874682519E-09	0.476764075806015	0.466	0.497	1.83833159929654E-05	1	EGLN1
5.46644464819956E-11	0.473336991222261	0.195	0.271	9.16066794145282E-07	1	EBAG9
8.16411038370489E-35	0.473286750425779	0.621	0.626	1.36814161810127E-30	1	TAPBP
9.99127195616994E-103	0.470191090571131	0.85	0.699	1.67433735441496E-98	1	ASPH
5.82950207468602E-13	0.469529401912723	0.177	0.252	9.76907957675883E-09	1	PPA2
5.06717224760729E-08	0.468890396475061	0.202	0.269	0.00084915672525403	1	NUDT21
2.79075476726719E-62	0.467609898058884	0.67	0.576	4.67674683898636E-58	1	DNM2
1.0622521638334E-09	0.467089218035856	0.494	0.531	1.78012217615201E-05	1	GJB1
6.86261473466663E-08	0.46632756252015	0.469	0.518	0.00115003697723543	1	PGRMC2
1.74096193921169E-19	0.465949159545336	0.547	0.571	2.91750401773096E-15	1	HABP2
9.51759368897658E-142	0.465669504712967	0.95	0.918	1.59495835039869E-137	1	GC
4.09424071280057E-14	0.46513759810615	0.501	0.504	6.8611285865112E-10	1	ATRN
1.08755637721406E-08	0.462315368493635	0.189	0.254	0.000182252697693532	1	SEPTIN2
4.17303878021873E-35	0.462068310428692	0.633	0.64	6.99317838789054E-31	1	RNASE4
1.16721308937322E-225	0.46182344352161	0.977	0.922	1.95601569517163E-221	1	SELENOP
2.67625058373579E-09	0.460744098752653	0.202	0.273	4.48486072822443E-05	1	SH3GL1
7.72758713457352E-09	0.460638965324713	0.222	0.297	0.000129498905201183	1	EXOSC4
2.66779390914014E-07	0.460558310267611	0.219	0.288	0.00447068903293705	1	DEPRECATED-ENSG00000073578
7.15766619843602E-08	0.460348279908393	0.247	0.325	0.00119948170153391	1	DCAF7
1.33683322719599E-06	0.458223360591132	0.229	0.299	0.0224026512213504	1	MCUR1
5.26446965635279E-14	0.456175599280665	0.183	0.262	8.822198250116E-10	1	LAMC1
2.80525770078093E-13	0.455816867295215	0.176	0.251	4.70105085496868E-09	1	GMNN
1.02125059723185E-12	0.455564515291127	0.198	0.28	1.71141175084113E-08	1	LARP1
2.31756309264075E-49	0.455365881125347	0.687	0.701	3.88377223064737E-45	1	NUCB1
1.13777678388046E-07	0.455126887138024	0.245	0.327	0.00190668633442688	1	OGDH
1.75400674413386E-07	0.454079927098066	0.238	0.313	0.00293936450181952	1	HDAC1
9.83594674176108E-07	0.452132644513483	0.455	0.504	0.0164830795498432	1	SSR2
3.72705002108356E-07	0.449380716759902	0.467	0.52	0.00624579042533183	1	CAST
1.58519039010224E-14	0.449100062308914	0.174	0.252	2.65646205573334E-10	1	LARP4B
5.25037567595848E-67	0.448449609235551	0.703	0.656	8.79857955777122E-63	1	NFKBIA
6.43973234064608E-10	0.446380721788607	0.21	0.287	1.07917034564547E-05	1	SKAP2
5.94595887225869E-08	0.446378024016225	0.213	0.283	0.000996423787813111	1	TCP1
7.4957448540311E-238	0.446212136539611	0.983	0.948	1.25613692263853E-233	1	MT-CO1
4.28332223295162E-09	0.443926157396688	0.455	0.465	7.17799139798032E-05	1	CLDN7
3.55746078836747E-10	0.443572948286787	0.2	0.273	5.9615927891462E-06	1	SUSD3
1.39786734442008E-11	0.443501937361394	0.2	0.276	2.34254609577917E-07	1	BROX
7.40493832002833E-12	0.443076769031617	0.22	0.308	1.24091956367035E-07	1	MAPK1IP1L
1.92236186294889E-13	0.442889300148531	0.19	0.269	3.22149400992974E-09	1	MORC4
1.18215336663832E-67	0.442870222332777	0.708	0.712	1.98105261181249E-63	1	GPX4
7.06851835092966E-07	0.442753796102887	0.254	0.333	0.0118454230524879	1	UBE2Z
8.12815876923227E-15	0.4426282430742	0.185	0.268	1.36211684654794E-10	1	PAFAH1B2
1.59728048028409E-09	0.439993793991151	0.484	0.511	2.67672262886007E-05	1	CTSH
4.65903145126535E-08	0.437935994537991	0.188	0.251	0.000780760490603047	1	COPS7A
1.77087545010174E-11	0.437378124971882	0.198	0.275	2.9676330792805E-07	1	BCAP29
1.27573194885058E-07	0.437329666285939	0.222	0.293	0.0021378715998838	1	SAP30L
2.27935798762593E-12	0.437206416459414	0.178	0.25	3.81974811566353E-08	1	NUDT3
4.94850720437063E-07	0.437072566308462	0.199	0.26	0.00829270837308431	1	ILF2
6.6848806102188E-08	0.435387301969419	0.471	0.499	0.00112025229266047	1	INF2
1.88810069721037E-12	0.434804062599456	0.232	0.324	3.16407914838514E-08	1	TGFBR2
6.27891381458486E-176	0.4343365413936	0.967	0.921	1.05222037704813E-171	1	FN1
7.34942669828842E-09	0.432298916248792	0.445	0.445	0.000123161692609917	1	DEGS1

3.56377559286321E-13	0.431772789621957	0.207	0.29	5.97217513852017E-09	1	IGF2BP3
7.0435493109086E-13	0.429260073698659	0.177	0.251	1.18035799352206E-08	1	RANBP3
1.37988097155889E-11	0.429013140673001	0.497	0.536	2.31240453213839E-07	1	SDC2
3.65264368754869E-11	0.42650536228124	0.215	0.295	6.12110029159409E-07	1	MEGF9
6.14900243462955E-08	0.426166026294243	0.225	0.297	0.00103044982799522	1	CMIP
9.73085272945508E-08	0.424428942297055	0.193	0.256	0.00163069630040208	1	WBP2
7.30419265734044E-11	0.424393952845422	0.188	0.259	1.22403660551711E-06	1	WDTC1
1.23174381892922E-08	0.424048256980891	0.253	0.339	0.000206415629176158	1	SEC23B
4.27972366736481E-08	0.421951443442636	0.195	0.259	0.000717196092176996	1	CPTP
1.88030135669078E-13	0.420464599722882	0.175	0.25	3.1510090135424E-09	1	NELFB
9.47828160001157E-07	0.420263728157815	0.21	0.274	0.0158837043052994	1	DEPRECATED-ENSG00000213246
2.55269877137776E-09	0.418843561821088	0.252	0.339	4.27781260107486E-05	1	MECP2
3.00272952812946E-103	0.418477289339727	0.711	0.647	5.03197414323934E-99	1	STARD10
2.75347544305923E-09	0.418123082721434	0.482	0.522	4.61427414747865E-05	1	UBB
1.07460865126839E-103	0.418059681582559	0.755	0.705	1.80082917779556E-99	1	EEF2
9.08809406802468E-13	0.417816752279931	0.187	0.265	1.52298280391958E-08	1	ZMYND11
1.95567636194655E-12	0.417613287925063	0.179	0.251	3.27732244735003E-08	1	NOP14
4.00435397233839E-11	0.416760828932426	0.224	0.31	6.71049638684467E-07	1	ARHGAP1
4.5145205360531E-15	0.414460469537325	0.19	0.274	7.56543351431779E-11	1	CNOT11
3.28952076411608E-11	0.412578245548928	0.202	0.28	5.51257889650572E-07	1	GNAI3
5.46706164864421E-07	0.411742198664484	0.213	0.278	0.00916170191079796	1	DEPRECATED-ENSG00000181061
6.09758746336499E-12	0.411318010663226	0.187	0.261	1.02183370711071E-07	1	AGTRAP
6.22010427795148E-18	0.411241810315507	0.167	0.251	1.04236507489911E-13	1	TULP4
3.29082877380793E-10	0.411141806149504	0.24	0.325	5.51477085914733E-06	1	CANT1
1.39695637297948E-09	0.41060751915583	0.246	0.331	2.34101948983902E-05	1	SSX2IP
4.30375061666403E-12	0.408562167802534	0.198	0.275	7.21222528340558E-08	1	WRNIP1
8.94835879368646E-09	0.408475431084175	0.193	0.259	0.000149956596664598	1	NDUFB2
2.10798144483788E-06	0.407757806309313	0.221	0.286	0.0353255530525932	1	STUB1
7.91603910075744E-07	0.406829181779367	0.28	0.367	0.0132656983250493	1	TAF7
1.16731336978402E-10	0.406683934451998	0.185	0.254	1.95618374508407E-06	1	PIN1
1.83731086369807E-08	0.405889429262932	0.248	0.33	0.000307896554538523	1	IDS
7.28555337031567E-11	0.405443265046708	0.201	0.275	1.2209130337975E-06	1	LRRC41
4.76652569025742E-15	0.40528648184865	0.205	0.294	7.98774375173339E-11	1	MSH6
1.79373802805293E-10	0.404012283137414	0.204	0.277	3.00594618741111E-06	1	GLA
6.48418843501254E-14	0.403812688469229	0.194	0.275	1.0866202979394E-09	1	POMGNT1
2.61614134959313E-11	0.403519530295414	0.197	0.273	4.38412967364816E-07	1	TMEM189
3.80758036840471E-08	0.403173950623563	0.199	0.264	0.000638074318137261	1	IRF2BPL
4.76433792214624E-07	0.402833762870209	0.235	0.308	0.00798407748993267	1	FLNB
3.16787792460777E-15	0.402770806321715	0.182	0.264	5.3087298260577E-11	1	ZC3H4
1.92202849925001E-18	0.402181743222443	0.187	0.278	3.22093535904317E-14	1	MGAT4A
2.50016335679833E-12	0.401736973070372	0.185	0.259	4.18977375332264E-08	1	STRAP
1.5270522421911E-34	0.401466278577109	0.638	0.656	2.55903414746384E-30	1	SOD1
1.04892474919649E-09	0.401089791692767	0.229	0.308	1.75778809470347E-05	1	CHAMP1
1.48980721735124E-19	0.400482520843715	0.172	0.262	2.49661893483721E-15	1	CNNM3
4.86278596948301E-09	0.399434290271453	0.5	0.547	8.14905672765962E-05	1	EIF1
9.23336719648339E-11	0.399328720572478	0.211	0.288	1.54732767478669E-06	1	PSMD7
4.9193549394592E-07	0.399061288468385	0.279	0.365	0.00824385500754572	1	PPP3R1
6.12667384578452E-17	0.39905647341201	0.206	0.301	1.02670800307657E-12	1	SNX6
5.98626965786707E-07	0.398187301447235	0.207	0.27	0.0100317906926536	1	HPCAL1
4.97721000698301E-09	0.397664567488853	0.194	0.261	8.34080852970213E-05	1	MEPCE
3.71185828981226E-08	0.397324045547592	0.242	0.322	0.000622033212206738	1	CRKL
2.29127249617935E-07	0.396755686567744	0.217	0.285	0.00383971444909735	1	ABCF1
1.33478533121619E-16	0.39674899107465	0.207	0.301	2.23683325805209E-12	1	RC3H2
1.52293109824879E-08	0.396541110254439	0.202	0.269	0.000255212793444533	1	TSC22D4
3.9067489887956E-14	0.396010529016694	0.177	0.254	6.54692995542367E-10	1	PITRM1
3.05790174721228E-09	0.395998193582605	0.219	0.295	5.12443174797834E-05	1	ATP6V0B
6.13798341637504E-64	0.395471866322747	0.758	0.693	1.02860326091613E-59	1	SERPINA7
1.66094736226534E-07	0.3954101943178	0.226	0.298	0.00278341558968426	1	TMED2
1.00192263477759E-09	0.394792526481102	0.184	0.25	1.67902195136029E-05	1	MED29
9.36056337331969E-09	0.39306880572271	0.253	0.342	0.000156864321010091	1	PSMB5

2.05656703705563E-16	0.390539900818596	0.182	0.265	3.44639504069783E-12	1	EDEM2
1.30832996892694E-10	0.389930513884052	0.19	0.26	2.19249936192777E-06	1	PRRC1
5.29194807939466E-17	0.389462295856078	0.204	0.297	8.86824659144957E-13	1	ARHGAP5
5.78863122498044E-14	0.388863679854467	0.194	0.277	9.70058820682221E-10	1	SCAF4
9.76611891485385E-08	0.38804366568692	0.278	0.366	0.00163660620775121	1	CYB5B
4.65941176368586E-07	0.387564545399058	0.277	0.36	0.00780824223358477	1	LRRC8A
1.19033716244439E-06	0.38482347075813	0.232	0.302	0.0199476701682431	1	RAC1
2.66704535791056E-12	0.384505262661138	0.188	0.263	4.46943461078651E-08	1	VPS26B
4.56809234122803E-12	0.384486031726701	0.206	0.286	7.65520914542993E-08	1	C6orf120
5.023548509998E-07	0.38350830770838	0.216	0.279	0.00841846259305464	1	KHDRBS1
1.8941085066501E-08	0.382411474964524	0.209	0.279	0.000317414703544425	1	PET100
4.58459844358881E-14	0.381947030278347	0.189	0.269	7.68287007176612E-10	1	TOMM6
1.49015355461448E-08	0.380937803303205	0.233	0.31	0.000249719932682295	1	NUP62
8.99094295403261E-09	0.380460460922111	0.22	0.294	0.000150670222023679	1	IRF2
2.13908478269987E-18	0.380452263915379	0.183	0.272	3.58467827884844E-14	1	NBN
2.06001988485939E-10	0.380006027774837	0.229	0.312	3.45218132304736E-06	1	PPP1R10
1.57617977207721E-12	0.379892398276787	0.245	0.341	2.64136206204699E-08	1	SOS 2
6.5767407890068E-07	0.379852681937302	0.264	0.342	0.0110213022142176	1	KCNJ3
1.48285274764222E-18	0.379622177118098	0.175	0.263	2.48496463449884E-14	1	CASP3
1.95211683021972E-07	0.378167189004661	0.218	0.284	0.0032713573840822	1	DHFR
1.21396758950736E-07	0.377596668062262	0.241	0.316	0.00203436688649644	1	FARSA
1.08798524404972E-11	0.377299948092913	0.52	0.533	1.82324567197852E-07	1	KCTD3
2.60392421300632E-10	0.376784823728843	0.229	0.312	4.363656196156E-06	1	UBE2E3
1.16151140654829E-16	0.375803629827198	0.183	0.269	1.94646081509362E-12	1	COLGALT1
7.54520515256073E-11	0.37566147399226	0.207	0.283	1.26442547946613E-06	1	CHMP1B
8.07946701570313E-07	0.375432293384491	0.21	0.273	0.0135395708249153	1	TXN2
2.79277780273468E-15	0.375081384982895	0.202	0.29	4.68013704182278E-11	1	TIMMDC1
1.95179493443521E-15	0.374341085737723	0.536	0.574	3.27081795112653E-11	1	NUCKS1
8.91605020715209E-13	0.373777404224452	0.217	0.303	1.49415169371455E-08	1	CDC42SE2
1.6708484134988E-09	0.373312760634579	0.255	0.347	2.80000777134129E-05	1	UBE2V1
1.71578996548357E-06	0.373043223947809	0.27	0.352	0.0287532082415736	1	PAK2
7.25650981420326E-12	0.372752049003897	0.205	0.283	1.21604591466418E-07	1	NDUFA9
3.73916724381866E-10	0.372450318622544	0.193	0.262	6.26609646719131E-06	1	PDK2
1.98384733704225E-08	0.37234837438867	0.199	0.265	0.00033245313674154	1	DCTN3
1.67859035285771E-15	0.372138831094779	0.205	0.295	2.81298171331895E-11	1	ATP11A
1.16739701593795E-15	0.371412548788516	0.587	0.612	1.95632391930881E-11	1	TXNRD1
4.30186851233391E-22	0.371361952217486	0.173	0.267	7.20907125296917E-18	1	WDR44
2.36443840150072E-14	0.370856601637696	0.186	0.266	3.9623258732349E-10	1	SCAMP4
2.2156915986997E-06	0.370828568642276	0.211	0.271	0.0371305598110096	1	PARK7
1.03216947763755E-15	0.370722274147922	0.199	0.285	1.72970961062501E-11	1	CLDN14
5.08907897603582E-09	0.368975581782293	0.264	0.354	8.52827854804083E-05	1	RBM10
3.6784332822439E-09	0.368210100602397	0.249	0.334	6.16431849438433E-05	1	CDC42BPB
2.28250801307072E-12	0.367771595236785	0.264	0.364	3.82502692830392E-08	1	CD47
3.36431378104548E-10	0.367745129105647	0.222	0.3	5.63791703427602E-06	1	SAMHD1
1.44013019594986E-10	0.367291123777025	0.231	0.317	2.41337018237278E-06	1	POLR2K
3.21942021185124E-08	0.36616726724353	0.24	0.319	0.000539510439102031	1	ARCN1
2.91638131328177E-06	0.365019772877416	0.24	0.31	0.048872718047976	1	SMDT1
4.69602581446262E-12	0.362276360224326	0.554	0.599	7.86960005987647E-08	1	CREG1
3.72869137252834E-15	0.362167843567822	0.197	0.284	6.24854100208299E-11	1	ABHD10
8.69625352806681E-15	0.361970177349543	0.194	0.277	1.45731816623344E-10	1	SLAIN2
2.27047725143142E-09	0.361571332068681	0.196	0.263	3.80486577794877E-05	1	SNAP29
4.00721168691368E-15	0.360202054684948	0.179	0.261	6.71528534492994E-11	1	ATIC
4.43631179034315E-11	0.360125624448645	0.188	0.26	7.43437129825704E-07	1	TIMM50
3.37911758399346E-13	0.360016031192692	0.215	0.302	5.66272524725624E-09	1	CLN3
8.99282679033379E-08	0.359286028536292	0.262	0.344	0.00150701791352414	1	TCIRG1
1.8751093159714E-06	0.358651236986959	0.281	0.366	0.0314230819170487	1	AGPAT3
1.22157029285154E-09	0.358643614401273	0.219	0.294	2.0471074967606E-05	1	CFDP1
2.35348853702417E-15	0.357341939615571	0.192	0.277	3.9439760903451E-11	1	HIPK1
7.26988834030425E-08	0.357068513318114	0.304	0.402	0.00121828788806819	1	HUWE1
3.52934996363806E-13	0.356800201450069	0.243	0.341	5.91448466906466E-09	1	LMBR1

4.92125744867818E-09	0.356519800182393	0.216	0.288	8.24704323249489E-05	1	SNF8
3.41036658134164E-16	0.356183262272441	0.198	0.288	5.71509231701232E-12	1	STIM1
1.15095607953893E-12	0.355814595052093	0.215	0.301	1.92877219809134E-08	1	TTC1
4.27000860720636E-21	0.353665481150103	0.161	0.25	7.15568042395642E-17	1	POFUT1
2.65713860256567E-13	0.353457830420505	0.197	0.278	4.45283287017954E-09	1	CNIH1
1.1577245726266E-08	0.353354212661788	0.233	0.308	0.000194011483880766	1	TMUB1
5.86235587774085E-65	0.353063787200843	0.748	0.638	9.82413597991812E-61	1	SELENBP1
1.297661174485E-16	0.352444248014858	0.171	0.251	2.17462059620197E-12	1	GALNT1
6.70408620994453E-13	0.352424695756593	0.229	0.319	1.1234707670625E-08	1	TMEM129
1.21436487622922E-18	0.352131418489239	0.199	0.295	2.03503265958492E-14	1	LRRC58
1.9539248740904E-151	0.351577350939989	0.956	0.91	3.2743873040007E-147	1	MT-CO3
1.32059193226239E-89	0.351390042394434	0.992	0.985	2.21304796008531E-85	1	ALB
8.69996851155891E-08	0.351317900106294	0.291	0.387	0.00145794072316704	1	KTN1
9.98384234017963E-11	0.350555703695473	0.393	0.364	1.6730922993673E-06	1	FXYD3
8.76448848170657E-16	0.350546811937906	0.208	0.3	1.46875297976439E-11	1	ATP2C1
6.93231173680438E-09	0.35001980123725	0.196	0.262	0.000116171680085368	1	COX17
6.60035589211577E-17	0.349611583054445	0.179	0.263	1.10608764040076E-12	1	IPO8
1.91687854205804E-16	0.34765970275416	0.179	0.263	3.21230506078086E-12	1	RAB5B
2.47945272120825E-10	0.347587327260251	0.259	0.353	4.15506687020079E-06	1	APEX1
3.61406011062815E-16	0.346975062992366	0.19	0.278	6.05644193339066E-12	1	COMMD3
1.39617973018556E-16	0.346521697896054	0.172	0.253	2.33971799184495E-12	1	TEX264
1.08929096116946E-11	0.346141926584588	0.242	0.332	1.82543379272778E-07	1	PDCD6
1.0946464603374E-12	0.345578657194876	0.208	0.29	1.83440853823342E-08	1	IFT27
1.3881333936175E-28	0.345352213720167	0.736	0.75	2.32623394102421E-24	1	METTL7A
1.23517938214542E-06	0.345339930110907	0.305	0.397	0.0206991360859929	1	ZBTB33
2.77160190106296E-10	0.345031405917406	0.207	0.281	4.6446504658013E-06	1	NDUFS3
8.54780177288249E-09	0.345023639643075	0.269	0.36	0.000143244062109965	1	PSMD12
2.2125006396367E-07	0.344773105617407	0.233	0.304	0.00370770857190318	1	DNAJA3
4.37325612673051E-17	0.343954816616533	0.24	0.345	7.32870261717498E-13	1	IL10RB
2.21993186491016E-07	0.343881114394512	0.286	0.374	0.00372016181921644	1	TRAPPC4
4.10937670114661E-12	0.343858959795943	0.198	0.274	6.88649347578149E-08	1	BCL2L1
4.1000846360893E-18	0.34384082929055	0.169	0.254	6.87092183315845E-14	1	CNOT8
9.46427119998252E-12	0.343554340867283	0.248	0.341	1.58602256769307E-07	1	SMARCA5
7.65949259677514E-15	0.343381628630269	0.178	0.257	1.28357776936758E-10	1	KIF3B
1.18822968910524E-12	0.342831128184521	0.577	0.626	1.99123531300257E-08	1	ADI1
1.11630334358937E-11	0.342137073783398	0.204	0.281	1.87070114318707E-07	1	HAX1
1.87915887371529E-09	0.342097814056944	0.247	0.331	3.14909444057208E-05	1	CKAP4
1.71487044856175E-12	0.341878889812541	0.227	0.314	2.87377989769978E-08	1	ZBTB7A
2.41870445626422E-09	0.341842059428843	0.543	0.599	4.05326492780757E-05	1	ACOX1
2.29706071935714E-15	0.34034759063027	0.172	0.25	3.8494143534987E-11	1	BABAM1
5.39729724108077E-13	0.339765185806382	0.231	0.324	9.04479071660315E-09	1	TOR1AIP1
1.55408340723191E-08	0.339421245840751	0.266	0.353	0.000260433297383924	1	MOCS2
2.13583345107053E-08	0.337655795810344	0.207	0.273	0.000357922969730399	1	SET
5.12361679870233E-07	0.337464568061731	0.196	0.253	0.00858615703126537	1	SHISA5
1.16618399114871E-11	0.337183111328577	0.2	0.276	1.95429113236701E-07	1	ARL 1.00
1.58341748080928E-06	0.336650685102234	0.313	0.405	0.0265349101434019	1	DGAT1
7.46101058595543E-10	0.336637668489284	0.211	0.284	1.25031615399441E-05	1	CDK4
3.0113381291973E-11	0.335686399244922	0.215	0.295	5.04640043690883E-07	1	RAP1A
6.66529895121625E-09	0.335266447631113	0.263	0.352	0.000111697079824482	1	GPI
5.34420809365212E-16	0.335113297431005	0.224	0.323	8.95582392334222E-12	1	NCKAP1
6.26428200964968E-15	0.33466929316736	0.203	0.29	1.04976837917709E-10	1	TXLNA
4.43786379171482E-10	0.333268426002778	0.248	0.335	7.4369721421557E-06	1	FOXO3
5.56214732571028E-23	0.332788435184066	0.169	0.263	9.32104648842528E-19	1	EIF2S3
7.4454784549341E-92	0.332290195926971	0.802	0.63	1.24771327947786E-87	1	SGK1
3.44232749824135E-09	0.332120851793449	0.225	0.301	5.76865242155285E-05	1	MDH2
8.46849268401815E-07	0.331685683311583	0.228	0.295	0.0141915000398776	1	ARPC1A
1.03616135759998E-06	0.331383888523314	0.289	0.378	0.0173639920306604	1	AP1M1
1.76462536799947E-07	0.330577057469032	0.288	0.38	0.00295715919169351	1	SNRNP200
1.15670945252311E-12	0.330458187062555	0.182	0.254	1.93841370053822E-08	1	ATXN1
4.74612651186546E-17	0.330411095477962	0.194	0.284	7.95355880858414E-13	1	PCMT1

2.8165980372528E-16	0.330071268639693	0.219	0.316	4.72005499082824E-12	1	RRP36
5.28233446348808E-24	0.329940050803036	0.165	0.259	8.85213609391333E-20	1	SUSD6
1.07882075321221E-16	0.329737273422704	0.202	0.294	1.80788781823301E-12	1	UBA1
1.31806651186787E-12	0.329189206706446	0.205	0.286	2.20881586058818E-08	1	CNPY2
2.56179551371925E-09	0.328921488868941	0.2	0.268	4.29305692189072E-05	1	NR1H2
1.39311506161043E-10	0.328504861422001	0.22	0.301	2.33458222024676E-06	1	SCAF1
1.58182682456948E-14	0.327987290687221	0.18	0.257	2.65082539261354E-10	1	GBE1
6.32478722245904E-12	0.32751576633436	0.236	0.323	1.05990784273969E-07	1	DNPEP
1.64838817130545E-12	0.326417987149193	0.198	0.275	2.76236889747368E-08	1	AAMDC
4.99094111997361E-07	0.326337399644313	0.22	0.283	0.00836381912885178	1	CCNE2
3.71068403527334E-07	0.324450593310151	0.288	0.378	0.00621836430631107	1	SRPRB
9.47021984231309E-17	0.324167212005739	0.222	0.321	1.58701944117483E-12	1	MSL1
8.79921238798251E-18	0.323469982942565	0.208	0.304	1.47457201197811E-13	1	AGGF1
1.00603719571333E-09	0.322649202194066	0.279	0.376	1.68591713257639E-05	1	SLC39A7
7.12227868567811E-23	0.322538751304619	0.195	0.3	1.19355146214594E-18	1	LMBRD2
7.45806360311038E-13	0.322534882932849	0.192	0.27	1.24982229860924E-08	1	DEPRECATED-ENSG00000196821
9.74722404558764E-15	0.322227588850883	0.594	0.638	1.63343980555958E-10	1	FBP1
1.82006482470152E-10	0.322208205293534	0.227	0.309	3.0500646332348E-06	1	ALG3
5.93426522827266E-11	0.321952603763391	0.256	0.352	9.94464166953932E-07	1	RNF130
8.15869089616915E-12	0.321854732545748	0.272	0.373	1.36723342038003E-07	1	SPPL2A
1.62301217328973E-13	0.321300075173477	0.215	0.303	2.71984379999893E-09	1	UBP1
2.81666015693448E-13	0.321105248301165	0.547	0.579	4.7201590909908E-09	1	MGAT4B
8.05091884228164E-10	0.320918322892805	0.284	0.383	1.34917297958956E-05	1	POLR1D
3.10010301847611E-11	0.319569669212396	0.227	0.306	5.19515263836226E-07	1	MGAM2
3.08796652392761E-07	0.319153097884407	0.268	0.351	0.00517481430079789	1	NAA20
9.86141794748104E-10	0.318983735053722	0.279	0.375	1.65257641963887E-05	1	IVD
1.87117850547644E-16	0.316310047741873	0.187	0.273	3.13572093947741E-12	1	MCMBP
5.13505430660075E-07	0.315204646174501	0.231	0.297	0.00860532400700154	1	POLD2
1.26441014939763E-24	0.314998951236667	0.18	0.281	2.11889852836054E-20	1	TMEM185A
5.96859240107596E-17	0.314693960341715	0.2	0.289	1.00021671457231E-12	1	NOP56
3.47331511996489E-11	0.314623673843733	0.23	0.316	5.82058147803716E-07	1	TMEM179B
3.51788990666121E-10	0.314115456558379	0.216	0.291	5.89527990558285E-06	1	CAPZA2
5.70517383747575E-08	0.313532340947445	0.289	0.38	0.000956073031684187	1	ESYT2
1.0601879015706E-17	0.313241927995496	0.218	0.318	1.77666288545201E-13	1	GNPAT
8.93513458449883E-13	0.312979529623742	0.232	0.32	1.49734985367031E-08	1	AATF
5.9913734370049E-15	0.31270646494071	0.179	0.255	1.00403436057328E-10	1	DNAJC15
5.37383105705348E-11	0.312295799892358	0.221	0.3	9.00546608541022E-07	1	CWC15
1.54432742323164E-10	0.311930571270325	0.231	0.312	2.58798389585158E-06	1	HMG20B
8.9864298255929E-18	0.311613148434983	0.234	0.339	1.50594591017286E-13	1	LNX2
1.2619040926683E-10	0.311393553942774	0.24	0.327	2.11469887849353E-06	1	ATXN7L3B
2.65186339808888E-06	0.310644614845877	0.306	0.397	0.0444399268251734	1	HNRNPL
3.23563738238037E-10	0.309570557694464	0.278	0.376	5.42228112539302E-06	1	SSR3
1.16580491945305E-15	0.309433822981426	0.177	0.256	1.95365588401942E-11	1	RCN1
4.46644025884189E-16	0.3090585936464	0.232	0.331	7.48486058576723E-12	1	PRKAB1
5.84689525003227E-22	0.308798259554279	0.182	0.28	9.79822706000407E-18	1	KDM3B
1.07671941209557E-15	0.308638540282148	0.221	0.316	1.80436639078976E-11	1	GAA
6.08002821374829E-11	0.307950642271888	0.268	0.364	1.01889112805994E-06	1	PSMC5
1.68251721568294E-17	0.307782098684267	0.177	0.261	2.81956235004146E-13	1	ATP13A1
3.9098315175449E-13	0.307544509966815	0.238	0.332	6.55209565710174E-09	1	TRIQK
3.56428437149083E-07	0.307191757426085	0.331	0.435	0.00597302774974434	1	NCSTN
2.11894999320734E-13	0.306993354876555	0.255	0.355	3.55093639861686E-09	1	NUDT9
2.61179965332138E-11	0.306376872358258	0.185	0.254	4.37685385903597E-07	1	RARA
8.53935064218674E-26	0.306284123477864	0.156	0.251	1.43102438061765E-21	1	CUL3
4.05379565819487E-19	0.306264743848594	0.178	0.268	6.79335076400296E-15	1	COMMD5
1.45855935540185E-08	0.306068046084226	0.263	0.349	0.000244425376778243	1	NUDT5
6.01187980807733E-10	0.305607882030582	0.289	0.389	1.0074708182376E-05	1	NECTIN3
1.17345455397656E-15	0.305409906738131	0.194	0.279	1.96647514155392E-11	1	GNL1
1.1768613826101E-08	0.304598268203559	0.258	0.344	0.0001972184304978	1	PLEC
3.35893148356146E-16	0.304143181329826	0.212	0.305	5.62889738015229E-12	1	PRPF6
6.07759840366535E-07	0.302934739432718	0.194	0.25	0.0101848394048624	1	PPDPF

1.0077987997308E-11	0.302736769479864	0.211	0.291	1.68886922858887E-07	1	GPS1
9.88991737622879E-19	0.302659321765549	0.17	0.253	1.65735235390842E-14	1	METAP2
1.63560722730911E-16	0.302446236697772	0.183	0.265	2.7409505915246E-12	1	GOLGA5
4.30936144738476E-10	0.302406166216145	0.268	0.36	7.22162791352737E-06	1	FZR1
2.13007192091645E-06	0.302086266266724	0.282	0.364	0.0356957452507179	1	AGPAT2
1.14870394478902E-14	0.301139123590746	0.245	0.345	1.92499807067744E-10	1	ATF6B
2.28116610126505E-14	0.300622002268449	0.239	0.336	3.82277815249998E-10	1	EEF2K
2.43333009554487E-11	0.300396494839364	0.204	0.28	4.07777457411409E-07	1	ODC1
2.43632395307304E-07	0.300265460880251	0.283	0.369	0.00408279168055979	1	NDUFAB1
5.58302458766584E-12	0.299772553295972	0.255	0.349	9.35603260401041E-08	1	DNAJB9
1.18515497982553E-13	0.299758826416419	0.211	0.296	1.98608271519162E-09	1	DDX24
1.85549298862222E-14	0.299507040019409	0.224	0.316	3.10943515033311E-10	1	C18orf32
1.03368427751042E-13	0.298136000917027	0.247	0.345	1.73224811225196E-09	1	CXorf56
2.10145580281443E-17	0.297453839276813	0.212	0.308	3.52161963435642E-13	1	RNF4
5.85736682697331E-26	0.296671071032734	0.722	0.756	9.81577532864187E-22	1	CERS2
5.95967457882233E-20	0.296475408209896	0.191	0.287	9.98722265919046E-16	1	CERS6
3.88079115849936E-14	0.295884517327087	0.26	0.366	6.50342982341323E-10	1	CD2AP
2.4878851999531E-09	0.295180393239727	0.193	0.258	4.1691980180814E-05	1	MFN2
5.75756853550159E-10	0.294766144331907	0.233	0.313	9.64853335179357E-06	1	EI24
3.2472655573618E-07	0.293705242070182	0.281	0.366	0.0054417676210269	1	TXNDC5
1.01429623221937E-17	0.293052071798553	0.177	0.261	1.69975762595322E-13	1	XRN2
2.27299095374909E-07	0.292364470081333	0.246	0.322	0.00380907824029272	1	DEPRECATED-ENSG00000067064
3.80503947348618E-07	0.290728837517629	0.194	0.251	0.00637648514966815	1	HINT2
2.11518133903709E-15	0.290694512700962	0.201	0.287	3.54462088795835E-11	1	RTCB
3.50894600185771E-23	0.290458322409698	0.697	0.716	5.88029170991315E-19	1	IGFBP4
4.74064286342345E-11	0.287584601419894	0.274	0.37	7.94436931052502E-07	1	PAXX
4.2114659775165E-15	0.287350739990418	0.188	0.269	7.05757468512214E-11	1	SEC61B
3.77142409406066E-14	0.286806545258658	0.219	0.308	6.32015249682686E-10	1	DYNC1I2
4.46636472190677E-12	0.2865349972239	0.23	0.319	7.48473400097137E-08	1	VAPA
8.30719475971686E-16	0.285023437358178	0.214	0.305	1.39211969783335E-11	1	YAP1
4.33516725109928E-13	0.284855936756007	0.178	0.252	7.26487327939217E-09	1	DEPRECATED-ENSG00000205937
1.07848037875621E-16	0.284553940459317	0.229	0.328	1.80731741871966E-12	1	ERAP1
3.71115352856195E-15	0.284316796468518	0.204	0.292	6.21915108316412E-11	1	SUPT16H
2.83180867702928E-08	0.284032833928289	0.293	0.386	0.000474554498096566	1	ENAH
4.16305822852279E-22	0.283683192319165	0.182	0.277	6.97645297935849E-18	1	UNC50
3.63806370113138E-11	0.283618380251385	0.24	0.328	6.09666715035597E-07	1	CDIPT
1.67223559191721E-12	0.282928103818135	0.235	0.324	2.80233240493487E-08	1	EIF4E2
7.39191849510442E-14	0.282719872389692	0.232	0.326	1.2387377014096E-09	1	MTFR1
1.30982628494651E-07	0.282233232876945	0.215	0.283	0.00219500688831336	1	PSMD4
2.1147943344949E-09	0.281563442893724	0.261	0.351	3.54397234574656E-05	1	DEPRECATED-ENSG00000174827
8.89372676312449E-13	0.280465316856089	0.247	0.342	1.4904107309644E-08	1	PPP4R3B
2.94498814218032E-08	0.278421211555943	0.496	0.53	0.000493521112866578	1	MIF
1.37291120820738E-09	0.278116164019322	0.295	0.393	2.30072460271393E-05	1	CCNG1
1.93541075829591E-09	0.277903728912514	0.303	0.401	3.24336134875229E-05	1	GRB10
7.72102063099344E-14	0.277701650800961	0.206	0.289	1.29388863734188E-09	1	MLLT1
7.83610144820568E-19	0.277559189152034	0.169	0.254	1.31317388069031E-14	1	MTA2
1.33295081509207E-15	0.277042839959208	0.235	0.333	2.2337589759313E-11	1	NUB1
1.0819276889518E-20	0.276259346959326	0.168	0.257	1.81309442114542E-16	1	SLC39A11
4.20771016290398E-07	0.276040498523039	0.276	0.356	0.00705128069099449	1	CDK16
1.83158279168435E-13	0.274849576270342	0.246	0.344	3.06936644230464E-09	1	PLSCR1
4.14668994491931E-12	0.274644768145487	0.213	0.293	6.94902300969578E-08	1	KIAA1671
5.19498809591738E-07	0.274553694974025	0.304	0.398	0.00870576105113835	1	ECI1
9.34060700091884E-12	0.272243166981747	0.227	0.311	1.56529892121398E-07	1	TRIOBP
1.36157676088344E-06	0.271710508154513	0.339	0.438	0.0228173033588847	1	CPEB4
5.36561589788443E-16	0.271597040433573	0.279	0.396	8.99169912167473E-12	1	MBNL1
1.45787084122776E-08	0.270635331798144	0.318	0.424	0.000244309995572949	1	EMC2
2.48432700234458E-13	0.269178713978863	0.246	0.339	4.16323519052904E-09	1	MYO19
6.06693417862072E-08	0.269039867356013	0.242	0.317	0.00101669682965326	1	NDUFB6
3.35245398903443E-14	0.268295741370029	0.2	0.283	5.6180423948239E-10	1	WIPF2
1.31770724771568E-14	0.266908790495885	0.265	0.372	2.20821380572193E-10	1	AHCYL1

2.38845082259839E-19	0.266759913571377	0.177	0.266	4.00256588851039E-15	1	PAPOLA
3.01044714177896E-23	0.265831868223595	0.181	0.278	5.04490732019318E-19	1	FAM8A1
2.82739454256848E-08	0.265523814155036	0.258	0.339	0.000473814777443626	1	PPP4C
8.45005085301229E-19	0.265015752131358	0.222	0.325	1.4160595219478E-14	1	STX7
6.20702342843917E-13	0.264348013604896	0.273	0.38	1.04017298613784E-08	1	UHMK1
6.51798946341375E-26	0.263627406526648	0.159	0.254	1.09228467427888E-21	1	TOR1B
1.20173650867529E-26	0.262792039156298	0.184	0.291	2.01387004123804E-22	1	TERF1
1.54194125580152E-11	0.262768372828657	0.185	0.254	2.58398515647218E-07	1	GALE
3.17337792266937E-17	0.262464246286214	0.19	0.277	5.31794672280932E-13	1	CENPV
1.89196987812246E-11	0.262130744462336	0.273	0.371	3.17056312175761E-07	1	NFIA
9.9878060783368E-28	0.261717850793645	0.189	0.299	1.67375654260768E-23	1	GET1
1.38316229859236E-08	0.260600812689776	0.286	0.378	0.000231790337998107	1	NAA60
3.9810454716707E-26	0.260188421609535	0.185	0.291	6.67143600142576E-22	1	SCFD1
1.56894518472394E-16	0.260128210961822	0.177	0.257	2.62923834056037E-12	1	YKT6
1.87127012956745E-20	0.259884202124003	0.176	0.265	3.13587448312913E-16	1	RO60
6.90348780853886E-18	0.259482951843653	0.222	0.322	1.15688648695494E-13	1	RBMX
3.43733522216642E-28	0.259274185698883	0.175	0.282	5.76028636530649E-24	1	PGAP3
9.54866454803809E-18	0.258108714315033	0.192	0.28	1.60016520496022E-13	1	ITGB5
3.21146182032481E-12	0.258069146367736	0.297	0.405	5.38176771850032E-08	1	ADD1
8.82827080009411E-10	0.257434368912057	0.254	0.341	1.47944162067977E-05	1	COX7A2
1.2214350072269E-14	0.256582363491961	0.197	0.281	2.04688078511084E-10	1	PAF1
6.1816024323275E-13	0.256323744155371	0.26	0.359	1.03591293560944E-08	1	NUP58
8.93081904367282E-24	0.256073478543322	0.165	0.259	1.49662665533869E-19	1	EAPP
2.40064929979062E-09	0.256005025410779	0.251	0.334	4.02300809658913E-05	1	SERBP1
1.92343539533255E-08	0.255496544259323	0.282	0.373	0.000322329303549829	1	CTDSP1
4.15504998428063E-07	0.254705953196746	0.222	0.285	0.00696303276365747	1	SUMO3
3.84002715432594E-14	0.254682762970906	0.198	0.278	6.4351175052194E-10	1	SPCS2
4.74418558601753E-23	0.254354628409512	0.186	0.284	7.95030620504818E-19	1	MAEA
2.09727425425553E-70	0.253779551540263	0.981	0.969	3.51461219528142E-66	1	CLU
1.91948017926924E-15	0.253751179768645	0.257	0.363	3.21666488441939E-11	1	C5orf24
4.3893568459342E-11	0.253134875911893	0.248	0.337	7.35568420241653E-07	1	RAB21
4.65461096570266E-26	0.252562297920369	0.179	0.284	7.80019705632452E-22	1	ATP6V1C1
2.27686714742625E-12	0.252478789271777	0.317	0.432	3.81557396565691E-08	1	DERL1
4.7024526135458E-17	0.252013298272981	0.207	0.3	7.88037008978005E-13	1	EPPK1
7.19638814031768E-18	0.251806478110085	0.676	0.718	1.20597072455444E-13	1	HSP90AB1
1.95684734573215E-16	0.251759876077782	0.223	0.321	3.27928478197793E-12	1	SDHA
8.24841538570565E-18	0.251040347175793	0.194	0.284	1.38226945033655E-13	1	FAM102A
1.89440146473828E-28	0.250800469861328	0.182	0.291	3.17463797460841E-24	1	MTMR1
0	3.22301442114606	0.484	0.047	0	2	ARHGEF38
0	2.96528326080719	0.451	0.055	0	2	DUSP21
0	2.73927522913806	0.349	0.048	0	2	LRP4
0	2.69442315930995	0.336	0.036	0	2	TRPV6
0	2.68682942211218	0.533	0.061	0	2	ATP2C2
0	2.67915790492559	0.581	0.073	0	2	B3GALT5
0	2.67910970366318	0.495	0.076	0	2	MUC1
0	2.56224481993196	0.519	0.063	0	2	MYO7B
0	2.54614397064985	0.369	0.056	0	2	SLC5A1
0	2.46117154360724	0.307	0.046	0	2	GPR153
0	2.43400585543116	0.467	0.08	0	2	CHST6
0	2.38272237184168	0.447	0.072	0	2	XKR9
0	2.35856748132033	0.3	0.063	0	2	HILPDA
0	2.33980203073043	0.789	0.175	0	2	PCSK5
0	2.2893243692596	0.512	0.096	0	2	DNM1
0	2.26994527613129	0.397	0.064	0	2	C6orf141
0	2.22719573660197	0.306	0.041	0	2	CALCR
0	2.20757420067847	0.338	0.046	0	2	FAM3D
0	2.19884045894362	0.322	0.056	0	2	PCDHGB7
0	2.19776269998044	0.332	0.06	0	2	PCDHGB6
0	2.17294952570966	0.631	0.153	0	2	AGR2
0	2.16762747140217	0.687	0.14	0	2	SHOX2

0	2.14938589811241	0.953	0.344	0	2	SULT1C2
0	2.14484105428782	0.384	0.069	0	2	FXYD2
0	2.11461599099721	0.933	0.237	0	2	GABRE
0	2.11099788669627	0.327	0.053	0	2	SLC4A7
0	2.09667457684998	0.511	0.115	0	2	POU5F1
0	2.07462664631886	0.268	0.042	0	2	GCNT3
0	2.04689610288691	0.319	0.063	0	2	TTLL6
0	2.03437576475062	0.66	0.13	0	2	NR3C2
0	2.02098164575282	0.335	0.063	0	2	PCDHGA8
0	2.01345047647016	0.297	0.054	0	2	PCDHGA6
0	2.00236085297556	0.36	0.057	0	2	ANKRD29
0	1.97766629527436	0.786	0.193	0	2	MGAM2
0	1.97089195422712	0.25	0.037	0	2	HTR4
0	1.9607475563722	0.742	0.173	0	2	TMC5
0	1.95111270131443	0.598	0.103	0	2	MMP28
0	1.94282222013747	0.427	0.088	0	2	PCDHGA5
0	1.93252014681529	0.412	0.067	0	2	MECOM
0	1.920912730708	0.293	0.054	0	2	SLC38A11
0	1.92031216665113	0.656	0.141	0	2	HSD11B2
0	1.91812840630713	0.937	0.372	0	2	SLC6A19
0	1.91496426460587	0.526	0.092	0	2	MROH6
0	1.90602009840854	0.254	0.035	0	2	CTBP2
0	1.90010328362948	0.863	0.272	0	2	FXYD3
0	1.88032612016185	0.535	0.106	0	2	TEP1
0	1.8731490643023	0.901	0.347	0	2	MUC5B
0	1.86339576395856	0.536	0.104	0	2	FZD8
0	1.86194338026505	0.449	0.081	0	2	EPB41L4A
0	1.85780370452776	0.584	0.124	0	2	VIPR1
0	1.85571299781035	0.715	0.17	0	2	POF1B
0	1.84066358079699	0.435	0.093	0	2	FSD1L
0	1.84006722095343	0.454	0.102	0	2	KCNMB3
0	1.83574513774679	0.73	0.181	0	2	ZMAT1
0	1.83159188385939	0.332	0.06	0	2	SLC44A4
0	1.82011200898851	0.264	0.04	0	2	PLCE1
0	1.81690993074932	0.89	0.293	0	2	SLC15A1
0	1.8136877687574	0.575	0.129	0	2	BTNL3
0	1.81033972391758	0.419	0.065	0	2	PPP1R1B
0	1.80377797792421	0.618	0.15	0	2	LAMA3
0	1.79427509366871	0.268	0.051	0	2	SYTL2
0	1.77232332822543	0.453	0.102	0	2	CCDC141
0	1.77138494056366	0.683	0.162	0	2	STXBP5
0	1.76398136593575	0.268	0.053	0	2	PCDHGA7
0	1.74319495801152	0.841	0.257	0	2	B3GNT3
0	1.74145207488514	0.567	0.123	0	2	SLC45A3
0	1.74118863373917	0.314	0.054	0	2	IQSEC2
0	1.7372691227468	0.983	0.569	0	2	MUC3A
0	1.73339348057761	0.752	0.219	0	2	SI
0	1.72475382070237	0.837	0.228	0	2	CCDC14
0	1.71805952095084	0.485	0.088	0	2	C1orf116
0	1.71385829230417	0.89	0.285	0	2	BTNL8
0	1.71360722057339	0.267	0.051	0	2	SWT1
0	1.71271263101272	0.832	0.248	0	2	KCNK5
0	1.7119198108699	0.516	0.106	0	2	CHST5
0	1.70653964400775	0.369	0.079	0	2	ZNF117
0	1.69083435130011	0.951	0.374	0	2	IVNS1ABP
0	1.69000434891612	0.383	0.085	0	2	HCN3
0	1.68640838151217	0.34	0.075	0	2	PPP1R3E
0	1.68261051012691	0.314	0.057	0	2	EPHB6
0	1.67841296042251	0.357	0.085	0	2	RPA4
0	1.67025107453136	0.674	0.177	0	2	CCL28

0	1.66998569560259	0.426	0.083	0	2	KRT15
0	1.66875555262701	0.327	0.051	0	2	GLIS3
0	1.66178143938891	0.614	0.139	0	2	SLC26A6
0	1.6603321173467	0.631	0.15	0	2	FRAS1
0	1.66000020025219	0.57	0.139	0	2	LAMB1
0	1.6563384801607	0.387	0.09	0	2	TTN
0	1.64640795811348	0.466	0.113	0	2	TMEM44
0	1.64620203700833	0.617	0.137	0	2	SLC1A5
0	1.6395160439572	0.958	0.397	0	2	ARGLU1
3.19704492231732E-305	1.63482332138215	0.263	0.061	5.35760788081936E-301	2	MPP3
0	1.62715034811788	0.324	0.068	0	2	ARSJ
0	1.62046205794491	0.942	0.376	0	2	SLC6A8
0	1.61495504879259	0.455	0.092	0	2	FBLIM1
0	1.61130424804979	0.638	0.154	0	2	ZFYVE28
0	1.60707411418573	0.263	0.052	0	2	BICD1
0	1.60569603423495	0.255	0.05	0	2	SLC25A12
0	1.60335259385546	0.621	0.159	0	2	TTLL3
0	1.60122374655751	0.535	0.118	0	2	TNFRSF11A
0	1.59278200627448	0.301	0.06	0	2	NCR3LG1
0	1.59253599866038	0.447	0.093	0	2	TMEM171
0	1.59231432534603	0.97	0.436	0	2	CDH17
0	1.58512953332224	0.263	0.041	0	2	MYO1A
0	1.58417161046893	0.748	0.203	0	2	CRACR2B
0	1.58245822640939	0.389	0.081	0	2	GALNT4
0	1.5674944276324	0.569	0.134	0	2	GALNS
0	1.56509577289136	0.585	0.15	0	2	CCDC198
0	1.55768997169053	0.273	0.053	0	2	BCL11A
0	1.5546609714112	0.395	0.087	0	2	SPATA6L
0	1.55384577379983	0.288	0.046	0	2	HTR1D
0	1.5530627219594	0.624	0.163	0	2	SLC2A13
0	1.54697746375866	0.34	0.056	0	2	GUCY2C
0	1.54428621639711	0.456	0.101	0	2	SLC2A1
0	1.54380929925392	0.266	0.056	0	2	MAP3K6
0	1.5432810692521	0.987	0.637	0	2	PIGR
0	1.54215479265358	0.598	0.15	0	2	FBXL8
0	1.54159147781439	0.39	0.082	0	2	FSTL1
0	1.53934358603456	0.47	0.098	0	2	B4GALNT2
0	1.53497443152986	0.414	0.085	0	2	RALGPS1
0	1.5327535501745	0.793	0.229	0	2	PDX1
0	1.52968455889404	0.327	0.071	0	2	PPP1R9A
0	1.5170057584888	0.722	0.212	0	2	MS4A8
0	1.50711608630732	0.687	0.175	0	2	PRR15L
0	1.50507022107397	0.632	0.157	0	2	ZNF91
0	1.50351678584186	0.463	0.12	0	2	NOL3
0	1.50013616563522	0.282	0.062	0	2	RIBC2
0	1.49873548754185	0.31	0.063	0	2	MYB
0	1.49651074610665	0.383	0.078	0	2	NAALADL2
0	1.4959694403217	0.351	0.069	0	2	PDE4D
0	1.48802584124369	0.685	0.185	0	2	QSOX1
0	1.48591049249181	0.414	0.079	0	2	FUT6
0	1.48477102542695	0.452	0.119	0	2	PDE7A
0	1.48397889691743	0.665	0.172	0	2	SAMD12
0	1.4824224444049	0.257	0.057	0	2	ZNF558
0	1.48165171592081	0.683	0.186	0	2	FRYL
0	1.47950730172515	0.297	0.056	0	2	SLC22A3
0	1.47902281694824	0.337	0.075	0	2	SMYD4
0	1.46773607518888	0.783	0.224	0	2	MICALL2
0	1.46452866794105	0.667	0.186	0	2	GSAP
0	1.46151055817884	0.702	0.193	0	2	ZNF75D
0	1.46043376151253	0.922	0.38	0	2	KLF5

0	1.45737723359541	0.651	0.19	0	2	WDYHV1
0	1.45359298073298	0.414	0.112	0	2	ANKRD36B
6.51509746957645E-299	1.44458630610073	0.271	0.066	1.09180003395162E-294	2	TRIM45
0	1.44440606007214	0.895	0.307	0	2	SLC17A4
0	1.44097768344106	0.494	0.096	0	2	CNTFR
0	1.43552174499291	0.934	0.432	0	2	TFRC
0	1.43346608547281	0.405	0.088	0	2	ZNF862
0	1.43084723381563	0.948	0.419	0	2	PTPRK
0	1.43048513252486	0.565	0.146	0	2	MFSD3
9.006980996817E-291	1.42774464524252	0.281	0.072	1.50938987544659E-286	2	GOLGA8A
0	1.42716227391112	0.359	0.08	0	2	ADAMTS10
1.43820841417114E-276	1.42668471626302	0.25	0.06	2.41014966046799E-272	2	ARSF
0	1.42362146446126	0.433	0.083	0	2	RAPGEFL1
0	1.42205214435411	0.46	0.106	0	2	PLXNA3
0	1.41863095517333	0.302	0.061	0	2	TRPV4
0	1.41541645813672	0.34	0.073	0	2	IGSF3
0	1.41402552726831	0.774	0.236	0	2	STK39
0	1.41058732467967	0.429	0.098	0	2	CLRN3
0	1.41028012605679	0.513	0.121	0	2	CASZ1
0	1.40948072472616	0.912	0.374	0	2	CLDN7
0	1.40794244839236	0.504	0.133	0	2	KNTC1
0	1.40471281986271	0.904	0.328	0	2	IKBKB
0	1.40453766109321	0.29	0.063	0	2	PDE5A
0	1.40351057869451	0.881	0.317	0	2	TMEM63A
0	1.39675289136609	0.514	0.125	0	2	TMEM168
0	1.39477358504725	0.396	0.096	0	2	ZNF26
0	1.39424926491765	0.882	0.32	0	2	MATN2
0	1.39270087109465	0.708	0.202	0	2	RBM12B
0	1.39237252106788	0.456	0.096	0	2	GRAMD1B
0	1.38705782999876	0.553	0.154	0	2	DGKE
0	1.38490198315196	0.515	0.123	0	2	TMEM8B
0	1.38408909077562	0.786	0.259	0	2	PKDCC
0	1.38360280825292	0.553	0.136	0	2	TRIM56
6.81154085582912E-286	1.38221209356656	0.259	0.062	1.14147801661984E-281	2	C14orf180
0	1.37943329863179	0.547	0.148	0	2	ZNF337
0	1.37712386097899	0.736	0.214	0	2	INAVA
0	1.37611916785972	0.814	0.248	0	2	VPS13B
0	1.37492614456331	0.977	0.602	0	2	LYZ
0	1.37320284055912	0.727	0.222	0	2	KDM6A
0	1.37319733049019	0.395	0.079	0	2	NTN4
0	1.37170317582145	0.664	0.177	0	2	SPIRE2
0	1.37031134866708	0.682	0.192	0	2	LRBA
0	1.37012013942581	0.514	0.113	0	2	C6orf132
0	1.36921151324614	0.651	0.183	0	2	LYST
0	1.36865499316	0.431	0.117	0	2	DEPRECATED-ENSG00000135976
0	1.36846367849608	0.656	0.181	0	2	TARBP1
0	1.36722008778292	0.603	0.17	0	2	GLI4
0	1.36560388342104	0.362	0.092	0	2	RAD51B
0	1.36535310492474	0.579	0.138	0	2	PLXNA2
0	1.36358967709765	0.435	0.117	0	2	POLE2
0	1.36184494218422	0.562	0.154	0	2	TMEM43
0	1.36142091240284	0.546	0.142	0	2	SLC25A29
0	1.35847107926691	0.532	0.148	0	2	USP53
0	1.35478761471613	0.391	0.099	0	2	CD58
3.65893281026006E-257	1.35257834627468	0.262	0.07	6.13163960343381E-253	2	ZBED8
0	1.35238608757042	0.404	0.103	0	2	TTC13
0	1.3522457699409	0.541	0.142	0	2	CES3
0	1.35099943878882	0.467	0.125	0	2	RAVER2
0	1.34934267544056	0.37	0.084	0	2	LTB4R
0	1.34841070101691	0.566	0.14	0	2	FAM221A

0	1.345748052797	0.941	0.446	0	2	CLDN4
0	1.34370423535166	0.702	0.195	0	2	RAPGEF5
0	1.34301750547075	0.329	0.074	0	2	DLG3
0	1.34128599495792	0.86	0.354	0	2	CLDN3
0	1.3388230817151	0.616	0.176	0	2	BCL2L2
0	1.33664686151732	0.832	0.279	0	2	TAZ
4.11376613527979E-215	1.33627260831514	0.26	0.079	6.89384928950188E-211	2	FSBP
0	1.33125873535692	0.291	0.058	0	2	DEPRECATED-ENSG00000242515
0	1.32697196974818	0.426	0.122	0	2	PPP1R15A
0	1.32341426955166	0.727	0.218	0	2	TBC1D8B
0	1.32162393522736	0.766	0.236	0	2	NIPAL2
0	1.31943884477648	0.767	0.24	0	2	KCNJ3
3.58998645328581E-295	1.31943336638792	0.256	0.059	6.01609929841635E-291	2	MAMLD1
0	1.31868592064079	0.357	0.088	0	2	AP002495.1
0	1.31626959573294	0.674	0.197	0	2	OTOP3
0	1.31581101504785	0.582	0.156	0	2	MAP3K21
0	1.31478819629269	0.334	0.066	0	2	CREB3L1
0	1.31429473523933	0.29	0.062	0	2	CLVS1
0	1.3135671144784	0.273	0.06	0	2	LYPD6B
0	1.30962055779238	0.69	0.184	0	2	MISP
0	1.30767483524031	0.798	0.306	0	2	DUSP9
0	1.30629362284182	0.912	0.353	0	2	DEGS1
0	1.30350037506408	0.875	0.324	0	2	MLPH
5.50584379877065E-257	1.30337686756254	0.268	0.072	9.22669303797986E-253	2	FHOD3
0	1.30174798116725	0.61	0.164	0	2	SIPA1L1
0	1.29591152086684	0.404	0.103	0	2	MFSD11
0	1.29368787279702	0.812	0.265	0	2	DIAPH2
0	1.2920539957987	0.261	0.057	0	2	C5orf30
0	1.29073939962806	0.339	0.083	0	2	VMAC
0	1.28845303070701	0.484	0.144	0	2	ANKS1B
0	1.28612050592623	0.367	0.105	0	2	ANKRD36C
0	1.28394073780776	0.349	0.094	0	2	PCDHGA3
0	1.28096651143983	0.93	0.334	0	2	CEP70
0	1.27584099156595	0.969	0.485	0	2	ADA
0	1.27573371610988	0.34	0.091	0	2	ANKRD26
0	1.27351567831638	0.487	0.124	0	2	NCEH1
0	1.27233497357604	0.36	0.079	0	2	LPCAT4
0	1.27173646507202	0.571	0.161	0	2	UGGT2
0	1.27172452684319	0.39	0.108	0	2	ZNF700
0	1.27056900970934	0.484	0.119	0	2	NPIPB15
0	1.27048319764548	0.528	0.149	0	2	FZD6
0	1.2676447915125	0.499	0.134	0	2	NBEAL1
0	1.26729106378881	0.937	0.4	0	2	ZBTB16
0	1.26726547607925	0.522	0.166	0	2	ITGA3
0	1.26487135181072	0.528	0.147	0	2	DMXL2
0	1.26183524294472	0.363	0.099	0	2	RBL1
0	1.26168106296199	0.577	0.153	0	2	NOTCH1
0	1.25908276609589	0.633	0.188	0	2	USP54
0	1.25631032365523	0.334	0.089	0	2	PPM1L
0	1.25565526152164	0.522	0.13	0	2	CDC42EP5
0	1.25552938558539	0.554	0.16	0	2	PRPF39
0	1.25232834674777	0.513	0.137	0	2	LRIG3
0	1.25066274100074	0.377	0.099	0	2	ZNF224
0	1.25001813362297	0.3	0.068	0	2	CNNM4
0	1.24847019388974	0.606	0.169	0	2	SUSD6
0	1.24792619628517	0.757	0.233	0	2	RBM26
0	1.24305035598717	0.917	0.383	0	2	MEP1A
0	1.24282298078394	0.36	0.074	0	2	KIAA1211
0	1.2423179426157	0.488	0.135	0	2	BRCC3
0	1.24186750676413	0.406	0.093	0	2	HSPG2

0	1.24063689244836	0.843	0.299	0	2	PVR
0	1.23935364592634	0.269	0.051	0	2	TMEM150B
0	1.23864112576728	0.517	0.129	0	2	ACE
0	1.23834182691386	0.418	0.102	0	2	C1GALT1
0	1.23590609224132	0.756	0.239	0	2	IL10RB
0	1.23277513520722	0.342	0.09	0	2	DEPRECATED-ENSG00000185619
1.01257084256562E-301	1.23269677920984	0.321	0.089	1.69686621797146E-297	2	CPLANE1
6.36772106976398E-199	1.23215343259443	0.399	0.164	1.06710269687105E-194	2	DUOXA2
0	1.23205713762112	0.772	0.247	0	2	TRMT2B
5.47412251599896E-238	1.23204675387235	0.255	0.07	9.17353451231106E-234	2	DEPRECATED-ENSG00000198105
0	1.23106969774339	0.359	0.078	0	2	PFKFB2
0	1.23019775721443	0.952	0.412	0	2	ALG13
0	1.22959982395571	0.361	0.084	0	2	MTCL1
0	1.22919921926697	0.726	0.226	0	2	PIEZO2
0	1.22321818170573	0.458	0.131	0	2	ZMYM5
0	1.22221059585727	0.781	0.265	0	2	CDS1
0	1.2219577652131	0.296	0.073	0	2	OBI1
0	1.22033302767316	0.847	0.308	0	2	NEB
0	1.21919196452393	0.541	0.138	0	2	DGKQ
0	1.21910213267589	0.366	0.096	0	2	ZNF839
4.5696089754827E-284	1.21837100056137	0.321	0.092	7.65775072111391E-280	2	NAIP
0	1.21816584976919	0.482	0.131	0	2	ZSWIM5
0	1.21809175388927	0.462	0.125	0	2	NVL
0	1.21482574607097	0.348	0.098	0	2	CDC7
0	1.21426227233745	0.421	0.106	0	2	TP53I3
0	1.2140773183443	0.486	0.14	0	2	ACYP1
0	1.21325145283687	0.946	0.418	0	2	ANKRD10
0	1.21323232034788	0.425	0.105	0	2	LPCAT1
0	1.21294697207835	0.446	0.124	0	2	DCUN1D2
5.98246756509866E-233	1.21149790296857	0.262	0.075	1.00254191455923E-228	2	ZNF782
0	1.2102984196574	0.291	0.058	0	2	HPSE
0	1.20999357401882	0.846	0.309	0	2	SYTL4
0	1.20918387716657	0.763	0.244	0	2	CLMN
0	1.20765377223472	0.975	0.508	0	2	MUC13
0	1.20745755318708	0.641	0.195	0	2	MTCP1
3.02964956714263E-302	1.20607286210721	0.264	0.061	5.07708674461762E-298	2	FUT8
0	1.20607107946337	0.447	0.112	0	2	PLXNC1
0	1.20552870676968	0.396	0.088	0	2	SLC44A3
0	1.20447863586951	0.563	0.161	0	2	TFF1
1.10822715024419E-233	1.2028241427508	0.255	0.071	1.85716705837921E-229	2	ZNF234
0	1.20250341553761	0.3	0.068	0	2	CTSE
0	1.20038395091852	0.402	0.11	0	2	ZNF266
0	1.1984642029864	0.659	0.191	0	2	ASRGL1
0	1.19798181500234	0.778	0.263	0	2	OGT
3.00943331469859E-257	1.19797656895554	0.29	0.083	5.0432083487719E-253	2	CCDC7
0	1.19752884011165	0.446	0.121	0	2	GTF2IRD1
4.29448043957686E-305	1.19715237512768	0.271	0.063	7.19669032064291E-301	2	ENTPD2
0	1.19660505116306	0.374	0.09	0	2	CASD1
1.60855627444702E-270	1.19554507894155	0.265	0.068	2.69561860471832E-266	2	LAMA4
0	1.19395129630293	0.473	0.134	0	2	GOLGA8B
0	1.19103030704396	0.714	0.262	0	2	PFKFB3
0	1.19010230626974	0.282	0.062	0	2	DOP1B
0	1.18962890551872	0.348	0.092	0	2	ABCA7
0	1.18891084072286	0.538	0.155	0	2	TFF3
0	1.18854381086348	0.654	0.184	0	2	SLC37A1
0	1.18836908540116	0.946	0.453	0	2	ATP1A1
0	1.18819100036679	0.499	0.15	0	2	ATG16L2
0	1.18785134847657	0.521	0.145	0	2	FANCL
0	1.18761477987061	0.321	0.079	0	2	MYLIP
0	1.18592407401999	0.703	0.219	0	2	DLG5

**0**	1.18546060466837	0.593	0.157	0	2	KCTD12
**0**	1.1841190276036	0.518	0.16	0	2	CACNA1H
**0**	1.18314091530867	0.944	0.415	0	2	ABCD1
**5.88596800617708E-284**	1.18303906619561	0.28	0.072	9.86370518475155E-280	2	FAM122C
**0**	1.1825638706059	0.601	0.171	0	2	DMTF1
**0**	1.18166183636614	0.321	0.069	0	2	HID1
**0**	1.18038650931291	0.283	0.063	0	2	LUZP1
**0**	1.17337011896955	0.627	0.186	0	2	PCF11
**6.72170253369795E-228**	1.17181388661778	0.279	0.085	1.1264229105971E-223	2	CEACAM19
**4.19263886688483E-247**	1.17148443049192	0.501	0.207	7.02602421312561E-243	2	DUOX2
**0**	1.16781932432472	0.38	0.092	0	2	FLVCR2
**0**	1.16679661608277	0.473	0.123	0	2	VPS13A
**0**	1.16673753096605	0.442	0.112	0	2	ARHGAP26
**0**	1.16627298008933	0.479	0.144	0	2	ZNF75A
**0**	1.16560472197693	0.537	0.141	0	2	ABCC4
**2.17828280800278E-277**	1.16390557862731	0.301	0.084	3.65036632965106E-273	2	MFSD8
**0**	1.16189271960555	0.477	0.113	0	2	PPP1R14D
**0**	1.1615510222972	0.36	0.088	0	2	B4GALT4
**0**	1.16147365987134	0.496	0.138	0	2	GEMIN7
**0**	1.15877847725751	0.79	0.25	0	2	EHBP1L1
**0**	1.15827979808464	0.785	0.274	0	2	PCDH1
**0**	1.15827557283482	0.805	0.287	0	2	KCNK1
**0**	1.15793112077826	0.947	0.552	0	2	VEGFA
**0**	1.15487571923475	0.79	0.258	0	2	SEL1L3
**0**	1.15416416518381	0.867	0.339	0	2	EHF
**8.70626437812206E-292**	1.15387590051939	0.293	0.077	1.45899578448569E-287	2	MBD5
**0**	1.15346881575545	0.409	0.107	0	2	SLC1A1
**0**	1.1528225498762	0.438	0.114	0	2	THBS3
**0**	1.15195839292797	0.533	0.162	0	2	PTEN
**0**	1.14961589377976	0.814	0.287	0	2	SH3RF2
**0**	1.14927346548604	0.365	0.096	0	2	ATXN7L1
**1.47400369773856E-286**	1.14795860298529	0.278	0.07	2.47013539667029E-282	2	WNT5B
**0**	1.14781842394804	0.636	0.182	0	2	SLC38A1
**0**	1.14747559047113	0.931	0.391	0	2	PUM1
**0**	1.14675229405285	0.679	0.21	0	2	DEPRECATED-ENSG00000134250
**0**	1.14606169325754	0.328	0.084	0	2	TRMT61A
**0**	1.14587194173781	0.643	0.189	0	2	HERC3
**6.26561659605342E-160**	1.14367452960207	0.382	0.165	1.04999202916663E-155	2	LCN2
**0**	1.14266996496787	0.522	0.153	0	2	UVSSA
**0**	1.1410386319277	0.636	0.196	0	2	ZBTB1
**0**	1.14082362160931	0.328	0.09	0	2	XRCC3
**0**	1.13973179092246	0.773	0.276	0	2	GMDS
**0**	1.13896802010921	0.566	0.158	0	2	DIP2A
**0**	1.13896570664913	0.551	0.141	0	2	URGCP
**0**	1.13865574467438	0.5	0.15	0	2	ART4
**0**	1.13788638139344	0.67	0.207	0	2	KLHDC4
**0**	1.13772072343111	0.355	0.085	0	2	MIGA2
**0**	1.13692167992065	0.933	0.434	0	2	ZKSCAN1
**0**	1.13635862886608	0.935	0.404	0	2	ERO1B
**0**	1.13566111924114	0.704	0.224	0	2	ZDHHC14
**0**	1.13525191674617	0.449	0.115	0	2	CHD7
**1.81404988964517E-254**	1.13519197610927	0.295	0.086	3.03998480506737E-250	2	ZNF552
**0**	1.13467007268881	0.576	0.16	0	2	ITPR3
**0**	1.13376147166377	0.788	0.289	0	2	GPT
**0**	1.13349715133908	0.434	0.121	0	2	PTPRD
**1.11350607536548E-149**	1.13084423628493	0.286	0.112	1.86601348109747E-145	2	CCL20
**6.06651646499598E-287**	1.13029064259931	0.316	0.089	1.01662682920403E-282	2	LIG4
**0**	1.13019764765218	0.65	0.232	0	2	IGFBP3
**0**	1.12893268321182	0.608	0.189	0	2	PRELID2
**0**	1.12849367474969	0.819	0.285	0	2	INTS8

0	1.12705301725157	0.919	0.422	0	2	ATRN
0	1.12576755700175	0.366	0.096	0	2	NAAA
0	1.12575964821384	0.283	0.068	0	2	ELOVL7
0	1.12515590973812	0.746	0.237	0	2	FGD4
0	1.12513487611972	0.942	0.413	0	2	F2RL1
0	1.12468890093663	0.424	0.105	0	2	DEPRECATED-ENSG00000147804
0	1.12290578835225	0.419	0.122	0	2	MYSM1
0	1.12195479945935	0.459	0.134	0	2	TMEM39A
0	1.12191201196735	0.395	0.113	0	2	DPY19L3
0	1.1207015308566	0.414	0.12	0	2	MSTO1
0	1.11928740708114	0.649	0.193	0	2	CERS6
9.1838975950209E-203	1.11851691185807	0.25	0.076	1.5390375589736E-198	2	PHOSPHO2
0	1.1175201633746	0.521	0.152	0	2	SLC7A1
0	1.11611845854094	0.909	0.37	0	2	PAG1
1.29040969442983E-244	1.11583560206995	0.257	0.069	2.16246856592552E-240	2	POLI
0	1.11578033902964	0.58	0.179	0	2	DCAF16
0	1.11457980203228	0.344	0.09	0	2	WDR19
0	1.11175634727825	0.444	0.107	0	2	LGALS9
3.31207877274683E-255	1.11094004605102	0.258	0.067	5.55038160736914E-251	2	SLC18B1
0	1.11061966302818	0.749	0.248	0	2	PRKAG2
1.12415421527823E-250	1.11024072560611	0.273	0.076	1.88385763396325E-246	2	ZNF587B
0	1.10962895128657	0.843	0.34	0	2	EVL
0	1.10939385999939	0.579	0.16	0	2	BAIAP2L2
0	1.1082468132815	0.645	0.197	0	2	LRRC14
0	1.10665027895349	0.331	0.088	0	2	ZNF248
0	1.10602597933283	0.318	0.08	0	2	IQCB1
0	1.10602349765342	0.528	0.162	0	2	DPH6
0	1.10590005819263	0.396	0.112	0	2	SPATA33
6.51120893464178E-297	1.10479106188144	0.332	0.095	1.09114839326727E-292	2	IFFO1
0	1.10313080211524	0.411	0.109	0	2	GLCE
4.21391350686904E-276	1.1026033256812	0.271	0.069	7.06167625481114E-272	2	LBHD1
0	1.10245809175167	0.866	0.343	0	2	PARM1
0	1.09994087229557	0.303	0.077	0	2	PTPRU
0	1.09943430042993	0.336	0.082	0	2	DCBLD1
0	1.09935945464459	0.877	0.345	0	2	NR1I2
0	1.09931171699282	0.474	0.137	0	2	CSPP1
0	1.09841239383651	0.431	0.122	0	2	P4HTM
0	1.09650109673137	0.358	0.084	0	2	FAM171A1
0	1.09481558707703	0.549	0.162	0	2	ENPP4
0	1.09461155675828	0.33	0.083	0	2	ERAP2
0	1.09411127773908	0.505	0.149	0	2	HSF4
0	1.09312702033629	0.52	0.146	0	2	TRADD
0	1.09300151812061	0.688	0.219	0	2	NT5DC3
0	1.09292264357125	0.564	0.178	0	2	L3HYPDH
0	1.09235123425791	0.649	0.202	0	2	GTF2H2C
0	1.09222754440142	0.42	0.118	0	2	YEATS2
0	1.09217629272312	0.405	0.113	0	2	CTPS2
0	1.09020331106063	0.386	0.097	0	2	ABR
0	1.08973685231468	0.61	0.187	0	2	SPIDR
0	1.08972430495781	0.312	0.074	0	2	TMEM87B
0	1.08915114978792	0.475	0.132	0	2	C2CD5
0	1.08684436365393	0.75	0.251	0	2	INTS3
0	1.08669725333397	0.384	0.101	0	2	SLC22A5
0	1.08571155053119	0.709	0.27	0	2	CLDN15
0	1.08564506058066	0.298	0.06	0	2	TLCD2
0	1.08212483624049	0.372	0.092	0	2	NEO1
0	1.0805778441618	0.72	0.242	0	2	BDH2
0	1.07998857723269	0.376	0.104	0	2	AP3M2
0	1.078519665497	0.393	0.102	0	2	OSBPL10
1.4472116161208E-284	1.07828147247355	0.294	0.079	2.42523722629524E-280	2	EFNA5

0	1.07696671418056	0.683	0.223	0	2	RBM41
0	1.07651225119371	0.582	0.172	0	2	WDR20
0	1.07593577112912	0.372	0.103	0	2	ITM2C
0	1.0758995269508	0.426	0.114	0	2	ATP11C
0	1.0757190787858	0.692	0.224	0	2	RALGAPA2
0	1.0754408453428	0.393	0.089	0	2	VDR
0	1.07537551577111	0.446	0.134	0	2	FAM76B
0	1.07463342480401	0.384	0.105	0	2	ZNF346
0	1.07434513799308	0.394	0.112	0	2	ZNF449
0	1.07327280447605	0.523	0.144	0	2	ERMP1
0	1.07326411737353	0.454	0.112	0	2	MTMR11
0	1.06915556743199	0.355	0.092	0	2	IL17RA
0	1.06772974018448	0.92	0.39	0	2	HNF4G
0	1.06729844339992	0.379	0.112	0	2	RADX
0	1.06723170851521	0.427	0.122	0	2	VPS8
0	1.06657807511018	0.79	0.28	0	2	GPAT4
0	1.06646788876748	0.522	0.154	0	2	SLC10A5
1.64364424694269E-212	1.06637425834128	0.265	0.08	2.75441902902656E-208	2	IL2RG
0	1.06584466724309	0.332	0.088	0	2	MSH5
0	1.06421453647083	0.501	0.15	0	2	AHI1
0	1.06385295195779	0.772	0.267	0	2	CYP4F12
0	1.06335791396797	0.397	0.107	0	2	THADA
0	1.06266790737757	0.294	0.067	0	2	C11orf49
0	1.06027855611529	0.723	0.25	0	2	TSPAN1
0	1.05909646521544	0.969	0.52	0	2	DNM2
0	1.05897052388591	0.589	0.176	0	2	HOXA13
0	1.05842001665329	0.569	0.17	0	2	RELCH
0	1.05797962293895	0.662	0.222	0	2	NUP210
0	1.05772983221694	0.567	0.166	0	2	SEMA6A
0	1.05696411908807	0.719	0.233	0	2	BIVM
0	1.05652599451366	0.519	0.155	0	2	ANO8
2.43984430837297E-307	1.05593972811953	0.286	0.069	4.08869109197142E-303	2	FAM118B
0	1.05578301993132	0.785	0.268	0	2	TMEM164
0	1.05573140421402	0.346	0.085	0	2	COG5
0	1.05562350690419	0.372	0.096	0	2	DHRS11
0	1.05543485006474	0.481	0.127	0	2	ASAP2
0	1.05504178871543	0.367	0.098	0	2	DEPRECATED-ENSG00000186204
0	1.05501266376863	0.891	0.361	0	2	IRF3
1.40802546029872E-221	1.05417911063984	0.273	0.083	2.3595690663686E-217	2	ZNF619
0	1.0540972747179	0.421	0.099	0	2	SLC12A9
1.30442834058156E-293	1.05399145372444	0.333	0.095	2.18596101314657E-289	2	ZFP41
0	1.05380003444965	0.975	0.599	0	2	ATP1B1
0	1.0533770416932	0.763	0.262	0	2	PARP4
0	1.05285477553834	0.693	0.226	0	2	PARP12
0	1.05148141437636	0.629	0.213	0	2	EPPK1
0	1.05018895912273	0.424	0.111	0	2	BTBD3
0	1.04893026100948	0.544	0.155	0	2	GNL3L
0	1.04790966141094	0.389	0.102	0	2	SLC2A5
0	1.04776809842183	0.44	0.121	0	2	PRR14L
0	1.04627695151207	0.44	0.125	0	2	IRAK4
0	1.04597577106234	0.534	0.161	0	2	NSMAF
0	1.04594529859527	0.499	0.142	0	2	ATAD2B
0	1.04536500284132	0.483	0.141	0	2	PIGG
0	1.04419227794447	0.814	0.343	0	2	BIRC3
7.87510982269556E-259	1.04399718910498	0.282	0.078	1.31971090408732E-254	2	JRKL
0	1.04389021456007	0.498	0.158	0	2	ERMARD
0	1.04274192120469	0.706	0.229	0	2	NOTCH2
0	1.04252103861511	0.658	0.192	0	2	EPS8L3
2.90848182751362E-268	1.04213642492012	0.317	0.094	4.87403384654733E-264	2	STXBP4
2.50681805335761E-296	1.04207459603397	0.3	0.079	4.20092569381669E-292	2	ZDHHC23

0	1.04107862593599	0.509	0.142	0	2	VSIG10
9.85313066850433E-242	1.04074251750595	0.289	0.086	1.65118763742796E-237	2	SENP7
0	1.04046224918565	0.701	0.231	0	2	CANT1
0	1.04013242195453	0.519	0.153	0	2	ALG6
0	1.03980497039864	0.626	0.2	0	2	CCNE2
0	1.03963467162327	0.517	0.149	0	2	TMEM50B
1.85503222916901E-238	1.03915977568203	0.255	0.07	3.10866300964143E-234	2	CASP10
7.31389147332501E-256	1.03772407737822	0.283	0.08	1.2256619330998E-251	2	RASA2
0	1.03641016947719	0.94	0.477	0	2	CAPN2
1.52716353978945E-287	1.03623654465283	0.28	0.071	2.55922065997915E-283	2	CHST12
0	1.03611968818561	0.829	0.365	0	2	HPGD
0	1.03454367971561	0.484	0.131	0	2	FBXL12
0	1.03413255202934	0.349	0.099	0	2	EXOC1
0	1.0338282385813	0.741	0.255	0	2	ZMYM2
0	1.03378963457557	0.793	0.28	0	2	ADAM9
0	1.03372996990866	0.754	0.254	0	2	ADAM15
8.02612014359026E-243	1.03296453094105	0.3	0.091	1.34501721366286E-238	2	FBF1
0	1.03238080828272	0.412	0.102	0	2	BTG2
0	1.03238075593652	0.459	0.139	0	2	ZNF251
0	1.03132169145669	0.433	0.13	0	2	POLA1
0	1.03071538461084	0.56	0.181	0	2	CKB
0	1.03035904075655	0.698	0.232	0	2	INTS6
0	1.03018804952576	0.8	0.284	0	2	MROH1
1.49596560460469E-288	1.03018312324202	0.272	0.067	2.50693916019655E-284	2	LRRC26
0	1.02970892397482	0.406	0.11	0	2	NT5C3A
0	1.02965059117037	0.855	0.35	0	2	TMPRSS2
0	1.02887562816122	0.539	0.147	0	2	EFNB1
0	1.0279595386932	0.542	0.145	0	2	LHFPL2
0	1.02767089246862	0.85	0.343	0	2	ARHGEF26
5.9932876298697E-277	1.02719889444257	0.336	0.101	1.00435514101356E-272	2	MELTF
2.02754450496815E-211	1.02693413093504	0.256	0.076	3.39775908142563E-207	2	PPM1K
0	1.02608030255036	0.369	0.099	0	2	ATP6V0A2
0	1.02508020011393	0.533	0.172	0	2	GTF2H2
0	1.02502202093059	0.87	0.352	0	2	NBL1
1.31513959660837E-246	1.02451522577139	0.265	0.073	2.2039109359963E-242	2	ZNF823
0	1.02429634395439	0.475	0.118	0	2	PIK3C2B
0	1.0241231138619	0.481	0.133	0	2	SSTR1
0	1.02406499837265	0.763	0.259	0	2	RNF157
0	1.0232693302834	0.542	0.177	0	2	S100P
0	1.02309198309655	0.451	0.126	0	2	LNPEP
3.53229991107344E-271	1.02302668927368	0.278	0.074	5.91942819097687E-267	2	PIGL
0	1.02259605086352	0.898	0.396	0	2	PYGB
0	1.02235214965488	0.438	0.106	0	2	ST14
0	1.02223820040545	0.473	0.139	0	2	TRIP11
0	1.01854702405478	0.349	0.093	0	2	TMEM260
1.97331931053832E-291	1.01711419195033	0.277	0.069	3.30688850060011E-287	2	DGKH
3.71883885738274E-242	1.01590189810275	0.302	0.093	6.232030157202E-238	2	MIPOL1
0	1.01590058904541	0.589	0.183	0	2	DOCK9
0	1.01368482355413	0.465	0.141	0	2	UPF3B
0	1.01302751106072	0.389	0.116	0	2	PRIM1
0	1.01250189003047	0.849	0.345	0	2	SLC35D2
0	1.01217394720061	0.42	0.118	0	2	SHPRH
0	1.01095982740842	0.735	0.249	0	2	ACSS2
8.63514958540614E-271	1.01046789075286	0.35	0.111	1.44707836752236E-266	2	NRXN3
0	1.01041225691726	0.782	0.28	0	2	IMPA1
3.3414537058245E-274	1.00950660148614	0.274	0.071	5.5996081202207E-270	2	DEPRECATED-ENSG00000130021
0	1.00918815850089	0.686	0.222	0	2	MOCOS
0	1.00903120594156	0.389	0.111	0	2	SOCS4
0	1.00890552530989	0.364	0.107	0	2	POLD3
6.62894208850663E-291	1.00881900684414	0.293	0.076	1.11087811519194E-286	2	CAPS

5.26712266561516E-301	1.0086372234591	0.351	0.103	8.82664416303789E-297	2	NFXL1
0	1.00734350843133	0.33	0.089	0	2	LPAR1
0	1.0073068395308	0.324	0.083	0	2	IL7
2.4600057131395E-294	1.00656888178312	0.308	0.083	4.12247757407917E-290	2	ME3
0	1.00644035898147	0.529	0.157	0	2	KIFC2
0	1.00569510367254	0.307	0.076	0	2	PDE3A
5.29311646113425E-276	1.00518570268645	0.292	0.079	8.87020456556877E-272	2	SGSH
0	1.00432981300288	0.512	0.159	0	2	MTERF3
0	1.00355249422913	0.412	0.119	0	2	FBXO4
0	1.00310272294937	0.747	0.257	0	2	TRAPPC9
0	1.00295453780835	0.545	0.171	0	2	ELF2
0	1.00277937371923	0.418	0.12	0	2	KLHL42
0	1.00247058202996	0.815	0.306	0	2	SIPA1L2
0	1.00213781115597	0.485	0.149	0	2	ATM
7.60733894260171E-307	1.00178589186251	0.391	0.123	1.2748378600012E-302	2	AC253572.1
0	1.00158185086873	0.811	0.297	0	2	PRKDC
0	1.00102150470525	0.935	0.491	0	2	OCLN
0	1.00084233015684	0.645	0.2	0	2	IGF2BP3
0	1.0005019622237	0.438	0.119	0	2	CDKL1
1.63453827826313E-306	1.00048662083738	0.293	0.073	2.73915924671335E-302	2	BTBD9
2.62860399981697E-268	0.999998004639009	0.311	0.091	4.40501458289328E-264	2	ANKMY1
4.06252972589534E-285	0.999655613387262	0.269	0.066	6.80798731465542E-281	2	TMEM41A
0	0.99930090044338	0.477	0.142	0	2	CPSF1
0	0.998681626041513	0.698	0.226	0	2	IDUA
0	0.996599128390821	0.977	0.506	0	2	FKBP5
0	0.995812477193769	0.384	0.103	0	2	ZZEF1
0	0.994573288217361	0.618	0.191	0	2	TMEM185A
9.70926191173464E-278	0.994529125054105	0.288	0.077	1.62707811116849E-273	2	PDSS1
0	0.993636430273671	0.662	0.211	0	2	NCKIPSD
3.61906401074669E-255	0.993380021077784	0.302	0.089	6.0648274692093E-251	2	NEDD9
0	0.993151098287129	0.622	0.21	0	2	FAIM
0	0.992697832205881	0.457	0.135	0	2	GAS2L3
0	0.99254453822782	0.583	0.165	0	2	PPP1R16A
0	0.992494896713497	0.822	0.295	0	2	TBC1D8
0	0.991791209727873	0.677	0.227	0	2	PDLIM5
0	0.991500093211133	0.614	0.206	0	2	ASB9
0	0.990869279378972	0.426	0.121	0	2	SCMH1
0	0.98954704712855	0.459	0.132	0	2	FARSB
0	0.989497241208722	0.677	0.223	0	2	EXOC3L4
0	0.989199452669671	0.449	0.137	0	2	NSD2
0	0.988521769224254	0.553	0.18	0	2	CD177
0	0.987979208155142	0.352	0.1	0	2	SMYD3
0	0.987806601786185	0.932	0.437	0	2	TMEM45B
0	0.986863962948818	0.573	0.184	0	2	DMXL1
0	0.986608068325849	0.921	0.534	0	2	GOLM1
0	0.986102648606636	0.973	0.505	0	2	NOTCH2NLC
0	0.985972145549771	0.526	0.151	0	2	XPR1
0	0.985707842595311	0.309	0.08	0	2	NAPEPLD
2.55632757507413E-306	0.985265898343048	0.306	0.079	4.28389375030923E-302	2	SCFD2
0	0.98517730976220	0.691	0.234	0	2	WDFY2
0	0.985103634563005	0.498	0.148	0	2	ADCK5
0	0.983738570801654	0.634	0.207	0	2	HELLS
0	0.983722418566701	0.564	0.173	0	2	PLD1
0	0.982123172226235	0.519	0.152	0	2	ZNF655
0	0.982049976587002	0.842	0.323	0	2	ATAD2
0	0.981245691112964	0.648	0.212	0	2	RAB3IP
0	0.981174169165391	0.508	0.151	0	2	STIM2
0	0.980689299078498	0.643	0.195	0	2	CORO2A
0	0.979446206408014	0.441	0.128	0	2	ARMCX5
0	0.97897838494894	0.442	0.122	0	2	MFSD10

1.86290558188041E-248	0.978976016814174	0.295	0.087	3.12185717411519E-244	2	CNEP1R1
0	0.978533335567726	0.453	0.138	0	2	RBMX2
0	0.97725787961	0.371	0.104	0	2	PRMT3
0	0.976839258676338	0.536	0.161	0	2	ARFGEF1
1.19310802837331E-230	0.974772973190411	0.251	0.069	1.99941043394799E-226	2	ZNF460
0	0.974737150172709	0.315	0.079	0	2	PLEKHG6
1.82567325018334E-281	0.974576898832328	0.299	0.082	3.05946323265724E-277	2	SLC16A7
0	0.973718102405749	0.563	0.181	0	2	ANAPC4
0	0.973411870146237	0.41	0.125	0	2	GRAMD2B
0	0.972494258708385	0.61	0.199	0	2	ACACA
0	0.97244611993263	0.916	0.382	0	2	NOTCH2NLB
0	0.972260362317426	0.873	0.364	0	2	SMOC1
0	0.971285347841865	0.491	0.138	0	2	SLC37A3
0	0.970975911077345	0.42	0.133	0	2	SDCBP2
0	0.97027571598513	0.834	0.309	0	2	THOC2
1.992675889954E-210	0.969157761626099	0.261	0.079	3.33932625638491E-206	2	JRK
0	0.968744618589	0.408	0.115	0	2	SLC35E2B
0	0.968135150354395	0.748	0.271	0	2	LRRC31
2.21932028484446E-236	0.967997065670395	0.251	0.068	3.71913693334234E-232	2	RMI2
1.84077164709353E-306	0.967928962973827	0.364	0.108	3.08476512619933E-302	2	ATR
0	0.967675646473776	0.425	0.13	0	2	PTGR2
0	0.96723383611906	0.703	0.237	0	2	MALL
6.0500594317901E-303	0.966408494447494	0.41	0.134	1.01386895957939E-298	2	UGT1A3
0	0.965687412104678	0.4	0.124	0	2	ENDOV
0	0.96478781909365	0.541	0.16	0	2	PLPP2
0	0.963733941578177	0.461	0.158	0	2	TBC1D1
1.09156786457712E-237	0.961789986573985	0.274	0.079	1.82924942745834E-233	2	JAK2
0	0.960922914918425	0.476	0.144	0	2	SULF2
0	0.960835108057058	0.815	0.305	0	2	HGSNAT
7.34776685245612E-302	0.960733544858475	0.406	0.133	1.2313387691346E-297	2	FRY
0	0.960074377574737	0.763	0.28	0	2	ETNK1
0	0.959146156257104	0.593	0.187	0	2	DDR1
0	0.958871654152567	0.489	0.149	0	2	TNFAIP2
1.14036351139881E-245	0.95864217889008	0.292	0.086	1.91102117240212E-241	2	WDR90
2.26283509636285E-286	0.95844003217951	0.346	0.103	3.79205905448486E-282	2	SLC49A4
0	0.958003855807585	0.968	0.598	0	2	SELENBP1
2.25542412374216E-265	0.958001287486863	0.264	0.068	3.77963974656712E-261	2	FP565260.6
4.62346211199762E-270	0.95799845496074	0.306	0.087	7.74799780728562E-266	2	SYDE2
1.15335544305508E-304	0.957355888695647	0.329	0.091	1.93279305147171E-300	2	KLF2
0	0.956025223083364	0.375	0.097	0	2	FKBP9
0	0.955157744739557	0.37	0.107	0	2	ZNF398
6.63141673310853E-303	0.954916942587362	0.362	0.108	1.11129281613433E-298	2	ARV1
0	0.95487313146501	0.338	0.086	0	2	TAPBPL
0	0.954216983043392	0.325	0.081	0	2	TRANK1
0	0.952964463546102	0.594	0.193	0	2	PAN3
4.93712752005753E-252	0.952886454569953	0.296	0.086	8.27363829811241E-248	2	MFSD4B
0	0.951924795295834	0.368	0.104	0	2	LRRC8E
2.34310698525138E-295	0.95150364295323	0.344	0.101	3.92657868588427E-291	2	XYLT2
6.06976994150038E-235	0.951176622291099	0.277	0.082	1.01717204679663E-230	2	MIOS
7.72206188843903E-255	0.950636822651666	0.277	0.077	1.29406313126461E-250	2	TOGARAM1
0	0.950158715300709	0.753	0.265	0	2	MICAL3
0	0.949439806228296	0.629	0.209	0	2	MBOAT1
0	0.949087457625987	0.518	0.158	0	2	MAP4K5
0	0.948470424454838	0.779	0.287	0	2	DIDO1
0	0.947933844399264	0.536	0.17	0	2	TTC14
0	0.947348662643578	0.929	0.442	0	2	C2orf72
0	0.945866644766801	0.375	0.103	0	2	USP37
0	0.94513665441911	0.806	0.311	0	2	OPLAH
0	0.94466569506385	0.735	0.253	0	2	PKP2
0	0.944469135774228	0.431	0.129	0	2	GK5

0	0.943542790016695	0.783	0.289	0	2	KAT6A
0	0.943359955772219	0.475	0.142	0	2	ARL4A
0	0.94309761104665	0.551	0.165	0	2	CDK13
8.0832551634117E-206	0.942446440918374	0.251	0.075	1.35459190028453E-201	2	TMEM104
0	0.942232381244461	0.617	0.206	0	2	MLH3
0	0.942182594798606	0.648	0.213	0	2	THOP1
0	0.941871866513279	0.789	0.286	0	2	MAVS
0	0.941695995051152	0.64	0.19	0	2	DTX4
9.84298324499208E-290	0.939312576941654	0.352	0.106	1.64948713219577E-285	2	MFSD6
0	0.939185703446966	0.431	0.122	0	2	PEX6
4.65484961040893E-296	0.938670274963356	0.352	0.105	7.80059697712328E-292	2	FPGT
6.71362227685468E-219	0.938153324345196	0.269	0.08	1.12506882115531E-214	2	HELQ
0	0.938086837372951	0.747	0.264	0	2	BIRC6
2.84050524381221E-262	0.937798690951274	0.313	0.093	4.76011868758051E-258	2	AASDH
0	0.937319015771545	0.401	0.109	0	2	PCSK7
0	0.937318484828258	0.416	0.129	0	2	ZFP62
4.13746867316298E-292	0.9370488665127	0.348	0.103	6.93357000248652E-288	2	MCM9
0	0.935750462394543	0.433	0.117	0	2	NIPAL1
0	0.935410522928289	0.413	0.126	0	2	SLC10A7
0	0.935376911811065	0.397	0.112	0	2	GCNT2
0	0.934567286631672	0.387	0.112	0	2	OFD1
0	0.934545565023268	0.486	0.155	0	2	PPIP5K2
2.96903834533401E-217	0.933977272465452	0.255	0.074	4.97551445911073E-213	2	RAC3
4.50645068452521E-263	0.933295488057244	0.284	0.078	7.55191005712734E-259	2	RALGAPA1
0	0.93294595102026	0.785	0.289	0	2	RABGAP1L
2.12438769710638E-274	0.932358639139861	0.315	0.091	3.56004890281088E-270	2	NHLRC3
0	0.932214799303665	0.486	0.14	0	2	CYTH3
0	0.931837154856385	0.644	0.213	0	2	ARL17A
0	0.931790627641811	0.836	0.318	0	2	PWWP3A
6.11557036397006E-213	0.929135151510696	0.281	0.089	1.0248472815941E-208	2	DOCK10
8.16998781452654E-295	0.928805690203204	0.286	0.072	1.36912655795836E-290	2	RNF145
3.4886102924858E-258	0.928308024535382	0.31	0.092	5.84621312814771E-254	2	C3orf85
0	0.928172141759698	0.535	0.167	0	2	ATG2B
0	0.92768693959089	0.415	0.123	0	2	HLCS
0	0.927122089191941	0.568	0.183	0	2	RCOR3
0	0.925125255426917	0.644	0.211	0	2	PHF6
1.22150278364385E-244	0.924826644517066	0.281	0.081	2.04699436483037E-240	2	POP1
1.02271496531424E-249	0.923793847795138	0.313	0.096	1.71386573887361E-245	2	ANKRA2
0	0.923788582028222	0.476	0.141	0	2	KRIT1
1.83723842159449E-254	0.923544358315658	0.305	0.09	3.07884414690805E-250	2	ATG10
0	0.923133647979709	0.397	0.113	0	2	FGD6
0	0.922239574466629	0.436	0.131	0	2	ABHD18
0	0.922184959339351	0.448	0.135	0	2	SMG1
0	0.922162852078528	0.42	0.13	0	2	XPO4
4.52332436562653E-269	0.921924295173586	0.344	0.107	7.58018697191694E-265	2	GPR180
0	0.92078185077572	0.513	0.145	0	2	ANKRD9
0	0.919751403439664	0.658	0.22	0	2	ADCY6
7.88533474644577E-235	0.919522097024704	0.252	0.069	1.32142439680938E-230	2	PPP2R1A
0	0.919104173344737	0.417	0.122	0	2	APAF1
0	0.917719323992866	0.711	0.253	0	2	EDNRB
0	0.917261644844509	0.411	0.116	0	2	FCHO2
0	0.916678752295955	0.543	0.174	0	2	DNAH14
0	0.915507337336643	0.502	0.141	0	2	DEPRECATED-ENSG00000122642
0	0.915452284147495	0.411	0.127	0	2	METTL15
0	0.915376906754392	0.625	0.198	0	2	CEP170B
0	0.91519174171291	0.903	0.454	0	2	VNN 1.00
0	0.914581260723485	0.632	0.219	0	2	CCT8
0	0.914414227257604	0.673	0.227	0	2	HECTD4
0	0.91439184484292	0.701	0.243	0	2	GATAD1
2.4336420129996E-272	0.913549632868103	0.278	0.073	4.07829728538473E-268	2	TMX3

0	0.91350491335904	0.394	0.109	0	2	PRKCD
0	0.912890724634755	0.459	0.132	0	2	PFN2
0	0.912123004257231	0.42	0.125	0	2	FAM126A
0	0.911153400871373	0.463	0.142	0	2	USPL1
0	0.910993025101757	0.375	0.112	0	2	RAP1GDS1
9.23259258641933E-274	0.910922207303136	0.32	0.094	1.54719786563215E-269	2	DRGX
0	0.910684536808658	0.348	0.099	0	2	DEPDC5
8.27430527273754E-237	0.909936189136574	0.304	0.094	1.38660807760536E-232	2	CEP95
0	0.909552976181557	0.348	0.092	0	2	TP53I11
0	0.90893293411085	0.414	0.127	0	2	SPRY2
0	0.908074054864293	0.46	0.139	0	2	TMEM165
0	0.907645550657297	0.854	0.362	0	2	CPD
7.8471549189175E-298	0.907347522583949	0.354	0.105	1.31502622131219E-293	2	LRIG2
1.50574141320207E-223	0.907342639321236	0.272	0.081	2.52332146024403E-219	2	CIITA
0	0.907040669008981	0.454	0.141	0	2	LTN1
2.27468105012407E-268	0.906998680067564	0.266	0.068	3.81191050379792E-264	2	LYPD6
0	0.906794571211206	0.508	0.158	0	2	ATPSCKMT
0	0.906295832825896	0.607	0.182	0	2	TRRAP
0	0.906223913470574	0.635	0.223	0	2	MMP14
2.98050176503185E-284	0.906096878000075	0.298	0.08	4.99472485784037E-280	2	CPT1B
0	0.905892976795954	0.857	0.336	0	2	N4BP2L2
0	0.904952210654703	0.39	0.11	0	2	MRI1
0	0.903616615387032	0.456	0.142	0	2	SMC5
6.92958337288636E-248	0.903580544783047	0.276	0.078	1.1612595816283E-243	2	DDHD1
4.1136228638469E-306	0.903493221659181	0.327	0.089	6.89360919523463E-302	2	B4GALT6
0	0.903384849111428	0.651	0.226	0	2	CPM
0	0.903149298968827	0.66	0.218	0	2	SH3RF1
6.31985618797931E-279	0.901960492648758	0.387	0.127	1.05908149998157E-274	2	SRGAP1
0	0.900597321727188	0.817	0.327	0	2	EDEM3
1.02252855517875E-306	0.900526233148748	0.348	0.1	1.71355335276855E-302	2	ORC2
0	0.899503908282322	0.45	0.138	0	2	VEZT
0	0.899112405693437	0.533	0.165	0	2	BAMBI
6.70585383661269E-243	0.898777402494782	0.258	0.07	1.12376698593956E-238	2	DGLUCY
0	0.898321745688093	0.973	0.536	0	2	NDRG1
2.42488573675448E-283	0.898081649511825	0.272	0.068	4.06362351765316E-279	2	MANSC1
0	0.898000824330197	0.435	0.118	0	2	CLIC5
0	0.8979810819332	0.53	0.163	0	2	DYRK2
0	0.89755317597281	0.393	0.114	0	2	ABHD15
0	0.89752346797424	0.395	0.119	0	2	ABHD13
0	0.897122237120266	0.839	0.322	0	2	XIAP
2.67144841272861E-282	0.896775145400732	0.308	0.085	4.47681325005061E-278	2	DEPRECATED-ENSG00000197302
0	0.896507464967913	0.545	0.178	0	2	MTERF4
0	0.89625575064403	0.828	0.328	0	2	FAT1
0	0.896131391627659	0.916	0.419	0	2	ZBTB7B
0	0.896022238801536	0.387	0.118	0	2	ENGASE
4.57334080129229E-242	0.895345673394498	0.298	0.09	7.66400451480562E-238	2	B3GLCT
0	0.895063590891092	0.572	0.192	0	2	PDE8A
1.58464376728567E-212	0.895013929499624	0.259	0.078	2.65554602521732E-208	2	NAPB
0	0.89467853440702	0.684	0.232	0	2	TRIO
0	0.894537356655151	0.4	0.126	0	2	THSD4
0	0.894533801553792	0.786	0.276	0	2	SUGP2
1.5149621419652E-244	0.894353098345865	0.292	0.086	2.53877355750529E-240	2	KMT5C
7.47800782693049E-269	0.893615977572017	0.311	0.09	1.25316455163701E-264	2	ZDHHC17
0	0.893536567660948	0.486	0.134	0	2	ARRDC1
1.01242027717928E-206	0.892836242654164	0.279	0.09	1.69661390049703E-202	2	TUBGCP5
0	0.892759660278942	0.483	0.155	0	2	POLK
3.95425245075024E-256	0.892158271850536	0.285	0.08	6.62653625696725E-252	2	TFB1M
2.3560398853407E-302	0.89183908296833	0.356	0.105	3.94825163985395E-298	2	ABCG1
4.22718767001068E-293	0.891430235053474	0.386	0.123	7.08392109740389E-289	2	FANCA
2.18111951234723E-267	0.890705068175939	0.347	0.109	3.65512007879148E-263	2	ZNF606

2.95274117740081E-215	0.89068215734996	0.256	0.075	4.94820366508828E-211	2	ZNF557
0	0.890399184590139	0.473	0.148	0	2	RAB28
0	0.890232354628174	0.538	0.173	0	2	KLHL22
6.55831064911439E-300	0.890026951229223	0.358	0.107	1.09904169857859E-295	2	ZNF654
0	0.889750550504803	0.457	0.136	0	2	PTPRJ
0	0.88952341835063	0.563	0.174	0	2	PHLPP2
0	0.889426932734623	0.466	0.145	0	2	ZNF507
3.08155774524176E-251	0.889354271658259	0.313	0.095	5.16407446947614E-247	2	TNK2
8.71643094336261E-265	0.88894825402579	0.329	0.1	1.46069949748871E-260	2	SMARCAD1
1.99027882096521E-263	0.888251131240066	0.285	0.078	3.3353092481735E-259	2	BCLAF3
2.98864868116478E-305	0.88806703543433	0.358	0.105	5.00837745989594E-301	2	NAA40
0	0.887878430175713	0.608	0.201	0	2	UBE2G2
0	0.887206975327095	0.567	0.178	0	2	MCM6
0	0.886971284710288	0.803	0.306	0	2	ACVR1B
5.77288054575675E-274	0.886952764671883	0.365	0.116	9.67419321857917E-270	2	POLA2
0	0.886648243880998	0.602	0.192	0	2	RSBN1L
0	0.886264730003999	0.641	0.211	0	2	STK38
0	0.885533130938546	0.375	0.107	0	2	UBN2
0	0.885180188526321	0.637	0.204	0	2	TJP3
0	0.884703686832054	0.68	0.226	0	2	SH3GLB2
0	0.884269707400427	0.568	0.18	0	2	LRP10
0	0.883532697180671	0.617	0.196	0	2	ATP8B1
0	0.882444229126658	0.532	0.174	0	2	S100PBP
1.04242666987011E-197	0.882104210004857	0.281	0.093	1.74689861336832E-193	2	WDR5B
1.40896934698418E-296	0.881573446333848	0.353	0.105	2.36115083167608E-292	2	BRAF
0	0.881051070497919	0.457	0.149	0	2	EPM2AIP1
0	0.881011093051457	0.924	0.435	0	2	PGM2
0	0.880883032570829	0.409	0.119	0	2	KCTD13
0	0.879887132070857	0.503	0.155	0	2	PWWP2A
0	0.879858172045302	0.774	0.282	0	2	DDX39B
4.09476310255959E-300	0.879102103972638	0.378	0.116	6.86200400726936E-296	2	CEP192
0	0.878477576193818	0.713	0.255	0	2	SGK3
6.07230563744992E-264	0.877267729479864	0.4	0.138	1.01759697872386E-259	2	TSPYL4
1.09901567287266E-238	0.877204177447658	0.277	0.079	1.8417304646E-234	2	ARAP2
7.16251274042359E-236	0.877104469980285	0.292	0.088	1.20029388504018E-231	2	ALMS1
0	0.876927641332395	0.922	0.443	0	2	TMEM37
0	0.876448310573331	0.71	0.258	0	2	MYO15B
0	0.876018774722959	0.524	0.169	0	2	C8orf76
2.91882903003252E-264	0.875683799573347	0.342	0.107	4.8913736885285E-260	2	TRIM52
0	0.875215287640902	0.804	0.298	0	2	NKTR
1.71452420174739E-284	0.87519833675982	0.317	0.089	2.87319965728828E-280	2	CPSF4
0	0.874647439166526	0.61	0.203	0	2	MTMR1
0	0.874286044588891	0.673	0.224	0	2	ARRB1
0	0.873689577370474	0.432	0.128	0	2	PKD2
0	0.873222426640401	0.407	0.123	0	2	PLCG1
0	0.87299188408324	0.467	0.15	0	2	MGAT5
0	0.871750013886215	0.631	0.211	0	2	KHDC4
0	0.871482086454032	0.441	0.136	0	2	TBCK
0	0.871402043089945	0.633	0.205	0	2	SPPL2B
0	0.871144959662301	0.663	0.226	0	2	ABCA5
0	0.871091287356379	0.589	0.195	0	2	APPL2
6.19852396238216E-239	0.870307885492575	0.324	0.105	1.038748645616E-234	2	PCNX4
0	0.869949966690371	0.655	0.232	0	2	ABRACL
0	0.869499380935303	0.586	0.19	0	2	PHF14
0	0.869039984183742	0.429	0.126	0	2	HIF1A
0	0.868047554187158	0.697	0.248	0	2	SMYD2
1.96394094989607E-279	0.867798677547265	0.292	0.078	3.29117224383583E-275	2	PARP8
0	0.866343890007595	0.525	0.168	0	2	SETX
0	0.865385142462423	0.417	0.133	0	2	ACTR3C
0	0.865123353856857	0.456	0.136	0	2	RIC8B

6.00108640234171E-288	0.864101602914538	0.351	0.106	1.00566205930442E-283	2	EID3
1.00602498421669E-235	0.863187911928425	0.288	0.086	1.68589666855033E-231	2	FAM120C
0	0.862890418728424	0.895	0.414	0	2	INF2
0	0.861812079024633	0.647	0.232	0	2	PRCP
0	0.861603430388912	0.634	0.216	0	2	COL5A3
0	0.861447117891136	0.894	0.399	0	2	FRK
6.29477155171759E-245	0.860752619124867	0.301	0.091	1.05487781663683E-240	2	SETD4
1.11412628120198E-231	0.860457965242132	0.304	0.096	1.86705282203828E-227	2	ZNF791
0	0.858997775267135	0.787	0.302	0	2	DEPRECATED-ENSG00000057294
0	0.858876484949681	0.62	0.204	0	2	GBA2
0	0.858301297481305	0.609	0.204	0	2	C19orf25
0	0.858196818162086	0.483	0.153	0	2	D2HGDH
0	0.857246649422119	0.663	0.226	0	2	PCED1A
5.06008661964729E-284	0.856451435118071	0.361	0.111	8.47969315720493E-280	2	PDK1
0	0.856286228312487	0.791	0.297	0	2	FAM122B
0	0.856210421704641	0.788	0.299	0	2	STX16
0	0.855814088065163	0.617	0.214	0	2	TMEM175
0	0.855082071250248	0.724	0.265	0	2	TMEM68
0	0.854718996726928	0.787	0.291	0	2	DLG1
0	0.854593632370191	0.381	0.102	0	2	B4GAT1
0	0.853970355717905	0.382	0.108	0	2	EPDR1
3.25896288695091E-231	0.853820921669655	0.316	0.103	5.46137000595234E-227	2	VCPKMT
0	0.852680397819351	0.788	0.312	0	2	NDFIP2
0	0.850109599389666	0.537	0.166	0	2	LMO4
3.40614460205888E-212	0.849760808932533	0.27	0.083	5.70801712413027E-208	2	SZT2
4.29643863067335E-298	0.849521583645854	0.309	0.081	7.1999718572824E-294	2	BMP2
0	0.84951198420336	0.547	0.175	0	2	TMEM131
0	0.849437418076861	0.353	0.091	0	2	PTCD1
0	0.849051728214119	0.694	0.245	0	2	NREP
0	0.848866934346344	0.634	0.214	0	2	DENND4C
0	0.848854575245411	0.823	0.343	0	2	FOSL2
0	0.847310342538279	0.604	0.192	0	2	SGCE
0	0.846854089971482	0.415	0.122	0	2	SPTLC2
9.66464327267482E-242	0.846597984253187	0.316	0.099	1.61960091963485E-237	2	SDCCAG8
0	0.846559699769259	0.402	0.123	0	2	PIGN
0	0.846202803884041	0.544	0.172	0	2	FAM172A
0	0.846168570323435	0.733	0.268	0	2	CD47
0	0.845263691668574	0.536	0.17	0	2	WWP2
0	0.844388071756339	0.436	0.138	0	2	MTERF2
0	0.842791672801269	0.456	0.151	0	2	SUSD4
7.85794962944367E-239	0.842551993654574	0.33	0.108	1.31683519890217E-234	2	ITGB3BP
0	0.842344668190178	0.442	0.139	0	2	KIAA1109
0	0.841654005553259	0.405	0.117	0	2	CMTM4
0	0.84096926179572	0.563	0.173	0	2	ELF4
0	0.840488202191267	0.586	0.179	0	2	ITGA6
1.94310093638128E-240	0.840163599694671	0.277	0.079	3.25624854918775E-236	2	ZNF696
0	0.83945086243991	0.686	0.258	0	2	CTSC
1.86865083632298E-234	0.839414745192366	0.298	0.092	3.13148507151005E-230	2	BTN3A1
3.84503869631515E-300	0.839321526587844	0.347	0.1	6.44351584728493E-296	2	FAHD2A
0	0.838564278303974	0.681	0.244	0	2	HELZ
0	0.838289488998743	0.349	0.095	0	2	PKD1
0	0.838234220168215	0.565	0.187	0	2	LEMD2
0	0.837516972616495	0.649	0.225	0	2	SNX5
1.83311597407467E-202	0.836314063633633	0.308	0.107	3.07193574935433E-198	2	OTUD3
0	0.836185838659763	0.922	0.447	0	2	NFIB
2.76593331022807E-254	0.836105228263414	0.323	0.1	4.6351510412802E-250	2	TMEM161B
1.45203638791264E-267	0.835873485739541	0.351	0.111	2.433322578864E-263	2	CSGALNACT2
0	0.835833501502239	0.358	0.097	0	2	CHPF2
0	0.835319994346105	0.604	0.211	0	2	LRRC58
0	0.835220575658745	0.969	0.545	0	2	RNF128

**0**	0.835005914945234	0.484	0.161	0	2	ZNF580
**0**	0.834574602503384	0.601	0.2	0	2	HIP1R
**3.04684988075367E-261**	0.834552663118734	0.325	0.099	5.105911030167E-257	2	ZNF397
**0**	0.834518697648716	0.543	0.17	0	2	SGMS1
**0**	0.834489416887374	0.422	0.123	0	2	NOP9
**4.77630856308558E-285**	0.833354934716674	0.334	0.098	8.00413789001882E-281	2	FAM53B
**1.69848863792285E-303**	0.83273087688572	0.366	0.109	2.84632725943112E-299	2	DNAJB14
**0**	0.832696381379593	0.343	0.089	0	2	SSH3
**3.95899563561824E-275**	0.832656796254138	0.349	0.108	6.63448488616905E-271	2	PARD3B
**3.12248491673512E-284**	0.832222995583711	0.374	0.119	5.23266022346471E-280	2	MALT1
**0**	0.830271097549698	0.477	0.156	0	2	ZNF608
**0**	0.829552970333281	0.488	0.159	0	2	LRRC45
**0**	0.829530497067414	0.447	0.138	0	2	MSH2
**0**	0.829324621739686	0.399	0.124	0	2	TCP11L1
**0**	0.828538132579856	0.418	0.122	0	2	FARP2
**0**	0.828536732123036	0.862	0.362	0	2	IL6R
**1.32074021368069E-175**	0.828209579639834	0.253	0.084	2.2132964500861E-171	2	TEFM
**7.91421654991897E-254**	0.827524679074673	0.298	0.087	1.32626440943542E-249	2	RNPC3
**1.12795626795024E-298**	0.827132521661588	0.375	0.115	1.890229113831E-294	2	ZNF44
**1.38751026443416E-275**	0.827089367826945	0.421	0.147	2.32518970113876E-271	2	PIK3C2G
**1.42417607759752E-232**	0.826992618156293	0.319	0.103	2.38663427083792E-228	2	TTC21B
**4.36942541503603E-231**	0.826431527778906	0.309	0.098	7.32228311051738E-227	2	ADGRG2
**0**	0.825975082265742	0.466	0.155	0	2	FAM193A
**0**	0.825840743524887	0.611	0.211	0	2	CXADR
**0**	0.825458965907563	0.697	0.25	0	2	ANKRD17
**0**	0.825305666246013	0.531	0.175	0	2	ATP6V0A1
**0**	0.825090496139544	0.514	0.158	0	2	CASP2
**0**	0.825000328407048	0.404	0.12	0	2	CLSTN1
**5.48886477144863E-297**	0.824806603166771	0.358	0.107	9.19823958399361E-293	2	ATP9B
**0**	0.824805843171852	0.573	0.19	0	2	DICER1
**0**	0.824798008229184	0.737	0.275	0	2	STK3
**0**	0.824353389156294	0.796	0.314	0	2	ACMSD
**0**	0.82301152282936	0.914	0.451	0	2	PNKD
**0**	0.822948501730022	0.669	0.233	0	2	PI4KB
**0**	0.822860281916084	0.341	0.092	0	2	BAZ1A
**2.7679869702757E-286**	0.82242411454963	0.377	0.12	4.63859256478802E-282	2	AMMECR1
**0**	0.822179206393779	0.606	0.206	0	2	TASOR2
**2.58579554781551E-249**	0.821734529957473	0.287	0.082	4.33327617902923E-245	2	ATG14
**4.42709099918433E-185**	0.821496013396371	0.293	0.104	7.41891909643311E-181	2	TCAF2
**2.58464554912004E-265**	0.8212653032473	0.267	0.069	4.33134901121537E-261	2	CCDC3
**0**	0.82087371468088	0.921	0.499	0	2	TSPAN8
**1.41829982896609E-193**	0.820841380389684	0.26	0.083	2.37678685338138E-189	2	ANXA2R
**2.15193905100505E-303**	0.820700872251963	0.37	0.112	3.60621946167426E-299	2	SCYL3
**3.02315447070091E-251**	0.82065727009087	0.288	0.082	5.06620226200059E-247	2	COA1
**0**	0.820538422790283	0.572	0.181	0	2	TTC17
**0**	0.820525681849037	0.556	0.187	0	2	BDP1
**7.86988661197796E-246**	0.819846918076269	0.255	0.067	1.31883559843527E-241	2	KCTD1
**0**	0.819719204613858	0.855	0.37	0	2	PLOD3
**6.2881955594179E-268**	0.819586889845278	0.3	0.084	1.05377581184725E-263	2	SDC3
**0**	0.819457577201737	0.417	0.117	0	2	GBP3
**0**	0.819250020512911	0.469	0.151	0	2	RABGAP1
**0**	0.818817529227125	0.434	0.138	0	2	MRNIP
**0**	0.818621970600485	0.568	0.19	0	2	ELMOD2
**0**	0.818571639438918	0.877	0.386	0	2	SLC38A10
**0**	0.817805744838343	0.855	0.349	0	2	NHSL1
**2.29830403656672E-233**	0.817153401783405	0.315	0.101	3.85149790447851E-229	2	ZNF33B
**7.30128827168416E-302**	0.816991490240915	0.368	0.11	1.22354988856883E-297	2	ZFP64
**7.96965152457451E-290**	0.8164628619455	0.376	0.118	1.3355542024882E-285	2	MFHAS1
**1.90213883516552E-268**	0.814752480052074	0.325	0.097	3.18760425997038E-264	2	KDM3A
**0**	0.814130674765879	0.591	0.194	0	2	NEMF

1.714905388821E-206	0.813979318724325	0.297	0.099	2.87383845058624E-202	2	CENPC
0	0.813130697503708	0.374	0.109	0	2	MANBA
0	0.812888484934703	0.611	0.205	0	2	SKAP2
0	0.812440598344524	0.492	0.155	0	2	SLC9A6
0	0.812310708741604	0.759	0.275	0	2	UPK3BL1
1.4584005115281E-243	0.81203091985461	0.275	0.077	2.44398757721879E-239	2	URB1
2.21122883908227E-282	0.810438718886298	0.321	0.091	3.70557728853407E-278	2	TCTEX1D2
0	0.810189490080786	0.392	0.115	0	2	TDRD7
5.95053639197343E-207	0.809921721138316	0.259	0.079	9.97190888566907E-203	2	CGRRF1
4.12531161478393E-208	0.809913092217908	0.257	0.077	6.91319720405492E-204	2	ZNF441
1.65019090660128E-196	0.809791070094823	0.255	0.079	2.76538992128243E-192	2	THEM4
0	0.808937467159644	0.926	0.481	0	2	SLC37A4
0	0.808535598164998	0.464	0.153	0	2	MAFG
0	0.807751853903573	0.296	0.073	0	2	TTC22
2.45193932712009E-227	0.80694976800494	0.265	0.076	4.10895992438785E-223	2	ZC3H6
0	0.806625473095642	0.585	0.207	0	2	DONSON
0	0.806621746985963	0.907	0.382	0	2	LENG8
0	0.806454480708426	0.485	0.148	0	2	MYO1D
0	0.806197818957441	0.492	0.148	0	2	TMEM181
0	0.806190273333296	0.494	0.16	0	2	MAP3K4
8.83646484364241E-242	0.806120121361898	0.324	0.104	1.4808147784976E-237	2	ZNF133
0	0.805893121138499	0.46	0.143	0	2	PKN2
1.04369059487585E-182	0.805822518871497	0.261	0.087	1.74901669889295E-178	2	SLF2
0	0.805456338441464	0.519	0.171	0	2	CBR4
0	0.804560050799446	0.379	0.114	0	2	TANC1
0	0.804466416177206	0.37	0.109	0	2	GPATCH2
0	0.80369984096671	0.902	0.387	0	2	OCRL
0	0.803676940403185	0.839	0.349	0	2	DBNL
0	0.803285312669402	0.433	0.123	0	2	DEPRECATED-ENSG00000026297
0	0.80296552709089	0.533	0.168	0	2	RALGAPB
0	0.802953049432377	0.428	0.13	0	2	DPH7
0	0.802760211468973	0.508	0.15	0	2	KIF13B
1.80892716083626E-276	0.802556609183749	0.397	0.134	3.03140013612941E-272	2	TINAG
0	0.802186300410341	0.361	0.102	0	2	COMTD1
0	0.801256581168717	0.964	0.503	0	2	TSPAN3
0	0.801182340803232	0.418	0.116	0	2	UNC93B1
1.15167178830178E-265	0.800915246795996	0.329	0.1	1.92997158283613E-261	2	TTI1
0	0.800880950441043	0.75	0.277	0	2	HECTD1
8.34744992184184E-243	0.800735915397092	0.286	0.083	1.39886565790226E-238	2	SLX4IP
0	0.800677774070559	0.551	0.188	0	2	ATG12
0	0.80051163634092	0.567	0.18	0	2	ZNF800
0	0.800473840763436	0.43	0.123	0	2	C7orf26
0	0.800109763980854	0.873	0.382	0	2	CTPS1
2.46001948340128E-280	0.799951816619647	0.346	0.104	4.12250065028387E-276	2	ARHGEF37
0	0.798945407290795	0.416	0.125	0	2	GYS1
2.04494383653424E-241	0.798839818804551	0.256	0.069	3.42691688126408E-237	2	LPIN3
0	0.798704282481162	0.768	0.288	0	2	ATP9A
4.29629444132446E-269	0.798397225600988	0.36	0.115	7.19973022477153E-265	2	FAN1
0	0.797332508408831	0.406	0.125	0	2	TRNT1
1.37463355825803E-222	0.797108091243878	0.27	0.08	2.3036109169288E-218	2	CYP2R1
0	0.796684878140999	0.539	0.176	0	2	TOR1B
0	0.796585977802794	0.36	0.102	0	2	C9orf152
0	0.796548784230038	0.374	0.101	0	2	PUS7
4.00985728152797E-262	0.796363115217135	0.269	0.07	6.71971883238458E-258	2	GPR155
3.47738349080371E-229	0.796130044631457	0.276	0.081	5.82739925388886E-225	2	WDR75
0	0.796106400421952	0.414	0.128	0	2	GTPBP3
0	0.795571712711826	0.498	0.159	0	2	CLN5
0	0.795341038520079	0.595	0.197	0	2	GSKIP
0	0.795258407398256	0.521	0.172	0	2	TBCE.1
5.20731548382369E-269	0.794473199171644	0.361	0.116	8.72641928779173E-265	2	CNTLN

0	0.794088141566277	0.405	0.121	0	2	TMCC3
0	0.792948378693151	0.492	0.169	0	2	IPMK
0	0.792698096744075	0.378	0.112	0	2	EXD3
2.33290164586795E-292	0.792689170765116	0.424	0.143	3.9094765781455E-288	2	RBBP6
0	0.791911812334319	0.799	0.309	0	2	ASH1L
0	0.791444815640035	0.406	0.128	0	2	CEP83
0	0.791141920098438	0.524	0.171	0	2	CHIC2
6.1560398131094E-292	0.790642534073211	0.325	0.091	1.03162915188087E-287	2	TRIM2
1.14805936164176E-283	0.790220089896173	0.388	0.126	1.92391787823926E-279	2	GANC
0	0.790174786930862	0.467	0.153	0	2	SMPD4
1.6148419024107E-207	0.790125809839103	0.253	0.076	2.70615206005985E-203	2	ZNF142
0	0.789496055817295	0.69	0.255	0	2	DPP4
1.63340415767428E-196	0.789439013655721	0.277	0.091	2.73725868743056E-192	2	NUP62CL
0	0.789241112430008	0.725	0.282	0	2	C1GALT1C1
0	0.788364826690313	0.699	0.249	0	2	TTC37
0	0.788089988991037	0.772	0.319	0	2	ANXA13
0	0.787405632737943	0.533	0.168	0	2	NCOA6
1.97497113042183E-284	0.78738558266522	0.315	0.088	3.30965662036089E-280	2	DOCK6
0	0.787339352929599	0.615	0.204	0	2	FAM49B
0	0.787199933208803	0.422	0.126	0	2	NOM1
0	0.787171853470924	0.688	0.252	0	2	SRSF5
0	0.786823956418231	0.383	0.114	0	2	TMEM94
1.79903176420628E-244	0.786496062300706	0.334	0.108	3.01481743045688E-240	2	MYCBP2
0	0.786368032732881	0.723	0.271	0	2	UBR5
0	0.786343350324807	0.765	0.291	0	2	NBPF26
1.40309988164711E-232	0.785289220362675	0.304	0.096	2.35131478166422E-228	2	ZNF562
1.15997943571139E-228	0.784854335104366	0.273	0.08	1.94389353836515E-224	2	AGO4
3.9868122405763E-227	0.784279941526326	0.295	0.091	6.68109995275776E-223	2	GUF1
0	0.783585394257121	0.447	0.141	0	2	PIBF1
1.1738501633733E-258	0.783419950010386	0.326	0.099	1.96713810378097E-254	2	WDFY3
7.95336627483172E-257	0.782959093781944	0.363	0.12	1.3328251203363E-252	2	FAM200B
2.05611964560359E-222	0.782807156351389	0.293	0.092	3.4456453021025E-218	2	ZNF57
0	0.782609420908842	0.488	0.158	0	2	OTUD6B
0	0.782534640023726	0.429	0.126	0	2	TSPAN7
0	0.78218604660462	0.588	0.205	0	2	DNASE1L1
0	0.781640455761015	0.5	0.171	0	2	UPF3A
0	0.781217048948877	0.911	0.445	0	2	MAT2A
0	0.780874738196389	0.657	0.223	0	2	KDM4C
7.00369806151888E-283	0.780679146700275	0.331	0.096	1.17367972114933E-278	2	PAXIP1
2.46850327071117E-256	0.780240576193675	0.252	0.064	4.13671778105778E-252	2	FCSK
9.73612911323062E-228	0.779996501379347	0.315	0.103	1.63158051679519E-223	2	ADSSL1
0	0.779809610960619	0.965	0.581	0	2	HSD17B11
0	0.779669292771919	0.582	0.204	0	2	KBTBD2
0	0.779587618415936	0.362	0.098	0	2	C4orf33
0	0.779064151958431	0.789	0.368	0	2	DDIT4
0	0.779038798021431	0.584	0.205	0	2	CLDN14
1.40034467874854E-275	0.778689031489747	0.286	0.076	2.3466976126468E-271	2	CHFR
0	0.77840546962152	0.619	0.214	0	2	AP3B1
0	0.778397119404027	0.589	0.207	0	2	TERF1
0	0.778302723893981	0.755	0.28	0	2	KMT2C
0	0.777584748933363	0.395	0.119	0	2	ZNF282
0	0.777514066241746	0.422	0.137	0	2	GNPTAB
1.96480590283623E-264	0.777017032772707	0.367	0.12	3.29262173197296E-260	2	SLC36A1
0	0.776709813174147	0.42	0.128	0	2	ADNP2
0	0.776407969050392	0.675	0.239	0	2	FBXO9
0	0.776402457464241	0.4	0.12	0	2	ANO10
0	0.776266137879955	0.518	0.168	0	2	SNX13
0	0.77558228142867	0.467	0.144	0	2	SMPDL3A
0	0.775541874655357	0.716	0.26	0	2	ENTPD6
0	0.775509493447319	0.472	0.154	0	2	ZFYVE16

0	0.773472074751212	0.461	0.146	0	2	CADPS2
0	0.772187601206355	0.69	0.25	0	2	SOS 2
0	0.771937954979765	0.478	0.157	0	2	MYO5C
0	0.771549422419281	0.67	0.241	0	2	PRKAB1
0	0.771529765795146	0.452	0.14	0	2	GPD2
5.4226995831854E-303	0.771505985030321	0.412	0.133	9.08735996150209E-299	2	FAM120B
0	0.771182910282158	0.403	0.124	0	2	LRRCC1
0	0.771125187210386	0.482	0.155	0	2	KMT2A
0	0.771084319124474	0.421	0.12	0	2	SYNJ2
2.70674975457646E-247	0.770900854534913	0.361	0.122	4.53597123871923E-243	2	PIAS1
0	0.770825081806619	0.556	0.187	0	2	MTR
0	0.770777318234072	0.484	0.169	0	2	ZBTB25
0	0.770325254226671	0.432	0.139	0	2	PSMG4
0	0.770143944813144	0.436	0.138	0	2	RNF170
0	0.769816698418213	0.697	0.254	0	2	TUBGCP3
3.32572714064707E-252	0.769720554584182	0.301	0.089	5.57325354229636E-248	2	WDR27
0	0.76857390346209	0.454	0.15	0	2	TOPBP1
0	0.767832213080661	0.932	0.482	0	2	IMMP2L
0	0.767350006097244	0.678	0.245	0	2	PPP4R3A
0	0.767035853056807	0.976	0.764	0	2	HSPA1B
0	0.76699006905301	0.482	0.159	0	2	ZCCHC2
0	0.766939022696734	0.676	0.241	0	2	MOGS
7.01849925280939E-215	0.765630749961147	0.327	0.113	1.1761601047858E-210	2	CEP44
0	0.765012704251475	0.773	0.306	0	2	PATJ
1.57695077750203E-224	0.764689780195181	0.261	0.075	2.6426541129379E-220	2	PMS2
0	0.764617114930394	0.668	0.233	0	2	BCAR1
2.58280536677735E-221	0.764538821126977	0.28	0.085	4.32826523364548E-217	2	B3GALNT2
1.92934486943325E-220	0.764034007338693	0.304	0.099	3.23319613219625E-216	2	MOSMO
8.54670871088836E-265	0.763994074089386	0.31	0.09	1.43225744577067E-260	2	E2F3
0	0.76344645500437	0.563	0.187	0	2	WDR44
9.90072823048377E-197	0.763267501251796	0.278	0.091	1.65916403686447E-192	2	TXNDC16
3.37798222390452E-273	0.762497939881513	0.286	0.076	5.66082261081919E-269	2	DOLPP1
0	0.761639376550687	0.501	0.165	0	2	CDKAL1
0	0.761615988374209	0.731	0.271	0	2	MAN2A1
0	0.761583085169226	0.817	0.332	0	2	LONRF3
3.65237173981486E-299	0.760878387993432	0.321	0.087	6.12064456158174E-295	2	ANTXR2
5.67530134498781E-268	0.760062681948842	0.303	0.085	9.51066999393058E-264	2	GALNT10
0	0.760061989674666	0.615	0.198	0	2	GRAMD4
0	0.759616802775331	0.635	0.214	0	2	ZFYVE21
0	0.759411949987522	0.47	0.149	0	2	MCM3AP
1.27496886914752E-225	0.759329312316799	0.266	0.077	2.13659283091741E-221	2	PDK3
0	0.759157854902894	0.418	0.122	0	2	CISD1
0	0.7585833046543	0.683	0.249	0	2	MYO19
6.78455737919866E-252	0.758318487760866	0.303	0.089	1.13695612560611E-247	2	ZBTB48
0	0.757840937136691	0.781	0.307	0	2	RNF19A
0	0.756988854847694	0.593	0.205	0	2	DPY19L4
0	0.756770714039473	0.341	0.09	0	2	SULT1B1
0	0.756642649009145	0.834	0.393	0	2	ETHE1
0	0.755979991643772	0.914	0.472	0	2	C6
0	0.755734406836558	0.461	0.149	0	2	CNOT10
3.07994068156223E-291	0.755083997123737	0.301	0.079	5.16136459416198E-287	2	BCAR3
0	0.754887493385326	0.512	0.169	0	2	TRAPPC10
0	0.754861006848809	0.37	0.103	0	2	PTPN18
0	0.754049032343925	0.432	0.137	0	2	RAD50
0	0.753997921089377	0.424	0.133	0	2	TMTC4
0	0.75390832270316	0.694	0.253	0	2	FBXL17
4.00066460907702E-248	0.753392356732871	0.35	0.115	6.70431375189127E-244	2	DDX60
2.14959663752502E-284	0.753163502099206	0.364	0.112	3.60229404516444E-280	2	COX19
0	0.753157754668364	0.906	0.46	0	2	ABCC3
0	0.753099510432746	0.477	0.15	0	2	YTHDC2

5.14291889544825E-241	0.753043676128712	0.315	0.098	8.61850348499218E-237	2	LIAS
0	0.75304035200566	0.555	0.181	0	2	ADGRA3
0	0.752747050250484	0.52	0.168	0	2	TRMT1L
5.05985004709294E-166	0.752218712942973	0.258	0.09	8.47929670891836E-162	2	ZXDB
0	0.752127590803604	0.512	0.177	0	2	CBFA2T2
2.78217513966399E-240	0.75176966688392	0.325	0.104	4.66236909904891E-236	2	ZNF263
0	0.751171714054605	0.636	0.217	0	2	ERCC5
3.83915708424851E-238	0.750689617455119	0.298	0.09	6.43365944178365E-234	2	PUS7L
0	0.750177452691175	0.771	0.299	0	2	MCTS1
0	0.749935182421654	0.551	0.181	0	2	MARCKSL1
0	0.749915954485203	0.683	0.246	0	2	NCLN
0	0.749776392277615	0.94	0.48	0	2	SERINC5
0	0.749757630923167	0.65	0.22	0	2	PTPRH
2.99866137728062E-216	0.749466904534992	0.276	0.085	5.02515673604686E-212	2	CAPRIN2
4.21820534802462E-217	0.749462996339537	0.3	0.098	7.06886852221967E-213	2	ZNF236
0	0.749425104148962	0.696	0.241	0	2	SIPA1L3
0	0.749373769012736	0.444	0.143	0	2	DNAAF2
7.78877032646549E-299	0.749001197479144	0.974	0.74	1.30524213130909E-294	2	FMO5
0	0.748952570395809	0.5	0.159	0	2	VIL1
6.91764222447136E-184	0.748924791267376	0.279	0.096	1.15925848397691E-179	2	HAUS3
0	0.748830669409307	0.947	0.628	0	2	CES2
7.26233734809259E-296	0.748234751844752	0.409	0.133	1.21702249279336E-291	2	PHYHIPL
0	0.748041542359296	0.738	0.283	0	2	SLC51A
0	0.747697711161471	0.43	0.139	0	2	IFI44
0	0.747679335834558	0.557	0.189	0	2	MON2
0	0.747402077663047	0.483	0.158	0	2	CHM
0	0.746825735115223	0.636	0.224	0	2	CAMK2D
1.23240609673531E-263	0.746272995671291	0.36	0.116	2.06526613690903E-259	2	FER
1.30122258730521E-207	0.746225882532558	0.284	0.091	2.18058881180608E-203	2	ALDH16A1
0	0.746177824143253	0.504	0.164	0	2	CEP250
0	0.745994850338545	0.91	0.443	0	2	WNK1
7.08473913551172E-275	0.745447810838226	0.318	0.092	1.18726058432905E-270	2	SLC35E2A
0	0.745424199970656	0.563	0.199	0	2	USP48
0	0.74528691192266	0.503	0.172	0	2	TTLL5
0	0.745122076618927	0.715	0.283	0	2	PDCD4
0	0.744985016269512	0.579	0.197	0	2	MGAT4A
5.36488259147626E-282	0.744354938841621	0.299	0.08	8.99047024679592E-278	2	LRRC8B
0	0.744201412436323	0.652	0.232	0	2	TPP2
0	0.744028639137368	0.583	0.195	0	2	AKAP9
0	0.743695226577664	0.421	0.123	0	2	PTPN6
0	0.743677124643582	0.602	0.212	0	2	MSH6
7.55198215424507E-285	0.743622898201936	0.366	0.114	1.26556116940839E-280	2	ATG7
0	0.74341838744021	0.79	0.303	0	2	PILRB
0	0.743111818042706	0.482	0.156	0	2	ERCC6L2
0	0.743042225472817	0.508	0.168	0	2	NEK9
4.6761742205115E-208	0.742906294885798	0.283	0.09	7.83633275873316E-204	2	UFSP2
2.31562273161337E-235	0.742839802928182	0.305	0.095	3.88052057363768E-231	2	CLASP2
2.25999303445721E-232	0.742755138005877	0.283	0.084	3.78729632714339E-228	2	TOP3B
6.34825949545725E-243	0.74178265548097	0.283	0.081	1.06384132624873E-238	2	EXD2
3.12417280947127E-250	0.741101797081735	0.287	0.082	5.23548879411195E-246	2	ATP2B1
0	0.741020945612461	0.568	0.195	0	2	MTM1
0	0.740877774360107	0.543	0.155	0	2	LAD1
0	0.740229559470111	0.431	0.134	0	2	ANKRD13A
0	0.74003918456427	0.737	0.275	0	2	PAXX
6.07288605692986E-212	0.739813514879731	0.258	0.076	1.01769424542031E-207	2	ZDHHC2
3.91634219899533E-302	0.73978728425595	0.394	0.123	6.56300625707638E-298	2	XRCC5
0	0.73948790034182	0.613	0.208	0	2	SLC22A23
5.13896212338249E-234	0.739188437499199	0.324	0.105	8.61187272636438E-230	2	ZSCAN26
3.69892094101665E-307	0.739128522872777	0.404	0.127	6.1986517129557E-303	2	CLGN
0	0.738679141774006	0.574	0.203	0	2	ITPKC

0	0.738586407619885	0.476	0.153	0	2	PTPN12
7.41890984106058E-284	0.737943374845403	0.334	0.097	1.24326091116493E-279	2	RNF121
1.21165565649533E-222	0.737752496766044	0.308	0.101	2.03049254915487E-218	2	CCNH
0	0.73766213670772	0.658	0.24	0	2	ERAP1
0	0.737342117559909	0.55	0.19	0	2	ADNP
0	0.736920317949016	0.556	0.186	0	2	PIP4P1
0	0.736567704943805	0.829	0.366	0	2	SYNE2
0	0.736314381142306	0.565	0.201	0	2	SERPINB6
0	0.735189238021658	0.38	0.11	0	2	TPCN1
0	0.735073397128767	0.474	0.153	0	2	KHNYN
0	0.734602833849425	0.486	0.138	0	2	MMP15
0	0.734492548799299	0.874	0.406	0	2	GRTP1
6.49116492759867E-289	0.7344380577456	0.388	0.124	1.08778941856699E-284	2	XPO5
0	0.734278029368254	0.509	0.17	0	2	EZH2
0	0.734007967483848	0.721	0.279	0	2	KIAA0232
0	0.733800712979511	0.696	0.253	0	2	KMT2B
0	0.733793522591139	0.596	0.214	0	2	HOOK3
8.43407869202153E-275	0.733304749325573	0.311	0.088	1.41338290720897E-270	2	NSUN3
0	0.733215287087168	0.374	0.109	0	2	C1orf131
1.56716052069398E-302	0.730758926461477	0.359	0.105	2.62624760057897E-298	2	ZNF692
5.38452811842193E-253	0.730567951683464	0.335	0.106	9.02339222085147E-249	2	FAM184A
0	0.730530223221749	0.604	0.206	0	2	PSEN1
2.02690825688382E-295	0.730349085596895	0.395	0.126	3.3966928568859E-291	2	IPP
0	0.730199915504352	0.605	0.215	0	2	DEDD2
4.23306081863445E-184	0.729893982057499	0.253	0.082	7.09376331986761E-180	2	MAP7D3
8.569713560026E-304	0.729097964133777	0.446	0.151	1.43611259838916E-299	2	ZNF121
9.90812717174141E-225	0.728970518777628	0.28	0.085	1.66040395144043E-220	2	GPR89A
0	0.728944208419145	0.661	0.24	0	2	ENOSF1
3.37245458618836E-249	0.728784419241732	0.31	0.094	5.65155939553446E-245	2	GALC
0	0.728208327491789	0.616	0.22	0	2	SLC35A3
0	0.728185155139523	0.516	0.163	0	2	LIMA1
0	0.728170483051012	0.516	0.164	0	2	EIF4G3
0	0.727878731759353	0.593	0.204	0	2	SMC6
0	0.727877083174891	0.533	0.177	0	2	HPS3
3.42732554376092E-259	0.727424925525912	0.347	0.11	5.74351214623456E-255	2	CMTR2
0	0.727172208702354	0.576	0.206	0	2	AKAP8
1.51847367747024E-249	0.727107150789239	0.28	0.079	2.54465818870462E-245	2	H2AFY2
0	0.727056983899647	0.844	0.359	0	2	STAU2
4.25660166656907E-275	0.72690932852916	0.374	0.121	7.13321307283645E-271	2	MLLT10
0	0.726193495113108	0.394	0.12	0	2	CDYL
1.50450983908907E-234	0.726018140006733	0.374	0.132	2.52125758834547E-230	2	KANSL1L
0	0.725973831328428	0.568	0.196	0	2	CLCC1
0	0.725031488462164	0.482	0.163	0	2	PLEKHA5
3.70533902566935E-303	0.724242810087628	0.401	0.127	6.20940713921669E-299	2	STOX2
6.32834675640108E-293	0.724139132956456	0.381	0.12	1.06050434943769E-288	2	GPCPD1
2.84079985992375E-263	0.723872962425524	0.313	0.091	4.76061240526021E-259	2	DTNBP1
0	0.723724551636218	0.758	0.338	0	2	SERPINB1
0	0.723441948633397	0.694	0.242	0	2	STXBP2
5.53726976894737E-293	0.723368783710755	0.415	0.137	9.279356678802E-289	2	DMAC2L
0	0.722692075781688	0.681	0.25	0	2	MBOAT7
2.05345292311493E-277	0.722248777821068	0.283	0.074	3.44117640855599E-273	2	GAL3ST1
7.42261340482474E-240	0.72213176058775	0.255	0.068	1.24388155438053E-235	2	ARSD
2.29794137661928E-219	0.721996459838445	0.295	0.094	3.85089015893858E-215	2	RAPGEF6
1.20017941167615E-270	0.721968378731386	0.332	0.099	2.0112606580869E-266	2	PELI1
0	0.721873079850014	0.49	0.162	0	2	PAQR7
1.03181532663101E-263	0.721196243851093	0.349	0.11	1.72911612436825E-259	2	GDAP2
0	0.721121680259279	0.681	0.237	0	2	SUN1
0	0.721095896281911	0.529	0.183	0	2	SCAF8
0	0.720848022570219	0.668	0.238	0	2	TRPC4AP
0	0.720403273080748	0.557	0.194	0	2	SLC25A32

0	0.720207059236717	0.378	0.105	0	2	PLAAT2
0	0.72015404537376	0.807	0.348	0	2	MCM3
0	0.719390567730273	0.535	0.184	0	2	POLR2B
0	0.719245605717418	0.506	0.172	0	2	DEPRECATED-ENSG00000134597
0	0.719135611283211	0.555	0.19	0	2	SCAMP4
0	0.718898963454623	0.674	0.254	0	2	PARP10
0	0.718446339998798	0.456	0.154	0	2	PDS5B
1.80917872729852E-198	0.718427685139009	0.297	0.101	3.03182171120686E-194	2	SPAG1
0	0.718312492083113	0.916	0.461	0	2	ATP6AP1
1.02872742990523E-286	0.718110591040666	0.34	0.099	1.72394142703518E-282	2	ARL4C
0	0.717676044360828	0.494	0.171	0	2	ZNF7
0	0.717660424912674	0.634	0.21	0	2	ESRRA
1.00242776439007E-254	0.717047931377632	0.28	0.077	1.67986844756488E-250	2	ARL 15.00
2.14073769008272E-154	0.716426675663992	0.315	0.127	3.58744822104062E-150	2	DEPRECATED-ENSG00000125675
3.42451546521027E-279	0.715970602632935	0.407	0.136	5.73880301659937E-275	2	PDP1
0	0.715942979109183	0.471	0.153	0	2	KIAA2026
0	0.715834039438144	0.415	0.132	0	2	PAXBP1
0	0.715596390895144	0.733	0.278	0	2	REPS1
2.40051341559412E-256	0.715581830796997	0.334	0.105	4.02278038185263E-252	2	RPE
1.45969644158017E-270	0.715482068382757	0.338	0.103	2.44615929680005E-266	2	TTC39B
4.25796144176278E-212	0.715424458000184	0.298	0.097	7.13549178410606E-208	2	CYP20A1
0	0.715246995755792	0.587	0.212	0	2	ZMYND8
0	0.714416013811736	0.538	0.18	0	2	MYO9B
0	0.714401914748623	0.839	0.351	0	2	RAP2C
0	0.714084594306316	0.424	0.138	0	2	FNBP1L
0	0.713804573778184	0.533	0.177	0	2	TM2D2
0	0.713709787283015	0.663	0.246	0	2	GALK2
0	0.713187425237414	0.787	0.312	0	2	MARCH6
6.06540938221261E-251	0.713133120275403	0.281	0.079	1.01644130427119E-246	2	DGKD
0	0.713096958911115	0.725	0.303	0	2	C15orf48
2.01197350631865E-202	0.712812383721602	0.284	0.093	3.3716652018888E-198	2	SETDB2
0	0.71236530861441	0.471	0.16	0	2	PCGF3
0	0.712348749195099	0.453	0.147	0	2	NIPA1
0	0.712196997004612	0.446	0.146	0	2	RPTOR
3.78325130205819E-224	0.711483448347664	0.327	0.109	6.33997253198911E-220	2	FANCC
0	0.711259205974509	0.899	0.46	0	2	ENTPD5
1.41582761003892E-263	0.711021479553225	0.389	0.132	2.37264390890323E-259	2	SEC22A
0	0.710783590033594	0.774	0.329	0	2	ERO1A
3.06833962060211E-257	0.709864028759772	0.326	0.1	5.14192353620502E-253	2	RASA1
0	0.709349052349246	0.45	0.132	0	2	C19orf33
0	0.709211996494208	0.587	0.212	0	2	TUT4
0	0.709148994401663	0.539	0.179	0	2	NR2F1
0	0.708947989042188	0.419	0.131	0	2	GABPB2
0	0.708922899623266	0.421	0.127	0	2	CALML4
5.55473118470827E-265	0.708829599748995	0.309	0.088	9.30861851933412E-261	2	RNF43
0	0.708798416667228	0.563	0.183	0	2	PTGES2
0	0.708525970366486	0.598	0.207	0	2	GPR107
0	0.708248280065612	0.596	0.214	0	2	NFS1
0	0.708167641632537	0.59	0.201	0	2	LMF2
0	0.708041100714121	0.643	0.229	0	2	SLC10A3
0	0.70763920755665	0.447	0.15	0	2	IFT88
0	0.707457722480385	0.712	0.268	0	2	CEP57
9.48191174689416E-239	0.707441519559366	0.277	0.079	1.58897877054452E-234	2	TRMT61B
0	0.706692001279757	0.659	0.238	0	2	MPHOSPH8
1.64330100111411E-226	0.706549792944283	0.296	0.093	2.75384381766703E-222	2	TBC1D2
0	0.706058447798709	0.562	0.192	0	2	PMM2
2.85498476625959E-204	0.70592191352367	0.263	0.081	4.78438347129782E-200	2	ZCCHC7
1.12764694195255E-254	0.705752216933684	0.515	0.207	1.88971074532408E-250	2	HAO2
2.41922962459623E-275	0.705689539310906	0.413	0.141	4.05414500489836E-271	2	SEPTIN7
0	0.70533518897196	0.493	0.168	0	2	SMCHD1

0	0.705055911172561	0.714	0.268	0	2	RAB11FIP1
0	0.704617611684555	0.737	0.291	0	2	PRPF3
0	0.704505419517468	0.498	0.172	0	2	MED23
0	0.704450909935872	0.517	0.177	0	2	MED16
4.30443847487345E-280	0.70394900331017	0.293	0.078	7.21337799619293E-276	2	ATP1B3
0	0.703780273209864	0.628	0.232	0	2	DAZAP1
0	0.70350826791722	0.458	0.148	0	2	FHL1
0	0.703002149904522	0.885	0.455	0	2	TPM1
0	0.702916230023368	0.676	0.252	0	2	LMBR1
0	0.702902515241401	0.774	0.311	0	2	DGAT1
0	0.702652935687584	0.632	0.23	0	2	PARP14
1.57305499615189E-157	0.702203748900816	0.267	0.098	2.63612556255133E-153	2	ARL17B
0	0.702082470848839	0.583	0.2	0	2	MANEAL
2.77065507500909E-249	0.70181275584633	0.329	0.103	4.64306377470024E-245	2	FBXL18
0	0.701547204181157	0.523	0.181	0	2	RNF19B
0	0.701172520069774	0.412	0.128	0	2	TRAF3
1.19881728873796E-205	0.701061393387457	0.294	0.097	2.00897801246707E-201	2	PRIM2
0	0.701037167738105	0.488	0.158	0	2	RCOR1
0	0.700976592773379	0.716	0.269	0	2	FAM193B
0	0.700853573703737	0.51	0.175	0	2	OSGEP
0	0.700139197298798	0.429	0.136	0	2	RAB3GAP2
4.922963915971E-306	0.699700573428273	0.445	0.15	8.24990293038421E-302	2	AKAP17A
0	0.699347999633569	0.618	0.212	0	2	TNRC18
0	0.69893892658689	0.831	0.345	0	2	MDM4
0	0.698735893349204	0.769	0.306	0	2	GRB10
0	0.69756361015623	0.456	0.148	0	2	NAGLU
0	0.697186866708434	0.618	0.211	0	2	ATP11A
5.0149734382168E-294	0.696679406338066	0.384	0.121	8.40409248776371E-290	2	CMPK2
0	0.696632135751223	0.482	0.157	0	2	SNX19
0	0.69623805872177	0.927	0.452	0	2	KCTD3
3.87845404266771E-286	0.695890883301503	0.369	0.115	6.49951328470255E-282	2	DDX55
0	0.695826397716635	0.62	0.218	0	2	BRI3BP
0	0.695443586567028	0.843	0.394	0	2	CYP4F11
9.86531676654634E-253	0.694787842980794	0.274	0.074	1.65322978373784E-248	2	TPRN
0	0.694715414883744	0.466	0.157	0	2	HHAT
2.85826171246421E-227	0.694470238764721	0.352	0.122	4.78987497774753E-223	2	CHML
0	0.694459051448136	0.459	0.142	0	2	EDARADD
0	0.694414537599513	0.932	0.452	0	2	ELL2
0	0.694238677889744	0.529	0.181	0	2	PLCB1
0	0.693879480944131	0.589	0.213	0	2	CNST
3.58246032597754E-183	0.693460806715302	0.25	0.08	6.00348701427316E-179	2	INVS
0	0.693272579772475	0.486	0.162	0	2	EXOSC2
3.1053960414712E-208	0.69304155369439	0.933	0.659	5.20402268629743E-204	2	DNAJB1
0	0.692648213788562	0.518	0.176	0	2	ABCB10
0	0.692566422811152	0.499	0.164	0	2	CCDC125
0	0.692518990070741	0.692	0.261	0	2	SENP6
0	0.692439472786238	0.597	0.213	0	2	TAF2
0	0.692206219303991	0.513	0.178	0	2	GTF2H1
8.35473275593003E-257	0.692165012646842	0.312	0.092	1.40008611523876E-252	2	KNOP1
1.95955467798695E-124	0.692049037940162	0.271	0.113	3.28382172937053E-120	2	TSPOAP1
0	0.691942985616444	0.5	0.158	0	2	P4HA2
0	0.691931090387687	0.683	0.249	0	2	PTK2
0	0.691827234869621	0.686	0.26	0	2	UGT2B11
3.98971331001091E-240	0.69112871657757	0.34	0.111	6.68596156491628E-236	2	EED
0	0.690884210741462	0.802	0.33	0	2	FAM3A
0	0.690838771817007	0.605	0.216	0	2	TCERG1
3.3501784389687E-208	0.690731959999249	0.302	0.101	5.61422902802375E-204	2	SNAPC3
0	0.690548020219946	0.772	0.311	0	2	SHROOM1
0	0.690153216482511	0.566	0.206	0	2	ABHD3
0	0.690029975352669	0.474	0.158	0	2	FZD5

1.08893532286568E-215	0.689922890215758	0.312	0.104	1.82483781405831E-211	2	ST3GAL3
0	0.689203356585118	0.613	0.226	0	2	FAM13A
0	0.688688706965172	0.493	0.164	0	2	BORCS8
0	0.688594923639166	0.459	0.155	0	2	NUP155
4.57350241717439E-292	0.688594781427779	0.383	0.119	7.66427535070083E-288	2	DEPRECATED-ENSG00000249915
0	0.687862484822045	0.532	0.182	0	2	SPATS2
4.69984357138425E-213	0.687800215996852	0.294	0.095	7.87599785692573E-209	2	DPP8
1.96741049276538E-196	0.687768496565398	0.273	0.088	3.29698650377622E-192	2	AHCTF1
0	0.687252354525167	0.854	0.385	0	2	PLEKHA6
0	0.686999684075358	0.535	0.187	0	2	EIF2S3
0	0.686858900641935	0.462	0.15	0	2	TFCP2
0	0.686511975284458	0.646	0.243	0	2	CHKA
0	0.686357987427197	0.733	0.289	0	2	KLF3
0	0.68628105593515	0.55	0.192	0	2	ZNF623
0	0.686268664919728	0.757	0.3	0	2	POGK
0	0.685681279005468	0.466	0.144	0	2	CPLX1
7.08936524115649E-196	0.685421173256101	0.275	0.089	1.188035827113E-191	2	ELAC1
4.17099939273228E-190	0.685417361947805	0.271	0.089	6.98976078234075E-186	2	C15orf41
7.67660931231578E-251	0.685197234433513	0.355	0.117	1.28644618855788E-246	2	THOC1
5.30077882813765E-255	0.685191942710003	0.341	0.108	8.88304516019308E-251	2	TM2D1
2.24448602141326E-302	0.685056683143734	0.378	0.115	3.76130967468435E-298	2	POM121
4.61314468340572E-217	0.684823002172049	0.294	0.094	7.7307078604513E-213	2	CYB5RL
1.09765572622655E-231	0.684570246049284	0.28	0.083	1.83945146601044E-227	2	NIPAL3
4.31129887714926E-245	0.684396922322758	0.355	0.119	7.22487465832673E-241	2	TRAPPC11
0	0.684344022062565	0.875	0.389	0	2	CFLAR
0	0.684274958468135	0.543	0.176	0	2	DIS3
8.36688876887656E-301	0.684002794737602	0.35	0.101	1.40212321988833E-296	2	LRRC75B
0	0.683262954934521	0.611	0.208	0	2	PNP
0	0.682973169280667	0.702	0.266	0	2	VPS45
3.33183178832024E-238	0.682636378125914	0.346	0.116	5.58348371086705E-234	2	TONSL
0	0.682555863598966	0.616	0.211	0	2	PLEKHJ1
0	0.682538614964548	0.594	0.207	0	2	METTL17
0	0.6823580470485	0.771	0.32	0	2	TMEM64
0	0.682163484723765	0.598	0.216	0	2	UQCC1
0	0.682009132936713	0.719	0.283	0	2	LYPLAL1
0	0.681670628492881	0.482	0.159	0	2	STARD3
7.81106022218777E-165	0.681273983597435	0.252	0.087	1.30897747203423E-160	2	FAM219B
0	0.680995666376285	0.812	0.341	0	2	RNF103
3.00815113938596E-272	0.680946949206346	0.565	0.235	5.041059679383E-268	2	FADS2
0	0.679852183527647	0.399	0.121	0	2	MYORG
2.30687091368043E-283	0.679787440083681	0.359	0.111	3.86585427714566E-279	2	SLC35B3
7.31760840871922E-222	0.679677807338323	0.251	0.07	1.22628481713317E-217	2	TMEM243
0	0.679471393266852	0.537	0.181	0	2	FBXO21
0	0.679270163515396	0.773	0.312	0	2	LIMK2
0	0.67892605259382	0.615	0.221	0	2	MMGT1
0	0.678610012524853	0.654	0.237	0	2	ARHGEF40
5.42834768241222E-277	0.678418106596898	0.362	0.113	9.0968250461864E-273	2	ARID2
0	0.677756419239275	0.404	0.126	0	2	HEATR3
1.01401095875774E-285	0.677576941466065	0.346	0.103	1.69927956468622E-281	2	TMEM209
0	0.676841286017248	0.401	0.123	0	2	SFT2D1
1.54170190915353E-249	0.675930066959788	0.358	0.119	2.58358405935948E-245	2	PPWD1
1.1946027626078E-209	0.67585734623773	0.258	0.077	2.00191530957814E-205	2	CUL9
2.2430438412074E-255	0.675559296296146	0.286	0.079	3.75889286909537E-251	2	PKM
0	0.675404451080938	0.653	0.251	0	2	ACACB
2.71961629509326E-214	0.675319968343478	0.291	0.092	4.55753298731729E-210	2	CHAF1B
0	0.675253886359724	0.715	0.27	0	2	SLC66A2
0	0.675163692352265	0.767	0.316	0	2	NEDD4L
1.77006118095262E-201	0.674804544772122	0.27	0.086	2.9662685270404E-197	2	NEPRO
3.68564529603719E-255	0.674252117227848	0.347	0.111	6.17640438709912E-251	2	L2HGDH
4.93137920735274E-289	0.674196999151938	0.352	0.105	8.26400527568172E-285	2	HEATR5B

0	0.674020840242361	0.447	0.149	0	2	GPAT3
0	0.67375432649689	0.509	0.177	0	2	UBA6
0	0.673630740716879	0.706	0.27	0	2	FNTA
0	0.672939784198043	0.435	0.135	0	2	RIN2
3.33179984422081E-259	0.672542440857254	0.356	0.115	5.58343017894524E-255	2	MBTPS2
0	0.672354174416923	0.706	0.275	0	2	PLOD2
2.54406712399032E-263	0.672121447605372	0.382	0.127	4.26334768638298E-259	2	ZFX
0	0.672080444150478	0.615	0.214	0	2	TAF1D
9.28186210515889E-247	0.671673599195983	0.278	0.078	1.55545445158253E-242	2	ATG9A
1.41235856635473E-190	0.671607289004446	0.253	0.079	2.36683048549726E-186	2	GGPS1
0	0.671596510719146	0.539	0.183	0	2	BCAT2
0	0.671450785230083	0.685	0.257	0	2	BPTF
0	0.671379826532243	0.86	0.375	0	2	CYP4V2
0	0.671086611083022	0.583	0.2	0	2	IFNGR1
0	0.670990689781193	0.758	0.314	0	2	SAFB2
0	0.670959573509023	0.607	0.228	0	2	DCUN1D4
0	0.670754590070985	0.801	0.327	0	2	AGFG2
0	0.670647250567796	0.492	0.159	0	2	ANKRD27
1.1674092226303E-225	0.669890133639983	0.307	0.098	1.95634437528385E-221	2	SRSF10
0	0.669855502537892	0.454	0.142	0	2	SLC35B4
0	0.669714410900429	0.707	0.293	0	2	TAP1
0	0.669681727995848	0.568	0.204	0	2	MTA1
0	0.669483407825576	0.67	0.26	0	2	GFPT1
4.27127165215432E-237	0.669363141180464	0.319	0.101	7.15779703468021E-233	2	IRGQ
0	0.6678923467238	0.54	0.181	0	2	CEMIP2
1.59320437624997E-252	0.66762258578461	0.357	0.117	2.66989189371971E-248	2	MTMR6
1.21394217826718E-304	0.667402781088702	0.447	0.151	2.03432430234014E-300	2	INPP1
1.12240649219709E-222	0.667335816438013	0.285	0.087	1.88092879962388E-218	2	SMAGP
6.84407102455692E-223	0.666929756563279	0.296	0.093	1.14692942229525E-218	2	NUDCD1
0	0.666414396612232	0.561	0.2	0	2	GPATCH2L
2.39963029706882E-274	0.666179264799832	0.375	0.12	4.02130045182793E-270	2	NOL9
0	0.665951007292692	0.471	0.156	0	2	NUP214
0	0.665705083851899	0.538	0.197	0	2	RUNX1
1.89291409473372E-226	0.665343973562597	0.309	0.099	3.17214543995477E-222	2	TBP
1.69338342600579E-306	0.665020241914221	0.444	0.148	2.8377719453005E-302	2	WFS1
0	0.664808193117051	0.404	0.12	0	2	EPHB4
0	0.664659909203596	0.516	0.171	0	2	NUP42
2.03869050607935E-295	0.664459722982194	0.446	0.153	3.41643755008778E-291	2	ACSF2
1.68986035211669E-204	0.664097715291478	0.307	0.103	2.83186797807715E-200	2	FAM111B
0	0.664054666973427	0.569	0.198	0	2	COP 1
0	0.664022160911176	0.46	0.145	0	2	RAB4B
0	0.664019757698425	0.433	0.13	0	2	WLS
3.96474372361843E-247	0.663854812313407	0.321	0.1	6.64411753203977E-243	2	PEX1
0	0.663751641974788	0.465	0.15	0	2	SLC25A37
2.63695272783674E-208	0.663698656688328	0.287	0.092	4.41900538130881E-204	2	ZNF292
0	0.662985955263214	0.728	0.276	0	2	AGRN
0	0.662628257149492	0.717	0.276	0	2	ILVBL
0	0.66248512853823	0.921	0.518	0	2	MAOA
0	0.661776723583557	0.468	0.161	0	2	CHD9
3.67097229015685E-268	0.661756493436655	0.422	0.148	6.15181536384486E-264	2	TMEM65
0	0.661603598409532	0.867	0.385	0	2	WWP1
0	0.661585538722467	0.697	0.265	0	2	CYBC1
7.76097320556076E-287	0.66151850878547	0.389	0.124	1.30058388978787E-282	2	ALKBH6
0	0.661501127747759	0.607	0.216	0	2	DCAF5
3.46716232337812E-289	0.661257984141127	0.442	0.152	5.81027062151706E-285	2	DSN1
0	0.66110633441608	0.482	0.152	0	2	CCNB1IP1
5.31804823089624E-244	0.660829656750826	0.325	0.103	8.91198522533592E-240	2	METTL27
1.01866194575095E-301	0.660795501968172	0.388	0.12	1.70707368868945E-297	2	ZNF445
0	0.660761986174541	0.548	0.17	0	2	MVP
0	0.660761816942882	0.564	0.198	0	2	TENT2

1.47821119940421E-211	0.660733946311222	0.282	0.089	2.47718632796158E-207	2	TRIM32
6.48708680739329E-218	0.660585794219075	0.286	0.089	1.08710600718297E-213	2	CXorf40B
0	0.660553026702833	0.572	0.196	0	2	HUS1
0	0.660544691546189	0.568	0.197	0	2	DHX16
3.46899693034795E-264	0.660243109251513	0.339	0.105	5.8133450558771E-260	2	DDX31
0	0.660190488494455	0.651	0.238	0	2	TGFBR2
0	0.659817576668178	0.692	0.259	0	2	ZNF638
1.03750088894899E-242	0.659289366005855	0.332	0.106	1.73864398970072E-238	2	RAD51D
0	0.65926785794288	0.641	0.234	0	2	ZNF410
0	0.659223332476114	0.819	0.368	0	2	ACSL5
0	0.659185435112233	0.508	0.178	0	2	RASGEF1B
0	0.659090560255926	0.773	0.311	0	2	DHX15
0	0.658445472192849	0.649	0.234	0	2	MTIF3
3.20674166067312E-251	0.658086310265863	0.406	0.145	5.37385767495601E-247	2	GUCY1A1
4.91792430722421E-272	0.657710705956565	0.337	0.101	8.24145755404633E-268	2	FAM222A
0	0.657612415292145	0.776	0.309	0	2	FAM199X
3.28383467234872E-273	0.657330963815608	0.39	0.13	5.50305014392198E-269	2	ZNF407
0	0.657044798584717	0.725	0.284	0	2	SPG7
0	0.657039631495274	0.599	0.211	0	2	GOLGB1
4.8368681041714E-238	0.65676723181323	0.334	0.108	8.10562356897043E-234	2	MED20
0	0.656495524418012	0.584	0.199	0	2	BTBD7
4.11198721820414E-162	0.65630547336402	0.263	0.093	6.8908681802665E-158	2	KIAA1328
0	0.656065109707548	0.541	0.188	0	2	CASK
0	0.65605312992976	0.434	0.133	0	2	BAK1
0	0.656013610242017	0.52	0.171	0	2	P2RX4
0	0.655720324439639	0.52	0.18	0	2	TMLHE
6.99859738787179E-285	0.65566941547581	0.527	0.203	1.17282495025956E-280	2	DEPRECATED-ENSG00000183747
0	0.655471407307331	0.482	0.166	0	2	GTPBP2
5.15122464149717E-249	0.654873223139134	0.271	0.074	8.63242225422096E-245	2	WDR60
0	0.654803751037602	0.444	0.145	0	2	KIDINS220
3.46804298279289E-246	0.654633114572043	0.291	0.085	5.81174643056433E-242	2	DAGLB
0	0.654326256646485	0.628	0.23	0	2	GOSR2
4.63906362799144E-197	0.653956036138391	0.31	0.108	7.77414282778805E-193	2	GRK3
3.22890885582592E-284	0.653929937459663	0.399	0.13	5.41100546059308E-280	2	KLHL8
0	0.65363910573867	0.856	0.386	0	2	HNRNPU
0	0.652979228936201	0.599	0.217	0	2	LMBRD2
0	0.652754427166395	0.57	0.2	0	2	PGAP3
1.8213217811765E-263	0.652648838791516	0.395	0.134	3.05217104089557E-259	2	OMA1
3.97356680663304E-228	0.652297786269029	0.955	0.606	6.65890325455565E-224	2	SGK1
0	0.65225978067121	0.806	0.345	0	2	GPRC5C
0	0.652215079169791	0.707	0.274	0	2	PHF3
2.23279365260193E-297	0.652171988875592	0.449	0.154	3.74171560303032E-293	2	PAN2
2.24355226353244E-188	0.652144579446869	0.26	0.084	3.75974488322767E-184	2	SETD6
1.12736052842255E-246	0.65190715897864	0.369	0.125	1.88923077353052E-242	2	RBM12
0	0.651161685027746	0.495	0.16	0	2	GPD1L
0	0.650953466437086	0.497	0.168	0	2	TOR1A
4.14122415651147E-242	0.650796389705138	0.346	0.114	6.93986344148193E-238	2	LCORL
0	0.650739106610926	0.76	0.321	0	2	CYP4F2
0	0.650433773352939	0.682	0.253	0	2	AGFG1
0	0.65021113243525	0.44	0.138	0	2	VOPP1
0	0.650062190416441	0.748	0.298	0	2	PEX2
3.48523679547908E-264	0.649979820889641	0.333	0.101	5.84055982186385E-260	2	ZMYND19
2.87315740684231E-273	0.649273735711637	0.333	0.099	4.81483718238634E-269	2	MICA
0	0.64902250323389	0.403	0.116	0	2	TTYH3
0	0.648949242801025	0.551	0.189	0	2	VIRMA
1.9469670520629E-286	0.648921134234245	0.334	0.096	3.262727385847E-282	2	RNASET2
1.32192313854334E-209	0.648669347910285	0.275	0.086	2.21527879557092E-205	2	GAPVD1
2.73794367218206E-230	0.648467349085535	0.337	0.113	4.58824600584269E-226	2	PPIL4
5.04549985449606E-215	0.648371711152034	0.256	0.074	8.4552486561645E-211	2	CD44
0	0.647669520811976	0.675	0.257	0	2	FBXL5

0	0.647222854412268	0.653	0.245	0	2	PSPC1
0	0.646890450513393	0.927	0.529	0	2	TM9SF2
0	0.646696231310431	0.742	0.269	0	2	BAIAP2L1
0	0.646012370004517	0.464	0.158	0	2	GART
0	0.645732478992516	0.803	0.338	0	2	PLEKHF2
0	0.644850807814427	0.726	0.274	0	2	SCRIB
1.51583627402342E-182	0.644799358870733	0.269	0.09	2.54023842800844E-178	2	RWDD4
2.43777536382795E-295	0.644500475682585	0.38	0.118	4.08522395470287E-291	2	PLCB3
0	0.64448219586934	0.507	0.18	0	2	CSTF3
2.89635800705895E-299	0.644443844600643	0.461	0.16	4.85371674822938E-295	2	SLC30A5
0	0.644420585219448	0.735	0.289	0	2	ESYT2
0	0.644380114628096	0.709	0.28	0	2	IPO9
0	0.644216206484915	0.733	0.285	0	2	ANKHD1
1.52808923443383E-220	0.643942362063172	0.306	0.099	2.56077193906421E-216	2	UIMC1
1.58564752320405E-228	0.643779961231481	0.312	0.1	2.65722811938535E-224	2	FAM133B
0	0.643655248564318	0.551	0.188	0	2	COQ4
2.84979943266843E-282	0.643619898622141	0.403	0.133	4.77569388926576E-278	2	ANAPC7
0	0.643577198170502	0.662	0.251	0	2	LNX2
0	0.64356375351121	0.456	0.154	0	2	ETAA1
0	0.643235253314462	0.748	0.299	0	2	SAMD5
0	0.643159767551799	0.888	0.44	0	2	PON2
0	0.642763364811776	0.54	0.188	0	2	CASP3
4.93448337400213E-249	0.642726073010401	0.343	0.111	8.26920723815276E-245	2	EXOC6B
0	0.642208961587327	0.847	0.37	0	2	SETD5
5.95374075374266E-283	0.642081941550032	0.384	0.123	9.97727875512195E-279	2	AKAP11
0	0.64205519464119	0.52	0.177	0	2	SLC35D1
0	0.642035688169329	0.605	0.215	0	2	ARHGAP5
0	0.64192710165997	0.513	0.173	0	2	SLC25A43
0	0.641747006757014	0.585	0.211	0	2	IP6K2
0	0.64171515437718	0.535	0.192	0	2	REV1
4.00044194220421E-250	0.641417344689329	0.322	0.099	6.70394060674582E-246	2	TWSG1
0	0.641033931287034	0.603	0.224	0	2	RBM14
7.4644656725269E-176	0.64101049983392	0.265	0.09	1.25089515740206E-171	2	SLC30A6
5.44240092349731E-183	0.640897712648558	0.38	0.156	9.1203754675968E-179	2	SPHK1
5.70648596090825E-264	0.640544351293489	0.336	0.103	9.56292917329005E-260	2	HEATR1
0	0.640255460702161	0.596	0.216	0	2	SLC46A1
0	0.640163659005261	0.368	0.105	0	2	CARD10
3.17476401788104E-291	0.639930150183615	0.463	0.164	5.32026954116504E-287	2	MYNN
8.87099539914677E-231	0.63958740416244	0.317	0.101	1.48660140898902E-226	2	PKN3
1.58753299500276E-268	0.639287777316753	0.345	0.107	2.66038779302562E-264	2	NSD3
0	0.639020753322301	0.493	0.171	0	2	SFPQ
1.13319781315977E-265	0.638839575993946	0.394	0.133	1.89901289529314E-261	2	USP33
3.40379472310308E-259	0.638703244781664	0.387	0.131	5.70407919697614E-255	2	ZDHHC6
0	0.63856760592304	0.783	0.336	0	2	ACSL3
0	0.638386139977358	0.489	0.168	0	2	ARHGAP17
0	0.63834974087419	0.481	0.159	0	2	ZNF687
0	0.63763991116353	0.521	0.168	0	2	BCL9L
0	0.636183657600193	0.503	0.177	0	2	NF1
0	0.635975813025494	0.848	0.365	0	2	STAG2
0	0.635790269277504	0.513	0.175	0	2	SLC30A9
0	0.635506172289676	0.499	0.172	0	2	FAF2
2.97484477134848E-280	0.634677260383157	0.372	0.117	4.98524486782578E-276	2	PAM
0	0.634433292460717	0.628	0.23	0	2	KIF13A
0	0.63436112267014	0.772	0.316	0	2	STT3B
8.77304198816141E-278	0.634120283915758	0.409	0.138	1.47018637637609E-273	2	ZHX3
3.35180942013643E-268	0.634001770061309	0.356	0.113	5.61696222626462E-264	2	WDR5
7.21037343622437E-233	0.633936189194972	0.339	0.113	1.20831438044248E-228	2	UHRF1BP1L
5.1011421115093E-305	0.633906679265987	0.469	0.162	8.54849395046729E-301	2	RNF139
0	0.63331237529953	0.564	0.204	0	2	TAP2
0	0.633270215304497	0.548	0.192	0	2	ATXN2L

3.77290062748404E-216	0.633201127122732	0.278	0.085	6.32262687153775E-212	2	TRMT13
1.72166759517898E-306	0.633008766803863	0.422	0.137	2.88517055600093E-302	2	TP53I13
2.72944202662833E-282	0.632931950430939	0.423	0.144	4.57399894822376E-278	2	CUL2
0	0.632288023520114	0.521	0.169	0	2	TAX1BP3
1.42263964749822E-292	0.631713965457533	0.426	0.143	2.38405952127751E-288	2	NPHP3
8.17620703165386E-178	0.631688415060063	0.295	0.107	1.37016877436455E-173	2	MGA
6.92016806784865E-292	0.631496954595507	0.357	0.107	1.15968176481008E-287	2	IL15RA
0	0.631003958602044	0.31	0.075	0	2	TMEM54
0	0.630872064738802	0.488	0.165	0	2	MAML1
0	0.630656231734943	0.588	0.218	0	2	NSMCE2
0	0.630587352628908	0.516	0.162	0	2	LMTK2
9.55737996616914E-272	0.630349014100296	0.327	0.096	1.60162573473063E-267	2	TES
1.34913890092912E-286	0.630132905184568	0.394	0.127	2.26088697017701E-282	2	ABL2
0	0.629331571155535	0.582	0.212	0	2	KDM4B
1.85482669865318E-301	0.629161800245248	0.411	0.132	3.108318581603E-297	2	PIAS3
0	0.628829725199831	0.621	0.227	0	2	PHKA2
1.65724835347575E-295	0.628806107221618	0.329	0.092	2.77721679075466E-291	2	EVI5L
1.24059843255672E-238	0.62861162081273	0.305	0.093	2.07899485327854E-234	2	DEPRECATED-ENSG00000197714
2.27938298674941E-202	0.628490401467685	0.278	0.089	3.81979000919466E-198	2	SMUG1
1.48973158667067E-276	0.628289373599742	0.408	0.138	2.49649219294271E-272	2	TMEM170A
1.25597493321054E-219	0.627984084256414	0.312	0.103	2.10476279307423E-215	2	THAP12
0	0.627724198741089	0.674	0.26	0	2	TCIRG1
0	0.627259682981278	0.54	0.183	0	2	NCAPG2
0	0.627061519585496	0.7	0.272	0	2	COBLL1
2.24901866288112E-293	0.626883028397121	0.421	0.14	3.76890547525618E-289	2	PDS5A
0	0.626175819932798	0.482	0.165	0	2	EPC1
0	0.62589120131067	0.503	0.176	0	2	NUP133
4.55997059371894E-247	0.625855582565199	0.815	0.437	7.6415987209542E-243	2	DEPRECATED-ENSG00000132002
3.82722558410203E-182	0.625702908942872	0.25	0.08	6.41366463383818E-178	2	ATAD3B
0	0.625145884294228	0.672	0.257	0	2	CXorf56
0	0.624746131031878	0.379	0.112	0	2	MXD1
6.20254723128553E-221	0.624745767006151	0.282	0.086	1.03942286501883E-216	2	PPL
9.75730518018311E-213	0.624335657459477	0.309	0.103	1.63512920209509E-208	2	TMEM184C
0	0.623916304740457	0.749	0.31	0	2	SFT2D2
8.36761046460246E-241	0.623894993424935	0.975	0.575	1.40224416165808E-236	2	TSC22D3
3.41385303103508E-189	0.62388048960153	0.265	0.086	5.72093490940859E-185	2	ZFP90
1.28929571776277E-232	0.623815351512754	0.312	0.099	2.16060176382685E-228	2	ACOX3
3.85047666729053E-244	0.623740927808063	0.309	0.094	6.45262879904547E-240	2	ARMC10
0	0.623585261699014	0.789	0.338	0	2	SLC44A1
0	0.623234251444358	0.898	0.434	0	2	LIPG
0	0.623056975880402	0.698	0.258	0	2	UBE3C
9.66190540536151E-308	0.622710159469043	0.473	0.164	1.61914210783048E-303	2	OTUD4
3.09256480523427E-212	0.622529575209305	0.325	0.112	5.18252010061158E-208	2	SPATA5L1
3.38915361334012E-277	0.622406829099532	0.434	0.152	5.67954362523537E-273	2	ORMDL1
2.62498506751345E-287	0.62154149657439	0.431	0.147	4.39894997613905E-283	2	TMX1
9.06173495312443E-205	0.621177517980999	0.285	0.092	1.51856554344459E-200	2	JKAMP
7.98776340145035E-237	0.621062401496794	0.293	0.088	1.33858939081505E-232	2	C6orf47
0	0.620879829957567	0.709	0.273	0	2	TMCO3
0	0.619650871117857	0.449	0.138	0	2	KIF12
2.20982700280607E-296	0.618982372326561	0.494	0.18	3.70322809130242E-292	2	SIN3B
5.58845032356809E-213	0.618825259758761	0.279	0.087	9.36512505223541E-209	2	C2CD2
1.4341227263375E-274	0.618604091584025	0.398	0.133	2.40330286479639E-270	2	RBM5
1.78381237303893E-198	0.618441985614431	0.312	0.108	2.98931277473864E-194	2	TMEM187
1.31714835511751E-238	0.618413380304862	0.264	0.073	2.20727721350592E-234	2	SYNE3
0	0.618360791381738	0.443	0.141	0	2	ADCK2
1.02731036573084E-299	0.618174008679619	0.414	0.134	1.72156671089174E-295	2	MAN1B1
0	0.617846127397413	0.445	0.143	0	2	SNTB2
8.93873630019814E-180	0.617815216305234	0.277	0.096	1.4979534291872E-175	2	SRCIN1
2.09242393592035E-278	0.617617508250585	0.357	0.11	3.50648403181532E-274	2	AMBRA1
0	0.617218201482037	0.407	0.128	0	2	PIK3R4

9.70551532570824E-171	0.616778573000691	0.264	0.091	1.62645025828219E-166	2	PHTF2
0	0.616735844511383	0.692	0.27	0	2	NUP58
0	0.616371990256447	0.583	0.203	0	2	RBM15
9.46128334409938E-213	0.615756607749626	0.266	0.08	1.58552186280417E-208	2	SGMS2
0	0.615499495933083	0.599	0.222	0	2	KMT2D
0	0.615290145700698	0.557	0.196	0	2	IFNAR2
3.39067817954538E-304	0.615181561583679	0.424	0.139	5.68209849328215E-300	2	AGAP1
0	0.614883131187673	0.682	0.266	0	2	ELOVL1
0	0.61484640649244	0.702	0.256	0	2	NIBAN2
0	0.614681485502427	0.746	0.319	0	2	C11orf54
3.49376516847212E-250	0.614547439223766	0.312	0.093	5.85485166932558E-246	2	CDCA7L
3.39776011085659E-243	0.614466082891058	0.332	0.106	5.69396639377347E-239	2	EXT2
3.52200276042756E-257	0.614430539077619	0.393	0.135	5.9021722259245E-253	2	TBCE
8.33013889211536E-265	0.614355031907424	0.398	0.136	1.39596467554069E-260	2	RECQL5
0	0.613474671467513	0.574	0.21	0	2	WDR33
2.14158708120314E-190	0.61329775060897	0.271	0.089	3.58887163068022E-186	2	ADAT2
0	0.613157051745005	0.715	0.286	0	2	TRAPPC4
0	0.613111173982325	0.642	0.236	0	2	RBMX
3.91323325027016E-222	0.612633946400297	0.32	0.106	6.55779628080274E-218	2	TUBGCP4
3.32302548783873E-282	0.612594498051956	0.455	0.162	5.56872611252014E-278	2	PLEKHH3
0	0.612209556711518	0.561	0.193	0	2	GORASP1
5.53171468194788E-273	0.61202015277508	0.344	0.105	9.27004746400826E-269	2	SLC52A2
0	0.611939149998167	0.665	0.255	0	2	TDP2
0	0.611831675164544	0.703	0.275	0	2	ZMIZ2
0	0.611558804799555	0.531	0.179	0	2	SOAT1
1.07063048081768E-290	0.61147453214204	0.442	0.152	1.79416255975426E-286	2	SLC25A46
6.53413151188507E-212	0.611427037534136	0.29	0.093	1.0949897587617E-207	2	INPP4A
0	0.611171247354109	0.599	0.215	0	2	VHL
0	0.610930188825693	0.766	0.316	0	2	DEPRECATED-ENSG00000213759
2.07914288804179E-216	0.610627924549167	0.289	0.091	3.48422765178043E-212	2	ALS2
4.11774765692182E-217	0.610467170688453	0.297	0.095	6.90052152346959E-213	2	ZNF12
0	0.61019088977321	0.849	0.426	0	2	DHRS7
0	0.609778148264079	0.493	0.167	0	2	PPP1CC
5.39129689974459E-206	0.609195767484767	0.331	0.117	9.03473534459199E-202	2	RFC4
0	0.609107881844245	0.608	0.214	0	2	UBE4A
1.0687613372822E-306	0.609008806271762	0.482	0.169	1.79103024901751E-302	2	RMDN1
0	0.608772275133576	0.417	0.129	0	2	UCHL3
3.91089655389577E-193	0.608321651035566	0.302	0.105	6.55388044501853E-189	2	VCL
4.86351257331976E-236	0.608113820861139	0.367	0.127	8.15027437036925E-232	2	RNF217
0	0.607749045821285	0.823	0.366	0	2	PDZD8
3.82537490409127E-294	0.607704980737943	0.435	0.147	6.41056326427615E-290	2	CMTR1
0	0.607638990722005	0.922	0.466	0	2	GDI1
1.77601113147695E-234	0.607432707000405	0.303	0.093	2.97623945412907E-230	2	TMEM30B
2.45570279855592E-296	0.607357892184699	0.416	0.136	4.11526674982001E-292	2	MINDY1
0	0.607085132707639	0.442	0.143	0	2	C6orf136
1.1033731819542E-298	0.606397411988312	0.489	0.175	1.84903277831885E-294	2	FKBP3
0	0.606231695530665	0.648	0.245	0	2	ABCB6
4.14224287843784E-221	0.606104156240063	0.359	0.128	6.94157061568614E-217	2	CDHR2
0	0.606059915745917	0.651	0.25	0	2	DNMT1
0	0.606014994016056	0.505	0.177	0	2	TRMT11
1.23180919949423E-208	0.605773592546741	0.303	0.101	2.06426585651244E-204	2	MTF2
0	0.605636397132373	0.674	0.251	0	2	CDC16
0	0.605200614269324	0.699	0.273	0	2	SEPTIN8
0	0.604777956416208	0.685	0.259	0	2	TTC3
1.09502006705434E-265	0.60467874822643	0.422	0.148	1.83503462836966E-261	2	ACAP3
7.11876790565696E-206	0.604578713630664	0.307	0.103	1.19296312562999E-201	2	RCC2
2.86316951872615E-159	0.604244543842228	0.253	0.089	4.79809947948128E-155	2	FUNDC2
0	0.603982769616882	0.508	0.176	0	2	CYHR1
0	0.603861693530191	0.507	0.178	0	2	TAPT1
1.38404133541564E-294	0.603812510441181	0.406	0.131	2.31937646988954E-290	2	MIGA1

0	0.603641553413382	0.738	0.297	0	2	NECTIN3
0	0.603228483060886	0.762	0.297	0	2	CLK1
6.8938506686901E-180	0.603217636223124	0.254	0.083	1.15527149505909E-175	2	FAM126B
0	0.603152920912069	0.403	0.122	0	2	PITPNM1
0	0.60312363466584	0.89	0.443	0	2	PLXNB2
0	0.602943346313809	0.542	0.17	0	2	RASSF7
0	0.60285519067165	0.636	0.24	0	2	STX7
9.72717945585186E-212	0.602651228037668	0.267	0.081	1.63008073321165E-207	2	MSH3
0	0.602454708063736	0.667	0.252	0	2	CARS2
0	0.602253330141077	0.676	0.266	0	2	IL17RB
0	0.601424206304965	0.45	0.15	0	2	SLC30A7
0	0.601342455077048	0.633	0.242	0	2	KIF1B
4.27856640985754E-305	0.601271144295371	0.452	0.153	7.17002158963926E-301	2	VPS54
0	0.600616683667788	0.409	0.127	0	2	PLEKHA7
0	0.600589408213402	0.63	0.235	0	2	TFDP1
0	0.600323076949335	0.587	0.219	0	2	FUCA2
6.53678262200345E-273	0.599852540133756	0.293	0.079	1.09543403179534E-268	2	TMEM120B
8.96345628237031E-288	0.599677555965694	0.416	0.138	1.50209600379962E-283	2	PPP1R13B
0	0.598962824418985	0.809	0.361	0	2	DNAJC3
1.08363088761277E-183	0.598772032195024	0.272	0.091	1.81594864146148E-179	2	MINDY3
1.88984899231655E-274	0.598705613944519	0.372	0.119	3.16700894132407E-270	2	ARID4A
1.30951080915021E-302	0.598636878435838	0.517	0.191	2.19447821397393E-298	2	FAM136A
0	0.59856940458527	0.497	0.175	0	2	VCPIP1
0	0.598535365654741	0.472	0.156	0	2	CXorf40A
1.56940842838315E-254	0.598504971702137	0.394	0.137	2.63001464428448E-250	2	COG1
0	0.598017124357493	0.619	0.235	0	2	GNPAT
3.14389084491477E-221	0.597994312963751	0.269	0.08	5.26853227790817E-217	2	METTL2B
0	0.59790123292676	0.682	0.262	0	2	SLC35F6
0	0.596932940688439	0.777	0.317	0	2	BIN1
0	0.59684521607458	0.591	0.22	0	2	DYM
0	0.596346616327391	0.613	0.232	0	2	MARVELD2
8.22535571241466E-222	0.596235911104303	0.326	0.11	1.37840511028645E-217	2	KIAA0355
0	0.596221138832436	0.615	0.221	0	2	AIMP1
6.47662749202338E-208	0.596145875067646	0.292	0.094	1.08535323511328E-203	2	VAMP4
0	0.595581501973739	0.9	0.452	0	2	MIA2
1.01160758370632E-203	0.595519778923881	0.28	0.09	1.69525198877506E-199	2	ZC3H11A
0	0.595032017336541	0.664	0.251	0	2	RAPH1
5.01575260021109E-308	0.595028998405475	0.478	0.166	8.40539820743374E-304	2	SPG11
2.77149020890682E-289	0.594157993786098	0.423	0.141	4.64446329208605E-285	2	ITSN1
0	0.593938614697826	0.609	0.228	0	2	TOB2
0	0.593255958600823	0.519	0.182	0	2	PRPF4B
7.47493617270026E-251	0.592829129293464	0.365	0.121	1.25264980382111E-246	2	PIGF
2.23225989194314E-293	0.592677862506206	0.428	0.143	3.74082112691831E-289	2	KLHL23
0	0.592561882331046	0.807	0.358	0	2	LACTB2
3.35767777886229E-257	0.592558116003503	0.382	0.128	5.62679642181743E-253	2	SLC25A40
3.27985683014776E-237	0.592532159671718	0.301	0.091	5.49638407596161E-233	2	BICRAL
0	0.592467498208077	0.56	0.191	0	2	COLGALT1
0	0.592467307619011	0.526	0.175	0	2	LYN
0	0.591681942583378	0.524	0.174	0	2	OSBPL2
0	0.591541557611335	0.625	0.238	0	2	TMEM129
0	0.590943206634143	0.533	0.188	0	2	ZNF33A
1.00544360940674E-299	0.590690190952664	0.383	0.118	1.68492240064382E-295	2	PIGS
6.99064368978926E-240	0.590575387505087	0.374	0.129	1.17149206953488E-235	2	TRAPPC8
3.56261945608698E-306	0.589843931045163	0.451	0.151	5.97023768451056E-302	2	CLCN3
4.80515193420418E-235	0.589747807327335	0.979	0.678	8.05247361133937E-231	2	ASPH
0	0.589697582385523	0.448	0.146	0	2	ADD3
0	0.589680841097862	0.396	0.083	0	2	EMP1
0	0.589187647827388	0.465	0.155	0	2	TMEM98
4.08691937432931E-258	0.589116791342404	0.3	0.085	6.84885948750106E-254	2	SLC7A9
0	0.588965465808368	0.595	0.216	0	2	TUBGCP6

2.12138514768119E-209	0.588848607470455	0.28	0.088	3.55501723048414E-205	2	POLG2
0	0.588477592522176	0.729	0.284	0	2	INSR
2.71006538374776E-216	0.588277345610253	0.258	0.075	4.54152757008449E-212	2	TCN2
0	0.588048002854271	0.713	0.313	0	2	RDH10
0	0.58802342435062	0.536	0.178	0	2	CYREN
4.19915814640046E-298	0.587934182509869	0.444	0.15	7.03694922173789E-294	2	RUSC1
0	0.587811175764556	0.424	0.131	0	2	APEX2
7.91268218743828E-255	0.587589761339692	0.347	0.11	1.32600728097091E-250	2	POLM
0	0.587573761032644	0.736	0.299	0	2	NUMB
0	0.587505754272521	0.861	0.398	0	2	AFF1
3.84267989733828E-304	0.587458671470343	0.394	0.123	6.43956297195949E-300	2	SLC16A13
2.96979431856317E-277	0.587223362305071	0.404	0.135	4.97678131904815E-273	2	NCOA3
2.68252379773197E-246	0.587014075293864	0.352	0.115	4.49537338023923E-242	2	MAML3
1.0092369632813E-290	0.586654517235947	0.39	0.123	1.6912793030668E-286	2	KRCC1
9.19765465117896E-231	0.58656819762808	0.364	0.127	1.54134296644457E-226	2	PMS1
0	0.586022192763641	0.635	0.244	0	2	SCML1
0	0.585902115667302	0.56	0.201	0	2	BRF1
8.7668877815541E-187	0.58554952043004	0.276	0.093	1.46915505443284E-182	2	PPM1H
0	0.585536614291941	0.604	0.223	0	2	NIPBL
0	0.585377610049206	0.506	0.179	0	2	LETMD1
1.40909436712941E-255	0.584930276113075	0.4	0.139	2.36136034043546E-251	2	RBPJ
0	0.584879610341759	0.5	0.176	0	2	SGPP1
0	0.584853825664157	0.867	0.408	0	2	TBC1D9B
7.70554116562213E-291	0.584463269772283	0.459	0.162	1.29129458853496E-286	2	FBH1
1.31022618561857E-305	0.584346936459389	0.847	0.414	2.19567704185961E-301	2	IL13RA1
0	0.584021005295253	0.505	0.171	0	2	STK4
5.2875023873646E-231	0.583718328569457	0.329	0.108	8.8607965007456E-227	2	HCFC2
0	0.583632823200745	0.452	0.152	0	2	PHKB
8.85295400465654E-273	0.583611132995139	0.38	0.124	1.48357803210034E-268	2	CCDC93
1.02944248904962E-234	0.583387349835584	0.382	0.136	1.72513972314935E-230	2	MNAT1
8.60604271053769E-224	0.583370175414918	0.339	0.116	1.44220063743191E-219	2	CHD6
1.0555576574498E-224	0.583346374258791	0.372	0.134	1.76890352235438E-220	2	NAA30
0	0.583332225455552	0.524	0.178	0	2	FAM111A
1.58800623132808E-202	0.582898904702731	0.292	0.096	2.66118084245959E-198	2	NDUFAF5
3.84234120995476E-216	0.582792390848359	0.355	0.127	6.43899539964218E-212	2	LATS2
3.71118283625211E-255	0.582699158524446	0.414	0.147	6.21920019699129E-251	2	PIGM
1.22785418042368E-286	0.582329075421472	0.941	0.59	2.057638035554E-282	2	GOLIM4
1.06999161049277E-227	0.582252588289481	0.317	0.102	1.79309194086379E-223	2	CLK2
1.6806590477543E-286	0.582151640388865	0.36	0.109	2.81644843222665E-282	2	UMAD1
0	0.581936528667138	0.878	0.426	0	2	LAPTM4B
0	0.581853200604965	0.623	0.225	0	2	FBXL6
0	0.581775087490557	0.429	0.136	0	2	RABL6
0	0.581369747727746	0.493	0.171	0	2	CAMSAP2
6.10127318290576E-179	0.581327790137044	0.274	0.094	1.02245135999135E-174	2	THAP5
1.28350813492135E-296	0.581303645462548	0.382	0.118	2.1509029325012E-292	2	MAN2B2
1.02342568860311E-289	0.581160209164549	0.426	0.143	1.71505676896108E-285	2	PHF11
4.82356971816896E-280	0.58086532501029	0.444	0.157	8.08333813370754E-276	2	TGS1
3.11719556303102E-175	0.580824400370437	0.277	0.096	5.22379632452738E-171	2	BIRC2
1.70729750543222E-299	0.580314312864716	0.407	0.13	2.86108915960331E-295	2	HDAC10
4.92410714867883E-217	0.580267430979022	0.277	0.085	8.25181875975598E-213	2	PRDM4
6.53609096478604E-220	0.580085396888277	0.345	0.119	1.09531812387884E-215	2	MOB3B
0	0.579904960425312	0.636	0.238	0	2	HACD2
1.86880701351553E-217	0.579709865091488	0.358	0.129	3.13174679324932E-213	2	MBTD1
4.00903011334213E-196	0.579673178984495	0.353	0.133	6.71833266393874E-192	2	RWDD3
0	0.579513881163967	0.637	0.235	0	2	LRCH3
1.56057584350335E-298	0.579469577314501	0.456	0.157	2.61521299854291E-294	2	KLC1
0	0.579289441054205	0.509	0.182	0	2	MTFR1L
7.16758413495655E-255	0.579257012548613	0.369	0.122	1.20114374933602E-250	2	PIGO
0	0.578867568652739	0.687	0.266	0	2	PHF20L1
2.28765093754426E-298	0.578638402179818	0.464	0.161	3.83364544113668E-294	2	PIGX

5.41102360464568E-288	0.578628052946384	0.384	0.121	9.06779335666523E-284	2	RELL1
0	0.578351869904045	0.523	0.185	0	2	RIPK1
1.88015762184495E-192	0.578000881831305	0.254	0.079	3.15076814268777E-188	2	CXorf38
5.26393525944915E-308	0.577912122347654	0.457	0.155	8.82130270778489E-304	2	INPPL1
2.11375181245139E-243	0.57763420482956	0.352	0.117	3.54222528730603E-239	2	HERC2
0	0.577082057419371	0.743	0.301	0	2	ZDHHC9
0	0.577061433910917	0.543	0.179	0	2	SLC39A11
6.5998057365587E-307	0.576532280586176	0.487	0.171	1.10599544533251E-302	2	CASP6
1.06615289144239E-232	0.576523919934623	0.357	0.122	1.78665901547915E-228	2	WDR76
0	0.576338783237344	0.497	0.174	0	2	DGCR2
0	0.57612377595276	0.734	0.313	0	2	TCIM
0	0.575965291813775	0.516	0.182	0	2	C6orf62
4.60517083273258E-268	0.575835484380025	0.385	0.128	7.71734528149325E-264	2	MPP6
2.77780002179431E-245	0.575426023043873	0.347	0.113	4.6550372765229E-241	2	PAPSS1
1.60049667506666E-274	0.575405402574004	0.431	0.15	2.68211232807671E-270	2	YTHDF2
2.71401890379312E-277	0.57536795225923	0.409	0.137	4.54815287897651E-273	2	DEPRECATED-ENSG00000178950
4.69642597066509E-263	0.575286058515779	0.845	0.466	7.87027064164056E-259	2	SLPI
8.42120549072198E-302	0.575262016324949	0.448	0.151	1.41122561613519E-297	2	FLVCR1
1.53442498039538E-281	0.575203058576661	0.898	0.488	2.57138938214657E-277	2	ZFP36
1.03967971882006E-163	0.57491310057165	0.265	0.094	1.74229527279866E-159	2	FAM13B
0	0.574440119099466	0.493	0.169	0	2	ZNF384
4.06065969497492E-301	0.574269967069599	0.475	0.166	6.80485351683898E-297	2	RHOT1
0	0.573968688681598	0.696	0.266	0	2	SHROOM3
4.47972677749865E-245	0.573926000849816	0.318	0.099	7.50712613373224E-241	2	DUS3L
0	0.573313216407977	0.568	0.209	0	2	HMGXB4
1.64000329134351E-202	0.573313057893343	0.271	0.085	2.74831751563346E-198	2	SPIN3
0	0.573110461956036	0.696	0.294	0	2	TM9SF1
0	0.573086180082661	0.69	0.268	0	2	CREBZF
1.61147408730576E-206	0.572953432966974	0.255	0.075	2.700508275507E-202	2	TRIM11
1.69794057241719E-225	0.572767335702935	0.279	0.083	2.84540881125672E-221	2	NUDT8
9.30933148826062E-298	0.572605321615637	0.399	0.127	1.56005777080271E-293	2	ZDHHC20
7.65197600661102E-215	0.572206379699545	0.949	0.595	1.28231813918788E-210	2	HGD
0	0.572125211328448	0.462	0.151	0	2	TEAD3
1.1260661452066E-232	0.572107719831066	0.352	0.12	1.88706164613722E-228	2	RGL2
8.64029754813316E-186	0.571588677235626	0.346	0.132	1.44794106311615E-181	2	SLC35E3
0	0.571325603151337	0.549	0.203	0	2	BCAP29
0	0.571020844557104	0.731	0.293	0	2	U2SURP
0	0.570718447035135	0.885	0.432	0	2	IDH3G
5.39185720690159E-276	0.570643769746819	0.421	0.144	9.03567430732569E-272	2	RSPRY1
0	0.570593249137086	0.76	0.325	0	2	SMARCA2
1.04817322623587E-257	0.570568503670939	0.366	0.12	1.75652869252606E-253	2	NPRL3
0	0.56980848691082	0.573	0.199	0	2	GIGYF1
0	0.569706447733238	0.57	0.212	0	2	DDX3X
0	0.569115748679324	0.797	0.338	0	2	ERBIN
2.36730261334638E-191	0.568878337209545	0.297	0.103	3.96712571944587E-187	2	TSNARE1
2.72811673266999E-297	0.567411175582857	0.476	0.168	4.57177802060837E-293	2	EPS15L1
0	0.567366925622199	0.561	0.201	0	2	AEBP2
8.6261026542111E-216	0.567141632559732	0.317	0.106	1.4455622827927E-211	2	WRAP73
0	0.567140905521685	0.626	0.244	0	2	NRP1
1.92001406392295E-184	0.56701705242921	0.272	0.091	3.21755956832208E-180	2	DSTYK
0	0.566672665544101	0.573	0.209	0	2	NPAS2
0	0.566447079064517	0.686	0.273	0	2	PCMTD1
0	0.566330918961312	0.783	0.337	0	2	PCCA
9.93206879937228E-237	0.566187246642061	0.352	0.119	1.66441608939881E-232	2	DESI2
4.1137386973147E-265	0.56617215086914	0.381	0.126	6.89380330895998E-261	2	BCL10
0	0.56606493148169	0.777	0.347	0	2	CAPN1
0	0.566041243189324	0.757	0.311	0	2	ADD1
1.47665935252777E-274	0.5658269450658	0.381	0.123	2.47458574296604E-270	2	COA8
0	0.565459884187526	0.779	0.319	0	2	LRP5
0	0.564698426956987	0.506	0.178	0	2	WASHC5

4.35809059038691E-269	0.564134629252776	0.363	0.115	7.30328821137039E-265	2	SVIL
2.89506036471153E-270	0.563959508683111	0.351	0.108	4.85154215918358E-266	2	SDHAF3
5.63703618415812E-212	0.563737508081861	0.336	0.118	9.44654523741217E-208	2	PIEZO1
4.49221169353536E-212	0.563341387713057	0.255	0.074	7.52804835602656E-208	2	CDK18
0	0.56324060315224	0.549	0.198	0	2	EBAG9
0	0.563087169160392	0.713	0.278	0	2	TAF7
2.7517236314732E-183	0.562950853289827	0.349	0.134	4.6113384616228E-179	2	PSD3
0	0.562903540893405	0.688	0.257	0	2	MINK1
2.74969323482259E-179	0.562884671091058	0.268	0.09	4.6079359229157E-175	2	EPG5
5.82153991064575E-246	0.562500301521105	0.411	0.148	9.75573658226015E-242	2	HIBCH
4.28306013099728E-226	0.562197089632811	0.295	0.091	7.17755216752524E-222	2	TMEM47
3.63756018503528E-183	0.56176411239362	0.259	0.084	6.09582335808212E-179	2	SENP1
9.77213543052534E-265	0.56141801654932	0.435	0.156	1.63761445544744E-260	2	VEZF1
0	0.560897268491893	0.652	0.244	0	2	TRIM25
2.0348797747238E-294	0.560625431325121	0.832	0.417	3.41005152648214E-290	2	CTSS
1.42747583584865E-215	0.56059749674132	0.341	0.119	2.39216400571517E-211	2	ITFG2
0	0.560550122882541	0.424	0.132	0	2	ITPKA
5.36558293086627E-216	0.560007250927145	0.252	0.072	8.99164387554569E-212	2	ZNF276
0	0.559955505702313	0.433	0.136	0	2	TRIM14
1.32560366091846E-267	0.559255600113575	0.409	0.14	2.22144661496715E-263	2	FBXO38
0	0.559161705511936	0.509	0.176	0	2	UGGT1
8.31938111719584E-261	0.559036115064944	0.38	0.127	1.39416188761968E-256	2	JADE2
0	0.558895738264069	0.582	0.213	0	2	ARHGAP12
3.24641038937229E-299	0.558854255869652	0.383	0.118	5.44033453051008E-295	2	CLIP2
9.63977317135773E-199	0.558736698103953	0.305	0.104	1.61543318805613E-194	2	CAMKMT
9.44945902418932E-246	0.558728354826528	0.422	0.155	1.58354034327365E-241	2	POLB
0	0.558679111038375	0.364	0.105	0	2	GLTP
5.60309594747567E-288	0.558355660283088	0.857	0.438	9.38966818877972E-284	2	TMBIM1
8.82892222295227E-248	0.558335655130657	0.367	0.124	1.47955078612234E-243	2	WDR41
6.40869542518315E-293	0.558177559404857	0.421	0.14	1.07396917935219E-288	2	GALNT11
9.05446364222047E-294	0.55799101749364	0.442	0.151	1.51734701716331E-289	2	STARD4
0	0.557917284849705	0.581	0.211	0	2	NSD1
4.20881742241261E-260	0.557894422417518	0.415	0.146	7.05313623647905E-256	2	NHLRC2
0	0.557880170967944	0.635	0.24	0	2	SELENON
1.57558096724287E-254	0.557745278241893	0.409	0.144	2.64035858490561E-250	2	ZADH2
1.11166824489258E-217	0.557710744187805	0.323	0.109	1.86293364479099E-213	2	GGA1
0	0.557636030368869	0.498	0.174	0	2	RBM24
0	0.557093850455506	0.726	0.287	0	2	UBR2
0	0.556941929242056	0.678	0.268	0	2	NUDT9
3.01783895083284E-307	0.556793234224839	0.58	0.221	5.05729451380567E-303	2	APOOL
4.54832385070044E-264	0.556540617730578	0.373	0.121	7.6220811090038E-260	2	METTL9
0	0.556495673115793	0.814	0.371	0	2	EFHD2
0	0.556123683305086	0.531	0.185	0	2	TM9SF4
1.31238111637944E-284	0.556048998402255	0.386	0.122	2.19928827482867E-280	2	SRGAP2B
7.78605635770484E-187	0.556030284731802	0.252	0.08	1.30478732442418E-182	2	ZNF207
5.83580089439144E-206	0.555874480474299	0.275	0.086	9.77963513882118E-202	2	TRAM2
1.7075765062276E-272	0.555351439681568	0.33	0.097	2.86155670913621E-268	2	PRLR
1.51570998415776E-161	0.554608030327503	0.265	0.095	2.54002679145158E-157	2	NICN1
1.59055423330524E-255	0.55456015518591	0.385	0.131	2.66545078417292E-251	2	CHD8
0	0.554304535567028	0.747	0.306	0	2	HNRNPL
0	0.553859796548946	0.643	0.252	0	2	AGO2
0	0.553707620220983	0.867	0.419	0	2	NAPRT
3.84193795589623E-226	0.553628510956904	0.346	0.118	6.4383196264909E-222	2	USP40
1.43485768181839E-233	0.553602520063275	0.986	0.699	2.40453450319125E-229	2	S100A10
1.86258037541104E-188	0.553382797162269	0.257	0.082	3.12131219311382E-184	2	PLA2G4B
2.28093447405804E-251	0.552840089217044	0.409	0.145	3.82238999162647E-247	2	MBIP
0	0.552425040845364	0.569	0.216	0	2	MORC3
1.05310767990176E-226	0.552320224340186	0.359	0.126	1.76479784997936E-222	2	DIP2C
9.92518892591383E-259	0.551853650763263	0.386	0.13	1.66326316020464E-254	2	KIF2A
5.64968748732519E-250	0.551769012521445	0.396	0.138	9.46774629125956E-246	2	PCNT

**0**	0.551510401120319	0.805	0.352	0	2	DPYD
**1.63272528685994E-170**	0.550928877193535	0.251	0.084	2.73612103571988E-166	2	RAB23
**1.98367469902557E-253**	0.550706768931074	0.344	0.109	3.32424206062705E-249	2	ANGEL1
**3.73889612486941E-219**	0.550637937890613	0.277	0.084	6.26564212605616E-215	2	RUBCN
**1.22728577491856E-291**	0.549946995721549	0.438	0.149	2.05668550160852E-287	2	ABCD4
**1.24830169276273E-226**	0.549746903287657	0.321	0.105	2.09190397673178E-222	2	PGS1
**2.29836936162336E-260**	0.54973595221625	0.411	0.143	3.85160737620842E-256	2	ELL
**5.02879552559701E-292**	0.549616296398318	0.462	0.162	8.42725554179547E-288	2	NAA15
**1.96410974453566E-286**	0.549587021943475	0.476	0.173	3.29145510989286E-282	2	NAE1
**2.44001390848845E-285**	0.549566532400593	0.534	0.204	4.08897530784494E-281	2	SUSD3
**0**	0.54953613711926	0.824	0.374	0	2	LLGL2
**0**	0.549478008229278	0.483	0.16	0	2	RAVER1
**1.06629435380332E-209**	0.549302849431336	0.338	0.119	1.7868960781036E-205	2	EXOC2
**8.98907334100163E-268**	0.548860028419083	0.384	0.126	1.50638891048505E-263	2	PIGH
**2.64185915941981E-258**	0.548694245918351	0.367	0.12	4.42722757935572E-254	2	PDCL3
**0**	0.548503538459992	0.538	0.196	0	2	ARID4B
**2.68514315374719E-274**	0.548367248950731	0.384	0.125	4.49976289704955E-270	2	DPY19L1
**2.03745918675514E-275**	0.547860308388773	0.497	0.187	3.41437410516426E-271	2	UTP23
**0**	0.547859200229705	0.5	0.172	0	2	PGK 1.00
**1.08174730695367E-306**	0.547799880199799	0.499	0.178	1.81279213699296E-302	2	PURB
**0**	0.547698859482365	0.815	0.358	0	2	KLHDC2
**3.5941091938744E-278**	0.547033303464009	0.865	0.448	6.02300818709472E-274	2	BTG1
**0**	0.546918615149639	0.692	0.274	0	2	PACSIN2
**0**	0.546113102638569	0.715	0.291	0	2	RNF115
**0**	0.545869700046374	0.548	0.202	0	2	FAM8A1
**0**	0.545579232915699	0.792	0.347	0	2	HNRNPD
**0**	0.545155072801187	0.716	0.287	0	2	CUL4B
**0**	0.545116704527622	0.638	0.249	0	2	SRPK1
**0**	0.54499325042959	0.481	0.16	0	2	NUDT22
**0**	0.544811134214985	0.815	0.364	0	2	PYCR2
**1.76019745356113E-251**	0.544674329568193	0.387	0.132	2.94973889267774E-247	2	MGME1
**0**	0.544248510589973	0.737	0.299	0	2	SPECC1L
**1.24325144267602E-296**	0.544084039040564	0.87	0.463	2.08344076763647E-292	2	RRBP1
**6.65196675220977E-286**	0.54401644652288	0.442	0.153	1.11473658833531E-281	2	CPSF2
**0**	0.543650390243297	0.519	0.178	0	2	UQCRC2
**2.85247530413713E-297**	0.543443755114591	0.497	0.18	4.780178114673E-293	2	TSPAN4
**9.04616519961708E-260**	0.54330310244016	0.339	0.105	1.51595636415183E-255	2	DHRS4L2
**2.91698814649906E-213**	0.543298788329344	0.323	0.11	4.88828873590312E-209	2	USP28
**1.94930245075328E-224**	0.543227678485108	0.339	0.115	3.26664104697234E-220	2	MEIS2
**0**	0.543207871180835	0.609	0.229	0	2	SNRPA
**1.86922744801167E-185**	0.54291809197008	0.282	0.096	3.13245135737796E-181	2	PACRG
**0**	0.542828366145164	0.501	0.176	0	2	HTT
**1.78356533333908E-250**	0.542474363553619	0.348	0.112	2.98889878560963E-246	2	SPTLC1
**2.01042391992486E-292**	0.542398244282871	0.474	0.168	3.36906840501009E-288	2	SNX24
**0**	0.542339097119859	0.615	0.232	0	2	CNOT6L
**1.13849297969452E-288**	0.542236513673256	0.327	0.092	1.90788653537208E-284	2	PTGFRN
**0**	0.542150377763665	0.616	0.231	0	2	DOT1L
**1.25145449347481E-218**	0.542012591597296	0.253	0.072	2.09718744016508E-214	2	EFNA2
**0**	0.541966956786502	0.741	0.302	0	2	NCOA2
**2.09721777071743E-257**	0.541948560498819	0.449	0.164	3.51451754016827E-253	2	MPHOSPH9
**8.02293025639127E-187**	0.54156797555411	0.253	0.08	1.34448265236605E-182	2	ACTR5
**1.38340912893402E-222**	0.541555531206401	0.308	0.099	2.31831701826763E-218	2	GGA3
**0**	0.541338865170069	0.566	0.213	0	2	STRN3
**1.34715075091118E-222**	0.540860095821234	0.337	0.115	2.25755522837696E-218	2	STAM2
**1.29420590312649E-194**	0.540840051586378	0.289	0.097	2.16883025245938E-190	2	MAP4K3
**7.65927318493923E-232**	0.540709253933623	0.359	0.123	1.28354100033212E-227	2	COG4
**5.43794433887862E-265**	0.540309364287892	0.558	0.224	9.11290712309279E-261	2	ZBTB10
**5.286576311527E-257**	0.540087981654925	0.398	0.137	8.85924458285695E-253	2	SUCO
**7.13571777743387E-166**	0.539597122598518	0.277	0.1	1.19580358514237E-161	2	ERCC6
**0**	0.539545282181014	0.7	0.28	0	2	ECPAS

4.65423559086134E-227	0.539532588455726	0.358	0.125	7.79956800316543E-223	2	SEPTIN10
0	0.539421231609392	0.482	0.167	0	2	CAMSAP3
7.29845710233936E-239	0.539324221443475	0.313	0.097	1.22307544121003E-234	2	BRWD3
2.12132915364534E-241	0.539101485137709	0.961	0.552	3.55492339567886E-237	2	MBNL3
0	0.538854134371795	0.5	0.175	0	2	MED13L
5.03746799080371E-302	0.538663044438571	0.549	0.208	8.44178885898886E-298	2	BTD
0	0.538349091517447	0.583	0.215	0	2	USP24
4.47058887931366E-220	0.538282329267446	0.318	0.105	7.49181284395383E-216	2	SMURF2
3.43231398325218E-175	0.538221018814958	0.27	0.092	5.75187177313401E-171	2	CBLL1
1.39225270965532E-184	0.53799551804138	0.255	0.082	2.33313709084039E-180	2	DEPRECATED-ENSG00000056050
1.80410183648531E-162	0.537643657590149	0.264	0.093	3.02331385758209E-158	2	C9orf85
0	0.537365204683919	0.465	0.15	0	2	UTRN
7.78396507801326E-305	0.537262042681013	0.649	0.259	1.30443686777346E-300	2	RAB33B
2.72631386856202E-246	0.537015832412627	0.351	0.115	4.56875678093623E-242	2	NT5C
0	0.536932864011367	0.483	0.165	0	2	PRKD2
1.08678567844936E-215	0.536789318407149	0.54	0.234	1.82123543994544E-211	2	GRIA3
1.45754063610307E-248	0.536786436794165	0.31	0.093	2.44254659798152E-244	2	HNF1B
4.89707236602887E-251	0.53663866220837	0.334	0.105	8.20651387099119E-247	2	ABCB1
1.78120307338234E-196	0.536437467078942	0.282	0.093	2.98494011037413E-192	2	NINL
2.36359444492051E-196	0.536434859587843	0.314	0.109	3.96091157079779E-192	2	CCDC88A
3.10314672538223E-233	0.536390458231586	0.344	0.115	5.20025328239554E-229	2	C3orf38
7.5459937966137E-307	0.536227778451512	0.8	0.347	1.26455764043652E-302	2	TM7SF3
2.46820921974504E-287	0.535739939931593	0.419	0.14	4.13622501044874E-283	2	PPP1R12A
1.34337837972229E-283	0.535672203034533	0.462	0.165	2.25123348873861E-279	2	TELO2
1.69902031005118E-247	0.535556868957745	0.378	0.13	2.84721823558377E-243	2	USP15
6.44448257367988E-234	0.53547166802469	0.331	0.108	1.07996638969727E-229	2	STARD3NL
6.23716827054457E-298	0.53530916937514	0.521	0.194	1.04522465877786E-293	2	RO60
3.40223782650677E-299	0.53511995201961	0.689	0.295	5.70147014966005E-295	2	CTSO
1.69390375092807E-281	0.534745466276854	0.413	0.138	2.83864390580527E-277	2	BOP 1.00
1.32044529597248E-258	0.534661956618907	0.353	0.112	2.21280222690069E-254	2	TATDN2
0	0.534637895480787	0.788	0.348	0	2	EML4
9.57530086359649E-278	0.534632012123866	0.482	0.177	1.6046289187215E-273	2	LCLAT1
9.22111960315029E-208	0.534559666453815	0.285	0.091	1.54527522309593E-203	2	G6PC3
0	0.534412170640956	0.561	0.202	0	2	KDM3B
0	0.534253004191461	0.663	0.255	0	2	ITPRID2
2.40204899795503E-256	0.534061156179707	0.381	0.128	4.02535371077304E-252	2	RIF1
0	0.533919556767016	0.812	0.349	0	2	LUC7L3
6.38190001897321E-220	0.533422342892726	0.272	0.081	1.06947880517953E-215	2	NOMO1
0	0.533344027220587	0.496	0.174	0	2	VARS
0	0.533165041023896	0.589	0.217	0	2	KIAA0319L
2.9673193474746E-240	0.533039436112042	0.393	0.14	4.97263376249794E-236	2	TBPL1
0	0.532900743795135	0.642	0.247	0	2	MARCH7
3.65564682255818E-297	0.532818944468297	0.413	0.134	6.126132945243E-293	2	TOR3A
0	0.532788933310872	0.671	0.27	0	2	CORO1B
0	0.532747405632228	0.591	0.215	0	2	GET1
5.26870174945176E-268	0.532058239273724	0.316	0.091	8.82929039173126E-264	2	GPRIN3
6.39967684137938E-234	0.532040656864915	0.332	0.108	1.07245784507836E-229	2	TBC1D7
1.0594870226985E-230	0.532024103509614	0.36	0.125	1.77548835263814E-226	2	ESCO1
1.34553605771185E-305	0.531819320022337	0.571	0.217	2.25484932551352E-301	2	SUPT16H
3.83117644854648E-182	0.531349991608435	0.263	0.087	6.42028549247418E-178	2	IFT81
0	0.5313121936276	0.527	0.193	0	2	ACBD6
0	0.530993398524186	0.539	0.197	0	2	ZBTB41
2.31172331128215E-289	0.530744545555999	0.698	0.298	3.87398592504663E-285	2	NID1
1.58765334267838E-250	0.530665186325097	0.361	0.119	2.66058947166042E-246	2	MED12
0	0.530645046212405	0.624	0.242	0	2	CPSF6
0	0.53046417475886	0.456	0.149	0	2	AVL9
3.14695251270855E-306	0.530379705437944	0.533	0.198	5.27366302079698E-302	2	PTAR1
0	0.530293813618682	0.737	0.302	0	2	SLC35F5
0	0.530160811696996	0.605	0.229	0	2	MAPK1IP1L
1.79457049026795E-282	0.53006882996028	0.508	0.19	3.00734122759103E-278	2	VPS13C

2.7125870162109E-199	0.530050452793684	0.334	0.12	4.54575332176622E-195	2	ZNF561
2.3906333688605E-266	0.529873321741531	0.399	0.135	4.00622339953642E-262	2	LDLRAD4
0	0.529381349469417	0.557	0.208	0	2	GSK3B
1.39316824031835E-145	0.529371017602484	0.263	0.099	2.33467133712548E-141	2	PPCDC
1.04425330338247E-300	0.529349313432582	0.666	0.276	1.74995968580834E-296	2	TIMM8A
4.19312192895784E-203	0.529251228875218	0.305	0.103	7.02683372854755E-199	2	NCBP3
5.3385085457124E-278	0.529095548786986	0.464	0.166	8.94627262090484E-274	2	INTS7
0	0.52899894809724	0.748	0.321	0	2	PDCD6IP
2.92642622947662E-288	0.528963626652765	0.436	0.148	4.90410507535691E-284	2	SLC44A2
1.44396767858375E-283	0.528838994258766	0.412	0.137	2.41980103577064E-279	2	FAM98C
7.08316647694844E-220	0.528357979848519	0.36	0.127	1.18699703820702E-215	2	TGDS
0	0.528067072957079	0.509	0.18	0	2	TULP4
1.30514991072674E-289	0.527743585154792	0.873	0.437	2.18717022039587E-285	2	MIDN
6.98844601341705E-191	0.527577435938448	0.311	0.111	1.17112378292843E-186	2	ADPGK
0	0.527517381922423	0.573	0.209	0	2	GOPC
8.32226412656875E-307	0.527436988312559	0.516	0.186	1.39464502233039E-302	2	RNF6
4.73771164053373E-223	0.527381219820077	0.295	0.092	7.93945716720642E-219	2	HMGXB3
4.10712206540605E-304	0.527173090054202	0.539	0.201	6.88271515720745E-300	2	RBCK1
3.07372681288873E-231	0.526941706723628	0.986	0.695	5.15095139303894E-227	2	ANXA4
3.39252072925899E-253	0.526536105867275	0.971	0.552	5.68518623809222E-249	2	MPP1
2.85652246593267E-182	0.525920151602776	0.258	0.084	4.78696034840997E-178	2	ZNF84
2.0846046850752E-238	0.525890129350186	0.275	0.078	3.49338053124902E-234	2	CAMK2G
3.23921137932567E-276	0.525790146418828	0.33	0.096	5.42827042947396E-272	2	JDP2
0	0.525634024347814	0.658	0.254	0	2	PCID2
3.63390751057647E-229	0.525568595637769	0.372	0.132	6.08970220622405E-225	2	TIPARP
1.30416525613951E-236	0.525454089386198	0.354	0.12	2.18552013623859E-232	2	RNF31
1.08422217086803E-234	0.525452610654073	0.341	0.112	1.81693951394064E-230	2	CDK8
8.16741653812969E-261	0.525233015165518	0.42	0.149	1.36869566345977E-256	2	ANKLE2
4.37072327762983E-257	0.524842459787762	0.395	0.135	7.32445806865207E-253	2	ANKS1A
2.52366483854345E-177	0.524732519860176	0.314	0.117	4.22915753643111E-173	2	ZBTB37
0	0.524470855189213	0.595	0.226	0	2	SEC24B
1.06388515547601E-279	0.524364424716861	0.523	0.198	1.7828587435467E-275	2	CLDND1
8.78632770014823E-264	0.5241620365061	0.361	0.115	1.47241279599084E-259	2	DSG2
0	0.523847079047355	0.713	0.297	0	2	TRIM27
7.64733103156378E-217	0.52363008652712	0.309	0.101	1.28153973426946E-212	2	TMEM185B
0	0.523594813237942	0.58	0.217	0	2	UBA1
2.90197402658902E-245	0.523175345080965	0.335	0.106	4.86312807375788E-241	2	COPG2
2.0599912195916E-211	0.522890916654427	0.329	0.113	3.4521332857916E-207	2	TAF5L
1.15526219953658E-294	0.522851787136129	0.724	0.312	1.93598839398339E-290	2	DST
0	0.522579591136621	0.646	0.249	0	2	IDS
2.55662382464158E-218	0.522553572094396	0.416	0.16	4.28439020533437E-214	2	PLCXD1
1.76593732937092E-247	0.522530209243681	0.363	0.121	2.95935777655979E-243	2	CNOT6
2.81446688493491E-256	0.522357432263548	0.364	0.119	4.71648360577392E-252	2	MEST
2.3964364074306E-291	0.52233347568994	0.409	0.133	4.0159481315722E-287	2	PNPLA6
0	0.522329749129603	0.756	0.319	0	2	TRA2A
8.23112715232264E-246	0.522302291456097	0.4	0.142	1.37937228818623E-241	2	TBC1D22B
8.56340602696036E-242	0.522140729148857	0.391	0.139	1.43505558199802E-237	2	TRAPPC6B
7.4186993697304E-169	0.522062497941434	0.253	0.085	1.24322564037942E-164	2	STK17B
0	0.522010774905864	0.6	0.231	0	2	ZFAND1
3.58682972570861E-280	0.521947794712544	0.387	0.124	6.0108092543425E-276	2	UBA7
2.4751739416209E-285	0.521910608286765	0.489	0.179	4.1478964913683E-281	2	SLC5A3
6.30648410550379E-188	0.521729810413038	0.38	0.15	1.05684060640033E-183	2	CENPM
2.33862845162377E-259	0.521694752888834	0.462	0.172	3.91907355923112E-255	2	WBP1
1.15795934948701E-283	0.521679287239686	0.386	0.123	1.94050827787033E-279	2	HOXA10
6.34588583132328E-294	0.521631188754158	0.459	0.159	1.06344354761316E-289	2	ME2
0	0.52102039231905	0.669	0.268	0	2	APEX1
1.511901936249E-187	0.520886982829278	0.33	0.122	2.53364526476607E-183	2	NSRP1
7.82495482784168E-183	0.520748215112679	0.264	0.087	1.31130593004971E-178	2	ZBTB45
4.22221357795297E-274	0.520101426315191	0.319	0.09	7.07558551393358E-270	2	TOX3
1.4158936603105E-270	0.519926766385718	0.449	0.16	2.37275459594834E-266	2	PCNX1

1.82862565219976E-268	0.519888051068625	0.938	0.523	3.06441086795635E-264	2	BCAP31
1.02171032829323E-181	0.51981903605926	0.271	0.091	1.7121821681538E-177	2	RAB2B
0	0.51978687467157	0.667	0.257	0	2	DNAJC22
0	0.519679589301983	0.558	0.208	0	2	TPM4
1.43853971681194E-169	0.51951074804979	0.279	0.099	2.41070485743344E-165	2	ACTR6
5.06355032780943E-304	0.519440779280148	0.508	0.183	8.48549763934305E-300	2	LARP4B
0	0.519413837151364	0.593	0.214	0	2	AP2A2
2.62184140723143E-170	0.519398743929075	0.262	0.09	4.39368183023843E-166	2	TADA2A
2.88264243135478E-218	0.51914899791729	0.3	0.096	4.83073218646434E-214	2	TXLNG
3.16812433951386E-272	0.518986214330219	0.385	0.126	5.30914276815732E-268	2	PLCL2
4.67924789222262E-265	0.518473895830322	0.488	0.185	7.84148361778666E-261	2	MOAP1
5.55397281072125E-210	0.518371131922518	0.26	0.077	9.30734763620667E-206	2	CCDC32
0	0.518361035751311	0.527	0.191	0	2	TYK2
2.14243585014147E-264	0.518052602251497	0.436	0.156	3.59029399766707E-260	2	MDC 1.00
1.01717281891682E-231	0.517966570538786	0.32	0.103	1.70457820994081E-227	2	ZNFX1
9.19816743870462E-297	0.517936385292255	0.457	0.158	1.54142889937812E-292	2	NFATC2
1.80828824408904E-235	0.517761734694496	0.351	0.118	3.03032943944441E-231	2	CLEC16A
3.33776904643937E-265	0.51772653601499	0.389	0.13	5.59343336802309E-261	2	CYB561
4.8301293083714E-303	0.517281325693165	0.708	0.303	8.09433069496879E-299	2	SEL1L
3.08024082782259E-247	0.517239160740128	0.38	0.13	5.16186757926509E-243	2	TBC1D10B
1.99561075091179E-221	0.516746534267936	0.326	0.109	3.34424449637798E-217	2	PACS2
2.17865661884565E-278	0.516700214886162	0.458	0.164	3.65099276186154E-274	2	RESF1
6.94158454640984E-212	0.515716929732182	0.305	0.1	1.16327073828736E-207	2	TGFBR1
0	0.515559342435817	0.46	0.156	0	2	SMAD1
1.94407683983415E-277	0.515531543173406	0.429	0.147	3.25788396819407E-273	2	STRN
1.48896689034423E-250	0.515170509878583	0.335	0.105	2.49521071483885E-246	2	HFE
0	0.514792147932312	0.603	0.221	0	2	MARK3
6.75949448282351E-191	0.514656912768102	0.299	0.103	1.13275608543156E-186	2	SMG9
1.61259724359012E-218	0.514199702856747	0.376	0.137	2.70239046080831E-214	2	GUCY1B1
1.9615041016579E-226	0.514086770403698	0.279	0.083	3.28708857355831E-222	2	SIRT6
3.16635925667587E-227	0.514045014718374	0.344	0.116	5.30618484233743E-223	2	PET117
3.43334499154463E-233	0.513928726970714	0.386	0.138	5.75359953683048E-229	2	UPF2
0	0.513675860377146	0.617	0.231	0	2	AP1S1
0	0.513567038850522	0.511	0.183	0	2	WASHC1
0	0.513357381905526	0.651	0.262	0	2	LGALS8
9.86607546353241E-182	0.513185736631426	0.253	0.081	1.65335692617876E-177	2	NIFK
2.00597891363824E-284	0.513123304703605	0.511	0.191	3.36161946347495E-280	2	MED13
0	0.51284123900479	0.622	0.22	0	2	TNFRSF14
9.92764761615893E-282	0.512745118398663	0.528	0.202	1.66367518751591E-277	2	GMPS
0	0.511848705771469	0.417	0.13	0	2	SIRPA
1.20584459130234E-266	0.511638608044967	0.418	0.145	2.02075436610447E-262	2	ARFRP1
4.55677429134562E-272	0.511402301872735	0.43	0.15	7.63624235743698E-268	2	OGFOD2
0	0.511229528802287	0.565	0.195	0	2	TSC22D4
0	0.510517794530849	0.779	0.332	0	2	POGZ
1.42992604103338E-279	0.50980246364167	0.441	0.154	2.39627005956374E-275	2	MOSPD2
3.00786443221361E-253	0.509582911099379	0.901	0.488	5.04057921550358E-249	2	TPD52
3.24860478592334E-288	0.509476942300198	0.466	0.165	5.44401190025034E-284	2	DNAJC13
6.87577036598854E-295	0.509094537388255	0.474	0.167	1.15224159793236E-290	2	SNX14
2.19494028567807E-250	0.508937105988533	0.334	0.105	3.6782809307393E-246	2	DNAAF5
3.47618623637486E-286	0.508782330473325	0.452	0.158	5.825392894917E-282	2	DRAM2
8.24405693828581E-306	0.508506161894638	0.557	0.211	1.38153906171794E-301	2	SAMD4B
1.33172927109238E-305	0.508492468954774	0.45	0.151	2.23171191249661E-301	2	VPS13D
0	0.508355445282931	0.472	0.16	0	2	ARFGAP1
1.16018791338894E-214	0.508276842633774	0.329	0.113	1.94424290525718E-210	2	PIGQ
9.93491538557981E-185	0.508147866107455	0.272	0.091	1.66489312031546E-180	2	WDR70
1.06973957389931E-281	0.508145556795412	0.885	0.435	1.79266957794047E-277	2	GPAA1
5.07749887772849E-282	0.508040255215363	0.463	0.165	8.50887261929741E-278	2	RAB3GAP1
9.14889031038945E-289	0.507723795134326	0.497	0.181	1.53317103821506E-284	2	NUP153
0	0.507621391400804	0.511	0.173	0	2	SCAP
2.61497992049895E-301	0.507505944899414	0.753	0.327	4.38218335077213E-297	2	MDM2

1.57210374139758E-271	0.50739271566941	0.435	0.153	2.63453144983407E-267	2	SETDB1
0	0.507365861095598	0.473	0.162	0	2	PHKG2
3.68663929360267E-276	0.507029075177903	0.473	0.173	6.17807012821935E-272	2	BTAF1
2.45398749014827E-285	0.506922339004447	0.857	0.397	4.11239223599046E-281	2	PICALM
0	0.506888500034103	0.631	0.243	0	2	DNAJC1
0	0.506567435008998	0.541	0.19	0	2	CC2D1A
1.45593220895533E-298	0.506519379949888	0.445	0.151	2.43985119576734E-294	2	SLC35E1
5.5144422349804E-302	0.50650461483852	0.458	0.156	9.24110229738016E-298	2	EXOC4
2.31238468956279E-223	0.506390242963553	0.321	0.105	3.87509426276933E-219	2	CUEDC1
1.67524574302864E-162	0.506091758840878	0.257	0.09	2.80737681616739E-158	2	SMU1
0	0.50606651681465	0.536	0.197	0	2	MED14
1.03635616891578E-262	0.505927381260259	0.395	0.135	1.73672566786906E-258	2	DMWD
2.8837922897813E-290	0.505859520931427	0.476	0.169	4.8326591192155E-286	2	MTX2
8.42387711532502E-225	0.505616511170573	0.363	0.128	1.41167332698617E-220	2	PIGC
0	0.505583687003977	0.653	0.257	0	2	EHMT1
4.0255499433531E-239	0.505375435578369	0.354	0.118	6.74601659507112E-235	2	SETBP1
8.18230283181251E-244	0.505366463597661	0.352	0.116	1.37119030855514E-239	2	CRYBG1
2.69646616511727E-287	0.50489032457256	0.451	0.157	4.51873799950352E-283	2	AAK1
3.75869675121464E-262	0.504859902311022	0.384	0.128	6.29882401568549E-258	2	C4orf19
2.69224883187031E-271	0.504758800851035	0.482	0.178	4.51167059244827E-267	2	NFAT5
2.76096843415688E-291	0.50440362716099	0.571	0.221	4.6268309019601E-287	2	NUP85
1.368016223238E-249	0.504395803744367	0.349	0.113	2.29252158690224E-245	2	MED22
0	0.504207035350579	0.757	0.32	0	2	LIMD1
1.53589921184669E-253	0.503691199453044	0.346	0.11	2.57385989921268E-249	2	CROT
9.95285051192871E-308	0.503615487831894	0.501	0.178	1.66789868878901E-303	2	ATG4B
4.23070731827173E-201	0.503381107876575	0.321	0.113	7.08981932395977E-197	2	UBE2K
0	0.503333393666603	0.834	0.386	0	2	RNF213
6.12127657385767E-220	0.503217352615154	0.33	0.111	1.02580352824707E-215	2	TAF1
1.15423867942563E-282	0.502866865579116	0.447	0.156	1.93427317898148E-278	2	BOD1L1
8.6019146913081E-272	0.502697237619855	0.809	0.388	1.44150886396941E-267	2	STT3A
0	0.502375748715589	0.524	0.184	0	2	DDX42
7.57203849806042E-302	0.502270442440144	0.539	0.199	1.26892221150496E-297	2	COX11
3.67845139538076E-232	0.502142850131998	0.361	0.124	6.16434884837908E-228	2	DIPK2A
8.11181214746187E-298	0.501887899051157	0.54	0.203	1.35937747967166E-293	2	UNC50
7.5566603365726E-186	0.501669476737995	0.256	0.082	1.26634513920284E-181	2	NCOA5
2.28055114284931E-223	0.50164450851579	0.335	0.113	3.82174760518687E-219	2	NBAS
6.6645427950139E-249	0.501498922506011	0.421	0.152	1.11684408158843E-244	2	DAPK2
1.02384406799057E-307	0.50148632514658	0.636	0.251	1.7157578891386E-303	2	SSX2IP
4.7054643001226E-274	0.501113947404021	0.594	0.242	7.88541707414546E-270	2	PARP9
0	0.501099345543071	0.69	0.275	0	2	SRRM2
7.90899053084166E-289	0.501064285447419	0.536	0.204	1.32538863315845E-284	2	HSPA4L
3.14417210059746E-174	0.500979979668497	0.252	0.083	5.26900360618122E-170	2	CBX7
1.13174892628952E-286	0.500968411644058	0.5	0.183	1.89658485067598E-282	2	TAF8
1.92535239868596E-207	0.500925365280495	0.297	0.097	3.22650554971793E-203	2	DEPRECATED-ENSG00000050130
1.4331044505761E-197	0.500894467016736	0.326	0.116	2.40159643827543E-193	2	TRNAU1AP
2.09781827098161E-226	0.500810712482328	0.351	0.12	3.51552385851099E-222	2	NUDT16L1
2.36341006692789E-223	0.500589875452506	0.344	0.118	3.96060259015775E-219	2	SLC2A8
3.87530570126004E-244	0.500570179699104	0.361	0.122	6.49423729417158E-240	2	COG7
3.32891814990889E-272	0.500518234106675	0.436	0.153	5.57860103561732E-268	2	DHRSX
7.16500903576891E-278	0.500468669624614	0.431	0.148	1.20071221421415E-273	2	RBM4
9.99339871062117E-216	0.499727702099144	0.283	0.088	1.6746937559259E-211	2	TCFL5
1.21974490063805E-203	0.499538891259335	0.328	0.115	2.04404850448925E-199	2	C5orf51
1.36389114717264E-283	0.49899317860677	0.398	0.129	2.28560878443192E-279	2	TCF3
7.88192322437287E-289	0.498660355642083	0.414	0.136	1.32085269394041E-284	2	TIMM10B
1.65039218102931E-297	0.498641364527248	0.488	0.174	2.76572721696892E-293	2	ARHGEF11
0	0.498318100375582	0.662	0.253	0	2	NCOR1
9.46791283875988E-233	0.497750531904571	0.367	0.128	1.58663283351938E-228	2	MTREX
0	0.497346354633818	0.472	0.143	0	2	ABHD11
2.69419636122509E-173	0.496878903373897	0.25	0.082	4.51493426214101E-169	2	THAP6
9.62108157578239E-211	0.496402561118386	0.322	0.11	1.61230085046961E-206	2	DENND1B

1.9569925512857E-187	0.496379417033547	0.275	0.092	3.27952811744458E-183	2	CNOT1
5.16199954585063E-274	0.495919729171489	0.431	0.15	8.65047883893648E-270	2	ATF7
3.85973190206608E-296	0.495877608762041	0.5	0.182	6.46813872148234E-292	2	MTMR4
2.37406229224713E-215	0.495647554957433	0.318	0.106	3.97845358934774E-211	2	INO80D
0	0.495539167696136	0.644	0.25	0	2	PDCD6
2.09749017749319E-273	0.495515544211534	0.386	0.126	3.51497403944309E-269	2	ACIN1
3.48406773245161E-197	0.495397130127675	0.3	0.102	5.83860070604241E-193	2	RTL10
0	0.495374649246221	0.616	0.236	0	2	HDAC1
1.2686854090727E-288	0.495318901226924	0.401	0.13	2.12606300852403E-284	2	SYNJ2BP
1.07793164967104E-269	0.495287655062607	0.389	0.129	1.80639785851873E-265	2	ACVR1
7.3623857477711E-250	0.495262691809279	0.428	0.156	1.23378860361148E-245	2	ASCC3
5.07958954575757E-211	0.494725310887076	0.347	0.124	8.51237616078054E-207	2	SUPT20H
1.2496582189915E-296	0.49463333636308	0.519	0.191	2.09417724338595E-292	2	PHF12
0	0.494523818625246	0.686	0.274	0	2	IGF2R
5.34860084309256E-237	0.494437947288382	0.345	0.114	8.96318529285452E-233	2	ACOT8
2.41835221977805E-249	0.49429693305594	0.429	0.156	4.05267464990406E-245	2	CUL5
5.7423466483035E-166	0.494104329186162	0.259	0.09	9.623024513227E-162	2	DEPRECATED-ENSG00000165948
0	0.494080157617657	0.576	0.218	0	2	LRP11
2.8650185573971E-271	0.493130084518129	0.5	0.189	4.80119809848607E-267	2	NDUFB4
6.5228568387248E-210	0.492994912402565	0.324	0.112	1.0931003490335E-205	2	STX2
4.59836715469571E-214	0.49284161483581	0.289	0.091	7.70594367783907E-210	2	RABGEF1
8.35876582764701E-299	0.492003282588544	0.742	0.321	1.40076197739709E-294	2	PER1
4.16466132610174E-237	0.491926168904182	0.384	0.136	6.97913945028129E-233	2	PSKH1
3.85547275987733E-255	0.491871232135522	0.447	0.165	6.46100125100242E-251	2	FBXO28
0	0.491730686297791	0.685	0.273	0	2	CAPN5
0	0.491702854611199	0.828	0.375	0	2	RBM33
0	0.491621851018078	0.536	0.192	0	2	EDEM2
0	0.491467638184267	0.598	0.227	0	2	TRIM24
6.60258542495508E-199	0.49112536660002	0.299	0.101	1.10646126551397E-194	2	KIZ
6.90168250157193E-201	0.491025347126234	0.313	0.108	1.15658395361342E-196	2	DGCR8
1.25709168919538E-184	0.490456417362383	0.27	0.09	2.10663425275362E-180	2	RICTOR
6.69014511503631E-199	0.489924573498599	0.293	0.097	1.12113451837779E-194	2	SYT1
1.92776006448216E-237	0.489887844796282	0.347	0.116	3.2305403160592E-233	2	CYCS
2.50887582151918E-208	0.489745669747581	0.268	0.082	4.20437410170184E-204	2	STK17A
2.81392664523274E-281	0.48895180374639	0.856	0.415	4.71557827208102E-277	2	NUDT16
1.11868166127863E-244	0.488864969581452	0.887	0.533	1.87468672797072E-240	2	APP
1.13188498936707E-304	0.488686600046032	0.497	0.176	1.89681286518134E-300	2	CDC42BPA
4.51391538696901E-188	0.48858273058765	0.258	0.082	7.56441940548267E-184	2	LRSAM1
1.19194031287877E-245	0.488486090793586	0.342	0.111	1.99745357632224E-241	2	LEMD3
3.0974592332332E-247	0.488378211056672	0.86	0.436	5.19072218305219E-243	2	CTSH
1.09767176245394E-205	0.488229434609228	0.443	0.18	1.83947833952031E-201	2	GREB1L
0	0.488177055052463	0.548	0.202	0	2	NFATC2IP
0	0.487739950658777	0.708	0.29	0	2	DDX17
5.31582676037777E-222	0.48771223915529	0.359	0.127	8.90826248504106E-218	2	SEPSECS
2.92810811386499E-244	0.487455423080903	0.385	0.133	4.90692357721496E-240	2	MTPAP
2.32643119582243E-289	0.487391906962952	0.791	0.376	3.89863339795923E-285	2	CYSTM1
1.5656610958917E-227	0.486879115362031	0.309	0.097	2.6237348644953E-223	2	DUSP22
5.70781838414982E-257	0.486036866981786	0.371	0.122	9.56516204815827E-253	2	NCBP1
0	0.485920377004536	0.702	0.289	0	2	PHF10
0	0.485860651943949	0.774	0.336	0	2	POLR2H
4.63922157425339E-188	0.485809474156929	0.273	0.091	7.77440751413384E-184	2	TOM1L2
2.25945200048211E-218	0.485437270882121	0.327	0.11	3.78638966240792E-214	2	UBXN2A
0	0.485412141894013	0.508	0.177	0	2	SYPL1
0	0.485405777020275	0.531	0.193	0	2	TNPO3
7.72222499024747E-243	0.485259015175604	0.383	0.134	1.29409046386567E-238	2	KYAT1
5.26157885017304E-199	0.485238120578996	0.273	0.087	8.81735383711997E-195	2	RFK
5.86299174809988E-202	0.485180497129802	0.306	0.104	9.82520157146578E-198	2	HARS2
2.13075239788609E-233	0.485156341127519	0.318	0.101	3.57071486837751E-229	2	TMED8
2.02172702017944E-258	0.48503626864759	0.355	0.113	3.38801014041671E-254	2	TBRG1
9.57519803021732E-222	0.484861884623196	0.374	0.135	1.60461168590382E-217	2	USP16

1.03194030094442E-282	0.48482252331329	0.461	0.163	1.72932555632266E-278	2	BPNT1
6.57860756137763E-239	0.484512863601745	0.403	0.145	1.10244305513566E-234	2	CPOX
9.17485112338317E-285	0.484382752014653	0.458	0.161	1.53752155125655E-280	2	TASOR
8.705931860114E-259	0.484264042715772	0.439	0.16	1.4589400611179E-254	2	MLH1
8.86357171130964E-280	0.48375954305296	0.388	0.125	1.48535734738127E-275	2	ABCG8
1.4624468980529E-299	0.483099306786838	0.49	0.174	2.45076851175704E-295	2	SYNRG
2.32882424731677E-294	0.483077180530648	0.543	0.206	3.90264367365345E-290	2	BROX
1.46799745239707E-260	0.482987134042173	0.391	0.132	2.46007013072701E-256	2	PDXDC1
7.82392922329429E-257	0.482875195631863	0.378	0.126	1.31113405923966E-252	2	CPEB2
2.84413462198647E-241	0.482729324834969	0.397	0.141	4.76620079952492E-237	2	SLC25A28
4.61267425105778E-202	0.482637770295985	0.307	0.104	7.72991950992262E-198	2	XPA
9.8109449404987E-224	0.48260707054465	0.253	0.07	1.64411815312877E-219	2	NECTIN1
2.82433210136386E-216	0.482580479553165	0.323	0.109	4.73301573546555E-212	2	ZNF131
6.0917621128972E-302	0.482447868816732	0.6	0.235	1.02085749487931E-297	2	PPP1R10
8.59126864705962E-215	0.482207770047296	0.957	0.635	1.43972479987425E-210	2	ANPEP
0	0.481915659283165	0.617	0.234	0	2	ZBTB7A
0	0.481829752010687	0.582	0.22	0	2	UBA2
6.28583806195882E-199	0.480923432890272	0.287	0.094	1.05338074242306E-194	2	RNMT
5.36028355087302E-276	0.480378754661358	0.851	0.424	8.982763174553E-272	2	CTNND1
4.24220249327037E-282	0.480219806542546	0.451	0.158	7.10908293822248E-278	2	CAMK1D
2.56471956133766E-276	0.480202147532879	0.467	0.169	4.29795704088964E-272	2	ZC3H14
1.71029579906776E-256	0.47999490519172	0.905	0.479	2.86611370007776E-252	2	FAM50A
1.90364347993166E-156	0.479644094784796	0.355	0.149	3.19012574366948E-152	2	ZNF436
0	0.479478512757351	0.566	0.211	0	2	UGT1A4
8.42814022130903E-263	0.47936311121128	0.371	0.121	1.41238773828697E-258	2	TBC1D5
0	0.479199187908006	0.719	0.296	0	2	GNAQ
0	0.47917593027422	0.66	0.263	0	2	PRRC2C
3.7905835671903E-174	0.479052280584965	0.266	0.09	6.3522599418975E-170	2	ZNF322
4.65888705271959E-259	0.479023066881285	0.368	0.121	7.80736292294748E-255	2	TANGO2
0	0.479021694590688	0.65	0.259	0	2	DHX9
0	0.47878835983066	0.704	0.293	0	2	RTL8C
4.32223505302938E-237	0.478209986746606	0.375	0.13	7.24320150186663E-233	2	DYNC1LI1
3.09485848948988E-164	0.478194420102275	0.253	0.087	5.18636385668714E-160	2	TIMM21
3.60754488844241E-261	0.478086636684833	0.39	0.131	6.04552372405179E-257	2	BRAT1
3.32091152867931E-201	0.478077668614231	0.298	0.099	5.56518353976079E-197	2	ZBED3
1.26840533153865E-246	0.477367052904794	0.32	0.098	2.12559365459247E-242	2	TRMT1
2.40235873212758E-305	0.477183181785249	0.55	0.207	4.02587276329939E-301	2	HDDC2
0	0.477122807708848	0.671	0.26	0	2	NUDCD3
4.2740737524021E-205	0.476946890300848	0.291	0.095	7.16249279427544E-201	2	USP30
1.18871900080791E-282	0.476940977933505	0.556	0.218	1.9920553015539E-278	2	ODR4
4.60653582528757E-239	0.476838311425058	0.38	0.132	7.71963273601691E-235	2	MRS2
0	0.476828521616422	0.804	0.342	0	2	LBR
0	0.476635392682481	0.661	0.256	0	2	ATP11B
1.74343295416951E-304	0.476533498030037	0.686	0.283	2.92164494459726E-300	2	TM4SF20
0	0.476412695127383	0.537	0.198	0	2	LSM 7.00
5.04810084072948E-306	0.476251023516554	0.624	0.245	8.45960738889446E-302	2	PDE4DIP
1.89107680647356E-286	0.476155932804847	0.55	0.21	3.16906651228839E-282	2	ZFHX3
2.43544568229421E-302	0.476095678784025	0.593	0.232	4.08131987438863E-298	2	WWOX
2.28687195334298E-281	0.475896859064323	0.425	0.145	3.83234001941217E-277	2	ZFC3H1
2.89941667001717E-277	0.47587058222577	0.434	0.15	4.85884245561478E-273	2	NUP205
5.72107226296548E-271	0.475604277740203	0.397	0.133	9.58737289827755E-267	2	CSNK1A1
0	0.475564789098888	0.722	0.3	0	2	SRP54
4.07826406578352E-213	0.475361062158774	0.302	0.098	6.83435492144003E-209	2	PIGU
9.66629460428485E-238	0.475328085214845	0.385	0.136	1.61987764978606E-233	2	SQLE
2.28544439309043E-288	0.475230248936402	0.533	0.201	3.82994771394095E-284	2	MAPK9
0	0.475146993738749	0.662	0.226	0	2	ADGRE5
0	0.475040984468223	0.622	0.236	0	2	EFCAB14
1.45942377616692E-210	0.474550933400152	0.361	0.131	2.44570236410052E-206	2	NUP188
6.70993393588529E-241	0.474456642894372	0.37	0.127	1.12445072897566E-236	2	EIF2AK4
7.87967741880214E-290	0.474396529375986	0.492	0.178	1.32047634184286E-285	2	PIK3CB

0	0.47435196502566	0.644	0.248	0	2	CYP2C18
0	0.474321202911946	0.584	0.203	0	2	HECTD3
6.36808777762548E-187	0.474166951580175	0.455	0.194	1.06716414977448E-182	2	RNF125
2.89264703381953E-251	0.474084803153675	0.457	0.172	4.84749789927477E-247	2	MICU2
2.73817152834407E-185	0.474004169291581	0.263	0.086	4.58862784719899E-181	2	SFI1
2.62057781259067E-226	0.473949594631822	0.362	0.128	4.39156429833945E-222	2	CLCN5
5.25329676223082E-263	0.473935564544271	0.415	0.144	8.80347471414641E-259	2	CCDC130
1.10576841133565E-255	0.473438409899307	0.386	0.131	1.85304670371628E-251	2	GPATCH8
6.71492727217978E-262	0.473146015618888	0.376	0.124	1.12528751227189E-257	2	DYNC1H1
1.05127673524746E-283	0.473050651546931	0.463	0.164	1.7617295529277E-279	2	ZC3H12A
0	0.472977253813428	0.601	0.232	0	2	ARHGAP1
2.12505387350066E-181	0.472792192640415	0.284	0.098	3.5611652812124E-177	2	EZH1
6.87198574638045E-304	0.472638000715511	0.501	0.18	1.15160737137844E-299	2	POFUT1
1.9219950308081E-226	0.472364900943046	0.352	0.122	3.22087927262821E-222	2	JMJD4
2.37365478633484E-172	0.472142587684385	0.28	0.099	3.97777069093992E-168	2	TBCCD1
0	0.472133587115501	0.578	0.218	0	2	PHYKPL
1.08521823364261E-284	0.472085066079314	0.476	0.171	1.81860871593828E-280	2	PAWR
5.44563954783981E-154	0.472019566315427	0.286	0.109	9.12580275426995E-150	2	RHOQ
4.04773594727049E-187	0.47150904001735	0.377	0.149	6.78319590043589E-183	2	UHRF1
1.80469629495479E-192	0.471342140929963	0.371	0.143	3.02431005108524E-188	2	EXOC8
2.07217322018248E-301	0.471282713106566	0.778	0.357	3.4725478823818E-297	2	ATP6V0D1
3.86250967553379E-307	0.470987882063795	0.668	0.273	6.47279371425953E-303	2	XRN1
1.43887337674004E-230	0.470884795310896	0.406	0.15	2.41126400474096E-226	2	UNK
1.75941631825766E-227	0.470630273049245	0.333	0.111	2.94842986613619E-223	2	FBXO34
5.51355035043625E-256	0.470464910018516	0.364	0.119	9.23960767726106E-252	2	IGBP1
1.21515107826569E-220	0.470456459352264	0.585	0.257	2.03635017695765E-216	2	ZFAND2A
2.19948450181611E-204	0.470379630096754	0.301	0.1	3.68589612814344E-200	2	RUSC2
0	0.470045055784616	0.639	0.254	0	2	EEF2K
2.79884035223905E-290	0.469964531541971	0.508	0.186	4.69029666228221E-286	2	PIKFYVE
4.14434267563256E-234	0.469880893281685	0.307	0.095	6.94508945582504E-230	2	VPS37B
5.37500918076853E-289	0.469875363846728	0.76	0.345	9.0074403851319E-285	2	MGAT2
8.051767354221E-254	0.469606258000102	0.476	0.181	1.34931517322036E-249	2	SRD5A3
5.85906813113939E-213	0.469284019595921	0.332	0.114	9.81862637416339E-209	2	TMEM30A
3.07005936342093E-223	0.469205803974958	0.976	0.661	5.1448054812208E-219	2	LAMP2
0	0.46857360593441	0.603	0.227	0	2	GNA11
1.2206636162662E-184	0.468310777898212	0.287	0.098	2.0455880881389E-180	2	SLC9A8
0	0.468272607157079	0.625	0.244	0	2	LGMN
8.36776523421644E-206	0.468167481058108	0.274	0.086	1.40227009794999E-201	2	SUMF1
1.39173061945295E-263	0.467879059060568	0.352	0.11	2.33226217207925E-259	2	ADCY9
2.67269392665259E-200	0.467646048406691	0.28	0.09	4.47890048228441E-196	2	RCHY1
5.67580924716824E-217	0.467623884170401	0.288	0.09	9.51152113640453E-213	2	MAPK8IP3
0	0.467513026523509	0.573	0.21	0	2	DNAJC5
0	0.46733302331929	0.514	0.182	0	2	RNF44
8.57184630943965E-283	0.467307860821299	0.829	0.401	1.4364700045359E-278	2	RSRP1
7.21770236481163E-182	0.467234691592747	0.266	0.088	1.20954256229513E-177	2	KANSL2
6.40367088834164E-261	0.467007832932699	0.404	0.138	1.07312716746829E-256	2	UEVLD
0	0.466929311964779	0.496	0.17	0	2	EEF1AKNMT
1.78505010410185E-294	0.466652263534042	0.579	0.225	2.99138696445388E-290	2	HAUS4
1.53045333253003E-172	0.466578303118684	0.257	0.086	2.56473369465383E-168	2	RAB24
6.7025187548467E-303	0.466152295041773	0.451	0.151	1.12320809293721E-298	2	NCK2
3.9096069814926E-249	0.465970516464792	0.346	0.111	6.5517193795853E-245	2	SATB2
0	0.465938460334492	0.554	0.205	0	2	MPPE1
1.48483775137084E-289	0.465890177214455	0.514	0.19	2.48829110374725E-285	2	C19orf48
5.89647865562931E-266	0.46582541895301	0.442	0.158	9.88131893110359E-262	2	STRBP
4.53450144782486E-233	0.465502823952188	0.353	0.12	7.5989175262649E-229	2	TRMU
1.14700542385671E-300	0.465292376131148	0.675	0.286	1.92215168929907E-296	2	CD164
2.24182977593491E-197	0.465265280646069	0.298	0.101	3.75685833851173E-193	2	PTRH2
4.50428272232791E-301	0.465245722514678	0.699	0.29	7.5482769860771E-297	2	SRSF3
1.05774368319507E-213	0.465175663789534	0.32	0.108	1.7725668642983E-209	2	RABL3
4.14041410006437E-234	0.465015860905452	0.363	0.124	6.93850594888787E-230	2	ARFIP1

3.25783825317885E-271	0.464973151875624	0.474	0.175	5.45948534467712E-267	2	PHC3
3.8368285265608E-301	0.464722868615524	0.628	0.247	6.42975724481059E-297	2	MAP3K5
3.28129654652362E-251	0.464719346269532	0.337	0.106	5.49879675266429E-247	2	CBLB
0	0.464640796344956	0.663	0.267	0	2	HPRT1
4.90065822710551E-233	0.464573994624822	0.343	0.114	8.21252305698341E-229	2	PSMG1
0	0.464067479170865	0.525	0.189	0	2	CNNM3
1.20800203734404E-292	0.464057086412798	0.845	0.384	2.02436981418114E-288	2	AMOTL1
1.64653763690563E-230	0.463894982570691	0.347	0.118	2.75926777192645E-226	2	CREBRF
6.74997797732977E-249	0.463835351430716	0.407	0.145	1.13116130944092E-244	2	PUS1
2.58192176018117E-263	0.463698576494189	0.463	0.17	4.3267844857116E-259	2	PBX3
3.4354852202466E-261	0.463541947339827	0.414	0.145	5.75718613208925E-257	2	CLK4
7.59359815092834E-287	0.463121344122519	0.489	0.177	1.27253517813257E-282	2	CCHCR1
0	0.462970576861417	0.61	0.236	0	2	RBM25
6.31941338265681E-254	0.462938797963771	0.414	0.146	1.05900729466563E-249	2	HSPA13
8.10453049035762E-306	0.462908010261814	0.702	0.296	1.35815721957413E-301	2	MCM4
1.25523257039693E-188	0.462879263489974	0.253	0.079	2.10351874147117E-184	2	RGMB
2.2422696400652E-188	0.462822140518155	0.298	0.104	3.75759546282127E-184	2	GABPA
1.4614117589438E-191	0.462587284190398	0.297	0.102	2.44903382563801E-187	2	WNT4
0	0.462571368588529	0.566	0.211	0	2	GLB1
2.91791730286513E-296	0.462570289126664	0.588	0.229	4.88984581614139E-292	2	GINM1
1.51823772651351E-284	0.462471114419667	0.446	0.155	2.54426278209133E-280	2	SLC17A5
1.92367055407802E-257	0.462300090367273	0.401	0.139	3.22368711452395E-253	2	ABL1
4.12520532937211E-244	0.462206603380358	0.361	0.12	6.91301909096178E-240	2	AP5M1
2.87002626392163E-216	0.462184766071836	0.366	0.132	4.80959001307987E-212	2	ERGIC2
1.07081418005232E-215	0.461972892720406	0.309	0.101	1.79447040293168E-211	2	POT1
4.76905499928932E-228	0.461803391359087	0.335	0.112	7.99198236780905E-224	2	COX18
1.18502367711448E-275	0.46163592130947	0.465	0.168	1.98586267810845E-271	2	SMAP1
0	0.4615455025107	0.563	0.212	0	2	SF3B3
7.85901716527229E-245	0.461386337283101	0.464	0.176	1.31701409655633E-240	2	DEPRECATED-ENSG00000188313
8.27544283744141E-205	0.461287080607863	0.32	0.11	1.38679871069843E-200	2	UBE3B
2.38420617013312E-295	0.461222022025565	0.734	0.317	3.99545269990909E-291	2	FARP1
3.633392600185E-244	0.460923856798538	0.363	0.121	6.08883931939002E-240	2	ZMAT3
3.87610033172678E-287	0.460805186865301	0.819	0.37	6.49556893590773E-283	2	CD99L2
5.95465230506036E-231	0.460549760417887	0.371	0.13	9.97880633282015E-227	2	PRPF18
1.29107572854713E-241	0.460511243886277	0.336	0.108	2.16358470589929E-237	2	SNX7
0	0.4604002911741	0.481	0.162	0	2	TBL2
6.44762118755388E-267	0.460348732092407	0.389	0.129	1.08049235861028E-262	2	SH2B1
0	0.460295446831006	0.522	0.181	0	2	MAPK3
1.24736122921282E-160	0.460188091126064	0.294	0.11	2.09032794791485E-156	2	DTL
8.3134758098118E-267	0.459782288990368	0.408	0.139	1.39317227620826E-262	2	THEM6
2.13187828665167E-301	0.459764120900116	0.699	0.296	3.57260163277086E-297	2	CALU
0	0.459541261491878	0.376	0.105	0	2	ARHGEF16
6.05833916820045E-256	0.459514640112237	0.317	0.094	1.01525647780703E-251	2	AKNA
4.56398957365948E-263	0.45908833325861	0.298	0.084	7.64833372753856E-259	2	MARVELD3
9.91457161240083E-283	0.459078554063504	0.473	0.169	1.66148391080613E-278	2	EXOSC9
1.71598874284595E-286	0.458731902133983	0.494	0.179	2.87565393526124E-282	2	LARP7
1.73423790389653E-213	0.45869645016736	0.379	0.141	2.90623587934981E-209	2	WASHC4
1.99653102533093E-176	0.458606084464195	0.343	0.135	3.34578669224958E-172	2	SH3TC1
7.09411555140529E-255	0.458579849224374	0.411	0.144	1.1888318841045E-250	2	BAG5
0	0.458073239311356	0.571	0.209	0	2	PI4KA
2.02004834133205E-304	0.457961740587339	0.76	0.336	3.38519701040425E-300	2	AKAP1
1.94061694966215E-249	0.457947426880133	0.397	0.138	3.25208588424383E-245	2	LATS1
6.2280921562355E-213	0.45779714821316	0.35	0.124	1.04370368354195E-208	2	RMI1
0	0.457767286169042	0.386	0.114	0	2	TSPAN13
4.56826198816314E-260	0.457713053758098	0.871	0.456	7.6554934397638E-256	2	GUSB
1.82115391910974E-167	0.457593043823666	0.261	0.09	3.0518897376441E-163	2	STRADB
2.15971512864122E-267	0.457423553041765	0.799	0.37	3.61925061257696E-263	2	IL1R1
0	0.45741368420588	0.637	0.251	0	2	GPR108
2.48639254346356E-202	0.456841935261431	0.316	0.109	4.16669662433624E-198	2	TTL
7.04900190039827E-286	0.456528018483341	0.524	0.198	1.18127173846874E-281	2	AP1G1

1.9254546636338E-171	0.456517793705213	0.974	0.709	3.22667692531753E-167	2	LGALS4
2.10954047987266E-281	0.456468895589405	0.8	0.36	3.5351679361706E-277	2	VMA21
3.48074905135858E-231	0.45607695587405	0.556	0.234	5.83303926026671E-227	2	FBXO42
0	0.456043859268336	0.554	0.207	0	2	MID1
5.51481493458716E-237	0.455918890449128	0.321	0.101	9.24172686738115E-233	2	TMEM184A
3.75844961466713E-244	0.455886433145943	0.405	0.145	6.29840986425918E-240	2	USP2
2.4980606510349E-210	0.455559384405714	0.356	0.128	4.18625003900429E-206	2	RIC1
3.51455506591044E-290	0.455196777495692	0.501	0.185	5.88969137945272E-286	2	MBTPS1
6.80671910361652E-209	0.455093226283535	0.259	0.077	1.14066998738406E-204	2	LGALSL
4.65704523553907E-278	0.454848968004431	0.502	0.187	7.80427640571637E-274	2	UBR3
2.80746522724783E-201	0.454489981859493	0.31	0.106	4.70475022782191E-197	2	SNW1
2.38202555713189E-165	0.454343624942783	0.261	0.09	3.99179842864162E-161	2	FAM76A
3.72856197383848E-212	0.454335412670298	0.32	0.109	6.24832415575852E-208	2	CSNK1G3
3.64196713468554E-159	0.454085742774513	0.271	0.098	6.10320852430603E-155	2	LDAH
1.87438384560652E-303	0.453994991796835	0.652	0.265	3.14109244846741E-299	2	MCM7
1.86998964672049E-171	0.453644998099798	0.269	0.093	3.13372864997419E-167	2	ZNF335
2.1351847100016E-274	0.453439416786608	0.471	0.17	3.57814253702068E-270	2	CFAP97
0	0.453395036239591	0.534	0.197	0	2	STK25
0	0.453332932055365	0.538	0.197	0	2	ZC3H3
0	0.453197437837234	0.55	0.196	0	2	DDX56
0	0.452507490056938	0.702	0.292	0	2	STAT1
4.99449333642682E-295	0.452448306447558	0.661	0.277	8.36977193318406E-291	2	PLS1
7.72440285725006E-215	0.45243564530654	0.3	0.097	1.29445543081797E-210	2	BFAR
5.34705964458292E-200	0.452273024102038	0.41	0.162	8.96060255239206E-196	2	COG2
0	0.45223741141692	0.662	0.265	0	2	TBC1D2B
7.13168673525927E-292	0.452084067267192	0.516	0.19	1.19512806309475E-287	2	USP34
7.29788227659093E-266	0.451883432584348	0.456	0.165	1.22297911191111E-261	2	APBB2
1.23665504662172E-290	0.45179633718494	0.456	0.158	2.07238652712868E-286	2	HECA
9.05210576303059E-215	0.451387249422701	0.322	0.108	1.51695188376867E-210	2	HIRA
4.16710825686345E-230	0.451319451176233	0.378	0.134	6.98324001685177E-226	2	NIPA2
1.28142782719866E-271	0.451269148616197	0.496	0.185	2.14741675281952E-267	2	ZYG11B
0	0.451213362989586	0.65	0.262	0	2	PEPD
3.545655895446E-260	0.451198423470464	0.309	0.089	5.94181014958842E-256	2	STYXL1
4.25159274661049E-241	0.451100018870568	0.356	0.119	7.12481912476986E-237	2	SPOUT1
1.01633156601148E-293	0.45104654501396	0.474	0.168	1.70316843832205E-289	2	TMEM9B
8.5258040341165E-242	0.450924893896897	0.369	0.125	1.42875424003724E-237	2	TRMT2A
0	0.450603297105038	0.506	0.18	0	2	CYP2C19
3.22441321986324E-173	0.450454721337288	0.285	0.102	5.40347167384681E-169	2	BBS2
0	0.450359796646417	0.623	0.245	0	2	NR5A2
6.33656315856986E-260	0.450042038809628	0.418	0.146	1.06188125411314E-255	2	SNHG32
4.82969946628114E-179	0.450040054773586	0.335	0.127	8.09361036559394E-175	2	TAF9
0	0.45002287309362	0.593	0.228	0	2	SYMPK
5.55170701416629E-268	0.449971829724084	0.37	0.119	9.30355061433986E-264	2	MIB2
2.18742847414563E-297	0.44984412720655	0.52	0.192	3.66569263697324E-293	2	LAMC1
5.43927200114493E-300	0.449838630222731	0.458	0.157	9.11513201951868E-296	2	MPZL1
1.83638228805402E-275	0.449645910160191	0.462	0.166	3.07740943832093E-271	2	IRS1
1.60944192081733E-288	0.449118025001758	0.716	0.309	2.69710277090568E-284	2	TMEM38B
9.3660855651774E-241	0.448960837147259	0.422	0.156	1.56956861901243E-236	2	MAGEH1
6.7558076462464E-202	0.448898797079991	0.325	0.114	1.13213824535797E-197	2	DENND6A
2.68047003702979E-233	0.448754179529015	0.394	0.142	4.49193168805451E-229	2	TMEM138
1.39750065343796E-193	0.448248301622593	0.311	0.108	2.34193159503134E-189	2	BICRA
2.46436672414539E-246	0.448030151509813	0.817	0.413	4.12978575632284E-242	2	SEC61A1
9.10358817228915E-227	0.448019241913723	0.305	0.096	1.52557930591222E-222	2	SRGAP2
4.6559555749418E-247	0.447728721866982	0.599	0.253	7.80245035248746E-243	2	WSB1
7.94757560939343E-308	0.447659500308813	0.58	0.223	1.33185472062215E-303	2	ZRANB2
2.1478264691059E-198	0.447093023189897	0.286	0.094	3.59932759692767E-194	2	DIS3L2
3.27345128445503E-218	0.44671234042061	0.424	0.164	5.48564966248975E-214	2	MCEE
3.00328438880997E-280	0.445930825947693	0.468	0.168	5.03290397876775E-276	2	TNS2
3.71587557741066E-228	0.445365837789665	0.365	0.127	6.22706429262479E-224	2	ATG4A
2.37873124894798E-293	0.445289027742651	0.487	0.174	3.98627782698703E-289	2	ABI1

0	0.445258831483159	0.678	0.278	0	2	MKLN1
5.84022708109137E-261	0.445082404153895	0.479	0.181	9.78705254249292E-257	2	DNMT3A
6.49777510463662E-171	0.444935904829292	0.277	0.098	1.088897152035E-166	2	CEP170
9.20734222291181E-199	0.444924996962102	0.285	0.093	1.54296640971556E-194	2	LTV1
1.12991720799054E-293	0.444917254725899	0.49	0.176	1.89351525715054E-289	2	KCTD5
0	0.444815815630888	0.565	0.205	0	2	FAAH
1.25504229237378E-302	0.444791981425663	0.642	0.26	2.10319987355999E-298	2	TMUB2
2.2532029634289E-199	0.444603948649635	0.908	0.509	3.77591752611415E-195	2	DECR1
5.21011534102641E-223	0.44456248669471	0.921	0.514	8.73111128849205E-219	2	SSR4
1.3597043083147E-282	0.444284026280723	0.479	0.173	2.27859247987377E-278	2	PER2
1.14062524319678E-259	0.444182477202357	0.877	0.426	1.91145978254916E-255	2	UBE2A
1.2005221080729E-181	0.444145436201686	0.297	0.105	2.01183494870857E-177	2	FBXO11
2.1194748489112E-224	0.444068525604598	0.329	0.109	3.55181595180539E-220	2	GIT2
1.87973731487915E-234	0.444039481950855	0.387	0.138	3.15006379227449E-230	2	IPO5
2.78425991946608E-233	0.443949769472835	0.545	0.228	4.66586277304125E-229	2	UGT1A9
1.55666171899298E-291	0.443486921035754	0.534	0.2	2.60865370868843E-287	2	NBN
1.158420779337E-203	0.443413773535328	0.287	0.093	1.94128154201295E-199	2	SPRED2
4.7143545908424E-252	0.443413001974271	0.787	0.376	7.9003154233337E-248	2	CITED2
1.46105320275655E-166	0.442932969129735	0.276	0.097	2.44843295717942E-162	2	MIS18BP1
0	0.442871971460621	0.668	0.271	0	2	FAM20C
2.95181742097286E-296	0.442840213056643	0.543	0.201	4.94665563406631E-292	2	TMEM19
2.0276269660647E-272	0.442815532325714	0.433	0.151	3.39789726973123E-268	2	NUDT19
1.60413292228644E-287	0.442561640275092	0.674	0.288	2.68820595116762E-283	2	GDA
0	0.442333249200688	0.641	0.253	0	2	TNRC6A
1.39136139783182E-248	0.442328836487459	0.469	0.178	2.33164343048657E-244	2	ATP6V1H
2.2803331959197E-141	0.44229579309327	0.264	0.101	3.82138236972223E-137	2	KPTN
2.76533207593853E-170	0.442268339457746	0.274	0.096	4.63414349285779E-166	2	TLK2
0	0.441982325712031	0.652	0.257	0	2	MECP2
1.67844285313633E-250	0.441970235611547	0.364	0.12	2.81273453328586E-246	2	PSME2
5.24237106541556E-293	0.441910894870443	0.565	0.218	8.7851654314234E-289	2	PCGF2
1.74721204320262E-205	0.441865417134681	0.309	0.104	2.92797794199895E-201	2	NR2C1
7.67570645675783E-219	0.441158737060183	0.301	0.096	1.28629488802348E-214	2	FUNDC1
1.13316249537558E-197	0.441144288290878	0.291	0.097	1.8989537097504E-193	2	TARDBP
1.42028946575762E-276	0.441112685685679	0.434	0.151	2.38012108671662E-272	2	SMARCC2
5.04001971711573E-285	0.441061127402851	0.518	0.193	8.44606504194254E-281	2	NELFA
1.84450960899259E-182	0.441059952511911	0.274	0.092	3.09102920274979E-178	2	DRG1
1.70042177926637E-216	0.440992898775338	0.347	0.121	2.84956681769458E-212	2	PARD6B
9.047371933289E-177	0.440982906389181	0.263	0.087	1.51615858858057E-172	2	FAM102B
9.56547167631776E-214	0.440629025468747	0.276	0.084	1.60298174351733E-209	2	UTP25
2.10943293404746E-280	0.440471333554484	0.556	0.217	3.53498771087673E-276	2	TIMMDC1
8.44260701148901E-293	0.440007810826131	0.517	0.191	1.41481208298533E-288	2	MYRF
2.27780824220027E-306	0.439903542895472	0.707	0.298	3.8171510522792E-302	2	CPNE3
0	0.439819018461458	0.723	0.29	0	2	COL27A1
6.49584088577416E-224	0.439779545203335	0.369	0.131	1.08857301563803E-219	2	PRSS23
1.27375834778678E-216	0.439660304224848	0.368	0.132	2.13456423922108E-212	2	CHAF1A
0	0.439537232019996	0.585	0.222	0	2	GAK
0	0.439253239102275	0.427	0.137	0	2	TBRG4
5.01172474398847E-273	0.439060216640689	0.541	0.21	8.39864832597587E-269	2	WWC2
2.90038829995376E-283	0.438829624884231	0.517	0.194	4.8604707130625E-279	2	DYRK1A
3.12452139444994E-222	0.438635629904812	0.843	0.434	5.23607295281921E-218	2	GCLC
7.27232492176215E-301	0.438541687764698	0.567	0.216	1.2186962103889E-296	2	ZNF326
0	0.438532407054902	0.553	0.203	0	2	CENPV
4.69232199433956E-289	0.438437787490301	0.757	0.331	7.86339319811424E-285	2	PA2G4
8.70031241199946E-258	0.438393813262548	0.36	0.116	1.45799835400287E-253	2	XPNPEP1
0	0.437941751542099	0.567	0.205	0	2	MAN2B1
1.66521680100377E-281	0.437898088418356	0.72	0.316	2.79057031512211E-277	2	OCIAD1
1.88212782924995E-186	0.437782513894179	0.331	0.123	3.15406981625707E-182	2	CCDC82
3.35067413358668E-217	0.43773284117366	0.367	0.131	5.61505971306456E-213	2	POLD1
8.83360157120123E-234	0.437716476763354	0.377	0.132	1.4803349513019E-229	2	ZC3H7A
1.42602145379109E-214	0.437143273290304	0.36	0.129	2.3897267522631E-210	2	MKKS

4.81359973132708E-193	0.437094210675026	0.284	0.094	8.06663042975791E-189	2	PIP5K1C
3.35900386744735E-229	0.437084274532048	0.397	0.145	5.62901868106826E-225	2	DNM1L
5.1118863056531E-241	0.436986365396012	0.384	0.134	8.56649907101347E-237	2	DEPRECATED-ENSG00000232119
1.73074733750893E-237	0.436945384178646	0.358	0.122	2.90038638819746E-233	2	DROSHA
3.81575325615094E-216	0.436873294319578	0.3	0.096	6.39443930665774E-212	2	PCNX3
1.90591909072114E-201	0.436763989915852	0.301	0.101	3.19393921223049E-197	2	APC
9.86968790969336E-260	0.436567888222293	0.932	0.488	1.65396229990641E-255	2	CCNL2
2.51869030111076E-294	0.436520410652921	0.639	0.262	4.22082120660142E-290	2	DNASE2
3.60364315938835E-183	0.436387347161614	0.946	0.637	6.03898520650299E-179	2	UPB1
0	0.436223440856621	0.603	0.23	0	2	CLIC1
1.55836574438104E-177	0.435979843631686	0.269	0.091	2.61150931443374E-173	2	MED9
1.1558711912403E-245	0.435936863776012	0.457	0.172	1.9370089422805E-241	2	LYSMD3
3.56637851571282E-193	0.435912859100214	0.31	0.109	5.97653711663154E-189	2	HOOK2
6.25628818242543E-194	0.435890364552223	0.336	0.123	1.04842877361085E-189	2	SOCS5
1.29492567569042E-294	0.43578719524201	0.516	0.189	2.170036447322E-290	2	PRRC2B
1.23935844057811E-252	0.435507375213912	0.345	0.109	2.0769168747208E-248	2	NACC2
5.64543853542233E-205	0.435388333967825	0.345	0.124	9.46062589766075E-201	2	WDR37
5.67772486398017E-270	0.435193157620164	0.42	0.144	9.51473132705797E-266	2	PTPRA
3.89191738979462E-296	0.435142743278116	0.537	0.2	6.52207516181783E-292	2	PSPH
3.20795039208409E-173	0.434913810391977	0.261	0.088	5.37588326705451E-169	2	PIK3CA
7.46985670405975E-223	0.434858071863729	0.264	0.076	1.25179858646633E-218	2	SLC66A3
1.72261201631278E-174	0.434730686269296	0.284	0.1	2.88675321693696E-170	2	DNAJC10
0	0.434568202311068	0.717	0.298	0	2	LRRFIP2
9.76803130615954E-196	0.434194002613668	0.287	0.095	1.63692668628622E-191	2	ALG1L
1.06589973606395E-298	0.434082734673641	0.596	0.234	1.78623477769596E-294	2	MED28
7.41840129674269E-217	0.434022807320637	0.33	0.112	1.24317568930814E-212	2	BACE1
1.10436323023879E-304	0.433695593815406	0.524	0.19	1.85069190123416E-300	2	ATIC
4.86617575597908E-214	0.433557057331548	0.319	0.106	8.15473733186974E-210	2	DEPRECATED-ENSG00000022267
2.25039317814032E-227	0.433129943487906	0.367	0.129	3.77120888792755E-223	2	REST
9.87133742688287E-190	0.433015741736252	0.318	0.114	1.65423872599703E-185	2	DEPRECATED-ENSG00000116489
3.80809602585679E-261	0.432696096857903	0.823	0.401	6.38160732013081E-257	2	RTN4
1.20222154271751E-285	0.432595897527194	0.69	0.293	2.014682861286E-281	2	SLC25A5
3.49417197590299E-306	0.432140357174666	0.671	0.273	5.85553339721823E-302	2	DKC1
1.69282347639372E-281	0.431892918143997	0.498	0.184	2.83683358174059E-277	2	NELFB
2.8039443114845E-280	0.431743047323889	0.599	0.242	4.69884987718573E-276	2	SLC33A1
1.85792345318698E-288	0.431709901660684	0.523	0.196	3.11350812285075E-284	2	RARS
3.53443290393549E-305	0.431477378473757	0.514	0.185	5.9230026604151E-301	2	CHD4
1.61298068959568E-213	0.431443534439384	0.292	0.093	2.70303303962444E-209	2	MAP3K10
4.49803089777023E-272	0.431422414310149	0.538	0.209	7.53780017848335E-268	2	ZBED6
1.67495759320518E-253	0.431406046236534	0.387	0.13	2.80689393469325E-249	2	LMAN2L
0	0.431276095239758	0.559	0.207	0	2	PRKCA
6.4196204243479E-250	0.431246922937434	0.809	0.381	1.07579999071222E-245	2	ABCA1
0	0.4312157034063	0.546	0.188	0	2	MPRIP
6.41046925543901E-308	0.431074982870512	0.685	0.283	1.07426643782647E-303	2	TRIP12
6.94396520356698E-161	0.430495027697512	0.251	0.086	1.16366968881375E-156	2	SIRT3
1.93976838970321E-266	0.430300387482542	0.873	0.441	3.25066386746464E-262	2	CDK5RAP3
4.63157765755842E-235	0.429692372345956	0.331	0.106	7.7615978385364E-231	2	DBNDD2
8.12626104341257E-280	0.429250743987048	0.55	0.214	1.36179882565508E-275	2	CAND1
1.80293581687396E-300	0.42923672803759	0.483	0.169	3.02135984191738E-296	2	RAB15
3.79360392208442E-274	0.429195337320353	0.522	0.199	6.35732145262907E-270	2	TSNAX
3.61309831086577E-193	0.429190354218908	0.297	0.102	6.05483014934885E-189	2	AACS
5.64215610730999E-241	0.429138513263729	0.875	0.453	9.45512520463009E-237	2	AFDN
8.70552979826557E-235	0.429121839909127	0.417	0.155	1.45887268359334E-230	2	QSER1
2.95365904778022E-273	0.429074394262691	0.771	0.356	4.94974183227009E-269	2	SPSB1
2.1524406105056E-178	0.428823419249468	0.309	0.113	3.60705997508528E-174	2	ASF1A
1.91334233327785E-265	0.428811028064481	0.449	0.162	3.20637908210702E-261	2	CCNQ
0	0.428515973249455	0.558	0.205	0	2	PURA
4.89616263400363E-277	0.42845827043623	0.578	0.232	8.20498934206329E-273	2	PCMTD2
1.82475058859513E-303	0.428317759888398	0.607	0.238	3.05791703636772E-299	2	KDM5C
2.25574812834337E-291	0.428310422507207	0.478	0.169	3.78018271347782E-287	2	UBAC2

**9.21977725538528E-227**	0.428074231869678	0.425	0.162	1.54505027245746E-222	2	MED6
**1.50699897180863E-162**	0.428032811316286	0.252	0.086	2.52542887695691E-158	2	WBP1L
**1.23322529217627E-284**	0.428000131971048	0.668	0.281	2.066638944629E-280	2	ECE1
**8.90683685657956E-286**	0.427964540005742	0.521	0.196	1.4926077204256E-281	2	PGAP2
**1.4651593861926E-153**	0.427879048181587	0.26	0.094	2.45531409938156E-149	2	FBXO30
**1.29279462962217E-206**	0.427620498376104	0.362	0.133	2.16646524032083E-202	2	SKP2
**6.80154167219339E-246**	0.427223478467402	0.36	0.12	1.13980235342617E-241	2	PSD4
**3.86566253996035E-300**	0.427176528190538	0.49	0.174	6.47807728446555E-296	2	KLHL2
**2.26423174360266E-300**	0.427104351195051	0.429	0.14	3.79439955592933E-296	2	VPS41
**2.42845308015753E-238**	0.426803785315367	0.421	0.156	4.06960167172799E-234	2	STXBP3
**1.48938538386167E-300**	0.426382641078893	0.63	0.251	2.49591202627538E-296	2	TCEAL1
**9.07697148917399E-201**	0.426310315761799	0.314	0.108	1.52111888215578E-196	2	ZNF318
**2.92027532065906E-216**	0.426271682499568	0.331	0.113	4.89379738236046E-212	2	STRIP1
**1.59754291247025E-222**	0.426167663370678	0.356	0.125	2.67716241271764E-218	2	CYLD
**1.38764650574268E-221**	0.426084782959014	0.348	0.12	2.32541801432358E-217	2	B4GALT7
**1.2014119060763E-264**	0.426052565470699	0.679	0.296	2.01332607220266E-260	2	SLC25A33
**0**	0.425917307653228	0.622	0.232	0	2	INTS1
**0**	0.425810437814979	0.715	0.298	0	2	SMARCA4
**3.59588994975719E-203**	0.425727914577265	0.304	0.102	6.02599237780309E-199	2	RABGGTA
**6.78862372969868E-185**	0.425533573085427	0.295	0.102	1.1376375646229E-180	2	LMBR1L
**8.24651547153973E-262**	0.425473168365865	0.522	0.201	1.38195106272063E-257	2	MORC4
**1.85350605200175E-138**	0.42539457115057	0.255	0.096	3.10610544194453E-134	2	TSPYL2
**2.20149156855581E-233**	0.425282683758308	0.32	0.102	3.68925957058583E-229	2	DEPRECATED-ENSG00000168924
**6.27954613025615E-302**	0.425029412384585	0.673	0.279	1.05232634050833E-297	2	LRRC8A
**7.95767989283788E-295**	0.424917751833053	0.544	0.204	1.33354799644177E-290	2	YWHAB
**6.12508140066381E-276**	0.424792509898992	0.456	0.163	1.02644114112324E-271	2	MAPKAPK5
**4.88497644820239E-299**	0.424757748214014	0.783	0.352	8.18624353189757E-295	2	LPP
**5.74590913383652E-197**	0.424692406232524	0.335	0.121	9.62899452648325E-193	2	SBNO1
**1.17881408207486E-291**	0.424530362362053	0.71	0.311	1.97545663874105E-287	2	CD68
**7.39771214197973E-289**	0.424506276137187	0.732	0.323	1.23970860075296E-284	2	MTUS1
**5.79422960944515E-256**	0.424358901808009	0.481	0.183	9.70996997950818E-252	2	PBX2
**4.73420317648033E-266**	0.424168124489145	0.354	0.11	7.93357768314574E-262	2	CORO7
**7.79003887069665E-258**	0.424009086875102	0.432	0.154	1.30545471395135E-253	2	CCT4
**8.5196244545068E-304**	0.42384383343522	0.644	0.259	1.42771866608625E-299	2	SMARCA5
**1.19337921539436E-242**	0.423755532325068	0.458	0.175	1.99986488915788E-238	2	XAF1
**3.10701923114376E-271**	0.42374916420349	0.558	0.221	5.20674282755071E-267	2	FKBP1A
**1.39707437955297E-230**	0.423417806680328	0.333	0.109	2.34121724525487E-226	2	ZDHHC5
**1.22905462955963E-223**	0.423391357283257	0.321	0.105	2.05964974821602E-219	2	MAP3K14
**0**	0.423291671090345	0.665	0.261	0	2	PLEC
**5.40221637874093E-262**	0.423150271957858	0.416	0.145	9.05303420749404E-258	2	MPP5
**2.704203999288E-226**	0.423005219775149	0.347	0.119	4.53170506200683E-222	2	ANKFY1
**1.97805001842302E-242**	0.422831188945074	0.413	0.15	3.3148162208733E-238	2	METTL3
**0**	0.42275296530768	0.497	0.171	0	2	PBDC1
**1.17020218115445E-147**	0.42253993669061	0.255	0.093	1.96102481517862E-143	2	SFXN2
**9.64098797028916E-241**	0.42242690974274	0.37	0.127	1.61563676406106E-236	2	ANAPC2
**6.65838582793718E-190**	0.422145835725944	0.287	0.097	1.11581229704571E-185	2	C7orf25
**1.31048962867999E-292**	0.422141120643361	0.481	0.171	2.19611851974193E-288	2	MVB12A
**8.87635962509022E-231**	0.422087517373741	0.369	0.129	1.48750034597262E-226	2	PRR12
**5.82148639385002E-196**	0.422020721557757	0.332	0.119	9.75564689881386E-192	2	SPAG16
**1.13414286158114E-215**	0.421956040945698	0.386	0.143	1.90059660743768E-211	2	COX20
**2.47677339574753E-295**	0.421929155024633	0.494	0.176	4.15057685659371E-291	2	REPIN1
**0**	0.421828882587965	0.628	0.249	0	2	GTF2I
**6.87648390743917E-185**	0.421409145151797	0.265	0.087	1.15236117320866E-180	2	ZNF274
**6.75022804357576E-281**	0.421386531206189	0.829	0.398	1.13120321554243E-276	2	SREBF2
**1.52918187977268E-230**	0.421267126507019	0.395	0.143	2.56260299412306E-226	2	NR2C2
**4.01843231953537E-220**	0.421090787145531	0.34	0.117	6.73408888107737E-216	2	SIX5
**0**	0.421048717868129	0.468	0.157	0	2	TNIP2
**1.17197404355086E-259**	0.421047129402793	0.463	0.172	1.96399410218253E-255	2	CBWD1
**1.74187816799044E-260**	0.420677555860827	0.513	0.2	2.91903943391839E-256	2	PSMC6
**2.9435352201929E-184**	0.420070308491464	0.265	0.087	4.93277632199926E-180	2	CRAMP1

1.24888231911483E-238	0.419905615518563	0.378	0.131	2.09287699037263E-234	2	FRS2
4.83838174582223E-272	0.419841955907323	0.782	0.35	8.1081601296489E-268	2	PANK3
1.12178174911029E-177	0.419613520430131	0.269	0.091	1.87988185515903E-173	2	OPA3
9.82507754569786E-185	0.419180051732593	0.884	0.531	1.64648649510805E-180	2	LSR
6.14792412463852E-293	0.419064766602351	0.748	0.32	1.03026912480692E-288	2	SYBU
6.12493419755368E-201	0.41870013995661	0.347	0.126	1.02641647282605E-196	2	ACYP2
3.7145223240331E-179	0.418609422027956	0.28	0.097	6.22479651061467E-175	2	TJAP1
2.76837158569318E-278	0.418525367966269	0.563	0.22	4.63923710330462E-274	2	YIPF4
3.64852839266202E-247	0.418309410882846	0.843	0.405	6.11420388042301E-243	2	PPP1R15B
7.28518743499541E-269	0.418197411470924	0.496	0.188	1.22085171035653E-264	2	SNX27
1.27001893055264E-234	0.418123310265754	0.353	0.119	2.12829772382011E-230	2	PARN
8.34993911234287E-170	0.41807496385863	0.257	0.087	1.39928279644642E-165	2	ALG14
1.63414705588968E-142	0.418068916940162	0.293	0.117	2.73850363625993E-138	2	C15orf40
2.90942849694115E-282	0.417486473216404	0.585	0.234	4.87562027517398E-278	2	EPRS
0	0.416992010407557	0.569	0.2	0	2	PGPEP1
2.67102540099539E-305	0.416916551779607	0.666	0.268	4.47610436698807E-301	2	MSI2
2.29865351308438E-226	0.416674950685488	0.383	0.137	3.8520835572268E-222	2	TMEM251
3.97954892449679E-175	0.416481333735895	0.263	0.088	6.66892808767173E-171	2	ABHD17A
1.04858834585653E-202	0.416464940388978	0.328	0.115	1.75722434998638E-198	2	ZDHHC16
9.34541457168566E-203	0.416442094473112	0.326	0.114	1.56610457392308E-198	2	RTCA
4.99276267333445E-212	0.416271416282372	0.335	0.116	8.36687168797387E-208	2	ZNF672
6.90982793169845E-193	0.416246391162681	0.344	0.127	1.15794896479403E-188	2	CEP85
2.4052286907623E-206	0.415969019735084	0.304	0.101	4.03068223997946E-202	2	SGCB
4.74434894842167E-204	0.415707676123606	0.328	0.115	7.95057996776503E-200	2	CCNYL1
1.70425137172968E-288	0.415669162080578	0.542	0.205	2.8559844487446E-284	2	SCAF4
1.83613569039028E-263	0.415535084749868	0.486	0.183	3.07699618995604E-259	2	BTF3L4
0	0.415450779125282	0.569	0.214	0	2	EIF2B5
8.13503099682784E-228	0.41542027505847	0.414	0.155	1.36326849444841E-223	2	SLC25A16
5.30656272696716E-221	0.415189907074153	0.35	0.122	8.89273781785156E-217	2	DVL2
1.17608293438126E-282	0.415145864218246	0.587	0.236	1.97087978143612E-278	2	FLNB
8.17466729012957E-225	0.414990389356036	0.346	0.118	1.36991074447991E-220	2	TAOK2
1.13930709411219E-225	0.41498212084784	0.387	0.141	1.90925082831321E-221	2	ACAP2
1.11786485455443E-303	0.414655675833533	0.556	0.21	1.87331792326231E-299	2	MXD4
2.46677935022066E-246	0.414226432489714	0.467	0.178	4.13382883509978E-242	2	CA2
9.96736572604071E-282	0.414050311841391	0.573	0.225	1.6703311483699E-277	2	ATP2C1
2.31041023111897E-253	0.41403028533579	0.491	0.189	3.87178546530917E-249	2	FGF13
2.63123118449979E-174	0.413754538179864	0.264	0.089	4.40941721898475E-170	2	SPAST
4.4549238426083E-198	0.413752510708338	0.34	0.123	7.46556137544299E-194	2	INPP5E
5.72111120827619E-204	0.413013382730743	0.293	0.096	9.58743816282924E-200	2	RIOX1
1.13348938431773E-243	0.412941334701389	0.389	0.135	1.89950151023966E-239	2	SDHAF4
5.72626171104258E-292	0.412659555000215	0.647	0.266	9.59606937536515E-288	2	RABGGTB
1.01498370079667E-238	0.412445579331231	0.435	0.163	1.70090968579506E-234	2	SOX9
5.07840431004803E-218	0.412434087290829	0.337	0.115	8.5103899427785E-214	2	ZMYM4
1.24118509854586E-252	0.411934002741605	0.725	0.337	2.07997798814316E-248	2	KDELR2
1.06839945865563E-229	0.411658467522676	0.356	0.122	1.79042381281511E-225	2	SFSWAP
4.01490368948989E-193	0.411420092806289	0.313	0.11	6.72817560284716E-189	2	ARHGAP21
1.98589686491788E-269	0.411313809291412	0.487	0.181	3.32796596622939E-265	2	ADSS
7.39730896541051E-254	0.41119439508763	0.425	0.152	1.23964103642349E-249	2	THUMPD3
7.84617848303304E-175	0.410733661822252	0.291	0.104	1.31486259018668E-170	2	PRMT7
2.6775262912933E-303	0.410643770894124	0.539	0.199	4.48699855894931E-299	2	NMRAL1
1.11104850755099E-267	0.410636981217485	0.301	0.083	1.86189508895395E-263	2	NOL4L
7.62035272589387E-236	0.41051989570148	0.861	0.415	1.27701870980529E-231	2	ZHX1
4.21590877057464E-231	0.410283285776671	0.409	0.151	7.06501991772898E-227	2	NUP160
2.16147436423639E-248	0.410270012164425	0.385	0.132	3.62219873958734E-244	2	SLC12A8
1.55644749507168E-304	0.410252597491745	0.558	0.21	2.60829471224112E-300	2	TBC1D14
6.80314764045991E-223	0.410221119290985	0.318	0.104	1.14007148158827E-218	2	DEPRECATED-ENSG00000272391
1.0158791745595E-287	0.410139327710258	0.566	0.221	1.70241032072682E-283	2	RPRD2
1.03393980616097E-179	0.409813368261331	0.314	0.114	1.73267632716455E-175	2	BLZF1
8.23086539751848E-194	0.409804624663941	0.285	0.094	1.37932842331615E-189	2	MFSD14B
8.75443257525672E-241	0.409604086032999	0.418	0.153	1.46706781096152E-236	2	NDRG3

1.48325575863143E-272	0.409508327430052	0.555	0.219	2.48564000031454E-268	2	TLE1
3.82338950289874E-277	0.409439789064311	0.477	0.173	6.40723612895771E-273	2	DARS
4.37400635935516E-180	0.40938670873707	0.276	0.094	7.32995985700737E-176	2	PPP1R14B
9.2173789892335E-270	0.409320542209511	0.516	0.199	1.54464837101575E-265	2	CALM1
2.62773492850865E-188	0.408887846722928	0.325	0.119	4.4035581931948E-184	2	RASSF8
1.62076903527538E-283	0.408859334774554	0.453	0.158	2.71608474931449E-279	2	SESN2
5.81580082990094E-179	0.408839520591414	0.309	0.112	9.74611903074799E-175	2	ZNF362
1.49026500797584E-224	0.408722239386541	0.942	0.492	2.49738610036591E-220	2	ERICH5
4.79267542086671E-220	0.408674745322215	0.391	0.145	8.03156547028843E-216	2	SOS 1
1.46321584007013E-203	0.408622906958626	0.329	0.115	2.45205710478952E-199	2	USP3
3.49340716693092E-228	0.408307605139901	0.371	0.13	5.85425173034284E-224	2	TTC33
3.8709279580166E-287	0.408281844855779	0.66	0.274	6.48690107204421E-283	2	EGFR
3.75291303336355E-269	0.407988109104616	0.477	0.176	6.28913166131064E-265	2	HERC4
1.19845459730715E-264	0.407805769976366	0.565	0.227	2.00837021416732E-260	2	RFC1
1.07862472964362E-154	0.407758887004392	0.947	0.601	1.80755932193678E-150	2	F5
4.95560538506526E-159	0.407593127826632	0.29	0.109	8.30460350429236E-155	2	LIN52
1.81854120707645E-160	0.407556438950434	0.253	0.087	3.04751135481872E-156	2	ASH2L
1.05602814529436E-262	0.40746938493502	0.677	0.29	1.76969196588429E-258	2	SPPL2A
0	0.407016293003421	0.618	0.237	0	2	MYO6
6.25098506063385E-186	0.406967411137266	0.28	0.094	1.04754007646102E-181	2	VKORC1L1
3.70410434731781E-246	0.406956224034293	0.91	0.506	6.20733806523519E-242	2	SLC12A7
4.11979709950336E-245	0.406922397618383	0.775	0.37	6.90395597934773E-241	2	SLC25A4
6.44735053814239E-300	0.40691930408284	0.595	0.235	1.0804470031819E-295	2	TSPO
2.98837650467049E-292	0.406624264732915	0.756	0.328	5.0079213465268E-288	2	ARHGAP35
1.99196459680797E-197	0.406232693135267	0.346	0.127	3.33813427133079E-193	2	NCAPD3
0	0.406226684682075	0.621	0.241	0	2	SEC24A
6.36397243962471E-247	0.406137948653236	0.368	0.124	1.06647450143231E-242	2	CSF1
1.61656194318619E-300	0.406115781691521	0.578	0.223	2.70903450439142E-296	2	ITFG1
6.56437918378329E-183	0.40599414236974	0.286	0.098	1.1000586636184E-178	2	TRAPPC13
4.57784617884679E-171	0.405908866115289	0.259	0.088	7.67155462651145E-167	2	ZNF512
6.54633335894496E-267	0.405853764136993	0.444	0.157	1.097034544292E-262	2	SGSM3
4.12874618653446E-239	0.405116632985524	0.387	0.136	6.91895285939445E-235	2	JAGN1
8.59328546866441E-307	0.40511505620492	0.67	0.273	1.44006277883878E-302	2	VPS52
2.29459400493457E-201	0.405069362491929	0.313	0.107	3.84528063346935E-197	2	RB1
6.43851746493749E-229	0.404915145916477	0.375	0.132	1.07896675677422E-224	2	ENOX2
2.06419989240535E-222	0.404373842200046	0.532	0.224	3.45918617969289E-218	2	RNF138
1.69508049004591E-282	0.404209141471933	0.432	0.147	2.84061588521893E-278	2	UCK2
1.5298904140193E-284	0.404120821005459	0.445	0.152	2.56379035581354E-280	2	SLCO2B1
1.03387060925275E-185	0.403822753864666	0.282	0.096	1.73256036698576E-181	2	VPS53
1.09375285489436E-258	0.403775713753142	0.491	0.187	1.83291103423197E-254	2	DELE1
0	0.403615702053506	0.489	0.17	0	2	RNF126
1.42558161009342E-295	0.403437604300982	0.618	0.248	2.38898966219454E-291	2	CLPTM1L
2.57858848073116E-269	0.403121481243926	0.448	0.159	4.32119857600928E-265	2	GGCT
2.48761171378557E-184	0.403006583325565	0.262	0.085	4.16873970996185E-180	2	POLR1B
3.01521881027863E-242	0.402862207159001	0.338	0.108	5.05290368226494E-238	2	GPR137B
1.94242873390976E-291	0.402749904500717	0.558	0.211	3.25512207228598E-287	2	ACTR10
4.26020744010344E-217	0.402561480336331	0.389	0.144	7.13925562812534E-213	2	ALG2
3.62921830033779E-228	0.402280216270391	0.856	0.464	6.08184402770607E-224	2	SUMF2
6.14536611998454E-191	0.402267237185366	0.904	0.563	1.02984045438701E-186	2	APLP2
1.6222418281553E-206	0.40224964775811	0.292	0.094	2.71855285562265E-202	2	CHMP4C
9.39848225225535E-249	0.402248314944616	0.465	0.176	1.57499765583295E-244	2	ELF1
6.70090244080579E-241	0.402226990779113	0.38	0.132	1.12293723103023E-236	2	SESTD1
8.39187767742606E-201	0.402089450530426	0.371	0.139	1.40631086118306E-196	2	XPO1
8.67458218087814E-262	0.402087419582835	0.513	0.199	1.45368648187156E-257	2	KPNA1
3.27017909086099E-186	0.401938493176736	0.892	0.517	5.48016612046484E-182	2	TRAM1
5.30246426199073E-270	0.401496358534979	0.543	0.214	8.88586961024407E-266	2	ZNF146
5.24685927550572E-295	0.401461038024177	0.64	0.261	8.79268677389249E-291	2	CDC42SE1
4.17142145342265E-288	0.401355718523648	0.555	0.213	6.99046807164567E-284	2	PPIC
4.47896953291854E-234	0.401313189272508	0.388	0.138	7.50585714326489E-230	2	DHX8
1.31617898507257E-284	0.40122423898669	0.704	0.309	2.20565274318462E-280	2	PAPSS2

5.86603289421628E-206	0.401145483289111	0.341	0.121	9.83029792412764E-202	2	SSR1
4.84844266585683E-274	0.401085559633277	0.616	0.254	8.12502021944287E-270	2	TRIQK
1.98351940942096E-191	0.400849288348857	0.329	0.119	3.32398182630765E-187	2	EXOSC6
2.52388944072071E-276	0.400747122580536	0.514	0.192	4.22953392475976E-272	2	OXR1
1.10729574666667E-291	0.400399019235361	0.629	0.255	1.85560621226401E-287	2	ST5
2.66750753839822E-184	0.399594380617943	0.304	0.107	4.47020913284774E-180	2	FAM241A
6.92622927092846E-261	0.399425754744166	0.53	0.209	1.16069750122219E-256	2	LARS
7.01786276342655E-288	0.399372691270919	0.61	0.243	1.17605344189502E-283	2	NCKAP1
7.80793212312867E-287	0.399167297239776	0.559	0.218	1.3084532651939E-282	2	BRWD1
1.46986158338935E-290	0.398764578927573	0.442	0.149	2.46319404144387E-286	2	ABCB8
2.63868771876875E-200	0.398746864904718	0.954	0.67	4.42191287911268E-196	2	GSTK1
4.09618775578896E-299	0.398653891000782	0.673	0.281	6.86439144115115E-295	2	PRKAA1
4.24621079421043E-165	0.398645021589888	0.277	0.099	7.11580004893783E-161	2	WASHC3
1.97573267479504E-251	0.398639215321774	0.406	0.142	3.31093281642153E-247	2	COQ8B
5.69448067761791E-200	0.398485511191721	0.342	0.124	9.54281071955209E-196	2	CRBN
1.8071606735828E-305	0.398398783731905	0.783	0.353	3.02843985679005E-301	2	KIAA1522
3.84797330369667E-269	0.39828120239213	0.707	0.307	6.44843366233488E-265	2	MBNL1
1.58849664826822E-256	0.398224590994173	0.418	0.146	2.66200268316789E-252	2	KDM5B
5.64111019293379E-253	0.39821691929795	0.561	0.229	9.45337246131844E-249	2	ASAP1
6.65970684950321E-142	0.39791989013671	0.258	0.097	1.11603367383975E-137	2	PPOX
5.89488588919422E-239	0.397887767781574	0.828	0.412	9.87864977311168E-235	2	SEC31A
6.90283956034311E-168	0.397862220906762	0.295	0.108	1.1567778535223E-163	2	FASTKD2
2.02470139381113E-211	0.397723504156076	0.889	0.476	3.39299459574869E-207	2	TSPAN6
9.18801355850326E-181	0.397629186753615	0.305	0.11	1.53972731213398E-176	2	DEPRECATED-ENSG00000072803
4.0885807762269E-258	0.397590120573465	0.452	0.166	6.85164366480104E-254	2	BRD1
2.3430377939573E-274	0.397493394617688	0.56	0.22	3.92646273511365E-270	2	SEMA4B
1.25669943616818E-196	0.397483322803328	0.338	0.122	2.10597691513064E-192	2	AGO3
3.52995391687686E-209	0.397250787189197	0.332	0.115	5.91549677390224E-205	2	CLTC
3.72353157692404E-294	0.396902607297081	0.703	0.3	6.23989421660931E-290	2	ATP2A2
6.7277665319142E-261	0.396891380598212	0.444	0.16	1.12743911541818E-256	2	C12orf75
0	0.396746470592827	0.53	0.192	0	2	GOLGA3
2.08632976252513E-258	0.396579630023013	0.43	0.153	3.49627141603961E-254	2	PPP5C
2.94334081247657E-273	0.396530245543837	0.734	0.329	4.93245053354823E-269	2	SLC25A44
1.4580049581077E-211	0.396484810647867	0.394	0.15	2.44332470879688E-207	2	KLHL12
6.74399608178673E-253	0.396341825750136	0.422	0.151	1.13015886338582E-248	2	PHLPP1
1.49751636822651E-247	0.396193795270768	0.825	0.395	2.50953792987398E-243	2	EZR
1.18496351007153E-173	0.395813499982538	0.28	0.099	1.98576185017788E-169	2	PPP1R21
3.40841532544429E-163	0.395549841392416	0.258	0.09	5.71182240237953E-159	2	RBIS
2.68112936835581E-167	0.395490791890302	0.257	0.088	4.49303659549067E-163	2	AMD 1.00
4.96183978972031E-187	0.395395174337726	0.28	0.094	8.31505111961329E-183	2	SBF2
1.93536009103467E-187	0.394879746965083	0.265	0.085	3.24327644055589E-183	2	FOXJ2
1.50670515476738E-306	0.394676062182869	0.6	0.233	2.52493649835918E-302	2	MYLK
5.55712517462096E-203	0.394624026457048	0.441	0.178	9.31263036762981E-199	2	TIGD2
5.46736403436395E-274	0.394491427196986	0.759	0.342	9.16220864878711E-270	2	COPS5
4.58152380032852E-300	0.39439897534228	0.618	0.244	7.67771758459053E-296	2	EGLN2
7.78258419045812E-168	0.394042950228857	0.816	0.457	1.30420545863697E-163	2	HSPH1
5.01999411709115E-198	0.393983118440129	0.303	0.103	8.41250614142134E-194	2	ASAH1
2.45289999447599E-256	0.393908935615625	0.414	0.146	4.11056981074286E-252	2	RMC1
4.76996075116477E-307	0.393712710867241	0.523	0.189	7.99350022680193E-303	2	TMEM250
2.80672518531355E-255	0.393677243193352	0.806	0.397	4.70351006554844E-251	2	CLINT1
1.23743369255329E-275	0.393321710377936	0.569	0.222	2.07369138198081E-271	2	NUP43
5.01937629147288E-259	0.393252161863927	0.442	0.159	8.41147078925025E-255	2	SMIM20
0	0.393231925083904	0.708	0.296	0	2	INTS11
2.14879113592327E-231	0.393178926955947	0.289	0.086	3.60094418558022E-227	2	TSPAN12
3.85176335275351E-265	0.392311478870067	0.707	0.308	6.45478502654433E-261	2	NUFIP2
2.10068141995323E-162	0.392056922087727	0.275	0.098	3.52032192355763E-158	2	MCM8
8.49251117818794E-186	0.391851474737317	0.403	0.164	1.42317502324074E-181	2	SLC4A4
7.2934384482656E-291	0.391832574051299	0.612	0.244	1.22223441516035E-286	2	NKAP
9.08615265535904E-257	0.39154559431881	0.383	0.128	1.52265746198507E-252	2	DNMBP
6.18104555413962E-166	0.391255058030235	0.963	0.627	1.03581961396272E-161	2	TDO2

4.17623223435215E-279	0.3911519430272	0.67	0.285	6.99852997832734E-275	2	FAM20B
4.75077458717023E-186	0.391123146522906	0.275	0.092	7.96134805317988E-182	2	NUDT12
1.00116375111176E-258	0.391107026752396	0.527	0.206	1.67775021411308E-254	2	MAP3K2
1.58796859986695E-254	0.390821277262545	0.444	0.161	2.66111777965704E-250	2	PSMC1
1.93046311266094E-264	0.390538237534118	0.433	0.153	3.2350700841972E-260	2	TTC7A
2.08538332265328E-291	0.390430707401867	0.656	0.273	3.49468537210236E-287	2	RIOK3
3.51291536719924E-297	0.390430242770975	0.541	0.202	5.88694357235248E-293	2	CNOT11
8.22331100811972E-228	0.39030164445859	0.351	0.12	1.3780624587407E-223	2	ELP1
0	0.390260982202244	0.525	0.185	0	2	YKT6
4.19293805825343E-283	0.390200172046372	0.699	0.295	7.0265255980211E-279	2	UBE2D3
1.58589943626235E-243	0.389922169344369	0.409	0.147	2.65765027528844E-239	2	UBE2O
9.39570030446838E-267	0.389514278245837	0.598	0.247	1.57453145702281E-262	2	SEC23A
1.95441598483231E-206	0.389468135619252	0.395	0.151	3.27521030738198E-202	2	PIGP
2.15732579798645E-169	0.389398537167226	0.292	0.107	3.61524657226569E-165	2	PRKRA
1.25722349359217E-197	0.389220718326521	0.359	0.134	2.10685513056176E-193	2	RECQL
5.49299792769959E-255	0.38885755205265	0.733	0.338	9.20516592723898E-251	2	PTTG1IP
1.06582021608741E-196	0.388645153348678	0.31	0.106	1.78610151811929E-192	2	DEPRECATED-ENSG00000099810
1.04315902436541E-183	0.388597365707633	0.306	0.109	1.74812589303156E-179	2	EMC1
1.45966229819763E-288	0.388436104101621	0.611	0.245	2.44610207931959E-284	2	KMT5B
1.92314944330654E-245	0.388381846234875	0.455	0.172	3.2228138370931E-241	2	GOLPH3L
2.95475652114497E-224	0.388343161300592	0.388	0.141	4.95158097813475E-220	2	PATZ1
8.0120128086197E-254	0.388298136060942	0.617	0.26	1.34265310646849E-249	2	FOXO3
1.06334181205745E-160	0.38806757642638	0.254	0.088	1.78194820864588E-156	2	RUFY2
3.68251646957371E-268	0.387880714479919	0.43	0.15	6.17116109971163E-264	2	KLHDC10
5.33057925651474E-289	0.387520249021971	0.549	0.209	8.9329847180674E-285	2	VPS35L
1.79625263326519E-257	0.387503065196397	0.406	0.14	3.0101601628258E-253	2	GMPR2
4.49351588276142E-269	0.387361959474848	0.573	0.23	7.53023391633158E-265	2	CDC42SE2
5.40633705767339E-252	0.387179865090937	0.42	0.149	9.05993964124907E-248	2	CCNK
4.25575573419896E-263	0.38715792336286	0.425	0.149	7.13179545937061E-259	2	USF3
2.24477312884684E-270	0.386820465840473	0.497	0.186	3.76179080932153E-266	2	PPA2
1.5102171974493E-284	0.386584007986057	0.69	0.296	2.53082197948554E-280	2	SEPTIN11
2.39065486694927E-237	0.386460410447286	0.416	0.153	4.00625942603358E-233	2	ITPRIPL2
4.2768563677574E-187	0.386242811348183	0.252	0.079	7.16715590108786E-183	2	RAB26
4.94684564961462E-303	0.386103965147317	0.554	0.207	8.28992393962418E-299	2	LARP1
2.83229885494464E-254	0.385533864262426	0.639	0.276	4.74636642111623E-250	2	SMAD3
7.26103621972287E-268	0.385389951733782	0.775	0.345	1.21680444970116E-263	2	CHTOP
2.12772941630169E-210	0.385187974177281	0.47	0.193	3.56564895583838E-206	2	GCH1
1.87510941390798E-193	0.385098337132815	0.299	0.102	3.14230835582699E-189	2	DEPRECATED-ENSG00000164164
1.51603347116271E-180	0.385068665894147	0.348	0.135	2.54056889097447E-176	2	SOCS7
1.84906069899918E-293	0.384993457095676	0.579	0.224	3.09865591938283E-289	2	SNX6
7.07418535636725E-291	0.384991955378176	0.584	0.229	1.18549198202002E-286	2	URI1
3.38163838263792E-294	0.384932450833002	0.586	0.229	5.66694960162462E-290	2	YAP1
3.77754850122513E-180	0.384832698155606	0.295	0.104	6.33041577835308E-176	2	PPP1R13L
2.23807943162079E-247	0.384825558277722	0.436	0.16	3.75057351151013E-243	2	PARD3
1.27524894347951E-213	0.384626131073972	0.364	0.131	2.13706217948296E-209	2	EXOC6
4.37950476185332E-221	0.38419051209001	0.406	0.153	7.33917407991379E-217	2	FAM98B
4.83637025306406E-250	0.383824079889839	0.407	0.144	8.10478927008475E-246	2	TBK1
5.89056463309021E-259	0.383682977947215	0.516	0.202	9.87140821213257E-255	2	PIP5K1A
1.42892469685531E-289	0.383590636585681	0.631	0.256	2.39459200699013E-285	2	UBE2Z
9.79287717339962E-266	0.383465091393902	0.393	0.131	1.64109035671831E-261	2	ARMH3
4.42687421635755E-205	0.383208976492924	0.338	0.12	7.41855581177198E-201	2	CARD19
5.90641206868703E-276	0.383042362897942	0.571	0.225	9.89796534470572E-272	2	RC3H2
0	0.382630877285031	0.564	0.211	0	2	RCC1L
6.17417396328451E-175	0.382552293702281	0.993	0.92	1.03466807276722E-170	2	SELENOP
3.03346125249503E-294	0.382530393885773	0.645	0.265	5.08347436693118E-290	2	CHP2
2.46401056920703E-208	0.382387272302805	0.318	0.108	4.12918891187715E-204	2	RRNAD1
9.21542117917231E-304	0.38210783701996	0.568	0.216	1.5443202812057E-299	2	NT5E
1.26450988273175E-246	0.381670541457946	0.397	0.138	2.11906566148186E-242	2	ICMT
1.9237197522915E-275	0.381617208600844	0.52	0.197	3.2237695608901E-271	2	COMMD5
6.23566358529876E-204	0.381442528628695	0.272	0.085	1.04497250362437E-199	2	MYBBP1A

1.38834569885796E-192	0.381436987488983	0.302	0.104	2.32658972214618E-188	2	CLASP1
1.56580702217543E-217	0.381425808395682	0.802	0.398	2.62397940776159E-213	2	DGAT2
2.60242629232161E-286	0.381014071090069	0.623	0.254	4.36114598067255E-282	2	DPP9
2.67767661044099E-273	0.380874759959678	0.501	0.187	4.487250463777E-269	2	CLYBL
1.51346642062157E-233	0.380862165267787	0.876	0.476	2.53626702767763E-229	2	CD151
9.15558169441422E-249	0.380784276191075	0.397	0.138	1.53429238034993E-244	2	DPAGT1
1.29870628614391E-266	0.380677150577134	0.716	0.328	2.17637199431996E-262	2	TM9SF3
9.37304678252038E-195	0.380516794370542	0.31	0.108	1.57073517981477E-190	2	RPAP1
7.49243738856901E-264	0.380318592068876	0.7	0.308	1.2555826575764E-259	2	SCAMP2
5.60776756430521E-272	0.380309561732584	0.66	0.275	9.39749688426267E-268	2	ERN 1.00
8.19761205429189E-156	0.380224359419463	0.254	0.09	1.37375582805823E-151	2	SLC25A17
1.61463685542387E-261	0.379917264546628	0.44	0.158	2.70580844231932E-257	2	B4GALT5
4.34386635862344E-154	0.379700436573365	0.271	0.1	7.27945124378116E-150	2	IFT80
2.91057382895585E-224	0.379213898352439	0.572	0.249	4.87753962256421E-220	2	ME1
9.45242438660923E-185	0.378968523397877	0.836	0.475	1.58403727870798E-180	2	MDK
7.72580435998138E-288	0.378662566888044	0.522	0.193	1.29469029464568E-283	2	SON
7.21970236967214E-234	0.378525326751444	0.372	0.13	1.20987772310966E-229	2	TIGD5
3.45114032028129E-189	0.378243378714054	0.349	0.131	5.78342094872739E-185	2	APPL1
2.30668676644217E-252	0.377983028558583	0.349	0.111	3.86554568320378E-248	2	SMURF1
4.02047354370144E-209	0.377876177143547	0.346	0.121	6.73750956453486E-205	2	SAP30
9.27036528785283E-220	0.377744305226666	0.297	0.094	1.55352781493838E-215	2	MICAL2
5.43774990052908E-187	0.377724021154368	0.409	0.169	9.11258128330663E-183	2	CD9
3.6185563024802E-278	0.377552267468839	0.6	0.243	6.06397665169632E-274	2	SDHA
1.24188814370267E-233	0.377541421509292	0.389	0.139	2.08115615121694E-229	2	HDAC8
2.65997995421583E-236	0.377479551354356	0.389	0.137	4.4575944072749E-232	2	MAP1S
8.91898222812448E-182	0.377471862396349	0.275	0.092	1.4946430417891E-177	2	SERGEF
4.8090019832971E-185	0.377424594544646	0.322	0.118	8.05892552360929E-181	2	PCBP2
2.24961850145899E-138	0.377272828102431	0.281	0.11	3.76991068474497E-134	2	FLNA
3.47289634519803E-201	0.377260144089743	0.346	0.126	5.81987969528285E-197	2	TMEM192
1.1534988020791E-243	0.377081684990864	0.437	0.161	1.93303329252415E-239	2	STX17
1.83201487184898E-174	0.376804873075608	0.291	0.104	3.07009052224452E-170	2	ZSWIM9
1.07740041090783E-264	0.376771062509598	0.512	0.198	1.80550760859935E-260	2	API5
8.06236056482247E-200	0.376738814997254	0.357	0.132	1.35109038345295E-195	2	C17orf75
2.89685094894197E-164	0.376708972247491	0.284	0.104	4.85454282023695E-160	2	CD320
1.08812619475669E-255	0.376690294488861	0.721	0.324	1.82348187717327E-251	2	SMC1A
1.53815878417106E-252	0.376444663360798	0.392	0.134	2.57764649051386E-248	2	ACTR3
1.04875940011078E-231	0.376230002796455	0.348	0.117	1.75751100270565E-227	2	UXS1
2.83295164617595E-168	0.376210732802131	0.275	0.097	4.74746036866166E-164	2	CEP104
7.63417247856919E-207	0.375801286402934	0.287	0.092	1.27933462395863E-202	2	GCC2
3.61077273494919E-180	0.3757004866975	0.336	0.127	6.05093294922785E-176	2	AARS2
1.89413258738788E-270	0.375553553548569	0.732	0.325	3.1741873899446E-266	2	CSNK1D
8.01615099338064E-281	0.375482697328143	0.549	0.212	1.34334658347073E-276	2	CCNY
3.92961620145081E-283	0.37536928825588	0.682	0.291	6.58525083039127E-279	2	ONECUT2
2.33416210841815E-246	0.375341263842858	0.46	0.173	3.91158886128713E-242	2	DLST
1.1121923179285E-197	0.375270643148307	0.325	0.115	1.86381188638459E-193	2	TFDP2
6.48308993592868E-269	0.375212081131864	0.653	0.274	1.08643621146293E-264	2	RBM10
2.3735178861639E-201	0.375081845573176	0.369	0.138	3.97754127363346E-197	2	BTBD1
7.95692298164307E-265	0.374906943752566	0.761	0.35	1.33342115326374E-260	2	EIF2AK1
4.09212828373701E-264	0.374525929061553	0.471	0.173	6.85758857788648E-260	2	CERT1
9.77711640252271E-303	0.374176509455853	0.564	0.213	1.63844916673476E-298	2	ARPC4
5.46683899742168E-285	0.374142361840046	0.504	0.186	9.16132879187925E-281	2	CHMP1A
5.85544362635628E-218	0.374048716425026	0.415	0.158	9.81255242904785E-214	2	CBX3
1.31354716312105E-260	0.373802287929734	0.402	0.137	2.20124233595825E-256	2	TAF4
3.85212466024523E-179	0.373658017151753	0.297	0.106	6.45539050563895E-175	2	ASXL2
2.33114283009254E-248	0.373534139384113	0.825	0.402	3.90652915466908E-244	2	RER1
9.78796285065265E-240	0.373231271108885	0.42	0.154	1.64026681451237E-235	2	BMPR2
3.78413559492221E-265	0.372969534021104	0.674	0.295	6.34145442997064E-261	2	SLC39A7
4.72315971737648E-273	0.372214442552404	0.533	0.206	7.91507105437951E-269	2	SLC16A2
8.97405331090597E-282	0.372075727137232	0.632	0.258	1.50387185384162E-277	2	ZFR
1.53395742420388E-165	0.371869447183898	0.981	0.699	2.57060585148086E-161	2	AKR1C3

7.04161805759137E-292	0.371841496548251	0.538	0.203	1.18003435409116E-287	2	ZC3HAV1
4.64416020285287E-220	0.371601116744223	0.396	0.147	7.78268366794083E-216	2	NUP54
1.69508464319542E-251	0.371507876875178	0.415	0.148	2.84062284506689E-247	2	CCNT2
1.2222880519956E-259	0.37126389819312	0.48	0.18	2.04831031753423E-255	2	ATL3
3.56093703517065E-244	0.371251783923236	0.827	0.405	5.96741828353898E-240	2	MARC2
6.76802730980146E-272	0.371160706882335	0.6	0.245	1.13418601657653E-267	2	CUL4A
1.08910848461636E-274	0.371097250638154	0.695	0.299	1.8251279985201E-270	2	YTHDF3
2.69972098388346E-257	0.3710638095724	0.42	0.147	4.5241924247919E-253	2	NT5DC1
3.51240265763357E-273	0.370999986048878	0.595	0.241	5.88608437366234E-269	2	ZNF664
9.17186886342074E-237	0.37097885236419	0.41	0.148	1.53702178413205E-232	2	FBRS
1.0064251534086E-193	0.370921989431107	0.314	0.11	1.68656727208213E-189	2	USP21
6.46634713221872E-197	0.370787725408783	0.575	0.264	1.08363045241721E-192	2	AKR1C8P
8.45302765033234E-187	0.370694468862537	0.299	0.104	1.41655837364269E-182	2	C5orf22
2.62853306273356E-191	0.370557817696921	0.329	0.119	4.40489570652889E-187	2	WDR36
4.36057251551539E-235	0.370160001846456	0.359	0.121	7.30744742150069E-231	2	SOWAHB
9.77270000069024E-204	0.370052997579856	0.805	0.432	1.63770906611567E-199	2	BSG
1.54463966665276E-214	0.37001438194284	0.826	0.433	2.5885071533767E-210	2	CMBL
4.47472570024091E-266	0.369986788992358	0.528	0.205	7.49874532846371E-262	2	NFKB1
2.28171443336873E-268	0.369763010362559	0.728	0.322	3.82369704743932E-264	2	FAM91A1
0	0.369502347451693	0.572	0.205	0	2	ABCA2
9.45620368732409E-272	0.369291884267927	0.501	0.188	1.58467061392177E-267	2	PTDSS1
5.22511762570881E-243	0.369268925519998	0.465	0.178	8.75625211716283E-239	2	KAT2A
9.65698147996442E-274	0.369202465356101	0.58	0.23	1.61831695641244E-269	2	MED25
3.28937019527272E-171	0.369124433028101	0.259	0.087	5.51232657323803E-167	2	TMA16
2.16875165340123E-266	0.368672023415332	0.753	0.341	3.63439402076978E-262	2	TMEM259
6.372084370567E-168	0.368599056424786	0.259	0.088	1.06783389881962E-163	2	CACTIN
1.81698301841839E-196	0.368588789303032	0.316	0.111	3.04490014226554E-192	2	VTA1
3.34382394793742E-216	0.368490111836569	0.379	0.139	5.60358017195353E-212	2	FGFR1OP2
5.79405745995925E-205	0.368489152822037	0.387	0.147	9.7096814913997E-201	2	IREB2
4.9706997766375E-271	0.3680097824563	0.521	0.2	8.32989868568913E-267	2	GATD1
2.71941028234853E-193	0.367814269819717	0.35	0.131	4.55718775115967E-189	2	RBFA
1.22110292648529E-276	0.367664072620495	0.596	0.24	2.04632428420406E-272	2	TMEM214
9.044465901142E-223	0.367513713738133	0.82	0.422	1.51567159571338E-218	2	SYNGR2
1.37507776739115E-285	0.367448794304065	0.679	0.285	2.30435532259409E-281	2	UPF1
1.23714138069628E-188	0.367287447589734	0.902	0.556	2.07320152577083E-184	2	ELF3
1.44411899641029E-273	0.367128455924759	0.722	0.316	2.42005461418436E-269	2	SP3
1.14597662233792E-177	0.367081980877903	0.294	0.105	1.92042762371389E-173	2	BRMS1L
1.6485373894854E-195	0.367005411935636	0.296	0.1	2.76261895729963E-191	2	KRAS
9.32164853033896E-240	0.366598245240184	0.558	0.23	1.5621218607142E-235	2	NLK
2.39073122232253E-277	0.366591668022729	0.631	0.257	4.0063873823681E-273	2	VAMP7
3.27813182811031E-268	0.36638334515176	0.491	0.183	5.49349331754726E-264	2	FIP1L1
8.19256685133486E-207	0.36621424411023	0.369	0.136	1.3729103529467E-202	2	COMMD2
7.13673288520475E-258	0.366150416933346	0.448	0.163	1.19597369690261E-253	2	TRPT1
1.63190817764768E-152	0.36596947244699	0.265	0.097	2.73475172410199E-148	2	HLX
2.14889874169312E-249	0.365946919179706	0.562	0.231	3.60112451132933E-245	2	SORBS1
1.30026844370354E-187	0.365727148202971	0.32	0.116	2.17898985795839E-183	2	PPARG
9.51636314831993E-238	0.365526093246925	0.461	0.178	1.59475213639545E-233	2	RFNG
4.7916063168729E-179	0.365347348795481	0.888	0.539	8.0297738658156E-175	2	CDHR5
4.61068462985639E-185	0.365263141129216	0.345	0.13	7.72658530271333E-181	2	ITPR1
1.94461155065828E-168	0.365249666776428	0.273	0.096	3.25878003659314E-164	2	EXOSC3
9.96135976067163E-236	0.365196937052484	0.722	0.334	1.66932466869335E-231	2	LSS
4.32250774612585E-258	0.365162108289166	0.522	0.206	7.2436584809577E-254	2	ZXDC
1.01858736757975E-283	0.364962249163243	0.599	0.242	1.70694871059014E-279	2	GALNT2
3.83405439352345E-277	0.36492067603343	0.585	0.233	6.4251083526666E-273	2	SLC39A1
6.74483285958916E-224	0.364902689784726	0.41	0.153	1.13029909060995E-219	2	NSMCE4A
9.13680235297303E-219	0.364878964981692	0.316	0.103	1.53114533831122E-214	2	KLHL7
1.19537159633981E-226	0.364357942297017	0.262	0.073	2.00320372114626E-222	2	ACY3
1.04931700491182E-286	0.364220772522771	0.633	0.257	1.75844543683123E-282	2	MAX
9.99417681971706E-274	0.363990618649596	0.527	0.202	1.67482415144818E-269	2	TMBIM4
4.08351383355485E-216	0.363879260122021	0.408	0.155	6.84315248227122E-212	2	ZNF704

0	0.363602555183364	0.494	0.166	0	2	PDGFA
2.04638179965235E-274	0.363544826356805	0.731	0.328	3.42932661985741E-270	2	STAP2
1.90717453022576E-163	0.363186845186894	0.251	0.085	3.19604307775233E-159	2	METTL2A
1.00718053433316E-226	0.363071107161557	0.418	0.156	1.68783313943551E-222	2	MIB1
2.78751741787757E-184	0.363045828526563	0.295	0.103	4.67132168887924E-180	2	HELZ2
5.7164017296465E-282	0.363027363332942	0.598	0.24	9.5795460185416E-278	2	PLA2G12A
9.03206244615022E-211	0.362681662199463	0.305	0.1	1.51359302472585E-206	2	PEX26
1.75475097687658E-296	0.362482093124992	0.579	0.224	2.94061168704977E-292	2	EXOSC4
1.04407469716722E-196	0.362044181584448	0.33	0.118	1.74966037751283E-192	2	AP5S1
8.12385323255328E-273	0.361885708275118	0.413	0.138	1.36139532471128E-268	2	GATA4
5.70400328984657E-190	0.361787631804424	0.303	0.105	9.55876871312488E-186	2	ALG1
1.38701010362816E-153	0.361170763184833	0.259	0.093	2.32435153166006E-149	2	PRKD3
4.84681133680771E-269	0.361159143172992	0.514	0.197	8.12228643822235E-265	2	PPP1R12B
2.30998545468491E-250	0.361130569601189	0.728	0.347	3.87107362496098E-246	2	RETSAT
9.66906715196519E-186	0.36103124360852	0.322	0.117	1.62034227332633E-181	2	KIFAP3
1.76192100720842E-294	0.361008645426762	0.541	0.203	2.95262722387987E-290	2	POMGNT1
2.69762213372181E-250	0.360682050241359	0.676	0.302	4.52067517169102E-246	2	TMEM109
2.5107978435098E-230	0.360613530548914	0.286	0.084	4.20759502615372E-226	2	PRKCZ
3.49890551436008E-271	0.360479305548883	0.489	0.182	5.86346586096463E-267	2	PRPF38B
2.23090012713165E-299	0.360441891479045	0.649	0.262	3.73854243304722E-295	2	ATF6B
7.03888927428603E-200	0.360238146309869	0.303	0.102	1.17957706458485E-195	2	STAMBP
0	0.360119515258823	0.538	0.194	0	2	ZNF219
6.79426375193745E-307	0.360101582070918	0.599	0.231	1.13858271954968E-302	2	NME3
1.19839384590063E-181	0.359550307876465	0.329	0.122	2.00826840696027E-177	2	DHX29
6.02749714313907E-200	0.359163333271439	0.34	0.123	1.01008797124724E-195	2	ZKSCAN8
2.1492219158081E-213	0.359108603638943	0.33	0.112	3.60166608651121E-209	2	BMS1
6.74654088381977E-223	0.35874394892119	0.783	0.372	1.13058532131052E-218	2	CHPT1
4.86407290895886E-157	0.358712261393455	0.294	0.111	8.15121338083327E-153	2	MCF2L
3.8413243802234E-193	0.358684714520164	0.255	0.079	6.43729139637837E-189	2	ABCF2
3.87470875329832E-235	0.35864800687694	0.815	0.403	6.49323692877732E-231	2	CD46
8.95552631667446E-252	0.358637386132564	0.532	0.214	1.50076710014831E-247	2	AFTPH
2.89181839772775E-196	0.358609328691579	0.282	0.092	4.84610927091216E-192	2	RPIA
9.47364977045172E-179	0.358537976275033	0.302	0.109	1.5875942285323E-174	2	ELAC2
3.40238981315592E-275	0.358404724563166	0.491	0.182	5.70172484888669E-271	2	CHCHD7
2.0293487354091E-286	0.358382570517849	0.517	0.19	3.40078261079856E-282	2	ATP13A1
2.59178816227199E-152	0.358225039426625	0.945	0.624	4.34331860233539E-148	2	IDH1
1.76657896298592E-273	0.358158176445579	0.491	0.183	2.96043302617181E-269	2	C2orf68
5.6517993860155E-282	0.358009284370505	0.691	0.294	9.47128541108477E-278	2	CHD2
1.55951872768771E-275	0.357954140236198	0.607	0.246	2.61344148385906E-271	2	MCM5
5.16992804425308E-228	0.357722662157921	0.369	0.129	8.66376541655931E-224	2	ARID1A
1.82752755384121E-192	0.357701092905374	0.854	0.47	3.0625706747271E-188	2	PCK2
1.82683579997276E-172	0.357684859098311	0.262	0.088	3.06141143359436E-168	2	NHEJ1
8.61143599688615E-259	0.357664440435752	0.72	0.324	1.44310444435818E-254	2	MAGED2
1.15828272337442E-228	0.357549047997223	0.396	0.144	1.94105018783085E-224	2	KDM5A
5.4317071662195E-251	0.357161095866149	0.794	0.368	9.10245486915063E-247	2	ZNRF2
1.67837915873115E-272	0.357107401260965	0.665	0.282	2.81262779420166E-268	2	CD2AP
8.93062381892669E-281	0.357062571357084	0.355	0.106	1.49659393957574E-276	2	SEMA4G
2.45362190596753E-231	0.357062429833896	0.424	0.159	4.11177959002038E-227	2	AAAS
9.62211984879496E-266	0.356816448559087	0.774	0.36	1.61247484426106E-261	2	SLC47A1
1.14999227985059E-257	0.35678521426461	0.472	0.176	1.92715706257362E-253	2	HMGN4
1.22554884732526E-256	0.356735505285802	0.714	0.313	2.05377475834767E-252	2	ZBTB33
6.38282294247698E-265	0.356471792642165	0.605	0.247	1.06963346870029E-260	2	TOR1AIP1
4.84012176285223E-270	0.356056699444666	0.643	0.271	8.11107605018776E-266	2	MPV17
5.24115551946548E-284	0.355822324397486	0.674	0.28	8.78312841952025E-280	2	TMEM245
1.95036054325636E-177	0.355774920602126	0.285	0.1	3.26841419838901E-173	2	STK35
7.54249181419563E-176	0.355769370420178	0.355	0.14	1.2639707782229E-171	2	NOL11
1.97635609524073E-167	0.355764524777846	0.279	0.099	3.31197754440441E-163	2	OXLD1
8.2475622583638E-214	0.355707401642532	0.371	0.135	1.38212648325661E-209	2	AK3
2.82990281883588E-287	0.355370159922228	0.735	0.32	4.74235114380517E-283	2	NAA10
5.13380335456157E-288	0.355285118785124	0.513	0.189	8.60322766157428E-284	2	GCN1

9.59806139510666E-190	0.355039475240977	0.34	0.126	1.60844312859197E-185	2	TMEM18
1.65904516068435E-239	0.355005957101132	0.434	0.161	2.78022788027483E-235	2	LNPK
2.99374432075468E-205	0.354989965930765	0.83	0.449	5.0169167327207E-201	2	SDF4
1.66962478441066E-244	0.354941356524349	0.358	0.118	2.79795721371538E-240	2	PTPRG
7.78787465152522E-283	0.354760462165835	0.53	0.199	1.3050920341026E-278	2	TOMM6
4.51232660226529E-261	0.354705612782088	0.52	0.202	7.56175692007617E-257	2	RRP1B
3.44101869092968E-286	0.354542490134455	0.609	0.244	5.76645912225996E-282	2	NTPCR
3.18537606264779E-255	0.354266910195617	0.548	0.216	5.33805320578517E-251	2	DHFR
7.10918762504835E-249	0.354242718179364	0.7	0.31	1.1913576622056E-244	2	CCNG1
3.30693259267197E-222	0.35421125865589	0.349	0.12	5.54175763879969E-218	2	DCBLD2
1.90940994642018E-264	0.353883094182091	0.547	0.218	3.19978918821094E-260	2	NOP56
3.6965471687398E-241	0.353535804143698	0.438	0.163	6.19467374537416E-237	2	RNF14
2.23879135180499E-205	0.353311049289891	0.361	0.132	3.7517665473548E-201	2	ZNF618
2.98334223847401E-178	0.353010717798412	0.332	0.126	4.99948492323475E-174	2	BCAS3
3.6471834228308E-155	0.352908377704707	0.256	0.091	6.11194997997985E-151	2	ARNTL2
2.05770608593739E-254	0.352884789490006	0.437	0.158	3.44830385881388E-250	2	AKAP8L
3.39160123920222E-207	0.35283392739496	0.288	0.092	5.68364535665509E-203	2	CCDC25
3.1039406724698E-277	0.352449758607246	0.549	0.213	5.20158377892489E-273	2	AZIN1
3.79303090717864E-256	0.352333365010107	0.521	0.204	6.35636119424996E-252	2	MCMBP
1.20106547965057E-133	0.351907088397015	0.256	0.099	2.01274553079843E-129	2	DIS3L
1.93286995269685E-261	0.351583887390169	0.493	0.187	3.23910346672938E-257	2	BLCAP
1.68816841546287E-274	0.351446221819795	0.689	0.294	2.82903263063267E-270	2	UHMK1
5.38380326944339E-217	0.351442135629535	0.347	0.121	9.02217751893324E-213	2	ANKRD52
9.16661021356781E-199	0.351371659464418	0.424	0.17	1.53614053958969E-194	2	RCAN1
9.59353484206546E-218	0.351323806266732	0.446	0.176	1.60768456883333E-213	2	FBXO46
8.68456507340741E-191	0.351259281664136	0.294	0.1	1.45535941500161E-186	2	LMF1
4.21012523667463E-241	0.351200049644534	0.737	0.347	7.05532787161935E-237	2	SURF4
5.10459489585302E-185	0.351130963957092	0.344	0.13	8.55428012647049E-181	2	WDR6
1.06120201494304E-277	0.350993015861969	0.539	0.205	1.77836233664155E-273	2	WRNIP1
0	0.350580228471856	0.633	0.25	0	2	MOV10
0	0.350415385267241	0.65	0.259	0	2	IGSF8
2.45132644854501E-270	0.350344430289565	0.578	0.234	4.10793286247173E-266	2	AKR7A2
3.46398364454386E-244	0.350287681558428	0.366	0.122	5.8049437915266E-240	2	SIRT7
1.97931606378302E-240	0.350220645606979	0.627	0.267	3.31693785968759E-236	2	KLB
2.10799162499504E-268	0.349907861652876	0.725	0.326	3.53257236516669E-264	2	AMFR
1.18742312309117E-257	0.34962199208822	0.773	0.363	1.98988366967618E-253	2	NIT1
2.87798629614668E-222	0.349112815834088	0.63	0.282	4.8229294350826E-218	2	TENT5A
1.89776872001103E-254	0.34895781414728	0.534	0.212	3.18028082099449E-250	2	MAEA
1.4917303753741E-148	0.348547908557914	0.945	0.675	2.49984176305191E-144	2	TM4SF4
1.89254538578686E-218	0.348524728843665	0.424	0.162	3.17152755750161E-214	2	WAC
1.07514952431764E-233	0.348406110553339	0.384	0.136	1.8017355728515E-229	2	RAB22A
1.66104609237354E-240	0.348390276530817	0.729	0.339	2.78358104159957E-236	2	SLC50A1
1.03900889539319E-253	0.348316488282607	0.696	0.314	1.74117110689991E-249	2	SRI
2.69525054182458E-286	0.348241779119619	0.621	0.252	4.51670085798962E-282	2	GIPC1
2.19359831250992E-257	0.347955152031012	0.673	0.294	3.67603205210412E-253	2	IVD
4.67662236815599E-253	0.34768812048326	0.462	0.172	7.83708376455581E-249	2	NIN
1.26199337224734E-299	0.347423424943831	0.505	0.181	2.11484849321209E-295	2	PPP1R35
1.55357302474106E-224	0.347404406941156	0.792	0.389	2.60347767486107E-220	2	CPQ
3.56475933944826E-228	0.347381907785067	0.373	0.131	5.9738237010474E-224	2	CCDC61
1.01917411935287E-252	0.347363981157431	0.627	0.272	1.70793198921154E-248	2	OCIAD2
7.03396839910004E-212	0.347353696596937	0.312	0.103	1.17875242432118E-207	2	YBEY
1.89914684187258E-227	0.347234560605173	0.423	0.158	3.18259027761007E-223	2	SPCS3
8.36994849718059E-190	0.347226307053217	0.434	0.179	1.40263596915752E-185	2	MID2
4.0616476860983E-243	0.3471822760587	0.539	0.22	6.80650919236354E-239	2	SRP19
5.74360183179536E-233	0.34664628678958	0.568	0.241	9.62512794972266E-229	2	MCM2
3.92996535814458E-284	0.346465930612441	0.574	0.225	6.58583594717869E-280	2	PEX13
5.13932155744388E-275	0.346401292916916	0.581	0.233	8.61247506596446E-271	2	WDR26
1.24770467473132E-180	0.346315175023797	0.285	0.099	2.09090349391474E-176	2	MRTFB
1.29078697524096E-234	0.346272139915479	0.705	0.318	2.16310081310879E-230	2	PPM1B
2.36988437123084E-153	0.346271596737417	0.261	0.094	3.97145222930865E-149	2	KATNA1

4.34746439776371E-145	0.346143152824046	0.305	0.122	7.28548083777243E-141	2	POLH
2.82107737988538E-167	0.34557437126261	0.265	0.092	4.72756147321191E-163	2	ZBTB4
1.7166206003165E-180	0.345574337242789	0.329	0.123	2.87671280201038E-176	2	SLC1A4
1.91828000853701E-239	0.34532594412798	0.412	0.15	3.21465363830633E-235	2	NANS
1.77543270836572E-240	0.344910318289547	0.738	0.345	2.97527013267928E-236	2	DERL1
3.16754789965737E-210	0.344642839446185	0.374	0.138	5.30817677024582E-206	2	DENR
4.61102962859373E-179	0.344220311161374	0.309	0.112	7.72716345159737E-175	2	DEPRECATED-ENSG00000188529
6.19422787080726E-244	0.344129734251604	0.588	0.246	1.03802870658988E-239	2	FBXL3
1.17154439460714E-256	0.343494698879259	0.656	0.281	1.96327409648264E-252	2	C5orf24
2.91492831909073E-159	0.343399410906739	0.282	0.104	4.88483687713224E-155	2	ZNF621
1.54430417727132E-226	0.343390441509554	0.42	0.158	2.58794494027128E-222	2	COPS4
2.54317704505414E-252	0.343357003826906	0.455	0.169	4.26185609210173E-248	2	CDK14
7.5232158920381E-194	0.343285576405099	0.343	0.126	1.26074051918774E-189	2	CLOCK
5.68301094540769E-220	0.343236233340912	0.555	0.237	9.52358974231421E-216	2	AQP3
1.12659537152968E-225	0.343194645304422	0.423	0.159	1.88794852360944E-221	2	CCT5
1.86751130137518E-212	0.342712678851125	0.403	0.154	3.12957543884453E-208	2	TIMM9
1.16899216417174E-300	0.34258379573211	0.601	0.235	1.958997068719E-296	2	KMT2E
7.44121832459785E-233	0.342418839800387	0.47	0.182	1.24699936683611E-228	2	DEPRECATED-ENSG00000078668
3.59163768549985E-262	0.342396522205219	0.671	0.291	6.01886643336065E-258	2	TMEM41B
1.57446274105191E-189	0.342286820092381	0.27	0.088	2.6384846614548E-185	2	TSPAN17
1.98862399554962E-271	0.342240247659878	0.486	0.18	3.33253609174206E-267	2	CYFIP1
1.43289544412059E-248	0.342210142209796	0.402	0.141	2.40124618525728E-244	2	CTNNBIP1
3.41388956442005E-160	0.342098218714668	0.252	0.088	5.72099613205512E-156	2	LCMT1
3.73226328360148E-216	0.341983549002189	0.457	0.182	6.25452681065936E-212	2	PCNA
6.56577419443086E-260	0.341961161588487	0.666	0.298	1.10029243950272E-255	2	PSMB10
2.33357159545458E-240	0.341449176220861	0.685	0.314	3.91059927966278E-236	2	FUCA1
1.95710559776273E-271	0.341330657559262	0.508	0.192	3.27971756073078E-267	2	CNOT9
1.14468344578636E-252	0.341309660651656	0.436	0.158	1.91826051844879E-248	2	MFN1
1.76720463741433E-206	0.341286299514277	0.379	0.142	2.96148153137893E-202	2	DENND4B
3.62430344705752E-183	0.34126932896463	0.309	0.111	6.073607716579E-179	2	MIER3
1.22652962567783E-277	0.341110430087686	0.529	0.202	2.05541834671091E-273	2	USF1
7.83687886014132E-198	0.341009307571477	0.314	0.109	1.31330415938248E-193	2	RIPK2
2.64941506187512E-228	0.340219938508473	0.435	0.166	4.43988976069032E-224	2	ILKAP
3.23730869290197E-256	0.340051529864236	0.554	0.222	5.42508190756513E-252	2	ZNF652
0	0.340040408884639	0.619	0.243	0	2	ARSA
1.16027621502959E-242	0.339563872467721	0.402	0.142	1.94439088114658E-238	2	TEF
1.20614137616677E-208	0.339223452890799	0.361	0.132	2.02125171818026E-204	2	PGGT1B
8.29110141338658E-227	0.338988718613077	0.379	0.135	1.38942277485532E-222	2	CHD1
2.98816540900563E-217	0.338832815893827	0.432	0.167	5.00756759241164E-213	2	NUP98
2.21575894524723E-217	0.338706788011394	0.376	0.137	3.7131688404453E-213	2	KDM4A
4.20331153214905E-278	0.338698387312739	0.676	0.288	7.04390946557538E-274	2	HGS
3.43874477226569E-201	0.338469783986392	0.393	0.152	5.76264848936284E-197	2	LIG3
1.23278633994942E-284	0.33808281917001	0.765	0.342	2.06590334848723E-280	2	AKT1
2.75515156101041E-171	0.337983692415908	0.3	0.11	4.61708298594125E-167	2	AQR
1.42386719365538E-244	0.337933685524878	0.467	0.179	2.38611664312769E-240	2	COMMD6
3.20080084449915E-217	0.337720121226878	0.497	0.206	5.36390205521167E-213	2	SDR39U1
1.45379736888007E-198	0.337688567466726	0.274	0.087	2.43627363076923E-194	2	BAHD1
1.60616020236091E-199	0.337401769764328	0.853	0.447	2.6916032671164E-195	2	AIFM1
1.99833698083238E-181	0.337252026603787	0.324	0.119	3.3488131124789E-177	2	SPIN4
1.52158702141893E-283	0.3371926699822	0.592	0.236	2.54987553049384E-279	2	KANSL1
3.13019883896027E-155	0.337137867048154	0.27	0.099	5.24558721432963E-151	2	MTX3
7.41408324636678E-211	0.336502770959371	0.364	0.132	1.24245207042615E-206	2	RGP1
1.16090640722056E-251	0.336335190569894	0.329	0.1	1.94544695722022E-247	2	GIPC2
3.63557462109902E-304	0.336067761015819	0.71	0.297	6.09249595003774E-300	2	POLR2J2
1.90897865245175E-241	0.335641514834459	0.472	0.181	3.19906642577864E-237	2	GMFB
2.0950421228998E-182	0.335442312735365	0.891	0.511	3.51087158955548E-178	2	ACTN4
1.55213831691779E-279	0.335430876097843	0.643	0.266	2.60107339149084E-275	2	MAPK14
2.70733433427211E-269	0.335221976367269	0.631	0.262	4.53695087737321E-265	2	RB1CC1
2.0486683963434E-193	0.334768075825739	0.28	0.092	3.43315849859227E-189	2	TRAFD1
1.32028011479344E-253	0.334320227037968	0.446	0.163	2.21252541637084E-249	2	GANAB

1.41195484587119E-208	0.33423167130156	0.37	0.136	2.36615393071094E-204	2	SUDS3
3.49545789979947E-228	0.334197434975333	0.383	0.136	5.85768834848395E-224	2	DEPRECATED-ENSG00000196313
3.20579654955552E-221	0.334040522840057	0.433	0.167	5.37227385774513E-217	2	UBQLN4
1.52258522410115E-239	0.334022733983442	0.452	0.172	2.5515483185487E-235	2	C5orf15
1.8094468322205E-145	0.333894797532872	0.274	0.103	3.03227100143511E-141	2	CENPJ
2.63809663434513E-280	0.333783433851053	0.586	0.229	4.42092233983558E-276	2	CYTH2
9.86690353403597E-231	0.333681756197628	0.36	0.123	1.65349569423375E-226	2	UBN1
2.40123275503808E-182	0.333613643052316	0.322	0.117	4.02398585089282E-178	2	NFYA
2.50703056789983E-187	0.333487325319122	0.299	0.104	4.20128182568653E-183	2	ANKZF1
1.02077127683476E-172	0.333297399579285	0.259	0.087	1.71060850571969E-168	2	AGPAT5
2.78287813092343E-217	0.333048044707407	0.359	0.127	4.66354717180149E-213	2	AASDHPPT
6.85640054748965E-257	0.333045069937977	0.432	0.154	1.14899560374832E-252	2	SLC35C2
0	0.332990245350558	0.581	0.208	0	2	ARPC1B
3.6900943542522E-261	0.332990065119257	0.537	0.21	6.18386011885584E-257	2	HCFC1
3.19059622746528E-146	0.332959933058395	0.254	0.093	5.34680115798632E-142	2	PIP4K2A
3.58107704653989E-253	0.332820803875723	0.782	0.355	6.00116891459155E-249	2	TMPO
2.36541992184232E-147	0.332598381379884	0.252	0.091	3.96397070502336E-143	2	DEPRECATED-ENSG00000104442
7.31400000491401E-232	0.332596532695861	0.663	0.298	1.22568012082349E-227	2	SGK2
6.14333767625378E-231	0.33228478540832	0.48	0.19	1.02950052778661E-226	2	KAT7
3.04173538378941E-239	0.332256651566493	0.439	0.164	5.0973401561543E-235	2	STAT5B
1.31914882647556E-196	0.332182626254224	0.404	0.16	2.21062960340775E-192	2	LY6E
9.60117323173206E-267	0.331810608579361	0.564	0.225	1.60896461017366E-262	2	IL17RC
1.67545494829849E-68	0.331669000650737	0.801	0.542	2.8077274023586E-64	2	AKR1C2
3.12408338349255E-230	0.331632776654726	0.409	0.15	5.23533893405682E-226	2	PPHLN1
1.24816221585302E-255	0.331593884265653	0.505	0.195	2.0916702413265E-251	2	UBE2J2
3.73670707515492E-264	0.331106243514535	0.532	0.207	6.26197371654461E-260	2	COMMD3
2.40606168046417E-189	0.330837829701334	0.344	0.128	4.03207816412186E-185	2	CAD
1.78708424642799E-255	0.330742974526536	0.508	0.197	2.99479578016403E-251	2	ZC3H4
5.73969646199852E-293	0.330514941733395	0.591	0.229	9.61858333101712E-289	2	EP300
1.66273304365853E-134	0.33046693265474	0.337	0.147	2.78640803456296E-130	2	ADRB2
3.1394852701297E-167	0.330344781400463	0.323	0.123	5.26114941568335E-163	2	COQ2
2.65750682796786E-168	0.330300687173778	0.864	0.508	4.45344994230853E-164	2	HABP2
2.09549431892369E-285	0.329853210963782	0.515	0.192	3.51162937965232E-281	2	WDR13
1.83501872357576E-236	0.329523303367289	0.394	0.139	3.07512437696826E-232	2	POLE
1.65887371050986E-237	0.329404108826464	0.435	0.162	2.77994056407243E-233	2	NRIP1
3.57523332815446E-243	0.329279445657233	0.542	0.22	5.99137601132124E-239	2	H2AFY
7.87364184966583E-156	0.329239908999357	0.273	0.1	1.319464901167E-151	2	KCTD9
1.99121741793732E-237	0.329166431543572	0.747	0.347	3.33688214897937E-233	2	MLEC
2.1032370509529E-249	0.328705613699689	0.533	0.213	3.52460464998688E-245	2	UBE3A
6.66796575411634E-162	0.328606958902676	0.25	0.085	1.11741770107482E-157	2	VGLL4
1.7500549391389E-291	0.328585874995711	0.557	0.212	2.93274206700897E-287	2	GSS
3.60042293442406E-268	0.328549040987219	0.634	0.261	6.03358875350784E-264	2	DEK
3.59071046386389E-217	0.328545866688708	0.359	0.127	6.0173125953431E-213	2	GLE1
7.19793000192516E-244	0.328260346997613	0.305	0.091	1.20622910972262E-239	2	PITX1
1.21201289657483E-151	0.32819828162221	0.264	0.097	2.0310912120801E-147	2	XRRA1
3.30270740298216E-217	0.328147530271391	0.374	0.135	5.5346770659175E-213	2	TMEM128
3.8177239649268E-158	0.327769127976315	0.911	0.554	6.39774182042433E-154	2	NDUFA1
9.68497390130571E-300	0.327721764384344	0.484	0.168	1.62300792638081E-295	2	AMPD2
2.68089937462531E-215	0.327717097072885	0.378	0.138	4.4926511719971E-211	2	ANKRD46
5.35397042140772E-191	0.327700217251206	0.358	0.136	8.97218363219506E-187	2	PITPNC1
5.37520524902891E-209	0.327652750707055	0.392	0.148	9.00776895632265E-205	2	TRIM33
1.25877673807425E-249	0.327520620349819	0.446	0.164	2.10945805766483E-245	2	UNG
2.7582600255973E-229	0.327366746921158	0.778	0.379	4.62229215089595E-225	2	ABCD3
2.83322725017137E-205	0.327268130814885	0.332	0.116	4.74792222583718E-201	2	PDE6D
8.26855943746368E-125	0.327031810052705	0.887	0.53	1.38564519053016E-120	2	ACSM2A
8.26721367495821E-192	0.326930408123167	0.301	0.104	1.3854196676495E-187	2	METTL21A
6.61289829711515E-181	0.326866478430612	0.312	0.113	1.10818949663056E-176	2	SRC
1.07210219630845E-250	0.326752775271038	0.469	0.177	1.7966288605737E-246	2	PPCS
1.07820827759514E-271	0.326309375513832	0.751	0.339	1.80686143159393E-267	2	UBC
2.7634419860051E-205	0.325952258864416	0.337	0.119	4.63097608014734E-201	2	ATP5F1C

2.49986932896321E-241	0.325827811011935	0.449	0.169	4.18928102147654E-237	2	BRPF3
2.47512874562854E-273	0.325551769640795	0.552	0.215	4.14782075192431E-269	2	SRRT
4.0477459205226E-225	0.325319694319106	0.387	0.139	6.78321261361177E-221	2	GNPDA1
1.33824162673043E-263	0.325153867180611	0.614	0.256	2.24262531807486E-259	2	TEX2
8.0556144279966E-245	0.324397445137191	0.531	0.213	1.34995986584367E-240	2	DEPRECATED-ENSG00000137575
3.28769375562843E-267	0.32433054196793	0.479	0.178	5.50951719568212E-263	2	STX6
2.18818231505755E-250	0.324126656392374	0.489	0.188	3.66695592357344E-246	2	SP1
3.12896741326714E-216	0.324110913194397	0.366	0.132	5.24352359115308E-212	2	MFSD14A
1.95172172947668E-281	0.324024499432948	0.64	0.26	3.27069527425701E-277	2	FOXP4
2.5898097837473E-243	0.324017699494503	0.499	0.197	4.34000323560372E-239	2	AGTRAP
4.74919810655917E-239	0.324002952264371	0.378	0.13	7.95870618697185E-235	2	MKRN1
1.13387547424043E-195	0.323792511747606	0.334	0.12	1.90014851973211E-191	2	DDX18
1.14082248865448E-261	0.323767787454931	0.569	0.229	1.91179032648717E-257	2	CLN3
6.63532050236299E-235	0.323542872172927	0.419	0.154	1.11194700978599E-230	2	CEBPZ
1.7569378338107E-176	0.323415220353095	0.258	0.085	2.94427642189998E-172	2	NOP2
2.02467735291461E-249	0.323290832021402	0.483	0.184	3.3929543080143E-245	2	USP25
2.08933409386732E-235	0.323222525749399	0.476	0.186	3.50130607450286E-231	2	CKLF
1.1481408057742E-247	0.323101246671516	0.64	0.278	1.92405436231641E-243	2	VDAC1
7.71371157833512E-175	0.322806919542182	0.267	0.09	1.2926637862974E-170	2	ATP5MPL
1.121400388119E-156	0.322740227547638	0.904	0.502	1.87924277040983E-152	2	CIRBP
2.51837494184121E-178	0.322498990777263	0.306	0.111	4.2202927275375E-174	2	DOCK7
3.90670987763797E-246	0.322441939432298	0.667	0.29	6.5468644129457E-242	2	USP12
1.15127086181463E-250	0.32197965301631	0.564	0.23	1.92929971022895E-246	2	EPB41L4B
2.24802840918438E-283	0.321683826439391	0.617	0.25	3.76724600811118E-279	2	OGDH
1.532444000196E-201	0.3216543203535	0.462	0.193	2.56806965552845E-197	2	SKIL
2.84646706677917E-280	0.321554438581686	0.655	0.273	4.77010951050854E-276	2	TIA1
3.2399223311479E-274	0.321279072794701	0.476	0.172	5.42946184253764E-270	2	C1orf122
1.01390652639361E-216	0.321254149407645	0.387	0.142	1.69910455693042E-212	2	GLRX2
2.74722640973671E-203	0.321182988275822	0.865	0.432	4.60380201743678E-199	2	CMC4
3.3476995320202E-202	0.321001951124389	0.771	0.375	5.61007487575945E-198	2	STBD1
1.34671447407936E-205	0.320943979533098	0.329	0.114	2.2568241156622E-201	2	PIAS2
3.95473182121597E-274	0.320936822500126	0.627	0.261	6.62733958599373E-270	2	SLC23A2
2.97626706371498E-277	0.320626969130096	0.561	0.221	4.98762834537356E-273	2	DOCK5
4.58944805700856E-196	0.320459093625305	0.834	0.433	7.69099705393494E-192	2	SEC16B
1.02116930480749E-271	0.32004234275548	0.524	0.199	1.7112755209964E-267	2	TGIF1
6.55188991477343E-212	0.319920126590526	0.38	0.14	1.09796571191773E-207	2	OSER1
1.07649490851984E-173	0.319652855307643	0.316	0.117	1.80399016769755E-169	2	TIMELESS
1.38636838600869E-220	0.318705451612556	0.743	0.35	2.32327614127337E-216	2	FDPS
4.2161494376508E-279	0.318349283108526	0.595	0.235	7.06542322761521E-275	2	NUP62
2.91603998690713E-267	0.318309937590866	0.682	0.296	4.88669981005896E-263	2	MKNK2
1.82035376374093E-170	0.318176793961158	0.794	0.434	3.05054883727705E-166	2	PRG4
8.67624833476294E-136	0.318083405831215	0.262	0.101	1.45396569593957E-131	2	GSTA4
7.23745178619002E-156	0.318035166353208	0.905	0.546	1.21285217032972E-151	2	TMCO1
1.34852107532129E-247	0.317733652544818	0.787	0.378	2.25985161802342E-243	2	SPTAN1
1.67266427967324E-282	0.317371874721631	0.738	0.328	2.80305079987641E-278	2	TLE5
2.2913419172576E-275	0.317330746238751	0.698	0.306	3.83983078494028E-271	2	EIF3B
3.78924813428919E-173	0.317297332204118	0.292	0.105	6.35002202344182E-169	2	ZNF280D
1.41990316962242E-160	0.317223250849427	0.259	0.09	2.37947373165326E-156	2	WASF3
7.96154513184775E-177	0.317195722236328	0.332	0.126	1.33419573319505E-172	2	LRRC57
2.71280146258884E-294	0.317025122164593	0.577	0.223	4.54611269100637E-290	2	FASTK
6.49337852181648E-188	0.316825055232796	0.336	0.124	1.08816037268601E-183	2	ST3GAL2
2.64562958189121E-196	0.316776348605435	0.386	0.149	4.4335460533333E-192	2	FAM110B
4.50014425361425E-267	0.316541682584201	0.674	0.289	7.54134174020675E-263	2	GPBP1
2.33665065279093E-244	0.316437975245919	0.537	0.218	3.91575916394704E-240	2	SHC2
9.83281123806014E-271	0.316379264202336	0.623	0.26	1.64778250727412E-266	2	SYVN1
1.63805383810052E-224	0.316204778077317	0.396	0.145	2.74505062188886E-220	2	GTPBP10
7.4695075023135E-274	0.316071327785097	0.633	0.263	1.2517400672377E-269	2	CRAT
1.07498726201208E-157	0.316054225844429	0.313	0.122	1.80146365367984E-153	2	KATNAL1
1.30019980081434E-156	0.315992683377178	0.288	0.108	2.17887482620466E-152	2	NFKBIL1
1.35334769094374E-264	0.315908121863969	0.555	0.221	2.26794006048351E-260	2	ABHD16A

5.12496812462037E-200	0.315537257940144	0.858	0.456	8.58842158323881E-196	2	DAP
6.64728531552047E-201	0.315519578234681	0.363	0.135	1.11395207317492E-196	2	SIAH1
1.74342483912471E-256	0.315365280318274	0.508	0.197	2.9216313454052E-252	2	JMJD6
1.47034530174291E-287	0.315075776703367	0.576	0.224	2.46400465666077E-283	2	EXOC3
4.02231852634862E-241	0.314993724411282	0.471	0.181	6.74060138645501E-237	2	DHX36
6.00626581974265E-194	0.314937173797191	0.251	0.076	1.00653002607247E-189	2	ZNF394
1.7416304432886E-202	0.314929356331117	0.384	0.146	2.91862429686304E-198	2	UBE2W
1.49395256875652E-168	0.314902261342759	0.259	0.088	2.50356571472218E-164	2	ABHD17C
2.54147716427241E-179	0.314818380743481	0.33	0.124	4.2590074318877E-175	2	FBXW11
6.47962501698434E-222	0.31463489757219	0.552	0.236	1.08585556034623E-217	2	SLC27A3
9.47841404146835E-188	0.314582163053351	0.43	0.179	1.58839262506927E-183	2	FNIP1
1.12261285535124E-274	0.314579521941734	0.5	0.186	1.88127462299761E-270	2	GON4L
1.30226815546286E-235	0.314351058855278	0.458	0.176	2.18234097492466E-231	2	THNSL2
2.12138182950592E-193	0.314312155985852	0.399	0.159	3.55501166988602E-189	2	MTMR10
1.50436764600288E-257	0.314170948324786	0.734	0.335	2.52101930117162E-253	2	TMED4
5.52889073354044E-241	0.314159278247462	0.568	0.233	9.26531509126706E-237	2	RBBP7
2.6788702187104E-250	0.314010064683885	0.548	0.221	4.48925071251488E-246	2	ATP6AP2
5.00345970271547E-190	0.313341664944407	0.331	0.121	8.38479776981058E-186	2	KLHL20
2.5156150680193E-243	0.313337765236458	0.441	0.163	4.21566773098674E-239	2	CNIH4
7.70904262886547E-168	0.313187773342028	0.294	0.107	1.29188136374527E-163	2	COG8
3.55208138674884E-176	0.313161438918602	0.331	0.125	5.95257798791371E-172	2	RNF38
1.34709266788955E-254	0.313121561982055	0.529	0.209	2.2574578928493E-250	2	VPS26C
2.07193591504256E-252	0.313015251177534	0.522	0.206	3.47215020642831E-248	2	MIER1
2.34798117007831E-205	0.312942298969452	0.834	0.423	3.93474684481723E-201	2	EGLN1
2.66351480321989E-174	0.312874267567469	0.908	0.505	4.46351810723589E-170	2	RBM47
5.06472442620584E-179	0.312812778873893	0.275	0.094	8.48746519343575E-175	2	APOO
3.74046026241254E-218	0.312782150828853	0.412	0.156	6.26826330775093E-214	2	PREPL
2.94691674092873E-180	0.312746627949522	0.268	0.089	4.93844307444837E-176	2	TTC19
1.87321226618809E-199	0.312739303321543	0.347	0.127	3.13912911567801E-195	2	N4BP1
3.59442630656313E-278	0.312590499596062	0.604	0.245	6.02353960453849E-274	2	PMPCB
4.35363962041695E-231	0.312532367399795	0.738	0.344	7.29582927589473E-227	2	KANK1
1.17637225161231E-246	0.312148337629789	0.631	0.274	1.97136461925191E-242	2	NEU1
3.54920695800873E-236	0.312040592891753	0.424	0.157	5.94776102023103E-232	2	METTL14
5.22268624698556E-208	0.311967592453157	0.809	0.41	8.7521776126984E-204	2	PDIA5
8.46625724813224E-265	0.311829858273939	0.68	0.291	1.418775389642E-260	2	NRDC
1.15462804801903E-254	0.311795475605433	0.546	0.218	1.93492568287029E-250	2	DERA
1.66666144178763E-183	0.311493715443185	0.419	0.175	2.79299124414771E-179	2	TMEM87A
3.7704688243126E-241	0.311402287209369	0.391	0.137	6.31855165578306E-237	2	RXRB
1.79435074794558E-218	0.311203975232862	0.404	0.152	3.0069729834072E-214	2	RETREG2
9.12771026491824E-202	0.310637191229169	0.384	0.145	1.529621686195E-197	2	ARMC1
3.01942658715399E-257	0.310619959038055	0.636	0.269	5.05995507475266E-253	2	UBE2V1
1.39923661395749E-215	0.310592316762003	0.422	0.163	2.34484071766997E-211	2	ALG12
1.74772555436238E-174	0.310537174825436	0.298	0.108	2.92883848400048E-170	2	POLR3C
6.41140634420366E-248	0.309995024834196	0.683	0.301	1.07442347516165E-243	2	UBL3
2.39348878715154E-182	0.309908219261894	0.787	0.413	4.01100850950855E-178	2	MSMO1
2.35221510214724E-176	0.309869212946744	0.342	0.131	3.94184206817835E-172	2	DDX59
3.18602640728528E-291	0.30945601466296	0.536	0.201	5.33914305332868E-287	2	GNG12
3.05082522570203E-187	0.309318579401505	0.31	0.11	5.11257291323147E-183	2	FNBP1
4.51245097122868E-230	0.309260036818114	0.365	0.126	7.56196533758502E-226	2	PRPF8
1.54856245149145E-249	0.308848904354705	0.78	0.349	2.59508095620937E-245	2	NECAB3
1.91863127169496E-249	0.30883061705311	0.537	0.215	3.21524228510642E-245	2	EIF2S1
6.66771422338196E-215	0.308797293629608	0.921	0.509	1.11737554955435E-210	2	VMP1
6.80471464386089E-254	0.308718270675687	0.69	0.304	1.14033408001821E-249	2	NDUFV3
1.43725105852636E-232	0.308515132806127	0.453	0.173	2.40854532387848E-228	2	ICK
4.78629193265897E-200	0.308276247331409	0.327	0.115	8.02086802074991E-196	2	COPS7B
2.5229386694792E-158	0.307767212933029	0.26	0.092	4.22794062231325E-154	2	MARF1
9.23878409537912E-144	0.307399027422816	0.275	0.106	1.54823543870363E-139	2	HPS4
4.85611009646393E-211	0.307155807053338	0.699	0.335	8.13786929965426E-207	2	FDFT1
1.20033155045362E-267	0.306984768416734	0.599	0.239	2.01151561225018E-263	2	FAM214A
6.08328206622237E-185	0.306958437132058	0.273	0.091	1.01943640865754E-180	2	MOB2

4.07934578428838E-241	0.306915720524893	0.385	0.134	6.83616766531046E-237	2	CIZ1
1.88958758696506E-192	0.306707660017184	0.773	0.395	3.16657087823606E-188	2	TMEM33
1.88727157388054E-156	0.30652633317038	0.279	0.104	3.16268970350901E-152	2	ELP4
2.51400697351909E-231	0.306429365511312	0.511	0.208	4.21297288622329E-227	2	MSL2
3.71168315726689E-177	0.306423000181183	0.258	0.085	6.22003863494786E-173	2	LARGE1
7.89784041185172E-231	0.306180715205932	0.652	0.292	1.32352009621811E-226	2	AHCYL1
1.03002187616923E-246	0.3058597325921	0.604	0.254	1.72611066008439E-242	2	BACH1
1.82052213666116E-210	0.305796000269349	0.36	0.13	3.05083099661676E-206	2	CYB561A3
1.17817486432631E-233	0.305493236272873	0.483	0.19	1.97438543763803E-229	2	ARFGEF2
4.25740205761126E-260	0.305368486391649	0.629	0.27	7.13455436814495E-256	2	HEBP1
7.16103425181762E-193	0.305128782620259	0.391	0.154	1.2000461199196E-188	2	USP38
5.60250533666924E-247	0.305014956783744	0.569	0.235	9.38867844319032E-243	2	YTHDC1
7.81622715370407E-225	0.304984745642484	0.4	0.147	1.30984334641773E-220	2	TCF20
2.11186376363797E-247	0.304921620406602	0.427	0.155	3.53906129510451E-243	2	DAG1
4.50038082882162E-231	0.304851041910692	0.394	0.142	7.54173819293928E-227	2	MBP
4.5934377608761E-176	0.304696327746853	0.322	0.12	7.69768299967618E-172	2	TNKS2
1.51451561362385E-215	0.304693207548118	0.421	0.163	2.53802526531084E-211	2	LARP1B
1.23519011263832E-242	0.304524851761424	0.674	0.302	2.06993159075929E-238	2	CCT6A
1.22994811710603E-247	0.304230512780355	0.576	0.239	2.06114705464628E-243	2	LIME1
9.06026927876237E-271	0.303955395938231	0.671	0.282	1.518319925735E-266	2	PYGO2
7.83216156641945E-167	0.303893097792131	0.278	0.099	1.31251363530057E-162	2	P3H1
1.77937096110217E-186	0.303592838701706	0.299	0.104	2.98186985661502E-182	2	RIOK2
1.16047053551611E-240	0.303565592316048	0.469	0.18	1.94471652341789E-236	2	TAOK3
5.05362963869191E-222	0.30300900486404	0.393	0.144	8.46887254851991E-218	2	RPUSD3
1.21610510776186E-272	0.302877694017817	0.599	0.243	2.03794893958733E-268	2	CLIP1
2.69168728042472E-262	0.302846922242342	0.552	0.219	4.51072954453575E-258	2	ANAPC5
1.72869709373118E-218	0.301994287518516	0.795	0.382	2.89695058967471E-214	2	VBP1
6.82986979498802E-268	0.301717304252089	0.671	0.289	1.14454958024409E-263	2	MLX
3.78264607914335E-212	0.30163571977202	0.431	0.169	6.33895829942843E-208	2	TBC1D16
5.69274674790645E-224	0.301590569414123	0.44	0.17	9.53990500014163E-220	2	LSG1
2.06562453411579E-236	0.301416595917258	0.625	0.272	3.46157359427124E-232	2	DYNLT1
1.40955121538549E-266	0.301083226382335	0.432	0.151	2.362125926743E-262	2	CASP4
2.26950851513967E-158	0.301047591788237	0.271	0.097	3.80324236967106E-154	2	OSGIN2
7.18277555498899E-204	0.300863560186388	0.437	0.177	1.20368952750505E-199	2	TNKS
2.6676739390712E-259	0.300465817168509	0.576	0.234	4.47048798709552E-255	2	DYNC1I2
5.60279944577202E-220	0.300425999703384	0.44	0.171	9.38917131122475E-216	2	ACTL6A
1.4522267189954E-186	0.300323036610897	0.341	0.126	2.43364153569248E-182	2	CDT1
1.66494961219122E-217	0.300299170400637	0.385	0.142	2.79012256011004E-213	2	GTPBP1
1.15471795560575E-235	0.300298806485383	0.309	0.095	1.93507635000412E-231	2	FRMD4A
2.27388416197869E-185	0.300228190587271	0.313	0.112	3.81057507864389E-181	2	AIFM2
2.44115351433566E-182	0.300116516329796	0.348	0.133	4.0908850593237E-178	2	MAP3K3
2.3140827871445E-271	0.300089275913987	0.496	0.184	3.87793993469675E-267	2	UBTD2
1.19555838512231E-163	0.300058292636145	0.273	0.097	2.00351674178796E-159	2	TAB1
1.63418711165969E-236	0.299936062749183	0.544	0.224	2.73857076171932E-232	2	RBM17
3.10555184400806E-228	0.299873162309078	0.766	0.366	5.2042837801887E-224	2	SLBP
4.52528986587617E-164	0.299837099392609	0.27	0.096	7.58348075723529E-160	2	ZFYVE1
6.99395847081488E-258	0.299644015181815	0.336	0.102	1.17204756053916E-253	2	IQCE
5.02895089923086E-253	0.29937450412193	0.618	0.262	8.42751591693107E-249	2	FPGS
1.80178592054031E-256	0.299349523351209	0.543	0.214	3.01943284564145E-252	2	TEAD1
8.6375964666247E-200	0.298952118043915	0.313	0.107	1.44748841587697E-195	2	RBM28
1.17175946828363E-240	0.298669507589213	0.736	0.338	1.96363451694971E-236	2	ACBD3
5.55577722433478E-260	0.298403779530537	0.55	0.219	9.31037147254022E-256	2	HGH1
8.58912964779279E-140	0.298333294413354	0.264	0.1	1.43936634637712E-135	2	RPAP3
8.0364502767148E-165	0.298131184411117	0.274	0.097	1.34674833737187E-160	2	RNF214
6.46293650177788E-263	0.298122218101089	0.591	0.242	1.08305889896794E-258	2	LRPPRC
8.96010054800821E-260	0.298001787561678	0.577	0.233	1.50153364983522E-255	2	RNF4
2.05779227463481E-226	0.297964645178105	0.407	0.15	3.44844829383302E-222	2	HERPUD2
1.27502242051172E-259	0.29774695405432	0.492	0.187	2.13668257229354E-255	2	DEAF1
1.39768310720762E-186	0.297556956839728	0.344	0.129	2.34223735105854E-182	2	SNRPG
2.60114281391859E-231	0.297529563081934	0.364	0.125	4.35899512756477E-227	2	THOC6

1.29064473472649E-192	0.297507622588571	0.335	0.122	2.16286244645465E-188	2	ZFYVE9
3.45094114657567E-179	0.297272587766955	0.271	0.091	5.78308717343151E-175	2	POLR1A
1.28560188149338E-259	0.297023879529444	0.536	0.21	2.15441163300661E-255	2	ATP6V1C1
3.78188298296216E-176	0.296977396839952	0.89	0.525	6.337679502848E-172	2	TGOLN2
7.39552404473384E-206	0.296666840402975	0.405	0.156	1.2393419194165E-201	2	VPS37A
4.75692084599772E-247	0.296502038347009	0.732	0.342	7.97164795372299E-243	2	MMP24OS
2.7485090452499E-213	0.296177092102453	0.373	0.136	4.60595145802979E-209	2	DIP2B
3.49411053039995E-170	0.29613716886122	0.277	0.097	5.85543042684423E-166	2	PRELID3B
7.06672567747825E-201	0.29609198242197	0.321	0.111	1.1842418890318E-196	2	PRPF4
3.31065733123273E-242	0.296088370155761	0.487	0.19	5.54799955567982E-238	2	RNF13
8.60711427698923E-246	0.29598452195469	0.467	0.176	1.44238021053786E-241	2	CRELD1
5.96669939956773E-276	0.295693855827121	0.544	0.211	9.9989948537956E-272	2	SIAE
1.46561802125816E-238	0.295533431687551	0.577	0.241	2.45608268002443E-234	2	TBCD
3.93810334477842E-230	0.295482793752246	0.556	0.227	6.59947358517968E-226	2	ZNF189
6.93263249999744E-236	0.295478814455908	0.473	0.185	1.16177055434957E-231	2	FBXW7
5.07742477743054E-193	0.295478319310458	0.315	0.11	8.5087484420181E-189	2	POFUT2
2.2696418090462E-169	0.29539580944613	0.3	0.11	3.80346574359963E-165	2	GCFC2
1.64258587175587E-200	0.295289784979213	0.758	0.378	2.75264540388849E-196	2	MCFD2
2.90743628474766E-242	0.295072691459808	0.626	0.266	4.87228172598013E-238	2	PLSCR1
1.23231087041382E-196	0.294994048390333	0.323	0.113	2.06510655663948E-192	2	RSF1
4.62375059705909E-253	0.294961633419381	0.663	0.288	7.74848125055163E-249	2	FAM177A1
2.221636308989E-185	0.294934940036243	0.296	0.102	3.72301812660377E-181	2	MRRF
7.15643911872193E-180	0.29490630312508	0.319	0.117	1.19927606751542E-175	2	PRUNE1
3.57776205261691E-135	0.29488218106325	0.268	0.105	5.99561364777542E-131	2	GKAP1
1.61432966855083E-229	0.294814001138119	0.39	0.139	2.70529365855748E-225	2	CCND3
2.05004669540107E-123	0.294803840634203	0.966	0.597	3.43546825215311E-119	2	ACSL4
8.65526610712401E-255	0.2947044160566	0.704	0.311	1.45044949423184E-250	2	LGR4
1.9212153684503E-262	0.294440821235344	0.721	0.316	3.21957271444902E-258	2	HUWE1
1.67557469674469E-279	0.294357912395487	0.68	0.291	2.80792807680475E-275	2	CTDSP1
4.48733015009867E-238	0.294147409412629	0.722	0.331	7.51986786553535E-234	2	IMPAD1
9.16743250420542E-223	0.294067085149351	0.409	0.152	1.53627833905474E-218	2	ZNF275
1.87587070598426E-265	0.293993354656495	0.509	0.195	3.14358412908843E-261	2	KIAA2013
1.38447880188302E-190	0.293619902225362	0.318	0.113	2.32010957619556E-186	2	LRRC8D
4.26681704184295E-241	0.293584846224005	0.474	0.182	7.15033199872042E-237	2	RNF113A
7.42821233819669E-215	0.29343326807565	0.464	0.187	1.244819823635E-210	2	CUL3
5.16463997869038E-260	0.293389617576678	0.537	0.212	8.65490367628934E-256	2	AFG3L2
1.21974578469976E-196	0.293341444026102	0.301	0.102	2.04404998599986E-192	2	GLG1
3.25262060021584E-240	0.293227701201674	0.47	0.18	5.4507416018417E-236	2	LRP6
7.72737164953553E-176	0.293053834235378	0.298	0.106	1.29495294102916E-171	2	EIF4EBP3
1.04269262379484E-158	0.292676178160359	0.305	0.117	1.7473442989554E-154	2	SIMC1
1.5971868809711E-214	0.292638101073642	0.427	0.166	2.67656577513136E-210	2	NMI
2.50860505000412E-125	0.292362680729129	0.98	0.735	4.20392034279691E-121	2	CYP3A5
4.47124432116382E-158	0.2922930778356	0.283	0.105	7.49291123340633E-154	2	DAAM1
1.63344559142468E-212	0.292091601186029	0.451	0.178	2.73732812210948E-208	2	USP1
8.70395753205022E-170	0.29186718314857	0.825	0.455	1.45860920322098E-165	2	MGST2
1.61348136873214E-158	0.291665855758279	0.874	0.537	2.70387207772133E-154	2	TSTA3
1.28522987931601E-198	0.291372182590414	0.361	0.135	2.15378823175776E-194	2	E4F1
7.0791586898683E-191	0.291288794017908	0.349	0.13	1.18632541324813E-186	2	BRIX1
6.24073473566679E-206	0.29079343839262	0.295	0.096	1.04582232700304E-201	2	BBX
3.74588862018865E-239	0.290580744455923	0.579	0.244	6.27736014971213E-235	2	SELENOS
3.4948992915848E-131	0.290511926293075	0.27	0.106	5.8567522328378E-127	2	FANCI
1.77618790580173E-202	0.290434833413523	0.367	0.136	2.97653569254254E-198	2	PLEKHA3
8.19730934636599E-242	0.290405919680577	0.683	0.304	1.37370510026401E-237	2	NDUFS1
4.9771839987765E-214	0.290085381750501	0.377	0.139	8.34076494514966E-210	2	ARIH2
1.82431327877329E-243	0.290040673623581	0.452	0.17	3.05718419256827E-239	2	ITSN2
6.92401547877429E-235	0.289875162706415	0.615	0.266	1.160326513933E-230	2	PPP4R3B
2.62407036606412E-279	0.289808071848147	0.542	0.207	4.39741711945025E-275	2	CTDSPL
4.26642427891649E-193	0.289766511545141	0.814	0.43	7.14967380660825E-189	2	GRN
1.39683486461528E-236	0.289650231220228	0.447	0.168	2.34081586612228E-232	2	TNRC6B
8.82093567763381E-201	0.289567676170511	0.808	0.413	1.47821240085787E-196	2	PDZK1

2.03541431299635E-189	0.28909858331105	0.305	0.106	3.41094730571928E-185	2	C1orf52
3.46531054908464E-248	0.289062738051869	0.47	0.178	5.80716741815605E-244	2	VAPB
2.21781722973653E-230	0.289013007713172	0.443	0.17	3.71661811359248E-226	2	FBXW4
9.92321526628265E-196	0.289008451449705	0.373	0.141	1.66293241432365E-191	2	BTG3
1.05743698295388E-188	0.288798900942657	0.379	0.149	1.77205289603412E-184	2	SLC23A1
1.99948756708448E-242	0.288697295445622	0.515	0.204	3.35074126492018E-238	2	ELK4
2.27004251471496E-181	0.288299199620846	0.306	0.109	3.80413724615933E-177	2	CNTNAP2
6.56500989478922E-197	0.288238057635397	0.366	0.137	1.10016435816878E-192	2	MOB4
2.98095406762258E-251	0.288086933583699	0.58	0.241	4.99548282652192E-247	2	TXNDC15
6.30346380110502E-251	0.287682835715245	0.561	0.228	1.05633446378918E-246	2	RUFY1
3.31936599796546E-158	0.287579614034565	0.268	0.096	5.56259353939051E-154	2	BRD9
1.1489987783811E-166	0.287576871038718	0.293	0.107	1.92549215281105E-162	2	PIGV
1.51579270359641E-169	0.287516439179392	0.329	0.127	2.54016541268686E-165	2	RAPGEF4
1.40731102245534E-218	0.28744676216494	0.369	0.132	2.35837181143065E-214	2	KAT2B
3.38375424658349E-160	0.287429791192567	0.298	0.113	5.67049536642462E-156	2	PKNOX1
3.89280320495563E-259	0.286960983048389	0.569	0.23	6.52355961086464E-255	2	AGGF1
6.87669766955301E-223	0.286899396385803	0.398	0.146	1.15239699546369E-218	2	ATXN2
5.63304824273966E-222	0.286821300888579	0.46	0.182	9.43986224518312E-218	2	TBC1D15
1.01594395660459E-189	0.286665832326691	0.297	0.102	1.70251888247797E-185	2	WDR43
3.5914995641909E-253	0.28655390012394	0.592	0.245	6.01863496967111E-249	2	USO1
1.13273074302764E-241	0.286222919889471	0.397	0.139	1.89823017916571E-237	2	NF2
1.01309947950669E-154	0.286067968510026	0.254	0.09	1.69775210775732E-150	2	PEX5
1.90803014616886E-265	0.286044964491967	0.599	0.243	3.19747691894978E-261	2	MSL1
4.80164744980531E-216	0.286040444514417	0.736	0.359	8.04660079638374E-212	2	RNPEP
1.53964358786568E-246	0.285808998377395	0.637	0.273	2.58013472454531E-242	2	DEPRECATED-ENSG00000133142
7.33004499521043E-210	0.285474551234904	0.798	0.389	1.22836894029736E-205	2	TCEAL4
6.36924315520364E-261	0.285414566536058	0.534	0.21	1.06735776794903E-256	2	GRIPAP1
3.00045364325959E-211	0.285298069829749	0.4	0.151	5.02816021537442E-207	2	SLC39A3
1.72047778865716E-244	0.285221099637531	0.522	0.207	2.88317667823167E-240	2	SH3GL1
5.47952354946363E-205	0.28485585205632	0.419	0.165	9.18258556419115E-201	2	SLC20A2
1.21907817717537E-222	0.284772299675861	0.796	0.375	2.04293120931048E-218	2	FMR1
1.80312586764061E-202	0.284770678513137	0.324	0.112	3.02167832899214E-198	2	HS2ST1
3.07417827310704E-254	0.284709456808908	0.452	0.165	5.15170795007278E-250	2	ARHGEF18
1.82181647752807E-178	0.284438860360392	0.252	0.081	3.05300005304153E-174	2	RCBTB1
1.8283830384174E-251	0.283828431797145	0.466	0.173	3.06400429577987E-247	2	CENPX
7.35876548290489E-145	0.283735316512786	0.253	0.093	1.2331819196252E-140	2	KLC2
2.73415843744742E-151	0.283321691232759	0.262	0.095	4.58190270947439E-147	2	BICDL1
9.85664570278996E-151	0.283114344784831	0.277	0.104	1.65177668687354E-146	2	ZNF414
4.08618314341219E-261	0.28309552333395	0.482	0.18	6.84762571173015E-257	2	ACO2
1.17413090318104E-149	0.283092578291625	0.277	0.104	1.96760856755078E-145	2	ZCCHC8
6.52084380145514E-189	0.28302377859015	0.838	0.441	1.09276300424785E-184	2	YWHAZ
1.49076807749913E-210	0.282925430473443	0.765	0.372	2.49822914427304E-206	2	PBLD
3.79079420370252E-188	0.28280476922316	0.319	0.115	6.35261292656468E-184	2	DHX34
1.8042447174437E-167	0.282722996448618	0.256	0.087	3.02355329749215E-163	2	PRDM2
4.05288503857417E-223	0.282475394727801	0.402	0.149	6.7918247476426E-219	2	INO80C
4.39537533532503E-188	0.282226272486623	0.321	0.115	7.36576998693768E-184	2	ADAT1
2.76953138024468E-171	0.282168687245645	0.33	0.126	4.64118068701403E-167	2	SNRNP48
5.01883221200781E-215	0.282140013169998	0.796	0.385	8.41055902088268E-211	2	ILF3
7.7169145307381E-211	0.282059567754357	0.801	0.395	1.29320053706109E-206	2	FAM114A1
2.22903228886727E-236	0.281747137587105	0.495	0.197	3.73541230968377E-232	2	ZNF444
1.55807732726658E-178	0.281549849219857	0.904	0.518	2.61102598503333E-174	2	TMEM106C
4.77943700671823E-170	0.281542542194693	0.257	0.086	8.00938053585842E-166	2	INAFM1
4.27672281116392E-236	0.281271357642842	0.468	0.181	7.1669320869485E-232	2	SNX9
4.35624815346736E-223	0.281262307346393	0.549	0.233	7.30020065558061E-219	2	PRKAB2
2.59247824353391E-290	0.28121417186898	0.529	0.196	4.34447504051413E-286	2	EEPD1
6.16499948327045E-157	0.281209619353494	0.301	0.115	1.03313061340646E-152	2	RNASEH1
1.14073757700701E-118	0.281082173067579	0.987	0.75	1.91164803154834E-114	2	UGDH
3.47796879872337E-275	0.28108164376374	0.627	0.257	5.82838011290062E-271	2	CDC42BPB
2.18071029363215E-281	0.281075204255883	0.667	0.279	3.65443431006875E-277	2	FAM83H
1.81346277664768E-218	0.281068592501383	0.354	0.124	3.03900092110618E-214	2	EHD1

5.36756646819593E-224	0.280996095827659	0.439	0.168	8.99496788740274E-220	2	GON7
2.48513468847007E-225	0.280704960805782	0.424	0.159	4.16458871093814E-221	2	RPRD1B
1.07311418737757E-215	0.280703975777471	0.391	0.144	1.79832475520733E-211	2	AKIRIN1
1.60667817692237E-222	0.280022688065146	0.438	0.168	2.6924712888865E-218	2	GRSF1
2.22098014635202E-194	0.278882713686227	0.333	0.12	3.72191852925672E-190	2	FOXK1
3.75968579806505E-147	0.278566193501392	0.265	0.099	6.30048146039741E-143	2	ING5
6.22933769529471E-231	0.278363969637991	0.472	0.185	1.04391241097749E-226	2	DOK4
5.68341935160171E-193	0.278264959696506	0.315	0.111	9.52427414941414E-189	2	UBLCP1
2.12923483834931E-270	0.278192714367929	0.555	0.218	3.56817174210577E-266	2	ANKRD11
3.0386292232454E-270	0.278107044452596	0.589	0.237	5.09213485231465E-266	2	PTBP3
1.48109598560647E-249	0.27806606989168	0.567	0.233	2.48202065267933E-245	2	SLC39A9
5.59805009604427E-168	0.277994964546679	0.256	0.086	9.38121235095098E-164	2	TMEM218
6.55388487040321E-234	0.277991794055017	0.499	0.199	1.09830002658217E-229	2	UBR7
4.91107956477101E-175	0.277984671722347	0.872	0.445	8.22998713464326E-171	2	MORF4L2
9.75768059107427E-251	0.277950459718564	0.662	0.292	1.63519211345223E-246	2	VASP
3.49251899961056E-239	0.277874927892921	0.49	0.191	5.85276333954738E-235	2	ATRAID
6.04834722368129E-246	0.277476433810834	0.62	0.267	1.01358202774451E-241	2	LIX1L
1.02302646229606E-226	0.277351156097545	0.429	0.163	1.71438774551574E-222	2	GTF3A
4.28013511877329E-195	0.277096895848932	0.381	0.147	7.17265043204028E-191	2	CNOT2
4.05875094759968E-163	0.27684262417365	0.277	0.1	6.80165483798755E-159	2	ATAD1
4.45965410629137E-225	0.276826393583219	0.731	0.34	7.47348835132309E-221	2	DCAF6
2.15466021628184E-169	0.276732310295432	0.25	0.083	3.6107795904451E-165	2	DEPRECATED-ENSG00000130305
2.27890115111274E-157	0.276271406941935	0.313	0.122	3.81898254903472E-153	2	HAUS2
1.11713422701681E-175	0.276237427617872	0.273	0.093	1.87209353763476E-171	2	HDAC4
8.27365610964834E-254	0.276179843149051	0.659	0.28	1.38649929085487E-249	2	FZR1
8.76533150172896E-190	0.276164883213308	0.378	0.146	1.46889425305974E-185	2	PCIF1
1.65188004372071E-246	0.275982137926526	0.7	0.311	2.76822057726717E-242	2	EIF4A2
1.00081057112744E-180	0.275671949871346	0.33	0.123	1.67715835509537E-176	2	SRSF1
2.47034296530412E-193	0.275637460072936	0.428	0.175	4.13980074125665E-189	2	PGM3
2.70280258027596E-203	0.275628391197654	0.392	0.149	4.52935656402645E-199	2	RBX1
3.76829810922984E-248	0.275610654950319	0.594	0.248	6.31491397144737E-244	2	TAOK1
2.16806205573337E-190	0.275348584907044	0.3	0.103	3.63323839299798E-186	2	CASP9
8.8042704127896E-244	0.275287792167908	0.519	0.206	1.47541963577528E-239	2	RSRC1
3.97994931773582E-169	0.275181209231308	0.263	0.09	6.6695990666617E-165	2	PFDN1
3.25788060901775E-214	0.275063452601234	0.558	0.236	5.45955632459194E-210	2	ST3GAL6
1.89305599159241E-213	0.27469895567593	0.7	0.331	3.17238323071056E-209	2	HEXB
2.05635914125412E-229	0.274637169112455	0.436	0.166	3.44604664891365E-225	2	GTF3C3
2.95118775340775E-221	0.274600871978921	0.697	0.317	4.94560043716071E-217	2	DTX3L
1.64251506888615E-205	0.274176147556202	0.385	0.145	2.75252675243941E-201	2	IFI27L1
8.14732022399943E-205	0.273691950477679	0.428	0.17	1.36532792313782E-200	2	ST7
3.99190874346524E-238	0.273658192726918	0.476	0.184	6.68964067229905E-234	2	ROCK1
4.68243122823569E-241	0.273504903480045	0.458	0.174	7.84681825227737E-237	2	SRCAP
1.79417980926676E-224	0.273428959627491	0.591	0.257	3.00668652436924E-220	2	EMC3
4.30379422115432E-201	0.273410699670583	0.349	0.126	7.21229835581041E-197	2	ARFIP2
3.65815929709212E-242	0.273361490916405	0.618	0.269	6.13034335006697E-238	2	APRT
8.67960758755197E-185	0.273094565834441	0.387	0.154	1.45452863952196E-180	2	ITGB1BP1
2.23089070002817E-266	0.27296839446162	0.521	0.2	3.73852663510721E-262	2	HDHD5
9.16355542869152E-149	0.272892816243818	0.251	0.09	1.53562861874012E-144	2	FAM210A
1.78990049177718E-230	0.272878066934401	0.658	0.293	2.9995152441202E-226	2	PSMD1
5.30948181285678E-260	0.272682318726608	0.598	0.245	8.89762962198539E-256	2	PAFAH1B1
1.14877650534924E-179	0.272410002457136	0.352	0.136	1.92511966766425E-175	2	STYX
1.85123140640748E-210	0.272391249978538	0.509	0.213	3.10229359085765E-206	2	SUZ12
9.01947899180353E-245	0.27236606451876	0.529	0.213	1.51148428944644E-240	2	DCTD
9.17640691597119E-149	0.272078971837833	0.266	0.098	1.53778227097845E-144	2	CTDP1
1.5876383721222E-246	0.271690446562823	0.59	0.247	2.66056438400238E-242	2	NPEPPS
1.69647120337749E-246	0.271401080918013	0.588	0.244	2.84294644262E-242	2	CEP350
1.37042608053345E-193	0.27134987173683	0.351	0.13	2.29656002575795E-189	2	DCAKD
1.32950411649871E-180	0.271334989479917	0.322	0.119	2.22798299842853E-176	2	ZNF76
3.7159259680418E-197	0.2712223561127	0.42	0.168	6.22714873724446E-193	2	TAF11
5.6063937699168E-207	0.271046936289626	0.365	0.133	9.39519467962657E-203	2	HNRNPLL

6.2462602794483E-269	0.270944532234039	0.541	0.21	1.04674829762995E-264	2	ARHGEF7
3.33235352220851E-163	0.270627304973035	0.367	0.151	5.58435803251703E-159	2	PM20D2
2.34411284428587E-177	0.270537428073536	0.304	0.109	3.92826430445427E-173	2	TMEM126B
7.36168154226775E-181	0.270369160526646	0.32	0.117	1.23367059285323E-176	2	RWDD1
3.46308110973352E-228	0.269992552997865	0.605	0.263	5.80343132369144E-224	2	SKP1
2.23729258725979E-242	0.269978031772796	0.549	0.226	3.74925491772995E-238	2	IRF2
3.75025399411759E-226	0.269757918608642	0.669	0.3	6.28467564334226E-222	2	DDX6
1.46355180595379E-259	0.269742739149392	0.465	0.171	2.45262011641736E-255	2	PXN
1.27267111652817E-188	0.269552953751316	0.39	0.154	2.13274225707791E-184	2	TRIM47
1.58057805092284E-241	0.269282895648606	0.548	0.224	2.6487326977365E-237	2	KCTD20
1.44428099056021E-141	0.269080140259077	0.297	0.119	2.4203260839808E-137	2	EDC3
5.53530600166059E-261	0.268330225293595	0.508	0.194	9.27606579758282E-257	2	WDTC1
1.85199956933427E-236	0.268282477598313	0.518	0.21	3.10358087829037E-232	2	HIPK1
3.89490135502616E-216	0.268149876008928	0.397	0.147	6.52707569075284E-212	2	RFC2
1.98782141864928E-139	0.268032517145032	0.921	0.574	3.33119113337246E-135	2	CRYL1
1.65909948622844E-176	0.267927780221442	0.301	0.108	2.78031891902162E-172	2	ATXN1L
2.47221559593618E-210	0.26784140258645	0.404	0.154	4.14293889566985E-206	2	RBM22
4.87761099522173E-165	0.267742719378988	0.256	0.087	8.17390050579258E-161	2	FAM189B
2.61869770971265E-174	0.267553851521201	0.85	0.45	4.38841362193646E-170	2	MTDH
1.6393780879923E-248	0.267430191819209	0.471	0.178	2.7472697998575E-244	2	CNPPD1
4.90493089854488E-248	0.267311108146942	0.411	0.145	8.21968319978152E-244	2	ARL6IP5
5.07262576850626E-167	0.267248297638367	0.254	0.085	8.50070626286278E-163	2	SLC35A5
4.17792331117524E-221	0.267066326527926	0.406	0.152	7.00136388486747E-217	2	TICAM1
3.59746133042363E-176	0.266463642101957	0.298	0.106	6.02862569752392E-172	2	RAE1
1.09581854610471E-207	0.266313502625462	0.323	0.111	1.83637271956227E-203	2	KLF16
1.69493504261117E-166	0.266218384427096	0.288	0.105	2.8403721444078E-162	2	WARS
1.09332715010294E-208	0.266200047855588	0.411	0.159	1.83219763814251E-204	2	INTS4
3.73145975139526E-248	0.266040663071043	0.658	0.283	6.25318025138818E-244	2	DDX46
2.0536383269484E-223	0.265971865299068	0.451	0.175	3.44148710830013E-219	2	EHMT2
2.31107173931768E-272	0.265676089236325	0.475	0.172	3.87289402074856E-268	2	TRAK1
4.40075638263264E-239	0.265638693258401	0.503	0.199	7.37478754601579E-235	2	ELOVL6
1.291526958442E-169	0.265596083001721	0.861	0.477	2.1643408769571E-165	2	TMEM205
9.62066294795794E-271	0.265368107186427	0.683	0.294	1.61223069681879E-266	2	KIAA0100
1.73191594965437E-199	0.265264680825281	0.453	0.186	2.90234474843079E-195	2	PLEKHG3
3.20368187598906E-252	0.265214695054918	0.727	0.329	5.36873008778246E-248	2	MTCH1
6.27050132185725E-218	0.265153260868536	0.76	0.367	1.05081061151684E-213	2	AK4
3.03638384280446E-245	0.265107236757027	0.53	0.214	5.08837204377171E-241	2	EIF2D
0	0.265100587668615	0.701	0.288	0	2	ERBB3
3.13560592284794E-158	0.26494800586779	0.312	0.121	5.25464840550857E-154	2	DDX52
2.54950054897965E-200	0.264817140919147	0.3	0.1	4.2724530199801E-196	2	ZPR1
2.58756315874382E-217	0.264686768133369	0.605	0.266	4.33623834142289E-213	2	MAST4
8.96706743595378E-238	0.264639270124458	0.504	0.201	1.50270116091713E-233	2	RASSF4
6.34346357719213E-162	0.26463055578971	0.283	0.103	1.06303762626586E-157	2	RRP15
9.23251558646959E-189	0.264543230885084	0.367	0.141	1.54718496198057E-184	2	NAGA
4.20428265551871E-198	0.264387936873549	0.95	0.543	7.04553687411826E-194	2	SMARCA1
4.03688041362705E-216	0.264144382090259	0.746	0.343	6.76500419715621E-212	2	RREB1
1.02692292576727E-238	0.263985941276405	0.416	0.151	1.72091743900079E-234	2	ARF5
4.66806232727314E-228	0.263819599376124	0.728	0.339	7.82273884804433E-224	2	EMC2
3.74368192062032E-143	0.263705395539902	0.856	0.509	6.27366216257552E-139	2	MGST3
2.1086046787329E-204	0.263232040379987	0.379	0.142	3.5335997206206E-200	2	FBXO3
1.14140356121996E-256	0.263121644292359	0.592	0.245	1.91276408789241E-252	2	PSMC2
2.27252793815124E-239	0.263085397646968	0.468	0.18	3.80830231875384E-235	2	AKIP1
1.96915605692793E-228	0.262954366868783	0.511	0.207	3.29991172019983E-224	2	EIF3H
8.18377055325478E-162	0.262732798808291	0.257	0.089	1.37143626931444E-157	2	F8
2.64389618792884E-174	0.262713242664508	0.312	0.115	4.43064123173115E-170	2	CCNT1
1.49233447503626E-221	0.262701317538252	0.332	0.111	2.50085411326577E-217	2	CSNK1E
5.41894203126563E-166	0.262606932143983	0.293	0.107	9.08106305599494E-162	2	ACP6
4.37839065515162E-246	0.262272681696705	0.461	0.174	7.33730705990308E-242	2	ROCK2
9.0428765443419E-218	0.262032230667181	0.432	0.167	1.51540525130082E-213	2	THUMPD1
1.33650070418694E-214	0.262025534609609	0.415	0.158	2.23970788007648E-210	2	CSTF2

5.7658169154233E-176	0.261754551864859	0.29	0.102	9.66235598686637E-172	2	TOMM40
3.53451935274325E-194	0.261423847252039	0.334	0.121	5.92314753132714E-190	2	TINF2
6.25300455498805E-194	0.261158269305827	0.331	0.119	1.0478785033249E-189	2	RBBP5
7.30685452817814E-179	0.260966890860646	0.355	0.137	1.22448268183209E-174	2	ING2
2.15647420019937E-230	0.260852514999067	0.326	0.105	3.6138194646941E-226	2	ARHGEF5
1.65809079500947E-247	0.260783311496617	0.506	0.198	2.77862855427687E-243	2	RETREG3
9.92291334723503E-143	0.260771951855108	0.25	0.092	1.66288181872965E-138	2	NIPSNAP2
1.64230451984822E-205	0.260705783798558	0.393	0.149	2.75217391436165E-201	2	MTRR
1.23182444851991E-223	0.260425934476146	0.466	0.184	2.06429141082966E-219	2	EPS8
5.37153572551685E-146	0.260035984577089	0.263	0.098	9.00161956882113E-142	2	CREB1
1.97689479046688E-193	0.259987292439199	0.443	0.183	3.31288028986439E-189	2	FNIP2
6.51477670484744E-189	0.259896783771061	0.338	0.124	1.09174628019833E-184	2	C1orf21
1.63091719442112E-176	0.259493638903243	0.321	0.119	2.73309103441091E-172	2	PSMA1
6.82759915891536E-240	0.259225229271746	0.653	0.285	1.14416906705104E-235	2	TACC1
1.24864476779673E-213	0.258913898433483	0.405	0.154	2.09247890187376E-209	2	TMEM115
7.29064403443085E-270	0.258699612514297	0.512	0.193	1.22176612728992E-265	2	CUX1
5.28450952533529E-183	0.258446851861873	0.372	0.146	8.85578106255689E-179	2	ZBTB44
1.09965836181243E-271	0.258425452105492	0.57	0.226	1.84280748272526E-267	2	CMIP
8.32581041266539E-226	0.258381122992168	0.415	0.154	1.39523930895447E-221	2	MTMR12
4.33095871990345E-243	0.258363639987531	0.509	0.201	7.2578206228142E-239	2	IRF2BPL
4.62011508039137E-248	0.258065934837427	0.45	0.166	7.74238885171987E-244	2	ARID1B
6.54685296628573E-111	0.257784067429687	0.967	0.726	1.09712162009016E-106	2	HSP90AA1
1.34709664627106E-209	0.257631224911608	0.72	0.342	2.25746455982105E-205	2	HADH
2.02782171213391E-198	0.257456400623498	0.434	0.177	3.398223625194E-194	2	ATF6
8.13887695881322E-185	0.257356848340745	0.378	0.148	1.36391300075792E-180	2	NDEL1
1.72675197208904E-176	0.257314948744312	0.288	0.101	2.89369095482682E-172	2	GTF3C2
3.27000571552471E-215	0.256928065264749	0.487	0.198	5.47987557807631E-211	2	RANBP9
3.86594713166921E-243	0.256371964142149	0.538	0.217	6.47855420325126E-239	2	SCFD1
5.90372204951102E-198	0.256318051856488	0.392	0.151	9.89345741057057E-194	2	LAMTOR3
3.62091208535537E-222	0.255708624844079	0.475	0.19	6.06792447263853E-218	2	TRAF2
1.36305541351242E-182	0.255590450807545	0.296	0.103	2.28420826196411E-178	2	NIPSNAP3A
1.86178158718067E-210	0.25520947013233	0.491	0.204	3.11997358379736E-206	2	AASS
4.12955742088009E-277	0.25507617919899	0.55	0.213	6.92031232591086E-273	2	KIAA1217
1.65578592913625E-197	0.255014258669779	0.679	0.325	2.77476606004653E-193	2	ARRDC2
2.18004499081701E-125	0.255011346056542	0.257	0.102	3.65331939561114E-121	2	MED21
2.83914102119021E-181	0.254894913201236	0.888	0.507	4.75783252331055E-177	2	OAF
3.51825842534325E-210	0.254766381025665	0.621	0.278	5.89589746919021E-206	2	UBXN2B
2.95206150747893E-253	0.25474125351409	0.536	0.21	4.9470646742332E-249	2	PRRC2A
8.1659096067967E-218	0.254542507481279	0.616	0.274	1.36844313190699E-213	2	DNAJB9
1.62907500414042E-237	0.254535688954475	0.747	0.349	2.73000389193851E-233	2	NCSTN
1.81903983906158E-179	0.254249099366625	0.84	0.454	3.04834696229939E-175	2	CALM2
4.22993523492689E-223	0.2541635105903	0.695	0.321	7.08852546669048E-219	2	WDR83OS
2.2262039703901E-180	0.25403904193537	0.308	0.111	3.73067261357974E-176	2	AP3S1
4.30505887442355E-222	0.253934391180761	0.462	0.182	7.21441766175899E-218	2	C6orf89
2.49864037846043E-195	0.253797483999447	0.597	0.279	4.18722154622398E-191	2	FAM162A
7.66352529733737E-221	0.253705866516609	0.528	0.221	1.2842535693278E-216	2	POMP
6.59150495540334E-251	0.25334746274984	0.627	0.266	1.10460440042649E-246	2	PPM1A
2.04297680496231E-221	0.253347270141339	0.435	0.167	3.42362052975584E-217	2	ZNF768
2.61521767351769E-234	0.253331588307568	0.568	0.239	4.38258177728094E-230	2	ELOVL2
3.04916251172646E-224	0.253156655003136	0.449	0.175	5.10978653715121E-220	2	MUS81
2.02031552869769E-217	0.253136824977253	0.766	0.365	3.38564476299159E-213	2	NPLOC4
8.30508674396281E-181	0.253132320456927	0.766	0.402	1.39176643655329E-176	2	PERP
8.17264638677439E-192	0.252795955498756	0.278	0.091	1.36957208149565E-187	2	SAMD4A
1.13577379115592E-261	0.252623339956332	0.609	0.252	1.90332971921908E-257	2	UGCG
2.51341021745527E-246	0.252099462722838	0.565	0.232	4.21197284241154E-242	2	CAMK1
7.8251660421876E-149	0.251204598709818	0.886	0.517	1.3113413253498E-144	2	MYL6
8.00166131208932E-229	0.251092016580999	0.499	0.202	1.34091840267993E-224	2	IMMT
3.58103614641214E-213	0.250948236935132	0.749	0.349	6.00110037415747E-209	2	PI4K2B
1.07610480225817E-222	0.250755058543996	0.459	0.179	1.80333642762425E-218	2	TEX261
3.5288159907774E-219	0.250678918769576	0.468	0.186	5.91358983734477E-215	2	NXT2

1.15231998486621E-135	0.250475844900505	0.876	0.562	1.9310578306388E-131	2	S100A16
1.07098808455102E-202	0.250348778086674	0.428	0.172	1.79476183209059E-198	2	NDUFAF6
1.04799497120384E-225	0.2501471536328	0.373	0.132	1.75622997274339E-221	2	MARK2
0	3.2868141356406	0.528	0.077	0	3	CES4A
0	2.79268341782228	0.443	0.098	0	3	TTN
0	2.73343432955618	0.465	0.071	0	3	PTPRM
0	2.70409534891857	0.613	0.126	0	3	ZNF436
0	2.688529483108	0.966	0.563	0	3	SMARCA1
0	2.61536217070622	0.485	0.117	0	3	CCDC141
0	2.59816825656794	0.795	0.199	0	3	MTCP1
0	2.50675874430887	0.819	0.229	0	3	FAM214A
0	2.41466278400946	0.652	0.167	0	3	PTEN
0	2.36109896195353	0.475	0.08	0	3	ALPK2
3.20613178849776E-248	2.21275152992388	0.262	0.065	5.37283565116455E-244	3	TAS2R14
0	2.1990670462347	0.944	0.399	0	3	CTPS1
0	2.18522063661763	0.38	0.088	0	3	LINS1
0	2.15792397448532	0.36	0.084	0	3	CEACAM19
0	2.13969566539531	0.338	0.064	0	3	PAPLN
9.58722367447577E-285	2.12317980246505	0.305	0.078	1.60662694336865E-280	3	PHOSPHO2
0	2.12151724768053	0.895	0.357	0	3	NECAB3
0	2.10929917281085	0.715	0.171	0	3	PLGLB2
0	2.10184692677454	0.366	0.099	0	3	NAIP
0	2.09318651573357	0.436	0.1	0	3	TSPOAP1
0	2.06909105290449	0.756	0.227	0	3	ST3GAL6
0	2.06770743800361	0.485	0.141	0	3	TSPYL4
0	2.05044164365509	0.479	0.116	0	3	DEPRECATED-ENSG00000125675
0	2.03360375034764	0.338	0.083	0	3	ADCY10
0	2.03359300561285	0.923	0.276	0	3	GABRE
0	2.03030209478678	0.345	0.091	0	3	ZNF552
0	2.02846942100141	0.525	0.134	0	3	UGT1A3
0	2.02544802857768	0.514	0.147	0	3	GOLGA8B
0	1.97842277420224	0.797	0.16	0	3	ETNK2
0	1.96836877987272	0.733	0.225	0	3	GRIA3
0	1.93854128286723	0.586	0.17	0	3	CACNA1H
0	1.89245640418439	0.616	0.171	0	3	GREB1L
0	1.88570362546692	0.864	0.318	0	3	TBC1D8
7.6649448862913E-305	1.85739366143061	0.307	0.073	1.28449146640447E-300	3	FZD4
0	1.8572508855225	0.669	0.215	0	3	ZBTB1
4.69550960665911E-186	1.84855037246008	0.261	0.08	7.86873499883934E-182	3	ZNF234
0	1.84214664889904	0.761	0.249	0	3	WDFY2
0	1.8393600291084	0.735	0.224	0	3	ARL17A
0	1.82969407795337	0.761	0.243	0	3	RHOBTB3
0	1.82344838702351	0.727	0.227	0	3	RBM12B
1.0102691977284E-288	1.82095987441783	0.268	0.058	1.69300912155326E-284	3	SYNE4
0	1.81806431688913	0.82	0.248	0	3	AKR1C8P
8.78790871768653E-213	1.79530165371651	0.285	0.085	1.47267774290991E-208	3	FSBP
0	1.79142978009376	0.664	0.203	0	3	DEPRECATED-ENSG00000183747
0	1.78155736682318	0.545	0.163	0	3	TBC1D1
0	1.77385022866695	0.582	0.173	0	3	TIGD2
0	1.77334798155032	0.56	0.175	0	3	DGKE
2.44501478988933E-210	1.76770118806334	0.298	0.094	4.09735578489653E-206	3	CCDC7
2.15019563181771E-249	1.76717708985072	0.327	0.098	3.60329783980013E-245	3	WDR5B
6.79500095073514E-223	1.76288448738475	0.28	0.079	1.1387062593242E-218	3	CUX2
0	1.76189178296891	0.664	0.213	0	3	WDYHV1
0	1.74849369468897	0.661	0.231	0	3	ZNF189
0	1.74621794171041	0.884	0.341	0	3	SLC17A4
0	1.74498956550846	0.444	0.132	0	3	RWDD3
6.41499327967605E-253	1.74258151275412	0.331	0.099	1.07502457380811E-248	3	LIG4
2.85421908830004E-188	1.72941257239421	0.269	0.084	4.7831003481732E-184	3	ZNF782
0	1.7193723266195	0.534	0.165	0	3	ART4

0	1.70883327281071	0.668	0.221	0	3	MLH3
0	1.7080475007444	0.498	0.16	0	3	ANKS1B
0	1.70043613903649	0.493	0.152	0	3	PIK3C2G
0	1.70035002909068	0.934	0.366	0	3	CEP70
0	1.70033667051322	0.671	0.225	0	3	ZNF75D
5.6847275585061E-243	1.67665493888591	0.358	0.116	9.52646644254451E-239	3	CHST6
0	1.67522284653904	0.662	0.22	0	3	ZMAT1
0	1.67431253228946	0.447	0.133	0	3	PSD3
0	1.66838633669868	0.591	0.19	0	3	RNF125
0	1.66774252257247	0.707	0.256	0	3	INTS6
9.11328354970918E-194	1.66749248398176	0.277	0.087	1.52720405726026E-189	3	JRK
0	1.6635518919219	0.725	0.251	0	3	TRIO
0	1.65131618455562	0.602	0.212	0	3	HAO2
0	1.64841117013376	0.961	0.672	0	3	SERPINA7
0	1.64023400313937	0.725	0.242	0	3	ABCA5
0	1.63000117784926	0.48	0.154	0	3	ZNF251
9.85285555862458E-204	1.62915859594508	0.301	0.097	1.65114153451431E-199	3	DOCK10
1.13951399778142E-183	1.62806523448058	0.269	0.085	1.9095975574821E-179	3	GOLGA8A
0	1.61860976179149	0.554	0.177	0	3	ZBTB25
2.5602715669703E-299	1.61257107664889	0.445	0.151	4.29050309192883E-295	3	ZMYM5
0	1.60647304240925	0.964	0.531	0	3	NOTCH2NLC
0	1.59522273944434	0.89	0.372	0	3	NR1I2
1.59465359264811E-268	1.58061861279791	0.362	0.111	2.67232049055971E-264	3	OTUD3
2.39089295396828E-224	1.57390428106028	0.336	0.11	4.00665841226004E-220	3	PCDHGA3
1.91764570310094E-277	1.57053407368748	0.461	0.168	3.21359066925656E-273	3	BTNL3
5.22758909095016E-171	1.56802685728199	0.262	0.085	8.76039379861427E-167	3	MAP10
2.83880474762441E-160	1.56632507115979	0.29	0.106	4.75726899606899E-156	3	C3orf85
0	1.56440600623641	0.593	0.199	0	3	DCAF16
3.92595854746113E-203	1.55918183195054	0.319	0.107	6.57912133383536E-199	3	ANKRD26
3.45883414751565E-228	1.55767796529823	0.32	0.099	5.79631426440672E-224	3	TTLL7
1.05320983383574E-285	1.55064696351323	0.443	0.154	1.76496903954193E-281	3	TMEM39A
0	1.54711000069453	0.588	0.203	0	3	BCL2L2
2.08168613863429E-223	1.5341469629255	0.354	0.121	3.48848963112334E-219	3	ZNF606
0	1.52664480159311	0.968	0.644	0	3	ERRFI1
0	1.51988396769983	0.912	0.414	0	3	OCRL
5.1479430426743E-216	1.51798378918897	0.293	0.088	8.62692295091359E-212	3	TEFM
1.64215415712434E-189	1.51784277008435	0.33	0.119	2.75192193650897E-185	3	PCDHGA5
3.19375655489571E-288	1.51413218984193	0.42	0.138	5.35209723469423E-284	3	THSD4
0	1.51256962496254	0.732	0.274	0	3	BPTF
0	1.50764518840126	0.685	0.235	0	3	FBXO42
3.61051190479642E-205	1.50568507376365	0.313	0.103	6.05049585005784E-201	3	MIPOL1
1.75859745759874E-301	1.50018471481047	0.467	0.164	2.94705761944397E-297	3	ZNF75A
1.29872892139919E-241	1.49394182600701	0.328	0.099	2.17640992648077E-237	3	PPCDC
3.32517202512496E-240	1.49110602353397	0.346	0.11	5.5723232797044E-236	3	VCPKMT
0	1.49005124368883	0.539	0.183	0	3	PRPF39
4.6829123241256E-194	1.48777848214574	0.313	0.106	7.84762447276969E-190	3	STXBP4
0	1.4840556539676	0.671	0.228	0	3	UGT1A9
2.30077047394274E-256	1.47975027694413	0.394	0.131	3.85563116023324E-252	3	ANKRD36B
0	1.47646197266249	0.639	0.225	0	3	TUT4
2.1514141535762E-277	1.47498513111725	0.284	0.066	3.60533983856299E-273	3	CALHM6
0	1.47035713069233	0.889	0.39	0	3	SMOC1
0	1.46123864572564	0.721	0.274	0	3	KLB
0	1.46107739849707	0.855	0.364	0	3	TM7SF3
1.90032979738661E-162	1.45674953089952	0.262	0.088	3.18457267446049E-158	3	ZNF587B
0	1.45297108809325	0.51	0.175	0	3	ERMARD
3.58619391767385E-203	1.451441100919	0.363	0.133	6.00974376723783E-199	3	KCNMB3
5.71341260891086E-249	1.45035951607098	0.364	0.117	9.57453685001282E-245	3	TRIM52
1.12128966789358E-245	1.44987565627128	0.397	0.139	1.87905722545607E-241	3	SRGAP1
0	1.44939010181283	0.619	0.203	0	3	C1RL
7.30023181952267E-154	1.44823833562522	0.275	0.099	1.22337284831561E-149	3	MFSD8

6.78479221664599E-255	1.44463182471822	0.403	0.139	1.13699547966553E-250	3	GANC
1.56997119844879E-247	1.44053170058474	0.417	0.149	2.63095773436048E-243	3	POU5F1
1.78436959903923E-283	1.44002924477383	0.479	0.176	2.99024657406995E-279	3	USP53
0	1.43574510432213	0.873	0.362	0	3	N4BP2L2
7.63808483739763E-135	1.43373720500922	0.253	0.093	1.27999025705109E-130	3	HELQ
0	1.43151961644042	0.599	0.216	0	3	LYST
1.01673899496211E-183	1.42459474835846	0.305	0.105	1.7038512077575E-179	3	CEP95
0	1.42381460176074	0.932	0.43	0	3	ZBTB16
0	1.41878367565176	0.951	0.625	0	3	SGK1
1.54917105887966E-236	1.4166462106508	0.342	0.108	2.59610086047053E-232	3	TCAF2
0	1.41500341254438	0.919	0.411	0	3	NOTCH2NLB
7.32766546422681E-164	1.41310883929922	0.293	0.105	1.22797017849513E-159	3	CPLANE1
1.92121754052417E-260	1.4111490779631	0.419	0.144	3.2195763544104E-256	3	NOL3
0	1.40984140924457	0.52	0.183	0	3	DPH6
0	1.40919300114063	0.499	0.177	0	3	SLC10A5
0	1.40889524166619	0.588	0.218	0	3	USP54
0	1.40887066127823	0.756	0.32	0	3	ACSM1
0	1.40676311731145	0.487	0.145	0	3	PLA2G4C
0	1.40431572669398	0.503	0.168	0	3	ATG16L2
0	1.40102100873709	0.616	0.226	0	3	TASOR2
1.75950788649309E-149	1.39849001162428	0.265	0.094	2.94858331618512E-145	3	ZNF619
9.03887039041331E-244	1.39814483031502	0.395	0.137	1.51473390002546E-239	3	AC253572.1
0	1.39802061032527	0.778	0.269	0	3	CCDC14
0	1.39759024745441	0.62	0.227	0	3	LRBA
3.51280188540573E-302	1.39735687620831	0.488	0.173	5.88675339956292E-298	3	DMXL2
0	1.39648307925849	0.671	0.253	0	3	TBC1D8B
4.58599884349942E-213	1.39386135723356	0.361	0.128	7.68521686193633E-209	3	ZNF700
1.36940338603337E-215	1.39108942566919	0.344	0.117	2.29484619431473E-211	3	ADPGK
0	1.38591058489044	0.645	0.24	0	3	CCT8
9.74159802811054E-249	1.38381362534228	0.461	0.179	1.63249699755076E-244	3	FZD6
0	1.38304598874019	0.512	0.176	0	3	TMEM87A
6.09826152296252E-250	1.37550730546417	0.4	0.139	1.02194666601806E-245	3	TRAPPC8
0	1.37449205438649	0.679	0.261	0	3	MAP3K5
0	1.37067855334419	0.717	0.267	0	3	RBM26
0	1.36870924000057	0.63	0.228	0	3	RNF138
0	1.36721095288741	0.727	0.277	0	3	CLMN
0	1.36715378725087	0.625	0.229	0	3	GTF2H2C
4.1895840494467E-175	1.36712328619889	0.3	0.105	7.02090495006279E-171	3	ZNF248
0	1.36333170474026	0.721	0.288	0	3	ERN 1.00
0	1.36271616363122	0.526	0.163	0	3	KCNT2
8.62594338183265E-176	1.36149296205811	0.289	0.099	1.44553559192752E-171	3	MFSD4B
3.1209150958873E-223	1.3613351121906	0.389	0.14	5.23002951768794E-219	3	DEPRECATED-ENSG00000135976
0	1.35907652025471	0.926	0.478	0	3	ELL2
0	1.35782934081768	0.543	0.204	0	3	ANAPC4
0	1.3574237702041	0.769	0.285	0	3	VPS13B
0	1.35428451132945	0.549	0.201	0	3	GLI4
0	1.35414609579554	0.834	0.33	0	3	PVR
0	1.35375998842086	0.589	0.215	0	3	TARBP1
0	1.35296173879018	0.875	0.396	0	3	ABCA1
0	1.35269055987101	0.948	0.541	0	3	ACSM2A
0	1.35202266653307	0.559	0.206	0	3	DMXL1
9.76240931479203E-172	1.35087899548827	0.323	0.121	1.63598455297285E-167	3	ZNF224
6.41793073100285E-308	1.34976322510118	0.521	0.192	1.07551683190146E-303	3	TTC14
6.73989326505022E-283	1.34951222733518	0.482	0.178	1.12947131335712E-278	3	ZNF337
0	1.3487423820875	0.918	0.435	0	3	ERO1B
4.06465876728278E-248	1.34815247967276	0.402	0.14	6.81155516221248E-244	3	MYSM1
3.91259158213207E-258	1.3480598170667	0.371	0.117	6.55672097333691E-254	3	C15orf40
8.78211448411662E-230	1.34627819243421	0.415	0.155	1.47170674524826E-225	3	FAM76B
0	1.34275210254682	0.697	0.252	0	3	PLGLB1
0	1.34223868299141	0.723	0.27	0	3	RAB33B

0	1.34029363811767	0.696	0.266	0	3	HELZ
7.63226065901045E-205	1.33676686828278	0.336	0.115	1.27901424123697E-200	3	ZSCAN26
3.58749095505594E-264	1.33330306508989	0.436	0.154	6.01191734248274E-260	3	MTERF2
1.1948468056646E-239	1.33192950231618	0.384	0.132	2.00232427693274E-235	3	PIAS1
0	1.33050735188697	0.69	0.257	0	3	NOTCH2
0	1.3283254071341	0.632	0.241	0	3	SEC24B
0	1.32780485800692	0.487	0.154	0	3	SPHK1
0	1.32728841846623	0.684	0.259	0	3	PIEZO2
3.89203129171883E-283	1.32639608485551	0.469	0.167	6.52226603866241E-279	3	PLCXD1
0	1.32096801202451	0.477	0.163	0	3	SLC19A2
0	1.31904826215165	0.827	0.323	0	3	NKTR
2.09385390144735E-155	1.31762344331697	0.297	0.111	3.50888036804546E-151	3	AP002495.1
0	1.31732801184588	0.646	0.236	0	3	NLK
1.22195657459369E-276	1.31725731415029	0.474	0.173	2.04775482770411E-272	3	D2HGDH
6.17700116470665E-120	1.31638153630067	0.257	0.101	1.03514185518154E-115	3	PPP1R3E
2.17785024065877E-265	1.31374785239954	0.423	0.145	3.64964143329596E-261	3	FRY
0	1.31264050602824	0.639	0.241	0	3	DEPRECATED-ENSG00000134250
0	1.31245029740248	0.881	0.387	0	3	IL6R
0	1.31230834340383	0.692	0.263	0	3	BIVM
5.20941696651225E-206	1.30713095283219	0.314	0.101	8.72994095248122E-202	3	SRCIN1
8.39725279758227E-211	1.30689329234702	0.347	0.12	1.40721162381884E-206	3	LRIG2
0	1.30496788219951	0.571	0.212	0	3	PDE8A
0	1.30384997895856	0.686	0.252	0	3	RORA
1.80591023598449E-289	1.30192479949762	0.507	0.191	3.02634437346281E-285	3	C8orf76
0	1.30109584712086	0.933	0.443	0	3	ALG13
1.11411787452901E-211	1.30084476636731	0.392	0.148	1.86703873413571E-207	3	ZFP62
0	1.3002145515132	0.763	0.317	0	3	NBPF26
3.64132612946444E-177	1.29784496354793	0.329	0.122	6.1021343277565E-173	3	GPR180
1.07026317263562E-240	1.29573000813893	0.446	0.17	1.79354702470276E-236	3	MGAT5
0	1.29112148987713	0.684	0.267	0	3	PPP4R3A
0	1.28902995445533	0.5	0.176	0	3	ZNF580
2.55163018609838E-216	1.2889943256425	0.355	0.122	4.27602186586367E-212	3	ZBTB37
0	1.2883878610375	0.486	0.166	0	3	COG2
1.0734838712743E-175	1.28604630546148	0.279	0.092	1.79894427148147E-171	3	FAM219B
7.22603063855721E-195	1.28377616812194	0.346	0.124	1.21093821440942E-190	3	NRXN3
7.70410945109358E-201	1.28219271833247	0.376	0.142	1.29105466181426E-196	3	ENDOV
4.32435457586463E-296	1.28115235422274	0.469	0.162	7.24675339823394E-292	3	SLC25A16
1.21050333970392E-257	1.27841489323961	0.472	0.179	2.02856149667583E-253	3	UVSSA
1.27191029533568E-189	1.27640135240164	0.381	0.15	2.13146727292353E-185	3	DCUN1D2
1.72607749536542E-163	1.27634058827249	0.317	0.121	2.89256066673337E-159	3	MCM9
0	1.27450465541902	0.653	0.25	0	3	EXOC3L4
1.47189311276583E-278	1.2726661255419	0.487	0.181	2.46659847837298E-274	3	EPC1
4.8628201384523E-137	1.27263219358118	0.292	0.116	8.14911398801836E-133	3	ZNF33B
7.22725032570491E-299	1.27085910507064	0.557	0.221	1.21114260958163E-294	3	APPL2
1.07188474908691E-191	1.26953755012999	0.355	0.132	1.79626446251984E-187	3	ZNF44
1.186464225037E-292	1.26934939227566	0.519	0.197	1.988276748317E-288	3	DNAH14
0	1.26848495799476	0.738	0.284	0	3	FTCD
1.4409461322165E-172	1.26718011954023	0.346	0.134	2.41473752836841E-168	3	DPY19L3
3.45729550841302E-182	1.26427813847702	0.32	0.114	5.79373581299853E-178	3	VCL
0	1.26401887080643	0.715	0.28	0	3	SGK3
0	1.26394463558193	0.701	0.271	0	3	BDH2
5.34330485589559E-156	1.26359147049792	0.293	0.109	8.95431027750983E-152	3	ZNF791
0	1.26256087379795	0.513	0.167	0	3	IL1RAP
9.03585383703194E-164	1.26012192650426	0.292	0.104	1.51422838600981E-159	3	ARL17B
5.0561540272114E-303	1.25978922516572	0.537	0.203	8.47310291880086E-299	3	L3HYPDH
1.14358784342324E-134	1.25973211142183	0.281	0.109	1.91642450800867E-130	3	RPA4
0	1.25904698152857	0.881	0.449	0	3	SEC16B
3.11261716175237E-196	1.2589549845685	0.354	0.13	5.21612383966461E-192	3	THOC1
6.89128372283618E-149	1.25887208692469	0.288	0.108	1.15484132627289E-144	3	AASDH
0	1.25746504823026	0.746	0.295	0	3	OGT

4.47626265531778E-234	1.25618071899122	0.422	0.158	7.50132095778153E-230	3	RBBP6
1.28547264684329E-180	1.25444601443219	0.337	0.123	2.15419506157999E-176	3	ANKRD36C
0	1.25199511852767	0.878	0.463	0	3	BHMT
3.65166299287014E-163	1.24980846627041	0.285	0.1	6.11945684345177E-159	3	C9orf85
1.69242136332583E-208	1.24971842765898	0.374	0.138	2.83615972066143E-204	3	RBM12
1.51369145543394E-234	1.24842990251059	0.94	0.703	2.5366441410162E-230	3	GLUL
6.0522999123141E-226	1.24834211737565	0.425	0.163	1.0142444193056E-221	3	NBEAL1
1.37418476564946E-204	1.24748508286468	0.422	0.167	2.30285883027537E-200	3	ACYP1
0	1.24283939055045	0.711	0.284	0	3	UBXN2B
0	1.24235302180681	0.858	0.368	0	3	LUC7L3
3.12820477043341E-275	1.24173119710846	0.509	0.196	5.24224555429232E-271	3	RASGEF1B
0	1.24117247372713	0.913	0.457	0	3	LIPG
4.14773671742399E-233	1.2411146237176	0.363	0.12	6.95077719105912E-229	3	EDC3
0	1.24088636705233	0.643	0.255	0	3	PARP9
3.60295605449519E-292	1.24006857188416	0.555	0.218	6.03783375612303E-288	3	PRELID2
6.81424201355225E-283	1.23997304137861	0.49	0.179	1.14193067663109E-278	3	MFSD3
1.9858815395936E-168	1.23875716436746	0.325	0.123	3.32794028405096E-164	3	MFSD6
0	1.23817464866032	0.786	0.319	0	3	SAMD5
0	1.23769646419214	0.591	0.222	0	3	GSAP
0	1.23712160708684	0.823	0.379	0	3	RAP2C
9.97725861661742E-285	1.23589903510289	0.503	0.189	1.67198899897275E-280	3	MED23
0	1.23409895845215	0.851	0.368	0	3	MDM4
1.67719615900903E-146	1.23262228771309	0.293	0.111	2.81064532326734E-142	3	MOSMO
7.92884768238674E-159	1.23197098629734	0.302	0.112	1.32871629461437E-154	3	ZFP41
0	1.23191305334185	0.79	0.338	0	3	CNKSR3
5.67109204720511E-273	1.23086805378591	0.481	0.18	9.50361605270633E-269	3	ATPSCKMT
0	1.23008404904066	0.62	0.235	0	3	TMEM175
0	1.22882699736521	0.51	0.182	0	3	MID2
0	1.22785722439022	0.937	0.608	0	3	BAAT
3.19652264630608E-155	1.22590876875492	0.285	0.103	5.35673265067974E-151	3	SETDB2
1.77776915529246E-173	1.22423205484356	0.322	0.118	2.97918555043911E-169	3	GRK3
8.1710814582173E-195	1.22165131328768	0.356	0.132	1.36930983076805E-190	3	SOCS5
1.95166861654869E-234	1.22161234136263	0.4	0.144	3.2706062676123E-230	3	SEC22A
4.00628344162443E-181	1.22124376780736	0.353	0.135	6.71372979147421E-177	3	FAM200B
0	1.2211299392043	0.531	0.192	0	3	SIN3B
2.4039950910564E-215	1.21945155366393	0.264	0.07	4.02861497359231E-211	3	FES
0	1.21912756488639	0.45	0.139	0	3	FAXDC2
0	1.2186848695651	0.785	0.332	0	3	RNF19A
1.3997889143279E-219	1.21665882953076	0.438	0.173	2.3457662626307E-215	3	ATM
2.77376883107435E-235	1.21495122279244	0.451	0.174	4.6482818071144E-231	3	FANCL
5.60968799511109E-110	1.2127763585383	0.265	0.112	9.40071514220716E-106	3	PPM1L
1.92790996769183E-190	1.21223031156804	0.268	0.08	3.23079152385797E-186	3	OXER1
3.30255997685274E-160	1.20991693631087	0.337	0.134	5.53443000920981E-156	3	ZNF266
4.12659325193628E-306	1.20942095005982	0.546	0.208	6.91534497159481E-302	3	ATG12
2.63955517945178E-157	1.20900001526872	0.354	0.145	4.42336656972529E-153	3	POLE2
7.22277554136353E-272	1.20814713235465	0.507	0.195	1.2103927252217E-267	3	GTF2H2
2.84391349647116E-202	1.2078450355956	0.374	0.139	4.76583023738638E-198	3	SLC35E3
9.86549668491184E-176	1.20726759892713	0.294	0.1	1.65325993445753E-171	3	NICN1
1.01683110829805E-125	1.20522386065404	0.261	0.101	1.70400557128586E-121	3	SENP7
0	1.20508064997019	0.689	0.272	0	3	CDC16
1.10769247904502E-133	1.20507088127553	0.265	0.1	1.85627105638364E-129	3	RWDD4
5.21098439675967E-206	1.20411289299133	0.36	0.129	8.73256765208985E-202	3	NSRP1
1.03236392145106E-152	1.20404522951638	0.325	0.13	1.73003545956769E-148	3	TTC13
1.72143633104221E-166	1.20169952487088	0.361	0.146	2.88478300356054E-162	3	TMEM44
5.41263748888944E-218	1.20139961229509	0.347	0.115	9.07049790388093E-214	3	CDC14B
1.61513649580773E-220	1.20087011016211	0.392	0.143	2.7066457396746E-216	3	GRAMD2B
1.23965261606349E-236	1.20049068156017	0.422	0.155	2.0774098539992E-232	3	TBCK
2.75823100639581E-265	1.19907188771062	0.492	0.189	4.62224352051811E-261	3	UPF3A
0	1.1957982706291	0.589	0.232	0	3	ZMYND8
4.53767059598332E-234	1.19455941724967	0.397	0.139	7.60422838474885E-230	3	SH3TC1

0	1.19448762120442	0.947	0.429	0	3	ARGLU1
1.0795455855238E-185	1.19433174164126	0.374	0.145	1.80910249222078E-181	3	VPS8
3.62697234841809E-140	1.19397672054722	0.289	0.112	6.07808026147904E-136	3	ADGRG2
0	1.18929229884009	0.682	0.276	0	3	NIPAL2
0	1.18821741917597	0.883	0.436	0	3	ZHX1
0	1.18700222552046	0.679	0.269	0	3	ACACB
2.36965066645321E-244	1.18666161739928	0.427	0.155	3.97106058684229E-240	3	PM20D2
0	1.1857095917412	0.91	0.411	0	3	IVNS1ABP
3.06524808616387E-263	1.18366527155313	0.422	0.144	5.13674274279341E-259	3	PAQR9
0	1.18271033180207	0.899	0.424	0	3	PUM1
1.65423495791944E-219	1.18263677913268	0.382	0.137	2.7721669424814E-215	3	ITPR1
0	1.18215216628688	0.837	0.352	0	3	ATAD2
0	1.18186786897114	0.638	0.243	0	3	PCMTD2
0	1.18139725336868	0.674	0.267	0	3	GALK2
3.91819580768645E-195	1.18096974468687	0.371	0.139	6.56611253452095E-191	3	RNF217
2.96327195283373E-252	1.18062229299524	0.482	0.188	4.96585113855876E-248	3	NSMAF
1.49525454565503E-216	1.17974719237336	0.397	0.148	2.5057475676087E-212	3	OMA1
0	1.17917017649335	0.696	0.287	0	3	ZMYM2
0	1.17915969301613	0.676	0.276	0	3	N4BP2L1
0	1.17849073054282	0.659	0.274	0	3	RAPH1
3.36687487349289E-273	1.17796813838918	0.528	0.209	5.64220891299939E-269	3	RCOR3
1.27372754866199E-144	1.1767401547959	0.305	0.12	2.13451262604776E-140	3	SMYD3
9.43946845073676E-139	1.17638280818897	0.303	0.121	1.58186612297447E-134	3	RBL1
3.14098017140649E-138	1.17610335154855	0.299	0.119	5.26365457124299E-134	3	ZNF839
9.96692903478615E-135	1.17431587101473	0.252	0.091	1.67025796764946E-130	3	FITM2
1.5013055693371E-166	1.17281320504529	0.314	0.116	2.51588787309511E-162	3	TTC21B
0	1.17227902640799	0.605	0.235	0	3	MBOAT1
6.14723899214481E-128	1.17209324877901	0.266	0.102	1.03015431030363E-123	3	TUBGCP5
4.0710496227161E-128	1.1713740084003	0.281	0.112	6.82226495774763E-124	3	DEPRECATED-ENSG00000185619
0	1.16855391134342	0.673	0.287	0	3	TLCD4
0	1.16846912585457	0.579	0.216	0	3	SUSD3
1.29506639447374E-192	1.16782445346785	0.377	0.145	2.17027226385909E-188	3	PTPRD
2.27280294762423E-264	1.16700649981762	0.527	0.213	3.80876317962868E-260	3	ADNP
3.29456811539502E-291	1.16671411069516	0.592	0.241	5.52103724777897E-287	3	EPPK1
4.52221973477021E-179	1.16591011415039	0.351	0.135	7.57833583152791E-175	3	CHML
0	1.16403273394169	0.591	0.229	0	3	STRN3
3.67683813195177E-220	1.16291256732778	0.42	0.161	6.16164534152478E-216	3	HIBCH
5.96210657972705E-164	1.16283167512304	0.294	0.104	9.99129820630658E-160	3	PACRG
7.32273499108551E-187	1.16229021514009	0.35	0.129	1.22714392980611E-182	3	FAN1
2.33336002670503E-134	1.16091249283657	0.282	0.11	3.9102447327523E-130	3	CYP20A1
0	1.16036133166369	0.583	0.225	0	3	ACACA
3.35743174038457E-143	1.15975386882886	0.282	0.106	5.62638411053645E-139	3	ZDHHC17
1.15106188174312E-237	1.15627424878586	0.425	0.156	1.92894950142512E-233	3	GUCY1A1
1.25474019611034E-224	1.15583863891886	0.438	0.171	2.10269362064171E-220	3	RAB28
8.25455461729788E-230	1.15255426648282	0.48	0.195	1.38329826276678E-225	3	UGGT2
9.63272247393717E-95	1.15018210309459	0.251	0.11	1.61425163218239E-90	3	ZNF117
1.38783720955615E-172	1.15013098142824	0.34	0.129	2.32573759577419E-168	3	MAML3
3.46501934222501E-282	1.1478509607446	0.538	0.212	5.80667941370067E-278	3	MON2
1.5915579764443E-302	1.14620992367045	0.61	0.25	2.66713285692536E-298	3	ADCY6
2.73564784989557E-153	1.1458587328197	0.298	0.112	4.584398666855E-149	3	ZNF621
1.84236657734183E-128	1.14538614012274	0.253	0.095	3.08743791030945E-124	3	SZT2
8.63570173701307E-178	1.14485267046439	0.357	0.139	1.44717089708865E-173	3	ABHD13
1.52429963236066E-202	1.14156085484835	0.384	0.144	2.55442132391E-198	3	KANSL1L
0	1.14107270781504	0.822	0.352	0	3	XIAP
5.00727469224471E-201	1.14003466738688	0.412	0.164	8.39119092926369E-197	3	CSPP1
6.46477677299518E-192	1.13687985992327	0.352	0.129	1.08336729161853E-187	3	ZNF561
1.02519778678494E-299	1.13672008666072	0.573	0.227	1.7180264510942E-295	3	AKAP8
0	1.13473293603042	0.973	0.751	0	3	ACSL1
8.90396024953563E-266	1.13319433539158	0.567	0.234	1.49212565861718E-261	3	ASB9
0	1.13192012255956	0.624	0.253	0	3	PARP14

0	1.13137815108441	0.627	0.239	0	3	SAMHD1
0	1.13101552508057	0.846	0.423	0	3	ONECUT1
6.02977071323478E-122	1.13085995333245	0.277	0.112	1.01046897612388E-117	3	CENPC
1.30535158911033E-151	1.13082341565259	0.319	0.125	2.18750819303109E-147	3	PARD3B
4.14904506565302E-246	1.12887447807176	0.472	0.183	6.95296972102133E-242	3	MTERF3
0	1.12679183254743	0.691	0.279	0	3	KCNJ3
2.71773374185944E-172	1.12677269650182	0.343	0.131	4.55437820460805E-168	3	USP40
1.05653404352017E-143	1.12456336023433	0.284	0.107	1.7705397501311E-139	3	KPTN
1.18353102067721E-238	1.12447261192247	0.449	0.171	1.98336128445087E-234	3	YTHDC2
2.38546376021232E-153	1.12176446662702	0.32	0.125	3.99756016936381E-149	3	CEP44
0	1.12086546681929	0.703	0.307	0	3	REPS1
0	1.12066504107194	0.801	0.341	0	3	THOC2
0	1.11946724727209	0.874	0.404	0	3	PAG1
1.41689188427102E-141	1.1181521334488	0.289	0.11	2.37442741966138E-137	3	TNK2
3.39883350881123E-238	1.11804662608367	0.501	0.204	5.69576519406586E-234	3	SLC2A13
0	1.11795607543628	0.847	0.429	0	3	PPP1R15B
0	1.11747706501965	0.594	0.234	0	3	NFS1
1.53779687500451E-290	1.11670917683563	0.609	0.255	2.57704000313256E-286	3	CPM
9.5645107476034E-276	1.11490086649702	0.572	0.237	1.60282071108338E-271	3	HOOK3
4.33296314227045E-251	1.11371693823507	0.485	0.189	7.26117963381681E-247	3	ZNF7
2.01638084565865E-282	1.11357188879261	0.555	0.22	3.37905102115477E-278	3	PAN3
8.05590390716971E-244	1.11336104217877	0.474	0.182	1.3500083767635E-239	3	PAQR7
4.08582323163337E-233	1.11312701413496	0.494	0.201	6.84702257157121E-229	3	TTLL3
3.13584651835749E-270	1.11273023675288	0.58	0.243	5.25505159546348E-266	3	PHF6
1.97723934335489E-117	1.11257888720885	0.262	0.105	3.31345769159412E-113	3	SYDE2
0	1.11176697037948	0.798	0.354	0	3	POGZ
0	1.11101384488567	0.853	0.411	0	3	ABHD2
8.10724436060209E-164	1.11097968648892	0.37	0.152	1.3586120099497E-159	3	XPO4
4.48747828649825E-183	1.11043578413216	0.368	0.143	7.52011611251376E-179	3	ZFX
5.87206327965947E-137	1.11015668954928	0.293	0.116	9.84040364405334E-133	3	TMEM161B
0	1.1098153840803	0.641	0.255	0	3	HECTD4
1.45057829001971E-124	1.10973545449003	0.308	0.132	2.43087909841503E-120	3	MFSD11
0	1.10685460856429	0.926	0.449	0	3	ANKRD10
0	1.10485147331077	0.575	0.213	0	3	UGT2B15
0	1.10352373459286	0.614	0.234	0	3	SLC46A1
1.18286247825644E-113	1.10310858910607	0.254	0.102	1.98224094106215E-109	3	FAM120C
2.08953155742803E-154	1.10290477782784	0.333	0.133	3.50163698393788E-150	3	MOB3B
1.32126045823148E-297	1.10224382757554	0.619	0.256	2.21416827590431E-293	3	RBM41
0	1.10180555406592	0.694	0.294	0	3	TMEM68
5.26593032764229E-143	1.10148927765774	0.273	0.1	8.82464604306295E-139	3	LRP12
1.04787220195223E-108	1.10141699669424	0.273	0.117	1.75602423603154E-104	3	TMEM260
2.86530573066404E-205	1.10041142797764	0.426	0.169	4.8016793434468E-201	3	ZNF121
0	1.09960908704028	0.635	0.257	0	3	PDLIM5
3.53450871302325E-185	1.09939655681125	0.377	0.147	5.92312970128437E-181	3	SLC10A7
0	1.09812967903143	0.957	0.646	0	3	TDO2
3.11975018539954E-125	1.09546051748147	0.259	0.099	5.22807736069255E-121	3	ZXDB
9.16077917201196E-142	1.09504963580165	0.318	0.129	1.53516337364576E-137	3	ZNF346
3.27783520147547E-142	1.09366186457912	0.303	0.119	5.49299623063259E-138	3	PCNX4
7.5532602972616E-261	1.0917733327922	0.519	0.206	1.2657753606151E-256	3	VPS13C
0	1.08864297867971	0.735	0.326	0	3	SH3RF2
1.64501801659974E-195	1.08771476687005	0.382	0.146	2.75672119221785E-191	3	METTL15
7.15584566978704E-101	1.08712835887672	0.273	0.121	1.19917661734291E-96	3	CDC7
0	1.08630954706221	0.953	0.619	0	3	F5
2.00685720237068E-203	1.08630834323516	0.47	0.198	3.36309129973278E-199	3	FRAS1
1.37414786678131E-178	1.0862622513673	0.384	0.155	2.30279699515213E-174	3	ABHD18
6.51185812648245E-173	1.08613134731098	0.352	0.136	1.09125718483593E-168	3	TAF9
1.06094352143586E-164	1.08447176236998	0.308	0.112	1.77792915322222E-160	3	TSNARE1
0	1.08242692774592	0.913	0.474	0	3	NFIB
4.3048135619026E-131	1.08217708764808	0.285	0.113	7.21400656703637E-127	3	SPAG1
5.09435644996292E-198	1.08205672181748	0.394	0.153	8.53712253884785E-194	3	PDP1

4.18692157595096E-162	1.08151778215201	0.37	0.154	7.01644317697862E-158	3	NPIPB15
4.29366332197884E-124	1.07922007779248	0.266	0.104	7.19532099497214E-120	3	THAP5
5.45473033964385E-234	1.07916916278708	0.528	0.223	9.14103710317516E-230	3	PCF11
3.62283585516628E-149	1.07910098977558	0.297	0.112	6.07114832608766E-145	3	SNAPC3
2.69976984184852E-175	1.07874883416362	0.377	0.152	4.52427430096975E-171	3	PTGR2
1.83566250511611E-280	1.07817924756139	0.554	0.22	3.07620322607358E-276	3	GPATCH2L
0	1.07766229674009	0.785	0.336	0	3	HGSNAT
0	1.07706559618731	0.681	0.274	0	3	MAST4
0	1.07575073100245	0.828	0.374	0	3	ARHGEF26
0	1.07539076451024	0.913	0.501	0	3	TOR1AIP2
9.59683250464884E-159	1.07358779483856	0.283	0.1	1.60823719112905E-154	3	MINDY3
5.92109204206127E-98	1.0721065353443	0.285	0.131	9.92256604408627E-94	3	FSD1L
7.68298925689281E-266	1.07171494635313	0.515	0.202	1.2875153396701E-261	3	MTERF4
2.40823577556033E-127	1.07030102071563	0.268	0.103	4.03572151268401E-123	3	TSPYL2
0	1.07007526860289	0.709	0.294	0	3	FAM193B
2.36622818725954E-168	1.06778840461725	0.34	0.132	3.96532519620954E-164	3	MRI1
0	1.0662056346664	0.846	0.367	0	3	IKBKB
0	1.06598802197859	0.724	0.306	0	3	LYPLAL1
1.51416077037138E-121	1.06574335796011	0.271	0.109	2.53743061898836E-117	3	DPP8
1.34604469924064E-114	1.06534388078148	0.292	0.126	2.25570170698746E-110	3	ZNF26
8.09078147438345E-195	1.06496914135	0.433	0.179	1.35585315947718E-190	3	SLC25A29
5.44687362337478E-180	1.06271934854377	0.396	0.163	9.12787081805145E-176	3	RBMX2
0	1.06258009280948	0.619	0.253	0	3	GOSR2
0	1.06232773373144	0.863	0.39	0	3	DEGS1
0	1.06222129427926	0.809	0.391	0	3	CHPT1
0	1.06204077491879	0.727	0.306	0	3	DIAPH2
2.01010009466008E-135	1.06055004296259	0.307	0.125	3.36852573863137E-131	3	TM2D1
7.443169390107E-167	1.0602567193705	0.343	0.134	1.24732632639413E-162	3	PPWD1
2.84165479774208E-120	1.06018183655272	0.293	0.124	4.76204511005618E-116	3	NFXL1
3.32027349693238E-174	1.05883135852401	0.382	0.156	5.56411432615929E-170	3	DMAC2L
0	1.05861136442443	0.908	0.464	0	3	PGM2
2.7088867315907E-121	1.05855181259917	0.312	0.135	4.53955238479969E-117	3	RADX
0	1.05639634751138	0.847	0.396	0	3	IRF3
1.34655551993687E-119	1.05606324798451	0.259	0.102	2.2565577403102E-115	3	ELAC1
2.57719140921462E-136	1.05441888141914	0.303	0.122	4.31885736356186E-132	3	SLC49A4
3.92938284264159E-178	1.05314243625899	0.391	0.16	6.58485976769878E-174	3	MRNIP
1.10208539826516E-116	1.05267449277852	0.286	0.121	1.84687471041276E-112	3	EXOC1
1.1933908949332E-156	1.05195860553187	0.278	0.097	1.99988446172906E-152	3	TMEM116
7.38014471510595E-208	1.05194703080493	0.443	0.178	1.23676465135746E-203	3	POLK
1.57296983358258E-291	1.05157696664307	0.475	0.165	2.63598284711769E-287	3	PEX11A
3.25621503463793E-113	1.05153139762543	0.272	0.114	5.45676515504625E-109	3	WDR19
0	1.05102674674632	0.947	0.634	0	3	PROX1
3.44430864816425E-123	1.05095952278717	0.269	0.106	5.77197243259364E-119	3	SETD4
1.34136659756538E-101	1.05081636644212	0.25	0.105	2.24786214420007E-97	3	DEPRECATED-ENSG00000197302
0	1.05077142343103	0.913	0.542	0	3	AKR1C2
3.8404679071909E-263	1.0505344694601	0.554	0.228	6.4358561188705E-259	3	ZFHX3
0	1.05027634434341	0.801	0.371	0	3	GPRC5C
1.01813146543982E-92	1.04970536646894	0.25	0.11	1.70618470978405E-88	3	HAUS3
6.3253840384643E-153	1.04970236243334	0.309	0.118	1.06000785716585E-148	3	ZNF133
6.21047347112089E-248	1.04930542030799	0.526	0.218	1.04075114429044E-243	3	SLC25A32
1.10967507879876E-218	1.04881127892471	0.447	0.178	1.85959349705097E-214	3	ERCC6L2
1.60408002454091E-196	1.04876732792473	0.364	0.133	2.68811730512565E-192	3	WDR37
8.27978271804101E-113	1.04836558577076	0.262	0.107	1.38752598788931E-108	3	ZNF57
6.73021409748939E-112	1.04692783659056	0.28	0.119	1.12784927845727E-107	3	SPATA6L
0	1.04640080385106	0.82	0.412	0	3	STOM
1.13878643544923E-144	1.04591861745395	0.334	0.138	1.90837830852582E-140	3	PRIM1
1.20220393368173E-212	1.04526413901915	0.433	0.169	2.01465335206384E-208	3	EPM2AIP1
4.15779021829916E-256	1.04489399394097	0.503	0.199	6.96762484782573E-252	3	MTMR4
2.03133886016445E-204	1.043867918479	0.418	0.166	3.40411766186359E-200	3	RPTOR
0	1.04325528372442	0.774	0.371	0	3	CPEB4

**0**	1.0431718886074	0.757	0.319	0	3	TAZ
**1.30525446622339E-258**	1.04121886525348	0.577	0.245	2.18734543449716E-254	3	RAB3IP
**1.31521751634524E-132**	1.03844310992805	0.266	0.1	2.20404151389136E-128	3	ADAT2
**2.3197500393582E-169**	1.03780588059801	0.374	0.153	3.88743711595647E-165	3	PHYHIPL
**3.4681152701596E-299**	1.03762221136006	0.623	0.254	5.81186756973347E-295	3	DAZAP1
**2.40498934562951E-184**	1.03709789755966	0.384	0.152	4.03028114540594E-180	3	RECQL5
**7.47440114597973E-138**	1.03612728896659	0.25	0.087	1.25256014404328E-133	3	TUBG2
**2.32154399287385E-238**	1.03564314043466	0.499	0.203	3.890443423258E-234	3	UTP23
**0**	1.03550729967597	0.688	0.284	0	3	PRKAG2
**4.41764952136577E-293**	1.03534484967662	0.565	0.223	7.40309706790476E-289	3	ZBED6
**0**	1.03341013780813	0.664	0.279	0	3	SRSF5
**1.08575577088067E-307**	1.03232127542056	0.661	0.281	1.81950952084183E-303	3	FBXL5
**5.57707638943077E-297**	1.03199929570952	0.692	0.304	9.34606461340808E-293	3	LRRC31
**0**	1.03188205469785	0.863	0.436	0	3	KLF15
**1.27927583263983E-205**	1.03167312043428	0.44	0.178	2.14381044033782E-201	3	ZNF608
**4.72848248118878E-154**	1.03155851303534	0.335	0.134	7.92399094197615E-150	3	TRAPPC11
**7.55997198946148E-159**	1.03060279795097	0.294	0.105	1.26690010599396E-154	3	GSTA4
**2.35844280629621E-209**	1.03059658804972	0.466	0.194	3.95227845479119E-205	3	ENPP4
**3.40257934504843E-255**	1.03042218631093	0.49	0.186	5.70204246643215E-251	3	XAF1
**2.96363680617291E-279**	1.03035049441336	0.497	0.181	4.96646255978456E-275	3	KIFC2
**9.80310485075743E-213**	1.02987002445176	0.418	0.16	1.64280431088993E-208	3	RANBP10
**0**	1.02835948608086	0.928	0.544	0	3	PCSK6
**2.58955264008936E-147**	1.02735495041634	0.364	0.157	4.33957231426174E-143	3	RAVER2
**1.65494094318116E-112**	1.02620502291999	0.272	0.113	2.77335003258299E-108	3	ANKRA2
**0**	1.02568699563096	0.821	0.361	0	3	MLPH
**0**	1.02451713030179	0.698	0.304	0	3	CYP4F12
**0**	1.02433042797277	0.666	0.285	0	3	TTC3
**0**	1.02397949597399	0.95	0.535	0	3	FKBP5
**5.51759751753902E-177**	1.02325424333807	0.444	0.192	9.2463899198919E-173	3	CCDC198
**1.8000347521238E-190**	1.0221444807867	0.404	0.163	3.01649823760907E-186	3	PIBF1
**1.68793876017014E-134**	1.02160437960721	0.294	0.117	2.82864777429313E-130	3	SMARCAD1
**5.72572136159056E-193**	1.01983980069106	0.433	0.178	9.59516385775345E-189	3	AHI1
**0**	1.0197091389893	0.883	0.424	0	3	HNF4G
**2.77638760122176E-101**	1.01943199667439	0.261	0.112	4.65267034212743E-97	3	CLASP2
**8.60660581606298E-211**	1.01815668786554	0.472	0.198	1.44229500265583E-206	3	TBCE.1
**3.00553662777156E-238**	1.01751372721854	0.54	0.229	5.03667828081958E-234	3	ABHD3
**2.84761729449046E-152**	1.01679197451043	0.377	0.164	4.77203706210711E-148	3	ZSWIM5
**0**	1.01571869556561	0.666	0.277	0	3	SOS 2
**1.91047455154321E-127**	1.01535722076573	0.32	0.138	3.2015732534761E-123	3	ZNF449
**1.95597555135723E-251**	1.01450895762177	0.57	0.239	3.27782382896445E-247	3	FAIM
**7.50482920371608E-117**	1.01383216553278	0.253	0.099	1.25765927795874E-112	3	TRMT13
**8.83926510909908E-268**	1.01336147789352	0.595	0.25	1.48128404698282E-263	3	DCUN1D4
**5.27137895552535E-225**	1.01291569745336	0.456	0.181	8.83377685366938E-221	3	RESF1
**8.8806096447703E-301**	1.01254839989726	0.648	0.275	1.48821256427061E-296	3	LNX2
**1.47371175851444E-182**	1.0104507581458	0.38	0.15	2.4696461649185E-178	3	MNAT1
**1.11186809478076E-180**	1.00937359224166	0.38	0.151	1.86326855323361E-176	3	TBCE
**1.08922058965838E-219**	1.00900816229891	0.48	0.199	1.82531586414951E-215	3	UBA6
**4.5283928909611E-130**	1.00726805043887	0.324	0.139	7.58868080667261E-126	3	CTPS2
**4.18307054250788E-294**	1.00695282375274	0.741	0.352	7.00998961513471E-290	3	LSS
**3.85959872610274E-304**	1.00654383555982	0.608	0.247	6.46791554520298E-300	3	AKAP13
**6.08838221236669E-192**	1.00509682276125	0.393	0.15	1.02029109114841E-187	3	ADRB2
**0**	1.00489996013583	0.95	0.614	0	3	HGD
**1.52388778605455E-159**	1.00487944443292	0.39	0.169	2.55373115187021E-155	3	UPF3B
**3.00411068384738E-114**	1.0045506304552	0.262	0.106	5.03428868399144E-110	3	GUF1
**7.07407970571516E-113**	1.00356935358046	0.305	0.135	1.18547427708375E-108	3	THADA
**1.64457406908502E-306**	1.002215375778	0.715	0.317	2.75597722497268E-302	3	DDX39B
**0**	1.00133539600968	0.722	0.315	0	3	IMPA1
**1.44174343483206E-264**	1.00116889542484	0.572	0.241	2.41607364809157E-260	3	APOOL
**9.09332995964592E-106**	1.00099817829951	0.259	0.108	1.52386023463746E-101	3	ATG10
**7.43179631089687E-208**	1.00095739871173	0.428	0.169	1.2454204257801E-203	3	POLB

**6.91870924718708E-296**	1.0000585724144	0.558	0.215	1.15943729564361E-291	3	MAN2A2
**1.2073178980688E-155**	0.999670204651958	0.351	0.142	2.02322333358369E-151	3	MBTD1
**0**	0.999535604161712	0.754	0.323	0	3	CLK1
**0**	0.999043289587476	0.95	0.62	0	3	SELENBP1
**0**	0.998988430709257	0.799	0.371	0	3	PANK3
**0**	0.99821050023964	0.782	0.36	0	3	RREB1
**7.63382985714925E-168**	0.998147500698898	0.395	0.168	1.27927720746107E-163	3	TRIP11
**2.87501464062462E-206**	0.997101056164557	0.261	0.071	4.81794953475874E-202	3	MICU3
**8.00524845604557E-144**	0.996701385559269	0.339	0.142	1.34151953626412E-139	3	TGDS
**0**	0.995207283448021	0.935	0.517	0	3	ERICH5
**1.62921201600378E-156**	0.994633340326892	0.366	0.154	2.73023349641913E-152	3	GK5
**2.23883788233159E-217**	0.992606671127748	0.454	0.183	3.75184452321127E-213	3	RAB3GAP1
**0**	0.991688755740709	0.701	0.306	0	3	KIAA0232
**1.94158368343295E-260**	0.991661365093494	0.535	0.213	3.25370593669695E-256	3	AASS
**1.45016002806555E-248**	0.991020974257906	0.539	0.222	2.43017817503225E-244	3	USP48
**0**	0.990943374612406	0.923	0.507	0	3	SERINC5
**6.99934251065181E-281**	0.989658989001064	0.584	0.239	1.17294981793503E-276	3	UQCC1
**0**	0.989605450131424	0.752	0.327	0	3	MCTS1
**1.1991421231225E-268**	0.98926934694106	0.594	0.252	2.00952236992869E-264	3	ZFAND1
**7.41143088003411E-134**	0.988887817592206	0.289	0.115	1.24200758687612E-129	3	FAM133B
**0**	0.988627258193733	0.697	0.299	0	3	UBR5
**1.03625162648971E-211**	0.987805298771992	0.471	0.196	1.73655047567146E-207	3	ATG2B
**8.96567542414249E-154**	0.987721763100829	0.287	0.103	1.5024678875778E-149	3	NINL
**6.21643261318983E-111**	0.987564377452363	0.25	0.1	1.04174977731835E-106	3	SMU1
**0**	0.986688467165882	0.667	0.279	0	3	TTPA
**1.23154356488974E-106**	0.985878842204433	0.293	0.131	2.06382070604223E-102	3	ARV1
**1.83832596757609E-228**	0.98562887231676	0.496	0.204	3.08066665646401E-224	3	GCH1
**6.87867202027958E-268**	0.985182855176618	0.568	0.236	1.15272785715845E-263	3	MORC3
**1.28249343192537E-243**	0.984171865739897	0.494	0.196	2.14920249322054E-239	3	NF1
**7.64593081135552E-222**	0.983506821162121	0.478	0.196	1.28130508536696E-217	3	CBR4
**7.01317660841314E-254**	0.983113714419166	0.555	0.23	1.17526813603787E-249	3	CLDN14
**3.32160803022183E-204**	0.982973172810154	0.476	0.201	5.56635073704575E-200	3	GTF2H1
**1.90369968931135E-185**	0.982339089583053	0.438	0.188	3.19021993934797E-181	3	MAP4K5
**8.98347446856089E-175**	0.982172219594938	0.451	0.198	1.50545065144143E-170	3	LAMA3
**4.27146898550112E-288**	0.982065540475936	0.621	0.264	7.15812772590278E-284	3	KIF1B
**2.07997223508659E-178**	0.981766066291793	0.432	0.187	3.4856174715581E-174	3	MAP3K4
**1.1583542939475E-144**	0.981217625113487	0.343	0.145	1.94117012579723E-140	3	SHPRH
**8.47306715343246E-224**	0.980951558269888	0.469	0.188	1.41991659357221E-219	3	FNIP1
**1.42768092493904E-94**	0.980056624084542	0.254	0.112	2.39250769401284E-90	3	NHLRC3
**3.7693130916036E-266**	0.979871460366582	0.653	0.291	6.31661487890932E-262	3	INTS3
**1.93239961040679E-280**	0.978834825433458	0.615	0.254	3.2383152671197E-276	3	NT5DC3
**1.13703379602313E-136**	0.978524805621469	0.31	0.127	1.90544123537556E-132	3	SIMC1
**1.54737047617887E-213**	0.977531458438678	0.475	0.198	2.59308344398055E-209	3	SLC5A3
**2.12651159031902E-206**	0.977104132159989	0.443	0.177	3.56360812305662E-202	3	HSF4
**3.44035227906412E-279**	0.976772938063218	0.572	0.231	5.76534234925566E-275	3	SHC2
**1.34747554814885E-155**	0.976419430749395	0.269	0.091	2.25809952358785E-151	3	ADAMTSL3
**0**	0.97640366740979	0.774	0.333	0	3	BTNL8
**2.19979900308975E-139**	0.976200142092649	0.262	0.094	3.68642316937781E-135	3	TTC28
**2.0688243116661E-165**	0.975947450624005	0.284	0.096	3.46693578149004E-161	3	BBOX1
**0**	0.975307009276065	0.898	0.472	0	3	C2orf72
**0**	0.974894776030374	0.742	0.331	0	3	ZBTB33
**1.31365372569756E-138**	0.97453954794085	0.357	0.157	2.20142091352396E-134	3	NVL
**1.27095675317825E-225**	0.974411692639762	0.497	0.206	2.12986932697611E-221	3	SCAF8
**1.74641566167635E-127**	0.974220461792775	0.297	0.122	2.92664336583723E-123	3	DEPRECATED-ENSG00000186204
**0**	0.971610012264476	0.733	0.312	0	3	COL27A1
**4.59794421170517E-295**	0.97149874602255	0.685	0.298	7.70523490997552E-291	3	BIRC6
**3.74998939078247E-121**	0.971456586552671	0.287	0.118	6.28423222107327E-117	3	MGA
**5.80351371311659E-203**	0.9700332698598	0.453	0.188	9.72552828044079E-199	3	SNX24
**0**	0.969045468531662	0.794	0.352	0	3	PWWP3A
**2.03458910575201E-107**	0.968893012557742	0.288	0.127	3.40956442341921E-103	3	BRAF

1.13955721270496E-200	0.96878118429833	0.431	0.172	1.90966997705097E-196	3	PAN2
3.76627027420063E-233	0.967133184684215	0.54	0.232	6.31151572550542E-229	3	UBE2G2
1.38103844351726E-296	0.966931519324997	0.731	0.326	2.31434422364623E-292	3	INTS8
4.23594509195154E-194	0.966807244836251	0.43	0.177	7.0985967850924E-190	3	ZFYVE16
6.59254289398487E-113	0.965779087993377	0.278	0.118	1.10477833817398E-108	3	WDFY3
0	0.964618304782864	0.959	0.508	0	3	CCNL2
0	0.96434124201695	0.871	0.468	0	3	TP53INP1
1.62224824727396E-148	0.96397474270274	0.361	0.153	2.71856361278171E-144	3	IRAK4
1.42694822508131E-186	0.96393595444858	0.347	0.126	2.39127983559126E-182	3	LRRC28
8.47345300748352E-226	0.963570440641937	0.539	0.234	1.41998125499409E-221	3	WDR33
8.3739549014705E-289	0.961691262845876	0.663	0.289	1.40330736238843E-284	3	SENP6
4.73629277319208E-136	0.96138630752852	0.314	0.129	7.93707942931528E-132	3	CSGALNACT2
7.98940021288301E-141	0.959990085344277	0.312	0.126	1.33886368767493E-136	3	TAF1
7.69739422995004E-162	0.959045206277503	0.381	0.16	1.28992932505503E-157	3	SEPTIN7
0	0.958669709346695	0.911	0.452	0	3	PTPRK
4.28940496522204E-164	0.958576927223938	0.349	0.138	7.18818484071909E-160	3	ENGASE
1.05563288578223E-147	0.958521724752512	0.376	0.164	1.76902958999386E-143	3	NSD2
3.25886692305965E-146	0.958235938185302	0.335	0.138	5.46120918966337E-142	3	SLC36A1
2.16867700825924E-289	0.957194407791265	0.654	0.282	3.63426893044083E-285	3	KMT2B
0	0.956655045494753	0.893	0.472	0	3	TMEM37
2.51054147363522E-159	0.956339923934729	0.361	0.148	4.20716540151791E-155	3	NAA30
2.73163290566255E-139	0.955957880194362	0.257	0.091	4.5776704233093E-135	3	SNX29
7.89795717481354E-120	0.955883685386737	0.289	0.12	1.32353966335525E-115	3	SMURF2
3.20023559378059E-115	0.95554612042819	0.293	0.126	5.36295480805751E-111	3	FPGT
7.10127893886409E-231	0.955120716349076	0.496	0.205	1.19003232457484E-226	3	UBR3
3.4699174057088E-176	0.95495423460662	0.408	0.171	5.8148875884868E-172	3	GPAT3
4.07493074449808E-175	0.954748824428705	0.411	0.174	6.82876894162989E-171	3	MCM3AP
3.20801367356382E-251	0.954488732413246	0.536	0.221	5.37598931415824E-247	3	CLCC1
1.66996488710472E-261	0.953929998604191	0.566	0.237	2.79852715781009E-257	3	CNST
0	0.953857254193994	0.743	0.331	0	3	STX16
8.7701340411181E-133	0.953220513132907	0.296	0.118	1.46969906261057E-128	3	FASTKD2
1.4829486509753E-301	0.952366509564816	0.647	0.275	2.48512534930441E-297	3	FOXO3
0	0.952340475653921	0.844	0.414	0	3	WWP1
2.77477528793198E-209	0.947529488696862	0.432	0.171	4.64996842751641E-205	3	CUL5
5.35344805316061E-149	0.94749936400819	0.363	0.152	8.97130824748655E-145	3	TINAG
1.94984568511387E-163	0.947239322228908	0.39	0.165	3.26755139911382E-159	3	PIGM
0	0.946975186159691	0.738	0.322	0	3	KAT6A
5.33414777771068E-156	0.94616830198694	0.393	0.171	8.93896484588756E-152	3	PIGG
5.40843207238237E-127	0.946126668185094	0.271	0.105	9.06345046689837E-123	3	DIS3L
5.82824248436078E-243	0.94541109042825	0.546	0.231	9.76696875529179E-239	3	GSTM4
5.37775493878899E-122	0.944406487908298	0.254	0.097	9.01204172642259E-118	3	HERC5
0	0.943912679398123	0.864	0.389	0	3	SULT1C2
1.11490999926514E-136	0.943623214418674	0.312	0.127	1.86836617676852E-132	3	EXOC6B
3.19267798857231E-245	0.942998169689442	0.589	0.256	5.35028977324947E-241	3	SNX5
7.4771764717273E-131	0.941944882277836	0.291	0.116	1.25302523313206E-126	3	FBXO11
2.26389805838722E-246	0.941324821609278	0.706	0.339	3.7938403662453E-242	3	TCIM
3.45676348990923E-113	0.941228463180276	0.258	0.104	5.79284425638989E-109	3	RNPC3
2.53917629589249E-266	0.940576732450355	0.625	0.273	4.25515163665663E-262	3	SSX2IP
3.71490133068731E-223	0.940426851728317	0.49	0.202	6.2254316499658E-219	3	TMLHE
0	0.940026748067587	0.699	0.303	0	3	TM4SF20
6.28349737451843E-170	0.939723314503515	0.372	0.149	1.0529884900218E-165	3	STK38L
1.36629552671388E-204	0.939291709950309	0.447	0.182	2.28963804366712E-200	3	MYNN
3.85854084938115E-128	0.93856449042836	0.264	0.1	6.46614275539293E-124	3	SFXN2
1.15850286186908E-108	0.937093958090136	0.263	0.109	1.9414190959202E-104	3	MTX3
1.2352823611492E-253	0.936002193294939	0.631	0.282	2.07008618081384E-249	3	SMYD2
0	0.934720729519633	0.823	0.388	0	3	STAU2
4.85682863958139E-168	0.934357533444738	0.411	0.176	8.13907343421049E-164	3	RABGAP1
5.08441317348635E-106	0.933109792917505	0.26	0.109	8.52045959612843E-102	3	VAMP4
6.31402291212564E-143	0.931347591734291	0.352	0.15	1.05810395961401E-138	3	CEP83
0	0.930202656904231	0.717	0.312	0	3	CHD2

5.87390124215198E-225	0.930116651643953	0.563	0.248	9.84348370159829E-221	3	THOP1
2.84880073345144E-183	0.929163670041129	0.438	0.186	4.77402026911793E-179	3	PLEKHA5
4.34051512244669E-201	0.929132518739573	0.427	0.169	7.27383524219617E-197	3	NSUN6
1.86954443004047E-119	0.928244006549146	0.265	0.105	3.13298255586182E-115	3	RLIM
2.11607680743932E-178	0.927823863133672	0.449	0.196	3.54612151390681E-174	3	DIP2A
5.24586320534783E-225	0.927816227658408	0.524	0.225	8.79101755952189E-221	3	NEMF
1.07745088718041E-177	0.927775675537025	0.435	0.188	1.80559219673693E-173	3	APOL2
4.10278923539582E-116	0.927378199570828	0.299	0.129	6.87545420067632E-112	3	EED
2.42034630737358E-230	0.927223014055786	0.487	0.196	4.05601634189664E-226	3	DNMT3A
3.29587596165219E-158	0.926387302552273	0.345	0.138	5.52322893653674E-154	3	MLLT10
2.61529438008286E-127	0.925240829180742	0.343	0.152	4.38271032214285E-123	3	GTF2IRD1
3.02561428217715E-220	0.923108243800227	0.483	0.2	5.07032441407247E-216	3	MED16
2.72632987179127E-163	0.923055945747221	0.266	0.086	4.5687835991478E-159	3	GLDC
4.49537248280502E-107	0.92237822077373	0.275	0.118	7.53334520668465E-103	3	ZNF397
2.73941809548587E-124	0.921362243217973	0.319	0.138	4.59071684441521E-120	3	GCNT2
2.00445544101827E-239	0.919425311278285	0.548	0.231	3.35906642805843E-235	3	DNASE1L1
0	0.918165330786279	0.835	0.404	0	3	CYP4V2
2.1124929909813E-188	0.917689324095616	0.425	0.176	3.54011575428647E-184	3	MED6
9.41051643464091E-172	0.915589472271283	0.366	0.145	1.57701434411712E-167	3	ABL2
3.04752845221481E-150	0.915215504921047	0.334	0.135	5.10704818022158E-146	3	AGO3
1.48738899165948E-210	0.915206386648419	0.432	0.168	2.49256647222296E-206	3	APBB3
0	0.914938818272204	0.962	0.754	0	3	FMO5
6.47666629865411E-200	0.912308761012141	0.454	0.189	1.08535973832846E-195	3	WBP1
2.90024504669295E-238	0.909541715145463	0.46	0.174	4.86023064924805E-234	3	TM2D3
7.49607939526933E-205	0.908462931498617	0.466	0.192	1.25619298505923E-200	3	FBXL8
1.57417330617367E-300	0.908101575782198	0.696	0.306	2.63799962648584E-296	3	USP12
5.24551919697337E-149	0.907871252988844	0.389	0.172	8.79044107028798E-145	3	GEMIN7
1.13096394267619E-211	0.907450376731366	0.513	0.224	1.89526937513676E-207	3	TENT2
1.03781710852661E-200	0.906854446902585	0.501	0.22	1.7391739104689E-196	3	ELMOD2
2.79449087982671E-218	0.906478650414697	0.511	0.217	4.68300781641359E-214	3	CLDND1
1.35947368487871E-158	0.905035207833505	0.395	0.171	2.27820600111974E-154	3	WFS1
5.84975494350445E-274	0.903291892685708	0.78	0.381	9.80301933432476E-270	3	SLC35D2
2.08001789323926E-284	0.903247740944089	0.7	0.309	3.48569398549035E-280	3	HECTD1
6.69355814677794E-211	0.90311429903458	0.351	0.115	1.12170647423705E-206	3	PFKFB1
1.18754876890332E-179	0.90191897854572	0.364	0.139	1.99009422692819E-175	3	WDR6
1.59216783251889E-295	0.90125065382522	0.672	0.285	2.66815485373515E-291	3	UGT2B11
3.89704416780116E-110	0.901119298226324	0.255	0.103	6.53066661640119E-106	3	RAB2B
3.42892367723253E-121	0.900345313593634	0.269	0.107	5.74619029830627E-117	3	LDAH
1.19831984013712E-168	0.899481973517335	0.413	0.178	2.00814438810178E-164	3	KIAA2026
6.00768096531803E-239	0.898559117169149	0.616	0.279	1.00676717616799E-234	3	GATAD1
1.83521914627874E-243	0.898410364082453	0.573	0.245	3.07546024533391E-239	3	ASAP1
1.95381672036011E-153	0.897814377054421	0.37	0.155	3.27420605997947E-149	3	ACTR3C
1.50405626141517E-274	0.897786646179766	0.645	0.28	2.52049748287955E-270	3	TTC37
3.00107776967119E-132	0.897734836129444	0.355	0.155	5.02920612641498E-128	3	HAL
4.85474893044517E-268	0.89609170648597	0.627	0.27	8.13558825764002E-264	3	MARCH7
2.48768958629181E-135	0.895234603151062	0.318	0.131	4.16887020870782E-131	3	CHD6
2.5873392075574E-174	0.8946242157218	0.409	0.172	4.33586304402469E-170	3	SLC25A46
1.23372143430652E-124	0.894510271109516	0.295	0.122	2.06747037961086E-120	3	DEPRECATED-ENSG00000072803
1.49972415140615E-266	0.8943211462206	0.611	0.261	2.51323773292643E-262	3	TMEM129
3.28133217936281E-161	0.893220016621404	0.339	0.132	5.4988564661762E-157	3	ITFG2
8.66257211301688E-198	0.893196715565069	0.477	0.205	1.45167383469937E-193	3	TMEM131
1.56141197367188E-184	0.892861140509769	0.447	0.191	2.61661418547933E-180	3	CDKAL1
1.39474242620201E-124	0.892820788302379	0.33	0.146	2.33730935782932E-120	3	FBXO4
3.08353710697618E-210	0.892100591483086	0.537	0.234	5.16739148387068E-206	3	DONSON
6.47447914600068E-137	0.891947367718974	0.376	0.171	1.08499321528679E-132	3	PKN2
2.79559803138145E-237	0.891863110600679	0.561	0.242	4.68486318098904E-233	3	NSMCE2
5.95207833068533E-151	0.891624282009107	0.377	0.162	9.97449286656247E-147	3	RNF170
3.33540596298393E-297	0.891135157383755	0.754	0.345	5.58947331276848E-293	3	OPLAH
6.58223779504614E-119	0.89043877588503	0.296	0.125	1.10305140969383E-114	3	MYCBP2
3.79608014275806E-196	0.890433630812905	0.46	0.195	6.36147110323395E-192	3	FNIP2

1.21345425739897E-269	0.888448352871873	0.669	0.302	2.03350664454919E-265	3	TACC1
5.45255627377194E-188	0.885991369515593	0.452	0.194	9.13739380358701E-184	3	NEK9
1.84337246720224E-161	0.885822533751285	0.348	0.138	3.08912358053752E-157	3	ACYP2
2.27065977397943E-194	0.885785845717328	0.472	0.202	3.80517164923472E-190	3	KLHL22
1.08810443691938E-103	0.885423364110136	0.274	0.119	1.8234454153895E-99	3	RASA1
1.42454568625551E-181	0.885258136961476	0.417	0.172	2.38725366102699E-177	3	ASCC3
1.29955672671586E-174	0.884751246351381	0.426	0.182	2.17779716263044E-170	3	PCGF3
8.1011670829373E-103	0.884657511986829	0.289	0.129	1.35759357975863E-98	3	PRMT3
6.78152926040921E-142	0.884395235863438	0.382	0.172	1.13644867345938E-137	3	KRIT1
2.5023555500553E-299	0.884312800419961	0.792	0.39	4.19344743078267E-295	3	ACSM2B
6.57761277622298E-121	0.884165081300049	0.3	0.127	1.10227634903945E-116	3	ZNF692
6.65455919338671E-302	0.884145204717403	0.749	0.349	1.11517102962775E-297	3	CRY2
1.22741215871663E-263	0.883379990317467	0.64	0.281	2.05689729557733E-259	3	PARP10
1.84846456753805E-202	0.883010483808486	0.493	0.211	3.09765692228026E-198	3	FAM136A
1.48611730704116E-181	0.882924812569063	0.368	0.139	2.49043538313958E-177	3	ZNF512B
1.56957726050417E-161	0.882643135503247	0.376	0.155	2.63029757315288E-157	3	EXOC8
2.80970896020305E-265	0.882415620017115	0.747	0.366	4.70851027550827E-261	3	TUT7
4.39852550417989E-93	0.88224329603317	0.279	0.13	7.37104903990466E-89	3	CD58
7.29066564468056E-122	0.882182103074097	0.37	0.171	1.22176974873557E-117	3	KNTC1
6.69560418959949E-115	0.879250036431216	0.327	0.148	1.12204935009308E-110	3	MSTO1
2.09351369664675E-107	0.87905175658888	0.293	0.13	3.50831025284062E-103	3	ZZEF1
3.80310148156825E-94	0.878947873470154	0.256	0.113	6.37323746281208E-90	3	DEPRECATED-ENSG00000050130
4.20340425605568E-109	0.878510443923615	0.313	0.142	7.0440648522981E-105	3	AMMECR1
9.15232605431115E-154	0.877713934618496	0.414	0.186	1.53374680018146E-149	3	OTUD6B
1.16970098699057E-260	0.877443543441471	0.628	0.282	1.9601849139988E-256	3	DHTKD1
0	0.876586978696864	0.824	0.406	0	3	ITPR2
2.35090700878958E-94	0.87644480738651	0.253	0.11	3.93964996532959E-90	3	TBC1D2
1.29937703468269E-191	0.875486356968461	0.49	0.217	2.17749603472125E-187	3	WDR44
1.29021763901107E-186	0.873773294287144	0.458	0.197	2.16214671945476E-182	3	LCLAT1
5.80909195161802E-167	0.873171080865534	0.393	0.165	9.73487629252148E-163	3	PIGP
1.49957142702333E-193	0.872332942088553	0.468	0.201	2.51298179740569E-189	3	TAPT1
1.90915881392774E-251	0.872254680304589	0.6	0.262	3.19936834038011E-247	3	SLC33A1
7.21863851598126E-102	0.872111447620435	0.28	0.123	1.20969944250814E-97	3	TMEM187
3.75626384242989E-235	0.871428655496515	0.596	0.265	6.29474694714401E-231	3	IDUA
1.40245854368596E-296	0.871299643629917	0.625	0.256	2.35024002750893E-292	3	GPC6
1.42217555283057E-191	0.871040795884489	0.452	0.188	2.38328179143347E-187	3	ZNF680
3.74639643726314E-160	0.870952792365503	0.387	0.163	6.27821114956557E-156	3	CLK4
7.60921660069444E-276	0.870799286935804	0.762	0.364	1.27515251794437E-271	3	PCCA
9.19788337659332E-280	0.870532420208333	0.751	0.346	1.54138129624951E-275	3	ACMSD
2.97592133815333E-205	0.870501347746019	0.523	0.232	4.98704897847735E-201	3	GSK3B
1.32874912567758E-208	0.870170047777321	0.541	0.242	2.22671778481049E-204	3	CXADR
1.31466781968124E-84	0.870087843330873	0.265	0.124	2.20312033222182E-80	3	ATXN7L1
7.23701807088243E-105	0.869594764407759	0.256	0.107	1.21277948831848E-100	3	WDR27
1.04510753331777E-94	0.869541072407421	0.278	0.127	1.75139120433392E-90	3	ITGB3BP
4.26171763523686E-133	0.86856132922724	0.357	0.158	7.14178641312994E-129	3	TBPL1
5.63848849452244E-126	0.865956128397072	0.288	0.116	9.44897901912071E-122	3	ZNF280D
4.12831664727649E-124	0.864804133260898	0.318	0.136	6.91823303750594E-120	3	OFD1
6.50725631693392E-92	0.864761092280024	0.262	0.118	1.09048601359179E-87	3	MTF2
2.14676538075587E-125	0.864021478320646	0.349	0.157	3.59754942507069E-121	3	LNPEP
1.22044195921806E-134	0.863215357097337	0.263	0.094	2.04521663525762E-130	3	CYP17A1
3.70146281572597E-152	0.862306027435552	0.349	0.141	6.20291138659357E-148	3	CDHR2
7.90400547220442E-218	0.86211840329286	0.586	0.265	1.32455323703202E-213	3	ZDHHC14
7.67622255819973E-192	0.861369170540809	0.425	0.171	1.28638137630311E-187	3	TRPM7
1.04069089305955E-169	0.861175636376605	0.416	0.178	1.7439897985892E-165	3	ADCK5
5.24073833885245E-198	0.861125592516685	0.462	0.195	8.78242930824893E-194	3	HERC4
5.04949912632351E-198	0.859962219644466	0.47	0.2	8.46195063589294E-194	3	TRMT11
7.28096007527434E-229	0.859863515752542	0.586	0.261	1.22014328941447E-224	3	MOCOS
2.75355644949335E-117	0.857772974112029	0.308	0.134	4.61440989806096E-113	3	DDX60
3.55608659391046E-150	0.857608657408871	0.381	0.164	5.95928991407515E-146	3	VEZT
4.4933123944349E-235	0.857441324784684	0.593	0.262	7.52989291059401E-231	3	TPP2

8.21972582100533E-234	0.857408061366809	0.54	0.228	1.37746165308407E-229	3	RSAD1
6.9357890284229E-114	0.857232971539259	0.309	0.137	1.16229952538311E-109	3	TANC1
1.78534547258696E-146	0.857232583678951	0.382	0.168	2.99188194296123E-142	3	LTN1
3.61510393389518E-177	0.856923525724475	0.474	0.211	6.05819117242155E-173	3	DMTF1
7.53263770390086E-263	0.856872861606057	0.711	0.328	1.26231942641971E-258	3	DLG1
6.01615387239232E-211	0.856260709114578	0.543	0.241	1.00818706593551E-206	3	LRRC58
8.89782550710577E-107	0.856259015001151	0.311	0.141	1.49109759848079E-102	3	MALT1
6.47793628833396E-140	0.855958736402315	0.424	0.2	1.08557256319901E-135	3	MAP3K21
5.0268851895655E-123	0.855013999259384	0.277	0.11	8.42405420067387E-119	3	ERCC6
4.8197908789833E-261	0.854795137895788	0.673	0.306	8.07700555500021E-257	3	ECPAS
3.93171596420305E-292	0.853750555181704	0.799	0.385	6.58876961281147E-288	3	KLHDC2
3.42063966803187E-301	0.853533923130787	0.899	0.447	5.7323079556878E-297	3	F2RL1
1.60022252622321E-95	0.852657881772733	0.273	0.123	2.68165290944485E-91	3	ZNF263
6.81583671659138E-113	0.851790482117714	0.266	0.108	1.14219791696638E-108	3	USP30
8.55101302651361E-153	0.851473730501538	0.375	0.161	1.43297876298315E-148	3	SOS 1
3.3468725626389E-222	0.851458341085078	0.593	0.27	5.60868904047027E-218	3	SCML1
7.78933869554833E-105	0.850926114297334	0.272	0.117	1.30533737859999E-100	3	CCNH
1.36953897760102E-206	0.85035672899233	0.543	0.245	2.29507341866379E-202	3	KHDC4
7.04071869699217E-222	0.850179622770149	0.582	0.261	1.17988363924195E-217	3	ARRB1
1.2707633436674E-180	0.850116238560401	0.46	0.202	2.12954521131782E-176	3	ABCB10
1.94630796334657E-129	0.849939571228046	0.373	0.171	3.26162288497618E-125	3	USPL1
3.82396234309213E-138	0.849927305672452	0.298	0.117	6.40819609455379E-134	3	NR2C1
7.1973615417791E-105	0.849862624436593	0.276	0.12	1.20613384717134E-100	3	RHOQ
1.66108236846708E-162	0.849628243436701	0.44	0.199	2.78364183307713E-158	3	DEPRECATED-ENSG00000134597
2.55198623951456E-258	0.849432540992961	0.686	0.322	4.2766185401785E-254	3	PRPF3
9.98970433006171E-176	0.848331460003036	0.44	0.192	1.67407465163174E-171	3	ARHGAP17
9.98904059094979E-101	0.848152094184071	0.289	0.13	1.67396342223137E-96	3	AP3M2
1.54819448617186E-212	0.847699313879678	0.534	0.235	2.59446431992681E-208	3	TERF1
8.82732135473307E-215	0.847614507932118	0.538	0.237	1.47928251262617E-210	3	SRP19
3.14426522963326E-95	0.847529916093821	0.28	0.127	5.26915967181942E-91	3	FANCC
9.42520767381652E-114	0.84739376836238	0.282	0.118	1.57947630197817E-109	3	XPA
5.81140221424516E-215	0.84704276266578	0.575	0.262	9.73874783063204E-211	3	PLPP1
3.49630342673434E-119	0.846719901930413	0.343	0.158	5.85910528252141E-115	3	ARMCX5
5.4452182702424E-155	0.846091025182656	0.294	0.105	9.12509677727221E-151	3	CYP7A1
6.06841346186506E-176	0.845179138633529	0.415	0.173	1.01694472793935E-171	3	RAPGEF2
2.49320848867184E-186	0.844959149657322	0.255	0.074	4.17811878531627E-182	3	COL6A1
3.56152342469722E-134	0.844718809939283	0.338	0.143	5.9684009551076E-130	3	PMS1
4.58656796480777E-157	0.8445549417831	0.412	0.183	7.68617059542487E-153	3	SLC30A5
1.85729749589905E-269	0.844179000105978	0.735	0.335	3.11245914362762E-265	3	PRKDC
9.42275894779868E-252	0.843718416728797	0.636	0.283	1.5790659444721E-247	3	ANKRD17
2.47737764981694E-259	0.843313811161641	0.724	0.343	4.15158946556322E-255	3	ACVR1B
3.23284871784506E-182	0.843296414708556	0.454	0.197	5.41760788136476E-178	3	SETX
7.26134663531679E-121	0.843183719606846	0.315	0.136	1.21685646914639E-116	3	FER
9.98786740492772E-288	0.843121670655824	0.705	0.318	1.67376681971779E-283	3	ZBTB20
8.78995068986498E-97	0.843087533828352	0.264	0.116	1.47301993660757E-92	3	UIMC1
3.37199990175477E-209	0.842860586729493	0.504	0.219	5.65079743536064E-205	3	TSNAX
1.48316509991175E-141	0.84223954965243	0.357	0.153	2.48548807443211E-137	3	RBM5
7.30695164910537E-142	0.841969276967862	0.395	0.18	1.22449895735708E-137	3	NUP155
2.27210394032449E-125	0.8417359802704	0.33	0.143	3.80759178319578E-121	3	SEPSECS
4.79250412486358E-127	0.839636678724156	0.351	0.158	8.03127841244638E-123	3	SUCO
2.11790701943514E-200	0.839321079433773	0.524	0.234	3.5491885831694E-196	3	SMC6
9.33484438879048E-100	0.839046325029405	0.303	0.141	1.56433322267351E-95	3	CEP192
5.17504507638371E-252	0.838788775983853	0.56	0.232	8.67234053900382E-248	3	ABCG5
7.46729499668851E-135	0.838513621013613	0.397	0.185	1.25136929554506E-130	3	TMEM50B
3.22397470340594E-201	0.83827306627853	0.511	0.226	5.40273680796768E-197	3	MAP3K2
1.41121170732654E-116	0.838006786813592	0.321	0.142	2.36490857913781E-112	3	SLC35E2B
1.18866925845062E-116	0.837345528475983	0.373	0.177	1.99197194331155E-112	3	VIPR1
2.96026433831872E-148	0.837093234659341	0.338	0.137	4.96081097815451E-144	3	BCAS3
2.43289063838835E-146	0.836120926599576	0.37	0.159	4.0770381318112E-142	3	GNPTAB
4.44327397412483E-146	0.835962526271	0.35	0.146	7.44603852583839E-142	3	SOCS7

1.07028605189727E-90	0.835644667354225	0.26	0.117	1.79358536576945E-86	3	TBP
1.35481536344684E-135	0.834297568649158	0.325	0.136	2.27039958606422E-131	3	RGL2
5.22542986496248E-104	0.834287583881605	0.289	0.129	8.75677536770412E-100	3	SLC22A5
1.14368047728315E-253	0.833697343181009	0.699	0.33	1.9165797438311E-249	3	MBNL1
7.0882364110469E-126	0.833538720938315	0.292	0.119	1.18784665776324E-121	3	LIN52
1.85519485366232E-155	0.833421110801125	0.318	0.119	3.10893553576731E-151	3	TAB3
2.8518698381098E-267	0.833308619280055	0.68	0.31	4.7791634747044E-263	3	SPPL2A
1.74347672492788E-191	0.833167051930044	0.407	0.161	2.92171829563414E-187	3	AP3S2
3.20640539992154E-112	0.83254808451068	0.317	0.143	5.37329416918851E-108	3	GPCPD1
1.60058230833598E-190	0.831778153041177	0.522	0.234	2.68225583230944E-186	3	FRYL
1.23424709694504E-157	0.831442043964336	0.41	0.181	2.0683512850605E-153	3	PCNX1
1.0398660141154E-88	0.830346972519626	0.255	0.115	1.74260746645459E-84	3	ZNF236
1.31515708786551E-154	0.829734786682071	0.408	0.18	2.20394024784502E-150	3	FAM193A
9.48054580826191E-233	0.829310598959578	0.559	0.242	1.58874986654853E-228	3	TUBGCP6
5.56197447621429E-147	0.829254758542529	0.385	0.169	9.3207568272399E-143	3	SMC5
2.71796930484449E-240	0.829053087862985	0.62	0.277	4.5547729610584E-236	3	EEF2K
4.004499647929E-141	0.82856639635396	0.36	0.155	6.71074050999942E-137	3	FAM120B
2.96156553119162E-110	0.828115329376078	0.297	0.131	4.96299151717091E-106	3	L2HGDH
1.47641452153771E-181	0.828101511117239	0.464	0.203	2.4741754551929E-177	3	ATP6V0A1
1.11591631803586E-291	0.828014456190019	0.79	0.359	1.8700525657645E-287	3	TMEM63A
1.29151544704251E-207	0.827478696914798	0.51	0.22	2.16432158615383E-203	3	SDR39U1
4.13421707684052E-151	0.827375972999792	0.401	0.177	6.92812097736934E-147	3	ETAA1
6.69408823460886E-148	0.827304286746673	0.388	0.17	1.12179530635575E-143	3	NSMCE4A
4.55225471613349E-105	0.826974189392469	0.274	0.118	7.6286684532965E-101	3	GABPA
1.16917012620067E-145	0.826770392462277	0.365	0.155	1.95929529748708E-141	3	WASHC4
2.75274879358043E-105	0.826392750563431	0.272	0.116	4.61305642828208E-101	3	HELZ2
6.67217115969125E-200	0.825486288242285	0.476	0.2	1.11812244294106E-195	3	CBFA2T2
1.32661247117335E-121	0.825321246852105	0.335	0.15	2.2231371791923E-117	3	TRNT1
8.58984230316537E-150	0.825250816737886	0.392	0.173	1.43948577316445E-145	3	NAGLU
3.91191111686123E-192	0.825097889816835	0.52	0.233	6.55558064963605E-188	3	KBTBD2
8.78032784688474E-92	0.824450654002703	0.262	0.117	1.47140734058094E-87	3	ERI2
1.06789420254961E-98	0.824228487019851	0.289	0.132	1.78957710463264E-94	3	ATR
2.64839389143908E-164	0.823704146322654	0.452	0.204	4.43817848327361E-160	3	ELF2
1.51105117291688E-247	0.823529849585294	0.637	0.289	2.53221955557411E-243	3	ZNF638
8.20190353722428E-236	0.823155483628859	0.659	0.304	1.37447499476804E-231	3	MICAL3
1.09096125857822E-232	0.822857265057632	0.637	0.292	1.82823287712538E-228	3	PKP2
1.21392705430017E-258	0.822295002833082	0.711	0.327	2.03429895759622E-254	3	GPT
1.07662134007621E-246	0.821902045688337	0.656	0.291	1.80420204169971E-242	3	TRMT2B
2.96213302563597E-152	0.821584255597003	0.381	0.165	4.96394252436076E-148	3	CUL2
3.61300438921812E-176	0.821544561676506	0.415	0.174	6.05467275545173E-172	3	COPS2
2.06157852804361E-223	0.82079690065724	0.562	0.247	3.45479329729549E-219	3	KMT2D
1.07815031843286E-238	0.819696530253612	0.646	0.3	1.80676430362978E-234	3	PCMTD1
1.92374748137764E-132	0.818431373610074	0.363	0.163	3.22381602929265E-128	3	SMG1
7.21749721876343E-115	0.816727014042074	0.297	0.127	1.20950818392038E-110	3	SPATA5L1
5.60970577078249E-150	0.816542608884227	0.377	0.162	9.40074493067729E-146	3	ELL
3.69539206375034E-130	0.816476066658048	0.37	0.169	6.19273802043282E-126	3	DNAAF2
1.33472108063601E-235	0.81623798826857	0.645	0.297	2.23672558692983E-231	3	VPS45
2.39542681057642E-205	0.815789503417321	0.585	0.268	4.01425624916397E-201	3	KDM6A
1.04836181947661E-202	0.81555026492976	0.485	0.205	1.7568447370789E-198	3	SKIL
6.86444090933561E-143	0.815270829539916	0.377	0.166	1.15034300758646E-138	3	NHLRC2
3.06674471346651E-210	0.815261586424937	0.546	0.242	5.13925079082718E-206	3	DOCK5
4.589620132294E-198	0.814970923026472	0.534	0.242	7.69128541769828E-194	3	TAF2
1.41811612399457E-118	0.814557793390639	0.291	0.122	2.37647900059009E-114	3	TUBGCP4
5.11063214108691E-107	0.814441855373512	0.294	0.13	8.56439734203345E-103	3	CMTR2
8.95531991978158E-256	0.814407688151156	0.889	0.499	1.500732512157E-251	3	C6
1.37443919463962E-190	0.814025276743857	0.522	0.238	2.30328520237708E-186	3	LRRC14
3.91037487875536E-175	0.81395060118698	0.433	0.185	6.55300622181823E-171	3	TELO2
2.65089418430468E-255	0.813358520304887	0.805	0.395	4.44236847405778E-251	3	CPD
6.42085635026152E-114	0.81328885577908	0.282	0.118	1.07600710717683E-109	3	IRGQ
1.10188716148961E-156	0.812898160114215	0.415	0.185	1.84654250522429E-152	3	CHD9

4.8610841320102E-206	0.812421928719683	0.487	0.207	8.14620478842269E-202	3	PIKFYVE
5.69960831296947E-151	0.811664673435167	0.428	0.195	9.55140361087423E-147	3	IPMK
1.18015834517239E-181	0.810882701709794	0.487	0.217	1.97770935483989E-177	3	REV1
5.0404960313169E-95	0.809325331837933	0.257	0.112	8.44686324928087E-91	3	MAP4K3
1.33419341036613E-242	0.809031377856988	0.638	0.292	2.23584131709156E-238	3	MPV17
2.74030985331916E-143	0.807131695677481	0.369	0.16	4.59221125219224E-139	3	FBXO38
1.83833648213361E-186	0.806990426107135	0.436	0.181	3.0806842767595E-182	3	PLEKHH3
2.3180367136074E-116	0.806590987774061	0.364	0.171	3.88456592466328E-112	3	BRCC3
5.64097305293713E-277	0.806415375382014	0.89	0.482	9.45314264211204E-273	3	KCTD3
2.96933241734081E-181	0.80634904640717	0.489	0.22	4.97600726497973E-177	3	ATXN2L
1.553654599208E-276	0.806326709289634	0.767	0.37	2.60361437735277E-272	3	CHTOP
4.88254733977627E-141	0.805827665945591	0.335	0.137	8.18217283199708E-137	3	RAPGEF4
2.96319748885031E-209	0.805643466024382	0.549	0.243	4.96572635181535E-205	3	ZBTB10
7.15282163443366E-118	0.805480184584446	0.355	0.165	1.19866984949839E-113	3	IFI44
3.12545548984221E-233	0.805249067636304	0.578	0.251	5.23763830987758E-229	3	AQP3
8.07993437741283E-97	0.804804630880794	0.275	0.123	1.35403540296684E-92	3	XYLT2
1.1687887148825E-261	0.803729805917068	0.73	0.35	1.95865612840009E-257	3	SLC25A15
1.87604692376548E-195	0.803665117488306	0.445	0.183	3.1438794348462E-191	3	NDUFAF6
3.16051804143079E-202	0.803251366081913	0.53	0.236	5.29639613382972E-198	3	METTL17
2.74685562697282E-126	0.802978526408393	0.361	0.165	4.60318065968105E-122	3	ITSN1
1.45419471314649E-241	0.802737408674948	0.657	0.304	2.43693950029088E-237	3	PHF3
1.72954556525393E-148	0.802587704483877	0.395	0.176	2.89837245825254E-144	3	ATAD2B
1.10680493485142E-210	0.802089247129418	0.537	0.235	1.85478370982401E-206	3	C19orf25
2.278609310927E-104	0.801884051293367	0.3	0.135	3.81849348325146E-100	3	SCYL3
3.39901049987724E-148	0.801355111639125	0.407	0.183	5.69606179569428E-144	3	PPIP5K2
1.66808901642629E-212	0.800751270183824	0.474	0.192	2.79538357372718E-208	3	KAT2A
5.49491498675474E-110	0.800713269615277	0.272	0.113	9.20837853480359E-106	3	GKAP1
3.01514580079415E-225	0.800065541477141	0.591	0.268	5.05278133297084E-221	3	TOR1AIP1
1.26613265423884E-138	0.799402545942846	0.358	0.155	2.12178510197345E-134	3	UPF2
3.2577785150987E-177	0.799391745599644	0.425	0.178	5.4593852356024E-173	3	MCEE
2.1774499743896E-171	0.798870340480574	0.473	0.214	3.64897066708209E-167	3	EIF2S3
5.33854494045334E-169	0.798759686971198	0.441	0.194	8.94633361121171E-165	3	DEPRECATED-ENSG00000188313
1.65516323535236E-186	0.798630683902789	0.501	0.224	2.77372254980348E-182	3	SPIDR
8.99644202856873E-190	0.798616273480178	0.449	0.189	1.50762375514755E-185	3	TNKS
1.55828919703956E-134	0.79827132330445	0.346	0.15	2.6113810363989E-130	3	ZNF407
9.97663997116816E-157	0.796983988184974	0.437	0.199	1.67188532636836E-152	3	FKBP3
6.82600721660079E-242	0.796301325795055	0.647	0.297	1.14390228935796E-237	3	XRN1
1.04312465150595E-231	0.796158017817667	0.704	0.349	1.74806829099368E-227	3	NSDHL
1.0307517310877E-100	0.795878501148895	0.283	0.126	1.72733375095677E-96	3	HCFC2
2.21720884196922E-146	0.795872367458937	0.406	0.185	3.71559857737202E-142	3	SNX19
3.82554120860046E-145	0.795746329156859	0.383	0.167	6.41084195737265E-141	3	UNK
7.27368363081802E-214	0.795523144189932	0.546	0.242	1.21892390285248E-209	3	PHYKPL
1.63518457516861E-269	0.795145204947188	0.837	0.427	2.74024231106755E-265	3	AFF1
8.05107821339942E-124	0.795100043752495	0.327	0.142	1.34919968700147E-119	3	ESCO1
9.28708106128139E-202	0.794966156115117	0.541	0.245	1.55632904424953E-197	3	ARHGAP5
1.82255976305173E-166	0.794935193672553	0.431	0.19	3.05424565092209E-162	3	ZC3H14
1.03712654059506E-238	0.79413885361697	0.641	0.285	1.7380166567292E-234	3	TUBGCP3
4.98996256615071E-98	0.793781623003761	0.298	0.139	8.36217926835537E-94	3	SOCS4
4.99378369971549E-125	0.793742639495112	0.32	0.136	8.36858272398323E-121	3	ZFYVE9
9.36766142635415E-284	0.793734477455809	0.805	0.398	1.56983270182843E-279	3	GSTA2
1.03169860611435E-233	0.792666879957931	0.643	0.302	1.72892052412643E-229	3	TM7SF2
3.29845168615425E-237	0.792202785153486	0.664	0.304	5.5275453356573E-233	3	SERPINA5
7.62895907796472E-245	0.791889363832022	0.649	0.293	1.27846096228533E-240	3	PHF20L1
1.63615789489255E-253	0.791851795577677	0.63	0.282	2.74187340026094E-249	3	FOXP4
2.2963697773371E-84	0.791022111862453	0.265	0.124	3.84825647286151E-80	3	ORC2
4.50463223022607E-245	0.78998481462581	0.758	0.374	7.54886269141284E-241	3	RNF103
1.52764691488683E-277	0.7899378432073	0.813	0.401	2.56003069996736E-273	3	SETD5
6.35181053262564E-129	0.789720876057667	0.388	0.181	1.0644364090574E-124	3	SMPD4
1.17112467430931E-231	0.789577919327135	0.586	0.26	1.96257072920755E-227	3	EPB41L5
1.79478042673602E-103	0.788310344577722	0.291	0.131	3.00769303912423E-99	3	PPIL4

7.70884782089615E-112	0.788122689934479	0.325	0.147	1.29184871782578E-107	3	THBS3
1.41933854293353E-100	0.787330035883974	0.292	0.133	2.37852753024801E-96	3	DNAJB14
5.97639237315372E-123	0.787080746221482	0.327	0.143	1.0015238338931E-118	3	DIP2C
2.22456026504382E-134	0.786723539482457	0.355	0.156	3.72791809216043E-130	3	NCOA3
1.41548232254077E-92	0.786452888655914	0.282	0.13	2.37206527611382E-88	3	ZNF654
3.91055591339436E-87	0.786357605577708	0.26	0.118	6.55330959966627E-83	3	SDCCAG8
3.52721951766848E-287	0.785610976108193	0.755	0.341	5.91091446770883E-283	3	DEPRECATED-ENSG00000213759
3.43618794498403E-87	0.785326494702467	0.276	0.13	5.75836375820424E-83	3	NAA40
1.17703935142158E-218	0.785030803657534	0.59	0.269	1.97248254511228E-214	3	TGFBR2
1.9694869034845E-137	0.784525526460483	0.33	0.137	3.30046615285932E-133	3	CRBN
2.14482636621409E-138	0.784438120537888	0.372	0.165	3.59430002450158E-134	3	MBIP
6.75575531792027E-202	0.783705332114075	0.461	0.191	1.13212947617708E-197	3	THNSL2
4.22592792309588E-231	0.782373226182442	0.604	0.273	7.08181001352408E-227	3	BACH1
2.97264589860416E-125	0.780656970442262	0.375	0.174	4.98155999688086E-121	3	ZNF507
7.80275935363575E-267	0.779755166138956	0.735	0.346	1.30758641248228E-262	3	LIMD1
7.28854097033094E-172	0.779626967659602	0.458	0.203	1.22141369580806E-167	3	LETMD1
2.62611715192357E-233	0.779210310481058	0.672	0.321	4.40084712319351E-229	3	UBE2D3
1.48348906042277E-210	0.778995968406643	0.648	0.305	2.48603096745648E-206	3	PARP4
1.64476607549375E-293	0.777596664431529	0.577	0.22	2.75629898931242E-289	3	LTBP3
1.40619641967112E-136	0.777035592090665	0.386	0.175	2.35650396008487E-132	3	INPP1
3.07801049180301E-220	0.77690510818079	0.56	0.246	5.15812998216348E-216	3	RFC1
8.79272749930445E-152	0.776077314984641	0.368	0.154	1.47348527433344E-147	3	GOLGA1
1.73843791995876E-146	0.775478312169825	0.397	0.177	2.91327426626689E-142	3	VPS54
4.62417368779761E-121	0.775168267848429	0.306	0.13	7.74919026601123E-117	3	C5orf51
9.28756562346497E-257	0.774986508736186	0.856	0.465	1.55641024718026E-252	3	NDUFV2
5.62850539537908E-133	0.773414887160877	0.375	0.17	9.43224934157626E-129	3	TMEM65
4.51362352564869E-112	0.772143085095925	0.305	0.134	7.56393030428207E-108	3	WDR36
2.33646709881771E-134	0.771561398841609	0.417	0.195	3.91545156419872E-130	3	TMEM43
6.44160832216856E-243	0.77140145821644	0.68	0.317	1.07948472262901E-238	3	UBR2
3.2050522823757E-169	0.770981458546908	0.433	0.189	5.3710266148052E-165	3	RHOT1
5.89946520074797E-219	0.77089445317006	0.611	0.282	9.88632378341345E-215	3	PCID2
8.56338674170219E-219	0.770413842532492	0.752	0.379	1.43505235017445E-214	3	EVL
1.18346515183966E-186	0.769923204959146	0.458	0.197	1.98325090145291E-182	3	LRP6
2.43081328273895E-256	0.769431179051678	0.772	0.381	4.07355689921394E-252	3	DPYD
7.37825018097146E-243	0.768149157182478	0.819	0.427	1.2364471653272E-238	3	PICALM
1.14663935626269E-166	0.768066334394359	0.475	0.218	1.92153823322502E-162	3	BDP1
1.5190444939738E-116	0.767402801757022	0.349	0.162	2.54561476300129E-112	3	RAB3GAP2
9.90273442000718E-157	0.767310400030781	0.394	0.17	1.6595002341048E-152	3	ITPRIPL2
2.00931517647715E-128	0.767233386442317	0.359	0.161	3.36721037274041E-124	3	TBC1D22B
1.1784636219168E-159	0.766475199996734	0.442	0.2	1.97486933760817E-155	3	VCPIP1
2.40393364630676E-86	0.765449621353141	0.252	0.114	4.02851200448087E-82	3	ZNF562
2.95421753223604E-83	0.764912836922111	0.26	0.121	4.95067774052115E-79	3	CLASP1
9.64834309660885E-259	0.764876612266324	0.658	0.293	1.61686933612971E-254	3	TIA1
5.58389353807252E-117	0.764269723083329	0.318	0.14	9.35748879110192E-113	3	SUPT20H
3.04445102395195E-161	0.763898321416797	0.427	0.189	5.10189102593868E-157	3	ANKRD40
5.3041595411857E-80	0.763880542585058	0.262	0.125	8.88871055911899E-76	3	FAHD2A
2.36396747361811E-152	0.763848688838306	0.455	0.211	3.96153669228923E-148	3	WDR20
2.95187612639572E-131	0.763319530295717	0.415	0.198	4.94675401261396E-127	3	ARFGEF1
8.02674908115709E-170	0.762701453044441	0.469	0.213	1.34512261102031E-165	3	ADGRA3
6.6145058837916E-97	0.76186304377244	0.297	0.138	1.1084588960058E-92	3	CLEC16A
5.66865372149731E-125	0.76182997616718	0.318	0.135	9.49952990648519E-121	3	MEF2A
1.85148257284751E-220	0.761659022807954	0.621	0.286	3.10271449557786E-216	3	FBXL17
1.66351737020502E-243	0.761092623678899	0.693	0.322	2.78772240898957E-239	3	U2SURP
7.95666727470123E-228	0.760849217058947	0.646	0.297	1.33337830189443E-223	3	TRAPPC9
1.16560569354706E-146	0.760788732374386	0.419	0.192	1.95332202124616E-142	3	BORCS8
0	0.760689915018503	0.883	0.414	0	3	LENG8
1.29142962046163E-231	0.760564494718258	0.714	0.338	2.16417775796959E-227	3	DEPRECATED-ENSG00000057294
3.99258393757686E-141	0.760171385060445	0.356	0.152	6.6907721625913E-137	3	BTBD1
2.47127867175779E-277	0.758804137520108	0.948	0.619	4.1413687981317E-273	3	ACSL4
3.84463177432346E-124	0.758234417011565	0.342	0.152	6.44283392741125E-120	3	USP16

1.4982179692225E-295	0.757688492614596	0.961	0.732	2.51071367282306E-291	3	UGT2B4
1.02694317692811E-148	0.757098924294037	0.366	0.155	1.72095137589613E-144	3	COG1
4.25709614476824E-132	0.757000842195372	0.384	0.176	7.13404171940261E-128	3	CLCN3
2.48549172934701E-82	0.756509127465329	0.269	0.127	4.16518704003972E-78	3	FAM184A
2.14455312486232E-95	0.756076816308139	0.312	0.149	3.59384212664427E-91	3	YEATS2
8.21884141624301E-103	0.756057149495696	0.287	0.128	1.377313444534E-98	3	UBE2K
1.07390219026799E-252	0.756053508404653	0.811	0.396	1.79964529045109E-248	3	STAG2
2.50813466130568E-124	0.755841308497647	0.397	0.189	4.20313206541606E-120	3	ALG6
8.91204991997373E-129	0.755694372129647	0.392	0.185	1.4934813255892E-124	3	KMT2A
1.5314382289102E-119	0.755277966284696	0.347	0.158	2.56638418400772E-115	3	TRAPPC6B
2.41845497926844E-143	0.75510107943736	0.418	0.193	4.05284685425806E-139	3	CCDC125
2.90429462744445E-97	0.754833724730458	0.253	0.107	4.8670169366714E-93	3	XRRA1
9.32896498724234E-259	0.754564661273336	0.84	0.441	1.56334795256207E-254	3	NUDT16
6.55014469234006E-187	0.754376854911102	0.521	0.236	1.09767324754235E-182	3	DPY19L4
2.1333059924159E-132	0.754013531609441	0.382	0.174	3.57499418209057E-128	3	ORMDL1
6.16132580016486E-204	0.753943412803405	0.573	0.265	1.03251497759163E-199	3	LRCH3
8.9469708555026E-192	0.753678130309755	0.504	0.224	1.49933337596513E-187	3	RBCK1
1.37498007801933E-111	0.753181544823729	0.316	0.142	2.3041916147448E-107	3	UMPS
1.86126940923875E-192	0.752663355932687	0.558	0.254	3.1191152760023E-188	3	FADS2
1.96352749695225E-160	0.752312476783128	0.462	0.213	3.29047937939258E-156	3	POLR2B
2.73628475517672E-186	0.752256924185595	0.52	0.237	4.58546599272514E-182	3	MTMR1
6.06528097805769E-131	0.752075901163261	0.343	0.15	1.01641978630291E-126	3	GYS1
8.11701818561799E-127	0.751777818091784	0.346	0.153	1.36024990754586E-122	3	NOL11
1.96010508147237E-218	0.751771536129684	0.601	0.274	3.2847440955314E-214	3	MOGS
6.24223873500019E-179	0.75165979359537	0.519	0.242	1.04607436721133E-174	3	TLE1
2.55331176028665E-249	0.751508347323937	0.803	0.394	4.27883984788836E-245	3	SYNE2
4.89986555785825E-238	0.751470823200067	0.776	0.395	8.21119470185885E-234	3	STBD1
1.44132278369592E-172	0.751110612299657	0.381	0.151	2.41536872091761E-168	3	KHDRBS3
1.28315724584167E-158	0.7506484050055	0.403	0.174	2.15031491258147E-154	3	SMARCE1
3.43457172388539E-218	0.750524853357173	0.68	0.322	5.75565529488714E-214	3	GPAT4
9.44224790823601E-162	0.749557354451705	0.477	0.221	1.58233190446219E-157	3	ZNF623
5.41694042774878E-112	0.748742170320945	0.297	0.129	9.0777087688214E-108	3	COX18
2.02653400404679E-177	0.748729857366282	0.532	0.242	3.39606568398162E-173	3	PCSK5
6.40035524546022E-217	0.748680231461487	0.641	0.304	1.07257153203422E-212	3	SRRM2
1.14269029442942E-246	0.748503433449834	0.956	0.599	1.91492039540482E-242	3	TSC22D3
2.06916670935516E-154	0.747026561425251	0.375	0.156	3.46750957153737E-150	3	DENND10
4.318383457456E-108	0.745944500523662	0.375	0.183	7.23674699800477E-104	3	GALNS
5.0474861534303E-174	0.74575830090222	0.51	0.237	8.4585772591185E-170	3	CAND1
4.28209975564707E-128	0.745513512734775	0.384	0.177	7.17594277051336E-124	3	ACSF2
4.85461724468937E-125	0.745243254646862	0.278	0.109	8.13536757865044E-121	3	DEPRECATED-ENSG00000122912
2.00398751865703E-111	0.744922524427558	0.378	0.185	3.35828228376545E-107	3	TRADD
1.96232531854567E-154	0.744742883497005	0.451	0.208	3.28846476881884E-150	3	RELCH
7.63574974832107E-179	0.744109055535726	0.515	0.238	1.27959894282364E-174	3	DDX3X
9.3087944904029E-105	0.742923165179195	0.331	0.156	1.55996778070172E-100	3	GUCY1B1
8.35432330813063E-102	0.742864957615571	0.301	0.137	1.40001749997653E-97	3	CNTLN
1.45279204575806E-92	0.742779573781358	0.276	0.126	2.43458891028135E-88	3	EXT2
1.16651044456782E-119	0.742455321796069	0.38	0.181	1.95483820300675E-115	3	PDS5B
1.05745198315832E-151	0.741064876818739	0.369	0.154	1.77207803337671E-147	3	ARPP19
9.31552445183511E-144	0.740575184894877	0.373	0.162	1.56109558763853E-139	3	IREB2
6.78959483959464E-83	0.740194589380652	0.274	0.131	1.13780030321927E-78	3	USP37
1.55183609153607E-137	0.740112370046679	0.384	0.172	2.60056692219614E-133	3	VPS37A
2.65514570977267E-126	0.738951566900455	0.348	0.155	4.44949318043704E-122	3	TMEM251
9.76746277061515E-172	0.738914524882796	0.497	0.231	1.63683141109969E-167	3	LARS
2.02024717168675E-151	0.738698800840033	0.402	0.176	3.38553021031265E-147	3	SLC25A37
6.99068363773336E-171	0.737690208877338	0.394	0.158	1.17149876401136E-166	3	FBXO17
6.68321487423039E-158	0.73746871898248	0.443	0.201	1.11997314862353E-153	3	NUP133
8.0602032578514E-181	0.737312390929228	0.519	0.24	1.35072886195074E-176	3	NSD1
5.29860039753103E-85	0.736947033734768	0.273	0.129	8.8793945461825E-81	3	HEATR5B
4.31960927763251E-214	0.736794860013253	0.655	0.311	7.23880122745655E-210	3	STK3
9.76271979664611E-150	0.736481907463172	0.468	0.221	1.63603658352196E-145	3	DOCK9

8.07319346444475E-135	0.736347125924862	0.347	0.149	1.35290576077165E-130	3	MRS2
1.35853743483904E-97	0.735936514846926	0.304	0.142	2.27663703330327E-93	3	RMI1
5.67538335898243E-171	0.735913764959515	0.477	0.217	9.51080743298276E-167	3	DYRK1A
1.00191358795665E-141	0.735072277729728	0.389	0.174	1.67900679069776E-137	3	IFT88
4.08390784304708E-291	0.734626951776429	0.86	0.443	6.8438127633783E-287	3	ECHDC2
4.10647476434628E-130	0.734602480128598	0.4	0.186	6.88163041009149E-126	3	MPHOSPH9
4.79610169334305E-129	0.734529123450991	0.361	0.162	8.03730721770428E-125	3	UBE2W
1.13943088345292E-231	0.734056114807994	0.693	0.33	1.90945827449041E-227	3	PEX2
1.72640942938454E-158	0.733677843287166	0.357	0.142	2.89311692176262E-154	3	ZNF22
6.62136684597532E-114	0.733209836469451	0.294	0.125	1.10960865604854E-109	3	ZNF362
1.33380016702706E-152	0.732177625312361	0.464	0.219	2.23518231990395E-148	3	MTR
5.53500855795403E-132	0.731839816005891	0.343	0.148	9.27556734141936E-128	3	USP15
2.69464582181661E-187	0.731237395627942	0.539	0.25	4.51568746820028E-183	3	DENND4C
3.18897498053464E-89	0.730864639927087	0.316	0.155	5.34408427237995E-85	3	PDE7A
7.82023529605766E-98	0.730793268641056	0.283	0.128	1.31051503091334E-93	3	MED20
3.59510866254392E-166	0.730782833391225	0.458	0.205	6.0246830966911E-162	3	CSTF3
0	0.730695171947651	0.969	0.815	0	3	PGRMC1
7.63227931630567E-133	0.729715061548699	0.359	0.159	1.2790173678265E-128	3	RBPJ
5.62348148508194E-236	0.729685260073276	0.688	0.317	9.42383027270032E-232	3	SUGP2
4.14297815542432E-117	0.72916003795608	0.294	0.124	6.94280279286007E-113	3	CCSER2
1.1085205661751E-91	0.72816637512279	0.272	0.123	1.85765876479624E-87	3	CEP120
3.99278391178339E-136	0.727765093053992	0.305	0.12	6.69110727936661E-132	3	MCF2L
9.46682712579464E-161	0.727631602064255	0.436	0.195	1.58645088974067E-156	3	NAE1
1.70155568101604E-231	0.727564037227118	0.56	0.24	2.85146701024668E-227	3	AUTS2
9.38388603024256E-121	0.72663523047969	0.364	0.169	1.57255162094805E-116	3	TMEM165
4.0125747545034E-235	0.726451905291609	0.676	0.32	6.7242727735968E-231	3	SLC25A30
4.11475134105836E-164	0.72620072913051	0.471	0.215	6.89550029734561E-160	3	ZNF33A
7.13082901739524E-177	0.724970145002773	0.459	0.202	1.19498432673509E-172	3	PBX2
2.6337878724736E-131	0.724767628561876	0.459	0.227	4.41370171669126E-127	3	SAMD12
2.27596699782988E-106	0.724266925335901	0.35	0.168	3.8140654949633E-102	3	RIC8B
6.98244701442024E-280	0.723985668833615	0.572	0.219	1.17011847067654E-275	3	SLCO1B1
1.06326082560254E-168	0.72367183672287	0.49	0.229	1.78181249154474E-164	3	RSBN1L
1.10011045474054E-207	0.723591457962939	0.639	0.301	1.8435651000542E-203	3	CEP57
1.41686476476113E-208	0.723541581449985	0.662	0.321	2.3743819727867E-204	3	RNF115
3.35135972941225E-154	0.723488397909505	0.448	0.207	5.61620863454904E-150	3	TAF8
1.07024552459628E-234	0.723008763690258	0.697	0.331	1.79351745011845E-230	3	NUFIP2
4.52456182535306E-172	0.723002914002724	0.494	0.229	7.58226070692665E-168	3	AEBP2
9.4117721779926E-146	0.722823683491776	0.436	0.203	1.577224781588E-141	3	PLEKHG3
5.58436739762509E-78	0.722351743286417	0.262	0.127	9.35828288494013E-74	3	RPE
3.66532889503153E-187	0.722300545186521	0.57	0.268	6.14235816229383E-183	3	PARP12
3.53437154651348E-94	0.72196995803001	0.278	0.127	5.92289983764729E-90	3	UBE3B
4.67422739560326E-164	0.721622038411857	0.519	0.249	7.83307026955194E-160	3	DCAF5
4.4863691896088E-153	0.721329740533565	0.469	0.221	7.51825748794642E-149	3	LEMD2
3.99697235321248E-197	0.721182601225654	0.58	0.273	6.69812626951348E-193	3	CHKA
2.31943559404864E-103	0.721061804539604	0.321	0.149	3.8869101685067E-99	3	KLHL42
4.09891797797554E-177	0.720392125997628	0.511	0.236	6.86896674749142E-173	3	HMGXB4
8.283940641242E-117	0.720240282607643	0.357	0.167	1.38822277265934E-112	3	KIAA1109
7.48546298182395E-116	0.719983189463993	0.337	0.153	1.25441388649406E-111	3	TMEM128
4.47358030369805E-116	0.719920397376294	0.345	0.159	7.4968258729372E-112	3	TMTC4
5.29719899165265E-158	0.719499483660197	0.406	0.176	8.8770460702115E-154	3	AAAS
2.93868527782888E-88	0.719460029340757	0.265	0.121	4.92464878858564E-84	3	PRKRA
1.62256165436937E-221	0.718770506045972	0.715	0.347	2.71908882039219E-217	3	ASH1L
8.16253129460009E-134	0.718240041531057	0.304	0.121	1.36787699434908E-129	3	SCAMP5
1.75372994377326E-244	0.718102751080769	0.666	0.308	2.93890063977524E-240	3	COG3
1.18536726308882E-153	0.718052493996154	0.391	0.167	1.98643845948424E-149	3	SHLD2
1.46632922623539E-87	0.718033769328533	0.251	0.113	2.45727451732527E-83	3	KCTD9
1.50687828048149E-213	0.717118946900277	0.745	0.371	2.52522662243088E-209	3	PLEKHF2
1.95631738439596E-180	0.716443798378778	0.558	0.267	3.27839667277075E-176	3	ZNF410
5.89217754728371E-100	0.716408549470688	0.284	0.127	9.87411113373804E-96	3	KIAA0355
2.41750365461311E-120	0.716346430839374	0.355	0.164	4.05125262440065E-116	3	PDS5A

6.51070347658505E-111	0.715622165998479	0.364	0.175	1.09106368860612E-106	3	CPSF1
9.74615113059871E-140	0.715022522819095	0.407	0.187	1.63326000646573E-135	3	RNF139
1.7711336138639E-93	0.714156988342115	0.266	0.117	2.96806571011312E-89	3	PRMT7
1.49645275492701E-158	0.713943872812133	0.459	0.211	2.50775552670668E-154	3	NDUFB4
8.01506421978106E-178	0.713794610960164	0.513	0.237	1.34316446195091E-173	3	UGT1A4
1.02054147725E-208	0.713728309040939	0.646	0.307	1.71022340757554E-204	3	MAN2A1
2.84120266712427E-105	0.713697119462798	0.317	0.146	4.76128742956685E-101	3	RIC1
4.11424913897261E-102	0.713569125393704	0.299	0.136	6.8946587070903E-98	3	MBTPS2
6.04535333847313E-242	0.713465006407479	0.809	0.419	1.01308031246133E-237	3	DGAT2
1.46413843015056E-209	0.713397580245649	0.628	0.297	2.45360318124631E-205	3	NUDT9
2.18703123637966E-91	0.713323282587219	0.307	0.146	3.66502694592503E-87	3	DNM1
1.50114708085382E-97	0.711640221169258	0.265	0.115	2.51562227809483E-93	3	BBS2
3.90419797279429E-142	0.710836266413924	0.377	0.165	6.54265496280867E-138	3	KLF13
7.34419719793751E-152	0.709983385177031	0.394	0.17	1.23074056643037E-147	3	DAPK2
5.9769935344983E-221	0.709303344630027	0.598	0.27	1.00162457651122E-216	3	TRPC4AP
1.70963813530846E-236	0.70915582471879	0.826	0.422	2.86501158714992E-232	3	CFLAR
3.69543644500856E-82	0.708735010659391	0.268	0.127	6.19281239454535E-78	3	ATP6V0A2
1.17074783870211E-81	0.708717871771135	0.287	0.14	1.96193922809699E-77	3	POLA2
5.228194908446E-136	0.708107540333135	0.461	0.226	8.76140902757381E-132	3	RUNX1
5.4442020748583E-106	0.707958394338	0.306	0.138	9.12339383704754E-102	3	FBXW11
1.26598479437857E-145	0.70768392639147	0.328	0.13	2.12153731841961E-141	3	GSTM1
2.63008498149533E-185	0.707522419504489	0.671	0.349	4.40749641198988E-181	3	SAFB2
3.62378439372953E-212	0.707421318971042	0.705	0.345	6.07273788701194E-208	3	DHX15
1.44157861669402E-144	0.706983143655971	0.432	0.201	2.41579744585583E-140	3	NCOA6
7.44547400776573E-72	0.706527419760032	0.25	0.122	1.24771253422138E-67	3	TMEM184C
9.19882789243935E-103	0.706407161367349	0.314	0.146	1.54153957821499E-98	3	AKAP11
4.33218252171355E-131	0.706119519434498	0.398	0.187	7.25987146988757E-127	3	CHM
6.87065272029992E-249	0.705531387284496	0.937	0.532	1.15138398286786E-244	3	TSPAN3
2.02212991373462E-197	0.704728866282211	0.67	0.328	3.38868530943648E-193	3	DIDO1
1.50815934324131E-91	0.704607732083579	0.302	0.145	2.52737342740378E-87	3	DEPRECATED-ENSG00000249915
7.37288600168493E-164	0.704482789285927	0.457	0.206	1.23554823616236E-159	3	MOAP1
9.76657642662172E-101	0.704415334348437	0.307	0.142	1.63668287757327E-96	3	PIGF
1.77475408887889E-125	0.703092384877563	0.371	0.171	2.97413290214324E-121	3	CMTR1
1.72818259930172E-218	0.70239208179109	0.671	0.316	2.89608839990982E-214	3	CUL4B
2.37760428908222E-234	0.702380024651177	0.677	0.314	3.98438926764398E-230	3	SPG7
5.29387039351983E-205	0.70172865454327	0.545	0.247	8.87146800546054E-201	3	AS3MT
2.9685842439216E-123	0.701369068817122	0.329	0.142	4.97475347596382E-119	3	LATS2
7.07085388408731E-99	0.701216192210852	0.271	0.118	1.18493369389535E-94	3	FOXO1
2.45724288902097E-92	0.701144898192053	0.256	0.112	4.11784763342135E-88	3	TBCCD1
1.62328471819138E-89	0.700187756397977	0.284	0.133	2.72030053074511E-85	3	UHRF1BP1L
3.12465891399266E-164	0.699998615204366	0.492	0.232	5.2363034080689E-160	3	BTBD7
9.76032741480504E-107	0.699785926049336	0.381	0.187	1.63563566817303E-102	3	LAMB1
2.22499722955565E-246	0.699351688558404	0.923	0.599	3.72865035728936E-242	3	HPN
1.59301631649106E-212	0.699336578332477	0.711	0.351	2.66957674317572E-208	3	CYP4F2
3.37439167951128E-138	0.697679130259544	0.404	0.186	5.654805576525E-134	3	FBXO28
4.7727613494277E-130	0.697521448391233	0.434	0.212	7.99819346937093E-126	3	PLD1
3.10175748142326E-187	0.697494081635683	0.595	0.289	5.19792518736691E-183	3	PLSCR1
2.6825975578533E-111	0.697191184128805	0.263	0.105	4.49549698745056E-107	3	PLA2G6
1.56826145698698E-96	0.697013791235207	0.287	0.13	2.62809254961877E-92	3	FEM1B
1.85540418151264E-235	0.696690249664321	0.899	0.525	3.10928632737888E-231	3	CIRBP
2.49297809135589E-203	0.696334968324984	0.567	0.26	4.1777326854942E-199	3	KIF13A
4.03626890527161E-190	0.696255539059076	0.564	0.263	6.76397943145416E-186	3	PCED1A
4.53600270553488E-78	0.69615090358305	0.274	0.133	7.60143333393536E-74	3	GDAP2
1.25919448506845E-178	0.69590054252335	0.601	0.298	2.11015811807771E-174	3	MSI2
8.27796020514842E-130	0.695682034084734	0.403	0.19	1.38722057117877E-125	3	CLN5
1.38483350564183E-82	0.695617031022983	0.279	0.135	2.32070398875458E-78	3	UBN2
2.94795340302253E-166	0.69530902839914	0.485	0.225	4.94018031278516E-162	3	NBN
5.853023017073E-238	0.69528834388204	0.719	0.338	9.80849597201094E-234	3	PILRB
3.03265286332661E-87	0.69504516310729	0.312	0.154	5.08211966836274E-83	3	P4HTM
2.84329696866379E-106	0.694709822763333	0.366	0.178	4.76479706008679E-102	3	CNOT10

1.13766583764263E-184	0.694404864485332	0.486	0.214	1.90650041072153E-180	3	ZNF444
8.24428336826797E-220	0.694374892966371	0.685	0.336	1.38157700685435E-215	3	CYP4F3
5.04086744908954E-143	0.693485727920014	0.427	0.197	8.44748567118426E-139	3	PHC3
2.62806410211759E-172	0.692461901261593	0.533	0.253	4.40410982232866E-168	3	NIPBL
9.37203569050089E-158	0.691862252192265	0.413	0.18	1.57056574101414E-153	3	SLC20A2
1.20112209719373E-106	0.691448595763922	0.328	0.152	2.01284041047725E-102	3	CHD8
1.59863974662497E-151	0.689979452132283	0.443	0.203	2.67900048739412E-147	3	CDK12
1.77842120488158E-137	0.689831248458069	0.431	0.205	2.98027825514054E-133	3	SGMS1
2.64093012728242E-170	0.689546127080089	0.53	0.252	4.42567070729988E-166	3	PRKAB2
2.31814325232613E-117	0.688708639590692	0.362	0.168	3.88474446224814E-113	3	GFOD2
1.16406625285841E-128	0.688493239376291	0.314	0.129	1.95074222654012E-124	3	U2AF1L4
1.56002793903521E-201	0.688481139853523	0.608	0.285	2.6142948202352E-197	3	TMUB2
3.44531424273791E-155	0.688176619828852	0.442	0.203	5.77365760798019E-151	3	CYHR1
1.48278936578247E-176	0.687838571956773	0.52	0.241	2.48485841917826E-172	3	ODR4
2.38782513818529E-115	0.687600745694215	0.338	0.154	4.0015173665709E-111	3	USP33
1.77457421909345E-179	0.686057464963436	0.534	0.248	2.9738314763568E-175	3	DYM
2.30568106009828E-212	0.685897627215141	0.843	0.461	3.86386032051269E-208	3	CTSH
3.41645386549093E-104	0.685634421250414	0.322	0.15	5.7252933877897E-100	3	IPP
4.77980957959126E-203	0.685461691297725	0.583	0.271	8.01000489347903E-199	3	PDE4DIP
6.04107937148804E-151	0.685448390399078	0.449	0.209	1.01236408107397E-146	3	WASHC1
3.13274685812526E-183	0.685370155461372	0.523	0.241	5.24985718484631E-179	3	ZNF326
2.62975148055316E-213	0.685189032082357	0.694	0.34	4.40693753111099E-209	3	PPM1B
1.80211399088357E-79	0.68513657240271	0.273	0.132	3.01998262592268E-75	3	PIGQ
1.64450188456023E-138	0.685103701205139	0.449	0.217	2.75585625814604E-134	3	ZNF800
1.10974355261528E-196	0.684587047088233	0.722	0.368	1.85970824547268E-192	3	ACSL3
1.39014399211188E-253	0.683835024441611	0.821	0.412	2.32960330198109E-249	3	AMOTL1
7.05824243510715E-98	0.683607705280387	0.317	0.15	1.18282026727526E-93	3	EXOC6
3.6035328733203E-197	0.683472519857319	0.632	0.303	6.03880038911016E-193	3	FNTA
4.94039882612033E-216	0.683322991506997	0.708	0.348	8.27912035281245E-212	3	PER1
1.62001616774076E-175	0.683224254201144	0.547	0.261	2.71482309389997E-171	3	KDM4C
1.04145429742747E-175	0.68314405612674	0.49	0.222	1.74526911162896E-171	3	RC3H1
1.9818720464445E-212	0.682871207391398	0.72	0.359	3.32122117543169E-208	3	PA2G4
1.22860861548253E-192	0.682316931474697	0.62	0.302	2.05890231782562E-188	3	TENT5A
4.59168641128687E-144	0.682316788772702	0.42	0.192	7.69474808803453E-140	3	NFYB
4.43225495477677E-141	0.681894475067497	0.409	0.187	7.42757285321491E-137	3	APBB2
2.14568600396628E-231	0.681699770857343	0.678	0.321	3.59574060544669E-227	3	SORBS2
4.37433628764956E-180	0.681661870584944	0.553	0.261	7.33051275084314E-176	3	CNOT6L
6.45738132863631E-150	0.681632441667755	0.607	0.309	1.08212796305287E-145	3	KCNK5
1.60334695529636E-105	0.681553116883398	0.346	0.164	2.68688882768564E-101	3	NDEL1
1.38268947983775E-132	0.680776746152849	0.491	0.241	2.3171110303121E-128	3	CCNE2
5.60925754769476E-181	0.680355305483943	0.49	0.219	9.39999379842687E-177	3	RASSF4
6.03441285064532E-194	0.680054896675603	0.544	0.25	1.01124690551114E-189	3	PYGL
2.7413457943265E-227	0.679559319519579	0.815	0.422	4.59394728213235E-223	3	CYP4F11
4.83979578984041E-88	0.67937276425781	0.305	0.149	8.11052978461455E-84	3	ALKBH6
5.56935827684603E-85	0.679160842770534	0.263	0.121	9.33313060033857E-81	3	CAMKMT
1.042588134816E-74	0.678766504624626	0.256	0.123	1.74716919632466E-70	3	ST3GAL3
2.7380759657086E-213	0.678061751638055	0.731	0.374	4.58846770333448E-209	3	USP9X
1.0963097343061E-233	0.677388354051187	0.89	0.469	1.83719585275017E-229	3	TMEM45B
7.69663948745652E-110	0.677344091657507	0.289	0.124	1.28980284530796E-105	3	ZBED6CL
4.98031808029416E-149	0.676640175468986	0.436	0.201	8.34601703895695E-145	3	DHX36
7.5236571690696E-85	0.676610224912084	0.281	0.134	1.26081446839268E-80	3	STAM2
7.03542726055333E-144	0.675824649650493	0.421	0.194	1.17899690032353E-139	3	FBXO46
1.37716740953758E-231	0.67560246179972	0.796	0.424	2.30785714490307E-227	3	IL6ST
2.97304813533438E-80	0.67519072674598	0.279	0.136	4.98223406519336E-76	3	GPATCH2
2.14240759524651E-218	0.673214808751476	0.865	0.48	3.5902466481141E-214	3	MIA2
1.25723233849761E-89	0.672729479600956	0.276	0.127	2.10686995285429E-85	3	ZC3H13
5.65015097685094E-100	0.671217501789079	0.292	0.132	9.4685230070068E-96	3	USP3
1.93394238814961E-162	0.670911919578162	0.453	0.205	3.24090065406111E-158	3	USP25
3.64908558803912E-210	0.670415958820003	0.765	0.398	6.11513762843596E-206	3	IL1R1
1.92885615737516E-266	0.67019962530483	0.598	0.24	3.23237714852929E-262	3	HLF

1.23015027785222E-77	0.669730711209441	0.275	0.135	2.06148583562475E-73	3	TRNAU1AP
4.80231787902167E-214	0.669465716799216	0.853	0.454	8.04772430166452E-210	3	ZBTB7B
5.83632042964669E-118	0.669329687470227	0.396	0.191	9.78050577600192E-114	3	ANO8
1.19232663473436E-123	0.669103219565195	0.337	0.148	1.99810097448785E-119	3	ZNF618
3.54119911490628E-112	0.66896044873894	0.419	0.211	5.93434147675994E-108	3	ZNF91
1.53014782855406E-221	0.668633069545677	0.733	0.38	2.56422173109089E-217	3	MAF
6.25060705027472E-101	0.668519437186136	0.261	0.11	1.04747672948504E-96	3	AGTR1
2.83689883905886E-159	0.668467031847164	0.479	0.223	4.75407507449484E-155	3	PTAR1
8.09626288972586E-121	0.66819156019365	0.37	0.173	1.35677173506026E-116	3	YTHDF2
1.46003588945213E-97	0.668098116173498	0.328	0.158	2.44672814354389E-93	3	ANAPC7
4.69109696782779E-98	0.667831550666471	0.3	0.138	7.86134029868581E-94	3	MTMR6
2.97080631405484E-205	0.666901440005944	0.645	0.305	4.97847722109311E-201	3	CD47
4.52201640395619E-77	0.666748211622989	0.304	0.156	7.57799508974978E-73	3	FAM126A
1.28045081378494E-104	0.666340697055872	0.314	0.145	2.1457794737408E-100	3	ZNF445
2.25963398218081E-98	0.666178527771221	0.323	0.154	3.7866946273386E-94	3	PIK3R4
4.8032509697355E-95	0.666102518662216	0.336	0.166	8.04928797508276E-91	3	FARSB
3.60653984948058E-153	0.665981121271086	0.522	0.257	6.04383947975956E-149	3	TRIM24
2.12978295167231E-179	0.665575230259824	0.539	0.252	3.56909027041246E-175	3	SORBS1
9.61290860703141E-102	0.665123484190765	0.301	0.137	1.61093122436632E-97	3	HERC2
7.17286128139268E-131	0.664783506731176	0.392	0.179	1.20202809353578E-126	3	MAFG
4.51224697821184E-184	0.664749555095368	0.494	0.219	7.5616234860874E-180	3	KPNA1
1.57539950423967E-99	0.663835891563078	0.339	0.165	2.64005448920484E-95	3	RAD50
6.92368145517158E-121	0.663395533375883	0.378	0.178	1.16027053825765E-116	3	VEZF1
1.47477035964515E-122	0.663027569904499	0.416	0.204	2.47142016869334E-118	3	CDK13
1.26881524464657E-108	0.66263675143259	0.343	0.161	2.12628058697872E-104	3	TMEM170A
5.31685678498498E-139	0.662043791621286	0.425	0.2	8.90998860027782E-135	3	SYNRG
2.6753522790802E-97	0.661824529804042	0.323	0.155	4.4833553492826E-93	3	KLHL8
1.72019754526119E-94	0.660810399932767	0.34	0.169	2.8827070463487E-90	3	TMEM168
2.18997253709358E-217	0.660628600684622	0.693	0.339	3.66995597766143E-213	3	DST
4.27971879732187E-149	0.659840589666721	0.45	0.21	7.171952760552E-145	3	KAT7
1.72051274516251E-193	0.659835837871354	0.609	0.288	2.88323525834333E-189	3	CXorf56
1.82740985457685E-202	0.659714961012669	0.637	0.308	3.06237343429989E-198	3	OTULIN
1.7541076947419E-226	0.659661484911407	0.677	0.319	2.93953367484847E-222	3	RTL8C
3.21767587846385E-85	0.659610325493847	0.271	0.126	5.39218123712973E-81	3	DDX31
1.54060233820879E-74	0.659403836881844	0.254	0.122	2.5817413983703E-70	3	PIGV
5.01937822922664E-172	0.658646309813326	0.526	0.247	8.411474036538E-168	3	TAF1D
1.82251753927709E-62	0.658571619373885	0.272	0.144	3.05417489232054E-58	3	SPATA33
1.14104586483696E-149	0.656736867168737	0.474	0.224	1.91216466029378E-145	3	DICER1
2.25225935830237E-199	0.656272434064511	0.782	0.429	3.77433623264312E-195	3	PIK3R1
1.44630570327786E-154	0.656268378331663	0.485	0.231	2.42371909755304E-150	3	PGAP3
2.03689311257177E-174	0.656100083797934	0.519	0.24	3.41342547804778E-170	3	GBA2
6.9986027229059E-157	0.65595105057639	0.451	0.205	1.17282584430457E-152	3	SEC24D
5.80962975025336E-109	0.655780922000924	0.382	0.185	9.73577753547458E-105	3	CES3
1.19081663621531E-122	0.655615223240792	0.379	0.178	1.99557051896961E-118	3	PHKB
1.03931508558328E-154	0.655515688836383	0.474	0.224	1.74168422042046E-150	3	ZBTB41
1.63223883655923E-152	0.655435957042255	0.427	0.192	2.73530584230596E-148	3	ATF6
3.47365208620747E-201	0.6553495593805	0.602	0.284	5.82114616606648E-197	3	SLC23A2
8.52297197841333E-141	0.655209392753359	0.467	0.224	1.42827964414251E-136	3	ARID4B
1.14439117528535E-149	0.655068921925889	0.429	0.195	1.9177707315432E-145	3	BTAF1
1.26025844622541E-201	0.65491056857432	0.65	0.32	2.11194110418455E-197	3	MAPK6
9.92199186322005E-124	0.654634325276983	0.314	0.132	1.66272739643842E-119	3	MPI
2.26573653308708E-100	0.654306595963622	0.262	0.109	3.79692128214732E-96	3	SLC22A1
1.90992625375489E-87	0.654240474275326	0.279	0.129	3.20065441604244E-83	3	EID3
1.22956980992243E-206	0.654150848937106	0.674	0.331	2.06051308746802E-202	3	SPECC1L
3.39235442541619E-197	0.653907460061688	0.592	0.278	5.68490754611245E-193	3	IDS
5.88799935655138E-195	0.65384285425312	0.862	0.476	9.8671093217088E-191	3	MAT2A
2.30088795814297E-207	0.652050105977443	0.688	0.328	3.85582804025599E-203	3	RABGAP1L
1.88682079627807E-102	0.651812441443351	0.325	0.153	3.16193429040278E-98	3	PEX6
4.89675634601097E-154	0.651455684108135	0.512	0.249	8.20598428464518E-150	3	NUP43
1.96430069230867E-129	0.651355743501698	0.391	0.181	3.29177510017086E-125	3	LARP1B

5.10964006702011E-100	0.651110225082595	0.306	0.142	8.56273482431231E-96	3	YAF2
6.03533006828651E-110	0.650966647232815	0.339	0.158	1.01140061284345E-105	3	DPH7
2.30365847331522E-120	0.649785099022328	0.366	0.17	3.86047086958164E-116	3	USP38
3.16039016308877E-223	0.649719409568651	0.721	0.347	5.29618183530416E-219	3	SYBU
3.06538521709644E-165	0.649699221413508	0.538	0.258	5.13697254681022E-161	3	MAPK1IP1L
2.05305791781896E-168	0.649432812382988	0.563	0.275	3.44051445868102E-164	3	ENOSF1
1.52530915244137E-75	0.648673829007446	0.269	0.132	2.55611307766125E-71	3	ATP9B
1.90070053190712E-198	0.648612271639107	0.623	0.3	3.18519395136995E-194	3	CREBZF
6.07265807983081E-201	0.648414236499329	0.704	0.349	1.01765604101805E-196	3	NEDD4L
3.42078967986152E-84	0.648385825910937	0.331	0.17	5.73255934551194E-80	3	C2CD5
4.38554509823231E-87	0.648306136884211	0.287	0.137	7.3492964756177E-83	3	ARID2
4.39717717287429E-170	0.648103082243225	0.601	0.288	7.36878950630273E-166	3	STK39
8.9131211610046E-85	0.647723070619275	0.297	0.145	1.49366084416115E-80	3	NOL9
1.24684918205597E-118	0.647622064886363	0.361	0.167	2.0894698592894E-114	3	NPHP3
6.37338056733149E-193	0.647566530370547	0.59	0.28	1.06805111547341E-188	3	ST5
2.22955020620471E-196	0.647230900663878	0.59	0.277	3.73628023555785E-192	3	TCEAL1
3.81928324135708E-161	0.646661309081779	0.473	0.218	6.40035485586619E-157	3	ACBD6
3.82405619819662E-85	0.645275441859658	0.26	0.119	6.40835337693789E-81	3	RTL10
1.0824689988508E-80	0.64447243029058	0.257	0.12	1.81400154827417E-76	3	PEX1
8.66336175651332E-142	0.644120812794088	0.408	0.185	1.4518061631565E-137	3	FBH1
2.25318320480024E-167	0.643351484207497	0.478	0.218	3.77588441460424E-163	3	VIRMA
6.5148064717907E-111	0.643273085413268	0.39	0.192	1.09175126854269E-106	3	INTS7
1.48968725055203E-161	0.64315943692548	0.488	0.228	2.4964178944751E-157	3	UNC50
2.06433964419147E-81	0.642336038421924	0.303	0.151	3.45942037573607E-77	3	PIGN
1.08086089544622E-216	0.641747931265711	0.663	0.323	1.81130668858878E-212	3	FURIN
1.61894300579802E-150	0.641713301026851	0.46	0.215	2.71302468911633E-146	3	MED13
9.7155780338209E-137	0.641652737052129	0.42	0.195	1.62813656690771E-132	3	RMDN1
3.73465093854497E-224	0.641481910928155	0.741	0.352	6.25852804281367E-220	3	MDM2
2.95425309753543E-96	0.641478695352148	0.339	0.167	4.95073734084987E-92	3	PSMG4
1.00164245960051E-173	0.641371208642048	0.544	0.257	1.67855243379854E-169	3	FBXL6
7.40120945452002E-86	0.641240349946216	0.274	0.128	1.24029468038847E-81	3	USP28
6.37648712902396E-162	0.640954844682122	0.512	0.245	1.06857171308184E-157	3	SEMA4B
1.67948098048483E-96	0.640149588965091	0.32	0.152	2.81447422709647E-92	3	ACVR1
2.21444964624193E-136	0.639860012941559	0.412	0.192	3.71097471717222E-132	3	SPG11
8.5524935809312E-188	0.639605920002962	0.555	0.259	1.43322687429245E-183	3	MED28
1.12555588972599E-133	0.639259924531036	0.388	0.173	1.88620656000282E-129	3	MTMR10
1.07029813650546E-168	0.639247899454314	0.486	0.221	1.79360561715586E-164	3	PSMC6
8.00830088014282E-151	0.639068528375394	0.45	0.21	1.34203106149433E-146	3	PIP4K2B
2.00464835771917E-102	0.638648027081343	0.347	0.169	3.35938971786579E-98	3	PHF11
9.54016533150301E-138	0.638625822242165	0.436	0.207	1.59874090625327E-133	3	FAM172A
3.26248868328206E-216	0.638514696836136	0.747	0.379	5.46727853544408E-212	3	PPARA
8.00581874533758E-205	0.638513004715868	0.596	0.278	1.34161510534367E-200	3	ZFAND5
1.18664598185932E-114	0.638476463389838	0.359	0.167	1.98858133639984E-110	3	KLHL23
2.05212623201266E-97	0.638379250997739	0.338	0.165	3.43895313960681E-93	3	FNBP1L
1.52690528601227E-200	0.637797934173104	0.624	0.298	2.55878787829936E-196	3	RBM10
2.4211412077475E-200	0.637607177430265	0.616	0.293	4.05734843594325E-196	3	TBC1D2B
3.20821994406551E-118	0.637310204555874	0.399	0.195	5.37633498226497E-114	3	LYSMD3
1.05509788716478E-93	0.63729083416816	0.316	0.152	1.76813303931074E-89	3	RECQL
7.2080315802383E-101	0.637050494357056	0.322	0.152	1.20792193221633E-96	3	STOX2
3.33392652986678E-161	0.635828696021823	0.555	0.275	5.58699407875075E-157	3	MPHOSPH8
3.81338847714543E-120	0.635820629016544	0.432	0.214	6.39047641000031E-116	3	SPATS2
4.37036748545015E-76	0.635548401099962	0.256	0.122	7.32386183211736E-72	3	TTI1
1.39352917888929E-185	0.634770943146571	0.6	0.285	2.33527619798267E-181	3	LMBR1
2.86741679791192E-97	0.634634156543043	0.27	0.118	4.80521706994079E-93	3	WDR45
4.93887912854905E-120	0.634428974578124	0.423	0.208	8.2765736436225E-116	3	S100PBP
5.51221294207243E-137	0.634287514056986	0.264	0.094	9.23736644832498E-133	3	DEPRECATED-ENSG00000178445
8.62114252065884E-203	0.633729750524015	0.629	0.297	1.44473106361201E-198	3	SMAD3
3.12422552814009E-204	0.63349943371756	0.659	0.319	5.23557714005717E-200	3	DDX17
3.04409102082429E-145	0.633095603996727	0.479	0.231	5.10128773269735E-141	3	MGAT4A
1.28901016718783E-177	0.632738293913562	0.565	0.27	2.16012323817336E-173	3	CPSF6

2.04301144992615E-95	0.631980782389853	0.278	0.125	3.42367858778623E-91	3	BLOC1S4
7.03314375238567E-137	0.631406755085011	0.428	0.201	1.17861423002479E-132	3	ARL8B
1.35194535802805E-176	0.630913200194249	0.549	0.261	2.26559003098341E-172	3	KANSL1
7.49578391877701E-102	0.63073326444967	0.338	0.162	1.25614346910865E-97	3	ZHX3
2.3958069002072E-297	0.630545903939206	0.663	0.25	4.01489320336723E-293	3	AQP9
2.95090797548316E-96	0.630268249586679	0.288	0.131	4.94513158531469E-92	3	MAPK8
4.1760172776596E-186	0.630081135412791	0.572	0.27	6.99816975390196E-182	3	R3HDM2
1.5951784855932E-167	0.629375958692755	0.558	0.264	2.67320010615708E-163	3	DLG5
2.0100425089571E-201	0.629236487573641	0.68	0.329	3.36842923651032E-197	3	NECTIN3
1.34376109232101E-155	0.628821437493593	0.485	0.228	2.25187483851155E-151	3	ZXDC
4.03730094289468E-209	0.628682067745839	0.815	0.421	6.76570892010291E-205	3	SLC38A10
4.84983403742439E-113	0.628432049602892	0.353	0.165	8.1273518799158E-109	3	TBK1
2.84266037671457E-86	0.628205828334594	0.25	0.111	4.76373025929828E-82	3	FGFRL1
8.68983889934387E-68	0.62776495212893	0.257	0.129	1.45624320275205E-63	3	HIRA
1.4443112397913E-134	0.627109732368393	0.408	0.187	2.42037677564226E-130	3	FRRS1
3.14514262758472E-229	0.627078129284563	0.864	0.455	5.27063001530647E-225	3	CMC4
2.18588038223776E-220	0.627047228535303	0.673	0.325	3.66309834455403E-216	3	SLC38A2
1.04156870274996E-95	0.626776321085254	0.289	0.132	1.74546083206838E-91	3	ZDHHC16
2.1400042434811E-125	0.626585514829568	0.419	0.203	3.58621911122563E-121	3	RALGAPB
3.94112816211735E-119	0.626462050132333	0.365	0.169	6.60454257407625E-115	3	LIG3
2.07735968606054E-179	0.625888769940825	0.553	0.265	3.48123936190025E-175	3	HIGD1A
1.38496579302307E-79	0.625043922436315	0.272	0.131	2.32092567594805E-75	3	ANGEL1
6.04375094503819E-194	0.625033423723127	0.66	0.309	1.0128117833695E-189	3	CDS1
4.24004932932081E-96	0.624829849208401	0.343	0.169	7.10547466607581E-92	3	RSPRY1
9.91333112458592E-189	0.624829381599883	0.646	0.312	1.66127602985811E-184	3	IPO9
4.09024847363303E-167	0.624117276971708	0.595	0.289	6.85443839211423E-163	3	IL10RB
3.95770758656625E-89	0.623782749427565	0.291	0.138	6.63232637356772E-85	3	ATG7
1.41782630393202E-87	0.622624042414354	0.315	0.155	2.37599332012928E-83	3	ZDHHC6
9.60217548387963E-121	0.622519304070782	0.347	0.156	1.60913256758855E-116	3	TTLL12
7.1662376095926E-83	0.62218348163379	0.262	0.121	1.20091809861553E-78	3	ETNPPL
1.01937156097398E-161	0.621944178195129	0.483	0.223	1.70826286188019E-157	3	PIP5K1A
3.26780704228565E-199	0.621844560708934	0.762	0.409	5.47619104146228E-195	3	HACD3
4.66393960543483E-112	0.620776958623336	0.376	0.181	7.81582999078769E-108	3	TGS1
1.36135518252615E-182	0.620512926749164	0.648	0.316	2.28135901487732E-178	3	C1GALT1C1
1.02467605232633E-188	0.620477312874417	0.626	0.301	1.71715212848846E-184	3	EHBP1L1
4.80322083416941E-109	0.620475368837565	0.383	0.188	8.0492374739011E-105	3	NAA15
2.89254033531893E-160	0.620185646812904	0.533	0.259	4.84731909392747E-156	3	TOB2
5.60152289481046E-126	0.619892840082	0.406	0.194	9.38703206712338E-122	3	MICU2
2.29327747360721E-158	0.619869040325484	0.496	0.234	3.84307439027096E-154	3	WWC2
6.0611842531221E-123	0.618199385880809	0.386	0.181	1.0157332571382E-118	3	TNFAIP2
6.36762851167161E-167	0.618004371433261	0.465	0.209	1.06708718598593E-162	3	ANKH
1.95979574489696E-87	0.617855804316915	0.283	0.133	3.28422570929832E-83	3	KIFAP3
3.09029884989947E-167	0.617619254430508	0.611	0.304	5.17872281266153E-163	3	TIMM8A
7.65989443209525E-154	0.617491602582997	0.547	0.273	1.28364510893052E-149	3	PI4KB
2.6802290636761E-125	0.617449483892027	0.427	0.208	4.49152786490841E-121	3	BTF3L4
1.6194456347794E-201	0.616892680766048	0.982	0.748	2.71386699476333E-197	3	CYP3A5
1.37207616192577E-164	0.616509167117544	0.601	0.304	2.29932523215521E-160	3	MARC1
4.7973677273135E-143	0.615657143613698	0.503	0.25	8.03942883743196E-139	3	UBA2
2.15504133702748E-122	0.6153417771856	0.379	0.177	3.61141827259065E-118	3	FLVCR1
3.03817222998744E-75	0.615235779228495	0.256	0.122	5.09136902301296E-71	3	CLK2
3.30837336034415E-142	0.61457496094603	0.415	0.188	5.54417207726472E-138	3	OTUD4
4.63819158151561E-181	0.614505199333225	0.64	0.316	7.77268145230385E-177	3	PDCD4
2.55785500152795E-148	0.61436251377889	0.454	0.21	4.28645341156055E-144	3	FGF13
2.45886630502945E-77	0.613835010973949	0.252	0.118	4.12056815396835E-73	3	DAAM1
6.34763345656389E-100	0.61382842074915	0.363	0.18	1.06373641465098E-95	3	TFCP2
9.55482808511872E-137	0.613635294736924	0.413	0.191	1.6011980905042E-132	3	PGM3
1.57853684402268E-106	0.613628819689937	0.339	0.159	2.6453120432132E-102	3	HDAC8
6.42436572955487E-189	0.613553089617633	0.576	0.274	1.07659520895881E-184	3	SIK2
9.64506286408095E-135	0.613147782615187	0.372	0.165	1.61631963476269E-130	3	CAP2
7.28813969531174E-201	0.612878807065498	0.753	0.395	1.22134645014034E-196	3	PBLD

5.31139970576137E-115	0.612542553994914	0.386	0.187	8.9008436269149E-111	3	ILKAP
5.6588585968092E-123	0.612340520607001	0.411	0.198	9.48311523653285E-119	3	CASP6
5.14271091081252E-149	0.611927251789925	0.491	0.236	8.61815494433963E-145	3	HIP1R
6.75242743962895E-88	0.611738208239272	0.25	0.11	1.13157179033302E-83	3	RBMXL1
7.65030554026406E-208	0.610995668082944	0.936	0.577	1.28203820243745E-203	3	MBNL3
5.82103748032418E-75	0.610608408327329	0.279	0.139	9.75489460952726E-71	3	RAP1GDS1
4.21693452356258E-84	0.610590865157753	0.309	0.153	7.06673887458618E-80	3	TIPARP
2.90190551671221E-108	0.609569481920624	0.384	0.189	4.86301326490632E-104	3	ZNF655
5.81220246378051E-88	0.609321405878378	0.295	0.141	9.74008888880337E-84	3	ZKSCAN8
2.65935908798169E-135	0.609259615694338	0.453	0.219	4.45655395963971E-131	3	CASK
2.85195745646528E-180	0.609176774630985	0.589	0.285	4.77931030554451E-176	3	CARS2
2.17396728879361E-106	0.609166510039715	0.35	0.167	3.64313438256032E-102	3	NUP54
8.00839689341371E-79	0.609116038869578	0.295	0.148	1.34204715139827E-74	3	FCHO2
5.07113695820355E-83	0.609055237649739	0.277	0.132	8.49821131455751E-79	3	TAF5L
7.64343760720077E-184	0.608955693491734	0.621	0.305	1.2808872742147E-179	3	C5orf24
9.16592109430597E-193	0.608769925714123	0.656	0.319	1.53602505698379E-188	3	KMT2C
3.78703719908932E-172	0.608656495278704	0.51	0.235	6.34631693823388E-168	3	MXD4
3.5125582584674E-142	0.608577760799553	0.501	0.249	5.88634512953968E-138	3	KIAA0319L
1.72361516244403E-103	0.608221986503912	0.355	0.173	2.88843428922371E-99	3	USF3
2.64356824064656E-199	0.608152379493102	0.665	0.328	4.4300916576755E-195	3	NDUFV3
2.15495590739778E-91	0.607528492957459	0.312	0.15	3.6112751096172E-87	3	EEF1AKMT2
2.30070109346717E-83	0.60740193263068	0.255	0.116	3.85551489243228E-79	3	GORAB
2.00096061313313E-72	0.606977122672857	0.273	0.136	3.35320979548851E-68	3	ZFP64
4.11174694406429E-192	0.606720391471476	0.728	0.373	6.89046552886294E-188	3	PI4K2B
1.03944893377411E-186	0.606628283223046	0.51	0.228	1.74190852321865E-182	3	DEPRECATED-ENSG00000146416
5.14608877974637E-185	0.606570806922947	0.881	0.511	8.62381557709897E-181	3	SLC37A4
6.45887580284912E-63	0.606443609143251	0.262	0.136	1.08237840704146E-58	3	ZNF398
4.7384036008308E-110	0.606295092824798	0.346	0.162	7.94061675427225E-106	3	SLC25A28
3.5974578845064E-101	0.606230448426633	0.332	0.158	6.02861992285583E-97	3	ANKS1A
5.1018045193671E-105	0.605642877974333	0.316	0.144	8.54960401355539E-101	3	ATRX
7.58569481441628E-144	0.605393058192471	0.51	0.255	1.27121073699988E-139	3	EPB41L4B
1.15183727943509E-179	0.605088625424197	0.611	0.298	1.93024891287733E-175	3	APEX1
9.09920774960455E-100	0.605061462133836	0.325	0.155	1.52484523467873E-95	3	MTPAP
5.37607223585241E-80	0.604224794088162	0.276	0.133	9.00922185284147E-76	3	CCNYL1
7.76333630343408E-189	0.603765367960332	0.893	0.533	1.30097989772948E-184	3	DECR1
2.93760118339541E-175	0.603586586356383	0.632	0.32	4.92283206313403E-171	3	IVD
1.93956997068537E-101	0.60345070678337	0.409	0.21	3.25033135687455E-97	3	ZFYVE28
5.80991963597238E-149	0.603331385727595	0.479	0.229	9.73626332596251E-145	3	DHX16
4.45996813101718E-127	0.602838118394701	0.431	0.21	7.47401459395858E-123	3	DIS3
1.64387598116383E-82	0.602625619337452	0.287	0.139	2.75480736923435E-78	3	SBNO1
6.48104964094671E-101	0.602357009053883	0.322	0.151	1.08609429882985E-96	3	ERGIC2
2.43638006722863E-190	0.602217082738082	0.537	0.246	4.08288571666175E-186	3	PID1
1.6315940351786E-104	0.602167764818879	0.282	0.121	2.73422528415229E-100	3	ZRANB1
6.17502849785702E-104	0.601132493750336	0.365	0.179	1.03481127567088E-99	3	MOSPD2
5.77288024589864E-184	0.600859511024048	0.586	0.282	9.67419271607694E-180	3	SH3BP4
1.51897582544885E-131	0.600476594455357	0.533	0.271	2.54549968828718E-127	3	ABRACL
2.9602316363034E-81	0.599615014977496	0.307	0.155	4.96075617611724E-77	3	ZC3H7A
6.54374766053647E-90	0.599594457151565	0.318	0.156	1.0966012329527E-85	3	GTPBP3
7.47045599488027E-102	0.598673438730108	0.308	0.141	1.25189901562204E-97	3	NPRL3
1.75214869683105E-178	0.598659329590465	0.651	0.329	2.93625078614947E-174	3	LRRFIP2
3.29603508411049E-226	0.598413541495175	0.695	0.333	5.52349559395235E-222	3	ADHFE1
2.82626157920778E-132	0.597821611374795	0.466	0.229	4.7362491544364E-128	3	MTM1
1.57443028983344E-123	0.597738699693066	0.393	0.185	2.63843027970288E-119	3	TAF11
1.22493693216026E-134	0.597710697904368	0.375	0.166	2.05274931091417E-130	3	ZNF281
3.40861397677813E-146	0.597597829854318	0.474	0.227	5.71215530228479E-142	3	MAPK9
6.60773603054604E-187	0.59759781611521	0.941	0.624	1.1073244039989E-182	3	ATP1B1
1.59849625683164E-83	0.597546012705165	0.303	0.15	2.67876002719847E-79	3	XRCC5
4.41849121835435E-127	0.597460628013317	0.391	0.183	7.40450758371823E-123	3	STX17
2.52058749501874E-112	0.597327657367391	0.452	0.234	4.22400052415241E-108	3	AKAP9
2.78822627492892E-175	0.59680356429554	0.564	0.27	4.67250959152588E-171	3	BCAR1

7.90293705846172E-125	0.59656229802907	0.364	0.163	1.32437419225702E-120	3	SLC23A1
1.62550165464669E-116	0.5965349929961	0.413	0.204	2.72401567285693E-112	3	MED13L
1.4137022281131E-205	0.596498880285094	0.719	0.366	2.36908219387194E-201	3	ELOVL5
3.2382453673674E-99	0.596433072986855	0.332	0.159	5.42665158663429E-95	3	PAXBP1
1.85085903396303E-193	0.595962289924192	0.871	0.489	3.10166956911524E-189	3	ABCC3
1.39002808091132E-166	0.595760780021587	0.488	0.222	2.32940905799118E-162	3	SPATA20
6.88978263414683E-201	0.595730815941716	0.598	0.279	1.15458977383033E-196	3	TNRC6A
4.31087704957865E-110	0.595287965542888	0.361	0.173	7.2241677596839E-106	3	KIDINS220
2.73338096822417E-225	0.59436967442062	0.711	0.352	4.58059982655006E-221	3	C19orf12
2.21363097771034E-187	0.594302452915522	0.682	0.343	3.70960279244699E-183	3	PATJ
5.69801509230841E-199	0.593949894005035	0.707	0.348	9.54873369169044E-195	3	MARCH6
1.75913551662382E-129	0.593898154776813	0.485	0.243	2.94795929875819E-125	3	PSEN1
1.68207979185136E-203	0.593766583443554	0.88	0.452	2.81882931518451E-199	3	ABCD1
1.17773048698778E-89	0.593722833575801	0.289	0.136	1.97364075009413E-85	3	PET117
1.96180646767898E-82	0.593628087974555	0.293	0.144	3.28759527853644E-78	3	TMEM18
7.98771936392334E-200	0.593394367299897	0.8	0.432	1.33858201100627E-195	3	MARC2
2.63106227157404E-135	0.59302728047526	0.429	0.202	4.40913415470378E-131	3	NFAT5
2.83353133739445E-194	0.592020347663771	0.706	0.357	4.74843181520561E-190	3	ARHGAP35
5.71740974712166E-92	0.591471338894291	0.266	0.119	9.58123525422648E-88	3	USP47
7.49317314175553E-182	0.591403162619005	0.634	0.313	1.25570595509539E-177	3	EMP2
1.27027135052761E-135	0.590603073744655	0.45	0.217	2.12872072921417E-131	3	USP34
5.06883360036172E-234	0.590486551237322	0.99	0.941	8.49435134748616E-230	3	AGT
9.10647230142089E-128	0.590198447540496	0.437	0.208	1.52606262827211E-123	3	FAM111A
7.64537247478542E-93	0.590112134562456	0.305	0.144	1.28121151932454E-88	3	SEPTIN10
9.13063866717117E-92	0.589607047580178	0.315	0.153	1.53011242784454E-87	3	HEATR3
5.97178705552707E-123	0.589575959082876	0.43	0.212	1.00075207476523E-118	3	C6orf62
2.082949069483E-182	0.589483150072545	0.725	0.354	3.4906060506396E-178	3	NEB
9.13629930977069E-97	0.589376427980706	0.32	0.152	1.53106103833137E-92	3	SH2B1
3.84042852741613E-123	0.589297150994569	0.399	0.191	6.43579012624395E-119	3	MESD
8.36349235693525E-134	0.589061666653609	0.432	0.206	1.40155404917521E-129	3	CUL3
2.48418820230411E-140	0.589011942081867	0.459	0.217	4.16300258942123E-136	3	IRF9
8.09827846856424E-94	0.588719688494902	0.346	0.173	1.357109505762E-89	3	TMX1
2.53613039296841E-167	0.588441645537431	0.537	0.258	4.25004731253646E-163	3	WDR26
8.07544352870565E-95	0.588386515197582	0.314	0.15	1.35328282654049E-90	3	C17orf75
2.76176639433268E-190	0.588322873453699	0.654	0.319	4.62816812362271E-186	3	ANKHD1
1.3421302923728E-74	0.588098479295263	0.284	0.142	2.24914194395833E-70	3	GLCE
9.9838002341244E-74	0.58768025863346	0.274	0.137	1.67308524323457E-69	3	MANBA
5.39391553287995E-167	0.587622346785845	0.557	0.271	9.03912365000021E-163	3	TAOK1
2.47871408934317E-170	0.587607493024094	0.582	0.289	4.15382907092128E-166	3	CDC42SE1
2.62142309685277E-101	0.587603357288689	0.309	0.142	4.39298082570587E-97	3	MKNK1
1.66206745545836E-79	0.587383598937511	0.284	0.139	2.78529264185712E-75	3	DHX29
9.62448468807018E-80	0.587316665040576	0.343	0.18	1.6128711440268E-75	3	LRIG3
4.90916278834406E-99	0.587313829992864	0.363	0.18	8.22677500070697E-95	3	SUSD4
3.04186289727696E-192	0.586623502201482	0.693	0.343	5.09755384325673E-188	3	ADD1
3.98254006458525E-108	0.586569690973305	0.32	0.145	6.67394064023196E-104	3	RAI14
6.1905551932919E-181	0.586399088492326	0.621	0.305	1.03741323929186E-176	3	COBLL1
8.08004808571097E-206	0.586168006905678	0.962	0.697	1.35405445820345E-201	3	ASPH
1.10377864828509E-84	0.585603933727406	0.264	0.121	1.84971225879615E-80	3	NFKBIL1
5.93843564394678E-158	0.58480067398098	0.53	0.256	9.95163045212601E-154	3	GINM1
2.33592875821554E-89	0.584797097717159	0.262	0.117	3.9145494130176E-85	3	APTX
8.57872848090787E-121	0.584653373475806	0.356	0.162	1.43762331883054E-116	3	ALG2
4.63683355816537E-86	0.5844221022901	0.26	0.117	7.77040567677353E-82	3	NBPF12
4.52367824372458E-97	0.584277619828305	0.342	0.168	7.58078000083366E-93	3	ZADH2
2.17104758753798E-162	0.584205155620438	0.539	0.258	3.63824154719614E-158	3	SARDH
1.61149654436959E-191	0.583941534599423	0.689	0.345	2.70054590905456E-187	3	FARP1
1.17848740044773E-73	0.583353445283611	0.276	0.136	1.9749091856703E-69	3	POLH
5.70111398137366E-87	0.583043327201384	0.284	0.134	9.55392680998599E-83	3	PCBP2
7.45142355128259E-113	0.582882931178723	0.355	0.165	1.24870955872394E-108	3	USP2
4.51259871628405E-67	0.582870651504745	0.284	0.15	7.56221292874881E-63	3	ATP11C
1.90536671162036E-156	0.582852866522404	0.519	0.249	3.19301353533339E-152	3	AP3B1

1.05906827123355E-178	0.582110570436543	0.655	0.332	1.77478660893318E-174	3	SRP54
8.06065656262842E-159	0.581765338751156	0.479	0.222	1.35080482676527E-154	3	USP32
6.28458595666286E-105	0.580998458541374	0.363	0.176	1.05317091461756E-100	3	NIPA1
9.7951510056858E-76	0.580682123828405	0.262	0.126	1.64147140553283E-71	3	REPS2
2.22828753403543E-47	0.58048943053603	0.262	0.15	3.73416424953658E-43	3	BTBD3
1.10000836173872E-178	0.579758635102556	0.842	0.477	1.84339401260175E-174	3	WNK1
4.68567510178191E-90	0.579576392183012	0.336	0.167	7.85225433556613E-86	3	GTPBP10
8.28296273825685E-129	0.57944608248272	0.366	0.164	1.38805889567708E-124	3	SIK3
1.52961768537434E-140	0.579279693795857	0.473	0.229	2.56333331715032E-136	3	MSL2
7.31188316404616E-99	0.57924251853857	0.35	0.17	1.22532538063085E-94	3	KDM5B
8.95909959898799E-113	0.579216650509041	0.396	0.196	1.50136591079841E-108	3	MTX2
1.15057610836432E-176	0.579045264209451	0.558	0.265	1.92813544239693E-172	3	SELENOS
1.55341929702581E-132	0.578400370128198	0.427	0.204	2.60322005795585E-128	3	PIK3CB
1.73701769278767E-113	0.57814644548336	0.399	0.196	2.91089424957358E-109	3	CFAP97
2.5523828481037E-147	0.578125455730612	0.46	0.217	4.27728317685218E-143	3	PHF12
3.62272955080522E-111	0.577971390062781	0.398	0.195	6.07097018123939E-107	3	EPS15L1
3.45128947463392E-80	0.577091855620048	0.304	0.152	5.78367090159152E-76	3	PLCG1
5.51992858177314E-182	0.576970041815484	0.657	0.327	9.25029631733542E-178	3	TRIM27
2.17703117134858E-180	0.576855092451166	0.666	0.329	3.64826883694595E-176	3	MAVS
4.57579030828582E-130	0.576560892901045	0.476	0.235	7.66810939862538E-126	3	HERC3
1.05521571469171E-173	0.576311643671737	0.691	0.352	1.76833049468036E-169	3	STT3B
1.45197336388358E-102	0.575674527686447	0.286	0.125	2.4332169631961E-98	3	SPRYD7
2.80786409260493E-101	0.575339796584671	0.352	0.173	4.70541864638734E-97	3	NUP160
4.71848533800361E-149	0.575147532214313	0.469	0.221	7.90723772942644E-145	3	ZCCHC9
1.81670734457308E-197	0.575140434518944	0.774	0.405	3.04443816803556E-193	3	LLGL2
1.88518646725749E-113	0.575000331681422	0.413	0.206	3.15919548183011E-109	3	DEPRECATED-ENSG00000078668
6.66661904124402E-117	0.57492909462182	0.376	0.176	1.11719201893167E-112	3	SULF2
2.0160921447677E-77	0.574799232892768	0.275	0.133	3.37856721620171E-73	3	POLM
1.08695730411145E-153	0.574772419951399	0.515	0.251	1.82152305022997E-149	3	PEX13
4.38427621307072E-105	0.574767154113137	0.354	0.171	7.34717007786391E-101	3	ITGB1BP1
7.06224583202783E-92	0.574213175199131	0.291	0.135	1.18349115653122E-87	3	TNKS2
2.8222968956596E-158	0.5741849062292	0.513	0.246	4.72960513774635E-154	3	YIPF4
5.0688501726781E-180	0.574131350782865	0.528	0.246	8.49437911937395E-176	3	DEXI
6.56131877911951E-87	0.574105968147665	0.266	0.121	1.09954580100485E-82	3	COG8
4.49573654403772E-161	0.573842244997882	0.702	0.377	7.53395530049841E-157	3	FDPS
8.98541787609591E-89	0.573807452744023	0.276	0.127	1.50577632767615E-84	3	GGA1
4.18690634639024E-115	0.573723038286551	0.337	0.151	7.01641765528076E-111	3	PITPNC1
2.0374691764627E-141	0.573629553225248	0.509	0.247	3.41439084591619E-137	3	HELLS
6.02023050187212E-147	0.573609190488289	0.5	0.244	1.00887022750373E-142	3	ARHGAP12
2.63358094514656E-203	0.57345425743112	0.838	0.457	4.4133549478766E-199	3	HBS1L
1.96313010464945E-127	0.572717617752444	0.463	0.232	3.28981342937155E-123	3	FAM8A1
1.46135312036028E-169	0.57232905498017	0.563	0.27	2.44893555909976E-165	3	SEC23A
3.93996843860428E-90	0.571930013326021	0.284	0.132	6.60259910941305E-86	3	NBAS
2.14554356678115E-122	0.571875252530599	0.38	0.177	3.59550190921185E-118	3	METTL14
1.9211013163055E-95	0.57163450223197	0.295	0.136	3.21938158586476E-91	3	RNF146
6.19276429844701E-82	0.571495085595377	0.294	0.143	1.03778344113375E-77	3	COG7
2.48481442845612E-62	0.57145434221102	0.271	0.143	4.16405201920677E-58	3	TP53I3
2.45585184572774E-204	0.571352855359813	0.953	0.681	4.11551652307055E-200	3	LAMP2
1.79264076513483E-153	0.571114201922259	0.508	0.243	3.00410739421294E-149	3	BRWD1
2.02840112960411E-136	0.571030659512684	0.456	0.22	3.39919461299057E-132	3	RO60
1.22199040803347E-127	0.570344697637599	0.435	0.211	2.04781152578249E-123	3	DELE1
8.75438185073206E-115	0.57033589641935	0.388	0.189	1.46705931054568E-110	3	BPNT1
1.63597376992617E-180	0.569577388785975	0.734	0.379	2.74156484364228E-176	3	EML4
8.94595589220647E-164	0.569558565797552	0.558	0.274	1.49916328841596E-159	3	EWSR1
7.77990659175167E-76	0.569154150024899	0.315	0.165	1.30375674664575E-71	3	UEVLD
2.55573603959107E-84	0.568951419028033	0.333	0.17	4.28290245514672E-80	3	MSH2
9.0606720759458E-115	0.568906045521708	0.433	0.221	1.518387426487E-110	3	TTC17
3.01631499545116E-117	0.568513414266805	0.374	0.176	5.05474066937705E-113	3	SETDB1
9.6814319715377E-76	0.568471960589903	0.269	0.131	1.62241436979029E-71	3	CCNT1
5.20882152413159E-69	0.568327229667732	0.26	0.13	8.72894311013973E-65	3	ASF1A

1.10461041135355E-198	0.567896141445026	0.784	0.396	1.85110612734627E-194	3	PDZD8
3.89636580113258E-181	0.567535433859443	0.688	0.346	6.52952980953798E-177	3	FAM199X
8.13518580134088E-161	0.566774719014104	0.52	0.249	1.3632944365887E-156	3	IL17RC
2.90133070011737E-166	0.566698019703204	0.561	0.271	4.86204998725668E-162	3	KMT5B
1.412026987863E-133	0.565825664366321	0.499	0.25	2.36627482626082E-129	3	NUP85
2.17704452434369E-171	0.565619502678001	0.581	0.283	3.64829121389515E-167	3	ZNF644
6.70673316547902E-122	0.565611322335453	0.365	0.166	1.12391434387097E-117	3	KIF12
9.16760783581297E-142	0.565584461723243	0.387	0.169	1.53630772112554E-137	3	HEXIM1
3.60249725982278E-172	0.565493512871728	0.591	0.291	6.03706490801102E-168	3	ANAPC16
1.42694833581155E-143	0.563979574959002	0.446	0.209	2.39128002115299E-139	3	ADH5
2.42638613073563E-99	0.5630363280626	0.334	0.161	4.06613787788676E-95	3	LATS1
8.9398507577143E-113	0.562853929172622	0.402	0.198	1.49814018997776E-108	3	SMCHD1
3.90623107802645E-70	0.562829850265546	0.296	0.155	6.54606204055673E-66	3	HLCS
1.30705401671607E-174	0.562806379891609	0.684	0.357	2.19036112121279E-170	3	SLC25A44
4.94870957957939E-99	0.562461947615462	0.348	0.171	8.29304751345914E-95	3	CCNT2
6.88928202228712E-119	0.56230553229558	0.404	0.194	1.15450588129488E-114	3	AK6
1.48794411288973E-137	0.562277981056551	0.457	0.219	2.49349674438062E-133	3	PIP4P1
5.41386575132971E-169	0.562093848331251	0.677	0.332	9.07255622607832E-165	3	KCNK1
6.8849868754676E-165	0.562051373078768	0.718	0.372	1.15378610059086E-160	3	LONRF3
3.89258362031355E-89	0.560504623634337	0.3	0.144	6.52319163092144E-85	3	TMEM192
4.79729365122432E-75	0.55962912451059	0.26	0.126	8.03930470072172E-71	3	AQR
2.56907653966456E-79	0.559503033216964	0.278	0.135	4.30525846516987E-75	3	PSMA1
6.1505807861663E-111	0.559400864359155	0.428	0.216	1.03071432814575E-106	3	SUSD6
1.03848630392309E-126	0.559337039567847	0.419	0.2	1.74029534811432E-122	3	FAF2
3.91354839919971E-92	0.559089116889848	0.371	0.192	6.55832440737887E-88	3	XPR1
4.76624599436652E-121	0.559003563173522	0.417	0.205	7.98727503735941E-117	3	HTT
1.27647902639249E-159	0.559001504081679	0.535	0.26	2.13912355242853E-155	3	MACF1
3.32370213043317E-123	0.558350410211305	0.452	0.226	5.5698600301799E-119	3	MED14
1.21232086316811E-69	0.558276527410077	0.304	0.16	2.03160730249713E-65	3	XPO1
7.43726536393771E-135	0.557959398773825	0.502	0.249	1.24633692968868E-130	3	RAPGEF5
1.12460917629347E-184	0.55794452546121	0.668	0.329	1.8846200576326E-180	3	MROH1
1.00314244445093E-185	0.557416008278183	0.636	0.316	1.68106610841086E-181	3	SPATA13
7.08942325380019E-69	0.557274497914581	0.288	0.15	1.18804554887184E-64	3	ANO10
5.58958830195432E-125	0.557270294702622	0.423	0.203	9.36703207641504E-121	3	SCAP
5.92555977514778E-165	0.55706617322086	0.588	0.294	9.93005307119265E-161	3	RABGGTB
3.76560912256965E-109	0.557040969298236	0.402	0.201	6.31040776760221E-105	3	CAMSAP2
1.81445514737988E-189	0.556839862609563	0.785	0.399	3.0406639359792E-185	3	CD99L2
1.40316270536123E-160	0.556458416886484	0.512	0.245	2.35142006164436E-156	3	AP2A2
2.08464136768375E-115	0.556295779970837	0.365	0.172	3.49344200396442E-111	3	FAM98B
3.76757716162208E-121	0.555725934505318	0.425	0.208	6.31370580744628E-117	3	SLC35D1
3.52628638853922E-185	0.555616253068056	0.613	0.298	5.90935072991403E-181	3	TP53BP2
1.23357943882156E-171	0.555114686161747	0.662	0.336	2.06723242357717E-167	3	NCOA2
3.61796008121707E-89	0.55465214604587	0.353	0.18	6.06297750410357E-85	3	TOPBP1
4.83862856004384E-112	0.554578675903568	0.382	0.184	8.10857374092147E-108	3	HHAT
1.12083458338952E-114	0.554537888779738	0.375	0.178	1.87829459484415E-110	3	KLHL24
1.37523819134971E-139	0.554403104527658	0.502	0.247	2.30462416106385E-135	3	MSH6
9.6360871302602E-102	0.55407210545105	0.355	0.173	1.614815481289E-97	3	RBM4
3.34056812420264E-83	0.553669267986237	0.305	0.149	5.59812406253879E-79	3	RBFA
3.27132763199904E-128	0.553604663116097	0.379	0.173	5.482090845704E-124	3	BMPR2
1.15013960117746E-83	0.553351290580992	0.291	0.141	1.92740394365319E-79	3	TMEM94
1.28010591794007E-176	0.553321263347646	0.67	0.341	2.14520149728397E-172	3	DTX3L
9.09761498499409E-102	0.552688334442073	0.374	0.186	1.52457831918531E-97	3	GART
2.52486388536447E-117	0.552558617630867	0.408	0.2	4.23116689909379E-113	3	SFPQ
7.51336134623536E-71	0.552398633456214	0.294	0.152	1.25908909440212E-66	3	TCP11L1
6.27122014971277E-111	0.552087540284181	0.497	0.263	1.05093107268887E-106	3	SH3RF1
2.99827138819938E-117	0.551552905338367	0.414	0.205	5.02450319234452E-113	3	SGPP1
7.2936587252681E-107	0.551543243204807	0.408	0.205	1.22227132918043E-102	3	CHIC2
4.43413056274741E-146	0.551438064575415	0.552	0.279	7.4307159970521E-142	3	FBXO9
4.60088782808704E-186	0.551103438209703	0.657	0.326	7.71016782230825E-182	3	YTHDF3
7.37388886150432E-84	0.551048889129222	0.293	0.142	1.23571629541089E-79	3	SNRNP48

8.76330282730771E-91	0.550444138024007	0.258	0.113	1.46855428780023E-86	3	FLCN
6.72701794001534E-156	0.549970062780262	0.467	0.215	1.12731366638777E-151	3	SELENOO
1.96130590814897E-167	0.549863901598663	0.58	0.288	3.28675644087605E-163	3	COPB1
9.24353647687866E-125	0.549420881286393	0.526	0.265	1.54903184279533E-120	3	MS4A8
3.11262446352739E-89	0.549319524028931	0.337	0.168	5.2161360759792E-85	3	TMEM8B
1.21963809669948E-164	0.549154051255558	0.576	0.285	2.04386952244898E-160	3	ZFR
1.14776399129278E-135	0.548460355921281	0.454	0.219	1.92342289660844E-131	3	NELFA
3.2797644034577E-126	0.548311323719715	0.361	0.157	5.49622918731442E-122	3	SLC28A1
1.68890760411637E-69	0.548213094633339	0.251	0.122	2.83027136297821E-65	3	ASXL2
3.58282104403078E-68	0.547942826199539	0.258	0.128	6.00409150558678E-64	3	CSNK1G3
6.13353639795444E-101	0.54774105193048	0.389	0.196	1.0278580295692E-96	3	GNL3L
2.59109039244944E-213	0.547609735644295	0.8	0.415	4.34214927966677E-209	3	RNF213
3.85277792179339E-166	0.546667397064448	0.557	0.267	6.45648524134137E-162	3	CADM1
2.08840272994217E-76	0.546656136372876	0.3	0.152	3.49974529483709E-72	3	NUP188
9.07208062296081E-128	0.546024972139421	0.389	0.179	1.52029927079577E-123	3	PXMP4
3.57262219944235E-124	0.545859203354208	0.448	0.223	5.98700028182549E-120	3	API5
1.05196482582109E-92	0.545840596934119	0.29	0.133	1.76288265511098E-88	3	BCS1L
3.24172954868439E-160	0.545650302463093	0.546	0.266	5.4324903776853E-156	3	PLA2G12A
6.92162364694851E-246	0.545623394008248	0.947	0.584	1.15992569075563E-241	3	PAH
6.98045378344065E-149	0.545251366117776	0.516	0.252	1.16978444502898E-144	3	BCL6
4.56487697987805E-160	0.545077457323891	0.556	0.274	7.64982084287964E-156	3	RAB20
9.53315613307461E-94	0.544498904661617	0.311	0.148	1.59756630478064E-89	3	PIGC
8.01957755965556E-88	0.544026637307198	0.311	0.151	1.34392080744708E-83	3	PRPF18
3.25687483628337E-149	0.543945169369815	0.489	0.234	5.45787085064367E-145	3	NR3C1
1.84582828981719E-98	0.543676253435802	0.43	0.224	3.09323904807564E-94	3	STXBP5
1.86700274663285E-78	0.543643256353549	0.285	0.141	3.12872320280733E-74	3	B4GALT7
1.58066719865815E-85	0.543415040519283	0.266	0.122	2.64888209151132E-81	3	KIAA1958
2.6695185409323E-98	0.543296035898234	0.353	0.172	4.47357917089434E-94	3	ACAP3
3.44310782142396E-145	0.542994008233643	0.481	0.231	5.76996008714228E-141	3	COMMD3
2.22550086674505E-160	0.542617590231276	0.455	0.204	3.72949435249135E-156	3	GHR
2.5242873595611E-159	0.542523497392006	0.56	0.276	4.2302007571525E-155	3	CMC2
1.03078930494624E-95	0.542352746339291	0.297	0.137	1.72739671722891E-91	3	KLHL20
9.01844329856367E-199	0.54160326088826	0.752	0.398	1.5113107279733E-194	3	F11
3.78220918386541E-132	0.541406174510559	0.372	0.163	6.33822615032165E-128	3	BNIP2
1.37607765707042E-162	0.541210384834243	0.559	0.272	2.3060309377186E-158	3	STX7
4.01243299338965E-125	0.540892426746155	0.429	0.208	6.72403521032238E-121	3	CYP2C19
1.00310052502219E-164	0.540803691903268	0.538	0.259	1.68099585983218E-160	3	WWOX
1.2306911499772E-72	0.540770299663509	0.278	0.14	2.0623922291318E-68	3	COQ2
1.71363289146678E-95	0.54075651630525	0.302	0.14	2.87170599952003E-91	3	INPP5E
7.20115377611468E-145	0.540640846642212	0.468	0.224	1.2067693498013E-140	3	STK25
3.04939720808401E-76	0.540245628799411	0.286	0.143	5.11017984130718E-72	3	AP5M1
1.10432441400206E-95	0.539924475768743	0.324	0.155	1.85062685298465E-91	3	DEPRECATED-ENSG00000232119
1.23385684567518E-156	0.539282396337421	0.578	0.289	2.06769730198246E-152	3	RB1CC1
1.77515273806887E-109	0.539151149869516	0.375	0.18	2.97480095845582E-105	3	APBB1IP
3.20807202269969E-134	0.538505984986963	0.438	0.21	5.37608709564014E-130	3	ZYG11B
8.72127860397835E-152	0.538490652715722	0.543	0.27	1.46151186845469E-147	3	USO1
2.55296091507221E-71	0.538439117253968	0.25	0.121	4.27825190147801E-67	3	RIOK2
3.86890331552074E-161	0.538319934459993	0.613	0.31	6.48350817614965E-157	3	TMEM245
1.21497992648509E-123	0.537986517855026	0.422	0.203	2.03606336080372E-119	3	TCAIM
7.35648463039315E-73	0.537926548473088	0.293	0.15	1.23279969436128E-68	3	KCTD13
8.17474775758939E-262	0.537911531092862	0.986	0.925	1.36992422921683E-257	3	SELENOP
8.18529237070002E-101	0.537540908688312	0.331	0.157	1.37169129548191E-96	3	DENR
2.11305363640682E-72	0.53718215900507	0.284	0.144	3.54105528389055E-68	3	MFHAS1
1.65892954229722E-110	0.537133129550502	0.397	0.196	2.78003412698167E-106	3	EXOSC9
3.24226525956798E-70	0.536548519959773	0.275	0.139	5.43338812198402E-66	3	EXOC2
2.57881907343529E-146	0.536368891564492	0.458	0.216	4.32158500326286E-142	3	CCS
6.91026316670147E-70	0.536210550589564	0.276	0.141	1.15802190147583E-65	3	DESI2
3.42194893681985E-64	0.535857237904661	0.268	0.139	5.73450202832271E-60	3	COX19
2.38836303182005E-108	0.535598072138696	0.355	0.168	4.00241876872404E-104	3	KLHL12
3.71437143481679E-77	0.535458700276093	0.291	0.146	6.22454365046597E-73	3	PIGO

8.87246457136755E-143	0.535196041470182	0.49	0.241	1.48684761286977E-138	3	SCFD1
5.02887948790067E-88	0.535178202762475	0.36	0.186	8.42739624582394E-84	3	ERMP1
2.51708656550904E-88	0.535101101322557	0.336	0.169	4.21813366648005E-84	3	PTPRJ
4.37307408872796E-85	0.534907980400036	0.294	0.142	7.32839755789032E-81	3	JMJD4
1.17309998441185E-127	0.534869419699204	0.431	0.208	1.96588095387737E-123	3	TULP4
5.1672210334506E-64	0.534631122745357	0.258	0.133	8.65922900785652E-60	3	FBXO34
2.90573150630734E-81	0.534041957585448	0.313	0.158	4.86942485826984E-77	3	MIGA1
6.37268478215205E-131	0.53390601380916	0.436	0.209	1.06793451579304E-126	3	MTFR1L
8.21285763577572E-94	0.533455720025157	0.333	0.164	1.37631068260329E-89	3	DCAF10
2.06151623405299E-63	0.532607402505554	0.293	0.157	3.454688905026E-59	3	PRR14L
1.10062031522226E-74	0.532423158108459	0.298	0.153	1.84441952424946E-70	3	TTC33
1.93722313983454E-165	0.532233293167894	0.632	0.321	3.24639853773473E-161	3	PHF10
2.99037682540766E-164	0.532154151277849	0.612	0.308	5.01127348401815E-160	3	MKLN1
7.66869929176438E-133	0.531666107996636	0.434	0.206	1.28512062731387E-128	3	TSPAN4
1.87380770234914E-99	0.531586383235112	0.303	0.139	3.14012694759669E-95	3	SIKE1
3.51706504095022E-84	0.530719010144778	0.284	0.135	5.89389759562437E-80	3	DDX52
2.19154445595145E-170	0.530607472445907	0.559	0.27	3.67259019928344E-166	3	NPEPPS
1.06095852234928E-68	0.53038780855317	0.264	0.132	1.77795429175293E-64	3	LRRC8E
6.13042521970609E-180	0.529956592719661	0.942	0.705	1.02733665831835E-175	3	HSD17B6
7.92139935846103E-85	0.529587791064754	0.325	0.164	1.3274681044909E-80	3	ANKRD13A
2.07717353645892E-78	0.5295700153005	0.315	0.16	3.48092741239786E-74	3	GABPB2
4.62895443512266E-103	0.529011577062245	0.359	0.175	7.75720184237856E-99	3	ABCD4
9.69725358498136E-67	0.528782249750898	0.278	0.145	1.62506575577118E-62	3	FGD6
8.28581657148185E-95	0.528698445057437	0.331	0.162	1.38853714104893E-90	3	PATZ1
4.91973699115049E-116	0.528493702574145	0.418	0.207	8.24449524977E-112	3	ATG4B
7.62674808374287E-87	0.528236359862289	0.303	0.147	1.27809044387363E-82	3	COQ6
5.05659915038786E-134	0.528003366176422	0.47	0.232	8.47384885621998E-130	3	COP 1
1.03109134735058E-143	0.527483653593087	0.557	0.28	1.7279028798901E-139	3	PRKAB1
8.3259210148567E-124	0.527414593766444	0.451	0.223	1.39525784366969E-119	3	PPP1R12B
1.63405866183121E-107	0.527402364202573	0.353	0.168	2.73835550549674E-103	3	BZW1
2.96438082107803E-150	0.52730556649426	0.591	0.301	4.96770937996256E-146	3	ENTPD6
7.69573565334027E-101	0.527273786673428	0.392	0.199	1.28965138078676E-96	3	PAWR
2.60508867889826E-126	0.527239102435192	0.476	0.241	4.36560760809771E-122	3	TEAD1
6.95292723388609E-94	0.527035041649682	0.343	0.171	1.16517154585463E-89	3	ZFC3H1
1.02788926357676E-93	0.526908581766741	0.31	0.146	1.72253682790194E-89	3	TRMT2A
3.12340776109512E-179	0.526878212551284	0.907	0.533	5.23420672604321E-175	3	VMP1
2.30088174356923E-72	0.526481846801119	0.273	0.136	3.85581762587331E-68	3	ZMYM4
1.08819466732179E-158	0.526460584894382	0.633	0.323	1.82359662349785E-154	3	SLC25A33
7.14977543760634E-87	0.526352339234111	0.282	0.132	1.19815936783407E-82	3	RTCA
6.81735268807384E-84	0.52531097434429	0.296	0.144	1.14245196346741E-79	3	CLOCK
1.51541770191048E-141	0.525237941433604	0.49	0.238	2.53953698486158E-137	3	SAMD4B
8.68481964540529E-71	0.525178855958723	0.28	0.14	1.45540207617702E-66	3	PITPNM2
3.07136433083416E-100	0.525017375576286	0.333	0.158	5.14699234561188E-96	3	FGFR1OP2
1.21154173070888E-149	0.524942493393229	0.497	0.237	2.03030163232194E-145	3	PNISR
2.43253048270029E-144	0.524461759782362	0.504	0.246	4.07643458290915E-140	3	ZNF652
4.7694790461534E-180	0.524149855380424	0.613	0.299	7.99269298554387E-176	3	ASB13
9.75401814275868E-163	0.524027834704528	0.602	0.293	1.6345783603635E-158	3	EDNRB
1.35462225520813E-103	0.523994275700425	0.393	0.199	2.27007597527779E-99	3	TMEM220
7.72882776567937E-112	0.523721640048666	0.372	0.178	1.29519695697255E-107	3	RBFOX2
1.07108708947357E-112	0.523613296106517	0.425	0.215	1.79492774453981E-108	3	MYO9B
1.20477062370122E-66	0.523426244378979	0.259	0.13	2.0189546111985E-62	3	DENND1B
9.50046683778681E-99	0.523177690918547	0.317	0.149	1.59208823267631E-94	3	TSC2
1.27893254268116E-103	0.522987507416754	0.339	0.16	2.14323515502508E-99	3	MED1
2.72332684842315E-149	0.52286922616114	0.509	0.246	4.56375113258751E-145	3	UBA1
2.77077915193718E-107	0.52284887139029	0.449	0.232	4.64327170281632E-103	3	SPIRE2
3.08755389813984E-110	0.522248329681668	0.458	0.235	5.17412282250274E-106	3	TMEM185A
5.47288548084756E-137	0.522201723330875	0.494	0.244	9.17146148880435E-133	3	IP6K2
1.37852635456402E-64	0.522151843975133	0.255	0.129	2.31013446497838E-60	3	ZNF131
1.42459953355845E-126	0.521580165356834	0.489	0.247	2.38734389833726E-122	3	GOLGB1
1.68223668775931E-129	0.521055708091847	0.477	0.236	2.81909224134706E-125	3	BTD

1.18794620451404E-79	0.521016839041103	0.264	0.125	1.99076024952462E-75	3	RPAP1
3.04725657941202E-198	0.520782812874842	0.446	0.177	5.10659257577866E-194	3	WHRN
1.36595829565249E-160	0.519445548526501	0.655	0.337	2.28907291185445E-156	3	SLC35F5
4.29874773938677E-102	0.519359803757738	0.331	0.156	7.20384146166434E-98	3	PSKH1
6.57829299094264E-74	0.519266148600766	0.3	0.155	1.10239033942217E-69	3	FRS2
3.23709971890538E-143	0.519156173694913	0.544	0.272	5.42473170894163E-139	3	HACD2
1.13233488184387E-69	0.518311600261735	0.271	0.137	1.89756679499396E-65	3	LCORL
2.51694951043045E-149	0.518309865332642	0.497	0.24	4.21790398957935E-145	3	KLHL5
2.71943559761161E-152	0.517939521525726	0.494	0.236	4.55723017447754E-148	3	EIF2D
6.83886717267664E-80	0.517706065560578	0.251	0.115	1.14605736079715E-75	3	OSBPL11
1.43804597311377E-166	0.517441418972338	0.788	0.452	2.40987744174406E-162	3	A1CF
3.2333145884991E-96	0.517411873287231	0.377	0.191	5.4183885874068E-92	3	ZCCHC2
4.23603699285461E-174	0.516993855308566	0.966	0.723	7.09875079262575E-170	3	CFH
3.93153201438091E-105	0.516821879761022	0.411	0.21	6.58846134969952E-101	3	NUP153
1.05447559618521E-166	0.516619298253018	0.661	0.336	1.76709020408718E-162	3	CCNG1
2.55341569668562E-142	0.516533533798822	0.505	0.248	4.27901402450576E-138	3	RPRD2
1.24509478906252E-81	0.516512408036996	0.291	0.142	2.08652984751097E-77	3	RNF38
3.94611904901758E-159	0.516376758493886	0.609	0.308	6.61290630234366E-155	3	SLC66A2
2.95954057610256E-132	0.516330673301072	0.454	0.222	4.95959809743266E-128	3	DDX1
3.12437839401747E-106	0.515983719345557	0.409	0.207	5.23583331269448E-102	3	SLC30A9
1.92416184259964E-94	0.515887840815673	0.388	0.201	3.22451041582848E-90	3	PLPP2
1.1733757296194E-81	0.514633688006545	0.26	0.12	1.96634304769618E-77	3	UFL1
5.98269933720412E-110	0.514489110211178	0.412	0.206	1.00258075492867E-105	3	OSGEP
3.81383133354922E-106	0.513901740085489	0.363	0.175	6.39121854876178E-102	3	NDRG3
6.78418087362795E-124	0.513847302599322	0.499	0.257	1.13689303080257E-119	3	NCKIPSD
1.20096813686479E-92	0.51373749164251	0.352	0.177	2.01258240375801E-88	3	THUMPD3
1.5965202772129E-170	0.51359859623947	0.674	0.34	2.67544868055339E-166	3	HNRNPL
3.45182950335594E-138	0.513360089784603	0.524	0.261	5.78457588172389E-134	3	CAMK2D
2.49825710838896E-146	0.513258767312561	0.551	0.275	4.18657926223822E-142	3	NRP1
5.47766970123289E-88	0.513182839432291	0.307	0.149	9.17947888532608E-84	3	APPL1
3.17119192306103E-121	0.513178558138906	0.447	0.224	5.31428342466568E-117	3	RARS
1.41630771444526E-74	0.513177771075862	0.272	0.134	2.37344846786736E-70	3	TMEM30A
1.06348630396601E-149	0.513110300859455	0.645	0.34	1.78219034818625E-145	3	LGR4
3.3197407842702E-73	0.513086737112034	0.25	0.119	5.56322160628E-69	3	LMBR1L
6.58416085198145E-177	0.512939941272912	0.852	0.471	1.10337367557505E-172	3	MORF4L2
1.04846793944068E-156	0.512924390778317	0.535	0.257	1.7570225729147E-152	3	FAM13A
8.6256847769994E-101	0.512812451962829	0.385	0.195	1.44549225492956E-96	3	SNX14
2.59278098532341E-97	0.51217856011911	0.365	0.184	4.34498237520498E-93	3	AAK1
4.2813930872331E-84	0.511360053971795	0.309	0.153	7.17475853558522E-80	3	DYNC1LI1
1.49108171598753E-131	0.511327513394789	0.464	0.228	2.4987547396519E-127	3	TMBIM4
9.23149683618453E-75	0.511258385861021	0.284	0.142	1.5470142398078E-70	3	RNF31
7.87111786577388E-84	0.510536879485005	0.314	0.157	1.31904193194639E-79	3	KYAT1
7.05481113143362E-153	0.510378330477828	0.632	0.321	1.18224524940565E-148	3	SRSF3
1.13323339975453E-149	0.510084959835511	0.496	0.239	1.89907253130865E-145	3	FBXW2
9.57674414499592E-149	0.509546524611209	0.925	0.657	1.60487078381842E-144	3	ANPEP
8.49341818497654E-59	0.509301257549817	0.287	0.156	1.42332701943837E-54	3	SCMH1
7.08969321623376E-98	0.509197628623136	0.363	0.18	1.18809078917645E-93	3	MDC 1.00
1.42692046585332E-136	0.507554738098584	0.339	0.139	2.39123331667699E-132	3	DENND4A
1.12747692045721E-76	0.507362536485227	0.282	0.138	1.8894258233022E-72	3	DDX18
3.28951461152429E-84	0.506848233398801	0.259	0.118	5.51256858599241E-80	3	HPS4
5.18782685445065E-124	0.50655841060707	0.458	0.23	8.6937602426884E-120	3	MFF
9.7904652644692E-147	0.505597583361494	0.547	0.27	1.64068616901975E-142	3	ME1
3.50688315610669E-102	0.505570410730856	0.396	0.201	5.87683479300359E-98	3	DYRK2
2.91068039682961E-157	0.505427122058633	0.579	0.291	4.87771820900706E-153	3	AFF4
2.6437102506981E-165	0.505288733557097	0.612	0.307	4.43032963811987E-161	3	PSMD12
4.60733071858594E-125	0.504370714110149	0.666	0.362	7.72096481820632E-121	3	SYTL4
1.74474537726043E-66	0.504350247212576	0.286	0.15	2.92384430321303E-62	3	DDX59
2.95583911185813E-144	0.504343746430542	0.491	0.238	4.95339518365186E-140	3	CCNY
1.14838765244935E-71	0.504258250806141	0.299	0.155	1.92446802797462E-67	3	NOP9
2.53985238982488E-96	0.503639071614865	0.362	0.181	4.25628463486854E-92	3	SPCS3

1.72898258324573E-128	0.503575728632357	0.497	0.251	2.89742901300319E-124	3	LMBRD2
5.02866774842009E-151	0.503539910601743	0.614	0.315	8.42704141280238E-147	3	TAF7
9.2085967973299E-80	0.503435425559662	0.295	0.145	1.54317665129655E-75	3	ARFIP2
1.73704596963074E-133	0.502606014373437	0.425	0.199	2.91094163590719E-129	3	EPB41
6.42318443674095E-165	0.502568846871576	0.622	0.321	1.07639724790905E-160	3	TJP1
2.68002161805725E-60	0.50249482909999	0.256	0.133	4.49118022754034E-56	3	DEPRECATED-ENSG00000116489
2.49985130259184E-120	0.502423822392252	0.461	0.234	4.1892508128834E-116	3	BRF1
2.68172339044681E-166	0.502249380375727	0.671	0.345	4.49403205771077E-162	3	OCIAD1
4.64065661317458E-97	0.502196776775625	0.356	0.176	7.77681235235795E-93	3	CEBPZ
4.88429653622403E-66	0.502019027143667	0.281	0.146	8.18510413540423E-62	3	COG4
1.33479493521022E-81	0.501833220236946	0.292	0.142	2.23684935242529E-77	3	DVL2
3.12601350309517E-122	0.501507277430762	0.465	0.237	5.23857342848688E-118	3	ATP6V1C1
1.18524425302693E-129	0.501089511735046	0.52	0.268	1.98623231922253E-125	3	RBM25
3.77648834258985E-116	0.50084454939326	0.388	0.187	6.32863916451206E-112	3	GTF3C3
1.32988805551218E-136	0.500527583772551	0.543	0.271	2.22862640342731E-132	3	RBMX
1.78467095044921E-146	0.500436152446553	0.541	0.269	2.99075157876278E-142	3	SH3GLB2
1.12228722521972E-131	0.500312908734953	0.47	0.233	1.88072893202321E-127	3	ZSWIM8
2.44785698502238E-99	0.500066275995221	0.324	0.152	4.10211873550051E-95	3	MAIP1
1.02196718448218E-83	0.499719322681711	0.303	0.149	1.71261260775523E-79	3	EIF2AK4
2.17133898886185E-59	0.499685495450116	0.284	0.155	3.6387298775347E-55	3	SPTLC2
6.04898282192052E-169	0.499674601443914	0.809	0.441	1.01368854129744E-164	3	TBC1D9B
3.33201389381078E-141	0.499361170972044	0.451	0.214	5.5837888832481E-137	3	HEXA
1.46548084186621E-105	0.498990854295294	0.377	0.186	2.45585279479939E-101	3	RNF14
3.0217020953392E-81	0.498686555659424	0.29	0.141	5.06376837136942E-77	3	TRMU
4.60526891922386E-79	0.498407217203046	0.282	0.138	7.71750965483534E-75	3	SPAG16
3.34944541542479E-59	0.497900720610633	0.272	0.146	5.61300062716887E-55	3	CNOT6
1.06326108241938E-108	0.497765819252889	0.409	0.205	1.78181292191841E-104	3	ATL3
2.84310749396751E-143	0.497291816424939	0.466	0.222	4.76447953839076E-139	3	RUFY3
4.17084743640417E-80	0.496657289559753	0.277	0.133	6.98950613392611E-76	3	PPARG
8.38055319836051E-158	0.496653989485141	0.67	0.344	1.40441310498125E-153	3	GRB10
2.49516199749842E-103	0.49655956763646	0.383	0.192	4.18139247540785E-99	3	TNRC6B
5.79392085334416E-86	0.496542016298714	0.292	0.14	9.70945256603414E-82	3	SSR1
4.42262979861558E-125	0.495657619903144	0.415	0.198	7.41144301651999E-121	3	ELP2
9.40503508251461E-124	0.495551309146223	0.549	0.289	1.5760957791278E-119	3	NREP
2.026712099208E-161	0.495497745725866	0.654	0.348	3.39636413585276E-157	3	TMEM147
2.75627440478282E-161	0.495341996055428	0.524	0.25	4.61896464753505E-157	3	ZRANB2
4.06058361479151E-124	0.495277875721546	0.423	0.205	6.80472602166761E-120	3	PSMA4
1.49761368979173E-128	0.494611308477778	0.418	0.197	2.50970102135299E-124	3	GOLT1B
3.71621426452372E-98	0.494517613444114	0.372	0.189	6.22763186448885E-94	3	TASOR
6.15866789301262E-122	0.49445970269101	0.458	0.226	1.03206956551106E-117	3	MORC4
8.30802116250424E-112	0.494162390259177	0.358	0.167	1.39225818641246E-107	3	EIF3J
1.12954265791478E-96	0.494010074636607	0.368	0.185	1.8928875861336E-92	3	DRAM2
5.69975953595889E-94	0.493984452645934	0.357	0.179	9.55165703035991E-90	3	NXF1
4.69173618804661E-173	0.493349729560389	0.743	0.407	7.86241150392851E-169	3	LONP2
1.89762025592805E-149	0.493046756813543	0.415	0.181	3.18003202488423E-145	3	SMO
4.28288535149396E-176	0.492988813547757	0.696	0.361	7.17725927203357E-172	3	SETD7
7.42024259631161E-92	0.492975752895391	0.37	0.19	1.2434842542899E-87	3	MAPKAPK5
3.59192176791094E-76	0.492965177979778	0.313	0.161	6.01934249866515E-72	3	PKD2
1.02522626458154E-140	0.491726146918052	0.483	0.233	1.71807417418575E-136	3	SUZ12
2.35255733330735E-89	0.491358573932435	0.309	0.149	3.94241557915645E-85	3	MTREX
1.22973920181242E-89	0.4912293095216	0.387	0.204	2.06079695439725E-85	3	TRAPPC10
5.60263347782699E-142	0.490490267395652	0.561	0.284	9.38889318214247E-138	3	AGO2
4.16500727636814E-168	0.490064034788468	0.628	0.319	6.97971919373773E-164	3	NRDC
5.7694149839537E-128	0.490033200232582	0.456	0.225	9.66838563010961E-124	3	AP1G1
5.7252403333154E-163	0.489993065042798	0.716	0.367	9.59435775056994E-159	3	EDEM3
2.17145500841104E-91	0.489441717493057	0.359	0.183	3.63892430309523E-87	3	BOD1L1
7.83502301033514E-90	0.48938574660068	0.317	0.154	1.31299315607196E-85	3	CYB561
1.1440365933583E-168	0.488727622831405	0.68	0.343	1.91717652314985E-164	3	SLC6A1
7.61611723404607E-147	0.488402504734079	0.541	0.271	1.27630892608144E-142	3	C16orf72
4.42823638269058E-167	0.488240531096559	0.799	0.439	7.42083853011287E-163	3	SEC31A

4.70087220245382E-94	0.486493927115455	0.326	0.158	7.87772163687211E-90	3	ANKRD46
2.2038198200711E-166	0.486303517721114	0.729	0.382	3.69316125447515E-162	3	LPP
3.20555070794963E-126	0.48607212172568	0.411	0.195	5.37186187638198E-122	3	PELO
4.15143489334128E-152	0.486061229931535	0.871	0.491	6.95697459426131E-148	3	ATP6AP1
4.93433974104672E-106	0.485804161615066	0.358	0.172	8.26896653804609E-102	3	SEC22C
1.0557280167152E-80	0.485486719224503	0.3	0.149	1.76918901041133E-76	3	ATG4A
8.7835801636935E-129	0.485444135261379	0.472	0.236	1.47195236383176E-124	3	ATL2
8.81889078218366E-70	0.485408977524609	0.268	0.135	1.47786971727834E-65	3	SPTLC1
5.16564244961369E-66	0.485356566352596	0.291	0.154	8.65658361706261E-62	3	APAF1
1.67833162384083E-127	0.48529745915409	0.538	0.281	2.81254813523247E-123	3	UGCG
8.50036338785406E-85	0.48528183876178	0.329	0.167	1.42449089653658E-80	3	PPP1R12A
4.07395504801426E-139	0.485275723172226	0.504	0.25	6.8271338694623E-135	3	UBA5
1.20069500678286E-65	0.484947622559802	0.276	0.143	2.01212469236671E-61	3	PAM
6.20662725376296E-68	0.48491791047377	0.271	0.138	1.0401065951856E-63	3	AP5S1
4.53783027572857E-76	0.484820284421124	0.306	0.157	7.60449597606594E-72	3	ARMH3
2.34917426252261E-86	0.484811022184146	0.264	0.12	3.9367462291354E-82	3	HEXD
5.2540822694205E-88	0.484625831663045	0.319	0.158	8.80479106709487E-84	3	SDHAF4
2.4675799885022E-121	0.484536133979176	0.525	0.271	4.13517054473198E-117	3	RALGAPA2
3.61468320037281E-69	0.484339785102061	0.301	0.158	6.05748610718475E-65	3	MOB4
1.97847043297628E-151	0.483699878767734	0.883	0.555	3.31552075158165E-147	3	SCCPDH
5.44017923877135E-109	0.482766970453027	0.424	0.214	9.11665236833303E-105	3	PLCB1
7.04650201493736E-111	0.482436348929342	0.39	0.19	1.1808528076632E-106	3	GON7
6.54630583441433E-147	0.482328449318394	0.526	0.26	1.09702993173115E-142	3	SENP2
1.45484937954253E-113	0.482252297912637	0.38	0.181	2.43803659023738E-109	3	INPPL1
4.68274308821588E-135	0.482108534490425	0.47	0.225	7.84734086723217E-131	3	E2F1
1.62752840490881E-74	0.4820614233637	0.273	0.134	2.72741210094618E-70	3	AMBRA1
3.02090622869055E-90	0.481987867149678	0.372	0.191	5.06243465803962E-86	3	PWWP2A
2.15193398172863E-104	0.481936158056214	0.321	0.146	3.60621096658084E-100	3	BCHE
2.7245757116726E-134	0.481726201811377	0.485	0.241	4.56584397762095E-130	3	GOPC
3.50704172212808E-80	0.481112933221081	0.278	0.134	5.87710051794223E-76	3	CLTC
4.74996036760316E-105	0.48064494813116	0.404	0.203	7.95998358402938E-101	3	SELENOI
8.27259282748577E-80	0.480573423825692	0.308	0.155	1.38632110603006E-75	3	GPATCH8
7.25731282292227E-148	0.480545141334231	0.537	0.265	1.21618048286531E-143	3	TXNDC15
2.76936426910313E-119	0.480261078155674	0.402	0.194	4.64090064216302E-115	3	C5orf15
3.52752234906035E-136	0.480119307186938	0.555	0.282	5.91142195255533E-132	3	SRPK1
2.3305667222204E-112	0.480045933507325	0.424	0.213	3.90556371309695E-108	3	PPA2
1.23564208530586E-74	0.479432777243901	0.286	0.143	2.07068900655556E-70	3	GAR1
3.20761613939726E-67	0.479339862739092	0.335	0.182	5.37532312640192E-63	3	DSN1
1.93976106403737E-140	0.478364613563824	0.51	0.254	3.25065159111382E-136	3	MARK3
1.37019828582902E-138	0.478143125023331	0.463	0.222	2.29617828739228E-134	3	ITGAV
2.75768601463339E-159	0.47806407718424	0.938	0.689	4.62133022332263E-155	3	GATM
2.03352185804872E-170	0.477580190631125	0.736	0.391	3.40777592971805E-166	3	NPLOC4
8.04288257264912E-72	0.477403666310909	0.343	0.187	1.34782626152454E-67	3	DGKQ
9.9853318968849E-113	0.477271611455731	0.451	0.23	1.67334191927997E-108	3	EBAG9
7.93549820975062E-60	0.477157960881668	0.332	0.187	1.32983078999001E-55	3	SLC4A4
9.55436725687698E-66	0.477117442148589	0.305	0.164	1.60112086490744E-61	3	VPS13A
6.20214881277988E-103	0.476962413695502	0.339	0.16	1.03935609804565E-98	3	FBXO3
1.38090884340377E-162	0.476657968768288	0.767	0.423	2.31412703977604E-158	3	RAB4A
1.08497976087095E-106	0.47659202206669	0.318	0.142	1.81820908326754E-102	3	ARIH1
1.36824899518114E-110	0.47653439116009	0.424	0.212	2.29291166612455E-106	3	TNPO2
1.18326204658574E-53	0.476507372025818	0.25	0.134	1.98291053766838E-49	3	BLZF1
3.75251643035927E-91	0.476501677655947	0.321	0.155	6.28846703399607E-87	3	CTBS
4.4133598302978E-68	0.476393937773203	0.258	0.127	7.39590840361305E-64	3	DOCK7
8.29179159500312E-79	0.475636894613113	0.265	0.125	1.38953843549062E-74	3	GCFC2
1.68791796401895E-165	0.474935311192915	0.83	0.466	2.82861292410295E-161	3	MIDN
1.89085144961563E-122	0.474630531906482	0.414	0.199	3.16868885926587E-118	3	RFNG
5.27862099408195E-124	0.47455062053594	0.477	0.24	8.84591306188253E-120	3	ABHD10
1.27168480464356E-81	0.474335032624561	0.301	0.149	2.13108939562168E-77	3	CLCN5
1.08631831705621E-101	0.473936967654285	0.377	0.187	1.82045223572279E-97	3	NMI
3.92901655886937E-79	0.472827083035427	0.321	0.165	6.5842459493533E-75	3	TMEM138

2.02355228424518E-62	0.472511755511213	0.259	0.134	3.39106891793808E-58	3	DENND6A
1.58246879495337E-80	0.47216097079342	0.314	0.159	2.65190120658286E-76	3	PIAS3
1.58180701518578E-159	0.472140448033966	0.608	0.307	2.65079219604833E-155	3	NUS1
3.86123073827726E-101	0.472043137219768	0.405	0.207	6.47065047120504E-97	3	LARP7
2.80787583294538E-148	0.471872257764205	0.926	0.573	4.70543832084986E-144	3	RNF128
3.34059973510093E-96	0.471650347893182	0.366	0.183	5.59817703608213E-92	3	CREM
2.58414222351233E-161	0.471525474796996	0.643	0.334	4.33050553816197E-157	3	ITCH
4.47881407908219E-107	0.47087274639226	0.386	0.189	7.50559663372593E-103	3	LEPROT
8.1109865429294E-102	0.470838988612325	0.373	0.185	1.35923912486411E-97	3	OSTC
7.95246667476734E-141	0.469434530531797	0.846	0.473	1.33267436535751E-136	3	ZKSCAN1
2.95918514273169E-79	0.46918819047461	0.292	0.144	4.95900246218977E-75	3	ARID4A
1.55842611951063E-87	0.469170485424924	0.325	0.161	2.61161049107592E-83	3	GNPDA1
8.50501801123095E-158	0.46914647948772	0.582	0.291	1.42527091832208E-153	3	NPTN
1.17117199234454E-92	0.468891653992143	0.386	0.201	1.96265002477098E-88	3	ZNF384
1.21907884695202E-68	0.468328228985075	0.259	0.129	2.0429323317222E-64	3	ARHGAP21
8.57790941453315E-152	0.468092987356817	0.473	0.216	1.43748605968746E-147	3	PPARGC1A
8.34339518841736E-102	0.467905943821687	0.35	0.168	1.39818616567498E-97	3	CLN6
2.89080981414667E-75	0.467829900719656	0.257	0.122	4.84441908654699E-71	3	MPV17L
2.66343942649481E-73	0.467790268014164	0.275	0.136	4.46339179092E-69	3	ZNF76
4.45210318058634E-118	0.46775986200693	0.436	0.217	7.46083451002659E-114	3	WIZ
3.13016869829693E-98	0.467547809015803	0.346	0.167	5.245536704606E-94	3	PUS1
8.37112771286758E-147	0.467399029813304	0.591	0.302	1.40283358212235E-142	3	RIOK3
1.81256320165489E-161	0.467106386630102	0.489	0.221	3.03749341333326E-157	3	SOX5
1.82424108189006E-141	0.466794260142676	0.554	0.284	3.05706320503137E-137	3	TEX2
5.51325268320027E-90	0.466573999775422	0.355	0.18	9.23910884650701E-86	3	CPSF2
1.4241883003665E-82	0.466366905600331	0.361	0.19	2.38665475375418E-78	3	MYO5C
6.77447386226881E-156	0.466348039973096	0.632	0.322	1.13526632983901E-151	3	SLC51A
1.16485084302521E-96	0.466133413868016	0.298	0.136	1.95205704274165E-92	3	MORF4L1
2.67451225287476E-147	0.465632636046846	0.6	0.309	4.48194763336753E-143	3	PACSIN2
1.74139370095066E-62	0.465599903204417	0.271	0.142	2.91822756405312E-58	3	CCDC82
2.10388019108021E-84	0.465229675868973	0.368	0.194	3.52568242421222E-80	3	ANKRD27
1.35147494417512E-193	0.464393313504788	0.781	0.418	2.26480171144867E-189	3	SNX1
1.67877887986908E-128	0.463879912593271	0.524	0.269	2.8132976468846E-124	3	KDM5C
1.16146084963635E-162	0.463785224809854	0.705	0.35	1.9463760918206E-158	3	SLC15A1
1.28204641758531E-69	0.463625269528793	0.257	0.126	2.14845338658947E-65	3	RNGTT
1.01029976674023E-143	0.463602784268957	0.613	0.319	1.69306034910327E-139	3	PSMD1
1.83982499544078E-53	0.463165892743649	0.257	0.138	3.08317872735966E-49	3	RFC4
1.75622612599338E-121	0.462987582334595	0.485	0.248	2.94308374193971E-117	3	USP24
1.96649113337181E-87	0.462935739526285	0.329	0.164	3.29544584130448E-83	3	ZBTB44
2.47871340589908E-91	0.462719935220373	0.34	0.169	4.15382792560568E-87	3	RBX1
1.02281863271092E-92	0.462356348343519	0.336	0.166	1.71403946469697E-88	3	FOXK2
3.44673009291675E-92	0.462007090002025	0.397	0.202	5.7760302897099E-88	3	ITGA3
1.60621197026009E-120	0.461949418598881	0.469	0.238	2.69169001976186E-116	3	MAEA
2.58524300854023E-89	0.461631772091513	0.326	0.16	4.33235023371172E-85	3	KPNA3
1.7776748218524E-76	0.461504468176493	0.284	0.141	2.97902746646026E-72	3	TNPO1
3.30829250529581E-125	0.460846593766178	0.47	0.236	5.54403658037472E-121	3	TAP2
4.4500238241333E-56	0.460563124224852	0.268	0.145	7.45734992448259E-52	3	NT5C3A
1.10424399783963E-154	0.459666458973138	0.65	0.333	1.85049209157966E-150	3	SEL1L
2.56744668735588E-128	0.459605706045508	0.433	0.208	4.30252715867098E-124	3	GOSR1
9.54402443473772E-107	0.459582815597703	0.346	0.163	1.59938761477335E-102	3	SECISBP2L
1.96978653047413E-76	0.459424556247918	0.341	0.181	3.30096826776854E-72	3	CCT4
1.03068806999684E-191	0.459148408225261	0.519	0.218	1.7272270677007E-187	3	F13B
3.21924699613418E-157	0.458651574403795	0.65	0.339	5.39481411612166E-153	3	EIF4A2
4.02888713919915E-93	0.458392414813382	0.297	0.137	6.75160906786994E-89	3	ABCC6
7.0835785204268E-126	0.458311465683178	0.51	0.262	1.18706608845312E-121	3	DNAJC8
2.63571151006227E-121	0.458235593381008	0.481	0.246	4.41692534856236E-117	3	KDM4B
1.42203883645837E-82	0.458110033848281	0.366	0.194	2.38305268213693E-78	3	DNAJC13
1.03781623069365E-98	0.457889951920572	0.412	0.214	1.73917243939642E-94	3	LARP4B
5.38575250476582E-88	0.457855540454429	0.332	0.165	9.02544404748656E-84	3	RIN2
3.78759309076924E-134	0.457434555296181	0.533	0.273	6.3472485015111E-130	3	CUL4A

3.78794444334837E-116	0.457406168725092	0.43	0.214	6.3478372981632E-112	3	RNF13
8.50772566912342E-119	0.457048728773282	0.436	0.217	1.4257246676317E-114	3	SNX2
2.44368286343774E-63	0.457040812899742	0.251	0.127	4.09512374254897E-59	3	LRRC75B
1.33640838977801E-85	0.45703744238961	0.336	0.17	2.23955317959E-81	3	ARFRP1
3.17102265568001E-97	0.456714035554723	0.352	0.173	5.31399976638856E-93	3	CDC23
1.90628326010662E-116	0.454823674664562	0.47	0.243	3.19454948728668E-112	3	LANCL1
2.57487096582574E-134	0.45429917331819	0.535	0.273	4.31496876453077E-130	3	NCKAP1
3.28639833199938E-85	0.454187462898136	0.286	0.135	5.50734632476457E-81	3	CREB3L2
6.55240219876641E-129	0.45401744708498	0.431	0.206	1.09805156046928E-124	3	DOK4
5.47647689335475E-119	0.453924517888084	0.471	0.242	9.17747997788389E-115	3	ACTR10
2.32732569614005E-82	0.453900069515578	0.348	0.181	3.9001324015915E-78	3	INTS4
2.45771534414637E-140	0.453124350394676	0.806	0.437	4.11863937372048E-136	3	FRK
1.79269489748059E-125	0.452388729439367	0.523	0.268	3.00419810919797E-121	3	GNPAT
2.99503244034502E-125	0.452385508287007	0.388	0.177	5.01907536353018E-121	3	APOL3
3.09167944832462E-126	0.452271660905407	0.398	0.185	5.1810364195024E-122	3	TOX4
1.33841915576083E-75	0.452132865950916	0.309	0.158	2.242922821224E-71	3	CALML4
3.0518663212018E-90	0.451657896364996	0.364	0.186	5.11431758106998E-86	3	NRIP1
2.26484662381255E-81	0.451274067181324	0.263	0.122	3.79542997218507E-77	3	TUT1
1.116874653971E-95	0.451085967842994	0.334	0.161	1.87165854512461E-91	3	DENND4B
6.18419507028415E-118	0.451083695985698	0.451	0.228	1.03634740987822E-113	3	ZC3H3
9.64073926170381E-135	0.450749093442193	0.538	0.273	1.61559508547632E-130	3	NKAP
4.01780406772729E-90	0.45066998253633	0.362	0.185	6.73303605669739E-86	3	MLH1
3.45963716055283E-99	0.450553193150802	0.491	0.269	5.79765995365442E-95	3	LIME1
1.30728610457457E-62	0.450483731719025	0.306	0.168	2.19075005404606E-58	3	NCEH1
1.49916158635716E-96	0.450117562546803	0.409	0.215	2.51229498641733E-92	3	PRPF4B
6.19400495907356E-152	0.450018532019946	0.691	0.365	1.03799135104155E-147	3	EMC2
6.64931090954102E-59	0.449977723542011	0.271	0.146	1.11429152222088E-54	3	TDRD7
7.43280181058252E-58	0.449824106078645	0.289	0.159	1.24558892741742E-53	3	MFSD10
2.43650672122645E-78	0.449475892459224	0.283	0.139	4.08309796343128E-74	3	CREBRF
2.3247881749466E-79	0.449373400862292	0.341	0.179	3.89588002357551E-75	3	SMPDL3A
1.34909513757949E-106	0.449321773742192	0.431	0.224	2.26081363155571E-102	3	RETREG3
2.13661451283616E-104	0.449031435441725	0.465	0.246	3.58053860061084E-100	3	FAM49B
1.84684555890183E-101	0.448404168255038	0.503	0.278	3.09494378760769E-97	3	IFI6
7.7391907436945E-139	0.448167986864788	0.519	0.259	1.29693358482832E-134	3	ASPSCR1
3.0031132637306E-73	0.44738007846809	0.276	0.137	5.03261720735974E-69	3	TBRG1
1.78408267430122E-152	0.447361698560416	0.633	0.324	2.98976574559399E-148	3	CTSO
5.32866779854667E-65	0.447332741344471	0.29	0.154	8.92978149680451E-61	3	RIF1
1.3107556200654E-118	0.446767902012918	0.432	0.213	2.19656426810559E-114	3	ARFGEF2
9.03569685442504E-78	0.446368902128077	0.44	0.244	1.51420207886455E-73	3	TMC5
2.81320448631848E-125	0.446355595078762	0.461	0.23	4.71436807817252E-121	3	ADIPOR2
8.48644188889638E-113	0.446312718367838	0.406	0.2	1.42215793174126E-108	3	ELF1
2.26632002833003E-91	0.446115655838498	0.37	0.191	3.79789910347546E-87	3	ARFGAP1
9.26145571619693E-144	0.445965911100573	0.524	0.261	1.55203474892028E-139	3	NME3
5.57082601413972E-93	0.445496241795529	0.447	0.244	9.33559023449534E-89	3	CEP170B
2.76939105315149E-113	0.445415108660491	0.414	0.203	4.64094552687127E-109	3	TBC1D15
3.74061674241307E-66	0.445407994730919	0.27	0.138	6.26852553693583E-62	3	WDR5
9.13104522004937E-87	0.445312034807428	0.349	0.178	1.53018055797587E-82	3	QSER1
3.41145907572459E-96	0.44517006253305	0.438	0.234	5.71692311909927E-92	3	HUS1
8.0356928977348E-39	0.444822517760606	0.271	0.164	1.3466214158024E-34	3	TEP1
4.27758800300762E-150	0.444719364349556	0.658	0.347	7.16838197544017E-146	3	ARRDC2
1.05391642211649E-83	0.444638481426156	0.346	0.179	1.76615314018281E-79	3	PREPL
7.83722589345254E-89	0.444347883470766	0.342	0.171	1.31336231522478E-84	3	LAMTOR3
3.55583573810689E-62	0.444010175258509	0.252	0.129	5.95886952991953E-58	3	POLR2D
3.77073311714155E-70	0.443903047057577	0.283	0.145	6.31899455770581E-66	3	SFSWAP
1.19964147853577E-111	0.443653604809059	0.444	0.227	2.01035918973024E-107	3	GATD1
6.66097560905024E-70	0.443644610562062	0.258	0.127	1.11624629256464E-65	3	RRNAD1
6.62590311703278E-88	0.443603390894981	0.313	0.153	1.11036884435235E-83	3	STYX
9.48911352424556E-58	0.443523494299987	0.271	0.145	1.59018564439307E-53	3	ABHD15
1.21022141565105E-76	0.443355802933893	0.298	0.15	2.02808904834803E-72	3	MKKS
1.61760855843086E-81	0.44335301355085	0.325	0.165	2.71078842221843E-77	3	TP53I13

8.38750608990995E-64	0.44278563010266	0.269	0.138	1.40557827054711E-59	3	TONSL
5.6186684602565E-77	0.442556792779991	0.307	0.155	9.41576460569784E-73	3	ING2
4.77869395432199E-141	0.442404541362362	0.48	0.229	8.00813532865279E-137	3	SLC7A2
5.66671667033558E-80	0.44125230211165	0.328	0.169	9.49628379614836E-76	3	DDX19A
3.62762247199739E-102	0.441193615983994	0.402	0.203	6.07916973857322E-98	3	ATP6V1H
6.6259583090065E-166	0.44058834859604	0.681	0.348	1.11037809342331E-161	3	SHROOM1
3.90946774672944E-117	0.440575648493564	0.466	0.238	6.5514860499692E-113	3	LARP1
8.25489674932619E-142	0.440147811759691	0.506	0.25	1.38335559725208E-137	3	MEGF9
8.10410603718836E-161	0.439975412464457	0.758	0.408	1.35808608971203E-156	3	RBM33
3.05896762019508E-47	0.439344203814368	0.252	0.142	5.12621793792292E-43	3	TPCN1
7.900159374755E-122	0.439302081330591	0.464	0.235	1.32390870802144E-117	3	KCMF1
1.19015172490687E-65	0.439136844416503	0.276	0.143	1.99445626059893E-61	3	MED12
3.62492751037831E-68	0.439094410684981	0.311	0.166	6.07465352189197E-64	3	COX20
3.22409762628154E-93	0.438905560891652	0.384	0.198	5.4029428021226E-89	3	CEP250
1.92273453796955E-97	0.438075186797938	0.36	0.179	3.22211853872937E-93	3	DHRSX
7.76996461041276E-126	0.43781929865387	0.564	0.295	1.30209066941297E-121	3	LGALS8
2.05706954318172E-126	0.437593143382906	0.613	0.319	3.44723714046393E-122	3	TMEM164
2.07218227576825E-86	0.437582876757447	0.356	0.184	3.47256305773244E-82	3	AKAP8L
1.18962197116817E-136	0.437476589334487	0.761	0.423	1.99356849928361E-132	3	PLEKHA6
1.0141417680283E-76	0.437240862038024	0.273	0.133	1.69949877486183E-72	3	LEMD3
5.7356699292478E-113	0.437110606366537	0.473	0.245	9.61183566743346E-109	3	GPR107
4.99945565766266E-69	0.436984769336758	0.251	0.122	8.37808779111109E-65	3	HARS2
9.09766981428414E-67	0.436650317405361	0.271	0.138	1.52458750747774E-62	3	EXOSC6
2.51718454380306E-90	0.436471623267092	0.34	0.169	4.21829785850518E-86	3	OSBPL8
3.16652384097666E-97	0.436070350911072	0.355	0.174	5.30646065270869E-93	3	BRD8
8.63935669134849E-69	0.435683145760429	0.293	0.152	1.44778339433618E-64	3	JADE2
2.44382960023764E-142	0.435678265000025	0.938	0.684	4.09536964407824E-138	3	SULT2A1
2.4880137006214E-156	0.435417156280887	0.769	0.415	4.16941335950133E-152	3	TCEAL4
5.57558473101701E-69	0.435265874672559	0.284	0.144	9.34356489223831E-65	3	WDR76
4.3547441453922E-93	0.434935988160434	0.355	0.18	7.29768023884825E-89	3	VPS13D
4.72950440623293E-61	0.433713517521944	0.265	0.138	7.92570348396514E-57	3	RASSF8
2.08068409084688E-79	0.433621740782764	0.324	0.166	3.48681039944121E-75	3	PPP1R13B
7.72077913312187E-83	0.433427547928502	0.323	0.163	1.29384816712856E-78	3	MINDY1
1.94352003583779E-124	0.433380149016168	0.478	0.24	3.25695087605696E-120	3	ZNF146
9.58208515904796E-133	0.433134079254706	0.533	0.273	1.60576583095326E-128	3	PAFAH1B1
1.01248603099631E-106	0.431987433815228	0.432	0.223	1.69672409074362E-102	3	SON
1.8202370059979E-103	0.431668078248093	0.452	0.239	3.05035317465129E-99	3	MTA1
4.18240848147488E-78	0.431558008457263	0.345	0.181	7.0088801332556E-74	3	CBX3
9.21668501718227E-153	0.431510936182272	0.724	0.392	1.5445320751794E-148	3	NIT1
6.37474795588207E-64	0.431268133484904	0.257	0.13	1.06828026244672E-59	3	AC118549.1
1.61352686371322E-83	0.431004325725751	0.297	0.143	2.70394831821062E-79	3	AARS2
3.81906948373398E-116	0.430805901012715	0.53	0.285	6.3999966408414E-112	3	CYP2C18
4.08916162329459E-114	0.430632425519199	0.49	0.254	6.85261704831708E-110	3	STK38
7.90111776508461E-131	0.429994256714023	0.536	0.275	1.32406931507288E-126	3	EGLN2
3.02445170981987E-77	0.429912761172185	0.354	0.19	5.06837617531613E-73	3	ME2
1.61017267148977E-153	0.428823937378705	0.755	0.403	2.69832736288256E-149	3	FMR1
7.04943944017596E-157	0.428778504088451	0.633	0.328	1.18134506138469E-152	3	SLC17A9
1.0425466433985E-62	0.428251919793607	0.262	0.135	1.74709966500721E-58	3	PIAS2
1.46332741615706E-94	0.427699262817148	0.348	0.172	2.45224408399601E-90	3	RDH14
5.59547960985981E-71	0.427571445022112	0.289	0.147	9.37690473020306E-67	3	CAD
1.47715073920869E-63	0.427216555219831	0.286	0.152	2.47540920876593E-59	3	XPO5
1.42638954360044E-143	0.427094916880353	0.669	0.355	2.39034359716561E-139	3	PDCD6IP
2.35901830966449E-57	0.42620246941996	0.274	0.148	3.95324288333575E-53	3	DIPK2A
4.13953941527066E-129	0.426183167600634	0.478	0.238	6.93704015211057E-125	3	UBE3A
6.27660116318118E-144	0.425986190367247	0.643	0.331	1.0518328229259E-139	3	ATP9A
1.52679317190386E-80	0.425963660638642	0.305	0.152	2.55859999747648E-76	3	RGP1
3.21231022910609E-148	0.425796948672766	0.691	0.35	5.38318948193598E-144	3	SIPA1L2
2.96496301216248E-104	0.425710765334488	0.443	0.231	4.96868501578189E-100	3	GMPS
8.29527894972041E-109	0.425660279046194	0.391	0.191	1.39012284639415E-104	3	FBXW4
1.75585917285199E-61	0.425493093162211	0.253	0.129	2.94246880186537E-57	3	MIER3

4.10159909509796E-99	0.425136169286386	0.4	0.204	6.87345976356516E-95	3	DGCR2
3.14302309527218E-76	0.424918294981084	0.334	0.175	5.26707810305712E-72	3	ARMC8
3.70270039589478E-91	0.424445478444343	0.39	0.204	6.20498532344048E-87	3	SNX13
9.88448046032087E-89	0.424383056643059	0.34	0.169	1.65644123554057E-84	3	SCRN2
1.62442527605064E-51	0.424280888212428	0.283	0.159	2.72221187760567E-47	3	SPRY2
5.56624081181218E-123	0.42350291729903	0.509	0.259	9.32790635243485E-119	3	RBBP7
2.25432699255274E-107	0.423451880424153	0.444	0.23	3.77780117411988E-103	3	IFNAR2
1.67132105564929E-76	0.423281664917373	0.263	0.126	2.80079982505709E-72	3	PRKACB
2.4634925956798E-109	0.423119405694463	0.493	0.258	4.12832089184021E-105	3	ERCC5
1.15834344770556E-105	0.423055929748346	0.393	0.195	1.94115194966499E-101	3	PBX3
2.52433847884377E-94	0.422961850514773	0.404	0.211	4.2302864228464E-90	3	CKLF
2.72435592399958E-138	0.422697013869104	0.704	0.383	4.5654756574385E-134	3	HNRNPD
8.80307186996442E-84	0.422300916952078	0.348	0.179	1.47521878396864E-79	3	AKAP17A
5.64606153300827E-136	0.422111402678049	0.779	0.455	9.46166991701526E-132	3	SHC1
8.66693096068185E-76	0.421982423210917	0.298	0.15	1.45240429039106E-71	3	ZNF32
1.67218488461464E-125	0.421754275933	0.491	0.248	2.80224742963721E-121	3	RBM17
6.35087733141652E-70	0.421375421250899	0.294	0.152	1.06428002319878E-65	3	PPID
2.15558385200728E-134	0.421252197295707	0.773	0.423	3.61232741919381E-130	3	HNRNPU
1.56461507791077E-120	0.420762331211188	0.304	0.124	2.62198194756287E-116	3	LAMA5
1.43176748705612E-72	0.420607838560675	0.322	0.17	2.39935595480864E-68	3	TRIM33
4.91171166059202E-91	0.420479197438565	0.336	0.166	8.2310464008201E-87	3	CNOT2
4.53501903054296E-48	0.420474072668074	0.271	0.152	7.59978489138389E-44	3	FANCA
9.42504839914102E-133	0.420215953462228	0.865	0.548	1.57944961072805E-128	3	SMIM14
8.63237975352863E-80	0.419940176307257	0.323	0.165	1.44661419909633E-75	3	UCHL5
1.10102861563942E-134	0.419390626785792	0.53	0.271	1.84510375408853E-130	3	MSL1
1.23078067165232E-118	0.418803971499193	0.516	0.27	2.06254224955495E-114	3	ZNF664
7.16279729962903E-69	0.418768344380064	0.289	0.148	1.20034157147183E-64	3	GTF2E2
1.21243975592064E-127	0.418344561979712	0.528	0.274	2.03180654297182E-123	3	SEC24A
1.1657247224394E-41	0.418229334134836	0.257	0.151	1.95352148986395E-37	3	MROH6
1.63775509959384E-71	0.418119056727865	0.302	0.156	2.74454999589935E-67	3	SESTD1
2.73405004591204E-137	0.417550040132812	0.531	0.267	4.58172106693939E-133	3	GAA
1.2074769532174E-70	0.417384074732442	0.284	0.145	2.02348987820172E-66	3	ZMAT3
2.56323747304978E-63	0.416972063763236	0.329	0.182	4.29547335733681E-59	3	SLMAP
6.6215166145982E-125	0.416956382154393	0.498	0.254	1.10963375427437E-120	3	SNX6
4.28182372101335E-120	0.416682870431639	0.504	0.262	7.17548019167417E-116	3	YTHDC1
8.34955480340438E-143	0.416409914189687	0.681	0.366	1.39921839395451E-138	3	SLC50A1
7.85568240596145E-88	0.415724535301655	0.353	0.18	1.31645525759102E-83	3	SLC35C2
1.34820912963835E-122	0.415264974978652	0.838	0.513	2.25932885944794E-118	3	SPTSSA
3.05879957739208E-58	0.415194405222428	0.276	0.149	5.12593633179364E-54	3	WDR41
3.16444860656734E-58	0.415003940443651	0.282	0.152	5.30298297488556E-54	3	DCAKD
2.77586560329267E-60	0.414989978608778	0.269	0.142	4.65179557799786E-56	3	DDX55
1.65981899818697E-93	0.414930657328985	0.401	0.206	2.78152467716173E-89	3	BAIAP2L2
1.05671467856694E-110	0.41443474033004	0.401	0.196	1.77084245834247E-106	3	CBWD1
1.42366760274012E-80	0.414345852854101	0.354	0.187	2.38578216867189E-76	3	CAMK1D
1.56135303109664E-86	0.41339613744167	0.358	0.185	2.61651540951176E-82	3	WAC
2.65280566606306E-104	0.413221487028325	0.441	0.228	4.44557173518848E-100	3	PMM2
1.34317166082045E-62	0.413114161775842	0.344	0.193	2.25088706920291E-58	3	FAM221A
9.7940100117551E-107	0.412894783858317	0.441	0.228	1.64128019776992E-102	3	NSL1
1.98306261890062E-110	0.41268535010233	0.434	0.22	3.32321633675367E-106	3	MYRF
1.98775531638767E-110	0.412575263609585	0.442	0.225	3.33108035920245E-106	3	NIT2
1.40718351729056E-79	0.412101425793743	0.35	0.185	2.35815813827552E-75	3	STRBP
4.09490072970564E-88	0.412054599311041	0.31	0.15	6.86223464284072E-84	3	USP6NL
4.02464240910092E-105	0.411987315201138	0.432	0.222	6.74449574917132E-101	3	TYK2
3.90165445066154E-132	0.411596799704257	0.692	0.389	6.53839252841861E-128	3	CRYZ
3.33011822812856E-132	0.411581083766328	0.497	0.249	5.58061212669784E-128	3	TMEM123
3.13102204609178E-96	0.411162587704179	0.384	0.195	5.2469667448406E-92	3	ITSN2
1.70764958903048E-77	0.410977265575046	0.309	0.157	2.86167918129728E-73	3	SUDS3
5.05974773952788E-73	0.41090860803353	0.282	0.141	8.47912526190082E-69	3	SNUPN
8.22154255220605E-60	0.410563927228719	0.264	0.139	1.37776610089869E-55	3	C3orf38
9.49216634543567E-143	0.409924671908024	0.731	0.4	1.59069723616811E-138	3	UGT1A1

5.00611209915655E-130	0.409269170737729	0.609	0.326	8.38924265576655E-126	3	UHMK1
1.1569467424134E-80	0.408772812652316	0.316	0.158	1.93881135093637E-76	3	ITGA1
7.97822671039274E-139	0.40839900471425	0.639	0.335	1.33699123212761E-134	3	NUMB
1.04368184744211E-128	0.408389918884544	0.829	0.461	1.74900203994348E-124	3	ATRN
7.00376551915353E-115	0.407963641301288	0.52	0.271	1.17369102569975E-110	3	TFDP1
1.33032152222496E-153	0.407711361037662	0.714	0.386	2.22935280694459E-149	3	AIG1
8.33508273605857E-99	0.407607569443577	0.38	0.19	1.3967931649087E-94	3	TBC1D16
1.86361595951514E-121	0.407258513167571	0.461	0.231	3.12304762495547E-117	3	MIER1
3.62130050234562E-111	0.407007938495223	0.506	0.266	6.06857538183079E-107	3	DOT1L
1.5686845429584E-66	0.40638472192978	0.296	0.157	2.62880155708969E-62	3	LMAN2L
9.07955798491853E-88	0.406040759840106	0.346	0.175	1.52155232711265E-83	3	TIMM9
1.08558989166486E-177	0.405710682158491	0.941	0.597	1.81923154045198E-173	3	MUC3A
2.21041690198317E-62	0.405675967690227	0.282	0.149	3.70421664434339E-58	3	CMTM4
1.79600522968826E-57	0.40565090152367	0.329	0.189	3.00974556391159E-53	3	TRIM56
2.09390522112208E-90	0.405388660704329	0.41	0.218	3.50896636955637E-86	3	RIPK1
1.32694759712343E-106	0.405202749798126	0.488	0.259	2.22369878325944E-102	3	RBM14
3.36897535843636E-103	0.404997509383274	0.472	0.25	5.64572890566764E-99	3	SPPL2B
2.26476472730229E-81	0.40493912797306	0.309	0.153	3.79529273001317E-77	3	KLHL36
2.86878020348774E-138	0.404398775955022	0.61	0.322	4.80750186500475E-134	3	ATP6V1A
1.51908311714191E-70	0.403857555233524	0.298	0.154	2.54567948770641E-66	3	SKP2
8.44548640311856E-99	0.403833851990299	0.392	0.2	1.41529461143461E-94	3	CERT1
2.25916198062855E-85	0.403588422338962	0.384	0.205	3.78590364713732E-81	3	STK4
7.31425682830363E-103	0.403527408087066	0.503	0.265	1.22572315928712E-98	3	CRACR2B
4.53240621171802E-83	0.403435509733029	0.325	0.164	7.59540632959705E-79	3	MAP2K1
1.40070788315285E-83	0.403407274314527	0.35	0.18	2.34730627058754E-79	3	MAGEH1
1.99833882219577E-82	0.403295544023456	0.385	0.207	3.34881619823568E-78	3	NUP42
3.88809259562738E-84	0.40307999333055	0.39	0.208	6.51566557175237E-80	3	SLC25A43
4.67697703307662E-132	0.402776192867751	0.66	0.36	7.8376781120298E-128	3	LRP5
4.97430665472616E-153	0.402229718796654	0.721	0.393	8.3359430919901E-149	3	PJA2
5.54740656675532E-90	0.402020381273432	0.38	0.197	9.29634392456856E-86	3	MAML1
1.84682105503389E-84	0.401787357589805	0.364	0.192	3.0949027240258E-80	3	ZNF687
2.70416766834373E-148	0.401562488261109	0.637	0.33	4.53164417861043E-144	3	CPNE3
6.31227474889545E-114	0.401484284656855	0.408	0.2	1.0578110024199E-109	3	ULK1
1.959253724531E-119	0.401224199931911	0.524	0.273	3.28331739156905E-115	3	FBXL3
5.46745679290731E-149	0.40120075353949	0.618	0.322	9.16236409355407E-145	3	SNRNP70
1.90963176861276E-95	0.401094822357175	0.387	0.199	3.20016091784127E-91	3	DLST
1.50159214949362E-61	0.400651269222866	0.257	0.131	2.51636812412141E-57	3	RNASEH1
7.78820474641216E-131	0.400545428192296	0.688	0.384	1.30514735140375E-126	3	DCAF8
1.73374669137169E-125	0.400458025030179	0.512	0.262	2.90541270540068E-121	3	PHKA2
1.27910000126842E-159	0.400364682992969	0.831	0.468	2.14351578212562E-155	3	NR1H4
3.47093109301262E-108	0.400282649985452	0.414	0.208	5.81658632567055E-104	3	FBXW7
8.7382066708965E-85	0.40025974954063	0.338	0.171	1.46434867390884E-80	3	UBE2O
4.8756948189268E-98	0.400140791773494	0.415	0.215	8.17068937755754E-94	3	SNX27
1.09110243192891E-118	0.399728903847044	0.515	0.266	1.82846945542647E-114	3	PPP1R10
5.15890661050075E-79	0.39959793744946	0.298	0.148	8.64529569787716E-75	3	ERCC2
8.15658998191947E-135	0.399207871340126	0.418	0.191	1.36688134917006E-130	3	ZNF592
4.72798829620611E-155	0.398927075654405	0.641	0.331	7.9231627867822E-151	3	LARP4
6.56044749664092E-116	0.398513599244703	0.465	0.236	1.09939979148708E-111	3	ITGB5
1.0537121019407E-65	0.39838107726198	0.293	0.153	1.76581074043222E-61	3	PGGT1B
1.84027418849603E-78	0.398199137595496	0.295	0.146	3.08393148508165E-74	3	PLEKHA1
5.06443764584608E-97	0.397934682179633	0.368	0.184	8.48698460690887E-93	3	PRPF38A
1.00400348445967E-97	0.397818987781542	0.554	0.308	1.68250903925752E-93	3	PFKFB3
9.26048364348753E-67	0.39725968913942	0.274	0.14	1.55187184897564E-62	3	EXD3
2.26708953341119E-130	0.397197373906932	0.559	0.287	3.79918864009048E-126	3	VAMP7
1.8076123148729E-66	0.396392350117196	0.308	0.165	3.029196717264E-62	3	DEPRECATED-ENSG00000178950
7.22579459098877E-80	0.39536621393246	0.387	0.21	1.2108986575579E-75	3	WWP2
5.05863320309706E-70	0.395126752454537	0.264	0.13	8.47725752175005E-66	3	SRC
1.63791190339751E-62	0.395126147761694	0.269	0.14	2.74481276771354E-58	3	CDC27
6.093919929529E-114	0.394880710521281	0.471	0.242	1.02121910179047E-109	3	NPAS2
2.00665781211774E-128	0.394302628455529	0.729	0.406	3.3627571615469E-124	3	CITED2

8.54460087624092E-68	0.394133234651358	0.3	0.159	1.43190421484045E-63	3	CHD1
3.27724026773577E-83	0.393798152202038	0.354	0.185	5.4919992406716E-79	3	MFN1
4.51123413088739E-117	0.39308976878779	0.492	0.255	7.55992615654108E-113	3	RC3H2
3.70329280779455E-84	0.392782758305614	0.329	0.165	6.20597808730211E-80	3	SPIN1
6.63142391234046E-86	0.392217177849714	0.413	0.219	1.11129401923001E-81	3	MCM6
4.90339020461787E-80	0.391723274485406	0.377	0.202	8.21710130489862E-76	3	USP1
1.31564366623517E-106	0.391451708719089	0.507	0.267	2.2047556558769E-102	3	NUP210
5.61690950582131E-61	0.391178763832674	0.284	0.153	9.41281694985535E-57	3	REST
1.91205507902585E-70	0.391024198997604	0.322	0.172	3.20422190143152E-66	3	HSPA13
3.30890408057607E-65	0.390553184766836	0.289	0.153	5.54506145822938E-61	3	PIGH
1.39288853329551E-75	0.390126234335414	0.337	0.178	2.33420260409661E-71	3	OGFOD2
2.86481580217912E-143	0.389995430707004	0.686	0.368	4.80085832129176E-139	3	DCAF6
6.73733950743544E-104	0.389960582539053	0.389	0.193	1.12904335465603E-99	3	FOXN3
1.57851377412317E-96	0.389289306767655	0.451	0.234	2.64527338267561E-92	3	CCL28
7.11322624814915E-74	0.38918810207653	0.285	0.142	1.19203445466483E-69	3	NUDT16L1
1.07980739443284E-108	0.388950928077941	0.45	0.233	1.80954123159055E-104	3	TMEM189
8.20647453031131E-115	0.388318914925005	0.741	0.439	1.37524100178957E-110	3	MSMO1
3.24006907670596E-121	0.38787053962345	0.616	0.329	5.42970775874385E-117	3	KLF3
6.04940280608733E-116	0.387499611641116	0.512	0.268	1.01375892224412E-111	3	TBCD
5.96992387951084E-77	0.387013329266915	0.309	0.156	1.00043984372843E-72	3	AK3
7.38506487757934E-126	0.386276497626082	0.589	0.313	1.23758917218475E-121	3	CD2AP
9.5065353362522E-70	0.385983504205905	0.28	0.142	1.59310519164914E-65	3	ELP1
3.40772588986428E-124	0.3858479092764	0.634	0.342	5.71066704623455E-120	3	TMEM38B
1.00587504886599E-78	0.385755131780405	0.384	0.209	1.68564540688962E-74	3	P2RX4
6.03395994895572E-67	0.385551795225999	0.286	0.149	1.011171008246E-62	3	AASDHPPT
2.85927339660201E-117	0.385349073240352	0.516	0.27	4.79157035802565E-113	3	TMEM214
2.38760651150111E-77	0.385268871621484	0.357	0.19	4.00115099197356E-73	3	STIM2
1.48011011526302E-59	0.385173576084303	0.305	0.168	2.48036853115777E-55	3	NR2C2
1.35749714078379E-65	0.383494577041116	0.298	0.159	2.27489370852548E-61	3	DIP2B
3.709243198383E-54	0.383481596097828	0.285	0.16	6.21594975185023E-50	3	DEPRECATED-ENSG00000026297
1.4959745434076E-60	0.383241391926219	0.26	0.136	2.50695413984246E-56	3	NFRKB
3.37820920572237E-90	0.383067837860854	0.419	0.223	5.66120298694955E-86	3	CASP3
9.16778816567407E-145	0.38271809779197	0.66	0.347	1.53633794080366E-140	3	HUWE1
4.1070296231547E-219	0.382499951916765	0.618	0.271	6.88256024248265E-215	3	PLIN5
1.3486665351804E-123	0.382342087891828	0.649	0.356	2.26009537965532E-119	3	MTUS1
8.29622841878034E-133	0.382248855377851	0.629	0.333	1.39028195841921E-128	3	VDAC3
3.09088673248329E-122	0.381983135130183	0.631	0.34	5.1797079862955E-118	3	ZDHHC9
2.37933335606184E-120	0.381701811341196	0.475	0.24	3.98728683808843E-116	3	TBC1D14
2.08530958598155E-91	0.381698163277159	0.372	0.19	3.49456180418788E-87	3	UBQLN4
2.13565787767963E-62	0.381304030135108	0.295	0.159	3.57893547141553E-58	3	TRAF3
2.86804805492719E-77	0.380990603028941	0.368	0.199	4.80627493044698E-73	3	CAMSAP3
6.20414147825758E-74	0.380882626896931	0.323	0.168	1.03969002892641E-69	3	DNM1L
3.74965296980702E-87	0.380527419160279	0.351	0.179	6.28366844680261E-83	3	STARD4
6.90242268124145E-67	0.38039972547363	0.299	0.158	1.15670799292244E-62	3	PDXDC1
1.88186699349918E-104	0.380274567024499	0.509	0.262	3.15363270770593E-100	3	MGAM2
2.10712271279336E-114	0.38020950473666	0.458	0.233	3.53111624209911E-110	3	DENND1A
4.59945222370521E-133	0.380095704437756	0.829	0.457	7.7077620364852E-129	3	UBE2A
2.20211552023028E-83	0.380071968455147	0.37	0.195	3.6903051888019E-79	3	CDK14
1.1844172061319E-69	0.380013386849485	0.283	0.145	1.98484635403584E-65	3	PDCL3
7.53831973173794E-114	0.37996378749292	0.462	0.237	1.26327162064464E-109	3	PURA
1.92989511656714E-60	0.379649500993697	0.301	0.164	3.23411823634321E-56	3	MAN1B1
1.5862746100474E-123	0.379556209078832	0.712	0.398	2.65827899151743E-119	3	DNAJC3
1.24809135810544E-119	0.379533624961528	0.431	0.209	2.0915514979131E-115	3	SLC41A2
3.03086835661566E-125	0.379431672555059	0.849	0.504	5.07912919201653E-121	3	TSPAN6
1.96431282930162E-104	0.379373137644481	0.443	0.227	3.29179543934365E-100	3	FADS1
6.01431739434854E-78	0.378873957516083	0.285	0.139	1.00787930894493E-73	3	TBC1D24
1.22106199082874E-119	0.378835733615045	0.41	0.196	2.0462556842308E-115	3	IBTK
5.49131871094889E-72	0.378625256874148	0.315	0.165	9.20235189580816E-68	3	FAM98C
6.29249172563099E-141	0.378480331309201	0.547	0.277	1.05449576338124E-136	3	SLTM
4.10133812586834E-67	0.378408687071187	0.326	0.177	6.87302243133017E-63	3	STRN

3.21394381279308E-113	0.378297090347544	0.963	0.754	5.38592704147865E-109	3	MT-CYB
1.23143657387353E-52	0.37818905361454	0.299	0.171	2.06364141049726E-48	3	CASZ1
2.90324833312593E-95	0.377946852250561	0.409	0.212	4.86526355665243E-91	3	PPP2R5C
3.64900488135817E-66	0.37787378310894	0.316	0.17	6.11500238018003E-62	3	CPOX
8.21941191530994E-77	0.377774586719191	0.299	0.15	1.37740904876764E-72	3	TBC1D23
2.56635896116209E-77	0.376802265129498	0.361	0.194	4.30070434711542E-73	3	BRD1
6.19831432373154E-81	0.376622802768108	0.298	0.146	1.03871351437093E-76	3	NMRK1
2.90587200974805E-78	0.376499159553886	0.329	0.169	4.86966031393579E-74	3	CEPT1
1.33632131162937E-107	0.376399524022986	0.931	0.606	2.2394072540285E-103	3	HSD17B11
1.2462297824554E-75	0.375145079319273	0.314	0.158	2.08843186943876E-71	3	CENPN
3.15439159840339E-70	0.374793511892255	0.344	0.187	5.2861294406044E-66	3	KHNYN
7.84095912164318E-140	0.374786876487503	0.631	0.328	1.31398792960496E-135	3	ASXL1
7.38057537746529E-89	0.374468572139137	0.324	0.158	1.23683682175563E-84	3	ARFGAP2
2.24832354309196E-56	0.374453069070389	0.318	0.181	3.7677405935135E-52	3	ARL4A
4.6431854670557E-77	0.374452336785365	0.393	0.217	7.78105020569194E-73	3	SOAT1
4.34556899895095E-101	0.37444367326945	0.422	0.219	7.282304528442E-97	3	GCN1
1.92063312821483E-143	0.374351045511569	0.589	0.304	3.21859699626241E-139	3	UBR4
9.16474344312902E-110	0.374257377228024	0.972	0.841	1.53582770619956E-105	3	ALDH1A1
6.96026840685878E-68	0.37396425749723	0.336	0.185	1.16640177962139E-63	3	SLC17A5
6.44924948911558E-99	0.37369203715525	0.341	0.163	1.08076522938599E-94	3	GNE
2.14743433012649E-46	0.373617162554959	0.275	0.159	3.59867045042597E-42	3	CLGN
7.40074014204966E-103	0.373560504992134	0.445	0.233	1.24021603300468E-98	3	DCUN1D1
3.49782128249965E-116	0.373227295670337	0.506	0.265	5.86164890521291E-112	3	YPEL5
3.27401404957118E-80	0.37276085096321	0.27	0.126	5.48659274427138E-76	3	TPST1
5.94083131347802E-131	0.37219286916	0.581	0.307	9.95564511512646E-127	3	VPS52
1.9996936641716E-126	0.371950854491316	0.822	0.457	3.35108664241876E-122	3	LAPTM4B
9.1860700673928E-56	0.371579732864523	0.259	0.139	1.53940162189369E-51	3	ACOT8
7.8749517192808E-125	0.371572528660636	0.545	0.286	1.31968440911708E-120	3	RAF1
1.23177470988008E-87	0.371234279041256	0.329	0.162	2.06420805881704E-83	3	MED17
4.65719499082959E-69	0.37121584481934	0.295	0.153	7.80452736563222E-65	3	TIGD5
6.59089848005887E-114	0.371202792697757	0.533	0.284	1.10450276728827E-109	3	TRIQK
4.1802154376448E-120	0.370723821657347	0.511	0.264	7.00520503040515E-116	3	PRPSAP1
1.02181412550699E-57	0.370608600102562	0.291	0.16	1.71235611152461E-53	3	CEP63
3.97530165069161E-138	0.37050320549473	0.673	0.368	6.66181050622901E-134	3	DIO1
3.28861521958669E-64	0.370222219593511	0.262	0.134	5.51106138498337E-60	3	STRIP1
2.09730438659273E-133	0.369254491573152	0.631	0.339	3.5146626910521E-129	3	TFG
9.40039019719652E-92	0.368498783151508	0.435	0.231	1.57531738924619E-87	3	PHF14
6.58086296707014E-94	0.368354447829744	0.411	0.215	1.10282101602161E-89	3	GON4L
1.07222599479657E-104	0.367873938271482	0.469	0.247	1.79683632208011E-100	3	TIMMDC1
1.61841587151152E-39	0.367869650161295	0.268	0.162	2.712141317479E-35	3	CHST5
3.09752285228511E-73	0.367718041700575	0.31	0.161	5.19082879585939E-69	3	METTL5
8.93928722433652E-49	0.367470683123088	0.253	0.14	1.49804575305431E-44	3	HAUS2
1.28886734580867E-124	0.367464416269671	0.543	0.281	2.15988389810617E-120	3	ABCB6
5.44143197516209E-94	0.367278867026311	0.41	0.214	9.11875170397662E-90	3	MBTPS1
1.80380004784017E-66	0.367012390408952	0.301	0.16	3.02280812017055E-62	3	SDF2
1.62043395602604E-128	0.3663003522646	0.662	0.341	2.71552322350844E-124	3	FAM122B
3.55796426885936E-72	0.366193922470802	0.353	0.192	5.96243652175451E-68	3	THUMPD1
7.12665931913545E-107	0.365912661066255	0.452	0.23	1.19428556870072E-102	3	PNPLA3
1.29031007979437E-90	0.365425077221193	0.432	0.232	2.16230163171941E-86	3	COX11
7.43225707636627E-128	0.365001392996608	0.764	0.432	1.24549764085746E-123	3	CD46
4.6093511284071E-112	0.364878528150793	0.725	0.419	7.72435062098462E-108	3	IKBKG
3.33886379031269E-62	0.364367068247695	0.294	0.158	5.59526793980601E-58	3	C7orf26
9.3657118707661E-101	0.364216988447703	0.488	0.259	1.56950599530298E-96	3	MMGT1
5.78638554413919E-99	0.364006660741441	0.429	0.224	9.69682489486846E-95	3	FXR1
1.44470375304599E-63	0.363493381216533	0.353	0.2	2.42103454935446E-59	3	GTPBP2
3.9216304957506E-81	0.363203235282811	0.355	0.187	6.57186838477885E-77	3	SPOP
1.38506239013413E-95	0.362992442951926	0.425	0.223	2.32108755338677E-91	3	LAMC1
7.35669426081223E-51	0.362952054310747	0.283	0.16	1.23283482422691E-46	3	MGME1
1.09384855750777E-91	0.362803907182029	0.383	0.197	1.83307141267152E-87	3	GOLPH3L
5.40188874390427E-51	0.362577248584505	0.25	0.137	9.05248515703478E-47	3	CDK8

2.62310095035979E-125	0.36229602068614	0.616	0.331	4.39579257261294E-121	3	UBL3
7.17956075912602E-80	0.362259743800088	0.331	0.17	1.20315079201434E-75	3	SNRPE
1.53858016205852E-117	0.361651234613433	0.791	0.471	2.57835263557767E-113	3	PGRMC2
1.5252147852665E-117	0.361640975045682	0.555	0.29	2.55595493714961E-113	3	MYO19
4.88653545907191E-93	0.360693977859497	0.879	0.587	8.1888561223127E-89	3	ALDH3A2
1.65611487934729E-73	0.36065468640239	0.274	0.135	2.77531731481018E-69	3	UBA3
6.78139900950099E-92	0.360520856519355	0.383	0.198	1.13642684601218E-87	3	UBE4B
1.42054491923549E-113	0.360412364433042	0.495	0.258	2.38054917565484E-109	3	METTL23
5.15455597797996E-96	0.360014598021645	0.448	0.239	8.63800490789882E-92	3	IFNGR1
1.62926319591427E-109	0.35964649315546	0.571	0.309	2.73031926371314E-105	3	NUP58
1.11724040959185E-88	0.359610588660077	0.325	0.159	1.87227147839402E-84	3	GFM2
1.79078008775375E-82	0.359404784050171	0.345	0.177	3.00098927105773E-78	3	CPLX1
1.60802320257452E-99	0.358949358510132	0.487	0.261	2.69472528287438E-95	3	MED25
2.49145493017128E-54	0.358898621048101	0.257	0.138	4.17518017198103E-50	3	MEIS2
7.04499144740836E-108	0.358220428926276	0.582	0.323	1.18059966675669E-103	3	TMEM41B
4.0654371039305E-122	0.358190474501429	0.663	0.369	6.81285949876674E-118	3	ACBD3
8.48239478982947E-125	0.358105961411269	0.659	0.36	1.42147971887962E-120	3	IMPAD1
1.80918290742306E-71	0.357910479956025	0.368	0.203	3.03182871625956E-67	3	EIF4G3
4.36992183491865E-95	0.357520278270934	0.399	0.206	7.32311501095667E-91	3	MAN1A2
1.69122575025178E-152	0.35715203208658	0.826	0.471	2.83415611227193E-148	3	CDK5RAP3
1.59731696915469E-110	0.357072636313786	0.288	0.118	2.67678377690943E-106	3	RGL3
1.16653322557454E-67	0.356798907313817	0.33	0.18	1.95487637941781E-63	3	SMARCC2
2.5205860797244E-94	0.356709150289266	0.404	0.211	4.22399815240216E-90	3	C2orf68
2.14290091405705E-98	0.356050433238757	0.43	0.226	3.59107335177681E-94	3	ZMYND11
2.95998629041228E-124	0.355399879989435	0.599	0.315	4.96034502547289E-120	3	SCRIB
8.55058851778255E-64	0.354724291236978	0.289	0.152	1.43290762381E-59	3	GRAMD1A
1.81438117334314E-127	0.354651415726011	0.284	0.105	3.04053997028844E-123	3	CYP21A2
8.01869115328346E-123	0.35461914383737	0.554	0.291	1.34377226346724E-118	3	DHCR7
1.3701092318623E-63	0.35445287360839	0.283	0.148	2.29602905075485E-59	3	MTMR3
4.96329570831725E-73	0.354363213809462	0.341	0.182	8.31749094799805E-69	3	COPS4
2.22647902494805E-113	0.354352821933143	0.582	0.314	3.73113355000795E-109	3	PLOD2
1.46605170316208E-119	0.354329640360962	0.621	0.34	2.45680944415901E-115	3	SCAMP2
1.19561054493639E-95	0.354217743257447	0.368	0.183	2.0036041512044E-91	3	EXOSC7
3.2367845506221E-81	0.354159204848502	0.307	0.151	5.42420354993251E-77	3	SLC38A7
3.23851183362644E-114	0.353615294908084	0.551	0.294	5.42709813079119E-110	3	DHX9
1.36921968168975E-79	0.35337631155094	0.353	0.186	2.29453834257568E-75	3	ALG12
1.37699145939244E-111	0.353336362275029	0.505	0.265	2.30756228764985E-107	3	ARHGAP1
2.5887633183276E-73	0.353271297183972	0.311	0.16	4.3382495688534E-69	3	ZNF639
6.13476547660446E-89	0.353245378522667	0.347	0.175	1.02806399856937E-84	3	PDHX
2.9664566256804E-100	0.353134837868182	0.928	0.67	4.97118801331522E-96	3	HSD17B4
5.06707446527717E-106	0.353114735900881	0.478	0.252	8.49140338891149E-102	3	PHACTR4
2.21868526336086E-65	0.352756385034336	0.29	0.152	3.71807276434013E-61	3	PRR12
2.73584059718266E-96	0.351690801498713	0.469	0.25	4.5847216727587E-92	3	SUPT16H
4.93542838641666E-90	0.3505939346319	0.462	0.254	8.27079088995704E-86	3	VHL
3.71650563145771E-113	0.350166733122285	0.41	0.2	6.22812013719683E-109	3	CRELD1
1.80422512981706E-64	0.350086500622787	0.303	0.162	3.02352047254743E-60	3	IPO5
1.89803292164732E-65	0.349862462990412	0.272	0.139	3.18072357009658E-61	3	ATP5F1C
7.84349006105656E-86	0.349763976700122	0.258	0.115	1.31441206443186E-81	3	HMG20A
3.89900686162649E-99	0.349404399143913	0.52	0.285	6.53395569871367E-95	3	PSPC1
1.42664436702068E-86	0.34924420943594	0.397	0.212	2.39077063025326E-82	3	FIP1L1
3.99369531559835E-60	0.348873133953839	0.323	0.181	6.69263460987972E-56	3	CADPS2
1.13807417945289E-126	0.348623561485916	0.727	0.394	1.90718470992716E-122	3	VMA21
5.62023317533936E-52	0.348467297359085	0.251	0.137	9.41838675523369E-48	3	COPS7B
9.42619954195492E-95	0.348139907131827	0.429	0.229	1.57964251924081E-90	3	PLRG1
5.72019235527633E-66	0.347837066513117	0.384	0.218	9.58589834897208E-62	3	SIPA1L1
1.92808360183388E-106	0.347749970911891	0.463	0.241	3.23108249995322E-102	3	PCMT1
2.05768384532667E-82	0.34757356005505	0.368	0.195	3.44826658799843E-78	3	BRPF3
4.92791889226911E-126	0.34743411212211	0.755	0.44	8.25820647966457E-122	3	CIAO2A
7.56721582128969E-73	0.347257667353137	0.32	0.168	1.26811402733173E-68	3	COQ8B
8.78496833764904E-99	0.347132435877741	0.449	0.239	1.47218499402323E-94	3	ADAM10

1.52803520938974E-110	0.346801786626705	0.553	0.301	2.56068140389532E-106	3	DNAJB9
1.89982376184045E-120	0.34656134255785	0.758	0.439	3.18372466009223E-116	3	HM13
1.64611061927901E-82	0.346117246413997	0.435	0.242	2.75855217578776E-78	3	VPS35L
1.48256826756143E-136	0.346088499319105	0.659	0.354	2.48448790277944E-132	3	AR
7.08931998452325E-73	0.345496502750951	0.36	0.196	1.18802824300641E-68	3	IRS1
6.71064768227379E-129	0.345323745430237	0.499	0.25	1.12457033859544E-124	3	PALLD
2.31221797437718E-101	0.345175747581677	0.429	0.218	3.87481488146128E-97	3	MLIP
3.8034594477872E-103	0.345172882041891	0.399	0.2	6.3738373426018E-99	3	CASC4
2.12415919196056E-109	0.345125994385179	0.849	0.5	3.55966597388751E-105	3	GDI1
3.39491260237961E-147	0.344650208217	0.72	0.395	5.68919453906776E-143	3	ABCC2
2.11324427602606E-112	0.344548821537465	0.642	0.358	3.54137475776447E-108	3	TRA2A
5.28045432898644E-98	0.344485576773209	0.564	0.316	8.84898536451547E-94	3	ECE1
5.6832707750113E-117	0.344431699768773	0.549	0.29	9.52402516476394E-113	3	PTK2
6.00422118543189E-107	0.344367883256411	0.992	0.948	1.00618738625468E-102	3	TF
3.63560752580004E-123	0.344200067928809	0.648	0.358	6.09255109173571E-119	3	CSNK1D
1.10174962194938E-113	0.343899725119409	0.588	0.32	1.84631201646277E-109	3	AHCYL1
8.0505016651695E-126	0.343666162893207	0.563	0.297	1.3491030690491E-121	3	MAPK14
6.56672567061269E-113	0.342873405007663	0.748	0.443	1.10045188788127E-108	3	PDZK1
2.51386612237658E-73	0.342561913893712	0.371	0.203	4.21273684787867E-69	3	TOR1A
5.36575253876052E-50	0.342320590873599	0.271	0.152	8.99192810445487E-46	3	ARHGAP26
1.33806200321686E-69	0.342127249431908	0.272	0.136	2.24232430499081E-65	3	PPTC7
1.49052895699218E-62	0.342098647897122	0.302	0.162	2.49782842612749E-58	3	LDLRAD4
3.0656804954309E-130	0.341008237078212	0.682	0.348	5.1374673742431E-126	3	DUSP9
3.1050803905977E-82	0.340632722277286	0.405	0.22	5.20349371856363E-78	3	BCAT2
2.39157851613992E-107	0.340149247425178	0.559	0.303	4.00780727734728E-103	3	MYO15B
5.59171087812259E-71	0.339586518651598	0.308	0.16	9.37058908955784E-67	3	SQLE
4.67800428731004E-79	0.339104973776676	0.361	0.193	7.83939958467417E-75	3	GRSF1
5.08231181755142E-110	0.338769496844622	0.537	0.291	8.51693814385267E-106	3	HIPK3
3.377263158013E-125	0.338288160476289	0.573	0.301	5.6596176001982E-121	3	DEPRECATED-ENSG00000133142
1.715872997201E-92	0.338132974210487	0.404	0.212	2.87545996870944E-88	3	UQCRC2
2.41072053354793E-105	0.337199551256875	0.484	0.256	4.03988547011962E-101	3	GAK
6.5020050219404E-83	0.337038897995571	0.344	0.177	1.08960600157677E-78	3	ATF7
1.25909924504152E-115	0.336905336383151	0.55	0.293	2.10999851484058E-111	3	SMARCA5
1.23770605251138E-114	0.336846326266708	0.524	0.278	2.07414780279856E-110	3	DEPRECATED-ENSG00000067064
2.56137069465778E-94	0.336832015701758	0.431	0.23	4.2923450101075E-90	3	LSM 7.00
8.41052909983039E-58	0.33635871924828	0.325	0.182	1.40943646654958E-53	3	TMEM131L
9.71758241983297E-115	0.336185827155834	0.487	0.253	1.62847246191561E-110	3	TMEM161A
2.86583160646528E-126	0.335882991218195	0.615	0.324	4.80256060611451E-122	3	SLC25A5
1.51698607033038E-88	0.335803339709776	0.337	0.167	2.54216525665965E-84	3	KANSL3
1.20563766937337E-91	0.335413317906393	0.53	0.296	2.02040760633589E-87	3	AGFG1
1.58131241333096E-121	0.335353308696259	0.721	0.404	2.64996334226003E-117	3	MCFD2
4.94491858956822E-75	0.335088039155823	0.318	0.166	8.28669457239843E-71	3	ARL 16.00
4.46913577236223E-113	0.334687309800514	0.722	0.421	7.48937772732463E-109	3	IRF2BP2
2.09762748811221E-96	0.334604381206344	0.39	0.198	3.51520414457844E-92	3	GATD3B
1.90918319058277E-70	0.334306518699023	0.391	0.22	3.1994091907786E-66	3	CEMIP2
1.98625654433322E-87	0.334063750788977	0.436	0.237	3.3285687169936E-83	3	SCAF4
1.63059217323133E-71	0.333997819155016	0.358	0.196	2.73254636390107E-67	3	EXOSC2
1.04255617230702E-113	0.333875680793807	0.748	0.432	1.7471156335521E-109	3	SREBF2
2.30133479802207E-118	0.33374400711428	0.519	0.272	3.85657685452539E-114	3	SMAD4
7.06125720652678E-84	0.333475865388216	0.776	0.499	1.18332548266976E-79	3	TOB1
9.22600949017971E-72	0.332785280650142	0.314	0.164	1.54609467036432E-67	3	ACAP2
2.90791551937561E-142	0.33248786511408	0.634	0.333	4.87308482736965E-138	3	TTC39C
3.237292864349E-116	0.332461895000959	0.828	0.464	5.42505538207605E-112	3	IDH3G
1.69611454197568E-93	0.332014627989485	0.452	0.246	2.84234874944284E-89	3	ZGPAT
1.41048885875236E-51	0.331987374039456	0.257	0.14	2.3636972294972E-47	3	COMMD10
4.77552918991823E-107	0.331898117255075	0.591	0.328	8.00283181646497E-103	3	SGK2
2.40066815874622E-96	0.331703129385889	0.438	0.231	4.02303970042691E-92	3	ERLEC1
5.4146602257845E-59	0.331352201597987	0.361	0.209	9.07388760636967E-55	3	TTLL5
2.17650696061011E-58	0.331331723134984	0.259	0.135	3.64739036459042E-54	3	RPAIN
1.77717512595112E-115	0.331142617055389	0.646	0.355	2.97819007606889E-111	3	FAM91A1

8.0407407421384E-62	0.33111291378833	0.28	0.148	1.34746733356755E-57	3	SENP5
3.05173919998859E-89	0.331076886830375	0.332	0.163	5.11410455134087E-85	3	POLR2C
2.4449150017273E-120	0.330752567039179	0.594	0.317	4.0971885598946E-116	3	TRIP12
3.11005479976063E-115	0.330294473196025	0.573	0.31	5.21182983343887E-111	3	CMTM8
6.43723441179148E-77	0.330273478456014	0.353	0.187	1.07875174272802E-72	3	NARF
6.73897777187131E-68	0.330196783736874	0.342	0.188	1.12931789501019E-63	3	KLC1
3.03827046876438E-48	0.330036215929546	0.265	0.15	5.09153365155534E-44	3	NCBP1
1.00728930035574E-91	0.329960708142182	0.386	0.199	1.68801540953614E-87	3	SRCAP
3.15443992616284E-102	0.329922194096111	0.548	0.301	5.28621042826369E-98	3	UBE3C
5.84480260770159E-92	0.329875043846136	0.964	0.766	9.79472020998633E-88	3	UGDH
4.21298533758541E-60	0.329322611811208	0.258	0.134	7.06012082872563E-56	3	ZMYM3
1.29015611339127E-80	0.329179278869291	0.358	0.188	2.16204361482109E-76	3	NUP214
1.38232453120437E-85	0.329070185318219	0.406	0.218	2.31649944939228E-81	3	FBXO21
1.38125903172794E-94	0.328903698537537	0.388	0.197	2.31471388536968E-90	3	CEBPZOS
1.62787203079148E-110	0.328834315381172	0.81	0.487	2.72798794920036E-106	3	PLS3
5.96916176449078E-101	0.328397996883951	0.531	0.291	1.00031212849336E-96	3	MAX
3.43485821687366E-83	0.32833970668056	0.374	0.194	5.75613539983688E-79	3	VIL1
4.20868836123318E-58	0.327723183370846	0.316	0.177	7.05291995575457E-54	3	SLC35B4
2.0875977568383E-66	0.327300337399077	0.379	0.213	3.49839632090962E-62	3	SEMA6A
7.08453665065768E-93	0.327267720945418	0.433	0.232	1.18722665191721E-88	3	ELK4
1.91253685203236E-60	0.326861252282882	0.259	0.133	3.20502925663583E-56	3	BACE1
8.68908845611934E-108	0.326751130095754	0.503	0.266	1.45611744347648E-103	3	MYLK
1.24793178605824E-79	0.326532989809453	0.298	0.147	2.0912840870764E-75	3	SSH1
6.1362325384127E-93	0.326461317119284	0.475	0.261	1.0283098487872E-88	3	AIMP1
3.51385532120862E-85	0.325787407795174	0.42	0.226	5.8885187472814E-81	3	ELOVL6
2.59527099070081E-106	0.325545097553981	0.466	0.244	4.34915512621641E-102	3	DNAJC5
1.37629459146043E-121	0.32494536311259	0.723	0.394	2.30639447636939E-117	3	NHSL1
3.80811662841681E-88	0.324443444890747	0.472	0.258	6.38164184590089E-84	3	KLHDC4
3.31654360955291E-123	0.324392729109098	0.597	0.32	5.55786378088877E-119	3	HGS
3.78696792548695E-117	0.324361662759301	0.58	0.314	6.34620084953103E-113	3	DDX46
1.18641341181121E-113	0.323915868353237	0.537	0.286	1.98819159551322E-109	3	DPP9
5.65358601786551E-70	0.323878979372367	0.307	0.16	9.47427944873902E-66	3	UTP6
1.04526898083677E-85	0.323810797873525	0.448	0.246	1.75166175808627E-81	3	SF3B3
1.20817486767712E-102	0.32379526617143	0.417	0.212	2.02465944325332E-98	3	NUDT3
6.54339659583606E-47	0.323735036024703	0.264	0.15	1.09654240153021E-42	3	ARFIP1
2.28453315387783E-137	0.323637596263041	0.634	0.336	3.82842065926846E-133	3	DYNC1LI2
3.28097578485512E-75	0.323236406429187	0.384	0.211	5.49825922026021E-71	3	UGGT1
5.77330703911808E-118	0.322929678372187	0.635	0.353	9.67490793615408E-114	3	SMIM19
8.59630778371725E-55	0.322735906280368	0.266	0.144	1.44056925839534E-50	3	SPOUT1
1.53481372812618E-65	0.322526715160442	0.29	0.152	2.57204084559385E-61	3	ABCG8
5.96607230576362E-94	0.322258617733223	0.418	0.219	9.99794396999867E-90	3	NOP58
3.25193626798747E-61	0.321576060068485	0.306	0.168	5.4495947978934E-57	3	ARMC1
8.89108018064643E-120	0.321098895504842	0.549	0.289	1.48996721667273E-115	3	SKP1
2.35864110372886E-77	0.320974450143749	0.382	0.207	3.95261076162883E-73	3	RBM24
8.28438175513286E-122	0.320974023643933	0.746	0.425	1.38829669452516E-117	3	FAM114A1
4.24623327986443E-109	0.320214147026849	0.442	0.226	7.11583773039681E-105	3	XAB2
2.9347552610416E-62	0.320155186321033	0.292	0.157	4.91806286645351E-58	3	ENOX2
1.04476213802952E-115	0.320054152242314	0.552	0.291	1.75081239090987E-111	3	MECP2
5.68577750150063E-55	0.319941516744275	0.263	0.141	9.52822593701475E-51	3	CARD19
1.5961778377235E-107	0.31981410470832	0.562	0.307	2.67487482045704E-103	3	PAK2
6.40117762400187E-65	0.319694664270735	0.267	0.136	1.07270934623023E-60	3	SF3B1
3.53761416313722E-66	0.319479045495563	0.266	0.135	5.92833381458535E-62	3	MAGIX
8.07847383454739E-126	0.319411800135288	0.592	0.309	1.35379064519345E-121	3	FZR1
7.51141645737556E-114	0.318972871446387	0.567	0.305	1.258763169927E-109	3	CGGBP1
5.6992443223808E-95	0.318963855030213	0.509	0.284	9.55079363544574E-91	3	HNRNPDL
8.11307596052926E-76	0.318952465507127	0.303	0.153	1.35958926946549E-71	3	MFSD14A
1.04154276369094E-101	0.318876220157388	0.4	0.202	1.74541736339327E-97	3	SCPEP1
8.86740732564385E-75	0.31886951092323	0.327	0.17	1.4860001196314E-70	3	FAM110B
3.92128306218534E-75	0.318543172301862	0.303	0.154	6.57128615561019E-71	3	HNRNPLL
1.65215899308244E-97	0.318500723521295	0.476	0.255	2.76868804060755E-93	3	TCERG1

6.63725548924209E-52	0.318427008498207	0.259	0.14	1.11227127488719E-47	3	PIEZO1
4.06646972839207E-86	0.318286162264335	0.923	0.644	6.81458997083944E-82	3	IDH1
4.11538147621438E-71	0.317953807636163	0.327	0.175	6.89655627784005E-67	3	PPHLN1
2.78472172637516E-75	0.317817027798358	0.408	0.228	4.6666366690595E-71	3	PGAP2
4.67359382954104E-76	0.31761917237107	0.269	0.129	7.83200853954487E-72	3	PACS1
7.49342404078908E-94	0.317238186897815	0.481	0.263	1.25574800075543E-89	3	SYMPK
3.49328214504009E-57	0.317184602095993	0.282	0.154	5.85404221865818E-53	3	PLCL2
8.41909700242956E-142	0.317025803954125	0.557	0.278	1.41087227566715E-137	3	WSB1
2.80154148515395E-105	0.316893332478709	0.397	0.197	4.69482322082099E-101	3	FBXO31
8.28890512871755E-89	0.316546064833464	0.422	0.226	1.38905472147049E-84	3	EDEM2
1.36188441552289E-107	0.316268100610555	0.363	0.168	2.28224590353326E-103	3	CYP2D6
2.91680963381936E-109	0.316198649254609	0.6	0.329	4.88798958435447E-105	3	PUM2
2.21765693915288E-110	0.316064212810118	0.561	0.301	3.7163494986324E-106	3	SLC35F6
6.12446975520506E-108	0.315782497834032	0.492	0.262	1.02633864157726E-103	3	EP300
1.32250029216013E-55	0.315091526630921	0.26	0.139	2.21624598960195E-51	3	SIX5
4.72943862901466E-92	0.315083937266919	0.408	0.214	7.92559325450276E-88	3	TRAF2
6.22340667748547E-85	0.314916622082142	0.395	0.21	1.04291849101301E-80	3	TERF2
8.8474586406241E-77	0.314747700900051	0.331	0.172	1.48265711899579E-72	3	KRR1
2.11351079695916E-85	0.314689012595074	0.441	0.242	3.54182139354415E-81	3	LMF2
3.46971830186821E-58	0.314106110951533	0.264	0.138	5.81455393027075E-54	3	ACAD8
1.45466071117197E-84	0.313641286893933	0.364	0.19	2.43772041978199E-80	3	TRPT1
7.72671578293831E-109	0.313472823776137	0.777	0.452	1.2948430309048E-104	3	ADAR
9.19503193684342E-99	0.31336280429231	0.835	0.529	1.54090345197622E-94	3	NFE2L1
2.66253921711732E-103	0.313319306964133	0.514	0.28	4.4618832200452E-99	3	LGMN
1.95542676327554E-95	0.313230443366074	0.461	0.252	3.27690416989715E-91	3	TPD52L2
3.41829304149627E-97	0.313020844496026	0.85	0.517	5.72837547893945E-93	3	IMMP2L
3.07512254910228E-65	0.312849237988852	0.269	0.137	5.15329036778561E-61	3	DEPRECATED-ENSG00000186591
5.33804066328626E-102	0.312840532024408	0.682	0.386	8.94548854353511E-98	3	BIRC3
4.19265882567438E-54	0.312094605993166	0.268	0.145	7.02605766006513E-50	3	LRRC57
9.80094400371791E-58	0.312032357423901	0.276	0.149	1.64244219614305E-53	3	SNRPG
2.77680966217258E-89	0.311811701563959	0.458	0.246	4.65337763186882E-85	3	DHFR
3.42024910148701E-102	0.311385780695714	0.557	0.309	5.73165344427194E-98	3	EGFR
1.65315096092286E-71	0.31108630117975	0.331	0.177	2.77035038031453E-67	3	RBM22
4.50497973164758E-71	0.311055466549672	0.331	0.176	7.54944503429501E-67	3	EDARADD
6.82669497064166E-49	0.311038360786817	0.251	0.138	1.14401754318013E-44	3	TATDN2
5.43963135485548E-63	0.310892151464342	0.292	0.155	9.11573422446681E-59	3	KAT2B
8.96984485023605E-49	0.310847143774889	0.253	0.139	1.50316660000256E-44	3	DCP2
1.80706405608605E-84	0.310364610717039	0.358	0.185	3.0282779451 89E-80	3	TMEM230
1.03319887940825E-100	0.310325256251163	0.768	0.448	1.73143468211235E-96	3	IL13RA1
3.90264996767766E-64	0.310054217517911	0.259	0.131	6.54006081583422E-60	3	IMPACT
9.20803495969979E-65	0.309785643498165	0.298	0.158	1.54308249854649E-60	3	PSME4
1.18555007580905E-110	0.309761977940141	0.422	0.209	1.98674481704081E-106	3	INSIG2
5.32611696794343E-61	0.309702699447828	0.269	0.141	8.9255068148796E-57	3	WTAP
7.54618225668475E-72	0.309607482780104	0.392	0.22	1.26458922257523E-67	3	RNF6
1.62343137557741E-107	0.30897734275767	0.614	0.331	2.72054629919262E-103	3	NID1
4.61615840506496E-112	0.308432173775798	0.541	0.288	7.73575825520786E-108	3	UBE2Z
5.27345970237706E-95	0.308028779033847	0.474	0.258	8.83726376924347E-91	3	RUFY1
9.02242716617065E-107	0.307995033975767	0.661	0.376	1.51197834450688E-102	3	KANK1
1.7337812735362E-56	0.307439844062483	0.25	0.131	2.90547065819196E-52	3	PACS2
1.23873391541347E-116	0.307139775540346	0.518	0.269	2.07587029544989E-112	3	PAM16
8.14137942274159E-104	0.307122133861841	0.679	0.378	1.36433236366303E-99	3	ERBIN
1.91543168301169E-92	0.306321174574661	0.357	0.177	3.20988041439099E-88	3	CLSTN3
7.9673848064088E-69	0.305586186493306	0.845	0.591	1.33517434585799E-64	3	SCD
1.3186959260483E-48	0.304942658998121	0.275	0.157	2.20987063287174E-44	3	FARP2
8.26907508792388E-92	0.304609795325271	0.423	0.224	1.38573160323428E-87	3	TBC1D17
5.46747902375154E-63	0.304564139943145	0.278	0.145	9.16240134800282E-59	3	MMD
4.34628350693413E-39	0.304485489984041	0.257	0.152	7.28350190092022E-35	3	CMPK2
1.64674475584393E-120	0.304375432105839	0.691	0.373	2.75961486184326E-116	3	COPS5
3.16439265759567E-103	0.304289742490329	0.539	0.291	5.30288921559883E-99	3	NCOR1
2.70416733646573E-119	0.303880133630025	0.559	0.298	4.53164362244926E-115	3	MINK1

2.86754647510941E-96	0.303503771991628	0.496	0.269	4.80543438298835E-92	3	MARVELD2
6.23411312108954E-101	0.303325677147417	0.481	0.256	1.04471267683218E-96	3	ATP2C1
2.32356065232622E-107	0.303192530656497	0.827	0.502	3.89382294116827E-103	3	TMEM205
4.31575815351513E-101	0.303145089863486	0.424	0.218	7.23234751366065E-97	3	FEM1C
6.47114577595566E-116	0.302174726128741	0.786	0.452	1.08443460913465E-111	3	EGLN1
9.25927755633634E-74	0.302046463187203	0.321	0.168	1.55166973289084E-69	3	KDM5A
1.88751146870142E-123	0.301750674687493	0.703	0.391	3.16309171924983E-119	3	UBAP2L
3.58829792194947E-58	0.301577745296403	0.277	0.148	6.01326965760291E-54	3	N4BP1
5.98831959946841E-87	0.301187632536803	0.452	0.249	1.00352259847892E-82	3	SRP72
1.40372535055861E-73	0.301096399756563	0.341	0.181	2.35236294246612E-69	3	STXBP3
1.35400819339677E-100	0.300916766003557	0.45	0.235	2.26904693049431E-96	3	SPAG9
1.51245609985392E-108	0.300907202632039	0.754	0.443	2.5345739321352E-104	3	NECTIN2
3.73330377964048E-57	0.300662888724835	0.254	0.133	6.25627047392153E-53	3	RAB29
7.66450709948196E-53	0.300602932381219	0.262	0.144	1.28441809973119E-48	3	PARN
5.6100969508978E-75	0.300529310177027	0.35	0.186	9.40140047031454E-71	3	PARD3
7.21707817313259E-53	0.300453526774063	0.285	0.159	1.20943796025356E-48	3	COMMD2
3.18405985679646E-55	0.300294915819185	0.271	0.148	5.33584750801951E-51	3	KAT8
1.8562264920028E-98	0.299831412938263	0.524	0.287	3.11066435529829E-94	3	PDCD6
6.08249046103166E-68	0.299593159116129	0.312	0.166	1.01930375145969E-63	3	SAV1
3.0142778055724E-101	0.299373433081952	0.531	0.288	5.05132674657824E-97	3	SIPA1L3
5.52669238955689E-81	0.299368183917568	0.322	0.161	9.26163110641943E-77	3	FGFR3
3.83105033405815E-42	0.299288487025596	0.346	0.215	6.42007414981465E-38	3	SHOX2
2.04408824164971E-99	0.298839621026493	0.74	0.436	3.42548307535659E-95	3	RSRP1
5.46177670498091E-113	0.29865351509431	0.646	0.353	9.15284540220702E-109	3	DGAT1
1.66574846112244E-106	0.298524273084161	0.527	0.283	2.79146127114898E-102	3	TRIM25
3.81141052697011E-79	0.298516203607145	0.345	0.18	6.3871617610965E-75	3	CHORDC1
1.1593679293353E-70	0.298359937039593	0.37	0.207	1.9428687759801E-66	3	ABI1
6.66056625808866E-60	0.298205913197996	0.373	0.217	1.1161776935305E-55	3	HPS3
1.90494697684947E-90	0.298138242543016	0.476	0.262	3.19231014380434E-86	3	CDC42SE2
4.42648748247296E-91	0.297947105393728	0.438	0.236	7.41790772312818E-87	3	POMGNT1
6.16264954815443E-96	0.297934034614	0.772	0.465	1.03273681127972E-91	3	GCLC
2.19368364100785E-50	0.297016976581156	0.253	0.139	3.67617504560096E-46	3	PAPSS1
2.76641827938066E-122	0.296613691505577	0.461	0.227	4.6359637525861E-118	3	C16orf70
1.78700747550586E-85	0.29644652327058	0.415	0.223	2.99466712745273E-81	3	RANBP9
1.14178965608158E-54	0.296183470900169	0.25	0.132	1.91341110566151E-50	3	TMEM135
1.56362149496772E-117	0.296153873321385	0.583	0.31	2.62031690126691E-113	3	IGF2R
6.65654567130808E-64	0.295982196286698	0.294	0.156	1.11550392359781E-59	3	E4F1
8.96619763353002E-98	0.295486939256586	0.496	0.27	1.50255539942696E-93	3	USP14
4.60856038271421E-88	0.29540465497101	0.452	0.25	7.72302548935247E-84	3	ANAPC5
1.41564418990329E-80	0.295378676181619	0.391	0.212	2.37233653343993E-76	3	POFUT1
8.48493780664425E-96	0.29531236786022	0.402	0.206	1.42190587763744E-91	3	GMPPA
1.49488312390185E-54	0.294854245617095	0.292	0.163	2.50512513903472E-50	3	JADE1
9.53745850575061E-117	0.294759532241014	0.594	0.321	1.59828729639369E-112	3	GPBP1
3.27629988309521E-80	0.294754219448071	0.387	0.208	5.49042334409095E-76	3	CDC42BPA
1.68799334084509E-95	0.294673663614711	0.465	0.25	2.82873924058821E-91	3	PCGF2
4.96015586859689E-89	0.294514066904854	0.464	0.253	8.31222920459467E-85	3	GET1
9.37306302662263E-87	0.294509848323515	0.438	0.238	1.57073790200142E-82	3	KDM3B
9.58282513889737E-90	0.29360537775144	0.391	0.204	1.60588983677642E-85	3	VAPB
6.6427601143E-75	0.293190050003085	0.33	0.173	1.11319373995439E-70	3	YIPF6
1.02333227235238E-73	0.292072655461013	0.357	0.193	1.71490022200813E-69	3	EIF2AK2
2.83145052436026E-48	0.291859388969991	0.251	0.139	4.74494478872292E-44	3	PSMG1
2.29503807177988E-111	0.290704038232932	0.817	0.486	3.84602480068872E-107	3	PEG10
2.00226941382266E-80	0.290696518288627	0.331	0.168	3.35540308368402E-76	3	CAMLG
4.32726840206887E-97	0.29051477663961	0.468	0.252	7.25163638818701E-93	3	KCTD20
4.57074024056317E-98	0.290435170592203	0.925	0.566	7.65964649513576E-94	3	NDRG1
5.27462022044262E-81	0.290305987437488	0.394	0.213	8.83920856541774E-77	3	PPP2R5E
1.11765523119208E-61	0.290261960057517	0.321	0.177	1.87296663643169E-57	3	ANKLE2
9.79085891146207E-66	0.290160337191542	0.309	0.166	1.64075213638281E-61	3	GLRX2
9.31998843573531E-57	0.289952220177383	0.265	0.141	1.56184366206052E-52	3	SEPHS1
1.09968232613238E-104	0.289518409937006	0.594	0.329	1.84284764213264E-100	3	LMBRD1

9.80190482534505E-107	0.289196250854014	0.594	0.326	1.64260321063132E-102	3	INSR
5.88933857276409E-95	0.289173360932964	0.421	0.221	9.86935358023806E-91	3	CNOT9
6.40018347313092E-72	0.289054526621023	0.354	0.192	1.07254274642728E-67	3	NUP98
5.71176828034227E-61	0.28892883588894	0.325	0.18	9.57178128419757E-57	3	NUDT19
4.96336094763118E-82	0.288799891811855	0.418	0.232	8.31760027604033E-78	3	TOMM6
3.95728963573939E-57	0.288796597791627	0.259	0.137	6.63162597157208E-53	3	MTG2
3.49712593729798E-60	0.288732759318176	0.283	0.151	5.86048364572395E-56	3	CLNS1A
1.57035621729155E-90	0.28829414654638	0.485	0.268	2.63160294893718E-86	3	CHAMP1
2.84761806884822E-87	0.288127328439664	0.379	0.198	4.77203835977585E-83	3	KIAA1191
3.34489292168099E-76	0.287451936951846	0.377	0.205	5.60537155815301E-72	3	TEX261
1.79805199981035E-81	0.287447006765742	0.399	0.216	3.01317554128218E-77	3	BLCAP
2.80600616197485E-96	0.285860090753759	0.426	0.224	4.70230512623745E-92	3	GOLGA3
2.45273057746083E-57	0.285789580854806	0.312	0.172	4.11028590170886E-53	3	GAS2L3
1.72733517834225E-118	0.285336001096097	0.619	0.333	2.89466829186594E-114	3	SMARCA4
2.37148573322867E-61	0.284829951162005	0.296	0.158	3.9741357917446E-57	3	SLC12A8
1.97816613735939E-51	0.284572337564943	0.262	0.145	3.31501081298687E-47	3	PARD6B
1.50489039864456E-119	0.2831946470894	0.831	0.509	2.52189533004855E-115	3	SLC39A14
4.66696900428742E-108	0.283014719438111	0.399	0.192	7.82090665738487E-104	3	CYP7B1
2.67170205666429E-48	0.282973878242725	0.287	0.166	4.47723830655801E-44	3	OSER1
1.04893258625439E-73	0.282084696105874	0.37	0.201	1.7578012280451E-69	3	EIF4E
3.7622852848991E-80	0.281974213681796	0.346	0.18	6.30483768043391E-76	3	SLC35E1
1.89824957690264E-42	0.28185939737639	0.259	0.15	3.18108664097344E-38	3	CEP85
1.73780448503533E-116	0.281478570599731	0.55	0.292	2.91221275602221E-112	3	HDHD3
7.6424551471131E-86	0.28129658720281	0.434	0.235	1.28072263355321E-81	3	HSPA4L
4.03982856157383E-52	0.281186351886346	0.273	0.15	6.76994470348543E-48	3	RNF169
1.82730803183787E-62	0.281096548605123	0.346	0.192	3.06220279975389E-58	3	SLC9A6
5.12047517432595E-104	0.280929930159105	0.539	0.293	8.58089229713543E-100	3	ITPRID2
3.33372085025613E-92	0.280616381544074	0.493	0.273	5.58664940085922E-88	3	CPSF7
7.44129401508494E-47	0.280253612090223	0.26	0.146	1.24701205104793E-42	3	MEST
4.18525917496193E-56	0.280217799793872	0.309	0.172	7.01365732540121E-52	3	PER3
2.36595697510051E-102	0.279733748044547	0.754	0.439	3.96487069887344E-98	3	PDIA5
6.01137715786363E-106	0.279369614570503	0.578	0.315	1.00738658411479E-101	3	PRKAA1
4.3039448956365E-69	0.279315568887481	0.27	0.134	7.21255085610766E-65	3	FBRSL1
7.72966180275478E-96	0.2793079946552	0.575	0.311	1.29533672490565E-91	3	RNF157
5.2964316801813E-82	0.279113048041988	0.935	0.58	8.87576020964783E-78	3	MPP1
6.85708129187909E-86	0.278331478257282	0.46	0.254	1.1491096828931E-81	3	ATP11A
1.13779138120154E-58	0.277010962583656	0.341	0.193	1.90671079661753E-54	3	FNBP4
6.03798600918313E-106	0.276876746045661	0.484	0.256	1.01184569541891E-101	3	CYTH1
4.15811493098345E-105	0.276391748759759	0.603	0.337	6.96816900134207E-101	3	KTN1
3.53978685560368E-98	0.275824445306518	0.75	0.448	5.93197481262065E-94	3	SCARB2
2.69260186637191E-74	0.275699408407935	0.335	0.176	4.51226220766605E-70	3	NUDT14
2.61540268887553E-89	0.275526358703011	0.454	0.248	4.38289182601762E-85	3	EIF2B5
1.51422206257045E-52	0.275484606376888	0.278	0.153	2.53753333245556E-48	3	TMCC3
6.66769196308749E-98	0.275321498245246	0.445	0.234	1.1173718191742E-93	3	NFKB1
9.37085370475048E-65	0.275119721208015	0.333	0.184	1.57036766384208E-60	3	UTRN
5.42080981988565E-69	0.274982421667937	0.351	0.193	9.08419309616437E-65	3	PPP2CA
4.70823030855811E-96	0.274967721330671	0.51	0.28	7.89005235108168E-92	3	NR5A2
1.67722018723476E-96	0.274924441698813	0.543	0.3	2.81068558976801E-92	3	PRRC2C
1.4121654198988E-71	0.274898972023124	0.298	0.151	2.36650681066641E-67	3	FERMT2
7.13793506932563E-100	0.274835084672073	0.529	0.29	1.19617515891759E-95	3	CDC42BPB
1.32787589568766E-110	0.274733216199867	0.641	0.356	2.22525442599338E-106	3	MAGED2
1.01479211982742E-49	0.27472353442813	0.318	0.187	1.70058863440678E-45	3	VSIG10
9.41133989899629E-151	0.274534036001532	0.498	0.231	1.5771523402738E-146	3	ECHDC3
1.90513373155592E-85	0.274425848650771	0.408	0.218	3.19262310734141E-81	3	RARA
2.14967273669845E-82	0.273990902859002	0.407	0.221	3.60242157215926E-78	3	TM9SF4
1.84309304582953E-125	0.273854609991628	0.614	0.331	3.08865532620112E-121	3	INTS11
2.71532839648113E-44	0.273402845393704	0.287	0.166	4.55034732682309E-40	3	POLA1
9.80890333528431E-66	0.273303228373497	0.349	0.194	1.64377602092695E-61	3	STARD3
6.56012221068368E-93	0.273238566432221	0.479	0.261	1.09934528006637E-88	3	CAMK1
2.72848033592948E-74	0.273083730961707	0.899	0.553	4.57238734695062E-70	3	DNM2

8.93751696891079E-55	0.272944252193653	0.33	0.19	1.49774909365007E-50	3	ABCC4
1.68553988271411E-60	0.272882258054121	0.278	0.147	2.8246277354523E-56	3	RMND1
1.16831498428982E-66	0.272796091471373	0.3	0.158	1.95786225067288E-62	3	ZNF106
7.70764536351528E-86	0.27262183994499	0.43	0.236	1.29164721001789E-81	3	ZC3HAV1
1.97247177956085E-105	0.271185806098591	0.675	0.385	3.30546820818808E-101	3	DCAF11
1.94896777298825E-66	0.270847195868207	0.312	0.165	3.2660801939737E-62	3	VPS39
4.96707072927087E-68	0.270400695629256	0.321	0.172	8.32381712811213E-64	3	MARCH8
3.2778805317541E-95	0.270165395827435	0.446	0.239	5.49307219511352E-91	3	GRIPAP1
1.42904815457322E-71	0.269857079061708	0.358	0.195	2.3947988974338E-67	3	ZMPSTE24
2.96203694090877E-97	0.269571652346237	0.49	0.265	4.96378150557492E-93	3	EPRS
3.81786024418546E-88	0.26929287733233	0.474	0.262	6.397970197206E-84	3	EBPL
1.56940808511888E-117	0.26904454541751	0.639	0.352	2.63001406904221E-113	3	QKI
4.83742236896264E-88	0.268893386349588	0.432	0.233	8.1065524059076E-84	3	USF1
5.6313668640512E-55	0.268791946859648	0.269	0.145	9.437044590777E-51	3	PSD4
7.43846050185968E-39	0.268455604382482	0.264	0.158	1.24653721090164E-34	3	CHD7
1.55174534477045E-69	0.268254878227684	0.381	0.213	2.60041484876631E-65	3	NXT2
4.05533690432295E-87	0.268134617579606	0.403	0.214	6.7959335842644E-83	3	NFYC
5.62839151914275E-77	0.267918082131185	0.396	0.219	9.43205850777941E-73	3	DDX42
2.97011709056472E-66	0.267742846055347	0.332	0.182	4.97732222036835E-62	3	MIB1
7.88734015243126E-87	0.267584721844189	0.477	0.267	1.32176046274443E-82	3	NAP1L4
7.95907557552728E-114	0.267499902006633	0.677	0.384	1.33378188494686E-109	3	RBM39
2.97700515750107E-94	0.267395052267813	0.833	0.501	4.98886524294029E-90	3	HNRNPH2
1.95819604522284E-77	0.267150272430245	0.429	0.239	3.28154493258443E-73	3	BROX
5.77666253610575E-100	0.266825390836135	0.455	0.24	9.68053107800601E-96	3	IFNAR1
3.0669282701361E-77	0.266554797935557	0.374	0.203	5.13955839509407E-73	3	HARS
1.43144555597574E-56	0.266437789637479	0.278	0.15	2.39881646270415E-52	3	TBC1D13
8.21023201946211E-106	0.266315556398332	0.661	0.376	1.37587068182146E-101	3	RERE
1.84768505014238E-63	0.266169510392663	0.289	0.151	3.0963506070286E-59	3	UBA7
1.87951260187018E-66	0.26611845737189	0.323	0.174	3.14968721821405E-62	3	FBRS
1.35408899403026E-81	0.265854654379246	0.92	0.674	2.26918233619592E-77	3	BST2
5.97263637511554E-50	0.265827202935215	0.266	0.148	1.00089440374186E-45	3	TBC1D5
1.66923834986085E-65	0.265728894829413	0.298	0.157	2.79730962669682E-61	3	FOXN2
6.37555816060476E-91	0.265618053857496	0.448	0.242	1.06841603655415E-86	3	TLK1
3.1483667991433E-61	0.265584326480471	0.344	0.193	5.27603308200434E-57	3	PCSK9
1.96215933570215E-120	0.265471110018026	0.619	0.341	3.28818661476966E-116	3	PPP6R2
1.92152956187386E-103	0.265339049072298	0.455	0.24	3.22009923978821E-99	3	ARHGEF7
7.78577898549282E-59	0.265319298322117	0.277	0.149	1.30474084238889E-54	3	ATE1
3.33764108685489E-40	0.265149626280119	0.273	0.164	5.59321893335142E-36	3	UCHL3
3.0878155129996E-38	0.265045019615672	0.264	0.159	5.17456123668473E-34	3	ZDHHC20
2.20591596607449E-39	0.265007179226776	0.265	0.159	3.69667397594763E-35	3	TBC1D10B
2.08207493719804E-96	0.265002492888679	0.474	0.255	3.48914117975647E-92	3	MMAB
9.5591434270353E-67	0.264602214250097	0.926	0.697	1.60192125550257E-62	3	AKR1B10
2.51515500973524E-65	0.264552139542125	0.336	0.185	4.21489676531431E-61	3	RPRD1B
7.73668696210008E-70	0.264406121324052	0.943	0.703	1.29651400110873E-65	3	IFI27
1.82128970071119E-79	0.264150618405131	0.412	0.226	3.05211728045181E-75	3	ZC3H4
4.91720090833979E-55	0.26362401590522	0.287	0.159	8.24024528219582E-51	3	PLEKHA7
2.42989689996572E-83	0.263452469298045	0.38	0.2	4.07202122496255E-79	3	TADA2B
1.38486779229444E-84	0.263385829864727	0.81	0.495	2.32076144632702E-80	3	ENTPD5
1.71877334714682E-103	0.263359808578235	0.649	0.358	2.88032037514864E-99	3	NUCB2
8.02269651395225E-111	0.263101898132511	0.596	0.329	1.34444348180812E-106	3	LPIN2
8.39750700960791E-48	0.263035581551915	0.331	0.197	1.40725422467009E-43	3	SLC7A1
1.47880879083936E-44	0.262963702153277	0.251	0.141	2.4781877716886E-40	3	SPNS1
6.72875376166846E-93	0.262794342667395	0.457	0.245	1.1276045553804E-88	3	PPIC
2.28250963193872E-60	0.262625444026531	0.27	0.141	3.8250296412029E-56	3	TCF12
1.94440523800654E-89	0.26228966632953	0.865	0.49	3.25843429785135E-85	3	ATP1A1
3.7644289667902E-94	0.2621047725458	0.531	0.295	6.30843006254701E-90	3	ATP11B
1.50858291178337E-59	0.262026678917156	0.303	0.167	2.52808324356657E-55	3	PHAX
1.38987603090639E-61	0.261949777880227	0.275	0.144	2.32915425259293E-57	3	ST3GAL2
2.04413853768084E-98	0.261757597367717	0.598	0.336	3.42556736144555E-94	3	NDUFS1
2.50974740021001E-76	0.261391874543506	0.397	0.218	4.20583469327194E-72	3	CHD4

3.03490813154534E-70	0.260978950425959	0.38	0.212	5.08589904684367E-66	3	WASHC5
7.10843241604457E-93	0.260463452295685	0.665	0.376	1.19123110428075E-88	3	FAT1
9.88677665100892E-78	0.260086237918611	0.368	0.197	1.65682603117607E-73	3	FAM168A
1.37161917636533E-95	0.259772957184842	0.85	0.549	2.29855941575303E-91	3	CPB2
8.65512655862483E-64	0.259767456289153	0.363	0.204	1.45042610869435E-59	3	YY1AP1
4.35852264044423E-48	0.259696114498012	0.263	0.147	7.30401224085644E-44	3	DROSHA
5.22458402603626E-105	0.259661263407272	0.695	0.393	8.75535791083156E-101	3	HNRNPA3
3.1422312033825E-101	0.259465541010701	0.727	0.419	5.2657510506284E-97	3	CPQ
1.06780130741209E-126	0.25945731220911	0.337	0.134	1.78942143096118E-122	3	ACOT12
4.02491297482871E-74	0.259290085323722	0.406	0.228	6.74494916321795E-70	3	TNPO3
4.11274743744867E-98	0.259131376189931	0.536	0.295	6.89214215567649E-94	3	PPP4R3B
1.45688142349627E-85	0.259068742470336	0.417	0.224	2.44144188949506E-81	3	AGTRAP
5.90442652120924E-50	0.258910846613918	0.27	0.152	9.89463796424245E-46	3	COA8
1.9121158139256E-75	0.258712458650894	0.406	0.226	3.20432368097651E-71	3	IPO8
6.53361563566545E-99	0.258647116997983	0.598	0.336	1.09490330822482E-94	3	ATP2A2
3.26624514063336E-84	0.258488952103887	0.368	0.191	5.47357360667339E-80	3	ZNF768
1.15475496615642E-102	0.257878764880303	0.599	0.335	1.93513837228493E-98	3	GNAQ
8.13810388405888E-57	0.257443950765301	0.276	0.148	1.36378344889059E-52	3	CYLD
2.43237593881083E-85	0.2572783271936	0.799	0.498	4.07617559825918E-81	3	PCK2
3.53701717724673E-85	0.257239486454703	0.494	0.277	5.92733338563008E-81	3	MTIF3
3.15314932170543E-81	0.256741979178389	0.411	0.224	5.28404763331395E-77	3	UBE2J2
3.95962754573807E-53	0.256247178201942	0.304	0.173	6.63554384114786E-49	3	CCDC130
2.23033037690318E-68	0.255780648327252	0.347	0.191	3.73758764561434E-64	3	TMEM167A
4.8269379049612E-112	0.254970223216518	0.615	0.34	8.08898254113398E-108	3	TOM1L1
6.6774858993259E-74	0.25496471708848	0.329	0.172	1.11901308700903E-69	3	MRTFA
1.23001129774562E-101	0.25461578321163	0.543	0.295	2.06125293276211E-97	3	PSMB5
1.92028722361674E-81	0.254594564131807	0.458	0.255	3.21801732933694E-77	3	HLTF
5.4209576365389E-94	0.254459636751631	0.498	0.274	9.08444080731189E-90	3	LRPPRC
1.03567526922281E-68	0.253676913646057	0.32	0.169	1.73558461616359E-64	3	ARRB2
1.14605478549979E-36	0.253658313845323	0.26	0.157	1.92055860954055E-32	3	NIPAL1
2.49712795689857E-64	0.253196291955536	0.315	0.171	4.18468703017062E-60	3	ATXN2
6.807844101282E-76	0.253081857004676	0.377	0.205	1.14085851449284E-71	3	ARHGEF11
1.25250885038664E-113	0.253031215085791	0.51	0.267	2.09895433147793E-109	3	RHOT2
2.70587754299232E-111	0.253027613819807	0.464	0.236	4.53450958654653E-107	3	LUC7L
6.52034728951704E-67	0.252994843242317	0.361	0.201	1.09267979877727E-62	3	UBAC2
4.96208085720728E-41	0.252440968992336	0.329	0.203	8.31545510050796E-37	3	SLC26A6
2.69842635090799E-105	0.252426309692184	0.66	0.376	4.52202287885161E-101	3	TMEM259
2.17700866377424E-90	0.252405294731037	0.447	0.241	3.64823111875287E-86	3	PRKCA
8.3259980682447E-92	0.251719375932336	0.554	0.315	1.39527075627645E-87	3	GNS
1.02521338636144E-99	0.251675505988986	0.391	0.197	1.7180525928645E-95	3	PHF8
3.84232238468391E-111	0.251263652272538	0.578	0.312	6.43896385225329E-107	3	FAM83H
5.87851184808546E-73	0.25125657020246	0.32	0.165	9.85121015502161E-69	3	KLC4
1.3837757760556E-102	0.251128609744675	0.508	0.274	2.31893144551397E-98	3	CLIP1
1.27785009660097E-60	0.250759429206653	0.38	0.219	2.1414211918839E-56	3	PHLPP2
0	5.00267394187444	0.324	0.012	0	4	WFDC2
0	4.03990804764234	0.781	0.288	0	4	SPP1
0	3.76767401117732	0.273	0.023	0	4	MSC
0	3.71747556739404	0.444	0.082	0	4	SELENOM
0	3.6770498112806	0.935	0.568	0	4	PLA2G2A
0	3.65219574147479	0.453	0.098	0	4	SAA1
0	3.38621303832053	0.64	0.19	0	4	CRP
0	2.99675146458357	0.584	0.225	0	4	DEPRECATED-ENSG00000175899
0	2.9758068652143	0.301	0.045	0	4	SDS
0	2.93029938972347	0.437	0.107	0	4	ICAM1
0	2.91765940984851	0.344	0.067	0	4	KYNU
1.80813904125712E-189	2.86815834626145	0.267	0.074	3.03007940533868E-185	4	TM4SF1
0	2.73226550047816	0.673	0.328	0	4	A2M
0	2.53614899920736	0.341	0.072	0	4	TIMP1
0	2.53168466625349	0.538	0.179	0	4	ARG1
0	2.24758799776292	0.429	0.116	0	4	CPS1

2.4925123029879E-169	2.19857899330793	0.507	0.272	4.17695211734711E-165	4	TYMP
8.00082707675344E-198	2.10030734322156	0.353	0.116	1.34077860152234E-193	4	TNFSF14
0	1.98926829475642	0.721	0.373	0	4	LBP
0	1.95671534270483	0.926	0.786	0	4	SOD2
0	1.95121451583987	0.708	0.274	0	4	SERPINA3
2.4253022594477E-121	1.91534581871657	0.457	0.258	4.06432152638245E-117	4	ROBO1
2.84853641947016E-72	1.90824610709772	0.281	0.141	4.7735773317481E-68	4	RGS14
3.4126703597644E-86	1.9024365298442	0.366	0.202	5.71895298889318E-82	4	ISG15
3.39271244468574E-140	1.90003762586297	0.557	0.36	5.68550751480437E-136	4	UBD
1.45688052316066E-227	1.89179444203267	0.39	0.12	2.44144038071263E-223	4	MBL2
0	1.88609099581469	0.799	0.38	0	4	APOE
8.7531910862053E-55	1.87790372503948	0.326	0.202	1.46685976222628E-50	4	CD24
0	1.8216325417055	0.995	0.963	0	4	C3
9.38503393649537E-151	1.81748928608529	0.531	0.307	1.57274398707789E-146	4	CREB3L3
9.05931325780297E-190	1.81695994534918	0.381	0.133	1.51815971574262E-185	4	SLC38A4
0	1.80766873974426	0.989	0.901	0	4	ORM1
2.81304510514591E-138	1.76316220252034	0.331	0.126	4.71410098720352E-134	4	SLC25A18
0	1.76102514238283	0.75	0.369	0	4	ITIH2
8.88612391520808E-294	1.66115941598294	0.8	0.557	1.48913664571057E-289	4	FGL1
6.15446736402861E-109	1.65786398623785	0.504	0.316	1.03136564086391E-104	4	DEPP1
5.41891437979805E-53	1.65015697139657	0.253	0.135	9.08101671766557E-49	4	RIOX2
3.95518309481767E-38	1.62162401200161	0.259	0.159	6.62809583029545E-34	4	VWA1
4.22291396633443E-184	1.61732857541515	0.381	0.129	7.07675922478324E-180	4	MASP2
1.50998002347817E-179	1.61539675517263	0.38	0.133	2.53042452334472E-175	4	C9
2.56362956229166E-85	1.61535895055973	0.51	0.362	4.29613042048836E-81	4	LGALS3BP
6.00710498534882E-50	1.61502718073418	0.277	0.159	1.00667065344476E-45	4	TRIM55
0	1.61287133657692	0.953	0.821	0	4	CFB
1.69845277678552E-108	1.56594664767842	0.304	0.124	2.84626716333718E-104	4	RND1
8.57130870908756E-252	1.56076789111253	0.685	0.363	1.43637991346889E-247	4	NNMT
0	1.52495702786988	0.926	0.863	0	4	IL32
0	1.47893963289338	0.89	0.831	0	4	IFITM3
1.58209052875131E-38	1.43745421777544	0.395	0.297	2.65126730808145E-34	4	DEPRECATED-ENSG00000163931
6.5194345285965E-194	1.40471668555135	0.77	0.635	1.0925268383022E-189	4	LRG1
1.03568881036984E-75	1.39269620809616	0.595	0.49	1.73560730841777E-71	4	KRT18
1.25256856810982E-105	1.3774098822399	0.267	0.097	2.09905440643844E-101	4	ALDH8A1
0	1.35909399095905	0.969	0.909	0	4	APOC2
2.3352354062839E-93	1.35652788510843	0.394	0.206	3.91338749385055E-89	4	TM4SF5
0	1.33897311514666	0.917	0.806	0	4	ORM2
1.21492831888671E-158	1.31595680377497	0.663	0.427	2.03597687679036E-154	4	APOC1
3.21237066131105E-50	1.31537452999683	0.408	0.28	5.38329075422506E-46	4	REEP6
1.42543201471057E-118	1.28168878305628	0.43	0.205	2.38873897025197E-114	4	SLC38A3
2.61346371716255E-49	1.26726039244123	0.496	0.402	4.379642497221E-45	4	TFPI
5.24180082616621E-44	1.26689379114924	0.383	0.27	8.78420982448933E-40	4	NCOA7
8.58153781969548E-201	1.25570685775447	0.735	0.474	1.43809410782457E-196	4	ITIH1
7.53150543598927E-26	1.25406262105258	0.382	0.311	1.26212968096308E-21	4	GLRX
2.66442589578671E-47	1.24824598150775	0.419	0.306	4.46504491615936E-43	4	NRBP2
1.94110180494923E-21	1.23235439225967	0.37	0.307	3.25289840473392E-17	4	MANF
5.90828399386109E-135	1.23100014018162	0.663	0.486	9.90110231691241E-131	4	SERPINF2
5.34360045161129E-55	1.22826517943629	0.548	0.464	8.9548056368102E-51	4	PNRC1
3.17650645760774E-241	1.22577217032032	0.686	0.311	5.32318952165904E-237	4	HPX
3.71931934876026E-28	1.20905061558276	0.317	0.225	6.23283536465245E-24	4	ALDH4A1
4.14242218183867E-55	1.20820272275024	0.317	0.18	6.94187109232524E-51	4	DUSP10
0	1.20780071831515	0.973	0.926	0	4	FN1
1.25119390107524E-208	1.20253369231261	0.804	0.746	2.09675073942189E-204	4	CFH
0	1.20023270974944	0.934	0.688	0	4	FGB
1.60989777911164E-41	1.1917268901069	0.439	0.335	2.69786669823528E-37	4	BCL3
2.62633736091994E-89	1.1766654423709	0.444	0.244	4.40121614942964E-85	4	IGFBP2
3.74305753205537E-169	1.16279710088959	0.768	0.571	6.27261581221838E-165	4	CP
4.76770101569892E-120	1.16054094163062	0.405	0.176	7.98971336210825E-116	4	SAA4
1.27462859719114E-28	1.1280129707096	0.426	0.347	2.13602260317291E-24	4	DUSP23

2.44889174006594E-44	1.09949034460489	0.49	0.393	4.1038527780025E-40	4	IFITM2
1.01996773982276E-12	1.09899178769019	0.265	0.212	1.70926193839498E-08	4	JUP
8.93361913047662E-19	1.07915311186552	0.318	0.25	1.49709589388527E-14	4	RGS3
1.04914497581805E-22	1.07876070520141	0.315	0.236	1.75815715047588E-18	4	TGM2
0	1.07613156167193	0.95	0.826	0	4	FGG
9.76129128924162E-264	1.07557295440112	0.907	0.842	1.63579719425111E-259	4	CD74
2.1475555678152E-21	1.06523098028281	0.356	0.283	3.59887362054471E-17	4	COMT
3.30274016949162E-86	1.06066786219108	0.49	0.291	5.53473197603406E-82	4	CFHR2
2.5856493540834E-70	1.06021709217072	0.7	0.645	4.33303118757297E-66	4	ALDH1L1
4.79329019687313E-27	1.04873368805666	0.442	0.375	8.03259571192E-23	4	CD59
3.21468883596454E-38	1.03853900799481	0.576	0.519	5.38717555130937E-34	4	MIF
6.98993400918367E-64	1.03266838210018	0.477	0.326	1.171373141259E-59	4	C5
1.46457523380432E-23	1.02879781122551	0.357	0.276	2.45433517680928E-19	4	NFIL3
2.43690667398047E-12	1.02188557248518	0.286	0.235	4.08376820425647E-08	4	SELENOK
8.88228159860239E-145	1.01916136211809	0.916	0.905	1.48849275029379E-140	4	SQSTM1
7.00921433166609E-54	1.01867675912755	0.413	0.271	1.1746041377006E-49	4	MTSS2
1.15538314515592E-34	1.0165118200709	0.352	0.237	1.93619107465229E-30	4	SLC9A3R2
9.24920617307062E-25	1.00082482128057	0.502	0.468	1.54998197048317E-20	4	SEC11C
9.1008034528157E-41	0.999938861530296	0.568	0.516	1.52511264262285E-36	4	CTSA
2.15762517842714E-18	0.991679595468398	0.27	0.198	3.6157482740082E-14	4	IRS2
5.54987347167693E-09	0.988067448269444	0.269	0.229	9.30047796383619E-05	4	METTL7B
7.34549164842876E-13	0.986810314772552	0.27	0.215	1.23095749044369E-08	4	UBE2L6
3.0141364907652E-263	0.985358953289285	0.974	0.925	5.05108993122432E-259	4	RBP4
1.63201452700762E-59	0.984365460828323	0.656	0.603	2.73492994435937E-55	4	CTSB
6.83784710539935E-37	0.981143133748664	0.542	0.48	1.14588641792282E-32	4	ITM2B
8.54259104894445E-15	0.970477277369422	0.409	0.367	1.43156740798211E-10	4	TMED9
7.14020949739898E-170	0.969973453473704	0.812	0.658	1.19655630757412E-165	4	C1S
3.23128057624435E-304	0.964437032525198	0.974	0.854	5.41497998967029E-300	4	FGA
2.60584428660078E-09	0.95937543673092	0.27	0.228	4.36687385548559E-05	4	MGLL
3.06896925238134E-126	0.954918585348136	0.822	0.769	5.14297867314065E-122	4	PRAP1
6.84761344285321E-26	0.946265981938621	0.502	0.462	1.14752306075334E-21	4	CALR
5.13035931783211E-21	0.931135955789203	0.469	0.419	8.59745614482305E-17	4	GRHPR
1.92254910852915E-237	0.92981233553974	0.986	0.972	3.22180779607314E-233	4	FTL
9.29434091428022E-14	0.928868642858763	0.387	0.341	1.55754565041508E-09	4	ASS1
7.49313600168876E-12	0.921652502601925	0.407	0.375	1.255699731163E-07	4	SELENOW
2.40342402980175E-08	0.916915037678404	0.368	0.342	0.000402765798914177	4	TMSB10
4.10297775237274E-102	0.90038235842258	0.824	0.816	6.87577011742623E-98	4	FTH1
2.83309102156227E-202	0.895545691232873	0.658	0.282	4.74769393393405E-198	4	HRG
1.55654894316432E-13	0.880368483128932	0.48	0.458	2.60846471895477E-09	4	CEBPB
2.65442702300695E-16	0.872158689286806	0.471	0.436	4.44828880515505E-12	4	PLAAT4
3.46736932544334E-22	0.870955697529612	0.474	0.419	5.81061751557794E-18	4	F10
3.18184452049405E-158	0.86931598065421	0.756	0.483	5.33213504744393E-154	4	SERPING1
3.99908808158697E-104	0.869056044521678	0.775	0.725	6.70167180712344E-100	4	LAMP1
1.03479465193536E-10	0.867566224248807	0.429	0.414	1.73410887771328E-06	4	HSPA5
4.44192378997451E-25	0.862871452984922	0.546	0.518	7.44377588723929E-21	4	MPST
2.19298333350543E-271	0.861051353618543	0.818	0.35	3.67500147028839E-267	4	HP
7.32661580687517E-58	0.849098199069382	0.676	0.644	1.22779427691614E-53	4	XBP1
5.89593081822462E-154	0.845180749707805	0.569	0.249	9.88040086518082E-150	4	HPR
2.56809732505399E-56	0.835475192962083	0.672	0.622	4.30361749732548E-52	4	PAH
4.49401999735608E-29	0.834875829746427	0.569	0.524	7.53107871156933E-25	4	GAMT
4.01924113348201E-42	0.83417588507702	0.624	0.593	6.73544429148916E-38	4	OS9
4.4108789198503E-15	0.822933308138564	0.498	0.481	7.39175089388513E-11	4	PSAP
2.06101576365627E-49	0.820135264937414	0.342	0.197	3.45385021673517E-45	4	APOM
4.38001308909568E-243	0.819283806948123	0.978	0.957	7.34002593470654E-239	4	AMBP
5.00293439414664E-25	0.818072248483271	0.623	0.622	8.38391745771093E-21	4	SDC4
1.83908849911354E-07	0.816804257136692	0.333	0.303	0.00308194450681447	4	GGH
5.75446261105047E-93	0.813376875765147	0.339	0.144	9.64332844359837E-89	4	HPD
1.94036476384428E-20	0.804962009741108	0.491	0.454	3.25166327125025E-16	4	ABAT
4.53197098967061E-76	0.796895795430717	0.52	0.327	7.59467698449001E-72	4	ITIH4
2.82116060386229E-07	0.787320188201152	0.385	0.368	0.00472770093995242	4	PDIA6

7.52707487958826E-86	0.766040050863534	0.765	0.748	1.2613872083214E-81	4	ASGR2
2.01927460339908E-25	0.762637467497054	0.47	0.39	3.38390038037618E-21	4	JUNB
3.34456795588727E-31	0.756820974441692	0.675	0.678	5.60482698047589E-27	4	ETFB
1.07548393163643E-08	0.754098007293096	0.418	0.404	0.000180229597263632	4	HSP90B1
3.40368183277682E-20	0.738638088788429	0.493	0.446	5.70389001536739E-16	4	DPYS
4.0636474834721E-36	0.737394549888732	0.661	0.644	6.80986045280254E-32	4	GRINA
3.12935879845777E-11	0.735786473470357	0.451	0.426	5.24417947445552E-07	4	SERP1
7.03447360384338E-08	0.734434062807626	0.261	0.223	0.00117883708653207	4	GNL3
6.64822920303677E-279	0.732635475606352	0.996	0.986	1.1141102498449E-274	4	VTN
2.42443135413727E-09	0.732021696760839	0.457	0.448	4.06286206326323E-05	4	NUDC
5.33605801161634E-52	0.731061469896232	0.742	0.712	8.94216601586666E-48	4	EEF2
6.83604350512281E-38	0.72529955662626	0.686	0.676	1.14558417058848E-33	4	CST3
2.37214508640078E-12	0.718320284926342	0.484	0.476	3.97524073579042E-08	4	PDIA3
9.64906710528291E-10	0.712138722660873	0.385	0.355	1.61699066550331E-05	4	SHFL
2.03646445493153E-273	0.707712996080278	0.982	0.991	3.41270713357427E-269	4	SERPINA1
6.05974794270027E-13	0.699231802197904	0.421	0.386	1.01549256023771E-08	4	LRP1
8.68390461065032E-14	0.699157316764617	0.512	0.487	1.45524873465278E-09	4	PCBD1
6.42842564029106E-27	0.68439816483783	0.682	0.688	1.07727556879998E-22	4	DHCR24
1.06596097164685E-10	0.683447388717028	0.447	0.422	1.7863373962858E-06	4	TCEA3
3.8176851854553E-98	0.677702867301076	0.887	0.82	6.39767683378598E-94	4	TTR
3.92650739537432E-13	0.677130356502058	0.523	0.516	6.58004109316829E-09	4	SNTB1
2.75225364272968E-139	0.677103872467827	0.976	0.954	4.6122266544864E-135	4	CES1
9.24980683612604E-217	0.672444148237539	0.981	0.97	1.550082629598E-212	4	CLU
2.30823254387034E-07	0.670862095938797	0.444	0.447	0.00386813609701792	4	DNAJB11
6.04143960138732E-38	0.670443905679307	0.681	0.679	1.01242444840049E-33	4	C2
9.61750374333501E-11	0.664080580307504	0.531	0.547	1.61170127730808E-06	4	LPGAT1
5.76023679089417E-08	0.658155703560956	0.501	0.517	0.000965300481418046	4	RIDA
5.83273676705465E-13	0.64920729982305	0.296	0.234	9.77450027423018E-09	4	PLIN4
7.05348277326888E-23	0.648014005953915	0.537	0.482	1.1820226431444E-18	4	SERPINF1
2.34938363906736E-14	0.645115884219915	0.576	0.592	3.93709710234909E-10	4	CREG1
5.81696322851553E-17	0.64421675451888	0.607	0.633	9.74806697834632E-13	4	DDT
4.27276540144326E-104	0.641605352144196	0.924	0.923	7.16030025973862E-100	4	FABP1
2.03780255952002E-18	0.638722193406316	0.627	0.625	3.41494952924366E-14	4	TAPBP
1.45785458644292E-08	0.634817772005813	0.421	0.394	0.000244307271596105	4	CYP27A1
1.78392731479321E-29	0.629900853515081	0.665	0.672	2.98950539413046E-25	4	LMAN2
3.00891582420067E-07	0.616042212806392	0.287	0.25	0.00504234113819549	4	F13B
7.25352114614656E-11	0.6108445569969	0.349	0.297	1.21554507367124E-06	4	DEPRECATED-ENSG00000115738
1.97637762663667E-06	0.586325261028998	0.496	0.517	0.0331201362671774	4	NDUFS2
5.27509947009504E-07	0.585156532764684	0.507	0.516	0.00884001169198527	4	MYDGF
4.85484314838775E-13	0.577956590612701	0.332	0.27	8.1357461480682E-09	4	ID2
2.23392343778419E-09	0.57333947318868	0.586	0.617	3.74360889703874E-05	4	AADAC
3.8002840860467E-07	0.546424006514875	0.491	0.493	0.00636851607139706	4	PRG4
2.14103715647088E-104	0.54534200237188	0.891	0.864	3.5879500668139E-100	4	APOB
6.04257348166257E-08	0.545227844283521	0.338	0.301	0.00101261446405701	4	EBNA1BP2
8.35433499918026E-70	0.542577979089057	0.665	0.471	1.40001945916263E-65	4	SERPINC1
2.96156958451972E-100	0.52235078693835	0.74	0.506	4.96299830973815E-96	4	ITIH3
4.96022198926061E-20	0.52159330359767	0.649	0.652	8.31234000960293E-16	4	TMEM176A
8.06823167397245E-11	0.517793947471894	0.577	0.58	1.3520742639243E-06	4	AOX1
6.37571237469325E-40	0.510023495094085	0.657	0.568	1.0684418797511E-35	4	F2
5.20819069206308E-09	0.50789133307002	0.483	0.461	8.72788596175931E-05	4	ASGR1
1.88292278523115E-90	0.499361964037375	0.48	0.231	3.15540200349036E-86	4	APCS
5.7891383492425E-09	0.47619990534078	0.56	0.583	9.70143804566059E-05	4	F11R
4.26719779279083E-17	0.47417991424881	0.668	0.701	7.15097006115887E-13	4	NUCB1
8.01905246671155E-53	0.469848060533235	0.802	0.834	1.34383281237152E-48	4	C4BPA
2.57602984730995E-07	0.465416990712856	0.357	0.323	0.00431691081812201	4	ORMDL3
1.4339438511194E-73	0.460203448465759	0.721	0.557	2.40300310570589E-69	4	C1R
4.85055915068303E-14	0.437606132044878	0.258	0.186	8.12856702471462E-10	4	RDH16
3.44681979632452E-11	0.43460462814111	0.692	0.705	5.77618061468063E-07	4	LMNA
5.73398124520413E-12	0.432680883644125	0.674	0.713	9.60900577071309E-08	4	HSP90AB1
5.11974509977211E-41	0.424744457209063	0.771	0.736	8.5796688381981E-37	4	GPX2

1.27336972897222E-22	0.414464702803732	0.732	0.771	2.13391299181164E-18	4	ST6GAL1
1.16042705867376E-38	0.388603546300711	0.871	0.902	1.94464366492549E-34	4	B2M
6.57066851715365E-57	0.388380788093764	0.926	0.949	1.10111263010461E-52	4	ALDOB
9.88707363159295E-07	0.383316631735074	0.193	0.257	0.0165687579918235	4	RASSF4
2.55969290354993E-40	0.36712843990277	0.846	0.872	4.28953336776897E-36	4	TMEM176B
7.70523952058594E-08	0.352068156426424	0.252	0.335	0.00129124403885979	4	PLEC
1.02176593562098E-22	0.337986843494481	0.414	0.306	1.71227535491363E-18	4	TAT
7.31692698817635E-13	0.335080848757993	0.297	0.224	1.22617062467859E-08	4	TFR2
2.92114150101931E-29	0.334843614882713	0.731	0.714	4.89524892740815E-25	4	PLG
3.17524541392951E-07	0.328614534582255	0.199	0.266	0.00532107626466308	4	FN3K
9.13541649546489E-23	0.327451338920592	0.762	0.674	1.53091309631001E-18	4	NQO1
2.03527622773687E-11	0.30799770693939	0.678	0.7	3.41071590244144E-07	4	ALDH2
8.29859259535607E-17	0.299466166720867	0.836	0.839	1.39067814712977E-12	4	PTGR1
7.59767478244745E-07	0.269416268110534	0.224	0.292	0.0127321834004254	4	SURF1
2.17793436125463E-07	0.26583008264236	0.205	0.275	0.00364978240259051	4	EPS8L3
1.34645113206736E-06	0.257280388442432	0.228	0.295	0.0225638280711847	4	CHMP4B
1.95586285152298E-06	0.256420948930341	0.217	0.284	0.032776349665822	4	KIAA1671
0	5.27702875208288	0.331	0.024	0	5	DEPRECATED-ENSG00000117399
0	4.96208687841774	0.392	0.034	0	5	CDC20
0	4.77744588642569	0.405	0.04	0	5	CCNB2
0	4.59378256364451	0.454	0.064	0	5	CCNB1
0	4.55045360083941	0.349	0.033	0	5	KIF20A
0	4.52031881330754	0.458	0.051	0	5	NEK2
0	4.50578716658692	0.44	0.046	0	5	PLK1
0	4.44657415775119	0.658	0.097	0	5	PTTG1
0	4.38045923028032	0.298	0.031	0	5	PRR11
0	4.25938590770085	0.39	0.045	0	5	CDCA8
0	4.20520032042377	0.378	0.042	0	5	DLGAP5
0	4.18865554222306	0.753	0.161	0	5	HIST1H1B
0	4.18304390306852	0.292	0.032	0	5	CENPA
0	4.13999604262865	0.356	0.053	0	5	AURKA
0	4.12968117725185	0.324	0.037	0	5	CDCA3
0	4.04888910985388	0.341	0.042	0	5	KIF2C
0	4.04271123747887	0.644	0.098	0	5	TPX2
0	4.00533136406789	0.411	0.049	0	5	BUB1
0	3.98629168568146	0.32	0.038	0	5	KIF4A
0	3.95219261282511	0.658	0.134	0	5	CENPF
0	3.90851140867499	0.301	0.036	0	5	GTSE1
0	3.82264040689462	0.508	0.073	0	5	BIRC5
0	3.81502887447255	0.68	0.127	0	5	TOP2A
0	3.79934758934076	0.32	0.051	0	5	ARHGAP11A
0	3.76294046810191	0.514	0.069	0	5	ANLN
0	3.71997525659076	0.519	0.075	0	5	KIFC1
0	3.71810177965578	0.517	0.091	0	5	HMMR
0	3.71546223351346	0.384	0.074	0	5	CCNA2
0	3.70686478991621	0.443	0.07	0	5	FOXM1
0	3.64138110449716	0.396	0.075	0	5	MAD2L1
8.47045533788023E-277	3.62223418287302	0.295	0.051	1.41947890552197E-272	5	PBK
0	3.60695003986334	0.418	0.093	0	5	CDKN3
0	3.56478513149017	0.39	0.077	0	5	HIST1H2AH
3.40190095611006E-301	3.5548305585139	0.262	0.036	5.70090562224924E-297	5	AURKB
7.39080181893691E-262	3.55430611574433	0.294	0.053	1.23855056881745E-257	5	CDKN2D
0	3.54768665249982	0.314	0.044	0	5	KIF14
1.67512334076549E-290	3.54483961822467	0.32	0.058	2.80717169445481E-286	5	KIF23
0	3.53890289200658	0.499	0.065	0	5	TACC3
0	3.53296823384389	0.514	0.083	0	5	SPC24
0	3.53187352448347	0.337	0.056	0	5	NUF2
0	3.50616146665203	0.383	0.075	0	5	UBE2C
8.03741880383541E-225	3.48282568039111	0.271	0.052	1.34691064314674E-220	5	CKAP2
0	3.47868987643495	0.519	0.111	0	5	CDK1

3.34226926980147E-285	3.47390566500913	0.295	0.049	5.6009748423333E-281	5	SKA1
0	3.46869014001638	0.422	0.075	0	5	PRC1
9.29778847349465E-264	3.45570053044681	0.254	0.039	1.55812339238823E-259	5	OIP5
0	3.44187013396537	0.437	0.085	0	5	UBE2S
2.24177108477692E-266	3.40767687779977	0.277	0.046	3.75675998386917E-262	5	CENPE
6.62567959039535E-209	3.40569674168381	0.276	0.058	1.11033138575845E-204	5	GPSM2
0	3.38976049055221	0.304	0.045	0	5	CIT
3.13750241850272E-294	3.38160792942076	0.344	0.066	5.25782655292686E-290	5	DEPRECATED-ENSG00000188486
0	3.37776318610759	0.306	0.045	0	5	HJURP
0	3.35620973570835	0.435	0.094	0	5	KNSTRN
0	3.34958685301923	0.349	0.058	0	5	NDC80
0	3.34863534494128	0.49	0.104	0	5	NCAPD2
3.62276444363367E-240	3.34495532995178	0.305	0.063	6.0710286546413E-236	5	DEPDC1
1.6288389893515E-284	3.3150885860702	0.437	0.115	2.72960837835524E-280	5	KPNA2
0	3.30355984239533	0.499	0.109	0	5	ECT2
0	3.29104735727578	0.817	0.319	0	5	HIST1H1C
0	3.26182107180481	0.462	0.106	0	5	CENPW
4.0685134462393E-298	3.23737324425093	0.328	0.059	6.81801483320782E-294	5	ASPM
0	3.19498661537655	0.481	0.124	0	5	HIST1H1E
0	3.19199265362222	0.52	0.097	0	5	MKI67
0	3.19005145294948	0.477	0.124	0	5	CKS2
0	3.1811847236042	0.394	0.075	0	5	FAM83D
9.42582576763623E-257	3.16894231916637	0.355	0.08	1.57957988214048E-252	5	H2AFX
0	3.11958868341263	0.627	0.164	0	5	NUSAP1
0	3.10015756311959	0.712	0.241	0	5	HMGB2
7.42126466625013E-173	3.09431064911198	0.257	0.059	1.2436555327702E-168	5	KIF11
0	3.01882026257498	0.535	0.155	0	5	NEURL1B
0	2.99575152606719	0.446	0.104	0	5	INCENP
0	2.95571984661668	0.437	0.101	0	5	SPAG5
2.51626744133807E-298	2.95005029340372	0.519	0.156	4.21676097819433E-294	5	HIST1H4C
6.38704906855977E-254	2.94928706285082	0.288	0.051	1.07034168290925E-249	5	ESPL1
1.81008783792424E-191	2.9341675271348	0.306	0.076	3.03334519879344E-187	5	CENPL
0	2.83264481523936	0.636	0.224	0	5	CDKN2C
2.19445206415619E-273	2.76389272120382	0.47	0.135	3.67746276911294E-269	5	KIF22
4.69587838907766E-299	2.76276291736588	0.393	0.084	7.86935300441634E-295	5	NCAPG
8.02920337983835E-138	2.74743313671156	0.258	0.07	1.34553390239331E-133	5	MND1
9.9177011419934E-162	2.66866676338516	0.345	0.108	1.66200835737525E-157	5	TUBA1B
2.63789550762226E-271	2.65652061639503	0.528	0.176	4.42058529167338E-267	5	CDC25B
0	2.61339709035015	0.399	0.063	0	5	IQGAP3
1.31328431123579E-266	2.60333160651545	0.525	0.176	2.20080184876894E-262	5	UBE2T
1.87107189342567E-179	2.57594806564372	0.285	0.068	3.13554227900273E-175	5	MXD3
0	2.57592635671437	0.692	0.293	0	5	H2AFZ
9.31745670788273E-222	2.52741480059109	0.442	0.14	1.56141939510699E-217	5	STMN1
8.96338428907863E-202	2.48566012884972	0.467	0.169	1.5020839391638E-197	5	TUBB4B
1.83815632701768E-298	2.47476834233815	0.458	0.112	3.08038237281623E-294	5	RRM2
1.09278344202269E-156	2.46258792755106	0.319	0.094	1.83128649214163E-152	5	MIS18A
0	2.44927878291358	0.694	0.28	0	5	LMNB1
6.62772846450932E-166	2.41544571579619	0.461	0.191	1.11067473608247E-161	5	CKAP5
2.67655136152558E-102	2.3975676130312	0.272	0.093	4.48536477164457E-98	5	MELK
1.43084731578524E-239	2.38989307122491	0.589	0.253	2.39781393179291E-235	5	RAD21
2.14760204165173E-177	2.37914550520349	0.507	0.223	3.59895150139996E-173	5	ARL6IP1
2.23480363084153E-255	2.37425216058407	0.592	0.231	3.74508392456424E-251	5	LMNB2
1.2426844957131E-170	2.37088715165749	0.453	0.178	2.08249067791601E-166	5	MZT1
9.03807474751597E-214	2.35712210292547	0.592	0.279	1.51460056618873E-209	5	ADGRE5
2.47042844263228E-146	2.348217585974	0.423	0.174	4.13994398416317E-142	5	HMGB3
4.31653649322725E-124	2.33003718083477	0.359	0.139	7.23365185535023E-120	5	RNF26
2.53622247872075E-198	2.31798475243947	0.504	0.199	4.25020162984024E-194	5	SMC4
2.43042981525165E-188	2.30486718217357	0.453	0.163	4.07291428439872E-184	5	DTYMK
1.40426914903626E-226	2.29222101200681	0.598	0.267	2.35327423995496E-222	5	TUBA1C
1.53075199575263E-141	2.25155556055079	0.403	0.157	2.56523419448225E-137	5	EMC9

1.80888426694737E-119	2.2403642624872	0.353	0.136	3.03132825455041E-115	5	NDE1
2.46884709036703E-245	2.24006005486511	0.632	0.28	4.13729395403706E-241	5	CKS1B
2.75586349233842E-121	2.22920989902785	0.319	0.111	4.61827604046072E-117	5	SMC2
1.89749490843247E-157	2.21658481372486	0.413	0.153	3.17982196755114E-153	5	PHF19
1.33069955854428E-211	2.19332459351325	0.538	0.209	2.2299863202085E-207	5	TK1
3.43368878068244E-305	2.15188034528198	0.474	0.117	5.75417565866764E-301	5	CENPI
8.11969094967296E-156	2.11763099265021	0.439	0.169	1.36069780934619E-151	5	ZWINT
3.63106935909757E-93	2.06981640319374	0.364	0.167	6.08494603197571E-89	5	SOGA1
1.6127365735042E-87	2.02042555407312	0.289	0.113	2.70262394987833E-83	5	JPT1
1.12116117248444E-114	1.98646082865751	0.315	0.11	1.87884189284943E-110	5	ASF1B
8.4100719814798E-117	1.97837841806591	0.389	0.163	1.40935986265638E-112	5	CENPN
1.74885488646366E-203	1.97295351900935	0.63	0.302	2.9307310187358E-199	5	TUBB
1.56817093666794E-83	1.9434231531986	0.281	0.11	2.62794085566814E-79	5	NDC1
4.14720601727104E-111	1.881426019665	0.314	0.112	6.94988784374281E-107	5	NAV2
1.5082426035868E-66	1.88005896155284	0.308	0.145	2.52751295509076E-62	5	SEPHS1
2.73226610267606E-63	1.87519785711749	0.34	0.179	4.57873153486454E-59	5	GAS2L3
1.40448995332962E-62	1.85742638021362	0.252	0.107	2.35364426378977E-58	5	FBLN1
1.85930287915074E-131	1.83890164652404	0.423	0.177	3.11581976488081E-127	5	RANGAP1
2.88908994935298E-189	1.79421685838879	0.648	0.331	4.84153693712573E-185	5	H1FX
2.70231307351107E-86	1.78755151897863	0.35	0.156	4.52853624858985E-82	5	ALDH3A1
6.18641813420562E-70	1.74176840525635	0.329	0.154	1.03671995093018E-65	5	PCLAF
6.63025186364088E-65	1.72043519180382	0.311	0.148	1.11109760730894E-60	5	SAP30
1.16963390078413E-83	1.71281462456576	0.386	0.186	1.96007249093405E-79	5	CENPU
1.80370273486608E-42	1.69925232661818	0.259	0.133	3.02264504308857E-38	5	DEPRECATED-ENSG00000022267
8.85762887784127E-77	1.67360129546907	0.427	0.247	1.48436144734864E-72	5	TMEM19
4.45457279760998E-125	1.66552200956916	0.501	0.235	7.46497309423481E-121	5	TYMS
1.30681906058863E-273	1.66292292512064	0.821	0.519	2.18996738173442E-269	5	CALM3
6.9252940629662E-49	1.62055817938696	0.259	0.125	1.16054077907187E-44	5	DARS2
8.05586034417433E-87	1.58614237949255	0.448	0.241	1.35000107647673E-82	5	ANP32E
2.52547347763E-42	1.56195762308142	0.278	0.149	4.23218845381235E-38	5	DEPRECATED-ENSG00000120802
3.95761551406774E-89	1.54183193439194	0.443	0.229	6.63217207847471E-85	5	NCAPG2
4.47289286090922E-54	1.54027022406938	0.363	0.2	7.49567385631168E-50	5	DCK
1.07595152945557E-107	1.48744741207489	0.554	0.327	1.80307957306165E-103	5	CDK16
1.09632665343985E-94	1.48438792706699	0.579	0.408	1.83722420583451E-90	5	LBR
5.48183254997316E-106	1.47580477545327	0.584	0.359	9.18645498724502E-102	5	KIF5B
3.56155879704233E-39	1.47308987586897	0.259	0.137	5.96846023208354E-35	5	UBALD2
1.74513525840866E-51	1.4688130107569	0.266	0.125	2.92449766604123E-47	5	BCL2L12
1.17194822313679E-178	1.46575514050071	0.743	0.503	1.96395083233263E-174	5	CALM2
8.86635866818454E-42	1.46475120349348	0.326	0.191	1.48582438561437E-37	5	FHL1
9.27247014517005E-66	1.44942214002805	0.368	0.194	1.5538805469276E-61	5	RUVBL2
8.59649045558343E-61	1.44216737641661	0.424	0.253	1.44059987054667E-56	5	GLA
1.55341404554952E-39	1.4406298341366	0.295	0.163	2.60321125753189E-35	5	WDR34
4.86943964348699E-50	1.4235241161728	0.333	0.183	8.16020695455549E-46	5	DDX39A
1.49057692652207E-31	1.42031064882963	0.275	0.161	2.49790881346568E-27	5	AGBL5
3.14355627352055E-100	1.39534422900912	0.52	0.291	5.26797160316574E-96	5	GGH
9.5539406477577E-31	1.39151377838966	0.26	0.15	1.60104937375123E-26	5	COQ2
2.843313649596E-58	1.39031136164967	0.407	0.238	4.76482501399298E-54	5	ILF2
5.81159752372024E-70	1.38919385588425	0.43	0.248	9.73907513025038E-66	5	MARCKS
2.48093332045832E-51	1.38796503607397	0.42	0.261	4.15754805842406E-47	5	RRM1
2.82951817329E-105	1.38151335468794	0.576	0.358	4.74170655479939E-101	5	PSMD10
1.56608546174463E-71	1.36544720302843	0.484	0.301	2.62444601679166E-67	5	NFIX
1.59188782417594E-65	1.35509190203034	0.358	0.177	2.66768561575404E-61	5	CENPM
1.80715258440545E-55	1.34127237129192	0.402	0.238	3.02842630094665E-51	5	MEPCE
3.81848102564817E-116	1.33994580442662	0.632	0.413	6.3990105027812E-112	5	TMPO
8.94916903407024E-32	1.30975277281567	0.275	0.159	1.49970174672949E-27	5	HOXA10
1.06088402777581E-42	1.29861290432778	0.265	0.135	1.7778294537467E-38	5	DDAH2
8.34644748948802E-51	1.2950352115322	0.445	0.294	1.3986976702884E-46	5	RBMX
1.46327958572774E-169	1.29139586959272	0.774	0.553	2.45216392976255E-165	5	NUCKS1
6.56312357130892E-27	1.28476599927034	0.275	0.17	1.09984824807995E-22	5	TSPAN7
5.70357552899687E-32	1.28468843522571	0.256	0.144	9.55805187149296E-28	5	THOC3

5.98086677193564E-46	1.28438696257349	0.356	0.21	1.00227365364097E-41	5	PLIN3
5.12648769047116E-63	1.27286235878945	0.437	0.255	8.59096807169156E-59	5	YWHAH
1.75102317405507E-33	1.27244837726005	0.26	0.144	2.93436463508148E-29	5	HMGN5
8.46870496903035E-47	1.26863515367746	0.349	0.201	1.41918557871011E-42	5	BUB3
1.02526260958137E-31	1.26532883426799	0.319	0.202	1.71813508113645E-27	5	CNIH4
2.931292307945E-33	1.25433653504157	0.323	0.199	4.91225964965423E-29	5	C12orf75
2.05287997069565E-46	1.25299972554986	0.384	0.231	3.44021625489176E-42	5	SAE1
6.22719605887061E-74	1.24242529039691	0.562	0.384	1.04355351554554E-69	5	CDKN1B
6.11625105757872E-53	1.24091861334992	0.417	0.245	1.02496135222904E-48	5	TCF19
4.18292844195003E-77	1.22806851333008	0.559	0.375	7.00975148301987E-73	5	TUBB2A
5.39055157371082E-42	1.22363181450784	0.358	0.216	9.03348632722459E-38	5	DBP
2.50472406536695E-81	1.22350627531307	0.588	0.387	4.19741658874193E-77	5	FLOT2
2.22502709904653E-42	1.21195563774068	0.388	0.245	3.72870041258217E-38	5	PPP2R5D
3.8664568845689E-38	1.20767612126225	0.333	0.199	6.47940844716056E-34	5	CBX5
1.39161450100074E-39	1.20638458032866	0.39	0.256	2.33206758077704E-35	5	PRRC2A
5.40811623797347E-31	1.20287913703067	0.303	0.188	9.06292119159595E-27	5	BRD8
3.84996597373136E-35	1.2014686007391	0.372	0.24	6.45177297877901E-31	5	TUBG1
1.6164447324517E-32	1.19685761537892	0.387	0.264	2.70883808264256E-28	5	DHFR
1.92890435736168E-23	1.1892619162077	0.278	0.178	3.23245792206671E-19	5	FEN1
3.00126443951934E-39	1.18801386407258	0.358	0.221	5.02951894774652E-35	5	KCTD12
9.63605252454961E-25	1.18676230864853	0.266	0.164	1.61480968206402E-20	5	OAS3
2.52394648028103E-35	1.18653374600337	0.341	0.213	4.22962951165495E-31	5	EIF4E
3.69847247712208E-25	1.17956283411102	0.282	0.178	6.19790017716119E-21	5	FDXR
5.35588284804425E-24	1.16425722704916	0.258	0.16	8.97538847675256E-20	5	PDE3B
4.57922836881628E-54	1.16399216392745	0.503	0.341	7.67387090046232E-50	5	LSM 4.00
4.1085750063092E-89	1.1554578831317	0.582	0.359	6.88514999557296E-85	5	C15orf48
1.00238550785572E-44	1.15478729568852	0.443	0.302	1.67979763406462E-40	5	DCAF7
1.11214145909216E-21	1.14844180463323	0.266	0.17	1.86372665714665E-17	5	GUCY1B1
1.40796479886654E-18	1.14800337976917	0.251	0.165	2.35946740994055E-14	5	LRRCC1
1.21328430850443E-50	1.14327280633927	0.475	0.313	2.03322184419172E-46	5	DEK
4.64763347683877E-179	1.1353380245647	0.868	0.694	7.78850418048641E-175	5	LMNA
3.49012231348888E-164	1.13418410613313	0.857	0.604	5.84874697294466E-160	5	MBNL3
1.31182988948505E-104	1.13002679085555	0.692	0.468	2.19836452879904E-100	5	HTATSF1
2.92563908636562E-87	1.12881700939627	0.639	0.438	4.9027859809315E-83	5	VBP1
1.04653330316226E-29	1.12659792161551	0.339	0.219	1.75378050943931E-25	5	EZH2
2.44186137279092E-28	1.12441901498666	0.299	0.187	4.09207128852303E-24	5	MED30
1.26794328976869E-27	1.12368361821705	0.275	0.165	2.12481936499437E-23	5	BCL7C
2.30336808998629E-106	1.12047058411594	0.692	0.504	3.85998424519902E-102	5	MORF4L2
1.19040961617155E-40	1.1159459372742	0.399	0.256	1.99488843478028E-36	5	PAF1
2.95299967901443E-51	1.10629301581409	0.493	0.328	4.94863686209239E-47	5	TMEM238
2.18638630297507E-40	1.09522885185772	0.465	0.334	3.66394616652562E-36	5	FZR1
2.36066941428947E-129	1.09141772649476	0.764	0.537	3.9560098044663E-125	5	ANXA2
5.24739061769068E-30	1.09124740040792	0.366	0.245	8.79357719712604E-26	5	KIF16B
8.75988450382103E-38	1.0904504806706	0.403	0.268	1.46798144515033E-33	5	YWHAQ
1.38639780514445E-27	1.08136241922104	0.342	0.23	2.32332544186107E-23	5	TNPO2
7.28290499538559E-28	1.0780919627114	0.342	0.226	1.22046921912672E-23	5	DEPRECATED-ENSG00000099797
3.82438660580693E-113	1.07649792767801	0.721	0.528	6.40890707401125E-109	5	HNRNPH2
1.04381649759816E-20	1.0760367565747	0.269	0.176	1.749227686675E-16	5	PAK4
5.09297277826892E-36	1.07548559553496	0.423	0.29	8.53480378182306E-32	5	SF3A2
8.19415663412075E-73	1.07283442971977	0.597	0.414	1.37317676874596E-68	5	HNRNPUL1
5.26402627807753E-33	1.06933787288111	0.37	0.244	8.82145523680233E-29	5	DCTN3
1.39974661012492E-60	1.0679485106753	0.519	0.347	2.34569536924733E-56	5	MAF 1.00
1.06886916054463E-24	1.06753873331918	0.349	0.248	1.79121093924069E-20	5	MORC4
2.21256515125784E-36	1.06558844986325	0.383	0.247	3.70781668047788E-32	5	PSPH
7.95455139164925E-110	1.06519728403588	0.699	0.494	1.33302372221258E-105	5	YWHAZ
2.10877012001878E-31	1.06502919885286	0.374	0.252	3.53387696712748E-27	5	TXNDC12
2.4594746910037E-31	1.0604011274499	0.27	0.153	4.12158768718399E-27	5	IER5
6.44336318668327E-39	1.06023076282503	0.424	0.283	1.07977880282438E-34	5	CLIC1
1.00540287095632E-35	1.0584753422596	0.38	0.249	1.68485413114859E-31	5	DEPRECATED-ENSG00000265241
2.23073880229366E-50	1.05836165102116	0.461	0.295	3.73827208488371E-46	5	MZT2B

1.83270848486254E-19	1.05602562467818	0.273	0.181	3.07125287893265E-15	5	MZT2A
9.58130829027659E-105	1.05444128512897	0.719	0.523	1.60563564328455E-100	5	NPC2
2.10313067574315E-20	1.05181722898248	0.299	0.203	3.52442638641036E-16	5	RFX5
7.83840113203621E-27	1.04954548825233	0.284	0.175	1.31355926170663E-22	5	TCOF1
1.88740303994733E-22	1.04144956787259	0.276	0.177	3.16291001434374E-18	5	COX20
4.55487337355602E-24	1.04064257866906	0.306	0.2	7.63305679940518E-20	5	GET3
6.86143475931976E-22	1.03936348577421	0.327	0.227	1.14983923696681E-17	5	NXT2
2.01756236225906E-18	1.03910127325879	0.273	0.186	3.38103100667374E-14	5	CTCF
2.74307554884625E-28	1.03694916470155	0.332	0.219	4.59684600475655E-24	5	POLR2A
5.41789938740521E-48	1.02634542008119	0.483	0.336	9.07931579341365E-44	5	RBM42
7.17366608280113E-58	1.01527736511663	0.506	0.334	1.20216296215581E-53	5	HDGF
7.432708504891E-19	1.01502226602232	0.251	0.164	1.24557329124963E-14	5	JADE2
9.77440026628621E-32	1.01124819271121	0.407	0.281	1.63799399662424E-27	5	RBBP7
8.66449347095275E-17	1.01121793770937	0.267	0.183	1.45199581586226E-12	5	LIG1
6.80013905620283E-36	1.01121197595811	0.424	0.294	1.13956730303847E-31	5	HNRNPUL2
4.78386870377603E-25	1.01104860441275	0.303	0.196	8.01680717378787E-21	5	CCT5
3.57055268392781E-34	1.00625686488823	0.444	0.317	5.98353218772623E-30	5	CDKN2A
9.84343268530469E-30	1.00249803480954	0.405	0.288	1.64956244940336E-25	5	NUP62
4.76052708937544E-37	1.00183039501595	0.424	0.287	7.97769129637536E-33	5	TRIOBP
3.10410321565806E-91	0.997084206818286	0.737	0.574	5.20185616879977E-87	5	ACTG1
1.90946360310767E-37	0.988732835184769	0.45	0.317	3.19987910608784E-33	5	LIX1L
9.37560320884383E-60	0.987801500220426	0.606	0.434	1.57116358573805E-55	5	SIVA1
4.46053195038069E-31	0.987179471121134	0.396	0.273	7.47495944244795E-27	5	FOXA1
1.24250752206336E-28	0.987172993768741	0.336	0.222	2.08219410547378E-24	5	CORO1C
2.90172648369996E-18	0.986908789297048	0.295	0.208	4.86271324138439E-14	5	RAVER1
3.27317780647426E-46	0.985093055982964	0.521	0.384	5.48519136808957E-42	5	NUCB2
8.70812482304503E-21	0.98393062458517	0.287	0.19	1.45930755784589E-16	5	SLC44A2
1.56716852261097E-36	0.981310155710378	0.455	0.329	2.62626101019147E-32	5	NNT
7.34802645568471E-40	0.972778937547659	0.482	0.346	1.23138227344364E-35	5	TMX4
5.63457295334405E-25	0.972177859748802	0.395	0.297	9.44241735521396E-21	5	SUN2
7.76750857115529E-26	0.970089634461819	0.36	0.249	1.3016790863542E-21	5	RAP2A
8.49996122334913E-16	0.96841008147131	0.258	0.177	1.42442350180885E-11	5	RALY
3.95164529433236E-79	0.966423898780359	0.648	0.455	6.62216718424217E-75	5	EZR
7.34741265176419E-17	0.96639983358732	0.308	0.223	1.23127941218264E-12	5	REPIN1
1.61922316228485E-25	0.961100001692332	0.363	0.249	2.71349417535695E-21	5	KLHDC3
3.88641426892613E-18	0.960815790151842	0.306	0.217	6.51285303186642E-14	5	USP1
5.64719436604351E-42	0.959623420449303	0.541	0.422	9.46356831861572E-38	5	LMO7
8.61206596844819E-20	0.953250631269894	0.289	0.194	1.44321001499255E-15	5	CSTF2
1.1567880012942E-51	0.950215011919709	0.718	0.597	1.93854533256883E-47	5	H1F0
4.59941037537683E-21	0.949435328265213	0.281	0.184	7.70769190705649E-17	5	FOPNL
8.6307466294223E-34	0.947791737516238	0.443	0.318	1.44634052015859E-29	5	PPIA
2.33297348661261E-88	0.942123227323519	0.7	0.514	3.90959696886541E-84	5	GUSB
2.70245886241448E-15	0.94154830358967	0.262	0.183	4.52878056163419E-11	5	KDM1A
2.75845049749706E-40	0.938344413392868	0.468	0.319	4.62261134370557E-36	5	CHP2
5.41670392608651E-26	0.93668942815485	0.388	0.282	9.07731243933577E-22	5	CYTH2
9.37566794701697E-45	0.936670990156881	0.545	0.396	1.5711744345611E-40	5	HADH
4.1619364526557E-17	0.935059485049007	0.302	0.216	6.97457310736042E-13	5	PPP1CC
8.85607676621538E-38	0.929651194615525	0.501	0.37	1.48410134448237E-33	5	FAM122B
4.66391201841493E-25	0.929165371655406	0.361	0.254	7.81578376045974E-21	5	AAMDC
6.79527888808309E-24	0.92915992427365	0.393	0.29	1.13875283606496E-19	5	HMG20B
5.87768127059365E-20	0.928291272848556	0.339	0.247	9.84981827326084E-16	5	ERH
2.76501333693504E-90	0.926456894111535	0.723	0.557	4.63360935003574E-86	5	EMD
1.14329516281779E-30	0.924716276279628	0.452	0.331	1.91593403385005E-26	5	DKC1
3.04251249349486E-22	0.92445552700446	0.362	0.261	5.09864243659868E-18	5	KHDRBS1
4.63456387427178E-18	0.923533786766621	0.368	0.278	7.76660214050465E-14	5	HAUS4
1.46821944154109E-13	0.920670309803629	0.308	0.235	2.46044214013456E-09	5	FGF13
6.6688165898954E-30	0.918449556837944	0.412	0.291	1.11756028413467E-25	5	PPM1G
2.77234636485948E-24	0.915542315353938	0.391	0.283	4.64589803823151E-20	5	GNA11
1.52888196750652E-13	0.913277885339676	0.282	0.204	2.56210040114742E-09	5	CCNQ
2.41290605332566E-17	0.911975622519402	0.272	0.183	4.04354796416315E-13	5	RALBP1

1.07290128388209E-12	0.910512099347831	0.265	0.192	1.79796797152961E-08	5	ADD3
9.39829674128062E-23	0.909623226177827	0.38	0.28	1.57496656790381E-18	5	MMGT1
3.12735925515796E-18	0.906023658926702	0.308	0.215	5.2408286397937E-14	5	YIF1B
5.72730998093963E-52	0.905277419356978	0.577	0.418	9.59782606605864E-48	5	PGD
2.39524958358036E-24	0.904278781651657	0.406	0.3	4.01395925216397E-20	5	CRKL
7.98786009325268E-24	0.900358185231471	0.379	0.273	1.33860559442728E-19	5	SAP30L
2.78475087060149E-50	0.900261390184882	0.52	0.359	4.66668550895397E-46	5	HNRNPAB
3.93034449159228E-22	0.899494271696159	0.341	0.238	6.58647129901034E-18	5	TMEM250
7.61656809012442E-15	0.898279669677013	0.311	0.232	1.27638448054305E-10	5	GMNN
2.19843462495095E-37	0.898117798350342	0.497	0.362	3.6841367444928E-33	5	TXNDC17
3.09944550360215E-32	0.897081238603599	0.462	0.334	5.19405077493649E-28	5	RNF157
2.61740177713309E-14	0.896675554084974	0.28	0.203	4.38624189811963E-10	5	PRMT2
3.74340248124969E-11	0.895717313900185	0.298	0.231	6.27319387807822E-07	5	FAM111A
7.99071150640198E-08	0.892093602541814	0.254	0.201	0.00133908343424284	5	PXMP4
1.13324931599467E-40	0.891142715042478	0.516	0.38	1.89909920374386E-36	5	NAA10
2.12807002452719E-124	0.890416099659647	0.831	0.647	3.56621974710266E-120	5	H3F3A
2.37407688786005E-44	0.889138143895029	0.543	0.393	3.97847804867587E-40	5	GCHFR
5.41925102840455E-64	0.888611721596678	0.625	0.467	9.08158087340035E-60	5	HNRNPA0
7.65336543857754E-73	0.888258992097533	0.678	0.507	1.28255098019682E-68	5	BTG1
1.71849809750096E-25	0.887997807217276	0.399	0.285	2.8798591117921E-21	5	PEA15
1.8572836514659E-22	0.886896178580714	0.359	0.253	3.11243594312656E-18	5	PGLS
3.47697302040977E-16	0.883487079173244	0.297	0.214	5.82671138760269E-12	5	AK6
1.56611520864259E-16	0.883234467759112	0.349	0.261	2.62449586664325E-12	5	TPM4
3.87437587285065E-15	0.882369643347606	0.301	0.22	6.49267908772313E-11	5	TEX261
1.03006469468291E-21	0.88217766254526	0.319	0.217	1.72618241534962E-17	5	TADA3
1.80724733192376E-29	0.881682309643708	0.332	0.211	3.02858507883783E-25	5	TFF3
7.98509057060524E-107	0.878696901139361	0.778	0.629	1.33814147782203E-102	5	HNRNPA2B1
2.87054868412139E-33	0.878535315365538	0.481	0.36	4.81046548485062E-29	5	EIF1B
1.0827227561827E-13	0.876970291110104	0.263	0.186	1.81442679481096E-09	5	TEX30
2.42400159412512E-13	0.876952911580758	0.254	0.18	4.06214187143487E-09	5	KRI1
4.86536080693968E-47	0.87637716512426	0.574	0.424	8.15337164026952E-43	5	VMA21
4.09998810766254E-29	0.875114211259856	0.449	0.325	6.87076007082089E-25	5	HPRT1
5.60134340567799E-22	0.872662740633354	0.346	0.246	9.38673127923518E-18	5	TGIF1
1.60137555284506E-17	0.871104617057284	0.346	0.254	2.68358515145775E-13	5	SH3GL1
8.82184527867517E-13	0.871062483529685	0.268	0.197	1.47836483180038E-08	5	HP1BP3
5.49312349049993E-22	0.870582249560806	0.405	0.305	9.20537634537979E-18	5	SMS
7.31396238083153E-29	0.869330386153568	0.436	0.32	1.22567381577975E-24	5	HDGFL2
5.22631260888848E-28	0.867270844625912	0.476	0.361	8.75825466997531E-24	5	PSMB9
6.86178692310246E-13	0.864857701265931	0.282	0.208	1.14989825257351E-08	5	RCOR1
3.54186226726979E-16	0.862737456546873	0.315	0.232	5.93545278749071E-12	5	DR1
7.63866069796106E-17	0.862402555274936	0.341	0.255	1.28008675976432E-12	5	WRNIP1
6.1574968469777E-13	0.860796005688903	0.26	0.186	1.03187332161652E-08	5	THOC7
3.7626811957641E-43	0.86017132650796	0.554	0.415	6.30550114786149E-39	5	ANAPC11
2.55034233757928E-67	0.859739067123919	0.66	0.49	4.27386368931537E-63	5	UBE2A
1.72210948852119E-15	0.859373283742738	0.253	0.171	2.88591108086381E-11	5	TPGS2
5.70367854649538E-160	0.858380234215272	0.934	0.734	9.55822450821697E-156	5	AKR1C3
2.13075360429365E-09	0.857968946887106	0.268	0.208	3.5707168900753E-05	5	PPP2CA
3.98623484654462E-23	0.857230996187693	0.415	0.313	6.68013235583947E-19	5	VAMP7
2.61714252997451E-17	0.856423993828341	0.322	0.232	4.38580745173129E-13	5	MTA2
6.28125939100692E-31	0.855515982879042	0.437	0.311	1.05261344874494E-26	5	SNRPB
1.84342042249181E-21	0.855147043679435	0.402	0.303	3.08920394401177E-17	5	CMC2
3.26900753448585E-20	0.853254242456408	0.382	0.286	5.47820282629139E-16	5	STK11
5.73442020724327E-18	0.850308259733873	0.336	0.245	9.60974138329827E-14	5	TIPRL
1.63393411911585E-32	0.849700581743061	0.43	0.302	2.73814679681434E-28	5	FLOT1
9.60220837405029E-26	0.848817537642884	0.443	0.331	1.60913807932335E-21	5	SSRP1
2.35999511135755E-165	0.847553734226524	0.984	0.876	3.95487980761298E-161	5	MT-CO2
2.18640999714986E-22	0.845959944359796	0.327	0.219	3.66398587322373E-18	5	CA2
3.63740709752079E-17	0.844699635397354	0.34	0.253	6.09556681402534E-13	5	RSRC1
1.91038584297659E-35	0.844359398523535	0.519	0.388	3.20142459566017E-31	5	IMPA2
7.2594166526538E-29	0.843009752463387	0.472	0.354	1.21653304265172E-24	5	SEPTIN11

1.76975456370837E-25	0.841998053392078	0.383	0.275	2.96575469786248E-21	5	RHEB
4.90584016187089E-180	0.841971264302757	0.97	0.732	8.22120694326323E-176	5	S100A10
2.59032838622435E-13	0.840305982052646	0.309	0.23	4.34087230963477E-09	5	COMMD4
2.71210591060596E-20	0.838977104933882	0.358	0.26	4.54494708499347E-16	5	CHMP3
2.28359604601971E-35	0.837951697189619	0.513	0.385	3.82685025391982E-31	5	SMIM15
1.42795435911253E-22	0.836796597463863	0.366	0.259	2.39296591500078E-18	5	ARPC5
8.70727255893677E-11	0.836614773667469	0.27	0.203	1.45916473542662E-06	5	C9orf64
1.16743211938127E-09	0.836540181682937	0.265	0.203	1.95638274565913E-05	5	RPA1
4.63221308555658E-29	0.836044901147743	0.469	0.354	7.76266268877572E-25	5	EIF4A3
3.0187045397101E-10	0.835612817522557	0.251	0.188	5.05874506764618E-06	5	SLC39A3
3.0470459435966E-24	0.835458740306007	0.261	0.158	5.10623959227918E-20	5	H3F3B
1.54500352810956E-18	0.834402307835148	0.289	0.198	2.589116912406E-14	5	TAF6
5.82713315672116E-59	0.83285036090132	0.56	0.358	9.76510974403331E-55	5	FXYD3
4.28836541404398E-37	0.832572515095253	0.494	0.362	7.1864427608549E-33	5	ISOC2
1.97944728605855E-45	0.831973686193741	0.582	0.435	3.31715776197691E-41	5	CD99L2
2.63303044499469E-69	0.831749367541964	0.673	0.458	4.4124324197221E-65	5	MEP1A
3.3834986207361E-55	0.830395590157671	0.653	0.52	5.67006698862955E-51	5	SDC2
2.07537444067463E-11	0.829243674872622	0.315	0.244	3.47791248768254E-07	5	UBR7
3.19016340820202E-25	0.829038176445531	0.32	0.211	5.34607583946494E-21	5	TAGLN2
1.96348275823183E-47	0.828203666566025	0.582	0.462	3.2904044062449E-43	5	MLF 2.00
7.48245122589316E-19	0.828194485164172	0.326	0.23	1.25390917643518E-14	5	YY1
2.60271830065034E-23	0.827754061007648	0.462	0.36	4.36163532822984E-19	5	BAX
2.6213397681001E-27	0.82721251914005	0.471	0.368	4.39284118338214E-23	5	CCNG1
1.58344506420945E-65	0.827102985821083	0.751	0.571	2.6535372386022E-61	5	TMEM106C
1.87274294881318E-39	0.826815495191296	0.547	0.421	3.13834263362112E-35	5	HNRNPA3
4.96130260385838E-19	0.826698738475674	0.352	0.257	8.31415090354588E-15	5	FAM32A
5.63526515763045E-33	0.824972260156453	0.498	0.385	9.44357735115711E-29	5	HEXB
1.52026165438328E-12	0.82368207819864	0.31	0.236	2.5476544804155E-08	5	PIN1
1.88303313535046E-17	0.823635347719985	0.349	0.26	3.15558692822029E-13	5	KHSRP
1.13322897231508E-18	0.821372887601667	0.349	0.259	1.8990651118056E-14	5	HCFC1
1.94130838060011E-13	0.821007621444024	0.284	0.208	3.25324458420966E-09	5	MBD3
8.36747508442612E-14	0.820924284207975	0.288	0.211	1.40222147464813E-09	5	ACTL6A
1.21124961413557E-12	0.819816787912368	0.273	0.199	2.02981210336839E-08	5	SHKBP1
7.54134890239043E-18	0.819446744124465	0.324	0.232	1.26377924906259E-13	5	DEPRECATED-ENSG00000171234
4.35293376028442E-12	0.815178490706011	0.268	0.196	7.29464639548462E-08	5	CBX3
5.44819762804096E-10	0.812990504088946	0.291	0.224	9.13008958507104E-06	5	CCHCR1
2.12153080481329E-31	0.811364101878771	0.516	0.396	3.55526132270611E-27	5	MAPK1
6.55370958555176E-126	0.811134055368821	0.876	0.605	1.09827065234676E-121	5	MPP1
1.44493657709363E-13	0.809512598606971	0.276	0.2	2.42142471589351E-09	5	VEGFB
2.62551041108225E-12	0.8094156140086	0.298	0.223	4.39983034689163E-08	5	KCTD5
1.09007179042559E-23	0.808868535952003	0.445	0.342	1.8267423063952E-19	5	FAM20B
1.27054655766785E-14	0.808787256281541	0.355	0.274	2.12918192133978E-10	5	PALLD
1.37130013137822E-31	0.807488292314308	0.524	0.397	2.29802476016362E-27	5	SRXN1
1.98412549791485E-70	0.807333121612884	0.728	0.576	3.3249975094057E-66	5	SMIM14
3.0139646988567E-13	0.804907541518874	0.326	0.248	5.05080204234406E-09	5	FADS1
7.45504598460285E-13	0.803143118672194	0.358	0.282	1.24931660609974E-08	5	NCAPH2
1.07436898423699E-18	0.802185735603701	0.368	0.28	1.80042754378435E-14	5	DEPRECATED-ENSG00000049860
1.40238126188392E-21	0.802126510412906	0.411	0.307	2.35011051866507E-17	5	GNB1
1.73067078962574E-14	0.801816038243203	0.302	0.222	2.90025810925482E-10	5	LMO4
2.76654831896842E-39	0.801721500066155	0.53	0.406	4.63618167292727E-35	5	MGAT2
8.61439128113468E-32	0.800031665171578	0.491	0.373	1.44359969089255E-27	5	SDCBP
1.35075898210744E-18	0.799799197908261	0.339	0.246	2.26360190221566E-14	5	IRF2BPL
2.47292017839279E-13	0.798107912460523	0.271	0.194	4.14411963495063E-09	5	CLIC4
1.27118563936233E-27	0.798035548722087	0.496	0.378	2.1302528944434E-23	5	RNF187
1.90553833172713E-19	0.79759786654353	0.411	0.321	3.19330113630833E-15	5	ATF6B
1.22821863655736E-30	0.79744408551328	0.466	0.345	2.05824879114282E-26	5	DEPRECATED-ENSG00000179010
1.08977866300429E-21	0.796223372618245	0.411	0.31	1.82625108346258E-17	5	NACC1
1.13759096719219E-19	0.794353134402014	0.368	0.27	1.90637494282067E-15	5	JPT2
7.43761297773987E-45	0.794099396499347	0.6	0.464	1.24639518280965E-40	5	STK24
4.07726662703251E-15	0.793187182251852	0.365	0.282	6.83268341358108E-11	5	CDC42SE2

1.37591458801288E-19	0.793150456445403	0.356	0.258	2.30575766659198E-15	5	COA6
9.81601917046151E-19	0.792459017297602	0.348	0.252	1.64496849258594E-14	5	ALKBH5
6.27023745328164E-18	0.789881883802291	0.352	0.261	1.05076639242094E-13	5	ELOC
9.56577044094255E-11	0.78464175726597	0.295	0.229	1.60303181049315E-06	5	HNRNPR
2.9380454088756E-87	0.784140332698356	0.771	0.58	4.92357649619373E-83	5	BCAP31
1.24897717638346E-28	0.783844301793651	0.486	0.371	2.09303595218341E-24	5	PRPS1
3.07536075168329E-30	0.783492009859073	0.349	0.22	5.15368954767085E-26	5	TFF1
4.4541741389359E-10	0.783393143245954	0.298	0.234	7.46430502202878E-06	5	CNOT8
3.9899918349818E-35	0.783168483851042	0.504	0.369	6.6864283170625E-31	5	PPP1CA
5.77488888435482E-14	0.78298545017049	0.349	0.272	9.67755879240181E-10	5	GBA
1.02265729724019E-14	0.782760809301174	0.386	0.307	1.71376909871511E-10	5	RAB1B
1.83091700048999E-32	0.782387853245067	0.49	0.367	3.06825070942113E-28	5	HMGN1
2.91494809103026E-17	0.781194026466987	0.369	0.282	4.88487001094851E-13	5	MED25
2.13394796572432E-26	0.780265428986198	0.497	0.382	3.57607000096081E-22	5	SMC1A
1.47622131904424E-17	0.780016941818613	0.317	0.225	2.47385168645434E-13	5	BANF1
1.15343692193718E-29	0.779098546675749	0.464	0.342	1.93292959378233E-25	5	CETN2
1.03249424761249E-59	0.778851535171248	0.644	0.476	1.73025386014902E-55	5	IRAK1
6.11764513787797E-21	0.778686725136108	0.377	0.28	1.02519497220559E-16	5	GSK3A
4.90718337753759E-30	0.778464533324344	0.5	0.379	8.2234579040775E-26	5	AGPAT1
1.44339556157285E-09	0.777592134305717	0.286	0.223	2.41884228208378E-05	5	AKIP1
7.7721295388448E-26	0.776570095840893	0.371	0.255	1.30245346811961E-21	5	AP2S1
6.66928321628767E-17	0.776056881662773	0.349	0.26	1.11763848138549E-12	5	DEPRECATED-ENSG00000137575
5.72720184645983E-43	0.775096602026369	0.604	0.466	9.59764485429738E-39	5	TRIR
8.88929619797925E-11	0.774909626940305	0.276	0.209	1.48966825685736E-06	5	PDLIM1
2.10316280799391E-35	0.77391228805392	0.578	0.467	3.5244802336362E-31	5	GSR
2.7505461999262E-10	0.772796506885774	0.251	0.187	4.60936532183633E-06	5	SSTR1
1.74788769926221E-30	0.771182669677021	0.524	0.433	2.92911020642361E-26	5	ZNRF2
3.87163207249018E-12	0.770768628106055	0.299	0.226	6.48808102707904E-08	5	DEPRECATED-ENSG00000078668
1.51195697705143E-13	0.770507347919809	0.346	0.267	2.53373750214279E-09	5	TAF15
4.31092784961242E-19	0.769750732420589	0.393	0.299	7.2242528903805E-15	5	NKAP
5.30682366312844E-99	0.769111436287511	0.87	0.651	8.89317509467064E-95	5	LYZ
2.61191550915016E-26	0.768075225831551	0.463	0.351	4.37704801023384E-22	5	SLC25A5
7.43924639352763E-10	0.766875111732717	0.292	0.228	1.24666891062736E-05	5	GLO1
3.7508713356981E-12	0.766836968194802	0.337	0.264	6.28571018436287E-08	5	ASRGL1
1.1032736655399E-13	0.765110396086719	0.349	0.269	1.84886600871176E-09	5	H2AFY
1.20240506258557E-17	0.764675335582221	0.311	0.219	2.0149904038809E-13	5	TIMM10
1.12690117764683E-12	0.762956567759691	0.295	0.22	1.88846099350055E-08	5	ZCCHC3
9.22243781952927E-42	0.762613919537123	0.55	0.413	1.54549612979672E-37	5	RNPEP
2.15228394584061E-11	0.761433253222598	0.293	0.225	3.60679743643969E-07	5	GMFB
2.65737273808429E-53	0.761325153436594	0.674	0.526	4.45322523448166E-49	5	MDK
2.05963944020974E-17	0.761093504648506	0.373	0.284	3.45154377390349E-13	5	RNF4
7.27658433415408E-24	0.760741478386416	0.461	0.358	1.21941000271754E-19	5	ADAM9
1.09323083834258E-32	0.759957284940378	0.547	0.425	1.8320362388945E-28	5	NENF
8.48741890691874E-30	0.759882759038343	0.538	0.438	1.42232166042144E-25	5	FMR1
1.66927158795962E-13	0.759641759115957	0.306	0.23	2.79736532710273E-09	5	BABAM1
5.42493699836663E-15	0.759133661790727	0.279	0.197	9.0911094218628E-11	5	AKR1C1
6.69936034935268E-10	0.757305315367346	0.323	0.263	1.12267880734452E-05	5	PCMT1
4.16192930020107E-14	0.757244285945145	0.399	0.321	6.97456112127696E-10	5	CXorf56
4.55235956407671E-22	0.75630931339326	0.373	0.267	7.62884415747974E-18	5	PSENEN
7.0935108505723E-15	0.754615434709749	0.427	0.366	1.18873054833891E-10	5	NECTIN3
7.50816653277464E-09	0.754550885542999	0.282	0.223	0.000125821854756237	5	ATP6V1H
2.74823700131301E-13	0.753622426873992	0.334	0.256	4.60549556680034E-09	5	HIPK1
1.81074928831726E-18	0.751454982753985	0.328	0.232	3.03445365736207E-14	5	COPRS
1.39550620385353E-13	0.75132241484798	0.311	0.232	2.33858929641774E-09	5	PSMG2
7.98513447863269E-12	0.750409426807254	0.309	0.235	1.33814883592927E-07	5	TSR2
1.5254564973439E-15	0.750382459061965	0.324	0.239	2.5563599982489E-11	5	AIP
3.2630303752415E-21	0.749829332486332	0.448	0.352	5.46818630282971E-17	5	KEAP1
3.08691965784249E-07	0.749389555282432	0.257	0.207	0.00517305996261244	5	SLC9A6
3.39844718889284E-10	0.749358242679885	0.305	0.242	5.69511779914662E-06	5	WDTC1
4.94617947536134E-09	0.748501605391984	0.253	0.193	8.28880756481053E-05	5	ZNF704

1.08056581248045E-61	0.747032535658653	0.682	0.524	1.81081218855474E-57	5	SAP18
1.47354245789205E-48	0.746934797381397	0.636	0.495	2.46936245093549E-44	5	PSMB8
9.18661749216509E-23	0.746687411307679	0.45	0.347	1.53949335933703E-18	5	VASP
2.26401122044128E-32	0.745972718567888	0.542	0.427	3.7940300032155E-28	5	SRP9
3.98514577131119E-10	0.745309156042708	0.263	0.198	6.67830728356328E-06	5	ERAL1
2.06644963104916E-11	0.745279155330382	0.303	0.233	3.46295629171218E-07	5	FIBP
2.30413131551235E-10	0.74316349850539	0.276	0.212	3.8612632585356E-06	5	GPD1L
4.26081319120613E-30	0.742148131777141	0.509	0.399	7.14027074582323E-26	5	SNRPC
2.31466779845675E-52	0.741733480870199	0.403	0.221	3.87892029665383E-48	5	AGR2
1.53413483646364E-18	0.741442344098813	0.418	0.332	2.57090315894576E-14	5	CMTM6
4.06179118534438E-10	0.739533086216095	0.294	0.232	6.8067496684001E-06	5	PPP2R5C
5.51616411975444E-12	0.739511708013836	0.267	0.195	9.24398783188449E-08	5	NAA38
1.33981368166548E-14	0.739379411990607	0.346	0.264	2.24525976773501E-10	5	TECR
1.39341060970025E-14	0.738911033736312	0.361	0.286	2.33507749973568E-10	5	NUMA1
8.4598422751013E-08	0.737404483501493	0.272	0.216	0.00141770036846148	5	HSPB11
3.29266169947706E-30	0.736697591101039	0.499	0.383	5.51784247598365E-26	5	ARF6
7.54972385744319E-25	0.736341985161684	0.469	0.357	1.26518272403033E-20	5	NID1
2.14715250297343E-26	0.735488245884833	0.475	0.364	3.59819816448287E-22	5	RAB13
1.98435468084212E-13	0.735049605530709	0.267	0.19	3.32538157415523E-09	5	CDC42EP5
3.46766031947587E-09	0.732773585969815	0.276	0.215	5.81110516337767E-05	5	ACOT7
5.38350193024578E-15	0.73179280380855	0.301	0.22	9.02167253470588E-11	5	WEE1
2.38446719761398E-19	0.729331378086921	0.352	0.254	3.99589012976151E-15	5	SET
9.68698667354399E-41	0.727813233981227	0.636	0.531	1.6233452267525E-36	5	EIF1
1.5579634623663E-13	0.726746271582734	0.363	0.283	2.61083517023344E-09	5	SLC25A11
4.89981878535006E-16	0.726667420860461	0.358	0.271	8.21111632048964E-12	5	CLTA
1.09515145475451E-10	0.726072701617441	0.379	0.312	1.83525480787761E-06	5	DNMT1
5.54629217232109E-12	0.726050095874682	0.271	0.2	9.29447642237568E-08	5	GTF2F1
6.3162836033633E-09	0.726020305518042	0.314	0.258	0.000105848280625162	5	KDM3B
9.13713032127165E-12	0.725993860199669	0.286	0.215	1.5312002992387E-07	5	ICK
8.50799175469434E-26	0.725773431591145	0.485	0.375	1.42576925825168E-21	5	RAB8A
3.90475553951826E-09	0.7254362962919	0.303	0.242	6.5435893331247E-05	5	CCDC117
4.91987811275527E-13	0.725317077305147	0.349	0.274	8.24473174135528E-09	5	PHACTR4
5.00923099170509E-33	0.725053032509347	0.578	0.458	8.39446929589939E-29	5	RTN3
1.50098310426907E-14	0.724260688133421	0.327	0.245	2.5153474861341E-10	5	CTNNB1
4.0039120757191E-11	0.72365448033486	0.328	0.258	6.70975585649006E-07	5	SUZ12
1.03270332785402E-12	0.723630843162284	0.333	0.255	1.73060423681777E-08	5	TMEM258
4.55953854982067E-14	0.723239371441826	0.286	0.206	7.64087470178948E-10	5	CDK5RAP2
2.09506751449587E-28	0.722401766770197	0.514	0.404	3.51091414079218E-24	5	ELAVL1
4.33061567038054E-31	0.722164627749427	0.544	0.437	7.25724574042371E-27	5	KRTCAP2
6.3621224546098E-20	0.722010379167987	0.449	0.358	1.06616448094351E-15	5	CCT6A
2.30524598667813E-53	0.721825203318498	0.651	0.458	3.86313122447521E-49	5	KLF5
1.09305512134739E-28	0.721589887885449	0.521	0.41	1.83174177235396E-24	5	SHARPIN
3.2628232734777E-48	0.721205647826196	0.652	0.5	5.46783924169394E-44	5	GPAA1
1.09325301020704E-12	0.720520491464665	0.257	0.184	1.83207339450495E-08	5	GTF2A1
1.22266552997002E-21	0.719764057067873	0.426	0.323	2.04894289512376E-17	5	CTSC
8.36151300525772E-22	0.719289575731226	0.432	0.329	1.40122234942109E-17	5	ACTR1B
5.68066953158487E-32	0.718493927554491	0.625	0.522	9.51966600102993E-28	5	SLPI
3.33743761752459E-14	0.718420926003271	0.328	0.243	5.59287795944771E-10	5	PGP
1.60955405376418E-10	0.718118664898023	0.3	0.232	2.69729068329802E-06	5	TM2D2
7.34765868145658E-43	0.717718717732595	0.608	0.486	1.23132064183849E-38	5	BSG
1.97010106846175E-19	0.7174420497409	0.42	0.322	3.3014953705282E-15	5	PKN1
3.58166788126979E-28	0.717371314727492	0.547	0.438	6.00215903543192E-24	5	STAG2
6.53935135119861E-21	0.717268025798865	0.421	0.321	1.09586449943386E-16	5	GFPT1
2.69480949059611E-11	0.717006547099877	0.347	0.279	4.51596174434096E-07	5	PPP1R7
2.33148447236996E-17	0.716800224725679	0.447	0.363	3.90710167879757E-13	5	VDAC3
1.13889837609764E-18	0.715391948793895	0.369	0.273	1.90856589866442E-14	5	NUDCD2
7.29443740844536E-17	0.715269556245658	0.39	0.302	1.22240182090727E-12	5	EIF4E2
2.22396611452119E-68	0.713909537685074	0.744	0.583	3.72692241471461E-64	5	PFN1
7.53840346715068E-09	0.712801006289366	0.336	0.276	0.000126328565302511	5	FUCA2
6.65452942268765E-17	0.712720174215966	0.308	0.218	1.115166040654E-12	5	CNN2

3.23922697423325E-14	0.711929218731973	0.32	0.237	5.42829656342008E-10	5	PYCARD
3.00351216193868E-94	0.711708871770024	0.828	0.548	5.03328568097684E-90	5	ADA
3.2371745357448E-11	0.711181038334919	0.298	0.228	5.42485708700114E-07	5	DAXX
6.12603853649734E-32	0.710603824012124	0.533	0.406	1.02660153794622E-27	5	TALDO1
8.32282057568482E-12	0.709971808000689	0.275	0.204	1.39473827207326E-07	5	CD9
1.41775903044243E-18	0.709017307424765	0.376	0.279	2.37588058321543E-14	5	PSMD8
3.42008877012074E-12	0.707883699955261	0.297	0.222	5.73138476096834E-08	5	PSMD2
7.03073420076308E-11	0.706277880717382	0.294	0.226	1.17821043736388E-06	5	PSMA4
1.91928816378431E-16	0.705974604288827	0.421	0.34	3.21634310486975E-12	5	TMCO3
2.14575431126322E-17	0.705436529132988	0.388	0.299	3.59585507481491E-13	5	NTPCR
5.66130578582761E-23	0.702726268344843	0.496	0.408	9.48721623588991E-19	5	MLEC
8.40905617375687E-10	0.701745174954437	0.332	0.267	1.40918963359818E-05	5	ARPC4
1.78550599627624E-09	0.70082150139767	0.351	0.292	2.99215094855972E-05	5	PPP1R10
2.00493043867439E-41	0.700801798545596	0.586	0.435	3.35986242913054E-37	5	EFHD2
1.1878237598607E-08	0.7006576198733	0.295	0.237	0.000199055505677455	5	RNF6
1.08861679007958E-12	0.700495821252824	0.303	0.225	1.82430401681536E-08	5	CCDC28A
9.73911287953543E-74	0.700218984791283	0.884	0.744	1.63208053635255E-69	5	MT-ND3
2.70577527086585E-12	0.699449060772584	0.301	0.225	4.534338198917E-08	5	SF3B4
2.33927058180396E-66	0.699218121555456	0.768	0.605	3.92014964098708E-62	5	BEX3
6.46086175071118E-21	0.697904885203659	0.47	0.373	1.08271121218418E-16	5	PIR
1.23375228240561E-10	0.696681996099475	0.342	0.274	2.06752207485532E-06	5	PLEKHJ1
6.52628941872177E-11	0.696623028830566	0.361	0.297	1.0936755807894E-06	5	CEP350
3.72522092784521E-15	0.696046314954698	0.405	0.324	6.24272523088301E-11	5	ZFP91
3.56596663553492E-18	0.695775482064753	0.396	0.303	5.97584688782942E-14	5	GRB2
1.42555003035674E-24	0.694404280294207	0.487	0.384	2.38893674087183E-20	5	GOT1
7.12015177151581E-17	0.694325952181222	0.304	0.212	1.19319503387062E-12	5	AP2M1
3.13349806913142E-31	0.693680168972294	0.56	0.447	5.25111606425043E-27	5	RNF5
4.70105202564309E-17	0.692059230034516	0.37	0.277	7.87802298457269E-13	5	ARPC1A
2.22735070889583E-15	0.690873612929453	0.342	0.256	3.73259431796763E-11	5	AURKAIP1
1.83117312823315E-16	0.690711297612337	0.352	0.26	3.06867992829312E-12	5	CENPB
1.41298977533842E-08	0.690541211113461	0.284	0.224	0.000236788826551212	5	PCNA
8.65388834160467E-08	0.689405252160406	0.274	0.219	0.00145021860828611	5	MVB12A
3.9731234893064E-14	0.68905613030042	0.276	0.195	6.65816034337967E-10	5	LY6E
5.75403362184742E-10	0.687705203263928	0.291	0.227	9.6426095434919E-06	5	AIMP2
9.24128032734606E-18	0.686906526402415	0.437	0.353	1.54865375725665E-13	5	SRSF3
2.1668296971827E-28	0.686855409764563	0.534	0.429	3.63117320653877E-24	5	PCYT2
1.18011897326593E-17	0.686836317318008	0.438	0.352	1.97764337539905E-13	5	TRAPPC4
1.23944320542415E-62	0.686524152436081	0.799	0.649	2.07705892364979E-58	5	MT-ND5
3.22662520276744E-28	0.685434161646233	0.524	0.401	5.40717851479768E-24	5	CDKN1A
3.47790489492772E-12	0.685433843098135	0.339	0.263	5.82827302291987E-08	5	UQCC2
4.78419936563622E-12	0.684960737643773	0.308	0.234	8.01736129693318E-08	5	COPS7A
1.65375865090512E-09	0.683555396548981	0.324	0.263	2.77136874718681E-05	5	HAX1
4.62168658276808E-08	0.683172260572725	0.282	0.226	0.000774502237540274	5	ATL3
4.30850088320464E-15	0.68309535345423	0.333	0.25	7.22018578007434E-11	5	SZRD1
2.24234968109155E-15	0.682563639054959	0.405	0.32	3.75772959557321E-11	5	AP2A1
2.82771848631554E-13	0.682528241962529	0.342	0.264	4.73869063936758E-09	5	PRELID1
3.37913579366303E-33	0.681757918588109	0.617	0.508	5.66275576302051E-29	5	UBB
1.67680243778432E-09	0.681003139914985	0.32	0.257	2.80998552523896E-05	5	BROX
3.78848615646155E-24	0.680136253978556	0.343	0.228	6.34874510099827E-20	5	ATF5
2.94222774181981E-07	0.679808083237074	0.282	0.229	0.00493058524974164	5	SEMA6A
1.79868577534505E-16	0.679710969055132	0.412	0.325	3.01423762232323E-12	5	DYNLT1
2.57154402792903E-23	0.679472122696799	0.454	0.34	4.30939348200346E-19	5	COPS6
2.82861791993744E-14	0.679208663874249	0.345	0.265	4.74019791023116E-10	5	TCP1
1.26928215034744E-17	0.678863373630227	0.371	0.274	2.12706302755224E-13	5	CLPP
2.63483928741581E-44	0.678187590510959	0.635	0.471	4.41546367785141E-40	5	LAGE3
5.74949994687187E-15	0.677951521204459	0.263	0.182	9.63501201096788E-11	5	RUVBL1
1.92516298750522E-07	0.677547763409064	0.26	0.208	0.00322618813446125	5	DUT
2.88131394252843E-36	0.67707159490494	0.595	0.486	4.82850590488914E-32	5	ENY2
6.42033743248991E-07	0.676976145698364	0.285	0.234	0.0107592014693666	5	C6orf62
6.90455457159177E-11	0.676012994430749	0.311	0.243	1.15706525510735E-06	5	NUP50

2.51495372097412E-21	0.675601425084205	0.473	0.376	4.21455944560843E-17	5	DYNLRB1
5.63746953211284E-48	0.674945756755195	0.695	0.572	9.44727144191469E-44	5	TRAM1
3.65465211859427E-11	0.67420858398535	0.367	0.3	6.12446602034027E-07	5	HACD2
6.09556971400137E-20	0.673219064540142	0.472	0.376	1.02149557267235E-15	5	ENSA
8.6429912106794E-19	0.672847692393752	0.383	0.289	1.44839246708565E-14	5	GLRX5
2.50939735918867E-09	0.672435129345723	0.314	0.254	4.20524809452837E-05	5	EIF3H
2.31479710485124E-26	0.67217190044616	0.542	0.432	3.8791369883097E-22	5	RAMP1
1.4766628809109E-16	0.67163651330894	0.427	0.343	2.47459165583048E-12	5	TMEM9
1.44112689710424E-15	0.671134618468027	0.285	0.199	2.41504045416729E-11	5	MAD2L2
1.99597079978018E-08	0.670721791630374	0.254	0.196	0.000334484786627162	5	UBL4A
4.115521519802E-30	0.670673641307172	0.525	0.407	6.8967909628842E-26	5	COX8A
1.0530370315275E-15	0.67024078058202	0.431	0.345	1.76467945743379E-11	5	EMP2
3.46135914999983E-16	0.669537211460515	0.35	0.262	5.80054566356972E-12	5	H2AFJ
1.56392387321887E-06	0.669029772017439	0.274	0.225	0.0262082362674017	5	ITPR3
2.07478358956265E-09	0.668761435546815	0.338	0.274	3.47692233938909E-05	5	LRP11
1.98842825917987E-14	0.668717585671707	0.298	0.216	3.33220807673363E-10	5	CARM1
1.33778173458499E-16	0.668088515281058	0.376	0.286	2.24185463081753E-12	5	PTBP1
1.11632980580176E-10	0.667652371174021	0.334	0.265	1.87074548856259E-06	5	ARPC1B
6.48681367612502E-20	0.666370248748427	0.454	0.362	1.08706023584503E-15	5	RHOA
1.45217944438177E-17	0.66632670933513	0.46	0.377	2.43356231289497E-13	5	MICOS10
2.13299182148629E-11	0.664766896781739	0.287	0.216	3.57446769444673E-07	5	MUS81
4.97066711819242E-13	0.664448298476796	0.315	0.235	8.32984395666685E-09	5	HINT2
1.81924141436194E-19	0.663637914920733	0.463	0.357	3.04868476218774E-15	5	MCM4
6.65748789186972E-16	0.663238409584515	0.391	0.3	1.11566182091953E-11	5	MT-ND6
1.1502353621105E-18	0.66268832170617	0.462	0.374	1.92756441982477E-14	5	HNRNPL
4.60860644657129E-69	0.661358195556378	0.761	0.573	7.72310268316417E-65	5	MAOA
2.11593230671694E-10	0.66133725685305	0.314	0.248	3.54587935959624E-06	5	CLDN12
9.00187211653053E-54	0.660923444832574	0.708	0.566	1.50853372928819E-49	5	ACTN4
5.13429376423239E-19	0.660255789141493	0.418	0.32	8.60404949010064E-15	5	NDUFS6
4.58238721357825E-13	0.66025267276728	0.251	0.177	7.67916449251443E-09	5	BRMS1
6.11085442776559E-18	0.659757430322607	0.431	0.337	1.02405698500496E-13	5	RABAC1
7.33379509039654E-27	0.659702347821108	0.538	0.419	1.22899738124865E-22	5	EHF
1.99413487836174E-14	0.659177753144865	0.36	0.28	3.3417712291586E-10	5	CLN3
1.47650297851188E-08	0.658665094973976	0.294	0.233	0.000247432369139022	5	CYREN
1.31861739834819E-09	0.657906210652915	0.338	0.276	2.2097390361519E-05	5	PID1
1.30817370169393E-14	0.657896272337752	0.301	0.215	2.19223748929869E-10	5	TSR3
5.57083843955499E-72	0.657570828641138	0.918	0.77	9.33561105700625E-68	5	MT-CYB
2.36757950662067E-11	0.656111040645587	0.319	0.248	3.96758973719492E-07	5	PAFAH1B2
5.5061901702688E-39	0.654814258096886	0.602	0.457	9.22727348733646E-35	5	ETHE1
7.09628755815723E-13	0.6547362125611	0.391	0.32	1.18919586899599E-08	5	DCTN1
6.02285638708922E-09	0.654650234709289	0.338	0.279	0.000100931027334841	5	RC3H2
1.17636343807165E-30	0.654516216001084	0.557	0.445	1.97134984952047E-26	5	ZNF706
3.03958616723391E-13	0.654203231932978	0.421	0.357	5.09373849905059E-09	5	DDX6
1.18618111706472E-22	0.653770460473801	0.493	0.402	1.98780231597706E-18	5	DAZAP2
2.02658793348338E-15	0.652884235177336	0.426	0.341	3.39615605893144E-11	5	ATP5PD
6.19861482638545E-11	0.652044456347089	0.376	0.308	1.03876387260567E-06	5	NDUFB1
7.84877951186355E-07	0.650885162712647	0.28	0.229	0.0131529847059809	5	SYPL1
2.33753881552915E-10	0.650451551081067	0.295	0.227	3.91724754706374E-06	5	ZCRB1
1.95390314858766E-12	0.649709208905734	0.371	0.291	3.2743508964032E-08	5	RNASEK
8.08614622238976E-09	0.649296382351312	0.346	0.287	0.000135507638394808	5	FBXL6
3.17821313408042E-42	0.649040411193262	0.655	0.494	5.32604957009197E-38	5	CMC4
2.74488094194318E-12	0.648948509631749	0.342	0.265	4.59987148250839E-08	5	ATF2
1.1686555105499E-11	0.648863985013708	0.408	0.336	1.95843290457953E-07	5	NUP58
5.47419858499145E-17	0.648849130022807	0.423	0.334	9.17366198872867E-13	5	CSTB
5.18678504743701E-12	0.648034057477119	0.363	0.285	8.69201438249495E-08	5	DEPRECATED-ENSG00000132646
5.48771299124001E-46	0.647755908070146	0.684	0.548	9.19630943072E-42	5	TPD52
2.34661694204343E-14	0.646740616133395	0.373	0.289	3.93246067147638E-10	5	FUS
3.81073933783657E-10	0.646674955677205	0.254	0.189	6.38603698234652E-06	5	RETREG2
4.56166771243879E-13	0.646120057969741	0.399	0.319	7.64444275250493E-09	5	MEA1
2.65776403500901E-55	0.646008810262465	0.793	0.655	4.45388096986809E-51	5	MT-ND2

7.74997824235876E-15	0.645675249068087	0.28	0.199	1.29874135385448E-10	5	C1orf198
3.16106058328651E-08	0.644634759226324	0.255	0.198	0.000529730532547153	5	MDC 1.00
3.16158346278561E-38	0.644312747005992	0.633	0.507	5.29818156693613E-34	5	AIFM1
1.26102326025834E-10	0.644070638048255	0.283	0.216	2.11322277954093E-06	5	SNRPD2
4.19094551320734E-12	0.64288934359862	0.31	0.234	7.02318649103286E-08	5	NSF
1.68833209766302E-101	0.642667128580614	0.943	0.779	2.82930692926369E-97	5	UGDH
6.37396840275088E-10	0.642070420076664	0.342	0.281	1.06814962493299E-05	5	SMARCD2
3.34871134793233E-07	0.640990368918911	0.313	0.262	0.005611770476865	5	MAEA
1.02983396681467E-17	0.640575230675964	0.422	0.337	1.72579576158802E-13	5	ATP5F1B
2.78167618714755E-08	0.640530264633737	0.292	0.233	0.000466153295442186	5	MAPK3
4.51260482952113E-80	0.640164659773964	0.822	0.657	7.56222317331151E-76	5	CFL1
3.4676206294246E-10	0.639121856533935	0.323	0.257	5.81103865078975E-06	5	YWHAB
4.57041941110381E-12	0.638447731992095	0.328	0.253	7.65910884912777E-08	5	TLN1
6.99163227089172E-54	0.636952341405497	0.746	0.604	1.17165773595603E-49	5	TMEM14A
1.75640821721545E-07	0.636641508540983	0.282	0.228	0.00294338889040964	5	ELP5
1.99852255007284E-11	0.634519057213379	0.39	0.32	3.34912408941207E-07	5	CRAT
2.07771935653876E-11	0.634475570273815	0.384	0.318	3.48184209768766E-07	5	MECP2
9.7790077775363E-12	0.634106084820783	0.265	0.193	1.63876612335953E-07	5	TUBA4A
1.26441617853746E-11	0.634011904879665	0.32	0.245	2.11890863199308E-07	5	FBL
1.4601997291849E-13	0.633142436054624	0.447	0.372	2.44700270616805E-09	5	TMEM38B
2.86867847727005E-08	0.632846553233345	0.307	0.251	0.000480733139220914	5	DPY30
1.8658390975301E-47	0.632624293744571	0.67	0.531	3.12677315964093E-43	5	PRDX1
3.15703302722977E-07	0.632349848120066	0.251	0.199	0.00529055594703166	5	PDZD11
6.09025742482834E-38	0.632157929902549	0.628	0.493	1.02060533925273E-33	5	ENO1
3.20883475202527E-54	0.63160906287486	0.722	0.534	5.37736527744395E-50	5	G6PD
1.86018373288632E-96	0.631188218355202	0.913	0.709	3.1172958995709E-92	5	SPINK1
2.08693620858402E-18	0.631183607220627	0.44	0.347	3.4972876983451E-14	5	HIGD2A
3.44541755096725E-23	0.63115915408208	0.513	0.411	5.77383073191091E-19	5	MAP2K2
2.94043159080537E-07	0.631077823286648	0.282	0.229	0.00492757525987164	5	CNP
1.66118902260634E-12	0.630380825705177	0.374	0.3	2.7838205640837E-08	5	LSM 6.00
8.2993656748401E-15	0.630257692964237	0.41	0.324	1.3908076997897E-10	5	MCM7
2.61011615228503E-08	0.63017587747795	0.293	0.234	0.000437403264799926	5	DEPRECATED-ENSG00000205937
9.6502429264825E-08	0.63005221370071	0.279	0.223	0.00161718770961994	5	MAP4K4
1.29590025281231E-11	0.62973470043192	0.373	0.304	2.17166964366287E-07	5	CANT1
9.08144366462449E-28	0.628108908838701	0.582	0.487	1.52186832931777E-23	5	ACBD5
1.76860670448968E-12	0.627956303665186	0.32	0.248	2.96383111538381E-08	5	PLEKHB2
2.1618952132243E-08	0.627913071488901	0.251	0.194	0.000362290399832129	5	ABCB8
2.82885559523177E-10	0.626943920258983	0.383	0.32	4.7405962064894E-06	5	AGFG1
1.38746264408083E-09	0.626576909496066	0.33	0.27	2.32510989895066E-05	5	IGF2BP3
1.40921607873689E-07	0.625989112261536	0.316	0.265	0.00236156430474728	5	ATF4
5.01779980489431E-10	0.625677259793672	0.285	0.221	8.40882891304188E-06	5	HMGN4
5.67637448529176E-09	0.623807456494203	0.292	0.229	9.51246836245193E-05	5	C14orf119
3.2332582860551E-10	0.623477657293919	0.345	0.279	5.41829423577113E-06	5	NDUFA7
2.81583428580526E-10	0.62340467071859	0.338	0.27	4.71877509615245E-06	5	PNP
1.8929458318133E-09	0.622343897172107	0.351	0.284	3.17219862495273E-05	5	SLC27A3
5.80316072819888E-18	0.622260660104917	0.478	0.384	9.72493674831568E-14	5	SIPA1L2
3.82572708335701E-08	0.621887338166021	0.273	0.216	0.000641115344628967	5	VCP
3.29380378740805E-13	0.621102868865403	0.41	0.333	5.51975638693841E-09	5	VDAC1
1.76492873122816E-13	0.61968141188691	0.41	0.338	2.95766756779215E-09	5	TMEM50A
4.9809439396004E-17	0.619320123700862	0.441	0.353	8.34706585398236E-13	5	GOLGA7
1.06870330732532E-06	0.618240784688981	0.294	0.241	0.0179093300241578	5	CKB
1.23741371532669E-07	0.618234127953107	0.285	0.229	0.00207365790414446	5	CUL3
3.93371008733415E-20	0.618230999473647	0.515	0.426	6.59211136435457E-16	5	LACTB2
9.06088053886313E-09	0.616991027922602	0.31	0.249	0.000151842236070268	5	PSMA2
8.08735680711068E-36	0.616928999314702	0.677	0.543	1.35527925373561E-31	5	SPTSSA
4.9384627167424E-24	0.616689943652182	0.543	0.449	8.27587582071692E-20	5	UBE2Q1
6.92442617652613E-10	0.616621380410369	0.382	0.319	1.16039533866225E-05	5	TDP2
1.59109929673396E-38	0.616161399463867	0.655	0.546	2.66636420146677E-34	5	NDUFA2
1.86792340387559E-13	0.616013284258655	0.443	0.374	3.13026604021471E-09	5	UBE2R2
3.6286670195256E-09	0.61543345822952	0.28	0.219	6.080920191321E-05	5	C19orf53

2.27694843968947E-15	0.615274356416642	0.462	0.392	3.81571019523162E-11	5	HNRNPC
6.64177164549658E-10	0.614646634651705	0.309	0.242	1.11302809235232E-05	5	GADD45GIP1
7.61134426791803E-08	0.614590199927087	0.307	0.251	0.0012755090724177	5	TAB2
5.46214120742201E-58	0.614313417393878	0.778	0.636	9.1534562353978E-54	5	HSD17B11
2.86254139313601E-09	0.613646283710903	0.39	0.331	4.79704686661733E-05	5	ELOVL1
1.58165195189397E-22	0.613639920173588	0.563	0.472	2.65053234098391E-18	5	CIAO2A
3.25275872706234E-13	0.612348475821715	0.449	0.378	5.45097307481107E-09	5	PPM1B
6.8079039788683E-14	0.611340962325559	0.399	0.318	1.14086854877875E-09	5	ESD
6.67782614795259E-11	0.611331095223216	0.421	0.359	1.1190701058739E-06	5	SNRNP200
6.23897125012993E-17	0.611236985327012	0.46	0.375	1.04552680209677E-12	5	G3BP2
5.9850020101123E-10	0.610569381022034	0.318	0.25	1.00296663685462E-05	5	DDR1
6.08766199880432E-10	0.609880398166365	0.357	0.292	1.02017039775963E-05	5	GDI2
7.67739350305861E-102	0.609845538093582	0.907	0.798	1.28657760324256E-97	5	OAZ1
1.69337361296707E-10	0.608290932656258	0.333	0.265	2.83775550061021E-06	5	GSS
6.49214339004161E-16	0.608145486694023	0.461	0.384	1.08795338930317E-11	5	FAM91A1
2.44297843967781E-07	0.607825470337752	0.287	0.234	0.00409394326921208	5	RNF19B
1.36492615865051E-07	0.607592736309793	0.289	0.234	0.00228734325666652	5	ELF4
1.1776007958168E-87	0.606553696612111	0.982	0.904	1.97342341362979E-83	5	MT-ATP6
3.35526081868439E-16	0.606451488387869	0.454	0.369	5.62274607995129E-12	5	PMF1
2.22981643884912E-06	0.606164617887181	0.251	0.202	0.0373672638822335	5	MAGOH
2.43693758534138E-15	0.604170267513956	0.403	0.315	4.08382000551508E-11	5	RASSF3
5.3227000268971E-10	0.604041093653702	0.399	0.333	8.91978070507416E-06	5	SHROOM3
1.65363848483079E-29	0.603908480245635	0.567	0.429	2.77116737287943E-25	5	NBL1
1.25487243313005E-18	0.603399620984063	0.507	0.428	2.10291522343933E-14	5	FAM107B
5.6749682767516E-07	0.60328707549877	0.276	0.226	0.00951011183818033	5	SGPP1
3.18332283506005E-20	0.603083876416649	0.471	0.374	5.33461240699363E-16	5	ANXA5
4.79745957719138E-09	0.603044407255953	0.251	0.19	8.03958275945731E-05	5	NANS
3.7281169559908E-12	0.602737180032689	0.261	0.187	6.24757839484939E-08	5	AK1
6.88013570793866E-15	0.602016286269568	0.386	0.304	1.15297314193636E-10	5	YWHAE
2.12236910496806E-21	0.601996173694347	0.491	0.389	3.55666614610547E-17	5	TLE5
3.86174887419327E-09	0.601753289971088	0.347	0.29	6.47151876337309E-05	5	TMX2
1.16562246691056E-06	0.601496684347924	0.279	0.228	0.0195335013004872	5	ADSS
1.36847350220515E-19	0.600989164388352	0.465	0.374	2.29328789499539E-15	5	GNB2
1.54536041400418E-13	0.600944084986194	0.41	0.336	2.58971498178821E-09	5	APH1A
1.89194426186771E-09	0.600290388380603	0.325	0.263	3.17052019403791E-05	5	WIPF2
3.01944742992046E-08	0.598312309732956	0.333	0.276	0.00050599900030607	5	HLTF
3.32292132675773E-11	0.598039135389452	0.318	0.247	5.56855155938061E-07	5	CALM1
1.30749312181683E-60	0.595788644014884	0.777	0.602	2.19109697354065E-56	5	NDUFA1
2.04187896912185E-21	0.594836550161298	0.554	0.481	3.42178077645439E-17	5	LAMTOR4
1.83816561972659E-13	0.59435006288264	0.465	0.402	3.08039794553782E-09	5	IARS2
1.85623507502698E-10	0.594340133228881	0.417	0.359	3.11067873873021E-06	5	KLF3
6.62281041782037E-21	0.594272614259529	0.481	0.383	1.10985056981834E-16	5	UQCR10
5.01816959578772E-07	0.593823242554029	0.268	0.216	0.00840944860862106	5	TSN
1.11326101124579E-12	0.592700616752877	0.431	0.354	1.8656028026457E-08	5	CISD2
1.04642034660207E-06	0.592608103004393	0.349	0.3	0.0175359121683574	5	PAFAH1B1
6.18531088029919E-07	0.590741617379131	0.333	0.281	0.0103653439732054	5	BRI3BP
2.01882583432174E-35	0.59068793690847	0.636	0.518	3.38314833315637E-31	5	PLS3
1.7002183527963E-20	0.589909364131463	0.531	0.433	2.84922591561604E-16	5	PYCR2
8.96167463059021E-08	0.589826600201973	0.295	0.236	0.00150179743459431	5	PSMA3
3.85856732318681E-09	0.589204016156533	0.344	0.282	6.46618712019646E-05	5	UBP1
1.15981475094437E-14	0.58895565080422	0.44	0.357	1.94361755963257E-10	5	JTB
3.25442016642942E-08	0.588502407030903	0.313	0.255	0.000545375731490242	5	HSPA4L
8.50657385710006E-11	0.58843811579011	0.354	0.283	1.42553164697283E-06	5	SLC35B2
2.8210189242571E-15	0.587992432401764	0.473	0.394	4.72746351327004E-11	5	SYAP1
3.10888553520655E-12	0.586924371412131	0.36	0.281	5.20987037989914E-08	5	C7orf50
1.13404813044573E-06	0.585564564490603	0.32	0.269	0.0190043785700095	5	SKAP2
2.29159387212615E-10	0.584912626423003	0.326	0.257	3.840253010909E-06	5	DEPRECATED-ENSG00000213246
8.32954339256669E-07	0.584336543659071	0.291	0.237	0.0139586488172633	5	NDUFB4
1.28505079262674E-10	0.584196575474014	0.405	0.336	2.15348811828389E-06	5	DNPH1
1.45861728083674E-12	0.58382533900573	0.358	0.284	2.44435083922621E-08	5	RAC1

2.5187823071732E-09	0.583671217665601	0.376	0.316	4.22097539036084E-05	5	MAT2B
5.81069014691477E-11	0.583325063886829	0.343	0.27	9.73755454819978E-07	5	GABARAP
1.74923759349664E-07	0.583136254856924	0.314	0.258	0.00293137235918166	5	RNF167
6.30906676287786E-14	0.582830768116527	0.437	0.356	1.05727340812307E-09	5	TAP1
1.15402104323987E-06	0.582574801765003	0.275	0.223	0.0193390846426137	5	PGK 1.00
2.80218041564062E-08	0.582574031909	0.298	0.238	0.000469589394053055	5	KIF3B
4.50755300604638E-07	0.581568104728217	0.285	0.233	0.00755375732753252	5	UBTD2
7.40436236276817E-12	0.581559792155378	0.412	0.346	1.24082304475269E-07	5	CCNI
6.79930384501185E-27	0.581423685591395	0.554	0.448	1.13942733834709E-22	5	SF3B5
1.05056814447912E-06	0.581370331315081	0.279	0.227	0.0176054209651811	5	NAA50
8.14789433713743E-10	0.581169325306051	0.361	0.298	1.36542413301749E-05	5	VAPA
1.3262052880193E-14	0.581103143349707	0.481	0.412	2.22245482166275E-10	5	EFR3A
2.38478223982098E-10	0.580394061734419	0.407	0.342	3.99641807749199E-06	5	PYGO2
8.52664340887431E-07	0.580254824935935	0.289	0.238	0.0142889490245916	5	SNX12
1.64446759652968E-56	0.578871266348756	0.896	0.79	2.75579879826444E-52	5	MT-ND4L
7.88744446329371E-07	0.578860911719382	0.296	0.243	0.0132177794315876	5	TIMM50
1.77025227202538E-09	0.577627174418008	0.432	0.374	2.96658875746013E-05	5	MCTS1
6.95043971584743E-07	0.577497830255876	0.27	0.218	0.0116475468758171	5	LIMA1
1.24697214343213E-06	0.57717956694807	0.283	0.231	0.0208967591796357	5	ZNF358
3.99856381170109E-10	0.576599965630018	0.406	0.347	6.70079323564869E-06	5	TAF7
3.92980610732205E-09	0.576593614330778	0.339	0.279	6.58556907465028E-05	5	SNX6
4.99784935904364E-10	0.576528365723644	0.388	0.319	8.37539595588533E-06	5	NET1
4.05956309381155E-12	0.576435551027225	0.429	0.362	6.8030158326094E-08	5	NEDD8
4.18439299417876E-10	0.575944343094816	0.361	0.296	7.01220577964477E-06	5	POLR2K
2.44181763039805E-14	0.575566977798549	0.4	0.317	4.09199798502106E-10	5	STOML2
1.33358269108751E-06	0.574592935602085	0.339	0.288	0.0223481787372446	5	SLC39A1
7.02568795591223E-07	0.574266254542226	0.294	0.244	0.0117736478765177	5	AGTRAP
2.90340304479877E-08	0.574122371979792	0.396	0.34	0.000486552282247377	5	SEPTIN8
4.69802010322316E-32	0.573887551231796	0.639	0.519	7.87294208898138E-28	5	IFI30
8.30381157791531E-11	0.573282099772532	0.364	0.299	1.39155274422705E-06	5	FBXL3
2.90962530566923E-08	0.573263523826827	0.329	0.273	0.000487595008724049	5	KDELR1
7.37083373806187E-39	0.573144788887601	0.649	0.533	1.23520431782441E-34	5	ARF1
1.62059254486111E-08	0.572846459276282	0.323	0.261	0.000271578898667825	5	GNAI3
4.69300304959905E-97	0.572338490117469	0.958	0.863	7.86453451051808E-93	5	ACTB
5.29996132454368E-10	0.5713523247457	0.333	0.267	8.88167518767029E-06	5	POMP
1.31966995519592E-08	0.571166670826513	0.35	0.289	0.000221150291091731	5	ELOVL2
7.1630650310481E-26	0.571033788677938	0.633	0.538	1.20038643790304E-21	5	TSPAN6
1.23617450314671E-10	0.57054688007034	0.329	0.258	2.07158123237326E-06	5	DEPRECATED-ENSG00000117691
4.75579899916108E-78	0.570457848720816	0.994	0.952	7.96976796279414E-74	5	MT-CO1
6.48426034965702E-07	0.569613371130414	0.335	0.287	0.0108663234939552	5	LAPTM4A
1.04349934152476E-09	0.569551376599612	0.336	0.269	1.74869619652719E-05	5	PSMD7
2.10550906041044E-83	0.568974753928109	0.97	0.882	3.52841208343582E-79	5	MT-ND4
1.10664257960955E-17	0.567866399883983	0.459	0.367	1.85451163490969E-13	5	RAD23A
2.88791238052459E-09	0.566847168347311	0.373	0.314	4.8395635672831E-05	5	UBE2Z
1.89156495828899E-16	0.56682103023599	0.491	0.413	3.16988455710069E-12	5	EIF2AK1
1.25838974735691E-16	0.566299244414868	0.509	0.437	2.10880953862071E-12	5	PDZD8
2.9716971324658E-06	0.56582147779749	0.355	0.311	0.0497997005458618	5	TRIQK
9.75177262976897E-24	0.565712258884312	0.547	0.451	1.63420205729668E-19	5	CHCHD2
3.22630426267997E-26	0.565519823950138	0.599	0.501	5.4066406833991E-22	5	IDH3G
4.14770124476767E-07	0.565388016232722	0.324	0.274	0.00695071774598166	5	UBA1
2.47325345557603E-09	0.565325567850066	0.254	0.191	4.14467814085431E-05	5	TMEM8A
2.58410032290651E-14	0.565178856866332	0.421	0.334	4.33043532112673E-10	5	NDUFB11
7.87852746633581E-14	0.565128411434451	0.462	0.388	1.32028363280856E-09	5	AMFR
4.29575194215036E-08	0.564885299511478	0.403	0.355	0.000719882110465557	5	CUL4B
1.443348490908E-06	0.564841997856212	0.315	0.266	0.0241876340106362	5	ACTR10
5.47809078532096E-14	0.564793395208371	0.427	0.347	9.18018453804086E-10	5	CAPNS1
1.33144907255134E-07	0.564206104973862	0.252	0.197	0.00223124235578153	5	LSM14A
2.08296969363818E-12	0.564136049255233	0.393	0.321	3.49064061259886E-08	5	UBE2I
1.05743553529436E-16	0.564086726358094	0.49	0.406	1.7720504700463E-12	5	PMVK
1.42606081799152E-30	0.563274485458189	0.623	0.492	2.38979271879018E-26	5	LAPTM4B

7.07499036755256E-13	0.561520549273944	0.38	0.301	1.18562688579446E-08	5	NDUFAF3
1.89757491225252E-06	0.560806241174675	0.292	0.245	0.0317995603795278	5	ZC3H4
9.92545834131807E-07	0.559551093279642	0.332	0.284	0.0166330830883808	5	CAMK1
1.10627125039043E-12	0.559206493295986	0.459	0.398	1.85388936140429E-08	5	HNRNPK
1.03667058859083E-07	0.558794700255578	0.291	0.234	0.00173725257236051	5	MED29
1.72801044019268E-26	0.558791783786301	0.594	0.505	2.89579989567489E-22	5	RAB2A
3.89949946653153E-10	0.557516959615185	0.305	0.238	6.53478120601354E-06	5	GBE1
5.82201537157142E-39	0.557464268852241	0.837	0.761	9.75653335967938E-35	5	NUPR1
1.26801191667195E-19	0.55631491409875	0.534	0.455	2.12493436995885E-15	5	RAB5C
4.47964498686092E-08	0.556284363161031	0.289	0.234	0.000750698906898153	5	RNF44
4.08265833867542E-11	0.555794459157172	0.389	0.32	6.84171884395226E-07	5	PSMB5
5.03761638514717E-21	0.55509603201449	0.532	0.436	8.44203753822962E-17	5	CDC37
5.61267996901276E-09	0.554068362180486	0.357	0.294	9.40572909207159E-05	5	PSMB2
3.70258140377989E-28	0.553662638026547	0.68	0.602	6.20478591645433E-24	5	TXNRD1
2.63806022090614E-07	0.553355925921859	0.386	0.342	0.00442086131819451	5	CD47
2.28061338703415E-13	0.553004462624376	0.49	0.421	3.82185191399184E-09	5	RAB10
1.24122135790465E-09	0.552644713973326	0.334	0.27	2.08003875157661E-05	5	HGH1
7.85230615958803E-22	0.551980436504315	0.553	0.455	1.31588946622376E-17	5	SLC25A39
1.47283180461284E-07	0.551670705034568	0.342	0.29	0.00246817153817019	5	PRPSAP1
2.17787930629237E-10	0.550359079876364	0.417	0.357	3.64969014148476E-06	5	PUM2
2.41782842035434E-14	0.549969420091177	0.424	0.342	4.0517968668298E-10	5	GUCD1
3.89222502377013E-07	0.549964848079995	0.278	0.223	0.00652259069483399	5	CHERP
3.7150529199467E-07	0.549205344232929	0.26	0.206	0.00622568568324669	5	DEPRECATED-ENSG00000147123
2.07397220893844E-12	0.549186035210864	0.257	0.18	3.47556262773904E-08	5	DEPRECATED-ENSG00000167797
6.39806764768112E-16	0.548817099918246	0.471	0.396	1.0721881763984E-11	5	NONO
5.13967103845643E-33	0.548518914909148	0.638	0.515	8.61306072624528E-29	5	DAP
2.10401897796042E-20	0.548515508448656	0.511	0.412	3.52591500326608E-16	5	CAPN1
1.04907458433341E-06	0.547691862772353	0.316	0.262	0.0175803918842593	5	DCTD
2.07283640954277E-09	0.546556514564063	0.327	0.263	3.47365925511178E-05	5	MRFAP1L1
6.41700084692767E-07	0.546502057049064	0.314	0.266	0.0107536100192814	5	SKI
7.53168192383221E-12	0.543466146262061	0.47	0.409	1.2621592567958E-07	5	SLC44A1
7.60259974205861E-44	0.543361699661266	0.765	0.65	1.27404366477418E-39	5	MPC2
4.55838113345411E-07	0.543218305641568	0.32	0.268	0.0076389351034424	5	STRN4
1.45850877958113E-19	0.543022178534335	0.514	0.423	2.44416901282206E-15	5	EIF4H
1.95984443370698E-09	0.542930227680919	0.374	0.309	3.28430730200615E-05	5	GIPC1
5.35861385689782E-07	0.541940756547282	0.286	0.232	0.00897996510138937	5	VAMP3
2.1804695235851E-09	0.541825739100538	0.371	0.307	3.65403082762391E-05	5	CDIPT
4.72139704475087E-08	0.540829748203667	0.292	0.234	0.00079121171675935	5	MCRS1
4.6572945861088E-14	0.540173836342461	0.469	0.395	7.80469426740112E-10	5	PSMD3
2.81532102740607E-14	0.539291136072358	0.386	0.299	4.71791497772709E-10	5	IFI6
2.00622510201004E-07	0.538975758561733	0.335	0.281	0.00336203202594843	5	TTC1
9.16153592926308E-07	0.538842243526153	0.253	0.201	0.0153529019102591	5	SLC4A4
3.51403590690977E-16	0.538745233422497	0.507	0.433	5.8888213727994E-12	5	ACTN1
4.55372424663822E-12	0.538158286476804	0.474	0.408	7.63113109251632E-08	5	AKT1
3.42514677967437E-12	0.537147354565871	0.465	0.407	5.7398609733783E-08	5	COPS5
2.06415440320136E-06	0.537138054399249	0.276	0.225	0.0345910994888484	5	C6orf89
1.82434848228816E-08	0.536925230115742	0.337	0.278	0.00030572431866185	5	C9orf78
1.28003057716731E-12	0.536841282659619	0.346	0.268	2.14507524121697E-08	5	SUMO3
1.83341318524773E-09	0.536711910859161	0.304	0.236	3.07243381583814E-05	5	C8orf33
2.13188076147697E-09	0.536608770914177	0.355	0.291	3.57260578008311E-05	5	TSPO
9.77472295407197E-08	0.53645923847913	0.342	0.287	0.00163804807264338	5	DYNC1I2
1.31377826429975E-66	0.536206481401173	0.904	0.624	2.20162961531353E-62	5	TSC22D3
9.17564295581192E-10	0.535958328469163	0.44	0.379	1.53765424653496E-05	5	CRTAP
8.83332640953224E-33	0.535687752254832	0.719	0.619	1.48028883970941E-28	5	MT-ND1
4.267984879986E-11	0.535486376994905	0.471	0.41	7.15228906188053E-07	5	ERBIN
2.60084274944365E-06	0.535284462985882	0.342	0.295	0.0435849227951767	5	SRA1
3.86888926676759E-11	0.534884920548027	0.348	0.275	6.48348463324914E-07	5	SGTA
1.54774540736619E-11	0.534612426122284	0.364	0.287	2.59371175366426E-07	5	NME1
1.00413018535452E-06	0.53214223710866	0.319	0.271	0.016827213646171	5	IFT27
3.14141568735171E-29	0.530923609481417	0.623	0.514	5.264384408864E-25	5	NDUFB9

1.20538634945189E-06	0.530443735928564	0.296	0.246	0.0201998644441147	5	FAM168B
9.65072579733479E-08	0.530042893214465	0.33	0.272	0.00161726862911736	5	TJP3
1.37063489573346E-08	0.529597390267965	0.301	0.24	0.000229690995827013	5	LRP10
1.37197345997272E-92	0.529130213595605	0.913	0.681	2.29915312422228E-88	5	PIGR
1.8979366801287E-07	0.528626225951695	0.28	0.224	0.00318056228855967	5	NDUFS8
9.24807497642309E-44	0.528424523841205	0.738	0.612	1.54979240454898E-39	5	PRDX2
1.68352769437696E-25	0.527044600917122	0.655	0.542	2.82125571023691E-21	5	FAM50A
6.3395514514144E-08	0.526853948892055	0.33	0.276	0.00106238203222803	5	TNRC18
5.00901167779914E-07	0.526637594631112	0.295	0.239	0.00839410176965579	5	MARCKSL1
1.64428041219026E-11	0.526636556992169	0.414	0.345	2.75548511474843E-07	5	PPP3R1
1.87006894201116E-18	0.526088438005399	0.535	0.439	3.13386153302231E-14	5	CYSTM1
5.00139874108548E-07	0.526003594263289	0.27	0.216	0.00838134401031105	5	RRAGA
1.06015152994755E-07	0.525775581489847	0.284	0.225	0.0017766019338861	5	DEPRECATED-ENSG00000134049
7.46651136671402E-10	0.525500096327075	0.404	0.345	1.25123797483394E-05	5	SCRIB
2.7311033643377E-17	0.525312398115907	0.533	0.453	4.57678301795712E-13	5	STT3A
3.05115221582325E-77	0.525171641505016	0.938	0.859	5.1131208832766E-73	5	DEPRECATED-ENSG00000106538
6.42350955205123E-20	0.524671809893263	0.567	0.49	1.07645173073275E-15	5	SSR2
2.46049646056578E-15	0.5239674004371	0.46	0.381	4.12329996861613E-11	5	AAMP
1.80601604789643E-20	0.520512339418487	0.528	0.433	3.02652169306483E-16	5	LASP1
4.40786434188588E-13	0.519384714645748	0.453	0.382	7.38669906413236E-09	5	PSMC3
1.60234851764899E-07	0.519370850249675	0.315	0.26	0.00268521564587618	5	GSKIP
7.03831159743487E-08	0.519333090187985	0.312	0.254	0.00117948025749814	5	CSDE1
2.85383981800567E-27	0.519163715699606	0.632	0.545	4.78246476701391E-23	5	PUF60
9.56630817954598E-08	0.51894443889054	0.315	0.256	0.00160312192472831	5	MYH9
4.83468200894365E-07	0.517077598482133	0.352	0.297	0.00810196011058777	5	TFDP1
2.33948034655763E-07	0.516889039806891	0.333	0.277	0.00392050116476128	5	KIF1C
1.90598417114885E-07	0.51668236804705	0.307	0.25	0.00319404827401124	5	ATP5MF
1.76989444831082E-10	0.515174319342019	0.445	0.382	2.96598911647927E-06	5	FAM199X
1.94820561263567E-06	0.514834725542121	0.323	0.274	0.0326480296565486	5	FKBP1A
3.76511926112806E-08	0.514558512360502	0.389	0.329	0.000630958685779841	5	DEPRECATED-ENSG00000133142
1.18251204365713E-10	0.5142330825984	0.392	0.325	1.98165368276062E-06	5	CAV2
1.23753678694678E-09	0.513902040362323	0.294	0.226	2.07386414756541E-05	5	SFN
1.42474205372421E-06	0.512741009703688	0.317	0.267	0.0238758273363103	5	SF3B3
1.22139593000169E-08	0.512606594359613	0.369	0.308	0.000204681529949683	5	PYURF
3.81129978330265E-10	0.510095028979118	0.442	0.378	6.38697617685858E-06	5	TXNL4A
6.47746926522771E-09	0.510067475717321	0.33	0.269	0.000108549429946686	5	ATP6V0C
4.78220805153724E-12	0.50944646467811	0.44	0.371	8.01402425276611E-08	5	GCLM
1.92703277682698E-06	0.508202776038502	0.267	0.215	0.0322932152740666	5	TMOD3
1.09061412352491E-06	0.507956611919702	0.29	0.239	0.0182765114820304	5	SEPTIN2
1.94669603179878E-07	0.507925674228917	0.268	0.213	0.00326227321008839	5	YME1L1
3.06718799659573E-08	0.507349441093741	0.359	0.303	0.000513999364469513	5	BTBD2
4.63822712654628E-15	0.505466425367842	0.481	0.388	7.77274101866625E-11	5	ANXA13
4.57244221320366E-62	0.503896808860632	0.976	0.915	7.6624986608867E-58	5	MT-CO3
3.23137327382134E-09	0.503500913509233	0.393	0.331	5.41513533226981E-05	5	C1orf43
1.57495010252775E-09	0.503436813217193	0.277	0.212	2.639301381816E-05	5	SYF2
7.52599797431868E-07	0.502553328085492	0.261	0.21	0.0126120674053632	5	ATP6V1E1
9.63316361970643E-09	0.502207579408444	0.294	0.231	0.00016143255593904	5	PPP6R1
2.38783256705063E-11	0.5012156830713	0.426	0.359	4.00152981586344E-07	5	TBL1XR1
2.07896948537935E-07	0.501087356256562	0.36	0.309	0.00348393706359871	5	HMGN3
1.39554376078943E-23	0.50105895781729	0.597	0.49	2.33865223433093E-19	5	DHRS7
4.56193207628224E-07	0.500351936200196	0.344	0.294	0.00764488577343378	5	DAZAP1
2.19429467807257E-10	0.500279738617806	0.329	0.26	3.67719902151401E-06	5	PRCC
1.00824192651144E-13	0.500105535673801	0.554	0.498	1.68961182044787E-09	5	HBS1L
1.62345071274063E-30	0.499872423991456	0.658	0.551	2.72057870441074E-26	5	NDUFC2
4.66946722977858E-32	0.499356603845933	0.689	0.587	7.82509318366294E-28	5	TSTA3
8.57161252612081E-07	0.499343122808846	0.35	0.3	0.0143643082712733	5	COQ8A
6.50371912608264E-09	0.499054580402181	0.431	0.38	0.000108989325114893	5	CPNE1
1.20194939798444E-11	0.498230359121477	0.425	0.356	2.01422680114233E-07	5	TM9SF1
2.38039734044065E-09	0.497262733416045	0.435	0.38	3.98906986311044E-05	5	WDR83OS
1.53559576764602E-30	0.496859724967152	0.661	0.519	2.5733513874212E-26	5	CLDN4

6.47380990042425E-08	0.496470651200619	0.363	0.309	0.0010848810631131	5	DDB1
3.58145780392298E-17	0.495655438698193	0.545	0.459	6.00180698781413E-13	5	DSTN
3.23395290448812E-18	0.494643591929551	0.552	0.467	5.41945827734119E-14	5	PRDX4
6.86857280671611E-07	0.494569771365247	0.364	0.311	0.0115103543094949	5	SSX2IP
2.7526035858573E-07	0.494403509708751	0.362	0.306	0.00461281308917966	5	ZMAT2
3.59524150782663E-12	0.494251682315334	0.314	0.233	6.02490571881586E-08	5	S100P
1.7013468430316E-10	0.493695137112162	0.46	0.39	2.85111703955235E-06	5	TMEM64
3.3611541956681E-10	0.493689504041889	0.387	0.323	5.6326222011006E-06	5	HNRNPM
3.96118000369038E-11	0.493332591060776	0.341	0.265	6.63814545018434E-07	5	TRMT112
8.50763481602416E-16	0.493033674810258	0.507	0.438	1.42570944246933E-11	5	DEPRECATED-ENSG00000163463
1.88950872557384E-19	0.492868388100626	0.557	0.478	3.16643872231664E-15	5	DSP
8.98217508001085E-08	0.492456687202197	0.407	0.356	0.00150523289990822	5	CLTB
1.00589409308351E-06	0.492348952594713	0.278	0.226	0.0168567732118935	5	NDUFC1
1.01926045809504E-08	0.491030696558078	0.386	0.319	0.000170807667567567	5	DNAJC4
6.49977711360717E-14	0.490614789954468	0.524	0.446	1.08923264869829E-09	5	HACD3
4.32116847979143E-23	0.490573888775043	0.638	0.535	7.24141413843448E-19	5	GDI1
2.28035244999187E-06	0.490519564117704	0.276	0.224	0.0382141463569638	5	TAX1BP3
1.28455433119146E-17	0.487551573299528	0.588	0.525	2.15265614821065E-13	5	SUMF2
4.4960573300427E-17	0.486629509275564	0.513	0.425	7.53449287368556E-13	5	CHMP2A
1.12088512094594E-11	0.486544869656537	0.462	0.398	1.87837928568121E-07	5	KDELR2
2.4913726948221E-10	0.485824203506292	0.487	0.431	4.17504236198287E-06	5	FH
7.09858356640447E-14	0.485809592781187	0.468	0.392	1.18958063405806E-09	5	HSF1
1.32327802376099E-06	0.48530143837921	0.358	0.309	0.0221754931221867	5	RNPS1
7.47128830330252E-08	0.484211603496067	0.413	0.361	0.00125203849386744	5	UBL3
2.41197270886508E-09	0.48419697967751	0.423	0.364	4.04198386551609E-05	5	ENAH
7.42396973260212E-15	0.484089704962491	0.534	0.455	1.24410884778946E-10	5	STOM
2.53362241694744E-09	0.48371642066082	0.429	0.371	4.24584444632051E-05	5	COX16
4.21326814059003E-12	0.483194385405159	0.39	0.313	7.06059475000078E-08	5	PCBP1
1.0461168651651E-06	0.483051557304235	0.329	0.277	0.0175308264264368	5	CFDP1
9.60004588881359E-08	0.48304588180826	0.355	0.3	0.00160877569004738	5	ARCN1
1.53872917581726E-12	0.482615871677941	0.499	0.422	2.57860235283457E-08	5	ATP6V0D1
1.04647607451387E-15	0.482431425895937	0.522	0.448	1.75368460567034E-11	5	ILF3
1.43084234983085E-06	0.481707210189631	0.31	0.257	0.0239780560984653	5	RNF168
3.9669982330372E-14	0.480342981058071	0.455	0.377	6.64789563892375E-10	5	UFC1
7.38169865066405E-40	0.480021580918173	0.735	0.571	1.23702505987828E-35	5	SSR4
3.50654743938314E-12	0.479970551402566	0.399	0.321	5.87627219891826E-08	5	YBX1
7.52251847775982E-08	0.479507421534933	0.412	0.357	0.00126062364650299	5	AP1M1
3.63412529943359E-07	0.479490453333495	0.297	0.241	0.0060900671767908	5	STRAP
1.95248998696955E-14	0.479378431626058	0.493	0.416	3.27198272016356E-10	5	DBI
4.28503707854815E-11	0.478340488848108	0.473	0.411	7.18086513623098E-07	5	PI4K2B
6.4821849915554E-07	0.477240779406974	0.426	0.382	0.0108628456088485	5	ADD1
1.39655575756805E-25	0.475940201756032	0.629	0.524	2.34034813853254E-21	5	COX6C
2.27480074677822E-10	0.475904025464029	0.371	0.3	3.81211109145093E-06	5	FKBP8
6.79509243597607E-70	0.475900547362902	0.946	0.73	1.13872159042087E-65	5	ANXA4
1.15185562525131E-08	0.475805888039825	0.39	0.333	0.000193027965679614	5	PAK2
1.76317862540405E-07	0.475473053320827	0.421	0.373	0.00295473474045211	5	POGK
7.23197481649893E-10	0.474980992811426	0.424	0.363	1.21193433974889E-05	5	RPN1
1.48553219296244E-07	0.474646717566562	0.326	0.267	0.00248945484896646	5	POLDIP3
1.75249100994044E-06	0.472406763038463	0.386	0.338	0.029368244344582	5	PLS1
3.93289110956844E-22	0.47104610689284	0.645	0.557	6.5907389214148E-18	5	CNN3
1.34588801722619E-16	0.46995683084213	0.517	0.441	2.25543913926765E-12	5	SPTAN1
5.90508294463554E-08	0.46969490366061	0.385	0.33	0.000989573799862024	5	MSI2
1.36578014252326E-06	0.469252976355173	0.376	0.33	0.0228877436284049	5	LAP3
2.81944657931149E-07	0.468771821744187	0.304	0.245	0.0047248285776102	5	PAPOLA
4.13578131950952E-12	0.468323850308156	0.385	0.308	6.93074233523405E-08	5	EIF4A1
1.89072093748881E-26	0.467943392420258	0.681	0.574	3.16847014704375E-22	5	HNRNPF
1.62882010257787E-06	0.467932824608921	0.369	0.321	0.027295767279	5	PPP4C
4.99669352679236E-12	0.466428262388788	0.315	0.237	8.37345901219863E-08	5	CD177
7.87523826588967E-08	0.466128057285181	0.366	0.305	0.00131973242859779	5	POLR2I
5.91975412780969E-08	0.465206967443729	0.414	0.355	0.000992032396738348	5	ISOC1

3.2755298493469E-08	0.463843769292058	0.488	0.428	0.000548913292153554	5	SLBP
1.06993529426914E-09	0.463628975938139	0.44	0.381	1.79299756613623E-05	5	NARS
3.68326579656544E-08	0.463382663155691	0.43	0.375	0.000617241682188436	5	POLR2E
1.36686899250624E-08	0.463016079108271	0.446	0.387	0.000229059905764196	5	SYBU
3.5696648296818E-10	0.46296112230934	0.348	0.278	5.98204432158076E-06	5	CCDC124
6.19963986850229E-12	0.462953976146608	0.514	0.451	1.03893564916361E-07	5	CPQ
6.71086108880064E-13	0.461935493339056	0.457	0.39	1.12460610126121E-08	5	PTPA
3.72892983423155E-07	0.461321939291781	0.345	0.29	0.00624894061620523	5	CHAMP1
9.66520183552314E-16	0.461053068879052	0.532	0.457	1.61969452359697E-11	5	PERP
2.073793993605E-22	0.459265967401524	0.638	0.545	3.47526397448325E-18	5	IDH2
2.65479155229593E-09	0.459265051088946	0.439	0.383	4.44889968333751E-05	5	BAG6
1.09197132836693E-20	0.458259631920267	0.632	0.541	1.8299255520773E-16	5	PRKACA
1.69084800843969E-19	0.457081915669142	0.595	0.519	2.83352309254323E-15	5	NDFIP1
9.38690061260907E-08	0.456675576424839	0.364	0.306	0.00157305680466103	5	PIK3R2
7.84435555266799E-20	0.456202675280113	0.625	0.53	1.3145571035161E-15	5	ATP6AP1
1.14611890852291E-08	0.456050533116693	0.301	0.239	0.00019206660669027	5	TRIM8
1.69670803288934E-10	0.455580196918068	0.436	0.366	2.84334332151596E-06	5	NDUFA13
5.86883443852462E-11	0.455262582748649	0.454	0.391	9.83499275207956E-07	5	DIAPH1
4.71002394867625E-32	0.454949535846185	0.711	0.609	7.89305813319166E-28	5	CYC1
2.46257214327214E-07	0.454605507486009	0.336	0.28	0.00412677839769545	5	TRAPPC5
1.16574388601729E-06	0.454449950905033	0.355	0.304	0.0195355360418778	5	MTFR1
1.45974011269284E-07	0.454326014101186	0.337	0.283	0.00244623248085067	5	PSMC4
1.01104671862989E-07	0.453403963282211	0.406	0.354	0.00169431209107997	5	LONP1
1.73881595991837E-07	0.451230032614551	0.432	0.383	0.00291390778563121	5	SMIM19
1.39605759079086E-06	0.450983613165883	0.279	0.227	0.0233951331064732	5	NSMCE1
2.9543610048853E-35	0.45055624648438	0.771	0.605	4.95091817198678E-31	5	RNF128
3.68914515255161E-12	0.450328337412914	0.481	0.415	6.18226944664599E-08	5	DCAF11
5.14489552856724E-09	0.449970152198104	0.301	0.237	8.62181592677299E-05	5	BUD31
4.73809016668366E-08	0.448208687156548	0.385	0.326	0.000794009150132847	5	RPN2
7.33350697783478E-07	0.447922066284234	0.348	0.294	0.0122894909934555	5	SLC10A3
5.18823562261893E-27	0.447199370966946	0.689	0.564	8.69444525638481E-23	5	RBM47
1.44019221467403E-16	0.446768101133499	0.579	0.507	2.41347411335074E-12	5	AKR1A1
5.04415762954207E-07	0.446192334530506	0.344	0.291	0.00845299935558661	5	CDC34
5.10259793759731E-18	0.445130893320506	0.541	0.431	8.55093362382557E-14	5	DDIT4
1.49321128946825E-07	0.444986131896017	0.324	0.267	0.0025023234788909	5	SRRT
1.16958042301859E-09	0.444829661070309	0.323	0.256	1.95998287289455E-05	5	ZFP36L2
2.6321448630738E-07	0.444334644965544	0.412	0.365	0.00441094836153908	5	KTN1
1.74374051552686E-07	0.443710166690859	0.276	0.221	0.00292216035591991	5	STIP1
9.6119352042815E-11	0.443662009996429	0.455	0.389	1.61076810153349E-06	5	CAPZB
5.32020501267578E-10	0.442764819439588	0.384	0.31	8.91559956024207E-06	5	MALL
2.21851559991307E-44	0.442254216332097	0.83	0.647	3.71778844233432E-40	5	ACSL4
2.26285778768572E-18	0.442120750611668	0.571	0.488	3.79209708060372E-14	5	GRN
1.31393383222001E-07	0.441344669641798	0.289	0.232	0.00220189031603429	5	ALKBH7
3.4060578000613E-10	0.440812396661535	0.397	0.33	5.70787166134273E-06	5	ARHGDIA
5.08600343301326E-09	0.440764925564787	0.516	0.469	8.52312455304362E-05	5	RER1
3.7065990897128E-09	0.440736674370659	0.375	0.314	6.21151875454071E-05	5	PRPF19
1.44581452499705E-09	0.440465649028055	0.441	0.379	2.42289598099005E-05	5	SUB1
9.2131370055963E-26	0.440327540142166	0.714	0.644	1.54393749939783E-21	5	GOLIM4
9.28255059950439E-07	0.438737122344453	0.32	0.267	0.0155556982946495	5	NHP2
1.32785051864197E-14	0.438466015302254	0.522	0.46	2.22521189914021E-10	5	EIF5
1.08413662694757E-06	0.437949083535155	0.381	0.332	0.0181679615943874	5	ACADS
1.82334924391277E-06	0.437593096377425	0.347	0.3	0.0305556866294902	5	AATF
2.38109138329228E-09	0.436893696494525	0.39	0.327	3.9902329401212E-05	5	MSN
4.27165291917912E-07	0.436784237852801	0.296	0.244	0.00715843596196037	5	KIAA2013
2.00183368052094E-06	0.436614479531156	0.325	0.276	0.0335467288181698	5	DDX24
2.0121002227227E-07	0.436267480328522	0.427	0.381	0.00337187755323871	5	HUWE1
1.14035300689003E-06	0.435890572519091	0.372	0.318	0.0191100356894632	5	MBOAT7
1.355820998349E-22	0.435749322474375	0.702	0.631	2.27208482903325E-18	5	RARRES2
1.35826049349747E-08	0.435621787862588	0.404	0.347	0.000227617293500307	5	RNF10
1.61360683464483E-13	0.43544206180339	0.512	0.443	2.70408233349781E-09	5	LAMTOR2

3.06237752150023E-58	0.435412527411882	0.883	0.668	5.13193225053008E-54	5	NQO1
2.21503760168159E-09	0.434923319041056	0.431	0.371	3.71196001289801E-05	5	PAPSS2
3.0285025281494E-66	0.434139701529901	0.97	0.849	5.07516453667276E-62	5	ALDH1A1
1.50609007644693E-24	0.433911261368899	0.618	0.518	2.52390575010977E-20	5	MIF
5.47950845673332E-16	0.433858013934815	0.563	0.476	9.1825602717937E-12	5	SLC25A1
5.78645435179895E-07	0.432403085079028	0.415	0.361	0.00969694020274468	5	TMEM109
2.17353941957569E-07	0.431162106887206	0.367	0.308	0.00364241735932494	5	POLR3GL
2.85271084093314E-35	0.429093736170573	0.749	0.646	4.78057282723575E-31	5	PSME1
2.0103021435938E-09	0.425472457307502	0.452	0.391	3.36886433223448E-05	5	MTCH1
1.56723719604994E-06	0.425157992263721	0.347	0.299	0.026263760931405	5	FARSA
7.95132098842931E-12	0.424142261598524	0.523	0.461	1.33248237124098E-07	5	ADIPOR1
9.02651202371774E-09	0.422867563753293	0.451	0.393	0.000151266288493462	5	RTF2
2.5432278467122E-09	0.421647943157187	0.458	0.4	4.26194122552031E-05	5	PSMB4
3.37385409525563E-12	0.421354527373468	0.538	0.488	5.65390469282938E-08	5	AHSA1
6.59797031421448E-07	0.420790179254925	0.409	0.361	0.0110568786525606	5	POLR1D
4.01203881685454E-20	0.420659508255451	0.645	0.561	6.72337464928485E-16	5	MGST3
4.678812157566E-07	0.420156887531736	0.398	0.35	0.0078407534136491	5	RNF114
1.33726680639284E-06	0.419945863447945	0.37	0.319	0.0224099171415311	5	PDX1
6.79540951973805E-07	0.419897100437281	0.4	0.349	0.011387747273177	5	NDUFAB1
1.90633769380189E-11	0.41738459400915	0.465	0.395	3.19464070727321E-07	5	HSD17B10
2.79536666908644E-10	0.415843564017823	0.526	0.471	4.68447546405505E-06	5	ARL6IP4
6.02202887504949E-11	0.415207146931238	0.509	0.454	1.00917159888079E-06	5	TMEM33
1.37367702592306E-06	0.413854334130033	0.382	0.335	0.0230200796004186	5	TMBIM6
1.15689779233887E-10	0.413711958834358	0.455	0.402	1.93872932040147E-06	5	HSP90B1
2.30344893322939E-11	0.412921591043734	0.532	0.48	3.86011972230581E-07	5	SCARB2
3.01330100286446E-11	0.411649224820093	0.411	0.339	5.04968982060026E-07	5	SNX17
3.55106551411013E-08	0.410461801462292	0.371	0.309	0.000595087558854576	5	MANF
1.4792528180645E-24	0.409976509647604	0.811	0.741	2.47893187251249E-20	5	SERPINA6
2.47295077332867E-22	0.409807322579483	0.673	0.59	4.14417090594418E-18	5	TM9SF2
1.09953502187435E-09	0.408592300304714	0.52	0.454	1.84260078965704E-05	5	CCL15
2.22406513436848E-09	0.406838511253233	0.467	0.406	3.7270883521747E-05	5	ROMO1
1.54245616787893E-26	0.404628073244234	0.714	0.642	2.58484804613151E-22	5	HSPA8
1.29431473409976E-07	0.404589342731745	0.341	0.282	0.00216901263140438	5	NOP53
1.05871314283615E-08	0.403728996805723	0.355	0.29	0.000177419148476482	5	AP1S1
5.76040572797983E-07	0.402225218736608	0.332	0.273	0.00965328791894859	5	DEPRECATED-ENSG00000170421
2.1403666672366E-07	0.40176734292424	0.438	0.395	0.00358682646095509	5	MDM2
1.25684995998092E-12	0.400349539478752	0.543	0.479	2.10622916293603E-08	5	RACK1
2.19780193410629E-12	0.399335591866584	0.562	0.494	3.68307648117532E-08	5	EBP
6.53636368804836E-11	0.399155809824294	0.458	0.39	1.09536382684314E-06	5	ATP5MG
8.85954223519763E-07	0.398957765687557	0.432	0.381	0.0148468208777442	5	TMEM147
6.26041108548759E-10	0.398200971090126	0.529	0.474	1.04911968970601E-05	5	ALDH9A1
1.0491682956459E-11	0.398190754032726	0.515	0.451	1.75819622984341E-07	5	IKBKG
1.78986932219235E-08	0.397948661987271	0.479	0.428	0.000299946301012995	5	VPS28
2.65490367672604E-13	0.396218764516137	0.424	0.339	4.44908758145749E-09	5	TMSB10
1.50213600021169E-24	0.394308065878628	0.724	0.625	2.51727950915476E-20	5	CRYL1
3.71223899941869E-12	0.393614906068704	0.515	0.424	6.22097011522585E-08	5	PARM1
2.20097822824621E-08	0.392828036509395	0.5	0.462	0.0003688399314895	5	HNRNPU
5.07508661697352E-13	0.391137656495572	0.57	0.513	8.50483015272423E-09	5	MTDH
2.34622557865152E-06	0.390490479092087	0.397	0.349	0.0393180482470421	5	AHCYL1
2.57126027419822E-06	0.390188725026643	0.317	0.267	0.0430891796750137	5	UBL5
3.08091854694208E-07	0.388428586448498	0.423	0.374	0.00516300330096554	5	SRI
1.06847836137202E-25	0.388378248400159	0.726	0.585	1.79055603798722E-21	5	DNM2
4.81048381222764E-08	0.386356371340244	0.421	0.366	0.000806140877253109	5	PNPO
2.57000387429479E-09	0.386278793850779	0.441	0.378	4.30681249254321E-05	5	LAMTOR1
2.46232188488439E-08	0.385582869334649	0.374	0.312	0.000412635901468926	5	ATP5F1A
4.64607732003219E-53	0.384582416245858	0.954	0.896	7.78589637290994E-49	5	B2M
2.18995244267577E-67	0.383792169707447	0.992	0.953	3.66992230343605E-63	5	EPHX1
5.04328287334315E-14	0.38306814935542	0.655	0.585	8.45153343914844E-10	5	LSR
1.77045438790629E-07	0.382956259424717	0.402	0.345	0.00296692746325336	5	NAXE
8.27177487307308E-08	0.381682370577617	0.426	0.373	0.00138618403322959	5	SLC25A3

2.55749364157333E-16	0.381535065936564	0.651	0.586	4.28584784454858E-12	5	CREG1
1.04666617114897E-08	0.377326146038451	0.402	0.333	0.000175400316961145	5	PFKFB3
6.14670840065695E-08	0.375839224944861	0.514	0.47	0.00103006539378209	5	PICALM
2.72011674369488E-08	0.375774218223201	0.514	0.484	0.000455837163908387	5	STAT3
3.05802289826708E-08	0.375469606812407	0.463	0.415	0.000512463477291597	5	ADRM1
8.95332763590676E-10	0.373761169646581	0.498	0.447	1.50039864522525E-05	5	COX6A1
1.53990945385713E-11	0.373068560341544	0.528	0.474	2.58058026277377E-07	5	TMED5
3.38362935028529E-30	0.370942101106531	0.686	0.515	5.67028606520808E-26	5	PEG10
3.69370748197953E-07	0.370709380254965	0.481	0.44	0.00618991499830129	5	ITPK1
1.07460304906715E-14	0.368459208844837	0.667	0.601	1.80081978962673E-10	5	TMCO1
3.99500159398513E-31	0.366096558195207	0.703	0.513	6.69482367120029E-27	5	CDH17
6.10476320629299E-07	0.36586636629337	0.45	0.401	0.0102303621811058	5	EMC4
1.28796775879349E-08	0.364991718651263	0.513	0.461	0.000215837637018613	5	DEPRECATED-ENSG00000089248
2.45488318715192E-07	0.361712285919046	0.385	0.331	0.00411389324502919	5	NAA20
1.2033910582481E-06	0.361094660625921	0.361	0.309	0.0201664273541217	5	PLAAT3
2.41448398720762E-42	0.360964726315587	0.934	0.842	4.04619226576254E-38	5	CD74
2.24920617087824E-09	0.360764730309869	0.473	0.392	3.76921970115776E-05	5	NEB
6.4353441200888E-11	0.360734380320014	0.493	0.403	1.07843496764448E-06	5	ATAD2
1.85001522216997E-11	0.359244039101365	0.566	0.521	3.10025550931243E-07	5	HINT1
2.04528818926303E-07	0.358528769943088	0.468	0.417	0.00342749394756699	5	RAB5IF
1.12087949779116E-06	0.35762655351051	0.35	0.297	0.0187836986239843	5	IK
4.0078316038711E-08	0.35752435944818	0.491	0.438	0.000671632420176718	5	SEC13
4.69945195479545E-10	0.357286964778915	0.493	0.427	7.87534158584621E-06	5	NDUFB10
1.27268454915356E-09	0.357087998079137	0.533	0.479	2.13276476747154E-05	5	GSDMD
2.00199850055032E-15	0.354944470712979	0.723	0.657	3.35494908722223E-11	5	DUSP1
3.16604125849009E-09	0.354125324388997	0.496	0.434	5.30565194097768E-05	5	CLDN3
4.1460815376769E-07	0.35019011480057	0.355	0.297	0.00694800344083894	5	IGFBP3
1.87206307098785E-07	0.349938693499802	0.401	0.345	0.00313720329436144	5	TOMM7
1.57748117167997E-06	0.345460052323082	0.475	0.429	0.0264354294750129	5	AK4
3.89513478798177E-11	0.343821214999638	0.566	0.512	6.52746687769985E-07	5	MYDGF
3.71437316165763E-08	0.342771691136025	0.402	0.343	0.000622454654430586	5	COPE
2.59270776547966E-08	0.342357023629416	0.454	0.399	0.000434485967339082	5	PFDN2
1.12073926291704E-27	0.34218651938588	0.81	0.746	1.87813485679637E-23	5	ECH1
2.47379095140782E-13	0.341889242225216	0.575	0.514	4.14557887636923E-09	5	UBA52
2.33401041400084E-19	0.341671156285357	0.667	0.546	3.91133465178261E-15	5	CAPN2
1.85800710593035E-06	0.341500573267301	0.424	0.381	0.0311364830811808	5	USF2
5.91862582527636E-08	0.34062320993628	0.503	0.454	0.000991843315799813	5	TCEAL4
2.25099170044924E-07	0.340166140081356	0.461	0.415	0.00377221189161284	5	PIGT
7.11031398119517E-12	0.339922365156408	0.519	0.463	1.19154641696869E-07	5	CTNNA1
2.87767627467751E-09	0.339341073886574	0.51	0.466	4.82240990110457E-05	5	ATP5IF1
4.16970733831013E-09	0.3385855285718	0.527	0.481	6.98759555754012E-05	5	ATP5MC2
1.17812217180917E-10	0.337642289278997	0.564	0.506	1.97429713551781E-06	5	CAST
2.16241436543524E-17	0.337183004742577	0.62	0.538	3.62377399359638E-13	5	UQCRQ
5.90474061825295E-11	0.335605966421221	0.56	0.494	9.89516432806829E-07	5	CMBL
8.79591088853068E-09	0.332631513962022	0.509	0.46	0.000147401874669997	5	BLOC1S1
6.67987142913923E-10	0.331667057373609	0.507	0.438	1.11941285409515E-05	5	HPGD
2.55279306991936E-06	0.331493627635646	0.478	0.439	0.0427797062657086	5	ATP5ME
3.58005188214046E-30	0.330604631869185	0.842	0.732	5.99945094409099E-26	5	GPX2
6.3967437675396E-08	0.330245428420606	0.494	0.446	0.00107196632056429	5	LRPAP1
6.90689824248368E-15	0.330091376867068	0.626	0.524	1.15745800747542E-10	5	KCTD3
3.92504376992601E-23	0.329606431531194	0.716	0.644	6.577588349642E-19	5	SLC9A3R1
4.11856249439132E-10	0.324558674175991	0.536	0.481	6.90188702810097E-06	5	EEF1G
5.70611547666393E-08	0.321127103915048	0.489	0.434	0.000956230831579342	5	NDUFS7
8.42297552300693E-09	0.321109807846583	0.528	0.485	0.00014115222381455	5	SYNGR2
1.16840906431477E-27	0.320975969805508	0.838	0.705	1.9580199099787E-23	5	LAMP2
3.49197222315527E-08	0.319868355772365	0.55	0.502	0.00058518470515636	5	CUTA
2.28973502140558E-11	0.316236346163716	0.515	0.439	3.83713794887148E-07	5	CITED2
6.86675154983E-09	0.31600733954829	0.573	0.525	0.000115073022472051	5	NAPA
3.33522446019402E-19	0.31489598174232	0.69	0.592	5.58916915039314E-15	5	GOLM1
7.01452805795548E-07	0.313282195219204	0.557	0.532	0.0117549461195218	5	AHNAK

1.85577119653169E-13	0.311093657778621	0.615	0.493	3.10990137114781E-09	5	F2RL1
2.35439026531122E-11	0.30974288414527	0.397	0.316	3.94548720660854E-07	5	SPTBN1
2.05422398646157E-13	0.309283678833216	0.646	0.576	3.44246855651229E-09	5	BLVRB
4.12762148806829E-24	0.308483529225982	0.76	0.653	6.91706808970484E-20	5	STARD10
1.10777675690664E-19	0.307880060393674	0.759	0.659	1.85641228922415E-15	5	NFKBIA
3.30770115944099E-26	0.307576512774681	0.885	0.751	5.54304560299121E-22	5	UGT2B4
3.45838272424856E-09	0.304084911568499	0.55	0.487	5.79555776929573E-05	5	EGLN1
9.3139575451146E-08	0.303937836163348	0.512	0.47	0.0015608330054103	5	LUC7L2
2.60366728348231E-07	0.302177328686825	0.486	0.434	0.00436322563365965	5	SLC25A4
1.26064770194363E-28	0.301259013478956	0.794	0.596	2.11259341891714E-24	5	NDRG1
1.5551349125521E-16	0.30091665257451	0.73	0.64	2.60609508645481E-12	5	DEPRECATED-ENSG00000198074
5.61196247355562E-20	0.300427645968225	0.772	0.707	9.40452671318451E-16	5	GPX4
1.3940399408562E-21	0.300166912406876	0.757	0.568	2.33613213288682E-17	5	TSPAN3
6.31558383910209E-15	0.298998178081472	0.606	0.509	1.05836553975673E-10	5	TFRC
5.59588272172002E-07	0.298076731235729	0.526	0.492	0.00937758026505841	5	HADHA
8.8709963461929E-08	0.297480123231847	0.544	0.514	0.00148660156769501	5	HSPH1
5.24424189833307E-13	0.296582407194792	0.648	0.596	8.78830057322655E-09	5	SDC1
4.48891618244037E-12	0.295747277210338	0.581	0.522	7.52252573853358E-08	5	NDUFB7
6.31647687580891E-08	0.293380146067586	0.559	0.518	0.00105851519484806	5	PSMB1
5.76206616603939E-39	0.293170774150057	0.945	0.826	9.65607048104881E-35	5	PGRMC1
8.1194426016859E-20	0.292647607637723	0.859	0.719	1.36065619119052E-15	5	ASPH
5.36051630041578E-08	0.290261801097663	0.565	0.508	0.000898315321623677	5	SDF4
2.25515295311439E-38	0.287671071681378	0.9	0.743	3.77918531882909E-34	5	LGALS4
1.42338753121857E-33	0.286049415550335	0.989	0.954	2.38531282481608E-29	5	CES1
4.07237659291709E-07	0.282708738069531	0.55	0.514	0.00682448869441045	5	ERGIC3
3.1052451924633E-08	0.280563323148094	0.561	0.51	0.000520376989353	5	NFIC
3.9653331124833E-07	0.274810228750364	0.628	0.605	0.00664510522989951	5	GOLPH3
4.80132300993654E-44	0.269608553168894	0.978	0.928	8.04605710005166E-40	5	HSPB1
1.48431236698332E-07	0.267599937021628	0.531	0.477	0.00248741066459066	5	FRK
9.57243298783908E-10	0.266134876275028	0.668	0.616	1.60414832010207E-05	5	APLP2
3.39965717548827E-22	0.265816065176069	0.899	0.818	5.69714549468325E-18	5	MGST1
2.02311986629077E-08	0.262203678675257	0.623	0.578	0.000339034427193007	5	COPG1
5.00953018531084E-08	0.261491505235095	0.494	0.42	0.000839497068454391	5	MCM3
2.7759112964722E-16	0.25751969389	0.703	0.65	4.65187215062812E-12	5	SOD1
3.3754932454531E-21	0.257329455509666	0.787	0.71	5.65665158073031E-17	5	EEF2
1.83368862170288E-06	0.254713394373868	0.511	0.465	0.0307289539224969	5	SLC2A4RG
3.68332769095951E-07	0.253967859270882	0.545	0.505	0.00617252054450995	5	NDUFA3
1.66409441491351E-09	0.250809858604792	0.579	0.526	2.78868942051206E-05	5	POLR2L
0	2.68472211703936	1	0.971	0	6	FTL
9.78288196138583E-279	2.49139332099535	0.716	0.385	1.63941535908904E-274	6	SRXN1
4.12547958836218E-109	2.38278077997713	0.432	0.224	6.91347869417734E-105	6	SLC25A23
1.96688987455793E-116	2.34608912821701	0.473	0.253	3.29611405178418E-112	6	CENPB
9.67229875234173E-76	2.34001940482393	0.363	0.194	1.62088382491743E-71	6	VEGFB
3.91125488840368E-83	2.2984317817666	0.415	0.24	6.55448094198689E-79	6	ZNF219
8.52407133611691E-154	2.27761084693401	0.488	0.219	1.42846387450647E-149	6	ETNK2
3.13129529612847E-114	2.26580838478652	0.492	0.28	5.24742465725209E-110	6	LRP3
2.78218761175433E-281	2.22678144616225	0.764	0.468	4.66238999977791E-277	6	IRAK1
0	2.18391197202857	0.907	0.644	0	6	STARD10
3.46096098937964E-134	2.13385619079379	0.545	0.314	5.7998784260024E-130	6	PKN1
1.47540302449565E-210	2.1313126562104	0.709	0.453	2.47248038844982E-206	6	SLC2A4RG
7.77809789612704E-85	2.12324739223294	0.45	0.27	1.30345364543297E-80	6	KIF1C
1.2042666293296E-76	2.11887776730813	0.396	0.224	2.01811001743054E-72	6	ZNF358
2.85008163793289E-110	2.08386247668076	0.517	0.311	4.77616680884794E-106	6	RNPEPL1
3.67510733211663E-92	2.07582674393387	0.429	0.234	6.15874486716105E-88	6	NME4
2.68537556094954E-127	2.05926095585556	0.55	0.331	4.50015236503923E-123	6	GUK1
6.49591840559582E-84	2.0039154446809	0.453	0.276	1.08858600640975E-79	6	C7orf50
4.89142639513831E-51	1.99719849958264	0.326	0.191	8.19705235297278E-47	6	UBL4A
2.37782868661343E-31	1.94843975807137	0.278	0.179	3.98476531302679E-27	6	C19orf33
2.46591965727387E-99	1.93335512486287	0.493	0.298	4.13238816165955E-95	6	PIK3R2
1.62901884663677E-49	1.9327687649003	0.38	0.246	2.7299097831939E-45	6	E2F1

2.45323793248276E-155	1.926913727551	0.622	0.381	4.11113612725461E-151	6	TLE5
2.64516453296765E-46	1.88461207408727	0.341	0.214	4.43276672434719E-42	6	PRSS3
4.68046158918564E-81	1.87427229798862	0.453	0.275	7.84351753115729E-77	6	NOP53
1.9861382939484E-35	1.85869889238644	0.322	0.217	3.32837055299874E-31	6	TADA3
3.54414828856693E-50	1.84677637422326	0.388	0.26	5.93928370198045E-46	6	KANK2
1.36986484890169E-24	1.83096442674062	0.319	0.239	2.29561951378946E-20	6	C19orf48
1.08100844760712E-46	1.82390170409799	0.43	0.313	1.81155395650001E-42	6	RASSF3
9.47648123935184E-120	1.82355446183635	0.589	0.39	1.58806872609058E-115	6	TMEM219
6.33297138692057E-153	1.81030566487035	0.66	0.448	1.06127934502015E-148	6	SLC25A39
1.28188442582386E-51	1.80862907225667	0.379	0.242	2.14818192079563E-47	6	SYNPO
9.91012574861605E-64	1.79405456140885	0.455	0.312	1.66073887295308E-59	6	WSB2
6.87648979966914E-48	1.78544673274444	0.398	0.275	1.15236216062855E-43	6	ARPC1A
3.48269404943586E-43	1.78148796470924	0.339	0.216	5.83629868804461E-39	6	CSNK1G2
3.7905641344511E-56	1.78081077649253	0.402	0.261	6.35222737651315E-52	6	PRELID1
3.23649754555709E-110	1.7736254731556	0.548	0.345	5.42372258684457E-106	6	MAF 1.00
6.95357229891237E-22	1.77283952932747	0.273	0.194	1.16527964585174E-17	6	JCAD
1.08112703349705E-125	1.77112707242198	0.6	0.405	1.81175268273436E-121	6	MAP2K2
3.20763139921007E-29	1.77044141505483	0.321	0.227	5.37534869879624E-25	6	DOK4
1.05355547086337E-27	1.76316513017044	0.306	0.214	1.76554825807284E-23	6	TSR3
2.70321123810534E-25	1.7624504618057	0.293	0.207	4.53004139281693E-21	6	MBD3
8.98646142785497E-38	1.75973820591865	0.308	0.194	1.50595120607994E-33	6	HDAC5
1.600438728252E-57	1.73160559917458	0.459	0.322	2.68201522080471E-53	6	C1orf115
6.0537684574013E-56	1.73104616569957	0.431	0.298	1.01449051809131E-51	6	BTBD2
2.24906667226659E-60	1.7268045211875	0.439	0.296	3.76898592938435E-56	6	FKBP8
1.86509012281735E-58	1.7206866567422	0.452	0.315	3.12551802781731E-54	6	DNAJC4
4.39526317495603E-39	1.69166274156027	0.363	0.251	7.36558202859132E-35	6	ALKBH5
8.84956074646502E-20	1.68415890401314	0.27	0.197	1.48300938989261E-15	6	NCK2
1.72500260330396E-80	1.67411399051009	0.644	0.459	2.89075936261677E-76	6	SLC6A8
9.8443590696791E-22	1.67288916019455	0.25	0.169	1.64971769289682E-17	6	DGCR6L
3.0465835229904E-93	1.6633130807332	0.568	0.402	5.10546466782731E-89	6	AKT1
3.98681753879209E-33	1.65630763004921	0.349	0.249	6.68110883150779E-29	6	SZRD1
4.15059416912253E-68	1.65559764504965	0.492	0.346	6.95556570861554E-64	6	UBXN6
2.78531336213264E-64	1.64693073754284	0.489	0.352	4.66762813226187E-60	6	DUS1L
1.94781515476604E-18	1.63379309263732	0.299	0.234	3.26414863635693E-14	6	DEAF1
1.21357995563836E-80	1.61405870680589	0.559	0.414	2.03371728965876E-76	6	NR2F6
9.7583516349848E-52	1.60753295525493	0.45	0.329	1.63530456699075E-47	6	ASB13
3.41548811862093E-50	1.60697226932234	0.449	0.329	5.72367498918495E-46	6	EPN1
4.08613965668603E-70	1.60571091188315	0.488	0.334	6.84755283667444E-66	6	SLC35A4
3.64106370630432E-124	1.60356292113444	0.666	0.486	6.10169455902478E-120	6	MAPKAPK2
6.50420517066021E-93	1.59424962714524	0.568	0.402	1.08997470249924E-88	6	PMVK
4.25661996915818E-18	1.57961056136577	0.333	0.275	7.13324374431527E-14	6	DEXI
2.75833087244316E-85	1.57157360450818	0.561	0.408	4.62241087604026E-81	6	SHARPIN
1.21360711847463E-99	1.56353401727743	0.599	0.429	2.03376280913979E-95	6	LASP1
2.2790573591269E-87	1.56080138311284	0.575	0.423	3.81924432242486E-83	6	SEPTIN9
2.88635644080069E-88	1.55094748820656	0.545	0.371	4.8369561234938E-84	6	LAMTOR1
1.05223967698185E-22	1.54549243354132	0.292	0.211	1.76334325068619E-18	6	MAP3K11
9.64473979486886E-26	1.54548872979675	0.347	0.265	1.61626549482412E-21	6	ARPC1B
1.23955390550851E-25	1.53644722063954	0.356	0.275	2.07724443485116E-21	6	ESRRA
3.5074934452136E-20	1.53193418540851	0.325	0.258	5.87785751548895E-16	6	CTDSPL
1.29835833861362E-148	1.52922595589683	0.73	0.563	2.1757889038487E-144	6	MGAT4B
2.48947663072108E-273	1.51200940094516	0.904	0.728	4.17186493776238E-269	6	GPX2
0	1.51056715267861	0.976	0.902	0	6	SQSTM1
7.73530096068041E-32	1.50585455098348	0.357	0.258	1.29628173499082E-27	6	TRAF7
1.54500307864044E-15	1.50309121038141	0.266	0.205	2.58911615918565E-11	6	TNIP2
1.72814964345479E-43	1.49960475877952	0.44	0.331	2.89603317250154E-39	6	SHROOM3
4.84623300782472E-39	1.49750777770992	0.415	0.31	8.12131727451266E-35	6	NACC1
8.11959897993868E-51	1.48609695501513	0.468	0.35	1.36068239705812E-46	6	KEAP1
6.28908665817917E-49	1.48351386240497	0.469	0.353	1.05392514217767E-44	6	NAA60
3.3410466911969E-37	1.48079118278331	0.415	0.316	5.59892604510776E-33	6	NCOR2
4.77668468392597E-19	1.47605503133118	0.305	0.236	8.00476819332313E-15	6	C8orf33

4.58579129668602E-14	1.47509628526708	0.293	0.241	7.68486905498643E-10	6	TMEM250
2.26223938480875E-14	1.47247492951815	0.262	0.204	3.7910607610625E-10	6	MPND
2.55893508562972E-67	1.46935490309924	0.553	0.427	4.28826341649829E-63	6	TAF10
7.13365661877494E-23	1.46421405214248	0.324	0.245	1.1954581761743E-18	6	SLC25A10
4.20648302940351E-42	1.45955385512085	0.431	0.323	7.04922426067441E-38	6	NIBAN2
4.87129122358347E-194	1.45698326894456	0.784	0.531	8.16330983248117E-190	6	G6PD
7.22362620849882E-64	1.44827877766051	0.539	0.417	1.21053528002023E-59	6	KIAA1522
3.00060870888938E-55	1.4448476190379	0.495	0.372	5.02842007435682E-51	6	TIMM13
9.96594215605543E-236	1.44197311900334	0.881	0.704	1.67009258651177E-231	6	EEF2
7.99336500474558E-18	1.44054159726246	0.301	0.236	1.33952810749526E-13	6	ATXN1
2.86368581687518E-51	1.43523502003969	0.455	0.324	4.79896469191943E-47	6	FBXW5
1.2589136643706E-61	1.43200141505079	0.521	0.388	2.10968751875224E-57	6	IMPA2
5.53470128538116E-61	1.43110938562319	0.502	0.363	9.27505241404175E-57	6	TNIP1
3.14100768636226E-54	1.42585097250372	0.516	0.395	5.26370068080588E-50	6	GCHFR
6.35976523312673E-29	1.42337932013574	0.415	0.339	1.06576945776738E-24	6	FAM83H
1.80201642684136E-25	1.42323750590747	0.353	0.271	3.01981912810074E-21	6	PCGF2
5.34277785380705E-36	1.41797970423518	0.443	0.35	8.95342712740985E-32	6	CTDSP1
1.26994704756959E-12	1.41469495921422	0.269	0.217	2.12817726231712E-08	6	RAB15
4.43399469057494E-09	1.41345671604374	0.257	0.22	7.43048830246548E-05	6	SNAP47
1.87767368194764E-10	1.41242711131324	0.257	0.214	3.14660555620786E-06	6	PPP1R12C
5.31829241190997E-33	1.40834264239572	0.402	0.306	8.91239442387872E-29	6	NFIX
9.80097871658066E-40	1.40448410929483	0.418	0.31	1.64244801332459E-35	6	VAT1
1.57836286629904E-19	1.40284689628021	0.321	0.252	2.64502049134393E-15	6	KLHDC3
2.84147389283748E-16	1.39898873779094	0.324	0.27	4.76174194961706E-12	6	HGH1
3.31800426978675E-24	1.39443551791788	0.333	0.25	5.56031155530864E-20	6	DEPRECATED-ENSG00000196821
9.24091051662393E-245	1.39309259651046	0.916	0.81	1.54859178437584E-240	6	FTH1
2.17076291815208E-29	1.39005717985115	0.368	0.274	3.63776449823926E-25	6	PALLD
3.22431257860884E-11	1.38956678145113	0.255	0.209	5.4033030192327E-07	6	ARHGEF18
7.37497171126451E-11	1.38388665714175	0.304	0.266	1.23589775937371E-06	6	DNASE1L1
1.25139552610786E-17	1.38262103002247	0.318	0.256	2.09708862265155E-13	6	BCL2L1
4.74235344016562E-20	1.37960401970941	0.338	0.27	7.94723589502955E-16	6	STUB1
2.75661400789163E-116	1.37891552486647	0.658	0.469	4.61953375442479E-112	6	LAGE3
8.46341722257686E-34	1.37764875439497	0.409	0.313	1.41829945815943E-29	6	TMEM141
6.05643029162224E-16	1.37603283171575	0.3	0.238	1.01493658827006E-11	6	PYCARD
4.79530023316498E-78	1.37117992930208	0.582	0.435	8.03596413073787E-74	6	EFHD2
2.36664495256265E-16	1.37092075999288	0.278	0.213	3.96602361150448E-12	6	GTPBP6
1.26535267725119E-21	1.36977934068363	0.374	0.307	2.12047801653754E-17	6	MLLT6
2.68386526984444E-40	1.3625232218361	0.471	0.376	4.49762141920531E-36	6	HIPK2
1.10067102538007E-08	1.35888941246359	0.272	0.237	0.000184450450433193	6	CHMP1A
6.73258332996135E-55	1.35260029582131	0.509	0.386	1.12824631443492E-50	6	BIN1
5.26551923817674E-14	1.35086512902891	0.308	0.254	8.82395713933658E-10	6	HPCAL1
6.18334080343726E-13	1.34605015382472	0.273	0.219	1.03620425184002E-08	6	NABP2
1.3993760899965E-27	1.34488773426432	0.391	0.308	2.34507445161613E-23	6	GIPC1
8.93494463904915E-43	1.34233061187005	0.451	0.34	1.49731802261186E-38	6	GUCD1
1.71540365796845E-15	1.34184091106381	0.304	0.245	2.87467345002352E-11	6	FAM168B
9.49401407904672E-12	1.34070196218994	0.297	0.254	1.59100687936665E-07	6	TSC22D4
7.32417271715237E-38	1.34002426870664	0.483	0.398	1.22738486394039E-33	6	NBR1
1.2523345138768E-13	1.33980786479651	0.289	0.234	2.09866217835475E-09	6	FIBP
3.70491999315023E-08	1.3373534153558	0.265	0.233	0.000620870492452115	6	PPP1R16A
8.72191529333061E-11	1.33634381176076	0.278	0.235	1.46161856485634E-06	6	MED29
5.89560567616247E-113	1.33312227668812	0.675	0.513	9.87985599211307E-109	6	DAP
8.56925474138686E-10	1.33216838468519	0.297	0.265	1.43603570956161E-05	6	MXD4
3.61454003773836E-17	1.32689961956689	0.32	0.256	6.05724619524194E-13	6	PGLS
1.04190769465386E-12	1.32511259334689	0.274	0.222	1.74602891470094E-08	6	DBP
1.69004355622563E-39	1.32208618034615	0.434	0.321	2.83217499152291E-35	6	CHP2
3.74984469030293E-09	1.31848061942938	0.258	0.216	6.28398973200965E-05	6	SNRNP25
2.86600309356308E-28	1.31746184449477	0.408	0.331	4.80284798419301E-24	6	ACTR1B
1.29862039516324E-11	1.31567790032922	0.283	0.235	2.17622805821455E-07	6	DEPRECATED-ENSG00000205937
1.33138117016966E-19	1.31522785465944	0.335	0.263	2.23112856497031E-15	6	MISP
9.89150175889407E-43	1.30869361586224	0.48	0.378	1.65761786475547E-38	6	USF2

7.0094213617479E-111	1.30858665083039	0.673	0.513	1.17463883180171E-106	6	ATP5F1D
1.94950709709422E-73	1.30692631614801	0.595	0.467	3.26698399331049E-69	6	ARL6IP4
3.53614200022623E-21	1.30191934171946	0.378	0.313	5.92586676397912E-17	6	DNAJB2
3.6262472382743E-15	1.29702796282495	0.331	0.278	6.07686512190007E-11	6	FASTK
1.76859645177251E-11	1.29654420737609	0.307	0.267	2.96381393388037E-07	6	DNAJC5
1.37161443042668E-38	1.28549683188157	0.471	0.38	2.29855146250903E-34	6	RNF187
3.02927288217896E-16	1.28168451660048	0.353	0.303	5.0764554959555E-12	6	EGLN2
5.50046636435133E-23	1.28146491961282	0.387	0.318	9.21768153337996E-19	6	MECP2
3.01930782446521E-14	1.28001949558973	0.325	0.273	5.0597560522388E-10	6	DEPRECATED-ENSG00000170421
1.18557954572626E-64	1.27950917442219	0.576	0.452	1.98679420272806E-60	6	AMOTL1
1.01762625830104E-22	1.27599446327969	0.429	0.376	1.70533808366089E-18	6	CISD3
3.99340444703391E-15	1.27179113592972	0.315	0.26	6.69214717233942E-11	6	FAM32A
3.07475999338818E-08	1.26636005268374	0.303	0.278	0.00051526827969199	6	SAP30L
1.85997815623952E-13	1.26262714776863	0.332	0.287	3.11695139422618E-09	6	GNA11
2.8021213883508E-13	1.25658247093386	0.313	0.264	4.69579502259827E-09	6	BOK
2.8212532007083E-104	1.2561337682811	0.678	0.523	4.72785611374696E-100	6	CBR1
4.20344795658538E-08	1.25495428422212	0.269	0.237	0.000704413808564577	6	NR2F1
1.09680148147066E-16	1.25459086932972	0.346	0.288	1.83801992264854E-12	6	STK11
1.4895194554076E-10	1.25427657228123	0.299	0.259	2.49613670337206E-06	6	E2F4
9.61030153634587E-48	1.25393642599321	0.519	0.421	1.61049433146084E-43	6	ILRUN
8.07179295624969E-17	1.24965219850218	0.356	0.304	1.35267106360832E-12	6	RAB20
1.03126884599148E-46	1.24887586759519	0.529	0.437	1.72820033211252E-42	6	DYNLL2
1.09430029205354E-39	1.2457626540163	0.481	0.389	1.83382842942332E-35	6	PTPA
2.11597013606502E-13	1.24504244899	0.332	0.285	3.54594275401776E-09	6	SLC25A11
9.8990092432989E-23	1.24448963033254	0.377	0.304	1.65887596899203E-18	6	GRB2
2.32682975196248E-15	1.23970812184578	0.365	0.32	3.89930129833873E-11	6	FOXP4
4.51103496956866E-77	1.23811969329768	0.661	0.555	7.55959240200316E-73	6	CAMK2N1
4.13200752837723E-33	1.23785054399047	0.442	0.354	6.92441821605455E-29	6	CLTB
4.08640985942512E-14	1.23756472117346	0.33	0.283	6.84800564242461E-10	6	GSK3A
2.79708487610817E-51	1.23649284986639	0.523	0.401	4.68735483538208E-47	6	CDKN1A
1.04095083355788E-11	1.23619191889535	0.314	0.273	1.74442540687629E-07	6	JPT2
2.40668146579816E-10	1.23409035741454	0.287	0.244	4.03311680038457E-06	6	CPTP
2.12595596276657E-178	1.22951970141169	0.835	0.704	3.56267700240422E-174	6	GPX4
6.27827352910898E-20	1.22518327626087	0.366	0.304	1.05211307800808E-15	6	PREB
9.79632238650098E-10	1.2242841658879	0.259	0.217	1.64166770552983E-05	6	JOSD1
1.82738993528554E-13	1.22356619420486	0.313	0.263	3.06234005355151E-09	6	FAM102A
2.93032246702585E-23	1.21897659816405	0.405	0.34	4.91063439024191E-19	6	SNX17
1.1467266612247E-21	1.21831230368081	0.417	0.357	1.92168453888035E-17	6	MKNK2
1.04702363878913E-249	1.21553700413395	0.922	0.666	1.75460221388282E-245	6	NQO1
1.12235697508639E-66	1.21539045279968	0.575	0.446	1.88084581884976E-62	6	DEPRECATED-ENSG00000159228
9.292011080251E-41	1.20804648391313	0.501	0.402	1.55715521682846E-36	6	FAM3A
6.06337285110274E-13	1.2040673768678	0.314	0.264	1.0161000223878E-08	6	SRF
1.96356900690979E-06	1.20361969760334	0.256	0.229	0.0329054894177943	6	ELP5
1.59671307139859E-14	1.20077820005274	0.356	0.31	2.67577176504976E-10	6	PHC2
1.15493230618628E-11	1.19733850091893	0.317	0.277	1.93543555870696E-07	6	SGTA
3.68884636848397E-12	1.19718885009727	0.333	0.294	6.18176874430544E-08	6	HMG20B
8.88217259752268E-07	1.19656991036092	0.29	0.27	0.0148847448389285	6	STRN4
2.19597143222411E-09	1.19606852896171	0.269	0.23	3.68000892612116E-05	6	DAXX
4.95283203545174E-08	1.19344475413994	0.295	0.267	0.000829995592501003	6	SKI
2.29252540783001E-09	1.19239863253077	0.262	0.223	3.84181407844153E-05	6	CERK
3.19008688107157E-26	1.18311295855942	0.439	0.368	5.34594759529974E-22	6	RAD23A
2.27706413206398E-50	1.17946464091834	0.547	0.439	3.81590407251281E-46	6	GSTA2
4.91730905239296E-17	1.17887310940746	0.359	0.302	8.24042651000012E-13	6	MZT2B
6.86530145632476E-35	1.1776649884464	0.472	0.385	1.1504872180509E-30	6	SYBU
4.54954797913208E-07	1.17755225640027	0.309	0.293	0.00762413250342954	6	TMUB1
4.38006647121754E-139	1.17591604389166	0.804	0.67	7.34011539246635E-135	6	KRT8
4.70225829001556E-11	1.17437320665183	0.342	0.31	7.88004444240807E-07	6	RAB1B
3.11209312172494E-116	1.17437013090746	0.748	0.605	5.21524565338665E-112	6	S100A16
3.17445502981588E-167	1.17396040272822	0.839	0.634	5.31975173896545E-163	6	DEPRECATED-ENSG00000198074
2.4558489985258E-65	1.1645906068506	0.632	0.533	4.11551175172954E-61	6	RXRA

2.77779652211195E-06	1.16023431125428	0.257	0.232	0.0465503141175521	6	SIRT2
5.61925000941409E-62	1.15863689210168	0.595	0.485	9.41673916577613E-58	6	EGLN1
1.82156601183112E-09	1.15643310347237	0.279	0.24	3.0525803226266E-05	6	UBTF
5.8228347900429E-15	1.15440358436858	0.363	0.316	9.75790654115389E-11	6	NME2
3.98777825146882E-09	1.15317583422307	0.282	0.244	6.68271879381146E-05	6	GADD45GIP1
1.55434095787455E-07	1.15296630230388	0.266	0.232	0.00260476457720617	6	TMEM134
2.15820917045513E-20	1.15225653869823	0.393	0.335	3.61672692784871E-16	6	DUSP3
7.05949979533069E-32	1.15105994761355	0.481	0.402	1.18303097570152E-27	6	MMP24OS
7.7895333839044E-21	1.14984219485712	0.402	0.343	1.3053700044747E-16	6	PYGO2
9.03358547875304E-24	1.14978856687923	0.416	0.346	1.51384825452943E-19	6	LSM 4.00
1.01199537562942E-08	1.14304923128039	0.311	0.281	0.000169590185047978	6	CCDC124
1.31274943487492E-32	1.14304092934956	0.476	0.39	2.19990550296339E-28	6	RHOC
2.88022801047894E-07	1.14243473432064	0.286	0.254	0.0048266860999606	6	TCF19
7.27140586195565E-63	1.14134493263868	0.615	0.506	1.21854219434653E-58	6	NFIC
5.44098435907658E-139	1.1394167741046	0.847	0.76	9.11800158894054E-135	6	NUPR1
6.7650906051815E-51	1.13697364272643	0.589	0.496	1.13369388361632E-46	6	ENO1
2.60351471964071E-06	1.13511590438188	0.286	0.267	0.0436296996717391	6	TBC1D14
1.7870043506353E-23	1.13484420011646	0.446	0.386	2.99466189079463E-19	6	FGGY
2.05666513989117E-26	1.13186016773886	0.446	0.375	3.44655944142962E-22	6	POLR2E
1.60200948121236E-240	1.12939396065778	0.921	0.798	2.68464748861567E-236	6	OAZ1
4.08273368729307E-72	1.12905580525452	0.637	0.522	6.84184511316572E-68	6	POLR2L
1.70997005016652E-21	1.12870740679017	0.401	0.335	2.86556781006905E-17	6	CSTB
4.49399914805533E-29	1.12812943549561	0.45	0.374	7.53104377231113E-25	6	PIR
6.87511224897624E-45	1.12482145174309	0.541	0.449	1.15213131068344E-40	6	SF3B5
5.04396215686247E-10	1.1237460170543	0.325	0.292	8.45267178247013E-06	6	FAM174C
8.18636999571398E-08	1.11715702428782	0.269	0.235	0.00137187188388175	6	RNF44
4.11623622179276E-27	1.11136347782282	0.453	0.382	6.8979886604803E-23	6	AGPAT1
4.57472977872839E-26	1.10999921553717	0.45	0.384	7.66633216319303E-22	6	NAA10
9.15291021611827E-08	1.1061863232718	0.291	0.26	0.0015338446940171	6	AURKAIP1
7.95390988265909E-45	1.10512275972619	0.533	0.437	1.33291621813601E-40	6	ITPK1
8.96850042205986E-190	1.09795151458713	0.901	0.712	1.50294130072879E-185	6	AKR1B10
2.00522520466929E-47	1.09447953312572	0.561	0.429	3.36035639798479E-43	6	NBL1
3.19107263961243E-11	1.09190071800006	0.342	0.306	5.34759952946251E-07	6	POLR2I
3.06431203712977E-17	1.09122142826169	0.389	0.337	5.13517411182207E-13	6	CDK16
1.88102207469996E-19	1.0906332825845	0.404	0.347	3.15221679278219E-15	6	RNF10
8.89263572947222E-90	1.089552425511	0.708	0.514	1.49022789554496E-85	6	PEG10
3.07890879226757E-09	1.08735733849485	0.301	0.263	5.159635354082E-05	6	ODC1
5.93942301615125E-247	1.08275098330242	0.951	0.832	9.95328509046626E-243	6	PTGR1
3.99794606358866E-57	1.08072675509542	0.613	0.497	6.69975801336187E-53	6	CMC4
7.04161912305746E-08	1.07756129606103	0.274	0.24	0.00118003453264197	6	CITED4
1.05662543762566E-08	1.07512726890115	0.339	0.317	0.000177069290837309	6	CDC42BPB
4.15313754229601E-80	1.07347374112262	0.724	0.643	6.95982789337965E-76	6	BRI3
6.72843736163935E-36	1.07170945330319	0.518	0.448	1.12755153306352E-31	6	PDXK
7.36384364559525E-13	1.06992196914408	0.345	0.301	1.23403291812885E-08	6	PXDC1
4.57134527120722E-32	1.0617367581489	0.554	0.484	7.66066040548906E-28	6	NUDT16
3.83106330294024E-16	1.06167686654516	0.395	0.35	6.42009588306725E-12	6	HIGD2A
3.17888592244912E-16	1.06075143102153	0.411	0.368	5.32717702884023E-12	6	ISOC2
1.5217867391724E-19	1.0598753158051	0.439	0.392	2.55021021750511E-15	6	MTCH1
1.74387012118829E-86	1.05873896050237	0.727	0.608	2.92237754908734E-82	6	BEX3
1.6017134445328E-12	1.05776574554591	0.304	0.247	2.68415139034806E-08	6	CSRP1
8.69463608823069E-13	1.05673255316772	0.352	0.303	1.4570471156657E-08	6	DEPRECATED-ENSG00000163931
1.61791604737851E-06	1.0559768463251	0.279	0.252	0.027113037121969	6	HMOX2
6.38209875880464E-32	1.05576942551023	0.499	0.417	1.06951211000048E-27	6	FOSL2
2.19646454177089E-18	1.05477491149642	0.413	0.361	3.68083527909965E-14	6	PIK3AP1
3.5249663750848E-10	1.04987481824327	0.343	0.313	5.9071386513671E-06	6	IDS
1.86072820906535E-20	1.04606223677857	0.443	0.39	3.11820833275172E-16	6	UQCR11
5.90900605340819E-08	1.04414085920606	0.331	0.311	0.000990231234430145	6	RNPS1
7.56841929753304E-30	1.04271019809226	0.492	0.419	1.26831570588059E-25	6	ANAPC11
1.6938087636265E-32	1.04250313232319	0.538	0.479	2.83848472608529E-28	6	RACK1
2.66794082025531E-37	1.04170620280843	0.526	0.45	4.47093522658385E-33	6	UBE2Q1

5.48540913777045E-20	1.04007556568441	0.421	0.365	9.19244863307573E-16	6	TTC39C
7.20829925653428E-48	1.03867347161493	0.597	0.503	1.20796678941002E-43	6	MIDN
2.06668066129502E-06	1.03830829361049	0.251	0.223	0.0346334345219819	6	DDX49
6.5220142584846E-18	1.0367993487841	0.408	0.359	1.09295914943685E-13	6	JTB
1.04862702843088E-16	1.03036757795618	0.403	0.353	1.75728917424447E-12	6	KXD1
1.14405915709691E-08	1.02901915916279	0.316	0.285	0.0001917214335463	6	MIEN1
4.80915557634747E-11	1.02879307377448	0.366	0.338	8.05918291484309E-07	6	DNPH1
7.57258030990591E-32	1.0270490303635	0.543	0.479	1.26901300833403E-27	6	DSP
1.66360313319516E-35	1.02684065453349	0.535	0.432	2.78786613060844E-31	6	DDIT4
3.49772409077801E-11	1.02674247261526	0.356	0.322	5.86148603132579E-07	6	CRAT
5.67060506146637E-28	1.02525366465581	0.496	0.437	9.50279996200534E-24	6	SERF2
6.4585432977955E-21	1.02367415583422	0.454	0.41	1.08232268584457E-16	6	COA3
1.35321027134486E-34	1.01748798083349	0.543	0.477	2.26770977271972E-30	6	SLC25A1
1.73753016402418E-138	1.01341023924408	0.847	0.705	2.91175304887173E-134	6	GSTA1
1.01741266576294E-21	1.01044657699009	0.435	0.371	1.70498014528553E-17	6	GCLM
9.98220559613756E-08	1.00940034001938	0.335	0.314	0.00167281801380073	6	ZFAND5
4.05224124986195E-18	1.00924566990309	0.425	0.374	6.79074588651865E-14	6	PPP1CA
6.60267913679057E-10	1.00880463937034	0.318	0.282	1.10647696974336E-05	6	POLD2
1.24215350800095E-12	1.00121766914975	0.389	0.36	2.08160084870799E-08	6	CTTN
5.25137832124954E-08	1.00018464257293	0.341	0.321	0.000880025979074999	6	IFI35
6.64119689885306E-22	1.00003019088085	0.464	0.416	1.11293177630979E-17	6	DCAF11
6.06545953771171E-07	0.9934717907313	0.29	0.262	0.0101644970932973	6	TOLLIP
3.83882281789696E-09	0.992840033960083	0.332	0.299	6.43309927823173E-05	6	KLHL21
2.00521500876289E-11	0.991798664138095	0.391	0.366	3.36033931168485E-07	6	AP3D1
7.15584574047366E-21	0.988284483771244	0.484	0.445	1.19917662918858E-16	6	LAMTOR2
1.18425013746815E-77	0.981141456709816	0.705	0.601	1.98456638036913E-73	6	TXNRD1
1.48102386272984E-06	0.979183893180334	0.291	0.269	0.0248189978916266	6	POLDIP3
6.03558104437228E-33	0.977772884690484	0.502	0.42	1.01144267141591E-28	6	HNRNPUL1
1.93527874697053E-50	0.975143520760688	0.636	0.559	3.24314012417322E-46	6	JUND
1.44930269726534E-07	0.973653581524801	0.333	0.315	0.00242874146007726	6	SIPA1L3
9.94591167045289E-18	0.971123780772937	0.431	0.387	1.6667358777345E-13	6	PIM3
6.97996217508862E-11	0.969408181884658	0.348	0.31	1.16970206130135E-06	6	PLAAT3
6.72731811295757E-13	0.963694513055452	0.417	0.396	1.12736396936943E-08	6	HSF1
1.56271344619907E-07	0.962441876706657	0.273	0.232	0.0026187951931404	6	ATF5
1.90007579150255E-12	0.960801993955252	0.369	0.329	3.18414701139998E-08	6	FAM162A
1.19947419490723E-06	0.955995388517416	0.308	0.289	0.0201007885582554	6	NADK
5.79676856493179E-15	0.955679594921441	0.407	0.368	9.71422476111269E-11	6	NDUFA13
9.67179030211656E-25	0.948130380751321	0.507	0.462	1.62079861882869E-20	6	ATOX1
1.44683830790572E-06	0.946463435913056	0.281	0.251	0.024246116363884	6	LMNB2
7.24478712704556E-73	0.942797862386356	0.669	0.529	1.21408142675029E-68	6	CALM3
7.72156343447359E-39	0.939642931909851	0.597	0.529	1.29397960034908E-34	6	AHNAK
7.37973817533226E-20	0.9370686234656	0.461	0.417	1.23669652342218E-15	6	RAB5IF
1.72774734298401E-14	0.937044253375878	0.499	0.485	2.8953589973726E-10	6	LAMTOR4
4.2882818359486E-13	0.93663154762313	0.422	0.401	7.18630270068267E-09	6	SMG5
1.96343777470443E-13	0.936550254292817	0.405	0.371	3.29032902284968E-09	6	SIAH2
2.02736465947074E-53	0.935382699391899	0.646	0.545	3.39745769634106E-49	6	ANXA2
7.4698689082785E-08	0.935017145004899	0.345	0.325	0.00125180063164931	6	POLD4
4.64351766155227E-20	0.93368016488388	0.47	0.422	7.7816068972293E-16	6	SPSB1
2.20868374882839E-11	0.932551373128577	0.358	0.322	3.70131222628661E-07	6	MEA1
6.17061562866897E-29	0.92620746420782	0.571	0.521	1.03407176705235E-24	6	MIF
4.98614276005846E-20	0.921246061412456	0.476	0.439	8.35577803730596E-16	6	ATP5ME
4.02089002541163E-50	0.919166470393184	0.646	0.576	6.73820750458481E-46	6	BLVRB
5.91288161148535E-08	0.9179399167131	0.386	0.379	0.000990880700452714	6	ECI1
1.15770634802514E-08	0.916032067902302	0.356	0.333	0.000194008429802053	6	ARHGDIA
6.17624537671343E-33	0.913978953218998	0.561	0.494	1.03501520022964E-28	6	CMBL
6.99447599495867E-72	0.912589244602799	0.72	0.644	1.17213428723517E-67	6	SLC9A3R1
7.10956887601419E-214	0.912051034261221	0.956	0.85	1.19142155224246E-209	6	ALDH1A1
1.15295403882848E-06	0.911936692921358	0.357	0.35	0.0193212037826876	6	CCNI
1.21618512696124E-08	0.91028890576154	0.335	0.308	0.000203808303576164	6	FLOT1
1.01767935341114E-17	0.907265250355225	0.482	0.457	1.70542706044639E-13	6	POLDIP2

1.34913471610457E-06	0.907148762979945	0.314	0.293	0.0226087995724804	6	CDC34
1.37007070824502E-43	0.906300944676505	0.615	0.529	2.295964492877E-39	6	SAP18
1.65477512442945E-155	0.905895278636826	0.909	0.743	2.77307215351888E-151	6	LGALS4
4.95351488886716E-10	0.903716427006767	0.396	0.381	8.30110025076358E-06	6	TXNL4A
2.0591849957771E-44	0.902738500664975	0.609	0.535	3.45078221592326E-40	6	SEPHS2
3.24216422535239E-68	0.902313990509954	0.709	0.609	5.43321880884553E-64	6	CYC1
1.65742770314519E-26	0.897210275388248	0.518	0.459	2.7775173449307E-22	6	FAM114A1
3.064819055887E-11	0.894898015599526	0.424	0.396	5.13602377385543E-07	6	LSS
7.35129081087475E-07	0.892617831797311	0.355	0.349	0.0123192931408639	6	NAXE
1.37096590269511E-10	0.892055840116681	0.401	0.381	2.29746465973647E-06	6	ENSA
3.72514459155388E-17	0.890357457934281	0.5	0.483	6.24259730652599E-13	6	EEF1G
5.69992181670588E-12	0.889649089935805	0.413	0.387	9.55192898043572E-08	6	UQCR10
4.11394980810337E-08	0.889091567267017	0.375	0.363	0.000689415708841963	6	POLR1D
5.77175417009027E-12	0.887655190391316	0.399	0.374	9.67230563823727E-08	6	HSD3B7
4.28965117716456E-13	0.883286428137578	0.433	0.409	7.18859744269237E-09	6	ROMO1
5.84306224156178E-73	0.88081170764899	0.731	0.625	9.79180370440923E-69	6	CRYL1
1.21097868552374E-23	0.88054354777995	0.521	0.481	2.02935808120069E-19	6	ATP5MC2
7.24734402585729E-08	0.878923209571039	0.349	0.325	0.00121450991185316	6	NDUFS6
2.73825605631936E-17	0.875493728531885	0.461	0.419	4.58876949917999E-13	6	MAF
2.42458251431139E-28	0.873090529414547	0.546	0.478	4.06311537748302E-24	6	HTATSF1
1.0896115954042E-14	0.873002019399179	0.456	0.431	1.82597111157835E-10	6	NENF
1.08201220393935E-06	0.87200966044832	0.352	0.343	0.0181323605136157	6	PACSIN2
7.19777533813664E-11	0.865187288370801	0.382	0.352	1.20620319116494E-06	6	VASP
3.5060169959881E-14	0.864017418476672	0.468	0.443	5.87538328187687E-10	6	SIVA1
4.60946367962967E-08	0.863567453147853	0.366	0.351	0.000772453923432341	6	MLX
1.80107880240896E-49	0.859583486433558	0.634	0.533	3.01824785707693E-45	6	PRDX1
9.20813474812066E-81	0.859212629896548	0.741	0.605	1.54309922109006E-76	6	NDUFA1
3.55791666649051E-21	0.858795205504532	0.516	0.475	5.9623567497048E-17	6	CIAO2A
6.06910016132589E-18	0.856699120977198	0.459	0.414	1.01705980503499E-13	6	NEK6
1.38005267522432E-06	0.85131262899463	0.311	0.29	0.0231269227314092	6	NME1
2.38250673711276E-06	0.850249731815902	0.348	0.336	0.0399260479005357	6	SPR
2.44401729333577E-130	0.848654643686901	0.922	0.735	4.09568418017208E-126	6	S100A10
3.5249268523E-23	0.848357771447237	0.505	0.44	5.90707241908434E-19	6	CITED2
1.7670272353E-52	0.847426735726719	0.645	0.543	2.96118424091573E-48	6	FAM50A
5.74255506300484E-43	0.844624366639111	0.661	0.577	9.6233737745835E-39	6	TMEM106C
3.62068451295615E-20	0.843886122152101	0.491	0.45	6.06754310681191E-16	6	ZNF706
1.12055194545876E-10	0.842598431933234	0.441	0.428	1.87782095019979E-06	6	SERP1
4.59300847489783E-13	0.838257829813744	0.45	0.43	7.69696360223379E-09	6	VPS28
1.68727263243603E-28	0.836443264839565	0.565	0.523	2.8275314774363E-24	6	NDUFB7
4.34981688350399E-08	0.835327862058825	0.368	0.349	0.000728942313337598	6	CETN2
3.438267693449E-11	0.832735241774367	0.438	0.42	5.76184900068183E-07	6	SELENOH
2.1857822382449E-15	0.830477401200373	0.493	0.48	3.66293387485081E-11	6	ZFAND3
2.66504445967142E-18	0.82965032015538	0.477	0.425	4.46608150551736E-14	6	PGD
5.25471921390027E-08	0.824114896000973	0.407	0.4	0.000880585845865406	6	SETD7
4.35249449820281E-13	0.821976447452589	0.44	0.416	7.29391028008827E-09	6	ADRM1
1.46427255869605E-21	0.819893913759165	0.521	0.474	2.45382795386283E-17	6	HNRNPA0
4.87982485343602E-08	0.819371430752207	0.401	0.393	0.000817761048938808	6	C4orf3
1.12154146570169E-42	0.818549914214938	0.634	0.562	1.87947918822289E-38	6	EMD
2.10960570246986E-46	0.817993780832478	0.658	0.579	3.535277236199E-42	6	ACTG1
1.454213995456E-10	0.817724315987407	0.417	0.389	2.43697181358517E-06	6	CNKSR3
4.88119709022971E-12	0.817580512552565	0.389	0.349	8.17991008380695E-08	6	CAPNS1
7.01658508966405E-42	0.816836266052674	0.653	0.589	1.1758393293259E-37	6	TSTA3
3.0781191056568E-11	0.816555826672918	0.446	0.437	5.15831199725967E-07	6	NDUFS7
7.99864149459948E-07	0.816542363858741	0.373	0.366	0.0134041234166498	6	NEDD8
5.4450806149027E-08	0.814506539294267	0.4	0.396	0.000912486609445394	6	RTF2
2.18567347172706E-06	0.81390280991199	0.385	0.386	0.0366275160392022	6	SMIM19
3.85401793218235E-50	0.813654218255793	0.656	0.566	6.45856325075118E-46	6	RBM47
3.32031659006771E-28	0.811140161082772	0.556	0.5	5.56418654163547E-24	6	PSMB8
3.0569621508867E-34	0.810462577940485	0.613	0.549	5.12285717245593E-30	6	NDUFA2
3.54409796959272E-08	0.809681403733282	0.332	0.301	0.000593919937744347	6	ME1

1.14952241280658E-15	0.808549502968347	0.456	0.427	1.92636965938127E-11	6	EIF4H
1.17881849942708E-08	0.806362418734568	0.401	0.389	0.000197546404133989	6	ARF6
4.92759903908849E-10	0.805650270237549	0.416	0.405	8.2576704697045E-06	6	NMT1
2.63489319073259E-24	0.798655084185589	0.519	0.469	4.41555400902968E-20	6	STK24
1.37034344408856E-07	0.797092678418404	0.409	0.407	0.00229642154360362	6	FAM120A
7.26985094107164E-08	0.793888934081464	0.394	0.383	0.00121828162070478	6	ARRDC2
6.25335041642247E-16	0.787929129435525	0.483	0.442	1.04793646278408E-11	6	CYSTM1
3.68503808490091E-62	0.78634414874965	0.776	0.597	6.17538682267695E-58	6	NDRG1
3.26448634960437E-11	0.785915955465459	0.424	0.385	5.470626224667E-07	6	DUSP9
2.68038139085883E-07	0.783064176054756	0.391	0.386	0.00449178313480123	6	PSMC3
4.93365939634539E-13	0.78273763584532	0.439	0.412	8.26782641639561E-09	6	TALDO1
1.61685502632521E-14	0.781494706795579	0.464	0.435	2.70952565311579E-10	6	EDF1
1.34133347923879E-21	0.780854556041908	0.566	0.545	2.24780664450837E-17	6	PRKACA
1.41943550249062E-141	0.77898001644125	0.97	0.945	2.37869001507378E-137	6	ALDOB
1.14768212373618E-47	0.778842336611894	0.67	0.588	1.92328570295709E-43	6	PFN1
1.60338106235425E-13	0.77672448554962	0.476	0.456	2.68694598429325E-09	6	TCEAL4
1.20299804770728E-10	0.764640560458826	0.434	0.418	2.01598412834786E-06	6	PHYH
3.69411130237247E-07	0.763482344770994	0.402	0.404	0.00619059172051579	6	PSMB4
6.40648309577088E-07	0.762911468631617	0.371	0.364	0.0107359843718928	6	UBL3
1.81576078385863E-09	0.76149117795951	0.443	0.438	3.04285192159029E-05	6	COX7C
5.37815238203829E-15	0.761334415195996	0.504	0.483	9.01270776181977E-11	6	TBC1D9B
2.97982352929673E-10	0.758066093325622	0.439	0.43	4.99358827039546E-06	6	NDUFB10
6.27402958165583E-24	0.756118773198531	0.552	0.497	1.05140187729388E-19	6	UBE2A
2.38576114600289E-11	0.751319919890496	0.441	0.414	3.99805852847164E-07	6	MYH14
1.64183181110849E-09	0.748228655094529	0.441	0.435	2.75138174905561E-05	6	PCYT2
1.92974365421833E-07	0.74736605487591	0.384	0.37	0.00323386441573908	6	PSMD10
3.38107949490199E-08	0.744428011313253	0.412	0.388	0.000566601301755676	6	SIPA1L2
1.46196751065202E-08	0.73899215106074	0.392	0.373	0.000244996515435066	6	PAPSS2
2.07969699032657E-28	0.738688686849868	0.547	0.461	3.48515621638926E-24	6	ETHE1
1.06285264718559E-29	0.736886679075194	0.6	0.523	1.78112846615361E-25	6	PNKD
6.00421495887501E-13	0.735838615107244	0.456	0.425	1.00618634280827E-08	6	ATP6V0D1
1.39362985946037E-73	0.735650376043119	0.787	0.699	2.33544491848368E-69	6	LMNA
3.25720611051864E-09	0.734497896455322	0.406	0.386	5.45842600000713E-05	6	GALK1
8.02116116937183E-42	0.73392603059918	0.673	0.616	1.34418618876333E-37	6	PRDX2
3.93230149624301E-17	0.729695474413519	0.537	0.498	6.58975084740403E-13	6	ZBTB7B
2.95360447862313E-21	0.729017593565373	0.562	0.529	4.94965038527663E-17	6	COX6C
2.94133441692424E-52	0.728844536950991	0.728	0.653	4.92908821588164E-48	6	H3F3A
8.17872390305771E-17	0.728026667548421	0.528	0.511	1.37059055167441E-12	6	AKR1A1
1.53491443372775E-06	0.72752655798437	0.412	0.418	0.0257220960804097	6	OPTN
5.38342814871482E-118	0.723955636868152	0.903	0.782	9.02154889161629E-114	6	UGDH
7.16253957486684E-08	0.72364549344532	0.434	0.432	0.00120029838195619	6	NHSL1
1.1283433635035E-06	0.72309007056703	0.387	0.383	0.0189087780855916	6	SUB1
1.4274942787793E-14	0.721721888545253	0.481	0.458	2.39219491237836E-10	6	RAB5C
1.33106519845793E-55	0.72158536149866	0.733	0.663	2.23059905957581E-51	6	CFL1
3.9384890082148E-19	0.716927601843495	0.496	0.447	6.60011987996637E-15	6	VBP1
2.3090390245762E-18	0.708658437291434	0.548	0.519	3.86948759738479E-14	6	NDUFB9
2.29116445403701E-07	0.703318591430383	0.45	0.464	0.00383953339207522	6	BLOC1S1
1.65316927734454E-06	0.702151977198726	0.436	0.439	0.0277038107497399	6	PLAAT4
2.22820306897995E-11	0.698965633886454	0.495	0.493	3.7340227029966E-07	6	SHC1
7.99737774427941E-12	0.69637832595376	0.484	0.475	1.34020056238634E-07	6	EIF6
4.63422009380427E-07	0.689795247736466	0.415	0.416	0.0077660260331972	6	RNH1
1.28237440004699E-20	0.688885311819755	0.543	0.504	2.14900301959875E-16	6	YWHAZ
2.23852704537709E-08	0.676902297007443	0.407	0.392	0.000375132362264293	6	SMIM15
8.52894116585186E-92	0.674685489362532	0.869	0.738	1.42927996057345E-87	6	AKR1C3
2.2208613599464E-15	0.673271198295783	0.525	0.506	3.72171946699818E-11	6	IDH3G
1.69629196864806E-07	0.67034097362116	0.456	0.464	0.00284264608106043	6	RAB4A
4.97707345617155E-28	0.670025326581116	0.641	0.582	8.34057969785228E-24	6	SMIM14
1.30636131646537E-07	0.669763610162569	0.435	0.432	0.00218920029413266	6	AK4
5.47377885256097E-13	0.668947187443885	0.489	0.473	9.17295860112168E-09	6	GSR
1.3502333519414E-06	0.668335177064234	0.38	0.37	0.022627210511834	6	TXNDC17

1.6792928917529E-06	0.666954735498037	0.417	0.414	0.028141590279995	6	COX8A
1.49169364938428E-39	0.666323072012833	0.706	0.676	2.49978021763817E-35	6	ETFB
7.50202247014885E-18	0.665235739479358	0.54	0.496	1.25718892554754E-13	6	KRT18
1.62977778447946E-23	0.658013491204974	0.636	0.602	2.73118161123068E-19	6	H1F0
1.21751368848355E-07	0.657893925155791	0.432	0.431	0.00204030943916073	6	CHMP2A
1.00490948705404E-07	0.656831622742891	0.466	0.475	0.00168402731840517	6	TRIR
3.85699588320693E-69	0.654164059964716	0.796	0.611	6.46355370107817E-65	6	MPP1
4.61056401638046E-43	0.652800906817976	0.718	0.653	7.72638317865037E-39	6	MPC2
7.71364569324811E-18	0.645341234210685	0.528	0.476	1.29265274527452E-13	6	PYGB
1.9823220950546E-10	0.643780522185304	0.467	0.456	3.3219753668925E-06	6	CHCHD2
1.43161295768431E-56	0.6377795235	0.798	0.746	2.39909699448737E-52	6	ECH1
2.96711499851117E-06	0.635934164529742	0.399	0.391	0.0497229131450502	6	NSDHL
2.99502032298662E-47	0.634711533969553	0.745	0.67	5.01905505726098E-43	6	PTMS
5.4184219852078E-07	0.633076164546782	0.482	0.5	0.00908019156281124	6	BRD2
1.20894777712484E-16	0.630821635449751	0.563	0.549	2.0259546849058E-12	6	PUF60
1.75372235569998E-07	0.62721565110524	0.45	0.454	0.00293888792368203	6	RNF5
4.33632736177867E-13	0.622153812685963	0.543	0.543	7.2668173928687E-09	6	UQCRQ
5.23671722054098E-15	0.618256044027326	0.555	0.532	8.77569071818257E-11	6	PCK2
4.62300206588912E-09	0.61725035719929	0.509	0.529	7.74722686201699E-05	6	NAPA
4.85085520973148E-35	0.613489855352015	0.716	0.694	8.12906316046801E-31	6	TMSB4X
5.92849001037454E-07	0.612925926589187	0.446	0.453	0.00993496355938565	6	COX4I1
2.86461806077308E-13	0.610758044017075	0.498	0.464	4.80052694624352E-09	6	EZR
1.54709934314235E-07	0.610168744184944	0.491	0.507	0.00259262907923795	6	HNF4A
3.43344722783889E-09	0.608200855974957	0.504	0.505	5.75377086441241E-05	6	CUTA
6.81737246859158E-16	0.603462980293001	0.547	0.507	1.14245527828658E-11	6	GPAA1
7.75937177914034E-22	0.603238455440834	0.606	0.567	1.30031552274834E-17	6	CIRBP
1.91209166714068E-06	0.590367664952142	0.469	0.485	0.0320428321579435	6	EIF4EBP2
3.06328592882622E-17	0.588617071880649	0.602	0.579	5.13345455952698E-13	6	HNRNPF
6.54675412783115E-83	0.58318851186005	0.912	0.732	1.09710505674194E-78	6	ANXA4
3.37207259669465E-14	0.574014257209559	0.547	0.537	5.6509192575409E-10	6	EIF1
8.84360573290235E-47	0.570814096056893	0.819	0.725	1.48201144871978E-42	6	IFI27
8.06620002794294E-31	0.556141574951168	0.703	0.663	1.35173380068268E-26	6	NFKBIA
2.48822340049681E-16	0.55408018667383	0.613	0.616	4.16976477455256E-12	6	ADI1
2.78942874429955E-16	0.553990928609589	0.583	0.554	4.67452468969718E-12	6	TPD52
2.55690338690388E-07	0.550616278209493	0.448	0.423	0.00428485869577352	6	MCM3
4.09490702487979E-16	0.545346692488053	0.582	0.55	6.86224519229354E-12	6	SPTSSA
2.36342876296133E-25	0.543927392445227	0.665	0.652	3.96063392097059E-21	6	SOD1
6.97363947443234E-58	0.542847557960012	0.852	0.627	1.16864250312537E-53	6	TSC22D3
6.30278389817153E-21	0.528184267172061	0.648	0.561	1.05622052565558E-16	6	ERICH5
3.2183365245608E-30	0.524459439463386	0.749	0.653	5.39328834785899E-26	6	SELENBP1
1.21953436252192E-11	0.521257390981285	0.571	0.57	2.04369568471423E-07	6	OAF
1.85414972496897E-30	0.512318054177333	0.679	0.579	3.107184109103E-26	6	AKR1C2
4.2333472461493E-08	0.509122138464662	0.509	0.516	0.0007094243315097	6	MORF4L2
6.3648796212729E-14	0.503457239127812	0.611	0.607	1.06662652693291E-09	6	ACAA2
1.96543903891613E-31	0.493086319528291	0.752	0.652	3.29368274141566E-27	6	ACSL4
3.94750124446361E-18	0.476954537895075	0.645	0.611	6.61522258547212E-14	6	TMEM14A
2.7894661774515E-17	0.471127743409026	0.649	0.56	4.67458742017322E-13	6	ADA
2.84460292700986E-20	0.464888150456827	0.675	0.651	4.76698558508312E-16	6	PSME1
8.08641291241198E-18	0.46292644029534	0.701	0.669	1.355121075862E-13	6	PROX1
1.99100409552796E-07	0.46276594703209	0.524	0.541	0.00333652466328576	6	ARF1
1.25524567243673E-18	0.459765026405837	0.658	0.59	2.10354069786948E-14	6	DNM2
1.78001801809544E-08	0.452498757165612	0.543	0.521	0.000298295419472434	6	C2orf72
2.04789267141563E-22	0.45156324171877	0.661	0.576	3.43185853875832E-18	6	SSR4
5.63507914045435E-08	0.442933240729034	0.544	0.54	0.000944326562357339	6	HNRNPH2
3.35054047637716E-14	0.435854850551143	0.691	0.661	5.61483573031284E-10	6	MT-ND2
1.02522491806363E-06	0.435251552329933	0.544	0.541	0.0171807191769103	6	GDI1
1.47086561943218E-06	0.434397478842651	0.17	0.252	0.0246487660504445	6	NELFA
6.53952071895497E-07	0.432107555294166	0.618	0.648	0.0109589288208247	6	XBP1
1.69586861549719E-13	0.421503857137584	0.764	0.751	2.84193662585019E-09	6	MT-ND3
8.80370482827575E-14	0.408706025701657	0.682	0.676	1.47532485512245E-09	6	IDH1

2.01804144276867E-10	0.401622718845201	0.658	0.658	3.38183384979173E-06	6	MT-ND5
8.96781852091679E-48	0.393144672752038	0.904	0.866	1.50282702773524E-43	6	IL32
3.1439996240328E-27	0.381652021392495	0.912	0.88	5.26871456995417E-23	6	MT-CO2
6.02999229177678E-08	0.369624034131845	0.619	0.645	0.00101050610825595	6	PEBP1
2.32535820388662E-13	0.359301866195814	0.746	0.766	3.89683527807319E-09	6	CEBPD
2.4105569913263E-06	0.352566218822401	0.174	0.256	0.0403961140606461	6	RASSF4
8.6933629320524E-10	0.339970813260727	0.62	0.582	1.45683376015334E-05	6	MAOA
1.43274566780337E-07	0.322944006295073	0.2	0.302	0.00240099519010489	6	KIF13A
3.91630691489769E-07	0.31882870028317	0.178	0.266	0.00656294712798555	6	KDM3B
6.03033358512419E-25	0.313050335756987	0.893	0.867	1.01056330219511E-20	6	ACTB
2.93722097452259E-06	0.31181870829982	0.185	0.268	0.0492219490910496	6	BTBD7
1.70008731939213E-08	0.297573070765449	0.18	0.277	0.000284900632983733	6	LRRC14
2.60321388420509E-06	0.296343323357322	0.196	0.278	0.0436246582715089	6	PSENEN
1.41391867700381E-06	0.289847417535652	0.66	0.713	0.0236944491892298	6	HSP90AB1
5.45141452246207E-07	0.289799647345526	0.212	0.306	0.00913548045674194	6	EFCAB14
2.12872224261305E-08	0.284932456915941	0.163	0.254	0.000356731273417096	6	ACBD6
2.37843859077457E-07	0.282590659456735	0.227	0.331	0.00398578739042002	6	PPM1A
1.0685316970519E-08	0.27418889667387	0.189	0.289	0.000179064541791958	6	SNX6
1.75662811616807E-06	0.273227223449893	0.189	0.275	0.0294375739707445	6	MTMR1
1.72978731016272E-06	0.271353480750721	0.257	0.363	0.0289877757437068	6	TJP1
3.04123672646522E-08	0.268381318809896	0.163	0.253	0.000509650450621041	6	PIP4P1
1.63971095198345E-10	0.264670816319884	0.717	0.702	2.74782761333387E-06	6	BST2
3.24447372910389E-09	0.261855233541671	0.163	0.257	5.4370890752323E-05	6	PSMC6
1.71106524321618E-06	0.254508109686058	0.186	0.269	0.0286740313458167	6	NR3C1
4.13788721964933E-07	0.251647594066358	0.198	0.291	0.00693427140268835	6	AGGF1
3.19743025644048E-07	0.25108269130774	0.186	0.275	0.00535825362374296	6	CNPY2
0	4.41932339778135	0.284	0.028	0	7	CCDC187
3.758000852113E-197	3.68791040426442	0.441	0.124	6.29765782797097E-193	7	TNFSF14
9.60298549547932E-156	3.34338521630383	0.328	0.081	1.60926830933242E-151	7	KYNU
1.34655988443079E-84	3.18136000776701	0.299	0.109	2.25656505432912E-80	7	MUC20
2.3590960028659E-85	2.84466592169689	0.503	0.308	3.95337308160268E-81	7	NRBP2
2.57652285193401E-44	2.78543872377249	0.344	0.202	4.31773699527102E-40	7	WHRN
4.67529408336814E-40	2.41103212162271	0.251	0.121	7.83485782490833E-36	7	CYP21A2
2.73198003432949E-29	2.3508633629378	0.273	0.161	4.57825214152935E-25	7	HKDC1
9.15812686942197E-34	2.27465052432048	0.273	0.151	1.53471890077773E-29	7	NSMF
8.63134348548676E-48	2.22746573901462	0.419	0.275	1.44644054129787E-43	7	PABPC1L
2.79183422911118E-45	2.21214829666907	0.438	0.307	4.67855580114452E-41	7	PLIN5
4.11687868549724E-60	2.17443534610482	0.452	0.276	6.89906530115628E-56	7	MTSS2
1.73731076440093E-35	2.15878335847954	0.361	0.234	2.91138537898307E-31	7	PLIN4
1.551885511917E-13	2.1536204553464	0.275	0.216	2.6006497408705E-09	7	KIFC2
5.16242557619496E-52	2.10742233990818	0.545	0.466	8.65119278058751E-48	7	LENG8
7.18780279822758E-42	2.08679418240313	0.289	0.148	1.20453199292698E-37	7	RGS14
2.35119725457768E-25	2.08131381321027	0.352	0.262	3.94013635922128E-21	7	ABCA2
4.79022693932431E-66	1.9700916676263	0.327	0.136	8.02746230491968E-62	7	CPS1
9.53354008017836E-46	1.89337395602029	0.278	0.128	1.59763064663629E-41	7	GCKR
2.95765579367465E-19	1.87753177430651	0.265	0.176	4.95643957903998E-15	7	NFKB2
6.02401915573339E-12	1.86582643400933	0.298	0.252	1.0095051301178E-07	7	ESRP2
1.00768047084255E-18	1.81579680005902	0.282	0.198	1.68867093303794E-14	7	PLXNB1
2.36282113427145E-21	1.81018244670624	0.379	0.3	3.95961565681209E-17	7	IRF1
1.91395255686411E-38	1.79300803455588	0.276	0.138	3.20740169479287E-34	7	SLC25A18
1.68852014330669E-19	1.73855159311914	0.382	0.32	2.82962205615335E-15	7	CHDH
7.86093768405234E-40	1.70821189546194	0.638	0.626	1.31733593709349E-35	7	PAH
3.03379743438623E-95	1.70740487003419	0.837	0.797	5.08403774054445E-91	7	SOD2
8.85287846480173E-17	1.69670519692796	0.289	0.21	1.48356537313147E-12	7	ZC3H12A
6.19089935105436E-32	1.6673044071319	0.633	0.651	1.03747091324969E-27	7	ALDH1L1
3.31118771376899E-09	1.64677471507398	0.313	0.283	5.54888837073408E-05	7	STAT2
3.03735014121392E-13	1.61589333786767	0.386	0.356	5.08999136664629E-09	7	SHFL
1.77389491005032E-11	1.59267071919694	0.361	0.333	2.97269309026232E-07	7	IL17RB
4.52433361090269E-33	1.58609720970456	0.261	0.131	7.58187826515073E-29	7	ICAM1
6.03408589808349E-10	1.57978462356602	0.282	0.238	1.01119211480083E-05	7	DENND11

1.74450549715521E-17	1.56527011408107	0.485	0.484	2.9234423121327E-13	7	GLYCTK
3.93557781064616E-17	1.56221011628708	0.403	0.362	6.59524129508084E-13	7	SLC17A9
2.53366063923067E-44	1.54209118753175	0.594	0.493	4.24590849922275E-40	7	ITIH1
4.70070655184557E-12	1.52732947584377	0.359	0.326	7.87744403958281E-08	7	PLEC
1.46305291309048E-12	1.51502729593257	0.382	0.356	2.45178407175703E-08	7	SNRNP70
1.12660024668055E-16	1.50925654477659	0.396	0.342	1.88795669338727E-12	7	BCL3
1.06886035359018E-18	1.49133330299969	0.364	0.289	1.79119618054642E-14	7	TYMP
1.25562647766398E-07	1.48399803199158	0.257	0.219	0.0021041788512693	7	CCDC159
9.52698947032187E-20	1.46916942735211	0.479	0.46	1.59653289543654E-15	7	RNF213
1.96674997591733E-06	1.4629183826831	0.312	0.301	0.0329587960964226	7	H6PD
2.29505510595722E-08	1.46024386992335	0.25	0.202	0.000384605334656311	7	IRS2
4.05571074303318E-07	1.45064580494737	0.287	0.261	0.006796560063175	7	LTBP3
3.9586043523209E-12	1.44056442915129	0.428	0.433	6.63382917361936E-08	7	ABCC2
7.69531495857865E-07	1.43936300017486	0.291	0.269	0.0128958088075861	7	SBNO2
4.25736309270114E-25	1.4385607651694	0.534	0.515	7.13448907074857E-21	7	TP53INP1
2.14889851172905E-13	1.43258537643561	0.332	0.273	3.60112412595555E-09	7	ROBO1
6.09241033768392E-15	1.38724210501956	0.419	0.388	1.02096612438907E-10	7	LRP1
3.91725574046947E-08	1.36527172516972	0.383	0.388	0.000656453716987874	7	LIMK2
2.68717872049802E-18	1.34485388839616	0.405	0.329	4.50317409981058E-14	7	DEPP1
1.77883436913085E-19	1.33717630472015	0.503	0.498	2.98097063578947E-15	7	EPS8L2
5.12872203524786E-07	1.33510040409006	0.365	0.369	0.00859471238666837	7	MROH1
8.19935422523412E-13	1.30702126222666	0.454	0.45	1.37404778106473E-08	7	DPYS
2.12299352599507E-09	1.29626046789731	0.408	0.42	3.55771255086254E-05	7	DCAF8
1.10525860659337E-31	1.2676210223246	0.346	0.205	1.85219237292917E-27	7	ARG1
2.29891354706788E-14	1.25301881719414	0.498	0.513	3.85251932217635E-10	7	CDK5RAP3
8.24671809924605E-12	1.23068324019715	0.373	0.324	1.38198501907165E-07	7	CREB3L3
6.30826349421243E-09	1.23049535401185	0.322	0.285	0.000105713879636012	7	NFKBIZ
9.34935293386266E-14	1.22420603000417	0.507	0.537	1.5667645646567E-09	7	BDH1
5.54932285944212E-11	1.21656603538451	0.479	0.512	9.29955524785311E-07	7	NR1H4
2.81771929485372E-06	1.21505786808383	0.365	0.374	0.0472193399431586	7	PPP6R2
4.31864599980968E-09	1.16482335654504	0.423	0.433	7.23718696648106E-05	7	FNDC3B
1.07552573719221E-07	1.15083576552025	0.453	0.493	0.0018023660303867	7	ECHDC2
2.00399180176797E-13	1.14963255520148	0.457	0.452	3.35828946140276E-09	7	MYO18A
1.2787037632854E-18	1.13545645835134	0.545	0.562	2.14285176651367E-14	7	CCNL2
2.70678089588197E-18	1.11479058891173	0.323	0.22	4.536023425319E-14	7	SLC38A3
4.42476333266801E-07	1.10427037314204	0.416	0.436	0.00741501839288505	7	ARHGEF10L
3.95949391472139E-14	1.08110038093224	0.486	0.493	6.6353199022901E-10	7	NAPRT
1.41357163065024E-14	1.06988262605354	0.512	0.501	2.36886333864368E-10	7	SERPINF2
1.50401573576752E-06	1.06669633283861	0.392	0.41	0.0252042956999921	7	TMEM259
7.04285485314002E-07	1.05470829114413	0.446	0.473	0.011802416162892	7	ONECUT1
1.17253620263497E-21	1.04123458225206	0.543	0.516	1.96493616837569E-17	7	PLXNB2
6.95956847011296E-11	1.04012500339637	0.347	0.289	1.16628448422153E-06	7	MST1
1.01380454064E-18	1.01972597371764	0.605	0.614	1.69893364920451E-14	7	ELF3
1.25033440743201E-11	1.00500592514475	0.395	0.342	2.09531039997456E-07	7	ITIH4
7.25052974471417E-07	0.99608506222542	0.332	0.301	0.012150437746192	7	DEPRECATED-ENSG00000115738
2.93885650306968E-11	0.988164638452041	0.49	0.518	4.92493572784417E-07	7	SNTB1
4.20172143151959E-09	0.973179007339368	0.322	0.273	7.04124477494053E-05	7	PROC
3.92192698667105E-19	0.8960876455161	0.589	0.614	6.57236524426334E-15	7	ACADVL
2.26319142318334E-07	0.851057880352415	0.479	0.522	0.00379265618697063	7	MPST
9.16212357377051E-10	0.826946513935082	0.493	0.524	1.53538866849246E-05	7	AFDN
9.36872561570336E-09	0.808729610991275	0.527	0.575	0.000157001103867957	7	SLC12A7
2.56096927461113E-06	0.75809746406897	0.49	0.535	0.0429167231039333	7	AHNAK
3.30102470409772E-28	0.753005142299514	0.451	0.326	5.53185719912696E-24	7	SPP1
8.78615345372151E-09	0.742827487478076	0.504	0.535	0.000147238359577465	7	ABCC3
1.53932504656876E-62	0.735577224337128	0.923	0.906	2.57960091303993E-58	7	SQSTM1
4.85071485869404E-17	0.697787838708135	0.382	0.263	8.12882796019948E-13	7	COL18A1
9.57449604608078E-10	0.640869334775002	0.561	0.59	1.60449404740222E-05	7	ACSM2A
1.94802343525383E-12	0.629351962916039	0.582	0.619	3.26449767279836E-08	7	VEGFA
3.8115464501653E-77	0.628499894319103	0.98	0.928	6.38738954118701E-73	7	FN1
3.77274421014606E-70	0.607912699092059	0.986	0.965	6.32236474736278E-66	7	C3

6.14755697374091E-08	0.583474267563979	0.447	0.401	0.0010302075976595	7	LBP
4.13183516893383E-10	0.569480143139395	0.561	0.525	6.92412937609932E-06	7	ITIH3
1.0457389876646E-06	0.563135755105782	0.16	0.265	0.0175244939552833	7	SPAG9
1.40475911470658E-07	0.558547112166957	0.146	0.25	0.00235409532442528	7	PHF12
3.22494093811854E-08	0.544863069222693	0.148	0.257	0.000540435602409905	7	STK25
2.10535543082019E-07	0.51893595030819	0.162	0.269	0.00352815463096847	7	LMF2
1.46871529847591E-06	0.515358507093554	0.155	0.253	0.0246127309718594	7	HDHD2
7.97332144351209E-10	0.514164099059909	0.135	0.251	1.33616920750376E-05	7	ZNF444
1.84121005284899E-07	0.507442006387502	0.157	0.263	0.00308549980656435	7	TMEM189
7.50472405055364E-30	0.505725211239778	0.874	0.837	1.25764165639178E-25	7	PTGR1
3.28331424829558E-09	0.498705010686693	0.144	0.261	5.50217801729373E-05	7	RBCK1
1.93724321976848E-07	0.498656859984515	0.173	0.284	0.00324643218768802	7	DEPRECATED-ENSG00000062485
9.96334164828331E-14	0.492737441060211	0.76	0.798	1.66965679341932E-09	7	MT-ND4L
1.32262175393253E-06	0.49166694222731	0.185	0.297	0.0221644953524013	7	ALG3
7.81104848316882E-07	0.47650293194376	0.221	0.351	0.0130897550480943	7	MICAL3
1.52696673611297E-08	0.472999492531429	0.706	0.768	0.000255889085637811	7	NUPR1
2.06079273795914E-08	0.471487662658367	0.162	0.279	0.000345347647027193	7	ODR4
1.65930823919119E-31	0.469597485440858	0.867	0.868	2.78066874723659E-27	7	IL32
2.89671630544979E-09	0.459981567169798	0.164	0.29	4.85431718467276E-05	7	NCKIPSD
1.64487455597911E-08	0.457230230889396	0.182	0.309	0.00027564807809098	7	IDUA
2.17482704965189E-08	0.443668263789098	0.147	0.258	0.000364457516980664	7	ZBTB41
2.66707184680441E-06	0.441849294961088	0.197	0.312	0.0446947900087484	7	NR5A2
1.60446148098109E-08	0.437274246591125	0.147	0.257	0.000268875654982811	7	PMM2
1.08334925895613E-07	0.434876706225327	0.156	0.264	0.00181547668815869	7	AKAP9
4.23620021841025E-09	0.431649667237121	0.14	0.25	7.0990243260119E-05	7	TIMM50
3.96541645133522E-09	0.430368233444612	0.143	0.256	6.64524488914756E-05	7	QARS
6.52651640850579E-10	0.427986529149289	0.152	0.277	1.0937136197374E-05	7	FAM49B
1.31916787426823E-06	0.427021318532489	0.177	0.286	0.0221066152369869	7	EHBP1
8.78025725372602E-08	0.426980848657626	0.15	0.257	0.00147139551057941	7	COLGALT1
2.99804525887506E-09	0.423595492596584	0.149	0.266	5.02412424482283E-05	7	SLC37A1
7.94505139848502E-10	0.421892737648136	0.189	0.332	1.33143171335812E-05	7	ATF6B
3.86908033492024E-07	0.417804080530388	0.185	0.301	0.00648380482525934	7	USP14
2.97935966705012E-08	0.415872060531599	0.172	0.292	0.000499281093004259	7	PRPF6
9.25201785291517E-07	0.411872969572321	0.193	0.308	0.0155045315179152	7	NPEPPS
1.26906212254299E-08	0.411024452190693	0.154	0.267	0.000212669430495754	7	GNL1
3.10155578516644E-08	0.409589061373899	0.157	0.268	0.000519758718478192	7	GLA
1.9941809166174E-07	0.407861368549391	0.165	0.276	0.00334184838006743	7	GGA2
5.50921613111937E-07	0.405068744001823	0.166	0.271	0.00923234439252984	7	WIPF2
1.16934978570889E-10	0.403989375230555	0.152	0.281	1.95959637089095E-06	7	USP24
1.63601024038248E-14	0.402000095258264	0.797	0.725	2.74162596083295E-10	7	ASPH
1.00200548884302E-06	0.401894737714371	0.166	0.269	0.0167916079820313	7	NSUN2
2.63762508627745E-07	0.400678735387903	0.214	0.342	0.00442013211958375	7	DNAJC21
8.15366092014767E-11	0.399653958480939	0.67	0.645	1.36639049699835E-06	7	DEPRECATED-ENSG00000198074
2.13216296924896E-11	0.399340864913639	0.146	0.278	3.5730787038674E-07	7	NSD1
7.94887387267514E-08	0.399115903796957	0.171	0.286	0.0013320722835829	7	FASTK
2.87355854307788E-06	0.394613052450256	0.193	0.303	0.0481550940648991	7	ESYT1
1.48147421040602E-07	0.394511797601466	0.159	0.267	0.0024826544817984	7	FAF1
6.10359391643683E-08	0.39412798918456	0.152	0.259	0.00102284026851648	7	DDX56
3.2540739760683E-09	0.393558230948811	0.142	0.256	5.45317716909526E-05	7	RASSF4
7.51358748064836E-08	0.39010974673387	0.168	0.283	0.00125912699000705	7	SLC46A1
1.32856805744653E-08	0.389702651841965	0.182	0.31	0.000222641435066889	7	PI4KB
9.19025378463838E-07	0.389374507464389	0.174	0.282	0.015401027292297	7	DHPS
1.07268910009555E-09	0.388906807902836	0.166	0.294	1.79761239394013E-05	7	PCMTD2
1.03667864534689E-08	0.388688336590248	0.147	0.258	0.000173726607387233	7	HDHD5
7.619058730177E-09	0.387845965567414	0.184	0.314	0.000127680186200306	7	TRPC4AP
1.16448902985655E-10	0.386515665238225	0.138	0.257	1.95145071623361E-06	7	USP32
4.63661230340919E-07	0.386075136549361	0.16	0.264	0.00777003489805312	7	RNF168
1.02858465140648E-07	0.38360243343776	0.18	0.298	0.00172370215882697	7	PRPSAP1
1.45643759838308E-07	0.381967915102643	0.147	0.25	0.00244069812737037	7	MFN2
7.0229113350174E-08	0.381245363193908	0.208	0.341	0.00117689948152222	7	RIOK3

4.56782774447213E-08	0.381243268834754	0.187	0.311	0.000765476573418639	7	R3HDM2
1.71273400905601E-07	0.379944955350911	0.154	0.257	0.00287019965237606	7	PTAR1
4.9883790994525E-09	0.372875217447059	0.17	0.296	8.3595256948625E-05	7	FBXL6
7.00679964820442E-07	0.372538080717607	0.213	0.342	0.011741994850461	7	CYBC1
1.80170852547767E-10	0.371654400790695	0.148	0.273	3.01930314699547E-06	7	UBE2G2
2.224269061046E-07	0.370318790226749	0.205	0.335	0.00372743009250088	7	TTC3
2.08538675174163E-08	0.368401916894572	0.16	0.278	0.000349469111856862	7	GBA2
4.91994689619955E-08	0.367884027370057	0.201	0.331	0.00082448470086512	7	KMT2B
9.56969014659442E-07	0.365630303194983	0.177	0.285	0.0160368867476629	7	ATP6V0B
3.46143151567736E-07	0.362938157325717	0.189	0.308	0.00580066693397212	7	AATF
2.26527344487235E-07	0.361917297468046	0.177	0.293	0.00379614523891709	7	PSMD13
2.3179224042237E-08	0.361909531254441	0.174	0.293	0.000388437436499807	7	VAV2
1.69867347986822E-06	0.361889677490663	0.158	0.255	0.0284663701756316	7	EEPD1
1.27052806620648E-06	0.359845345874544	0.181	0.287	0.0212915093334883	7	BCL6
1.03149633087947E-06	0.355674907221311	0.185	0.295	0.0172858155128781	7	NUMA1
3.75429535283785E-09	0.354810857471134	0.146	0.258	6.29144815228568E-05	7	GORASP1
5.54481725526254E-08	0.353966080807331	0.179	0.297	0.000929200475636897	7	DNAJC7
8.25544464653026E-07	0.352948204625679	0.221	0.349	0.0138344741386554	7	LRRC8A
5.04206181185025E-07	0.352239555006536	0.167	0.272	0.00844948718429865	7	CEP170B
2.07715801325966E-06	0.352080478932194	0.2	0.316	0.0348090139862054	7	RBL2
2.67526383277731E-09	0.350929664711357	0.173	0.3	4.48320713096821E-05	7	KANSL1
1.03601899021752E-06	0.34918670339762	0.234	0.371	0.0173616062380652	7	GNAQ
6.82877636191851E-08	0.347849183701332	0.168	0.28	0.0011443663427303	7	EXOC7
1.2048223306177E-06	0.347414271950475	0.167	0.27	0.0201904126164914	7	FAM102A
4.08452305557426E-07	0.347044631837196	0.208	0.333	0.00684484373653135	7	NUDCD3
1.55115104650212E-07	0.345837382886417	0.229	0.372	0.00259941892372825	7	SLC38A2
5.05098851748808E-09	0.345216267591112	0.141	0.251	8.46444655760653E-05	7	UBE2J2
1.63540005834493E-08	0.344028707626173	0.154	0.265	0.000274060341777443	7	FN3K
1.10165581010195E-08	0.343463316023738	0.17	0.291	0.000184615480656885	7	UBP1
1.02272321368201E-11	0.343368134050976	0.752	0.722	1.71387956148832E-07	7	AKR1B10
1.14935364671777E-07	0.342961338644601	0.204	0.33	0.00192608684116964	7	ATN1
1.59131914112829E-08	0.340509745171319	0.176	0.3	0.000266673261670279	7	PTBP3
5.88613280338479E-07	0.340474921247316	0.197	0.316	0.00986398135191222	7	FBXO9
3.01133338818727E-07	0.340061621000021	0.19	0.306	0.00504639249192423	7	HNRNPUL2
2.99106718329795E-09	0.338142444934298	0.162	0.283	5.0124303857707E-05	7	VHL
3.77560790493338E-09	0.338097814664327	0.148	0.264	6.32716372708736E-05	7	USP48
6.68634624202084E-07	0.337201208078336	0.188	0.299	0.0112049790323785	7	CDC34
1.29567294807385E-08	0.336735307730536	0.23	0.381	0.000217128872638217	7	EIF4A2
3.32407070562285E-07	0.335945369357977	0.188	0.303	0.00557047768848277	7	CPSF7
1.29819991048779E-10	0.335526069596129	0.146	0.269	2.17552340999544E-06	7	HCFC1
1.01953797644726E-07	0.333624463016691	0.173	0.283	0.00170854174093032	7	TNRC18
6.22113071179306E-07	0.331225973268889	0.275	0.429	0.0104253708468228	7	LPP
5.86758905889438E-08	0.330747404569852	0.2	0.329	0.00098329057448952	7	PPP4C
5.6901260788543E-12	0.329194696220848	0.15	0.288	9.53551328294404E-08	7	ITFG1
1.88054432190076E-09	0.32718369914201	0.221	0.374	3.15141617464129E-05	7	ENAH
2.62294281973847E-09	0.326686672281669	0.177	0.307	4.39552757731772E-05	7	LRCH3
1.01704526839043E-08	0.326090084492851	0.193	0.326	0.000170436446076868	7	CARS2
3.21890886151619E-10	0.32597101649881	0.147	0.269	5.39424747012883E-06	7	MTA1
3.6622543707738E-09	0.324889365606032	0.142	0.255	6.13720587454274E-05	7	ITGAV
9.30425308327696E-08	0.324356595089582	0.197	0.323	0.00155920673169555	7	NCLN
2.5757477372545E-06	0.321727229124647	0.214	0.332	0.0431643805809109	7	HDGFL2
5.62597940347372E-09	0.318849197131726	0.187	0.318	9.42801628434126E-05	7	DDB1
1.25869694982797E-07	0.3174777238937	0.209	0.339	0.00210932434852171	7	PNN
1.4757355867642E-06	0.315631543842158	0.192	0.304	0.0247303769629945	7	TRAP1
1.38010781699595E-10	0.315585397985346	0.137	0.257	2.31278467972182E-06	7	TIAL1
5.94883906906734E-07	0.31396743465608	0.165	0.269	0.00996906451194304	7	RNF40
5.82412108995792E-10	0.31347107646095	0.14	0.256	9.76006212255148E-06	7	XAB2
2.83756809010852E-06	0.312962680945575	0.247	0.381	0.0475519660540386	7	SLC25A3
4.53569629521197E-09	0.312898032818062	0.16	0.279	7.60091985151621E-05	7	ARHGAP12
9.38886915350898E-09	0.310778135301826	0.197	0.329	0.000157338669274503	7	PARP10

1.83382954425071E-06	0.310257704050451	0.22	0.341	0.0307313155025534	7	CORO1B
1.99290290634092E-07	0.309867405268229	0.222	0.355	0.00333970669044611	7	AGPAT3
7.17677586487786E-10	0.307592869443687	0.182	0.318	1.20268409943623E-05	7	MOGS
2.85274530133401E-09	0.306756319970264	0.203	0.342	4.78063057597553E-05	7	TIA1
8.1773080405004E-08	0.305640166635645	0.157	0.265	0.00137035328142706	7	LRRC41
9.37353136422659E-07	0.30525757266565	0.221	0.346	0.0157081638601709	7	CAPN5
5.10055838121413E-11	0.304846192965255	0.151	0.282	8.54751573523865E-07	7	AP2A2
4.55593357417533E-07	0.302226312993759	0.171	0.277	0.00763483348360301	7	MLLT1
1.11792687333588E-07	0.301210457191898	0.187	0.305	0.00187342185433627	7	HHEX
1.1010025276764E-10	0.301044072420717	0.165	0.3	1.84506003588012E-06	7	DPM1
9.36328426727356E-07	0.300904988690478	0.251	0.392	0.015690991775097	7	QKI
1.4358613688931E-07	0.30056015471898	0.162	0.269	0.00240621648199106	7	MAN2B1
7.77105509202543E-10	0.299854056527987	0.147	0.266	1.30227341232162E-05	7	RASL11A
3.28252956352429E-08	0.29981785511302	0.221	0.366	0.000550086304255401	7	CHD2
1.19707098531029E-09	0.298907082603293	0.207	0.351	2.00605155718299E-05	7	TMEM245
1.41585200795699E-08	0.297296646319679	0.206	0.348	0.000237268479493433	7	IGF2R
7.63112862035117E-07	0.295791577097812	0.228	0.357	0.0127882453419845	7	PAXX
7.6913137024414E-09	0.295437505184381	0.158	0.273	0.000128891035025513	7	PCYT1A
2.50512469413277E-09	0.29517600345072	0.152	0.269	4.1980879624277E-05	7	NR3C1
1.36206020743593E-08	0.292561047804965	0.175	0.296	0.000228254049562113	7	VPS4A
3.56134709553553E-10	0.29169917116202	0.155	0.28	5.96810546269844E-06	7	GPS1
2.33781356394913E-07	0.291447003814713	0.195	0.317	0.00391770797046595	7	PSPC1
2.44526120565626E-08	0.290155562549504	0.164	0.279	0.000409776872843877	7	ANAPC5
2.28527847673801E-07	0.289118454935891	0.182	0.296	0.00382966967131756	7	PTBP1
8.78232746587504E-12	0.288144381716888	0.163	0.303	1.47174243673134E-07	7	GAA
2.20289796568064E-11	0.288116902523788	0.179	0.327	3.69161641088762E-07	7	CDC16
7.51135223674312E-09	0.287721998892378	0.142	0.251	0.000125875240783341	7	MPRIP
3.85986673462991E-11	0.286174973893349	0.138	0.262	6.4683646738928E-07	7	SPIDR
6.2638250046793E-08	0.286011557008483	0.221	0.358	0.00104969179428416	7	GNPTG
7.2155911718042E-10	0.285993104303773	0.155	0.276	1.20918876857095E-05	7	STRN4
8.27226128055165E-07	0.285763987185314	0.247	0.391	0.0138626554539484	7	NARS
1.88178586263282E-09	0.285092141084302	0.18	0.312	3.15349674860008E-05	7	CHID1
7.16067588976083E-13	0.283213949371656	0.124	0.252	1.19998606560612E-08	7	NDUFB2
1.55185291006177E-10	0.28310922648557	0.149	0.276	2.60059510668151E-06	7	ABCG5
3.99732157329098E-10	0.281694349865314	0.135	0.25	6.69871149252102E-06	7	DEPRECATED-ENSG00000124831
1.3097765054951E-06	0.281101781762662	0.206	0.322	0.0219492346790868	7	GLRX
3.96596136180246E-10	0.279011007274135	0.172	0.306	6.64615805010856E-06	7	HECTD4
9.81870719716703E-11	0.278729300681991	0.154	0.281	1.64541895210125E-06	7	ABHD16A
2.96024523009977E-06	0.278353003646882	0.283	0.437	0.049607789566012	7	NIT1
6.69561292359052E-12	0.278045164593155	0.163	0.304	1.1220508137353E-07	7	HDAC1
1.66241038158406E-06	0.27766106059794	0.197	0.311	0.0278586731745856	7	DNPEP
6.35875602565348E-07	0.277163967622724	0.258	0.405	0.0106560033477901	7	NMD3
9.74958699767184E-10	0.276656409472097	0.198	0.339	1.63383578906985E-05	7	DEPRECATED-ENSG00000174827
8.44441085910123E-10	0.276029330978988	0.139	0.253	1.41511437176818E-05	7	ACBD6
7.16115625735541E-09	0.275908641723811	0.167	0.288	0.000120006656560762	7	RUFY1
1.88226526762803E-09	0.275712645173182	0.157	0.276	3.15430013549105E-05	7	ABCF1
5.66732462343347E-07	0.275277215974834	0.235	0.373	0.00949730260394981	7	ATP2A2
7.89262450857211E-10	0.274141575436428	0.185	0.322	1.32264601514651E-05	7	SIPA1L3
5.25493295156053E-10	0.274013966733213	0.216	0.365	8.80621664022514E-06	7	DDX17
2.01489386523842E-07	0.27348491001529	0.159	0.263	0.00337655913936655	7	BCL2L1
9.96968766497554E-10	0.273392345122662	0.192	0.334	1.6707202588966E-05	7	RABGGTB
2.08034009301322E-07	0.273358923077182	0.229	0.366	0.00348623392787156	7	AP1M1
6.45994976539895E-08	0.273257020869741	0.208	0.338	0.00108255838168556	7	MYO15B
1.89480343482918E-08	0.272529629485599	0.172	0.291	0.000317531159608673	7	BPHL
1.36733394684524E-11	0.272455624137321	0.15	0.283	2.29137822812326E-07	7	RPRD2
2.75843971830193E-08	0.271605173845173	0.149	0.257	0.000462259327993037	7	SEC63
1.0523541511979E-07	0.271201433226956	0.159	0.266	0.00176353508657743	7	SERPINB6
8.97702982877007E-07	0.269422083285592	0.159	0.258	0.0150437065870529	7	BCL2L13
6.89279763278636E-09	0.267377120448106	0.221	0.366	0.000115509502730234	7	LPIN2
1.88120461556048E-11	0.266881371014407	0.163	0.301	3.15252269475625E-07	7	GOSR2

6.08782110266142E-08	0.266767249743784	0.209	0.342	0.001020197060384	7	SMAD3
5.30350991615716E-11	0.265070845253092	0.135	0.257	8.88762191749617E-07	7	SLC29A1
1.53512424033024E-07	0.264830975566763	0.176	0.289	0.00257256120194542	7	PITPNB
1.64501938718221E-08	0.262979167678546	0.181	0.304	0.000275672348903995	7	KDM5C
7.36776373089332E-09	0.262505777299709	0.209	0.351	0.00012346898460231	7	CD2AP
1.56437397622646E-08	0.262453750409165	0.21	0.349	0.00026215779093603	7	SEPTIN8
1.8231637024496E-11	0.262170116195283	0.139	0.265	3.05525773256503E-07	7	COP 1
3.77367501395609E-09	0.260980992580528	0.171	0.296	6.32392458838761E-05	7	SLC39A1
1.90731819418313E-06	0.260805696221956	0.25	0.387	0.0319628382981209	7	SEC22B
5.2333046287539E-14	0.259550458153879	0.125	0.262	8.76997189686579E-10	7	SLC38A1
1.00384148543613E-09	0.259416466778974	0.165	0.29	1.68223756129387E-05	7	PRKAB2
3.64467266329322E-10	0.257790929393235	0.163	0.291	6.10774244914679E-06	7	SCAF1
1.36012788814357E-09	0.256724143382978	0.16	0.284	2.279302314951E-05	7	UQCC1
3.99677794245548E-07	0.255702888720884	0.788	0.835	0.00669780047596689	7	CFB
5.29882317528396E-10	0.254957747337056	0.207	0.358	8.87976787714085E-06	7	MLX
7.12209820737728E-07	0.253399498663691	0.209	0.331	0.0119352121759228	7	RNF149
4.63336081625552E-10	0.253247518093286	0.185	0.326	7.764586055881E-06	7	SYVN1
1.11519110910176E-10	0.252709251928269	0.14	0.261	1.86883726063273E-06	7	USF1
1.70855165881961E-07	0.252489521741134	0.179	0.294	0.0028631908698499	7	BUD23
9.69603264274945E-09	0.251883259946378	0.197	0.33	0.000162486115027195	7	DNAJC22
2.79135200610754E-12	0.251097042692826	0.176	0.327	4.67774769183501E-08	7	MGAT1
2.66265609728834E-12	0.250751369978257	0.142	0.277	4.4620790878358E-08	7	GPR107
9.10679085993896E-09	0.250712310736364	0.198	0.332	0.000152611601230857	7	TXNDC11
0	5.58803070583508	0.838	0.025	0	8	FBXO43
0	5.47006059117045	0.65	0.02	0	8	AC091057.6
0	5.3173722474052	0.72	0.016	0	8	POLQ
0	5.14569194313352	0.253	0.005	0	8	ARHGAP11B
0	5.13387168012947	0.292	0.006	0	8	BLM
0	4.86612334808792	0.965	0.109	0	8	CENPI
0	4.79311540447026	0.794	0.032	0	8	NEIL3
0	4.73549343874383	0.334	0.007	0	8	RTKN2
0	4.59232567204804	0.82	0.057	0	8	IQGAP3
0	4.43855698912987	0.711	0.028	0	8	KNL1
0	4.37470419489321	0.807	0.049	0	8	ASPM
0	4.36752140259247	0.575	0.025	0	8	RAD54L
0	4.34206727459625	0.539	0.016	0	8	KIF15
0	4.31766573287505	0.533	0.024	0	8	XRCC2
0	4.31589856964026	0.796	0.035	0	8	HJURP
0	4.27850434517614	0.743	0.041	0	8	BRCA2
0	4.26514193878915	0.517	0.02	0	8	FANCD2
0	4.26322876354015	0.513	0.02	0	8	PARPBP
0	4.24358651435379	0.266	0.007	0	8	EME1
0	4.24088316440007	0.426	0.013	0	8	TICRR
0	4.23658784213549	0.803	0.039	0	8	ESPL1
0	4.20102968360494	0.768	0.035	0	8	CIT
0	4.18763107665907	0.598	0.02	0	8	CDC25C
0	4.16780815823171	0.549	0.017	0	8	DIAPH3
0	4.15609418850443	0.933	0.073	0	8	NCAPG
0	4.14453218210772	0.368	0.009	0	8	C17orf53
0	4.13929952756875	0.522	0.02	0	8	TTK
0	4.1315638578138	0.684	0.026	0	8	WDR62
0	4.12804017070721	0.698	0.058	0	8	ZGRF1
0	4.12131320333954	0.779	0.043	0	8	MYBL1
0	4.10925033270379	0.521	0.026	0	8	FANCB
0	4.10242909742192	0.298	0.012	0	8	KIF24
0	4.01938352619894	0.901	0.062	0	8	TACC3
0	4.018041590028	0.742	0.035	0	8	SPC25
0	3.99653727010913	0.381	0.012	0	8	ERCC6L
0	3.99522232932986	0.77	0.044	0	8	SKA3
0	3.9916680404772	0.803	0.073	0	8	RAD54B

0	3.97974717663763	0.53	0.022	0	8	PLK4
0	3.9706854722928	0.397	0.011	0	8	HASPIN
0	3.96709979846817	0.672	0.037	0	8	EXO1
0	3.95152881691415	0.652	0.046	0	8	ATAD5
0	3.94205366987024	0.578	0.027	0	8	FAM72C
0	3.93136233968701	0.726	0.041	0	8	DEPDC1B
0	3.92574410028819	0.619	0.023	0	8	PKMYT1
0	3.91877145753406	0.514	0.018	0	8	BUB1B
0	3.89881781966459	0.483	0.017	0	8	NCAPH
0	3.88769021219098	0.915	0.094	0	8	MKI67
0	3.88124511109172	0.29	0.009	0	8	DDIAS
0	3.87987610948971	0.695	0.037	0	8	KIF14
0	3.87856550613459	0.614	0.032	0	8	SGO2
0	3.87608171169247	0.606	0.032	0	8	ARHGEF39
0	3.84736461738848	0.662	0.028	0	8	AURKB
0	3.8266020944542	0.57	0.029	0	8	CIP2A
0	3.82367977275951	0.594	0.041	0	8	CENPE
0	3.8213865353913	0.815	0.049	0	8	NDC80
0	3.80717624510726	0.628	0.028	0	8	CKAP2L
0	3.79812681274124	0.722	0.041	0	8	STIL
0	3.78984863017177	0.903	0.067	0	8	ANLN
0	3.78063008857063	0.551	0.027	0	8	TROAP
0	3.77534450809485	0.654	0.028	0	8	KIF18B
0	3.77459079775786	0.893	0.104	0	8	CENPJ
0	3.75541645898036	0.379	0.014	0	8	MTFR2
0	3.7334056231407	0.706	0.044	0	8	ORC6
0	3.73105127768273	0.505	0.023	0	8	MCM10
0	3.72962834994759	0.334	0.014	0	8	RAD51
0	3.70992916069108	0.716	0.04	0	8	SKA1
0	3.61969879159094	0.674	0.045	0	8	CENPO
0	3.61471622104998	0.736	0.049	0	8	NUF2
0	3.61102043701578	0.434	0.032	0	8	CEP152
0	3.60359883056797	0.779	0.074	0	8	RECQL4
0	3.59472176151787	0.912	0.073	0	8	KIFC1
0	3.59251952762647	0.658	0.054	0	8	CDC45
0	3.58860467769578	0.497	0.031	0	8	CCDC18
0	3.5842621430194	0.575	0.045	0	8	CENPK
0	3.57405454423963	0.85	0.078	0	8	MELK
0	3.56731973627589	0.931	0.105	0	8	RRM2
0	3.56591563155025	0.53	0.029	0	8	KIF20B
0	3.54762820440515	0.719	0.051	0	8	KIF23
0	3.52893311921081	0.891	0.081	0	8	SPC24
0	3.52176722097458	0.704	0.061	0	8	C21orf58
0	3.51509952288723	0.557	0.039	0	8	LIN9
0	3.50969039790327	0.776	0.076	0	8	ARHGAP33
0	3.50875537904552	0.627	0.034	0	8	CDCA3
0	3.49258058737474	0.764	0.046	0	8	BUB1
0	3.48817125075893	0.501	0.027	0	8	PSRC1
0	3.4772798131778	0.34	0.022	0	8	E2F2
0	3.47499496318221	0.574	0.028	0	8	CEP55
0	3.45801457359828	0.341	0.019	0	8	KIF18A
0	3.45074026285548	0.415	0.017	0	8	SGO1
0	3.43997054243201	0.589	0.033	0	8	GTSE1
0	3.42468418502637	0.752	0.071	0	8	UBE2C
0	3.42187742976785	0.658	0.05	0	8	KIF11
0	3.41221890754486	0.433	0.016	0	8	TRIP13
0	3.4114563857185	0.453	0.029	0	8	REXO5
0	3.4082726068612	0.59	0.032	0	8	OIP5
0	3.4056017703518	0.558	0.036	0	8	E2F8
0	3.39974160145216	0.529	0.032	0	8	CEP128

0	3.39930620100525	0.692	0.066	0	8	BRCA1
0	3.39538738042753	0.694	0.045	0	8	ARHGAP11A
0	3.37511579316591	0.847	0.108	0	8	FANCI
0	3.33726381483803	0.553	0.024	0	8	CDCA5
0	3.334059994611	0.562	0.035	0	8	CHTF18
0	3.32487069773825	0.68	0.062	0	8	GEN1
0	3.3218048396703	0.467	0.023	0	8	CDCA2
0	3.32154008825864	0.619	0.042	0	8	DLGAP5
0	3.30666172774612	0.338	0.016	0	8	APOLD1
0	3.30586269098338	0.734	0.058	0	8	MND1
0	3.28958213463755	0.768	0.065	0	8	CENPL
0	3.28682992547912	0.49	0.032	0	8	CENPA
0	3.28288500424763	0.559	0.04	0	8	CCDC15
0	3.28156143901599	0.94	0.165	0	8	NUSAP1
0	3.27371842411321	0.603	0.044	0	8	GINS4
0	3.26849899462142	0.834	0.091	0	8	HMMR
0	3.26017937212184	0.896	0.134	0	8	TOP2A
0	3.25901202301959	0.716	0.074	0	8	HIST1H2AH
0	3.25674669333648	0.816	0.07	0	8	PRC1
0	3.22403507877976	0.551	0.029	0	8	SHCBP1
0	3.20838102347842	0.308	0.018	0	8	GPR137C
0	3.20700023353477	0.858	0.173	0	8	HIST1H1B
0	3.1959928285071	0.651	0.039	0	8	KIF2C
0	3.18807687528879	0.518	0.032	0	8	RAD51AP1
0	3.18557937935126	0.875	0.109	0	8	CDK1
0	3.1750728371301	0.803	0.102	0	8	MIS18BP1
0	3.14165017528576	0.668	0.056	0	8	DEPDC1
0	3.13380125145767	0.272	0.011	0	8	HIST1H2BM
0	3.13213272939037	0.26	0.013	0	8	TEDC2
0	3.126001491643	0.622	0.046	0	8	PBK
0	3.12532456410936	0.495	0.032	0	8	ZWILCH
0	3.10484357554654	0.905	0.129	0	8	KIF22
0	3.09966724656543	0.862	0.107	0	8	ECT2
0	3.09804436473017	0.951	0.191	0	8	SMC4
0	3.09553091472665	0.37	0.03	0	8	ORC1
0	3.09229286218031	0.382	0.03	0	8	ZNF165
0	3.0881935323576	0.782	0.085	0	8	NEMP1
0	3.08801642133696	0.668	0.061	0	8	MXD3
0	3.08504037130191	0.478	0.037	0	8	FANCM
0	3.0843030681199	0.511	0.049	0	8	MTBP
0	3.07887071528657	0.529	0.045	0	8	CEP135
0	3.07519360253058	0.722	0.086	0	8	MASTL
0	3.05930881694144	0.847	0.095	0	8	SPAG5
0	3.04740446816873	0.916	0.172	0	8	CENPU
0	3.04577854474821	0.558	0.04	0	8	CHEK2
0	3.04570766589913	0.474	0.029	0	8	MYBL2
0	3.03842831544235	0.631	0.058	0	8	RFC3
0	3.03583001295283	0.369	0.025	0	8	ESCO2
0	3.026792691584	0.487	0.04	0	8	E2F7
0	3.01277993791369	0.31	0.021	0	8	DDX11
0	3.00819715893772	0.86	0.141	0	8	FANCA
0	3.00157832215724	0.883	0.14	0	8	CENPF
0	2.97415324348542	0.602	0.059	0	8	DSCC1
0	2.97174405236055	0.763	0.094	0	8	CENPP
0	2.96742464648385	0.286	0.012	0	8	HIST1H2BN
0	2.96051463264328	0.912	0.162	0	8	CENPM
0	2.96017079940762	0.397	0.028	0	8	C4orf46
0	2.9519137293177	0.77	0.069	0	8	FOXM1
0	2.95133695062215	0.804	0.125	0	8	ANKRD36C
0	2.94701025049553	0.573	0.054	0	8	CCNF

0	2.94245108361891	0.771	0.075	0	8	BIRC5
0	2.9327507474148	0.342	0.016	0	8	HIST1H2AE
0	2.93178583730678	0.477	0.039	0	8	SASS6
0	2.92002684854395	0.415	0.028	0	8	RACGAP1
0	2.90395212205144	0.843	0.105	0	8	TPX2
0	2.88262845777844	0.51	0.044	0	8	CDCA4
0	2.88190791280394	0.559	0.066	0	8	BRIP1
0	2.87625771851518	0.368	0.031	0	8	DBF4B
0	2.85337957801179	0.387	0.018	0	8	HIST1H2BB
0	2.85227910341448	0.615	0.052	0	8	AURKA
0	2.84436913569617	0.674	0.081	0	8	BARD1
0	2.84309109278724	0.803	0.139	0	8	ANKRD36B
0	2.84187662457044	0.603	0.061	0	8	FBXO5
0	2.8340812172749	0.638	0.045	0	8	CDCA8
0	2.83336787244169	0.542	0.049	0	8	CKAP2
0	2.83205908169707	0.574	0.037	0	8	KIF4A
0	2.83055811443984	0.691	0.101	0	8	NUP62CL
0	2.82551633036591	0.394	0.033	0	8	PSMC3IP
0	2.82272688777252	0.763	0.105	0	8	MCM8
0	2.82219145426101	0.289	0.016	0	8	APOBEC3B
0	2.80731974413849	0.784	0.081	0	8	C1orf112
0	2.80651721714022	0.69	0.071	0	8	CCNA2
0	2.79438261610489	0.551	0.054	0	8	GPSM2
0	2.77874148438447	0.559	0.05	0	8	CDKN2D
0	2.77203025673293	0.907	0.211	0	8	FAM111A
0	2.77052145346015	0.746	0.124	0	8	HIST1H1E
0	2.76962781736172	0.877	0.153	0	8	NEURL1B
0	2.75002595471595	0.466	0.059	0	8	BORA
0	2.74726882504481	0.346	0.035	0	8	CLSPN
0	2.74472411510605	0.796	0.146	0	8	DEPRECATED-ENSG00000135976
0	2.73871553360368	0.664	0.101	0	8	WDR90
0	2.72253387289865	0.734	0.099	0	8	TDP1
0	2.72125710371665	0.944	0.217	0	8	NCAPG2
0	2.71900752066104	0.494	0.041	0	8	TEDC1
0	2.7151375105751	0.398	0.045	0	8	DCLRE1C
0	2.70718190938493	0.895	0.33	0	8	HIST1H1C
0	2.69609696597909	0.487	0.037	0	8	KIF20A
0	2.69274248648242	0.594	0.061	0	8	DBF4
0	2.68822251318522	0.437	0.033	0	8	PRR11
0	2.66525882602791	0.501	0.031	0	8	POC1A
0	2.65333651171428	0.679	0.085	0	8	RAD18
0	2.64601490808393	0.764	0.104	0	8	NCAPD2
3.0769964335155E-260	2.64600724828901	0.341	0.046	5.15643062328528E-256	8	FOXN4
0	2.63437661338075	0.884	0.173	0	8	UBE2T
0	2.63233893307009	0.256	0.02	0	8	SPDL1
0	2.62900924032755	0.662	0.074	0	8	VRK1
0	2.62666207519719	0.792	0.129	0	8	POLH
0	2.6261041641068	0.398	0.025	0	8	DEPRECATED-ENSG00000090889
0	2.62538399558664	0.866	0.144	0	8	PHF19
0	2.62443491752805	0.55	0.053	0	8	PLK1
0	2.62186725025791	0.662	0.122	0	8	DTL
0	2.62105786627674	0.59	0.072	0	8	CENPH
0	2.61310233970037	0.413	0.053	0	8	CDH24
0	2.60602563226005	0.759	0.102	0	8	INCENP
0	2.6049134877113	0.686	0.094	0	8	SCLT1
0	2.59764598487594	0.644	0.055	0	8	NEK2
0	2.59610235938849	0.489	0.056	0	8	FIGNL1
0	2.59494382572049	0.639	0.077	0	8	H2AFX
1.15545771407349E-276	2.58379153415616	0.333	0.041	1.93631603724435E-272	8	ABCA12
0	2.58327665219907	0.663	0.085	0	8	TBC1D31

0	2.57854609409438	0.751	0.1	0	8	ASF1B
0	2.57463569448577	0.276	0.023	0	8	TESMIN
0	2.56702523054899	0.439	0.058	0	8	SYNE1
0	2.56014290852039	0.422	0.056	0	8	FIBCD1
0	2.5568021599448	0.779	0.122	0	8	CKS2
1.05298241969283E-290	2.55262870556507	0.285	0.028	1.76458793892125E-286	8	RTTN
0	2.53421891356404	0.634	0.075	0	8	MAD2L1
0	2.53343321779826	0.835	0.139	0	8	NCAPD3
0	2.53076868956554	0.557	0.073	0	8	G2E3
0	2.52994872439201	0.811	0.166	0	8	GAS2L3
0	2.51692851155995	0.479	0.056	0	8	CENPQ
0	2.51511642800374	0.628	0.078	0	8	TTF2
0	2.51443779907384	0.605	0.064	0	8	DEPRECATED-ENSG00000188486
0	2.50110288695251	0.747	0.101	0	8	SMC2
0	2.49433579356101	0.842	0.258	0	8	HELLS
0	2.48893058974092	0.453	0.066	0	8	PRTFDC1
0	2.48489711574942	0.92	0.202	0	8	EZH2
0	2.48257274054008	0.74	0.149	0	8	POLE2
0	2.46358476466468	0.663	0.095	0	8	KNSTRN
0	2.46260713451153	0.635	0.119	0	8	FAM111B
1.09772496820635E-185	2.45388206250913	0.252	0.034	1.8395675017202E-181	8	ANKRD36
0	2.43987317480191	0.71	0.104	0	8	GINS1
0	2.43900246484598	0.831	0.172	0	8	KNTC1
0	2.43581798802452	0.764	0.108	0	8	CEP72
0	2.43034769815563	0.744	0.158	0	8	HIST1H4C
0	2.42685250222721	0.47	0.052	0	8	PPP2R3B
0	2.42320575571744	0.506	0.046	0	8	CCNB2
0	2.42154510795794	0.732	0.106	0	8	CENPW
0	2.40077315276006	0.511	0.072	0	8	CCNB1
0	2.3584392250083	0.682	0.135	0	8	TONSL
0	2.35429746453835	0.433	0.047	0	8	EFCAB11
0	2.35341654811974	0.543	0.08	0	8	METTL4
0	2.35332919386128	0.483	0.041	0	8	CDC20
0	2.34064902205294	0.683	0.102	0	8	CEP78
5.51001006485476E-298	2.33830267958521	0.402	0.055	9.2336748666836E-294	8	CEP295
0	2.33130477022888	0.567	0.09	0	8	FAM122C
0	2.32949183003406	0.342	0.032	0	8	REEP4
5.77333874512818E-243	2.31405449146631	0.252	0.026	9.6749610690858E-239	8	CTSV
0	2.31323622561593	0.728	0.137	0	8	POLA2
1.77743711838811E-280	2.31102016145132	0.443	0.072	2.9786291229948E-276	8	MMS22L
0	2.30505502028397	0.398	0.047	0	8	TMEM107
1.26637086994655E-286	2.28777755618165	0.467	0.078	2.12218430385642E-282	8	ATP8B3
0	2.28370248749673	0.948	0.307	0	8	CCDC14
0	2.26368589404654	0.672	0.131	0	8	POLD3
0	2.26139285234168	0.594	0.109	0	8	FBF1
0	2.26053614307695	0.635	0.094	0	8	CDKN3
0	2.25990956971705	0.57	0.065	0	8	SUV39H1
2.42359634099198E-288	2.25637383275409	0.326	0.037	4.06146274823435E-284	8	HAUS8
0	2.2546982328112	0.601	0.077	0	8	FAM83D
0	2.24736303747352	0.674	0.118	0	8	PKN3
0	2.24048203787633	0.628	0.103	0	8	KIAA1328
0	2.23888160539324	0.866	0.206	0	8	TK1
6.12362554727678E-286	2.2361386004567	0.345	0.042	1.02619716921264E-281	8	RNASEH2A
0	2.23355092195526	0.603	0.09	0	8	DNA2
1.97513375108212E-268	2.2276423534513	0.296	0.033	3.30992914006341E-264	8	YPEL1
0	2.22715755738455	0.667	0.11	0	8	PRIM2
0	2.22629240532364	0.498	0.063	0	8	GSTCD
0	2.22169600144951	0.53	0.076	0	8	CHEK1
0	2.22125466668358	0.915	0.246	0	8	HMGB2
5.04532366480041E-271	2.21311661293332	0.374	0.052	8.45495339747252E-267	8	DNAJC9

0	2.20752626991044	0.774	0.168	0	8	NSD2
0	2.2069172500576	0.538	0.078	0	8	FANCG
2.60822238129786E-204	2.20451274759447	0.297	0.044	4.37085906657895E-200	8	TRIM7
0	2.18301470260916	0.779	0.187	0	8	ATG16L2
6.35716881665081E-202	2.18210390185076	0.265	0.035	1.06533435029434E-197	8	STRIP2
0	2.18184913667175	0.758	0.158	0	8	SOGA1
0	2.1814116878372	0.587	0.089	0	8	LRR1
0	2.17796984615998	0.78	0.176	0	8	ACYP1
1.64185818040409E-239	2.177555530238	0.399	0.067	2.75142593872117E-235	8	PASK
1.12909814153218E-228	2.17754713271644	0.348	0.053	1.89214266557962E-224	8	SAPCD1
1.08295920566412E-301	2.17119091463881	0.686	0.16	1.81482303685194E-297	8	ADRB2
0	2.16803600599355	0.389	0.03	0	8	DEPRECATED-ENSG00000117399
1.54028118087679E-150	2.16366726951903	0.254	0.042	2.58120320291332E-146	8	TERC
0	2.15377005872657	0.664	0.102	0	8	RFWD3
0	2.15174700811466	0.537	0.07	0	8	NRM
0	2.13856304320368	0.695	0.121	0	8	KMT5A
0	2.1375695403646	0.518	0.083	0	8	RFC5
0	2.1308845187785	0.831	0.163	0	8	ZWINT
9.05477955056026E-298	2.12800641339005	0.527	0.094	1.51739995708289E-293	8	FSBP
0	2.12707266465207	0.759	0.144	0	8	PCLAF
0	2.12591067224743	0.636	0.1	0	8	SPATA5
0	2.12437639887191	0.747	0.142	0	8	CEP85
0	2.12369897193666	0.659	0.121	0	8	MELTF
0	2.12034188720367	0.944	0.282	0	8	LMNB1
0	2.11757683917256	0.711	0.151	0	8	CHAF1A
4.05373333377725E-167	2.11576553453666	0.27	0.043	6.79324632074391E-163	8	C19orf57
1.08992170930419E-265	2.11002238934232	0.565	0.119	1.82649080045196E-261	8	HCN3
0	2.10707198993993	0.61	0.109	0	8	HAUS3
0	2.10316090769303	0.282	0.024	0	8	NRGN
0	2.1015920249792	0.625	0.119	0	8	RAD51B
0	2.09586337081969	0.634	0.089	0	8	MIS18A
0	2.0955371097022	0.779	0.167	0	8	LIG1
0	2.09090575382432	0.634	0.121	0	8	CDC7
0	2.08196367272482	0.626	0.114	0	8	CENPC
0	2.0784791771733	0.609	0.112	0	8	XRCC3
5.75400741467732E-273	2.07740766208434	0.374	0.052	9.64256562551625E-269	8	DEPRECATED-ENSG00000145241
0	2.07601168584715	0.822	0.189	0	8	MPHOSPH9
0	2.07418311790555	0.675	0.138	0	8	RADX
1.20259463994698E-284	2.06912499431543	0.461	0.075	2.01530809762314E-280	8	WDHD1
1.33816083953153E-259	2.06582317819551	0.406	0.064	2.24248993488694E-255	8	SLF1
0	2.06360405256171	0.885	0.233	0	8	TCF19
0	2.05812879222296	0.71	0.111	0	8	PTTG1
0	2.05794126602449	0.876	0.228	0	8	TYMS
1.22587163580947E-205	2.054202797878	0.383	0.07	2.05431568728951E-201	8	MCOLN3
3.74572423304192E-285	2.04755080139592	0.458	0.074	6.27708466973164E-281	8	PROSER3
0	2.04368648792618	0.963	0.377	0	8	MDM2
1.10816332727862E-257	2.03792194636102	0.356	0.05	1.85706010385351E-253	8	DDB2
0	2.03586394437454	0.724	0.141	0	8	ZNF367
3.86442436465324E-223	2.03162351343283	0.365	0.059	6.4760023502859E-219	8	ZBTB8A
0	2.02262358865441	0.69	0.139	0	8	SAP30
0	2.01759643453146	0.542	0.094	0	8	TMEM201
0	2.01535101851043	0.708	0.14	0	8	CEP192
0	2.01463292763604	0.88	0.244	0	8	RBM15
0	2.01066083266796	0.65	0.134	0	8	RFC4
0	2.00250855307763	0.758	0.16	0	8	PCNT
1.23073693291463E-291	1.99687307552839	0.586	0.119	2.06246895217833E-287	8	KPNA2
8.37713418348833E-247	1.99251964896383	0.563	0.124	1.40384014646897E-242	8	TSPOAP1
0	1.98788029677924	0.546	0.087	0	8	CLEC2D
0	1.98784692876093	0.646	0.113	0	8	PARP2
6.14631576508505E-177	1.98257048453229	0.309	0.053	1.02999595591295E-172	8	SLC22A15

6.02108199309812E-302	1.98211493702731	0.537	0.096	1.00901292040338E-297	8	ZMYM1
6.91678767996864E-274	1.97620367208056	0.558	0.113	1.15911527940915E-269	8	MSH5
0	1.9724631264349	0.582	0.095	0	8	C9orf40
5.74394860168345E-306	1.96583685542435	0.538	0.095	9.62570906670112E-302	8	VANGL1
1.72428303721274E-270	1.96376300604314	0.513	0.097	2.88955351376111E-266	8	CDC6
0	1.9608559929021	0.953	0.389	0	8	ATAD2
7.3115828647433E-231	1.95836356240035	0.282	0.035	1.22527505647368E-226	8	POC5
0	1.9546162765867	0.631	0.128	0	8	FANCC
9.20218230128786E-259	1.95278736433659	0.819	0.28	1.54210171004982E-254	8	ADGRE5
0	1.9527457559646	0.71	0.151	0	8	POLD1
4.18186858103895E-251	1.94579460428145	0.466	0.086	7.00797536810507E-247	8	RIBC2
0	1.94438188460094	0.628	0.102	0	8	NDC1
0	1.94260828886545	0.84	0.228	0	8	CDKN2C
2.18237404750283E-215	1.94075396674512	0.421	0.08	3.65722242880525E-211	8	KIAA1841
4.56300126268387E-201	1.93646026434668	0.429	0.088	7.64667751600563E-197	8	TRIM45
1.10471798583882E-294	1.93444010849356	0.96	0.424	1.8512864006687E-290	8	SYNE2
1.25782179522832E-258	1.93312195691365	0.389	0.059	2.10785776444361E-254	8	GINS3
0	1.92732923757728	0.477	0.065	0	8	CCDC34
6.13477625954336E-295	1.92620372711877	0.34	0.039	1.02806580557428E-290	8	C1QL1
1.01352253990802E-214	1.92477875703636	0.382	0.067	1.69846107237786E-210	8	FAM49A
1.82664713622386E-296	1.92393603300537	0.489	0.081	3.06109527088394E-292	8	CEP97
0	1.92097565646109	0.688	0.134	0	8	SPIN4
1.4275927745179E-229	1.92063885713179	0.375	0.061	2.39235997153709E-225	8	RAD51C
9.17586322751677E-217	1.92053760791285	0.755	0.253	1.53769115966726E-212	8	CCNE2
5.30048297266775E-295	1.91433869529931	0.266	0.024	8.88254936559662E-291	8	MNS1
7.16668977116331E-267	1.91317981010847	0.621	0.142	1.20099387185155E-262	8	SPATA33
9.42862293499045E-293	1.91246941098321	0.477	0.077	1.5800486314457E-288	8	HAUS5
0	1.90536350462693	0.85	0.25	0	8	DHFR
2.25125082789038E-264	1.90492388004576	0.587	0.127	3.77264613737869E-260	8	RBL1
1.25462995680676E-268	1.90097014562582	0.676	0.169	2.10250888161678E-264	8	UHRF1
1.96787762580408E-201	1.89170002583788	0.401	0.077	3.29776932532247E-197	8	ACTRT3
0	1.89055949428502	0.859	0.231	0	8	LMNB2
0	1.88706548514108	0.819	0.189	0	8	CDK5RAP2
8.22866010089634E-280	1.88173747987005	0.503	0.09	1.37895885970821E-275	8	C11orf80
4.57790733216714E-298	1.88071220484093	0.493	0.082	7.6716571072457E-294	8	STMND1
1.8237906070764E-286	1.87142249364611	0.498	0.086	3.05630829933863E-282	8	IFT122
0	1.8672404850888	0.671	0.114	0	8	BCL2L12
9.96368281431207E-233	1.86586005499014	0.634	0.164	1.66971396602242E-228	8	POLA1
1.543565922118E-225	1.86288304635741	0.434	0.082	2.58670777228534E-221	8	CCNG2
0	1.86268291795892	0.651	0.117	0	8	NUP107
0	1.85583451607466	0.71	0.155	0	8	MGME1
0	1.85417104700014	0.949	0.339	0	8	SUGP2
0	1.85206205802137	0.766	0.18	0	8	DSN1
9.69322717939105E-288	1.85014003767925	0.483	0.081	1.62439101072235E-283	8	KIAA0586
1.77991113575666E-268	1.84357109947166	0.553	0.11	2.98277508130102E-264	8	TTLL7
4.01356463612338E-163	1.84112537212149	0.252	0.038	6.72593161721556E-159	8	SLC25A35
0	1.82911432310919	0.694	0.139	0	8	STMN1
0	1.82875374012207	0.627	0.126	0	8	CCDC88A
8.75835802678226E-301	1.82486460949755	0.74	0.188	1.46772563812817E-296	8	CKAP5
8.86675461806498E-298	1.81757250493909	0.893	0.277	1.48589073889533E-293	8	DLG5
0	1.81337268118254	0.688	0.134	0	8	KATNAL1
4.51317361278722E-144	1.80629513195927	0.257	0.044	7.56317634030882E-140	8	C3orf52
3.12262239230371E-212	1.7986961849276	0.29	0.04	5.23289060502256E-208	8	LSM 11.00
0	1.79838065101065	0.9	0.297	0	8	H2AFZ
0	1.7971554029484	0.561	0.093	0	8	RCCD1
2.80361531647405E-306	1.79640645779238	0.74	0.18	4.69829854734722E-302	8	FHL1
3.38597046910979E-123	1.79589135293464	0.292	0.063	5.67420931213418E-119	8	PRR36
9.0827487491271E-281	1.79552444425161	0.613	0.132	1.52208703537872E-276	8	NDE1
7.70302331826941E-278	1.78920980959348	0.603	0.127	1.29087264767559E-273	8	FAM184A
6.50911244480076E-173	1.78327902439224	0.333	0.061	1.09079706349971E-168	8	PTPDC1

2.5845629349395E-304	1.78230043751229	0.706	0.165	4.33121056637161E-300	8	FEN1
3.02794640347226E-214	1.78162096737316	0.356	0.058	5.07423258293881E-210	8	ILDR2
0	1.78160432322164	0.754	0.19	0	8	DCK
9.19134291032402E-236	1.77993572559515	0.515	0.108	1.5402852449121E-231	8	BICDL1
1.48966299815514E-161	1.77611008260279	0.27	0.044	2.49637725230838E-157	8	DEPRECATED-ENSG00000204516
1.72445582891924E-192	1.7757917135749	0.557	0.146	2.88984307810286E-188	8	KCNMB3
5.65022904625939E-291	1.7712778695497	0.787	0.208	9.46865383572148E-287	8	LAMA3
2.22555590189778E-143	1.77113537296777	0.282	0.053	3.7295865804003E-139	8	MAATS1
0	1.76693599381947	0.574	0.105	0	8	LIN54
5.6852654704434E-287	1.75993692263993	0.539	0.101	9.52736787536905E-283	8	FBLN1
3.43027240648225E-223	1.75954401261339	0.426	0.079	5.74845049878295E-219	8	CCP110
5.07822209208229E-271	1.75340801283745	0.96	0.414	8.51008458191151E-267	8	CEP70
0	1.75171405651036	0.851	0.242	0	8	GLA
0	1.7501824755173	0.76	0.172	0	8	DDX39A
0	1.74820043481181	0.756	0.17	0	8	TEX30
1.03331131589171E-210	1.74667106949345	0.503	0.112	1.73162310317133E-206	8	ARL17B
4.26210882155785E-259	1.74547045643237	0.498	0.094	7.14244196316664E-255	8	TP73
3.41179947683687E-199	1.74446250808866	0.401	0.077	5.71749356328323E-195	8	SLX4
0	1.74110987474579	0.513	0.085	0	8	NUDT1
2.04441686219699E-272	1.73890493884999	0.85	0.264	3.42603377766972E-268	8	ARL17A
8.84922624819999E-236	1.73814723600101	0.601	0.145	1.48295333467335E-231	8	WDR76
1.98336190577143E-287	1.73049524768521	0.831	0.251	3.32371788169176E-283	8	RRM1
7.20468647178184E-174	1.73028919898492	0.421	0.093	1.2073613589412E-169	8	FHOD3
7.20329115715692E-236	1.72990009388142	0.418	0.073	1.20712753211636E-231	8	C2orf69
1.71223500674855E-286	1.72931959379973	0.726	0.181	2.86936342430922E-282	8	TOPBP1
1.17034024323751E-129	1.7290852694505	0.278	0.056	1.96125617961743E-125	8	ZFP3
8.59062434863931E-213	1.72848488473363	0.603	0.156	1.43961682834498E-208	8	TMEM44
2.94241698935927E-242	1.7195517581635	0.461	0.086	4.93090239076827E-238	8	SUV39H2
5.30969328148822E-261	1.7152836870147	0.569	0.119	8.89798400111796E-257	8	ABCA7
0	1.7140861674212	0.735	0.157	0	8	CENPN
7.10498089692559E-222	1.7133799234326	0.458	0.091	1.19065269870679E-217	8	DGKH
1.24274780774122E-272	1.71334861751967	0.706	0.177	2.08259677621273E-268	8	BRD8
1.48899168592191E-175	1.71107463672026	0.305	0.052	2.49525226726793E-171	8	PTPN14
1.05746604449827E-186	1.71107069897367	0.462	0.105	1.7721015973702E-182	8	CCDC7
0	1.70891682391016	0.554	0.09	0	8	UBE2S
7.28368742497759E-202	1.70677436711389	0.39	0.072	1.22060033867774E-197	8	PRIMPOL
8.63417804455633E-172	1.70401721548975	0.366	0.074	1.44691555670675E-167	8	RNASE7
3.53093165235193E-167	1.70160523784057	0.354	0.07	5.91713526301137E-163	8	CCDC138
6.26115190293284E-201	1.69902064914759	0.329	0.053	1.04924383589349E-196	8	ZNF92
0	1.69734393687762	0.747	0.16	0	8	DTYMK
1.12844483744679E-245	1.6913477782106	0.586	0.132	1.89104785859332E-241	8	CEP44
3.3301354790622E-228	1.69065297361433	0.585	0.14	5.58064103581243E-224	8	ZNF700
1.82416813057785E-267	1.68893488201144	0.767	0.208	3.05694095322236E-263	8	TTLL5
2.26230494796746E-113	1.68872087708858	0.27	0.058	3.79117063180386E-109	8	C2orf92
4.34572829383548E-209	1.68421794339199	0.349	0.057	7.2825714748095E-205	8	CCSAP
1.80149741155157E-136	1.6795151317777	0.278	0.054	3.01894936227812E-132	8	PRDM5
8.40608188826493E-239	1.67866217891609	0.455	0.084	1.40869120283544E-234	8	CSE1L
1.89148264271694E-277	1.67864489874535	0.874	0.275	3.16974661266505E-273	8	NT5DC3
0	1.67761294203541	0.658	0.135	0	8	DCP2
1.48974727673127E-236	1.67581972466618	0.506	0.103	2.49651848634627E-232	8	EFCAB2
4.60445740196573E-290	1.67376720438725	0.917	0.306	7.71614971421417E-286	8	TUBGCP3
3.9510193960291E-157	1.6712394754061	0.39	0.088	6.62111830386556E-153	8	SLC4A7
1.13960404473168E-237	1.66913516781752	0.461	0.087	1.90974845816135E-233	8	HAUS7
1.16528526947559E-267	1.66824213769816	0.434	0.07	1.9527850545872E-263	8	IKBIP
3.71435078766027E-254	1.66584089632273	0.619	0.145	6.22450904996108E-250	8	CDT1
6.02374461994347E-268	1.6654967928715	0.83	0.25	1.00945912341013E-263	8	DONSON
3.81358234962881E-172	1.66083940967694	0.257	0.038	6.39080130150796E-168	8	ATRIP
8.57350932646133E-179	1.65829120860759	0.352	0.066	1.43674869292839E-174	8	PIDD1
2.07347416773982E-176	1.65613049276652	0.398	0.083	3.47472801029839E-172	8	DEPRECATED-ENSG00000196757
4.05025369213584E-161	1.65570636179424	0.366	0.077	6.78741513728124E-157	8	ALPK1

1.95549609080539E-282	1.6523778788866	0.715	0.179	3.27702034897167E-278	8	CDC25B
6.05726009248613E-210	1.65139952154788	0.49	0.107	1.01507564629883E-205	8	KMT5C
1.60805187012422E-299	1.6492154378274	0.877	0.281	2.69477332395417E-295	8	CKS1B
2.39221757977864E-300	1.64875981161388	0.648	0.139	4.00887822019304E-296	8	COQ2
1.51347472321663E-216	1.64850783496776	0.406	0.074	2.53628094116643E-212	8	CEP89
8.33127965055279E-159	1.64547878034559	0.362	0.076	1.39615584383964E-154	8	FAM185A
1.93274978068114E-220	1.64430855372472	0.509	0.111	3.23890208246546E-216	8	CHAF1B
1.07136929824844E-258	1.63986530628666	0.86	0.303	1.79540067000474E-254	8	TUBB
5.11814501146279E-199	1.63930629122296	0.487	0.111	8.57698741020934E-195	8	B3GLCT
7.16785041732692E-266	1.63863226404597	0.949	0.399	1.20118837293564E-261	8	LBR
8.1302228941194E-283	1.63702408685418	0.281	0.027	1.36246275259653E-278	8	SMTN
2.13844045122719E-210	1.63698784316387	0.559	0.135	3.58359850816652E-206	8	FSD1L
1.04643689112374E-291	1.63445860409165	0.561	0.107	1.75361894214516E-287	8	TUBA1B
3.24623986511376E-261	1.63172270387509	0.595	0.129	5.44004876595764E-257	8	ITGB3BP
5.38960312021815E-263	1.62914678297622	0.499	0.092	9.03189690886157E-259	8	RGS12
9.90106622494089E-154	1.62315100528604	0.324	0.064	1.65922067797559E-149	8	SLC25A14
1.97231902334575E-135	1.62241395493735	0.35	0.081	3.30521221932281E-131	8	MAP3K6
1.98216097611456E-281	1.62166468410103	0.84	0.253	3.32170536377278E-277	8	RAD21
2.46214537238972E-230	1.6102780950179	0.517	0.109	4.12606321505069E-226	8	ALDH16A1
3.53290399008757E-259	1.6041280169273	0.644	0.153	5.92044050658876E-255	8	LRRCC1
1.25366147008915E-203	1.60289079124637	0.324	0.05	2.1008858915754E-199	8	NUP35
4.17801458096516E-269	1.60146146415763	0.726	0.186	7.00151683478142E-265	8	NUP155
6.14909693374447E-222	1.59715173149299	0.591	0.146	1.0304656641569E-217	8	PRIM1
1.73331893249772E-277	1.59413977274623	0.692	0.169	2.90469586707968E-273	8	TUBB4B
8.06265159201912E-221	1.59300639483299	0.814	0.276	1.35113915379056E-216	8	NUP210
1.81287351333565E-196	1.59150535157139	0.346	0.059	3.03801343364789E-192	8	CDAN1
1.47878804750322E-185	1.58880085150427	0.362	0.067	2.47815301000589E-181	8	SPRTN
1.51549187379161E-276	1.58682986436582	0.703	0.176	2.53966128209998E-272	8	MZT1
7.40625361616743E-272	1.58639376223873	0.573	0.117	1.24113998099734E-267	8	DARS2
7.63778885188592E-153	1.58560216703138	0.377	0.084	1.27994065579904E-148	8	TFCP2L1
2.15021275872665E-271	1.58401562535744	0.614	0.134	3.60332654107413E-267	8	TIMELESS
1.07858482320255E-146	1.58239170546597	0.372	0.085	1.80749244672283E-142	8	MDGA1
8.05567919053417E-258	1.58200340718108	0.682	0.171	1.34997071874972E-253	8	HMGB3
6.2288050322156E-177	1.57758334398744	0.333	0.06	1.04382314729869E-172	8	TLN2
4.98218007896622E-189	1.57523698939928	0.399	0.079	8.34913737633159E-185	8	TMEM143
6.46578367562185E-274	1.57418546817719	0.927	0.321	1.08353602836071E-269	8	RNF157
2.23737365774299E-298	1.56619752234318	0.848	0.238	3.7493907756457E-294	8	NFATC2IP
1.51992348774548E-234	1.56553195687358	0.635	0.157	2.54708778076387E-230	8	PDE7A
2.80140044004068E-263	1.56484055328894	0.82	0.245	4.69458685742018E-259	8	LMF2
1.13405175737958E-157	1.56073183956989	0.318	0.061	1.9004439350167E-153	8	MTURN
5.09362724354665E-258	1.55968139355648	0.818	0.247	8.53590053473547E-254	8	KBTBD2
2.02497664607195E-178	1.55863038206404	0.423	0.093	3.39345586348738E-174	8	MYB
9.56173168345127E-246	1.55816744115126	0.563	0.123	1.60235499551276E-241	8	FLNA
2.58931276822929E-159	1.5572676596255	0.425	0.101	4.33917033699865E-155	8	ANXA2R
8.46689747243094E-169	1.55665426750825	0.399	0.087	1.41888267842998E-164	8	RMI2
5.27558186512659E-159	1.55615313782493	0.317	0.06	8.84082008957914E-155	8	WDR53
2.91637946801407E-225	1.55613437574939	0.57	0.134	4.88726871249798E-221	8	EED
2.6467710744394E-194	1.55590658276104	0.625	0.177	4.43545896654554E-190	8	PIK3C2G
1.10192349515913E-112	1.55436289793085	0.292	0.067	1.84660339318767E-108	8	AKAP6
3.48191364090697E-251	1.55380694613758	0.844	0.266	5.83499087943191E-247	8	RBM12B
2.39580355273756E-208	1.55361468097613	0.401	0.074	4.0148875936776E-204	8	GAB1
2.87477252651813E-157	1.55083282400459	0.462	0.118	4.81754379993909E-153	8	NAIP
5.66372396356097E-222	1.54993832058787	0.502	0.106	9.49126861813548E-218	8	PHTF2
2.76636349189365E-249	1.54813144533947	0.75	0.21	4.63587193971538E-245	8	ITPR3
1.75955844147663E-279	1.54672592259344	0.664	0.152	2.94866803622653E-275	8	FGFR1OP
1.7684739968932E-127	1.54251860181846	0.258	0.049	2.96360872399362E-123	8	RASEF
1.15397933167192E-271	1.5398553454406	0.855	0.258	1.9338385640158E-267	8	NUP85
9.08521468691839E-207	1.53905073391357	0.258	0.032	1.52250027723378E-202	8	WRAP53
5.44605824564688E-262	1.53128240953487	0.611	0.135	9.12650440805504E-258	8	TRNAU1AP
6.03850316907263E-105	1.53071615288189	0.377	0.11	1.01193236107319E-100	8	PPP1R3E

3.89166473768595E-213	1.53071498680837	0.483	0.102	6.52165176741412E-209	8	SLF2
4.13574504446426E-118	1.52750504508732	0.288	0.063	6.9306815455132E-114	8	ZNF620
1.42711846804216E-266	1.52538108625569	0.591	0.125	2.39156512874505E-262	8	CEP120
1.64257792725106E-239	1.5237221196408	0.819	0.252	2.75263209048733E-235	8	ZMAT1
8.10068853991123E-150	1.52336580420587	0.341	0.071	1.35751338551832E-145	8	CARD8
1.73446191430408E-113	1.5233115756487	0.3	0.07	2.90661127599078E-109	8	DNAH11
6.11739798355174E-293	1.5231216830684	0.621	0.127	1.0251535540836E-288	8	DCAF15
2.4668616315987E-248	1.52162979331443	0.538	0.11	4.1339667222331E-244	8	NAV2
5.99782152764325E-187	1.52043935257442	0.493	0.117	1.00511493160246E-182	8	RPA4
1.15067545595122E-203	1.51818655309329	0.595	0.154	1.92830192908305E-199	8	SRGAP1
8.132912025094E-240	1.51310353587222	0.63	0.153	1.36291339716525E-235	8	SLC25A40
1.33363228511975E-280	1.51202285763732	0.692	0.163	2.23490098340368E-276	8	POLE
8.02271162152949E-257	1.51170196512097	0.746	0.204	1.34444601353591E-252	8	USP1
1.1765695012108E-147	1.50865858517914	0.321	0.065	1.97169517012906E-143	8	ADCY3
2.2468903807472E-216	1.50838815558654	0.796	0.27	3.76533890005615E-212	8	CYTH2
2.97146527688172E-232	1.50829211217583	0.84	0.275	4.97958151099839E-228	8	DOT1L
4.20393043447538E-233	1.50807681960603	0.744	0.215	7.04494662209384E-229	8	S100PBP
2.23461281651275E-149	1.50155320507588	0.27	0.047	3.74476415791206E-145	8	CNTRL
9.64803004411597E-227	1.50117342173606	0.55	0.126	1.61681687479295E-222	8	DEPRECATED-ENSG00000022267
5.56687397066304E-158	1.50110800212511	0.296	0.053	9.32896740003712E-154	8	TMEM237
1.91109324647934E-162	1.49901568727149	0.433	0.103	3.20261006245007E-158	8	ZNF619
9.98083709160808E-234	1.49598842908153	0.892	0.345	1.67258867981168E-229	8	NID1
1.81785025503438E-137	1.49395086326027	0.356	0.082	3.04635345738661E-133	8	ARSF
1.83790252644599E-135	1.49386364274004	0.308	0.064	3.0799570538182E-131	8	NDOR1
4.98827784616317E-232	1.492304109696	0.603	0.144	8.35935601460024E-228	8	IER5
5.84295493366917E-131	1.49089274888255	0.316	0.069	9.79162387784279E-127	8	HECTD2
1.16231938502245E-220	1.4907950714409	0.543	0.124	1.94781482542063E-216	8	ST3GAL3
8.6599665128662E-195	1.49007530566071	0.374	0.069	1.45123718822612E-190	8	ZNF184
6.6101100240536E-178	1.48670130693861	0.525	0.137	1.1077222378309E-173	8	OSBPL10
1.51845643843473E-250	1.48528357621502	0.667	0.167	2.54462929952892E-246	8	CDK2
4.76690264683221E-176	1.48164243635422	0.587	0.166	7.98837545556141E-172	8	POU5F1
6.26979526602335E-173	1.48155586776239	0.383	0.078	1.05069229068019E-168	8	ODF2
3.58310163964031E-220	1.48145748354422	0.699	0.195	6.00456172770924E-216	8	FZD6
9.12974922315463E-207	1.48009679489619	0.603	0.157	1.52996337481625E-202	8	KANSL1L
8.07889671653471E-156	1.47775445885617	0.394	0.09	1.35386151175689E-151	8	PDE5A
4.68660042699361E-208	1.47307018080383	0.7	0.205	7.85380499555588E-204	8	CCDC198
1.26614691356065E-121	1.47268325044546	0.329	0.078	2.12180899774494E-117	8	BICD1
5.05181307257471E-230	1.47070948912108	0.451	0.086	8.4658283470207E-226	8	RCC1
3.04053568253631E-241	1.46881822187076	0.381	0.059	5.09532969679436E-237	8	RASSF1
3.22053188854741E-171	1.46842282119791	0.418	0.093	5.39696733882776E-167	8	RAC3
8.44023441592353E-236	1.46714514946155	0.928	0.331	1.41441448342046E-231	8	GABRE
2.28596925075478E-232	1.46282517950124	0.739	0.211	3.83082727041486E-228	8	UGGT2
3.11612658553465E-123	1.46175366556236	0.401	0.109	5.22200493203897E-119	8	ARHGEF38
1.6172359566596E-231	1.46029528036582	0.386	0.063	2.71016401617017E-227	8	CNTROB
9.9844005208091E-230	1.46004784519056	0.462	0.089	1.67318583927719E-225	8	RHNO1
1.07809992530029E-231	1.45912589247272	0.77	0.225	1.80667985481823E-227	8	ANAPC4
2.01071466825904E-244	1.45841723584209	0.699	0.183	3.36955564106849E-240	8	HP1BP3
4.40562341510463E-258	1.45563718701132	0.726	0.187	7.38294371903233E-254	8	SMPD4
2.50035422902093E-135	1.45367929448777	0.362	0.086	4.19009361699327E-131	8	RHPN1
7.28203206926316E-260	1.45330343268872	0.638	0.148	1.22032293416712E-255	8	SRGAP2B
6.92965948603283E-233	1.45299763497914	0.916	0.359	1.16127233666938E-228	8	FAM122B
1.21120344191744E-168	1.45247660591602	0.433	0.1	2.02973472796525E-164	8	PDSS1
2.3591139532991E-279	1.45206274642083	0.712	0.173	3.95340316293864E-275	8	RANGAP1
5.51700523874126E-140	1.45091498041639	0.314	0.065	9.2453973790826E-136	8	CYP3A43
9.41700854694448E-219	1.44957931829494	0.694	0.196	1.57810229229696E-214	8	SLC7A1
2.49653459065752E-197	1.44848185344509	0.453	0.096	4.18369266702388E-193	8	PHTF1
5.24048143713108E-223	1.4436929701045	0.619	0.156	8.78199879234427E-219	8	SCMH1
1.44630295881841E-260	1.44339400906621	0.704	0.178	2.42371449838788E-256	8	NUP205
4.46333972851758E-200	1.43920542116569	0.826	0.311	7.47966471704976E-196	8	MCM7
4.5366044687593E-141	1.4387660200401	0.411	0.105	7.60244176874683E-137	8	ZDHHC23

4.66935447026358E-147	1.43864118496673	0.458	0.121	7.82490422126771E-143	8	ADGRG2
2.10038643335273E-231	1.43473297206848	0.594	0.142	3.51982758501251E-227	8	SLC1A4
7.1236748802924E-229	1.43191805260229	0.838	0.278	1.1937854364394E-224	8	DAZAP1
5.47571076465831E-175	1.43155400199248	0.366	0.072	9.1761960994144E-171	8	NEDD1
1.18817534598889E-87	1.4313679816277	0.257	0.065	1.99114424480818E-83	8	CTBP2
5.63566674320855E-161	1.43022728331237	0.375	0.08	9.4442503282689E-157	8	MDM1
1.23694973281438E-165	1.42962945780357	0.36	0.073	2.07288036225033E-161	8	TUBD1
6.36793642989729E-197	1.42882749625611	0.486	0.109	1.06713878692219E-192	8	ALMS1
2.23192815501712E-190	1.42880013662898	0.356	0.064	3.74026520217769E-186	8	CCDC134
9.20096974074936E-103	1.42836517677609	0.286	0.07	1.54189850915478E-98	8	PLCE1
2.00348760174429E-209	1.42827141336008	0.744	0.228	3.35744452300308E-205	8	DCAF16
8.78082765103941E-249	1.42714796908562	0.71	0.186	1.47149109776118E-244	8	PDS5B
2.06184876748173E-126	1.42487478813724	0.272	0.054	3.45524616454588E-122	8	NEMP2
4.91467110594936E-209	1.42306544302726	0.497	0.109	8.23600583934994E-205	8	FAM13B
1.48295374617598E-234	1.42301166516855	0.57	0.128	2.48513388784171E-230	8	TTL
8.63095957235455E-160	1.41788386936146	0.422	0.099	1.44637620513518E-155	8	OBI1
8.24536537288332E-192	1.41719860659408	0.36	0.064	1.38175832918779E-187	8	PPIH
3.42567718809745E-208	1.41271911162036	0.762	0.241	5.7407498318137E-204	8	PCF11
2.05126287055142E-259	1.4089982947174	0.756	0.206	3.43750631847008E-255	8	WEE1
1.44848881814977E-133	1.40830160425104	0.411	0.109	2.42737756145538E-129	8	TUBE1
6.09969073541751E-237	1.40683725524798	0.712	0.192	1.02218617344127E-232	8	MYO5C
5.89082756711965E-219	1.40592747804792	0.866	0.297	9.87184883697911E-215	8	MICALL2
1.93113077313436E-177	1.40363083990716	0.442	0.1	3.23618894961856E-173	8	DEPRECATED-ENSG00000132570
1.44871561935819E-164	1.40227882666942	0.372	0.078	2.42775763492045E-160	8	SACS
9.03172686386236E-220	1.40002229237282	0.571	0.138	1.51353678784605E-215	8	RNF26
4.7272955676209E-165	1.39892041302452	0.338	0.065	7.92200191221911E-161	8	SRGAP2C
9.30910637615468E-193	1.39706093319606	0.884	0.352	1.560020046516E-188	8	FXYD3
4.36220891006195E-150	1.39686647976758	0.321	0.064	7.31018969148181E-146	8	TOE1
3.76894899911916E-211	1.39486808907111	0.67	0.183	6.31600473272388E-207	8	ANKS1B
2.06557582914718E-100	1.39334964320302	0.257	0.058	3.46149197448485E-96	8	MME
7.16290579850827E-132	1.39273419692491	0.32	0.07	1.20035975371402E-127	8	ARHGAP4
1.26490117944971E-226	1.3924546950051	0.708	0.196	2.11972139652183E-222	8	LRRC45
3.70250343433289E-209	1.39045146547558	0.585	0.146	6.20465525525506E-205	8	MALT1
2.40189162938191E-215	1.38983564651102	0.451	0.089	4.02508999251821E-211	8	PHIP
4.67593336987452E-156	1.38639416056086	0.462	0.118	7.83592914123572E-152	8	PPM1L
5.98474634793217E-234	1.38625833421563	0.61	0.145	1.00292379298647E-229	8	RMI1
1.72875166666353E-223	1.38087915044936	0.8	0.249	2.89704204299474E-219	8	FRYL
3.18132454165602E-213	1.37989701948126	0.66	0.178	5.33126366690716E-209	8	UPF3B
4.2684700899229E-190	1.37974580146373	0.632	0.178	7.1531021766928E-186	8	ACAP3
1.43386779026713E-102	1.37947099673143	0.309	0.079	2.40287564292965E-98	8	EML5
2.54673903933409E-227	1.37942440531014	0.808	0.256	4.26782528211607E-223	8	SUPT16H
1.18549590952963E-230	1.37844795733624	0.554	0.122	1.98665404518975E-226	8	RCC2
6.04527406873582E-192	1.37522851519238	0.767	0.269	1.01306702843875E-187	8	TUBA1C
5.23354577594625E-253	1.37341592095286	0.762	0.209	8.77037601133073E-249	8	CCHCR1
3.46155734734239E-187	1.37270238833006	0.519	0.128	5.80087780267638E-183	8	TMEM187
4.28925819548082E-166	1.37041386439726	0.497	0.127	7.18793888398676E-162	8	OTUD3
1.4575501891561E-241	1.36919042991375	0.776	0.225	2.44256260698779E-237	8	C19orf48
7.61257845696323E-236	1.36903694773447	0.759	0.218	1.2757158978179E-231	8	TNPO2
1.99083516658977E-144	1.36901681141577	0.27	0.048	3.33624157217114E-140	8	TCF7
1.04167053104212E-233	1.36682645841527	0.77	0.23	1.74563147592038E-229	8	TUBG1
1.86234258301197E-82	1.36629989948497	0.25	0.064	3.12091370061147E-78	8	SALL4
3.07926830909669E-217	1.36449131735669	0.613	0.155	5.16023783238424E-213	8	SKP2
2.35154535944894E-218	1.36247414152708	0.724	0.208	3.94071971336453E-214	8	DIP2A
6.8345826479122E-181	1.36219597166955	0.389	0.078	1.14533936013713E-176	8	ERI1
3.87997336848892E-156	1.3605606021524	0.819	0.347	6.50205937091373E-152	8	MCM4
2.80666933234163E-225	1.36051597565224	0.82	0.268	4.7034164671381E-221	8	NCAPH2
1.95069000933343E-141	1.35996374824293	0.333	0.071	3.26896631764096E-137	8	TOP3A
6.5301778431033E-208	1.35794584397489	0.449	0.091	1.09432720294725E-203	8	CTNNAL1
8.34502468568387E-158	1.35702271212476	0.447	0.11	1.3984592368269E-153	8	TUBGCP5
9.26882578278507E-202	1.35646569598765	0.434	0.087	1.55326982467912E-197	8	MRE11

6.45357632809256E-234	1.35488306528489	0.628	0.153	1.08149032106175E-229	8	NUP188
4.07215568983526E-233	1.35344616364147	0.644	0.161	6.82411850502592E-229	8	XPO1
1.85400235085995E-254	1.35303643919179	0.774	0.215	3.1069371395711E-250	8	CKLF
1.48282521774094E-215	1.35286072345685	0.695	0.196	2.48491849989026E-211	8	TELO2
7.49030227962929E-258	1.34982755285129	0.654	0.154	1.25522485602028E-253	8	WDR34
5.95124366966996E-195	1.34635165516619	0.498	0.116	9.97309414163292E-191	8	CDCA7L
4.56869348110777E-196	1.34530107105557	0.566	0.144	7.6562165356404E-192	8	ZNF266
7.57727248824897E-182	1.34263477735515	0.39	0.078	1.26979932358076E-177	8	ACVR2B
1.24548219102687E-190	1.34190238737014	0.578	0.152	2.08717905572283E-186	8	SLC35E3
3.26324562360085E-161	1.34035371257412	0.549	0.156	5.4685470160303E-157	8	KLHL42
4.17093353650159E-185	1.33709167796169	0.459	0.104	6.98965042046936E-181	8	FAM102B
1.666396858547E-204	1.33454190389655	0.84	0.293	2.79254785555306E-200	8	PSPC1
4.07734725116335E-264	1.33400718023032	0.68	0.164	6.83281852349954E-260	8	TCOF1
3.11670350397302E-163	1.33133877890634	0.566	0.163	5.22297173195799E-159	8	DCUN1D2
2.61163098297437E-176	1.33116334067986	0.609	0.175	4.37657120126844E-172	8	RBMX2
6.95022993981869E-183	1.32860735081203	0.434	0.094	1.16471953331482E-178	8	CTDSPL2
9.44974446937531E-138	1.32786072302245	0.382	0.093	1.58358817817792E-133	8	POGLUT3
8.777528147491E-120	1.32636061552414	0.318	0.075	1.47093816695654E-115	8	NPAT
2.49645168028818E-200	1.32604728668861	0.823	0.28	4.18355372582693E-196	8	RBM41
5.91506860623758E-199	1.32590131438418	0.558	0.139	9.91247197033294E-195	8	HAUS2
2.99340513708088E-105	1.32442507902763	0.29	0.069	5.01634832872014E-101	8	MFSD14C
5.50217548081268E-178	1.32342428269545	0.762	0.265	9.22054567074588E-174	8	ZNF189
3.31268894234513E-212	1.32341822127798	0.398	0.071	5.55140412958197E-208	8	FBLN7
1.02562908250876E-118	1.32319840558743	0.306	0.07	1.71874921646818E-114	8	KIAA1586
8.95167968231623E-123	1.32229149953731	0.403	0.109	1.50012248116255E-118	8	C6orf141
1.14549889719437E-232	1.32196626713555	0.87	0.288	1.91962705191833E-228	8	SCML1
1.24452898207424E-157	1.32008332231621	0.418	0.1	2.08558166816001E-153	8	GINS2
1.88969737598553E-75	1.31967748893976	0.292	0.087	3.16675486267655E-71	8	MPP3
2.19970305055459E-184	1.31860148300481	0.575	0.153	3.68626237211938E-180	8	AC253572.1
1.00004391386315E-185	1.31653584229828	0.535	0.133	1.67587359085187E-181	8	STX2
9.12309433207006E-178	1.31493972755973	0.525	0.134	1.5288481481683E-173	8	CD58
4.12872198837335E-120	1.31406205037804	0.379	0.101	6.91891230811605E-116	8	ARSJ
2.54995048787179E-135	1.3129183185042	0.34	0.076	4.27320702757554E-131	8	OSBPL3
1.87413594550498E-257	1.31139718586893	0.652	0.153	3.14067701747724E-253	8	OAS3
1.81332881360037E-147	1.30986186714611	0.385	0.088	3.0387764258315E-143	8	MYO9A
5.35198544787124E-218	1.30810890054482	0.754	0.222	8.96885721354262E-214	8	SPATS2
1.37977203238586E-203	1.30758755396257	0.766	0.24	2.31222197187223E-199	8	PRELID2
3.12976193349812E-171	1.30295491238655	0.545	0.146	5.24485504815615E-167	8	CHML
7.81136238065009E-210	1.30258361634775	0.679	0.194	1.30902810774934E-205	8	SLC9A6
1.82138667287858E-146	1.30080799287263	0.387	0.091	3.05227978640992E-142	8	LRRC26
2.34160168469342E-214	1.30056468543999	0.707	0.2	3.92405610320923E-210	8	GTPBP2
1.4024586306383E-204	1.30020167034855	0.929	0.37	2.35024017322366E-200	8	HGSNAT
9.29197897514755E-117	1.30008514766431	0.289	0.064	1.55714983665523E-112	8	CARMIL1
9.16084911983484E-215	1.29950568995282	0.712	0.204	1.53517509550192E-210	8	SMCHD1
9.2671667910023E-170	1.29553453772845	0.401	0.086	1.55299181083617E-165	8	CENPS
5.73793685590328E-178	1.29443908747883	0.815	0.303	9.61563458312272E-174	8	MYO19
6.47538873920135E-185	1.29422488241636	0.543	0.139	1.08514564491536E-180	8	LCORL
4.72588160826371E-180	1.29181376006567	0.632	0.184	7.91963239912833E-176	8	ZNF75A
8.99646864533412E-174	1.28900666832267	0.927	0.443	1.50762821558509E-169	8	STOM
8.0853869309219E-151	1.28814420857192	0.425	0.104	1.35494914188389E-146	8	NOCT
1.35162692075526E-109	1.28751083224319	0.35	0.093	2.26505639380166E-105	8	ZBED8
1.28399827872442E-94	1.2863500140231	0.25	0.058	2.15172431548638E-90	8	MINDY4
2.24998302164876E-224	1.28414019637875	0.818	0.26	3.77052154767898E-220	8	NUP43
6.7092078764345E-134	1.28369197122493	0.328	0.072	1.12432905593289E-129	8	ORC3
3.34345523590829E-209	1.28325020371067	0.483	0.102	5.60296228433511E-205	8	EXOSC8
2.6999198653965E-182	1.2808027237836	0.601	0.166	4.52452571043145E-178	8	NVL
4.1507287082216E-178	1.27863146009831	0.921	0.379	6.95579116923775E-174	8	SYTL4
1.53336585286845E-233	1.27398567358616	0.778	0.23	2.56961449623694E-229	8	PGP
7.6894949753396E-169	1.27376050680801	0.662	0.212	1.28860556796741E-164	8	PCNA
1.51591645669711E-220	1.27042517745455	0.527	0.116	2.54037279813302E-216	8	SRGAP2

4.82960129799389E-216	1.26938740106143	0.63	0.164	8.09344585517817E-212	8	BTG3
7.77807433204361E-191	1.2693335139083	0.756	0.243	1.30344969656387E-186	8	LYST
1.22219322604117E-221	1.26705778421341	0.839	0.286	2.0481514081998E-217	8	GGH
2.51330804583838E-190	1.26633650433792	0.582	0.153	4.21180162321596E-186	8	PLXNC1
4.5957978432191E-208	1.26629493417555	0.826	0.284	7.70163802566657E-204	8	RBMX
1.02720693664973E-107	1.26566529859981	0.298	0.071	1.72139338443761E-103	8	MED7
2.77234477512361E-127	1.26457477711751	0.411	0.11	4.64589537415215E-123	8	DOCK10
1.18673736194399E-106	1.26393268816582	0.302	0.074	1.98873447114574E-102	8	ZNF280C
3.41059704401043E-160	1.26059489476961	0.483	0.124	5.71547852635269E-156	8	ZNF33B
1.18277338711096E-195	1.26015119723205	0.517	0.121	1.98209164212054E-191	8	MTF2
1.33647502133485E-93	1.25986297298659	0.276	0.069	2.23966484075293E-89	8	KIF21B
2.02679031143250E-210	1.2590812167223	0.621	0.162	3.39649520389859E-206	8	JADE1
1.65930903645417E-98	1.25795513762352	0.3	0.076	2.7806700832899E-94	8	SLC25A12
6.6661748870537E-178	1.25677522896361	0.569	0.155	1.11711758757246E-173	8	EEF1AKMT2
6.87802811078195E-122	1.25654294975688	0.368	0.093	1.15261995080484E-117	8	AP4B1
6.57666682927042E-186	1.25631675119654	0.779	0.27	1.10211782724914E-181	8	SLC27A3
1.29432616383255E-64	1.25536500751501	0.257	0.077	2.16903178535059E-60	8	PNMT
5.69320825519473E-158	1.25413199294946	0.74	0.274	9.54067839405533E-154	8	DEPRECATED-ENSG00000132646
1.61487675414575E-220	1.25373923519252	0.617	0.156	2.70621046459744E-216	8	EMC9
3.33735945328583E-186	1.25303932613533	0.816	0.299	5.59274697181639E-182	8	DNMT1
4.75534061772839E-183	1.25090302026557	0.591	0.162	7.96899980718924E-179	8	ZFP62
1.23943099022179E-103	1.24971506534736	0.26	0.058	2.07703845341368E-99	8	STON2
5.9289477785453E-158	1.24962069944018	0.541	0.153	9.93573068728622E-154	8	PMS1
3.89776372838492E-196	1.24869271986979	0.917	0.351	6.53187245602744E-192	8	TAZ
2.4639806753797E-228	1.24833833979139	0.751	0.218	4.12913881580131E-224	8	GMNN
7.2071876505256E-174	1.24817805474439	0.742	0.255	1.20778050647508E-169	8	SLC3A2
6.17294414162997E-143	1.24732287062704	0.383	0.09	1.03446197925435E-138	8	LYPD6
6.55726322252132E-96	1.24687781244012	0.262	0.062	1.09886617083012E-91	8	ZNF532
1.17008314066417E-149	1.24457868677284	0.375	0.084	1.96082532712502E-145	8	MCUB
1.21192633946116E-183	1.24380035666087	0.659	0.192	2.030946159669E-179	8	AHI1
5.46991293569951E-173	1.24295314862683	0.415	0.091	9.16648009764525E-169	8	NT5DC2
6.61903244829027E-177	1.24152568079527	0.546	0.143	1.10921745768448E-172	8	CCDC82
1.30642815099333E-208	1.24077865957314	0.822	0.266	2.18931229543463E-204	8	THOP1
1.18271670857784E-160	1.24065439282004	0.485	0.125	1.98199666023475E-156	8	TTI1
3.66796313301617E-167	1.24058482738292	0.663	0.206	6.1467726183085E-163	8	ZBTB25
1.27402885541102E-199	1.24042977958524	0.421	0.083	2.13501755589778E-195	8	HAUS6
1.72592098300599E-185	1.2397026199058	0.525	0.13	2.89229838332143E-181	8	PKNOX1
4.48702402022689E-192	1.2389246930278	0.98	0.568	7.51935485309622E-188	8	NOTCH2NLC
2.19133559109917E-166	1.23836571292507	0.374	0.077	3.67224018356399E-162	8	SCAPER
7.65118872881202E-190	1.23806006910562	0.783	0.259	1.28218620717432E-185	8	ZNF75D
4.12700199421681E-257	1.23728852319782	0.752	0.196	6.91602994190853E-253	8	CENPT
2.45760525113134E-159	1.23723795009852	0.417	0.096	4.1184548798459E-155	8	PPM1D
8.06377741449465E-194	1.23706362941975	0.679	0.199	1.35132781912101E-189	8	INTS7
2.69289334554352E-190	1.23695755340769	0.771	0.26	4.51275066846183E-186	8	FAIM
3.16712520839386E-178	1.23684417908563	0.808	0.289	5.30746842422642E-174	8	TRIO
1.72972877671874E-169	1.23665285344595	0.573	0.161	2.89867948402526E-165	8	GTPBP3
1.20165963157822E-187	1.23516000814108	0.703	0.217	2.01374121059878E-183	8	KLHL22
6.76090499487189E-164	1.2344996595554	0.438	0.103	1.13299245904063E-159	8	SFI1
8.30860399672757E-202	1.23354603645343	0.862	0.3	1.39235585777161E-197	8	RBM26
3.21620034289651E-119	1.2321834245935	0.325	0.076	5.38970853462597E-115	8	GLMN
6.58623632353786E-160	1.23108200785627	0.599	0.18	1.10372148309847E-155	8	SMC5
9.78342862453854E-178	1.23058832692843	0.442	0.1	1.63950696890017E-173	8	DHRS13
6.97323805530062E-119	1.23010665662171	0.284	0.061	1.16857523330728E-114	8	ZNF101
1.03398796602989E-157	1.22877279433772	0.49	0.127	1.73275703347289E-153	8	KRT15
1.67762620737528E-164	1.2255504418271	0.59	0.171	2.8113659983195E-160	8	FAM76B
7.23329779657951E-101	1.22500430369172	0.308	0.078	1.2121560447508E-96	8	PLAGL2
1.43844120721137E-120	1.22485895270878	0.427	0.123	2.41053977504482E-116	8	B4GALT4
5.48294515351741E-165	1.22466373891476	0.586	0.167	9.18831948826447E-161	8	RAVER2
1.80027736328189E-175	1.2239540799064	0.531	0.137	3.0169048053878E-171	8	CSGALNACT2
2.74509558747942E-170	1.22373220699849	0.497	0.125	4.60023118549801E-166	8	RHOQ

3.50958842030269E-198	1.22250231513071	0.888	0.332	5.88136827474324E-194	8	IPO9
1.0676790319917E-203	1.21858760645823	0.699	0.203	1.7892165218117E-199	8	CEP250
1.65963674685029E-148	1.21835630897552	0.45	0.116	2.78121926037172E-144	8	ANKMY1
7.44090307045953E-169	1.21831152160315	0.489	0.123	1.24694653654761E-164	8	SMG9
3.16192748918153E-170	1.21804550130123	0.562	0.155	5.29875808637041E-166	8	TCP11L1
9.37895701375955E-130	1.21748778111295	0.372	0.091	1.57172561636582E-125	8	EMSY
8.3504085439078E-170	1.21635813429069	0.459	0.11	1.39936146378807E-165	8	KATNA1
1.9738847071983E-173	1.21543521666056	0.975	0.472	3.30783599232291E-169	8	ARGLU1
3.5852663798622E-124	1.21354434481853	0.403	0.108	6.00818939937307E-120	8	SMYD4
1.55263225799526E-176	1.21329816594905	0.461	0.106	2.60190113794845E-172	8	ZBTB5
3.56065089068567E-161	1.21299461028803	0.445	0.107	5.96693876261104E-157	8	TMEM116
4.38920230864218E-196	1.21187226628618	0.716	0.217	7.35542522882256E-192	8	CSTF3
5.06491161837504E-109	1.21111453675654	0.302	0.072	8.4877788900729E-105	8	DHRS4
2.37442486966349E-164	1.21066120636192	0.877	0.364	3.97906119658208E-160	8	PRKDC
1.22204376850765E-139	1.21034858740185	0.282	0.053	2.04790094726512E-135	8	ULK4
1.30474880370706E-155	1.20969863685505	0.437	0.106	2.18649804525229E-151	8	DEPRECATED-ENSG00000165948
4.82722159928162E-160	1.20908224958576	0.457	0.112	8.08945795607614E-156	8	PPOX
1.15802046742116E-222	1.20855038913555	0.748	0.222	1.94061069930438E-218	8	SAE1
3.81325080116846E-202	1.20846919464822	0.933	0.408	6.3902456925981E-198	8	TMPO
1.56043990573692E-188	1.2072060982611	0.626	0.173	2.61498519403393E-184	8	C2CD5
2.86491219983942E-180	1.20471366792858	0.454	0.102	4.8010198644909E-176	8	TIMM21
1.6690757836983E-206	1.20327981354945	0.748	0.236	2.79703719832161E-202	8	ANP32E
2.05962640639951E-165	1.20301288739219	0.425	0.097	3.4515219318443E-161	8	DEPRECATED-ENSG00000179051
7.1016262601315E-95	1.20252837535534	0.321	0.088	1.19009052867284E-90	8	ZUP1
2.94298272843063E-116	1.20016047626916	0.413	0.117	4.93185045630406E-112	8	CPLANE1
8.68662455694418E-171	1.19985147263476	0.509	0.129	1.4557045432527E-166	8	DGCR8
5.95751195291432E-211	1.19938443391769	0.609	0.153	9.98359853069382E-207	8	MAP3K3
3.55237404658368E-195	1.19928671189582	0.885	0.326	5.95306842726493E-191	8	MICAL3
4.00794747277162E-100	1.19879411220423	0.297	0.074	6.71651837487069E-96	8	CCDC171
1.53712669765024E-149	1.19803095117151	0.381	0.086	2.57591691992226E-145	8	DTNB
9.23280546833891E-140	1.1977377007172	0.451	0.12	1.54723354038423E-135	8	LTB4R
3.19045076590245E-207	1.19706842389492	0.808	0.269	5.34655739349933E-203	8	MED25
7.25823132236629E-68	1.19610637141745	0.272	0.083	1.21633440500214E-63	8	GPR153
1.90820201403293E-194	1.19592124270001	0.759	0.244	3.19776493511638E-190	8	SUZ12
4.35887055143518E-147	1.19351871679979	0.413	0.1	7.30459527009508E-143	8	RAB23
3.59901811213636E-193	1.19335327587711	0.635	0.177	6.03123455231811E-189	8	SNTB2
2.41953135376287E-165	1.19235204856157	0.391	0.084	4.05465064263582E-161	8	SPICE1
1.94028640760822E-176	1.19220568250413	0.663	0.199	3.25153196186985E-172	8	ANO8
3.07572700556828E-143	1.19101042373497	0.342	0.074	5.15430331593132E-139	8	FOXRED2
7.0065034934501E-185	1.19086665260628	0.491	0.115	1.17414985543237E-180	8	IFT80
2.72390258330584E-170	1.18983984615897	0.439	0.101	4.56471594910393E-166	8	STK17B
3.85512017205806E-175	1.18950337905705	0.566	0.153	6.4604103843349E-171	8	DPY19L1
4.90540798923403E-178	1.18942193062904	0.613	0.174	8.22048270835839E-174	8	CLN6
3.53344887766348E-112	1.18916825412658	0.314	0.076	5.92135362918846E-108	8	POMT2
3.43551591459392E-147	1.1880962964175	0.441	0.112	5.7572375696765E-143	8	RLIM
5.98169339817408E-173	1.1877347492847	0.569	0.157	1.00241217966601E-168	8	CDKL1
1.3433912865514E-191	1.18667833341561	0.831	0.289	2.25125511800284E-187	8	NOTCH2
2.66784032509421E-112	1.18655754185363	0.34	0.087	4.47076681679288E-108	8	WNT3
3.35394266633223E-199	1.18578440067233	0.736	0.228	5.62053712023956E-195	8	OXR1
2.39906873807023E-161	1.18502016496721	0.477	0.121	4.0203593912581E-157	8	SRSF10
4.60399904350017E-193	1.18434457560385	0.804	0.269	7.71538159709758E-189	8	DEPRECATED-ENSG00000134250
4.08520961638239E-174	1.18400752148358	0.832	0.333	6.8459942751336E-170	8	H1FX
1.83538184633328E-173	1.18186314030641	0.771	0.258	3.0757328980853E-169	8	PCSK5
3.24582793247118E-158	1.18097466553489	0.868	0.366	5.43935844923521E-154	8	PILRB
4.33377074471177E-110	1.18082873195474	0.41	0.12	7.26253301398799E-106	8	CES4A
3.81436153282957E-120	1.18045026314949	0.352	0.087	6.39210705671579E-116	8	INPP5F
4.93323136794822E-151	1.18012444660438	0.558	0.164	8.26710912640763E-147	8	FRY
6.65962711047968E-200	1.1801201138722	0.566	0.14	1.11602031117419E-195	8	CRYBG1
7.60260115887346E-185	1.18008789228691	0.529	0.131	1.27404390220401E-180	8	HRCT1
1.22366760639433E-120	1.17990367442503	0.417	0.116	2.05062217479561E-116	8	ZNF117

4.42504288215001E-131	1.1796482148138	0.362	0.087	7.41548686190699E-127	8	HPSE
4.45162441515696E-167	1.17818657989527	0.618	0.184	7.46003219492003E-163	8	LRIG3
9.3208433512396E-161	1.17746230645931	0.451	0.11	1.56198692880073E-156	8	TXNDC16
1.82462470316876E-180	1.17723662151193	0.603	0.17	3.0577060775702E-176	8	PRRG4
8.09995543390421E-111	1.1751976571582	0.31	0.075	1.35739053161367E-106	8	RNF144A
4.4048692884994E-123	1.17475617450941	0.345	0.084	7.38167995366729E-119	8	MTHFD1L
4.85643196770657E-193	1.17440368456541	0.561	0.143	8.13840869148267E-189	8	PLP2
7.01878387460898E-169	1.17395401557449	0.961	0.453	1.17620780170697E-164	8	NOTCH2NLB
4.6027949750655E-154	1.17393618974345	0.603	0.188	7.71336381921476E-150	8	MDC 1.00
2.1288285889365E-94	1.17302991404138	0.321	0.088	3.56749094933979E-90	8	PAPLN
7.30642935165653E-195	1.17231632304643	0.586	0.153	1.2244114307506E-190	8	ARID1A
2.68966605380977E-125	1.17214498844214	0.467	0.137	4.50734237297442E-121	8	AP3M2
8.04337481754981E-75	1.1715548757122	0.316	0.099	1.347908751925E-70	8	PPM1K
5.00260408047962E-162	1.17138574034638	0.968	0.488	8.38336391806775E-158	8	ANKRD10
2.10343396196474E-205	1.1703498186235	0.774	0.241	3.52493463346051E-201	8	USP48
2.54530698050184E-94	1.16967969843509	0.276	0.068	4.26542543792498E-90	8	SCAI
6.92828202882393E-225	1.16961780582791	0.634	0.159	1.16104150239031E-220	8	TPGS2
2.38246968576406E-114	1.16959399422362	0.301	0.069	3.99254269940341E-110	8	ARHGEF28
1.54376619601668E-172	1.16833521079047	0.574	0.158	2.58704339128475E-168	8	KIF2A
8.7532498172186E-205	1.16818949837449	0.401	0.073	1.46686960436949E-200	8	UBXN11
7.02337182268438E-174	1.16750280622856	0.686	0.216	1.17697665004545E-169	8	SEMA6A
1.00446507571263E-183	1.16737932627854	0.69	0.211	1.68328257387922E-179	8	DEPRECATED-ENSG00000134597
8.30491430799975E-197	1.16714440337148	0.501	0.115	1.3917375397346E-192	8	XRCC1
4.90316356710529E-174	1.16643774898589	0.61	0.177	8.21672150575504E-170	8	UNK
8.39011246362916E-141	1.16594214530914	0.352	0.079	1.40601504665497E-136	8	SLC25A19
5.63761664262245E-190	1.16580985210044	0.912	0.35	9.44751796970671E-186	8	MROH1
4.52207589879464E-114	1.16579656713822	0.298	0.068	7.57809479120005E-110	8	KCTD7
8.78498511853083E-163	1.16576751516014	0.602	0.179	1.4721878061634E-158	8	CSPP1
8.94744794228615E-121	1.16561124405023	0.354	0.088	1.49941332616831E-116	8	EFHC1
4.14039063527331E-173	1.16413528471501	0.639	0.191	6.93846662659101E-169	8	FAM193A
5.50368709618413E-149	1.16270786312424	0.502	0.138	9.22307883578536E-145	8	ATR
6.09237740198291E-181	1.16247535889922	0.866	0.323	1.0209606050243E-176	8	VPS13B
1.64721227279401E-161	1.16235574337224	0.602	0.179	2.76039832674821E-157	8	BRCC3
2.08082588206195E-187	1.16200038280467	0.772	0.261	3.48704801315941E-183	8	PHACTR4
6.96483741495344E-192	1.1605880853047	0.799	0.264	1.1671674539979E-187	8	PHF6
2.00704471893931E-177	1.16022216407409	0.542	0.141	3.3634055399985E-173	8	SEPHS1
3.61787611204635E-187	1.16004840483009	0.634	0.178	6.06283678856727E-183	8	NUP160
4.3446470437187E-231	1.15887590780171	0.585	0.135	7.2807595158638E-227	8	THOC3
7.40429972693196E-147	1.15616244043937	0.475	0.128	1.24081254823926E-142	8	DEPDC5
1.53864445085169E-147	1.15598506168961	0.957	0.472	2.57846037073725E-143	8	ZBTB16
1.91796179889173E-196	1.15538366872537	0.839	0.295	3.21412038258277E-192	8	SRPK1
3.23214221938797E-104	1.15409838629162	0.361	0.1	5.41642393125036E-100	8	JRK
3.31113373081823E-164	1.15377613026936	0.615	0.186	5.54879790610519E-160	8	CNOT10
4.96595633352826E-130	1.15279579390554	0.439	0.122	8.32194962372665E-126	8	FSTL1
7.32197341992302E-169	1.15165496660737	0.565	0.157	1.2270163057107E-164	8	RECQL
1.99002123547686E-102	1.15103523167447	0.276	0.065	3.33487758641212E-98	8	HAUS1
9.6256873517159E-131	1.1508028001112	0.366	0.088	1.61307268640055E-126	8	RBBP8
6.33651389577541E-142	1.15077892811444	0.403	0.098	1.06187299865404E-137	8	SNX16
2.76424272897882E-184	1.14802167726928	0.625	0.176	4.6323179652227E-180	8	GPD2
5.55608860081745E-173	1.1476041258314	0.767	0.261	9.31089327724989E-169	8	MBOAT1
1.92866995111725E-119	1.14720998732287	0.399	0.11	3.23206510408229E-115	8	SDC3
2.87230151564193E-131	1.14627429511235	0.401	0.103	4.81340287991275E-127	8	RASA2
1.00287205921048E-95	1.14571735980416	0.288	0.073	1.68061299682492E-91	8	ZC3H12C
4.29697979380754E-137	1.14509362729085	0.413	0.105	7.20087873846268E-133	8	PDE4D
3.43039224670888E-174	1.14480935614711	0.471	0.112	5.74865132703474E-170	8	ATF7IP
7.48273793685078E-114	1.14277965702798	0.356	0.092	1.25395722345745E-109	8	TRPV4
3.40859983015084E-177	1.14203577091121	0.823	0.29	5.71213159536678E-173	8	PIEZO2
1.59853837718151E-167	1.14176985732694	0.538	0.144	2.67883061248077E-163	8	SOCS4
2.41146426947663E-112	1.14003951834899	0.284	0.063	4.04113182278893E-108	8	PIK3R3
8.26733538615839E-226	1.1394070836191	0.704	0.192	1.38544006401242E-221	8	BUB3

3.31506428239399E-146	1.13934535161205	0.543	0.159	5.55538472443585E-142	8	P4HTM
2.92665397660711E-178	1.13867813888406	0.771	0.255	4.9044867339982E-174	8	LRBA
3.02181003832181E-124	1.13845151254541	0.36	0.088	5.06394926221969E-120	8	LRP4
5.8344484866881E-129	1.13821410691878	0.482	0.141	9.77736877399191E-125	8	TTC13
1.06916926003606E-101	1.13794169124356	0.318	0.082	1.79171384596843E-97	8	C5orf30
7.88704790944282E-132	1.1376528959089	0.366	0.088	1.32171148866443E-127	8	CEP68
2.40005023179053E-172	1.13537767717835	0.605	0.172	4.02200417843456E-168	8	SMG1
5.90889196323422E-178	1.13507414177236	0.627	0.182	9.9021211519879E-174	8	ZNF704
8.97705024954105E-189	1.13480625217222	0.715	0.219	1.50437408081809E-184	8	HPS3
2.31049340032921E-195	1.13380301193273	0.586	0.151	3.8719248402717E-191	8	PDE3B
1.39814444486201E-149	1.13353772877587	0.558	0.165	2.34301046069976E-145	8	PKD2
1.99359133558471E-176	1.13310115570637	0.846	0.323	3.34086036017286E-172	8	CEP57
2.58842306862265E-144	1.13182005053731	0.487	0.135	4.33767937839784E-140	8	NAA40
3.10695957596133E-153	1.13166791179264	0.461	0.116	5.206642857396E-149	8	NHLRC3
2.32406145607408E-178	1.13141152761701	0.782	0.266	3.89466218808894E-174	8	ASAP1
5.60967934202213E-135	1.13055590272823	0.406	0.103	9.40070064136068E-131	8	SLC38A9
6.72242838741416E-158	1.12994139649895	0.554	0.158	1.12654454916286E-153	8	HLCS
4.14600326690209E-148	1.12868182583631	0.587	0.18	6.94787227467452E-144	8	NBEAL1
4.14341361277903E-118	1.12793459481495	0.579	0.205	6.94353253229509E-114	8	NR3C2
4.89163958018586E-126	1.12791323136134	0.34	0.08	8.19740960847546E-122	8	CEP131
2.40285213283707E-131	1.1276457403899	0.356	0.084	4.02669960420837E-127	8	SH3BP2
2.31682588281796E-203	1.12711414905174	0.808	0.265	3.88253681442633E-199	8	HAUS4
4.76252617508134E-170	1.12640324880634	0.715	0.237	7.98104136420131E-166	8	MORC4
3.12379707653128E-126	1.12617551891206	0.337	0.078	5.23485914085112E-122	8	RAD1
4.12178744747529E-184	1.12607564228919	0.9	0.371	6.9072914044791E-180	8	SMC1A
9.23500781153766E-175	1.12470598862404	0.775	0.266	1.54760260905748E-170	8	KLHDC4
4.37324436479702E-134	1.12317109478772	0.349	0.08	7.32868290652684E-130	8	BMP2K
6.72155268110669E-174	1.12305153256195	0.618	0.181	1.12639779829986E-169	8	SLC44A2
4.90521037726576E-186	1.12269699599868	0.798	0.268	8.22015155022197E-182	8	RBM14
5.15911757598727E-185	1.12195809652867	0.636	0.182	8.64564923383947E-181	8	VPS37A
3.79458104688659E-189	1.12129594943285	0.639	0.178	6.35895891837254E-185	8	ANKLE2
3.33835343267555E-172	1.12102209817364	0.881	0.337	5.59441268247768E-168	8	DIAPH2
2.36743349298198E-189	1.12087368348739	0.79	0.262	3.96734504753921E-185	8	TCERG1
1.17174821191799E-184	1.11979674445964	0.529	0.131	1.96361565353218E-180	8	DEPRECATED-ENSG00000093000
1.65314414663862E-171	1.11914262817201	0.63	0.189	2.770338960937E-167	8	PXMP4
6.88910708016586E-190	1.11860449506315	0.51	0.121	1.15447656449419E-185	8	ZW10
1.19387161234805E-175	1.11829103661302	0.525	0.134	2.00069004797285E-171	8	FBXO34
1.6273832021627E-109	1.11818043488679	0.34	0.088	2.72716877018425E-105	8	GLIS3
2.79693304839081E-136	1.11756128196881	0.491	0.14	4.68710040249332E-132	8	MFSD11
2.02653503607993E-125	1.1174230517154	0.409	0.109	3.39606741346275E-121	8	FAM120C
1.73668087426059E-146	1.11694831550006	0.465	0.125	2.9103298090859E-142	8	CAMK2N2
6.12946373566808E-122	1.11675841700789	0.372	0.095	1.02717553282326E-117	8	ZNF823
1.72291248661655E-87	1.11652173872439	0.268	0.068	2.88725674507202E-83	8	HYKK
6.03409193284231E-134	1.11633072187632	0.639	0.221	1.01119312610571E-129	8	TTLL3
2.16720009938102E-184	1.11512890577963	0.61	0.169	3.63179392654271E-180	8	FDXR
4.45809662082689E-178	1.11453415360327	0.81	0.282	7.4708783171817E-174	8	HECTD4
1.20131426643075E-136	1.1144742170111	0.618	0.206	2.01316244768466E-132	8	DGKE
3.22589797820223E-146	1.11429367769158	0.573	0.175	5.4059598318713E-142	8	MSH2
2.0271522906976E-193	1.1136352063726	0.563	0.143	3.39710180875103E-189	8	DEPRECATED-ENSG00000120802
2.07940822312741E-180	1.1135952745223	0.486	0.115	3.48467230031691E-176	8	FOXRED1
2.65385224092208E-204	1.11284323858072	0.704	0.204	4.44732558533721E-200	8	FN3KRP
1.23572746015601E-134	1.11227882019712	0.587	0.189	2.07083207772944E-130	8	BTNL3
2.23766716965769E-131	1.1114675681037	0.41	0.108	3.74988264291236E-127	8	ARNTL2
3.04739534277827E-149	1.11095906521277	0.915	0.407	5.10682511542783E-145	8	MDM4
5.90764407399052E-117	1.11084888453977	0.358	0.092	9.90002993919332E-113	8	STS
1.49254130973868E-135	1.10998847640856	0.593	0.193	2.50120072686008E-131	8	FANCL
1.31463977733572E-194	1.10991958117554	0.893	0.344	2.2030733388592E-190	8	ASXL1
2.89173185494072E-83	1.10695861511075	0.276	0.074	4.84596424250966E-79	8	CASP8AP2
3.38220398578349E-166	1.10615944738958	0.422	0.095	5.66789743937598E-162	8	MAZ
8.28671611513365E-142	1.1061036081208	0.479	0.132	1.3886878865741E-137	8	ARHGEF37

5.13771135009571E-133	1.10550622852623	0.479	0.138	8.6097766804904E-129	8	L2HGDH
1.50828000518414E-115	1.10513170704676	0.369	0.097	2.52757563268759E-111	8	ZNF441
3.44055609024568E-164	1.1030630266765	0.562	0.156	5.76568389603371E-160	8	APAF1
5.96101811168888E-185	1.10071882601145	0.625	0.175	9.98947415156823E-181	8	SNHG32
7.86502290035959E-199	1.10053397036704	0.818	0.276	1.31802053764226E-194	8	TRIOBP
5.98759844300663E-78	1.10046543602812	0.285	0.082	1.00340174707905E-73	8	C8orf37
2.6672486854898E-131	1.10022686588572	0.445	0.122	4.46977534714381E-127	8	SPAG1
7.02037629710512E-86	1.09962844541762	0.31	0.088	1.17647465986888E-81	8	ZNF324
5.45777993565636E-123	1.09949303469405	0.63	0.23	9.14614761617292E-119	8	CKB
1.3293785472738E-186	1.09922926283683	0.507	0.12	2.22777256952144E-182	8	ERI2
2.39663839165887E-135	1.09902954594898	0.928	0.453	4.01628661674194E-131	8	CFLAR
1.18826278485762E-180	1.09738403490983	0.647	0.191	1.99129077486441E-176	8	LNPK
7.86844767874072E-156	1.09714071525655	0.699	0.242	1.31859446200337E-151	8	TMEM19
1.09211468488796E-109	1.09678133724238	0.314	0.077	1.83016578893524E-105	8	TRIM37
5.15463965526742E-181	1.09577818722672	0.682	0.204	8.63814513429714E-177	8	FNIP1
6.71745504165069E-141	1.09343350410654	0.417	0.105	1.12571111587982E-136	8	IFT81
5.44540184755745E-171	1.09341513146892	0.692	0.217	9.12540441613677E-167	8	ELF2
2.70892006545761E-138	1.09326094903662	0.495	0.142	4.53960824569386E-134	8	PIEZO1
4.13884847851359E-106	1.0929764726579	0.297	0.071	6.93588228029308E-102	8	TNFAIP8L1
3.02097991438947E-184	1.09278642272298	0.707	0.217	5.06255814053387E-180	8	NUP153
8.55942390023834E-106	1.09261713189132	0.288	0.068	1.43438825720194E-101	8	EXO5
1.31084543500063E-186	1.09185192645694	0.726	0.229	2.19671477997405E-182	8	RANBP9
2.15436068673923E-186	1.09160768268952	0.736	0.229	3.61027763883759E-182	8	RCOR3
7.09908819828368E-106	1.09126761571262	0.285	0.067	1.18966520026838E-101	8	SH3PXD2B
1.41507839259473E-180	1.09089135450359	0.788	0.262	2.37138837031025E-176	8	TMEM175
2.01605056857144E-162	1.08986675538021	0.832	0.313	3.37849754281202E-158	8	SGK3
1.43786746946832E-164	1.0894562894442	0.518	0.136	2.40957830533501E-160	8	ADAT1
9.78035321086104E-150	1.0882507762784	0.581	0.175	1.63899159107609E-145	8	RIC8B
1.4534641621256E-124	1.08802679805819	0.397	0.104	2.43571524289008E-120	8	EFNA5
1.01520611964041E-151	1.0876621173101	0.798	0.316	1.7012824152934E-147	8	HPRT1
3.23735178939488E-150	1.08735657686086	0.45	0.114	5.42515412866795E-146	8	MFSD14B
1.21417883032426E-190	1.08613810049754	0.747	0.23	2.0347208838574E-186	8	BDP1
5.98343985402185E-182	1.08605800767848	0.615	0.172	1.00270485073698E-177	8	CPOX
1.99045896932771E-175	1.08585701693941	0.571	0.155	3.33561114079938E-171	8	RIF1
5.41969596936978E-179	1.08516612140806	0.688	0.209	9.08232650546989E-175	8	TRAPPC10
1.8454080964757E-138	1.08448682693359	0.467	0.128	3.09253488807398E-134	8	ZNF133
9.7196152806871E-86	1.08351365754565	0.336	0.1	1.62881312873754E-81	8	NAPB
3.30952929248125E-107	1.08271568107129	0.27	0.06	5.54610918834008E-103	8	SNORC
1.02926341795333E-136	1.08243663761473	0.361	0.084	1.72483963580618E-132	8	CABLES2
2.52138554410329E-135	1.08241039695239	0.461	0.126	4.2253378948083E-131	8	VCPKMT
6.54233558273286E-121	1.08238695376214	0.362	0.091	1.09636459695437E-116	8	FP565260.6
7.79590799370142E-155	1.08207944214954	0.539	0.152	1.30643826158448E-150	8	DDX59
1.51335848513102E-128	1.08147817611383	0.455	0.128	2.53608614938256E-124	8	FLVCR2
2.7316059368568E-170	1.08140185423578	0.659	0.201	4.57762522898462E-166	8	CASP2
7.91164856799933E-75	1.08104469946809	0.29	0.086	1.32583406702533E-70	8	SLC38A11
5.9541309385218E-170	1.08014711532466	0.636	0.189	9.97793262677484E-166	8	ATM
1.66702867451593E-98	1.07961951694969	0.356	0.101	2.79360665275379E-94	8	MAP7D3
2.53124740757135E-162	1.07865376312157	0.928	0.427	4.24186440560807E-158	8	FMR1
1.46386206942661E-177	1.07836998209142	0.856	0.322	2.45314005594511E-173	8	NUP58
8.89679918900976E-159	1.07789602149015	0.643	0.204	1.49092560809426E-154	8	EXOSC9
6.7001263762123E-103	1.0776371625783	0.353	0.097	1.12280717812566E-98	8	MAP10
3.40483319056821E-135	1.0763750453644	0.542	0.167	5.70581946075421E-131	8	LNPEP
1.93306867800591E-121	1.07630212409405	0.447	0.131	3.23943649060231E-117	8	GRK3
3.2909033808149E-107	1.07612027478838	0.328	0.083	5.51489588556962E-103	8	MSANTD2
6.97693725010701E-142	1.07542228060177	0.478	0.132	1.16919514437293E-137	8	RAD51D
2.84816614117519E-158	1.07397650549541	0.71	0.241	4.77295681938139E-154	8	SPIDR
2.52687976473838E-78	1.07359580720812	0.304	0.09	4.23454510974858E-74	8	TBC1D19
3.18852999579758E-100	1.07178065636465	0.316	0.082	5.34333856695759E-96	8	SP4
3.1282022822615E-137	1.07114708372229	0.511	0.149	5.24224138461382E-133	8	PIAS1
3.0220790313653E-121	1.07004314175568	0.418	0.116	5.06440004076198E-117	8	PPCDC

8.78152739688267E-148	1.06941457728885	0.465	0.122	1.4716083611696E-143	8	AACS
1.26174733252957E-89	1.06856319181585	0.337	0.098	2.11443617985305E-85	8	ZNF782
3.41925854110722E-124	1.06778622041032	0.463	0.136	5.72999346318748E-120	8	EID3
1.70420557161708E-172	1.06774670180345	0.699	0.223	2.8559076969159E-168	8	PLCB1
8.40952366010255E-163	1.06774183353797	0.839	0.316	1.40926797495999E-158	8	IL17RB
3.12844759942208E-131	1.0671971850609	0.545	0.175	5.24265248711152E-127	8	RFC2
4.87827529506242E-145	1.06704648160842	0.486	0.132	8.17501373946561E-141	8	ADPGK
2.94617340738338E-157	1.06704530447227	0.461	0.115	4.93719739609307E-153	8	TIFA
2.69511131207643E-98	1.06657481125716	0.381	0.113	4.51646753677769E-94	8	LINS1
1.13252144952017E-182	1.06553351088809	0.83	0.305	1.8978794451059E-178	8	DEK
1.94916873745519E-158	1.06232796285546	0.527	0.144	3.26641697022741E-154	8	DESI2
1.22988569525853E-177	1.0623204651121	0.759	0.25	2.06104244811424E-173	8	SMC6
3.04280660353955E-180	1.06165373338197	0.615	0.171	5.09913530621158E-176	8	AGAP1
4.91040161288469E-134	1.06154005610486	0.597	0.196	8.22885102287217E-130	8	DMXL2
1.06456780296046E-156	1.06120843311359	0.771	0.283	1.78400272420114E-152	8	TFDP1
2.16664529913658E-188	1.06031510342217	0.808	0.281	3.63086419229308E-184	8	TPR
1.88015195727323E-164	1.06021661323765	0.485	0.122	3.15075864999848E-160	8	SGCB
1.28952064925556E-138	1.06017240770565	0.579	0.183	2.16097870402246E-134	8	ARL4A
1.26747685530711E-144	1.05961700504925	0.478	0.13	2.12403771412366E-140	8	TTC39B
1.7634001360846E-138	1.05765813834353	0.864	0.368	2.95510594805058E-134	8	BTNL8
7.00191579782819E-106	1.05654511723386	0.356	0.097	1.17338104940005E-101	8	TTLL6
6.6338341522955E-111	1.05631601680712	0.342	0.088	1.11169792724168E-106	8	ZNF250
7.43666063796778E-90	1.05574861795386	0.296	0.08	1.24623558971064E-85	8	LLGL1
2.34470800420618E-155	1.05525986398603	0.65	0.208	3.92926167344871E-151	8	NOTCH1
5.12158806345044E-107	1.05346825506806	0.407	0.119	8.58275727673025E-103	8	CEP95
1.08152573242276E-175	1.053336748255	0.792	0.279	1.81242082239406E-171	8	TBCD
3.25838334250535E-139	1.05329330412632	0.505	0.144	5.46039880537047E-135	8	THOC1
6.14819758161668E-145	1.05318787452324	0.553	0.164	1.03031495072732E-140	8	ANAPC7
2.80691693491698E-142	1.05259183756492	0.639	0.211	4.70383139953388E-138	8	MAP3K21
3.37466816280375E-140	1.05257745800987	0.526	0.155	5.65526890722653E-136	8	YEATS2
1.5571617188613E-173	1.0517605878618	0.844	0.308	2.60949160846776E-169	8	KMT2B
9.19745056680692E-169	1.05169852320198	0.82	0.291	1.5413087659855E-164	8	WDFY2
2.55426823001945E-137	1.0513916073371	0.437	0.115	4.2804426998666E-133	8	CPSF4
1.57437951533822E-197	1.05122747264229	0.671	0.19	2.63834519180378E-193	8	CBX5
3.81970373607185E-90	1.05107775687542	0.338	0.098	6.4010595209092E-86	8	GOLGA8A
1.00786382875303E-102	1.05070213261187	0.328	0.086	1.68897820422433E-98	8	AGTPBP1
2.59711644447555E-165	1.05029553943934	0.9	0.363	4.35224773765213E-161	8	NKTR
7.04909649058536E-88	1.04977438794847	0.308	0.086	1.1812875898923E-83	8	AC093323.1
2.4565680248093E-136	1.04958289030417	0.467	0.129	4.11671669597543E-132	8	WRAP73
3.01188888295008E-143	1.04921940767992	0.521	0.151	5.04732339004774E-139	8	CMPK2
3.40405352799677E-99	1.0482986391052	0.309	0.08	5.70451290221698E-95	8	TNRC6C
2.2541629290436E-190	1.04657243297373	0.664	0.189	3.77752623649127E-186	8	MLH1
2.90696849750599E-100	1.04585746027238	0.321	0.085	4.87149780812054E-96	8	HSDL1
1.83657102130244E-94	1.0454663229808	0.41	0.13	3.07772571749863E-90	8	ATP2C2
4.5901574696827E-126	1.04392713316325	0.353	0.085	7.69218588769427E-122	8	CERS5
4.68365949860266E-101	1.04378913611492	0.357	0.1	7.84887658775834E-97	8	ATAD3B
1.38823939853336E-100	1.04353729797258	0.258	0.058	2.3264115840622E-96	8	ARL13B
8.06209399695914E-151	1.04291089639038	0.69	0.232	1.35104571201041E-146	8	STXBP5
4.5588578566351E-156	1.04288016926291	0.907	0.377	7.6397339961491E-152	8	THOC2
6.88161963107309E-155	1.04265325156558	0.7	0.237	1.15322181777523E-150	8	ELMOD2
1.39215009295422E-133	1.04253340289727	0.383	0.094	2.33296512577269E-129	8	NRF1
2.83162062225938E-123	1.04221132370356	0.401	0.107	4.74522983878226E-119	8	SLX4IP
9.65555810451347E-106	1.04204684570789	0.373	0.104	1.61807842715437E-101	8	POP1
1.52319430283791E-169	1.04150141059785	0.591	0.169	2.55256901269576E-165	8	UCHL5
7.54126438879139E-116	1.04099190009061	0.441	0.13	1.26376508627366E-111	8	ATXN7L1
2.92443243699795E-100	1.04012315802428	0.403	0.122	4.90076387792116E-96	8	ANKRD26
5.64891811770424E-158	1.0394145565369	0.559	0.159	9.46645698164877E-154	8	CLGN
6.19781098858229E-146	1.03849329859498	0.541	0.16	1.03862916546662E-141	8	ING2
3.32087484621675E-159	1.03816506538393	0.583	0.17	5.56512206729003E-155	8	IFI27L1
1.14973077978638E-121	1.03740392089461	0.47	0.14	1.92671884076601E-117	8	ZNF346

1.4888754128628E-74	1.0372192947926	0.25	0.068	2.49505741687548E-70	8	ANKS6
5.6439382276026E-161	1.03681222036393	0.507	0.133	9.45811168181643E-157	8	NSD3
2.26265029058177E-133	1.03672016394195	0.443	0.12	3.79174935695694E-129	8	ZNF236
9.16070588603127E-184	1.03649243378113	0.852	0.318	1.53515109238112E-179	8	NNT
3.5530071366674E-162	1.03639465300971	0.771	0.267	5.95412935962722E-158	8	RAB3IP
2.76988455379905E-144	1.03541592022486	0.622	0.199	4.64177253525645E-140	8	ZNF337
2.91207906875778E-153	1.03419708571188	0.83	0.312	4.8800621034243E-149	8	CLMN
3.55698620191177E-153	1.03411976409565	0.735	0.255	5.96079747716375E-149	8	LRRC14
9.01871485738195E-143	1.03370700437941	0.447	0.116	1.51135623580007E-138	8	RAPGEF6
1.13965377217489E-99	1.03314238727676	0.325	0.086	1.90983179141067E-95	8	FUT8
2.44193440781398E-136	1.03297328545763	0.796	0.321	4.09219368061467E-132	8	PFKFB3
1.26655548481801E-92	1.03138514157405	0.294	0.076	2.12249368145802E-88	8	TMEM80
3.44717915776156E-87	1.03129500824812	0.288	0.077	5.77678283257682E-83	8	ACER3
9.19400218091012E-130	1.03128583811179	0.634	0.226	1.54073088547692E-125	8	ARL6IP1
1.0130154099041E-142	1.03111676516656	0.595	0.186	1.69761122391729E-138	8	EPM2AIP1
8.06497205360953E-149	1.03052722522094	0.929	0.405	1.35152801674389E-144	8	IKBKB
1.32277918784196E-180	1.02987701006031	0.664	0.197	2.21671336298556E-176	8	PHKG2
1.72709575632209E-101	1.0296957482207	0.328	0.086	2.89426706844456E-97	8	PTBP2
4.19650626333536E-137	1.0293557743841	0.485	0.137	7.0325051960974E-133	8	TAF5L
5.48320152828772E-116	1.02898523999906	0.369	0.096	9.18874912110456E-112	8	PPP2R3C
4.83206367982523E-95	1.02890428548588	0.286	0.072	8.09757231465111E-91	8	MAP2
7.4292324560419E-157	1.02882530246435	0.748	0.268	1.2449907749835E-152	8	BRI3BP
6.49798550122278E-118	1.02829695078723	0.494	0.156	1.08893241029491E-113	8	ALDH3A1
3.76524691481511E-156	1.02807845190826	0.607	0.185	6.30980077984716E-152	8	IFT88
4.32252573016414E-140	1.02797783753299	0.868	0.381	7.24368861860907E-136	8	NEB
7.47800537701495E-178	1.02788210032233	0.798	0.276	1.25316414108017E-173	8	FUS
9.80990526857741E-123	1.02750570182323	0.403	0.108	1.6439439249082E-118	8	TADA2A
2.82501694518643E-150	1.02738602758223	0.462	0.119	4.73416339674341E-146	8	CREB3L4
1.16953706537511E-76	1.0272359067924	0.257	0.07	1.9599102141556E-72	8	ANK2
1.52089954844397E-137	1.02674236759557	0.904	0.404	2.5487234632824E-133	8	BIRC3
3.34019232670116E-122	1.02497770683701	0.434	0.121	5.5974943010858E-118	8	ANKRA2
2.33905080888971E-83	1.02415943142889	0.265	0.069	3.91978134553738E-79	8	EVA1C
3.93904443785017E-170	1.02385179794649	0.77	0.262	6.60105066894931E-166	8	NSMCE2
5.42682810324151E-160	1.0234978624328	0.525	0.142	9.09427853541212E-156	8	CDC27
1.31167733428625E-119	1.02322888426217	0.328	0.078	2.1981088767969E-115	8	PLEK2
7.38629473557117E-115	1.02304866760622	0.365	0.096	1.23779527178702E-110	8	TRIM11
9.07422686271862E-158	1.02301208557337	0.884	0.351	1.52065893765439E-153	8	NBPF26
6.24833314014153E-69	1.02288234416541	0.264	0.077	1.04709566762492E-64	8	PCNX2
2.24817584577038E-97	1.02248709605461	0.341	0.095	3.767493082342E-93	8	ELOVL7
9.62335890802086E-131	1.02230834491858	0.539	0.168	1.61268248580614E-126	8	EXOC8
2.94992680768974E-81	1.0222927608197	0.257	0.067	4.94348734432646E-77	8	DEPRECATED-ENSG00000196693
7.62095558207206E-146	1.02212434208567	0.418	0.102	1.27711973644364E-141	8	C19orf47
1.86408739718156E-137	1.02179431603138	0.666	0.238	3.12383766019686E-133	8	TGIF1
3.91752303270871E-172	1.02004560160276	0.754	0.248	6.56498509821326E-168	8	ABHD3
1.38123697246623E-151	1.01989151968599	0.585	0.175	2.31467691845891E-147	8	RBBP6
7.02840282424204E-119	1.01951780119993	0.458	0.135	1.17781974528648E-114	8	GPR180
4.84508712260572E-127	1.01884658706485	0.586	0.194	8.11939700006267E-123	8	TBC1D1
1.1222271125966E-150	1.01798306278839	0.433	0.106	1.88062819528939E-146	8	FNTB
2.49225370194636E-140	1.0178142273716	0.667	0.227	4.17651875372171E-136	8	GLI4
2.39790920966227E-167	1.01781154192436	0.771	0.271	4.01841625355202E-163	8	RBBP7
1.50961988772519E-117	1.01734122046694	0.417	0.117	2.52982100784987E-113	8	KPTN
4.28183446212889E-88	1.01673879141682	0.289	0.077	7.1754981916356E-84	8	SLC38A6
1.55336686118345E-167	1.01442868394773	0.68	0.214	2.60313218597122E-163	8	NUP133
3.57257763794829E-159	1.01435569821943	0.529	0.143	5.98692560567374E-155	8	CNTLN
3.11582679667947E-111	1.01351775609681	0.266	0.057	5.22150254587545E-107	8	AKAP12
3.50998693955833E-130	1.01349233822372	0.514	0.155	5.88203611331185E-126	8	FBXO4
6.8662687516022E-152	1.01167082157652	0.625	0.198	1.1506493173935E-147	8	ZCCHC2
1.67493046307845E-109	1.01155495005429	0.458	0.144	2.80684847002687E-105	8	THADA
3.91325809909188E-143	1.0115258977963	0.57	0.174	6.55783792245818E-139	8	ZADH2
1.48231207190556E-111	1.01084436232416	0.446	0.135	2.48405857009934E-107	8	ZNF26

7.12042452571916E-159	1.00989306428837	0.583	0.17	1.19324074202002E-154	8	NR2C2
2.42101280534364E-135	1.00887224494629	0.427	0.112	4.05713325919487E-131	8	DRG1
3.9134544343861E-101	1.00863860638399	0.324	0.085	6.55816694114422E-97	8	PXYLP1
5.26223925616753E-134	1.00846084893952	0.605	0.199	8.81846054548554E-130	8	ALG6
4.17198536266547E-160	1.00819928722634	0.823	0.305	6.99141307075479E-156	8	RAB33B
2.56662585516183E-157	1.00566022247857	0.718	0.243	4.3011516080802E-153	8	GPATCH2L
4.00677884712226E-165	1.00541405154201	0.618	0.183	6.71455999200748E-161	8	QSER1
7.38541467384549E-139	1.00539431215968	0.399	0.099	1.23764779104303E-134	8	DEPRECATED-ENSG00000102007
2.92675356747736E-168	1.00525940649007	0.655	0.204	4.90465362837856E-164	8	HSPB11
1.7035706898331E-102	1.00515728562197	0.354	0.097	2.85484376202231E-98	8	ZDHHC2
2.2968737934046E-97	1.0040109423191	0.333	0.092	3.84910110298743E-93	8	RARRES1
6.31568282677512E-146	1.00322520061313	0.643	0.21	1.05838212811098E-141	8	UPF3A
4.06641015787928E-186	1.00166102259311	0.779	0.256	6.8144901425741E-182	8	H2AFY
1.45095601534786E-154	1.00091563007833	0.772	0.278	2.43151209051994E-150	8	SNX5
2.27358316130339E-83	1.00062937918587	0.294	0.082	3.81007066171222E-79	8	LY6G5B
9.1381753395697E-110	1.00035262817865	0.308	0.074	1.53137542340509E-105	8	ANKRD44
2.7178554031623E-92	1.00001987245072	0.33	0.094	4.55458208461938E-88	8	BMPR1A
1.56174797964224E-125	0.999724525533574	0.529	0.165	2.61717726428446E-121	8	XPO4
1.38267957410717E-149	0.9995943245808	0.899	0.394	2.3170944302888E-145	8	PCCA
2.65568594300633E-156	0.999279235276876	0.601	0.179	4.45039850329001E-152	8	TMEM65
2.61067093871942E-149	0.99859094683414	0.623	0.198	4.37496235910601E-145	8	PWWP2A
1.05116817181008E-105	0.998482044777845	0.385	0.11	1.76154762231933E-101	8	IQCB1
1.85426868471684E-115	0.998235639369482	0.341	0.085	3.10738346184847E-111	8	STK26
1.52790429532046E-121	0.997915577054232	0.478	0.144	2.56046201809803E-117	8	RAP1GDS1
1.67077935286693E-98	0.997665518394349	0.405	0.124	2.7998920395344E-94	8	IFFO1
2.93658009124337E-167	0.997621282259604	0.868	0.34	4.92112091690564E-163	8	SRSF3
8.60575728039171E-152	0.995730024041331	0.68	0.226	1.44215280504804E-147	8	DMTF1
8.20038479068436E-129	0.994861073040985	0.618	0.21	1.37422048322288E-124	8	GREB1L
1.161329873283E-156	0.994803484461329	0.782	0.284	1.94615660164765E-152	8	TPP2
7.63501325830129E-113	0.993431367661384	0.338	0.085	1.27947552182613E-108	8	CMTM7
4.24426292084612E-104	0.991981874187336	0.391	0.114	7.11253580275392E-100	8	ERAP2
3.45324109101443E-121	0.99175345530747	0.658	0.244	5.78694142032199E-117	8	HAO2
1.55305535054925E-141	0.99111008284201	0.625	0.203	2.60261015645044E-137	8	MAP4K5
4.61112445576921E-86	0.990624126044642	0.253	0.063	7.72732236297804E-82	8	HACE1
9.03197131387601E-139	0.990520528467394	0.652	0.222	1.51357775277934E-134	8	SIPA1L1
1.16178756164339E-133	0.990413789878299	0.523	0.156	1.946923595802E-129	8	RWDD3
9.52317629169023E-109	0.989857154818368	0.285	0.065	1.59589388296145E-104	8	YEATS4
4.66816174539831E-144	0.988201374340539	0.461	0.122	7.82290545293849E-140	8	RPA2
9.5099720585439E-110	0.988082120831233	0.365	0.098	1.59368111757079E-105	8	ZC3H6
7.31546156485822E-185	0.987180586518472	0.783	0.263	1.22592504903894E-180	8	RHEB
1.57292543288784E-178	0.987161350899437	0.374	0.073	2.63590844043345E-174	8	NUAK2
3.56925715333792E-162	0.986705094644237	0.807	0.291	5.98136113756369E-158	8	ENOSF1
6.59731306430113E-102	0.986554516329305	0.459	0.148	1.10557772331558E-97	8	DPY19L3
6.41352186576479E-91	0.98624235874598	0.35	0.103	1.07477799426486E-86	8	ARAP2
4.33440050083692E-157	0.983159156191812	0.704	0.233	7.26358835930 25E-153	8	ADNP
4.28162104848462E-117	0.982993169366417	0.86	0.409	7.17514055305053E-113	8	MCM3
1.71222594804792E-86	0.98210296527361	0.276	0.072	2.8693482437387E-82	8	ZFYVE26
7.58693864284686E-129	0.981948943439952	0.368	0.09	1.27141917776828E-124	8	UHRF2
1.49758063691525E-125	0.981682637762001	0.549	0.176	2.50964563134257E-121	8	ZSWIM5
1.14074939163768E-121	0.981373339815025	0.372	0.094	1.91166783050642E-117	8	ABCC5
5.24627855555597E-116	0.981342753697757	0.473	0.145	8.79171360340069E-112	8	FER
1.294917450813E-136	0.981204446817831	0.427	0.111	2.17002266407242E-132	8	CNOT1
1.38748456380884E-125	0.980994624224805	0.397	0.103	2.32514663203085E-121	8	SENP1
2.55602171576733E-152	0.98064081537953	0.595	0.18	4.2833811912829E-148	8	STRN
1.27400741686544E-149	0.980638281776934	0.818	0.307	2.1349816291831E-145	8	STK39
3.11651557236548E-160	0.979572784603194	0.776	0.274	5.22265679617007E-156	8	WWOX
2.00088076171728E-143	0.979449768394896	0.433	0.11	3.35307598048582E-139	8	PSAT1
1.30119036823787E-82	0.97938191433089	0.274	0.073	2.18053481909302E-78	8	NEK1
3.59181067047111E-137	0.979008581458506	0.879	0.364	6.01915632157548E-133	8	TBC1D8
2.31020803229384E-177	0.978466782203736	0.747	0.245	3.87144662051802E-173	8	PCM1

1.80646478219684E-149	0.977344843176745	0.887	0.369	3.02727368200547E-145	8	DHX15
1.99270240795657E-106	0.977332240714368	0.375	0.105	3.33937069525361E-102	8	PTPRU
7.87201738079071E-114	0.977142153945182	0.43	0.126	1.31919267267291E-109	8	TMEM161B
1.06605641950414E-111	0.976513682498882	0.471	0.148	1.78649734780504E-107	8	ZNF449
1.80359849037054E-110	0.976156030997321	0.447	0.137	3.02247035016296E-106	8	USP37
4.17532117783976E-141	0.975935190039478	0.461	0.123	6.99700322982386E-137	8	PAXIP1
3.04087378794206E-137	0.975866051103049	0.533	0.161	5.09589629383331E-133	8	NIPA2
5.53748867135847E-169	0.975314555320951	0.78	0.277	9.27972351546253E-165	8	NUP62
3.96547012712797E-92	0.975232335224143	0.377	0.115	6.64533483904104E-88	8	MECOM
3.29955273287728E-116	0.975170666504412	0.636	0.24	5.52939046975574E-112	8	E2F1
9.97716467143956E-112	0.974834422759614	0.407	0.117	1.67197325563984E-107	8	NUDCD1
7.50284795130163E-134	0.974776355216808	0.525	0.157	1.25732725967913E-129	8	SOCS7
2.26967313643064E-176	0.974467973107453	0.74	0.241	3.80351824203047E-172	8	RSRC1
1.97708389645479E-138	0.974420819793186	0.36	0.082	3.31319719367893E-134	8	USP13
1.25611062628886E-147	0.974108025302042	0.867	0.353	2.10499018753486E-143	8	RABGAP1L
4.64839852612305E-154	0.973624499031187	0.586	0.172	7.78978625007701E-150	8	SEPTIN7
3.311620280979E-161	0.97356491782838	0.502	0.131	5.5496132668646E-157	8	UBALD2
2.17662619540477E-157	0.973099809630801	0.574	0.167	3.64759017825932E-153	8	ICMT
2.22810200498575E-139	0.972448854994294	0.917	0.428	3.73385333995512E-135	8	STAG2
1.1177383536307E-83	0.972137621369664	0.32	0.094	1.87310593301433E-79	8	ITGAM
2.05441088779268E-148	0.971912904353879	0.613	0.193	3.44278176576298E-144	8	HHAT
3.38440317697191E-138	0.971671742195549	0.711	0.25	5.67158284396953E-134	8	WDYHV1
1.26236118447647E-138	0.969709873175885	0.603	0.197	2.11546487294567E-134	8	PIGX
4.02850389624841E-147	0.969340582013712	0.779	0.286	6.75096682933309E-143	8	RHOBTB3
3.03711164934819E-111	0.969160455281294	0.361	0.096	5.0895917019777E-107	8	ZNF142
7.15672984439741E-163	0.96826481614483	0.71	0.233	1.19932478732412E-158	8	PPP1R12B
4.94690382656539E-137	0.967808910278323	0.711	0.253	8.29002143255828E-133	8	ZBTB1
1.94716970466873E-146	0.966862103879663	0.702	0.24	3.26306699108386E-142	8	GMPS
6.96392194645508E-135	0.966696699098618	0.609	0.201	1.16701403978694E-130	8	SLC10A5
2.00486746707246E-156	0.966562009054923	0.534	0.148	3.35975690132002E-152	8	KPNB1
2.17664118387053E-161	0.966376988957225	0.925	0.397	3.64761529593024E-157	8	PI4K2B
7.89658813199836E-117	0.966359321310656	0.403	0.11	1.32331023916028E-112	8	LRP12
5.70489256128665E-162	0.964964342265876	0.719	0.236	9.56025895420416E-158	8	PTAR1
7.10342489508494E-136	0.963937459004233	0.514	0.151	1.19039194391833E-131	8	DIPK2A
5.22274961279988E-130	0.963309826479593	0.587	0.192	8.75228380113005E-126	8	ANKRD9
1.48412313112514E-104	0.963190260854906	0.429	0.132	2.48709354313951E-100	8	EPB41L4A
1.63660915554603E-157	0.962705392647486	0.746	0.259	2.74262962286403E-153	8	SPPL2B
7.08504300776285E-123	0.962529915585617	0.445	0.126	1.1873115072409E-118	8	PRKRA
2.1136960955277E-154	0.962348116002557	0.49	0.129	3.54213191688532E-150	8	PNRC2
1.55980755406594E-164	0.962262627306238	0.726	0.24	2.6139254991037E-160	8	MAPK9
4.0622209430665E-138	0.961895124422828	0.543	0.163	6.80746985639084E-134	8	ADNP2
2.05039028740622E-85	0.961325549273573	0.289	0.079	3.43604404363534E-81	8	ARL6IP6
3.64063175313997E-130	0.961297865290642	0.603	0.202	6.10097069191196E-126	8	PLEKHA5
4.48643618337586E-168	0.96101323309372	0.647	0.194	7.51836975610127E-164	8	KMT2A
6.72216914007729E-136	0.960150624631271	0.674	0.233	1.12650110449415E-131	8	BCL2L2
1.30718532398132E-130	0.960036263087402	0.443	0.121	2.19058116592789E-126	8	GKAP1
1.20000578983127E-89	0.959727790346942	0.394	0.125	2.01096970259925E-85	8	XKR9
7.90944919832751E-117	0.958845085860666	0.383	0.102	1.32546549665572E-112	8	JAK2
2.96777770446338E-130	0.958526276036188	0.531	0.164	4.97340187713972E-126	8	GUCY1B1
1.63000163152097E-164	0.957593022092445	0.806	0.283	2.73155673410284E-160	8	SH3GLB2
3.57535796445811E-108	0.957207213613858	0.386	0.109	5.9915848768389E-104	8	AHCTF1
9.93961913213814E-183	0.957144765833847	0.724	0.23	1.66568137416371E-178	8	NUP50
1.65602920874472E-144	0.95669261882553	0.692	0.238	2.77517374801441E-140	8	DDR1
1.38075856427414E-83	0.956346640718257	0.258	0.066	2.31387520201061E-79	8	VAMP1
1.37136245248224E-96	0.955212809784484	0.293	0.074	2.29812919786974E-92	8	REV3L
1.1548329199001E-94	0.953383566312862	0.258	0.061	1.93526900716858E-90	8	CD83
2.94610477042983E-104	0.953129879465179	0.433	0.133	4.93708237428631E-100	8	MCM9
1.11970950939969E-107	0.952986065005694	0.352	0.093	1.876409195852E-103	8	NLRC5
4.57342434862097E-124	0.952316265897141	0.332	0.077	7.66414452341902E-120	8	POMGNT2
2.13929221558459E-151	0.952262210051471	0.726	0.256	3.58502589487666E-147	8	NOP56

1.20567897374231E-158	0.95160736638919	0.656	0.208	2.02047682419736E-154	8	CBFB
2.98727516814589E-71	0.951458977973836	0.277	0.081	5.00607572677888E-67	8	TAS2R14
1.23403339165758E-143	0.951427556008348	0.726	0.26	2.06799315773977E-139	8	LRRC58
6.18363470628063E-112	0.951315421446533	0.325	0.08	1.03625350407851E-107	8	FAS
5.99012772472767E-138	0.950261907317992	0.719	0.258	1.00382560410986E-133	8	MLH3
1.21807002133892E-124	0.950178063528876	0.426	0.116	2.04124174175977E-120	8	CYB5RL
3.69355668562874E-162	0.950151930082852	0.538	0.146	6.18966229377664E-158	8	C1orf21
3.81918705053225E-127	0.950135830532498	0.406	0.106	6.40019365928194E-123	8	COA1
7.44784376652537E-133	0.949193164171541	0.441	0.119	1.24810965839432E-128	8	STK35
6.66460961351967E-127	0.948531716308667	0.571	0.186	1.11685527903363E-122	8	ZNF121
5.63009396828265E-130	0.9482805687649	0.352	0.082	9.43491147204807E-126	8	AKAP7
8.9044929446039E-113	0.948245412626258	0.349	0.09	1.49221492765672E-108	8	PKP4
1.10401115342906E-173	0.948146849949576	0.602	0.169	1.85010189091642E-169	8	KRI1
6.72824686711593E-94	0.947998396906818	0.34	0.097	1.12751960999129E-89	8	TRAF5
1.84111637328928E-165	0.947414513041516	0.543	0.147	3.08534281835817E-161	8	MAST2
1.33676304364291E-159	0.946532650817839	0.7	0.232	2.24014750853678E-155	8	UBR7
1.07006913113518E-127	0.945654260854403	0.599	0.202	1.79322184995634E-123	8	ATPSCKMT
2.10945478265985E-116	0.94446640585952	0.385	0.103	3.53502432478137E-112	8	CIITA
1.83266313380694E-78	0.944239310969485	0.429	0.156	3.07117687963367E-74	8	DNM1
4.67205694730195E-133	0.944112237766034	0.407	0.104	7.8294330322886E-129	8	ASH2L
3.56608013864045E-90	0.944108499631992	0.282	0.073	5.97603709633366E-86	8	ING3
4.66472300932524E-105	0.944070780797244	0.434	0.133	7.81714281902723E-101	8	NFXL1
1.47750095758206E-115	0.942966358101683	0.486	0.151	2.47599610471602E-111	8	BTBD3
1.64564340789215E-122	0.942641586107903	0.493	0.15	2.75776922294567E-118	8	TAF9
1.5308757838053E-148	0.942306145354137	0.583	0.176	2.56544163850092E-144	8	MRTFA
6.86622389997612E-100	0.942006554337361	0.401	0.121	1.150641801158E-95	8	STXBP4
3.18775396967663E-99	0.941874253893108	0.334	0.09	5.3420381023841E-95	8	ZBED4
1.68364971442693E-157	0.941768654246504	0.795	0.288	2.82146019143664E-153	8	ARHGEF40
9.63306090727451E-147	0.941690471318791	0.594	0.185	1.61430834684106E-142	8	RUSC1
1.54239036148005E-140	0.941275204594228	0.838	0.338	2.58473776776826E-136	8	HECTD1
9.58940167858769E-87	0.941232301008031	0.37	0.117	1.60699193329772E-82	8	ZNF57
2.69611996546649E-102	0.940487820861256	0.375	0.107	4.51815783812874E-98	8	DLG3
1.77097132074567E-138	0.940458352864535	0.81	0.326	2.9677937393056E-134	8	FZR1
6.96447483114017E-134	0.940364156052941	0.497	0.143	1.16710669220247E-129	8	ATG7
6.92837306117263E-157	0.940270154129146	0.75	0.257	1.16105675759131E-152	8	ZMYND8
3.3592086847646E-89	0.939697571520068	0.258	0.063	5.62936191392852E-85	8	KIF1BP
2.04138069159888E-142	0.939477687632656	0.426	0.106	3.4209457629814E-138	8	TRAM2
7.13850435203835E-108	0.938917008966211	0.375	0.104	1.19627055931459E-103	8	HSPA14.1
1.7676610112676E-118	0.938234432984875	0.434	0.124	2.96224632268224E-114	8	SDCCAG8
8.60840181959165E-153	0.93811241512017	0.718	0.243	1.44259597692717E-148	8	PAN3
2.13012266146858E-144	0.938086450532208	0.598	0.183	3.56965955608905E-140	8	ZNF507
5.15776230880189E-141	0.937685165527486	0.656	0.217	8.64337807709021E-137	8	GTF2H2
2.90257103968916E-135	0.937524529580649	0.526	0.156	4.8641285483111E-131	8	PIGN
2.5171822315935E-141	0.937455921966915	0.598	0.191	4.21829398370438E-137	8	STRBP
1.290088169767E-135	0.937439973528247	0.659	0.224	2.16192975489554E-131	8	SLC2A13
1.46385127811201E-149	0.937059818037141	0.542	0.154	2.4531219718601E-145	8	CYB561A3
4.75881305721601E-118	0.935714299575263	0.399	0.109	7.97481892128259E-114	8	MYLIP
1.8102778781902E-128	0.935539095460518	0.591	0.196	3.03366366827114E-124	8	PPIP5K2
2.07548809906411E-108	0.934612457332936	0.348	0.091	3.47810295641163E-104	8	TTBK2
4.94949026997026E-153	0.934171359508066	0.64	0.2	8.29435579441616E-149	8	MAP3K4
8.98064378679438E-170	0.933996169119477	0.738	0.242	1.504976285791E-165	8	HSPA4L
5.36843071195979E-149	0.933946385671982	0.828	0.319	8.99641618710221E-145	8	ADAM15
2.51967077383379E-159	0.933912594664753	0.594	0.174	4.22246428279067E-155	8	NUP54
1.05723423599706E-133	0.933312990987816	0.373	0.089	1.77171313268387E-129	8	MAP3K9
1.15417169981758E-165	0.933057258815459	0.605	0.177	1.9341609345543E-161	8	CTCF
2.72095367063756E-140	0.932407984864065	0.71	0.252	4.55977416125443E-136	8	AKAP8
3.39863297399757E-148	0.932147051868415	0.877	0.352	5.69542913782512E-144	8	KAT6A
1.55033207566324E-123	0.931766721797668	0.423	0.117	2.59804649239645E-119	8	MED21
1.33030179045462E-161	0.931425730085066	0.687	0.221	2.22931974044386E-157	8	HAT1
1.0057217599931E-132	0.931383178518129	0.455	0.126	1.68538852539644E-128	8	TMEM60

2.83492562070135E-158	0.931246440831817	0.708	0.232	4.75076835517133E-154	8	REV1
3.8839098784281E-120	0.930931199336474	0.609	0.21	6.50865617426982E-116	8	PTEN
2.9874866761002E-142	0.930060203129293	0.563	0.169	5.00643017180872E-138	8	TMEM170A
4.82973485507947E-121	0.927167551720701	0.353	0.087	8.09366967014217E-117	8	VPS29
4.06996525269168E-135	0.926639304361691	0.554	0.17	6.82044777046072E-131	8	SLC25A28
3.73616600797068E-124	0.926604842662244	0.851	0.373	6.26106699615727E-120	8	SIPA1L2
1.27399773012062E-139	0.92639486750961	0.804	0.311	2.13496539613614E-135	8	BPTF
1.7572994573643E-90	0.925355605786997	0.25	0.059	2.94488243065109E-86	8	RTEL1
1.3644001891597E-132	0.924557530296028	0.897	0.398	2.28646183699382E-128	8	PLEKHF2
5.96353044312054E-157	0.924329060190201	0.638	0.198	9.99368431658141E-153	8	RESF1
2.44289462541013E-116	0.923947007052319	0.569	0.195	4.09380281326229E-112	8	HSF4
2.96527703878738E-145	0.923668536361856	0.739	0.27	4.96921126159989E-141	8	ERCC5
3.77738312745479E-133	0.923495075634371	0.675	0.239	6.33013864498874E-129	8	RUNX1
4.19245485793946E-123	0.92327265198083	0.51	0.158	7.02571585093495E-119	8	PLCG1
1.34648310206975E-149	0.922784521254807	0.652	0.211	2.25643638244849E-145	8	STK4
4.2091524238048E-85	0.922129824719835	0.312	0.088	7.05369763181209E-81	8	INTS2
2.90584383394189E-167	0.921094047760449	0.767	0.262	4.86961309691983E-163	8	HLTF
2.79901624910901E-97	0.920306613448187	0.353	0.1	4.69059143025688E-93	8	CGRRF1
8.87051732121032E-83	0.919931756945178	0.379	0.124	1.48652129268842E-78	8	AP002495.1
6.60953203876967E-83	0.918922297033745	0.312	0.09	1.10762537905702E-78	8	ENTPD2
1.69292637868294E-98	0.918859150407205	0.322	0.086	2.83700602539687E-94	8	NUP37
9.18693893655124E-138	0.91846436597971	0.475	0.132	1.53954722698726E-133	8	DBNDD2
8.64956345181934E-136	0.918352540354265	0.635	0.213	1.44949384325588E-131	8	LCLAT1
2.71979397403848E-93	0.918346673602999	0.67	0.292	4.55783074169369E-89	8	MCM5
9.04785369283572E-135	0.918025620432598	0.591	0.188	1.51623932184541E-130	8	RAB28
2.02706505309737E-181	0.917923148765483	0.79	0.263	3.39695561598058E-177	8	PABPC1L
2.2417081973073E-73	0.917340837069493	0.352	0.116	3.75665459704757E-69	8	WDR5B
1.27433601062688E-125	0.917245238132452	0.522	0.164	2.13553228660853E-121	8	TSPAN7
1.31939399589491E-126	0.916513846959054	0.446	0.125	2.2110404583207E-122	8	POT1
2.16895232550958E-142	0.916012650808973	0.635	0.206	3.63473030708895E-138	8	CDKAL1
4.7495127139911E-148	0.915943357979682	0.743	0.262	7.95923340610628E-144	8	RAPGEF5
3.33745635131432E-126	0.915886893463279	0.578	0.189	5.59290935353254E-122	8	PLCXD1
3.20337764689164E-94	0.915732640512975	0.362	0.107	5.36822026066101E-90	8	ZFP90
1.02792612677189E-142	0.915702234325098	0.799	0.3	1.72259860324433E-138	8	ACACB
1.84747160482694E-157	0.915363065244292	0.636	0.198	3.09599291536899E-153	8	RAVER1
3.71929833065603E-144	0.914667401814916	0.834	0.321	6.23280014251338E-140	8	EHBP1L1
4.47664222899428E-150	0.914122596573214	0.431	0.105	7.50195704734862E-146	8	PIGK
3.73625895514028E-113	0.914028122614503	0.446	0.134	6.26122275702408E-109	8	HSPG2
6.95685717273555E-111	0.913674935474129	0.593	0.212	1.16583012500702E-106	8	PRPF39
9.82511817401215E-84	0.913275693408361	0.352	0.109	1.64649330360096E-79	8	TOP3B
1.26635001291174E-115	0.912659522003302	0.682	0.262	2.12214935163749E-111	8	MSH6
1.92193886913894E-81	0.911597284122284	0.284	0.078	3.22078515690304E-77	8	C17orf80
8.62169718273458E-127	0.911532410657422	0.913	0.423	1.44482401388266E-122	8	STAU2
2.33336075715454E-75	0.911438482233453	0.305	0.093	3.91024595683958E-71	8	NCR3LG1
1.55728013846816E-102	0.911253261075026	0.372	0.105	2.60969005604495E-98	8	PRKCI
3.57163222989729E-84	0.910921934776447	0.282	0.076	5.98534129086188E-80	8	IPPK
3.29681055812908E-137	0.910808016248267	0.597	0.193	5.52479513331271E-133	8	ZFYVE16
8.25939092800031E-143	0.910736517662793	0.631	0.204	1.38410873171429E-138	8	BORCS8
7.0759852587232E-157	0.910552971546843	0.744	0.253	1.18579360965683E-152	8	MTMR1
3.98417862371946E-130	0.910377127364473	0.398	0.101	6.67668653762907E-126	8	CBX7
1.21439735395746E-95	0.910280785593687	0.405	0.126	2.03508708576191E-91	8	TMEM260
5.50974328308699E-104	0.910160417017607	0.386	0.112	9.23322779379718E-100	8	TRIM32
1.97734566903126E-62	0.909306489696015	0.256	0.078	3.31363587216259E-58	8	ST7L
1.52980146798999E-149	0.90861938290032	0.698	0.236	2.56364130005763E-145	8	CLDND1
2.63700163671358E-119	0.90830989778338	0.338	0.082	4.41908734280463E-115	8	PIM2
2.91301449976538E-162	0.907502173683297	0.768	0.274	4.88162969870682E-158	8	NUMA1
3.67500556976793E-68	0.90675880488266	0.28	0.086	6.15857433381709E-64	8	STX18
1.35298065333318E-82	0.906332603552586	0.29	0.081	2.26732497885574E-78	8	DYRK3
1.85523679971543E-72	0.905854932169563	0.29	0.088	3.10900582896311E-68	8	VRK2
6.69424446749699E-113	0.905796845494901	0.699	0.274	1.12182148786315E-108	8	ST3GAL6

2.2598463283538E-184	0.905318832514046	0.69	0.202	3.78705047705529E-180	8	ZNF680
1.55345467486803E-111	0.905190147419174	0.696	0.281	2.60327934414384E-107	8	MCM2
4.09257982643865E-131	0.905135448738952	0.356	0.084	6.85834527314589E-127	8	HIRIP3
2.50726563108623E-138	0.905054531644887	0.658	0.22	4.20167574457431E-134	8	DNAH14
1.33859823425851E-153	0.904236697394538	0.648	0.205	2.24322292097041E-149	8	FBXO46
5.98410666434426E-120	0.904131313777642	0.511	0.161	1.00281659481081E-115	8	FAM126A
2.81897102619864E-75	0.903433437627432	0.269	0.075	4.72403164570368E-71	8	PPP4R4
2.45316502259159E-141	0.903092198308611	0.652	0.218	4.11101394485898E-137	8	ABCB10
3.99869072268421E-103	0.903008149066925	0.332	0.087	6.7010059130742E-99	8	SRBD1
1.83694377145766E-104	0.902247435422188	0.37	0.104	3.07835037220874E-100	8	ATP1B3
1.56492732426049E-115	0.902237453892411	0.332	0.081	2.62250520999573E-111	8	ZCCHC17
1.68156999839249E-118	0.901476334982227	0.332	0.079	2.81797500330613E-114	8	RPA3
2.82700455376562E-143	0.901450878985109	0.738	0.259	4.73749423120042E-139	8	GTF2H2C
1.82966325156769E-125	0.90143222371593	0.493	0.146	3.06614967697714E-121	8	PPWD1
1.62256799176002E-99	0.901178662960154	0.543	0.197	2.71909944059145E-95	8	SLC25A29
1.86372335571375E-148	0.900740670490662	0.678	0.227	3.1232275995051E-144	8	PSMG3
2.51036841744035E-148	0.8997670336307	0.784	0.29	4.20687539394654E-144	8	NRP1
1.58288280846702E-79	0.899749934011046	0.329	0.101	2.65259501042903E-75	8	ZNF554
1.88313719753546E-80	0.899103553629049	0.352	0.111	3.15576131562993E-76	8	ADAT2
1.26672307287424E-94	0.89908185793408	0.361	0.105	2.12277452552266E-90	8	TFB1M
1.00615373399875E-121	0.898245602827703	0.447	0.127	1.68611242743511E-117	8	MGA
2.1440617586548E-144	0.898244664480967	0.838	0.333	3.59301869515371E-140	8	EMP2
2.1472585289115E-138	0.897981274142483	0.866	0.383	3.59837584274989E-134	8	FLOT2
1.37328622819793E-113	0.89778998031899	0.51	0.165	2.30135306121409E-109	8	IRAK4
2.25654065412609E-131	0.897699279635674	0.522	0.158	3.78151082818451E-127	8	SPTLC2
1.18771558443074E-84	0.897203505521875	0.317	0.091	1.99037377638904E-80	8	EPHB6
3.40663665274838E-162	0.896229400487204	0.706	0.235	5.70884170267574E-158	8	SLC29A1
3.68547142828354E-136	0.896224304180134	0.601	0.196	6.17611301951755E-132	8	FZD5
1.53817762294424E-136	0.895898685203739	0.912	0.428	2.57767806052995E-132	8	SIVA1
1.65300020957032E-92	0.895642780575226	0.318	0.087	2.77009775119794E-88	8	MEX3C
2.34418367597898E-165	0.895484478454981	0.72	0.231	3.92838300420557E-161	8	MTR
9.15387879427854E-157	0.894342524567535	0.591	0.174	1.5340070083452E-152	8	BAG5
8.01928706045839E-150	0.894030418040658	0.72	0.253	1.34387212559162E-145	8	TEAD1
2.71328863996736E-107	0.894029719504598	0.313	0.077	4.5469291028573E-103	8	CSNK1G1
3.00822279777552E-113	0.893599889025715	0.486	0.154	5.04117976451221E-109	8	FCHO2
5.50920383416222E-126	0.893513007073692	0.593	0.196	9.23232378528905E-122	8	STIM2
1.13257385487257E-129	0.893511616124148	0.727	0.272	1.89796726599545E-125	8	FBXO42
3.47424883660849E-132	0.893467719398871	0.585	0.19	5.82214620038851E-128	8	ETAA1
1.29913148565863E-96	0.893459286506916	0.73	0.329	2.17708454366673E-92	8	CLDN15
9.37676714629127E-140	0.893354303573848	0.535	0.158	1.57135863837549E-135	8	TBC1D10B
8.7423449159521E-99	0.892985336633956	0.357	0.101	1.46504216101525E-94	8	ZCCHC4
2.41806421367913E-142	0.89288139817269	0.529	0.154	4.05219200928349E-138	8	DCAKD
1.60529468362948E-147	0.89278014525659	0.698	0.237	2.69015283082629E-143	8	GATD1
2.08294841683283E-93	0.892418008629374	0.349	0.1	3.49060495692846E-89	8	DUSP12
1.46752929919621E-126	0.892186505862806	0.515	0.158	2.45928559959301E-122	8	IPP
2.54873572805433E-135	0.89121686288987	0.858	0.378	4.27117133307345E-131	8	CDKN1B
1.05393875100868E-108	0.8909130206971	0.314	0.077	1.76619055894035E-104	8	DEPRECATED-ENSG00000189366
4.94079689470375E-129	0.890489876094767	0.519	0.157	8.27978743614454E-125	8	GANC
3.1016286613717E-87	0.890263778069732	0.273	0.07	5.19770931072669E-83	8	BTN2A1
9.48612032717519E-144	0.889936056058742	0.656	0.217	1.58968404442802E-139	8	TAPT1
1.00221463856326E-116	0.889878235880425	0.951	0.485	1.67951129130432E-112	8	ALG13
9.84748999294185E-98	0.88913331759509	0.397	0.12	1.65024237301719E-93	8	MTCL1
2.04501396470648E-139	0.888855626228586	0.824	0.323	3.42703440205512E-135	8	TIMM8A
4.59513034047494E-144	0.888615258934359	0.73	0.26	7.70051942456791E-140	8	TAF2
1.21619981994579E-142	0.888449010712503	0.731	0.264	2.03810765826516E-138	8	UBA2
4.27862555068788E-113	0.888419864763859	0.354	0.091	7.17012069784275E-109	8	ZBED5
1.96113320418587E-113	0.888208590016813	0.341	0.086	3.28646702357468E-109	8	FRMD4B
1.08902990277521E-77	0.888045170257808	0.277	0.078	1.8249963110707E-73	8	ZSWIM1
4.05149240402758E-115	0.887294148561411	0.438	0.127	6.78949097066942E-111	8	SMARCAD1
1.1317269143285E-144	0.886677514339934	0.349	0.076	1.8965479630317E-140	8	SAC3D1

1.01916430608483E-112	0.886395994486285	0.354	0.092	1.70791554413695E-108	8	AMDHD2
3.13718550341393E-131	0.885907142545982	0.481	0.137	5.25729546662107E-127	8	DENND6A
9.47671847857424E-90	0.884837137716271	0.304	0.082	1.58810848263947E-85	8	N4BP2
1.61037221531883E-112	0.88415927225045	0.502	0.162	2.6986617584313E-108	8	SPRY2
5.6672766868831E-139	0.883968253086675	0.915	0.421	9.49722227187869E-135	8	ZNRF2
7.57993372861575E-151	0.883952587547147	0.764	0.276	1.27024529424143E-146	8	CHAMP1
2.62543068571459E-134	0.883400020229336	0.57	0.178	4.39969674312051E-130	8	PIGM
3.48044072815867E-118	0.883248089031871	0.454	0.134	5.8325225722483E-114	8	ABR
1.62026904347394E-142	0.882912542464032	0.406	0.098	2.71524686305363E-138	8	POU2F1
3.21549660641686E-141	0.882612432932124	0.901	0.383	5.38852921303337E-137	8	PA2G4
9.04852956635719E-112	0.882599846834145	0.453	0.136	1.51635258473014E-107	8	SPATA5L1
5.05852920725557E-132	0.882332955426676	0.551	0.172	8.47708324551889E-128	8	ARMC1
1.4938197251362E-91	0.881569784492938	0.375	0.115	2.50334309538325E-87	8	DCBLD1
6.10240968460271E-141	0.880400291906041	0.575	0.176	1.02264181494572E-136	8	HSPA13
7.4790249490649E-153	0.879687886631565	0.545	0.154	1.2533350009643E-148	8	AGBL5
6.0330276880929E-96	0.879683363355894	0.333	0.092	1.01101477997061E-91	8	IMMP1L
5.25436111090831E-122	0.879293369204039	0.802	0.336	8.80525834966015E-118	8	KIAA0232
2.25243584235075E-162	0.879095866665182	0.628	0.188	3.77463198461138E-158	8	DUOXA2
5.8317271136239E-131	0.878711031427357	0.646	0.222	9.77280829701093E-127	8	RELCH
4.4229897951932E-108	0.878707975404576	0.602	0.217	7.41204629878477E-104	8	SHOX2
3.36040782982861E-136	0.8779933616393	0.554	0.169	5.63137144122679E-132	8	ZHX3
2.93769556681423E-70	0.87780947165741	0.253	0.071	4.92299023086729E-66	8	TRIM66
1.19264904776195E-151	0.877167554260496	0.772	0.277	1.99864127423948E-147	8	STAG1
2.31162676142191E-121	0.877156084148552	0.453	0.13	3.87382412679084E-117	8	ORC2
2.44008371295652E-60	0.876841016100361	0.317	0.11	4.08909228617253E-56	8	PTPRM
8.64489329083619E-129	0.876655643976238	0.602	0.198	1.44871121767833E-124	8	USP53
2.3017122355231E-79	0.876296607266851	0.366	0.119	3.8572093642896E-75	8	MIPOL1
9.87859267600816E-150	0.875901340217783	0.519	0.144	1.65545456064545E-145	8	CPSF3
1.82604758134038E-133	0.875174118390214	0.37	0.088	3.06009053681021E-129	8	NUP93
1.88580930175866E-133	0.875094001155825	0.574	0.181	3.16023922788717E-129	8	USPL1
6.40779726912597E-71	0.874874633569008	0.274	0.08	1.07381866636013E-66	8	SWT1
2.87063675678919E-135	0.874645138441845	0.831	0.328	4.81061307702732E-131	8	PARP4
5.88043740828027E-81	0.874452496612923	0.465	0.171	9.85443700879608E-77	8	TSPYL4
4.70563444576799E-134	0.873862129794484	0.636	0.214	7.885702204218E-130	8	VCPIP1
8.66969096458851E-133	0.873600414643974	0.83	0.34	1.45286681184574E-128	8	PHF10
3.12816697474015E-118	0.873131880398933	0.491	0.153	5.24218221626954E-114	8	DIP2C
2.73233091650658E-83	0.873104792018791	0.375	0.121	4.57884014988172E-79	8	CLASP2
4.0724822798396E-107	0.872474794097255	0.405	0.118	6.8246658045552E-103	8	DTNBP1
3.8934039955926E-113	0.872230357260483	0.479	0.149	6.52456641581407E-109	8	FAM200B
2.9905977766475E-120	0.86935979774271	0.419	0.116	5.01164375410588E-116	8	ALG1L
1.69046586367104E-142	0.869117536666546	0.558	0.166	2.83288269433993E-138	8	OSER1
8.47594260190858E-93	0.868795732656068	0.378	0.115	1.42039846122784E-88	8	NEDD9
2.66289902985954E-131	0.868509414662411	0.551	0.171	4.46248619423861E-127	8	GNPTAB
1.12361549028594E-160	0.867775900208436	0.688	0.221	1.88295483862118E-156	8	PSMG2
1.49710376255549E-76	0.867699539926571	0.33	0.104	2.50884648529049E-72	8	HELQ
5.24547493952514E-156	0.867660693594118	0.418	0.097	8.79036690365623E-152	8	PAFAH1B3
1.76074387853488E-140	0.867257060033554	0.598	0.19	2.95065459164876E-136	8	RUVBL2
1.9949440287332E-134	0.867238574471296	0.586	0.187	3.3431272033511E-130	8	CBX3
1.32021460366082E-141	0.866210585052923	0.501	0.141	2.2124156328148E-137	8	ARPIN
7.95045692614787E-123	0.865739702922875	0.503	0.154	1.33233757168386E-118	8	RIC1
6.72759761349036E-138	0.865696243923619	0.672	0.231	1.12741080806871E-133	8	ITGA6
3.14420145415938E-147	0.865338076399833	0.666	0.217	5.26905279688028E-143	8	UBA6
1.59175790903443E-148	0.865320066475472	0.663	0.219	2.6674679039599E-144	8	COMMD4
8.55815299589995E-139	0.865297507982049	0.687	0.235	1.43417527905291E-134	8	DOCK9
1.81595126652575E-141	0.865174029836028	0.732	0.264	3.04317113244384E-137	8	LMBRD2
9.08301869516038E-106	0.865137704701122	0.429	0.13	1.52213227293498E-101	8	EXOC1
3.38404718874672E-141	0.86484179318289	0.676	0.232	5.67098627890176E-137	8	DYRK1A
2.90938530526755E-106	0.864506609006866	0.421	0.125	4.87554789456737E-102	8	IL17RA
1.4579136740712E-111	0.863630535732058	0.293	0.067	2.44317173500852E-107	8	SORT1
8.13451338628636E-142	0.863499635602605	0.611	0.196	1.36318175327387E-137	8	NUP98

1.71336753470244E-134	0.862951078026059	0.642	0.218	2.87126131465434E-130	8	CBFA2T2
2.35483120142236E-135	0.86242594896932	0.403	0.101	3.94622612734359E-131	8	ANP32B
8.3501603672585E-98	0.862335673217178	0.438	0.14	1.39931987434518E-93	8	NRXN3
2.99510973494248E-127	0.862283299413257	0.402	0.105	5.0192048938166E-123	8	PHF13
9.42810044231406E-139	0.861915349774132	0.771	0.291	1.57996107212299E-134	8	DNAJC1
3.80174029023607E-122	0.861075357094522	0.527	0.167	6.37095637837761E-118	8	ARPP19
1.30947866748281E-97	0.860619307982881	0.285	0.07	2.1944243509677E-93	8	CHD3
1.58629184708722E-133	0.860375776331508	0.575	0.182	2.65830787734876E-129	8	PKN2
1.09338519471463E-75	0.860320858364208	0.301	0.09	1.83229490930278E-71	8	CRYZL1
2.15806424683977E-103	0.859795109599435	0.379	0.108	3.61648406485408E-99	8	DEPRECATED-ENSG00000104442
6.68695002830096E-118	0.859315693144684	0.469	0.142	1.12059908574268E-113	8	RASSF8
2.70831841467262E-112	0.858702229958592	0.349	0.09	4.53859999930838E-108	8	SFMBT1
7.23048317348166E-138	0.858269138566562	0.622	0.205	1.21168437021206E-133	8	AK6
7.29344689722083E-125	0.858144015722208	0.664	0.241	1.22223583103627E-120	8	COX11
3.08789718892986E-97	0.857720343556588	0.312	0.081	5.17469810920866E-93	8	CWF19L2
2.00682473516202E-117	0.857192764818244	0.598	0.209	3.36303689118451E-113	8	IPMK
1.00242071075701E-88	0.857140610706767	0.324	0.091	1.6798566270866E-84	8	ZNF451
4.40372053721604E-126	0.857005930932565	0.499	0.149	7.37975487626664E-122	8	MFHAS1
1.07916747145349E-149	0.856654074483329	0.654	0.211	1.80846884866175E-145	8	POLR2A
1.11207342398033E-117	0.856591828507964	0.435	0.124	1.86361264390624E-113	8	CCNH
1.5614994989313E-104	0.856346012867299	0.447	0.139	2.61676086030907E-100	8	ARV1
6.60668597709129E-113	0.856074884971066	0.446	0.133	1.10714843604096E-108	8	ZNF131
2.2213796597571E-79	0.855642676893656	0.354	0.113	3.72258803382095E-75	8	CNEP1R1
2.07715770597184E-73	0.855185742633965	0.298	0.091	3.48090088366761E-69	8	WHAMM
2.85968171282978E-97	0.85420609500295	0.269	0.063	4.79225461436014E-93	8	PEAK1
6.06746088393054E-95	0.854177638901578	0.296	0.076	1.01678509492908E-90	8	PHACTR2
5.80909426288651E-153	0.85401949325756	0.622	0.191	9.73488016574521E-149	8	PSIP1
2.55681606520588E-131	0.85373220445966	0.513	0.152	4.28471236207202E-127	8	RELL1
3.40685069346632E-102	0.853137751285628	0.489	0.163	5.70920039211085E-98	8	METTL15
4.59705463787742E-126	0.852411588531648	0.373	0.092	7.70374416215499E-122	8	UACA
6.10934838860725E-130	0.852215022311382	0.578	0.187	1.0238046029628E-125	8	FLVCR1
7.02717698066725E-97	0.851807345998345	0.397	0.12	1.17761431842022E-92	8	AASDH
3.11047265212991E-120	0.851643846060239	0.454	0.132	5.21253007043931E-116	8	POLR2D
1.63399202091739E-84	0.851380790423509	0.306	0.086	2.73824382865336E-80	8	WDSUB1
3.1208435885814E-90	0.851211705139159	0.365	0.11	5.22990968574471E-86	8	PIP4K2A
5.94678456383064E-134	0.851139497407899	0.767	0.285	9.96562157206738E-130	8	TBC1D8B
5.74581953970203E-88	0.850786204139565	0.318	0.089	9.62884438463265E-84	8	IARS
1.56237685719573E-100	0.850558431298897	0.475	0.158	2.6182311372886E-96	8	MSTO1
5.83773945007044E-145	0.850536155952249	0.714	0.245	9.78288377042804E-141	8	KDM3B
9.81241628583169E-125	0.850393594222984	0.455	0.13	1.64436472117967E-120	8	CNTNAP2
6.97363353003443E-100	0.850373991367393	0.402	0.121	1.16864150696317E-95	8	ZNF791
1.0956757595717E-105	0.849358645555973	0.397	0.115	1.83613343789025E-101	8	TMEM47
6.61182576285215E-69	0.848470059009403	0.334	0.112	1.10800976133876E-64	8	MFSD8
1.2407566206596E-137	0.847743462773318	0.573	0.177	2.07925994490136E-133	8	NHLRC2
5.33536334028347E-87	0.847576917481365	0.981	0.793	8.94100188564705E-83	8	HSPA1B
9.24604851699493E-129	0.846528206121013	0.571	0.183	1.54945281047801E-124	8	KRIT1
4.49589202465218E-135	0.845409970403465	0.609	0.199	7.53421585491212E-131	8	RCOR1
4.2783537522592E-108	0.843462745067499	0.344	0.09	7.16966521803597E-104	8	ELP6
1.48544367474269E-111	0.843438617027608	0.479	0.15	2.4893065101338E-107	8	SLC36A1
8.87926270887573E-113	0.843436353202278	0.415	0.119	1.48798684475339E-108	8	TBCCD1
1.73940056080824E-78	0.842088102985685	0.332	0.102	2.91488745980246E-74	8	THAP6
3.32946552263245E-55	0.840701434250826	0.285	0.098	5.57951832282746E-51	8	PHOSPHO2
7.15264182399863E-147	0.840634762268218	0.613	0.189	1.19863971686569E-142	8	TGS1
1.78791476636247E-114	0.840419311842349	0.422	0.121	2.99618756547022E-110	8	PIGU
4.30817120382082E-128	0.8402406657227	0.811	0.321	7.21963330336293E-124	8	TRAPPC9
2.69304440399095E-129	0.84015051700599	0.622	0.21	4.51300381220804E-125	8	SFPQ
9.52899711152003E-155	0.839485055535694	0.652	0.207	1.59686933594853E-150	8	SMC3
2.65086738464576E-127	0.838919039634301	0.497	0.148	4.44232356318936E-123	8	LRRC57
1.16115526882588E-89	0.838171550355874	0.321	0.089	1.94586399949841E-85	8	NAA35
9.24753555608818E-129	0.837037792894258	0.7	0.256	1.54970200848926E-124	8	METTL17

5.09335692599747E-89	0.836973045416207	0.445	0.151	8.53544753658656E-85	8	CTPS2
7.51052824158836E-137	0.836764079715588	0.831	0.327	1.25861432272538E-132	8	CD47
2.98430484820537E-116	0.835843095481628	0.383	0.102	5.00109806462256E-112	8	IPO11
1.54735730146169E-78	0.835752121424529	0.338	0.106	2.59306136578951E-74	8	FAM219B
3.64427931212588E-142	0.835583848111735	0.727	0.26	6.10708327126055E-138	8	SEMA4B
1.05457785319895E-104	0.835497647732899	0.344	0.091	1.76726156639079E-100	8	MAP3K1
8.32030259955601E-105	0.833419302054442	0.47	0.151	1.3943163096336E-100	8	AMMECR1
5.28755769714814E-116	0.83341221295226	0.601	0.209	8.86088918888085E-112	8	DPH6
1.95150530592495E-128	0.833051653086861	0.527	0.161	3.27033259166902E-124	8	ZDHHC6
2.46475568932248E-120	0.832972518089335	0.473	0.141	4.13043758416662E-116	8	ZNF76
1.91134413337708E-144	0.832434153401065	0.615	0.194	3.20303049871331E-140	8	RFX5
5.50521984966861E-150	0.831595614834354	0.755	0.264	9.22564742407466E-146	8	AP3B1
1.69904933801367E-148	0.831345221314202	0.498	0.134	2.84726688064331E-144	8	INIP
1.70271074681177E-131	0.831170076090645	0.506	0.149	2.85340266950716E-127	8	CNOT6
3.71418205549588E-119	0.830777427615014	0.752	0.293	6.2242262886E-115	8	INTS6
5.76301963739754E-101	0.830367876927434	0.324	0.084	9.6576683083508E-97	8	KLHL15
1.23913011219577E-93	0.830047789104425	0.398	0.124	2.07653424201767E-89	8	TBP
1.16628432445069E-55	0.829805021022399	0.292	0.101	1.95445927091447E-51	8	PCDHGA8
3.33331780110986E-129	0.829756899131393	0.92	0.436	5.58597397109991E-125	8	ILF3
1.08206796378009E-96	0.829692602062823	0.296	0.075	1.81332949370268E-92	8	GNG4
3.28188615363695E-137	0.829435679989974	0.82	0.328	5.49978481626481E-133	8	TMEM245
5.0966654496649E-85	0.829300437147897	0.356	0.109	8.54099196054844E-81	8	ZXDB
4.1993585698295E-138	0.828991827364165	0.826	0.32	7.03728509132027E-134	8	CREBZF
1.81773816446909E-102	0.828929282965292	0.615	0.24	3.0461656160173E-98	8	FADS1
9.53981631476978E-116	0.828152972805218	0.832	0.366	1.59868241802912E-111	8	PPM1B
1.81817336837813E-124	0.828089743887466	0.807	0.336	3.04689493072807E-120	8	PAXX
1.2568507577274E-124	0.827350038113502	0.445	0.124	2.10623049779958E-120	8	FAM133B
3.0903534213673E-68	0.825491063340346	0.341	0.116	5.17881426352731E-64	8	SYDE2
1.5904071776027E-131	0.825395843162908	0.546	0.168	2.66520434822661E-127	8	VPS13A
1.82319667668966E-131	0.825018595033404	0.563	0.178	3.05531299079653E-127	8	ADCK2
5.88369120902504E-129	0.825011034291728	0.783	0.305	9.85988972808416E-125	8	CDC16
3.87177960125617E-63	0.824816848982056	0.265	0.082	6.4883282557851E-59	8	KLHL28
1.40449150915952E-134	0.824775556663079	0.531	0.159	2.35364687104953E-130	8	ZDHHC20
3.83784722448754E-81	0.824236805452421	0.298	0.085	6.43146437879622E-77	8	DEPRECATED-ENSG00000107863
1.4694355544116E-70	0.823937615212758	0.309	0.098	2.46248010208296E-66	8	IGIP
1.80203652023812E-89	0.823663412572053	0.391	0.124	3.01985280061503E-85	8	UIMC1
1.23601315245697E-121	0.823618799180311	0.485	0.146	2.07131084088739E-117	8	TNPO1
3.58612008391592E-138	0.823441572822023	0.792	0.3	6.0096200366263E-134	8	AGO2
4.31611974190504E-146	0.822293605885953	0.707	0.244	7.23295346348446E-142	8	BCAP29
8.01138906572165E-95	0.822111491999301	0.375	0.111	1.34254857963363E-90	8	SENP7
1.40367741916188E-110	0.821808687292147	0.442	0.132	2.35228261903148E-106	8	RPE
3.15358180238799E-92	0.821528894064363	0.407	0.129	5.28477238444179E-88	8	TTC21B
1.05966670204367E-54	0.821391067389446	0.285	0.098	1.77578945928479E-50	8	PCDHGB6
4.58806629163744E-130	0.821022826245002	0.855	0.351	7.68868149152602E-126	8	DIDO1
2.9155327528752E-93	0.820687311495632	0.477	0.164	4.88584978726827E-89	8	GTF2IRD1
4.52867464895997E-105	0.820290895610231	0.434	0.131	7.58915297672712E-101	8	SMYD3
2.58509008945081E-122	0.820252786562566	0.379	0.097	4.33209397190167E-118	8	CABIN1
8.69641100315591E-132	0.820130328074737	0.483	0.137	1.45734455590887E-127	8	PIGQ
1.67719810209554E-117	0.819369365389933	0.514	0.162	2.8106485794917E-113	8	SEC22A
3.74107308784554E-121	0.819341949221628	0.846	0.357	6.26929028061156E-117	8	NUFIP2
4.30397948431292E-96	0.819312333411712	0.304	0.079	7.21260881981159E-92	8	IQGAP1
2.65539427175333E-120	0.818968189253523	0.885	0.393	4.44990972060423E-116	8	FAT1
1.69481983574355E-98	0.818921736238684	0.382	0.112	2.84017908073904E-94	8	FBXO30
3.06462245795245E-105	0.818614538782757	0.493	0.161	5.13569431503671E-101	8	PTPRD
6.67788193015923E-110	0.818052946658377	0.407	0.118	1.11907945385608E-105	8	CEP170
1.11683208223681E-80	0.817932415716959	0.281	0.077	1.87158720341244E-76	8	RAI1
3.13543959301366E-166	0.817713190843789	0.611	0.175	5.25436966997228E-162	8	SIN3A
2.20296591016916E-120	0.817260677310568	0.518	0.163	3.69173027226149E-116	8	KYAT1
2.82723125133355E-134	0.816647927465872	0.446	0.12	4.73787413098476E-130	8	AKNA
1.45875802373457E-91	0.815980363660733	0.378	0.115	2.44458669617439E-87	8	WDR27

8.34603340180472E-117	0.815513059332982	0.526	0.171	1.39862827747443E-112	8	ANKRD13A
1.96906091788529E-130	0.8154228635138	0.656	0.227	3.29975228619217E-126	8	POLR2B
1.29388457992266E-132	0.815411998934519	0.708	0.253	2.16829177903439E-128	8	ASRGL1
2.79034415722765E-108	0.815310787450958	0.317	0.078	4.6760587386821E-104	8	LSM 5.00
9.83115588124307E-119	0.815233102022246	0.836	0.357	1.64750510257871E-114	8	SAMD5
1.83380992375943E-97	0.815200856009589	0.377	0.111	3.07309867023605E-93	8	SMAGP
1.47205679497253E-131	0.815197497401733	0.64	0.216	2.46687277701496E-127	8	LARP7
1.33835626861092E-107	0.814868545026057	0.53	0.178	2.24281743493817E-103	8	GOLGA8B
1.46157716575594E-114	0.814409184107732	0.566	0.192	2.44931101437381E-110	8	TRIM56
3.40287949411713E-126	0.814125592480518	0.455	0.13	5.70254545624148E-122	8	MAPRE1
7.36448246728909E-85	0.813610775311974	0.313	0.089	1.23413997186831E-80	8	YOD1
2.82108642549412E-134	0.813550041429105	0.521	0.153	4.72757663184304E-130	8	COQ6
1.70159308139848E-99	0.813481712305744	0.427	0.134	2.85152968580757E-95	8	ARHGAP21
1.51681764518189E-112	0.812444085967913	0.487	0.153	2.54188300979582E-108	8	ZNF445
1.26305501871012E-77	0.81226667524587	0.306	0.091	2.11662760035442E-73	8	GFOD1
7.07302285618506E-125	0.811893147167912	0.458	0.13	1.18529717023949E-120	8	RB1
6.49196021835762E-105	0.81086728815757	0.493	0.162	1.08792269339237E-100	8	PRR14L
2.48113817473472E-121	0.810860732553575	0.859	0.373	4.15789135322044E-117	8	ASH1L
1.92316693047465E-112	0.810430860103167	0.573	0.196	3.22284314208942E-108	8	POLK
1.74251182607307E-125	0.810422093091918	0.691	0.254	2.92010131813325E-121	8	DPY19L4
1.20212757273665E-128	0.810188087785652	0.81	0.328	2.01452538639208E-124	8	TMCO3
1.67012937131751E-121	0.810007946511456	0.79	0.317	2.79880280045388E-117	8	TRMT2B
4.31422602083127E-125	0.809588023145843	0.537	0.168	7.22977996570905E-121	8	TMTC4
8.42330628617111E-124	0.809348951297699	0.662	0.241	1.41157766743655E-119	8	PSPH
5.09740827834753E-105	0.809216898571523	0.421	0.127	8.54223679285479E-101	8	CLASP1
6.55134888871551E-115	0.809107526831744	0.947	0.569	1.09787504677094E-110	8	TMEM106C
2.34679174440891E-79	0.808952056542789	0.288	0.081	3.93275360528045E-75	8	GRK6
7.46870356133106E-122	0.808937580329597	0.419	0.115	1.25160534280786E-117	8	VKORC1L1
7.19746046368117E-112	0.808778714026476	0.911	0.439	1.20615042450369E-107	8	IKBKG
6.99929840405482E-76	0.808553686485192	0.258	0.07	1.17294242655151E-71	8	ASCC1
4.84327176254859E-94	0.808349918375879	0.348	0.1	8.11635481967893E-90	8	PDK3
7.65987094819616E-107	0.807974443329152	0.45	0.139	1.28364117349871E-102	8	CCNYL1
1.21382912861942E-125	0.807621406519767	0.525	0.162	2.03413485374042E-121	8	PANK4
3.52741811640774E-121	0.807553045100296	0.449	0.128	5.9112472794761E-117	8	LIN52
3.56555674938383E-131	0.807165665444735	0.674	0.235	5.97516000061743E-127	8	LEMD2
1.91161890243831E-115	0.806515307758282	0.554	0.185	3.20349095670612E-111	8	NIPA1
1.95759586690286E-139	0.806407823970819	0.834	0.321	3.28053915375581E-135	8	VPS45
9.05357807295756E-65	0.806224154142299	0.288	0.092	1.51719861346623E-60	8	DOP1B
7.05996672999436E-128	0.806110187343547	0.51	0.152	1.18310922461245E-123	8	WDR41
1.21031817585481E-124	0.80608438227282	0.623	0.214	2.02825119909749E-120	8	NCOA6
2.89536759778615E-104	0.805541601626169	0.952	0.506	4.85205702037003E-100	8	MAT2A
7.94916644178419E-92	0.805179012311383	0.284	0.072	1.33212131231419E-87	8	PFAS
9.05085497767274E-102	0.805138985820845	0.364	0.102	1.5167422771584E-97	8	NCOA5
1.09173971532839E-120	0.804735799709226	0.775	0.312	1.82953741494732E-116	8	TCIRG1
8.46862974165957E-85	0.804583943031197	0.341	0.103	1.41917297210731E-80	8	FAM126B
2.79718106759804E-99	0.804064876021189	0.481	0.16	4.68751603308079E-95	8	THSD4
3.17681509904489E-95	0.803660670499874	0.337	0.094	5.32370674297942E-91	8	BUD13
5.85031492730957E-59	0.803461666122557	0.285	0.095	9.80395775518538E-55	8	ZNF234
2.42043834782279E-82	0.803345329912956	0.278	0.075	4.05617058328143E-78	8	GTF2E1
7.54682373619083E-139	0.80268385844104	0.72	0.252	1.26469672171086E-134	8	HMGXB4
4.42614389566344E-145	0.801735460249926	0.636	0.205	7.4173319403528E-141	8	NIN
5.05476489366921E-92	0.801649111949085	0.39	0.122	8.47077500881086E-88	8	TNK2
8.48034265976127E-73	0.801309313228094	0.282	0.083	1.42113582292279E-68	8	AREL1
2.57063165762131E-100	0.801115772310378	0.389	0.114	4.3078645318418E-96	8	THAP5
3.57190486973523E-87	0.800974285758527	0.35	0.106	5.9857981807023E-83	8	TTC28
1.75592055786469E-110	0.798358822318569	0.429	0.126	2.94257167086965E-106	8	FAM53B
2.23307164843712E-121	0.79815726079641	0.577	0.193	3.74218146845093E-117	8	ERMP1
4.75316439992517E-60	0.798070853034754	0.25	0.077	7.96535290139461E-56	8	ERCC8
2.1413360707861E-137	0.798049902072111	0.402	0.099	3.58845098742335E-133	8	CBX1
3.41523581962306E-103	0.797983947396012	0.501	0.168	5.72325218652433E-99	8	FAM120B

1.76665715638268E-110	0.797715826970652	0.507	0.167	2.96056406266609E-106	8	KHDRBS3
4.42059740931119E-85	0.796967180252325	0.341	0.102	7.40803713852369E-81	8	SGMS2
8.69614700430164E-66	0.796332535311904	0.266	0.08	1.45730031498087E-61	8	CCDC57
4.46786416158685E-108	0.796284295816086	0.427	0.127	7.48724676198724E-104	8	PKD1
6.45279606910124E-99	0.796272733982291	0.534	0.193	1.08135956525999E-94	8	MAFG
6.13199257257301E-112	0.796052074593425	0.505	0.162	1.02759931531178E-107	8	CEP83
4.01876377769502E-121	0.796048385207434	0.708	0.265	6.73464433866132E-117	8	KHDC4
7.17148064340641E-112	0.795925915923135	0.513	0.166	1.20179672622205E-107	8	DMWD
5.34814138433828E-122	0.795744739095707	0.699	0.257	8.96241533187408E-118	8	TASOR2
1.3991482208846E-150	0.794426181291994	0.7	0.233	2.34469258855841E-146	8	TNPO3
2.36818560532309E-109	0.794198139433924	0.405	0.116	3.96860543740044E-105	8	INPP4A
1.99785257707263E-147	0.794024098391322	0.787	0.292	3.34800134865832E-143	8	CMC2
4.34802030416701E-56	0.79387342468388	0.298	0.105	7.28641242572307E-52	8	JRKL
1.5154113353925E-114	0.793413813994649	0.843	0.357	2.53952631585075E-110	8	SH3RF2
2.43236020278383E-134	0.793360306174251	0.597	0.193	4.07614922782514E-130	8	C9orf64
2.98313777976628E-136	0.793317962994027	0.698	0.249	4.99914229133233E-132	8	PRRC2A
5.26631763461222E-119	0.79228369588397	0.362	0.091	8.82529509208315E-115	8	SLC44A5
9.66457427132828E-91	0.792270381655976	0.889	0.453	1.61958935638919E-86	8	CYP4F11
2.58748307499556E-131	0.792206744767412	0.619	0.207	4.33610413707755E-127	8	CENPX
3.3776290296861E-111	0.792165161436869	0.687	0.265	5.66023072794796E-107	8	ZBTB10
2.7997789079476E-158	0.792092758585104	0.743	0.252	4.69186949393859E-154	8	BAZ1B
5.36127970195012E-152	0.791920214481232	0.74	0.255	8.98443252452801E-148	8	TAF15
3.04129943462548E-100	0.79172167996982	0.374	0.107	5.09660959254538E-96	8	FUNDC2
2.40589139068244E-93	0.791651410004661	0.362	0.107	4.03179279250563E-89	8	HERC5
9.60219899273557E-85	0.791064884772335	0.399	0.131	1.60913650720263E-80	8	ZNF839
9.42298007645413E-127	0.790940146991159	0.676	0.242	1.57910300121218E-122	8	MTM1
1.18055753469862E-120	0.790533784870363	0.932	0.432	1.97837831664795E-116	8	SETD5
1.47557052669607E-91	0.790469540453886	0.672	0.279	2.47276108863727E-87	8	ADCY6
9.06217162690945E-85	0.790124460174932	0.288	0.077	1.51863872123749E-80	8	CLDN11
1.39596354335449E-101	0.78986494856887	0.483	0.16	2.33935570595346E-97	8	MYSM1
4.2891964648301E-127	0.78967147580514	0.581	0.188	7.18783543576228E-123	8	VEZF1
2.86702686955188E-129	0.789248234551259	0.451	0.125	4.80456362799504E-125	8	PPP1R13L
7.82259237363047E-108	0.788954266572591	0.885	0.406	1.31091002997299E-103	8	EVL
9.2878651946303E-118	0.788952990287093	0.571	0.193	1.55646044931615E-113	8	GGCT
1.75269641456762E-99	0.78889697372226	0.377	0.109	2.93716865153242E-95	8	DAGLB
4.41823922032702E-119	0.788686099041128	0.59	0.202	7.40408528542402E-115	8	EXOSC2
1.74492159534364E-129	0.788446781821755	0.598	0.196	2.92413960947687E-125	8	ILKAP
5.84033912819237E-102	0.7883532842108	0.522	0.177	9.78724031102477E-98	8	TMEM168
1.36057481597747E-97	0.788268526024159	0.435	0.138	2.28005127661504E-93	8	PARD3B
6.40656476515422E-117	0.787932595784541	0.57	0.19	1.07361212334454E-112	8	RABGAP1
1.46503778821017E-119	0.787145681421287	0.804	0.324	2.45511032548261E-115	8	TMEM68
3.26095913791706E-117	0.786730927138913	0.638	0.228	5.46471532332141E-113	8	L3HYPDH
1.38159171788875E-97	0.786367469944221	0.587	0.221	2.31527140083797E-93	8	ZFYVE28
6.01417223458063E-127	0.785029006537403	0.523	0.16	1.00785498307102E-122	8	ZNF407
4.03082349655854E-78	0.784829665102333	0.34	0.106	6.75485401553281E-74	8	SZT2
4.93984145301756E-127	0.78461559975058	0.509	0.152	8.27818630696683E-123	8	RNF169
7.85386981564695E-69	0.784482311579571	0.258	0.074	1.31615150370612E-64	8	TRMT10A
2.42765122017036E-134	0.784480198743648	0.704	0.247	4.06825791476149E-130	8	BTBD7
8.8916744592395E-143	0.784228799726599	0.764	0.274	1.49006680587935E-138	8	DCUN1D4
2.27559593612873E-130	0.784009536466861	0.598	0.198	3.81344366976453E-126	8	DUT
2.65298305703007E-108	0.783302433413946	0.443	0.134	4.44586900697099E-104	8	ASF1A
5.42950366942495E-88	0.783040704897115	0.358	0.108	9.09876224922233E-84	8	CAPRIN2
1.02062339342924E-109	0.782859949906645	0.912	0.415	1.71036068270872E-105	8	NR1I2
7.32139041418554E-115	0.782674801382199	0.349	0.088	1.22691860560921E-110	8	ING1
4.47771914555659E-124	0.782510421109026	0.68	0.247	7.50376174412373E-120	8	TMEM185A
5.06186915148216E-90	0.782171890213276	0.358	0.108	8.48268032405381E-86	8	MAGI3
1.61866229166164E-121	0.781611220477707	0.856	0.355	2.71255426836658E-117	8	DLG1
9.63811746873626E-152	0.781453121559008	0.69	0.227	1.61515572541082E-147	8	NAGK
1.88836203303823E-97	0.780463175337899	0.534	0.19	3.16451709496546E-93	8	MGAT5
1.21915173321764E-121	0.779915154634564	0.483	0.144	2.04305447452611E-117	8	DSG2

4.72882598820496E-128	0.779741802737056	0.498	0.148	7.92456659103387E-124	8	TLNRD1
3.02199956763583E-117	0.779567836914659	0.485	0.148	5.06426687544413E-113	8	SNRNP48
6.72573051094093E-119	0.779165323748728	0.692	0.259	1.12709791902348E-114	8	IGF2BP3
9.01758892179681E-74	0.778999552288094	0.314	0.098	1.51116755151471E-69	8	ZNF557
1.97285274613427E-127	0.778767720127555	0.485	0.141	3.3061066319718E-123	8	TRIM28
2.20714886871438E-118	0.778738255668127	0.806	0.329	3.69874007419156E-114	8	PKDCC
7.65053966583027E-139	0.778436390908007	0.81	0.324	1.28207743719984E-134	8	CDK16
2.06839917205895E-113	0.778311248977569	0.907	0.428	3.46622333253638E-109	8	CD99L2
3.28968143096962E-132	0.777811703826931	0.636	0.215	5.51284814201889E-128	8	ATL3
2.67384044724439E-105	0.77751918545221	0.39	0.111	4.48082182149215E-101	8	PRKD3
7.97700205334499E-117	0.7770959934505	0.451	0.132	1.33678600409955E-112	8	TMEM209
2.74094319017725E-104	0.77688609456018	0.463	0.147	4.59327259809904E-100	8	TANC1
2.28994083055313E-129	0.776784418449071	0.589	0.193	3.83748284384094E-125	8	RPA1
8.48120827422866E-93	0.776712821114507	0.358	0.105	1.42128088259524E-88	8	PAICS
6.80529252347789E-97	0.776186939412082	0.969	0.57	1.14043092108443E-92	8	FKBP5
4.09833543160087E-92	0.775772780875091	0.338	0.096	6.86799051627674E-88	8	RAD17
1.83765626065664E-82	0.775743137405957	0.812	0.418	3.0795443616084E-78	8	SLBP
2.95913127304462E-125	0.775478881900081	0.501	0.15	4.95891218736817E-121	8	CLOCK
3.88021605831112E-89	0.775286875568961	0.433	0.143	6.50246607051778E-85	8	BTG2
5.10088078395308E-103	0.774904635216016	0.36	0.099	8.54805601774857E-99	8	RGMB
2.38627393053146E-144	0.774753459788482	0.672	0.222	3.99891785278462E-140	8	SP1
9.51688382382193E-84	0.774482301747565	0.373	0.118	1.59483939119608E-79	8	MTX3
3.04377338650127E-118	0.774363319056268	0.863	0.375	5.10075544109883E-114	8	STT3B
6.99337007818932E-118	0.774085642078976	0.559	0.184	1.17194895770297E-113	8	CPSF1
3.79642809430323E-141	0.773923015874102	0.76	0.282	6.36205420043335E-137	8	SF3A2
2.0375180756694E-84	0.773902558916201	0.478	0.172	3.41447279120679E-80	8	ZNF436
3.81221101940262E-92	0.773744934918505	0.478	0.165	6.3885032263149E-88	8	FZD8
1.43350693856842E-117	0.772969537649091	0.455	0.134	2.40227092765295E-113	8	DEPRECATED-ENSG00000188529
1.40192755988066E-84	0.772185007260631	0.393	0.129	2.34935020484801E-80	8	ETNPPL
3.61671927292731E-155	0.772182841723333	0.78	0.274	6.06089815757159E-151	8	NAP1L4
5.0618952974242E-117	0.772002024637429	0.502	0.156	8.48272413942348E-113	8	XPO5
1.37986527644949E-100	0.771942247487961	0.422	0.13	2.31237823027406E-96	8	METTL27
6.59395668964971E-125	0.771924015013024	0.566	0.182	1.1050152620515E-120	8	CMTR1
2.72974761380155E-115	0.771873427698859	0.513	0.163	4.57451105120864E-111	8	SUDS3
3.15878218826011E-137	0.771712622135518	0.726	0.254	5.2934871910863E-133	8	TERF1
4.24047504504463E-101	0.771612853517065	0.381	0.11	7.10618808048579E-97	8	SLC30A6
2.52018628031468E-108	0.77082279017453	0.386	0.108	4.22332816855134E-104	8	GMEB2
2.36836063892804E-150	0.770524483352755	0.679	0.221	3.96889875871561E-146	8	TRAF2
6.8886890814854E-116	0.76935504385212	0.652	0.237	1.15440651627532E-111	8	ZNF623
6.52619793343632E-132	0.769348607849095	0.563	0.178	1.09366024968526E-127	8	LSM 2.00
1.5654097746152E-92	0.768915658052196	0.301	0.079	2.62331370030016E-88	8	ZNF182
3.74504926393956E-103	0.76884090791594	0.368	0.102	6.27595355650992E-99	8	TMCO4
1.82673813757828E-108	0.768145293966707	0.627	0.238	3.06124777095368E-104	8	CLDN12
4.94562387917998E-104	0.768048625797092	0.94	0.523	8.28787649672981E-100	8	CALM3
6.42971460596802E-129	0.767874027426899	0.631	0.217	1.07749157366812E-124	8	ADSS
6.35406816507817E-76	0.767673448616496	0.281	0.08	1.0648147431038E-71	8	PARP16
3.44822431152027E-85	0.767598782930119	0.378	0.12	5.77853430124566E-81	8	LPAR1
6.70009300203555E-84	0.767416821407682	0.41	0.137	1.12280158528112E-79	8	MFSD6
4.0397568090703E-100	0.767386909126091	0.31	0.079	6.76982446064001E-96	8	USP18
2.96445032468123E-68	0.766960897144721	0.272	0.081	4.9678258541008E-64	8	ZNF496
3.43455312456539E-108	0.765968072199261	0.491	0.157	5.75562412614667E-104	8	JADE2
3.18224518820889E-119	0.765893317590104	0.806	0.33	5.33280648640046E-115	8	SEL1L3
1.40137171062483E-119	0.76571299853177	0.751	0.292	2.34841871266509E-115	8	CANT1
3.16590074341469E-136	0.765082081145162	0.714	0.25	5.30541646581434E-132	8	TPM4
7.67409773550407E-131	0.764190100437355	0.636	0.215	1.28602529851577E-126	8	CHIC2
4.21470831096357E-130	0.76408923120366	0.803	0.309	7.06300818751276E-126	8	FBXL5
1.27602304142324E-122	0.763894296407898	0.714	0.267	2.13835941281706E-118	8	STK38
5.68299228982203E-92	0.763756390744096	0.342	0.098	9.52355847928375E-88	8	TCHP
2.61748740909959E-117	0.763673639169566	0.663	0.242	4.3863854001691E-113	8	SLC38A1
1.55359551250658E-90	0.763538591284274	0.25	0.059	2.60351535985852E-86	8	USP11

2.2778437243906E-116	0.761980174926311	0.619	0.217	3.81721051333377E-112	8	NF1
1.21049196537377E-65	0.761916779239557	0.282	0.088	2.02854243557336E-61	8	HSF2
3.09830747146517E-120	0.761287733502564	0.526	0.169	5.19214366068133E-116	8	PRPS2
1.96117339577875E-135	0.761047509455928	0.527	0.154	3.28653437664603E-131	8	CCDC93
7.72904041307121E-97	0.76104499847433	0.467	0.155	1.29523259242247E-92	8	RBM12
1.1525942222357E-123	0.7609272068529	0.473	0.138	1.93151739762258E-119	8	C12orf65
7.057360467074E-140	0.760790878838162	0.621	0.199	1.18267246707226E-135	8	APBB2
4.59613668643486E-121	0.760772284684516	0.802	0.325	7.70220585912754E-117	8	FNTA
7.20678664610722E-122	0.759830301584216	0.851	0.362	1.20771330615465E-117	8	HNRNPL
2.17179992453845E-119	0.75950168294359	0.692	0.256	3.63950231354153E-115	8	C19orf25
2.46429146974579E-113	0.758807575232454	0.455	0.138	4.129659645E-109	8	PLAA
4.72428833625633E-130	0.758218939815769	0.617	0.205	7.91696239389835E-126	8	NFYB
9.3925707855808E-81	0.758158376950703	0.364	0.116	1.57400701224763E-76	8	ZNF292
1.20135543589613E-105	0.758106406957909	0.462	0.145	2.01323143947474E-101	8	NSRP1
1.69792854942889E-134	0.758085950284005	0.7	0.249	2.84538866313294E-130	8	HCFC1
2.56384797157227E-126	0.757991456195292	0.65	0.234	4.29649643076082E-122	8	MEPCE
1.69070071413038E-82	0.757875567293013	0.377	0.122	2.83327625673969E-78	8	CYP20A1
2.86336102263658E-51	0.757852443448649	0.257	0.087	4.79842040173438E-47	8	TDG
9.74449862655444E-124	0.757750777786589	0.846	0.345	1.63298307983799E-119	8	IMPA1
8.13177598482786E-116	0.757485761208922	0.455	0.135	1.36272301953745E-111	8	UBXN2A
6.69090847032278E-98	0.757299694574233	0.565	0.205	1.12126244145669E-93	8	MTERF3
1.47401233976755E-120	0.75697592359943	0.583	0.195	2.47014987898246E-116	8	NUP214
2.23127752282336E-79	0.7567613007665	0.312	0.093	3.73917487274739E-75	8	TMEM243
6.20016961637064E-133	0.75657151786335	0.595	0.191	1.03902442431139E-128	8	AAK1
1.07598562981831E-101	0.75646833156387	0.414	0.125	1.80313671844952E-97	8	TMEM185B
3.32896189490567E-122	0.755936640110445	0.454	0.131	5.57867434348292E-118	8	ZNF318
1.09183353574827E-82	0.7558967583762	0.321	0.094	1.82969463920695E-78	8	CDK17
3.86395898403086E-137	0.755867961795368	0.786	0.294	6.47522246543891E-133	8	SUN1
8.21954099676808E-140	0.755515471778074	0.55	0.163	1.3774306802384E-135	8	ITGA1
1.75701004551769E-120	0.755225678033201	0.621	0.214	2.94439743427854E-116	8	RALGAPB
2.40454785998468E-91	0.754970354507648	0.333	0.094	4.02954130376233E-87	8	DDX50
1.17169714240454E-118	0.754142387513692	0.816	0.335	1.96353007124153E-114	8	STK3
6.51726628274655E-101	0.753593940887049	0.328	0.086	1.09216348366267E-96	8	DGKZ
2.82239278887928E-78	0.753093140633018	0.356	0.115	4.7297658356039E-74	8	PPM1H
3.74528835735373E-101	0.752782165824108	0.411	0.124	6.27635422925337E-97	8	LIAS
3.20774106238113E-112	0.752572271813235	0.875	0.396	5.37553247233829E-108	8	LONRF3
2.77149255996286E-122	0.752572266525797	0.799	0.329	4.64446723198575E-118	8	DDX46
3.235149941534E-123	0.752478069646759	0.381	0.097	5.42146427202268E-119	8	RP2
1.54945944536991E-95	0.752249184774731	0.434	0.14	2.5965841385509E-91	8	CDK8
3.97246593379562E-123	0.752172517573886	0.754	0.285	6.65705841118547E-119	8	PDLIM5
2.5168585235681E-119	0.752159363941919	0.666	0.24	4.21775151379543E-115	8	PDE8A
1.28430739662988E-90	0.752097147455122	0.445	0.15	2.15224233527235E-86	8	ABHD15
5.99962024998179E-152	0.752065250839913	0.518	0.14	1.00541636149195E-147	8	DEPRECATED-ENSG00000140365
1.53010351812104E-111	0.751682549248613	0.808	0.333	2.56414747566724E-107	8	LRRC31
3.92928250571446E-101	0.750814945944346	0.443	0.14	6.58469162307629E-97	8	UHRF1BP1L
1.81917401261901E-129	0.750810760659182	0.76	0.289	3.04857181034693E-125	8	CPSF6
8.65810057259432E-118	0.750746255741806	0.863	0.389	1.45092449395536E-113	8	HADH
2.24423454003966E-117	0.750595781324351	0.534	0.174	3.76088824219845E-113	8	DNM1L
1.72382886921131E-93	0.750357513665997	0.386	0.117	2.88879241902432E-89	8	BIRC2
6.6051035156882E-70	0.750200757869514	0.292	0.089	1.10688324715903E-65	8	SLC22A3
1.32827546146463E-101	0.750085261558171	0.747	0.318	2.22592401832243E-97	8	TLCD4
3.55088802659182E-108	0.749849420373836	0.475	0.15	5.95057815496258E-104	8	AARS2
4.90042672619498E-128	0.749739703250201	0.834	0.329	8.21213510775756E-124	8	UBR5
1.2565378819451E-132	0.749704058602648	0.683	0.238	2.1057061825636E-128	8	ZC3H3
2.34847563448592E-98	0.749550102565496	0.454	0.147	3.9355754682715E-94	8	LPCAT1
8.81075343256678E-66	0.749430260461681	0.374	0.135	1.47650606022954E-61	8	PCDHGA5
9.09531280750166E-92	0.748844731289644	0.537	0.199	1.52419252028113E-87	8	PLEKHH3
5.24373055295796E-117	0.748293144683671	0.49	0.15	8.78744366064696E-113	8	COG4
7.81421860999736E-63	0.747267463250646	0.262	0.08	1.30950675466336E-58	8	DEPRECATED-ENSG00000243135
1.68170048620436E-124	0.747157272459498	0.726	0.285	2.81819367478127E-120	8	SLC20A1

2.32494669609512E-114	0.746780042631085	0.375	0.099	3.8961456733162E-110	8	INPP5B
1.51544280482762E-58	0.746631699929595	0.277	0.091	2.53957905233012E-54	8	PAQR3
8.89397937633961E-101	0.746433053750858	0.582	0.212	1.49045306388699E-96	8	FBXL8
1.77283408850208E-94	0.745939367993924	0.409	0.128	2.97091536551178E-90	8	THAP12
2.60046943885755E-74	0.74588843493076	0.393	0.137	4.35786668563749E-70	8	TRIM52
1.50647943845514E-116	0.744886825448337	0.718	0.27	2.52455824296313E-112	8	NCKIPSD
3.37284147144786E-84	0.744864236743265	0.471	0.169	5.65220773785232E-80	8	UGT1A3
3.05957537284194E-80	0.744717812816583	0.324	0.097	5.12723640980853E-76	8	UHRF1BP1
1.73982153604934E-75	0.744625505327806	0.302	0.09	2.91559293011149E-71	8	STARD13
1.18755285467889E-138	0.744520074593516	0.75	0.273	1.99010107387089E-134	8	RNF4
1.68680037377322E-131	0.744452056098126	0.582	0.187	2.82674006636916E-127	8	CSTF2
1.55221025862382E-81	0.74440929560244	0.391	0.13	2.60119395140179E-77	8	SLC44A3
6.99983490725995E-79	0.744122628122632	0.277	0.076	1.17303233375862E-74	8	HTRA2
3.51091923549592E-111	0.74407661697752	0.932	0.447	5.88359845484405E-107	8	HNRNPU
1.46040340284714E-134	0.74399897785535	0.638	0.213	2.44734402249124E-130	8	LSM 8.00
1.49214807912686E-126	0.743967380700725	0.766	0.296	2.5005417510008E-122	8	MARCH7
2.53610408994764E-126	0.743823936900573	0.668	0.236	4.25000323393426E-122	8	SCAMP4
1.1280926905311E-133	0.742267164084641	0.561	0.175	1.89045773079202E-129	8	GTF2A1
5.59832288925459E-76	0.741915694305489	0.293	0.086	9.38166949781284E-72	8	TOPORS
8.58037303311601E-67	0.741740811340374	0.269	0.081	1.43789891288958E-62	8	HERC6
1.36550155875759E-102	0.741433212742106	0.933	0.456	2.28830751216596E-98	8	OCRL
2.47099769785426E-113	0.741365179572411	0.659	0.243	4.14089794206416E-109	8	CLCC1
1.3307357056927E-112	0.740932030343327	0.405	0.114	2.23004689559983E-108	8	JPT1
1.27858590290432E-51	0.740747762869962	0.253	0.085	2.14265425608705E-47	8	ZNF558
5.64979867710046E-105	0.740172938722158	0.913	0.428	9.46793262308495E-101	8	SULT1C2
6.67282835712074E-117	0.73956067040364	0.569	0.189	1.11823257608629E-112	8	VSIG10
1.28498587070271E-108	0.739237062549822	0.491	0.158	2.15337932212361E-104	8	SYNJ2
5.34212832283295E-111	0.739183160590573	0.842	0.356	8.95233864340346E-107	8	INTS8
2.36072417078089E-94	0.738884468836973	0.956	0.49	3.95610156539461E-90	8	PTPRK
1.49358582187138E-109	0.738126244343341	0.466	0.143	2.50295112029206E-105	8	EXOSC6
9.84316700401997E-86	0.73791882090323	0.373	0.117	1.64951792653367E-81	8	ATG10
3.14134650933048E-76	0.737549496874865	0.402	0.14	5.26426848033602E-72	8	ZNF606
3.20613984677495E-123	0.737424360302242	0.772	0.298	5.37284915522546E-119	8	SSX2IP
6.93032302386868E-93	0.737228597026043	0.399	0.124	1.16138353233991E-88	8	ZNF621
3.78761269899307E-114	0.737130636673598	0.855	0.376	6.34728136097259E-110	8	LIMD1
7.20109948198632E-90	0.736858750548728	0.336	0.096	1.20676025119127E-85	8	CD44
1.82737203276128E-144	0.736781410978195	0.67	0.222	3.06231005250135E-140	8	MTA2
6.88510326933757E-89	0.736689116184563	0.417	0.136	1.15380560587559E-84	8	BRAF
7.47980692997752E-77	0.736222193126532	0.296	0.086	1.25346604532563E-72	8	GXYLT1
6.60629434410568E-116	0.736197992467643	0.618	0.217	1.10708280618523E-111	8	OSGEP
4.70623564907663E-122	0.736154039394347	0.437	0.123	7.88670970072262E-118	8	ENKD1
8.5551248757705E-109	0.736083269001531	0.519	0.171	1.43366782668162E-104	8	RBPJ
1.48125765795328E-123	0.735859198878664	0.678	0.244	2.48229158319811E-119	8	DHX16
1.42281425694447E-131	0.735744491767866	0.581	0.185	2.38435213178754E-127	8	PPP5C
1.44335761310907E-107	0.735700511716692	0.535	0.181	2.41877868804818E-103	8	LTN1
3.78864091850657E-119	0.735693617870333	0.593	0.202	6.34900445123331E-115	8	OTUD4
1.87076926137892E-93	0.735492980191821	0.437	0.143	3.1350351282188E-89	8	CHD6
2.42120729305555E-81	0.735438000705041	0.505	0.189	4.0574591817025E-77	8	VIPR1
9.45644000432361E-123	0.735275556359804	0.545	0.175	1.58471021592455E-118	8	KDM1A
7.01311122401308E-112	0.734859031362834	0.607	0.213	1.17525717892011E-107	8	SETX
7.46392459096867E-87	0.734633158873192	0.365	0.112	1.25080448295453E-82	8	DSTYK
1.25118497590779E-77	0.734611714897664	0.312	0.094	2.09673578262627E-73	8	MANSC1
4.28668886308882E-87	0.734604741750758	0.431	0.144	7.18363319676424E-83	8	C1GALT1
1.89070205935776E-101	0.73421948392196	0.454	0.145	3.16843851107174E-97	8	EXD3
2.87002650674124E-117	0.734102692393021	0.614	0.215	4.80959041999696E-113	8	REPIN1
1.46027962555699E-94	0.734030228059226	0.478	0.162	2.44713659650841E-90	8	NAA30
3.52651983361477E-72	0.733725135443548	0.546	0.231	5.90974193717163E-68	8	MCM6
1.85521076887415E-88	0.733674412336651	0.349	0.104	3.1089622064793E-84	8	TRMT61B
8.35889832794147E-85	0.73362987977231	0.377	0.119	1.40078418179643E-80	8	NAALADL2
2.10957685553148E-122	0.733589739324136	0.511	0.159	3.53522889449965E-118	8	CHRAC1

1.72160711894373E-65	0.733203184996744	0.417	0.162	2.88506920992591E-61	8	CHD7
3.61088728834091E-134	0.732945729127161	0.686	0.238	6.05112491780169E-130	8	NSL1
3.99269704298321E-115	0.732939144110966	0.798	0.331	6.69096170463127E-111	8	MAN2A1
8.27181071643049E-122	0.732824411304884	0.598	0.201	1.38619003985942E-117	8	DNAJC13
3.57297091746713E-125	0.732643894252957	0.712	0.264	5.98758466349141E-121	8	FUCA2
7.58876260763424E-62	0.732177425644806	0.26	0.08	1.27172483778735E-57	8	TBCEL
1.85609545483858E-69	0.731533929606874	0.306	0.097	3.11044476321849E-65	8	PGBD2
5.30246043423367E-116	0.731382025547637	0.65	0.235	8.88586319568879E-112	8	WDR44
1.31532096895547E-97	0.731241210501773	0.485	0.164	2.20421487977557E-93	8	NOM1
5.99040088818137E-108	0.731065496239503	0.535	0.18	1.00387138084143E-103	8	TMEM165
1.2845267380474E-101	0.730775135269386	0.679	0.272	2.15260990761984E-97	8	MMGT1
3.76408898387731E-119	0.730349459741363	0.502	0.155	6.30786031918159E-115	8	CLCN5
2.15664466228112E-100	0.730214699276843	0.466	0.153	3.61410512505071E-96	8	CAD
5.8075292640125E-106	0.730124735910574	0.519	0.174	9.73225754063214E-102	8	PDS5A
7.79586716395478E-71	0.72992010036252	0.308	0.096	1.30643141933554E-66	8	ZSCAN9
1.99700195630746E-114	0.729675376371606	0.61	0.216	3.34657587838004E-110	8	DEPRECATED-ENSG00000078668
3.48783505181533E-129	0.729630460861412	0.577	0.184	5.84491397983214E-125	8	SMARCC2
1.95867943028454E-87	0.729290073969317	0.302	0.083	3.28235498927083E-83	8	RFX1
3.45861630238774E-92	0.729197053908804	0.373	0.112	5.79594919954137E-88	8	ZC3H11A
2.02940770699515E-77	0.72803480565281	0.345	0.11	3.40088143538247E-73	8	SPAST
3.97944166226293E-123	0.727985468577999	0.671	0.238	6.66874833762022E-119	8	DICER1
1.11800496576907E-108	0.727175643525048	0.491	0.159	1.8735527216358E-104	8	CCDC61
9.10493240565808E-152	0.727072860713872	0.611	0.185	1.52580457254018E-147	8	TENT4A
4.04646628487912E-67	0.726968549687169	0.252	0.072	6.78106820020042E-63	8	ADAM17
4.82848395486045E-119	0.726794237061132	0.495	0.153	8.09157341155515E-115	8	GLE1
9.40205768566919E-78	0.726514610073951	0.256	0.068	1.57559682696444E-73	8	MIER2
1.4811741232628E-92	0.726508154962825	0.487	0.17	2.4821515957638E-88	8	PDP1
9.30137488106769E-115	0.725980578997276	0.39	0.105	1.55872440256932E-110	8	AGPAT5
3.01902222564352E-118	0.725745171605825	0.802	0.327	5.05927744573342E-114	8	SRRM2
5.38612546586035E-121	0.72531751051635	0.758	0.293	9.02606905568877E-117	8	ERAP1
8.33921079254313E-124	0.725201443486567	0.842	0.344	1.39748494461438E-119	8	RNF115
7.23648678197298E-89	0.725180336561825	0.43	0.143	1.21269045492303E-84	8	GPATCH2
1.14835061248652E-125	0.725066064751357	0.884	0.383	1.92440595640491E-121	8	NONO
4.24544660322416E-107	0.72502128805515	0.658	0.247	7.11451941768305E-103	8	USP54
3.60019246629237E-122	0.725004619383432	0.852	0.346	6.03320253501276E-118	8	DDX39B
7.41887736754933E-118	0.72490526017232	0.563	0.186	1.24325546925392E-113	8	ORMDL1
1.05488991890736E-113	0.724758241211829	0.387	0.105	1.76778452610495E-109	8	PBRM1
2.03678807269531E-106	0.724251556440224	0.429	0.128	3.4132494522228E-102	8	WAPL
2.86756946244075E-149	0.724213389955083	0.716	0.24	4.80547290515821E-145	8	ABHD12
4.86406882378009E-118	0.724045660541837	0.667	0.244	8.15120653489068E-114	8	ZXDC
1.15793866158287E-93	0.723975032338443	0.491	0.172	1.94047360908057E-89	8	THEM6
2.04746392174083E-115	0.723714570180481	0.557	0.184	3.43114004005328E-111	8	SLC37A3
8.34819970963132E-106	0.723332918207486	0.466	0.148	1.39899130734002E-101	8	INPP5E
2.11828607401145E-99	0.723081984883806	0.33	0.088	3.5498238028284E-95	8	TMCO6
1.31897309607915E-120	0.722914769596155	0.703	0.259	2.21033511440944E-116	8	TUT4
1.52574660763585E-117	0.722840560438288	0.818	0.344	2.55684616507616E-113	8	STAT1
2.55662149367413E-100	0.722245080188424	0.361	0.101	4.28438629909911E-96	8	THOC5
4.39090966636259E-105	0.72213426741352	0.439	0.135	7.35828641889043E-101	8	USP28
4.10518557287875E-81	0.721920568436425	0.318	0.094	6.87946998303022E-77	8	DRAM1
1.05249608800139E-125	0.721823306301914	0.778	0.308	1.76377294427272E-121	8	CDKN2A
6.68788253329815E-88	0.72180186619548	0.411	0.134	1.1207553549301E-83	8	ATP6V0A2
4.63556963396747E-101	0.721657849635754	0.459	0.148	7.76828759260269E-97	8	RNF31
1.15908780385576E-83	0.721477165768673	0.333	0.099	1.94239934170149E-79	8	RNF145
6.52665497222278E-70	0.721078248176222	0.304	0.096	1.09373684024509E-65	8	GUCY2C
6.64256419935745E-110	0.720992021897032	0.582	0.204	1.11316090852832E-105	8	EPC1
9.41237857822528E-124	0.720018333076995	0.719	0.267	1.57732640213899E-119	8	RFC1
1.8078279062133E-104	0.719818096648123	0.413	0.122	3.02955800523224E-100	8	ZCCHC8
1.45226832055203E-107	0.719506243398116	0.774	0.311	2.4337112515811E-103	8	KCNJ3
3.59597466278697E-72	0.719427804629618	0.294	0.088	6.02613433989841E-68	8	LYPD6B
1.84884662933819E-126	0.719284615908789	0.648	0.225	3.09829718144493E-122	8	MYO9B

4.99827010771466E-111	0.719201499959882	0.514	0.169	8.37610104650822E-107	8	CCND3
9.79741396707401E-115	0.719068388893897	0.694	0.264	1.64185063260226E-110	8	TUBGCP6
2.72285631931423E-127	0.718964346495462	0.566	0.18	4.56296261990679E-123	8	CDC23
2.66347219636561E-87	0.718777540556155	0.375	0.116	4.46344670666948E-83	8	SETD4
2.37843959880601E-104	0.718384723787473	0.431	0.131	3.98578907967912E-100	8	RNGTT
1.42206573019211E-124	0.718222774954157	0.632	0.216	2.38309775065593E-120	8	SLC5A3
1.82385213968473E-103	0.717810103943569	0.636	0.24	3.05641141568368E-99	8	SAMD12
1.03049214566164E-67	0.71740923473322	0.286	0.089	1.72689873769978E-63	8	BCORL1
1.59964404247484E-100	0.717363007860785	0.656	0.255	2.68068348637933E-96	8	MTCP1
4.66472246825309E-137	0.717343602898674	0.734	0.261	7.81714191229853E-133	8	RBM17
3.98143598823816E-87	0.71685069367989	0.936	0.485	6.67209042908951E-83	8	F2RL1
2.45598096186205E-104	0.716102080799776	0.585	0.213	4.11573289588842E-100	8	FAF2
1.13682788783868E-108	0.716080869631183	0.566	0.195	1.90509617444005E-104	8	TRADD
1.80453422156821E-109	0.716006764101493	0.666	0.252	3.024038448504E-105	8	ZFHX3
4.31526266261064E-109	0.715998643239448	0.724	0.295	7.23151717000292E-105	8	DCAF7
1.01622994646821E-75	0.715696468909557	0.391	0.136	1.70299814429142E-71	8	COMTD1
2.76095047484029E-100	0.715613017263591	0.318	0.082	4.62680080573735E-96	8	R3HDM1
2.38216466351469E-117	0.715568649959455	0.688	0.255	3.99203154311791E-113	8	WDR33
1.11019639724603E-112	0.715135699129031	0.711	0.272	1.8604671225049E-108	8	CCT8
8.22049069439687E-80	0.715002532764803	0.313	0.092	1.37758983056703E-75	8	ABHD5
7.60377875099199E-80	0.714685474458161	0.378	0.124	1.27424124309124E-75	8	MOSMO
2.05962283243272E-93	0.714678557944836	0.543	0.199	3.45151594259076E-89	8	PLXNA2
1.15774597039051E-59	0.714421333689885	0.418	0.168	1.94015069718041E-55	8	NOL3
5.18491496255276E-95	0.713866458415419	0.402	0.125	8.68888049424592E-91	8	NIPSNAP3A
5.2124840086216E-77	0.713770616969586	0.265	0.072	8.73508070164809E-73	8	SNRPD1
2.84849143954176E-113	0.713389795338058	0.639	0.23	4.77350195438409E-109	8	EIF2S3
3.39033293218307E-84	0.712413943598203	0.491	0.179	5.68151992775238E-80	8	KLHL23
1.46841412830325E-101	0.712346017112781	0.401	0.119	2.46076839621058E-97	8	TRAPPC13
2.97294580236304E-99	0.712345869499742	0.333	0.089	4.98206257559998E-95	8	GMEB1
7.28870618351167E-91	0.712310985791756	0.405	0.129	1.22144138223289E-86	8	NEO1
6.98106374728538E-120	0.712238483026012	0.559	0.183	1.16988666277008E-115	8	NSMCE4A
6.64960112904916E-121	0.711745006817662	0.529	0.166	1.11434015720606E-116	8	IPO5
1.08171344971522E-52	0.711683197260902	0.264	0.089	1.81273539903276E-48	8	SLC25A27
6.68219944550619E-117	0.71132527682169	0.766	0.307	1.11980298307793E-112	8	TMUB2
1.30799287867513E-126	0.711300451787068	0.727	0.266	2.19193446608378E-122	8	DYM
2.07748895972546E-85	0.711264249941769	0.393	0.127	3.48145599870793E-81	8	GABPA
1.60151252680136E-92	0.710733890495062	0.541	0.199	2.68381469241372E-88	8	FAM221A
1.39440821420327E-96	0.709104535049135	0.431	0.137	2.33674928536184E-92	8	ABCG1
2.5554918284451E-95	0.708938032362395	0.386	0.116	4.2824932061083E-91	8	PDIK1L
1.72888661249916E-112	0.70873480683466	0.419	0.12	2.89726818522609E-108	8	NUP88
4.01685442874303E-42	0.70871898488204	0.254	0.095	6.73144465168757E-38	8	PCDHGB7
2.2298664595699E-97	0.708508831543746	0.414	0.128	3.73681021294724E-93	8	MICA
2.95624225061821E-115	0.708228016218419	0.387	0.105	4.954070763586E-111	8	TPRKB
1.22384185513912E-89	0.708217278891994	0.332	0.094	2.05091418084214E-85	8	PRKCE
1.25559001507599E-65	0.707755184423801	0.324	0.108	2.10411774726434E-61	8	NIPAL3
5.40532015199935E-54	0.707733774672987	0.281	0.097	9.05823551072052E-50	8	TMEM104
3.99748306637946E-92	0.707469837429521	0.483	0.168	6.69898212263869E-88	8	RECQL5
1.05050576943874E-129	0.707253648397791	0.754	0.285	1.76043756842544E-125	8	CEP350
7.06142208304573E-120	0.707137866240647	0.632	0.223	1.1833531126768E-115	8	CYREN
6.16245140623654E-73	0.707059652150822	0.3	0.091	1.03270360665712E-68	8	ZBTB14
1.47772732649856E-104	0.706655677283554	0.393	0.112	2.47637545374629E-100	8	CBLL1
1.13420325956333E-57	0.706603930605069	0.253	0.08	1.90069782237623E-53	8	TIRAP
3.20739646964401E-126	0.706207677915366	0.671	0.239	5.37495500382943E-122	8	PPP2R5D
2.94618362207125E-102	0.706014307954371	0.628	0.234	4.93721451386701E-98	8	DMXL1
1.08207303596811E-123	0.705850798651495	0.824	0.332	1.81333799367536E-119	8	SCRIB
1.37239107890724E-75	0.705708850546735	0.32	0.098	2.29985297003276E-71	8	EIF4ENIF1
2.49320404803353E-98	0.70550505350449	0.565	0.202	4.17811134369459E-94	8	ERMARD
7.76799728771428E-98	0.705033817517737	0.527	0.186	1.30176098547516E-93	8	PIGG
3.93878837206292E-98	0.704572809282089	0.501	0.172	6.60062155390304E-94	8	COX20
6.21399565637845E-80	0.704390909642148	0.338	0.104	1.0413413920959E-75	8	CDKN2AIPNL

5.8938006972682E-115	0.704141157814439	0.775	0.308	9.87683120848205E-111	8	SRSF5
3.91540267347507E-112	0.704098176224387	0.602	0.213	6.56143180020953E-108	8	ATP6V1H
5.03309312032462E-107	0.703985266200952	0.455	0.141	8.43445745103999E-103	8	PCBP2
6.27767862795339E-111	0.703839300639357	0.567	0.195	1.05201338447243E-106	8	PCNX1
1.96633436179164E-77	0.703468184181368	0.394	0.136	3.29518312349044E-73	8	STARD3NL
3.91668030729005E-77	0.703302092719774	0.344	0.109	6.56357285895667E-73	8	B3GALNT2
1.01070306905204E-134	0.703162875558369	0.611	0.196	1.69373620311741E-130	8	TASOR
1.64972970492053E-114	0.701938972762457	0.49	0.152	2.76461703950582E-110	8	GPCPD1
1.40493444898348E-109	0.701879456432168	0.618	0.224	2.35438914960652E-105	8	C6orf62
4.74411110461578E-104	0.701851596750907	0.724	0.288	7.95018138911512E-100	8	ZDHHC14
7.04577138705905E-96	0.701784371503176	0.43	0.137	1.18073036904336E-91	8	UBE2K
1.23687490592916E-110	0.701439923924803	0.458	0.139	2.07275496735609E-106	8	TFDP2
3.11478389244454E-142	0.701326531153098	0.706	0.244	5.21975484695856E-138	8	RNF168
4.65764088864521E-96	0.701322434495685	0.405	0.124	7.80527460119164E-92	8	DAAM1
3.80037726420061E-109	0.701316418449825	0.582	0.202	6.36867221934737E-105	8	TMEM87A
1.00789695995948E-103	0.701245258175985	0.459	0.145	1.6890337255001E-99	8	HERC2
1.16292480581346E-98	0.701006722477835	0.478	0.161	1.94882938958219E-94	8	TRNT1
1.17516735044644E-125	0.70098445671208	0.758	0.291	1.96934544587815E-121	8	CRKL
1.49167641099073E-118	0.700768688988811	0.818	0.335	2.49975132953827E-114	8	USP12
2.59283493242564E-98	0.700530568111457	0.457	0.147	4.34507277975889E-94	8	AGO3
1.52518797948909E-77	0.700005329085202	0.314	0.094	2.55591001602781E-73	8	CDK5RAP1
1.82304795649577E-73	0.699876973005173	0.276	0.079	3.0550637654956E-69	8	VPS50
2.68865851812944E-83	0.699726981939576	0.35	0.108	4.50565394468132E-79	8	CPT1B
1.98352528702028E-114	0.699719817761255	0.525	0.171	3.32399167598859E-110	8	SAV1
2.28850645211207E-111	0.699701766124992	0.847	0.381	3.8350791124494E-107	8	SMARCA2
1.40451530659255E-89	0.699661766850978	0.356	0.106	2.3536867507878E-85	8	FOXJ2
6.10345723253002E-80	0.69939058839395	0.274	0.074	1.02281736302738E-75	8	BGLAP
2.95703746022959E-87	0.699363014675126	0.418	0.138	4.95540337585274E-83	8	PRMT3
8.88099028793577E-121	0.699054725165951	0.656	0.232	1.48827635245228E-116	8	TYK2
1.17514948452124E-84	0.698686914336324	0.402	0.132	1.9693155061607E-80	8	XYLT2
1.00606747587499E-112	0.698654201583163	0.803	0.332	1.68596787607131E-108	8	OGT
1.71117178899265E-134	0.698338099607267	0.648	0.218	2.86758168399388E-130	8	HNRNPR
5.05556888856988E-90	0.69826184824674	0.349	0.102	8.47212234346541E-86	8	ARID5B
1.1362446092275E-121	0.69813996222155	0.557	0.181	1.90411871614344E-117	8	SEC22C
1.01932273835333E-97	0.698040310472028	0.554	0.198	1.7081810449325E-93	8	LAMB1
2.32360403631053E-132	0.698016259692549	0.707	0.253	3.89389564404918E-128	8	NAB1
8.07389334714309E-61	0.698004144928566	0.273	0.086	1.35302304711424E-56	8	ZNF333
2.94697715345014E-105	0.697768744458352	0.893	0.416	4.93854431375175E-101	8	VMA21
1.05875396396578E-117	0.697529504419017	0.562	0.185	1.77425989281385E-113	8	CADPS2
2.74513404802418E-104	0.69726637957201	0.53	0.181	4.60029563767893E-100	8	TMX1
7.17383615075049E-91	0.697248812800187	0.369	0.111	1.20219146214277E-86	8	EPG5
6.49370150591459E-77	0.697029601622909	0.322	0.099	1.08821449836117E-72	8	CIART
1.00942761019309E-100	0.697018890019171	0.344	0.093	1.69159878916158E-96	8	MYH10
2.59489690705458E-92	0.696969116216409	0.33	0.092	4.34852823684207E-88	8	SPSB2
4.86885767884503E-112	0.696736311989613	0.827	0.348	8.1592316982085E-108	8	ADAM9
5.56105930724099E-75	0.696605352348427	0.31	0.095	9.31922318707445E-71	8	TMEM62
6.23682501575897E-113	0.696546841735989	0.692	0.263	1.04516713614089E-108	8	DOCK5
1.5073499647218E-108	0.696400941522321	0.498	0.161	2.52601707088079E-104	8	ZC3H7A
2.73640245973405E-99	0.696145932405577	0.638	0.244	4.58566324202232E-95	8	SPIRE2
4.8323216026241E-99	0.695958988946458	0.507	0.174	8.09800454167747E-95	8	ASAP2
6.72251094864617E-88	0.695713193834471	0.401	0.128	1.12655838477412E-83	8	XPOT
3.34408371825178E-99	0.695687737787595	0.423	0.131	5.60401549504633E-95	8	INO80D
1.12738147089891E-111	0.695379351517947	0.617	0.224	1.8892658689324E-107	8	RNF19B
4.88103641226051E-71	0.694970984884714	0.342	0.114	8.17964081966617E-67	8	RNPC3
5.02852007358768E-113	0.694524852692047	0.463	0.141	8.42679393931823E-109	8	SAP130
1.15036808365011E-59	0.694323769583507	0.25	0.077	1.92778683458086E-55	8	BAHCC1
1.84749882736513E-120	0.694187558933717	0.678	0.251	3.09603853489848E-116	8	YWHAH
1.80769035212146E-91	0.693935997743796	0.936	0.464	3.02932749208515E-87	8	PUM1
1.27784313880998E-101	0.693591022867456	0.483	0.159	2.14140953201777E-97	8	MPP6
1.5946454625553E-109	0.693267457157741	0.844	0.376	2.67230686615017E-105	8	NUCB2

1.28772001605113E-99	0.693109117082305	0.379	0.11	2.15796120289848E-95	8	DBR1
3.5108860182726E-113	0.692876852205882	0.61	0.215	5.88354278942122E-109	8	CDC42BPA
5.18616004629389E-107	0.69267310197484	0.824	0.348	8.6909670055793E-103	8	GPAT4
2.94895361249596E-94	0.692370641440673	0.916	0.447	4.94185646382074E-90	8	CTPS1
2.75899671702386E-102	0.692168417910842	0.375	0.106	4.62352669838859E-98	8	SARS
9.94668796411543E-82	0.692085034267894	0.342	0.105	1.66686596902646E-77	8	LARGE1
1.16702588262129E-84	0.692072931098615	0.402	0.132	1.95570197409675E-80	8	PCNX4
6.14117263839469E-91	0.691646584422695	0.292	0.076	1.02913771074218E-86	8	DEPRECATED-ENSG00000153487
7.45902726010136E-87	0.691472281655696	0.903	0.443	1.24998378824779E-82	8	PAG1
5.95735606343126E-78	0.691345516878781	0.39	0.133	9.98333729109811E-74	8	TUBGCP4
8.55835247129876E-54	0.69076534142945	0.252	0.082	1.43420870714025E-49	8	MEIS1
6.77940719105967E-55	0.690387811625437	0.277	0.094	1.13609305707778E-50	8	DEPRECATED-ENSG00000198105
2.34623613243744E-102	0.689796573731698	0.891	0.405	3.93182251073867E-98	8	N4BP2L2
3.04432139780288E-94	0.689380364984189	0.702	0.296	5.10167379843806E-90	8	WSB1
8.29648232798118E-107	0.68769853064855	0.501	0.164	1.39032450852309E-102	8	USP33
8.25315619986035E-104	0.687646881216898	0.866	0.397	1.3830639159726E-99	8	ERBIN
4.91344670006235E-101	0.687479059067877	0.581	0.209	8.23395397996448E-97	8	NSMAF
2.40572677801746E-111	0.687145402409377	0.879	0.389	4.03151693460166E-107	8	POGZ
1.58167302154368E-88	0.68706411394882	0.475	0.165	2.6505676495029E-84	8	FAXDC2
7.02116889461809E-91	0.686737897177846	0.429	0.139	1.1766074833601E-86	8	ZBTB37
2.37186173172591E-116	0.68621191753406	0.621	0.218	3.97476589002628E-112	8	ATG4B
3.90159017333581E-79	0.685787689553605	0.285	0.08	6.53828481247615E-75	8	CHST14
8.64365109130405E-114	0.685723978760002	0.722	0.276	1.44850304988073E-109	8	MMP14
1.09294908574353E-75	0.685128014179083	0.393	0.135	1.83156407788901E-71	8	SLC49A4
2.1653591633142E-106	0.685085855926486	0.381	0.106	3.62870888588194E-102	8	PPIL3
1.68530340337896E-98	0.68503291160711	0.557	0.201	2.82423144338246E-94	8	MYNN
1.12724250903886E-133	0.68502000979838	0.719	0.255	1.88903299664732E-129	8	SRRT
1.61541472386556E-96	0.684908160910132	0.499	0.172	2.7071119942539E-92	8	TMEM138
2.3099823850961E-125	0.684543371568901	0.543	0.171	3.87106848094405E-121	8	RABL6
1.30918175708088E-121	0.684432128059655	0.613	0.208	2.19392678851614E-117	8	ZNF384
8.05072820073647E-103	0.684256087043289	0.549	0.191	1.34914103187942E-98	8	SLC4A4
5.75559116447538E-75	0.683924869690466	0.357	0.118	9.64521967342783E-71	8	E2F3
3.44167657694429E-93	0.683876176202152	0.505	0.177	5.76756160764324E-89	8	GUCY1A1
7.88423311832164E-109	0.683604282059982	0.79	0.328	1.32123978596834E-104	8	SEPTIN8
2.85331200689264E-112	0.683303420568605	0.494	0.156	4.78158026115068E-108	8	MKKS
5.44065628164305E-78	0.68300531717697	0.317	0.095	9.11745179677743E-74	8	NAA16
6.00805011382273E-108	0.682964306969533	0.647	0.239	1.00682903807441E-103	8	ZBTB41
9.40594674151294E-94	0.682535494903248	0.348	0.099	1.57624855494274E-89	8	GAS6
6.79531304081694E-76	0.682411890356468	0.469	0.175	1.1387585593801E-71	8	ZMYM5
9.60091615276225E-113	0.682210706228505	0.505	0.161	1.6089215288799E-108	8	SESTD1
4.84228528558374E-82	0.68220667026861	0.356	0.111	8.11470168158124E-78	8	ZNF322
5.53633745261212E-115	0.68202444194894	0.393	0.106	9.27779430308739E-111	8	AJUBA
1.05375269659908E-112	0.681959181351306	0.78	0.323	1.76587876896074E-108	8	DKC1
6.4507778209053E-114	0.681266865536457	0.571	0.192	1.08102134722731E-109	8	MED6
4.46146098260943E-124	0.681154590567485	0.435	0.121	7.47651631465689E-120	8	ZDHHC12
3.13008384130204E-112	0.680846999418254	0.751	0.296	5.24539450125396E-108	8	PRKAB1
2.95158031092016E-96	0.680784412283175	0.43	0.137	4.94625828504E-92	8	ITM2C
1.91140986616053E-110	0.680743886635351	0.814	0.345	3.20314065371181E-106	8	TAP1
1.21973020397753E-94	0.680697418368803	0.414	0.129	2.04402387582554E-90	8	KIAA1958
3.54046006307896E-85	0.680668754761996	0.435	0.149	5.93310297370772E-81	8	NT5C3A
4.05189561432783E-114	0.680663766528596	0.77	0.3	6.79016667049058E-110	8	HELZ
2.88950930400284E-106	0.680358742813485	0.505	0.165	4.84223969164795E-102	8	MNAT1
2.8187107092907E-120	0.680302828872084	0.838	0.349	4.72359540662935E-116	8	U2SURP
1.36637839509972E-109	0.680183033342459	0.881	0.409	2.28977691450811E-105	8	HNRNPUL1
4.77409174326187E-72	0.680179154813681	0.369	0.126	8.00042294335825E-68	8	SNAPC3
5.97600899530928E-77	0.680124100579572	0.348	0.111	1.00145958743393E-72	8	SFXN2
4.22649086347084E-77	0.679601282150458	0.345	0.11	7.08275338900444E-73	8	NAPEPLD
6.4826335426969E-136	0.67956785236853	0.628	0.205	1.08635972908515E-131	8	MUS81
3.15441258332083E-94	0.679497615231829	0.393	0.12	5.28616460712905E-90	8	TCTEX1D2
1.9594052114976E-99	0.679495189403383	0.435	0.139	3.28357125342768E-95	8	SP2

1.27549004072826E-65	0.679437220761582	0.305	0.099	2.13746621025242E-61	8	CNNM4
8.007430522472E-131	0.679109062925563	0.543	0.165	1.34188520695586E-126	8	NAGA
5.65035652255689E-111	0.678698150550566	0.621	0.224	9.46886746050084E-107	8	KAT7
2.31767888267497E-120	0.678618233904546	0.732	0.275	3.88396627158672E-116	8	FBXL6
2.59451734609608E-109	0.678579868381726	0.466	0.145	4.34789216858782E-105	8	TAOK2
2.04661868118036E-93	0.678492983179283	0.342	0.097	3.42972358592204E-89	8	UBAP2
1.72609008307645E-105	0.678203423292557	0.562	0.197	2.89258176121951E-101	8	ERCC6L2
4.92193686020087E-113	0.678091516534935	0.605	0.214	8.24818179032462E-109	8	TBC1D15
6.51932455727616E-117	0.677596177129574	0.824	0.341	1.09250840930834E-112	8	SRSF2
1.77525093515765E-57	0.676953820422977	0.27	0.088	2.97496551713718E-53	8	RBM45
2.24331492702348E-95	0.6767817804456	0.364	0.106	3.75934715470595E-91	8	INAFM1
8.85572800003135E-104	0.676226227551069	0.465	0.149	1.48404289824525E-99	8	DVL2
8.31146453695072E-77	0.676082710457253	0.314	0.095	1.3928352271022E-72	8	ZNF707
1.17887125385334E-76	0.675831288789829	0.366	0.121	1.97555244720742E-72	8	SLC9A8
1.63268844497749E-57	0.675751018030134	0.328	0.119	2.73605929609327E-53	8	ADAMTS10
1.84696693264586E-132	0.675341484110714	0.646	0.215	3.09514718572793E-128	8	PLEKHG3
3.70038226029718E-75	0.675118223713185	0.289	0.084	6.20110059180602E-71	8	ZFYVE19
8.27679984779355E-102	0.674843435832761	0.451	0.143	1.38702611849324E-97	8	WDR5
3.40702043485168E-90	0.67467305665486	0.387	0.122	5.70948484472444E-86	8	SMIM13
4.34183747870579E-50	0.674606625188888	0.953	0.695	7.27605124681516E-46	8	DNAJB1
2.83918515832319E-116	0.674526385512822	0.574	0.192	4.757906488318E-112	8	WAC
1.27530244345833E-67	0.67449022759606	0.312	0.101	2.13715183474747E-63	8	ACTR5
1.94038950358591E-107	0.673777356171886	0.664	0.251	3.25170473010927E-103	8	GSAP
3.0459260868038E-97	0.673772822494804	0.394	0.119	5.10436293626581E-93	8	RIOX1
1.24824883763838E-97	0.67369667766047	0.429	0.134	2.09181540211439E-93	8	GGA1
4.22976073851617E-97	0.673514817292846	0.949	0.494	7.0882330456054E-93	8	GPAA1
4.50761671011699E-88	0.673469825001013	0.433	0.146	7.55386408281405E-84	8	SVIL
3.79751717988646E-80	0.673456999571587	0.405	0.136	6.36387929005374E-76	8	FPGT
1.9992740725039E-122	0.673375039931208	0.68	0.243	3.35038349070204E-118	8	MSL2
3.00057012506777E-86	0.673338530272614	0.916	0.511	5.02835541558857E-82	8	GUSB
3.34032573876982E-115	0.673326680470188	0.545	0.181	5.59771787303047E-111	8	MED30
4.54743916525105E-112	0.673046709885119	0.752	0.305	7.62059855312771E-108	8	NCOR1
4.39483692117684E-119	0.673035779827639	0.651	0.23	7.36486771250814E-115	8	HOXA13
2.21962892874097E-120	0.672726909079636	0.455	0.132	3.71965415878412E-116	8	DDAH2
2.75559242189606E-113	0.672345427231077	0.526	0.171	4.61782178061341E-109	8	ZBTB44
2.24318571895939E-93	0.671158792609554	0.467	0.157	3.75913062783215E-89	8	XRCC5
9.87730182730508E-119	0.670973107760477	0.575	0.191	1.65523824021979E-114	8	BOD1L1
1.85870716927129E-69	0.670654277628227	0.27	0.079	3.11482147426482E-65	8	CHUK
5.53600444926963E-118	0.670540966227123	0.667	0.245	9.27723625608604E-114	8	TXNDC12
2.4565733244288E-110	0.670527384781278	0.565	0.193	4.11672557707778E-106	8	PTPN12
5.55355057430505E-115	0.670519853688083	0.667	0.246	9.30664005242041E-111	8	BRF1
7.01937344673736E-70	0.670351243582416	0.304	0.095	1.17630660220425E-65	8	POLG
1.6063931915125E-84	0.670130776844013	0.43	0.147	2.69199371033666E-80	8	DDX55
4.16169617803896E-87	0.669740798930816	0.382	0.121	6.97417045515769E-83	8	SYT1
1.10920251159779E-107	0.669547793699753	0.486	0.154	1.85880156893558E-103	8	ATP11C
1.43639554148816E-86	0.669385815868512	0.397	0.128	2.40711164842586E-82	8	ASXL2
2.32888844365693E-104	0.669326417438114	0.336	0.087	3.90275125388028E-100	8	FBXO45
3.42362252641753E-86	0.669038509844969	0.896	0.458	5.7373066297705E-82	8	PICALM
3.27853014738888E-109	0.668979447401446	0.471	0.147	5.49416082099428E-105	8	DCBLD2
1.49926862648628E-93	0.668955160240411	0.423	0.135	2.51247436426572E-89	8	KIAA0355
1.08876485151067E-92	0.668690268851563	0.493	0.17	1.82455213816158E-88	8	ABHD18
4.72488217850584E-85	0.668274746322174	0.457	0.159	7.91795755474009E-81	8	ENDOV
5.28417883733554E-105	0.667900263945222	0.413	0.122	8.8552268956069E-101	8	AARS
7.48151089176601E-108	0.66734487115568	0.573	0.199	1.25375159524215E-103	8	EIF2AK2
3.3689008273645E-119	0.667193775300323	0.727	0.272	5.64560400649743E-115	8	TRIM24
2.71223372520727E-109	0.666684791005224	0.598	0.211	4.54516127670235E-105	8	ZNF7
5.14029101437382E-95	0.666624243283928	0.477	0.161	8.61409968188764E-91	8	STOX2
1.28097104022526E-98	0.666425827370127	0.593	0.221	2.14665126920948E-94	8	GTF2H1
3.43016660724931E-66	0.666334888826114	0.257	0.075	5.74827320042839E-62	8	R3HCC1L
1.61017774382375E-116	0.666214779643401	0.387	0.103	2.69833586309984E-112	8	RNASEH2C

2.96427396942646E-108	0.66619702204535	0.553	0.189	4.96753031796486E-104	8	MOSPD2
2.50910913214109E-98	0.665913497629845	0.449	0.144	4.20476508364204E-94	8	SLC2A8
1.01538747804859E-62	0.665858150890593	0.269	0.083	1.70158633571383E-58	8	BCL11A
4.10912532873579E-97	0.665413173312032	0.768	0.339	6.88607222589543E-93	8	TMX4
4.95230153051898E-60	0.66534103577644	0.317	0.11	8.29906690484371E-56	8	SLC16A7
3.78597378278119E-88	0.664593893296676	0.463	0.159	6.34453486518472E-84	8	PSD3
1.85557767737663E-54	0.664165179334976	0.25	0.081	3.10957707174776E-50	8	SYTL2
2.7597765187939E-88	0.663568697635037	0.485	0.172	4.62483349019482E-84	8	LAS1L
3.28593642068467E-106	0.663014933780286	0.475	0.152	5.50657225378337E-102	8	NCBP1
3.32585115730731E-104	0.662538771724999	0.523	0.174	5.57346136941558E-100	8	TNFRSF11A
4.39007538640972E-76	0.662099535694099	0.395	0.136	7.35688833254541E-72	8	ZNF224
3.08838705765421E-89	0.661890528672088	0.366	0.111	5.17551903121693E-85	8	TCFL5
2.56398025317038E-91	0.661757587618753	0.427	0.138	4.29671810826292E-87	8	ZNF654
2.67456596389013E-108	0.661205174018026	0.394	0.111	4.48203764228708E-104	8	GTF3C5
6.60452415668236E-107	0.660462768312726	0.459	0.143	1.10678615817683E-102	8	RBBP5
3.97879052678472E-102	0.66042783023843	0.61	0.224	6.66765716478583E-98	8	RNF125
1.58551377298089E-78	0.660309554713588	0.334	0.104	2.65700398076137E-74	8	H2AFY2
4.68794078813307E-134	0.659690093294679	0.719	0.26	7.8560511727534E-130	8	JPT2
1.16344871197592E-88	0.659661976181323	0.419	0.137	1.94970735152924E-84	8	PTPN18
5.19271642247012E-110	0.659609753440567	0.517	0.168	8.70195418077543E-106	8	TOR3A
5.4668603247418E-105	0.659508624996172	0.506	0.167	9.16136453220231E-101	8	PAXBP1
1.62345869929581E-91	0.659409254847261	0.434	0.142	2.72059208827992E-87	8	PSMG1
3.02069414216352E-97	0.659348303811277	0.495	0.167	5.06207924343762E-93	8	GK5
5.54783216996925E-83	0.659179550136085	0.489	0.18	9.29705715043446E-79	8	SSTR1
1.0760095432202E-69	0.659153125018673	0.403	0.146	1.80317679252842E-65	8	FAN1
7.80145284764776E-71	0.659012844206456	0.288	0.086	1.30736746820881E-66	8	SARNP
8.34751521815975E-91	0.658838722580454	0.494	0.173	1.39887660025921E-86	8	NCEH1
1.02252650550626E-107	0.65876833887088	0.551	0.189	1.71354991792739E-103	8	TEAD3
1.18576827365492E-104	0.658466018622722	0.59	0.21	1.98711047299091E-100	8	ARFGEF1
1.39319793954914E-105	0.65775121410861	0.852	0.374	2.33472110709645E-101	8	NEDD4L
4.82048507900507E-72	0.657643082629807	0.358	0.121	8.0781688953967E-68	8	NDUFAF5
2.08635296182248E-83	0.657202668881127	0.431	0.148	3.49631029342211E-79	8	SOCS5
4.07903925663119E-109	0.65718324769426	0.489	0.155	6.83565398626254E-105	8	CLSTN1
1.73042829287077E-94	0.657039604052669	0.325	0.088	2.89985173319284E-90	8	DEPRECATED-ENSG00000134453
9.4640561690156E-89	0.656490124253831	0.899	0.429	1.58598653280364E-84	8	IL6R
2.21337434342221E-108	0.65647287468126	0.481	0.152	3.70917272470695E-104	8	METTL9
5.30578713070427E-90	0.656440023371893	0.414	0.134	8.89143807363422E-86	8	HIRA
1.67885209203131E-118	0.656275477796019	0.752	0.288	2.81342033582607E-114	8	HACD2
3.38493219971042E-78	0.656153776850803	0.337	0.106	5.67246938027473E-74	8	PKM
4.61856910466504E-100	0.655825099911201	0.84	0.369	7.73979810559768E-96	8	RNF19A
3.79931726608233E-102	0.655805412582042	0.706	0.289	6.36689587450077E-98	8	SUN2
7.46806401991571E-69	0.655407672866331	0.34	0.114	1.25149816845747E-64	8	C9orf85
4.89766793393793E-111	0.6546300949439	0.43	0.125	8.20751192369318E-107	8	NPC1
2.108115019503E-100	0.654620377599941	0.459	0.148	3.53277914968313E-96	8	PARD6B
5.80074651396696E-111	0.654512213358556	0.391	0.108	9.72089100810583E-107	8	SNTA1
3.68028674924076E-113	0.654452955499514	0.67	0.249	6.16742453437766E-109	8	TAP2
7.86251023496064E-96	0.65425692936801	0.437	0.141	1.3175994651747E-91	8	NFYA
9.36918122930525E-106	0.653827227318316	0.718	0.282	1.57008739040697E-101	8	PARP14
4.47416466984065E-103	0.65373082927723	0.606	0.22	7.49780515371896E-99	8	MED16
7.41429380023878E-78	0.65319675314357	0.945	0.515	1.24248735504401E-73	8	KCTD3
9.24130433549241E-81	0.652954041270026	0.446	0.158	1.54865778054182E-76	8	SHPRH
4.88295649062893E-112	0.652855900369431	0.523	0.171	8.18285848695596E-108	8	NFATC3
1.15684788781451E-107	0.652454531939916	0.578	0.205	1.93864569039955E-103	8	PBX3
4.81151459867855E-58	0.652280960960071	0.272	0.088	8.06313616446552E-54	8	ZNF605
2.93595286605289E-116	0.652183688740173	0.791	0.317	4.92006981293143E-112	8	MSI2
9.51424355989125E-113	0.651851868400653	0.844	0.366	1.59439693576658E-108	8	SP3
2.2568129046623E-70	0.65131995104336	0.308	0.096	3.78196706563308E-66	8	TRIP4
2.97040481006611E-101	0.651304092945654	0.92	0.439	4.97780438070879E-97	8	ITPR2
2.72777348198186E-130	0.65119687934938	0.68	0.237	4.5712028011052E-126	8	KIF16B
1.27822796105824E-108	0.651058099511932	0.566	0.196	2.1420544171414E-104	8	CNIH4

**1.50909803361158E-99**	0.651046289033467	0.391	0.115	2.52894648472628E-95	8	BRD9
**1.33994108536874E-69**	0.650632672162646	0.309	0.098	2.24547327086093E-65	8	CCDC90B
**3.7164559602605E-54**	0.650427367633987	0.266	0.09	6.22803689820454E-50	8	CSNK2A1
**2.04454014551141E-96**	0.650419216052262	0.507	0.177	3.42624037584802E-92	8	PER3
**6.19496913751861E-69**	0.650248220925558	0.29	0.089	1.03815292806537E-64	8	TTC32
**8.73406785220816E-113**	0.650080788002498	0.694	0.262	1.46365509067304E-108	8	ZNF652
**3.51047162688277E-99**	0.649979195098233	0.928	0.432	5.88284835233015E-95	8	IRF3
**1.79734561467744E-116**	0.649973545788023	0.671	0.246	3.01199178107645E-112	8	GIGYF1
**4.99274523395724E-125**	0.649891549474196	0.639	0.221	8.36684246306554E-121	8	PPP2R5C
**1.6985513025622E-103**	0.649663280889179	0.663	0.253	2.84643227283373E-99	8	ACACA
**8.36897447478005E-121**	0.649538206998908	0.703	0.256	1.40247274248364E-116	8	GPR107
**1.57683066709248E-89**	0.649330832408726	0.349	0.102	2.64245283191358E-85	8	KAT6B
**4.17108113453955E-83**	0.648904887977445	0.905	0.456	6.98989776526138E-79	8	MEP1A
**1.75305680896347E-83**	0.64885895385089	0.483	0.175	2.93777260046099E-79	8	FARSB
**6.79021036403076E-84**	0.648845656526719	0.373	0.118	1.13790345280427E-79	8	LDAH
**5.93114760275527E-113**	0.648500417354262	0.642	0.231	9.93941715269729E-109	8	TTC17
**8.05909786462394E-107**	0.648365340625985	0.674	0.254	1.35054362015368E-102	8	DNASE1L1
**6.52343520126087E-123**	0.647741768860671	0.691	0.249	1.0931972710273E-118	8	MID1
**8.44145221449748E-111**	0.647683104447341	0.597	0.208	1.41461856210549E-106	8	RMDN1
**2.82179269403543E-94**	0.647585676388861	0.462	0.154	4.72876019666458E-90	8	CDYL
**1.67173125798311E-86**	0.647169151069953	0.525	0.195	2.8014872421281E-82	8	GALNS
**5.28393170691275E-117**	0.646819770573826	0.642	0.23	8.85481275444438E-113	8	CNOT9
**4.19235271214548E-85**	0.646799906135415	0.345	0.103	7.0255446750134E-81	8	RAB11FIP2
**1.46320391885123E-105**	0.646706388001405	0.601	0.215	2.4520371272109E-101	8	CDK13
**1.51843557982712E-90**	0.646106785330872	0.884	0.424	2.54459434467429E-86	8	CHPT1
**1.8226271112641E-79**	0.646062323417615	0.387	0.128	3.05435851305639E-75	8	RASA1
**9.321858502845E-76**	0.646027981048779	0.349	0.112	1.56215704790676E-71	8	ELAC1
**4.26532038015928E-129**	0.645822900730431	0.65	0.221	7.14782389307092E-125	8	NELFB
**1.12246483536042E-100**	0.645772130665477	0.399	0.119	1.881026571097E-96	8	USP31
**2.56024515815045E-75**	0.64559189678587	0.332	0.104	4.29045883602852E-71	8	AGO4
**3.49698318037767E-83**	0.645500285005803	0.407	0.138	5.8602444136769E-79	8	NR2C2AP
**2.38093151318055E-103**	0.645340767031948	0.771	0.32	3.98996502978796E-99	8	PHF20L1
**3.08678679170333E-112**	0.645317691908943	0.692	0.261	5.17283730553644E-108	8	USP24
**5.49305469697948E-84**	0.645311700745617	0.952	0.526	9.20526106119821E-80	8	GDI1
**2.43582008424649E-90**	0.64517908382148	0.399	0.126	4.08194729718027E-86	8	ZSWIM9
**1.29263104747604E-93**	0.645073839508897	0.782	0.349	2.16619110936034E-89	8	TMEM109
**2.25765525469781E-91**	0.645031606711967	0.86	0.405	3.7833786758226E-87	8	HNRNPD
**1.39333957748451E-55**	0.644933829824532	0.258	0.084	2.33495846394853E-51	8	KYAT3
**4.70800387993747E-124**	0.644890476148526	0.688	0.244	7.8896729019992E-120	8	COP 1
**1.06540066231801E-53**	0.644716733777806	0.274	0.094	1.78539842991252E-49	8	CASP10
**1.66227853926966E-70**	0.644585490019143	0.274	0.08	2.7856463761081E-66	8	ANKMY2
**5.31604487711482E-118**	0.644270751397561	0.551	0.18	8.90862800506902E-114	8	PPHLN1
**4.37241918156374E-108**	0.644249728869791	0.808	0.346	7.32730006446451E-104	8	SNRNP200
**2.69145174949467E-108**	0.644161542238991	0.586	0.208	4.51033484180317E-104	8	SRCAP
**2.80875193473749E-96**	0.64414725546458	0.407	0.126	4.70690649223308E-92	8	BRMS1L
**1.35558748349003E-115**	0.643934558723975	0.455	0.135	2.2716935048326E-111	8	PRPF4
**1.69845832657067E-97**	0.643420818557982	0.619	0.235	2.84627646366714E-93	8	ATG12
**1.87958234915775E-102**	0.643404140660065	0.601	0.22	3.14980410071855E-98	8	RASGEF1B
**1.28888231262613E-57**	0.643241733738328	0.293	0.1	2.15990897949887E-53	8	TMX3
**3.47786964197188E-106**	0.64302563619242	0.473	0.151	5.82821394601647E-102	8	GPC1
**4.475319174896E-79**	0.642993468533538	0.352	0.111	7.49973987329072E-75	8	C15orf41
**5.74154536988804E-107**	0.642709473966961	0.407	0.119	9.62168173085837E-103	8	GIT1
**2.76733774952839E-122**	0.642665646711861	0.731	0.274	4.63750460065967E-118	8	GNA11
**7.91391662649145E-97**	0.642628218735724	0.458	0.151	1.32621414826744E-92	8	PLCB3
**1.21914306524193E-78**	0.642096712519352	0.455	0.164	2.04303994873243E-74	8	SLC10A7
**2.44948623640786E-96**	0.641531049086752	0.66	0.253	4.10484903497229E-92	8	TMC5
**1.22601946265668E-80**	0.64126539967822	0.928	0.553	2.05456341552007E-76	8	NUCKS1
**1.26640833726475E-128**	0.640684896182695	0.51	0.152	2.12224709158827E-124	8	C1orf174
**1.24545225289834E-79**	0.640599543638551	0.282	0.078	2.08712888540704E-75	8	GABPB1
**1.11963127174024E-93**	0.640561059925802	0.912	0.458	1.87627808518229E-89	8	GSR

2.44704267893073E-106	0.640353948649	0.593	0.21	4.10075412135211E-102	8	CASP6
1.00069724372415E-76	0.640309650100419	0.418	0.148	1.67696844103292E-72	8	OFD1
1.97745558213135E-63	0.640179972560517	0.501	0.209	3.31382006453572E-59	8	CACNA1H
6.87197792751033E-97	0.64013555274761	0.421	0.131	1.15160606109218E-92	8	FBXL18
5.70011038293327E-111	0.640122721448288	0.714	0.277	9.55224497971958E-107	8	EPRS
1.68998395360641E-92	0.640075512467207	0.446	0.148	2.83207510945362E-88	8	TRAPPC11
1.15863263231964E-71	0.63985487551305	0.318	0.101	1.94163656524125E-67	8	SLC19A1
4.01446439038964E-76	0.639474035082072	0.298	0.088	6.72743942541496E-72	8	FASTKD3
2.62868112658401E-95	0.639366493180451	0.437	0.141	4.40514383192949E-91	8	PRUNE1
1.74085253591286E-90	0.639082319686342	0.332	0.094	2.91732067968277E-86	8	POGLUT2
2.74943366617963E-103	0.639071387340266	0.523	0.177	4.60750093778382E-99	8	TCF20
7.22003823945461E-97	0.638894991502729	0.309	0.08	1.2099340081678E-92	8	CDK19
5.78211296460783E-111	0.638789258022637	0.639	0.232	9.6896649060898E-107	8	CASK
1.43846662056281E-114	0.638685093747574	0.587	0.202	2.41058236273916E-110	8	ACTL6A
2.19703968741067E-101	0.638600462548438	0.473	0.153	3.6817991081628E-97	8	SNRPG
9.73948888444829E-110	0.638561699958294	0.68	0.254	1.63214354725584E-105	8	CERS6
4.10822343049688E-91	0.638244108380603	0.466	0.158	6.88456082482667E-87	8	SH3TC1
8.50889757488573E-127	0.638142366470781	0.686	0.244	1.42592105559935E-122	8	SH3GL1
2.36781553750048E-103	0.637821947580832	0.551	0.194	3.96798527774331E-99	8	C12orf75
6.0809490072089E-67	0.637603060999947	0.25	0.072	1.01904543462807E-62	8	C19orf54
1.44120543855246E-118	0.637550904750198	0.704	0.262	2.41517207392621E-114	8	TRA2B
1.47832083601432E-108	0.637448625554065	0.523	0.172	2.4773700569928E-104	8	TBC1D22B
1.6236723118125E-79	0.637359122709057	0.373	0.121	2.72095006013539E-75	8	MIGA2
1.14721027643221E-100	0.63706428107379	0.68	0.27	1.9224949812451E-96	8	KMT2D
3.73995064224083E-133	0.636510683260662	0.738	0.267	6.26740928626718E-129	8	RUFY1
1.00335763049962E-45	0.636442518283209	0.26	0.094	1.68142671719126E-41	8	ZNF460
5.92056826662429E-82	0.636308011134305	0.293	0.082	9.92168830120898E-78	8	MAPK7
4.59006932698593E-108	0.635985900662349	0.764	0.308	7.69203817816302E-104	8	ANKRD17
8.71524949074549E-107	0.6359425310087	0.806	0.343	1.46050150965913E-102	8	KMT2C
4.70687406588492E-70	0.63593218879296	0.33	0.109	7.88777955960995E-66	8	LRRC8B
1.19404600837304E-118	0.635788994541954	0.684	0.252	2.00098230083154E-114	8	PCMT1
6.73837556593307E-108	0.63566062692661	0.413	0.119	1.12921697733906E-103	8	KCTD9
7.13420970355597E-125	0.635475367173505	0.617	0.209	1.19555086212191E-120	8	EDC4
6.40590210957923E-99	0.635446594622361	0.426	0.131	1.07350107552329E-94	8	AQR
7.60852880454384E-78	0.635267794083236	0.313	0.094	1.27503725706546E-73	8	USP39
2.38838487853362E-114	0.635247539640643	0.724	0.277	4.00245537944663E-110	8	DEPRECATED-ENSG00000161547
5.91499774634164E-80	0.635199641030625	0.41	0.14	9.91235322331932E-76	8	PPARG
1.23722112454933E-82	0.634971536389076	0.36	0.112	2.07333516051976E-78	8	MED9
6.98221012088801E-81	0.634821962332677	0.352	0.109	1.17007877205841E-76	8	EPC2
2.11997712646885E-110	0.634752578624399	0.688	0.258	3.55265766853649E-106	8	NSD1
3.54316726320279E-103	0.634726946496203	0.61	0.224	5.93763969967523E-99	8	ELF4
2.2265904817597E-76	0.634292843932716	0.344	0.109	3.7313203293329E-72	8	GAPVD1
7.26640403356205E-96	0.633977258358333	0.434	0.138	1.21770398794433E-91	8	SIMC1
3.99556001481842E-95	0.633629919202689	0.543	0.196	6.69575947283271E-91	8	GART
8.13013797996166E-100	0.633218686522579	0.559	0.2	1.36244852268198E-95	8	CHD9
2.01925630598402E-92	0.633194157590596	0.443	0.146	3.38386971756801E-88	8	SBNO1
1.22928524893843E-87	0.633123735906381	0.505	0.182	2.06003622017102E-83	8	DNAAF2
2.44545706606052E-83	0.632850348343507	0.37	0.116	4.09809695130421E-79	8	DIS3L
5.022371500944E-92	0.632633609620182	0.917	0.446	8.41649016128196E-88	8	PLEKHA6
1.18454159779519E-60	0.632482401414323	0.256	0.078	1.98505480958518E-56	8	POGLUT1
1.60153912093672E-101	0.632442222455403	0.461	0.147	2.68385925886576E-97	8	ANKRD52
1.57394081766782E-104	0.632337856767917	0.525	0.176	2.63761002224774E-100	8	TRIM33
3.86281845522402E-95	0.631448094204973	0.876	0.391	6.47331116726441E-91	8	XIAP
2.21370084995376E-104	0.631326251432802	0.517	0.174	3.70971988435251E-100	8	PPP1R12A
1.57866463798573E-106	0.631301686860393	0.703	0.274	2.64552620033648E-102	8	GINM1
1.00576777169311E-130	0.631253016783816	0.527	0.158	1.68546563180332E-126	8	UBAC1
1.49749270807423E-78	0.631188078856956	0.411	0.141	2.5094982801908E-74	8	ZNF398
3.13993818079286E-104	0.630890299361646	0.66	0.249	5.26190840337267E-100	8	HERC3
9.31510779637181E-111	0.630881778281888	0.397	0.11	1.56102576451599E-106	8	DEPRECATED-ENSG00000084463
8.88491684236108E-75	0.630296663460775	0.365	0.12	1.48893436444287E-70	8	EZH1

1.80070246638869E-101	0.629935552615914	0.594	0.215	3.01761719317417E-97	8	C8orf76
1.07437240812146E-62	0.629897987936483	0.266	0.082	1.80043328152994E-58	8	TANGO6
1.17246074341811E-109	0.629732022731291	0.816	0.344	1.96480971382007E-105	8	PUM2
9.17108392104958E-89	0.62948669893752	0.415	0.136	1.53689024348949E-84	8	SNX7
1.44205609918774E-108	0.628943441256676	0.743	0.297	2.41659761101882E-104	8	BACH1
1.24813102618704E-76	0.62865179725816	0.308	0.092	2.09161797368424E-72	8	ZNF518A
1.57944382783406E-86	0.62822737694273	0.398	0.128	2.64683196668432E-82	8	CLK2
1.49372720674463E-107	0.628224829297115	0.732	0.286	2.50318805306266E-103	8	RALGAPA2
6.9679763800331E-89	0.627293086089263	0.301	0.08	1.16769348176595E-84	8	HSPA2
9.12933941036058E-67	0.626897295124777	0.276	0.084	1.52989469838823E-62	8	BICC1
1.26448670018496E-112	0.626713413326906	0.522	0.168	2.11902681216996E-108	8	SUCO
3.77897822279844E-80	0.626607167273281	0.61	0.247	6.33281170576562E-76	8	POF1B
1.41471612737179E-40	0.626508308206193	0.272	0.107	2.37078128624965E-36	8	SETD6
8.62301099619031E-109	0.62649582045607	0.712	0.277	1.44504418274157E-104	8	SNRPA
4.67545228464614E-55	0.626309512698664	0.341	0.128	7.83512293861E-51	8	TCAF2
1.2912301090452E-108	0.625467808015475	0.762	0.305	2.16384341673794E-104	8	PCID2
3.76743888979786E-90	0.625422818783823	0.364	0.108	6.31347409152325E-86	8	SLC25A17
1.07334360906254E-61	0.625256893727219	0.313	0.106	1.79870922006701E-57	8	MBD5
1.38752884645806E-79	0.624842719445115	0.848	0.412	2.32522084089442E-75	8	EHF
1.19854580741244E-110	0.624468949672921	0.66	0.245	2.00852306406176E-106	8	NEMF
4.28184082991059E-120	0.624257972743336	0.8	0.322	7.17550886276416E-116	8	EGFR
2.62547195482892E-99	0.624179853344558	0.767	0.317	4.3997659019023E-95	8	PKP2
4.24887097150471E-113	0.624090805688606	0.736	0.287	7.1202579740476E-109	8	FBXL3
6.40120715916831E-104	0.623660790510144	0.76	0.323	1.07271429573342E-99	8	SSRP1
1.22933898557455E-83	0.623483847269919	0.326	0.096	2.06012627202583E-79	8	MED26
1.66766271858485E-109	0.62320778745454	0.832	0.348	2.79466918380449E-105	8	PRPF3
1.7322452243058E-73	0.622753103016424	0.282	0.082	2.90289654689166E-69	8	TULP3
5.51011914250065E-72	0.622740888406605	0.31	0.097	9.23385765900259E-68	8	SYNE3
1.23751781610166E-52	0.622485031619672	0.292	0.104	2.07383235622316E-48	8	DDHD1
5.94839126543911E-70	0.621866850915314	0.277	0.082	9.96831408262286E-66	8	BRAP
2.51712810690348E-86	0.621352052041591	0.441	0.15	4.21820328154885E-82	8	TDRD7
7.82406284074064E-100	0.621303020259868	0.487	0.161	1.31115645085132E-95	8	ENOX2
9.11242190800595E-107	0.621144307462324	0.754	0.301	1.52705966334364E-102	8	PPP4R3A
1.59998515502755E-87	0.620978550518566	0.57	0.217	2.68125512279516E-83	8	CBR4
4.73098789997391E-91	0.620792444254928	0.449	0.151	7.92818952277627E-87	8	TMEM18
5.12535461717359E-87	0.620368034570023	0.421	0.139	8.5890692674595E-83	8	ATP9B
1.7371429479063E-73	0.620121995571286	0.375	0.13	2.91110415210138E-69	8	TSEN54
5.62261394754425E-101	0.619786442586444	0.549	0.194	9.42237645329465E-97	8	KCNT2
3.95755889528032E-90	0.619656200898205	0.394	0.124	6.63207719671075E-86	8	CD320
2.42362811188655E-102	0.61898219298825	0.639	0.238	4.06151598989948E-98	8	TRRAP
2.68892732197966E-57	0.61882729477816	0.27	0.089	4.50610440617351E-53	8	GPR89B
1.50751284370049E-112	0.618466473004763	0.475	0.145	2.52629002347328E-108	8	ANKFY1
6.66363122990629E-96	0.618231717112136	0.614	0.232	1.1166913215077E-91	8	ZNF33A
1.02974593545751E-110	0.617250233045412	0.726	0.281	1.72564823863969E-106	8	KIF13A
7.00273299833041E-94	0.617073697165949	0.421	0.134	1.17351799586021E-89	8	USP21
1.64880518224835E-101	0.616829540731705	0.674	0.261	2.76306772441179E-97	8	RNF138
2.92428224687211E-90	0.616789584945669	0.562	0.209	4.90051218930827E-86	8	TIGD2
1.30319819285346E-93	0.616454937492313	0.475	0.161	2.18389953158383E-89	8	NOP9
1.93744310520656E-98	0.616180724151946	0.462	0.15	3.24676715570516E-94	8	MMD
1.05849706760637E-110	0.616122689883184	0.581	0.199	1.77382938589476E-106	8	CHM
1.72928683830109E-99	0.616050079137002	0.875	0.393	2.89793888362497E-95	8	RREB1
7.86104922480154E-108	0.615865895733999	0.598	0.212	1.31735462909224E-103	8	ARHGEF11
1.2488296521064E-120	0.615693734235968	0.663	0.233	2.0927887309999E-116	8	ZC3H4
5.96314852060191E-91	0.615603987076541	0.537	0.196	9.99304429082468E-87	8	MCEE
3.69638260194257E-95	0.615331846643133	0.398	0.121	6.19439796433536E-91	8	VARS2
1.20639006888825E-94	0.615172856180213	0.814	0.353	2.02166847744293E-90	8	MAVS
1.00865354365936E-83	0.614849529941212	0.431	0.147	1.69030160846436E-79	8	MTMR6
4.15666043075707E-116	0.614755259184855	0.589	0.2	6.96573154986271E-112	8	BRD1
3.45193924041959E-101	0.61448223578569	0.743	0.307	5.78475977909515E-97	8	MECP2
4.49004311714919E-108	0.614384511159216	0.642	0.237	7.52441425571861E-104	8	KPNA1

5.96044563282668E-87	0.613997858744825	0.375	0.116	9.98851479149095E-83	8	CREB1
1.2303695421593E-92	0.613912650011408	0.655	0.266	2.06185327875056E-88	8	DEDD2
3.84477473539086E-118	0.613847458532996	0.686	0.25	6.443073501568E-114	8	MAEA
6.47472040060634E-91	0.613736298750271	0.446	0.149	1.08503364473361E-86	8	RNF38
7.02992083963029E-114	0.613683919564779	0.602	0.208	1.17807413430524E-109	8	EIF4E
3.97598549478515E-92	0.613338692677719	0.739	0.31	6.66295649216096E-88	8	IL10RB
1.2612869202373E-63	0.613333345182451	0.281	0.088	2.11366462093366E-59	8	TRMT5
2.20276356818755E-98	0.61310990795201	0.597	0.223	3.6913911875687E-94	8	TAF8
4.25301353913821E-125	0.612973616059718	0.566	0.178	7.12720008888781E-121	8	CCNT2
3.49962821908749E-108	0.61243836019262	0.622	0.226	5.86467696954682E-104	8	FBXO21
2.35109730633692E-64	0.612382501587806	0.957	0.607	3.93996886595942E-60	8	MBNL3
1.46930889259673E-79	0.612337366486082	0.362	0.116	2.4622678422136E-75	8	SETDB2
9.21898840753839E-111	0.612250717914988	0.273	0.058	1.54491807733528E-106	8	PHGDH
8.94662202344872E-102	0.611862962885929	0.393	0.114	1.49927491868954E-97	8	APOO
1.41833066835813E-50	0.611606488382252	0.374	0.153	2.37683853403456E-46	8	CCDC141
1.87529774057044E-75	0.611585456085467	0.375	0.127	3.14262395364795E-71	8	RTL10
1.57339655979614E-91	0.611493375506602	0.503	0.177	2.63669795490637E-87	8	CCDC130
4.53092882336051E-71	0.611413591508947	0.373	0.128	7.59293052218754E-67	8	CASD1
4.32350965779759E-51	0.611188143307824	0.254	0.086	7.2453374845372E-47	8	MAMLD1
2.3416785480032E-93	0.61094140613501	0.875	0.416	3.92418491074376E-89	8	LACTB2
4.63304200926662E-79	0.610593110055542	0.47	0.173	7.76405179912901E-75	8	DMAC2L
3.33604477693958E-77	0.610585328689105	0.341	0.108	5.59054383719535E-73	8	SAYSD1
1.32905281099303E-95	0.609552491152415	0.529	0.188	2.22722670066213E-91	8	SLC19A2
1.1988620564093E-108	0.609474397128348	0.667	0.246	2.00905303413071E-104	8	MGAT4A
9.4506262936703E-80	0.60946744709282	0.36	0.114	1.58373595429327E-75	8	TSPYL2
7.07777618898996E-89	0.609063492735253	0.495	0.176	1.18609373375094E-84	8	MRNIP
2.53650880730793E-58	0.608569964892653	0.352	0.131	4.25068145928664E-54	8	ZMYND19
5.60997324453625E-63	0.60850112538838	0.305	0.101	9.40119316319385E-59	8	GPC3
9.57079023793173E-94	0.608480692218553	0.328	0.09	1.6038730280726E-89	8	ZNF544
6.62353051581409E-89	0.608104394689455	0.401	0.127	1.10997124384012E-84	8	NCBP3
3.89559059309694E-113	0.607708690296718	0.605	0.213	6.52823071591186E-109	8	KLHL2
9.39696903830048E-109	0.607653917044734	0.651	0.238	1.57474407143839E-104	8	MED14
3.77339924823083E-73	0.60757923342277	0.397	0.138	6.32346246018523E-69	8	ZNF692
5.6936849657479E-104	0.607525803315231	0.818	0.35	9.54147726560033E-100	8	SLC38A2
6.19952452102733E-88	0.607523395723822	0.364	0.11	1.03891631923376E-83	8	BMP2
3.29428690080352E-72	0.60751842770611	0.94	0.516	5.52056598836654E-68	8	ELL2
1.85413889557836E-92	0.607440962880937	0.706	0.297	3.10716596121022E-88	8	ABCB6
1.85283529646436E-102	0.60736941685798	0.828	0.354	3.10498138981498E-98	8	NECTIN3
8.29767958193387E-109	0.607356357511325	0.611	0.217	1.39052514434048E-104	8	TTC14
1.17554059959197E-77	0.607337544584742	0.37	0.121	1.96997093679623E-73	8	DEPRECATED-ENSG00000050130
1.0559661722201E-86	0.606852005342398	0.792	0.36	1.76958811140644E-82	8	CCNG1
2.5067092147952E-96	0.606474953418877	0.541	0.191	4.2007433021538E-92	8	MYO1D
6.2474560642374E-78	0.60634316080686	0.439	0.157	1.0469486872449E-73	8	KCTD13
1.75951540541435E-89	0.606130801873793	0.407	0.131	2.94859591639337E-85	8	SLC39A8
1.31038309227242E-112	0.604578513510104	0.573	0.194	2.19593998603012E-108	8	NRIP1
3.52435831613843E-71	0.604541701960765	0.309	0.096	5.90611966618477E-67	8	ICE2
3.09213721715774E-104	0.604338392522136	0.666	0.252	5.18180354851293E-100	8	GSK3B
1.01598843500138E-96	0.604188628754348	0.461	0.151	1.70259341937532E-92	8	UBN1
5.45301402898344E-109	0.60416126876169	0.638	0.231	9.13816090977045E-105	8	PHF12
3.06487704470372E-99	0.60395473182453	0.854	0.378	5.1361209515145E-95	8	TMEM64
6.70548714688699E-102	0.603885079248611	0.742	0.304	1.12370553607532E-97	8	VAMP7
1.23079196754454E-104	0.603883532623139	0.467	0.149	2.06256117921114E-100	8	ALYREF
2.21875552386599E-72	0.603849691321522	0.36	0.121	3.71819050689463E-68	8	SLC35E2A
7.99466950350396E-80	0.603672206926166	0.422	0.145	1.33974671539719E-75	8	DDX60
1.86362268969239E-94	0.603637891584831	0.437	0.142	3.1230589033865E-90	8	ZNF672
2.35098363361316E-107	0.603607825291942	0.585	0.207	3.93977837320893E-103	8	JOSD1
1.51879709860597E-103	0.603353086612589	0.571	0.2	2.54520017784388E-99	8	XPR1
3.2179208246846E-109	0.603086732399622	0.68	0.255	5.39259171800646E-105	8	SF3B3
8.58370545799203E-124	0.602865313338766	0.467	0.135	1.43845736065030E-119	8	TMEM160
1.83508146182203E-100	0.602398303561555	0.391	0.115	3.07522951372136E-96	8	ABI2

3.83422999776882E-64	0.602107924334098	0.278	0.087	6.425402630261E-60	8	TRIM35
1.09736930934682E-98	0.601664055996975	0.834	0.363	1.83897148860339E-94	8	STX16
1.79753777516021E-92	0.60145819098987	0.652	0.267	3.01231380361347E-88	8	FOXA1
9.40032147183356E-98	0.60136610471724	0.597	0.225	1.57530587224987E-93	8	DEAF1
1.1972935927298E-59	0.601325195282221	0.278	0.09	2.0064246026966E-55	8	RUNDC1
1.04937940765921E-77	0.601239057282695	0.341	0.108	1.7585500113553E-73	8	RUBCN
1.34598489085645E-71	0.600964527555781	0.322	0.102	2.25560148009724E-67	8	TOGARAM1
3.55994678778783E-58	0.600963119266572	0.28	0.092	5.96575882697485E-54	8	SLC18B1
1.20359745096079E-64	0.600883910306151	0.265	0.08	2.01698860832009E-60	8	BOLA1
8.44670186981321E-74	0.600598314707528	0.337	0.109	1.4154982993433E-69	8	PRDM4
5.91108527808139E-110	0.600137661322878	0.663	0.245	9.9057967090088E-106	8	AKAP9
1.35221382590001E-111	0.600048424209426	0.82	0.338	2.26603992944323E-107	8	UPK3BL1
4.45337550555751E-78	0.600028824512432	0.346	0.109	7.46296667221327E-74	8	GPR89A
1.0499404597992E-95	0.599819583611744	0.35	0.099	1.7594902225315E-91	8	BTBD6
1.32157376089713E-108	0.59930745400636	0.489	0.154	2.21469330851141E-104	8	COA8
2.484763622692E-100	0.59925858345054	0.754	0.311	4.16396687890726E-96	8	CXorf56
5.66398112141707E-125	0.599227656134546	0.575	0.186	9.49169956327073E-121	8	TMEM131L
6.29259170392268E-102	0.599193019586786	0.759	0.307	1.05451251774336E-97	8	SOS 2
6.15931756150376E-90	0.599090853575885	0.526	0.191	1.0321784369568E-85	8	PAN2
4.79703476943872E-86	0.599001256883462	0.553	0.209	8.03887086662541E-82	8	TMEM43
1.06517833747117E-67	0.598928848108391	0.503	0.206	1.78502585793419E-63	8	SLC1A5
2.3040617111949E-65	0.598506717302517	0.253	0.074	3.86114661562041E-61	8	ORC5
1.5871787085966E-75	0.598143982186115	0.792	0.372	2.65979407986618E-71	8	DUSP9
9.88386162621666E-83	0.598086657911351	0.407	0.136	1.65633753132139E-78	8	TBC1D7
2.68525293941891E-103	0.597862037040281	0.478	0.153	4.49994687587821E-99	8	ZNF282
1.59885275771683E-87	0.597827008446434	0.434	0.145	2.67935745138186E-83	8	EXOC2
7.19320103683916E-98	0.59773991997216	0.57	0.207	1.20543662975351E-93	8	EPS15L1
1.54032975078431E-83	0.597653499194177	0.86	0.412	2.58128459636434E-79	8	ARHGEF26
1.70709926870582E-111	0.597628253878102	0.539	0.178	2.86075695449722E-107	8	KLHL12
7.01337926317632E-83	0.59759669345347	0.401	0.132	1.17530209692309E-78	8	HEATR1
2.56953464218602E-105	0.597343373386365	0.563	0.197	4.30602615337533E-101	8	GANAB
2.24011396670748E-75	0.597255391248101	0.439	0.16	3.7539829854084E-71	8	ZFX
2.43892603195481E-103	0.597188461073442	0.662	0.256	4.08715224434987E-99	8	ATF2
1.15290311676683E-87	0.596661854236782	0.854	0.422	1.93203504307786E-83	8	KLF6
2.22776265124168E-96	0.596635502838994	0.611	0.231	3.73328465095081E-92	8	FAM136A
1.29241842824491E-107	0.5963810441906	0.598	0.211	2.16583480205282E-103	8	CAMSAP2
1.58930263134778E-51	0.59634774823776	0.336	0.129	2.66335334961261E-47	8	FBXO11
1.75527828711782E-114	0.59552122998826	0.565	0.187	2.94149535355204E-110	8	NXF1
8.0247353591325E-96	0.595416437126626	0.778	0.328	1.34478515148342E-91	8	BIRC6
3.53708429941165E-112	0.595233896618157	0.788	0.335	5.92744586895404E-108	8	LSM 4.00
1.62143842150621E-58	0.595189339055566	0.297	0.101	2.71720650676011E-54	8	INVS
1.13557986537951E-94	0.595122052846813	0.783	0.343	1.90300473840299E-90	8	MAPK6
5.37323980782968E-80	0.594694270839155	0.394	0.131	9.00447526996098E-76	8	PGS1
7.79457710971987E-109	0.59454660428454	0.668	0.251	1.30621523204686E-104	8	VPS35L
1.26148311625051E-99	0.594490089945229	0.674	0.259	2.1139934062126E-95	8	ASB9
3.54848565040977E-103	0.594161591049098	0.802	0.343	5.94655225295669E-99	8	ANKHD1
4.76573045692979E-93	0.593827770147225	0.356	0.102	7.98641109972293E-89	8	SUPT7L
1.53058704680796E-89	0.593494751034937	0.386	0.119	2.56495777304077E-85	8	WASHC3
1.7025231124497E-72	0.593414201194589	0.372	0.126	2.85308823184321E-68	8	TSNARE1
8.62702277444254E-57	0.593106655638945	0.262	0.085	1.44571647654108E-52	8	E2F6
2.44154631085439E-62	0.593074949140801	0.342	0.12	4.09154330772979E-58	8	C3orf85
3.00207730247639E-97	0.592929718860705	0.513	0.175	5.03088114348994E-93	8	PM20D2
1.93057818708387E-51	0.592671853810102	0.313	0.117	3.23526292591514E-47	8	PPP1R1B
7.78018076969658E-73	0.592504451424035	0.306	0.093	1.30380269338575E-68	8	DGLUCY
1.48385428304397E-96	0.59250184127318	0.626	0.238	2.48664300752509E-92	8	ATXN2L
4.17997130331065E-83	0.592400251763138	0.545	0.207	7.00479591008798E-79	8	GNL3L
9.64853142655128E-109	0.592328493620521	0.687	0.263	1.61690089646146E-104	8	LRP11
3.93945605140409E-98	0.592281129887837	0.824	0.358	6.60174045094297E-94	8	NCOA2
3.90731750553824E-120	0.591995250719626	0.656	0.234	6.54788267578098E-116	8	ILF2
4.82035664124668E-77	0.59196526880362	0.358	0.115	8.07795365940119E-73	8	JKAMP

4.64921110725944E-99	0.591843334342218	0.803	0.348	7.79114797354537E-95	8	ZBTB20
1.03982254952401E-86	0.591744398287776	0.63	0.256	1.74253462849233E-82	8	EPS8L3
2.23158168595609E-74	0.591462473431006	0.413	0.147	3.73968458932521E-70	8	MOB3B
1.53775873421916E-86	0.591461876608082	0.372	0.115	2.57697608680446E-82	8	RABGEF1
6.37201011255495E-103	0.591405267437887	0.822	0.352	1.06782145466196E-98	8	VDAC3
7.34779549440992E-95	0.591190484107959	0.796	0.347	1.23134356895321E-90	8	KLF3
1.37350675598583E-59	0.591070003659957	0.264	0.084	2.30172262168105E-55	8	FAAP100
5.88978056576299E-96	0.591038292729653	0.887	0.409	9.87009427210562E-92	8	LUC7L3
1.3650783633638E-97	0.590969890566449	0.534	0.188	2.28759832132506E-93	8	CPSF2
1.02631001408045E-99	0.590745873144778	0.59	0.214	1.71989032159602E-95	8	FKBP3
8.30715162432448E-82	0.590685508569854	0.45	0.158	1.3921124692043E-77	8	ALKBH6
2.64894150288706E-87	0.590634145016682	0.322	0.091	4.43909617053814E-83	8	BRPF1
1.50634430314844E-106	0.590453458461647	0.506	0.168	2.52433178321616E-102	8	DHX8
1.62311695091786E-113	0.589696066680742	0.626	0.224	2.72001938634816E-109	8	CNOT8
6.42334411895087E-91	0.589692016950978	0.346	0.1	1.07642400745379E-86	8	MYCBP
8.70525900212747E-61	0.589268262540777	0.35	0.127	1.45882730357652E-56	8	ZFP41
5.55528802676371E-107	0.588704428243493	0.591	0.209	9.30955167525063E-103	8	BTAF1
4.12371660737672E-75	0.588633951358923	0.382	0.129	6.9105242906419E-71	8	VCL
2.22407549611136E-96	0.588584602841652	0.471	0.157	3.72710571638341E-92	8	APPL1
5.35577369647764E-89	0.588491938172459	0.418	0.137	8.97520556055722E-85	8	ZDHHC5
1.06921911173504E-90	0.588357857318492	0.498	0.176	1.79179738744558E-86	8	OXSR1
4.78790556612539E-120	0.588267651681471	0.687	0.247	8.02357214771292E-116	8	NFX1
2.95615773515149E-96	0.587964943525903	0.836	0.37	4.95392913256687E-92	8	FARP1
3.47821622561696E-105	0.587433396010903	0.774	0.321	5.8287947508889E-101	8	CYBC1
1.27900111583874E-105	0.587009107642893	0.759	0.308	2.14335006992256E-101	8	AGFG1
9.36050910704585E-94	0.586744459847548	0.678	0.271	1.56863411615874E-89	8	SLC35A3
5.03646308398961E-80	0.586641147551634	0.433	0.151	8.44010483614979E-76	8	FGD6
2.7765963174383E-97	0.586540321378286	0.395	0.119	4.65302010876311E-93	8	BLOC1S5
1.3550260156387E-98	0.586390022454744	0.457	0.146	2.27075259700733E-94	8	CLEC16A
3.02133382084528E-105	0.586380251881438	0.695	0.271	5.06315121697252E-101	8	SORBS1
2.17848144093026E-75	0.586084014290191	0.34	0.108	3.65069919871092E-71	8	ATP2B1
4.43139647264551E-29	0.585824814454347	0.269	0.122	7.42613420885935E-25	8	ALPK2
7.83312711506102E-91	0.585817174155158	0.399	0.125	1.31267544194193E-86	8	HPS4
1.45220779963353E-104	0.585440682812676	0.631	0.234	2.43360983062587E-100	8	PIP4P1
1.82157953719784E-60	0.585027072295067	0.278	0.09	3.05260298843614E-56	8	PDE12
4.34802213919521E-104	0.584807216543343	0.834	0.357	7.28641550086334E-100	8	MBNL1
1.74594017156698E-90	0.58469553785065	0.646	0.255	2.92584653951195E-86	8	CLDN14
7.84007189331419E-94	0.584621608474429	0.407	0.127	1.31383924788159E-89	8	CETN3
5.69473535393729E-66	0.584015847342302	0.301	0.097	9.54323750612811E-62	8	MPHOSPH6
1.36528865977735E-92	0.583231991793314	0.565	0.205	2.28795073605489E-88	8	SLC26A6
1.38226896213889E-110	0.583124373398838	0.423	0.122	2.31640632675235E-106	8	GNAI1
5.46050158146869E-91	0.582868860579056	0.43	0.14	9.15070855022523E-87	8	PPIL4
2.74375015872367E-88	0.582749390947003	0.59	0.227	4.59797651598912E-84	8	WDR20
9.63975610270415E-88	0.582624845415591	0.443	0.15	1.61543032769116E-83	8	BCAS3
2.13985127354384E-100	0.582370895365575	0.494	0.164	3.58596276420477E-96	8	CHD1
7.61377366482285E-116	0.582254811563647	0.531	0.169	1.27591619075101E-111	8	ACAP2
3.36532561531691E-108	0.582246235667105	0.743	0.297	5.63961266614807E-104	8	RNPS1
4.76336341075614E-100	0.581785799343543	0.832	0.359	7.98244440374514E-96	8	POGK
1.49090158709948E-79	0.581744377234346	0.485	0.178	2.49845287966131E-75	8	TMEM39A
5.11874926416815E-98	0.581493427440806	0.521	0.177	8.57800001689299E-94	8	KIAA1109
1.63012009089697E-84	0.581393369810296	0.457	0.16	2.73175524832514E-80	8	USP15
3.40815566943597E-88	0.581094967138321	0.474	0.164	5.71138727084081E-84	8	USP16
8.27977399998668E-59	0.581013733131917	0.284	0.094	1.38752452691777E-54	8	FBXO32
2.99027278300503E-68	0.58082853114068	0.993	0.718	5.01109912975982E-64	8	ASPH
1.34803804039222E-78	0.580767168738344	0.298	0.086	2.25904214808928E-74	8	PARG
8.30256276092247E-48	0.580042274301475	0.289	0.107	1.39134346747539E-43	8	WDR75
1.40720388697712E-51	0.57974586676689	0.292	0.105	2.35819227379627E-47	8	CYP2R1
1.11451126030545E-82	0.57972698009663	0.411	0.138	1.86769797001988E-78	8	TAF1
9.30857552171551E-56	0.578980516380335	0.253	0.081	1.55993108592909E-51	8	TICAM2
6.0868776012991E-45	0.578576040415214	0.296	0.115	1.0200389484257E-40	8	MFSD4B

5.55906882276651E-98	0.578229390052194	0.63	0.237	9.31588753319213E-94	8	MON2
1.20273770660812E-78	0.578219419639858	0.925	0.485	2.01554784873389E-74	8	UBE2A
1.58257402106376E-81	0.578086449402717	0.51	0.193	2.65207754449865E-77	8	YTHDC2
1.38987531810696E-74	0.57782213310289	0.344	0.11	2.32915305808365E-70	8	FAM76A
1.41721198724308E-115	0.577797582711303	0.615	0.213	2.37496384822195E-111	8	EXOC5
2.47996051343827E-69	0.577706022322638	0.31	0.098	4.15591782841986E-65	8	INTS12
1.11477252297664E-96	0.577527541561276	0.581	0.214	1.86813579400425E-92	8	LMO4
1.0571091367587E-79	0.577459921968252	0.601	0.244	1.77150349138022E-75	8	DEPRECATED-ENSG00000183747
3.39222489182525E-113	0.577374043461382	0.864	0.374	5.68469047372076E-109	8	TRA2A
7.69595610093403E-115	0.577333860201108	0.732	0.286	1.28968832339452E-110	8	HNRNPUL2
3.52234615532676E-109	0.57719872475116	0.759	0.31	5.90274768709658E-105	8	AP2A1
2.31224500293711E-110	0.577168901805216	0.651	0.24	3.87486017592201E-106	8	RRP1B
6.76203885914302E-76	0.576969636036352	0.36	0.117	1.13318247201519E-71	8	LPCAT4
3.22858590075992E-60	0.576593452198348	0.292	0.097	5.41046425249347E-56	8	C15orf39
8.02131931428452E-81	0.576585609997437	0.383	0.125	1.3442126906878E-76	8	TGFBR1
1.23828691884489E-79	0.57643830922111	0.507	0.19	2.07512121860026E-75	8	SUSD4
1.11076469960925E-92	0.576363978602036	0.756	0.329	1.86141948360519E-88	8	LRRC8A
2.33415994839652E-65	0.576299902612698	0.278	0.086	3.9115852415229E-61	8	BLOC1S3
6.33359083589407E-74	0.575677035977586	0.297	0.089	1.06138315227913E-69	8	DHX33
3.98210562584283E-102	0.57494806005866	0.655	0.254	6.67321260778742E-98	8	ABHD10
1.64410941991072E-59	0.574929923571771	0.268	0.085	2.75519856588638E-55	8	EDRF1
1.29479073037679E-98	0.574782833821066	0.795	0.346	2.16981030596543E-94	8	SEPTIN11
6.12479112163322E-93	0.574023034411101	0.453	0.151	1.0263924961633E-88	8	PPP1R37
2.05652754552121E-91	0.573957798746816	0.397	0.124	3.44632886078445E-87	8	PPP4R1
3.66511848201504E-99	0.57349966664161	0.561	0.2	6.1420055521608E-95	8	ZNF687
1.25177967206366E-62	0.573438472092554	0.341	0.12	2.09773237444427E-58	8	DPP8
4.66954080569952E-71	0.573170595647272	0.843	0.423	7.82521648219126E-67	8	DDIT4
1.52966759860052E-50	0.572725494568892	0.254	0.086	2.56341696173475E-46	8	KAT14
2.47988626777985E-96	0.57253028789408	0.57	0.209	4.15579340754547E-92	8	PAWR
4.10133887636154E-100	0.572516515930241	0.663	0.26	6.87302368900667E-96	8	TLE1
1.96825941594192E-82	0.571936860291613	0.467	0.168	3.29840912923548E-78	8	UPF2
4.35641155336038E-63	0.571910456832803	0.919	0.538	7.30047448112132E-59	8	SPTSSA
1.27312725187249E-81	0.571855589865393	0.345	0.106	2.13350664868792E-77	8	TMEM218
1.01548469105059E-95	0.571777629379513	0.786	0.344	1.70174924526257E-91	8	DDX17
7.27709514643445E-71	0.571607954154782	0.322	0.103	1.21949560463948E-66	8	DEPRECATED-ENSG00000056050
1.13986878900941E-58	0.570652023803246	0.256	0.08	1.91019211662197E-54	8	TMEM69
7.77154608458295E-110	0.570391949910763	0.695	0.264	1.30235569285441E-105	8	KIAA0319L
3.55994250818116E-116	0.570295287088424	0.55	0.18	5.96575165520998E-112	8	SNRNP40
2.0089169031922E-72	0.570272931556336	0.382	0.132	3.36654294636949E-68	8	CUEDC1
1.47225673358615E-75	0.570063034513537	0.447	0.162	2.46720783414367E-71	8	PPP1R15A
1.32482098378708E-83	0.570040966415281	0.381	0.121	2.22013500463038E-79	8	ZNF562
2.68977420893132E-75	0.569676935243041	0.3	0.089	4.50752361932711E-71	8	EIPR1
7.24734600327567E-112	0.569629679598912	0.748	0.298	1.21451024322894E-107	8	CYP2C18
1.93766123039649E-99	0.569601428706216	0.756	0.307	3.24713268989844E-95	8	SMYD2
1.56885325944352E-62	0.56919674702212	0.288	0.092	2.62908429217544E-58	8	FKTN
3.93149025167713E-109	0.569151765215795	0.397	0.111	6.58839136376054E-105	8	PRADC1
2.76404532439218E-115	0.569084136866199	0.779	0.313	4.63198715461642E-111	8	ZFP91
5.20919845595122E-57	0.569055183203079	0.304	0.106	8.72957477248305E-53	8	BCLAF3
6.05778628840828E-76	0.568727233529783	0.34	0.108	1.01516382621146E-71	8	AMD 1.00
9.98844583755512E-109	0.568304684204175	0.708	0.271	1.67386375345749E-104	8	NIPBL
3.70128890886532E-79	0.567717077697736	0.36	0.115	6.2026199534765E-75	8	TNIK
2.20377543256823E-69	0.567484973281802	0.411	0.15	3.69308686989784E-65	8	SLC2A1
7.97911052395772E-111	0.567446806434622	0.787	0.32	1.33713934160483E-106	8	TIA1
3.05946948204363E-86	0.567204866612509	0.559	0.212	5.12705895800871E-82	8	NEK9
1.4709495421438E-101	0.566967597013189	0.762	0.321	2.46501724272458E-97	8	CGGBP1
2.60232861996685E-84	0.566586315236078	0.478	0.17	4.36098230134045E-80	8	ACTR3C
1.87756531592475E-70	0.566445045008277	0.36	0.122	3.14642395642669E-66	8	DNAJC10
1.52205027083598E-74	0.566352828049469	0.326	0.102	2.55065184386694E-70	8	CWC27
4.31865702131402E-84	0.566250870734933	0.502	0.185	7.23720543631803E-80	8	RPTOR
2.44070188056263E-105	0.565976235603114	0.715	0.281	4.09012821144686E-101	8	CNOT6L

2.29463693592344E-89	0.565895817699176	0.487	0.171	3.84535257722051E-85	8	ABL1
3.02891032532785E-87	0.56573526288142	0.559	0.211	5.07584792318441E-83	8	PLPP2
6.58368987526126E-99	0.565697021951453	0.792	0.333	1.10329474929628E-94	8	ECPAS
1.61940774103963E-92	0.565656416159578	0.47	0.158	2.71380349243421E-88	8	C17orf75
5.31523849163691E-48	0.565574989185892	0.258	0.091	8.90727666428513E-44	8	POMT1
4.18404993183931E-95	0.565538162074153	0.471	0.156	7.01163087577631E-91	8	MBTD1
7.26114483418896E-83	0.565495089860358	0.419	0.141	1.21682265131339E-78	8	CORO7
1.06671600054279E-111	0.565451520864081	0.839	0.35	1.78760267370961E-107	8	SMARCA4
1.83888125019412E-100	0.565180913149222	0.598	0.219	3.0815971990753E-96	8	SYPL1
2.11320417395648E-94	0.564932141685657	0.461	0.153	3.54130755471627E-90	8	SEPTIN10
2.90909263398138E-103	0.564842972993566	0.615	0.226	4.8750574360226E-99	8	PTDSS1
4.03779187667713E-116	0.56480808990962	0.586	0.196	6.76653162693554E-112	8	TAF11
1.31630929133742E-77	0.564745258682812	0.905	0.454	2.20587111042325E-73	8	KLF5
1.83882557102775E-90	0.564301039542105	0.647	0.255	3.08150389192831E-86	8	UBE2G2
3.06738616245245E-95	0.564190951590577	0.562	0.205	5.14032573103782E-91	8	SNX14
1.50476644796281E-110	0.563908998326648	0.736	0.288	2.52168761349608E-106	8	PAFAH1B1
4.71028146885042E-103	0.563880041076729	0.658	0.25	7.89348968549954E-99	8	MTA1
5.96002197161842E-101	0.563859892970444	0.692	0.277	9.98780482003815E-97	8	PTBP1
2.56352079157155E-47	0.563559065773646	0.282	0.104	4.29594814251561E-43	8	URB1
3.70209165965926E-81	0.563498683311195	0.466	0.168	6.20396520325698E-77	8	GABPB2
1.40324145039793E-62	0.5633331663122	0.364	0.131	2.35155202257685E-58	8	RALGPS1
1.64679803214192E-57	0.563066826998853	0.301	0.104	2.75970414226344E-53	8	CHFR
5.58597989157915E-61	0.562829164514115	0.348	0.124	9.36098510230834E-57	8	DEPRECATED-ENSG00000185619
4.05577799657596E-80	0.562794678396063	0.971	0.626	6.796672766662E-76	8	HNRNPA2B1
4.14852451884292E-73	0.562687518789126	0.403	0.143	6.95209738867697E-69	8	DNAJB14
2.53034299303838E-106	0.562564872900815	0.786	0.316	4.24034878773372E-102	8	SENP6
3.12121626886917E-90	0.562348536451523	0.252	0.059	5.23053422337095E-86	8	IGFLR1
2.04578432877816E-95	0.560354386038876	0.941	0.452	3.42832537816645E-91	8	LENG8
6.46979756765865E-96	0.560130172194888	0.453	0.148	1.08420867638824E-91	8	SPOUT1
4.45664882355456E-111	0.560123563536091	0.621	0.22	7.46845209851274E-107	8	C2orf68
1.20057698233972E-55	0.560014540218093	0.854	0.487	2.0119269070049E-51	8	DEPRECATED-ENSG00000132002
2.13809548528335E-84	0.559595619213065	0.712	0.303	3.58302041423783E-80	8	EEF2K
5.97416647834001E-96	0.559098935888068	0.754	0.31	1.00115081844022E-91	8	FBXL17
2.47741454531999E-70	0.559095029067449	0.334	0.108	4.15165129504724E-66	8	NEPRO
3.19439848093991E-69	0.558969032350239	0.33	0.109	5.3531729743591E-65	8	CRAMP1
5.92804763860081E-117	0.558891201617719	0.82	0.329	9.93422223276724E-113	8	OTULIN
4.91567834314997E-110	0.557495905398982	0.617	0.219	8.23769376745072E-106	8	CUL3
2.05700611491729E-96	0.557172802398492	0.443	0.142	3.44713084737839E-92	8	MEIS2
2.31949707698804E-107	0.557031262177783	0.607	0.217	3.88701320161656E-103	8	HTT
3.72920239025862E-103	0.556845522721954	0.609	0.22	6.24939736559539E-99	8	BTF3L4
4.25505551094988E-88	0.55657775549224	0.787	0.34	7.13062202524981E-84	8	C1GALT1C1
2.95051073777243E-86	0.556524175852554	0.413	0.135	4.94446589435904E-82	8	UBE3B
2.60223358822149E-108	0.556196177662208	0.593	0.207	4.36082304714157E-104	8	DLST
7.24689053970285E-91	0.554954920447153	0.441	0.144	1.2144339166434E-86	8	ARID2
4.74837303903477E-93	0.554676929343707	0.561	0.205	7.95732353881447E-89	8	RHOT1
5.9584508146344E-81	0.554529349732393	0.443	0.156	9.98517187516433E-77	8	AKAP11
1.16200916074376E-103	0.554437384394571	0.728	0.292	1.94729495157439E-99	8	EWSR1
1.4750197089893E-68	0.554350495228838	0.269	0.079	2.47183802832427E-64	8	TP53
5.34043489863126E-92	0.554182159216923	0.726	0.299	8.94950080312626E-88	8	BIVM
4.67812454333797E-90	0.554109708566051	0.481	0.168	7.83960110972577E-86	8	CHKB
1.14634511043998E-45	0.55393948942629	0.298	0.114	1.92104513607531E-41	8	ZNF552
2.22970526151968E-108	0.553684978782404	0.702	0.274	3.73654007725467E-104	8	ENPP1
3.62031309709164E-116	0.553505156655084	0.567	0.188	6.06692068810617E-112	8	INTS4
3.30965476027325E-66	0.553274262416976	0.252	0.072	5.54631944726591E-62	8	ZC3H8
1.78146912354088E-77	0.553268536361489	0.361	0.117	2.98538595722981E-73	8	DEPRECATED-ENSG00000054118
2.16286088676507E-65	0.552964766208908	0.293	0.093	3.6245222740409E-61	8	FMO4
4.38770438301053E-60	0.552476458752085	0.345	0.125	7.35291500504905E-56	8	WDR19
1.84451289094712E-96	0.552402161754698	0.647	0.246	3.09103470264919E-92	8	APPL2
1.12572166724105E-86	0.552266554443591	0.593	0.228	1.88648436996254E-82	8	SUSD6
3.90477498324662E-92	0.552233870651445	0.702	0.297	6.54362191692469E-88	8	SMS

3.30912624766761E-89	0.551949437103669	0.585	0.221	5.54543376584138E-85	8	FAM172A
4.77837499661628E-86	0.55172412281393	0.377	0.117	8.00760081932956E-82	8	DIS3L2
1.61719169811428E-91	0.5515187648521	0.674	0.268	2.71008984769991E-87	8	EPPK1
5.4316610575898E-51	0.551175283993896	0.964	0.629	9.10237760030899E-47	8	TSC22D3
1.07704249475826E-97	0.550867961607119	0.671	0.263	1.80490781271589E-93	8	HOOK3
1.50253462724215E-99	0.550865953554741	0.611	0.224	2.5179475283324E-95	8	PRPF4B
2.60683983681853E-95	0.550783991417247	0.79	0.351	4.3685421985405E-91	8	BAX
7.65894587837428E-69	0.550196811476976	0.433	0.163	1.28348615029796E-64	8	VPS8
9.12818762624539E-91	0.549554176939773	0.83	0.361	1.5297016824062E-86	8	MCTS1
5.18548967840539E-73	0.549413060271318	0.932	0.501	8.68984360307175E-69	8	MORF4L2
2.22909045000928E-86	0.54939229002053	0.531	0.197	3.73550977612556E-82	8	ZNF608
2.86758981112338E-91	0.549215875942392	0.664	0.265	4.80550700548056E-87	8	UQCC1
6.50782798836248E-62	0.549086367530945	0.43	0.168	1.09058181428978E-57	8	TEP1
5.32383114982414E-89	0.549007039875053	0.393	0.124	8.92167624087529E-85	8	ACD
1.07125642960105E-85	0.548116802940849	0.759	0.344	1.79521152472545E-81	8	SGK2
1.7225888942421E-84	0.548076978397943	0.41	0.135	2.88671446897091E-80	8	HCFC2
3.65680684300273E-80	0.54806633824759	0.454	0.163	6.12807690750397E-76	8	CHD8
1.05925750449505E-92	0.548039227219778	0.473	0.16	1.77510372603281E-88	8	FARP2
6.36783112151558E-103	0.547974189829573	0.65	0.247	1.06712113934358E-98	8	WRNIP1
1.01469704060017E-77	0.547560240509347	0.313	0.093	1.70042930063776E-73	8	DEPRECATED-ENSG00000071894
1.74449703269817E-86	0.547341557401916	0.668	0.278	2.9234281273956E-82	8	MAPK1IP1L
1.14770001232587E-58	0.547242757006674	0.294	0.099	1.92331568065569E-54	8	RSBN1
9.37508244660558E-99	0.546896827193606	0.771	0.323	1.57107631640216E-94	8	MARC1
4.55193061157784E-99	0.546895827851609	0.537	0.188	7.62812531888215E-95	8	NSUN6
4.30516996810003E-103	0.546578474009906	0.505	0.167	7.21460383254203E-99	8	MAP1S
6.12314814130947E-100	0.546356448086279	0.76	0.323	1.02611716552064E-95	8	TMEM238
8.48329065667008E-93	0.546317820086926	0.796	0.344	1.42162984824477E-88	8	SLC25A33
5.65099673905086E-111	0.546266150882705	0.651	0.237	9.46994033530143E-107	8	IMMT
3.32604272676669E-84	0.546238474613691	0.369	0.115	5.57378240151561E-80	8	PPP1R14B
6.50916653746765E-70	0.545782869721759	0.306	0.096	1.09080612834883E-65	8	EIF2AK3
4.72439899259811E-55	0.545703120456783	0.284	0.097	7.91714783179592E-51	8	MEGF8
5.99044588870716E-98	0.545606660567341	0.57	0.205	1.00387892202955E-93	8	ZC3H14
2.89651422024184E-101	0.545466651541428	0.606	0.22	4.85397853028127E-97	8	LETMD1
1.84374204083353E-78	0.545430509721378	0.523	0.201	3.08974291202883E-74	8	RCAN1
1.93948766452165E-68	0.545291433333973	0.41	0.15	3.25019342820538E-64	8	GLCE
8.69489953289364E-99	0.545216607437596	0.652	0.255	1.45709126372232E-94	8	TECR
2.27760559939861E-100	0.545150737770898	0.462	0.148	3.81681146347218E-96	8	LRRC40
4.06314743846521E-90	0.544701188731561	0.66	0.266	6.80902247737999E-86	8	ARHGAP5
1.53478010567991E-58	0.544328451110696	0.26	0.082	2.57198450109839E-54	8	DCAF17
7.38304731201808E-95	0.544166842325714	0.706	0.284	1.23725106854799E-90	8	PCED1A
6.20769229642963E-85	0.543965902621149	0.378	0.119	1.04028507503568E-80	8	CMSS1
6.39457557809991E-62	0.543834622197463	0.316	0.107	1.07160297537798E-57	8	MIOS
7.94079147237372E-92	0.543570960346905	0.442	0.145	1.33071783494039E-87	8	FOXK1
1.40013635302091E-71	0.543512343909033	0.398	0.14	2.34634850039244E-67	8	ZZEF1
3.59947219296865E-93	0.543401579285263	0.839	0.37	6.03199550097686E-89	8	FAM199X
1.31810471361267E-64	0.543357820727238	0.25	0.073	2.20887987907212E-60	8	EFL1
3.11906101720294E-69	0.543168606952237	0.425	0.157	5.22692245262869E-65	8	RNF217
4.58735181088847E-71	0.543143313176979	0.316	0.099	7.6874841646869E-67	8	REXO1
1.07106293863131E-105	0.543022321407785	0.794	0.327	1.79488727255835E-101	8	FAM193B
9.0102379528783E-72	0.542793903719686	0.364	0.123	1.50993567614335E-67	8	OSBPL11
6.1342096044373E-53	0.54243766232185	0.304	0.109	1.0279708455116E-48	8	SPIN3
5.30091684683333E-66	0.542059620665124	0.336	0.113	8.8832764519233E-62	8	WDR70
6.36319833600556E-88	0.541897754463006	0.402	0.129	1.06634477714781E-83	8	KLHL7
8.64725777388746E-90	0.541807661414001	0.515	0.185	1.44910745774806E-85	8	YTHDF2
3.57520859547724E-89	0.54131941547515	0.623	0.245	5.99133456430076E-85	8	TENT2
3.50554809413948E-95	0.541266275713185	0.507	0.176	5.87459749615894E-91	8	MBD4
8.44523877006962E-71	0.540521720285278	0.899	0.429	1.41525311308827E-66	8	DEGS1
1.19797145862567E-80	0.540123980259593	0.469	0.168	2.00756057036491E-76	8	PTGR2
1.23512623217527E-79	0.54011041381607	0.487	0.179	2.06982453987932E-75	8	CUL2
4.76396696116991E-92	0.540001486922394	0.53	0.191	7.98345583352853E-88	8	RPRD1B

1.27123040958268E-69	0.539991351311607	0.932	0.485	2.13032792037866E-65	8	ZBTB7B
2.4776244102725E-79	0.539981750009602	0.481	0.177	4.15200298673466E-75	8	MTERF2
2.84156924398288E-94	0.539847225581357	0.711	0.287	4.76190173906651E-90	8	ZNF410
3.26256883098235E-98	0.539759027369431	0.615	0.23	5.46741284696023E-94	8	ADGRA3
2.29123416703897E-77	0.538993959038007	0.442	0.159	3.8396502171239E-73	8	PLCL2
8.11116670430166E-87	0.538694028536222	0.51	0.183	1.35926931630687E-82	8	TRIP11
4.8175201172801E-63	0.538612928819889	0.313	0.105	8.07320021253798E-59	8	RALGAPA1
6.06382363960362E-89	0.538404204951416	0.585	0.225	1.01617556552477E-84	8	SOAT1
1.41979673254558E-83	0.538327843149011	0.519	0.195	2.37929536439988E-79	8	ADCK5
1.77651262288842E-102	0.538041880070292	0.602	0.218	2.97707985343641E-98	8	PURB
4.59123754844167E-73	0.537770227977525	0.369	0.124	7.69399588367856E-69	8	ELP4
6.72650387388199E-91	0.537711047391447	0.546	0.2	1.12722751918514E-86	8	ZNF655
5.94367770295858E-81	0.537537734647467	0.454	0.161	9.96041509461798E-77	8	ERGIC2
2.91316694399724E-87	0.537149237497005	0.521	0.189	4.88188516475058E-83	8	INPP1
2.08645306070828E-64	0.537081499673816	0.314	0.104	3.49647803913493E-60	8	MSH3
2.37275390605704E-74	0.536962196330222	0.389	0.132	3.9762609957704E-70	8	ZNF263
6.70647408417278E-60	0.536814495676609	0.963	0.581	1.12387092702568E-55	8	DNM2
5.84182484033903E-113	0.536528402698245	0.622	0.219	9.78973006744015E-109	8	ROCK1
2.27954415066627E-97	0.535807667717203	0.726	0.29	3.82006008768654E-93	8	PI4KB
8.12838153508354E-67	0.53574282089471	0.415	0.155	1.3621541776493E-62	8	LATS2
7.83163466140946E-115	0.53571531101939	0.517	0.163	1.312425336559E-110	8	CEP63
1.23704150216374E-119	0.5356972183917	0.734	0.277	2.07303414932599E-115	8	PRPSAP1
1.6861000469143E-82	0.53550529180645	0.557	0.214	2.82556645861899E-78	8	MED23
1.93001834713E-56	0.53498702486933	0.258	0.083	3.23432474612045E-52	8	ZBTB8OS
2.76617414959311E-63	0.53476899941312	0.368	0.134	4.63555463988814E-59	8	DEPRECATED-ENSG00000072803
1.13613668892795E-100	0.534614488847256	0.586	0.21	1.90393786330546E-96	8	TOR1A
1.01874618801504E-89	0.53441306283532	0.82	0.367	1.70721486187561E-85	8	SFT2D2
1.69756020240072E-109	0.534222228813645	0.614	0.218	2.84477138718313E-105	8	TERF2
3.05882229026044E-70	0.534159766094	0.394	0.14	5.12597439401844E-66	8	CMTR2
5.34211121775001E-96	0.533770142735828	0.756	0.315	8.95230997870547E-92	8	ZNF638
4.17332377881102E-63	0.533696578546274	0.277	0.087	6.9936559885315E-59	8	FES
3.32489967080956E-86	0.533575605441758	0.538	0.199	5.57186686834266E-82	8	PCGF3
1.03955685211913E-88	0.53351441637141	0.867	0.413	1.74208937278125E-84	8	HNRNPA3
3.22342499597614E-88	0.533014579831771	0.421	0.137	5.40181560825681E-84	8	SS18L2
3.80284419718308E-67	0.532653772019296	0.26	0.076	6.3728063056394E-63	8	LRRC20
1.30005746824192E-69	0.532652008515012	0.481	0.187	2.1786363052798E-65	8	GEMIN7
2.5957870836049E-104	0.532511682116908	0.585	0.206	4.35001999470509E-100	8	SNX24
1.79353820974249E-111	0.532447054045492	0.652	0.236	3.00561133188646E-107	8	RUFY3
1.37948702669343E-107	0.531932611480997	0.727	0.281	2.31174435933284E-103	8	USP14
6.27847827863884E-97	0.531617467432833	0.519	0.181	1.0521473899343E-92	8	SLC39A3
5.30650791060047E-90	0.531452504763504	0.752	0.319	8.89264595658427E-86	8	INTS3
1.22964358997252E-100	0.531433315252681	0.438	0.136	2.06063672807595E-96	8	MEX3D
4.04366113945827E-74	0.531419898946714	0.306	0.093	6.77636733750417E-70	8	FASTKD5
1.0029290724732E-95	0.531331181034262	0.463	0.154	1.68070853965058E-91	8	UBE2M
1.04076973175957E-68	0.531275496616385	0.366	0.127	1.74412191648268E-64	8	KDM3A
1.76025924951555E-82	0.531262324141736	0.437	0.152	2.94984245033816E-78	8	TANGO2
2.3870795790572E-82	0.531148386493914	0.87	0.403	4.00026795858405E-78	8	RNF103
2.09888715074885E-77	0.531014934228217	0.387	0.13	3.51731508722492E-73	8	POLR3C
6.75537081197435E-49	0.530873855071686	0.27	0.096	1.13206504067066E-44	8	PRRG1
1.02065362510967E-112	0.530668160645627	0.654	0.234	1.71041134495879E-108	8	ACBD6
4.23669526784148E-95	0.530489034410649	0.663	0.264	7.09985392984876E-91	8	GET1
3.7671983389057E-59	0.530041641051403	0.928	0.536	6.31307097633817E-55	8	TOR1AIP2
4.22052250526653E-101	0.529843754915758	0.403	0.119	7.07275161432564E-97	8	P3H1
2.38928458153861E-81	0.529778180256838	0.381	0.123	4.0039631017424E-77	8	ZBED3
2.56547850329452E-73	0.529548106670521	0.893	0.432	4.29922887582096E-69	8	SMOC1
3.03193780660697E-63	0.529367971747677	0.306	0.101	5.08092137631196E-59	8	GGPS1
1.30140799642588E-110	0.529248785215385	0.579	0.199	2.18089952041048E-106	8	MBD3
3.53155065789814E-57	0.528770951006567	0.286	0.096	5.91817259250571E-53	8	ELMOD3
4.10493660279259E-98	0.528349966673959	0.619	0.234	6.87905275895982E-94	8	NELFA
3.43376073018106E-87	0.528089402314419	0.43	0.142	5.75429623163742E-83	8	STAM2

2.45094479735648E-75	0.528067064170558	0.923	0.496	4.10729329140999E-71	8	NDUFV2
2.4132813618198E-62	0.527940978355931	0.33	0.114	4.04417690613762E-58	8	UFSP2
7.25462402461442E-71	0.527924345565514	0.405	0.145	1.21572989404488E-66	8	COX19
3.38196004392414E-76	0.527680347018031	0.431	0.153	5.66748864160808E-72	8	MLLT10
2.6083817215677E-99	0.527478383440585	0.605	0.223	4.37112608900316E-95	8	ZYG11B
2.65549775896828E-90	0.527419491768926	0.581	0.22	4.45008314447905E-86	8	SIN3B
1.23598642698531E-84	0.52709321469843	0.426	0.143	2.07126605434199E-80	8	C3orf38
2.50321459279733E-92	0.527021232507445	0.458	0.153	4.19488701460977E-88	8	ATE1
1.72493662622047E-99	0.526652299007682	0.561	0.199	2.89064879822026E-95	8	UBQLN4
8.17669384030271E-110	0.526600957214969	0.551	0.185	1.37025035375793E-105	8	MTMR12
9.80972375914376E-89	0.526538454475334	0.577	0.218	1.64391350755731E-84	8	TBCE.1
5.95553690277671E-92	0.526425778141711	0.752	0.325	9.98028874167321E-88	8	TENT5A
7.67169994207745E-78	0.526364962829732	0.547	0.218	1.28562347629334E-73	8	SRD5A3
4.78274995076183E-83	0.526135060624653	0.352	0.108	8.01493236748668E-79	8	COIL
1.58790787985854E-74	0.526121652800154	0.317	0.098	2.66101602506694E-70	8	MOB1A
1.57179637195496E-52	0.525854294742489	0.272	0.093	2.63401636012213E-48	8	TMEM41A
1.27647853575554E-93	0.525394879389464	0.82	0.375	2.13912273021913E-89	8	ARF6
2.4297931109443E-99	0.524966384126662	0.635	0.243	4.07184729532045E-95	8	MCMBP
2.1167615432794E-105	0.524700678621759	0.591	0.21	3.54726899422762E-101	8	YY1AP1
7.16855240542273E-82	0.524553163066389	0.495	0.183	1.20130601210074E-77	8	SLC35B4
1.63828370452943E-44	0.52448690532771	0.308	0.122	2.74543583205042E-40	8	ZNF248
6.71569168154151E-52	0.52427419532483	0.322	0.12	1.12541561199273E-47	8	TJAP1
1.99765401858911E-59	0.524086454809118	0.26	0.081	3.34766860435162E-55	8	SMARCAL1
2.12468597166363E-82	0.524029835816007	0.361	0.113	3.5605487513139E-78	8	CRY1
8.79982637696239E-99	0.523885558255882	0.654	0.251	1.47467490425136E-94	8	WWC2
3.37449556121822E-89	0.523459680172823	0.454	0.153	5.65497966148949E-85	8	MTOR
9.50026719705284E-96	0.523414929657147	0.647	0.25	1.59205477688211E-91	8	IFNGR1
2.44858044510494E-69	0.523383908941435	0.318	0.102	4.10333110990686E-65	8	BORCS5
4.18022845370388E-93	0.52313683624884	0.676	0.266	7.00522684271696E-89	8	APOOL
3.0174792820891E-100	0.523091482853085	0.599	0.216	5.05669178092492E-96	8	SGPP1
3.28001232303721E-66	0.522949196792057	0.368	0.13	5.49664465094575E-62	8	NR2C1
8.23013445174936E-95	0.522937425886347	0.742	0.304	1.37920593142416E-90	8	NREP
1.44711773957041E-105	0.522492147430971	0.648	0.242	2.4250799079721E-101	8	NUDT21
4.99753610073214E-125	0.522105283445576	0.704	0.253	8.37487099760692E-121	8	CIC
6.65985917729416E-92	0.52165488821944	0.505	0.178	1.11605920093096E-87	8	FOPNL
3.27890232132216E-65	0.521515406590837	0.278	0.086	5.49478451007168E-61	8	ETV1
1.10428732307745E-64	0.521468590925117	0.421	0.161	1.85056469601319E-60	8	TRAPPC8
9.02350926692233E-92	0.521347838644189	0.714	0.291	1.51215968295084E-87	8	KDM6A
1.02452332965397E-86	0.521197675001841	0.498	0.179	1.71689619583413E-82	8	NPHP3
8.62462288924899E-101	0.52114566965304	0.76	0.315	1.44531430378035E-96	8	UBE3C
1.20180757402946E-84	0.520742030359498	0.47	0.165	2.01398913255857E-80	8	NOL11
1.15101189050673E-64	0.520608174195564	0.304	0.099	1.92886572611118E-60	8	RFT1
2.87235517681718E-76	0.520452947326054	0.389	0.131	4.81349280531023E-72	8	SMURF2
4.46538972948338E-103	0.519994643148225	0.567	0.2	7.48310010866825E-99	8	ST7
1.66340401074825E-96	0.519909687315508	0.495	0.168	2.78753244121191E-92	8	APEX2
4.29762787800273E-79	0.519840509794618	0.381	0.125	7.20196479795697E-75	8	GGA3
2.74589337370319E-82	0.519627667609602	0.819	0.367	4.6015681156518E-78	8	DEPRECATED-ENSG00000057294
1.56734455832983E-79	0.519375953772368	0.418	0.144	2.62655601084913E-75	8	MANBA
6.84269747729993E-76	0.519147344726968	0.447	0.163	1.14669924324592E-71	8	LMAN2L
7.87094101054426E-71	0.519068499247784	0.395	0.14	1.31901229454701E-66	8	EDC3
3.12405552867526E-95	0.518990531475865	0.378	0.111	5.235292254954E-91	8	GPN3
3.26945596022256E-106	0.518762150557913	0.465	0.145	5.47895429814096E-102	8	EIF4EBP1
7.13128859912724E-100	0.518602913942762	0.545	0.189	1.19506134344174E-95	8	ASCC3
4.84177832050052E-106	0.518359070794204	0.66	0.247	8.11385210949476E-102	8	HIPK1
3.83242636932896E-85	0.518329077853493	0.569	0.219	6.42238010972147E-81	8	UGGT1
4.67152501733089E-114	0.517615903787567	0.7	0.259	7.8285416240431E-110	8	ANAPC5
1.5034867958995E-70	0.517556058355156	0.503	0.2	2.51954317256839E-66	8	TMEM50B
4.38433174223441E-65	0.517265649522682	0.258	0.077	7.34726313363642E-61	8	DEPRECATED-ENSG00000150867
5.09625268929089E-69	0.517116923595181	0.373	0.129	8.54030025671367E-65	8	PELI1
2.48290429471949E-100	0.516700704914062	0.707	0.276	4.16085101709092E-96	8	SENP2

4.93940619133491E-72	0.516652071371509	0.389	0.136	8.27745689543905E-68	8	C9orf152
6.91933157621632E-114	0.516488901641916	0.635	0.223	1.15954158554233E-109	8	DR1
2.73961088160001E-88	0.516438557438864	0.692	0.285	4.5910399153853E-84	8	ABCA5
3.37292191777721E-101	0.516092589203627	0.473	0.152	5.65234254981105E-97	8	SENP5
8.77350643532665E-95	0.516057326525394	0.498	0.17	1.47026420843204E-90	8	MED1
7.77850592704572E-89	0.515978098915322	0.419	0.137	1.30352202325432E-84	8	RNF8
6.8622104676875E-105	0.515938415992359	0.558	0.193	1.14996923017507E-100	8	EXOC4
9.58632773113904E-70	0.515693103950402	0.357	0.121	1.60647680118428E-65	8	TARDBP
3.42737727491004E-111	0.51542176892757	0.622	0.222	5.74359883729424E-107	8	HDAC6
6.28660163951983E-89	0.515401688018936	0.85	0.393	1.05350870275073E-84	8	KANK1
4.90238087354718E-97	0.515119982094553	0.478	0.159	8.21540986789037E-93	8	C4orf19
1.65978925980168E-95	0.515065524681195	0.497	0.169	2.78147484157566E-91	8	HDAC8
1.25778381193559E-107	0.514825121270904	0.451	0.137	2.10779411204166E-103	8	PDCL
2.3040934686918E-51	0.514649283168971	0.318	0.119	3.86119983483373E-47	8	SRCIN1
3.30989383808573E-64	0.514574569252858	0.269	0.082	5.54672009386406E-60	8	RAD9A
3.89004686213553E-103	0.514343540253443	0.578	0.206	6.51894053156673E-99	8	ACOT7
4.08824474152996E-84	0.51428484805564	0.727	0.31	6.8510805378559E-80	8	DHX9
5.36698304166448E-64	0.514003467912727	0.32	0.107	8.99399018122133E-60	8	PRDM2
9.17734782193672E-90	0.513971686630976	0.602	0.232	1.53793994800016E-85	8	CNNM3
2.19324637391573E-80	0.513902004073746	0.889	0.467	3.67544227340798E-76	8	HTATSF1
4.62929861356097E-93	0.513704402036742	0.566	0.21	7.75777861660547E-89	8	CERT1
1.08197970513676E-77	0.513667508728099	0.345	0.108	1.81318158986819E-73	8	ZNF274
1.70229967580183E-101	0.51351162181005	0.594	0.216	2.85271379670871E-97	8	CORO1C
1.67653057996646E-61	0.513387378629547	0.94	0.511	2.80952994590779E-57	8	NFIB
1.07609967691937E-68	0.513291040531824	0.329	0.108	1.80332783858149E-64	8	RUFY2
3.31326081710289E-104	0.513247456165407	0.644	0.242	5.55236247730102E-100	8	RAP2A
3.87442751596999E-81	0.513022361948506	0.294	0.082	6.4927656312625E-77	8	TEX10
8.28018770679435E-62	0.512989023077574	0.301	0.1	1.3875938559046E-57	8	CUL9
1.57055562014916E-71	0.512951634409768	0.943	0.553	2.63193710824597E-67	8	EMD
7.07800216073993E-95	0.512673187790124	0.694	0.276	1.1861316020968E-90	8	SH3RF1
8.57267984588243E-83	0.512641524094932	0.887	0.434	1.43660968857298E-78	8	VBP1
6.21186428662927E-97	0.512626485740319	0.581	0.21	1.04098421715333E-92	8	NAE1
4.72256898840366E-84	0.512551415627428	0.786	0.338	7.91408111076686E-80	8	TMEM164
1.24946480164837E-88	0.512532575692722	0.483	0.17	2.09385311460233E-84	8	PAK4
3.43043772354987E-89	0.512140065474163	0.858	0.372	5.74872753712488E-85	8	PVR
1.23934029145736E-79	0.512054247014956	0.365	0.117	2.07688646042425E-75	8	HLX
4.0806839565273E-88	0.511956011276555	0.703	0.295	6.83841017434845E-84	8	TRPC4AP
5.22947832049224E-98	0.511565231346186	0.59	0.216	8.76355976948089E-94	8	TCAIM
1.50022262179599E-99	0.51153801888149	0.682	0.264	2.51407306960573E-95	8	VHL
6.46405589052505E-75	0.511401240068905	0.382	0.13	1.08324648613419E-70	8	ZNF830
1.24311994872702E-93	0.510786560147505	0.791	0.347	2.08322041007673E-89	8	TBL1XR1
2.44240379833007E-102	0.510685389160309	0.683	0.265	4.09298028524153E-98	8	TNRC18
6.66670706887661E-80	0.510491365571428	0.459	0.165	1.11720677060234E-75	8	KLHL8
1.08355089636275E-80	0.510394414909838	0.561	0.219	1.8158145921247E-76	8	WWP2
5.59926312649121E-63	0.510037797390049	0.293	0.095	9.38324514737397E-59	8	DEPRECATED-ENSG00000068305
1.58620047667853E-97	0.510005428660668	0.582	0.213	2.65815475881789E-93	8	HMGN4
1.08058586655541E-53	0.50944470370074	0.294	0.104	1.81084579517355E-49	8	FITM2
1.25517254706561E-70	0.509185229455142	0.366	0.125	2.10341815437255E-66	8	RUSC2
1.07684959986793E-84	0.508967703819749	0.45	0.155	1.80458455945867E-80	8	SEPSECS
1.58466103995042E-118	0.508966680034898	0.699	0.256	2.65557497074891E-114	8	STRN4
3.38593171614786E-82	0.508319711227613	0.55	0.212	5.67414436992059E-78	8	PHC3
1.71589076363843E-62	0.508305189097833	0.368	0.134	2.87548974170527E-58	8	BICRA
7.87611946494376E-87	0.50828239877586	0.389	0.123	1.31988009993528E-82	8	GTF3C2
1.13389423012824E-77	0.5077352655015	0.365	0.118	1.90017995084891E-73	8	RNF214
7.62171911950794E-82	0.50762024418626	0.502	0.187	1.27724769004714E-77	8	CEBPZ
2.83536016889105E-88	0.507284700734533	0.723	0.312	4.75149657102763E-84	8	ATF6B
1.1159345909222E-85	0.50720461843105	0.726	0.309	1.87008318746742E-81	8	TTC37
5.89092813153423E-113	0.50645572625975	0.636	0.227	9.87201736282507E-109	8	PIN1
5.07199582555878E-104	0.506126051594937	0.414	0.122	8.4996506044714E-100	8	NT5C2
2.20325493616743E-91	0.505765926250448	0.381	0.115	3.69221462202938E-87	8	ZFYVE1

3.00375316900275E-86	0.505678405681185	0.625	0.249	5.03368956061481E-82	8	SLC37A1
1.51790373095076E-90	0.505642178308119	0.593	0.224	2.54370307232729E-86	8	LARP4B
1.53888583456677E-88	0.505621212206597	0.784	0.354	2.57886488156699E-84	8	HNRNPAB
4.63953571359691E-49	0.505573462483285	0.31	0.118	7.77493394884571E-45	8	TRMT61A
2.45796961321823E-80	0.505519034444925	0.406	0.135	4.11906547783111E-76	8	DENND1B
2.22240762579616E-78	0.505327056299335	0.383	0.128	3.72431069930921E-74	8	TMED8
3.68612368986953E-68	0.505122748068994	0.84	0.423	6.17720607948335E-64	8	NBL1
2.29592314234871E-97	0.505031595071564	0.541	0.191	3.84750800194798E-93	8	CCT5
1.00146656689518E-64	0.504989523730316	0.269	0.082	1.67825767280294E-60	8	WDR48
2.3539511216484E-94	0.504608143359191	0.723	0.293	3.94475128965838E-90	8	PDE4DIP
2.18619170943709E-72	0.5043983232935	0.318	0.1	3.66362006667467E-68	8	RABIF
8.81246974489039E-94	0.504370094325556	0.554	0.202	1.47679367984873E-89	8	P4HA2
9.07269126647294E-89	0.503938512411599	0.826	0.373	1.52040160243554E-84	8	FAM91A1
2.02089374387021E-71	0.503528011475812	0.933	0.453	3.3866137359777E-67	8	IVNS1ABP
6.53790261033133E-83	0.503428050427728	0.431	0.147	1.09562171943932E-78	8	MEF2A
8.81525029575491E-105	0.503272025794362	0.667	0.25	1.47725964456261E-100	8	NR3C1
3.1805845182434E-74	0.503103813169927	0.904	0.451	5.33002353567229E-70	8	SLC38A10
1.79464965084492E-58	0.503094776647899	0.261	0.083	3.00747388488591E-54	8	ANKIB1
1.3886432988116E-99	0.50271844849767	0.594	0.22	2.32708844014848E-95	8	DEPRECATED-ENSG00000099797
5.0539043103241E-80	0.502252867545883	0.627	0.259	8.46933284324113E-76	8	STRN3
1.84691441489837E-79	0.50147186744013	0.454	0.162	3.09505917648669E-75	8	FRS2
2.27892583682963E-98	0.501461569112168	0.59	0.215	3.81902391735909E-94	8	NUP42
9.77052880133005E-66	0.501421770379966	0.955	0.564	1.63734521652689E-61	8	VMP1
2.52578188402453E-96	0.501402933825398	0.565	0.203	4.23270528124831E-92	8	CDK14
2.91534529712925E-78	0.50129468409883	0.41	0.142	4.8855356489292E-74	8	SPTLC1
2.64730356165495E-96	0.500667431032399	0.651	0.254	4.43635130862136E-92	8	SAMD4B
4.49311929996955E-93	0.500393437255236	0.756	0.321	7.52956932288898E-89	8	SMAD3
4.10092795266267E-101	0.500236216969514	0.598	0.217	6.87233506307211E-97	8	PIK3CB
5.2806118598605E-85	0.500179525475656	0.486	0.173	8.84924935475423E-81	8	RAB3GAP2
4.33015183595333E-101	0.499932697437786	0.543	0.188	7.25646844669058E-97	8	LSM14A
7.6894967457864E-73	0.499867607773012	0.395	0.138	1.28860586465889E-68	8	BLZF1
1.37413197107686E-85	0.49948628190061	0.429	0.144	2.3027703571306E-81	8	USP5
2.1959188372007E-123	0.49930335367989	0.437	0.12	3.67992078738094E-119	8	EPB41L2
1.33186064696182E-103	0.499261835376244	0.69	0.263	2.23193207217861E-99	8	RPRD2
4.74667656165587E-118	0.499083433164299	0.482	0.145	7.9544805820229E-114	8	MCOLN1
1.67054727255381E-68	0.498803087368265	0.26	0.074	2.79950311934568E-64	8	RNFT1
1.92926023776536E-94	0.498784164331881	0.658	0.255	3.23305430644719E-90	8	CAND1
2.54955769769605E-104	0.498619054428876	0.71	0.274	4.27254878979905E-100	8	SYMPK
2.05465004723719E-63	0.498496033051173	0.269	0.083	3.44318254916008E-59	8	HERC1
3.97259730891614E-76	0.498082434573974	0.423	0.149	6.65727857028166E-72	8	MED12
1.45797923072949E-85	0.498067804920045	0.621	0.25	2.44328159485647E-81	8	PURA
2.44902422342447E-65	0.497965159649896	0.353	0.123	4.10407479361473E-61	8	DRGX
5.78603405124622E-56	0.497577016063336	0.36	0.137	9.69623586307842E-52	8	MUC1
1.36794388879812E-52	0.497510842853119	0.383	0.156	2.29240036884789E-48	8	MROH6
1.86550261238599E-67	0.497494585448926	0.368	0.128	3.12620927783644E-63	8	ZNF397
2.28462419173305E-45	0.497478588150869	0.281	0.106	3.82857322050625E-41	8	CAPS
4.54961855179762E-62	0.497376030749169	0.262	0.081	7.62425076910245E-58	8	FAM222B
5.03080480096427E-78	0.497342395610543	0.804	0.357	8.43062268545593E-74	8	KCNK1
8.32984009689045E-60	0.497316444431815	0.288	0.095	1.3959146034369E-55	8	HOMEZ
4.5476989675404E-80	0.49729209856144	0.418	0.143	7.6210339298042E-76	8	DDX52
2.11865036702882E-54	0.49726097637387	0.337	0.125	3.5504342850669E-50	8	GALNT4
1.34291490315251E-73	0.49718460539875	0.395	0.136	2.25045679470297E-69	8	MYCBP2
7.89723723658998E-56	0.497140526900796	0.3	0.104	1.32341901610775E-51	8	ZCCHC7
2.72730036834906E-99	0.497092748223654	0.848	0.371	4.57040995727936E-95	8	LIMK2
7.04618238779772E-88	0.496957940857398	0.426	0.141	1.18079924454714E-83	8	ZDHHC16
1.07081241874149E-53	0.496853185887812	0.301	0.107	1.79446745132699E-49	8	PPP1R9A
2.37207657120086E-79	0.496787745357278	0.366	0.118	3.9751259180184E-75	8	ING5
1.63334814261644E-78	0.496786038972327	0.413	0.143	2.73716481739664E-74	8	PAPSS1
7.62412839277445E-90	0.496548524359901	0.471	0.163	1.27765143606114E-85	8	BRAT1
6.90175801647988E-73	0.495980571141803	0.334	0.107	1.1565966084017E-68	8	LCMT1

2.00052323786166E-79	0.495949312604447	0.33	0.1	3.35247684200858E-75	8	KCTD2
7.04133634409369E-63	0.495300370745972	0.269	0.083	1.17998714454322E-58	8	TIMM29
8.05764846094195E-58	0.49524584870541	0.506	0.222	1.35030072908465E-53	8	AGR2
1.73665492342527E-76	0.495173810609877	0.575	0.234	2.91028632067607E-72	8	IRF9
1.04880906445669E-80	0.495125551671825	0.395	0.131	1.75759423021651E-76	8	SNW1
4.26134397553738E-60	0.495030106028479	0.361	0.132	7.14116023420554E-56	8	ZNF862
6.83760025037704E-74	0.494748800597747	0.417	0.148	1.14584504995818E-69	8	SLC1A1
8.82354579446099E-83	0.494679044467185	0.531	0.199	1.47864980423577E-78	8	SLC30A5
2.67468470180314E-73	0.494654968278186	0.387	0.134	4.48223662328171E-69	8	DNAAF5
3.33996444348856E-103	0.493896251756659	0.574	0.202	5.59711241439813E-99	8	BRPF3
2.29804512408616E-87	0.49364135320768	0.595	0.231	3.85106401894358E-83	8	PRRC2B
6.07444714232398E-90	0.492872076982858	0.82	0.381	1.01795585211065E-85	8	IMPA2
8.41273044413035E-63	0.492550121760168	0.32	0.109	1.40980536782736E-58	8	ABHD17C
7.73798689640096E-108	0.492293846363301	0.655	0.24	1.29673184409887E-103	8	LSM 7.00
1.01470779896437E-64	0.492225713285705	0.332	0.113	1.70044732950449E-60	8	OPA3
1.92262682758057E-61	0.492115665941001	0.308	0.104	3.22193803765951E-57	8	ATG9A
1.09385134242E-84	0.491914177988233	0.743	0.316	1.83307607962744E-80	8	PRKAG2
1.78113442990456E-90	0.491622793927712	0.844	0.388	2.98482507763406E-86	8	MAPK1
3.17402530592898E-89	0.490869496826201	0.621	0.245	5.31903160767579E-85	8	MIER1
3.69267528361331E-95	0.490699652012812	0.621	0.235	6.18818524027919E-91	8	VIRMA
4.08389811646554E-65	0.490513066283907	0.289	0.092	6.84379646357296E-61	8	DTD1
9.31277023235475E-96	0.490274251292588	0.822	0.358	1.56063403553801E-91	8	CLK1
3.66074311942743E-83	0.490130331945842	0.486	0.175	6.13467331953648E-79	8	SOS 1
1.60576803778679E-93	0.490068698307245	0.718	0.29	2.6909460777231E-89	8	NKAP
3.16955862641454E-99	0.490020461567928	0.676	0.26	5.31154634614549E-95	8	MORC3
1.11151827392881E-80	0.489841676942317	0.391	0.129	1.8626823234499E-76	8	NFKBIL1
7.51646583829125E-72	0.489577323008188	0.353	0.117	1.25960934518085E-67	8	EXOSC3
1.6770317814947E-111	0.489462134597514	0.688	0.258	2.81036985942883E-107	8	TXLNA
3.54490148857569E-90	0.489242081702849	0.867	0.4	5.94054591455514E-86	8	CHTOP
9.1505163388561E-70	0.489149062373107	0.309	0.097	1.5334435280655E-65	8	TGFBRAP1
1.78316414240708E-102	0.489145919437925	0.627	0.233	2.98822646984579E-98	8	MPRIP
1.66405626069684E-100	0.488497668897274	0.557	0.195	2.78862548167576E-96	8	NFATC2
1.28330214134888E-61	0.488377973726839	0.333	0.115	2.15055772847245E-57	8	NICN1
9.78856255830737E-64	0.488288508051789	0.29	0.092	1.64036731352115E-59	8	ZNF780A
3.29311799362208E-66	0.487552624925583	0.298	0.095	5.51860713371188E-62	8	HILPDA
5.40434844061617E-76	0.487403766318107	0.474	0.176	9.05660711678457E-72	8	PFN2
6.39067048526261E-80	0.487187847228928	0.738	0.331	1.07094855992031E-75	8	ECE1
5.19979176298314E-91	0.487043341480575	0.494	0.173	8.71381103640715E-87	8	PCIF1
2.63164005479888E-66	0.48691609458849	0.385	0.139	4.41010240383196E-62	8	SLC22A5
2.47373007314629E-78	0.486742884506793	0.405	0.137	4.14547685657855E-74	8	SRC
4.69556988500558E-95	0.486682148929083	0.739	0.308	7.86883601329235E-91	8	EHMT1
3.56971547332717E-98	0.486605645411573	0.441	0.14	5.98212919020167E-94	8	EIF1AD
1.53516346495515E-92	0.486523096058942	0.569	0.208	2.57262693457184E-88	8	PPP1CC
2.50388588928685E-65	0.48647973278014	0.31	0.102	4.1960119732669E-61	8	IBA57
5.10331961047445E-72	0.486135189928834	0.847	0.428	8.55214300323309E-68	8	ACSL5
2.11928088014686E-82	0.486110806599503	0.851	0.397	3.5514908989501E-78	8	SLC44A1
8.14044087312511E-81	0.485637104481602	0.577	0.224	1.36417508151831E-76	8	ZNF91
1.29832046179719E-105	0.48560807546833	0.602	0.217	2.17572542987973E-101	8	SFN
3.96108994496519E-67	0.485228126709151	0.927	0.508	6.63799452977267E-63	8	AFDN
2.19809890803084E-71	0.485120223096827	0.387	0.136	3.68357415007808E-67	8	PACS2
1.63609179000073E-95	0.484962485501162	0.631	0.241	2.74176262168323E-91	8	SDR39U1
9.47638425267331E-86	0.484893381852598	0.455	0.157	1.58805247306299E-81	8	RBFA
5.5739532424622E-87	0.484730323249374	0.648	0.259	9.34083084371816E-83	8	FAM49B
1.77837708954561E-92	0.484674293591257	0.782	0.33	2.98020432666054E-88	8	PHF3
1.74093787077769E-71	0.484625016555208	0.318	0.1	2.91746368384925E-67	8	ZEB1
6.98385629875677E-57	0.484420699195018	0.88	0.504	1.17035463854566E-52	8	BTG1
1.28270108905728E-86	0.484125255115617	0.836	0.375	2.14955048504219E-82	8	SYBU
7.44407319024186E-103	0.48412150087313	0.644	0.238	1.24747778522073E-98	8	STK25
8.27790295850941E-72	0.483829723463487	0.366	0.124	1.38721097778701E-67	8	BBS2
5.4239067526557E-74	0.483739685760914	0.423	0.152	9.08938293610042E-70	8	YAF2

5.14495316833793E-79	0.483700294740655	0.438	0.153	8.62191251950071E-75	8	SLC35E2B
1.43762192901771E-103	0.483347234457476	0.628	0.232	2.40916682864788E-99	8	WDTC1
2.66821636805137E-63	0.483098359785529	0.277	0.087	4.47139698958049E-59	8	RRP7A
4.20391140973729E-106	0.482434596952143	0.615	0.221	7.04491474043774E-102	8	ZC3H18
3.13708192532248E-91	0.482423582643253	0.493	0.169	5.25712189045541E-87	8	TRAPPC6B
5.16343096743735E-81	0.482379654955596	0.754	0.347	8.6528776152315E-77	8	EIF4A3
1.73783456864949E-78	0.482290760120266	0.386	0.129	2.91226317014281E-74	8	RAE1
1.21302606888458E-85	0.482286414311535	0.666	0.273	2.03278908623678E-81	8	SEC24B
2.31675642195956E-96	0.482087758213773	0.582	0.214	3.88242041191982E-92	8	VAPB
2.04348336402335E-77	0.482081094242215	0.439	0.157	3.42446942143032E-73	8	ARHGAP26
1.36847816350871E-102	0.482032542260838	0.599	0.217	2.29329570640789E-98	8	MBD6
4.58381671879932E-45	0.481955114951864	0.282	0.106	7.6815600573639E-41	8	SLC25A45
1.04394745905965E-81	0.481950615933226	0.329	0.098	1.74944715189216E-77	8	SNRNP27
1.24479453425538E-63	0.48180286870292	0.288	0.092	2.08602668050517E-59	8	KCTD1
2.04115810622566E-90	0.481671576984928	0.663	0.264	3.42057275441296E-86	8	PLEKHJ1
1.18838580602896E-71	0.481476284141396	0.36	0.12	1.99149693374333E-67	8	ACTR6
1.08395971659928E-62	0.481384477073391	0.302	0.099	1.81649969307707E-58	8	UBOX5
2.27092305125046E-82	0.481160161450453	0.482	0.175	3.80561284928552E-78	8	KDM5A
5.24778711513798E-92	0.481083567085386	0.743	0.306	8.79424164754823E-88	8	CARS2
3.06820075301479E-94	0.481073110884089	0.57	0.211	5.14169082190219E-90	8	RAB27A
5.49454842330229E-56	0.481060356133746	0.284	0.096	9.20776424776998E-52	8	PDPR
8.60393167223301E-81	0.481022249777417	0.531	0.201	1.44184686963281E-76	8	RNF139
9.20945142522222E-94	0.48074962082452	0.487	0.168	1.54331986983874E-89	8	HAL
7.18538762147546E-104	0.480747900546807	0.664	0.253	1.20412725760686E-99	8	KHDRBS1
4.75340415719385E-85	0.480719878229959	0.402	0.131	7.96575468662546E-81	8	DEPRECATED-ENSG00000099810
9.93956605085074E-80	0.480338704355876	0.726	0.313	1.66567247880157E-75	8	GFPT1
7.53034095117282E-54	0.480330163615065	0.302	0.109	1.26193453659754E-49	8	NUDT8
1.55556718365241E-91	0.480132305493111	0.51	0.182	2.60681948636471E-87	8	SAMD1
2.07173558320237E-72	0.480077750045763	0.382	0.131	3.47181449033053E-68	8	FAM241A
6.28965065904377E-85	0.479920554764892	0.465	0.162	1.05401965744256E-80	8	GPATCH8
1.9787389249533E-96	0.479877215489719	0.707	0.284	3.31597069043674E-92	8	HDAC1
2.75767700431767E-99	0.479608440180902	0.655	0.254	4.62131512383555E-95	8	ATF4
4.32564912809459E-86	0.479511382801877	0.643	0.261	7.24892280886091E-82	8	PNP
1.58532390154978E-107	0.479013462745795	0.555	0.189	2.65668579421713E-103	8	GMPPB
4.94797828806823E-83	0.478609450633954	0.872	0.416	8.29182201514474E-79	8	NPLOC4
7.79251595776966E-79	0.478241179958097	0.47	0.172	1.30586982420304E-74	8	UEVLD
3.41903054584169E-71	0.478123049377777	0.469	0.179	5.7296113887215E-67	8	PTPRJ
2.42046731368973E-73	0.477955662905449	0.353	0.115	4.05621912428124E-69	8	ALS2
3.43514798153273E-72	0.477280970153668	0.859	0.427	5.75662098745255E-68	8	HPGD
8.61324255889353E-49	0.477245182242196	0.317	0.121	1.44340718801938E-44	8	DEPRECATED-ENSG00000197714
4.23121085182188E-60	0.477231231439588	0.252	0.077	7.09066314548311E-56	8	ANGEL2
1.31281058901728E-75	0.476748231345626	0.718	0.31	2.20000798507515E-71	8	NIPAL2
4.08346661471396E-96	0.476618659663435	0.529	0.185	6.84307335293765E-92	8	MED24
5.11162710813246E-101	0.476450895939322	0.589	0.214	8.56606470780838E-97	8	CHERP
1.42419008002722E-72	0.4755231176991	0.305	0.093	2.38665773610962E-68	8	TDRKH
9.90072219745601E-83	0.475259115205071	0.435	0.15	1.65916302584968E-78	8	AP5M1
1.1251552161433E-59	0.474848105336191	0.35	0.127	1.88553511121294E-55	8	PRMT7
6.90143484805835E-93	0.474807242002239	0.469	0.157	1.15654245183762E-88	8	NBPF15
6.13910382160144E-61	0.474520806582364	0.32	0.11	1.02879101842397E-56	8	SMU1
1.9667507541029E-73	0.47376524760762	0.387	0.133	3.29588091372564E-69	8	C4orf33
5.87374942633996E-42	0.473661579275286	0.269	0.102	9.8432292886605E-38	8	ZNF587B
4.93074579261432E-82	0.473450914839495	0.731	0.315	8.26294379926308E-78	8	TTC3
5.20410116920417E-64	0.473289982549751	0.298	0.096	8.72103273935235E-60	8	EYA3
1.25467151530449E-84	0.473288783881274	0.297	0.081	2.10257852534727E-80	8	LIMS1
1.78609766309582E-90	0.473213756250887	0.631	0.248	2.99314246381597E-86	8	BROX
5.87253738056634E-55	0.472915916471611	0.937	0.587	9.84119814235307E-51	8	SDC1
2.46200886559126E-67	0.472732669152517	0.913	0.489	4.12583445695783E-63	8	CMC4
4.31145477046676E-97	0.472285950851314	0.663	0.26	7.22513590434819E-93	8	ANKRD11
1.28167350374614E-82	0.472075928021966	0.514	0.19	2.14782845757778E-78	8	PHKB
2.34919320826092E-72	0.471897749635636	0.36	0.12	3.93677797840364E-68	8	TMEM30B

3.34451372783789E-111	0.471628339221873	0.644	0.233	5.60473610511073E-107	8	ACAD9
4.08904698939954E-87	0.471591294799283	0.691	0.285	6.85242494483575E-83	8	MOCOS
1.60337434956243E-89	0.471332899542537	0.45	0.15	2.68693473499672E-85	8	PIP4K2C
2.34650375658806E-76	0.471212596759098	0.446	0.161	3.93227099529028E-72	8	DYNC1LI1
7.77062962044807E-82	0.471114379503531	0.656	0.27	1.30220211179469E-77	8	DENND4C
4.60297249566389E-66	0.471083293142239	0.402	0.149	7.71366130823355E-62	8	PCSK7
3.5411635540267E-63	0.470911997752391	0.364	0.131	5.93428188383794E-59	8	ARL4C
4.6237426254702E-79	0.470836476594432	0.446	0.159	7.74846789176296E-75	8	TSC2
7.02552504352374E-83	0.470677525261766	0.467	0.167	1.17733748679371E-78	8	SIRPA
8.30635998882616E-109	0.470168165362432	0.491	0.155	1.39197980692749E-104	8	PITHD1
6.20666960018093E-59	0.469985907606804	0.892	0.505	1.04011369159832E-54	8	CALM2
3.33795382457377E-101	0.469950926196073	0.587	0.211	5.59374301922073E-97	8	ZCCHC3
8.61033804775545E-98	0.469095291736279	0.561	0.199	1.44292045004286E-93	8	THUMPD1
3.02474348601825E-69	0.469087641392065	0.273	0.08	5.06886513386938E-65	8	JADE3
1.01391705772574E-76	0.468904382258316	0.437	0.156	1.69912220533679E-72	8	ANO10
1.39898257664843E-45	0.46888867288888	0.272	0.1	2.34441500194744E-41	8	PMS2
9.66207025736137E-99	0.468835334946559	0.752	0.304	1.61916973372862E-94	8	ZFR
5.86452604775901E-111	0.468563462501449	0.473	0.145	9.82777275083456E-107	8	MAP3K20
1.37693965436965E-54	0.468526274506969	0.274	0.092	2.30747547279266E-50	8	IFT57
5.17630063581153E-77	0.468502077833362	0.413	0.142	8.67444460549297E-73	8	UBN2
2.11386466642848E-78	0.468441392421497	0.523	0.2	3.54241440800085E-74	8	LHFPL2
1.27205494128167E-78	0.468350830221607	0.462	0.167	2.13170967059983E-74	8	RBM5
1.74578782198373E-77	0.46811372771475	0.438	0.156	2.92559123208034E-73	8	HOXA10
1.19061171068234E-74	0.467925324554924	0.66	0.278	1.99522710476147E-70	8	MGAM2
9.668730018757E-81	0.467917777884437	0.326	0.097	1.6202857765433E-76	8	MYCL
2.15843076575257E-86	0.467744771694698	0.482	0.17	3.61709827724815E-82	8	DPAGT1
1.28429841846466E-63	0.467701212154228	0.326	0.111	2.15222728966308E-59	8	PPL
1.1189387557311E-89	0.467685910824696	0.648	0.263	1.87511756685417E-85	8	YWHAQ
1.86842208782659E-88	0.467626514702717	0.594	0.228	3.1311017347798E-84	8	RIPK1
7.61409036326187E-54	0.467152606311778	0.305	0.109	1.27596926307542E-49	8	ZBTB45
5.83471900198664E-87	0.466917550079387	0.822	0.369	9.77782210352921E-83	8	ADD1
3.00592060571814E-68	0.466913807363797	0.391	0.141	5.03732175106247E-64	8	EXOC6B
8.44917886017121E-99	0.466355951116326	0.603	0.221	1.41591339338749E-94	8	FIP1L1
1.27120777147614E-97	0.466168491517719	0.61	0.227	2.13028998343971E-93	8	DDX42
6.76880811424983E-78	0.465589235042902	0.369	0.121	1.13431686378599E-73	8	RIC8A
6.11822838165189E-91	0.465539088207219	0.519	0.184	1.02529271219722E-86	8	ADD3
1.39257973349641E-68	0.465533402937959	0.34	0.113	2.33368511739329E-64	8	TRANK1
5.44433572771208E-75	0.465450125130124	0.391	0.134	9.1236178124999E-71	8	DOCK7
1.70133265671823E-91	0.465330529198262	0.738	0.308	2.85109326612841E-87	8	TDP2
3.14347631810066E-100	0.465190390639833	0.73	0.295	5.26783761387309E-96	8	GGNBP2
6.1027657454915E-86	0.465173847252279	0.543	0.202	1.02270148362947E-81	8	ANKRD27
6.08162496526508E-74	0.46500975753709	0.449	0.165	1.01915871167912E-69	8	MTPAP
1.07996906907448E-46	0.464893651431584	0.302	0.115	1.80981216595501E-42	8	DEPRECATED-ENSG00000197302
1.07505266314896E-64	0.464876672646404	0.45	0.176	1.80157325290503E-60	8	CASZ1
2.22899722708659E-74	0.464625293155162	0.507	0.198	3.73535355315171E-70	8	CD9
6.8141726542926E-97	0.464479029494662	0.571	0.207	1.14191905340635E-92	8	UBE4B
7.44607826690818E-88	0.46394873571559	0.483	0.169	1.24781379596847E-83	8	KPNA3
2.95278265990408E-93	0.463831326457341	0.517	0.181	4.94827318146726E-89	8	ITGB1BP1
9.89036406274561E-75	0.463519821551135	0.289	0.084	1.65742720963491E-70	8	DEPRECATED-ENSG00000172977
6.30815295749786E-54	0.463499635172478	0.313	0.113	1.05712027261749E-49	8	SMUG1
2.10102073478827E-85	0.463429080787525	0.449	0.154	3.52089054735818E-81	8	GNB5
9.86536585610443E-89	0.463294559130215	0.515	0.186	1.65323801016598E-84	8	ABCB8
1.2309963577132E-65	0.462806480719187	0.434	0.165	2.06290369625579E-61	8	CHST5
7.14586538132271E-50	0.462705414651836	0.262	0.09	1.19750412060206E-45	8	PLEKHA8
5.18483429243564E-91	0.462523142948255	0.714	0.291	8.68874530726364E-87	8	TOR1AIP1
1.71822271335757E-86	0.462420898697775	0.587	0.226	2.87939762304462E-82	8	GCH1
9.6612078925469E-74	0.462121452083689	0.814	0.379	1.61902521863301E-69	8	ACMSD
2.68517379164825E-101	0.46137228934664	0.557	0.195	4.49981424004413E-97	8	HECA
3.44696030612857E-76	0.460932287189042	0.383	0.128	5.77641608101026E-72	8	ZDHHC18
9.68557089314903E-85	0.460906960293406	0.474	0.169	1.62310797027391E-80	8	SALL1

6.38692144358031E-94	0.460639885981048	0.55	0.2	1.07032029551519E-89	8	ARHGEF18
7.06052194498158E-89	0.460473021940428	0.619	0.242	1.18320226754001E-84	8	NBN
3.98332792082721E-88	0.460469031692284	0.513	0.184	6.67526092972224E-84	8	PHLPP1
9.68007895184908E-74	0.460228121903801	0.499	0.193	1.62218763075087E-69	8	COG2
1.45780841659891E-77	0.459998726098508	0.455	0.165	2.44299534453646E-73	8	MOB4
1.75268272178885E-83	0.459822697406002	0.748	0.328	2.93714570517375E-79	8	IGF2R
7.07635078959388E-74	0.459788547439314	0.356	0.116	1.18585486532014E-69	8	FBXO22
2.31821445000702E-72	0.459382241054318	0.505	0.197	3.88486377532176E-68	8	D2HGDH
1.09595892474634E-87	0.459358060557695	0.726	0.308	1.83660796608992E-83	8	MAST4
1.03872285486939E-78	0.459170496088016	0.513	0.196	1.74069176019013E-74	8	SESN2
1.03654625326321E-65	0.458775697322083	0.373	0.133	1.73704421121849E-61	8	FAHD2A
1.66045351556084E-79	0.458279673193197	0.372	0.119	2.78258800137686E-75	8	RPAP3
5.8133448972879E-81	0.458156652501896	0.47	0.169	9.74200337887506E-77	8	DPH7
4.94387082631978E-69	0.457995056957692	0.438	0.165	8.28493873074669E-65	8	TRAF3
4.25979863611963E-84	0.457862526337139	0.589	0.228	7.13857055440927E-80	8	SCAF8
7.06456605689692E-85	0.457785312714874	0.575	0.225	1.18387997981479E-80	8	DEPRECATED-ENSG00000171234
2.64519986286243E-88	0.457438417851171	0.759	0.331	4.43282593018487E-84	8	PYGO2
3.820502613154E-57	0.4573831072204	0.925	0.502	6.40239827912348E-53	8	ZKSCAN1
9.00486118192382E-85	0.457317161180378	0.462	0.161	1.50903463686679E-80	8	SH2B1
8.24063244893441E-75	0.457131537435008	0.326	0.102	1.38096518579243E-70	8	RCBTB1
3.97275080891377E-51	0.456905686540452	0.276	0.096	6.65753580557769E-47	8	APPBP2
1.8989582920721E-75	0.456852993836576	0.875	0.425	3.18227430585442E-71	8	IL1R1
3.23745430028994E-70	0.456789437305652	0.336	0.11	5.42532591642589E-66	8	NIPSNAP2
8.39769790712982E-76	0.456645236844832	0.378	0.126	1.40728621527681E-71	8	SHOC2
1.03934853880243E-47	0.455849323032623	0.265	0.094	1.74174028132511E-43	8	LUZP1
1.35822770928106E-54	0.455844724179997	0.289	0.1	2.2761179952132E-50	8	PXK
4.50437305849514E-57	0.455687251872522	0.387	0.15	7.54842837142616E-53	8	TMEM94
2.69222276997756E-84	0.455618850816909	0.471	0.166	4.5116269179284E-80	8	KDM4A
6.17488488403171E-73	0.455534751633748	0.422	0.152	1.03478720886603E-68	8	ARID4A
2.04470151433267E-91	0.455463168390671	0.498	0.173	3.42651079771868E-87	8	TP53I13
4.59218577964055E-88	0.455276589006407	0.374	0.114	7.69558492952164E-84	8	BAP1
3.39706328620337E-68	0.455145353928236	0.883	0.457	5.69279865501961E-64	8	STK24
3.00389012191621E-88	0.455049868559594	0.627	0.25	5.03391906630719E-84	8	GNL1
1.1523008658206E-103	0.45486107019247	0.703	0.269	1.93102579094216E-99	8	MARK3
2.80970583278312E-42	0.454797093884286	0.913	0.573	4.70850503457796E-38	8	AKR1C2
1.70879603764581E-67	0.454520057023663	0.419	0.156	2.86360039988684E-63	8	SSH1
3.16597204902162E-74	0.454248684714158	0.515	0.2	5.30553595975042E-70	8	NAA15
1.08019828036964E-67	0.454226645430578	0.333	0.11	1.81019627824344E-63	8	KANSL2
2.73201326876531E-69	0.454134590351141	0.692	0.309	4.5783078357969E-65	8	PDX1
3.08173204288846E-44	0.453972812903469	0.985	0.771	5.16436655747247E-40	8	FMO5
6.99746741485159E-63	0.453965744030424	0.346	0.123	1.17263558938083E-58	8	PTGFRN
4.04492203047209E-85	0.453954827662529	0.458	0.158	6.77848033866513E-81	8	PRR12
5.73522980598337E-87	0.453762318548937	0.659	0.269	9.61109810886694E-83	8	ATP2C1
3.63238763178008E-89	0.453595083858153	0.651	0.262	6.08715519333705E-85	8	DPYSL2
2.58729403167195E-92	0.453325666895561	0.625	0.242	4.33578733827585E-88	8	MAN2A2
6.90233594028239E-60	0.452991735338702	0.317	0.109	1.15669345687252E-55	8	CACTIN
1.46655526125427E-72	0.452990577573135	0.828	0.395	2.45765330680991E-68	8	ACSL3
2.10784845320736E-81	0.452962187494633	0.507	0.189	3.53233243788489E-77	8	LY6E
1.49139895901308E-92	0.452927758548324	0.675	0.268	2.49928637551413E-88	8	PEX13
9.70827646274675E-54	0.452857828055141	0.314	0.114	1.6269129696271E-49	8	MINDY3
3.3059444758701E-78	0.452691635260988	0.382	0.126	5.54010175266311E-74	8	DEPRECATED-ENSG00000164164
2.53822648168909E-85	0.452676116771268	0.454	0.158	4.25355993801457E-81	8	EEA1
1.59448912474745E-62	0.452422117999347	0.446	0.177	2.67204487525178E-58	8	TBCK
1.78376399511828E-90	0.452383982280533	0.529	0.189	2.98923170301921E-86	8	TRPM7
1.68391274525191E-78	0.452369461259691	0.502	0.189	2.82190097849315E-74	8	MAGEH1
6.0328639693754E-89	0.452366048293381	0.503	0.176	1.01098734398793E-84	8	PSMG4
1.48945211555958E-72	0.452257948768606	0.342	0.111	2.49602385525475E-68	8	GATC
4.20767732442418E-67	0.452232621961404	0.583	0.247	7.05122566027005E-63	8	TARBP1
2.94330648816059E-59	0.45221613348046	0.31	0.106	4.93239301285952E-55	8	PARP8
3.26389585255302E-101	0.452043091316108	0.611	0.224	5.46963666970835E-97	8	RANBP3

2.19948359402469E-65	0.451917019800339	0.349	0.12	3.68589460686658E-61	8	COG5
7.33299678115919E-87	0.451791057331004	0.533	0.195	1.22886360058666E-82	8	DRAM2
3.94299194100697E-92	0.451603531099478	0.64	0.246	6.60766589473947E-88	8	RSBN1L
2.25827335836072E-92	0.45157003006369	0.439	0.143	3.78441449394089E-88	8	ERC1
1.68655527229251E-69	0.451532876359427	0.625	0.272	2.82632932530779E-65	8	NLK
2.18108852212484E-88	0.451258279828093	0.731	0.304	3.65506814537681E-84	8	RAPH1
1.20871646503888E-74	0.451000940209205	0.856	0.405	2.02556705211216E-70	8	EML4
1.79403949045794E-97	0.450857312343261	0.575	0.208	3.00645137810941E-93	8	EHMT2
3.41888139398065E-88	0.45072900467562	0.752	0.322	5.72936144003277E-84	8	SHROOM3
3.92803989523783E-81	0.450693271113556	0.766	0.335	6.58260925643956E-77	8	TAF7
1.65656846688998E-61	0.450196022534984	0.434	0.17	2.77607743681423E-57	8	NPIPB15
1.33745441158995E-79	0.450079460743533	0.547	0.215	2.24130610294244E-75	8	CA2
1.69199465660331E-77	0.449881197521203	0.88	0.423	2.83544464553583E-73	8	PYCR2
1.72629154601474E-94	0.449824494778655	0.715	0.287	2.8929193728115E-90	8	SDHA
3.28175152736908E-81	0.449763477142553	0.475	0.171	5.49955920956511E-77	8	TAF4
2.47520129749612E-108	0.449567596808597	0.577	0.198	4.147942334344E-104	8	PPP6R3
5.55109347201874E-67	0.449500552027459	0.366	0.128	9.302522440409E-63	8	RNF121
1.23022520080322E-59	0.449381217277733	0.28	0.091	2.06161139150604E-55	8	DHX58
1.0213366170917E-49	0.448965508799406	0.935	0.541	1.71155590292227E-45	8	SLC37A4
8.82731930536605E-72	0.448915471254775	0.9	0.451	1.47928216919324E-67	8	EZR
6.08243368084136E-83	0.448484126546951	0.364	0.113	1.0192942362354E-78	8	DEPRECATED-ENSG00000109536
1.04773713271739E-61	0.448129369566018	0.851	0.451	1.75579788700781E-57	8	RTN3
6.82348209704399E-75	0.447738070351384	0.45	0.164	1.14347912982263E-70	8	COMMD2
5.16085832147099E-72	0.447291732407937	0.422	0.152	8.64856637512109E-68	8	SFSWAP
8.0975415597203E-84	0.447187599987374	0.354	0.108	1.35698601457793E-79	8	FAM210A
6.36075202293465E-81	0.44712848881306	0.486	0.177	1.06593482400339E-76	8	ATXN2
3.57849902599052E-90	0.446709908212602	0.509	0.18	5.99684866775491E-86	8	ZFC3H1
1.97568514594576E-82	0.446659723541669	0.718	0.311	3.3108531675759E-78	8	ATN1
2.00473413564274E-93	0.446650384991886	0.807	0.344	3.35953346451011E-89	8	UBR2
3.39954921595403E-73	0.446563414548876	0.362	0.12	5.69696457609576E-69	8	PPP1R21
2.11988899278885E-88	0.446526670895557	0.652	0.258	3.55250997411556E-84	8	PSEN1
1.28161812063981E-77	0.446469880318091	0.839	0.403	2.14773564656819E-73	8	EIF2AK1
4.26706534287411E-73	0.446412968857116	0.478	0.181	7.15074810158843E-69	8	PIBF1
1.66566806551296E-52	0.446178604909733	0.302	0.109	2.79132654418662E-48	8	ZNF512
2.36631475833718E-73	0.446169506315383	0.611	0.26	3.96547027202145E-69	8	DERA
2.18771130317769E-104	0.445868756137122	0.643	0.237	3.66616660186517E-100	8	ZNF219
1.44707612091519E-80	0.445835173838742	0.373	0.12	2.42501016342968E-76	8	GPRIN3
1.77062779926847E-69	0.445572344296344	0.433	0.161	2.96721806601411E-65	8	ZNF618
2.21545856486055E-91	0.445203570777323	0.498	0.175	3.7126654629933E-87	8	FOXK2
1.29042998130256E-82	0.444572128256291	0.493	0.178	2.16250256266683E-78	8	MBIP
1.01561634685844E-98	0.44413567377782	0.679	0.26	1.70196987406538E-94	8	ODR4
8.75373952839009E-45	0.443800997338823	0.292	0.111	1.46695167016761E-40	8	IGSF3
7.98859946746899E-66	0.443575213818205	0.399	0.146	1.33872949875845E-61	8	MBTPS2
2.16658288916734E-67	0.443549381591347	0.839	0.406	3.63075960566663E-63	8	PANK3
1.77446201449413E-98	0.443539452555956	0.581	0.21	2.97364344388926E-94	8	EFTUD2
2.50523283489418E-79	0.443355670123345	0.7	0.302	4.19826918471567E-75	8	AKR1C8P
4.95774372123558E-69	0.443239756307337	0.397	0.143	8.30818692804658E-65	8	TATDN2
1.08791398454304E-39	0.443186583957034	0.278	0.111	1.82312625529723E-35	8	TRMT13
3.44179635317011E-87	0.443039374695076	0.499	0.178	5.76776232864248E-83	8	MPP5
1.08835940340249E-89	0.442919038099636	0.732	0.303	1.8238726882219E-85	8	UBE2Z
1.77640193064074E-66	0.442743752841861	0.88	0.445	2.97689435536776E-62	8	RNF213
9.57958206652886E-59	0.442372651619928	0.354	0.13	1.60534636270891E-54	8	CAMKMT
5.4463882627014E-48	0.442137744602805	0.281	0.102	9.12705745063501E-44	8	THEM4
2.48850733842443E-77	0.4420225630858	0.375	0.124	4.17024059773166E-73	8	PELP1
2.51589225039201E-105	0.441843875539876	0.656	0.244	4.21613223320693E-101	8	ZC3HAV1
2.69800195906792E-77	0.441658023471309	0.503	0.191	4.52131168300602E-73	8	ATAD2B
1.99863379288769E-84	0.441400857858983	0.491	0.177	3.3493105101212E-80	8	VOPP1
2.91554586971298E-71	0.440836788691932	0.522	0.208	4.88587176846502E-67	8	MAML1
1.27363827467817E-77	0.440819352850562	0.431	0.151	2.13436302070567E-73	8	SUPT20H
1.23348665329098E-78	0.440535187550269	0.503	0.19	2.06707693358503E-74	8	CLCN3

2.64767464961993E-75	0.440318480950606	0.788	0.358	4.43697317783308E-71	8	PEX2
3.47610821975224E-43	0.440311319110367	0.256	0.094	5.8252621546608E-39	8	SUPT3H
8.62657092936629E-72	0.440242872950501	0.421	0.152	1.4456407563432E-67	8	PIGF
1.09450683091556E-74	0.440102007620238	0.438	0.157	1.8341745472483E-70	8	PIGC
3.05475508555733E-124	0.439907481903036	0.625	0.21	5.11915857237697E-120	8	PHF1
7.82218008536753E-93	0.439884712333176	0.545	0.194	1.31084093870589E-88	8	KLC1
6.58941738869264E-66	0.439849692774867	0.304	0.097	1.10425456599711E-61	8	DHX35
2.2165332584483E-82	0.439579700122383	0.704	0.296	3.71446643450765E-78	8	MAP3K5
5.84389033519065E-94	0.439539520938169	0.646	0.254	9.79319142371248E-90	8	UQCC2
3.30081057641245E-73	0.439526641638442	0.373	0.126	5.53149836395198E-69	8	JDP2
9.73834699306388E-85	0.439104769994861	0.502	0.183	1.63195218909764E-80	8	UCK2
4.4743616842325E-84	0.438642521056097	0.636	0.255	7.49813531043683E-80	8	CEP170B
1.03676940127326E-73	0.438469012682527	0.419	0.15	1.73741816265374E-69	8	GAR1
1.95802687945928E-75	0.438438562344399	0.565	0.224	3.28126144459786E-71	8	TMLHE
1.58069680526739E-97	0.437992082376571	0.586	0.214	2.64893170626709E-93	8	AKIP1
7.35515680909335E-69	0.437506784799774	0.344	0.114	1.23257717806786E-64	8	MADD
1.62470321042012E-77	0.437263058544331	0.383	0.128	2.72267764002204E-73	8	GCA
1.29947566886326E-90	0.437021929427145	0.65	0.256	2.17766132588105E-86	8	FBXW2
1.57159533035619E-74	0.436904735219235	0.618	0.262	2.6336794546109E-70	8	ENOPH1
6.12213426417444E-86	0.436772243543792	0.623	0.241	1.02594725999035E-81	8	SLC25A32
1.08156027339208E-79	0.436341014050394	0.513	0.193	1.81247870615044E-75	8	BCLAF1
2.08929992648713E-89	0.436288694275631	0.71	0.296	3.50124881680713E-85	8	RBM23
4.29467710089003E-66	0.436153409173369	0.344	0.118	7.19701988567151E-62	8	PIP5K1C
6.55125888673532E-74	0.436116524607115	0.768	0.357	1.0978599642391E-69	8	PSMD10
4.16314065849517E-96	0.436085589377949	0.613	0.226	6.9765911155062E-92	8	UBR3
1.45093183225878E-68	0.436032550803064	0.41	0.149	2.43147156449926E-64	8	DEPRECATED-ENSG00000147804
6.80185790807902E-65	0.435945357795155	0.465	0.185	1.13985534823588E-60	8	RBM4
6.4841061926584E-79	0.435851364046124	0.607	0.246	1.08660651576569E-74	8	HUS1
5.79035123773808E-68	0.435622276591571	0.465	0.18	9.70347060420147E-64	8	FBXL12
1.80914701179578E-83	0.435585404298642	0.607	0.24	3.03176856236737E-79	8	ARID4B
2.5971541628705E-92	0.435326989470126	0.743	0.308	4.35231094613839E-88	8	RB1CC1
8.94731800925219E-61	0.435166676252537	0.313	0.107	1.49939155199048E-56	8	SIPA1
1.93404749005052E-53	0.433775270559853	0.312	0.112	3.24107678382666E-49	8	RWDD4
1.0336583716082E-59	0.433691357673167	0.308	0.105	1.73220469914102E-55	8	NDUFAF7
2.04893752204835E-77	0.433488691933755	0.455	0.164	3.43360949944862E-73	8	AK3
1.38738328955418E-79	0.432634594004805	0.771	0.338	2.3249769166349E-75	8	REPS1
2.06284639046205E-85	0.43172886094026	0.553	0.207	3.4569179811363E-81	8	LYSMD3
2.26214751304266E-85	0.431678263941424	0.728	0.306	3.79090680235689E-81	8	FGD4
2.53658958270368E-92	0.431657123780411	0.663	0.26	4.25081682269483E-88	8	BRWD1
5.55698815968729E-94	0.431370281563096	0.566	0.207	9.31240075800396E-90	8	TSN
1.57608195238858E-71	0.43122970358908	0.391	0.137	2.64119813581278E-67	8	HEATR5B
5.93932037715213E-79	0.431045197015776	0.76	0.338	9.95311308803154E-75	8	GPBP1
3.70940106938853E-64	0.430989782056319	0.454	0.18	6.2162143120813E-60	8	VEZT
1.3081264460194E-87	0.430964336923808	0.573	0.217	2.19215829823931E-83	8	NAA50
2.22663748366003E-87	0.430931177821406	0.542	0.201	3.73139909511747E-83	8	ARID1B
2.18267036603023E-80	0.430740440211322	0.78	0.343	3.65771899939346E-76	8	CUL4B
1.7422677441792E-66	0.430739845545488	0.345	0.118	2.9196922856955E-62	8	OSGIN2
8.98948261373994E-48	0.430500781173448	0.274	0.099	1.50645749641054E-43	8	CHST12
8.50694903263531E-78	0.430291835650659	0.442	0.157	1.42559451888902E-73	8	EIF2AK4
3.78571585021637E-79	0.430124487898905	0.774	0.348	6.3441026217926E-75	8	CALU
1.43592183273742E-83	0.43009958713917	0.706	0.292	2.40631780730136E-79	8	KIF1B
2.95632676278004E-81	0.429880799096616	0.509	0.188	4.95421238906679E-77	8	POLB
2.8786922335285E-56	0.429787450362958	0.893	0.466	4.82411244494706E-52	8	FRK
8.49776560889302E-94	0.429412472502856	0.72	0.297	1.42405556073829E-89	8	NFIX
4.07777475492303E-79	0.429276992937504	0.794	0.363	6.83353493430001E-75	8	PRPS1
1.20450739989119E-87	0.428754128894744	0.434	0.144	2.01851350073766E-83	8	C1orf35
4.96735644168294E-91	0.428640568061185	0.526	0.188	8.32429592497227E-87	8	MIB1
2.72210390930986E-76	0.428511003695196	0.407	0.141	4.56170173122146E-72	8	SLC27A4
1.75443700927202E-90	0.428466633136501	0.831	0.364	2.94008554013806E-86	8	DST
3.14764560673024E-100	0.428400100075368	0.656	0.25	5.27482450775853E-96	8	PAF1

7.58594423842365E-83	0.42833784414236	0.783	0.343	1.27125253547504E-78	8	SLC25A5
2.86687001862292E-84	0.427890386179276	0.71	0.293	4.80430077720829E-80	8	MPHOSPH8
2.45272197825323E-76	0.427881986885716	0.405	0.139	4.11027149115676E-72	8	FEM1B
2.17482872619654E-63	0.427868744854027	0.908	0.487	3.64457797936017E-59	8	HBS1L
4.2259283122607E-66	0.427778793958144	0.418	0.157	7.08181066568647E-62	8	CDHR2
2.77618925504926E-69	0.427370908465373	0.534	0.215	4.65233795361155E-65	8	ENPP4
2.17760122023507E-70	0.427360977674875	0.51	0.202	3.64922412486993E-66	8	OTUD6B
2.29850411080532E-89	0.427341897269392	0.565	0.211	3.85183318888756E-85	8	EEF1AKNMT
5.14347857923964E-67	0.427176346363582	0.301	0.095	8.61944140308978E-63	8	GEMIN6
4.54657558326616E-82	0.426914289320166	0.545	0.206	7.61915136243744E-78	8	SPG11
1.85361162623584E-63	0.426760164866578	0.322	0.109	3.10628236324602E-59	8	F8
2.10053046237811E-67	0.426451590414071	0.348	0.118	3.52006894885324E-63	8	TLK2
1.00792095767185E-57	0.426414319826016	0.332	0.119	1.68907394086649E-53	8	VPS53
2.47064213473545E-84	0.426308082164671	0.621	0.25	4.14030208938967E-80	8	MPPE1
1.00361175525517E-73	0.42611904960737	0.487	0.184	1.68185257945661E-69	8	FAM98B
1.26729991080943E-93	0.426000633262794	0.694	0.281	2.12374119053444E-89	8	PTBP3
3.45872403682593E-72	0.425967127315339	0.341	0.111	5.7961297409129E-68	8	MARVELD3
3.16917112881436E-54	0.425641593019272	0.266	0.089	5.3108969776671E-50	8	RLF
3.18848306702491E-90	0.425466696649158	0.622	0.242	5.34325992372034E-86	8	IFNAR2
2.57616349191727E-81	0.425210382769603	0.836	0.383	4.31713477975496E-77	8	ARHGAP35
5.52875028282354E-74	0.425105274865467	0.336	0.107	9.26507972395568E-70	8	TBCC
4.87020429531597E-67	0.425033238544559	0.356	0.122	8.1614883580905E-63	8	IMPDH2
1.72596349542415E-80	0.425032994846727	0.591	0.234	2.89236962563178E-76	8	CASP3
1.15047468899823E-88	0.425024299258754	0.796	0.344	1.92796548382323E-84	8	CHD2
1.8607815185523E-60	0.424285990225462	0.357	0.129	3.11829766878994E-56	8	WDFY3
1.67530519891817E-100	0.424209220905948	0.636	0.239	2.80747645234707E-96	8	PLRG1
1.10801815685338E-82	0.423906197734938	0.469	0.167	1.8568168272549E-78	8	UTP6
4.3231402687711E-80	0.423890030107723	0.353	0.11	7.24471846240661E-76	8	MPZL2
1.66877214439225E-89	0.423862412542412	0.607	0.234	2.79652835957253E-85	8	IPO8
8.91963429192679E-56	0.423772371177572	0.27	0.089	1.49475231464109E-51	8	DEPRECATED-ENSG00000147905
1.27481456811319E-86	0.423615458730715	0.589	0.225	2.13633425324408E-82	8	MBTPS1
2.92465479263193E-68	0.423539428896384	0.334	0.111	4.90113650149258E-64	8	HIP1
1.17836453160051E-67	0.423333629830424	0.45	0.171	1.97470328205614E-63	8	LATS1
3.73210829601088E-73	0.423226150709946	0.82	0.372	6.25426708245503E-69	8	ACVR1B
5.99191514766848E-91	0.423125094877758	0.601	0.226	1.00412514044628E-86	8	MTERF4
3.32623965968461E-68	0.422954714989159	0.644	0.28	5.57411242169946E-64	8	CRACR2B
1.28288879378351E-82	0.422308142196067	0.487	0.177	2.14986504062241E-78	8	TBK1
6.39868538245831E-75	0.421972027171203	0.386	0.13	1.07229169639236E-70	8	DEPRECATED-ENSG00000272391
7.26904501150628E-79	0.421919916265557	0.481	0.175	1.21814656302822E-74	8	FNBP1L
1.63118513077092E-79	0.421595997068103	0.778	0.36	2.73354004214591E-75	8	HMGN1
6.92301512140412E-95	0.421332936398739	0.585	0.213	1.1601588740449E-90	8	FNIP2
3.76232827464965E-93	0.421038902528863	0.457	0.151	6.30490972265789E-89	8	ALG9
4.19409395963571E-79	0.420846829940423	0.55	0.214	7.02846265755752E-75	8	SNX13
4.06193193793578E-50	0.420438226806157	0.302	0.112	6.80698554159278E-46	8	STRADB
1.55122709990708E-95	0.420241646128416	0.631	0.243	2.59954637402428E-91	8	KLHDC3
2.28010724055915E-75	0.420114470539435	0.423	0.15	3.82100371372902E-71	8	ST3GAL2
4.68342004696073E-61	0.420042514350095	0.341	0.121	7.84847531469679E-57	8	ZNF12
4.7538479441718E-60	0.419937359157771	0.308	0.104	7.9664983848431E-56	8	ANKRD12
1.01304462614576E-74	0.419780222306438	0.421	0.148	1.69766018449506E-70	8	RGL2
9.54462617050062E-67	0.419737576781733	0.461	0.177	1.59948845365249E-62	8	RNF170
2.75987010694325E-62	0.419561006492062	0.345	0.123	4.6249903252155E-58	8	KLF2
5.00451998601146E-77	0.419531303335194	0.483	0.182	8.38657459255801E-73	8	NT5DC1
1.29008769381421E-84	0.419448996925382	0.691	0.285	2.16192895729386E-80	8	ZNF664
8.95296799519144E-87	0.419004088220378	0.519	0.189	1.50033837663418E-82	8	DHRSX
4.86654059166926E-88	0.418832116390266	0.643	0.258	8.15534872351935E-84	8	GGA2
1.61171752140681E-76	0.418828079337795	0.763	0.339	2.70091622237353E-72	8	SPPL2A
4.89284151335936E-66	0.418666357387041	0.369	0.13	8.19942380808762E-62	8	RGL3
1.93370564890539E-88	0.418276950846342	0.605	0.235	3.24050392643565E-84	8	CC2D1A
7.38909520598217E-94	0.418120909778552	0.728	0.299	1.23826457461849E-89	8	MOV10
1.02301672603922E-88	0.418068712120524	0.497	0.174	1.71437142949652E-84	8	TBRG4

8.54126167680724E-88	0.417700746087497	0.51	0.183	1.43134463179936E-83	8	CCNK
9.59392073382439E-71	0.417609123865259	0.885	0.462	1.60774923657429E-66	8	HNRNPA0
4.79258220081382E-68	0.417425313413477	0.418	0.154	8.03140925212379E-64	8	DEPRECATED-ENSG00000249915
1.87906073732742E-68	0.417294298501424	0.839	0.4	3.14892998361328E-64	8	MLPH
4.35521425670461E-76	0.417114180798	0.487	0.182	7.29846805138558E-72	8	C6orf136
6.83254087056792E-96	0.417085084890217	0.578	0.206	1.14499719908977E-91	8	MICU2
1.48212839892124E-83	0.417018539699696	0.659	0.272	2.48375077091221E-79	8	DEPRECATED-ENSG00000049860
1.87536864722946E-86	0.417006086864297	0.676	0.278	3.14274277902713E-82	8	DNAJC8
1.71242742251724E-71	0.416573875827868	0.514	0.201	2.86968587465439E-67	8	FBXO28
2.14581308858396E-79	0.41635644273934	0.435	0.152	3.595953573849E-75	8	UMPS
1.94954457674664E-75	0.416246659951774	0.541	0.212	3.26704680171202E-71	8	DEPRECATED-ENSG00000188313
2.80368186669276E-76	0.416212477264501	0.453	0.164	4.69841007220373E-72	8	PLEKHA7
6.12133060855385E-83	0.416152101337386	0.694	0.291	1.02581258338145E-78	8	TAOK1
6.51601925885852E-98	0.416022860897688	0.589	0.213	1.09195450739951E-93	8	CNPPD1
1.17557333354605E-78	0.41589983560447	0.778	0.343	1.97002579235648E-74	8	GMDS
1.03684383046978E-68	0.415655507655644	0.696	0.316	1.73754289110125E-64	8	CTSC
3.17007216819753E-72	0.415623545594048	0.362	0.121	5.31240693946541E-68	8	APIP
1.75062355180114E-83	0.415302659781312	0.756	0.335	2.93369494810835E-79	8	CYB5B
1.86060768890943E-73	0.415295323907838	0.451	0.167	3.11800636507442E-69	8	SDF2
2.70073583191866E-86	0.414737387212367	0.589	0.223	4.52589310712929E-82	8	TOR1B
2.07990420921108E-84	0.414560846629348	0.655	0.269	3.48550347379593E-80	8	GAK
3.38198126784963E-42	0.414446321727773	0.25	0.092	5.66752420866241E-38	8	SNX33
1.23104097260696E-60	0.414326824203438	0.454	0.183	2.06297846189474E-56	8	ZNF251
7.89593372145294E-55	0.414131877958955	0.353	0.134	1.32320057304108E-50	8	ZSCAN26
1.98350994728668E-51	0.413871440794551	0.368	0.145	3.32396596966302E-47	8	CLRN3
1.18013453930821E-42	0.413810409614312	0.328	0.135	1.9776694609727E-38	8	DHRS11
7.95158504729158E-80	0.413595518969945	0.734	0.316	1.33252662222512E-75	8	TBC1D2B
2.1109146153799E-100	0.413568358773916	0.487	0.161	3.53747071245364E-96	8	BCL7C
1.82276996421496E-79	0.4134348058514	0.506	0.189	3.05459790603143E-75	8	CCT4
2.51979288926136E-54	0.413281608691062	0.375	0.146	4.22266892382418E-50	8	SCYL3
6.2108064556569E-86	0.413183952870667	0.61	0.239	1.04080694583898E-81	8	COLGALT1
5.92197252350235E-49	0.412410721596325	0.288	0.105	9.92404155488524E-45	8	TRIM23
1.23135074723961E-74	0.412355980399558	0.877	0.406	2.06349758222414E-70	8	TM7SF3
3.95841129017948E-66	0.412313935448768	0.369	0.13	6.63350564008277E-62	8	ZNFX1
3.95388627946244E-74	0.412269537852458	0.756	0.343	6.62592262712315E-70	8	TRAPPC4
8.2589191279854E-79	0.41225621989752	0.422	0.146	1.38402966746779E-74	8	UXS1
1.48064719635804E-72	0.412194045513546	0.393	0.136	2.4812685716568E-68	8	SCNM1
1.12408479045818E-71	0.412120496614993	0.389	0.136	1.88374129184982E-67	8	COPG2
3.98894922113362E-65	0.412114393928074	0.294	0.094	6.68468110477573E-61	8	WDR1
5.31531022441742E-84	0.411889179955289	0.776	0.338	8.90739687407872E-80	8	SPATA13
1.02349185206416E-84	0.41164678596794	0.478	0.17	1.71516764568913E-80	8	ITPKA
7.56263885060117E-96	0.411574844343801	0.642	0.247	1.26734701858374E-91	8	SPAG9
2.29596062434963E-66	0.410783275501549	0.414	0.154	3.84757081428512E-62	8	N4BP1
3.23045607659593E-52	0.410692699282027	0.895	0.492	5.41359829315946E-48	8	CTSH
1.92433131102482E-66	0.410393301156375	0.354	0.122	3.22479441101539E-62	8	FLCN
4.05403993684685E-96	0.41027910629348	0.527	0.184	6.79376012616795E-92	8	CD82
3.69671921741203E-63	0.410039817278725	0.876	0.428	6.19496206453908E-59	8	CPD
2.05577931644894E-50	0.41000996466509	0.268	0.093	3.44507497850513E-46	8	TAF1C
2.54323099791415E-44	0.409721062825379	0.258	0.094	4.26194650630454E-40	8	ZNF784
2.77383317803936E-75	0.409485753407172	0.425	0.15	4.64838963975836E-71	8	IGBP1
8.0527123446549E-65	0.409388709398911	0.86	0.426	1.34947353471727E-60	8	PDZD8
1.83931559357782E-76	0.409370365083841	0.399	0.136	3.0823250717177E-72	8	GIT2
4.90795811778232E-37	0.409369593170736	0.257	0.102	8.22475621377961E-33	8	PIGL
4.98534523810341E-84	0.409353993517529	0.587	0.228	8.3544415500137E-80	8	RNF6
7.76764061626927E-84	0.40879014704003	0.782	0.343	1.3017012144744E-79	8	INSR
1.45537427234224E-95	0.408736703559794	0.554	0.197	2.43891620559113E-91	8	KIF13B
4.4346699242342E-64	0.408688309033446	0.441	0.17	7.43161985903167E-60	8	ARMCX5
1.37308554062497E-55	0.4086310892833	0.923	0.476	2.30101674897932E-51	8	ERO1B
4.10782734480577E-88	0.408395934799335	0.51	0.182	6.88389706442551E-84	8	METTL3
1.48927520481334E-91	0.407774722883158	0.622	0.236	2.4957273882262E-87	8	API5

2.80882476511952E-81	0.407655442453584	0.575	0.226	4.70702854138729E-77	8	GON4L
5.72623627910038E-69	0.40722675930164	0.403	0.145	9.59602675651642E-65	8	PET117
4.92203656404608E-93	0.406990907655057	0.562	0.202	8.24834887402843E-89	8	RAB3GAP1
5.96102561645968E-65	0.406985900577698	0.411	0.154	9.98948672806313E-61	8	PIGO
9.34926531848957E-108	0.406959379277664	0.599	0.21	1.56674988207248E-103	8	CNN2
5.3963654783259E-78	0.406713961342869	0.563	0.223	9.04322926857854E-74	8	MTMR4
2.70741452870741E-70	0.406709854344237	0.344	0.113	4.53708526720788E-66	8	RPP30
5.77764846163006E-72	0.406705967162666	0.502	0.195	9.68218329199965E-68	8	TMEM98
6.3594168167325E-93	0.406427064848778	0.617	0.235	1.06571107014803E-88	8	JMJD6
3.93223319450838E-86	0.406146185193201	0.73	0.304	6.58963638735715E-82	8	GATAD1
2.96998204428675E-63	0.406043924801721	0.332	0.114	4.97709590981573E-59	8	SLC39A6
6.65067401078295E-69	0.405982505929565	0.449	0.17	1.11451995072701E-64	8	COG1
6.21862567574732E-88	0.405949749023368	0.429	0.14	1.04211729074174E-83	8	COPS7B
6.1853676115614E-78	0.405717267884602	0.398	0.134	1.03654390434546E-73	8	TTC9C
1.52743334739245E-51	0.405599852325581	0.334	0.128	2.55967280356026E-47	8	TWSG1
1.20992711913519E-63	0.405006431555531	0.385	0.142	2.02759586624674E-59	8	AMBRA1
5.27327260142844E-91	0.404420396492342	0.636	0.249	8.83695022547379E-87	8	FAF1
1.45377857314691E-78	0.404412336940417	0.329	0.1	2.43624213287959E-74	8	NELFE
1.28048805488391E-74	0.404329750260153	0.806	0.364	2.14584188237446E-70	8	ZBTB33
4.33019587403734E-76	0.404252944284195	0.487	0.181	7.25654224571178E-72	8	HIBCH
2.80850701457611E-83	0.403782824132459	0.461	0.161	4.70649605502664E-79	8	HNRNPLL
1.04571563711596E-89	0.403779020337388	0.675	0.278	1.75241026467893E-85	8	PEA15
1.68154914736891E-79	0.403737632275764	0.454	0.162	2.81794006116082E-75	8	STYX
4.46406965822824E-85	0.40360907248525	0.625	0.249	7.48088793325888E-81	8	LARS
1.06009906430879E-79	0.403520637095963	0.337	0.102	1.77651401196867E-75	8	MBD1
1.6950681322772E-70	0.40336935628911	0.491	0.191	2.84059517607013E-66	8	UTRN
3.38062442731476E-83	0.403335671895784	0.394	0.127	5.66525041529407E-79	8	METTL21A
1.06911462074944E-98	0.403108478189387	0.547	0.191	1.79162228145192E-94	8	TAF6
1.43572179561854E-68	0.403097180575636	0.373	0.129	2.40598258509754E-64	8	PIGV
7.87439037263743E-82	0.402760495382365	0.565	0.218	1.31959033864658E-77	8	GMFB
7.11877311791255E-63	0.402531556331767	0.676	0.309	1.19296399909978E-58	8	BDH2
1.24775059092953E-80	0.402508289959048	0.775	0.34	2.0909804402797E-76	8	NRDC
2.38061359743448E-84	0.402299400439479	0.577	0.224	3.9894322665807E-80	8	YY1
3.35150259449585E-69	0.402166758209011	0.461	0.175	5.61644804785615E-65	8	FBXO38
8.61768085514211E-65	0.401696631101025	0.26	0.077	1.44415095770472E-60	8	NSUN5
5.22459685914549E-76	0.40142856784772	0.502	0.191	8.755379416556E-72	8	RAPGEF2
2.64735356236887E-62	0.40133521316337	0.668	0.312	4.43643509981775E-58	8	TTPA
2.64445665689074E-73	0.401267480602228	0.441	0.16	4.43158046561751E-69	8	EXOC6
2.2079425432369E-89	0.401210028217969	0.755	0.322	3.7000701139564E-85	8	UBR4
1.24127019836435E-47	0.401075174804195	0.877	0.488	2.08012059841898E-43	8	GCLC
5.30846594018452E-81	0.400770358642413	0.847	0.392	8.89592722256121E-77	8	ELOVL5
1.92659331892579E-78	0.400556430420478	0.836	0.396	3.22858508385584E-74	8	ELAVL1
5.44111744145239E-46	0.400485211065676	0.302	0.117	9.11822460838592E-42	8	IL7
1.43894588568497E-65	0.400207113563837	0.33	0.111	2.41138551523087E-61	8	HRAS
1.2410161303476E-94	0.400190164736652	0.658	0.255	2.07969483123651E-90	8	PI4KA
3.39892647699774E-89	0.399939973588401	0.614	0.236	5.6959209901528E-85	8	PGAP2
9.57633901972907E-78	0.399887452199021	0.699	0.302	1.6048028929262E-73	8	ZFAND5
2.60212003276839E-90	0.39968625168184	0.723	0.304	4.36063275091327E-86	8	ZNF644
7.79855624463203E-87	0.399611341186367	0.615	0.241	1.30688205547544E-82	8	RC3H1
8.33010121748298E-90	0.399226440682671	0.678	0.269	1.3959583620258E-85	8	SNX6
8.09094347130468E-77	0.399011420474764	0.635	0.264	1.35588030692124E-72	8	NFS1
7.95825446443551E-77	0.398979181418285	0.423	0.148	1.3336442831501E-72	8	PARN
8.89032302927659E-87	0.39896867297068	0.694	0.287	1.48984033324617E-82	8	ZNF217
1.22815702622469E-65	0.398671910451813	0.848	0.431	2.05814554454733E-61	8	SPTAN1
8.52427455611292E-73	0.398547848441831	0.458	0.17	1.4284979301134E-68	8	WASHC4
2.20173645686385E-47	0.398484021323455	0.265	0.094	3.68966995441244E-43	8	CLVS1
6.78057181373691E-73	0.398475344846213	0.557	0.222	1.13628822454603E-68	8	ATP6V0A1
1.83692744550162E-80	0.397858068920441	0.566	0.221	3.07832301317162E-76	8	NXT2
7.54948560695743E-46	0.397703397406905	0.293	0.112	1.26514279801393E-41	8	PLEKHG6
1.01342824218916E-92	0.397549912857903	0.814	0.358	1.69830304826059E-88	8	PMF1

1.86360889338441E-78	0.397154383875791	0.723	0.313	3.12303578353359E-74	8	NUDCD3
5.37588633250385E-83	0.396734713176033	0.357	0.109	9.00891031600995E-79	8	ATXN7
5.8486351917109E-63	0.396673420275895	0.346	0.121	9.80114285426913E-59	8	DENND5A
5.71042467676612E-90	0.396392994285573	0.477	0.164	9.56952967332467E-86	8	ZNF106
4.68829564867099E-76	0.396195624026565	0.788	0.356	7.85664584804284E-72	8	SPECC1L
3.80076961452361E-66	0.396159594159639	0.353	0.121	6.36932972001866E-62	8	MAP4K3
2.45055974836418E-68	0.395787004204454	0.511	0.207	4.10664802630869E-64	8	TNS2
1.51416665003406E-88	0.395719092275002	0.601	0.232	2.53744047212709E-84	8	TMEM250
3.59611272678741E-52	0.39565654551809	0.261	0.088	6.02636570755034E-48	8	REL
1.84664446550578E-80	0.395569263276288	0.399	0.133	3.09460679529458E-76	8	COPS3
1.22129994215899E-69	0.395481209806042	0.827	0.396	2.04665444307003E-65	8	COPS5
1.56852932175189E-82	0.39530911374846	0.703	0.301	2.62854143739182E-78	8	CKAP4
3.1546570885796E-82	0.395228654572259	0.538	0.204	5.28657434904169E-78	8	MESD
5.96929998826417E-72	0.39505232689417	0.427	0.156	1.00033529203331E-67	8	DYNC1H1
4.3923601202718E-50	0.394971251856515	0.256	0.087	7.36071708955148E-46	8	GEMIN5
2.63304231443805E-49	0.394966580651719	0.352	0.14	4.41245231053528E-45	8	LRRC8E
1.72842592865364E-67	0.394904038123389	0.651	0.284	2.89649617123777E-63	8	CPM
5.2867252672293E-61	0.394885874034088	0.399	0.153	8.85949420282285E-57	8	MAGEF1
1.52882709053444E-63	0.394716818688693	0.437	0.169	2.56200843831761E-59	8	LDLRAD4
3.53564212718144E-77	0.394513382888485	0.451	0.162	5.92502907673065E-73	8	SIAH1
1.74717518001233E-49	0.394492803300826	0.286	0.104	2.92791616666467E-45	8	LMNTD2
1.11518306552139E-80	0.394410523802892	0.543	0.208	1.86882378120075E-76	8	UBAC2
2.35016430042464E-46	0.394316250802162	0.322	0.127	3.93840533465161E-42	8	WNT4
3.80395450450406E-60	0.394313315489934	0.381	0.142	6.37466695864791E-56	8	C5orf51
1.18101541509032E-52	0.394067727883047	0.923	0.505	1.97914563260835E-48	8	WNK1
9.86126739066318E-47	0.394056958441113	0.304	0.115	1.65255118932734E-42	8	KIAA1211
5.921592616355E-62	0.394024940509311	0.34	0.119	9.92340490648772E-58	8	TBC1D2
3.9577533984452E-86	0.393349373025625	0.547	0.205	6.63240314511447E-82	8	QRICH1
1.64148602129156E-64	0.393300465843849	0.374	0.134	2.75080227448039E-60	8	HOOK2
5.80335099827883E-84	0.39316533352387	0.638	0.255	9.72525560291567E-80	8	AZIN1
1.81479325625988E-74	0.393056662673264	0.639	0.272	3.04123053884031E-70	8	SAMHD1
1.26822943574234E-74	0.392838235915538	0.447	0.163	2.12529888841702E-70	8	AGA
1.28583892839502E-59	0.392807121812322	0.325	0.113	2.15480887620438E-55	8	RAB2B
1.79099191451907E-80	0.392564143372021	0.565	0.218	3.00134425035106E-76	8	SLC25A43
4.45502118996816E-63	0.39242495974127	0.356	0.127	7.46572451014864E-59	8	CSTF1
5.23759133810916E-37	0.392383079350883	0.312	0.132	8.77715556440332E-33	8	MYO7B
1.21318587266348E-85	0.392294488728611	0.482	0.171	2.03305688540945E-81	8	NF2
2.77466161162916E-88	0.39228747541095	0.617	0.244	4.64977792876814E-84	8	DEPRECATED-ENSG00000265241
1.59767164011071E-91	0.391920060319048	0.519	0.185	2.67737813449753E-87	8	ISG20L2
1.08742766404836E-59	0.391819682087617	0.333	0.117	1.82231127941224E-55	8	NSUN3
1.71813111713683E-97	0.391781152291378	0.601	0.221	2.87924412609789E-93	8	PPP1R35
1.3004870405528E-71	0.391347234762283	0.414	0.148	2.17935618255838E-67	8	SRSF1
5.64154130543474E-79	0.39126754156436	0.663	0.278	9.45409491964754E-75	8	AKR7A2
3.26965384596218E-84	0.391248812412493	0.67	0.273	5.47928591506341E-80	8	CDC42SE2
3.61963913720451E-72	0.39108344568271	0.421	0.152	6.06579126612732E-68	8	DROSHA
1.35278978083699E-77	0.3908507000601	0.455	0.164	2.26700511472663E-73	8	PARP1
2.82123052567533E-70	0.39075664525842	0.531	0.213	4.72781811492672E-66	8	ELF1
4.12199470040956E-86	0.39074563222298	0.585	0.225	6.90763871894634E-82	8	DEPRECATED-ENSG00000205937
1.19038249720279E-60	0.390702895063487	0.342	0.122	1.99484298881243E-56	8	TXLNG
3.3167893694033E-82	0.390643789642838	0.395	0.129	5.55827562524604E-78	8	ZFYVE27
4.49277697282824E-63	0.390448569654243	0.897	0.48	7.52899565106556E-59	8	HADHA
3.42270586755961E-80	0.390173205646728	0.776	0.342	5.7357704928564E-76	8	SPG7
3.00492038828414E-63	0.390139839671418	0.329	0.112	5.03564558668656E-59	8	RICTOR
6.5597310970095E-70	0.39013507702878	0.434	0.162	1.09927973723685E-65	8	KAT2B
8.48618027177423E-64	0.390117155716546	0.325	0.11	1.42211408994392E-59	8	RFK
3.04268994376874E-52	0.390099677726107	0.296	0.105	5.09893980776766E-48	8	FBXL4
2.87337759279646E-62	0.38991266907261	0.381	0.14	4.81520617000831E-58	8	STRIP1
4.2315711710037E-70	0.389466410460058	0.324	0.103	7.091266968368E-66	8	TTPAL
8.74681356850222E-91	0.389321458488166	0.683	0.273	1.4657910178096E-86	8	CAMK1
8.92214324564554E-87	0.389115509052855	0.79	0.358	1.49517276510528E-82	8	KIF5B

2.44962317449608E-88	0.389091015337702	0.581	0.219	4.10507851582053E-84	8	CDK12
9.855512200534E-70	0.389084289738936	0.447	0.168	1.65158673456549E-65	8	TMEM251
6.25651462790831E-79	0.389060654515711	0.666	0.279	1.04846672134487E-74	8	ZFAND1
8.5958780630098E-39	0.388585906182861	0.959	0.633	1.44049724579918E-34	8	HSD17B11
2.27571422074983E-97	0.388538164530242	0.686	0.271	3.81364189113256E-93	8	MCUR1
8.97944133824912E-71	0.388521540024536	0.803	0.376	1.50477477946379E-66	8	MARCH6
1.51856313970471E-43	0.388249057537331	0.265	0.098	2.54480810951715E-39	8	DEPRECATED-ENSG00000130021
3.95084499468064E-69	0.387508809572801	0.511	0.203	6.62082604208582E-65	8	UVSSA
2.50696760639923E-66	0.387327272798544	0.387	0.14	4.20117631480384E-62	8	SDHAF3
2.19726325610703E-78	0.38724726438228	0.398	0.134	3.68217376458417E-74	8	RRNAD1
3.39977441230278E-58	0.387057784159685	0.899	0.46	5.697341960137E-54	8	AFF1
1.56216868884545E-54	0.386370378344655	0.302	0.107	2.61788228876721E-50	8	WBP1L
2.32147688637391E-79	0.386364878561792	0.621	0.253	3.8903309661854E-75	8	PRKCA
5.24916340378406E-35	0.38630708573181	0.939	0.651	8.79654803206132E-31	8	DUSP1
1.88782341102522E-68	0.38611270747197	0.663	0.289	3.16361447219607E-64	8	ABRACL
5.14285696178257E-95	0.385979544519587	0.648	0.25	8.61839969655523E-91	8	ATL2
4.16953830242475E-77	0.385957222414133	0.385	0.128	6.9873122872034E-73	8	CASP7
7.96839060754805E-70	0.385825112492955	0.399	0.143	1.3353428980129E-65	8	PDE6D
6.63205602588428E-52	0.385715346219064	0.3	0.108	1.11139994881769E-47	8	WDR11
1.99671599541098E-83	0.385601869712167	0.505	0.184	3.34609666510972E-79	8	KIDINS220
2.86088221531226E-78	0.38529492203222	0.533	0.204	4.79426641642028E-74	8	LSG1
9.71679454773299E-65	0.384896805901692	0.366	0.13	1.62834043030909E-60	8	ACP6
9.96995183503634E-84	0.38488178871371	0.767	0.333	1.67076452851539E-79	8	FAM20B
2.4676479107003E-100	0.384803188393557	0.477	0.154	4.13528436875157E-96	8	THOC6
1.38939660844373E-83	0.384457892109665	0.743	0.313	2.32835083643001E-79	8	MINK1
1.59008481674985E-47	0.384338489326032	0.293	0.109	2.66466413590939E-43	8	PLGRKT
9.97100693691229E-77	0.38432157605732	0.71	0.307	1.67094134248776E-72	8	MBOAT7
2.06315862810142E-74	0.384319178404444	0.764	0.34	3.45744122897236E-70	8	PDCD4
3.42577676860326E-48	0.384237773092665	0.309	0.118	5.74091670882533E-44	8	PUS7L
1.37097565352937E-67	0.384143410467438	0.374	0.132	2.29748100018452E-63	8	ATXN1L
4.99508684465283E-81	0.383991646516031	0.558	0.217	8.37076653426921E-77	8	BRD7
1.55052671608287E-79	0.383616962187816	0.407	0.136	2.59837267081168E-75	8	PPIL1
1.33497254248251E-73	0.383037422932647	0.794	0.365	2.23714698669219E-69	8	DTX3L
8.3149555117095E-31	0.382978429353858	0.951	0.653	1.39342024465228E-26	8	SGK1
5.72656784279408E-68	0.382918901309108	0.655	0.29	9.59658239095433E-64	8	ME1
5.36722713935791E-65	0.382913751006597	0.318	0.105	8.99439924013599E-61	8	UBE2N
1.07324540680645E-76	0.38222551305031	0.76	0.346	1.79854465272624E-72	8	AP1M1
2.94528865928602E-60	0.382075302766158	0.27	0.086	4.9357147352315E-56	8	ZNF574
9.31073927340595E-76	0.381683321862725	0.543	0.212	1.56029368743737E-71	8	DYRK2
1.40374868152333E-78	0.381558142085797	0.739	0.322	2.3524020404968E-74	8	RBM10
1.90570469296374E-73	0.381204438745784	0.425	0.152	3.19357992446864E-69	8	FCHSD2
8.33285730361096E-58	0.380715997749945	0.361	0.135	1.39642022693912E-53	8	ABCB1
1.20994867099648E-81	0.380548874000325	0.47	0.168	2.0276319828559E-77	8	ZNF639
5.28269004045469E-75	0.380212451368285	0.449	0.163	8.85273196979397E-71	8	PITPNC1
6.75591953971043E-63	0.380116325050443	0.417	0.16	1.13215699646467E-58	8	SFT2D1
3.04497494820396E-58	0.379969469464937	0.36	0.134	5.1027690182002E-54	8	REPS2
2.1349846063588E-56	0.379912576655657	0.289	0.099	3.57780720333608E-52	8	MTAP
7.97164181979191E-74	0.379546113747835	0.511	0.198	1.33588773616073E-69	8	NMI
3.12276649753013E-66	0.37921979962168	0.67	0.294	5.23313209656099E-62	8	SI
8.96997153652202E-51	0.379040826033785	0.286	0.103	1.50318783009036E-46	8	BTBD9
5.31232576484595E-49	0.378638533291603	0.277	0.099	8.90239551672884E-45	8	FAM118B
1.38669518508396E-79	0.378493454301141	0.719	0.304	2.3238237911637E-75	8	FOXO3
9.75835340654998E-87	0.378412998431833	0.723	0.301	1.63530486386965E-82	8	DPP9
2.584643854535E-102	0.378264725605722	0.76	0.306	4.33134617142976E-98	8	NCOR2
1.5540640232072E-67	0.378258430170653	0.639	0.283	2.60430049009063E-63	8	PPP1R10
1.10509088990021E-65	0.378188372889645	0.325	0.108	1.85191131329478E-61	8	PHF20
9.114335552668E-68	0.378034202201428	0.397	0.143	1.5273803519161E-63	8	SLC35B3
1.78696544867945E-81	0.377549590130771	0.688	0.288	2.99459669889703E-77	8	LRCH3
3.97510647968957E-73	0.377300319881978	0.334	0.106	6.66148343866379E-69	8	VTI1A
3.5765923065949E-57	0.377153540941012	0.34	0.124	5.99365338739174E-53	8	NTN4

2.26400143965053E-62	0.377102965194485	0.31	0.103	3.79401361256636E-58	8	RNF111
1.56306494093674E-58	0.376412123942129	0.366	0.137	2.61938422802179E-54	8	ZNF362
1.45769954665378E-86	0.37639636324113	0.638	0.256	2.44281290028241E-82	8	SKI
1.51649407246377E-62	0.376247967722975	0.382	0.141	2.54134076663478E-58	8	LEMD3
4.17828574452993E-80	0.376090446692877	0.766	0.335	7.00197125068326E-76	8	UPF1
2.94284852292331E-77	0.375334404773586	0.406	0.139	4.93162555471489E-73	8	ABLIM1
9.56602341685774E-152	0.375118636815363	0.732	0.239	1.60307420419702E-147	8	RGS3
1.50813175946857E-73	0.37502307714223	0.474	0.178	2.52732720251742E-69	8	RBM27
1.10348035372441E-64	0.374600879565635	0.344	0.119	1.84921237677137E-60	8	SMARCD1
5.917742904888E-71	0.374470086639518	0.755	0.341	9.91695356001131E-67	8	LYPLAL1
9.69233324377249E-56	0.374427347453302	0.301	0.105	1.62424120499139E-51	8	AP2B1
1.12706813352447E-57	0.374252676670399	0.431	0.173	1.88874077816031E-53	8	TBPL1
3.09183613591693E-49	0.373937251740876	0.284	0.102	5.18129899656959E-45	8	TRAF6
1.16866984887379E-80	0.373685461150928	0.526	0.2	1.95845693274269E-76	8	UFD1
9.9905340736878E-78	0.373645117698492	0.843	0.408	1.6742137000686E-73	8	SRSF4
2.24970875949553E-55	0.373638667021995	0.848	0.444	3.7700619391626E-51	8	STT3A
4.36498287908096E-71	0.373158786849759	0.348	0.115	7.31483830876387E-67	8	HDAC4
5.00704655608235E-49	0.373018602812585	0.505	0.228	8.3908086186828E-45	8	S100P
2.71380979644296E-79	0.372633670521645	0.71	0.302	4.54780245687911E-75	8	SIPA1L3
1.57893372632898E-61	0.372261249456283	0.321	0.11	2.64597713858211E-57	8	ENC1
4.35716361296093E-97	0.372227822358473	0.518	0.177	7.30173478259993E-93	8	PHF2
7.62786611315185E-64	0.372162776941177	0.374	0.136	1.27827780324199E-59	8	CCSER2
6.60990308290437E-97	0.372154046458739	0.531	0.186	1.10768755863311E-92	8	TUBA4A
1.39519693187364E-47	0.372139797857909	0.288	0.107	2.33807101843384E-43	8	SESN1
8.53770954666671E-76	0.372050891862254	0.814	0.372	1.43074936583041E-71	8	SAFB2
7.88812691812482E-81	0.372007086508329	0.686	0.29	1.32189230893936E-76	8	CUL4A
2.8881983163452E-68	0.371805056351693	0.364	0.125	4.84004273853129E-64	8	BAZ1A
1.11748939396839E-52	0.371447311228327	0.358	0.139	1.87268872641224E-48	8	C15orf40
4.69517247716728E-74	0.371322792165892	0.795	0.369	7.86817003723693E-70	8	HUWE1
9.42664949118324E-76	0.37110398167157	0.679	0.287	1.57971792173249E-71	8	PARP9
3.76944826924327E-68	0.371090622646239	0.426	0.159	6.31684140959788E-64	8	VRK3
2.14167947609105E-53	0.370818555880436	0.368	0.143	3.58902646603338E-49	8	TMEM171
1.91664670018754E-60	0.370781463844913	0.379	0.141	3.21191654017428E-56	8	GDAP2
8.74442801753878E-92	0.370543655810559	0.683	0.272	1.46539124717915E-87	8	AGGF1
3.7435422152107E-79	0.370391276088282	0.782	0.352	6.27342804425009E-75	8	LRRFIP2
1.6831455998969E-66	0.370314267920667	0.782	0.378	2.82061539630722E-62	8	HEXB
1.01403219863973E-68	0.370248782282269	0.447	0.169	1.69931515848046E-64	8	FGFR1OP2
5.4129476571139E-68	0.369743234375796	0.527	0.217	9.07101768379147E-64	8	C6orf89
1.18547987097217E-82	0.36933965852908	0.691	0.289	1.98662716777516E-78	8	AATF
6.73035108923776E-77	0.369134690113105	0.694	0.297	1.12787223553446E-72	8	HNRNPDL
3.42568323701972E-73	0.368586572979736	0.798	0.373	5.74075996859765E-69	8	SHROOM1
3.31585758872933E-83	0.368540541674281	0.555	0.21	5.55671414719261E-79	8	TMEM220
1.88927902230374E-83	0.368478731413334	0.563	0.214	3.16605378557661E-79	8	HERC4
2.70354987734025E-58	0.368266424731102	0.346	0.126	4.53060888444679E-54	8	NBPF12
1.21120685982235E-73	0.368241618329894	0.715	0.315	2.0297404556903E-69	8	PRRC2C
7.84559010216841E-80	0.368134331974391	0.434	0.151	1.31476398932138E-75	8	PSME2
4.51693518378171E-69	0.367946460256968	0.88	0.458	7.56947998098138E-65	8	LUC7L2
8.17797616859198E-85	0.367858228202888	0.678	0.278	1.37046524633264E-80	8	MACF1
1.94597638893027E-73	0.367611209917696	0.493	0.188	3.26106723256934E-69	8	AKAP17A
1.03008338945753E-79	0.367551009431395	0.437	0.153	1.72621374405293E-75	8	EEFSEC
9.43460292464768E-80	0.367515916664105	0.558	0.217	1.58105075811246E-75	8	NFAT5
2.32311254469109E-64	0.367406266067131	0.322	0.108	3.89307200239332E-60	8	RRAGC
2.17530248264546E-97	0.367401978567023	0.595	0.219	3.64537190041726E-93	8	SLC16A1
1.35499303089587E-51	0.367231386260296	0.273	0.094	2.2706973211753E-47	8	MITD1
3.8965559240122E-61	0.367153152737137	0.329	0.114	6.52984841745964E-57	8	ZNF335
2.03020924749145E-48	0.366759677462157	0.919	0.536	3.40222465694617E-44	8	FAM50A
5.6372576330456E-64	0.366713226821179	0.86	0.435	9.44691634145781E-60	8	RBM33
5.73495878789188E-67	0.366605240839113	0.786	0.376	9.61064393674922E-63	8	PER1
2.51330744999749E-74	0.366541599053492	0.776	0.35	4.2118006247058E-70	8	YTHDF3
2.46947146188311E-63	0.366530709187409	0.39	0.144	4.13834027582371E-59	8	PDK1

2.72749606273431E-39	0.366462128393083	0.25	0.095	4.57073790193016E-35	8	POLI
6.65522226118393E-69	0.36636730146025	0.417	0.152	1.1152821465292E-64	8	COG7
6.15341294004899E-67	0.366167670078174	0.342	0.115	1.03118894049341E-62	8	SCYL2
1.83885710520557E-46	0.366032358344962	0.308	0.119	3.0815567369035E-42	8	HMGXB3
1.60409010899482E-58	0.365917156289268	0.285	0.094	2.68813420465352E-54	8	DEPRECATED-ENSG00000144228
2.16253975522315E-87	0.365685853012294	0.672	0.272	3.62398412180296E-83	8	SCAF1
2.33656688583478E-42	0.365362219288196	0.28	0.108	3.91561878728192E-38	8	TMEM87B
7.82872828763079E-75	0.365332519911902	0.565	0.227	1.31193828644117E-70	8	SNX27
7.42789375352553E-77	0.365283966722432	0.68	0.289	1.24476643521581E-72	8	RORA
3.28125831164751E-78	0.365042118731594	0.73	0.316	5.4987326786589E-74	8	DYNLT1
1.34720609107383E-78	0.36482869306639	0.767	0.342	2.25764796742152E-74	8	SAFB
1.60431584421616E-53	0.364529868459534	0.294	0.104	2.68851249173744E-49	8	SF3A3
7.12869035360118E-73	0.364524413443244	0.348	0.113	1.19462592945648E-68	8	COQ7
2.58149643487518E-86	0.364450513965426	0.531	0.195	4.32607172556383E-82	8	NARF
2.88401994008611E-42	0.364167162855274	0.936	0.54	4.83304061559631E-38	8	CAPN2
8.99313165706296E-72	0.36409536144794	0.581	0.236	1.50706900309061E-67	8	RO60
3.99718624412176E-72	0.364080839801374	0.659	0.286	6.69848470789924E-68	8	FAM214A
6.79577444729243E-63	0.363926378665004	0.348	0.122	1.13883588187727E-58	8	TRIM2
1.81237742017783E-46	0.363683345143747	0.301	0.115	3.03718208073401E-42	8	C6orf47
9.16709513368467E-96	0.363356194147284	0.679	0.265	1.53622180250288E-91	8	SAP30L
1.79954078392806E-81	0.363095436044068	0.471	0.168	3.01567044570665E-77	8	TTLL12
9.16415771066981E-84	0.363000690137144	0.678	0.282	1.53572954915405E-79	8	SPEN
5.3188730579626E-76	0.362733447162422	0.743	0.321	8.91336747053373E-72	8	ZMYM2
1.21754005957595E-74	0.362606652449558	0.577	0.231	2.04035363183737E-70	8	VPS13C
1.73561736491198E-70	0.362517350525743	0.469	0.179	2.90854758011949E-66	8	RBX1
3.81029210707139E-87	0.362021276332001	0.766	0.328	6.38528751303023E-83	8	FAM83H
9.32037085816491E-84	0.361977895672966	0.678	0.277	1.56190774841128E-79	8	DYNC1I2
2.89888938652177E-60	0.36169302881086	0.32	0.11	4.85795883393319E-56	8	MEN1
1.30921920597733E-41	0.36148086534776	0.94	0.542	2.19398954537681E-37	8	SERINC5
3.54240406435434E-71	0.361401896887982	0.563	0.227	5.936360731045E-67	8	FGF13
7.26509115619066E-83	0.36136358192961	0.712	0.303	1.21748397595443E-78	8	RAF1
3.15582283206446E-86	0.360807212392623	0.719	0.3	5.28852790197362E-82	8	THRAP3
3.13677815589077E-84	0.360787721873736	0.437	0.148	5.25661283364176E-80	8	MRM2
9.92917770169927E-76	0.360707083273252	0.659	0.285	1.66393159925076E-71	8	PPM1G
2.15617176150377E-42	0.360405679305027	0.975	0.759	3.61331263792802E-38	8	HSP90AA1
1.27647982985441E-70	0.360391843065338	0.386	0.135	2.13912489887001E-66	8	MIER3
1.23195378184611E-86	0.359977023442242	0.618	0.241	2.06450814761771E-82	8	PIP5K1A
1.14700043555083E-79	0.35964820327899	0.509	0.189	1.92214332989608E-75	8	STXBP3
2.28700155420032E-77	0.359608853254849	0.425	0.148	3.83255720452889E-73	8	SNUPN
1.38024357015017E-74	0.359467292075836	0.445	0.16	2.31301217485766E-70	8	PRPF18
1.81070583486343E-78	0.359204955944082	0.477	0.174	3.03438083806414E-74	8	GMPR2
4.65509764785013E-59	0.359095274388751	0.324	0.113	7.80101263826724E-55	8	MARF1
5.8509956640726E-50	0.359018216778213	0.318	0.12	9.80509853385286E-46	8	VAMP4
1.5572393405073E-70	0.35879388372869	0.254	0.071	2.60962168682214E-66	8	WBP11
3.78104528960765E-76	0.358789244850524	0.479	0.177	6.33627569632449E-72	8	IFI44
2.19259972516948E-66	0.358639739792333	0.361	0.125	3.67435861943901E-62	8	KIZ
2.6806128591775E-31	0.358041501525795	0.957	0.636	4.49217102940965E-27	8	BAAT
1.05170607617857E-109	0.357897993240343	0.514	0.165	1.76244904246005E-105	8	CCM2
5.82901236582908E-93	0.357853521952408	0.477	0.16	9.76825892265638E-89	8	KIF21A
5.10832810863014E-65	0.35751353445741	0.387	0.141	8.5605362444424E-61	8	MAPK8
1.58731505765929E-76	0.357459272997969	0.603	0.248	2.66002257362544E-72	8	SCAF4
1.1243980963982E-59	0.35739868716497	0.298	0.101	1.8842663299441E-55	8	MTA3
3.61360406059266E-85	0.357171625782821	0.537	0.197	6.05567768474118E-81	8	CCNQ
8.6177346585306E-47	0.357048197583503	0.253	0.088	1.44415997407656E-42	8	VIPAS39
1.15265956969768E-77	0.356982016187269	0.55	0.215	1.93162690689937E-73	8	SYNRG
4.40899569409761E-72	0.356969520749594	0.756	0.354	7.38859498416877E-68	8	PSMB9
2.03553392479664E-78	0.35678638809482	0.546	0.214	3.4111477511742E-74	8	LCOR
3.42044119054108E-53	0.356681560526217	0.298	0.106	5.73197534710874E-49	8	CNOT4
1.76193078700442E-68	0.356342070252241	0.443	0.169	2.95264361286201E-64	8	SLC41A3
2.04057584200381E-73	0.35583536872875	0.389	0.134	3.41959699602998E-69	8	ORAI1

1.31489553263205E-59	0.355486233729285	0.746	0.366	2.20350193358478E-55	8	RDH10
2.13810750788929E-69	0.35540807775266	0.425	0.156	3.58304056172087E-65	8	TBC1D13
3.16473539271519E-76	0.355323379810832	0.763	0.339	5.30346357111211E-72	8	PSMD1
4.60010832073279E-65	0.355199362515931	0.546	0.226	7.708861523884E-61	8	TMEM131
1.12317267637893E-71	0.35495005745102	0.527	0.209	1.88221277107581E-67	8	CEBPZOS
1.21617585181963E-76	0.354597145248648	0.338	0.105	2.03806749247934E-72	8	CWC25
1.81482935784009E-93	0.354493058724715	0.571	0.207	3.04129103786842E-89	8	ATF6
3.14334609429661E-68	0.354308752562558	0.41	0.149	5.26761938482226E-64	8	POM121
1.05980111680351E-51	0.354293553267966	0.33	0.125	1.77601471153932E-47	8	DUSP22
1.21475817907613E-59	0.353862077288763	0.972	0.546	2.03569175649577E-55	8	CCNL2
1.89129649748092E-33	0.35341777369249	0.25	0.103	3.16943467047852E-29	8	FMO1
3.49962364498457E-83	0.353368348358924	0.628	0.252	5.86466930426514E-79	8	FAAH
9.457838212752E-78	0.353207814367138	0.257	0.067	1.58494452769298E-73	8	RBM8A
8.77400275443147E-58	0.353167277842053	0.855	0.438	1.47034738158763E-53	8	ABCA1
1.13188873132901E-73	0.353119727976293	0.511	0.198	1.89681913596115E-69	8	ME2
1.49986472279471E-70	0.353027904583186	0.406	0.145	2.51347330245938E-66	8	TAF12
7.6149183069055E-79	0.352934897504134	0.525	0.2	1.27610800987122E-74	8	ZNF768
1.32989069343485E-77	0.352551554205196	0.422	0.146	2.22863082405812E-73	8	WTAP
2.606585265399E-58	0.352531947324874	0.511	0.216	4.36811558775565E-54	8	HSD11B2
1.70110409363196E-58	0.352415692109222	0.25	0.077	2.85071024010844E-54	8	SVBP
5.80686048339383E-71	0.352106062384372	0.868	0.445	9.73113679807138E-67	8	SLC43A3
2.47917672716153E-48	0.351944739645407	0.253	0.087	4.1546043593773E-44	8	METTL22
1.90229943465418E-64	0.35175448089062	0.892	0.468	3.18787339259348E-60	8	SHMT1
5.57233742977524E-92	0.351754181007404	0.474	0.16	9.33812306481735E-88	8	GTF2H4
9.09833019932868E-86	0.351751090479707	0.743	0.313	1.5246981748035E-81	8	MAPK14
1.73609325051096E-85	0.351431534262881	0.648	0.261	2.90934506920626E-81	8	PPIG
2.26953109753105E-44	0.351372021186745	0.901	0.527	3.80328021324254E-40	8	HNRNPH2
7.97374241673691E-58	0.35118421148177	0.344	0.126	1.33623975419677E-53	8	LRIF1
7.11913459569186E-74	0.351131992934834	0.332	0.105	1.19302457554604E-69	8	FAM53C
8.58688181331478E-52	0.35053076973269	0.285	0.1	1.43898965427529E-47	8	SPATA2
2.37030807535612E-67	0.350504671146218	0.37	0.13	3.97216227268178E-63	8	AIDA
7.85434708926797E-81	0.350283886695827	0.541	0.206	1.31623148521953E-76	8	FAM168A
1.346884672098E-38	0.350254603986224	0.953	0.566	2.25710933350183E-34	8	TSPAN3
7.80051686852875E-72	0.350193358777562	0.467	0.176	1.30721061682805E-67	8	GTPBP10
1.07487715574529E-74	0.350114283098719	0.465	0.172	1.80127913759796E-70	8	MED17
3.95570969515645E-70	0.349946506363554	0.747	0.349	6.62897830714318E-66	8	TMEM97
1.11257537425837E-71	0.349841994634927	0.557	0.225	1.86445381218217E-67	8	TM2D2
1.87120623191993E-61	0.34978647234812	0.478	0.196	3.13576740345141E-57	8	KIAA2026
3.11532355127859E-53	0.349746759903388	0.269	0.091	5.22065920723267E-49	8	PSMD14
4.1210054208856E-68	0.349742978709102	0.462	0.178	6.90598088432008E-64	8	DDX19A
2.93667925901176E-77	0.349529429139382	0.523	0.2	4.92128710225191E-73	8	PPP2CA
1.80358871992168E-77	0.349190231860074	0.531	0.204	3.02245397684475E-73	8	BAG2
2.1117537636455E-86	0.348885226669381	0.537	0.197	3.53887695711713E-82	8	STAT5B
1.99736462826554E-53	0.348769467360826	0.276	0.094	3.3471836440474E-49	8	NECTIN1
5.35814805385787E-62	0.348705413728079	0.79	0.377	8.97918450865502E-58	8	NDFIP2
3.1555133753406E-56	0.348676438353967	0.329	0.119	5.28800931439578E-52	8	BICRAL
7.12168072603333E-70	0.348626832563169	0.457	0.172	1.19345125606866E-65	8	DEPRECATED-ENSG00000178950
9.41211517633482E-47	0.348479803031307	0.346	0.139	1.57728226125019E-42	8	LRIG2
1.11667942317699E-60	0.348448493102289	0.402	0.154	1.87133137736E-56	8	NOL9
1.18182639968398E-66	0.348426843218994	0.395	0.143	1.98050468059041E-62	8	MED22
9.59862406560712E-85	0.348233780515201	0.578	0.22	1.60853742091444E-80	8	PRPF38B
1.48476773144373E-71	0.348105971264146	0.723	0.327	2.48817376435341E-67	8	APH1A
4.98772196219588E-58	0.348089893307354	0.338	0.121	8.35842446424785E-54	8	B4GALT6
1.16278413901224E-80	0.348006857467733	0.569	0.22	1.9485936601567E-76	8	WASHC5
1.69596226141094E-59	0.347803255662245	0.824	0.398	2.84209355767245E-55	8	MATN2
2.1438656146839E-59	0.347770812194502	0.35	0.128	3.59268999708728E-55	8	RABGGTA
5.04769986546397E-73	0.347672562312996	0.497	0.19	8.45893543454453E-69	8	COPS4
9.45988049551471E-92	0.347638482520422	0.567	0.207	1.58528677343836E-87	8	TNKS
1.38759754062961E-85	0.347633005323606	0.443	0.15	2.32533595858711E-81	8	PPIF
6.05917110877304E-71	0.34757167655487	0.382	0.132	1.01539589440819E-66	8	POLE4

4.28183817654456E-46	0.347553931735433	0.308	0.119	7.17550441625337E-42	8	VMAC
2.34074173600464E-77	0.347366351130672	0.679	0.284	3.92261500119657E-73	8	SLC10A3
3.45655522911412E-58	0.347292271827336	0.394	0.152	5.79249525294944E-54	8	WDR37
1.97630881368527E-76	0.347186043246325	0.634	0.26	3.31189830997378E-72	8	SKAP2
2.76248633848598E-73	0.347141797709384	0.698	0.304	4.62937460603481E-69	8	SH3BP4
4.26750715256107E-91	0.347137884602768	0.738	0.305	7.15148848626183E-87	8	HIPK3
1.14462311950021E-81	0.34595431279798	0.601	0.238	1.91815942365845E-77	8	ITGAV
7.53816624073228E-75	0.345649334131798	0.784	0.352	1.26324589862192E-70	8	ATP2A2
3.64551908085837E-71	0.344948734076148	0.728	0.317	6.10916087570245E-67	8	UGT2B11
6.19405560042199E-72	0.34487571198842	0.786	0.366	1.03799983751872E-67	8	RHPN2
3.13569435483697E-80	0.344725637096006	0.545	0.209	5.2547965998358E-76	8	YIF1B
1.33695617693779E-83	0.344554427082074	0.573	0.22	2.24047116131234E-79	8	FBXW7
4.17173692572615E-67	0.344539562015544	0.462	0.178	6.99099674013189E-63	8	ITSN1
7.11064359060437E-63	0.344333611964119	0.836	0.42	1.19160165291348E-58	8	SRP9
2.39991297702981E-67	0.344049396458128	0.502	0.201	4.02177416690655E-63	8	FBH1
5.09336955405343E-41	0.343999770451328	0.931	0.577	8.53546869868275E-37	8	LSR
5.75997827863101E-73	0.343997256423915	0.497	0.19	9.65257159932984E-69	8	VPS13D
2.01617785569757E-83	0.343624071984748	0.668	0.278	3.37871085057799E-79	8	STK11
3.00717509192163E-73	0.343617576230837	0.695	0.305	5.03942401904227E-69	8	SNRPB
2.80418751689349E-58	0.343498366638333	0.391	0.15	4.6992574408101E-54	8	TP53I3
3.42384969891129E-57	0.343252841075454	0.475	0.199	5.73768732543554E-53	8	CES3
1.68456264057662E-87	0.343137177458049	0.482	0.167	2.82299007307831E-83	8	ARIH2
6.72752485562167E-75	0.343077864978903	0.569	0.226	1.12739861530508E-70	8	UTP23
1.85091780597175E-56	0.342976135305542	0.63	0.289	3.10176805924745E-52	8	INAVA
4.22707175901376E-57	0.34288794028343	0.305	0.105	7.08372685375525E-53	8	VGLL4
5.59487216769009E-74	0.342715439236283	0.563	0.226	9.37588677861505E-70	8	WASHC1
2.52015873420079E-72	0.342530861538073	0.451	0.167	4.22328200677368E-68	8	MIGA1
1.07610353924498E-59	0.342103529850078	0.394	0.15	1.80333431106673E-55	8	ZKSCAN8
8.23797881744192E-64	0.341946416442813	0.521	0.215	1.38052049022692E-59	8	BAMBI
7.96072390813596E-70	0.341882243983422	0.631	0.273	1.33405811252542E-65	8	CLN3
7.6264741925778E-77	0.341863934021606	0.494	0.184	1.27804454519219E-72	8	HDAC3
4.62460985246965E-83	0.341737846161187	0.518	0.193	7.74992119076863E-79	8	CREM
2.2391762586337E-65	0.341552643398741	0.441	0.169	3.75241157421835E-61	8	TBCE
8.23088551500789E-65	0.341226645215383	0.842	0.405	1.37933179460502E-60	8	NECAB3
4.62000219870761E-72	0.341209831402337	0.334	0.106	7.74219968459421E-68	8	PPP3CB
5.06486440582474E-72	0.340506350757559	0.391	0.136	8.4876997712811E-68	8	SLC52A2
1.92975707210295E-79	0.340484615843078	0.563	0.22	3.23388690143013E-75	8	GLO1
6.39697638624291E-67	0.340450155948559	0.83	0.398	1.07200530280659E-62	8	MLEC
4.41168096131137E-78	0.340306591384298	0.712	0.308	7.39309495549656E-74	8	ITPRID2
1.04935374764945E-80	0.340219173661536	0.611	0.245	1.75850701031095E-76	8	TLN1
3.4801151524029E-76	0.340119439020238	0.581	0.232	5.83197697239678E-72	8	USP34
8.46334376653485E-74	0.340093879805442	0.755	0.339	1.41828714839591E-69	8	TMEM41B
7.72046756833286E-73	0.340010093581578	0.71	0.305	1.29379595510122E-68	8	LNX2
2.16303863114555E-71	0.339871284675429	0.599	0.25	3.62482013807371E-67	8	VPS26C
8.40058485955019E-75	0.339797709964611	0.443	0.159	1.40777001076342E-70	8	ACIN1
4.44412343252585E-65	0.339434612746811	0.398	0.146	7.44746204822682E-61	8	SPAG16
1.27132907645226E-65	0.33933578266248	0.704	0.321	2.1304932663187E-61	8	ELOVL1
9.52298931469026E-59	0.339313252918765	0.309	0.106	1.59586254935579E-54	8	SLC35A5
3.50681863697595E-71	0.339252106231042	0.401	0.142	5.8767266718443E-67	8	HMGN5
1.49022713850842E-77	0.339231868925073	0.45	0.162	2.49732263871242E-73	8	MARK2
1.01627929201217E-65	0.33903767045501	0.501	0.2	1.703080837554E-61	8	SNX19
1.00283303475786E-73	0.338970790918691	0.337	0.107	1.68054759964722E-69	8	CCDC97
1.84383284465185E-73	0.338641001489666	0.454	0.167	3.08989508106757E-69	8	CSNK1A1
3.54444036329673E-51	0.338588545790307	0.908	0.474	5.93977316081266E-47	8	ZHX1
4.00935839392065E-85	0.338513103880703	0.614	0.242	6.71888279653222E-81	8	TAB2
4.67683676827074E-56	0.338492498961261	0.389	0.151	7.83744305626811E-52	8	PAM
3.01959572986676E-66	0.338404727917748	0.35	0.121	5.06023852411071E-62	8	BBX
2.81215298960739E-58	0.338274633744523	0.281	0.092	4.71260597998406E-54	8	KATNB1
1.82877504727735E-57	0.338202917325342	0.366	0.138	3.06466122422739E-53	8	MED20
4.52802604957698E-72	0.338031910594192	0.353	0.117	7.5880660538811E-68	8	UBXN7

4.56866944822976E-50	0.337894010476611	0.284	0.102	7.65617626134343E-46	8	FBXO25
8.6035506053199E-80	0.337889478964808	0.602	0.24	1.44178301043951E-75	8	PAFAH1B2
2.19681082511983E-52	0.337689167450455	0.369	0.145	3.68141558073582E-48	8	ZFP64
1.64916376910014E-47	0.337606923140414	0.936	0.561	2.76366864425801E-43	8	ACTN4
9.90580237702324E-76	0.337439962584144	0.553	0.218	1.66001436234155E-71	8	RNF113A
1.24605089989053E-80	0.337383704649514	0.503	0.186	2.08813209803656E-76	8	OGFOD2
9.86714037721565E-67	0.337224696930608	0.462	0.177	1.6535353844138E-62	8	IREB2
2.42822867171158E-56	0.337075960146844	0.458	0.189	4.06922560805427E-52	8	GPAT3
9.26880961687048E-87	0.336904559001624	0.533	0.194	1.55326711559515E-82	8	PARD3
1.47711055333597E-51	0.336773119625107	0.55	0.253	2.47534186528041E-47	8	MANEAL
6.40793301362521E-67	0.336755772039235	0.844	0.389	1.07384141442331E-62	8	SLC17A4
6.23015264118221E-71	0.336015505558271	0.547	0.22	1.04404897960931E-66	8	SGMS1
1.5901829789079E-58	0.335937033449957	0.258	0.081	2.66482863605386E-54	8	CCDC84
4.29159474544958E-68	0.335821594742356	0.819	0.388	7.1918544744244E-64	8	ERO1A
9.54282459920302E-77	0.335091774219512	0.597	0.24	1.59918654633444E-72	8	AP1G1
2.05192590587166E-70	0.334959113857587	0.471	0.18	3.43861743305973E-66	8	PHF11
1.06310277101661E-48	0.334820206935685	0.92	0.51	1.78154762366964E-44	8	CDH17
1.10825887481188E-62	0.334785536176459	0.344	0.12	1.85722022240974E-58	8	RBBP9
7.57154236896984E-60	0.334501168409466	0.503	0.211	1.26883907019197E-55	8	MID2
5.65550504577078E-33	0.334117458000795	0.964	0.647	9.47749535570268E-29	8	F5
9.91391317315346E-72	0.334082966829335	0.583	0.242	1.66137356955706E-67	8	RPRD1A
6.67004059902624E-85	0.334067756636233	0.647	0.26	1.11776540358482E-80	8	ATPAF1
2.35335542981564E-70	0.334010205623078	0.545	0.22	3.94375302928505E-66	8	SLC30A9
2.35367400298657E-69	0.333750698931012	0.338	0.111	3.94428689420489E-65	8	TSPAN12
1.66762000906694E-76	0.333719767411099	0.437	0.155	2.79459761119438E-72	8	PRR14
1.0504714312849E-65	0.333649284465687	0.551	0.229	1.76038002454724E-61	8	NDUFB4
5.66125228706163E-59	0.333453525050168	0.417	0.164	9.48712658265788E-55	8	GYS1
7.00620860349969E-77	0.332885835739767	0.638	0.263	1.17410043777448E-72	8	PCGF2
2.55730116446527E-69	0.332885168234567	0.482	0.187	4.2855252914109E-65	8	CYTH3
4.17081687795387E-78	0.332707260215258	0.498	0.186	6.9894549240751E-74	8	ABCD4
7.11442723531824E-74	0.332555277773064	0.566	0.228	1.19223571609463E-69	8	CHD4
2.34590621277235E-59	0.332500577195071	0.407	0.157	3.93126963136391E-55	8	TGDS
8.17886028626428E-65	0.33237059778075	0.324	0.108	1.37061340677217E-60	8	OPN3
2.29297304018448E-69	0.33236512562151	0.84	0.4	3.84256422074116E-65	8	FDPS
1.50136952123222E-73	0.3321169584341	0.438	0.158	2.51599504368095E-69	8	REST
8.69694121384465E-68	0.332071572284601	0.394	0.142	1.45743340861609E-63	8	SMURF1
1.39797918110194E-84	0.332017782339421	0.742	0.314	2.34273351169062E-80	8	SETD3
1.48715740912118E-78	0.331997612748953	0.562	0.219	2.49217838620527E-74	8	EPS8
1.21609403441602E-73	0.33194572657086	0.808	0.368	2.03793038287437E-69	8	PATJ
9.10887435078768E-42	0.331637760287048	0.951	0.605	1.526465163705E-37	8	VEGFA
9.71407807240813E-81	0.331509091253329	0.449	0.156	1.62788520337416E-76	8	BRIX1
2.10753755247636E-47	0.331480651422836	0.256	0.09	3.53181143043989E-43	8	CCDC126
5.75864761992083E-55	0.331326790603467	0.301	0.106	9.65034168146333E-51	8	XPO7
1.32503334234127E-77	0.331302635399116	0.618	0.253	2.2204908750955E-73	8	AFG3L2
8.64570712143983E-58	0.331148009380396	0.365	0.136	1.44884759941089E-53	8	CSNK1G3
4.81125426892826E-70	0.330561261528912	0.446	0.167	8.06269990386997E-66	8	DENR
6.9723871729436E-62	0.330487884789546	0.471	0.19	1.16843264244189E-57	8	SLC30A7
7.37379641995365E-89	0.330381632016531	0.554	0.204	1.23570080405583E-84	8	FOXN3
3.66345095337125E-70	0.330302677436446	0.782	0.368	6.13921110765953E-66	8	MICOS10
2.26673707434734E-57	0.330131326106826	0.915	0.498	3.79859798919127E-53	8	NFIC
2.33295799028853E-84	0.329836520629222	0.671	0.272	3.90957100012552E-80	8	MED15
5.34118995585301E-64	0.329554773444956	0.509	0.209	8.95076612801847E-60	8	MTX2
6.94190184506279E-65	0.329336853445075	0.601	0.259	1.16332391119562E-60	8	DDX3X
2.44588068740223E-74	0.329149686091476	0.543	0.215	4.09880685594866E-70	8	PHRF1
1.0632035012128E-62	0.328961674824868	0.866	0.46	1.78171642733241E-58	8	TRIR
1.49439064546075E-91	0.328848898705719	0.438	0.142	2.50429984366313E-87	8	PATL1
1.25805759631168E-70	0.328639522051304	0.762	0.35	2.10825291989912E-66	8	CCT6A
3.67579141705979E-61	0.328361892230839	0.276	0.087	6.1598912567088E-57	8	B4GALT2
1.92687990293713E-64	0.328347986863339	0.77	0.362	3.22906534134205E-60	8	TMEM38B
1.28557814561987E-41	0.328270067256562	0.264	0.099	2.15437185642978E-37	8	SLC44A4

1.15014542848929E-73	0.328165281978502	0.678	0.289	1.92741370906235E-69	8	MTIF3
1.21269246687557E-55	0.327811491371409	0.281	0.094	2.03223003599008E-51	8	MUL1
6.06869592641855E-59	0.327561360160886	0.808	0.422	1.01699206334922E-54	8	PCYT2
1.46018643240132E-75	0.327224691328257	0.675	0.287	2.44698042341814E-71	8	SMAD4
6.87416299515703E-78	0.327186592456095	0.668	0.28	1.15197223472842E-73	8	ELOVL2
1.79759684395542E-49	0.327073416327011	0.296	0.108	3.0124127911005E-45	8	SGSH
2.03575380851024E-52	0.326885817022682	0.257	0.086	3.41151623230146E-48	8	KCTD10
2.87625981231277E-42	0.326453709523622	0.293	0.115	4.82003619347375E-38	8	FXYD2
5.73992515133381E-64	0.326386330723731	0.397	0.147	9.6189665686052E-60	8	CARD19
4.549033210823E-70	0.326362077091298	0.411	0.148	7.62326985469719E-66	8	SETBP1
1.10553984194655E-61	0.326326236078831	0.385	0.142	1.85266366713403E-57	8	TMEM30A
3.05674657896978E-73	0.325728076971658	0.503	0.194	5.12249591703756E-69	8	INPPL1
1.04795308278489E-65	0.325637813182297	0.601	0.257	1.75615977613092E-61	8	CORO2A
3.00429669319834E-79	0.32559217169819	0.724	0.306	5.03460039846178E-75	8	PTK2
7.11217766756813E-68	0.32544273914162	0.35	0.119	1.19185873353107E-63	8	STAM
9.40842987406159E-75	0.325153638681801	0.439	0.158	1.57666467829524E-70	8	MTREX
7.58089417303779E-70	0.324896505760061	0.57	0.237	1.27040624551767E-65	8	COQ4
4.76400603174144E-49	0.324709202203091	0.92	0.538	7.98352130799231E-45	8	ANXA2
6.72494802558947E-77	0.324541569331674	0.523	0.2	1.12696679012828E-72	8	MAPKAPK5
1.95080576622281E-69	0.324498498658337	0.486	0.189	3.26916030303618E-65	8	SETDB1
8.45778738788928E-82	0.324454506559216	0.628	0.258	1.41735601046249E-77	8	POLDIP3
4.45828170061694E-79	0.324276347154596	0.494	0.184	7.47118847389388E-75	8	STXBP6
2.05066325252205E-35	0.324214210503507	0.967	0.696	3.43650147857646E-31	8	SERPINA7
2.91488253437108E-64	0.324208153784725	0.438	0.167	4.88476015109905E-60	8	BTBD1
5.39688735566732E-71	0.324177686107955	0.695	0.313	9.04410383062729E-67	8	PPIA
6.19445298829718E-80	0.324132984311909	0.589	0.232	1.03806643177884E-75	8	GATAD2B
8.98950317937652E-72	0.32400775212889	0.293	0.088	1.50646094279992E-67	8	GARS
4.82359725959683E-66	0.323128293938555	0.346	0.118	8.08338428763237E-62	8	RBMXL1
2.94589931918841E-65	0.323098146837985	0.467	0.183	4.93673807909593E-61	8	DNTTIP2
8.23256857703893E-67	0.323060335638742	0.522	0.21	1.37961384214018E-62	8	ARHGAP17
1.30318799772856E-80	0.322775871422933	0.675	0.283	2.18388244659352E-76	8	INTS1
2.78055937516814E-72	0.322727289839355	0.692	0.3	4.65966140090678E-68	8	TRIQK
7.07278576702912E-73	0.322551057632896	0.618	0.256	1.18525743883874E-68	8	ACTR10
7.77937793213911E-53	0.32252954421379	0.828	0.426	1.30366815386787E-48	8	STBD1
7.7403862439753E-67	0.322461833220954	0.557	0.233	1.29713392676538E-62	8	ZNF800
3.3577230736337E-84	0.322283531051166	0.638	0.254	5.62687232679535E-80	8	CCNY
4.642444885752E-87	0.322015408978268	0.541	0.199	7.7798091395432E-83	8	FNBP4
7.88176145095619E-77	0.321796949462754	0.617	0.251	1.32082558395124E-72	8	ZBED6
7.07348952747799E-75	0.321752211159336	0.487	0.183	1.18537537501476E-70	8	NANS
1.92730017977976E-68	0.321649501419127	0.31	0.098	3.22976964127492E-64	8	AKTIP
2.07904069696979E-73	0.321389928972242	0.708	0.309	3.48405639998197E-69	8	ATP11B
9.53425469318975E-74	0.321297306292677	0.71	0.311	1.59775040148474E-69	8	AFF4
6.938265029448E-71	0.321217813804783	0.399	0.141	1.1627144536349E-66	8	PIAS2
2.44998141012918E-68	0.320958693401387	0.435	0.163	4.10567884709449E-64	8	WASF2
1.56406033923421E-62	0.320875847367867	0.353	0.125	2.6210523164887E-58	8	MTMR14
4.60007091624207E-55	0.320721263335658	0.32	0.116	7.70879884143846E-51	8	CCT2
6.32935158470483E-71	0.320629503729492	0.674	0.289	1.06067273856483E-66	8	TMEM129
7.32930031004762E-74	0.320609771058421	0.529	0.207	1.22824414595778E-69	8	PLIN3
1.19059922336096E-82	0.32038313512975	0.656	0.263	1.9952061785083E-78	8	TPD52L2
2.86275623883639E-67	0.320057934010324	0.451	0.173	4.79740690504203E-63	8	GLRX2
1.73428984406326E-60	0.319957067707004	0.386	0.145	2.90632292068122E-56	8	SIX5
5.3350779733439E-59	0.319794991691019	0.374	0.14	8.94052366772971E-55	8	RSF1
5.6257905527243E-77	0.319318643817921	0.611	0.248	9.42769980825538E-73	8	CENPV
8.8568763026003E-69	0.318938114259936	0.497	0.194	1.48423533078976E-64	8	TMEM181
2.63689396762782E-71	0.318852178270218	0.401	0.142	4.41890691095071E-67	8	NFRKB
6.85084262594893E-53	0.318767111279644	0.375	0.148	1.14806420725652E-48	8	USP40
3.91151456697859E-81	0.318716269453648	0.45	0.157	6.55491611134273E-77	8	OGG1
2.23472241833158E-82	0.318632006109015	0.491	0.178	3.74494782864006E-78	8	OSBPL8
3.50968885572111E-58	0.318553936871012	0.403	0.157	5.88153658441744E-54	8	UNC93B1
5.78535448814104E-67	0.318364372330101	0.513	0.203	9.69509705122675E-63	8	ZNF580

**1.27437058211293E-45**	0.318216828434777	0.257	0.092	2.13559022150485E-41	8	DEPRECATED-ENSG00000103512
**3.19253053505345E-72**	0.318195074154544	0.523	0.205	5.35004267064258E-68	8	GPD1L
**3.10724793569353E-69**	0.317945141816728	0.615	0.26	5.20712609063522E-65	8	SRP19
**2.07184592538529E-68**	0.317729989432832	0.494	0.195	3.47199940176067E-64	8	LARP1B
**5.88320852044695E-91**	0.317040932990977	0.67	0.264	9.859080838565E-87	8	KCTD20
**1.01813881213793E-75**	0.316947951217292	0.463	0.168	1.70619702138074E-71	8	ANKS1A
**1.49660242407198E-35**	0.316152668912557	0.261	0.105	2.50800634225982E-31	8	PLA2G4B
**3.91823192523356E-69**	0.316077118021945	0.47	0.18	6.56617306030641E-65	8	RSPRY1
**1.16679886339398E-90**	0.315583384430218	0.699	0.281	1.95532153527564E-86	8	RHOT2
**2.58629894356734E-76**	0.315317128296762	0.726	0.314	4.33411976963016E-72	8	CHP2
**3.99592083473527E-86**	0.315299850528366	0.676	0.275	6.69636413484937E-82	8	TNFSF10
**1.32136044375715E-51**	0.31508413340029	0.292	0.104	2.21433583164823E-47	8	DOLPP1
**4.20971760231871E-59**	0.315014112772249	0.352	0.128	7.05464475796569E-55	8	GTF2H3
**3.71598327761997E-83**	0.31498269955247	0.555	0.21	6.22724477663555E-79	8	DEPRECATED-ENSG00000163374
**3.72345899027301E-44**	0.314740829914628	0.28	0.105	6.23977257589951E-40	8	ZNF696
**6.79551730489525E-72**	0.314740618388027	0.546	0.219	1.13879278995435E-67	8	PSMA4
**1.37874880518808E-65**	0.314604852776511	0.527	0.217	2.31050724773419E-61	8	ARL8B
**6.59894955084092E-37**	0.31450368509479	0.945	0.576	1.10585196572992E-32	8	ACSM2A
**1.60768409734177E-83**	0.314417368429368	0.626	0.249	2.69415701032534E-79	8	KCMF1
**5.03511402773666E-77**	0.314350831337229	0.691	0.293	8.4378440876811E-73	8	LGMN
**1.694340588142E-52**	0.31423700335371	0.301	0.108	2.83937595760836E-48	8	EID2
**2.28167945551494E-79**	0.314092856965196	0.674	0.28	3.82363843155194E-75	8	MED28
**2.22129917550682E-62**	0.314011897780729	0.609	0.265	3.72245315831433E-58	8	CXADR
**1.99419077508569E-78**	0.313885378763195	0.457	0.164	3.3418649008886E-74	8	HCFC1R1
**5.70058011690345E-80**	0.313793100099297	0.493	0.18	9.55303215990679E-76	8	INO80C
**6.64971366618833E-66**	0.313767740241362	0.317	0.104	1.11435901617984E-61	8	ACOT9
**1.94729288485981E-81**	0.313755768302487	0.675	0.281	3.26327341644807E-77	8	BMI1
**1.49807092647945E-90**	0.313622472276191	0.582	0.217	2.51046725859425E-86	8	CNPY3
**6.60363294678362E-74**	0.313508110387725	0.486	0.183	1.106636809222E-69	8	RETREG2
**4.31742424847668E-76**	0.313398996231881	0.415	0.144	7.23513955559722E-72	8	CAPRIN1
**2.77702379796547E-62**	0.313369741139944	0.39	0.145	4.65373648063054E-58	8	TNKS2
**3.25270190383533E-43**	0.313277641286005	0.368	0.157	5.45087785044725E-39	8	PLXNA3
**1.14764015252989E-48**	0.313184021661197	0.306	0.115	1.92321536760959E-44	8	RCHY1
**4.4951565455724E-61**	0.313023377625756	0.344	0.122	7.53298333907022E-57	8	PCNX3
**2.85297124748353E-46**	0.312949245165775	0.265	0.094	4.7810092165329E-42	8	LAMA4
**3.48221289708124E-42**	0.312382011623121	0.875	0.503	5.83549237292875E-38	8	HSPH1
**8.21773621119582E-70**	0.312315439272618	0.399	0.143	1.37712823427219E-65	8	NXT1
**1.01514789310112E-76**	0.31190253278535	0.674	0.284	1.70118483925886E-72	8	RBM25
**3.19260635356654E-71**	0.311834341499535	0.515	0.202	5.35016972730681E-67	8	BPNT1
**3.3464996721116E-30**	0.311379910678811	0.294	0.133	5.60806415052462E-26	8	TTN
**1.30866254715588E-76**	0.311037959743331	0.494	0.184	2.19305669652382E-72	8	TIMM9
**1.85702974808359E-66**	0.311006224684694	0.529	0.215	3.11201045183848E-62	8	TRMT1L
**6.08911385340223E-81**	0.310767651597204	0.561	0.214	1.02041369955315E-76	8	ABI1
**7.90255793904316E-77**	0.310600918919922	0.534	0.205	1.32431065942485E-72	8	ANKRD40
**9.19870559787107E-71**	0.310528570289304	0.493	0.19	1.54151908409123E-66	8	SMARCE1
**2.19683064063431E-54**	0.309820839466489	0.395	0.157	3.68144878757498E-50	8	WDR6
**8.44530886689323E-62**	0.309763534719704	0.567	0.245	1.41526485991397E-57	8	RBCK1
**7.33206135016389E-56**	0.309688475039245	0.354	0.133	1.22870684106047E-51	8	ELAC2
**3.96771085923881E-69**	0.309426945559477	0.642	0.279	6.64908985791239E-65	8	CLIC1
**1.1584649359271E-46**	0.309376084440468	0.309	0.118	1.94135553962663E-42	8	PFKFB2
**2.24209537593229E-91**	0.309196642535301	0.671	0.265	3.75730343098732E-87	8	SGTA
**2.10607892645782E-61**	0.309169042951494	0.28	0.089	3.52936706495801E-57	8	EIF2B2
**5.56893363400367E-83**	0.308936544849513	0.589	0.23	9.33241898386335E-79	8	GBE1
**3.20408316047944E-70**	0.308232177426671	0.565	0.231	5.36940256033145E-66	8	GCN1
**9.91624094062941E-75**	0.308075664786933	0.762	0.344	1.66176365683068E-70	8	BRD4
**9.25535826636241E-66**	0.307539847339347	0.447	0.171	1.55101293827701E-61	8	MAN1B1
**3.99481091321271E-74**	0.307508904602797	0.455	0.166	6.69450412836186E-70	8	U2AF2
**6.69470986117322E-59**	0.307485619386357	0.349	0.127	1.12189947853541E-54	8	APC
**2.50744994870876E-42**	0.307448850525196	0.276	0.106	4.20198462404615E-38	8	BEND7
**6.56658892249893E-55**	0.307173375224371	0.306	0.108	1.10042897163237E-50	8	VPS35

6.13881782603103E-51	0.306984027458186	0.337	0.128	1.02874309128628E-46	8	RNASET2
4.20204567067024E-69	0.306981380036713	0.764	0.348	7.04178813490919E-65	8	UBE2D3
5.20736784689219E-77	0.306901246968946	0.614	0.246	8.72650703782193E-73	8	MAP3K2
1.0511517526416E-76	0.306876833571319	0.491	0.183	1.7615201070768E-72	8	ARMC8
6.31801472123349E-72	0.306636419532719	0.499	0.191	1.05877290698431E-67	8	MCM3AP
3.21037165922085E-67	0.306467544065595	0.831	0.395	5.37994082652231E-63	8	DERL1
4.34719625578129E-70	0.306377179841773	0.707	0.319	7.28503148543829E-66	8	NUDT5
5.76177554613191E-73	0.306365004391052	0.502	0.192	9.65558346020785E-69	8	SLC17A5
1.79729353453793E-68	0.306359614180267	0.775	0.355	3.01190450517866E-64	8	SRP54
2.92781996468412E-47	0.306226133565777	0.912	0.5	4.90644069681765E-43	8	TFRC
1.74251763712904E-73	0.306171522755194	0.506	0.195	2.92011105630084E-69	8	MANBAL
2.14784896472636E-71	0.306130916468175	0.505	0.198	3.59936529508843E-67	8	TNIP2
7.09469190869947E-52	0.306124314885337	0.338	0.129	1.18892847005986E-47	8	C18orf25
2.31695402500603E-62	0.305716867717503	0.542	0.227	3.88275155510511E-58	8	PLD1
1.00007035831963E-49	0.305697091949924	0.28	0.1	1.67591790647204E-45	8	LLPH
1.78951831702178E-81	0.305230233493382	0.562	0.216	2.99887479566511E-77	8	ZNF148
4.79746153786446E-59	0.305223350292246	0.407	0.158	8.03958604515326E-55	8	ANAPC2
1.41694525390727E-40	0.3049434208275	0.937	0.551	2.37451685649781E-36	8	OCLN
2.82757410543204E-54	0.304909053477305	0.369	0.142	4.73844868588301E-50	8	ARHGEF16
5.14656982286179E-77	0.304475771845734	0.545	0.211	8.62462170915179E-73	8	EIF4G3
5.64865052242164E-83	0.304235837290745	0.594	0.233	9.46600854547419E-79	8	CCDC117
3.79528490032291E-43	0.304212261022837	0.896	0.463	6.36013843596112E-39	8	HNF4G
2.75664983385039E-67	0.304106702280457	0.409	0.149	4.61959379156649E-63	8	FBXW11
1.13580179069152E-46	0.303761031892649	0.348	0.141	1.90337664084084E-42	8	ANGEL1
2.44786755292166E-93	0.303640620902472	0.7	0.276	4.10213644518612E-89	8	YTHDC1
4.08968426396573E-35	0.303055220441225	0.935	0.58	6.85349288955378E-31	8	BCAP31
1.67767132052688E-74	0.30300344891059	0.66	0.281	2.81144159893895E-70	8	KANSL1
2.68562768732944E-69	0.30286412285012	0.768	0.353	4.50057487842667E-65	8	NDUFS1
2.655670172054E-68	0.302831220050148	0.422	0.155	4.45037207432809E-64	8	ARFIP1
4.860859983274E-41	0.302644614879473	0.262	0.099	8.14582915997057E-37	8	AC073111.4
1.59291091976373E-72	0.302420122151562	0.634	0.267	2.66940011934006E-68	8	IRF2
2.62950504917053E-73	0.302377442899254	0.491	0.189	4.40652456139997E-69	8	STARD4
2.61263304996936E-76	0.302259345865212	0.47	0.172	4.37825046513866E-72	8	GTPBP1
9.8647800917144E-57	0.30223353455425	0.269	0.088	1.6531398477695E-52	8	DNAJB6
1.7531837131205E-78	0.301760128747184	0.543	0.209	2.93798526644734E-74	8	RAB15
4.73027802990051E-50	0.301751094929758	0.26	0.089	7.92699992250727E-46	8	CAMSAP1
8.56466138947296E-91	0.301663699129909	0.535	0.192	1.43526595564788E-86	8	BRD3
6.62439309957814E-66	0.301599143722054	0.748	0.343	1.1101157956273E-61	8	SLC39A7
1.08808920668924E-46	0.301525463695962	0.302	0.115	1.82341989256983E-42	8	CXorf40B
5.07166505152106E-50	0.301204247601369	0.3	0.11	8.499096293339E-46	8	PIK3CA
3.18009885687821E-57	0.301061071118665	0.305	0.105	5.3292096643565E-53	8	CTDNEP1
4.86923322157258E-77	0.300999171469402	0.656	0.273	8.15986103271133E-73	8	METTL23
1.68069651766132E-73	0.300530306753396	0.411	0.145	2.81651122429684E-69	8	NR1D2
6.55167097825679E-64	0.300494430462288	0.425	0.161	1.09792902253627E-59	8	TTC33
1.76323240050351E-80	0.300406679495341	0.387	0.126	2.95482485676378E-76	8	ARMC6
3.47017721525392E-46	0.300214753281302	0.451	0.199	5.81532297732252E-42	8	ART4
1.93187072618829E-65	0.299993854479975	0.431	0.165	3.23742896294633E-61	8	ARMH3
1.03120140625779E-69	0.299889747079812	0.611	0.253	1.7280873166068E-65	8	ATP8B1
6.5158765222583E-71	0.299856640389608	0.66	0.285	1.09193058760005E-66	8	GAA
2.17556732741182E-73	0.299818518433205	0.716	0.312	3.64581572727672E-69	8	HNRNPM
8.98011405751431E-68	0.29970365167485	0.482	0.189	1.50488751375825E-63	8	TTC7A
2.43067728362137E-64	0.299286179311797	0.439	0.168	4.07332899189269E-60	8	SACM1L
7.03906512374156E-63	0.299159953232898	0.61	0.266	1.17960653343661E-58	8	ATP11A
1.14015351011422E-51	0.298737120722017	0.29	0.103	1.91066925224941E-47	8	ATF1
2.4948113039689E-73	0.298542173210438	0.783	0.356	4.18080478319109E-69	8	DYNC1LI2
3.35584134064523E-45	0.298144224652807	0.286	0.108	5.62371891865328E-41	8	PDE3A
4.38106550504951E-79	0.297810598029248	0.732	0.309	7.34178957336196E-75	8	PARP10
9.68717443496118E-73	0.297752890282245	0.795	0.372	1.6233766918108E-68	8	QKI
6.73253136964157E-82	0.297751749566427	0.648	0.261	1.12823760692453E-77	8	ZNF326
4.65729179684378E-75	0.297715149652151	0.529	0.205	7.80468959315081E-71	8	PPP1R12C

2.82764975729309E-87	0.29732690472385	0.526	0.193	4.73857546327175E-83	8	MAD2L2
5.27663507390376E-32	0.297322866896158	0.948	0.624	8.84258505684792E-28	8	RARRES2
2.63842014327084E-72	0.29712062896341	0.731	0.32	4.42146447609328E-68	8	UBXN2B
7.18469141908067E-76	0.297052119095689	0.796	0.36	1.20401058800954E-71	8	DVL3
2.26372701039725E-46	0.296654663091787	0.916	0.492	3.79355372402372E-42	8	IDH3G
6.66650280442553E-78	0.296623975356748	0.586	0.234	1.11717253996563E-73	8	SRSF7
1.83946221631491E-87	0.296459715496838	0.589	0.222	3.08257078210053E-83	8	BABAM1
1.37941871249107E-47	0.296427689200303	0.378	0.157	2.31162987839254E-43	8	ABHD13
1.33787717800419E-61	0.296402358960386	0.446	0.178	2.24201457489943E-57	8	ARRB2
4.22646742853005E-65	0.296386727113372	0.425	0.161	7.08271411673066E-61	8	TIGD5
5.49289678566349E-60	0.296343961376825	0.518	0.22	9.20499643341487E-56	8	DNMT3A
9.02743859550827E-66	0.296056974769372	0.719	0.329	1.51281815983528E-61	8	PSMD12
8.36493592343756E-77	0.295791895664918	0.394	0.133	1.40179596204967E-72	8	PRKACB
6.23903858854134E-74	0.295726172281577	0.663	0.279	1.04553808666776E-69	8	CAMK2D
7.39465751092966E-64	0.2957255448588	0.419	0.16	1.23919670568159E-59	8	ARL4D
2.08596541868362E-79	0.295339107859058	0.704	0.295	3.49566084863E-75	8	CHKA
1.05194180730164E-66	0.295234737421666	0.774	0.357	1.76284408067608E-62	8	NUMB
3.58274624780902E-66	0.294883984650007	0.403	0.147	6.00396616207836E-62	8	CDS2
1.09279439826869E-54	0.29478843614301	0.364	0.138	1.83130485261867E-50	8	RNASEH1
4.93792587042041E-65	0.294443003729191	0.33	0.111	8.27497617365053E-61	8	PFDN1
2.26117882412935E-56	0.294303827408046	0.373	0.142	3.78928347347597E-52	8	RWDD1
4.35320633552588E-73	0.294302160418244	0.533	0.209	7.29510317707427E-69	8	CBWD1
1.94033293335877E-51	0.29429815687967	0.332	0.125	3.25160992972263E-47	8	ZNF414
1.20031344695229E-55	0.294188355893379	0.798	0.394	2.01148527440265E-51	8	EDEM3
9.64166020598288E-85	0.294173883684442	0.549	0.206	1.61574941731861E-80	8	TAGLN2
1.83519408882433E-72	0.29402370958441	0.74	0.33	3.07541825405181E-68	8	FUBP1
1.73278682565097E-49	0.293919164462222	0.284	0.102	2.90380416242589E-45	8	ZNF207
1.82796028560149E-85	0.293873500761021	0.573	0.217	3.06329584661098E-81	8	SF3B4
9.78566046071419E-68	0.293638983847109	0.401	0.145	1.63988098000648E-63	8	AP5S1
7.28626732513761E-46	0.293599989271769	0.273	0.1	1.22103267834656E-41	8	ZSCAN29
1.90984000690336E-64	0.293331901541462	0.652	0.286	3.20050988356865E-60	8	ARRB1
3.46050739678036E-78	0.293057758083006	0.704	0.298	5.79911829552452E-74	8	RAB1B
2.5584319932887E-69	0.292903003413391	0.646	0.277	4.28742033435321E-65	8	AKAP13
8.88183542222166E-61	0.292402293146763	0.623	0.277	1.48841798005591E-56	8	FADS2
9.19518804754082E-78	0.292374273103676	0.646	0.264	1.54092961300689E-73	8	YIPF4
1.94852667306842E-41	0.292293923767183	0.908	0.488	3.26534099872806E-37	8	ABCD1
7.72876251011306E-39	0.29224605365484	0.285	0.114	1.29518602144475E-34	8	ME3
7.39754395853198E-71	0.292165086306953	0.742	0.331	1.23968041657079E-66	8	CD2AP
9.01781424743488E-66	0.292077398302222	0.439	0.167	1.51120531158514E-61	8	RAB22A
8.80124499903661E-76	0.291881777939764	0.418	0.145	1.47491263693855E-71	8	MORF4L1
1.09294192570775E-57	0.291881315101868	0.855	0.416	1.83155207910105E-53	8	RAP2C
6.08354904987504E-59	0.291880525756594	0.334	0.119	1.01948114977806E-54	8	PRELID3B
5.34949733809934E-62	0.291076701761902	0.379	0.139	8.96468763918688E-58	8	CCNT1
1.25398450760901E-78	0.291068699717143	0.487	0.179	2.10142723785119E-74	8	MARCH8
1.17722919743697E-47	0.290938980861728	0.274	0.099	1.97280068906488E-43	8	INTS14
3.02370274467398E-49	0.290745738005533	0.884	0.476	5.06712105952466E-45	8	ADAR
2.10779358947129E-56	0.290679377116712	0.807	0.416	3.53224049723598E-52	8	LMO7
4.9893547994052E-39	0.290646817795111	0.915	0.531	8.36116077284324E-35	8	TSPAN6
4.62150372663887E-112	0.290463735148503	0.438	0.126	7.74471594510142E-108	8	PIM1
2.79606759841496E-77	0.290288376212153	0.632	0.26	4.68565008142378E-73	8	EIF2B5
4.38345308223242E-65	0.290258287150985	0.286	0.089	7.3457906752051E-61	8	RAP2B
8.29156787565141E-57	0.289840013994485	0.345	0.126	1.38950094460166E-52	8	ELP3
1.24645457828087E-63	0.289829026414917	0.394	0.145	2.08880858228307E-59	8	EPDR1
1.9048175116859E-75	0.288624595641763	0.615	0.254	3.19209318608322E-71	8	TLK1
6.1903769056276E-82	0.288616466736645	0.648	0.263	1.03738336184507E-77	8	GRK2
1.53842791605745E-53	0.288597103124409	0.274	0.093	2.57809750172907E-49	8	LDB1
5.26306336136323E-71	0.28850372382137	0.485	0.186	8.8198415809725E-67	8	KLHDC10
9.83768609880589E-71	0.288494706029045	0.676	0.291	1.64859943643789E-66	8	NCKAP1
1.2450502611108E-66	0.288486849922295	0.381	0.135	2.08645522756949E-62	8	MYO1E
2.10796664194818E-36	0.288376603800988	0.92	0.567	3.53253049857675E-32	8	TRAM1

5.33199277844827E-76	0.28803202393575	0.606	0.245	8.93535349812361E-72	8	CREBBP
1.18087444626129E-78	0.287970973655421	0.534	0.205	1.97890939704468E-74	8	CMAS
3.54033164634605E-60	0.287900027801653	0.399	0.152	5.93288777294671E-56	8	SOWAHB
5.1839928175366E-52	0.287873711259183	0.413	0.169	8.68733516362784E-48	8	OMA1
6.09522970403974E-86	0.287680610111698	0.531	0.194	1.02143859380298E-81	8	PAGR1
2.30156395685433E-75	0.287566133896726	0.463	0.171	3.85696087889649E-71	8	IRF2BP1
3.71629655738971E-62	0.28754789242074	0.578	0.248	6.22776977087367E-58	8	FAM8A1
1.19736094393101E-50	0.287361097635377	0.375	0.15	2.00653746983959E-46	8	TRMU
1.06102327778327E-96	0.287074712873984	0.446	0.141	1.7780628089092E-92	8	TTC31
2.29288211193925E-65	0.28666563739562	0.758	0.352	3.8424118431878E-61	8	TRIM27
9.66326144414496E-75	0.28656377838363	0.656	0.276	1.61936935280981E-70	8	PCMTD2
2.67248524566776E-61	0.286360003037145	0.379	0.139	4.47855077469002E-57	8	DEPRECATED-ENSG00000116489
1.1942827359475E-73	0.286103454944285	0.433	0.155	2.00137900890082E-69	8	ASNSD1
1.0533236147975E-58	0.285977083108559	0.338	0.121	1.76515971367766E-54	8	C7orf25
4.00840398478583E-52	0.285911129276794	0.358	0.139	6.71728339770409E-48	8	TM2D1
1.72927771090524E-74	0.285888873097496	0.674	0.285	2.89792358793501E-70	8	PLPP1
7.20760840314671E-74	0.285711808237596	0.79	0.35	1.20785101619933E-69	8	GNAQ
3.28298591887603E-51	0.285630508111227	0.285	0.101	5.50162780285245E-47	8	TMCC1
1.71175840188211E-51	0.285463995656212	0.313	0.116	2.86856472987403E-47	8	GPALPP1
8.90064994321139E-41	0.285422284185251	0.3	0.12	1.49157091748337E-36	8	ZDHHC17
4.31068490939216E-67	0.285404992187432	0.732	0.339	7.22384577115938E-63	8	NFIA
4.67593476489995E-72	0.285255317409316	0.522	0.206	7.83593147901934E-68	8	ITSN2
2.59432482132005E-35	0.285135837086323	0.9	0.532	4.34756953556814E-31	8	C6
9.47196585366934E-51	0.284868932739449	0.27	0.093	1.58731203775791E-46	8	TTC23
1.5692125950066E-77	0.284850238664345	0.582	0.232	2.62968646671206E-73	8	SELENOO
1.05121813588379E-60	0.284663666112278	0.276	0.088	1.76163135211406E-56	8	ABHD17B
1.22991733294549E-66	0.284600049776971	0.333	0.111	2.06109546655005E-62	8	CNOT3
8.78667064424754E-61	0.284486366704484	0.446	0.176	1.472470266563E-56	8	UBE2W
2.15285824630972E-50	0.284236117815496	0.333	0.127	3.60775984916583E-46	8	TP53I11
1.84516851773772E-64	0.284203671398935	0.679	0.302	3.09213340202487E-60	8	GALK2
8.28629140507291E-71	0.28411869838025	0.434	0.158	1.38861671366212E-66	8	MRGBP
2.8929802957197E-65	0.284087439713308	0.541	0.224	4.84805637956708E-61	8	PPP1R16A
3.48435937569143E-40	0.283262346468656	0.274	0.107	5.83908944178369E-36	8	ZNF84
2.40359735880551E-69	0.283040305352441	0.362	0.124	4.02794845388627E-65	8	TADA1
2.62419953055091E-76	0.2823953720309	0.575	0.23	4.39763357329722E-72	8	SEPTIN2
1.032795344964E-65	0.282182379764606	0.654	0.286	1.73075843909067E-61	8	PLA2G12A
1.2999293068309E-60	0.281970007312196	0.411	0.159	2.17842153238723E-56	8	COPS8
3.62162719942772E-76	0.281629311865262	0.518	0.195	6.06912286080097E-72	8	CAMK1D
3.10281381365576E-49	0.281386403893143	0.372	0.15	5.19969538892432E-45	8	ZNF44
2.32199438585575E-55	0.281233586432015	0.301	0.105	3.89119819181706E-51	8	RAB8B
8.30082758556625E-56	0.280741847005142	0.349	0.129	1.39105268678919E-51	8	TMEM184C
7.0740202594148E-73	0.280516828225353	0.551	0.22	1.18546431507273E-68	8	CNP
4.15278073828344E-82	0.28034048604777	0.65	0.261	6.95922996121538E-78	8	DEPRECATED-ENSG00000073578
4.51832097378415E-64	0.280057120045233	0.631	0.279	7.57180228786748E-60	8	SLC39A1
3.48104641926135E-78	0.279901110136356	0.771	0.343	5.83353758939817E-74	8	KEAP1
1.88552003349667E-65	0.279791429920503	0.378	0.135	3.15975447213372E-61	8	AP3S1
2.59822747139217E-81	0.279768926822815	0.607	0.24	4.354109596559E-77	8	USP32
4.17716736304987E-69	0.279735089289685	0.59	0.247	7.00009706699897E-65	8	NFKB1
2.42083605546525E-59	0.279690464906671	0.446	0.178	4.05683706174866E-55	8	ELL
1.26373480476327E-64	0.279617904340558	0.289	0.092	2.11776678582229E-60	8	SEC14L1
5.89758899111933E-69	0.279437577776788	0.521	0.208	9.88317963131777E-65	8	ICK
3.01258442335572E-47	0.27894530852073	0.832	0.437	5.04848897665951E-43	8	HACD3
2.21041368845213E-74	0.278899308907714	0.395	0.135	3.70421125910807E-70	8	PPME1
1.46716521060053E-45	0.278898175582491	0.34	0.137	2.45867545992437E-41	8	EXT2
5.18491311616876E-75	0.278684256310704	0.485	0.18	8.6888774000756E-71	8	RMC1
2.60782295173929E-81	0.278643993263288	0.719	0.305	4.3701897025247E-77	8	DCTN2
2.48919590500667E-53	0.278517652975963	0.285	0.098	4.17139449761018E-49	8	ZNF689
9.5754541166452E-48	0.278442488650967	0.772	0.406	1.6046546008674E-43	8	CPEB4
1.21005001751924E-74	0.278323378750298	0.423	0.15	2.02780181935874E-70	8	DDA1
6.05128491172773E-76	0.27818991363107	0.609	0.246	1.01407432550733E-71	8	TMEM189

2.51967013688258E-55	0.278109713845331	0.305	0.107	4.22246321538783E-51	8	IPO13
1.07635260398475E-50	0.277880610967794	0.862	0.464	1.80375169375765E-46	8	CIAO2A
5.59507067375494E-40	0.277495209715304	0.345	0.149	9.37621943507853E-36	8	MRI1
5.44023917082554E-58	0.277405674234906	0.553	0.241	9.11675280246944E-54	8	PSMC6
1.48086944785529E-66	0.277403385577451	0.434	0.164	2.48164102071589E-62	8	TCF3
4.75317919553449E-61	0.277392981875396	0.438	0.172	7.9653776958767E-57	8	PATZ1
1.80146106415268E-65	0.277314274631651	0.76	0.346	3.01888845130706E-61	8	TM9SF1
9.02493487021005E-73	0.277255260802165	0.494	0.189	1.5123985855498E-68	8	NAA38
1.34292539858826E-77	0.277049067269894	0.626	0.253	2.25047438295421E-73	8	AFTPH
1.28821331586391E-49	0.276807557930725	0.322	0.122	2.15878787472474E-45	8	ARMC10
4.44341658622299E-80	0.276729298172044	0.513	0.19	7.44627751519248E-76	8	METTL14
2.5390627528828E-76	0.276692859420832	0.617	0.251	4.25496136128099E-72	8	ATP6V1C1
3.21605398028103E-74	0.276634036076414	0.746	0.334	5.38946326015496E-70	8	HNRNPH3
9.61559769806307E-51	0.276416113695066	0.306	0.112	1.61138186224141E-46	8	KLC2
9.02296251819396E-61	0.276319094101352	0.77	0.368	1.51206805879894E-56	8	GRB10
3.14314080725374E-65	0.276270496270703	0.438	0.168	5.26727536479582E-61	8	DEPRECATED-ENSG00000196313
5.63732436261401E-78	0.276265236184995	0.513	0.194	9.44702816686855E-74	8	MAGOH
4.88828419019346E-52	0.276099210734215	0.338	0.129	8.1917866459262E-48	8	ZNF280D
1.37154744685643E-47	0.276088389596296	0.282	0.103	2.298439211442E-43	8	QPCTL
2.98272633222316E-74	0.276043394035832	0.394	0.134	4.99845278753957E-70	8	MCRIP1
2.03073358768049E-32	0.275941183711645	0.961	0.654	3.40310334623496E-28	8	LYZ
8.86967578818388E-62	0.275744929005813	0.57	0.245	1.48638026858385E-57	8	EBAG9
3.63100993244798E-57	0.275387121338317	0.403	0.158	6.08484644479632E-53	8	ATG4A
1.48991176639024E-51	0.275242563648749	0.808	0.421	2.49679413811676E-47	8	FH
4.55604869926885E-52	0.275211363587929	0.337	0.128	7.63502641023475E-48	8	EVI5
3.55324066966977E-51	0.274156982603513	0.368	0.146	5.95452071423261E-47	8	TBRG1
7.04289262966414E-59	0.274130323689411	0.461	0.188	1.18024794687912E-54	8	SLC25A16
7.3994683203531E-81	0.27392877231891	0.663	0.271	1.24000290112477E-76	8	AGPS
2.95888882188059E-58	0.273665272604387	0.423	0.168	4.95850588770749E-54	8	SDHAF4
5.19245474498169E-53	0.273658303194559	0.352	0.134	8.70151566164031E-49	8	RPAP1
8.61421484390334E-61	0.272544524809168	0.28	0.089	1.44357012354132E-56	8	MCU
2.17969744798605E-62	0.272522438566746	0.621	0.276	3.65273698333503E-58	8	SLC35B2
1.75235621062469E-58	0.272258609047144	0.39	0.148	2.93659853776485E-54	8	DHX29
1.41166973359089E-82	0.271930544118685	0.513	0.189	2.36567613955162E-78	8	OARD1
5.72317477349143E-56	0.271717693620823	0.839	0.415	9.59089628541694E-52	8	DBNL
1.27982857798064E-77	0.271596032417772	0.566	0.222	2.14473673097996E-73	8	CYP2C19
3.36661884347066E-47	0.271410338006473	0.81	0.42	5.64177985788812E-43	8	TMPRSS2
8.74315048020319E-72	0.27136424111298	0.605	0.248	1.46517715747245E-67	8	AEBP2
5.69700908695635E-76	0.271352345919273	0.577	0.23	9.54704782792145E-72	8	SRRM1
1.99130985030569E-35	0.271241480556609	0.278	0.116	3.33703704714227E-31	8	GALNT10
6.0047820018031E-76	0.271040462151002	0.561	0.221	1.00628136786216E-71	8	POFUT1
1.23429937420953E-79	0.270713411947043	0.589	0.233	2.06843889130033E-75	8	DEPRECATED-ENSG00000124831
3.9014513260785E-65	0.270652364348428	0.525	0.217	6.53805213224235E-61	8	TFF1
1.93643711995361E-70	0.270624944286467	0.439	0.163	3.24508132561826E-66	8	GPS2
1.68579116765091E-56	0.270295502821269	0.337	0.122	2.8250488387494E-52	8	MRTFB
6.4506434594423E-50	0.270069646345706	0.333	0.127	1.08099883093334E-45	8	HELZ2
4.89150826201328E-67	0.269966899441519	0.426	0.158	8.19718954548186E-63	8	ZNF22
5.7041380383541E-63	0.269912396560266	0.603	0.263	9.5589945246738E-59	8	ARHGAP12
1.03694825181004E-30	0.269712064847762	0.935	0.562	1.73771788038327E-26	8	DECR1
4.30860472031133E-69	0.269708509000729	0.724	0.324	7.22035979029772E-65	8	XRN1
6.70110507910161E-74	0.269428987367315	0.609	0.253	1.12297118915585E-69	8	ARHGEF7
1.63666482878031E-74	0.269282195121403	0.406	0.141	2.74272292007005E-70	8	RPAIN
2.05931310288267E-61	0.269072235495657	0.454	0.18	3.45099689781078E-57	8	AP3S2
1.96727150630148E-89	0.268947190484535	0.566	0.209	3.29675359026003E-85	8	HYI
3.75789203905802E-52	0.268835037672928	0.415	0.17	6.29747547905342E-48	8	PHYHIPL
2.72794769592925E-59	0.2683461448136	0.346	0.124	4.57149474883825E-55	8	KRAS
1.06800365123721E-48	0.268238453694025	0.304	0.113	1.78976051874332E-44	8	POLG2
1.69884771096269E-73	0.267970076055283	0.71	0.314	2.84692899403127E-69	8	HDGFL2
1.47775905068658E-68	0.267947445257971	0.756	0.335	2.47642861714056E-64	8	ILVBL
7.19366679970171E-74	0.267926114800376	0.615	0.257	1.20551468229401E-69	8	RMDN3

**1.99670238827569E-81**	0.267416231561425	0.678	0.278	3.3460738622724E-77	8	FOXP1
**4.17890022753702E-54**	0.267198806864388	0.79	0.396	7.00300100130655E-50	8	TMEM259
**1.49702517030365E-70**	0.266919563121187	0.652	0.277	2.50871478039486E-66	8	FAM13A
**4.410430585194E-41**	0.266916043894384	0.3	0.12	7.3909995746681E-37	8	TAB1
**4.81383513546558E-60**	0.266900171538051	0.786	0.373	8.06702492001322E-56	8	DGAT1
**7.42567444123807E-72**	0.266810566476419	0.64	0.269	1.24439452286268E-67	8	ITFG1
**7.66837773383615E-79**	0.266705057695128	0.69	0.29	1.28506674063626E-74	8	NPEPPS
**1.35690477821964E-48**	0.266702838540122	0.27	0.096	2.27390102734047E-44	8	JARID2
**1.89970446282984E-64**	0.266657867359326	0.482	0.192	3.18352473881024E-60	8	SPCS3
**7.82230122500854E-54**	0.266456257163636	0.439	0.184	1.31086123928693E-49	8	ARRDC1
**1.61940226248007E-67**	0.266399107739405	0.611	0.261	2.7137943114641E-63	8	GBA2
**4.6856945776797E-67**	0.266259131665556	0.638	0.274	7.85228697327565E-63	8	AIMP1
**8.6316353740673E-78**	0.266154987367249	0.678	0.286	1.4464894559862E-73	8	ERF
**6.48376922352683E-66**	0.266147812405374	0.354	0.121	1.08655004647863E-61	8	ATAD1
**2.15206918067339E-78**	0.265993925915961	0.663	0.272	3.60643753297246E-74	8	SMARCD2
**2.41477072686296E-73**	0.265969968115153	0.755	0.336	4.04667278407696E-69	8	FAM177A1
**8.69230106730223E-61**	0.265848230844408	0.541	0.227	1.45665581285851E-56	8	PHLPP2
**3.4288596165826E-73**	0.265582805194567	0.635	0.266	5.74608294546912E-69	8	NUDCD2
**7.61466228601911E-42**	0.265497133873353	0.298	0.118	1.27606510589108E-37	8	ZBTB48
**2.48369551876993E-75**	0.265329300654351	0.647	0.273	4.16217695035464E-71	8	UBP1
**7.98098694111351E-67**	0.265281527433538	0.348	0.119	1.3374537915918E-62	8	RALA
**3.51366890704001E-65**	0.265189025755709	0.358	0.125	5.88820635441764E-61	8	TOMM40
**4.17566554477987E-65**	0.264895189955138	0.726	0.332	6.99758031994211E-61	8	PLOD2
**7.49804207094736E-60**	0.264740076775407	0.732	0.345	1.25652189024936E-55	8	CISD2
**2.08712250924063E-54**	0.264737176966675	0.431	0.177	3.49759990098545E-50	8	COQ8B
**1.35241272876033E-65**	0.264582461988776	0.486	0.192	2.26637325085656E-61	8	TFCP2
**2.80072751943424E-75**	0.264560635136087	0.639	0.267	4.69345917706791E-71	8	TAF1D
**2.78056812657253E-58**	0.264549648541859	0.377	0.141	4.65967606651024E-54	8	NBAS
**3.85928598374487E-63**	0.264440956295682	0.447	0.175	6.46739145155965E-59	8	SPIN1
**4.67348863853512E-65**	0.264440519239731	0.667	0.3	7.83183226045715E-61	8	NDUFB1
**2.96931218373069E-75**	0.264220873727258	0.479	0.178	4.97597335749588E-71	8	SIK3
**2.16629267614851E-78**	0.264084253366547	0.39	0.129	3.63027326668967E-74	8	ILK
**2.53831516205959E-67**	0.264067479438308	0.547	0.223	4.25370854857946E-63	8	SLC35D1
**3.02329900153582E-67**	0.263843443091308	0.601	0.253	5.06644446677373E-63	8	BTD
**2.43403975507314E-39**	0.263292484723249	0.828	0.45	4.07896382155157E-35	8	CLDN7
**3.14105873179606E-74**	0.262747221489225	0.722	0.321	5.26378622274384E-70	8	ACTR1B
**5.33000978886189E-72**	0.262560134682322	0.567	0.229	8.93203040417476E-68	8	RCN1
**2.18229073661604E-52**	0.262388485205535	0.84	0.451	3.65708281642115E-48	8	EIF5
**7.51438315996171E-55**	0.262359521107076	0.735	0.345	1.25926032994638E-50	8	PCDH1
**2.31663654605836E-55**	0.262216769556176	0.519	0.225	3.8822195238846E-51	8	PLGLB2
**2.4101008842553E-50**	0.261901994606332	0.382	0.155	4.03884706183503E-46	8	ENGASE
**3.53555434800089E-96**	0.261684657949562	0.479	0.156	5.92488197637989E-92	8	CSAD
**7.65189686204704E-73**	0.261674728166638	0.618	0.257	1.28230487614184E-68	8	GSS
**3.55675213257308E-51**	0.2614776840737	0.34	0.13	5.96040522376597E-47	8	GIPC2
**2.60937703691226E-72**	0.261427027732233	0.437	0.158	4.37279403845757E-68	8	TBC1D23
**6.54796825554509E-53**	0.261235495328782	0.429	0.177	1.09730852026425E-48	8	RAD50
**2.49029207075493E-72**	0.260816613659874	0.531	0.209	4.17323145217111E-68	8	SNRPD2
**1.41083403966612E-66**	0.260790757932614	0.375	0.133	2.36427568367249E-62	8	L3MBTL2
**2.89041085038251E-46**	0.260692675768978	0.257	0.091	4.84375050307102E-42	8	QTRT2
**9.80674039678464E-61**	0.260502031812117	0.393	0.147	1.64341355569317E-56	8	TCF12
**1.65103581628636E-59**	0.260497027996104	0.296	0.098	2.76680582093269E-55	8	CCDC59
**2.70825439857124E-72**	0.26042814203387	0.585	0.238	4.53849272112568E-68	8	ZMYND11
**2.75178788761402E-76**	0.260098476845858	0.565	0.224	4.61144614206357E-72	8	UBTD2
**9.8111847252739E-61**	0.259892244067869	0.856	0.424	1.6441583362614E-56	8	UGT1A1
**1.46915578313118E-63**	0.259688208621693	0.711	0.327	2.46201126137123E-59	8	TOP 1.00
**2.52647205656606E-49**	0.259625572621632	0.304	0.112	4.23386187239341E-45	8	SRD5A1
**1.24906379406501E-64**	0.25956545325146	0.723	0.331	2.09318110609415E-60	8	MKLN1
**1.0478022987788E-53**	0.259195116543208	0.77	0.378	1.75590709229352E-49	8	PDCD6IP
**3.43035926404395E-58**	0.258804755577999	0.672	0.313	5.74859605468485E-54	8	PLSCR1
**3.00633539087573E-55**	0.25818712236976	0.787	0.376	5.03801684802955E-51	8	DEPRECATED-ENSG00000213759

**3.04357084854036E-41**	0.258116272447226	0.373	0.162	5.10041602798394E-37	8	NIPAL1
**2.64825534963814E-69**	0.25790298420982	0.439	0.163	4.43794631492359E-65	8	PTPN6
**1.82911230429193E-48**	0.257631411617656	0.858	0.449	3.06522639953242E-44	8	ABHD2
**7.50613664682962E-61**	0.257608468984398	0.387	0.144	1.25787837927571E-56	8	ACOT8
**4.70911947926174E-72**	0.257160361002697	0.626	0.264	7.89154242334683E-68	8	GBA
**9.27975600801823E-63**	0.257065361003591	0.415	0.158	1.5551015118237E-58	8	GTF3C1
**4.04228505879831E-106**	0.256862285265418	0.758	0.3	6.7740613015342E-102	8	NRBP2
**8.53070055420438E-85**	0.256303519500339	0.597	0.233	1.42957479887357E-80	8	INO80E
**4.88336167679821E-74**	0.256250808906432	0.606	0.25	8.18353749797844E-70	8	CTDSPL
**1.17277517766965E-19**	0.256050256233852	0.967	0.77	1.96533664273879E-15	8	ACSL1
**1.44591729117792E-57**	0.256042903128672	0.326	0.116	2.42306819655595E-53	8	PRORP
**6.30822536889749E-49**	0.25601222038922	0.844	0.441	1.05713240731984E-44	8	CPQ
**2.0781745708352E-62**	0.255975121588014	0.586	0.251	3.48260494580562E-58	8	GSKIP
**9.58149186927438E-77**	0.255943482255304	0.575	0.226	1.605666407453E-72	8	STX10
**1.25719386495882E-65**	0.255927756422603	0.662	0.293	2.10680547889798E-61	8	EGLN2
**4.55424340473336E-52**	0.255877844661709	0.277	0.095	7.63200109765216E-48	8	NMNAT1
**5.82134366827011E-65**	0.255802776992395	0.511	0.207	9.75540771928705E-61	8	SMAP1
**2.24172661615345E-28**	0.255799078875561	0.925	0.583	3.75668546334994E-24	8	TM9SF2
**2.18921913891566E-63**	0.255584462674513	0.457	0.18	3.66869343299486E-59	8	PTPRA
**3.64703296067536E-56**	0.255459698780809	0.814	0.396	6.11169783549977E-52	8	TMEM63A
**1.42296494358759E-80**	0.255386363080711	0.778	0.342	2.38460465246408E-76	8	SNRNP70
**2.22796274604239E-74**	0.255331236307097	0.57	0.226	3.73361996981784E-70	8	PPA2
**7.16348867951154E-66**	0.255088828530943	0.423	0.158	1.20045743291254E-61	8	EPHB4
**2.27217048272232E-58**	0.255014201441814	0.386	0.148	3.80770329494606E-54	8	TINF2
**1.34010508522105E-63**	0.254817249605983	0.537	0.224	2.24574810181344E-59	8	TULP4
**4.9938681641876E-64**	0.254598168622918	0.534	0.218	8.36872426954559E-60	8	KCTD12
**1.20558267587751E-42**	0.254459344441788	0.905	0.503	2.02031544823552E-38	8	PLXNB2
**6.53481663390492E-68**	0.2544589405001	0.595	0.254	1.09510457150979E-63	8	ARPC5
**3.54876404999378E-64**	0.2543604217987	0.386	0.141	5.94701879497958E-60	8	EXOSC10
**3.76473030521085E-57**	0.254257884085141	0.465	0.192	6.30893504547234E-53	8	ACSF2
**3.07260782982895E-63**	0.253921611585834	0.337	0.116	5.14907620122735E-59	8	NOA1
**1.00068962291598E-62**	0.253751539960974	0.414	0.157	1.67695567008259E-58	8	PRPF8
**1.76845355183496E-36**	0.253689321707912	0.268	0.109	2.96357446216502E-32	8	POLR1B
**1.10547306920312E-79**	0.253142393646872	0.442	0.154	1.85255176937059E-75	8	LRRFIP1
**5.55291475972018E-57**	0.252855613766102	0.81	0.414	9.30557455433908E-53	8	PGD
**5.68009723084273E-70**	0.252631259792699	0.594	0.246	9.51870693944625E-66	8	CNOT11
**8.97051127731363E-70**	0.252388148999418	0.748	0.338	1.50327827985222E-65	8	HGS
**2.23726197672278E-43**	0.252223883686048	0.269	0.101	3.74920362059204E-39	8	CIR1
**7.20257966080526E-51**	0.251887239683296	0.292	0.104	1.20700829955775E-46	8	CAMK2G
**1.35379852036784E-66**	0.251452907640311	0.358	0.123	2.26869556043243E-62	8	L3MBTL4
**2.72490239431207E-41**	0.251379850970345	0.304	0.122	4.56639143238817E-37	8	KNOP1
**6.26647883958351E-68**	0.251330196103692	0.732	0.325	1.0501365239374E-63	8	VPS52
**1.38330753012557E-83**	0.251320933262072	0.635	0.25	2.31814675898443E-79	8	ADAM10
**1.39084624480493E-72**	0.250989173167994	0.684	0.296	2.33078013704411E-68	8	FLOT1
**6.2759437209375E-72**	0.250243500025936	0.652	0.27	1.05172264875471E-67	8	ZFYVE21

SupTable5D_C1_Hep_HepatoSubtype

p_val	avg_log2FC	pct.1	pct.2	p_val_adj	cluster	gene
0	3.47188747247864	0.91	0.124	0	Mature metabolic hepatocytes	HRG
0	4.42006153320789	0.871	0.091	0	Mature metabolic hepatocytes	AHSG
0	4.8524806341748	0.851	0.078	0	Mature metabolic hepatocytes	IGFBP1
0	3.78560089764188	0.975	0.203	0	Mature metabolic hepatocytes	HP
0	4.40251726834359	0.912	0.186	0	Mature metabolic hepatocytes	CYP 20.00
0	2.64216466761143	0.964	0.24	0	Mature metabolic hepatocytes	APOE
0	3.7050061541184	0.805	0.107	0	Mature metabolic hepatocytes	HPR
0	5.15454444128964	0.738	0.041	0	Mature metabolic hepatocytes	ADH4
0	2.98726084569162	0.854	0.179	0	Mature metabolic hepatocytes	HPX
0	5.99065984302081	0.707	0.034	0	Mature metabolic hepatocytes	HAMP
0	2.72354002986523	0.816	0.15	0	Mature metabolic hepatocytes	SERPINA3
0	3.93192662029806	0.755	0.092	0	Mature metabolic hepatocytes	APCS
0	3.41962121424923	0.758	0.11	0	Mature metabolic hepatocytes	COL18A1
0	2.57661445808761	0.875	0.25	0	Mature metabolic hepatocytes	ITIH2
0	5.51804604623758	0.622	0.027	0	Mature metabolic hepatocytes	SERPINA11
0	4.50420160411523	0.997	0.404	0	Mature metabolic hepatocytes	APOA2
0	3.0939146638466	0.934	0.396	0	Mature metabolic hepatocytes	ITIH3
0	3.51728601390597	0.829	0.305	0	Mature metabolic hepatocytes	AZGP1
0	6.10695764369728	0.54	0.02	0	Mature metabolic hepatocytes	CHI3L1
0	3.40628591940817	0.59	0.07	0	Mature metabolic hepatocytes	SAA4
0	3.98365234117729	0.741	0.237	0	Mature metabolic hepatocytes	ALAS1
0	3.06465556276818	0.885	0.385	0	Mature metabolic hepatocytes	SERPING1
0	3.98385740840665	0.541	0.043	0	Mature metabolic hepatocytes	APOC4
0	3.06991423321961	0.862	0.368	0	Mature metabolic hepatocytes	SERPINC1
0	3.89468850172383	0.551	0.076	0	Mature metabolic hepatocytes	CD14
0	3.70115394255033	0.633	0.177	0	Mature metabolic hepatocytes	ADH1B
0	2.4584270002139	0.556	0.118	0	Mature metabolic hepatocytes	SLC38A3
0	2.9373623367603	0.618	0.187	0	Mature metabolic hepatocytes	MST1
0	2.51137213676432	0.664	0.241	0	Mature metabolic hepatocytes	ITIH4
0	4.08201247074454	0.464	0.041	0	Mature metabolic hepatocytes	AFM
0	2.66627238784646	0.627	0.206	0	Mature metabolic hepatocytes	CFHR2
0	3.29572614206587	0.523	0.109	0	Mature metabolic hepatocytes	APOM
0	3.31470398999986	0.47	0.062	0	Mature metabolic hepatocytes	HPD
0	1.89686801715495	0.805	0.398	0	Mature metabolic hepatocytes	ITIH1
0	2.93806616334196	0.462	0.056	0	Mature metabolic hepatocytes	C9
0	3.84053768279081	0.597	0.192	0	Mature metabolic hepatocytes	G0S2
0	2.8839184474898	0.876	0.473	0	Mature metabolic hepatocytes	C1R
0	4.05719269443966	0.432	0.029	0	Mature metabolic hepatocytes	NR1I3
0	2.34117733368075	0.892	0.491	0	Mature metabolic hepatocytes	CP
0	2.17338194218731	0.75	0.35	0	Mature metabolic hepatocytes	APOC1
0	4.51844080781512	0.44	0.04	0	Mature metabolic hepatocytes	ID1
0	4.75563140156198	0.406	0.018	0	Mature metabolic hepatocytes	HSD11B1
0	2.50584215367281	0.609	0.221	0	Mature metabolic hepatocytes	TAT
0	1.73574248528551	0.696	0.309	0	Mature metabolic hepatocytes	LBP
0	2.50381706260316	0.626	0.247	0	Mature metabolic hepatocytes	C5
0	2.6968097396088	0.434	0.06	0	Mature metabolic hepatocytes	MASP2
0	1.38207506973283	0.493	0.12	0	Mature metabolic hepatocytes	ARG1
0	2.34557069480905	0.434	0.065	0	Mature metabolic hepatocytes	SLC38A4
0	2.42781305319805	0.539	0.172	0	Mature metabolic hepatocytes	IGFBP2
0	3.68901201776324	0.401	0.034	0	Mature metabolic hepatocytes	CDO1
0	4.9152896108564	0.414	0.05	0	Mature metabolic hepatocytes	HSD17B13
0	5.40316341820322	0.374	0.01	0	Mature metabolic hepatocytes	SLC25A47
0	2.67645109480356	0.739	0.378	0	Mature metabolic hepatocytes	BHLHE40
0	3.77951644023456	0.507	0.146	0	Mature metabolic hepatocytes	CYP2C9
0	4.01849250128218	0.394	0.056	0	Mature metabolic hepatocytes	ABCB4
0	2.98838707604036	0.965	0.627	0	Mature metabolic hepatocytes	FGB
0	2.15111963343509	0.646	0.31	0	Mature metabolic hepatocytes	NNMT
0	2.86928124906437	0.852	0.518	0	Mature metabolic hepatocytes	PCK1

0	2.830089247943	0.444	0.111	0	Mature metabolic hepatocytes	RDH16
0	1.23341444588849	0.48	0.149	0	Mature metabolic hepatocytes	CRP
0	3.38247701412746	0.402	0.073	0	Mature metabolic hepatocytes	EGR1
0	3.67617774053231	0.402	0.073	0	Mature metabolic hepatocytes	SLC22A7
0	3.15148781367621	0.373	0.047	0	Mature metabolic hepatocytes	FGF21
0	2.4034395293114	0.389	0.065	0	Mature metabolic hepatocytes	MBL2
0	4.531334939801	0.342	0.02	0	Mature metabolic hepatocytes	GCGR
0	2.72158606332783	0.627	0.306	0	Mature metabolic hepatocytes	G6PC
0	2.74506458186924	0.825	0.506	0	Mature metabolic hepatocytes	LEAP2
0	3.26411378500151	0.45	0.133	0	Mature metabolic hepatocytes	MT2A
0	4.451031741687	0.336	0.022	0	Mature metabolic hepatocytes	ANGPTL8
0	2.66293737629854	0.467	0.154	0	Mature metabolic hepatocytes	TFR2
0	2.36146989223793	0.512	0.199	0	Mature metabolic hepatocytes	PROC
0	2.77708174123718	0.52	0.21	0	Mature metabolic hepatocytes	ATF3
0	5.18464703301105	0.321	0.011	0	Mature metabolic hepatocytes	F9
0	4.25826783693719	0.329	0.024	0	Mature metabolic hepatocytes	HJV
0	1.3342136763913	0.584	0.286	0	Mature metabolic hepatocytes	A2M
0	1.96564767213367	0.8	0.507	0	Mature metabolic hepatocytes	FGL1
0	4.51392106471295	0.373	0.082	0	Mature metabolic hepatocytes	DEPRECATEI
0	2.27998548255519	0.435	0.154	0	Mature metabolic hepatocytes	TM4SF5
0	1.43839897718446	0.354	0.076	0	Mature metabolic hepatocytes	CPS1
0	2.46632841067976	0.658	0.381	0	Mature metabolic hepatocytes	TXNIP
0	3.67758584225275	0.309	0.036	0	Mature metabolic hepatocytes	ANGPTL3
0	4.21952463781051	0.302	0.029	0	Mature metabolic hepatocytes	CYP8B1
0	2.33294985107272	0.453	0.182	0	Mature metabolic hepatocytes	SLC9A3R2
0	2.04662733462859	0.78	0.51	0	Mature metabolic hepatocytes	F2
0	3.55095926589769	0.302	0.033	0	Mature metabolic hepatocytes	HGFAC
0	3.78302433566495	0.298	0.031	0	Mature metabolic hepatocytes	CPED1
0	2.66252819853178	0.5	0.235	0	Mature metabolic hepatocytes	GADD45A
0	3.92040792624057	0.292	0.031	0	Mature metabolic hepatocytes	SERPINA4
0	5.59749494229615	0.265	0.007	0	Mature metabolic hepatocytes	LECT2
0	3.7149022889219	0.316	0.06	0	Mature metabolic hepatocytes	CFHR5
0	2.53881807367164	0.306	0.05	0	Mature metabolic hepatocytes	ALDH8A1
0	2.57651699116216	0.752	0.496	0	Mature metabolic hepatocytes	CYP2C8
0	1.85243378162508	0.865	0.609	0	Mature metabolic hepatocytes	C1S
0	3.02109622601667	0.304	0.05	0	Mature metabolic hepatocytes	CHRD
0	4.89492998968792	0.266	0.016	0	Mature metabolic hepatocytes	MAFB
0	2.29916552613109	0.603	0.355	0	Mature metabolic hepatocytes	PLPP3
0	4.20653914589873	0.288	0.042	0	Mature metabolic hepatocytes	SLC10A1
0	2.67987652868777	0.465	0.222	0	Mature metabolic hepatocytes	ADH6
0	4.32144270785511	0.252	0.014	0	Mature metabolic hepatocytes	EVA1A
0	2.76644129611402	0.302	0.064	0	Mature metabolic hepatocytes	MASP1
0	2.11109055554008	0.474	0.247	0	Mature metabolic hepatocytes	DEPRECATEI
0	2.97208047222595	0.323	0.098	0	Mature metabolic hepatocytes	IL1RN
0	2.26502753560668	0.304	0.08	0	Mature metabolic hepatocytes	GCKR
0	4.06414812248031	0.251	0.03	0	Mature metabolic hepatocytes	DEPRECATEI
0	4.37788961767029	0.231	0.014	0	Mature metabolic hepatocytes	PGLYRP2
0	4.35211417708514	0.226	0.012	0	Mature metabolic hepatocytes	ADRA1A
0	2.37125802866174	0.302	0.088	0	Mature metabolic hepatocytes	RND1
0	2.1307044362875	0.636	0.425	0	Mature metabolic hepatocytes	SERPINA10
0	2.36743478724806	0.571	0.362	0	Mature metabolic hepatocytes	PON3
0	0.992096429650732	0.784	0.576	0	Mature metabolic hepatocytes	PAH
0	3.59169467061288	0.227	0.022	0	Mature metabolic hepatocytes	CCND1
0	3.85603451639526	0.239	0.035	0	Mature metabolic hepatocytes	CFHR3
0	3.46199609738373	0.24	0.04	0	Mature metabolic hepatocytes	INHBE
0	1.42293362292335	0.651	0.453	0	Mature metabolic hepatocytes	SERPINF2
0	2.74250231404884	0.24	0.047	0	Mature metabolic hepatocytes	CPN2
0	3.9053624562031	0.26	0.068	0	Mature metabolic hepatocytes	CYP3A4
0	3.46108476416046	0.211	0.022	0	Mature metabolic hepatocytes	APOF
0	1.90769472051594	0.79	0.603	0	Mature metabolic hepatocytes	JUN

0	2.05788534031237	0.694	0.507	0	Mature metabolic hepatocytes	POR
0	1.90644297752623	0.537	0.352	0	Mature metabolic hepatocytes	JUNB
0	3.53136968242737	0.22	0.037	0	Mature metabolic hepatocytes	SPP2
0	3.12868794384874	0.229	0.049	0	Mature metabolic hepatocytes	ACKR3
0	1.96355940185654	0.231	0.052	0	Mature metabolic hepatocytes	TIMP1
0	2.39667758554408	0.97	0.793	0	Mature metabolic hepatocytes	FGG
0	3.78648327837116	0.195	0.019	0	Mature metabolic hepatocytes	GLYATL1
0	2.44186844481872	0.218	0.043	0	Mature metabolic hepatocytes	FAM20A
0	2.03371556201904	0.848	0.673	0	Mature metabolic hepatocytes	PLG
0	3.9831946094417	0.184	0.011	0	Mature metabolic hepatocytes	MPDZ
0	2.06317243647587	0.552	0.38	0	Mature metabolic hepatocytes	PPP2R1B
0	3.25331070254156	0.225	0.056	0	Mature metabolic hepatocytes	CYP4A11
0	2.42255501717972	0.213	0.044	0	Mature metabolic hepatocytes	AMDHD1
0	3.88449005407359	0.198	0.03	0	Mature metabolic hepatocytes	DEPRECATEI
0	3.53710283333054	0.195	0.031	0	Mature metabolic hepatocytes	TGFBI
0	5.5835281942707	0.167	0.003	0	Mature metabolic hepatocytes	DTX1
0	1.62809226517377	0.953	0.794	0	Mature metabolic hepatocytes	CFB
0	2.00637848323657	0.932	0.774	0	Mature metabolic hepatocytes	KNG1
0	2.59504430063025	0.983	0.826	0	Mature metabolic hepatocytes	FGA
0	3.66278638073569	0.187	0.03	0	Mature metabolic hepatocytes	MT1X
0	3.34990076972463	0.171	0.016	0	Mature metabolic hepatocytes	PCOLCE
0	4.43975943193427	0.163	0.009	0	Mature metabolic hepatocytes	DAO
0	3.19910171028332	0.181	0.03	0	Mature metabolic hepatocytes	CPN1
0	2.04347001042985	0.692	0.544	0	Mature metabolic hepatocytes	INSIG1
0	1.85097816020709	0.615	0.471	0	Mature metabolic hepatocytes	DCXR
0	1.72391481458044	0.653	0.51	0	Mature metabolic hepatocytes	C8A
0	1.51601341315804	0.722	0.58	0	Mature metabolic hepatocytes	SLC2A2
0	5.23009265776532	0.142	0.002	0	Mature metabolic hepatocytes	MPPED1
0	1.79083598974403	0.915	0.783	0	Mature metabolic hepatocytes	ORM2
0	2.00025989413021	0.965	0.835	0	Mature metabolic hepatocytes	APOB
0	4.22876251912899	0.135	0.007	0	Mature metabolic hepatocytes	CLDN2
0	3.22971850893092	0.141	0.015	0	Mature metabolic hepatocytes	ACSS3
0	1.32348186787066	0.741	0.616	0	Mature metabolic hepatocytes	LRG1
0	4.49499584539377	0.126	0.005	0	Mature metabolic hepatocytes	ATOH8
0	1.77654633672447	0.79	0.669	0	Mature metabolic hepatocytes	ALDH2
0	3.30572904353095	0.134	0.014	0	Mature metabolic hepatocytes	IGLC2
0	1.65274214732432	0.917	0.797	0	Mature metabolic hepatocytes	TTR
0	4.52010791052618	0.126	0.007	0	Mature metabolic hepatocytes	ABCA8
0	4.24722605412073	0.124	0.007	0	Mature metabolic hepatocytes	ADIRF
0	1.37746438217207	0.818	0.703	0	Mature metabolic hepatocytes	GLUL
0	4.1374762359001	0.12	0.006	0	Mature metabolic hepatocytes	GYS2
0	1.55617453121116	0.808	0.696	0	Mature metabolic hepatocytes	MLXIPL
0	1.19066610524484	0.858	0.749	0	Mature metabolic hepatocytes	CYP3A5
0	4.36935875018433	0.112	0.005	0	Mature metabolic hepatocytes	THRSP
0	1.64247483365702	0.628	0.526	0	Mature metabolic hepatocytes	ACAT1
0	1.34336775295729	0.903	0.803	0	Mature metabolic hepatocytes	AGXT
0	1.86257665817808	0.984	0.885	0	Mature metabolic hepatocytes	ORM1
0	4.287657801824	0.102	0.004	0	Mature metabolic hepatocytes	UROC1
0	1.37890682306017	0.735	0.655	0	Mature metabolic hepatocytes	PTMS
0	1.43457580339459	0.972	0.896	0	Mature metabolic hepatocytes	APOC2
0	2.2839484434308	0.977	0.902	0	Mature metabolic hepatocytes	APOA1
0	2.29775181790857	0.981	0.912	0	Mature metabolic hepatocytes	RBP4
0	1.26923208885763	0.767	0.705	0	Mature metabolic hepatocytes	EFNA1
0	1.38349734776688	0.873	0.818	0	Mature metabolic hepatocytes	C4BPA
0	1.34346124828694	0.815	0.76	0	Mature metabolic hepatocytes	PRAP1
0	1.28351829834245	0.696	0.642	0	Mature metabolic hepatocytes	ECHS1
0	1.20746844870361	0.756	0.703	0	Mature metabolic hepatocytes	RHOB
0	1.76376413763975	0.94	0.918	0	Mature metabolic hepatocytes	FABP1
0	0.984486668793443	0.965	0.949	0	Mature metabolic hepatocytes	TF
0	0.550988123725184	0.959	0.975	0	Mature metabolic hepatocytes	CLU

0	1.09309344190935	0.833	0.818	0	Mature metabolic hepatocytes	CYB5A
0	1.73066776511055	0.983	0.969	0	Mature metabolic hepatocytes	APOC3
0	1.35138591890647	0.953	0.961	0	Mature metabolic hepatocytes	AMBP
0	0.93411073640776	0.851	0.843	0	Mature metabolic hepatocytes	APOH
0	2.38105808286699	0.994	0.989	0	Mature metabolic hepatocytes	SERPINA1
0	0.921891274235433	0.869	0.869	0	Mature metabolic hepatocytes	TMEM176B
3.9346543155526E-306	1.87988757568988	0.461	0.246	6.59369370200306E-302	Mature metabolic hepatocytes	AQP9
6.97321802826781E-305	1.67885546708165	0.578	0.426	1.16857187717712E-300	Mature metabolic hepatocytes	ASGR1
3.98399153650677E-302	3.13344201668017	0.358	0.158	6.67637301687805E-298	Mature metabolic hepatocytes	SERPINE1
2.86633039077084E-300	2.10275590779742	0.563	0.43	4.80339646885378E-296	Mature metabolic hepatocytes	DEPRECATEI
1.70165149041068E-296	3.4619092917422	0.14	0.018	2.85162756763022E-292	Mature metabolic hepatocytes	THPO
1.5049821795473E-295	4.38374745050116	0.136	0.016	2.52204913648536E-291	Mature metabolic hepatocytes	CCN1
6.32597227389845E-294	2.60486587299368	0.325	0.129	1.0601064336599E-289	Mature metabolic hepatocytes	FXYD1
2.51325118899855E-293	3.13248858895009	0.127	0.013	4.21170634252377E-289	Mature metabolic hepatocytes	F12
3.36757967289891E-293	1.46805834770749	0.602	0.451	5.64339001584399E-289	Mature metabolic hepatocytes	SERPINF1
1.0946765766966E-292	2.75506273450722	0.186	0.037	1.83445900722816E-288	Mature metabolic hepatocytes	FOXA3
1.09365233245161E-291	0.755108643538613	0.877	0.898	1.8327425787224E-287	Mature metabolic hepatocytes	DEPRECATEI
2.78522770681681E-291	2.98505233472912	0.298	0.112	4.6674845910836E-287	Mature metabolic hepatocytes	CIDEB
7.06805562970126E-278	1.24749339173826	0.673	0.617	1.18446476242534E-273	Mature metabolic hepatocytes	DDT
6.72385599457104E-275	3.640488741915	0.109	0.009	1.12678378757022E-270	Mature metabolic hepatocytes	LRRC3
4.95080399259341E-271	2.68592185250606	0.239	0.073	8.29655733078804E-267	Mature metabolic hepatocytes	ABCA6
9.44631258138312E-271	2.94990420926803	0.192	0.045	1.58301306238818E-266	Mature metabolic hepatocytes	SLC13A5
1.30450943373454E-270	2.32711261701686	0.289	0.106	2.18609690905234E-266	Mature metabolic hepatocytes	TMEM140
7.13227412674996E-267	2.84324458407749	0.146	0.023	1.19522649816076E-262	Mature metabolic hepatocytes	SOCS3
4.20387907827801E-264	3.31740296188212	0.177	0.039	7.04486055937828E-260	Mature metabolic hepatocytes	DEPRECATEI
5.77672017049112E-261	2.36181888464501	0.399	0.22	9.68062766170902E-257	Mature metabolic hepatocytes	ACADSB
3.43161736981496E-255	1.54734990572722	0.581	0.453	5.75070438833591E-251	Mature metabolic hepatocytes	GLYCTK
3.77319585154641E-248	1.32857557750349	0.639	0.558	6.32312160802148E-244	Mature metabolic hepatocytes	ETS2
2.56046162246958E-244	2.36202504440607	0.339	0.16	4.29082158693452E-240	Mature metabolic hepatocytes	HES1
2.90920135846784E-241	3.21372096323571	0.127	0.019	4.87523963652041E-237	Mature metabolic hepatocytes	PPP1R1A
1.3018345593814E-235	2.29987762475819	0.201	0.056	2.18161435461134E-231	Mature metabolic hepatocytes	LGALS3
1.63310077722239E-230	2.99403462458353	0.202	0.06	2.73675028246929E-226	Mature metabolic hepatocytes	SLC27A2
2.90726587212632E-230	1.14820833856607	0.67	0.623	4.87199614850929E-226	Mature metabolic hepatocytes	SERPIND1
1.81163453160633E-228	2.29549458964463	0.347	0.175	3.03593714806589E-224	Mature metabolic hepatocytes	SLC27A5
2.70442869455831E-226	2.49388180480214	0.36	0.192	4.53208160634081E-222	Mature metabolic hepatocytes	GBP1
4.73590557867333E-225	2.8617714329717	0.207	0.064	7.93643056874077E-221	Mature metabolic hepatocytes	VNN 3.00
1.9332020326238E-224	2.19020007078947	0.354	0.182	3.23965996627096E-220	Mature metabolic hepatocytes	RORC
5.38048678718028E-220	2.08136307671827	0.151	0.032	9.01661975795672E-216	Mature metabolic hepatocytes	FETUB
4.17161695946658E-219	2.55652127623799	0.127	0.022	6.99079570067409E-215	Mature metabolic hepatocytes	UGT3A1
5.09581763279499E-214	1.31040087015774	0.619	0.552	8.53957118903784E-210	Mature metabolic hepatocytes	BHMT2
5.51808737685972E-214	2.44109474324745	0.138	0.027	9.24721082614152E-210	Mature metabolic hepatocytes	TMPRSS6
6.06707133979797E-213	1.55106541536597	0.564	0.469	1.01671981512334E-208	Mature metabolic hepatocytes	ADH1A
2.7588409932328E-212	2.12251927641978	0.224	0.076	4.62326573645952E-208	Mature metabolic hepatocytes	OGDHL
5.36310376937428E-211	1.72818356946177	0.539	0.435	8.98748929671742E-207	Mature metabolic hepatocytes	CCNL1
7.32125849223126E-211	3.15552829166323	0.167	0.044	1.22689649812811E-206	Mature metabolic hepatocytes	CFHR4
1.97464618287278E-208	1.5061822280326	0.531	0.415	3.3091120732582E-204	Mature metabolic hepatocytes	C8B
2.27121592532854E-208	1.88666749557057	0.404	0.243	3.80610364766558E-204	Mature metabolic hepatocytes	F7
1.65232298340143E-206	2.5605701797063	0.111	0.017	2.76896285558412E-202	Mature metabolic hepatocytes	DNALI1
4.21895095304764E-206	2.06586891177012	0.427	0.271	7.07011800711723E-202	Mature metabolic hepatocytes	CXCL2
6.28648240077874E-206	1.0264409212243	0.703	0.683	1.0534887207225E-201	Mature metabolic hepatocytes	ADH1C
8.78322534772123E-205	1.09966583312637	0.679	0.664	1.47189290377112E-200	Mature metabolic hepatocytes	FMO3
1.44463406404614E-204	1.38991599441844	0.562	0.465	2.42091776452853E-200	Mature metabolic hepatocytes	PCBD1
2.74037252399713E-204	1.04445558299134	0.396	0.212	4.59231627571439E-200	Mature metabolic hepatocytes	DEPRECATEI
9.95582456397386E-196	2.67293442248454	0.139	0.031	1.66839708043074E-191	Mature metabolic hepatocytes	CCL16
1.06668570863827E-193	1.70601720541905	0.506	0.403	1.78755191053601E-189	Mature metabolic hepatocytes	SLC30A1
1.57422902841129E-192	1.76302522348668	0.19	0.058	2.63809300581164E-188	Mature metabolic hepatocytes	MYOM1
3.78437100706834E-192	0.503680056435433	0.916	0.957	6.34184893364513E-188	Mature metabolic hepatocytes	ALDOB
5.24388349511115E-189	2.79145138388498	0.191	0.063	8.78769996110726E-185	Mature metabolic hepatocytes	FYB2
7.40269745006236E-189	2.85612912446433	0.247	0.105	1.24054403868145E-184	Mature metabolic hepatocytes	APOA5
7.581180468876E-188	3.05014693299428	0.173	0.052	1.27045422297424E-183	Mature metabolic hepatocytes	SLC17A2

6.77310690464403E-185	1.8742327996493	0.389	0.238	1.13503725508025E-180	Mature metabolic hepatocytes	ID2
4.59029415071047E-183	2.04814265264358	0.145	0.036	7.69241493776061E-179	Mature metabolic hepatocytes	FNDC4
9.02023548130102E-181	2.44437725490889	0.257	0.113	1.51161106195643E-176	Mature metabolic hepatocytes	FOS
3.59868999176775E-180	1.52598728477082	0.259	0.107	6.0306846882044E-176	Mature metabolic hepatocytes	SLC25A18
1.85912963441949E-179	1.23219765785924	0.62	0.584	3.11552944136019E-175	Mature metabolic hepatocytes	ANG
3.30108879170388E-178	0.301381517026251	0.964	0.967	5.53196459713737E-174	Mature metabolic hepatocytes	C3
1.57862828713915E-176	1.57206074054467	0.522	0.442	2.6454652835878E-172	Mature metabolic hepatocytes	MPC1
1.67356711359406E-176	1.36451958005768	0.405	0.244	2.80456376896092E-172	Mature metabolic hepatocytes	MTSS2
5.92290947549252E-176	2.05898295221092	0.162	0.047	9.92561169903037E-172	Mature metabolic hepatocytes	ANXA6
5.13358513149282E-174	0.833451708906237	0.736	0.738	8.60286196335567E-170	Mature metabolic hepatocytes	C4BPB
1.8126100948471E-173	1.27234882084699	0.586	0.516	3.03757199694478E-169	Mature metabolic hepatocytes	MAT1A
1.08730566258695E-172	1.79648556256959	0.425	0.294	1.82210682936321E-168	Mature metabolic hepatocytes	ORMDL3
1.75688311276303E-172	3.04609945248018	0.133	0.033	2.94418472036829E-168	Mature metabolic hepatocytes	SUGCT
3.22318115300282E-171	0.960025756934714	0.663	0.648	5.40140697620213E-167	Mature metabolic hepatocytes	TMEM176A
2.18826019231498E-167	2.92142779640991	0.289	0.152	3.66708643028145E-163	Mature metabolic hepatocytes	SERPINH1
1.63233592023303E-166	1.2326591678174	0.593	0.55	2.7354685351265E-162	Mature metabolic hepatocytes	CFI
2.98572695787572E-165	2.09108918637473	0.394	0.268	5.00348123600814E-161	Mature metabolic hepatocytes	SLC30A10
7.53240816967126E-164	2.94064022536172	0.117	0.026	1.26228096107351E-159	Mature metabolic hepatocytes	ASPDH
5.11102965376394E-163	1.58430620176883	0.476	0.371	8.56506349377762E-159	Mature metabolic hepatocytes	CYP27A1
2.72303377476032E-162	2.68906263262043	0.189	0.069	4.56325999974335E-158	Mature metabolic hepatocytes	ACOX2
3.44044351014496E-161	1.55303457674225	0.488	0.39	5.76549523430093E-157	Mature metabolic hepatocytes	PON1
5.83875850401762E-161	3.07022270863856	0.101	0.019	9.78459150103273E-157	Mature metabolic hepatocytes	ANGPTL4
3.00754722654172E-160	1.55731249795721	0.15	0.042	5.04004764223861E-156	Mature metabolic hepatocytes	FST
2.27144366819793E-159	2.47387750854425	0.208	0.084	3.80648529916608E-155	Mature metabolic hepatocytes	CISH
5.6178364837358E-159	1.50442532734358	0.504	0.423	9.41437037944446E-155	Mature metabolic hepatocytes	FUOM
8.52795147773639E-157	1.35080116586815	0.569	0.532	1.42911410863906E-152	Mature metabolic hepatocytes	NFE2L2
8.47822299502911E-156	1.72695720523877	0.442	0.334	1.42078060950698E-151	Mature metabolic hepatocytes	RGN
5.55836644438501E-154	2.41415394371599	0.14	0.04	9.3147104875004E-150	Mature metabolic hepatocytes	CD81
1.28108944426849E-153	0.450270760847952	0.797	0.848	2.14684969070513E-149	Mature metabolic hepatocytes	IFITM3
1.76304036887207E-153	1.92719417573354	0.125	0.031	2.95450305015582E-149	Mature metabolic hepatocytes	ATP2B2
5.71705274954309E-153	2.05921894206642	0.352	0.223	9.58063699768432E-149	Mature metabolic hepatocytes	ARRDC3
1.9307019933626E-149	2.25237353562347	0.214	0.091	3.23547040047705E-145	Mature metabolic hepatocytes	LEPR
8.53623092692085E-149	2.46460054176729	0.151	0.048	1.4305015787334E-144	Mature metabolic hepatocytes	ADGRG6
3.98875263833662E-142	1.16607067591097	0.571	0.524	6.68435167132451E-138	Mature metabolic hepatocytes	BDH1
4.88180976088945E-142	3.56626543533464	0.142	0.045	8.18093679729855E-138	Mature metabolic hepatocytes	MFSD2A
2.11688749436868E-139	1.90469945661849	0.383	0.267	3.54748006306304E-135	Mature metabolic hepatocytes	SLC25A25
1.68225366161407E-136	2.00221921535904	0.103	0.023	2.81912068613285E-132	Mature metabolic hepatocytes	MAGED1
2.16504827032364E-133	2.67166624397715	0.168	0.065	3.62818789140836E-129	Mature metabolic hepatocytes	CD302
9.46642010579451E-131	2.78759603567137	0.116	0.032	1.58638268132904E-126	Mature metabolic hepatocytes	SLC6A12
1.65389844728106E-128	2.11368342777724	0.278	0.157	2.77160301795359E-124	Mature metabolic hepatocytes	TRIP6
3.82263191082655E-128	1.7762808558821	0.168	0.063	6.40596655616314E-124	Mature metabolic hepatocytes	IER5L
3.84888625154345E-127	1.4780480812262	0.381	0.262	6.44996358033652E-123	Mature metabolic hepatocytes	REEP6
8.81979457106856E-127	2.31276396146602	0.113	0.031	1.47802117421967E-122	Mature metabolic hepatocytes	NLRP1
1.40526408131476E-126	2.34093677912784	0.228	0.113	2.35494154746728E-122	Mature metabolic hepatocytes	GADD45G
7.8406836131825E-126	1.70092512695096	0.287	0.161	1.31394175989712E-121	Mature metabolic hepatocytes	DUSP10
2.64882317336623E-124	1.98006263087854	0.303	0.186	4.43889787392712E-120	Mature metabolic hepatocytes	KLF10
9.83150543270519E-124	2.0700013316177	0.247	0.129	1.64756368041274E-119	Mature metabolic hepatocytes	ACOT12
1.41808005443221E-123	2.97490559673485	0.107	0.029	2.3764185552175E-119	Mature metabolic hepatocytes	MOGAT2
6.54276356508436E-123	1.62950455839323	0.368	0.256	1.09643631823684E-118	Mature metabolic hepatocytes	NFIL3
1.60120890154564E-121	1.9280312475981	0.378	0.28	2.68330587721018E-117	Mature metabolic hepatocytes	MTTP
4.96853336810276E-119	2.70604719781769	0.125	0.04	8.3262682182666E-115	Mature metabolic hepatocytes	IFIT1
5.83780606049151E-116	2.81714527995687	0.143	0.052	9.78299539617167E-112	Mature metabolic hepatocytes	HSPA6
5.93884890813623E-116	1.40961393110443	0.493	0.449	9.95232300025469E-112	Mature metabolic hepatocytes	EHHADH
8.26665686285674E-116	1.81745824087051	0.27	0.154	1.38532635707753E-111	Mature metabolic hepatocytes	ACSM5
1.26144186021654E-115	1.46821922740576	0.485	0.425	2.11392426935088E-111	Mature metabolic hepatocytes	F11
1.27536943590309E-115	1.97689519359512	0.221	0.11	2.13726410068639E-111	Mature metabolic hepatocytes	CDH2
6.66842466746324E-114	2.46760477420971	0.222	0.115	1.11749460577349E-109	Mature metabolic hepatocytes	KLHL25
1.19352188806187E-113	0.46663498172743	0.77	0.745	2.00010398001408E-109	Mature metabolic hepatocytes	CFH
1.57007825120306E-113	1.28415013047527	0.478	0.406	2.63113713336608E-109	Mature metabolic hepatocytes	F10
1.29981919567364E-112	2.55219916733115	0.265	0.156	2.17823700810988E-108	Mature metabolic hepatocytes	DEFB1

2.13194242246494E-112	1.30941108868765	0.495	0.445	3.57270911156675E-108	Mature metabolic hepatocytes	ABAT
3.17492879650113E-111	2.07545957669763	0.241	0.132	5.32054567717659E-107	Mature metabolic hepatocytes	GOLT1A
8.92958507428278E-111	1.5462803781961	0.34	0.229	1.49641986674831E-106	Mature metabolic hepatocytes	RGS3
2.63548614881 47E-108	1.7827082472339	0.512	0.482	4.41654768818368E-104	Mature metabolic hepatocytes	HMGCS1
4.33282367218963E-107	1.93544743352834	0.329	0.226	7.26094590985539E-103	Mature metabolic hepatocytes	DCPS
7.08298699186524E-105	2.26211454863947	0.279	0.176	1.18696696009678E-100	Mature metabolic hepatocytes	PDK4
8.90816797728803E-105	1.43452651637999	0.476	0.423	1.49283078963393E-100	Mature metabolic hepatocytes	ARHGEF10L
1.89416109906726E-104	0.585594840262668	0.718	0.759	3.17423516981692E-100	Mature metabolic hepatocytes	ASGR2
6.88345466907237E-103	0.900080838947607	0.602	0.609	1.15352933344315E-98	Mature metabolic hepatocytes	CTSB
7.06454685071145E-103	1.25815400820633	0.536	0.525	1.18387676124223E-98	Mature metabolic hepatocytes	ACAA1
2.07589824322271E-101	1.68815837765504	0.139	0.053	3.47879027599262E-97	Mature metabolic hepatocytes	SLC22A25
3.62795023161812E-101	1.89047084725774	0.114	0.038	6.07971899814565E-97	Mature metabolic hepatocytes	IFIT2
4.94590641030434E-101	0.710975334005552	0.653	0.649	8.28834996238801E-97	Mature metabolic hepatocytes	ALDH1L1
1.37340573599669E-100	1.84883457750973	0.277	0.171	2.30155333238325E-96	Mature metabolic hepatocytes	RBP5
3.41712772652443E-91	1.02446149285178	0.496	0.436	5.72642264410963E-87	Mature metabolic hepatocytes	DPYS
3.3683580280117E-89	3.01187094927141	0.107	0.038	5.644694383342E-85	Mature metabolic hepatocytes	IGSF9
7.73349397845809E-87	1.16214386901984	0.395	0.304	1.29597892091001E-82	Mature metabolic hepatocytes	CREB3L3
4.73404862047013E-86	2.44352130107197	0.137	0.059	7.93331867818384E-82	Mature metabolic hepatocytes	INHBA
2.62278351526311E-82	2.44946684106554	0.125	0.051	4.39526061487792E-78	Mature metabolic hepatocytes	TBX3
3.69355986243336E-82	1.26572933402499	0.351	0.258	6.18966761746583E-78	Mature metabolic hepatocytes	PABPC1L
5.08971621571139E-81	0.534593717322209	0.746	0.812	8.52934643428915E-77	Mature metabolic hepatocytes	SCP2
3.43253662401465E-80	0.288466681328719	0.141	0.303	5.75224487452376E-76	Mature metabolic hepatocytes	ZBED6
1.03625777263108E-79	1.97386611163013	0.258	0.171	1.73656077537516E-75	Mature metabolic hepatocytes	CYP2J2
1.47871171090503E-77	1.70579033622493	0.369	0.306	2.47802508513464E-73	Mature metabolic hepatocytes	SEC14L2
3.72101828554825E-76	1.69555075738076	0.307	0.223	6.23568244292176E-72	Mature metabolic hepatocytes	PPP1R3C
3.62028349791064E-75	1.88019972493016	0.284	0.202	6.06687108579865E-71	Mature metabolic hepatocytes	PEMT
5.11423833782022E-75	2.13457886492557	0.236	0.151	8.57044060651913E-71	Mature metabolic hepatocytes	P4HA1
1.1787196641644E-74	2.21948267936039	0.136	0.063	1.9752984132067E-70	Mature metabolic hepatocytes	ADAMTSL4
4.56090159070513E-74	0.707776661749098	0.681	0.725	7.64315888570366E-70	Mature metabolic hepatocytes	SULT2A1
4.94486141313395E-74	1.61497486974977	0.342	0.268	8.28659875612988E-70	Mature metabolic hepatocytes	NFKBIZ
1.04922203536316E-73	1.01877106797033	0.56	0.58	1.75828628686158E-69	Mature metabolic hepatocytes	HSPD1
7.35559056762147E-73	0.273874402338125	0.12	0.263	1.23264986732201E-68	Mature metabolic hepatocytes	LRP6
2.56713254720147E-72	1.19351319069521	0.449	0.415	4.30200072260022E-68	Mature metabolic hepatocytes	GRHPR
2.88385577641888E-72	0.263897366296569	0.146	0.301	4.83276551012277E-68	Mature metabolic hepatocytes	ATL2
1.62591794726699E-71	1.30333718602641	0.424	0.378	2.72471329603002E-67	Mature metabolic hepatocytes	LRP1
2.40052076673438E-71	1.038514590757	0.495	0.482	4.02279270089348E-67	Mature metabolic hepatocytes	ITM2B
9.65072464673731E-71	0.699349589075401	0.208	0.113	1.61726843630024E-66	Mature metabolic hepatocytes	ICAM1
1.98534533891738E-70	1.74553454889437	0.156	0.078	3.32704171895775E-66	Mature metabolic hepatocytes	CBS
3.96085377318812E-70	1.86787281841914	0.18	0.1	6.63759875310865E-66	Mature metabolic hepatocytes	LDHD
5.29059789597978E-70	0.2543773489533	0.089	0.21	8.86598395408291E-66	Mature metabolic hepatocytes	HDAC8
3.44806365340177E-69	1.87906407727984	0.168	0.09	5.77826507037068E-65	Mature metabolic hepatocytes	IER3
1.67746223252605E-68	0.339683729466128	0.189	0.364	2.81109120926715E-64	Mature metabolic hepatocytes	SLC23A2
4.79135291470109E-67	0.404578568180787	0.078	0.191	8.02934921445609E-63	Mature metabolic hepatocytes	GPCPD1
7.91426790002286E-67	0.29360255466411	0.104	0.23	1.32627301468583E-62	Mature metabolic hepatocytes	SLC19A2
4.13463424183619E-66	2.41020686360188	0.135	0.066	6.92882006246908E-62	Mature metabolic hepatocytes	PHLDA1
4.94995123884135E-66	2.09487961702947	0.15	0.078	8.29512828605034E-62	Mature metabolic hepatocytes	TIMP3
8.02701586215486E-66	2.42284426221617	0.155	0.083	1.34516731817991E-61	Mature metabolic hepatocytes	ZNF595
1.49142552551021E-65	0.293828223781247	0.108	0.235	2.49933089565E-61	Mature metabolic hepatocytes	BCLAF1
1.57834309264934E-65	0.268618545027976	0.06	0.159	2.64498735466177E-61	Mature metabolic hepatocytes	FAM133B
1.03523950153399E-64	0.330256724298973	0.114	0.244	1.73485435667066E-60	Mature metabolic hepatocytes	MAST3
1.97643586837547E-64	1.10936844896567	0.487	0.489	3.31211122822362E-60	Mature metabolic hepatocytes	HAO1
2.1068420295958E-64	0.268991102634962	0.062	0.162	3.53064587319664E-60	Mature metabolic hepatocytes	MICA
2.81306505766265E-64	0.414757799983529	0.078	0.188	4.71413442363106E-60	Mature metabolic hepatocytes	ARID4A
4.01739854625736E-64	1.50255742892853	0.141	0.069	6.73235648381809E-60	Mature metabolic hepatocytes	DTNA
4.21265707353665E-64	2.04668620508452	0.151	0.079	7.05957072383272E-60	Mature metabolic hepatocytes	LIPC
7.19847672647841E-64	0.337597176746098	0.127	0.263	1.20632072982325E-59	Mature metabolic hepatocytes	SLC5A3
7.81688148698007E-64	0.293463775061907	0.162	0.315	1.30995299958812E-59	Mature metabolic hepatocytes	PHYKPL
1.19463688800634E-63	0.28421329350552	0.151	0.298	2.00197249692102E-59	Mature metabolic hepatocytes	PCM1
1.55558071457042E-63	0.97664602939341	0.508	0.486	2.60684216147711E-59	Mature metabolic hepatocytes	ECHDC2
1.62217268495059E-63	0.251994523561792	0.081	0.19	2.7184369854402E-59	Mature metabolic hepatocytes	ATRX

1.97939623248685E-63	0.314632518883328	0.189	0.357	3.31707220640147E-59	Mature metabolic hepatocytes	TNRC6A
2.13130924136356E-63	0.463118073029799	0.058	0.155	3.57164802667706E-59	Mature metabolic hepatocytes	LTB4R
2.69854168232778E-63	1.47643695069814	0.54	0.569	4.5222161512449E-59	Mature metabolic hepatocytes	FASN
1.47359887407547E-61	2.27187420796737	0.132	0.066	2.46945699317567E-57	Mature metabolic hepatocytes	GABARAPL1
1.72254822374033E-61	0.351255209922294	0.192	0.358	2.88664631334404E-57	Mature metabolic hepatocytes	ZNF644
2.35891908026167E-61	0.301735550431656	0.144	0.283	3.95307659470251E-57	Mature metabolic hepatocytes	PPP1R12B
3.58696086171671E-61	0.293086353413503	0.187	0.349	6.01102901206486E-57	Mature metabolic hepatocytes	CHKA
4.33873414979594E-61	0.264313638537178	0.09	0.201	7.27085068822804E-57	Mature metabolic hepatocytes	ARMH3
1.56528794156787E-60	1.65023909224075	0.157	0.085	2.62310953247943E-56	Mature metabolic hepatocytes	ALDH1B1
2.27723230709741E-60	0.32011886255648	0.127	0.257	3.81618590023384E-56	Mature metabolic hepatocytes	KAT2A
2.55847321689768E-60	0.505032412580633	0.76	0.823	4.28748941687714E-56	Mature metabolic hepatocytes	HSPA1A
5.59578751016076E-60	1.13496761880149	0.427	0.393	9.3774207095274E-56	Mature metabolic hepatocytes	IFITM2
1.37429850103638E-59	0.28559774331728	0.083	0.19	2.30304942803677E-55	Mature metabolic hepatocytes	PLEKHA1
1.6239714563846E-59	0.391129843670696	0.062	0.157	2.72145136660932E-55	Mature metabolic hepatocytes	DAAM1
2.59941212011995E-59	0.275674531905263	0.098	0.213	4.35609483089702E-55	Mature metabolic hepatocytes	SECISBP2L
3.78164288715646E-59	0.431645481717226	0.113	0.235	6.33727715029679E-55	Mature metabolic hepatocytes	KCNT2
4.00334239799516E-59	0.328206866182887	0.114	0.235	6.7088011905603E-55	Mature metabolic hepatocytes	EXOC4
4.83799253097188E-59	0.538166219714007	0.68	0.731	8.10750788340268E-55	Mature metabolic hepatocytes	GATM
8.29440414654625E-59	0.374555542930472	0.102	0.219	1.38997624687822E-54	Mature metabolic hepatocytes	ZNF281
9.82794719063392E-59	0.256967258957874	0.126	0.254	1.64696739020643E-54	Mature metabolic hepatocytes	BTAF1
1.80026785879602E-58	0.272526413231158	0.074	0.175	3.01688887777036E-54	Mature metabolic hepatocytes	DENND5B
7.22580412224198E-58	0.258085627394838	0.181	0.336	1.21090025480531E-53	Mature metabolic hepatocytes	CPSF7
7.23822700175598E-58	0.298526942891274	0.19	0.35	1.21298208095427E-53	Mature metabolic hepatocytes	UGCG
9.86569172638071E-58	0.359953735529507	0.096	0.208	1.65329261950688E-53	Mature metabolic hepatocytes	BCL2L11
2.54067532072031E-57	0.31078217338266	0.181	0.335	4.25766370246309E-53	Mature metabolic hepatocytes	RBM25
3.5337355958555E-57	0.41529545028039	0.154	0.295	5.92183411153464E-53	Mature metabolic hepatocytes	ZSWIM8
7.2078708930531E-56	1.59101709519931	0.308	0.247	1.20789500425784E-51	Mature metabolic hepatocytes	AKIRIN2
1.97439944594156E-55	0.475298035570527	0.104	0.217	3.30869859150886E-51	Mature metabolic hepatocytes	CLK4
3.44741424679114E-55	0.502133284432505	0.125	0.25	5.77717679477259E-51	Mature metabolic hepatocytes	TNKS
1.79548184315857E-54	0.314412979318813	0.049	0.13	3.00886847276512E-50	Mature metabolic hepatocytes	RNF111
2.49961876566042E-54	0.28184541999684	0.105	0.216	4.18886112749373E-50	Mature metabolic hepatocytes	VPS26A
3.08092174331466E-54	2.16953637054949	0.173	0.105	5.1630086574467E-50	Mature metabolic hepatocytes	CFL2
3.64209383736985E-54	0.728762638159308	0.661	0.697	6.1034208526644E-50	Mature metabolic hepatocytes	UPB1
4.86838474341454E-54	0.342811877677727	0.14	0.269	8.15843915301409E-50	Mature metabolic hepatocytes	SKIL
5.05322724188141E-54	2.1706329106035	0.11	0.053	8.46819821194486E-50	Mature metabolic hepatocytes	SMLR1
7.33575710917373E-54	1.46645229074583	0.415	0.399	1.22932617635533E-49	Mature metabolic hepatocytes	HSPE1
1.03588417786954E-53	0.476167724367929	0.144	0.276	1.73593470527377E-49	Mature metabolic hepatocytes	SELENOO
1.09934194812211E-53	1.33565199600083	0.4	0.373	1.84227723666304E-49	Mature metabolic hepatocytes	ECI2
1.15588103433997E-53	0.282812308697585	0.054	0.139	1.93702543734693E-49	Mature metabolic hepatocytes	ZBTB5
3.23838562986785E-53	0.263978596576212	0.087	0.188	5.42688663853254E-49	Mature metabolic hepatocytes	MMADHC
7.27757022918911E-53	0.407802764512787	0.136	0.263	1.21957521900751E-48	Mature metabolic hepatocytes	FBXW7
1.15186495754237E-52	0.319373292875087	0.112	0.226	1.9302952958495E-48	Mature metabolic hepatocytes	MTMR10
1.67689118438998E-52	0.262766833236688	0.116	0.231	2.81013424680073E-48	Mature metabolic hepatocytes	TCF7L2
1.69360822883726E-52	0.314438385194618	0.066	0.156	2.83814866988548E-48	Mature metabolic hepatocytes	WDR43
3.47619543217574E-52	0.365617821051404	0.078	0.174	5.82540830524011E-48	Mature metabolic hepatocytes	USP3
4.10666771364447E-52	0.303312140946762	0.245	0.423	6.88195375452541E-48	Mature metabolic hepatocytes	DST
4.11890830492445E-52	0.387244271190527	0.091	0.194	6.90246653739239E-48	Mature metabolic hepatocytes	PPID
5.3368731285906E-52	0.270961916544874	0.129	0.249	8.94353198889212E-48	Mature metabolic hepatocytes	GSE1
1.13014472896962E-51	0.4665669669966	0.178	0.324	1.89389653680728E-47	Mature metabolic hepatocytes	TKFC
1.2532704502473E-51	0.428250567180948	0.09	0.192	2.10023062052442E-47	Mature metabolic hepatocytes	CPEB2
1.41515572040275E-51	0.429114699123849	0.121	0.239	2.37151795625094E-47	Mature metabolic hepatocytes	GTF3C3
1.68151647740101E-51	0.288161230242239	0.096	0.2	2.81788531282861E-47	Mature metabolic hepatocytes	CEP63
1.90779604071454E-51	0.391935840260882	0.147	0.278	3.19708460502943E-47	Mature metabolic hepatocytes	SRSF7
2.56455345271863E-51	0.306699218898363	0.059	0.144	4.29767867606587E-47	Mature metabolic hepatocytes	MOCS1
2.71880751635331E-51	0.267330464652706	0.136	0.259	4.55617763590488E-47	Mature metabolic hepatocytes	ZNF24
4.33746451318761E-51	0.526639464235064	0.08	0.177	7.26872303119979E-47	Mature metabolic hepatocytes	TBRG1
1.02878001576841E-50	0.434537586322075	0.116	0.231	1.7240295504247E-46	Mature metabolic hepatocytes	PAN2
1.11010731197916E-50	0.305734637167908	0.066	0.153	1.86031783341468E-46	Mature metabolic hepatocytes	PPP2R2A
1.12996553460932E-50	0.253210044910861	0.091	0.191	1.8935962428983E-46	Mature metabolic hepatocytes	BZW2
3.03136328129979E-50	0.449000102508343	0.111	0.22	5.07995858680219E-46	Mature metabolic hepatocytes	ARAP1

3.34581824260856E-50	0.330366053649588	0.168	0.305	5.60692221096342E-46	Mature metabolic hepatocytes	SC5D
5.34563361977085E-50	0.374696000498943	0.064	0.15	8.95821282001198E-46	Mature metabolic hepatocytes	KCTD9
7.43248502957536E-50	0.386941063579665	0.094	0.195	1.24553584125624E-45	Mature metabolic hepatocytes	GTF2H4
8.16631022568868E-50	0.544298671929894	0.108	0.219	1.36851026762091E-45	Mature metabolic hepatocytes	CCNT2
9.02333887289201E-50	0.289634818349039	0.067	0.154	1.51213112831924E-45	Mature metabolic hepatocytes	USP36
1.6533465371038E-49	0.268235088529899	0.071	0.159	2.77067812687855E-45	Mature metabolic hepatocytes	PDSS2
2.04876772620101E-49	0.350442365162078	0.089	0.188	3.43332495556766E-45	Mature metabolic hepatocytes	NMRK1
3.00757501989885E-49	0.31880465346134	0.126	0.241	5.0400942183465E-45	Mature metabolic hepatocytes	MEF2D
3.61101517560491E-49	1.9387546539027	0.164	0.098	6.05133923127871E-45	Mature metabolic hepatocytes	GDF15
4.52214294891501E-49	0.409509575142942	0.097	0.199	7.57820715379177E-45	Mature metabolic hepatocytes	AK3
1.02950155938935E-48	0.887862488794428	0.378	0.317	1.72523871322468E-44	Mature metabolic hepatocytes	DEPP1
1.06911515181218E-48	2.48968494619693	0.124	0.067	1.79162317140685E-44	Mature metabolic hepatocytes	PLIN2
1.63312483287973E-48	0.373731271286161	0.041	0.113	2.73679059493986E-44	Mature metabolic hepatocytes	DEPRECATEI
1.83726785817037E-48	1.56913966330974	0.319	0.27	3.07889347672191E-44	Mature metabolic hepatocytes	HLF
3.07149459722807E-48	0.460679988815772	0.134	0.254	5.1472106460348E-44	Mature metabolic hepatocytes	LCOR
4.54133334595739E-48	0.27042114252968	0.186	0.329	7.6103664211554E-44	Mature metabolic hepatocytes	STAG1
7.49750865838706E-48	0.399573183446295	0.08	0.172	1.2564325009725E-43	Mature metabolic hepatocytes	PIAS2
9.39246760168848E-48	0.340127331193609	0.156	0.282	1.57398972069096E-43	Mature metabolic hepatocytes	C1RL
1.11222011523154E-47	0.698554295848028	0.587	0.634	1.86385846910501E-43	Mature metabolic hepatocytes	SDC4
1.66231909560255E-47	0.401777274467813	0.162	0.294	2.78571434041076E-43	Mature metabolic hepatocytes	NFX1
2.02260545498304E-47	0.364115788138249	0.171	0.308	3.38948222146058E-43	Mature metabolic hepatocytes	PPIG
2.06660220407666E-47	2.3721437941709	0.104	0.051	3.46321197359166E-43	Mature metabolic hepatocytes	SLC22A10
2.10544950113873E-47	0.581697972681234	0.117	0.228	3.52831227400828E-43	Mature metabolic hepatocytes	METTL14
2.27196693096988E-47	0.335842170440712	0.105	0.209	3.80736218291932E-43	Mature metabolic hepatocytes	NISCH
3.04129695956759E-47	0.315749105829281	0.066	0.149	5.09660544484336E-43	Mature metabolic hepatocytes	CACFD1
4.13792853808611E-47	0.32873700137794	0.082	0.174	6.9343406441247E-43	Mature metabolic hepatocytes	LRRC28
6.52066368527528E-47	0.528783306137619	0.6	0.549	1.09273282037843E-42	Mature metabolic hepatocytes	CCNL2
7.69723088958468E-47	0.427935421596686	0.093	0.191	1.2899019524766E-42	Mature metabolic hepatocytes	DPY19L1
9.68282885186002E-47	0.274613879580295	0.081	0.172	1.6226484589947E-42	Mature metabolic hepatocytes	RPAIN
1.047070512264E-46	0.294322249098501	0.063	0.144	1.75468076445201E-42	Mature metabolic hepatocytes	ABI2
1.10554799668377E-46	0.29921152986216	0.228	0.389	1.85267733284266E-42	Mature metabolic hepatocytes	COG3
1.65899193072839E-46	0.445640294356286	0.174	0.311	2.78013867751464E-42	Mature metabolic hepatocytes	DOCK5
1.68984811926716E-46	0.450672929856751	0.142	0.263	2.83184747826791E-42	Mature metabolic hepatocytes	CYP2C19
1.88569055482659E-46	0.259703488839031	0.062	0.143	3.16004023177841E-42	Mature metabolic hepatocytes	NECAP1
2.14891077678561E-46	0.736626930485787	0.525	0.53	3.60114467973732E-42	Mature metabolic hepatocytes	GAMT
2.27483890843811E-46	0.281590450711763	0.048	0.12	3.81217504276058E-42	Mature metabolic hepatocytes	FOXJ3
2.58465367112507E-46	0.299157652577526	0.103	0.203	4.3313626220714E-42	Mature metabolic hepatocytes	SUGT1
4.01264041038512E-46	0.347130439328394	0.099	0.199	6.72438279972338E-42	Mature metabolic hepatocytes	PSME4
5.51988951970225E-46	0.258740137793364	0.071	0.156	9.25023085711702E-42	Mature metabolic hepatocytes	EVI5
7.0560319070486E-46	0.30477039257007	0.066	0.149	1.1824498269832E-41	Mature metabolic hepatocytes	PPP1R21
8.40507376096595E-46	0.508201661786651	0.102	0.203	1.40852226086267E-41	Mature metabolic hepatocytes	GFM2
9.56964181651392E-46	1.50060509324178	0.161	0.097	1.6036805756114E-41	Mature metabolic hepatocytes	RTN4RL2
1.01993774111622E-45	0.379343880007149	0.084	0.176	1.70921166656255E-41	Mature metabolic hepatocytes	COMMD10
1.17977120116937E-45	0.414008619009615	0.091	0.185	1.97706057891963E-41	Mature metabolic hepatocytes	RMND1
1.3267074938147E-45	0.437249261898053	0.149	0.271	2.22329641813468E-41	Mature metabolic hepatocytes	HEXA
1.6974890008137E-45	0.253617880081648	0.049	0.121	2.8446520675636E-41	Mature metabolic hepatocytes	RIT1
2.08551735515683E-45	0.288632925958129	0.213	0.364	3.49490998377182E-41	Mature metabolic hepatocytes	GPBP1L1
2.46744343443521E-45	0.270744507724111	0.037	0.104	4.13494170742652E-41	Mature metabolic hepatocytes	SCAPER
3.81546144537299E-45	0.35750531597439	0.057	0.134	6.39395029015605E-41	Mature metabolic hepatocytes	RUFY2
3.97821143604633E-45	0.363927016489565	0.223	0.383	6.66668672452644E-41	Mature metabolic hepatocytes	MKLN1
4.61033456051763E-45	0.386224595289497	0.137	0.252	7.72599865651544E-41	Mature metabolic hepatocytes	CRELD1
8.58145279575453E-45	0.453197223069545	0.092	0.187	1.43807985951254E-40	Mature metabolic hepatocytes	LRRFIP1
1.23044665719164E-44	0.333513458045404	0.272	0.451	2.06198250812174E-40	Mature metabolic hepatocytes	ELOVL5
1.2896074955604E-44	0.470122899131579	0.134	0.249	2.16112424106012E-40	Mature metabolic hepatocytes	IBTK
1.341861593225E-44	0.398674364018063	0.149	0.272	2.24869165792646E-40	Mature metabolic hepatocytes	PTS
1.83593521816978E-44	0.307391633277889	0.159	0.281	3.07666023860892E-40	Mature metabolic hepatocytes	SLC29A1
2.17153639664084E-44	1.631062488912	0.273	0.218	3.63906069349072E-40	Mature metabolic hepatocytes	DEPRECATEI
2.7039888074638E-44	0.311468151200728	0.145	0.262	4.53134444354783E-40	Mature metabolic hepatocytes	GHR
2.86268844067907E-44	1.44968484202788	0.273	0.216	4.79729328888999E-40	Mature metabolic hepatocytes	ACY1
2.93441831213797E-44	0.255135357361011	0.044	0.113	4.9174982074808E-40	Mature metabolic hepatocytes	DTNB

3.22426246522377E-44	0.351592337104742	0.152	0.274	5.403219039222E-40	Mature metabolic hepatocytes	ERGIC1
4.00762829715188E-44	0.441319677017699	0.103	0.203	6.71598350036713E-40	Mature metabolic hepatocytes	SH3D19
5.89791972207135E-44	0.392455267872204	0.087	0.178	9.88373387024716E-40	Mature metabolic hepatocytes	ABCC6
6.8606428658942E-44	0.399959635637799	0.174	0.308	1.14970653146655E-39	Mature metabolic hepatocytes	IWS1
7.77393281447691E-44	0.315650400855594	0.071	0.153	1.30275566105004E-39	Mature metabolic hepatocytes	GNAI1
9.08903595317504E-44	0.252263942313904	0.285	0.467	1.52314064503307E-39	Mature metabolic hepatocytes	SRSF4
9.4691466086532E-44	0.281642409282122	0.204	0.351	1.5868395886781E-39	Mature metabolic hepatocytes	SEC11A
1.04679934008246E-43	0.412703605468437	0.061	0.138	1.75422633411019E-39	Mature metabolic hepatocytes	SFXN2
1.30582908552328E-43	0.522879051184948	0.073	0.156	2.18830838151991E-39	Mature metabolic hepatocytes	ZNF280D
1.39176727136174E-43	0.461330664556399	0.049	0.12	2.33232359334801E-39	Mature metabolic hepatocytes	CLEC2D
1.49792236084507E-43	1.28542646355111	0.393	0.374	2.51021829230417E-39	Mature metabolic hepatocytes	GADD45B
1.61393515469477E-43	0.328488780585118	0.18	0.314	2.70463253223749E-39	Mature metabolic hepatocytes	RHEB
1.73717447988912E-43	0.392559875616763	0.219	0.374	2.91115699339819E-39	Mature metabolic hepatocytes	TIA1
2.36317564915052E-43	0.416868172759886	0.078	0.164	3.96020975284644E-39	Mature metabolic hepatocytes	DOCK7
3.35735272983507E-43	0.4322775377121	0.146	0.264	5.62625170465761E-39	Mature metabolic hepatocytes	DUSP16
3.54907634265412E-43	0.478505584036707	0.047	0.116	5.94754213501978E-39	Mature metabolic hepatocytes	ZNF518A
3.9975028805623E-43	2.01078080405352	0.211	0.153	6.6990153272463E-39	Mature metabolic hepatocytes	RYBP
5.14407321550909E-43	0.252384330292065	0.158	0.28	8.62043789455013E-39	Mature metabolic hepatocytes	LIPA
5.36926428524308E-43	0.28948927593545	0.089	0.178	8.99781308921035E-39	Mature metabolic hepatocytes	CTR9
7.68128786354072E-43	1.96620856211296	0.25	0.198	1.28723022017215E-38	Mature metabolic hepatocytes	ACAD11
8.052567424479E-43	0.414077275047444	0.114	0.216	1.34944924899419E-38	Mature metabolic hepatocytes	UCKL1
1.00920134791565E-42	0.28501944752472	0.063	0.139	1.69121961883705E-38	Mature metabolic hepatocytes	CYB5R4
1.14550050198331E-42	0.366499826913761	0.077	0.16	1.91962974122363E-38	Mature metabolic hepatocytes	ZRANB1
1.60035699522805E-42	0.556152180090166	0.111	0.212	2.68187825260317E-38	Mature metabolic hepatocytes	ATXN2
2.08656029617051E-42	0.250903524429066	0.057	0.13	3.49665774432254E-38	Mature metabolic hepatocytes	CRTC3
2.13173707544324E-42	0.545513982093589	0.068	0.147	3.57236499102779E-38	Mature metabolic hepatocytes	RINT1
2.29296567634471E-42	1.49997668289945	0.203	0.141	3.84255188041847E-38	Mature metabolic hepatocytes	GPD1
2.85932804369388E-42	0.35628379460572	0.197	0.338	4.79166193562221E-38	Mature metabolic hepatocytes	PMPCB
2.97357185016856E-42	0.418115287020937	0.14	0.254	4.98311170651246E-38	Mature metabolic hepatocytes	SELENOI
3.92312996075442E-42	0.322120320831745	0.132	0.24	6.57438118823225E-38	Mature metabolic hepatocytes	MCCC1
4.81509934717931E-42	0.387876803482014	0.044	0.111	8.06914348600309E-38	Mature metabolic hepatocytes	PTBP2
1.14029548682108E-41	0.410730545247144	0.074	0.155	1.91090717681477E-37	Mature metabolic hepatocytes	PRLR
1.19404608449099E-41	0.304901975690932	0.248	0.412	2.00098242839E-37	Mature metabolic hepatocytes	TOM1L1
1.26546707947993E-41	0.394515051937235	0.193	0.332	2.12066973179247E-37	Mature metabolic hepatocytes	EPB41L5
1.84003115102433E-41	0.34310361498261	0.057	0.129	3.08352420288658E-37	Mature metabolic hepatocytes	LRSAM1
1.91275621914221E-41	0.399397476861	0.111	0.209	3.20539687203852E-37	Mature metabolic hepatocytes	C11orf24
3.33827645727523E-41	1.71265611977574	0.13	0.075	5.59428368710183E-37	Mature metabolic hepatocytes	DOCK4
3.86358119498523E-41	0.480033965484816	0.156	0.277	6.47458936655625E-37	Mature metabolic hepatocytes	FXR1
4.57031265521451E-41	0.404135559265088	0.197	0.339	7.65892994760847E-37	Mature metabolic hepatocytes	RALGPS2
7.64996329230392E-41	1.74380160422294	0.132	0.077	1.28198084852429E-36	Mature metabolic hepatocytes	GPT2
7.79179314697726E-41	0.504158212295216	0.07	0.148	1.30574869557045E-36	Mature metabolic hepatocytes	DEPRECATEI
7.97797657440775E-41	0.527360547899494	0.085	0.172	1.33694931433925E-36	Mature metabolic hepatocytes	SF3B1
1.23106221540001E-40	0.404688340808233	0.125	0.23	2.06301406056734E-36	Mature metabolic hepatocytes	CREM
1.27800624658798E-40	0.413141838948998	0.058	0.13	2.14168286803213E-36	Mature metabolic hepatocytes	CNOT4
1.29662855919631E-40	1.47015016575756	0.364	0.338	2.17289013950117E-36	Mature metabolic hepatocytes	LDLR
1.32000811028621E-40	1.64279198825436	0.136	0.08	2.21206959121763E-36	Mature metabolic hepatocytes	WWC1
1.40453253369012E-40	0.290846186435152	0.051	0.119	2.3537156199579E-36	Mature metabolic hepatocytes	GPR155
1.73252114105008E-40	0.301319685224792	0.131	0.235	2.90335892817172E-36	Mature metabolic hepatocytes	NTMT1
2.16291667084503E-40	1.82813929951429	0.128	0.074	3.62461575700209E-36	Mature metabolic hepatocytes	TMOD1
4.58984496499666E-40	1.17515491077098	0.283	0.226	7.69166219234141E-36	Mature metabolic hepatocytes	PLIN4
5.02893718242439E-40	1.36524332727246	0.216	0.155	8.42749293030678E-36	Mature metabolic hepatocytes	SPON2
5.34297719178893E-40	0.495857825729068	0.074	0.154	8.95376117799989E-36	Mature metabolic hepatocytes	MRRF
9.57176808381031E-40	1.81219070053568	0.197	0.14	1.60403689548493E-35	Mature metabolic hepatocytes	DNAJB4
1.09954333243048E-39	0.551049574770375	0.167	0.291	1.842614716487E-35	Mature metabolic hepatocytes	DEPRECATEI
1.56288722292822E-39	0.255940453181491	0.076	0.156	2.61908640818312E-35	Mature metabolic hepatocytes	C18orf25
1.82770697039712E-39	0.42499261460107	0.161	0.28	3.0628713409915E-35	Mature metabolic hepatocytes	MTG1
2.50503036303406E-39	0.563950489987502	0.097	0.187	4.19792988237247E-35	Mature metabolic hepatocytes	SEPSECS
3.08407771977866E-39	0.495522144250257	0.168	0.293	5.16829744280508E-35	Mature metabolic hepatocytes	CUL1
4.25353960633167E-39	0.432438814463112	0.214	0.358	7.12808167229061E-35	Mature metabolic hepatocytes	N4BP2L1
4.67626407743972E-39	0.427118831873636	0.087	0.171	7.83648334097348E-35	Mature metabolic hepatocytes	SMNDC1

5.5474100420234E-39	0.288379024549137	0.045	0.109	9.29634974842281E-35	Mature metabolic hepatocytes	SH3BP2
6.74106335826625E-39	0.410676137621446	0.053	0.12	1.12966739757826E-34	Mature metabolic hepatocytes	SLC35A1
6.89803980708847E-39	0.410817233674468	0.14	0.248	1.15597351087189E-34	Mature metabolic hepatocytes	HYI
7.1452086981431E-39	0.298631907215197	0.051	0.117	1.19739407363482E-34	Mature metabolic hepatocytes	FRA10AC1
7.6136571071726E-39	0.476725288779694	0.124	0.226	1.27589665801998E-34	Mature metabolic hepatocytes	ENTR1
9.64948633051079E-39	0.350679810830342	0.078	0.158	1.617060919267E-34	Mature metabolic hepatocytes	ZFYVE27
1.16818698886814E-38	1.07799722233447	0.345	0.302	1.95764775594524E-34	Mature metabolic hepatocytes	PLIN5
1.22266925173899E-38	0.295736685670116	0.117	0.214	2.0489491320642E-34	Mature metabolic hepatocytes	PLA2G12B
1.25589546872112E-38	0.396351974072643	0.092	0.177	2.10462962648286E-34	Mature metabolic hepatocytes	MXD1
1.86837021860851E-38	0.300877079369125	0.056	0.124	3.13101481234413E-34	Mature metabolic hepatocytes	SCN8A
1.96297144518153E-38	0.454179702082471	0.096	0.184	3.28954754783521E-34	Mature metabolic hepatocytes	ARIH1
2.15314517830047E-38	1.62770822473907	0.271	0.225	3.60824068979593E-34	Mature metabolic hepatocytes	AHR
2.88564331427618E-38	0.280065507310586	0.068	0.143	4.83576106606403E-34	Mature metabolic hepatocytes	FBXO22
3.92692513762716E-38	0.367895481698614	0.086	0.169	6.5807411456356E-34	Mature metabolic hepatocytes	EPS15
5.0293929998506E-38	0.341099682215991	0.071	0.146	8.42825678914963E-34	Mature metabolic hepatocytes	DEPRECATEI
6.80604868411921E-38	0.426676101286493	0.066	0.139	1.1405576384847E-33	Mature metabolic hepatocytes	CRY1
6.86018138102051E-38	0.343774564704853	0.057	0.126	1.14962919583142E-33	Mature metabolic hepatocytes	FAM160B2
7.23028332496277E-38	0.304240825874605	0.089	0.173	1.21165087959726E-33	Mature metabolic hepatocytes	ACAD8
7.29916250667895E-38	0.628047211601341	0.176	0.303	1.22319365286926E-33	Mature metabolic hepatocytes	DEPRECATEI
7.38662072734395E-38	0.411498102327093	0.126	0.226	1.2378499014883E-33	Mature metabolic hepatocytes	PLPP5
1.00454962482967E-37	0.4387228440836	0.132	0.236	1.68342426128957E-33	Mature metabolic hepatocytes	FNBP4
1.17830079407305E-37	1.85597767654376	0.128	0.076	1.97459647070762E-33	Mature metabolic hepatocytes	NAB2
1.21817300641633E-37	0.551303668896021	0.155	0.271	2.04141432415249E-33	Mature metabolic hepatocytes	NOP58
1.28822086403375E-37	0.413719442282436	0.131	0.232	2.15880052394775E-33	Mature metabolic hepatocytes	IL1RAP
1.32896061440392E-37	0.640425093356053	0.165	0.285	2.22707219761809E-33	Mature metabolic hepatocytes	MAN2A2
1.49020940341159E-37	0.456482049105003	0.079	0.159	2.49729291823714E-33	Mature metabolic hepatocytes	TENT4B
1.8642586102673E-37	0.563787719971242	0.044	0.106	3.12412457908594E-33	Mature metabolic hepatocytes	ENTPD4
1.939240536979E-37	0.285159085024378	0.321	0.498	3.24977929186941E-33	Mature metabolic hepatocytes	ITPR2
2.21860682581974E-37	0.353122695029105	0.203	0.339	3.71794131870872E-33	Mature metabolic hepatocytes	DEPRECATEI
3.64579281737429E-37	0.339192774639928	0.077	0.154	6.10961960335583E-33	Mature metabolic hepatocytes	FOXO1
3.82649348477016E-37	0.272646741015456	0.11	0.201	6.41243778177784E-33	Mature metabolic hepatocytes	NR1H3
3.83546861845885E-37	0.321217273679198	0.247	0.401	6.42747831081334E-33	Mature metabolic hepatocytes	SORBS2
7.08686853932609E-37	0.675321016604849	0.123	0.223	1.18761742982027E-32	Mature metabolic hepatocytes	STARD4
8.45462887071683E-37	0.4638269628271	0.173	0.294	1.41682670615473E-32	Mature metabolic hepatocytes	NSUN2
8.88646550969496E-37	1.56096245131753	0.312	0.281	1.48919389011468E-32	Mature metabolic hepatocytes	ENPP1
1.17125148245138E-36	0.490226655381882	0.098	0.185	1.96278323429202E-32	Mature metabolic hepatocytes	ZBTB11
1.4826368940474E-36	0.543182035686781	0.119	0.216	2.48460290704463E-32	Mature metabolic hepatocytes	HERPUD2
1.50218813645701E-36	0.576041572004927	0.123	0.221	2.51736687907466E-32	Mature metabolic hepatocytes	MTO1
2.30217721463528E-36	0.765612842051404	0.519	0.551	3.85798857628581E-32	Mature metabolic hepatocytes	CAT
2.6719986727033E-36	0.460765509117883	0.066	0.136	4.4777353757162E-32	Mature metabolic hepatocytes	NOL4L
2.93897115215578E-36	0.48591099400319	0.124	0.222	4.92512785678266E-32	Mature metabolic hepatocytes	CLSTN3
3.27159011438001E-36	0.47317994098926	0.069	0.141	5.48253071367803E-32	Mature metabolic hepatocytes	TNIK
3.75980521818965E-36	0.438447509718652	0.074	0.15	6.30068158464222E-32	Mature metabolic hepatocytes	L3MBTL4
4.64586219987955E-36	0.602240198234671	0.125	0.224	7.78553587455815E-32	Mature metabolic hepatocytes	NSUN6
4.92219909887359E-36	1.45280181540169	0.223	0.169	8.24862124989236E-32	Mature metabolic hepatocytes	DDIT3
5.92216164497562E-36	0.48385559760256	0.074	0.148	9.92435848465014E-32	Mature metabolic hepatocytes	NUP88
6.93255200188897E-36	0.385579896629539	0.099	0.184	1.16175706447655E-31	Mature metabolic hepatocytes	FKBP4
7.47163335521971E-36	0.376746952816381	0.047	0.108	1.25209631766772E-31	Mature metabolic hepatocytes	TMEM242
9.9032576807084E-36	0.304559445230927	0.073	0.146	1.65958792213311E-31	Mature metabolic hepatocytes	COQ10B
1.04391217323902E-35	0.520780732899927	0.07	0.142	1.74938801991395E-31	Mature metabolic hepatocytes	ZFAND6
1.21614514434924E-35	0.62408381709906	0.14	0.246	2.03801603290045E-31	Mature metabolic hepatocytes	CBWD1
1.35012953409655E-35	0.257649612180149	0.233	0.371	2.262547073239E-31	Mature metabolic hepatocytes	FTCD
1.39130722022157E-35	0.430279968744991	0.203	0.336	2.33155263964731E-31	Mature metabolic hepatocytes	B4GALT1
1.59913977722265E-35	0.355193948512856	0.165	0.279	2.67983843866972E-31	Mature metabolic hepatocytes	CLDN12
1.77157424965623E-35	0.273239477818509	0.062	0.13	2.96880412757391E-31	Mature metabolic hepatocytes	ZNHIT6
1.99074166378554E-35	0.297588575220284	0.231	0.373	3.33608488017181E-31	Mature metabolic hepatocytes	MARC1
3.26462472128059E-35	0.370818190599489	0.079	0.155	5.47085810792201E-31	Mature metabolic hepatocytes	NGEF
6.52721267268647E-35	1.25422672087987	0.109	0.06	1.0938302996888E-30	Mature metabolic hepatocytes	PALM2-AKAI
6.66640326235376E-35	0.435977649371728	0.073	0.145	1.11715585870524E-30	Mature metabolic hepatocytes	CAPN7
7.07955296467601E-35	0.510850515702524	0.123	0.218	1.18639148582041E-30	Mature metabolic hepatocytes	CRCP

7.67919466923888E-35	0.318654290635159	0.06	0.126	1.28687944267105E-30	Mature metabolic hepatocytes	TYW3
7.69101316466648E-35	0.464587572946508	0.09	0.17	1.28885998613481E-30	Mature metabolic hepatocytes	U2AF1L4
7.99100502290907E-35	0.453208981011256	0.125	0.22	1.3391326217391E-30	Mature metabolic hepatocytes	TRIM38
8.68582196557395E-35	2.07317123803484	0.159	0.109	1.45557004499088E-30	Mature metabolic hepatocytes	HSD17B7
1.20423323006297E-34	0.369879586723121	0.092	0.172	2.01805404693953E-30	Mature metabolic hepatocytes	PDK1
1.27429392716515E-34	0.618189342174862	0.087	0.166	2.13546176314335E-30	Mature metabolic hepatocytes	INIP
1.3162384523035E-34	0.33803068351784	0.101	0.186	2.20575239837021E-30	Mature metabolic hepatocytes	RDX
1.32699700071345E-34	0.287344249253116	0.05	0.111	2.2237815737956E-30	Mature metabolic hepatocytes	DEPRECATEI
1.47063306603909E-34	0.291843296843291	0.072	0.143	2.46448689206831E-30	Mature metabolic hepatocytes	DIS3L2
1.59584045518273E-34	0.373717804135895	0.109	0.197	2.67430943479522E-30	Mature metabolic hepatocytes	ESF1
4.47923767509333E-34	0.32490461300509	0.064	0.13	7.50630649592141E-30	Mature metabolic hepatocytes	PPP3CB
5.23240836397695E-34	0.47196677964846	0.073	0.144	8.76846993635257E-30	Mature metabolic hepatocytes	CYP7A1
7.93364968033581E-34	0.475913346268821	0.066	0.134	1.32952101343068E-29	Mature metabolic hepatocytes	PNPLA8
8.97049152114567E-34	0.353936672516076	0.245	0.392	1.50327496911359E-29	Mature metabolic hepatocytes	TJP1
9.45330620810002E-34	0.555134753076578	0.126	0.223	1.5841850543534E-29	Mature metabolic hepatocytes	CHORDC1
1.00559998135404E-33	0.62005481031236	0.053	0.115	1.6851844487531E-29	Mature metabolic hepatocytes	FMO4
1.01278674929709E-33	0.642676424448422	0.054	0.116	1.69722803447207E-29	Mature metabolic hepatocytes	DGLUCY
1.21030898289227E-33	0.583174804028261	0.087	0.165	2.02823579353086E-29	Mature metabolic hepatocytes	TMEM135
1.30484633766666E-33	0.25525121617031	0.051	0.111	2.18666149266178E-29	Mature metabolic hepatocytes	KDM6B
1.36779654329453E-33	0.308672432227243	0.073	0.143	2.29215344725297E-29	Mature metabolic hepatocytes	CCDC107
1.49723952407436E-33	0.422673729249529	0.17	0.283	2.50907399444382E-29	Mature metabolic hepatocytes	PNPLA3
1.5696973549035E-33	0.269416716940931	0.056	0.118	2.63049882734729E-29	Mature metabolic hepatocytes	ZNF3
2.34251038465942E-33	0.382210358696623	0.058	0.121	3.92557890261226E-29	Mature metabolic hepatocytes	PDP2
2.36049754401582E-33	0.594416603852777	0.145	0.249	3.95572178426171E-29	Mature metabolic hepatocytes	ELP2
2.74503157890231E-33	0.408366160073991	0.16	0.267	4.60012391992449E-29	Mature metabolic hepatocytes	TNFAIP3
3.01826539059974E-33	0.467272241137208	0.118	0.209	5.05800914156704E-29	Mature metabolic hepatocytes	ZNF330
3.92105636011598E-33	0.446471456328973	0.104	0.188	6.57090624828237E-29	Mature metabolic hepatocytes	PCGF5
4.67355864529568E-33	0.255282700752408	0.099	0.179	7.8319495777865E-29	Mature metabolic hepatocytes	GLMP
4.83107376936077E-33	0.287945198027169	0.161	0.268	8.09591342269477E-29	Mature metabolic hepatocytes	NINJ1
4.97724738920945E-33	0.499045856692663	0.077	0.149	8.3408711748372E-29	Mature metabolic hepatocytes	HMG20A
5.29050386357105E-33	0.319384148756169	0.196	0.32	8.86582637457237E-29	Mature metabolic hepatocytes	TMEM120A
5.64524125040477E-33	0.390687784571945	0.055	0.117	9.46029528742831E-29	Mature metabolic hepatocytes	TSC1
6.90403250601363E-33	0.636601546402488	0.129	0.225	1.15697776735776E-28	Mature metabolic hepatocytes	PEX11A
8.09582441645157E-33	0.57174905199221	0.137	0.236	1.35669825570895E-28	Mature metabolic hepatocytes	XYLB
8.86537952748842E-33	0.518985566851866	0.129	0.225	1.48566030121651E-28	Mature metabolic hepatocytes	VPS36
8.97719182654037E-33	0.25674338160157	0.063	0.128	1.50439780629164E-28	Mature metabolic hepatocytes	TEN1
9.61211882291765E-33	0.552200242944207	0.143	0.243	1.61079887234454E-28	Mature metabolic hepatocytes	ATP2B4
1.16432226752861E-32	0.415517610124958	0.092	0.17	1.95117125592444E-28	Mature metabolic hepatocytes	TTC31
1.91152441251069E-32	0.612258630883861	0.079	0.151	3.20333261048542E-28	Mature metabolic hepatocytes	NBPF12
2.18618094638686E-32	0.387137284413188	0.053	0.113	3.6636020299551E-28	Mature metabolic hepatocytes	SFMBT1
2.22722286689599E-32	0.282989455740201	0.141	0.238	3.73238008034429E-28	Mature metabolic hepatocytes	HOMER2
3.04473057468685E-32	0.570033945266954	0.079	0.152	5.10235949706022E-28	Mature metabolic hepatocytes	LMBR1L
3.14542232390308E-32	0.542778831894064	0.156	0.262	5.27109873039678E-28	Mature metabolic hepatocytes	HDAC6
3.20124848874964E-32	0.460516188707382	0.13	0.224	5.36465221744664E-28	Mature metabolic hepatocytes	MARCH5
5.12750242518806E-32	1.81971771941148	0.185	0.136	8.59266856413015E-28	Mature metabolic hepatocytes	RCL1
5.13579290853989E-32	0.485129155634328	0.096	0.175	8.60656175613115E-28	Mature metabolic hepatocytes	MAP3K20
8.15026473341577E-32	0.687477060117949	0.166	0.278	1.36582136402581E-27	Mature metabolic hepatocytes	RUFY3
1.12826243277384E-31	0.418443977756338	0.148	0.249	1.89074218484239E-27	Mature metabolic hepatocytes	ALDH7A1
1.47436282999173E-31	0.53039076058127	0.095	0.173	2.47073723050015E-27	Mature metabolic hepatocytes	NR1D2
1.5768933285239E-31	0.353136051727823	0.249	0.394	2.64255783994036E-27	Mature metabolic hepatocytes	SGK2
2.31635978526213E-31	0.716681188838313	0.147	0.251	3.88175572814227E-27	Mature metabolic hepatocytes	HERC4
2.99557833849695E-31	0.314497615984245	0.058	0.119	5.01999017965319E-27	Mature metabolic hepatocytes	ACY3
3.00624146536815E-31	0.392768878629945	0.194	0.315	5.03785944766394E-27	Mature metabolic hepatocytes	BPHL
3.26583873121386E-31	0.296221940687987	0.071	0.137	5.47289254576819E-27	Mature metabolic hepatocytes	GPN3
3.4476273814628E-31	0.510010378001572	0.111	0.196	5.77753396585537E-27	Mature metabolic hepatocytes	GNPNAT1
3.66720962925637E-31	0.40774896911983	0.047	0.103	6.14550989670782E-27	Mature metabolic hepatocytes	RPUSD4
4.22420363086103E-31	0.454580847117168	0.097	0.175	7.07892044459692E-27	Mature metabolic hepatocytes	CDS2
4.24190523321489E-31	0.630909278984894	0.114	0.199	7.10858478982151E-27	Mature metabolic hepatocytes	CHKB
4.54325595423771E-31	0.420636134363214	0.173	0.115	7.61358832811156E-27	Mature metabolic hepatocytes	SAA1
6.72123507792255E-31	1.40551406964259	0.302	0.271	1.12634457435826E-26	Mature metabolic hepatocytes	IFRD1

6.84400529720938E-31	0.369943226754037	0.115	0.199	1.14691840770635E-26	Mature metabolic hepatocytes	LRMDA
6.8900048221429E-31	0.319577305473435	0.086	0.159	1.15462700809471E-26	Mature metabolic hepatocytes	MXI1
8.45552501339827E-31	0.26467217692421	0.266	0.416	1.41697688174528E-26	Mature metabolic hepatocytes	RMND5A
1.11403348952349E-30	0.462014397904301	0.192	0.314	1.86689732174347E-26	Mature metabolic hepatocytes	PITPNB
1.16002474309236E-30	0.605079439087396	0.153	0.256	1.94396946447418E-26	Mature metabolic hepatocytes	LRCH4
1.4787617354712E-30	1.62662925279454	0.212	0.165	2.47810891630264E-26	Mature metabolic hepatocytes	PPFIBP2
1.64191365100287E-30	0.373942833561794	0.246	0.391	2.75151889635062E-26	Mature metabolic hepatocytes	FAM3C
1.6955568822022E-30	0.43068703123615	0.07	0.135	2.84141422319444E-26	Mature metabolic hepatocytes	SYS1
2.08307242525932E-30	0.457375639697897	0.118	0.204	3.49081277024956E-26	Mature metabolic hepatocytes	RAB5A
2.44956921047835E-30	0.477043626916125	0.15	0.25	4.10498808291963E-26	Mature metabolic hepatocytes	AUP1
2.68340451367406E-30	0.345146318889736	0.054	0.112	4.496849284015E-26	Mature metabolic hepatocytes	DEPRECATEI
2.77608232627227E-30	1.63580002535655	0.158	0.109	4.65215876236706E-26	Mature metabolic hepatocytes	DHODH
3.06335877531517E-30	1.14704612460148	0.385	0.387	5.13357663567317E-26	Mature metabolic hepatocytes	ALDH6A1
4.97352622671948E-30	0.31553875650906	0.062	0.124	8.33463525073651E-26	Mature metabolic hepatocytes	SLC19A1
5.59348359922787E-30	0.426361610993601	0.095	0.17	9.37355981558607E-26	Mature metabolic hepatocytes	ACOT2
7.8294393716177E-30	0.335062709512073	0.134	0.225	1.31205744989569E-25	Mature metabolic hepatocytes	NUDT7
8.45309394841914E-30	0.71636473448466	0.056	0.115	1.41656948387608E-25	Mature metabolic hepatocytes	SLC44A5
8.48496650244476E-30	0.2935955341658	0.049	0.104	1.42191068647969E-25	Mature metabolic hepatocytes	AQP11
8.80947215173216E-30	2.1705989818258	0.113	0.07	1.47629134318728E-25	Mature metabolic hepatocytes	DUSP6
1.20460905530128E-29	0.646695257551154	0.102	0.181	2.01868385487388E-25	Mature metabolic hepatocytes	USP8
1.42088788731646E-29	0.614399217018403	0.064	0.126	2.38112392156492E-25	Mature metabolic hepatocytes	PLA2G4B
1.74259808157391E-29	0.532817723619213	0.156	0.257	2.92024586510155E-25	Mature metabolic hepatocytes	SARAF
2.48683713910151E-29	0.385004507906771	0.24	0.379	4.16744167770631E-25	Mature metabolic hepatocytes	GGCX
2.80533765696345E-29	0.379498915786146	0.21	0.333	4.70118484553936E-25	Mature metabolic hepatocytes	COA4
3.49590132332314E-29	0.284248209065008	0.164	0.265	5.85843143762492E-25	Mature metabolic hepatocytes	BUD31
4.88637388134099E-29	0.369494446579042	0.136	0.227	8.18858535035124E-25	Mature metabolic hepatocytes	GJB2
6.37019489690238E-29	0.593769703338615	0.552	0.61	1.0675172608229E-24	Mature metabolic hepatocytes	OS9
6.49856219426891E-29	1.97712135074398	0.161	0.116	1.08902905251558E-24	Mature metabolic hepatocytes	PTPN3
6.734786371904E-29	0.622721986792524	0.071	0.136	1.12861550020367E-24	Mature metabolic hepatocytes	CBLC
7.66753221399315E-29	0.3748293277847	0.129	0.216	1.28492504842097E-24	Mature metabolic hepatocytes	TRAPPC6A
1.69520493678519E-28	1.88369912089388	0.157	0.112	2.84082443306462E-24	Mature metabolic hepatocytes	BCKDHB
1.70568299459912E-28	0.403691720899934	0.087	0.156	2.8583835623492E-24	Mature metabolic hepatocytes	CFAP20
1.95009925261511E-28	0.280206068167233	0.147	0.239	3.2679763275324E-24	Mature metabolic hepatocytes	JUP
2.03873073637826E-28	0.500172644511471	0.082	0.15	3.41650496802268E-24	Mature metabolic hepatocytes	CAAP1
2.59820868897648E-28	0.385752756413416	0.103	0.179	4.35407812098678E-24	Mature metabolic hepatocytes	PPIF
3.10658799610621E-28	0.392837459696283	0.256	0.403	5.20602016387479E-24	Mature metabolic hepatocytes	LARP4
3.27645687059032E-28	0.492669086166028	0.076	0.142	5.49068642373525E-24	Mature metabolic hepatocytes	SP140L
3.52026736929269E-28	0.440353270823846	0.184	0.295	5.89926405746068E-24	Mature metabolic hepatocytes	NDUFB8
3.54009781814697E-28	0.883670938162173	0.452	0.477	5.93249592365069E-24	Mature metabolic hepatocytes	PNRC1
3.62852679264526E-28	0.558672481954781	0.122	0.207	6.08068519911493E-24	Mature metabolic hepatocytes	GTPBP10
3.94797408786666E-28	0.254098668114024	0.281	0.43	6.61601497644695E-24	Mature metabolic hepatocytes	IQGAP2
4.1398285448259E-28	0.287280041064004	0.122	0.204	6.93752467541923E-24	Mature metabolic hepatocytes	KIFC3
4.17510703695056E-28	0.644545216077943	0.061	0.12	6.99664437252174E-24	Mature metabolic hepatocytes	C1orf112
4.63284572174532E-28	0.63909843187256	0.054	0.11	7.76372286050081E-24	Mature metabolic hepatocytes	TMCO6
5.89004340674038E-28	0.452459846702989	0.1	0.174	9.87053474101553E-24	Mature metabolic hepatocytes	PITPNM2
6.25591012317439E-28	0.647264723906261	0.084	0.152	1.04836541844156E-23	Mature metabolic hepatocytes	HMBOX1
8.61605973687702E-28	1.52167623662775	0.165	0.118	1.44387929070585E-23	Mature metabolic hepatocytes	ABLIM3
9.48060358175008E-28	0.674021846366408	0.482	0.504	1.58875954822968E-23	Mature metabolic hepatocytes	KRT18
9.57579761892724E-28	0.500914223843359	0.053	0.107	1.60471216497983E-23	Mature metabolic hepatocytes	DCAF1
9.99761139572108E-28	0.358145599850551	0.171	0.273	1.67539971769494E-23	Mature metabolic hepatocytes	MACROD1
1.01560340181025E-27	0.358612594717343	0.236	0.365	1.70194818075362E-23	Mature metabolic hepatocytes	CDH1
1.17634455637244E-27	1.0478463156142	0.438	0.468	1.97131820756893E-23	Mature metabolic hepatocytes	TNFRSF1A
1.3976664249935E-27	0.659553304583883	0.111	0.191	2.34220939500411E-23	Mature metabolic hepatocytes	DEPRECATEI
1.4132703329239E-27	0.372620633608717	0.051	0.104	2.36835842391388E-23	Mature metabolic hepatocytes	HEMK1
1.59295491859564E-27	0.625264693391554	0.123	0.208	2.66947385258258E-23	Mature metabolic hepatocytes	CEPT1
1.6558700318011E-27	0.374023173433019	0.134	0.221	2.77490699929228E-23	Mature metabolic hepatocytes	CCDC9
1.82205123969731E-27	0.385237381699035	0.057	0.113	3.05339346748476E-23	Mature metabolic hepatocytes	NPEPL1
1.8914853602343E-27	0.391135701561293	0.179	0.284	3.16975116668064E-23	Mature metabolic hepatocytes	ARL8A
1.96425922318386E-27	0.573993686396024	0.134	0.223	3.29170560621152E-23	Mature metabolic hepatocytes	NUDT4
2.10413257828256E-27	0.627709766951602	0.096	0.169	3.52610537468592E-23	Mature metabolic hepatocytes	AZI2

2.41362279443186E-27	0.483365914538286	0.065	0.124	4.04474907890891E-23	Mature metabolic hepatocytes	ATG5
3.29095734073008E-27	0.252961434829136	0.317	0.483	5.51498631159547E-23	Mature metabolic hepatocytes	HADHB
3.43692616527825E-27	0.688608988849093	0.063	0.122	5.7596008677733E-23	Mature metabolic hepatocytes	ORC4
3.48132372501782E-27	0.314709879191108	0.247	0.38	5.83400229838486E-23	Mature metabolic hepatocytes	TOMM7
3.75561669239104E-27	0.306277295402847	0.078	0.142	6.2936624531089E-23	Mature metabolic hepatocytes	NGRN
3.90162068246955E-27	1.20049977720094	0.356	0.352	6.53833593968248E-23	Mature metabolic hepatocytes	ACADM
3.93645485708826E-27	0.473863130095362	0.258	0.406	6.59671104950851E-23	Mature metabolic hepatocytes	ITCH
4.93774201123625E-27	0.282156138885291	0.052	0.104	8.2746680624297E-23	Mature metabolic hepatocytes	ST3GAL4
5.16765599401342E-27	0.316314446571259	0.069	0.128	8.65995791476769E-23	Mature metabolic hepatocytes	TP53RK
5.22065456299683E-27	0.530822545034751	0.087	0.155	8.74877291667009E-23	Mature metabolic hepatocytes	RNF123
5.37907779753815E-27	0.424717759386193	0.084	0.151	9.01425857311444E-23	Mature metabolic hepatocytes	BET1
6.31779303214763E-27	0.656202645444144	0.066	0.126	1.0587357563273E-22	Mature metabolic hepatocytes	FBXL4
6.93905233765353E-27	1.6868397928432	0.203	0.161	1.16284639074398E-22	Mature metabolic hepatocytes	PANK1
7.24669760152593E-27	0.658854052485558	0.116	0.196	1.21440158406372E-22	Mature metabolic hepatocytes	ITGA1
7.48520433881242E-27	0.414991213670946	0.328	0.492	1.25437054309819E-22	Mature metabolic hepatocytes	SREBF1
7.96160139973996E-27	0.690116476078535	0.109	0.187	1.33420516256842E-22	Mature metabolic hepatocytes	NBPF15
8.04269127508478E-27	0.314454366571877	0.89	0.944	1.34779420387871E-22	Mature metabolic hepatocytes	HSPB1
9.19005007070473E-27	0.488741490467752	0.055	0.109	1.5400685908487E-22	Mature metabolic hepatocytes	ZNRF3
9.46734683614186E-27	0.572720512656881	0.05	0.102	1.58653798280065E-22	Mature metabolic hepatocytes	CWF19L2
1.10516934207451E-26	0.516810937471484	0.172	0.278	1.85204278344846E-22	Mature metabolic hepatocytes	SEC63
1.18535228807064E-26	0.625173433567722	0.097	0.169	1.98641336434878E-22	Mature metabolic hepatocytes	CLK3
1.19728603621445E-26	0.868424761576357	0.07	0.131	2.00641193948817E-22	Mature metabolic hepatocytes	CPT1B
1.77313149326972E-26	1.24525912506754	0.227	0.182	2.97141375642139E-22	Mature metabolic hepatocytes	QPRT
1.81862999940454E-26	0.55088289263679	0.072	0.134	3.04766015300212E-22	Mature metabolic hepatocytes	MPZL2
2.87546407866401E-26	0.503287058925825	0.066	0.125	4.81870270302514E-22	Mature metabolic hepatocytes	KAT6B
3.0425904174449E-26	0.520328421634272	0.118	0.197	5.09877302155416E-22	Mature metabolic hepatocytes	INO80B
4.16144287167083E-26	0.596276533942896	0.081	0.146	6.97374596434597E-22	Mature metabolic hepatocytes	SHPK
4.30807303250914E-26	0.99217072078781	0.437	0.47	7.21946878787882E-22	Mature metabolic hepatocytes	MTHFD1
4.34967022177213E-26	0.944923768176501	0.06	0.117	7.28917735764574E-22	Mature metabolic hepatocytes	NAA16
4.84688952895822E-26	1.73970629656755	0.267	0.239	8.12241747262819E-22	Mature metabolic hepatocytes	PPARGC1A
5.47519852259962E-26	0.418234511935804	0.218	0.34	9.17533768417244E-22	Mature metabolic hepatocytes	WSB1
7.61685794894326E-26	1.06382527041561	0.371	0.371	1.27643305508391E-21	Mature metabolic hepatocytes	ZNHIT1
9.94622988069904E-26	0.375786933812372	0.054	0.106	1.66678920340754E-21	Mature metabolic hepatocytes	FES
1.24432890788081E-25	0.760545667435217	0.054	0.107	2.08524638382667E-21	Mature metabolic hepatocytes	REL
1.40762641460597E-25	0.486167640437865	0.073	0.133	2.35890034559668E-21	Mature metabolic hepatocytes	UROS
1.48970608351091E-25	0.395292290275209	0.185	0.291	2.49644945474759E-21	Mature metabolic hepatocytes	SIAE
1.50097159938003E-25	0.55680698498656	0.112	0.189	2.51532820624106E-21	Mature metabolic hepatocytes	PARL
1.55054286985049E-25	1.60736695168511	0.229	0.192	2.59839974129545E-21	Mature metabolic hepatocytes	PLXNB1
1.64246245572617E-25	0.254094737611354	0.217	0.331	2.75243858330591E-21	Mature metabolic hepatocytes	PXDC1
1.9048398674379E-25	0.514248394513565	0.082	0.146	3.19213064985243E-21	Mature metabolic hepatocytes	C16orf87
2.22344517253676E-25	0.508418251142674	0.196	0.309	3.7260494201371E-21	Mature metabolic hepatocytes	EHBP1
2.82001783484197E-25	0.555483285004879	0.084	0.148	4.72578588762818E-21	Mature metabolic hepatocytes	DAPK1
3.42630016796732E-25	0.413044664983217	0.078	0.139	5.74179382147963E-21	Mature metabolic hepatocytes	PLA2G6
3.74454520677621E-25	0.511483224417831	0.236	0.366	6.27510885751557E-21	Mature metabolic hepatocytes	PNN
3.81446537007727E-25	0.499569982343125	0.12	0.199	6.39228106717548E-21	Mature metabolic hepatocytes	GALM
3.82330694667506E-25	0.439005455006594	0.053	0.104	6.40709778123807E-21	Mature metabolic hepatocytes	FAM204A
4.23441321070357E-25	0.282269180952861	0.068	0.124	7.09602965849704E-21	Mature metabolic hepatocytes	SH3PXD2A
4.34186306788187E-25	0.553984548924659	0.227	0.354	7.27609412915644E-21	Mature metabolic hepatocytes	HDHD3
4.57180729136892E-25	1.22210840693056	0.354	0.351	7.66143465887603E-21	Mature metabolic hepatocytes	CGN
6.59442695471611E-25	0.53037708645717	0.098	0.168	1.10509406907133E-20	Mature metabolic hepatocytes	WDR59
7.00719041999245E-25	0.699416532854689	0.147	0.238	1.17426497058233E-20	Mature metabolic hepatocytes	FAM160A2
7.30437425104573E-25	0.422315920017135	0.061	0.115	1.22406703699024E-20	Mature metabolic hepatocytes	MINPP1
8.80327927859351E-25	1.43289405589888	0.329	0.325	1.4752535415067E-20	Mature metabolic hepatocytes	RNF149
8.96698391736459E-25	0.927362110920917	0.357	0.34	1.50268716487196E-20	Mature metabolic hepatocytes	BCL3
1.07403792949683E-24	0.59649117350595	0.118	0.195	1.79987276225078E-20	Mature metabolic hepatocytes	CCNC
1.08789619580921E-24	0.541604013786485	0.11	0.185	1.82309644493708E-20	Mature metabolic hepatocytes	CDR2
1.3041162165502E-24	1.53048177828316	0.237	0.203	2.18543795569482E-20	Mature metabolic hepatocytes	KLF11
1.48959889626891E-24	0.424618563125686	0.151	0.24	2.49626983036745E-20	Mature metabolic hepatocytes	OAS1
1.5355870716458E-24	0.54471528012522	0.086	0.15	2.57333681466404E-20	Mature metabolic hepatocytes	ALCAM
1.74953643310787E-24	0.527073595101616	0.156	0.249	2.93187315460217E-20	Mature metabolic hepatocytes	AMACR

1.92605622984858E-24	0.342380138763782	0.272	0.412	3.22768502998025E-20	Mature metabolic hepatocytes	TMEM248
2.61136506255834E-24	0.542568004562104	0.132	0.213	4.37612557183527E-20	Mature metabolic hepatocytes	MAFK
2.71170614198914E-24	0.979241094911594	0.422	0.444	4.54427715274541E-20	Mature metabolic hepatocytes	UGT1A1
3.76569817480024E-24	0.71902164349485	0.139	0.225	6.31055700133023E-20	Mature metabolic hepatocytes	COBL
3.89171599195569E-24	0.448322788915166	0.255	0.389	6.52173765931934E-20	Mature metabolic hepatocytes	SNRNP70
3.95932216689028E-24	0.448883382881293	0.23	0.354	6.63503208727474E-20	Mature metabolic hepatocytes	SLC43A1
4.70674086225787E-24	0.344776374938957	0.06	0.113	7.88755633697174E-20	Mature metabolic hepatocytes	DCUN1D3
6.09845618991248E-24	0.522367344855848	0.096	0.164	1.02197928830553E-19	Mature metabolic hepatocytes	SEMA4G
8.62070001281068E-24	0.67978954407348	0.117	0.192	1.44465690814681E-19	Mature metabolic hepatocytes	CSRNP1
9.09199662789188E-24	0.587253428725829	0.073	0.131	1.52363679490212E-19	Mature metabolic hepatocytes	ATXN7
9.22054860278588E-24	0.370806162216776	0.085	0.146	1.54517953485486E-19	Mature metabolic hepatocytes	ZNF609
2.23021829833554E-23	0.49846521155428	0.125	0.201	3.7373998243507E-19	Mature metabolic hepatocytes	PCTP
3.01963921123595E-23	0.699152515922167	0.075	0.133	5.06031139018921E-19	Mature metabolic hepatocytes	TUBE1
3.55225434743423E-23	0.510021001328911	0.074	0.13	5.95286783543029E-19	Mature metabolic hepatocytes	SH2B3
3.56632737132238E-23	0.642375909632646	0.078	0.138	5.97645140886205E-19	Mature metabolic hepatocytes	SNIP1
3.56999128496461E-23	1.83311044473589	0.138	0.098	5.98259139534369E-19	Mature metabolic hepatocytes	DMGDH
4.3682629155751E-23	0.461549124276375	0.283	0.426	7.32033499392074E-19	Mature metabolic hepatocytes	TRA2A
7.6677955579159E-23	0.279761959961392	0.366	0.541	1.28496917959555E-18	Mature metabolic hepatocytes	EPS8L2
9.13431424100246E-23	0.510354489707707	0.171	0.266	1.53072838050719E-18	Mature metabolic hepatocytes	MLIP
1.87966850561849E-22	0.543221240115221	0.275	0.421	3.14994848171546E-18	Mature metabolic hepatocytes	CELF1
2.96886517122735E-22	0.544648114141093	0.157	0.246	4.97522425394279E-18	Mature metabolic hepatocytes	FNDC3A
3.62700196303727E-22	0.683365658836184	0.119	0.194	6.07812988965785E-18	Mature metabolic hepatocytes	ZBTB18
3.89632606478186E-22	0.597088946459638	0.177	0.276	6.52946321936143E-18	Mature metabolic hepatocytes	TIAL1
3.92371823247124E-22	0.533632232519828	0.081	0.139	6.5753670139753E-18	Mature metabolic hepatocytes	SIRT5
4.89022540569368E-22	0.427371105959431	0.132	0.209	8.19503973486147E-18	Mature metabolic hepatocytes	SHTN1
5.66319186393502E-22	0.720681826015714	0.112	0.183	9.49037692558231E-18	Mature metabolic hepatocytes	IFT43
7.44011035042901E-22	0.511263804141181	0.184	0.282	1.24681369252489E-17	Mature metabolic hepatocytes	SLC7A2
7.56236081374191E-22	0.779108902704681	0.152	0.239	1.26730042516687E-17	Mature metabolic hepatocytes	ZNF592
1.07864584166586E-21	0.563625125905409	0.089	0.15	1.80759470146365E-17	Mature metabolic hepatocytes	GRB14
1.20280581105177E-21	0.653800627772666	0.555	0.584	2.01566197816055E-17	Mature metabolic hepatocytes	VMP1
1.25479755082258E-21	0.65283871040024	0.095	0.158	2.10278973566847E-17	Mature metabolic hepatocytes	TRIM4
1.8267702194023E-21	0.663384277057157	0.054	0.101	3.06130153367438E-17	Mature metabolic hepatocytes	CDC40
1.97635273235442E-21	0.656163962439223	0.086	0.146	3.31197190887953E-17	Mature metabolic hepatocytes	SULT1B1
1.99182302810829E-21	0.49670425470835	0.139	0.218	3.33789703050388E-17	Mature metabolic hepatocytes	ATP6V1G1
2.31953677977525E-21	1.22741481911011	0.231	0.196	3.88707973554737E-17	Mature metabolic hepatocytes	IRS2
2.37707347988688E-21	0.582615088873374	0.152	0.237	3.983499973759444E-17	Mature metabolic hepatocytes	CLASRP
2.49692087022828E-21	0.795707010075383	0.122	0.196	4.18433999432855E-17	Mature metabolic hepatocytes	TNFRSF10B
2.56138840146412E-21	0.502287775373385	0.568	0.63	4.29237468317357E-17	Mature metabolic hepatocytes	AADAC
2.81893825337298E-21	0.396098199984863	0.067	0.117	4.72397672500244E-17	Mature metabolic hepatocytes	WDR74
2.85151884332044E-21	0.344152706174839	0.278	0.414	4.77857527763639E-17	Mature metabolic hepatocytes	SEM1
3.40731128351296E-21	0.449019867973088	0.094	0.155	5.70997224891102E-17	Mature metabolic hepatocytes	SLC39A8
3.60031640118998E-21	1.18075188680315	0.356	0.36	6.03341022511417E-17	Mature metabolic hepatocytes	RAB17
3.79377571742539E-21	0.754891150843707	0.077	0.133	6.35760934726146E-17	Mature metabolic hepatocytes	SRD5A1
6.77515513891224E-21	0.333278034535327	0.789	0.894	1.13538049817891E-16	Mature metabolic hepatocytes	IL32
7.57006460516155E-21	1.40713796557134	0.119	0.082	1.26859142653297E-16	Mature metabolic hepatocytes	DNAH1
9.49560031086152E-21	1.38913631418904	0.19	0.152	1.59127270009417E-16	Mature metabolic hepatocytes	STEAP3
1.16500314556616E-20	0.507198187690524	0.197	0.299	1.95231227133978E-16	Mature metabolic hepatocytes	NT5E
1.32744780451222E-20	0.602102944103696	0.086	0.145	2.22453703080159E-16	Mature metabolic hepatocytes	MAP3K7
1.39482211563707E-20	0.50440674110565	0.23	0.347	2.3374429013846E-16	Mature metabolic hepatocytes	UBE2H
1.67198950491403E-20	0.382683220204409	0.092	0.151	2.80192001233493E-16	Mature metabolic hepatocytes	DEPRECATEI
1.82308724495227E-20	0.493836554094472	0.077	0.13	3.05512960509102E-16	Mature metabolic hepatocytes	LHPP
2.88548067921214E-20	1.26869265771654	0.329	0.331	4.8354885222237E-16	Mature metabolic hepatocytes	KLKB1
3.87898214699584E-20	0.606786875393697	0.07	0.122	6.50039828193564E-16	Mature metabolic hepatocytes	MANEA
4.1072019834287E-20	0.551553258286076	0.251	0.378	6.88284908382982E-16	Mature metabolic hepatocytes	SND1
4.11354857402352E-20	0.843688382437726	0.178	0.275	6.89348470034862E-16	Mature metabolic hepatocytes	EEPD1
4.3716296030146E-20	0.477184280784869	0.236	0.348	7.32597688873187E-16	Mature metabolic hepatocytes	SPTBN1
4.44642858800413E-20	0.439905899550272	0.148	0.228	7.45132502777732E-16	Mature metabolic hepatocytes	POP7
7.10858161737662E-20	1.25778941903283	0.202	0.164	1.19125610743997E-15	Mature metabolic hepatocytes	CGNL1
7.83130231662114E-20	0.852056398457392	0.149	0.234	1.31236964221937E-15	Mature metabolic hepatocytes	FRRS1
9.66267869119778E-20	1.19961203903545	0.367	0.378	1.61927169507092E-15	Mature metabolic hepatocytes	ADHFE1

9.67285515906084E-20	0.50482056930628	0.089	0.145	1.62097706755542E-15	Mature metabolic hepatocytes	RBM19
1.04647372391233E-19	0.589052153023324	0.159	0.243	1.75368066653228E-15	Mature metabolic hepatocytes	UFM1
1.31004442064148E-19	0.253070150226203	0.075	0.126	2.19537244011099E-15	Mature metabolic hepatocytes	CYP17A1
1.53498572418427E-19	0.609201372058346	0.218	0.328	2.572329076588E-15	Mature metabolic hepatocytes	H6PD
1.97659708721585E-19	1.72384895556484	0.191	0.159	3.31238139875633E-15	Mature metabolic hepatocytes	TSC22D1
2.16184704014606E-19	0.400868648217442	0.067	0.116	3.62282326987677E-15	Mature metabolic hepatocytes	RAC3
2.20193570892036E-19	0.457122641855438	0.209	0.314	3.69000386100875E-15	Mature metabolic hepatocytes	TNFSF10
2.42406386629853E-19	0.456291240165683	0.267	0.398	4.06224622714308E-15	Mature metabolic hepatocytes	RAB1A
2.47633353093335E-19	0.840706202467879	0.139	0.218	4.14983973113811E-15	Mature metabolic hepatocytes	TMEM131L
2.65240925795432E-19	0.560065696763409	0.074	0.126	4.44490743447986E-15	Mature metabolic hepatocytes	GCDH
4.52837912963734E-19	2.21180158867599	0.111	0.078	7.58865774544625E-15	Mature metabolic hepatocytes	ID3
8.52403669559773E-19	1.42899133512833	0.272	0.256	1.42845806944827E-14	Mature metabolic hepatocytes	SLCO1B1
8.5715758222749E-19	1.04932998804796	0.33	0.319	1.43642467629683E-14	Mature metabolic hepatocytes	OSGIN1
9.6500507355615E-19	0.645877535526748	0.094	0.151	1.6171555022654E-14	Mature metabolic hepatocytes	TMEM184A
1.03600450742201E-18	0.487940976641413	0.095	0.152	1.7361363535378E-14	Mature metabolic hepatocytes	DEPRECATEI
2.02927734151748E-18	0.574447350355463	0.066	0.113	3.400662968915E-14	Mature metabolic hepatocytes	ATXN3
2.07218921648947E-18	0.405959656763257	0.317	0.464	3.47257468899306E-14	Mature metabolic hepatocytes	SLC47A1
2.08521255757253E-18	1.70696024728661	0.163	0.13	3.49439920398005E-14	Mature metabolic hepatocytes	CTH
3.16763749032911E-18	1.64634447404701	0.163	0.13	5.30832690629352E-14	Mature metabolic hepatocytes	PRODH2
3.26754221792503E-18	1.8042975938825	0.115	0.082	5.47574724879876E-14	Mature metabolic hepatocytes	NKIRAS1
3.39235787255579E-18	0.771212804116682	0.142	0.218	5.68491332282899E-14	Mature metabolic hepatocytes	TENT4A
4.68185196525987E-18	0.431480232118505	0.233	0.344	7.84584752338249E-14	Mature metabolic hepatocytes	CTSL
5.14728888465937E-18	0.472159775206636	0.588	0.667	8.62582671291218E-14	Mature metabolic hepatocytes	BRI3
5.318158409676E-18	0.797285817943925	0.078	0.129	8.91216986293505E-14	Mature metabolic hepatocytes	ENC1
7.43977003356938E-18	0.279914862373911	0.27	0.394	1.24675666222556E-13	Mature metabolic hepatocytes	RAB7A
8.14532866426072E-18	0.403366868675897	0.241	0.353	1.36499417755681E-13	Mature metabolic hepatocytes	PMM1
9.90948411540042E-18	0.350117444310807	0.673	0.752	1.66063134805588E-13	Mature metabolic hepatocytes	HSD17B6
1.22343955991911E-17	0.68447072552132	0.109	0.17	2.05024001451244E-13	Mature metabolic hepatocytes	YPEL2
2.17309828681789E-17	0.740948537957094	0.214	0.321	3.64167810904941E-13	Mature metabolic hepatocytes	KMT2E
2.27439709375939E-17	0.643159646894265	0.074	0.122	3.81143464972199E-13	Mature metabolic hepatocytes	SFXN4
2.32546064510263E-17	0.583068193125413	0.213	0.316	3.89700694906298E-13	Mature metabolic hepatocytes	HOOK1
2.3533479643968E-17	1.327601952973	0.309	0.303	3.94374051873616E-13	Mature metabolic hepatocytes	EBNA1BP2
2.4236936893065E-17	0.597189356817061	0.51	0.563	4.06162588453984E-13	Mature metabolic hepatocytes	IDH2
3.18683560819443E-17	0.402889975151524	0.131	0.196	5.34049911221223E-13	Mature metabolic hepatocytes	NFKB2
3.91180091859333E-17	0.714281023931634	0.189	0.282	6.55539597937871E-13	Mature metabolic hepatocytes	MEAF6
5.28767640839398E-17	1.61268045182882	0.189	0.161	8.86108812518664E-13	Mature metabolic hepatocytes	CSAD
5.80217967352895E-17	0.742703860017231	0.127	0.195	9.72329269689981E-13	Mature metabolic hepatocytes	BLNK
6.88405256116032E-17	0.495228067059919	0.289	0.413	1.15362952819925E-12	Mature metabolic hepatocytes	PILRB
7.40318920732909E-17	0.535623598162128	0.276	0.407	1.24062644736421E-12	Mature metabolic hepatocytes	RSRC2
8.44561335277194E-17	0.894722100220454	0.493	0.564	1.41531588565752E-12	Mature metabolic hepatocytes	SLC39A14
8.85734183891702E-17	0.560322974541522	0.267	0.39	1.48431334536571E-12	Mature metabolic hepatocytes	CHD2
1.02228299324185E-16	0.89467615372943	0.074	0.122	1.71314184007469E-12	Mature metabolic hepatocytes	LMNTD2
1.21664403922503E-16	1.21334809021266	0.348	0.363	2.03885208093331E-12	Mature metabolic hepatocytes	TNS3
2.10564928746434E-16	0.426749588150512	0.094	0.147	3.52864707593275E-12	Mature metabolic hepatocytes	YPEL3
2.15314012761491E-16	0.500866543278381	0.254	0.373	3.60823222585706E-12	Mature metabolic hepatocytes	TMEM106B
2.15938058948594E-16	0.565067199621407	0.291	0.427	3.61868991860653E-12	Mature metabolic hepatocytes	TSC22D2
2.4260607178915E-16	0.628396338998381	0.137	0.207	4.06559255104257E-12	Mature metabolic hepatocytes	MICU1
2.55535684139046E-16	0.599045034686296	0.062	0.104	4.28226699480214E-12	Mature metabolic hepatocytes	MMAA
2.62402531027458E-16	0.790739780728314	0.086	0.138	4.39734161495815E-12	Mature metabolic hepatocytes	TMF1
2.76865960792582E-16	0.874608721204646	0.177	0.264	4.63971977096209E-12	Mature metabolic hepatocytes	FEM1C
2.8554786006145E-16	1.34201483503524	0.107	0.075	4.78521103890978E-12	Mature metabolic hepatocytes	BCAM
3.12750751195047E-16	1.84418019281506	0.116	0.085	5.24107708852659E-12	Mature metabolic hepatocytes	KLF12
3.99399233534349E-16	0.866970164657502	0.129	0.196	6.69313235556862E-12	Mature metabolic hepatocytes	FGFR3
3.99690928274216E-16	0.586256466370769	0.08	0.128	6.69802057601932E-12	Mature metabolic hepatocytes	GSTZ1
8.08073557706113E-16	0.764641752113702	0.062	0.104	1.3541696680039E-11	Mature metabolic hepatocytes	BAZ2B
1.11480987062941E-15	0.588312927735005	0.139	0.207	1.86819838120077E-11	Mature metabolic hepatocytes	CYP2D6
1.32114031039942E-15	0.70856468821571	0.076	0.122	2.21396693216734E-11	Mature metabolic hepatocytes	CNGA1
1.45910845852457E-15	0.456987909295771	0.261	0.377	2.44517395479548E-11	Mature metabolic hepatocytes	TMEM263
1.4631533923E-15	1.79148347989105	0.113	0.083	2.45195245481634E-11	Mature metabolic hepatocytes	AVPI1
3.35356929293588E-15	0.731716156829407	0.088	0.137	5.61991142110195E-11	Mature metabolic hepatocytes	LRCH1

3.36725390150169E-15	0.641289574196814	0.079	0.125	5.64284408813654E-11	Mature metabolic hepatocytes	DBT
5.60865742185573E-15	1.75511815475089	0.239	0.226	9.39898810754583E-11	Mature metabolic hepatocytes	SORL1
6.34892687017659E-15	0.62256860374069	0.219	0.319	1.06395316490419E-10	Mature metabolic hepatocytes	RAB6A
7.26301773199136E-15	0.628196795743403	0.258	0.378	1.21713651152711E-10	Mature metabolic hepatocytes	HNRNPH3
7.5411653152713E-15	0.859096963883161	0.164	0.24	1.26374848353317E-10	Mature metabolic hepatocytes	XAF1
1.11360598213335E-14	0.937498182539133	0.088	0.138	1.86618090485906E-10	Mature metabolic hepatocytes	RBMXL1
1.12482808629485E-14	0.942125202791846	0.405	0.442	1.8849869070129E-10	Mature metabolic hepatocytes	FNDC3B
1.66184503241166E-14	0.632232205443015	0.082	0.128	2.78491990531546E-10	Mature metabolic hepatocytes	FAM174A
1.70617556064072E-14	0.585567802394781	0.212	0.307	2.85920900452172E-10	Mature metabolic hepatocytes	STAT2
1.82988235145414E-14	1.35222335364446	0.226	0.207	3.06651684456685E-10	Mature metabolic hepatocytes	PDHB
2.13853735545719E-14	0.536264821124376	0.14	0.204	3.58376090027515E-10	Mature metabolic hepatocytes	TSHZ2
2.14992143939031E-14	1.02702250225959	0.381	0.415	3.60283834813028E-10	Mature metabolic hepatocytes	ETFA
2.27027301749391E-14	0.445667668460646	0.393	0.555	3.8045235227163E-10	Mature metabolic hepatocytes	HSPH1
2.57079893580654E-14	0.77105710752934	0.429	0.469	4.3081448566246E-10	Mature metabolic hepatocytes	CEBPB
2.58206857984951E-14	0.537882171615523	0.18	0.261	4.3270305261118E-10	Mature metabolic hepatocytes	DEPRECATEI
2.6645441985426E-14	0.600022929766657	0.359	0.342	4.46524316791769E-10	Mature metabolic hepatocytes	SERPINA5
2.72048969021468E-14	0.647702431415068	0.489	0.561	4.55899662286176E-10	Mature metabolic hepatocytes	CALD1
3.4644193872885E-14	0.483402346246839	0.646	0.723	5.80567400921808E-10	Mature metabolic hepatocytes	DNAJB1
4.11079862947645E-14	0.554489430767102	0.083	0.128	6.88887634327663E-10	Mature metabolic hepatocytes	MAPRE2
4.11157210841917E-14	0.39603687325893	0.282	0.4	6.89017253928885E-10	Mature metabolic hepatocytes	HAAO
4.3701567962621E-14	0.518752109749352	0.284	0.41	7.32350875917603E-10	Mature metabolic hepatocytes	MIA3
4.45886595327447E-14	1.03889176952811	0.303	0.297	7.47216756449736E-10	Mature metabolic hepatocytes	PIPOX
5.40264702504546E-14	0.776261878893035	0.199	0.289	9.05375588457118E-10	Mature metabolic hepatocytes	PNISR
8.19582798489974E-14	0.432660263435486	0.255	0.358	1.3734568537095E-09	Mature metabolic hepatocytes	TLCD4
9.29885389838369E-14	0.803492251335235	0.104	0.157	1.55830193629114E-09	Mature metabolic hepatocytes	POFUT2
9.89833574251537E-14	0.877558342923426	0.115	0.173	1.65876310373072E-09	Mature metabolic hepatocytes	LRRC40
1.07615830211135E-13	0.803410189770367	0.094	0.142	1.8034260826782E-09	Mature metabolic hepatocytes	ABTB2
1.16617531503317E-13	0.5211608236436	0.296	0.426	1.95427659293258E-09	Mature metabolic hepatocytes	UBE2B
1.51097911175951E-13	0.699454985028317	0.091	0.138	2.53209879548658E-09	Mature metabolic hepatocytes	INTS10
1.6272098926883E-13	0.474341098854423	0.221	0.312	2.72687833816706E-09	Mature metabolic hepatocytes	NIPSNAP1
1.68400721266533E-13	1.07039131940035	0.081	0.126	2.82205928698457E-09	Mature metabolic hepatocytes	PBRM1
2.02125603523004E-13	0.433967784372721	0.111	0.163	3.38722086383849E-09	Mature metabolic hepatocytes	BAG1
2.05225691871911E-13	0.481808104749794	0.552	0.621	3.43917214438949E-09	Mature metabolic hepatocytes	H1F0
2.07300676205528E-13	0.624017572651259	0.244	0.353	3.47394473185224E-09	Mature metabolic hepatocytes	TXNDC11
2.21050981718023E-13	0.259657621651979	0.293	0.404	3.70437235163064E-09	Mature metabolic hepatocytes	UBD
2.31209749884686E-13	1.61475495599641	0.101	0.074	3.87461298856758E-09	Mature metabolic hepatocytes	TNS1
3.28091409817836E-13	1.16893995633724	0.254	0.239	5.4981558457273E-09	Mature metabolic hepatocytes	TGM2
4.37617739637863E-13	0.641338385838149	0.233	0.334	7.33359808085131E-09	Mature metabolic hepatocytes	SIK2
5.62456124811283E-13	1.7774994979148	0.153	0.128	9.42563973958747E-09	Mature metabolic hepatocytes	DEPDC7
7.07809620705474E-13	0.419643080880114	0.214	0.297	1.18614736237823E-08	Mature metabolic hepatocytes	TSKU
8.1611024432867E-13	1.66763760336736	0.201	0.182	1.36763754744599E-08	Mature metabolic hepatocytes	GPAM
8.73419050780169E-13	0.668920790067031	0.124	0.18	1.46367564529741E-08	Mature metabolic hepatocytes	TMEM11
1.05816046819948E-12	0.869520574258006	0.37	0.386	1.7732653126087E-08	Mature metabolic hepatocytes	SLC6A1
1.21561756897605E-12	1.4136411775727	0.101	0.075	2.03713192209006E-08	Mature metabolic hepatocytes	PALM3
2.06426038522321E-12	0.530423666992602	0.3	0.424	3.45928755355705E-08	Mature metabolic hepatocytes	C19orf12
2.26818397250123E-12	0.758897074468723	0.083	0.125	3.80102270111757E-08	Mature metabolic hepatocytes	AUH
2.71455662799193E-12	0.527430659841867	0.288	0.405	4.54905399718888E-08	Mature metabolic hepatocytes	CYP4F3
2.73361790253063E-12	0.278458810695042	0.372	0.52	4.58099688106083E-08	Mature metabolic hepatocytes	EIF4EBP2
2.75189949709134E-12	0.436461526117393	0.356	0.503	4.61163317722567E-08	Mature metabolic hepatocytes	IL6ST
4.35931647071853E-12	0.816195402793998	0.091	0.135	7.30534254163012E-08	Mature metabolic hepatocytes	CCDC91
6.07696453686021E-12	0.591154839757897	0.087	0.13	1.01837771708703E-07	Mature metabolic hepatocytes	EAF1
6.43531654884186E-12	1.48921047988804	0.199	0.182	1.07843034725492E-07	Mature metabolic hepatocytes	MOGAT3
7.41989412097254E-12	1.52349974160327	0.145	0.121	1.24342585679258E-07	Mature metabolic hepatocytes	TSPAN9
7.4575664994645E-12	0.749164851365783	0.078	0.118	1.24973899398026E-07	Mature metabolic hepatocytes	AHCYL2
7.63427038498406E-12	1.12410658885905	0.283	0.279	1.27935103111563E-07	Mature metabolic hepatocytes	NCOA7
8.31319517591034E-12	0.826806894533277	0.143	0.205	1.39312524757905E-07	Mature metabolic hepatocytes	DDI2
1.16045476816793E-11	0.914268892093574	0.097	0.143	1.94469010049581E-07	Mature metabolic hepatocytes	ARL5B
1.24763616836454E-11	0.897893735338699	0.166	0.237	2.09078869094529E-07	Mature metabolic hepatocytes	PHF8
1.2670102061119E-11	0.739423226806348	0.088	0.13	2.12325570340233E-07	Mature metabolic hepatocytes	DEPRECATEI
1.30035855334814E-11	0.778697623569143	0.064	0.1	2.17914086370082E-07	Mature metabolic hepatocytes	SLC25A26

**1.36186788001108E-11**	0.797911235481934	0.077	0.116	2.28221819332256E-07	Mature metabolic hepatocytes	RPF2
**1.5316687671759E-11**	0.449309924589591	0.356	0.501	2.56677052003337E-07	Mature metabolic hepatocytes	CEBPG
**1.86227636376174E-11**	0.452579997408905	0.265	0.369	3.12080273039193E-07	Mature metabolic hepatocytes	PCCB
**2.25427485266339E-11**	0.56629809080601	0.316	0.451	3.77771379809332E-07	Mature metabolic hepatocytes	RBM39
**2.30677313121581E-11**	0.723580471397225	0.103	0.149	3.86569041329146E-07	Mature metabolic hepatocytes	RGL3
**2.48379126637554E-11**	0.840383804139905	0.09	0.133	4.16233740419212E-07	Mature metabolic hepatocytes	ABCB7
**2.6500372216472E-11**	0.698958802661078	0.099	0.144	4.44093237603637E-07	Mature metabolic hepatocytes	TMEM35B
**2.71796897723429E-11**	0.820975370609476	0.2	0.283	4.55477241204923E-07	Mature metabolic hepatocytes	LUC7L
**3.35239893168884E-11**	0.853203432119957	0.073	0.11	5.61795012972415E-07	Mature metabolic hepatocytes	UACA
**3.63784612206807E-11**	0.735862411013407	0.067	0.102	6.09630253136167E-07	Mature metabolic hepatocytes	NEK4
**3.80653996320616E-11**	1.2678013690527	0.454	0.546	6.37899967034089E-07	Mature metabolic hepatocytes	CYP2B6
**3.85429780544138E-11**	0.443237075002317	0.166	0.229	6.45903226235866E-07	Mature metabolic hepatocytes	ZC3H12A
**4.10048305441415E-11**	0.542506920230076	0.184	0.257	6.87158950258723E-07	Mature metabolic hepatocytes	SELENOK
**5.55890766398026E-11**	0.381306626782922	0.267	0.37	9.31561746329812E-07	Mature metabolic hepatocytes	ASS1
**6.12961524332654E-11**	0.460810224821197	0.293	0.412	1.02720092247666E-06	Mature metabolic hepatocytes	PEX19
**6.17211463933785E-11**	0.888062161593926	0.127	0.182	1.03432297126024E-06	Mature metabolic hepatocytes	FERMT2
**6.95867743059307E-11**	0.683075217673303	0.24	0.339	1.16613516381879E-06	Mature metabolic hepatocytes	SPG21
**1.08342886814176E-10**	0.730827341625536	0.117	0.166	1.81561009723197E-06	Mature metabolic hepatocytes	THYN1
**1.45060246924765E-10**	1.01905153142196	0.122	0.097	2.43091961796522E-06	Mature metabolic hepatocytes	TNFRSF12A
**1.46910198663231E-10**	1.45856474209608	0.116	0.092	2.46192110919842E-06	Mature metabolic hepatocytes	ALS2CL
**1.63233380070141E-10**	0.695008753712677	0.172	0.239	2.73546498321542E-06	Mature metabolic hepatocytes	IRF7
**1.68476281958247E-10**	0.852337243358852	0.143	0.201	2.8233255330563E-06	Mature metabolic hepatocytes	ZCCHC14
**1.8259171938648E-10**	0.62536302678109	0.206	0.286	3.05987203347863E-06	Mature metabolic hepatocytes	CBX4
**1.84606390473095E-10**	0.817362563196661	0.093	0.135	3.09363389154813E-06	Mature metabolic hepatocytes	SPRYD4
**1.91315214464575E-10**	0.875482619704828	0.143	0.202	3.20606036399735E-06	Mature metabolic hepatocytes	BNIP2
**2.07931211379235E-10**	1.58585782238556	0.123	0.101	3.48451124029322E-06	Mature metabolic hepatocytes	DNAJC25
**2.27155912510059E-10**	0.684951705812087	0.299	0.423	3.80667878184357E-06	Mature metabolic hepatocytes	MAP3K13
**2.48610078008249E-10**	0.787053415402117	0.083	0.121	4.16620768726223E-06	Mature metabolic hepatocytes	HABP4
**2.9922794361322E-10**	0.676755132873732	0.121	0.171	5.01446187907034E-06	Mature metabolic hepatocytes	CCDC12
**3.41641991872483E-10**	1.77696565469112	0.1	0.078	5.72523649979907E-06	Mature metabolic hepatocytes	RTP4
**3.69709935707646E-10**	0.906929500137883	0.147	0.208	6.19559910258873E-06	Mature metabolic hepatocytes	ZNF277
**3.8586855790183E-10**	1.09619882863636	0.258	0.252	6.46638529331887E-06	Mature metabolic hepatocytes	F13B
**4.01267926213927E-10**	0.652389961826538	0.196	0.27	6.72444790749299E-06	Mature metabolic hepatocytes	SOX5
**4.62584339305242E-10**	0.732531882109149	0.441	0.504	7.75198835807724E-06	Mature metabolic hepatocytes	FAH
**5.34696303872641E-10**	0.702157266281075	0.196	0.274	8.96044066029771E-06	Mature metabolic hepatocytes	C16orf70
**6.46140724288657E-10**	0.804944992695258	0.169	0.236	1.08280262576293E-05	Mature metabolic hepatocytes	HBP1
**6.56619124309443E-10**	1.07919948455367	0.081	0.118	1.10036232851777E-05	Mature metabolic hepatocytes	INPP5B
**7.98678838042927E-10**	0.406652096365823	0.255	0.349	1.33842599679234E-05	Mature metabolic hepatocytes	POLD4
**8.10937484293801E-10**	0.707360315804434	0.192	0.266	1.35896903617955E-05	Mature metabolic hepatocytes	TTC38
**8.80646279578832E-10**	1.27514156416595	0.282	0.29	1.47578703531821E-05	Mature metabolic hepatocytes	CEBPA
**9.42032137845687E-10**	0.886950369928369	0.126	0.177	1.5786574566018E-05	Mature metabolic hepatocytes	HNMT
**1.01857302574033E-09**	1.3629795296196	0.259	0.264	1.70692467653564E-05	Mature metabolic hepatocytes	AGMO
**1.09400432202446E-09**	1.0071420817931	0.154	0.216	1.83333244284858E-05	Mature metabolic hepatocytes	TRPM7
**1.3946493859688E-09**	1.33318309529877	0.132	0.11	2.33715344100652E-05	Mature metabolic hepatocytes	RABEPK
**1.84830161849087E-09**	0.654904709882936	0.181	0.249	3.09738385226699E-05	Mature metabolic hepatocytes	APOL1
**2.13244248542724E-09**	0.410194667437139	0.296	0.403	3.57354711707898E-05	Mature metabolic hepatocytes	CARHSP1
**2.32505819421512E-09**	0.847863658344003	0.173	0.241	3.89633252186569E-05	Mature metabolic hepatocytes	MET
**3.52755213031708E-09**	0.819523623322617	0.12	0.167	5.91147185998535E-05	Mature metabolic hepatocytes	MAP2K3
**3.80309637123671E-09**	0.675696496673518	0.288	0.401	6.37322889891847E-05	Mature metabolic hepatocytes	CLK1
**3.81225457600686E-09**	0.738433951375186	0.162	0.223	6.3885762184723E-05	Mature metabolic hepatocytes	RBM6
**5.67850407737787E-09**	0.743332922841404	0.135	0.185	9.51603713286983E-05	Mature metabolic hepatocytes	ZCCHC24
**6.01191534481483E-09**	1.34838712611081	0.313	0.338	0.000100747677348407	Mature metabolic hepatocytes	HMGCR
**7.37176283379162E-09**	0.735254564467947	0.186	0.256	0.00012353600156868	Mature metabolic hepatocytes	RNF11
**7.47643487681657E-09**	0.481482277196512	0.557	0.653	0.000125290095665692	Mature metabolic hepatocytes	FBP1
**7.69910730977998E-09**	0.67066926485513	0.137	0.188	0.000129021640297293	Mature metabolic hepatocytes	FAXDC2
**1.02278048571521E-08**	0.378794888732005	0.601	0.704	0.000171397553796155	Mature metabolic hepatocytes	C2
**1.03106902539064E-08**	0.483485824707738	0.31	0.425	0.000172786547274963	Mature metabolic hepatocytes	CIB1
**1.42976815123337E-08**	1.13303517678227	0.278	0.285	0.000239600546783688	Mature metabolic hepatocytes	BCL6
**2.10136288049828E-08**	0.782878647841779	0.077	0.109	0.000352146391513902	Mature metabolic hepatocytes	ADGRL2
**2.52350377976177E-08**	0.402888082581786	0.344	0.468	0.000422888763412478	Mature metabolic hepatocytes	C8G

3.11714115966888E-08	0.656578200623707	0.195	0.265	0.00052237051553731	Mature metabolic hepatocytes	RAD23B
4.16856879598369E-08	0.785806400633494	0.073	0.103	0.000698568758830947	Mature metabolic hepatocytes	RILP
4.646392331678E-08	0.715567168384114	0.113	0.154	0.000778642426942599	Mature metabolic hepatocytes	HTRA1
4.74231194962384E-08	0.921411804170111	0.341	0.372	0.000794716636517962	Mature metabolic hepatocytes	SAT2
4.74603615565004E-08	0.510622938287592	0.176	0.234	0.000795340738963834	Mature metabolic hepatocytes	UBE2L6
5.00482761376117E-08	0.803536902389719	0.13	0.177	0.000838709011514096	Mature metabolic hepatocytes	SORBS3
5.68146342006005E-08	0.44262245143921	0.56	0.67	0.000952099639933663	Mature metabolic hepatocytes	PEBP1
7.14130252343203E-08	0.585781724173816	0.123	0.166	0.00119673947687674	Mature metabolic hepatocytes	NSMF
1.04369169660919E-07	0.778623695574422	0.081	0.113	0.00174901854517768	Mature metabolic hepatocytes	PSTPIP2
1.22683719635141E-07	1.02878229977914	0.093	0.129	0.0020559337736457	Mature metabolic hepatocytes	CBWD3
1.27439639152637E-07	0.661164462661602	0.32	0.443	0.00213563347291989	Mature metabolic hepatocytes	NDST1
1.28100652342113E-07	0.381156414856574	0.223	0.292	0.00214671073194913	Mature metabolic hepatocytes	ROBO1
1.67546057015963E-07	0.581289055364247	0.325	0.45	0.00280773682347351	Mature metabolic hepatocytes	SLC25A13
1.6788180309883E-07	1.01923072962157	0.313	0.339	0.00281336325633019	Mature metabolic hepatocytes	GOT2
2.2816917370276E-07	0.725672085149821	0.239	0.326	0.00382365901291084	Mature metabolic hepatocytes	PLGLB1
2.89059063993499E-07	0.991941071255703	0.282	0.292	0.00484405179440306	Mature metabolic hepatocytes	COMT
2.95770755949899E-07	1.50619015120132	0.147	0.133	0.00495652632820841	Mature metabolic hepatocytes	NAGS
3.06603436862758E-07	1.24291960055394	0.176	0.164	0.00513806039494609	Mature metabolic hepatocytes	ODF3B
3.31060737589022E-07	0.898089491034014	0.144	0.193	0.00554791584051684	Mature metabolic hepatocytes	SLC28A1
3.31450927150853E-07	0.481421537227844	0.355	0.48	0.005554454637194	Mature metabolic hepatocytes	RBM33
3.31798933093977E-07	1.00833908534333	0.345	0.39	0.00556028652078887	Mature metabolic hepatocytes	APMAP
3.47493205046047E-07	0.880934570358986	0.125	0.169	0.00582329113016165	Mature metabolic hepatocytes	BORCS7
3.91941686006016E-07	0.592947614575156	0.099	0.133	0.00656815877408881	Mature metabolic hepatocytes	NAV2
3.93481453913454E-07	1.27473579227947	0.204	0.197	0.00659396220468166	Mature metabolic hepatocytes	SHB
4.39257321123932E-07	0.502919123707647	0.343	0.47	0.00736107418739484	Mature metabolic hepatocytes	PPP2R5A
5.33651723560035E-07	1.6737176788568	0.207	0.206	0.00894293558341907	Mature metabolic hepatocytes	SLC5A6
5.88472117983782E-07	0.976813025998355	0.35	0.399	0.00986161575317221	Mature metabolic hepatocytes	PECR
6.47790855825529E-07	1.21603126086974	0.274	0.289	0.0108556791619242	Mature metabolic hepatocytes	IER2
7.23794750490755E-07	0.824748665658698	0.435	0.513	0.0121293524287241	Mature metabolic hepatocytes	ASL
7.26168511528167E-07	0.443090806268749	0.572	0.658	0.012169131916189	Mature metabolic hepatocytes	HPN
8.9305078798902E-07	0.540123930836534	0.202	0.263	0.01496574510512	Mature metabolic hepatocytes	ALDOC
9.2714035903507E-07	0.483613546751896	0.262	0.344	0.0155370181367097	Mature metabolic hepatocytes	TTPA
9.32879282551491E-07	0.53832064256189	0.292	0.396	0.0156331910169979	Mature metabolic hepatocytes	MALSU1
1.02692232252457E-06	0.570694216681768	0.307	0.416	0.0172091642808667	Mature metabolic hepatocytes	TRAF4
1.13227090725032E-06	0.791765865574752	0.126	0.168	0.0189745958637008	Mature metabolic hepatocytes	SLC25A20
1.38342182494201E-06	1.08914372792692	0.186	0.25	0.0231833829423781	Mature metabolic hepatocytes	ATF5
1.43224998347323E-06	0.887739863575335	0.107	0.143	0.0240016452230444	Mature metabolic hepatocytes	UGT2B7
1.8007646372873E-06	0.641558808934972	0.257	0.344	0.0301772137916606	Mature metabolic hepatocytes	CCDC50
1.90530164942485E-06	0.952834503514839	0.085	0.115	0.0319290450410617	Mature metabolic hepatocytes	PPFIBP1
2.0551698181149E-06	0.77020263603354	0.112	0.148	0.0344405358119695	Mature metabolic hepatocytes	STIMATE
2.45400364653669E-06	1.18693839721239	0.105	0.14	0.0411241931086618	Mature metabolic hepatocytes	NT5C2
0	2.1935941733899	0.845	0.346	0	Ductular-like hepatocytes	CDH17
0	2.58155221190637	0.756	0.262	0	Ductular-like hepatocytes	MUC5B
0	1.86040975705863	0.88	0.389	0	Ductular-like hepatocytes	ADA
0	1.60089978016006	0.882	0.42	0	Ductular-like hepatocytes	MUC13
0	0.978328404755445	0.927	0.481	0	Ductular-like hepatocytes	TSC22D3
0	2.11887530147361	0.748	0.307	0	Ductular-like hepatocytes	SLC6A19
0	1.83016709456701	0.754	0.314	0	Ductular-like hepatocytes	MEP1A
0	1.6149053592244	0.749	0.314	0	Ductular-like hepatocytes	SLC6A8
0	1.1431188852241	0.899	0.467	0	Ductular-like hepatocytes	MPP1
0	1.29270341052345	0.883	0.455	0	Ductular-like hepatocytes	NDRG1
0	1.27882211900158	0.854	0.426	0	Ductular-like hepatocytes	TSPAN3
0	1.61447309129504	0.807	0.382	0	Ductular-like hepatocytes	ATP1A1
0	1.78794906544325	0.739	0.319	0	Ductular-like hepatocytes	KLF5
0	1.03146726264359	0.845	0.438	0	Ductular-like hepatocytes	FKBP5
0	1.49646428860624	0.697	0.292	0	Ductular-like hepatocytes	NBL1
0	1.59750925684811	0.782	0.385	0	Ductular-like hepatocytes	CLDN4
0	1.27589350516191	0.754	0.359	0	Ductular-like hepatocytes	F2RL1
0	2.34675972366191	0.947	0.554	0	Ductular-like hepatocytes	PIGR
0	1.25588973300308	0.864	0.476	0	Ductular-like hepatocytes	RNF128

0	1.18566457393208	0.83	0.442	0	Ductular-like hepatocytes	SSR4
0	1.76579697957744	0.762	0.377	0	Ductular-like hepatocytes	TFRC
0	1.13463859059315	0.772	0.388	0	Ductular-like hepatocytes	PEG10
0	1.62659945816123	0.675	0.293	0	Ductular-like hepatocytes	PARM1
0	2.13923983818868	0.597	0.217	0	Ductular-like hepatocytes	B3GNT3
0	1.06583492161233	0.788	0.41	0	Ductular-like hepatocytes	G6PD
0	2.8766076036357	0.475	0.097	0	Ductular-like hepatocytes	AGR2
0	1.78853348261276	0.61	0.236	0	Ductular-like hepatocytes	FXYD3
0	1.98593249816789	0.904	0.531	0	Ductular-like hepatocytes	LYZ
0	0.893162537111373	0.804	0.433	0	Ductular-like hepatocytes	ERICH5
0	1.33246226781678	0.806	0.437	0	Ductular-like hepatocytes	TSPAN8
0	1.14226638306706	0.828	0.46	0	Ductular-like hepatocytes	BCAP31
0	0.792010827234123	0.915	0.551	0	Ductular-like hepatocytes	NQO1
0	1.2684690232036	0.734	0.373	0	Ductular-like hepatocytes	ABCD1
0	1.26461564722139	0.749	0.39	0	Ductular-like hepatocytes	TMEM45B
0	1.65029362052875	0.643	0.285	0	Ductular-like hepatocytes	MATN2
0	1.08738926104122	0.746	0.388	0	Ductular-like hepatocytes	PGM2
0	1.38722894987873	0.705	0.353	0	Ductular-like hepatocytes	PYGB
0	1.29375225873396	0.972	0.621	0	Ductular-like hepatocytes	S100A10
0	1.27215139638042	0.967	0.618	0	Ductular-like hepatocytes	ANXA4
0	1.29318753273211	0.777	0.429	0	Ductular-like hepatocytes	CAPN2
0	1.1894451906641	0.808	0.46	0	Ductular-like hepatocytes	MAOA
0	0.771360983366632	0.843	0.495	0	Ductular-like hepatocytes	MBNL3
0	1.63478941979699	0.66	0.314	0	Ductular-like hepatocytes	CLDN3
0	1.82571593006052	0.549	0.205	0	Ductular-like hepatocytes	TSPAN1
0	1.38804849466162	0.686	0.343	0	Ductular-like hepatocytes	ETHE1
0	1.21913661534853	0.88	0.539	0	Ductular-like hepatocytes	ATP1B1
0	1.11075092701239	0.719	0.378	0	Ductular-like hepatocytes	LAPTM4B
0	1.25717518214155	0.64	0.304	0	Ductular-like hepatocytes	MCM3
0	1.12574292861755	0.659	0.324	0	Ductular-like hepatocytes	EFHD2
0	1.26294860402659	0.66	0.325	0	Ductular-like hepatocytes	DEGS1
0	1.32057101877024	0.641	0.307	0	Ductular-like hepatocytes	EHF
0	1.07532149716208	0.808	0.475	0	Ductular-like hepatocytes	DNM2
0	2.08604735072797	0.521	0.188	0	Ductular-like hepatocytes	SI
0	1.10266930230155	0.69	0.36	0	Ductular-like hepatocytes	HNF4G
0	0.6601898526397	0.873	0.547	0	Ductular-like hepatocytes	SGK1
0	1.95635946631649	0.468	0.143	0	Ductular-like hepatocytes	PRR15L
0	0.88174849525049	0.928	0.605	0	Ductular-like hepatocytes	SPINK1
0	1.03807025646224	0.743	0.42	0	Ductular-like hepatocytes	ATP6AP1
0	0.950870010827693	0.699	0.376	0	Ductular-like hepatocytes	ERO1B
0	1.37262698338894	0.601	0.278	0	Ductular-like hepatocytes	ANXA13
0	0.662334589545224	0.865	0.542	0	Ductular-like hepatocytes	ACSL4
0	1.58738783305732	0.551	0.229	0	Ductular-like hepatocytes	KCNK5
0	1.21241774063233	0.703	0.381	0	Ductular-like hepatocytes	DHRS7
0	1.51844127905416	0.65	0.331	0	Ductular-like hepatocytes	SULT1C2
0	1.37828060326215	0.59	0.271	0	Ductular-like hepatocytes	BTNL8
0	1.73262801034564	0.472	0.153	0	Ductular-like hepatocytes	MISP
0	0.896771841746359	0.776	0.458	0	Ductular-like hepatocytes	RBM47
0	1.36643776281216	0.666	0.349	0	Ductular-like hepatocytes	CLDN7
0	1.30105626736585	0.601	0.284	0	Ductular-like hepatocytes	NEB
0	1.69895007317335	0.517	0.201	0	Ductular-like hepatocytes	MALL
0	1.60048841438562	0.524	0.209	0	Ductular-like hepatocytes	PDX1
0	0.99186638517326	0.668	0.354	0	Ductular-like hepatocytes	EZR
0	1.32976085646578	0.573	0.261	0	Ductular-like hepatocytes	C15orf48
0	0.911923951955037	0.812	0.502	0	Ductular-like hepatocytes	NDUFA1
0	1.06526595324072	0.726	0.416	0	Ductular-like hepatocytes	PNKD
0	1.65967441605516	0.499	0.19	0	Ductular-like hepatocytes	INAVA
0	1.99591562018957	0.474	0.165	0	Ductular-like hepatocytes	OTOP3
0	1.52135223025039	0.548	0.24	0	Ductular-like hepatocytes	CDS1
0	0.719863375624364	0.88	0.572	0	Ductular-like hepatocytes	TDO2

0	1.39993278164636	0.796	0.488	0	Ductular-like hepatocytes	GOLM1
0	2.04665041162706	0.458	0.15	0	Ductular-like hepatocytes	POF1B
0	0.955945019024302	0.754	0.446	0	Ductular-like hepatocytes	TPD52
0	1.43647165070014	0.543	0.236	0	Ductular-like hepatocytes	SEL1L3
0	1.54749888399129	0.589	0.282	0	Ductular-like hepatocytes	SLC15A1
0	1.4717549529479	0.834	0.527	0	Ductular-like hepatocytes	MUC3A
0	0.996164721663338	0.655	0.35	0	Ductular-like hepatocytes	PAG1
0	0.825042902249579	0.677	0.373	0	Ductular-like hepatocytes	HTATSF1
0	1.34019682008355	0.581	0.278	0	Ductular-like hepatocytes	DUSP9
0	0.730419931939428	0.698	0.395	0	Ductular-like hepatocytes	CMC4
0	1.84880476736682	0.485	0.183	0	Ductular-like hepatocytes	MGAM2
0	1.89734587724396	0.423	0.121	0	Ductular-like hepatocytes	KCTD12
0	1.80531423011389	0.49	0.188	0	Ductular-like hepatocytes	MS4A8
0	1.93042063364644	0.461	0.16	0	Ductular-like hepatocytes	QSOX1
0	0.937012818376185	0.906	0.606	0	Ductular-like hepatocytes	LAMP2
0	2.16916055353157	0.42	0.12	0	Ductular-like hepatocytes	TFF1
0	0.995308740915922	0.837	0.537	0	Ductular-like hepatocytes	HSD17B11
0	0.995900807682877	0.636	0.337	0	Ductular-like hepatocytes	CITED2
0	1.35114586384996	0.565	0.266	0	Ductular-like hepatocytes	KCNK1
0	1.20721070952502	0.581	0.283	0	Ductular-like hepatocytes	DEPRECATEI
0	1.07851322909975	0.944	0.646	0	Ductular-like hepatocytes	LGALS4
0	0.885844661531709	0.735	0.438	0	Ductular-like hepatocytes	TSPAN6
0	0.908690187093	0.85	0.553	0	Ductular-like hepatocytes	SELENBP1
0	1.33263965119396	0.543	0.247	0	Ductular-like hepatocytes	TMEM164
0	1.34870016101029	0.632	0.336	0	Ductular-like hepatocytes	HPGD
0	0.837396982944855	0.739	0.443	0	Ductular-like hepatocytes	FAM50A
0	1.13062624230686	0.694	0.398	0	Ductular-like hepatocytes	ATRN
0	0.91679800972621	0.935	0.64	0	Ductular-like hepatocytes	AKR1C3
0	0.815146937003608	0.669	0.375	0	Ductular-like hepatocytes	LAGE3
0	2.49767849573675	0.374	0.08	0	Ductular-like hepatocytes	MMP28
0	0.799147191594803	0.648	0.355	0	Ductular-like hepatocytes	AMOTL1
0	1.08872755279091	0.577	0.285	0	Ductular-like hepatocytes	SIPA1L2
0	1.25376690791918	0.551	0.26	0	Ductular-like hepatocytes	ADAM9
0	1.0649313561191	0.713	0.425	0	Ductular-like hepatocytes	SLPI
0	0.933013829271628	0.665	0.378	0	Ductular-like hepatocytes	FRK
0	0.983766849436777	0.649	0.362	0	Ductular-like hepatocytes	DGAT2
0	3.1505378493191	0.341	0.054	0	Ductular-like hepatocytes	B3GALT5
0	1.02760297319424	0.629	0.342	0	Ductular-like hepatocytes	CYSTM1
0	0.91477728424793	0.617	0.33	0	Ductular-like hepatocytes	VMA21
0	0.803685106190749	0.693	0.407	0	Ductular-like hepatocytes	GPAA1
0	1.02345423620612	0.61	0.324	0	Ductular-like hepatocytes	ATP6V0D1
0	1.0746835427442	0.601	0.316	0	Ductular-like hepatocytes	CAPN1
0	0.716135440727426	0.91	0.626	0	Ductular-like hepatocytes	ASPH
0	0.840690540753835	0.689	0.405	0	Ductular-like hepatocytes	IDH3G
0	1.13874539404811	0.651	0.368	0	Ductular-like hepatocytes	IVNS1ABP
0	0.830264675477824	0.912	0.63	0	Ductular-like hepatocytes	IFI27
0	1.98103476418951	0.423	0.141	0	Ductular-like hepatocytes	CD177
0	1.07574635850732	0.542	0.263	0	Ductular-like hepatocytes	MCM4
0	0.974981959252085	0.587	0.308	0	Ductular-like hepatocytes	FAM3A
0	1.39944914760012	0.507	0.229	0	Ductular-like hepatocytes	EDNRB
0	0.934522661497205	0.611	0.333	0	Ductular-like hepatocytes	NHSL1
0	0.884533992477253	0.676	0.399	0	Ductular-like hepatocytes	CMBL
0	0.913884798963521	0.616	0.339	0	Ductular-like hepatocytes	DDIT4
0	1.81040940296449	0.437	0.161	0	Ductular-like hepatocytes	CCL28
0	1.00372379589479	0.706	0.43	0	Ductular-like hepatocytes	VNN 1.00
0	0.881231917359215	0.702	0.426	0	Ductular-like hepatocytes	GUSB
0	0.893969272724935	0.771	0.496	0	Ductular-like hepatocytes	TM9SF2
0	1.69014118315524	0.444	0.169	0	Ductular-like hepatocytes	TMC5
0	1.11534633849811	0.567	0.292	0	Ductular-like hepatocytes	NDFIP2
0	0.744061472386317	0.718	0.443	0	Ductular-like hepatocytes	GDI1

0	0.794291594919035	0.677	0.403	0	Ductular-like hepatocytes	ALG13
0	0.668846758283218	0.756	0.484	0	Ductular-like hepatocytes	TMEM106C
0	1.1662980326055	0.855	0.583	0	Ductular-like hepatocytes	CES2
0	0.771053680386886	0.933	0.662	0	Ductular-like hepatocytes	UGT2B4
0	1.2465908290118	0.532	0.261	0	Ductular-like hepatocytes	PCDH1
0	1.08372379031784	0.503	0.232	0	Ductular-like hepatocytes	NIBAN2
0	0.854851134195963	0.713	0.442	0	Ductular-like hepatocytes	PRDX1
0	0.763019588847487	0.675	0.405	0	Ductular-like hepatocytes	ENO1
0	0.893301124188748	0.667	0.397	0	Ductular-like hepatocytes	INF2
0	1.05763832200165	0.568	0.298	0	Ductular-like hepatocytes	TMEM64
0	0.901525452114545	0.611	0.343	0	Ductular-like hepatocytes	PYCR2
0	1.97284629888003	0.389	0.122	0	Ductular-like hepatocytes	TFF3
0	0.773200768968039	0.75	0.484	0	Ductular-like hepatocytes	TRAM1
0	1.16951370188355	0.499	0.233	0	Ductular-like hepatocytes	CHP2
0	1.10305001100224	0.52	0.255	0	Ductular-like hepatocytes	TM4SF20
0	1.33637658701444	0.471	0.206	0	Ductular-like hepatocytes	IGFBP3
0	1.2355882128304	0.496	0.233	0	Ductular-like hepatocytes	KCNJ3
0	1.06749614552772	0.536	0.273	0	Ductular-like hepatocytes	ATP9A
0	0.816409908952952	0.789	0.527	0	Ductular-like hepatocytes	SCD
0	0.713181501404606	0.78	0.519	0	Ductular-like hepatocytes	TMEM14A
0	0.802565494107058	0.684	0.424	0	Ductular-like hepatocytes	BTG1
0	1.16996283880706	0.525	0.265	0	Ductular-like hepatocytes	GMDS
0	1.20393619051217	0.496	0.236	0	Ductular-like hepatocytes	CTSC
0	0.88772754942805	0.6	0.34	0	Ductular-like hepatocytes	TMPRSS2
0	0.827606722393341	0.674	0.415	0	Ductular-like hepatocytes	TMBIM1
0	1.19457212498588	0.492	0.235	0	Ductular-like hepatocytes	DPP4
0	0.811477458328385	0.853	0.596	0	Ductular-like hepatocytes	ANPEP
0	1.78540940603838	0.391	0.134	0	Ductular-like hepatocytes	HSD11B2
0	1.77442259041207	0.394	0.138	0	Ductular-like hepatocytes	SHOX2
0	0.712147199247042	0.796	0.54	0	Ductular-like hepatocytes	CRYL1
0	1.71936858186907	0.38	0.124	0	Ductular-like hepatocytes	SLC1A5
0	1.50794897718997	0.401	0.146	0	Ductular-like hepatocytes	S100P
0	1.21729133294771	0.494	0.24	0	Ductular-like hepatocytes	UGT2B11
0	1.39028294253682	0.431	0.178	0	Ductular-like hepatocytes	ASRGL1
0	1.30448791035489	0.43	0.179	0	Ductular-like hepatocytes	CORO2A
0	0.73714287317483	0.864	0.613	0	Ductular-like hepatocytes	BST2
0	1.10274582376186	0.506	0.256	0	Ductular-like hepatocytes	PKDCC
0	1.18545510061513	0.485	0.235	0	Ductular-like hepatocytes	STK39
0	1.49954732189873	0.402	0.153	0	Ductular-like hepatocytes	MARCKSL1
0	2.76609709075296	0.299	0.05	0	Ductular-like hepatocytes	ATP2C2
0	0.771363114247035	0.948	0.7	0	Ductular-like hepatocytes	UGDH
0	0.709526375540046	0.873	0.625	0	Ductular-like hepatocytes	GSTA1
0	0.783790592419813	0.826	0.578	0	Ductular-like hepatocytes	CFL1
0	1.21867740998735	0.434	0.186	0	Ductular-like hepatocytes	PNP
0	0.817826266367701	0.688	0.44	0	Ductular-like hepatocytes	SUMF2
0	1.71426476504058	0.353	0.106	0	Ductular-like hepatocytes	CDC42EP5
0	1.53246991019349	0.434	0.188	0	Ductular-like hepatocytes	PCSK5
0	1.63413328728571	0.375	0.13	0	Ductular-like hepatocytes	NR3C2
0	1.28904124260042	0.457	0.213	0	Ductular-like hepatocytes	PRCP
0	0.728824415773838	0.768	0.528	0	Ductular-like hepatocytes	S100A16
0	2.99253101768552	0.299	0.059	0	Ductular-like hepatocytes	MUC1
0	0.893778462655738	0.66	0.421	0	Ductular-like hepatocytes	PTPRK
0	0.500739066988251	0.928	0.696	0	Ductular-like hepatocytes	MT-CYB
0	1.3388111109374	0.392	0.161	0	Ductular-like hepatocytes	CKB
0	2.31431334509225	0.287	0.056	0	Ductular-like hepatocytes	MYO7B
0	1.34917192595892	0.376	0.146	0	Ductular-like hepatocytes	ITPR3
0	0.821448640102297	0.712	0.482	0	Ductular-like hepatocytes	OCLN
0	0.765624433947292	0.795	0.566	0	Ductular-like hepatocytes	GOLIM4
0	2.1472228269142	0.3	0.072	0	Ductular-like hepatocytes	C1orf116
0	0.702228413754633	0.798	0.571	0	Ductular-like hepatocytes	PSME1

0	1.36026089896349	0.383	0.156	0	Ductular-like hepatocytes	ELF4
0	1.84952385245033	0.337	0.112	0	Ductular-like hepatocytes	ACE
0	0.811406136979288	0.735	0.511	0	Ductular-like hepatocytes	APP
0	1.5364166731689	0.373	0.15	0	Ductular-like hepatocytes	FRAS1
0	1.81085588547139	0.305	0.082	0	Ductular-like hepatocytes	CNTFR
0	0.746819350149547	0.762	0.539	0	Ductular-like hepatocytes	APLP2
0	1.94059349413832	0.315	0.098	0	Ductular-like hepatocytes	FZD8
0	2.58813781907119	0.261	0.046	0	Ductular-like hepatocytes	PPP1R1B
0	1.66876067392685	0.363	0.148	0	Ductular-like hepatocytes	ITGA3
0	1.62801825918255	0.31	0.097	0	Ductular-like hepatocytes	PPP1R14D
0	1.6055302411642	0.334	0.125	0	Ductular-like hepatocytes	VIPR1
0	1.51614991668073	0.313	0.105	0	Ductular-like hepatocytes	C6orf132
0	2.723969163501	0.252	0.045	0	Ductular-like hepatocytes	ARHGEF38
0	1.9608728835415	0.289	0.087	0	Ductular-like hepatocytes	B4GALNT2
0	2.76376865070162	0.248	0.051	0	Ductular-like hepatocytes	FXYD2
0	0.633484386317907	0.846	0.649	0	Ductular-like hepatocytes	TM4SF4
0	0.685123523187255	0.96	0.763	0	Ductular-like hepatocytes	PGRMC1
0	3.03170451999872	0.21	0.029	0	Ductular-like hepatocytes	FAM3D
0	0.657958871687969	0.832	0.652	0	Ductular-like hepatocytes	GSTK1
0	2.3132347386784	0.235	0.057	0	Ductular-like hepatocytes	DUSP21
0	1.9890406939173	0.236	0.061	0	Ductular-like hepatocytes	MECOM
0	2.86430280130029	0.219	0.045	0	Ductular-like hepatocytes	SLC5A1
0	2.91406008418827	0.184	0.03	0	Ductular-like hepatocytes	TRPV6
0	0.534432500219975	0.975	0.83	0	Ductular-like hepatocytes	MT-CO2
0	0.56831822508258	0.984	0.903	0	Ductular-like hepatocytes	SELENOP
0	0.576385431278916	0.987	0.937	0	Ductular-like hepatocytes	EPHX1
9.5649106013431E-307	0.974242899970393	0.552	0.288	1.60288771857308E-302	Ductular-like hepatocytes	DEPRECATEI
2.96327378830001E-304	1.06743161358603	0.449	0.208	4.96585421443316E-300	Ductular-like hepatocytes	AP1S1
1.88560915415886E-303	1.28287928773825	0.412	0.181	3.15990382053942E-299	Ductular-like hepatocytes	MANEAL
3.77677922510027E-302	0.752732748286376	0.682	0.412	6.32912662542303E-298	Ductular-like hepatocytes	MIDN
5.8335486994168E-302	1.68753358023739	0.28	0.09	9.77586091048267E-298	Ductular-like hepatocytes	DEPRECATEI
3.87949346769736E-301	1.63443426875549	0.258	0.075	6.50125515316724E-297	Ductular-like hepatocytes	RAPGEFL1
6.93126586042928E-301	1.33681512165284	0.349	0.134	1.16154153289074E-296	Ductular-like hepatocytes	LHFPL2
2.2038836869226E-300	1.13735428207112	0.451	0.211	3.6932682825449E-296	Ductular-like hepatocytes	SLC10A3
1.44273515651933E-299	1.14500286465116	0.444	0.208	2.4177355752951E-295	Ductular-like hepatocytes	CRACR2B
7.43749647212384E-298	0.734978870290956	0.682	0.399	1.24637565879851E-293	Ductular-like hepatocytes	UBE2A
2.58757804200119E-297	0.565858613933409	0.967	0.794	4.33626328278559E-293	Ductular-like hepatocytes	ALDH1A1
6.17851948404707E-297	0.889978542446941	0.589	0.329	1.03539629513661E-292	Ductular-like hepatocytes	FOSL2
6.5617379053974E-297	0.97931204567606	0.537	0.287	1.0996160381865E-292	Ductular-like hepatocytes	CD68
1.44241545156312E-296	0.795760621848917	0.655	0.403	2.41719981372948E-292	Ductular-like hepatocytes	BSG
4.6416427091915E-296	1.72593568164298	0.254	0.075	7.77846485206311E-292	Ductular-like hepatocytes	VDR
1.92302262416847E-295	1.06457342204072	0.476	0.235	3.22260131358152E-291	Ductular-like hepatocytes	MBOAT7
5.18557306231054E-295	0.758390565815373	0.713	0.467	8.68998333782001E-291	Ductular-like hepatocytes	SLC37A4
8.66226119865179E-295	0.704304696032905	0.721	0.439	1.45162173167007E-290	Ductular-like hepatocytes	HNRNPH2
1.01567623582221E-293	0.724639094918764	0.708	0.431	1.70207023599086E-289	Ductular-like hepatocytes	KCTD3
2.24183164279243E-293	0.49218996519284	0.669	0.383	3.75686146699155E-289	Ductular-like hepatocytes	IRAK1
1.00909055526463E-292	0.972520715906597	0.501	0.253	1.69103395251247E-288	Ductular-like hepatocytes	RAB11FIP1
2.04516861225788E-292	0.81359936784469	0.621	0.346	3.42729356042175E-288	Ductular-like hepatocytes	CD99L2
2.75305628246563E-292	0.707342686285355	0.691	0.429	4.6135717181559E-288	Ductular-like hepatocytes	DAP
3.51477215025491E-291	0.717097793824117	0.689	0.429	5.89005516939718E-287	Ductular-like hepatocytes	C2orf72
4.95567462169682E-289	1.55178937696496	0.314	0.116	8.30471953103953E-285	Ductular-like hepatocytes	TNFRSF11A
1.79400458380114E-288	1.13514289416239	0.478	0.239	3.00639288153395E-284	Ductular-like hepatocytes	IL10RB
1.52156755497213E-287	1.12515132326533	0.423	0.193	2.5498429086223E-283	Ductular-like hepatocytes	TJP3
4.38166238774063E-287	0.953423326541709	0.559	0.323	7.34278982937575E-283	Ductular-like hepatocytes	SERPINB1
9.54017380122487E-286	1.13975731829859	0.452	0.217	1.59874232560926E-281	Ductular-like hepatocytes	ABRACL
2.15480272862934E-285	0.777928679143251	0.607	0.363	3.61101841263704E-281	Ductular-like hepatocytes	PLOD3
2.4934162406953E-285	1.13697841608062	0.446	0.211	4.17846693615718E-281	Ductular-like hepatocytes	MCM2
5.08094014162322E-284	0.897126227602005	0.577	0.323	8.5146394893322E-280	Ductular-like hepatocytes	MGAT2
7.26854841716996E-284	0.771411145568227	0.681	0.421	1.21806334374934E-279	Ductular-like hepatocytes	AIFM1
9.82611657212806E-284	0.638561828561563	0.815	0.6	1.64666061515722E-279	Ductular-like hepatocytes	IDH1

9.28865700530473E-283	0.923149759044044	0.513	0.275	1.55659314094897E-278	Ductular-like hepatocytes	ETNK1
1.28200887990771E-282	0.775453980517574	0.655	0.403	2.14839048094935E-278	Ductular-like hepatocytes	GRN
2.09304092646934E-281	0.751198376706154	0.667	0.425	3.50751798457732E-277	Ductular-like hepatocytes	SDF4
2.76994105494489E-281	1.29127430587169	0.388	0.17	4.64186721987665E-277	Ductular-like hepatocytes	STXBP5
6.31819221287008E-281	0.760403644728241	0.717	0.482	1.05880265103277E-276	Ductular-like hepatocytes	MGST3
1.84054269580194E-280	0.992261072145781	0.508	0.261	3.08438144962488E-276	Ductular-like hepatocytes	LRRC31
1.82322990211195E-277	1.23744559288043	0.325	0.124	3.0553686699592E-273	Ductular-like hepatocytes	SLC45A3
2.53095709971785E-276	0.797778001838649	0.553	0.304	4.24137790770717E-272	Ductular-like hepatocytes	BIN1
3.23587981490328E-276	0.726025014633905	0.679	0.447	5.42268739381492E-272	Ductular-like hepatocytes	RRBP1
2.37216937329091E-275	0.759217151063375	0.698	0.452	3.9752814357609E-271	Ductular-like hepatocytes	TMEM205
5.72200457331487E-275	0.725846691659591	0.695	0.444	9.58893526396106E-271	Ductular-like hepatocytes	SAP18
6.91818804879597E-275	0.875510459360884	0.48	0.245	1.15934995321723E-270	Ductular-like hepatocytes	ACSS2
1.48251771871912E-274	1.00007052163072	0.457	0.221	2.4844031930295E-270	Ductular-like hepatocytes	MCM5
6.71368251171882E-274	0.499142146242356	0.944	0.826	1.12507891531384E-269	Ductular-like hepatocytes	ACTB
1.38142022049835E-273	0.8570805835801	0.618	0.368	2.31498400551114E-269	Ductular-like hepatocytes	STT3A
1.74930875618686E-273	1.25317382247608	0.393	0.176	2.93149161361794E-269	Ductular-like hepatocytes	DDR1
2.36431155405199E-273	0.969792407994788	0.44	0.209	3.96211330228033E-269	Ductular-like hepatocytes	CLIC1
1.15511669616853E-272	1.53148643892991	0.291	0.103	1.93574455943923E-268	Ductular-like hepatocytes	CLIC5
1.23641969759318E-272	1.18102655022121	0.38	0.165	2.07199212922665E-268	Ductular-like hepatocytes	MCM6
2.14482630656006E-272	0.914728154901338	0.491	0.251	3.59429992453334E-268	Ductular-like hepatocytes	SHROOM3
3.36726809560425E-272	1.6235287537917	0.266	0.088	5.6428678746136E-268	Ductular-like hepatocytes	FBLIM1
3.42498864987052E-272	0.801014946664867	0.637	0.401	5.73959597945301E-268	Ductular-like hepatocytes	CTSS
4.44192150789516E-272	0.734161767570201	0.688	0.449	7.44377206293071E-268	Ductular-like hepatocytes	MDK
9.30794875407679E-271	1.02008453911683	0.492	0.251	1.55982605220819E-266	Ductular-like hepatocytes	PFKFB3
1.14095132747999E-270	0.938255358083786	0.511	0.263	1.91200623459097E-266	Ductular-like hepatocytes	GABRE
2.41239392158805E-270	1.9413835399065	0.2	0.049	4.04268973379725E-266	Ductular-like hepatocytes	GUCY2C
8.70843126534983E-270	1.03962580411253	0.448	0.215	1.45935891144732E-265	Ductular-like hepatocytes	ARRB1
4.44225281144357E-269	1.5154036702819	0.294	0.107	7.44432726141713E-265	Ductular-like hepatocytes	CHST5
9.67753987047374E-267	0.650525084295713	0.73	0.487	1.62176213149399E-262	Ductular-like hepatocytes	ACTN4
1.13890618312834E-266	0.851927542348332	0.57	0.325	1.90857898168647E-262	Ductular-like hepatocytes	SLC44A1
3.40611226504664E-265	0.679728856661414	0.556	0.306	5.70796293376515E-261	Ductular-like hepatocytes	TLE5
4.39289837128701E-265	0.825628820514628	0.616	0.369	7.36161909060278E-261	Ductular-like hepatocytes	CCL15
1.77585348934062E-264	3.00245127958079	0.132	0.017	2.975975277437E-260	Ductular-like hepatocytes	PRDM16
1.81627740827616E-264	0.674824521547303	0.731	0.515	3.04371768078918E-260	Ductular-like hepatocytes	TSTA3
2.80915676059098E-263	0.836325900851907	0.554	0.299	4.70758489939837E-259	Ductular-like hepatocytes	SYBU
6.82875485842441E-263	0.998400680633055	0.473	0.24	1.14436273917476E-258	Ductular-like hepatocytes	NIPAL2
5.78434810077439E-262	0.967195811848618	0.485	0.247	9.69341054727772E-258	Ductular-like hepatocytes	PKP2
7.33049999530966E-262	0.871392747511152	0.413	0.189	1.22844518921399E-257	Ductular-like hepatocytes	EPS8L3
2.42531207283291E-261	3.3768607569195	0.139	0.021	4.06433797165339E-257	Ductular-like hepatocytes	GP2
3.14728230587079E-261	0.655015016705463	0.725	0.462	5.27421568817827E-257	Ductular-like hepatocytes	SERINC5
2.88966969520047E-260	1.04720613897845	0.427	0.205	4.84250847521694E-256	Ductular-like hepatocytes	BRI3BP
3.50484367776812E-260	0.853791433584758	0.594	0.35	5.87341703520381E-256	Ductular-like hepatocytes	SLC25A4
6.22145669215643E-260	0.702914391975166	0.695	0.445	1.04259171247157E-255	Ductular-like hepatocytes	NPC2
3.91575934217008E-258	1.26364642763072	0.332	0.134	6.56202950560862E-254	Ductular-like hepatocytes	DGKQ
1.03761756683615E-257	0.531818713207092	0.828	0.571	1.73883951850403E-253	Ductular-like hepatocytes	MT-ND2
8.60859255496574E-257	0.833267187130543	0.552	0.312	1.44262794036116E-252	Ductular-like hepatocytes	SYTL4
1.30950237875054E-256	0.948928479027655	0.465	0.233	2.19446408631015E-252	Ductular-like hepatocytes	SIPA1L3
2.0839453920534E-256	1.01769025398323	0.453	0.228	3.49227568800309E-252	Ductular-like hepatocytes	CANT1
3.1268553597741E-256	0.768544396449672	0.616	0.357	5.23998421190943E-252	Ductular-like hepatocytes	VBP1
3.50360271973957E-256	1.44997539572181	0.336	0.14	5.87133743773958E-252	Ductular-like hepatocytes	LAMB1
9.175100436435E-256	0.999177473264369	0.45	0.225	1.53756333113778E-251	Ductular-like hepatocytes	SELENON
8.0414086228273E-255	2.33665224718764	0.189	0.046	1.3475792570134E-250	Ductular-like hepatocytes	LRP4
9.12419587759515E-255	0.525681141938985	0.713	0.44	1.5290327451674E-250	Ductular-like hepatocytes	CALM3
2.61092305899292E-254	1.07461689098108	0.432	0.211	4.37538486226033E-250	Ductular-like hepatocytes	DEPRECATEI
2.72768274161841E-254	0.707115813291996	0.687	0.448	4.57105073840413E-250	Ductular-like hepatocytes	PCK2
3.05502718610505E-254	1.35892083966479	0.295	0.111	5.11961455847484E-250	Ductular-like hepatocytes	TEP1
8.34764810626122E-254	0.658718465239351	0.667	0.416	1.39889886964725E-249	Ductular-like hepatocytes	YWHAZ
2.43546145107182E-253	1.01039449461393	0.398	0.185	4.08134629970616E-249	Ductular-like hepatocytes	SLC37A1
3.71847867145139E-253	0.57496052098977	0.799	0.579	6.23142655761824E-249	Ductular-like hepatocytes	H3F3A
1.61415514497562E-252	0.967559109510164	0.513	0.281	2.70500119195014E-248	Ductular-like hepatocytes	TMEM109

2.21071264097804E-252	1.20723493206124	0.349	0.15	3.704712243751E-248	Ductular-like hepatocytes	LIMA1
4.96932778054121E-252	0.962165486448142	0.481	0.251	8.32759949463096E-248	Ductular-like hepatocytes	TRMT2B
5.07679446373462E-252	2.32647146066871	0.171	0.037	8.50769216232648E-248	Ductular-like hepatocytes	CALCR
5.74270847902299E-252	1.78217293735445	0.222	0.066	9.62363086914672E-248	Ductular-like hepatocytes	KIAA1211
1.05145409383295E-251	0.657621267414067	0.749	0.525	1.76202677044526E-247	Ductular-like hepatocytes	TMCO1
1.42259708824506E-251	0.852256878710328	0.543	0.298	2.38398820048108E-247	Ductular-like hepatocytes	FAM199X
1.68033070052122E-251	0.800663352511944	0.595	0.359	2.81589818793346E-247	Ductular-like hepatocytes	CPD
2.61592227583541E-251	0.988289893422885	0.508	0.279	4.38376254984497E-247	Ductular-like hepatocytes	TM9SF1
2.81097032575747E-251	1.78546044861536	0.207	0.057	4.71062407190437E-247	Ductular-like hepatocytes	CREB3L1
1.07986293947059E-250	1.36179732135521	0.268	0.093	1.80963431396481E-246	Ductular-like hepatocytes	GRAMD1B
1.09392828154946E-250	0.785835212803272	0.351	0.146	1.83320501422059E-246	Ductular-like hepatocytes	LAD1
1.2461352838317E-250	0.853607993771206	0.533	0.295	2.08827350864517E-246	Ductular-like hepatocytes	G3BP2
1.63978771216729E-250	1.54303843764043	0.297	0.115	2.74795624804994E-246	Ductular-like hepatocytes	SDCBP2
5.81663750789329E-250	2.09022999407553	0.198	0.053	9.74752113572757E-246	Ductular-like hepatocytes	SLC44A4
1.39966440588679E-249	0.869558001582216	0.481	0.255	2.34555761138508E-245	Ductular-like hepatocytes	CORO1B
3.90497312855725E-248	0.796504535464651	0.498	0.269	6.54395396883624E-244	Ductular-like hepatocytes	AGRN
8.12507479255684E-248	0.834242803059364	0.495	0.257	1.36160003373667E-243	Ductular-like hepatocytes	RNF157
1.56642401626687E-247	0.763786826825532	0.59	0.344	2.62501336646002E-243	Ductular-like hepatocytes	SLBP
2.08238000196616E-247	0.636421751988982	0.736	0.515	3.48965240729489E-243	Ductular-like hepatocytes	CTSD
2.41753196835116E-247	1.09741642892614	0.376	0.171	4.05130007256288E-243	Ductular-like hepatocytes	HOXA13
4.91434092392465E-247	1.56220216817034	0.248	0.083	8.23545252031292E-243	Ductular-like hepatocytes	EPB41L4A
9.1934184377766E-247	0.846097141262875	0.508	0.275	1.5406330618026E-242	Ductular-like hepatocytes	SLC51A
1.05234084179001E-246	0.677990321388593	0.578	0.314	1.7635127826717E-242	Ductular-like hepatocytes	ATAD2
1.70969870371275E-246	1.31771419893217	0.276	0.1	2.86511308768182E-242	Ductular-like hepatocytes	LGALS9
2.88300256834388E-246	1.34860850661523	0.311	0.124	4.83133570403068E-242	Ductular-like hepatocytes	SSTR1
7.88674625165665E-246	1.91069855192169	0.172	0.038	1.32166093685262E-241	Ductular-like hepatocytes	HTR1D
5.14255814996416E-245	1.5835250480272	0.245	0.081	8.61789894770993E-241	Ductular-like hepatocytes	KRT15
6.21682686016645E-245	1.20580163101459	0.303	0.118	1.04181584522669E-240	Ductular-like hepatocytes	C19orf33
7.14226609950618E-245	1.04946154869269	0.358	0.157	1.19690095295525E-240	Ductular-like hepatocytes	BAIAP2L2
7.82202333533553E-245	0.842605827431311	0.501	0.271	1.31081467053553E-240	Ductular-like hepatocytes	VASP
1.01500111543296E-244	1.3292211768874	0.304	0.12	1.70093886924255E-240	Ductular-like hepatocytes	CASZ1
4.11297386273879E-244	1.11158081768336	0.374	0.171	6.89252159917767E-240	Ductular-like hepatocytes	LRP10
8.09598103246401E-244	0.951077028656853	0.47	0.248	1.35672450142032E-239	Ductular-like hepatocytes	GFPT1
6.80396317489746E-243	1.0928834303574	0.396	0.188	1.14020814884932E-238	Ductular-like hepatocytes	GSKIP
2.0961017925085E-242	0.773457124006977	0.598	0.354	3.51264738388575E-238	Ductular-like hepatocytes	PDZD8
8.78628953788352E-242	1.08129175621042	0.336	0.142	1.47240640075852E-237	Ductular-like hepatocytes	EFNB1
2.32107447614729E-241	0.828441619412614	0.514	0.281	3.88965660712763E-237	Ductular-like hepatocytes	GPAT4
1.95265490091177E-240	1.08674651373391	0.427	0.216	3.27225908294794E-236	Ductular-like hepatocytes	MMP14
5.20830104375197E-240	1.26027271078757	0.339	0.146	8.72807088911956E-236	Ductular-like hepatocytes	VIL1
7.32487321233804E-240	0.666404654209014	0.657	0.414	1.22750225292361E-235	Ductular-like hepatocytes	ZBTB7B
1.11618788783094E-239	1.00097227484097	0.402	0.194	1.8705076624271E-235	Ductular-like hepatocytes	CERS6
2.05318522136425E-239	0.686401090939712	0.672	0.454	3.44072779396221E-235	Ductular-like hepatocytes	ENTPD5
9.99617648789834E-239	0.553953843932676	0.802	0.576	1.675159255842E-234	Ductular-like hepatocytes	MPC2
1.44451007635829E-238	0.955899626320488	0.408	0.194	2.42070998596123E-234	Ductular-like hepatocytes	CCNE2
1.77694400415054E-238	0.691330539021529	0.653	0.42	2.97780276215547E-234	Ductular-like hepatocytes	PSMB8
2.6268927184887E-238	1.41146196744313	0.302	0.121	4.40214681764336E-234	Ductular-like hepatocytes	PFN2
1.18559165183951E-237	0.866940686151132	0.494	0.268	1.98681449015265E-233	Ductular-like hepatocytes	ILVBL
9.78873773363034E-237	1.00033794190565	0.409	0.199	1.64039666940177E-232	Ductular-like hepatocytes	IGF2BP3
1.01632421234071E-236	0.859227628549004	0.468	0.247	1.70315611504057E-232	Ductular-like hepatocytes	PEPD
2.81486199183265E-236	0.520667362379343	0.814	0.571	4.71714572591316E-232	Ductular-like hepatocytes	MT-ND5
2.93457073530129E-236	0.703019155598168	0.408	0.192	4.9177536382179E-232	Ductular-like hepatocytes	ARPC1B
8.44333236417652E-236	0.916874307543677	0.505	0.276	1.4149336375887E-231	Ductular-like hepatocytes	SLC25A5
9.29591628026209E-236	0.756921762637129	0.563	0.32	1.55780965024632E-231	Ductular-like hepatocytes	XIAP
1.03649887305634E-235	1.56364044399558	0.275	0.104	1.73696481146781E-231	Ductular-like hepatocytes	DNM1
2.24841444234725E-235	0.763106204686051	0.569	0.339	3.76789292248552E-231	Ductular-like hepatocytes	RNPEP
4.00693895113712E-235	0.666272225870828	0.728	0.523	6.71482829431559E-231	Ductular-like hepatocytes	CDHR5
1.59509942415643E-234	1.1358982183052	0.369	0.17	2.67306761500135E-230	Ductular-like hepatocytes	SUSD6
6.84726254878826E-234	1.9749914444982	0.197	0.056	1.14746425792594E-229	Ductular-like hepatocytes	CTSE
1.63474701598151E-233	0.820422496600454	0.48	0.257	2.73950904938182E-229	Ductular-like hepatocytes	ADAM15
3.10879726086987E-233	1.01152938079943	0.423	0.209	5.20972244976573E-229	Ductular-like hepatocytes	MMGT1

1.39055005247452E-232	0.687427729415398	0.614	0.369	2.33028377793681E-228	Ductular-like hepatocytes	TCEAL4
4.51377925064925E-232	0.991720341451727	0.397	0.192	7.56419126823801E-228	Ductular-like hepatocytes	ATP8B1
4.54320579254854E-232	0.698799277843285	0.619	0.387	7.61350426715284E-228	Ductular-like hepatocytes	RTN4
6.10976596431596E-232	1.02726051497852	0.384	0.181	1.02387458030007E-227	Ductular-like hepatocytes	SAMD12
6.20982381174984E-232	0.604282822131564	0.651	0.373	1.04064227437304E-227	Ductular-like hepatocytes	OCRL
7.92505764628198E-232	1.25690173377398	0.346	0.154	1.32808116036393E-227	Ductular-like hepatocytes	PLPP2
9.46806200826341E-232	0.445808963088663	0.893	0.674	1.58665783134478E-227	Ductular-like hepatocytes	MT-ND3
2.18909996778896E-231	0.926430376037018	0.419	0.208	3.66849372602074E-227	Ductular-like hepatocytes	ZFYVE21
3.0813840849867E-231	0.887492691125686	0.469	0.251	5.16378344962071E-227	Ductular-like hepatocytes	HEBP1
4.26597104501667E-231	0.828190660703383	0.49	0.264	7.14891427723894E-227	Ductular-like hepatocytes	TMCO3
2.33393045128887E-230	1.06231502367953	0.364	0.166	3.91120065026989E-226	Ductular-like hepatocytes	MAPK3
2.29943986120909E-229	0.877806677978299	0.456	0.241	3.85340131941419E-225	Ductular-like hepatocytes	NCLN
6.28160074769428E-229	0.906121003449357	0.498	0.276	1.05267065329861E-224	Ductular-like hepatocytes	C1GALT1C1
1.18896418355977E-228	0.57193871778985	0.894	0.76	1.99246617880947E-224	Ductular-like hepatocytes	HMGCS2
1.82880150581627E-228	0.474131393051062	0.848	0.663	3.0647055634469E-224	Ductular-like hepatocytes	LAMP1
4.40943176551723E-228	0.902915901403509	0.492	0.27	7.38932575265377E-224	Ductular-like hepatocytes	CETN2
6.08489359193593E-228	0.4780677698309	0.446	0.216	1.01970646813662E-223	Ductular-like hepatocytes	ADGRE5
2.78163468487623E-227	0.74560175836051	0.523	0.294	4.66146340491559E-223	Ductular-like hepatocytes	POGK
3.8425690307675E-227	0.617167409339335	0.692	0.463	6.43937718176017E-223	Ductular-like hepatocytes	C6
4.43573762904668E-227	1.04768242433744	0.339	0.15	7.43340911875642E-223	Ductular-like hepatocytes	SLC26A6
4.9824983004442E-227	0.715717066794423	0.575	0.336	8.34967065188439E-223	Ductular-like hepatocytes	GPRC5C
2.48346260845936E-225	0.66653649960258	0.607	0.36	4.16178663925619E-221	Ductular-like hepatocytes	SMOC1
4.05460629268069E-225	0.843989969982018	0.476	0.253	6.7947092252743E-221	Ductular-like hepatocytes	HPRT1
5.52207071501621E-225	1.43809456938677	0.266	0.1	9.25388610422416E-221	Ductular-like hepatocytes	SLC2A1
1.20011844416325E-224	0.860040840028906	0.429	0.22	2.01115848872878E-220	Ductular-like hepatocytes	TSPO
5.93505241208828E-224	2.21866858537994	0.155	0.034	9.94596083217755E-220	Ductular-like hepatocytes	MYO1A
9.07962077617887E-224	0.809838545635265	0.513	0.291	1.52156284967205E-219	Ductular-like hepatocytes	TXNDC17
1.68443358659012E-223	0.675004401193058	0.607	0.354	2.82277380440772E-219	Ductular-like hepatocytes	IL6R
5.02933570458987E-223	0.768723034586597	0.459	0.244	8.42816077375171E-219	Ductular-like hepatocytes	FGD4
4.1186023230412E-222	1.02750771017528	0.35	0.158	6.90195377295245E-218	Ductular-like hepatocytes	LMO4
6.73196695453924E-222	0.793323154934552	0.485	0.265	1.12814302224169E-217	Ductular-like hepatocytes	SEPTIN8
1.06619620130849E-221	0.860258575782382	0.48	0.26	1.78673159415277E-217	Ductular-like hepatocytes	DKC1
1.44297325713884E-221	0.740176130696076	0.518	0.294	2.41813458431327E-217	Ductular-like hepatocytes	ZDHHC9
2.04055106332788E-221	0.739608855978264	0.583	0.352	3.41955547192486E-217	Ductular-like hepatocytes	DNAJC3
3.15660676029165E-221	0.825268134471123	0.493	0.271	5.28984160889674E-217	Ductular-like hepatocytes	CYP4F12
1.87199170281841E-220	1.06402263642132	0.357	0.165	3.13708369558308E-216	Ductular-like hepatocytes	ZFYVE28
4.94784603771852E-220	1.51738121183504	0.23	0.079	8.29160039000869E-216	Ductular-like hepatocytes	FSTL1
5.96444117407796E-220	0.795096906189774	0.526	0.302	9.99521051951984E-216	Ductular-like hepatocytes	SFT2D2
8.063882757939E-220	1.048808547436	0.423	0.216	1.35134547257542E-215	Ductular-like hepatocytes	FAM13A
6.9266100496261E-218	1.07163447616161	0.268	0.101	1.16076131211634E-213	Ductular-like hepatocytes	ST14
2.33900469892775E-217	1.50791263892145	0.22	0.073	3.91970407446313E-213	Ductular-like hepatocytes	LPCAT4
3.89122595668593E-217	0.714262995179488	0.578	0.35	6.52091645821427E-213	Ductular-like hepatocytes	LACTB2
5.20270101097055E-217	0.66952509236302	0.536	0.306	8.71868635418445E-213	Ductular-like hepatocytes	NAA10
5.95459415663113E-217	2.3106646360809	0.128	0.022	9.97870887768246E-213	Ductular-like hepatocytes	CDX2
7.26124634628485E-217	0.73799243004345	0.537	0.307	1.21683966271042E-212	Ductular-like hepatocytes	SMC1A
7.84822683414651E-217	0.468241542891489	0.87	0.666	1.31520585286627E-212	Ductular-like hepatocytes	GPX2
6.87807811288473E-216	0.627112440590922	0.624	0.387	1.15262833015722E-211	Ductular-like hepatocytes	STK24
8.1883940546836E-216	0.856503497103549	0.459	0.248	1.37221107568388E-211	Ductular-like hepatocytes	TDP2
3.08455609728233E-215	0.614757178701391	0.719	0.517	5.16909910782574E-211	Ductular-like hepatocytes	LSR
1.50451104066394E-214	0.476561764100483	0.867	0.707	2.52125960194462E-210	Ductular-like hepatocytes	KHK
2.04009937456703E-214	0.547570947691456	0.589	0.355	3.41879853189943E-210	Ductular-like hepatocytes	LASP1
5.03970241426654E-214	1.5091795678366	0.254	0.096	8.44553330582787E-210	Ductular-like hepatocytes	CLRN3
1.04343045588677E-213	0.732949828939341	0.526	0.301	1.74858075797505E-209	Ductular-like hepatocytes	PRKDC
1.04923528246312E-213	0.669296860410365	0.602	0.356	1.75830848635169E-209	Ductular-like hepatocytes	STAG2
4.52711841035503E-213	0.664816378461553	0.656	0.438	7.58654503207297E-209	Ductular-like hepatocytes	MGST2
1.30125544446144E-212	0.909355734781785	0.383	0.187	2.18064387382848E-208	Ductular-like hepatocytes	SLC38A1
1.88388585359571E-212	0.57927514064054	0.659	0.437	3.1570159134557E-208	Ductular-like hepatocytes	PLXNB2
2.75053156467188E-212	1.23933059642	0.317	0.139	4.60934079607713E-208	Ductular-like hepatocytes	BTNL3
4.46965889864219E-212	0.740135736660648	0.514	0.296	7.49025438234458E-208	Ductular-like hepatocytes	PAPSS2
1.17292275869208E-211	0.654772924603885	0.571	0.35	1.96558395901618E-207	Ductular-like hepatocytes	DBNL

1.191470441295E-211	1.08009572033227	0.342	0.157	1.99666616552216E-207	Ductular-like hepatocytes	NOTCH1
2.538376310315E-211	0.785835446837226	0.452	0.241	4.25381102082587E-207	Ductular-like hepatocytes	MICALL2
4.30461680809931E-211	1.14630054020578	0.275	0.109	7.21367684701282E-207	Ductular-like hepatocytes	MTMR11
5.44287433193894E-211	0.760443377931516	0.493	0.283	9.12116880546328E-207	Ductular-like hepatocytes	PSMB10
9.99684270568894E-211	0.622934674931385	0.657	0.421	1.67527090061935E-206	Ductular-like hepatocytes	CTSH
1.38502479410023E-210	1.00047382194174	0.359	0.17	2.32102454995317E-206	Ductular-like hepatocytes	PHLPP2
8.23541971102734E-210	1.24688796294774	0.252	0.095	1.38009163517396E-205	Ductular-like hepatocytes	TMEM171
3.10493118725869E-209	0.678471786554434	0.619	0.391	5.20324368360811E-205	Ductular-like hepatocytes	PRDX4
6.79339027656356E-209	0.696862396936378	0.569	0.345	1.13843634254652E-204	Ductular-like hepatocytes	SPSB1
7.04794338933568E-209	0.699528480110579	0.554	0.328	1.18109435318487E-204	Ductular-like hepatocytes	CDKN1A
8.59618022030474E-209	1.50120270367029	0.229	0.081	1.44054788131867E-204	Ductular-like hepatocytes	XKR9
8.92941750679309E-209	0.856304993092038	0.329	0.146	1.49639178578839E-204	Ductular-like hepatocytes	PLXNA2
1.5076442064588E-208	0.637038934460967	0.588	0.356	2.52651016118365E-204	Ductular-like hepatocytes	RAMP1
1.82514206100491E-208	0.869388132821618	0.473	0.262	3.05857306583203E-204	Ductular-like hepatocytes	VDAC1
5.26708280918837E-208	0.920543163474799	0.421	0.22	8.82657737163786E-204	Ductular-like hepatocytes	CAMK2D
8.76392020605191E-208	0.620530738718495	0.667	0.447	1.46865774813018E-203	Ductular-like hepatocytes	IFI30
1.46584130950463E-207	0.712172524303803	0.633	0.415	2.45645686646786E-203	Ductular-like hepatocytes	PRG4
2.46443802079046E-207	0.615076987441669	0.622	0.406	4.12990523524066E-203	Ductular-like hepatocytes	GRTP1
2.71031083472787E-207	2.96653509703893	0.1	0.012	4.54193889683696E-203	Ductular-like hepatocytes	EPHA8
8.30523312423572E-207	0.957103124749621	0.343	0.159	1.39179096695942E-202	Ductular-like hepatocytes	PBDC1
9.15498615314371E-207	1.12811636729496	0.331	0.151	1.53419257954382E-202	Ductular-like hepatocytes	GPD1L
1.44137738128132E-206	0.77462879196407	0.518	0.297	2.41546021555123E-202	Ductular-like hepatocytes	TMEM38B
2.76125405712403E-206	0.704863088337676	0.51	0.294	4.62730954892845E-202	Ductular-like hepatocytes	GPT
3.06619351314648E-206	1.56443833141013	0.248	0.094	5.13832708933088E-202	Ductular-like hepatocytes	SLC2A5
4.2666339358472E-206	0.568167781514619	0.713	0.461	7.15002514969274E-202	Ductular-like hepatocytes	SPTSSA
5.52400015456216E-206	1.07152507562251	0.344	0.161	9.25711945901528E-202	Ductular-like hepatocytes	PGK 1.00
6.41122046424655E-206	0.75022924172252	0.521	0.297	1.07439232539844E-201	Ductular-like hepatocytes	MCTS1
1.1129056301555E-205	0.582683652320165	0.674	0.45	1.86500725501459E-201	Ductular-like hepatocytes	COX6C
1.56745653603782E-205	0.531836681475378	0.665	0.436	2.62674366309218E-201	Ductular-like hepatocytes	CALM2
1.92566462727719E-205	0.727055792741229	0.536	0.314	3.22702878239112E-201	Ductular-like hepatocytes	ACMSD
2.81824824888729E-205	1.53738448686931	0.242	0.09	4.72282041548532E-201	Ductular-like hepatocytes	CHST6
6.18851381360841E-205	1.15728541511144	0.295	0.125	1.0370711448845E-200	Ductular-like hepatocytes	ASAP2
6.44584514583126E-205	0.713934042052871	0.468	0.251	1.0801947295384E-200	Ductular-like hepatocytes	MCM7
9.21176612837641E-205	1.07439818918421	0.333	0.153	1.54370776779332E-200	Ductular-like hepatocytes	CASP2
1.40159753011405E-204	0.809817830762825	0.406	0.207	2.34879714096513E-200	Ductular-like hepatocytes	PLEKHJ1
1.55054072595534E-204	0.64888073492648	0.609	0.38	2.59839614855596E-200	Ductular-like hepatocytes	FAM114A1
2.21519064103179E-204	0.712298683781452	0.545	0.334	3.71221647624107E-200	Ductular-like hepatocytes	RETSAT
8.08004321361285E-204	0.658748387466795	0.609	0.385	1.35405364173724E-199	Ductular-like hepatocytes	RTN3
1.73207456950739E-203	2.19465565038193	0.153	0.038	2.90261056358049E-199	Ductular-like hepatocytes	GCNT3
1.7803183251206E-203	0.806273358314256	0.474	0.266	2.9834574492371E-199	Ductular-like hepatocytes	PLS1
2.74850197707417E-203	0.807144708333708	0.452	0.241	4.6059396131809E-199	Ductular-like hepatocytes	DNMT1
5.09297787196433E-203	1.08934260454871	0.307	0.135	8.53481231783782E-199	Ductular-like hepatocytes	CYTH3
8.02535811973888E-203	0.589761229515194	0.665	0.442	1.34488951370584E-198	Ductular-like hepatocytes	TMEM37
1.42745280677548E-202	0.718107282945648	0.539	0.325	2.39212541359434E-198	Ductular-like hepatocytes	KDELR2
2.71642226545868E-202	0.747173480057294	0.353	0.165	4.55218043245566E-198	Ductular-like hepatocytes	MVP
3.67908211206653E-202	1.15225769671534	0.331	0.152	6.16540580340109E-198	Ductular-like hepatocytes	PAQR7
8.12545115010216E-202	0.830863963348959	0.48	0.269	1.36166310373412E-197	Ductular-like hepatocytes	CD47
1.02475672352774E-201	0.559362144311578	0.718	0.504	1.71728731728778E-197	Ductular-like hepatocytes	HNRNPF
1.07297191786993E-201	0.803242033328539	0.467	0.26	1.79808633996642E-197	Ductular-like hepatocytes	ELOVL1
1.13665227979236E-201	0.559275021567235	0.631	0.381	1.90480189047604E-197	Ductular-like hepatocytes	NOTCH2NLE
1.15077274662785E-201	0.655426270531579	0.588	0.362	1.92846496879895E-197	Ductular-like hepatocytes	ITPK1
1.65692620925654E-201	0.766310498476585	0.465	0.257	2.77667694147212E-197	Ductular-like hepatocytes	SLC35F6
5.26404961586403E-201	0.664663134894125	0.531	0.315	8.82149434626494E-197	Ductular-like hepatocytes	AMFR
6.18313190839258E-201	0.993326491519713	0.32	0.143	1.03616924520843E-196	Ductular-like hepatocytes	ABCC4
7.16772867548704E-201	0.776906814911907	0.445	0.239	1.20116797143812E-196	Ductular-like hepatocytes	GIPC1
2.58501533388612E-200	0.536357032551492	0.682	0.423	4.33196869652637E-196	Ductular-like hepatocytes	MORF4L2
3.29857673742747E-200	0.700233849421468	0.525	0.31	5.52775489658095E-196	Ductular-like hepatocytes	DGAT1
5.48254614248674E-200	0.92162549213004	0.372	0.184	9.18765082557928E-196	Ductular-like hepatocytes	CALM1
9.35515248269132E-200	1.03820808679766	0.351	0.167	1.56773645304941E-195	Ductular-like hepatocytes	SEMA6A
1.36861220794804E-199	0.873704129486578	0.386	0.195	2.29352033807932E-195	Ductular-like hepatocytes	HERC3

2.87737101443807E-199	1.51052165973413	0.221	0.078	4.82189834599532E-195	Ductular-like hepatocytes	FAM171A1
4.29535972390075E-199	1.23888719860193	0.281	0.118	7.19816382531288E-195	Ductular-like hepatocytes	TSPAN7
5.3001121399891E-199	1.27237075319016	0.308	0.139	8.88192792419373E-195	Ductular-like hepatocytes	SULF2
5.90989369636067E-199	0.737172720310442	0.479	0.27	9.9037998563612E-195	Ductular-like hepatocytes	PARP4
1.62160784209078E-198	0.741340652015289	0.475	0.267	2.71749042177573E-194	Ductular-like hepatocytes	CAPN5
2.67349261599019E-198	0.732515846659085	0.542	0.336	4.48023892587637E-194	Ductular-like hepatocytes	TMEM63A
3.5526137926182E-198	1.92169037565609	0.177	0.052	5.95347019366957E-194	Ductular-like hepatocytes	HPSE
4.11824970639086E-198	0.600144381618959	0.695	0.48	6.90136285796981E-194	Ductular-like hepatocytes	NDUFC2
5.38588132499996E-198	1.54633941887129	0.181	0.054	9.02565992443493E-194	Ductular-like hepatocytes	TLCD2
1.20722509501513E-197	1.45317555647733	0.192	0.06	2.02306781422635E-193	Ductular-like hepatocytes	ANKRD29
1.21339890939408E-197	0.756126251584235	0.491	0.28	2.0334138923626E-193	Ductular-like hepatocytes	DIAPH2
4.01750801918686E-197	0.635515282865903	0.622	0.409	6.73253993855335E-193	Ductular-like hepatocytes	IL13RA1
7.62190315252674E-197	0.522614239925094	0.707	0.464	1.27727853030043E-192	Ductular-like hepatocytes	ANXA2
9.79727614422104E-197	2.60847684242601	0.106	0.016	1.64182753624856E-192	Ductular-like hepatocytes	FAM83E
1.01489835273204E-196	0.753748280787153	0.483	0.273	1.70076665950834E-192	Ductular-like hepatocytes	FAM20B
1.07617615211276E-196	0.787369617115853	0.389	0.196	1.80345599571056E-192	Ductular-like hepatocytes	MAN2B1
4.79121554582714E-196	1.04087785895933	0.252	0.098	8.02911901169713E-192	Ductular-like hepatocytes	BTG2
7.14899363039493E-196	0.755173472681228	0.495	0.282	1.19802835258158E-191	Ductular-like hepatocytes	CUL4B
8.21606627334944E-196	1.46423521632196	0.243	0.094	1.3768483860879E-191	Ductular-like hepatocytes	ITM2C
2.32955376019116E-195	1.13592285152114	0.266	0.108	3.90386619132835E-191	Ductular-like hepatocytes	PLXNA3
2.83647001151471E-195	0.516933176682885	0.72	0.503	4.75335644529635E-191	Ductular-like hepatocytes	BLVRB
6.312697428872E-195	0.805982971225133	0.433	0.232	1.05788183513037E-190	Ductular-like hepatocytes	NTPCR
9.68317394448333E-195	0.862568180719352	0.395	0.204	1.62270628961652E-190	Ductular-like hepatocytes	SKAP2
1.54782903479624E-194	0.600638216230862	0.546	0.327	2.59385189651154E-190	Ductular-like hepatocytes	AGFG2
1.75001670452004E-194	1.27974958633206	0.234	0.087	2.93267799343468E-190	Ductular-like hepatocytes	SLC44A3
2.06467235053478E-194	0.573456051995816	0.582	0.347	3.45997792502618E-190	Ductular-like hepatocytes	RAP2C
3.91673589860775E-194	1.1870420327939	0.302	0.134	6.56366601888687E-190	Ductular-like hepatocytes	EDARADD
4.18033043885253E-194	0.576611078258375	0.626	0.411	7.00539774942908E-190	Ductular-like hepatocytes	SYNGR2
5.28906531692801E-194	0.693125107566054	0.488	0.276	8.86341565810796E-190	Ductular-like hepatocytes	PAXX
6.08523184563762E-194	0.709800263760801	0.595	0.377	1.01976315269195E-189	Ductular-like hepatocytes	CPQ
6.57545571241693E-194	0.563796858561133	0.538	0.317	1.10191486828683E-189	Ductular-like hepatocytes	IMPA2
9.78753725757402E-194	0.657921501889367	0.523	0.31	1.64019549362425E-189	Ductular-like hepatocytes	AGPAT1
1.08008992663769E-193	0.976412181702278	0.381	0.194	1.81001469905945E-189	Ductular-like hepatocytes	RASL11A
1.98044127016243E-193	0.518201250773034	0.772	0.528	3.31882348053821E-189	Ductular-like hepatocytes	BEX3
2.18362355514541E-193	0.818317887344999	0.422	0.224	3.65931635371267E-189	Ductular-like hepatocytes	LIME1
3.26780960745628E-193	0.512298766891822	0.718	0.482	5.47619534017523E-189	Ductular-like hepatocytes	EMD
4.42655546419446E-193	0.577311711570036	0.637	0.406	7.41802164689708E-189	Ductular-like hepatocytes	ZBTB16
9.41896834431842E-193	0.88761655016744	0.265	0.107	1.57843071514088E-188	Ductular-like hepatocytes	MROH6
3.22361910887362E-192	0.653291431534306	0.541	0.315	5.40214090265042E-188	Ductular-like hepatocytes	THOC2
3.38589909263987E-192	1.01936332832801	0.411	0.222	5.6740896994459E-188	Ductular-like hepatocytes	FADS2
4.06157287949559E-192	0.910786257314752	0.382	0.194	6.80638383145887E-188	Ductular-like hepatocytes	TMEM185A
5.52239849745105E-192	2.49947112003452	0.11	0.018	9.25443540202847E-188	Ductular-like hepatocytes	PLEKHS1
9.75369831230547E-192	0.515094017962819	0.741	0.512	1.63452476317615E-187	Ductular-like hepatocytes	SCCPDH
9.78016864235172E-192	0.525598629046094	0.565	0.346	1.63896066108853E-187	Ductular-like hepatocytes	KIAA1522
2.73010251866363E-191	0.72397052251254	0.444	0.243	4.57510580077651E-187	Ductular-like hepatocytes	GPR108
9.54249597367374E-191	1.12418721431949	0.315	0.145	1.59913147526825E-186	Ductular-like hepatocytes	TMEM98
1.56847593669216E-190	1.64459156004408	0.176	0.053	2.62845197470872E-186	Ductular-like hepatocytes	IQSEC2
1.70523423257791E-190	0.588982348703236	0.674	0.441	2.85763152695407E-186	Ductular-like hepatocytes	PLS3
2.97403909652235E-190	0.730645237390248	0.511	0.303	4.98389471795215E-186	Ductular-like hepatocytes	SRI
3.01191140782203E-190	0.824841538891774	0.464	0.26	5.04736113722816E-186	Ductular-like hepatocytes	DEPRECATEI
3.39663657424402E-190	1.45201997921238	0.195	0.064	5.69208357111813E-186	Ductular-like hepatocytes	HID1
3.57773446901935E-190	0.708787324494826	0.529	0.317	5.99556742318263E-186	Ductular-like hepatocytes	CYP4F2
3.60283898004452E-190	1.08321970022734	0.341	0.165	6.0376375627586E-186	Ductular-like hepatocytes	PCNA
4.20274078011138E-190	1.29674855676269	0.268	0.111	7.04295299931065E-186	Ductular-like hepatocytes	PLXNC1
1.01368277719751E-189	0.701028950166136	0.524	0.314	1.69872959802758E-185	Ductular-like hepatocytes	PVR
1.09397886640308E-189	0.671398525536377	0.512	0.293	1.83328978431828E-185	Ductular-like hepatocytes	PSMD10
2.66532338596546E-189	1.17323374558554	0.247	0.097	4.46654893020092E-185	Ductular-like hepatocytes	PLAAT2
5.27658595071949E-189	0.67496028483533	0.56	0.34	8.84250273621572E-185	Ductular-like hepatocytes	RNF103
6.11421924675216E-189	0.882942167560797	0.342	0.164	1.02462086137073E-184	Ductular-like hepatocytes	TAX1BP3
6.11425439194192E-189	0.731345726557697	0.502	0.287	1.02462675100163E-184	Ductular-like hepatocytes	NID1

1.02152965227184E-188	0.742946969849457	0.482	0.279	1.71187939127715E-184	Ductular-like hepatocytes	CAPNS1
3.83345165142087E-188	0.808203106767279	0.428	0.229	6.4240982774511E-184	Ductular-like hepatocytes	TFDP1
4.27144636081293E-188	2.51270681849879	0.109	0.018	7.15808981145032E-184	Ductular-like hepatocytes	ACE2
6.34153498644311E-188	1.02135204157817	0.287	0.124	1.06271443302814E-183	Ductular-like hepatocytes	BAK1
6.57245563393752E-188	0.632224875995408	0.446	0.245	1.10141211513525E-183	Ductular-like hepatocytes	STXBP2
7.48983364512666E-188	0.951570177140557	0.354	0.174	1.25514632225032E-183	Ductular-like hepatocytes	NR2F1
8.05887611379569E-188	0.603892436647285	0.622	0.393	1.35050645914988E-183	Ductular-like hepatocytes	AFF1
1.38852492714112E-187	1.14030090714419	0.199	0.066	2.32689007290309E-183	Ductular-like hepatocytes	TMEM54
2.93111928144344E-187	0.72018842867767	0.495	0.286	4.91196969184292E-183	Ductular-like hepatocytes	RTL8C
3.09583882276985E-187	0.668779980732619	0.548	0.333	5.18800669919772E-183	Ductular-like hepatocytes	ACSL3
4.60064940837084E-187	1.10834852675456	0.323	0.151	7.70976827854785E-183	Ductular-like hepatocytes	SLC7A1
5.02953832796502E-187	0.940930892592645	0.338	0.163	8.42850033000379E-183	Ductular-like hepatocytes	CA2
8.70231293887587E-187	0.881505375818328	0.226	0.083	1.45833360229682E-182	Ductular-like hepatocytes	FUT6
2.07737029097577E-186	1.87787884435645	0.139	0.033	3.48125713361719E-182	Ductular-like hepatocytes	CTBP2
2.08233619796898E-186	1.03114551849492	0.34	0.165	3.48957900055641E-182	Ductular-like hepatocytes	LAMA3
5.55457478444237E-186	1.03840432931237	0.317	0.148	9.30835642376852E-182	Ductular-like hepatocytes	CES3
1.67436748859558E-185	1.04561760070024	0.319	0.149	2.80590503738848E-181	Ductular-like hepatocytes	UNG
3.74582934769667E-185	0.633976413077176	0.596	0.387	6.27726082087008E-181	Ductular-like hepatocytes	PERP
4.70591662760559E-185	0.704658050446213	0.449	0.248	7.88617508454145E-181	Ductular-like hepatocytes	RASSF3
1.39950455964101E-184	1.63544833792424	0.178	0.055	2.3452897410464E-180	Ductular-like hepatocytes	EPHB6
1.54163509951804E-184	0.657596837379638	0.596	0.369	2.58347209977234E-180	Ductular-like hepatocytes	HACD3
2.07801087639219E-184	0.640938916430274	0.494	0.289	3.48233062665803E-180	Ductular-like hepatocytes	ESYT2
8.6712700628863E-184	0.913477631470013	0.402	0.215	1.45313143713849E-179	Ductular-like hepatocytes	EPPK1
1.00592081852569E-183	0.486360713843783	0.752	0.554	1.68572210768536E-179	Ductular-like hepatocytes	TAPBP
1.29546470773409E-183	0.655632680922142	0.518	0.306	2.17093975722078E-179	Ductular-like hepatocytes	GRB10
1.29835440632514E-183	1.58973024054888	0.204	0.072	2.17578231411968E-179	Ductular-like hepatocytes	C6orf141
1.32638146228155E-183	0.606715998377837	0.641	0.429	2.22275005449142E-179	Ductular-like hepatocytes	ANKRD10
1.68688967950911E-183	1.01196238901469	0.292	0.129	2.82688972492136E-179	Ductular-like hepatocytes	TMEM8B
2.40242786335495E-183	0.469890689634552	0.928	0.764	4.02598861341023E-179	Ductular-like hepatocytes	MGST1
2.95779825479208E-183	0.572932292320737	0.597	0.391	4.95667831538056E-179	Ductular-like hepatocytes	CLINT1
3.15175689413462E-183	0.755367815784491	0.508	0.307	5.2817142031908E-179	Ductular-like hepatocytes	RDH10
4.08509398204997E-183	1.18157905761222	0.237	0.093	6.84580049511933E-179	Ductular-like hepatocytes	HSPG2
5.05242210810715E-183	0.537297042406157	0.693	0.496	8.46684896876597E-179	Ductular-like hepatocytes	HABP2
7.1510780097344E-183	1.11023248239563	0.301	0.137	1.19837765287129E-178	Ductular-like hepatocytes	SLC44A2
2.08391891729523E-182	1.19116602135318	0.242	0.096	3.49223132160335E-178	Ductular-like hepatocytes	GLTP
2.44901086462574E-182	0.627169930509797	0.545	0.335	4.10405240693981E-178	Ductular-like hepatocytes	FAT1
2.76409284762618E-182	0.754916763207506	0.414	0.222	4.63206679405195E-178	Ductular-like hepatocytes	SH3RF1
5.55225518079044E-182	0.82306555814722	0.384	0.2	9.30446923196862E-178	Ductular-like hepatocytes	TPM4
5.65598370205707E-182	0.517872873924144	0.551	0.334	9.47829748790723E-178	Ductular-like hepatocytes	AKT1
5.81479771235131E-182	1.78509286432766	0.173	0.053	9.74443800635833E-178	Ductular-like hepatocytes	DEPRECATEI
9.63928895232303E-182	0.966094897631014	0.349	0.173	1.61535204263029E-177	Ductular-like hepatocytes	TM2D2
1.48004074003304E-181	0.846158389220051	0.351	0.174	2.48025227214737E-177	Ductular-like hepatocytes	SIPA1L1
1.52643193267048E-181	0.629065279577157	0.515	0.302	2.55799463276919E-177	Ductular-like hepatocytes	FAM122B
2.07624587470616E-181	1.11511914582638	0.286	0.127	3.47937283683258E-177	Ductular-like hepatocytes	NCEH1
3.45806435504522E-181	0.534280251937295	0.636	0.411	5.79502424618478E-177	Ductular-like hepatocytes	EGLN1
4.24745033801011E-181	0.544101908688002	0.531	0.318	7.11787727643735E-177	Ductular-like hepatocytes	MTCH1
5.95443246702765E-181	0.70980723594465	0.474	0.27	9.97843792824494E-177	Ductular-like hepatocytes	PACSIN2
8.18070687199918E-181	0.741849485504895	0.378	0.194	1.37092285760962E-176	Ductular-like hepatocytes	SGCE
9.45911989855634E-181	1.17471114100165	0.174	0.053	1.58515931260007E-176	Ductular-like hepatocytes	GLIS3
1.04749774146828E-180	0.493979276178016	0.724	0.52	1.75539671515254E-176	Ductular-like hepatocytes	PFN1
1.11385277509892E-180	2.41427884378286	0.112	0.021	1.86659448051078E-176	Ductular-like hepatocytes	EDN2
1.88280138306426E-180	0.62996784396286	0.54	0.329	3.15519855773908E-176	Ductular-like hepatocytes	SLC17A4
4.1500709223792E-180	1.23098898300254	0.251	0.103	6.95468885172307E-176	Ductular-like hepatocytes	PRKCD
4.77689070280635E-180	0.941228213692252	0.379	0.196	8.00511343976289E-176	Ductular-like hepatocytes	USP54
6.67638905940698E-180	0.735273128913611	0.318	0.148	1.11882927857542E-175	Ductular-like hepatocytes	URGCP
8.7315442654487E-180	1.06827820583741	0.263	0.111	1.46323218800389E-175	Ductular-like hepatocytes	UNC93B1
1.17139337746606E-179	0.670739027879744	0.513	0.302	1.96302102195762E-175	Ductular-like hepatocytes	PRPS1
2.09651262087028E-179	0.865275558904355	0.378	0.195	3.51333585005441E-175	Ductular-like hepatocytes	YWHAB
2.28545993874112E-179	0.581550628694067	0.655	0.455	3.82997376534236E-175	Ductular-like hepatocytes	TPM1
8.30344232394079E-179	0.900356592676325	0.306	0.142	1.391490864646E-174	Ductular-like hepatocytes	DEPRECATEI

1.13339207821211E-178	0.7061903511582	0.487	0.282	1.89933844466786E-174	Ductular-like hepatocytes	ATP6V1A
3.99086440754076E-178	0.488276527988597	0.856	0.684	6.6878905741568E-174	Ductular-like hepatocytes	SERPINA6
4.72977573151587E-178	2.08990318206028	0.138	0.035	7.92615817087429E-174	Ductular-like hepatocytes	NOX1
5.4573183434367E-178	0.782992951187038	0.45	0.251	9.14537407993122E-174	Ductular-like hepatocytes	SOS 2
5.56386745732961E-178	1.24324779843628	0.236	0.094	9.32392908499296E-174	Ductular-like hepatocytes	ABR
5.99269288310448E-178	0.822823893170548	0.278	0.12	1.00425547335065E-173	Ductular-like hepatocytes	PIK3C2B
6.35531516239175E-178	0.627762492250923	0.548	0.344	1.06502371491361E-173	Ductular-like hepatocytes	PIGT
1.44111159202535E-177	0.551505814298324	0.572	0.37	2.41501480591608E-173	Ductular-like hepatocytes	ACSL5
1.62923944769004E-177	1.90833868259814	0.144	0.038	2.73027946643897E-173	Ductular-like hepatocytes	ANK2
2.31985934516372E-177	0.882664377352346	0.336	0.164	3.88762029062535E-173	Ductular-like hepatocytes	BAMBI
2.33867415729407E-177	0.504212892612462	0.698	0.491	3.91915015279341E-173	Ductular-like hepatocytes	CYB5R3
2.50184190026057E-177	2.54961879051957	0.117	0.024	4.19258665645667E-173	Ductular-like hepatocytes	SLC26A3
2.98965206164816E-177	0.740113125512074	0.443	0.249	5.01005892490998E-173	Ductular-like hepatocytes	SNRPB
3.34736490386994E-177	0.712572746339545	0.477	0.28	5.60951410590525E-173	Ductular-like hepatocytes	CD164
4.92371857144216E-177	1.00970063058743	0.351	0.177	8.25116758202277E-173	Ductular-like hepatocytes	SLC2A13
9.09027732764194E-177	0.683527552373186	0.45	0.254	1.52334867456624E-172	Ductular-like hepatocytes	CRAT
1.20873109573757E-176	0.970917998255165	0.302	0.139	2.02559157023703E-172	Ductular-like hepatocytes	CPLX1
1.27308025882948E-176	0.696753554967295	0.375	0.189	2.13342789774645E-172	Ductular-like hepatocytes	TCF19
4.11953870322385E-176	1.12975692719042	0.257	0.108	6.90352295886252E-172	Ductular-like hepatocytes	CLIP2
6.85561985044636E-176	0.690921809701925	0.515	0.316	1.1488647745378E-171	Ductular-like hepatocytes	GALK1
7.3368288398215E-176	0.551301152033982	0.666	0.46	1.22950577697729E-171	Ductular-like hepatocytes	CD63
4.88162241327697E-175	0.488123530449027	0.764	0.564	8.18062284016954E-171	Ductular-like hepatocytes	RARRES2
4.9851775630149E-175	0.992782886987362	0.291	0.132	8.35416056010037E-171	Ductular-like hepatocytes	TMEM168
5.23104611067983E-175	0.86952754113914	0.375	0.198	8.76618707227726E-171	Ductular-like hepatocytes	SERPINB6
5.57772045647634E-175	0.584144269648487	0.55	0.336	9.34714394096306E-171	Ductular-like hepatocytes	MLEC
6.80026433571311E-175	0.956082910786954	0.31	0.146	1.1395882973788E-170	Ductular-like hepatocytes	TMEM181
1.05465739166417E-174	0.874483261907839	0.311	0.146	1.76739485695081E-170	Ductular-like hepatocytes	TRIM56
1.07429219130797E-174	0.550246145315817	0.576	0.352	1.8002988541939E-170	Ductular-like hepatocytes	NR1I2
1.64604075185217E-174	0.694173141795462	0.447	0.251	2.75843509195386E-170	Ductular-like hepatocytes	MYO19
1.82313088985423E-174	1.02935901942861	0.263	0.112	3.05520274521772E-170	Ductular-like hepatocytes	GBP3
1.93737116262152E-174	0.78392827061897	0.347	0.174	3.24664659432115E-170	Ductular-like hepatocytes	ZNF91
2.82848968530216E-174	0.788763673685557	0.457	0.264	4.73998301462936E-170	Ductular-like hepatocytes	NEU1
3.27849265322158E-174	0.560030869943437	0.592	0.378	5.49409798826872E-170	Ductular-like hepatocytes	UBE2Q1
5.58795581647577E-174	0.686139082911601	0.527	0.316	9.3642963572501E-170	Ductular-like hepatocytes	NSDHL
8.09004141612533E-174	0.668500794347692	0.469	0.272	1.35572914051428E-169	Ductular-like hepatocytes	RABAC1
8.70071867387732E-174	0.593079037345157	0.434	0.24	1.45806643536836E-169	Ductular-like hepatocytes	PIK3R2
9.4568284488272E-174	0.94214050987611	0.281	0.125	1.58477531145446E-169	Ductular-like hepatocytes	ITPKA
5.7192978371078E-173	0.572200981905473	0.536	0.334	9.58439931542525E-169	Ductular-like hepatocytes	MMP24OS
1.58744971796524E-172	0.647401563037216	0.478	0.273	2.66024823736616E-168	Ductular-like hepatocytes	PYGO2
1.9095950627843E-172	0.936805543951368	0.306	0.144	3.20009940621393E-168	Ductular-like hepatocytes	VSIG10
2.4122776787578E-172	0.730085553801612	0.422	0.234	4.04249493406232E-168	Ductular-like hepatocytes	RALGAPA2
3.03964019869079E-172	0.719936933739968	0.501	0.305	5.09382904496602E-168	Ductular-like hepatocytes	FUCA1
4.39243673738185E-172	0.668877188061267	0.52	0.318	7.3608454845045E-168	Ductular-like hepatocytes	ATP6V0E1
4.46061966537799E-172	0.683812431244077	0.501	0.298	7.47510643524043E-168	Ductular-like hepatocytes	TAZ
8.0966209460507E-172	0.673822830199713	0.503	0.301	1.35683173813918E-167	Ductular-like hepatocytes	INTS8
1.38771890999996E-171	0.470055874874038	0.746	0.542	2.32553934937793E-167	Ductular-like hepatocytes	CYC1
1.61635426839199E-171	0.823665230756792	0.353	0.18	2.70868648297129E-167	Ductular-like hepatocytes	PYCARD
1.97981212260896E-171	1.35329640537907	0.228	0.091	3.3177691550681E-167	Ductular-like hepatocytes	NAAA
2.43628514509459E-171	2.50485627040688	0.119	0.026	4.08272664614952E-167	Ductular-like hepatocytes	CDH26
3.7503361917341E-171	0.579886002049705	0.55	0.338	6.284813390108E-167	Ductular-like hepatocytes	ERBIN
1.46902403704395E-170	0.424684451484358	0.79	0.613	2.46179048127826E-166	Ductular-like hepatocytes	CST3
1.64321669079651E-170	0.677822155199847	0.494	0.291	2.75370253043679E-166	Ductular-like hepatocytes	UBL3
2.44804381934193E-170	0.470938783718273	0.668	0.472	4.10243183245321E-166	Ductular-like hepatocytes	CD151
3.79672250698317E-170	1.7118374075902	0.177	0.059	6.3625475772024E-166	Ductular-like hepatocytes	LRRC26
4.04776509231647E-170	0.553560214555567	0.564	0.347	6.78324474170394E-166	Ductular-like hepatocytes	HNRNPUL1
5.22405960922788E-170	0.584067327030399	0.589	0.376	8.75447909314409E-166	Ductular-like hepatocytes	ZNF706
8.79788370967134E-170	0.806490450793553	0.352	0.18	1.47434935206672E-165	Ductular-like hepatocytes	BCAT2
9.87320331915992E-170	0.582857130427601	0.524	0.322	1.65455141222482E-165	Ductular-like hepatocytes	STAP2
3.62724281839245E-169	1.05414415033354	0.272	0.12	6.07853351506206E-165	Ductular-like hepatocytes	MFSD10
3.79574481144127E-169	2.33672375271635	0.127	0.031	6.36090915501328E-165	Ductular-like hepatocytes	MME

3.96783715533527E-169	1.13274228541614	0.268	0.118	6.64930150491084E-165	Ductular-like hepatocytes	CDKL1
4.03367054568731E-169	0.661562533308115	0.452	0.259	6.7596251004628E-165	Ductular-like hepatocytes	TCIRG1
4.37564057406932E-169	0.566445949027852	0.599	0.388	7.33269847402537E-165	Ductular-like hepatocytes	HNRNPU
4.7263740512175E-169	1.67281303162204	0.178	0.06	7.92045763503029E-165	Ductular-like hepatocytes	CCDC3
4.8766091256176E-169	0.613092335037113	0.465	0.264	8.17222157270997E-165	Ductular-like hepatocytes	ASB13
5.05533749118136E-169	0.524651514762087	0.486	0.288	8.47173456772173E-165	Ductular-like hepatocytes	CLTB
5.43177742312985E-169	0.614979918259655	0.41	0.225	9.102572605681E-165	Ductular-like hepatocytes	PTPRH
5.76620798682027E-169	0.550879050849358	0.38	0.199	9.66301134431341E-165	Ductular-like hepatocytes	DTX4
5.8763674265416E-169	0.579115029675482	0.504	0.302	9.84761653339841E-165	Ductular-like hepatocytes	SH3RF2
8.99719081037326E-169	0.532386969684978	0.671	0.467	1.50774923600235E-164	Ductular-like hepatocytes	ARF1
1.10037957078064E-168	0.735380634570364	0.416	0.229	1.84401608471419E-164	Ductular-like hepatocytes	CPM
1.92151894119427E-168	0.721343282522788	0.396	0.212	3.22008144165336E-164	Ductular-like hepatocytes	ATP11A
2.10211227593263E-168	0.640040198248104	0.484	0.286	3.5227197520079E-164	Ductular-like hepatocytes	TRAPPC4
3.15285609415088E-168	0.930450174815444	0.334	0.166	5.28355624257805E-164	Ductular-like hepatocytes	MAP3K21
3.77014826490754E-168	0.789857790149359	0.393	0.213	6.31801446233205E-164	Ductular-like hepatocytes	LRP11
4.45811551550722E-168	0.771706837816836	0.423	0.234	7.47090998088701E-164	Ductular-like hepatocytes	TBC1D8B
4.57570977222828E-168	0.512176346473497	0.562	0.353	7.66797443630016E-164	Ductular-like hepatocytes	PGD
5.18342701891176E-168	0.555752242406061	0.647	0.434	8.68638699829232E-164	Ductular-like hepatocytes	RAB2A
8.29988540413693E-168	0.650144410606087	0.48	0.278	1.39089479602527E-163	Ductular-like hepatocytes	TAF7
2.58563293664402E-167	1.62789431824331	0.18	0.061	4.33300367522804E-163	Ductular-like hepatocytes	MYB
2.68168035382217E-167	0.454552342931105	0.648	0.433	4.49395993693519E-163	Ductular-like hepatocytes	P4HB
2.93854297968717E-167	0.717392378134408	0.419	0.232	4.92441032535976E-163	Ductular-like hepatocytes	MOCOS
3.22360855136735E-167	0.731876739627457	0.434	0.244	5.40212321038141E-163	Ductular-like hepatocytes	PRKAB1
7.81714790657882E-167	0.537464663027043	0.639	0.437	1.30997764618448E-162	Ductular-like hepatocytes	OTC
8.73245311752423E-167	0.741272728965842	0.436	0.245	1.46338449343471E-162	Ductular-like hepatocytes	TCEAL1
8.89166198263337E-167	1.05232413457531	0.298	0.14	1.4900647150497E-162	Ductular-like hepatocytes	ARL4A
1.03723011709493E-166	0.73861756114585	0.431	0.241	1.73819023022768E-162	Ductular-like hepatocytes	PIEZO2
1.06322864760718E-166	0.717920883612327	0.452	0.263	1.78175856766011E-162	Ductular-like hepatocytes	OCIAD2
1.75567976268318E-166	0.6660133532333	0.463	0.268	2.94216814630447E-162	Ductular-like hepatocytes	SSRP1
2.42646626080928E-166	0.498792957241911	0.698	0.486	4.06627215986419E-162	Ductular-like hepatocytes	CNN3
3.16465189328748E-166	2.11650014397676	0.113	0.024	5.30332364277116E-162	Ductular-like hepatocytes	PNCK
3.28631430804908E-166	2.67212481102725	0.1	0.018	5.50720551742864E-162	Ductular-like hepatocytes	AGR3
3.41032052131581E-166	0.702244828980847	0.408	0.223	5.71501512962104E-162	Ductular-like hepatocytes	GNA11
4.79591546276794E-166	0.566158418901441	0.594	0.392	8.03699513250652E-162	Ductular-like hepatocytes	DSTN
8.76131564731104E-166	0.71459027844571	0.443	0.255	1.46822127617638E-161	Ductular-like hepatocytes	LMBR1
8.98525650361864E-166	0.712261967123317	0.298	0.138	1.50574928487641E-161	Ductular-like hepatocytes	MMP15
9.40824350423086E-166	0.749518703309002	0.433	0.244	1.57663344643901E-161	Ductular-like hepatocytes	ABCB6
1.73890045089293E-165	0.723581445962572	0.486	0.291	2.91404937560637E-161	Ductular-like hepatocytes	TAP1
1.93912553419283E-165	0.678186770261187	0.403	0.22	3.24958657020034E-161	Ductular-like hepatocytes	NCKIPSD
2.07127253737534E-165	1.15893411479588	0.363	0.196	3.4710385181336E-161	Ductular-like hepatocytes	DUOX2
2.7422599070863E-165	1.2032519467287	0.217	0.085	4.59547915229523E-161	Ductular-like hepatocytes	GALNT4
3.44497437688697E-165	0.572005990668565	0.511	0.311	5.77308806078719E-161	Ductular-like hepatocytes	CRTAP
3.57131585637397E-165	0.475019590756309	0.619	0.406	5.98481111211149E-161	Ductular-like hepatocytes	GSDMD
4.62076506790489E-165	0.574031110484119	0.397	0.211	7.74347810079502E-161	Ductular-like hepatocytes	HELLS
5.26242653407813E-165	1.15144938391162	0.261	0.116	8.81877438580814E-161	Ductular-like hepatocytes	TMCC3
6.99743216480922E-165	0.606509415401984	0.557	0.36	1.17262968217873E-160	Ductular-like hepatocytes	CHMP2A
7.97467770437516E-165	0.822263719547365	0.217	0.083	1.33639648969919E-160	Ductular-like hepatocytes	NTN4
2.55419632100247E-164	0.595283355496287	0.477	0.277	4.28032219473594E-160	Ductular-like hepatocytes	SCRIB
3.53775740735581E-164	0.644402763129596	0.54	0.346	5.92857386324686E-160	Ductular-like hepatocytes	MLPH
3.61020376048911E-164	0.535156619155182	0.603	0.387	6.04997946182764E-160	Ductular-like hepatocytes	WWP1
4.27505931025772E-164	0.704738272670717	0.444	0.253	7.16414439212988E-160	Ductular-like hepatocytes	BDH2
5.02260199520668E-164	0.761065963817236	0.411	0.228	8.41687642356736E-160	Ductular-like hepatocytes	MYLK
1.22073466736634E-163	0.865766798337323	0.349	0.179	2.04570715557251E-159	Ductular-like hepatocytes	FBXO21
1.37766630823612E-163	0.707900022761737	0.445	0.257	2.30869319934209E-159	Ductular-like hepatocytes	DNASE2
1.7614013522893E-163	0.74019030193115	0.386	0.207	2.95175638616641E-159	Ductular-like hepatocytes	ARPC4
2.23678712486242E-163	0.568205606765374	0.548	0.346	3.74840786384445E-159	Ductular-like hepatocytes	EIF2AK1
2.51469640030346E-163	1.7706022902428	0.136	0.037	4.21412822762853E-159	Ductular-like hepatocytes	HTR4
3.28601595647494E-163	0.846084917085672	0.404	0.223	5.50670553986071E-159	Ductular-like hepatocytes	SLC35B2
3.6012908488065E-163	0.780340350453937	0.408	0.226	6.03504320442994E-159	Ductular-like hepatocytes	TOB2
4.10614575437449E-163	1.27087257062597	0.263	0.119	6.88107905518078E-159	Ductular-like hepatocytes	PRSS23

4.30641846698354E-163	0.606326367073765	0.37	0.193	7.21669606697102E-159	Ductular-like hepatocytes	TSC22D4
4.3785745885676E-163	0.971403873278141	0.313	0.154	7.33761529552159E-159	Ductular-like hepatocytes	PIGX
5.35214438192457E-163	0.508803160910663	0.655	0.444	8.9691235552292E-159	Ductular-like hepatocytes	WNK1
8.77935304541422E-163	0.737357176707241	0.391	0.211	1.47124398335052E-158	Ductular-like hepatocytes	PCGF2
1.05401032895122E-162	0.766355169861633	0.381	0.205	1.76631050925645E-158	Ductular-like hepatocytes	FRYL
1.76465024082347E-162	0.876554724723718	0.244	0.102	2.95720087357198E-158	Ductular-like hepatocytes	SLC12A9
2.78402357900921E-162	0.79861690598005	0.411	0.231	4.66546671370363E-158	Ductular-like hepatocytes	MARVELD2
3.91348903312389E-162	0.614779893927841	0.537	0.332	6.55822492170902E-158	Ductular-like hepatocytes	IARS2
4.66815761820939E-162	0.689145631726666	0.427	0.243	7.82289853659529E-158	Ductular-like hepatocytes	ERAP1
4.77462410288214E-162	0.667910971957334	0.452	0.26	8.00131507160989E-158	Ductular-like hepatocytes	CCDC14
8.31070873176919E-162	0.75585614071997	0.394	0.216	1.39270856926988E-157	Ductular-like hepatocytes	FUCA2
1.49443574930276E-161	0.746337757012341	0.314	0.153	2.50437542868157E-157	Ductular-like hepatocytes	FAM221A
2.26233570840386E-161	0.750988396347258	0.424	0.238	3.79122218014318E-157	Ductular-like hepatocytes	HACD2
3.96486455279072E-161	0.568863447440255	0.536	0.332	6.64432001756669E-157	Ductular-like hepatocytes	HADH
7.38756603018811E-161	0.741410952944138	0.378	0.203	1.23800831533892E-156	Ductular-like hepatocytes	PRKCA
8.41179666552245E-161	0.45328082384015	0.734	0.502	1.40964888520825E-156	Ductular-like hepatocytes	SMIM14
8.62265450195739E-161	0.473944070300697	0.625	0.412	1.44498444143802E-156	Ductular-like hepatocytes	NUDT16
1.07428141156591E-160	0.560988790640527	0.582	0.37	1.80028078950215E-156	Ductular-like hepatocytes	GSTA2
1.54435136920504E-160	1.70361534103157	0.143	0.04	2.58802402451381E-156	Ductular-like hepatocytes	PLCE1
2.36251252459439E-160	1.94806710284968	0.108	0.022	3.95909848871529E-156	Ductular-like hepatocytes	RHOV
3.75265942743005E-160	0.767578404756476	0.382	0.207	6.28870666848727E-156	Ductular-like hepatocytes	PPIC
3.90450941869489E-160	0.606180995910606	0.488	0.293	6.5431768838489E-156	Ductular-like hepatocytes	KLF3
3.97912268006126E-160	0.630117406279712	0.55	0.349	6.66821378724666E-156	Ductular-like hepatocytes	BIRC3
4.6536113434401E-160	0.370586116631482	0.928	0.827	7.79852188933692E-156	Ductular-like hepatocytes	DEPRECATEI
5.4602224911969E-160	0.748611602433056	0.406	0.227	9.15024085074777E-156	Ductular-like hepatocytes	AKR7A2
5.84376958767571E-160	0.740116669739598	0.425	0.24	9.79298907502695E-156	Ductular-like hepatocytes	TGFBR2
1.23210360444192E-159	0.507566451520762	0.563	0.359	2.06475922032377E-155	Ductular-like hepatocytes	AK4
1.27527369905423E-159	0.907376446471607	0.333	0.169	2.13710366487507E-155	Ductular-like hepatocytes	RBM24
1.56839905302335E-159	0.670603032782321	0.465	0.276	2.62832313305654E-155	Ductular-like hepatocytes	SNX17
3.74229645017069E-159	1.44071603879402	0.196	0.073	6.27134039119604E-155	Ductular-like hepatocytes	IGSF3
1.40931402965034E-158	0.540387239171783	0.606	0.409	2.36172845088804E-154	Ductular-like hepatocytes	SEC61A1
2.9027173754766E-158	0.786276403916994	0.365	0.194	4.86437377782369E-154	Ductular-like hepatocytes	NMRAL1
4.17556974180134E-158	0.655211162140575	0.369	0.196	6.99741977331069E-154	Ductular-like hepatocytes	SPIRE2
4.49837796254772E-158	0.52408314864	0.649	0.449	7.53838178963747E-154	Ductular-like hepatocytes	NDUFB9
6.10304929152085E-158	1.03770884952475	0.247	0.108	1.02274900027306E-153	Ductular-like hepatocytes	LPCAT1
6.38403082157322E-158	0.721449122112763	0.407	0.226	1.06983588507924E-153	Ductular-like hepatocytes	SLC39A1
9.13815342519306E-158	0.535652855145876	0.493	0.295	1.53137175099385E-153	Ductular-like hepatocytes	BAX
9.61700001598146E-158	0.709248018271213	0.353	0.184	1.61161686267817E-153	Ductular-like hepatocytes	TMEM250
1.15667281222323E-157	0.923095514287628	0.324	0.164	1.93835229872368E-153	Ductular-like hepatocytes	TMEM43
1.27509967740064E-157	0.485482010675857	0.379	0.203	2.13681203938799E-153	Ductular-like hepatocytes	CENPB
1.77792746689706E-157	1.30381628573475	0.194	0.072	2.9794508490261E-153	Ductular-like hepatocytes	PDE4D
1.95544819937575E-157	1.24520194468589	0.155	0.048	3.27694009251388E-153	Ductular-like hepatocytes	TMEM150B
2.21052673265433E-157	0.774288205995613	0.385	0.211	3.70440069858213E-153	Ductular-like hepatocytes	ASB9
5.81151239789299E-157	1.20987535932325	0.231	0.097	9.73893247638907E-153	Ductular-like hepatocytes	C9orf152
6.05486989870564E-157	0.298250780663561	0.961	0.846	1.01467509762509E-152	Ductular-like hepatocytes	MT-ND4
6.95910839844069E-157	0.840817254991032	0.337	0.172	1.16620738541069E-152	Ductular-like hepatocytes	SGMS1
7.47386253649374E-157	0.905081252990752	0.3	0.146	1.25246988386562E-152	Ductular-like hepatocytes	NCK2
1.3977246460181E-156	1.03592758934074	0.236	0.1	2.34230696179713E-152	Ductular-like hepatocytes	B4GAT1
1.58100951617519E-156	0.68601980238566	0.385	0.208	2.64945574720638E-152	Ductular-like hepatocytes	LRRC14
2.0872131740231E-156	0.607355486038749	0.52	0.326	3.49775183702792E-152	Ductular-like hepatocytes	FDFT1
2.28201546260781E-156	0.708606715585353	0.374	0.2	3.82420151223817E-152	Ductular-like hepatocytes	CTDSPL
2.36659983158988E-156	0.391211035601105	0.447	0.254	3.96594799777832E-152	Ductular-like hepatocytes	RNPEPL1
3.40719777508576E-156	0.99777670501703	0.206	0.08	5.70978203148872E-152	Ductular-like hepatocytes	PFKFB2
4.49295043945905E-156	0.559487557725683	0.503	0.307	7.52928634644548E-152	Ductular-like hepatocytes	PPP1CA
5.09377955773975E-156	1.80353760741901	0.171	0.059	8.53615578286028E-152	Ductular-like hepatocytes	CCL25
1.24117893935922E-155	0.795005745760527	0.337	0.175	2.07996766657818E-151	Ductular-like hepatocytes	LYN
1.28056635215226E-155	0.828200222872549	0.346	0.18	2.14597309293733E-151	Ductular-like hepatocytes	WDR20
1.34162748391912E-155	0.87793123184041	0.271	0.125	2.24829933755167E-151	Ductular-like hepatocytes	DCTPP1
1.84954625717824E-155	1.42404742255581	0.194	0.073	3.0994696177793E-151	Ductular-like hepatocytes	DLG3
1.89463852196516E-155	0.663062213468072	0.45	0.262	3.17503523510922E-151	Ductular-like hepatocytes	CLMN

2.12907636170209E-155	0.9042037387395	0.297	0.144	3.56790616694037E-151	Ductular-like hepatocytes	LRIG3
3.0429562360808E-155	1.22330137669701	0.217	0.088	5.09938606042421E-151	Ductular-like hepatocytes	TP53I11
3.45081170951295E-155	0.309689709348745	0.695	0.505	5.7828702628018E-151	Ductular-like hepatocytes	MGAT4B
4.05178970264768E-155	0.629438245610456	0.431	0.244	6.78998918369699E-151	Ductular-like hepatocytes	SMS
4.87226791229804E-155	0.581402702846613	0.486	0.292	8.16494656742905E-151	Ductular-like hepatocytes	SEPTIN11
5.44021376321857E-155	1.15173280694233	0.223	0.092	9.11671022440169E-151	Ductular-like hepatocytes	ARL4C
8.91126982030746E-155	0.574488256714845	0.509	0.306	1.49335059648712E-150	Ductular-like hepatocytes	HNRNPL
9.83453827348889E-155	1.32197870473138	0.191	0.071	1.64807192387127E-150	Ductular-like hepatocytes	PDE3A
1.95858041742521E-154	0.799366425284969	0.321	0.162	3.28218906352116E-150	Ductular-like hepatocytes	GNL3L
4.24185205698314E-154	0.68623627870472	0.425	0.245	7.10849567709235E-150	Ductular-like hepatocytes	CLPTM1L
4.53495970085678E-154	0.708767446056475	0.356	0.188	7.59968546669579E-150	Ductular-like hepatocytes	CC2D1A
5.52023720460456E-154	0.60593632323956	0.496	0.3	9.25081350747632E-150	Ductular-like hepatocytes	NECTIN3
6.13319368411753E-154	0.645625194896742	0.423	0.24	1.02780059758442E-149	Ductular-like hepatocytes	MT-ND6
6.64675801884802E-154	0.940219859045857	0.344	0.181	1.11386370879855E-149	Ductular-like hepatocytes	FGF13
8.00562051700333E-154	0.743003317828283	0.365	0.194	1.34158188623942E-149	Ductular-like hepatocytes	PSPH
1.00914677009507E-153	0.647785284581776	0.497	0.304	1.69112815732531E-149	Ductular-like hepatocytes	SCAMP2
1.05723813675518E-153	1.25467034012655	0.247	0.11	1.77171966957433E-149	Ductular-like hepatocytes	PAM
1.36146257583654E-153	0.50469985785069	0.395	0.216	2.28153898458688E-149	Ductular-like hepatocytes	ESRRA
2.24819991251662E-153	0.471706347231857	0.706	0.502	3.76753341339536E-149	Ductular-like hepatocytes	DECR1
2.37906771927208E-153	0.779965688978931	0.388	0.215	3.98684168395616E-149	Ductular-like hepatocytes	MSH6
6.38374741444636E-153	1.63403037409128	0.157	0.051	1.06978839171292E-148	Ductular-like hepatocytes	GPR153
6.47694412307987E-153	0.735092230089893	0.343	0.178	1.08540629614572E-148	Ductular-like hepatocytes	CYREN
6.54175006389053E-153	0.757214234318907	0.344	0.18	1.09626647570678E-148	Ductular-like hepatocytes	CHMP1A
6.92676692869848E-153	0.661576827616549	0.479	0.294	1.16078760191129E-148	Ductular-like hepatocytes	CTSO
8.50014653759554E-153	0.854530784252766	0.326	0.167	1.42445455677026E-148	Ductular-like hepatocytes	DYRK2
9.40502215281505E-153	0.580393969870175	0.399	0.221	1.57609361236875E-148	Ductular-like hepatocytes	COL5A3
1.03876125093974E-152	0.625089428813101	0.444	0.255	1.74075610432481E-148	Ductular-like hepatocytes	DEK
1.23834763295477E-152	0.725968113458878	0.419	0.24	2.0752229633056E-148	Ductular-like hepatocytes	LSM 6.00
1.24570049062028E-152	0.321942119944028	0.938	0.783	2.08754488218147E-148	Ductular-like hepatocytes	PTGR1
1.31752809674637E-152	1.56100424002185	0.171	0.06	2.20791358452757E-148	Ductular-like hepatocytes	CLVS1
2.01666889178515E-152	0.899733291603084	0.322	0.164	3.37953372885355E-148	Ductular-like hepatocytes	PPP1CC
2.34138908324887E-152	0.481939337635147	0.601	0.4	3.92369982570845E-148	Ductular-like hepatocytes	RER1
2.44997226183113E-152	0.593806885862779	0.341	0.175	4.10566351637661E-148	Ductular-like hepatocytes	PPP1R16A
2.46279151636388E-152	0.500164072323445	0.656	0.453	4.12714602312259E-148	Ductular-like hepatocytes	NFIB
2.81887271542611E-152	0.582189709150294	0.516	0.323	4.72386689651107E-148	Ductular-like hepatocytes	HEXB
3.59698558389229E-152	0.554072810814116	0.439	0.253	6.02782844148669E-148	Ductular-like hepatocytes	WSB2
1.20840881870256E-151	0.520968911921966	0.544	0.351	2.02505149838176E-147	Ductular-like hepatocytes	RAB5IF
1.38878240225265E-151	0.645281165972099	0.419	0.238	2.32732154969499E-147	Ductular-like hepatocytes	ZDHHC14
1.44694560953744E-151	0.900092265274091	0.287	0.137	2.42479145246285E-147	Ductular-like hepatocytes	ADCK2
1.82978226496436E-151	1.28568411950044	0.183	0.067	3.06634911962727E-147	Ductular-like hepatocytes	GAL3ST1
3.09466686910969E-151	0.626767451530566	0.474	0.288	5.18604273925401E-147	Ductular-like hepatocytes	PDCD4
4.15450111119638E-151	0.968205956246114	0.246	0.109	6.96211296214289E-147	Ductular-like hepatocytes	TP53I3
4.78546019163732E-151	0.819942267555975	0.389	0.216	8.01947418914581E-147	Ductular-like hepatocytes	FAIM
1.24003711060404E-150	0.621113557798972	0.432	0.249	2.07805418995025E-146	Ductular-like hepatocytes	IDS
1.84873683321671E-150	0.761123227332527	0.37	0.202	3.09811318510456E-146	Ductular-like hepatocytes	VPS26C
2.12830782042538E-150	0.607044767985413	0.526	0.334	3.56661824546885E-146	Ductular-like hepatocytes	ERO1A
3.09818156455064E-150	0.770422252137084	0.38	0.209	5.19193266587396E-146	Ductular-like hepatocytes	MTMR1
5.06627996597561E-150	0.78199266886617	0.316	0.159	8.49007196698192E-146	Ductular-like hepatocytes	MFSD3
5.41882431527952E-150	0.479925116340034	0.446	0.262	9.08086578754542E-146	Ductular-like hepatocytes	PLEC
5.89753141588193E-150	0.652109953055137	0.423	0.242	9.88308314673494E-146	Ductular-like hepatocytes	LGMN
9.70512760117662E-150	0.607161209078695	0.487	0.299	1.62638528340518E-145	Ductular-like hepatocytes	CPNE3
1.64590790547692E-149	0.45676152716818	0.615	0.411	2.75821246799823E-145	Ductular-like hepatocytes	TBC1D9B
2.03649469062277E-149	0.521476198093931	0.605	0.41	3.41275780254564E-145	Ductular-like hepatocytes	PUM1
2.26913186078008E-149	0.761239136575318	0.356	0.191	3.80261117229526E-145	Ductular-like hepatocytes	DOCK9
2.34322407532555E-149	0.979374144249525	0.295	0.146	3.92677490543056E-145	Ductular-like hepatocytes	MAGEH1
2.88375962426156E-149	1.48933535812234	0.188	0.072	4.83260437833753E-145	Ductular-like hepatocytes	PPP1R9A
3.3550433838619E-149	1.71288584645132	0.15	0.048	5.62238170267578E-145	Ductular-like hepatocytes	CLDN11
3.55861411886343E-149	0.778619906774672	0.367	0.201	5.96352554039134E-145	Ductular-like hepatocytes	MGAT4A
4.77977321080436E-149	2.15231947820419	0.116	0.029	8.00994394666595E-145	Ductular-like hepatocytes	PHGR1
1.4412077445133E-148	0.53070351070016	0.491	0.299	2.41517593825539E-144	Ductular-like hepatocytes	PSMB9

1.77810825606865E-148	0.693734803768139	0.412	0.232	2.97975381551985E-144	Ductular-like hepatocytes	NT5DC3
2.45868185748101E-148	0.638026780591622	0.413	0.235	4.12025905676668E-144	Ductular-like hepatocytes	HDAC1
3.2073368817303E-148	0.672889800825181	0.383	0.212	5.37485514640364E-144	Ductular-like hepatocytes	HGH1
4.13722350471046E-148	0.37839495331569	0.805	0.587	6.93315914919379E-144	Ductular-like hepatocytes	NFKBIA
4.48666026360145E-148	0.367698551152104	0.727	0.501	7.51874526974331E-144	Ductular-like hepatocytes	NOTCH2NLC
6.01430217303672E-148	0.781470357014518	0.377	0.207	1.00787675815749E-143	Ductular-like hepatocytes	CLDN14
6.22194654606091E-148	0.736598446988187	0.357	0.192	1.04267380218889E-143	Ductular-like hepatocytes	SCAMP4
6.68609689420718E-148	0.512389406780232	0.378	0.205	1.12045611753124E-143	Ductular-like hepatocytes	GRAMD4
1.17390347014902E-147	0.53876602013779	0.49	0.302	1.96722743527572E-143	Ductular-like hepatocytes	MAVS
1.17656953921485E-147	0.620989051793229	0.389	0.217	1.97169523381625E-143	Ductular-like hepatocytes	RAPGEF5
1.19496290360979E-147	0.811132718483448	0.386	0.215	2.00251883386929E-143	Ductular-like hepatocytes	LRRC58
1.23195234664787E-147	0.955472045973762	0.256	0.116	2.06450574251251E-143	Ductular-like hepatocytes	HOXA10
1.23264254794401E-147	1.02980980227326	0.244	0.109	2.06566238184457E-143	Ductular-like hepatocytes	SLC1A1
1.36492005181264E-147	0.317834151792186	0.905	0.736	2.28733302282763E-143	Ductular-like hepatocytes	MT-ND4L
1.54354455416113E-147	1.09716271082983	0.271	0.128	2.58667196386322E-143	Ductular-like hepatocytes	ARMCX5
1.71535743411003E-147	0.752771473102349	0.313	0.158	2.87459598808159E-143	Ductular-like hepatocytes	XPR1
1.86979540421417E-147	0.959816656200387	0.285	0.139	3.13340313838211E-143	Ductular-like hepatocytes	GPD2
2.03251242148421E-147	0.601101711387715	0.435	0.255	3.40608431592324E-143	Ductular-like hepatocytes	NREP
2.42948605280266E-147	1.57196995280168	0.134	0.038	4.0713327272867E-143	Ductular-like hepatocytes	XPNPEP2
3.26092698895398E-147	0.578021832272352	0.519	0.326	5.46466144808908E-143	Ductular-like hepatocytes	IMPAD1
3.64418051244793E-147	0.896858021351767	0.296	0.145	6.10691770276025E-143	Ductular-like hepatocytes	FHL1
4.49130320851196E-147	0.585523627334783	0.506	0.314	7.52652591682435E-143	Ductular-like hepatocytes	OCIAD1
4.93364007357231E-147	0.909752276995075	0.331	0.173	8.26779403529247E-143	Ductular-like hepatocytes	SRD5A3
5.01638161774478E-147	1.42691441783003	0.189	0.073	8.4064523150167E-143	Ductular-like hepatocytes	PTPRU
1.47729378513632E-146	0.933439786505201	0.255	0.117	2.47564892513145E-142	Ductular-like hepatocytes	ARHGAP26
1.65160070060131E-146	1.14354037254305	0.209	0.086	2.76775245406768E-142	Ductular-like hepatocytes	MTCL1
2.48033534169173E-146	0.852299371807801	0.294	0.145	4.15654596560701E-142	Ductular-like hepatocytes	JCAD
2.76545492014854E-146	0.588462229818387	0.503	0.314	4.63434935518492E-142	Ductular-like hepatocytes	MICOS10
3.10541905155412E-146	0.908250924423084	0.265	0.124	5.2040612465944E-142	Ductular-like hepatocytes	DEPRECATED
4.32979219248477E-146	0.542915297846105	0.555	0.363	7.25586575616599E-142	Ductular-like hepatocytes	REEP5
4.35462763110445E-146	0.709553099531622	0.388	0.215	7.29748498420483E-142	Ductular-like hepatocytes	ZNF75D
5.8850191819724E-146	0.660400600812908	0.373	0.206	9.86211514514934E-142	Ductular-like hepatocytes	CHMP3
6.22274174571266E-146	0.900006176288057	0.281	0.136	1.04280706174653E-141	Ductular-like hepatocytes	VOPP1
1.04618418062257E-145	0.550338776261643	0.449	0.268	1.75319544988731E-141	Ductular-like hepatocytes	MYO15B
1.4453195997839E-145	0.579740862032635	0.494	0.304	2.42206658531785E-141	Ductular-like hepatocytes	PEX2
1.83911773741893E-145	0.350880747309568	0.786	0.617	3.08199350436665E-141	Ductular-like hepatocytes	KRT8
1.88085021610223E-145	0.544750413757445	0.604	0.407	3.15192879214412E-141	Ductular-like hepatocytes	ALDH9A1
2.25929731412585E-145	0.580678485362945	0.46	0.273	3.7861304390121E-141	Ductular-like hepatocytes	NUP58
2.5831620003066E-145	0.526645920958426	0.526	0.342	4.3288628801138E-141	Ductular-like hepatocytes	EDEM3
4.04087607302234E-145	0.662241227379209	0.442	0.261	6.77170012317083E-141	Ductular-like hepatocytes	FBXL17
4.33351363112411E-145	0.545732104606031	0.51	0.315	7.26210214303778E-141	Ductular-like hepatocytes	ADD1
5.79541608945154E-145	0.64813182288234	0.357	0.194	9.71195828270288E-141	Ductular-like hepatocytes	COLGALT1
8.02121992354673E-145	0.664397985956093	0.416	0.24	1.34419603478796E-140	Ductular-like hepatocytes	PARP12
2.30487329417036E-144	0.626094048025915	0.377	0.208	3.8625066663707E-140	Ductular-like hepatocytes	CEP170B
2.41109684109891E-144	0.841160533921084	0.307	0.156	4.04051608631355E-140	Ductular-like hepatocytes	STIM2
2.52105484959376E-144	1.06263922821808	0.225	0.097	4.22478371694923E-140	Ductular-like hepatocytes	FKBP9
3.73195673997889E-144	0.554055226128622	0.421	0.241	6.25401310485663E-140	Ductular-like hepatocytes	ARHGEF40
8.11702571071068E-144	0.635366968352296	0.367	0.197	1.3602511686009E-139	Ductular-like hepatocytes	TMEM19
9.79992553557361E-144	1.00552435305376	0.278	0.136	1.64227152125143E-139	Ductular-like hepatocytes	UHRF1
2.16245072580304E-143	0.470157668342172	0.591	0.398	3.62383492630073E-139	Ductular-like hepatocytes	CTNNA1
2.31435735309981E-143	0.548305717392927	0.516	0.318	3.87840005232466E-139	Ductular-like hepatocytes	HGSNAT
2.81846289261588E-143	0.638222627529118	0.415	0.244	4.72318011544568E-139	Ductular-like hepatocytes	KDM6A
3.56357622553997E-143	1.10092580134989	0.261	0.123	5.97184103875988E-139	Ductular-like hepatocytes	CLGN
3.90417348365482E-143	0.465931292482771	0.646	0.452	6.54261392390875E-139	Ductular-like hepatocytes	MAT2A
6.47082943491988E-143	0.934449994081454	0.307	0.156	1.08438159670387E-138	Ductular-like hepatocytes	SLC9A6
8.66803729371792E-143	0.621633041419288	0.437	0.259	1.45258969968125E-138	Ductular-like hepatocytes	SMYD2
1.18455392431718E-142	0.510705943204374	0.566	0.373	1.98507546637073E-138	Ductular-like hepatocytes	CDC37
1.40473530809831E-142	0.660579544786976	0.422	0.244	2.35405542931115E-138	Ductular-like hepatocytes	PDE4DIP
1.57563249180454E-142	0.705322452837163	0.38	0.212	2.64044492976604E-138	Ductular-like hepatocytes	ZMAT1
1.75728694672913E-142	0.616042596621443	0.443	0.261	2.94486146532868E-138	Ductular-like hepatocytes	CXorf56

1.77286802864466E-142	0.591248392511585	0.44	0.263	2.97097224240273E-138	Ductular-like hepatocytes	APRT
2.1298700825829E-142	0.665071152842347	0.475	0.294	3.56923628439243E-138	Ductular-like hepatocytes	CISD2
2.30723808291389E-142	0.563819060998225	0.518	0.333	3.8664695793471E-138	Ductular-like hepatocytes	SYAP1
4.28036811517214E-142	0.421144191050174	0.648	0.453	7.17304088740547E-138	Ductular-like hepatocytes	ATP5F1D
5.56992211282049E-142	0.53598014252916	0.56	0.37	9.33407547666458E-138	Ductular-like hepatocytes	COX7C
5.8865677696195E-142	0.430561382003768	0.672	0.477	9.86471026832837E-138	Ductular-like hepatocytes	PRKACA
8.81371746015677E-142	0.425268181066891	0.571	0.376	1.47700277197307E-137	Ductular-like hepatocytes	SPTAN1
1.58373462406177E-141	0.745744753395784	0.312	0.16	2.65402248300271E-137	Ductular-like hepatocytes	P4HA2
9.69098371344981E-141	1.36626760376981	0.187	0.073	1.62401505069992E-136	Ductular-like hepatocytes	PKM
1.37714254556316E-140	0.568728729028195	0.468	0.284	2.30781547785474E-136	Ductular-like hepatocytes	UPF1
1.88433654657563E-140	0.601578581949506	0.47	0.285	3.15777118475143E-136	Ductular-like hepatocytes	STK3
2.97177825637769E-140	0.564075192308137	0.358	0.193	4.98010600203773E-136	Ductular-like hepatocytes	TRRAP
3.11019825234726E-140	0.493444416910397	0.451	0.27	5.21207023128354E-136	Ductular-like hepatocytes	ENTPD6
3.23728284366751E-140	0.543975229611975	0.454	0.273	5.42503858941802E-136	Ductular-like hepatocytes	TRAPPC9
5.28144283666566E-140	0.540720652790759	0.466	0.28	8.85064190568431E-136	Ductular-like hepatocytes	MAN2A1
5.55110989257461E-140	0.652004428412996	0.442	0.27	9.30254995797654E-136	Ductular-like hepatocytes	FAM162A
5.59735897467636E-140	0.575617214399196	0.498	0.314	9.38005416976265E-136	Ductular-like hepatocytes	SDCBP
5.70040123085301E-140	0.498478757442622	0.477	0.292	9.55273238266347E-136	Ductular-like hepatocytes	INSR
8.98970454791225E-140	0.305414011824916	0.901	0.751	1.50649468813914E-135	Ductular-like hepatocytes	OAZ1
9.83332022899436E-140	1.88876920565632	0.103	0.024	1.64786780397488E-135	Ductular-like hepatocytes	FERMT3
1.0859291144736E-139	0.46300680529663	0.452	0.271	1.81980001003485E-135	Ductular-like hepatocytes	EPN1
1.12149649476705E-139	1.18238175626291	0.229	0.102	1.87940382593062E-135	Ductular-like hepatocytes	LRRC8E
1.36949183374353E-139	0.693644893939798	0.389	0.218	2.29499441498741E-135	Ductular-like hepatocytes	ARHGAP5
1.97986666854156E-139	0.386365586265434	0.547	0.345	3.31786056314194E-135	Ductular-like hepatocytes	MAP2K2
1.99076280427732E-139	0.882420724874047	0.258	0.122	3.33612030740794E-135	Ductular-like hepatocytes	CISD1
2.90005021387469E-139	0.365158201440747	0.467	0.28	4.85990414841121E-135	Ductular-like hepatocytes	BAIAP2L1
2.98814231152157E-139	0.770072626230494	0.353	0.191	5.00752888564785E-135	Ductular-like hepatocytes	WDR44
3.92310624576057E-139	0.80386512736047	0.269	0.129	6.57434144664557E-135	Ductular-like hepatocytes	APEX2
4.31946665608445E-139	0.733761385423321	0.324	0.171	7.23856222226633E-135	Ductular-like hepatocytes	TRMT1L
4.39571283007093E-139	1.32005919993426	0.194	0.078	7.36633556063287E-135	Ductular-like hepatocytes	SDC3
4.40304180524947E-139	0.403816363735401	0.983	0.927	7.37861745723707E-135	Ductular-like hepatocytes	AGT
4.88217252819464E-139	0.466672118157857	0.614	0.428	8.18154472274857E-135	Ductular-like hepatocytes	CTNND1
7.81665577346527E-139	0.755131411094608	0.304	0.155	1.30991517451731E-134	Ductular-like hepatocytes	CXorf40A
7.87489691043631E-139	0.799248400802846	0.32	0.167	1.31967522425092E-134	Ductular-like hepatocytes	CCDC198
8.18048221458868E-139	0.560906847374243	0.468	0.288	1.37088520952077E-134	Ductular-like hepatocytes	MLX
9.0780864367601E-139	0.849724016361056	0.311	0.161	1.52130572507226E-134	Ductular-like hepatocytes	CLN5
1.13332946176155E-138	0.601054005217654	0.38	0.212	1.89923351202E-134	Ductular-like hepatocytes	FAM49B
1.32854967786255E-138	0.58375601164126	0.441	0.262	2.22638355016206E-134	Ductular-like hepatocytes	PPIA
2.06369345937494E-138	0.526484551081102	0.513	0.326	3.45833749922053E-134	Ductular-like hepatocytes	OPLAH
2.35940604764642E-138	0.740045734795685	0.302	0.153	3.95389265464587E-134	Ductular-like hepatocytes	CCNB1IP1
2.80777190402853E-138	0.57571834690692	0.36	0.198	4.705264156771E-134	Ductular-like hepatocytes	GNG12
2.88318347192084E-138	0.476920201389822	0.488	0.305	4.83163886224494E-134	Ductular-like hepatocytes	EIF3B
2.92598508207874E-138	0.538272365845378	0.507	0.321	4.90336580054755E-134	Ductular-like hepatocytes	ACVR1B
3.26687159950224E-138	0.669117205095129	0.343	0.184	5.47462342644586E-134	Ductular-like hepatocytes	SNX12
3.91712333760632E-138	0.848054662432254	0.27	0.131	6.56431528916067E-134	Ductular-like hepatocytes	CCND3
4.22097655851013E-138	1.25884580352531	0.166	0.06	7.07351251675128E-134	Ductular-like hepatocytes	SLC4A7
4.24408275213218E-138	0.557502921518198	0.455	0.276	7.1122338760231E-134	Ductular-like hepatocytes	DNPH1
4.81115437831595E-138	0.563704019051309	0.41	0.237	8.06253250718187E-134	Ductular-like hepatocytes	DLG5
7.08598609420944E-138	0.479230293676191	0.574	0.372	1.18746954966762E-133	Ductular-like hepatocytes	SIVA1
8.24964460989445E-138	0.862029107978958	0.223	0.097	1.38247544372611E-133	Ductular-like hepatocytes	C4orf33
8.60920255929023E-138	0.619083806344315	0.373	0.208	1.44273016488586E-133	Ductular-like hepatocytes	KANK2
9.55626617603296E-138	0.543244580037323	0.515	0.324	1.6014390857796E-133	Ductular-like hepatocytes	NUCB2
9.71483713407767E-138	0.566079049929594	0.341	0.183	1.62801240692874E-133	Ductular-like hepatocytes	SLC39A11
1.5060215763331E-137	0.845094040456744	0.273	0.133	2.52379095761901E-133	Ductular-like hepatocytes	THEM6
1.72692959414113E-137	0.642730242021195	0.376	0.21	2.8939886138617E-133	Ductular-like hepatocytes	GSS
1.79520986745678E-137	0.726262046746933	0.375	0.209	3.00841269588406E-133	Ductular-like hepatocytes	ACTR10
2.97015622195443E-137	0.558922057929907	0.428	0.252	4.97738779675123E-133	Ductular-like hepatocytes	PDCD6
4.34782537629207E-137	0.660735793990309	0.392	0.221	7.28608576559025E-133	Ductular-like hepatocytes	PHF6
6.72851011822243E-137	0.570949663632292	0.455	0.28	1.12756372561171E-132	Ductular-like hepatocytes	CLDN15
9.06916427015308E-137	0.762914038570788	0.301	0.154	1.51981054839225E-132	Ductular-like hepatocytes	C12orf75

9.71905663298321E-137	0.62187467663884	0.443	0.268	1.62871951055533E-132	Ductular-like hepatocytes	DYNLT1
1.30131884617252E-136	0.57089229536566	0.319	0.166	2.18075012241592E-132	Ductular-like hepatocytes	LMTK2
2.02389372520285E-136	1.80735195871923	0.12	0.034	3.39164110469494E-132	Ductular-like hepatocytes	DISP2
2.31005550681168E-136	0.680307577151101	0.336	0.181	3.87119101831502E-132	Ductular-like hepatocytes	AP1B1
3.1279474306118E-136	0.411662768502618	0.558	0.352	5.24181430421926E-132	Ductular-like hepatocytes	TMPO
4.07530519258593E-136	0.632255863483957	0.389	0.222	6.8293964417355E-132	Ductular-like hepatocytes	STK38
4.43794225579752E-136	1.18937100682443	0.174	0.065	7.43710363226548E-132	Ductular-like hepatocytes	C11orf49
4.56441451916968E-136	0.515564277873687	0.444	0.264	7.64904585122456E-132	Ductular-like hepatocytes	MINK1
4.71628242722963E-136	1.00450713588071	0.27	0.132	7.90354609155142E-132	Ductular-like hepatocytes	RAVER2
5.75656986657909E-136	0.523817586973509	0.419	0.244	9.64685978241323E-132	Ductular-like hepatocytes	BTBD2
6.70075548757602E-136	1.42142823935797	0.162	0.059	1.12291260460799E-131	Ductular-like hepatocytes	LYPD6B
7.38956265413578E-136	0.486917684600394	0.557	0.365	1.23834290958007E-131	Ductular-like hepatocytes	CANX
7.78184837643598E-136	0.63568845900264	0.37	0.207	1.30408215092314E-131	Ductular-like hepatocytes	KHSRP
8.47263233019492E-136	0.651164639910702	0.367	0.206	1.41984372589406E-131	Ductular-like hepatocytes	HDDC2
1.0528907190658E-135	0.615378449763913	0.318	0.167	1.76443426701047E-131	Ductular-like hepatocytes	PRSS3
1.18631370988484E-135	1.4998170065785	0.122	0.035	1.98802451502501E-131	Ductular-like hepatocytes	MICAL1
1.33901644265045E-135	0.685015757572987	0.397	0.227	2.24392375459363E-131	Ductular-like hepatocytes	DEPRECATEI
1.38552073807796E-135	0.575574828930505	0.437	0.258	2.32185565287105E-131	Ductular-like hepatocytes	MECP2
1.41968663019558E-135	0.698842218911151	0.381	0.217	2.37911085488175E-131	Ductular-like hepatocytes	ABHD16A
1.75202461090683E-135	0.785170825794739	0.274	0.135	2.93604284295766E-131	Ductular-like hepatocytes	RIN2
2.03898244271128E-135	0.513888456843981	0.458	0.279	3.41692677749556E-131	Ductular-like hepatocytes	SLC35A4
2.13371654736105E-135	0.596515805270984	0.419	0.245	3.57568219006765E-131	Ductular-like hepatocytes	MPHOSPH8
2.22129427493922E-135	0.525947132608971	0.511	0.327	3.72244494594315E-131	Ductular-like hepatocytes	CAPZB
2.41789342371851E-135	1.41377732226636	0.181	0.072	4.05190579946748E-131	Ductular-like hepatocytes	IL2RG
2.65618950133381E-135	0.577623618386515	0.448	0.271	4.4512423663352E-131	Ductular-like hepatocytes	APEX1
3.15232091706203E-135	1.50982381791187	0.174	0.067	5.28265939281255E-131	Ductular-like hepatocytes	TTLL6
3.2648873247423E-135	0.727310542892108	0.363	0.203	5.47129817880314E-131	Ductular-like hepatocytes	SLC16A2
5.1493613274605E-135	0.742598020203775	0.323	0.172	8.6292997125583E-131	Ductular-like hepatocytes	ENPP4
5.59843801129137E-135	0.770705226855879	0.34	0.184	9.38186241932207E-131	Ductular-like hepatocytes	RNF6
7.08335131430929E-135	0.423813871537386	0.514	0.329	1.18702801325195E-130	Ductular-like hepatocytes	RHOC
8.13299403564375E-135	0.906921006457151	0.263	0.127	1.36292714049318E-130	Ductular-like hepatocytes	HIF1A
1.5231485270968E-134	0.860425314807303	0.3	0.155	2.55249230170881E-130	Ductular-like hepatocytes	RPA1
1.76546624682678E-134	0.719002568439119	0.334	0.181	2.95856833643231E-130	Ductular-like hepatocytes	TOR1B
1.93001776215315E-134	0.546476168813197	0.407	0.237	3.23432376581625E-130	Ductular-like hepatocytes	ZBTB7A
1.95984143822628E-134	0.563178601525	0.414	0.241	3.28430228217961E-130	Ductular-like hepatocytes	MTIF3
2.05588514468354E-134	0.690184301912949	0.297	0.151	3.44525232546068E-130	Ductular-like hepatocytes	MYO1D
2.43567944382839E-134	0.703511368185015	0.315	0.165	4.08171161196761E-130	Ductular-like hepatocytes	ACOT7
2.97251858204876E-134	1.37016870062118	0.181	0.071	4.98134663979732E-130	Ductular-like hepatocytes	ARSJ
3.85997388497151E-134	0.673661565896762	0.284	0.141	6.46854423643526E-130	Ductular-like hepatocytes	ARRDC1
4.72009290274559E-134	0.418273363328618	0.68	0.478	7.90993168642106E-130	Ductular-like hepatocytes	PUF60
5.04006679671671E-134	0.960534978803062	0.283	0.143	8.44614393793786E-130	Ductular-like hepatocytes	BRCC3
6.8963720089549E-134	0.541182242066456	0.459	0.279	1.15569402126066E-129	Ductular-like hepatocytes	LRRC8A
7.65100817465479E-134	0.630653085812771	0.411	0.241	1.28215594990865E-129	Ductular-like hepatocytes	TMEM129
1.0227914972354E-133	0.953427914922584	0.278	0.139	1.71399399106709E-129	Ductular-like hepatocytes	MSH2
1.19341395562862E-133	1.10549252899916	0.206	0.088	1.99992310684245E-129	Ductular-like hepatocytes	KLF2
1.42004718058801E-133	0.897995512500973	0.264	0.128	2.37971506522939E-129	Ductular-like hepatocytes	UCHL3
1.59799570890282E-133	0.638891890993047	0.432	0.256	2.67792120897934E-129	Ductular-like hepatocytes	LNX2
1.62844199046339E-133	0.573712766749556	0.45	0.272	2.72894308761855E-129	Ductular-like hepatocytes	NUDT9
1.66375206800205E-133	0.699429507303599	0.358	0.199	2.78811571555783E-129	Ductular-like hepatocytes	EBAG9
2.97085544701403E-133	1.11096801151065	0.226	0.103	4.97855955810611E-129	Ductular-like hepatocytes	ABCG1
3.03531509668265E-133	0.502129409406564	0.56	0.375	5.08658103902079E-129	Ductular-like hepatocytes	MCFD2
3.92963112067848E-133	0.59490134252985	0.432	0.254	6.585275832033E-129	Ductular-like hepatocytes	SH3BP4
4.96238415927168E-133	0.645730974178902	0.374	0.212	8.31596337410749E-129	Ductular-like hepatocytes	SF3B3
5.10781863381954E-133	0.504608166420508	0.437	0.26	8.55968246655479E-129	Ductular-like hepatocytes	DNAJC4
6.78841542649912E-133	0.497180034558581	0.51	0.321	1.13760265717272E-128	Ductular-like hepatocytes	FAM91A1
7.20593349569789E-133	0.539223611665842	0.454	0.277	1.20757033520905E-128	Ductular-like hepatocytes	APH1A
7.43652586248511E-133	0.393623082924674	0.989	0.937	1.24621300403525E-128	Ductular-like hepatocytes	CES1
7.56076414988349E-133	0.580209680720424	0.416	0.24	1.26703285623747E-128	Ductular-like hepatocytes	RBMX
8.81313864164411E-133	0.977575432716856	0.251	0.12	1.47690577356672E-128	Ductular-like hepatocytes	CLSTN1
1.4135010104251E-132	0.783206215535384	0.328	0.175	2.36874499327039E-128	Ductular-like hepatocytes	RNF113A

1.43343596222147E-132	0.944272866245152	0.22	0.098	2.40215198549074E-128	Ductular-like hepatocytes	DHRS11
1.48332781480148E-132	0.758641981562601	0.284	0.143	2.48576075204431E-128	Ductular-like hepatocytes	UCK2
1.64019346747279E-132	0.692981824201045	0.305	0.157	2.7486362127909E-128	Ductular-like hepatocytes	TMEM50B
3.76137383701774E-132	0.777810224749793	0.256	0.122	6.30331027607433E-128	Ductular-like hepatocytes	SYNJ2
4.00096786782762E-132	0.497650664993196	0.491	0.305	6.70482195290552E-128	Ductular-like hepatocytes	SPECC1L
4.41126335490796E-132	0.622914864929979	0.427	0.255	7.39239513015475E-128	Ductular-like hepatocytes	VAT1
6.46508020871924E-132	0.534129702693274	0.382	0.217	1.08341814137717E-127	Ductular-like hepatocytes	SPPL2B
1.0801994730591E-131	0.354485414938991	0.609	0.428	1.81019827695243E-127	Ductular-like hepatocytes	NAPRT
1.22836134911231E-131	0.369561465836115	0.546	0.354	2.05848794884241E-127	Ductular-like hepatocytes	NR2F6
1.23264020105625E-131	0.584120580016271	0.402	0.235	2.06565844893006E-127	Ductular-like hepatocytes	NUP210
1.55831045194308E-131	1.23782084064433	0.167	0.063	2.61141665536621E-127	Ductular-like hepatocytes	NCR3LG1
1.64721374349761E-131	0.764274731474397	0.313	0.165	2.7604007913533E-127	Ductular-like hepatocytes	TMEM9B
1.98344141251613E-131	0.446487066073135	0.635	0.438	3.32385111909452E-127	Ductular-like hepatocytes	PGRMC2
2.03246234962737E-131	0.638972878954749	0.39	0.225	3.40600040550554E-127	Ductular-like hepatocytes	HAUS4
2.57940435307986E-131	0.514576284662165	0.437	0.263	4.32256581489122E-127	Ductular-like hepatocytes	AP2A1
3.38198098095728E-131	0.79635265397838	0.33	0.178	5.66752372788821E-127	Ductular-like hepatocytes	ABCB10
3.43191440377613E-131	0.501936775188859	0.491	0.309	5.75120215784804E-127	Ductular-like hepatocytes	SLC35F5
5.04503950848022E-131	0.762016407967134	0.301	0.156	8.45447720831115E-127	Ductular-like hepatocytes	TRADD
5.39262067044221E-131	0.433583363621152	0.781	0.57	9.03695371952706E-127	Ductular-like hepatocytes	DEPRECATEI
5.78529475950155E-131	0.527653598552581	0.407	0.237	9.69499695797269E-127	Ductular-like hepatocytes	EXOC3L4
6.2948427664729E-131	0.539264929394737	0.478	0.3	1.05488975080553E-126	Ductular-like hepatocytes	CCT6A
6.38604342807282E-131	0.597123690701739	0.299	0.154	1.07017315767644E-126	Ductular-like hepatocytes	GALNS
8.47512302712644E-131	0.544193765290446	0.401	0.231	1.42026111688585E-126	Ductular-like hepatocytes	ADCY6
1.06870200056996E-130	0.653811080435785	0.398	0.232	1.79093081255514E-126	Ductular-like hepatocytes	MAPK1IP1L
1.31738627771672E-130	0.727719366056364	0.309	0.163	2.20767592419768E-126	Ductular-like hepatocytes	ANO8
1.61823224094064E-130	0.709001692722556	0.365	0.207	2.71183358936833E-126	Ductular-like hepatocytes	COA6
1.76782627847484E-130	0.624672492084777	0.419	0.246	2.96252327746814E-126	Ductular-like hepatocytes	NOTCH2
1.9029650222153E-130	0.44649420077315	0.291	0.145	3.1889887842284E-126	Ductular-like hepatocytes	ABHD11
2.05097230995379E-130	0.373717843373785	0.443	0.263	3.43701939702056E-126	Ductular-like hepatocytes	PKN1
2.27313690191888E-130	0.403918861011509	0.643	0.446	3.80932282023566E-126	Ductular-like hepatocytes	MTDH
2.51715889442544E-130	0.47624878565115	0.33	0.176	4.21825487527816E-126	Ductular-like hepatocytes	RASSF7
3.90649865715282E-130	0.829671753297507	0.288	0.147	6.54651044965669E-126	Ductular-like hepatocytes	SMPDL3A
5.01902820000399E-130	0.328738488430212	0.521	0.335	8.41088745756668E-126	Ductular-like hepatocytes	TMEM219
7.00009882321446E-130	0.394071686742481	0.63	0.41	1.17307656079428E-125	Ductular-like hepatocytes	ZHX1
7.01670571471565E-130	0.470942218349137	0.661	0.46	1.17585954367205E-125	Ductular-like hepatocytes	CBR1
7.50573581852101E-130	0.50919906359835	0.334	0.182	1.25781120846775E-125	Ductular-like hepatocytes	SLC25A23
8.50023566886314E-130	0.443286797520802	0.451	0.271	1.42446949338809E-125	Ductular-like hepatocytes	ACTR1B
1.55299340046862E-129	0.585766052644938	0.406	0.238	2.60250634050532E-125	Ductular-like hepatocytes	PPM1G
1.81755985314408E-129	0.468169734401628	0.538	0.346	3.04586680189885E-125	Ductular-like hepatocytes	PLEKHF2
2.37105003620177E-129	0.451789256871044	0.852	0.652	3.97340565066693E-125	Ductular-like hepatocytes	AKR1B10
2.54017209793463E-129	0.794720438646424	0.325	0.178	4.25682040171885E-125	Ductular-like hepatocytes	SFN
3.13590245099069E-129	0.442232211346954	0.627	0.441	5.2551453273702E-125	Ductular-like hepatocytes	NDUFA3
3.37329191902952E-129	0.587842235066422	0.463	0.285	5.65296259790968E-125	Ductular-like hepatocytes	TMEM9
3.98716914878101E-129	0.6316968154502	0.376	0.214	6.68169805952722E-125	Ductular-like hepatocytes	DERA
1.37548682065375E-128	0.628373374894367	0.377	0.215	2.30504081405155E-124	Ductular-like hepatocytes	LRBA
1.83827934338726E-128	0.86703948527479	0.235	0.109	3.08058852364837E-124	Ductular-like hepatocytes	C1GALT1
1.88062430188032E-128	0.60032068063758	0.503	0.318	3.15155020509104E-124	Ductular-like hepatocytes	TMEM147
2.44342716719679E-128	0.777718563762453	0.211	0.092	4.09469524678837E-124	Ductular-like hepatocytes	PTCD1
2.57368480437745E-128	0.633192660384972	0.372	0.213	4.31298099517573E-124	Ductular-like hepatocytes	GLB1
3.07675812709393E-128	0.554444006937946	0.428	0.256	5.156031269384E-124	Ductular-like hepatocytes	UBE2Z
3.57769451641511E-128	0.672122446260132	0.217	0.096	5.99550047060844E-124	Ductular-like hepatocytes	RALGPS1
3.59447490994857E-128	0.678334981506381	0.36	0.204	6.02362105409181E-124	Ductular-like hepatocytes	BCAP29
3.64258860132188E-128	0.576716612704026	0.413	0.245	6.10424997809521E-124	Ductular-like hepatocytes	STX7
3.93144979427895E-128	0.752151949811778	0.347	0.193	6.58832356525266E-124	Ductular-like hepatocytes	UBR7
4.11570801659081E-128	0.607228710489885	0.403	0.24	6.89710349420289E-124	Ductular-like hepatocytes	GNPAT
4.58542162509833E-128	0.698551072898579	0.394	0.229	7.68424955933978E-124	Ductular-like hepatocytes	RBBP7
5.81852901210267E-128	0.610011340425212	0.426	0.253	9.75069091848166E-124	Ductular-like hepatocytes	SSX2IP
6.82757845510629E-128	0.686953981388024	0.344	0.192	1.14416559750671E-123	Ductular-like hepatocytes	CASP3
6.84619544581002E-128	0.741011483320716	0.327	0.177	1.14728543280884E-123	Ductular-like hepatocytes	SLC30A9
6.86163703092905E-128	1.15753795760523	0.168	0.064	1.14987313364309E-123	Ductular-like hepatocytes	TRPV4

7.35878729502607E-128	0.64343005193622	0.369	0.21	1.23318557490047E-123	Ductular-like hepatocytes	MXD4
8.23403939323327E-128	0.693782578103293	0.376	0.216	1.37986032151803E-123	Ductular-like hepatocytes	H2AFY
9.9410138617796E-128	0.740445512033916	0.306	0.161	1.66591510295703E-123	Ductular-like hepatocytes	ALG6
1.21554732874942E-127	1.02301913936145	0.23	0.106	2.03701421351828E-123	Ductular-like hepatocytes	OSBPL10
1.3693067098481E-127	0.605663650215937	0.403	0.239	2.29468418436345E-123	Ductular-like hepatocytes	MAL2
1.62599254612668E-127	0.439503613179497	0.522	0.336	2.72483830879909E-123	Ductular-like hepatocytes	GCHFR
1.91598555797534E-127	0.584969820924989	0.322	0.173	3.21080859805508E-123	Ductular-like hepatocytes	BCL9L
2.38756432715931E-127	0.518796642542007	0.431	0.259	4.00108029945357E-123	Ductular-like hepatocytes	CARS2
2.46809792752764E-127	1.29185461214865	0.146	0.051	4.13603850695081E-123	Ductular-like hepatocytes	CYP2U1
2.66442165447717E-127	1.00830615347438	0.189	0.078	4.46503780857285E-123	Ductular-like hepatocytes	PLEKHG6
3.05337602370328E-127	0.399681382534827	0.74	0.552	5.11684754052195E-123	Ductular-like hepatocytes	PRDX2
4.03493460146235E-127	0.719672870475327	0.261	0.128	6.76174340513061E-123	Ductular-like hepatocytes	CALML4
4.49850253984112E-127	0.594988444826818	0.398	0.235	7.53859055626574E-123	Ductular-like hepatocytes	ARHGAP1
5.24610234519517E-127	0.574785839993637	0.44	0.268	8.79141831007807E-123	Ductular-like hepatocytes	RPN2
5.84910911361525E-127	0.582129224688926	0.43	0.257	9.80193705259643E-123	Ductular-like hepatocytes	VAMP7
6.0840283974916E-127	0.607193201724133	0.409	0.242	1.01956147885164E-122	Ductular-like hepatocytes	PDLIM5
7.79851856902885E-127	0.453181177661131	0.559	0.375	1.30687574179785E-122	Ductular-like hepatocytes	ATP5ME
8.53483672709058E-127	0.420443501997015	0.626	0.445	1.43026793872584E-122	Ductular-like hepatocytes	CAST
1.15437926148625E-126	0.672363552798895	0.254	0.122	1.93450876639865E-122	Ductular-like hepatocytes	NIPAL1
1.27880437901554E-126	0.95268084957553	0.193	0.081	2.14302037835423E-122	Ductular-like hepatocytes	TRANK1
2.11803343146261E-126	0.699970310509847	0.203	0.087	3.54940042444504E-122	Ductular-like hepatocytes	NAALADL2
2.2031501527921E-126	1.46099971048505	0.145	0.05	3.692039026049E-122	Ductular-like hepatocytes	RNASE7
2.72180319939106E-126	0.544850218732986	0.36	0.203	4.56119780153954E-122	Ductular-like hepatocytes	TARBP1
3.74335522869849E-126	0.452772122872914	0.529	0.344	6.27311469225294E-122	Ductular-like hepatocytes	COPS5
3.81243828066511E-126	0.576807115131312	0.39	0.227	6.38888407073859E-122	Ductular-like hepatocytes	DEPRECATEI
3.89902603740384E-126	0.449689713702788	0.639	0.46	6.53398783348136E-122	Ductular-like hepatocytes	GJB1
3.95160316874342E-126	0.358417835558302	0.672	0.452	6.62209659018023E-122	Ductular-like hepatocytes	ELL2
4.2348252410588E-126	0.404225011877434	0.451	0.277	7.09672013896633E-122	Ductular-like hepatocytes	EHBP1L1
5.11175327750056E-126	0.728616801235694	0.337	0.187	8.56627614243543E-122	Ductular-like hepatocytes	ATRAID
6.29242867329395E-126	0.462330278030033	0.584	0.402	1.0544851970706E-121	Ductular-like hepatocytes	SLC38A10
8.60545451757491E-126	0.832228935364484	0.284	0.147	1.4421020680552E-121	Ductular-like hepatocytes	ADD3
8.80410104293905E-126	0.606447089443597	0.406	0.239	1.47539125277573E-121	Ductular-like hepatocytes	RBM41
1.00368774353493E-125	0.7001966527296	0.305	0.161	1.68197992061584E-121	Ductular-like hepatocytes	ARHGEF18
1.04879345854274E-125	0.601190178203595	0.295	0.152	1.75756807782592E-121	Ductular-like hepatocytes	SHKBP1
1.62202383889618E-125	0.485710204935255	0.497	0.318	2.71818754922222E-121	Ductular-like hepatocytes	DYNLRB1
1.67793595149965E-125	0.37809770517896	0.508	0.324	2.81188506752311E-121	Ductular-like hepatocytes	PIM3
1.77982136298246E-125	0.494479734912283	0.241	0.113	2.98262464008601E-121	Ductular-like hepatocytes	TSPAN13
1.92403480074422E-125	0.892117590968295	0.266	0.133	3.22429751908716E-121	Ductular-like hepatocytes	ICMT
2.19599051152643E-125	0.483528589041731	0.516	0.337	3.68004089921599E-121	Ductular-like hepatocytes	TMED4
2.41624235790045E-125	0.478230320715439	0.492	0.311	4.04913894336957E-121	Ductular-like hepatocytes	HSD3B7
2.58398783407863E-125	1.83502532760783	0.106	0.028	4.33024681234897E-121	Ductular-like hepatocytes	GPR35
2.74741108322687E-125	0.604874150809839	0.35	0.195	4.6041114932716E-121	Ductular-like hepatocytes	TYMS
2.81410784041541E-125	0.664676678595808	0.306	0.162	4.71588191896814E-121	Ductular-like hepatocytes	LRRC45
2.9270030229625E-125	0.475503262253437	0.518	0.333	4.90507166588055E-121	Ductular-like hepatocytes	FLOT2
4.34951578876398E-125	0.489769773957511	0.498	0.317	7.28891855881067E-121	Ductular-like hepatocytes	TXNL4A
4.62229838206175E-125	0.504506218874576	0.464	0.291	7.74604762865908E-121	Ductular-like hepatocytes	HIGD2A
5.02182423348111E-125	0.581243800106934	0.451	0.279	8.41557305046764E-121	Ductular-like hepatocytes	CMTM8
7.03452453501868E-125	0.460917976381043	0.53	0.345	1.17884562157843E-120	Ductular-like hepatocytes	ELAVL1
8.27491133583684E-125	0.539472969767119	0.468	0.287	1.38670964165954E-120	Ductular-like hepatocytes	KIAA0232
9.02009750808005E-125	0.615184870382243	0.378	0.219	1.51158794040405E-120	Ductular-like hepatocytes	KDELR1
9.16513149616913E-125	0.5539645288208	0.387	0.225	1.53589273612802E-120	Ductular-like hepatocytes	EXOSC4
1.01767131370372E-124	0.516901767310923	0.43	0.26	1.70541358750469E-120	Ductular-like hepatocytes	PTK2
1.10639820958827E-124	0.699775572964051	0.292	0.151	1.85410211962803E-120	Ductular-like hepatocytes	UTRN
1.13767393721913E-124	0.507158671683795	0.498	0.319	1.90651398399183E-120	Ductular-like hepatocytes	WDR83OS
1.75711342539195E-124	0.704571089329756	0.371	0.214	2.94457067827183E-120	Ductular-like hepatocytes	TERF1
1.77906054733401E-124	0.62576401718854	0.366	0.21	2.98134966522233E-120	Ductular-like hepatocytes	DCTD
2.30610145025399E-124	0.566584992303533	0.389	0.225	3.86456481033563E-120	Ductular-like hepatocytes	DENND4C
2.63849563070021E-124	0.626699765629839	0.36	0.202	4.42159097792741E-120	Ductular-like hepatocytes	PCF11
3.84217336355364E-124	0.670219039969616	0.366	0.209	6.43871412264319E-120	Ductular-like hepatocytes	ARPC5
4.81895869404397E-124	0.545723159030478	0.398	0.236	8.07561097947888E-120	Ductular-like hepatocytes	FLNB

5.47622287264259E-124	0.94942544925096	0.254	0.125	9.17705428997445E-120	Ductular-like hepatocytes	TMEM44
5.6004358674847E-124	0.835516593704544	0.222	0.102	9.38521042673087E-120	Ductular-like hepatocytes	COMTD1
6.31120855107428E-124	0.409032490352576	0.438	0.266	1.05763232898903E-119	Ductular-like hepatocytes	NUDCD3
7.35056655787569E-124	0.355910730845605	0.694	0.514	1.23180794376881E-119	Ductular-like hepatocytes	MYL6
9.56192346881953E-124	0.848553721913462	0.275	0.14	1.60238713490478E-119	Ductular-like hepatocytes	ZSWIM5
1.53241449262325E-123	0.541281602348905	0.415	0.247	2.56802020673805E-119	Ductular-like hepatocytes	RAB20
1.59521979015564E-123	0.72585053684906	0.133	0.043	2.67326932434283E-119	Ductular-like hepatocytes	ST6GALNAC
1.69127468343627E-123	0.472758975682375	0.434	0.262	2.83423811450251E-119	Ductular-like hepatocytes	AGFG1
1.71191056878762E-123	0.62714879528052	0.285	0.147	2.8688197311743E-119	Ductular-like hepatocytes	SLC37A3
1.78836771563635E-123	0.613785214565526	0.375	0.218	2.9969466178634E-119	Ductular-like hepatocytes	TMEM14C
1.84228614961975E-123	0.62167969183188	0.379	0.22	3.08730312953278E-119	Ductular-like hepatocytes	CXADR
2.1270827274776E-123	0.495874126808871	0.345	0.192	3.56456523470695E-119	Ductular-like hepatocytes	ITGA6
2.38537906282905E-123	0.438497614843306	0.562	0.377	3.99741823348892E-119	Ductular-like hepatocytes	KRTCAP2
3.27454615628522E-123	0.863546612294742	0.242	0.116	5.48748444870277E-119	Ductular-like hepatocytes	PLCB3
3.49569860337305E-123	0.447667715874117	0.469	0.289	5.85809171953256E-119	Ductular-like hepatocytes	REPS1
3.81830176437222E-123	0.660057336536632	0.306	0.162	6.39871009673497E-119	Ductular-like hepatocytes	RAVER1
4.49263305509678E-123	0.624283690548338	0.335	0.186	7.52875447373119E-119	Ductular-like hepatocytes	PTDSS1
5.08215370281889E-123	0.793999572916659	0.285	0.148	8.5166731751839E-119	Ductular-like hepatocytes	CPSF1
5.27737355073146E-123	1.00800733267637	0.22	0.101	8.84382259631579E-119	Ductular-like hepatocytes	DBNDD2
5.34594168350767E-123	0.407327222961048	0.645	0.471	8.95872907322216E-119	Ductular-like hepatocytes	ABCC3
5.81640570219097E-123	0.633435530742734	0.356	0.203	9.74713267573163E-119	Ductular-like hepatocytes	EIF3H
5.8792410155268E-123	0.480031821544679	0.521	0.333	9.85243209381982E-119	Ductular-like hepatocytes	SETD7
6.15479978735902E-123	0.527560064933502	0.433	0.265	1.03142134836563E-118	Ductular-like hepatocytes	LIX1L
7.43099940741037E-123	0.598346385594343	0.334	0.184	1.24528688069383E-118	Ductular-like hepatocytes	PLD1
7.76217136394328E-123	0.653412677104278	0.381	0.223	1.30078467716961E-118	Ductular-like hepatocytes	LMBRD2
8.34375065123222E-123	0.493271317577924	0.431	0.263	1.3982457341335E-118	Ductular-like hepatocytes	NET1
1.02456611662229E-122	0.938544679766909	0.252	0.124	1.71696789823563E-118	Ductular-like hepatocytes	LRRCC1
1.39408798121307E-122	0.460106329171529	0.553	0.369	2.33621263891686E-118	Ductular-like hepatocytes	FH
1.53336397425322E-122	0.574478139725398	0.4	0.237	2.56961134805354E-118	Ductular-like hepatocytes	PPP1R10
2.12284073108699E-122	0.44979817494701	0.525	0.348	3.55745649715559E-118	Ductular-like hepatocytes	SURF4
2.31927987036951E-122	0.640016710211444	0.322	0.175	3.88664920676522E-118	Ductular-like hepatocytes	KCTD5
3.04169684806747E-122	0.600276210307572	0.446	0.276	5.09727557799147E-118	Ductular-like hepatocytes	CMTM6
3.78510626649476E-122	0.523397685593284	0.417	0.252	6.34308108139192E-118	Ductular-like hepatocytes	OGDH
3.97873564151392E-122	0.492593000397724	0.297	0.155	6.66756518804902E-118	Ductular-like hepatocytes	ANKRD9
4.08182048256625E-122	1.64471432654335	0.128	0.042	6.84031476468452E-118	Ductular-like hepatocytes	TMEM246
5.044103782699E-122	0.418715107809517	0.596	0.4	8.45290911904698E-118	Ductular-like hepatocytes	PIK3R1
5.69250370529424E-122	0.590711843598018	0.41	0.246	9.53949770933209E-118	Ductular-like hepatocytes	NKAP
6.46432072417431E-122	0.624569959016988	0.296	0.156	1.08329086695713E-117	Ductular-like hepatocytes	ERMP1
6.90499417873409E-122	0.452389746602569	0.557	0.366	1.15713892447226E-117	Ductular-like hepatocytes	SRP9
8.47339482079558E-122	0.754229245677731	0.287	0.15	1.41997150406892E-117	Ductular-like hepatocytes	UBL4A
8.87136821303333E-122	0.504427558304577	0.502	0.326	1.48666388514012E-117	Ductular-like hepatocytes	MAGED2
1.13784991011854E-121	0.650790801798765	0.35	0.199	1.90680887937665E-117	Ductular-like hepatocytes	DICER1
1.23898691922238E-121	0.532515375026485	0.422	0.253	2.07629427923287E-117	Ductular-like hepatocytes	BIVM
1.42067374366306E-121	0.441239593962548	0.573	0.392	2.38076505963056E-117	Ductular-like hepatocytes	CHCHD2
1.65304374257948E-121	0.576147822262489	0.369	0.213	2.77017070381469E-117	Ductular-like hepatocytes	DNASE1L1
1.96835738520724E-121	0.651421477357085	0.295	0.156	3.29857330613029E-117	Ductular-like hepatocytes	KHNYN
2.06379071475313E-121	0.686314584983487	0.319	0.174	3.45850047978329E-117	Ductular-like hepatocytes	KLHL2
2.30627183155733E-121	1.38086868671922	0.15	0.056	3.86485033532377E-117	Ductular-like hepatocytes	BCL11A
2.32086109132045E-121	0.734389123084369	0.322	0.177	3.8892990168348E-117	Ductular-like hepatocytes	UGGT2
2.35384301385053E-121	0.848521339237383	0.311	0.169	3.94457012261072E-117	Ductular-like hepatocytes	DGKE
2.68416841141286E-121	0.621162652088054	0.316	0.171	4.49812942384568E-117	Ductular-like hepatocytes	C1orf122
3.58503905356665E-121	0.957483070166959	0.255	0.128	6.00780844596699E-117	Ductular-like hepatocytes	PDE7A
3.96363021873188E-121	0.371660697472077	0.387	0.224	6.64225152055088E-117	Ductular-like hepatocytes	ARPC1A
4.59595868847623E-121	0.600402827276038	0.403	0.24	7.70190757014846E-117	Ductular-like hepatocytes	TMEM214
5.3762041287772E-121	1.17287399501496	0.203	0.091	9.00944287900482E-117	Ductular-like hepatocytes	DRGX
6.14957139018623E-121	0.648863676942296	0.324	0.179	1.03054517356741E-116	Ductular-like hepatocytes	WWP2
6.80795525088343E-121	0.401277918524241	0.464	0.287	1.14087714094305E-116	Ductular-like hepatocytes	LSM 4.00
8.19648148319024E-121	0.500378138616174	0.537	0.36	1.37356636695302E-116	Ductular-like hepatocytes	EVL
9.12749583447019E-121	1.42130835948562	0.145	0.052	1.52958575194051E-116	Ductular-like hepatocytes	TLR3
9.2439629505493E-121	0.407650025378322	0.503	0.32	1.54910331125305E-116	Ductular-like hepatocytes	RNF187

1.1518925365512E-120	0.516089445115074	0.431	0.263	1.9303415127525E-116	Ductular-like hepatocytes	TTC37
1.33118291785494E-120	0.39280548213159	0.462	0.287	2.2307963337413E-116	Ductular-like hepatocytes	AGPAT3
1.67689924460119E-120	0.713032417112166	0.227	0.106	2.81014775410267E-116	Ductular-like hepatocytes	PHLDA2
1.81586947879559E-120	0.846727435318525	0.286	0.15	3.04303407256566E-116	Ductular-like hepatocytes	GPAT3
1.95878980139342E-120	0.774354104356021	0.266	0.135	3.28253994917509E-116	Ductular-like hepatocytes	RALY
2.16374878251102E-120	0.558889806647258	0.385	0.226	3.62601020973197E-116	Ductular-like hepatocytes	NDUFA7
5.27087209623306E-120	0.96119800258746	0.214	0.099	8.83292745886736E-116	Ductular-like hepatocytes	FAM222A
5.95170731869819E-120	0.493190476247662	0.572	0.394	9.97387112467443E-116	Ductular-like hepatocytes	TMEM33
5.98401727503786E-120	0.537398797897779	0.365	0.208	1.00280161495084E-115	Ductular-like hepatocytes	PRRC2A
6.00396196642209E-120	1.09992871789555	0.122	0.038	1.00614394633301E-115	Ductular-like hepatocytes	ARHGAP27
6.08796867863679E-120	0.5480997904648	0.352	0.2	1.02022179116595E-115	Ductular-like hepatocytes	E2F1
1.12131450274594E-119	0.704255577880977	0.345	0.196	1.87909884370165E-115	Ductular-like hepatocytes	ELOVL6
1.30146764007436E-119	0.508273949739186	0.431	0.263	2.18099947123662E-115	Ductular-like hepatocytes	SMARCA5
1.69056964269866E-119	0.507217335117878	0.3	0.159	2.83305660723441E-115	Ductular-like hepatocytes	KIF13B
2.00954862115874E-119	0.444500241342703	0.588	0.401	3.36760157933781E-115	Ductular-like hepatocytes	CYP4F11
2.28899054469833E-119	0.455819149644051	0.506	0.33	3.83589035480546E-115	Ductular-like hepatocytes	UQCR11
2.90013210416531E-119	0.615608128351793	0.354	0.202	4.86004138016023E-115	Ductular-like hepatocytes	MTM1
3.59095184233044E-119	0.67681649074323	0.327	0.181	6.01771709737735E-115	Ductular-like hepatocytes	DEPRECATEI
4.51248282040922E-119	0.586194239249721	0.364	0.211	7.56201871044177E-115	Ductular-like hepatocytes	AFG3L2
4.52766241340312E-119	0.833119811694685	0.255	0.128	7.58745667238094E-115	Ductular-like hepatocytes	PRR14L
5.9204774852232E-119	0.428029357919343	0.597	0.415	9.92153616973704E-115	Ductular-like hepatocytes	SLC22A18
6.70040256656336E-119	0.465052945285217	0.506	0.326	1.12285346210469E-114	Ductular-like hepatocytes	NEDD4L
7.18221378440127E-119	1.32034523583646	0.142	0.05	1.20359538598996E-114	Ductular-like hepatocytes	ARHGAP18
9.59263363169593E-119	1.31706302857936	0.136	0.047	1.6075335439996E-114	Ductular-like hepatocytes	SH3BP1
1.11587320766148E-118	0.474900329587474	0.474	0.3	1.8699803213991E-114	Ductular-like hepatocytes	IMPA1
1.1334196668911E-118	0.639843931428013	0.295	0.157	1.89938467777611E-114	Ductular-like hepatocytes	MPZL1
1.40830920941863E-118	0.584979234478872	0.276	0.142	2.36004457314374E-114	Ductular-like hepatocytes	FBXL12
1.51462760716533E-118	0.649670766453609	0.314	0.172	2.53821294408766E-114	Ductular-like hepatocytes	MVB12A
1.61139953413873E-118	0.459219727922987	0.376	0.221	2.70038333930968E-114	Ductular-like hepatocytes	DEPRECATEI
1.62936332376917E-118	0.588377584444642	0.345	0.196	2.73048705797237E-114	Ductular-like hepatocytes	EDEM2
1.78143935602699E-118	0.498507361456553	0.418	0.254	2.98533607283003E-114	Ductular-like hepatocytes	DDB1
1.8108834788279E-118	0.825537090488582	0.264	0.134	3.03467853381979E-114	Ductular-like hepatocytes	PRPS2
2.2439009384988E-118	0.596780602354039	0.354	0.202	3.76032919273628E-114	Ductular-like hepatocytes	SPIDR
2.32879079595039E-118	0.566476303303771	0.377	0.221	3.90258761585366E-114	Ductular-like hepatocytes	UBA1
2.65794054702267E-118	0.594570342851015	0.322	0.178	4.45417676870059E-114	Ductular-like hepatocytes	SLC25A43
2.8567413556527E-118	0.592366193501748	0.325	0.18	4.78732716380279E-114	Ductular-like hepatocytes	SYPL1
3.50817404779133E-118	0.576559898169749	0.332	0.185	5.87899806928872E-114	Ductular-like hepatocytes	FIBP
4.44801624487522E-118	0.52527691433818	0.388	0.229	7.4539856231619E-114	Ductular-like hepatocytes	GSK3A
4.86843091402126E-118	1.14620641711535	0.128	0.042	8.15851652571683E-114	Ductular-like hepatocytes	SLC9A1
5.24084787214606E-118	0.643235116160683	0.374	0.22	8.78261286414236E-114	Ductular-like hepatocytes	DPYSL2
5.3383108103069E-118	1.05803213769896	0.181	0.076	8.94594125591231E-114	Ductular-like hepatocytes	ATP1B3
5.36553066734573E-118	0.845923192691725	0.253	0.128	8.99155629233798E-114	Ductular-like hepatocytes	P4HTM
5.43520621486845E-118	0.71076865983944	0.286	0.151	9.10831857487656E-114	Ductular-like hepatocytes	SLC35E1
6.1931764212339E-118	0.624918023069699	0.287	0.152	1.03785250467038E-113	Ductular-like hepatocytes	LY6E
6.93176812901849E-118	0.87917467937899	0.241	0.118	1.16162570306092E-113	Ductular-like hepatocytes	CDT1
7.65461422453754E-118	0.608513575918723	0.353	0.202	1.282760251748E-113	Ductular-like hepatocytes	RAP2A
8.41596880503381E-118	0.459413744688582	0.547	0.363	1.41034805234757E-113	Ductular-like hepatocytes	LMO7
9.93772593978085E-118	0.45993158889168	0.511	0.329	1.66536411298848E-113	Ductular-like hepatocytes	SLC25A15
1.11772999061151E-117	0.539154832346761	0.53	0.364	1.87309191826678E-113	Ductular-like hepatocytes	IKBKB
1.226733079529E-117	0.860525621014572	0.212	0.097	2.0557592946747E-113	Ductular-like hepatocytes	NEO1
1.32087675826487E-117	0.593711639774057	0.394	0.236	2.21352527150027E-113	Ductular-like hepatocytes	ELOVL2
1.33706485747516E-117	0.775347199716153	0.244	0.12	2.24065328815687E-113	Ductular-like hepatocytes	CMTM4
1.43707276125611E-117	0.3598876660232	0.465	0.294	2.40824653331299E-113	Ductular-like hepatocytes	CTDSP1
1.92414390540733E-117	0.667160086555762	0.369	0.217	3.2244803566816E-113	Ductular-like hepatocytes	POMP
2.47452067065506E-117	0.541063319895233	0.372	0.214	4.14680173988374E-113	Ductular-like hepatocytes	RRM1
2.49009991728101E-117	0.484775590692985	0.451	0.283	4.17290944137952E-113	Ductular-like hepatocytes	TMEM50A
2.71575018500429E-117	0.481123080475752	0.245	0.119	4.55105416003018E-113	Ductular-like hepatocytes	TTYH3
3.12015665939677E-117	0.591789908999329	0.374	0.22	5.22875852981711E-113	Ductular-like hepatocytes	ATP6AP2
3.64294085060046E-117	0.554934247791546	0.319	0.176	6.10484027743625E-113	Ductular-like hepatocytes	VARS
3.71732163946125E-117	0.393495843688866	0.613	0.425	6.22948760340917E-113	Ductular-like hepatocytes	ENY2

4.79405798713331E-117	0.508026281476744	0.45	0.282	8.033882374838E-113	Ductular-like hepatocytes	ATP5F1B
5.24884890330557E-117	0.466004828266417	0.4	0.236	8.79602099215947E-113	Ductular-like hepatocytes	NUP62
5.58095642366941E-117	0.679778074024749	0.293	0.156	9.3525667747852E-113	Ductular-like hepatocytes	TNFAIP2
5.90812354123383E-117	0.503780090586516	0.451	0.281	9.90083343039965E-113	Ductular-like hepatocytes	FNTA
6.5462944884362E-117	0.490464193997939	0.455	0.286	1.09702803037214E-112	Ductular-like hepatocytes	COPS6
7.57740247741154E-117	0.506419636221058	0.35	0.199	1.26982110716463E-112	Ductular-like hepatocytes	GORASP1
7.64278467453892E-117	0.327449818936183	0.598	0.423	1.28077785575923E-112	Ductular-like hepatocytes	LAMTOR4
1.08071143932742E-116	0.76184973350138	0.273	0.142	1.81105623002488E-112	Ductular-like hepatocytes	C2CD5
1.18320144749187E-116	0.56559142669214	0.362	0.21	1.98280898570688E-112	Ductular-like hepatocytes	LARP1
1.19028813462662E-116	0.507774408027362	0.41	0.247	1.99468485600729E-112	Ductular-like hepatocytes	PI4KB
1.23770625783658E-116	0.624161328455013	0.355	0.204	2.07414814688255E-112	Ductular-like hepatocytes	NFKB1
1.86061086824334E-116	0.725096506108958	0.273	0.142	3.11801169300219E-112	Ductular-like hepatocytes	PTPRJ
1.97830671461391E-116	0.576824003937653	0.303	0.163	3.31524639235E-112	Ductular-like hepatocytes	MYO5B
2.38159582385428E-116	0.515360339826011	0.371	0.216	3.991078281615E-112	Ductular-like hepatocytes	PSEN1
2.52556826970437E-116	0.550730320530939	0.424	0.261	4.23234730637058E-112	Ductular-like hepatocytes	MAT2B
2.56583195164187E-116	0.616216916374336	0.302	0.163	4.29982118456144E-112	Ductular-like hepatocytes	PDLIM1
2.98175910890756E-116	0.886169123780341	0.238	0.117	4.9968319147073E-112	Ductular-like hepatocytes	ABHD15
3.94315634433377E-116	0.426015060976533	0.337	0.188	6.60794140183453E-112	Ductular-like hepatocytes	YKT6
4.22636524649574E-116	0.702121466929186	0.273	0.142	7.08254288007757E-112	Ductular-like hepatocytes	ARL6IP5
4.57101923588909E-116	0.565441432991268	0.248	0.122	7.66011403550294E-112	Ductular-like hepatocytes	EPHB4
5.15438775755357E-116	1.18910317382766	0.138	0.049	8.63772300410827E-112	Ductular-like hepatocytes	RAB27B
5.95703149409716E-116	0.432108460810473	0.583	0.396	9.98279337780802E-112	Ductular-like hepatocytes	RAB4A
6.64626441970933E-116	0.457853828494554	0.408	0.247	1.11378099145489E-111	Ductular-like hepatocytes	BCAR1
7.73129204640555E-116	0.457188265665212	0.367	0.214	1.29560992113664E-111	Ductular-like hepatocytes	PI4KA
8.5994211540517E-116	0.412619207389986	0.479	0.304	1.44109099699598E-111	Ductular-like hepatocytes	SMARCA4
9.08860325378064E-116	0.686312612268491	0.39	0.234	1.52306813326856E-111	Ductular-like hepatocytes	AQP3
1.63600317854854E-115	0.621643894374919	0.358	0.207	2.74161412661164E-111	Ductular-like hepatocytes	BROX
1.80761327429425E-115	0.512811570467177	0.298	0.159	3.0291983250623E-111	Ductular-like hepatocytes	TNIP2
1.85008980069185E-115	0.841297652581553	0.253	0.128	3.10038048799939E-111	Ductular-like hepatocytes	CYB561
2.32977524673406E-115	0.508798066217655	0.424	0.258	3.90423735847694E-111	Ductular-like hepatocytes	EEF2K
2.5106568842271E-115	0.461340313716099	0.384	0.227	4.20735880658778E-111	Ductular-like hepatocytes	CMIP
2.53310500069343E-115	0.527331390274789	0.403	0.243	4.24497736016205E-111	Ductular-like hepatocytes	PPP1CB
2.83554083647814E-115	0.632921551537428	0.314	0.173	4.75179933377007E-111	Ductular-like hepatocytes	ARFGEF1
3.04349063804628E-115	0.528396535085851	0.43	0.265	5.10028161123796E-111	Ductular-like hepatocytes	ANKRD17
3.05080315886278E-115	0.681721976818003	0.202	0.091	5.11253593362224E-111	Ductular-like hepatocytes	EVI5L
3.34015058630872E-115	0.617146711878058	0.375	0.222	5.59742435253615E-111	Ductular-like hepatocytes	FKBP1A
3.55943602672977E-115	0.685486355872029	0.338	0.192	5.96490289359374E-111	Ductular-like hepatocytes	EIF2S3
3.67243083422162E-115	0.699303344955183	0.324	0.182	6.15425959198859E-111	Ductular-like hepatocytes	TSPAN4
3.72974931088792E-115	0.480413641288467	0.467	0.292	6.25031389518598E-111	Ductular-like hepatocytes	HECTD1
4.2883904730271E-115	0.66765562839846	0.354	0.206	7.18648475469881E-111	Ductular-like hepatocytes	SUSD3
5.0087664851998E-115	0.608707985853449	0.357	0.207	8.39369087589783E-111	Ductular-like hepatocytes	PGAP3
5.40212841150289E-115	0.426913128567112	0.499	0.324	9.05288679199654E-111	Ductular-like hepatocytes	BAG6
6.6143447784827E-115	0.381993076741881	0.567	0.382	1.10843189797813E-110	Ductular-like hepatocytes	LLGL2
7.44807848762999E-115	0.479045909166725	0.349	0.2	1.24814899295703E-110	Ductular-like hepatocytes	ATP5MF
7.57032540727755E-115	0.822086359668653	0.259	0.132	1.26863513175157E-110	Ductular-like hepatocytes	MICOS13
7.94977341280698E-115	1.2685506868528	0.177	0.075	1.33222302851819E-110	Ductular-like hepatocytes	H2AFY2
7.96397334580645E-115	0.500945343260051	0.431	0.265	1.33460265329025E-110	Ductular-like hepatocytes	DCTN1
1.01767963048225E-114	0.383326846681949	0.548	0.352	1.70542752476215E-110	Ductular-like hepatocytes	TM7SF3
1.12292890894733E-114	0.548161457854619	0.384	0.23	1.88180426561393E-110	Ductular-like hepatocytes	SLC35A3
1.1585538793492E-114	1.02679920358564	0.2	0.09	1.94150459101338E-110	Ductular-like hepatocytes	CDCA7L
1.79296801706026E-114	0.666565390015898	0.318	0.177	3.00465580298959E-110	Ductular-like hepatocytes	AKIP1
2.04920716464952E-114	0.560275897022425	0.363	0.211	3.43406136651966E-110	Ductular-like hepatocytes	DEPRECATEI
2.1424559426505E-114	0.704714029495362	0.282	0.149	3.59032766869371E-110	Ductular-like hepatocytes	GEMIN7
2.50198448562172E-114	0.423996640829607	0.502	0.323	4.19282560100487E-110	Ductular-like hepatocytes	DHX15
2.51829593906032E-114	1.32139300751465	0.134	0.047	4.22016033467728E-110	Ductular-like hepatocytes	FAM234B
2.52849077991404E-114	0.575501223797082	0.329	0.184	4.23724484897995E-110	Ductular-like hepatocytes	UBTD2
2.6107059673564E-114	0.350358797204921	0.313	0.169	4.37502106009586E-110	Ductular-like hepatocytes	PDGFA
2.70405139341829E-114	0.445660116847866	0.516	0.341	4.53144932509037E-110	Ductular-like hepatocytes	ACBD3
3.4697586501359E-114	0.517647647137511	0.315	0.174	5.81462154589775E-110	Ductular-like hepatocytes	MYO1C
3.9888415683388E-114	0.443521659975183	0.562	0.372	6.68450070022216E-110	Ductular-like hepatocytes	STBD1

4.01456314178474E-114	0.436438112286342	0.404	0.243	6.72760491300287E-110	Ductular-like hepatocytes	SH3GLB2
4.86701376156112E-114	0.417289771609888	0.551	0.374	8.15614166162413E-110	Ductular-like hepatocytes	NDUFS7
5.09058143447223E-114	0.666584042930358	0.253	0.128	8.53079636788857E-110	Ductular-like hepatocytes	PTPN6
8.52135472153135E-114	0.876148110702956	0.224	0.107	1.42800862423422E-109	Ductular-like hepatocytes	SDHAF3
1.11748619747174E-113	0.688640688168139	0.292	0.158	1.87268336972314E-109	Ductular-like hepatocytes	SMIM20
1.177914320147E-113	0.685945384078623	0.324	0.182	1.97394881770234E-109	Ductular-like hepatocytes	NXT2
1.19307003321627E-113	1.51249271790534	0.133	0.047	1.99934676166382E-109	Ductular-like hepatocytes	ZG16B
1.25216321070527E-113	0.496677747906602	0.417	0.256	2.09837510849989E-109	Ductular-like hepatocytes	GNB1
3.39643206515199E-113	0.627493438436568	0.262	0.134	5.69174085478171E-109	Ductular-like hepatocytes	WLS
3.87349800065232E-113	0.432755916698441	0.423	0.259	6.49120794949315E-109	Ductular-like hepatocytes	CDC42BPB
3.89146774628736E-113	0.743593759244876	0.202	0.091	6.52132164922836E-109	Ductular-like hepatocytes	SSH3
4.05628088563443E-113	1.51599424038658	0.128	0.044	6.79751550814618E-109	Ductular-like hepatocytes	ADCY3
4.70722088536504E-113	0.358901028709606	0.597	0.417	7.88836075969473E-109	Ductular-like hepatocytes	SLC25A1
6.21784559356766E-113	0.702585309933971	0.311	0.173	1.04198656457007E-108	Ductular-like hepatocytes	NSMAF
6.54134538058885E-113	0.370428112533645	0.38	0.223	1.09619865887908E-108	Ductular-like hepatocytes	TNRC18
7.03115784198126E-113	0.3747543388349	0.514	0.333	1.17828143115922E-108	Ductular-like hepatocytes	LSS
8.05360565280731E-113	0.559047260588585	0.357	0.208	1.34962323529745E-108	Ductular-like hepatocytes	KDM3B
9.15982287501573E-113	0.708571069048046	0.229	0.111	1.53500311739514E-108	Ductular-like hepatocytes	EPDR1
9.775488612205E-113	0.460194111546086	0.357	0.206	1.63817638163331E-108	Ductular-like hepatocytes	CENPV
9.84796446709404E-113	0.524194326863465	0.379	0.224	1.65032188539562E-108	Ductular-like hepatocytes	UBE4A
1.09874899284851E-112	1.1121927971412	0.14	0.051	1.84128356221553E-108	Ductular-like hepatocytes	CLCN2
1.11834979777806E-112	0.632228840133722	0.362	0.213	1.87413059111647E-108	Ductular-like hepatocytes	WDYHV1
1.12564230751208E-112	0.328250621073232	0.665	0.495	1.88635137892874E-108	Ductular-like hepatocytes	ZFP36
1.26334057969806E-112	0.783016893512596	0.285	0.153	2.117106143458E-108	Ductular-like hepatocytes	CSTF2
1.28320258409688E-112	0.650732453417931	0.328	0.186	2.15039089042956E-108	Ductular-like hepatocytes	LARP4B
1.3719598527907E-112	0.563714939976142	0.283	0.149	2.29913032130665E-108	Ductular-like hepatocytes	RAB4B
1.40131196630848E-112	0.476419627060443	0.449	0.281	2.34831859313975E-108	Ductular-like hepatocytes	TMEM68
1.47546586511901E-112	0.808012769680714	0.293	0.161	2.47258569676644E-108	Ductular-like hepatocytes	CD9
1.63511759370782E-112	1.17919555112502	0.284	0.158	2.74013006353556E-108	Ductular-like hepatocytes	DUOXA2
1.9118028860616E-112	0.404780597514706	0.537	0.356	3.20379927646203E-108	Ductular-like hepatocytes	HNRNPD
1.97087432300305E-112	0.577783700814257	0.356	0.207	3.30279119048851E-108	Ductular-like hepatocytes	LYST
2.43668346460682E-112	0.721097280365242	0.298	0.163	4.08339414998811E-108	Ductular-like hepatocytes	USP53
2.56077476487283E-112	0.568539905252214	0.395	0.238	4.29134635097389E-108	Ductular-like hepatocytes	UBQLN2
2.74033603187752E-112	0.465064450130233	0.494	0.316	4.59225512222034E-108	Ductular-like hepatocytes	ZBTB33
3.03367792296931E-112	0.501863888734858	0.385	0.23	5.08383746331196E-108	Ductular-like hepatocytes	AHCY
3.17137396204903E-112	0.520323115580154	0.373	0.221	5.31458848560177E-108	Ductular-like hepatocytes	GOLGB1
4.1130357363356E-112	1.05486857692073	0.198	0.09	6.8926252869512E-108	Ductular-like hepatocytes	B4GALT6
4.27375628030406E-112	0.566947681913872	0.38	0.227	7.16196077453354E-108	Ductular-like hepatocytes	CCT3
4.61333577186283E-112	0.622580997392091	0.366	0.215	7.73102808648774E-108	Ductular-like hepatocytes	TECR
4.62535875824187E-112	0.652632027816692	0.276	0.147	7.75117620706172E-108	Ductular-like hepatocytes	NANS
4.67895401548369E-112	0.440450339924506	0.436	0.273	7.84099113914757E-108	Ductular-like hepatocytes	PRKAG2
5.08954094853028E-112	0.434964274158801	0.416	0.254	8.52905272154704E-108	Ductular-like hepatocytes	RAB1B
6.59169924188004E-112	0.4082928372376	0.487	0.312	1.10463695895426E-107	Ductular-like hepatocytes	NCOA2
7.13144484329274E-112	0.539919293433312	0.374	0.22	1.195087526839E-107	Ductular-like hepatocytes	SUPT16H
7.55924399014047E-112	0.707085515441229	0.314	0.175	1.26677810786774E-107	Ductular-like hepatocytes	TEX261
8.75076486725653E-112	1.34827592875849	0.133	0.047	1.46645317645485E-107	Ductular-like hepatocytes	SLC45A4
1.03230536629243E-111	0.750465722614836	0.175	0.073	1.72993733283286E-107	Ductular-like hepatocytes	TTC22
1.0382815539839E-111	0.501964434512359	0.392	0.234	1.73995222816622E-107	Ductular-like hepatocytes	FBXL6
1.25101318435507E-111	0.912671179686287	0.223	0.108	2.09644789434223E-107	Ductular-like hepatocytes	FSD1L
1.26858300868576E-111	0.655064423524454	0.283	0.152	2.12589140595559E-107	Ductular-like hepatocytes	RUSC1
1.29498336282435E-111	0.495019821553612	0.47	0.3	2.17013311942105E-107	Ductular-like hepatocytes	DDX6
1.37491702714987E-111	0.724896355269105	0.331	0.189	2.30408595409776E-107	Ductular-like hepatocytes	NDUFB4
1.59356310923747E-111	0.526846927406706	0.338	0.193	2.67049305846016E-107	Ductular-like hepatocytes	ATIC
1.79716662909179E-111	0.698559570915488	0.283	0.152	3.01169183703202E-107	Ductular-like hepatocytes	TTC7A
2.00792142352409E-111	0.628694082852941	0.325	0.184	3.36487472154167E-107	Ductular-like hepatocytes	TULP4
2.1519420685907E-111	0.428223883781274	0.478	0.308	3.60622451854429E-107	Ductular-like hepatocytes	HNRNPAB
2.67684713188595E-111	1.20099896915652	0.163	0.067	4.48586042361448E-107	Ductular-like hepatocytes	MANSC1
3.0276693150097E-111	0.315541872845422	0.364	0.212	5.07376823809326E-107	Ductular-like hepatocytes	HECTD3
3.18939246156915E-111	0.419973856580494	0.47	0.299	5.34478388709759E-107	Ductular-like hepatocytes	STAT1
3.8843691532335E-111	0.55295409585139	0.356	0.207	6.5094258269887E-107	Ductular-like hepatocytes	APPL2

3.90491717864264E-111	0.646132482763105	0.401	0.248	6.54386020796933E-107	Ductular-like hepatocytes	ME1
4.24735678979864E-111	0.837248009547442	0.268	0.142	7.11772050834457E-107	Ductular-like hepatocytes	RNF170
5.28760391751976E-111	1.13850847412899	0.211	0.1	8.86096664497961E-107	Ductular-like hepatocytes	MELTF
5.95336913118011E-111	0.500389001438842	0.425	0.261	9.97665599003163E-107	Ductular-like hepatocytes	RBM26
6.26918740243843E-111	0.433194477821009	0.497	0.32	1.05059042490063E-106	Ductular-like hepatocytes	HUWE1
6.63954168822045E-111	0.392938669300029	0.407	0.247	1.11265439611198E-106	Ductular-like hepatocytes	FKBP8
8.37745225198027E-111	0.391855326740565	0.597	0.403	1.40389344838685E-106	Ductular-like hepatocytes	PICALM
8.59744291355298E-111	0.643077466929771	0.268	0.14	1.44075948345321E-106	Ductular-like hepatocytes	GALNT11
8.93430507969759E-111	1.25968688854829	0.144	0.055	1.49721084525572E-106	Ductular-like hepatocytes	SYTL2
9.33574388461656E-111	0.392523710175506	0.427	0.266	1.56448396018404E-106	Ductular-like hepatocytes	PRKCSH
9.47294136830321E-111	0.527092282657268	0.339	0.196	1.58747551450025E-106	Ductular-like hepatocytes	KIAA2013
9.50819050390088E-111	0.608538419134745	0.339	0.195	1.59338256464371E-106	Ductular-like hepatocytes	LAMC1
9.57434498066353E-111	0.377143474709072	0.521	0.344	1.6044687318596E-106	Ductular-like hepatocytes	FAM120A
1.80266293827871E-110	0.494401503233288	0.444	0.277	3.02090255196745E-106	Ductular-like hepatocytes	RBM10
1.90016773250342E-110	0.671174631697779	0.254	0.13	3.18430108612923E-106	Ductular-like hepatocytes	SYNJ2BP
1.95296044092462E-110	0.575701255475929	0.367	0.218	3.27277110690148E-106	Ductular-like hepatocytes	TMEM59
2.10061968678193E-110	1.37247083978812	0.132	0.047	3.52021847110916E-106	Ductular-like hepatocytes	ENDOD1
2.41512474154162E-110	0.775492728443492	0.235	0.117	4.04726604187545E-106	Ductular-like hepatocytes	NT5C3A
2.46634267691209E-110	1.09360382630059	0.183	0.081	4.13309705796929E-106	Ductular-like hepatocytes	SLC16A7
2.99480442643918E-110	0.626827857346113	0.277	0.147	5.01869325782678E-106	Ductular-like hepatocytes	SLC35B4
3.08116783189262E-110	0.469271584029548	0.385	0.229	5.16342105268566E-106	Ductular-like hepatocytes	THOP1
3.28527190047464E-110	0.681702217158961	0.315	0.178	5.50545865081541E-106	Ductular-like hepatocytes	DGCR2
3.31710036815551E-110	0.417731612228435	0.431	0.265	5.558796796955E-106	Ductular-like hepatocytes	CDKN2A
3.59050062899803E-110	0.331623208485069	0.555	0.364	6.0169609540749E-106	Ductular-like hepatocytes	CEP70
3.78331759906203E-110	0.500821932823682	0.356	0.206	6.34008363250814E-106	Ductular-like hepatocytes	PGLS
4.08923729015596E-110	0.402478942845273	0.464	0.295	6.85274385084336E-106	Ductular-like hepatocytes	ONECUT2
4.11453930565282E-110	0.435536701663655	0.431	0.267	6.89514496841299E-106	Ductular-like hepatocytes	ATF6B
4.64596204378893E-110	0.51022404763968	0.446	0.281	7.78570319298148E-106	Ductular-like hepatocytes	TIMM8A
5.84905707109824E-110	0.448369192284805	0.454	0.289	9.80184983974644E-106	Ductular-like hepatocytes	SCAMP3
6.04608535241341E-110	0.406524818726334	0.366	0.215	1.01320298335744E-105	Ductular-like hepatocytes	PRELID1
6.51096299225535E-110	0.69623157718359	0.261	0.136	1.09110717824215E-105	Ductular-like hepatocytes	POLA1
7.16225836160661E-110	0.437185298847404	0.47	0.302	1.20025125623803E-105	Ductular-like hepatocytes	CALU
8.41624375841788E-110	0.930271915297848	0.213	0.101	1.41039412903567E-105	Ductular-like hepatocytes	CNTNAP2
8.68853887576692E-110	0.598738138063249	0.334	0.191	1.45602534480102E-105	Ductular-like hepatocytes	GLI4
1.04851297867389E-109	0.523273706829523	0.465	0.3	1.7570980496617E-105	Ductular-like hepatocytes	GOLGA7
1.21647179710233E-109	0.68724873479704	0.311	0.174	2.03856343758408E-105	Ductular-like hepatocytes	CASP6
1.27075793185297E-109	0.639194968037125	0.24	0.12	2.1295361421992E-105	Ductular-like hepatocytes	BTBD3
1.34132972582318E-109	0.510568004731287	0.354	0.206	2.24780035453448E-105	Ductular-like hepatocytes	POMGNT1
1.37894596600683E-109	0.547658743610112	0.367	0.218	2.31083764983425E-105	Ductular-like hepatocytes	GPR107
1.41309362957738E-109	0.610733897817282	0.353	0.205	2.36806230444578E-105	Ductular-like hepatocytes	PRELID2
1.47622790649974E-109	0.780421985213756	0.204	0.095	2.47386272571226E-105	Ductular-like hepatocytes	TES
1.50773681963866E-109	1.1033603097694	0.162	0.066	2.52666536235047E-105	Ductular-like hepatocytes	PDE5A
1.51308897239968E-109	0.725116159772653	0.254	0.13	2.53563449994738E-105	Ductular-like hepatocytes	GTF2IRD1
1.55746739917605E-109	0.473958634148761	0.489	0.317	2.61000386753922E-105	Ductular-like hepatocytes	PIR
1.72733018112095E-109	1.21319953803857	0.15	0.059	2.89465991752249E-105	Ductular-like hepatocytes	MAMLD1
1.87307693486961E-109	0.709074718248108	0.298	0.164	3.1389023274545E-105	Ductular-like hepatocytes	FZD6
2.15291591051745E-109	0.391064978712857	0.473	0.305	3.60785648284515E-105	Ductular-like hepatocytes	GNAQ
2.29381616101541E-109	0.48108668395053	0.475	0.307	3.84397712262963E-105	Ductular-like hepatocytes	NDUFS1
3.06077549829128E-109	0.553495115166798	0.366	0.215	5.12924758003653E-105	Ductular-like hepatocytes	DPY19L4
3.36149111473504E-109	0.376174664610509	0.597	0.406	5.63318681007297E-105	Ductular-like hepatocytes	GSR
3.65845922814703E-109	0.43038680368152	0.501	0.331	6.13084597452879E-105	Ductular-like hepatocytes	PDCD6IP
3.88581977563557E-109	0.358572614169035	0.502	0.328	6.51185678001009E-105	Ductular-like hepatocytes	MARCH6
4.38704097224139E-109	0.465560768783193	0.444	0.281	7.35180326128212E-105	Ductular-like hepatocytes	CSTB
4.57188438318511E-109	0.452061201956497	0.484	0.317	7.66156384934161E-105	Ductular-like hepatocytes	IRF6
4.57371187196343E-109	1.66268361936679	0.163	0.068	7.66462635503632E-105	Ductular-like hepatocytes	HILPDA
5.06528311594517E-109	0.967322630024569	0.259	0.136	8.48840144570092E-105	Ductular-like hepatocytes	POU5F1
5.24821885828474E-109	1.13908960692364	0.152	0.06	8.79496516271357E-105	Ductular-like hepatocytes	SLC38A11
6.69038236483372E-109	0.600717128488673	0.3	0.164	1.12117427669883E-104	Ductular-like hepatocytes	RCOR1
8.24067211044812E-109	0.38293827819249	0.491	0.32	1.3809718322689E-104	Ductular-like hepatocytes	LAMTOR1
8.26359355383037E-109	0.700386272612686	0.234	0.117	1.38481300775089E-104	Ductular-like hepatocytes	GLCE

9.54886335996636E-109	0.353517204633075	0.71	0.537	1.60019852186316E-104	Ductular-like hepatocytes	SDC1
9.67624187058174E-109	0.487302850463245	0.37	0.216	1.62154461267209E-104	Ductular-like hepatocytes	DHFR
1.15165645131404E-108	0.569457786074514	0.365	0.217	1.92994588111207E-104	Ductular-like hepatocytes	UBL5
1.31838900530295E-108	0.778431046993591	0.206	0.096	2.20935629508669E-104	Ductular-like hepatocytes	RNASET2
1.37916464273919E-108	0.389694858468867	0.413	0.254	2.31120410830234E-104	Ductular-like hepatocytes	GTF2I
1.56547952443898E-108	0.717871879631152	0.238	0.12	2.62343058705484E-104	Ductular-like hepatocytes	PIGS
1.80341700203853E-108	0.563174919992112	0.355	0.208	3.02216621201617E-104	Ductular-like hepatocytes	TSPAN33
2.00093942547803E-108	0.62043940513605	0.294	0.162	3.35317428921608E-104	Ductular-like hepatocytes	CCNQ
2.01054368932049E-108	0.56107396196274	0.311	0.173	3.36926911456327E-104	Ductular-like hepatocytes	NABP2
2.3220715709988E-108	0.649390403497505	0.2	0.092	3.89132753867979E-104	Ductular-like hepatocytes	PTGFRN
2.67707604727134E-108	0.388564799522663	0.395	0.238	4.48624404001732E-104	Ductular-like hepatocytes	TMUB1
2.7426292030134E-108	0.50187952286067	0.405	0.248	4.59609801840986E-104	Ductular-like hepatocytes	STMP1
2.96475539075359E-108	0.347009448629774	0.405	0.247	4.96833708382487E-104	Ductular-like hepatocytes	IDUA
3.78316084635007E-108	0.537795514777945	0.352	0.205	6.33982094631345E-104	Ductular-like hepatocytes	CNOT11
3.83259808436545E-108	0.40362060805993	0.443	0.28	6.42266786977962E-104	Ductular-like hepatocytes	TMEM238
5.10153263901226E-108	0.679089136074471	0.345	0.201	8.54914839645675E-104	Ductular-like hepatocytes	FADS1
5.87905201068878E-108	0.405967314508887	0.571	0.398	9.85211535951225E-104	Ductular-like hepatocytes	DDRGK1
5.90490851738861E-108	0.898181103018174	0.254	0.134	9.89544569343984E-104	Ductular-like hepatocytes	TINAG
7.05125551544941E-108	0.651485074168876	0.244	0.123	1.18164939927901E-103	Ductular-like hepatocytes	MYORG
7.90693440041916E-108	0.262757214579159	0.358	0.207	1.32504406682224E-103	Ductular-like hepatocytes	PGPEP1
7.93006733525425E-108	0.446795543396474	0.481	0.314	1.32892068404191E-103	Ductular-like hepatocytes	COX16
8.10834612059267E-108	0.793933987786405	0.275	0.148	1.35879664288892E-103	Ductular-like hepatocytes	TMX1
8.81803964220259E-108	0.470031717323307	0.386	0.235	1.47772708324031E-103	Ductular-like hepatocytes	CHMP4B
8.84436581714396E-108	0.405423772767162	0.372	0.221	1.48213882363698E-103	Ductular-like hepatocytes	SLC22A23
9.3912317054669E-108	0.470251287410993	0.457	0.291	1.57378260920214E-103	Ductular-like hepatocytes	CCNI
9.98642716881766E-108	0.769915435072195	0.274	0.147	1.67352546495046E-103	Ductular-like hepatocytes	DNAAF2
1.54482558802673E-107	0.796940918603503	0.284	0.154	2.5888187204152E-103	Ductular-like hepatocytes	CLCN3
1.58323081897614E-107	0.680323957485602	0.306	0.171	2.65317820644021E-103	Ductular-like hepatocytes	ICK
1.75320709505851E-107	0.486558322491349	0.424	0.265	2.93802444989904E-103	Ductular-like hepatocytes	IFI35
1.91742498393743E-107	0.62316025236414	0.266	0.14	3.21322078808235E-103	Ductular-like hepatocytes	TEF
2.17678525620278E-107	0.99551174365798	0.184	0.082	3.64785673234462E-103	Ductular-like hepatocytes	MYLIP
2.31621928521239E-107	0.309073987514546	0.345	0.198	3.88152027815893E-103	Ductular-like hepatocytes	ZNF219
2.83863098137238E-107	0.562572259300534	0.326	0.185	4.75697779858383E-103	Ductular-like hepatocytes	HPS3
2.90865746527823E-107	0.466091098934015	0.395	0.239	4.87432818031325E-103	Ductular-like hepatocytes	SNX5
3.76567187792076E-107	0.911641865310664	0.229	0.114	6.3105129330196E-103	Ductular-like hepatocytes	RAP1GDS1
4.07116183156808E-107	0.430203838046512	0.458	0.291	6.82245299734179E-103	Ductular-like hepatocytes	IPO9
4.45084587880605E-107	0.639900187434304	0.319	0.181	7.45872752370318E-103	Ductular-like hepatocytes	OSGEP
4.75531217966174E-107	0.630487070385103	0.194	0.088	7.96895215067714E-103	Ductular-like hepatocytes	PITX1
4.80692129992409E-107	0.396299871361975	0.502	0.329	8.05543871441278E-103	Ductular-like hepatocytes	STT3B
4.94209461067416E-107	0.606110807367041	0.381	0.23	8.28196214856776E-103	Ductular-like hepatocytes	GRIA3
7.02809633348779E-107	0.852425430277957	0.215	0.104	1.17776838356588E-102	Ductular-like hepatocytes	PCDHGA5
7.2151263147434E-107	0.353317252554239	0.695	0.517	1.2091108678247E-102	Ductular-like hepatocytes	COPG1
7.48239824057611E-107	1.01595057733087	0.16	0.066	1.25390029715574E-102	Ductular-like hepatocytes	LUZP1
8.29783079082691E-107	0.449317711155221	0.395	0.24	1.39055048392677E-102	Ductular-like hepatocytes	CNOT6L
9.07969303791166E-107	0.679817214202288	0.298	0.166	1.52157495929324E-102	Ductular-like hepatocytes	GON7
9.12125062421486E-107	0.351403393686593	0.562	0.375	1.52853917960593E-102	Ductular-like hepatocytes	SYNE2
9.99263700695655E-107	0.693510883917328	0.187	0.083	1.67456610962578E-102	Ductular-like hepatocytes	SLC7A9
1.01489577586456E-106	0.797210528520718	0.254	0.133	1.70076234119383E-102	Ductular-like hepatocytes	NOL3
1.13745094947683E-106	0.535712357982709	0.355	0.208	1.90614030113327E-102	Ductular-like hepatocytes	SCAF4
1.25046645815886E-106	0.652117049040259	0.304	0.169	2.09553169058262E-102	Ductular-like hepatocytes	MAP4K5
1.34173126656368E-106	0.913142468575884	0.224	0.111	2.24847325650742E-102	Ductular-like hepatocytes	HMGN5
1.58798512889188E-106	0.459314589246068	0.462	0.299	2.66114547899701E-102	Ductular-like hepatocytes	SLC39A7
1.6861998252074E-106	1.1644121238687	0.148	0.058	2.82573366708256E-102	Ductular-like hepatocytes	C5orf30
2.14709777180883E-106	0.505798016974405	0.413	0.256	3.59810644599724E-102	Ductular-like hepatocytes	NDUFB1
2.49556993925073E-106	0.914249504303674	0.178	0.078	4.18207610419638E-102	Ductular-like hepatocytes	TMEM87B
2.87122527811492E-106	1.17534245992529	0.16	0.067	4.81159932106499E-102	Ductular-like hepatocytes	TMEM243
3.48595072217568E-106	0.460004353270144	0.201	0.092	5.841756220222E-102	Ductular-like hepatocytes	SULT1B1
3.64010357819073E-106	0.507516814005159	0.355	0.208	6.10008557633202E-102	Ductular-like hepatocytes	WRNIP1
5.028692524923E-106	0.726956066555999	0.26	0.137	8.42708293326596E-102	Ductular-like hepatocytes	TMTC4
5.15435115970103E-106	1.22920145053837	0.141	0.054	8.63766167342698E-102	Ductular-like hepatocytes	SMIM31

5.97742442661436E-106	0.40840400422885	0.411	0.255	1.00169678541203E-101	Ductular-like hepatocytes	FBXO9
6.48875344557564E-106	0.462374152860785	0.424	0.265	1.08738530240957E-101	Ductular-like hepatocytes	ITPRID2
6.64200796525044E-106	1.37780505873898	0.177	0.079	1.11306769481667E-101	Ductular-like hepatocytes	EFNA5
7.79663570687399E-106	0.453027690504793	0.376	0.224	1.30656021175794E-101	Ductular-like hepatocytes	SLC46A1
8.39050229038487E-106	1.16940607805059	0.16	0.067	1.4060803738227E-101	Ductular-like hepatocytes	LAMA4
1.42250088442337E-105	0.624855562245629	0.321	0.184	2.38382698211669E-101	Ductular-like hepatocytes	ELF2
1.59491817318162E-105	0.722140051911823	0.263	0.139	2.67276387461775E-101	Ductular-like hepatocytes	BOP 1.00
1.64094691106518E-105	0.835597500748631	0.252	0.132	2.74989883356303E-101	Ductular-like hepatocytes	DEPRECATEI
1.69874100937951E-105	0.526929673657516	0.411	0.257	2.84675018351819E-101	Ductular-like hepatocytes	EMC3
1.77160301162803E-105	0.310664287800775	0.762	0.585	2.96885232688625E-101	Ductular-like hepatocytes	SLC9A3R1
2.21974152156809E-105	0.524674186653842	0.374	0.224	3.7198428418438E-101	Ductular-like hepatocytes	GET1
2.23797781593751E-105	1.05206382331527	0.164	0.07	3.75040322394807E-101	Ductular-like hepatocytes	ELOVL7
3.08112944938253E-105	0.409561495592334	0.493	0.332	5.16335673127524E-101	Ductular-like hepatocytes	C11orf54
3.12674294160316E-105	0.795991828440445	0.213	0.103	5.23979582153857E-101	Ductular-like hepatocytes	DEPRECATEI
3.40686006585474E-105	0.57683243296464	0.335	0.194	5.70921609835937E-101	Ductular-like hepatocytes	CNNM3
4.04717282923294E-105	0.440188504793065	0.386	0.233	6.78225222722855E-101	Ductular-like hepatocytes	YAP1
4.34537845104808E-105	0.447046766704831	0.489	0.319	7.28198520826638E-101	Ductular-like hepatocytes	DTX3L
4.46937244021481E-105	0.523561538797493	0.327	0.187	7.48977433531198E-101	Ductular-like hepatocytes	C6orf62
5.38363573697573E-105	0.417933257280844	0.337	0.19	9.02189676802393E-101	Ductular-like hepatocytes	NCAPG2
5.48736073642624E-105	0.694803584203294	0.293	0.162	9.1957191221031E-101	Ductular-like hepatocytes	ME2
5.48984080462341E-105	0.473760659624628	0.314	0.177	9.19987522038792E-101	Ductular-like hepatocytes	DIP2A
5.64135999323309E-105	1.13440655464054	0.16	0.067	9.45379107666002E-101	Ductular-like hepatocytes	PCDHGB7
6.74265992594794E-105	0.372425269906415	0.465	0.297	1.12993495039036E-100	Ductular-like hepatocytes	KEAP1
7.32391846650954E-105	0.393159542373879	0.413	0.256	1.22734225661767E-100	Ductular-like hepatocytes	EIF4A1
7.34939780220358E-105	0.450533319491414	0.332	0.192	1.23161208369328E-100	Ductular-like hepatocytes	TM9SF4
9.14808527329326E-105	0.331204885714295	0.467	0.301	1.53303613009848E-100	Ductular-like hepatocytes	MKNK2
9.52393617007692E-105	0.425411050536974	0.501	0.333	1.59602122338149E-100	Ductular-like hepatocytes	SMIM15
1.29137488468385E-104	0.979160961564339	0.167	0.071	2.1640860317532E-100	Ductular-like hepatocytes	FAM118B
1.32657893497589E-104	0.569826332934209	0.324	0.186	2.22308097923259E-100	Ductular-like hepatocytes	NELFB
1.37940565949459E-104	0.760512786417144	0.239	0.122	2.31160800418103E-100	Ductular-like hepatocytes	CMPK2
1.39360948335446E-104	0.661035719283055	0.251	0.13	2.3354107722054E-100	Ductular-like hepatocytes	TCF3
1.40521881086077E-104	0.663548148767864	0.25	0.13	2.35486568324048E-100	Ductular-like hepatocytes	PEX6
2.20741054576499E-104	1.119489574572	0.166	0.071	3.69917859259297E-100	Ductular-like hepatocytes	PCDHGB6
2.48820698382106E-104	0.743478110752007	0.184	0.083	4.16973726348733E-100	Ductular-like hepatocytes	BMP2
2.50773442632963E-104	0.418875894396083	0.481	0.313	4.20246135164319E-100	Ductular-like hepatocytes	DLG1
2.55315974978616E-104	0.465559675304529	0.426	0.267	4.27858510869165E-100	Ductular-like hepatocytes	MEA1
2.57040370226623E-104	0.387700058521082	0.528	0.352	4.30748252425774E-100	Ductular-like hepatocytes	FDPS
2.59285200602923E-104	0.378772473974849	0.632	0.458	4.34510139170378E-100	Ductular-like hepatocytes	PSMB1
3.39060410905817E-104	0.387473991500548	0.514	0.346	5.68197436595968E-100	Ductular-like hepatocytes	AKAP1
3.56674352508351E-104	0.675045903049575	0.304	0.172	5.97714879933494E-100	Ductular-like hepatocytes	GOLPH3L
3.8488098919597E-104	0.639090183583053	0.245	0.126	6.44983561694606E-100	Ductular-like hepatocytes	SFT2D1
4.14446855342026E-104	0.433570534440945	0.476	0.308	6.94530040182167E-100	Ductular-like hepatocytes	VDAC3
4.76996432083781E-104	0.591062625262301	0.292	0.161	7.99350620886E-100	Ductular-like hepatocytes	PSMC1
5.47797105886793E-104	0.502677160264243	0.383	0.233	9.17998390045088E-100	Ductular-like hepatocytes	CAMK1
7.99045686387122E-104	0.423104414435231	0.375	0.225	1.33904076124754E-99	Ductular-like hepatocytes	VHL
8.74358202849217E-104	0.4903111031364	0.346	0.202	1.46524947633472E-99	Ductular-like hepatocytes	TOMM6
9.12828109481044E-104	0.564261948596124	0.29	0.16	1.52971734586833E-99	Ductular-like hepatocytes	GGCT
9.85925526737641E-104	1.13836365825463	0.16	0.067	1.65221399770694E-99	Ductular-like hepatocytes	LYPD6
1.18307566030018E-103	0.625896676669047	0.302	0.17	1.98259819153104E-99	Ductular-like hepatocytes	SMAP1
1.27006336258449E-103	0.820900673360881	0.256	0.135	2.12837218301909E-99	Ductular-like hepatocytes	PHYHIPL
1.31523641317862E-103	0.430590998167007	0.351	0.206	2.20407318120474E-99	Ductular-like hepatocytes	HUS1
1.41384077706763E-103	0.416449225431801	0.398	0.245	2.36931437420993E-99	Ductular-like hepatocytes	EFCAB14
1.44151798325554E-103	0.699282250527227	0.254	0.134	2.41569583633963E-99	Ductular-like hepatocytes	JAGN1
1.48663310035779E-103	0.515830761714199	0.31	0.176	2.49129974957959E-99	Ductular-like hepatocytes	FBXL8
1.50396423825765E-103	0.498577923496305	0.303	0.169	2.52034327047217E-99	Ductular-like hepatocytes	PRKD2
1.55485788131991E-103	1.04372886993146	0.191	0.088	2.6056308375159E-99	Ductular-like hepatocytes	TMEM47
1.61767436462356E-103	0.498635388931562	0.344	0.202	2.71089870023617E-99	Ductular-like hepatocytes	PSMA2
1.92437568439137E-103	0.420202977879639	0.401	0.246	3.22486877190306E-99	Ductular-like hepatocytes	ZNF410
1.94600203433933E-103	0.497564850355953	0.457	0.296	3.26111020914585E-99	Ductular-like hepatocytes	TMX4
2.10285959060617E-103	0.822975868626241	0.233	0.119	3.52397210193782E-99	Ductular-like hepatocytes	KCNMB3

2.10614200704766E-103	0.678218635457391	0.246	0.128	3.52947277541046E-99	Ductular-like hepatocytes	SPTLC2
2.14046532918813E-103	0.369540523347541	0.482	0.307	3.58699179865346E-99	Ductular-like hepatocytes	NBPF26
2.2475275727555E-103	0.408271045928769	0.477	0.312	3.76640670642367E-99	Ductular-like hepatocytes	NUMB
2.67725434168737E-103	0.45544207994752	0.431	0.273	4.48654282579969E-99	Ductular-like hepatocytes	UBE2V1
2.77776429962088E-103	0.560588527497251	0.325	0.188	4.65497741330467E-99	Ductular-like hepatocytes	TMEM131
2.89955118061547E-103	0.368064782285052	0.419	0.26	4.85906786847541E-99	Ductular-like hepatocytes	NACC1
3.27805100232491E-103	0.699361793731157	0.206	0.098	5.49335786969609E-99	Ductular-like hepatocytes	CASD1
3.28846127730733E-103	0.762107511040806	0.219	0.108	5.51080340851163E-99	Ductular-like hepatocytes	SLC22A5
3.81145930084786E-103	0.857539691634461	0.243	0.126	6.38724349636084E-99	Ductular-like hepatocytes	FBXO4
3.81749233345432E-103	0.390178530294283	0.448	0.289	6.39735365240275E-99	Ductular-like hepatocytes	PLOD2
4.1663466505783E-103	0.505204225831783	0.329	0.19	6.98196371703911E-99	Ductular-like hepatocytes	MYO9B
4.30306040531807E-103	0.939837998252264	0.258	0.138	7.21106862723203E-99	Ductular-like hepatocytes	PDP1
5.75640850459682E-103	1.01642706073436	0.169	0.073	9.64658937200335E-99	Ductular-like hepatocytes	RNF145
6.15813518821498E-103	0.419158522772691	0.395	0.244	1.03198029484107E-98	Ductular-like hepatocytes	ESYT1
6.98934362342024E-103	0.519693359502319	0.375	0.228	1.17127420441276E-98	Ductular-like hepatocytes	AP3B1
7.09813084087899E-103	0.363003932880798	0.555	0.383	1.1895047663145E-98	Ductular-like hepatocytes	SLC40A1
7.3172958454415E-103	1.26938910713851	0.157	0.066	1.22623243777909E-98	Ductular-like hepatocytes	RIBC2
8.36781151082679E-103	0.720098142945137	0.24	0.123	1.40227785298435E-98	Ductular-like hepatocytes	DYNC1H1
9.18291520945819E-103	0.451523025502961	0.488	0.324	1.538872930801E-98	Ductular-like hepatocytes	SUB1
9.38617011476639E-103	0.456957789736663	0.298	0.165	1.57293438783255E-98	Ductular-like hepatocytes	NUDT22
9.43405095128674E-103	0.715017429270633	0.202	0.096	1.58095825841663E-98	Ductular-like hepatocytes	BAZ1A
1.03180946375603E-102	0.440202196145097	0.461	0.299	1.72910629936235E-98	Ductular-like hepatocytes	IVD
1.23380815154376E-102	0.593559918485356	0.274	0.148	2.06761570035703E-98	Ductular-like hepatocytes	SNTB2
1.29604900540267E-102	0.597106588130141	0.25	0.13	2.1719189232538E-98	Ductular-like hepatocytes	DEPRECATEI
1.32650269096025E-102	0.510842911009018	0.418	0.265	2.22295320951118E-98	Ductular-like hepatocytes	SKP1
1.47423057465127E-102	0.403410609676436	0.465	0.3	2.47051559700059E-98	Ductular-like hepatocytes	UHMK1
1.4892802861012E-102	0.903677042434496	0.186	0.085	2.49573590344838E-98	Ductular-like hepatocytes	GALNT10
1.52949828931341E-102	0.584525692147002	0.309	0.176	2.56313323323141E-98	Ductular-like hepatocytes	DARS
1.93667669917464E-102	0.608621908657152	0.296	0.165	3.24548281247686E-98	Ductular-like hepatocytes	CHM
2.19977044085364E-102	1.20067837577696	0.107	0.034	3.68637530478253E-98	Ductular-like hepatocytes	ADGRG7
2.34780109495099E-102	0.522860246554198	0.378	0.23	3.93444507491887E-98	Ductular-like hepatocytes	SNX6
2.60773507709278E-102	0.529281498702474	0.323	0.186	4.37004244219209E-98	Ductular-like hepatocytes	TTLL3
2.66513383570575E-102	0.6857828642437	0.283	0.156	4.4662312818757E-98	Ductular-like hepatocytes	SUSD4
3.12890207855478E-102	0.482768422673963	0.325	0.187	5.2434141032421E-98	Ductular-like hepatocytes	DIS3
3.39273978065733E-102	0.439851816986569	0.438	0.278	5.68555332442555E-98	Ductular-like hepatocytes	ZMYM2
3.41366541446122E-102	0.60604459402103	0.278	0.152	5.72062050155411E-98	Ductular-like hepatocytes	NUDT19
3.52787973228249E-102	1.22030289784052	0.131	0.049	5.91202085535899E-98	Ductular-like hepatocytes	ABCG2
3.70276517991327E-102	0.694363721257738	0.263	0.14	6.20509388849865E-98	Ductular-like hepatocytes	GMPR2
4.84258316758837E-102	0.956555995371865	0.158	0.066	8.11520087224459E-98	Ductular-like hepatocytes	DOP1B
4.84280401397821E-102	0.888661978811727	0.168	0.073	8.11557096662469E-98	Ductular-like hepatocytes	SLC66A3
4.91360157609827E-102	0.413589254734428	0.608	0.465	8.23421352122548E-98	Ductular-like hepatocytes	ZKSCAN1
5.64252268351655E-102	0.473238072888645	0.311	0.177	9.45573951303704E-98	Ductular-like hepatocytes	CERK
5.96622757791801E-102	0.590603292015254	0.318	0.184	9.998204175075E-98	Ductular-like hepatocytes	CNP
6.16383430651507E-102	0.515164431949308	0.392	0.242	1.03293535308579E-97	Ductular-like hepatocytes	GDI2
6.31379508921954E-102	0.556408635490724	0.252	0.132	1.05806578105141E-97	Ductular-like hepatocytes	NOM1
6.59106328569861E-102	0.611833489502525	0.243	0.126	1.10453038541737E-97	Ductular-like hepatocytes	SLC16A13
7.31054715584165E-102	0.635202762337353	0.315	0.181	1.22510149237594E-97	Ductular-like hepatocytes	ATL3
7.35977407123569E-102	0.467079320786485	0.412	0.259	1.23335093885768E-97	Ductular-like hepatocytes	TRIQK
7.96061747732149E-102	0.452872833320076	0.494	0.33	1.33404027684954E-97	Ductular-like hepatocytes	UQCR10
8.44044239499669E-102	0.485754021547134	0.402	0.249	1.41444933655355E-97	Ductular-like hepatocytes	NCKAP1
1.01755869092482E-101	0.570648018439271	0.338	0.198	1.70522485425181E-97	Ductular-like hepatocytes	RETREG3
1.03415966320056E-101	0.49528577885188	0.21	0.101	1.73304476359149E-97	Ductular-like hepatocytes	ZNF862
1.09140939303619E-101	0.602023695934038	0.29	0.16	1.82898386085004E-97	Ductular-like hepatocytes	FANCL
1.13202938441758E-101	1.23410908674276	0.152	0.063	1.89705484240698E-97	Ductular-like hepatocytes	FUT8
1.18435811741759E-101	0.437687221577787	0.3	0.167	1.9847473331684E-97	Ductular-like hepatocytes	TBL2
1.22087023178823E-101	0.754573571413144	0.213	0.105	2.04593433443072E-97	Ductular-like hepatocytes	DHRS4L2
1.44871283922104E-101	0.553897874151499	0.298	0.167	2.42775297596662E-97	Ductular-like hepatocytes	ANKRD27
1.51531308232242E-101	0.527520263382302	0.394	0.242	2.53936166335591E-97	Ductular-like hepatocytes	PARP14
2.08225380345056E-101	1.04720681540465	0.148	0.06	3.48944092382244E-97	Ductular-like hepatocytes	SOWAHA
2.15847991203879E-101	0.347487222717746	0.591	0.416	3.61718063659461E-97	Ductular-like hepatocytes	PDZK1

2.32082232822805E-101	0.466180506104679	0.336	0.195	3.88923405764457E-97	Ductular-like hepatocytes	WDTC1
3.83971680576255E-101	0.595005051673213	0.24	0.124	6.43459742309688E-97	Ductular-like hepatocytes	ATP11C
3.99194205408731E-101	0.330457162223014	0.702	0.517	6.68969649423951E-97	Ductular-like hepatocytes	ACTG1
4.16083347437037E-101	0.373324430787584	0.592	0.412	6.97272473634986E-97	Ductular-like hepatocytes	HNRNPA0
4.25061015521952E-101	0.343265680248988	0.572	0.401	7.12317249811687E-97	Ductular-like hepatocytes	PLEKHA6
4.53980590243002E-101	0.373945691142657	0.59	0.414	7.60780673129223E-97	Ductular-like hepatocytes	HM13
5.009848425938E-101	0.415404851096315	0.473	0.31	8.3955039921869E-97	Ductular-like hepatocytes	DIDO1
5.63795066607099E-101	0.420138298809649	0.557	0.388	9.44807772620177E-97	Ductular-like hepatocytes	LRPAP1
5.78673531311267E-101	0.65106605449602	0.234	0.119	9.69741103771421E-97	Ductular-like hepatocytes	TDRD7
6.07569379277675E-101	0.842789854958382	0.173	0.076	1.01816476579353E-96	Ductular-like hepatocytes	DOLPP1
6.34677081069943E-101	0.464549895015433	0.434	0.273	1.06359185245701E-96	Ductular-like hepatocytes	SGK3
8.35165659436818E-101	0.863288900919424	0.196	0.093	1.39957061208422E-96	Ductular-like hepatocytes	TRIM2
8.40605071147853E-101	0.436000831409722	0.397	0.243	1.40868597822957E-96	Ductular-like hepatocytes	SF3A2
9.98705114755479E-101	0.532900443070387	0.383	0.236	1.67363003130723E-96	Ductular-like hepatocytes	SLC39A9
1.02611845647256E-100	0.371468912280386	0.523	0.354	1.71956930935671E-96	Ductular-like hepatocytes	NCSTN
1.05910950441113E-100	0.871598215043332	0.247	0.13	1.77485570749218E-96	Ductular-like hepatocytes	STOX2
1.1788833733059E-100	0.465707031207479	0.305	0.172	1.97557275698602E-96	Ductular-like hepatocytes	PXN
1.22444689882192E-100	0.432331270590886	0.424	0.268	2.05192811304578E-96	Ductular-like hepatocytes	DHX9
1.24526697936368E-100	0.613537066127493	0.194	0.091	2.08681840401766E-96	Ductular-like hepatocytes	TAPBPL
1.3114128477705E-100	0.375445236020831	0.503	0.338	2.1976656502938E-96	Ductular-like hepatocytes	SMARCA2
1.3889238854016E-100	0.665260016457051	0.239	0.124	2.32755864715599E-96	Ductular-like hepatocytes	CDYL
1.49566271022142E-100	0.576485145964954	0.325	0.189	2.50643156978906E-96	Ductular-like hepatocytes	PPA2
1.73924405609337E-100	0.547038761306956	0.368	0.223	2.91462518920127E-96	Ductular-like hepatocytes	ENOPH1
2.20826320284146E-100	0.505865417006352	0.381	0.234	3.70060747532171E-96	Ductular-like hepatocytes	CDC42SE2
2.21789519963403E-100	0.444092698021214	0.342	0.201	3.7167487755467E-96	Ductular-like hepatocytes	IRF2BPL
2.52928643950853E-100	0.498285666382821	0.39	0.244	4.23857821532839E-96	Ductular-like hepatocytes	RNASEK
2.5466923842364E-100	0.376523327447405	0.527	0.354	4.26774709750337E-96	Ductular-like hepatocytes	EFR3A
2.56658585052445E-100	0.544901162618426	0.319	0.185	4.30108456830887E-96	Ductular-like hepatocytes	ATP6V0A1
3.31208862032358E-100	0.418948873347924	0.417	0.264	5.55039810993825E-96	Ductular-like hepatocytes	TMEM141
3.58688347911912E-100	0.456254176311186	0.41	0.259	6.01089933430782E-96	Ductular-like hepatocytes	PROS1
4.91215960458299E-100	1.14365340122661	0.138	0.054	8.23179706536017E-96	Ductular-like hepatocytes	SLC25A12
5.02231572693344E-100	0.536724810104134	0.351	0.208	8.41639669519506E-96	Ductular-like hepatocytes	FAM8A1
5.11626832214309E-100	0.477082991189312	0.418	0.263	8.5738424542474E-96	Ductular-like hepatocytes	FOXO3
5.63181153183107E-100	0.381748976687859	0.448	0.283	9.4377897650425E-96	Ductular-like hepatocytes	FZR1
6.39152933115983E-100	0.542291773398503	0.365	0.221	1.07109248531576E-95	Ductular-like hepatocytes	TIMMDC1
6.73817996893233E-100	0.446831231211956	0.382	0.233	1.12918419919368E-95	Ductular-like hepatocytes	ERCC5
7.39433546674493E-100	0.338127157006721	0.336	0.196	1.23914273751711E-95	Ductular-like hepatocytes	PTGES2
8.91015596849636E-100	0.480978143185374	0.383	0.231	1.49316393720062E-95	Ductular-like hepatocytes	RBM12B
9.08585984240831E-100	0.33709528340084	0.51	0.343	1.52260839239078E-95	Ductular-like hepatocytes	EMC10
9.88430912994641E-100	0.481672922842633	0.459	0.295	1.65641252399642E-95	Ductular-like hepatocytes	SPPL2A
1.05758102942745E-99	0.570772719852518	0.154	0.064	1.77229428911451E-95	Ductular-like hepatocytes	SLC22A3
1.07473443423793E-99	0.427233487363956	0.441	0.282	1.80103996489592E-95	Ductular-like hepatocytes	VPS52
1.09633389435056E-99	0.313288973626827	0.792	0.646	1.83723634015267E-95	Ductular-like hepatocytes	NUCB1
1.14632386204667E-99	0.370249884666138	0.494	0.335	1.92100952801781E-95	Ductular-like hepatocytes	TM9SF3
1.27955567249984E-99	0.382033288536326	0.418	0.263	2.14427939597524E-95	Ductular-like hepatocytes	GATAD1
1.30518394577777E-99	0.464471645836764	0.316	0.182	2.18722725633439E-95	Ductular-like hepatocytes	ACO2
1.34526248759976E-99	0.931586459085711	0.166	0.073	2.25439087671968E-95	Ductular-like hepatocytes	CNNM4
1.74796844630534E-99	1.04451942479839	0.203	0.099	2.92924552231848E-95	Ductular-like hepatocytes	PCDHGA3
1.81293110195934E-99	0.469566184276868	0.386	0.238	3.03810994066346E-95	Ductular-like hepatocytes	GORASP2
1.9237057071827E-99	0.506699260416612	0.376	0.23	3.22374602409678E-95	Ductular-like hepatocytes	ATP2C1
1.96190461006121E-99	0.617822791438419	0.208	0.101	3.28775974554058E-95	Ductular-like hepatocytes	FLVCR2
2.17778932049603E-99	0.363792043109887	0.435	0.277	3.64953934328725E-95	Ductular-like hepatocytes	INTS3
2.3336753431796E-99	1.1025313050223	0.164	0.071	3.91077314010037E-95	Ductular-like hepatocytes	WNT5B
2.34719767021081E-99	0.6451418181404	0.239	0.124	3.93343385573928E-95	Ductular-like hepatocytes	RELL1
2.59162018134111E-99	0.631127950114638	0.279	0.154	4.34303709989143E-95	Ductular-like hepatocytes	NAGLU
2.59237950471822E-99	0.92300606396005	0.188	0.088	4.34430957400679E-95	Ductular-like hepatocytes	ERAP2
2.61784070151751E-99	0.668752074364473	0.321	0.187	4.38697744760305E-95	Ductular-like hepatocytes	MOAP1
3.31557735806816E-99	0.410423085018106	0.432	0.277	5.55624453665062E-95	Ductular-like hepatocytes	LAP3
3.66280740989911E-99	0.561524579221644	0.336	0.198	6.13813265750892E-95	Ductular-like hepatocytes	MTR
4.93757463611316E-99	0.443516224402602	0.289	0.161	8.27438757519844E-95	Ductular-like hepatocytes	SESN2

5.32340437606106E-99	0.41057263226133	0.446	0.283	8.92096105340312E-95	Ductular-like hepatocytes	CEP57
5.62690114103018E-99	0.415062414216983	0.482	0.314	9.42956093213837E-95	Ductular-like hepatocytes	CCNG1
5.77714216860943E-99	0.48762919023731	0.355	0.211	9.68133484615567E-95	Ductular-like hepatocytes	BTBD7
6.28039831209153E-99	0.964763470856083	0.194	0.093	1.0524691491403E-94	Ductular-like hepatocytes	LPAR1
6.38445510468212E-99	0.640023147666121	0.31	0.178	1.06990698644263E-94	Ductular-like hepatocytes	HMGN4
6.49886105441465E-99	0.548486383309647	0.318	0.185	1.08907913549881E-94	Ductular-like hepatocytes	POFUT1
6.57696261305799E-99	0.374191269660256	0.357	0.213	1.10216739469626E-94	Ductular-like hepatocytes	FAAH
6.94774235196629E-99	0.399051483568707	0.371	0.224	1.16430266334251E-94	Ductular-like hepatocytes	AP2A2
7.23685548620289E-99	0.499951719969847	0.364	0.222	1.21275224237788E-94	Ductular-like hepatocytes	ATP6V0C
7.46533499749217E-99	0.517589675166828	0.313	0.181	1.25104083887974E-94	Ductular-like hepatocytes	RALGAPB
7.5427673418182E-99	0.838404834913942	0.241	0.127	1.26401695114189E-94	Ductular-like hepatocytes	XRCC5
7.65674677299566E-99	0.952013353316376	0.191	0.091	1.28311762421861E-94	Ductular-like hepatocytes	ALG1L
8.28181641244739E-99	0.42959565777167	0.447	0.289	1.38786679439793E-94	Ductular-like hepatocytes	CD2AP
9.27330938170622E-99	0.703229633489032	0.235	0.122	1.55402118618633E-94	Ductular-like hepatocytes	EHD1
9.28974980046057E-99	0.404991437061271	0.512	0.343	1.55677627156118E-94	Ductular-like hepatocytes	MAPK1
1.10848094861931E-98	0.726960588263252	0.277	0.153	1.85759237369624E-94	Ductular-like hepatocytes	WFS1
1.18362444710662E-98	0.467799745586755	0.381	0.234	1.98351784846127E-94	Ductular-like hepatocytes	PSMC4
1.19628093868992E-98	1.63555486714504	0.103	0.033	2.00472759705657E-94	Ductular-like hepatocytes	UGT1A10
1.23976324013773E-98	0.699936190936713	0.2	0.096	2.0775952378228E-94	Ductular-like hepatocytes	B4GALT4
1.28078749622592E-98	0.425061957289102	0.255	0.134	2.14634368617539E-94	Ductular-like hepatocytes	SIRPA
1.37069007218039E-98	0.372657182854292	0.361	0.218	2.29700242295989E-94	Ductular-like hepatocytes	NPAS2
1.54282207017495E-98	0.548215707867729	0.345	0.205	2.58546122519918E-94	Ductular-like hepatocytes	NBN
1.6305883584794E-98	0.455554945278675	0.307	0.174	2.73253997113978E-94	Ductular-like hepatocytes	CENPX
1.76857267306989E-98	1.04251279190247	0.141	0.057	2.96377408553052E-94	Ductular-like hepatocytes	BICD1
1.86340387548413E-98	0.326809589863941	0.425	0.265	3.12269221453631E-94	Ductular-like hepatocytes	FOXP4
1.93879051118342E-98	0.953787823154097	0.168	0.074	3.24902513864117E-94	Ductular-like hepatocytes	PCDHGA8
2.04729768596364E-98	0.571594869131835	0.287	0.161	3.43086146213787E-94	Ductular-like hepatocytes	SMAD1
2.09623289387413E-98	0.497330325298818	0.314	0.181	3.51286708355426E-94	Ductular-like hepatocytes	CCHCR1
2.93180187271802E-98	0.325487276547057	0.76	0.594	4.91311357830085E-94	Ductular-like hepatocytes	HGD
3.19246527858837E-98	0.75839362465491	0.232	0.12	5.34993331385839E-94	Ductular-like hepatocytes	MEST
3.57515324001083E-98	0.793417292819821	0.25	0.133	5.99124179961016E-94	Ductular-like hepatocytes	KLHL8
3.67453139595397E-98	0.68733147881985	0.224	0.113	6.15777971333966E-94	Ductular-like hepatocytes	C1orf131
3.8447962337147E-98	0.434656831110352	0.372	0.228	6.44310952845909E-94	Ductular-like hepatocytes	IRF2
4.09428184066224E-98	0.364700559972414	0.551	0.382	6.86119750858179E-94	Ductular-like hepatocytes	DEPRECATEI
4.94561719952062E-98	0.422509266515625	0.468	0.309	8.28786530295665E-94	Ductular-like hepatocytes	NEDD8
5.05237171211881E-98	1.03191628616984	0.155	0.066	8.46676451516871E-94	Ductular-like hepatocytes	ENTPD2
5.12081959833641E-98	0.438804565134926	0.334	0.194	8.58146948289215E-94	Ductular-like hepatocytes	ATP13A1
5.43531290515945E-98	0.51490545077961	0.339	0.2	9.10849736646621E-94	Ductular-like hepatocytes	LEMD2
6.39940695916781E-98	0.64169627614216	0.247	0.13	1.07241261821734E-93	Ductular-like hepatocytes	HEATR3
6.51178837469194E-98	0.460736414769019	0.404	0.251	1.09124549583087E-93	Ductular-like hepatocytes	TOR1AIP1
6.77521855100389E-98	1.19056067626121	0.13	0.05	1.13539112477723E-93	Ductular-like hepatocytes	CCNE1
7.67261306309051E-98	0.409185383984852	0.475	0.311	1.28577649711271E-93	Ductular-like hepatocytes	SRP54
1.02595264070073E-97	0.485141725083589	0.363	0.22	1.71929143528628E-93	Ductular-like hepatocytes	C19orf25
1.07879495447104E-97	0.342601772423246	0.361	0.218	1.80784458470257E-93	Ductular-like hepatocytes	RCC1L
1.16791319050159E-97	0.504220132383972	0.35	0.209	1.95718892464256E-93	Ductular-like hepatocytes	SH3GL1
1.19565001839888E-97	0.408868502288732	0.305	0.173	2.00367030083285E-93	Ductular-like hepatocytes	RAB15
1.26602987329128E-97	1.05400421390575	0.119	0.043	2.12161286166153E-93	Ductular-like hepatocytes	NDUFA4L2
1.26970798786478E-97	0.547233414136927	0.359	0.217	2.12777664606379E-93	Ductular-like hepatocytes	EIF2S1
1.56260710877828E-97	0.56860796824677	0.294	0.166	2.61861699289065E-93	Ductular-like hepatocytes	ZNF687
1.61050325360873E-97	0.446105401104531	0.29	0.163	2.69888135239751E-93	Ductular-like hepatocytes	SOX9
1.63431664373292E-97	0.57941508102255	0.274	0.15	2.73878783156763E-93	Ductular-like hepatocytes	KNTC1
1.80846577451261E-97	0.525100673401933	0.335	0.199	3.03062694492823E-93	Ductular-like hepatocytes	RO60
1.93606240731803E-97	0.579625143195871	0.313	0.18	3.24445338218356E-93	Ductular-like hepatocytes	DEPRECATEI
1.98562422463132E-97	0.423286545364343	0.437	0.276	3.32750907563716E-93	Ductular-like hepatocytes	MSI2
2.09732339488335E-97	0.59557081005209	0.572	0.444	3.51469454514552E-93	Ductular-like hepatocytes	ARGLU1
2.27017103409697E-97	0.314377412699321	0.402	0.251	3.8043526189397E-93	Ductular-like hepatocytes	ARSA
2.44903605334108E-97	0.437169070575747	0.382	0.236	4.10409461818898E-93	Ductular-like hepatocytes	SLC25A11
2.59182560766753E-97	0.422312667487985	0.409	0.259	4.34338135332925E-93	Ductular-like hepatocytes	MOGS
2.86426782635271E-97	0.35644566079988	0.509	0.344	4.79994002340187E-93	Ductular-like hepatocytes	PTTG1IP
2.95321167047402E-97	0.470310102224413	0.338	0.199	4.94899211738037E-93	Ductular-like hepatocytes	FAM168B

3.19785271370146E-97	0.568932888113144	0.316	0.185	5.35896157762091E-93	Ductular-like hepatocytes	GLO1
3.28674412516286E-97	0.487257010838102	0.357	0.215	5.50792580494793E-93	Ductular-like hepatocytes	YWHAH
4.50473293164233E-97	0.480798646450156	0.206	0.1	7.54903144684621E-93	Ductular-like hepatocytes	GIPC2
4.6219303438524E-97	0.54730387041471	0.287	0.161	7.74543087022786E-93	Ductular-like hepatocytes	CAMK1D
4.99248264247106E-97	0.58297896787292	0.296	0.168	8.366402412253E-93	Ductular-like hepatocytes	ZNF337
5.16449491842413E-97	0.399647165069421	0.405	0.253	8.65466058429515E-93	Ductular-like hepatocytes	DNAJC1
5.72333846933877E-97	0.348184957335995	0.551	0.386	9.59117060691791E-93	Ductular-like hepatocytes	CYP51A1
6.2486829109386E-97	0.724807843130515	0.254	0.137	1.04715428221509E-92	Ductular-like hepatocytes	ATP6V1D
7.03667122905802E-97	1.09370613706571	0.155	0.067	1.17920536456554E-92	Ductular-like hepatocytes	KCTD1
1.14772089705603E-96	0.645865258528257	0.254	0.137	1.92335067928649E-92	Ductular-like hepatocytes	PKD2
1.42190172730188E-96	0.348158978260804	0.61	0.437	2.38282291461249E-92	Ductular-like hepatocytes	SERINC1
1.49680127550973E-96	0.43851396522988	0.367	0.225	2.50833957749921E-92	Ductular-like hepatocytes	POLR2F
1.60748843684999E-96	1.04086223944703	0.193	0.094	2.69382912247321E-92	Ductular-like hepatocytes	GALC
1.67715961983296E-96	0.415362328236031	0.424	0.272	2.81058409091608E-92	Ductular-like hepatocytes	NDUFS6
1.87625802572951E-96	0.411720692366558	0.397	0.249	3.14423319951751E-92	Ductular-like hepatocytes	SDHA
1.90284585928901E-96	0.63111001766689	0.237	0.124	3.18878909099652E-92	Ductular-like hepatocytes	ZNF282
1.93133360594683E-96	0.340495155830874	0.494	0.325	3.2365288568457E-92	Ductular-like hepatocytes	TUBB2A
2.01195818018942E-96	0.478228253403602	0.318	0.186	3.37163951836144E-92	Ductular-like hepatocytes	CHCHD7
2.07892111358331E-96	0.471324497003183	0.319	0.186	3.48385600214291E-92	Ductular-like hepatocytes	FAM172A
3.09462298100545E-96	0.371554638486215	0.466	0.305	5.18596919156893E-92	Ductular-like hepatocytes	SNRNP200
3.12035002278243E-96	0.390338617342	0.387	0.239	5.22908256817879E-92	Ductular-like hepatocytes	SNRPA
3.20660892100065E-96	0.518757356056219	0.256	0.137	5.37363522981289E-92	Ductular-like hepatocytes	TOR3A
3.54503070984633E-96	0.317401282183331	0.681	0.532	5.94076246356048E-92	Ductular-like hepatocytes	TGOLN2
4.2787520318468E-96	0.36502929866651	0.592	0.422	7.17033265496887E-92	Ductular-like hepatocytes	DSP
4.7337405522455E-96	0.376293941843908	0.568	0.399	7.93280241745301E-92	Ductular-like hepatocytes	RAB5C
5.18761521499603E-96	0.403974196599952	0.476	0.312	8.69340557729035E-92	Ductular-like hepatocytes	KAT6A
5.72640785293607E-96	0.472963293302555	0.403	0.254	9.59631427995027E-92	Ductular-like hepatocytes	NRP1
6.2624413040542E-96	0.325507531126692	0.558	0.385	1.0494599137334E-91	Ductular-like hepatocytes	KLF9
6.44130773741608E-96	0.399106794420743	0.547	0.381	1.07943435063619E-91	Ductular-like hepatocytes	CLPTM1
6.44199851508352E-96	0.454136794596085	0.378	0.233	1.0795501111577E-91	Ductular-like hepatocytes	CLN3
6.81279716514727E-96	0.508913795831807	0.263	0.142	1.14168854893538E-91	Ductular-like hepatocytes	TRIM14
7.18570545535349E-96	0.501384710129817	0.37	0.229	1.20418052020814E-91	Ductular-like hepatocytes	CLPP
7.31462760121646E-96	0.473977607264745	0.311	0.179	1.22578529341185E-91	Ductular-like hepatocytes	PSMD2
1.10937963586991E-95	0.374757876474062	0.446	0.29	1.85909839379079E-91	Ductular-like hepatocytes	PRKAA1
1.38968812953025E-95	0.929459187466595	0.149	0.063	2.32883936746679E-91	Ductular-like hepatocytes	PCDHGA6
1.68437913180791E-95	0.484521857517663	0.34	0.203	2.82268254908369E-91	Ductular-like hepatocytes	CPT1A
2.02444997622291E-95	0.582228818372532	0.343	0.204	3.39257327015435E-91	Ductular-like hepatocytes	MORC4
2.43482757450136E-95	0.470835682659582	0.313	0.181	4.08028404934937E-91	Ductular-like hepatocytes	NCOA6
2.96885563703965E-95	0.353340421925473	0.498	0.33	4.97520827655105E-91	Ductular-like hepatocytes	PER1
3.10546250287783E-95	0.600501212375028	0.177	0.081	5.20413406232267E-91	Ductular-like hepatocytes	BCAR3
3.30779434515646E-95	0.489611761878411	0.273	0.151	5.5432017636132E-91	Ductular-like hepatocytes	ARF5
3.31564075349213E-95	0.56205007805335	0.309	0.181	5.55635077470211E-91	Ductular-like hepatocytes	COMMD6
4.12860235673085E-95	0.519869075551018	0.375	0.231	6.91871182940956E-91	Ductular-like hepatocytes	RC3H2
4.37370347483358E-95	0.526171821148538	0.26	0.141	7.32945228312611E-91	Ductular-like hepatocytes	CTNNBIP1
4.47561811763029E-95	0.778253143652588	0.208	0.103	7.50024084152484E-91	Ductular-like hepatocytes	DEPRECATEI
4.53099819861506E-95	0.672556055165269	0.19	0.09	7.59304678123912E-91	Ductular-like hepatocytes	ADAMTS10
4.78906963254853E-95	0.53768272389892	0.336	0.201	8.02552289022483E-91	Ductular-like hepatocytes	CTNNB1
5.56334275599432E-95	0.307970452119388	0.534	0.369	9.32304979049529E-91	Ductular-like hepatocytes	EIF4H
5.57167246715339E-95	0.527130542505005	0.292	0.165	9.33700872045564E-91	Ductular-like hepatocytes	SNX19
5.85025187497137E-95	0.318576958796749	0.623	0.454	9.80385209207702E-91	Ductular-like hepatocytes	UBB
5.90901827392864E-95	0.952171245597085	0.201	0.099	9.90233282344961E-91	Ductular-like hepatocytes	TWSG1
6.32947566158975E-95	0.523215792339131	0.294	0.167	1.06069353136921E-90	Ductular-like hepatocytes	PWWP2A
6.84483887354787E-95	0.388036298938372	0.53	0.367	1.14705809842915E-90	Ductular-like hepatocytes	RAB10
7.1704046570686E-95	0.362893002645644	0.496	0.331	1.20161641243156E-90	Ductular-like hepatocytes	ARF6
7.17408484342266E-95	0.425655198707487	0.355	0.214	1.20223313806077E-90	Ductular-like hepatocytes	MID1
7.6055313490124E-95	0.269369677576805	0.377	0.232	1.2745349434675E-90	Ductular-like hepatocytes	SH3BGRL3
7.64754263728412E-95	0.377320181727077	0.344	0.205	1.28157519515607E-90	Ductular-like hepatocytes	DDX56
7.78213478584112E-95	0.836257303495405	0.158	0.069	1.30413014741125E-90	Ductular-like hepatocytes	NECTIN1
8.62847235998168E-95	0.787428911939778	0.252	0.136	1.44595939808573E-90	Ductular-like hepatocytes	ANKRD46
9.04209276568685E-95	0.473554087939093	0.345	0.207	1.5152739056738E-90	Ductular-like hepatocytes	KLHDC3

1.02333551634068E-94	0.287378097706254	0.87	0.76	1.71490565828371E-90	Ductular-like hepatocytes	HSPA1B
1.1677189005492E-94	0.498506693753487	0.35	0.213	1.95686333354034E-90	Ductular-like hepatocytes	DEPRECATEI
1.43261694830104E-94	0.37074434235466	0.51	0.35	2.40077948196288E-90	Ductular-like hepatocytes	DAZAP2
1.53479449839556E-94	0.841279314557809	0.231	0.121	2.57200862041127E-90	Ductular-like hepatocytes	WDR76
1.62695655946361E-94	0.399525911462923	0.38	0.234	2.72645380234912E-90	Ductular-like hepatocytes	AIMP1
1.6769519179817E-94	0.334773516844913	0.385	0.24	2.81023602415374E-90	Ductular-like hepatocytes	VPS4A
1.69748148152064E-94	0.517310783629002	0.328	0.194	2.84463946673229E-90	Ductular-like hepatocytes	OST4
1.85017126395277E-94	0.518587916152812	0.347	0.21	3.10051700413206E-90	Ductular-like hepatocytes	UNC50
2.60857625145615E-94	0.52764350659735	0.228	0.118	4.37145208219022E-90	Ductular-like hepatocytes	PCSK7
2.68412334056503E-94	1.0016591895396	0.157	0.068	4.49805389411888E-90	Ductular-like hepatocytes	RARRES1
2.86773942466874E-94	0.446778619424643	0.287	0.163	4.80575772785988E-90	Ductular-like hepatocytes	HECA
3.16102362358784E-94	0.37508907452952	0.329	0.195	5.2972433884085E-90	Ductular-like hepatocytes	ADGRA3
3.27783108789067E-94	0.292589882270348	0.408	0.257	5.49298933708718E-90	Ductular-like hepatocytes	SUN1
3.38602023598134E-94	0.40992781132605	0.318	0.187	5.67429271145753E-90	Ductular-like hepatocytes	DEPTOR
3.64694946755635E-94	0.574680371641239	0.267	0.148	6.11155791773093E-90	Ductular-like hepatocytes	INO80C
3.98410843241273E-94	0.992187506918102	0.14	0.057	6.67656891103726E-90	Ductular-like hepatocytes	SWT1
4.09516871112918E-94	1.18680102853304	0.127	0.049	6.86268372611027E-90	Ductular-like hepatocytes	PAQR4
4.23461570262379E-94	0.467154625819154	0.367	0.225	7.09636899445695E-90	Ductular-like hepatocytes	YWHAQ
4.28661365644934E-94	0.441366223533607	0.323	0.189	7.18350716547781E-90	Ductular-like hepatocytes	RNF44
4.30158679450965E-94	0.492298696691947	0.289	0.164	7.20859915023928E-90	Ductular-like hepatocytes	SLC25A29
4.86172891944547E-94	0.404551162429537	0.449	0.293	8.14728532320672E-90	Ductular-like hepatocytes	CYB5B
5.47751411366703E-94	0.368509566678321	0.432	0.28	9.1792181516832E-90	Ductular-like hepatocytes	GPI
5.68342545977774E-94	0.664787024977432	0.248	0.133	9.52428438549554E-90	Ductular-like hepatocytes	SPRY2
6.36188535428249E-94	0.644220079148335	0.266	0.147	1.06612474767066E-89	Ductular-like hepatocytes	VEZT
7.43385819110442E-94	0.493226541069096	0.201	0.098	1.24576595566528E-89	Ductular-like hepatocytes	JDP2
7.69236884711989E-94	0.337288098063095	0.491	0.328	1.28908717140035E-89	Ductular-like hepatocytes	PSMC3
7.93819600453369E-94	0.546413918728701	0.288	0.164	1.33028288643976E-89	Ductular-like hepatocytes	GART
8.35586125901305E-94	0.266809358695966	0.528	0.355	1.40027522978541E-89	Ductular-like hepatocytes	SHARPIN
9.24223274643836E-94	0.4750264149111	0.325	0.192	1.54881336364814E-89	Ductular-like hepatocytes	PSMA3
9.84519968343717E-94	0.57685089989412	0.242	0.128	1.6498585629504E-89	Ductular-like hepatocytes	COX5B
9.99592632119177E-94	0.469385512029005	0.359	0.219	1.67511733290532E-89	Ductular-like hepatocytes	GOPC
1.03042256048539E-93	0.412254291068892	0.343	0.206	1.72678212686141E-89	Ductular-like hepatocytes	PHF14
1.08917135524395E-93	0.492600508094256	0.26	0.142	1.8252333571178E-89	Ductular-like hepatocytes	RABL6
1.16227215995062E-93	0.336747709961145	0.551	0.387	1.94773568564525E-89	Ductular-like hepatocytes	FCGRT
1.17941837166726E-93	0.981238833770907	0.144	0.06	1.97646930723999E-89	Ductular-like hepatocytes	MAP3K6
1.18289869242736E-93	0.278073771879147	0.807	0.659	1.98230162876976E-89	Ductular-like hepatocytes	IGFBP4
1.22577128546431E-93	0.387102980751456	0.441	0.286	2.0541475201811E-89	Ductular-like hepatocytes	VPS13B
1.45164733053764E-93	0.698237269119403	0.266	0.148	2.43267059651498E-89	Ductular-like hepatocytes	RBMX2
1.67648351107588E-93	0.351758853020781	0.427	0.274	2.80945106786095E-89	Ductular-like hepatocytes	PRRC2C
1.7795098408654E-93	0.41315713058903	0.326	0.193	2.98210259132224E-89	Ductular-like hepatocytes	CEMIP2
1.78244953428947E-93	0.315610824250193	0.331	0.195	2.9870289295623E-89	Ductular-like hepatocytes	C19orf48
2.25093171814921E-93	0.46534049989296	0.342	0.205	3.77211137327445E-89	Ductular-like hepatocytes	PPP2R5D
2.63496183010407E-93	0.688879351063319	0.185	0.087	4.4156690348884E-89	Ductular-like hepatocytes	CRB3
2.92595054239172E-93	0.439668981284515	0.477	0.316	4.90330791894004E-89	Ductular-like hepatocytes	MBNL1
3.01192588962079E-93	0.422493027703405	0.331	0.196	5.04738540582652E-89	Ductular-like hepatocytes	AIP
3.0561479573296E-93	0.330760689236389	0.527	0.359	5.12149274689294E-89	Ductular-like hepatocytes	DCAF11
3.06527176681281E-93	1.00067054129033	0.277	0.158	5.1367824268249E-89	Ductular-like hepatocytes	LCN2
3.72070817676997E-93	0.478776240861845	0.35	0.212	6.23516276263111E-89	Ductular-like hepatocytes	RNF167
4.00016439557221E-93	0.34352114703978	0.471	0.31	6.7034754940999E-89	Ductular-like hepatocytes	ENAH
5.04296506574828E-93	0.30567287231139	0.356	0.215	8.45100085718097E-89	Ductular-like hepatocytes	SIAE
6.32320668412083E-93	0.522654463883446	0.287	0.163	1.05964297612497E-88	Ductular-like hepatocytes	NFATC2
6.89036226060714E-93	0.380875540824437	0.552	0.392	1.15468690763255E-88	Ductular-like hepatocytes	IRF3
8.6100262192252E-93	0.688961425175905	0.162	0.072	1.44286819381776E-88	Ductular-like hepatocytes	CD44
1.04326429296975E-92	0.66756902492853	0.192	0.093	1.74830230215871E-88	Ductular-like hepatocytes	GPRIN3
1.11987794724819E-92	0.386058810422799	0.384	0.239	1.87669146399852E-88	Ductular-like hepatocytes	DYNC1I2
1.1886280682611E-92	0.38509396926451	0.445	0.29	1.99190291679195E-88	Ductular-like hepatocytes	ECE1
1.42502046519183E-92	0.324733021572806	0.47	0.31	2.38804929556848E-88	Ductular-like hepatocytes	AP3D1
1.67030413281566E-92	0.409790968188874	0.442	0.287	2.79909566577248E-88	Ductular-like hepatocytes	GNS
2.34176643490875E-92	0.576394638403302	0.248	0.134	3.92433219162009E-88	Ductular-like hepatocytes	GLYR1
2.3530536335477E-92	0.413602739058333	0.371	0.229	3.94324727909924E-88	Ductular-like hepatocytes	SGTA

2.49953900908622E-92	0.344860029028197	0.484	0.325	4.18872747142668E-88	Ductular-like hepatocytes	RAB8A
2.78068688128641E-92	0.783810504915783	0.199	0.098	4.65987507565976E-88	Ductular-like hepatocytes	HCN3
2.9828962043762E-92	0.336861894872277	0.397	0.248	4.99873745929364E-88	Ductular-like hepatocytes	HNRNPUL2
3.087504919729E-92	0.428531568706251	0.374	0.233	5.17404074448186E-88	Ductular-like hepatocytes	TRAPPC5
3.09121914770613E-92	0.377300629343079	0.378	0.234	5.18026504772594E-88	Ductular-like hepatocytes	UBP1
3.10771771405736E-92	0.68563947068667	0.269	0.151	5.20791334521733E-88	Ductular-like hepatocytes	TRIM47
3.32532743667065E-92	0.804490675044878	0.216	0.111	5.57258371837268E-88	Ductular-like hepatocytes	AP3M2
4.03370781974351E-92	1.02526415728564	0.203	0.103	6.75968756432618E-88	Ductular-like hepatocytes	FLNA
4.07362047961431E-92	0.308312714855465	0.306	0.177	6.82657319973765E-88	Ductular-like hepatocytes	RNF126
4.20801452426984E-92	0.38704113645404	0.345	0.209	7.0517907397714E-88	Ductular-like hepatocytes	ALKBH5
4.21452213915722E-92	0.547773417939808	0.326	0.193	7.06269620079966E-88	Ductular-like hepatocytes	SAE1
4.64726800650205E-92	0.650354926904289	0.166	0.075	7.78789172529614E-88	Ductular-like hepatocytes	TPRN
4.99937716520597E-92	0.485345122386118	0.302	0.174	8.37795625345216E-88	Ductular-like hepatocytes	UBAC2
6.09792645308742E-92	0.543088372838134	0.327	0.195	1.02189051500839E-87	Ductular-like hepatocytes	CITED4
6.17481491243958E-92	0.622081321006464	0.288	0.164	1.03477548302663E-87	Ductular-like hepatocytes	CNIH4
6.33007210151552E-92	0.379681324945796	0.414	0.264	1.06079348277197E-87	Ductular-like hepatocytes	PRPF19
7.92783054566455E-92	0.325960170476695	0.688	0.502	1.32854584284247E-87	Ductular-like hepatocytes	CIRBP
8.72952882701259E-92	1.05035616418398	0.111	0.039	1.46289444083077E-87	Ductular-like hepatocytes	AP4M1
8.77621809312235E-92	0.461997925192403	0.27	0.15	1.47071862804544E-87	Ductular-like hepatocytes	C6orf136
9.04990871681838E-92	0.399194083763606	0.435	0.282	1.51658370276442E-87	Ductular-like hepatocytes	PAK2
9.71985025382627E-92	0.351080503269069	0.507	0.345	1.62885250553621E-87	Ductular-like hepatocytes	EMC2
1.00479488130571E-91	0.564760703921484	0.285	0.163	1.6838352620921E-87	Ductular-like hepatocytes	C9orf64
1.0728754083963E-91	0.280157036369357	0.434	0.282	1.79792460939051E-87	Ductular-like hepatocytes	FAM20C
1.14282086128557E-91	0.788196357085802	0.135	0.054	1.91513919934236E-87	Ductular-like hepatocytes	TNFRSF21
1.16840520150189E-91	0.673843135236847	0.255	0.14	1.95801343667686E-87	Ductular-like hepatocytes	NVL
1.29645977270054E-91	0.764569285044091	0.223	0.117	2.17260728709156E-87	Ductular-like hepatocytes	ATP5F1C
1.38523406733261E-91	0.437641820149222	0.469	0.315	2.32137525003598E-87	Ductular-like hepatocytes	SEL1L
1.40821427635538E-91	0.593809126697501	0.286	0.164	2.35988548431634E-87	Ductular-like hepatocytes	HHAT
1.60230211483358E-91	0.271657941281778	0.963	0.864	2.68513788403811E-87	Ductular-like hepatocytes	B2M
1.62885099118844E-91	0.406297189560211	0.301	0.173	2.72962849103359E-87	Ductular-like hepatocytes	MYD88
1.97858770036485E-91	0.805623665562843	0.183	0.087	3.31571726827142E-87	Ductular-like hepatocytes	C6orf47
2.02482608203288E-91	0.480469397037071	0.286	0.162	3.3932035482707E-87	Ductular-like hepatocytes	PTPN12
2.05831989815603E-91	0.542890993858817	0.191	0.093	3.44933248532988E-87	Ductular-like hepatocytes	COG5
2.59514643444819E-91	1.03034594955811	0.161	0.072	4.34894639484828E-87	Ductular-like hepatocytes	DEPRECATEI
2.84209168617449E-91	0.335016943562294	0.613	0.445	4.7627772476912E-87	Ductular-like hepatocytes	CUTA
3.39607328180816E-91	0.851120271278168	0.237	0.127	5.69113960565412E-87	Ductular-like hepatocytes	ATG4A
3.61388575204179E-91	0.540964755381615	0.349	0.214	6.05614974327164E-87	Ductular-like hepatocytes	CPT2
4.08818570497636E-91	0.594847352843219	0.292	0.168	6.85098160439938E-87	Ductular-like hepatocytes	BPNT1
5.12042778848473E-91	0.393279321224705	0.275	0.153	8.58081288794271E-87	Ductular-like hepatocytes	ABCB8
5.5839319739775E-91	0.452191927219492	0.313	0.184	9.3575532019915E-87	Ductular-like hepatocytes	CYFIP1
5.88588555256732E-91	0.437982817280179	0.239	0.127	9.86356700899231E-87	Ductular-like hepatocytes	KRCC1
6.56809376592178E-91	0.431395961863466	0.339	0.205	1.10068115329317E-86	Ductular-like hepatocytes	ERH
7.39453080158267E-91	0.411839505902818	0.412	0.263	1.23917547172922E-86	Ductular-like hepatocytes	AGO2
7.4587798280875E-91	0.435643048336262	0.402	0.257	1.2499423235909E-86	Ductular-like hepatocytes	GRB2
7.59596932824966E-91	0.509141677342458	0.278	0.157	1.27293254002808E-86	Ductular-like hepatocytes	VPS13D
7.9005338463717E-91	0.582549101939456	0.278	0.157	1.32397146197497E-86	Ductular-like hepatocytes	CPSF2
8.02319182274377E-91	0.446378738911613	0.292	0.168	1.3445264856554E-86	Ductular-like hepatocytes	STARD3
8.37085135118593E-91	0.640795181091707	0.287	0.164	1.40278726943174E-86	Ductular-like hepatocytes	ART4
8.42180227172438E-91	0.430649713421794	0.365	0.226	1.41132562469557E-86	Ductular-like hepatocytes	JPT2
8.59063995095553E-91	0.380185730097542	0.306	0.179	1.43961944298113E-86	Ductular-like hepatocytes	LDLRAP1
9.61637025434548E-91	0.421613664323531	0.26	0.141	1.61151132722322E-86	Ductular-like hepatocytes	GATA4
1.17846250712797E-90	0.449321874989244	0.348	0.212	1.97486746944506E-86	Ductular-like hepatocytes	COMMD3
1.23622025085276E-90	0.363126141647798	0.475	0.317	2.07165789637905E-86	Ductular-like hepatocytes	RDH11
1.25198911532091E-90	0.506426625298315	0.331	0.199	2.09808335945478E-86	Ductular-like hepatocytes	CASK
1.29982996998999E-90	0.380919894791269	0.419	0.27	2.17825506370922E-86	Ductular-like hepatocytes	PARP10
1.32310736737504E-90	1.42089006602555	0.115	0.043	2.21726332624709E-86	Ductular-like hepatocytes	FIBCD1
1.40483032316473E-90	0.413398514784657	0.443	0.294	2.35421465555945E-86	Ductular-like hepatocytes	HSD17B2
1.44060248342954E-90	0.419860163542565	0.316	0.186	2.41416164173123E-86	Ductular-like hepatocytes	DDX23
1.56249642661281E-90	0.802534190511146	0.24	0.13	2.61843151171775E-86	Ductular-like hepatocytes	KLHL42
1.57194354266253E-90	0.690200886771276	0.288	0.166	2.63426298879387E-86	Ductular-like hepatocytes	SLC30A5

1.64161695540355E-90	0.313605070621303	0.568	0.402	2.75102169386527E-86	Ductular-like hepatocytes	PGM1
1.64847165473893E-90	0.767254753871607	0.199	0.1	2.7625087990115E-86	Ductular-like hepatocytes	IL17RA
1.85351086034702E-90	1.22596504593457	0.134	0.055	3.10611349976953E-86	Ductular-like hepatocytes	RNF144A
1.86421851041549E-90	0.398141103921178	0.423	0.273	3.12405737975427E-86	Ductular-like hepatocytes	HNRNPM
1.99733410161765E-90	0.80907357400996	0.198	0.099	3.34713248749085E-86	Ductular-like hepatocytes	ABCA7
2.35725180251012E-90	0.637677222228408	0.279	0.159	3.95028257064646E-86	Ductular-like hepatocytes	MGAT5
2.71649083491869E-90	0.427360946326042	0.35	0.214	4.55229534115674E-86	Ductular-like hepatocytes	FAM32A
2.86888879302002E-90	0.492033729009391	0.331	0.2	4.80768383934295E-86	Ductular-like hepatocytes	AGTRAP
3.1078857942749E-90	0.799852950271845	0.135	0.055	5.20819501404587E-86	Ductular-like hepatocytes	SPTLC3
3.40678173596239E-90	0.449225609786512	0.374	0.232	5.70908483312577E-86	Ductular-like hepatocytes	PPP6C
3.46952316361056E-90	0.542387744311531	0.312	0.184	5.81422691757857E-86	Ductular-like hepatocytes	GMFB
3.4767351822816E-90	0.447586791636625	0.326	0.195	5.82631281846751E-86	Ductular-like hepatocytes	SEPTIN2
3.61192055303195E-90	0.493460899608913	0.351	0.216	6.05285646277094E-86	Ductular-like hepatocytes	BTD
4.0893648404867E-90	0.829584370301381	0.164	0.075	6.85295759968761E-86	Ductular-like hepatocytes	LGALSL
4.22689386508171E-90	0.400441907836234	0.455	0.3	7.08342873910392E-86	Ductular-like hepatocytes	LYPLAL1
4.25727347502106E-90	0.449238816438031	0.33	0.198	7.13433888944029E-86	Ductular-like hepatocytes	MYRF
4.37266927576009E-90	0.526264007490712	0.305	0.179	7.32771917231875E-86	Ductular-like hepatocytes	NEK9
4.450393934078E-90	0.40373136570947	0.396	0.249	7.45797015472791E-86	Ductular-like hepatocytes	MSL1
4.86631976179198E-90	0.759598756450239	0.212	0.109	8.15497865681101E-86	Ductular-like hepatocytes	SNX7
5.07937371831503E-90	0.453091437613251	0.336	0.203	8.51201447715232E-86	Ductular-like hepatocytes	COMMD5
5.15619751185056E-90	0.432140243946956	0.24	0.128	8.64075579035917E-86	Ductular-like hepatocytes	PITPNM1
5.33481227585111E-90	0.542682721077799	0.265	0.147	8.94007841187129E-86	Ductular-like hepatocytes	PTPRA
5.67806271575744E-90	0.488906445705024	0.295	0.171	9.51529749906631E-86	Ductular-like hepatocytes	EXOSC2
5.68562002989581E-90	0.436678988334044	0.443	0.292	9.5279620460994E-86	Ductular-like hepatocytes	ATP5PD
5.97927962078497E-90	0.381933906278301	0.415	0.266	1.00200767885114E-85	Ductular-like hepatocytes	MAX
6.09176174134898E-90	0.568365347183482	0.253	0.139	1.02085743261526E-85	Ductular-like hepatocytes	LNPEP
6.42683139177303E-90	0.40120555522095	0.315	0.186	1.07700840463332E-85	Ductular-like hepatocytes	TMEM127
6.69402890481351E-90	0.756622073957321	0.202	0.102	1.12178536386865E-85	Ductular-like hepatocytes	DEPRECATEI
9.81711517341049E-90	0.651336189573395	0.262	0.146	1.64515216076013E-85	Ductular-like hepatocytes	USP2
1.0588432615409E-89	0.407594321725748	0.311	0.183	1.77440953769025E-85	Ductular-like hepatocytes	P2RX4
1.06267871336122E-89	1.11334808734458	0.126	0.05	1.78083698785073E-85	Ductular-like hepatocytes	CCDC9B
1.17600970616304E-89	0.722900720434638	0.265	0.148	1.97075706558803E-85	Ductular-like hepatocytes	HSPA13
1.22871998838921E-89	0.550427493867938	0.316	0.188	2.05908895654264E-85	Ductular-like hepatocytes	MED16
1.31727380884477E-89	0.321788756602568	0.603	0.434	2.20748744886206E-85	Ductular-like hepatocytes	SSR2
1.6365760846929E-89	0.517892524372712	0.319	0.19	2.74257420272836E-85	Ductular-like hepatocytes	TMLHE
1.67200190261595E-89	0.444235218924312	0.249	0.135	2.80194078840381E-85	Ductular-like hepatocytes	HDAC10
1.67609609613921E-89	0.287553870679298	0.461	0.304	2.80880183791009E-85	Ductular-like hepatocytes	NAA60
1.85687158172342E-89	0.421009045436371	0.419	0.272	3.1117453966521E-85	Ductular-like hepatocytes	PSMB5
2.38591911498632E-89	0.476847145314562	0.271	0.153	3.99832325289407E-85	Ductular-like hepatocytes	ARL2BP
2.42787449532045E-89	0.572908590824907	0.303	0.178	4.068632079258E-85	Ductular-like hepatocytes	TOR1A
2.54682731916492E-89	0.619248033085087	0.228	0.12	4.26797322145657E-85	Ductular-like hepatocytes	GCNT2
2.63231544589391E-89	0.39848349041079	0.331	0.199	4.41123422422902E-85	Ductular-like hepatocytes	NDUFB2
3.08480909855006E-89	0.440896590529533	0.36	0.223	5.1695230873502E-85	Ductular-like hepatocytes	NOP56
3.51297871794455E-89	0.408279127418236	0.4	0.255	5.88704973553148E-85	Ductular-like hepatocytes	CRKL
3.64389629552661E-89	0.617024842525786	0.273	0.155	6.10644141204349E-85	Ductular-like hepatocytes	ACYP1
3.71287608712565E-89	0.596648630356752	0.274	0.155	6.22203774680517E-85	Ductular-like hepatocytes	NIPA1
4.90547603548474E-89	0.885766675152521	0.162	0.074	8.22059674026533E-85	Ductular-like hepatocytes	TCN2
5.10331059268896E-89	0.70068459423076	0.223	0.117	8.55212789122816E-85	Ductular-like hepatocytes	UXS1
6.45734357790326E-89	0.414918554567622	0.355	0.219	1.08212163678503E-84	Ductular-like hepatocytes	NDUFA9
6.45813229682946E-89	0.444308127957759	0.364	0.226	1.08225381030268E-84	Ductular-like hepatocytes	CNST
7.51953255928812E-89	0.318107036840939	0.33	0.196	1.2601232662855E-84	Ductular-like hepatocytes	ZNF800
8.25550654222075E-89	0.310711373617364	0.488	0.337	1.38345778634535E-84	Ductular-like hepatocytes	VAMP8
8.30205561737129E-89	0.422357603726058	0.365	0.226	1.39125848035908E-84	Ductular-like hepatocytes	GTF2H2C
8.94736322384079E-89	0.627247798569286	0.306	0.182	1.49939912905124E-84	Ductular-like hepatocytes	ATP6V1H
9.08495592623665E-89	0.288902339623353	0.481	0.323	1.52245691411874E-84	Ductular-like hepatocytes	GNB2
9.25611843573168E-89	0.59435135101929	0.213	0.11	1.55114032745991E-84	Ductular-like hepatocytes	MEX3D
9.75716212802189E-89	0.390656638109697	0.441	0.288	1.63510522941391E-84	Ductular-like hepatocytes	C5orf24
1.03334136651179E-88	0.391133298508327	0.445	0.291	1.73167346200046E-84	Ductular-like hepatocytes	UBR5
1.08593795559567E-88	0.605684259862052	0.277	0.157	1.81981482598723E-84	Ductular-like hepatocytes	DSN1
1.12322941823913E-88	0.641407928718095	0.246	0.135	1.88230785908514E-84	Ductular-like hepatocytes	PDXDC1

1.17287322822966E-88	0.561789024952135	0.267	0.15	1.96550095586727E-84	Ductular-like hepatocytes	TMEM165
1.20183802324568E-88	0.490041512959071	0.332	0.201	2.0140401593551E-84	Ductular-like hepatocytes	ZC3H4
1.26797744339772E-88	0.61633463426173	0.23	0.122	2.1248765996459E-84	Ductular-like hepatocytes	PSME2
1.29066468812069E-88	0.31679614684846	0.832	0.7	2.16289588435265E-84	Ductular-like hepatocytes	METTL7A
1.504782636387E-88	0.625591085211263	0.308	0.183	2.52171474205733E-84	Ductular-like hepatocytes	LCLAT1
1.51711907601818E-88	0.339118534555348	0.436	0.284	2.54238814759126E-84	Ductular-like hepatocytes	DUSP3
1.56403515367899E-88	0.611979470440067	0.253	0.14	2.62101011053525E-84	Ductular-like hepatocytes	GNPDA1
1.58808419353076E-88	0.524611841714176	0.374	0.236	2.66131149151885E-84	Ductular-like hepatocytes	PSMD8
2.04574621946236E-88	0.605031072751093	0.154	0.068	3.42826151457502E-84	Ductular-like hepatocytes	PRXL2B
2.20682242153917E-88	0.339094907025425	0.443	0.29	3.69819301401534E-84	Ductular-like hepatocytes	PHF3
2.40445311801764E-88	0.566542186127319	0.229	0.122	4.02938253517397E-84	Ductular-like hepatocytes	SOWAHB
2.48078423062942E-88	0.360188082880169	0.495	0.335	4.15729821368879E-84	Ductular-like hepatocytes	CSNK1D
2.52461587019946E-88	0.584086534272199	0.212	0.109	4.23075127528025E-84	Ductular-like hepatocytes	GPR137B
2.60156806803023E-88	0.413531385410586	0.41	0.265	4.35970776840507E-84	Ductular-like hepatocytes	CYB5R1
2.76645423290173E-88	0.588291260536329	0.25	0.137	4.63602400349673E-84	Ductular-like hepatocytes	PIAS3
3.25409651708832E-88	0.657742908386432	0.256	0.143	5.4532149433366E-84	Ductular-like hepatocytes	MBP
3.5150792390754E-88	0.379430705036193	0.323	0.193	5.89056978884255E-84	Ductular-like hepatocytes	CHD4
3.66584221202998E-88	0.531641756101464	0.284	0.164	6.14321837891983E-84	Ductular-like hepatocytes	GET3
3.66811034453292E-88	0.619810965602307	0.228	0.122	6.14701931536826E-84	Ductular-like hepatocytes	BLVRA
3.79523983027194E-88	0.329950260080441	0.483	0.328	6.36006290756972E-84	Ductular-like hepatocytes	PATJ
4.22279063428605E-88	0.46813708202711	0.214	0.111	7.07655254493656E-84	Ductular-like hepatocytes	IL15RA
4.64528853375273E-88	0.389232182147451	0.529	0.37	7.78457452486283E-84	Ductular-like hepatocytes	ARHGEF26
5.5654715467921E-88	0.433476815707108	0.243	0.13	9.32661721811421E-84	Ductular-like hepatocytes	CHD7
5.65064487617279E-88	0.527647220208568	0.367	0.231	9.46935068349036E-84	Ductular-like hepatocytes	PALLD
5.78867601263081E-88	0.466888134269593	0.333	0.201	9.70066326196671E-84	Ductular-like hepatocytes	BCL2L2
5.80778552628328E-88	0.54433165621366	0.255	0.142	9.73268698494552E-84	Ductular-like hepatocytes	FAM98C
5.8325452677589E-88	0.451432770997136	0.326	0.195	9.77417935971037E-84	Ductular-like hepatocytes	POLR2B
6.03205783126207E-88	0.390760935341027	0.512	0.354	1.0108522513629E-83	Ductular-like hepatocytes	DERL1
6.85184848635955E-88	1.20991569004403	0.124	0.049	1.14823276934413E-83	Ductular-like hepatocytes	EVA1C
7.20100705406834E-88	0.580438483514295	0.241	0.131	1.20674476212077E-83	Ductular-like hepatocytes	PLCG1
7.62080845991046E-88	0.390795823993761	0.305	0.178	1.27709508171179E-83	Ductular-like hepatocytes	EEF1AKNMT
8.65364838588326E-88	0.487561659461788	0.309	0.183	1.45017839650632E-83	Ductular-like hepatocytes	SNX9
8.69936930244249E-88	0.946674377951543	0.174	0.083	1.45784030770331E-83	Ductular-like hepatocytes	NIPAL3
9.25813198477416E-88	0.342942650400874	0.441	0.289	1.55147775800845E-83	Ductular-like hepatocytes	BIRC6
1.17638753464651E-87	0.323525710082712	0.384	0.24	1.97139023056063E-83	Ductular-like hepatocytes	PTBP1
1.32253436956753E-87	0.359865117462845	0.282	0.161	2.21630309652126E-83	Ductular-like hepatocytes	VEGFB
1.36789248977168E-87	0.331252128751887	0.444	0.291	2.29231423435938E-83	Ductular-like hepatocytes	MICAL3
1.52567277015114E-87	0.420547818214857	0.351	0.218	2.55672242821929E-83	Ductular-like hepatocytes	GSAP
1.54042471075664E-87	0.637568207834911	0.276	0.159	2.58144373028598E-83	Ductular-like hepatocytes	COPS4
1.57462504389182E-87	0.350065520965552	0.468	0.316	2.63875664855391E-83	Ductular-like hepatocytes	RABGAP1L
1.71602330201998E-87	0.579304530257323	0.291	0.169	2.87571184952507E-83	Ductular-like hepatocytes	RNF139
1.7349191780022E-87	0.625035176792507	0.302	0.178	2.90737755849609E-83	Ductular-like hepatocytes	IPMK
1.7592918730021E-87	1.54409827495358	0.117	0.045	2.94822132077692E-83	Ductular-like hepatocytes	IL1RAPL1
1.8123698326912E-87	0.388817998887603	0.392	0.248	3.0371693656239E-83	Ductular-like hepatocytes	ZNF664
1.91387987875584E-87	0.351505518628529	0.593	0.422	3.20727990081904E-83	Ductular-like hepatocytes	SCARB2
2.09756275452705E-87	0.395271391873135	0.433	0.281	3.51509566403643E-83	Ductular-like hepatocytes	PHF20L1
2.88296172090652E-87	0.417590796775736	0.373	0.234	4.83126725189514E-83	Ductular-like hepatocytes	RAB3IP
3.33424258172583E-87	0.352739221852881	0.425	0.272	5.58752371845615E-83	Ductular-like hepatocytes	TUBGCP3
3.40192395151484E-87	0.893964036989544	0.233	0.128	5.70094415794858E-83	Ductular-like hepatocytes	CDHR2
3.76925984625775E-87	0.437013047367806	0.418	0.273	6.31652565035873E-83	Ductular-like hepatocytes	NDUFA5
4.25985917100883E-87	0.362107706641285	0.416	0.265	7.13867199877659E-83	Ductular-like hepatocytes	RAPH1
4.73350642218988E-87	0.586249611487087	0.297	0.175	7.9324100623058E-83	Ductular-like hepatocytes	CACNA1H
5.01594252334625E-87	0.612540267969727	0.229	0.122	8.40571648062364E-83	Ductular-like hepatocytes	CCDC141
5.78281909877907E-87	0.61529658218355	0.184	0.089	9.69084824573396E-83	Ductular-like hepatocytes	IL7
6.11635664880417E-87	0.406971521998374	0.35	0.215	1.0249790472066E-82	Ductular-like hepatocytes	TAP2
6.41749089283741E-87	0.308607486911527	0.546	0.383	1.07544312382169E-82	Ductular-like hepatocytes	SEC13
7.02672922985903E-87	0.910020791974956	0.184	0.091	1.17753928433978E-82	Ductular-like hepatocytes	NEDD9
7.06997208982361E-87	0.297837279927565	0.632	0.466	1.18478592281264E-82	Ductular-like hepatocytes	NDUFB7
7.56071803584166E-87	0.629810620535312	0.24	0.13	1.26702512844635E-82	Ductular-like hepatocytes	POLE2
9.18955218878102E-87	0.417516067790104	0.282	0.161	1.53998515579592E-82	Ductular-like hepatocytes	SGSM3

9.81380432278037E-87	0.820342255796163	0.2	0.102	1.64459732841153E-82	Ductular-like hepatocytes	FAM53B
9.94259592042467E-87	0.901240453263826	0.211	0.111	1.66618022434477E-82	Ductular-like hepatocytes	MFSD6
1.04109426998018E-86	0.545985742767239	0.236	0.127	1.74466577763278E-82	Ductular-like hepatocytes	ANO10
1.09106213448662E-86	0.325335254926531	0.421	0.272	1.82840192497267E-82	Ductular-like hepatocytes	PPM1A
1.0954119389015E-86	0.330044795908632	0.399	0.254	1.83569132721114E-82	Ductular-like hepatocytes	DNPEP
1.10618076245487E-86	0.293187571201178	0.536	0.373	1.85373772172187E-82	Ductular-like hepatocytes	VPS28
1.16780272592381E-86	0.400031481719701	0.313	0.186	1.95700380810313E-82	Ductular-like hepatocytes	UGGT1
1.22850265668946E-86	0.400876772543188	0.332	0.202	2.0587247520802E-82	Ductular-like hepatocytes	VIRMA
1.33750431241515E-86	0.6270241771612	0.291	0.171	2.24138972674531E-82	Ductular-like hepatocytes	ZNF580
1.35083656017429E-86	0.336784043176461	0.472	0.317	2.26373190754008E-82	Ductular-like hepatocytes	RAD23A
1.61891145768256E-86	0.644834838840366	0.24	0.131	2.71297182078443E-82	Ductular-like hepatocytes	OAS3
1.65190445830725E-86	0.352064849664559	0.431	0.283	2.76826149123129E-82	Ductular-like hepatocytes	SPR
1.71830004061211E-86	0.440985398270066	0.341	0.209	2.87952720805777E-82	Ductular-like hepatocytes	MCMBP
1.86693053328249E-86	0.372602961746725	0.362	0.225	3.1286021876748E-82	Ductular-like hepatocytes	IFT27
2.04628376697955E-86	0.328228637985617	0.361	0.224	3.42916233670432E-82	Ductular-like hepatocytes	KDM4B
2.09203218930176E-86	0.465684666614966	0.314	0.188	3.50582754283189E-82	Ductular-like hepatocytes	C14orf119
2.2386433348978E-86	0.431766648028902	0.384	0.243	3.75151850062173E-82	Ductular-like hepatocytes	CHAMP1
2.32309579924641E-86	0.565466003535549	0.306	0.182	3.89304394037713E-82	Ductular-like hepatocytes	FKBP3
2.37909745267887E-86	1.46501071540929	0.112	0.042	3.98689151119925E-82	Ductular-like hepatocytes	TMEM51
2.47638356535541E-86	0.374089405157979	0.421	0.272	4.1499235788226E-82	Ductular-like hepatocytes	PLSCR1
2.55254683550591E-86	0.714373118808456	0.177	0.085	4.2775579869408E-82	Ductular-like hepatocytes	SMYD4
2.63921892477349E-86	0.331015550353555	0.533	0.37	4.42280307413541E-82	Ductular-like hepatocytes	SPATS2L
2.73046382565619E-86	0.541733955299222	0.269	0.152	4.57571127903464E-82	Ductular-like hepatocytes	STX12
2.74433896373831E-86	0.632983623802142	0.239	0.131	4.59896323543266E-82	Ductular-like hepatocytes	TIGD5
2.75394536729283E-86	0.392119893282813	0.38	0.239	4.61506164650932E-82	Ductular-like hepatocytes	RNF4
2.8531968955492E-86	0.623730058295759	0.271	0.155	4.78138735756135E-82	Ductular-like hepatocytes	NDRG3
3.07561585881445E-86	0.633100842042935	0.269	0.153	5.15411705620126E-82	Ductular-like hepatocytes	UPF3B
3.07677508750819E-86	0.454610559439612	0.238	0.129	5.15605969164622E-82	Ductular-like hepatocytes	ABCG8
3.17981948256554E-86	0.401196195057074	0.357	0.219	5.32874148888333E-82	Ductular-like hepatocytes	ZBTB1
3.24963386667234E-86	0.360670060545306	0.391	0.25	5.44573643376952E-82	Ductular-like hepatocytes	GALNT2
3.32689123203971E-86	0.376837002250466	0.409	0.262	5.57520432665215E-82	Ductular-like hepatocytes	GALK2
3.36354575434617E-86	0.403220899022685	0.246	0.134	5.63662997513331E-82	Ductular-like hepatocytes	C7orf26
3.66369779859147E-86	0.637116321537697	0.185	0.091	6.13962477087959E-82	Ductular-like hepatocytes	TRMT61A
4.50850154929011E-86	0.369611083954546	0.411	0.263	7.55534689630037E-82	Ductular-like hepatocytes	PPP4R3A
4.71369551077441E-86	0.376714758074904	0.395	0.249	7.89921093695575E-82	Ductular-like hepatocytes	TPP2
5.49822359096403E-86	0.710399480600352	0.174	0.083	9.21392309373751E-82	Ductular-like hepatocytes	NUDT8
7.01346901496329E-86	0.696954751171009	0.191	0.095	1.17531713752755E-81	Ductular-like hepatocytes	TCTEX1D2
7.18484379238276E-86	0.444733886544609	0.394	0.253	1.2040361227275E-81	Ductular-like hepatocytes	COQ8A
7.23122075243887E-86	0.46482425387105	0.332	0.202	1.21180797369371E-81	Ductular-like hepatocytes	TUBG1
7.48873031801055E-86	0.33536078184008	0.447	0.296	1.25496142669221E-81	Ductular-like hepatocytes	ATP5F1E
8.59980758221284E-86	0.475140014838239	0.262	0.147	1.44115575462723E-81	Ductular-like hepatocytes	RFC2
8.72627476941305E-86	0.337271544976818	0.36	0.224	1.46234912585824E-81	Ductular-like hepatocytes	TBCB
9.64292552494735E-86	0.844071783646923	0.121	0.048	1.61596145947068E-81	Ductular-like hepatocytes	IL22RA1
1.12815629414246E-85	0.374034754027077	0.487	0.329	1.89056431772393E-81	Ductular-like hepatocytes	SMIM19
1.22557379131316E-85	0.312530712506916	0.314	0.187	2.05381655948259E-81	Ductular-like hepatocytes	OSBPL2
1.23566953542774E-85	0.598297090763343	0.22	0.117	2.0707350074698E-81	Ductular-like hepatocytes	NT5C
1.32210848053616E-85	0.402681675223993	0.312	0.187	2.21558939168249E-81	Ductular-like hepatocytes	WASHC5
1.3498763945898E-85	0.368207938726662	0.393	0.251	2.26212286205358E-81	Ductular-like hepatocytes	FARSA
1.37954550996649E-85	0.492601345384587	0.258	0.145	2.31184236560184E-81	Ductular-like hepatocytes	FARSB
1.40473215221144E-85	0.415373711294046	0.306	0.181	2.35405014067594E-81	Ductular-like hepatocytes	SNX13
1.57857434424617E-85	0.638920313250415	0.231	0.125	2.64537488608774E-81	Ductular-like hepatocytes	METTL9
1.59517892104219E-85	0.730281328874188	0.216	0.114	2.67320083588251E-81	Ductular-like hepatocytes	ZNF398
1.60572050904474E-85	0.508427906701814	0.202	0.102	2.69086642905718E-81	Ductular-like hepatocytes	SPATA6L
1.6313325658432E-85	0.422838525618104	0.319	0.192	2.73378711384003E-81	Ductular-like hepatocytes	RNF19B
1.65972557626566E-85	0.731327691100091	0.209	0.108	2.781368120706E-81	Ductular-like hepatocytes	COPG2
1.87761206417658E-85	0.434198019853669	0.286	0.166	3.14650229714711E-81	Ductular-like hepatocytes	STAT5B
1.99038727784546E-85	0.351996485962668	0.513	0.357	3.33549100021342E-81	Ductular-like hepatocytes	COA3
2.2584287511965E-85	0.323841723364241	0.455	0.302	3.78467490125509E-81	Ductular-like hepatocytes	LONP1
2.32567678085504E-85	1.00311927293291	0.185	0.092	3.89736914935687E-81	Ductular-like hepatocytes	CHAF1B
2.36297438277295E-85	0.444684960245899	0.318	0.19	3.95987247065092E-81	Ductular-like hepatocytes	YY1

2.42150582144292E-85	0.386400988529764	0.355	0.22	4.05795945557404E-81	Ductular-like hepatocytes	AZIN1
2.69146597206986E-85	0.403787388256782	0.375	0.236	4.51035867599466E-81	Ductular-like hepatocytes	AGGF1
2.74940861198267E-85	0.654006790602701	0.298	0.176	4.60745895196056E-81	Ductular-like hepatocytes	NIN
3.0599302320604E-85	0.487999542197775	0.317	0.192	5.12783108288682E-81	Ductular-like hepatocytes	HAT1
3.08694498328151E-85	0.820607561666088	0.178	0.086	5.17310240298316E-81	Ductular-like hepatocytes	PPP1R3E
3.31896650549746E-85	0.334273932781283	0.353	0.218	5.56192406991265E-81	Ductular-like hepatocytes	HIP1R
3.6690204965829E-85	0.423336596890133	0.29	0.169	6.14854454817362E-81	Ductular-like hepatocytes	MBD3
4.62212293135643E-85	0.436393869444612	0.33	0.201	7.74575360836711E-81	Ductular-like hepatocytes	PIP4P1
5.36420739412495E-85	0.619842917866927	0.29	0.17	8.98933875107459E-81	Ductular-like hepatocytes	APBB2
5.53490340270637E-85	0.254815803576746	0.568	0.402	9.27539112225534E-81	Ductular-like hepatocytes	CNDP2
5.67536590660221E-85	0.700182692212143	0.242	0.134	9.51077818628399E-81	Ductular-like hepatocytes	ENOX2
5.70634230784413E-85	0.709620592663219	0.188	0.094	9.56268843948519E-81	Ductular-like hepatocytes	TMEM30B
5.76769120077009E-85	0.50045822799483	0.308	0.184	9.66549691425051E-81	Ductular-like hepatocytes	C6orf89
6.36244579166114E-85	0.308475629054696	0.499	0.339	1.06621866576657E-80	Ductular-like hepatocytes	HNRNPC
6.90053597801869E-85	0.585258413696473	0.234	0.128	1.15639181919637E-80	Ductular-like hepatocytes	COA8
7.25871296825762E-85	0.275730480656295	0.969	0.899	1.21641511922061E-80	Ductular-like hepatocytes	GC
8.13380542945731E-85	0.571583184840524	0.225	0.121	1.36306311386846E-80	Ductular-like hepatocytes	TMEM94
8.29509451128958E-85	0.295840888170238	0.388	0.243	1.39009193820191E-80	Ductular-like hepatocytes	TRIOBP
8.47644225835431E-85	0.307648922040666	0.429	0.281	1.42048219365501E-80	Ductular-like hepatocytes	DEPRECATEI
8.52541729111146E-85	0.387442441473377	0.19	0.093	1.42868942964446E-80	Ductular-like hepatocytes	TOX3
9.09511476993268E-85	0.295682310947535	0.324	0.195	1.52415933314532E-80	Ductular-like hepatocytes	GALE
9.45995365915404E-85	0.3188396025869	0.353	0.216	1.58529903420103E-80	Ductular-like hepatocytes	LMF2
1.04797568553818E-84	0.358419991439742	0.446	0.296	1.75619765382488E-80	Ductular-like hepatocytes	TRIP12
1.12218805691004E-84	0.4668639141633	0.341	0.211	1.88056274576985E-80	Ductular-like hepatocytes	TLN1
1.13244498494513E-84	0.593404976038538	0.251	0.141	1.89775130577105E-80	Ductular-like hepatocytes	CDC42EP4
1.18058834833062E-84	0.323220358215018	0.476	0.319	1.97842995413244E-80	Ductular-like hepatocytes	LGR4
1.2562025769594E-84	0.580991770993947	0.22	0.117	2.10514427846857E-80	Ductular-like hepatocytes	CYCS
1.27330009252745E-84	0.734508249178474	0.134	0.057	2.1337962950575E-80	Ductular-like hepatocytes	ELMO3
1.3095576342141E-84	0.494729587332392	0.338	0.208	2.19455668341598E-80	Ductular-like hepatocytes	SLC25A32
1.73681555448175E-84	0.408219453641474	0.36	0.226	2.91055550620051E-80	Ductular-like hepatocytes	DEPRECATEI
1.78243380120559E-84	0.347292469801923	0.316	0.188	2.98700256406032E-80	Ductular-like hepatocytes	UQCRC2
1.91154717540557E-84	0.307743693496808	0.214	0.112	3.20337075654466E-80	Ductular-like hepatocytes	CARD10
1.95567523417618E-84	0.625519268111629	0.218	0.116	3.27732055743243E-80	Ductular-like hepatocytes	MFSD11
1.95650243116942E-84	0.374740414266585	0.417	0.272	3.27870677415371E-80	Ductular-like hepatocytes	PPP4R3B
2.02646151077733E-84	0.275568692314835	0.547	0.389	3.39594419976065E-80	Ductular-like hepatocytes	ABCD3
2.49770743080679E-84	0.330017499862826	0.507	0.349	4.18565811254602E-80	Ductular-like hepatocytes	SNRPC
2.61032582963068E-84	1.22027172427588	0.124	0.05	4.37438402529509E-80	Ductular-like hepatocytes	SIX1
2.82987042793503E-84	1.32407152783127	0.112	0.043	4.74229686313353E-80	Ductular-like hepatocytes	ABCA3
2.84556789077945E-84	0.487713964760885	0.186	0.092	4.7686026713682E-80	Ductular-like hepatocytes	ANTXR2
2.87628803222132E-84	0.89781917168909	0.188	0.094	4.82008348843965E-80	Ductular-like hepatocytes	C3orf85
3.35771375020722E-84	0.596304407442696	0.184	0.091	5.62685670259726E-80	Ductular-like hepatocytes	DCBLD1
3.59776372666562E-84	0.602978889677307	0.228	0.123	6.02913245314625E-80	Ductular-like hepatocytes	CTPS2
4.19311675977777E-84	0.543531094352078	0.253	0.143	7.02682506603559E-80	Ductular-like hepatocytes	ABL1
4.27955079438086E-84	0.653954255990135	0.187	0.093	7.17167122122344E-80	Ductular-like hepatocytes	ZNF117
4.31793692757516E-84	0.40666271349743	0.42	0.275	7.23599870323045E-80	Ductular-like hepatocytes	EMC7
5.09927273192796E-84	0.608101639080997	0.184	0.091	8.54536124416488E-80	Ductular-like hepatocytes	CCDC25
5.96521390373596E-84	0.528454270629288	0.225	0.12	9.99650545988072E-80	Ductular-like hepatocytes	PTPRG
6.14463175310043E-84	0.296956941154608	0.393	0.251	1.02971739918457E-79	Ductular-like hepatocytes	PSMC2
6.32814456241072E-84	0.811747257968707	0.222	0.12	1.06047046576879E-79	Ductular-like hepatocytes	RADX
6.96697187112405E-84	0.288364586444247	0.426	0.277	1.16752514616297E-79	Ductular-like hepatocytes	UBE3C
7.29475994176817E-84	0.333006090402866	0.302	0.178	1.22245587104151E-79	Ductular-like hepatocytes	EIF4G3
8.09415798810982E-84	0.489579187780998	0.286	0.168	1.35641899564744E-79	Ductular-like hepatocytes	MYO5C
8.37704825724875E-84	0.262910173243857	0.463	0.308	1.40382574694975E-79	Ductular-like hepatocytes	DDX39B
8.45708792366608E-84	0.399166944204817	0.345	0.214	1.41723879424796E-79	Ductular-like hepatocytes	PINK1
8.66041979140555E-84	0.27805899331372	0.286	0.164	1.45131314864374E-79	Ductular-like hepatocytes	MPND
9.18754763671262E-84	0.485354710847859	0.305	0.182	1.5396492329603E-79	Ductular-like hepatocytes	FAF2
9.35648628047568E-84	0.347730951877126	0.513	0.355	1.56795997088211E-79	Ductular-like hepatocytes	SRPRA
9.60286797031591E-84	0.314541267608364	0.405	0.26	1.60924861446554E-79	Ductular-like hepatocytes	RNPS1
1.06794562491309E-83	0.382366180491288	0.394	0.255	1.78966327822935E-79	Ductular-like hepatocytes	SEC23A
1.15690058672033E-83	0.34413383409389	0.354	0.22	1.93873400322594E-79	Ductular-like hepatocytes	TBC1D14

**1.29983439688078E-83**	0.646618146310742	0.25	0.14	2.17826248229281E-79	Ductular-like hepatocytes	BTG3
**1.33143556985924E-83**	1.16536385897051	0.154	0.071	2.23121972797012E-79	Ductular-like hepatocytes	WDR1
**1.35651447784411E-83**	0.454278569457148	0.216	0.114	2.27324696197116E-79	Ductular-like hepatocytes	CORO7
**1.50655155717799E-83**	0.40201858700352	0.376	0.238	2.52467909951888E-79	Ductular-like hepatocytes	CCT8
**1.50676138525242E-83**	0.315207480343829	0.578	0.419	2.52503072940601E-79	Ductular-like hepatocytes	PDIA5
**1.60859666213632E-83**	0.516143732795853	0.307	0.184	2.69568628640805E-79	Ductular-like hepatocytes	REEP3
**1.72080603772597E-83**	0.595533011842624	0.285	0.167	2.88372675802117E-79	Ductular-like hepatocytes	PPIP5K2
**1.7759078920967E-83**	0.386677438418634	0.303	0.18	2.97606644557565E-79	Ductular-like hepatocytes	PER2
**2.14327210619909E-83**	0.584363338972102	0.275	0.159	3.59169539556843E-79	Ductular-like hepatocytes	TOPBP1
**2.20324820701448E-83**	0.304833205193634	0.547	0.381	3.69220334531486E-79	Ductular-like hepatocytes	IL1R1
**2.55880897735835E-83**	0.603806221357664	0.3	0.18	4.28805208425712E-79	Ductular-like hepatocytes	CASC4
**2.5597821019376E-83**	0.534779052449988	0.241	0.133	4.28968284642702E-79	Ductular-like hepatocytes	HLCS
**3.12841592839085E-83**	0.323863345430647	0.5	0.347	5.24259941279739E-79	Ductular-like hepatocytes	SNU13
**3.34542143710801E-83**	0.542428580786039	0.282	0.165	5.60625724430561E-79	Ductular-like hepatocytes	LNPK
**3.59066250855557E-83**	0.615665888329604	0.204	0.105	6.01723223183742E-79	Ductular-like hepatocytes	ATXN7L1
**3.63325824706333E-83**	0.393797986880205	0.306	0.182	6.08861417042873E-79	Ductular-like hepatocytes	STK4
**3.66546436928198E-83**	0.616576260389493	0.228	0.124	6.14258519004275E-79	Ductular-like hepatocytes	PDCL3
**3.67010063795567E-83**	0.778939918627698	0.228	0.125	6.15035464908611E-79	Ductular-like hepatocytes	AMMECR1
**3.73079404375515E-83**	0.671620092616516	0.236	0.13	6.25206465852488E-79	Ductular-like hepatocytes	BCL10
**5.51987269847131E-83**	0.751541674483086	0.203	0.106	9.25020266809822E-79	Ductular-like hepatocytes	DTL
**5.61324320037096E-83**	0.326429802611514	0.495	0.338	9.40667295518166E-79	Ductular-like hepatocytes	C4orf3
**5.78950883492584E-83**	0.395486127871571	0.371	0.236	9.70205890556873E-79	Ductular-like hepatocytes	TTC1
**6.06843813351699E-83**	0.280761994582422	0.446	0.297	1.01694886241478E-78	Ductular-like hepatocytes	FAM177A1
**7.51488915011704E-83**	0.561020685047872	0.222	0.119	1.25934512377661E-78	Ductular-like hepatocytes	DSG2
**8.61686790085411E-83**	0.271624465932909	0.365	0.229	1.44401472282513E-78	Ductular-like hepatocytes	KIAA1671
**9.41021039388399E-83**	0.702697080155315	0.225	0.122	1.57696305780708E-78	Ductular-like hepatocytes	ZNF449
**9.73714495341767E-83**	0.265646427995078	0.473	0.321	1.63175075129373E-78	Ductular-like hepatocytes	SLC25A3
**9.99656948522805E-83**	0.731432297419695	0.199	0.102	1.67522511433452E-78	Ductular-like hepatocytes	FAM111B
**1.05034669324437E-82**	0.430050247932961	0.376	0.24	1.76017098853891E-78	Ductular-like hepatocytes	GINM1
**1.09462771895577E-82**	0.392909243235611	0.313	0.189	1.83437713142609E-78	Ductular-like hepatocytes	KLHL22
**1.21107263842734E-82**	0.469046458479441	0.353	0.221	2.02951552747653E-78	Ductular-like hepatocytes	HMGXB4
**1.47775705122056E-82**	0.28460833762256	0.621	0.456	2.47642526643542E-78	Ductular-like hepatocytes	MYDGF
**1.60517165415507E-82**	0.495263500421528	0.298	0.177	2.68994665803307E-78	Ductular-like hepatocytes	DLST
**2.0536757656752E-82**	0.302568351423151	0.405	0.26	3.44154984811851E-78	Ductular-like hepatocytes	NFIX
**2.142367242604E-82**	0.319602187345356	0.54	0.38	3.59017902515578E-78	Ductular-like hepatocytes	PBLD
**2.27980169588018E-82**	0.511716865551065	0.294	0.174	3.82049168195601E-78	Ductular-like hepatocytes	MTERF3
**2.39366490383764E-82**	0.520319944389155	0.306	0.185	4.01130364585112E-78	Ductular-like hepatocytes	VCPIP1
**2.50391299674982E-82**	0.505756484760964	0.259	0.148	4.19605739995336E-78	Ductular-like hepatocytes	IFI44
**2.63120350387162E-82**	0.358122717803798	0.501	0.349	4.40937083178805E-78	Ductular-like hepatocytes	PSMB4
**2.80331565972754E-82**	0.592588362369597	0.247	0.138	4.69779638257141E-78	Ductular-like hepatocytes	SDHAF4
**3.12505709233614E-82**	0.280376938183499	0.399	0.256	5.2369706753369E-78	Ductular-like hepatocytes	PREB
**3.5005384338198E-82**	0.276631673361112	0.422	0.275	5.86620230739522E-78	Ductular-like hepatocytes	HDGFL2
**3.63890984862761E-82**	0.305372870362271	0.385	0.243	6.09808512433015E-78	Ductular-like hepatocytes	DOT1L
**3.823489051669E-82**	1.30634445693382	0.1	0.036	6.40740295278691E-78	Ductular-like hepatocytes	TNFSF13
**4.06798890812145E-82**	0.58884321153112	0.208	0.109	6.81713581222992E-78	Ductular-like hepatocytes	SLC52A2
**4.08559319952004E-82**	0.308587118299474	0.464	0.313	6.84663708375568E-78	Ductular-like hepatocytes	MAGT1
**4.61035122567267E-82**	0.528713292129237	0.309	0.186	7.72602658398227E-78	Ductular-like hepatocytes	TBCE.1
**4.69501210609094E-82**	0.633880136106024	0.192	0.097	7.8679012873872E-78	Ductular-like hepatocytes	ARL 2.00
**4.79638412058921E-82**	0.364637343286729	0.366	0.232	8.0377805092834E-78	Ductular-like hepatocytes	RAP1A
**4.85434301094478E-82**	0.332806517689459	0.518	0.366	8.13490801774126E-78	Ductular-like hepatocytes	DBI
**5.13383122095967E-82**	0.498896983788077	0.156	0.072	8.60327436008421E-78	Ductular-like hepatocytes	EFNA2
**5.86315526324409E-82**	0.345904398766139	0.456	0.302	9.82547559014444E-78	Ductular-like hepatocytes	PHF10
**6.14697759845503E-82**	0.459649979893324	0.319	0.195	1.03011050594909E-77	Ductular-like hepatocytes	PLCB1
**7.57021834129993E-82**	0.264368739405843	0.41	0.268	1.26861718963504E-77	Ductular-like hepatocytes	NME2
**7.87498738498958E-82**	0.30256979720337	0.442	0.297	1.31969038597655E-77	Ductular-like hepatocytes	NAXE
**7.97397397255242E-82**	0.512890523284288	0.37	0.237	1.33627855832033E-77	Ductular-like hepatocytes	SMARCD2
**8.1711221959726E-82**	0.280487231903058	0.465	0.315	1.36931665760109E-77	Ductular-like hepatocytes	MROH1
**8.20413862720854E-82**	0.464929775869768	0.266	0.154	1.37484955114761E-77	Ductular-like hepatocytes	TICAM1
**9.52481293293704E-82**	0.494215854943909	0.304	0.183	1.59616815130159E-77	Ductular-like hepatocytes	VAPB
**9.58689938180596E-82**	0.349523355021859	0.366	0.231	1.60657259840304E-77	Ductular-like hepatocytes	SAP30L

9.83442617620383E-82	0.366330656587516	0.316	0.192	1.64805313860824E-77	Ductular-like hepatocytes	RELCH
1.07262162641783E-81	0.389530302287899	0.339	0.209	1.79749932155101E-77	Ductular-like hepatocytes	COX11
1.08757779233578E-81	0.615537221072537	0.206	0.108	1.82256286439629E-77	Ductular-like hepatocytes	TMEM209
1.09014799227227E-81	0.692873936350055	0.21	0.112	1.82687000544988E-77	Ductular-like hepatocytes	CD58
1.10578004904964E-81	0.548603232313011	0.253	0.143	1.85306620619739E-77	Ductular-like hepatocytes	NF2
1.24599476481456E-81	0.500529206867258	0.256	0.145	2.08803802687624E-77	Ductular-like hepatocytes	CDK2
1.30850891402707E-81	0.333080759442589	0.348	0.216	2.19279923812656E-77	Ductular-like hepatocytes	PURA
1.32005911704693E-81	0.569007283907484	0.214	0.114	2.21215506834725E-77	Ductular-like hepatocytes	ADCY9
1.32470324554383E-81	0.491231884297553	0.272	0.158	2.21993769888235E-77	Ductular-like hepatocytes	TIMM17B
1.33316008007611E-81	0.302249859869273	0.412	0.27	2.23410966219155E-77	Ductular-like hepatocytes	FPGS
1.43983897373477E-81	0.767511540799323	0.215	0.116	2.41288215218472E-77	Ductular-like hepatocytes	CLTC
1.47022312275813E-81	0.250971911493925	0.491	0.322	2.46379990911807E-77	Ductular-like hepatocytes	TBC1D8
1.56502725998067E-81	0.551735722050979	0.233	0.128	2.62267268227561E-77	Ductular-like hepatocytes	PRPF8
1.59967291158458E-81	0.304746861668987	0.342	0.212	2.68073186523344E-77	Ductular-like hepatocytes	ZC3HAV1
1.75064087201302E-81	0.738427809314708	0.196	0.101	2.93372397331942E-77	Ductular-like hepatocytes	PAXIP1
1.88495476369445E-81	0.299004681634909	0.328	0.2	3.15880719299916E-77	Ductular-like hepatocytes	TIMM50
1.94420730869483E-81	0.358553327457298	0.371	0.238	3.2581026079108E-77	Ductular-like hepatocytes	MCUR1
2.00783494464192E-81	0.562914393734953	0.238	0.133	3.36472980023093E-77	Ductular-like hepatocytes	APAF1
2.01046722685227E-81	0.366674538755006	0.375	0.239	3.36914097875903E-77	Ductular-like hepatocytes	SLC27A3
2.16255983336893E-81	0.66049462886877	0.207	0.109	3.62401776875965E-77	Ductular-like hepatocytes	ARHGEF37
2.4475551338543E-81	0.25987476796642	0.455	0.305	4.10161289331303E-77	Ductular-like hepatocytes	KIAA0100
2.67587221310008E-81	0.322614486973752	0.343	0.212	4.48422665471312E-77	Ductular-like hepatocytes	BCL2L1
2.89254462358409E-81	0.524833526147544	0.297	0.178	4.84732628020221E-77	Ductular-like hepatocytes	EHMT2
2.96676456908415E-81	0.285790897592911	0.674	0.48	4.97170406487122E-77	Ductular-like hepatocytes	TOR1AIP2
3.43413951463352E-81	0.291750274967949	0.561	0.404	5.75493099862285E-77	Ductular-like hepatocytes	HTATIP2
3.48758592889974E-81	0.446639681718983	0.244	0.136	5.84449649965018E-77	Ductular-like hepatocytes	TRAF3
3.92668076778454E-81	0.257950186482139	0.342	0.211	6.58033163065334E-77	Ductular-like hepatocytes	HPCAL1
4.530519841516E-81	0.566166138025606	0.252	0.142	7.59224515041252E-77	Ductular-like hepatocytes	PATZ1
5.18372587691934E-81	0.296217597058478	0.332	0.202	8.68688782454142E-77	Ductular-like hepatocytes	MEPCE
5.43040863142467E-81	0.474833456782641	0.302	0.181	9.10027878454146E-77	Ductular-like hepatocytes	CAMSAP2
5.94513799326277E-81	0.610753861852939	0.206	0.108	9.96286224910975E-77	Ductular-like hepatocytes	HFE
6.38901002114586E-81	0.2702285046446	0.477	0.325	1.07067029934362E-76	Ductular-like hepatocytes	TIMM13
6.74648780444615E-81	0.552517677700709	0.218	0.117	1.13057642626909E-76	Ductular-like hepatocytes	MANBA
7.03623805783553E-81	0.308921045660531	0.344	0.213	1.17913277373208E-76	Ductular-like hepatocytes	RSBN1L
7.34980996761666E-81	0.51206890560374	0.267	0.154	1.2316811543732E-76	Ductular-like hepatocytes	KIDINS220
7.94074907474117E-81	1.15203609899319	0.113	0.044	1.33071072994513E-76	Ductular-like hepatocytes	ALDH1A2
8.79561151394591E-81	0.346637401510931	0.357	0.219	1.47396857750706E-76	Ductular-like hepatocytes	MTCP1
8.99810551682023E-81	0.421135749399875	0.306	0.184	1.50790252250873E-76	Ductular-like hepatocytes	ATG2B
9.06982513113519E-81	0.715904061009662	0.192	0.099	1.51992129547563E-76	Ductular-like hepatocytes	DEPRECATEI
9.59157731468396E-81	0.860705954208932	0.188	0.096	1.60735652639474E-76	Ductular-like hepatocytes	XRCC3
1.02745804447556E-80	0.321908770505496	0.353	0.221	1.72181419093214E-76	Ductular-like hepatocytes	PCYT1A
1.07081416892335E-80	0.350944649270107	0.426	0.283	1.79447038428174E-76	Ductular-like hepatocytes	ACADS
1.10064513184011E-80	0.329642158142338	0.37	0.235	1.84446111193765E-76	Ductular-like hepatocytes	PPP1R7
1.24143955453282E-80	0.532128505998381	0.226	0.124	2.08040440548609E-76	Ductular-like hepatocytes	PBXIP1
1.2747199156529E-80	0.274107429869054	0.5	0.343	2.13617563465112E-76	Ductular-like hepatocytes	PSMD3
1.28775860485744E-80	0.301985782506596	0.331	0.202	2.15802587002011E-76	Ductular-like hepatocytes	ELMO1
1.30020812021618E-80	0.681285958793544	0.189	0.097	2.17888876785827E-76	Ductular-like hepatocytes	FUNDC1
1.31999327557296E-80	0.316489976454697	0.373	0.236	2.21204473120517E-76	Ductular-like hepatocytes	SCAF1
1.32550843890616E-80	0.582963761094884	0.315	0.193	2.22128704191894E-76	Ductular-like hepatocytes	ADH5
1.42280090855378E-80	0.336873163176824	0.321	0.195	2.38432976255442E-76	Ductular-like hepatocytes	ATXN1
1.43348789717229E-80	0.492879458138098	0.308	0.186	2.40223901808133E-76	Ductular-like hepatocytes	DEPRECATEI
1.47261753250681E-80	0.583325409013883	0.212	0.112	2.46781246097491E-76	Ductular-like hepatocytes	USP37
1.57714318764187E-80	0.649879068174368	0.227	0.124	2.64297655385025E-76	Ductular-like hepatocytes	ZNF367
1.59016025990212E-80	0.771756439179664	0.233	0.13	2.66479056354398E-76	Ductular-like hepatocytes	BCHE
1.79075297280425E-80	0.382686249956896	0.402	0.258	3.00094383182536E-76	Ductular-like hepatocytes	MAP3K5
1.79483748032707E-80	0.322906130606574	0.375	0.24	3.00778864953211E-76	Ductular-like hepatocytes	RAC1
1.85172276804486E-80	0.310395515055173	0.388	0.25	3.10311701468958E-76	Ductular-like hepatocytes	TMEM179B
1.88555345403024E-80	0.285992434920593	0.348	0.217	3.15981047826388E-76	Ductular-like hepatocytes	ITPKC
2.0649026093173E-80	0.609937850327469	0.264	0.152	3.46036379269393E-76	Ductular-like hepatocytes	SLC39A3
2.22266465792647E-80	0.541390851793218	0.205	0.108	3.72474143375317E-76	Ductular-like hepatocytes	DNAAF5

**2.23243288866067E-80**	0.413011397865663	0.319	0.195	3.74111103481754E-76	Ductular-like hepatocytes	PITRM1
**2.24412274067919E-80**	0.269789237013652	0.369	0.235	3.76070088883018E-76	Ductular-like hepatocytes	KLHDC4
**2.37301982684962E-80**	0.419548499045954	0.241	0.134	3.97670662583459E-76	Ductular-like hepatocytes	NOP9
**2.72590094754824E-80**	0.458177086753399	0.273	0.159	4.56806480790134E-76	Ductular-like hepatocytes	LYPLA1
**2.83947988980518E-80**	0.272512397337762	0.538	0.376	4.75840039933552E-76	Ductular-like hepatocytes	PTOV1
**2.92242458035024E-80**	0.463877644022241	0.272	0.158	4.89739911175093E-76	Ductular-like hepatocytes	PPP5C
**3.10041694598045E-80**	0.305005315387743	0.591	0.414	5.19567871807403E-76	Ductular-like hepatocytes	CIAO2A
**3.92903607126553E-80**	0.316600547326135	0.329	0.203	6.58427864822678E-76	Ductular-like hepatocytes	COQ4
**4.02288129548499E-80**	0.338885669315125	0.395	0.256	6.74154447497374E-76	Ductular-like hepatocytes	MZT2B
**4.3455963547006E-80**	0.382210985075817	0.307	0.185	7.28235037120726E-76	Ductular-like hepatocytes	MED13L
**4.57690207155755E-80**	0.605313153716359	0.238	0.132	7.66997249151614E-76	Ductular-like hepatocytes	POLD1
**5.06758829971467E-80**	1.08971856225513	0.12	0.049	8.49226447266185E-76	Ductular-like hepatocytes	VSIR
**5.32186502977732E-80**	0.507905814966001	0.283	0.168	8.91838141690083E-76	Ductular-like hepatocytes	ZNF608
**5.49857722515881E-80**	0.39904475517043	0.307	0.185	9.21451571392113E-76	Ductular-like hepatocytes	CHIC2
**5.9149207932292E-80**	0.439428148980596	0.307	0.186	9.91222426529349E-76	Ductular-like hepatocytes	SGPP1
**6.07034502217543E-80**	0.5292290917508	0.242	0.136	1.01726841881616E-75	Ductular-like hepatocytes	PARP1
**6.15803906410044E-80**	0.406579979799543	0.315	0.192	1.03196418636195E-75	Ductular-like hepatocytes	ANO6
**6.20088875988611E-80**	0.380820284190215	0.414	0.272	1.03914493838171E-75	Ductular-like hepatocytes	SEC23B
**6.7551090252093E-80**	0.327279041763303	0.463	0.313	1.13202117044457E-75	Ductular-like hepatocytes	RPN1
**7.18630813062095E-80**	0.497235401839094	0.299	0.18	1.20428151652946E-75	Ductular-like hepatocytes	THNSL2
**7.30494121598601E-80**	0.403165557372105	0.316	0.193	1.22416204897494E-75	Ductular-like hepatocytes	MBTPS1
**8.01275099764116E-80**	0.416519452433345	0.343	0.215	1.3427768121847E-75	Ductular-like hepatocytes	HIPK1
**8.03887849950649E-80**	0.661895135647579	0.253	0.145	1.3471552589473E-75	Ductular-like hepatocytes	FEN1
**8.19482784090049E-80**	0.673647992392516	0.137	0.06	1.3732892495781E-75	Ductular-like hepatocytes	PHETA1
**8.71942850539072E-80**	0.480965104616871	0.306	0.185	1.46120182893338E-75	Ductular-like hepatocytes	C8orf76
**8.7312815593031E-80**	0.557842547175258	0.225	0.123	1.46318816370801E-75	Ductular-like hepatocytes	IGBP1
**8.98872797667946E-80**	0.334280892444459	0.355	0.222	1.50633103433194E-75	Ductular-like hepatocytes	DNAJC5
**9.13598003842575E-80**	0.758664195610499	0.194	0.101	1.53100753483939E-75	Ductular-like hepatocytes	SGCB
**9.40309931647366E-80**	0.307867975230173	0.429	0.285	1.57577138345466E-75	Ductular-like hepatocytes	CYBC1
**9.55024694014281E-80**	0.537545511807272	0.252	0.143	1.60043038222913E-75	Ductular-like hepatocytes	MINDY1
**9.61473373395161E-80**	0.342171087150548	0.368	0.232	1.61123707913561E-75	Ductular-like hepatocytes	FOXA1
**9.70211442896027E-80**	0.334970531740926	0.304	0.181	1.62588033600516E-75	Ductular-like hepatocytes	DBP
**1.04853217396024E-79**	0.28617874007205	0.543	0.386	1.75713021712257E-75	Ductular-like hepatocytes	DYNLL2
**1.05075169391882E-79**	0.295096382969075	0.317	0.193	1.76084968866916E-75	Ductular-like hepatocytes	SOAT1
**1.08324899288001E-79**	0.307684707161041	0.334	0.208	1.81530866226831E-75	Ductular-like hepatocytes	PMM2
**1.09853797142846E-79**	0.45246530721179	0.337	0.21	1.84092993251982E-75	Ductular-like hepatocytes	GMPS
**1.18970123327162E-79**	0.474070067977925	0.317	0.195	1.99370132671658E-75	Ductular-like hepatocytes	MTERF4
**1.25678516459872E-79**	0.898162668260718	0.205	0.109	2.10612057883453E-75	Ductular-like hepatocytes	SLC49A4
**1.28853786979419E-79**	0.372036376109295	0.269	0.156	2.15933176220111E-75	Ductular-like hepatocytes	KLHDC10
**1.64333911270961E-79**	0.280726816270085	0.571	0.415	2.75390768507876E-75	Ductular-like hepatocytes	CD46
**1.92353148757548E-79**	0.570206818615973	0.272	0.16	3.22345406687898E-75	Ductular-like hepatocytes	CDC123
**1.94753895714738E-79**	0.60667155036186	0.212	0.114	3.26368578438758E-75	Ductular-like hepatocytes	RHOG
**1.97270870361537E-79**	1.13788478319266	0.132	0.057	3.30586524551864E-75	Ductular-like hepatocytes	ACER3
**2.05584763515745E-79**	0.480546351671721	0.254	0.145	3.44518946699685E-75	Ductular-like hepatocytes	RAB3GAP2
**2.17046543664091E-79**	0.485743540066141	0.297	0.179	3.63726597872284E-75	Ductular-like hepatocytes	CARM1
**2.29315735488115E-79**	0.437805496720828	0.35	0.221	3.84287309530983E-75	Ductular-like hepatocytes	ACACA
**2.85382022913042E-79**	0.32169028083651	0.487	0.329	4.78243193997675E-75	Ductular-like hepatocytes	RNF19A
**2.89802493718149E-79**	0.329847582053201	0.434	0.289	4.85651018972874E-75	Ductular-like hepatocytes	PCMTD1
**2.97511091156071E-79**	0.687485186204938	0.208	0.111	4.98569086559344E-75	Ductular-like hepatocytes	CTNNBL1
**2.9797541337193E-79**	0.293319839390531	0.615	0.458	4.99347197728681E-75	Ductular-like hepatocytes	MAOB
**3.1243685968118E-79**	0.371494015230622	0.306	0.182	5.23581689453721E-75	Ductular-like hepatocytes	EZH2
**3.74019521692922E-79**	0.639711768624885	0.215	0.117	6.26781914452998E-75	Ductular-like hepatocytes	PDE6D
**3.79179695842905E-79**	0.423620623628385	0.238	0.132	6.35429334293539E-75	Ductular-like hepatocytes	DNMBP
**4.35167503884339E-79**	0.529609587569785	0.242	0.136	7.29253703009375E-75	Ductular-like hepatocytes	SEC61G
**4.85239018534585E-79**	0.617729857251093	0.235	0.132	8.13163547260258E-75	Ductular-like hepatocytes	JADE2
**4.88842817211867E-79**	1.22281398172946	0.132	0.057	8.19202793083646E-75	Ductular-like hepatocytes	LPCAT2
**4.96276135892302E-79**	0.729523709258602	0.182	0.092	8.31659548528319E-75	Ductular-like hepatocytes	NDUFA6
**5.5400482835053E-79**	1.24324017230112	0.131	0.057	9.28401291349818E-75	Ductular-like hepatocytes	PNMT
**5.69228038440562E-79**	0.380430497768729	0.384	0.247	9.53912346818694E-75	Ductular-like hepatocytes	KIF13A
**5.78541665536053E-79**	0.409048573438462	0.294	0.176	9.69520123105317E-75	Ductular-like hepatocytes	CSNK2B

6.13279348202017E-79	0.837888708658257	0.173	0.085	1.02773353171694E-74	Ductular-like hepatocytes	F8
6.22960547190524E-79	0.559419027432985	0.245	0.139	1.04395728498188E-74	Ductular-like hepatocytes	MIGA1
6.29550548704107E-79	0.283104518630468	0.399	0.259	1.05500080951834E-74	Ductular-like hepatocytes	TRPC4AP
6.62311948055252E-79	0.426908285565125	0.348	0.221	1.10990236255099E-74	Ductular-like hepatocytes	ELOC
6.95820338132354E-79	0.445880414999128	0.334	0.208	1.1660557226422E-74	Ductular-like hepatocytes	KIF16B
7.02721558227833E-79	0.522636044009279	0.294	0.176	1.1776207872782E-74	Ductular-like hepatocytes	PBX3
7.05277109367946E-79	0.512084278548022	0.271	0.159	1.1819033798788E-74	Ductular-like hepatocytes	UQCRFS1
7.91357127957039E-79	0.592349496949655	0.258	0.149	1.32615627503041E-74	Ductular-like hepatocytes	BAG5
8.06930049027559E-79	0.424063795122774	0.31	0.19	1.35225337616038E-74	Ductular-like hepatocytes	DOK4
8.47416347353217E-79	0.351427179579118	0.327	0.202	1.42010031489452E-74	Ductular-like hepatocytes	CPTP
9.15253385022911E-79	0.375639921140399	0.444	0.3	1.5337816226214E-74	Ductular-like hepatocytes	AHCYL1
9.2077170964952E-79	0.306937354817059	0.383	0.246	1.54302923103067E-74	Ductular-like hepatocytes	PTBP3
9.49706195244704E-79	0.372197863789184	0.336	0.208	1.59151764199108E-74	Ductular-like hepatocytes	RRP1B
1.0138097661848E-78	0.598553067346862	0.173	0.085	1.6989424061725E-74	Ductular-like hepatocytes	LRRC8B
1.16783378854905E-78	0.689574204119732	0.151	0.071	1.9570558628505E-74	Ductular-like hepatocytes	TMEM41A
1.19574261605594E-78	0.370599489617986	0.297	0.178	2.00382547598654E-74	Ductular-like hepatocytes	DDOST
1.19905558461593E-78	0.26545434213855	0.539	0.381	2.00937734869937E-74	Ductular-like hepatocytes	ACTN1
1.37773527972801E-78	0.40483171382371	0.363	0.231	2.3088087817682E-74	Ductular-like hepatocytes	MBOAT1
1.48643732167414E-78	0.53374475526963	0.239	0.135	2.49097166366152E-74	Ductular-like hepatocytes	PTPRD
1.52701569728408E-78	0.902386748178061	0.138	0.061	2.55897290550865E-74	Ductular-like hepatocytes	PCDHGA7
1.53194059053255E-78	0.546074237793627	0.237	0.133	2.56722604161445E-74	Ductular-like hepatocytes	C4orf19
1.53895995032617E-78	0.419276936911465	0.353	0.223	2.5789890847566E-74	Ductular-like hepatocytes	DONSON
1.62972128305739E-78	0.26182300642553	0.454	0.305	2.73108692614758E-74	Ductular-like hepatocytes	KMT2C
1.77856039234166E-78	0.269038303117647	0.41	0.269	2.98051150548616E-74	Ductular-like hepatocytes	DCTN2
2.59137309658541E-78	0.389659217432866	0.335	0.207	4.34262303525783E-74	Ductular-like hepatocytes	ELMOD2
2.59876231530588E-78	0.36604624206977	0.422	0.28	4.35500588798959E-74	Ductular-like hepatocytes	DNAJB9
2.92362218506223E-78	0.672315317015133	0.271	0.16	4.89940605772728E-74	Ductular-like hepatocytes	ANKS1B
3.245783995078E-78	0.396975192298281	0.303	0.184	5.43928481895171E-74	Ductular-like hepatocytes	CHERP
3.36666997549526E-78	0.464720772021018	0.3	0.182	5.64186554493496E-74	Ductular-like hepatocytes	YIPF5
3.48191627914376E-78	0.386883519212953	0.248	0.14	5.83499530058911E-74	Ductular-like hepatocytes	PNPLA6
3.54056500429978E-78	0.397637626867258	0.399	0.263	5.93327883420556E-74	Ductular-like hepatocytes	PYURF
3.56902769661508E-78	0.550890358992663	0.238	0.134	5.98097661398755E-74	Ductular-like hepatocytes	WDR34
3.88896060387344E-78	0.290493669152237	0.251	0.142	6.51712017997111E-74	Ductular-like hepatocytes	VPS13A
4.11355609884686E-78	0.599260864329629	0.218	0.119	6.89349731044757E-74	Ductular-like hepatocytes	SCYL3
4.21074407255033E-78	0.366587891877903	0.32	0.197	7.05636491677985E-74	Ductular-like hepatocytes	PIN1
4.91096213971477E-78	0.517738734896647	0.296	0.179	8.22979035373401E-74	Ductular-like hepatocytes	RMDN1
5.39377549122783E-78	0.40974852061697	0.318	0.197	9.0388889681996E-74	Ductular-like hepatocytes	DGUOK
5.53794215790896E-78	0.390750018345568	0.312	0.19	9.28048346822384E-74	Ductular-like hepatocytes	SLC35D1
5.55890610379796E-78	0.645332694964869	0.215	0.117	9.31561484874462E-74	Ductular-like hepatocytes	PAPSS1
6.93135923750799E-78	0.482669861370447	0.317	0.195	1.16155718102159E-73	Ductular-like hepatocytes	NSF
8.46220442112167E-78	0.578432997221714	0.245	0.14	1.41809621689157E-73	Ductular-like hepatocytes	XPO4
8.86878294363495E-78	0.305576914967947	0.501	0.349	1.48623064569434E-73	Ductular-like hepatocytes	SLC50A1
9.41017690175057E-78	0.278999953645323	0.457	0.307	1.57695744519536E-73	Ductular-like hepatocytes	AP1M1
9.72623448233234E-78	0.569414816090272	0.222	0.122	1.62992237454925E-73	Ductular-like hepatocytes	MRM2
1.13782983137354E-77	0.418395855192765	0.269	0.157	1.90677523141578E-73	Ductular-like hepatocytes	PIGG
1.16902356393785E-77	0.740562534276201	0.237	0.134	1.95904968844705E-73	Ductular-like hepatocytes	CHRAC1
1.26362096024152E-77	0.477091440735094	0.315	0.194	2.11757600517275E-73	Ductular-like hepatocytes	PSMG2
1.37947700359157E-77	0.357169175720692	0.397	0.26	2.31172756261875E-73	Ductular-like hepatocytes	YWHAE
1.51532491130719E-77	0.260620802860638	0.473	0.326	2.53938148636859E-73	Ductular-like hepatocytes	ANXA11
1.54772692540405E-77	0.306380404117837	0.321	0.196	2.5936807815921E-73	Ductular-like hepatocytes	DNAJC15
1.8024066337765E-77	0.461355840448044	0.361	0.232	3.02047303688265E-73	Ductular-like hepatocytes	TMEM123
1.91165843651862E-77	0.409820228357804	0.328	0.203	3.20355720791791E-73	Ductular-like hepatocytes	DMXL1
1.97182935642685E-77	0.458735850704105	0.28	0.166	3.30439163550011E-73	Ductular-like hepatocytes	CBX5
1.98177390807374E-77	0.487049410536855	0.301	0.183	3.32105671514997E-73	Ductular-like hepatocytes	PPCS
2.50085470772972E-77	0.393581774074147	0.266	0.155	4.19093231921347E-73	Ductular-like hepatocytes	TRIP11
2.60563882665262E-77	0.261222030712153	0.597	0.43	4.36652954570446E-73	Ductular-like hepatocytes	ADAR
2.69446909387315E-77	0.490048453894299	0.2	0.105	4.51539130751262E-73	Ductular-like hepatocytes	ZMYND19
2.95032828874307E-77	0.703760250853404	0.243	0.139	4.94416014627563E-73	Ductular-like hepatocytes	GUCY1B1
2.97721816147596E-77	0.414106750359573	0.269	0.158	4.98922219500141E-73	Ductular-like hepatocytes	CADPS2
3.39357931320121E-77	0.376265445175651	0.347	0.218	5.68696021306258E-73	Ductular-like hepatocytes	HCFC1

4.11773567173353E-77	0.284547646372496	0.293	0.176	6.90050143869106E-73	Ductular-like hepatocytes	RAB11FIP4
4.51028558760471E-77	0.316964004340221	0.452	0.31	7.55833658770798E-73	Ductular-like hepatocytes	SRPRB
4.73692204498567E-77	0.38818742320141	0.306	0.186	7.93813396298698E-73	Ductular-like hepatocytes	CDC42BPA
4.89663039983857E-77	0.534514571726549	0.234	0.132	8.20577322404948E-73	Ductular-like hepatocytes	UQCRH
4.96079758334724E-77	0.498931619081158	0.25	0.144	8.3133045901733E-73	Ductular-like hepatocytes	DEPRECATEI
5.01453135501248E-77	0.297107081481433	0.323	0.199	8.40335164472991E-73	Ductular-like hepatocytes	PRRC2B
5.69124930370163E-77	0.592619904832405	0.276	0.164	9.53739558314319E-73	Ductular-like hepatocytes	PDS5B
6.16069000698374E-77	0.64426671128408	0.184	0.094	1.03240843137033E-72	Ductular-like hepatocytes	CPSF4
6.69538334080243E-77	0.582671424229733	0.214	0.117	1.12201234025167E-72	Ductular-like hepatocytes	DNAJB14
6.82444202064879E-77	0.285581108579706	0.502	0.345	1.14363999382032E-72	Ductular-like hepatocytes	PA2G4
7.48428406234188E-77	0.334367558181082	0.521	0.376	1.25421632316725E-72	Ductular-like hepatocytes	SLC35D2
7.6401644223474E-77	0.40577090668576	0.302	0.184	1.28033875389698E-72	Ductular-like hepatocytes	CNPPD1
8.44538864409383E-77	0.385161549844301	0.343	0.215	1.41527822897724E-72	Ductular-like hepatocytes	SLAIN2
8.67128363654008E-77	0.375520025727893	0.318	0.196	1.45313371181139E-72	Ductular-like hepatocytes	C8orf33
8.72197745735392E-77	0.590125601340655	0.21	0.113	1.46162898230337E-72	Ductular-like hepatocytes	PRMT3
9.16962522972684E-77	0.879205926581577	0.207	0.112	1.53664579599762E-72	Ductular-like hepatocytes	GPR180
9.3004127775605E-77	0.413395206522951	0.346	0.217	1.55856317326359E-72	Ductular-like hepatocytes	GLA
1.00588944127967E-76	0.275580020602967	0.532	0.377	1.68566952569647E-72	Ductular-like hepatocytes	NENF
1.04256731723636E-76	0.381922468886494	0.313	0.192	1.74713431022469E-72	Ductular-like hepatocytes	IFNGR2
1.07563644943999E-76	0.654534103747031	0.186	0.096	1.80255156197154E-72	Ductular-like hepatocytes	DTNBP1
1.10791340490328E-76	0.451960528539401	0.272	0.16	1.85664128393692E-72	Ductular-like hepatocytes	CNOT10
1.20023355432556E-76	0.651814462297524	0.184	0.094	2.01135139033878E-72	Ductular-like hepatocytes	E2F3
1.20422200749033E-76	0.29003020691731	0.552	0.399	2.01803524015229E-72	Ductular-like hepatocytes	RNF5
1.29880810533135E-76	0.509481260108076	0.26	0.151	2.17654262291428E-72	Ductular-like hepatocytes	KLHL23
1.30650086771566E-76	0.548078905177968	0.268	0.158	2.1894341541179E-72	Ductular-like hepatocytes	BMPR2
1.32244063096107E-76	0.31528472553218	0.329	0.204	2.21614600936456E-72	Ductular-like hepatocytes	VPS26B
1.5010212774631E-76	0.304819139990222	0.479	0.331	2.51541145677266E-72	Ductular-like hepatocytes	ARRDC2
1.56677543463045E-76	0.294790408834811	0.368	0.235	2.62560227335371E-72	Ductular-like hepatocytes	GAK
1.60277755062631E-76	0.388394662481207	0.308	0.189	2.68593461933957E-72	Ductular-like hepatocytes	ZCRB1
1.64897312111793E-76	0.705863139439367	0.216	0.119	2.76334915636943E-72	Ductular-like hepatocytes	MPI
1.79560396796005E-76	0.469530745334271	0.254	0.147	3.00907312950745E-72	Ductular-like hepatocytes	FNBP1L
1.84171706165541E-76	0.38633539537961	0.337	0.211	3.08634945192213E-72	Ductular-like hepatocytes	NUDT21
2.09804001453019E-76	0.473015410339074	0.306	0.187	3.5158954563497E-72	Ductular-like hepatocytes	NUP133
2.18589237905789E-76	0.462529389843584	0.302	0.185	3.6631184488252E-72	Ductular-like hepatocytes	EXOC5
2.25211213224514E-76	0.479668060156341	0.257	0.149	3.7740895112164E-72	Ductular-like hepatocytes	PSMG4
2.33015940899896E-76	0.319970637362691	0.407	0.267	3.90488113760046E-72	Ductular-like hepatocytes	ACACB
2.34845579161661E-76	0.507721445568835	0.236	0.133	3.93554221559112E-72	Ductular-like hepatocytes	MPP6
2.54707887303935E-76	0.746638975859521	0.126	0.054	4.26839477543934E-72	Ductular-like hepatocytes	SHD
2.63141572104553E-76	0.276134744582669	0.511	0.359	4.40972646532809E-72	Ductular-like hepatocytes	TALDO1
2.77599493116664E-76	0.751621951366586	0.184	0.095	4.65201230564906E-72	Ductular-like hepatocytes	CHMP4C
2.86069647805294E-76	0.611086382838698	0.2	0.107	4.79395515792112E-72	Ductular-like hepatocytes	ZNF839
3.01974349908884E-76	0.464285872983168	0.265	0.155	5.06048615577309E-72	Ductular-like hepatocytes	NBEAL1
3.10011305399438E-76	0.766218878616277	0.174	0.088	5.19516945588378E-72	Ductular-like hepatocytes	PPL
3.21132342512749E-76	0.821601483838639	0.169	0.084	5.38153579582865E-72	Ductular-like hepatocytes	EXD2
4.01304197465096E-76	0.47399196161983	0.263	0.154	6.72505574112007E-72	Ductular-like hepatocytes	RETREG2
4.56741418611151E-76	0.606450281795367	0.246	0.141	7.65407269308567E-72	Ductular-like hepatocytes	MAP1S
4.65208872585609E-76	0.573997171026488	0.232	0.131	7.79597028678964E-72	Ductular-like hepatocytes	ALDH3A1
4.75985700181741E-76	1.18259096798556	0.14	0.064	7.97656836364562E-72	Ductular-like hepatocytes	ARSF
4.99343982782885E-76	0.355760052586419	0.382	0.248	8.36800646347559E-72	Ductular-like hepatocytes	UBE2E3
5.39889178897285E-76	0.491026118236944	0.145	0.067	9.04746285996071E-72	Ductular-like hepatocytes	FCSK
7.06990191106435E-76	0.545549925959141	0.256	0.15	1.18477416225616E-71	Ductular-like hepatocytes	CPOX
7.25145639199414E-76	0.331094065102479	0.422	0.284	1.21519906217038E-71	Ductular-like hepatocytes	RNF130
7.5473780372487E-76	0.43513054877435	0.183	0.093	1.26478961148214E-71	Ductular-like hepatocytes	DOCK6
7.83059309746894E-76	0.399989139440505	0.4	0.269	1.31225079127384E-71	Ductular-like hepatocytes	ATP5F1A
8.83737728810931E-76	0.362994690210174	0.359	0.229	1.48096768594136E-71	Ductular-like hepatocytes	USP24
1.18097794540262E-75	0.414613619240285	0.235	0.133	1.97908284090571E-71	Ductular-like hepatocytes	RAPGEF1
1.35306319972096E-75	0.383284234324963	0.301	0.183	2.26746331009238E-71	Ductular-like hepatocytes	TRAPPC10
1.42451845345841E-75	0.532201104543963	0.267	0.158	2.3872080243056E-71	Ductular-like hepatocytes	ZNF704
1.59052851113247E-75	0.306439205367016	0.21	0.112	2.6654076789558E-71	Ductular-like hepatocytes	PTPN18
1.59385406279856E-75	0.426300662720638	0.294	0.179	2.67098063843783E-71	Ductular-like hepatocytes	SNRPD2

1.66257260763735E-75	0.456659392157206	0.251	0.144	2.78613917587867E-71	Ductular-like hepatocytes	UEVLD
1.68149548716605E-75	0.345283470194384	0.315	0.194	2.81785013739286E-71	Ductular-like hepatocytes	MTA2
1.77253129765985E-75	0.372662006693244	0.237	0.134	2.97040794861837E-71	Ductular-like hepatocytes	FARP2
1.89820783106648E-75	0.389428073701444	0.314	0.194	3.1810166833012E-71	Ductular-like hepatocytes	RANBP3
1.93511996911921E-75	0.443259328178029	0.273	0.162	3.24287404424998E-71	Ductular-like hepatocytes	PDZD11
1.938028552288E-75	0.330508405442611	0.313	0.192	3.24774824792423E-71	Ductular-like hepatocytes	VAMP3
2.16816999600238E-75	0.415595715962154	0.316	0.197	3.63341927930078E-71	Ductular-like hepatocytes	TSR2
2.24282007126987E-75	0.538198687099303	0.275	0.165	3.75851787543406E-71	Ductular-like hepatocytes	ANAPC13
2.38969220595383E-75	0.442131483012738	0.296	0.179	4.00464619873743E-71	Ductular-like hepatocytes	ARHGAP17
2.52529603982093E-75	0.310679499674069	0.403	0.266	4.23189110353192E-71	Ductular-like hepatocytes	CKAP4
2.75188346225573E-75	0.255323162730266	0.526	0.363	4.61160630604815E-71	Ductular-like hepatocytes	PANK3
2.83486254548359E-75	0.627385653975343	0.204	0.11	4.7506626537214E-71	Ductular-like hepatocytes	RAD51D
2.88432820919366E-75	0.415619948416526	0.286	0.172	4.83355721296674E-71	Ductular-like hepatocytes	ZCCHC2
2.89621645025426E-75	0.382160181188785	0.332	0.208	4.85347952733609E-71	Ductular-like hepatocytes	MED14
2.92973489514992E-75	0.356449049898289	0.297	0.181	4.90964973729223E-71	Ductular-like hepatocytes	ZNF384
2.98093012642749E-75	0.293714594870501	0.389	0.253	4.99544270586719E-71	Ductular-like hepatocytes	CLIP1
3.00053263183624E-75	0.490459746047644	0.234	0.132	5.02829258443118E-71	Ductular-like hepatocytes	TCP11L1
3.08710378269929E-75	0.262095628133256	0.386	0.25	5.17336851904747E-71	Ductular-like hepatocytes	PRDX5
3.17135810003928E-75	0.394619156394072	0.299	0.181	5.31456190404583E-71	Ductular-like hepatocytes	RAB27A
3.20977097814616E-75	0.28875698540759	0.641	0.488	5.37893420517734E-71	Ductular-like hepatocytes	UQCRQ
3.44029144448121E-75	0.495693476304916	0.274	0.163	5.7652404026616E-71	Ductular-like hepatocytes	CCT5
3.48298716233098E-75	0.41265602402641	0.32	0.199	5.83678988663425E-71	Ductular-like hepatocytes	UBTF
3.48447019626815E-75	0.563562521463363	0.191	0.1	5.83927515490616E-71	Ductular-like hepatocytes	BRWD3
3.52276042551976E-75	0.37151203098249	0.231	0.129	5.90344192108601E-71	Ductular-like hepatocytes	DEPRECATEI
3.58482720983294E-75	0.478423277983759	0.293	0.178	6.00745343823803E-71	Ductular-like hepatocytes	VCP
4.546772216583E-75	0.34859038676109	0.382	0.25	7.6194808805498E-71	Ductular-like hepatocytes	PSMB2
4.73734951824527E-75	0.335518434400427	0.359	0.23	7.93885032267543E-71	Ductular-like hepatocytes	CLTA
5.39245648127551E-75	0.388580807111218	0.331	0.207	9.03667857132151E-71	Ductular-like hepatocytes	ZNF623
5.40439095118603E-75	0.493371935273717	0.316	0.197	9.05667835599755E-71	Ductular-like hepatocytes	ANKH
5.93568995745127E-75	0.381068721252451	0.315	0.194	9.94702923069684E-71	Ductular-like hepatocytes	NOP14
5.96714440024879E-75	0.274905653939895	0.474	0.325	9.99974058593693E-71	Ductular-like hepatocytes	UBE2R2
6.11994924827268E-75	0.7012518321068	0.22	0.122	1.02558109502553E-70	Ductular-like hepatocytes	SOCS4
7.01586200451554E-75	0.463111697925418	0.252	0.146	1.17571815471671E-70	Ductular-like hepatocytes	PPP1R13B
7.18823842900478E-75	0.559029484998725	0.233	0.132	1.20460499593262E-70	Ductular-like hepatocytes	CYB561A3
7.71843254495413E-75	0.481413201408603	0.27	0.161	1.29345492588341E-70	Ductular-like hepatocytes	DHRSX
7.93468733228278E-75	0.445262225397717	0.302	0.185	1.32969490314395E-70	Ductular-like hepatocytes	CCNDBP1
8.43497320647989E-75	0.404490183440576	0.33	0.206	1.4135328099419E-70	Ductular-like hepatocytes	ZMYND11
8.48208343161721E-75	0.607984463897983	0.205	0.111	1.42142754147041E-70	Ductular-like hepatocytes	TBC1D7
8.99892566075538E-75	0.539592022292595	0.283	0.169	1.50803996222939E-70	Ductular-like hepatocytes	DCK
9.74180383528446E-75	0.658976736033337	0.217	0.121	1.63253148671697E-70	Ductular-like hepatocytes	MBTPS2
1.19378877307273E-74	0.282347926954347	0.305	0.186	2.00055122591529E-70	Ductular-like hepatocytes	NUP42
1.22404407999474E-74	0.395416235331889	0.338	0.215	2.05125306925518E-70	Ductular-like hepatocytes	TMEM189
1.44512946491776E-74	0.768272473932004	0.179	0.092	2.42174795730918E-70	Ductular-like hepatocytes	G6PC3
1.54747873865277E-74	0.423760816160527	0.313	0.194	2.59326487023431E-70	Ductular-like hepatocytes	NUDT3
1.67538013329057E-74	0.549494767260985	0.242	0.139	2.80760202736834E-70	Ductular-like hepatocytes	CHCHD3
1.74140076633066E-74	0.394558264495338	0.346	0.219	2.91823940421692E-70	Ductular-like hepatocytes	MAEA
1.76943093156076E-74	0.456135187635372	0.248	0.143	2.96521235510952E-70	Ductular-like hepatocytes	DPAGT1
1.8314313318039E-74	0.357084912004924	0.317	0.197	3.06911262583698E-70	Ductular-like hepatocytes	SPATS2
1.94930610132381E-74	0.880831266130645	0.21	0.117	3.26664716459844E-70	Ductular-like hepatocytes	NRXN3
1.96927906715656E-74	0.577332412891709	0.228	0.129	3.30011786074096E-70	Ductular-like hepatocytes	UBE2M
2.22881598282498E-74	0.46400309821477	0.269	0.159	3.7350498240181E-70	Ductular-like hepatocytes	ATP5PB
2.24550599113053E-74	0.468624756880571	0.208	0.112	3.76301893993654E-70	Ductular-like hepatocytes	PARVA
2.42852624922023E-74	0.31690649311918	0.188	0.097	4.06972428844327E-70	Ductular-like hepatocytes	MIGA2
2.49760401433379E-74	0.397402860304106	0.277	0.165	4.18548480722056E-70	Ductular-like hepatocytes	CHMP6
2.61241321143206E-74	0.539932801817519	0.244	0.141	4.37788205971784E-70	Ductular-like hepatocytes	SLC41A3
2.75310240951945E-74	0.264671015809632	0.319	0.197	4.61364901787269E-70	Ductular-like hepatocytes	RARA
2.91384394983481E-74	0.727858611825655	0.149	0.07	4.88301969113317E-70	Ductular-like hepatocytes	DTD1
2.93367675408593E-74	0.351176396428962	0.295	0.179	4.9162555044972E-70	Ductular-like hepatocytes	ATG3
3.01532369492696E-74	0.664566806612893	0.19	0.1	5.0530794479586E-70	Ductular-like hepatocytes	ZBED3
3.39017722153678E-74	0.362030417835957	0.323	0.203	5.68125898785133E-70	Ductular-like hepatocytes	GADD45GIP

**3.81688066420137E-74**	0.423014351903781	0.252	0.146	6.39632861706866E-70	Ductular-like hepatocytes	TP53I13
**3.89700510258821E-74**	0.400093206678235	0.442	0.299	6.53060115091733E-70	Ductular-like hepatocytes	EMP2
**4.02005726005044E-74**	0.33351124800056	0.295	0.178	6.73681195639252E-70	Ductular-like hepatocytes	CEP250
**4.1656295593801E-74**	0.345302988544573	0.2	0.107	6.98076201560916E-70	Ductular-like hepatocytes	CHPF2
**4.6010533733674E-74**	0.394542087778537	0.324	0.204	7.71044524308908E-70	Ductular-like hepatocytes	RAB5B
**4.79798527779822E-74**	0.4855343870837	0.2	0.107	8.04046372853425E-70	Ductular-like hepatocytes	MAP3K14
**4.89797775432667E-74**	0.483687942289543	0.238	0.135	8.20803112070064E-70	Ductular-like hepatocytes	PPP1R15A
**4.9269710333263E-74**	0.379114735521696	0.333	0.209	8.2566180576482E-70	Ductular-like hepatocytes	ZBTB41
**5.15849129119016E-74**	0.367367789847528	0.277	0.166	8.64459970577646E-70	Ductular-like hepatocytes	ADCK5
**5.40613889554392E-74**	0.345490328580528	0.364	0.233	9.0596075611525E-70	Ductular-like hepatocytes	UBA2
**5.66243355799329E-74**	0.582618233353907	0.216	0.119	9.48910615648515E-70	Ductular-like hepatocytes	PET117
**5.99325825168625E-74**	0.468430172188237	0.175	0.088	1.00435021781758E-69	Ductular-like hepatocytes	TSPAN12
**6.26653713072668E-74**	0.421887145486979	0.273	0.162	1.05014629236718E-69	Ductular-like hepatocytes	MCM3AP
**6.37293616728241E-74**	0.468570306090844	0.297	0.183	1.06797664291319E-69	Ductular-like hepatocytes	ZNF7
**6.5540240771252E-74**	0.273893157916038	0.391	0.259	1.09832335484464E-69	Ductular-like hepatocytes	DEPRECATEI
**7.43627973840859E-74**	0.458088935521782	0.271	0.161	1.24617175856251E-69	Ductular-like hepatocytes	CCT4
**7.76195224809887E-74**	0.564856235297292	0.15	0.071	1.30074795773641E-69	Ductular-like hepatocytes	ARSD
**8.02899107068154E-74**	0.261966223279361	0.566	0.414	1.34549832362481E-69	Ductular-like hepatocytes	ATP5IF1
**9.80269769572401E-74**	0.444905165536479	0.265	0.157	1.64273607984943E-69	Ductular-like hepatocytes	PHLPP1
**9.83488328906145E-74**	0.582714767184452	0.23	0.131	1.64812974158092E-69	Ductular-like hepatocytes	MXRA7
**1.03221930577546E-73**	0.644617728208376	0.213	0.118	1.72979311261851E-69	Ductular-like hepatocytes	PSMG1
**1.03756541479085E-73**	0.358894179347578	0.374	0.244	1.7387521221065E-69	Ductular-like hepatocytes	LAPTM4A
**1.07381385563404E-73**	0.38718811552067	0.303	0.186	1.79949725927153E-69	Ductular-like hepatocytes	TAOK3
**1.08622840188073E-73**	0.302956609274263	0.405	0.267	1.82030155587173E-69	Ductular-like hepatocytes	HELZ
**1.15060021250161E-73**	0.704911372905049	0.109	0.044	1.9281758361102E-69	Ductular-like hepatocytes	NPAS3
**1.23818565550259E-73**	0.398740995154349	0.249	0.143	2.07495152149124E-69	Ductular-like hepatocytes	PAK4
**1.29251602650521E-73**	0.483871232559507	0.263	0.155	2.16599835721744E-69	Ductular-like hepatocytes	STRN
**1.29258254399012E-73**	0.33848087524918	0.27	0.16	2.16610982721864E-69	Ductular-like hepatocytes	SLC30A7
**1.38662915505166E-73**	0.263363743518054	0.456	0.308	2.32371313803558E-69	Ductular-like hepatocytes	PUM2
**1.47148273523072E-73**	0.29541625009085	0.341	0.215	2.46591076769964E-69	Ductular-like hepatocytes	BRF1
**1.48952715058093E-73**	0.298904999791957	0.294	0.177	2.49614959894353E-69	Ductular-like hepatocytes	CAMSAP3
**1.56626341243673E-73**	0.35989428225451	0.243	0.139	2.62474422656147E-69	Ductular-like hepatocytes	U2AF2
**1.57227686913319E-73**	0.608473742827184	0.244	0.141	2.63482157729341E-69	Ductular-like hepatocytes	TMEM251
**1.74525678936447E-73**	0.39696519467804	0.276	0.165	2.92470132761697E-69	Ductular-like hepatocytes	INPPL1
**1.7707898456345E-73**	0.303968446162121	0.39	0.255	2.9674896233143E-69	Ductular-like hepatocytes	PAFAH1B1
**1.83865104223484E-73**	0.669565173760019	0.197	0.105	3.08121141657714E-69	Ductular-like hepatocytes	EIF4EBP3
**2.03832035609584E-73**	0.569657545105191	0.226	0.127	3.4158172527454E-69	Ductular-like hepatocytes	NOL9
**2.08374205395091E-73**	0.296028068673035	0.286	0.172	3.49193493401094E-69	Ductular-like hepatocytes	AKT1S1
**2.25616204936428E-73**	0.359485421320296	0.31	0.192	3.78087636232466E-69	Ductular-like hepatocytes	FIP1L1
**2.27347795287564E-73**	0.365608994350576	0.326	0.203	3.809894353429E-69	Ductular-like hepatocytes	IPO8
**2.31151594532234E-73**	0.395419552849796	0.244	0.14	3.87363842117118E-69	Ductular-like hepatocytes	IRAK4
**2.51654377892711E-73**	0.397957486288636	0.243	0.14	4.21722406472606E-69	Ductular-like hepatocytes	ACTR3
**3.34985307442867E-73**	0.548940951630323	0.208	0.114	5.61368378212757E-69	Ductular-like hepatocytes	BACE1
**3.53449808138603E-73**	0.380285858737522	0.433	0.292	5.92311188478671E-69	Ductular-like hepatocytes	TM7SF2
**3.6393041962763E-73**	0.40738451533561	0.261	0.154	6.09874597211983E-69	Ductular-like hepatocytes	HSD17B12
**3.74228319500402E-73**	0.290973956041835	0.402	0.266	6.27131817818774E-69	Ductular-like hepatocytes	NUB1
**4.40452227396222E-73**	0.50577089873947	0.227	0.128	7.3810984267059E-69	Ductular-like hepatocytes	ACTR2
**4.73131719595189E-73**	0.252612272982635	0.53	0.372	7.92874135697618E-69	Ductular-like hepatocytes	HNRNPA3
**4.74726981138769E-73**	0.329828005028061	0.317	0.198	7.95547474992349E-69	Ductular-like hepatocytes	RIPK1
**4.80573484889516E-73**	0.483108054567476	0.184	0.096	8.0534504597785E-69	Ductular-like hepatocytes	MICAL2
**5.18088037851338E-73**	1.31337115541657	0.101	0.039	8.68211933831273E-69	Ductular-like hepatocytes	PRDM5
**5.55741569977202E-73**	0.480999843859402	0.274	0.164	9.31311722967795E-69	Ductular-like hepatocytes	ETAA1
**5.65824613195501E-73**	0.392153847912135	0.292	0.178	9.4820888679302E-69	Ductular-like hepatocytes	MAML1
**6.60754383247822E-73**	0.49839933745373	0.244	0.141	1.1072921954467E-68	Ductular-like hepatocytes	PEF1
**6.70124186288396E-73**	0.471028700779337	0.239	0.137	1.12299411138209E-68	Ductular-like hepatocytes	FAM126A
**6.84773069494834E-73**	0.623493784519046	0.198	0.107	1.14754270985944E-68	Ductular-like hepatocytes	METTL27
**7.12172240906121E-73**	0.389306153199038	0.398	0.261	1.19345824131048E-68	Ductular-like hepatocytes	DCAF7
**7.22359625339894E-73**	0.500455172829201	0.276	0.165	1.21053026014459E-68	Ductular-like hepatocytes	NUP155
**7.27548782658207E-73**	0.298679632108591	0.542	0.382	1.21922624997862E-68	Ductular-like hepatocytes	STAU2
**7.35438571392447E-73**	0.462127499177321	0.3	0.186	1.23244795793946E-68	Ductular-like hepatocytes	COX7B

8.98577649708785E-73	0.396051369369057	0.173	0.087	1.50583642538198E-68	Ductular-like hepatocytes	PRKCZ
9.15839425136969E-73	0.266306203810284	0.483	0.338	1.53476370864453E-68	Ductular-like hepatocytes	MTUS1
9.68085602422765E-73	0.489866617840535	0.243	0.14	1.62231785254007E-68	Ductular-like hepatocytes	SQLE
1.12288257936092E-72	0.843268994539313	0.16	0.08	1.88172662649303E-68	Ductular-like hepatocytes	PDK3
1.26027028435955E-72	0.926208157053236	0.117	0.05	2.11196094252974E-68	Ductular-like hepatocytes	ANKS6
1.33792282575838E-72	0.700984439896171	0.175	0.09	2.24209107140589E-68	Ductular-like hepatocytes	SMAGP
1.40810763914437E-72	0.404893862970148	0.269	0.161	2.35970678167814E-68	Ductular-like hepatocytes	DAG1
1.94542100871753E-72	0.572159055984179	0.206	0.112	3.26013652640883E-68	Ductular-like hepatocytes	ZNF26
1.97258805848439E-72	0.261629922136723	0.352	0.224	3.30566306840814E-68	Ductular-like hepatocytes	UGT1A4
2.05822186199693E-72	0.96555755130179	0.111	0.046	3.44916819633446E-68	Ductular-like hepatocytes	ZNF532
2.06669038628962E-72	0.502460337204266	0.238	0.137	3.46335974934414E-68	Ductular-like hepatocytes	CHD8
2.1724552771315E-72	0.290940774117436	0.452	0.305	3.64060055341696E-68	Ductular-like hepatocytes	SRSF3
2.21978256558246E-72	0.355974368125107	0.21	0.115	3.71991162340309E-68	Ductular-like hepatocytes	CROT
2.33219859606507E-72	0.476672288965989	0.218	0.121	3.90829840728585E-68	Ductular-like hepatocytes	MRI1
2.4135078705912E-72	0.270590291972024	0.441	0.301	4.04455648953673E-68	Ductular-like hepatocytes	GDA
2.43218613600588E-72	0.666217657118983	0.169	0.085	4.07585752671865E-68	Ductular-like hepatocytes	ZDHHC23
2.66550488115542E-72	0.266562201382645	0.414	0.278	4.46685307984025E-68	Ductular-like hepatocytes	LGALS8
2.74384258104836E-72	0.759019035450703	0.17	0.087	4.59813139732084E-68	Ductular-like hepatocytes	ABHD17C
2.81511571505309E-72	0.859952564283212	0.131	0.059	4.71757091528598E-68	Ductular-like hepatocytes	DPP3
3.00082422953077E-72	0.359367650097807	0.344	0.219	5.02878124384767E-68	Ductular-like hepatocytes	ATP6V1C1
3.00374254964413E-72	0.42102014002675	0.304	0.188	5.03367176469363E-68	Ductular-like hepatocytes	CCDC28A
3.04330071387737E-72	0.510868131263944	0.297	0.182	5.0999633363157E-68	Ductular-like hepatocytes	DEPRECATEI
3.05553311124354E-72	0.425639326212675	0.363	0.234	5.12046238782192E-68	Ductular-like hepatocytes	APOOL
3.21682282102611E-72	0.276706129049958	0.462	0.314	5.39075168347556E-68	Ductular-like hepatocytes	LRRFIP2
3.23238583769296E-72	0.325725736572006	0.282	0.17	5.41683218680587E-68	Ductular-like hepatocytes	DMXL2
3.49857481675192E-72	0.798155749117261	0.127	0.056	5.86291167791288E-68	Ductular-like hepatocytes	XKR8
3.74599123141021E-72	0.933514757897148	0.14	0.066	6.27753210559723E-68	Ductular-like hepatocytes	KAT14
3.8481552872495E-72	0.517251270832932	0.237	0.136	6.44873863037271E-68	Ductular-like hepatocytes	KIF2A
4.59288145332795E-72	0.277930284980731	0.429	0.285	7.69675073948699E-68	Ductular-like hepatocytes	NNT
5.01753871041668E-72	0.472846894260514	0.264	0.158	8.40839137091628E-68	Ductular-like hepatocytes	ITPRIPL2
5.23677830266795E-72	0.324721882536418	0.382	0.25	8.77579307961094E-68	Ductular-like hepatocytes	RRP36
5.45226950641571E-72	0.501404306218793	0.234	0.134	9.13691323885145E-68	Ductular-like hepatocytes	IPP
5.56169848270929E-72	0.35124617678833	0.266	0.158	9.32029431732422E-68	Ductular-like hepatocytes	ISG20L2
5.58550408513485E-72	0.468177074805468	0.233	0.133	9.36018774586897E-68	Ductular-like hepatocytes	PIGH
5.58760392216839E-72	0.260258687925696	0.304	0.186	9.36370665276979E-68	Ductular-like hepatocytes	CDK13
5.70887046187949E-72	0.690867811739843	0.254	0.151	9.56692512001764E-68	Ductular-like hepatocytes	GUCY1A1
6.51353017373774E-72	0.262898936727507	0.637	0.511	1.09153738651497E-67	Ductular-like hepatocytes	IMMP2L
6.91264583417155E-72	0.263580894377535	0.353	0.226	1.15842118889047E-67	Ductular-like hepatocytes	STRN4
7.83326831583708E-72	0.815114261278362	0.15	0.073	1.31269910436798E-67	Ductular-like hepatocytes	FBXO32
9.21619873066661E-72	0.516049427474892	0.172	0.088	1.54445058328511E-67	Ductular-like hepatocytes	NAPEPLD
1.1016676424781E-71	0.258664841404587	0.549	0.399	1.8461746352648E-67	Ductular-like hepatocytes	PDHA1
1.12913245783399E-71	0.36652168724866	0.257	0.15	1.8922001728382E-67	Ductular-like hepatocytes	GAS2L3
1.24064367243315E-71	0.478247513310446	0.185	0.097	2.07907066626348E-67	Ductular-like hepatocytes	BBX
1.40631393951519E-71	0.587355494063618	0.204	0.111	2.35670089983956E-67	Ductular-like hepatocytes	STARD3NL
1.48180370305964E-71	0.336773899985378	0.218	0.121	2.48320664558735E-67	Ductular-like hepatocytes	XPNPEP1
1.51023482894801E-71	0.437918510439917	0.303	0.188	2.53085152635108E-67	Ductular-like hepatocytes	NDUFC1
1.52369065325085E-71	0.799425784894537	0.182	0.096	2.55340079671777E-67	Ductular-like hepatocytes	PACRG
1.61673728845891E-71	0.604963447862683	0.194	0.104	2.70932834799944E-67	Ductular-like hepatocytes	RAD51B
1.68216861494556E-71	0.26889400534662	0.524	0.365	2.81897816492577E-67	Ductular-like hepatocytes	MAF
1.71600355508169E-71	0.29251153072664	0.379	0.248	2.87567875760589E-67	Ductular-like hepatocytes	BMI1
1.72669984563551E-71	0.559396740130125	0.213	0.119	2.89360360131599E-67	Ductular-like hepatocytes	DEPRECATEI
1.75220973943597E-71	0.330869016453284	0.34	0.217	2.93635308134681E-67	Ductular-like hepatocytes	LRRC41
1.78803663084629E-71	0.664865133931112	0.174	0.089	2.99639178597221E-67	Ductular-like hepatocytes	IQCB1
2.08646684863014E-71	0.839607870034738	0.119	0.051	3.49650114493439E-67	Ductular-like hepatocytes	PCDHGB2
2.1730362088936E-71	0.258638316895663	0.364	0.235	3.6415740788639E-67	Ductular-like hepatocytes	IL17RC
2.18177783640394E-71	0.42622882631586	0.27	0.161	3.65622329824572E-67	Ductular-like hepatocytes	INPP1
2.36406113704298E-71	0.718821508715889	0.162	0.081	3.96169365345662E-67	Ductular-like hepatocytes	OBI1
2.37617829960729E-71	0.417972310157339	0.223	0.126	3.9819995944819E-67	Ductular-like hepatocytes	PIP4K2C
2.55736279678683E-71	0.537496068971053	0.18	0.094	4.28562857485537E-67	Ductular-like hepatocytes	VMAC
2.57825132037938E-71	0.350626223564141	0.279	0.169	4.32063356269177E-67	Ductular-like hepatocytes	NUP214

**3.02214499192997E-71**	0.447756218091535	0.305	0.191	5.06451057747624E-67	Ductular-like hepatocytes	GTF2H2
**3.07413469862623E-71**	0.272429522861956	0.438	0.294	5.15163492795784E-67	Ductular-like hepatocytes	TACC1
**3.15998463524698E-71**	0.367814855684337	0.335	0.213	5.29550225174689E-67	Ductular-like hepatocytes	MAPK9
**3.72047646514601E-71**	0.464203072443012	0.239	0.138	6.23477446029168E-67	Ductular-like hepatocytes	HCFC1R1
**3.74193252970681E-71**	0.395529959936835	0.323	0.203	6.27073053328267E-67	Ductular-like hepatocytes	DCAF16
**3.94545079557819E-71**	0.315607841527189	0.217	0.12	6.61178644322993E-67	Ductular-like hepatocytes	TPCN1
**3.99845747376218E-71**	0.424527346752387	0.269	0.161	6.70061503453067E-67	Ductular-like hepatocytes	NR2F2
**4.46552349718178E-71**	0.41677175076802	0.27	0.162	7.48332427657723E-67	Ductular-like hepatocytes	FLVCR1
**4.54681115400921E-71**	0.268400641160608	0.351	0.225	7.61954613188864E-67	Ductular-like hepatocytes	CDK4
**4.94004555222997E-71**	0.68569907356848	0.21	0.117	8.27852833642699E-67	Ductular-like hepatocytes	ARV1
**4.96144285929311E-71**	0.401922376884187	0.287	0.175	8.3143859436034E-67	Ductular-like hepatocytes	BRPF3
**5.12631221122752E-71**	0.879217856713647	0.15	0.073	8.59067400357508E-67	Ductular-like hepatocytes	CASP10
**5.29675826280521E-71**	0.469736700285083	0.168	0.085	8.87630749680896E-67	Ductular-like hepatocytes	SIRT6
**5.32719081905847E-71**	0.408419705952642	0.278	0.167	8.92730637457819E-67	Ductular-like hepatocytes	OSTC
**5.37182392096036E-71**	0.356654638655461	0.296	0.182	9.00210252674536E-67	Ductular-like hepatocytes	EFTUD2
**5.69014734581239E-71**	0.360791772287408	0.359	0.231	9.5355489221124E-67	Ductular-like hepatocytes	ZNF652
**5.77070268421018E-71**	0.345471682621484	0.347	0.223	9.67054355819942E-67	Ductular-like hepatocytes	CCNY
**6.2692381967931E-71**	0.29483883368608	0.49	0.344	1.05059893701859E-66	Ductular-like hepatocytes	RTF2
**6.66386097324195E-71**	0.47071517561483	0.252	0.148	1.11672982189589E-66	Ductular-like hepatocytes	RPUSD3
**6.82500091268213E-71**	0.552384041078681	0.156	0.077	1.14373365294727E-66	Ductular-like hepatocytes	CHST12
**7.21616231022347E-71**	0.498161428144578	0.235	0.136	1.20928447994725E-66	Ductular-like hepatocytes	BNIP3L
**7.31798873675876E-71**	0.378949922348684	0.331	0.211	1.22634855250603E-66	Ductular-like hepatocytes	HMOX2
**7.67334994545451E-71**	0.298462567129105	0.709	0.55	1.28589998385927E-66	Ductular-like hepatocytes	ACAA2
**8.33970984885813E-71**	1.11860781901055	0.12	0.053	1.39756857647165E-66	Ductular-like hepatocytes	GPR160
**9.4879882638936E-71**	0.253953409155122	0.3	0.185	1.58999707326329E-66	Ductular-like hepatocytes	ABI1
**1.01784796795256E-70**	0.273875592044594	0.453	0.31	1.7057096246949E-66	Ductular-like hepatocytes	EIF4A3
**1.21280619519703E-70**	0.412863682937453	0.33	0.209	2.03242062191119E-66	Ductular-like hepatocytes	TSNAX
**1.24616871129102E-70**	0.404333886722001	0.264	0.158	2.08832952638149E-66	Ductular-like hepatocytes	DAD1
**1.31522475863565E-70**	0.436890347218332	0.17	0.087	2.20405365052163E-66	Ductular-like hepatocytes	SCFD2
**1.32368673257612E-70**	0.892208677928194	0.142	0.068	2.21823422645107E-66	Ductular-like hepatocytes	MCUB
**1.40477982748019E-70**	0.429341888238452	0.256	0.152	2.3541300348913E-66	Ductular-like hepatocytes	KIAA1109
**1.61163174284051E-70**	0.41188408837062	0.32	0.202	2.70077247465212E-66	Ductular-like hepatocytes	MED13
**1.82162600632017E-70**	0.474535227938745	0.259	0.154	3.05268086139134E-66	Ductular-like hepatocytes	PIBF1
**1.88382129414378E-70**	0.350946756806483	0.24	0.139	3.15690772472615E-66	Ductular-like hepatocytes	GTPBP3
**1.92851038839878E-70**	0.377245869880325	0.324	0.206	3.23179770887867E-66	Ductular-like hepatocytes	CHMP2B
**1.98746632911434E-70**	0.251116168687529	0.359	0.231	3.33059607432981E-66	Ductular-like hepatocytes	TPD52L2
**2.02602124153931E-70**	0.431238926282709	0.22	0.124	3.39520639657158E-66	Ductular-like hepatocytes	POM121
**2.0703029968164E-70**	0.360137970186686	0.358	0.232	3.46941376206492E-66	Ductular-like hepatocytes	YIPF4
**2.55141971659238E-70**	0.370162531030255	0.135	0.062	4.27566916106552E-66	Ductular-like hepatocytes	MLKL
**2.59662674529117E-70**	0.364787620717854	0.225	0.127	4.35142709975894E-66	Ductular-like hepatocytes	TBC1D5
**2.83388515492879E-70**	0.258111191274556	0.579	0.424	4.74902474262966E-66	Ductular-like hepatocytes	NECTIN2
**2.91234633922927E-70**	0.900714242270893	0.141	0.067	4.88050999528041E-66	Ductular-like hepatocytes	CTNND2
**3.0681787043034E-70**	0.3351577868776	0.324	0.205	5.14165387267164E-66	Ductular-like hepatocytes	ATP5PF
**3.07905326096885E-70**	0.418714512492864	0.256	0.152	5.15987745473159E-66	Ductular-like hepatocytes	RIC8B
**3.07961494373619E-70**	0.718204106005168	0.168	0.086	5.16081872271311E-66	Ductular-like hepatocytes	FAM189B
**3.17993879206125E-70**	1.03823485400378	0.125	0.056	5.32894142773624E-66	Ductular-like hepatocytes	ADAMTSL1
**3.20657352492442E-70**	0.597072463347363	0.201	0.11	5.37357591306835E-66	Ductular-like hepatocytes	RABL3
**3.61877059439132E-70**	0.344913640987761	0.362	0.236	6.06433576208097E-66	Ductular-like hepatocytes	DYM
**3.91719225907802E-70**	0.330197479569505	0.347	0.224	6.56443078776294E-66	Ductular-like hepatocytes	SAMD4B
**3.92717529212332E-70**	0.555771198229976	0.165	0.083	6.58116035454026E-66	Ductular-like hepatocytes	NOMO1
**3.9923244742011E-70**	0.437964806019187	0.262	0.157	6.69033735386621E-66	Ductular-like hepatocytes	SMC5
**4.0702107724621E-70**	0.250017437134683	0.2	0.108	6.82085921249198E-66	Ductular-like hepatocytes	ARHGEF5
**4.11580295919278E-70**	0.371813144219599	0.237	0.137	6.89726259901527E-66	Ductular-like hepatocytes	GYS1
**4.18050932365575E-70**	0.487578872034552	0.243	0.143	7.00569752458231E-66	Ductular-like hepatocytes	GK5
**5.12316901205945E-70**	0.518444347403225	0.138	0.064	8.58540663040922E-66	Ductular-like hepatocytes	TBC1D4
**5.46310716599856E-70**	0.535054990776354	0.243	0.142	9.15507498878039E-66	Ductular-like hepatocytes	PTGR2
**5.47371889593283E-70**	0.455379153763606	0.173	0.089	9.17285812580423E-66	Ductular-like hepatocytes	RRAS
**5.6013686003068E-70**	0.381242055013093	0.244	0.142	9.38677350039413E-66	Ductular-like hepatocytes	GABPB2
**6.12693306758821E-70**	0.602070165332818	0.158	0.079	1.02675144346643E-65	Ductular-like hepatocytes	THRA
**6.58658753517771E-70**	0.69724231838175	0.217	0.123	1.10378033914508E-65	Ductular-like hepatocytes	SLC1A4

6.66425901108029E-70	0.430662218598174	0.286	0.174	1.11679652507683E-65	Ductular-like hepatocytes	OGFOD3
6.93714245071497E-70	0.256115716253816	0.459	0.318	1.16252633189082E-65	Ductular-like hepatocytes	RNF7
6.9975733003096E-70	1.07669657180726	0.104	0.043	1.17265333366588E-65	Ductular-like hepatocytes	CDYL2
7.4170492960835E-70	0.411012249453552	0.209	0.116	1.24294912103767E-65	Ductular-like hepatocytes	ZZEF1
7.94063705634331E-70	0.312501670572521	0.315	0.197	1.33069195790201E-65	Ductular-like hepatocytes	CLYBL
8.06485610839446E-70	0.622521835911752	0.229	0.132	1.35150858664474E-65	Ductular-like hepatocytes	PIGC
8.12322827711919E-70	0.268582051325436	0.342	0.218	1.36129059467963E-65	Ductular-like hepatocytes	GNL1
8.75237534051338E-70	0.354572257036984	0.322	0.204	1.46672305956323E-65	Ductular-like hepatocytes	NDUFA8
9.33659650998913E-70	0.348386349355455	0.307	0.191	1.56462684314398E-65	Ductular-like hepatocytes	S100PBP
9.79277839576589E-70	0.645644878322503	0.203	0.112	1.64107380356245E-65	Ductular-like hepatocytes	VTA1
9.88968757707503E-70	0.724397888219171	0.12	0.053	1.65731384416623E-65	Ductular-like hepatocytes	NOD1
1.06979515515663E-69	0.670235387310457	0.198	0.109	1.79276272101148E-65	Ductular-like hepatocytes	XYLT2
1.19121909211913E-69	0.294880194022872	0.284	0.173	1.99624495457323E-65	Ductular-like hepatocytes	PEX11B
1.22456125591288E-69	0.67633371687069	0.168	0.087	2.05211975265881E-65	Ductular-like hepatocytes	ATP2B1
1.36959500639992E-69	0.329233659977234	0.497	0.354	2.29516731172498E-65	Ductular-like hepatocytes	CCDC47
1.52776485236374E-69	0.553003265528528	0.174	0.09	2.56022833959116E-65	Ductular-like hepatocytes	ME3
1.58017844905361E-69	0.812679036628822	0.142	0.068	2.64806304492404E-65	Ductular-like hepatocytes	C14orf180
1.69463501031748E-69	0.282384092407983	0.427	0.289	2.83986935029003E-65	Ductular-like hepatocytes	VPS45
1.76141815541449E-69	0.371628937967568	0.312	0.196	2.9517845448436E-65	Ductular-like hepatocytes	CNOT8
1.94058702805034E-69	0.254340759229248	0.486	0.343	3.25203574160677E-65	Ductular-like hepatocytes	ATP5MG
1.98268865876178E-69	0.749421930994997	0.147	0.072	3.32258965435299E-65	Ductular-like hepatocytes	PPP2R1A
2.24730145637146E-69	0.5981444886905	0.257	0.155	3.76602778058729E-65	Ductular-like hepatocytes	PIGP
2.51204257868461E-69	0.529995251280895	0.214	0.12	4.20968095335966E-65	Ductular-like hepatocytes	SIX5
2.58523598253616E-69	0.768410488875378	0.178	0.094	4.33233845953409E-65	Ductular-like hepatocytes	B3GLCT
2.66978265284452E-69	0.403258946582646	0.352	0.229	4.47402176963684E-65	Ductular-like hepatocytes	ATPAF1
2.74758969570836E-69	0.329964862697497	0.333	0.212	4.60441081206807E-65	Ductular-like hepatocytes	TMBIM4
2.90923750751515E-69	0.314306917036305	0.312	0.196	4.87530021509388E-65	Ductular-like hepatocytes	PRPF4B
3.11628409903092E-69	0.581797165146401	0.211	0.118	5.22226889315602E-65	Ductular-like hepatocytes	ZNF346
3.21824483600529E-69	0.779389201969246	0.149	0.073	5.39313469617766E-65	Ductular-like hepatocytes	FP565260.6
3.44169347053114E-69	0.369432975563537	0.411	0.277	5.76758991791608E-65	Ductular-like hepatocytes	NPTN
3.60665273091705E-69	0.634601091860078	0.138	0.065	6.04402864647079E-65	Ductular-like hepatocytes	LIMK1
3.74580785194883E-69	0.336070336316975	0.35	0.226	6.27722479829585E-65	Ductular-like hepatocytes	C6orf120
4.30710298549401E-69	0.325387578709011	0.253	0.15	7.21784318309087E-65	Ductular-like hepatocytes	EIF3K
4.82366211534701E-69	0.363344188712299	0.331	0.211	8.08349297289852E-65	Ductular-like hepatocytes	RUNX1
5.15932482805139E-69	0.411099761976744	0.27	0.163	8.64599654684852E-65	Ductular-like hepatocytes	MOSPD2
5.44275508896675E-69	0.364103076942621	0.328	0.21	9.12096897809048E-65	Ductular-like hepatocytes	AP1G1
5.46110643669765E-69	0.529054663986092	0.234	0.135	9.15172216661792E-65	Ductular-like hepatocytes	CHAF1A
5.7006612767904E-69	0.275856258634077	0.453	0.31	9.55316816764536E-65	Ductular-like hepatocytes	RNF115
5.89869078541158E-69	0.415131436049789	0.272	0.164	9.88502601819272E-65	Ductular-like hepatocytes	RPRD1B
6.12433303636783E-69	0.419565188415054	0.286	0.176	1.02631573023452E-64	Ductular-like hepatocytes	ACTL6A
6.49244893670821E-69	0.383844539235481	0.346	0.224	1.08800459281356E-64	Ductular-like hepatocytes	ABHD10
6.72310891287486E-69	0.530027315260743	0.269	0.164	1.12665859161957E-64	Ductular-like hepatocytes	VEZF1
7.76446760365844E-69	0.424774289212228	0.294	0.182	1.30116948102108E-64	Ductular-like hepatocytes	DPH6
8.0035502461437E-69	1.12760585020436	0.112	0.048	1.34123495024876E-64	Ductular-like hepatocytes	SYNE1
8.45815534127767E-69	0.383666560115545	0.271	0.163	1.41741767209131E-64	Ductular-like hepatocytes	TFCP2
8.5296027710839E-69	0.547164655745743	0.218	0.123	1.42939083237824E-64	Ductular-like hepatocytes	DEPRECATEI
1.07931744091384E-68	0.77623089118573	0.133	0.062	1.80872016748341E-64	Ductular-like hepatocytes	EML5
1.08176135993136E-68	0.359544716932081	0.291	0.179	1.81281568697297E-64	Ductular-like hepatocytes	MTX2
1.25505331826152E-68	0.821687847146828	0.146	0.071	2.10321835074265E-64	Ductular-like hepatocytes	RMI2
1.25570807880156E-68	0.277453582161496	0.565	0.41	2.10431559845566E-64	Ductular-like hepatocytes	ATP13A3
1.27352891749513E-68	0.387658592116107	0.268	0.162	2.13417975993834E-64	Ductular-like hepatocytes	SLC25A46
1.3552593586038E-68	0.417483864407885	0.222	0.126	2.27114363314826E-64	Ductular-like hepatocytes	FGD6
1.42271448542183E-68	0.266965602301354	0.329	0.21	2.3841849346699E-64	Ductular-like hepatocytes	STK25
1.48747067737567E-68	0.278843734078475	0.305	0.19	2.49270336114616E-64	Ductular-like hepatocytes	PURB
1.51789006202146E-68	0.27419026683006	0.591	0.438	2.54368016593557E-64	Ductular-like hepatocytes	ACBD5
1.79010612445056E-68	0.586710323120502	0.25	0.148	2.99985984335425E-64	Ductular-like hepatocytes	ARMC1
1.90423682489046E-68	0.726458600873598	0.203	0.113	3.19112007115143E-64	Ductular-like hepatocytes	MYCBP2
2.12228117321236E-68	1.00407554313569	0.11	0.047	3.55651879006928E-64	Ductular-like hepatocytes	CBX6
2.15248749050263E-68	0.263378900497326	0.186	0.099	3.60713853658431E-64	Ductular-like hepatocytes	FRMD4A
2.28390990521552E-68	0.387402453926633	0.282	0.172	3.82737621916017E-64	Ductular-like hepatocytes	PPP2CA

2.32870996705831E-68	0.657638512838344	0.2	0.111	3.90245216279632E-64	Ductular-like hepatocytes	RBL1
2.39504614468757E-68	0.430560422231704	0.26	0.156	4.01361832926742E-64	Ductular-like hepatocytes	CSPP1
2.47599434202294E-68	0.268571293808251	0.337	0.216	4.14927131836204E-64	Ductular-like hepatocytes	MYH9
2.56347952448519E-68	0.462661576033252	0.179	0.094	4.29587898713229E-64	Ductular-like hepatocytes	NSUN3
2.67457637254062E-68	0.374785969928816	0.288	0.178	4.48205508510356E-64	Ductular-like hepatocytes	FAM234A
2.9040572062904E-68	0.401593363682421	0.315	0.201	4.86661906630146E-64	Ductular-like hepatocytes	L3HYPDH
2.91051702671149E-68	0.615364100934651	0.209	0.118	4.87744443336311E-64	Ductular-like hepatocytes	TMEM30A
3.00269179313852E-68	0.505036269436215	0.171	0.088	5.03191090694153E-64	Ductular-like hepatocytes	BAHD1
3.06346700677802E-68	0.47188340681031	0.212	0.119	5.1337580099586E-64	Ductular-like hepatocytes	TTC13
3.14750124163811E-68	0.38218606290831	0.231	0.134	5.27458258073714E-64	Ductular-like hepatocytes	PLCL2
3.23156324306725E-68	0.479646357079499	0.261	0.158	5.41545368273209E-64	Ductular-like hepatocytes	STXBP6
3.29530621859714E-68	0.405649051217108	0.277	0.169	5.52227416112509E-64	Ductular-like hepatocytes	RNF14
3.4890081039733E-68	0.285215549446908	0.365	0.239	5.84687978063846E-64	Ductular-like hepatocytes	TMED2
3.57399933975161E-68	0.418833502003475	0.194	0.106	5.98930809355576E-64	Ductular-like hepatocytes	YBEY
3.6139940582785E-68	0.411089102208966	0.286	0.176	6.05633124286311E-64	Ductular-like hepatocytes	ATPSCKMT
3.71789708605469E-68	0.360492147194051	0.345	0.222	6.23045193681045E-64	Ductular-like hepatocytes	ZFHX3
4.02801622102335E-68	0.463269808477144	0.193	0.105	6.75014958319093E-64	Ductular-like hepatocytes	RNF121
4.12161070232739E-68	0.873486314544738	0.104	0.042	6.90699521496024E-64	Ductular-like hepatocytes	C1orf226
4.54039508257574E-68	0.349138029232186	0.213	0.119	7.60879407938043E-64	Ductular-like hepatocytes	RRS1
4.8327540542763E-68	0.56536067036151	0.254	0.152	8.09872924415622E-64	Ductular-like hepatocytes	FAM110B
5.01904127167147E-68	0.3908292923455	0.345	0.224	8.41090936306704E-64	Ductular-like hepatocytes	UQCC2
5.40513057481882E-68	0.284279617887638	0.454	0.313	9.05791781728138E-64	Ductular-like hepatocytes	POLR1D
5.58647366360716E-68	0.391535552773841	0.277	0.169	9.36181256547288E-64	Ductular-like hepatocytes	PRMT2
5.89074459971362E-68	0.269453638252297	0.551	0.401	9.87170980020008E-64	Ductular-like hepatocytes	UBXN4
6.41412233775384E-68	0.263176165732428	0.45	0.31	1.07487862136079E-63	Ductular-like hepatocytes	NQO2
6.71565938276535E-68	0.320231440116606	0.238	0.138	1.12541019936382E-63	Ductular-like hepatocytes	BRAT1
6.96447456490172E-68	0.253852223773079	0.302	0.187	1.16710664758623E-63	Ductular-like hepatocytes	REPIN1
7.59250656108045E-68	0.368797287682418	0.223	0.126	1.27235224950586E-63	Ductular-like hepatocytes	DEPRECATEI
7.78991194474724E-68	0.362885532371885	0.259	0.155	1.30543344370074E-63	Ductular-like hepatocytes	NT5DC1
7.99992584716501E-68	0.42581305208107	0.232	0.135	1.34062757346791E-63	Ductular-like hepatocytes	RIF1
8.2348649520062E-68	0.394730324439404	0.281	0.173	1.3799986686572E-63	Ductular-like hepatocytes	ZNF768
8.52326053284274E-68	0.573629047053047	0.162	0.082	1.42832800009379E-63	Ductular-like hepatocytes	ATG9A
8.68867040587693E-68	0.373007566884158	0.291	0.18	1.45604738661686E-63	Ductular-like hepatocytes	JOSD1
1.16381756098761E-67	0.80279197328236	0.137	0.065	1.95032546870304E-63	Ductular-like hepatocytes	UPP1
1.17355933315673E-67	0.267038158302641	0.27	0.163	1.96665073050404E-63	Ductular-like hepatocytes	ATAD2B
1.27745969633256E-67	0.336489136770568	0.312	0.198	2.14076695911411E-63	Ductular-like hepatocytes	SNX27
1.34019096048273E-67	1.22868348526315	0.1	0.041	2.24589201157696E-63	Ductular-like hepatocytes	ARFGEF3
1.34099950908374E-67	0.753286441727242	0.195	0.108	2.24724697732253E-63	Ductular-like hepatocytes	CDC7
1.37301639480584E-67	0.259624023697495	0.192	0.104	2.30090087441563E-63	Ductular-like hepatocytes	PRLR
1.37806065104318E-67	0.297883940677043	0.228	0.13	2.30935403901817E-63	Ductular-like hepatocytes	FCHO2
1.55526822152955E-67	0.436023770974007	0.184	0.098	2.60631848563922E-63	Ductular-like hepatocytes	HNF1B
1.63064280963083E-67	0.574235667469844	0.189	0.103	2.73263122037935E-63	Ductular-like hepatocytes	WNT4
1.64073498495492E-67	0.274377367113859	0.391	0.262	2.74954368778746E-63	Ductular-like hepatocytes	POLR2I
1.68585853214599E-67	0.279251580068419	0.446	0.311	2.82516172817024E-63	Ductular-like hepatocytes	LMBRD1
1.73834102469383E-67	0.368886057235646	0.356	0.233	2.91311188918191E-63	Ductular-like hepatocytes	NFS1
1.81493954905173E-67	0.514619210334592	0.208	0.116	3.04147569630088E-63	Ductular-like hepatocytes	POLD3
1.82218606066436E-67	0.393659479122635	0.288	0.178	3.05361940046134E-63	Ductular-like hepatocytes	RHOT1
1.82282409391518E-67	0.400551533744114	0.271	0.165	3.05468861658305E-63	Ductular-like hepatocytes	SH3GLB1
1.90355492803747E-67	0.478538707606429	0.225	0.13	3.1899773484052E-63	Ductular-like hepatocytes	ARFIP1
1.97071363369022E-67	0.625680540397179	0.213	0.122	3.30252190733807E-63	Ductular-like hepatocytes	ARID2
2.14759962926066E-67	0.468584461345652	0.254	0.152	3.59894745871502E-63	Ductular-like hepatocytes	TMEM39A
2.30713570067055E-67	0.28392004014666	0.362	0.236	3.86629800718371E-63	Ductular-like hepatocytes	TMEM161A
2.46669634246435E-67	0.606892842288315	0.219	0.126	4.13368973070177E-63	Ductular-like hepatocytes	DPY19L3
2.47826669183851E-67	0.32948358317804	0.304	0.191	4.15307932218297E-63	Ductular-like hepatocytes	ELP5
2.64359081390756E-67	0.466828535432885	0.212	0.119	4.4301294859463E-63	Ductular-like hepatocytes	ZFP64
2.85285788644027E-67	0.39811384642226	0.23	0.133	4.7808192460966E-63	Ductular-like hepatocytes	YEATS2
2.97115727400065E-67	0.29000548225758	0.362	0.238	4.97906535977028E-63	Ductular-like hepatocytes	ITFG1
3.13414884513559E-67	0.357278041865982	0.302	0.19	5.25220663467822E-63	Ductular-like hepatocytes	PIK3CB
3.30653865790216E-67	0.458585040645606	0.178	0.094	5.54109748291244E-63	Ductular-like hepatocytes	C11orf52
3.41374540999035E-67	0.930791452746325	0.118	0.052	5.72075455806183E-63	Ductular-like hepatocytes	PDE9A

**3.5763313483922E-67**	0.337700291176307	0.209	0.116	5.99321607363565E-63	Ductular-like hepatocytes	SATB2
**3.60537088875145E-67**	0.533837370346546	0.234	0.137	6.04188053536968E-63	Ductular-like hepatocytes	GRAMD2B
**3.6961029891034E-67**	0.254185414845386	0.421	0.286	6.19392938913948E-63	Ductular-like hepatocytes	C1orf43
**3.93303343523699E-67**	0.413255077198289	0.362	0.238	6.59097743077014E-63	Ductular-like hepatocytes	RFC1
**3.95760765327365E-67**	0.328295509438315	0.201	0.111	6.63215890535598E-63	Ductular-like hepatocytes	ATP6V0E2
**4.22330443862932E-67**	0.804744437727803	0.154	0.078	7.07741357825502E-63	Ductular-like hepatocytes	MOB1A
**4.25946632195512E-67**	0.773879750280804	0.133	0.063	7.13801366233238E-63	Ductular-like hepatocytes	STK10
**4.2816916688901E-67**	0.270696595098302	0.381	0.253	7.17525889872603E-63	Ductular-like hepatocytes	PLA2G12A
**4.34200307257224E-67**	0.273144463679793	0.24	0.141	7.27632874901656E-63	Ductular-like hepatocytes	ADNP2
**4.39444360106395E-67**	0.251282959090763	0.398	0.267	7.36420858666296E-63	Ductular-like hepatocytes	SRPK1
**4.84832849797977E-67**	0.269386278331527	0.369	0.243	8.1248288969145E-63	Ductular-like hepatocytes	URI1
**4.97352521104448E-67**	0.273353905633888	0.308	0.194	8.33463354866834E-63	Ductular-like hepatocytes	BET1L
**5.67873275645634E-67**	0.42435399095988	0.294	0.185	9.51642035326953E-63	Ductular-like hepatocytes	ELF1
**5.72116438923454E-67**	0.854616902176092	0.138	0.067	9.58752728347924E-63	Ductular-like hepatocytes	NEU4
**5.83149883801944E-67**	0.351763382466778	0.204	0.113	9.77242575275297E-63	Ductular-like hepatocytes	KLF16
**6.04456600922141E-67**	0.445000482836841	0.284	0.177	1.01294837182532E-62	Ductular-like hepatocytes	BAG2
**6.39382988399834E-67**	0.272863258807745	0.457	0.316	1.07147801196044E-62	Ductular-like hepatocytes	EIF1B
**6.62324478614189E-67**	0.282147741995098	0.493	0.35	1.10992336126166E-62	Ductular-like hepatocytes	HNRNPK
**7.11161693087527E-67**	0.283283770428208	0.386	0.258	1.19176476527608E-62	Ductular-like hepatocytes	ARCN1
**7.14663171114144E-67**	0.35408002829489	0.322	0.205	1.19763254215308E-62	Ductular-like hepatocytes	NELFA
**7.15225719141912E-67**	0.516979720900487	0.192	0.105	1.19857526013802E-62	Ductular-like hepatocytes	TMED8
**8.06810151749496E-67**	0.251487817112521	0.626	0.474	1.35205245230181E-62	Ductular-like hepatocytes	POLR2L
**8.1508825170156E-67**	0.435094172352711	0.211	0.119	1.36592489220147E-62	Ductular-like hepatocytes	LANCL2
**8.56092123023713E-67**	0.560829485292747	0.259	0.157	1.43463917976314E-62	Ductular-like hepatocytes	TMEM65
**8.84043284072425E-67**	0.58933791017729	0.221	0.128	1.48147973544857E-62	Ductular-like hepatocytes	PRIM1
**1.03229998800829E-66**	0.534577482567074	0.237	0.139	1.7299283199043E-62	Ductular-like hepatocytes	NIPA2
**1.18147225054441E-66**	0.75018009974789	0.161	0.083	1.97991119746233E-62	Ductular-like hepatocytes	SGMS2
**1.21413924382524E-66**	0.404652137123017	0.227	0.131	2.03465454480234E-62	Ductular-like hepatocytes	CAPZA1
**1.22448418735037E-66**	0.349254017748604	0.361	0.236	2.05199060116175E-62	Ductular-like hepatocytes	TMEM175
**1.23555023847654E-66**	0.370641203464055	0.185	0.099	2.07053508963898E-62	Ductular-like hepatocytes	VPS37B
**1.34721179266373E-66**	0.341942244791293	0.345	0.223	2.25765752214588E-62	Ductular-like hepatocytes	KBTBD2
**1.463706722335E-66**	0.780962910480839	0.188	0.103	2.45287972528899E-62	Ductular-like hepatocytes	CD320
**1.49986873928422E-66**	0.53782441277444	0.291	0.182	2.5134800332925E-62	Ductular-like hepatocytes	EIF4E
**1.53353869045172E-66**	1.11194759237837	0.123	0.057	2.569904137459E-62	Ductular-like hepatocytes	MCOLN3
**1.54445481501494E-66**	0.31599259416832	0.251	0.149	2.58819737900203E-62	Ductular-like hepatocytes	RAD50
**1.60332932358843E-66**	0.402466280979699	0.261	0.158	2.68685928046949E-62	Ductular-like hepatocytes	ZNF275
**1.71617032606149E-66**	0.26753270977343	0.429	0.293	2.87595823241384E-62	Ductular-like hepatocytes	LRRC59
**1.76158697773603E-66**	0.433433019595392	0.242	0.144	2.95206745729004E-62	Ductular-like hepatocytes	IPO5
**1.80108624880196E-66**	0.250837341852467	0.551	0.399	3.01826033574233E-62	Ductular-like hepatocytes	DEPRECATEI
**1.83292330120108E-66**	0.30110756157425	0.355	0.232	3.07161286815278E-62	Ductular-like hepatocytes	TAF2
**1.94739319736109E-66**	0.568501107804182	0.219	0.127	3.26344152013771E-62	Ductular-like hepatocytes	LRRC57
**2.01115541474859E-66**	0.302855606939035	0.183	0.097	3.37029424403569E-62	Ductular-like hepatocytes	SLC25A6
**2.04285751953862E-66**	0.531198376509373	0.233	0.136	3.42342063124282E-62	Ductular-like hepatocytes	DYNC1LI1
**2.04355552030366E-66**	0.667132900324702	0.17	0.09	3.42459034092488E-62	Ductular-like hepatocytes	C2CD2
**2.15452366970268E-66**	0.436946043722901	0.219	0.125	3.61055076568775E-62	Ductular-like hepatocytes	SPATA33
**2.63976516107559E-66**	0.673312452743586	0.123	0.056	4.42371845693047E-62	Ductular-like hepatocytes	RGS2
**2.99679967553052E-66**	0.627056210970834	0.177	0.094	5.02203689625404E-62	Ductular-like hepatocytes	APOO
**3.27808183903821E-66**	1.04649472743585	0.101	0.042	5.49340954586023E-62	Ductular-like hepatocytes	CACHD1
**4.03317482215574E-66**	0.268142829138302	0.172	0.09	6.75879436696859E-62	Ductular-like hepatocytes	MARVELD3
**4.28885430619938E-66**	0.395299229755722	0.289	0.18	7.18726204632892E-62	Ductular-like hepatocytes	RRAGA
**4.65566994001252E-66**	0.326335457216769	0.304	0.191	7.80197168547297E-62	Ductular-like hepatocytes	ADSS
**5.02738400804991E-66**	0.275774612175896	0.342	0.222	8.42489012069003E-62	Ductular-like hepatocytes	TMEM70
**5.89153912696046E-66**	0.292060144864878	0.346	0.226	9.87304126896034E-62	Ductular-like hepatocytes	CAND1
**6.8354353528459E-66**	0.271891956359633	0.344	0.223	1.14548225642992E-61	Ductular-like hepatocytes	GSK3B
**7.05171583575553E-66**	0.306886810999217	0.2	0.111	1.18172653975591E-61	Ductular-like hepatocytes	IDH3A
**7.46452541068755E-66**	0.5413781910143	0.199	0.111	1.25090516832302E-61	Ductular-like hepatocytes	DEPRECATEI
**7.64696785906086E-66**	0.788608218597582	0.161	0.083	1.28147887382142E-61	Ductular-like hepatocytes	RAB23
**7.93915659014996E-66**	0.33179758262242	0.238	0.14	1.33044386137733E-61	Ductular-like hepatocytes	CIZ1
**8.39550848707872E-66**	0.635719998068035	0.22	0.128	1.40691931226465E-61	Ductular-like hepatocytes	SLC36A1
**9.71855009781017E-66**	0.332109193536366	0.326	0.211	1.62863462539103E-61	Ductular-like hepatocytes	PSMC6

9.73665668196823E-66	0.31063716764022	0.234	0.137	1.63166892676424E-61	Ductular-like hepatocytes	SCMH1
9.85324368172018E-66	0.32624049580351	0.342	0.223	1.65120657618267E-61	Ductular-like hepatocytes	AFTPH
1.08320974895796E-65	0.338447893772468	0.281	0.174	1.81524289730375E-61	Ductular-like hepatocytes	OSTF1
1.17037970537943E-65	0.608793120255207	0.21	0.12	1.96132231027485E-61	Ductular-like hepatocytes	TRIM28
1.21245567825137E-65	0.608726073087363	0.224	0.131	2.03183322561364E-61	Ductular-like hepatocytes	SNRPG
1.2500948356563E-65	0.446729397059439	0.218	0.125	2.09490892559283E-61	Ductular-like hepatocytes	NUDT16L1
1.2542750625215E-65	0.422650608684265	0.224	0.129	2.10191414977353E-61	Ductular-like hepatocytes	DEPRECATEI
1.49117847541631E-65	0.613292001995227	0.154	0.078	2.49891688910266E-61	Ductular-like hepatocytes	TRIM11
1.51115422619123E-65	0.508119824193542	0.186	0.101	2.53239225225127E-61	Ductular-like hepatocytes	ZDHHC12
1.57130563026852E-65	0.338692086418608	0.348	0.227	2.63319397520399E-61	Ductular-like hepatocytes	SCFD1
1.73941644163185E-65	0.369759741270569	0.258	0.156	2.91491407288665E-61	Ductular-like hepatocytes	RSU1
1.7706690778835E-65	0.49951254169322	0.195	0.107	2.96728724071716E-61	Ductular-like hepatocytes	TTN
1.80615342301057E-65	0.335770706384609	0.311	0.197	3.02675190628112E-61	Ductular-like hepatocytes	SP1
1.96972041187239E-65	0.615254771233731	0.21	0.121	3.30085746621576E-61	Ductular-like hepatocytes	RASSF8
2.10989027071414E-65	0.370552292721584	0.264	0.161	3.53575411566275E-61	Ductular-like hepatocytes	ZNF507
2.11816108989383E-65	0.571016554340271	0.125	0.057	3.54961435444409E-61	Ductular-like hepatocytes	NAGPA
2.17529309451278E-65	0.289040727552325	0.339	0.221	3.64535616778451E-61	Ductular-like hepatocytes	ITGB5
2.5201627433868E-65	0.713293252710182	0.166	0.087	4.22328872536759E-61	Ductular-like hepatocytes	FOXJ2
2.64240763020951E-65	0.52960985424541	0.205	0.116	4.4281467067051E-61	Ductular-like hepatocytes	ZNF654
2.707133089773E-65	0.551153190755643	0.156	0.079	4.53661363184159E-61	Ductular-like hepatocytes	CDK5
2.71282856969017E-65	0.265296353294078	0.381	0.255	4.54615811708678E-61	Ductular-like hepatocytes	HSD17B8
2.82673819235144E-65	0.274997825896218	0.394	0.266	4.73704786274254E-61	Ductular-like hepatocytes	PLAAT3
2.87599936107845E-65	0.328739353381638	0.26	0.157	4.81959972929527E-61	Ductular-like hepatocytes	CCNK
2.97656856785797E-65	0.337718302527689	0.302	0.191	4.98813360601639E-61	Ductular-like hepatocytes	EPS8
3.54699726093666E-65	0.435723607064374	0.28	0.173	5.94405800987765E-61	Ductular-like hepatocytes	FBXO28
3.60638744886814E-65	0.33113986432461	0.296	0.185	6.04358408681323E-61	Ductular-like hepatocytes	PRPF39
4.00655108904628E-65	0.525159142362293	0.221	0.129	6.71417831502375E-61	Ductular-like hepatocytes	GPC1
4.39779174170468E-65	0.735009502820992	0.169	0.09	7.3698194007487E-61	Ductular-like hepatocytes	WASF3
4.57924796932604E-65	0.287210276882397	0.353	0.229	7.67390374699657E-61	Ductular-like hepatocytes	MLH3
4.68089615649683E-65	0.316588570029058	0.302	0.19	7.84424577905739E-61	Ductular-like hepatocytes	PCYOX1
5.09312564207802E-65	0.377094403312638	0.291	0.183	8.53505995099435E-61	Ductular-like hepatocytes	TADA2B
5.69995062328855E-65	0.633342086366066	0.138	0.067	9.55197725450695E-61	Ductular-like hepatocytes	ETV1
6.31483257699623E-65	0.437180065101163	0.219	0.126	1.05823964325303E-60	Ductular-like hepatocytes	BAD
8.56862421826352E-65	0.355511184576513	0.253	0.153	1.4359300464966E-60	Ductular-like hepatocytes	PHF2
8.58739735788299E-65	0.286290771344825	0.227	0.131	1.43907604923403E-60	Ductular-like hepatocytes	SRGAP2B
8.84558648660198E-65	0.264696571852845	0.444	0.311	1.48234338342476E-60	Ductular-like hepatocytes	ISOC1
1.01771087772972E-64	0.427798764106203	0.158	0.081	1.70547988889947E-60	Ductular-like hepatocytes	ABCF2
1.13553230710046E-64	0.483211609293273	0.198	0.11	1.90292504023896E-60	Ductular-like hepatocytes	HEATR1
1.41529574312339E-64	0.307541118388787	0.297	0.188	2.37175260632618E-60	Ductular-like hepatocytes	SETX
1.61131276258875E-64	0.469547611540276	0.267	0.165	2.70023792754623E-60	Ductular-like hepatocytes	SNRPB2
1.87804298088984E-64	0.47288638741459	0.198	0.111	3.14722442737519E-60	Ductular-like hepatocytes	ORAI1
1.94378860630785E-64	0.885035790189319	0.102	0.043	3.25740094645069E-60	Ductular-like hepatocytes	SIDT2
2.09324417682906E-64	0.283320848220071	0.206	0.116	3.50785859153015E-60	Ductular-like hepatocytes	NACC2
2.22840521393605E-64	0.611621842690492	0.157	0.081	3.73436145751403E-60	Ductular-like hepatocytes	MYCBP
2.40024969269843E-64	0.277883873529556	0.35	0.23	4.02233843502403E-60	Ductular-like hepatocytes	SRP72
2.47919373246529E-64	0.551109638241631	0.182	0.099	4.15463285686533E-60	Ductular-like hepatocytes	KNOP1
2.48681130915191E-64	0.680100224268424	0.157	0.081	4.16739839187677E-60	Ductular-like hepatocytes	DEPRECATEI
2.52137578831437E-64	0.510199440449859	0.219	0.127	4.22532154605721E-60	Ductular-like hepatocytes	JMJD4
2.61069372910018E-64	0.49255127906111	0.129	0.061	4.37500055122608E-60	Ductular-like hepatocytes	OSBPL3
2.72028563563928E-64	0.26052224356377	0.264	0.161	4.5586546682043E-60	Ductular-like hepatocytes	TSEN34
2.74520420177765E-64	0.630333929606073	0.15	0.076	4.60041320133899E-60	Ductular-like hepatocytes	LBHD1
2.88541648399656E-64	0.450689264499267	0.178	0.095	4.83538094388144E-60	Ductular-like hepatocytes	RNF43
3.00750637547124E-64	0.429578060053154	0.225	0.131	5.03997918401471E-60	Ductular-like hepatocytes	GLE1
3.28316035334142E-64	0.418678608167975	0.357	0.237	5.50192012012955E-60	Ductular-like hepatocytes	UGT1A9
3.57174698623034E-64	0.287859472958812	0.453	0.316	5.9855335995248E-60	Ductular-like hepatocytes	YTHDF3
3.65049319510995E-64	0.251204781442113	0.282	0.175	6.11749649636526E-60	Ductular-like hepatocytes	SRSF9
3.79277440359659E-64	0.50906603279503	0.119	0.054	6.35593134554717E-60	Ductular-like hepatocytes	ARHGEF28
3.81230818257748E-64	0.397836408146234	0.184	0.1	6.38866605236334E-60	Ductular-like hepatocytes	SRGAP2
3.94746782495223E-64	0.417716188130085	0.199	0.111	6.61516658105494E-60	Ductular-like hepatocytes	ABCB1
4.52230042398178E-64	0.293423162039117	0.233	0.137	7.57847105050867E-60	Ductular-like hepatocytes	ZDHHC20

**4.57494469480447E-64**	0.423295323029163	0.231	0.136	7.66669231955333E-60	Ductular-like hepatocytes	SLC25A40
**4.61494312928222E-64**	0.337636903284827	0.318	0.203	7.73372169605114E-60	Ductular-like hepatocytes	ANAPC4
**5.08465075322691E-64**	0.501415216279389	0.183	0.099	8.52085773225766E-60	Ductular-like hepatocytes	ARMC10
**5.42455854203804E-64**	0.842173573591082	0.127	0.06	9.09047520474735E-60	Ductular-like hepatocytes	DEPRECATEI
**5.60262616836998E-64**	0.638052460477443	0.209	0.12	9.38888093295442E-60	Ductular-like hepatocytes	RFC4
**5.81207914818119E-64**	0.297508797556902	0.26	0.157	9.73988223652203E-60	Ductular-like hepatocytes	USF3
**5.90950818762674E-64**	0.689361216309042	0.134	0.064	9.90315382082489E-60	Ductular-like hepatocytes	ZNF16
**6.13419333857551E-64**	0.448934325439953	0.249	0.151	1.02796811967848E-59	Ductular-like hepatocytes	FAM76B
**6.28369775883944E-64**	0.270423863509001	0.239	0.142	1.05302207042631E-59	Ductular-like hepatocytes	CSNK1A1
**6.65325085486027E-64**	0.487851188218497	0.229	0.135	1.11495177825748E-59	Ductular-like hepatocytes	PRR12
**6.68525719555345E-64**	0.441387842305616	0.248	0.149	1.12031540083085E-59	Ductular-like hepatocytes	IFI27L1
**6.90809930394039E-64**	0.385264322016749	0.256	0.155	1.15765928135433E-59	Ductular-like hepatocytes	LSM 2.00
**7.39258928788916E-64**	0.314351115572669	0.324	0.208	1.23885011286447E-59	Ductular-like hepatocytes	ADNP
**7.55017198046866E-64**	0.263387018745287	0.294	0.184	1.26525782048694E-59	Ductular-like hepatocytes	CHMP5
**7.71824930115873E-64**	0.350821809348573	0.16	0.083	1.29342421788818E-59	Ductular-like hepatocytes	CAMK2G
**8.12749753433937E-64**	0.45076825332882	0.184	0.1	1.36200603680459E-59	Ductular-like hepatocytes	DEPRECATEI
**8.21763797965385E-64**	0.361911175081742	0.205	0.116	1.37711177263039E-59	Ductular-like hepatocytes	ANGEL1
**8.4593059884247E-64**	0.254890335770823	0.392	0.264	1.41761049754021E-59	Ductular-like hepatocytes	ATXN7L3B
**9.46539399004552E-64**	0.530759472768376	0.212	0.122	1.58621072485183E-59	Ductular-like hepatocytes	USP5
**9.64914426637748E-64**	0.475271257853035	0.247	0.149	1.61700359615954E-59	Ductular-like hepatocytes	GNPTAB
**1.02053487859241E-63**	0.350089430802363	0.356	0.236	1.71021234954516E-59	Ductular-like hepatocytes	NUDCD2
**1.10670651397694E-63**	0.310048164606184	0.381	0.255	1.85461877612255E-59	Ductular-like hepatocytes	POLR2K
**1.27726886749975E-63**	0.305228726034937	0.253	0.152	2.14044716815608E-59	Ductular-like hepatocytes	ITSN1
**1.29774499556513E-63**	0.320466767123236	0.303	0.191	2.17476106356804E-59	Ductular-like hepatocytes	LARP7
**1.327474861205E-63**	0.288416054731146	0.278	0.172	2.22458237240733E-59	Ductular-like hepatocytes	GANAB
**1.42762437948516E-63**	0.584877945633408	0.161	0.084	2.39241293514124E-59	Ductular-like hepatocytes	PDSS1
**1.51838604570232E-63**	0.570256457628042	0.269	0.167	2.54451133538794E-59	Ductular-like hepatocytes	PXMP4
**1.77120881540174E-63**	0.663479800697919	0.173	0.093	2.96819173285024E-59	Ductular-like hepatocytes	CXorf40B
**1.79539502574203E-63**	0.525849381462523	0.247	0.15	3.0087229841385E-59	Ductular-like hepatocytes	FDXR
**1.80683614727941E-63**	0.281883563593237	0.409	0.279	3.02789601561083E-59	Ductular-like hepatocytes	UBE2I
**1.93306342896272E-63**	0.405226761466764	0.204	0.116	3.23942769425572E-59	Ductular-like hepatocytes	XPO6
**1.95669985645265E-63**	0.364882177775299	0.272	0.169	3.27903761944335E-59	Ductular-like hepatocytes	MANBAL
**1.98375365023917E-63**	0.606415996009773	0.211	0.122	3.3243743670708E-59	Ductular-like hepatocytes	DDX60
**2.0024146550967E-63**	0.3846214329582	0.214	0.123	3.35564647901106E-59	Ductular-like hepatocytes	SVIL
**2.04332424825711E-63**	0.255572598042568	0.425	0.293	3.42420277522926E-59	Ductular-like hepatocytes	TOP 1.00
**2.04781327516102E-63**	0.254968001316799	0.301	0.19	3.43172548651484E-59	Ductular-like hepatocytes	HTT
**2.05501321336857E-63**	0.261919707153118	0.31	0.198	3.44379114296306E-59	Ductular-like hepatocytes	STX10
**2.24941435307539E-63**	0.413938147953671	0.25	0.15	3.76956857288374E-59	Ductular-like hepatocytes	ZWINT
**2.25652829075506E-63**	0.34527415780293	0.235	0.138	3.78149010964733E-59	Ductular-like hepatocytes	TMEM184B
**2.5195885542507E-63**	0.486591856513522	0.252	0.154	4.22232649921333E-59	Ductular-like hepatocytes	MBIP
**2.56445197515974E-63**	0.338586205013682	0.292	0.184	4.2975086199727E-59	Ductular-like hepatocytes	C19orf53
**2.5838291028005E-63**	0.448027770533257	0.174	0.094	4.32998081047308E-59	Ductular-like hepatocytes	TBC1D9
**3.00057735327144E-63**	0.435432948862391	0.229	0.135	5.02836752861228E-59	Ductular-like hepatocytes	MSTO1
**3.15724106725666E-63**	0.731688687712085	0.177	0.097	5.29090458050871E-59	Ductular-like hepatocytes	ZNF57
**3.29128523630746E-63**	0.365822310396044	0.277	0.172	5.51553579900405E-59	Ductular-like hepatocytes	DEPRECATEI
**3.79594327764714E-63**	0.496557913746166	0.199	0.113	6.36124174468107E-59	Ductular-like hepatocytes	EXT2
**3.88113112947742E-63**	0.475416814402422	0.233	0.139	6.50399954677827E-59	Ductular-like hepatocytes	CAB39
**4.16871761893527E-63**	0.264042883264826	0.292	0.185	6.98593698581172E-59	Ductular-like hepatocytes	TADA3
**4.22855757634174E-63**	0.429636447281232	0.196	0.11	7.08621678643348E-59	Ductular-like hepatocytes	FAHD2A
**4.37526564780975E-63**	0.462851093434677	0.253	0.153	7.33207017259958E-59	Ductular-like hepatocytes	RBX1
**4.44011253952021E-63**	0.693370734733156	0.144	0.072	7.44074059372796E-59	Ductular-like hepatocytes	FAM149A
**5.08806803677321E-63**	0.314689111840794	0.258	0.157	8.52658441602455E-59	Ductular-like hepatocytes	SAMD1
**5.08841710687757E-63**	0.323600790571664	0.274	0.17	8.52716938770543E-59	Ductular-like hepatocytes	DRAM2
**5.10088661220338E-63**	0.474630567285859	0.201	0.114	8.54806578473042E-59	Ductular-like hepatocytes	AIFM2
**5.1572795172247E-63**	1.14908702479624	0.1	0.042	8.64256901496515E-59	Ductular-like hepatocytes	BTC
**5.28413852012449E-63**	1.00655539930496	0.103	0.044	8.85515933202462E-59	Ductular-like hepatocytes	CCDC88B
**5.85137417033189E-63**	0.352736312219506	0.162	0.084	9.80573283464218E-59	Ductular-like hepatocytes	DGKD
**6.4599052375457E-63**	0.366185618460562	0.287	0.181	1.08255091970791E-58	Ductular-like hepatocytes	SNX24
**6.79143453521235E-63**	0.570960097788823	0.197	0.112	1.13810859941089E-58	Ductular-like hepatocytes	RPE
**6.98825822220325E-63**	0.339042588080618	0.307	0.196	1.17109231287682E-58	Ductular-like hepatocytes	GALNT1

6.99148844342509E-63	0.329131767678987	0.27	0.167	1.17163363334918E-58	Ductular-like hepatocytes	BOD1L1
7.21730581116393E-63	0.253131273563167	0.301	0.188	1.20947610783485E-58	Ductular-like hepatocytes	TK1
7.85685880054629E-63	0.396238064436317	0.271	0.169	1.31665239779555E-58	Ductular-like hepatocytes	MLH1
8.2198531051708E-63	0.381250556482144	0.325	0.212	1.37748298336452E-58	Ductular-like hepatocytes	PAFAH1B2
8.5583817217584E-63	0.522709959508144	0.23	0.138	1.43421360893227E-58	Ductular-like hepatocytes	SKP2
1.03376381577324E-62	0.473244629387264	0.224	0.131	1.7323814024728E-58	Ductular-like hepatocytes	ABHD13
1.185541365646E-62	0.308040809327756	0.337	0.22	1.98673022054956E-58	Ductular-like hepatocytes	AP2S1
1.19522246936583E-62	0.314977292540463	0.228	0.134	2.00295381416325E-58	Ductular-like hepatocytes	ALKBH6
1.2654804980229E-62	0.435160058411859	0.309	0.199	2.12069221858677E-58	Ductular-like hepatocytes	UTP23
1.28522806812856E-62	0.445330901211124	0.255	0.156	2.15378519656984E-58	Ductular-like hepatocytes	LAMTOR3
1.32695147692576E-62	0.422095106157257	0.245	0.148	2.22370528503219E-58	Ductular-like hepatocytes	GLRX2
1.39422190477043E-62	0.261961496096321	0.233	0.137	2.33643706801429E-58	Ductular-like hepatocytes	MARK2
1.53135936724754E-62	0.404596425880273	0.202	0.114	2.56625202763344E-58	Ductular-like hepatocytes	HEATR5B
1.53684271196052E-62	0.74478076656313	0.154	0.08	2.57544101670344E-58	Ductular-like hepatocytes	ZDHHC2
1.54310879336944E-62	0.480699377391987	0.189	0.105	2.58594171592851E-58	Ductular-like hepatocytes	ACLY
1.60626420033221E-62	0.309319209870928	0.37	0.248	2.69177754691672E-58	Ductular-like hepatocytes	EPRS
1.75646344810118E-62	0.316678036281393	0.187	0.103	2.94348144632796E-58	Ductular-like hepatocytes	ZPR1
1.76326539002553E-62	0.392909417216792	0.221	0.129	2.95488014060479E-58	Ductular-like hepatocytes	UBN1
1.78999846587521E-62	0.255448142642648	0.35	0.23	2.99967942911368E-58	Ductular-like hepatocytes	MLLT1
1.86764957088768E-62	0.29096312594726	0.282	0.177	3.12980715089357E-58	Ductular-like hepatocytes	VTI1B
1.98470776968405E-62	0.364169222302744	0.252	0.152	3.32597328043653E-58	Ductular-like hepatocytes	CENPM
2.04517749453405E-62	0.447853542683565	0.219	0.128	3.42730844534016E-58	Ductular-like hepatocytes	CNOT6
2.08894435568737E-62	0.586780802873568	0.233	0.14	3.5006529512609E-58	Ductular-like hepatocytes	ING2
2.16671009153849E-62	0.320740807536932	0.311	0.201	3.63097277140021E-58	Ductular-like hepatocytes	RCN1
2.47072219753569E-62	0.774829883060514	0.112	0.05	4.14043625863031E-58	Ductular-like hepatocytes	DNASE1
2.57127344309365E-62	0.395751122447705	0.234	0.14	4.30894003593634E-58	Ductular-like hepatocytes	PRKAR2A
2.59336599059211E-62	0.569245921270944	0.232	0.139	4.34596272703426E-58	Ductular-like hepatocytes	AGA
2.75905380114729E-62	0.506928688830505	0.262	0.162	4.62362235996262E-58	Ductular-like hepatocytes	QSER1
3.08481578932935E-62	0.372551807288385	0.222	0.131	5.16953429975813E-58	Ductular-like hepatocytes	MAP7D1
3.31505784049727E-62	0.258699304227234	0.262	0.16	5.55537392910532E-58	Ductular-like hepatocytes	NUP205
3.58029295379379E-62	0.343917517591194	0.228	0.134	5.99985493196763E-58	Ductular-like hepatocytes	VRK3
3.87470246888276E-62	0.366527538705593	0.248	0.151	6.49322639735374E-58	Ductular-like hepatocytes	KDM5A
4.04814535266759E-62	0.271843988287977	0.245	0.148	6.78388198200035E-58	Ductular-like hepatocytes	ANKRD13A
4.6589664336225E-62	0.263315231422599	0.376	0.251	7.80749594946458E-58	Ductular-like hepatocytes	GOSR2
4.83129188749759E-62	0.308425205751387	0.259	0.159	8.09627894506846E-58	Ductular-like hepatocytes	USPL1
4.99004329953983E-62	0.60197978938384	0.184	0.102	8.36231456136884E-58	Ductular-like hepatocytes	PIGU
5.3652255007765E-62	0.357263409494427	0.225	0.132	8.99104489420126E-58	Ductular-like hepatocytes	AASDHPPT
5.57252529824599E-62	0.400283586492111	0.275	0.173	9.33843789480063E-58	Ductular-like hepatocytes	POLK
5.68913686921048E-62	0.266112833337892	0.372	0.249	9.53385556542292E-58	Ductular-like hepatocytes	YPEL5
5.85531711949345E-62	0.448253206249345	0.244	0.148	9.81234042884713E-58	Ductular-like hepatocytes	RBPJ
5.86032455892032E-62	0.335281397726602	0.227	0.134	9.82073189583867E-58	Ductular-like hepatocytes	MRGBP
7.1230246578631E-62	0.879709397925521	0.144	0.073	1.1936764721647E-57	Ductular-like hepatocytes	IFT57
8.83934715828939E-62	0.411427398739673	0.204	0.116	1.48129779678614E-57	Ductular-like hepatocytes	MTX1
9.21937764817574E-62	0.81866852974126	0.122	0.057	1.54498330628129E-57	Ductular-like hepatocytes	RTL8A
9.36653486565004E-62	0.351200009487708	0.303	0.195	1.56964391278563E-57	Ductular-like hepatocytes	M6PR
9.56497187140104E-62	0.596949108771961	0.17	0.092	1.60289798620939E-57	Ductular-like hepatocytes	ATP5MPL
1.15734563770853E-61	0.435406572964605	0.188	0.104	1.93947981967195E-57	Ductular-like hepatocytes	PEX26
1.18015802121669E-61	0.488565673915145	0.222	0.132	1.97770881195494E-57	Ductular-like hepatocytes	WDR41
1.23957082948129E-61	0.268807410875086	0.443	0.309	2.07727279604474E-57	Ductular-like hepatocytes	SSR3
1.26391654336369E-61	0.405160622112776	0.139	0.068	2.11807134336887E-57	Ductular-like hepatocytes	GIMAP2
1.31738336749206E-61	0.273320747193734	0.262	0.161	2.20767104724319E-57	Ductular-like hepatocytes	SMARCC2
1.44303069296567E-61	0.356955692472705	0.279	0.175	2.41823083527186E-57	Ductular-like hepatocytes	EIF2AK2
1.55333633540513E-61	0.512680804361394	0.214	0.125	2.60308103087191E-57	Ductular-like hepatocytes	MTMR6
1.79351129783467E-61	0.546803137318524	0.163	0.086	3.00556623291134E-57	Ductular-like hepatocytes	PARP8
1.83503475951658E-61	0.268064918857587	0.374	0.251	3.07515124999788E-57	Ductular-like hepatocytes	GLRX5
1.97836703152588E-61	0.374004334445455	0.298	0.191	3.31534747143107E-57	Ductular-like hepatocytes	SF3B4
2.07961097465688E-61	0.978797860859717	0.117	0.054	3.48501207132999E-57	Ductular-like hepatocytes	KALRN
2.24653448053573E-61	0.832242409026898	0.106	0.047	3.76474248248178E-57	Ductular-like hepatocytes	MRM1
2.27195521243895E-61	0.585510962244187	0.194	0.11	3.80734254500519E-57	Ductular-like hepatocytes	SNW1
2.44641473391763E-61	0.476876401150463	0.252	0.154	4.09970181109916E-57	Ductular-like hepatocytes	SNRPE

2.79330513567437E-61	0.585214110648766	0.187	0.104	4.68102074636312E-57	Ductular-like hepatocytes	TGFBR1
2.83973520675235E-61	0.311016676205906	0.311	0.201	4.75882825947559E-57	Ductular-like hepatocytes	EAPP
2.84177015542331E-61	0.390191213659327	0.286	0.181	4.76223842645839E-57	Ductular-like hepatocytes	PLIN3
2.84488370959362E-61	0.726058776758925	0.166	0.089	4.76745612053699E-57	Ductular-like hepatocytes	FAM102B
2.84567461000792E-61	0.354663706217275	0.215	0.126	4.76878151145127E-57	Ductular-like hepatocytes	DCBLD2
3.00819610112632E-61	0.382167665746409	0.247	0.15	5.04113502626749E-57	Ductular-like hepatocytes	ZMYM5
3.10032296906208E-61	0.781545200402125	0.158	0.084	5.19552123155423E-57	Ductular-like hepatocytes	GINS2
3.11248514171726E-61	0.264390019626715	0.445	0.312	5.21590260048978E-57	Ductular-like hepatocytes	SLC25A33
3.6619688854155E-61	0.328657755060004	0.287	0.182	6.13672745817929E-57	Ductular-like hepatocytes	HSPB11
3.77331810361728E-61	0.424230376888587	0.235	0.141	6.32332647804185E-57	Ductular-like hepatocytes	TMEM128
4.43697034508181E-61	0.377981763606583	0.269	0.168	7.43547490428809E-57	Ductular-like hepatocytes	TGS1
4.88454436913003E-61	0.416679499602385	0.231	0.138	8.1855194537881E-57	Ductular-like hepatocytes	YIPF2
5.00042032967678E-61	0.427857684825487	0.202	0.115	8.37970438847235E-57	Ductular-like hepatocytes	ZDHHC5
5.07564131038826E-61	0.552982389105002	0.252	0.156	8.50575970794865E-57	Ductular-like hepatocytes	KLHL12
5.10093777336075E-61	0.808415365205764	0.161	0.086	8.54815152059795E-57	Ductular-like hepatocytes	UBE2N
5.49880460013931E-61	0.291076495697108	0.427	0.296	9.21489674891345E-57	Ductular-like hepatocytes	PSMD12
5.80086898695698E-61	0.579533245866107	0.231	0.138	9.7210962483425E-57	Ductular-like hepatocytes	THSD4
5.8426128095717E-61	0.264951546292517	0.332	0.216	9.79105054628025E-57	Ductular-like hepatocytes	CLCC1
5.88499289313172E-61	0.268247400648385	0.365	0.244	9.86207109031014E-57	Ductular-like hepatocytes	HSPA9
5.94372592035503E-61	0.352747557824754	0.326	0.214	9.96049589733095E-57	Ductular-like hepatocytes	DPY30
6.42362127579579E-61	0.790559326579257	0.145	0.074	1.07647045339786E-56	Ductular-like hepatocytes	HAUS7
7.12061005248993E-61	0.444875605196598	0.293	0.188	1.19327183259626E-56	Ductular-like hepatocytes	FNIP2
7.28292175476752E-61	0.72607252668086	0.158	0.083	1.22047202766394E-56	Ductular-like hepatocytes	PAK1
8.02324908760505E-61	0.283889014724759	0.302	0.194	1.34453608210085E-56	Ductular-like hepatocytes	HNRNPR
8.94223205253164E-61	0.298705935912676	0.166	0.088	1.49853924736325E-56	Ductular-like hepatocytes	UTP25
9.00076175982948E-61	0.363420706505573	0.222	0.13	1.50834765571222E-56	Ductular-like hepatocytes	NCBP1
9.13716308457223E-61	0.44144383054238	0.229	0.136	1.53120578971261E-56	Ductular-like hepatocytes	PIGN
9.77575962964388E-61	0.36224454902317	0.286	0.182	1.63822179873572E-56	Ductular-like hepatocytes	TSN
1.00734622819598E-60	0.55076947610426	0.209	0.122	1.68811080921082E-56	Ductular-like hepatocytes	SPIN4
1.13368440184209E-60	0.409547355143846	0.227	0.135	1.89982832060698E-56	Ductular-like hepatocytes	REST
1.14847653887591E-60	0.665742433946531	0.158	0.083	1.92461698384824E-56	Ductular-like hepatocytes	DEPRECATEI
1.23398956696199E-60	0.675361322581168	0.126	0.06	2.06791971631491E-56	Ductular-like hepatocytes	PCNX2
1.28249571473452E-60	0.319612136371307	0.268	0.166	2.14920631875211E-56	Ductular-like hepatocytes	SLC17A5
1.34922753645599E-60	0.358403290284833	0.231	0.138	2.26103550559295E-56	Ductular-like hepatocytes	TBC1D10B
1.46530590300921E-60	0.29646839297908	0.377	0.256	2.45555963226283E-56	Ductular-like hepatocytes	SELENOS
1.48197174380116E-60	0.397419534282	0.239	0.146	2.48348824826198E-56	Ductular-like hepatocytes	TCTA
1.6493921063123E-60	0.290718878196839	0.383	0.259	2.76405129175815E-56	Ductular-like hepatocytes	NPEPPS
1.66738882069853E-60	0.485510753423376	0.208	0.121	2.7942101857266E-56	Ductular-like hepatocytes	ZNF672
1.73220267816538E-60	0.767849208564376	0.112	0.051	2.90282524806954E-56	Ductular-like hepatocytes	ARHGEF35
1.84607996517282E-60	0.347357987536816	0.192	0.108	3.09366080563662E-56	Ductular-like hepatocytes	PELI1
2.02982507209452E-60	0.266211691633164	0.419	0.29	3.40158085581599E-56	Ductular-like hepatocytes	MOCS2
2.03011985712504E-60	0.507022781573229	0.171	0.093	3.40207485657013E-56	Ductular-like hepatocytes	CHCHD4
2.09011996809969E-60	0.727589131841656	0.134	0.066	3.50262304254146E-56	Ductular-like hepatocytes	ZNF558
2.33773022101643E-60	0.34941579154667	0.208	0.121	3.91756830437933E-56	Ductular-like hepatocytes	MED22
3.36693942405638E-60	0.553852830168336	0.2	0.114	5.64231708683369E-56	Ductular-like hepatocytes	XRCC6
3.48183882609903E-60	0.269897620780675	0.372	0.25	5.83486550477675E-56	Ductular-like hepatocytes	MED28
3.94825095316259E-60	0.368913236893083	0.291	0.184	6.61647894730988E-56	Ductular-like hepatocytes	USP1
4.13525814774344E-60	0.414744646818606	0.199	0.114	6.92986560398846E-56	Ductular-like hepatocytes	ZNF224
4.60575869383944E-60	0.547265947214784	0.131	0.064	7.71833041913614E-56	Ductular-like hepatocytes	DEPRECATEI
4.64571703498585E-60	0.323191071966076	0.223	0.132	7.78529260722929E-56	Ductular-like hepatocytes	ZNF445
4.70511006936009E-60	0.459266476222279	0.218	0.129	7.88482345423364E-56	Ductular-like hepatocytes	MFHAS1
5.0787262222572E-60	0.258729132152112	0.306	0.197	8.51092940325861E-56	Ductular-like hepatocytes	DNAJC11
5.22465733201752E-60	0.422747482355531	0.245	0.15	8.75548075699495E-56	Ductular-like hepatocytes	ALG2
5.2570998611821E-60	0.455078968507836	0.285	0.182	8.80984794736896E-56	Ductular-like hepatocytes	MICU2
5.44676688856562E-60	0.275323818192492	0.455	0.32	9.12769195185827E-56	Ductular-like hepatocytes	KTN1
5.59254800976168E-60	0.47469843560801	0.222	0.132	9.37199195475863E-56	Ductular-like hepatocytes	CAD
5.73015941137566E-60	0.543807231255317	0.19	0.107	9.60260114158333E-56	Ductular-like hepatocytes	WDFY3
5.80245764126796E-60	0.308951543764298	0.283	0.178	9.72375851523684E-56	Ductular-like hepatocytes	MAP3K4
6.2439854505222E-60	1.17080814512614	0.109	0.05	1.04636708179851E-55	Ductular-like hepatocytes	PRR36
7.00678845102699E-60	0.440811895971378	0.202	0.116	1.1741976086231E-55	Ductular-like hepatocytes	NAA40

7.59792755686987E-60	0.890912299877043	0.124	0.06	1.27326069998025E-55	Ductular-like hepatocytes	GNG4
7.73677637817261E-60	0.508097266738579	0.204	0.118	1.29652898545417E-55	Ductular-like hepatocytes	COX18
8.53211054733517E-60	0.577122351084985	0.191	0.109	1.42981108552243E-55	Ductular-like hepatocytes	ALG1
9.74852417756E-60	0.2692412881396	0.348	0.232	1.6336576816755E-55	Ductular-like hepatocytes	TXLNA
9.90916717446197E-60	0.342297284511804	0.263	0.164	1.66057823509634E-55	Ductular-like hepatocytes	MIB1
1.03332656826489E-59	0.773418459333301	0.131	0.065	1.7316486630983E-55	Ductular-like hepatocytes	DEPRECATEI
1.08917401720048E-59	0.410614443501907	0.216	0.127	1.82523781802456E-55	Ductular-like hepatocytes	DDA1
1.10003025742545E-59	0.332324480360261	0.269	0.169	1.84343070539357E-55	Ductular-like hepatocytes	GTF2F1
1.16467192910252E-59	0.280367666285893	0.208	0.121	1.95175721879E-55	Ductular-like hepatocytes	SLC35B3
1.18105951984328E-59	0.314798980703697	0.278	0.175	1.97921954335337E-55	Ductular-like hepatocytes	CHD9
1.18191009807794E-59	0.375953195765305	0.217	0.128	1.980644942359E-55	Ductular-like hepatocytes	AP5M1
1.21966622753845E-59	0.332147553953267	0.211	0.124	2.04391666410893E-55	Ductular-like hepatocytes	MAD2L1BP
1.23765195538499E-59	0.395345389013401	0.201	0.115	2.07405714683417E-55	Ductular-like hepatocytes	NSD3
1.2517765669497E-59	0.300705024526707	0.275	0.172	2.0977271708943E-55	Ductular-like hepatocytes	EMC8
1.42752930268058E-59	0.257771135650642	0.252	0.155	2.39225360543211E-55	Ductular-like hepatocytes	MPP5
1.53807551493297E-59	0.347560961577556	0.237	0.143	2.57750694792468E-55	Ductular-like hepatocytes	DCUN1D2
1.5589979733836E-59	0.534084489522912	0.2	0.116	2.61256880379624E-55	Ductular-like hepatocytes	UBXN2A
1.58951339366889E-59	0.28407788654419	0.242	0.147	2.66370654511032E-55	Ductular-like hepatocytes	TAF4
1.76765584921587E-59	0.342209912330872	0.208	0.12	2.96223767211596E-55	Ductular-like hepatocytes	TATDN2
1.78655521135273E-59	0.463467763965971	0.199	0.115	2.9939092231849E-55	Ductular-like hepatocytes	PACS2
1.9481630934247E-59	0.456852020509474	0.219	0.13	3.26473171196111E-55	Ductular-like hepatocytes	PIGF
2.08862461437092E-59	0.350438973126822	0.204	0.117	3.50011712876279E-55	Ductular-like hepatocytes	BMS1
2.1620083213788E-59	0.264879987780288	0.322	0.209	3.6230935449666E-55	Ductular-like hepatocytes	GOLGA5
2.26499925437737E-59	0.52130507556384	0.18	0.1	3.7956857504856E-55	Ductular-like hepatocytes	GIT1
2.32248802615873E-59	0.599316861167857	0.176	0.098	3.8920254342368E-55	Ductular-like hepatocytes	SLC48A1
2.40838015354432E-59	0.254293247042773	0.411	0.28	4.03596346130957E-55	Ductular-like hepatocytes	KLB
2.49614190004279E-59	0.358187911589815	0.221	0.131	4.18303459609171E-55	Ductular-like hepatocytes	SLC35E2B
3.06599580288721E-59	0.341680541356608	0.219	0.129	5.13799576647839E-55	Ductular-like hepatocytes	ZMAT3
3.09716261580209E-59	0.321993297939773	0.266	0.166	5.19022511156114E-55	Ductular-like hepatocytes	RAB28
3.70433176552876E-59	0.542094132013304	0.206	0.12	6.2077191726731E-55	Ductular-like hepatocytes	PRUNE1
3.8932151185626E-59	0.263094603126174	0.407	0.276	6.5242498956872E-55	Ductular-like hepatocytes	RAB33B
4.34055567090408E-59	0.794702704246363	0.1	0.043	7.27390319330106E-55	Ductular-like hepatocytes	APBA1
4.57822468283786E-59	0.390675045905987	0.241	0.147	7.67218892349969E-55	Ductular-like hepatocytes	NRAS
4.8827138065112E-59	0.287267930353376	0.118	0.055	8.18245179695147E-55	Ductular-like hepatocytes	PPARGC1B
5.09097885308376E-59	0.449468725471977	0.195	0.112	8.53146236199777E-55	Ductular-like hepatocytes	TTC39B
5.58456059270937E-59	0.437052697386008	0.252	0.156	9.35860664126236E-55	Ductular-like hepatocytes	CUL2
5.67374624725571E-59	0.39761766805581	0.244	0.149	9.50806396115112E-55	Ductular-like hepatocytes	DMAC2L
5.77910827579023E-59	0.635349032182558	0.176	0.098	9.68462964856926E-55	Ductular-like hepatocytes	PIP5K1C
6.04821366982492E-59	0.459888097074508	0.237	0.145	1.01355964678926E-54	Ductular-like hepatocytes	NAGA
6.07386706597708E-59	0.561854273280808	0.181	0.101	1.01785864291644E-54	Ductular-like hepatocytes	PPM1L
6.18029785547217E-59	0.360278624171471	0.239	0.146	1.03569431462003E-54	Ductular-like hepatocytes	DHX8
6.89257756810164E-59	0.626552847297073	0.153	0.081	1.15505814886247E-54	Ductular-like hepatocytes	CIART
7.20683307526817E-59	1.11297781439989	0.1	0.044	1.20772108675344E-54	Ductular-like hepatocytes	B3GNT9
7.25460466184189E-59	0.485330020881489	0.162	0.087	1.21572664923146E-54	Ductular-like hepatocytes	SGSH
7.98191485863535E-59	0.253997142158614	0.543	0.403	1.33760929201011E-54	Ductular-like hepatocytes	TMED7
8.34829082128851E-59	0.363968484937924	0.252	0.156	1.39900657583153E-54	Ductular-like hepatocytes	KLF13
8.49170307788042E-59	0.32290924939423	0.279	0.177	1.4230396017912E-54	Ductular-like hepatocytes	ZMPSTE24
9.65857223899238E-59	0.435873679113975	0.255	0.159	1.61858353581034E-54	Ductular-like hepatocytes	RDH14
1.27760212137953E-58	0.565925432728597	0.153	0.081	2.14100563500782E-54	Ductular-like hepatocytes	MDP1
1.34253064037284E-58	0.487597045665455	0.142	0.072	2.24981284713681E-54	Ductular-like hepatocytes	DEPRECATEI
1.42147193009239E-58	0.468704252799625	0.166	0.091	2.38210266044883E-54	Ductular-like hepatocytes	RFK
1.5259360409716E-58	0.463767088894862	0.209	0.122	2.5571636174602E-54	Ductular-like hepatocytes	CAPRIN1
1.69954829490096E-58	0.484356644786864	0.179	0.1	2.84810303259503E-54	Ductular-like hepatocytes	TMEM203
1.80355719039042E-58	0.307932062848169	0.261	0.162	3.02240113965626E-54	Ductular-like hepatocytes	THUMPD3
1.80631784835304E-58	0.40502704452931	0.234	0.142	3.02702745027003E-54	Ductular-like hepatocytes	MTPAP
1.83688744352234E-58	0.273578675540202	0.245	0.15	3.07825597785474E-54	Ductular-like hepatocytes	DEPRECATEI
1.86633402024082E-58	0.349703658081667	0.242	0.148	3.12760255111956E-54	Ductular-like hepatocytes	TBPL1
2.50920978691759E-58	0.296217958149203	0.276	0.174	4.2049337609165E-54	Ductular-like hepatocytes	EIF5B
2.60350121729581E-58	0.35461797485545	0.208	0.122	4.36294733994431E-54	Ductular-like hepatocytes	ACOT8
2.72142234279562E-58	0.843362682795931	0.106	0.048	4.5605595620569E-54	Ductular-like hepatocytes	MFSD5

2.73785854040756E-58	0.307199357981772	0.28	0.178	4.58810334201498E-54	Ductular-like hepatocytes	DNAJC13
2.75112636151442E-58	0.352638019290825	0.262	0.165	4.61033755662586E-54	Ductular-like hepatocytes	STXBP3
2.79540981674661E-58	0.821120283290165	0.148	0.078	4.68454777090397E-54	Ductular-like hepatocytes	POGLUT3
2.80866114622814E-58	0.271030770211657	0.31	0.202	4.70675434884912E-54	Ductular-like hepatocytes	SCAF8
2.85134133362332E-58	0.465740409073168	0.256	0.16	4.77827780688596E-54	Ductular-like hepatocytes	FAM98B
2.89416686696821E-58	0.87435609402672	0.137	0.07	4.85004483566533E-54	Ductular-like hepatocytes	CMTM7
2.94783122597251E-58	0.751565175542061	0.172	0.096	4.93997556848474E-54	Ductular-like hepatocytes	PPM1H
3.12971330808736E-58	0.285591372905909	0.239	0.145	5.2447735616928E-54	Ductular-like hepatocytes	LDLRAD4
3.24977977357854E-58	0.399053643900363	0.23	0.139	5.44598094456292E-54	Ductular-like hepatocytes	WASF2
3.51208407824928E-58	0.737024570842566	0.112	0.052	5.88555049833015E-54	Ductular-like hepatocytes	TFEB
3.63345201811953E-58	0.308560243691534	0.373	0.256	6.08893889196471E-54	Ductular-like hepatocytes	GAA
3.87245756067494E-58	0.340302840856996	0.207	0.121	6.48946438017907E-54	Ductular-like hepatocytes	FADD
3.96013196403214E-58	0.302609073675882	0.319	0.209	6.63638914532505E-54	Ductular-like hepatocytes	ILF2
4.16564981209089E-58	0.876047128943318	0.128	0.063	6.98079595510191E-54	Ductular-like hepatocytes	B3GNT5
4.89414883225554E-58	0.487530235872939	0.158	0.084	8.20161461309383E-54	Ductular-like hepatocytes	ERP29
4.98875368616785E-58	0.650445345954557	0.123	0.059	8.36015342728008E-54	Ductular-like hepatocytes	TMEM198
5.0946486133168E-58	0.467466255341953	0.189	0.108	8.5376121461963E-54	Ductular-like hepatocytes	CAMK2N2
5.6192022221856E-58	0.386503861489236	0.188	0.106	9.41665908393863E-54	Ductular-like hepatocytes	TMEM260
5.69844144495946E-58	0.500529346869995	0.205	0.12	9.54944817346306E-54	Ductular-like hepatocytes	L2HGDH
6.42137561975461E-58	0.456478421884746	0.169	0.092	1.07609412635848E-53	Ductular-like hepatocytes	TCFL5
6.45019604911404E-58	0.419182784547652	0.188	0.107	1.08092385391053E-53	Ductular-like hepatocytes	POT1
6.46690042668323E-58	0.27676045703894	0.247	0.152	1.08372317350358E-53	Ductular-like hepatocytes	PDS5A
7.68967155105998E-58	0.609473598933533	0.187	0.106	1.28863515852663E-53	Ductular-like hepatocytes	WARS
8.3482605129348E-58	0.324787958460524	0.225	0.135	1.39900149675761E-53	Ductular-like hepatocytes	KCTD13
8.72208454221741E-58	0.442420562698939	0.227	0.137	1.46164692758479E-53	Ductular-like hepatocytes	ENDOV
9.46570879477381E-58	0.699161797157795	0.144	0.075	1.58626347982819E-53	Ductular-like hepatocytes	TRIM45
9.52083732825073E-58	0.25982173750771	0.339	0.225	1.59550191946826E-53	Ductular-like hepatocytes	ODC1
9.92422210427994E-58	0.34087511474184	0.23	0.139	1.66310114023523E-53	Ductular-like hepatocytes	SH2B1
1.03202616984638E-57	0.267575151350438	0.347	0.23	1.72946945542856E-53	Ductular-like hepatocytes	TASOR2
1.19354142048977E-57	0.270541494125155	0.321	0.212	2.00013671245676E-53	Ductular-like hepatocytes	IMMT
1.25862841319719E-57	0.414690672371417	0.194	0.112	2.10920949483585E-53	Ductular-like hepatocytes	RB1
1.30729608602444E-57	0.376908225925861	0.262	0.165	2.19076678095976E-53	Ductular-like hepatocytes	NAA38
1.4421030858435E-57	0.455664560042192	0.193	0.111	2.41667635125654E-53	Ductular-like hepatocytes	POLE4
1.54035379325289E-57	0.523098060644144	0.22	0.133	2.58132488673319E-53	Ductular-like hepatocytes	LATS2
1.59938095606681E-57	0.470497949936266	0.179	0.101	2.68024260617677E-53	Ductular-like hepatocytes	RIOX1
1.60711139143864E-57	0.376819942243654	0.206	0.12	2.69319726977287E-53	Ductular-like hepatocytes	SPTLC1
1.61314678442321E-57	0.325339094608345	0.291	0.187	2.70331138133641E-53	Ductular-like hepatocytes	ZCCHC3
1.61457141808174E-57	0.396742434042494	0.189	0.108	2.70569878242139E-53	Ductular-like hepatocytes	GCA
1.61990653225093E-57	0.646454938980284	0.217	0.13	2.71463936674611E-53	Ductular-like hepatocytes	RMI1
1.96551515990588E-57	0.260323009498743	0.28	0.179	3.29381030497028E-53	Ductular-like hepatocytes	FLII
2.02933618370751E-57	0.384500762096045	0.29	0.187	3.40076157665704E-53	Ductular-like hepatocytes	VPS25
2.03347777578466E-57	0.283853853817348	0.198	0.114	3.40770205665994E-53	Ductular-like hepatocytes	PPP3CA
2.40362870250683E-57	0.379332120408179	0.23	0.139	4.02800097966094E-53	Ductular-like hepatocytes	SMPD1
2.40657383631407E-57	0.283904323786365	0.257	0.16	4.03293643489513E-53	Ductular-like hepatocytes	RBM4
2.43279298222564E-57	0.392392406971537	0.21	0.125	4.07687447961373E-53	Ductular-like hepatocytes	ATG7
2.63391165515028E-57	0.4230032859536	0.168	0.092	4.41390915170084E-53	Ductular-like hepatocytes	HIP1
2.63795515237421E-57	0.428131446019262	0.223	0.134	4.4206852443487E-53	Ductular-like hepatocytes	ERCC2
2.66517505061301E-57	0.522220107160315	0.174	0.097	4.46630034981728E-53	Ductular-like hepatocytes	OLA1
2.66998071565189E-57	0.300031090343586	0.247	0.152	4.47435368328943E-53	Ductular-like hepatocytes	PPP1R12A
2.73281319444313E-57	0.26974809317869	0.105	0.046	4.57964835124779E-53	Ductular-like hepatocytes	SLC22A18A
2.76033596880925E-57	0.307548363309681	0.211	0.124	4.62577101653054E-53	Ductular-like hepatocytes	FOXK1
3.23783037834106E-57	0.386477094298576	0.257	0.161	5.42595614802394E-53	Ductular-like hepatocytes	TIMM9
3.54119147235228E-57	0.560631551966903	0.149	0.079	5.93432866936795E-53	Ductular-like hepatocytes	RSPH3
3.86974718022775E-57	0.863413690484456	0.102	0.045	6.48492232462567E-53	Ductular-like hepatocytes	RTN4RL1
3.89370231334689E-57	0.325424550031228	0.241	0.148	6.52506633670672E-53	Ductular-like hepatocytes	ABHD18
4.28640313585556E-57	0.332981785910477	0.222	0.134	7.18315437506674E-53	Ductular-like hepatocytes	TBC1D13
4.28694243683019E-57	0.341317687912686	0.252	0.157	7.18405813564003E-53	Ductular-like hepatocytes	SNHG32
4.28812257472961E-57	0.787619377158682	0.101	0.045	7.18603581073188E-53	Ductular-like hepatocytes	LMCD1
4.7656464597796E-57	0.250765012000794	0.251	0.155	7.98627033729866E-53	Ductular-like hepatocytes	RSPRY1
4.77153002048751E-57	0.573349747647769	0.195	0.114	7.99613000833297E-53	Ductular-like hepatocytes	SCAMP5

5.32518593589117E-57	0.604435675305478	0.159	0.085	8.92394659136642E-53	Ductular-like hepatocytes	ZNF696
5.85928755020716E-57	0.571428593874698	0.159	0.086	9.81899407663715E-53	Ductular-like hepatocytes	ARAP2
5.93766262018752E-57	0.400012530330459	0.239	0.146	9.95033501891025E-53	Ductular-like hepatocytes	PSMD11
6.61409190221154E-57	0.305312554250489	0.158	0.084	1.10838952097261E-52	Ductular-like hepatocytes	CHFR
6.83481698257781E-57	0.402833175187085	0.181	0.102	1.14537862994039E-52	Ductular-like hepatocytes	BFAR
7.50289857170505E-57	0.687713421900168	0.177	0.1	1.25733574264633E-52	Ductular-like hepatocytes	CPLANE1
7.91890326100628E-57	0.449070033056981	0.238	0.146	1.32704980847943E-52	Ductular-like hepatocytes	ENDOG
9.06612142561016E-57	0.25216631776625	0.537	0.401	1.51930062850375E-52	Ductular-like hepatocytes	COX6A1
9.10057926696971E-57	0.253170506599292	0.318	0.209	1.52507507355878E-52	Ductular-like hepatocytes	BDP1
9.11627926492141E-57	0.537044016132187	0.173	0.096	1.52770607921553E-52	Ductular-like hepatocytes	SYDE2
9.36336294816791E-57	0.617530866972905	0.187	0.108	1.56911236285398E-52	Ductular-like hepatocytes	BRMS1L
9.64084895192361E-57	0.251228488056764	0.284	0.181	1.61561346736336E-52	Ductular-like hepatocytes	GTPBP2
1.03014690308001E-56	0.436790183768148	0.212	0.126	1.72632018018148E-52	Ductular-like hepatocytes	ANKFY1
1.03873978208028E-56	0.289636973767449	0.209	0.123	1.74072012681014E-52	Ductular-like hepatocytes	COX19
1.1028444988249E-56	0.538524006055938	0.159	0.087	1.84814681113076E-52	Ductular-like hepatocytes	MBD5
1.25247378636958E-56	0.276423469937647	0.342	0.229	2.09889557119814E-52	Ductular-like hepatocytes	TCP1
1.35459789819266E-56	0.481909552323029	0.222	0.134	2.27003515779127E-52	Ductular-like hepatocytes	TGDS
1.54886180520344E-56	0.340786484769754	0.256	0.161	2.59558261315992E-52	Ductular-like hepatocytes	HDAC3
1.56600058104276E-56	0.339141677503437	0.268	0.17	2.62430377371146E-52	Ductular-like hepatocytes	ALG12
1.59252677113069E-56	0.25707881269562	0.212	0.125	2.66875636306081E-52	Ductular-like hepatocytes	EXD3
1.7010452057894E-56	0.347027347922032	0.231	0.14	2.85061155586187E-52	Ductular-like hepatocytes	LMAN2L
1.70958741516138E-56	0.390679318154881	0.2	0.117	2.86492659032744E-52	Ductular-like hepatocytes	HS2ST1
1.7230403565448E-56	0.308362945436164	0.206	0.121	2.88747102949777E-52	Ductular-like hepatocytes	UBN2
1.78645002379248E-56	0.377821879043839	0.246	0.153	2.99373294987143E-52	Ductular-like hepatocytes	WDR45B
1.82829445048332E-56	0.334065305628247	0.258	0.162	3.06385584011994E-52	Ductular-like hepatocytes	YTHDF2
1.83948097414467E-56	0.564272669284896	0.185	0.106	3.08260221647163E-52	Ductular-like hepatocytes	APC
2.22092566212431E-56	0.273283612238127	0.317	0.21	3.72182722458792E-52	Ductular-like hepatocytes	ACBD6
2.37203951239978E-56	0.403934676832417	0.193	0.112	3.97506381487954E-52	Ductular-like hepatocytes	MAPRE1
2.5034806574257E-56	0.332345254817379	0.208	0.123	4.19533288571399E-52	Ductular-like hepatocytes	C3orf38
2.52837452875538E-56	0.409499452444102	0.231	0.141	4.23705003528826E-52	Ductular-like hepatocytes	ZDHHC6
3.21998274315074E-56	0.394123804454694	0.153	0.081	5.39604708097201E-52	Ductular-like hepatocytes	CCDC32
3.9202955471376E-56	0.286371128905643	0.222	0.134	6.56963127789318E-52	Ductular-like hepatocytes	GPR137
4.11070725357009E-56	0.451401619423949	0.178	0.101	6.88872321553276E-52	Ductular-like hepatocytes	C7orf25
4.17763440650608E-56	0.269294324607837	0.274	0.176	7.00087973842288E-52	Ductular-like hepatocytes	FBH1
4.94052806986359E-56	0.272709758594673	0.288	0.187	8.27933693947741E-52	Ductular-like hepatocytes	NAE1
5.13105202574992E-56	0.55889490792045	0.104	0.047	8.59861698475171E-52	Ductular-like hepatocytes	CD83
5.68002284129036E-56	0.543166505510676	0.175	0.099	9.51858227743439E-52	Ductular-like hepatocytes	MFSD14B
5.97095997929076E-56	0.264197580079717	0.259	0.162	1.00061347332955E-51	Ductular-like hepatocytes	ABCD4
6.0015907153237E-56	0.275732335102763	0.395	0.271	1.00574657207395E-51	Ductular-like hepatocytes	ZFAND5
6.1889876173266E-56	0.273469062304855	0.197	0.114	1.03715054491159E-51	Ductular-like hepatocytes	ATP6V0A2
6.58192226699069E-56	0.307629070901521	0.296	0.193	1.1029985335023E-51	Ductular-like hepatocytes	NAA50
6.75078099618945E-56	0.473635813700876	0.189	0.109	1.13129587934143E-51	Ductular-like hepatocytes	TTI1
6.94975951243135E-56	0.428410331829938	0.265	0.17	1.16464069909325E-51	Ductular-like hepatocytes	SLC4A4
7.18323611296416E-56	1.12312390206322	0.134	0.07	1.20376670781053E-51	Ductular-like hepatocytes	PLA2G1B
7.38211494508611E-56	0.363453880866249	0.23	0.14	1.23709482249753E-51	Ductular-like hepatocytes	FRS2
8.42212312396712E-56	0.69902771446437	0.108	0.05	1.41137939311441E-51	Ductular-like hepatocytes	CTTNBP2NL
9.79546537829383E-56	0.423210664442723	0.194	0.113	1.64152408809448E-51	Ductular-like hepatocytes	COPS3
9.86549989466302E-56	0.312695015468479	0.19	0.109	1.65326047234763E-51	Ductular-like hepatocytes	EIF3I
9.89080876876043E-56	0.527957377198017	0.144	0.075	1.65750173346887E-51	Ductular-like hepatocytes	HDAC11
1.09280799453719E-55	0.275031587963604	0.304	0.2	1.83132763724542E-51	Ductular-like hepatocytes	TEX264
1.19392376606769E-55	0.290162876746387	0.224	0.135	2.00077744717624E-51	Ductular-like hepatocytes	JAZF1
1.21199468040303E-55	0.305909644803419	0.21	0.124	2.0310606854194E-51	Ductular-like hepatocytes	TSPAN31
1.6353533902737E-55	0.340311592806305	0.19	0.11	2.74052521142066E-51	Ductular-like hepatocytes	ZNFX1
1.67413332503041E-55	0.415931108885397	0.164	0.09	2.80551262608596E-51	Ductular-like hepatocytes	COA1
1.7643506938371E-55	0.548805374181232	0.211	0.126	2.95669889273221E-51	Ductular-like hepatocytes	MOB3B
1.7804301676713E-55	0.320575690385235	0.202	0.118	2.98364487498357E-51	Ductular-like hepatocytes	PGAM5
1.94048886917818E-55	0.340529224177878	0.213	0.127	3.25187124696879E-51	Ductular-like hepatocytes	B4GALT7
1.95814832383485E-55	0.532203670491817	0.152	0.081	3.28146496108244E-51	Ductular-like hepatocytes	SPATA2
2.09137842844193E-55	0.672587200850239	0.142	0.075	3.50473197038298E-51	Ductular-like hepatocytes	SLC18B1
2.18130879285221E-55	0.338057752676782	0.327	0.219	3.65543727506173E-51	Ductular-like hepatocytes	HAO2

2.18325131241034E-55	0.272657539876993	0.314	0.208	3.65869254933725E-51	Ductular-like hepatocytes	PRRC1
2.24774759152946E-55	0.399757954767169	0.181	0.103	3.76677541388507E-51	Ductular-like hepatocytes	STARD7
2.44447217434752E-55	0.504555166258303	0.2	0.118	4.09644646977158E-51	Ductular-like hepatocytes	DEPRECATEI
2.71358553016227E-55	0.256810755171762	0.183	0.104	4.54742663144594E-51	Ductular-like hepatocytes	WDR19
3.0258582562931E-55	0.427309263330885	0.179	0.102	5.07073326589597E-51	Ductular-like hepatocytes	SYT1
3.07934440047575E-55	0.490343449231766	0.152	0.081	5.16036534631725E-51	Ductular-like hepatocytes	PDIA4
3.17649789036777E-55	0.75009997569384	0.139	0.073	5.32317516467831E-51	Ductular-like hepatocytes	ZNF250
3.18166904180154E-55	0.373854915495478	0.224	0.137	5.33184098025102E-51	Ductular-like hepatocytes	XPO5
3.36305003288427E-55	0.453584351136658	0.188	0.108	5.63579924510747E-51	Ductular-like hepatocytes	TMEM184C
3.51741442457484E-55	0.61235884579269	0.138	0.072	5.89448309270252E-51	Ductular-like hepatocytes	BCORL1
3.56828992823972E-55	0.502809210733319	0.177	0.1	5.97974026174412E-51	Ductular-like hepatocytes	USP22
3.60270996254896E-55	0.520721105539432	0.152	0.082	6.03742135523954E-51	Ductular-like hepatocytes	ZNF524
3.62669987108567E-55	0.306478879858341	0.291	0.189	6.07762364396536E-51	Ductular-like hepatocytes	MED23
3.65991079313876E-55	0.311626064075055	0.239	0.148	6.13327850714194E-51	Ductular-like hepatocytes	BSCL2
4.00670976870388E-55	0.285115616241016	0.301	0.197	6.71444423039395E-51	Ductular-like hepatocytes	SEC24D
4.28168386561748E-55	0.310767830909945	0.206	0.122	7.17524582200177E-51	Ductular-like hepatocytes	NXT1
5.34086377704712E-55	0.338743776465603	0.242	0.15	8.95021951757556E-51	Ductular-like hepatocytes	LAS1L
5.61163667272095E-55	0.289121033757288	0.149	0.079	9.40398073614577E-51	Ductular-like hepatocytes	SYNE3
5.91986040069158E-55	0.595344934384952	0.109	0.051	9.92050205947895E-51	Ductular-like hepatocytes	CARMIL1
5.92389813721255E-55	0.398026801883669	0.215	0.129	9.92726849834079E-51	Ductular-like hepatocytes	C1orf21
6.26702952628392E-55	0.39297787912328	0.188	0.108	1.05022880801466E-50	Ductular-like hepatocytes	PJA1
6.44755656847618E-55	0.318860750556788	0.192	0.111	1.08048152974524E-50	Ductular-like hepatocytes	DEPDC5
6.77805600604613E-55	0.322431418621096	0.254	0.159	1.13586662549321E-50	Ductular-like hepatocytes	LTN1
7.88097667867764E-55	0.395395077530276	0.298	0.196	1.3206940718128E-50	Ductular-like hepatocytes	CUL3
8.20849679119906E-55	0.519198873000144	0.138	0.071	1.37557989226914E-50	Ductular-like hepatocytes	EXOSC5
8.23942806522635E-55	0.515451265852825	0.192	0.112	1.38076335517063E-50	Ductular-like hepatocytes	WRAP73
8.2539357898837E-55	0.297816186134746	0.235	0.144	1.38319455966871E-50	Ductular-like hepatocytes	RAB22A
9.3984850995411E-55	0.487659151535061	0.186	0.107	1.5749981329811E-50	Ductular-like hepatocytes	TMEM185B
9.4663864726282E-55	0.463621695912598	0.185	0.106	1.58637704508303E-50	Ductular-like hepatocytes	TSHZ1
1.02782743358725E-54	0.376235739069209	0.21	0.126	1.72243321320552E-50	Ductular-like hepatocytes	TINF2
1.03682842866344E-54	0.251462261547856	0.258	0.163	1.7375170807542E-50	Ductular-like hepatocytes	OGFOD2
1.06951122538145E-54	0.466463762549397	0.211	0.127	1.79228691149424E-50	Ductular-like hepatocytes	DESI2
1.10867411726856E-54	0.586353851780081	0.175	0.1	1.85791608571866E-50	Ductular-like hepatocytes	NUDCD1
1.10952570209728E-54	0.485990296630914	0.192	0.112	1.85934317157463E-50	Ductular-like hepatocytes	ELAC2
1.27914435588533E-54	0.408417256033685	0.175	0.099	2.14359011159263E-50	Ductular-like hepatocytes	TRAPPC1
1.30913919189401E-54	0.297688374114858	0.216	0.13	2.19385545777598E-50	Ductular-like hepatocytes	PPP1R37
1.374172950166E-54	0.396121777680003	0.198	0.117	2.30283902988819E-50	Ductular-like hepatocytes	FPGT
1.40156884668381E-54	0.50749113866673	0.183	0.105	2.34874907327272E-50	Ductular-like hepatocytes	PTRH2
1.45873352253544E-54	0.332005572878271	0.172	0.096	2.44454563706488E-50	Ductular-like hepatocytes	RPIA
1.48562152218165E-54	0.272578083124347	0.263	0.167	2.48960454687201E-50	Ductular-like hepatocytes	PHKB
1.65564059588049E-54	0.753428607243338	0.124	0.062	2.77452251057653E-50	Ductular-like hepatocytes	MMS22L
1.6840422842314E-54	0.450932775937773	0.173	0.098	2.82211805991499E-50	Ductular-like hepatocytes	TOMM34
1.73432570421204E-54	0.274891172338261	0.303	0.199	2.90638301511853E-50	Ductular-like hepatocytes	ZYG11B
1.73681442362351E-54	0.729841039380495	0.136	0.071	2.91055361110828E-50	Ductular-like hepatocytes	EIF3CL
1.76522884635612E-54	0.682108749712006	0.116	0.056	2.95817050072359E-50	Ductular-like hepatocytes	IFT140
1.81006015604514E-54	0.435198034215185	0.193	0.113	3.03329880950044E-50	Ductular-like hepatocytes	SMYD3
2.14094383783586E-54	0.278307208545165	0.248	0.155	3.58779368344534E-50	Ductular-like hepatocytes	LIG1
2.16518442015196E-54	0.302634784683055	0.214	0.128	3.62841605129066E-50	Ductular-like hepatocytes	MED12
2.29481214635578E-54	0.320941528693469	0.268	0.171	3.84564619486301E-50	Ductular-like hepatocytes	PARD3
2.31659923613678E-54	0.267569592793749	0.256	0.161	3.88215699991802E-50	Ductular-like hepatocytes	TMEM115
2.32983029585862E-54	0.390216076844302	0.268	0.171	3.90432960979988E-50	Ductular-like hepatocytes	TMEM230
2.6189193204507E-54	0.331970642778548	0.214	0.129	4.38878499721128E-50	Ductular-like hepatocytes	LSM14B
2.72515763829661E-54	0.396427466592471	0.165	0.092	4.56681917025745E-50	Ductular-like hepatocytes	TSPAN17
2.90432461613934E-54	0.386430974527838	0.223	0.135	4.86706719172631E-50	Ductular-like hepatocytes	NGDN
2.90802927856124E-54	0.300378931912215	0.153	0.082	4.87327546501293E-50	Ductular-like hepatocytes	RAB26
3.02473282257856E-54	0.52647594852049	0.147	0.078	5.06884726407715E-50	Ductular-like hepatocytes	SCN1B
3.30369881239796E-54	0.847669843493361	0.149	0.08	5.5363384698165E-50	Ductular-like hepatocytes	FHOD3
3.52566829406515E-54	0.542857991552883	0.196	0.117	5.90831492719438E-50	Ductular-like hepatocytes	USP28
3.65221158165122E-54	0.333990483572218	0.211	0.127	6.12037616853112E-50	Ductular-like hepatocytes	BECN1
3.75619744468702E-54	0.586969597334918	0.145	0.077	6.29463567780651E-50	Ductular-like hepatocytes	TUSC1

3.82680297693345E-54	0.446501256986057	0.161	0.089	6.41295642874508E-50	Ductular-like hepatocytes	INAFM1
3.935906539533E-54	0.441437078251175	0.156	0.085	6.5957921789494E-50	Ductular-like hepatocytes	DEPRECATEI
4.16649580642342E-54	0.359563728005413	0.268	0.171	6.98221367240438E-50	Ductular-like hepatocytes	WAC
4.92414593648609E-54	0.299236334390558	0.156	0.085	8.25188376036338E-50	Ductular-like hepatocytes	ARL 15.00
5.56429618622324E-54	0.339904880076044	0.28	0.181	9.3246475488729E-50	Ductular-like hepatocytes	CMAS
5.97706955471123E-54	0.381785835167222	0.212	0.128	1.00163731597851E-49	Ductular-like hepatocytes	ANKRD52
6.20103221974662E-54	0.464301283255487	0.227	0.14	1.03916897938514E-49	Ductular-like hepatocytes	ERGIC2
6.28404570500926E-54	0.633494574775983	0.144	0.077	1.05308037924545E-49	Ductular-like hepatocytes	DEPRECATEI
7.42621042839988E-54	0.636210684424144	0.159	0.088	1.24448434359125E-49	Ductular-like hepatocytes	WBP1L
7.70789453503804E-54	0.350576403598337	0.209	0.125	1.29168896618167E-49	Ductular-like hepatocytes	CRYBG1
8.14396649984253E-54	0.256935491317849	0.31	0.205	1.36476590604361E-49	Ductular-like hepatocytes	NAGK
8.4121280466652E-54	0.406889300547152	0.183	0.106	1.40970441806015E-49	Ductular-like hepatocytes	ACD
9.48939623704862E-54	0.819605803975204	0.136	0.071	1.59023302140461E-49	Ductular-like hepatocytes	TFCP2L1
1.08016298698569E-53	0.346234836539802	0.277	0.18	1.81013713359062E-49	Ductular-like hepatocytes	ERMARD
1.09301680195054E-53	0.42308637754437	0.182	0.105	1.83167755670871E-49	Ductular-like hepatocytes	FLYWCH2
1.10100922841436E-53	0.504859239332254	0.252	0.161	1.84507126497678E-49	Ductular-like hepatocytes	SEC22C
1.16697775349295E-53	0.543689203085927	0.188	0.11	1.95562131930348E-49	Ductular-like hepatocytes	SMARCAD1
1.16938420979351E-53	0.411472211720561	0.234	0.146	1.95965405877196E-49	Ductular-like hepatocytes	GTF2H5
1.37872065302888E-53	0.326308060415264	0.336	0.226	2.31046007034579E-49	Ductular-like hepatocytes	ABHD3
1.5070481138477E-53	0.285734557898193	0.267	0.171	2.52551122918598E-49	Ductular-like hepatocytes	SMPD4
1.78179272103734E-53	0.346466472750314	0.22	0.134	2.98592824191437E-49	Ductular-like hepatocytes	UBE2G1
1.92512159605183E-53	0.350205530554338	0.303	0.199	3.22611877066366E-49	Ductular-like hepatocytes	GMNN
2.00173718593325E-53	0.253444702303688	0.214	0.129	3.35451117618695E-49	Ductular-like hepatocytes	GM2A
2.07624500652632E-53	0.326464627833231	0.273	0.177	3.4793713819368E-49	Ductular-like hepatocytes	UBQLN4
2.34439843959933E-53	0.277459808890925	0.341	0.232	3.92874290508055E-49	Ductular-like hepatocytes	DNAJA1
2.45888781624333E-53	0.256347491318579	0.26	0.165	4.12060420246058E-49	Ductular-like hepatocytes	MTLN
2.54859940081971E-53	0.352814173652863	0.173	0.098	4.27094287589366E-49	Ductular-like hepatocytes	SPRED2
2.59791343739425E-53	0.547194140989599	0.183	0.106	4.35358333838529E-49	Ductular-like hepatocytes	RPA2
2.89465279065843E-53	0.903520439419765	0.108	0.052	4.85085914658539E-49	Ductular-like hepatocytes	GPR135
2.96352447286406E-53	0.575979619703852	0.156	0.086	4.96627431162559E-49	Ductular-like hepatocytes	NCOA5
3.03277212329502E-53	0.258767908583127	0.366	0.251	5.0823195242178E-49	Ductular-like hepatocytes	PEA15
3.11656953488931E-53	0.850820618872408	0.124	0.063	5.2227472265675E-49	Ductular-like hepatocytes	DEPRECATEI
3.22486764790065E-53	0.27519307938476	0.221	0.135	5.40423320435191E-49	Ductular-like hepatocytes	AKAP11
3.85344940670517E-53	0.511035184996314	0.14	0.074	6.45761051575653E-49	Ductular-like hepatocytes	DCAF13
3.90814094112667E-53	0.784534472281768	0.159	0.089	6.54926258914008E-49	Ductular-like hepatocytes	PAICS
3.99939712798511E-53	0.416651787555429	0.191	0.112	6.70218970707744E-49	Ductular-like hepatocytes	DEPRECATEI
4.29775972950967E-53	0.896666782004512	0.106	0.05	7.20218575471231E-49	Ductular-like hepatocytes	SALL4
4.42289742549582E-53	0.373120605025514	0.236	0.148	7.41189150564589E-49	Ductular-like hepatocytes	COG1
4.56178840650505E-53	0.352080757642124	0.179	0.102	7.64464501162117E-49	Ductular-like hepatocytes	REXO4
4.62778399443906E-53	0.357708459338917	0.248	0.156	7.75524041788098E-49	Ductular-like hepatocytes	RPP21
4.72211139516793E-53	0.766437625711104	0.135	0.071	7.91331427602242E-49	Ductular-like hepatocytes	CABLES2
4.80932484739697E-53	0.280121802282814	0.229	0.141	8.05946657926784E-49	Ductular-like hepatocytes	GPATCH8
5.20252654720465E-53	0.335819051355893	0.149	0.081	8.71839398780555E-49	Ductular-like hepatocytes	DEPRECATEI
5.26174533581166E-53	0.259812641257056	0.254	0.162	8.81763283375318E-49	Ductular-like hepatocytes	CMTR1
6.12370157430416E-53	0.303626101780664	0.162	0.09	1.02620990982189E-48	Ductular-like hepatocytes	MYBBP1A
6.57761401665528E-53	0.41466929700246	0.15	0.081	1.10227655691109E-48	Ductular-like hepatocytes	PMS2
6.89861242327958E-53	0.446085151224788	0.194	0.115	1.15606946989319E-48	Ductular-like hepatocytes	AP3S1
6.90586356418251E-53	0.475940572504565	0.206	0.124	1.1572846160857E-48	Ductular-like hepatocytes	TONSL
7.18595468563463E-53	0.513583708025328	0.215	0.132	1.20422228621865E-48	Ductular-like hepatocytes	MAGEF1
8.1407386440349E-53	0.304127398898695	0.215	0.131	1.36422498196737E-48	Ductular-like hepatocytes	DROSHA
9.59078663362744E-53	0.56779181698385	0.175	0.101	1.60722402406329E-48	Ductular-like hepatocytes	TIFA
9.89850189978976E-53	0.265657373400758	0.299	0.197	1.65879094836677E-48	Ductular-like hepatocytes	GTF2H1
1.04801893296716E-52	0.256064752797019	0.275	0.177	1.75627012786637E-48	Ductular-like hepatocytes	GRSF1
1.04874272306783E-52	0.43720129701444	0.25	0.159	1.75748305531707E-48	Ductular-like hepatocytes	LIG3
1.06339465786705E-52	0.483755898643816	0.196	0.117	1.7820367676536E-48	Ductular-like hepatocytes	KIAA0355
1.09082193525614E-52	0.336271172175588	0.17	0.096	1.82799939910224E-48	Ductular-like hepatocytes	TRAFD1
1.13928280239156E-52	0.520185738117469	0.171	0.097	1.90921012024777E-48	Ductular-like hepatocytes	NOA1
1.15334814427428E-52	0.29273517471278	0.251	0.159	1.93278082017483E-48	Ductular-like hepatocytes	ZNF251
1.18158011836204E-52	0.281632243044482	0.205	0.123	1.9800919623511E-48	Ductular-like hepatocytes	GPATCH2
1.35974147472778E-52	0.374091726434516	0.188	0.11	2.27865476334881E-48	Ductular-like hepatocytes	ADSSL1

**1.38013948649146E-52**	0.386935916847709	0.251	0.16	2.31283775146239E-48	Ductular-like hepatocytes	USP38
**1.49243809618799E-52**	0.49501081765673	0.172	0.098	2.50102776159183E-48	Ductular-like hepatocytes	GUF1
**1.70012089668736E-52**	0.700304568697994	0.126	0.065	2.84906259866868E-48	Ductular-like hepatocytes	IQGAP1
**1.70792559539468E-52**	0.512467636669096	0.19	0.112	2.8621417127624E-48	Ductular-like hepatocytes	FAM241A
**2.01179862590259E-52**	0.276889015403697	0.185	0.107	3.37137213728756E-48	Ductular-like hepatocytes	NIPSNAP3A
**2.08265262676004E-52**	0.585705889235478	0.114	0.056	3.49010927192447E-48	Ductular-like hepatocytes	PPP1R26
**2.12889291819499E-52**	0.292200073384737	0.173	0.098	3.56759875231117E-48	Ductular-like hepatocytes	HMGXB3
**2.38529923209233E-52**	0.289598295544754	0.227	0.14	3.99728445314033E-48	Ductular-like hepatocytes	ABL2
**2.39316107390696E-52**	0.346253244197914	0.231	0.144	4.01045932765329E-48	Ductular-like hepatocytes	TK2
**2.48422469319483E-52**	0.286613399970011	0.253	0.161	4.1630637408559E-48	Ductular-like hepatocytes	RMND5B
**2.81244732966827E-52**	0.415225468594387	0.211	0.128	4.71309923505808E-48	Ductular-like hepatocytes	SSR1
**2.86315561626001E-52**	0.286526012677958	0.206	0.124	4.79807618172853E-48	Ductular-like hepatocytes	WDR5
**2.89245318497693E-52**	0.790367332040448	0.103	0.048	4.84717304738434E-48	Ductular-like hepatocytes	S100A14
**2.97375113953619E-52**	0.261561093019982	0.276	0.178	4.98341215963475E-48	Ductular-like hepatocytes	ATP6V1E1
**3.03274957642153E-52**	0.282665039800743	0.208	0.125	5.0822817401672E-48	Ductular-like hepatocytes	SLC2A8
**3.10783577680111E-52**	0.381166375714524	0.198	0.118	5.20811119476331E-48	Ductular-like hepatocytes	LRRC8D
**3.51347627449213E-52**	0.255660917127714	0.239	0.15	5.88788354079392E-48	Ductular-like hepatocytes	RAB35
**3.80910104127977E-52**	0.366169513227767	0.192	0.113	6.38329152497664E-48	Ductular-like hepatocytes	CUEDC1
**4.14147797438957E-52**	0.372203930745954	0.214	0.131	6.94028878948204E-48	Ductular-like hepatocytes	NOP10
**4.96398513959644E-52**	0.501063296167881	0.205	0.124	8.31864629693572E-48	Ductular-like hepatocytes	LCORL
**5.95329724152287E-52**	0.353031226489285	0.206	0.124	9.97653551734403E-48	Ductular-like hepatocytes	C1orf35
**6.39162956308419E-52**	0.379791006946101	0.128	0.066	1.07110928218165E-47	Ductular-like hepatocytes	FASTKD1
**6.76027924060413E-52**	0.296189289274369	0.171	0.097	1.13288759514044E-47	Ductular-like hepatocytes	PRORP
**6.91420936966362E-52**	0.523155546780062	0.157	0.087	1.15868320616823E-47	Ductular-like hepatocytes	RALGAPA1
**7.13391574665286E-52**	0.375515973997478	0.295	0.194	1.19550160082409E-47	Ductular-like hepatocytes	PSMA4
**7.96914795052071E-52**	0.261401180488545	0.272	0.177	1.33546981354826E-47	Ductular-like hepatocytes	SUCLG2
**8.08362574730181E-52**	0.427598658305709	0.207	0.125	1.35465400273284E-47	Ductular-like hepatocytes	SPAG16
**9.47870322990385E-52**	0.621182497514525	0.168	0.096	1.58844108726729E-47	Ductular-like hepatocytes	DEPRECATEI
**9.93665142555992E-52**	0.390818885767403	0.176	0.101	1.66518404589533E-47	Ductular-like hepatocytes	ZNF248
**1.14002895614643E-51**	0.475406018019231	0.127	0.065	1.91046052471019E-47	Ductular-like hepatocytes	MAP2K6
**1.15680811953817E-51**	0.590522912112251	0.165	0.094	1.93857904672207E-47	Ductular-like hepatocytes	MFSD8
**1.1748371740754E-51**	0.50171289223665	0.218	0.135	1.96879213631556E-47	Ductular-like hepatocytes	PCLAF
**1.22272812370834E-51**	0.298008112547202	0.222	0.136	2.04904778971044E-47	Ductular-like hepatocytes	EIF2AK4
**1.26064081673231E-51**	0.53076420063913	0.185	0.109	2.11258188068001E-47	Ductular-like hepatocytes	PPP1R13L
**1.31840704555719E-51**	0.263674813261992	0.174	0.099	2.20938652694473E-47	Ductular-like hepatocytes	BICRAL
**1.56216202137014E-51**	0.31759829825388	0.182	0.105	2.61787111541209E-47	Ductular-like hepatocytes	SSBP1
**1.67759830320979E-51**	0.280159379082378	0.299	0.198	2.81131923651897E-47	Ductular-like hepatocytes	CKLF
**1.78490467450146E-51**	0.355360929285735	0.161	0.09	2.99114325352955E-47	Ductular-like hepatocytes	ATG14
**1.85470569901659E-51**	0.776443721964145	0.144	0.078	3.108115810412E-47	Ductular-like hepatocytes	ZBED8
**1.96739852724854E-51**	0.533080082552641	0.177	0.103	3.2969664519631E-47	Ductular-like hepatocytes	RPA4
**2.54743000410109E-51**	0.283419848977967	0.35	0.24	4.26898320087261E-47	Ductular-like hepatocytes	ZBTB10
**2.5489535009184E-51**	0.482055124283332	0.178	0.103	4.27153627683906E-47	Ductular-like hepatocytes	DEPRECATEI
**2.60200357361877E-51**	0.290929687349279	0.178	0.103	4.36043758867033E-47	Ductular-like hepatocytes	PCNX3
**3.28037616279633E-51**	0.345541550139338	0.234	0.147	5.49725437361409E-47	Ductular-like hepatocytes	TBCE
**3.77919243178173E-51**	0.704941836130411	0.105	0.05	6.33317067717982E-47	Ductular-like hepatocytes	C11orf98
**3.8080786927967E-51**	0.264355877415002	0.28	0.182	6.3815782733887E-47	Ductular-like hepatocytes	FAM168A
**4.54354692031483E-51**	0.363241512490631	0.194	0.115	7.61407592906358E-47	Ductular-like hepatocytes	MCM9
**4.5826066206793E-51**	0.280254350435039	0.211	0.129	7.67953217493437E-47	Ductular-like hepatocytes	ZNF266
**4.77481920954625E-51**	0.345258578875028	0.233	0.146	8.00164203135761E-47	Ductular-like hepatocytes	PSKH1
**4.84537448757208E-51**	0.397733532604052	0.199	0.12	8.1198785662733E-47	Ductular-like hepatocytes	PDCL
**4.90562730708275E-51**	0.319615967221454	0.269	0.174	8.22085024120927E-47	Ductular-like hepatocytes	PCNX1
**5.52913618836133E-51**	0.428269320753616	0.167	0.095	9.26572642445591E-47	Ductular-like hepatocytes	TOM1L2
**5.90051980548454E-51**	0.336506199084308	0.235	0.148	9.88809109003099E-47	Ductular-like hepatocytes	POLR2G
**6.27856764922643E-51**	0.74873660762361	0.125	0.064	1.05216236665737E-46	Ductular-like hepatocytes	ARL6IP6
**6.87721883626747E-51**	0.765340583735178	0.121	0.062	1.1524843325817E-46	Ductular-like hepatocytes	ZNF526
**7.3592587943209E-51**	0.456940693474171	0.16	0.09	1.2332645887523E-46	Ductular-like hepatocytes	TMA16
**8.02049952597926E-51**	0.454637076197327	0.201	0.122	1.3440753105636E-46	Ductular-like hepatocytes	RWDD1
**8.11783720795685E-51**	0.382634199923745	0.202	0.122	1.36038715930941E-46	Ductular-like hepatocytes	TIMELESS
**8.22997049157795E-51**	0.632494641474465	0.139	0.075	1.37917845497863E-46	Ductular-like hepatocytes	PAQR3
**8.36831992566832E-51**	0.410869870524749	0.223	0.139	1.4023630531435E-46	Ductular-like hepatocytes	C17orf75

8.88426757841084E-51	0.34732910828768	0.138	0.073	1.48882556079009E-46	Ductular-like hepatocytes	PSEN2
9.21888822217289E-51	0.393803674809405	0.243	0.156	1.54490128827173E-46	Ductular-like hepatocytes	SLC23A1
1.02568579346521E-50	0.419842315923239	0.191	0.114	1.71884425268901E-46	Ductular-like hepatocytes	RPAP1
1.0344179157648E-50	0.280138940588393	0.191	0.113	1.73347754323865E-46	Ductular-like hepatocytes	RBM28
1.21311382455584E-50	0.430205119055498	0.182	0.107	2.03293614719067E-46	Ductular-like hepatocytes	AACS
1.2885295157593E-50	0.277289716036915	0.217	0.133	2.15931776250943E-46	Ductular-like hepatocytes	PIGO
1.31244725521047E-50	0.38756683781216	0.239	0.152	2.1993991102817E-46	Ductular-like hepatocytes	COX20
1.4020059089603E-50	0.800359811355908	0.182	0.109	2.34948150223568E-46	Ductular-like hepatocytes	TCAF2
1.42447595514008E-50	0.484001506342738	0.198	0.119	2.38713680562374E-46	Ductular-like hepatocytes	RTRAF
1.4834025625108E-50	0.316540243128549	0.377	0.264	2.48588601425561E-46	Ductular-like hepatocytes	IFI6
1.595255135433E-50	0.466480308986845	0.204	0.125	2.67332855595863E-46	Ductular-like hepatocytes	ARPIN
1.81448440372207E-50	0.510584355455067	0.156	0.088	3.04071296375745E-46	Ductular-like hepatocytes	CIITA
1.94459734996069E-50	0.53239230427001	0.115	0.057	3.25875623906412E-46	Ductular-like hepatocytes	CAVIN1
1.94556831348794E-50	0.442778194426141	0.227	0.143	3.26038337974309E-46	Ductular-like hepatocytes	SOX13
2.5600957161733E-50	0.428121414590328	0.162	0.092	4.29020840116322E-46	Ductular-like hepatocytes	SLX4IP
2.6239564186873E-50	0.384869335513959	0.156	0.088	4.39722616643618E-46	Ductular-like hepatocytes	AGO4
2.80540746390134E-50	0.25172196614547	0.21	0.128	4.70130182800587E-46	Ductular-like hepatocytes	PARN
3.09125945027383E-50	0.371419712961249	0.11	0.054	5.18033258676888E-46	Ductular-like hepatocytes	ENPP2
3.23859743421288E-50	0.422101167047294	0.202	0.122	5.42724158025394E-46	Ductular-like hepatocytes	KIFAP3
3.3302386946999E-50	0.304823598442783	0.189	0.111	5.5808140045781E-46	Ductular-like hepatocytes	DEPRECATEI
3.71470420154866E-50	0.728597553717923	0.137	0.073	6.22510130095524E-46	Ductular-like hepatocytes	CCDC127
3.71657721511626E-50	0.362401771769138	0.2	0.12	6.22824009709183E-46	Ductular-like hepatocytes	STRIP1
3.99579285525129E-50	0.353850533086221	0.239	0.151	6.69614966683011E-46	Ductular-like hepatocytes	SAV1
4.18209284962819E-50	0.441511335636775	0.208	0.128	7.00835119740693E-46	Ductular-like hepatocytes	NFU1
4.86353662910675E-50	0.346633893345744	0.172	0.1	8.1503146830571E-46	Ductular-like hepatocytes	BRK1
5.1480596233356E-50	0.375257064176815	0.162	0.092	8.6271183167858E-46	Ductular-like hepatocytes	CCAR2
5.92328868912644E-50	0.545512867662775	0.12	0.061	9.92624718523809E-46	Ductular-like hepatocytes	PPP4R4
6.29436867094846E-50	0.466914026915452	0.158	0.088	1.05481030187754E-45	Ductular-like hepatocytes	SLC35A5
7.03731572657155E-50	0.446496752478213	0.229	0.144	1.17931336945886E-45	Ductular-like hepatocytes	SEC22A
8.93047306256999E-50	0.366459196334392	0.208	0.127	1.49656867582548E-45	Ductular-like hepatocytes	TBC1D24
9.36947453258346E-50	0.429820953209546	0.144	0.079	1.57013654217034E-45	Ductular-like hepatocytes	ZNF317
1.03696702560516E-49	0.525180159008653	0.262	0.171	1.73774934150912E-45	Ductular-like hepatocytes	COG2
1.09757126810668E-49	0.368133130876276	0.187	0.111	1.83930993109318E-45	Ductular-like hepatocytes	AIDA
1.33034058865793E-49	0.375042422418548	0.209	0.128	2.22938475847296E-45	Ductular-like hepatocytes	POLA2
1.40521107961766E-49	0.328730223439521	0.243	0.156	2.35485272722328E-45	Ductular-like hepatocytes	IREB2
1.4110658488751E-49	0.274770078312541	0.3	0.201	2.3646641495449E-45	Ductular-like hepatocytes	SRP68
1.6135916305125E-49	0.32962100960757	0.173	0.1	2.70405685441284E-45	Ductular-like hepatocytes	TBC1D2
1.61412356850139E-49	0.327498334343863	0.258	0.167	2.70494827609462E-45	Ductular-like hepatocytes	ZNF75A
1.62673223669056E-49	0.503481148035832	0.153	0.086	2.72607788224604E-45	Ductular-like hepatocytes	NIFK
1.78362794886327E-49	0.273467099597959	0.111	0.054	2.98900371670508E-45	Ductular-like hepatocytes	SORT1
1.98638592177418E-49	0.307218555078499	0.193	0.115	3.32878552770917E-45	Ductular-like hepatocytes	RIPK2
1.99724670382332E-49	0.275092318610381	0.292	0.194	3.34698602626711E-45	Ductular-like hepatocytes	BRD7
2.05719779860781E-49	0.294879331107764	0.164	0.093	3.44745207090697E-45	Ductular-like hepatocytes	DAGLB
2.16007460921549E-49	0.33017140956145	0.198	0.119	3.61985303012332E-45	Ductular-like hepatocytes	NR2C2AP
2.16688004952386E-49	0.37507643046638	0.155	0.087	3.63125758699208E-45	Ductular-like hepatocytes	RALB
2.20866984545102E-49	0.42204276722888	0.158	0.089	3.70128892700682E-45	Ductular-like hepatocytes	DPM2
2.41830013600553E-49	0.934743676750315	0.11	0.055	4.05258736791806E-45	Ductular-like hepatocytes	MAN1C1
2.78333803041322E-49	0.379317123025652	0.223	0.14	4.66431787136647E-45	Ductular-like hepatocytes	NDUFB3
2.85161349683513E-49	0.659954240000899	0.119	0.061	4.77873389799631E-45	Ductular-like hepatocytes	UTP14A
3.03177636126539E-49	0.280534975721009	0.252	0.163	5.08065082620853E-45	Ductular-like hepatocytes	NUDT14
3.12741335985145E-49	0.340578049952791	0.259	0.169	5.24091930843907E-45	Ductular-like hepatocytes	CUL5
3.64812219281072E-49	0.282530674736533	0.278	0.183	6.11352317071221E-45	Ductular-like hepatocytes	TAGLN2
3.87140648352559E-49	0.368976627259265	0.193	0.116	6.48770298509218E-45	Ductular-like hepatocytes	NFXL1
4.46238414266151E-49	0.501972984920053	0.18	0.107	7.47806334627216E-45	Ductular-like hepatocytes	PAIP2
4.5815010070256E-49	0.252790688182962	0.18	0.106	7.67767938757349E-45	Ductular-like hepatocytes	AP002495.1
4.70930964911405E-49	0.399406940098463	0.167	0.096	7.89186110998533E-45	Ductular-like hepatocytes	RCHY1
4.84178772112386E-49	0.648905606287773	0.164	0.095	8.11386786305936E-45	Ductular-like hepatocytes	SENP7
5.45822585123101E-49	0.3755937059738	0.174	0.102	9.14689488149293E-45	Ductular-like hepatocytes	JOSD2
5.97863515461629E-49	0.298074693188578	0.203	0.124	1.0018996792106E-44	Ductular-like hepatocytes	MEIS2
6.97117983282841E-49	0.530765969766965	0.159	0.091	1.16823031638538E-44	Ductular-like hepatocytes	PLGRKT

7.12362199229429E-49	0.407856735248733	0.177	0.104	1.19377657346868E-44	Ductular-like hepatocytes	NDUFA12
7.79971358360678E-49	0.40248582641693	0.22	0.138	1.30707600234082E-44	Ductular-like hepatocytes	AGBL5
8.79342315120667E-49	0.499473187196323	0.186	0.111	1.47360185167921E-44	Ductular-like hepatocytes	RAE1
9.0608110296321E-49	0.29695644221721	0.263	0.172	1.51841071234575E-44	Ductular-like hepatocytes	MAGOH
9.4344960766605E-49	0.637409024162765	0.121	0.062	1.58103285252677E-44	Ductular-like hepatocytes	RNF2
9.49376743635819E-49	0.345939364092554	0.219	0.137	1.59096554698491E-44	Ductular-like hepatocytes	BRIX1
9.95421783184718E-49	0.281547447117984	0.291	0.194	1.66812782426095E-44	Ductular-like hepatocytes	MAN1A2
1.02470921955352E-48	0.52717931498485	0.148	0.083	1.71720771012779E-44	Ductular-like hepatocytes	CINP
1.03520835848499E-48	0.25263371141802	0.145	0.08	1.73480216714914E-44	Ductular-like hepatocytes	DGKH
1.31800812825783E-48	0.835884795204624	0.115	0.059	2.20871802133447E-44	Ductular-like hepatocytes	TECPR2
1.49122304054573E-48	0.253251424962359	0.228	0.143	2.49899157134654E-44	Ductular-like hepatocytes	SLC10A7
1.50974652179374E-48	0.489302714234447	0.109	0.053	2.53003322122195E-44	Ductular-like hepatocytes	ALKBH4
1.51072874621043E-48	0.255052524504833	0.331	0.228	2.53167923289943E-44	Ductular-like hepatocytes	GNAI3
1.52287819746582E-48	0.285432150993137	0.246	0.157	2.55203928331322E-44	Ductular-like hepatocytes	NSD2
1.67413235445209E-48	0.316207249222581	0.217	0.135	2.80551099959082E-44	Ductular-like hepatocytes	ASNSD1
1.70846904698267E-48	0.473229358934841	0.184	0.111	2.86305242893356E-44	Ductular-like hepatocytes	RCC2
1.72837789001758E-48	0.250780800176611	0.224	0.141	2.89641566809146E-44	Ductular-like hepatocytes	UBAC1
1.73468966392345E-48	0.567792105229249	0.142	0.078	2.90699293880291E-44	Ductular-like hepatocytes	HOMEZ
1.75628692978015E-48	0.290751532885919	0.244	0.157	2.94318563692558E-44	Ductular-like hepatocytes	NUP54
1.80188264407284E-48	0.472770163844284	0.118	0.061	3.01959493493727E-44	Ductular-like hepatocytes	ZNF627
1.91706979643908E-48	0.255999904270205	0.201	0.122	3.2126255648726E-44	Ductular-like hepatocytes	PPIE
1.91969434654115E-48	0.513309439166886	0.15	0.084	3.21702378593366E-44	Ductular-like hepatocytes	TP73
1.93453291092928E-48	0.50694413247123	0.149	0.083	3.24189025213529E-44	Ductular-like hepatocytes	RP2
1.94326754968006E-48	0.430534329909662	0.248	0.162	3.25652775975384E-44	Ductular-like hepatocytes	SPHK1
2.21041748317297E-48	0.491197814824746	0.19	0.115	3.70421761830126E-44	Ductular-like hepatocytes	FEM1A
2.26097693513327E-48	0.322003924037484	0.168	0.097	3.78894514789634E-44	Ductular-like hepatocytes	NUDT12
2.37287496757535E-48	0.501847721332458	0.19	0.115	3.97646387066277E-44	Ductular-like hepatocytes	TUBGCP4
2.66921184027646E-48	0.431059774716513	0.134	0.072	4.4730652019353E-44	Ductular-like hepatocytes	DGKZ
2.93566136542335E-48	0.484429085318042	0.116	0.059	4.91958131617646E-44	Ductular-like hepatocytes	NKRF
3.72142290905676E-48	0.358608725080578	0.153	0.086	6.23636051099732E-44	Ductular-like hepatocytes	URB1
3.9232391351284E-48	0.253988115842377	0.176	0.103	6.57456414264818E-44	Ductular-like hepatocytes	SLC35E2A
4.02824960017324E-48	0.344791853549116	0.24	0.153	6.75054067997032E-44	Ductular-like hepatocytes	PCIF1
4.33328474511225E-48	0.288921099503041	0.208	0.128	7.26171857585911E-44	Ductular-like hepatocytes	SRSF1
4.50531234927432E-48	0.465623388562863	0.159	0.091	7.55000243491391E-44	Ductular-like hepatocytes	OPN3
4.66268904538935E-48	0.439749546626403	0.155	0.087	7.81373430226347E-44	Ductular-like hepatocytes	JRKL
4.77798637306558E-48	0.284186577188188	0.224	0.14	8.00694956398329E-44	Ductular-like hepatocytes	TTC33
4.7827592056123E-48	0.37646284718156	0.189	0.114	8.01494787676509E-44	Ductular-like hepatocytes	L3MBTL2
4.91896179012716E-48	0.526680481654954	0.136	0.074	8.24319616789509E-44	Ductular-like hepatocytes	EIF3E
4.97261103443909E-48	0.300514324137512	0.229	0.144	8.33310157151303E-44	Ductular-like hepatocytes	COMMD2
5.01882828760211E-48	0.258310372722907	0.161	0.091	8.41055244436362E-44	Ductular-like hepatocytes	DEPRECATEI
5.2775244936599E-48	0.274102229773259	0.236	0.151	8.84407554647525E-44	Ductular-like hepatocytes	FBXO3
5.92888100492975E-48	0.348362754370064	0.19	0.115	9.93561878806127E-44	Ductular-like hepatocytes	HOOK2
6.02378648650527E-48	0.353316431561678	0.192	0.117	1.00946613940855E-43	Ductular-like hepatocytes	CSNK1G3
6.22416015900796E-48	0.396601433891791	0.181	0.108	1.04304475944655E-43	Ductular-like hepatocytes	FCF1
7.04944041219362E-48	0.42747738784273	0.192	0.116	1.18134522427541E-43	Ductular-like hepatocytes	MX1
7.23626632302304E-48	0.508433957937552	0.152	0.086	1.2126535104122E-43	Ductular-like hepatocytes	TOGARAM1
7.52437068371668E-48	0.397621026794808	0.199	0.122	1.26093403917724E-43	Ductular-like hepatocytes	PPIL4
7.88225435768175E-48	0.537623208126893	0.122	0.064	1.32090818526031E-43	Ductular-like hepatocytes	ZBTB42
7.93410111499494E-48	0.308531031464866	0.156	0.088	1.32959666485085E-43	Ductular-like hepatocytes	CAPS
8.29272033928228E-48	0.481916929723659	0.108	0.054	1.38969407445692E-43	Ductular-like hepatocytes	ZNF212
9.82992329102895E-48	0.452777322409961	0.172	0.101	1.64729854511063E-43	Ductular-like hepatocytes	LIG4
1.1431259267258E-47	0.659616947215288	0.15	0.085	1.91565042800709E-43	Ductular-like hepatocytes	CGRRF1
1.23087274673856E-47	0.345997697265252	0.22	0.138	2.06269654898448E-43	Ductular-like hepatocytes	MKKS
1.31068604614174E-47	0.526219929363132	0.179	0.107	2.19644767612432E-43	Ductular-like hepatocytes	ADGRG2
1.68652442948987E-47	0.368669489978289	0.181	0.108	2.82627763893913E-43	Ductular-like hepatocytes	IFFO1
1.86727975560363E-47	0.310514078948652	0.206	0.127	3.12918741444057E-43	Ductular-like hepatocytes	ADPRHL2
2.09365631702225E-47	0.825293921142563	0.11	0.056	3.50854925606589E-43	Ductular-like hepatocytes	COMMD8
2.09781039113747E-47	0.510124179545716	0.139	0.076	3.51551065346818E-43	Ductular-like hepatocytes	PTER
2.10559123564637E-47	0.465129660777986	0.161	0.093	3.52854979269619E-43	Ductular-like hepatocytes	B3GALNT2
2.16921386621865E-47	0.255237353650433	0.25	0.161	3.63516859700921E-43	Ductular-like hepatocytes	PPHLN1

2.31390862540841E-47	0.308846456956792	0.172	0.101	3.87764807445942E-43	Ductular-like hepatocytes	ABHD4
2.33585079784223E-47	0.410990411582574	0.153	0.087	3.91441876702401E-43	Ductular-like hepatocytes	DDHD1
2.45491732273909E-47	0.337282979149663	0.173	0.102	4.11395044944617E-43	Ductular-like hepatocytes	SNX8
2.52509878866116E-47	0.405472620847732	0.181	0.108	4.23156055003837E-43	Ductular-like hepatocytes	HELZ2
2.77923723420003E-47	0.353713152881247	0.203	0.126	4.6574457570724E-43	Ductular-like hepatocytes	AP5S1
3.04293414190891E-47	0.523195158729203	0.124	0.065	5.09934903501095E-43	Ductular-like hepatocytes	PLEK2
3.30401176050113E-47	0.627714034983466	0.188	0.114	5.5368629082478E-43	Ductular-like hepatocytes	CCL20
4.63142964985314E-47	0.292284673873768	0.222	0.14	7.7613498072239E-43	Ductular-like hepatocytes	ZFX
4.97439122014192E-47	0.582423162861909	0.135	0.074	8.33608480671382E-43	Ductular-like hepatocytes	MCU
5.47720872028365E-47	0.371843404632219	0.137	0.075	9.17870637345134E-43	Ductular-like hepatocytes	QTRT2
5.49686558878597E-47	0.299972306506992	0.223	0.141	9.21164735368753E-43	Ductular-like hepatocytes	RGP1
5.94013764561194E-47	0.439477784160886	0.187	0.113	9.95448266651649E-43	Ductular-like hepatocytes	TMEM101
6.01547108070267E-47	0.311448080366071	0.182	0.108	1.00807264370415E-42	Ductular-like hepatocytes	ZFP41
6.08744216178883E-47	0.31050469062372	0.242	0.156	1.02013355747257E-42	Ductular-like hepatocytes	MZT2A
6.2848928937359E-47	0.404012077254926	0.165	0.096	1.05322235113226E-42	Ductular-like hepatocytes	OPA3
6.39615511763153E-47	0.473323460550053	0.158	0.091	1.07186767461269E-42	Ductular-like hepatocytes	ALG14
6.56986441686434E-47	0.617135379167151	0.115	0.06	1.10097787897813E-42	Ductular-like hepatocytes	RTN4R
7.35864189546863E-47	0.683337541064672	0.1	0.049	1.23316120884263E-42	Ductular-like hepatocytes	TOR2A
8.47124002642418E-47	0.282909055431692	0.301	0.204	1.41961040362816E-42	Ductular-like hepatocytes	GCH1
9.69232567878058E-47	0.358160368263256	0.215	0.136	1.62423993725005E-42	Ductular-like hepatocytes	DIP2C
9.87593449128755E-47	0.595390972682539	0.137	0.076	1.65500910204997E-42	Ductular-like hepatocytes	ZBTB14
1.03969220952035E-46	0.612807954218365	0.126	0.067	1.7423162047142E-42	Ductular-like hepatocytes	CDKN2AIP
1.05693094272806E-46	0.479383195000658	0.163	0.095	1.77120487382368E-42	Ductular-like hepatocytes	ZNF322
1.07933755836652E-46	0.315906060889595	0.188	0.114	1.80875388031062E-42	Ductular-like hepatocytes	EXOC1
1.1018950377479E-46	0.770719848600302	0.116	0.06	1.84655570425793E-42	Ductular-like hepatocytes	PGAM1
1.2969303894814E-46	0.261776747267898	0.23	0.147	2.17339594669293E-42	Ductular-like hepatocytes	ZFP62
1.36230374630915E-46	0.315180963315003	0.207	0.128	2.28294861806487E-42	Ductular-like hepatocytes	TARS2
1.38607039232205E-46	0.253036109719744	0.242	0.156	2.32277676345329E-42	Ductular-like hepatocytes	DNM1L
1.41320227659519E-46	0.439211635003769	0.107	0.053	2.36824437511822E-42	Ductular-like hepatocytes	CABLES1
1.57183462951154E-46	0.419906428325548	0.16	0.093	2.63408047213543E-42	Ductular-like hepatocytes	GPR89A
1.58325379662061E-46	0.428412136053762	0.221	0.141	2.65321671237682E-42	Ductular-like hepatocytes	PIAS4
1.63533652274416E-46	0.274377224602567	0.2	0.122	2.74049694481467E-42	Ductular-like hepatocytes	CDK8
1.65464631811967E-46	0.504292574873668	0.211	0.133	2.77285629990494E-42	Ductular-like hepatocytes	KPNB1
1.66173673730456E-46	0.421516825802627	0.208	0.13	2.78473842437498E-42	Ductular-like hepatocytes	TRAPPC11
1.7196350654152E-46	0.872285403368948	0.149	0.085	2.88176444262279E-42	Ductular-like hepatocytes	FMO1
1.96338517633808E-46	0.477258581612759	0.156	0.09	3.29024087850736E-42	Ductular-like hepatocytes	POP1
2.11162962124554E-46	0.287209685519763	0.15	0.084	3.53866891928328E-42	Ductular-like hepatocytes	RGMB
2.24857656958058E-46	0.286103510904189	0.247	0.161	3.76816461530314E-42	Ductular-like hepatocytes	HIBCH
2.31352959491053E-46	0.281159672658529	0.18	0.107	3.87701289515106E-42	Ductular-like hepatocytes	TOMM40
2.43240383390009E-46	0.283951963503584	0.207	0.128	4.07622234484976E-42	Ductular-like hepatocytes	RABEP1
2.58961224524186E-46	0.962590467890334	0.127	0.068	4.33967220057631E-42	Ductular-like hepatocytes	CXCL9
2.80420131381524E-46	0.464499525225258	0.198	0.122	4.69928056169158E-42	Ductular-like hepatocytes	TAF5L
3.0144662385236E-46	0.792554192854284	0.126	0.068	5.05164252251784E-42	Ductular-like hepatocytes	C8orf37
3.11557236251089E-46	0.300058009793004	0.196	0.12	5.22107616509575E-42	Ductular-like hepatocytes	FBXO34
3.12158177649442E-46	0.597975455954731	0.123	0.065	5.23114674104934E-42	Ductular-like hepatocytes	ANKRD16
3.30558222569977E-46	0.418319863347844	0.144	0.081	5.53949469382767E-42	Ductular-like hepatocytes	ARPC3
3.35203006224543E-46	0.419955827402924	0.162	0.094	5.61733197831089E-42	Ductular-like hepatocytes	PFDN1
3.44307750149019E-46	0.353745063003892	0.215	0.135	5.76990927699726E-42	Ductular-like hepatocytes	ESCO1
3.48381405297527E-46	0.555666532334245	0.149	0.085	5.83817558997596E-42	Ductular-like hepatocytes	GGPS1
3.55277632537315E-46	0.474930378605544	0.1	0.049	5.95374256606032E-42	Ductular-like hepatocytes	DCAF4
3.96674796743482E-46	0.398811315414532	0.16	0.093	6.64747624382727E-42	Ductular-like hepatocytes	PEX5
4.35140796063418E-46	0.266937091981038	0.243	0.157	7.29208946043076E-42	Ductular-like hepatocytes	TRIM33
4.44892611909704E-46	0.32665062157217	0.183	0.11	7.45551039038282E-42	Ductular-like hepatocytes	RIOK2
4.61401748883177E-46	0.621804461490022	0.114	0.059	7.73217050778428E-42	Ductular-like hepatocytes	SERAC1
6.18581158612319E-46	0.424530705037172	0.159	0.092	1.03661830560252E-41	Ductular-like hepatocytes	ZBTB45
6.35129476442275E-46	0.254499435141401	0.197	0.121	1.06434997662196E-41	Ductular-like hepatocytes	PARD3B
6.90257732679869E-46	0.336766677388853	0.223	0.142	1.15673390842492E-41	Ductular-like hepatocytes	RECQL
7.04040153102946E-46	0.424530011070319	0.169	0.1	1.17983048856992E-41	Ductular-like hepatocytes	VKORC1L1
8.89512334040587E-46	0.263676025292075	0.239	0.154	1.49064476938522E-41	Ductular-like hepatocytes	FBXO38
9.38100865719121E-46	0.269407796374331	0.184	0.111	1.5720694307721E-41	Ductular-like hepatocytes	PKN3

1.01846701531123E-45	0.273124417275204	0.219	0.138	1.70674702425857E-41	Ductular-like hepatocytes	ZNF32
1.12070216680952E-45	0.351487307316203	0.179	0.107	1.87807269113939E-41	Ductular-like hepatocytes	RRP15
1.20219629898592E-45	0.52405127962348	0.164	0.097	2.01464055784061E-41	Ductular-like hepatocytes	NKIRAS2
1.2711185656931E-45	0.272662979249462	0.165	0.096	2.13014049238849E-41	Ductular-like hepatocytes	POLR1A
1.29675170623008E-45	0.333093498074706	0.246	0.161	2.17309650930036E-41	Ductular-like hepatocytes	PIGM
1.30348118471095E-45	0.292005848433163	0.192	0.118	2.18437376933861E-41	Ductular-like hepatocytes	HCFC2
1.45219216339218E-45	0.572897147677737	0.123	0.065	2.43358362741261E-41	Ductular-like hepatocytes	TIMM22
1.49935264490613E-45	0.397886364323734	0.208	0.131	2.51261516233369E-41	Ductular-like hepatocytes	PARD6B
1.77073830207851E-45	0.291788030617108	0.174	0.104	2.96740324662316E-41	Ductular-like hepatocytes	COX15
1.87288358563859E-45	0.539424586217449	0.108	0.055	3.13857831281315E-41	Ductular-like hepatocytes	SARM1
2.00223936700103E-45	0.392390484305203	0.203	0.127	3.35535273122033E-41	Ductular-like hepatocytes	EXOSC6
2.03600682190732E-45	0.601637616936899	0.104	0.052	3.41194023215229E-41	Ductular-like hepatocytes	C2CD2L
2.0427296989317E-45	0.3431073065315	0.229	0.147	3.42320642946974E-41	Ductular-like hepatocytes	BTBD1
2.08258768802791E-45	0.482611414713911	0.221	0.141	3.49000044759717E-41	Ductular-like hepatocytes	SRGAP1
2.22874407692938E-45	0.442697380993021	0.136	0.075	3.73492932411826E-41	Ductular-like hepatocytes	PLEKHA8
2.55728202113044E-45	0.253780895249987	0.214	0.135	4.28549321101039E-41	Ductular-like hepatocytes	SEPTIN10
2.58834528070108E-45	0.458566136259701	0.148	0.084	4.33754902139887E-41	Ductular-like hepatocytes	FAM122C
2.74353064783263E-45	0.457943783898204	0.229	0.149	4.59760865963793E-41	Ductular-like hepatocytes	FRY
2.79301566365778E-45	0.440772814308462	0.157	0.091	4.68053564915771E-41	Ductular-like hepatocytes	ASH2L
2.8655071993577E-45	0.571743899936957	0.165	0.098	4.80201696468364E-41	Ductular-like hepatocytes	CNEP1R1
2.86927984583854E-45	0.530075861521006	0.145	0.082	4.80833916565623E-41	Ductular-like hepatocytes	ZNF557
2.98487005480824E-45	0.44062126383953	0.195	0.12	5.00204523784765E-41	Ductular-like hepatocytes	UBE2K
3.71610968716292E-45	0.403827335357905	0.207	0.13	6.22745661374762E-41	Ductular-like hepatocytes	FBXW11
3.82416355522786E-45	0.2968325605739	0.165	0.096	6.40853328585085E-41	Ductular-like hepatocytes	ARL 3.00
3.91648966881389E-45	0.349788764937044	0.234	0.151	6.56325338699831E-41	Ductular-like hepatocytes	IRF2BP1
3.92980744579564E-45	0.355825123035477	0.173	0.103	6.58557131766433E-41	Ductular-like hepatocytes	ZNF12
4.07901068325638E-45	0.403523977093996	0.163	0.096	6.83560610300105E-41	Ductular-like hepatocytes	FAM120C
4.08635094667689E-45	0.251539199671251	0.204	0.126	6.84790691644113E-41	Ductular-like hepatocytes	MAML3
4.72807851904375E-45	0.666602301675299	0.144	0.081	7.92331398221351E-41	Ductular-like hepatocytes	TMEM104
5.26108774501183E-45	0.407766204119591	0.164	0.096	8.81653084309083E-41	Ductular-like hepatocytes	ZBTB4
6.17516393208854E-45	0.595581584885409	0.16	0.094	1.0348339717394E-40	Ductular-like hepatocytes	DEPRECATEI
6.52623395298327E-45	0.440029035271575	0.193	0.12	1.09366628584094E-40	Ductular-like hepatocytes	PPIL1
8.66979299448591E-45	0.505118541535846	0.166	0.099	1.45288391001595E-40	Ductular-like hepatocytes	PACSIN3
9.73758494632537E-45	0.281266077331135	0.142	0.08	1.6318244853052E-40	Ductular-like hepatocytes	CAMTA2
1.14788825610626E-44	0.547066350750636	0.1	0.049	1.92363113958287E-40	Ductular-like hepatocytes	PLCD1
1.18852627171942E-44	0.839486151034254	0.107	0.055	1.99173232614741E-40	Ductular-like hepatocytes	SIX4
1.20276255205182E-44	0.464132651506545	0.134	0.074	2.01558948472843E-40	Ductular-like hepatocytes	RHPN1
1.26058950205758E-44	0.575484770909235	0.166	0.099	2.11249588754809E-40	Ductular-like hepatocytes	ALDH16A1
1.26095708711442E-44	0.416652951379814	0.195	0.121	2.11311188658635E-40	Ductular-like hepatocytes	SS18L2
1.27672707757226E-44	0.288983668791718	0.192	0.118	2.13953923659559E-40	Ductular-like hepatocytes	SELENOT
1.42021367774158E-44	0.300596844376141	0.204	0.128	2.37999408115934E-40	Ductular-like hepatocytes	FAN1
1.53677790619931E-44	0.506634600080152	0.161	0.094	2.5753324152088E-40	Ductular-like hepatocytes	ARNTL2
1.66761886709111E-44	0.311023940001277	0.189	0.116	2.79459569747128E-40	Ductular-like hepatocytes	ZNF318
1.67822073008835E-44	0.316651109381706	0.162	0.095	2.81236229948206E-40	Ductular-like hepatocytes	TTC19
1.90782799597716E-44	0.493163585524889	0.157	0.091	3.19713815565853E-40	Ductular-like hepatocytes	EID2
2.03885121988416E-44	0.305888662936	0.202	0.126	3.41670687428188E-40	Ductular-like hepatocytes	UBQLN1
2.38742226877715E-44	0.567120434018994	0.14	0.079	4.00084223801676E-40	Ductular-like hepatocytes	MUL1
2.65906866470002E-44	0.337234455891213	0.119	0.063	4.45606726830429E-40	Ductular-like hepatocytes	CCDC115
2.72886025406672E-44	0.27864107664614	0.183	0.112	4.57302401376501E-40	Ductular-like hepatocytes	RASA1
2.85411161320662E-44	0.285557724885608	0.174	0.104	4.78292024141165E-40	Ductular-like hepatocytes	MRTFB
3.01176533180757E-44	0.558668725681469	0.132	0.074	5.04711634304313E-40	Ductular-like hepatocytes	MDGA1
3.12388339514047E-44	0.524366110510627	0.121	0.064	5.23500379357639E-40	Ductular-like hepatocytes	SURF6
3.27533120616573E-44	0.70466590984072	0.146	0.084	5.48880003529253E-40	Ductular-like hepatocytes	PPM1K
3.33221423564774E-44	0.500934538407456	0.188	0.116	5.58412461609847E-40	Ductular-like hepatocytes	TMEM187
3.68588614842238E-44	0.269256910263082	0.209	0.132	6.17680800752622E-40	Ductular-like hepatocytes	ALYREF
3.7433466455747E-44	0.270091456742003	0.217	0.138	6.27310030865408E-40	Ductular-like hepatocytes	LAMTOR5
3.95489971205617E-44	0.437645551023658	0.169	0.101	6.62762093746373E-40	Ductular-like hepatocytes	TMEM256
3.97190166713069E-44	0.383333337367794	0.183	0.112	6.6561128137776E-40	Ductular-like hepatocytes	IRGQ
4.08789244157143E-44	0.257891723038413	0.187	0.115	6.8504901535854E-40	Ductular-like hepatocytes	FBXL18
4.60836644561504E-44	0.372692183498737	0.15	0.086	7.72270048956169E-40	Ductular-like hepatocytes	MYL12A

**4.65337468209246E-44**	0.358220381016609	0.166	0.099	7.79812529225054E-40	Ductular-like hepatocytes	CCT2
**5.28688181170297E-44**	0.304002630731076	0.249	0.163	8.85975654005183E-40	Ductular-like hepatocytes	MED30
**5.4620015824957E-44**	0.515941031199465	0.161	0.096	9.15322225194629E-40	Ductular-like hepatocytes	C15orf41
**5.78603909387376E-44**	0.262195207831096	0.197	0.122	9.69624431351365E-40	Ductular-like hepatocytes	GDE1
**5.7879463896111E-44**	0.343483012619871	0.173	0.105	9.69944055971029E-40	Ductular-like hepatocytes	RIC8A
**5.81363685111949E-44**	0.402318138337964	0.153	0.089	9.74249263510605E-40	Ductular-like hepatocytes	VGLL4
**6.23156450034084E-44**	0.86437990373647	0.119	0.064	1.04428557896712E-39	Ductular-like hepatocytes	DEPRECATEI
**7.12211998961921E-44**	0.413176181047103	0.209	0.133	1.19352486786039E-39	Ductular-like hepatocytes	FAM200B
**7.83747115259161E-44**	0.483567977031058	0.226	0.147	1.3134034157513E-39	Ductular-like hepatocytes	NOL11
**8.33848599522818E-44**	0.431178100941612	0.175	0.106	1.39736348308034E-39	Ductular-like hepatocytes	STK35
**8.97545281635979E-44**	0.433981989542253	0.203	0.128	1.50410638296557E-39	Ductular-like hepatocytes	ZNF700
**1.0274887273551E-43**	0.363492390890415	0.15	0.087	1.72186560930167E-39	Ductular-like hepatocytes	RCBTB1
**1.11649947844578E-43**	0.457221772813544	0.158	0.093	1.87102982597943E-39	Ductular-like hepatocytes	PHF20
**1.20607821159302E-43**	0.480934472431062	0.141	0.08	2.02114586698758E-39	Ductular-like hepatocytes	BMPR1A
**1.325180109965E-43**	0.451652918968226	0.159	0.094	2.22073682827934E-39	Ductular-like hepatocytes	RBIS
**1.3581998574392E-43**	0.556883517950861	0.132	0.073	2.27607132109662E-39	Ductular-like hepatocytes	MPP3
**1.37039252702749E-43**	0.459684331265927	0.14	0.08	2.29650379679266E-39	Ductular-like hepatocytes	USP39
**1.37642727044708E-43**	0.717563770581387	0.105	0.054	2.30661681981522E-39	Ductular-like hepatocytes	FKBP14
**1.53687982572961E-43**	0.711044538469263	0.102	0.052	2.57550321195768E-39	Ductular-like hepatocytes	ZNF786
**1.67186879912536E-43**	0.725663431287384	0.143	0.082	2.80171773357428E-39	Ductular-like hepatocytes	COX7A1
**1.75240742611675E-43**	0.358969767623144	0.169	0.101	2.93668436468645E-39	Ductular-like hepatocytes	BTN3A1
**1.75443918990827E-43**	0.312424977811437	0.171	0.102	2.94008919444828E-39	Ductular-like hepatocytes	USP30
**1.7739066393437E-43**	0.375548449934518	0.159	0.093	2.97271274621217E-39	Ductular-like hepatocytes	NHEJ1
**2.44106136019389E-43**	0.389746879022468	0.144	0.082	4.09073062741293E-39	Ductular-like hepatocytes	EMC6
**2.74452601890893E-43**	0.46114939774839	0.164	0.097	4.59927670248758E-39	Ductular-like hepatocytes	TRIM32
**2.78353941927818E-43**	0.332178733762743	0.183	0.112	4.66465535882637E-39	Ductular-like hepatocytes	THAP12
**2.91513405581846E-43**	0.370391785190054	0.262	0.177	4.88518165074058E-39	Ductular-like hepatocytes	TBC1D1
**2.97331916172806E-43**	0.362731461679249	0.177	0.107	4.98268825122389E-39	Ductular-like hepatocytes	ZNF414
**3.2016586532071E-43**	0.385033031970154	0.103	0.052	5.36533957104446E-39	Ductular-like hepatocytes	DEPRECATEI
**3.56065130221593E-43**	0.323853959314748	0.189	0.117	5.96693945225345E-39	Ductular-like hepatocytes	BLOC1S4
**3.59420264278762E-43**	0.346301157129808	0.196	0.122	6.02316478878349E-39	Ductular-like hepatocytes	DENND6A
**3.9293532789599E-43**	0.418146136317873	0.113	0.06	6.584810224881E-39	Ductular-like hepatocytes	ZDHHC21
**4.23440674683627E-43**	0.490108767223601	0.18	0.111	7.09601882634823E-39	Ductular-like hepatocytes	SMG9
**4.41412225349745E-43**	0.295317515505203	0.155	0.091	7.39718607241102E-39	Ductular-like hepatocytes	MIEF1
**4.50309262512882E-43**	0.272908005951094	0.182	0.111	7.54628262119087E-39	Ductular-like hepatocytes	ATMIN
**4.5205096136213E-43**	0.308968679835542	0.208	0.132	7.57547001050658E-39	Ductular-like hepatocytes	ST3GAL2
**4.88490020699314E-43**	0.273152881954727	0.181	0.11	8.1861157668791E-39	Ductular-like hepatocytes	RABGGTA
**4.93854335272566E-43**	0.27090555279389	0.182	0.111	8.27601095049767E-39	Ductular-like hepatocytes	METTL21A
**4.99793424776342E-43**	0.434688324646789	0.21	0.134	8.37553821240194E-39	Ductular-like hepatocytes	PIAS1
**5.03600287996549E-43**	0.356967294866068	0.174	0.105	8.43933362624617E-39	Ductular-like hepatocytes	CLASP2
**5.20013577661782E-43**	0.314530145704918	0.239	0.157	8.71438753445614E-39	Ductular-like hepatocytes	SEPTIN7
**5.35465046430934E-43**	0.371913972680175	0.164	0.097	8.97332324808959E-39	Ductular-like hepatocytes	SF3B6
**5.61156654345148E-43**	0.483033760706233	0.144	0.082	9.40386321351599E-39	Ductular-like hepatocytes	GNG5
**5.75822930578449E-43**	0.607501198098952	0.125	0.069	9.64964067063365E-39	Ductular-like hepatocytes	PSME3
**5.86624321580594E-43**	0.593186766633334	0.104	0.053	9.8306503810476E-39	Ductular-like hepatocytes	PIK3R3
**6.45887706718657E-43**	0.504065253022016	0.119	0.064	1.08237861891913E-38	Ductular-like hepatocytes	POMT2
**6.65641895541374E-43**	0.84795483151614	0.108	0.056	1.11548268854823E-38	Ductular-like hepatocytes	MIER2
**7.97814515460983E-43**	0.374008948588382	0.128	0.071	1.33697756500952E-38	Ductular-like hepatocytes	COPS9
**8.21365562534838E-43**	0.339631956345496	0.162	0.097	1.37644440969588E-38	Ductular-like hepatocytes	KMT5C
**8.34597697324836E-43**	0.256195566717575	0.201	0.126	1.39861882117696E-38	Ductular-like hepatocytes	ZNF622
**9.30645212977773E-43**	0.256408403295139	0.183	0.113	1.55957524790815E-38	Ductular-like hepatocytes	COG8
**9.8738601765719E-43**	0.407446963962962	0.19	0.118	1.65466148838992E-38	Ductular-like hepatocytes	ARHGAP21
**1.03178794549282E-42**	0.294330256014273	0.277	0.188	1.72907023905686E-38	Ductular-like hepatocytes	CEBPZOS
**1.16067166799852E-42**	0.300918893170848	0.208	0.133	1.94505358123193E-38	Ductular-like hepatocytes	RAPGEF4
**1.20625196352904E-42**	0.284682492982741	0.115	0.061	2.02143704048196E-38	Ductular-like hepatocytes	CRPPA
**1.50136093829213E-42**	0.654274667997918	0.128	0.071	2.51598066038995E-38	Ductular-like hepatocytes	TTC5
**1.64246124566771E-42**	0.264672163410297	0.204	0.129	2.75243655548994E-38	Ductular-like hepatocytes	EIF4EBP1
**1.68453378498822E-42**	0.53977857418625	0.115	0.061	2.82294171688325E-38	Ductular-like hepatocytes	ZDHHC7
**1.89728495997466E-42**	0.358016315965891	0.197	0.123	3.17947013592553E-38	Ductular-like hepatocytes	SMIM10L1
**1.99989149037304E-42**	0.38509888972038	0.176	0.108	3.35141815956714E-38	Ductular-like hepatocytes	ANKRA2

**2.05465888636067E-42**	0.389023200523012	0.202	0.128	3.4431973617632E-38	Ductular-like hepatocytes	TSG101
**2.05545838591858E-42**	0.262952291926683	0.211	0.135	3.44453716312235E-38	Ductular-like hepatocytes	TMEM192
**2.1027569071269E-42**	0.280860381134226	0.178	0.109	3.52380002496325E-38	Ductular-like hepatocytes	RUSC2
**2.17086489652682E-42**	0.270444167887755	0.17	0.102	3.63793539359965E-38	Ductular-like hepatocytes	ZDHHC17
**2.20831253371566E-42**	0.515551908150082	0.147	0.086	3.7006901440007E-38	Ductular-like hepatocytes	KCTD2
**2.22343921341912E-42**	0.559421365283534	0.118	0.063	3.72603943384776E-38	Ductular-like hepatocytes	INPP5K
**2.56107732064022E-42**	0.263952429531966	0.182	0.111	4.29185337392887E-38	Ductular-like hepatocytes	C5orf22
**2.61931246765864E-42**	0.309028962447355	0.188	0.116	4.38944383330235E-38	Ductular-like hepatocytes	BICRA
**2.6313099063753E-42**	0.590751836498186	0.204	0.13	4.40954914110372E-38	Ductular-like hepatocytes	COQ2
**2.85868416947893E-42**	0.250550143930708	0.199	0.125	4.79058293121278E-38	Ductular-like hepatocytes	STAM2
**3.70566935422464E-42**	0.437937848684951	0.139	0.079	6.20996070380965E-38	Ductular-like hepatocytes	SCRN3
**3.79230283187107E-42**	0.57810110120708	0.186	0.117	6.35514108564954E-38	Ductular-like hepatocytes	PRKACB
**3.85304224734744E-42**	0.525550397033718	0.157	0.093	6.45692819810484E-38	Ductular-like hepatocytes	SAYSD1
**3.96992449652346E-42**	0.287136211901893	0.164	0.098	6.65279947127401E-38	Ductular-like hepatocytes	SAMM50
**4.15624670987976E-42**	0.456208413525563	0.138	0.079	6.9650382364165E-38	Ductular-like hepatocytes	CELSR1
**4.4977557845118E-42**	0.688725122416273	0.133	0.075	7.53733914368488E-38	Ductular-like hepatocytes	EIF2B2
**4.84021234108411E-42**	0.465367320443114	0.142	0.082	8.11122784118876E-38	Ductular-like hepatocytes	C15orf39
**5.32644876728147E-42**	0.407084190106706	0.127	0.07	8.92606284421029E-38	Ductular-like hepatocytes	C1QBP
**5.59958017097038E-42**	0.439894557746833	0.112	0.06	9.38377645051217E-38	Ductular-like hepatocytes	PASK
**5.82973645662679E-42**	0.447399185133945	0.164	0.098	9.76947235401517E-38	Ductular-like hepatocytes	UFSP2
**5.87698875260864E-42**	0.429910202359475	0.123	0.068	9.84865775162156E-38	Ductular-like hepatocytes	HERC6
**6.26827272135518E-42**	0.348625705949701	0.132	0.074	1.0504371426447E-37	Ductular-like hepatocytes	GPR89B
**6.51428129785356E-42**	0.256391753140836	0.197	0.124	1.0916632598943E-37	Ductular-like hepatocytes	TFDP2
**6.83636504517461E-42**	0.63731414348465	0.102	0.052	1.14563805427036E-37	Ductular-like hepatocytes	SNX25
**7.06206431861445E-42**	0.306054885544414	0.212	0.136	1.18346073851341E-37	Ductular-like hepatocytes	ATE1
**7.10043712213335E-42**	0.285826234615474	0.144	0.083	1.18989125292711E-37	Ductular-like hepatocytes	TGFBRAP1
**7.15747238140211E-42**	0.372125699217508	0.151	0.089	1.19944922167537E-37	Ductular-like hepatocytes	CBX7
**7.53183727579699E-42**	0.523431182443711	0.148	0.087	1.26218529067806E-37	Ductular-like hepatocytes	PAFAH1B3
**8.790490075339E-42**	0.411671811617065	0.145	0.084	1.47311032682531E-37	Ductular-like hepatocytes	KDM1B
**9.46943495357679E-42**	0.382329617256844	0.216	0.14	1.5868879095204E-37	Ductular-like hepatocytes	AC253572.1
**9.64948979800934E-42**	0.294415032337071	0.21	0.135	1.6170615003504E-37	Ductular-like hepatocytes	CLOCK
**1.0049942756764E-41**	0.489368379338778	0.133	0.075	1.68416940717851E-37	Ductular-like hepatocytes	DHX33
**1.05896032722682E-41**	0.52826046117988	0.135	0.077	1.7746057163667E-37	Ductular-like hepatocytes	RBBP8
**1.1243492392448E-41**	0.254234534030729	0.993	0.983	1.88418445512644E-37	Ductular-like hepatocytes	VTN
**1.18199815411521E-41**	0.440159033030967	0.194	0.123	1.98079250666628E-37	Ductular-like hepatocytes	SIMC1
**1.32154846208603E-41**	0.262767270091677	0.157	0.093	2.21465091276377E-37	Ductular-like hepatocytes	SOWAHC
**1.36467684612024E-41**	0.325101689527426	0.146	0.085	2.28692545872829E-37	Ductular-like hepatocytes	CUL9
**1.38952925925247E-41**	0.330876143418453	0.147	0.085	2.3285731326553E-37	Ductular-like hepatocytes	ZC3H6
**1.72187814618066E-41**	0.343968400944426	0.169	0.102	2.88552339736954E-37	Ductular-like hepatocytes	OSGIN2
**1.88919486770803E-41**	0.262886376756556	0.176	0.108	3.16591275930512E-37	Ductular-like hepatocytes	KRAS
**2.0480340498274E-41**	0.371116737989919	0.151	0.089	3.43209546070076E-37	Ductular-like hepatocytes	CYP2R1
**2.08051212553382E-41**	0.377941813482832	0.209	0.134	3.48652221996958E-37	Ductular-like hepatocytes	TMEM18
**2.08434238806689E-41**	0.365515493213393	0.172	0.105	3.4929409739225E-37	Ductular-like hepatocytes	TRAPPC13
**2.14208100086854E-41**	0.363430573546672	0.163	0.098	3.58969934125551E-37	Ductular-like hepatocytes	PRADC1
**2.47359818802279E-41**	0.278352937943094	0.128	0.07	4.1452558434886E-37	Ductular-like hepatocytes	AQP11
**2.53659237746321E-41**	0.746000057342048	0.1	0.051	4.25082150615285E-37	Ductular-like hepatocytes	ILDR2
**2.57478351690701E-41**	0.295147504748635	0.229	0.15	4.31482221763277E-37	Ductular-like hepatocytes	TTLL12
**2.7658495998336E-41**	0.323563515048672	0.178	0.109	4.63501075940114E-37	Ductular-like hepatocytes	LIAS
**2.90573390364955E-41**	0.287423001104298	0.127	0.071	4.86942887573591E-37	Ductular-like hepatocytes	EDRF1
**2.9091522160327E-41**	0.295397208380613	0.349	0.248	4.87515728362761E-37	Ductular-like hepatocytes	PYGL
**3.16062064470761E-41**	0.591412499327621	0.123	0.068	5.29656807640102E-37	Ductular-like hepatocytes	SACS
**3.46966638482062E-41**	0.619108680810582	0.162	0.098	5.81446692768239E-37	Ductular-like hepatocytes	DOCK10
**3.68553619170088E-41**	0.721345694213807	0.158	0.095	6.17622155005234E-37	Ductular-like hepatocytes	ZXDB
**3.78447481530855E-41**	0.265056705488117	0.153	0.09	6.34202289549407E-37	Ductular-like hepatocytes	BCLAF3
**3.90706288456095E-41**	0.444850335074862	0.138	0.079	6.54745598194723E-37	Ductular-like hepatocytes	SPSB2
**4.21676961781038E-41**	0.378989164411521	0.151	0.089	7.06646252552664E-37	Ductular-like hepatocytes	JAK2
**4.23899197465761E-41**	0.34163588659502	0.17	0.103	7.10370275113122E-37	Ductular-like hepatocytes	VAMP4
**4.35696530749283E-41**	0.566592336282171	0.121	0.066	7.30140246229649E-37	Ductular-like hepatocytes	TIRAP
**4.41191699569561E-41**	0.561260018118672	0.102	0.053	7.39349050138671E-37	Ductular-like hepatocytes	ZNF213
**4.83313671883429E-41**	0.677277008878663	0.119	0.065	8.0993705134225E-37	Ductular-like hepatocytes	DEPRECATEI

6.24909137440995E-41	0.635587580241862	0.126	0.071	1.04722273252362E-36	Ductular-like hepatocytes	BICC1
7.6224257720631E-41	0.282933122111756	0.194	0.122	1.27736611088233E-36	Ductular-like hepatocytes	KRT10
7.78494809682038E-41	0.508178909016522	0.116	0.063	1.30460160206516E-36	Ductular-like hepatocytes	DEPRECATEI
8.66118682492961E-41	0.286683938376454	0.167	0.101	1.4514416881217E-36	Ductular-like hepatocytes	PUS7L
9.3517067735946E-41	0.498006167982321	0.153	0.091	1.56715902111898E-36	Ductular-like hepatocytes	SESN1
9.358681130418E-41	0.331864224962304	0.166	0.1	1.56832778383545E-36	Ductular-like hepatocytes	JPT1
9.6651352067123E-41	0.321516202005566	0.244	0.163	1.61968335794085E-36	Ductular-like hepatocytes	ITGB1BP1
1.01454281026581E-40	0.454278745983394	0.125	0.07	1.70017084144344E-36	Ductular-like hepatocytes	BMP2K
1.0711373147026E-40	0.466641564050699	0.207	0.134	1.79501191197862E-36	Ductular-like hepatocytes	CHML
1.08186709161423E-40	0.339257844409284	0.15	0.088	1.81299287212713E-36	Ductular-like hepatocytes	SERTAD3
1.22199804035435E-40	0.369802327194088	0.19	0.12	2.04782431602583E-36	Ductular-like hepatocytes	ITGB3BP
1.35055063835599E-40	0.320896922407969	0.167	0.102	2.26325275975697E-36	Ductular-like hepatocytes	SCOC
1.43442132132505E-40	0.250108486035932	0.13	0.073	2.40380325027652E-36	Ductular-like hepatocytes	SGPL1
1.50567613647104E-40	0.323770911879295	0.201	0.128	2.52321206949817E-36	Ductular-like hepatocytes	TAF12
1.59973114067276E-40	0.272164180330214	0.154	0.092	2.68082944553941E-36	Ductular-like hepatocytes	TMEM218
1.75945970143107E-40	0.299784301297501	0.168	0.102	2.94850256765818E-36	Ductular-like hepatocytes	ATG10
1.79438205602972E-40	0.371256209737901	0.155	0.092	3.0070254494946E-36	Ductular-like hepatocytes	LCMT1
1.80952684990678E-40	0.447151154779513	0.143	0.084	3.03240509507378E-36	Ductular-like hepatocytes	UHRF1BP1
1.93111150898496E-40	0.433265160158316	0.129	0.073	3.23615666675699E-36	Ductular-like hepatocytes	TDG
2.10387681358624E-40	0.776215831836041	0.113	0.062	3.52567676420783E-36	Ductular-like hepatocytes	ZC3H12C
2.22579869127374E-40	0.4210403252337	0.156	0.093	3.72999344683653E-36	Ductular-like hepatocytes	ZFP90
2.2276730327506E-40	0.81107522272803	0.124	0.069	3.73313446828346E-36	Ductular-like hepatocytes	WDHD1
2.36569459979058E-40	0.30268475522039	0.172	0.106	3.96443101032906E-36	Ductular-like hepatocytes	ZNF562
2.54569551963598E-40	0.267408224324026	0.203	0.13	4.26607655180598E-36	Ductular-like hepatocytes	DHX29
2.65587798178348E-40	0.292361923239461	0.196	0.125	4.45072032187275E-36	Ductular-like hepatocytes	CSGALNACT
3.37993988500043E-40	0.306760011137987	0.188	0.118	5.66410325928371E-36	Ductular-like hepatocytes	TTL
3.68303991430301E-40	0.392266264937684	0.112	0.06	6.17203828838899E-36	Ductular-like hepatocytes	CAPN15
3.76733638395999E-40	0.336581088988626	0.18	0.112	6.31330231224015E-36	Ductular-like hepatocytes	UTP3
3.79558284782871E-40	0.509791928193756	0.138	0.08	6.36063773639135E-36	Ductular-like hepatocytes	DEPRECATEI
3.80227970422308E-40	0.447797892430895	0.183	0.115	6.37186032833703E-36	Ductular-like hepatocytes	C17orf58
4.07396877643645E-40	0.287210277112219	0.197	0.125	6.8271568755522E-36	Ductular-like hepatocytes	ZDHHC16
4.45805489519823E-40	0.600548909423634	0.105	0.055	7.47080839337319E-36	Ductular-like hepatocytes	RNF216
4.53519267058898E-40	0.351503152502035	0.116	0.063	7.60007587737301E-36	Ductular-like hepatocytes	ZNF564
4.53892238301055E-40	0.299919496725922	0.147	0.086	7.60632612944908E-36	Ductular-like hepatocytes	CXorf38
4.79918067636716E-40	0.316379024929344	0.166	0.102	8.04246697745609E-36	Ductular-like hepatocytes	HLX
4.892116108968E-40	0.372357727514838	0.196	0.125	8.19820817540858E-36	Ductular-like hepatocytes	UHRF1BP1L
5.03414117145662E-40	0.715392413809269	0.116	0.064	8.436213775127E-36	Ductular-like hepatocytes	MOSPD1
5.53298774480065E-40	0.570886533026404	0.117	0.065	9.27218086273693E-36	Ductular-like hepatocytes	NOXA1
6.09947817143464E-40	0.340351577995164	0.196	0.125	1.02215055196902E-35	Ductular-like hepatocytes	C5orf51
6.16313939708757E-40	0.275993617736537	0.222	0.146	1.03281890016394E-35	Ductular-like hepatocytes	NAA30
6.5132674053702E-40	0.37837843172985	0.115	0.063	1.09149335179194E-35	Ductular-like hepatocytes	CASP8AP2
6.90243366962378E-40	0.408370323482628	0.173	0.107	1.15670983435555E-35	Ductular-like hepatocytes	STXBP4
6.91608860179616E-40	0.375834455829049	0.132	0.076	1.158998127889E-35	Ductular-like hepatocytes	YARS2
7.28073194569744E-40	0.315277650890732	0.188	0.119	1.22010505945998E-35	Ductular-like hepatocytes	MIER3
7.31325582722448E-40	0.767879189259895	0.1	0.052	1.22555541152628E-35	Ductular-like hepatocytes	TIPIN
8.08483789488855E-40	0.384209783983919	0.153	0.092	1.35485713442542E-35	Ductular-like hepatocytes	MIOS
8.72421683395936E-40	0.387933806433814	0.189	0.12	1.46200425703491E-35	Ductular-like hepatocytes	ZNF362
9.49496312903439E-40	0.802611892402947	0.129	0.074	1.59116592116358E-35	Ductular-like hepatocytes	PXYLP1
1.07839305142919E-39	0.261779607597389	0.232	0.153	1.80717107558504E-35	Ductular-like hepatocytes	TSPYL4
1.13982400386334E-39	0.435013297973071	0.154	0.092	1.91011706567418E-35	Ductular-like hepatocytes	ALKBH2
1.21229538019737E-39	0.467695466662799	0.151	0.091	2.03156459813476E-35	Ductular-like hepatocytes	ANXA2R
1.23329187433255E-39	0.522317527754496	0.162	0.099	2.06675052300649E-35	Ductular-like hepatocytes	TXNDC16
1.58063791317197E-39	0.296883691700124	0.114	0.062	2.64883301489359E-35	Ductular-like hepatocytes	ATAT1
1.6071902446973E-39	0.324856102699489	0.118	0.065	2.69332941206374E-35	Ductular-like hepatocytes	SHH
1.63328079868492E-39	0.256086061516294	0.177	0.11	2.73705196243619E-35	Ductular-like hepatocytes	TBP
1.94763661118197E-39	0.348309022998187	0.153	0.091	3.26384943301875E-35	Ductular-like hepatocytes	SENP1
2.01416670861615E-39	0.282034001418602	0.163	0.099	3.37534057029894E-35	Ductular-like hepatocytes	CNOT1
2.05947407154488E-39	0.432003968779839	0.142	0.083	3.4512666490949E-35	Ductular-like hepatocytes	ZNF823
2.25663586439552E-39	0.36552155191832	0.161	0.098	3.78167038155401E-35	Ductular-like hepatocytes	UNC13B
2.52093309724642E-39	0.444225075251667	0.313	0.224	4.22457968436555E-35	Ductular-like hepatocytes	UGT2B15

**2.52342634397411E-39**	0.349573696607691	0.149	0.088	4.22875786723181E-35	Ductular-like hepatocytes	OCEL1
**2.54748146350134E-39**	0.438995791257799	0.158	0.096	4.26906943653554E-35	Ductular-like hepatocytes	DEPRECATEI
**2.55710035587927E-39**	0.362761525340938	0.165	0.101	4.28518877638249E-35	Ductular-like hepatocytes	JKAMP
**2.6922660567618E-39**	0.467730793393011	0.138	0.081	4.51169945792142E-35	Ductular-like hepatocytes	STS
**2.84960668222235E-39**	0.492580754405294	0.105	0.055	4.77537087806821E-35	Ductular-like hepatocytes	PRAG1
**2.85110633441592E-39**	0.388899782391248	0.157	0.094	4.77788399521419E-35	Ductular-like hepatocytes	EEF1AKMT1
**2.90339279379647E-39**	0.253102722931869	0.179	0.112	4.86550564384412E-35	Ductular-like hepatocytes	ZNF397
**2.97693265039347E-39**	0.476764119729592	0.146	0.086	4.98874373552938E-35	Ductular-like hepatocytes	MED11
**3.0648510865489E-39**	0.455927034470232	0.137	0.08	5.13607745083865E-35	Ductular-like hepatocytes	DEPRECATEI
**3.64232447192467E-39**	0.395999379377546	0.11	0.059	6.10380735005135E-35	Ductular-like hepatocytes	IFT22
**3.73044812801972E-39**	0.330719983041621	0.157	0.095	6.25148497293544E-35	Ductular-like hepatocytes	ABHD17A
**3.98092784631368E-39**	0.276400313386126	0.194	0.124	6.67123888485247E-35	Ductular-like hepatocytes	EED
**4.41080080139676E-39**	0.400884747616292	0.128	0.073	7.39161998298069E-35	Ductular-like hepatocytes	GEMIN5
**4.57006378656511E-39**	0.660683325912907	0.116	0.064	7.65851289352581E-35	Ductular-like hepatocytes	ISCA2
**4.99720425749355E-39**	0.537909059061969	0.121	0.068	8.37431489470769E-35	Ductular-like hepatocytes	LLGL1
**5.71533949934165E-39**	0.282527558995462	0.195	0.125	9.57776593299673E-35	Ductular-like hepatocytes	PHB
**6.14893637001764E-39**	0.325285035263463	0.189	0.12	1.03043875688756E-34	Ductular-like hepatocytes	UBE3B
**7.71277160439535E-39**	0.42927001039369	0.136	0.079	1.29250626546457E-34	Ductular-like hepatocytes	ZNF460
**8.18791562032378E-39**	0.373734747877945	0.171	0.106	1.37213089965386E-34	Ductular-like hepatocytes	AASDH
**8.27130027974048E-39**	0.32651066599696	0.15	0.089	1.38610450087891E-34	Ductular-like hepatocytes	NOL7
**8.41779059318801E-39**	0.299243379728351	0.189	0.12	1.41065334760645E-34	Ductular-like hepatocytes	DEPRECATEI
**8.58927444141139E-39**	0.591821511708203	0.132	0.076	1.43939061089172E-34	Ductular-like hepatocytes	FAM104B
**8.60546617445368E-39**	0.301403651993441	0.149	0.089	1.44210402151495E-34	Ductular-like hepatocytes	CIAPIN1
**8.74367327945518E-39**	0.620155478763712	0.203	0.134	1.4652647681711E-34	Ductular-like hepatocytes	DEPRECATEI
**1.00081244671532E-38**	0.326810794454409	0.174	0.109	1.67716149820553E-34	Ductular-like hepatocytes	AARS
**1.04294219437839E-38**	0.448869210132977	0.132	0.076	1.74776252933931E-34	Ductular-like hepatocytes	ZNF324
**1.07688905027966E-38**	0.506245575543192	0.142	0.084	1.80465067045866E-34	Ductular-like hepatocytes	ZNF689
**1.08509233792474E-38**	0.363251096581086	0.164	0.101	1.81839773989428E-34	Ductular-like hepatocytes	RPP38
**1.08934031966217E-38**	0.261071842114399	0.168	0.103	1.82551650768987E-34	Ductular-like hepatocytes	PRELID3B
**1.22860865047589E-38**	0.506689125395023	0.155	0.094	2.05890237646749E-34	Ductular-like hepatocytes	FUNDC2
**1.23986857043357E-38**	0.481611367019272	0.116	0.064	2.07777175033258E-34	Ductular-like hepatocytes	ANGEL2
**1.34579273089034E-38**	0.541564418393337	0.139	0.082	2.25527945842603E-34	Ductular-like hepatocytes	GPRASP2
**1.45655831936993E-38**	0.354818117819831	0.176	0.11	2.44090043160014E-34	Ductular-like hepatocytes	ENKD1
**1.71900471591912E-38**	0.385303267903854	0.161	0.099	2.88070810293726E-34	Ductular-like hepatocytes	GTF3C5
**2.56098473540602E-38**	0.531026408460952	0.159	0.097	4.29169821959341E-34	Ductular-like hepatocytes	RWDD4
**2.72046019325193E-38**	0.361219547078175	0.185	0.118	4.55894719185159E-34	Ductular-like hepatocytes	DEPRECATEI
**2.97330677522443E-38**	0.309302606995479	0.217	0.143	4.98266749392109E-34	Ductular-like hepatocytes	NUP188
**3.30314265624786E-38**	0.275978111387133	0.168	0.103	5.53540646334016E-34	Ductular-like hepatocytes	TRAPPC2L
**4.30601375610978E-38**	0.498618567076621	0.118	0.066	7.21601785248877E-34	Ductular-like hepatocytes	RXYLT1
**5.51675405635212E-38**	0.565563746743342	0.106	0.057	9.24497644763488E-34	Ductular-like hepatocytes	SRGAP2C
**5.82877185741545E-38**	0.284444709971804	0.212	0.139	9.76785587865681E-34	Ductular-like hepatocytes	RBM12
**6.60580584075428E-38**	0.365760516240345	0.15	0.09	1.1070009427936E-33	Ductular-like hepatocytes	HELQ
**6.92345732428802E-38**	0.316762668119126	0.139	0.082	1.16023297840419E-33	Ductular-like hepatocytes	FANCF
**7.04925135141444E-38**	0.419371091825273	0.168	0.105	1.18131354147003E-33	Ductular-like hepatocytes	MIPOL1
**8.88788284039743E-38**	0.28057683914664	0.169	0.105	1.4894314063938E-33	Ductular-like hepatocytes	DPP8
**1.03686065096389E-37**	0.316723663074973	0.156	0.095	1.73757107888528E-33	Ductular-like hepatocytes	GAPVD1
**1.09237740233943E-37**	0.401101005471257	0.121	0.069	1.83060605084041E-33	Ductular-like hepatocytes	ALPK1
**1.10544121328658E-37**	0.339036365705475	0.158	0.097	1.85249838522565E-33	Ductular-like hepatocytes	PAK1IP1
**1.13109406152268E-37**	0.480160887525861	0.161	0.099	1.89548742829971E-33	Ductular-like hepatocytes	NUP62CL
**1.21159372394018E-37**	0.366186923092217	0.177	0.112	2.03038876257895E-33	Ductular-like hepatocytes	PRMT7
**1.22663099048098E-37**	0.406977969638859	0.117	0.065	2.05558821384803E-33	Ductular-like hepatocytes	PHACTR2
**1.31352254986018E-37**	0.33305985263065	0.178	0.112	2.20120108905569E-33	Ductular-like hepatocytes	GTF2H3
**1.3800807349531E-37**	0.349821807268361	0.174	0.11	2.3127392956344E-33	Ductular-like hepatocytes	PELP1
**1.38010958952471E-37**	0.46055042574941	0.124	0.071	2.31278765012551E-33	Ductular-like hepatocytes	AREL1
**1.55935282820059E-37**	0.4854772283894	0.12	0.068	2.61316346949855E-33	Ductular-like hepatocytes	ATP5PO
**1.60814870687147E-37**	0.456590661492435	0.129	0.074	2.69493560297521E-33	Ductular-like hepatocytes	SCAMP1
**1.74506400898289E-37**	0.331544685662928	0.179	0.114	2.92437826625353E-33	Ductular-like hepatocytes	CETN3
**2.22527768138476E-37**	0.268394682234486	0.147	0.088	3.72912033846459E-33	Ductular-like hepatocytes	QPCTL
**2.43382439353988E-37**	0.36280079822719	0.186	0.119	4.07860291869413E-33	Ductular-like hepatocytes	POLR2D
**2.64690953327971E-37**	0.256288609542739	0.109	0.06	4.43569099587014E-33	Ductular-like hepatocytes	BTN2A1

2.87774225504898E-37	0.336700251175251	0.183	0.117	4.82252047101108E-33	Ductular-like hepatocytes	PCNX4
3.11392042654689E-37	0.303494952429417	0.132	0.077	5.21830785080728E-33	Ductular-like hepatocytes	WDR77
3.24272891408986E-37	0.351569722035201	0.172	0.108	5.43416511423179E-33	Ductular-like hepatocytes	SMIM13
4.01336822527703E-37	0.270343917923595	0.167	0.105	6.72560247191924E-33	Ductular-like hepatocytes	ANKMY1
4.88038844756634E-37	0.289862015120425	0.148	0.089	8.17855496043167E-33	Ductular-like hepatocytes	RAB32
5.02961469886251E-37	0.409636972478241	0.168	0.106	8.42862831235379E-33	Ductular-like hepatocytes	XRCC1
5.87661656734455E-37	0.287524219742208	0.169	0.106	9.848034043556E-33	Ductular-like hepatocytes	BLOC1S5
6.16959648762034E-37	0.393355712843373	0.179	0.115	1.03390097939542E-32	Ductular-like hepatocytes	VCL
7.11600300047795E-37	0.62048036482027	0.149	0.091	1.19249978282009E-32	Ductular-like hepatocytes	CDC6
7.61076117303001E-37	0.319248221589346	0.14	0.083	1.27541135737637E-32	Ductular-like hepatocytes	DEPRECATEI
7.67676756141786E-37	0.26723103800691	0.234	0.158	1.28647270794241E-32	Ductular-like hepatocytes	UBE2W
9.94743733543503E-37	0.574360640593575	0.109	0.06	1.6669915486722E-32	Ductular-like hepatocytes	PPAT
1.10202791262295E-36	0.284650751953714	0.161	0.1	1.84677837597355E-32	Ductular-like hepatocytes	ALMS1
1.1266945884775E-36	0.501747392821958	0.147	0.089	1.8881147913706E-32	Ductular-like hepatocytes	MAP7D3
1.15154715067503E-36	0.269332425093335	0.208	0.137	1.92976271510121E-32	Ductular-like hepatocytes	ANKRD36B
1.23361257992306E-36	0.35772538310883	0.131	0.077	2.06728796143506E-32	Ductular-like hepatocytes	RAP2B
1.27207686247669E-36	0.604894120307065	0.128	0.075	2.13174640613844E-32	Ductular-like hepatocytes	CERS5
1.36116389880168E-36	0.477672382453474	0.128	0.075	2.28103846161186E-32	Ductular-like hepatocytes	RHBDD3
1.41352723496344E-36	0.255432726716842	0.117	0.066	2.36878894035173E-32	Ductular-like hepatocytes	NRTN
1.59635451865099E-36	0.339192180035515	0.128	0.074	2.67517090235534E-32	Ductular-like hepatocytes	RRP7A
1.63370520957743E-36	0.274873418539987	0.192	0.124	2.73776319020986E-32	Ductular-like hepatocytes	ZNF606
1.66488202512335E-36	0.255541686749033	0.178	0.113	2.79000929770171E-32	Ductular-like hepatocytes	GABPA
1.71902445091421E-36	0.408130398299418	0.184	0.118	2.88074117484203E-32	Ductular-like hepatocytes	GRK3
1.8151869915182E-36	0.392038776087299	0.122	0.07	3.0418903603862E-32	Ductular-like hepatocytes	CRIP3
2.11169443084356E-36	0.358977871761168	0.195	0.127	3.53877752720764E-32	Ductular-like hepatocytes	TRNAU1AP
2.46121824057603E-36	0.349377591458468	0.158	0.097	4.12450952755732E-32	Ductular-like hepatocytes	SLC30A6
2.53609949047648E-36	0.288079870962293	0.115	0.065	4.24999552614048E-32	Ductular-like hepatocytes	CDC26
2.80901645455961E-36	0.321311795350689	0.238	0.162	4.707349774551E-32	Ductular-like hepatocytes	CLN6
3.15540568020385E-36	0.353701934243169	0.142	0.085	5.2878288388856E-32	Ductular-like hepatocytes	PPP2R3C
3.21677020424046E-36	0.258602991504293	0.11	0.061	5.39066350826616E-32	Ductular-like hepatocytes	WDR81
3.33102500412073E-36	0.346912014268731	0.157	0.097	5.58213170190552E-32	Ductular-like hepatocytes	AHCTF1
3.83666870031156E-36	0.251770783009757	0.184	0.118	6.42948940798211E-32	Ductular-like hepatocytes	LYSMD2
4.11362694532633E-36	0.491847944535123	0.125	0.073	6.89361603497786E-32	Ductular-like hepatocytes	KIAA1841
4.55376035204434E-36	0.255584219873831	0.183	0.117	7.6311915979559E-32	Ductular-like hepatocytes	UBE2D2
5.08440692350815E-36	0.730947753978521	0.104	0.057	8.52044912241495E-32	Ductular-like hepatocytes	SENP3
6.58824751598609E-36	0.270016502243571	0.126	0.073	1.10405851872895E-31	Ductular-like hepatocytes	RHBDD1
6.63785565653012E-36	0.313684952716843	0.146	0.089	1.11237185092132E-31	Ductular-like hepatocytes	DOLK
6.85693987229165E-36	0.449201508029194	0.142	0.086	1.14908598379863E-31	Ductular-like hepatocytes	TBL1X
7.16311848905218E-36	0.30312781302285	0.15	0.092	1.20039539639536E-31	Ductular-like hepatocytes	RASA2
7.66132176281476E-36	0.288843049713662	0.14	0.084	1.2838843010125E-31	Ductular-like hepatocytes	TMEM177
8.10636877052443E-36	0.515546505227913	0.144	0.088	1.35846527856448E-31	Ductular-like hepatocytes	DEPRECATEI
8.54238103784848E-36	0.382213994913292	0.154	0.095	1.43153221432265E-31	Ductular-like hepatocytes	KARS
8.54624806051637E-36	0.282879140372037	0.138	0.082	1.43218024998133E-31	Ductular-like hepatocytes	GEMIN6
8.69736455658415E-36	0.311153725679723	0.141	0.085	1.45750435239237E-31	Ductular-like hepatocytes	FBXO6
1.07798821788829E-35	0.349231381175004	0.126	0.073	1.8064926555372E-31	Ductular-like hepatocytes	CBY1
1.23418185196754E-35	0.265756872519129	0.184	0.119	2.0682419475272E-31	Ductular-like hepatocytes	DGCR8
1.35899038487871E-35	0.368742244223323	0.102	0.055	2.27739608697974E-31	Ductular-like hepatocytes	PYROXD1
1.40773311919627E-35	0.307229513193399	0.127	0.074	2.35907916114912E-31	Ductular-like hepatocytes	PAFAH2
1.5066776061333E-35	0.270493834326885	0.196	0.129	2.52489033235818E-31	Ductular-like hepatocytes	CREB3L2
1.53603176208377E-35	0.394477203601104	0.134	0.08	2.57408202689999E-31	Ductular-like hepatocytes	SUPT3H
2.18048941039215E-35	0.610971953351067	0.105	0.058	3.65406415393517E-31	Ductular-like hepatocytes	USP46
2.23664473550732E-35	0.294421746720148	0.168	0.105	3.74816924776316E-31	Ductular-like hepatocytes	SWAP70
2.44416615093744E-35	0.392359102618743	0.127	0.074	4.09593363574096E-31	Ductular-like hepatocytes	SLC39A13
2.88325142848396E-35	0.329971312492601	0.134	0.079	4.83175274385342E-31	Ductular-like hepatocytes	ZBED4
3.53428928618585E-35	0.299853676840721	0.149	0.091	5.92276198579025E-31	Ductular-like hepatocytes	DHRS13
3.87623090462963E-35	0.424787771325572	0.142	0.086	6.49578774997833E-31	Ductular-like hepatocytes	RSBN1
3.99616675262029E-35	0.416710979310979	0.119	0.068	6.69677624404108E-31	Ductular-like hepatocytes	FAM185A
4.8112224232061E-35	0.694124499871424	0.101	0.056	8.06264653680878E-31	Ductular-like hepatocytes	LRAT
5.3758696348733E-35	0.476404396361489	0.132	0.079	9.00888233412067E-31	Ductular-like hepatocytes	DZIP3
5.3898085266172E-35	0.418587626384112	0.112	0.063	9.0322411289051E-31	Ductular-like hepatocytes	MSANTD4

**5.87016585168892E-35**	0.370980301778794	0.151	0.094	9.83722393426029E-31	Ductular-like hepatocytes	TIMM21
**6.64612710297949E-35**	0.446990197004709	0.145	0.088	1.1137579799173E-30	Ductular-like hepatocytes	ZNF554
**6.85385369936563E-35**	0.347221847741569	0.11	0.062	1.14856880293969E-30	Ductular-like hepatocytes	ARMC7
**8.04325697274647E-35**	0.314923113217886	0.173	0.11	1.34788900349285E-30	Ductular-like hepatocytes	MOSMO
**8.42537500649358E-35**	0.299356159132642	0.236	0.161	1.41192434358819E-30	Ductular-like hepatocytes	SIK3
**1.02310022858641E-34**	0.292540518879675	0.125	0.073	1.7145113630651E-30	Ductular-like hepatocytes	CFAP410
**1.02966484188361E-34**	0.41909111643593	0.11	0.062	1.72551234202855E-30	Ductular-like hepatocytes	MTHFSD
**1.03470185047416E-34**	0.485351994409809	0.151	0.094	1.7339533610246E-30	Ductular-like hepatocytes	ZNF619
**1.04210970616483E-34**	0.403856547284471	0.153	0.095	1.74636744559101E-30	Ductular-like hepatocytes	PPT1
**1.25009636326253E-34**	0.335828282241089	0.109	0.061	2.09491148555536E-30	Ductular-like hepatocytes	ZNF446
**1.36079217917109E-34**	0.403461101160623	0.121	0.07	2.28041553385492E-30	Ductular-like hepatocytes	PRMT9
**1.62210039619465E-34**	0.27029152435688	0.15	0.092	2.718315843943E-30	Ductular-like hepatocytes	PTPN9
**1.69345561111435E-34**	0.482796716958954	0.107	0.06	2.83789291310543E-30	Ductular-like hepatocytes	SVIP
**1.77261830078732E-34**	0.477318912028384	0.131	0.078	2.9705537484594E-30	Ductular-like hepatocytes	RUNDC1
**1.9342944387666E-34**	0.33339891349853	0.12	0.07	3.24149062048507E-30	Ductular-like hepatocytes	KLHL28
**2.58043270771413E-34**	0.648411361902542	0.111	0.063	4.32428913158734E-30	Ductular-like hepatocytes	OSBPL1A
**2.95175451347777E-34**	0.382283481266243	0.1	0.055	4.94655021368604E-30	Ductular-like hepatocytes	EFNA4
**3.12930657331669E-34**	0.407632418301855	0.131	0.078	5.2440919555641E-30	Ductular-like hepatocytes	CEP68
**4.07997687649879E-34**	0.527098082278593	0.127	0.076	6.83722524963667E-30	Ductular-like hepatocytes	RBM45
**4.26577723001583E-34**	0.514006543716342	0.1	0.055	7.14858948206053E-30	Ductular-like hepatocytes	EXTL3
**4.26691811555015E-34**	0.322675445214047	0.115	0.066	7.15050137803895E-30	Ductular-like hepatocytes	NSUN5
**4.30168115117193E-34**	0.366093112961695	0.119	0.069	7.20875727313393E-30	Ductular-like hepatocytes	FAM222B
**4.63347585296157E-34**	0.293974540545521	0.177	0.114	7.764778834393E-30	Ductular-like hepatocytes	ZNF33B
**4.98592561990405E-34**	0.408396463681034	0.115	0.066	8.35541415383521E-30	Ductular-like hepatocytes	RAI1
**5.39676020944919E-34**	0.362515056893392	0.14	0.085	9.04389075899496E-30	Ductular-like hepatocytes	ZNF142
**6.1129037282582E-34**	0.266514564038967	0.137	0.083	1.02440040678151E-29	Ductular-like hepatocytes	NLRC5
**8.51325650261765E-34**	0.645015360058601	0.102	0.057	1.42665152470867E-29	Ductular-like hepatocytes	SLC25A14
**9.93388634951987E-34**	0.357544487490257	0.163	0.103	1.66472067445254E-29	Ductular-like hepatocytes	IP6K1
**1.18145244451929E-33**	0.411384074847192	0.106	0.06	1.97987800652543E-29	Ductular-like hepatocytes	RPUSD1
**1.23701955093269E-33**	0.409340846950038	0.119	0.069	2.072997363453E-29	Ductular-like hepatocytes	CHST14
**2.36035469835469E-33**	0.261848156239145	0.139	0.084	3.95548240350279E-29	Ductular-like hepatocytes	TSR1
**2.52793735550308E-33**	0.27294213310944	0.188	0.124	4.23631742035206E-29	Ductular-like hepatocytes	SPATA5L1
**2.58491439127749E-33**	0.304121675378568	0.155	0.098	4.33179953690281E-29	Ductular-like hepatocytes	STRADB
**3.67831820926015E-33**	0.515001427631796	0.106	0.06	6.16412565507816E-29	Ductular-like hepatocytes	MFSD14C
**4.09522612566535E-33**	0.309171306246301	0.114	0.066	6.86277994139E-29	Ductular-like hepatocytes	NPAT
**4.13229346700105E-33**	0.290423667429085	0.15	0.094	6.92489739200035E-29	Ductular-like hepatocytes	IPO13
**4.17750032908892E-33**	0.574967727108172	0.11	0.064	7.00065505148721E-29	Ductular-like hepatocytes	DHRS4
**4.34757294184575E-33**	0.313127977748572	0.117	0.068	7.2856627359451E-29	Ductular-like hepatocytes	YAE1
**4.6697827708502E-33**	0.566245637164948	0.106	0.061	7.82562196739077E-29	Ductular-like hepatocytes	SEMA5A
**5.76329315740163E-33**	0.293266147100583	0.17	0.109	9.65812667317364E-29	Ductular-like hepatocytes	CYP20A1
**5.97032087015982E-33**	0.254840689197087	0.164	0.105	1.00050637142138E-28	Ductular-like hepatocytes	CTDP1
**7.2842548070848E-33**	0.561550455448275	0.118	0.07	1.22069542057127E-28	Ductular-like hepatocytes	USP18
**7.39457495281629E-33**	0.251363539052524	0.132	0.08	1.23918287059295E-28	Ductular-like hepatocytes	KATNB1
**1.03612760339585E-32**	0.266056952426442	0.358	0.266	1.73634263777076E-28	Ductular-like hepatocytes	GPC6
**1.07344128859939E-32**	0.263503342823236	0.116	0.067	1.79887291143486E-28	Ductular-like hepatocytes	SLC66A1
**1.08924741077273E-32**	0.471767748523441	0.1	0.056	1.82536081097295E-28	Ductular-like hepatocytes	TLE2
**1.17178277575573E-32**	0.308203752081213	0.142	0.088	1.96367357561146E-28	Ductular-like hepatocytes	BTBD6
**1.28143495530995E-32**	0.510129043309953	0.125	0.075	2.14742869810841E-28	Ductular-like hepatocytes	BLOC1S3
**1.30233710334406E-32**	0.459512047225602	0.148	0.093	2.18245651778398E-28	Ductular-like hepatocytes	STK17B
**1.33390525662719E-32**	0.33615607244208	0.111	0.064	2.23535842905585E-28	Ductular-like hepatocytes	TRMT10A
**1.4598624322372E-32**	0.30982995945949	0.101	0.056	2.44643746394309E-28	Ductular-like hepatocytes	SSH2
**1.52046550080194E-32**	0.355611063371006	0.124	0.074	2.5479960862439E-28	Ductular-like hepatocytes	DEPRECATEI
**1.52914713067431E-32**	0.323704632778558	0.166	0.107	2.56254476158401E-28	Ductular-like hepatocytes	CMSS1
**1.55954587952374E-32**	0.435435584563984	0.151	0.095	2.61348698490588E-28	Ductular-like hepatocytes	RRAGC
**1.60313597882786E-32**	0.29458223116243	0.102	0.057	2.68653527331973E-28	Ductular-like hepatocytes	VPS16
**1.88893511368551E-32**	0.371900538935467	0.13	0.079	3.16547746351418E-28	Ductular-like hepatocytes	IARS
**2.00283167121541E-32**	0.366418385659526	0.118	0.069	3.35634531462279E-28	Ductular-like hepatocytes	KIAA1143
**2.05698486110131E-32**	0.346667700060029	0.163	0.105	3.44709523023357E-28	Ductular-like hepatocytes	FBF1
**2.451995806225E-32**	0.425879201645546	0.108	0.062	4.10905457207185E-28	Ductular-like hepatocytes	IFT52
**2.7227779635013E-32**	0.345833148047048	0.143	0.089	4.56283131123548E-28	Ductular-like hepatocytes	DEPRECATEI

3.60260558957115E-32	0.424556730989087	0.148	0.093	6.03724644700333E-28	Ductular-like hepatocytes	RNASEH2C
3.71880218419984E-32	0.322821984216753	0.148	0.093	6.2319687002821E-28	Ductular-like hepatocytes	XPO7
4.3664233893159E-32	0.47893765703542	0.133	0.081	7.31725231581559E-28	Ductular-like hepatocytes	TMEM42
5.08383718079833E-32	0.389002762557203	0.155	0.099	8.51949434758185E-28	Ductular-like hepatocytes	PIP4K2A
6.07578512314884E-32	0.3652038024306	0.154	0.098	1.01818007093728E-27	Ductular-like hepatocytes	SMIM30
7.28649622213996E-32	0.298397053024152	0.126	0.075	1.22107103690621E-27	Ductular-like hepatocytes	GXYLT1
7.57969561656688E-32	0.492495585551251	0.116	0.069	1.27020539142428E-27	Ductular-like hepatocytes	FICD
9.73061958279466E-32	0.577629701994514	0.121	0.072	1.63065722968473E-27	Ductular-like hepatocytes	KYAT3
1.03490320050193E-31	0.356745170826089	0.127	0.077	1.73429078340113E-27	Ductular-like hepatocytes	ZNF605
1.16826945898567E-31	0.39523723633428	0.127	0.077	1.95778595936819E-27	Ductular-like hepatocytes	FASTKD3
1.18085739656322E-31	0.407953708532598	0.103	0.058	1.97888082516064E-27	Ductular-like hepatocytes	KIAA0513
1.32000574727125E-31	1.00757839335338	0.106	0.062	2.21206563127716E-27	Ductular-like hepatocytes	PROKR1
1.41530060444049E-31	0.343362468031161	0.14	0.087	2.37176075292137E-27	Ductular-like hepatocytes	GOLGA8A
1.59618621593876E-31	0.314218759802374	0.153	0.097	2.67488886067018E-27	Ductular-like hepatocytes	FNTB
1.61133391446438E-31	0.457775549907934	0.103	0.059	2.70027337385941E-27	Ductular-like hepatocytes	MIPEP
1.71934192198611E-31	0.376889264507137	0.143	0.089	2.88127319286432E-27	Ductular-like hepatocytes	PHTF1
2.85704801367507E-31	0.297219495271139	0.139	0.087	4.78784106131669E-27	Ductular-like hepatocytes	ZSCAN29
3.35979105159281E-31	0.34107054926573	0.134	0.082	5.63033784425922E-27	Ductular-like hepatocytes	FAM114A2
3.42531244165245E-31	0.410363973473186	0.124	0.075	5.74013858972117E-27	Ductular-like hepatocytes	DEPRECATEI
3.72495254205293E-31	0.315221775198549	0.176	0.117	6.2422754699723E-27	Ductular-like hepatocytes	MGA
3.90305459621822E-31	0.30248415974949	0.142	0.089	6.5407388923425E-27	Ductular-like hepatocytes	NELFE
3.92378183642076E-31	0.560566602591046	0.108	0.063	6.57547360147391E-27	Ductular-like hepatocytes	ZFYVE26
3.96807806044498E-31	0.281643913421737	0.289	0.209	6.64970521369369E-27	Ductular-like hepatocytes	RNF125
4.46169971075442E-31	0.47883697308629	0.146	0.092	7.47691637528225E-27	Ductular-like hepatocytes	C9orf40
5.62389544376038E-31	0.527231406461796	0.136	0.085	9.42452398465364E-27	Ductular-like hepatocytes	CUX2
5.86675527394309E-31	0.431990756645118	0.148	0.094	9.83150848807382E-27	Ductular-like hepatocytes	HSPA14.1
6.47477980280311E-31	0.320027085440304	0.117	0.07	1.08504359935375E-26	Ductular-like hepatocytes	UNC119B
6.48006426599794E-31	0.436280671968397	0.144	0.091	1.08592916969593E-26	Ductular-like hepatocytes	ZBTB22
7.62618088081513E-31	0.276128886878264	0.132	0.082	1.277995392007E-26	Ductular-like hepatocytes	ZBED5
8.83893183180887E-31	0.328061173976708	0.103	0.059	1.48122819637453E-26	Ductular-like hepatocytes	GCC1
9.17635404167855E-31	0.421469292790193	0.134	0.083	1.53777341030449E-26	Ductular-like hepatocytes	NMNAT1
9.53926135351188E-31	0.277064511179806	0.157	0.102	1.59858941762152E-26	Ductular-like hepatocytes	COQ7
9.81888613821438E-31	0.622723510691712	0.118	0.072	1.64544893904197E-26	Ductular-like hepatocytes	ACTRT3
1.05981089900958E-30	0.269025944472137	0.156	0.101	1.77603110456025E-26	Ductular-like hepatocytes	DSTYK
1.24209578882089E-30	0.288068671649428	0.129	0.079	2.08150412290605E-26	Ductular-like hepatocytes	PSMD14
1.35784363526805E-30	0.300740834471029	0.231	0.162	2.2754743639822E-26	Ductular-like hepatocytes	PM20D2
1.60410242215927E-30	0.331749833147478	0.104	0.06	2.68815483905451E-26	Ductular-like hepatocytes	DDX60L
1.64455963769053E-30	0.552771797533481	0.108	0.063	2.75595304084178E-26	Ductular-like hepatocytes	LONRF2
2.08007984822797E-30	0.767322295585109	0.114	0.069	3.48579780966042E-26	Ductular-like hepatocytes	RCAN2
2.25710995528601E-30	0.27610381298052	0.199	0.135	3.7824648630683E-26	Ductular-like hepatocytes	SOCS5
2.85890228783039E-30	0.288604066828488	0.148	0.094	4.79094845394617E-26	Ductular-like hepatocytes	C19orf47
4.08960863811347E-30	0.44900284329708	0.123	0.075	6.85336615575055E-26	Ductular-like hepatocytes	ZNF574
4.21824632395309E-30	0.258647799832522	0.121	0.073	7.06893718968059E-26	Ductular-like hepatocytes	MDM1
4.48942501329204E-30	0.490611905922895	0.117	0.07	7.5233784372748E-26	Ductular-like hepatocytes	TICAM2
5.00979079068567E-30	0.32176692314203	0.156	0.101	8.39540740703104E-26	Ductular-like hepatocytes	MIF4GD
5.64510182839894E-30	0.332143505678582	0.146	0.093	9.46006164403094E-26	Ductular-like hepatocytes	IPO11
5.98615973729736E-30	0.497231257369275	0.108	0.064	1.00316064877629E-25	Ductular-like hepatocytes	DHX40
6.42050546962039E-30	0.389583990623161	0.164	0.107	1.07594830659898E-25	Ductular-like hepatocytes	GSTA4
6.69323560662498E-30	0.279963303444256	0.13	0.08	1.12165242295821E-25	Ductular-like hepatocytes	FBXL20
6.95407684076085E-30	0.437790528371334	0.144	0.092	1.1653641969747E-25	Ductular-like hepatocytes	VANGL1
7.02488918483186E-30	0.357538443201005	0.106	0.062	1.17723092959412E-25	Ductular-like hepatocytes	N6AMT1
7.53773590231134E-30	0.458919555777679	0.112	0.066	1.26317378250933E-25	Ductular-like hepatocytes	ERCC8
1.07740324146017E-29	0.26901303054611	0.154	0.1	1.80551235203895E-25	Ductular-like hepatocytes	DBR1
1.0899799529351E-29	0.621763270580901	0.107	0.063	1.82658840512864E-25	Ductular-like hepatocytes	SPATC1L
1.13817432996148E-29	0.376724535162899	0.117	0.07	1.90735254214945E-25	Ductular-like hepatocytes	BOLA1
1.27619099610736E-29	0.387259297148224	0.136	0.085	2.13864087127672E-25	Ductular-like hepatocytes	MOB3A
1.30395912146089E-29	0.313939008677235	0.159	0.104	2.18517469574416E-25	Ductular-like hepatocytes	THAP5
1.39626279822369E-29	0.321112654591328	0.121	0.074	2.33985719726327E-25	Ductular-like hepatocytes	PIM2
1.46241354776469E-29	0.282259616597651	0.137	0.086	2.45071262334407E-25	Ductular-like hepatocytes	UNC45A
1.50310534129372E-29	0.707100991713693	0.126	0.079	2.51890393094002E-25	Ductular-like hepatocytes	STMND1

1.57013610424817E-29	0.724650239731293	0.121	0.074	2.63123408349907E-25	Ductular-like hepatocytes	ISX
1.59928114191831E-29	0.406516434074247	0.101	0.058	2.68007533762671E-25	Ductular-like hepatocytes	KCTD18
1.84110004429303E-29	0.264599144392578	0.119	0.072	3.08531545422626E-25	Ductular-like hepatocytes	DYRK3
1.9023315130141E-29	0.269804862600141	0.13	0.081	3.18792714950903E-25	Ductular-like hepatocytes	BRPF1
1.96575372975059E-29	0.379300189246321	0.128	0.079	3.29421010031603E-25	Ductular-like hepatocytes	POMT1
2.00181123401776E-29	0.425223171723788	0.136	0.086	3.35463526596695E-25	Ductular-like hepatocytes	PGBD2
2.23647987904835E-29	0.425217191350082	0.116	0.07	3.74789298130922E-25	Ductular-like hepatocytes	SDAD1
2.70913478422947E-29	0.354770673447477	0.151	0.098	4.53996807141175E-25	Ductular-like hepatocytes	SMU1
3.56428168817904E-29	0.291504790788458	0.129	0.08	5.97302325305044E-25	Ductular-like hepatocytes	TBC1D19
4.32156412558752E-29	0.491520022471074	0.133	0.084	7.24207716165957E-25	Ductular-like hepatocytes	NT5DC2
4.56934751270408E-29	0.310727141878814	0.119	0.072	7.65731256178949E-25	Ductular-like hepatocytes	TULP3
4.82798256144759E-29	0.422166335419216	0.115	0.069	8.09073317647386E-25	Ductular-like hepatocytes	GAB1
5.17525492009134E-29	0.496120650621345	0.111	0.066	8.67269219508906E-25	Ductular-like hepatocytes	GTF2E1
5.89522298793832E-29	0.281440222359652	0.123	0.075	9.87921468318704E-25	Ductular-like hepatocytes	STX18
5.99761135671429E-29	0.281526819325287	0.17	0.113	1.00507971115818E-24	Ductular-like hepatocytes	MTMR14
7.64244770371715E-29	0.269817742084366	0.124	0.076	1.28072138618892E-24	Ductular-like hepatocytes	UTP11
8.750311668029E-29	0.360090388361679	0.12	0.073	1.4663772293283E-24	Ductular-like hepatocytes	SP4
1.12755768524637E-28	0.52234460890677	0.102	0.06	1.88956116893586E-24	Ductular-like hepatocytes	AKAP6
1.14337369547938E-28	0.552157829161674	0.117	0.072	1.91606563888435E-24	Ductular-like hepatocytes	RPA3
1.43497010561413E-28	0.534336636929326	0.1	0.058	2.40472290298816E-24	Ductular-like hepatocytes	DEPRECATEI
1.57625943949918E-28	0.261392528490179	0.112	0.067	2.64149556871272E-24	Ductular-like hepatocytes	DEPRECATEI
1.62279829745788E-28	0.482129173201575	0.109	0.066	2.71948538687991E-24	Ductular-like hepatocytes	ZNF280C
1.84668089486294E-28	0.39105018818417	0.133	0.084	3.09466784361132E-24	Ductular-like hepatocytes	ZNF234
1.88966090550961E-28	0.265194230677801	0.126	0.078	3.16669374545301E-24	Ductular-like hepatocytes	VRK2
1.93665769555777E-28	0.383367736155742	0.126	0.079	3.24545096621572E-24	Ductular-like hepatocytes	FBXO45
3.00393465695896E-28	0.323875602801158	0.22	0.155	5.03399369813183E-24	Ductular-like hepatocytes	KHDRBS3
3.03438181398914E-28	0.355864222131955	0.141	0.091	5.085017043883E-24	Ductular-like hepatocytes	IBA57
4.90338253603032E-28	0.280818507056557	0.142	0.091	8.2170884538796E-24	Ductular-like hepatocytes	TMEM201
5.93822399079391E-28	0.288835340406784	0.139	0.089	9.95127576377243E-24	Ductular-like hepatocytes	GZF1
6.22313628787407E-28	0.343239074272483	0.119	0.073	1.04287317912194E-23	Ductular-like hepatocytes	TIMM29
6.77117306008461E-28	0.298495952500075	0.152	0.099	1.13471318140898E-23	Ductular-like hepatocytes	FAM76A
8.98065801381111E-28	0.280120743722703	0.169	0.114	1.50497866995447E-23	Ductular-like hepatocytes	SPAG1
9.05409373199481E-28	0.257692100639516	0.127	0.08	1.51728502760769E-23	Ductular-like hepatocytes	ARMT1
1.14731600803845E-27	0.283391267841224	0.139	0.089	1.92267216627084E-23	Ductular-like hepatocytes	PPM1D
2.00892486485589E-27	0.375400099674842	0.116	0.071	3.36655628852551E-23	Ductular-like hepatocytes	PROSER3
2.34665983159462E-27	0.305719376586137	0.133	0.085	3.93253254578627E-23	Ductular-like hepatocytes	DRAM1
2.68642196409866E-27	0.538836465619624	0.113	0.069	4.50190592743653E-23	Ductular-like hepatocytes	ZSWIM1
3.08879010487061E-27	0.301461179427351	0.151	0.099	5.17619445774217E-23	Ductular-like hepatocytes	CCDC7
3.7984310387358E-27	0.299086395048635	0.144	0.094	6.36541073471345E-23	Ductular-like hepatocytes	CDKN2AIPNI
4.47538210379088E-27	0.43169275581819	0.11	0.067	7.49984532953276E-23	Ductular-like hepatocytes	FYCO1
4.76950314706842E-27	0.253773996643179	0.134	0.085	7.99273337385727E-23	Ductular-like hepatocytes	APPBP2
5.26691220528104E-27	0.271275083256174	0.117	0.072	8.82629147360996E-23	Ductular-like hepatocytes	BICD2
5.44187105108827E-27	0.406224285420537	0.133	0.085	9.11948750741373E-23	Ductular-like hepatocytes	BUD13
5.6015801780672E-27	0.430368344076804	0.149	0.098	9.38712806240502E-23	Ductular-like hepatocytes	FBLN1
9.12431537833692E-27	0.282769212718959	0.144	0.094	1.5290527711017E-22	Ductular-like hepatocytes	ANP32B
9.3267095388968E-27	0.301565628341639	0.112	0.069	1.56296998452832E-22	Ductular-like hepatocytes	SLC38A6
9.79744592221648E-27	0.343262888552029	0.155	0.103	1.64185598764504E-22	Ductular-like hepatocytes	LIN54
1.02437505711722E-26	0.335137075786658	0.132	0.084	1.71664772071703E-22	Ductular-like hepatocytes	AMZ2
1.44739573770226E-26	0.276627377482679	0.13	0.083	2.42554577724145E-22	Ductular-like hepatocytes	TRMT10C
1.72998542086903E-26	0.26989693918324	0.122	0.077	2.89910956829232E-22	Ductular-like hepatocytes	ZNF333
1.77697534569026E-26	0.307436757588893	0.109	0.066	2.97785528430774E-22	Ductular-like hepatocytes	CCDC171
1.99134976818934E-26	0.547819779820672	0.105	0.064	3.3371039415317E-22	Ductular-like hepatocytes	ARHGAP4
2.3163265474003E-26	0.392451720791278	0.107	0.066	3.88170002813343E-22	Ductular-like hepatocytes	TOP3A
2.48326286964397E-26	0.471958870571073	0.105	0.064	4.16145191694937E-22	Ductular-like hepatocytes	DNAL4
2.60762497579619E-26	0.250663992143768	0.159	0.106	4.36985793443926E-22	Ductular-like hepatocytes	GINS1
2.71243187051609E-26	0.326572780552219	0.129	0.082	4.54549332861087E-22	Ductular-like hepatocytes	DTWD2
3.14376484607418E-26	0.25444821876689	0.22	0.157	5.26832112905111E-22	Ductular-like hepatocytes	ARPP19
4.01806479728853E-26	0.464274225209467	0.119	0.074	6.73347298729613E-22	Ductular-like hepatocytes	KBTBD4
4.0544211828925E-26	0.305185317449146	0.122	0.077	6.79439901829124E-22	Ductular-like hepatocytes	TOPORS
4.80305781156101E-26	0.284120598300842	0.136	0.089	8.04896428061394E-22	Ductular-like hepatocytes	ZNF782

**5.9551212956505E-26**	0.643524705507845	0.12	0.076	9.9795922672511E-22	Ductular-like hepatocytes	CHEK1
**6.09683802686568E-26**	0.488212595828569	0.109	0.067	1.02170811654215E-21	Ductular-like hepatocytes	NEDD1
**6.29852017494134E-26**	0.335328967016334	0.123	0.078	1.05550601091667E-21	Ductular-like hepatocytes	DEPRECATEI
**8.34885217270022E-26**	0.401268813668	0.132	0.085	1.3991006471011E-21	Ductular-like hepatocytes	RHNO1
**1.01022461305242E-25**	0.276759684737969	0.159	0.107	1.69293440655324E-21	Ductular-like hepatocytes	PDIK1L
**1.03896077713007E-25**	0.4335004249529	0.117	0.073	1.74109047031457E-21	Ductular-like hepatocytes	TMEM267
**1.17893037883491E-25**	0.294362748739743	0.125	0.08	1.97565152885154E-21	Ductular-like hepatocytes	INTS2
**1.37559236045495E-25**	0.370246494847641	0.102	0.062	2.30521767765041E-21	Ductular-like hepatocytes	ZNF514
**1.46767486725697E-25**	0.256774366256058	0.147	0.097	2.45952954254923E-21	Ductular-like hepatocytes	TBCC
**1.49858828591494E-25**	0.268176809087016	0.126	0.081	2.51133424953626E-21	Ductular-like hepatocytes	CENPS
**1.50514162710935E-25**	0.439454345041093	0.123	0.078	2.52231633870985E-21	Ductular-like hepatocytes	STK26
**1.93160675027322E-25**	0.288813908872622	0.147	0.098	3.23698659210786E-21	Ductular-like hepatocytes	SLF2
**1.97197347272709E-25**	0.280400698070716	0.155	0.104	3.30463314559605E-21	Ductular-like hepatocytes	TSPYL2
**2.2805781212474E-25**	0.359154932116901	0.122	0.078	3.82179281558639E-21	Ductular-like hepatocytes	FANCG
**3.34181724540484E-25**	0.266800936736576	0.152	0.102	5.60021733984944E-21	Ductular-like hepatocytes	PHTF2
**4.9946126427349E-25**	0.314694935859315	0.107	0.066	8.36997186669514E-21	Ductular-like hepatocytes	TFPT
**7.14281801589983E-25**	0.318101875959641	0.122	0.078	1.19699344310449E-20	Ductular-like hepatocytes	VIPAS39
**1.43418080683388E-24**	0.299405013023604	0.125	0.081	2.40340019609222E-20	Ductular-like hepatocytes	CRYZL1
**1.58250080246479E-24**	0.258612686361609	0.108	0.067	2.6519548447705E-20	Ductular-like hepatocytes	DNAJC14
**1.59837943218858E-24**	0.30010768751907	0.172	0.119	2.67856425246163E-20	Ductular-like hepatocytes	RHOQ
**1.77492724832989E-24**	0.273048182857619	0.153	0.104	2.97442308275124E-20	Ductular-like hepatocytes	TUBGCP5
**2.60309835933281E-24**	0.269875939402069	0.111	0.069	4.36227223056992E-20	Ductular-like hepatocytes	ST7L
**4.07916784956039E-24**	0.364711448537621	0.154	0.105	6.8358694822933E-20	Ductular-like hepatocytes	ASF1B
**4.76094851940605E-24**	0.324217042582882	0.115	0.073	7.97839752882065E-20	Ductular-like hepatocytes	DNAJC19
**5.25313725920918E-24**	0.252727075316994	0.131	0.086	8.80320741898274E-20	Ductular-like hepatocytes	TMEM62
**9.9872174248884E-24**	0.374081824482362	0.149	0.101	1.6736578960628E-19	Ductular-like hepatocytes	BBOX1
**1.03732694958199E-23**	0.338041005254826	0.164	0.113	1.7383525021095E-19	Ductular-like hepatocytes	CREB3L4
**1.26983071583092E-23**	0.453442383594048	0.104	0.065	2.12798231358946E-19	Ductular-like hepatocytes	SNRPD1
**1.31743932519448E-23**	0.35343930674848	0.139	0.093	2.20776482116091E-19	Ductular-like hepatocytes	JRK
**1.73138125929201E-23**	0.412359065886572	0.115	0.073	2.90144871432156E-19	Ductular-like hepatocytes	SLC25A19
**2.92194368931237E-23**	0.379619908103874	0.14	0.094	4.89659323454968E-19	Ductular-like hepatocytes	FITM2
**3.18200374074454E-23**	0.356784992450528	0.159	0.11	5.3324018687397E-19	Ductular-like hepatocytes	KPTN
**3.33301996771981E-23**	0.341747548797954	0.662	0.592	5.58547486190485E-19	Ductular-like hepatocytes	VEGFA
**1.42732482857162E-22**	0.385801286751325	0.103	0.065	2.39191094772032E-18	Ductular-like hepatocytes	KIF3A
**2.02437765233769E-22**	0.420784876874111	0.137	0.093	3.3924520697875E-18	Ductular-like hepatocytes	FSBP
**3.36946145850395E-22**	0.485692618622613	0.1	0.063	5.64654351216092E-18	Ductular-like hepatocytes	KIF21B
**5.09175132292045E-22**	0.315699632081659	0.1	0.062	8.5327568669501E-18	Ductular-like hepatocytes	ZNF174
**5.62535654344219E-22**	0.430478585869642	0.119	0.078	9.42697249550042E-18	Ductular-like hepatocytes	HSDL1
**5.90747248011536E-22**	0.441029051153735	0.125	0.083	9.89974238217733E-18	Ductular-like hepatocytes	SYNM
**1.35274195457034E-21**	0.424024705786835	0.135	0.091	2.26692496746897E-17	Ductular-like hepatocytes	LRR1
**1.88364462131939E-21**	0.266989523815286	0.11	0.071	3.15661165640703E-17	Ductular-like hepatocytes	GLMN
**3.28847483443168E-21**	0.386663392566802	0.111	0.073	5.51082612754061E-17	Ductular-like hepatocytes	AKR1B15
**1.00598882140346E-20**	0.258448141064089	0.128	0.087	1.68583606690792E-16	Ductular-like hepatocytes	BARD1
**1.61319818658323E-20**	0.439385881032456	0.1	0.065	2.70339752107617E-16	Ductular-like hepatocytes	CHD3
**4.42337193477948E-20**	0.25068589085941	0.1	0.064	7.41268668830345E-16	Ductular-like hepatocytes	GNAZ
**5.09748117776839E-20**	0.444239793101462	0.127	0.087	8.54235895770427E-16	Ductular-like hepatocytes	ITGAM
**1.00907054644862E-19**	0.373447421584192	0.106	0.07	1.6910004217386E-15	Ductular-like hepatocytes	DEPRECATEI
**1.30781887540543E-19**	0.397508147384386	0.106	0.07	2.19164287140442E-15	Ductular-like hepatocytes	PRIMPOL
**1.35113959160288E-19**	0.361142812307384	0.106	0.07	2.26423972760811E-15	Ductular-like hepatocytes	BRIP1
**4.05298606195382E-19**	0.275476042400701	0.108	0.072	6.79199404262222E-15	Ductular-like hepatocytes	SAC3D1
**2.15511803058056E-18**	0.27838308587476	0.119	0.082	3.6115467956469E-14	Ductular-like hepatocytes	CEP97
**2.44469381151498E-17**	0.327952942303496	0.13	0.092	4.0968178893368E-13	Ductular-like hepatocytes	MAP10
**7.58713013220833E-17**	0.427506855131822	0.107	0.074	1.27145126755547E-12	Ductular-like hepatocytes	POMGNT2
**9.16719635525513E-17**	0.290078432400158	0.101	0.068	1.53623876521365E-12	Ductular-like hepatocytes	RCN2
**4.13281063498091E-16**	0.381439957620511	0.107	0.074	6.92576406210101E-12	Ductular-like hepatocytes	NRM
**5.74628143775147E-16**	0.303210607369729	0.11	0.076	9.62961843338392E-12	Ductular-like hepatocytes	SLX4
**5.9419196137937E-16**	0.270534907263944	0.121	0.086	9.95746888879549E-12	Ductular-like hepatocytes	MYO9A
**1.0600068125204E-15**	0.376464860787149	0.145	0.107	1.77635941642169E-11	Ductular-like hepatocytes	LRP12
**1.7975726747996E-15**	0.402428957471421	0.131	0.095	3.01237228842917E-11	Ductular-like hepatocytes	GPC3
**1.90595994434401E-15**	0.326915013481663	0.106	0.074	3.19400767473169E-11	Ductular-like hepatocytes	C17orf80

3.50989593758295E-15	0.254583425877171	0.106	0.073	5.88188361220151E-11	Ductular-like hepatocytes	TRIM37
1.25769453620641E-14	0.2810291583659	0.109	0.077	2.1076445037747E-10	Ductular-like hepatocytes	SYT7
5.41501612327677E-14	0.862167388457451	0.101	0.071	9.07448401938721E-10	Ductular-like hepatocytes	CXCL10
9.7986587198177E-08	0.659396600569174	0.105	0.084	0.00164205922826705	Ductular-like hepatocytes	CALHM6
0	2.03359300561285	0.923	0.276	0	Detoxification-associated hepat	GABRE
0	1.97842277420224	0.797	0.16	0	Detoxification-associated hepat	ETNK2
0	2.59816825656794	0.795	0.199	0	Detoxification-associated hepat	MTCP1
0	2.50675874430887	0.819	0.229	0	Detoxification-associated hepat	FAM214A
0	1.81806431688913	0.82	0.248	0	Detoxification-associated hepat	AKR1C8P
0	1.70035002909068	0.934	0.366	0	Detoxification-associated hepat	CEP70
0	1.88570362546692	0.864	0.318	0	Detoxification-associated hepat	TBC1D8
0	2.1990670462347	0.944	0.399	0	Detoxification-associated hepat	CTPS1
0	2.10929917281085	0.715	0.171	0	Detoxification-associated hepat	PLGLB2
0	1.74621794171041	0.884	0.341	0	Detoxification-associated hepat	SLC17A4
0	2.12151724768053	0.895	0.357	0	Detoxification-associated hepat	NECAB3
0	2.06909105290449	0.756	0.227	0	Detoxification-associated hepat	ST3GAL6
0	1.59522273944434	0.89	0.372	0	Detoxification-associated hepat	NR1I2
0	1.82969407795337	0.761	0.243	0	Detoxification-associated hepat	RHOBTB3
0	1.19448762120442	0.947	0.429	0	Detoxification-associated hepat	ARGLU1
0	1.84214664889904	0.761	0.249	0	Detoxification-associated hepat	WDFY2
0	1.43574510432213	0.873	0.362	0	Detoxification-associated hepat	N4BP2L2
0	1.8393600291084	0.735	0.224	0	Detoxification-associated hepat	ARL17A
0	1.39802061032527	0.778	0.269	0	Detoxification-associated hepat	CCDC14
0	1.41500341254438	0.919	0.411	0	Detoxification-associated hepat	NOTCH2NLE
0	1.96836877987272	0.733	0.225	0	Detoxification-associated hepat	GRIA3
0	1.31904826215165	0.827	0.323	0	Detoxification-associated hepat	NKTR
0	1.35414609579554	0.834	0.33	0	Detoxification-associated hepat	PVR
0	1.42381460176074	0.932	0.43	0	Detoxification-associated hepat	ZBTB16
0	1.82344838702351	0.727	0.227	0	Detoxification-associated hepat	RBM12B
0	1.1857095917412	0.91	0.411	0	Detoxification-associated hepat	IVNS1ABP
0	1.47035713069233	0.889	0.39	0	Detoxification-associated hepat	SMOC1
0	1.51988396769983	0.912	0.414	0	Detoxification-associated hepat	OCRL
0	1.31245029740248	0.881	0.387	0	Detoxification-associated hepat	IL6R
0	1.46107739849707	0.855	0.364	0	Detoxification-associated hepat	TM7SF3
0	1.30109584712086	0.933	0.443	0	Detoxification-associated hepat	ALG13
0	1.24235302180681	0.858	0.368	0	Detoxification-associated hepat	LUC7L3
0	2.70409534891857	0.613	0.126	0	Detoxification-associated hepat	ZNF436
0	1.18215216628688	0.837	0.352	0	Detoxification-associated hepat	ATAD2
0	2.41466278400946	0.652	0.167	0	Detoxification-associated hepat	PTEN
0	1.3574237702041	0.769	0.285	0	Detoxification-associated hepat	VPS13B
0	1.3487423820875	0.918	0.435	0	Detoxification-associated hepat	ERO1B
0	1.23409895845215	0.851	0.368	0	Detoxification-associated hepat	MDM4
0	1.64023400313937	0.725	0.242	0	Detoxification-associated hepat	ABCA5
0	1.0662056346664	0.846	0.367	0	Detoxification-associated hepat	IKBKB
0	1.35296173879018	0.875	0.396	0	Detoxification-associated hepat	ABCA1
0	1.10685460856429	0.926	0.449	0	Detoxification-associated hepat	ANKRD10
0	1.18271033180207	0.899	0.424	0	Detoxification-associated hepat	PUM1
0	0.943912679398123	0.864	0.389	0	Detoxification-associated hepat	SULT1C2
0	1.6635518919219	0.725	0.251	0	Detoxification-associated hepat	TRIO
0	1.06232773373144	0.863	0.39	0	Detoxification-associated hepat	DEGS1
0	1.11946724727209	0.874	0.404	0	Detoxification-associated hepat	PAG1
0	1.14107270781504	0.822	0.352	0	Detoxification-associated hepat	XIAP
0	0.760689915018503	0.883	0.414	0	Detoxification-associated hepat	LENG8
0	1.23817464866032	0.786	0.319	0	Detoxification-associated hepat	SAMD5
0	1.79142978009376	0.664	0.203	0	Detoxification-associated hepat	DEPRECATEI
0	1.12066504107194	0.801	0.341	0	Detoxification-associated hepat	THOC2
0	1.02568699563096	0.821	0.361	0	Detoxification-associated hepat	MLPH
0	0.958669709346695	0.911	0.452	0	Detoxification-associated hepat	PTPRK
0	1.0197091389893	0.883	0.424	0	Detoxification-associated hepat	HNF4G
0	1.51256962496254	0.732	0.274	0	Detoxification-associated hepat	BPTF

0	1.24117247372713	0.913	0.457	0	Detoxification-associated hepat	LIPG
0	1.8572508855225	0.669	0.215	0	Detoxification-associated hepat	ZBTB1
0	1.26848495799476	0.738	0.284	0	Detoxification-associated hepat	FTCD
0	1.07575073100245	0.828	0.374	0	Detoxification-associated hepat	ARHGEF26
0	1.2186848695651	0.785	0.332	0	Detoxification-associated hepat	RNF19A
0	1.34223868299141	0.723	0.27	0	Detoxification-associated hepat	RAB33B
0	1.23191305334185	0.79	0.338	0	Detoxification-associated hepat	CNKSR3
0	1.76189178296891	0.664	0.213	0	Detoxification-associated hepat	WDYHV1
0	3.2868141356406	0.528	0.077	0	Detoxification-associated hepat	CES4A
0	1.25746504823026	0.746	0.295	0	Detoxification-associated hepat	OGT
0	0.964618304782864	0.959	0.508	0	Detoxification-associated hepat	CCNL2
0	1.66774252257247	0.707	0.256	0	Detoxification-associated hepat	INTS6
0	1.05639634751138	0.847	0.396	0	Detoxification-associated hepat	IRF3
0	1.50764518840126	0.685	0.235	0	Detoxification-associated hepat	FBXO42
0	1.37067855334419	0.717	0.267	0	Detoxification-associated hepat	RBM26
0	1.36721095288741	0.727	0.277	0	Detoxification-associated hepat	CLMN
0	1.07766229674009	0.785	0.336	0	Detoxification-associated hepat	HGSNAT
0	1.35907652025471	0.926	0.478	0	Detoxification-associated hepat	ELL2
0	1.70883327281071	0.668	0.221	0	Detoxification-associated hepat	MLH3
0	1.18821741917597	0.883	0.436	0	Detoxification-associated hepat	ZHX1
0	1.46123864572564	0.721	0.274	0	Detoxification-associated hepat	KLB
0	1.70033667051322	0.671	0.225	0	Detoxification-associated hepat	ZNF75D
0	1.3002145515132	0.763	0.317	0	Detoxification-associated hepat	NBPF26
0	1.34275210254682	0.697	0.252	0	Detoxification-associated hepat	PLGLB1
0	1.89245640418439	0.616	0.171	0	Detoxification-associated hepat	GREB1L
0	1.11176697037948	0.798	0.354	0	Detoxification-associated hepat	POGZ
0	1.05861136442443	0.908	0.464	0	Detoxification-associated hepat	PGM2
0	1.23712160708684	0.823	0.379	0	Detoxification-associated hepat	RAP2C
0	1.4840556539676	0.671	0.228	0	Detoxification-associated hepat	UGT1A9
0	0.969045468531662	0.794	0.352	0	Detoxification-associated hepat	PWWP3A
0	1.67522284653904	0.662	0.22	0	Detoxification-associated hepat	ZMAT1
0	1.11101384488567	0.853	0.411	0	Detoxification-associated hepat	ABHD2
0	0.97640366740979	0.774	0.333	0	Detoxification-associated hepat	BTNL8
0	1.08242692774592	0.913	0.474	0	Detoxification-associated hepat	NFIB
0	1.0431718886074	0.757	0.319	0	Detoxification-associated hepat	TAZ
0	1.40887066127823	0.756	0.32	0	Detoxification-associated hepat	ACSM1
0	1.26401887080643	0.715	0.28	0	Detoxification-associated hepat	SGK3
0	0.934720729519633	0.823	0.388	0	Detoxification-associated hepat	STAU2
0	1.30384997895856	0.686	0.252	0	Detoxification-associated hepat	RORA
0	1.36333170474026	0.721	0.288	0	Detoxification-associated hepat	ERN 1.00
0	1.33050735188697	0.69	0.257	0	Detoxification-associated hepat	NOTCH2
0	1.60647304240925	0.964	0.531	0	Detoxification-associated hepat	NOTCH2NLC
0	1.25904698152857	0.881	0.449	0	Detoxification-associated hepat	SEC16B
0	0.999535604161712	0.754	0.323	0	Detoxification-associated hepat	CLK1
0	0.918165330786279	0.835	0.404	0	Detoxification-associated hepat	CYP4V2
0	1.74849369468897	0.661	0.231	0	Detoxification-associated hepat	ZNF189
0	1.05027634434341	0.801	0.371	0	Detoxification-associated hepat	GPRC5C
0	0.952340475653921	0.844	0.414	0	Detoxification-associated hepat	WWP1
0	1.26394463558193	0.701	0.271	0	Detoxification-associated hepat	BDH2
0	1.34029363811767	0.696	0.266	0	Detoxification-associated hepat	HELZ
0	1.31230834340383	0.692	0.263	0	Detoxification-associated hepat	BIVM
0	0.998988430709257	0.799	0.371	0	Detoxification-associated hepat	PANK3
0	1.03188205469785	0.863	0.436	0	Detoxification-associated hepat	KLF15
0	1.24283939055045	0.711	0.284	0	Detoxification-associated hepat	UBXN2B
0	0.975307009276065	0.898	0.472	0	Detoxification-associated hepat	C2orf72
0	1.32728841846623	0.684	0.259	0	Detoxification-associated hepat	PIEZO2
0	0.989605450131424	0.752	0.327	0	Detoxification-associated hepat	MCTS1
0	1.13101552508057	0.846	0.423	0	Detoxification-associated hepat	ONECUT1
0	0.99821050023964	0.782	0.36	0	Detoxification-associated hepat	RREB1
0	0.956655045494753	0.893	0.472	0	Detoxification-associated hepat	TMEM37

0	0.971610012264476	0.733	0.312	0	Detoxification-associated hepat	COL27A1
0	1.06204077491879	0.727	0.306	0	Detoxification-associated hepat	DIAPH2
0	1.37449205438649	0.679	0.261	0	Detoxification-associated hepat	MAP3K5
0	0.995207283448021	0.935	0.517	0	Detoxification-associated hepat	ERICH5
0	1.06222129427926	0.809	0.391	0	Detoxification-associated hepat	CHPT1
0	1.39648307925849	0.671	0.253	0	Detoxification-associated hepat	TBC1D8B
0	1.11795607543628	0.847	0.429	0	Detoxification-associated hepat	PPP1R15B
0	1.06598802197859	0.724	0.306	0	Detoxification-associated hepat	LYPLAL1
0	0.876586978696864	0.824	0.406	0	Detoxification-associated hepat	ITPR2
0	1.29112148987713	0.684	0.267	0	Detoxification-associated hepat	PPP4R3A
0	1.20508064997019	0.689	0.272	0	Detoxification-associated hepat	CDC16
0	0.990943374612406	0.923	0.507	0	Detoxification-associated hepat	SERINC5
0	0.946975186159691	0.738	0.322	0	Detoxification-associated hepat	KAT6A
0	1.44939010181283	0.619	0.203	0	Detoxification-associated hepat	C1RL
0	1.93854128286723	0.586	0.17	0	Detoxification-associated hepat	CACNA1H
0	1.25199511852767	0.878	0.463	0	Detoxification-associated hepat	BHMT
0	1.07007526860289	0.709	0.294	0	Detoxification-associated hepat	FAM193B
0	1.02397949597399	0.95	0.535	0	Detoxification-associated hepat	FKBP5
0	1.47646197266249	0.639	0.225	0	Detoxification-associated hepat	TUT4
0	1.07539076451024	0.913	0.501	0	Detoxification-associated hepat	TOR1AIP2
0	0.953857254193994	0.743	0.331	0	Detoxification-associated hepat	STX16
0	1.12679183254743	0.691	0.279	0	Detoxification-associated hepat	KCNJ3
0	0.974894776030374	0.742	0.331	0	Detoxification-associated hepat	ZBTB33
0	1.18700222552046	0.679	0.269	0	Detoxification-associated hepat	ACACB
0	1.31732801184588	0.646	0.236	0	Detoxification-associated hepat	NLK
0	1.77385022866695	0.582	0.173	0	Detoxification-associated hepat	TIGD2
0	1.08864297867971	0.735	0.326	0	Detoxification-associated hepat	SH3RF2
0	1.17917017649335	0.696	0.287	0	Detoxification-associated hepat	ZMYM2
0	1.04640080385106	0.82	0.412	0	Detoxification-associated hepat	STOM
0	1.07706559618731	0.681	0.274	0	Detoxification-associated hepat	MAST4
0	1.18139725336868	0.674	0.267	0	Detoxification-associated hepat	GALK2
0	1.00133539600968	0.722	0.315	0	Detoxification-associated hepat	IMPA1
0	1.35269055987101	0.948	0.541	0	Detoxification-associated hepat	ACSM2A
0	1.18929229884009	0.682	0.276	0	Detoxification-associated hepat	NIPAL2
0	1.38591058489044	0.645	0.24	0	Detoxification-associated hepat	CCT8
0	0.930202656904231	0.717	0.312	0	Detoxification-associated hepat	CHD2
0	1.03550729967597	0.688	0.284	0	Detoxification-associated hepat	PRKAG2
0	1.04325528372442	0.774	0.371	0	Detoxification-associated hepat	CPEB4
0	1.27450465541902	0.653	0.25	0	Detoxification-associated hepat	EXOC3L4
0	2.688529483108	0.966	0.563	0	Detoxification-associated hepat	SMARCA1
0	0.96434124201695	0.871	0.468	0	Detoxification-associated hepat	TP53INP1
0	1.36870924000057	0.63	0.228	0	Detoxification-associated hepat	RNF138
0	1.66838633669868	0.591	0.19	0	Detoxification-associated hepat	RNF125
0	1.17915969301613	0.676	0.276	0	Detoxification-associated hepat	N4BP2L1
0	1.10180555406592	0.694	0.294	0	Detoxification-associated hepat	TMEM68
0	1.31264050602824	0.639	0.241	0	Detoxification-associated hepat	DEPRECATEI
0	0.988627258193733	0.697	0.299	0	Detoxification-associated hepat	UBR5
0	1.36715378725087	0.625	0.229	0	Detoxification-associated hepat	GTF2H2C
0	0.940026748067587	0.699	0.303	0	Detoxification-associated hepat	TM4SF20
0	1.12086546681929	0.703	0.307	0	Detoxification-associated hepat	REPS1
0	1.18186786897114	0.638	0.243	0	Detoxification-associated hepat	PCMTD2
0	0.991688755740709	0.701	0.306	0	Detoxification-associated hepat	KIAA0232
0	2.36109896195353	0.475	0.08	0	Detoxification-associated hepat	ALPK2
0	2.73343432955618	0.465	0.071	0	Detoxification-associated hepat	PTPRM
0	1.56440600623641	0.593	0.199	0	Detoxification-associated hepat	DCAF16
0	1.02451713030179	0.698	0.304	0	Detoxification-associated hepat	CYP4F12
0	1.39759024745441	0.62	0.227	0	Detoxification-associated hepat	LRBA
0	2.02846942100141	0.525	0.134	0	Detoxification-associated hepat	UGT1A3
0	1.3283254071341	0.632	0.241	0	Detoxification-associated hepat	SEC24B
0	1.65131618455562	0.602	0.212	0	Detoxification-associated hepat	HAO2

0	1.40102100873709	0.616	0.226	0	Detoxification-associated hepat	TASOR2
0	1.01571869556561	0.666	0.277	0	Detoxification-associated hepat	SOS 2
0	1.24088636705233	0.643	0.255	0	Detoxification-associated hepat	PARP9
0	0.986688467165882	0.667	0.279	0	Detoxification-associated hepat	TTPA
0	1.13137815108441	0.627	0.239	0	Detoxification-associated hepat	SAMHD1
0	1.16855391134342	0.673	0.287	0	Detoxification-associated hepat	TLCD4
0	1.1098153840803	0.641	0.255	0	Detoxification-associated hepat	HECTD4
0	1.77334798155032	0.56	0.175	0	Detoxification-associated hepat	DGKE
0	1.17849073054282	0.659	0.274	0	Detoxification-associated hepat	RAPH1
0	1.23008404904066	0.62	0.235	0	Detoxification-associated hepat	TMEM175
0	1.03341013780813	0.664	0.279	0	Detoxification-associated hepat	SRSF5
0	1.54711000069453	0.588	0.203	0	Detoxification-associated hepat	BCL2L2
0	1.02835948608086	0.928	0.544	0	Detoxification-associated hepat	PCSK6
0	1.43151961644042	0.599	0.216	0	Detoxification-associated hepat	LYST
0	1.78155736682318	0.545	0.163	0	Detoxification-associated hepat	TBC1D1
0	1.02433042797277	0.666	0.285	0	Detoxification-associated hepat	TTC3
0	1.10352373459286	0.614	0.234	0	Detoxification-associated hepat	SLC46A1
0	1.09960908704028	0.635	0.257	0	Detoxification-associated hepat	PDLIM5
0	1.61860976179149	0.554	0.177	0	Detoxification-associated hepat	ZBTB25
0	1.35375998842086	0.589	0.215	0	Detoxification-associated hepat	TARBP1
0	1.13192012255956	0.624	0.253	0	Detoxification-associated hepat	PARP14
0	1.05077142343103	0.913	0.542	0	Detoxification-associated hepat	AKR1C2
0	1.17227902640799	0.605	0.235	0	Detoxification-associated hepat	MBOAT1
0	1.40889524166619	0.588	0.218	0	Detoxification-associated hepat	USP54
0	1.23769646419214	0.591	0.222	0	Detoxification-associated hepat	GSAP
0	1.7193723266195	0.534	0.165	0	Detoxification-associated hepat	ART4
0	2.61536217070622	0.485	0.117	0	Detoxification-associated hepat	CCDC141
0	2.02544802857768	0.514	0.147	0	Detoxification-associated hepat	GOLGA8B
0	1.06258009280948	0.619	0.253	0	Detoxification-associated hepat	GOSR2
0	1.36271616363122	0.526	0.163	0	Detoxification-associated hepat	KCNT2
0	1.16846912585457	0.579	0.216	0	Detoxification-associated hepat	SUSD3
0	2.05044164365509	0.479	0.116	0	Detoxification-associated hepat	DEPRECATEI
0	1.10485147331077	0.575	0.213	0	Detoxification-associated hepat	UGT2B15
0	1.16403273394169	0.591	0.229	0	Detoxification-associated hepat	STRN3
0	1.11747706501965	0.594	0.234	0	Detoxification-associated hepat	NFS1
0	1.30496788219951	0.571	0.212	0	Detoxification-associated hepat	PDE8A
0	1.16036133166369	0.583	0.225	0	Detoxification-associated hepat	ACACA
0	1.1957982706291	0.589	0.232	0	Detoxification-associated hepat	ZMYND8
0	1.49005124368883	0.539	0.183	0	Detoxification-associated hepat	PRPF39
0	1.35202266653307	0.559	0.206	0	Detoxification-associated hepat	DMXL1
0	1.35428451132945	0.549	0.201	0	Detoxification-associated hepat	GLI4
0	1.26256087379795	0.513	0.167	0	Detoxification-associated hepat	IL1RAP
0	2.79268341782228	0.443	0.098	0	Detoxification-associated hepat	TTN
0	2.06770743800361	0.485	0.141	0	Detoxification-associated hepat	TSPYL4
0	1.40676311731145	0.487	0.145	0	Detoxification-associated hepat	PLA2G4C
0	1.70043613903649	0.493	0.152	0	Detoxification-associated hepat	PIK3C2G
0	1.35782934081768	0.543	0.204	0	Detoxification-associated hepat	ANAPC4
0	1.2211299392043	0.531	0.192	0	Detoxification-associated hepat	SIN3B
0	1.7080475007444	0.498	0.16	0	Detoxification-associated hepat	ANKS1B
0	1.40984140924457	0.52	0.183	0	Detoxification-associated hepat	DPH6
0	1.38304598874019	0.512	0.176	0	Detoxification-associated hepat	TMEM87A
0	1.00489996013583	0.95	0.614	0	Detoxification-associated hepat	HGD
0	2.09318651573357	0.436	0.1	0	Detoxification-associated hepat	TSPOAP1
0	1.45297108809325	0.51	0.175	0	Detoxification-associated hepat	ERMARD
0	1.40431572669398	0.503	0.168	0	Detoxification-associated hepat	ATG16L2
0	1.08630954706221	0.953	0.619	0	Detoxification-associated hepat	F5
0	1.32780485800692	0.487	0.154	0	Detoxification-associated hepat	SPHK1
0	0.999043289587476	0.95	0.62	0	Detoxification-associated hepat	SELENBP1
0	1.22785722439022	0.937	0.608	0	Detoxification-associated hepat	BAAT
0	1.22882699736521	0.51	0.182	0	Detoxification-associated hepat	MID2

0	1.41878367565176	0.951	0.625	0	Detoxification-associated hepat	SGK1
0	1.63000117784926	0.48	0.154	0	Detoxification-associated hepat	ZNF251
0	1.28902995445533	0.5	0.176	0	Detoxification-associated hepat	ZNF580
0	1.52664480159311	0.968	0.644	0	Detoxification-associated hepat	ERRFI1
0	1.40919300114063	0.499	0.177	0	Detoxification-associated hepat	SLC10A5
0	1.2883878610375	0.486	0.166	0	Detoxification-associated hepat	COG2
0	1.67431253228946	0.447	0.133	0	Detoxification-associated hepat	PSD3
0	1.32096801202451	0.477	0.163	0	Detoxification-associated hepat	SLC19A2
0	1.05102674674632	0.947	0.634	0	Detoxification-associated hepat	PROX1
0	1.74498956550846	0.444	0.132	0	Detoxification-associated hepat	RWDD3
0	1.21912756488639	0.45	0.139	0	Detoxification-associated hepat	FAXDC2
0	1.09812967903143	0.957	0.646	0	Detoxification-associated hepat	TDO2
0	2.18522063661763	0.38	0.088	0	Detoxification-associated hepat	LINS1
0	1.64841117013376	0.961	0.672	0	Detoxification-associated hepat	SERPINA7
0	2.15792397448532	0.36	0.084	0	Detoxification-associated hepat	CEACAM19
0	2.13969566539531	0.338	0.064	0	Detoxification-associated hepat	PAPLN
0	2.10184692677454	0.366	0.099	0	Detoxification-associated hepat	NAIP
0	2.03360375034764	0.338	0.083	0	Detoxification-associated hepat	ADCY10
0	2.03030209478678	0.345	0.091	0	Detoxification-associated hepat	ZNF552
0	1.13473293603042	0.973	0.751	0	Detoxification-associated hepat	ACSL1
0	0.914938818272204	0.962	0.754	0	Detoxification-associated hepat	FMO5
0	0.730695171947651	0.969	0.815	0	Detoxification-associated hepat	PGRMC1
0	3.98437805535726	0.147	0.01	0	Detoxification-associated hepat	IAPP
6.41793073100285E-308	1.34976322510118	0.521	0.192	1.07551683190146E-303	Detoxification-associated hepat	TTC14
1.08575577088067E-307	1.03232127542056	0.661	0.281	1.81950952084183E-303	Detoxification-associated hepat	FBXL5
1.64457406908502E-306	1.002215375778	0.715	0.317	2.75597722497268E-302	Detoxification-associated hepat	DDX39B
4.12659325193628E-306	1.20942095005982	0.546	0.208	6.91534497159481E-302	Detoxification-associated hepat	ATG12
7.6649448862913E-305	1.85739366143061	0.307	0.073	1.2844914640447E-300	Detoxification-associated hepat	FZD4
3.85959872610274E-304	1.00654383555982	0.608	0.247	6.46791554520298E-300	Detoxification-associated hepat	AKAP13
5.0561540272114E-303	1.25978922516572	0.537	0.203	8.47310291880086E-299	Detoxification-associated hepat	L3HYPDH
1.5915579764443E-302	1.14620992367045	0.61	0.25	2.66713285692536E-298	Detoxification-associated hepat	ADCY6
3.51280188540573E-302	1.39735687620831	0.488	0.173	5.88675339956292E-298	Detoxification-associated hepat	DMXL2
6.65455919338671E-302	0.884145204717403	0.749	0.349	1.11517102962775E-297	Detoxification-associated hepat	CRY2
1.4829486509753E-301	0.952366509564816	0.647	0.275	2.48512534930441E-297	Detoxification-associated hepat	FOXO3
1.75859745759874E-301	1.50018471481047	0.467	0.164	2.94705761944397E-297	Detoxification-associated hepat	ZNF75A
3.42063966803187E-301	0.853533923130787	0.899	0.447	5.7323079556878E-297	Detoxification-associated hepat	F2RL1
8.8806096447703E-301	1.01254839989726	0.648	0.275	1.48821256427061E-296	Detoxification-associated hepat	LNX2
1.57417330617367E-300	0.908101575782198	0.696	0.306	2.63799962648584E-296	Detoxification-associated hepat	USP12
1.02519778678494E-299	1.13672008666072	0.573	0.227	1.7180264510942E-295	Detoxification-associated hepat	AKAP8
2.5023555500553E-299	0.884312800419961	0.792	0.39	4.19344743078267E-295	Detoxification-associated hepat	ACSM2B
2.5602715669703E-299	1.61257107664889	0.445	0.151	4.29050309192883E-295	Detoxification-associated hepat	ZMYM5
3.4681152701596E-299	1.03762221136006	0.623	0.254	5.81186756973347E-295	Detoxification-associated hepat	DAZAP1
7.22725032570491E-299	1.27085910507064	0.557	0.221	1.21114260958163E-294	Detoxification-associated hepat	APPL2
1.32126045823148E-297	1.10224382757554	0.619	0.256	2.21416827590431E-293	Detoxification-associated hepat	RBM41
2.3958069002072E-297	0.630545903939206	0.663	0.25	4.01489320336723E-293	Detoxification-associated hepat	AQP9
3.33540596298393E-297	0.891135157383755	0.754	0.345	5.58947331276848E-293	Detoxification-associated hepat	OPLAH
5.57707638943077E-297	1.03199929570952	0.692	0.304	9.34606461340808E-293	Detoxification-associated hepat	LRRC31
1.38103844351726E-296	0.966931519324997	0.731	0.326	2.31434422364623E-292	Detoxification-associated hepat	INTS8
1.40245854368596E-296	0.871299643629917	0.625	0.256	2.35024002750893E-292	Detoxification-associated hepat	GPC6
4.32435457586463E-296	1.28115235422274	0.469	0.162	7.24675339823394E-292	Detoxification-associated hepat	SLC25A16
6.91870924718708E-296	1.0000585724144	0.558	0.215	1.15943729564361E-291	Detoxification-associated hepat	MAN2A2
1.4982179692225E-295	0.757688492614596	0.961	0.732	2.51071367282306E-291	Detoxification-associated hepat	UGT2B4
1.59216783251889E-295	0.90125065382522	0.672	0.285	2.66815485373515E-291	Detoxification-associated hepat	UGT2B11
4.59794421170517E-295	0.97149874602255	0.685	0.298	7.70523490997552E-291	Detoxification-associated hepat	BIRC6
4.18307054250788E-294	1.00695282375274	0.741	0.352	7.00998961513471E-290	Detoxification-associated hepat	LSS
1.64476607549375E-293	0.777596664431529	0.577	0.22	2.75629898931242E-289	Detoxification-associated hepat	LTBP3
4.41764952136577E-293	1.03534484967662	0.565	0.223	7.40309706790476E-289	Detoxification-associated hepat	ZBED6
1.186464225037E-292	1.26934939227566	0.519	0.197	1.988276748317E-288	Detoxification-associated hepat	DNAH14
3.60295605449519E-292	1.24006857188416	0.555	0.218	6.03783375612303E-288	Detoxification-associated hepat	PRELID2
3.93171596420305E-292	0.853750555181704	0.799	0.385	6.58876961281147E-288	Detoxification-associated hepat	KLHDC2

1.11591631803586E-291	0.828014456190019	0.79	0.359	1.8700525657645E-287	Detoxification-associated hepat	TMEM63A
1.57296983358258E-291	1.05157696664307	0.475	0.165	2.63598284711769E-287	Detoxification-associated hepat	PEX11A
3.29456811539502E-291	1.16671411069516	0.592	0.241	5.52103724777897E-287	Detoxification-associated hepat	EPPK1
4.08390784304708E-291	0.734626951776429	0.86	0.443	6.8438127633783E-287	Detoxification-associated hepat	ECHDC2
1.53779687500451E-290	1.11670917683563	0.609	0.255	2.57704000313256E-286	Detoxification-associated hepat	CPM
1.80591023598449E-289	1.30192479949762	0.507	0.191	3.02634437346281E-285	Detoxification-associated hepat	C8orf76
2.16867700825924E-289	0.957194407791265	0.654	0.282	3.63426893044083E-285	Detoxification-associated hepat	KMT2B
8.3739549014705E-289	0.961691262845876	0.663	0.289	1.40330736238843E-284	Detoxification-associated hepat	SENP6
1.0102691977284E-288	1.82095987441783	0.268	0.058	1.69300912155326E-284	Detoxification-associated hepat	SYNE4
3.19375655489571E-288	1.51413218984193	0.42	0.138	5.35209723469423E-284	Detoxification-associated hepat	THSD4
4.27146898550112E-288	0.982065540475936	0.621	0.264	7.15812772590278E-284	Detoxification-associated hepat	KIF1B
9.98786740492772E-288	0.843121670655824	0.705	0.318	1.67376681971779E-283	Detoxification-associated hepat	ZBTB20
3.52721951766848E-287	0.785610976108193	0.755	0.341	5.91091446770883E-283	Detoxification-associated hepat	DEPRECATEI
1.05320983383574E-285	1.55064696351323	0.443	0.154	1.76496903954193E-281	Detoxification-associated hepat	TMEM39A
9.58722367447577E-285	2.12317980246505	0.305	0.078	1.60662694336865E-280	Detoxification-associated hepat	PHOSPHO2
9.97725861661742E-285	1.23589903510289	0.503	0.189	1.67198899897275E-280	Detoxification-associated hepat	MED23
2.08001789323926E-284	0.903247740944089	0.7	0.309	3.48569398549035E-280	Detoxification-associated hepat	HECTD1
9.36766142635415E-284	0.793734477455809	0.805	0.398	1.56983270182843E-279	Detoxification-associated hepat	GSTA2
1.78436959903923E-283	1.44002924477383	0.479	0.176	2.99024657406995E-279	Detoxification-associated hepat	USP53
3.89203129171883E-283	1.32639608485551	0.469	0.167	6.52226603866241E-279	Detoxification-associated hepat	PLCXD1
6.73989326505022E-283	1.34951222733518	0.482	0.178	1.12947131335712E-278	Detoxification-associated hepat	ZNF337
6.81424201355225E-283	1.23997304137861	0.49	0.179	1.14193067663109E-278	Detoxification-associated hepat	MFSD3
2.01638084565865E-282	1.11357188879261	0.555	0.22	3.37905102115477E-278	Detoxification-associated hepat	PAN3
3.46501934222501E-282	1.1478509607446	0.538	0.212	5.80667941370067E-278	Detoxification-associated hepat	MON2
6.99934251065181E-281	0.989658989001064	0.584	0.239	1.17294981793503E-276	Detoxification-associated hepat	UQCC1
1.83566250511611E-280	1.07817924756139	0.554	0.22	3.07620322607358E-276	Detoxification-associated hepat	GPATCH2L
1.93239961040679E-280	0.978834825433458	0.615	0.254	3.2383152671197E-276	Detoxification-associated hepat	NT5DC3
6.98244701442024E-280	0.723985668833615	0.572	0.219	1.17011847067654E-275	Detoxification-associated hepat	SLCO1B1
9.19788337659332E-280	0.870532420208333	0.751	0.346	1.54138129624951E-275	Detoxification-associated hepat	ACMSD
2.96363680617291E-279	1.03035049441336	0.497	0.181	4.96646255978456E-275	Detoxification-associated hepat	KIFC2
3.44035227906412E-279	0.976772938063218	0.572	0.231	5.76534234925566E-275	Detoxification-associated hepat	SHC2
1.47189311276583E-278	1.2726661255419	0.487	0.181	2.46659847837298E-274	Detoxification-associated hepat	EPC1
1.52764691488683E-277	0.7899378432073	0.813	0.401	2.56003069996736E-273	Detoxification-associated hepat	SETD5
1.91764570310094E-277	1.57053407368748	0.461	0.168	3.21359066925656E-273	Detoxification-associated hepat	BTNL3
2.1514141535762E-277	1.47498513111725	0.284	0.066	3.60533983856299E-273	Detoxification-associated hepat	CALHM6
2.47127867175779E-277	0.758804137520108	0.948	0.619	4.1413687981317E-273	Detoxification-associated hepat	ACSL4
5.64097305293713E-277	0.806415375382014	0.89	0.482	9.45314264211204E-273	Detoxification-associated hepat	KCTD3
1.22195657459369E-276	1.31725731415029	0.474	0.173	2.04775482770411E-272	Detoxification-associated hepat	D2HGDH
1.553654599208E-276	0.806326709289634	0.767	0.37	2.60361437735277E-272	Detoxification-associated hepat	CHTOP
7.60921660069444E-276	0.870799286935804	0.762	0.364	1.27515251794437E-271	Detoxification-associated hepat	PCCA
9.5645107476034E-276	1.11490086649702	0.572	0.237	1.60282071108338E-271	Detoxification-associated hepat	HOOK3
3.12820477043341E-275	1.24173119710846	0.509	0.196	5.24224555429232E-271	Detoxification-associated hepat	RASGEF1B
1.50405626141517E-274	0.897786646179766	0.645	0.28	2.52049748287955E-270	Detoxification-associated hepat	TTC37
5.84975494350445E-274	0.903291892685708	0.78	0.381	9.80301933432476E-270	Detoxification-associated hepat	SLC35D2
3.36687487349289E-273	1.17796813838918	0.528	0.209	5.64220891299939E-269	Detoxification-associated hepat	RCOR3
5.67109204720511E-273	1.23086805378591	0.481	0.18	9.50361605270633E-269	Detoxification-associated hepat	ATPSCKMT
7.22277554136353E-272	1.20814713235465	0.507	0.195	1.2103927252217E-267	Detoxification-associated hepat	GTF2H2
3.13584651835749E-270	1.11273023675288	0.58	0.243	5.25505159546348E-266	Detoxification-associated hepat	PHF6
1.21345425739897E-269	0.888448352871873	0.669	0.302	2.03350664454919E-265	Detoxification-associated hepat	TACC1
1.63518457516861E-269	0.795145204947188	0.837	0.427	2.74024231106755E-265	Detoxification-associated hepat	AFF1
1.85729749589905E-269	0.844179000105978	0.735	0.335	3.11245914362762E-265	Detoxification-associated hepat	PRKDC
1.1991421231225E-268	0.98926934694106	0.594	0.252	2.00952236992869E-264	Detoxification-associated hepat	ZFAND1
1.59465359264811E-268	1.58061861279791	0.362	0.111	2.67232049055971E-264	Detoxification-associated hepat	OTUD3
4.85474893044517E-268	0.89609170648597	0.627	0.27	8.13558825764002E-264	Detoxification-associated hepat	MARCH7
6.87867202027958E-268	0.985182855176618	0.568	0.236	1.15272785715845E-263	Detoxification-associated hepat	MORC3
8.83926510909908E-268	1.01336147789352	0.595	0.25	1.48128404698282E-263	Detoxification-associated hepat	DCUN1D4
2.8518698381098E-267	0.833308619280055	0.68	0.31	4.7791634747044E-263	Detoxification-associated hepat	SPPL2A
7.80275935363575E-267	0.779755166138956	0.735	0.346	1.30758641248228E-262	Detoxification-associated hepat	LIMD1
1.49972415140615E-266	0.8943211462206	0.611	0.261	2.51323773292643E-262	Detoxification-associated hepat	TMEM129
1.92885615737516E-266	0.67019962530483	0.598	0.24	3.23237714852929E-262	Detoxification-associated hepat	HLF

2.53917629589249E-266	0.940576732450355	0.625	0.273	4.25515163665663E-262	Detoxification-associated hepat	SSX2IP
3.7693130916036E-266	0.979871460366582	0.653	0.291	6.31661487890932E-262	Detoxification-associated hepat	INTS3
7.68298925689281E-266	1.07171494635313	0.515	0.202	1.2875153396701E-261	Detoxification-associated hepat	MTERF4
8.90396024953563E-266	1.13319433539158	0.567	0.234	1.49212565861718E-261	Detoxification-associated hepat	ASB9
2.17785024065877E-265	1.31374785239954	0.423	0.145	3.64964143329596E-261	Detoxification-associated hepat	FRY
2.75823100639581E-265	1.19907188771062	0.492	0.189	4.62224352051811E-261	Detoxification-associated hepat	UPF3A
2.80970896020305E-265	0.882415620017115	0.747	0.366	4.70851027550827E-261	Detoxification-associated hepat	TUT7
1.44174343483206E-264	1.00116889542484	0.572	0.241	2.41607364809157E-260	Detoxification-associated hepat	APOOL
2.27280294762423E-264	1.16700649981762	0.527	0.213	3.80876317962868E-260	Detoxification-associated hepat	ADNP
3.58749095505594E-264	1.33330306508989	0.436	0.154	6.01191734248274E-260	Detoxification-associated hepat	MTERF2
1.22741215871663E-263	0.883379990317467	0.64	0.281	2.05689729557733E-259	Detoxification-associated hepat	PARP10
3.06524808616387E-263	1.18366527155313	0.422	0.144	5.13674274279341E-259	Detoxification-associated hepat	PAQR9
3.8404679071909E-263	1.0505344694601	0.554	0.228	6.4358561188705E-259	Detoxification-associated hepat	ZFHX3
7.53263770390086E-263	0.856872861606057	0.711	0.328	1.26231942641971E-258	Detoxification-associated hepat	DLG1
8.17474775758939E-262	0.537911531092862	0.986	0.925	1.36992422921683E-257	Detoxification-associated hepat	SELENOP
1.1687887148825E-261	0.803729805917068	0.73	0.35	1.95865612840009E-257	Detoxification-associated hepat	SLC25A15
1.66996488710472E-261	0.953929998604191	0.566	0.237	2.79852715781009E-257	Detoxification-associated hepat	CNST
4.8197908789833E-261	0.854795137895788	0.673	0.306	8.07700555500021E-257	Detoxification-associated hepat	ECPAS
7.5532602972616E-261	1.0917733327922	0.519	0.206	1.2657753606151E-256	Detoxification-associated hepat	VPS13C
1.16970098699057E-260	0.877443543441471	0.628	0.282	1.9601849139988E-256	Detoxification-associated hepat	DHTKD1
1.92121754052417E-260	1.4111490779631	0.419	0.144	3.2195763544104E-256	Detoxification-associated hepat	NOL3
1.94158368343295E-260	0.991661365093494	0.535	0.213	3.25370593669695E-256	Detoxification-associated hepat	AASS
2.47737764981694E-259	0.843313811161641	0.724	0.343	4.15158946556322E-255	Detoxification-associated hepat	ACVR1B
9.32896498724234E-259	0.754564661273336	0.84	0.441	1.56334795256207E-254	Detoxification-associated hepat	NUDT16
9.64834309660885E-259	0.764876612266324	0.658	0.293	1.61686933612971E-254	Detoxification-associated hepat	TIA1
1.21392705430017E-258	0.822295002833082	0.711	0.327	2.03429895759622E-254	Detoxification-associated hepat	GPT
1.30525446622339E-258	1.04121886525348	0.577	0.245	2.18734543449716E-254	Detoxification-associated hepat	RAB3IP
2.55198623951456E-258	0.849432540992961	0.686	0.322	4.2766185401785E-254	Detoxification-associated hepat	PRPF3
3.91259158213207E-258	1.3480598170667	0.371	0.117	6.55672097333691E-254	Detoxification-associated hepat	C15orf40
1.21050333970392E-257	1.27841489323961	0.472	0.179	2.02856149667583E-253	Detoxification-associated hepat	UVSSA
1.55930224272983E-257	1.17880706151353	0.239	0.049	2.61307869836664E-253	Detoxification-associated hepat	OBSL1
9.28756562346497E-257	0.774986508736186	0.856	0.465	1.55641024718026E-252	Detoxification-associated hepat	NDUFV2
2.30077047394274E-256	1.47975027694413	0.394	0.131	3.85563116023324E-252	Detoxification-associated hepat	ANKRD36B
2.43081328273895E-256	0.769431179051678	0.772	0.381	4.07355689921394E-252	Detoxification-associated hepat	DPYD
4.15779021829916E-256	1.04489399394097	0.503	0.199	6.96762484782573E-252	Detoxification-associated hepat	MTMR4
8.95531991978158E-256	0.814407688151156	0.889	0.499	1.500732512157E-251	Detoxification-associated hepat	C6
2.65089418430468E-255	0.813358520304887	0.805	0.395	4.44236847405778E-251	Detoxification-associated hepat	CPD
3.40257934504843E-255	1.03042218631093	0.49	0.186	5.70204246643215E-251	Detoxification-associated hepat	XAF1
6.78479221664599E-255	1.44463182471822	0.403	0.139	1.13699547966553E-250	Detoxification-associated hepat	GANC
7.38954428157995E-255	2.04072357960676	0.246	0.055	1.23833983070717E-250	Detoxification-associated hepat	DNAH11
7.01317660841314E-254	0.983113714419166	0.555	0.23	1.17526813603787E-249	Detoxification-associated hepat	CLDN14
1.14368047728315E-253	0.833697343181009	0.699	0.33	1.9165797438311E-249	Detoxification-associated hepat	MBNL1
1.2352823611492E-253	0.936002193294939	0.631	0.282	2.07008618081384E-249	Detoxification-associated hepat	SMYD2
1.39014399211188E-253	0.683835024441611	0.821	0.412	2.32960330198109E-249	Detoxification-associated hepat	AMOTL1
1.63615789489255E-253	0.791851795577677	0.63	0.282	2.74187340026094E-249	Detoxification-associated hepat	FOXP4
6.41499327967605E-253	1.74258151275412	0.331	0.099	1.07502457380811E-248	Detoxification-associated hepat	LIG4
1.07390219026799E-252	0.756053508404653	0.811	0.396	1.79964529045109E-248	Detoxification-associated hepat	STAG2
2.96327195283373E-252	1.18062229299524	0.482	0.188	4.96585113855876E-248	Detoxification-associated hepat	NSMAF
5.17504507638371E-252	0.838788775983853	0.56	0.232	8.67234053900382E-248	Detoxification-associated hepat	ABCG5
9.42275894779868E-252	0.843718416728797	0.636	0.283	1.5790659444721E-247	Detoxification-associated hepat	ANKRD17
1.90915881392774E-251	0.872254680304589	0.6	0.262	3.19936834038011E-247	Detoxification-associated hepat	SLC33A1
1.95597555135723E-251	1.01450895762177	0.57	0.239	3.27782382896445E-247	Detoxification-associated hepat	FAIM
3.20801367356382E-251	0.954488732413246	0.536	0.221	5.37598931415824E-247	Detoxification-associated hepat	CLCC1
4.33296314227045E-251	1.11371693823507	0.485	0.189	7.26117963381681E-247	Detoxification-associated hepat	ZNF7
6.09826152296252E-250	1.37550730546417	0.4	0.139	1.02194666601806E-245	Detoxification-associated hepat	TRAPPC8
2.15019563181771E-249	1.76717708985072	0.327	0.098	3.60329783980013E-245	Detoxification-associated hepat	WDR5B
2.55331176028665E-249	0.751508347323937	0.803	0.394	4.27883984788836E-245	Detoxification-associated hepat	SYNE2
5.71341260891086E-249	1.45035951607098	0.364	0.117	9.57453685001282E-245	Detoxification-associated hepat	TRIM52
6.87065272029992E-249	0.705531387284496	0.937	0.532	1.15138398286786E-244	Detoxification-associated hepat	TSPAN3
9.74159802811054E-249	1.38381362534228	0.461	0.179	1.63249699755076E-244	Detoxification-associated hepat	FZD6

1.45016002806555E-248	0.991020974257906	0.539	0.222	2.43017817503225E-244	Detoxification-associated hepat	USP48
3.20613178849776E-248	2.21275152992388	0.262	0.065	5.37283565116455E-244	Detoxification-associated hepat	TAS2R14
4.06465876728278E-248	1.34815247967276	0.402	0.14	6.81155516221248E-244	Detoxification-associated hepat	MYSM1
6.21047347112089E-248	1.04930542030799	0.526	0.218	1.04075114429044E-243	Detoxification-associated hepat	SLC25A32
1.51105117291688E-247	0.823529849585294	0.637	0.289	2.53221955557411E-243	Detoxification-associated hepat	ZNF638
1.56997119844879E-247	1.44053170058474	0.417	0.149	2.63095773436048E-243	Detoxification-associated hepat	POU5F1
1.07662134007621E-246	0.821902045688337	0.656	0.291	1.80420204169971E-242	Detoxification-associated hepat	TRMT2B
1.14269029442942E-246	0.748503433449834	0.956	0.599	1.91492039540482E-242	Detoxification-associated hepat	TSC22D3
2.22499722955565E-246	0.699351688558404	0.923	0.599	3.72865035728936E-242	Detoxification-associated hepat	HPN
2.26389805838722E-246	0.941324821609278	0.706	0.339	3.7938403662453E-242	Detoxification-associated hepat	TCIM
4.14904506565302E-246	1.12887447807176	0.472	0.183	6.95296972102133E-242	Detoxification-associated hepat	MTERF3
6.92162364694851E-246	0.545623394008248	0.947	0.584	1.15992569075563E-241	Detoxification-associated hepat	PAH
1.12128966789358E-245	1.44987565627128	0.397	0.139	1.87905722545607E-241	Detoxification-associated hepat	SRGAP1
3.19267798857231E-245	0.942998169689442	0.589	0.256	5.35028977324947E-241	Detoxification-associated hepat	SNX5
4.50463223022607E-245	0.78998481462581	0.758	0.374	7.54886269141284E-241	Detoxification-associated hepat	RNF103
7.62895907796472E-245	0.791889363832022	0.649	0.293	1.27846096228533E-240	Detoxification-associated hepat	PHF20L1
1.75372994377326E-244	0.718102751080769	0.666	0.308	2.93890063977524E-240	Detoxification-associated hepat	COG3
2.36965066645321E-244	1.18666161739928	0.427	0.155	3.97106058684229E-240	Detoxification-associated hepat	PM20D2
8.05590390716971E-244	1.11336104217877	0.474	0.182	1.3500083767635E-239	Detoxification-associated hepat	PAQR7
9.03887039041331E-244	1.39814483031502	0.395	0.137	1.51473390002546E-239	Detoxification-associated hepat	AC253572.1
1.28249343192537E-243	0.984171865739897	0.494	0.196	2.14920249322054E-239	Detoxification-associated hepat	NF1
1.66351737020502E-243	0.761092623678899	0.693	0.322	2.78772240898957E-239	Detoxification-associated hepat	U2SURP
1.83521914627874E-243	0.898410364082453	0.573	0.245	3.07546024533391E-239	Detoxification-associated hepat	ASAP1
5.6847275585061E-243	1.67665493888591	0.358	0.116	9.52646644254451E-239	Detoxification-associated hepat	CHST6
5.82824248436078E-243	0.94541109042825	0.546	0.231	9.76696875529179E-239	Detoxification-associated hepat	GSTM4
6.44160832216856E-243	0.77140145821644	0.68	0.317	1.07948472262901E-238	Detoxification-associated hepat	UBR2
7.37825018097146E-243	0.768149157182478	0.819	0.427	1.2364471653272E-238	Detoxification-associated hepat	PICALM
1.33419341036613E-242	0.809031377856988	0.638	0.292	2.23584131709156E-238	Detoxification-associated hepat	MPV17
6.04535333847313E-242	0.713465006407479	0.809	0.419	1.01308031246133E-237	Detoxification-associated hepat	DGAT2
6.82600721660079E-242	0.796301325795055	0.647	0.297	1.14390228935796E-237	Detoxification-associated hepat	XRN1
1.29872892139919E-241	1.49394182600701	0.328	0.099	2.17640992648077E-237	Detoxification-associated hepat	PPCDC
1.45419471314649E-241	0.802737408674948	0.657	0.304	2.43693950029088E-237	Detoxification-associated hepat	PHF3
1.07026317263562E-240	1.29573000813893	0.446	0.17	1.79354702470276E-236	Detoxification-associated hepat	MGAT5
2.71796930484449E-240	0.829053087862985	0.62	0.277	4.5547729610584E-236	Detoxification-associated hepat	EEF2K
3.32517202512496E-240	1.49110602353397	0.346	0.11	5.5723232797044E-236	Detoxification-associated hepat	VCPKMT
1.1948468056646E-239	1.33192950231618	0.384	0.132	2.00232427693274E-235	Detoxification-associated hepat	PIAS1
2.00445544101827E-239	0.919425311278285	0.548	0.231	3.35906642805843E-235	Detoxification-associated hepat	DNASE1L1
6.00768096531803E-239	0.898559117169149	0.616	0.279	1.00676717616799E-234	Detoxification-associated hepat	GATAD1
1.03712654059506E-238	0.79413885361697	0.641	0.285	1.7380166567292E-234	Detoxification-associated hepat	TUBGCP3
1.07815031843286E-238	0.819696530253612	0.646	0.3	1.80676430362978E-234	Detoxification-associated hepat	PCMTD1
1.18353102067721E-238	1.12447261192247	0.449	0.171	1.98336128445087E-234	Detoxification-associated hepat	YTHDC2
2.32154399287385E-238	1.03564314043466	0.499	0.203	3.890443423258E-234	Detoxification-associated hepat	UTP23
2.90024504669295E-238	0.909541715145463	0.46	0.174	4.86023064924805E-234	Detoxification-associated hepat	TM2D3
3.00553662777156E-238	1.01751372721854	0.54	0.229	5.03667828081958E-234	Detoxification-associated hepat	ABHD3
3.39883350881123E-238	1.11804662608367	0.501	0.204	5.69576519406586E-234	Detoxification-associated hepat	SLC2A13
4.89986555785825E-238	0.751470823200067	0.776	0.395	8.21119470185885E-234	Detoxification-associated hepat	STBD1
5.853023017073E-238	0.69528834388204	0.719	0.338	9.80849597201094E-234	Detoxification-associated hepat	PILRB
1.15106188174312E-237	1.15627424878586	0.425	0.156	1.92894950142512E-233	Detoxification-associated hepat	GUCY1A1
2.79559803138145E-237	0.891863110600679	0.561	0.242	4.68486318098904E-233	Detoxification-associated hepat	NSMCE2
3.29845168615425E-237	0.792202785153486	0.664	0.304	5.5275453356573E-233	Detoxification-associated hepat	SERPINA5
1.23965261606349E-236	1.20049068156017	0.422	0.155	2.0774098539992E-232	Detoxification-associated hepat	TBCK
1.54917105887966E-236	1.4166462106508	0.342	0.108	2.59610086047053E-232	Detoxification-associated hepat	TCAF2
1.70963813530846E-236	0.70915582471879	0.826	0.422	2.86501158714992E-232	Detoxification-associated hepat	CFLAR
5.62348148508194E-236	0.729685260073276	0.688	0.317	9.42383027270032E-232	Detoxification-associated hepat	SUGP2
8.20190353722428E-236	0.823155483628859	0.659	0.304	1.37447499476804E-231	Detoxification-associated hepat	MICAL3
1.33472108063601E-235	0.81623798826857	0.645	0.297	2.23672558692983E-231	Detoxification-associated hepat	VPS45
1.85540418151264E-235	0.696690249664321	0.899	0.525	3.10928632737888E-231	Detoxification-associated hepat	CIRBP
2.77376883107435E-235	1.21495122279244	0.451	0.174	4.6482818071144E-231	Detoxification-associated hepat	FANCL
3.75626384242989E-235	0.871428655496515	0.596	0.265	6.29474694714401E-231	Detoxification-associated hepat	IDUA
4.0125747545034E-235	0.726451905291609	0.676	0.32	6.7242727735968E-231	Detoxification-associated hepat	SLC25A30

4.4933123944349E-235	0.857441324784684	0.593	0.262	7.52989291059401E-231	Detoxification-associated hepat	TPP2
6.68229867711616E-235	2.51220508002832	0.205	0.042	1.11981961231113E-230	Detoxification-associated hepat	SLCO6A1
1.07024552459628E-234	0.723008763690258	0.697	0.331	1.79351745011845E-230	Detoxification-associated hepat	NUFIP2
1.51369145543394E-234	1.24842990251059	0.94	0.703	2.5366441410162E-230	Detoxification-associated hepat	GLUL
1.95166861654869E-234	1.22161234136263	0.4	0.144	3.2706062676123E-230	Detoxification-associated hepat	SEC22A
2.37760428908222E-234	0.702380024651177	0.677	0.314	3.98438926764398E-230	Detoxification-associated hepat	SPG7
4.47626265531778E-234	1.25618071899122	0.422	0.158	7.50132095778153E-230	Detoxification-associated hepat	RBBP6
4.53767059598332E-234	1.19455941724967	0.397	0.139	7.60422838474885E-230	Detoxification-associated hepat	SH3TC1
5.06883360036172E-234	0.590486551237322	0.99	0.941	8.49435134748616E-230	Detoxification-associated hepat	AGT
5.45473033964385E-234	1.07916916278708	0.528	0.223	9.14103710317516E-230	Detoxification-associated hepat	PCF11
8.21972582100533E-234	0.857408061366809	0.54	0.228	1.37746165308407E-229	Detoxification-associated hepat	RSAD1
1.03169860611435E-233	0.792666879957931	0.643	0.302	1.72892052412643E-229	Detoxification-associated hepat	TM7SF2
1.0963097343061E-233	0.677388354051187	0.89	0.469	1.83719585275017E-229	Detoxification-associated hepat	TMEM45B
2.62611715192357E-233	0.779210310481058	0.672	0.321	4.40084712319351E-229	Detoxification-associated hepat	UBE2D3
3.12545548984221E-233	0.805249067636304	0.578	0.251	5.23763830987758E-229	Detoxification-associated hepat	AQP3
3.76627027420063E-233	0.967133184684215	0.54	0.232	6.31151572550542E-229	Detoxification-associated hepat	UBE2G2
4.08582323163337E-233	1.11312701413496	0.494	0.201	6.84702257157121E-229	Detoxification-associated hepat	TTLL3
4.14773671742399E-233	1.2411146237176	0.363	0.12	6.95077719105912E-229	Detoxification-associated hepat	EDC3
9.48054580826191E-233	0.829310598959578	0.559	0.242	1.58874986654853E-228	Detoxification-associated hepat	TUBGCP6
1.09096125857822E-232	0.822857265057632	0.637	0.292	1.82823287712538E-228	Detoxification-associated hepat	PKP2
1.04312465150595E-231	0.796158017817667	0.704	0.349	1.74806829099368E-227	Detoxification-associated hepat	NSDHL
1.13943088345292E-231	0.734056114807994	0.693	0.33	1.90945827449041E-227	Detoxification-associated hepat	PEX2
1.17112467430931E-231	0.789577919327135	0.586	0.26	1.96257072920755E-227	Detoxification-associated hepat	EPB41L5
1.29142962046163E-231	0.760564494718258	0.714	0.338	2.16417775796959E-227	Detoxification-associated hepat	DEPRECATEI
1.37716740953758E-231	0.67560246179972	0.796	0.424	2.30785714490307E-227	Detoxification-associated hepat	IL6ST
1.70155568101604E-231	0.727564037227118	0.56	0.24	2.85146701024668E-227	Detoxification-associated hepat	AUTS2
2.14568600396628E-231	0.681699770857343	0.678	0.321	3.59574060544669E-227	Detoxification-associated hepat	SORBS2
4.22592792309588E-231	0.782373226182442	0.604	0.273	7.08181001352408E-227	Detoxification-associated hepat	BACH1
7.10127893886409E-231	0.955120716349076	0.496	0.205	1.19003232457484E-226	Detoxification-associated hepat	UBR3
2.42034630737358E-230	0.927223014055786	0.487	0.196	4.05601634189664E-226	Detoxification-associated hepat	DNMT3A
8.25455461729788E-230	1.15255426648282	0.48	0.195	1.38329826276678E-225	Detoxification-associated hepat	UGGT2
8.78211448411662E-230	1.34627819243421	0.415	0.155	1.47170674524826E-225	Detoxification-associated hepat	FAM76B
3.14514262758472E-229	0.627078129284563	0.864	0.455	5.27063001530647E-225	Detoxification-associated hepat	CMC4
7.28096007527434E-229	0.859863515752542	0.586	0.261	1.22014328941447E-224	Detoxification-associated hepat	MOCOS
1.83832596757609E-228	0.98562887231676	0.496	0.204	3.08066665646401E-224	Detoxification-associated hepat	GCH1
3.45883414751565E-228	1.55767796529823	0.32	0.099	5.79631426440672E-224	Detoxification-associated hepat	TTLL7
7.95666727470123E-228	0.760849217058947	0.646	0.297	1.33337830189443E-223	Detoxification-associated hepat	TRAPPC9
2.7413457943265E-227	0.679559319519579	0.815	0.422	4.59394728213235E-223	Detoxification-associated hepat	CYP4F11
1.7541076947419E-226	0.659661484911407	0.677	0.319	2.93953367484847E-222	Detoxification-associated hepat	RTL8C
3.29603508411049E-226	0.598413541495175	0.695	0.333	5.52349559395235E-222	Detoxification-associated hepat	ADHFE1
6.0522999123141E-226	1.24834211737565	0.425	0.163	1.0142444193056E-221	Detoxification-associated hepat	NBEAL1
8.47345300748352E-226	0.963570440641937	0.539	0.234	1.41998125499409E-221	Detoxification-associated hepat	WDR33
1.27095675317825E-225	0.974411692639762	0.497	0.206	2.12986932697611E-221	Detoxification-associated hepat	SCAF8
2.73338096822417E-225	0.59436967442062	0.711	0.352	4.58059982655006E-221	Detoxification-associated hepat	C19orf12
3.01514580079415E-225	0.800065541477141	0.591	0.268	5.05278133297084E-221	Detoxification-associated hepat	TOR1AIP1
5.24586320534783E-225	0.927816227658408	0.524	0.225	8.79101755952189E-221	Detoxification-associated hepat	NEMF
5.27137895552535E-225	1.01291569745336	0.456	0.181	8.83377685366938E-221	Detoxification-associated hepat	RESF1
5.87390124215198E-225	0.930116651643953	0.563	0.248	9.84348370159829E-221	Detoxification-associated hepat	THOP1
1.25474019611034E-224	1.15583863891886	0.438	0.171	2.10269362064171E-220	Detoxification-associated hepat	RAB28
2.39089295396828E-224	1.57390428106028	0.336	0.11	4.00665841226004E-220	Detoxification-associated hepat	PCDHGA3
3.73465093854497E-224	0.641481910928155	0.741	0.352	6.25852804281367E-220	Detoxification-associated hepat	MDM2
8.47306715343246E-224	0.980951558269888	0.469	0.188	1.41991659357221E-219	Detoxification-associated hepat	FNIP1
2.06157852804361E-223	0.82079690065724	0.562	0.247	3.45479329729549E-219	Detoxification-associated hepat	KMT2D
2.08168613863429E-223	1.5341469629255	0.354	0.121	3.48848963112334E-219	Detoxification-associated hepat	ZNF606
3.1209150958873E-223	1.3613351121906	0.389	0.14	5.23002951768794E-219	Detoxification-associated hepat	DEPRECATEI
3.16039016308877E-223	0.649719409568651	0.721	0.347	5.29618183530416E-219	Detoxification-associated hepat	SYBU
3.71490133068731E-223	0.940426851728317	0.49	0.202	6.2254316499658E-219	Detoxification-associated hepat	TMLHE
6.79500095073514E-223	1.76288448738475	0.28	0.079	1.1387062593242E-218	Detoxification-associated hepat	CUX2
3.3468725626389E-222	0.851458341085078	0.593	0.27	5.60868904047027E-218	Detoxification-associated hepat	SCML1
7.04071869699217E-222	0.850179622770149	0.582	0.261	1.17988363924195E-217	Detoxification-associated hepat	ARRB1

7.64593081135552E-222	0.983506821162121	0.478	0.196	1.28130508536696E-217	Detoxification-associated hepat	CBR4
1.53014782855406E-221	0.668633069545677	0.733	0.38	2.56422173109089E-217	Detoxification-associated hepat	MAF
1.62256165436937E-221	0.718770506045972	0.715	0.347	2.71908882039219E-217	Detoxification-associated hepat	ASH1L
1.71575549994442E-221	2.88824690241359	0.154	0.024	2.87526306680686E-217	Detoxification-associated hepat	BRS3
5.9769935344983E-221	0.709303344630027	0.598	0.27	1.00162457651122E-216	Detoxification-associated hepat	TRPC4AP
1.61513649580773E-220	1.20087011016211	0.392	0.143	2.7066457396746E-216	Detoxification-associated hepat	GRAMD2B
1.85148257284751E-220	0.761659022807954	0.621	0.286	3.10271449557786E-216	Detoxification-associated hepat	FBXL17
2.18588038223776E-220	0.627047228535303	0.673	0.325	3.66309834455403E-216	Detoxification-associated hepat	SLC38A2
3.02561428217715E-220	0.923108243800227	0.483	0.2	5.07032441407247E-216	Detoxification-associated hepat	MED16
3.07801049180301E-220	0.77690510818079	0.56	0.246	5.15812998216348E-216	Detoxification-associated hepat	RFC1
3.67683813195177E-220	1.16291256732778	0.42	0.161	6.16164534152478E-216	Detoxification-associated hepat	HIBCH
8.24428336826797E-220	0.694374892966371	0.685	0.336	1.38157700685435E-215	Detoxification-associated hepat	CYP4F3
1.08922058965838E-219	1.00900816229891	0.48	0.199	1.82531586414951E-215	Detoxification-associated hepat	UBA6
1.3997889143279E-219	1.21665882953076	0.438	0.173	2.3457662626307E-215	Detoxification-associated hepat	ATM
1.65423495791944E-219	1.18263677913268	0.382	0.137	2.7721669424814E-215	Detoxification-associated hepat	ITPR1
4.1070296231547E-219	0.382499951916765	0.618	0.271	6.88256024248265E-215	Detoxification-associated hepat	PLIN5
5.89946520074797E-219	0.77089445317006	0.611	0.282	9.88632378341345E-215	Detoxification-associated hepat	PCID2
8.56338674170219E-219	0.770413842532492	0.752	0.379	1.43505235017445E-214	Detoxification-associated hepat	EVL
1.10967507879876E-218	1.04881127892471	0.447	0.178	1.85959349705097E-214	Detoxification-associated hepat	ERCC6L2
1.17703935142158E-218	0.785030803657534	0.59	0.269	1.97248254511228E-214	Detoxification-associated hepat	TGFBR2
1.72818259930172E-218	0.70239208179109	0.671	0.316	2.89608839990982E-214	Detoxification-associated hepat	CUL4B
1.96010508147237E-218	0.751771536129684	0.601	0.274	3.2847440955314E-214	Detoxification-associated hepat	MOGS
2.14240759524651E-218	0.673214808751476	0.865	0.48	3.5902466481141E-214	Detoxification-associated hepat	MIA2
2.79449087982671E-218	0.906478650414697	0.511	0.217	4.68300781641359E-214	Detoxification-associated hepat	CLDND1
3.43457172388539E-218	0.750524853357173	0.68	0.322	5.75565529488714E-214	Detoxification-associated hepat	GPAT4
5.41263748888944E-218	1.20139961229509	0.347	0.115	9.07049790388093E-214	Detoxification-associated hepat	CDC14B
7.90400547220442E-218	0.86211840329286	0.586	0.265	1.32455323703202E-213	Detoxification-associated hepat	ZDHHC14
2.18997253709358E-217	0.660628600684622	0.693	0.339	3.66995597766143E-213	Detoxification-associated hepat	DST
2.23883788233159E-217	0.992606671127748	0.454	0.183	3.75184452321127E-213	Detoxification-associated hepat	RAB3GAP1
6.40035524546022E-217	0.748680231461487	0.641	0.304	1.07257153203422E-212	Detoxification-associated hepat	SRRM2
1.08086089544622E-216	0.641747931265711	0.663	0.323	1.81130668858878E-212	Detoxification-associated hepat	FURIN
1.49525454565503E-216	1.17974719237336	0.397	0.148	2.5057475676087E-212	Detoxification-associated hepat	OMA1
2.55163018609838E-216	1.2889943256425	0.355	0.122	4.27602186586367E-212	Detoxification-associated hepat	ZBTB37
3.26248868328206E-216	0.638514696836136	0.747	0.379	5.46727853544408E-212	Detoxification-associated hepat	PPARA
4.94039882612033E-216	0.683322991506997	0.708	0.348	8.27912035281245E-212	Detoxification-associated hepat	PER1
5.1479430426743E-216	1.51798378918897	0.293	0.088	8.62692295091359E-212	Detoxification-associated hepat	TEFM
1.36940338603337E-215	1.39108942566919	0.344	0.117	2.29484619431473E-211	Detoxification-associated hepat	ADPGK
2.4039950910564E-215	1.21945155366393	0.264	0.07	4.02861497359231E-211	Detoxification-associated hepat	FES
5.81140221424516E-215	0.84704276266578	0.575	0.262	9.73874783063204E-211	Detoxification-associated hepat	PLPP1
8.82732135473307E-215	0.847614507932118	0.538	0.237	1.47928251262617E-210	Detoxification-associated hepat	SRP19
1.19378751370083E-214	1.64442306849592	0.177	0.033	2.00054911545985E-210	Detoxification-associated hepat	RTN1
4.31960927763251E-214	0.736794860013253	0.655	0.311	7.23880122745655E-210	Detoxification-associated hepat	STK3
4.80231787902167E-214	0.669465716799216	0.853	0.454	8.04772430166452E-210	Detoxification-associated hepat	ZBTB7B
7.27368363081802E-214	0.795523144189932	0.546	0.242	1.21892390285248E-209	Detoxification-associated hepat	PHYKPL
1.50687828048149E-213	0.717118946900277	0.745	0.371	2.52522662243088E-209	Detoxification-associated hepat	PLEKHF2
1.54737047617887E-213	0.977531458438678	0.475	0.198	2.59308344398055E-209	Detoxification-associated hepat	SLC5A3
2.11701649076879E-213	2.07485566948992	0.227	0.056	3.54769623523034E-209	Detoxification-associated hepat	ZNF546
2.59109039244944E-213	0.547609735644295	0.8	0.415	4.34214927966677E-209	Detoxification-associated hepat	RNF213
2.62975148055316E-213	0.685189032082357	0.694	0.34	4.40693753111099E-209	Detoxification-associated hepat	PPM1B
2.7380759657086E-213	0.678061751638055	0.731	0.374	4.58846770333448E-209	Detoxification-associated hepat	USP9X
4.58599884349942E-213	1.39386135723356	0.361	0.128	7.68521686193633E-209	Detoxification-associated hepat	ZNF700
8.78790871768653E-213	1.79530165371651	0.285	0.085	1.47267774290991E-208	Detoxification-associated hepat	FSBP
9.80310485075743E-213	1.02987002445176	0.418	0.16	1.64280431088993E-208	Detoxification-associated hepat	RANBP10
1.20220393368173E-212	1.04526413901915	0.433	0.169	2.01465335206384E-208	Detoxification-associated hepat	EPM2AIP1
1.54819448617186E-212	0.847699313879678	0.534	0.235	2.59446431992681E-208	Detoxification-associated hepat	TERF1
1.59301631649106E-212	0.699336578332477	0.711	0.351	2.66957674317572E-208	Detoxification-associated hepat	CYP4F2
1.66808901642629E-212	0.800751270183824	0.474	0.192	2.79538357372718E-208	Detoxification-associated hepat	KAT2A
1.9818720464445E-212	0.682871207391398	0.72	0.359	3.32122117543169E-208	Detoxification-associated hepat	PA2G4
2.30568106009828E-212	0.685897627215141	0.843	0.461	3.86386032051269E-208	Detoxification-associated hepat	CTSH
3.62378439372953E-212	0.707421318971042	0.705	0.345	6.07273788701194E-208	Detoxification-associated hepat	DHX15

**1.03625162648971E-211**	0.987805298771992	0.471	0.196	1.73655047567146E-207	Detoxification-associated hepat	ATG2B
**1.11411787452901E-211**	1.30084476636731	0.392	0.148	1.86703873413571E-207	Detoxification-associated hepat	ZFP62
**1.13096394267619E-211**	0.907450376731366	0.513	0.224	1.89526937513676E-207	Detoxification-associated hepat	TENT2
**6.01615387239232E-211**	0.856260709114578	0.543	0.241	1.00818706593551E-206	Detoxification-associated hepat	LRRC58
**6.69355814677794E-211**	0.90311429903458	0.351	0.115	1.12170647423705E-206	Detoxification-associated hepat	PFKFB1
**8.39725279758227E-211**	1.30689329234702	0.347	0.12	1.40721162381884E-206	Detoxification-associated hepat	LRIG2
**8.60660581606298E-211**	1.01815668786554	0.472	0.198	1.44229500265583E-206	Detoxification-associated hepat	TBCE.1
**1.10680493485142E-210**	0.802089247129418	0.537	0.235	1.85478370982401E-206	Detoxification-associated hepat	C19orf25
**1.48348906042277E-210**	0.778995968406643	0.648	0.305	2.48603096745648E-206	Detoxification-associated hepat	PARP4
**1.48738899165948E-210**	0.915206386648419	0.432	0.168	2.49256647222296E-206	Detoxification-associated hepat	APBB3
**2.44501478988933E-210**	1.76770118806334	0.298	0.094	4.09735578489653E-206	Detoxification-associated hepat	CCDC7
**3.06674471346651E-210**	0.815261586424937	0.546	0.242	5.13925079082718E-206	Detoxification-associated hepat	DOCK5
**3.08353710697618E-210**	0.892100591483086	0.537	0.234	5.16739148387068E-206	Detoxification-associated hepat	DONSON
**3.64908558803912E-210**	0.670415958820003	0.765	0.398	6.11513762843596E-206	Detoxification-associated hepat	IL1R1
**5.48310726234657E-210**	1.6901505234871	0.246	0.065	9.18859115024038E-206	Detoxification-associated hepat	DEPRECATEI
**5.56506991323275E-210**	1.98076241313722	0.243	0.065	9.32594416059544E-206	Detoxification-associated hepat	ZNF345
**1.46413843015056E-209**	0.713397580245649	0.628	0.297	2.45360318124631E-205	Detoxification-associated hepat	NUDT9
**2.35844280629621E-209**	1.03059658804972	0.466	0.194	3.95227845479119E-205	Detoxification-associated hepat	ENPP4
**2.77477528793198E-209**	0.947529488696862	0.432	0.171	4.64996842751641E-205	Detoxification-associated hepat	CUL5
**2.96319748885031E-209**	0.805643466024382	0.549	0.243	4.96572635181535E-205	Detoxification-associated hepat	ZBTB10
**3.37199990175477E-209**	0.842860586729493	0.504	0.219	5.65079743536064E-205	Detoxification-associated hepat	TSNAX
**4.03730094289468E-209**	0.628682067745839	0.815	0.421	6.76570892010291E-205	Detoxification-associated hepat	SLC38A10
**1.02054147725E-208**	0.713728309040939	0.646	0.307	1.71022340757554E-204	Detoxification-associated hepat	MAN2A1
**1.32874912567758E-208**	0.870170047777321	0.541	0.242	2.22671778481049E-204	Detoxification-associated hepat	CXADR
**1.41686476476113E-208**	0.723541581449985	0.662	0.321	2.3743819727867E-204	Detoxification-associated hepat	RNF115
**1.69242136332583E-208**	1.24971842765898	0.374	0.138	2.83615972066143E-204	Detoxification-associated hepat	RBM12
**6.89177411084799E-208**	2.16810314442336	0.197	0.043	1.15492350549591E-203	Detoxification-associated hepat	CATSPER2
**7.38014471510595E-208**	1.05194703080493	0.443	0.178	1.23676465135746E-203	Detoxification-associated hepat	POLK
**7.43179631089687E-208**	1.00095739871173	0.428	0.169	1.2454204257801E-203	Detoxification-associated hepat	POLB
**7.65030554026406E-208**	0.610995668082944	0.936	0.577	1.28203820243745E-203	Detoxification-associated hepat	MBNL3
**1.10011045474054E-207**	0.723591457962939	0.639	0.301	1.8435651000542E-203	Detoxification-associated hepat	CEP57
**1.29151544704251E-207**	0.827478696914798	0.51	0.22	2.16432158615383E-203	Detoxification-associated hepat	SDR39U1
**2.30088795814297E-207**	0.652050105977443	0.688	0.328	3.85582804025599E-203	Detoxification-associated hepat	RABGAP1L
**1.22956980992243E-206**	0.654150848937106	0.674	0.331	2.06051308746802E-202	Detoxification-associated hepat	SPECC1L
**1.36953897760102E-206**	0.85035672899233	0.543	0.245	2.29507341866379E-202	Detoxification-associated hepat	KHDC4
**2.12651159031902E-206**	0.977104132159989	0.443	0.177	3.56360812305662E-202	Detoxification-associated hepat	HSF4
**2.87501464062462E-206**	0.997101056164557	0.261	0.071	4.81794953475874E-202	Detoxification-associated hepat	MICU3
**4.8610841320102E-206**	0.812421928719683	0.487	0.207	8.14620478842269E-202	Detoxification-associated hepat	PIKFYVE
**5.20941696651225E-206**	1.30713095283219	0.314	0.101	8.72994095248122E-202	Detoxification-associated hepat	SRCIN1
**5.21098439675967E-206**	1.20411289299133	0.36	0.129	8.73256765208985E-202	Detoxification-associated hepat	NSRP1
**8.08004808571097E-206**	0.586168006905678	0.962	0.697	1.35405445820345E-201	Detoxification-associated hepat	ASPH
**1.27927583263983E-205**	1.03167312043428	0.44	0.178	2.14381044033782E-201	Detoxification-associated hepat	ZNF608
**1.4137022281131E-205**	0.596498880285094	0.719	0.366	2.36908219387194E-201	Detoxification-associated hepat	ELOVL5
**2.39542681057642E-205**	0.815789503417321	0.585	0.268	4.01425624916397E-201	Detoxification-associated hepat	KDM6A
**2.86530573066404E-205**	1.10041142797764	0.426	0.169	4.8016793434468E-201	Detoxification-associated hepat	ZNF121
**2.97080631405484E-205**	0.666901440005944	0.645	0.305	4.97847722109311E-201	Detoxification-associated hepat	CD47
**2.97592133815333E-205**	0.870501347746019	0.523	0.232	4.98704897847735E-201	Detoxification-associated hepat	GSK3B
**3.61051190479642E-205**	1.50568507376365	0.313	0.103	6.05049585005784E-201	Detoxification-associated hepat	MIPOL1
**5.29387039351983E-205**	0.70172865454327	0.545	0.247	8.87146800546054E-201	Detoxification-associated hepat	AS3MT
**7.49607939526933E-205**	0.908462931498617	0.466	0.192	1.25619298505923E-200	Detoxification-associated hepat	FBXL8
**7.63226065901045E-205**	1.33676686828278	0.336	0.115	1.27901424123697E-200	Detoxification-associated hepat	ZSCAN26
**8.00581874533758E-205**	0.638513004715868	0.596	0.278	1.34161510534367E-200	Detoxification-associated hepat	ZFAND5
**1.36629552671388E-204**	0.939291709950309	0.447	0.182	2.28963804366712E-200	Detoxification-associated hepat	MYNN
**1.37418476564946E-204**	1.24748508286468	0.422	0.167	2.30285883027537E-200	Detoxification-associated hepat	ACYP1
**2.03133886016445E-204**	1.043867918479	0.418	0.166	3.40411766186359E-200	Detoxification-associated hepat	RPTOR
**2.45585184572774E-204**	0.571352855359813	0.953	0.681	4.11551652307055E-200	Detoxification-associated hepat	LAMP2
**3.12422552814009E-204**	0.63349943371756	0.659	0.319	5.23557714005717E-200	Detoxification-associated hepat	DDX17
**3.32160803022183E-204**	0.982973172810154	0.476	0.201	5.56635073704575E-200	Detoxification-associated hepat	GTF2H1
**6.16132580016486E-204**	0.753943412803405	0.573	0.265	1.03251497759163E-199	Detoxification-associated hepat	LRCH3
**9.85285555862458E-204**	1.62915859594508	0.301	0.097	1.65114153451431E-199	Detoxification-associated hepat	DOCK10

1.68207979185136E-203	0.593766583443554	0.88	0.452	2.81882931518451E-199	Detoxification-associated hepat	ABCD1
2.00685720237068E-203	1.08630834323516	0.47	0.198	3.36309129973278E-199	Detoxification-associated hepat	FRAS1
2.49297809135589E-203	0.696334968324984	0.567	0.26	4.1777326854942E-199	Detoxification-associated hepat	KIF13A
2.63358094514656E-203	0.57345425743112	0.838	0.457	4.4133549478766E-199	Detoxification-associated hepat	HBS1L
3.58619391767385E-203	1.451441100919	0.363	0.133	6.00974376723783E-199	Detoxification-associated hepat	KCNMB3
3.92595854746113E-203	1.55918183195054	0.319	0.107	6.57912133383536E-199	Detoxification-associated hepat	ANKRD26
4.77980957959126E-203	0.685461691297725	0.583	0.271	8.01000489347903E-199	Detoxification-associated hepat	PDE4DIP
5.80351371311659E-203	0.9700332698598	0.453	0.188	9.72552828044079E-199	Detoxification-associated hepat	SNX24
8.62114252065884E-203	0.633729750524015	0.629	0.297	1.44473106361201E-198	Detoxification-associated hepat	SMAD3
1.04836181947661E-202	0.81555026492976	0.485	0.205	1.7568447370789E-198	Detoxification-associated hepat	SKIL
1.52429963236066E-202	1.14156085484835	0.384	0.144	2.55442132391E-198	Detoxification-associated hepat	KANSL1L
1.82740985457685E-202	0.659714961012669	0.637	0.308	3.06237343429989E-198	Detoxification-associated hepat	OTULIN
1.84846456753805E-202	0.883010483808486	0.493	0.211	3.09765692228026E-198	Detoxification-associated hepat	FAM136A
2.84391349647116E-202	1.2078450355956	0.374	0.139	4.76583023738638E-198	Detoxification-associated hepat	SLC35E3
3.16051804143079E-202	0.803251366081913	0.53	0.236	5.29639613382972E-198	Detoxification-associated hepat	METTL17
6.75575531792027E-202	0.783705332114075	0.461	0.191	1.13212947617708E-197	Detoxification-associated hepat	THNSL2
9.28708106128139E-202	0.794966156115117	0.541	0.245	1.55632904424953E-197	Detoxification-associated hepat	ARHGAP5
1.26025844622541E-201	0.65491056857432	0.65	0.32	2.11194110418455E-197	Detoxification-associated hepat	MAPK6
1.56002793903521E-201	0.688481139853523	0.608	0.285	2.6142948202352E-197	Detoxification-associated hepat	TMUB2
1.6194456347794E-201	0.616892680766048	0.982	0.748	2.71386699476333E-197	Detoxification-associated hepat	CYP3A5
2.0100425089571E-201	0.629236487573641	0.68	0.329	3.36842923651032E-197	Detoxification-associated hepat	NECTIN3
3.22397470340594E-201	0.83827306627853	0.511	0.226	5.40273680796768E-197	Detoxification-associated hepat	MAP3K2
3.47365208620747E-201	0.6553495593805	0.602	0.284	5.82114616606648E-197	Detoxification-associated hepat	SLC23A2
4.34051512244669E-201	0.929132518739573	0.427	0.169	7.27383524219617E-197	Detoxification-associated hepat	NSUN6
5.00727469224471E-201	1.14003466738688	0.412	0.164	8.39119092926369E-197	Detoxification-associated hepat	CSPP1
6.07265807983081E-201	0.648414236499329	0.704	0.349	1.01765604101805E-196	Detoxification-associated hepat	NEDD4L
6.88978263414683E-201	0.595730815941716	0.598	0.279	1.15458977383033E-196	Detoxification-associated hepat	TNRC6A
7.28813969531174E-201	0.612878807065498	0.753	0.395	1.22134645014034E-196	Detoxification-associated hepat	PBLD
7.70410945109358E-201	1.28219271833247	0.376	0.142	1.29105466181426E-196	Detoxification-associated hepat	ENDOV
1.03781710852661E-200	0.906854446902585	0.501	0.22	1.7391739104689E-196	Detoxification-associated hepat	ELMOD2
1.13955721270496E-200	0.96878118429833	0.431	0.172	1.90966997705097E-196	Detoxification-associated hepat	PAN2
1.52690528601227E-200	0.637797934173104	0.624	0.298	2.55878787829936E-196	Detoxification-associated hepat	RBM10
2.11790701943514E-200	0.839321079433773	0.524	0.234	3.5491885831694E-196	Detoxification-associated hepat	SMC6
2.4211412077475E-200	0.637607177430265	0.616	0.293	4.05734843594325E-196	Detoxification-associated hepat	TBC1D2B
5.12187696693342E-200	1.32815528308067	0.19	0.04	8.58324142118702E-196	Detoxification-associated hepat	SLC22A9
6.47666629865411E-200	0.912308761012141	0.454	0.189	1.08535973832846E-195	Detoxification-associated hepat	WBP1
6.67217115969125E-200	0.825486288242285	0.476	0.2	1.11812244294106E-195	Detoxification-associated hepat	CBFA2T2
7.98771936392334E-200	0.593394367299897	0.8	0.432	1.33858201100627E-195	Detoxification-associated hepat	MARC2
2.25225935830237E-199	0.656272434064511	0.782	0.429	3.77433623264312E-195	Detoxification-associated hepat	PIK3R1
2.64356824064656E-199	0.608152379493102	0.665	0.328	4.4300916576755E-195	Detoxification-associated hepat	NDUFV3
3.26780704228565E-199	0.621844560708934	0.762	0.409	5.47619104146228E-195	Detoxification-associated hepat	HACD3
3.49396972742851E-199	2.02412948458629	0.229	0.06	5.85519446922469E-195	Detoxification-associated hepat	CCDC138
5.69801509230841E-199	0.593949894005035	0.707	0.348	9.54873369169044E-195	Detoxification-associated hepat	MARCH6
9.01844329856367E-199	0.54160326088826	0.752	0.398	1.5113107279733E-194	Detoxification-associated hepat	F11
1.10461041135355E-198	0.567896141445026	0.784	0.396	1.85110612734627E-194	Detoxification-associated hepat	PDZD8
1.90070053190712E-198	0.648612271639107	0.623	0.3	3.18519395136995E-194	Detoxification-associated hepat	CREBZF
3.04725657941202E-198	0.520782812874842	0.446	0.177	5.10659257577866E-194	Detoxification-associated hepat	WHRN
4.589620132294E-198	0.814970923026472	0.534	0.242	7.69128541769828E-194	Detoxification-associated hepat	TAF2
5.04949912632351E-198	0.859962219644466	0.47	0.2	8.46195063589294E-194	Detoxification-associated hepat	TRMT11
5.09435644996292E-198	1.08205672181748	0.394	0.153	8.53712253884785E-194	Detoxification-associated hepat	PDP1
5.24073833885245E-198	0.861125592516685	0.462	0.195	8.78242930824893E-194	Detoxification-associated hepat	HERC4
8.66257211301688E-198	0.893196715565069	0.477	0.205	1.45167383469937E-193	Detoxification-associated hepat	TMEM131
1.81670734457308E-197	0.575140434518944	0.774	0.405	3.04443816803556E-193	Detoxification-associated hepat	LLGL2
2.02212991373462E-197	0.704728866282211	0.67	0.328	3.38868530943648E-193	Detoxification-associated hepat	DIDO1
3.39235442541619E-197	0.653907460061688	0.592	0.278	5.68490754611245E-193	Detoxification-associated hepat	IDS
3.6035328733203E-197	0.683472519857319	0.632	0.303	6.03880038911016E-193	Detoxification-associated hepat	FNTA
3.99697235321248E-197	0.721182601225654	0.58	0.273	6.69812626951348E-193	Detoxification-associated hepat	CHKA
1.10974355261528E-196	0.684587047088233	0.722	0.368	1.85970824547268E-192	Detoxification-associated hepat	ACSL3
1.60408002454091E-196	1.04876732792473	0.364	0.133	2.68811730512565E-192	Detoxification-associated hepat	WDR37
2.22955020620471E-196	0.647230900663878	0.59	0.277	3.73628023555785E-192	Detoxification-associated hepat	TCEAL1

3.11261716175237E-196	1.2589549845685	0.354	0.13	5.21612383966461E-192	Detoxification-associated hepat	THOC1
3.79608014275806E-196	0.890433630812905	0.46	0.195	6.36147110323395E-192	Detoxification-associated hepat	FNIP2
1.64501801659974E-195	1.08771476687005	0.382	0.146	2.75672119221785E-191	Detoxification-associated hepat	METTL15
1.87604692376548E-195	0.803665117488306	0.445	0.183	3.1438794348462E-191	Detoxification-associated hepat	NDUFAF6
3.91819580768645E-195	1.18096974468687	0.371	0.139	6.56611253452095E-191	Detoxification-associated hepat	RNF217
5.88799935655138E-195	0.65384285425312	0.862	0.476	9.8671093217088E-191	Detoxification-associated hepat	MAT2A
7.22603063855721E-195	1.28377616812194	0.346	0.124	1.21093821440942E-190	Detoxification-associated hepat	NRXN3
8.09078147438345E-195	1.06496914135	0.433	0.179	1.35585315947718E-190	Detoxification-associated hepat	SLC25A29
8.1710814582173E-195	1.22165131328768	0.356	0.132	1.36930983076805E-190	Detoxification-associated hepat	SOCS5
2.27065977397943E-194	0.885785845717328	0.472	0.202	3.80517164923472E-190	Detoxification-associated hepat	KLHL22
2.83353133739445E-194	0.592020347663771	0.706	0.357	4.74843181520561E-190	Detoxification-associated hepat	ARHGAP35
4.23594509195154E-194	0.966807244836251	0.43	0.177	7.0985967850924E-190	Detoxification-associated hepat	ZFYVE16
4.6829123241256E-194	1.48777848214574	0.313	0.106	7.84762447276969E-190	Detoxification-associated hepat	STXBP4
6.03441285064532E-194	0.680054896675603	0.544	0.25	1.01124690551114E-189	Detoxification-associated hepat	PYGL
6.04375094503819E-194	0.625033423723127	0.66	0.309	1.0128117833695E-189	Detoxification-associated hepat	CDS1
9.11328354970918E-194	1.66749248398176	0.277	0.087	1.52720405726026E-189	Detoxification-associated hepat	JRK
1.35147494417512E-193	0.464393313504788	0.781	0.418	2.26480171144867E-189	Detoxification-associated hepat	SNX1
1.49957142702333E-193	0.872332942088553	0.468	0.201	2.51298179740569E-189	Detoxification-associated hepat	TAPT1
1.72051274516251E-193	0.659835837871354	0.609	0.288	2.88323525834333E-189	Detoxification-associated hepat	CXorf56
1.85085903396303E-193	0.595962289924192	0.871	0.489	3.10166956911524E-189	Detoxification-associated hepat	ABCC3
5.72572136159056E-193	1.01983980069106	0.433	0.178	9.59516385775345E-189	Detoxification-associated hepat	AHI1
6.37338056733149E-193	0.647566530370547	0.59	0.28	1.06805111547341E-188	Detoxification-associated hepat	ST5
9.16592109430597E-193	0.608769925714123	0.656	0.319	1.53602505698379E-188	Detoxification-associated hepat	KMT2C
1.22860861548253E-192	0.682316931474697	0.62	0.302	2.05890231782562E-188	Detoxification-associated hepat	TENT5A
1.29506639447374E-192	1.16782445346785	0.377	0.145	2.17027226385909E-188	Detoxification-associated hepat	PTPRD
1.86126940923875E-192	0.752663355932687	0.558	0.254	3.1191152760023E-188	Detoxification-associated hepat	FADS2
3.04186289727696E-192	0.586623502201482	0.693	0.343	5.09755384325673E-188	Detoxification-associated hepat	ADD1
3.91191111686123E-192	0.825097889816835	0.52	0.233	6.55558064963605E-188	Detoxification-associated hepat	KBTBD2
4.11174694406429E-192	0.606720391471476	0.728	0.373	6.89046552886294E-188	Detoxification-associated hepat	PI4K2B
6.08838221236669E-192	1.00509682276125	0.393	0.15	1.02029109114841E-187	Detoxification-associated hepat	ADRB2
6.46477677299518E-192	1.13687985992327	0.352	0.129	1.08336729161853E-187	Detoxification-associated hepat	ZNF561
7.67622255819973E-192	0.861369170540809	0.425	0.171	1.28638137630311E-187	Detoxification-associated hepat	TRPM7
7.68821615166954E-192	1.81635152037269	0.135	0.021	1.28839126269678E-187	Detoxification-associated hepat	SLCO1B3
8.9469708555026E-192	0.753678130309755	0.504	0.224	1.49933337596513E-187	Detoxification-associated hepat	RBCK1
1.03068806999684E-191	0.459148408225261	0.519	0.218	1.7272270677007E-187	Detoxification-associated hepat	F13B
1.07188474908691E-191	1.26953755012999	0.355	0.132	1.79626446251984E-187	Detoxification-associated hepat	ZNF44
1.29937703468269E-191	0.875486356968461	0.49	0.217	2.17749603472125E-187	Detoxification-associated hepat	WDR44
1.42217555283057E-191	0.871040795884489	0.452	0.188	2.38328179143347E-187	Detoxification-associated hepat	ZNF680
1.61149654436959E-191	0.583941534599423	0.689	0.345	2.70054590905456E-187	Detoxification-associated hepat	FARP1
1.74347672492788E-191	0.833167051930044	0.407	0.161	2.92171829563414E-187	Detoxification-associated hepat	AP3S2
1.37443919463962E-190	0.814025276743857	0.522	0.238	2.30328520237708E-186	Detoxification-associated hepat	LRRC14
1.60058230833598E-190	0.831778153041177	0.522	0.234	2.68225583230944E-186	Detoxification-associated hepat	FRYL
1.8000347521238E-190	1.0221444807867	0.404	0.163	3.01649823760907E-186	Detoxification-associated hepat	PIBF1
1.92790996769183E-190	1.21223031156804	0.268	0.08	3.23079152385797E-186	Detoxification-associated hepat	OXER1
2.43638006722863E-190	0.602217082738082	0.537	0.246	4.08288571666175E-186	Detoxification-associated hepat	PID1
2.76176639433268E-190	0.588322873453699	0.654	0.319	4.62816812362271E-186	Detoxification-associated hepat	ANKHD1
4.03626890527161E-190	0.696255539059076	0.564	0.263	6.76397943145416E-186	Detoxification-associated hepat	PCED1A
8.99644202856873E-190	0.798616273480178	0.449	0.189	1.50762375514755E-185	Detoxification-associated hepat	TNKS
1.27191029533568E-189	1.27640135240164	0.381	0.15	2.13146727292353E-185	Detoxification-associated hepat	DCUN1D2
1.64215415712434E-189	1.51784277008435	0.33	0.119	2.75192193650897E-185	Detoxification-associated hepat	PCDHGA5
1.81445514737988E-189	0.556839862609563	0.785	0.399	3.0406639359792E-185	Detoxification-associated hepat	CD99L2
6.42436572955487E-189	0.613553089617633	0.576	0.274	1.07659520895881E-184	Detoxification-associated hepat	SIK2
7.76333630343408E-189	0.603765367960332	0.893	0.533	1.30097989772948E-184	Detoxification-associated hepat	DECR1
9.91333112458592E-189	0.624829381599883	0.646	0.312	1.66127602985811E-184	Detoxification-associated hepat	IPO9
1.02467605232633E-188	0.620477312874417	0.626	0.301	1.71715212848846E-184	Detoxification-associated hepat	EHBP1L1
2.1124929909813E-188	0.917689324095616	0.425	0.176	3.54011575428647E-184	Detoxification-associated hepat	MED6
2.85421908830004E-188	1.72941257239421	0.269	0.084	4.7831003481732E-184	Detoxification-associated hepat	ZNF782
5.45255627377194E-188	0.885991369515593	0.452	0.194	9.13739380358701E-184	Detoxification-associated hepat	NEK9
6.49741898996604E-188	1.59139924140718	0.189	0.043	1.08883747433851E-183	Detoxification-associated hepat	SNX22
8.5524935809312E-188	0.639605920002962	0.555	0.259	1.43322687429245E-183	Detoxification-associated hepat	MED28

2.21363097771034E-187	0.594302452915522	0.682	0.343	3.70960279244699E-183	Detoxification-associated hepat	PATJ
2.69464582181661E-187	0.731237395627942	0.539	0.25	4.51568746820028E-183	Detoxification-associated hepat	DENND4C
3.10175748142326E-187	0.697494081635683	0.595	0.289	5.1979251873691E-183	Detoxification-associated hepat	PLSCR1
3.66532889503153E-187	0.722300545186521	0.57	0.268	6.14235816229383E-183	Detoxification-associated hepat	PARP12
6.55014469234006E-187	0.754376854911102	0.521	0.236	1.09767324754235E-182	Detoxification-associated hepat	DPY19L4
6.60773603054604E-187	0.59759781611521	0.941	0.624	1.1073244039989E-182	Detoxification-associated hepat	ATP1B1
7.32273499108551E-187	1.16229021514009	0.35	0.129	1.22714392980611E-182	Detoxification-associated hepat	FAN1
1.03944893377411E-186	0.606628283223046	0.51	0.228	1.74190852321865E-182	Detoxification-associated hepat	DEPRECATEI
1.18346515183966E-186	0.769923204959146	0.458	0.197	1.98325090145291E-182	Detoxification-associated hepat	LRP6
1.29021763901107E-186	0.873773294287144	0.458	0.197	2.16214671945476E-182	Detoxification-associated hepat	LCLAT1
1.42694822508131E-186	0.96393595444858	0.347	0.126	2.39127983559126E-182	Detoxification-associated hepat	LRRC28
1.65516323535236E-186	0.798630683902789	0.501	0.224	2.77372254980348E-182	Detoxification-associated hepat	SPIDR
1.83833648213361E-186	0.806990426107135	0.436	0.181	3.0806842767595E-182	Detoxification-associated hepat	PLEKHH3
2.49320848867184E-186	0.844959149657322	0.255	0.074	4.17811878531627E-182	Detoxification-associated hepat	COL6A1
2.73628475517672E-186	0.752256924185595	0.52	0.237	4.58546599272514E-182	Detoxification-associated hepat	MTMR1
4.1760172776596E-186	0.630081135412791	0.572	0.27	6.99816975390196E-182	Detoxification-associated hepat	R3HDM2
4.60088782808704E-186	0.551103438209703	0.657	0.326	7.71016782230825E-182	Detoxification-associated hepat	YTHDF3
4.69550960665911E-186	1.84855037246008	0.261	0.08	7.86873499883934E-182	Detoxification-associated hepat	ZNF234
1.00314244445093E-185	0.557416008278183	0.636	0.316	1.68106610841086E-181	Detoxification-associated hepat	SPATA13
1.0795455855238E-185	1.19433174164126	0.374	0.145	1.80910249222078E-181	Detoxification-associated hepat	VPS8
1.39352917888929E-185	0.634770943146571	0.6	0.285	2.33527619798267E-181	Detoxification-associated hepat	LMBR1
1.90369968931135E-185	0.982339089583053	0.438	0.188	3.19021993934797E-181	Detoxification-associated hepat	MAP4K5
2.63008498149533E-185	0.707522419504489	0.671	0.349	4.40749641198988E-181	Detoxification-associated hepat	SAFB2
3.52628638853922E-185	0.555616253068056	0.613	0.298	5.90935072991403E-181	Detoxification-associated hepat	TP53BP2
3.53450871302325E-185	1.09939655681125	0.377	0.147	5.92312970128437E-181	Detoxification-associated hepat	SLC10A7
5.14608877974637E-185	0.606570806922947	0.881	0.511	8.62381557709897E-181	Detoxification-associated hepat	SLC37A4
1.12460917629347E-184	0.55794452546121	0.668	0.329	1.8846200576326E-180	Detoxification-associated hepat	MROH1
1.13766583764263E-184	0.694404864485332	0.486	0.214	1.90650041072153E-180	Detoxification-associated hepat	ZNF444
1.56141197367188E-184	0.892861140509769	0.447	0.191	2.61661418547933E-180	Detoxification-associated hepat	CDKAL1
2.40498934562951E-184	1.03709789755966	0.384	0.152	4.03028114540594E-180	Detoxification-associated hepat	RECQL5
4.51224697821184E-184	0.664749555095368	0.494	0.219	7.5616234860874E-180	Detoxification-associated hepat	KPNA1
5.77288024589864E-184	0.600859511024048	0.586	0.282	9.67419271607694E-180	Detoxification-associated hepat	SH3BP4
7.64343760720077E-184	0.608955693491734	0.621	0.305	1.2808872742147E-179	Detoxification-associated hepat	C5orf24
1.01673899496211E-183	1.42459474835846	0.305	0.105	1.7038512077575E-179	Detoxification-associated hepat	CEP95
1.13951399778142E-183	1.62806523448058	0.269	0.085	1.9095975574821E-179	Detoxification-associated hepat	GOLGA8A
2.84880073345144E-183	0.929163670041129	0.438	0.186	4.77402026911793E-179	Detoxification-associated hepat	PLEKHA5
3.13274685812526E-183	0.685370155461372	0.523	0.241	5.24985718484631E-179	Detoxification-associated hepat	ZNF326
4.48747828649825E-183	1.11043578413216	0.368	0.143	7.52011611251376E-179	Detoxification-associated hepat	ZFX
1.36135518252615E-182	0.620512926749164	0.648	0.316	2.28135901487732E-178	Detoxification-associated hepat	C1GALT1C1
1.47371175851444E-182	1.0104507581458	0.38	0.15	2.4696461649185E-178	Detoxification-associated hepat	MNAT1
2.082949069483E-182	0.589483150072545	0.725	0.354	3.4906060506396E-178	Detoxification-associated hepat	NEB
3.23284871784506E-182	0.843296414708556	0.454	0.197	5.41760788136476E-178	Detoxification-associated hepat	SETX
3.45729550841302E-182	1.26427813847702	0.32	0.114	5.79373581299853E-178	Detoxification-associated hepat	VCL
5.51992858177314E-182	0.576970041815484	0.657	0.327	9.25029631733542E-178	Detoxification-associated hepat	TRIM27
7.49317314175553E-182	0.591403162619005	0.634	0.313	1.25570595509539E-177	Detoxification-associated hepat	EMP2
1.18015834517239E-181	0.810882701709794	0.487	0.217	1.97770935483989E-177	Detoxification-associated hepat	REV1
1.42454568625551E-181	0.885258136961476	0.417	0.172	2.38725366102699E-177	Detoxification-associated hepat	ASCC3
1.47641452153771E-181	0.828101511117239	0.464	0.203	2.4741754551929E-177	Detoxification-associated hepat	ATP6V0A1
1.48611730704116E-181	0.882924812569063	0.368	0.139	2.49043538313958E-177	Detoxification-associated hepat	ZNF512B
2.96933241734081E-181	0.80634904640717	0.489	0.22	4.97600726497973E-177	Detoxification-associated hepat	ATXN2L
3.89636580113258E-181	0.567535433859443	0.688	0.346	6.52952980953798E-177	Detoxification-associated hepat	FAM199X
4.00628344162443E-181	1.22124376780736	0.353	0.135	6.71372979147421E-177	Detoxification-associated hepat	FAM200B
4.63819158151561E-181	0.614505199333225	0.64	0.316	7.77268145230385E-177	Detoxification-associated hepat	PDCD4
5.60925754769476E-181	0.680355305483943	0.49	0.219	9.39999379842687E-177	Detoxification-associated hepat	RASSF4
6.1905551932919E-181	0.586399088492326	0.621	0.305	1.03741323929186E-176	Detoxification-associated hepat	COBLL1
8.0602032578514E-181	0.737312390929228	0.519	0.24	1.35072886195074E-176	Detoxification-associated hepat	NSD1
1.11186809478076E-180	1.00937359224166	0.38	0.151	1.86326855323361E-176	Detoxification-associated hepat	TBCE
1.2707633436674E-180	0.850116238560401	0.46	0.202	2.12954521131782E-176	Detoxification-associated hepat	ABCB10
1.28547264684329E-180	1.25444601443219	0.337	0.123	2.15419506157999E-176	Detoxification-associated hepat	ANKRD36C
1.63597376992617E-180	0.569577388785975	0.734	0.379	2.74156484364228E-176	Detoxification-associated hepat	EML4

1.95631738439596E-180	0.716443798378778	0.558	0.267	3.27839667277075E-176	Detoxification-associated hepat	ZNF410
2.17703117134858E-180	0.576855092451166	0.666	0.329	3.64826883694595E-176	Detoxification-associated hepat	MAVS
2.85195745646528E-180	0.609176774630985	0.589	0.285	4.77931030554451E-176	Detoxification-associated hepat	CARS2
4.37433628764956E-180	0.681661870584944	0.553	0.261	7.33051275084314E-176	Detoxification-associated hepat	CNOT6L
4.7694790461534E-180	0.524149855380424	0.613	0.299	7.99269298554387E-176	Detoxification-associated hepat	ASB13
5.0688501726781E-180	0.574131350782865	0.528	0.246	8.49437911937395E-176	Detoxification-associated hepat	DEXI
5.44687362337478E-180	1.06271934854377	0.396	0.163	9.12787081805145E-176	Detoxification-associated hepat	RBMX2
6.13042521970609E-180	0.529956592719661	0.942	0.705	1.02733665831835E-175	Detoxification-associated hepat	HSD17B6
1.15183727943509E-179	0.605088625424197	0.611	0.298	1.93024891287733E-175	Detoxification-associated hepat	APEX1
1.18754876890332E-179	0.90191897854572	0.364	0.139	1.99009422692819E-175	Detoxification-associated hepat	WDR6
1.77457421909345E-179	0.686057464963436	0.534	0.248	2.9738314763568E-175	Detoxification-associated hepat	DYM
2.07735968606054E-179	0.625888769940825	0.553	0.265	3.48123936190025E-175	Detoxification-associated hepat	HIGD1A
2.12978295167231E-179	0.665575230259824	0.539	0.252	3.56909027041246E-175	Detoxification-associated hepat	SORBS1
3.12340776109512E-179	0.526878212551284	0.907	0.533	5.23420672604321E-175	Detoxification-associated hepat	VMP1
4.52221973477021E-179	1.16591011415039	0.351	0.135	7.57833583152791E-175	Detoxification-associated hepat	CHML
6.24223873500019E-179	0.75165979359537	0.519	0.242	1.04607436721133E-174	Detoxification-associated hepat	TLE1
7.63574974832107E-179	0.744109055535726	0.515	0.238	1.27959894282364E-174	Detoxification-associated hepat	DDX3X
1.05906827123355E-178	0.582110570436543	0.655	0.332	1.77478660893318E-174	Detoxification-associated hepat	SRP54
1.10000836173872E-178	0.579758635102556	0.842	0.477	1.84339401260175E-174	Detoxification-associated hepat	WNK1
1.25919448506845E-178	0.69590054252335	0.601	0.298	2.11015811807771E-174	Detoxification-associated hepat	MSI2
1.37414786678131E-178	1.0862622513673	0.384	0.155	2.30279699515213E-174	Detoxification-associated hepat	ABHD18
1.75214869683105E-178	0.598659329590465	0.651	0.329	2.93625078614947E-174	Detoxification-associated hepat	LRRFIP2
2.07997223508659E-178	0.981766066291793	0.432	0.187	3.4856174715581E-174	Detoxification-associated hepat	MAP3K4
2.11607680743932E-178	0.927823863133672	0.449	0.196	3.54612151390681E-174	Detoxification-associated hepat	DIP2A
2.85416706220773E-178	1.44154665901731	0.198	0.048	4.78301316284772E-174	Detoxification-associated hepat	NABP1
3.92938284264159E-178	1.05314243625899	0.391	0.16	6.58485976769878E-174	Detoxification-associated hepat	MRNIP
8.01506421978106E-178	0.713794610960164	0.513	0.237	1.34316446195091E-173	Detoxification-associated hepat	UGT1A4
8.63570173701307E-178	1.14485267046439	0.357	0.139	1.44717089708865E-173	Detoxification-associated hepat	ABHD13
1.07745088718041E-177	0.927775675537025	0.435	0.188	1.80559219673693E-173	Detoxification-associated hepat	APOL2
1.08558989166486E-177	0.405710682158491	0.941	0.597	1.81923154045198E-173	Detoxification-associated hepat	MUC3A
1.28901016718783E-177	0.632738293913562	0.565	0.27	2.16012323817336E-173	Detoxification-associated hepat	CPSF6
2.02653400404679E-177	0.748729857366282	0.532	0.242	3.39606568398162E-173	Detoxification-associated hepat	PCSK5
3.2577785150987E-177	0.799391745599644	0.425	0.178	5.4593852356024E-173	Detoxification-associated hepat	MCEE
3.61510393389518E-177	0.856923525724475	0.474	0.211	6.05819117242155E-173	Detoxification-associated hepat	DMTF1
3.64132612946444E-177	1.29784496354793	0.329	0.122	6.1021343277565E-173	Detoxification-associated hepat	GPR180
4.09891797797554E-177	0.720392125997628	0.511	0.236	6.86896674749142E-173	Detoxification-associated hepat	HMGXB4
5.51759751753902E-177	1.02325424333807	0.444	0.192	9.2463899198919E-173	Detoxification-associated hepat	CCDC198
6.58416085198145E-177	0.512939941272912	0.852	0.471	1.10337367557505E-172	Detoxification-associated hepat	MORF4L2
7.13082901739524E-177	0.724970145002773	0.459	0.202	1.19498432673509E-172	Detoxification-associated hepat	PBX2
1.15057610836432E-176	0.579045264209451	0.558	0.265	1.92813544239693E-172	Detoxification-associated hepat	SELENOS
1.28010591794007E-176	0.553321263347646	0.67	0.341	2.14520149728397E-172	Detoxification-associated hepat	DTX3L
1.35194535802805E-176	0.630913200194249	0.549	0.261	2.26559003098341E-172	Detoxification-associated hepat	KANSL1
1.48278936578247E-176	0.687838571956773	0.52	0.241	2.48485841917826E-172	Detoxification-associated hepat	ODR4
3.4699174057088E-176	0.95495423460662	0.408	0.171	5.8148875884868E-172	Detoxification-associated hepat	GPAT3
3.61300438921812E-176	0.821544561676506	0.415	0.174	6.05467275545173E-172	Detoxification-associated hepat	COPS2
4.28288535149396E-176	0.492988813547757	0.696	0.361	7.17725927203357E-172	Detoxification-associated hepat	SETD7
6.06841346186506E-176	0.845179138633529	0.415	0.173	1.01694472793935E-171	Detoxification-associated hepat	RAPGEF2
8.62594338183265E-176	1.36149296205811	0.289	0.099	1.44553559192752E-171	Detoxification-associated hepat	MFSD4B
9.86549668491184E-176	1.20726759892713	0.294	0.1	1.65325993445753E-171	Detoxification-associated hepat	NICN1
9.98970433006171E-176	0.848331460003036	0.44	0.192	1.67407465163174E-171	Detoxification-associated hepat	ARHGAP17
1.04145429742747E-175	0.68314405612674	0.49	0.222	1.74526911162896E-171	Detoxification-associated hepat	RC3H1
1.0734838712743E-175	1.28604630546148	0.279	0.092	1.79894427148147E-171	Detoxification-associated hepat	FAM219B
1.62001616774076E-175	0.683224254201144	0.547	0.261	2.71482309389997E-171	Detoxification-associated hepat	KDM4C
2.69976984184852E-175	1.07874883416362	0.377	0.152	4.52427430096975E-171	Detoxification-associated hepat	PTGR2
2.78822627492892E-175	0.59680356429554	0.564	0.27	4.67250959152588E-171	Detoxification-associated hepat	BCAR1
2.93760118339541E-175	0.603586586356383	0.632	0.32	4.92283206313403E-171	Detoxification-associated hepat	IVD
3.91037487875536E-175	0.81395060118698	0.433	0.185	6.55300622181823E-171	Detoxification-associated hepat	TELO2
4.07493074449808E-175	0.954748824428705	0.411	0.174	6.82876894162989E-171	Detoxification-associated hepat	MCM3AP
4.1895840494467E-175	1.36712328619889	0.3	0.105	7.02090495006279E-171	Detoxification-associated hepat	ZNF248
8.98347446856089E-175	0.982172219594938	0.451	0.198	1.50545065144143E-170	Detoxification-associated hepat	LAMA3

**1.29955672671586E-174**	0.884751246351381	0.426	0.182	2.17779716263044E-170	Detoxification-associated hepat	PCGF3
**1.30705401671607E-174**	0.562806379891609	0.684	0.357	2.19036112121279E-170	Detoxification-associated hepat	SLC25A44
**2.03689311257177E-174**	0.656100083797934	0.519	0.24	3.41342547804778E-170	Detoxification-associated hepat	GBA2
**2.5873392075574E-174**	0.8946242157218	0.409	0.172	4.33586304402469E-170	Detoxification-associated hepat	SLC25A46
**3.32027349693238E-174**	1.05883135852401	0.382	0.156	5.56411432615929E-170	Detoxification-associated hepat	DMAC2L
**4.23603699285461E-174**	0.516993855308566	0.966	0.723	7.09875079262575E-170	Detoxification-associated hepat	CFH
**5.0474861534303E-174**	0.74575830090222	0.51	0.237	8.4585772959185E-170	Detoxification-associated hepat	CAND1
**1.00164245960051E-173**	0.641371208642048	0.544	0.257	1.67855243379854E-169	Detoxification-associated hepat	FBXL6
**1.05521571469171E-173**	0.576311643671737	0.691	0.352	1.76833049468036E-169	Detoxification-associated hepat	STT3B
**1.77776915529246E-173**	1.22423205484356	0.322	0.118	2.97918555043911E-169	Detoxification-associated hepat	GRK3
**2.23970632812051E-173**	0.999240177793768	0.199	0.049	3.75329986466435E-169	Detoxification-associated hepat	CRIP2
**4.69173618804661E-173**	0.493349729560389	0.743	0.407	7.86241150392851E-169	Detoxification-associated hepat	LONP2
**6.51185812648245E-173**	1.08613134731098	0.352	0.136	1.09125718483593E-168	Detoxification-associated hepat	TAF9
**1.38783720955615E-172**	1.15013098142824	0.34	0.129	2.32573759577419E-168	Detoxification-associated hepat	MAML3
**1.4409461322165E-172**	1.26718011954023	0.346	0.134	2.41473752836841E-168	Detoxification-associated hepat	DPY19L3
**1.44132278369592E-172**	0.751110612299657	0.381	0.151	2.41536872091761E-168	Detoxification-associated hepat	KHDRBS3
**2.62806410211759E-172**	0.692461901261593	0.533	0.253	4.40410982232866E-168	Detoxification-associated hepat	NIPBL
**2.71773374185944E-172**	1.12677269650182	0.343	0.131	4.55437820460805E-168	Detoxification-associated hepat	USP40
**3.60249725982278E-172**	0.565493512871728	0.591	0.291	6.03706490801102E-168	Detoxification-associated hepat	ANAPC16
**3.78703719908932E-172**	0.608656495278704	0.51	0.235	6.34631693823388E-168	Detoxification-associated hepat	MXD4
**4.52456182535306E-172**	0.723002914002724	0.494	0.229	7.58226070692665E-168	Detoxification-associated hepat	AEBP2
**5.01937822922664E-172**	0.658646309813326	0.526	0.247	8.411474036538E-168	Detoxification-associated hepat	TAF1D
**6.10421104367415E-172**	1.78999103710476	0.22	0.062	1.02294368669891E-167	Detoxification-associated hepat	ZNF484
**7.28854097033094E-172**	0.779626967659602	0.458	0.203	1.22141369580806E-167	Detoxification-associated hepat	LETMD1
**9.41051643464091E-172**	0.915589472271283	0.366	0.145	1.57701434411712E-167	Detoxification-associated hepat	ABL2
**9.76240931479203E-172**	1.35087899548827	0.323	0.121	1.63598455297285E-167	Detoxification-associated hepat	ZNF224
**9.76746277061515E-172**	0.738914524882796	0.497	0.231	1.63683141109969E-167	Detoxification-associated hepat	LARS
**1.23357943882156E-171**	0.555114686161747	0.662	0.336	2.06723242357717E-167	Detoxification-associated hepat	NCOA2
**2.17704452434369E-171**	0.565619502678001	0.581	0.283	3.64829121389515E-167	Detoxification-associated hepat	ZNF644
**2.1774499743896E-171**	0.798870340480574	0.473	0.214	3.64897066708209E-167	Detoxification-associated hepat	EIF2S3
**5.22758909095016E-171**	1.56802685728199	0.262	0.085	8.76039379861427E-167	Detoxification-associated hepat	MAP10
**5.67538335898243E-171**	0.735913764959515	0.477	0.217	9.51080743298276E-167	Detoxification-associated hepat	DYRK1A
**6.99068363773336E-171**	0.737690208877338	0.394	0.158	1.17149876401136E-166	Detoxification-associated hepat	FBXO17
**1.5965202772129E-170**	0.51359859623947	0.674	0.34	2.67544868055339E-166	Detoxification-associated hepat	HNRNPL
**2.03352185804872E-170**	0.477580190631125	0.736	0.391	3.40777592971805E-166	Detoxification-associated hepat	NPLOC4
**2.19154445595145E-170**	0.530607472445907	0.559	0.27	3.67259019928344E-166	Detoxification-associated hepat	NPEPPS
**2.47871408934317E-170**	0.587607493024094	0.582	0.289	4.15382907092128E-166	Detoxification-associated hepat	CDC42SE1
**2.64093012728242E-170**	0.689546127080089	0.53	0.252	4.42567070729988E-166	Detoxification-associated hepat	PRKAB2
**4.39717717287429E-170**	0.648103082243225	0.601	0.288	7.36878950630273E-166	Detoxification-associated hepat	STK39
**6.28349737451843E-170**	0.939723314503515	0.372	0.149	1.0529884900218E-165	Detoxification-associated hepat	STK38L
**8.02674908115709E-170**	0.762701453044441	0.469	0.213	1.34512261102031E-165	Detoxification-associated hepat	ADGRA3
**1.04069089305955E-169**	0.861175636376605	0.416	0.178	1.7439897985892E-165	Detoxification-associated hepat	ADCK5
**1.46135312036028E-169**	0.57232905498017	0.563	0.27	2.44893555909976E-165	Detoxification-associated hepat	SEC23A
**2.3197500393582E-169**	1.03780588059801	0.374	0.153	3.88743711595647E-165	Detoxification-associated hepat	PHYHIPL
**3.2050522823757E-169**	0.770981458546908	0.433	0.189	5.3710266148052E-165	Detoxification-associated hepat	RHOT1
**5.33854494045334E-169**	0.798759686971198	0.441	0.194	8.94633361121171E-165	Detoxification-associated hepat	DEPRECATEI
**5.41386575132971E-169**	0.562093848331251	0.677	0.332	9.07255622607832E-165	Detoxification-associated hepat	KCNK1
**6.04898282192052E-169**	0.499674601443914	0.809	0.441	1.01368854129744E-164	Detoxification-associated hepat	TBC1D9B
**1.06326082560254E-168**	0.72367183672287	0.49	0.229	1.78181249154474E-164	Detoxification-associated hepat	RSBN1L
**1.07029813650546E-168**	0.639247899454314	0.486	0.221	1.79360561715586E-164	Detoxification-associated hepat	PSMC6
**1.1440365933583E-168**	0.488727622831405	0.68	0.343	1.91717652314985E-164	Detoxification-associated hepat	SLC6A1
**1.19831984013712E-168**	0.899481973517335	0.413	0.178	2.00814438810178E-164	Detoxification-associated hepat	KIAA2026
**1.9858815395936E-168**	1.23875716436746	0.325	0.123	3.32794028405096E-164	Detoxification-associated hepat	MFSD6
**2.05305791781896E-168**	0.649432812382988	0.563	0.275	3.44051445868102E-164	Detoxification-associated hepat	ENOSF1
**2.36622818725954E-168**	1.06778840461725	0.34	0.132	3.96532519620954E-164	Detoxification-associated hepat	MRI1
**4.16500727636814E-168**	0.490064034788468	0.628	0.319	6.97971919373773E-164	Detoxification-associated hepat	NRDC
**4.85682863958139E-168**	0.934357533444738	0.411	0.176	8.13907343421049E-164	Detoxification-associated hepat	RABGAP1
**7.63382985714925E-168**	0.998147500698898	0.395	0.168	1.27927720746107E-163	Detoxification-associated hepat	TRIP11
**1.5951784855932E-167**	0.629375958692755	0.558	0.264	2.67320010615708E-163	Detoxification-associated hepat	DLG5
**1.96130590814897E-167**	0.549863901598663	0.58	0.288	3.28675644087605E-163	Detoxification-associated hepat	COPB1

2.25318320480024E-167	0.643351484207497	0.478	0.218	3.77588441460424E-163	Detoxification-associated hepat	VIRMA
2.53613039296841E-167	0.588441645537431	0.537	0.258	4.25004731253646E-163	Detoxification-associated hepat	WDR26
3.09029884989947E-167	0.617619254430508	0.611	0.304	5.17872281266153E-163	Detoxification-associated hepat	TIMM8A
4.09024847363303E-167	0.624117276971708	0.595	0.289	6.85443839211423E-163	Detoxification-associated hepat	IL10RB
4.42823638269058E-167	0.488240531096559	0.799	0.439	7.42083853011287E-163	Detoxification-associated hepat	SEC31A
5.39391553287995E-167	0.587622346785845	0.557	0.271	9.03912365000021E-163	Detoxification-associated hepat	TAOK1
5.80909195161802E-167	0.873171080865534	0.393	0.165	9.73487629252148E-163	Detoxification-associated hepat	PIGP
6.36762851167161E-167	0.618004371433261	0.465	0.209	1.06708718598593E-162	Detoxification-associated hepat	ANKH
6.63347649797346E-167	1.5790178351431	0.186	0.046	1.11163799153039E-162	Detoxification-associated hepat	LYRM9
7.443169390107E-167	1.0602567193705	0.343	0.134	1.24732632639413E-162	Detoxification-associated hepat	PPWD1
1.05447559618521E-166	0.516619298253018	0.661	0.336	1.76709020408718E-162	Detoxification-associated hepat	CCNG1
1.14663935626269E-166	0.768066334394359	0.475	0.218	1.92153823322502E-162	Detoxification-associated hepat	BDP1
1.38142474281723E-166	1.83612626027162	0.241	0.075	2.31499158401312E-162	Detoxification-associated hepat	MPP3
1.39002808091132E-166	0.595760780021587	0.488	0.222	2.32940905799118E-162	Detoxification-associated hepat	SPATA20
1.43804597311377E-166	0.517441418972338	0.788	0.452	2.40987744174406E-162	Detoxification-associated hepat	A1CF
1.5013055693371E-166	1.17281320504529	0.314	0.116	2.51588787309511E-162	Detoxification-associated hepat	TTC21B
1.72143633104221E-166	1.20169952487088	0.361	0.146	2.88478300356054E-162	Detoxification-associated hepat	TMEM44
1.82255976305173E-166	0.794935193672553	0.431	0.19	3.05424565092209E-162	Detoxification-associated hepat	ZC3H14
2.2038198200711E-166	0.486303517721114	0.729	0.382	3.69316125447515E-162	Detoxification-associated hepat	LPP
2.68172339044681E-166	0.502249380375727	0.671	0.345	4.49403205771077E-162	Detoxification-associated hepat	OCIAD1
2.90133070011737E-166	0.566698019703204	0.561	0.271	4.86204998725668E-162	Detoxification-associated hepat	KMT5B
2.94795340302253E-166	0.69530902839914	0.485	0.225	4.94018031278516E-162	Detoxification-associated hepat	NBN
3.59510866254392E-166	0.730782833391225	0.458	0.205	6.0246830966911E-162	Detoxification-associated hepat	CSTF3
3.85277792179339E-166	0.546667397064448	0.557	0.267	6.45648524134137E-162	Detoxification-associated hepat	CADM1
4.59724612333865E-166	2.03045702211493	0.201	0.055	7.70406505349091E-162	Detoxification-associated hepat	LRAT
6.6259583090065E-166	0.44058834859604	0.681	0.348	1.11037809342331E-161	Detoxification-associated hepat	SHROOM1
1.68791796401895E-165	0.474935311192915	0.83	0.466	2.82861292410295E-161	Detoxification-associated hepat	MIDN
1.93722313983454E-165	0.532233293167894	0.632	0.321	3.24639853773473E-161	Detoxification-associated hepat	PHF10
2.0688243116661E-165	0.975947450624005	0.284	0.096	3.46693578149004E-161	Detoxification-associated hepat	BBOX1
2.6437102506981E-165	0.505288733557097	0.612	0.307	4.43032963811987E-161	Detoxification-associated hepat	PSMD12
3.06538521709644E-165	0.649699221413508	0.538	0.258	5.13697254681022E-161	Detoxification-associated hepat	MAPK1IP1L
5.92555977514778E-165	0.55706617322086	0.588	0.294	9.93005307119265E-161	Detoxification-associated hepat	RABGGTB
6.42318443674095E-165	0.502568846871576	0.622	0.321	1.07639724790905E-160	Detoxification-associated hepat	TJP1
6.8849868754676E-165	0.562051373078768	0.718	0.372	1.15378610059086E-160	Detoxification-associated hepat	LONRF3
1.00310052502219E-164	0.540803691903268	0.538	0.259	1.68099585983218E-160	Detoxification-associated hepat	WWOX
1.06094352143586E-164	1.08447176236998	0.308	0.112	1.77792915322222E-160	Detoxification-associated hepat	TSNARE1
1.21963809669948E-164	0.549154051255558	0.576	0.285	2.04386952244898E-160	Detoxification-associated hepat	ZFR
1.37207616192577E-164	0.616509167117544	0.601	0.304	2.29932523215521E-160	Detoxification-associated hepat	MARC1
2.64839389143908E-164	0.823704146322654	0.452	0.204	4.43817848327361E-160	Detoxification-associated hepat	ELF2
2.99037682540766E-164	0.532154151277849	0.612	0.308	5.01127348401815E-160	Detoxification-associated hepat	MKLN1
3.12465891399266E-164	0.699998615204366	0.492	0.232	5.2363034080689E-160	Detoxification-associated hepat	BTBD7
4.11475134105836E-164	0.72620072913051	0.471	0.215	6.89550029734561E-160	Detoxification-associated hepat	ZNF33A
4.28940496522204E-164	0.958576927223938	0.349	0.138	7.18818484071909E-160	Detoxification-associated hepat	ENGASE
4.67422739560326E-164	0.721622038411857	0.519	0.249	7.83307026955194E-160	Detoxification-associated hepat	DCAF5
5.96210657972705E-164	1.16283167512304	0.294	0.104	9.99129820630658E-160	Detoxification-associated hepat	PACRG
7.32766546422681E-164	1.41310883929922	0.293	0.105	1.22797017849513E-159	Detoxification-associated hepat	CPLANE1
7.37288600168493E-164	0.704482789285927	0.457	0.206	1.23554823616236E-159	Detoxification-associated hepat	MOAP1
8.10724436060209E-164	1.11097968648892	0.37	0.152	1.3586120099497E-159	Detoxification-associated hepat	XPO4
8.94595589220647E-164	0.569558565797552	0.558	0.274	1.49916328841596E-159	Detoxification-associated hepat	EWSR1
9.03585383703194E-164	1.26012192650426	0.292	0.104	1.51422838600981E-159	Detoxification-associated hepat	ARL17B
1.72607749536542E-163	1.27634058827249	0.317	0.121	2.89256066673337E-159	Detoxification-associated hepat	MCM9
1.94984568511387E-163	0.947239322228908	0.39	0.165	3.26755139911382E-159	Detoxification-associated hepat	PIGM
2.72632987179127E-163	0.923055945747221	0.266	0.086	4.5687835991478E-159	Detoxification-associated hepat	GLDC
3.65166299287014E-163	1.24980846627041	0.285	0.1	6.11945684345177E-159	Detoxification-associated hepat	C9orf85
5.7252403333154E-163	0.489993065042798	0.716	0.367	9.59435775056994E-159	Detoxification-associated hepat	EDEM3
9.75401814275868E-163	0.524027834704528	0.602	0.293	1.6345783603635E-158	Detoxification-associated hepat	EDNRB
1.16146084963635E-162	0.463785224809854	0.705	0.35	1.9463760918206E-158	Detoxification-associated hepat	SLC15A1
1.37607765707042E-162	0.541210384834243	0.559	0.272	2.3060309377186E-158	Detoxification-associated hepat	STX7
1.38090884340377E-162	0.476657968768288	0.767	0.423	2.31412703977604E-158	Detoxification-associated hepat	RAB4A
1.66108236846708E-162	0.849628243436701	0.44	0.199	2.78364183307713E-158	Detoxification-associated hepat	DEPRECATEI

1.90032979738661E-162	1.45674953089952	0.262	0.088	3.18457267446049E-158	Detoxification-associated hepat	ZNF587B
1.93394238814961E-162	0.670911919578162	0.453	0.205	3.24090065406111E-158	Detoxification-associated hepat	USP25
2.17104758753798E-162	0.584205155620438	0.539	0.258	3.63824154719614E-158	Detoxification-associated hepat	SARDH
4.18692157595096E-162	1.08151778215201	0.37	0.154	7.01644317697862E-158	Detoxification-associated hepat	NPIPB15
6.37648712902396E-162	0.640954844682122	0.512	0.245	1.06857171308184E-157	Detoxification-associated hepat	SEMA4B
7.69739422995004E-162	0.959045206277503	0.381	0.16	1.28992932505503E-157	Detoxification-associated hepat	SEPTIN7
9.44224790823601E-162	0.749557354451705	0.477	0.221	1.58233190446219E-157	Detoxification-associated hepat	ZNF623
1.01937156097398E-161	0.621944178195129	0.483	0.223	1.70826286188019E-157	Detoxification-associated hepat	PIP5K1A
1.48968725055203E-161	0.64315943692548	0.488	0.228	2.4964178944751E-157	Detoxification-associated hepat	UNC50
1.56957726050417E-161	0.882643135503247	0.376	0.155	2.63029757315288E-157	Detoxification-associated hepat	EXOC8
1.81256320165489E-161	0.467106386630102	0.489	0.221	3.03749341333326E-157	Detoxification-associated hepat	SOX5
1.84337246720224E-161	0.885822533751285	0.348	0.138	3.08912358053752E-157	Detoxification-associated hepat	ACYP2
2.026712099208E-161	0.495497745725866	0.654	0.348	3.39636413585276E-157	Detoxification-associated hepat	TMEM147
2.58414222351233E-161	0.471525474796996	0.643	0.334	4.33050553816197E-157	Detoxification-associated hepat	ITCH
2.75627440478282E-161	0.495341996055428	0.524	0.25	4.61896464753505E-157	Detoxification-associated hepat	ZRANB2
3.04445102395195E-161	0.763898321416797	0.427	0.189	5.10189102593868E-157	Detoxification-associated hepat	ANKRD40
3.28133217936281E-161	0.893220016621404	0.339	0.132	5.4988564661762E-157	Detoxification-associated hepat	ITFG2
3.33392652986678E-161	0.635828696021823	0.555	0.275	5.58699407875075E-157	Detoxification-associated hepat	MPHOSPH8
3.81928324135708E-161	0.646661309081779	0.473	0.218	6.40035485586619E-157	Detoxification-associated hepat	ACBD6
3.86890331552074E-161	0.538319934459993	0.613	0.31	6.48350817614965E-157	Detoxification-associated hepat	TMEM245
4.49573654403772E-161	0.573842244997882	0.702	0.377	7.53395530049841E-157	Detoxification-associated hepat	FDPS
8.10410603718836E-161	0.439975412464457	0.758	0.408	1.35808608971203E-156	Detoxification-associated hepat	RBM33
8.13518580134088E-161	0.566774719014104	0.52	0.249	1.3632944365887E-156	Detoxification-associated hepat	IL17RC
9.46682712579464E-161	0.727631602064255	0.436	0.195	1.58645088974067E-156	Detoxification-associated hepat	NAE1
1.36595829565249E-160	0.519445548526501	0.655	0.337	2.28907291185445E-156	Detoxification-associated hepat	SLC35F5
1.40316270536123E-160	0.556458416886484	0.512	0.245	2.35142006164436E-156	Detoxification-associated hepat	AP2A2
1.96352749695225E-160	0.752312476783128	0.462	0.213	3.29047937939258E-156	Detoxification-associated hepat	POLR2B
2.22550086674505E-160	0.542617590231276	0.455	0.204	3.72949435249135E-156	Detoxification-associated hepat	GHR
2.83880474762441E-160	1.56632507115979	0.29	0.106	4.75726899606899E-156	Detoxification-associated hepat	C3orf85
2.89254033531893E-160	0.620185646812904	0.533	0.259	4.84731909392747E-156	Detoxification-associated hepat	TOB2
3.24172954868439E-160	0.545650302463093	0.546	0.266	5.4324903776853E-156	Detoxification-associated hepat	PLA2G12A
3.30255997685274E-160	1.20991693631087	0.337	0.134	5.53443000920981E-156	Detoxification-associated hepat	ZNF266
3.74639643726314E-160	0.870952792365503	0.387	0.163	6.27821114956557E-156	Detoxification-associated hepat	CLK4
4.56487697987805E-160	0.545077457323891	0.556	0.274	7.64982084287964E-156	Detoxification-associated hepat	RAB20
1.1784636219168E-159	0.766475199996734	0.442	0.2	1.97486933760817E-155	Detoxification-associated hepat	VCPIP1
1.27647902639249E-159	0.559001504081679	0.535	0.26	2.13912355242853E-155	Detoxification-associated hepat	MACF1
1.27910000126842E-159	0.400364682992969	0.831	0.468	2.14351578212562E-155	Detoxification-associated hepat	NR1H4
1.52388778605455E-159	1.00487944443292	0.39	0.169	2.55373115187021E-155	Detoxification-associated hepat	UPF3B
1.58180701518578E-159	0.472140448033966	0.608	0.307	2.65079219604833E-155	Detoxification-associated hepat	NUS1
2.51054147363522E-159	0.956339923934729	0.361	0.148	4.20716540151791E-155	Detoxification-associated hepat	NAA30
2.5242873595611E-159	0.542523497392006	0.56	0.276	4.23020075715 25E-155	Detoxification-associated hepat	CMC2
2.75768601463339E-159	0.47806407718424	0.938	0.689	4.62133022332263E-155	Detoxification-associated hepat	GATM
2.83689883905886E-159	0.668467031847164	0.479	0.223	4.75407507449484E-155	Detoxification-associated hepat	PTAR1
3.94611904901758E-159	0.516376758493886	0.609	0.308	6.61290630234366E-155	Detoxification-associated hepat	SLC66A2
7.55997198946148E-159	1.03060279795097	0.294	0.105	1.26690010599396E-154	Detoxification-associated hepat	GSTA4
7.92884768238674E-159	1.23197098629734	0.302	0.112	1.32871629461437E-154	Detoxification-associated hepat	ZFP41
8.06065656262842E-159	0.581765338751156	0.479	0.222	1.35080482676527E-154	Detoxification-associated hepat	USP32
9.59683250464884E-159	1.07358779483856	0.283	0.1	1.60823719112905E-154	Detoxification-associated hepat	MINDY3
1.08819466732179E-158	0.526460584894382	0.633	0.323	1.82359662349785E-154	Detoxification-associated hepat	SLC25A33
1.28315724584167E-158	0.7506484050055	0.403	0.174	2.15031491258147E-154	Detoxification-associated hepat	SMARCE1
1.35947368487871E-158	0.905035207833505	0.395	0.171	2.27820600111974E-154	Detoxification-associated hepat	WFS1
1.49645275492701E-158	0.713943872812133	0.459	0.211	2.50775552670668E-154	Detoxification-associated hepat	NDUFB4
1.72640942938454E-158	0.733677843287166	0.357	0.142	2.89311692176262E-154	Detoxification-associated hepat	ZNF22
2.29327747360721E-158	0.619869040325484	0.496	0.234	3.84307439027096E-154	Detoxification-associated hepat	WWC2
2.8222968956596E-158	0.5741849062292	0.513	0.246	4.72960513774635E-154	Detoxification-associated hepat	YIPF4
3.29587596165219E-158	0.926387302552273	0.345	0.138	5.52322893653674E-154	Detoxification-associated hepat	MLLT10
5.29719899165265E-158	0.719499483660197	0.406	0.176	8.8770460702115E-154	Detoxification-associated hepat	AAAS
5.93843564394678E-158	0.58480067398098	0.53	0.256	9.95163045212601E-154	Detoxification-associated hepat	GINM1
6.68321487423039E-158	0.73746871898248	0.443	0.201	1.11997314862353E-153	Detoxification-associated hepat	NUP133
8.38055319836051E-158	0.496653989485141	0.67	0.344	1.40441310498125E-153	Detoxification-associated hepat	GRB10

8.50501801123095E-158	0.46914647948772	0.582	0.291	1.42527091832208E-153	Detoxification-associated hepat	NPTN
9.37203569050089E-158	0.691862252192265	0.413	0.18	1.57056574101414E-153	Detoxification-associated hepat	SLC20A2
1.23424709694504E-157	0.831442043964336	0.41	0.181	2.0683512850605E-153	Detoxification-associated hepat	PCNX1
2.63955517945178E-157	1.20900001526872	0.354	0.145	4.42336656972529E-153	Detoxification-associated hepat	POLE2
2.91068039682961E-157	0.505427122058633	0.579	0.291	4.87771820900706E-153	Detoxification-associated hepat	AFF4
3.21924699613418E-157	0.458651574403795	0.65	0.339	5.39481411612166E-153	Detoxification-associated hepat	EIF4A2
4.58656796480777E-157	0.8445549417831	0.412	0.183	7.68617059542487E-153	Detoxification-associated hepat	SLC30A5
6.9986027229059E-157	0.65595105057639	0.451	0.205	1.17282584430457E-152	Detoxification-associated hepat	SEC24D
7.04943944017596E-157	0.428778504088451	0.633	0.328	1.18134506138469E-152	Detoxification-associated hepat	SLC17A9
9.90273442000718E-157	0.767310400030781	0.394	0.17	1.6595002341048E-152	Detoxification-associated hepat	ITPRIPL2
9.97663997116816E-157	0.796983988184974	0.437	0.199	1.67188532636836E-152	Detoxification-associated hepat	FKBP3
1.04846793944068E-156	0.512924390778317	0.535	0.257	1.7570225729147E-152	Detoxification-associated hepat	FAM13A
1.10188716148961E-156	0.812898160114215	0.415	0.185	1.84654250522429E-152	Detoxification-associated hepat	CHD9
1.1933908949332E-156	1.05195860553187	0.278	0.097	1.99988446172906E-152	Detoxification-associated hepat	TMEM116
1.23385684567518E-156	0.539282396337421	0.578	0.289	2.06769730198246E-152	Detoxification-associated hepat	RB1CC1
1.62921201600378E-156	0.994633340326892	0.366	0.154	2.73023349641913E-152	Detoxification-associated hepat	GK5
1.90536671162036E-156	0.582852866522404	0.519	0.249	3.19301353533339E-152	Detoxification-associated hepat	AP3B1
2.34270046099681E-156	1.38725366999808	0.193	0.051	3.92589743253845E-152	Detoxification-associated hepat	ZNF493
2.4880137006214E-156	0.435417156280887	0.769	0.415	4.16941335950133E-152	Detoxification-associated hepat	TCEAL4
5.33414777771068E-156	0.94616830198694	0.393	0.171	8.93896484588756E-152	Detoxification-associated hepat	PIGG
5.34330485589559E-156	1.26359147049792	0.293	0.109	8.95431027750983E-152	Detoxification-associated hepat	ZNF791
6.72701794001534E-156	0.549970062780262	0.467	0.215	1.12731366638777E-151	Detoxification-associated hepat	SELENOO
6.77447386226881E-156	0.466348039973096	0.632	0.322	1.13526632983901E-151	Detoxification-associated hepat	SLC51A
1.2073178980688E-155	0.999670204651958	0.351	0.142	2.02322333358369E-151	Detoxification-associated hepat	MBTD1
1.34376109232101E-155	0.628821437493593	0.485	0.228	2.25187483851155E-151	Detoxification-associated hepat	ZXDC
1.34747554814885E-155	0.976419430749395	0.269	0.091	2.25809952358785E-151	Detoxification-associated hepat	ADAMTSL3
1.85519485366232E-155	0.833421110801125	0.318	0.119	3.10893553576731E-151	Detoxification-associated hepat	TAB3
2.09385390144735E-155	1.31762344331697	0.297	0.111	3.50888036804546E-151	Detoxification-associated hepat	AP002495.1
3.19652264630608E-155	1.22590876875492	0.285	0.103	5.35673265067974E-151	Detoxification-associated hepat	SETDB2
3.44531424273791E-155	0.688176619828852	0.442	0.203	5.77365760798019E-151	Detoxification-associated hepat	CYHR1
4.72798829620611E-155	0.398927075654405	0.641	0.331	7.9231627867822E-151	Detoxification-associated hepat	LARP4
5.4452182702424E-155	0.846091025182656	0.294	0.105	9.12509677727221E-151	Detoxification-associated hepat	CYP7A1
8.72014253159488E-155	1.49668002789583	0.214	0.063	1.46132148544467E-150	Detoxification-associated hepat	SLC26A1
1.03931508558328E-154	0.655515688836383	0.474	0.224	1.74168422042046E-150	Detoxification-associated hepat	ZBTB41
1.10424399783963E-154	0.459666458973138	0.65	0.333	1.85049209157966E-150	Detoxification-associated hepat	SEL1L
1.31515708786551E-154	0.829734786682071	0.408	0.18	2.20394024784502E-150	Detoxification-associated hepat	FAM193A
1.44630570327786E-154	0.656268378331663	0.485	0.231	2.42371909755304E-150	Detoxification-associated hepat	PGAP3
1.96232531854567E-154	0.744742883497005	0.451	0.208	3.28846476881884E-150	Detoxification-associated hepat	RELCH
2.06916670935516E-154	0.747026561425251	0.375	0.156	3.46750957153737E-150	Detoxification-associated hepat	DENND10
2.08953155742803E-154	1.10290477782784	0.333	0.133	3.50163698393788E-150	Detoxification-associated hepat	MOB3B
3.35135972941225E-154	0.723488397909505	0.448	0.207	5.61620863454904E-150	Detoxification-associated hepat	TAF8
4.72848248118878E-154	1.03155851303534	0.335	0.134	7.92399094197615E-150	Detoxification-associated hepat	TRAPPC11
4.89675634601097E-154	0.651455684108135	0.512	0.249	8.20598428464518E-150	Detoxification-associated hepat	NUP43
7.30023181952267E-154	1.44823833562522	0.275	0.099	1.22337284831561E-149	Detoxification-associated hepat	MFSD8
7.65989443209525E-154	0.617491602582997	0.547	0.273	1.28364510893052E-149	Detoxification-associated hepat	PI4KB
8.96567542414249E-154	0.987721763100829	0.287	0.103	1.5024678875778E-149	Detoxification-associated hepat	NINL
9.15232605431115E-154	0.877713934618496	0.414	0.186	1.53374680018146E-149	Detoxification-associated hepat	OTUD6B
1.08695730411145E-153	0.574772419951399	0.515	0.251	1.82152305022997E-149	Detoxification-associated hepat	PEX13
1.18536726308882E-153	0.718052493996154	0.391	0.167	1.98643845948424E-149	Detoxification-associated hepat	SHLD2
1.33032152222496E-153	0.407711361037662	0.714	0.386	2.22935280694459E-149	Detoxification-associated hepat	AIG1
1.35896580123326E-153	1.54029437662068	0.237	0.076	2.27735488970669E-149	Detoxification-associated hepat	C14orf180
1.61017267148977E-153	0.428823937378705	0.755	0.403	2.69832736288256E-149	Detoxification-associated hepat	FMR1
1.79264076513483E-153	0.571114201922259	0.508	0.243	3.00410739421294E-149	Detoxification-associated hepat	BRWD1
1.95381672036011E-153	0.897814377054421	0.37	0.155	3.27420605997947E-149	Detoxification-associated hepat	ACTR3C
2.38546376021232E-153	1.12176446662702	0.32	0.125	3.99756016936381E-149	Detoxification-associated hepat	CEP44
2.73564784989557E-153	1.1458587328197	0.298	0.112	4.584398666855E-149	Detoxification-associated hepat	ZNF621
3.60653984948058E-153	0.665981121271086	0.522	0.257	6.04383947975956E-149	Detoxification-associated hepat	TRIM24
4.4863691896088E-153	0.721329740533565	0.469	0.221	7.51825748794642E-149	Detoxification-associated hepat	LEMD2
4.97430665472616E-153	0.402229718796654	0.721	0.393	8.3359430919901E-149	Detoxification-associated hepat	PJA2
6.3253840384643E-153	1.04970236243334	0.309	0.118	1.06000785716585E-148	Detoxification-associated hepat	ZNF133

**7.05481113143362E-153**	0.510378330477828	0.632	0.321	1.18224524940565E-148	Detoxification-associated hepat	SRSF3
**8.55101302651361E-153**	0.851473730501538	0.375	0.161	1.43297876298315E-148	Detoxification-associated hepat	SOS 1
**9.21668501718227E-153**	0.431510936182272	0.724	0.392	1.5445320751794E-148	Detoxification-associated hepat	NIT1
**1.03236392145106E-152**	1.20404522951638	0.325	0.13	1.73003545956769E-148	Detoxification-associated hepat	TTC13
**1.33380016702706E-152**	0.732177625312361	0.464	0.219	2.23518231990395E-148	Detoxification-associated hepat	MTR
**1.63223883655923E-152**	0.655435957042255	0.427	0.192	2.73530584230596E-148	Detoxification-associated hepat	ATF6
**1.69122575025178E-152**	0.35715203208658	0.826	0.471	2.83415611227193E-148	Detoxification-associated hepat	CDK5RAP3
**1.78408267430122E-152**	0.447361698560416	0.633	0.324	2.98976574559399E-148	Detoxification-associated hepat	CTSO
**2.36396747361811E-152**	0.763848688838306	0.455	0.211	3.96153669228923E-148	Detoxification-associated hepat	WDR20
**2.71943559761161E-152**	0.517939521525726	0.494	0.236	4.55723017447754E-148	Detoxification-associated hepat	EIF2D
**2.84761729449046E-152**	1.01679197451043	0.377	0.164	4.77203706210711E-148	Detoxification-associated hepat	ZSWIM5
**2.96213302563597E-152**	0.821584255597003	0.381	0.165	4.96394252436076E-148	Detoxification-associated hepat	CUL2
**3.70146281572597E-152**	0.862306027435552	0.349	0.141	6.20291138659357E-148	Detoxification-associated hepat	CDHR2
**4.15143489334128E-152**	0.486061229931535	0.871	0.491	6.95697459426131E-148	Detoxification-associated hepat	ATP6AP1
**6.19400495907356E-152**	0.450018532019946	0.691	0.365	1.03799135104155E-147	Detoxification-associated hepat	EMC2
**7.34419719793751E-152**	0.709983385177031	0.394	0.17	1.23074056643037E-147	Detoxification-associated hepat	DAPK2
**8.57790941453315E-152**	0.468092987356817	0.473	0.216	1.43748605968746E-147	Detoxification-associated hepat	PPARGC1A
**8.72127860397835E-152**	0.538490652715722	0.543	0.27	1.46151186845469E-147	Detoxification-associated hepat	USO1
**8.79272749930445E-152**	0.776077314984641	0.368	0.154	1.47348527433344E-147	Detoxification-associated hepat	GOLGA1
**1.05745198315832E-151**	0.741064876818739	0.369	0.154	1.77207803337671E-147	Detoxification-associated hepat	ARPP19
**1.30535158911033E-151**	1.13082341565259	0.319	0.125	2.18750819303109E-147	Detoxification-associated hepat	PARD3B
**1.59863974662497E-151**	0.689979452132283	0.443	0.203	2.67900048739412E-147	Detoxification-associated hepat	CDK12
**1.97847043297628E-151**	0.483699878767734	0.883	0.555	3.31552075158165E-147	Detoxification-associated hepat	SCCPDH
**2.02024717168675E-151**	0.738698800840033	0.402	0.176	3.38553021031265E-147	Detoxification-associated hepat	SLC25A37
**4.13421707684052E-151**	0.827375972999792	0.401	0.177	6.92812097736934E-147	Detoxification-associated hepat	ETAA1
**5.02866774842009E-151**	0.503539910601743	0.614	0.315	8.42704141280238E-147	Detoxification-associated hepat	TAF7
**5.69960831296947E-151**	0.811664673435167	0.428	0.195	9.55140361087423E-147	Detoxification-associated hepat	IPMK
**5.95207833068533E-151**	0.891624282009107	0.377	0.162	9.97449286656247E-147	Detoxification-associated hepat	RNF170
**6.04107937148804E-151**	0.685448390399078	0.449	0.209	1.01236408107397E-146	Detoxification-associated hepat	WASHC1
**8.00830088014282E-151**	0.639068528375394	0.45	0.21	1.34203106149433E-146	Detoxification-associated hepat	PIP4K2B
**9.41133989899629E-151**	0.274534036001532	0.498	0.231	1.5771523402738E-146	Detoxification-associated hepat	ECHDC3
**1.61894300579802E-150**	0.641713301026851	0.46	0.215	2.71302468911633E-146	Detoxification-associated hepat	MED13
**2.96438082107803E-150**	0.52730556649426	0.591	0.301	4.96770937996256E-146	Detoxification-associated hepat	ENTPD6
**3.04752845221481E-150**	0.915215504921047	0.334	0.135	5.10704818022158E-146	Detoxification-associated hepat	AGO3
**3.55608659391046E-150**	0.857608657408871	0.381	0.164	5.95928991407515E-146	Detoxification-associated hepat	VEZT
**4.27758800300762E-150**	0.444719364349556	0.658	0.347	7.16838197544017E-146	Detoxification-associated hepat	ARRDC2
**5.60970577078249E-150**	0.816542608884227	0.377	0.162	9.40074493067729E-146	Detoxification-associated hepat	ELL
**6.45738132863631E-150**	0.681632441667755	0.607	0.309	1.08212796305287E-145	Detoxification-associated hepat	KCNK5
**8.58984230316537E-150**	0.825250816737886	0.392	0.173	1.43948577316445E-145	Detoxification-associated hepat	NAGLU
**9.76271979664611E-150**	0.736481907463172	0.468	0.221	1.63603658352196E-145	Detoxification-associated hepat	DOCK9
**1.06348630396601E-149**	0.513110300859455	0.645	0.34	1.78219034818625E-145	Detoxification-associated hepat	LGR4
**1.13323339975453E-149**	0.510084959835511	0.496	0.239	1.89907253130865E-145	Detoxification-associated hepat	FBXW2
**1.14104586483696E-149**	0.656736867168737	0.474	0.224	1.91216466029378E-145	Detoxification-associated hepat	DICER1
**1.14439117528535E-149**	0.655068921925889	0.429	0.195	1.9177707315432E-145	Detoxification-associated hepat	BTAF1
**1.21154173070888E-149**	0.524942493393229	0.497	0.237	2.03030163232194E-145	Detoxification-associated hepat	PNISR
**1.40000992781091E-149**	1.44019608169705	0.243	0.081	2.34613663702553E-145	Detoxification-associated hepat	POLI
**1.75950788649309E-149**	1.39849001162428	0.265	0.094	2.94858331618512E-145	Detoxification-associated hepat	ZNF619
**1.89762025592805E-149**	0.493046756813543	0.415	0.181	3.18003202488423E-145	Detoxification-associated hepat	SMO
**2.51694951043045E-149**	0.518309865332642	0.497	0.24	4.21790398957935E-145	Detoxification-associated hepat	KLHL5
**2.72332684842315E-149**	0.52286922616114	0.509	0.246	4.56375113258751E-145	Detoxification-associated hepat	UBA1
**3.25687483628337E-149**	0.543945169369815	0.489	0.234	5.45787085064367E-145	Detoxification-associated hepat	NR3C1
**3.62283585516628E-149**	1.07910098977558	0.297	0.112	6.07114832608766E-145	Detoxification-associated hepat	SNAPC3
**4.27971879732187E-149**	0.659840589666721	0.45	0.21	7.171952760552E-145	Detoxification-associated hepat	KAT7
**4.71848533800361E-149**	0.575147532214313	0.469	0.221	7.90723772942644E-145	Detoxification-associated hepat	ZCCHC9
**4.98031808029416E-149**	0.676640175468986	0.436	0.201	8.34601703895695E-145	Detoxification-associated hepat	DHX36
**5.14271091081252E-149**	0.611927251789925	0.491	0.236	8.61815494433963E-145	Detoxification-associated hepat	HIP1R
**5.24551919697337E-149**	0.907871252988844	0.389	0.172	8.79044107028798E-145	Detoxification-associated hepat	GEMIN7
**5.35344805316061E-149**	0.94749936400819	0.363	0.152	8.97130824748655E-145	Detoxification-associated hepat	TINAG
**5.46745679290731E-149**	0.40120075353949	0.618	0.322	9.16236409355407E-145	Detoxification-associated hepat	SNRNP70
**5.80991963597238E-149**	0.603331385727595	0.479	0.229	9.73626332596251E-145	Detoxification-associated hepat	DHX16

6.89128372283618E-149	1.25887208692469	0.288	0.108	1.15484132627289E-144	Detoxification-associated hepat	AASDH
6.98045378344065E-149	0.545251366117776	0.516	0.252	1.16978444502898E-144	Detoxification-associated hepat	BCL6
9.57674414499592E-149	0.509546524611209	0.925	0.657	1.60487078381842E-144	Detoxification-associated hepat	ANPEP
1.02694317692811E-148	0.757098924294037	0.366	0.155	1.72095137589613E-144	Detoxification-associated hepat	COG1
1.62224824727396E-148	0.96397474270274	0.361	0.153	2.71856361278171E-144	Detoxification-associated hepat	IRAK4
1.72954556525393E-148	0.802587704483877	0.395	0.176	2.89837245825254E-144	Detoxification-associated hepat	ATAD2B
2.55785500152795E-148	0.61436251377889	0.454	0.21	4.28645341156055E-144	Detoxification-associated hepat	FGF13
2.70416766834373E-148	0.401562488261109	0.637	0.33	4.53164417861043E-144	Detoxification-associated hepat	CPNE3
2.80787583294538E-148	0.471872257764205	0.926	0.573	4.70543832084986E-144	Detoxification-associated hepat	RNF128
2.96026433831872E-148	0.837093234659341	0.338	0.137	4.96081097815451E-144	Detoxification-associated hepat	BCAS3
3.21231022910609E-148	0.425796948672766	0.691	0.35	5.38318948193598E-144	Detoxification-associated hepat	SIPA1L2
3.39901049987724E-148	0.801355111639125	0.407	0.183	5.69606179569428E-144	Detoxification-associated hepat	PPIP5K2
6.69408823460886E-148	0.827304286746673	0.388	0.17	1.12179530635575E-143	Detoxification-associated hepat	NSMCE4A
7.25731282292227E-148	0.480545141334231	0.537	0.265	1.21618048286531E-143	Detoxification-associated hepat	TXNDC15
1.05563288578223E-147	0.958521724752512	0.376	0.164	1.76902958999386E-143	Detoxification-associated hepat	NSD2
2.5523828481037E-147	0.578125455730612	0.46	0.217	4.27728317685218E-143	Detoxification-associated hepat	PHF12
2.58955264008936E-147	1.02735495041634	0.364	0.157	4.33957231426174E-143	Detoxification-associated hepat	RAVER2
2.67451225287476E-147	0.465632636046846	0.6	0.309	4.48194763336753E-143	Detoxification-associated hepat	PACSIN2
3.39491260237961E-147	0.344650208217	0.72	0.395	5.68919453906776E-143	Detoxification-associated hepat	ABCC2
5.56197447621429E-147	0.829254758542529	0.385	0.169	9.3207568272399E-143	Detoxification-associated hepat	SMC5
6.02023050187212E-147	0.573609190488289	0.5	0.244	1.00887022750373E-142	Detoxification-associated hepat	ARHGAP12
6.54630583441433E-147	0.482328449318394	0.526	0.26	1.09702993173115E-142	Detoxification-associated hepat	SENP2
7.28140388642865E-147	1.59585600548158	0.237	0.079	1.22021766328771E-142	Detoxification-associated hepat	TBC1D19
7.61611723404607E-147	0.488402504734079	0.541	0.271	1.27630892608144E-142	Detoxification-associated hepat	C16orf72
7.84787729030616E-147	1.47238260249319	0.171	0.044	1.31514727630951E-142	Detoxification-associated hepat	ADPRM
8.37112771286758E-147	0.467399029813304	0.591	0.302	1.40283358212235E-142	Detoxification-associated hepat	RIOK3
9.7904652644692E-147	0.505597583361494	0.547	0.27	1.64068616901975E-142	Detoxification-associated hepat	ME1
1.16560569354706E-146	0.760788732374386	0.419	0.192	1.95332202124616E-142	Detoxification-associated hepat	BORCS8
1.67719615900903E-146	1.23262228771309	0.293	0.111	2.81064532326734E-142	Detoxification-associated hepat	MOSMO
1.73843791995876E-146	0.775478312169825	0.397	0.177	2.91327426626689E-142	Detoxification-associated hepat	VPS54
1.78467095044921E-146	0.500436152446553	0.541	0.269	2.99075157876278E-142	Detoxification-associated hepat	SH3GLB2
1.78534547258696E-146	0.857232583678951	0.382	0.168	2.99188194296123E-142	Detoxification-associated hepat	LTN1
2.21720884196922E-146	0.795872367458937	0.406	0.185	3.71559857737202E-142	Detoxification-associated hepat	SNX19
2.43289063838835E-146	0.836120926599576	0.37	0.159	4.0770381318112E-142	Detoxification-associated hepat	GNPTAB
2.49825710838896E-146	0.513258767312561	0.551	0.275	4.18657926223822E-142	Detoxification-associated hepat	NRP1
2.57881907343529E-146	0.536368891564492	0.458	0.216	4.32158500326286E-142	Detoxification-associated hepat	CCS
3.25886692305965E-146	0.958235938185302	0.335	0.138	5.46120918966337E-142	Detoxification-associated hepat	SLC36A1
3.40861397677813E-146	0.597597829854318	0.474	0.227	5.71215530228479E-142	Detoxification-associated hepat	MAPK9
3.5343531304756E-146	1.64431950224793	0.153	0.036	5.92286897605102E-142	Detoxification-associated hepat	CABYR
4.43413056274741E-146	0.551438064575415	0.552	0.279	7.4307159970521E-142	Detoxification-associated hepat	FBXO9
4.44327397412483E-146	0.835962526271	0.35	0.146	7.44603852583839E-142	Detoxification-associated hepat	SOCS7
9.4117721779926E-146	0.722823683491776	0.436	0.203	1.577224781588E-141	Detoxification-associated hepat	PLEKHG3
1.16917012620067E-145	0.826770392462277	0.365	0.155	1.95929529748708E-141	Detoxification-associated hepat	WASHC4
1.26598479437857E-145	0.70768392639147	0.328	0.13	2.12153731841961E-141	Detoxification-associated hepat	GSTM1
3.04409102082429E-145	0.633095603996727	0.479	0.231	5.10128773269735E-141	Detoxification-associated hepat	MGAT4A
3.44310782142396E-145	0.542994008233643	0.481	0.231	5.76996008714228E-141	Detoxification-associated hepat	COMMD3
3.82554120860046E-145	0.795746329156859	0.383	0.167	6.41084195737265E-141	Detoxification-associated hepat	UNK
7.20115377611468E-145	0.540640846642212	0.468	0.224	1.2067693498013E-140	Detoxification-associated hepat	STK25
9.16778816567407E-145	0.38271809779197	0.66	0.347	1.53633794080366E-140	Detoxification-associated hepat	HUWE1
1.13878643544923E-144	1.04591861745395	0.334	0.138	1.90837830852582E-140	Detoxification-associated hepat	PRIM1
1.1583542939475E-144	0.981217625113487	0.343	0.145	1.94117012579723E-140	Detoxification-associated hepat	SHPRH
1.27372754866199E-144	1.1767401547959	0.305	0.12	2.13451262604776E-140	Detoxification-associated hepat	SMYD3
1.44157861669402E-144	0.706983143655971	0.432	0.201	2.41579744585583E-140	Detoxification-associated hepat	NCOA6
2.43253048270029E-144	0.524461759782362	0.504	0.246	4.07643458290915E-140	Detoxification-associated hepat	ZNF652
2.95583911185813E-144	0.504343746430542	0.491	0.238	4.95339518365186E-140	Detoxification-associated hepat	CCNY
4.28120837886092E-144	2.09696872981339	0.174	0.047	7.17444900129513E-140	Detoxification-associated hepat	CFAP47
4.59168641128687E-144	0.682316788772702	0.42	0.192	7.69474808803453E-140	Detoxification-associated hepat	NFYB
6.27660116318118E-144	0.425986190367247	0.643	0.331	1.0518328229259E-139	Detoxification-associated hepat	ATP9A
7.03542726055333E-144	0.675824649650493	0.421	0.194	1.17899690032353E-139	Detoxification-associated hepat	FBXO46
7.58569481441628E-144	0.605393058192471	0.51	0.255	1.27121073699988E-139	Detoxification-associated hepat	EPB41L4B

8.00524845604557E-144	0.996701385559269	0.339	0.142	1.34151953626412E-139	Detoxification-associated hepat	TGDS
9.26145571619693E-144	0.445965911100573	0.524	0.261	1.55203474892028E-139	Detoxification-associated hepat	NME3
9.31552445183511E-144	0.740575184894877	0.373	0.162	1.56109558763853E-139	Detoxification-associated hepat	IREB2
1.01029976674023E-143	0.463602784268957	0.613	0.319	1.69306034910327E-139	Detoxification-associated hepat	PSMD1
1.03109134735058E-143	0.527483653593087	0.557	0.28	1.7279028798901E-139	Detoxification-associated hepat	PRKAB1
1.05653404352017E-143	1.12456336023433	0.284	0.107	1.7705397501311E-139	Detoxification-associated hepat	KPTN
1.42638954360044E-143	0.427094916880353	0.669	0.355	2.39034359716561E-139	Detoxification-associated hepat	PDCD6IP
1.42694833581155E-143	0.563979574959002	0.446	0.209	2.39128002115299E-139	Detoxification-associated hepat	ADH5
1.92063312821483E-143	0.374351045511569	0.589	0.304	3.21859699626241E-139	Detoxification-associated hepat	UBR4
2.41845497926844E-143	0.75510107943736	0.418	0.193	4.05284685425806E-139	Detoxification-associated hepat	CCDC125
2.74030985331916E-143	0.807131695677481	0.369	0.16	4.59221125219224E-139	Detoxification-associated hepat	FBXO38
2.84310749396751E-143	0.497291816424939	0.466	0.222	4.76447953839076E-139	Detoxification-associated hepat	RUFY3
2.86481580217912E-143	0.389995430707004	0.686	0.368	4.80085832129176E-139	Detoxification-associated hepat	DCAF6
3.23709971890538E-143	0.519156173694913	0.544	0.272	5.42473170894163E-139	Detoxification-associated hepat	HACD2
3.35743174038457E-143	1.15975386882886	0.282	0.106	5.62638411053645E-139	Detoxification-associated hepat	ZDHHC17
3.49525050381744E-143	1.70620560900236	0.112	0.02	5.85734079429726E-139	Detoxification-associated hepat	IGFALS
4.7973677273135E-143	0.615657143613698	0.503	0.25	8.03942883743196E-139	Detoxification-associated hepat	UBA2
5.04086744908954E-143	0.693485727920014	0.427	0.197	8.44748567118426E-139	Detoxification-associated hepat	PHC3
5.26593032764229E-143	1.10148927765774	0.273	0.1	8.82464604306295E-139	Detoxification-associated hepat	LRP12
6.31402291212564E-143	0.931347591734291	0.352	0.15	1.05810395961401E-138	Detoxification-associated hepat	CEP83
6.86444090933561E-143	0.815270829539916	0.377	0.166	1.15034300758646E-138	Detoxification-associated hepat	NHLRC2
8.34955480340438E-143	0.416409914189687	0.681	0.366	1.39921839395451E-138	Detoxification-associated hepat	SLC50A1
8.87246457136755E-143	0.535196041470182	0.49	0.241	1.48684761286977E-138	Detoxification-associated hepat	SCFD1
9.49216634543567E-143	0.409924671908024	0.731	0.4	1.59069723616811E-138	Detoxification-associated hepat	UGT1A1
2.44382960023764E-142	0.435678265000025	0.938	0.684	4.09536964407824E-138	Detoxification-associated hepat	SULT2A1
2.55341569668562E-142	0.516533533798822	0.505	0.248	4.27901402450576E-138	Detoxification-associated hepat	RPRD2
2.90791551937561E-142	0.33248786511408	0.634	0.333	4.87308482736965E-138	Detoxification-associated hepat	TTC39C
3.27783520147547E-142	1.09366186457912	0.303	0.119	5.49299623063259E-138	Detoxification-associated hepat	PCNX4
3.30837336034415E-142	0.61457496094603	0.415	0.188	5.54417207726472E-138	Detoxification-associated hepat	OTUD4
3.5125582584674E-142	0.608577760799553	0.501	0.249	5.88634512953968E-138	Detoxification-associated hepat	KIAA0319L
3.90419797279429E-142	0.710836266413924	0.377	0.165	6.54265496280867E-138	Detoxification-associated hepat	KLF13
5.60263347782699E-142	0.490490267395652	0.561	0.284	9.38889318214247E-138	Detoxification-associated hepat	AGO2
6.78152926040921E-142	0.884395235863438	0.382	0.172	1.13644867345938E-137	Detoxification-associated hepat	KRIT1
7.30695164910537E-142	0.841969276967862	0.395	0.18	1.22449895735708E-137	Detoxification-associated hepat	NUP155
8.02639234327836E-142	1.57109114893232	0.229	0.076	1.34506282888659E-137	Detoxification-associated hepat	RBM45
8.25489674932619E-142	0.440147811759691	0.506	0.25	1.38335559725208E-137	Detoxification-associated hepat	MEGF9
8.41909700242956E-142	0.317025803954125	0.557	0.278	1.41087227566715E-137	Detoxification-associated hepat	WSB1
8.6337077267895E-142	1.06083183174692	0.197	0.056	1.44683674085538E-137	Detoxification-associated hepat	TDRD10
8.66336175651332E-142	0.644120812794088	0.408	0.185	1.4518061631565E-137	Detoxification-associated hepat	FBH1
9.16077917201196E-142	1.09504963580165	0.318	0.129	1.53516337364576E-137	Detoxification-associated hepat	ZNF346
9.16760783581297E-142	0.565584461723243	0.387	0.169	1.53630772112554E-137	Detoxification-associated hepat	HEXIM1
1.00191358795665E-141	0.735072277729728	0.389	0.174	1.67900679069776E-137	Detoxification-associated hepat	IFT88
1.41689188427102E-141	1.1181521334488	0.289	0.11	2.37442741966138E-137	Detoxification-associated hepat	TNK2
1.48316509991175E-141	0.84223954965243	0.357	0.153	2.48548807443211E-137	Detoxification-associated hepat	RBM5
1.51541770191048E-141	0.525237941433604	0.49	0.238	2.53953698486158E-137	Detoxification-associated hepat	SAMD4B
1.82424108189006E-141	0.466794260142676	0.554	0.284	3.05706320503137E-137	Detoxification-associated hepat	TEX2
2.0374691764627E-141	0.573629553225248	0.509	0.247	3.41439084591619E-137	Detoxification-associated hepat	HELLS
3.33201389381078E-141	0.499361170972044	0.451	0.214	5.5837888832481E-137	Detoxification-associated hepat	HEXA
3.99258393757686E-141	0.760171385060445	0.356	0.152	6.6907721625913E-137	Detoxification-associated hepat	BTBD1
4.004499647929E-141	0.82856639635396	0.36	0.155	6.71074050999942E-137	Detoxification-associated hepat	FAM120B
4.43225495477677E-141	0.681894475067497	0.409	0.187	7.42757285321491E-137	Detoxification-associated hepat	APBB2
4.77869395432199E-141	0.442404541362362	0.48	0.229	8.00813532865279E-137	Detoxification-associated hepat	SLC7A2
4.88254733977627E-141	0.805827665945591	0.335	0.137	8.18217283199708E-137	Detoxification-associated hepat	RAPGEF4
6.29249172563099E-141	0.378480331309201	0.547	0.277	1.05449576338124E-136	Detoxification-associated hepat	SLTM
7.95246667476734E-141	0.469434530531797	0.846	0.473	1.33267436535751E-136	Detoxification-associated hepat	ZKSCAN1
7.98940021288301E-141	0.959990085344277	0.312	0.126	1.33886368767493E-136	Detoxification-associated hepat	TAF1
8.52297197841333E-141	0.655209392753359	0.467	0.224	1.42827964414251E-136	Detoxification-associated hepat	ARID4B
1.02522626458154E-140	0.491726146918052	0.483	0.233	1.71807417418575E-136	Detoxification-associated hepat	SUZ12
1.52961768537434E-140	0.579279693795857	0.473	0.229	2.56333331715032E-136	Detoxification-associated hepat	MSL2
1.93976106403737E-140	0.478364613563824	0.51	0.254	3.25065159111382E-136	Detoxification-associated hepat	MARK3

2.45771534414637E-140	0.453124350394676	0.806	0.437	4.11863937372048E-136	Detoxification-associated hepat	FRK
2.48418820230411E-140	0.589011942081867	0.459	0.217	4.16300258942123E-136	Detoxification-associated hepat	IRF9
3.13088483744705E-140	1.52443197610826	0.207	0.064	5.24673681059377E-136	Detoxification-associated hepat	KPNA5
3.62697234841809E-140	1.19397672054722	0.289	0.112	6.07808026147904E-136	Detoxification-associated hepat	ADGRG2
6.47793628833396E-140	0.855958736402315	0.424	0.2	1.08557256319901E-135	Detoxification-associated hepat	MAP3K21
7.84095912164318E-140	0.374786876487503	0.631	0.328	1.31398792960496E-135	Detoxification-associated hepat	ASXL1
9.74615113059871E-140	0.715022522819095	0.407	0.187	1.63326000646573E-135	Detoxification-associated hepat	RNF139
1.37523819134971E-139	0.554403104527658	0.502	0.247	2.30462416106385E-135	Detoxification-associated hepat	MSH6
1.41999160036241E-139	1.93013999543196	0.205	0.064	2.37962192388732E-135	Detoxification-associated hepat	ADAMTSL1
2.19979900308975E-139	0.976200142092649	0.262	0.094	3.68642316937781E-135	Detoxification-associated hepat	TTC28
2.73163290566255E-139	0.955957880194362	0.257	0.091	4.5776704233093E-135	Detoxification-associated hepat	SNX29
4.07395504801426E-139	0.485275723172226	0.504	0.25	6.8271338694623E-135	Detoxification-associated hepat	UBA5
5.31685678498498E-139	0.662043791621286	0.425	0.2	8.90998860027782E-135	Detoxification-associated hepat	SYNRG
7.7391907436945E-139	0.448167986864788	0.519	0.259	1.29693358482832E-134	Detoxification-associated hepat	ASPSCR1
7.97822671039274E-139	0.40839900471425	0.639	0.335	1.33699123212761E-134	Detoxification-associated hepat	NUMB
9.43946845073676E-139	1.17638280818897	0.303	0.121	1.58186612297447E-134	Detoxification-associated hepat	RBL1
1.26613265423884E-138	0.799402545942846	0.358	0.155	2.12178510197345E-134	Detoxification-associated hepat	UPF2
1.31365372569756E-138	0.97453954794085	0.357	0.157	2.20142091352396E-134	Detoxification-associated hepat	NVL
1.37019828582902E-138	0.478143125023331	0.463	0.222	2.29617828739228E-134	Detoxification-associated hepat	ITGAV
1.4131155705375E-138	1.45409261553449	0.246	0.087	2.36809907310674E-134	Detoxification-associated hepat	PPM1K
1.64450188456023E-138	0.685103701205139	0.449	0.217	2.75585625814604E-134	Detoxification-associated hepat	ZNF800
2.14482636621409E-138	0.784438120537888	0.372	0.165	3.59430002450158E-134	Detoxification-associated hepat	MBIP
2.72435592399958E-138	0.422697013869104	0.704	0.383	4.5654756574385E-134	Detoxification-associated hepat	HNRNPD
2.86878020348774E-138	0.404398775955022	0.61	0.322	4.80750186500475E-134	Detoxification-associated hepat	ATP6V1A
3.14098017140649E-138	1.17610335154855	0.299	0.119	5.26365457124299E-134	Detoxification-associated hepat	ZNF839
3.37439167951128E-138	0.697679130259544	0.404	0.186	5.654805576525E-134	Detoxification-associated hepat	FBXO28
3.45182950335594E-138	0.513360089784603	0.524	0.261	5.78457588172389E-134	Detoxification-associated hepat	CAMK2D
3.82396234309213E-138	0.849927305672452	0.298	0.117	6.40819609455379E-134	Detoxification-associated hepat	NR2C1
3.97530165069161E-138	0.37050320549473	0.673	0.368	6.66181050622901E-134	Detoxification-associated hepat	DIO1
7.47440114597973E-138	1.03612728896659	0.25	0.087	1.25256014404328E-133	Detoxification-associated hepat	TUBG2
7.88421033171178E-138	1.16356491477621	0.147	0.034	1.32123596738826E-133	Detoxification-associated hepat	ACKR2
9.54016533150301E-138	0.638625822242165	0.436	0.207	1.59874090625327E-133	Detoxification-associated hepat	FAM172A
1.48794411288973E-137	0.562277981056551	0.457	0.219	2.49349674438062E-133	Detoxification-associated hepat	PIP4P1
1.55183609153607E-137	0.740112370046679	0.384	0.172	2.60056692219614E-133	Detoxification-associated hepat	VPS37A
1.69631448085811E-137	1.13593447512127	0.238	0.082	2.84268380702202E-133	Detoxification-associated hepat	ZNF784
1.77842120488158E-137	0.689831248458069	0.431	0.205	2.98027825514054E-133	Detoxification-associated hepat	SGMS1
1.9694869034845E-137	0.784525526460483	0.33	0.137	3.30046615285932E-133	Detoxification-associated hepat	CRBN
2.28453315387783E-137	0.323637596263041	0.634	0.336	3.82842065926846E-133	Detoxification-associated hepat	DYNC1LI2
2.73405004591204E-137	0.417550040132812	0.531	0.267	4.58172106693939E-133	Detoxification-associated hepat	GAA
4.8628201384523E-137	1.27263219358118	0.292	0.116	8.14911398801836E-133	Detoxification-associated hepat	ZNF33B
5.47288548084756E-137	0.522201723330875	0.494	0.244	9.17146148880435E-133	Detoxification-associated hepat	IP6K2
5.51221294207243E-137	0.634287514056986	0.264	0.094	9.23736644832498E-133	Detoxification-associated hepat	DEPRECATEI
5.87206327965947E-137	1.11015668954928	0.293	0.116	9.84040364405334E-133	Detoxification-associated hepat	TMEM161B
6.47447914600068E-137	0.891947367718974	0.376	0.171	1.08499321528679E-132	Detoxification-associated hepat	PKN2
7.03314375238567E-137	0.631406755085011	0.428	0.201	1.17861423002479E-132	Detoxification-associated hepat	ARL8B
9.55482808511872E-137	0.613635294736924	0.413	0.191	1.6011980905042E-132	Detoxification-associated hepat	PGM3
9.7155780338209E-137	0.641652737052129	0.42	0.195	1.62813656690771E-132	Detoxification-associated hepat	RMDN1
1.11490999926514E-136	0.943623214418674	0.312	0.127	1.86836617676852E-132	Detoxification-associated hepat	EXOC6B
1.13703379602313E-136	0.978524805621469	0.31	0.127	1.90544123537556E-132	Detoxification-associated hepat	SIMC1
1.18962197116817E-136	0.437476589334487	0.761	0.423	1.99356849928361E-132	Detoxification-associated hepat	PLEKHA6
1.32988805551218E-136	0.500527583772551	0.543	0.271	2.22862640342731E-132	Detoxification-associated hepat	RBMX
1.40619641967112E-136	0.777035592090665	0.386	0.175	2.35650396008487E-132	Detoxification-associated hepat	INPP1
1.42692046585332E-136	0.507554738098584	0.339	0.139	2.39123331667699E-132	Detoxification-associated hepat	DENND4A
1.48256826756143E-136	0.346088499319105	0.659	0.354	2.48448790277944E-132	Detoxification-associated hepat	AR
2.02840112960411E-136	0.571030659512684	0.456	0.22	3.39919461299057E-132	Detoxification-associated hepat	RO60
2.21444964624193E-136	0.639860012941559	0.412	0.192	3.71097471717222E-132	Detoxification-associated hepat	SPG11
2.57719140921462E-136	1.05441888141914	0.303	0.122	4.31885736356186E-132	Detoxification-associated hepat	SLC49A4
3.52752234906035E-136	0.480119307186938	0.555	0.282	5.91142195255533E-132	Detoxification-associated hepat	SRPK1
3.99278391178339E-136	0.727765093053992	0.305	0.12	6.69110727936661E-132	Detoxification-associated hepat	MCF2L
4.73629277319208E-136	0.96138630752852	0.314	0.129	7.93707942931528E-132	Detoxification-associated hepat	CSGALNACT

5.19541851331237E-136	1.32281969049324	0.233	0.08	8.70648234460887E-132	Detoxification-associated hepat	DZIP3
5.228194908446E-136	0.708107540333135	0.461	0.226	8.76140902757381E-132	Detoxification-associated hepat	RUNX1
5.64606153300827E-136	0.422111402678049	0.779	0.455	9.46166991701526E-132	Detoxification-associated hepat	SHC1
1.14776399129278E-135	0.548460355921281	0.454	0.219	1.92342289660844E-131	Detoxification-associated hepat	NELFA
1.27027135052761E-135	0.590603073744655	0.45	0.217	2.12872072921417E-131	Detoxification-associated hepat	USP34
1.35481536344684E-135	0.834297568649158	0.325	0.136	2.27039958606422E-131	Detoxification-associated hepat	RGL2
2.01010009466008E-135	1.06055004296259	0.307	0.125	3.36852573863137E-131	Detoxification-associated hepat	TM2D1
2.48768958629181E-135	0.895234603151062	0.318	0.131	4.16887020870782E-131	Detoxification-associated hepat	CHD6
2.63106227157404E-135	0.59302728047526	0.429	0.202	4.40913415470378E-131	Detoxification-associated hepat	NFAT5
2.65935908798169E-135	0.609259615694338	0.453	0.219	4.45655395963971E-131	Detoxification-associated hepat	CASK
4.68274308821588E-135	0.482108534490425	0.47	0.225	7.84734086723217E-131	Detoxification-associated hepat	E2F1
7.43726536393771E-135	0.557959398773825	0.502	0.249	1.24633692968868E-130	Detoxification-associated hepat	RAPGEF5
7.46729499668851E-135	0.838513621013613	0.397	0.185	1.25136929554506E-130	Detoxification-associated hepat	TMEM50B
7.63808483739763E-135	1.43373720500922	0.253	0.093	1.27999025705109E-130	Detoxification-associated hepat	HELQ
8.07319346444475E-135	0.736347125924862	0.347	0.149	1.35290576077165E-130	Detoxification-associated hepat	MRS2
8.15658998191947E-135	0.399207871340126	0.418	0.191	1.36688134917006E-130	Detoxification-associated hepat	ZNF592
8.62772389030901E-135	1.2562744770542	0.218	0.071	1.44583396953798E-130	Detoxification-associated hepat	ZNF563
9.64073926170381E-135	0.450749093442193	0.538	0.273	1.61559508547632E-130	Detoxification-associated hepat	NKAP
9.64506286408095E-135	0.613147782615187	0.372	0.165	1.61631963476269E-130	Detoxification-associated hepat	CAP2
9.96692903478615E-135	1.17431587101473	0.252	0.091	1.67025796764946E-130	Detoxification-associated hepat	FITM2
1.10102861563942E-134	0.419390626785792	0.53	0.271	1.84510375408853E-130	Detoxification-associated hepat	MSL1
1.14358784342324E-134	1.25973211142183	0.281	0.109	1.91642450800867E-130	Detoxification-associated hepat	RPA4
1.22044195921806E-134	0.863215357097337	0.263	0.094	2.04521663525762E-130	Detoxification-associated hepat	CYP17A1
1.22493693216026E-134	0.597710697904368	0.375	0.166	2.05274931091417E-130	Detoxification-associated hepat	ZNF281
1.4443112397913E-134	0.627109732368393	0.408	0.187	2.42037677564226E-130	Detoxification-associated hepat	FRRS1
1.55828919703956E-134	0.79827132330445	0.346	0.15	2.6113810363989E-130	Detoxification-associated hepat	ZNF407
1.68793876017014E-134	1.02160437960721	0.294	0.117	2.82864777429313E-130	Detoxification-associated hepat	SMARCAD1
2.15558385200728E-134	0.421252197295707	0.773	0.423	3.61232741919381E-130	Detoxification-associated hepat	HNRNPU
2.22456026504382E-134	0.786723539482457	0.355	0.156	3.72791809216043E-130	Detoxification-associated hepat	NCOA3
2.33336002670503E-134	1.16091249283657	0.282	0.11	3.9102447327523E-130	Detoxification-associated hepat	CYP20A1
2.33646709881771E-134	0.771561398841609	0.417	0.195	3.91545156419872E-130	Detoxification-associated hepat	TMEM43
2.57487096582574E-134	0.45429917331819	0.535	0.273	4.31496876453077E-130	Detoxification-associated hepat	NCKAP1
2.7245757116726E-134	0.481726201811377	0.485	0.241	4.56584397762095E-130	Detoxification-associated hepat	GOPC
3.20807202269969E-134	0.538505984986963	0.438	0.21	5.37608709564014E-130	Detoxification-associated hepat	ZYG11B
3.56152342469722E-134	0.844718809939283	0.338	0.143	5.9684009551076E-130	Detoxification-associated hepat	PMS1
3.78759309076924E-134	0.457434555296181	0.533	0.273	6.3472485015111E-130	Detoxification-associated hepat	CUL4A
5.05659915038786E-134	0.528003366176422	0.47	0.232	8.47384885621998E-130	Detoxification-associated hepat	COP 1
7.41143088003411E-134	0.988887817592206	0.289	0.115	1.24200758687612E-129	Detoxification-associated hepat	FAM133B
8.16253129460009E-134	0.718240041531057	0.304	0.121	1.36787699434908E-129	Detoxification-associated hepat	SCAMP5
8.36349235693525E-134	0.589061666653609	0.432	0.206	1.40155404917521E-129	Detoxification-associated hepat	CUL3
1.10769247904502E-133	1.20507088127553	0.265	0.1	1.85627105638364E-129	Detoxification-associated hepat	RWDD4
1.12555588972599E-133	0.639259924531036	0.388	0.173	1.88620656000282E-129	Detoxification-associated hepat	MTMR10
1.412026987863E-133	0.565825664366321	0.499	0.25	2.36627482626082E-129	Detoxification-associated hepat	NUP85
1.73704596963074E-133	0.502606014373437	0.425	0.199	2.91094163590719E-129	Detoxification-associated hepat	EPB41
2.09730438659273E-133	0.369254491573152	0.631	0.339	3.5146626910521E-129	Detoxification-associated hepat	TFG
4.26171763523686E-133	0.86856132922724	0.357	0.158	7.14178641312994E-129	Detoxification-associated hepat	TBPL1
4.59945222370521E-133	0.380095704437756	0.829	0.457	7.7077620364852E-129	Detoxification-associated hepat	UBE2A
5.62850539537908E-133	0.773414887160877	0.375	0.17	9.43224934157626E-129	Detoxification-associated hepat	TMEM65
7.63227931630567E-133	0.729715061548699	0.359	0.159	1.2790173678265E-128	Detoxification-associated hepat	RBPJ
7.66869929176438E-133	0.531666107996636	0.434	0.206	1.28512062731387E-128	Detoxification-associated hepat	TSPAN4
8.29622841878034E-133	0.382248855377851	0.629	0.333	1.39028195841921E-128	Detoxification-associated hepat	VDAC3
8.7701340411181E-133	0.953220513132907	0.296	0.118	1.46969906261057E-128	Detoxification-associated hepat	FASTKD2
9.0943130056539E-133	1.68885194198187	0.245	0.09	1.52402497348748E-128	Detoxification-associated hepat	PCDHGA8
9.42504839914102E-133	0.420215953462228	0.865	0.548	1.57944961072805E-128	Detoxification-associated hepat	SMIM14
9.58208515904796E-133	0.433134079254706	0.533	0.273	1.60576583095326E-128	Detoxification-associated hepat	PAFAH1B1
1.2249384493137E-132	1.23683375351387	0.235	0.082	2.05275185335989E-128	Detoxification-associated hepat	TMEM42
1.31521751634524E-132	1.03844310992805	0.266	0.1	2.20404151389136E-128	Detoxification-associated hepat	ADAT2
1.38268947983775E-132	0.680776746152849	0.491	0.241	2.3171110303121E-128	Detoxification-associated hepat	CCNE2
1.55341929702581E-132	0.578400370128198	0.427	0.204	2.60322005795585E-128	Detoxification-associated hepat	PIK3CB
1.92374748137764E-132	0.818431373610074	0.363	0.163	3.22381602929265E-128	Detoxification-associated hepat	SMG1

2.1333059924159E-132	0.754013531609441	0.382	0.174	3.57499418209057E-128	Detoxification-associated hepat	ORMDL1
2.82626157920778E-132	0.597821611374795	0.466	0.229	4.7362491544364E-128	Detoxification-associated hepat	MTM1
2.95954057610256E-132	0.516330673301072	0.454	0.222	4.95959809743266E-128	Detoxification-associated hepat	DDX1
3.00107776967119E-132	0.897734836129444	0.355	0.155	5.02920612641498E-128	Detoxification-associated hepat	HAL
3.33011822812856E-132	0.411581083766328	0.497	0.249	5.58061212669784E-128	Detoxification-associated hepat	TMEM123
3.78220918386541E-132	0.541406174510559	0.372	0.163	6.33822615032165E-128	Detoxification-associated hepat	BNIP2
3.90165445066154E-132	0.411596799704257	0.692	0.389	6.53839252841861E-128	Detoxification-associated hepat	CRYZ
4.25709614476824E-132	0.757000842195372	0.384	0.176	7.13404171940261E-128	Detoxification-associated hepat	CLCN3
4.67697703307662E-132	0.402776192867751	0.66	0.36	7.8376781120298E-128	Detoxification-associated hepat	LRP5
5.05354755400283E-132	1.32994609542356	0.227	0.078	8.46873499099795E-128	Detoxification-associated hepat	CSNK2A1
5.53500855795403E-132	0.731839816005891	0.343	0.148	9.27556734141936E-128	Detoxification-associated hepat	USP15
9.94053475928439E-132	1.33330061667441	0.233	0.082	1.66583481496088E-127	Detoxification-associated hepat	SUPT3H
1.12228722521972E-131	0.500312908734953	0.47	0.233	1.88072893202321E-127	Detoxification-associated hepat	ZSWIM8
1.27478338769537E-131	1.98261516607697	0.107	0.02	2.13628200109989E-127	Detoxification-associated hepat	SFRP5
1.49108171598753E-131	0.511327513394789	0.464	0.228	2.4987547396519E-127	Detoxification-associated hepat	TMBIM4
1.51897582544885E-131	0.600476594455357	0.533	0.271	2.54549968828718E-127	Detoxification-associated hepat	ABRACL
2.6337878724736E-131	0.724767628561876	0.459	0.227	4.41370171669126E-127	Detoxification-associated hepat	SAMD12
2.95187612639572E-131	0.763319530295717	0.415	0.198	4.94675401261396E-127	Detoxification-associated hepat	ARFGEF1
4.3048135619026E-131	1.08217708764808	0.285	0.113	7.21400656703637E-127	Detoxification-associated hepat	SPAG1
4.33218252171355E-131	0.706119519434498	0.398	0.187	7.25987146988757E-127	Detoxification-associated hepat	CHM
4.70335923763108E-131	1.54831358705062	0.197	0.062	7.88188941042216E-127	Detoxification-associated hepat	N6AMT1
5.94083131347802E-131	0.37219286916	0.581	0.307	9.95564511512646E-127	Detoxification-associated hepat	VPS52
6.06528097805769E-131	0.752075901163261	0.343	0.15	1.01641978630291E-126	Detoxification-associated hepat	GYS1
6.37268478215205E-131	0.53390601380916	0.436	0.209	1.06793451579304E-126	Detoxification-associated hepat	MTFR1L
7.17286128139268E-131	0.664783506731176	0.392	0.179	1.20202809353578E-126	Detoxification-associated hepat	MAFG
7.4771764717273E-131	0.941944882277836	0.291	0.116	1.25302523313206E-126	Detoxification-associated hepat	FBXO11
7.78820474641216E-131	0.400545428192296	0.688	0.384	1.30514735140375E-126	Detoxification-associated hepat	DCAF8
7.90111776508461E-131	0.429994256714023	0.536	0.275	1.32406931507288E-126	Detoxification-associated hepat	EGLN2
1.18220250944612E-130	1.05023432439636	0.216	0.071	1.98113496532981E-126	Detoxification-associated hepat	HSPA2
1.69378822111042E-130	1.29021980277718	0.245	0.089	2.83845030093684E-126	Detoxification-associated hepat	NAPB
2.26708953341119E-130	0.397197373906932	0.559	0.287	3.79918864009048E-126	Detoxification-associated hepat	VAMP7
2.43867864909605E-130	1.20248334197008	0.24	0.086	4.08673768015517E-126	Detoxification-associated hepat	PGBD2
3.0656804954309E-130	0.341008237078212	0.682	0.348	5.1374673742431E-126	Detoxification-associated hepat	DUSP9
3.69539206375034E-130	0.816476066658048	0.37	0.169	6.19273802043282E-126	Detoxification-associated hepat	DNAAF2
3.96562663094303E-130	1.58275006830593	0.224	0.077	6.64559710813433E-126	Detoxification-associated hepat	ZNF605
4.10647476434628E-130	0.734602480128598	0.4	0.186	6.88163041009149E-126	Detoxification-associated hepat	MPHOSPH9
4.5283928909611E-130	1.00726805043887	0.324	0.139	7.58868080667261E-126	Detoxification-associated hepat	CTPS2
4.57579030828582E-130	0.576560892901045	0.476	0.235	7.66810939862538E-126	Detoxification-associated hepat	HERC3
4.7727613494277E-130	0.697521448391233	0.434	0.212	7.99819346937093E-126	Detoxification-associated hepat	PLD1
5.00611209915655E-130	0.409269170737729	0.609	0.326	8.38924265576655E-126	Detoxification-associated hepat	UHMK1
8.27796020514842E-130	0.695682034084734	0.403	0.19	1.38722057117877E-125	Detoxification-associated hepat	CLN5
1.18524425302693E-129	0.501089511735046	0.52	0.268	1.98623231922253E-125	Detoxification-associated hepat	RBM25
1.4417101773419E-129	1.309980716423	0.185	0.055	2.41601791518956E-125	Detoxification-associated hepat	PLD6
1.68223668775931E-129	0.521055708091847	0.477	0.236	2.81909224134706E-125	Detoxification-associated hepat	BTD
1.75913551662382E-129	0.593898154776813	0.485	0.243	2.94795929875819E-125	Detoxification-associated hepat	PSEN1
1.94630796334657E-129	0.849939571228046	0.373	0.171	3.26162288497618E-125	Detoxification-associated hepat	USPL1
1.96430069230867E-129	0.651355743501698	0.391	0.181	3.29177510017086E-125	Detoxification-associated hepat	LARP1B
4.13953941527066E-129	0.426183167600634	0.478	0.238	6.93704015211057E-125	Detoxification-associated hepat	UBE3A
4.79610169334305E-129	0.734529123450991	0.361	0.162	8.03730721770428E-125	Detoxification-associated hepat	UBE2W
6.35181053262564E-129	0.789720876057667	0.388	0.181	1.0644364090574E-124	Detoxification-associated hepat	SMPD4
6.55240219876641E-129	0.45401744708498	0.431	0.206	1.09805156046928E-124	Detoxification-associated hepat	DOK4
6.71064768227379E-129	0.345323745430237	0.499	0.25	1.12457033859544E-124	Detoxification-associated hepat	PALLD
8.28296273825685E-129	0.57944608248272	0.366	0.164	1.38805889567708E-124	Detoxification-associated hepat	SIK3
8.7835801636935E-129	0.485444135261379	0.472	0.236	1.47195236383176E-124	Detoxification-associated hepat	ATL2
8.91204991997373E-129	0.755694372129647	0.392	0.185	1.4934813255892E-124	Detoxification-associated hepat	KMT2A
9.57299827370048E-129	1.33766061674753	0.225	0.078	1.60424305070673E-124	Detoxification-associated hepat	SLC25A27
1.04368184744211E-128	0.408389918884544	0.829	0.461	1.74900203994348E-124	Detoxification-associated hepat	ATRN
1.16406625285841E-128	0.688493239376291	0.314	0.129	1.95074222654012E-124	Detoxification-associated hepat	U2AF1L4
1.49761368979173E-128	0.494611308477778	0.418	0.197	2.50970102135299E-124	Detoxification-associated hepat	GOLT1B
1.62043395602604E-128	0.3663003522646	0.662	0.341	2.71552322350844E-124	Detoxification-associated hepat	FAM122B

**1.67877887986908E-128**	0.463879912593271	0.524	0.269	2.8132976468846E-124	Detoxification-associated hepat	KDM5C
**1.72898258324573E-128**	0.503575728632357	0.497	0.251	2.89742901300319E-124	Detoxification-associated hepat	LMBRD2
**1.84236657734183E-128**	1.14538614012274	0.253	0.095	3.08743791030945E-124	Detoxification-associated hepat	SZT2
**2.00665781211774E-128**	0.394302628455529	0.729	0.406	3.3627571615469E-124	Detoxification-associated hepat	CITED2
**2.00931517647715E-128**	0.767233386442317	0.359	0.161	3.36721037274041E-124	Detoxification-associated hepat	TBC1D22B
**2.56744668735588E-128**	0.459605706045508	0.433	0.208	4.30252715867098E-124	Detoxification-associated hepat	GOSR1
**3.27132763199904E-128**	0.553604663116097	0.379	0.173	5.482090845704E-124	Detoxification-associated hepat	BMPR2
**3.85854084938115E-128**	0.93856449042836	0.264	0.1	6.46614275539293E-124	Detoxification-associated hepat	SFXN2
**4.0710496227161E-128**	1.1713740084003	0.281	0.112	6.82226495774763E-124	Detoxification-associated hepat	DEPRECATEI
**4.28209975564707E-128**	0.745513512734775	0.384	0.177	7.17594277051336E-124	Detoxification-associated hepat	ACSF2
**5.7694149839537E-128**	0.490033200232582	0.456	0.225	9.66838563010961E-124	Detoxification-associated hepat	AP1G1
**6.14723899214481E-128**	1.17209324877901	0.266	0.102	1.03015431030363E-123	Detoxification-associated hepat	TUBGCP5
**7.43225707636627E-128**	0.365001392996608	0.764	0.432	1.24549764085746E-123	Detoxification-associated hepat	CD46
**9.07208062296081E-128**	0.546024972139421	0.389	0.179	1.52029927079577E-123	Detoxification-associated hepat	PXMP4
**9.10647230142089E-128**	0.590198447540496	0.437	0.208	1.52606262827211E-123	Detoxification-associated hepat	FAM111A
**1.17309998441185E-127**	0.534869419699204	0.431	0.208	1.96588095387737E-123	Detoxification-associated hepat	TULP4
**1.21243975592064E-127**	0.418344561979712	0.528	0.274	2.03180654297182E-123	Detoxification-associated hepat	SEC24A
**1.22199040803347E-127**	0.570344697637599	0.435	0.211	2.04781152578249E-123	Detoxification-associated hepat	DELE1
**1.67833162384083E-127**	0.48529745915409	0.538	0.281	2.81254813523247E-123	Detoxification-associated hepat	UGCG
**1.74641566167635E-127**	0.974220461792775	0.297	0.122	2.92664336583723E-123	Detoxification-associated hepat	DEPRECATEI
**1.81438117334314E-127**	0.354651415726011	0.284	0.105	3.04053997028844E-123	Detoxification-associated hepat	CYP21A2
**1.91047455154321E-127**	1.01535722076573	0.32	0.138	3.2015732534761E-123	Detoxification-associated hepat	ZNF449
**1.96313010464945E-127**	0.572717617752444	0.463	0.232	3.28981342937155E-123	Detoxification-associated hepat	FAM8A1
**2.40823577556033E-127**	1.07030102071563	0.268	0.103	4.03572151268401E-123	Detoxification-associated hepat	TSPYL2
**2.61529438008286E-127**	0.925240829180742	0.343	0.152	4.38271032214285E-123	Detoxification-associated hepat	GTF2IRD1
**4.41849121835435E-127**	0.597460628013317	0.391	0.183	7.40450758371823E-123	Detoxification-associated hepat	STX17
**4.45996813101718E-127**	0.602838118394701	0.431	0.21	7.47401459395858E-123	Detoxification-associated hepat	DIS3
**4.79250412486358E-127**	0.839636678724156	0.351	0.158	8.03127841244638E-123	Detoxification-associated hepat	SUCO
**5.40843207238237E-127**	0.946126668185094	0.271	0.105	9.06345046689837E-123	Detoxification-associated hepat	DIS3L
**6.38240228577559E-127**	1.31577719741541	0.189	0.058	1.06956297505027E-122	Detoxification-associated hepat	ZNF573
**8.11701818561799E-127**	0.751777818091784	0.346	0.153	1.36024990754586E-122	Detoxification-associated hepat	NOL11
**1.03848630392309E-126**	0.559337039567847	0.419	0.2	1.74029534811432E-122	Detoxification-associated hepat	FAF2
**1.06780130741209E-126**	0.25945731220911	0.337	0.134	1.78942143096118E-122	Detoxification-associated hepat	ACOT12
**1.13807417945289E-126**	0.348623561485916	0.727	0.394	1.90718470992716E-122	Detoxification-associated hepat	VMA21
**1.42459953355845E-126**	0.521580165356834	0.489	0.247	2.38734389833726E-122	Detoxification-associated hepat	GOLGB1
**1.9996936641716E-126**	0.371950854491316	0.822	0.457	3.35108664241876E-122	Detoxification-associated hepat	LAPTM4B
**2.05706954318172E-126**	0.437593143382906	0.613	0.319	3.44723714046393E-122	Detoxification-associated hepat	TMEM164
**2.60508867889826E-126**	0.527239102435192	0.476	0.241	4.36560760809771E-122	Detoxification-associated hepat	TEAD1
**2.65514570977267E-126**	0.738951566900455	0.348	0.155	4.44949318043704E-122	Detoxification-associated hepat	TMEM251
**2.74685562697282E-126**	0.802978526408393	0.361	0.165	4.60318065968105E-122	Detoxification-associated hepat	ITSN1
**2.86583160646528E-126**	0.335882991218195	0.615	0.324	4.80256060611451E-122	Detoxification-associated hepat	SLC25A5
**3.09167944832462E-126**	0.452271660905407	0.398	0.185	5.1810364195024E-122	Detoxification-associated hepat	TOX4
**3.20555070794963E-126**	0.48607212172568	0.411	0.195	5.37186187638198E-122	Detoxification-associated hepat	PELO
**3.2797644034577E-126**	0.548311323719715	0.361	0.157	5.49622918731442E-122	Detoxification-associated hepat	SLC28A1
**3.31190585372175E-126**	1.2270539035096	0.23	0.082	5.55009182966691E-122	Detoxification-associated hepat	DTWD2
**4.92791889226911E-126**	0.34743411212211	0.755	0.44	8.25820647966457E-122	Detoxification-associated hepat	CIAO2A
**5.60152289481046E-126**	0.619892840082	0.406	0.194	9.38703206712338E-122	Detoxification-associated hepat	MICU2
**5.63848849452244E-126**	0.865956128397072	0.288	0.116	9.44897901912071E-122	Detoxification-associated hepat	ZNF280D
**7.0835785204268E-126**	0.458311465683178	0.51	0.262	1.18706608845312E-121	Detoxification-associated hepat	DNAJC8
**7.0882364110469E-126**	0.833538720938315	0.292	0.119	1.18784665776324E-121	Detoxification-associated hepat	LIN52
**7.38506487757934E-126**	0.386276497626082	0.589	0.313	1.23758917218475E-121	Detoxification-associated hepat	CD2AP
**7.76996461041276E-126**	0.43781929865387	0.564	0.295	1.30209066941297E-121	Detoxification-associated hepat	LGALS8
**8.0505016651695E-126**	0.343666162893207	0.563	0.297	1.3491030690491E-121	Detoxification-associated hepat	MAPK14
**8.07847383454739E-126**	0.319411800135288	0.592	0.309	1.35379064519345E-121	Detoxification-associated hepat	FZR1
**1.01683110829805E-125**	1.20522386065404	0.261	0.101	1.70400557128586E-121	Detoxification-associated hepat	SENP7
**1.01930539404061E-125**	1.48913832700877	0.153	0.04	1.70815197933325E-121	Detoxification-associated hepat	PGBD1
**1.67218488461464E-125**	0.421754275933	0.491	0.248	2.80224742963721E-121	Detoxification-associated hepat	RBM17
**1.73374669137169E-125**	0.400458025030179	0.512	0.262	2.90541270540068E-121	Detoxification-associated hepat	PHKA2
**1.77475408887889E-125**	0.703092384877563	0.371	0.171	2.97413290214324E-121	Detoxification-associated hepat	CMTR1
**1.79269489748059E-125**	0.452388729439367	0.523	0.268	3.00419810919797E-121	Detoxification-associated hepat	GNPAT

1.84309304582953E-125	0.273854609991628	0.614	0.331	3.08865532620112E-121	Detoxification-associated hepat	INTS11
2.1400042434811E-125	0.626585514829568	0.419	0.203	3.58621911122563E-121	Detoxification-associated hepat	RALGAPB
2.14676538075587E-125	0.864021478320646	0.349	0.157	3.59754942507069E-121	Detoxification-associated hepat	LNPEP
2.27210394032449E-125	0.8417359802704	0.33	0.143	3.80759178319578E-121	Detoxification-associated hepat	SEPSECS
2.32861183509646E-125	1.28866832421947	0.232	0.083	3.90228771325465E-121	Detoxification-associated hepat	DEPRECATEI
2.62310095035979E-125	0.36229602068614	0.616	0.331	4.39579257261294E-121	Detoxification-associated hepat	UBL3
2.6802290636761E-125	0.617449483892027	0.427	0.208	4.49152786490841E-121	Detoxification-associated hepat	BTF3L4
2.81320448631848E-125	0.446355595078762	0.461	0.23	4.71436807817252E-121	Detoxification-associated hepat	ADIPOR2
2.97264589860416E-125	0.780656970442262	0.375	0.174	4.98155999688086E-121	Detoxification-associated hepat	ZNF507
2.99503244034502E-125	0.452385508287007	0.388	0.177	5.01907536353018E-121	Detoxification-associated hepat	APOL3
3.03086835661566E-125	0.379431672555059	0.849	0.504	5.07912919201653E-121	Detoxification-associated hepat	TSPAN6
3.11975018539954E-125	1.09546051748147	0.259	0.099	5.22807736069255E-121	Detoxification-associated hepat	ZXDB
3.30829250529581E-125	0.460846593766178	0.47	0.236	5.54403658037472E-121	Detoxification-associated hepat	TAP2
3.377263158013E-125	0.338288160476289	0.573	0.301	5.6596176001982E-121	Detoxification-associated hepat	DEPRECATEI
4.01243299338965E-125	0.540892426746155	0.429	0.208	6.72403521032238E-121	Detoxification-associated hepat	CYP2C19
4.20660981022337E-125	1.40086861364796	0.238	0.087	7.04943671997232E-121	Detoxification-associated hepat	IGIP
4.42262979861558E-125	0.495657619903144	0.415	0.198	7.41144301651999E-121	Detoxification-associated hepat	ELP2
4.60733071858594E-125	0.504370714110149	0.666	0.362	7.72096481820632E-121	Detoxification-associated hepat	SYTL4
4.85461724468937E-125	0.745243254646862	0.278	0.109	8.13536757865044E-121	Detoxification-associated hepat	DEPRECATEI
4.99378369971549E-125	0.793742639495112	0.32	0.136	8.36858272398323E-121	Detoxification-associated hepat	ZFYVE9
5.58958830195432E-125	0.557270294702622	0.423	0.203	9.36703207641504E-121	Detoxification-associated hepat	SCAP
5.66865372149731E-125	0.76182997616718	0.318	0.135	9.49952990648519E-121	Detoxification-associated hepat	MEF2A
6.6215166145982E-125	0.416956382154393	0.498	0.254	1.10963375427437E-120	Detoxification-associated hepat	SNX6
7.8749517192808E-125	0.371572528660636	0.545	0.286	1.31968440911708E-120	Detoxification-associated hepat	RAF1
7.90293705846172E-125	0.59656229802907	0.364	0.163	1.32437419225702E-120	Detoxification-associated hepat	SLC23A1
8.48239478982947E-125	0.358105961411269	0.659	0.36	1.42147971887962E-120	Detoxification-associated hepat	IMPAD1
9.24353647687866E-125	0.549420881286393	0.526	0.265	1.54903184279533E-120	Detoxification-associated hepat	MS4A8
1.00532482580532E-124	1.21547144456866	0.242	0.089	1.68472334308456E-120	Detoxification-associated hepat	PIGL
1.23372143430652E-124	0.894510271109516	0.295	0.122	2.06747037961086E-120	Detoxification-associated hepat	DEPRECATEI
1.28886734580867E-124	0.367464416269671	0.543	0.281	2.15988389810617E-120	Detoxification-associated hepat	ABCB6
1.39474242620201E-124	0.892820788302379	0.33	0.146	2.33730935782932E-120	Detoxification-associated hepat	FBXO4
1.45057829001971E-124	1.10973545449003	0.308	0.132	2.43087909841503E-120	Detoxification-associated hepat	MFSD11
1.53170659536748E-124	1.27994699745759	0.248	0.093	2.56683391251682E-120	Detoxification-associated hepat	JRKL
1.94352003583779E-124	0.433380149016168	0.478	0.24	3.25695087605696E-120	Detoxification-associated hepat	ZNF146
2.50813466130568E-124	0.755841308497647	0.397	0.189	4.20313206541606E-120	Detoxification-associated hepat	ALG6
2.73941809548587E-124	0.921362243217973	0.319	0.138	4.59071684441521E-120	Detoxification-associated hepat	GCNT2
2.95998629041228E-124	0.355399879989435	0.599	0.315	4.96034502547289E-120	Detoxification-associated hepat	SCRIB
3.40772588986428E-124	0.3858479092764	0.634	0.342	5.71066704623455E-120	Detoxification-associated hepat	TMEM38B
3.57262219944235E-124	0.545859203354208	0.448	0.223	5.98700028182549E-120	Detoxification-associated hepat	API5
3.84463177432346E-124	0.758234417011565	0.342	0.152	6.44283392741125E-120	Detoxification-associated hepat	USP16
4.06058361479151E-124	0.495277875721546	0.423	0.205	6.80472602166761E-120	Detoxification-associated hepat	PSMA4
4.12831664727649E-124	0.864804133260898	0.318	0.136	6.91823303750594E-120	Detoxification-associated hepat	OFD1
4.29366332197884E-124	1.07922007779248	0.266	0.104	7.19532099497214E-120	Detoxification-associated hepat	THAP5
5.18782685445065E-124	0.50655841060707	0.458	0.23	8.6937602426884E-120	Detoxification-associated hepat	MFF
5.27862099408195E-124	0.47455062053594	0.477	0.24	8.84591306188253E-120	Detoxification-associated hepat	ABHD10
6.78418087362795E-124	0.513847302599322	0.499	0.257	1.13689303080257E-119	Detoxification-associated hepat	NCKIPSD
8.05107821339942E-124	0.795100043752495	0.327	0.142	1.34919968700147E-119	Detoxification-associated hepat	ESCO1
8.3259210148567E-124	0.527414593766444	0.451	0.223	1.39525784366969E-119	Detoxification-associated hepat	PPP1R12B
9.40503508251461E-124	0.495551309146223	0.549	0.289	1.5760957791278E-119	Detoxification-associated hepat	NREP
9.92199186322005E-124	0.654634325276983	0.314	0.132	1.66272739643842E-119	Detoxification-associated hepat	MPI
1.02620811671423E-123	0.969916168136976	0.229	0.081	1.7197195619897E-119	Detoxification-associated hepat	SYNM
1.19232663473436E-123	0.669103219565195	0.337	0.148	1.99810097448785E-119	Detoxification-associated hepat	ZNF618
1.21497992648509E-123	0.537986517855026	0.422	0.203	2.03606336080372E-119	Detoxification-associated hepat	TCAIM
1.3486665351804E-123	0.382342087891828	0.649	0.356	2.26009537965532E-119	Detoxification-associated hepat	MTUS1
1.57443028983344E-123	0.597738699693066	0.393	0.185	2.63843027970288E-119	Detoxification-associated hepat	TAF11
1.5862746100474E-123	0.379556209078832	0.712	0.398	2.65827899151743E-119	Detoxification-associated hepat	DNAJC3
1.88751146870142E-123	0.301750674687493	0.703	0.391	3.16309171924983E-119	Detoxification-associated hepat	UBAP2L
2.9685842439216E-123	0.701369068817122	0.329	0.142	4.97475347596382E-119	Detoxification-associated hepat	LATS2
3.31654360955291E-123	0.324392729109098	0.597	0.32	5.55786378088877E-119	Detoxification-associated hepat	HGS
3.32370213043317E-123	0.558350410211305	0.452	0.226	5.5698600301799E-119	Detoxification-associated hepat	MED14

3.44430864816425E-123	1.05095952278717	0.269	0.106	5.77197243259364E-119	Detoxification-associated hepat	SETD4
3.63560752580004E-123	0.344200067928809	0.648	0.358	6.09255109173571E-119	Detoxification-associated hepat	CSNK1D
3.84042852741613E-123	0.589297150994569	0.399	0.191	6.43579012624395E-119	Detoxification-associated hepat	MESD
4.02236721284891E-123	1.47533158872498	0.231	0.084	6.7406829752922E-119	Detoxification-associated hepat	ZBED8
5.0268851895655E-123	0.855013999259384	0.277	0.11	8.42405420067387E-119	Detoxification-associated hepat	ERCC6
5.53250716806607E-123	1.01407719823782	0.183	0.056	9.27137551224511E-119	Detoxification-associated hepat	GPER1
5.56624081181218E-123	0.42350291729903	0.509	0.259	9.32790635243485E-119	Detoxification-associated hepat	RBBP7
5.6588585968092E-123	0.612340520607001	0.411	0.198	9.48311523653285E-119	Detoxification-associated hepat	CASP6
5.97178705552707E-123	0.589575959082876	0.43	0.212	1.00075207476523E-118	Detoxification-associated hepat	C6orf62
5.97639237315372E-123	0.787080746221482	0.327	0.143	1.0015238338931E-118	Detoxification-associated hepat	DIP2C
6.0611842531221E-123	0.618199385880809	0.386	0.181	1.0157332571382E-118	Detoxification-associated hepat	TNFAIP2
8.01869115328346E-123	0.35461914383737	0.554	0.291	1.34377226346724E-118	Detoxification-associated hepat	DHCR7
1.00433001481118E-122	2.08993783188659	0.164	0.048	1.68305623882057E-118	Detoxification-associated hepat	ARMCX4
1.19081663621531E-122	0.655615223240792	0.379	0.178	1.99557051896961E-118	Detoxification-associated hepat	PHKB
1.34820912963835E-122	0.415264974978652	0.838	0.513	2.25932885944794E-118	Detoxification-associated hepat	SPTSSA
1.47477035964515E-122	0.663027569904499	0.416	0.204	2.47142016869334E-118	Detoxification-associated hepat	CDK13
1.84059478170748E-122	0.904645334672019	0.248	0.092	3.0844687351854E-118	Detoxification-associated hepat	PLPPR2
1.89085144961563E-122	0.474630531906482	0.414	0.199	3.16868885926587E-118	Detoxification-associated hepat	RFNG
2.14554356678115E-122	0.571875252530599	0.38	0.177	3.59550190921185E-118	Detoxification-associated hepat	METTL14
2.15504133702748E-122	0.6153417771856	0.379	0.177	3.61141827259065E-118	Detoxification-associated hepat	FLVCR1
2.76641827938066E-122	0.296613691505577	0.461	0.227	4.6359637525861E-118	Detoxification-associated hepat	C16orf70
3.09088673248329E-122	0.381983135130183	0.631	0.34	5.1797079862955E-118	Detoxification-associated hepat	ZDHHC9
3.12601350309517E-122	0.501507277430762	0.465	0.237	5.23857342848688E-118	Detoxification-associated hepat	ATP6V1C1
3.96608042094924E-122	1.53092486327827	0.218	0.077	6.64635756942674E-118	Detoxification-associated hepat	AC093323.1
4.0654371039305E-122	0.358190474501429	0.663	0.369	6.81285949876674E-118	Detoxification-associated hepat	ACBD3
4.61403341458474E-122	1.28236747208792	0.129	0.03	7.7321971961611E-118	Detoxification-associated hepat	FBXO27
5.37775493878899E-122	0.944406487908298	0.254	0.097	9.01204172642259E-118	Detoxification-associated hepat	HERC5
6.02977071323478E-122	1.13085995333245	0.277	0.112	1.01046897612388E-117	Detoxification-associated hepat	CENPC
6.15866789301262E-122	0.49445970269101	0.458	0.226	1.03206956551106E-117	Detoxification-associated hepat	MORC4
6.70673316547902E-122	0.565611322335453	0.365	0.166	1.12391434387097E-117	Detoxification-associated hepat	KIF12
7.29066564468056E-122	0.882182103074097	0.37	0.171	1.22176974873557E-117	Detoxification-associated hepat	KNTC1
7.900159374755E-122	0.439302081330591	0.464	0.235	1.32390870802144E-117	Detoxification-associated hepat	KCMF1
8.27164646890085E-122	0.766522664892148	0.24	0.087	1.3861625152584E-117	Detoxification-associated hepat	PPP2CB
8.28438175513286E-122	0.320974023643933	0.746	0.425	1.38829669452516E-117	Detoxification-associated hepat	FAM114A1
1.05343466058576E-121	1.18060741873034	0.236	0.087	1.76534580420962E-117	Detoxification-associated hepat	TRAF5
1.32661247117335E-121	0.825321246852105	0.335	0.15	2.2231371791923E-117	Detoxification-associated hepat	TRNT1
1.37629459146043E-121	0.32494536311259	0.723	0.394	2.30639447636939E-117	Detoxification-associated hepat	NHSL1
1.51416077037138E-121	1.06574335796011	0.271	0.109	2.53743061898836E-117	Detoxification-associated hepat	DPP8
1.58131241333096E-121	0.335353308696259	0.721	0.404	2.64996334226003E-117	Detoxification-associated hepat	MCFD2
1.75622612599338E-121	0.462987582334595	0.485	0.248	2.94308374193971E-117	Detoxification-associated hepat	USP24
1.86361595951514E-121	0.407258513167571	0.461	0.231	3.12304762495547E-117	Detoxification-associated hepat	MIER1
2.4675799885022E-121	0.484536133979176	0.525	0.271	4.13517054473198E-117	Detoxification-associated hepat	RALGAPA2
2.63571151006227E-121	0.458235593381008	0.481	0.246	4.41692534856236E-117	Detoxification-associated hepat	KDM4B
2.7088867315907E-121	1.05855181259917	0.312	0.135	4.53955238479969E-117	Detoxification-associated hepat	RADX
2.81593672036658E-121	1.19850418984425	0.241	0.09	4.71894675599032E-117	Detoxification-associated hepat	DNA2
3.17119192306103E-121	0.513178558138906	0.447	0.224	5.31428342466568E-117	Detoxification-associated hepat	RARS
3.24006907670596E-121	0.38787053962345	0.616	0.329	5.42970775874385E-117	Detoxification-associated hepat	KLF3
3.42892367723253E-121	0.900345313593634	0.269	0.107	5.74619029830627E-117	Detoxification-associated hepat	LDAH
3.74998939078247E-121	0.971456586552671	0.287	0.118	6.28423222107327E-117	Detoxification-associated hepat	MGA
3.76757716162208E-121	0.555725934505318	0.425	0.208	6.31370580744628E-117	Detoxification-associated hepat	SLC35D1
4.62417368779761E-121	0.775168267848429	0.306	0.13	7.74919026601123E-117	Detoxification-associated hepat	C5orf51
4.76624599436652E-121	0.559003563173522	0.417	0.205	7.98727503735941E-117	Detoxification-associated hepat	HTT
6.57761277622298E-121	0.884165081300049	0.3	0.127	1.10227634903945E-116	Detoxification-associated hepat	ZNF692
6.92368145517158E-121	0.663395533375883	0.378	0.178	1.16027053825765E-116	Detoxification-associated hepat	VEZF1
7.26134663531679E-121	0.843183719606846	0.315	0.136	1.21685646914639E-116	Detoxification-associated hepat	FER
8.09626288972586E-121	0.66819156019365	0.37	0.173	1.35677173506026E-116	Detoxification-associated hepat	YTHDF2
8.46477351851931E-121	1.3517418832354	0.248	0.096	1.41852674623347E-116	Detoxification-associated hepat	POP1
8.57872848090787E-121	0.584653373475806	0.356	0.162	1.43762331883054E-116	Detoxification-associated hepat	ALG2
9.3022624275706E-121	1.63889626294744	0.233	0.088	1.55887313761228E-116	Detoxification-associated hepat	PCDHGB6
9.38388603024256E-121	0.72663523047969	0.364	0.169	1.57255162094805E-116	Detoxification-associated hepat	TMEM165

**9.60217548387963E-121**	0.622519304070782	0.347	0.156	1.60913256758855E-116	Detoxification-associated hepat	TTLL12
**1.56461507791077E-120**	0.420762331211188	0.304	0.124	2.62198194756287E-116	Detoxification-associated hepat	LAMA5
**1.60621197026009E-120**	0.461949418598881	0.469	0.238	2.69169001976186E-116	Detoxification-associated hepat	MAEA
**1.64674475584393E-120**	0.304375432105839	0.691	0.373	2.75961486184326E-116	Detoxification-associated hepat	COPS5
**1.89982376184045E-120**	0.34656134255785	0.758	0.439	3.18372466009223E-116	Detoxification-associated hepat	HM13
**1.96215933570215E-120**	0.265471110018026	0.619	0.341	3.28818661476966E-116	Detoxification-associated hepat	PPP6R2
**2.30365847331522E-120**	0.649785099022328	0.366	0.17	3.86047086958164E-116	Detoxification-associated hepat	USP38
**2.37933335606184E-120**	0.381701811341196	0.475	0.24	3.98728683808843E-116	Detoxification-associated hepat	TBC1D14
**2.41750365461311E-120**	0.716346430839374	0.355	0.164	4.05125262440065E-116	Detoxification-associated hepat	PDS5A
**2.4449150017273E-120**	0.330752567039179	0.594	0.317	4.0971885598946E-116	Detoxification-associated hepat	TRIP12
**2.49985130259184E-120**	0.502423822392252	0.461	0.234	4.1892508128834E-116	Detoxification-associated hepat	BRF1
**2.84165479774208E-120**	1.06018183655272	0.293	0.124	4.76204511005618E-116	Detoxification-associated hepat	NFXL1
**2.87286738264205E-120**	1.06540087071077	0.207	0.069	4.81435115983155E-116	Detoxification-associated hepat	MYL5
**3.81338847714543E-120**	0.635820629016544	0.432	0.214	6.39047641000031E-116	Detoxification-associated hepat	SPATS2
**4.1802154376448E-120**	0.370723821657347	0.511	0.264	7.00520503040515E-116	Detoxification-associated hepat	PRPSAP1
**4.28182372101335E-120**	0.416682870431639	0.504	0.262	7.17548019167417E-116	Detoxification-associated hepat	YTHDC1
**4.93887912854905E-120**	0.634428974578124	0.423	0.208	8.2765736436225E-116	Detoxification-associated hepat	S100PBP
**6.17700116470665E-120**	1.31638153630067	0.257	0.101	1.03514185518154E-115	Detoxification-associated hepat	PPP1R3E
**7.50751727161564E-120**	1.12616384779267	0.241	0.091	1.25810974437735E-115	Detoxification-associated hepat	THEM4
**7.89795717481354E-120**	0.955883685386737	0.289	0.12	1.32353966335525E-115	Detoxification-associated hepat	SMURF2
**8.89108018064643E-120**	0.321098895504842	0.549	0.289	1.48996721667273E-115	Detoxification-associated hepat	SKP1
**1.16651044456782E-119**	0.742455321796069	0.38	0.181	1.95483820300675E-115	Detoxification-associated hepat	PDS5B
**1.22106199082874E-119**	0.378835733615045	0.41	0.196	2.0462556842308E-115	Detoxification-associated hepat	IBTK
**1.24809135810544E-119**	0.379533624961528	0.431	0.209	2.0915514979131E-115	Detoxification-associated hepat	SLC41A2
**1.34655551993687E-119**	1.05606324798451	0.259	0.102	2.2565577403102E-115	Detoxification-associated hepat	ELAC1
**1.41569152113032E-119**	0.962776450076569	0.243	0.09	2.37241585111018E-115	Detoxification-associated hepat	GPC3
**1.46605170316208E-119**	0.354329640360962	0.621	0.34	2.45680944415901E-115	Detoxification-associated hepat	SCAMP2
**1.50489039864456E-119**	0.28319464708940	0.831	0.509	2.52189533004855E-115	Detoxification-associated hepat	SLC39A14
**1.5314382289102E-119**	0.755277966284696	0.347	0.158	2.56638418400772E-115	Detoxification-associated hepat	TRAPPC6B
**1.56944247843316E-119**	0.755932586744505	0.235	0.085	2.63007170535829E-115	Detoxification-associated hepat	LZTS2
**1.86954443004047E-119**	0.928244006549146	0.265	0.105	3.13298255586182E-115	Detoxification-associated hepat	RLIM
**1.959253724531E-119**	0.401224199931911	0.524	0.273	3.28331739156905E-115	Detoxification-associated hepat	FBXL3
**2.70416733646573E-119**	0.303880133630025	0.559	0.298	4.53164362244926E-115	Detoxification-associated hepat	MINK1
**2.76936426910313E-119**	0.480261078155674	0.402	0.194	4.64090064216302E-115	Detoxification-associated hepat	C5orf15
**3.49630342673434E-119**	0.846719901930413	0.343	0.158	5.85910528252141E-115	Detoxification-associated hepat	ARMCX5
**3.94112816211735E-119**	0.626462050132333	0.365	0.169	6.60454257407625E-115	Detoxification-associated hepat	LIG3
**4.40537754031457E-119**	1.27720441194691	0.216	0.076	7.38253168205916E-115	Detoxification-associated hepat	ZNF333
**5.47647689335475E-119**	0.453924517888084	0.471	0.242	9.17747997783389E-115	Detoxification-associated hepat	ACTR10
**6.58223779504614E-119**	0.89043877588503	0.296	0.125	1.10305140969383E-114	Detoxification-associated hepat	MYCBP2
**6.88928202228712E-119**	0.56230553229558	0.404	0.194	1.15450588129488E-114	Detoxification-associated hepat	AK6
**8.50772566912342E-119**	0.457048728773282	0.436	0.217	1.4257246676317E-114	Detoxification-associated hepat	SNX2
**1.09110243192891E-118**	0.399728903847044	0.515	0.266	1.82846945542647E-114	Detoxification-associated hepat	PPP1R10
**1.23078067165232E-118**	0.418803971499193	0.516	0.27	2.06254224955495E-114	Detoxification-associated hepat	ZNF664
**1.24684918205597E-118**	0.647622064886363	0.361	0.167	2.0894698592894E-114	Detoxification-associated hepat	NPHP3
**1.3107556200654E-118**	0.446767902012918	0.432	0.213	2.19656426810559E-114	Detoxification-associated hepat	ARFGEF2
**1.35351455643634E-118**	1.16299469461796	0.24	0.091	2.26821969367602E-114	Detoxification-associated hepat	ZNF554
**1.41811612399457E-118**	0.814557793390639	0.291	0.122	2.37647900059009E-114	Detoxification-associated hepat	TUBGCP4
**1.72733517834225E-118**	0.285336001096097	0.619	0.333	2.89466829186594E-114	Detoxification-associated hepat	SMARCA4
**1.74364458699563E-118**	1.60739720467222	0.225	0.083	2.92199959888727E-114	Detoxification-associated hepat	PCDHGB7
**2.30133479802207E-118**	0.33374400711428	0.519	0.272	3.85657685452539E-114	Detoxification-associated hepat	SMAD4
**2.63874485410582E-118**	0.425270076841225	0.242	0.087	4.42200862651053E-114	Detoxification-associated hepat	SHANK2
**3.20821994406551E-118**	0.637310204555874	0.399	0.195	5.37633498226497E-114	Detoxification-associated hepat	LYSMD3
**3.5336297923307E-118**	1.21043292922323	0.246	0.095	5.92165680598779E-114	Detoxification-associated hepat	MBD5
**4.45210318058634E-118**	0.46775986200693	0.436	0.217	7.46083451002659E-114	Detoxification-associated hepat	WIZ
**5.77330703911808E-118**	0.322929678372187	0.635	0.353	9.67490793615408E-114	Detoxification-associated hepat	SMIM19
**5.83632042964669E-118**	0.669329687470227	0.396	0.191	9.78050577600192E-114	Detoxification-associated hepat	ANO8
**6.18419507028415E-118**	0.451083695985698	0.451	0.228	1.03634740987822E-113	Detoxification-associated hepat	ZC3H3
**7.15282163443366E-118**	0.805480184584446	0.355	0.165	1.19866984949839E-113	Detoxification-associated hepat	IFI44
**7.39783307015392E-118**	1.40124749865618	0.194	0.064	1.23972886589639E-113	Detoxification-associated hepat	LONRF2
**1.23371217740283E-117**	2.07820064953514	0.14	0.037	2.06745486689167E-113	Detoxification-associated hepat	ANKAR

**1.5252147852665E-117**	0.361640975045682	0.555	0.29	2.55595493714961E-113	Detoxification-associated hepat	MYO19
**1.53858016205852E-117**	0.361651234613433	0.791	0.471	2.57835263557767E-113	Detoxification-associated hepat	PGRMC2
**1.56362149496772E-117**	0.296153873321385	0.583	0.31	2.62031690126691E-113	Detoxification-associated hepat	IGF2R
**1.56940808511888E-117**	0.26904454541751	0.639	0.352	2.63001406904221E-113	Detoxification-associated hepat	QKI
**1.97723934335489E-117**	1.11257888720885	0.262	0.105	3.31345769159412E-113	Detoxification-associated hepat	SYDE2
**2.31814325232613E-117**	0.688708639590692	0.362	0.168	3.88474446224814E-113	Detoxification-associated hepat	GFOD2
**2.52486388536447E-117**	0.552558617630867	0.408	0.2	4.23116689909379E-113	Detoxification-associated hepat	SFPQ
**2.75355644949335E-117**	0.857772974112029	0.308	0.134	4.61440989806096E-113	Detoxification-associated hepat	DDX60
**2.85927339660201E-117**	0.385349073240352	0.516	0.27	4.79157035802565E-113	Detoxification-associated hepat	TMEM214
**2.99827138819938E-117**	0.551552905338367	0.414	0.205	5.02450319234452E-113	Detoxification-associated hepat	SGPP1
**3.01631499545116E-117**	0.568513414266805	0.374	0.176	5.05474066937705E-113	Detoxification-associated hepat	SETDB1
**3.6866676788538E-117**	1.58316702942576	0.171	0.052	6.1781176962232E-113	Detoxification-associated hepat	SGSM1
**3.78696792548695E-117**	0.324361662759301	0.58	0.314	6.34620084953103E-113	Detoxification-associated hepat	DDX46
**3.90946774672944E-117**	0.440575648493564	0.466	0.238	6.5514860499692E-113	Detoxification-associated hepat	LARP1
**4.14297815542432E-117**	0.72916003795608	0.294	0.124	6.94280279286007E-113	Detoxification-associated hepat	CCSER2
**4.51123413088739E-117**	0.39308976878779	0.492	0.255	7.55992615654108E-113	Detoxification-associated hepat	RC3H2
**5.58389353807252E-117**	0.764269723083329	0.318	0.14	9.35748879110192E-113	Detoxification-associated hepat	SUPT20H
**5.6832707750113E-117**	0.344431699768773	0.549	0.29	9.52402516476394E-113	Detoxification-associated hepat	PTK2
**6.66661904124402E-117**	0.57492909462182	0.376	0.176	1.11719201893167E-112	Detoxification-associated hepat	SULF2
**7.50482920371608E-117**	1.01383216553278	0.253	0.099	1.25765927795874E-112	Detoxification-associated hepat	TRMT13
**8.283940641242E-117**	0.720240282607643	0.357	0.167	1.38822277265934E-112	Detoxification-associated hepat	KIAA1109
**9.09457748683943E-117**	1.01450759725922	0.24	0.09	1.52406929524455E-112	Detoxification-associated hepat	FMO1
**9.53745850575061E-117**	0.294759532241014	0.594	0.321	1.59828729639369E-112	Detoxification-associated hepat	GPBP1
**1.10208539826516E-116**	1.05267449277852	0.286	0.121	1.84687471041276E-112	Detoxification-associated hepat	EXOC1
**1.18866925845062E-116**	0.837345528475983	0.373	0.177	1.99197194331155E-112	Detoxification-associated hepat	VIPR1
**1.23873391541347E-116**	0.307139775540346	0.518	0.269	2.07587029544989E-112	Detoxification-associated hepat	PAM16
**1.41121170732654E-116**	0.838006786813592	0.321	0.142	2.36490857913781E-112	Detoxification-associated hepat	SLC35E2B
**1.5190444939738E-116**	0.767402801757022	0.349	0.162	2.54561476300129E-112	Detoxification-associated hepat	RAB3GAP2
**1.62550165464669E-116**	0.5965349929961	0.413	0.204	2.72401567285693E-112	Detoxification-associated hepat	MED13L
**1.73780448503533E-116**	0.281478570599731	0.55	0.292	2.91221275602221E-112	Detoxification-associated hepat	HDHD3
**1.90628326010662E-116**	0.454823674664562	0.47	0.243	3.19454948728668E-112	Detoxification-associated hepat	LANCL1
**2.3180367136074E-116**	0.806590987774061	0.364	0.171	3.88456592466328E-112	Detoxification-associated hepat	BRCC3
**2.40617118459221E-116**	1.5071896107024	0.122	0.028	4.03226167113963E-112	Detoxification-associated hepat	GSTM2
**2.69132900738788E-116**	1.78198091084485	0.163	0.048	4.51012915058061E-112	Detoxification-associated hepat	ZBTB26
**3.237292864349E-116**	0.332461895000959	0.828	0.464	5.42505538207605E-112	Detoxification-associated hepat	IDH3G
**3.49782128249965E-116**	0.373227295670337	0.506	0.265	5.86164890521291E-112	Detoxification-associated hepat	YPEL5
**3.77648834258985E-116**	0.50084454939326	0.388	0.187	6.32863916451206E-112	Detoxification-associated hepat	GTF3C3
**3.78794444334837E-116**	0.457406168725092	0.43	0.214	6.3478372981632E-112	Detoxification-associated hepat	RNF13
**3.81906948373398E-116**	0.430805901012715	0.53	0.285	6.3999966408414E-112	Detoxification-associated hepat	CYP2C18
**4.10278923539582E-116**	0.927378199570828	0.299	0.129	6.87545420067632E-112	Detoxification-associated hepat	EED
**4.11672923549269E-116**	1.04343356132247	0.244	0.094	6.89881485283865E-112	Detoxification-associated hepat	DEPRECATEI
**4.47358030369805E-116**	0.719920397376294	0.345	0.159	7.4968258729372E-112	Detoxification-associated hepat	TMTC4
**4.91973699115049E-116**	0.528493702574145	0.418	0.207	8.24449524977E-112	Detoxification-associated hepat	ATG4B
**6.04940280608733E-116**	0.387499611641116	0.512	0.268	1.01375892224412E-111	Detoxification-associated hepat	TBCD
**6.47114577595566E-116**	0.302174726128741	0.786	0.452	1.08443460913465E-111	Detoxification-associated hepat	EGLN1
**6.56044749664092E-116**	0.398513599244703	0.465	0.236	1.09939979148708E-111	Detoxification-associated hepat	ITGB5
**7.48546298182395E-116**	0.719983189463993	0.337	0.153	1.25441388649406E-111	Detoxification-associated hepat	TMEM128
**1.04476213802952E-115**	0.320054152242314	0.552	0.291	1.75081239090987E-111	Detoxification-associated hepat	MECP2
**1.25909924504152E-115**	0.336905336383151	0.55	0.293	2.10999851484058E-111	Detoxification-associated hepat	SMARCA5
**1.77717512595112E-115**	0.331142617055389	0.646	0.355	2.97819007606889E-111	Detoxification-associated hepat	FAM91A1
**2.08464136768375E-115**	0.556295779970837	0.365	0.172	3.49344200396442E-111	Detoxification-associated hepat	FAM98B
**2.38782513818529E-115**	0.687600745694215	0.338	0.154	4.0015173665709E-111	Detoxification-associated hepat	USP33
**3.07880709608908E-115**	1.18278533888209	0.242	0.094	5.15946493162609E-111	Detoxification-associated hepat	CYP2R1
**3.11005479976063E-115**	0.330294473196025	0.573	0.31	5.21182983343887E-111	Detoxification-associated hepat	CMTM8
**3.20023559378059E-115**	0.95554612042819	0.293	0.126	5.36295480805751E-111	Detoxification-associated hepat	FPGT
**3.53726215258605E-115**	1.56292438023912	0.162	0.048	5.92774391530371E-111	Detoxification-associated hepat	C1QTNF6
**4.18690634639024E-115**	0.573723038286551	0.337	0.151	7.01641765528076E-111	Detoxification-associated hepat	PITPNC1
**5.07250195966043E-115**	1.61365474259207	0.133	0.034	8.50049878399895E-111	Detoxification-associated hepat	ZDHHC11
**5.31139970576137E-115**	0.612542553994914	0.386	0.187	8.9008436269149E-111	Detoxification-associated hepat	ILKAP
**6.69560418959949E-115**	0.879250036431216	0.327	0.148	1.12204935009308E-110	Detoxification-associated hepat	MSTO1

7.00376551915353E-115	0.407963641301288	0.52	0.271	1.17369102569975E-110	Detoxification-associated hepat	TFDP1
7.21749721876343E-115	0.816727014042074	0.297	0.127	1.20950818392038E-110	Detoxification-associated hepat	SPATA5L1
8.20647453031131E-115	0.388318914925005	0.741	0.439	1.37524100178957E-110	Detoxification-associated hepat	MSMO1
8.75438185073206E-115	0.57033589641935	0.388	0.189	1.46705931054568E-110	Detoxification-associated hepat	BPNT1
9.0606720759458E-115	0.568906045521708	0.433	0.221	1.518387426487E-110	Detoxification-associated hepat	TTC17
9.71758241983297E-115	0.336185827155834	0.487	0.253	1.62847246191561E-110	Detoxification-associated hepat	TMEM161A
1.12083458338952E-114	0.554537888779738	0.375	0.178	1.87829459484415E-110	Detoxification-associated hepat	KLHL24
1.18664598185932E-114	0.638476463389838	0.359	0.167	1.98858133639984E-110	Detoxification-associated hepat	KLHL23
1.23770605251138E-114	0.336846326266708	0.524	0.278	2.07414780279856E-110	Detoxification-associated hepat	DEPRECATEI
1.34604469924064E-114	1.06534388078148	0.292	0.126	2.25570170698746E-110	Detoxification-associated hepat	ZNF26
2.10712271279336E-114	0.38020950473666	0.458	0.233	3.53111624209911E-110	Detoxification-associated hepat	DENND1A
3.00411068384738E-114	1.0045506304552	0.262	0.106	5.03428868399144E-110	Detoxification-associated hepat	GUF1
3.23851183362644E-114	0.353615294908084	0.551	0.294	5.42709813079119E-110	Detoxification-associated hepat	DHX9
4.08916162329459E-114	0.430632425519199	0.49	0.254	6.85261704831708E-110	Detoxification-associated hepat	STK38
6.093919929529E-114	0.394880710521281	0.471	0.242	1.02121910179047E-109	Detoxification-associated hepat	NPAS2
6.31227474889545E-114	0.401484284656855	0.408	0.2	1.0578110024199E-109	Detoxification-associated hepat	ULK1
6.42085635026152E-114	0.81328885577908	0.282	0.118	1.07600710717683E-109	Detoxification-associated hepat	IRGQ
6.59089848005887E-114	0.371202792697757	0.533	0.284	1.10450276728827E-109	Detoxification-associated hepat	TRIQK
6.62136684597532E-114	0.733209836469451	0.294	0.125	1.10960865604854E-109	Detoxification-associated hepat	ZNF362
6.9357890284229E-114	0.857232971539259	0.309	0.137	1.16229952538311E-109	Detoxification-associated hepat	TANC1
7.51141645737556E-114	0.318972871446387	0.567	0.305	1.258763169927E-109	Detoxification-associated hepat	CGGBP1
7.53831973173794E-114	0.37996378749292	0.462	0.237	1.26327162064464E-109	Detoxification-associated hepat	PURA
7.95907557552728E-114	0.267499902006633	0.677	0.384	1.33378188494686E-109	Detoxification-associated hepat	RBM39
8.0061305055393E-114	1.90146050079749	0.148	0.041	1.34166735011828E-109	Detoxification-associated hepat	DSEL
8.5503822552296E-114	0.907265926448625	0.212	0.075	1.43287305833138E-109	Detoxification-associated hepat	ISX
9.42520767381652E-114	0.84739376836238	0.282	0.118	1.57947630197817E-109	Detoxification-associated hepat	XPA
1.04255617230702E-113	0.333875680793807	0.748	0.432	1.7471156335521E-109	Detoxification-associated hepat	SREBF2
1.10174962194938E-113	0.343899725119409	0.588	0.32	1.84631201646277E-109	Detoxification-associated hepat	AHCYL1
1.18286247825644E-113	1.10310858910607	0.254	0.102	1.98224094106215E-109	Detoxification-associated hepat	FAM120C
1.18641341181121E-113	0.323915868353237	0.537	0.286	1.98819159551322E-109	Detoxification-associated hepat	DPP9
1.25250885038664E-113	0.253031215085791	0.51	0.267	2.09895433147793E-109	Detoxification-associated hepat	RHOT2
1.42054491923549E-113	0.360412364433042	0.495	0.258	2.38054917565484E-109	Detoxification-associated hepat	METTL23
1.45484937954253E-113	0.482252297912637	0.38	0.181	2.43803659023738E-109	Detoxification-associated hepat	INPPL1
1.73701769278767E-113	0.57814644548336	0.399	0.196	2.91089424957358E-109	Detoxification-associated hepat	CFAP97
1.88518646725749E-113	0.575000331681422	0.413	0.206	3.15919548183011E-109	Detoxification-associated hepat	DEPRECATEI
2.22647902494805E-113	0.354352821933143	0.582	0.314	3.73113355000795E-109	Detoxification-associated hepat	PLOD2
2.76939105315149E-113	0.445415108660491	0.414	0.203	4.64094552687127E-109	Detoxification-associated hepat	TBC1D15
2.79910271224568E-113	0.746765400569046	0.143	0.038	4.6907363251813E-109	Detoxification-associated hepat	MCC
3.21394381279308E-113	0.378297090347544	0.963	0.754	5.38592704147865E-109	Detoxification-associated hepat	MT-CYB
3.25621503463793E-113	1.05153139762543	0.272	0.114	5.45676515504625E-109	Detoxification-associated hepat	WDR19
3.45676348990923E-113	0.941228463180276	0.258	0.104	5.79284425638989E-109	Detoxification-associated hepat	RNPC3
3.71650563145771E-113	0.350166733122285	0.41	0.2	6.22812013719683E-109	Detoxification-associated hepat	CRELD1
4.46913577236223E-113	0.334687309800514	0.722	0.421	7.48937772732463E-109	Detoxification-associated hepat	IRF2BP2
4.84983403742439E-113	0.628432049602892	0.353	0.165	8.1273518799158E-109	Detoxification-associated hepat	TBK1
5.46177670498091E-113	0.29865351509431	0.646	0.353	9.15284540220702E-109	Detoxification-associated hepat	DGAT1
5.7356699292478E-113	0.437110606366537	0.473	0.245	9.61183566743346E-109	Detoxification-associated hepat	GPR107
5.96570488998007E-113	1.00740516799232	0.245	0.095	9.9973282546286E-109	Detoxification-associated hepat	SLC25A45
6.56672567061269E-113	0.342873405007663	0.748	0.443	1.10045188788127E-108	Detoxification-associated hepat	PDZK1
6.59254289398487E-113	0.965779087993377	0.278	0.118	1.10477833817398E-108	Detoxification-associated hepat	WDFY3
6.81583671659138E-113	0.851790482117714	0.266	0.108	1.14219791696638E-108	Detoxification-associated hepat	USP30
7.07407970571516E-113	1.00356935358046	0.305	0.135	1.18547427708375E-108	Detoxification-associated hepat	THADA
7.45142355128259E-113	0.582882931178723	0.355	0.165	1.24870955872394E-108	Detoxification-associated hepat	USP2
8.27978271804101E-113	1.04836558577076	0.262	0.107	1.38752598788931E-108	Detoxification-associated hepat	ZNF57
8.48644188889638E-113	0.446312718367838	0.406	0.2	1.42215793174126E-108	Detoxification-associated hepat	ELF1
8.9398507577143E-113	0.562853929172622	0.402	0.198	1.49814018997776E-108	Detoxification-associated hepat	SMCHD1
8.95909959898799E-113	0.579216650509041	0.396	0.196	1.50136591079841E-108	Detoxification-associated hepat	MTX2
9.9853318968849E-113	0.477271611455731	0.451	0.23	1.67334191927997E-108	Detoxification-associated hepat	EBAG9
1.07108708947357E-112	0.523613296106517	0.425	0.215	1.79492774453981E-108	Detoxification-associated hepat	MYO9B
1.65494094318116E-112	1.02620502291999	0.272	0.113	2.77335003258299E-108	Detoxification-associated hepat	ANKRA2
2.11324427602606E-112	0.344548821537465	0.642	0.358	3.54137475776447E-108	Detoxification-associated hepat	TRA2A

2.23241182794426E-112	1.12483047030497	0.19	0.063	3.74107574126898E-108	Detoxification-associated hepat	FAM49A
2.3305667222204E-112	0.480045933507325	0.424	0.213	3.90556371309695E-108	Detoxification-associated hepat	PPA2
2.52058749501874E-112	0.597327657367391	0.452	0.234	4.22400052415241E-108	Detoxification-associated hepat	AKAP9
3.20640539992154E-112	0.83254808451068	0.317	0.143	5.37329416918851E-108	Detoxification-associated hepat	GPCPD1
3.54119911490628E-112	0.66896044873894	0.419	0.211	5.93434147675994E-108	Detoxification-associated hepat	ZNF91
4.51362352564869E-112	0.772143085095925	0.305	0.134	7.56393030428207E-108	Detoxification-associated hepat	WDR36
4.6093511284071E-112	0.364878528150793	0.725	0.419	7.72435062098462E-108	Detoxification-associated hepat	IKBKG
4.61615840506496E-112	0.308432173775798	0.541	0.288	7.73575825520786E-108	Detoxification-associated hepat	UBE2Z
4.66393960543483E-112	0.620776958623336	0.376	0.181	7.81582999078769E-108	Detoxification-associated hepat	TGS1
4.8269379049612E-112	0.254970223216518	0.615	0.34	8.08898254113398E-108	Detoxification-associated hepat	TOM1L1
4.83862856004384E-112	0.554578675903568	0.382	0.184	8.10857374092147E-108	Detoxification-associated hepat	HHAT
5.41694042774878E-112	0.748742170320945	0.297	0.129	9.0777087688214E-108	Detoxification-associated hepat	COX18
6.73021409748939E-112	1.04692783659056	0.28	0.119	1.12784927845727E-107	Detoxification-associated hepat	SPATA6L
7.25891385966368E-112	1.38396178049126	0.23	0.088	1.21644878460244E-107	Detoxification-associated hepat	FHOD3
7.47951772142768E-112	0.935020838998582	0.207	0.072	1.25341757975685E-107	Detoxification-associated hepat	AMT
7.70884782089615E-112	0.788122689934479	0.325	0.147	1.29184871782578E-107	Detoxification-associated hepat	THBS3
7.72882776567937E-112	0.523721640048666	0.372	0.178	1.29519695697255E-107	Detoxification-associated hepat	RBFOX2
8.30802116250424E-112	0.494162390259177	0.358	0.167	1.39225818641246E-107	Detoxification-associated hepat	EIF3J
1.19964147853577E-111	0.443653604809059	0.444	0.227	2.01035918973024E-107	Detoxification-associated hepat	GATD1
1.36183770393964E-111	0.883665680920101	0.227	0.085	2.28216762426204E-107	Detoxification-associated hepat	DEPRECATEI
1.37498007801933E-111	0.753181544823729	0.316	0.142	2.3041916147448E-107	Detoxification-associated hepat	UMPS
1.37699145939244E-111	0.353336362275029	0.505	0.265	2.30756228764985E-107	Detoxification-associated hepat	ARHGAP1
2.00398751865703E-111	0.744922524427558	0.378	0.185	3.35828228376545E-107	Detoxification-associated hepat	TRADD
2.29503807177988E-111	0.290704038232932	0.817	0.486	3.84602480068872E-107	Detoxification-associated hepat	PEG10
2.6825975578533E-111	0.697191184128805	0.263	0.105	4.49549698745056E-107	Detoxification-associated hepat	PLA2G6
2.70587754299232E-111	0.253027613819807	0.464	0.236	4.53450958654653E-107	Detoxification-associated hepat	LUC7L
3.62130050234562E-111	0.407007938495223	0.506	0.266	6.06857538183079E-107	Detoxification-associated hepat	DOT1L
3.62272955080522E-111	0.577971390062781	0.398	0.195	6.07097018123939E-107	Detoxification-associated hepat	EPS15L1
3.84232238468391E-111	0.251263652272538	0.578	0.312	6.43896385225329E-107	Detoxification-associated hepat	FAM83H
6.1505807861663E-111	0.559400864359155	0.428	0.216	1.03071432814575E-106	Detoxification-associated hepat	SUSD6
6.21643261318983E-111	0.987564377452363	0.25	0.1	1.04174977731835E-106	Detoxification-associated hepat	SMU1
6.27122014971277E-111	0.552087540284181	0.497	0.263	1.05093107268887E-106	Detoxification-associated hepat	SH3RF1
6.51070347658505E-111	0.715622165998479	0.364	0.175	1.09106368860612E-106	Detoxification-associated hepat	CPSF1
6.5148064717907E-111	0.643273085413268	0.39	0.192	1.09175126854269E-106	Detoxification-associated hepat	INTS7
7.04650201493736E-111	0.482436348929342	0.39	0.19	1.1808528076632E-106	Detoxification-associated hepat	GON7
8.02269651395225E-111	0.263101898132511	0.596	0.329	1.34444348180812E-106	Detoxification-associated hepat	LPIN2
1.05671467856694E-110	0.41443474033004	0.401	0.196	1.77084245834247E-106	Detoxification-associated hepat	CBWD1
1.18555007580905E-110	0.309761977940141	0.422	0.209	1.98674481704081E-106	Detoxification-associated hepat	INSIG2
1.32787589568766E-110	0.274733216199867	0.641	0.356	2.22525442599338E-106	Detoxification-associated hepat	MAGED2
1.36824899518114E-110	0.47653439116009	0.424	0.212	2.29291166612455E-106	Detoxification-associated hepat	TNPO2
1.52803520938974E-110	0.346801786626705	0.553	0.301	2.56068140389532E-106	Detoxification-associated hepat	DNAJB9
1.59731696915469E-110	0.357072636313786	0.288	0.118	2.67678377690943E-106	Detoxification-associated hepat	RGL3
1.62787203079148E-110	0.328834315381172	0.81	0.487	2.72798794920036E-106	Detoxification-associated hepat	PLS3
1.65892954229722E-110	0.537133129550502	0.397	0.196	2.78003412698167E-106	Detoxification-associated hepat	EXOSC9
1.98306261890062E-110	0.41268535010233	0.434	0.22	3.32321633675367E-106	Detoxification-associated hepat	MYRF
1.98775531638767E-110	0.412575263609585	0.442	0.225	3.33108035920245E-106	Detoxification-associated hepat	NIT2
2.21765693915288E-110	0.316064212810118	0.561	0.301	3.7163494986324E-106	Detoxification-associated hepat	SLC35F6
2.96156553119162E-110	0.828115329376078	0.297	0.131	4.96299151717091E-106	Detoxification-associated hepat	L2HGDH
3.08755389813984E-110	0.522248329681668	0.458	0.235	5.17412282250274E-106	Detoxification-associated hepat	TMEM185A
3.88140407175929E-110	1.39269946972155	0.186	0.063	6.50445694345421E-106	Detoxification-associated hepat	HECTD2
3.88362480619578E-110	1.08430892256534	0.24	0.095	6.50817845022289E-106	Detoxification-associated hepat	CDKN2AIPNI
3.89704416780116E-110	0.901119298226324	0.255	0.103	6.53066661640119E-106	Detoxification-associated hepat	RAB2B
4.31087704957865E-110	0.595287965542888	0.361	0.173	7.2241677596839E-106	Detoxification-associated hepat	KIDINS220
4.37164835580409E-110	1.54668351289355	0.185	0.062	7.32600831465649E-106	Detoxification-associated hepat	SEC61A2
4.7384036008308E-110	0.606295092824798	0.346	0.162	7.94061675427225E-106	Detoxification-associated hepat	SLC25A28
5.08231181755142E-110	0.338769496844622	0.537	0.291	8.51693814385267E-106	Detoxification-associated hepat	HIPK3
5.49491498675474E-110	0.800713269615277	0.272	0.113	9.20837853480359E-106	Detoxification-associated hepat	GKAP1
5.60968799511109E-110	1.2127763585383	0.265	0.112	9.40071514220716E-106	Detoxification-associated hepat	PPM1L
5.98269933720412E-110	0.514489110211178	0.412	0.206	1.00258075492867E-105	Detoxification-associated hepat	OSGEP
6.00502682596436E-110	1.07555255832616	0.243	0.097	1.00632239549511E-105	Detoxification-associated hepat	MIOS

6.03533006828651E-110	0.650966647232815	0.339	0.158	1.01140061284345E-105	Detoxification-associated hepat	DPH7
7.69663948745652E-110	0.677344091657507	0.289	0.124	1.28980284530796E-105	Detoxification-associated hepat	ZBED6CL
9.16474344312902E-110	0.374257377228024	0.972	0.841	1.53582770619956E-105	Detoxification-associated hepat	ALDH1A1
1.62926319591427E-109	0.35964649315546	0.571	0.309	2.73031926371314E-105	Detoxification-associated hepat	NUP58
1.77515273806887E-109	0.539151149869516	0.375	0.18	2.97480095845582E-105	Detoxification-associated hepat	APBB1IP
2.12415919196056E-109	0.345125994385179	0.849	0.5	3.55966597388751E-105	Detoxification-associated hepat	GDI1
2.4634925956798E-109	0.423119405694463	0.493	0.258	4.12832089184021E-105	Detoxification-associated hepat	ERCC5
2.91680963381936E-109	0.316198649254609	0.6	0.329	4.88798958435447E-105	Detoxification-associated hepat	PUM2
3.76560912256965E-109	0.557040969298236	0.402	0.201	6.31040776760221E-105	Detoxification-associated hepat	CAMSAP2
4.20340425605568E-109	0.878510443923615	0.313	0.142	7.0440648522981E-105	Detoxification-associated hepat	AMMECR1
4.24623327986443E-109	0.320214147026849	0.442	0.226	7.11583773039681E-105	Detoxification-associated hepat	XAB2
4.55667936848393E-109	1.29272520838497	0.248	0.101	7.63608328570537E-105	Detoxification-associated hepat	SMYD4
4.80138592184981E-109	1.22803787846608	0.187	0.063	8.04616252783591E-105	Detoxification-associated hepat	PUS10
4.80322083416941E-109	0.620475368837565	0.383	0.188	8.0492374739011E-105	Detoxification-associated hepat	NAA15
5.44017923877135E-109	0.482766970453027	0.424	0.214	9.11665236833303E-105	Detoxification-associated hepat	PLCB1
5.80962975025336E-109	0.655780922000924	0.382	0.185	9.73577753547458E-105	Detoxification-associated hepat	CES3
7.65325114153083E-109	0.633901528344745	0.201	0.069	1.28253182629774E-104	Detoxification-associated hepat	CHAD
7.72671578293831E-109	0.313472823776137	0.777	0.452	1.2948430309048E-104	Detoxification-associated hepat	ADAR
8.29527894972041E-109	0.425660279046194	0.391	0.191	1.39012284639415E-104	Detoxification-associated hepat	FBXW4
1.04787220195223E-108	1.10141699669424	0.273	0.117	1.75602423603154E-104	Detoxification-associated hepat	TMEM260
1.06326108241938E-108	0.497765819252889	0.409	0.205	1.78181292191841E-104	Detoxification-associated hepat	ATL3
1.07980739443284E-108	0.388950928077941	0.45	0.233	1.80954123159055E-104	Detoxification-associated hepat	TMEM189
1.15850286186908E-108	0.937093958090136	0.263	0.109	1.9414190959202E-104	Detoxification-associated hepat	MTX3
1.26881524464657E-108	0.66263675143259	0.343	0.161	2.12628058697872E-104	Detoxification-associated hepat	TMEM170A
1.51245609985392E-108	0.300907202632039	0.754	0.443	2.5345739321352E-104	Detoxification-associated hepat	NECTIN2
2.38836303182005E-108	0.535598072138696	0.355	0.168	4.00241876872404E-104	Detoxification-associated hepat	KLHL12
2.90190551671221E-108	0.609569481920624	0.384	0.189	4.86301326490632E-104	Detoxification-associated hepat	ZNF655
3.25411720012093E-108	0.960148677884489	0.237	0.093	5.45324960396266E-104	Detoxification-associated hepat	ZBTB22
3.47093109301262E-108	0.400282649985452	0.414	0.208	5.81658632567055E-104	Detoxification-associated hepat	FBXW7
3.65820944147509E-108	1.08885205334198	0.218	0.081	6.13042738202396E-104	Detoxification-associated hepat	SNX33
3.98254006458525E-108	0.586569690973305	0.32	0.145	6.67394064023196E-104	Detoxification-associated hepat	RAI14
4.318383457456E-108	0.745944500523662	0.375	0.183	7.23674699800477E-104	Detoxification-associated hepat	GALNS
4.66696900428742E-108	0.283014719438111	0.399	0.192	7.82090665738487E-104	Detoxification-associated hepat	CYP7B1
6.12446975520506E-108	0.315782497834032	0.492	0.262	1.02633864157726E-103	Detoxification-associated hepat	EP300
7.04499144740836E-108	0.358220428926276	0.582	0.323	1.18059966675669E-103	Detoxification-associated hepat	TMEM41B
8.68905211257106E-108	1.09159209778496	0.221	0.084	1.45611135302466E-103	Detoxification-associated hepat	ZNF740
8.68908845611934E-108	0.326751130095754	0.503	0.266	1.45611744347648E-103	Detoxification-associated hepat	MYLK
1.27555732343593E-107	1.22491156646173	0.22	0.083	2.13757896261393E-103	Detoxification-associated hepat	ZNF460
1.33632131162937E-107	0.376399524022986	0.931	0.606	2.2394072540285E-103	Detoxification-associated hepat	HSD17B11
1.36188441552289E-107	0.316268100610555	0.363	0.168	2.28224590353326E-103	Detoxification-associated hepat	CYP2D6
1.5961778377235E-107	0.31981410470832	0.562	0.307	2.67487482045704E-103	Detoxification-associated hepat	PAK2
1.62343137557741E-107	0.30897734275767	0.614	0.331	2.72054629919262E-103	Detoxification-associated hepat	NID1
1.63405866183121E-107	0.527402364202573	0.353	0.168	2.73835550549674E-103	Detoxification-associated hepat	BZW1
2.03458910575201E-107	0.968893012557742	0.288	0.127	3.40956442341921E-103	Detoxification-associated hepat	BRAF
2.09351369664675E-107	0.87905175658888	0.293	0.13	3.50831025284062E-103	Detoxification-associated hepat	ZZEF1
2.14324143583353E-107	1.23939976395473	0.216	0.081	3.59164399816982E-103	Detoxification-associated hepat	CRYZL1
2.25432699255274E-107	0.423451880424153	0.444	0.23	3.77780117411988E-103	Detoxification-associated hepat	IFNAR2
2.32356065232622E-107	0.303192530656497	0.827	0.502	3.89382294116827E-103	Detoxification-associated hepat	TMEM205
2.39157851613992E-107	0.340149247425178	0.559	0.303	4.00780727734728E-103	Detoxification-associated hepat	MYO15B
2.77077915193718E-107	0.52284887139029	0.449	0.232	4.64327170281632E-103	Detoxification-associated hepat	SPIRE2
4.47881407908219E-107	0.47087274639226	0.386	0.189	7.50559663372593E-103	Detoxification-associated hepat	LEPROT
4.49537248280502E-107	0.92237822077373	0.275	0.118	7.53334520668465E-103	Detoxification-associated hepat	ZNF397
4.77552918991823E-107	0.331898117255075	0.591	0.328	8.00283181646497E-103	Detoxification-associated hepat	SGK2
5.11063214108691E-107	0.814441855373512	0.294	0.13	8.56439734203345E-103	Detoxification-associated hepat	CMTR2
6.00422118543189E-107	0.344367883256411	0.992	0.948	1.00618738625468E-102	Detoxification-associated hepat	TF
7.12665931913545E-107	0.365912661066255	0.452	0.23	1.19428556870072E-102	Detoxification-associated hepat	PNPLA3
7.2936587252681E-107	0.551543243204807	0.408	0.205	1.22227132918043E-102	Detoxification-associated hepat	CHIC2
8.89782550710577E-107	0.856259015001151	0.311	0.141	1.49109759848079E-102	Detoxification-associated hepat	MALT1
9.02242716617065E-107	0.307995033975767	0.661	0.376	1.51197834450688E-102	Detoxification-associated hepat	KANK1
9.54402443473772E-107	0.459582815597703	0.346	0.163	1.59938761477335E-102	Detoxification-associated hepat	SECISBP2L

9.76032741480504E-107	0.699785926049336	0.381	0.187	1.63563566817303E-102	Detoxification-associated hepat	LAMB1
9.7940100117551E-107	0.412894783858317	0.441	0.228	1.64128019776992E-102	Detoxification-associated hepat	NSL1
9.80190482534505E-107	0.289196250854014	0.594	0.326	1.64260321063132E-102	Detoxification-associated hepat	INSR
1.01248603099631E-106	0.431987433815228	0.432	0.223	1.69672409074362E-102	Detoxification-associated hepat	SON
1.08497976087095E-106	0.47659202206669	0.318	0.142	1.81820908326754E-102	Detoxification-associated hepat	ARIH1
1.20112209719373E-106	0.691448595763922	0.328	0.152	2.01284041047725E-102	Detoxification-associated hepat	CHD8
1.23154356488974E-106	0.985878842204433	0.293	0.131	2.06382070604223E-102	Detoxification-associated hepat	ARV1
1.31564366623517E-106	0.391451708719089	0.507	0.267	2.2047556558769E-102	Detoxification-associated hepat	NUP210
1.32694759712343E-106	0.405202749798126	0.488	0.259	2.22369878325944E-102	Detoxification-associated hepat	RBM14
1.34909513757949E-106	0.449321773742192	0.431	0.224	2.26081363155571E-102	Detoxification-associated hepat	RETREG3
1.57853684402268E-106	0.613628819689937	0.339	0.159	2.6453120432132E-102	Detoxification-associated hepat	HDAC8
1.66574846112244E-106	0.298524273084161	0.527	0.283	2.79146127114898E-102	Detoxification-associated hepat	TRIM25
1.92808360183388E-106	0.347749970911891	0.463	0.241	3.23108249995322E-102	Detoxification-associated hepat	PCMT1
2.04848015283627E-106	1.70020740465567	0.118	0.029	3.43284304012302E-102	Detoxification-associated hepat	EGF
2.17396728879361E-106	0.609166510039715	0.35	0.167	3.64313438256032E-102	Detoxification-associated hepat	NUP54
2.25803534725809E-106	1.02520373738398	0.245	0.099	3.7840156349351E-102	Detoxification-associated hepat	CAPRIN2
2.27596699782988E-106	0.724266925335901	0.35	0.168	3.8140654949633E-102	Detoxification-associated hepat	RIC8B
2.59527099070081E-106	0.325545097553981	0.466	0.244	4.34915512621641E-102	Detoxification-associated hepat	DNAJC5
2.84329696866379E-106	0.694709822763333	0.366	0.178	4.76479706008679E-102	Detoxification-associated hepat	CNOT10
3.12437839401747E-106	0.515983719345557	0.409	0.207	5.23583331269448E-102	Detoxification-associated hepat	SLC30A9
3.81383133354922E-106	0.513901740085489	0.363	0.175	6.39121854876178E-102	Detoxification-associated hepat	NDRG3
4.93433974104672E-106	0.485804161615066	0.358	0.172	8.26896653804609E-102	Detoxification-associated hepat	SEC22C
5.06707446527717E-106	0.353114735900881	0.478	0.252	8.49140338891149E-102	Detoxification-associated hepat	PHACTR4
5.08441317348635E-106	0.933109792917505	0.26	0.109	8.52045959612843E-102	Detoxification-associated hepat	VAMP4
5.4442020748583E-106	0.707958394338	0.306	0.138	9.12339383704754E-102	Detoxification-associated hepat	FBXW11
6.01137715786363E-106	0.279369614570503	0.578	0.315	1.00738658411479E-101	Detoxification-associated hepat	PRKAA1
6.03798600918313E-106	0.276876746045661	0.484	0.256	1.01184569541891E-101	Detoxification-associated hepat	CYTH1
8.21023201946211E-106	0.266315556398332	0.661	0.376	1.37587068182146E-101	Detoxification-associated hepat	RERE
9.09332995964592E-106	1.00099817829951	0.259	0.108	1.52386023463746E-101	Detoxification-associated hepat	ATG10
1.15834344770556E-105	0.423055929748346	0.393	0.195	1.94115194966499E-101	Detoxification-associated hepat	PBX3
1.27769553848177E-105	0.823345194095247	0.194	0.067	2.14116218338776E-101	Detoxification-associated hepat	C9orf72
1.46548084186621E-105	0.498990854295294	0.377	0.186	2.45585279479939E-101	Detoxification-associated hepat	RNF14
1.60334695529636E-105	0.681553116883398	0.346	0.164	2.68688882768564E-101	Detoxification-associated hepat	NDEL1
1.97247177956085E-105	0.271185806098591	0.675	0.385	3.30546820818808E-101	Detoxification-associated hepat	DCAF11
2.41072053354793E-105	0.337199551256875	0.484	0.256	4.03988547011962E-101	Detoxification-associated hepat	GAK
2.69842635090799E-105	0.252426309692184	0.66	0.376	4.52202287885161E-101	Detoxification-associated hepat	TMEM259
2.75274879358043E-105	0.826392750563431	0.272	0.116	4.61305642828208E-101	Detoxification-associated hepat	HELZ2
2.80154148515395E-105	0.316893332478709	0.397	0.197	4.69482322082099E-101	Detoxification-associated hepat	FBXO31
2.84120266712427E-105	0.713697119462798	0.317	0.146	4.76128742956685E-101	Detoxification-associated hepat	RIC1
3.59830138892023E-105	0.902811848881249	0.191	0.066	6.03003346755252E-101	Detoxification-associated hepat	ASB7
3.93153201438091E-105	0.516821879761022	0.411	0.21	6.58846134969952E-101	Detoxification-associated hepat	NUP153
4.02464240910092E-105	0.411987315201138	0.432	0.222	6.74449574917132E-101	Detoxification-associated hepat	TYK2
4.15811493098345E-105	0.276391748759759	0.603	0.337	6.96816900134207E-101	Detoxification-associated hepat	KTN1
4.38427621307072E-105	0.574767154113137	0.354	0.171	7.34717007786391E-101	Detoxification-associated hepat	ITGB1BP1
4.55225471613349E-105	0.826974189392469	0.274	0.118	7.6286684532965E-101	Detoxification-associated hepat	GABPA
4.74996036760316E-105	0.48064494813116	0.404	0.203	7.95998358402938E-101	Detoxification-associated hepat	SELENOI
5.1018045193671E-105	0.605642877974333	0.316	0.144	8.54960401355539E-101	Detoxification-associated hepat	ATRX
5.22458402603626E-105	0.259661263407272	0.695	0.393	8.75535791083156E-101	Detoxification-associated hepat	HNRNPA3
6.28458595666286E-105	0.580998458541374	0.363	0.176	1.05317091461756E-100	Detoxification-associated hepat	NIPA1
7.1973615417791E-105	0.849862624436593	0.276	0.12	1.20613384717134E-100	Detoxification-associated hepat	RHOQ
7.23701807088243E-105	0.869594764407759	0.256	0.107	1.21277948831848E-100	Detoxification-associated hepat	WDR27
7.78933869554833E-105	0.850926114297334	0.272	0.117	1.30533737859999E-100	Detoxification-associated hepat	CCNH
8.06180097848976E-105	1.47138305297389	0.133	0.036	1.35099660797531E-100	Detoxification-associated hepat	TRIM71
8.98514172594312E-105	0.997447724515882	0.218	0.082	1.50573005043355E-100	Detoxification-associated hepat	WHAMM
9.3087944904029E-105	0.742923165179195	0.331	0.156	1.55996778070172E-100	Detoxification-associated hepat	GUCY1B1
1.07222599479657E-104	0.367873938271482	0.469	0.247	1.7968363220801E-100	Detoxification-associated hepat	TIMMDC1
1.09968232613238E-104	0.289518409937006	0.594	0.329	1.84284764213264E-100	Detoxification-associated hepat	LMBRD1
1.28045081378494E-104	0.666340697055872	0.314	0.145	2.1457794737408E-100	Detoxification-associated hepat	ZNF445
1.6315940351786E-104	0.602167764818879	0.282	0.121	2.73422528415229E-100	Detoxification-associated hepat	ZRANB1
1.88186699349918E-104	0.380274567024499	0.509	0.262	3.15363270770593E-100	Detoxification-associated hepat	MGAM2

1.89819554764835E-104	1.02027318664729	0.224	0.087	3.18099609874911E-100	Detoxification-associated hepat	ICE2
1.96431282930162E-104	0.379373137644481	0.443	0.227	3.29179543934365E-100	Detoxification-associated hepat	FADS1
2.13661451283616E-104	0.449031435441725	0.465	0.246	3.58053860061084E-100	Detoxification-associated hepat	FAM49B
2.15193398172863E-104	0.481936158056214	0.321	0.146	3.60621096658084E-100	Detoxification-associated hepat	BCHE
2.278609310927E-104	0.801884051293367	0.3	0.135	3.81849348325146E-100	Detoxification-associated hepat	SCYL3
2.45632142576959E-104	1.00703703005216	0.225	0.087	4.11630344530468E-100	Detoxification-associated hepat	MEGF8
2.65280566606306E-104	0.413221487028325	0.441	0.228	4.44557173518848E-100	Detoxification-associated hepat	PMM2
2.96496301216248E-104	0.425710765334488	0.443	0.231	4.96868501578189E-100	Detoxification-associated hepat	GMPS
3.41645386549093E-104	0.685634421250414	0.322	0.15	5.7252933877897E-100	Detoxification-associated hepat	IPP
5.12047517432595E-104	0.280929930159105	0.539	0.293	8.58089229713543E-100	Detoxification-associated hepat	ITPRID2
5.22542986496248E-104	0.834287583881605	0.289	0.129	8.75677536770412E-100	Detoxification-associated hepat	SLC22A5
5.2788542694418E-104	0.965581885267367	0.161	0.05	8.84630398473056E-100	Detoxification-associated hepat	SLC35G2
6.17502849785702E-104	0.601132493750336	0.365	0.179	1.03481127567088E-99	Detoxification-associated hepat	MOSPD2
6.73733950743544E-104	0.389960582539053	0.389	0.193	1.12904335465603E-99	Detoxification-associated hepat	FOXN3
8.14137942274159E-104	0.307122133861841	0.679	0.378	1.36433236366303E-99	Detoxification-associated hepat	ERBIN
1.08810443691938E-103	0.885423364110136	0.274	0.119	1.8234454153895E-99	Detoxification-associated hepat	RASA1
1.27893254268116E-103	0.522987507416754	0.339	0.16	2.14323515502508E-99	Detoxification-associated hepat	MED1
1.35462225520813E-103	0.523994275700425	0.393	0.199	2.27007597527779E-99	Detoxification-associated hepat	TMEM220
1.71877334714682E-103	0.263359808578235	0.649	0.358	2.88032037514864E-99	Detoxification-associated hepat	NUCB2
1.72361516244403E-103	0.608221986503912	0.355	0.173	2.88843428922371E-99	Detoxification-associated hepat	USF3
1.79478042673602E-103	0.788310344577722	0.291	0.131	3.00769303912423E-99	Detoxification-associated hepat	PPIL4
1.8202370059979E-103	0.431668078248093	0.452	0.239	3.05035317465129E-99	Detoxification-associated hepat	MTA1
1.92152956187386E-103	0.265339049072298	0.455	0.24	3.22009923978821E-99	Detoxification-associated hepat	ARHGEF7
1.94621435034974E-103	1.40034922026943	0.197	0.072	3.2614660083161E-99	Detoxification-associated hepat	PLAGL2
2.31943559404864E-103	0.721061804539604	0.321	0.149	3.8869101685067E-99	Detoxification-associated hepat	KLHL42
2.49516199749842E-103	0.49655956763646	0.383	0.192	4.18139247540785E-99	Detoxification-associated hepat	TNRC6B
2.66253921711732E-103	0.313319306964133	0.514	0.28	4.4618832200452E-99	Detoxification-associated hepat	LGMN
3.16439265759567E-103	0.304289742490329	0.539	0.291	5.30288921559883E-99	Detoxification-associated hepat	NCOR1
3.27590705341014E-103	1.03476451780721	0.203	0.074	5.48976504010471E-99	Detoxification-associated hepat	N4BP2
3.36897535843636E-103	0.404997509383274	0.472	0.25	5.64572890566764E-99	Detoxification-associated hepat	SPPL2B
3.49291542432487E-103	1.44599729835619	0.154	0.047	5.85342766808361E-99	Detoxification-associated hepat	TRIM13
3.8034594477872E-103	0.345172882041891	0.399	0.2	6.3738373426018E-99	Detoxification-associated hepat	CASC4
4.62895443512266E-103	0.529011577062245	0.359	0.175	7.75720184237856E-99	Detoxification-associated hepat	ABCD4
6.20214881277988E-103	0.476962413695502	0.339	0.16	1.03935609804565E-98	Detoxification-associated hepat	FBXO3
7.31425682830363E-103	0.403527408087066	0.503	0.265	1.22572315928712E-98	Detoxification-associated hepat	CRACR2B
7.40074014204966E-103	0.373560504992134	0.445	0.233	1.24021603300468E-98	Detoxification-associated hepat	DCUN1D1
8.1011670829373E-103	0.884657511986829	0.289	0.129	1.35759357975863E-98	Detoxification-associated hepat	PRMT3
8.21884141624301E-103	0.756057149495696	0.287	0.128	1.377313444534E-98	Detoxification-associated hepat	UBE2K
9.19882789243935E-103	0.706407161367349	0.314	0.146	1.54153957821499E-98	Detoxification-associated hepat	AKAP11
1.15475496615642E-102	0.257878764880303	0.599	0.335	1.93513837228493E-98	Detoxification-associated hepat	GNAQ
1.20817486767712E-102	0.32379526617143	0.417	0.212	2.02465944325332E-98	Detoxification-associated hepat	NUDT3
1.3837757760556E-102	0.251128609744675	0.508	0.274	2.31893144551397E-98	Detoxification-associated hepat	CLIP1
1.45197336388358E-102	0.575674527686447	0.286	0.125	2.4332169631961E-98	Detoxification-associated hepat	SPRYD7
1.88682079627807E-102	0.651812441443351	0.325	0.153	3.16193429040278E-98	Detoxification-associated hepat	PEX6
2.00464835771917E-102	0.638648027081343	0.347	0.169	3.35938971786579E-98	Detoxification-associated hepat	PHF11
2.22283461957553E-102	1.43476832140175	0.166	0.054	3.72502625548468E-98	Detoxification-associated hepat	CARF
2.36595697510051E-102	0.279733748044547	0.754	0.439	3.96487069887344E-98	Detoxification-associated hepat	PDIA5
3.15443992616284E-102	0.329922194096111	0.548	0.301	5.28621042826369E-98	Detoxification-associated hepat	UBE3C
3.42024910148701E-102	0.311385780695714	0.557	0.309	5.73165344427194E-98	Detoxification-associated hepat	EGFR
3.50688315610669E-102	0.505570410730856	0.396	0.201	5.87683479300359E-98	Detoxification-associated hepat	DYRK2
3.62762247199739E-102	0.441193615983994	0.402	0.203	6.07916973857322E-98	Detoxification-associated hepat	ATP6V1H
3.85522748331745E-102	0.95801726948221	0.246	0.101	6.46059021654338E-98	Detoxification-associated hepat	STRADB
4.11424913897261E-102	0.713569125393704	0.299	0.136	6.8946587070903E-98	Detoxification-associated hepat	MBTPS2
4.29874773938677E-102	0.519359803757738	0.331	0.156	7.20384146166434E-98	Detoxification-associated hepat	PSKH1
5.33804066328626E-102	0.312840532024408	0.682	0.386	8.94548854353511E-98	Detoxification-associated hepat	BIRC3
7.21863851598126E-102	0.872111447620435	0.28	0.123	1.20969944250814E-97	Detoxification-associated hepat	TMEM187
7.47045599488027E-102	0.598673438730108	0.308	0.141	1.25189901562204E-97	Detoxification-associated hepat	NPRL3
7.49578391877701E-102	0.63073326444967	0.338	0.162	1.25614346910865E-97	Detoxification-associated hepat	ZHX3
8.1109865429294E-102	0.470838988612325	0.373	0.185	1.35923912486411E-97	Detoxification-associated hepat	OSTC
8.34339518841736E-102	0.467905943821687	0.35	0.168	1.39818616567498E-97	Detoxification-associated hepat	CLN6

**8.35432330813063E-102**	0.742864957615571	0.301	0.137	1.40001749997653E-97	Detoxification-associated hepat	CNTLN
**9.09761498499409E-102**	0.552688334442073	0.374	0.186	1.52457831918531E-97	Detoxification-associated hepat	GART
**9.61290860703141E-102**	0.665123484190765	0.301	0.137	1.61093122436632E-97	Detoxification-associated hepat	HERC2
**9.6360871302602E-102**	0.55407210545105	0.355	0.173	1.614815481289E-97	Detoxification-associated hepat	RBM4
**1.04154276369094E-101**	0.318876220157388	0.4	0.202	1.74541736339327E-97	Detoxification-associated hepat	SCPEP1
**1.08631831705621E-101**	0.473936967654285	0.377	0.187	1.82045223572279E-97	Detoxification-associated hepat	NMI
**1.19130755310461E-101**	0.72656399067833	0.161	0.05	1.9963931974927E-97	Detoxification-associated hepat	TMEM86B
**1.23001129774562E-101**	0.25461578321163	0.543	0.295	2.06125293276211E-97	Detoxification-associated hepat	PSMB5
**1.34136659756538E-101**	1.05081636644212	0.25	0.105	2.24786214420007E-97	Detoxification-associated hepat	DEPRECATEI
**1.84684555890183E-101**	0.448404168255038	0.503	0.278	3.09494378760769E-97	Detoxification-associated hepat	IFI6
**1.93956997068537E-101**	0.60345070678337	0.409	0.21	3.25033135687455E-97	Detoxification-associated hepat	ZFYVE28
**2.31221797437718E-101**	0.345175747581677	0.429	0.218	3.87481488146128E-97	Detoxification-associated hepat	MLIP
**2.39308342297358E-101**	1.28853296812134	0.199	0.074	4.01032920021912E-97	Detoxification-associated hepat	PRMT9
**2.62142309685277E-101**	0.587603357288689	0.309	0.142	4.39298082570587E-97	Detoxification-associated hepat	MKNK1
**2.77638760122176E-101**	1.01943199667439	0.261	0.112	4.65267034212743E-97	Detoxification-associated hepat	CLASP2
**2.80786409260493E-101**	0.575339796584671	0.352	0.173	4.70541864638734E-97	Detoxification-associated hepat	NUP160
**3.0142778055724E-101**	0.299373433081952	0.531	0.288	5.05132674657824E-97	Detoxification-associated hepat	SIPA1L3
**3.1422312033825E-101**	0.259465541010701	0.727	0.419	5.2657510506284E-97	Detoxification-associated hepat	CPQ
**3.5974578845064E-101**	0.606230448426633	0.332	0.158	6.02861992285583E-97	Detoxification-associated hepat	ANKS1A
**3.86123073827726E-101**	0.472043137219768	0.405	0.207	6.47065047120504E-97	Detoxification-associated hepat	LARP7
**4.31575815351513E-101**	0.303145089863486	0.424	0.218	7.23234751366065E-97	Detoxification-associated hepat	FEM1C
**4.34556899895095E-101**	0.37444367326945	0.422	0.219	7.282304528442E-97	Detoxification-associated hepat	GCN1
**4.91746921161392E-101**	0.812460176128488	0.241	0.099	8.24069490482261E-97	Detoxification-associated hepat	ZFP90
**4.99330078024719E-101**	1.68304243854887	0.122	0.032	8.36777344753823E-97	Detoxification-associated hepat	C17orf113
**5.96916176449078E-101**	0.328397996883951	0.531	0.291	1.00031212849336E-96	Detoxification-associated hepat	MAX
**6.13353639795444E-101**	0.54774105193048	0.389	0.196	1.0278580295692E-96	Detoxification-associated hepat	GNL3L
**6.23411312108954E-101**	0.303325677147417	0.481	0.256	1.04471267683218E-96	Detoxification-associated hepat	ATP2C1
**6.25060705027472E-101**	0.668519437186136	0.261	0.11	1.04747672948504E-96	Detoxification-associated hepat	AGTR1
**6.48104964094671E-101**	0.602357009053883	0.322	0.151	1.08609429882985E-96	Detoxification-associated hepat	ERGIC2
**6.60874936883733E-101**	1.49062213678651	0.188	0.067	1.10749421922976E-96	Detoxification-associated hepat	CCDC171
**7.15584566978704E-101**	1.08712835887672	0.273	0.121	1.19917661734291E-96	Detoxification-associated hepat	CDC7
**7.2080315802383E-101**	0.637050494357056	0.322	0.152	1.20792193221633E-96	Detoxification-associated hepat	STOX2
**7.69573565334027E-101**	0.527273786673428	0.392	0.199	1.28965138078676E-96	Detoxification-associated hepat	PAWR
**8.18529237070002E-101**	0.537540908688312	0.331	0.157	1.37169129548191E-96	Detoxification-associated hepat	DENR
**8.6256847769994E-101**	0.512812451962829	0.385	0.195	1.44549225492956E-96	Detoxification-associated hepat	SNX14
**9.3657118707661E-101**	0.364216988447703	0.488	0.259	1.56950599530298E-96	Detoxification-associated hepat	MMGT1
**9.76657642662172E-101**	0.704415334348437	0.307	0.142	1.63668287757327E-96	Detoxification-associated hepat	PIGF
**9.89890037023024E-101**	0.8994749477403	0.244	0.101	1.65885772404318E-96	Detoxification-associated hepat	FAM76A
**9.98904059094979E-101**	0.848152094184071	0.289	0.13	1.67396342223137E-96	Detoxification-associated hepat	AP3M2
**1.0307517310877E-100**	0.795878501148895	0.283	0.126	1.72733375095677E-96	Detoxification-associated hepat	HCFC2
**1.03319887940825E-100**	0.310325256251163	0.768	0.448	1.73143468211235E-96	Detoxification-associated hepat	IL13RA1
**1.35400819339677E-100**	0.300916766003557	0.45	0.235	2.26904693049431E-96	Detoxification-associated hepat	SPAG9
**1.41933854293353E-100**	0.787330035883974	0.292	0.133	2.37852753024801E-96	Detoxification-associated hepat	DNAJB14
**2.26573653308708E-100**	0.654306595963622	0.262	0.109	3.79692128214732E-96	Detoxification-associated hepat	SLC22A1
**2.9664566256804E-100**	0.353134837868182	0.928	0.67	4.97118801331522E-96	Detoxification-associated hepat	HSD17B4
**3.07136433083416E-100**	0.525017375576286	0.333	0.158	5.14699234561188E-96	Detoxification-associated hepat	FGFR1OP2
**3.5318655370737E-100**	1.37722991057615	0.207	0.079	5.9187002670281E-96	Detoxification-associated hepat	DEPRECATEI
**5.10964006702011E-100**	0.651110225082595	0.306	0.142	8.56273482431231E-96	Detoxification-associated hepat	YAF2
**5.65015097685094E-100**	0.671217501789079	0.292	0.132	9.4685230070068E-96	Detoxification-associated hepat	USP3
**5.77666253610575E-100**	0.266825390836135	0.455	0.24	9.68053107800601E-96	Detoxification-associated hepat	IFNAR1
**5.89217754728371E-100**	0.716408549470688	0.284	0.127	9.87411113373804E-96	Detoxification-associated hepat	KIAA0355
**6.34763345656389E-100**	0.61382842074915	0.363	0.18	1.06373641465098E-95	Detoxification-associated hepat	TFCP2
**7.13793506932563E-100**	0.274835084672073	0.529	0.29	1.19617515891759E-95	Detoxification-associated hepat	CDC42BPB
**9.09920774960455E-100**	0.605061462133836	0.325	0.155	1.52484523467873E-95	Detoxification-associated hepat	MTPAP
**9.33484438879048E-100**	0.839046325029405	0.303	0.141	1.56433322267351E-95	Detoxification-associated hepat	CEP192
**1.02521338636144E-99**	0.251675505988986	0.391	0.197	1.7180525928645E-95	Detoxification-associated hepat	PHF8
**1.57539950423967E-99**	0.663835891563078	0.339	0.165	2.64005448920484E-95	Detoxification-associated hepat	RAD50
**1.60802320257452E-99**	0.358949358510132	0.487	0.261	2.69472528287438E-95	Detoxification-associated hepat	MED25
**1.87380770234914E-99**	0.531586383235112	0.303	0.139	3.14012694759669E-95	Detoxification-associated hepat	SIKE1
**1.89621551053104E-99**	0.737474784764023	0.183	0.064	3.17767795254791E-95	Detoxification-associated hepat	MS4A6A

2.04408824164971E-99	0.298839621026493	0.74	0.436	3.42548307535659E-95	Detoxification-associated hepat	RSRP1
2.42638613073563E-99	0.5630363280626	0.334	0.161	4.06613787788676E-95	Detoxification-associated hepat	LATS1
2.44785698502238E-99	0.500066275995221	0.324	0.152	4.10211873550051E-95	Detoxification-associated hepat	MAIP1
3.2382453673674E-99	0.596433072986855	0.332	0.159	5.42665158663429E-95	Detoxification-associated hepat	PAXBP1
3.4358121941143E-99	0.925607251342436	0.218	0.085	5.75773407489675E-95	Detoxification-associated hepat	CELSR1
3.45963716055283E-99	0.450553193150802	0.491	0.269	5.79765995365442E-95	Detoxification-associated hepat	LIME1
3.86068171094653E-99	1.12191487760065	0.197	0.073	6.46973041120419E-95	Detoxification-associated hepat	C1QTNF3
3.89900686162649E-99	0.349404399143913	0.52	0.285	6.53395569871367E-95	Detoxification-associated hepat	PSPC1
4.10159909509796E-99	0.425136169286386	0.4	0.204	6.87345976356516E-95	Detoxification-associated hepat	DGCR2
4.42614559190028E-99	1.40869273162875	0.162	0.053	7.41733478290649E-95	Detoxification-associated hepat	TATDN1
4.90916278834406E-99	0.587313829992864	0.363	0.18	8.22677500070697E-95	Detoxification-associated hepat	SUSD4
4.94870957957939E-99	0.562461947615462	0.348	0.171	8.29304751345914E-95	Detoxification-associated hepat	CCNT2
5.78638554413919E-99	0.364006660741441	0.429	0.224	9.69682489486846E-95	Detoxification-associated hepat	FXR1
6.22456931476777E-99	1.55843734910861	0.172	0.059	1.04311332576878E-94	Detoxification-associated hepat	ZNF302
6.44924948911558E-99	0.37369203715525	0.341	0.163	1.08076522938599E-94	Detoxification-associated hepat	GNE
6.53361563566545E-99	0.258647116997983	0.598	0.336	1.09490330822482E-94	Detoxification-associated hepat	ATP2A2
7.07085388408731E-99	0.701216192210852	0.271	0.118	1.18493369389535E-94	Detoxification-associated hepat	FOXO1
7.31188316404616E-99	0.57924251853857	0.35	0.17	1.22532538063085E-94	Detoxification-associated hepat	KDM5B
7.72673919834963E-99	1.12417381149206	0.214	0.083	1.29484695485943E-94	Detoxification-associated hepat	MYO9A
8.33508273605857E-99	0.407607569443577	0.38	0.19	1.3967931649087E-94	Detoxification-associated hepat	TBC1D16
8.44548640311856E-99	0.403833851990299	0.392	0.2	1.41529461143461E-94	Detoxification-associated hepat	CERT1
8.78496833764904E-99	0.347132435877741	0.449	0.239	1.47218499402323E-94	Detoxification-associated hepat	ADAM10
9.19503193684342E-99	0.31336280429231	0.835	0.529	1.54090345197622E-94	Detoxification-associated hepat	NFE2L1
9.50046683778681E-99	0.523177690918547	0.317	0.149	1.59208823267631E-94	Detoxification-associated hepat	TSC2
1.03781623069365E-98	0.457889951920572	0.412	0.214	1.73917243939642E-94	Detoxification-associated hepat	LARP4B
1.06789420254961E-98	0.824228487019851	0.289	0.132	1.78957710463264E-94	Detoxification-associated hepat	ATR
1.84582828981719E-98	0.543676253435802	0.43	0.224	3.09323904807564E-94	Detoxification-associated hepat	STXBP5
1.8562264920028E-98	0.299831412938263	0.524	0.287	3.11066435529829E-94	Detoxification-associated hepat	PDCD6
2.04413853768084E-98	0.261757597367717	0.598	0.336	3.42556736144555E-94	Detoxification-associated hepat	NDUFS1
2.14290091405705E-98	0.356050433238757	0.43	0.226	3.59107335177681E-94	Detoxification-associated hepat	ZMYND11
2.25963398218081E-98	0.666178527771221	0.323	0.154	3.7866946273386E-94	Detoxification-associated hepat	PIK3R4
2.6695185409323E-98	0.543296035898234	0.353	0.172	4.47357917089434E-94	Detoxification-associated hepat	ACAP3
3.13016869829693E-98	0.467547809015803	0.346	0.167	5.245536704606E-94	Detoxification-associated hepat	PUS1
3.53978685560368E-98	0.275824445306518	0.75	0.448	5.93197481262065E-94	Detoxification-associated hepat	SCARB2
3.71621426452372E-98	0.494517613444114	0.372	0.189	6.22763186448885E-94	Detoxification-associated hepat	TASOR
4.11274743744867E-98	0.259131376189931	0.536	0.295	6.89214215567649E-94	Detoxification-associated hepat	PPP4R3B
4.57074024056317E-98	0.290435170592203	0.925	0.566	7.65964649513576E-94	Detoxification-associated hepat	NDRG1
4.69109696782779E-98	0.667831550666471	0.3	0.138	7.86134029868581E-94	Detoxification-associated hepat	MTMR6
4.8756948189268E-98	0.400140791773494	0.415	0.215	8.17068937755754E-94	Detoxification-associated hepat	SNX27
4.98996256615071E-98	0.793781623003761	0.298	0.139	8.36217926835537E-94	Detoxification-associated hepat	SOCS4
5.28045432898644E-98	0.344485576773209	0.564	0.316	8.84898536451547E-94	Detoxification-associated hepat	ECE1
5.92109204206127E-98	1.0721065353443	0.285	0.131	9.92256604408627E-94	Detoxification-associated hepat	FSD1L
6.66769196308749E-98	0.275321498245246	0.445	0.234	1.1173718191742E-93	Detoxification-associated hepat	NFKB1
7.05824243510715E-98	0.683607705280387	0.317	0.15	1.18282026727526E-93	Detoxification-associated hepat	EXOC6
7.08969321623376E-98	0.509197628623136	0.363	0.18	1.18809078917645E-93	Detoxification-associated hepat	MDC 1.00
7.82023529605766E-98	0.730793268641056	0.283	0.128	1.31051503091334E-93	Detoxification-associated hepat	MED20
8.96619763353002E-98	0.295486939256586	0.496	0.27	1.50255539942696E-93	Detoxification-associated hepat	USP14
1.00400348445967E-97	0.397818987781542	0.554	0.308	1.68250903925752E-93	Detoxification-associated hepat	PFKFB3
1.06155790165051E-97	1.03761396356352	0.214	0.084	1.77895873158593E-93	Detoxification-associated hepat	FKTN
1.32720992333519E-97	1.1345585304317	0.193	0.071	2.22413838952511E-93	Detoxification-associated hepat	C17orf80
1.35853743483904E-97	0.735936514846926	0.304	0.142	2.27663703330327E-93	Detoxification-associated hepat	RMI1
1.46003588945213E-97	0.668098116173498	0.328	0.158	2.44672814354389E-93	Detoxification-associated hepat	ANAPC7
1.50114708085382E-97	0.711640221169258	0.265	0.115	2.51562227809483E-93	Detoxification-associated hepat	BBS2
1.65215899308244E-97	0.318500723521295	0.476	0.255	2.76868804060755E-93	Detoxification-associated hepat	TCERG1
1.92273453796955E-97	0.438075186797938	0.36	0.179	3.22211853872937E-93	Detoxification-associated hepat	DHRSX
2.05212623201266E-97	0.638379250997739	0.338	0.165	3.43895313960681E-93	Detoxification-associated hepat	FNBP1L
2.44905082124139E-97	0.986476752035313	0.21	0.081	4.10411936623633E-93	Detoxification-associated hepat	TTC32
2.59278098532341E-97	0.51217856011911	0.365	0.184	4.34498237520498E-93	Detoxification-associated hepat	AAK1
2.6753522790802E-97	0.661824529804042	0.323	0.155	4.4833553492826E-93	Detoxification-associated hepat	KLHL8
2.86741679791192E-97	0.634634156543043	0.27	0.118	4.80521706994079E-93	Detoxification-associated hepat	WDR45

2.90429462744445E-97	0.754833724730458	0.253	0.107	4.8670169366714E-93	Detoxification-associated hepat	XRRA1
2.96203694090877E-97	0.269571652346237	0.49	0.265	4.96378150557492E-93	Detoxification-associated hepat	EPRS
3.16652384097666E-97	0.436070350911072	0.355	0.174	5.30646065270869E-93	Detoxification-associated hepat	BRD8
3.17102265568001E-97	0.456714035554723	0.352	0.173	5.31399976638856E-93	Detoxification-associated hepat	CDC23
3.41829304149627E-97	0.313020844496026	0.85	0.517	5.72837547893945E-93	Detoxification-associated hepat	IMMP2L
4.32726840206887E-97	0.29051477663961	0.468	0.252	7.25163638818701E-93	Detoxification-associated hepat	KCTD20
4.52367824372458E-97	0.584277619828305	0.342	0.168	7.58078000083366E-93	Detoxification-associated hepat	ZADH2
4.64065661317458E-97	0.502196776775625	0.356	0.176	7.77681235235795E-93	Detoxification-associated hepat	CEBPZ
5.06443764584608E-97	0.397934682179633	0.368	0.184	8.48698460690887E-93	Detoxification-associated hepat	PRPF38A
6.6145058837916E-97	0.76186304377244	0.297	0.138	1.1084588960058E-92	Detoxification-associated hepat	CLEC16A
7.01960306419993E-97	0.965958332738359	0.232	0.096	1.17634508149862E-92	Detoxification-associated hepat	RALGAPA1
8.07993437741283E-97	0.804804630880794	0.275	0.123	1.35403540296684E-92	Detoxification-associated hepat	XYLT2
8.78995068986498E-97	0.843087533828352	0.264	0.116	1.47301993660757E-92	Detoxification-associated hepat	UIMC1
9.13629930977069E-97	0.589376427980706	0.32	0.152	1.53106103833137E-92	Detoxification-associated hepat	SH2B1
1.11453375991991E-96	1.32551731440045	0.19	0.07	1.86773567487379E-92	Detoxification-associated hepat	POGLUT1
1.12954265791478E-96	0.494010074636607	0.368	0.185	1.8928875861336E-92	Detoxification-associated hepat	DRAM2
1.16485084302521E-96	0.466133413868016	0.298	0.136	1.95205704274165E-92	Detoxification-associated hepat	MORF4L1
1.49916158635716E-96	0.450117562546803	0.409	0.215	2.51229498641733E-92	Detoxification-associated hepat	PRPF4B
1.56826145698698E-96	0.697013791235207	0.287	0.13	2.62809254961877E-92	Detoxification-associated hepat	FEM1B
1.57851377412317E-96	0.389289306767655	0.451	0.234	2.64527338267561E-92	Detoxification-associated hepat	CCL28
1.67722018723476E-96	0.274924441698813	0.543	0.3	2.81068558976801E-92	Detoxification-associated hepat	PRRC2C
1.67948098048483E-96	0.640149588965091	0.32	0.152	2.81447422709647E-92	Detoxification-associated hepat	ACVR1
2.08207493719804E-96	0.265002492888679	0.474	0.255	3.48914117975647E-92	Detoxification-associated hepat	MMAB
2.09762748811221E-96	0.334604381206344	0.39	0.198	3.51520414457844E-92	Detoxification-associated hepat	GATD3B
2.40066815874622E-96	0.331703129385889	0.438	0.231	4.02303970042691E-92	Detoxification-associated hepat	ERLEC1
2.53985238982488E-96	0.503639071614865	0.362	0.181	4.25628463486854E-92	Detoxification-associated hepat	SPCS3
2.73584059718266E-96	0.351690801498713	0.469	0.25	4.5847216727587E-92	Detoxification-associated hepat	SUPT16H
2.80600616197485E-96	0.285860090753759	0.426	0.224	4.70230512623745E-92	Detoxification-associated hepat	GOLGA3
2.86754647510941E-96	0.303503771991628	0.496	0.269	4.80543438298835E-92	Detoxification-associated hepat	MARVELD2
2.95090797548316E-96	0.630268249586679	0.288	0.131	4.94513158531469E-92	Detoxification-associated hepat	MAPK8
2.95425309753543E-96	0.641478695352148	0.339	0.167	4.95073734084987E-92	Detoxification-associated hepat	PSMG4
3.13102204609178E-96	0.411162587704179	0.384	0.195	5.2469667448406E-92	Detoxification-associated hepat	ITSN2
3.2333145884991E-96	0.517411873287231	0.377	0.191	5.4183885874068E-92	Detoxification-associated hepat	ZCCHC2
3.34059973510093E-96	0.471650347893182	0.366	0.183	5.59817703608213E-92	Detoxification-associated hepat	CREM
3.41145907572459E-96	0.44517006253305	0.438	0.234	5.71692311909927E-92	Detoxification-associated hepat	HUS1
4.24004932932081E-96	0.624829849208401	0.343	0.169	7.10547466607581E-92	Detoxification-associated hepat	RSPRY1
4.70823030855811E-96	0.274967721330671	0.51	0.28	7.89005235108168E-92	Detoxification-associated hepat	NR5A2
5.15455597797996E-96	0.360014598021645	0.448	0.239	8.63800490789882E-92	Detoxification-associated hepat	IFNGR1
5.22247856719889E-96	0.964555432735474	0.228	0.092	8.7518295829119E-92	Detoxification-associated hepat	INVS
6.16264954815443E-96	0.297934034614	0.772	0.465	1.03273681127972E-91	Detoxification-associated hepat	GCLC
7.72966180275478E-96	0.2793079946552	0.575	0.311	1.29533672490565E-91	Detoxification-associated hepat	RNF157
8.06423150576488E-96	1.0067643858417	0.199	0.075	1.35140391573608E-91	Detoxification-associated hepat	WDR25
8.48493780664425E-96	0.29531236786022	0.402	0.206	1.42190587763744E-91	Detoxification-associated hepat	GMPPA
1.03078930494624E-95	0.542352746339291	0.297	0.137	1.72739671722891E-91	Detoxification-associated hepat	KLHL20
1.04156870274996E-95	0.626776321085254	0.289	0.132	1.74546083206838E-91	Detoxification-associated hepat	ZDHHC16
1.07532590422881E-95	0.906027468337672	0.24	0.1	1.80203115030664E-91	Detoxification-associated hepat	EFCAB2
1.10432441400206E-95	0.539924475768743	0.324	0.155	1.85062685298465E-91	Detoxification-associated hepat	DEPRECATEI
1.116874653971E-95	0.451085967842994	0.334	0.161	1.87165854512461E-91	Detoxification-associated hepat	DENND4B
1.19561054493639E-95	0.354217743257447	0.368	0.183	2.0036041512044E-91	Detoxification-associated hepat	EXOSC7
1.37161917636533E-95	0.259772957184842	0.85	0.549	2.29855941575303E-91	Detoxification-associated hepat	CPB2
1.38506239013413E-95	0.362992442951926	0.425	0.223	2.32108755338677E-91	Detoxification-associated hepat	LAMC1
1.42335797063207E-95	1.82108156444274	0.129	0.037	2.38526328718523E-91	Detoxification-associated hepat	KANTR
1.59702800073174E-95	1.10649865744941	0.181	0.064	2.67629952362625E-91	Detoxification-associated hepat	PROKR1
1.60022252622321E-95	0.852657881772733	0.273	0.123	2.68165290944485E-91	Detoxification-associated hepat	ZNF263
1.68799334084509E-95	0.294673663614711	0.465	0.25	2.82873924058821E-91	Detoxification-associated hepat	PCGF2
1.71363289146678E-95	0.54075651630525	0.302	0.14	2.87170599952003E-91	Detoxification-associated hepat	INPP5E
1.90963176861276E-95	0.401094822357175	0.387	0.199	3.20016091784127E-91	Detoxification-associated hepat	DLST
1.9211013163055E-95	0.57163450223197	0.295	0.136	3.21938158586476E-91	Detoxification-associated hepat	RNF146
1.95542676327554E-95	0.313230443366074	0.461	0.252	3.27690416989715E-91	Detoxification-associated hepat	TPD52L2
2.04301144992615E-95	0.631980782389853	0.278	0.125	3.42367858778623E-91	Detoxification-associated hepat	BLOC1S4

2.14455312486232E-95	0.756076816308139	0.312	0.149	3.59384212664427E-91	Detoxification-associated hepat	YEATS2
2.72925546435313E-95	1.49060363480466	0.133	0.039	4.57368630716297E-91	Detoxification-associated hepat	KIF27
2.90324833312593E-95	0.377946852250561	0.409	0.212	4.86526355665243E-91	Detoxification-associated hepat	PPP2R5C
3.14426522963326E-95	0.847529916093821	0.28	0.127	5.26915967181942E-91	Detoxification-associated hepat	FANCC
3.2778805317541E-95	0.270165395827435	0.446	0.239	5.49307219511352E-91	Detoxification-associated hepat	GRIPAP1
3.3138959729899E-95	1.00721068553133	0.246	0.105	5.55342687153647E-91	Detoxification-associated hepat	KIAA1328
4.36992183491865E-95	0.357520278270934	0.399	0.206	7.32311501095667E-91	Detoxification-associated hepat	MAN1A2
4.8032509697355E-95	0.666102518662216	0.336	0.166	8.04928797508276E-91	Detoxification-associated hepat	FARSB
5.0404960313169E-95	0.809325331837933	0.257	0.112	8.44686324928087E-91	Detoxification-associated hepat	MAP4K3
5.27345970237706E-95	0.308028779033847	0.474	0.258	8.83726376924347E-91	Detoxification-associated hepat	RUFY1
5.63695764373424E-95	1.29141847605634	0.207	0.081	9.44641361936983E-91	Detoxification-associated hepat	ZNF324
5.6992443223808E-95	0.318963855030213	0.509	0.284	9.55079363544574E-91	Detoxification-associated hepat	HNRNPDL
5.88933857276409E-95	0.289173360932964	0.421	0.221	9.86935358023806E-91	Detoxification-associated hepat	CNOT9
6.45881207262788E-95	0.853679949267435	0.223	0.09	1.08236772713098E-90	Detoxification-associated hepat	INTS12
8.07544352870565E-95	0.588386515197582	0.314	0.15	1.35328282654049E-90	Detoxification-associated hepat	C17orf75
8.28581657148185E-95	0.528698445057437	0.331	0.162	1.38853714104893E-90	Detoxification-associated hepat	PATZ1
9.42619954195492E-95	0.348139907131827	0.429	0.229	1.57964251924081E-90	Detoxification-associated hepat	PLRG1
9.63272247393717E-95	1.15018210309459	0.251	0.11	1.61425163218239E-90	Detoxification-associated hepat	ZNF117
1.04510753331777E-94	0.869541072407421	0.278	0.127	1.75139120433392E-90	Detoxification-associated hepat	ITGB3BP
1.38125903172794E-94	0.328903698537537	0.388	0.197	2.31471388536968E-90	Detoxification-associated hepat	CEBPZOS
1.42768092493904E-94	0.980056624084542	0.254	0.112	2.39250769401284E-90	Detoxification-associated hepat	NHLRC3
1.46332741615706E-94	0.427699262817148	0.348	0.172	2.45224408399601E-90	Detoxification-associated hepat	RDH14
1.57200092019174E-94	0.879639961429014	0.197	0.074	2.63435914205732E-90	Detoxification-associated hepat	ZNF770
1.72019754526119E-94	0.660810399932767	0.34	0.169	2.8827070463487E-90	Detoxification-associated hepat	TMEM168
1.92416184259964E-94	0.515887840815673	0.388	0.201	3.22451041582848E-90	Detoxification-associated hepat	PLPP2
2.35090700878958E-94	0.87644480738651	0.253	0.11	3.93964996532959E-90	Detoxification-associated hepat	TBC1D2
2.5205860797244E-94	0.356709150289266	0.404	0.211	4.22399815240216E-90	Detoxification-associated hepat	C2orf68
2.52433847884377E-94	0.422961850514773	0.404	0.211	4.2302864228464E-90	Detoxification-associated hepat	CKLF
2.56137069465778E-94	0.336832015701758	0.431	0.23	4.2923450101075E-90	Detoxification-associated hepat	LSM 7.00
2.97700515750107E-94	0.267395052267813	0.833	0.501	4.98886524294029E-90	Detoxification-associated hepat	HNRNPH2
3.53437154651348E-94	0.72196995803001	0.278	0.127	5.92289983764729E-90	Detoxification-associated hepat	UBE3B
3.7644289667902E-94	0.2621047725458	0.531	0.295	6.30843006254701E-90	Detoxification-associated hepat	ATP11B
3.76832627674134E-94	1.20699431963032	0.158	0.052	6.31496117456314E-90	Detoxification-associated hepat	CCDC146
3.80310148156825E-94	0.878947873470154	0.256	0.113	6.37323746281208E-90	Detoxification-associated hepat	DEPRECATEI
4.70087220245382E-94	0.486493927115455	0.326	0.158	7.87772163687211E-90	Detoxification-associated hepat	ANKRD46
5.4209576365389E-94	0.254459636751631	0.498	0.274	9.08444080731189E-90	Detoxification-associated hepat	LRPPRC
5.44143197516209E-94	0.367278867026311	0.41	0.214	9.11875170397662E-90	Detoxification-associated hepat	MBTPS1
5.69975953595889E-94	0.493984452645934	0.357	0.179	9.55165703035991E-90	Detoxification-associated hepat	NXF1
5.96607230576362E-94	0.322258617733223	0.418	0.219	9.99794396999867E-90	Detoxification-associated hepat	NOP58
6.18182561629396E-94	1.46966770060249	0.144	0.045	1.03595033677854E-89	Detoxification-associated hepat	ZNF891
6.58086296707014E-94	0.368354447829744	0.411	0.215	1.10282101602161E-89	Detoxification-associated hepat	GON4L
6.79428247022809E-94	0.452564498738073	0.248	0.104	1.13858585636082E-89	Detoxification-associated hepat	CSRP2
6.95292723388609E-94	0.527035041649682	0.343	0.171	1.16517154585463E-89	Detoxification-associated hepat	ZFC3H1
7.49342404078908E-94	0.317238186897815	0.481	0.263	1.25574800075543E-89	Detoxification-associated hepat	SYMPK
7.7916839877999E-94	0.849339481406352	0.181	0.064	1.30573040267551E-89	Detoxification-associated hepat	FAM78B
8.09827846856424E-94	0.588719688494902	0.346	0.173	1.357109505762E-89	Detoxification-associated hepat	TMX1
8.21285763577572E-94	0.533455720025157	0.333	0.164	1.37631068260329E-89	Detoxification-associated hepat	DCAF10
9.21202683010334E-94	0.933782066447253	0.249	0.108	1.54375145618872E-89	Detoxification-associated hepat	PUS7L
9.53315613307461E-94	0.544498904661617	0.311	0.148	1.59756630478064E-89	Detoxification-associated hepat	PIGC
1.02788926357676E-93	0.526908581766741	0.31	0.146	1.72253682790194E-89	Detoxification-associated hepat	TRMT2A
1.05509788716478E-93	0.63729083416816	0.316	0.152	1.76813303931074E-89	Detoxification-associated hepat	RECQL
1.42227457366153E-93	0.959139253498451	0.232	0.097	2.38344773054199E-89	Detoxification-associated hepat	RASA2
1.5194003065582E-93	0.759540889219702	0.233	0.097	2.54621103373023E-89	Detoxification-associated hepat	SESN1
1.65981899818697E-93	0.414930657328985	0.401	0.206	2.78152467716173E-89	Detoxification-associated hepat	BAIAP2L2
1.69611454197568E-93	0.332014627989485	0.452	0.246	2.84234874944284E-89	Detoxification-associated hepat	ZGPAT
1.7711336138639E-93	0.714156988342115	0.266	0.117	2.96806571011312E-89	Detoxification-associated hepat	PRMT7
1.88471419739306E-93	0.474442169200731	0.222	0.088	3.1584040519913E-89	Detoxification-associated hepat	COX7A1
1.97510515600482E-93	1.01887102168459	0.237	0.1	3.30988122043288E-89	Detoxification-associated hepat	NEPRO
3.22409762628154E-93	0.438905560891652	0.384	0.198	5.4029428021226E-89	Detoxification-associated hepat	CEP250
4.02888713919915E-93	0.458392414813382	0.297	0.137	6.75160906786994E-89	Detoxification-associated hepat	ABCC6

4.3547441453922E-93	0.434935988160434	0.355	0.18	7.29768023884825E-89	Detoxification-associated hepat	VPS13D
4.39852550417989E-93	0.88224329603317	0.279	0.13	7.37104903990466E-89	Detoxification-associated hepat	CD58
4.88653545907191E-93	0.360693977859497	0.879	0.587	8.1888561223127E-89	Detoxification-associated hepat	ALDH3A2
5.57082601413972E-93	0.445496241795529	0.447	0.244	9.33559023449534E-89	Detoxification-associated hepat	CEP170B
6.04911152640693E-93	1.01528334171152	0.192	0.072	1.01371010959527E-88	Detoxification-associated hepat	TBCEL
6.1362325384127E-93	0.326461317119284	0.475	0.261	1.0283098487872E-88	Detoxification-associated hepat	AIMP1
6.56012221068368E-93	0.273238566432221	0.479	0.261	1.09934528006637E-88	Detoxification-associated hepat	CAMK1
6.72875376166846E-93	0.262794342667395	0.457	0.245	1.1276045553804E-88	Detoxification-associated hepat	PPIC
7.08453665065768E-93	0.327267720945418	0.433	0.232	1.18722665191721E-88	Detoxification-associated hepat	ELK4
7.10843241604457E-93	0.260463452295685	0.665	0.376	1.19123110428075E-88	Detoxification-associated hepat	FAT1
7.38711824244615E-93	0.686566116768678	0.249	0.107	1.23793327506913E-88	Detoxification-associated hepat	TNIK
7.64537247478542E-93	0.590112134562456	0.305	0.144	1.28121151932454E-88	Detoxification-associated hepat	SEPTIN10
9.1264957893519E-93	1.2094435675708	0.178	0.064	1.52941816437959E-88	Detoxification-associated hepat	ZNF283
1.01813146543982E-92	1.04970536646894	0.25	0.11	1.70618470978405E-88	Detoxification-associated hepat	HAUS3
1.02281863271092E-92	0.462356348343519	0.336	0.166	1.71403946469697E-88	Detoxification-associated hepat	FOXK2
1.05196482582109E-92	0.545840596934119	0.29	0.133	1.76288265511098E-88	Detoxification-associated hepat	BCS1L
1.17117199234454E-92	0.468891653992143	0.386	0.201	1.96265002477098E-88	Detoxification-associated hepat	ZNF384
1.20096813686479E-92	0.51373749164251	0.352	0.177	2.01258240375801E-88	Detoxification-associated hepat	THUMPD3
1.41548232254077E-92	0.786452888655914	0.282	0.13	2.37206527611382E-88	Detoxification-associated hepat	ZNF654
1.45279204575806E-92	0.742779573781358	0.276	0.126	2.43458891028135E-88	Detoxification-associated hepat	EXT2
1.715872997201E-92	0.338132974210487	0.404	0.212	2.87545996870944E-88	Detoxification-associated hepat	UQCRC2
1.91543168301169E-92	0.306321174574661	0.357	0.177	3.20988041439099E-88	Detoxification-associated hepat	CLSTN3
2.45724288902097E-92	0.701144898192053	0.256	0.112	4.11784763342135E-88	Detoxification-associated hepat	TBCCD1
3.33372085025613E-92	0.280616381544074	0.493	0.273	5.58664940085922E-88	Detoxification-associated hepat	CPSF7
3.44673009291675E-92	0.462007090002025	0.397	0.202	5.7760302897099E-88	Detoxification-associated hepat	ITGA3
3.91354839919971E-92	0.559089116889848	0.371	0.192	6.55832440737887E-88	Detoxification-associated hepat	XPR1
4.40853743808579E-92	0.91431373510215	0.196	0.075	7.38782703874417E-88	Detoxification-associated hepat	HERC1
4.72943862901466E-92	0.315083937266919	0.408	0.214	7.92559325450276E-88	Detoxification-associated hepat	TRAF2
5.71740974712166E-92	0.591471338894291	0.266	0.119	9.58123525422648E-88	Detoxification-associated hepat	USP47
5.84480260770159E-92	0.329875043846136	0.964	0.766	9.79472020998633E-88	Detoxification-associated hepat	UGDH
6.50725631693392E-92	0.864761092280024	0.262	0.118	1.09048601359179E-87	Detoxification-associated hepat	MTF2
6.78139900950099E-92	0.360520856519355	0.383	0.198	1.13642684601218E-87	Detoxification-associated hepat	UBE4B
7.06224583202783E-92	0.574213175199131	0.291	0.135	1.18349115653122E-87	Detoxification-associated hepat	TNKS2
7.42024259631161E-92	0.492975752895391	0.37	0.19	1.2434842542899E-87	Detoxification-associated hepat	MAPKAPK5
8.21017513178352E-92	2.30616703946423	0.101	0.025	1.37586114858428E-87	Detoxification-associated hepat	PTTG2
8.26907508792388E-92	0.304609795325271	0.423	0.224	1.38573160323428E-87	Detoxification-associated hepat	TBC1D17
8.3259980682447E-92	0.251719375932336	0.554	0.315	1.39527075627645E-87	Detoxification-associated hepat	GNS
8.78032784688474E-92	0.824450654002703	0.262	0.117	1.47140734058094E-87	Detoxification-associated hepat	ERI2
9.13063866717117E-92	0.589607047580178	0.315	0.153	1.53011242784454E-87	Detoxification-associated hepat	HEATR3
9.40039019719652E-92	0.368498783151508	0.435	0.231	1.57531738924619E-87	Detoxification-associated hepat	PHF14
1.00728930035574E-91	0.329960708142182	0.386	0.199	1.68801540953614E-87	Detoxification-associated hepat	SRCAP
1.09384855750777E-91	0.362803907182029	0.383	0.197	1.83307141267152E-87	Detoxification-associated hepat	GOLPH3L
1.1085205661751E-91	0.72816637512279	0.272	0.123	1.85765876479624E-87	Detoxification-associated hepat	CEP120
1.20563766937337E-91	0.335413317906393	0.53	0.296	2.02040760633589E-87	Detoxification-associated hepat	AGFG1
1.37725787192805E-91	0.843876066906991	0.199	0.076	2.30800874177703E-87	Detoxification-associated hepat	RILPL1
1.50815934324131E-91	0.704607732083579	0.302	0.145	2.52737342740378E-87	Detoxification-associated hepat	DEPRECATEI
2.08530958598155E-91	0.381698163277159	0.372	0.19	3.49456180418788E-87	Detoxification-associated hepat	UBQLN4
2.15495590739778E-91	0.607528492957459	0.312	0.15	3.6112751096172E-87	Detoxification-associated hepat	EEF1AKMT2
2.17145500841104E-91	0.489441717493057	0.359	0.183	3.63892430309523E-87	Detoxification-associated hepat	BOD1L1
2.18703123637966E-91	0.713323282587219	0.307	0.146	3.66502694592503E-87	Detoxification-associated hepat	DNM1
2.26632002833003E-91	0.446115655838498	0.37	0.191	3.79789910347546E-87	Detoxification-associated hepat	ARFGAP1
2.47871340589908E-91	0.462719935220373	0.34	0.169	4.15382792560568E-87	Detoxification-associated hepat	RBX1
3.42588892465607E-91	0.751747191638642	0.237	0.1	5.74110465993865E-87	Detoxification-associated hepat	ZNF512
3.70270039589478E-91	0.424445478444343	0.39	0.204	6.20498532344048E-87	Detoxification-associated hepat	SNX13
3.75251643035927E-91	0.476501677655947	0.321	0.155	6.28846703399607E-87	Detoxification-associated hepat	CTBS
4.42648748247296E-91	0.297947105393728	0.438	0.236	7.41790772312818E-87	Detoxification-associated hepat	POMGNT1
4.91171166059202E-91	0.420479197438565	0.336	0.166	8.2310464008201E-87	Detoxification-associated hepat	CNOT2
5.51274217572826E-91	0.886075671641156	0.209	0.083	9.23825333808542E-87	Detoxification-associated hepat	FBXL20
6.37555816060476E-91	0.265618053857496	0.448	0.242	1.06841603655415E-86	Detoxification-associated hepat	TLK1
8.76330282730771E-91	0.550444138024007	0.258	0.113	1.46855428780023E-86	Detoxification-associated hepat	FLCN

1.07028605189727E-90	0.835644667354225	0.26	0.117	1.79358536576945E-86	Detoxification-associated hepat	TBP
1.24762296471918E-90	1.00732977034912	0.167	0.058	2.09076656427641E-86	Detoxification-associated hepat	KBTBD7
1.29031007979437E-90	0.365425077221193	0.432	0.232	2.16230163171941E-86	Detoxification-associated hepat	COX11
1.57035621729155E-90	0.28829414654638	0.485	0.268	2.63160294893718E-86	Detoxification-associated hepat	CHAMP1
1.90494697684947E-90	0.298138242543016	0.476	0.262	3.19231014380434E-86	Detoxification-associated hepat	CDC42SE2
2.09390522112208E-90	0.405388660704329	0.41	0.218	3.50896636955637E-86	Detoxification-associated hepat	RIPK1
2.17700866377424E-90	0.252405294731037	0.447	0.241	3.64823111875287E-86	Detoxification-associated hepat	PRKCA
2.51718454380306E-90	0.436471623267092	0.34	0.169	4.21829785850518E-86	Detoxification-associated hepat	OSBPL8
2.8313469161875E-90	0.607912707903013	0.215	0.086	4.74477116214702E-86	Detoxification-associated hepat	HIF1AN
2.95239527140189E-90	1.37760228139956	0.169	0.06	4.94762399581528E-86	Detoxification-associated hepat	ZNF354B
3.02090622869055E-90	0.481987867149678	0.372	0.191	5.06243465803962E-86	Detoxification-associated hepat	PWWP2A
3.0518663212018E-90	0.451657896364996	0.364	0.186	5.11431758106998E-86	Detoxification-associated hepat	NRIP1
3.37820920572237E-90	0.383067837860854	0.419	0.223	5.66120298694955E-86	Detoxification-associated hepat	CASP3
3.93996843860428E-90	0.571930013326021	0.284	0.132	6.60259910941305E-86	Detoxification-associated hepat	NBAS
4.01780406772729E-90	0.45066998253633	0.362	0.185	6.73303605669739E-86	Detoxification-associated hepat	MLH1
4.52014859280838E-90	1.68771228650177	0.134	0.041	7.57486501182829E-86	Detoxification-associated hepat	GDPD5
4.68567510178191E-90	0.579576392183012	0.336	0.167	7.85225433556613E-86	Detoxification-associated hepat	GTPBP10
4.93542838641666E-90	0.3505939346319	0.462	0.254	8.27079088995704E-86	Detoxification-associated hepat	VHL
5.51325268320027E-90	0.466573999775422	0.355	0.18	9.23910884650701E-86	Detoxification-associated hepat	CPSF2
5.54740656675532E-90	0.402020381273432	0.38	0.197	9.29634392456856E-86	Detoxification-associated hepat	MAML1
6.12157811326652E-90	0.829804037457106	0.248	0.109	1.0258540602212E-85	Detoxification-associated hepat	VMAC
6.54374766053647E-90	0.599594457151565	0.318	0.156	1.0966012329527E-85	Detoxification-associated hepat	GTPBP3
6.67194789426442E-90	1.38518149114658	0.174	0.063	1.11808502812083E-85	Detoxification-associated hepat	KALRN
6.69645516114095E-90	1.56590845461216	0.108	0.028	1.122191955904E-85	Detoxification-associated hepat	ZNF341
7.83502301033514E-90	0.48938574660068	0.317	0.154	1.31299315607196E-85	Detoxification-associated hepat	CYB561
9.14381071072349E-90	0.849644076604965	0.205	0.081	1.53231979890304E-85	Detoxification-associated hepat	WNT3
9.58282513889737E-90	0.29360537775144	0.391	0.204	1.60588983677642E-85	Detoxification-associated hepat	VAPB
1.17773048698778E-89	0.593722833575801	0.289	0.136	1.97364075009413E-85	Detoxification-associated hepat	PET117
1.22973920181242E-89	0.4912293095216	0.387	0.204	2.06079695439725E-85	Detoxification-associated hepat	TRAPPC10
1.25723233849761E-89	0.672729479600956	0.276	0.127	2.10686995285429E-85	Detoxification-associated hepat	ZC3H13
1.60513694694946E-89	0.704413295570221	0.226	0.094	2.6898884956979E-85	Detoxification-associated hepat	PTPN4
1.62328471819138E-89	0.700187756397977	0.284	0.133	2.72030053074511E-85	Detoxification-associated hepat	UHRF1BP1L
1.94440523800654E-89	0.26228966632953	0.865	0.49	3.25843429785135E-85	Detoxification-associated hepat	ATP1A1
2.33592875821554E-89	0.584797097717159	0.262	0.117	3.9145494130176E-85	Detoxification-associated hepat	APTX
2.35255733330735E-89	0.491358573932435	0.309	0.149	3.94241557915645E-85	Detoxification-associated hepat	MTREX
2.58524300854023E-89	0.461631772091513	0.326	0.16	4.33235023371172E-85	Detoxification-associated hepat	KPNA3
2.61540268887553E-89	0.275526358703011	0.454	0.248	4.38289182601762E-85	Detoxification-associated hepat	EIF2B5
2.77680966217258E-89	0.311811701563959	0.458	0.246	4.65337763186882E-85	Detoxification-associated hepat	DHFR
3.05173919998859E-89	0.331076886830375	0.332	0.163	5.11410455134087E-85	Detoxification-associated hepat	POLR2C
3.11262446352739E-89	0.549319524028931	0.337	0.168	5.2161360759792E-85	Detoxification-associated hepat	TMEM8B
3.18897498053464E-89	0.730864639927087	0.316	0.155	5.34408427237995E-85	Detoxification-associated hepat	PDE7A
3.61796008121707E-89	0.55465214604587	0.353	0.18	6.06297750410357E-85	Detoxification-associated hepat	TOPBP1
3.89258362031355E-89	0.560504623634337	0.3	0.144	6.52319163092144E-85	Detoxification-associated hepat	TMEM192
3.95770758656625E-89	0.623782749427565	0.291	0.138	6.63232637356772E-85	Detoxification-associated hepat	ATG7
4.96015586859689E-89	0.294514066904854	0.464	0.253	8.31222920459467E-85	Detoxification-associated hepat	GET1
6.13476547660446E-89	0.353245378522667	0.347	0.175	1.02806399856937E-84	Detoxification-associated hepat	PDHX
7.38057537746529E-89	0.374468572139137	0.324	0.158	1.23683682175563E-84	Detoxification-associated hepat	ARFGAP2
7.83722589345254E-89	0.444347883470766	0.342	0.171	1.31336231522478E-84	Detoxification-associated hepat	LAMTOR3
8.28890512871755E-89	0.316546064833464	0.422	0.226	1.38905472147049E-84	Detoxification-associated hepat	EDEM2
8.98541787609591E-89	0.573807452744023	0.276	0.127	1.50577632767615E-84	Detoxification-associated hepat	GGA1
9.41771393445752E-89	0.91605310854261	0.228	0.096	1.57822050113639E-84	Detoxification-associated hepat	NDUFAF7
9.88448046032087E-89	0.424383056643059	0.34	0.169	1.65644123554057E-84	Detoxification-associated hepat	SCRN2
1.0398660141154E-88	0.830346972519626	0.255	0.115	1.74260746645459E-84	Detoxification-associated hepat	ZNF236
1.11724040959185E-88	0.359610588660077	0.325	0.159	1.87227147839402E-84	Detoxification-associated hepat	GFM2
1.51698607033038E-88	0.335803339709776	0.337	0.167	2.54216525665965E-84	Detoxification-associated hepat	KANSL3
1.91590922662779E-88	0.820691790095226	0.233	0.1	3.21068068198285E-84	Detoxification-associated hepat	RUFY2
2.51708656550904E-88	0.535101101322557	0.336	0.169	4.21813366648005E-84	Detoxification-associated hepat	PTPRJ
2.84599190275926E-88	0.841670695200541	0.246	0.108	4.76931323064396E-84	Detoxification-associated hepat	ZNF292
2.93868527782888E-88	0.719460029340757	0.265	0.121	4.92464878858564E-84	Detoxification-associated hepat	PRKRA
3.80811662841681E-88	0.324443444890747	0.472	0.258	6.38164184590089E-84	Detoxification-associated hepat	KLHDC4

**3.81786024418546E-88**	0.26929287733233	0.474	0.262	6.397970197206E-84	Detoxification-associated hepat	EBPL
**4.09490072970564E-88**	0.412054599311041	0.31	0.15	6.86223464284072E-84	Detoxification-associated hepat	USP6NL
**4.22227945193567E-88**	0.932409899615751	0.162	0.056	7.07569590555379E-84	Detoxification-associated hepat	KCNJ11
**4.31135894202113E-88**	0.723732931947136	0.215	0.088	7.22497531503901E-84	Detoxification-associated hepat	POLG
**4.60856038271421E-88**	0.29540465497101	0.452	0.25	7.72302548935247E-84	Detoxification-associated hepat	ANAPC5
**4.83742236896264E-88**	0.268893386349588	0.432	0.233	8.1065524059076E-84	Detoxification-associated hepat	USF1
**4.83979578984041E-88**	0.67937276425781	0.305	0.149	8.11052978461455E-84	Detoxification-associated hepat	ALKBH6
**5.02887948790067E-88**	0.535178202762475	0.36	0.186	8.42739624582394E-84	Detoxification-associated hepat	ERMP1
**5.2540822694205E-88**	0.484625831663045	0.319	0.158	8.80479106709487E-84	Detoxification-associated hepat	SDHAF4
**5.38575250476582E-88**	0.457855540454429	0.332	0.165	9.02544404748656E-84	Detoxification-associated hepat	RIN2
**5.47766970123289E-88**	0.513182839432291	0.307	0.149	9.17947888532608E-84	Detoxification-associated hepat	APPL1
**5.81220246378051E-88**	0.609321405878378	0.295	0.141	9.74008888880337E-84	Detoxification-associated hepat	ZKSCAN8
**6.62590311703278E-88**	0.443603390894981	0.313	0.153	1.11036884435235E-83	Detoxification-associated hepat	STYX
**6.75242743962895E-88**	0.611738208239272	0.25	0.11	1.13157179033302E-83	Detoxification-associated hepat	RBMXL1
**7.85568240596145E-88**	0.415724535301655	0.353	0.18	1.31645525759102E-83	Detoxification-associated hepat	SLC35C2
**7.91662587104366E-88**	0.728195615434155	0.165	0.057	1.3266681634695E-83	Detoxification-associated hepat	PREX1
**8.01957755965556E-88**	0.544026637307198	0.311	0.151	1.34392080744708E-83	Detoxification-associated hepat	PRPF18
**8.6033291899755E-88**	0.845149740340412	0.23	0.098	1.44174590565609E-83	Detoxification-associated hepat	WDR75
**9.06942319081912E-88**	1.4449307923553	0.19	0.075	1.51985393831747E-83	Detoxification-associated hepat	PCDHGA7
**9.07955798491853E-88**	0.406040759840106	0.346	0.175	1.52155232711265E-83	Detoxification-associated hepat	TIMM9
**1.07466815893812E-87**	0.905214630938518	0.241	0.106	1.8009289007485E-83	Detoxification-associated hepat	CNEP1R1
**1.23177470988008E-87**	0.371234279041256	0.329	0.162	2.06420805881704E-83	Detoxification-associated hepat	MED17
**1.41782630393202E-87**	0.622624042414354	0.315	0.155	2.37599332012928E-83	Detoxification-associated hepat	ZDHHC6
**1.46632922623539E-87**	0.718033769328533	0.251	0.113	2.45727451732527E-83	Detoxification-associated hepat	KCTD9
**1.55842611951063E-87**	0.469170485424924	0.325	0.161	2.61161049107592E-83	Detoxification-associated hepat	GNPDA1
**1.90992625375489E-87**	0.654240474275326	0.279	0.129	3.20065441604244E-83	Detoxification-associated hepat	EID3
**1.95979574489696E-87**	0.617855804316915	0.283	0.133	3.28422570929832E-83	Detoxification-associated hepat	KIFAP3
**1.96649113337181E-87**	0.462935739526285	0.329	0.164	3.29544584130448E-83	Detoxification-associated hepat	ZBTB44
**1.98625654433322E-87**	0.334063750788977	0.436	0.237	3.3285687169936E-83	Detoxification-associated hepat	SCAF4
**2.5987518212835E-87**	0.861306992684331	0.235	0.101	4.35498830210688E-83	Detoxification-associated hepat	TOP3B
**2.84761806884822E-87**	0.288127328439664	0.379	0.198	4.77203835977585E-83	Detoxification-associated hepat	KIAA1191
**3.03265286332661E-87**	0.69504516310729	0.312	0.154	5.08211966836274E-83	Detoxification-associated hepat	P4HTM
**3.43618794498403E-87**	0.785326494702467	0.276	0.13	5.75836375820424E-83	Detoxification-associated hepat	NAA40
**3.74965296980702E-87**	0.380527419160279	0.351	0.179	6.28366844680261E-83	Detoxification-associated hepat	STARD4
**3.91055591339436E-87**	0.786357605577708	0.26	0.118	6.55330959966627E-83	Detoxification-associated hepat	SDCCAG8
**4.05533690432295E-87**	0.268134617579606	0.403	0.214	6.7959335842644E-83	Detoxification-associated hepat	NFYC
**4.38554509823231E-87**	0.648306136884211	0.287	0.137	7.3492964756177E-83	Detoxification-associated hepat	ARID2
**5.39037525111326E-87**	0.80577445746243	0.229	0.097	9.03319084581561E-83	Detoxification-associated hepat	SETD6
**5.70111398137366E-87**	0.583043327201384	0.284	0.134	9.55392680998599E-83	Detoxification-associated hepat	PCBP2
**5.916002224686E-87**	0.795324437927015	0.241	0.106	9.9140365281288E-83	Detoxification-associated hepat	FBXO30
**5.98831959946841E-87**	0.301187632536803	0.452	0.249	1.00352259847892E-82	Detoxification-associated hepat	SRP72
**6.1030316730223E-87**	0.537751124075222	0.227	0.095	1.02274604776508E-82	Detoxification-associated hepat	PLA2G4B
**6.17441588348448E-87**	0.736626367591148	0.233	0.1	1.03470861375433E-82	Detoxification-associated hepat	C11orf71
**6.21757895850118E-87**	0.776816548065385	0.205	0.082	1.04194188186563E-82	Detoxification-associated hepat	TXNRD3
**6.56131877911951E-87**	0.574105968147665	0.266	0.121	1.09954580100485E-82	Detoxification-associated hepat	COG8
**7.14977543760634E-87**	0.526352339234111	0.282	0.132	1.19815936783407E-82	Detoxification-associated hepat	RTCA
**7.35385437180715E-87**	1.00368905017964	0.188	0.072	1.23235891562744E-82	Detoxification-associated hepat	FICD
**7.62674808374287E-87**	0.528236359862289	0.303	0.147	1.27809044387363E-82	Detoxification-associated hepat	COQ6
**7.88734015243126E-87**	0.267584721844189	0.477	0.267	1.32176046274443E-82	Detoxification-associated hepat	NAP1L4
**9.13104522004937E-87**	0.445312034807428	0.349	0.178	1.53018055797587E-82	Detoxification-associated hepat	QSER1
**9.37306302662263E-87**	0.294509848323515	0.438	0.238	1.57073790200142E-82	Detoxification-associated hepat	KDM3B
**1.05658943598419E-86**	1.11413401755104	0.162	0.057	1.7706325768223E-82	Detoxification-associated hepat	ZNF260
**1.06931028800936E-86**	0.925787020353794	0.235	0.104	1.79195018064609E-82	Detoxification-associated hepat	SLC30A6
**1.36754803156306E-86**	0.739139726810765	0.226	0.095	2.29173699129337E-82	Detoxification-associated hepat	ZNF197
**1.42664436702068E-86**	0.34924420943594	0.397	0.212	2.39077063025326E-82	Detoxification-associated hepat	FIP1L1
**1.56135303109664E-86**	0.41339613744167	0.358	0.185	2.61651540951176E-82	Detoxification-associated hepat	WAC
**1.95098151299781E-86**	0.978309204598124	0.214	0.089	3.26945481948173E-82	Detoxification-associated hepat	TMEM104
**2.07218227576825E-86**	0.437582876757447	0.356	0.184	3.47256305773244E-82	Detoxification-associated hepat	AKAP8L
**2.34917426252261E-86**	0.484811022184146	0.264	0.12	3.9367462291354E-82	Detoxification-associated hepat	HEXD
**2.38230751645427E-86**	1.22021982869766	0.172	0.064	3.99227093607406E-82	Detoxification-associated hepat	ZNF514

**2.40393364630676E-86**	0.765449621353141	0.252	0.114	4.02851200448087E-82	Detoxification-associated hepat	ZNF562
**2.84266037671457E-86**	0.628205828334594	0.25	0.111	4.76373025929828E-82	Detoxification-associated hepat	FGFRL1
**3.32696573679367E-86**	0.72517335172918	0.232	0.1	5.57532918171884E-82	Detoxification-associated hepat	AGL
**4.06646972839207E-86**	0.318286162264335	0.923	0.644	6.81458997083944E-82	Detoxification-associated hepat	IDH1
**4.63683355816537E-86**	0.5844221022901	0.26	0.117	7.77040567677353E-82	Detoxification-associated hepat	NBPF12
**4.99057979588035E-86**	1.37678765114724	0.197	0.08	8.3632136219363E-82	Detoxification-associated hepat	PCDHGA6
**5.56341085116596E-86**	0.79281481907911	0.242	0.108	9.32316390438392E-82	Detoxification-associated hepat	JKAMP
**5.79392085334416E-86**	0.496542016298714	0.292	0.14	9.70945256603414E-82	Detoxification-associated hepat	SSR1
**6.3347092933727E-86**	0.701637189103757	0.246	0.11	1.0615705833834E-81	Detoxification-associated hepat	INPP4A
**6.63142391234046E-86**	0.392217177849714	0.413	0.219	1.11129401923001E-81	Detoxification-associated hepat	MCM6
**6.85708129187909E-86**	0.278331478257282	0.46	0.254	1.1491096828931E-81	Detoxification-associated hepat	ATP11A
**7.40120945452002E-86**	0.641240349946216	0.274	0.128	1.24029468038847E-81	Detoxification-associated hepat	USP28
**7.6424551471131E-86**	0.28129658720281	0.434	0.235	1.28072263355321E-81	Detoxification-associated hepat	HSPA4L
**7.70764536351528E-86**	0.27262183994499	0.43	0.236	1.29164721001789E-81	Detoxification-associated hepat	ZC3HAV1
**7.84349006105656E-86**	0.349763976700122	0.258	0.115	1.31441206443186E-81	Detoxification-associated hepat	HMG20A
**8.09053835617224E-86**	1.26386513655191	0.127	0.038	1.35581241772734E-81	Detoxification-associated hepat	METTL25
**8.88507998832103E-86**	0.745908705913633	0.232	0.1	1.48896170444284E-81	Detoxification-associated hepat	NOCT
**1.04526898083677E-85**	0.323810797873525	0.448	0.246	1.75166175808627E-81	Detoxification-associated hepat	SF3B3
**1.1068399463159E-85**	0.648363560363325	0.225	0.095	1.85484238203619E-81	Detoxification-associated hepat	RNF111
**1.33640838977801E-85**	0.45703744238961	0.336	0.17	2.23955317959E-81	Detoxification-associated hepat	ARFRP1
**1.38232453120437E-85**	0.329070185318219	0.406	0.218	2.31649944939228E-81	Detoxification-associated hepat	FBXO21
**1.45688142349627E-85**	0.259068742470336	0.417	0.224	2.44144188949506E-81	Detoxification-associated hepat	AGTRAP
**1.58066719865815E-85**	0.543415040519283	0.266	0.122	2.64888209151132E-81	Detoxification-associated hepat	KIAA1958
**1.78700747550586E-85**	0.29644652327058	0.415	0.223	2.99466712745273E-81	Detoxification-associated hepat	RANBP9
**1.88480101933246E-85**	0.964862181470026	0.192	0.075	3.15854954819734E-81	Detoxification-associated hepat	TMEM267
**1.90513373155592E-85**	0.274425848650771	0.408	0.218	3.19262310734141E-81	Detoxification-associated hepat	RARA
**2.04072564175745E-85**	0.668557920552193	0.248	0.111	3.41984803045713E-81	Detoxification-associated hepat	TJAP1
**2.11351079695916E-85**	0.314689012595074	0.441	0.242	3.54182139354415E-81	Detoxification-associated hepat	LMF2
**2.25916198062855E-85**	0.403588422338962	0.384	0.205	3.78590364713732E-81	Detoxification-associated hepat	STK4
**2.36517774208501E-85**	0.766272210517518	0.228	0.098	3.96356486018607E-81	Detoxification-associated hepat	SIRT3
**2.43237593881083E-85**	0.2572783271936	0.799	0.498	4.07617559825918E-81	Detoxification-associated hepat	PCK2
**3.21767587846385E-85**	0.659610325493847	0.271	0.126	5.39218123712973E-81	Detoxification-associated hepat	DDX31
**3.28639833199938E-85**	0.454187462898136	0.286	0.135	5.50734632476457E-81	Detoxification-associated hepat	CREB3L2
**3.32045250969434E-85**	0.78512726153306	0.237	0.103	5.56441431574578E-81	Detoxification-associated hepat	SRD5A1
**3.51385532120862E-85**	0.325787407795174	0.42	0.226	5.8885187472814E-81	Detoxification-associated hepat	ELOVL6
**3.53701717724673E-85**	0.257239486454703	0.494	0.277	5.92733338563008E-81	Detoxification-associated hepat	MTIF3
**3.63544290756839E-85**	1.01866245241133	0.18	0.068	6.09227522450311E-81	Detoxification-associated hepat	RRAGB
**3.82405619819662E-85**	0.645275441859658	0.26	0.119	6.40835337693789E-81	Detoxification-associated hepat	RTL10
**3.84192417777168E-85**	0.763895868050847	0.248	0.111	6.43829653710978E-81	Detoxification-associated hepat	RAPGEF6
**4.37307408872796E-85**	0.534907980400036	0.294	0.142	7.32839755789032E-81	Detoxification-associated hepat	JMJD4
**4.54983079142645E-85**	0.736704985132076	0.193	0.075	7.62460644027244E-81	Detoxification-associated hepat	MINDY2
**5.29860039753103E-85**	0.736947033734768	0.273	0.129	8.8793945461825E-81	Detoxification-associated hepat	HEATR5B
**5.56935827684603E-85**	0.679160842770534	0.263	0.121	9.33313060033857E-81	Detoxification-associated hepat	CAMKMT
**6.22340667748547E-85**	0.314916622082142	0.395	0.21	1.04291849101301E-80	Detoxification-associated hepat	TERF2
**7.5236571690696E-85**	0.676610224912084	0.281	0.134	1.26081446839268E-80	Detoxification-associated hepat	STAM2
**7.5525354836148E-85**	1.1586971123351	0.145	0.048	1.26565389634417E-80	Detoxification-associated hepat	ZNF529
**7.92139935846103E-85**	0.529587791064754	0.325	0.164	1.3274681044909E-80	Detoxification-associated hepat	ANKRD13A
**8.50036338785406E-85**	0.48528183876178	0.329	0.167	1.42449089653658E-80	Detoxification-associated hepat	PPP1R12A
**8.7382066708965E-85**	0.40025974954063	0.338	0.171	1.46434867390884E-80	Detoxification-associated hepat	UBE2O
**8.9131211610046E-85**	0.647723070619275	0.297	0.145	1.49366084416115E-80	Detoxification-associated hepat	NOL9
**9.32102842759717E-85**	0.928942060277545	0.211	0.088	1.56201794389673E-80	Detoxification-associated hepat	APPBP2
**1.10377864828509E-84**	0.585603933727406	0.264	0.121	1.84971225879615E-80	Detoxification-associated hepat	NFKBIL1
**1.26816561889026E-84**	0.738643054718308	0.245	0.11	2.1251919441363E-80	Detoxification-associated hepat	TLK2
**1.28651554522505E-84**	0.663569735725442	0.248	0.111	2.15594275068814E-80	Detoxification-associated hepat	CYB5RL
**1.31466781968124E-84**	0.870087843330873	0.265	0.124	2.20312033222182E-80	Detoxification-associated hepat	ATXN7L1
**1.38486779229444E-84**	0.263385829864727	0.81	0.495	2.32076144632702E-80	Detoxification-associated hepat	ENTPD5
**1.45466071117197E-84**	0.313641286893933	0.364	0.19	2.43772041978199E-80	Detoxification-associated hepat	TRPT1
**1.55319194071052E-84**	1.58315611215878	0.123	0.037	2.60283905424268E-80	Detoxification-associated hepat	MOGAT1
**1.80706405608605E-84**	0.310364610717039	0.358	0.185	3.028277945189E-80	Detoxification-associated hepat	TMEM230
**1.84682105503389E-84**	0.401787357589805	0.364	0.192	3.0949027240258E-80	Detoxification-associated hepat	ZNF687

2.10388019108021E-84	0.465229675868973	0.368	0.194	3.52568242421222E-80	Detoxification-associated hepat	ANKRD27
2.2963697773371E-84	0.791022111862453	0.265	0.124	3.84825647286151E-80	Detoxification-associated hepat	ORC2
2.55573603959107E-84	0.568951419028033	0.333	0.17	4.28290245514672E-80	Detoxification-associated hepat	MSH2
3.26624514063336E-84	0.258488952103887	0.368	0.191	5.47357360667339E-80	Detoxification-associated hepat	ZNF768
3.28951461152429E-84	0.506848233398801	0.259	0.118	5.51256858599241E-80	Detoxification-associated hepat	HPS4
3.42078967986152E-84	0.648385825910937	0.331	0.17	5.73255934551194E-80	Detoxification-associated hepat	C2CD5
3.51706504095022E-84	0.530719010144778	0.284	0.135	5.89389759562437E-80	Detoxification-associated hepat	DDX52
3.70329280779455E-84	0.392782758305614	0.329	0.165	6.20597808730211E-80	Detoxification-associated hepat	SPIN1
3.88809259562738E-84	0.40307999333055	0.39	0.208	6.51566557175237E-80	Detoxification-associated hepat	SLC25A43
4.03268395767255E-84	0.877828766614277	0.211	0.088	6.75797177626767E-80	Detoxification-associated hepat	LBHD1
4.21693452356258E-84	0.610590865157753	0.309	0.153	7.06673887458618E-80	Detoxification-associated hepat	TIPARP
4.2813930872331E-84	0.511360053971795	0.309	0.153	7.17475853558522E-80	Detoxification-associated hepat	DYNC1LI1
6.81735268807384E-84	0.52531097434429	0.296	0.144	1.14245196346741E-79	Detoxification-associated hepat	CLOCK
7.06125720652678E-84	0.333475865388216	0.776	0.499	1.18332548266976E-79	Detoxification-associated hepat	TOB1
7.37388886150432E-84	0.551048889129222	0.293	0.142	1.23571629541089E-79	Detoxification-associated hepat	SNRNP48
7.87111786577388E-84	0.510536879485005	0.314	0.157	1.31904193194639E-79	Detoxification-associated hepat	KYAT1
8.80307186996442E-84	0.422300916952078	0.348	0.179	1.47521878396864E-79	Detoxification-associated hepat	AKAP17A
9.68405773089276E-84	1.33583490116035	0.127	0.039	1.62285439454301E-79	Detoxification-associated hepat	LRRK2
1.02196718448218E-83	0.499719322681711	0.303	0.149	1.71261260775523E-79	Detoxification-associated hepat	EIF2AK4
1.05391642211649E-83	0.444638481426156	0.346	0.179	1.76615314018281E-79	Detoxification-associated hepat	PREPL
1.15013960117746E-83	0.553351290580992	0.291	0.141	1.92740394365319E-79	Detoxification-associated hepat	TMEM94
1.40070788315285E-83	0.403407274314527	0.35	0.18	2.34730627058754E-79	Detoxification-associated hepat	MAGEH1
1.59849625683164E-83	0.597546012705165	0.303	0.15	2.67876002719847E-79	Detoxification-associated hepat	XRCC5
1.61352686371322E-83	0.431004325725751	0.297	0.143	2.70394831821062E-79	Detoxification-associated hepat	AARS2
1.70030739338601E-83	0.827563040624947	0.215	0.091	2.84937512983627E-79	Detoxification-associated hepat	CTDSPL2
1.87749832547469E-83	1.22295114497022	0.169	0.063	3.14631169383049E-79	Detoxification-associated hepat	TIGD7
1.95838628639924E-83	0.991466454842544	0.22	0.095	3.28186373874784E-79	Detoxification-associated hepat	MAP7D3
2.02143361732126E-83	0.918092177475188	0.219	0.094	3.38751845590698E-79	Detoxification-associated hepat	DUSP12
2.20211552023028E-83	0.380071968455147	0.37	0.195	3.6903051888019E-79	Detoxification-associated hepat	CDK14
2.30070109346717E-83	0.60740193263068	0.255	0.116	3.85551489243228E-79	Detoxification-associated hepat	GORAB
2.42989689996572E-83	0.263452469298045	0.38	0.2	4.07202122496255E-79	Detoxification-associated hepat	TADA2B
2.95421753223604E-83	0.764912836922111	0.26	0.121	4.95067774052115E-79	Detoxification-associated hepat	CLASP1
2.99345679814481E-83	1.12204565747911	0.165	0.06	5.01643490233107E-79	Detoxification-associated hepat	ZNF517
3.27724026773577E-83	0.393798152202038	0.354	0.185	5.4919992406716E-79	Detoxification-associated hepat	MFN1
3.34056812420264E-83	0.553669267986237	0.305	0.149	5.59812406253879E-79	Detoxification-associated hepat	RBFA
3.37804277782502E-83	0.838986532040744	0.182	0.07	5.66092408707916E-79	Detoxification-associated hepat	RNF215
3.43485821687366E-83	0.32833970668056	0.374	0.194	5.75613539983688E-79	Detoxification-associated hepat	VIL1
4.53240621171802E-83	0.403435509733029	0.325	0.164	7.59540632959705E-79	Detoxification-associated hepat	MAP2K1
5.07113695820355E-83	0.609055237649739	0.277	0.132	8.49821131455751E-79	Detoxification-associated hepat	TAF5L
5.35869316180396E-83	0.948670101004424	0.199	0.081	8.98009800055107E-79	Detoxification-associated hepat	TRMT5
6.5020050219404E-83	0.337038897995571	0.344	0.177	1.08960600157677E-78	Detoxification-associated hepat	ATF7
6.78959483959464E-83	0.740194589380652	0.274	0.131	1.13780030321927E-78	Detoxification-associated hepat	USP37
7.1662376095926E-83	0.62218348163379	0.262	0.121	1.20091809861553E-78	Detoxification-associated hepat	ETNPPL
7.72077913312187E-83	0.433427547928502	0.323	0.163	1.29384816712856E-78	Detoxification-associated hepat	MINDY1
1.28513033370132E-82	0.806528638272024	0.223	0.096	2.15362141321667E-78	Detoxification-associated hepat	TRIM23
1.38483350564183E-82	0.695617031022983	0.279	0.135	2.32070398875458E-78	Detoxification-associated hepat	UBN2
1.42203883645837E-82	0.458110033848281	0.366	0.194	2.38305268213693E-78	Detoxification-associated hepat	DNAJC13
1.4241883003665E-82	0.466366905600331	0.361	0.19	2.38665475375418E-78	Detoxification-associated hepat	MYO5C
1.64387598116383E-82	0.602625619337452	0.287	0.139	2.75480736923435E-78	Detoxification-associated hepat	SBNO1
1.64611061927901E-82	0.346117246413997	0.435	0.242	2.75855217578776E-78	Detoxification-associated hepat	VPS35L
1.79078008775375E-82	0.359404784050171	0.345	0.177	3.00098927105773E-78	Detoxification-associated hepat	CPLX1
1.96180646767898E-82	0.593628087974555	0.293	0.144	3.28759527853644E-78	Detoxification-associated hepat	TMEM18
1.99833882219577E-82	0.403295544023456	0.385	0.207	3.34881619823568E-78	Detoxification-associated hepat	NUP42
2.05768384532667E-82	0.34757356005505	0.368	0.195	3.44826658799843E-78	Detoxification-associated hepat	BRPF3
2.12461235772195E-82	0.775946143529017	0.246	0.112	3.56042538907044E-78	Detoxification-associated hepat	DEPRECATEI
2.14967273669845E-82	0.273990902859002	0.407	0.221	3.60242157215926E-78	Detoxification-associated hepat	TM9SF4
2.32732569614005E-82	0.453900069515578	0.348	0.181	3.9001324015915E-78	Detoxification-associated hepat	INTS4
2.48549172934701E-82	0.756509127465329	0.269	0.127	4.16518704003972E-78	Detoxification-associated hepat	FAM184A
2.52466551945917E-82	1.64587013183744	0.123	0.038	4.23083447750967E-78	Detoxification-associated hepat	ZNF555
2.61343647922339E-82	0.98179994384515	0.196	0.08	4.37959685188255E-78	Detoxification-associated hepat	TDG

2.94090714954836E-82	0.893316202774337	0.205	0.085	4.92837220121315E-78	Detoxification-associated hepat	ZNF518A
3.1050803905977E-82	0.340632722277286	0.405	0.22	5.20349371856363E-78	Detoxification-associated hepat	BCAT2
3.69543644500856E-82	0.708735010659391	0.268	0.127	6.19281239454535E-78	Detoxification-associated hepat	ATP6V0A2
4.87264744248954E-82	1.20878709896738	0.192	0.078	8.16558258412398E-78	Detoxification-associated hepat	MSANTD2
4.96336094763118E-82	0.288799891811855	0.418	0.232	8.31760027604033E-78	Detoxification-associated hepat	TOMM6
5.2964316801813E-82	0.279113048041988	0.935	0.58	8.87576020964783E-78	Detoxification-associated hepat	MPP1
6.19276429844701E-82	0.571495085595377	0.294	0.143	1.03778344113375E-77	Detoxification-associated hepat	COG7
8.25638060948351E-82	1.1324057274053	0.189	0.075	1.38360426253725E-77	Detoxification-associated hepat	C8orf37
8.8925877686645E-82	0.420641740160105	0.244	0.109	1.4902198582728E-77	Detoxification-associated hepat	FBXO22
9.23129667121828E-82	0.907668479306696	0.209	0.088	1.54698069616276E-77	Detoxification-associated hepat	TMEM62
1.17074783870211E-81	0.708717871771135	0.287	0.14	1.96193922809699E-77	Detoxification-associated hepat	POLA2
1.1733757296194E-81	0.514633688006545	0.26	0.12	1.96634304769618E-77	Detoxification-associated hepat	UFL1
1.24509478906252E-81	0.516512408036996	0.291	0.142	2.08652984751097E-77	Detoxification-associated hepat	RNF38
1.27168480464356E-81	0.474335032624561	0.301	0.149	2.13108939562168E-77	Detoxification-associated hepat	CLCN5
1.33479493521022E-81	0.501833220236946	0.292	0.142	2.23684935242529E-77	Detoxification-associated hepat	DVL2
1.35408899403026E-81	0.265854654379246	0.92	0.674	2.26918233619592E-77	Detoxification-associated hepat	BST2
1.61760855843086E-81	0.44335301355085	0.325	0.165	2.71078842221843E-77	Detoxification-associated hepat	TP53I13
1.79805199981035E-81	0.287447006765742	0.399	0.216	3.01317554128218E-77	Detoxification-associated hepat	BLCAP
1.92028722361674E-81	0.254594564131807	0.458	0.255	3.21801732933694E-77	Detoxification-associated hepat	HLTF
2.06433964419147E-81	0.642336038421924	0.303	0.151	3.45942037573607E-77	Detoxification-associated hepat	PIGN
2.26476472730229E-81	0.40493912797306	0.309	0.153	3.79529273001317E-77	Detoxification-associated hepat	KLHL36
2.26484662381255E-81	0.451274067181324	0.263	0.122	3.79542997218507E-77	Detoxification-associated hepat	TUT1
2.8176841170272E-81	0.814500425772351	0.212	0.09	4.72187504331418E-77	Detoxification-associated hepat	TMEM177
2.90573150630734E-81	0.534041957585448	0.313	0.158	4.86942485826984E-77	Detoxification-associated hepat	MIGA1
2.9602316363034E-81	0.599615014977496	0.307	0.155	4.96075617611724E-77	Detoxification-associated hepat	ZC3H7A
3.0217020953392E-81	0.498686555659424	0.29	0.141	5.06376837136942E-77	Detoxification-associated hepat	TRMU
3.15314932170543E-81	0.256741979178389	0.411	0.224	5.28404763331395E-77	Detoxification-associated hepat	UBE2J2
3.2367845506221E-81	0.354159204848502	0.307	0.151	5.42420354993251E-77	Detoxification-associated hepat	SLC38A7
3.9216304957506E-81	0.363203235282811	0.355	0.187	6.57186838477885E-77	Detoxification-associated hepat	SPOP
3.9979402370109E-81	0.759341049867794	0.244	0.111	6.69974824918287E-77	Detoxification-associated hepat	ANKMY1
4.51331309002661E-81	1.75376052886693	0.114	0.033	7.56341007626659E-77	Detoxification-associated hepat	TAS2R30
4.57785651176604E-81	0.983525515505927	0.208	0.088	7.67157194241753E-77	Detoxification-associated hepat	ELMOD3
5.27462022044262E-81	0.290305987437488	0.394	0.213	8.83920856541774E-77	Detoxification-associated hepat	PPP2R5E
5.52669238955689E-81	0.299368183917568	0.322	0.161	9.26163110641943E-77	Detoxification-associated hepat	FGFR3
6.19831432373154E-81	0.376622802768108	0.298	0.146	1.03871351437093E-76	Detoxification-associated hepat	NMRK1
9.3424000292686E-81	0.990546463107059	0.212	0.09	1.56559939690483E-76	Detoxification-associated hepat	WNT5B
1.0557280167152E-80	0.485486719224503	0.3	0.149	1.76918901041133E-76	Detoxification-associated hepat	ATG4A
1.0824689988508E-80	0.64447243029058	0.257	0.12	1.81400154827417E-76	Detoxification-associated hepat	PEX1
1.1569467424134E-80	0.408772812652316	0.316	0.158	1.93881135093637E-76	Detoxification-associated hepat	ITGA1
1.29015611339127E-80	0.329179278869291	0.358	0.188	2.16204361482109E-76	Detoxification-associated hepat	NUP214
1.37287862054593E-80	0.886882574165072	0.223	0.097	2.30066999231087E-76	Detoxification-associated hepat	CAPS
1.41564418990329E-80	0.295378676181619	0.391	0.212	2.37233653343993E-76	Detoxification-associated hepat	POFUT1
1.42366760274012E-80	0.414345852854101	0.354	0.187	2.38578216867189E-76	Detoxification-associated hepat	CAMK1D
1.47175032081006E-80	0.829290664454148	0.231	0.103	2.46635918761349E-76	Detoxification-associated hepat	TADA2A
1.52679317190386E-80	0.425963660638642	0.305	0.152	2.55859999747648E-76	Detoxification-associated hepat	RGP1
1.58246879495337E-80	0.47216097079342	0.314	0.159	2.65190120658286E-76	Detoxification-associated hepat	PIAS3
2.00226941382266E-80	0.290696518288627	0.331	0.168	3.35540308368402E-76	Detoxification-associated hepat	CAMLG
2.97304813533438E-80	0.67519072674598	0.279	0.136	4.98223406519336E-76	Detoxification-associated hepat	GPATCH2
3.27401404957118E-80	0.37276085096321	0.27	0.126	5.48659274427138E-76	Detoxification-associated hepat	TPST1
3.27629988309521E-80	0.294754219448071	0.387	0.208	5.49042334409095E-76	Detoxification-associated hepat	CDC42BPA
3.45128947463392E-80	0.577091855620048	0.304	0.152	5.78367090159152E-76	Detoxification-associated hepat	PLCG1
3.50704172212808E-80	0.481112933221081	0.278	0.134	5.87710051794223E-76	Detoxification-associated hepat	CLTC
3.70719328851963E-80	1.01405851701711	0.165	0.061	6.2125145129012E-76	Detoxification-associated hepat	CYP3A43
3.7622852848991E-80	0.281974213681796	0.346	0.18	6.30483768043391E-76	Detoxification-associated hepat	SLC35E1
3.96945259811489E-80	1.36834621707826	0.18	0.071	6.65200866392094E-76	Detoxification-associated hepat	PNMT
4.17084743640417E-80	0.496657289559753	0.277	0.133	6.98950613392611E-76	Detoxification-associated hepat	PPARG
4.66169360662857E-80	0.820652182435447	0.242	0.111	7.81206614598816E-76	Detoxification-associated hepat	BIRC2
4.90339020461787E-80	0.391723274485406	0.377	0.202	8.21710130489862E-76	Detoxification-associated hepat	USP1
5.3041595411857E-80	0.763880542585058	0.262	0.125	8.88871055911899E-76	Detoxification-associated hepat	FAHD2A
5.37607223585241E-80	0.604224794088162	0.276	0.133	9.00922185284147E-76	Detoxification-associated hepat	CCNYL1

5.66671667033558E-80	0.44125230211165	0.328	0.169	9.49628379614836E-76	Detoxification-associated hepat	DDX19A
6.83886717267664E-80	0.517706065560578	0.251	0.115	1.14605736079715E-75	Detoxification-associated hepat	OSBPL11
7.17956075912602E-80	0.362259743800088	0.331	0.17	1.20315079201434E-75	Detoxification-associated hepat	SNRPE
7.22579459098877E-80	0.39536621393246	0.387	0.21	1.2108986575579E-75	Detoxification-associated hepat	WWP2
8.27259282748577E-80	0.480573423825692	0.308	0.155	1.38632110603006E-75	Detoxification-associated hepat	GPATCH8
8.63237975352863E-80	0.419940176307257	0.323	0.165	1.44661419909633E-75	Detoxification-associated hepat	UCHL5
9.2085967973299E-80	0.503435425559662	0.295	0.145	1.54317665129655E-75	Detoxification-associated hepat	ARFIP2
9.62448468807018E-80	0.587316665040576	0.343	0.18	1.6128711440268E-75	Detoxification-associated hepat	LRIG3
9.66776266892686E-80	0.80004395643639	0.213	0.091	1.62012366805876E-75	Detoxification-associated hepat	AC073111.4
1.10339887227266E-79	0.763342021192276	0.246	0.113	1.84907583015452E-75	Detoxification-associated hepat	LPAR1
1.18794620451404E-79	0.521016839041103	0.264	0.125	1.99076024952462E-75	Detoxification-associated hepat	RPAP1
1.24793178605824E-79	0.326532989809453	0.298	0.147	2.0912840870764E-75	Detoxification-associated hepat	SSH1
1.36921968168975E-79	0.35337631155094	0.353	0.186	2.29453834257568E-75	Detoxification-associated hepat	ALG12
1.38496579302307E-79	0.625043922436315	0.272	0.131	2.32092567594805E-75	Detoxification-associated hepat	ANGEL1
1.40718351729056E-79	0.412101425793743	0.35	0.185	2.35815813827552E-75	Detoxification-associated hepat	STRBP
1.66206745545836E-79	0.587383598937511	0.284	0.139	2.78529264185712E-75	Detoxification-associated hepat	DHX29
1.80211399088357E-79	0.68513657240271	0.273	0.132	3.01998262592268E-75	Detoxification-associated hepat	PIGQ
1.82128970071119E-79	0.264150618405131	0.412	0.226	3.05211728045181E-75	Detoxification-associated hepat	ZC3H4
1.843575621568E-79	1.42415826552849	0.169	0.065	3.08946402662365E-75	Detoxification-associated hepat	CCDC9B
1.98173037348924E-79	0.83786770436454	0.223	0.098	3.32098375989327E-75	Detoxification-associated hepat	ZNF84
2.08068409084688E-79	0.433621740782764	0.324	0.166	3.48681039944121E-75	Detoxification-associated hepat	PPP1R13B
2.3247881749466E-79	0.449373400862292	0.341	0.179	3.89588002357551E-75	Detoxification-associated hepat	SMPDL3A
2.55560555006239E-79	0.77898504091503	0.246	0.113	4.28268378079456E-75	Detoxification-associated hepat	NDUFAF5
2.56907653966456E-79	0.559503033216964	0.278	0.135	4.30525846516987E-75	Detoxification-associated hepat	PSMA1
2.95918514273169E-79	0.46918819047461	0.292	0.144	4.95900246218977E-75	Detoxification-associated hepat	ARID4A
3.11471208601698E-79	0.672812831738757	0.233	0.104	5.21963451374725E-75	Detoxification-associated hepat	EPG5
3.30130851117366E-79	1.21725282293207	0.176	0.069	5.53233280302482E-75	Detoxification-associated hepat	ZNF280C
3.30240641843482E-79	1.11715959813923	0.151	0.054	5.53417267601307E-75	Detoxification-associated hepat	MTHFD2L
3.36998477878887E-79	0.926235265286841	0.224	0.099	5.64742049229439E-75	Detoxification-associated hepat	TFB1M
3.49865433183945E-79	1.08462629761868	0.194	0.08	5.86304492929656E-75	Detoxification-associated hepat	WDSUB1
3.81141052697011E-79	0.298516203607145	0.345	0.18	6.3871617610965E-75	Detoxification-associated hepat	CHORDC1
3.92901655886937E-79	0.472827083035427	0.321	0.165	6.5842459493533E-75	Detoxification-associated hepat	TMEM138
4.60526891922386E-79	0.498407217203046	0.282	0.138	7.71750965483534E-75	Detoxification-associated hepat	SPAG16
4.67800428731004E-79	0.339104973776676	0.361	0.193	7.83939958467417E-75	Detoxification-associated hepat	GRSF1
5.09533129734459E-79	1.34919596734211	0.143	0.049	8.53875618809006E-75	Detoxification-associated hepat	EFCAB7
5.15890661050075E-79	0.39959793744946	0.298	0.148	8.64529569787716E-75	Detoxification-associated hepat	ERCC2
8.00839689341371E-79	0.609116038869578	0.295	0.148	1.34204715139827E-74	Detoxification-associated hepat	FCHO2
8.29179159500312E-79	0.475636894613113	0.265	0.125	1.38953843549062E-74	Detoxification-associated hepat	GCFC2
1.00587504886599E-78	0.385755131780405	0.384	0.209	1.68564540688962E-74	Detoxification-associated hepat	P2RX4
1.11932603322288E-78	0.534179755975418	0.179	0.069	1.8757665664749E-74	Detoxification-associated hepat	ANKRD65
1.18076897172722E-78	1.20119339576727	0.177	0.07	1.97873264282048E-74	Detoxification-associated hepat	ERCC8
1.37392408815239E-78	1.43484693396796	0.15	0.054	2.30242198692577E-74	Detoxification-associated hepat	C3orf33
1.55056926916366E-78	0.415460101629018	0.194	0.078	2.59844398126446E-74	Detoxification-associated hepat	CD276
1.84027418849603E-78	0.398199137595496	0.295	0.146	3.08393148508165E-74	Detoxification-associated hepat	PLEKHA1
1.86700274663285E-78	0.543643256353549	0.285	0.141	3.12872320280733E-74	Detoxification-associated hepat	B4GALT7
2.07717353645892E-78	0.5295700153005	0.315	0.16	3.48092741239786E-74	Detoxification-associated hepat	GABPB2
2.43650672122645E-78	0.449475892459224	0.283	0.139	4.08309796343128E-74	Detoxification-associated hepat	CREBRF
2.5591318309976E-78	1.47570383209547	0.151	0.054	4.28859312238577E-74	Detoxification-associated hepat	C2orf92
2.78735383126693E-78	1.06373500381163	0.181	0.072	4.67104755043712E-74	Detoxification-associated hepat	GLMN
2.90587200974805E-78	0.376499159553886	0.329	0.169	4.86966031393579E-74	Detoxification-associated hepat	CEPT1
3.17851016660754E-78	0.815325610612016	0.21	0.09	5.32654733720091E-74	Detoxification-associated hepat	RAD17
3.4755566742338E-78	1.62990017965757	0.157	0.059	5.824337874681E-74	Detoxification-associated hepat	SALL4
3.58617946441528E-78	0.665862181502386	0.231	0.103	6.00971954646713E-74	Detoxification-associated hepat	C2CD2
4.18240848147488E-78	0.431558008457263	0.345	0.181	7.0088801332556E-74	Detoxification-associated hepat	CBX3
4.53600270553488E-78	0.69615090358305	0.274	0.133	7.60143333393536E-74	Detoxification-associated hepat	GDAP2
5.58436739762509E-78	0.722351743286417	0.262	0.127	9.35828288494013E-74	Detoxification-associated hepat	RPE
6.01431739434854E-78	0.378873957516083	0.285	0.139	1.00787930894493E-73	Detoxification-associated hepat	TBC1D24
6.09083065504727E-78	0.75218023940721	0.234	0.106	1.02070140117282E-73	Detoxification-associated hepat	ZC3H11A
7.95921890787606E-78	0.751402839240071	0.227	0.101	1.33380590458187E-73	Detoxification-associated hepat	ZBTB45
8.98090739170192E-78	0.758796825361056	0.244	0.113	1.50502046070141E-73	Detoxification-associated hepat	ACTR6

**9.03569685442504E-78**	0.446368902128077	0.44	0.244	1.51420207886455E-73	Detoxification-associated hepat	TMC5
**9.88677665100892E-78**	0.260086237918611	0.368	0.197	1.65682603117607E-73	Detoxification-associated hepat	FAM168A
**1.23015027785222E-77**	0.669730711209441	0.275	0.135	2.06148583562475E-73	Detoxification-associated hepat	TRNAU1AP
**1.45605325367163E-77**	1.68162553219898	0.119	0.037	2.44005404250292E-73	Detoxification-associated hepat	KATNBL1
**1.67095242792429E-77**	1.31066927047315	0.14	0.048	2.80018207871553E-73	Detoxification-associated hepat	ZNF181
**1.70764958903048E-77**	0.410977265575046	0.309	0.157	2.86167918129728E-73	Detoxification-associated hepat	SUDS3
**1.95791272868775E-77**	1.95665461630817	0.11	0.032	3.28107015073492E-73	Detoxification-associated hepat	SPTY2D1OS
**1.95819604522284E-77**	0.267150272430245	0.429	0.239	3.28154493258443E-73	Detoxification-associated hepat	BROX
**2.0160921447677E-77**	0.574799232892768	0.275	0.133	3.37856721620171E-73	Detoxification-associated hepat	POLM
**2.15686217060249E-77**	1.13978912799861	0.186	0.076	3.61446962549565E-73	Detoxification-associated hepat	LY6G5B
**2.35864110372886E-77**	0.320974450143749	0.382	0.207	3.95261076162883E-73	Detoxification-associated hepat	RBM24
**2.38546520816227E-77**	0.537934411380163	0.23	0.103	3.99756259583833E-73	Detoxification-associated hepat	SMIM30
**2.38760651150111E-77**	0.385268871621484	0.357	0.19	4.00115099197356E-73	Detoxification-associated hepat	STIM2
**2.45886630502945E-77**	0.613835010973949	0.252	0.118	4.12056815396835E-73	Detoxification-associated hepat	DAAM1
**2.56635896116209E-77**	0.376802265129498	0.361	0.194	4.30070434711542E-73	Detoxification-associated hepat	BRD1
**2.86804805492719E-77**	0.380990603028941	0.368	0.199	4.80627493044698E-73	Detoxification-associated hepat	CAMSAP3
**3.02445170981987E-77**	0.429912761172185	0.354	0.19	5.06837617531613E-73	Detoxification-associated hepat	ME2
**3.0669282701361E-77**	0.266554797935557	0.374	0.203	5.13955839509407E-73	Detoxification-associated hepat	HARS
**3.49649864066654E-77**	1.59961833465724	0.14	0.048	5.85943242202899E-73	Detoxification-associated hepat	PCDHGA10
**3.71437143481679E-77**	0.535458700276093	0.291	0.146	6.22454365046597E-73	Detoxification-associated hepat	PIGO
**3.91965648149428E-77**	0.883868441939608	0.216	0.095	6.56856033168811E-73	Detoxification-associated hepat	BORCS5
**4.52201640395619E-77**	0.666748211622989	0.304	0.156	7.57799508974978E-73	Detoxification-associated hepat	FAM126A
**4.6431854670557E-77**	0.374452336785365	0.393	0.217	7.78105020569194E-73	Detoxification-associated hepat	SOAT1
**4.97425098361044E-77**	0.455271321068908	0.244	0.112	8.33584979833437E-73	Detoxification-associated hepat	RBBP9
**4.99493350801395E-77**	0.939511015380582	0.173	0.068	8.37050957272977E-73	Detoxification-associated hepat	RCN2
**5.03839604778173E-77**	0.61508899478708	0.216	0.093	8.44334409687263E-73	Detoxification-associated hepat	SCN8A
**5.44161513610367E-77**	1.16933405085845	0.15	0.054	9.11905864508253E-73	Detoxification-associated hepat	ZNF567
**5.6186684602565E-77**	0.442556792779991	0.307	0.155	9.41576460569784E-73	Detoxification-associated hepat	ING2
**5.62839151914275E-77**	0.267918082131185	0.396	0.219	9.43205850777941E-73	Detoxification-associated hepat	DDX42
**5.91018432222484E-77**	0.537663876323019	0.153	0.054	9.90428688718439E-73	Detoxification-associated hepat	TMEM150A
**5.96992387951084E-77**	0.387013329266915	0.309	0.156	1.00043984372843E-72	Detoxification-associated hepat	AK3
**6.43723441179148E-77**	0.330273478456014	0.353	0.187	1.07875174272802E-72	Detoxification-associated hepat	NARF
**8.21941191530994E-77**	0.377774586719191	0.299	0.15	1.37740904876764E-72	Detoxification-associated hepat	TBC1D23
**8.8474586406241E-77**	0.314747700900051	0.331	0.172	1.48265711899579E-72	Detoxification-associated hepat	KRR1
**9.833455259549E-77**	0.865428363342661	0.229	0.104	1.64789043239522E-72	Detoxification-associated hepat	AHCTF1
**1.00219116121426E-76**	0.974278699174919	0.144	0.051	1.67947194796286E-72	Detoxification-associated hepat	PPIP5K1
**1.0141417680283E-76**	0.437240862038024	0.273	0.133	1.69949877486183E-72	Detoxification-associated hepat	LEMD3
**1.12747692045721E-76**	0.507362536485227	0.282	0.138	1.8894258233022E-72	Detoxification-associated hepat	DDX18
**1.15702244127519E-76**	0.86829435781496	0.219	0.097	1.93893820708897E-72	Detoxification-associated hepat	ZMYM1
**1.16912772092729E-76**	1.05417830443339	0.176	0.07	1.95922423472995E-72	Detoxification-associated hepat	TUBD1
**1.21022141565105E-76**	0.443355802933893	0.298	0.15	2.02808904834803E-72	Detoxification-associated hepat	MKKS
**1.21946980317724E-76**	0.712416877350927	0.23	0.104	2.04358749616442E-72	Detoxification-associated hepat	C15orf41
**1.67132105564929E-76**	0.423281664917373	0.263	0.126	2.80079982505709E-72	Detoxification-associated hepat	PRKACB
**1.7776748218524E-76**	0.461504468176493	0.284	0.141	2.97902746646026E-72	Detoxification-associated hepat	TNPO1
**1.82520148673294E-76**	0.699954180722146	0.206	0.089	3.05867265146707E-72	Detoxification-associated hepat	GPRASP2
**1.96978653047413E-76**	0.459424556247918	0.341	0.181	3.30096826776854E-72	Detoxification-associated hepat	CCT4
**2.01367647081549E-76**	1.47035464399562	0.103	0.029	3.3745190297926E-72	Detoxification-associated hepat	ZNF708
**2.06276336000385E-76**	0.781222241736985	0.226	0.102	3.45677883869446E-72	Detoxification-associated hepat	SPIN3
**2.08840272994217E-76**	0.546656136372876	0.3	0.152	3.49974529483709E-72	Detoxification-associated hepat	NUP188
**2.50974740021001E-76**	0.261391874543506	0.397	0.218	4.20583469327194E-72	Detoxification-associated hepat	CHD4
**3.04939720808401E-76**	0.540245628799411	0.286	0.143	5.11017984130718E-72	Detoxification-associated hepat	AP5M1
**3.14302309527218E-76**	0.424918294981084	0.334	0.175	5.26707810305712E-72	Detoxification-associated hepat	ARMC8
**3.34489292168099E-76**	0.287451936951846	0.377	0.205	5.60537155815301E-72	Detoxification-associated hepat	TEX261
**3.59192176791094E-76**	0.492965177979778	0.313	0.161	6.01934249866515E-72	Detoxification-associated hepat	PKD2
**3.74828435203864E-76**	0.776675270950183	0.201	0.086	6.28137491714636E-72	Detoxification-associated hepat	ZNF451
**4.37036748545015E-76**	0.635548401099962	0.256	0.122	7.32386183211736E-72	Detoxification-associated hepat	TTI1
**4.53783027572857E-76**	0.484820284421124	0.306	0.157	7.60449597606594E-72	Detoxification-associated hepat	ARMH3
**4.67359382954104E-76**	0.31761917237107	0.269	0.129	7.83200853954487E-72	Detoxification-associated hepat	PACS1
**5.28104976005489E-76**	1.08041291721575	0.165	0.063	8.84998318789998E-72	Detoxification-associated hepat	HYKK
**5.56221878780192E-76**	0.735717477186003	0.201	0.086	9.32116624459846E-72	Detoxification-associated hepat	TTC23

**5.80396777548963E-76**	0.749862826709518	0.219	0.097	9.72628919816553E-72	Detoxification-associated hepat	FAM126B
**6.807844101282E-76**	0.253081857004676	0.377	0.205	1.14085851449284E-71	Detoxification-associated hepat	ARHGEF11
**7.77990659175167E-76**	0.569154150024899	0.315	0.165	1.30375674664575E-71	Detoxification-associated hepat	UEVLD
**8.04000622076419E-76**	1.05530380546064	0.199	0.086	1.34734424247566E-71	Detoxification-associated hepat	SLC18B1
**8.11307596052926E-76**	0.318952465507127	0.303	0.153	1.35958926946549E-71	Detoxification-associated hepat	MFSD14A
**8.2446762430429E-76**	0.614557162715223	0.196	0.081	1.38164284480913E-71	Detoxification-associated hepat	ECM2
**8.66693096068185E-76**	0.421982423210917	0.298	0.15	1.45240429039106E-71	Detoxification-associated hepat	ZNF32
**9.6814319715377E-76**	0.568471960589903	0.269	0.131	1.62241436979029E-71	Detoxification-associated hepat	CCNT1
**9.7951510056858E-76**	0.580682123828405	0.262	0.126	1.64147140553283E-71	Detoxification-associated hepat	REPS2
**1.04780062036659E-75**	0.688206158078345	0.215	0.094	1.75590427961033E-71	Detoxification-associated hepat	ATAD3B
**1.05031165102154E-75**	1.26675420645854	0.144	0.051	1.7601122647819E-71	Detoxification-associated hepat	ZNF225
**1.19979962448261E-75**	0.797817252304125	0.155	0.057	2.01062421070795E-71	Detoxification-associated hepat	TPPP
**1.2462297824554E-75**	0.375145079319273	0.314	0.158	2.08843186943876E-71	Detoxification-associated hepat	CENPN
**1.33841915576083E-75**	0.452132865950916	0.309	0.158	2.242922821224E-71	Detoxification-associated hepat	CALML4
**1.39288853329551E-75**	0.390126234335414	0.337	0.178	2.33420260409661E-71	Detoxification-associated hepat	OGFOD2
**1.453505471433E-75**	0.635325406785602	0.208	0.09	2.43578446902742E-71	Detoxification-associated hepat	SLC35A1
**1.50470445733916E-75**	0.695788781046142	0.23	0.105	2.52158372960897E-71	Detoxification-associated hepat	SMUG1
**1.52530915244137E-75**	0.648673829007446	0.269	0.132	2.55611307766125E-71	Detoxification-associated hepat	ATP9B
**1.9121158139256E-75**	0.258712458650894	0.406	0.226	3.20432368097651E-71	Detoxification-associated hepat	IPO8
**2.19089206671052E-75**	0.750121627275975	0.214	0.094	3.67149692539348E-71	Detoxification-associated hepat	TRAF6
**2.78472172637516E-75**	0.317817027798358	0.408	0.228	4.6666366690595E-71	Detoxification-associated hepat	PGAP2
**2.89080981414667E-75**	0.467829900719656	0.257	0.122	4.84441908654699E-71	Detoxification-associated hepat	MPV17L
**3.03817222998744E-75**	0.615235779228495	0.256	0.122	5.09136902301296E-71	Detoxification-associated hepat	CLK2
**3.28097578485512E-75**	0.323236406429187	0.384	0.211	5.49825922026021E-71	Detoxification-associated hepat	UGGT1
**3.50228753357005E-75**	0.569443824457839	0.157	0.057	5.86913344875669E-71	Detoxification-associated hepat	SLC17A3
**3.92128306218534E-75**	0.318543172301862	0.303	0.154	6.57128615561019E-71	Detoxification-associated hepat	HNRNPLL
**4.79729365122432E-75**	0.55962912451059	0.26	0.126	8.03930470072172E-71	Detoxification-associated hepat	AQR
**4.94491858956822E-75**	0.335088039155823	0.318	0.166	8.28669457239843E-71	Detoxification-associated hepat	ARL 16.00
**5.38258099211671E-75**	0.886461083341826	0.197	0.084	9.02012922658919E-71	Detoxification-associated hepat	STARD13
**5.6100969508978E-75**	0.300529310177027	0.35	0.186	9.40140047031454E-71	Detoxification-associated hepat	PARD3
**5.82103748032418E-75**	0.610608408327329	0.279	0.139	9.75489460952726E-71	Detoxification-associated hepat	RAP1GDS1
**6.6427601143E-75**	0.293190050003085	0.33	0.173	1.11319373995439E-70	Detoxification-associated hepat	YIPF6
**8.74260782405725E-75**	0.625382015254945	0.219	0.097	1.46508621915551E-70	Detoxification-associated hepat	BEND7
**8.86740732564385E-75**	0.31886951092323	0.327	0.17	1.4860001196314E-70	Detoxification-associated hepat	FAM110B
**9.23149683618453E-75**	0.511258385861021	0.284	0.142	1.5470142398078E-70	Detoxification-associated hepat	RNF31
**1.042588134816E-74**	0.678766504624626	0.256	0.123	1.74716919632466E-70	Detoxification-associated hepat	ST3GAL3
**1.10062031522226E-74**	0.532423158108459	0.298	0.153	1.84441952424946E-70	Detoxification-associated hepat	TTC33
**1.23564208530586E-74**	0.479432777243901	0.286	0.143	2.07068900655556E-70	Detoxification-associated hepat	GAR1
**1.3421302923728E-74**	0.588098479295263	0.284	0.142	2.24914194395833E-70	Detoxification-associated hepat	GLCE
**1.41630771444526E-74**	0.513177771075862	0.272	0.134	2.37344846786736E-70	Detoxification-associated hepat	TMEM30A
**1.54060233820879E-74**	0.659403836881844	0.254	0.122	2.5817413983703E-70	Detoxification-associated hepat	PIGV
**1.62752840490881E-74**	0.4820614233637	0.273	0.134	2.72741210094618E-70	Detoxification-associated hepat	AMBRA1
**2.69260186637191E-74**	0.275699408407935	0.335	0.176	4.51226220766605E-70	Detoxification-associated hepat	NUDT14
**2.72848033592948E-74**	0.273083730961707	0.899	0.553	4.57238734695062E-70	Detoxification-associated hepat	DNM2
**3.54551244835348E-74**	0.928242297934817	0.208	0.091	5.94156976095076E-70	Detoxification-associated hepat	ZNF557
**3.71442903535336E-74**	1.13290470149314	0.142	0.051	6.22464017744515E-70	Detoxification-associated hepat	KIAA0895L
**4.02491297482871E-74**	0.259290085323722	0.406	0.228	6.74494916321795E-70	Detoxification-associated hepat	TNPO3
**5.31806583983954E-74**	1.20961086925163	0.113	0.035	8.9120147344031E-70	Detoxification-associated hepat	KLHDC1
**6.04077668398824E-74**	1.78430179550477	0.113	0.035	1.01231335670275E-69	Detoxification-associated hepat	WBP2NL
**6.20414147825758E-74**	0.380882626896931	0.323	0.168	1.03969002892641E-69	Detoxification-associated hepat	DNM1L
**6.57829299094264E-74**	0.519266148600766	0.3	0.155	1.10239033942217E-69	Detoxification-associated hepat	FRS2
**6.6774858993259E-74**	0.25496471708848	0.329	0.172	1.11901308700903E-69	Detoxification-associated hepat	MRTFA
**7.11322624814915E-74**	0.38918810207653	0.285	0.142	1.19203445466483E-69	Detoxification-associated hepat	NUDT16L1
**9.25927755633634E-74**	0.302046463187203	0.321	0.168	1.55166973289084E-69	Detoxification-associated hepat	KDM5A
**9.28392615454294E-74**	0.662999821823711	0.249	0.118	1.55580034497831E-69	Detoxification-associated hepat	IFFO1
**9.9838002341244E-74**	0.58768025863346	0.274	0.137	1.67308524323457E-69	Detoxification-associated hepat	MANBA
**1.02333227235238E-73**	0.292072655461013	0.357	0.193	1.71490022200813E-69	Detoxification-associated hepat	EIF2AK2
**1.04893258625439E-73**	0.282084696105874	0.37	0.201	1.7578012280451E-69	Detoxification-associated hepat	EIF4E
**1.17848740044773E-73**	0.583353445283611	0.276	0.136	1.9749091856703E-69	Detoxification-associated hepat	POLH
**1.3217514493566E-73**	1.09140231586695	0.176	0.072	2.21499107883178E-69	Detoxification-associated hepat	ST7L

1.33510845894271E-73	0.925360253758034	0.17	0.067	2.23737475549619E-69	Detoxification-associated hepat	MAP2
1.35804886012986E-73	0.561738667898262	0.248	0.118	2.27581827980563E-69	Detoxification-associated hepat	RUSC2
1.40372535055861E-73	0.301096399756563	0.341	0.181	2.35236294246612E-69	Detoxification-associated hepat	STXBP3
1.65611487934729E-73	0.36065468640239	0.274	0.135	2.77531731481018E-69	Detoxification-associated hepat	UBA3
1.66349142549098E-73	0.79181157614519	0.191	0.08	2.78767893083779E-69	Detoxification-associated hepat	TMEM242
2.19291212639498E-73	0.868182568682077	0.203	0.089	3.6748821414127E-69	Detoxification-associated hepat	CDK17
2.51386612237658E-73	0.342561913893712	0.371	0.203	4.21273684787867E-69	Detoxification-associated hepat	TOR1A
2.5887633183276E-73	0.353271297183972	0.311	0.16	4.3382495688534E-69	Detoxification-associated hepat	ZNF639
2.66343942649481E-73	0.467790268014164	0.275	0.136	4.46339179092E-69	Detoxification-associated hepat	ZNF76
3.0031132637306E-73	0.44738007846809	0.276	0.137	5.03261720735974E-69	Detoxification-associated hepat	TBRG1
3.09752285228511E-73	0.367718041700575	0.31	0.161	5.19082879585939E-69	Detoxification-associated hepat	METTL5
3.3197407842702E-73	0.513086737112034	0.25	0.119	5.56322160628E-69	Detoxification-associated hepat	LMBR1L
3.45766216433171E-73	0.562256613568588	0.247	0.117	5.79435025498707E-69	Detoxification-associated hepat	ELP4
3.59432616923037E-73	0.623687623132954	0.249	0.119	6.02337179439626E-69	Detoxification-associated hepat	WNT4
3.80885628380498E-73	0.861102601453628	0.198	0.086	6.38288136040038E-69	Detoxification-associated hepat	ZNF780A
4.96329570831725E-73	0.354363213809462	0.341	0.182	8.31749094799805E-69	Detoxification-associated hepat	COPS4
5.05974773952788E-73	0.41090860803353	0.282	0.141	8.47912526190082E-69	Detoxification-associated hepat	SNUPN
5.87851184808546E-73	0.25125657020246	0.32	0.165	9.85121015502161E-69	Detoxification-associated hepat	KLC4
5.9354495031222E-73	0.615029617426906	0.228	0.105	9.94662627733219E-69	Detoxification-associated hepat	PIP4K2A
7.08931998452325E-73	0.345496502750951	0.36	0.196	1.18802824300641E-68	Detoxification-associated hepat	IRS1
7.35648463039315E-73	0.537926548473088	0.293	0.15	1.23279969436128E-68	Detoxification-associated hepat	KCTD13
7.52178419450967E-73	0.94197683672809	0.189	0.079	1.26050059531593E-68	Detoxification-associated hepat	NEU4
7.56721582128969E-73	0.347257667353137	0.32	0.168	1.26811402733173E-68	Detoxification-associated hepat	COQ8B
7.96329552491824E-73	0.658826714657716	0.207	0.091	1.3344890640658E-68	Detoxification-associated hepat	INTS14
8.80903763792527E-73	0.517642802273676	0.238	0.111	1.47621852736352E-68	Detoxification-associated hepat	RNMT
8.91919151736962E-73	0.786021428888877	0.215	0.098	1.4946781144808E-68	Detoxification-associated hepat	ZNF696
1.10451875404677E-72	0.816557833343944	0.2	0.087	1.85095252803158E-68	Detoxification-associated hepat	TTBK2
1.2306911499772E-72	0.540770299663509	0.278	0.14	2.0623922291318E-68	Detoxification-associated hepat	COQ2
1.43176748705612E-72	0.420607838560675	0.322	0.17	2.39935595480864E-68	Detoxification-associated hepat	TRIM33
1.60668525825072E-72	0.650694405692996	0.215	0.097	2.69248315577655E-68	Detoxification-associated hepat	TTPAL
1.70680225104036E-72	0.771122475438998	0.135	0.046	2.86025921229343E-68	Detoxification-associated hepat	STARD8
1.72641867025953E-72	0.761676410231747	0.212	0.095	2.89313240762093E-68	Detoxification-associated hepat	SNX16
2.00096061313313E-72	0.606977122672857	0.273	0.136	3.35320979548851E-68	Detoxification-associated hepat	ZFP64
2.11305363640682E-72	0.53718215900507	0.284	0.144	3.54105528389055E-68	Detoxification-associated hepat	MFHAS1
2.30088174356923E-72	0.526481846801119	0.273	0.136	3.85581762587331E-68	Detoxification-associated hepat	ZMYM4
2.33727601921281E-72	1.14472423425782	0.155	0.059	3.91680715299683E-68	Detoxification-associated hepat	LYSMD4
2.60855048752271E-72	0.772305864030775	0.219	0.099	4.37140890699055E-68	Detoxification-associated hepat	HSPA14.1
2.94883479081879E-72	1.32513347319606	0.125	0.042	4.94165734245412E-68	Detoxification-associated hepat	DTWD1
3.55796426885936E-72	0.366193922470802	0.353	0.192	5.96243652175451E-68	Detoxification-associated hepat	THUMPD1
4.07595592249934E-72	1.56002630204727	0.105	0.031	6.8304869349244E-68	Detoxification-associated hepat	PCDHB14
5.03952091175467E-72	1.41860203734846	0.134	0.047	8.44522914391847E-68	Detoxification-associated hepat	ZNF587
5.49040518059836E-72	0.278346485142073	0.248	0.116	9.20082100164673E-68	Detoxification-associated hepat	ZNF609
5.49131871094889E-72	0.378625256874148	0.315	0.165	9.20235189580816E-68	Detoxification-associated hepat	FAM98C
6.40018347313092E-72	0.289054526621023	0.354	0.192	1.07254274642728E-67	Detoxification-associated hepat	NUP98
6.4418813863278E-72	0.829550742767162	0.208	0.093	1.07953048272081E-67	Detoxification-associated hepat	ZNF441
6.83882919264349E-72	0.800662825376785	0.192	0.082	1.1460509961032E-67	Detoxification-associated hepat	PTBP2
7.25023418860759E-72	1.12802989042248	0.117	0.037	1.21499424532686E-67	Detoxification-associated hepat	AMN1
7.44547400776573E-72	0.706527419760032	0.25	0.122	1.24771253422138E-67	Detoxification-associated hepat	TMEM184C
7.54618225668475E-72	0.309607482780104	0.392	0.22	1.26458922257523E-67	Detoxification-associated hepat	RNF6
8.04288257264912E-72	0.477403666310909	0.343	0.187	1.34782626152454E-67	Detoxification-associated hepat	DGKQ
9.22600949017971E-72	0.332785280650142	0.314	0.164	1.54609467036432E-67	Detoxification-associated hepat	ACAP2
1.14838765244935E-71	0.504258250806141	0.299	0.155	1.92446802797462E-67	Detoxification-associated hepat	NOP9
1.24700228666069E-71	0.577895602291199	0.233	0.109	2.08972643198598E-67	Detoxification-associated hepat	PPOX
1.4121654198988E-71	0.274898972023124	0.298	0.151	2.36650681066641E-67	Detoxification-associated hepat	FERMT2
1.419232705137E-71	0.559340387030137	0.233	0.109	2.37835016726858E-67	Detoxification-associated hepat	PPM1H
1.42904815457322E-71	0.269857079061708	0.358	0.195	2.3947988974338E-67	Detoxification-associated hepat	ZMPSTE24
1.50491376854349E-71	0.731521725100384	0.192	0.083	2.52193449332518E-67	Detoxification-associated hepat	DEPRECATEI
1.63059217323133E-71	0.333997819155016	0.358	0.196	2.73254636390107E-67	Detoxification-associated hepat	EXOSC2
1.63775509959384E-71	0.418119056727865	0.302	0.156	2.74454999589935E-67	Detoxification-associated hepat	SESTD1
1.65315096092286E-71	0.31108630117975	0.331	0.177	2.77035038031453E-67	Detoxification-associated hepat	RBM22

1.80918290742306E-71	0.357910479956025	0.368	0.203	3.03182871625956E-67	Detoxification-associated hepat	EIF4G3
1.90026440949865E-71	1.11813391304822	0.157	0.061	3.18446309743784E-67	Detoxification-associated hepat	HSPBAP1
2.09758887173165E-71	0.493631298490228	0.206	0.09	3.5151394312479E-67	Detoxification-associated hepat	UNC45A
2.42085462083538E-71	1.06542364570834	0.133	0.046	4.05686817359594E-67	Detoxification-associated hepat	CLTCL1
2.55296091507221E-71	0.538439117253968	0.25	0.121	4.27825190147801E-67	Detoxification-associated hepat	RIOK2
4.11538147621438E-71	0.317953807636163	0.327	0.175	6.89655627784005E-67	Detoxification-associated hepat	PPHLN1
4.50497973164758E-71	0.311055466549672	0.331	0.176	7.54944503429501E-67	Detoxification-associated hepat	EDARADD
4.63820686433432E-71	0.892090628476368	0.19	0.082	7.77270706325146E-67	Detoxification-associated hepat	KIAA0895
5.59171087812259E-71	0.339586518651598	0.308	0.16	9.37058908955784E-67	Detoxification-associated hepat	SQLE
5.59547960985981E-71	0.427571445022112	0.289	0.147	9.37690473020306E-67	Detoxification-associated hepat	CAD
6.54574804395843E-71	0.732516865444889	0.179	0.073	1.09693645720655E-66	Detoxification-associated hepat	PNPLA7
7.51336134623536E-71	0.552398633456214	0.294	0.152	1.25908909440212E-66	Detoxification-associated hepat	TCP11L1
8.44381587429294E-71	0.948469259044143	0.245	0.119	1.41501466421401E-66	Detoxification-associated hepat	HCN3
8.68481964540529E-71	0.525178855958723	0.28	0.14	1.45540207617702E-66	Detoxification-associated hepat	PITPNM2
8.84162612564568E-71	0.576708109701418	0.23	0.107	1.4816797061357E-66	Detoxification-associated hepat	RCHY1
9.0552331158552E-71	1.51469450502476	0.101	0.03	1.51747596555501E-66	Detoxification-associated hepat	ACSS1
1.05221875334244E-70	0.719211127048901	0.18	0.075	1.76330818685126E-66	Detoxification-associated hepat	PARP16
1.1593679293353E-70	0.298359937039593	0.37	0.207	1.9428687759801E-66	Detoxification-associated hepat	ABI1
1.2074769532174E-70	0.417384074732442	0.284	0.145	2.02348987820172E-66	Detoxification-associated hepat	ZMAT3
1.51908311714191E-70	0.403857555233524	0.298	0.154	2.54567948770641E-66	Detoxification-associated hepat	SKP2
1.54100920123186E-70	0.677316587175882	0.207	0.092	2.58242321942435E-66	Detoxification-associated hepat	RYK
1.58735988741495E-70	0.829480054302648	0.18	0.075	2.66009769932998E-66	Detoxification-associated hepat	POLL
1.73985082308299E-70	0.755950283350074	0.192	0.083	2.91564200932248E-66	Detoxification-associated hepat	SRBD1
1.90918319058277E-70	0.334306518699023	0.391	0.22	3.1994091907786E-66	Detoxification-associated hepat	CEMIP2
1.91205507902585E-70	0.391024198997604	0.322	0.172	3.20422190143152E-66	Detoxification-associated hepat	HSPA13
2.03988410236216E-70	0.74744753931179	0.202	0.089	3.4184377787385E-66	Detoxification-associated hepat	ZNF707
2.09441714551517E-70	1.09179486934576	0.169	0.068	3.50982425245432E-66	Detoxification-associated hepat	NEK1
2.79027415877146E-70	0.792015311973342	0.186	0.079	4.67594143526922E-66	Detoxification-associated hepat	E2F6
3.03490813154534E-70	0.260978950425959	0.38	0.212	5.08589904684367E-66	Detoxification-associated hepat	WASHC5
3.15439159840339E-70	0.374793511892255	0.344	0.187	5.2861294406044E-66	Detoxification-associated hepat	KHNYN
3.24226525956798E-70	0.536548519959773	0.275	0.139	5.43338812198402E-66	Detoxification-associated hepat	EXOC2
3.26701756837381E-70	0.947217077278444	0.165	0.066	5.47486804108084E-66	Detoxification-associated hepat	KIAA1586
3.29579426288655E-70	1.01374824711819	0.185	0.079	5.52309202574528E-66	Detoxification-associated hepat	TTC5
3.50319997396031E-70	0.626052680857437	0.167	0.066	5.87066251636268E-66	Detoxification-associated hepat	ZSCAN2
3.77073311714155E-70	0.443903047057577	0.283	0.145	6.31899455770581E-66	Detoxification-associated hepat	SFSWAP
3.90623107802645E-70	0.562829850265546	0.296	0.155	6.54606204055673E-66	Detoxification-associated hepat	HLCS
3.9822629145012E-70	0.714180606586697	0.212	0.097	6.6734761921211E-66	Detoxification-associated hepat	ARL 15.00
4.04934988224606E-70	0.513296552800631	0.24	0.114	6.78590053266795E-66	Detoxification-associated hepat	OXLD1
4.31079868662956E-70	0.818182615418064	0.22	0.102	7.22403643905382E-66	Detoxification-associated hepat	FUNDC2
5.05863320309706E-70	0.395126752454537	0.264	0.13	8.47725752175005E-66	Detoxification-associated hepat	SRC
5.11419351497386E-70	1.79538531166695	0.119	0.04	8.5703654923932E-66	Detoxification-associated hepat	TERC
5.65358601786551E-70	0.323878979372367	0.307	0.16	9.47427944873902E-66	Detoxification-associated hepat	UTP6
6.35087733141652E-70	0.421375421250899	0.294	0.152	1.06428002319878E-65	Detoxification-associated hepat	PPID
6.66097560905024E-70	0.443644610562062	0.258	0.127	1.11624629256464E-65	Detoxification-associated hepat	RRNAD1
6.91026316670147E-70	0.536210550589564	0.276	0.141	1.15802190147583E-65	Detoxification-associated hepat	DESI2
7.73668696210008E-70	0.264406121324052	0.943	0.703	1.29651400110873E-65	Detoxification-associated hepat	IFI27
7.79277196681644E-70	1.17181420096386	0.152	0.058	1.3059127261991E-65	Detoxification-associated hepat	ZNF34
7.88810797312704E-70	0.893547929411931	0.178	0.074	1.32188913413663E-65	Detoxification-associated hepat	ANKRD16
8.28322313767247E-70	0.60124862593463	0.219	0.101	1.38810253341115E-65	Detoxification-associated hepat	PRKCI
8.81889078218366E-70	0.485408977524609	0.268	0.135	1.47786971727834E-65	Detoxification-associated hepat	SPTLC1
9.5065353362522E-70	0.385983504205905	0.28	0.142	1.59310519164914E-65	Detoxification-associated hepat	ELP1
1.08637097483962E-69	1.4119212228425	0.138	0.051	1.82054047963623E-65	Detoxification-associated hepat	USP49
1.13233488184387E-69	0.518311600261735	0.271	0.137	1.89756679499396E-65	Detoxification-associated hepat	LCORL
1.1844172061319E-69	0.380013386849485	0.283	0.145	1.98484635403584E-65	Detoxification-associated hepat	PDCL3
1.19888461334146E-69	0.432718173232733	0.248	0.12	2.00909083503762E-65	Detoxification-associated hepat	ZSWIM9
1.21232086316811E-69	0.558276527410077	0.304	0.16	2.03160730249713E-65	Detoxification-associated hepat	XPO1
1.24400546517632E-69	0.552266600085099	0.202	0.088	2.08470435854247E-65	Detoxification-associated hepat	ITGAM
1.28204641758531E-69	0.463625269528793	0.257	0.126	2.14845338658947E-65	Detoxification-associated hepat	RNGTT
1.33806200321686E-69	0.342127249431908	0.272	0.136	2.24232430499081E-65	Detoxification-associated hepat	PPTC7
1.35625720377908E-69	0.993797151344151	0.192	0.085	2.27281582209297E-65	Detoxification-associated hepat	NAA35

**1.44706783101156E-69**	0.580895588040765	0.223	0.103	2.42499627120917E-65	Detoxification-associated hepat	EPC2
**1.55174534477045E-69**	0.268254878227684	0.381	0.213	2.60041484876631E-65	Detoxification-associated hepat	NXT2
**1.68890760411637E-69**	0.548213094633339	0.251	0.122	2.83027136297821E-65	Detoxification-associated hepat	ASXL2
**1.69054329213946E-69**	0.68583223613003	0.244	0.119	2.8330124489673E-65	Detoxification-associated hepat	LIAS
**1.89947003497488E-69**	1.37416473351111	0.157	0.063	3.1831318846109E-65	Detoxification-associated hepat	SIX4
**2.19849568676249E-69**	0.500382037531006	0.246	0.119	3.68423907187658E-65	Detoxification-associated hepat	APC
**2.52455373332682E-69**	1.52359125378826	0.142	0.053	4.23064714630909E-65	Detoxification-associated hepat	ZFP3
**2.5535129216663E-69**	0.682124707640946	0.203	0.09	4.27917695412839E-65	Detoxification-associated hepat	TRIP4
**3.61468320037281E-69**	0.484339785102061	0.301	0.158	6.05748610718475E-65	Detoxification-associated hepat	MOB4
**4.27493669693644E-69**	0.562606461075406	0.203	0.089	7.16393891672609E-65	Detoxification-associated hepat	DEPRECATEI
**4.3039448956365E-69**	0.279315568887481	0.27	0.134	7.21255085610766E-65	Detoxification-associated hepat	FBRSL1
**4.46622672681077E-69**	0.554075657615313	0.245	0.118	7.48450274878949E-65	Detoxification-associated hepat	PTRH2
**4.65719499082959E-69**	0.37121584481934	0.295	0.153	7.80452736563222E-65	Detoxification-associated hepat	TIGD5
**4.99945565766266E-69**	0.436984769336758	0.251	0.122	8.37808779111109E-65	Detoxification-associated hepat	HARS2
**5.10267630906814E-69**	1.03619528969933	0.147	0.056	8.55106495873638E-65	Detoxification-associated hepat	HEATR6
**5.20882152413159E-69**	0.568327229667732	0.26	0.13	8.72894311013973E-65	Detoxification-associated hepat	ASF1A
**5.36376318772583E-69**	1.00856084980847	0.103	0.031	8.98859434999095E-65	Detoxification-associated hepat	SPTB
**5.42080981988565E-69**	0.274982421667937	0.351	0.193	9.08419309616437E-65	Detoxification-associated hepat	PPP2CA
**5.57558473101701E-69**	0.435265874672559	0.284	0.144	9.34356489223831E-65	Detoxification-associated hepat	WDR76
**6.22719176280916E-69**	0.668074609182205	0.192	0.083	1.04355279561156E-64	Detoxification-associated hepat	RAB9A
**6.51216532677756E-69**	0.49883891848467	0.239	0.114	1.09130866546138E-64	Detoxification-associated hepat	EZH1
**6.56882952249083E-69**	0.822527461545937	0.167	0.067	1.10080445137901E-64	Detoxification-associated hepat	RBAK
**7.08942325380019E-69**	0.557274497914581	0.288	0.15	1.18804554887184E-64	Detoxification-associated hepat	ANO10
**7.16279729962903E-69**	0.418768344380064	0.289	0.148	1.20034157147183E-64	Detoxification-associated hepat	GTF2E2
**7.9673848064088E-69**	0.305586186493306	0.845	0.591	1.33517434585799E-64	Detoxification-associated hepat	SCD
**8.63935669134849E-69**	0.435683145760429	0.293	0.152	1.44778339433618E-64	Detoxification-associated hepat	JADE2
**1.03567526922281E-68**	0.253676913646057	0.32	0.169	1.73558461616359E-64	Detoxification-associated hepat	ARRB2
**1.06095852234928E-68**	0.53038780855317	0.264	0.132	1.77795429175293E-64	Detoxification-associated hepat	LRRC8E
**1.20948266918068E-68**	1.37605320901091	0.112	0.036	2.02685105701298E-64	Detoxification-associated hepat	PORCN
**1.21907884695202E-68**	0.468328228985075	0.259	0.129	2.0429323317222E-64	Detoxification-associated hepat	ARHGAP21
**1.33583056934486E-68**	1.01670344737484	0.173	0.073	2.23858486810811E-64	Detoxification-associated hepat	SLC38A6
**1.38356967070425E-68**	1.01211497153795	0.182	0.078	2.31858605416619E-64	Detoxification-associated hepat	ZNF558
**1.50839483565089E-68**	1.17624637473801	0.134	0.048	2.52776806558376E-64	Detoxification-associated hepat	APLF
**1.72872720623421E-68**	0.6318505873786	0.212	0.097	2.8970010522073E-64	Detoxification-associated hepat	NSUN4
**2.23033037690318E-68**	0.255780648327252	0.347	0.191	3.73758764561434E-64	Detoxification-associated hepat	TMEM167A
**2.61510376147559E-68**	1.19032976941866	0.111	0.035	4.3823908834808E-64	Detoxification-associated hepat	KSR1
**2.97103497259864E-68**	0.843961274717669	0.127	0.044	4.9788604070808E-64	Detoxification-associated hepat	ZNF25
**3.30337582227437E-68**	0.629992215685643	0.203	0.091	5.53579720296739E-64	Detoxification-associated hepat	DHX35
**3.58282104403078E-68**	0.547942826199539	0.258	0.128	6.00409150558678E-64	Detoxification-associated hepat	CSNK1G3
**3.62492751037831E-68**	0.439094410684981	0.311	0.166	6.07465352189197E-64	Detoxification-associated hepat	COX20
**4.17347377768455E-68**	1.14821281671612	0.167	0.069	6.99390735664378E-64	Detoxification-associated hepat	CARD8
**4.33636313398702E-68**	0.615695488662959	0.201	0.089	7.26687733993545E-64	Detoxification-associated hepat	CAMTA2
**4.4133598302978E-68**	0.476393937773203	0.258	0.127	7.39590840361305E-64	Detoxification-associated hepat	DOCK7
**4.48095163606932E-68**	0.589139523958428	0.237	0.115	7.50917875172497E-64	Detoxification-associated hepat	SLC35E2A
**4.96707072927087E-68**	0.270400695629256	0.321	0.172	8.32381712811213E-64	Detoxification-associated hepat	MARCH8
**5.05792071372672E-68**	0.264082993296052	0.246	0.117	8.47606353206324E-64	Detoxification-associated hepat	ARL5B
**5.90004590864204E-68**	1.92078391576025	0.113	0.038	9.88729693370233E-64	Detoxification-associated hepat	ATP5MGL
**6.08249046103166E-68**	0.299593159116129	0.312	0.166	1.01930375145969E-63	Detoxification-associated hepat	SAV1
**6.20662725376296E-68**	0.48491791047377	0.271	0.138	1.0401065951856E-63	Detoxification-associated hepat	AP5S1
**6.73897777187131E-68**	0.330196783736874	0.342	0.188	1.12931789501019E-63	Detoxification-associated hepat	KLC1
**6.96026840685878E-68**	0.37396425749723	0.336	0.185	1.16640177962139E-63	Detoxification-associated hepat	SLC17A5
**7.01074709063566E-68**	0.343904527588399	0.241	0.116	1.17486099744872E-63	Detoxification-associated hepat	VARS2
**7.14124259400145E-68**	0.806519457217063	0.202	0.091	1.19672943390276E-63	Detoxification-associated hepat	ZNF823
**7.32366535984292E-68**	0.650593581879485	0.211	0.097	1.22729984100248E-63	Detoxification-associated hepat	SF3A3
**8.54460087624092E-68**	0.394133234651358	0.3	0.159	1.43190421484045E-63	Detoxification-associated hepat	CHD1
**8.68983889934387E-68**	0.62776495212893	0.257	0.129	1.45624320275205E-63	Detoxification-associated hepat	HIRA
**1.15524734765453E-67**	0.427059828370052	0.224	0.105	1.93596350519946E-63	Detoxification-associated hepat	RICTOR
**1.16653322557454E-67**	0.356798907313817	0.33	0.18	1.95487637941781E-63	Detoxification-associated hepat	SMARCC2
**1.78299831728937E-67**	0.499864022222886	0.235	0.113	2.98794858011353E-63	Detoxification-associated hepat	MED21
**2.25470750397598E-67**	0.428100458297902	0.176	0.073	3.77843883516295E-63	Detoxification-associated hepat	RCAN2

**3.0078507398564E-67**	1.02852394102792	0.136	0.05	5.04055626985135E-63	Detoxification-associated hepat	ZNF510
**3.12388889434629E-67**	0.545252590164842	0.211	0.095	5.23501300914551E-63	Detoxification-associated hepat	SLC19A1
**3.20761613939726E-67**	0.479339862739092	0.335	0.182	5.37532312640192E-63	Detoxification-associated hepat	DSN1
**3.2896674522718E-67**	0.671653246273547	0.197	0.088	5.51282471651708E-63	Detoxification-associated hepat	SCRN3
**3.32202150936927E-67**	0.814700685707231	0.138	0.051	5.56704364540102E-63	Detoxification-associated hepat	GNG7
**4.10133812586834E-67**	0.378408687071187	0.326	0.177	6.87302243133017E-63	Detoxification-associated hepat	STRN
**4.3682810380206E-67**	0.46414180066485	0.234	0.112	7.32036536351492E-63	Detoxification-associated hepat	TAB1
**4.51259871628405E-67**	0.582870651504745	0.284	0.15	7.56221292874881E-63	Detoxification-associated hepat	ATP11C
**5.17977144210733E-67**	0.76187765220755	0.169	0.069	8.68026098268346E-63	Detoxification-associated hepat	RPH3AL
**5.22349812427715E-67**	0.815740060729066	0.232	0.112	8.75353815666365E-63	Detoxification-associated hepat	FBF1
**5.32992655218825E-67**	1.20745361488625	0.143	0.054	8.93189091615706E-63	Detoxification-associated hepat	SLC26A2
**5.44772401672996E-67**	0.629554985513964	0.219	0.103	9.12929590723607E-63	Detoxification-associated hepat	SCFD2
**6.03395994895572E-67**	0.385551795225999	0.286	0.149	1.011171008246E-62	Detoxification-associated hepat	AASDHPPT
**6.49732508243238E-67**	0.745292850778471	0.17	0.07	1.08882173731402E-62	Detoxification-associated hepat	REV3L
**6.52034728951704E-67**	0.252994843242317	0.361	0.201	1.09267979877727E-62	Detoxification-associated hepat	UBAC2
**6.65097566045045E-67**	1.12049473339129	0.139	0.052	1.11457050117829E-62	Detoxification-associated hepat	MIS12
**6.74915570230318E-67**	0.553526038301684	0.233	0.111	1.13102351259197E-62	Detoxification-associated hepat	BTN3A1
**6.90242268124145E-67**	0.38039972547363	0.299	0.158	1.15670799292244E-62	Detoxification-associated hepat	PDXDC1
**7.29014901494089E-67**	0.68985762291482	0.2	0.09	1.22168317192379E-62	Detoxification-associated hepat	ZSCAN9
**7.65964392931103E-67**	0.9868435737909	0.163	0.067	1.28360312967394E-62	Detoxification-associated hepat	KIF3A
**7.97275250076282E-67**	0.807426033980708	0.212	0.099	1.33607386407783E-62	Detoxification-associated hepat	ARAP2
**8.00627253602948E-67**	0.587078337220462	0.21	0.096	1.34169115158782E-62	Detoxification-associated hepat	ZCCHC4
**8.71794073024615E-67**	0.818003418215546	0.206	0.095	1.46095250757465E-62	Detoxification-associated hepat	GGPS1
**9.09766981428414E-67**	0.436650317405361	0.271	0.138	1.52458750747774E-62	Detoxification-associated hepat	EXOSC6
**9.26048364348753E-67**	0.39725968913942	0.274	0.14	1.55187184897564E-62	Detoxification-associated hepat	EXD3
**9.5591434270353E-67**	0.264602214250097	0.926	0.697	1.60192125550257E-62	Detoxification-associated hepat	AKR1B10
**9.69725358498136E-67**	0.528782249750898	0.278	0.145	1.62506575577118E-62	Detoxification-associated hepat	FGD6
**9.89921253159668E-67**	0.69032753544803	0.21	0.097	1.65891003604497E-62	Detoxification-associated hepat	THAP6
**1.16831498428982E-66**	0.272796091471373	0.3	0.158	1.95786225067288E-62	Detoxification-associated hepat	ZNF106
**1.20477062370122E-66**	0.523426244378979	0.259	0.13	2.0189546111985E-62	Detoxification-associated hepat	DENND1B
**1.35335859685244E-66**	1.13560707860282	0.109	0.035	2.26795833660532E-62	Detoxification-associated hepat	SLC16A10
**1.5686845429584E-66**	0.40638472192978	0.296	0.157	2.62880155708969E-62	Detoxification-associated hepat	LMAN2L
**1.59156813401163E-66**	1.02791139335368	0.171	0.072	2.66714987897669E-62	Detoxification-associated hepat	TMEM241
**1.62915215525149E-66**	0.454902248978972	0.236	0.113	2.73013318177045E-62	Detoxification-associated hepat	CAPN7
**1.74474537726043E-66**	0.504350247212576	0.286	0.15	2.92384430321303E-62	Detoxification-associated hepat	DDX59
**1.80380004784017E-66**	0.367012390408952	0.301	0.16	3.02280812017055E-62	Detoxification-associated hepat	SDF2
**1.8076123148729E-66**	0.396392350117196	0.308	0.165	3.029196717264E-62	Detoxification-associated hepat	DEPRECATEI
**1.87951260187018E-66**	0.26611845737189	0.323	0.174	3.14968721821405E-62	Detoxification-associated hepat	FBRS
**1.94896777298825E-66**	0.270847195868207	0.312	0.165	3.2660801939737E-62	Detoxification-associated hepat	VPS39
**2.0875977568383E-66**	0.327300337399077	0.379	0.213	3.49839632090962E-62	Detoxification-associated hepat	SEMA6A
**2.4451470670184E-66**	0.654777434897803	0.217	0.102	4.09757745490944E-62	Detoxification-associated hepat	PPIL3
**2.61741073063183E-66**	1.3723528122046	0.105	0.033	4.38625690239283E-62	Detoxification-associated hepat	ZNF771
**2.85118267276518E-66**	1.00617354339601	0.144	0.055	4.7780119230199E-62	Detoxification-associated hepat	POU5F2
**2.97011709056472E-66**	0.267742846055347	0.332	0.182	4.97732222036835E-62	Detoxification-associated hepat	MIB1
**3.14921285491204E-66**	0.453218044288965	0.211	0.097	5.2774509022616E-62	Detoxification-associated hepat	OCEL1
**3.15557530889713E-66**	0.979648466334596	0.177	0.076	5.28811310264981E-62	Detoxification-associated hepat	KLHL28
**3.16979609690528E-66**	0.667127827472772	0.237	0.115	5.31194429919387E-62	Detoxification-associated hepat	SLC9A8
**3.53761416313722E-66**	0.319479045495563	0.266	0.135	5.92833381458535E-62	Detoxification-associated hepat	MAGIX
**3.54165857340913E-66**	0.67907051089793	0.241	0.119	5.93511143731902E-62	Detoxification-associated hepat	SRSF10
**3.64900488135817E-66**	0.37787378310894	0.316	0.17	6.11500238018003E-62	Detoxification-associated hepat	CPOX
**3.74061674241307E-66**	0.445407994730919	0.27	0.138	6.26852553693583E-62	Detoxification-associated hepat	WDR5
**3.86065489670023E-66**	0.650109614540476	0.122	0.042	6.46968547589024E-62	Detoxification-associated hepat	TIAM1
**4.88429653622403E-66**	0.502019027143667	0.281	0.146	8.18510413540423E-62	Detoxification-associated hepat	COG4
**5.16564244961369E-66**	0.485356566352596	0.291	0.154	8.65658361706261E-62	Detoxification-associated hepat	APAF1
**5.72019235527633E-66**	0.347837066513117	0.384	0.218	9.58589834897208E-62	Detoxification-associated hepat	SIPA1L1
**5.98474521645185E-66**	1.26953930576899	0.127	0.046	1.002923603373E-61	Detoxification-associated hepat	EME2
**7.46873588587298E-66**	1.44028759246737	0.138	0.052	1.25161075975459E-61	Detoxification-associated hepat	CATSPERG
**8.92329946773113E-66**	0.401608940254028	0.241	0.117	1.49536652480238E-61	Detoxification-associated hepat	LMF1
**9.14062596000209E-66**	0.555749504648348	0.235	0.114	1.53178609837715E-61	Detoxification-associated hepat	WASHC3
**9.55436725687698E-66**	0.477117442148589	0.305	0.164	1.60112086490744E-61	Detoxification-associated hepat	VPS13A

9.79085891146207E-66	0.290160337191542	0.309	0.166	1.64075213638281E-61	Detoxification-associated hepat	GLRX2
9.80890333528431E-66	0.273303228373497	0.349	0.194	1.64377602092695E-61	Detoxification-associated hepat	STARD3
1.0537121019407E-65	0.39838107726198	0.293	0.153	1.76581074043222E-61	Detoxification-associated hepat	PGGT1B
1.08753354417531E-65	0.434059693994311	0.207	0.094	1.82248871332899E-61	Detoxification-associated hepat	ORC4
1.10822900835452E-65	0.687600427095677	0.187	0.082	1.85717017220051E-61	Detoxification-associated hepat	RHBDD3
1.17108993341219E-65	0.538897537353405	0.235	0.115	1.96251251041215E-61	Detoxification-associated hepat	ATAD1
1.19015172490687E-65	0.439136844416503	0.276	0.143	1.99445626059893E-61	Detoxification-associated hepat	MED12
1.20069500678286E-65	0.484947622559802	0.276	0.143	2.01212469236671E-61	Detoxification-associated hepat	PAM
1.24940132858157E-65	0.565616803620655	0.196	0.087	2.09374674643699E-61	Detoxification-associated hepat	WDR61
1.2642426044048E-65	0.539675872663934	0.243	0.12	2.11861775646157E-61	Detoxification-associated hepat	ACOX3
1.34397879956744E-65	0.679900608925516	0.195	0.087	2.25223967231511E-61	Detoxification-associated hepat	IMMP1L
1.35749714078379E-65	0.383494577041116	0.298	0.159	2.27489370852548E-61	Detoxification-associated hepat	DIP2B
1.38538341892612E-65	0.559332032497278	0.242	0.119	2.3216255334364E-61	Detoxification-associated hepat	KIZ
1.49169067379971E-65	0.519194437845872	0.197	0.088	2.49977523115356E-61	Detoxification-associated hepat	FRA10AC1
1.53481372812618E-65	0.322526715160442	0.29	0.152	2.57204084559385E-61	Detoxification-associated hepat	ABCG8
1.54054801253707E-65	0.581210134651021	0.221	0.105	2.58165035940962E-61	Detoxification-associated hepat	ZBTB5
1.57077525986655E-65	0.639169559273301	0.224	0.107	2.63230518048437E-61	Detoxification-associated hepat	TRIM32
1.66923834986085E-65	0.265728894829413	0.298	0.157	2.79730962669682E-61	Detoxification-associated hepat	FOXN2
1.89803292164732E-65	0.349862462990412	0.272	0.139	3.18072357009658E-61	Detoxification-associated hepat	ATP5F1C
2.21868526336086E-65	0.352756385034336	0.29	0.152	3.71807276434013E-61	Detoxification-associated hepat	PRR12
2.47698791775748E-65	0.517691884839963	0.223	0.106	4.15093635257798E-61	Detoxification-associated hepat	MARF1
2.51515500973524E-65	0.264552139542125	0.336	0.185	4.21489676531431E-61	Detoxification-associated hepat	RPRD1B
3.07512254910228E-65	0.312849237988852	0.269	0.137	5.15329036778561E-61	Detoxification-associated hepat	DEPRECATEI
3.30890408057607E-65	0.390553184766836	0.289	0.153	5.54506145822938E-61	Detoxification-associated hepat	PIGH
3.76734855895465E-65	0.67776245798252	0.209	0.097	6.3133227150962E-61	Detoxification-associated hepat	DDHD1
4.09406875368627E-65	0.57475304040534	0.228	0.11	6.86084041742745E-61	Detoxification-associated hepat	PPP1R14B
4.16508906707495E-65	1.61367661479486	0.1	0.031	6.97985625860421E-61	Detoxification-associated hepat	LRRC69
4.48844231132412E-65	0.355671607517189	0.233	0.111	7.52173162531696E-61	Detoxification-associated hepat	SIRT5
4.82933375045375E-65	0.413393091839738	0.211	0.097	8.0929974990104E-61	Detoxification-associated hepat	LMNTD2
5.32866779854667E-65	0.447332741344471	0.29	0.154	8.92978149680451E-61	Detoxification-associated hepat	RIF1
5.50323479960658E-65	0.785918056638912	0.228	0.11	9.2223208771807E-61	Detoxification-associated hepat	NEDD9
6.40117762400187E-65	0.319694664270735	0.267	0.136	1.07270934623023E-60	Detoxification-associated hepat	SF3B1
8.85658573800582E-65	0.72523864500272	0.16	0.065	1.48418663797502E-60	Detoxification-associated hepat	MAP2K5
9.20803495969979E-65	0.309785643498165	0.298	0.158	1.54308249854649E-60	Detoxification-associated hepat	PSME4
9.37085370475048E-65	0.275119721208015	0.333	0.184	1.57036766384208E-60	Detoxification-associated hepat	UTRN
1.20009023284566E-64	0.574036448175518	0.224	0.107	2.01111121220276E-60	Detoxification-associated hepat	DSTYK
1.37852635456402E-64	0.522151843975133	0.255	0.129	2.31013446497838E-60	Detoxification-associated hepat	ZNF131
1.73345074134342E-64	0.760189932755967	0.188	0.084	2.9049167523433E-60	Detoxification-associated hepat	INTS2
1.80422512981706E-64	0.350086500622787	0.303	0.162	3.02352047254743E-60	Detoxification-associated hepat	IPO5
2.1920209109718E-64	0.704355791916238	0.232	0.114	3.67338864260654E-60	Detoxification-associated hepat	MSH5
2.49712795689857E-64	0.253196291955536	0.315	0.171	4.18468703017062E-60	Detoxification-associated hepat	ATXN2
2.81769620887133E-64	0.913636587423446	0.192	0.087	4.72189530682658E-60	Detoxification-associated hepat	ZBED5
3.04727839309405E-64	0.509296414637171	0.215	0.101	5.106629131147E-60	Detoxification-associated hepat	RAB24
3.10470085703617E-64	0.536648950558146	0.233	0.114	5.20285769622121E-60	Detoxification-associated hepat	ZNF12
3.28861521958669E-64	0.370222219593511	0.262	0.134	5.51106138498337E-60	Detoxification-associated hepat	STRIP1
3.42194893681985E-64	0.535857237904661	0.268	0.139	5.73450202832271E-60	Detoxification-associated hepat	COX19
3.57593179198507E-64	0.738043772195702	0.209	0.098	5.99254649700858E-60	Detoxification-associated hepat	MSH3
3.90264996767766E-64	0.310054217517911	0.259	0.131	6.54006081583422E-60	Detoxification-associated hepat	IMPACT
4.08387759319096E-64	0.647078773598509	0.223	0.106	6.84376207066941E-60	Detoxification-associated hepat	ME3
4.46388039985498E-64	0.391294438771295	0.24	0.118	7.48057077407697E-60	Detoxification-associated hepat	PIK3C2A
5.1672210334506E-64	0.534631122745357	0.258	0.133	8.65922900785652E-60	Detoxification-associated hepat	FBXO34
6.26974732844672E-64	0.526222452131517	0.223	0.107	1.0506842573011E-59	Detoxification-associated hepat	ZBTB4
6.37474795588207E-64	0.431268133484904	0.257	0.13	1.06828026244672E-59	Detoxification-associated hepat	AC118549.1
6.65654567130808E-64	0.295982196286698	0.294	0.156	1.11550392359781E-59	Detoxification-associated hepat	E4F1
6.99922294809602E-64	0.841400120359464	0.205	0.096	1.17292978164193E-59	Detoxification-associated hepat	CGRRF1
7.08392963997724E-64	0.62540178173883	0.177	0.076	1.18712492906739E-59	Detoxification-associated hepat	ZNF496
7.44364456631336E-64	1.16941027888412	0.153	0.062	1.24740595642279E-59	Detoxification-associated hepat	DEPRECATEI
7.44864226817407E-64	1.06650775637622	0.155	0.063	1.24824347130061E-59	Detoxification-associated hepat	ANKRD49
7.58833276872363E-64	0.592518670511795	0.201	0.092	1.27165280538271E-59	Detoxification-associated hepat	RPAP2
7.67899561679913E-64	0.635299815454717	0.249	0.125	1.2868460854632E-59	Detoxification-associated hepat	DEPDC5

8.38750608990995E-64	0.44278563010266	0.269	0.138	1.40557827054711E-59	Detoxification-associated hepat	TONSL
8.55058851778255E-64	0.354724291236978	0.289	0.152	1.43290762381E-59	Detoxification-associated hepat	GRAMD1A
8.65512655862483E-64	0.259767456289153	0.363	0.204	1.45042610869435E-59	Detoxification-associated hepat	YY1AP1
1.12216097821392E-63	0.727095492148649	0.226	0.11	1.88051736729089E-59	Detoxification-associated hepat	B3GLCT
1.32324693129515E-63	0.592771372578113	0.2	0.092	2.21749720746441E-59	Detoxification-associated hepat	NAA25
1.3701092318623E-63	0.35445287360839	0.283	0.148	2.29602905075485E-59	Detoxification-associated hepat	MTMR3
1.44470375304599E-63	0.363493381216533	0.353	0.2	2.42103454935446E-59	Detoxification-associated hepat	GTPBP2
1.47715073920869E-63	0.427216555219831	0.286	0.152	2.47540920876593E-59	Detoxification-associated hepat	XPO5
1.57099992312968E-63	1.59704910238692	0.133	0.051	2.63268167118071E-59	Detoxification-associated hepat	PCDHGA12
1.84768505014238E-63	0.266169510392663	0.289	0.151	3.0963506070286E-59	Detoxification-associated hepat	UBA7
2.00327237447184E-63	0.442316807230846	0.219	0.105	3.35708384513992E-59	Detoxification-associated hepat	NIPSNAP2
2.06151623405299E-63	0.532607402505554	0.293	0.157	3.454688905026E-59	Detoxification-associated hepat	PRR14L
2.23618663862428E-63	0.655046175234224	0.223	0.108	3.74740156900656E-59	Detoxification-associated hepat	TXNDC16
2.44368286343774E-63	0.457040812899742	0.251	0.127	4.09512374254897E-59	Detoxification-associated hepat	LRRC75B
2.56323747304978E-63	0.416972063763236	0.329	0.182	4.29547335733681E-59	Detoxification-associated hepat	SLMAP
2.62867399859053E-63	0.398646058062512	0.248	0.125	4.40513188683802E-59	Detoxification-associated hepat	TMEM101
2.67199997088895E-63	0.715114415278309	0.178	0.078	4.4777375512157E-59	Detoxification-associated hepat	INTS13
2.69983883057352E-63	1.00858439493783	0.139	0.053	4.5243899122751E-59	Detoxification-associated hepat	USP45
2.70312888068974E-63	0.488098564472722	0.214	0.101	4.52990337825987E-59	Detoxification-associated hepat	IPO13
3.26956897719768E-63	0.684363917575332	0.212	0.101	5.47914369198788E-59	Detoxification-associated hepat	SGSH
4.78282332432889E-63	0.475270166434615	0.24	0.12	8.01505532691035E-59	Detoxification-associated hepat	GGA3
5.40793952387217E-63	0.684102309639733	0.187	0.083	9.06262505410497E-59	Detoxification-associated hepat	STRADA
5.43963135485548E-63	0.310892151464342	0.292	0.155	9.11573422446681E-59	Detoxification-associated hepat	KAT2B
5.46747902375154E-63	0.304564139943145	0.278	0.145	9.16240134800282E-59	Detoxification-associated hepat	MMD
6.45887580284912E-63	0.606443609143251	0.262	0.136	1.08237840704146E-58	Detoxification-associated hepat	ZNF398
7.72110313527848E-63	0.600688049438609	0.245	0.123	1.29390246340997E-58	Detoxification-associated hepat	THAP12
1.0425466433985E-62	0.428251919793607	0.262	0.135	1.74709966500721E-58	Detoxification-associated hepat	PIAS2
1.29028146461265E-62	1.06402611470406	0.162	0.068	2.16225367839787E-58	Detoxification-associated hepat	BBS9
1.30728610457457E-62	0.450483731719025	0.306	0.168	2.19075005404606E-58	Detoxification-associated hepat	NCEH1
1.34317166082045E-62	0.413114161775842	0.344	0.193	2.25088706920291E-58	Detoxification-associated hepat	FAM221A
1.47445373214538E-62	1.28686848347772	0.113	0.039	2.47088956432923E-58	Detoxification-associated hepat	ZNF155
1.49052895699218E-62	0.342098647897122	0.302	0.162	2.49782842612749E-58	Detoxification-associated hepat	LDLRAD4
1.63791190339751E-62	0.395126147761694	0.269	0.14	2.74481276771354E-58	Detoxification-associated hepat	CDC27
1.73817056058371E-62	0.565213276646013	0.219	0.105	2.91282622542619E-58	Detoxification-associated hepat	GATC
1.74139370095066E-62	0.465599903204417	0.271	0.142	2.91822756405312E-58	Detoxification-associated hepat	CCDC82
1.82251753927709E-62	0.658571619373885	0.272	0.144	3.05417489232054E-58	Detoxification-associated hepat	SPATA33
1.82730803183787E-62	0.281096548605123	0.346	0.192	3.06220279975389E-58	Detoxification-associated hepat	SLC9A6
2.02355228424518E-62	0.472511755511213	0.259	0.134	3.39106891793808E-58	Detoxification-associated hepat	DENND6A
2.13565787767963E-62	0.381304030135108	0.295	0.159	3.57893547141553E-58	Detoxification-associated hepat	TRAF3
2.21041690198317E-62	0.405675967690227	0.282	0.149	3.70421664434339E-58	Detoxification-associated hepat	CMTM4
2.48481442845612E-62	0.57145434221102	0.271	0.143	4.16405201920677E-58	Detoxification-associated hepat	TP53I3
2.72376812279364E-62	0.30543170471894	0.23	0.11	4.56449062017759E-58	Detoxification-associated hepat	SPRYD4
2.9347552610416E-62	0.320155186321033	0.292	0.157	4.91806286645351E-58	Detoxification-associated hepat	ENOX2
3.182583298828E-62	0.500294931545049	0.249	0.126	5.33337309217597E-58	Detoxification-associated hepat	PGS1
3.32577362278939E-62	0.749800656231545	0.213	0.102	5.57333143707046E-58	Detoxification-associated hepat	SLF2
3.33886379031269E-62	0.364367068247695	0.294	0.158	5.59526793980601E-58	Detoxification-associated hepat	C7orf26
3.55583573810689E-62	0.444010175258509	0.252	0.129	5.95886952991953E-58	Detoxification-associated hepat	POLR2D
4.28639596269625E-62	0.682461661274423	0.228	0.112	7.18314235428638E-58	Detoxification-associated hepat	ADAMTS10
4.54960382573369E-62	0.489738472858968	0.221	0.107	7.62422609116452E-58	Detoxification-associated hepat	POLG2
7.74285806020493E-62	1.45841825503721	0.117	0.042	1.29754815372914E-57	Detoxification-associated hepat	CTSK
7.85074731164102E-62	0.513888126743429	0.244	0.123	1.3156282344848E-57	Detoxification-associated hepat	NCBP3
8.0407407421384E-62	0.33111291378833	0.28	0.148	1.34746733356755E-57	Detoxification-associated hepat	SENP5
8.58069750745749E-62	0.674574067291329	0.182	0.081	1.43795328829973E-57	Detoxification-associated hepat	HSDL1
1.01095615691232E-61	0.699696033943323	0.222	0.109	1.69416032775366E-57	Detoxification-associated hepat	UFSP2
1.11765523119208E-61	0.290261960057517	0.321	0.177	1.87296663643169E-57	Detoxification-associated hepat	ANKLE2
1.20472783002829E-61	0.486616429012173	0.236	0.117	2.0188828975614E-57	Detoxification-associated hepat	CREB3L4
1.21562578457622E-61	0.783608524466654	0.175	0.077	2.03714568979282E-57	Detoxification-associated hepat	DYRK3
1.22510634202392E-61	0.421182773848642	0.222	0.107	2.05303320796369E-57	Detoxification-associated hepat	UNC13B
1.38987603090639E-61	0.261949777880227	0.275	0.144	2.32915425259293E-57	Detoxification-associated hepat	ST3GAL2
1.50159214949362E-61	0.400651269222866	0.257	0.131	2.51636812412141E-57	Detoxification-associated hepat	RNASEH1

**1.75585917285199E-61**	0.425493093162211	0.253	0.129	2.94246880186537E-57	Detoxification-associated hepat	MIER3
**1.77446276329071E-61**	1.22981014855209	0.142	0.057	2.97364469872257E-57	Detoxification-associated hepat	SHLD1
**1.78965534424434E-61**	1.40144969399758	0.102	0.033	2.99910442588466E-57	Detoxification-associated hepat	TLR5
**2.15276806521595E-61**	0.484695797465557	0.237	0.117	3.60760872368888E-57	Detoxification-associated hepat	L3MBTL4
**2.37148573322867E-61**	0.284829951162005	0.296	0.158	3.9741357917446E-57	Detoxification-associated hepat	SLC12A8
**2.55102081252935E-61**	0.388367614245179	0.244	0.122	4.27500067763669E-57	Detoxification-associated hepat	UTP3
**2.62252947524209E-61**	0.446431044554867	0.155	0.063	4.39483489461069E-57	Detoxification-associated hepat	ADGRG1
**2.77344718802665E-61**	0.644156629159879	0.222	0.109	4.64774279769506E-57	Detoxification-associated hepat	FAM13B
**3.1483667991433E-61**	0.265584326480471	0.344	0.193	5.27603308200434E-57	Detoxification-associated hepat	PCSK9
**3.25193626798747E-61**	0.321576060068485	0.306	0.168	5.4495947978934E-57	Detoxification-associated hepat	ARMC1
**4.14585333494755E-61**	0.378167362041171	0.229	0.113	6.9476210187051E-57	Detoxification-associated hepat	CEP104
**4.29114101729859E-61**	1.00243970088957	0.162	0.069	7.19109411678897E-57	Detoxification-associated hepat	DHRS4
**4.72950440623293E-61**	0.433713517521944	0.265	0.138	7.92570348396514E-57	Detoxification-associated hepat	RASSF8
**4.76895445585418E-61**	1.03267224183891	0.133	0.051	7.99181387712044E-57	Detoxification-associated hepat	ZNF23
**5.32611696794343E-61**	0.309702699447828	0.269	0.141	8.9255068148796E-57	Detoxification-associated hepat	WTAP
**5.61690950582131E-61**	0.391178763832674	0.284	0.153	9.41281694985535E-57	Detoxification-associated hepat	REST
**5.71176828034227E-61**	0.28892883588894	0.325	0.18	9.57178128419757E-57	Detoxification-associated hepat	NUDT19
**5.77217419313015E-61**	0.57985026545138	0.21	0.101	9.67300951284751E-57	Detoxification-associated hepat	SLC38A9
**5.81989732005351E-61**	0.574477590815015	0.203	0.096	9.75298392894567E-57	Detoxification-associated hepat	IBA57
**6.57385393450405E-61**	0.597600328259305	0.201	0.094	1.10164644234419E-56	Detoxification-associated hepat	TMX3
**6.62752596179565E-61**	1.15321976877467	0.142	0.057	1.11064080067772E-56	Detoxification-associated hepat	FAM47E
**1.08985899851786E-60**	0.665469475701659	0.227	0.113	1.82638570971623E-56	Detoxification-associated hepat	NUDCD1
**1.11989487866409E-60**	0.671638395118402	0.181	0.08	1.87671983766528E-56	Detoxification-associated hepat	METTL22
**1.13420681854034E-60**	0.589832726586865	0.226	0.111	1.9007037865099E-56	Detoxification-associated hepat	SBF2
**1.26294506825066E-60**	0.473545796316934	0.224	0.11	2.11644334537445E-56	Detoxification-associated hepat	NECAP1
**1.27785009660097E-60**	0.250759429206653	0.38	0.219	2.1414211918839E-56	Detoxification-associated hepat	PHLPP2
**1.30377249059387E-60**	0.743642198222645	0.208	0.1	2.18486193973721E-56	Detoxification-associated hepat	ANXA2R
**1.4959745434076E-60**	0.383241391926219	0.26	0.136	2.50695413984246E-56	Detoxification-associated hepat	NFRKB
**1.68553988271411E-60**	0.272882258054121	0.278	0.147	2.8246277354523E-56	Detoxification-associated hepat	RMND1
**1.91253685203236E-60**	0.326861252282882	0.259	0.133	3.20502925663583E-56	Detoxification-associated hepat	BACE1
**1.92989511656714E-60**	0.379649500993697	0.301	0.164	3.23411823634321E-56	Detoxification-associated hepat	MAN1B1
**1.99623352302605E-60**	0.614506502115754	0.182	0.082	3.34528813788705E-56	Detoxification-associated hepat	PXYLP1
**2.02616593721223E-60**	0.799322274456356	0.216	0.107	3.39544887758025E-56	Detoxification-associated hepat	IQCB1
**2.18504223668157E-60**	0.611310363496832	0.188	0.085	3.66169378023098E-56	Detoxification-associated hepat	DHX58
**2.28250963193872E-60**	0.262625444026531	0.27	0.141	3.8250296412029E-56	Detoxification-associated hepat	TCF12
**2.36828444828643E-60**	0.463322917607597	0.171	0.074	3.9687710784384E-56	Detoxification-associated hepat	SNAPC5
**2.68002161805725E-60**	0.50249482909999	0.256	0.133	4.49118022754034E-56	Detoxification-associated hepat	DEPRECATEI
**2.68318379998976E-60**	0.56123209901222	0.248	0.128	4.49647941202284E-56	Detoxification-associated hepat	DGCR8
**2.76141706082023E-60**	0.794196164737509	0.183	0.083	4.62758271052254E-56	Detoxification-associated hepat	HSF2
**2.77586560329267E-60**	0.414989978608778	0.269	0.142	4.65179557799786E-56	Detoxification-associated hepat	DDX55
**3.2941191385888E-60**	0.533903009755773	0.221	0.108	5.52028485244711E-56	Detoxification-associated hepat	WDR70
**3.30046973092293E-60**	0.408104267282787	0.244	0.123	5.53092717508065E-56	Detoxification-associated hepat	PDSS2
**3.31910955574678E-60**	0.633010016291503	0.194	0.09	5.56216379352045E-56	Detoxification-associated hepat	PDPR
**3.49712593729798E-60**	0.288732759318176	0.283	0.151	5.86048364572395E-56	Detoxification-associated hepat	CLNS1A
**3.99369531559835E-60**	0.348873133953839	0.323	0.181	6.69263460987972E-56	Detoxification-associated hepat	CADPS2
**4.21298533758541E-60**	0.329322611811208	0.258	0.134	7.06012082872563E-56	Detoxification-associated hepat	ZMYM3
**4.54846248114106E-60**	1.21439509340059	0.125	0.047	7.62231342589618E-56	Detoxification-associated hepat	ZNF77
**4.96981914357887E-60**	0.527784841908947	0.193	0.089	8.32842292080947E-56	Detoxification-associated hepat	NMNAT1
**6.66056625808866E-60**	0.298205913197996	0.373	0.217	1.1161776935305E-55	Detoxification-associated hepat	HPS3
**6.72566379856997E-60**	1.38637330612596	0.117	0.042	1.12708673936435E-55	Detoxification-associated hepat	DEPRECATEI
**7.93549820975062E-60**	0.477157960881668	0.332	0.187	1.32983078999001E-55	Detoxification-associated hepat	SLC4A4
**8.22154255220605E-60**	0.410563927228719	0.264	0.139	1.37776610089869E-55	Detoxification-associated hepat	C3orf38
**9.18019102366581E-60**	0.668678185098358	0.206	0.098	1.53841641174592E-55	Detoxification-associated hepat	ZCCHC7
**9.88988045919882E-60**	0.738508701955725	0.179	0.08	1.65734616735254E-55	Detoxification-associated hepat	PIK3C3
**1.05887774558868E-59**	0.82861135808021	0.157	0.066	1.77446732605752E-55	Detoxification-associated hepat	ASCC1
**1.24296804786869E-59**	0.292487338892995	0.245	0.124	2.08296585461835E-55	Detoxification-associated hepat	TSEN54
**1.4373601481298E-59**	1.14269732078083	0.135	0.053	2.40872813623593E-55	Detoxification-associated hepat	P2RY11
**1.48011011526302E-59**	0.385173576084303	0.305	0.168	2.48036853115777E-55	Detoxification-associated hepat	NR2C2
**1.50858291178337E-59**	0.262026678917156	0.303	0.167	2.52808324356657E-55	Detoxification-associated hepat	PHAX
**1.54478894807451E-59**	1.04280456630853	0.147	0.06	2.58875731918327E-55	Detoxification-associated hepat	PIGW

**1.62047161882458E-59**	0.567928201716742	0.181	0.081	2.71558633882623E-55	Detoxification-associated hepat	SLC39A13
**1.8472710968825E-59**	0.782587400168839	0.167	0.073	3.0956569041557E-55	Detoxification-associated hepat	DEPRECATEI
**1.96267264274702E-59**	0.685177196921515	0.124	0.046	3.28904681471546E-55	Detoxification-associated hepat	KLHDC9
**2.06246709763779E-59**	0.876873181552059	0.182	0.083	3.45628236222141E-55	Detoxification-associated hepat	FASTKD3
**2.17133898886185E-59**	0.499685495450116	0.284	0.155	3.6387298775347E-55	Detoxification-associated hepat	SPTLC2
**2.26393853907037E-59**	1.06557312629334	0.136	0.053	3.79390820377412E-55	Detoxification-associated hepat	TREH
**2.26838143863624E-59**	0.434141611996482	0.22	0.108	3.8013536148666E-55	Detoxification-associated hepat	SLC39A6
**2.61757125260395E-59**	0.646251241235698	0.204	0.097	4.38652590511371E-55	Detoxification-associated hepat	URB1
**2.71952817394212E-59**	0.752562348295293	0.158	0.066	4.5573853138922E-55	Detoxification-associated hepat	TRIM66
**3.0000337338888E-59**	1.5473131244647	0.111	0.039	5.02745653125086E-55	Detoxification-associated hepat	SHLD3
**3.34944541542479E-59**	0.497900720610633	0.272	0.146	5.61300062716887E-55	Detoxification-associated hepat	CNOT6
**3.49623924800113E-59**	0.724497575235514	0.187	0.086	5.85899773180029E-55	Detoxification-associated hepat	PAQR3
**3.61490186680805E-59**	1.2583067535701	0.148	0.062	6.05785254839693E-55	Detoxification-associated hepat	GPR135
**3.73493624320923E-59**	0.807277150116842	0.174	0.078	6.25900615637003E-55	Detoxification-associated hepat	ZBTB8OS
**4.64933147240561E-59**	0.58672996922184	0.175	0.077	7.79134968145732E-55	Detoxification-associated hepat	CAPN3
**5.34643327523333E-59**	0.712448760460916	0.239	0.123	8.95955288263601E-55	Detoxification-associated hepat	CASD1
**5.4146602257845E-59**	0.331352201597987	0.361	0.209	9.07388760636967E-55	Detoxification-associated hepat	TTLL5
**6.64931090954102E-59**	0.449977723542011	0.271	0.146	1.11429152222088E-54	Detoxification-associated hepat	TDRD7
**7.78577898549282E-59**	0.265319298322117	0.277	0.149	1.30474084238889E-54	Detoxification-associated hepat	ATE1
**8.49341818497654E-59**	0.509301257549817	0.287	0.156	1.42332701943837E-54	Detoxification-associated hepat	SCMH1
**8.65921712961478E-59**	1.27719027791911	0.146	0.061	1.45111160658085E-54	Detoxification-associated hepat	ZNF620
**1.12976789965051E-58**	0.68345627574251	0.205	0.099	1.89326504623433E-54	Detoxification-associated hepat	SUPT7L
**1.13779138120154E-58**	0.277010962583656	0.341	0.193	1.90671079661753E-54	Detoxification-associated hepat	FNBP4
**1.14101821488294E-58**	0.697329727807655	0.213	0.105	1.91211832450084E-54	Detoxification-associated hepat	B3GALNT2
**1.31684768588503E-58**	0.622646457983464	0.217	0.107	2.20677335200613E-54	Detoxification-associated hepat	PHTF2
**1.33480934154134E-58**	0.943183432922034	0.172	0.077	2.23687349455498E-54	Detoxification-associated hepat	SLX4
**1.40705030603012E-58**	0.389560182572962	0.232	0.117	2.35793490284527E-54	Detoxification-associated hepat	DNAJC10
**1.5093205597939E-58**	0.399619098453236	0.207	0.099	2.52931939410261E-54	Detoxification-associated hepat	RAB8B
**1.70733413863338E-58**	0.915543210762922	0.162	0.07	2.86115054952182E-54	Detoxification-associated hepat	ATAT1
**1.73480221702025E-58**	0.294288305137285	0.226	0.111	2.90718155528254E-54	Detoxification-associated hepat	SRRD
**1.94189618011515E-58**	1.20511576676455	0.134	0.053	3.25422961863697E-54	Detoxification-associated hepat	ZNF417
**2.17650696061011E-58**	0.331331723134984	0.259	0.135	3.64739036459042E-54	Detoxification-associated hepat	RPAIN
**2.41826144980239E-58**	0.733860262787182	0.16	0.069	4.05252253757885E-54	Detoxification-associated hepat	SPHK2
**2.65889414502443E-58**	0.392240030162409	0.214	0.103	4.45577480823193E-54	Detoxification-associated hepat	COIL
**2.67386169005163E-58**	1.05991619996988	0.165	0.072	4.48085742018852E-54	Detoxification-associated hepat	PRIMPOL
**3.05879957739208E-58**	0.415194405222428	0.276	0.149	5.12593633179364E-54	Detoxification-associated hepat	WDR41
**3.16444860656734E-58**	0.415003940443651	0.282	0.152	5.30298297488556E-54	Detoxification-associated hepat	DCAKD
**3.24619515640678E-58**	0.357517653546431	0.192	0.089	5.43997384310648E-54	Detoxification-associated hepat	FAM131A
**3.41847467744767E-58**	0.86835444109877	0.166	0.073	5.7286798644668E-54	Detoxification-associated hepat	DEPRECATEI
**3.46971830186821E-58**	0.314106110951533	0.264	0.138	5.81455393027075E-54	Detoxification-associated hepat	ACAD8
**3.58829792194947E-58**	0.301577745296403	0.277	0.148	6.01326965760291E-54	Detoxification-associated hepat	N4BP1
**3.82805991300157E-58**	0.369259524246794	0.227	0.113	6.41506280220803E-54	Detoxification-associated hepat	ING5
**4.04584959923576E-58**	0.667845337670854	0.174	0.078	6.78003475839935E-54	Detoxification-associated hepat	ASB3
**4.20868836123318E-58**	0.327723183370846	0.316	0.177	7.05291995575457E-54	Detoxification-associated hepat	SLC35B4
**4.42227661222939E-58**	0.623926625621767	0.221	0.111	7.41085114677402E-54	Detoxification-associated hepat	ALS2
**5.33286642621465E-58**	0.480895025361334	0.228	0.114	8.9368175570505E-54	Detoxification-associated hepat	CEP170
**6.44407162339115E-58**	0.836553559770434	0.14	0.056	1.07989752264789E-53	Detoxification-associated hepat	DEPRECATEI
**6.52613909044348E-58**	0.387937589829713	0.23	0.115	1.09365038877652E-53	Detoxification-associated hepat	DEPRECATEI
**6.99257294371972E-58**	0.792755036523658	0.185	0.086	1.17181537390855E-53	Detoxification-associated hepat	ZBTB14
**7.08986235878196E-58**	0.601137806733668	0.249	0.129	1.18811913408468E-53	Detoxification-associated hepat	MX1
**7.13816826809455E-58**	0.636431978204267	0.196	0.093	1.19621423836728E-53	Detoxification-associated hepat	UHRF1BP1
**7.43280181058252E-58**	0.449824106078645	0.289	0.159	1.24558892741742E-53	Detoxification-associated hepat	MFSD10
**7.64351920230305E-58**	0.5927032306704	0.223	0.112	1.28090094792195E-53	Detoxification-associated hepat	ZBTB48
**7.73602493025368E-58**	1.21799538685353	0.116	0.042	1.29640305781191E-53	Detoxification-associated hepat	AC015871.1
**7.74498422706556E-58**	0.418061170304388	0.231	0.117	1.29790445677165E-53	Detoxification-associated hepat	PPP1R8
**8.41052909983039E-58**	0.33635871924828	0.325	0.182	1.40943646654958E-53	Detoxification-associated hepat	TMEM131L
**9.12019211669841E-58**	0.681784740008354	0.156	0.067	1.52836179491632E-53	Detoxification-associated hepat	CHD3
**9.14392017062894E-58**	0.878800986379352	0.152	0.064	1.532338142194E-53	Detoxification-associated hepat	FKRP
**9.24089001907696E-58**	0.731744367953052	0.156	0.067	1.54858834939692E-53	Detoxification-associated hepat	ZNF805
**9.48911352424556E-58**	0.443523494299987	0.271	0.145	1.59018564439307E-53	Detoxification-associated hepat	ABHD15

**9.80094400371791E-58**	0.312032357423901	0.276	0.149	1.64244219614305E-53	Detoxification-associated hepat	SNRPG
**1.02181412550699E-57**	0.370608600102562	0.291	0.16	1.71235611152461E-53	Detoxification-associated hepat	CEP63
**1.09888684144383E-57**	0.983647846073683	0.154	0.066	1.84151456889157E-53	Detoxification-associated hepat	ZNF227
**1.13506932590429E-57**	0.672228022138315	0.196	0.093	1.9021491763504E-53	Detoxification-associated hepat	ZNF142
**1.20090549549029E-57**	0.390782465011333	0.23	0.116	2.01247742934263E-53	Detoxification-associated hepat	HSBP1L1
**1.25928953910488E-57**	0.563683395201446	0.212	0.104	2.11031740963196E-53	Detoxification-associated hepat	ABHD17A
**1.35209136247224E-57**	0.50221279995866	0.213	0.104	2.26583470523098E-53	Detoxification-associated hepat	SUMF1
**1.37724396851074E-57**	0.589486780848764	0.191	0.09	2.3079854424303E-53	Detoxification-associated hepat	CDK5RAP1
**1.79600522968826E-57**	0.40565090152367	0.329	0.189	3.00974556391159E-53	Detoxification-associated hepat	TRIM56
**1.98645056451104E-57**	0.83275503736174	0.122	0.046	3.32889385600759E-53	Detoxification-associated hepat	PDGFRB
**2.12814931320249E-57**	0.471322832854678	0.23	0.117	3.56635261906474E-53	Detoxification-associated hepat	TXLNG
**2.23219143431017E-57**	0.696232480640916	0.198	0.095	3.74070640561699E-53	Detoxification-associated hepat	MDP1
**2.2947694219695E-57**	0.540217068982769	0.244	0.128	3.84557459733649E-53	Detoxification-associated hepat	INO80D
**2.35901830966449E-57**	0.42620246941996	0.274	0.148	3.95324288333575E-53	Detoxification-associated hepat	DIPK2A
**2.4292141678889E-57**	0.900964694730341	0.158	0.069	4.07087710254821E-53	Detoxification-associated hepat	MED7
**2.45273057746083E-57**	0.285789580854806	0.312	0.172	4.11028590170886E-53	Detoxification-associated hepat	GAS2L3
**2.49416148129732E-57**	1.20316275482006	0.14	0.057	4.17971581035804E-53	Detoxification-associated hepat	ABRAXAS1
**2.50796369284643E-57**	0.45631860865468	0.23	0.116	4.20284555647204E-53	Detoxification-associated hepat	SYT1
**2.95683130172139E-57**	0.690286326171871	0.182	0.084	4.9550578954247E-53	Detoxification-associated hepat	ZUP1
**3.4727266866077E-57**	1.22962161318775	0.151	0.064	5.81959538141719E-53	Detoxification-associated hepat	AKAP6
**3.49328214504009E-57**	0.317184602095993	0.282	0.154	5.85404221865818E-53	Detoxification-associated hepat	PLCL2
**3.73330377964048E-57**	0.300662888724835	0.254	0.133	6.25627047392153E-53	Detoxification-associated hepat	RAB29
**3.95728963573939E-57**	0.288796597791627	0.259	0.137	6.63162597157208E-53	Detoxification-associated hepat	MTG2
**4.18326762066109E-57**	0.535765368906764	0.236	0.121	7.01031987870386E-53	Detoxification-associated hepat	DEPRECATEI
**4.62689222003703E-57**	0.504057985828027	0.177	0.08	7.75374598233806E-53	Detoxification-associated hepat	ENTPD4
**6.9438929801005E-57**	0.717809233073547	0.156	0.067	1.16365758560524E-52	Detoxification-associated hepat	GATB
**8.13810388405888E-57**	0.257443950765301	0.276	0.148	1.36378344889059E-52	Detoxification-associated hepat	CYLD
**8.49772148992305E-57**	0.420870805538377	0.176	0.08	1.4240481672813E-52	Detoxification-associated hepat	RAD54L2
**9.31998843573531E-57**	0.289952220177383	0.265	0.141	1.56184366206052E-52	Detoxification-associated hepat	SEPHS1
**9.65764415367205E-57**	0.378674436424419	0.169	0.074	1.61842800727236E-52	Detoxification-associated hepat	CUL7
**1.04044024579051E-56**	0.497484239577241	0.215	0.106	1.74356976389573E-52	Detoxification-associated hepat	KLC2
**1.18491758044901E-56**	0.618610436843818	0.197	0.094	1.98568488131645E-52	Detoxification-associated hepat	UBOX5
**1.43144555597574E-56**	0.266437789637479	0.278	0.15	2.39881646270415E-52	Detoxification-associated hepat	TBC1D13
**1.48697517402836E-56**	0.447761708705214	0.226	0.114	2.49187299663673E-52	Detoxification-associated hepat	RPAP3
**1.7337812735362E-56**	0.307439844062483	0.25	0.131	2.90547065819196E-52	Detoxification-associated hepat	PACS2
**1.75285741292993E-56**	0.955378311157338	0.152	0.065	2.93743845258797E-52	Detoxification-associated hepat	SCAI
**1.77283896003313E-56**	0.335784184909689	0.221	0.109	2.97092352922352E-52	Detoxification-associated hepat	MAPK8IP3
**1.84248495816512E-56**	0.427171500423807	0.195	0.092	3.08763629289311E-52	Detoxification-associated hepat	MDN1
**1.99849529011421E-56**	0.729012482491197	0.168	0.075	3.3490784071734E-52	Detoxification-associated hepat	TIRAP
**2.13375642308765E-56**	0.50049878675887	0.193	0.091	3.57574901381028E-52	Detoxification-associated hepat	EIF2AK3
**2.24832354309196E-56**	0.374453069070389	0.318	0.181	3.7677405935135E-52	Detoxification-associated hepat	ARL4A
**2.68910102491081E-56**	0.558933121464555	0.204	0.099	4.50639549754553E-52	Detoxification-associated hepat	RAB11FIP2
**2.78273682660312E-56**	0.860149034308313	0.148	0.062	4.66331037402151E-52	Detoxification-associated hepat	PRDM10
**2.81802711577925E-56**	0.648226656811273	0.21	0.105	4.72244984062286E-52	Detoxification-associated hepat	GPR89A
**2.88756488249905E-56**	0.446728859701421	0.204	0.099	4.83898123009191E-52	Detoxification-associated hepat	ACOT9
**3.13806845006306E-56**	0.716390420430504	0.166	0.074	5.25877510861567E-52	Detoxification-associated hepat	TRIM37
**3.59566927066205E-56**	0.484021973771595	0.228	0.116	6.02562256377547E-52	Detoxification-associated hepat	MRTFB
**3.63129732051028E-56**	0.62904518360096	0.192	0.091	6.08532804971113E-52	Detoxification-associated hepat	FOXJ3
**4.07414736667395E-56**	0.762942035438912	0.18	0.084	6.82745615707221E-52	Detoxification-associated hepat	VRK2
**4.18525917496193E-56**	0.280217799793872	0.309	0.172	7.01365732540121E-52	Detoxification-associated hepat	PER3
**4.28596610569442E-56**	0.684350658067747	0.117	0.043	7.18242199992271E-52	Detoxification-associated hepat	MAPT
**4.35932539796818E-56**	0.473214394940928	0.138	0.055	7.30535750191507E-52	Detoxification-associated hepat	FGFR2
**4.4500238241333E-56**	0.460563124224852	0.268	0.145	7.45734992448259E-52	Detoxification-associated hepat	NT5C3A
**6.44064769025331E-56**	0.419727818011725	0.237	0.122	1.07932373993265E-51	Detoxification-associated hepat	GTF2H3
**7.25487536368855E-56**	0.908091714731386	0.135	0.055	1.21577201344693E-51	Detoxification-associated hepat	ZNF584
**8.39181840556378E-56**	0.285945576094255	0.241	0.125	1.40630092840438E-51	Detoxification-associated hepat	ZNF830
**9.16745809428766E-56**	0.813135713357872	0.153	0.066	1.53628262744073E-51	Detoxification-associated hepat	KIF21B
**9.1860700673928E-56**	0.371579732864523	0.259	0.139	1.53940162189369E-51	Detoxification-associated hepat	ACOT8
**9.3180552145077E-56**	0.260304207914854	0.241	0.124	1.5615196928472E-51	Detoxification-associated hepat	LACTB
**9.45101339656837E-56**	0.282725013207507	0.233	0.119	1.58380082499693E-51	Detoxification-associated hepat	JMY

9.59932347611776E-56	0.368075858534506	0.204	0.099	1.60865462812781E-51	Detoxification-associated hepat	ZNF697
1.01312112633322E-55	0.636986370517804	0.122	0.046	1.69778838350921E-51	Detoxification-associated hepat	C11orf95
1.13319778420895E-55	1.13432238489295	0.152	0.066	1.89901284677735E-51	Detoxification-associated hepat	DDX60L
1.301743779858E-55	0.923427261565173	0.162	0.072	2.18146222628603E-51	Detoxification-associated hepat	ANGEL2
1.32250029216013E-55	0.315091526630921	0.26	0.139	2.21624598960195E-51	Detoxification-associated hepat	SIX5
1.95987773721931E-55	0.426906229854713	0.18	0.083	3.28436311203212E-51	Detoxification-associated hepat	SLC26A11
2.40691719746634E-55	0.501298771031265	0.196	0.094	4.03351183951409E-51	Detoxification-associated hepat	ZSCAN29
2.75851618792273E-55	0.305517264081536	0.205	0.098	4.62272142772091E-51	Detoxification-associated hepat	CNGA1
3.09366285368844E-55	0.847706515137604	0.142	0.059	5.18436021021109E-51	Detoxification-associated hepat	DDX58
3.18405985679646E-55	0.300294915819185	0.271	0.148	5.33584750801951E-51	Detoxification-associated hepat	KAT8
3.24609630724567E-55	0.580864750195805	0.199	0.096	5.43980819168229E-51	Detoxification-associated hepat	ACTR5
3.41292599264317E-55	0.388621061273825	0.208	0.103	5.71938137847143E-51	Detoxification-associated hepat	ALG14
3.78060357393521E-55	0.464711640758432	0.181	0.084	6.33553546920062E-51	Detoxification-associated hepat	ZNF343
4.35402527969171E-55	0.465089115006694	0.224	0.114	7.29647556370737E-51	Detoxification-associated hepat	RNF214
4.49701331626909E-55	0.576937404767578	0.17	0.077	7.53609491540375E-51	Detoxification-associated hepat	SCAPER
4.5593103604218E-55	0.835604751191444	0.126	0.049	7.64049230199485E-51	Detoxification-associated hepat	MREG
4.87771065962516E-55	1.14243510951783	0.116	0.043	8.17406752339985E-51	Detoxification-associated hepat	ZNF589
4.91720090833979E-55	0.26362401590522	0.287	0.159	8.24024528219582E-51	Detoxification-associated hepat	PLEKHA7
5.0855556724789E-55	1.08032637360906	0.139	0.058	8.52237419594014E-51	Detoxification-associated hepat	TYSND1
5.6313668640512E-55	0.268791946859648	0.269	0.145	9.437044590777E-51	Detoxification-associated hepat	PSD4
5.68577750150063E-55	0.319941516744275	0.263	0.141	9.52822593701475E-51	Detoxification-associated hepat	CARD19
5.70048846555404E-55	0.518086426177935	0.222	0.112	9.55287857057546E-51	Detoxification-associated hepat	CREB1
5.99444930085591E-55	0.453372126180938	0.211	0.105	1.00454981383743E-50	Detoxification-associated hepat	SPAST
8.59630778371725E-55	0.322735906280368	0.266	0.144	1.44056925839534E-50	Detoxification-associated hepat	SPOUT1
8.93751696891079E-55	0.272944252193653	0.33	0.19	1.49774909365007E-50	Detoxification-associated hepat	ABCC4
9.25163356325048E-55	0.476476620978284	0.235	0.122	1.55038875252952E-50	Detoxification-associated hepat	RABGGTA
9.28206218128607E-55	0.462324409785057	0.187	0.088	1.55548798033992E-50	Detoxification-associated hepat	TRMT10C
1.14178965608158E-54	0.296183470900169	0.25	0.132	1.91341110566151E-50	Detoxification-associated hepat	TMEM135
1.3544676514601E-54	0.910054205671051	0.144	0.061	2.26981689031684E-50	Detoxification-associated hepat	ZNF180
1.49488312390185E-54	0.294854245617095	0.292	0.163	2.50512513903472E-50	Detoxification-associated hepat	JADE1
1.8219797399833E-54	0.523075547288681	0.214	0.107	3.05327364826401E-50	Detoxification-associated hepat	ZNF322
2.1868533244628E-54	1.13785897378906	0.11	0.041	3.66472880113477E-50	Detoxification-associated hepat	TTC6
2.20804647291606E-54	0.351664678407512	0.241	0.127	3.70024427931274E-50	Detoxification-associated hepat	ATXN1L
2.24431581956723E-54	1.13383346457186	0.116	0.044	3.76102445043076E-50	Detoxification-associated hepat	MBLAC2
2.49145493017128E-54	0.358898621048101	0.257	0.138	4.17518017198103E-50	Detoxification-associated hepat	MEIS2
2.55184035495847E-54	1.26871299084104	0.104	0.037	4.2763740668394E-50	Detoxification-associated hepat	PTCD2
3.46950540372676E-54	0.403782753095239	0.235	0.122	5.81419715556531E-50	Detoxification-associated hepat	SMG9
3.709243198383E-54	0.383481596097828	0.285	0.16	6.21594975185023E-50	Detoxification-associated hepat	DEPRECATEI
3.89570376165491E-54	0.689831644645845	0.159	0.071	6.52842036378129E-50	Detoxification-associated hepat	ATG4C
4.05642867133498E-54	0.515585373975147	0.22	0.112	6.79776316742316E-50	Detoxification-associated hepat	LTV1
4.19265882567438E-54	0.312094605993166	0.268	0.145	7.02605766006513E-50	Detoxification-associated hepat	LRRC57
4.51889127262067E-54	0.479322432248424	0.216	0.109	7.57275799465772E-50	Detoxification-associated hepat	ZNF335
4.55775366547137E-54	0.948327886830481	0.151	0.066	7.63788359259693E-50	Detoxification-associated hepat	ZNF174
4.67200587716761E-54	0.469043315140279	0.215	0.109	7.82934744895748E-50	Detoxification-associated hepat	KATNA1
4.71989507874296E-54	0.278275870551818	0.23	0.118	7.90960017295745E-50	Detoxification-associated hepat	SNX21
5.5762072688749E-54	0.283588430201632	0.232	0.119	9.34460814118055E-50	Detoxification-associated hepat	PELP1
5.89451781022704E-54	1.59192524390861	0.1	0.035	9.87803294637847E-50	Detoxification-associated hepat	FAM92A
6.65607969425697E-54	0.402446021977668	0.187	0.087	1.11542583516358E-49	Detoxification-associated hepat	NPEPL1
7.67805443445974E-54	0.504413984504717	0.21	0.105	1.28668836212676E-49	Detoxification-associated hepat	RBIS
1.12615551334887E-53	0.509824169976397	0.209	0.105	1.88721140927003E-49	Detoxification-associated hepat	GAPVD1
1.16855291834384E-53	0.711620260740348	0.14	0.058	1.95826098056061E-49	Detoxification-associated hepat	PDCD7
1.18326204658574E-53	0.476507372025818	0.25	0.134	1.98291053766838E-49	Detoxification-associated hepat	BLZF1
1.23404202004164E-53	0.614498523539814	0.228	0.119	2.06800761718578E-49	Detoxification-associated hepat	LTB4R
1.33673917699715E-53	0.64954824826629	0.133	0.054	2.24010751281183E-49	Detoxification-associated hepat	NAT2
1.47370789363196E-53	0.452424203167604	0.226	0.117	2.46963968814843E-49	Detoxification-associated hepat	GALC
1.57678029688802E-53	0.354248395950764	0.244	0.129	2.64236842152495E-49	Detoxification-associated hepat	HOOK2
1.5791011141394E-53	0.300063283389253	0.244	0.128	2.6462576470748E-49	Detoxification-associated hepat	BICRA
1.61237420810884E-53	0.474350989402124	0.191	0.092	2.70201669794879E-49	Detoxification-associated hepat	DEPRECATEI
1.7443632291588E-53	0.975876147835973	0.163	0.074	2.92320389942431E-49	Detoxification-associated hepat	TRMT10B
1.77901669896779E-53	0.353881831435486	0.224	0.115	2.98127618413022E-49	Detoxification-associated hepat	FXR2

**1.83982499544078E-53**	0.463165892743649	0.257	0.138	3.08317872735966E-49	Detoxification-associated hepat	RFC4
**1.92418405304811E-53**	0.279732486535953	0.221	0.112	3.22454763609803E-49	Detoxification-associated hepat	ZFAND6
**2.32905006866121E-53**	0.537507776161363	0.217	0.111	3.90302210506246E-49	Detoxification-associated hepat	G6PC3
**2.52017657137856E-53**	0.404753166645038	0.233	0.123	4.22331189831619E-49	Detoxification-associated hepat	TWSG1
**2.52311803075229E-53**	0.391513412471935	0.2	0.098	4.22824119593469E-49	Detoxification-associated hepat	TYW3
**2.82881134302267E-53**	1.06609435925851	0.124	0.049	4.7405220486374E-49	Detoxification-associated hepat	SLC30A4
**3.36899057121246E-53**	0.490365000125152	0.191	0.092	5.64575439923784E-49	Detoxification-associated hepat	MPHOSPH6
**3.95962754573807E-53**	0.256247178201942	0.304	0.173	6.63554384114786E-49	Detoxification-associated hepat	CCDC130
**4.0386154905497E-53**	0.477608404497077	0.219	0.113	6.76791183906318E-49	Detoxification-associated hepat	HMGXB3
**4.30580243892211E-53**	0.771386225425176	0.184	0.088	7.21566372714568E-49	Detoxification-associated hepat	TRIM45
**4.49324577675505E-53**	0.44467365401268	0.208	0.104	7.52978127268611E-49	Detoxification-associated hepat	PIK3CA
**4.65737400248559E-53**	0.992695664136429	0.176	0.083	7.80482735336535E-49	Detoxification-associated hepat	TFCP2L1
**5.08761231011076E-53**	1.21472286768246	0.12	0.047	8.52582070928361E-49	Detoxification-associated hepat	TIAF1
**5.20987945847591E-53**	0.515048762468806	0.215	0.11	8.73071599651393E-49	Detoxification-associated hepat	ZMIZ1
**6.32791270176131E-53**	0.642991246475398	0.197	0.098	1.06043161056116E-48	Detoxification-associated hepat	BTBD9
**7.21707817313259E-53**	0.300453526774063	0.285	0.159	1.20943796025356E-48	Detoxification-associated hepat	COMMD2
**7.4011423745794E-53**	0.658618953457272	0.16	0.072	1.24028343913202E-48	Detoxification-associated hepat	TTC12
**7.66450709948196E-53**	0.300602932381219	0.262	0.144	1.28441809973119E-48	Detoxification-associated hepat	PARN
**7.67835355411534E-53**	0.64913276341333	0.166	0.076	1.28673848859865E-48	Detoxification-associated hepat	CRTC1
**7.71895684192172E-53**	0.617058881008918	0.151	0.066	1.29354278756924E-48	Detoxification-associated hepat	ZNF140
**8.14285734482367E-53**	0.484193285200663	0.176	0.082	1.36458003384555E-48	Detoxification-associated hepat	FAM160B1
**8.1509001989406E-53**	0.604159615837762	0.175	0.082	1.36592785533847E-48	Detoxification-associated hepat	TOPORS
**9.39889458286169E-53**	0.722016797606881	0.233	0.124	1.57506675419596E-48	Detoxification-associated hepat	RAD51B
**9.5167459766033E-53**	0.422608748454241	0.187	0.09	1.59481629075918E-48	Detoxification-associated hepat	AMZ2
**1.11433228120556E-52**	0.89615665364311	0.159	0.072	1.86739803684428E-48	Detoxification-associated hepat	ZNF564
**1.16767185037575E-52**	0.538928106315953	0.235	0.125	1.95678448685969E-48	Detoxification-associated hepat	NEO1
**1.23143657387353E-52**	0.37818905361454	0.299	0.171	2.06364141049726E-48	Detoxification-associated hepat	CASZ1
**1.51422206257045E-52**	0.275484606376888	0.278	0.153	2.53753333245556E-48	Detoxification-associated hepat	TMCC3
**1.53109625955108E-52**	0.303282886354102	0.217	0.11	2.5658111117557E-48	Detoxification-associated hepat	GPALPP1
**1.57453559610332E-52**	0.345949900119734	0.222	0.114	2.63860675194995E-48	Detoxification-associated hepat	ABHD4
**1.8080723880271E-52**	0.686759622885287	0.174	0.081	3.02996770785581E-48	Detoxification-associated hepat	STX18
**2.05368803525955E-52**	0.436681995148609	0.235	0.124	3.44157040948796E-48	Detoxification-associated hepat	TMEM60
**2.08230076990641E-52**	0.697391393502821	0.188	0.091	3.48951963020917E-48	Detoxification-associated hepat	AP4B1
**2.42103336758811E-52**	0.366415633822633	0.223	0.115	4.05716771740415E-48	Detoxification-associated hepat	PML
**2.89745113619928E-52**	0.388144228190111	0.208	0.105	4.85554861404275E-48	Detoxification-associated hepat	EEF1AKMT1
**2.93236889624766E-52**	0.870650363302344	0.131	0.054	4.91406379633183E-48	Detoxification-associated hepat	ANKRD6
**2.9683562463649E-52**	0.408303529272321	0.244	0.13	4.97437139765829E-48	Detoxification-associated hepat	USP21
**3.19466111175869E-52**	1.09324603571503	0.102	0.037	5.3536130910852E-48	Detoxification-associated hepat	ZNF235
**3.41862969734732E-52**	0.439365035788752	0.163	0.073	5.72893964681464E-48	Detoxification-associated hepat	DTD2
**4.03982856157383E-52**	0.281186351886346	0.273	0.15	6.76994470348543E-48	Detoxification-associated hepat	RNF169
**4.13401251334854E-52**	0.468109794106287	0.208	0.106	6.92777816986948E-48	Detoxification-associated hepat	DEPRECATEI
**5.35634380764966E-52**	0.861469400744997	0.169	0.079	8.97616095285929E-48	Detoxification-associated hepat	SP4
**5.54503240102242E-52**	0.432269853594608	0.228	0.12	9.29236529763338E-48	Detoxification-associated hepat	ZCCHC8
**5.62023317533936E-52**	0.348467297359085	0.251	0.137	9.41838675523369E-48	Detoxification-associated hepat	COPS7B
**5.93140858211668E-52**	0.339758108924781	0.194	0.094	9.93985450191114E-48	Detoxification-associated hepat	PDP2
**6.63725548924209E-52**	0.318427008498207	0.259	0.14	1.11227127488719E-47	Detoxification-associated hepat	PIEZO1
**6.81894382913484E-52**	0.74192212363701	0.196	0.098	1.14271860688642E-47	Detoxification-associated hepat	TOGARAM1
**8.16948603168259E-52**	0.90272550531016	0.135	0.056	1.36904246918937E-47	Detoxification-associated hepat	ARHGAP42
**9.1723739857776E-52**	0.547139038620079	0.217	0.113	1.53710643253661E-47	Detoxification-associated hepat	NSUN3
**1.0311819846398E-51**	0.676247369328893	0.153	0.068	1.72805476985937E-47	Detoxification-associated hepat	DNAJC24
**1.0783801541063E-51**	0.374760675352481	0.189	0.092	1.80714946225133E-47	Detoxification-associated hepat	NAA16
**1.13940463714547E-51**	0.563031988139594	0.201	0.101	1.90941429092838E-47	Detoxification-associated hepat	BCLAF3
**1.31606274619574E-51**	0.330010945818239	0.229	0.12	2.20545795007482E-47	Detoxification-associated hepat	LRIF1
**1.31764978726263E-51**	0.560064052042777	0.146	0.063	2.20811751349472E-47	Detoxification-associated hepat	EBLN2
**1.41048885875236E-51**	0.331987374039456	0.257	0.14	2.3636972294972E-47	Detoxification-associated hepat	COMMD10
**1.4707188639703E-51**	0.36734169526163	0.213	0.109	2.46463067224144E-47	Detoxification-associated hepat	TOM1L2
**1.51193690820494E-51**	0.71095832300451	0.14	0.06	2.53370387076984E-47	Detoxification-associated hepat	MAP2K4
**1.55599362025404E-51**	1.04790995786843	0.133	0.056	2.60753410882172E-47	Detoxification-associated hepat	THUMPD2
**1.60420024171101E-51**	0.380127036461772	0.201	0.1	2.68831876505931E-47	Detoxification-associated hepat	TMCO4
**1.62168703355325E-51**	0.37887932004579	0.245	0.131	2.71762313082854E-47	Detoxification-associated hepat	CBLB

1.62442527605064E-51	0.424280888212428	0.283	0.159	2.72221187760567E-47	Detoxification-associated hepat	SPRY2
1.6367326449371E-51	0.328809531862322	0.245	0.132	2.74283656638559E-47	Detoxification-associated hepat	SNX7
1.6619348898781E-51	0.89248436168165	0.145	0.063	2.78507048845771E-47	Detoxification-associated hepat	TXNDC9
1.70382119723896E-51	0.442901334849568	0.176	0.083	2.85526356233305E-47	Detoxification-associated hepat	CPNE8
1.97816613735939E-51	0.284572337564943	0.262	0.145	3.31501081298687E-47	Detoxification-associated hepat	PARD6B
2.00794987207488E-51	0.919769527593294	0.13	0.054	3.36492239562308E-47	Detoxification-associated hepat	CELSR3
2.10152458415463E-51	0.774296789936719	0.143	0.062	3.52173489812634E-47	Detoxification-associated hepat	SGSM2
2.73475865259541E-51	1.01426445657223	0.111	0.042	4.58290855001939E-47	Detoxification-associated hepat	RWDD2A
2.74210596494392E-51	0.682485789965927	0.199	0.101	4.59522117605301E-47	Detoxification-associated hepat	AGO4
2.8096303727156E-51	0.255362270536868	0.233	0.122	4.70837857859681E-47	Detoxification-associated hepat	TRMT1
3.36899238237025E-51	0.917986558164176	0.114	0.044	5.64575743437607E-47	Detoxification-associated hepat	BBS10
3.44180951966541E-51	0.336922825945517	0.224	0.116	5.7677843930553E-47	Detoxification-associated hepat	EIF2A
4.28743354497008E-51	0.496155120713584	0.17	0.079	7.18488113466086E-47	Detoxification-associated hepat	FAAP100
4.37496030816096E-51	0.430473104003616	0.207	0.105	7.33155848441614E-47	Detoxification-associated hepat	SLC25A17
4.47247720492653E-51	0.715383439340966	0.161	0.074	7.49497730001588E-47	Detoxification-associated hepat	TMEM80
4.7193876858455E-51	1.10041951124763	0.106	0.039	7.90874988393989E-47	Detoxification-associated hepat	HEATR5A
5.35925091619986E-51	0.294467371149216	0.232	0.122	8.98103268536772E-47	Detoxification-associated hepat	WARS
5.40188874390427E-51	0.362577248584505	0.25	0.137	9.05248515703478E-47	Detoxification-associated hepat	CDK8
5.6028461786654E-51	0.540118548505196	0.204	0.104	9.38924962620748E-47	Detoxification-associated hepat	AMD 1.00
6.6427555621876E-51	0.279935553675693	0.223	0.116	1.1131929771114E-46	Detoxification-associated hepat	USP31
6.68221819090226E-51	0.301038094849648	0.243	0.13	1.1198061244314E-46	Detoxification-associated hepat	PKNOX1
6.68979534469144E-51	1.37131197584255	0.108	0.041	1.12107590386339E-46	Detoxification-associated hepat	AOC2
6.88804413900135E-51	0.444272435419701	0.237	0.128	1.15429843681385E-46	Detoxification-associated hepat	FBXL18
7.23679060879219E-51	0.419750496047883	0.186	0.09	1.2127413702214E-46	Detoxification-associated hepat	BLOC1S6
7.35669426081223E-51	0.362952054310747	0.283	0.16	1.23283482422691E-46	Detoxification-associated hepat	MGME1
8.1127084712667E-51	0.569193258207977	0.162	0.075	1.35952768561487E-46	Detoxification-associated hepat	CSNK1G1
8.22426065453573E-51	0.689314228623295	0.167	0.078	1.3782216004871E-46	Detoxification-associated hepat	TANGO6
8.46164042117577E-51	0.368197410694108	0.153	0.067	1.41800170178064E-46	Detoxification-associated hepat	RCN3
9.04786795830212E-51	0.320811471944408	0.233	0.124	1.51624171245227E-46	Detoxification-associated hepat	C5orf22
1.03561562951096E-50	0.456746383916696	0.2	0.101	1.73548467193446E-46	Detoxification-associated hepat	XPO7
1.14297645241776E-50	0.973067474453869	0.105	0.039	1.91539993896169E-46	Detoxification-associated hepat	ETFBKMT
1.3909364697957E-50	0.735780452854669	0.144	0.063	2.33093133608363E-46	Detoxification-associated hepat	PLEKHM3
1.49062800070227E-50	0.899049311143131	0.125	0.051	2.49799440357686E-46	Detoxification-associated hepat	CHSY1
1.50305345027033E-50	0.683974351337423	0.184	0.091	2.51881697196302E-46	Detoxification-associated hepat	BMPR1A
1.60530999585807E-50	0.750004760237895	0.144	0.063	2.69017849105895E-46	Detoxification-associated hepat	SETMAR
1.69333509426312E-50	0.550433339721626	0.196	0.098	2.83769095096614E-46	Detoxification-associated hepat	CWC27
1.86269118213088E-50	0.338654680623599	0.22	0.114	3.12149788301493E-46	Detoxification-associated hepat	VPS53
2.19368364100785E-50	0.297016976581156	0.253	0.139	3.67617504560096E-46	Detoxification-associated hepat	PAPSS1
2.32936726273989E-50	0.530917353709884	0.191	0.095	3.90355365889951E-46	Detoxification-associated hepat	RFT1
2.4366042581296E-50	0.629646036673576	0.114	0.044	4.08326141577358E-46	Detoxification-associated hepat	CLCN4
2.79300719537012E-50	0.587550714867289	0.166	0.078	4.68052145800125E-46	Detoxification-associated hepat	DCAF17
3.85235593947017E-50	0.281736400689306	0.23	0.12	6.4557780833641E-46	Detoxification-associated hepat	IRAK2
3.86592037686518E-50	0.431226400331868	0.2	0.1	6.47850936755067E-46	Detoxification-associated hepat	MKRN2
4.11274374737477E-50	0.529132722925467	0.16	0.073	6.89213597185064E-46	Detoxification-associated hepat	PEX10
4.14543296525117E-50	0.357522413376634	0.182	0.088	6.9469165631679E-46	Detoxification-associated hepat	TAF1C
4.3089782715156E-50	0.822191475304036	0.174	0.084	7.22098578740585E-46	Detoxification-associated hepat	GPR89B
5.19133476694069E-50	0.259209252749524	0.231	0.122	8.6996388024392E-46	Detoxification-associated hepat	CSTF1
5.36575253876052E-50	0.342320590873599	0.271	0.152	8.99192810445487E-46	Detoxification-associated hepat	ARHGAP26
5.90442652120924E-50	0.258910846613918	0.27	0.152	9.89463796424245E-46	Detoxification-associated hepat	COA8
5.97263637511554E-50	0.265827202935215	0.266	0.148	1.00089440374186E-45	Detoxification-associated hepat	TBC1D5
7.77096115193273E-50	0.327099512330308	0.233	0.125	1.30225766984089E-45	Detoxification-associated hepat	RAE1
8.46833243507245E-50	0.854090309386833	0.108	0.041	1.41912314946944E-45	Detoxification-associated hepat	ZNF566
8.5044085351553E-50	0.39442556581147	0.221	0.116	1.42516878232132E-45	Detoxification-associated hepat	KNOP1
8.70583416666837E-50	0.586150020766388	0.182	0.089	1.45892368965028E-45	Detoxification-associated hepat	FASTKD5
8.79413182445687E-50	0.544267765118948	0.17	0.08	1.47372061114248E-45	Detoxification-associated hepat	TMEM143
8.87953521669095E-50	1.55393792716345	0.104	0.039	1.48803251161307E-45	Detoxification-associated hepat	PCDHGA11
9.92854455607924E-50	0.484565765852115	0.24	0.13	1.66382549670776E-45	Detoxification-associated hepat	CCDC88A
1.01479211982742E-49	0.27472353442813	0.318	0.187	1.70058863440678E-45	Detoxification-associated hepat	VSIG10
1.19637815317266E-49	0.497971229492065	0.158	0.072	2.00489050908674E-45	Detoxification-associated hepat	PWWP2B
1.21530265899998E-49	0.542153710693921	0.194	0.097	2.03660419595217E-45	Detoxification-associated hepat	ZNF207

1.27374709988051E-49	0.936203958185505	0.139	0.061	2.13454538997976E-45	Detoxification-associated hepat	WRN
1.35513424038841E-49	1.01906253294528	0.1	0.037	2.27093396004289E-45	Detoxification-associated hepat	PGAP1
1.38830417656908E-49	1.29723519852148	0.115	0.045	2.32652013909446E-45	Detoxification-associated hepat	LRRC37B
1.56292221759152E-49	0.4567312984059	0.155	0.07	2.61914505223986E-45	Detoxification-associated hepat	BRF2
1.64530049268171E-49	0.577662696462845	0.171	0.081	2.75719456563601E-45	Detoxification-associated hepat	TSTD2
1.75184604606837E-49	1.05678702451103	0.159	0.074	2.93574360400137E-45	Detoxification-associated hepat	POMT2
2.09943562253776E-49	0.266563663898808	0.23	0.122	3.51823421624877E-45	Detoxification-associated hepat	MRRF
2.25511117953749E-49	0.386622146010992	0.222	0.117	3.77911531466892E-45	Detoxification-associated hepat	TARDBP
2.33538170550012E-49	0.58648915998064	0.176	0.085	3.91363266207709E-45	Detoxification-associated hepat	VPS29
2.33969948226838E-49	0.551911022692366	0.213	0.111	3.92086839238534E-45	Detoxification-associated hepat	ALDH16A1
2.69080370637549E-49	0.61646354979818	0.177	0.086	4.50924885114404E-45	Detoxification-associated hepat	RUNDC1
3.11524625166471E-49	1.12698096201435	0.162	0.077	5.22052966853972E-45	Detoxification-associated hepat	SYTL2
3.34061061560469E-49	0.572850164780923	0.199	0.102	5.59819526963033E-45	Detoxification-associated hepat	PARP8
3.44943059444003E-49	0.74670757108035	0.101	0.037	5.7805557901626E-45	Detoxification-associated hepat	RAMAC
3.67417659325178E-49	0.314707908891898	0.24	0.13	6.15718513497133E-45	Detoxification-associated hepat	RABL3
3.74186540133943E-49	0.467953403420226	0.129	0.054	6.27061803956461E-45	Detoxification-associated hepat	MOB1B
3.9438049484844E-49	0.733115363143707	0.176	0.085	6.60902833267015E-45	Detoxification-associated hepat	METTL4
4.95279397763327E-49	0.553292726928905	0.178	0.086	8.29989214771783E-45	Detoxification-associated hepat	CSE1L
5.26527294494213E-49	0.654103935106446	0.179	0.088	8.82354440113402E-45	Detoxification-associated hepat	IFT57
5.45588827007607E-49	0.556065825524849	0.178	0.086	9.14297756299348E-45	Detoxification-associated hepat	NARS2
5.45781531898567E-49	0.324729204564959	0.223	0.118	9.14620691155619E-45	Detoxification-associated hepat	NUP88
5.82516736989582E-49	0.681137407156797	0.21	0.11	9.76181547847141E-45	Detoxification-associated hepat	NUP62CL
6.55220566468205E-49	0.398583377652708	0.165	0.077	1.09801862528742E-44	Detoxification-associated hepat	SUPV3L1
6.82669497064166E-49	0.311038360786817	0.251	0.138	1.14401754318013E-44	Detoxification-associated hepat	TATDN2
7.24833995996115E-49	0.608480433238882	0.188	0.094	1.21467681049029E-44	Detoxification-associated hepat	CCDC90B
8.05898155398817E-49	1.30048525481645	0.126	0.053	1.35052412881734E-44	Detoxification-associated hepat	ERV3-1
8.44469573648736E-49	0.867067968299167	0.132	0.056	1.41516211152055E-44	Detoxification-associated hepat	ZNF500
8.93928722433652E-49	0.367470683123088	0.253	0.14	1.49804575305431E-44	Detoxification-associated hepat	HAUS2
8.96984485023605E-49	0.310847143774889	0.253	0.139	1.50316660000256E-44	Detoxification-associated hepat	DCP2
1.04516102660153E-48	0.477921210563708	0.21	0.11	1.75148084837885E-44	Detoxification-associated hepat	CXorf40B
1.3186959260483E-48	0.304942658998121	0.275	0.157	2.20987063287174E-44	Detoxification-associated hepat	FARP2
1.55100860702423E-48	0.278023976532784	0.185	0.09	2.5991802236512E-44	Detoxification-associated hepat	MYH10
2.04365357307213E-48	0.304493603384258	0.226	0.121	3.42475465775427E-44	Detoxification-associated hepat	MTMR14
2.25866662943426E-48	0.347883212331683	0.182	0.089	3.78507353760594E-44	Detoxification-associated hepat	TMEM170B
2.46392200901391E-48	0.907922702644925	0.151	0.069	4.1290405027055E-44	Detoxification-associated hepat	ZDHHC21
2.46825046096985E-48	0.546763519470833	0.197	0.101	4.13629412249327E-44	Detoxification-associated hepat	STK17B
2.55392942330711E-48	1.41802532896228	0.104	0.039	4.27987492757806E-44	Detoxification-associated hepat	GIN1
2.67170205666429E-48	0.282973878242725	0.287	0.166	4.47723830655801E-44	Detoxification-associated hepat	OSER1
2.83145052436026E-48	0.291859388969991	0.251	0.139	4.74494478872292E-44	Detoxification-associated hepat	PSMG1
3.03246934394381E-48	0.773592934635228	0.162	0.076	5.08181212658104E-44	Detoxification-associated hepat	SDAD1
3.03827046876438E-48	0.330036215929546	0.265	0.15	5.09153365155534E-44	Detoxification-associated hepat	NCBP1
3.39875460140069E-48	0.573320566796828	0.162	0.076	5.69563296102728E-44	Detoxification-associated hepat	TMEM69
4.13262547577514E-48	0.306238419992909	0.232	0.125	6.92545377230397E-44	Detoxification-associated hepat	XPOT
4.35852264044423E-48	0.259696114498012	0.263	0.147	7.30401224085644E-44	Detoxification-associated hepat	DROSHA
4.53501903054296E-48	0.420474072668074	0.271	0.152	7.59978489138389E-44	Detoxification-associated hepat	FANCA
5.41352421060354E-48	0.580622159966819	0.233	0.127	9.07198387212942E-44	Detoxification-associated hepat	RALGPS1
5.84827052677356E-48	0.337289462599678	0.234	0.125	9.80053174876714E-44	Detoxification-associated hepat	TMEM184A
6.44981983054074E-48	0.460022058312601	0.203	0.104	1.08086080720202E-43	Detoxification-associated hepat	CPT1B
8.04558883836956E-48	0.522247495332305	0.133	0.057	1.34827977753397E-43	Detoxification-associated hepat	WDR73
8.33765343400691E-48	0.623493118607593	0.171	0.083	1.39722396247088E-43	Detoxification-associated hepat	GXYLT1
8.39750700960791E-48	0.263035581551915	0.331	0.197	1.40725422467009E-43	Detoxification-associated hepat	SLC7A1
8.41916946562671E-48	0.329279037508588	0.23	0.124	1.41088441904972E-43	Detoxification-associated hepat	METTL21A
1.02540121383619E-47	0.477498624590876	0.163	0.077	1.71836735414669E-43	Detoxification-associated hepat	ZSWIM7
1.12837476344674E-47	1.22969641420149	0.125	0.053	1.89093042858404E-43	Detoxification-associated hepat	ZC3H12B
1.59582271365048E-47	0.871036966512053	0.108	0.042	2.67427970353548E-43	Detoxification-associated hepat	TYW5
1.61542503073339E-47	0.363549312664319	0.19	0.095	2.70712926650301E-43	Detoxification-associated hepat	ANKRD13C
1.66213004772837E-47	0.472962354550173	0.171	0.082	2.7853975339832E-43	Detoxification-associated hepat	ARL14EP
1.97659695894835E-47	0.399415973810613	0.215	0.113	3.31238118380565E-43	Detoxification-associated hepat	DOCK6
2.01609284988108E-47	0.977915596271421	0.119	0.049	3.37856839783071E-43	Detoxification-associated hepat	SMIM8
2.22828753403543E-47	0.58048943053603	0.262	0.15	3.73416424953658E-43	Detoxification-associated hepat	BTBD3

**3.05896762019508E-47**	0.439344203814368	0.252	0.142	5.12621793792292E-43	Detoxification-associated hepat	TPCN1
**3.09687581347539E-47**	0.665798623417709	0.187	0.095	5.18974448822206E-43	Detoxification-associated hepat	CHST12
**3.66748779139459E-47**	0.6401359115455	0.158	0.074	6.14597604081905E-43	Detoxification-associated hepat	NOXA1
**4.00117112732314E-47**	0.455607617915528	0.181	0.089	6.70516257516812E-43	Detoxification-associated hepat	PPM1F
**4.64884994950216E-47**	0.607668394595854	0.196	0.101	7.79054274537573E-43	Detoxification-associated hepat	TRMT61B
**5.07022985753026E-47**	0.994871819194177	0.124	0.053	8.49669119524921E-43	Detoxification-associated hepat	MAATS1
**6.54339659583606E-47**	0.323735036024703	0.264	0.15	1.09654240153021E-42	Detoxification-associated hepat	ARFIP1
**6.66041884108195E-47**	0.267879171176576	0.248	0.137	1.11615298938851E-42	Detoxification-associated hepat	RWDD1
**6.76956538253745E-47**	0.385433124105748	0.157	0.073	1.13444376680563E-42	Detoxification-associated hepat	DEPRECATEI
**7.01100004030235E-47**	0.621844233008296	0.158	0.074	1.17490338675387E-42	Detoxification-associated hepat	DPH5
**7.44129401508494E-47**	0.280253612090223	0.26	0.146	1.24701205104793E-42	Detoxification-associated hepat	MEST
**9.10302588899556E-47**	0.558595954796249	0.149	0.068	1.52548507847788E-42	Detoxification-associated hepat	VPS51
**1.14636057127141E-46**	0.852415535769848	0.151	0.069	1.92107104533662E-42	Detoxification-associated hepat	PRTFDC1
**1.18263940587785E-46**	0.72580509254096	0.179	0.089	1.98186711637011E-42	Detoxification-associated hepat	FMO4
**1.19980604230334E-46**	0.262827216838515	0.241	0.132	2.01063496569194E-42	Detoxification-associated hepat	DEPRECATEI
**1.22614307726486E-46**	0.503932861818985	0.226	0.123	2.05477056888046E-42	Detoxification-associated hepat	IL17RA
**1.2743590101957E-46**	0.628128857389776	0.185	0.093	2.13557082928595E-42	Detoxification-associated hepat	DEPRECATEI
**1.3754578097339E-46**	0.289208744857596	0.244	0.135	2.30499219755206E-42	Detoxification-associated hepat	RSF1
**1.41571187519515E-46**	0.476588642453033	0.184	0.093	2.37244996045203E-42	Detoxification-associated hepat	EYA3
**1.42467550457298E-46**	0.264304139042093	0.236	0.129	2.38747121056339E-42	Detoxification-associated hepat	TMEM126B
**1.5387717497415E-46**	0.507440461632744	0.218	0.118	2.5786736982168E-42	Detoxification-associated hepat	TCTEX1D2
**1.57327768113318E-46**	0.344861244951008	0.215	0.114	2.63649873804298E-42	Detoxification-associated hepat	OSGIN2
**1.71704587060246E-46**	0.662727490640261	0.165	0.08	2.87742546995561E-42	Detoxification-associated hepat	KYAT3
**2.10473353312623E-46**	0.788942046858447	0.114	0.046	3.52711245481293E-42	Detoxification-associated hepat	SLC2A11
**2.14743433012649E-46**	0.373617162554959	0.275	0.159	3.59867045042597E-42	Detoxification-associated hepat	CLGN
**2.28331432990384E-46**	0.413408191011796	0.195	0.1	3.82637815405285E-42	Detoxification-associated hepat	ANKRD12
**2.29936722151506E-46**	0.400680552702563	0.204	0.106	3.85327958981494E-42	Detoxification-associated hepat	DEPRECATEI
**2.37994432157524E-46**	0.527735792025541	0.138	0.061	3.98831069409578E-42	Detoxification-associated hepat	DDTL
**2.42799298458891E-46**	0.612557455609499	0.174	0.086	4.0688306435741E-42	Detoxification-associated hepat	CCDC127
**2.46193207148408E-46**	0.481796148859968	0.217	0.117	4.12570576539302E-42	Detoxification-associated hepat	B4GALT6
**2.64714143841937E-46**	0.550474571811985	0.181	0.09	4.43607962250318E-42	Detoxification-associated hepat	CIPC
**2.75413275538953E-46**	0.419159820366178	0.196	0.101	4.61537567148177E-42	Detoxification-associated hepat	FBXL4
**2.89564036502969E-46**	0.501934561227174	0.141	0.063	4.85251412371676E-42	Detoxification-associated hepat	ANKS3
**3.41275965803216E-46**	0.875488547499441	0.138	0.062	5.7191026349303E-42	Detoxification-associated hepat	IL1RAPL1
**3.85477401932717E-46**	0.310481574684567	0.228	0.123	6.45983030158847E-42	Detoxification-associated hepat	ASAH1
**3.91807371184963E-46**	0.323353776005707	0.194	0.099	6.56590792631761E-42	Detoxification-associated hepat	ARID5B
**3.96744443920988E-46**	0.562844802397658	0.187	0.095	6.64864339122791E-42	Detoxification-associated hepat	RSBN1
**4.91311419280552E-46**	0.765239844839735	0.144	0.066	8.23339676430348E-42	Detoxification-associated hepat	USP46
**5.16317542107465E-46**	0.809754647655247	0.111	0.045	8.6524493706369E-42	Detoxification-associated hepat	ZFAND4
**5.37058903403141E-46**	0.708144933691008	0.151	0.07	9.00003310322984E-42	Detoxification-associated hepat	OSBPL1A
**5.67510606579297E-46**	0.703251842613613	0.126	0.054	9.51034274505586E-42	Detoxification-associated hepat	RFX3
**5.80082817394083E-46**	0.69403272443016	0.195	0.101	9.72102785389005E-42	Detoxification-associated hepat	CIITA
**5.93967137755493E-46**	0.5399000177466	0.15	0.069	9.95370129450655E-42	Detoxification-associated hepat	SPATC1L
**6.9699294888922E-46**	0.366948811630996	0.245	0.136	1.16802078374855E-41	Detoxification-associated hepat	SLC2A5
**7.90330672760971E-46**	0.414727271967192	0.215	0.115	1.32443614141284E-41	Detoxification-associated hepat	E2F3
**8.18460752057924E-46**	0.259694741136699	0.169	0.082	1.37157652829867E-41	Detoxification-associated hepat	CFAP410
**8.5665106699284E-46**	0.677674720697044	0.162	0.078	1.4355758580666E-41	Detoxification-associated hepat	MEIS1
**9.33754172774208E-46**	0.45383143013441	0.187	0.095	1.56478524273502E-41	Detoxification-associated hepat	LLPH
**1.04735332987373E-45**	0.280550885528344	0.214	0.114	1.75515471020239E-41	Detoxification-associated hepat	TMEM256
**1.06106776574483E-45**	0.307210529998817	0.214	0.114	1.77813736183519E-41	Detoxification-associated hepat	PDIK1L
**1.08910998150432E-45**	0.445982354582282	0.112	0.045	1.82513050700494E-41	Detoxification-associated hepat	SLC9B2
**1.13935236405601E-45**	0.296527983053018	0.189	0.095	1.90932669168507E-41	Detoxification-associated hepat	RALGDS
**1.50016408048902E-45**	1.17200799301892	0.119	0.051	2.5139749660835E-41	Detoxification-associated hepat	TMPPE
**1.68166470842071E-45**	0.324288403571509	0.23	0.126	2.81813371837142E-41	Detoxification-associated hepat	MICA
**1.88974656982394E-45**	0.67039522890032	0.2	0.105	3.16683730171096E-41	Detoxification-associated hepat	PRDM4
**1.89869093957916E-45**	0.522918776728175	0.193	0.1	3.18182627654675E-41	Detoxification-associated hepat	JAK2
**1.98354290690826E-45**	0.57506647535688	0.211	0.113	3.32402120339687E-41	Detoxification-associated hepat	TRMT61A
**2.13578666727167E-45**	0.329287863015751	0.181	0.09	3.57915129701387E-41	Detoxification-associated hepat	CCDC174
**2.20642214346795E-45**	0.624004558222521	0.168	0.082	3.69752222802359E-41	Detoxification-associated hepat	DEPRECATEI
**2.43301709734814E-45**	1.11596977885638	0.143	0.066	4.07725005173601E-41	Detoxification-associated hepat	PCDHGB2

**2.46743743270473E-45**	0.741173288379014	0.222	0.122	4.13493164972658E-41	Detoxification-associated hepat	XKR9
**2.55988341136838E-45**	0.280727136293261	0.241	0.134	4.28985262077113E-41	Detoxification-associated hepat	RNF8
**2.5702949003226E-45**	0.360989439153089	0.246	0.137	4.30730019396062E-41	Detoxification-associated hepat	TFDP2
**2.73782414394944E-45**	0.86343557056381	0.152	0.072	4.58804570043048E-41	Detoxification-associated hepat	ZNF627
**2.84814361295884E-45**	0.747642665940038	0.194	0.103	4.77291906659642E-41	Detoxification-associated hepat	PPP1R9A
**3.41992133648793E-45**	0.358363624448978	0.217	0.117	5.73110417568647E-41	Detoxification-associated hepat	TRAPPC13
**3.56615759717195E-45**	0.967090017192974	0.115	0.048	5.97616690134076E-41	Detoxification-associated hepat	DEPRECATEI
**3.65688318379615E-45**	0.532113421265775	0.159	0.076	6.12820483940559E-41	Detoxification-associated hepat	YAE1
**3.67035127388421E-45**	0.295590386207682	0.203	0.107	6.15077466477516E-41	Detoxification-associated hepat	WASF3
**4.03062406393161E-45**	0.975112345108203	0.138	0.063	6.75451980633659E-41	Detoxification-associated hepat	UBE2V2
**5.02654393699128E-45**	0.428265490020021	0.148	0.068	8.42348232960999E-41	Detoxification-associated hepat	SIRT1
**6.13930625069722E-45**	0.311470953331371	0.184	0.093	1.02882494149184E-40	Detoxification-associated hepat	MOB3A
**6.3981609368217E-45**	0.465446311664868	0.232	0.129	1.07220380979258E-40	Detoxification-associated hepat	TTC39B
**7.28854153424795E-45**	0.474407046372235	0.178	0.089	1.22141379030927E-40	Detoxification-associated hepat	MAP3K1
**7.31827659032385E-45**	0.785425063361781	0.19	0.099	1.22639679100647E-40	Detoxification-associated hepat	OBI1
**8.65233121778838E-45**	0.459902907202768	0.156	0.074	1.44995766547698E-40	Detoxification-associated hepat	SMG7
**8.68819527461556E-45**	0.343881616955363	0.233	0.129	1.45596776412007E-40	Detoxification-associated hepat	CUEDC1
**9.17072164821369E-45**	0.273627760905878	0.24	0.134	1.53682953380765E-40	Detoxification-associated hepat	LRRC8D
**1.11737183122368E-44**	0.925122033823559	0.13	0.058	1.87249171476465E-40	Detoxification-associated hepat	PIGB
**1.12546866697075E-44**	0.54707703509679	0.133	0.059	1.88606039210958E-40	Detoxification-associated hepat	ZNF205
**1.34497512423916E-44**	0.593033985219133	0.155	0.074	2.25390931319999E-40	Detoxification-associated hepat	TRIM39
**1.37563800479649E-44**	0.347279771417691	0.169	0.083	2.30529416843796E-40	Detoxification-associated hepat	ZC3HC1
**1.47880879083936E-44**	0.262963702153277	0.251	0.141	2.4781877716886E-40	Detoxification-associated hepat	SPNS1
**1.57973746396863E-44**	0.36190244565323	0.215	0.117	2.64732404211863E-40	Detoxification-associated hepat	IFT80
**1.6498645242005E-44**	0.550318187180914	0.137	0.062	2.7648429696552E-40	Detoxification-associated hepat	CEP164
**1.6629638088215E-44**	1.01663759675631	0.112	0.046	2.78679475082306E-40	Detoxification-associated hepat	NANP
**1.83735037508747E-44**	0.357031469617716	0.165	0.08	3.07903175857158E-40	Detoxification-associated hepat	KBTBD4
**1.89698674843943E-44**	0.332195493317199	0.232	0.128	3.17897039303479E-40	Detoxification-associated hepat	ELAC2
**2.16531584084474E-44**	0.373840409873987	0.132	0.058	3.62863628608762E-40	Detoxification-associated hepat	ST3GAL5
**2.40365536874663E-44**	0.68207424038698	0.159	0.077	4.0280456669456E-40	Detoxification-associated hepat	TICAM2
**2.67873724594396E-44**	0.702053707652295	0.149	0.07	4.48902787675288E-40	Detoxification-associated hepat	MCPH1
**2.67915221575642E-44**	0.347919886737587	0.236	0.131	4.4897232831646E-40	Detoxification-associated hepat	RAD51D
**2.69175965211978E-44**	0.657134535300024	0.12	0.051	4.51085082502233E-40	Detoxification-associated hepat	TCP11L2
**2.71532839648113E-44**	0.273402845393704	0.287	0.166	4.55034732682309E-40	Detoxification-associated hepat	POLA1
**2.83609138198886E-44**	0.674409712912154	0.176	0.089	4.75272193793693E-40	Detoxification-associated hepat	PPP2R1A
**3.35642785031042E-44**	0.693726175303964	0.177	0.09	5.6247017915502E-40	Detoxification-associated hepat	CASP10
**3.4390145832091E-44**	0.390110591124752	0.198	0.104	5.76310063854182E-40	Detoxification-associated hepat	TMA16
**3.66405535079284E-44**	0.740857990155408	0.151	0.071	6.14022395685864E-40	Detoxification-associated hepat	ORC3
**3.87783956377412E-44**	0.393460150745932	0.194	0.101	6.49848354097267E-40	Detoxification-associated hepat	CRTC3
**4.24095036270675E-44**	0.388745437109398	0.217	0.117	7.10698461782396E-40	Detoxification-associated hepat	PPP1R21
**4.41571970412758E-44**	0.937593243241763	0.145	0.068	7.399863080177E-40	Detoxification-associated hepat	MFSD14C
**4.72061513142197E-44**	0.279100272838966	0.199	0.104	7.91080683723694E-40	Detoxification-associated hepat	WDR11
**5.06173546447888E-44**	0.309702260500168	0.238	0.134	8.4824562913737E-40	Detoxification-associated hepat	NSD3
**6.75552545745087E-44**	0.257289591553126	0.233	0.13	1.13209095615962E-39	Detoxification-associated hepat	EMC1
**7.47301540602142E-44**	0.954535288380389	0.125	0.055	1.25232792174107E-39	Detoxification-associated hepat	KLHL9
**8.65881427581047E-44**	0.408314877414796	0.246	0.14	1.45104409634032E-39	Detoxification-associated hepat	CLRN3
**1.03754838388232E-43**	0.778825529580417	0.136	0.062	1.73872358170999E-39	Detoxification-associated hepat	DEPRECATEI
**1.05948953552916E-43**	0.503649488619337	0.129	0.057	1.77549256363976E-39	Detoxification-associated hepat	PLPP6
**1.21518170046942E-43**	0.46268789344458	0.151	0.071	2.03640149364666E-39	Detoxification-associated hepat	UBALD1
**1.25653946329689E-43**	0.594339074595858	0.18	0.092	2.10570883259293E-39	Detoxification-associated hepat	NLRC5
**1.26690695115676E-43**	0.458860339164396	0.198	0.105	2.1230826687485E-39	Detoxification-associated hepat	ZNF274
**1.36880313666523E-43**	0.785103173658822	0.129	0.057	2.2938402964236E-39	Detoxification-associated hepat	MTRF1L
**1.46441112376551E-43**	0.788362162550453	0.106	0.042	2.45406016120624E-39	Detoxification-associated hepat	GDPGP1
**1.5246549204935E-43**	0.481123290157535	0.178	0.09	2.555016715763E-39	Detoxification-associated hepat	MITD1
**1.76880650612999E-43**	0.368777171222845	0.205	0.109	2.96416594297263E-39	Detoxification-associated hepat	OPA3
**1.99854008737125E-43**	0.322638991191166	0.246	0.141	3.34915347841674E-39	Detoxification-associated hepat	C1orf131
**2.04734611862301E-43**	0.836467643355886	0.136	0.062	3.43094262558844E-39	Detoxification-associated hepat	PTPDC1
**2.33618250174989E-43**	0.293905184745337	0.2	0.106	3.91497463643247E-39	Detoxification-associated hepat	PEX5
**2.41717212522276E-43**	0.546534194211673	0.146	0.068	4.0506970474483E-39	Detoxification-associated hepat	DEPRECATEI
**2.44740462259888E-43**	0.331653271973118	0.23	0.128	4.10136066655121E-39	Detoxification-associated hepat	CHPF2

**2.51756494064736E-43**	0.462090171207815	0.118	0.05	4.21893532753684E-39	Detoxification-associated hepat	TRIM22
**2.6059151001218E-43**	0.707597919779483	0.122	0.053	4.36699252478411E-39	Detoxification-associated hepat	DCP1B
**2.88078391988692E-43**	0.311318143602065	0.236	0.133	4.82761769294651E-39	Detoxification-associated hepat	TBC1D7
**2.94809051342848E-43**	0.808096381517077	0.129	0.058	4.94041008240345E-39	Detoxification-associated hepat	KIN
**3.22394348441213E-43**	0.479899689084243	0.209	0.113	5.40268449117785E-39	Detoxification-associated hepat	BRD9
**5.08192109534207E-43**	0.355781037058219	0.219	0.121	8.51628337157424E-39	Detoxification-associated hepat	BRWD3
**5.38448333362406E-43**	0.310781779883928	0.197	0.104	9.0233171704872E-39	Detoxification-associated hepat	RRAGC
**5.64650406165099E-43**	0.33716800810432	0.244	0.139	9.46241150651472E-39	Detoxification-associated hepat	SLC35B3
**7.87944439010316E-43**	0.65979134163124	0.206	0.112	1.32043729089349E-38	Detoxification-associated hepat	IL7
**8.37220384084333E-43**	0.329451610150624	0.178	0.091	1.40301391964852E-38	Detoxification-associated hepat	FAM114A2
**8.7217119530214E-43**	0.363084635530602	0.235	0.132	1.46158448908733E-38	Detoxification-associated hepat	UBLCP1
**9.56890419261274E-43**	0.345494739803256	0.196	0.104	1.60355696459804E-38	Detoxification-associated hepat	EID2
**1.12496591028755E-42**	0.945828743338716	0.126	0.057	1.88521787245988E-38	Detoxification-associated hepat	ATP7A
**1.12991673317261E-42**	0.996531485700871	0.137	0.064	1.89351446145065E-38	Detoxification-associated hepat	SLC25A14
**1.19962949634078E-42**	1.05277331730027	0.135	0.063	2.01033910996788E-38	Detoxification-associated hepat	PRR36
**1.56527629599886E-42**	0.264310147129234	0.216	0.117	2.62309001683489E-38	Detoxification-associated hepat	DENND5A
**1.68607166903969E-42**	0.852913033243728	0.122	0.054	2.82551890297671E-38	Detoxification-associated hepat	MEAK7
**1.89824957690264E-42**	0.28185939737639	0.259	0.15	3.18108664097344E-38	Detoxification-associated hepat	CEP85
**1.93431094264762E-42**	1.10266311136382	0.101	0.041	3.24151827768888E-38	Detoxification-associated hepat	EXOG
**1.99747866986978E-42**	0.473799322510404	0.214	0.117	3.34737475496778E-38	Detoxification-associated hepat	C7orf25
**2.02832900611684E-42**	0.731460483208344	0.146	0.07	3.3990737484506E-38	Detoxification-associated hepat	TECPR2
**2.04653767127247E-42**	0.342813928655221	0.203	0.11	3.4295878295184E-38	Detoxification-associated hepat	PRKD3
**2.21964259891312E-42**	0.387225346439788	0.206	0.112	3.7196770672586E-38	Detoxification-associated hepat	SCYL2
**2.40927732417536E-42**	0.528130649228298	0.194	0.104	4.03746693985306E-38	Detoxification-associated hepat	EXD2
**2.63055741966675E-42**	1.03248131698527	0.142	0.067	4.40828812387754E-38	Detoxification-associated hepat	SLF1
**3.22659782975978E-42**	0.807363250778288	0.163	0.082	5.40713264311143E-38	Detoxification-associated hepat	KIAA1841
**3.36658534513286E-42**	0.282095285277671	0.219	0.121	5.64172372137365E-38	Detoxification-associated hepat	ZNF414
**3.83105033405815E-42**	0.299288487025596	0.346	0.215	6.42007414981465E-38	Detoxification-associated hepat	SHOX2
**4.44229625418947E-42**	0.530543601706129	0.178	0.092	7.44440006277072E-38	Detoxification-associated hepat	DRAM1
**5.27605761920373E-42**	0.392338842385384	0.142	0.066	8.84161735826162E-38	Detoxification-associated hepat	PRPF40B
**5.42017280061267E-42**	0.316433136182831	0.206	0.112	9.08312557926671E-38	Detoxification-associated hepat	INO80
**5.69808749884273E-42**	0.537069259210311	0.165	0.083	9.54885503056064E-38	Detoxification-associated hepat	GEMIN5
**5.80756990597819E-42**	0.535229830206781	0.158	0.078	9.73232564843824E-38	Detoxification-associated hepat	SMARCAL1
**6.24546335616377E-42**	1.08853784165802	0.101	0.04	1.04661474922592E-37	Detoxification-associated hepat	CHMP4A
**6.78226686950618E-42**	0.465648234287992	0.179	0.093	1.13657228199185E-37	Detoxification-associated hepat	PRKCE
**6.97702611538817E-42**	0.312599431759496	0.189	0.099	1.16921003641675E-37	Detoxification-associated hepat	DOLK
**7.03950192124378E-42**	0.434417756668034	0.18	0.093	1.17967973196203E-37	Detoxification-associated hepat	C15orf39
**7.13497921656475E-42**	0.430484401443228	0.204	0.111	1.19567981711192E-37	Detoxification-associated hepat	CNOT1
**8.07846917225766E-42**	0.543566473428839	0.227	0.129	1.35378986388694E-37	Detoxification-associated hepat	ZNF862
**8.23111274194768E-42**	0.597074610970489	0.176	0.091	1.37936987329559E-37	Detoxification-associated hepat	EMSY
**8.31218881698965E-42**	0.514525041736796	0.125	0.055	1.39295660195113E-37	Detoxification-associated hepat	IFT172
**8.61668215483626E-42**	0.275274447781814	0.224	0.125	1.44398359550746E-37	Detoxification-associated hepat	FAM53B
**9.01752909379284E-42**	0.629135782727606	0.171	0.088	1.5111575255378E-37	Detoxification-associated hepat	EFHC1
**9.35788270790073E-42**	0.713617978312673	0.143	0.068	1.56819398419E-37	Detoxification-associated hepat	ARHGAP32
**9.42269167444452E-42**	0.363041840644067	0.223	0.124	1.57905467080341E-37	Detoxification-associated hepat	NPC1
**9.73261078980037E-42**	1.08176817154212	0.118	0.052	1.63099091615475E-37	Detoxification-associated hepat	CYB561D1
**1.1657247224394E-41**	0.418229334134836	0.257	0.151	1.95352148986395E-37	Detoxification-associated hepat	MROH6
**1.17056694500568E-41**	0.47318197098486	0.205	0.111	1.96163608644051E-37	Detoxification-associated hepat	NUDT12
**1.17700361999226E-41**	0.535565200897981	0.105	0.042	1.97242266638303E-37	Detoxification-associated hepat	AQP7
**1.2098532447232E-41**	0.981194179758216	0.133	0.061	2.02747206750714E-37	Detoxification-associated hepat	ZNF786
**1.23573410052251E-41**	0.295018878702888	0.145	0.068	2.07084320565562E-37	Detoxification-associated hepat	SLC5A9
**1.42694033388058E-41**	0.385254674536696	0.175	0.089	2.39126661151708E-37	Detoxification-associated hepat	LDB1
**1.94082275645509E-41**	0.266699616569043	0.185	0.096	3.25243077526744E-37	Detoxification-associated hepat	TBL1X
**2.01186509324221E-41**	0.467037474484729	0.161	0.08	3.3714835232553E-37	Detoxification-associated hepat	CWF19L2
**2.0282516897683E-41**	0.465871917140773	0.184	0.097	3.39894418171371E-37	Detoxification-associated hepat	CUL9
**2.1702527297433E-41**	0.945231769329392	0.162	0.082	3.63690952450382E-37	Detoxification-associated hepat	ARSF
**2.3291927554244E-41**	0.286181068674645	0.247	0.143	3.9032612195402E-37	Detoxification-associated hepat	SLC2A8
**2.37499381964222E-41**	0.677443142382975	0.148	0.071	3.98001464295644E-37	Detoxification-associated hepat	ZDHHC7
**2.82606884716639E-41**	0.590163904064015	0.134	0.061	4.73592617408143E-37	Detoxification-associated hepat	RMDN2
**3.15854709330216E-41**	0.324840875554289	0.207	0.113	5.29309321895576E-37	Detoxification-associated hepat	ZFYVE1

3.21630857857274E-41	0.324108363013844	0.189	0.099	5.3898899159722E-37	Detoxification-associated hepat	THOC5
3.54681337488636E-41	0.873731743102122	0.113	0.049	5.94374985363457E-37	Detoxification-associated hepat	DUS4L
3.83344725625763E-41	0.865673770100558	0.126	0.057	6.42409091203653E-37	Detoxification-associated hepat	TET2
3.90565685891174E-41	0.757709778817426	0.166	0.085	6.54509976416429E-37	Detoxification-associated hepat	AGTPBP1
4.18316999027487E-41	0.257761700000954	0.235	0.133	7.01015626970264E-37	Detoxification-associated hepat	GIT2
4.6119025630063E-41	0.825518847792027	0.107	0.044	7.72862631508597E-37	Detoxification-associated hepat	AP4E1
4.96208085720728E-41	0.252440968992336	0.329	0.203	8.31545510050796E-37	Detoxification-associated hepat	SLC26A6
5.0379438962932E-41	0.953035715207952	0.12	0.053	8.44258638140815E-37	Detoxification-associated hepat	AP1S2
5.16899544850233E-41	0.493675495920023	0.122	0.054	8.66220257260021E-37	Detoxification-associated hepat	DGKG
5.62434807811333E-41	0.611961750155178	0.158	0.079	9.42528250930231E-37	Detoxification-associated hepat	FASTKD1
5.63077137852305E-41	0.308374089118209	0.248	0.144	9.43604667612893E-37	Detoxification-associated hepat	TAOK2
6.42528924496814E-41	0.669804533874	0.133	0.062	1.07674997167176E-36	Detoxification-associated hepat	TAF1B
6.53549403282085E-41	0.575201481830376	0.169	0.087	1.09521809002012E-36	Detoxification-associated hepat	ZNF250
7.39673958998282E-41	0.8466180149312	0.111	0.048	1.23954562048932E-36	Detoxification-associated hepat	ERCC4
7.68919863897366E-41	0.375310212078451	0.144	0.069	1.28855590791921E-36	Detoxification-associated hepat	TFB2M
9.26976283583407E-41	0.391799761397683	0.185	0.097	1.55342685602907E-36	Detoxification-associated hepat	HACL1
9.44468257181088E-41	0.462969119370251	0.115	0.05	1.58273990538407E-36	Detoxification-associated hepat	CTC1
1.03003265518858E-40	0.303455469491447	0.231	0.132	1.72612872356502E-36	Detoxification-associated hepat	DHRS4L2
1.05365626925966E-40	0.346072109358522	0.221	0.124	1.76571717602534E-36	Detoxification-associated hepat	TMEM185B
1.30227003643857E-40	0.520079266327631	0.144	0.068	2.18234412706376E-36	Detoxification-associated hepat	SEMA5A
1.30557958126419E-40	0.564499782334815	0.169	0.086	2.18789026228253E-36	Detoxification-associated hepat	RHPN1
1.33067683455316E-40	0.399738151482998	0.207	0.114	2.22994823934418E-36	Detoxification-associated hepat	EXOSC3
1.35126846308942E-40	0.762877764014392	0.106	0.044	2.26445569044526E-36	Detoxification-associated hepat	ZFP14
1.46348943160863E-40	0.411938676101474	0.176	0.091	2.45251558948975E-36	Detoxification-associated hepat	DGLUCY
1.57261735594129E-40	0.333608847875456	0.166	0.083	2.63539216508642E-36	Detoxification-associated hepat	PLA2G1B
1.76934121965549E-40	0.696405868900795	0.127	0.058	2.96506201589866E-36	Detoxification-associated hepat	ALKBH1
1.90883115180027E-40	0.648581452329245	0.181	0.096	3.1988192441869E-36	Detoxification-associated hepat	ZDHHC2
1.99613759583929E-40	0.645672321303717	0.128	0.058	3.34512738310749E-36	Detoxification-associated hepat	WDR89
2.08269479726948E-40	0.301431879273638	0.236	0.135	3.49017994126419E-36	Detoxification-associated hepat	ABCG1
2.35419633020526E-40	0.576975726185164	0.176	0.092	3.94516221015797E-36	Detoxification-associated hepat	PRRG1
2.46704120233517E-40	0.370492721543944	0.16	0.079	4.13426764687328E-36	Detoxification-associated hepat	ZSCAN31
2.65782652826003E-40	0.411651334144842	0.17	0.087	4.45398569605815E-36	Detoxification-associated hepat	POMT1
2.7293867993934E-40	0.635700802750627	0.149	0.072	4.57390639842347E-36	Detoxification-associated hepat	BGLAP
2.84039645476062E-40	0.395253021180869	0.174	0.09	4.75993637888784E-36	Detoxification-associated hepat	ABHD5
2.86131703936178E-40	0.400112489589302	0.161	0.08	4.79499509456247E-36	Detoxification-associated hepat	CXCL9
2.98188405533338E-40	0.602260440938767	0.12	0.053	4.99704129992767E-36	Detoxification-associated hepat	LYSMD1
3.18169914862797E-40	0.500704638027863	0.243	0.142	5.33189143327075E-36	Detoxification-associated hepat	C1GALT1
3.20139534494662E-40	0.264130495244235	0.234	0.134	5.36489831906154E-36	Detoxification-associated hepat	UBXN2A
3.33764108685489E-40	0.265149626280119	0.273	0.164	5.59321893335142E-36	Detoxification-associated hepat	UCHL3
3.63308823571829E-40	0.915477266289628	0.155	0.078	6.08832926541671E-36	Detoxification-associated hepat	ALPK1
3.97962403483232E-40	0.548393614853269	0.223	0.128	6.669053957572E-36	Detoxification-associated hepat	FLVCR2
4.10641661568595E-40	0.592440651400495	0.175	0.091	6.88153296456651E-36	Detoxification-associated hepat	HOMEZ
4.46623196306596E-40	0.267725184978037	0.233	0.133	7.48451152370593E-36	Detoxification-associated hepat	STARD3NL
4.73173183689288E-40	0.304470406894541	0.208	0.115	7.92943621226509E-36	Detoxification-associated hepat	RIOK1
5.04139998340178E-40	0.273645547798146	0.208	0.114	8.4483780921847E-36	Detoxification-associated hepat	SPRED2
5.42722476923156E-40	0.638583421320976	0.14	0.067	9.09494326827825E-36	Detoxification-associated hepat	EXO5
6.05102647289427E-40	0.397735504767169	0.172	0.089	1.01403101632762E-35	Detoxification-associated hepat	PTER
6.94798430142722E-40	0.258878322600314	0.194	0.105	1.16434320923317E-35	Detoxification-associated hepat	PPT1
6.96160037669208E-40	0.981609475178349	0.109	0.047	1.16662499112606E-35	Detoxification-associated hepat	EOGT
7.8637259070279E-40	0.633026865912528	0.136	0.064	1.31780318749974E-35	Detoxification-associated hepat	RAD52
8.27899555008716E-40	0.526497956733428	0.19	0.103	1.38739407428361E-35	Detoxification-associated hepat	SENP1
9.28812918935573E-40	0.314451483190627	0.113	0.048	1.55650468955223E-35	Detoxification-associated hepat	ABCC1
1.07887677137543E-39	0.353887765346845	0.158	0.078	1.80798169347094E-35	Detoxification-associated hepat	ZSCAN30
1.09378665465055E-39	0.534833487375494	0.131	0.061	1.83296767586339E-35	Detoxification-associated hepat	GRAMD1C
1.25662902053413E-39	0.819668439156729	0.11	0.047	2.1058589126111E-35	Detoxification-associated hepat	SREK1IP1
1.27065511309844E-39	0.749910037892942	0.135	0.063	2.12936383853037E-35	Detoxification-associated hepat	ATF7IP2
1.4402800700183E-39	1.01140888042385	0.116	0.052	2.41362134133667E-35	Detoxification-associated hepat	ZNF785
1.51544724749696E-39	0.326961008568465	0.185	0.098	2.53958649735541E-35	Detoxification-associated hepat	MMS19
1.61841587151152E-39	0.367869650161295	0.268	0.162	2.712141317479E-35	Detoxification-associated hepat	CHST5
1.7231932208159E-39	0.6446425105974	0.123	0.056	2.88772719944328E-35	Detoxification-associated hepat	DDHD2

**2.0486898155594E-39**	0.418300150453462	0.189	0.102	3.43319439291444E-35	Detoxification-associated hepat	AP2B1
**2.20591596607449E-39**	0.265007179226776	0.265	0.159	3.69667397594763E-35	Detoxification-associated hepat	TBC1D10B
**2.21228022612125E-39**	0.452839700193274	0.181	0.096	3.70733920293399E-35	Detoxification-associated hepat	EIF4ENIF1
**2.62075794802806E-39**	0.713561287876305	0.165	0.086	4.39186616930543E-35	Detoxification-associated hepat	CEP97
**3.13201585911287E-39**	0.630303971455781	0.128	0.059	5.24863217670135E-35	Detoxification-associated hepat	DEPRECATEI
**3.19019184769722E-39**	0.789308966070939	0.147	0.073	5.34612349837101E-35	Detoxification-associated hepat	CASP8AP2
**3.42827388483444E-39**	0.458711422711081	0.186	0.101	5.74510137620555E-35	Detoxification-associated hepat	SGMS2
**3.74293805449097E-39**	0.426004773386306	0.199	0.11	6.27241559171597E-35	Detoxification-associated hepat	MED9
**3.86551412798832E-39**	0.969779163478042	0.113	0.05	6.47782857568283E-35	Detoxification-associated hepat	ZNF30
**4.34628350693413E-39**	0.304485489984041	0.257	0.152	7.28350190092022E-35	Detoxification-associated hepat	CMPK2
**4.38435911100667E-39**	0.772686860129074	0.117	0.052	7.34730899822498E-35	Detoxification-associated hepat	DCLRE1A
**5.12981298168788E-39**	0.626020312756066	0.144	0.07	8.59654059471256E-35	Detoxification-associated hepat	ARHGAP4
**5.43615221357645E-39**	0.287951621921554	0.244	0.141	9.10990387951142E-35	Detoxification-associated hepat	KATNAL1
**5.43954632962526E-39**	0.917431919226793	0.131	0.062	9.11559173918601E-35	Detoxification-associated hepat	ZBTB8A
**6.27495918504635E-39**	0.511052237857778	0.165	0.086	1.05155766023007E-34	Detoxification-associated hepat	RLF
**6.99579770199558E-39**	0.260669563807453	0.167	0.085	1.17235577890042E-34	Detoxification-associated hepat	ECD
**7.43846050185968E-39**	0.268455604382482	0.264	0.158	1.24653721090164E-34	Detoxification-associated hepat	CHD7
**7.58156652230134E-39**	0.294483396907113	0.191	0.103	1.27051891780726E-34	Detoxification-associated hepat	PAICS
**8.00263805172035E-39**	0.432490996964922	0.2	0.111	1.3410820847073E-34	Detoxification-associated hepat	C6orf47
**8.0356928977348E-39**	0.444822517760606	0.271	0.164	1.3466214158024E-34	Detoxification-associated hepat	TEP1
**8.38343752486777E-39**	0.82460561441273	0.108	0.046	1.40489646041734E-34	Detoxification-associated hepat	C21orf91
**8.65047866957227E-39**	0.719330786578669	0.101	0.042	1.44964721544692E-34	Detoxification-associated hepat	BCAS4
**8.66650231217173E-39**	0.693146407076946	0.123	0.056	1.45233245747374E-34	Detoxification-associated hepat	DEPRECATEI
**8.93274565374317E-39**	0.475113652947619	0.133	0.062	1.49694951665428E-34	Detoxification-associated hepat	NBPF20
**1.00372843350513E-38**	0.616867101331182	0.135	0.064	1.6820481088679E-34	Detoxification-associated hepat	ZNF668
**1.06835366583938E-38**	0.641755597839348	0.138	0.066	1.79034707321364E-34	Detoxification-associated hepat	VSIR
**1.26736652254536E-38**	0.3438808697975	0.214	0.121	2.12385281848151E-34	Detoxification-associated hepat	KRAS
**1.26970439020862E-38**	1.02267618442666	0.145	0.072	2.1277706171116E-34	Detoxification-associated hepat	MCOLN3
**1.28046353083106E-38**	0.593811033417882	0.144	0.07	2.1458007849667E-34	Detoxification-associated hepat	MTHFSD
**1.35440660604559E-38**	0.780836335630418	0.13	0.061	2.26971459041119E-34	Detoxification-associated hepat	HACE1
**1.50882544886893E-38**	0.522240307494678	0.222	0.126	2.52848968721455E-34	Detoxification-associated hepat	FAM111B
**1.52300330050524E-38**	0.912940545554627	0.1	0.042	2.55224893098667E-34	Detoxification-associated hepat	CTNS
**1.59854185227152E-38**	0.77109755544852	0.133	0.063	2.67883643603662E-34	Detoxification-associated hepat	TAMM41
**1.81023404029677E-38**	0.416873708657036	0.163	0.084	3.03359020472933E-34	Detoxification-associated hepat	UTP4
**1.84878302312848E-38**	0.323612494870826	0.189	0.102	3.09819059015871E-34	Detoxification-associated hepat	VGLL4
**2.0122182720283E-38**	0.446693216918309	0.202	0.112	3.37207538026503E-34	Detoxification-associated hepat	CEP78
**2.29389446128393E-38**	0.31890906070675	0.147	0.072	3.84410833821962E-34	Detoxification-associated hepat	CNNM2
**2.31328554420713E-38**	0.760743836079006	0.153	0.077	3.87660391498231E-34	Detoxification-associated hepat	FAM185A
**2.37256169061288E-38**	0.440367587299625	0.19	0.104	3.97593888112907E-34	Detoxification-associated hepat	TIMM21
**2.51344600675335E-38**	0.398987631687737	0.137	0.066	4.21203281811726E-34	Detoxification-associated hepat	KIAA0513
**2.79153404168263E-38**	0.509419288372166	0.179	0.096	4.67805274705176E-34	Detoxification-associated hepat	PPP2R3C
**2.86457333768062E-38**	0.495335895783691	0.171	0.09	4.80045199928518E-34	Detoxification-associated hepat	UHRF2
**3.04650620165335E-38**	0.334864350989689	0.163	0.083	5.10533509273069E-34	Detoxification-associated hepat	SLC9A7
**3.0878155129996E-38**	0.265045019615672	0.264	0.159	5.17456123668473E-34	Detoxification-associated hepat	ZDHHC20
**3.63576754837852E-38**	0.676542398666076	0.153	0.077	6.09281925757273E-34	Detoxification-associated hepat	PAOX
**4.04634191149938E-38**	0.51779707585	0.166	0.087	6.78085977529066E-34	Detoxification-associated hepat	ACAD10
**4.23469108847072E-38**	0.844376905470713	0.135	0.065	7.09649532605923E-34	Detoxification-associated hepat	PEX3
**4.74935566821089E-38**	0.395178647177634	0.196	0.108	7.9589702287878E-34	Detoxification-associated hepat	SPATA5
**5.67494793557241E-38**	0.322053623032568	0.194	0.106	9.51007775043224E-34	Detoxification-associated hepat	MAGI3
**5.8978720186724E-38**	0.575500376958658	0.167	0.087	9.88365392889121E-34	Detoxification-associated hepat	PACRGL
**7.48887051038819E-38**	0.763809083966246	0.122	0.057	1.25498492013085E-33	Detoxification-associated hepat	TRIM65
**7.91246667406771E-38**	0.357705810288591	0.176	0.093	1.32597116524027E-33	Detoxification-associated hepat	TSC1
**8.68777674210601E-38**	0.659477125099909	0.123	0.056	1.45589762644212E-33	Detoxification-associated hepat	CLCN6
**9.12821476916935E-38**	0.362987658956388	0.236	0.138	1.5297062310174E-33	Detoxification-associated hepat	POLD3
**1.00733704431563E-37**	0.496321519826288	0.167	0.087	1.68809541886413E-33	Detoxification-associated hepat	TMCO6
**1.07477892727002E-37**	0.615430383312541	0.138	0.067	1.8011145263191E-33	Detoxification-associated hepat	SNAPC1
**1.21166602650677E-37**	0.580855206527278	0.159	0.082	2.03050992722005E-33	Detoxification-associated hepat	EDRF1
**1.24757458515577E-37**	0.628234705969285	0.165	0.087	2.09068548980404E-33	Detoxification-associated hepat	YARS2
**1.37549141371464E-37**	0.329189822424859	0.217	0.124	2.30504851110299E-33	Detoxification-associated hepat	KDM3A
**1.40502899275858E-37**	0.358040574575739	0.195	0.108	2.35454758606482E-33	Detoxification-associated hepat	PFDN1

1.43566913066216E-37	0.312709329649097	0.192	0.105	2.40589432916365E-33	Detoxification-associated hepat	SAYSD1
1.49721992266392E-37	0.411507116131186	0.218	0.125	2.5090411464002E-33	Detoxification-associated hepat	NUP107
1.55058885200252E-37	0.402700465679554	0.195	0.108	2.59847679818582E-33	Detoxification-associated hepat	ARNTL2
1.61179460957386E-37	0.406573639786453	0.215	0.124	2.70104540672388E-33	Detoxification-associated hepat	PAXIP1
1.70297559184078E-37	0.440924147029632	0.171	0.09	2.85384649680678E-33	Detoxification-associated hepat	LPIN3
1.72554487406509E-37	0.707807179140802	0.103	0.044	2.89166809995827E-33	Detoxification-associated hepat	GTDC1
1.7424082476281E-37	0.821196831727723	0.113	0.051	2.91992774137517E-33	Detoxification-associated hepat	ZNF778
1.81499911407966E-37	0.348924752054462	0.199	0.111	3.04157551537469E-33	Detoxification-associated hepat	CBLL1
2.40351130990433E-37	0.882598094519352	0.16	0.084	4.02780425313768E-33	Detoxification-associated hepat	SLC38A11
2.68022709903729E-37	0.861720792822374	0.118	0.054	4.49152457256668E-33	Detoxification-associated hepat	FBXO33
2.78601534832919E-37	0.587330456310411	0.15	0.076	4.66880452073005E-33	Detoxification-associated hepat	ZSWIM1
2.86371502628638E-37	0.584092757074059	0.169	0.089	4.79901364105072E-33	Detoxification-associated hepat	RFC5
3.10262404299528E-37	0.289953706093029	0.224	0.13	5.19937737125149E-33	Detoxification-associated hepat	WRAP73
3.21649040532787E-37	0.349162057086378	0.164	0.085	5.39019462124844E-33	Detoxification-associated hepat	MMAA
3.61466200183208E-37	0.748187932640915	0.113	0.051	6.0574505826702E-33	Detoxification-associated hepat	ZKSCAN4
4.0054872473537E-37	0.273163026521293	0.179	0.096	6.71239552911533E-33	Detoxification-associated hepat	KDM1B
4.40351889720121E-37	0.543258445971728	0.144	0.072	7.37941696792979E-33	Detoxification-associated hepat	CWC22
4.87239960282238E-37	0.275866809748242	0.185	0.101	8.16516725440974E-33	Detoxification-associated hepat	NOL7
4.9800636510466E-37	0.438696537121756	0.194	0.107	8.3455906664239E-33	Detoxification-associated hepat	CENPP
5.44918839788237E-37	0.793131789568439	0.108	0.048	9.13174991717127E-33	Detoxification-associated hepat	ZNF490
5.88283980062157E-37	0.408557377964776	0.217	0.125	9.85846293788162E-33	Detoxification-associated hepat	POT1
7.14359126220467E-37	0.514065552048259	0.129	0.062	1.19712302372026E-32	Detoxification-associated hepat	METTL16
7.59282641429046E-37	0.558007981595123	0.102	0.043	1.27240585050679E-32	Detoxification-associated hepat	BBS4
7.66533918091703E-37	0.407713751575735	0.173	0.092	1.28455753993808E-32	Detoxification-associated hepat	POGLUT2
7.78352335366278E-37	0.313425239077203	0.19	0.104	1.30436284360681E-32	Detoxification-associated hepat	PRDM2
8.15905991452656E-37	0.451271339126491	0.126	0.059	1.36729526047636E-32	Detoxification-associated hepat	FRG1
8.66071116903568E-37	0.348974147709354	0.164	0.085	1.451361977707E-32	Detoxification-associated hepat	WBP4
8.79258055776855E-37	0.439857638301803	0.191	0.106	1.47346064987085E-32	Detoxification-associated hepat	IFT81
1.08565928586042E-36	0.458705162368506	0.166	0.088	1.8193478312449E-32	Detoxification-associated hepat	GMEB1
1.14605478549979E-36	0.253658313845323	0.26	0.157	1.92055860954055E-32	Detoxification-associated hepat	NIPAL1
1.4441087329344E-36	0.430250879528516	0.189	0.104	2.42003741465147E-32	Detoxification-associated hepat	POLR1B
1.64594029409514E-36	0.860911097821508	0.106	0.047	2.75826674484464E-32	Detoxification-associated hepat	TSEN2
1.70988034146538E-36	0.250148184250448	0.219	0.128	2.86541747622768E-32	Detoxification-associated hepat	KLHL7
1.73167191186398E-36	0.446458985063954	0.144	0.071	2.90193578990166E-32	Detoxification-associated hepat	PISD
1.80114596719236E-36	0.784715860117457	0.12	0.056	3.01836041182096E-32	Detoxification-associated hepat	DNAJC9
2.13544711341287E-36	0.436004045261308	0.112	0.05	3.5785822726573E-32	Detoxification-associated hepat	UPRT
2.17053512653989E-36	0.35380944639653	0.221	0.129	3.63738276505555E-32	Detoxification-associated hepat	METTL27
2.40886856144075E-36	0.315842847172783	0.184	0.101	4.03678193526241E-32	Detoxification-associated hepat	CIAPIN1
2.63068951130467E-36	0.656713481046583	0.141	0.071	4.40850948304436E-32	Detoxification-associated hepat	ZFYVE26
3.18927386616235E-36	0.404287809754243	0.171	0.091	5.34458514491487E-32	Detoxification-associated hepat	DEPRECATEI
3.36059841998345E-36	0.572513878221957	0.129	0.062	5.63169083220826E-32	Detoxification-associated hepat	CRLF3
3.44106183396276E-36	0.31536267875661	0.174	0.093	5.76653142135479E-32	Detoxification-associated hepat	NAT10
3.59662451934514E-36	0.707068747791095	0.129	0.062	6.02722336951858E-32	Detoxification-associated hepat	AKAP10
3.60136506882776E-36	0.423888423874579	0.135	0.065	6.03516758234156E-32	Detoxification-associated hepat	SARM1
3.68638220439856E-36	0.257539018923213	0.188	0.104	6.17763929813111E-32	Detoxification-associated hepat	WBP1L
3.79400854510015E-36	0.354639819996837	0.142	0.07	6.35799951987883E-32	Detoxification-associated hepat	ZC3H8
3.91911151738495E-36	0.525578005105523	0.199	0.114	6.5676470808337E-32	Detoxification-associated hepat	ERAP2
5.96765015442553E-36	0.305510592234225	0.154	0.079	1.00005881287863E-31	Detoxification-associated hepat	HIVEP2
6.05145865221484E-36	0.492312807306409	0.146	0.074	1.01410344093816E-31	Detoxification-associated hepat	ZNF526
6.50282689010833E-36	0.363936921082734	0.11	0.049	1.08974373024435E-31	Detoxification-associated hepat	DEPRECATEI
7.07107797132255E-36	0.926369604463734	0.123	0.058	1.18497124643423E-31	Detoxification-associated hepat	DEPRECATEI
8.22197588237276E-36	0.516661855378227	0.187	0.105	1.37783871836803E-31	Detoxification-associated hepat	EFNA5
9.92823048371934E-36	0.774103796419611	0.125	0.06	1.66377286446169E-31	Detoxification-associated hepat	DDX10
1.05503439708021E-35	0.613255743137859	0.129	0.062	1.76802664262702E-31	Detoxification-associated hepat	UTP15
1.11364334705099E-35	0.306873677624888	0.16	0.083	1.86624352098804E-31	Detoxification-associated hepat	ING4
1.27309660272881E-35	0.443304474467164	0.177	0.097	2.13345528685293E-31	Detoxification-associated hepat	PMS2
1.69965624932715E-35	0.402424217241361	0.19	0.106	2.84828394262244E-31	Detoxification-associated hepat	ATG14
2.06135988224535E-35	0.656156920675042	0.119	0.056	3.45442689066676E-31	Detoxification-associated hepat	BTRC
2.22147081853531E-35	0.471436608513168	0.197	0.112	3.72274079770147E-31	Detoxification-associated hepat	ALMS1
2.28498520617994E-35	0.254328445205607	0.223	0.131	3.82917820851634E-31	Detoxification-associated hepat	DHRS11

2.30401983993752E-35	0.628952487700817	0.105	0.046	3.8610764477673E-31	Detoxification-associated hepat	TMEM223
2.61299456057218E-35	0.524451756743414	0.18	0.099	4.37885628460686E-31	Detoxification-associated hepat	PHTF1
3.10170188512612E-35	0.451108926779867	0.152	0.079	5.19783201909435E-31	Detoxification-associated hepat	RAD1
3.14373784187857E-35	0.611411353924588	0.126	0.061	5.26827587542011E-31	Detoxification-associated hepat	PTPN2
3.28928518795844E-35	0.67536135290861	0.147	0.076	5.51218411798076E-31	Detoxification-associated hepat	LPCAT2
4.44895165852314E-35	0.278593618312214	0.163	0.087	7.45555318935307E-31	Detoxification-associated hepat	ARL5A
5.54145908455664E-35	0.55167628419468	0.138	0.069	9.28637713390002E-31	Detoxification-associated hepat	ZMYM6
6.51636847104746E-35	0.927996399863676	0.11	0.05	1.09201302837813E-30	Detoxification-associated hepat	ADCY5
6.60970407244258E-35	0.769334264740126	0.103	0.045	1.10765420845993E-30	Detoxification-associated hepat	DFFB
7.0003013051881E-35	0.576639736024331	0.162	0.086	1.17311049272342E-30	Detoxification-associated hepat	MDGA1
7.13932168492455E-35	0.469978908117625	0.105	0.047	1.19640752795966E-30	Detoxification-associated hepat	RNF207
7.4065150235304E-35	0.557119425614247	0.189	0.107	1.24118378764322E-30	Detoxification-associated hepat	SLX4IP
7.51018563860847E-35	0.45809037384685	0.146	0.075	1.25855690931801E-30	Detoxification-associated hepat	TRIT1
8.53816021527667E-35	0.537147383903652	0.153	0.08	1.43082488887606E-30	Detoxification-associated hepat	ANKIB1
8.96632804319704E-35	0.843428184198388	0.13	0.064	1.50257725347896E-30	Detoxification-associated hepat	PYROXD1
9.5947984461827E-35	0.592825462696589	0.131	0.064	1.6078963236113E-30	Detoxification-associated hepat	SSH2
1.08717063963845E-34	0.490412438178311	0.183	0.102	1.82188055790611E-30	Detoxification-associated hepat	PDSS1
1.14479422235242E-34	0.725311704630192	0.127	0.062	1.91844615781819E-30	Detoxification-associated hepat	TIPIN
1.27885489955708E-34	0.258471092957647	0.178	0.098	2.14310504067776E-30	Detoxification-associated hepat	CCDC32
1.36217942599503E-34	0.502815072764855	0.145	0.075	2.28274028208246E-30	Detoxification-associated hepat	IPPK
1.45542726339976E-34	0.295578556232942	0.217	0.127	2.43900500800533E-30	Detoxification-associated hepat	PKD1
1.606996606093E-34	0.361553612920245	0.108	0.048	2.69300491249065E-30	Detoxification-associated hepat	LPXN
1.64643696411273E-34	0.552387951460691	0.156	0.083	2.75909906446011E-30	Detoxification-associated hepat	TDRD3
1.79226732103113E-34	0.599464502312825	0.172	0.094	3.00348157658397E-30	Detoxification-associated hepat	CLEC2D
1.88141609188148E-34	0.305914873404188	0.176	0.097	3.15287708677499E-30	Detoxification-associated hepat	CINP
2.76773219486293E-34	0.529612411916658	0.111	0.051	4.6381656121513E-30	Detoxification-associated hepat	TIGD3
2.84583513442468E-34	0.270617897537499	0.214	0.124	4.76905051826888E-30	Detoxification-associated hepat	ABCA7
3.47012732233202E-34	0.364465693990679	0.159	0.085	5.81523936676399E-30	Detoxification-associated hepat	PARG
3.51931158714717E-34	0.677590561595986	0.148	0.077	5.89766235774123E-30	Detoxification-associated hepat	VPS50
3.96551459882054E-34	0.299561220308747	0.192	0.109	6.64540936470346E-30	Detoxification-associated hepat	TTC19
4.12136811816613E-34	0.658769278931469	0.151	0.079	6.9065886924228E-30	Detoxification-associated hepat	EML5
4.46484673878249E-34	0.537002546647856	0.163	0.088	7.4821901648517E-30	Detoxification-associated hepat	CEP68
4.82378883556311E-34	0.540117731937577	0.148	0.077	8.08370533063667E-30	Detoxification-associated hepat	LZTR1
5.4942444210477E-34	0.824875791697773	0.123	0.06	9.20725480079174E-30	Detoxification-associated hepat	CEP295
5.96020940006608E-34	0.506232768494424	0.149	0.078	9.98811891263073E-30	Detoxification-associated hepat	ZNF182
6.07031688554981E-34	0.563283479718791	0.1	0.044	1.01726370368044E-29	Detoxification-associated hepat	TMEM99
6.77915562864199E-34	0.314237304307346	0.159	0.084	1.13605090024782E-29	Detoxification-associated hepat	BLOC1S3
7.11048390580552E-34	0.406598722638697	0.166	0.09	1.19157489293489E-29	Detoxification-associated hepat	TET3
8.75053340755357E-34	0.2595062214731	0.162	0.087	1.46641438843783E-29	Detoxification-associated hepat	CCDC186
1.01264912555628E-33	0.37465958454538	0.136	0.068	1.69699740460722E-29	Detoxification-associated hepat	MYPOP
1.01787099134621E-33	0.639781189997109	0.108	0.049	1.70574820729798E-29	Detoxification-associated hepat	WNK4
1.0648682242552E-33	0.272491390056083	0.184	0.104	1.78450617020686E-29	Detoxification-associated hepat	SLC35A5
1.11615847174689E-33	0.484599934053095	0.138	0.07	1.87045836695343E-29	Detoxification-associated hepat	ZNF446
1.61756544407188E-33	0.557611503791845	0.128	0.063	2.71071617117565E-29	Detoxification-associated hepat	DEPRECATEI
1.669037515106E-33	0.345742447598996	0.147	0.076	2.79697306781463E-29	Detoxification-associated hepat	SLC66A1
1.74748033896446E-33	0.527349299499446	0.181	0.102	2.92842755203663E-29	Detoxification-associated hepat	RAB23
2.11237153469537E-33	0.449702442406354	0.156	0.083	3.53991221784249E-29	Detoxification-associated hepat	FAM204A
2.21371280890259E-33	0.658665457108917	0.118	0.056	3.70973992515896E-29	Detoxification-associated hepat	PPP3CC
2.2440208123529E-33	0.301596700150741	0.166	0.09	3.760530077341E-29	Detoxification-associated hepat	MEMO1
2.55319261094937E-33	0.915397966639905	0.107	0.05	4.27864017742895E-29	Detoxification-associated hepat	ZNF41
2.61991026118395E-33	0.404771758999344	0.145	0.075	4.39044561569207E-29	Detoxification-associated hepat	HTRA2
2.71540456672138E-33	0.325541801941377	0.131	0.065	4.55047497291169E-29	Detoxification-associated hepat	YEATS4
2.7461584259361E-33	0.452863602917403	0.136	0.069	4.60201229018371E-29	Detoxification-associated hepat	NBEAL2
2.91222461410381E-33	0.333545288882774	0.144	0.074	4.88030600831517E-29	Detoxification-associated hepat	TOP2B
2.97555461709819E-33	0.855580930869949	0.107	0.049	4.98643442733314E-29	Detoxification-associated hepat	TAF1A
3.13213516093912E-33	0.320470429794254	0.19	0.108	5.24883210270178E-29	Detoxification-associated hepat	TSPAN17
3.13934203542675E-33	0.254816662101106	0.162	0.087	5.26090938296815E-29	Detoxification-associated hepat	PSEN2
3.15965324130147E-33	0.344339308701725	0.171	0.094	5.294946901773E-29	Detoxification-associated hepat	USP20
3.25552171861868E-33	0.641146224411259	0.106	0.048	5.45560329606118E-29	Detoxification-associated hepat	AMER1
3.65165082302058E-33	0.336500545130666	0.103	0.046	6.11943644921788E-29	Detoxification-associated hepat	CROCC

**3.94717080225157E-33**	0.419945826991676	0.182	0.103	6.61466883041318E-29	Detoxification-associated hepat	DGKD
**4.23887526232525E-33**	0.262757163483248	0.221	0.132	7.10350716460466E-29	Detoxification-associated hepat	FKBP9
**4.30368126401786E-33**	0.484262477993646	0.177	0.099	7.21210906224113E-29	Detoxification-associated hepat	MASTL
**5.04304089092438E-33**	0.319130862884883	0.142	0.073	8.45112792501107E-29	Detoxification-associated hepat	RNFT1
**6.83916187734777E-33**	0.404264379229287	0.147	0.077	1.14610674740594E-28	Detoxification-associated hepat	NME7
**6.96336346346237E-33**	0.433417130944465	0.186	0.106	1.16692044920702E-28	Detoxification-associated hepat	ATP2B1
**7.41633385988058E-33**	0.444182160512938	0.148	0.078	1.24282922823879E-28	Detoxification-associated hepat	ARL6IP6
**7.99087364750717E-33**	0.530230574218402	0.128	0.063	1.33911060584925E-28	Detoxification-associated hepat	AMMECR1L
**1.2417801085114E-32**	0.41998640767873	0.133	0.067	2.0809751058434E-28	Detoxification-associated hepat	MAU2
**1.36953487773147E-32**	1.18854513499504	0.107	0.05	2.2950665481024E-28	Detoxification-associated hepat	DCLRE1C
**1.52512038565281E-32**	0.267133559382938	0.237	0.145	2.55579674227698E-28	Detoxification-associated hepat	GRAMD1B
**1.58177381395511E-32**	0.641214851325327	0.115	0.055	2.65073655742597E-28	Detoxification-associated hepat	DHFR2
**1.8980684022009E-32**	0.335314826016378	0.114	0.054	3.18078302840828E-28	Detoxification-associated hepat	NEURL4
**2.23060466381473E-32**	0.35942279408565	0.1	0.045	3.73804729562072E-28	Detoxification-associated hepat	PRKN
**2.39297195479455E-32**	0.309286474443481	0.144	0.075	4.0101424018447E-28	Detoxification-associated hepat	TCTN1
**3.22910424408628E-32**	0.65064082720738	0.106	0.049	5.41133289223979E-28	Detoxification-associated hepat	XPNPEP3
**3.29231906371996E-32**	0.6627682650538	0.147	0.079	5.51726828698191E-28	Detoxification-associated hepat	B3GNT5
**3.87589097299893E-32**	0.26845212731627	0.128	0.064	6.4952180925516E-28	Detoxification-associated hepat	CCDC149
**3.92129966786032E-32**	0.731709842566472	0.127	0.064	6.57131398340032E-28	Detoxification-associated hepat	IQCG
**4.05048006998233E-32**	0.253497941819098	0.198	0.115	6.78779450127638E-28	Detoxification-associated hepat	RNF43
**4.39302199798501E-32**	0.323200421070215	0.154	0.083	7.36182626422329E-28	Detoxification-associated hepat	HAUS5
**5.03531667317691E-32**	1.11839319001289	0.102	0.047	8.43818368090987E-28	Detoxification-associated hepat	ZNF223
**5.51198279522241E-32**	0.573568606664767	0.121	0.059	9.23698076823372E-28	Detoxification-associated hepat	C19orf44
**5.68006768754699E-32**	0.771850482398333	0.189	0.11	9.51865743079125E-28	Detoxification-associated hepat	C6orf141
**6.34816015323784E-32**	0.287715281839993	0.169	0.093	1.0638246784796E-27	Detoxification-associated hepat	NAPG
**6.38238102664721E-32**	0.500728830151793	0.117	0.057	1.06955941244554E-27	Detoxification-associated hepat	BCDIN3D
**6.56747779473224E-32**	0.317235722822445	0.168	0.093	1.10057792884123E-27	Detoxification-associated hepat	DDX50
**6.93997818611362E-32**	0.453426935388247	0.104	0.047	1.16300154442892E-27	Detoxification-associated hepat	CYB5D2
**7.61255602629318E-32**	0.465889500829205	0.178	0.101	1.27571213888621E-27	Detoxification-associated hepat	VANGL1
**9.00918064429906E-32**	0.616081017122255	0.138	0.072	1.50975849237164E-27	Detoxification-associated hepat	ZC3H12C
**9.18951173868051E-32**	0.283335873634258	0.176	0.099	1.53997837716808E-27	Detoxification-associated hepat	ZEB1
**1.08455055498791E-31**	0.550792391663761	0.105	0.049	1.81748982004874E-27	Detoxification-associated hepat	SMCR8
**1.17250819746714E-31**	0.7477796534818	0.104	0.048	1.96488923731543E-27	Detoxification-associated hepat	MTMR9
**1.17743845919525E-31**	0.379434421655491	0.148	0.079	1.9731513699194E-27	Detoxification-associated hepat	FAM222B
**1.31875470084011E-31**	0.619482939406454	0.136	0.071	2.20996912766786E-27	Detoxification-associated hepat	SERAC1
**1.61947431266908E-31**	1.09933259897706	0.104	0.049	2.71391505317084E-27	Detoxification-associated hepat	ARSK
**1.96807533290828E-31**	0.46681274691819	0.114	0.055	3.29810064288769E-27	Detoxification-associated hepat	RFX7
**2.22464400912491E-31**	0.30512795197657	0.174	0.098	3.72805843049152E-27	Detoxification-associated hepat	PPM1D
**2.67767950649297E-31**	0.762629876612972	0.133	0.069	4.48725531698092E-27	Detoxification-associated hepat	SVIP
**2.97315208743631E-31**	0.262294589996312	0.205	0.122	4.98240826812578E-27	Detoxification-associated hepat	PIGU
**3.0344806396608E-31**	0.268335197338535	0.18	0.102	5.08518265594356E-27	Detoxification-associated hepat	LRSAM1
**3.41302425453138E-31**	0.661493180716461	0.114	0.055	5.71954604574368E-27	Detoxification-associated hepat	TRMT12
**4.06224943695374E-31**	0.501982043243435	0.151	0.082	6.80751760644708E-27	Detoxification-associated hepat	AREL1
**4.0989246560317E-31**	0.381929779619128	0.134	0.069	6.86897793857792E-27	Detoxification-associated hepat	C12orf45
**5.33074234655078E-31**	0.310907989015923	0.142	0.074	8.9332580243498E-27	Detoxification-associated hepat	MOSPD1
**6.80528957041066E-31**	0.285209868704564	0.149	0.08	1.14043042620942E-26	Detoxification-associated hepat	DNAJC19
**7.88088488808531E-31**	0.381961279367706	0.159	0.088	1.32067868954534E-26	Detoxification-associated hepat	DTNB
**8.514001839809E-31**	0.546083805654148	0.12	0.06	1.42677642831519E-26	Detoxification-associated hepat	SSBP2
**9.0248365855746E-31**	0.622904035693953	0.151	0.083	1.51238211501059E-26	Detoxification-associated hepat	CCP110
**9.12471141649686E-31**	0.467945706350027	0.159	0.088	1.52911913917654E-26	Detoxification-associated hepat	INPP5F
**9.71996228314911E-31**	0.551247060688774	0.156	0.086	1.62887127941013E-26	Detoxification-associated hepat	KIAA0586
**1.01344484682331E-30**	0.507051734367871	0.138	0.073	1.6983308743065E-26	Detoxification-associated hepat	TRMT10A
**1.15174705316252E-30**	0.614676044152204	0.106	0.051	1.93009771168974E-26	Detoxification-associated hepat	NME6
**1.33612480853062E-30**	0.280435146242422	0.187	0.108	2.23907795413562E-26	Detoxification-associated hepat	SLC16A7
**1.36070741991488E-30**	0.7569501237744	0.113	0.056	2.28027349429336E-26	Detoxification-associated hepat	HPF1
**1.93044203388457E-30**	0.417553629506148	0.15	0.081	3.23503476038376E-26	Detoxification-associated hepat	TIMM29
**2.18731292667946E-30**	0.30846758502731	0.12	0.06	3.66549900252944E-26	Detoxification-associated hepat	RWDD2B
**3.73405404145354E-30**	0.584664402142153	0.149	0.081	6.25752776266785E-26	Detoxification-associated hepat	ZNF16
**4.27127147977769E-30**	0.358973680433962	0.125	0.064	7.15779674581145E-26	Detoxification-associated hepat	FOCAD
**4.59025283495142E-30**	0.493322216918746	0.171	0.098	7.69234570081159E-26	Detoxification-associated hepat	CNNM4

**5.09482905337173E-30**	0.570849296444001	0.101	0.047	8.53791452764035E-26	Detoxification-associated hepat	INTU
**5.27059323852464E-30**	0.301576433234279	0.15	0.082	8.83246014911959E-26	Detoxification-associated hepat	NOL10
**5.73313671795321E-30**	0.321896073422472	0.165	0.093	9.60759051194599E-26	Detoxification-associated hepat	ZNF276
**6.88828175292682E-30**	0.38201104515921	0.148	0.08	1.15433825615548E-25	Detoxification-associated hepat	WDR48
**7.20631317800109E-30**	0.288152701166841	0.245	0.154	1.20763396236942E-25	Detoxification-associated hepat	PLXNA3
**7.45494521603538E-30**	0.625328660503447	0.104	0.05	1.24929971930321E-25	Detoxification-associated hepat	CDPF1
**7.69126468188231E-30**	0.804004476750707	0.102	0.048	1.28890213538984E-25	Detoxification-associated hepat	GLS
**7.72017849313995E-30**	0.294435176944011	0.103	0.048	1.29374751188039E-25	Detoxification-associated hepat	C20orf96
**8.38994170136847E-30**	0.824798986295368	0.117	0.059	1.40598643031533E-25	Detoxification-associated hepat	FAM135A
**9.2218500964966E-30**	0.597862576703077	0.146	0.08	1.5453976391709E-25	Detoxification-associated hepat	TNRC6C
**9.47656252603966E-30**	0.382863745561844	0.183	0.107	1.58808234811373E-25	Detoxification-associated hepat	COA1
**1.03153243856332E-29**	0.304317505056172	0.189	0.111	1.72864206054442E-25	Detoxification-associated hepat	KMT5C
**1.04169632214486E-29**	0.410254306890218	0.16	0.09	1.74567469665035E-25	Detoxification-associated hepat	RBBP8
**1.08627413029945E-29**	0.500663343438857	0.165	0.094	1.82037818755582E-25	Detoxification-associated hepat	GPR155
**1.21725212475654E-29**	0.282270737311497	0.177	0.102	2.03987111066701E-25	Detoxification-associated hepat	NCOA5
**1.23799785812317E-29**	0.695467310516483	0.141	0.076	2.0746368106428E-25	Detoxification-associated hepat	PCNX2
**1.449159785288E-29**	0.523923679640328	0.163	0.092	2.42850196818563E-25	Detoxification-associated hepat	SLC44A5
**1.52238733313599E-29**	0.565455594473551	0.171	0.098	2.5512166928693E-25	Detoxification-associated hepat	FAM122C
**1.52448527899312E-29**	0.258351989896328	0.158	0.088	2.55473243053667E-25	Detoxification-associated hepat	FAM104B
**1.64449964260966E-29**	0.369003349697483	0.105	0.05	2.75585250108527E-25	Detoxification-associated hepat	CDK20
**1.71162882361372E-29**	0.284860511927108	0.135	0.071	2.86834758261188E-25	Detoxification-associated hepat	TSEN15
**1.79469637387991E-29**	0.262855681499436	0.153	0.083	3.00755218334795E-25	Detoxification-associated hepat	AKAP7
**1.84123718367852E-29**	0.431312520997444	0.143	0.077	3.08554527240846E-25	Detoxification-associated hepat	RXYLT1
**1.90665512560329E-29**	0.537015282906573	0.128	0.066	3.19517265948599E-25	Detoxification-associated hepat	KCTD18
**2.25641691786746E-29**	0.363487820453541	0.14	0.075	3.78130347096229E-25	Detoxification-associated hepat	ISCA2
**2.36522721597438E-29**	0.555236319055605	0.129	0.067	3.96364776852987E-25	Detoxification-associated hepat	MIER2
**3.85239158892082E-29**	0.546483607998416	0.14	0.075	6.4558378247135E-25	Detoxification-associated hepat	NPAT
**4.45696940210616E-29**	0.534383190114938	0.104	0.049	7.4689893240495E-25	Detoxification-associated hepat	OSBPL6
**5.49476997344715E-29**	0.274343215751107	0.167	0.095	9.20813552150273E-25	Detoxification-associated hepat	RIT1
**5.4999002540415E-29**	0.323369875431881	0.2	0.12	9.21673284572275E-25	Detoxification-associated hepat	PRIM2
**6.53022817478352E-29**	0.512105069715087	0.119	0.061	1.09433563753022E-24	Detoxification-associated hepat	C18orf21
**6.84236210943283E-29**	0.620884707274966	0.108	0.053	1.14664304229875E-24	Detoxification-associated hepat	RANBP6
**7.56237318606649E-29**	0.381248405320547	0.118	0.06	1.26730249852102E-24	Detoxification-associated hepat	ZNF641
**7.60631346793309E-29**	0.267297194782911	0.175	0.102	1.27466601095623E-24	Detoxification-associated hepat	CHFR
**9.38796675354728E-29**	0.715080495181923	0.1	0.048	1.57323546855945E-24	Detoxification-associated hepat	UTP20
**9.82902462893283E-29**	0.59491509176761	0.145	0.08	1.64714794731656E-24	Detoxification-associated hepat	HERC6
**9.86016662016041E-29**	0.371887559845883	0.192	0.115	1.65236672220648E-24	Detoxification-associated hepat	DCBLD1
**1.12734864640123E-28**	0.317500572755641	0.199	0.121	1.88921086163919E-24	Detoxification-associated hepat	MIGA2
**1.27793310768184E-28**	0.25646872809746	0.178	0.104	2.14156030185323E-24	Detoxification-associated hepat	UBE2N
**1.60512883764532E-28**	0.371754226365142	0.116	0.058	2.68987490612603E-24	Detoxification-associated hepat	MOCS3
**1.78754104747207E-28**	0.299558682548352	0.194	0.116	2.99556128735369E-24	Detoxification-associated hepat	CHAF1B
**1.89423933847759E-28**	0.401962470223216	0.14	0.076	3.17436628342074E-24	Detoxification-associated hepat	SHH
**2.52831098219159E-28**	0.350988722133342	0.162	0.092	4.23694354395667E-24	Detoxification-associated hepat	LAMA4
**3.11926227956015E-28**	0.378364706740406	0.136	0.073	5.2272597280869E-24	Detoxification-associated hepat	MSANTD4
**3.19508163395793E-28**	0.494918088943241	0.124	0.064	5.3543178021867E-24	Detoxification-associated hepat	TLE2
**3.38388795583042E-28**	0.271193927381491	0.156	0.087	5.67071943638062E-24	Detoxification-associated hepat	UBE2E1
**4.84811518549064E-28**	0.660409539639954	0.137	0.075	8.12447142784521E-24	Detoxification-associated hepat	PPP4R4
**6.85594142521936E-28**	0.458760798182856	0.136	0.073	1.14891866403826E-23	Detoxification-associated hepat	TOP3A
**8.42677693717887E-28**	0.264220094707158	0.147	0.081	1.41215927913243E-23	Detoxification-associated hepat	WDR24
**8.63794151554192E-28**	0.32999561712116	0.192	0.116	1.44754623917452E-23	Detoxification-associated hepat	TMEM47
**8.88143125129131E-28**	0.420702214231234	0.13	0.069	1.4883502490914E-23	Detoxification-associated hepat	ZNF766
**9.26425319476059E-28**	0.253073230069987	0.155	0.087	1.55250355037798E-23	Detoxification-associated hepat	MEX3C
**9.46998922035004E-28**	0.339333975631413	0.133	0.071	1.58698079354626E-23	Detoxification-associated hepat	CAPN15
**9.59214398933212E-28**	0.374400342644317	0.154	0.087	1.60745148973228E-23	Detoxification-associated hepat	FCSK
**1.0335182869958E-27**	0.376111946165221	0.117	0.06	1.73196994534756E-23	Detoxification-associated hepat	ZFP1
**1.22321871961373E-27**	0.399509708076679	0.153	0.086	2.04986993032869E-23	Detoxification-associated hepat	SCAMP1
**1.48494627438326E-27**	0.490247902475198	0.136	0.074	2.48847296661148E-23	Detoxification-associated hepat	UTP14A
**1.60247839494782E-27**	0.460115061068183	0.135	0.073	2.68543329425356E-23	Detoxification-associated hepat	ZSCAN32
**1.63654029367381E-27**	0.561626415314385	0.121	0.063	2.74251422413857E-23	Detoxification-associated hepat	SNX25
**1.71338317313727E-27**	0.494946005995935	0.157	0.09	2.87128752154344E-23	Detoxification-associated hepat	RMI2

**1.72018374802364E-27**	0.527644425659759	0.109	0.054	2.88268392493802E-23	Detoxification-associated hepat	HIC2
**3.18360474195364E-27**	0.396103414300528	0.117	0.06	5.33508482656591E-23	Detoxification-associated hepat	FAM118A
**3.6082377088823E-27**	0.275134599262241	0.183	0.109	6.04668475254496E-23	Detoxification-associated hepat	BAHD1
**3.68458308050803E-27**	0.780417339511179	0.11	0.057	6.17462432631536E-23	Detoxification-associated hepat	DEPRECATEI
**4.51674110805011E-27**	0.387276897337121	0.153	0.087	7.56915474887037E-23	Detoxification-associated hepat	SPICE1
**5.4180468577477E-27**	0.343777335980814	0.129	0.069	9.0795629242136E-23	Detoxification-associated hepat	TTC27
**5.49843873822783E-27**	0.32725715064408	0.159	0.091	9.2142836375222E-23	Detoxification-associated hepat	ARSD
**5.78236673656744E-27**	0.26863860320231	0.105	0.052	9.69009017713971E-23	Detoxification-associated hepat	LTBP2
**6.825116967702E-27**	0.335345958068267	0.15	0.085	1.1437531014475E-22	Detoxification-associated hepat	KAT14
**7.65711944560068E-27**	0.258676125582111	0.196	0.12	1.28318007669376E-22	Detoxification-associated hepat	ARMC10
**7.84727850789778E-27**	0.376583486896678	0.186	0.113	1.31504693235351E-22	Detoxification-associated hepat	GALNT10
**9.07019256412812E-27**	0.375208909678038	0.164	0.096	1.51998286989659E-22	Detoxification-associated hepat	C11orf80
**1.12162222207591E-26**	0.854874671059195	0.105	0.053	1.87961451975481E-22	Detoxification-associated hepat	DEPRECATEI
**1.23263897804576E-26**	0.437863052995132	0.126	0.067	2.06565639940908E-22	Detoxification-associated hepat	SH3PXD2B
**1.239736172263E-26**	0.372069010429209	0.144	0.081	2.07754987747834E-22	Detoxification-associated hepat	ACVR2B
**1.38473594593888E-26**	0.278355075028151	0.145	0.081	2.32054049820437E-22	Detoxification-associated hepat	BRAP
**1.45356163903563E-26**	0.386812788023831	0.138	0.076	2.4358785946959E-22	Detoxification-associated hepat	BAHCC1
**1.47977648599506E-26**	0.333624850469675	0.109	0.055	2.47980943523052E-22	Detoxification-associated hepat	NHLRC1
**2.19604762790726E-26**	0.692583705769823	0.111	0.058	3.68013661484699E-22	Detoxification-associated hepat	C12orf4
**2.43040866725328E-26**	0.756024853151806	0.137	0.077	4.07287884458304E-22	Detoxification-associated hepat	ACER3
**2.64706686733886E-26**	0.267390817536995	0.152	0.087	4.43595465628647E-22	Detoxification-associated hepat	HAUS6
**3.03254509238056E-26**	0.340713771363522	0.126	0.067	5.08193906581134E-22	Detoxification-associated hepat	MAN1C1
**3.14599295523206E-26**	0.545861371884204	0.131	0.072	5.2720549943779E-22	Detoxification-associated hepat	IFT52
**3.77799851535821E-26**	0.359652512751593	0.115	0.06	6.3311699120373E-22	Detoxification-associated hepat	CASKIN2
**3.79198735845595E-26**	0.557469522203971	0.116	0.061	6.35461241530048E-22	Detoxification-associated hepat	MTMR2
**3.93510038871007E-26**	0.348951996630367	0.124	0.066	6.59444123140034E-22	Detoxification-associated hepat	PRAG1
**4.93305701700502E-26**	0.349708619291832	0.115	0.06	8.26681694909701E-22	Detoxification-associated hepat	MESP1
**5.13278379619141E-26**	0.741061501143856	0.102	0.052	8.60151908565756E-22	Detoxification-associated hepat	ZCCHC10
**5.29610371773592E-26**	0.456755724234378	0.153	0.089	8.87521061018186E-22	Detoxification-associated hepat	PLEKHA8
**5.90121400686158E-26**	0.327614122758235	0.128	0.069	9.88925443269864E-22	Detoxification-associated hepat	YTHDF1
**6.20880686544203E-26**	0.250891524223232	0.133	0.073	1.04047185451078E-21	Detoxification-associated hepat	TRMO
**6.3453063023428E-26**	0.588800706239527	0.105	0.053	1.06334643014661E-21	Detoxification-associated hepat	EML1
**6.5372188996909E-26**	0.52450297753314	0.11	0.057	1.0955071432102E-21	Detoxification-associated hepat	SCO1
**9.2415093659684E-26**	0.513498376248108	0.144	0.081	1.54869213954898E-21	Detoxification-associated hepat	WDHD1
**1.07163485644039E-25**	0.286410438146788	0.159	0.093	1.79584569242281E-21	Detoxification-associated hepat	DEPRECATEI
**1.18156577752036E-25**	0.357107944737416	0.14	0.079	1.98006792996862E-21	Detoxification-associated hepat	TIMM22
**1.43152058889688E-25**	0.405274079087124	0.143	0.08	2.3989422028734E-21	Detoxification-associated hepat	PHETA1
**2.22365286313677E-25**	0.417070597019119	0.128	0.07	3.72639746680446E-21	Detoxification-associated hepat	PPP1R26
**2.38916154207879E-25**	0.679771754216004	0.113	0.06	4.00375691221564E-21	Detoxification-associated hepat	WDR47
**2.51225353197604E-25**	0.623939245121884	0.112	0.058	4.21003446888545E-21	Detoxification-associated hepat	ZFAT
**2.62741497631239E-25**	0.285005199055542	0.185	0.113	4.40302201730431E-21	Detoxification-associated hepat	TDP1
**3.61208793042225E-25**	0.324055610716609	0.132	0.073	6.0531369538016E-21	Detoxification-associated hepat	WDR4
**4.65415866876981E-25**	0.488007784893888	0.118	0.063	7.79943909712445E-21	Detoxification-associated hepat	MTURN
**4.98224373277493E-25**	0.267136344279397	0.156	0.091	8.34924404738423E-21	Detoxification-associated hepat	SUV39H2
**6.34653241199441E-25**	0.356447874584348	0.118	0.063	1.06355190160202E-20	Detoxification-associated hepat	SAMD8
**6.57397281488907E-25**	0.495883401199553	0.124	0.067	1.10166636431911E-20	Detoxification-associated hepat	LONRF1
**7.04415567372823E-25**	0.461995194747547	0.164	0.098	1.18045960780338E-20	Detoxification-associated hepat	TTLL6
**7.09505284679707E-25**	0.517035610170494	0.115	0.061	1.18898895606625E-20	Detoxification-associated hepat	DEPRECATEI
**7.20513873407827E-25**	0.279748242617191	0.114	0.06	1.20743714905684E-20	Detoxification-associated hepat	PNPT1
**8.24517620720363E-25**	0.267294477055364	0.138	0.078	1.38172662880318E-20	Detoxification-associated hepat	ZBTB42
**1.28385599641032E-24**	0.295694084272439	0.183	0.112	2.15148587878441E-20	Detoxification-associated hepat	WDR90
**1.52822984990225E-24**	0.515971042132617	0.133	0.074	2.56100758246619E-20	Detoxification-associated hepat	ZGRF1
**1.57213917975153E-24**	0.461650413608652	0.133	0.075	2.63459083742761E-20	Detoxification-associated hepat	XKR8
**1.60249600882694E-24**	0.380120945965819	0.118	0.063	2.68546281159218E-20	Detoxification-associated hepat	INTS5
**1.67036798186782E-24**	0.5149519299589	0.115	0.062	2.7992026640141E-20	Detoxification-associated hepat	MRM1
**1.78620590960691E-24**	0.484725102921435	0.121	0.065	2.99332386331926E-20	Detoxification-associated hepat	FKBP14
**1.81821146240221E-24**	0.468992366938985	0.128	0.071	3.04695876869363E-20	Detoxification-associated hepat	SPRTN
**2.18699020457138E-24**	0.293431851504162	0.193	0.12	3.66495818482071E-20	Detoxification-associated hepat	XRCC3
**2.84047480868067E-24**	0.323947507893271	0.118	0.064	4.76006768438706E-20	Detoxification-associated hepat	MTF1
**3.23189060904071E-24**	0.411468137157753	0.119	0.065	5.41600228263042E-20	Detoxification-associated hepat	SLC31A2

3.51101744580847E-24	0.387332484826302	0.116	0.062	5.88376303568583E-20	Detoxification-associated hepat	WDR53
3.5498858914124E-24	0.290256516717115	0.117	0.063	5.94889877682889E-20	Detoxification-associated hepat	ZNF691
3.84239896932413E-24	0.250047790857685	0.167	0.101	6.43909219279338E-20	Detoxification-associated hepat	PDK3
3.93054019291922E-24	0.26764095759177	0.13	0.072	6.58679925529403E-20	Detoxification-associated hepat	ZNF184
4.24530517906986E-24	0.343118868093544	0.101	0.052	7.11428241908527E-20	Detoxification-associated hepat	TAF9B
4.61871501504691E-24	0.521193794808502	0.111	0.059	7.74004262221561E-20	Detoxification-associated hepat	PRR3
6.29953765884382E-24	0.334768068662633	0.114	0.061	1.05567652086905E-19	Detoxification-associated hepat	BTN2A2
6.40234780437295E-24	0.398134573270693	0.111	0.059	1.07290544505682E-19	Detoxification-associated hepat	LARS2
6.61947045331051E-24	0.307086682065006	0.14	0.08	1.10929085856577E-19	Detoxification-associated hepat	CCDC57
1.19023163026078E-23	0.413300355568801	0.113	0.061	1.99459016599101E-19	Detoxification-associated hepat	GEMIN4
1.19182907677238E-23	0.303366382202932	0.139	0.08	1.99726716685516E-19	Detoxification-associated hepat	ANKMY2
1.25349128387486E-23	0.45710671270983	0.136	0.078	2.10060069351748E-19	Detoxification-associated hepat	C2orf69
1.41326115055995E-23	0.325061577741362	0.115	0.062	2.36834303610837E-19	Detoxification-associated hepat	FOXO4
1.68419168448771E-23	0.294617309037708	0.108	0.057	2.8223684248645E-19	Detoxification-associated hepat	SMG8
1.75485946879156E-23	0.324592107418969	0.147	0.087	2.94079349780089E-19	Detoxification-associated hepat	CERS5
1.78874030918579E-23	0.369145753303855	0.12	0.066	2.99757101013355E-19	Detoxification-associated hepat	ACVR2A
1.89574899871961E-23	0.327883790167157	0.159	0.095	3.17689617205432E-19	Detoxification-associated hepat	POGLUT3
2.22481901448095E-23	0.276773228001518	0.113	0.06	3.72835170446718E-19	Detoxification-associated hepat	RP9
4.41896280922755E-23	0.308700207197414	0.174	0.108	7.40529787570354E-19	Detoxification-associated hepat	ZDHHC23
4.89093242009222E-23	0.609662984047237	0.1	0.052	8.19622454959054E-19	Detoxification-associated hepat	TCEANC2
4.9067305162895E-23	0.460655605664682	0.106	0.056	8.22269899919794E-19	Detoxification-associated hepat	NEMP2
5.62439349812763E-23	0.271919307215935	0.112	0.06	9.42535862416228E-19	Detoxification-associated hepat	DNAJC16
5.99744983401892E-23	0.356295995695053	0.153	0.091	1.00505264318489E-18	Detoxification-associated hepat	RAD54B
8.45164179699821E-23	0.450242243623226	0.118	0.065	1.41632613234096E-18	Detoxification-associated hepat	TBC1D12
9.81665871269044E-23	0.390286658667097	0.142	0.083	1.64507566707266E-18	Detoxification-associated hepat	R3HDM1
9.8713995981884E-23	0.326960573102697	0.103	0.054	1.65424914466441E-18	Detoxification-associated hepat	MAPKBP1
1.03278419109184E-22	0.277194121925861	0.119	0.066	1.73073974743171E-18	Detoxification-associated hepat	POLR1C
1.08496717957782E-22	0.541532621965542	0.127	0.072	1.81818799953652E-18	Detoxification-associated hepat	PASK
1.32090889498685E-22	0.309379554083337	0.162	0.099	2.21357912621896E-18	Detoxification-associated hepat	LRR1
1.40653112456005E-22	0.287859112145389	0.122	0.068	2.35706485853774E-18	Detoxification-associated hepat	SENP3
2.383071901162E-22	0.331031757651156	0.195	0.125	3.99355189196728E-18	Detoxification-associated hepat	B4GALT4
2.73568652741725E-22	0.594453049913688	0.124	0.071	4.58446348264582E-18	Detoxification-associated hepat	GPR160
2.80155138199754E-22	0.415696342710201	0.116	0.064	4.69483980595148E-18	Detoxification-associated hepat	KIF1BP
3.20742937947043E-22	0.349936840867958	0.112	0.061	5.37501015411654E-18	Detoxification-associated hepat	NEIL2
3.61536323952032E-22	0.265750752638028	0.117	0.064	6.05862571678815E-18	Detoxification-associated hepat	TMEM39B
4.03063490294147E-22	0.580356301426751	0.101	0.054	6.75453797034932E-18	Detoxification-associated hepat	ZBTB3
4.19361592873956E-22	0.373990478441134	0.118	0.066	7.02766157338176E-18	Detoxification-associated hepat	RAD51C
4.43276941187627E-22	0.26094082745398	0.14	0.082	7.42843498042225E-18	Detoxification-associated hepat	PSME3
4.54093164215891E-22	0.418894029955627	0.116	0.065	7.6096932459299E-18	Detoxification-associated hepat	PEAK1
4.56652298805087E-22	0.464201625858183	0.151	0.092	7.65257922337565E-18	Detoxification-associated hepat	DOP1B
6.47753208324155E-22	0.422211669332438	0.104	0.056	1.08550482650962E-17	Detoxification-associated hepat	DMPK
6.69104539771874E-22	0.355453369742952	0.1	0.052	1.12128538774971E-17	Detoxification-associated hepat	C22orf39
7.51572924199393E-22	0.366809449638486	0.11	0.06	1.25948590637334E-17	Detoxification-associated hepat	PUM3
7.63144106645583E-22	0.326403209520521	0.127	0.073	1.27887689391667E-17	Detoxification-associated hepat	ARMC7
9.5815867969655E-22	0.383003057200689	0.115	0.063	1.60568231543548E-17	Detoxification-associated hepat	TLN2
9.84364202780863E-22	0.446813439580374	0.143	0.085	1.64959753102017E-17	Detoxification-associated hepat	CHEK1
1.36025264536171E-21	0.567775777771785	0.124	0.071	2.27951138309715E-17	Detoxification-associated hepat	BTN2A1
1.37414540605211E-21	0.279912437996225	0.126	0.073	2.30279287146213E-17	Detoxification-associated hepat	PANX1
1.63513809285189E-21	0.409441174572872	0.161	0.1	2.7401644160012E-17	Detoxification-associated hepat	ZC3H6
1.92949957854645E-21	0.412841053258763	0.123	0.07	3.23345539372814E-17	Detoxification-associated hepat	KCTD7
2.3692718595824E-21	0.329283071342219	0.14	0.083	3.97042578228819E-17	Detoxification-associated hepat	G2E3
2.42219531183232E-21	0.425605157297732	0.133	0.079	4.05911490356861E-17	Detoxification-associated hepat	CEP89
2.61175825295058E-21	0.506348103049671	0.107	0.058	4.37678448029458E-17	Detoxification-associated hepat	SIDT2
2.61569890455204E-21	0.439410837463785	0.103	0.056	4.38338822424831E-17	Detoxification-associated hepat	URB2
3.05540247556869E-21	0.270569840691963	0.174	0.11	5.12024346855801E-17	Detoxification-associated hepat	IGSF3
3.14489778583294E-21	0.40107462258649	0.101	0.054	5.27021970949884E-17	Detoxification-associated hepat	GTF2IRD2
3.74878764823247E-21	0.296004547632505	0.12	0.069	6.28221834090797E-17	Detoxification-associated hepat	BTBD10
5.05287892592388E-21	0.529616663769988	0.123	0.071	8.46761450406324E-17	Detoxification-associated hepat	NOD1
5.1352722767913E-21	0.309373303905233	0.14	0.084	8.60568928144686E-17	Detoxification-associated hepat	UPP1
5.98267759592614E-21	0.319403456114375	0.113	0.063	1.0025771115253E-16	Detoxification-associated hepat	CDAN1

6.10328652844618E-21	0.467087417185895	0.111	0.062	1.02278875643701E-16	Detoxification-associated hepat	ABCA3
7.25637321936279E-21	0.30146025035467	0.119	0.067	1.21602302410082E-16	Detoxification-associated hepat	GMCL1
1.20805934495095E-20	0.40485519399413	0.102	0.055	2.02446585026879E-16	Detoxification-associated hepat	HSPA14
1.46530071834078E-20	0.271743451281316	0.115	0.065	2.45555094379548E-16	Detoxification-associated hepat	EFNA4
2.42006305954099E-20	0.251875044842008	0.129	0.076	4.05554167517879E-16	Detoxification-associated hepat	ANKRD44
3.20018356710713E-20	0.816525996924459	0.101	0.056	5.36286762175813E-16	Detoxification-associated hepat	ELL3
3.62716720031841E-20	0.408645139546249	0.139	0.084	6.07840679429359E-16	Detoxification-associated hepat	MDM1
4.89762904349093E-20	0.663736387449986	0.134	0.081	8.2074467510821E-16	Detoxification-associated hepat	SWT1
4.90632196830432E-20	0.282928339452091	0.103	0.057	8.22201435448438E-16	Detoxification-associated hepat	RRM2B
6.16776352910621E-20	0.2856804584257	0.143	0.087	1.03359381220762E-15	Detoxification-associated hepat	GIMAP2
6.66591118251708E-20	0.332110907465783	0.126	0.074	1.11707339596621E-15	Detoxification-associated hepat	RTL8A
1.35187817502353E-19	0.288654284463754	0.115	0.065	2.26547744570443E-15	Detoxification-associated hepat	KCTD6
2.1201823125754E-19	0.343268574581627	0.111	0.063	3.55300151941385E-15	Detoxification-associated hepat	TMEM234
2.58050148895573E-19	0.673402355362573	0.136	0.083	4.32440439519201E-15	Detoxification-associated hepat	GPR153
7.05149089535488E-19	0.534190253174112	0.12	0.071	1.18168884424357E-14	Detoxification-associated hepat	SIX1
8.12255404040087E-19	0.250765648371936	0.135	0.082	1.36117760609038E-14	Detoxification-associated hepat	ERI1
1.24655718862025E-18	0.350820265261512	0.108	0.062	2.08898053668981E-14	Detoxification-associated hepat	DCAF4
1.81466438816286E-18	0.650377085788669	0.102	0.058	3.04101458168332E-14	Detoxification-associated hepat	BTC
2.55877460907751E-18	0.568048823207951	0.102	0.058	4.2879944898921E-14	Detoxification-associated hepat	SAPCD1
2.63960675623207E-18	0.261768821867389	0.125	0.075	4.4234530020937E-14	Detoxification-associated hepat	PDCD11
2.93173158484731E-18	0.424775120379706	0.118	0.07	4.91299578988712E-14	Detoxification-associated hepat	PDE9A
3.91287845432307E-18	0.439351448757565	0.108	0.062	6.55720171375459E-14	Detoxification-associated hepat	DNHD1
8.78468477593841E-18	0.515239683787593	0.128	0.079	1.47213747475176E-13	Detoxification-associated hepat	MMS22L
2.02925722443329E-17	0.49078052816187	0.106	0.062	3.4006292567053E-13	Detoxification-associated hepat	CCSAP
6.09700208780964E-17	0.365794980956162	0.131	0.082	1.02173560987514E-12	Detoxification-associated hepat	SACS
9.92250015275941E-17	0.416444276793005	0.132	0.082	1.66281257559942E-12	Detoxification-associated hepat	BRCA1
1.55873107711893E-16	0.435075447107202	0.121	0.074	2.61212153903591E-12	Detoxification-associated hepat	ING3
2.26857715540683E-16	0.804005682921865	0.1	0.059	3.80168159703076E-12	Detoxification-associated hepat	CENPK
3.19563462724873E-16	0.291052873666336	0.135	0.086	5.35524450834342E-12	Detoxification-associated hepat	MAMLD1
4.91399708459928E-16	0.323054027263522	0.114	0.069	8.23487631437148E-12	Detoxification-associated hepat	ZSWIM6
7.02914570020785E-16	0.483600919487629	0.124	0.078	1.17794423644083E-11	Detoxification-associated hepat	BRIP1
7.45259792294394E-16	0.270178117644932	0.112	0.068	1.24890635992695E-11	Detoxification-associated hepat	ZNF653
7.76331938120056E-16	0.268980529834367	0.118	0.072	1.30097706190159E-11	Detoxification-associated hepat	GSTCD
8.54440052280491E-16	0.39534210482258	0.102	0.061	1.43187063961165E-11	Detoxification-associated hepat	RTN4RL1
2.656553593656E-15	0.305772059380344	0.122	0.076	4.45185251224872E-11	Detoxification-associated hepat	NEDD1
7.03711360279926E-15	0.264929278585463	0.114	0.071	1.1792794975571E-10	Detoxification-associated hepat	PIDD1
8.97547991866987E-15	0.335511988656663	0.147	0.098	1.5041109247707E-10	Detoxification-associated hepat	ELOVL7
1.23016852613138E-14	0.263290801085923	0.11	0.068	2.06151641609097E-10	Detoxification-associated hepat	ZNF212
1.30859963827921E-14	0.264338618566482	0.101	0.061	2.19295127382830E-10	Detoxification-associated hepat	DEPRECATEI
2.43249526779715E-14	0.395550034236811	0.129	0.084	4.07637556977447E-10	Detoxification-associated hepat	BCL11A
2.96658074313954E-14	0.276471977226899	0.142	0.094	4.97139600935323E-10	Detoxification-associated hepat	NCR3LG1
3.26322247789026E-14	0.283001094447793	0.108	0.067	5.4685082284485E-10	Detoxification-associated hepat	NUBPL
1.2031175832197E-13	0.301263720135276	0.153	0.105	2.01618444595957E-09	Detoxification-associated hepat	ARSJ
1.29613173897346E-13	0.417931207437907	0.165	0.115	2.17205756817173E-09	Detoxification-associated hepat	DUSP21
1.89754891859281E-13	0.295279797376546	0.129	0.085	3.17991247777784E-09	Detoxification-associated hepat	MAP3K6
8.2392560203743E-13	0.315405468993308	0.138	0.093	1.38073452389432E-08	Detoxification-associated hepat	RIBC2
2.01213031415865E-12	0.344550999964368	0.104	0.066	3.37192798046707E-08	Detoxification-associated hepat	FIGNL1
2.13613401943452E-12	0.350798776295361	0.11	0.071	3.57973338976837E-08	Detoxification-associated hepat	EVA1C
3.9207141566324E-11	0.384578812639223	0.102	0.067	6.57033278368457E-07	Detoxification-associated hepat	CENPQ
4.04260552368329E-11	0.327189632296947	0.121	0.082	6.77459833658845E-07	Detoxification-associated hepat	BICD1
6.67547468443608E-11	0.281486741725167	0.102	0.067	1.1186760476178E-06	Detoxification-associated hepat	HELB
8.62021627740733E-11	0.309765676212749	0.112	0.076	1.44457584376792E-06	Detoxification-associated hepat	ORC5
3.92911725837146E-10	0.320777534452616	0.116	0.08	6.58441470157889E-06	Detoxification-associated hepat	SLC25A12
0	3.84276967350677	0.676	0.28	0	Inflammatory reactive hepatocy	SPP1
0	1.47676408648773	0.718	0.372	0	Inflammatory reactive hepatocy	APOE
0	2.89548554430776	0.509	0.188	0	Inflammatory reactive hepatocy	CRP
0	2.47102334871194	0.477	0.172	0	Inflammatory reactive hepatocy	ARG1
0	2.50470921372575	0.396	0.107	0	Inflammatory reactive hepatocy	CPS1
0	4.12399610834491	0.339	0.054	0	Inflammatory reactive hepatocy	KYNU
0	2.96674036235202	0.381	0.1	0	Inflammatory reactive hepatocy	ICAM1

0	3.61684449626689	0.381	0.101	0	Inflammatory reactive hepatocy	TNFSF14
0	3.51186438894857	0.352	0.079	0	Inflammatory reactive hepatocy	SELENOM
0	3.2454574692068	0.829	0.566	0	Inflammatory reactive hepatocy	PLA2G2A
0	3.2106119395122	0.352	0.096	0	Inflammatory reactive hepatocy	SAA1
0	5.31160027375636	0.253	0.008	0	Inflammatory reactive hepatocy	WFDC2
0	3.49671554087828	0.278	0.037	0	Inflammatory reactive hepatocy	SDS
0	4.53501704714402	0.247	0.015	0	Inflammatory reactive hepatocy	MSC
0	4.0188353153732	0.187	0.017	0	Inflammatory reactive hepatocy	CCDC187
0	4.90865974069971	0.17	0.009	0	Inflammatory reactive hepatocy	CXCL8
0	5.51422800243122	0.14	0.002	0	Inflammatory reactive hepatocy	TMPRSS15
0	3.95391942427522	0.127	0.007	0	Inflammatory reactive hepatocy	KRT23
0	4.55197179326815	0.121	0.005	0	Inflammatory reactive hepatocy	TESC
0	2.13611150754115	0.898	0.784	0	Inflammatory reactive hepatocy	SOD2
0	1.36729667414579	0.901	0.823	0	Inflammatory reactive hepatocy	CFB
0	1.12512353949614	0.975	0.923	0	Inflammatory reactive hepatocy	FN1
0	1.34879137100667	0.954	0.903	0	Inflammatory reactive hepatocy	ORM1
0	1.34357021769106	0.907	0.863	0	Inflammatory reactive hepatocy	IL32
0	1.63727589707259	0.992	0.962	0	Inflammatory reactive hepatocy	C3
2.67615230477025E-288	1.77962274167943	0.634	0.369	4.48469603233399E-284	Inflammatory reactive hepatocy	LBP
3.29626628890814E-272	2.38966827766482	0.565	0.328	5.52388304695226E-268	Inflammatory reactive hepatocy	A2M
3.57760135347196E-270	2.62622144003544	0.475	0.225	5.99534434814832E-266	Inflammatory reactive hepatocy	DEPRECATEI
2.56099937881278E-262	1.00893179303112	0.941	0.911	4.29172275901446E-258	Inflammatory reactive hepatocy	APOC2
1.16244690471467E-251	4.18045107122764	0.103	0.008	1.94802852292084E-247	Inflammatory reactive hepatocy	SPATA21
4.275515339306E-251	3.21370263859968	0.129	0.014	7.164908605609E-247	Inflammatory reactive hepatocy	MPZ
1.62067501822652E-250	1.51593371671533	0.691	0.469	2.715927195544E-246	Inflammatory reactive hepatocy	ITIH1
1.59504654867281E-240	1.44252230805475	0.575	0.273	2.6729790062659E-236	Inflammatory reactive hepatocy	SERPINA3
1.80320597194351E-237	1.174273440088	0.842	0.835	3.02181256778293E-233	Inflammatory reactive hepatocy	IFITM3
4.93916071435562E-232	2.87761040238516	0.179	0.034	8.27704552511716E-228	Inflammatory reactive hepatocy	SRGN
9.69521383042444E-227	2.21871311415265	0.212	0.047	1.62472393370253E-222	Inflammatory reactive hepatocy	FST
1.01794526770811E-225	1.31633514045723	0.644	0.367	1.70587267962526E-221	Inflammatory reactive hepatocy	ITIH2
5.62619099865527E-218	1.01137309552616	0.918	0.905	9.4283708755465E-214	Inflammatory reactive hepatocy	SQSTM1
2.11695195149848E-202	3.03996488305855	0.116	0.015	3.54758808032116E-198	Inflammatory reactive hepatocy	IGF1
3.18038915855901E-202	2.05991022135161	0.252	0.073	5.32969615191318E-198	Inflammatory reactive hepatocy	TIMP1
6.58716254110325E-199	2.09145633301827	0.24	0.068	1.10387669863808E-194	Inflammatory reactive hepatocy	MYOM1
3.58600524099808E-193	0.713728174521162	0.838	0.69	6.00942758286459E-189	Inflammatory reactive hepatocy	FGB
5.48361799295272E-190	3.12795959518792	0.11	0.014	9.18944703259017E-186	Inflammatory reactive hepatocy	DUSP5
2.95828952826767E-189	1.6429338293781	0.324	0.117	4.95750159147097E-185	Inflammatory reactive hepatocy	MBL2
4.57358641280729E-183	0.52956632955594	0.932	0.854	7.66441611058245E-179	Inflammatory reactive hepatocy	FGA
1.32330555320642E-182	2.01848903384148	0.313	0.119	2.21759544606332E-178	Inflammatory reactive hepatocy	SLC25A18
2.08832056062475E-182	1.73325794887836	0.332	0.128	3.49960759549496E-178	Inflammatory reactive hepatocy	SLC38A4
6.2410888131755E-182	0.378464544272277	0.704	0.345	1.04588166331195E-177	Inflammatory reactive hepatocy	HP
1.10624636397438E-180	2.25182058766798	0.462	0.268	1.85384765674826E-176	Inflammatory reactive hepatocy	TYMP
5.68381651563917E-178	0.896635804230869	0.592	0.307	9.52493971690812E-174	Inflammatory reactive hepatocy	HPX
9.12511981855421E-178	0.924773558068549	0.841	0.812	1.52918757919331E-173	Inflammatory reactive hepatocy	ORM2
3.20062182978537E-176	0.653399145944363	0.892	0.828	5.36360206235432E-172	Inflammatory reactive hepatocy	FGG
4.17828864835835E-175	2.64813650202615	0.143	0.028	7.00197611691893E-171	Inflammatory reactive hepatocy	GLS2
8.13736566768208E-171	3.02355382351411	0.115	0.018	1.36365973859016E-166	Inflammatory reactive hepatocy	SAA2
2.05244110025673E-170	0.600839172794741	0.586	0.276	3.43948079581023E-166	Inflammatory reactive hepatocy	HRG
4.0999052911143E-170	0.59776098326332	0.948	0.926	6.87062128684935E-166	Inflammatory reactive hepatocy	RBP4
2.69666066167554E-163	1.26055162160689	0.683	0.563	4.51906393683588E-159	Inflammatory reactive hepatocy	FGL1
2.24806221012231E-161	0.799994635651834	0.858	0.846	3.76730265172297E-157	Inflammatory reactive hepatocy	CD74
1.71279089334787E-155	1.96626615848723	0.177	0.046	2.87029497907236E-151	Inflammatory reactive hepatocy	FNDC4
1.83118976944069E-155	2.08029999015607	0.173	0.044	3.06870781562871E-151	Inflammatory reactive hepatocy	FETUB
1.09173031182637E-150	1.82772738554735	0.481	0.303	1.82952165655863E-146	Inflammatory reactive hepatocy	CREB3L3
1.39884294728609E-148	0.992415911728757	0.755	0.751	2.34418101106203E-144	Inflammatory reactive hepatocy	CFH
3.3929787029101E-148	1.16100702994086	0.567	0.366	5.68595371033675E-144	Inflammatory reactive hepatocy	NNMT
1.54561624469298E-147	0.511398481473229	0.99	0.986	2.5901437028565E-143	Inflammatory reactive hepatocy	VTN
3.62476659633205E-147	2.65595625066613	0.215	0.072	6.07438386213325E-143	Inflammatory reactive hepatocy	TM4SF1
7.66232610148764E-143	1.29672883971828	0.615	0.485	1.2840526080873E-138	Inflammatory reactive hepatocy	SERPINF2
3.0951035673512E-141	0.579916893465103	0.974	0.953	5.18677455816715E-137	Inflammatory reactive hepatocy	CES1

**2.63353689334027E-138**	1.31097391706671	0.311	0.128	4.41328112585962E-134	Inflammatory reactive hepatocy	MASP2
**2.60453031674408E-135**	1.87527357649985	0.153	0.039	4.36467190479974E-131	Inflammatory reactive hepatocy	ATP2B2
**6.01389428173679E-135**	1.35300477787953	0.396	0.2	1.00780840373345E-130	Inflammatory reactive hepatocy	SLC38A3
**1.1308510218254E-134**	2.09362500069224	0.183	0.056	1.89508014237501E-130	Inflammatory reactive hepatocy	PALM2-AKAI
**1.00634930517347E-128**	1.98609873643361	0.417	0.254	1.6864401656097E-124	Inflammatory reactive hepatocy	ROBO1
**3.24713058726523E-126**	1.73977719942489	0.473	0.311	5.44154143813907E-122	Inflammatory reactive hepatocy	DEPP1
**7.03255073036543E-125**	2.29040425666044	0.445	0.297	1.17851485139464E-120	Inflammatory reactive hepatocy	NRBP2
**1.32879849848481E-123**	1.24652671190409	0.307	0.133	2.22680052376085E-119	Inflammatory reactive hepatocy	C9
**4.26277631138339E-119**	2.28112206650637	0.284	0.134	7.14356054261629E-115	Inflammatory reactive hepatocy	RGS14
**6.30746188015824E-119**	1.82892427580008	0.18	0.058	1.05700446187692E-114	Inflammatory reactive hepatocy	SLC22A25
**6.28328727710146E-118**	0.57085751242327	0.972	0.973	1.05295328189666E-113	Inflammatory reactive hepatocy	FTL
**1.88231099539543E-117**	1.46347923138234	0.2	0.069	3.15437676608367E-113	Inflammatory reactive hepatocy	FAM20A
**3.34981973627897E-117**	2.10295845414986	0.125	0.031	5.61362791405629E-113	Inflammatory reactive hepatocy	MAGED1
**3.23600287471995E-116**	0.274895356344009	0.981	0.991	5.4228936174557E-112	Inflammatory reactive hepatocy	SERPINA1
**2.11461223849928E-114**	0.724643191279484	0.742	0.661	3.5436671892771E-110	Inflammatory reactive hepatocy	C1S
**2.93387062483583E-114**	1.69786605667001	0.425	0.263	4.91658039309989E-110	Inflammatory reactive hepatocy	MTSS2
**5.62750743087683E-114**	0.900107262173437	0.689	0.574	9.4305769526634E-110	Inflammatory reactive hepatocy	CP
**1.34205534677998E-106**	1.43953935589658	0.678	0.646	2.2490163501339E-102	Inflammatory reactive hepatocy	ALDH1L1
**1.57433373450115E-104**	1.33473707281783	0.235	0.094	2.63826847227703E-100	Inflammatory reactive hepatocy	ALDH8A1
**4.87587362690222E-104**	0.580351930424272	0.683	0.504	8.17098902396273E-100	Inflammatory reactive hepatocy	ITIH3
**2.01869321803027E-99**	1.3130706116182	0.661	0.622	3.38292609477512E-95	Inflammatory reactive hepatocy	PAH
**2.86364317735483E-98**	1.48881560188304	0.264	0.122	4.79889323661122E-94	Inflammatory reactive hepatocy	RND1
**2.77969458398723E-97**	1.03601069134157	0.676	0.642	4.6582121838458E-93	Inflammatory reactive hepatocy	LRG1
**3.41955828362458E-97**	2.92492158201849	0.132	0.04	5.73049577169808E-93	Inflammatory reactive hepatocy	CAPN12
**1.79674744162696E-96**	1.63121251431173	0.48	0.362	3.01098936267845E-92	Inflammatory reactive hepatocy	UBD
**1.11905483661169E-93**	1.97880124846261	0.238	0.11	1.87531209519386E-89	Inflammatory reactive hepatocy	CYP21A2
**1.18554746805823E-92**	1.79687051762656	0.188	0.074	1.98674044697199E-88	Inflammatory reactive hepatocy	IER5L
**1.6789587158077E-92**	0.421258967106926	0.971	0.971	2.81359901595054E-88	Inflammatory reactive hepatocy	CLU
**4.30093720631668E-92**	2.85187503428827	0.148	0.051	7.20751057034549E-88	Inflammatory reactive hepatocy	PDZK1IP1
**3.17284898363922E-89**	0.629788315904396	0.303	0.141	5.31706032678261E-85	Inflammatory reactive hepatocy	HPD
**7.56199946268482E-88**	0.460040008556302	0.95	0.96	1.26723986995672E-83	Inflammatory reactive hepatocy	AMBP
**5.7277268586262E-87**	0.727222705033883	0.738	0.728	9.59852466968579E-83	Inflammatory reactive hepatocy	LAMP1
**1.40865643204063E-85**	0.94933534896572	0.481	0.324	2.36062644881369E-81	Inflammatory reactive hepatocy	ITIH4
**1.8902727312307E-85**	0.478781010998354	0.656	0.485	3.16771904299641E-81	Inflammatory reactive hepatocy	SERPING1
**1.56842890119082E-82**	0.396968772822751	0.463	0.25	2.62837315261558E-78	Inflammatory reactive hepatocy	HPR
**3.72821129975512E-81**	2.61755380737954	0.134	0.046	6.24773649612963E-77	Inflammatory reactive hepatocy	OASL
**4.94652537804385E-78**	0.910810955065634	0.558	0.431	8.28938722852588E-74	Inflammatory reactive hepatocy	APOC1
**2.18749460375657E-76**	1.44029471495084	0.117	0.037	3.66580345697526E-72	Inflammatory reactive hepatocy	UGT3A1
**2.78466908641137E-75**	1.34147405270595	0.241	0.119	4.66654845500817E-71	Inflammatory reactive hepatocy	GCKR
**1.31104373283352E-74**	2.02391925887207	0.246	0.131	2.19704708748242E-70	Inflammatory reactive hepatocy	RIOX2
**4.2786741529766E-72**	1.17183887736294	0.339	0.205	7.17020214555819E-68	Inflammatory reactive hepatocy	TM4SF5
**6.07978815440094E-72**	0.981686035672937	0.389	0.242	1.01885089891451E-67	Inflammatory reactive hepatocy	IGFBP2
**8.03321845753202E-71**	0.328274533105157	0.862	0.867	1.34620674911322E-66	Inflammatory reactive hepatocy	APOB
**1.16617427263772E-70**	1.47386473816977	0.173	0.073	1.9542748460863E-66	Inflammatory reactive hepatocy	AMDHD1
**1.24596787532634E-66**	1.56792319688051	0.154	0.063	2.08799296547188E-62	Inflammatory reactive hepatocy	ANXA6
**2.26828169955145E-64**	0.414033464375928	0.417	0.246	3.80118647210831E-60	Inflammatory reactive hepatocy	COL18A1
**3.87437089542466E-64**	0.601859789632767	0.729	0.752	6.49267074655264E-60	Inflammatory reactive hepatocy	ASGR2
**4.26266454215675E-63**	1.4373094756617	0.175	0.079	7.14337323974627E-59	Inflammatory reactive hepatocy	LGALS3
**4.54139973010796E-63**	0.669628427206897	0.778	0.822	7.61047766771491E-59	Inflammatory reactive hepatocy	FTH1
**8.86062967180963E-62**	1.43689214233776	0.448	0.365	1.48486432040186E-57	Inflammatory reactive hepatocy	LGALS3BP
**3.58353102088898E-61**	0.720711223612341	0.319	0.178	6.00528128480575E-57	Inflammatory reactive hepatocy	SAA4
**3.98761742146754E-61**	1.33604274660854	0.294	0.177	6.6824492748953E-57	Inflammatory reactive hepatocy	DUSP10
**8.96934962673135E-60**	1.45416180772759	0.425	0.332	1.50308361044764E-55	Inflammatory reactive hepatocy	BCL3
**1.68817988697767E-57**	1.40219356811983	0.164	0.075	2.82905185459718E-53	Inflammatory reactive hepatocy	DTNA
**4.54944375935806E-53**	1.45062136252079	0.115	0.045	7.62395785193224E-49	Inflammatory reactive hepatocy	TMPRSS6
**3.44621534989659E-50**	1.74120990554529	0.251	0.157	5.7751676833567E-46	Inflammatory reactive hepatocy	TRIM55
**5.45361025583305E-50**	1.58929368653715	0.198	0.108	9.13916006672502E-46	Inflammatory reactive hepatocy	GBP7
**1.19806455392741E-48**	2.19725522841108	0.19	0.106	2.00771657947155E-44	Inflammatory reactive hepatocy	MUC20
**2.18267398364362E-45**	1.83571466232595	0.289	0.201	3.65772506178998E-41	Inflammatory reactive hepatocy	CD24
**9.84150451281155E-45**	0.868370728459407	0.421	0.327	1.64923932625696E-40	Inflammatory reactive hepatocy	C5

2.05549550001082E-44	1.42631167241148	0.187	0.102	3.44459935891813E-40	Inflammatory reactive hepatocy	RTN4RL2
5.98506746507264E-43	0.398143885387866	0.848	0.837	1.00297760579687E-38	Inflammatory reactive hepatocy	PTGR1
7.1336982909457E-43	0.583955384551334	0.707	0.716	1.19546515959668E-38	Inflammatory reactive hepatocy	EEF2
2.28302062454422E-42	0.593640625097892	0.734	0.779	3.8258859626112E-38	Inflammatory reactive hepatocy	PRAP1
1.66282046074817E-41	1.32898367592388	0.351	0.27	2.78655452812178E-37	Inflammatory reactive hepatocy	NCOA7
4.10812174572958E-41	1.4710226048193	0.316	0.228	6.88439042149362E-37	Inflammatory reactive hepatocy	PLIN4
9.24074665075728E-41	1.61600138633903	0.155	0.082	1.5485643237339E-36	Inflammatory reactive hepatocy	DNAH1
3.25289228273309E-40	1.61610281644585	0.191	0.111	5.45119688740411E-36	Inflammatory reactive hepatocy	VNN 2.00
4.91536590950684E-40	1.54196186112678	0.29	0.205	8.23717019115157E-36	Inflammatory reactive hepatocy	ISG15
5.05318167421839E-40	1.47072994011685	0.163	0.088	8.46812184965518E-36	Inflammatory reactive hepatocy	CBS
2.56797907114812E-39	0.307812536763161	0.892	0.928	4.30341932743002E-35	Inflammatory reactive hepatocy	FABP1
7.17461607924051E-39	0.703619525174322	0.401	0.295	1.20232216255912E-34	Inflammatory reactive hepatocy	CFHR2
3.37927216156689E-38	0.798473149398887	0.598	0.609	5.6629842883538E-34	Inflammatory reactive hepatocy	CTSB
8.01037833522996E-35	0.593519053929361	0.652	0.683	1.34237920141784E-30	Inflammatory reactive hepatocy	C2
1.85440125213787E-34	0.766035333892424	0.192	0.111	3.10760561833265E-30	Inflammatory reactive hepatocy	MASP1
6.37263735348099E-34	1.07845303553038	0.513	0.497	1.06792656769634E-29	Inflammatory reactive hepatocy	KRT18
3.23373119212234E-33	1.0447652960573	0.481	0.446	5.41908673175862E-29	Inflammatory reactive hepatocy	DPYS
5.53452109438283E-33	1.02885000357216	0.187	0.111	9.27475044996675E-29	Inflammatory reactive hepatocy	LEPR
1.27061593539161E-32	0.655373386683011	0.621	0.65	2.12929818452925E-28	Inflammatory reactive hepatocy	XBP1
3.83654519309453E-32	0.935398156978772	0.525	0.52	6.42928243458781E-28	Inflammatory reactive hepatocy	MPST
5.36655926253822E-32	0.922483552798375	0.128	0.065	8.99328001216154E-28	Inflammatory reactive hepatocy	FOXA3
6.7519692768614E-32	0.638253873466032	0.624	0.649	1.13149501141643E-27	Inflammatory reactive hepatocy	GRINA
1.27670517140713E-31	1.04419736661925	0.485	0.469	2.13950252624406E-27	Inflammatory reactive hepatocy	PNRC1
1.36054923844187E-31	1.84879448869725	0.115	0.059	2.28000841378088E-27	Inflammatory reactive hepatocy	PHGDH
2.57957760592249E-31	0.737196972628668	0.577	0.598	4.32285615200492E-27	Inflammatory reactive hepatocy	OS9
3.28049854333659E-31	0.324849065134462	0.823	0.826	5.49745945892345E-27	Inflammatory reactive hepatocy	TTR
9.29905100875005E-31	1.71620883800787	0.225	0.156	1.55833496804633E-26	Inflammatory reactive hepatocy	HKDC1
4.62766043992472E-30	0.884665391438463	0.153	0.085	7.75503336522585E-26	Inflammatory reactive hepatocy	CPN2
3.28708857661634E-29	0.636583491694106	0.193	0.116	5.50850303669367E-25	Inflammatory reactive hepatocy	FGF21
4.7730353067448E-29	0.890969874202584	0.52	0.52	7.99865256704293E-25	Inflammatory reactive hepatocy	CTSA
6.19525386668168E-29	0.42806516211354	0.157	0.273	1.03820064297852E-24	Inflammatory reactive hepatocy	ZC3HAV1
9.83026234827952E-29	1.47115732173161	0.24	0.171	1.64735536432468E-24	Inflammatory reactive hepatocy	NFKB2
1.40784629041175E-28	0.98267601405659	0.171	0.103	2.35926881347201E-24	Inflammatory reactive hepatocy	OGDHL
4.5957088161647E-28	0.983121869282687	0.123	0.066	7.7014888341288E-24	Inflammatory reactive hepatocy	ADGRG6
1.07019240738586E-26	1.28961646048748	0.264	0.196	1.79342843629722E-22	Inflammatory reactive hepatocy	IRS2
1.11496900911785E-26	1.0580700824171	0.42	0.385	1.86846506547969E-22	Inflammatory reactive hepatocy	LRP1
1.44076769690108E-26	0.55082405114773	0.632	0.677	2.41443850646683E-22	Inflammatory reactive hepatocy	LMAN2
1.94885821501506E-24	0.599786945029614	0.652	0.692	3.26589659672224E-20	Inflammatory reactive hepatocy	DHCR24
2.50177304265197E-24	0.277396314797863	0.062	0.134	4.19247126487618E-20	Inflammatory reactive hepatocy	VMAC
4.4338054320701E-24	1.24186441946222	0.139	0.083	7.43017114306307E-20	Inflammatory reactive hepatocy	GPT2
7.44357754097275E-24	1.36706351912196	0.142	0.087	1.24739472431621E-19	Inflammatory reactive hepatocy	TMEM45A
8.19081645162114E-24	0.858070387945918	0.513	0.517	1.37261702096267E-19	Inflammatory reactive hepatocy	SNTB1
1.48141210817091E-23	0.735154124879671	0.593	0.627	2.48255041087281E-19	Inflammatory reactive hepatocy	SDC4
2.29054112627413E-23	1.02875047417997	0.137	0.082	3.83848881941019E-19	Inflammatory reactive hepatocy	DOCK4
3.69736713287851E-23	0.292401021729869	0.727	0.741	6.19604784127781E-19	Inflammatory reactive hepatocy	GPX2
1.04934091255747E-22	0.271260392207221	0.173	0.282	1.75848550126381E-18	Inflammatory reactive hepatocy	PI4KA
1.59618912916603E-22	0.786311329092686	0.166	0.104	2.67489374265644E-18	Inflammatory reactive hepatocy	CHRD
7.18567350300931E-22	1.00949140789177	0.34	0.284	1.2041751656343E-17	Inflammatory reactive hepatocy	REEP6
7.80675426386095E-22	0.687632214649869	0.255	0.183	1.30825587953782E-17	Inflammatory reactive hepatocy	RDH16
8.17170893735743E-22	1.49359547399344	0.217	0.16	1.36941498372236E-17	Inflammatory reactive hepatocy	VWA1
2.68322261594E-21	1.18511669731065	0.385	0.353	4.49654445979226E-17	Inflammatory reactive hepatocy	SHFL
4.73266589850364E-21	0.330127215611656	0.598	0.572	7.93100151271241E-17	Inflammatory reactive hepatocy	F2
6.14307176784768E-21	0.255477367509373	0.077	0.148	1.02945596685592E-16	Inflammatory reactive hepatocy	ZMYND19
1.36429026682407E-20	0.279677197165227	0.166	0.265	2.28627762914378E-16	Inflammatory reactive hepatocy	GATD1
1.88478747592E-20	0.291751165509094	0.067	0.134	3.15852685214673E-16	Inflammatory reactive hepatocy	DOCK6
3.33777823947197E-20	0.328975717020593	0.084	0.157	5.59344877370713E-16	Inflammatory reactive hepatocy	ZNF692
3.85042276826156E-20	0.263242400512623	0.103	0.181	6.45253847505273E-16	Inflammatory reactive hepatocy	MARK2
4.02971362958893E-20	1.60665047427139	0.185	0.132	6.75299410046513E-16	Inflammatory reactive hepatocy	DEPRECATEI
4.61742746218579E-20	0.614025701032837	0.6	0.629	7.73788494113094E-16	Inflammatory reactive hepatocy	TAPBP
7.49618240121127E-20	1.0572565564734	0.178	0.122	1.25621024679499E-15	Inflammatory reactive hepatocy	ABLIM3

1.77265635381597E-19	1.66119143966069	0.13	0.083	2.97061751772481E-15	Inflammatory reactive hepatocy	RFTN1
3.08843261957558E-19	1.75799044770533	0.122	0.076	5.17559538388475E-15	Inflammatory reactive hepatocy	SDSL
4.37609246903449E-19	1.28202317012489	0.195	0.142	7.333455759608E-15	Inflammatory reactive hepatocy	AL135905.2
6.09115321838565E-19	0.481264333376927	0.098	0.173	1.02075545633707E-14	Inflammatory reactive hepatocy	MROH6
7.34793447508738E-19	0.271282374229548	0.108	0.185	1.23136685933514E-14	Inflammatory reactive hepatocy	KYAT1
1.77701062687765E-18	0.324443192945985	0.149	0.241	2.97791440852156E-14	Inflammatory reactive hepatocy	SCAP
1.78024302309465E-18	0.253364267317853	0.139	0.223	2.98333125810201E-14	Inflammatory reactive hepatocy	AKT1S1
1.86173049161203E-18	0.301108806286516	0.157	0.25	3.11988795784344E-14	Inflammatory reactive hepatocy	STX10
1.89856312105807E-18	0.27190051704226	0.08	0.146	3.18161207826911E-14	Inflammatory reactive hepatocy	IRGQ
2.38421198962059E-18	0.740910001814388	0.122	0.074	3.99546245220618E-14	Inflammatory reactive hepatocy	SLC13A5
2.63273570402318E-18	1.11780805919116	0.297	0.25	4.41193849280204E-14	Inflammatory reactive hepatocy	RGS3
2.80283840663623E-18	0.87643414462497	0.201	0.143	4.696996601841E-14	Inflammatory reactive hepatocy	TMEM140
3.09891833855358E-18	1.35336438629917	0.146	0.097	5.19316735174809E-14	Inflammatory reactive hepatocy	TNFRSF12A
3.14879567692223E-18	0.326720860855501	0.146	0.232	5.27675179538627E-14	Inflammatory reactive hepatocy	SRCAP
4.11254102949758E-18	0.28312516616947	0.095	0.166	6.89179625723205E-14	Inflammatory reactive hepatocy	DDX55
5.46747028160539E-18	0.288137445454809	0.132	0.215	9.16238669791431E-14	Inflammatory reactive hepatocy	MAFG
8.08232453328177E-18	1.19680180654689	0.336	0.301	1.35443594528736E-13	Inflammatory reactive hepatocy	DEPRECATEI
8.26468905821646E-18	0.278016086138822	0.187	0.285	1.38499659237591E-13	Inflammatory reactive hepatocy	NOP56
9.09497021696686E-18	0.309457219673484	0.118	0.196	1.52413510895931E-13	Inflammatory reactive hepatocy	AGAP1
9.13324821076709E-18	0.270131436040361	0.051	0.106	1.53054973516035E-13	Inflammatory reactive hepatocy	ZBED4
9.65168650011167E-18	0.273482510261356	0.16	0.251	1.61742962368871E-13	Inflammatory reactive hepatocy	SLC39A11
1.11738636232926E-17	0.258632664956439	0.227	0.337	1.87251606599138E-13	Inflammatory reactive hepatocy	EHMT1
1.99104625284703E-17	0.998415238576171	0.416	0.409	3.33659531052105E-13	Inflammatory reactive hepatocy	TFPI
2.16667588922525E-17	0.278435753659789	0.117	0.194	3.63091545516367E-13	Inflammatory reactive hepatocy	KRI1
2.32340023277017E-17	0.261589068196733	0.075	0.137	3.89355411007625E-13	Inflammatory reactive hepatocy	SLC35E2A
2.75573627547496E-17	0.340108226069603	0.196	0.296	4.61806285044094E-13	Inflammatory reactive hepatocy	KLHDC4
3.00317260281879E-17	0.361226054365473	0.134	0.215	5.03271664780372E-13	Inflammatory reactive hepatocy	TNFAIP2
3.37087778453507E-17	0.81583275243004	0.344	0.296	5.64891699132387E-13	Inflammatory reactive hepatocy	DEPRECATEI
4.23687399157706E-17	1.41532531744396	0.173	0.126	7.10015343508484E-13	Inflammatory reactive hepatocy	RBM19
4.29165057246297E-17	0.250910204422732	0.09	0.157	7.19194802933345E-13	Inflammatory reactive hepatocy	IL15RA
4.39437300765714E-17	0.251809120399429	0.075	0.136	7.36409028623183E-13	Inflammatory reactive hepatocy	JOSD2
4.45301726529104E-17	0.421893458037214	0.166	0.258	7.46236633317473E-13	Inflammatory reactive hepatocy	TYK2
4.60073362092698E-17	0.268347115588467	0.086	0.151	7.70990940194944E-13	Inflammatory reactive hepatocy	DNAAF5
5.02630784100382E-17	0.257919014026282	0.095	0.164	8.4230866799542E-13	Inflammatory reactive hepatocy	FOXK1
5.7697012906393E-17	0.296896345639264	0.122	0.198	9.66886542285335E-13	Inflammatory reactive hepatocy	NLN
8.6517608648051E-17	0.372055060034932	0.073	0.133	1.44986208572404E-12	Inflammatory reactive hepatocy	HMGXB3
1.24412743497427E-16	0.259190906793853	0.089	0.153	2.08490875552987E-12	Inflammatory reactive hepatocy	XRCC6
1.25945122544383E-16	0.829030870180614	0.318	0.269	2.11058836359877E-12	Inflammatory reactive hepatocy	ID2
1.27241202445675E-16	0.787761834813763	0.298	0.24	2.13230807058461E-12	Inflammatory reactive hepatocy	SLC9A3R2
1.30203665890208E-16	0.65399593803159	0.427	0.393	2.1819530329881E-12	Inflammatory reactive hepatocy	JUNB
1.34312478569055E-16	0.327471391341274	0.133	0.212	2.25080851586023E-12	Inflammatory reactive hepatocy	ZER1
1.34908921565237E-16	0.253900427440122	0.055	0.108	2.26080370759025E-12	Inflammatory reactive hepatocy	JARID2
1.42521478189699E-16	0.413104501145849	0.148	0.233	2.38837493150298E-12	Inflammatory reactive hepatocy	EDC4
1.50955984092882E-16	1.04183015252758	0.134	0.088	2.52972038142851E-12	Inflammatory reactive hepatocy	WWC1
1.59696823752355E-16	0.265307815667716	0.063	0.119	2.67619937244197E-12	Inflammatory reactive hepatocy	CAPS
1.69251891067437E-16	1.0832032886226	0.275	0.227	2.8363231905081E-12	Inflammatory reactive hepatocy	ALDH4A1
1.81531913582286E-16	0.295350945670898	0.197	0.296	3.04211180781195E-12	Inflammatory reactive hepatocy	ASAP1
1.86002758016142E-16	0.330449774269419	0.266	0.384	3.11703421883451E-12	Inflammatory reactive hepatocy	MROH1
1.98356144604512E-16	0.279915244830771	0.221	0.326	3.32405227128241E-12	Inflammatory reactive hepatocy	BACH1
2.65748032883846E-16	0.397287275145787	0.686	0.707	4.45340553506749E-12	Inflammatory reactive hepatocy	LMNA
2.69787573537496E-16	0.324254580851876	0.069	0.126	4.52110015734136E-12	Inflammatory reactive hepatocy	MARVELD3
2.82806847684951E-16	0.357226982993443	0.088	0.153	4.73927715350441E-12	Inflammatory reactive hepatocy	SLC52A2
2.91214448895805E-16	0.408283124990976	0.192	0.29	4.8801717345959E-12	Inflammatory reactive hepatocy	SLC4A2
3.24455622812305E-16	0.271067506911909	0.095	0.161	5.4372273270886E-12	Inflammatory reactive hepatocy	ACOT8
3.53489701298833E-16	1.30740546626877	0.147	0.102	5.92378041436584E-12	Inflammatory reactive hepatocy	MYC
3.54426106562416E-16	0.280107594212643	0.223	0.329	5.93947269377297E-12	Inflammatory reactive hepatocy	DPP9
4.67318954661219E-16	0.333121133898	0.089	0.153	7.83133104221271E-12	Inflammatory reactive hepatocy	GGA1
4.79275616916765E-16	0.270106023049505	0.161	0.246	8.03170078829115E-12	Inflammatory reactive hepatocy	PPP6R1
5.16869029730976E-16	1.04443593643462	0.282	0.237	8.6616912002317E-12	Inflammatory reactive hepatocy	TGM2
9.37329543802975E-16	0.290310069273014	0.102	0.17	1.57077684950503E-11	Inflammatory reactive hepatocy	TANGO2

**1.28575604615155E-15**	0.264421439611012	0.142	0.22	2.15466998214077E-11	Inflammatory reactive hepatocy	GANAB
**1.34729973094209E-15**	0.413251208980341	0.068	0.124	2.25780488911275E-11	Inflammatory reactive hepatocy	TOP3B
**1.35833059578567E-15**	1.0582587267246	0.205	0.155	2.27629041241763E-11	Inflammatory reactive hepatocy	STEAP3
**1.39994618942788E-15**	0.312026057678992	0.072	0.129	2.34602982424325E-11	Inflammatory reactive hepatocy	MADD
**1.65262361100208E-15**	1.28807048610721	0.34	0.308	2.76946664731728E-11	Inflammatory reactive hepatocy	PLIN5
**1.68442904804909E-15**	0.365272824115118	0.066	0.121	2.82276619872066E-11	Inflammatory reactive hepatocy	SZT2
**1.81254947598362E-15**	0.289062738965452	0.131	0.206	3.03747041185334E-11	Inflammatory reactive hepatocy	ATF7
**1.83817865427759E-15**	0.314724774202693	0.216	0.316	3.08041978883838E-11	Inflammatory reactive hepatocy	CUL4A
**2.45479420893923E-15**	0.567995117166173	0.078	0.137	4.11374413534036E-11	Inflammatory reactive hepatocy	MIGA2
**2.50472826971999E-15**	0.389416622271931	0.267	0.201	4.19742363439676E-11	Inflammatory reactive hepatocy	APOM
**3.07825531975946E-15**	0.368792056074854	0.104	0.172	5.1585402648529E-11	Inflammatory reactive hepatocy	SLC35E2B
**3.3575694440133E-15**	0.314804413661659	0.173	0.259	5.62661487427749E-11	Inflammatory reactive hepatocy	COQ4
**3.39962748513844E-15**	0.309064682733292	0.124	0.196	5.697095739595E-11	Inflammatory reactive hepatocy	SLC23A1
**3.58767574192979E-15**	0.250511414303811	0.121	0.193	6.01222700832595E-11	Inflammatory reactive hepatocy	TMEM138
**3.61041780259903E-15**	0.278086926827042	0.135	0.209	6.05033815359546E-11	Inflammatory reactive hepatocy	SLC30A7
**3.82358361341026E-15**	0.371708697643807	0.154	0.237	6.40756141935291E-11	Inflammatory reactive hepatocy	TPRA1
**4.74630561590158E-15**	0.280850097304756	0.108	0.175	7.95385895112786E-11	Inflammatory reactive hepatocy	ANAPC2
**4.90399881558608E-15**	0.293517354169609	0.101	0.165	8.21812121515915E-11	Inflammatory reactive hepatocy	SETBP1
**5.38951464465737E-15**	0.344856636714825	0.159	0.24	9.03174864151682E-11	Inflammatory reactive hepatocy	ACO2
**7.14240741455931E-15**	0.290613246713482	0.099	0.164	1.19692463453185E-10	Inflammatory reactive hepatocy	DSG2
**7.35024634809475E-15**	0.279262748712654	0.095	0.158	1.23175428301372E-10	Inflammatory reactive hepatocy	FBRSL1
**7.63010164982169E-15**	0.255257639157309	0.147	0.225	1.27865243447712E-10	Inflammatory reactive hepatocy	MAP3K11
**8.02525297453885E-15**	0.384619633559333	0.083	0.142	1.34487189347322E-10	Inflammatory reactive hepatocy	DUS3L
**8.50945731404212E-15**	0.485369781759565	0.084	0.145	1.42601485668718E-10	Inflammatory reactive hepatocy	PKD1
**8.55930641995849E-15**	0.341348391403592	0.099	0.164	1.43436856985664E-10	Inflammatory reactive hepatocy	ABCC6
**8.79519855651746E-15**	0.317393063659885	0.064	0.117	1.4738993741012E-10	Inflammatory reactive hepatocy	CAMK2G
**9.1710190835723E-15**	0.259872544368688	0.117	0.186	1.53687937802505E-10	Inflammatory reactive hepatocy	UTP6
**9.76634775534307E-15**	1.4320856380479	0.193	0.15	1.63664455684039E-10	Inflammatory reactive hepatocy	NSMF
**1.02835298104643E-14**	0.325180803486319	0.082	0.141	1.72331392563761E-10	Inflammatory reactive hepatocy	TOMM40
**1.10970508938086E-14**	0.254908072708244	0.3	0.425	1.85964378878445E-10	Inflammatory reactive hepatocy	TMEM259
**1.14724925124897E-14**	1.70655164582986	0.111	0.073	1.92256029524302E-10	Inflammatory reactive hepatocy	UNC5CL
**1.3185361626209E-14**	0.396160007883712	0.137	0.211	2.2096029013201E-10	Inflammatory reactive hepatocy	TRAPPC12
**1.32851227363784E-14**	1.33244934229133	0.153	0.11	2.2263208681623E-10	Inflammatory reactive hepatocy	PC
**1.38767435561464E-14**	0.31055156173156	0.125	0.196	2.32546468513902E-10	Inflammatory reactive hepatocy	ARRB2
**1.4467078937412E-14**	0.254963326796739	0.158	0.237	2.42439308833151E-10	Inflammatory reactive hepatocy	VARS
**1.49404555320965E-14**	0.334010107426688	0.089	0.15	2.50372153806873E-10	Inflammatory reactive hepatocy	BICRA
**1.85447933864448E-14**	0.422327869569583	0.156	0.237	3.10773647570043E-10	Inflammatory reactive hepatocy	PSMD2
**1.91384266925712E-14**	0.254846709772298	0.192	0.282	3.20721754514108E-10	Inflammatory reactive hepatocy	RTCB
**1.92629239657971E-14**	0.357134966809336	0.185	0.272	3.22808079818828E-10	Inflammatory reactive hepatocy	FN3K
**1.99658356258265E-14**	0.270982080595056	0.102	0.165	3.34587473417601E-10	Inflammatory reactive hepatocy	CLEC16A
**2.00231908267716E-14**	0.301404046774257	0.115	0.182	3.35548631875038E-10	Inflammatory reactive hepatocy	DEPRECATEI
**2.06043226734932E-14**	0.315922440580045	0.171	0.258	3.452872393624E-10	Inflammatory reactive hepatocy	TBC1D17
**2.06893919799537E-14**	0.291375971213257	0.231	0.33	3.46712830800064E-10	Inflammatory reactive hepatocy	ANXA7
**2.18038441636045E-14**	0.257930481304926	0.087	0.146	3.65388820493685E-10	Inflammatory reactive hepatocy	TUT1
**2.33616664286785E-14**	0.41446290088607	0.177	0.262	3.91494806011793E-10	Inflammatory reactive hepatocy	RASSF4
**2.46393791796692E-14**	0.260600239752328	0.171	0.116	4.12906716292897E-10	Inflammatory reactive hepatocy	CDO1
**2.50657319041085E-14**	0.273018785107929	0.192	0.282	4.20051535249051E-10	Inflammatory reactive hepatocy	ZGPAT
**2.81904023334327E-14**	0.254066406823906	0.136	0.207	4.72414762303665E-10	Inflammatory reactive hepatocy	TSEN34
**3.25883485446385E-14**	0.318315544323295	0.129	0.201	5.46115544911052E-10	Inflammatory reactive hepatocy	CAMKK2
**3.70307760241794E-14**	0.531286931418869	0.622	0.685	6.20561744613199E-10	Inflammatory reactive hepatocy	CST3
**4.17340589768638E-14**	0.275523385229889	0.205	0.296	6.99379360334284E-10	Inflammatory reactive hepatocy	TMED2
**4.8688556648248E-14**	0.404984858693777	0.119	0.187	8.1592283231134E-10	Inflammatory reactive hepatocy	SLC41A3
**4.95387457155927E-14**	0.26814241016904	0.198	0.29	8.30170300701902E-10	Inflammatory reactive hepatocy	ATP11A
**5.08274452257825E-14**	0.293338501936366	0.247	0.35	8.51766327093662E-10	Inflammatory reactive hepatocy	DNAJC21
**5.19135799729583E-14**	0.343283666456807	0.084	0.142	8.69967773186835E-10	Inflammatory reactive hepatocy	TSNARE1
**5.21297077919893E-14**	0.650512640600565	0.18	0.131	8.73589643178157E-10	Inflammatory reactive hepatocy	CDH2
**5.35775905499304E-14**	0.262848845745959	0.259	0.366	8.97853262435733E-10	Inflammatory reactive hepatocy	GNPTG
**5.39340914032998E-14**	0.297672352443342	0.119	0.184	9.03827503736499E-10	Inflammatory reactive hepatocy	THAP11
**5.46486924883255E-14**	0.318852963361943	0.183	0.269	9.1580278871936E-10	Inflammatory reactive hepatocy	ZXDC
**5.46734910641357E-14**	1.23905395346654	0.309	0.277	9.16218363252786E-10	Inflammatory reactive hepatocy	PABPC1L

5.77003998653931E-14	0.38996838397894	0.151	0.228	9.66943300944257E-10	Inflammatory reactive hepatocy	C5orf15
6.10324298736733E-14	0.316978752032716	0.139	0.214	1.02278145982302E-09	Inflammatory reactive hepatocy	SGSM3
6.16741641177256E-14	0.457856746075514	0.28	0.223	1.03353564228485E-09	Inflammatory reactive hepatocy	TFR2
6.73763277974486E-14	0.405443507892693	0.071	0.123	1.12909250122964E-09	Inflammatory reactive hepatocy	CACTIN
7.47582137784863E-14	0.301704214607431	0.162	0.242	1.25279814649987E-09	Inflammatory reactive hepatocy	TRMT11
7.84097437658472E-14	0.265533266659703	0.09	0.149	1.31399048602807E-09	Inflammatory reactive hepatocy	ZNF513
7.91321284655402E-14	1.27194589536961	0.326	0.3	1.32609620882552E-09	Inflammatory reactive hepatocy	IRF1
8.46803253377689E-14	0.262357024145939	0.187	0.271	1.41907289201033E-09	Inflammatory reactive hepatocy	TXN2
9.83580592627108E-14	0.308741694736704	0.112	0.177	1.64828435712451E-09	Inflammatory reactive hepatocy	TSC2
1.19533652254356E-13	0.335058012993448	0.216	0.31	2.00314494447849E-09	Inflammatory reactive hepatocy	LRRC47
1.23853453224854E-13	0.338511527196719	0.093	0.153	2.0755361691421E-09	Inflammatory reactive hepatocy	GIT2
1.29996476569902E-13	0.319823996934406	0.195	0.283	2.17848095435841E-09	Inflammatory reactive hepatocy	SLC3A2
1.35167300632597E-13	0.274374340548191	0.232	0.327	2.26513362400106E-09	Inflammatory reactive hepatocy	GPR108
1.57445182393264E-13	0.462853819080635	0.173	0.257	2.63846636654632E-09	Inflammatory reactive hepatocy	PTGES2
1.63655562366627E-13	0.291337637227292	0.149	0.223	2.74253991413994E-09	Inflammatory reactive hepatocy	ZMPSTE24
1.74895948718663E-13	0.33041528432216	0.143	0.216	2.93090630862735E-09	Inflammatory reactive hepatocy	MPZL1
1.78938856933474E-13	0.313223011485184	0.062	0.11	2.99865736449116E-09	Inflammatory reactive hepatocy	C11orf80
2.70867627156962E-13	0.430543099848065	0.134	0.205	4.53919969589637E-09	Inflammatory reactive hepatocy	MMP15
3.18367420573437E-13	0.429788425701056	0.099	0.16	5.33520123396965E-09	Inflammatory reactive hepatocy	GPATCH2
3.18392725502552E-13	0.258612981872579	0.089	0.145	5.33562529397176E-09	Inflammatory reactive hepatocy	HARS2
3.21940585768889E-13	0.351938797570107	0.648	0.645	5.39508033631504E-09	Inflammatory reactive hepatocy	DEPRECATEI
3.24125325379193E-13	0.445204210906396	0.066	0.116	5.43169220270452E-09	Inflammatory reactive hepatocy	TTC22
4.13934516890922E-13	1.36501668137279	0.2	0.157	6.93671463405806E-09	Inflammatory reactive hepatocy	LGALS1
4.51378562461565E-13	0.505806741622069	0.101	0.063	7.5642019497309E-09	Inflammatory reactive hepatocy	APOF
4.52949943378802E-13	0.289677075486036	0.171	0.25	7.59053515114197E-09	Inflammatory reactive hepatocy	YIF1A
5.05626284631489E-13	0.300551373634791	0.112	0.175	8.47328527785449E-09	Inflammatory reactive hepatocy	PPP1R9B
5.0823478191087E-13	0.295059892944708	0.221	0.313	8.51699847526236E-09	Inflammatory reactive hepatocy	FARSA
5.12761182131677E-13	0.314769073818801	0.238	0.336	8.59285189016264E-09	Inflammatory reactive hepatocy	CDC42SE1
5.55392911941158E-13	0.394822475729836	0.094	0.152	9.30727441830993E-09	Inflammatory reactive hepatocy	CBLB
6.18311375824666E-13	0.253423794704172	0.152	0.224	1.03616620360698E-08	Inflammatory reactive hepatocy	FLII
7.11322777440763E-13	0.256609843504769	0.064	0.111	1.19203471043523E-08	Inflammatory reactive hepatocy	TRAF5
7.23144753858911E-13	0.285345322910825	0.072	0.122	1.21184597851676E-08	Inflammatory reactive hepatocy	UTP25
7.980722063379E-13	0.371731063630085	0.133	0.203	1.33740940338105E-08	Inflammatory reactive hepatocy	ANKLE2
8.23945520138698E-13	0.285476818640701	0.139	0.21	1.38076790264843E-08	Inflammatory reactive hepatocy	INTS4
1.00125940805784E-12	0.313755772756568	0.155	0.23	1.67791051602333E-08	Inflammatory reactive hepatocy	PDGFA
1.01717288711987E-12	0.277346322846036	0.108	0.169	1.70457832423548E-08	Inflammatory reactive hepatocy	CSTF2T
1.04314718233906E-12	0.29259526833054	0.056	0.101	1.74810604816379E-08	Inflammatory reactive hepatocy	SRBD1
1.13810108031074E-12	0.312062837413425	0.131	0.199	1.90722979038473E-08	Inflammatory reactive hepatocy	MRTFA
1.34925772049783E-12	0.259560446336024	0.107	0.166	2.26108608801026E-08	Inflammatory reactive hepatocy	FLAD1
1.37470795223686E-12	0.329356698453607	0.159	0.234	2.30373558635853E-08	Inflammatory reactive hepatocy	LDLRAP1
1.49224887147899E-12	0.392427089583525	0.639	0.707	2.50071065882449E-08	Inflammatory reactive hepatocy	NUCB1
1.5001429330076E-12	0.273560749904095	0.091	0.146	2.51393952713414E-08	Inflammatory reactive hepatocy	NDUFS5
1.50390876006419E-12	0.310459635231365	0.156	0.23	2.52025030011558E-08	Inflammatory reactive hepatocy	FBXO31
1.64473986799831E-12	0.421314264494491	0.338	0.285	2.75625507079157E-08	Inflammatory reactive hepatocy	MST1
1.80686063988913E-12	0.311428734121534	0.098	0.155	3.0279370603262E-08	Inflammatory reactive hepatocy	CSNK1E
1.84505541875382E-12	0.329863800067068	0.14	0.209	3.09194387074766E-08	Inflammatory reactive hepatocy	SLC35C2
1.97540491377586E-12	0.445003431237811	0.202	0.291	3.31038355450559E-08	Inflammatory reactive hepatocy	TAF1D
2.04408582337234E-12	0.314011415301365	0.217	0.306	3.42547902280737E-08	Inflammatory reactive hepatocy	FAM174C
2.08716966841982E-12	0.465044260534571	0.084	0.137	3.49767893033794E-08	Inflammatory reactive hepatocy	PTGFRN
2.21969138977413E-12	0.321580054933723	0.056	0.1	3.71975883098349E-08	Inflammatory reactive hepatocy	FCSK
2.39709565514402E-12	0.335478805334449	0.233	0.325	4.01705289889035E-08	Inflammatory reactive hepatocy	CYP2C18
2.39789056173854E-12	0.880115030644631	0.313	0.279	4.01838500336145E-08	Inflammatory reactive hepatocy	NFIL3
2.86100018332749E-12	0.336492779052713	0.191	0.273	4.79446410722021E-08	Inflammatory reactive hepatocy	MPPE1
3.07358712960035E-12	0.361925309015492	0.123	0.187	5.15071731178426E-08	Inflammatory reactive hepatocy	RXRB
3.11110609972912E-12	0.813709138726649	0.424	0.425	5.21359160192606E-08	Inflammatory reactive hepatocy	TCEA3
3.16591613318725E-12	0.367252017379414	0.132	0.199	5.30544225599519E-08	Inflammatory reactive hepatocy	UBE2O
3.28212089622661E-12	0.255491468950994	0.062	0.108	5.50017819789655E-08	Inflammatory reactive hepatocy	EFNA2
3.3330985920177E-12	0.631498204134276	0.465	0.463	5.58560662050325E-08	Inflammatory reactive hepatocy	ASGR1
3.41592736475933E-12	0.316586323669194	0.211	0.297	5.72441107786369E-08	Inflammatory reactive hepatocy	PSMC4
3.49132094380027E-12	0.376566393128417	0.125	0.189	5.8507556376205E-08	Inflammatory reactive hepatocy	MLXIP

**3.64502786404864E-12**	0.405101825741604	0.097	0.154	6.1083376945727E-08	Inflammatory reactive hepatocy	STX2	
**3.69420569007399E-12**	0.318719147187386	0.138	0.204	6.19074989542599E-08	Inflammatory reactive hepatocy	TMEM115	
**4.54920869338788E-12**	0.528759651946203	0.316	0.269	7.62356392837941E-08	Inflammatory reactive hepatocy	PROC	
**4.80096276744525E-12**	0.280339179224627	0.177	0.252	8.04545340568474E-08	Inflammatory reactive hepatocy	NINJ1	
**5.66827244962899E-12**	0.265818984638798	0.104	0.162	9.49889097108825E-08	Inflammatory reactive hepatocy	KIAA0930	
**5.77741988513621E-12**	0.38833365839123	0.119	0.18	9.68180024351126E-08	Inflammatory reactive hepatocy	TMEM184B	
**5.89645172135092E-12**	0.305858384176474	0.128	0.191	9.88127379463987E-08	Inflammatory reactive hepatocy	ACP2	
**6.00995771874977E-12**	0.34030859156498	0.26	0.364	1.00714871450809E-07	Inflammatory reactive hepatocy	AGRN	
**6.23774537218166E-12**	0.305390834483254	0.075	0.123	1.0453213694702E-07	Inflammatory reactive hepatocy	MPG	
**6.42945209722471E-12**	0.35869556913727	0.131	0.197	1.07744758245292E-07	Inflammatory reactive hepatocy	CCDC130	
**6.49132328421108E-12**	0.264275850014464	0.262	0.361	1.08781595596809E-07	Inflammatory reactive hepatocy	SCAMP3	
**6.71907720647261E-12**	0.293009977792158	0.17	0.246	1.12598295826068E-07	Inflammatory reactive hepatocy	HDAC6	
**7.06374214111596E-12**	0.802714880846644	0.454	0.46	1.18374190800821E-07	Inflammatory reactive hepatocy	CEBPB	
**7.33601393892242E-12**	0.363738949604974	0.213	0.303	1.22936921588462E-07	Inflammatory reactive hepatocy	DEPRECATEI	
**7.62190877248174E-12**	0.282145516292258	0.134	0.2	1.27727947209249E-07	Inflammatory reactive hepatocy	SART3	
**8.02307273873942E-12**	0.264635347648788	0.274	0.381	1.34450652955795E-07	Inflammatory reactive hepatocy	LARP4	
**1.02887625319856E-11**	0.402433511901682	0.264	0.371	1.72419082511014E-07	Inflammatory reactive hepatocy	SPG7	
**1.08856387117882E-11**	0.316347349495857	0.224	0.312	1.82421533532147E-07	Inflammatory reactive hepatocy	KLHL21	
**1.44972744459691E-11**	1.03188903562643	0.155	0.116	2.4294532516555E-07	Inflammatory reactive hepatocy	DHODH	
**1.47508738864752E-11**	0.385484634984588	0.122	0.184	2.47195144589552E-07	Inflammatory reactive hepatocy	KDM4A	
**1.5369015181509E-11**	0.506642912257131	0.169	0.247	2.57553956411727E-07	Inflammatory reactive hepatocy	TRAF2	
**1.54523678573807E-11**	0.361516544395878	0.085	0.135	2.58950780553985E-07	Inflammatory reactive hepatocy	EIF2B4	
**1.58868979643819E-11**	0.75019470418804	0.498	0.527	2.66232636087112E-07	Inflammatory reactive hepatocy	MIF	
**1.61430988686067E-11**	0.274309475581907	0.061	0.105	2.70526050840111E-07	Inflammatory reactive hepatocy	LDB1	
**1.61461035534267E-11**	0.280669955366407	0.27	0.371	2.70576403348325E-07	Inflammatory reactive hepatocy	KIAA0100	
**1.66363293960621E-11**	0.698533544467093	0.507	0.552	2.78791608019208E-07	Inflammatory reactive hepatocy	LPGAT1	
**1.72908755724048E-11**	0.369599944531533	0.134	0.199	2.8976049284236E-07	Inflammatory reactive hepatocy	UCKL1	
**1.79260096330745E-11**	0.373432905930862	0.126	0.188	3.00404069431063E-07	Inflammatory reactive hepatocy	PSMD9	
**1.80217813811077E-11**	0.270560760731824	0.114	0.172	3.02009012384603E-07	Inflammatory reactive hepatocy	H3F3B	
**1.80965230881747E-11**	0.415309042243012	0.062	0.105	3.03261533911631E-07	Inflammatory reactive hepatocy	MAP3K1	
**1.83061121180256E-11**	0.315861025412718	0.166	0.238	3.06773826873872E-07	Inflammatory reactive hepatocy	P2RX4	
**2.03687987007199E-11**	1.16156829710149	0.111	0.077	3.41340328626663E-07	Inflammatory reactive hepatocy	PALM3	
**2.14735906204969E-11**	0.377046845742831	0.184	0.262	3.59854431618287E-07	Inflammatory reactive hepatocy	QARS	
**2.16384228615102E-11**	0.424652863834097	0.096	0.15	3.62616690313188E-07	Inflammatory reactive hepatocy	PPARD	
**2.24155849688265E-11**	0.266507565022422	0.089	0.139	3.75640372907595E-07	Inflammatory reactive hepatocy	PDPK1	
**2.24381073430423E-11**	0.287593804481165	0.063	0.107	3.76017802854702E-07	Inflammatory reactive hepatocy	CCDC174	
**2.25048936819906E-11**	1.0841374693565	0.26	0.228	3.77137008322798E-07	Inflammatory reactive hepatocy	MGLL	
**2.42015586390754E-11**	0.370302409173058	0.106	0.161	4.05569719673625E-07	Inflammatory reactive hepatocy	TPRG1L	
**2.47975567307305E-11**	0.310751903015656	0.103	0.158	4.15557455693581E-07	Inflammatory reactive hepatocy	MTG2	
**2.49204167834216E-11**	0.258658681111175	0.224	0.313	4.1761634445658E-07	Inflammatory reactive hepatocy	ZNF217	
**2.4941842783617E-11**	0.786887455068785	0.329	0.301	4.17975401367853E-07	Inflammatory reactive hepatocy	EBNA1BP2	
**2.50100893574354E-11**	0.355327488316953	0.062	0.106	4.19119077451902E-07	Inflammatory reactive hepatocy	UBIAD1	
**2.50392164361992E-11**	0.257683549343818	0.138	0.201	4.19607189037826E-07	Inflammatory reactive hepatocy	RSU1	
**2.53011918595313E-11**	0.304886312354895	0.113	0.17	4.23997373182026E-07	Inflammatory reactive hepatocy	RNF20	
**2.55747774294073E-11**	0.363130780695355	0.158	0.23	4.28582120162007E-07	Inflammatory reactive hepatocy	AMPD2	
**2.65980790637435E-11**	0.292232541119489	0.074	0.121	4.45730608950213E-07	Inflammatory reactive hepatocy	VPS35	
**2.70159441526507E-11**	0.275839445138998	0.268	0.366	4.5273319211012E-07	Inflammatory reactive hepatocy	SPATA13	
**2.97131444755337E-11**	0.384992223319465	0.213	0.3	4.97932875120994E-07	Inflammatory reactive hepatocy	SYMPK	
**3.22341517286327E-11**	0.424996238974373	0.061	0.103	5.40179914668427E-07	Inflammatory reactive hepatocy	DEPRECATEI	
**3.28707222383759E-11**	0.284914377490661	0.062	0.105	5.50847563270704E-07	Inflammatory reactive hepatocy	GPR157	
**3.53496415136743E-11**	0.325982528344244	0.245	0.335	5.92389292486154E-07	Inflammatory reactive hepatocy	PPP4C	
**3.65816641823144E-11**	0.26328230997723	0.178	0.251	6.13035528367225E-07	Inflammatory reactive hepatocy	SRRM1	
**3.79419041240736E-11**	0.345911086304208	0.198	0.281	6.35830429311225E-07	Inflammatory reactive hepatocy	STIM1	
**3.90427653014439E-11**	0.427252387882023	0.106	0.161	6.54278660921597E-07	Inflammatory reactive hepatocy	SPNS1	
**3.92559458294375E-11**	0.254638671082481	0.088	0.138	6.57851140209714E-07	Inflammatory reactive hepatocy	CHCHD5	
**3.92880434882921E-11**	0.697323457846312	0.446	0.459	6.58389032776799E-07	Inflammatory reactive hepatocy	ABAT	
**4.09401860519837E-11**	0.281930624165075	0.175	0.247	6.86075637859142E-07	Inflammatory reactive hepatocy	COPRS	
**4.27240984925285E-11**	0.320455374742495	0.185	0.26	7.15970442537792E-07	Inflammatory reactive hepatocy	SNAP29	
**4.27285701760001E-11**	0.385802755659	0.076	0.123	7.1604537900941E-07	Inflammatory reactive hepatocy	DRG2	
**4.46099261092568E-11**	0.335731830341937	0.1	0.153	7.47573141738926E-07	Inflammatory reactive hepatocy	LRRC8D	

4.98785560904845E-11	0.314725078726026	0.097	0.149	8.35864842964339E-07	Inflammatory reactive hepatocy	PLEKHM2
5.02465590353996E-11	0.880947732187235	0.384	0.38	8.42031836315227E-07	Inflammatory reactive hepatocy	CD59
5.09538721296837E-11	0.445377924277246	0.216	0.303	8.53884989149239E-07	Inflammatory reactive hepatocy	NME3
5.15773641963004E-11	0.286236401604287	0.067	0.111	8.64333469201602E-07	Inflammatory reactive hepatocy	DEPRECATEI
5.4118910185235E-11	0.422440089772302	0.195	0.275	9.06924696884169E-07	Inflammatory reactive hepatocy	MAN2B1
5.51213743914778E-11	0.342775384457646	0.113	0.169	9.23723992052385E-07	Inflammatory reactive hepatocy	UQCC3
5.90571392677714E-11	0.454429906262819	0.148	0.214	9.89679539849313E-07	Inflammatory reactive hepatocy	EXOSC7
5.95746190784995E-11	0.331454765124067	0.079	0.126	9.98351466517494E-07	Inflammatory reactive hepatocy	CBWD3
5.97069271010855E-11	0.312875164505672	0.125	0.186	1.00056868435999E-06	Inflammatory reactive hepatocy	CHKB
5.97351909948845E-11	1.18948371021806	0.135	0.098	1.00104233069227E-06	Inflammatory reactive hepatocy	ALDH1B1
5.98600846227628E-11	0.285636369129252	0.165	0.234	1.00313529810826E-06	Inflammatory reactive hepatocy	EPB41
6.02306996129728E-11	0.482394468254343	0.094	0.148	1.0093460641142E-06	Inflammatory reactive hepatocy	IQCE
6.29053724254908E-11	0.335251869772514	0.099	0.151	1.05416823110637E-06	Inflammatory reactive hepatocy	UBLCP1
6.67668559170493E-11	0.369311374043046	0.103	0.156	1.11887897145791E-06	Inflammatory reactive hepatocy	RTCA
7.23905071684373E-11	0.355369461684318	0.159	0.228	1.21312011912867E-06	Inflammatory reactive hepatocy	PHF8
7.40070336597436E-11	0.579899208081682	0.075	0.122	1.24020987006998E-06	Inflammatory reactive hepatocy	RUBCN
7.60933249987648E-11	0.546246509409435	0.125	0.187	1.2751719403293E-06	Inflammatory reactive hepatocy	DPH7
7.71175561283899E-11	0.318727546562115	0.146	0.212	1.29233600559956E-06	Inflammatory reactive hepatocy	BRD3
7.7532029600299E-11	0.30839648800135	0.219	0.303	1.29928175204181E-06	Inflammatory reactive hepatocy	DNAJC7
8.25750297082875E-11	0.253769345367444	0.184	0.256	1.38379234785148E-06	Inflammatory reactive hepatocy	NDUFA8
8.86527181813951E-11	0.374150829936273	0.131	0.194	1.48564225128382E-06	Inflammatory reactive hepatocy	COASY
8.92748623597934E-11	0.254293436928858	0.121	0.179	1.49606814342542E-06	Inflammatory reactive hepatocy	GSPT1
9.1866480790394E-11	0.418900218655137	0.08	0.127	1.53949848508542E-06	Inflammatory reactive hepatocy	WDR70
9.58530958588229E-11	0.382703364857561	0.067	0.111	1.60630618040215E-06	Inflammatory reactive hepatocy	EIF4ENIF1
1.02641863436828E-10	0.51154882888969	0.148	0.108	1.72007234747436E-06	Inflammatory reactive hepatocy	ABCA6
1.05656893439441E-10	0.403271438552918	0.153	0.219	1.77059822025816E-06	Inflammatory reactive hepatocy	UFD1
1.0757147660017E-10	0.2935730790649	0.206	0.283	1.80268280486564E-06	Inflammatory reactive hepatocy	PSENEN
1.13478024256855E-10	0.304904455383088	0.123	0.183	1.90166473049637E-06	Inflammatory reactive hepatocy	DIP2B
1.14202081477698E-10	0.308186990150116	0.277	0.38	1.91379848140327E-06	Inflammatory reactive hepatocy	AP3D1
1.14615604503881E-10	0.483103917641801	0.117	0.175	1.92072830027604E-06	Inflammatory reactive hepatocy	RBFA
1.14940800452087E-10	0.270618145556058	0.064	0.106	1.92617793397607E-06	Inflammatory reactive hepatocy	ABHD8
1.17429268082918E-10	0.322219162444854	0.114	0.17	1.96787967453355E-06	Inflammatory reactive hepatocy	FKBP4
1.20054280327871E-10	0.507048397922232	0.103	0.158	2.01186962973446E-06	Inflammatory reactive hepatocy	CORO7
1.22917340733994E-10	0.320345163603216	0.079	0.125	2.05984879602027E-06	Inflammatory reactive hepatocy	NOL4L
1.30925993454439E-10	0.355793458915741	0.06	0.101	2.1940577983095E-06	Inflammatory reactive hepatocy	SLC22A3
1.37515519316571E-10	0.338691078282629	0.263	0.359	2.3044850727071E-06	Inflammatory reactive hepatocy	BAIAP2L1
1.51266200343035E-10	0.422406285204738	0.193	0.27	2.53491898534859E-06	Inflammatory reactive hepatocy	ZSWIM8
1.5898487280268E-10	0.315919531446757	0.095	0.144	2.66426849842732E-06	Inflammatory reactive hepatocy	ALG1
1.62614848768178E-10	0.357141827771458	0.101	0.153	2.72509963565713E-06	Inflammatory reactive hepatocy	PPIL2
1.63445115051411E-10	0.384664979361514	0.253	0.35	2.73901323803155E-06	Inflammatory reactive hepatocy	UBR4
1.65251065634949E-10	0.482202585806889	0.117	0.175	2.76927735791047E-06	Inflammatory reactive hepatocy	UBA7
1.6566226705688E-10	0.814444064780641	0.216	0.177	2.7761682713392E-06	Inflammatory reactive hepatocy	ACSM5
1.66906591524514E-10	0.391637301076677	0.163	0.232	2.79702066076781E-06	Inflammatory reactive hepatocy	CSNK1G2
1.77377243267177E-10	0.263095255666816	0.196	0.27	2.97248784267136E-06	Inflammatory reactive hepatocy	TIMM17A
1.88439452186363E-10	0.351762561697083	0.069	0.112	3.15786833973907E-06	Inflammatory reactive hepatocy	TUBG2
1.92244183989881E-10	0.381970777103549	0.17	0.238	3.22162803530243E-06	Inflammatory reactive hepatocy	IER3IP1
1.92747037267014E-10	0.255719952976548	0.084	0.132	3.23005485052062E-06	Inflammatory reactive hepatocy	ING5
1.94356405563689E-10	0.290944971679459	0.08	0.126	3.2570246444363E-06	Inflammatory reactive hepatocy	ATG16L1
1.94714086609332E-10	0.568884219284676	0.071	0.115	3.26301866339918E-06	Inflammatory reactive hepatocy	RCBTB1
1.96920227807577E-10	0.402034150805719	0.086	0.134	3.29998917759938E-06	Inflammatory reactive hepatocy	IFT80
2.05270940594374E-10	0.574496013729026	0.14	0.204	3.43993042248053E-06	Inflammatory reactive hepatocy	SETD1B
2.07563395610323E-10	0.348118616184716	0.164	0.231	3.47834738363779E-06	Inflammatory reactive hepatocy	HARS
2.08387573043373E-10	0.405530035300952	0.196	0.273	3.49215894906085E-06	Inflammatory reactive hepatocy	TAP2
2.18746687796485E-10	0.308910011577331	0.088	0.136	3.6657569940935E-06	Inflammatory reactive hepatocy	IMPDH2
2.2516864624645E-10	0.250874527951734	0.112	0.165	3.77337617379801E-06	Inflammatory reactive hepatocy	C11orf68
2.25368507133678E-10	0.43552579393464	0.173	0.245	3.77672544254617E-06	Inflammatory reactive hepatocy	AP1B1
2.39505872218362E-10	1.17108022797043	0.241	0.209	4.01363940663531E-06	Inflammatory reactive hepatocy	ZC3H12A
2.41609964993802E-10	0.467564076105047	0.167	0.236	4.04889979336613E-06	Inflammatory reactive hepatocy	NDUFS8
2.43346033302777E-10	0.299901840025625	0.079	0.125	4.07799282608793E-06	Inflammatory reactive hepatocy	CNOT3
2.48060915754972E-10	0.280169990568534	0.078	0.123	4.15700482622182E-06	Inflammatory reactive hepatocy	DUSP11

**2.51762725337822E-10**	0.33577206996832	0.093	0.142	4.21903975121122E-06	Inflammatory reactive hepatocy	CASP9
**2.69182283390519E-10**	0.346676373409813	0.109	0.162	4.51095670505832E-06	Inflammatory reactive hepatocy	WTAP
**2.9116825904685E-10**	0.252294705097782	0.171	0.238	4.87939768510712E-06	Inflammatory reactive hepatocy	NSMCE1
**2.98551744222409E-10**	0.318833443091588	0.131	0.191	5.0031301297073E-06	Inflammatory reactive hepatocy	NISCH
**3.08519102683366E-10**	0.306947770535886	0.187	0.259	5.17016312276785E-06	Inflammatory reactive hepatocy	FBL
**3.08852061340241E-10**	1.29891145950365	0.133	0.1	5.17574284393976E-06	Inflammatory reactive hepatocy	CDK18
**3.4136326484437E-10**	0.410200059043566	0.09	0.138	5.72056559226196E-06	Inflammatory reactive hepatocy	LMF1
**3.55212139291368E-10**	0.723243363358656	0.46	0.485	5.95264503024475E-06	Inflammatory reactive hepatocy	PSAP
**3.71565653380832E-10**	0.491128172674769	0.083	0.13	6.22669721935598E-06	Inflammatory reactive hepatocy	NINL
**3.7370565734736E-10**	0.50674395743616	0.061	0.101	6.26255940582705E-06	Inflammatory reactive hepatocy	FBXL15
**3.74372999128843E-10**	0.359482990010885	0.065	0.107	6.27374271940115E-06	Inflammatory reactive hepatocy	ZNF276
**3.77283904556345E-10**	0.368470583373667	0.23	0.317	6.32252367255522E-06	Inflammatory reactive hepatocy	KPNA4
**3.91032628155666E-10**	0.447907307084041	0.186	0.261	6.55292478263265E-06	Inflammatory reactive hepatocy	MTG1
**3.94749877373484E-10**	0.530992482956351	0.611	0.687	6.61521844502485E-06	Inflammatory reactive hepatocy	ETFB
**3.97524303976775E-10**	0.295157054878941	0.129	0.186	6.6617122860428E-06	Inflammatory reactive hepatocy	NR1H3
**4.50532931656615E-10**	0.43991355226187	0.13	0.189	7.55003086870155E-06	Inflammatory reactive hepatocy	GTPBP1
**4.66646119365988E-10**	0.315167973417094	0.065	0.105	7.82005566833523E-06	Inflammatory reactive hepatocy	BYSL
**4.75341084355489E-10**	0.269350318507678	0.235	0.318	7.96576589162928E-06	Inflammatory reactive hepatocy	HMCES
**5.06017226002603E-10**	0.449528502885631	0.165	0.233	8.47983667335162E-06	Inflammatory reactive hepatocy	MYO1C
**5.08847445090535E-10**	1.13467777322078	0.153	0.119	8.52726548482718E-06	Inflammatory reactive hepatocy	PLEKHF1
**5.24145740610825E-10**	0.762163301190236	0.203	0.165	8.7836343211562E-06	Inflammatory reactive hepatocy	SPON2
**5.34373476996722E-10**	0.26524310833764	0.117	0.172	8.95503072751107E-06	Inflammatory reactive hepatocy	RNF169
**5.37500177775436E-10**	0.487199591130889	0.097	0.147	9.00742797916076E-06	Inflammatory reactive hepatocy	CHPF2
**6.08301962954072E-10**	0.30663161860377	0.073	0.117	1.01939242951843E-05	Inflammatory reactive hepatocy	TMCO4
**6.46801726233748E-10**	0.272869766555929	0.275	0.37	1.08391033282251E-05	Inflammatory reactive hepatocy	DPP7
**6.64514387476131E-10**	0.357778763372125	0.061	0.101	1.1135932105325E-05	Inflammatory reactive hepatocy	ACAD10
**6.67379568100394E-10**	0.367701418192392	0.146	0.209	1.11839468022264E-05	Inflammatory reactive hepatocy	ASCC3
**6.6933766784881E-10**	0.799888115302907	0.308	0.283	1.12167606378104E-05	Inflammatory reactive hepatocy	NFKBIZ
**6.69520953010234E-10**	1.37840960126423	0.18	0.148	1.12198321305455E-05	Inflammatory reactive hepatocy	SLC35C1
**6.96835667449105E-10**	0.538476701462394	0.22	0.307	1.16775721151121E-05	Inflammatory reactive hepatocy	RHOT2
**7.53427644227802E-10**	0.3183773694574	0.13	0.188	1.26259404619695E-05	Inflammatory reactive hepatocy	MAN1B1
**7.82143289008717E-10**	0.457426168904677	0.164	0.234	1.31071572372081E-05	Inflammatory reactive hepatocy	KAT2A
**7.8759053724928E-10**	0.357486911329239	0.191	0.263	1.31984422232234E-05	Inflammatory reactive hepatocy	BCL2L13
**7.9466733945323E-10**	0.683324704953463	0.085	0.133	1.33170352745572E-05	Inflammatory reactive hepatocy	CYB5RL
**8.7349357809001E-10**	0.32552789322041	0.142	0.203	1.46380053816324E-05	Inflammatory reactive hepatocy	R3HDM4
**8.90838385642392E-10**	0.310004719710548	0.162	0.227	1.49286696665952E-05	Inflammatory reactive hepatocy	SART1
**9.7136138386246E-10**	0.778179372744876	0.4	0.401	1.62780740707671E-05	Inflammatory reactive hepatocy	IFITM2
**9.8471639602055E-10**	0.349788574141307	0.111	0.163	1.65018773645124E-05	Inflammatory reactive hepatocy	TBC1D24
**1.03459187494756E-09**	0.262193875257323	0.126	0.18	1.73376906403711E-05	Inflammatory reactive hepatocy	MAP1LC3A
**1.04055940641066E-09**	0.269935685661739	0.078	0.121	1.74376945326298E-05	Inflammatory reactive hepatocy	PPT1
**1.05555985048501E-09**	0.294662717120082	0.274	0.369	1.76890719744278E-05	Inflammatory reactive hepatocy	SSR3
**1.05975104204649E-09**	0.252356885151833	0.088	0.134	1.7759307962615E-05	Inflammatory reactive hepatocy	NGLY1
**1.06729183022644E-09**	0.351223237254291	0.143	0.203	1.78856764909346E-05	Inflammatory reactive hepatocy	ACTR1A
**1.13813096005977E-09**	0.362740670231707	0.118	0.172	1.90727986286816E-05	Inflammatory reactive hepatocy	C1orf174
**1.15306068358478E-09**	1.35564885768096	0.28	0.264	1.93229909355137E-05	Inflammatory reactive hepatocy	ABCA2
**1.25447612498423E-09**	0.263384603329599	0.199	0.273	2.10225109024858E-05	Inflammatory reactive hepatocy	TRAF7
**1.27223959947721E-09**	0.394028399676433	0.071	0.112	2.13201912080391E-05	Inflammatory reactive hepatocy	THAP4
**1.32229302986354E-09**	0.486540493677586	0.081	0.126	2.21589865944531E-05	Inflammatory reactive hepatocy	POLG2
**1.36096556471995E-09**	0.374658902296069	0.171	0.239	2.28070609335769E-05	Inflammatory reactive hepatocy	CNPY3
**1.39982958999308E-09**	0.256953484659016	0.068	0.108	2.3458344269104E-05	Inflammatory reactive hepatocy	DEPRECATEI
**1.59854882736898E-09**	1.06405071750878	0.326	0.316	2.67884812490494E-05	Inflammatory reactive hepatocy	GLRX
**1.71578660992578E-09**	0.412995306625737	0.278	0.376	2.87531520091363E-05	Inflammatory reactive hepatocy	INTS11
**1.71791342535273E-09**	0.356265270920806	0.207	0.282	2.87887931820611E-05	Inflammatory reactive hepatocy	TBCB
**1.77561572627749E-09**	0.392438770206963	0.164	0.231	2.97557683409581E-05	Inflammatory reactive hepatocy	OTUD7B
**1.8293050281738E-09**	0.286674488764681	0.163	0.227	3.06554936621365E-05	Inflammatory reactive hepatocy	ATP2B4
**1.90289095245871E-09**	0.250678397757573	0.147	0.207	3.1888646581303E-05	Inflammatory reactive hepatocy	CLIC4
**1.94765749309185E-09**	0.259351777385127	0.076	0.117	3.26388442692332E-05	Inflammatory reactive hepatocy	SDHAF1
**1.9549533178086E-09**	0.320668803464025	0.117	0.17	3.27611076998364E-05	Inflammatory reactive hepatocy	MTOR
**2.17630947174222E-09**	0.600387326866998	0.124	0.18	3.64705941274561E-05	Inflammatory reactive hepatocy	E4F1
**2.27016192295528E-09**	0.277107911832474	0.285	0.379	3.80433735048846E-05	Inflammatory reactive hepatocy	RPN1

2.2833292895871E-09	0.30136689560558	0.076	0.118	3.82640322349006E-05	Inflammatory reactive hepatocy	SETD6
2.28968027963429E-09	0.345748348140399	0.222	0.302	3.83704621261115E-05	Inflammatory reactive hepatocy	FOXP1
2.38339388422885E-09	0.397135183738598	0.138	0.197	3.99409147119071E-05	Inflammatory reactive hepatocy	CRTC2
2.43680073598962E-09	0.902639208409927	0.469	0.516	4.08359067337141E-05	Inflammatory reactive hepatocy	NR1H4
2.65476993673573E-09	0.312327534041907	0.112	0.163	4.44886345998174E-05	Inflammatory reactive hepatocy	TOMM40L
2.84917086627986E-09	0.384561447094428	0.151	0.213	4.77464053771179E-05	Inflammatory reactive hepatocy	B4GALT5
2.8758566709163E-09	0.378412936230986	0.154	0.216	4.81936060912153E-05	Inflammatory reactive hepatocy	NAXD
2.91011376780796E-09	0.470852486331884	0.219	0.301	4.87676865209259E-05	Inflammatory reactive hepatocy	SEC16A
2.98041256399845E-09	0.515857803439884	0.115	0.168	4.9945753747486E-05	Inflammatory reactive hepatocy	MKNK1
3.34875229658557E-09	0.273028164683617	0.13	0.185	5.6118390986181E-05	Inflammatory reactive hepatocy	INO80B
3.51359862299647E-09	0.345299632618148	0.082	0.124	5.88808857241748E-05	Inflammatory reactive hepatocy	HRAS
3.66527110908817E-09	0.459663971633261	0.081	0.125	6.14226132460995E-05	Inflammatory reactive hepatocy	BMP2
3.82561177075707E-09	0.414839365718242	0.263	0.355	6.41096020543469E-05	Inflammatory reactive hepatocy	FAM83H
4.08193435307927E-09	0.55019176972554	0.07	0.11	6.84050558889024E-05	Inflammatory reactive hepatocy	TCN2
4.17985039377336E-09	0.519913313436673	0.095	0.142	7.00459328988539E-05	Inflammatory reactive hepatocy	WDR45
4.21412051005652E-09	0.397811129791155	0.129	0.184	7.06202315075272E-05	Inflammatory reactive hepatocy	TACC2
4.36989175898057E-09	0.467273715045616	0.09	0.135	7.32306460969964E-05	Inflammatory reactive hepatocy	DEPRECATEI
4.48226177980674E-09	1.66000316593237	0.228	0.205	7.51137429060013E-05	Inflammatory reactive hepatocy	WHRN
4.70128031512733E-09	0.250115827492408	0.324	0.426	7.87840555209038E-05	Inflammatory reactive hepatocy	ATP5MC3
4.89484846121703E-09	0.258877610071928	0.085	0.127	8.20278705130749E-05	Inflammatory reactive hepatocy	ZNRD1
4.99151733345731E-09	0.270084124487687	0.102	0.149	8.36478474740775E-05	Inflammatory reactive hepatocy	NECAP2
5.40060752963807E-09	0.516501685794457	0.083	0.126	9.05033809816748E-05	Inflammatory reactive hepatocy	POLR1A
5.51590303599186E-09	0.80657988367553	0.086	0.132	9.24355030771516E-05	Inflammatory reactive hepatocy	ADAMTS10
5.57214350046153E-09	0.409261977274185	0.211	0.288	9.33779807807343E-05	Inflammatory reactive hepatocy	KIAA1671
6.08406587020403E-09	0.376271674426305	0.225	0.303	0.000101956775852879	Inflammatory reactive hepatocy	TMX2
6.3872797738194E-09	0.336167043966815	0.201	0.275	0.000107038034449665	Inflammatory reactive hepatocy	RNF40
6.43060604120365E-09	0.500301581233497	0.077	0.12	0.000107764096038491	Inflammatory reactive hepatocy	TBC1D22A
6.72850219781754E-09	0.54623448882759	0.115	0.167	0.000112756239831026	Inflammatory reactive hepatocy	MIB2
6.74882972718834E-09	0.257262409000904	0.324	0.428	0.000113096888568222	Inflammatory reactive hepatocy	NCSTN
6.76266880140559E-09	0.259005396363433	0.137	0.193	0.000113328803773955	Inflammatory reactive hepatocy	MOGAT3
6.78554303869766E-09	0.560123868177281	0.084	0.127	0.000113712130242495	Inflammatory reactive hepatocy	MOB2
6.93369187032874E-09	0.806073541375735	0.419	0.435	0.000116194808362969	Inflammatory reactive hepatocy	FNDC3B
7.02948060432368E-09	0.280707507288199	0.117	0.166	0.000117800035967256	Inflammatory reactive hepatocy	ATG13
7.05137275528519E-09	0.839422140179218	0.481	0.521	0.000118166904633069	Inflammatory reactive hepatocy	TP53INP1
7.44890818355224E-09	0.348861011671864	0.098	0.145	0.000124828803339968	Inflammatory reactive hepatocy	C1orf52
7.55399019095137E-09	1.10734039799065	0.136	0.105	0.000126589767619963	Inflammatory reactive hepatocy	IER3
7.6272205922928E-09	0.898952807053963	0.307	0.287	0.000127816962685643	Inflammatory reactive hepatocy	COMT
7.70371502411764E-09	0.959674480081567	0.148	0.115	0.000129098856374163	Inflammatory reactive hepatocy	LDHD
7.71025579816327E-09	0.311609075040836	0.109	0.157	0.00012920846666562	Inflammatory reactive hepatocy	PSMD6
7.85907460569388E-09	0.58629526546879	0.089	0.134	0.000131702372242218	Inflammatory reactive hepatocy	HNF1B
7.9890566342482E-09	0.483476376862672	0.178	0.247	0.000133880611076731	Inflammatory reactive hepatocy	GON4L
8.18596057852836E-09	0.506907126088898	0.119	0.172	0.000137180327374978	Inflammatory reactive hepatocy	WDR6
8.26907598205797E-09	0.451026509656911	0.114	0.165	0.000138573175307327	Inflammatory reactive hepatocy	TRMU
8.63733256031225E-09	0.399682213211219	0.088	0.131	0.000144744419045713	Inflammatory reactive hepatocy	GRWD1
8.75834939965607E-09	0.256485046378482	0.067	0.105	0.000146772419239436	Inflammatory reactive hepatocy	TDRKH
9.05489750768361E-09	0.31679602836632	0.066	0.104	0.000151741972433762	Inflammatory reactive hepatocy	MEMO1
9.09310424991179E-09	0.370976499959422	0.218	0.292	0.000152382241020022	Inflammatory reactive hepatocy	CCDC124
1.00482035833954E-08	0.284759740498912	0.068	0.106	0.00016838779565054	Inflammatory reactive hepatocy	ITGAM
1.01092442547873E-08	0.252175988640308	0.296	0.394	0.000169410715221726	Inflammatory reactive hepatocy	UFC1
1.08860452357225E-08	0.420020171177467	0.103	0.15	0.000182428346060238	Inflammatory reactive hepatocy	EIF2B3
1.09981725186686E-08	0.289379700402176	0.169	0.232	0.000184307375067849	Inflammatory reactive hepatocy	ULK1
1.11464953902858E-08	0.352404946085382	0.128	0.18	0.00018679296975041	Inflammatory reactive hepatocy	IFIH1
1.14842420260508E-08	0.342231352011117	0.069	0.107	0.000192452927872559	Inflammatory reactive hepatocy	FANCF
1.19611807639622E-08	0.319786217257165	0.171	0.233	0.000200445467242478	Inflammatory reactive hepatocy	PRPF31
1.21377698753565E-08	0.316942956174681	0.104	0.15	0.000203404747571224	Inflammatory reactive hepatocy	PPME1
1.33489137855001E-08	0.421907547886756	0.069	0.107	0.00022370109721741	Inflammatory reactive hepatocy	NAT10
1.33949393209907E-08	0.381143396498871	0.093	0.137	0.000224472393141162	Inflammatory reactive hepatocy	L3MBTL4
1.39530901452672E-08	0.520471631452522	0.107	0.156	0.000233825884654388	Inflammatory reactive hepatocy	EXOSC10
1.40784599465307E-08	0.373248596035848	0.078	0.118	0.000235926831783961	Inflammatory reactive hepatocy	CWC25
1.47000081463123E-08	0.294089439074079	0.201	0.268	0.000246342736515902	Inflammatory reactive hepatocy	PARK7

1.49192474956619E-08	0.515558035315192	0.123	0.175	0.000250016749532301	Inflammatory reactive hepatocy	GRAMD1A
1.50011510432524E-08	0.406707777014528	0.121	0.172	0.000251389289182824	Inflammatory reactive hepatocy	GBF1
1.50988666308298E-08	0.362227260530831	0.226	0.301	0.000253026806999446	Inflammatory reactive hepatocy	NME1
1.7382539361295E-08	0.29179307193366	0.082	0.123	0.000291296594616582	Inflammatory reactive hepatocy	CDK11B
1.73946627691358E-08	0.419324421758482	0.091	0.134	0.000291499758685178	Inflammatory reactive hepatocy	EZH1
1.80965984194747E-08	0.347034637819537	0.152	0.209	0.000303262796313557	Inflammatory reactive hepatocy	APOL3
1.88860532847308E-08	0.520681361712801	0.112	0.161	0.000316492480945519	Inflammatory reactive hepatocy	TPCN1
1.91241287730927E-08	0.294165217919255	0.131	0.182	0.000320482149979487	Inflammatory reactive hepatocy	ESF1
1.91450061135672E-08	0.441487468018595	0.089	0.132	0.000320832012451159	Inflammatory reactive hepatocy	VPS53
1.96765227617124E-08	0.342485675023529	0.249	0.33	0.000329739168440776	Inflammatory reactive hepatocy	DCTN2
1.98072317451224E-08	0.495970451902535	0.183	0.251	0.000331929589584761	Inflammatory reactive hepatocy	GCN1
2.12506967775936E-08	0.407159649466523	0.085	0.126	0.000356119176598914	Inflammatory reactive hepatocy	IDE
2.14287412394557E-08	1.39285612850824	0.142	0.113	0.000359102845690798	Inflammatory reactive hepatocy	RELB
2.24308100435797E-08	0.325748429947778	0.071	0.109	0.000375895514710308	Inflammatory reactive hepatocy	TSR1
2.24561221769262E-08	0.459679780193587	0.065	0.101	0.00037631969544093	Inflammatory reactive hepatocy	EIPR1
2.29113342540105E-08	0.373351035701252	0.195	0.266	0.000383948139428707	Inflammatory reactive hepatocy	SMAD5
2.3462579961244E-08	0.252279982445082	0.08	0.119	0.000393185914990528	Inflammatory reactive hepatocy	DBT
2.35075172438536E-08	0.397119985449186	0.123	0.174	0.000393938973972499	Inflammatory reactive hepatocy	WDFY1
2.36509837905993E-08	0.286824800720457	0.284	0.373	0.000396343186362864	Inflammatory reactive hepatocy	NCL
2.45676258463448E-08	0.537533816261627	0.122	0.174	0.000411704273933045	Inflammatory reactive hepatocy	GTF3C1
2.47124668060071E-08	0.275031100866076	0.097	0.141	0.000414131518735068	Inflammatory reactive hepatocy	XDH
2.53181799079854E-08	0.335312939219687	0.16	0.22	0.000424282058898019	Inflammatory reactive hepatocy	MEF2D
2.54084844162403E-08	0.502260237330202	0.17	0.235	0.000425795381847355	Inflammatory reactive hepatocy	PHRF1
2.57662080257804E-08	0.281124620520491	0.697	0.778	0.000431790114096028	Inflammatory reactive hepatocy	ST6GAL1
2.70835013945392E-08	0.279573591693552	0.157	0.214	0.000453865316369688	Inflammatory reactive hepatocy	IFRD2
2.86408873488758E-08	0.419228404173378	0.21	0.283	0.000479963990192461	Inflammatory reactive hepatocy	ANKRD11
2.87795409995544E-08	0.511355650297843	0.078	0.117	0.000482287548070532	Inflammatory reactive hepatocy	NOMO1
2.91171825197558E-08	0.395012943852938	0.16	0.218	0.000487945744666067	Inflammatory reactive hepatocy	PCSK9
3.18149554411242E-08	0.300587269491849	0.139	0.192	0.00053315502328236	Inflammatory reactive hepatocy	C11orf24
3.23829832736497E-08	0.630210729659311	0.152	0.212	0.000542674033699822	Inflammatory reactive hepatocy	DAPK3
3.30003985511966E-08	0.502695211419553	0.132	0.185	0.000553020678920952	Inflammatory reactive hepatocy	ARIH2
3.39235641304794E-08	0.687582913737065	0.459	0.489	0.000568491087698573	Inflammatory reactive hepatocy	ITM2B
3.51899470524943E-08	0.380492475287916	0.065	0.101	0.000589713132705699	Inflammatory reactive hepatocy	PDE12
3.53064539304145E-08	0.405037808668306	0.196	0.264	0.000591665554965887	Inflammatory reactive hepatocy	DUOX2
3.62099025432212E-08	0.356289298733332	0.123	0.173	0.0006068055468193	Inflammatory reactive hepatocy	JMJD8
3.75032653726826E-08	0.304815328100673	0.071	0.108	0.000628479721115416	Inflammatory reactive hepatocy	EYA3
3.78224930501764E-08	0.481408485521931	0.129	0.181	0.000633829338534857	Inflammatory reactive hepatocy	PANK4
4.0302119370832E-08	0.435599857098696	0.142	0.196	0.000675382916416403	Inflammatory reactive hepatocy	CAP2
4.37887051247193E-08	0.287836012156618	0.248	0.329	0.000733811120480045	Inflammatory reactive hepatocy	NSFL1C
4.52829518883477E-08	0.422939688708037	0.075	0.112	0.000758851707744932	Inflammatory reactive hepatocy	PSMB7
4.59249399681536E-08	0.322020507352256	0.115	0.162	0.000769610143986318	Inflammatory reactive hepatocy	CDS2
4.76275041927085E-08	0.434367569228028	0.164	0.224	0.000798141715261408	Inflammatory reactive hepatocy	DDX54
4.76992403715517E-08	0.330001170925248	0.077	0.116	0.000799343870146464	Inflammatory reactive hepatocy	ZCCHC7
4.90633140579285E-08	0.565807944920676	0.142	0.198	0.000822203016982766	Inflammatory reactive hepatocy	RANBP10
5.06784023668127E-08	0.625388413944771	0.478	0.52	0.000849268666863047	Inflammatory reactive hepatocy	NDUFS2
5.1220397953368E-08	0.417637190014605	0.151	0.206	0.000858351428902541	Inflammatory reactive hepatocy	SF3B2
5.18841370848008E-08	0.39050909186552	0.093	0.135	0.000869474369267092	Inflammatory reactive hepatocy	WASHC2A
5.3756709714452E-08	0.434167244876292	0.202	0.273	0.000900854941394787	Inflammatory reactive hepatocy	TXNRD2
5.53677572043472E-08	0.280601627531284	0.128	0.176	0.00092785287523045	Inflammatory reactive hepatocy	PARL
5.8467555878077E-08	0.264478992958132	0.105	0.149	0.000979799301404814	Inflammatory reactive hepatocy	MCRIP1
6.02898285780618E-08	0.396299551405194	0.1	0.143	0.00101033694731116	Inflammatory reactive hepatocy	EIF3I
6.09800638255454E-08	0.384625677228766	0.213	0.285	0.00102190390958849	Inflammatory reactive hepatocy	STX5
6.11222660727762E-08	0.322815111737843	0.066	0.101	0.00102428693484758	Inflammatory reactive hepatocy	MARCH2
6.21346831471882E-08	0.366022324874918	0.085	0.125	0.00104125302018058	Inflammatory reactive hepatocy	TRUB2
6.37987925596587E-08	0.376013147529266	0.263	0.348	0.00106914016571476	Inflammatory reactive hepatocy	FTCD
6.41633357384201E-08	0.399633735300454	0.111	0.157	0.00107524918030444	Inflammatory reactive hepatocy	WDR59
6.45432561693695E-08	0.294994874652483	0.216	0.288	0.00108161588688629	Inflammatory reactive hepatocy	IFRD1
6.48268583124625E-08	0.897195629003665	0.44	0.463	0.00108636849160025	Inflammatory reactive hepatocy	RNF213
6.54772681799457E-08	0.277144441778648	0.222	0.294	0.00109726806015953	Inflammatory reactive hepatocy	STX4
6.65049272974443E-08	0.607873295110634	0.071	0.108	0.00111448957165057	Inflammatory reactive hepatocy	LZTS2

6.68001166332344E-08	0.458557753393898	0.166	0.225	0.00111943635453974	Inflammatory reactive hepatocy	TAGLN2
6.89475260327653E-08	0.338283961956715	0.246	0.328	0.00115542264125708	Inflammatory reactive hepatocy	CYB5R1
6.9058661547352E-08	0.507688738274896	0.068	0.104	0.00115728505021052	Inflammatory reactive hepatocy	LPIN3
7.0099597390738E-08	0.401363519283222	0.083	0.122	0.00117472905307399	Inflammatory reactive hepatocy	HYAL2
7.19620481961545E-08	0.414249334810786	0.127	0.177	0.00120594000367116	Inflammatory reactive hepatocy	KIF21A
7.29379789785583E-08	0.465926791849302	0.119	0.167	0.00122229465172268	Inflammatory reactive hepatocy	PBXIP1
7.76429923357138E-08	0.363933903031691	0.103	0.147	0.00130114126556189	Inflammatory reactive hepatocy	SHMT2
7.88831780275738E-08	0.357001800163515	0.123	0.172	0.00132192429738608	Inflammatory reactive hepatocy	TFIP11
8.06366478283365E-08	1.20605845708365	0.2	0.173	0.00135130894430726	Inflammatory reactive hepatocy	TWF2
8.12964201267384E-08	0.38122510546146	0.134	0.185	0.00136236540848388	Inflammatory reactive hepatocy	SS18
8.48532526310751E-08	0.309525542350254	0.112	0.157	0.00142197080759156	Inflammatory reactive hepatocy	SF3B1
8.55495332502497E-08	0.313183493342791	0.08	0.119	0.00143363907820768	Inflammatory reactive hepatocy	NOP2
8.99145803281354E-08	0.347314675094373	0.133	0.184	0.00150678853713889	Inflammatory reactive hepatocy	SIRPA
9.20921611059062E-08	0.57764021462493	0.135	0.189	0.00154328043581278	Inflammatory reactive hepatocy	TRIM41
9.83348525235646E-08	0.335115720438929	0.145	0.198	0.0016478954585899	Inflammatory reactive hepatocy	RANGAP1
1.00920389274833E-07	0.40576652690937	0.093	0.135	0.00169122388346765	Inflammatory reactive hepatocy	DHX38
1.01967521025618E-07	0.397573077878094	0.187	0.254	0.00170877171734731	Inflammatory reactive hepatocy	INO80E
1.06685639626076E-07	0.277672655174369	0.187	0.247	0.00178783794885378	Inflammatory reactive hepatocy	PPDPF
1.08151227521374E-07	0.348216355837565	0.281	0.368	0.00181239827080319	Inflammatory reactive hepatocy	SLC39A7
1.0937708262508E-07	0.467650021370392	0.192	0.259	0.00183294115063109	Inflammatory reactive hepatocy	ITGAV
1.12206396028492E-07	0.450801039548562	0.21	0.282	0.00188035478464547	Inflammatory reactive hepatocy	IP6K2
1.1512127860944E-07	0.276923869546685	0.147	0.2	0.001929202386937	Inflammatory reactive hepatocy	EIF4B
1.24646124964104E-07	0.493730281569291	0.066	0.101	0.00208881976214846	Inflammatory reactive hepatocy	PSEN2
1.28672399356779E-07	0.348691720845165	0.134	0.184	0.0021562920684209	Inflammatory reactive hepatocy	UCK1
1.29566562306697E-07	1.35614907865576	0.137	0.11	0.00217127645113562	Inflammatory reactive hepatocy	NUAK1
1.36907732671337E-07	0.383627527484972	0.153	0.208	0.00229429978410626	Inflammatory reactive hepatocy	HDAC5
1.37353316167085E-07	0.641894537257324	0.397	0.396	0.00230176687232801	Inflammatory reactive hepatocy	CYP27A1
1.40578560196911E-07	0.28058317936301	0.344	0.444	0.00235581551177984	Inflammatory reactive hepatocy	VPS28
1.41462250959851E-07	0.715577954742414	0.085	0.126	0.00237062440158519	Inflammatory reactive hepatocy	WDR55
1.44580520669829E-07	0.487756502894926	0.179	0.24	0.00242288036538499	Inflammatory reactive hepatocy	MVP
1.53583281671153E-07	0.387295712475576	0.166	0.223	0.00257374863424518	Inflammatory reactive hepatocy	OTUB1
1.86058443714966E-07	0.373007067811359	0.192	0.253	0.0031179673997754	Inflammatory reactive hepatocy	NME4
1.94718044605917E-07	0.268357319354648	0.368	0.473	0.00326308499150596	Inflammatory reactive hepatocy	DDRGK1
1.9562553266871E-07	0.265775691541021	0.097	0.138	0.00327829267646225	Inflammatory reactive hepatocy	DAPK1
1.95713894623516E-07	0.388387117681024	0.161	0.217	0.00327977344610088	Inflammatory reactive hepatocy	NTMT1
2.00607465165699E-07	0.28676112185075	0.081	0.118	0.00336177990124679	Inflammatory reactive hepatocy	TRIM16
2.08721597924418E-07	0.575192779742373	0.497	0.533	0.00349775653801739	Inflammatory reactive hepatocy	GAMT
2.11413441868415E-07	0.432201338293217	0.067	0.101	0.00354286645883089	Inflammatory reactive hepatocy	PTRH1
2.18284815359908E-07	0.632539821590385	0.07	0.105	0.00365801693580133	Inflammatory reactive hepatocy	CELSR1
2.19206138572082E-07	0.426699194873418	0.152	0.207	0.00367345647019095	Inflammatory reactive hepatocy	DAG1
2.20251590730057E-07	0.287588357313002	0.08	0.117	0.0036909761574543	Inflammatory reactive hepatocy	TEN1
2.39346331986282E-07	0.875363846141624	0.373	0.379	0.00401096583142611	Inflammatory reactive hepatocy	SELENOW
2.42861675847221E-07	1.1444122375642	0.106	0.08	0.00406987596384773	Inflammatory reactive hepatocy	BCAM
2.71160934429131E-07	0.332044103023085	0.071	0.105	0.00454411493916337	Inflammatory reactive hepatocy	LSM 10.00
2.76662697882726E-07	0.720159593580012	0.436	0.469	0.00463631349111872	Inflammatory reactive hepatocy	CALR
2.87315760906508E-07	0.577197127313594	0.09	0.13	0.00481483752127126	Inflammatory reactive hepatocy	BRD9
2.88707841017199E-07	0.276523037915325	0.291	0.38	0.00483816599976622	Inflammatory reactive hepatocy	TTC39C
2.91263192797753E-07	0.487382614544176	0.111	0.156	0.00488098858490475	Inflammatory reactive hepatocy	U2AF1L4
3.29163030803306E-07	0.698491275393732	0.094	0.135	0.00551611407020179	Inflammatory reactive hepatocy	PCNX3
3.38042368796277E-07	0.357091722525153	0.278	0.36	0.00566491401628801	Inflammatory reactive hepatocy	SIGMAR1
3.42917500265318E-07	0.461448913982151	0.149	0.201	0.0057466114694462	Inflammatory reactive hepatocy	TMEM8A
3.71474061345228E-07	0.520235905239447	0.08	0.117	0.00622516232002333	Inflammatory reactive hepatocy	SLC25A45
3.8492208041375E-07	0.393215693685783	0.232	0.304	0.00645052422357363	Inflammatory reactive hepatocy	SPEN
3.85905814068318E-07	0.424168163335866	0.196	0.261	0.00646700963215687	Inflammatory reactive hepatocy	SEC63
3.87827983608696E-07	0.4200104966449	0.078	0.114	0.00649922134931453	Inflammatory reactive hepatocy	THOC5
3.88564960812606E-07	0.537778783220232	0.068	0.102	0.00651157161329765	Inflammatory reactive hepatocy	POLRMT
3.9317244230768E-07	0.443582630457861	0.532	0.588	0.00658878378819211	Inflammatory reactive hepatocy	F11R
4.10441509923067E-07	0.405029253446	0.3	0.394	0.00687817882329075	Inflammatory reactive hepatocy	C16orf58
4.19018694587618E-07	0.387081810991657	0.069	0.103	0.0070219152838993	Inflammatory reactive hepatocy	NOC4L
4.5067989979714E-07	0.471513942519603	0.164	0.218	0.00755249376080048	Inflammatory reactive hepatocy	MSRB1

4.55465954421793E-07	0.396624711498841	0.173	0.231	0.00763269846420041	Inflammatory reactive hepatocy	ORMDL2
4.92734435753381E-07	0.281845899197798	0.283	0.367	0.00825724367435516	Inflammatory reactive hepatocy	ERBB3
5.0976242241728E-07	0.579569273923806	0.073	0.107	0.00854259867486877	Inflammatory reactive hepatocy	RSL1D1
5.10091567694504E-07	0.347119863500107	0.204	0.269	0.0085481144914245	Inflammatory reactive hepatocy	LRRC41
5.10204820591163E-07	0.380002666584148	0.086	0.122	0.0085500123834667	Inflammatory reactive hepatocy	PLXND1
5.18381673723222E-07	0.274899957838353	0.142	0.19	0.00868704008825376	Inflammatory reactive hepatocy	CREB3
5.58645016750214E-07	0.365348781213363	0.414	0.527	0.00936177319070009	Inflammatory reactive hepatocy	CDK5RAP3
5.70583796662849E-07	0.464126707178956	0.08	0.116	0.00956184326447602	Inflammatory reactive hepatocy	PHF23
5.90018482173032E-07	0.478424150456442	0.229	0.303	0.00988752972425566	Inflammatory reactive hepatocy	SUPT6H
5.98885978322923E-07	1.12194180522834	0.33	0.334	0.0100361312247355	Inflammatory reactive hepatocy	IL17RB
6.05308713145657E-07	0.316145307153838	0.182	0.24	0.0101437634148949	Inflammatory reactive hepatocy	APOL1
6.21702671012212E-07	0.319613839535577	0.302	0.39	0.0104184933608227	Inflammatory reactive hepatocy	ANXA11
6.3138541735267E-07	0.395127042061668	0.148	0.2	0.0105807568239961	Inflammatory reactive hepatocy	CTDSP2
6.74402753891706E-07	0.256613719901009	0.173	0.228	0.0113016413497172	Inflammatory reactive hepatocy	IBTK
7.03271224809534E-07	0.305381425200728	0.178	0.235	0.0117854191853582	Inflammatory reactive hepatocy	GSDMB
7.26069754441075E-07	0.558817807054052	0.087	0.124	0.0121674769449235	Inflammatory reactive hepatocy	AXIN1
7.49084466001242E-07	0.42673288303783	0.175	0.233	0.0125531574812488	Inflammatory reactive hepatocy	AUP1
8.12820859877473E-07	0.41580483951741	0.192	0.25	0.0136212519698267	Inflammatory reactive hepatocy	PPP1R11
8.36272570719943E-07	0.346227334469591	0.263	0.341	0.0140142557401248	Inflammatory reactive hepatocy	MSN
8.57344822989054E-07	0.311259613855974	0.265	0.343	0.0143673845436506	Inflammatory reactive hepatocy	MFSD1
8.66594732147322E-07	0.538679875776669	0.179	0.237	0.0145223945213248	Inflammatory reactive hepatocy	GMPPA
8.71017056787648E-07	0.477920306446168	0.15	0.203	0.0145965038376474	Inflammatory reactive hepatocy	BAZ2A
9.08040109039418E-07	1.1798432977572	0.168	0.143	0.0152169361472826	Inflammatory reactive hepatocy	GPATCH4
9.72407594274592E-07	0.491937749789121	0.15	0.202	0.0162956064648536	Inflammatory reactive hepatocy	PMPCA
1.00922804367485E-06	0.308098443860122	0.087	0.122	0.0169126435559032	Inflammatory reactive hepatocy	ATG101
1.02212761797164E-06	0.297318237237196	0.299	0.392	0.0171288146219687	Inflammatory reactive hepatocy	MIA3
1.02736666373961E-06	0.409566774555024	0.258	0.336	0.0172166105509484	Inflammatory reactive hepatocy	PMM1
1.05184452588689E-06	0.38312225611319	0.1	0.139	0.0176268105648125	Inflammatory reactive hepatocy	APTX
1.06231965149383E-06	0.486384652089578	0.113	0.156	0.0178023527197337	Inflammatory reactive hepatocy	ZNRD2
1.10333535023418E-06	0.555769011183624	0.206	0.273	0.0184896937992245	Inflammatory reactive hepatocy	ARHGEF7
1.14394520992947E-06	0.517698036433075	0.091	0.129	0.019170233827998	Inflammatory reactive hepatocy	AP5Z1
1.15627645051702E-06	0.425668931248807	0.191	0.253	0.0193768807577641	Inflammatory reactive hepatocy	ITPA
1.17523556973551E-06	0.371641029620935	0.315	0.409	0.0196945976776276	Inflammatory reactive hepatocy	HSF1
1.20423933383238E-06	0.453222307721665	0.204	0.268	0.0201806427563631	Inflammatory reactive hepatocy	ZFP36L2
1.21152545694464E-06	1.62672199050865	0.169	0.147	0.0203027436074782	Inflammatory reactive hepatocy	ARHGEF16
1.21196963202724E-06	0.635480377303802	0.1	0.14	0.0203101870935124	Inflammatory reactive hepatocy	GLG1
1.22571600652588E-06	0.527478788441605	0.11	0.151	0.0205405488373606	Inflammatory reactive hepatocy	RNASEH1
1.24405642767509E-06	0.418488502071356	0.075	0.108	0.0208478976149791	Inflammatory reactive hepatocy	MPHOSPH6
1.24571958180378E-06	0.297087260095751	0.08	0.114	0.0208757687518677	Inflammatory reactive hepatocy	TYW3
1.26456419293427E-06	0.469775138364527	0.122	0.166	0.0211915667451925	Inflammatory reactive hepatocy	COPZ1
1.26871092167872E-06	0.466047657713623	0.185	0.245	0.021261057625492	Inflammatory reactive hepatocy	MCRS1
1.2719542246689E-06	0.388799490423779	0.306	0.396	0.0213154088970013	Inflammatory reactive hepatocy	NARS
1.32403235966924E-06	0.273917885565076	0.073	0.106	0.0221881342833371	Inflammatory reactive hepatocy	NAPG
1.35215214717633E-06	0.488940997197899	0.227	0.3	0.0226593656823809	Inflammatory reactive hepatocy	NADK
1.38265565132396E-06	0.472256219979721	0.08	0.115	0.0231705434048869	Inflammatory reactive hepatocy	PLA2G4B
1.40872827934114E-06	0.386053751401117	0.078	0.112	0.0236074685051988	Inflammatory reactive hepatocy	NCBP2AS2
1.45182881095281E-06	1.18649848314695	0.263	0.252	0.0243297472139472	Inflammatory reactive hepatocy	ESRP2
1.51882060999314E-06	0.411614204979779	0.173	0.228	0.0254523957822651	Inflammatory reactive hepatocy	YIF1B
1.54128891910633E-06	0.442596756804378	0.161	0.215	0.0258289197063839	Inflammatory reactive hepatocy	PDCD5
1.61012072231986E-06	0.515403104427271	0.123	0.168	0.0269824030646362	Inflammatory reactive hepatocy	SIRT7
1.64582676067383E-06	0.450954902755039	0.08	0.115	0.027580764855372	Inflammatory reactive hepatocy	PLPPR2
1.65966896648152E-06	0.280887541908901	0.079	0.113	0.0278127325402973	Inflammatory reactive hepatocy	ANAPC15
1.73205594385712E-06	0.593581219862746	0.144	0.195	0.0290257935071577	Inflammatory reactive hepatocy	PUS1
1.81725719510937E-06	0.913569269108056	0.398	0.419	0.0304535960756428	Inflammatory reactive hepatocy	MYH14
1.84433693608527E-06	0.35920572541761	0.22	0.285	0.030907398374917	Inflammatory reactive hepatocy	GBA
1.88478833421248E-06	0.593362530202901	0.336	0.325	0.0315852829047327	Inflammatory reactive hepatocy	ORMDL3
1.9445313048464E-06	0.387037322734866	0.118	0.16	0.032586455606616	Inflammatory reactive hepatocy	YPEL2
1.97355656763588E-06	0.315800817706233	0.234	0.302	0.0330728609604421	Inflammatory reactive hepatocy	SLC35B1
2.00480940541209E-06	0.563441244643999	0.111	0.154	0.0335965960158959	Inflammatory reactive hepatocy	CARD10
2.01153463660306E-06	0.496466344591943	0.134	0.179	0.0337092974401941	Inflammatory reactive hepatocy	RELA

2.01327135861753E-06	0.348646784530825	0.282	0.362	0.0337384014277126	Inflammatory reactive hepatocy	DEPRECATEI
2.01569752395836E-06	0.449513444438779	0.131	0.178	0.0337790591064943	Inflammatory reactive hepatocy	DVL1
2.04384341175778E-06	0.359204626364827	0.266	0.344	0.0342507278942369	Inflammatory reactive hepatocy	IQSEC1
2.04783768790866E-06	0.877073509508632	0.364	0.372	0.0343176639739734	Inflammatory reactive hepatocy	TNIP1
2.05206612229659E-06	0.470766119314449	0.191	0.253	0.0343885240774462	Inflammatory reactive hepatocy	WDR13
2.09508248562431E-06	0.573022356399205	0.209	0.275	0.0351093922940922	Inflammatory reactive hepatocy	HECTD3
2.16786647287534E-06	0.380375340335352	0.192	0.249	0.036329106352445	Inflammatory reactive hepatocy	SHISA5
2.17190827777025E-06	0.724016545980978	0.408	0.426	0.0363968389188739	Inflammatory reactive hepatocy	GRHPR
2.17934208694605E-06	0.559039789134531	0.084	0.118	0.0365214146930419	Inflammatory reactive hepatocy	SRFBP1
2.21601690808884E-06	0.429704268809818	0.081	0.115	0.0371360113457528	Inflammatory reactive hepatocy	ARID5B
2.38578046777526E-06	0.358100581648684	0.297	0.378	0.0399809090789779	Inflammatory reactive hepatocy	RHOA
2.44535471114962E-06	0.488080879451637	0.077	0.111	0.0409792542494453	Inflammatory reactive hepatocy	RALGDS
2.5575537465116E-06	0.926696245419676	0.219	0.198	0.0428594856840415	Inflammatory reactive hepatocy	PLXNB1
2.62733399081794E-06	1.15969182260346	0.205	0.186	0.044028863018127	Inflammatory reactive hepatocy	TSHZ2
2.68329498960559E-06	0.566474622687168	0.117	0.16	0.0449666574358104	Inflammatory reactive hepatocy	PIEZO1
2.68949974215589E-06	0.551852969591623	0.119	0.162	0.0450706366790485	Inflammatory reactive hepatocy	MCOLN1
2.75748413307919E-06	0.38860638518503	0.121	0.163	0.0462099191021411	Inflammatory reactive hepatocy	MSANTD3
2.91784776465914E-06	0.304473121501015	0.109	0.147	0.0488972928401579	Inflammatory reactive hepatocy	TAB3
2.96017930994472E-06	0.502375772619435	0.1	0.138	0.0496066848760536	Inflammatory reactive hepatocy	SF3A1
0	4.68939292876638	0.759	0.097	0	Proliferating hepatocytes	TOP2A
0	5.14671746985146	0.791	0.134	0	Proliferating hepatocytes	HIST1H1B
0	4.65586539911987	0.716	0.069	0	Proliferating hepatocytes	TPX2
0	4.62573845322375	0.74	0.106	0	Proliferating hepatocytes	CENPF
0	4.91791565235257	0.662	0.043	0	Proliferating hepatocytes	KIFC1
0	5.22496413662715	0.655	0.037	0	Proliferating hepatocytes	ANLN
0	5.20487674673692	0.645	0.033	0	Proliferating hepatocytes	TACC3
0	3.93743514965039	0.741	0.134	0	Proliferating hepatocytes	NUSAP1
0	4.56811200727453	0.676	0.074	0	Proliferating hepatocytes	PTTG1
0	4.60654776695176	0.651	0.052	0	Proliferating hepatocytes	SPC24
0	4.58771593373247	0.663	0.066	0	Proliferating hepatocytes	MKI67
0	3.24330956648692	0.786	0.215	0	Proliferating hepatocytes	HMGB2
0	4.57661043643351	0.632	0.063	0	Proliferating hepatocytes	HMMR
0	4.70655243207582	0.652	0.084	0	Proliferating hepatocytes	CENPI
0	4.2286958457815	0.648	0.082	0	Proliferating hepatocytes	CDK1
0	4.4201132387733	0.603	0.047	0	Proliferating hepatocytes	BIRC5
0	3.97662277374648	0.631	0.081	0	Proliferating hepatocytes	ECT2
0	3.60582737243136	0.63	0.081	0	Proliferating hepatocytes	RRM2
0	3.69254709582105	0.845	0.297	0	Proliferating hepatocytes	HIST1H1C
0	4.42705623519357	0.589	0.051	0	Proliferating hepatocytes	NCAPG
0	3.47605551435337	0.66	0.127	0	Proliferating hepatocytes	NEURL1B
0	2.63446215277264	0.785	0.255	0	Proliferating hepatocytes	LMNB1
0	3.47910716058661	0.628	0.106	0	Proliferating hepatocytes	KIF22
0	4.29432539178216	0.562	0.043	0	Proliferating hepatocytes	FOXM1
0	4.80188552257356	0.552	0.034	0	Proliferating hepatocytes	IQGAP3
0	4.27734503355044	0.565	0.047	0	Proliferating hepatocytes	PRC1
0	5.20675142528965	0.539	0.022	0	Proliferating hepatocytes	BUB1
0	3.61447606421463	0.586	0.073	0	Proliferating hepatocytes	SPAG5
0	3.7102240701863	0.589	0.078	0	Proliferating hepatocytes	NCAPD2
0	2.87571603161532	0.71	0.201	0	Proliferating hepatocytes	CDKN2C
0	3.03647402025746	0.656	0.149	0	Proliferating hepatocytes	UBE2T
0	5.02802516382341	0.526	0.028	0	Proliferating hepatocytes	NEK2
0	2.59403633101495	0.768	0.27	0	Proliferating hepatocytes	H2AFZ
0	3.12051050704856	0.666	0.17	0	Proliferating hepatocytes	SMC4
0	4.69878671878207	0.518	0.029	0	Proliferating hepatocytes	NDC80
0	3.50505092004407	0.587	0.099	0	Proliferating hepatocytes	CKS2
0	2.46690886406779	0.689	0.207	0	Proliferating hepatocytes	LMNB2
0	3.35509482666606	0.559	0.079	0	Proliferating hepatocytes	INCENP
0	3.4969026751144	0.56	0.082	0	Proliferating hepatocytes	CENPW
0	3.64013149239316	0.577	0.1	0	Proliferating hepatocytes	HIST1H1E
0	2.49502824598075	0.657	0.184	0	Proliferating hepatocytes	TK1

0	5.28165690745088	0.502	0.031	0	Proliferating hepatocytes	ASPM
0	5.4047499950357	0.484	0.016	0	Proliferating hepatocytes	HJURP
0	4.47162956490587	0.517	0.05	0	Proliferating hepatocytes	UBE2C
0	3.21544927916295	0.601	0.134	0	Proliferating hepatocytes	HIST1H4C
0	2.25618224178918	0.721	0.257	0	Proliferating hepatocytes	CKS1B
0	4.87574085521061	0.48	0.022	0	Proliferating hepatocytes	CDCA8
0	4.38721568134459	0.509	0.053	0	Proliferating hepatocytes	HIST1H2AH
0	5.24448130352694	0.472	0.017	0	Proliferating hepatocytes	CIT
0	5.03062106220408	0.48	0.027	0	Proliferating hepatocytes	PLK1
0	4.69432125679324	0.482	0.03	0	Proliferating hepatocytes	NUF2
0	4.71882437817131	0.475	0.023	0	Proliferating hepatocytes	ESPL1
0	2.73219462992625	0.577	0.126	0	Proliferating hepatocytes	PHF19
0	2.36735995550263	0.68	0.231	0	Proliferating hepatocytes	RAD21
0	5.3049626264071	0.466	0.02	0	Proliferating hepatocytes	DLGAP5
0	3.61080748490572	0.518	0.072	0	Proliferating hepatocytes	KNSTRN
0	4.1960636296481	0.495	0.051	0	Proliferating hepatocytes	CCNA2
0	2.60461123878535	0.596	0.156	0	Proliferating hepatocytes	CDC25B
0	2.38197196794322	0.581	0.144	0	Proliferating hepatocytes	ZWINT
0	4.93312342762571	0.454	0.019	0	Proliferating hepatocytes	KIF2C
0	5.02728652261986	0.452	0.019	0	Proliferating hepatocytes	KIF14
0	2.04283237082602	0.714	0.281	0	Proliferating hepatocytes	TUBB
0	4.63949331218884	0.465	0.033	0	Proliferating hepatocytes	KIF23
0	3.9386577014755	0.483	0.054	0	Proliferating hepatocytes	MAD2L1
0	4.80361480584506	0.456	0.027	0	Proliferating hepatocytes	ARHGAP11A
0	4.97213836950979	0.475	0.047	0	Proliferating hepatocytes	CCNB1
0	2.02329818486513	0.637	0.21	0	Proliferating hepatocytes	TYMS
0	3.76812470718391	0.474	0.049	0	Proliferating hepatocytes	CENPL
0	3.75110638315927	0.497	0.072	0	Proliferating hepatocytes	CDKN3
0	4.72615486316197	0.448	0.023	0	Proliferating hepatocytes	SKA1
0	2.34942339432396	0.625	0.202	0	Proliferating hepatocytes	NCAPG2
0	5.2432922534029	0.442	0.022	0	Proliferating hepatocytes	CCNB2
0	2.68043753744818	0.578	0.158	0	Proliferating hepatocytes	CENPU
0	2.32787889685743	0.56	0.141	0	Proliferating hepatocytes	DTYMK
0	4.73506697395993	0.45	0.031	0	Proliferating hepatocytes	AURKA
0	5.42641467791274	0.434	0.015	0	Proliferating hepatocytes	CDCA3
0	3.55096044692623	0.482	0.065	0	Proliferating hepatocytes	MELK
0	2.48130052852916	0.675	0.259	0	Proliferating hepatocytes	ADGRE5
0	2.5657364221639	0.533	0.119	0	Proliferating hepatocytes	STMN1
0	3.33427071365043	0.469	0.055	0	Proliferating hepatocytes	FAM83D
0	3.3449520302619	0.48	0.067	0	Proliferating hepatocytes	UBE2S
0	2.1945728411332	0.659	0.248	0	Proliferating hepatocytes	TUBA1C
0	2.41539092554311	0.559	0.149	0	Proliferating hepatocytes	CENPM
0	5.45522096018112	0.425	0.017	0	Proliferating hepatocytes	CDC20
0	1.72503257525775	0.715	0.312	0	Proliferating hepatocytes	H1FX
0	4.67391564898848	0.423	0.021	0	Proliferating hepatocytes	SPC25
0	2.43653970044476	0.549	0.149	0	Proliferating hepatocytes	TUBB4B
0	3.50695666423915	0.458	0.059	0	Proliferating hepatocytes	H2AFX
0	4.05060681849138	0.437	0.04	0	Proliferating hepatocytes	DEPDC1
0	4.9949489919963	0.407	0.012	0	Proliferating hepatocytes	AURKB
0	3.34010431736873	0.491	0.097	0	Proliferating hepatocytes	KPNA2
0	3.66393220308915	0.439	0.045	0	Proliferating hepatocytes	DEPRECATEI
0	4.25797594094291	0.421	0.027	0	Proliferating hepatocytes	DEPDC1B
0	4.52662225118286	0.412	0.018	0	Proliferating hepatocytes	KIF4A
0	4.15573771934327	0.424	0.031	0	Proliferating hepatocytes	SKA3
0	5.51246802939635	0.409	0.016	0	Proliferating hepatocytes	FBXO43
0	2.47152383303239	0.562	0.17	0	Proliferating hepatocytes	CKAP5
0	5.00036719594168	0.406	0.015	0	Proliferating hepatocytes	GTSE1
0	3.61573632565501	0.431	0.044	0	Proliferating hepatocytes	MND1
0	2.666957312841	0.474	0.087	0	Proliferating hepatocytes	SMC2
0	2.53995553635822	0.473	0.086	0	Proliferating hepatocytes	ASF1B

0	2.33787047051195	0.544	0.158	0	Proliferating hepatocytes	MZT1
0	4.3372250003471	0.413	0.029	0	Proliferating hepatocytes	PBK
0	5.15589002363162	0.399	0.016	0	Proliferating hepatocytes	KIF20A
0	3.31110117354178	0.425	0.046	0	Proliferating hepatocytes	MXD3
0	4.92621829002411	0.391	0.014	0	Proliferating hepatocytes	KIF18B
0	2.07880615498062	0.515	0.141	0	Proliferating hepatocytes	CENPN
0	1.86213586137423	0.528	0.156	0	Proliferating hepatocytes	RANGAP1
0	4.73965538054206	0.394	0.023	0	Proliferating hepatocytes	NEIL3
0	3.86549287626028	0.403	0.033	0	Proliferating hepatocytes	MYBL1
0	4.81630781266276	0.385	0.017	0	Proliferating hepatocytes	KNL1
0	4.02820107853677	0.403	0.036	0	Proliferating hepatocytes	KIF11
0	4.77255084554126	0.392	0.025	0	Proliferating hepatocytes	CENPE
0	1.75792129425527	0.587	0.221	0	Proliferating hepatocytes	TCF19
0	2.31721125390972	0.517	0.154	0	Proliferating hepatocytes	HMGB3
0	4.83172244740938	0.376	0.014	0	Proliferating hepatocytes	CKAP2L
0	2.37287718684129	0.507	0.145	0	Proliferating hepatocytes	SOGA1
0	1.48184669394583	0.864	0.503	0	Proliferating hepatocytes	CALM3
0	2.63249880021274	0.433	0.074	0	Proliferating hepatocytes	MIS18A
0	4.39670939409566	0.376	0.018	0	Proliferating hepatocytes	OIP5
0	1.95929526617169	0.55	0.193	0	Proliferating hepatocytes	EZH2
0	2.43467422615862	0.511	0.155	0	Proliferating hepatocytes	GAS2L3
0	4.00322732161162	0.39	0.034	0	Proliferating hepatocytes	CDKN2D
0	2.91290262133763	0.452	0.098	0	Proliferating hepatocytes	CENPJ
0	2.11182818082921	0.485	0.131	0	Proliferating hepatocytes	PCLAF
0	4.978947739945	0.366	0.013	0	Proliferating hepatocytes	CEP55
0	5.2273321062972	0.364	0.015	0	Proliferating hepatocytes	CENPA
0	4.52263142198667	0.365	0.016	0	Proliferating hepatocytes	WDR62
0	2.15743025929244	0.553	0.207	0	Proliferating hepatocytes	ARL6IP1
0	5.72565278705751	0.353	0.007	0	Proliferating hepatocytes	CDC25C
0	2.69648804061129	0.445	0.1	0	Proliferating hepatocytes	FANCI
0	2.70612609832599	0.438	0.094	0	Proliferating hepatocytes	MIS18BP1
0	4.69713226751521	0.356	0.013	0	Proliferating hepatocytes	FAM72C
0	5.7771447303221	0.352	0.01	0	Proliferating hepatocytes	DEPRECATEI
0	2.11843760918231	0.481	0.14	0	Proliferating hepatocytes	EMC9
0	2.96867273212577	0.404	0.066	0	Proliferating hepatocytes	RECQL4
0	3.86065347284326	0.376	0.039	0	Proliferating hepatocytes	GPSM2
0	3.96562026667194	0.369	0.034	0	Proliferating hepatocytes	CKAP2
0	4.91172549232909	0.349	0.015	0	Proliferating hepatocytes	PRR11
0	2.60777766155673	0.424	0.091	0	Proliferating hepatocytes	TUBA1B
0	4.80653152513051	0.347	0.014	0	Proliferating hepatocytes	TROAP
0	3.47189901943232	0.365	0.033	0	Proliferating hepatocytes	STIL
0	4.58003260414828	0.351	0.02	0	Proliferating hepatocytes	SGO2
0	2.317578755334	0.447	0.118	0	Proliferating hepatocytes	NDE1
0	1.68811600376952	0.714	0.387	0	Proliferating hepatocytes	LBR
0	4.25229519954639	0.339	0.016	0	Proliferating hepatocytes	SHCBP1
0	2.04127541593692	0.448	0.128	0	Proliferating hepatocytes	SAP30
0	3.58745471058891	0.353	0.035	0	Proliferating hepatocytes	BRCA2
0	2.15935441360517	0.407	0.09	0	Proliferating hepatocytes	NDC1
0	5.10238875596926	0.324	0.01	0	Proliferating hepatocytes	POLQ
0	2.23441511749777	0.436	0.123	0	Proliferating hepatocytes	RNF26
0	2.95232561367065	0.365	0.053	0	Proliferating hepatocytes	SUV39H1
0	2.54807848163842	0.386	0.076	0	Proliferating hepatocytes	TBC1D31
0	2.37265794139735	0.405	0.096	0	Proliferating hepatocytes	GINS1
0	1.79093324590444	0.413	0.104	0	Proliferating hepatocytes	BCL2L12
0	3.21285944833552	0.343	0.037	0	Proliferating hepatocytes	CENPO
0	4.02855924100509	0.328	0.023	0	Proliferating hepatocytes	ARHGEF39
0	2.19402928492883	0.384	0.08	0	Proliferating hepatocytes	NEMP1
0	3.30014587748139	0.338	0.035	0	Proliferating hepatocytes	GINS4
0	2.74168842477715	0.374	0.072	0	Proliferating hepatocytes	ARHGAP33
0	1.92279004853808	0.396	0.096	0	Proliferating hepatocytes	NAV2

0	2.22226597652475	0.393	0.093	0	Proliferating hepatocytes	TDP1
0	3.11877392306941	0.338	0.038	0	Proliferating hepatocytes	ORC6
0	4.29988338174899	0.314	0.014	0	Proliferating hepatocytes	CDCA5
0	4.0043568800832	0.311	0.015	0	Proliferating hepatocytes	PSRC1
0	3.47636266054866	0.326	0.031	0	Proliferating hepatocytes	EXO1
0	3.17998956149788	0.339	0.044	0	Proliferating hepatocytes	CCNF
0	5.46653611396654	0.302	0.008	0	Proliferating hepatocytes	BUB1B
0	2.17109484173485	0.393	0.1	0	Proliferating hepatocytes	MCM8
0	2.72273006214341	0.345	0.052	0	Proliferating hepatocytes	DBF4
0	2.90554340091638	0.363	0.07	0	Proliferating hepatocytes	RAD54B
0	1.88706001425598	0.37	0.078	0	Proliferating hepatocytes	C1orf112
0	2.29611252858048	0.378	0.087	0	Proliferating hepatocytes	SCLT1
0	2.3607613356589	0.356	0.067	0	Proliferating hepatocytes	VRK1
0	2.6497223190797	0.341	0.056	0	Proliferating hepatocytes	GEN1
0	2.81293040668747	0.338	0.053	0	Proliferating hepatocytes	FBXO5
0	4.46592112339787	0.296	0.012	0	Proliferating hepatocytes	CDCA2
0	3.70781461860623	0.299	0.017	0	Proliferating hepatocytes	PKMYT1
0	2.2278384061984	0.371	0.091	0	Proliferating hepatocytes	CENPP
0	5.09010588268654	0.291	0.011	0	Proliferating hepatocytes	TTK
0	2.78999284085373	0.332	0.057	0	Proliferating hepatocytes	C21orf58
0	2.97374738154017	0.321	0.049	0	Proliferating hepatocytes	CDC45
0	2.89484266085703	0.293	0.022	0	Proliferating hepatocytes	POC1A
0	3.69563541777551	0.293	0.022	0	Proliferating hepatocytes	CIP2A
0	4.84273919723049	0.279	0.011	0	Proliferating hepatocytes	PARPBP
0	4.59055916327406	0.278	0.01	0	Proliferating hepatocytes	DIAPH3
0	4.82089924651948	0.283	0.016	0	Proliferating hepatocytes	AC091057.6
0	4.87413616919713	0.274	0.009	0	Proliferating hepatocytes	KIF15
0	2.40690916766132	0.33	0.065	0	Proliferating hepatocytes	G2E3
0	3.38680483005419	0.296	0.032	0	Proliferating hepatocytes	LIN9
0	4.34421333184597	0.277	0.013	0	Proliferating hepatocytes	DEPRECATEI
0	3.6450792884241	0.284	0.021	0	Proliferating hepatocytes	KIF20B
0	4.76941413866587	0.266	0.009	0	Proliferating hepatocytes	NCAPH
0	4.06458402519285	0.274	0.017	0	Proliferating hepatocytes	RACGAP1
0	3.47699212880418	0.282	0.025	0	Proliferating hepatocytes	RAD51AP1
0	2.4870591778144	0.316	0.064	0	Proliferating hepatocytes	BRCA1
0	2.85949317822754	0.283	0.033	0	Proliferating hepatocytes	TEDC1
0	2.46707394964619	0.303	0.054	0	Proliferating hepatocytes	DSCC1
0	2.32918379390628	0.302	0.054	0	Proliferating hepatocytes	RFC3
0	2.90260473299646	0.304	0.057	0	Proliferating hepatocytes	ZGRF1
0	3.58260242596982	0.265	0.021	0	Proliferating hepatocytes	MYBL2
0	4.00544968194307	0.261	0.017	0	Proliferating hepatocytes	PLK4
0	4.91667193804628	0.251	0.008	0	Proliferating hepatocytes	HIST1H2BB
0	2.78401012323542	0.279	0.039	0	Proliferating hepatocytes	CEP135
0	4.3722121330115	0.248	0.008	0	Proliferating hepatocytes	SGO1
0	3.07484701198211	0.27	0.031	0	Proliferating hepatocytes	E2F8
0	3.76275930246769	0.259	0.021	0	Proliferating hepatocytes	RAD54L
0	2.93942240235871	0.286	0.05	0	Proliferating hepatocytes	BORA
0	2.87008420020295	0.28	0.045	0	Proliferating hepatocytes	ATAD5
0	4.10448178392251	0.24	0.009	0	Proliferating hepatocytes	TRIP13
0	2.4450361035812	0.265	0.036	0	Proliferating hepatocytes	CHEK2
0	2.37033881419861	0.256	0.032	0	Proliferating hepatocytes	CHTF18
0	2.86529110222411	0.25	0.027	0	Proliferating hepatocytes	ZWILCH
0	2.93067867531139	0.247	0.026	0	Proliferating hepatocytes	CCDC18
0	2.69323895349322	0.263	0.043	0	Proliferating hepatocytes	CENPK
0	2.60472566051857	0.258	0.04	0	Proliferating hepatocytes	CDCA4
0	3.53820693508644	0.231	0.017	0	Proliferating hepatocytes	FANCD2
0	2.57062255788259	0.243	0.029	0	Proliferating hepatocytes	CEP128
0	2.30487183850592	0.25	0.038	0	Proliferating hepatocytes	CCDC15
0	3.11662650344589	0.226	0.02	0	Proliferating hepatocytes	MCM10
0	3.25589556635588	0.226	0.023	0	Proliferating hepatocytes	ZNF165

0	3.25243117116772	0.223	0.023	0	Proliferating hepatocytes	XRCC2
0	2.82587973117339	0.223	0.023	0	Proliferating hepatocytes	REEP4
0	3.21575577681402	0.225	0.025	0	Proliferating hepatocytes	FANCB
0	5.01233183209464	0.205	0.006	0	Proliferating hepatocytes	HASPIN
0	3.04790229797431	0.219	0.022	0	Proliferating hepatocytes	C4orf46
0	4.63692883279548	0.199	0.006	0	Proliferating hepatocytes	ERCC6L
0	4.14943260872695	0.205	0.012	0	Proliferating hepatocytes	KIF18A
0	3.86477604339874	0.199	0.009	0	Proliferating hepatocytes	HIST1H2AE
0	4.14327018097748	0.199	0.009	0	Proliferating hepatocytes	TICRR
0	2.8141997257425	0.214	0.026	0	Proliferating hepatocytes	REXO5
0	3.85082263917413	0.195	0.009	0	Proliferating hepatocytes	MTFR2
0	4.37472184002762	0.193	0.008	0	Proliferating hepatocytes	APOBEC3B
0	2.64078310726243	0.208	0.026	0	Proliferating hepatocytes	DBF4B
0	2.14283175297811	0.2	0.019	0	Proliferating hepatocytes	SMTN
0	3.46683960214567	0.191	0.01	0	Proliferating hepatocytes	APOLD1
0	3.32969287853916	0.18	0.016	0	Proliferating hepatocytes	TESMIN
0	3.80973114444572	0.167	0.006	0	Proliferating hepatocytes	C17orf53
0	4.4563141364475	0.165	0.006	0	Proliferating hepatocytes	HIST1H2BN
0	4.57998760498296	0.153	0.006	0	Proliferating hepatocytes	HIST1H2BM
0	3.27790736957241	0.158	0.011	0	Proliferating hepatocytes	RAD51
0	4.80429869345289	0.15	0.005	0	Proliferating hepatocytes	RTKN2
0	5.21471274360167	0.146	0.003	0	Proliferating hepatocytes	FAM72D
0	3.92379802364112	0.141	0.006	0	Proliferating hepatocytes	DDIAS
0	3.54328322783388	0.141	0.009	0	Proliferating hepatocytes	KIF24
0	4.69084625678611	0.132	0.004	0	Proliferating hepatocytes	EME1
0	4.50571900312542	0.129	0.004	0	Proliferating hepatocytes	RDM1
0	5.13645821811191	0.12	0.002	0	Proliferating hepatocytes	PIF1
0	5.08992503109155	0.117	0.003	0	Proliferating hepatocytes	FAM72B
0	4.20400517459718	0.118	0.005	0	Proliferating hepatocytes	BLM
0	4.36538382275928	0.11	0.004	0	Proliferating hepatocytes	ARHGAP11E
0	3.86855966266899	0.107	0.004	0	Proliferating hepatocytes	CCDC163
6.57055257017109E-308	1.39559573304383	0.741	0.393	1.10109319970927E-303	Proliferating hepatocytes	TMPO
2.46215282495598E-306	2.22657429208737	0.231	0.035	4.12607570406124E-302	Proliferating hepatocytes	SASS6
1.19840676230147E-300	1.45937560983143	0.636	0.271	2.0082900522648E-296	Proliferating hepatocytes	GGH
7.97560808148421E-298	2.01082505447119	0.424	0.124	1.33655240229512E-293	Proliferating hepatocytes	POLH
1.62330622938876E-297	2.22599726697933	0.344	0.083	2.72033657920969E-293	Proliferating hepatocytes	MASTL
2.87796046640269E-297	1.71479300504871	0.579	0.23	4.82288614959763E-293	Proliferating hepatocytes	GLA
3.01272944432159E-295	1.70142773108185	0.488	0.161	5.04873200279412E-291	Proliferating hepatocytes	DDX39A
4.55555761816171E-295	2.40909250939453	0.31	0.069	7.63420345651539E-291	Proliferating hepatocytes	ATP8B3
2.26358482446925E-290	2.1472513498265	0.3	0.064	3.79331544884556E-286	Proliferating hepatocytes	NRM
1.04846587556447E-289	2.46348008911752	0.229	0.037	1.75701911427094E-285	Proliferating hepatocytes	E2F7
1.07650272867902E-289	2.09282318067929	0.34	0.082	1.80400327272031E-285	Proliferating hepatocytes	LRR1
2.93832130925229E-289	2.01766377030368	0.327	0.075	4.92403885004499E-285	Proliferating hepatocytes	NUDT1
3.47171852117179E-289	2.1420224993577	0.307	0.067	5.81790589777968E-285	Proliferating hepatocytes	CENPH
1.43939717407E-288	2.18465120916976	0.242	0.041	2.41214178430651E-284	Proliferating hepatocytes	EFCAB11
8.01838655689263E-287	3.27137374765864	0.107	0.005	1.34372121920407E-282	Proliferating hepatocytes	TRAIP
1.66910495842047E-285	2.00868733665688	0.356	0.091	2.79708608932102E-281	Proliferating hepatocytes	FBLN1
4.69062833134843E-285	1.96545665746598	0.321	0.073	7.86055495767369E-281	Proliferating hepatocytes	TTF2
5.84120696363726E-285	2.00663892763924	0.329	0.078	9.78869462966332E-281	Proliferating hepatocytes	BARD1
2.29558151445983E-284	3.80511017623527	0.116	0.007	3.84693550193177E-280	Proliferating hepatocytes	ACBD7
6.3176155989114E-283	1.7954891248806	0.505	0.179	1.05870602206557E-278	Proliferating hepatocytes	DCK
1.68985178534154E-281	2.05078471704101	0.519	0.205	2.83185362187536E-277	Proliferating hepatocytes	FAM111A
2.45793597674518E-281	2.74315997645235	0.179	0.022	4.11900910982957E-277	Proliferating hepatocytes	ESCO2
5.50807475730142E-279	2.01746254412124	0.346	0.087	9.23043167828573E-275	Proliferating hepatocytes	C9orf40
9.77277820522633E-279	3.02328277371833	0.152	0.015	1.63772217163183E-274	Proliferating hepatocytes	SPDL1
1.39327092057865E-277	1.97085840351825	0.28	0.058	2.3348434087057E-273	Proliferating hepatocytes	CCDC34
1.37441305729934E-270	1.57294106525936	0.557	0.221	2.30324140142223E-266	Proliferating hepatocytes	ANP32E
7.13808594651505E-270	1.80890883080536	0.361	0.096	1.19620044291699E-265	Proliferating hepatocytes	RFWD3
7.84617935528693E-268	2.28277128849293	0.306	0.072	1.31486273635898E-263	Proliferating hepatocytes	CCNG2
1.96759865471119E-265	1.66827879165836	0.569	0.239	3.29730182556502E-261	Proliferating hepatocytes	RRM1

2.11406605859705E-265	3.21125855675777	0.121	0.009	3.54275190099693E-261	Proliferating hepatocytes	C18orf54
8.61748069468155E-265	1.68930469024836	0.427	0.135	1.44411741481473E-260	Proliferating hepatocytes	NCAPD3
1.20432467733257E-264	1.69733792676907	0.393	0.115	2.01820729427391E-260	Proliferating hepatocytes	KMT5A
9.29948464132733E-262	2.29484887397952	0.214	0.035	1.55840763619363E-257	Proliferating hepatocytes	FANCM
2.09601385493386E-259	1.14995555230121	0.83	0.539	3.51250001809816E-255	Proliferating hepatocytes	NUCKS1
5.26119302341194E-258	1.8530031662265	0.326	0.082	8.81670726863373E-254	Proliferating hepatocytes	RAD18
9.2681352255403E-258	1.75214779551642	0.477	0.17	1.55315410109604E-253	Proliferating hepatocytes	FHL1
4.81150673941694E-254	1.76445105388807	0.245	0.047	8.06312299391491E-250	Proliferating hepatocytes	PPP2R3B
2.72167511711759E-252	1.99103829372377	0.423	0.139	4.56098316126565E-248	Proliferating hepatocytes	FANCA
1.52181784588229E-251	1.75803265792453	0.373	0.108	2.55026234612955E-247	Proliferating hepatocytes	DARS2
3.43646195185026E-251	1.72989431977429	0.355	0.098	5.75882293891067E-247	Proliferating hepatocytes	CEP78
2.89142676033562E-249	2.57580043427736	0.148	0.017	4.84545296497043E-245	Proliferating hepatocytes	DEPRECATEI
6.08969584558712E-249	2.29940619165179	0.204	0.034	1.02051122980349E-244	Proliferating hepatocytes	C1QL1
1.40921153393959E-246	1.34616154993532	0.647	0.308	2.36155668857597E-242	Proliferating hepatocytes	CDK16
1.58049885116265E-244	2.07817858959011	0.346	0.098	2.64859997477837E-240	Proliferating hepatocytes	NUP62CL
6.62605278650893E-243	2.36923336346883	0.237	0.047	1.11039392596317E-238	Proliferating hepatocytes	MTBP
2.14419448285775E-242	1.23516649022153	0.797	0.489	3.59324111437302E-238	Proliferating hepatocytes	CALM2
2.58489939880535E-241	1.64087027155109	0.555	0.241	4.331774412518E-237	Proliferating hepatocytes	DHFR
5.78552265991874E-241	1.64599175297601	0.453	0.16	9.69537887349183E-237	Proliferating hepatocytes	LIG1
2.15533544512648E-240	2.91148301280685	0.129	0.013	3.61191113894295E-236	Proliferating hepatocytes	TMEM173
9.64936473584765E-239	2.56778204797136	0.156	0.02	1.61704054243335E-234	Proliferating hepatocytes	NRGN
8.7667916236516E-237	1.41319894588505	0.364	0.106	1.46913894029154E-232	Proliferating hepatocytes	CEP72
3.45326450596369E-235	1.02116568948963	0.894	0.591	5.78698065909395E-231	Proliferating hepatocytes	MBNL3
1.2048180069064E-232	1.58921832679862	0.324	0.087	2.01903401597374E-228	Proliferating hepatocytes	RCCD1
1.05522082163109E-230	1.62930089993763	0.401	0.131	1.76833905288938E-226	Proliferating hepatocytes	COQ2
5.07026445302878E-230	1.94223241695392	0.262	0.06	8.49674917038563E-226	Proliferating hepatocytes	TNFAIP8L1
4.05809943580209E-229	1.8249842520642	0.26	0.059	6.80056303451714E-225	Proliferating hepatocytes	GSTCD
7.28015482615458E-226	2.25290697293831	0.186	0.031	1.22000834576699E-221	Proliferating hepatocytes	PSMC3IP
2.33917696536407E-225	1.52616228195442	0.426	0.145	3.91999275855711E-221	Proliferating hepatocytes	WDR34
1.76696867605167E-224	1.36435042701103	0.48	0.183	2.9610861073274E-220	Proliferating hepatocytes	CDK5RAP2
2.99972081863447E-224	1.68557129546937	0.393	0.128	5.02693214786764E-220	Proliferating hepatocytes	SPIN4
2.73918945065789E-223	1.54808075265351	0.405	0.135	4.5903336814125E-219	Proliferating hepatocytes	ZNF367
1.17264278233697E-220	2.73452043076477	0.15	0.02	1.9651147746403E-216	Proliferating hepatocytes	E2F2
2.20070670723614E-220	1.78337002869728	0.393	0.13	3.68794429998633E-216	Proliferating hepatocytes	SEPHS1
2.42977931537701E-220	2.69602504754071	0.119	0.012	4.07182417670879E-216	Proliferating hepatocytes	TEDC2
7.90632696630259E-220	1.31604129881192	0.478	0.182	1.32494227301299E-215	Proliferating hepatocytes	BUB3
1.32129116421391E-219	1.59975711516797	0.526	0.23	2.21421973298966E-215	Proliferating hepatocytes	TMEM19
1.90386507965687E-219	1.28068575694488	0.631	0.312	3.19049710048899E-215	Proliferating hepatocytes	RNF157
3.83833567523972E-219	1.14956188402205	0.678	0.338	6.43228292456672E-215	Proliferating hepatocytes	FXYD3
6.53606643875691E-218	1.44831594124315	0.378	0.12	1.09531401380688E-213	Proliferating hepatocytes	DCAF15
9.39093029606845E-218	1.68175549869833	0.368	0.116	1.57373209901515E-213	Proliferating hepatocytes	MELTF
2.26286195587691E-217	1.72567862708635	0.362	0.114	3.79210406565853E-213	Proliferating hepatocytes	PKN3
3.28595660617258E-217	1.33407818526844	0.516	0.212	5.50660608062401E-213	Proliferating hepatocytes	SAE1
4.84369824087671E-217	2.56008322777535	0.185	0.032	8.11706951206119E-213	Proliferating hepatocytes	CEP152
4.94357977308607E-216	1.54022443276581	0.449	0.168	8.28445098373764E-212	Proliferating hepatocytes	BRD8
1.00363367367423E-215	1.38823391418051	0.583	0.274	1.68188931034327E-211	Proliferating hepatocytes	RBMX
2.41133269127596E-215	1.56838428644295	0.433	0.158	4.04091132404026E-211	Proliferating hepatocytes	FEN1
1.41116799502869E-212	1.18903365362019	0.689	0.369	2.36483532606907E-208	Proliferating hepatocytes	FLOT2
1.51667168214488E-212	1.77756926576445	0.365	0.117	2.54163840493839E-208	Proliferating hepatocytes	DEPRECATEI
3.42364956087752E-210	1.50840938474307	0.385	0.128	5.73735193411855E-206	Proliferating hepatocytes	DCP2
2.94955176345276E-208	1.50177458667842	0.4	0.138	4.94285884519413E-204	Proliferating hepatocytes	CEP85
8.02238634900368E-208	1.36500109685567	0.442	0.164	1.34439150436604E-203	Proliferating hepatocytes	TEX30
2.43138254920907E-207	1.23685132389434	0.652	0.35	4.07451087596456E-203	Proliferating hepatocytes	FAM122B
2.56260562697947E-205	2.00377645534852	0.26	0.065	4.29441450969219E-201	Proliferating hepatocytes	BRIP1
1.5379209937937E-203	1.81476622083034	0.277	0.073	2.57724800139948E-199	Proliferating hepatocytes	CHEK1
1.08387411236198E-202	1.66493528530633	0.402	0.143	1.81635623749621E-198	Proliferating hepatocytes	ALDH3A1
1.1943345122275E-202	1.3690269878277	0.516	0.22	2.00146577559084E-198	Proliferating hepatocytes	TUBG1
4.62204049458959E-202	1.25328808427784	0.428	0.156	7.74561546083323E-198	Proliferating hepatocytes	TCOF1
2.36862839316188E-200	1.20030759201486	0.669	0.366	3.96934746126068E-196	Proliferating hepatocytes	CDKN1B
7.83994447597981E-200	1.76775324973173	0.331	0.102	1.3138178952847E-195	Proliferating hepatocytes	JPT1

1.42234215646353E-199	1.91888226595288	0.234	0.054	2.38356098580158E-195	Proliferating hepatocytes	CENPQ
3.68826164409224E-199	1.19777390131644	0.604	0.293	6.18078886316978E-195	Proliferating hepatocytes	DEK
1.07498930234206E-198	1.46529761689857	0.233	0.053	1.80146707286482E-194	Proliferating hepatocytes	RASSF1
1.24408612847945E-198	1.2208221693024	0.659	0.343	2.08483953410586E-194	Proliferating hepatocytes	KIF5B
3.01525744024775E-198	2.57234342449196	0.148	0.022	5.05296841836719E-194	Proliferating hepatocytes	CTSV
9.7343456749027E-198	2.35055227571527	0.132	0.017	1.63128164820019E-193	Proliferating hepatocytes	GPR137C
9.376703595095E-195	1.33498042946518	0.375	0.127	1.57134798846602E-190	Proliferating hepatocytes	THOC3
5.49049384135008E-194	1.33748769355472	0.391	0.135	9.20096957933446E-190	Proliferating hepatocytes	IER5
1.23718194403692E-193	1.33348746203027	0.406	0.145	2.07326950181707E-189	Proliferating hepatocytes	OAS3
1.52731820516461E-193	1.77007861632567	0.224	0.051	2.55947984821485E-189	Proliferating hepatocytes	SNORC
3.57573580031256E-193	0.81614861461392	0.848	0.616	5.99221805416379E-189	Proliferating hepatocytes	HNRNPA2B1
5.84237005801695E-193	1.34627159478422	0.451	0.178	9.7906437432248E-189	Proliferating hepatocytes	RUVBL2
6.31567641311116E-192	1.52004115105826	0.382	0.134	1.05838105330917E-187	Proliferating hepatocytes	DEPRECATEI
9.34945046444763E-192	1.42929371785378	0.361	0.12	1.56678090883213E-187	Proliferating hepatocytes	CCDC88A
2.41819637043671E-191	1.39376258801688	0.322	0.097	4.05241347757784E-187	Proliferating hepatocytes	SPATA5
5.38745699587884E-191	0.985269557307078	0.764	0.452	9.02830043369376E-187	Proliferating hepatocytes	HTATSF1
1.64533803169318E-190	1.81059506015578	0.352	0.119	2.75725747351143E-186	Proliferating hepatocytes	DTL
5.66260305998197E-190	1.37917821524348	0.349	0.113	9.48939020791779E-186	Proliferating hepatocytes	NUP107
1.98833752176237E-189	1.84577692592301	0.229	0.054	3.33205601896938E-185	Proliferating hepatocytes	SYNE1
3.35776784955942E-188	1.29077894591501	0.494	0.21	5.62694736229167E-184	Proliferating hepatocytes	TNPO2
1.4945767736627E-187	1.4931851000823	0.338	0.109	2.50461175730396E-183	Proliferating hepatocytes	PARP2
2.60721125654893E-187	2.92262498035738	0.116	0.014	4.3691646237247E-183	Proliferating hepatocytes	DEPRECATEI
5.96171232411681E-187	1.23681347461839	0.456	0.181	9.99063751275495E-183	Proliferating hepatocytes	CBX5
9.87646139614558E-186	1.11593266172812	0.567	0.266	1.65509740076608E-181	Proliferating hepatocytes	TRIOBP
1.13502190589772E-185	0.989663349778448	0.779	0.488	1.9020697099034E-181	Proliferating hepatocytes	MORF4L2
2.17378316178902E-185	1.15769069103365	0.646	0.342	3.64282582252604E-181	Proliferating hepatocytes	PSMD10
2.29603122823435E-184	2.00553033950134	0.204	0.044	3.84768913227512E-180	Proliferating hepatocytes	TMEM107
1.83515187109145E-183	1.80045264082981	0.22	0.051	3.07534750557506E-179	Proliferating hepatocytes	CCSAP
7.31261399721613E-183	0.896652911485708	0.821	0.523	1.22544785365348E-178	Proliferating hepatocytes	ANXA2
1.34132609513481E-181	1.02052457223447	0.717	0.416	2.24779427022691E-177	Proliferating hepatocytes	SIVA1
1.89158224013971E-181	1.23804214814849	0.66	0.382	3.16991351802613E-177	Proliferating hepatocytes	ATAD2
3.23391285950809E-181	1.55975556253374	0.318	0.101	5.41939116996365E-177	Proliferating hepatocytes	LIN54
5.43759395966302E-181	1.14504502484753	0.623	0.337	9.11231995760328E-177	Proliferating hepatocytes	NID1
6.77280720422169E-180	1.33722353863465	0.449	0.186	1.13498703128347E-175	Proliferating hepatocytes	MPHOSPH9
1.68698935358691E-179	1.67005592969992	0.261	0.071	2.82705675874095E-175	Proliferating hepatocytes	ODF2
2.22475993160942E-179	1.44890886338229	0.394	0.147	3.72825269339107E-175	Proliferating hepatocytes	LRRCC1
6.93869094110256E-179	0.999119404972602	0.7	0.396	1.16278582790997E-174	Proliferating hepatocytes	HNRNPUL1
2.90169759418092E-178	2.16449992447222	0.143	0.023	4.86266482832838E-174	Proliferating hepatocytes	RNFT2
4.26717375128864E-178	0.902116470111299	0.779	0.498	7.1509297724095E-174	Proliferating hepatocytes	GUSB
7.9977139952778E-178	1.32966698235411	0.387	0.142	1.34025691132865E-173	Proliferating hepatocytes	SRGAP2B
9.65862205178002E-178	1.54915022485585	0.277	0.08	1.6185918834373E-173	Proliferating hepatocytes	RCC1
1.07997642600863E-177	1.59808368781772	0.283	0.083	1.80982449470527E-173	Proliferating hepatocytes	RHNO1
1.0994924040272E-177	1.59273530275651	0.268	0.075	1.84252937066878E-173	Proliferating hepatocytes	FANCG
3.0920130597733E-177	1.50160650720767	0.33	0.108	5.1815954855681E-173	Proliferating hepatocytes	PRIM2
5.23235475140902E-177	1.28561811068855	0.466	0.197	8.76838009241124E-173	Proliferating hepatocytes	USP1
4.14729282841594E-176	1.8939155321913	0.164	0.03	6.95003332185943E-172	Proliferating hepatocytes	POC5
5.45161253385348E-176	1.50698129307142	0.297	0.091	9.13581228423167E-172	Proliferating hepatocytes	VANGL1
1.13393709848434E-175	1.07444414279557	0.599	0.309	1.90025178964005E-171	Proliferating hepatocytes	NNT
1.33735989538321E-175	1.40626009071393	0.39	0.147	2.24114771268319E-171	Proliferating hepatocytes	POLD1
3.06733617587715E-175	1.45756510181913	0.247	0.066	5.14024196353493E-171	Proliferating hepatocytes	IKBIP
2.39136861447542E-174	1.15295485576276	0.524	0.239	4.00745552413791E-170	Proliferating hepatocytes	YWHAH
6.33314271074238E-174	1.26950414136474	0.536	0.262	1.06130805546621E-169	Proliferating hepatocytes	CYTH2
1.04208197957433E-173	0.983891108357669	0.729	0.421	1.74632098137066E-169	Proliferating hepatocytes	VBP1
1.30878531466934E-173	2.12681971288867	0.159	0.029	2.19326243032287E-169	Proliferating hepatocytes	ORC1
1.60219967677414E-173	0.874996803827685	0.822	0.556	2.68496621833811E-169	Proliferating hepatocytes	TMEM106C
4.53585591763937E-173	1.42701327653884	0.417	0.165	7.60118734678006E-169	Proliferating hepatocytes	NSD2
2.14658087318195E-172	1.31231094434447	0.629	0.373	3.59724022727831E-168	Proliferating hepatocytes	MDM2
2.68169381206817E-172	1.94570090229171	0.235	0.061	4.49398249026384E-168	Proliferating hepatocytes	FAM49A
7.50081294716544E-172	1.54623330282384	0.269	0.078	1.25698623368598E-167	Proliferating hepatocytes	METTL4
1.74798040756331E-171	2.24363074664195	0.167	0.033	2.92926556699459E-167	Proliferating hepatocytes	CLSPN

3.04367050324187E-171	1.48617072913963	0.229	0.058	5.10058302933272E-167	Proliferating hepatocytes	CNTROB
1.07292648331684E-170	1.59851230437845	0.267	0.077	1.79801020074236E-166	Proliferating hepatocytes	CEP97
1.13956122266946E-170	1.27880691671765	0.405	0.157	1.90967669694947E-166	Proliferating hepatocytes	PCNT
1.33450304198414E-170	1.43444188319716	0.374	0.139	2.23636019775702E-166	Proliferating hepatocytes	CDT1
5.98415380766366E-170	1.33617500388991	0.394	0.151	1.00282449508828E-165	Proliferating hepatocytes	MGME1
9.49781588616624E-170	1.09538943206266	0.467	0.2	1.59164398620374E-165	Proliferating hepatocytes	WEE1
3.73182232325683E-168	2.25569699770723	0.113	0.015	6.2537878493138E-164	Proliferating hepatocytes	HIST1H2AG
3.9893252910738E-168	1.22651877571476	0.492	0.223	6.68531132278147E-164	Proliferating hepatocytes	MEPCE
4.79994470032435E-168	1.56430343332776	0.252	0.07	8.04374732880354E-164	Proliferating hepatocytes	PROSER3
1.41342295587824E-167	1.21066689042934	0.498	0.222	2.36861418946075E-163	Proliferating hepatocytes	ILF2
1.18449248244823E-166	0.833095701018129	0.803	0.542	1.98497250208674E-162	Proliferating hepatocytes	EMD
2.53089709476973E-166	1.40144185677699	0.347	0.123	4.24127735141512E-162	Proliferating hepatocytes	UBALD2
6.28014505920663E-166	2.72815396982817	0.113	0.015	1.05242670902185E-161	Proliferating hepatocytes	DEPRECATEI
1.89489186453481E-164	1.14282330668217	0.57	0.285	3.17545978658743E-160	Proliferating hepatocytes	NFIX
1.80392216582566E-163	1.3411349424211	0.274	0.082	3.02301276549063E-159	Proliferating hepatocytes	IFT122
9.83338718422337E-163	0.678309194149275	0.95	0.725	1.64787902433215E-158	Proliferating hepatocytes	AKR1C3
1.80025870738737E-161	0.893164985019174	0.787	0.514	3.01687354183976E-157	Proliferating hepatocytes	HNRNPH2
5.98172699786764E-161	1.43095699154209	0.381	0.149	1.00241781030266E-156	Proliferating hepatocytes	CHAF1A
2.0722486467802E-160	1.3060222134798	0.423	0.177	3.47267428227427E-156	Proliferating hepatocytes	DSN1
4.92438134463433E-160	1.51327813354234	0.36	0.136	8.2522782573382E-156	Proliferating hepatocytes	POLA2
5.63811743092216E-160	1.64468567488552	0.346	0.128	9.44835719073935E-156	Proliferating hepatocytes	ANKRD36C
3.16319721199321E-159	1.57344200414556	0.216	0.055	5.30088588785822E-155	Proliferating hepatocytes	FIGNL1
4.06352418841431E-159	1.56827313358734	0.296	0.097	6.8096538349447E-155	Proliferating hepatocytes	FAM102B
4.99368659575265E-159	1.23719914337531	0.335	0.116	8.36841999716228E-155	Proliferating hepatocytes	RCC2
5.37361973694279E-158	1.54140544350223	0.242	0.068	9.00511195516873E-154	Proliferating hepatocytes	C2orf69
5.61940412890124E-158	1.73400994717926	0.188	0.043	9.4169974392127E-154	Proliferating hepatocytes	DCLRE1C
6.46898918028372E-158	1.24032321320165	0.377	0.144	1.08407320683195E-153	Proliferating hepatocytes	PDE3B
1.84081065181349E-157	1.25928325216855	0.262	0.077	3.08483049030905E-153	Proliferating hepatocytes	KIAA0586
1.97122159396473E-157	1.56864933002636	0.262	0.078	3.3033731471661E-153	Proliferating hepatocytes	STMND1
2.5228196079168E-157	1.23534287084436	0.339	0.12	4.22774109894698E-153	Proliferating hepatocytes	CEP120
2.59081929178184E-157	1.28985613597706	0.322	0.11	4.341694969168E-153	Proliferating hepatocytes	SRGAP2
5.63203120692682E-157	1.08667094999843	0.539	0.262	9.43815789656797E-153	Proliferating hepatocytes	RBBP7
5.63588462265542E-157	1.22264023999914	0.358	0.132	9.44461545064595E-153	Proliferating hepatocytes	KATNAL1
1.01506783956315E-156	0.919854120883493	0.65	0.344	1.70105068553993E-152	Proliferating hepatocytes	C15orf48
1.55475841585736E-156	0.983690218320303	0.644	0.363	2.60546415329377E-152	Proliferating hepatocytes	SMC1A
1.73938336952594E-156	1.3653179798647	0.343	0.124	2.91485865065157E-152	Proliferating hepatocytes	HRCT1
2.51399401263828E-156	2.15912824233451	0.174	0.038	4.21295116637923E-152	Proliferating hepatocytes	ABCA12
7.24894140758455E-156	1.03756110976342	0.607	0.323	1.21477760108302E-151	Proliferating hepatocytes	LSM 4.00
7.99204139753845E-156	1.20358245493629	0.425	0.178	1.33930629739949E-151	Proliferating hepatocytes	HP1BP3
1.80883816857403E-155	1.54470080274891	0.302	0.101	3.03125100289635E-151	Proliferating hepatocytes	WDR90
2.11773395775433E-155	1.88065480114188	0.209	0.054	3.5488985664047E-151	Proliferating hepatocytes	FIBCD1
4.22456925607614E-155	1.19010726828206	0.662	0.419	7.07953315933239E-151	Proliferating hepatocytes	SYNE2
4.3134080458283E-155	0.960028271229256	0.528	0.255	7.22840920319907E-151	Proliferating hepatocytes	RHEB
9.11771226268177E-155	1.59049176888831	0.352	0.133	1.52794622098021E-150	Proliferating hepatocytes	TONSL
1.40137343923247E-154	1.06919386784372	0.382	0.147	2.34842160946577E-150	Proliferating hepatocytes	FGFR1OP
1.786468016077E-154	1.01767955032309	0.491	0.223	2.99376310134183E-150	Proliferating hepatocytes	PGP
2.4144893272933E-154	0.84076582175343	0.74	0.438	4.04620121467811E-150	Proliferating hepatocytes	EZR
3.1955656583098E-154	1.06292847513277	0.541	0.269	5.35512893019556E-150	Proliferating hepatocytes	NUP62
3.64229139579908E-154	1.24319426500681	0.401	0.162	6.1037519210801E-150	Proliferating hepatocytes	FDXR
1.0757732204698E-153	1.11335954224599	0.59	0.316	1.80278076286329E-149	Proliferating hepatocytes	FZR1
1.33042806404937E-153	1.11588086870375	0.471	0.212	2.22953134973394E-149	Proliferating hepatocytes	GMNN
1.41626985665997E-153	1.05356662307334	0.391	0.153	2.37338502579078E-149	Proliferating hepatocytes	TPGS2
2.085994193697E-153	1.26450177836735	0.331	0.118	3.49570906979744E-149	Proliferating hepatocytes	FLNA
9.07328621588267E-153	0.814793877876568	0.757	0.441	1.52050130405762E-148	Proliferating hepatocytes	MEP1A
2.95055641721906E-152	1.31055738443424	0.287	0.093	4.9445424439757E-148	Proliferating hepatocytes	ZMYM1
2.01144688426667E-151	1.45554768183102	0.385	0.157	3.37078268865409E-147	Proliferating hepatocytes	ADRB2
3.20959069758923E-151	1.12498774985369	0.462	0.204	5.37863209102002E-147	Proliferating hepatocytes	CCHCR1
6.08997783169645E-151	1.13762550641515	0.502	0.239	1.02055848503569E-146	Proliferating hepatocytes	PRRC2A
1.32097949055466E-150	1.18342860997618	0.359	0.137	2.2136974302715E-146	Proliferating hepatocytes	PLP2
1.68190111874144E-150	0.828405156054787	0.89	0.685	2.8185298947869E-146	Proliferating hepatocytes	LMNA

2.25441276703231E-150	1.71555210270217	0.126	0.021	3.77794491499274E-146	Proliferating hepatocytes	DDX11
2.2754810784559E-150	0.948651747311224	0.679	0.42	3.8132511912764E-146	Proliferating hepatocytes	FMR1
3.36183906271298E-150	0.838145875691793	0.763	0.479	5.63376990129441E-146	Proliferating hepatocytes	YWHAZ
1.99756545008374E-149	1.57376594517406	0.276	0.089	3.34752018125032E-145	Proliferating hepatocytes	FAM122C
3.87218003345397E-149	1.70300365045069	0.21	0.056	6.48899930006216E-145	Proliferating hepatocytes	GINS3
3.87720138485188E-149	1.29108080676348	0.265	0.083	6.49741408073479E-145	Proliferating hepatocytes	HAUS7
6.26833113008096E-149	1.01793439573257	0.529	0.262	1.05044693077897E-144	Proliferating hepatocytes	MED25
7.78852353513866E-149	1.0758868295329	0.528	0.258	1.30520077401854E-144	Proliferating hepatocytes	HAUS4
1.11757034017446E-148	0.710369400784107	0.866	0.636	1.87282437606436E-144	Proliferating hepatocytes	H3F3A
1.14303772321066E-148	1.1110323498883	0.491	0.229	1.91550261655642E-144	Proliferating hepatocytes	PPP2R5D
2.68408172300473E-148	1.39463542454028	0.279	0.091	4.49798415141132E-144	Proliferating hepatocytes	PAFAH1B3
2.9314764540068E-148	1.03501148954075	0.545	0.272	4.91256824162459E-144	Proliferating hepatocytes	SF3A2
7.57790076111179E-148	1.53679090233703	0.128	0.022	1.26990460954711E-143	Proliferating hepatocytes	MNS1
7.66879564677318E-148	1.3281195856859	0.373	0.146	1.28513677448625E-143	Proliferating hepatocytes	AGBL5
1.06365159116531E-146	1.90921352859689	0.173	0.04	1.78246733647482E-142	Proliferating hepatocytes	TRIM7
1.43108825437815E-146	0.808115067604397	0.756	0.474	2.39821769668691E-142	Proliferating hepatocytes	UBE2A
1.70360325540326E-146	1.11269702250957	0.526	0.262	2.85489833540478E-142	Proliferating hepatocytes	NCAPH2
1.73768050751302E-146	0.927437061765636	0.661	0.378	2.91200499449031E-142	Proliferating hepatocytes	HADH
5.80681624592778E-146	1.42522138859462	0.345	0.132	9.73106266492578E-142	Proliferating hepatocytes	RFC4
3.45531675779768E-145	1.16483876944091	0.334	0.123	5.79041982271735E-141	Proliferating hepatocytes	DDAH2
4.96965483206657E-145	1.17129706071867	0.482	0.23	8.32814756757716E-141	Proliferating hepatocytes	MORC4
2.01245312977069E-144	0.865668183223895	0.7	0.417	3.37246895486973E-140	Proliferating hepatocytes	CD99L2
2.28326004249067E-144	1.38549771465056	0.402	0.173	3.82628717920586E-140	Proliferating hepatocytes	KNTC1
8.33218889831258E-144	1.76775039109712	0.173	0.04	1.39630821557922E-139	Proliferating hepatocytes	RNASEH2A
1.142263100205E-143	1.75294971870585	0.162	0.035	1.91420450332354E-139	Proliferating hepatocytes	HAUS8
1.82825063782926E-143	1.24216058123039	0.39	0.163	3.06378241887428E-139	Proliferating hepatocytes	CDK2
6.37602087484503E-143	0.977743170866527	0.59	0.312	1.06849357820653E-138	Proliferating hepatocytes	TMEM238
7.64633480415115E-143	1.28611462565185	0.309	0.109	1.28137278647965E-138	Proliferating hepatocytes	XRCC1
8.26057755950724E-143	0.940145390652872	0.638	0.367	1.38430758742222E-138	Proliferating hepatocytes	NUCB2
9.89354697319687E-143	1.0265875960674	0.575	0.307	1.65796060176833E-138	Proliferating hepatocytes	HPRT1
2.38518691350795E-142	1.78805940460225	0.197	0.052	3.99709622965661E-138	Proliferating hepatocytes	CDH24
1.0911613305119E-141	1.61651592648397	0.145	0.029	1.82856815767184E-137	Proliferating hepatocytes	WRAP53
2.69577779459596E-141	1.23832567814989	0.314	0.113	4.51758442818392E-137	Proliferating hepatocytes	CENPC
5.18399816613973E-141	1.30565453224115	0.296	0.103	8.68734412681696E-137	Proliferating hepatocytes	KIAA1328
6.14351428992226E-141	0.864702708014215	0.69	0.406	1.02953012470517E-136	Proliferating hepatocytes	VMA21
1.49618776788845E-140	1.33092437810419	0.274	0.09	2.50731146142747E-136	Proliferating hepatocytes	DNA2
1.7570122897481E-140	0.646558046841209	0.906	0.592	2.94440119515987E-136	Proliferating hepatocytes	MPP1
1.91196927528357E-140	1.42050845517956	0.296	0.104	3.20407811152021E-136	Proliferating hepatocytes	KATNA1
2.39548987542731E-140	1.0720088943661	0.545	0.285	4.01436193324109E-136	Proliferating hepatocytes	DCAF7
3.11092582665525E-140	1.52740350431547	0.257	0.082	5.21328950030888E-136	Proliferating hepatocytes	RFC5
5.4367373431949E-140	1.03883421331729	0.449	0.202	9.11088443972602E-136	Proliferating hepatocytes	POLR2A
5.70424368261202E-140	1.11719723611623	0.558	0.303	9.55917156332122E-136	Proliferating hepatocytes	TUBGCP3
2.54640362596685E-139	1.53502567618027	0.335	0.13	4.26726319639525E-135	Proliferating hepatocytes	POLD3
4.77294205309486E-139	1.11354663944583	0.407	0.174	7.99849629257636E-135	Proliferating hepatocytes	SLC44A2
5.44867067529559E-139	1.05390961923451	0.561	0.305	9.13088231766035E-135	Proliferating hepatocytes	MCM7
1.20413131086366E-138	1.29638908114516	0.311	0.113	2.01788325074531E-134	Proliferating hepatocytes	ZDHHC12
2.12576183615194E-138	1.53893075446029	0.353	0.142	3.56235168502342E-134	Proliferating hepatocytes	ANKRD36B
2.14556259410241E-138	1.38325381490036	0.274	0.092	3.59553379519682E-134	Proliferating hepatocytes	TMEM201
8.96290521136711E-138	1.00123375199715	0.349	0.135	1.5020036553209E-133	Proliferating hepatocytes	CRYBG1
9.89165335267518E-138	1.23428574466787	0.303	0.108	1.65764326884131E-133	Proliferating hepatocytes	HAUS3
1.25286523611395E-137	1.04106292173079	0.485	0.231	2.09955156267975E-133	Proliferating hepatocytes	PSPH
2.10413838668265E-137	0.868768455286621	0.666	0.415	3.52611510840278E-133	Proliferating hepatocytes	ZNRF2
3.02747320719173E-137	1.32531441351235	0.298	0.105	5.0734396006119E-133	Proliferating hepatocytes	ALDH16A1
3.45554137388233E-137	1.26012378708059	0.256	0.082	5.79079623435201E-133	Proliferating hepatocytes	SUV39H2
4.7725803666707E-137	1.45502566557396	0.185	0.047	7.99789017846676E-133	Proliferating hepatocytes	NUP35
4.85705895075464E-137	1.31554478742948	0.275	0.092	8.13945938967462E-133	Proliferating hepatocytes	CBX1
8.48074189551619E-137	1.13567335196415	0.447	0.206	1.4212027268506E-132	Proliferating hepatocytes	ITPR3
1.26691265390243E-136	0.747983710187665	0.76	0.483	2.12309222540969E-132	Proliferating hepatocytes	GPAA1
2.02425265838745E-136	1.14524810263895	0.353	0.139	3.39224260492569E-132	Proliferating hepatocytes	CEP192
3.2521138000333E-136	0.55782286266812	0.97	0.723	5.4498923060958E-132	Proliferating hepatocytes	S100A10

**5.89846215443525E-136**	1.22090998329578	0.387	0.166	9.88464287840259E-132	Proliferating hepatocytes	UHRF1
**6.96779885694495E-136**	1.26848158304714	0.243	0.075	1.16766373244683E-131	Proliferating hepatocytes	HAUS5
**8.75765889556894E-136**	0.772548448496932	0.72	0.451	1.46760847771944E-131	Proliferating hepatocytes	HNRNPA0
**1.813387122314E-135**	1.26933602776496	0.328	0.124	3.0388741395738E-131	Proliferating hepatocytes	DBNDD2
**2.35407002751851E-135**	0.814796343078942	0.771	0.51	3.94495055211552E-131	Proliferating hepatocytes	NPC2
**2.67596178293247E-135**	1.04406667638023	0.406	0.175	4.48437675583823E-131	Proliferating hepatocytes	NUP205
**2.77111561611052E-135**	0.965815097525924	0.565	0.299	4.64383554947801E-131	Proliferating hepatocytes	CDKN2A
**1.950250549112E-134**	0.577223775067581	0.865	0.534	3.26822987020189E-130	Proliferating hepatocytes	ADA
**2.21456157964257E-134**	1.87999432047557	0.135	0.027	3.71116229516502E-130	Proliferating hepatocytes	RTTN
**2.35695773115062E-134**	1.51222509955163	0.274	0.094	3.94978976586221E-130	Proliferating hepatocytes	CDC6
**5.25268458593536E-134**	1.31978051503849	0.304	0.111	8.80244882911048E-130	Proliferating hepatocytes	XRCC3
**5.64373267868685E-134**	1.0026675784334	0.286	0.098	9.45776722294343E-130	Proliferating hepatocytes	AJUBA
**5.83784123793527E-134**	1.70298260295937	0.141	0.029	9.78305434653193E-130	Proliferating hepatocytes	TAF5
**1.5511517978234E-133**	0.872828394232267	0.682	0.42	2.59942018279245E-129	Proliferating hepatocytes	STAG2
**2.61006341825162E-133**	0.795221038605305	0.751	0.492	4.37394427630607E-129	Proliferating hepatocytes	BTG1
**3.12845165198054E-133**	1.4710224638644	0.268	0.091	5.242659278389E-129	Proliferating hepatocytes	TP73
**4.26063069548628E-133**	0.637311380055566	0.903	0.639	7.13996491949592E-129	Proliferating hepatocytes	LYZ
**5.28320422723769E-133**	0.964719285841158	0.456	0.211	8.85359364400493E-129	Proliferating hepatocytes	CKLF
**5.40850827927707E-133**	0.811190505366475	0.616	0.343	9.06357817441252E-129	Proliferating hepatocytes	HNRNPAB
**2.82446542853765E-132**	0.95614937095377	0.485	0.234	4.7332391651434E-128	Proliferating hepatocytes	RSRC1
**1.88288255241762E-131**	0.971783045850726	0.622	0.361	3.15533458134145E-127	Proliferating hepatocytes	TUBB2A
**2.73800462718676E-131**	1.53891144566229	0.31	0.119	4.58834815423957E-127	Proliferating hepatocytes	FAM111B
**3.5762550613775E-131**	1.00205419496935	0.48	0.229	5.99308823185642E-127	Proliferating hepatocytes	KIF16B
**4.88041642837232E-131**	1.27111124059595	0.25	0.081	8.17860185066633E-127	Proliferating hepatocytes	CSE1L
**6.23701954193545E-131**	0.917342961674764	0.536	0.277	1.04519973483754E-126	Proliferating hepatocytes	HNRNPUL2
**7.19613381853022E-131**	1.28863249005935	0.321	0.122	1.20592810530929E-126	Proliferating hepatocytes	CNTNAP2
**7.876419152256E-131**	1.61461616841191	0.145	0.031	1.31993032153506E-126	Proliferating hepatocytes	YPEL1
**5.55594435383259E-130**	1.18831093618153	0.342	0.135	9.31065154815266E-126	Proliferating hepatocytes	CDC27
**1.02583529902561E-129**	1.38724914372242	0.265	0.09	1.71909479410712E-125	Proliferating hepatocytes	MAZ
**1.12036845886631E-129**	1.69462609851549	0.14	0.029	1.87751346336816E-125	Proliferating hepatocytes	ARHGAP19
**1.30271669182883E-129**	1.05893951113715	0.486	0.244	2.18309263216676E-125	Proliferating hepatocytes	KBTBD2
**1.37243334468414E-129**	1.29011657683651	0.274	0.095	2.29992379902168E-125	Proliferating hepatocytes	ANP32B
**1.95443712979685E-129**	0.978210809613166	0.523	0.273	3.27524574211355E-125	Proliferating hepatocytes	DAZAP1
**2.1469356589034E-129**	1.06028920969908	0.436	0.199	3.59783477719032E-125	Proliferating hepatocytes	EIF4E
**2.57670296337272E-129**	1.07047619012988	0.393	0.17	4.31803882602001E-125	Proliferating hepatocytes	CTCF
**2.62394354932083E-129**	1.15296853306967	0.374	0.157	4.39720459995185E-125	Proliferating hepatocytes	JADE1
**3.43175233623614E-129**	1.07839948497917	0.421	0.188	5.75093056506453E-125	Proliferating hepatocytes	MYO5C
**4.15630178799689E-129**	1.17591424809628	0.343	0.138	6.96513053632518E-125	Proliferating hepatocytes	SLC1A4
**5.72207086789456E-129**	1.30192597996606	0.269	0.092	9.58904636041771E-125	Proliferating hepatocytes	DEPRECATEI
**7.52418549931799E-129**	0.916318914642469	0.505	0.249	1.26090300597571E-124	Proliferating hepatocytes	H2AFY
**1.04659238803857E-128**	0.864782232585055	0.593	0.333	1.75387952387503E-124	Proliferating hepatocytes	SRSF3
**1.06501383644199E-128**	1.17193191098427	0.402	0.179	1.78475018710948E-124	Proliferating hepatocytes	TOPBP1
**1.42544884680735E-128**	1.45009116045908	0.357	0.15	2.38876717747976E-124	Proliferating hepatocytes	POLE2
**1.93725347166374E-128**	0.705903408471037	0.744	0.441	3.24644936781409E-124	Proliferating hepatocytes	KLF5
**2.58178836203048E-128**	1.06672668421443	0.481	0.243	4.32656093709068E-124	Proliferating hepatocytes	RBM15
**3.8134742284457E-128**	0.946869458858616	0.492	0.241	6.39062011202931E-124	Proliferating hepatocytes	PAF1
**4.37843943546058E-128**	1.48672277177562	0.236	0.076	7.33738880594483E-124	Proliferating hepatocytes	PIM2
**4.82016119598188E-128**	1.08050624160441	0.475	0.236	8.07762613222643E-124	Proliferating hepatocytes	MARCKS
**8.32123261382396E-128**	0.966369202335474	0.502	0.255	1.39447216142462E-123	Proliferating hepatocytes	PHACTR4
**2.03267443351887E-127**	1.04392306691859	0.31	0.116	3.40635581569093E-123	Proliferating hepatocytes	ZW10
**2.8547070764499E-127**	1.1698450360409	0.324	0.124	4.78391811871475E-123	Proliferating hepatocytes	TTL
**2.60182706877392E-126**	1.03062202311734	0.592	0.339	4.36014180185133E-122	Proliferating hepatocytes	MCM4
**2.86664448578781E-126**	0.85365385675554	0.527	0.269	4.80392282928321E-122	Proliferating hepatocytes	FUS
**4.04523073183776E-126**	1.15839243013946	0.307	0.116	6.77899766041372E-122	Proliferating hepatocytes	AACS
**4.38980114270268E-126**	0.981840610668023	0.485	0.239	7.35642875494115E-122	Proliferating hepatocytes	SUZ12
**6.15174169513529E-126**	2.45824327201036	0.108	0.018	1.03090887327077E-121	Proliferating hepatocytes	CDC25A
**6.48740820734808E-126**	1.34379135198698	0.213	0.063	1.08715986738739E-121	Proliferating hepatocytes	SPRTN
**9.71492469561416E-126**	0.989591342052837	0.48	0.237	1.62802708049102E-121	Proliferating hepatocytes	TXNDC12
**1.63428661250636E-125**	0.887632512013144	0.542	0.285	2.73873750523815E-121	Proliferating hepatocytes	CMC2
**2.29352057700803E-125**	2.18491423547669	0.107	0.018	3.84348178295006E-121	Proliferating hepatocytes	SPA17

2.29813369662398E-125	0.890629868127314	0.676	0.437	3.85121244880246E-121	Proliferating hepatocytes	STOM
3.71577596540312E-125	0.657281591791684	0.83	0.567	6.22689736282255E-121	Proliferating hepatocytes	BCAP31
3.89300113716999E-125	1.25509906667967	0.272	0.096	6.52389130566947E-121	Proliferating hepatocytes	STK17B
4.56911580772708E-125	1.23656957725487	0.394	0.177	7.65692427058904E-121	Proliferating hepatocytes	ACYP1
8.58841510189636E-125	0.920950964081373	0.586	0.329	1.43924660277579E-120	Proliferating hepatocytes	TMX4
8.67645656900194E-125	1.07644014774255	0.514	0.273	1.45400059183335E-120	Proliferating hepatocytes	NT5DC3
8.6844317981675E-125	1.71408169857153	0.156	0.037	1.45533708073691E-120	Proliferating hepatocytes	LSM 11.00
1.8998624770935E-124	1.02714195490878	0.419	0.192	3.18378953911328E-120	Proliferating hepatocytes	RAVER1
2.52503244430024E-124	1.51071484521245	0.24	0.079	4.23144937015835E-120	Proliferating hepatocytes	STK26
2.97328615166632E-124	0.425033038089155	0.929	0.67	4.98263293296241E-120	Proliferating hepatocytes	PIGR
7.45335164152553E-123	1.24034349685496	0.365	0.156	1.24903266808685E-118	Proliferating hepatocytes	TSPAN7
1.23125294529323E-122	1.45909063009402	0.351	0.15	2.0633336857224E-118	Proliferating hepatocytes	DEPRECATEI
1.70244145342916E-122	1.27737601389	0.206	0.061	2.85295138765659E-118	Proliferating hepatocytes	PPIH
2.55444436498349E-122	1.29592739748728	0.337	0.137	4.28073786683933E-118	Proliferating hepatocytes	RADX
5.14024597569303E-122	1.51459590098805	0.478	0.257	8.61402420606638E-118	Proliferating hepatocytes	HELLS
9.76300079212115E-122	0.933252030702904	0.459	0.221	1.63608367274366E-117	Proliferating hepatocytes	C19orf48
1.16025520308859E-121	1.03989699872711	0.414	0.187	1.94435566933587E-117	Proliferating hepatocytes	RFX5
1.59490457850571E-121	0.965907078481808	0.38	0.164	2.67274109265987E-117	Proliferating hepatocytes	KRI1
1.98240718736427E-121	1.67959754565141	0.143	0.033	3.32211796458504E-117	Proliferating hepatocytes	GEM
3.96913588474599E-121	1.0504669461228	0.411	0.189	6.65147791565732E-117	Proliferating hepatocytes	SLC9A6
3.97348388976072E-121	0.89267233849336	0.571	0.314	6.65876430246101E-117	Proliferating hepatocytes	DKC1
4.16172637388813E-121	1.70430647837933	0.211	0.065	6.97422105736172E-117	Proliferating hepatocytes	PRTFDC1
8.93884834610135E-121	0.995427711131197	0.462	0.23	1.49797220583966E-116	Proliferating hepatocytes	TGIF1
9.21492697959016E-121	0.574120906599207	0.926	0.611	1.54423746323972E-116	Proliferating hepatocytes	TSC22D3
1.05613783957176E-120	1.01191388873898	0.538	0.293	1.76987579155435E-116	Proliferating hepatocytes	DNMT1
2.08231156340741E-120	0.993590421384424	0.493	0.252	3.48953771795814E-116	Proliferating hepatocytes	SUPT16H
3.56439052950281E-120	1.13251311297792	0.409	0.188	5.9732056493408E-116	Proliferating hepatocytes	CNIH4
5.83934530082171E-120	1.1924644401617	0.219	0.068	9.78557485511702E-116	Proliferating hepatocytes	FBLN7
7.57850241398281E-120	1.25194565659846	0.352	0.148	1.27000543453524E-115	Proliferating hepatocytes	PLXNC1
1.03063296780111E-119	0.991869975885376	0.265	0.092	1.72713472744111E-115	Proliferating hepatocytes	POU2F1
1.55389959130993E-119	0.83906415083669	0.461	0.224	2.60402493511718E-115	Proliferating hepatocytes	NUP50
1.66167528311766E-119	0.964009790026268	0.29	0.108	2.78463543944858E-115	Proliferating hepatocytes	ATF7IP
3.34298851110297E-119	0.846640657502367	0.476	0.237	5.60218014690636E-115	Proliferating hepatocytes	NFATC2IP
3.90423306498164E-119	1.11980720138507	0.406	0.186	6.54271377029624E-115	Proliferating hepatocytes	C12orf75
4.53374624917415E-119	0.879105019519676	0.571	0.316	7.59765196436604E-115	Proliferating hepatocytes	NUP58
7.79434538400715E-119	1.03402745007993	0.388	0.173	1.30617639945192E-114	Proliferating hepatocytes	MED30
7.97827341888067E-119	0.862691954915051	0.22	0.069	1.33699905953602E-114	Proliferating hepatocytes	NUAK2
9.36338487622119E-119	1.98006238204771	0.151	0.037	1.56911603755715E-114	Proliferating hepatocytes	LRRC37A3
1.0929571644756E-118	1.14613932311621	0.326	0.132	1.83157761622821E-114	Proliferating hepatocytes	TIMELESS
1.24985738641315E-118	1.19990131446433	0.281	0.104	2.09451100815116E-114	Proliferating hepatocytes	PSAT1
1.29565868095231E-118	1.27181323282762	0.309	0.121	2.17126481753988E-114	Proliferating hepatocytes	CDC7
1.32016074812948E-118	0.857547470832574	0.509	0.268	2.21232538171538E-114	Proliferating hepatocytes	NUMA1
2.14887903331637E-118	1.26995883586156	0.221	0.071	3.60109148403157E-114	Proliferating hepatocytes	SAC3D1
2.63329557355414E-118	0.956675202361334	0.356	0.15	4.41287672216202E-114	Proliferating hepatocytes	NUP188
2.82617219383261E-118	1.11209538161176	0.244	0.082	4.73609936242469E-114	Proliferating hepatocytes	NUP93
2.93294001751703E-118	0.885305859619256	0.534	0.283	4.91502088135505E-114	Proliferating hepatocytes	CRKL
3.09782460304171E-118	1.13257940481414	0.229	0.074	5.19133446977729E-114	Proliferating hepatocytes	ACVR2B
3.25934563961743E-118	0.768154363307254	0.663	0.404	5.46201142287089E-114	Proliferating hepatocytes	HNRNPA3
4.62977009805567E-118	1.31766658377178	0.222	0.071	7.7585687303217E-114	Proliferating hepatocytes	GAB1
1.09631179725106E-117	0.904770745321151	0.368	0.157	1.83719930983332E-113	Proliferating hepatocytes	XPO1
1.5520000497047E-117	1.20199450268791	0.237	0.08	2.60084168329514E-113	Proliferating hepatocytes	FRMD4B
3.92183351192517E-117	1.27975818595118	0.193	0.056	6.57220859928419E-113	Proliferating hepatocytes	CDAN1
4.67515335555155E-117	1.27205100906644	0.313	0.124	7.83462199323329E-113	Proliferating hepatocytes	TMEM187
6.75966116588267E-117	0.951915735582358	0.506	0.265	1.13278401817862E-112	Proliferating hepatocytes	SLC27A3
1.25071642753078E-116	1.18719299781849	0.318	0.127	2.09595058925608E-112	Proliferating hepatocytes	ITGB3BP
1.6495985738097E-116	0.939846240898002	0.492	0.253	2.7643972899903E-112	Proliferating hepatocytes	YWHAQ
2.95563329787899E-116	0.88335143251403	0.298	0.113	4.95305028058561E-112	Proliferating hepatocytes	EPB41L2
3.13012065365238E-116	0.57950052314071	0.984	0.872	5.24545619139066E-112	Proliferating hepatocytes	MT-CO2
4.00969058307357E-116	1.47320875378232	0.193	0.057	6.71943947911468E-112	Proliferating hepatocytes	TLN2
4.1977246451738E-116	0.871335917289793	0.515	0.266	7.03454696038226E-112	Proliferating hepatocytes	GNA11

5.44800303696476E-116	1.36805970033522	0.197	0.059	9.12976348934554E-112	Proliferating hepatocytes	RAD51C
5.52421240234009E-116	1.15744559523186	0.362	0.157	9.25747514384152E-112	Proliferating hepatocytes	GUCY1B1
6.3380460256147E-116	1.04689946165328	0.419	0.197	1.06212975297251E-111	Proliferating hepatocytes	PLIN3
8.0345607664107E-116	0.771172619285968	0.699	0.45	1.3464316932351E-111	Proliferating hepatocytes	GSR
1.45786050583936E-115	0.846472197507136	0.322	0.128	2.44308263568 56E-111	Proliferating hepatocytes	TMEM160
2.51833471368739E-115	0.848177727897208	0.448	0.215	4.22022531319733E-111	Proliferating hepatocytes	PSMG2
2.51889159546143E-115	1.08844077586482	0.26	0.093	4.22115853567426E-111	Proliferating hepatocytes	DEPRECATEI
3.59376200137831E-115	0.865442519095076	0.448	0.216	6.02242636190977E-111	Proliferating hepatocytes	MTA2
5.22478100781459E-115	1.11559807613856	0.34	0.142	8.75568801289569E-111	Proliferating hepatocytes	RMI1
6.20818260738503E-115	1.00703738020895	0.384	0.173	1.04036724134558E-110	Proliferating hepatocytes	SNTB2
7.58850494878051E-115	1.13318904315593	0.352	0.151	1.27168165931664E-110	Proliferating hepatocytes	SLC25A40
1.37927534997534E-114	1.83411625218977	0.114	0.022	2.31138963148868E-110	Proliferating hepatocytes	ALG10
1.4109546540351E-114	1.00906385248252	0.383	0.171	2.36447780923202E-110	Proliferating hepatocytes	SNHG32
3.14223961477643E-114	0.792423798708906	0.495	0.253	5.26576514644234E-110	Proliferating hepatocytes	JPT2
3.96888200740867E-114	0.91058033153681	0.351	0.149	6.65105246801544E-110	Proliferating hepatocytes	ARID1A
4.78927254064045E-114	2.34444014627161	0.132	0.03	8.02586292360527E-110	Proliferating hepatocytes	PCDHA6
1.68992480284114E-113	0.977303724681717	0.365	0.16	2.83197598460118E-109	Proliferating hepatocytes	BTG3
1.98316794848758E-113	1.26747023941164	0.285	0.109	3.32339284807549E-109	Proliferating hepatocytes	FBF1
2.50344987148677E-113	0.817284857272914	0.533	0.282	4.19528129463752E-109	Proliferating hepatocytes	MZT2B
2.77889360541122E-113	0.76619004551257	0.628	0.372	4.65686990394812E-109	Proliferating hepatocytes	IMPA2
5.30826516356307E-113	0.936035286614943	0.396	0.18	8.89559076109899E-109	Proliferating hepatocytes	CSTF2
5.89689122005456E-113	1.5465512120738	0.174	0.048	9.88201030656744E-109	Proliferating hepatocytes	DDB2
5.93945116958956E-113	0.978212747671126	0.3	0.116	9.95333226999818E-109	Proliferating hepatocytes	ERI2
8.33533957700893E-113	1.44074455359 84	0.213	0.068	1.39683620631516E-108	Proliferating hepatocytes	NEDD1
1.5830176048109E-112	0.891485603096051	0.466	0.235	2.65282090214211E-108	Proliferating hepatocytes	DEPRECATEI
1.67461252468E-112	1.19582792723377	0.239	0.082	2.80631566885874E-108	Proliferating hepatocytes	CENPS
1.8922880114396E-112	0.849824239034876	0.476	0.242	3.17109624957048E-108	Proliferating hepatocytes	HCFC1
3.71349674490065E-112	0.803349715383323	0.51	0.267	6.2230778451045E-108	Proliferating hepatocytes	RNF4
4.04254382708836E-112	0.974799780953419	0.559	0.339	6.77449494543467E-108	Proliferating hepatocytes	SUGP2
4.69698270313182E-112	1.15290046254284	0.277	0.104	7.8712036139083E-108	Proliferating hepatocytes	PHTF2
9.71869846383058E-112	1.25351551820778	0.311	0.127	1.62865948856873E-107	Proliferating hepatocytes	ARHGEF37
1.02800856601832E-111	0.96742116302622	0.389	0.177	1.7227367549335E-107	Proliferating hepatocytes	ZNF704
1.10885432292662E-111	1.02191254875737	0.352	0.152	1.85821807436043E-107	Proliferating hepatocytes	SKP2
1.18217457350213E-111	1.09571064089995	0.336	0.142	1.98108815027487E-107	Proliferating hepatocytes	TLNRD1
1.35652625427807E-111	0.809120971501496	0.578	0.327	2.2732666969192E-107	Proliferating hepatocytes	EMP2
1.00259063125655E-110	0.693808843302445	0.441	0.21	1.68014137985973E-106	Proliferating hepatocytes	AGR2
1.1735677705771E-110	1.06965510624893	0.279	0.106	1.96666486993311E-106	Proliferating hepatocytes	FAM13B
1.17468230545765E-110	0.829109151494839	0.489	0.249	1.96853260748592E-106	Proliferating hepatocytes	TAF15
1.54387584313899E-110	0.877367140518999	0.429	0.208	2.58722713793232E-106	Proliferating hepatocytes	CORO1C
2.89750610813909E-110	0.776169337980125	0.562	0.303	4.85564073601949E-106	Proliferating hepatocytes	CHP2
4.8459872662605E-110	0.716612922245001	0.703	0.448	8.12090546079935E-106	Proliferating hepatocytes	STK24
4.88885059821896E-110	1.02567991590196	0.224	0.074	8.19273583249533E-106	Proliferating hepatocytes	ERI1
5.94975470784875E-110	0.965930399491593	0.5	0.268	9.97059893941293E-106	Proliferating hepatocytes	DEPRECATEI
1.57257593619618E-109	0.731732869010074	0.662	0.403	2.63532275387756E-105	Proliferating hepatocytes	PGD
2.18440926107358E-109	1.4653962591404	0.174	0.05	3.66063303970711E-105	Proliferating hepatocytes	TMEM237
2.68126487822618E-109	0.721612837415498	0.637	0.392	4.49326368293143E-105	Proliferating hepatocytes	PI4K2B
2.83567834407934E-109	1.16282763540694	0.311	0.126	4.75202976900815E-105	Proliferating hepatocytes	PKNOX1
3.20488521231457E-109	0.933745594733364	0.455	0.227	5.37074663879675E-105	Proliferating hepatocytes	UBR7
4.05027618353223E-109	1.04203979162868	0.337	0.143	6.78745282836332E-105	Proliferating hepatocytes	KPNB1
4.23884921874264E-109	0.784050109678756	0.594	0.34	7.10346352076892E-105	Proliferating hepatocytes	ADAM9
4.5040184624831E-109	0.932749707118757	0.415	0.199	7.54783413942917E-105	Proliferating hepatocytes	AK6
4.87038161254973E-109	1.34761778493074	0.221	0.074	8.16178550631083E-105	Proliferating hepatocytes	ACTRT3
4.9084515085118E-109	0.794721152527	0.589	0.338	8.22558303796407E-105	Proliferating hepatocytes	SEPTIN11
6.46102017689461E-109	0.799370905180389	0.269	0.099	1.082737761244E-104	Proliferating hepatocytes	EXOSC8
6.4745295074397E-109	0.8953326174772	0.481	0.243	1.08500165485675E-104	Proliferating hepatocytes	TPM4
1.95177926160181E-108	0.908150910774614	0.489	0.259	3.27079168659232E-104	Proliferating hepatocytes	FOXA1
2.99928338237823E-108	0.906566310470627	0.438	0.213	5.02619909218944E-104	Proliferating hepatocytes	COMMD4
3.50434182183261E-108	0.947794890286495	0.352	0.153	5.87257602502709E-104	Proliferating hepatocytes	BCL7C
4.79404453545938E-108	0.941688510654331	0.433	0.212	8.03385983252283E-104	Proliferating hepatocytes	DEPRECATEI
7.35676606007755E-108	0.701231549028516	0.616	0.366	1.2328468563478E-103	Proliferating hepatocytes	ARF6

1.16595318591684E-107	0.870298119684742	0.416	0.197	1.95390434895945E-103	Proliferating hepatocytes	GTPBP2
1.22721485302276E-107	0.864766542090154	0.463	0.234	2.05656665069553E-103	Proliferating hepatocytes	RAP2A
1.32369512554463E-107	1.12401903188226	0.196	0.061	2.21824829138768E-103	Proliferating hepatocytes	CCDC134
1.40095239137351E-107	0.741929451217013	0.635	0.379	2.34771601746372E-103	Proliferating hepatocytes	MAPK1
2.40376263795267E-107	1.26987934429823	0.197	0.062	4.02822542868109E-103	Proliferating hepatocytes	SRGAP2C
2.66588851318309E-107	0.816974981328327	0.558	0.315	4.46749597039223E-103	Proliferating hepatocytes	SSRP1
3.9230107208256E-107	1.13678457803747	0.304	0.124	6.57418136595955E-103	Proliferating hepatocytes	MAPRE1
4.12850048074336E-107	1.10739661936269	0.176	0.051	6.91854110562972E-103	Proliferating hepatocytes	DEPRECATEI
8.27964617623487E-107	0.880053459251132	0.395	0.184	1.38750310621344E-102	Proliferating hepatocytes	PDS5B
1.2556808981125E-106	1.12973641287593	0.245	0.088	2.10427004905693E-102	Proliferating hepatocytes	CTNNAL1
4.9275665943506E-106	1.12607845567071	0.309	0.128	8.25761609881274E-102	Proliferating hepatocytes	FANCC
5.42188279012375E-106	0.776292141199386	0.583	0.334	9.08599117968937E-102	Proliferating hepatocytes	MAF 1.00
5.84256969244393E-106	0.620364509107672	0.749	0.478	9.79097829059753E-102	Proliferating hepatocytes	CMC4
6.85248385261964E-106	1.11588444192882	0.297	0.12	1.148339244022E-101	Proliferating hepatocytes	RAD51B
7.62084840227602E-106	1.00089070551222	0.421	0.208	1.27710177525342E-101	Proliferating hepatocytes	PCNA
9.3285974922989E-106	0.921271482180214	0.32	0.133	1.56328636775945E-101	Proliferating hepatocytes	TRNAU1AP
1.04268771330082E-105	0.837915774974242	0.47	0.237	1.74733606994951E-101	Proliferating hepatocytes	SH3GL1
2.49795623310664E-105	0.885467184914519	0.325	0.135	4.18607505544011E-101	Proliferating hepatocytes	DEPRECATEI
3.35468365018211E-105	0.744057196549738	0.603	0.356	5.62177886097518E-101	Proliferating hepatocytes	HNRNPL
3.48052251341912E-105	0.773308360650057	0.467	0.237	5.83265962798776E-101	Proliferating hepatocytes	HSPA4L
3.60404107976094E-105	0.961366349378723	0.405	0.193	6.03965204146338E-101	Proliferating hepatocytes	SLC7A1
3.73240176618321E-105	1.38135045028207	0.214	0.071	6.25475887976982E-101	Proliferating hepatocytes	DEPRECATEI
4.19612783331708E-105	0.791479977541094	0.613	0.366	7.03187102307276E-101	Proliferating hepatocytes	SIPA1L2
5.40869746426817E-105	1.16759406851076	0.216	0.073	9.06389521062059E-101	Proliferating hepatocytes	LSM 5.00
6.17446508670813E-105	1.00182552360465	0.305	0.125	1.03471685923055E-100	Proliferating hepatocytes	ZNF318
7.09087363730112E-105	0.916908377478542	0.508	0.282	1.18828860413892E-100	Proliferating hepatocytes	SUN2
8.46762973031315E-105	1.05141668552274	0.39	0.184	1.41900539020588E-100	Proliferating hepatocytes	PXMP4
1.04521359657597E-104	0.870496226427112	0.484	0.257	1.751568945142E-100	Proliferating hepatocytes	NUP85
1.26268889428907E-104	0.932440167202451	0.309	0.127	2.11601404904962E-100	Proliferating hepatocytes	DEPRECATEI
1.40218448691344E-104	1.0000335405078	0.456	0.238	2.34978076316954E-100	Proliferating hepatocytes	PCF11
1.69437059230463E-104	0.793977154563804	0.562	0.322	2.8394262385841E-100	Proliferating hepatocytes	TMCO3
3.04542069107417E-104	0.875456935141491	0.267	0.101	5.10351599410209E-100	Proliferating hepatocytes	PIGK
3.23830630681489E-104	0.835326686464967	0.25	0.091	5.42675370896039E-100	Proliferating hepatocytes	RGS12
3.42298861694896E-104	0.715941458344845	0.566	0.321	5.73624432428306E-100	Proliferating hepatocytes	HDGF
4.24710030745934E-104	0.941020906473635	0.428	0.212	7.11729069524037E-100	Proliferating hepatocytes	SEMA6A
4.65707251032839E-104	0.77356054413948	0.49	0.257	7.80432211280831E-100	Proliferating hepatocytes	HLTF
5.26046469594006E-104	0.865020688933855	0.52	0.29	8.81548673745635E-100	Proliferating hepatocytes	PSPC1
6.62846510806357E-104	1.01895121171278	0.327	0.139	1.11079818280929E-99	Proliferating hepatocytes	DESI2
6.73987422171777E-104	0.877584153858138	0.437	0.215	1.12946812207546E-99	Proliferating hepatocytes	HPS3
7.3974671808264E-104	1.21703147007307	0.219	0.075	1.23966755016289E-99	Proliferating hepatocytes	WDHD1
2.41823559347362E-103	0.809665818075164	0.346	0.15	4.05247920754309E-99	Proliferating hepatocytes	MAP3K3
2.85497945973215E-103	1.67712578709805	0.16	0.045	4.78437457861914E-99	Proliferating hepatocytes	FOXN4
3.05303976979097E-103	0.659151007685757	0.699	0.45	5.11628404621571E-99	Proliferating hepatocytes	TRIR
3.11788437706313E-103	0.659613526238251	0.667	0.43	5.22495063908239E-99	Proliferating hepatocytes	ILF3
3.82313115798229E-103	0.579542943086982	0.791	0.551	6.40680319454673E-99	Proliferating hepatocytes	ACTN4
3.83402881298322E-103	0.899955322691697	0.411	0.199	6.42506548479728E-99	Proliferating hepatocytes	HSPB11
5.53230198856328E-103	0.867511428059118	0.503	0.27	9.27103167243434E-99	Proliferating hepatocytes	CLIC1
6.33136673761578E-103	1.43243263751382	0.21	0.071	1.06101043788965E-98	Proliferating hepatocytes	RNASE7
9.48859899391159E-103	1.25426252905918	0.312	0.133	1.5900994193997E-98	Proliferating hepatocytes	OSBPL10
1.07922852673116E-102	0.829899729114898	0.526	0.291	1.80857116509607E-98	Proliferating hepatocytes	SRPK1
1.17835168034476E-102	1.16031433118653	0.173	0.051	1.97468174592174E-98	Proliferating hepatocytes	ZNF92
1.57655322032242E-102	1.11062722021495	0.28	0.111	2.64198788661631E-98	Proliferating hepatocytes	ALG1L
1.62069827016437E-102	1.17111048663974	0.182	0.055	2.71596616114145E-98	Proliferating hepatocytes	CEP295
5.77903282536182E-102	0.892019445993799	0.389	0.183	9.68450320874134E-98	Proliferating hepatocytes	CCT5
8.40494659222852E-102	0.93411808025246	0.419	0.207	1.40850094992566E-97	Proliferating hepatocytes	TTLL5
9.44743804944483E-102	0.55236342438553	0.729	0.46	1.58320166832596E-97	Proliferating hepatocytes	IRAK1
1.0170386846852E-101	0.654834657444848	0.621	0.377	1.70435342779546E-97	Proliferating hepatocytes	NONO
1.1066027101429E-101	1.11332713937601	0.282	0.113	1.85444482165748E-97	Proliferating hepatocytes	CDCA7L
1.31513399009332E-101	0.989857213440203	0.312	0.131	2.20390154059839E-97	Proliferating hepatocytes	CEP44
1.38451275729048E-101	0.696180985851567	0.594	0.352	2.32016647866739E-97	Proliferating hepatocytes	HMGN1

1.40920752855783E-101	0.826828168452121	0.46	0.235	2.36154997635721E-97	Proliferating hepatocytes	KLHDC3
1.54693985360683E-101	0.815467939436217	0.533	0.297	2.59236180667432E-97	Proliferating hepatocytes	VAMP7
1.71333146487114E-101	0.973713670429772	0.341	0.15	2.87120086883106E-97	Proliferating hepatocytes	CYB561A3
2.01624714273644E-101	1.03152137847984	0.244	0.089	3.37882696179772E-97	Proliferating hepatocytes	C11orf80
3.3508698662241E-101	0.819997095593636	0.472	0.245	5.61538772181836E-97	Proliferating hepatocytes	KHDRBS1
3.79934129223175E-101	1.71600291319591	0.113	0.025	6.36693613752197E-97	Proliferating hepatocytes	RIPPLY3
7.39763646715668E-101	0.703040925780922	0.303	0.124	1.23969591916612E-96	Proliferating hepatocytes	PNRC2
7.57202520881987E-101	1.05605859068011	0.343	0.153	1.26891998449403E-96	Proliferating hepatocytes	CDKL1
9.67035408661689E-101	0.969739206961493	0.413	0.205	1.62055793783526E-96	Proliferating hepatocytes	DBP
1.03557969369593E-100	0.900424559566885	0.488	0.265	1.73542445069564E-96	Proliferating hepatocytes	MMGT1
1.21411925725234E-100	0.864036571497968	0.479	0.257	2.03462105130348E-96	Proliferating hepatocytes	FAIM
1.31019725788662E-100	0.942782760373384	0.363	0.166	2.19562856476639E-96	Proliferating hepatocytes	PRRG4
4.47791250868172E-100	0.914761076281715	0.4	0.193	7.50408578204883E-96	Proliferating hepatocytes	RCOR1
4.76850658677403E-100	1.25226375753753	0.157	0.044	7.99106333811592E-96	Proliferating hepatocytes	CNTRL
6.09900181055492E-100	0.795357954650294	0.443	0.225	1.02207072341279E-95	Proliferating hepatocytes	RANBP9
7.7608013035143E-100	1.42987136619555	0.225	0.081	1.30055508244293E-95	Proliferating hepatocytes	MCUB
1.04685601289825E-99	0.958248654193718	0.327	0.142	1.75432130641489E-95	Proliferating hepatocytes	ALYREF
1.71300575321952E-99	0.951817703023173	0.422	0.209	2.87065504124527E-95	Proliferating hepatocytes	KCTD12
2.19438404392546E-99	0.564907317161481	0.844	0.624	3.67734878081029E-95	Proliferating hepatocytes	HSD17B11
2.22757039019048E-99	1.11399324218074	0.238	0.087	3.7329624598812E-95	Proliferating hepatocytes	NT5DC2
3.78316941795342E-99	1.10922760595516	0.334	0.149	6.33983531060633E-95	Proliferating hepatocytes	HOXA10
6.26566546768431E-99	0.941596227075178	0.419	0.208	1.05000021907454E-94	Proliferating hepatocytes	REPIN1
6.869828858847E-99	0.76286813598758	0.488	0.257	1.15124592016558E-94	Proliferating hepatocytes	SAP30L
7.55097560905594E-99	0.77454541232163	0.368	0.17	1.26539249256559E-94	Proliferating hepatocytes	GTF2A1
7.84074493413398E-99	0.902833508936414	0.387	0.186	1.31395203606217E-94	Proliferating hepatocytes	NUP155
9.34666611280614E-99	0.924194520775527	0.365	0.169	1.56631430718405E-94	Proliferating hepatocytes	KDM1A
1.06735205426232E-98	0.795443932290774	0.588	0.352	1.7886685725328E-94	Proliferating hepatocytes	CCNG1
1.4950477190596E-98	0.785533033860356	0.555	0.322	2.50540096760008E-94	Proliferating hepatocytes	RBM42
1.66755097107393E-98	0.856208666515687	0.381	0.179	2.79448191732569E-94	Proliferating hepatocytes	QSER1
2.50814665376563E-98	0.936574813997894	0.417	0.208	4.20315216238044E-94	Proliferating hepatocytes	LAMA3
3.38953948392159E-98	1.3771806921992	0.215	0.075	5.6801902671558E-94	Proliferating hepatocytes	GRK6
5.10562864427247E-98	0.984160713157364	0.357	0.165	8.5560124820718E-94	Proliferating hepatocytes	COX20
7.60900604178164E-98	0.643383341716045	0.571	0.341	1.27511723248177E-93	Proliferating hepatocytes	ASXL1
8.2284691284356E-98	0.890179483767155	0.383	0.181	1.37892685654324E-93	Proliferating hepatocytes	CBX3
9.06584862210512E-98	0.767151763058093	0.5	0.27	1.51925491209238E-93	Proliferating hepatocytes	PEA15
9.8702648118651E-98	0.775053113014994	0.581	0.343	1.65405897717235E-93	Proliferating hepatocytes	BAX
1.26266555143542E-97	0.926868505970142	0.497	0.278	2.11597493109548E-93	Proliferating hepatocytes	DLG5
1.34134519765469E-97	0.79763670066917	0.454	0.233	2.24782628222973E-93	Proliferating hepatocytes	DDR1
1.39582653538771E-97	0.98787961527013	0.338	0.151	2.33912610800272E-93	Proliferating hepatocytes	JADE2
1.43985873195268E-97	0.756363123621399	0.573	0.339	2.4129152630063E-93	Proliferating hepatocytes	EIF4A3
1.65010629752293E-97	0.922823890410344	0.382	0.183	2.76524813338893E-93	Proliferating hepatocytes	MDC 1.00
2.08943962088817E-97	0.96216611825207	0.241	0.09	3.5014829166844E-93	Proliferating hepatocytes	CD44
2.22150530384664E-97	0.84094961034417	0.338	0.152	3.7227985881862E-93	Proliferating hepatocytes	EEF1AKMT2
2.26288314786383E-97	0.726554483390418	0.412	0.201	3.7921395791902E-93	Proliferating hepatocytes	FN3KRP
2.28797137986988E-97	0.786056238678477	0.534	0.304	3.83418243838595E-93	Proliferating hepatocytes	PPIA
2.96716522575026E-97	1.02422715287933	0.21	0.072	4.97237548531229E-93	Proliferating hepatocytes	CEP89
2.96784995022955E-97	1.17030040721752	0.219	0.077	4.97352294659468E-93	Proliferating hepatocytes	CCP110
3.0052371817329E-97	1.01503018173764	0.257	0.099	5.036176469148E-93	Proliferating hepatocytes	TPRKB
4.50094376615109E-97	1.04216326725236	0.311	0.134	7.54268156331599E-93	Proliferating hepatocytes	HMGN5
4.9011203311732E-97	0.846723816672922	0.353	0.16	8.21329745098004E-93	Proliferating hepatocytes	NAGA
5.25507862678719E-97	0.836428088928569	0.472	0.247	8.80646076276998E-93	Proliferating hepatocytes	ASRGL1
6.68519980208039E-97	0.711556692553461	0.411	0.2	1.12030578283263E-92	Proliferating hepatocytes	MUS81
6.99057577776999E-97	0.784769366190855	0.638	0.407	1.1714806888387E-92	Proliferating hepatocytes	LMO7
9.96885436495432E-97	0.623538792654597	0.8	0.563	1.67058061447905E-92	Proliferating hepatocytes	SMIM14
1.0189237903078E-96	0.688858834464414	0.522	0.287	1.70751248779782E-92	Proliferating hepatocytes	FLOT1
1.76678616599728E-96	0.705130349506159	0.611	0.365	2.96078025697824E-92	Proliferating hepatocytes	NAA10
1.85579926874921E-96	0.899702888685201	0.358	0.166	3.10994841456993E-92	Proliferating hepatocytes	UCHL5
1.90993122953876E-96	1.09311263561263	0.321	0.142	3.20066275446106E-92	Proliferating hepatocytes	SPATA33
2.42969507673408E-96	0.83971769233255	0.446	0.232	4.07168300959098E-92	Proliferating hepatocytes	DCTN3
2.85891977453223E-96	0.675266764342784	0.502	0.278	4.7909777581611E-92	Proliferating hepatocytes	TPR

3.52892028747981E-96	1.35070975477825	0.192	0.063	5.91376461775866E-92	Proliferating hepatocytes	SLF1
3.73721490947241E-96	0.994098679467945	0.199	0.066	6.26282474529386E-92	Proliferating hepatocytes	ZNF184
3.84725770818581E-96	1.10113212715636	0.252	0.097	6.44723446737778E-92	Proliferating hepatocytes	DEPRECATEI
6.08519012874268E-96	0.71408633376832	0.786	0.562	1.0197561617747E-91	Proliferating hepatocytes	ACTG1
1.18674617391567E-95	1.07016918610422	0.251	0.097	1.98874923824788E-91	Proliferating hepatocytes	DHRS13
1.28653981179315E-95	0.699935979526256	0.598	0.355	2.15598341660297E-91	Proliferating hepatocytes	PRPS1
1.31385136614106E-95	0.787342435762696	0.431	0.217	2.20175211937919E-91	Proliferating hepatocytes	DR1
2.36482857672239E-95	0.957985639360499	0.314	0.136	3.96297972887138E-91	Proliferating hepatocytes	LCORL
2.40600409225213E-95	1.27087300784464	0.179	0.057	4.03198165779612E-91	Proliferating hepatocytes	ILDR2
2.49732185656754E-95	1.54188609808339	0.115	0.026	4.18501196723588E-91	Proliferating hepatocytes	CEP76
3.58642069964934E-95	0.775612167191153	0.501	0.277	6.01012380847236E-91	Proliferating hepatocytes	PPM1G
4.08691893602005E-95	0.791039048456648	0.504	0.279	6.8488587529824E-91	Proliferating hepatocytes	TFDP1
5.22961854263925E-95	0.824178121109659	0.459	0.24	8.76379475375486E-91	Proliferating hepatocytes	KDM3B
6.38663585504441E-95	0.723113278391136	0.531	0.296	1.07027243658834E-90	Proliferating hepatocytes	SNRPB
7.63831530648584E-95	0.819582062487766	0.484	0.263	1.2800288790609E-90	Proliferating hepatocytes	BRI3BP
8.14886764796335E-95	0.64565444520359	0.631	0.388	1.3655872404457E-90	Proliferating hepatocytes	ELAVL1
8.53807109979934E-95	1.0099380443864	0.26	0.102	1.43080995490437E-90	Proliferating hepatocytes	EFCAB2
8.57889929203378E-95	0.815953151691215	0.176	0.054	1.43765194335902E-90	Proliferating hepatocytes	PHGDH
8.95533377183037E-95	0.903682526935682	0.405	0.201	1.50073483348333E-90	Proliferating hepatocytes	CEP250
9.95469386670393E-95	0.697550600302249	0.618	0.38	1.66820759818224E-90	Proliferating hepatocytes	GCHFR
1.06107097712325E-94	0.520576992939904	0.82	0.56	1.77814274346315E-90	Proliferating hepatocytes	MAOA
1.14198627588173E-94	0.988757476531065	0.337	0.152	1.9137406011226E-90	Proliferating hepatocytes	SRGAP1
1.2039029446127E-94	0.937183567993557	0.358	0.166	2.01750055458196E-90	Proliferating hepatocytes	ARMC1
1.38039337494535E-94	0.855949660632865	0.224	0.081	2.31326321773342E-90	Proliferating hepatocytes	HAUS6
1.45802346941875E-94	0.688193832841778	0.579	0.335	2.44335573005194E-90	Proliferating hepatocytes	SLC25A5
1.51987196433976E-94	0.874292171539313	0.388	0.188	2.54700143784056E-90	Proliferating hepatocytes	C9orf64
1.60638501181422E-94	0.80062249878636	0.424	0.213	2.69198000279827E-90	Proliferating hepatocytes	HNRNPR
1.61304211578973E-94	0.909142296052493	0.462	0.249	2.70313597764043E-90	Proliferating hepatocytes	DONSON
1.76637069444138E-94	1.36776764089963	0.206	0.072	2.96008400974486E-90	Proliferating hepatocytes	MMS22L
2.12818918042075E-94	0.791637090655974	0.533	0.303	3.56641942854909E-90	Proliferating hepatocytes	LIX1L
2.78668619244423E-94	0.689364482640126	0.541	0.306	4.66992872129804E-90	Proliferating hepatocytes	ZFP91
2.97478701241763E-94	0.973947448974522	0.342	0.156	4.98514807540947E-90	Proliferating hepatocytes	NIPA2
3.11241789753712E-94	0.636653882132563	0.765	0.529	5.21578991269271E-90	Proliferating hepatocytes	SPTSSA
4.30777319921927E-94	0.686905659317405	0.403	0.198	7.21896632725165E-90	Proliferating hepatocytes	TAGLN2
8.59355873436996E-94	0.683485575234213	0.617	0.374	1.44010857270572E-89	Proliferating hepatocytes	NEB
1.02131782563309E-93	2.09311257829447	0.101	0.021	1.71152441219592E-89	Proliferating hepatocytes	TOX
1.51815015796956E-93	0.640606563336714	0.413	0.204	2.54411603472538E-89	Proliferating hepatocytes	CNN2
1.79537069456166E-93	0.806126694912508	0.513	0.29	3.00868220994643E-89	Proliferating hepatocytes	SMS
3.09946016847492E-93	0.686656880337289	0.651	0.403	5.19407535033027E-89	Proliferating hepatocytes	EHF
3.96331443019134E-93	0.860595962858521	0.386	0.188	6.64172232211466E-89	Proliferating hepatocytes	LNPK
6.22500914155585E-93	0.709607615325636	0.601	0.37	1.04318703194193E-88	Proliferating hepatocytes	HEXB
6.66496630073219E-93	0.862370896992659	0.312	0.136	1.1169150526767E-88	Proliferating hepatocytes	TRIM28
6.69952171060339E-93	0.72071029566862	0.477	0.259	1.12270584826292E-88	Proliferating hepatocytes	NUP43
1.22492568752779E-92	0.671577998425347	0.498	0.272	2.05273046715908E-88	Proliferating hepatocytes	CHAMP1
1.94759307876578E-92	0.74463701936371	0.504	0.28	3.2637764813957E-88	Proliferating hepatocytes	CEP350
2.06934225362993E-92	0.864246865736935	0.304	0.131	3.46780374863303E-88	Proliferating hepatocytes	FBXO34
2.57449495077242E-92	0.611034295179019	0.386	0.184	4.31433863850443E-88	Proliferating hepatocytes	DUOXA2
2.69425303334285E-92	0.975862170562262	0.217	0.078	4.51502923327594E-88	Proliferating hepatocytes	USP13
3.00159630400434E-92	0.978940682504614	0.339	0.155	5.03007508625047E-88	Proliferating hepatocytes	CLGN
4.4571711497509E-92	0.709045916482439	0.535	0.306	7.46932741275255E-88	Proliferating hepatocytes	HDGFL2
4.87966931859091E-92	0.850748538486682	0.329	0.148	8.17749848409465E-88	Proliferating hepatocytes	GLE1
5.00402028335606E-92	0.87041174512351	0.416	0.21	8.38573719084809E-88	Proliferating hepatocytes	NUP133
8.60682964896419E-92	1.00642732984209	0.258	0.105	1.44233251257342E-87	Proliferating hepatocytes	TUBE1
9.67395902065009E-92	1.04363126397072	0.285	0.121	1.62116205268054E-87	Proliferating hepatocytes	CAMK2N2
9.84067930659171E-92	0.852103723727518	0.362	0.172	1.64910103819864E-87	Proliferating hepatocytes	FOPNL
1.29102139391864E-91	0.712402687427282	0.583	0.345	2.16349365192885E-87	Proliferating hepatocytes	VDAC3
1.45395028975254E-91	1.05885902695036	0.285	0.12	2.43652989556731E-87	Proliferating hepatocytes	SMG9
1.84816835770175E-91	0.772776247681437	0.435	0.224	3.09716053383659E-87	Proliferating hepatocytes	TMEM250
2.10751016796869E-91	0.813518933449123	0.322	0.143	3.53176553948194E-87	Proliferating hepatocytes	MAST2
2.42930730718677E-91	0.727083827015194	0.383	0.185	4.07103318538358E-87	Proliferating hepatocytes	TAF6

2.56081575286957E-91	0.702256368136693	0.559	0.329	4.29141503865883E-87	Proliferating hepatocytes	IPO9
2.74029771734605E-91	0.68321709612951	0.534	0.303	4.59219091472851E-87	Proliferating hepatocytes	AP2A1
3.7487014721051E-91	0.837881246339562	0.453	0.245	6.28207392695373E-87	Proliferating hepatocytes	LMF2
3.84528016267593E-91	0.920136521908702	0.362	0.172	6.44392049661232E-87	Proliferating hepatocytes	CLN6
3.95817915054351E-91	0.739476648600595	0.496	0.275	6.63311662048081E-87	Proliferating hepatocytes	TBCD
4.06366116389289E-91	0.854519020416127	0.311	0.137	6.80988337845171E-87	Proliferating hepatocytes	HAUS2
5.38332000579445E-91	0.802504516298978	0.397	0.197	9.02136766571033E-87	Proliferating hepatocytes	ACTL6A
5.38426794657456E-91	0.653006986401031	0.646	0.409	9.02295622486964E-87	Proliferating hepatocytes	LACTB2
5.91914612205831E-91	0.702655103721232	0.562	0.326	9.91930507134531E-87	Proliferating hepatocytes	FAM20B
6.41695628492966E-91	0.844151731460787	0.489	0.274	1.07535353422851E-86	Proliferating hepatocytes	DOT1L
8.66963941421375E-91	1.04988749526167	0.261	0.105	1.45285817303394E-86	Proliferating hepatocytes	SMAGP
1.2840708911102E-90	0.798486421402221	0.453	0.24	2.15184599932248E-86	Proliferating hepatocytes	WRNIP1
2.11212766697511E-90	1.61549827149645	0.106	0.024	3.53950354431689E-86	Proliferating hepatocytes	FAM216A
2.28124379407025E-90	0.854572656444687	0.35	0.162	3.82290835010292E-86	Proliferating hepatocytes	OSER1
2.36884779342145E-90	0.740832894776172	0.588	0.36	3.96971513221567E-86	Proliferating hepatocytes	PPM1B
2.57389812218005E-90	1.01738747215179	0.269	0.11	4.31333847314933E-86	Proliferating hepatocytes	TTLL7
3.30807371264367E-90	0.862654699471759	0.362	0.172	5.54366992764827E-86	Proliferating hepatocytes	HSPA13
3.82228971051437E-90	0.583076653040776	0.752	0.519	6.40539309687998E-86	Proliferating hepatocytes	GDI1
4.71821878421336E-90	0.695462998806457	0.545	0.315	7.90679103858474E-86	Proliferating hepatocytes	PFKFB3
5.24603569135473E-90	0.692935163479401	0.385	0.186	8.79130661157226E-86	Proliferating hepatocytes	SMPD4
7.90227336620078E-90	0.736378004733341	0.578	0.346	1.32426297070793E-85	Proliferating hepatocytes	PSMB9
1.00343123215017E-89	0.744828680475156	0.228	0.085	1.68155005883726E-85	Proliferating hepatocytes	MAP3K9
1.38855942308779E-89	1.28892551430053	0.167	0.052	2.32694788121051E-85	Proliferating hepatocytes	DNAJC9
1.73392503706518E-89	0.77042916707747	0.349	0.163	2.90571157711383E-85	Proliferating hepatocytes	ICMT
1.86421802998236E-89	0.623063455377686	0.233	0.088	3.12405657464445E-85	Proliferating hepatocytes	PHIP
1.95863720545756E-89	1.11440594966484	0.449	0.252	3.28228422890577E-85	Proliferating hepatocytes	CCNE2
1.9752081052239E-89	0.664684471090434	0.677	0.443	3.31005374273421E-85	Proliferating hepatocytes	RTN3
2.0606371760286E-89	0.643064460602117	0.649	0.411	3.45321577958873E-85	Proliferating hepatocytes	SRP9
2.15386492393142E-89	0.838527311385645	0.256	0.103	3.60944683952427E-85	Proliferating hepatocytes	FNTB
2.6431237850204E-89	0.882025668293488	0.261	0.105	4.42934683893719E-85	Proliferating hepatocytes	DEPRECATEI
2.99203735807303E-89	0.601539398896011	0.666	0.413	5.01405620465879E-85	Proliferating hepatocytes	NBL1
3.104114517159E-89	0.711368711442343	0.511	0.283	5.20187510785506E-85	Proliferating hepatocytes	NKAP
3.50959299962012E-89	0.760342226103724	0.429	0.22	5.8813759487634E-85	Proliferating hepatocytes	SPATS2
4.45737245965478E-89	0.943079851834027	0.347	0.164	7.46966476788948E-85	Proliferating hepatocytes	PAK4
4.55216836591627E-89	0.867517559345077	0.382	0.188	7.62852374760249E-85	Proliferating hepatocytes	RPA1
5.40066078047241E-89	0.667970757969529	0.491	0.271	9.05042733591567E-85	Proliferating hepatocytes	PTBP1
5.73347282251933E-89	0.658103664991606	0.574	0.339	9.60815375597789E-85	Proliferating hepatocytes	TAP1
5.81660023019537E-89	0.615027872480214	0.746	0.513	9.74745866576141E-85	Proliferating hepatocytes	MDK
5.92233149695858E-89	0.937888400967973	0.249	0.099	9.92464312260318E-85	Proliferating hepatocytes	RNASEH2C
5.94718863771689E-89	0.852394534797508	0.277	0.116	9.96629871908596E-85	Proliferating hepatocytes	CREB3L4
7.35130506963678E-89	0.876363491634214	0.209	0.075	1.23193170356973E-84	Proliferating hepatocytes	CDK19
8.93651094124428E-89	0.724747049770024	0.51	0.287	1.49758050353372E-84	Proliferating hepatocytes	CANT1
1.02358303981026E-88	0.801083696194893	0.361	0.174	1.71532045811404E-84	Proliferating hepatocytes	LSM 2.00
1.11651145551994E-88	0.777927640324718	0.383	0.187	1.87104989716032E-84	Proliferating hepatocytes	MLH1
1.51381183274186E-88	0.887603216016781	0.336	0.156	2.53684586930881E-84	Proliferating hepatocytes	ING2
1.91182533343583E-88	0.668372868737518	0.385	0.187	3.20383689377177E-84	Proliferating hepatocytes	PSIP1
1.95820265458056E-88	0.595963797644985	0.717	0.481	3.2815560085461E-84	Proliferating hepatocytes	PSMB8
1.99834178846161E-88	0.819746796176005	0.448	0.238	3.34882116910397E-84	Proliferating hepatocytes	PRELID2
3.19694524415538E-88	0.640046216280919	0.454	0.242	5.35744084015559E-84	Proliferating hepatocytes	PCM1
4.1162202518708E-88	0.64583091170975	0.412	0.208	6.89796189808509E-84	Proliferating hepatocytes	TFF1
4.23833000452714E-88	0.525660087473232	0.822	0.593	7.10259342158658E-84	Proliferating hepatocytes	BEX3
4.67561324625269E-88	0.924054561980804	0.332	0.154	7.83539267807026E-84	Proliferating hepatocytes	CHRAC1
5.19491515291497E-88	0.761858575319138	0.391	0.194	8.70563881325491E-84	Proliferating hepatocytes	MBD3
5.32275960524154E-88	0.750491889477464	0.419	0.216	8.91988054646377E-84	Proliferating hepatocytes	PPP2R5C
1.06302638649681E-87	1.07376534219431	0.277	0.118	1.78141961849135E-83	Proliferating hepatocytes	B4GALT4
1.09993479230918E-87	1.08799934798784	0.209	0.076	1.84327072495172E-83	Proliferating hepatocytes	SLX4
1.21445509807876E-87	0.852071220498125	0.336	0.155	2.03518385336038E-83	Proliferating hepatocytes	KIF2A
1.82807934567935E-87	1.19818007675666	0.207	0.075	3.06349536748946E-83	Proliferating hepatocytes	SACS
1.91941355652678E-87	0.851943670940768	0.614	0.413	3.21655323802759E-83	Proliferating hepatocytes	CEP70
2.41288150023137E-87	1.13497649479373	0.314	0.145	4.04350681808773E-83	Proliferating hepatocytes	WDR76

2.47898483259549E-87	0.755648661647341	0.428	0.221	4.15428278246352E-83	Proliferating hepatocytes	PIN1
2.49430407999605E-87	0.911914968062588	0.267	0.111	4.17995477725738E-83	Proliferating hepatocytes	MFSD14B
3.24770169265843E-87	0.803592362958608	0.412	0.211	5.442498496557E-83	Proliferating hepatocytes	DEPRECATEI
3.92817125102349E-87	0.70876121568895	0.507	0.283	6.58282938246516E-83	Proliferating hepatocytes	HACD2
5.60674869371246E-87	0.932707042947448	0.367	0.179	9.39578946092335E-83	Proliferating hepatocytes	ARL4A
6.3186738577989E-87	0.823673306575404	0.308	0.137	1.05888336508994E-82	Proliferating hepatocytes	ARPIN
6.52754682362786E-87	0.900062031135532	0.361	0.173	1.09388629670356E-82	Proliferating hepatocytes	GPD2
7.5831588656762E-87	0.91088365241156	0.238	0.093	1.27078576271002E-82	Proliferating hepatocytes	RP2
8.03452666398677E-87	0.643175229937415	0.724	0.508	1.3464259783509E-82	Proliferating hepatocytes	SDC2
1.17787987813716E-86	0.73755460316178	0.524	0.305	1.97389109978224E-82	Proliferating hepatocytes	ATF6B
1.23755470220941E-86	0.631383801906752	0.557	0.327	2.07389416996253E-82	Proliferating hepatocytes	SCRIB
1.33527309464613E-86	0.601090752773728	0.454	0.24	2.23765065200799E-82	Proliferating hepatocytes	RNF168
1.93835299345889E-86	0.744780069108017	0.573	0.349	3.2482919464384E-82	Proliferating hepatocytes	NECTIN3
2.45010882764526E-86	0.910964618741894	0.35	0.167	4.10589237336793E-82	Proliferating hepatocytes	IFI27L1
2.96788716059475E-86	0.70699827658784	0.548	0.323	4.97358530372468E-82	Proliferating hepatocytes	CD47
3.13089665621385E-86	0.787326471101228	0.459	0.252	5.24675661648318E-82	Proliferating hepatocytes	SLC3A2
3.52034839970878E-86	0.572521718518014	0.777	0.558	5.89939984823198E-82	Proliferating hepatocytes	TRAM1
3.67078820621309E-86	0.76470059121528	0.411	0.21	6.1515068759719E-82	Proliferating hepatocytes	ATL3
3.70117702737338E-86	0.64314300537552	0.638	0.401	6.20243246247231E-82	Proliferating hepatocytes	ANAPC11
3.72248577055162E-86	0.751949363080823	0.407	0.207	6.23814165429041E-82	Proliferating hepatocytes	CA2
4.17847731932351E-86	0.756658982948572	0.232	0.09	7.00229229172234E-82	Proliferating hepatocytes	CLEC2D
4.27966676004606E-86	0.877658744923977	0.326	0.15	7.17186555648519E-82	Proliferating hepatocytes	SLC35E3
4.89214243822839E-86	0.734265194382066	0.533	0.321	8.19825229798313E-82	Proliferating hepatocytes	CEP57
5.66218715572371E-86	0.601136932737743	0.494	0.27	9.4886932355618E-82	Proliferating hepatocytes	NAP1L4
6.15959406741284E-86	0.649470014226412	0.404	0.204	1.03222477381704E-81	Proliferating hepatocytes	SMC3
6.5467075697557E-86	0.417378522921519	0.287	0.121	1.09709725453966E-81	Proliferating hepatocytes	PIM1
6.7337552631058E-86	0.878533472964107	0.414	0.214	1.12844270699127E-81	Proliferating hepatocytes	NXT2
1.05577951563485E-85	0.450283613872705	0.958	0.771	1.76927531230089E-81	Proliferating hepatocytes	UGDH
1.41278968031062E-85	0.603052791936543	0.343	0.16	2.36755294626454E-81	Proliferating hepatocytes	CCM2
1.42440715102203E-85	1.06593303409133	0.198	0.071	2.38702150368271E-81	Proliferating hepatocytes	FOXRED2
1.44700575909169E-85	0.64029968315396	0.521	0.295	2.42489225108585E-81	Proliferating hepatocytes	NACC1
1.47580519624284E-85	0.817337931064946	0.363	0.176	2.47315434786375E-81	Proliferating hepatocytes	STRN
1.55237622270758E-85	0.544225292994031	0.674	0.42	2.60147207401336E-81	Proliferating hepatocytes	EFHD2
1.9443447266251E-85	0.938268303375091	0.221	0.084	3.25833289287834E-81	Proliferating hepatocytes	ING1
2.02266949877564E-85	0.579913838662875	0.438	0.232	3.38958954604821E-81	Proliferating hepatocytes	SLC29A1
2.02777722368674E-85	0.570547007892976	0.468	0.25	3.39814907145423E-81	Proliferating hepatocytes	SRRT
2.29061309743371E-85	0.743963748667576	0.448	0.241	3.83860942867942E-81	Proliferating hepatocytes	AAMDC
2.61541328557594E-85	0.911468437123277	0.251	0.101	4.38290958396816E-81	Proliferating hepatocytes	SLF2
3.29298810641718E-85	0.726332801705492	0.384	0.193	5.5183894687339E-81	Proliferating hepatocytes	NUP98
3.79964967765355E-85	0.710169140576319	0.453	0.241	6.36745292981183E-81	Proliferating hepatocytes	HIPK1
5.33372643179512E-85	0.829504349645206	0.399	0.202	8.93825875440226E-81	Proliferating hepatocytes	PPP1CC
5.41415446889892E-85	0.671476298253339	0.573	0.345	9.07304005898081E-81	Proliferating hepatocytes	EIF1B
5.42607829856871E-85	0.893228923660501	0.346	0.166	9.09302201274144E-81	Proliferating hepatocytes	SAV1
5.78094557918311E-85	0.721353942267064	0.486	0.271	9.68770860159505E-81	Proliferating hepatocytes	STK11
7.68915153876515E-85	0.59808090651562	0.659	0.433	1.28854801486626E-80	Proliferating hepatocytes	IKBKG
8.18130428412952E-85	0.46730597833267	0.396	0.196	1.37102297193442E-80	Proliferating hepatocytes	CENPT
8.37392237865205E-85	1.07811410411883	0.252	0.104	1.40330191221451E-80	Proliferating hepatocytes	DEPRECATEI
8.61284856564429E-85	0.663306526287669	0.602	0.374	1.44334116263067E-80	Proliferating hepatocytes	THOC2
8.93220401524895E-85	0.765823124695099	0.472	0.259	1.49685874887542E-80	Proliferating hepatocytes	FUCA2
1.86637701971747E-84	1.80657097338242	0.109	0.026	3.12767460964254E-80	Proliferating hepatocytes	CHRNA5
2.3748001862002E-84	1.04800006696377	0.213	0.08	3.97969015203429E-80	Proliferating hepatocytes	HIRIP3
2.88055440223565E-84	0.97588978025227	0.206	0.076	4.8272330672665E-80	Proliferating hepatocytes	RPA3
3.99471114490362E-84	0.757347434998305	0.455	0.247	6.69433693662949E-80	Proliferating hepatocytes	PCMT1
4.26460464353521E-84	0.774880813046045	0.354	0.17	7.14662446163631E-80	Proliferating hepatocytes	CPOX
4.85813220492318E-84	1.1032433988019	0.151	0.046	8.14125794901027E-80	Proliferating hepatocytes	MYADM
7.61748348243499E-84	0.622803777403875	0.54	0.316	1.27653788198646E-79	Proliferating hepatocytes	ADAM15
8.33580573740497E-84	0.670690143230247	0.532	0.306	1.39691432547432E-79	Proliferating hepatocytes	GFPT1
8.82496787812204E-84	0.710212757392138	0.457	0.249	1.47888811701569E-79	Proliferating hepatocytes	TECR
1.01350007527768E-83	1.30045442168023	0.143	0.042	1.69842342615033E-79	Proliferating hepatocytes	DEPRECATEI
1.17157784573138E-83	0.743378566267796	0.528	0.305	1.96333015387665E-79	Proliferating hepatocytes	CXorf56

1.22064176182984E-83	0.488603581415437	0.841	0.557	2.04555146447445E-79	Proliferating hepatocytes	FKBP5
1.68419035688123E-83	0.819771700644046	0.362	0.177	2.82236620006156E-79	Proliferating hepatocytes	UPF3B
1.85671888025381E-83	0.728370900277328	0.462	0.254	3.11148949952934E-79	Proliferating hepatocytes	IGF2BP3
1.8648555704484E-83	0.7636639441513	0.383	0.194	3.12512496495743E-79	Proliferating hepatocytes	DUT
1.96810540078077E-83	0.73718828912558	0.418	0.218	3.29815103062842E-79	Proliferating hepatocytes	HAT1
2.09730252054967E-83	0.628013108586986	0.499	0.282	3.51465956393714E-79	Proliferating hepatocytes	FBXL3
2.13859305006023E-83	0.562369284878714	0.585	0.352	3.58385423329093E-79	Proliferating hepatocytes	PMF1
2.42788663824448E-83	0.790162259412692	0.238	0.095	4.06865242837011E-79	Proliferating hepatocytes	PHTF1
2.48340993130264E-83	1.21025068323242	0.223	0.087	4.16169836287697E-79	Proliferating hepatocytes	RARRES1
2.56860307418372E-83	0.624026586483384	0.565	0.336	4.30446503171708E-79	Proliferating hepatocytes	KEAP1
2.63025360406457E-83	1.03181204738852	0.196	0.071	4.40777898969141E-79	Proliferating hepatocytes	TUBD1
2.80823087373357E-83	0.864244287463038	0.36	0.176	4.70603329820271E-79	Proliferating hepatocytes	SEC22C
3.45235966328576E-83	0.722334003629126	0.417	0.217	5.78546432373428E-79	Proliferating hepatocytes	YY1
3.48852677211517E-83	0.708300963244301	0.343	0.164	5.84607316471061E-79	Proliferating hepatocytes	POLE
3.61273690934351E-83	0.546652004738037	0.774	0.562	6.05422451267785E-79	Proliferating hepatocytes	NOTCH2NLC
4.57221222814713E-83	0.8676730194577	0.431	0.234	7.66211325192896E-79	Proliferating hepatocytes	FADS1
4.9267671797715E-83	0.970703695646026	0.257	0.107	8.25627643986108E-79	Proliferating hepatocytes	GTF3C5
5.6057463863489E-83	0.944084315248155	0.257	0.108	9.39410979424348E-79	Proliferating hepatocytes	BICDL1
8.21749777917531E-83	1.02910042670607	0.279	0.122	1.3770882778342E-78	Proliferating hepatocytes	CETN3
8.60585743025277E-83	0.590872206782983	0.36	0.172	1.44216958816176E-78	Proliferating hepatocytes	SIN3A
9.04801353066302E-83	0.828119554916771	0.322	0.149	1.51626610746851E-78	Proliferating hepatocytes	DDX59
9.51630514697713E-83	1.05626167635185	0.495	0.311	1.59474241653043E-78	Proliferating hepatocytes	CCDC14
2.01673290597847E-82	0.708688267384293	0.474	0.266	3.37964100383873E-78	Proliferating hepatocytes	DEPRECATEI
2.15771728231857E-82	0.686984505076578	0.54	0.319	3.61590262170946E-78	Proliferating hepatocytes	TIMM8A
2.3859870960066E-82	0.766137482167686	0.403	0.208	3.99843717548787E-78	Proliferating hepatocytes	LMO4
3.09898951811546E-82	0.700594327938941	0.426	0.226	5.19328663445789E-78	Proliferating hepatocytes	OXR1
3.12586952590685E-82	0.701069484132768	0.486	0.27	5.2383321515147E-78	Proliferating hepatocytes	FBXL6
3.22281492064527E-82	1.30120104631372	0.157	0.05	5.40079324401735E-78	Proliferating hepatocytes	PTPN14
5.47581234512344E-82	0.88653141122883	0.257	0.108	9.17636632795786E-78	Proliferating hepatocytes	CNOT1
5.49715799964945E-82	0.505125352698345	0.813	0.592	9.21213737581255E-78	Proliferating hepatocytes	TMEM14A
5.78687361600636E-82	0.860919994866108	0.277	0.119	9.69764280570345E-78	Proliferating hepatocytes	SGCB
6.29178977171381E-82	0.905606842640967	0.256	0.107	1.0543781299438E-77	Proliferating hepatocytes	PRADC1
7.20481862752714E-82	0.757736665430203	0.417	0.219	1.207383505601E-77	Proliferating hepatocytes	CNOT8
9.82597243212015E-82	0.735332264841452	0.415	0.219	1.64663646017469E-77	Proliferating hepatocytes	DEPRECATEI
1.02339991202894E-81	0.595834192619452	0.537	0.314	1.7150135725781E-77	Proliferating hepatocytes	ACTR1B
1.08965943575098E-81	0.570611290032894	0.658	0.416	1.82605128243149E-77	Proliferating hepatocytes	PYCR2
1.1150454971283E-81	1.13660185130938	0.174	0.059	1.86859324408761E-77	Proliferating hepatocytes	ZBTB8A
1.29478781637803E-81	1.1447254383641	0.204	0.076	2.1698054226863E-77	Proliferating hepatocytes	HSPA2
1.74067492264547E-81	0.667889346051961	0.458	0.25	2.91702303536927E-77	Proliferating hepatocytes	ATF2
1.81077331028136E-81	0.699208475235269	0.501	0.287	3.0344939133695E-77	Proliferating hepatocytes	DNAJC1
1.8192260874687E-81	0.8409063408842	0.349	0.17	3.04865907738005E-77	Proliferating hepatocytes	MZT2A
2.19208408256814E-81	0.965808895044152	0.26	0.111	3.67349450556769E-77	Proliferating hepatocytes	CHAF1B
2.25972574958014E-81	0.801921250238323	0.465	0.262	3.78684841114639E-77	Proliferating hepatocytes	ZNF189
2.58609893761187E-81	1.01462643210227	0.251	0.105	4.33378459964997E-77	Proliferating hepatocytes	MYLIP
2.73513713346385E-81	0.718883789900827	0.417	0.218	4.58354280825872E-77	Proliferating hepatocytes	CYREN
3.77115655470191E-81	0.508929903488672	0.789	0.573	6.31970415436946E-77	Proliferating hepatocytes	PFN1
3.86048587372299E-81	0.698939667553127	0.401	0.206	6.46940222718498E-77	Proliferating hepatocytes	ZCCHC3
4.79195426850085E-81	0.824852703818408	0.2	0.074	8.03035696315373E-77	Proliferating hepatocytes	POMGNT2
5.24262516815081E-81	0.594507484598518	0.633	0.414	8.78559125678712E-77	Proliferating hepatocytes	PCYT2
6.05677182592581E-81	0.661816423293231	0.561	0.341	1.01499382258865E-76	Proliferating hepatocytes	SNRNP200
6.55959989966273E-81	0.703076475192932	0.392	0.199	1.09925775118548E-76	Proliferating hepatocytes	CASP2
6.89649442998434E-81	1.05963829108871	0.22	0.086	1.15571453657678E-76	Proliferating hepatocytes	RIBC2
7.2199626333284E-81	1.55243274910055	0.125	0.035	1.20992133809317E-76	Proliferating hepatocytes	FBXO48
7.98645191255151E-81	0.833163824451584	0.384	0.194	1.33836961150538E-76	Proliferating hepatocytes	FZD6
1.19453942235334E-80	0.68082590271833	0.582	0.369	2.00180916397972E-76	Proliferating hepatocytes	HGSNAT
1.4166268291756E-80	0.615318058061949	0.562	0.34	2.37398324033247E-76	Proliferating hepatocytes	PUM2
1.56464409212012E-80	0.623776800214538	0.499	0.288	2.6220305695749E-76	Proliferating hepatocytes	SCML1
1.6206506628426E-80	0.603784846070472	0.593	0.367	2.71588638079163E-76	Proliferating hepatocytes	FAM91A1
1.63678902792929E-80	0.8553449393307	0.339	0.164	2.7429310530039E-76	Proliferating hepatocytes	PRPS2
1.71559713863333E-80	1.08873445096943	0.177	0.062	2.87499768492173E-76	Proliferating hepatocytes	TOE1

2.12945157262794E-80	0.706963162708283	0.407	0.213	3.5685349454099E-76	Proliferating hepatocytes	ADSS
2.31238735275779E-80	0.749796851607691	0.471	0.263	3.87509872575151E-76	Proliferating hepatocytes	PHF6
2.38396943396632E-80	0.947652269981754	0.24	0.099	3.99505597744076E-76	Proliferating hepatocytes	IPO11
2.48968173377336E-80	0.891940313970972	0.274	0.12	4.1722086494574E-76	Proliferating hepatocytes	ABCA7
3.01363446508952E-80	0.885994470521998	0.296	0.135	5.05024863659701E-76	Proliferating hepatocytes	C12orf65
3.4169834349874E-80	0.795454272134534	0.374	0.189	5.72618084035188E-76	Proliferating hepatocytes	GET3
4.77081755313605E-80	0.647266559536751	0.598	0.372	7.99493605554538E-76	Proliferating hepatocytes	SMIM15
5.71068432794411E-80	0.746416302413001	0.427	0.233	9.56996479676875E-76	Proliferating hepatocytes	CLDN12
6.2165989391084E-80	0.498782528677918	0.938	0.762	1.04177765021579E-75	Proliferating hepatocytes	MT-CYB
7.1366583068034E-80	0.626886650642708	0.613	0.393	1.19596119905411E-75	Proliferating hepatocytes	MGAT2
9.38253728926661E-80	1.22565441323364	0.124	0.034	1.5723255989353E-75	Proliferating hepatocytes	STRIP2
1.03745214259681E-79	0.567811305780044	0.449	0.242	1.73856230056373E-75	Proliferating hepatocytes	AP2S1
1.28618359541117E-79	0.687786784089051	0.57	0.349	2.15538646919004E-75	Proliferating hepatocytes	TXNDC17
1.49620376645232E-79	0.760770425976772	0.394	0.203	2.5073382718208E-75	Proliferating hepatocytes	YIF1B
2.11079577516701E-79	0.6752171475408	0.467	0.26	3.53727156002487E-75	Proliferating hepatocytes	UBA2
3.24116336487298E-79	1.6090660456822	0.109	0.028	5.43154156685414E-75	Proliferating hepatocytes	DCLRE1B
3.50521253723156E-79	0.598390952544022	0.663	0.449	5.87403516989264E-75	Proliferating hepatocytes	MLF 2.00
4.38770483077874E-79	0.630126924511355	0.614	0.393	7.35291575541901E-75	Proliferating hepatocytes	ERBIN
4.9751665801228E-79	1.00703187028455	0.215	0.084	8.33738415496979E-75	Proliferating hepatocytes	HPSE
5.14101266713149E-79	0.743042554660655	0.295	0.134	8.61530902757895E-75	Proliferating hepatocytes	ADAT1
5.18433002486633E-79	0.935851967259136	0.263	0.114	8.687900255671E-75	Proliferating hepatocytes	MSH5
5.60847087010073E-79	0.572105476051719	0.372	0.186	9.3986754841148E-75	Proliferating hepatocytes	MAD2L2
5.62308297096762E-79	0.390537865168723	0.93	0.699	9.42316244274753E-75	Proliferating hepatocytes	SPINK1
6.16295678320312E-79	0.725478783203752	0.351	0.171	1.03278829772918E-74	Proliferating hepatocytes	BAG5
9.58029169754004E-79	0.680075000280152	0.449	0.246	1.60546528267376E-74	Proliferating hepatocytes	ARPC5
1.01223517226675E-78	0.87295840463343	0.324	0.155	1.69630370168462E-74	Proliferating hepatocytes	RECQL
1.06799739401515E-78	0.610386507183355	0.617	0.391	1.78975003289059E-74	Proliferating hepatocytes	MLEC
1.07041929816724E-78	0.63779915696815	0.454	0.247	1.79380865986866E-74	Proliferating hepatocytes	ELOC
1.09464205351321E-78	0.770829595113008	0.291	0.132	1.83440115327743E-74	Proliferating hepatocytes	MEX3D
1.36011399118295E-78	1.04724417770586	0.174	0.06	2.27927902642439E-74	Proliferating hepatocytes	PIK3R3
1.76272000491866E-78	0.748937275008541	0.399	0.209	2.95396618424268E-74	Proliferating hepatocytes	DEPRECATEI
1.7793953331341E-78	0.782101889749298	0.357	0.176	2.98191069926612E-74	Proliferating hepatocytes	TMEM65
1.92236277121907E-78	0.604145003482008	0.627	0.403	3.22149553200892E-74	Proliferating hepatocytes	MCM3
1.97902502273645E-78	0.577693025062933	0.484	0.273	3.31645013310175E-74	Proliferating hepatocytes	PRPSAP1
2.17752128279504E-78	0.752170733741701	0.23	0.092	3.64909016570793E-74	Proliferating hepatocytes	CTDSPL2
2.22650001075287E-78	0.5438889282527	0.696	0.471	3.73116871801965E-74	Proliferating hepatocytes	ENY2
2.312090938839E-78	0.665152790853648	0.515	0.302	3.87460199530639E-74	Proliferating hepatocytes	MECP2
2.75219490260224E-78	0.625282076668467	0.513	0.294	4.61212821778083E-74	Proliferating hepatocytes	SSX2IP
3.74593287357126E-78	0.649052853909785	0.519	0.303	6.27743430953072E-74	Proliferating hepatocytes	AGFG1
4.01158169467509E-78	0.737284910935856	0.483	0.277	6.72260860393652E-74	Proliferating hepatocytes	HMG20B
4.48140658026408E-78	0.782226379177272	0.398	0.208	7.50994114720655E-74	Proliferating hepatocytes	ATP6V1H
4.56128666321373E-78	0.608137996368472	0.251	0.104	7.64380419021357E-74	Proliferating hepatocytes	ZBTB5
5.04285801158686E-78	0.741814157282754	0.381	0.196	8.45082145581727E-74	Proliferating hepatocytes	LRRC45
5.25294077067257E-78	0.687421017060007	0.431	0.232	8.80287814349309E-74	Proliferating hepatocytes	TIPRL
7.0088370874692E-78	0.470921265123367	0.812	0.571	1.17454091911809E-73	Proliferating hepatocytes	DNM2
7.31954392136513E-78	1.04718586394456	0.193	0.072	1.22660917034237E-73	Proliferating hepatocytes	DEPRECATEI
7.99192943584071E-78	0.572203369075251	0.532	0.313	1.33928753485819E-73	Proliferating hepatocytes	MSI2
8.05717253953168E-78	0.649037291128907	0.524	0.308	1.35022097417472E-73	Proliferating hepatocytes	CTSC
9.35571209142895E-78	0.968187927376691	0.202	0.077	1.56783023228166E-73	Proliferating hepatocytes	CEP131
9.46423458428023E-78	0.626631054564418	0.57	0.35	1.58601643163368E-73	Proliferating hepatocytes	TAZ
1.02834614141153E-77	0.679517764449906	0.465	0.258	1.72330246377743E-73	Proliferating hepatocytes	LRP11
1.30869836374807E-77	0.616796426798557	0.443	0.241	2.19311671796901E-73	Proliferating hepatocytes	BCAP29
1.33706939040668E-77	0.870579663924491	0.416	0.226	2.24066088444352E-73	Proliferating hepatocytes	CKB
2.02466574901402E-77	0.941604322962238	0.308	0.146	3.3929348621977E-73	Proliferating hepatocytes	PRIM1
2.26359549211538E-77	1.20277577983499	0.237	0.099	3.79333332568695E-73	Proliferating hepatocytes	ANXA2R
2.86849711021043E-77	0.533729585189461	0.631	0.394	4.80702745729064E-73	Proliferating hepatocytes	SHARPIN
3.04377821978177E-77	0.855518168796533	0.289	0.131	5.10076354071029E-73	Proliferating hepatocytes	PPIL1
3.96867355063491E-77	0.669840846014191	0.449	0.247	6.65070313615398E-73	Proliferating hepatocytes	CHMP3
4.23257855602917E-77	0.830642784952532	0.285	0.129	7.09295514419368E-73	Proliferating hepatocytes	FAM184A
4.64185181852407E-77	0.523558432494801	0.317	0.149	7.77881527748263E-73	Proliferating hepatocytes	C1orf174

5.49662628140189E-77	1.16271683092835	0.123	0.035	9.21124632237328E-73	Proliferating hepatocytes	LRTOMT
6.44429709128649E-77	0.374060092452243	0.782	0.498	1.07993530655779E-72	Proliferating hepatocytes	CDH17
6.8933276682742E-77	0.696326881598351	0.278	0.124	1.15518385064939E-72	Proliferating hepatocytes	ST3GAL3
7.20254305051189E-77	0.694226164102547	0.423	0.227	1.20700216440478E-72	Proliferating hepatocytes	WDTC1
7.22062265319707E-77	0.772785452060041	0.264	0.116	1.21003194422277E-72	Proliferating hepatocytes	DEPRECATEI
7.58782926671132E-77	1.02447581145289	0.246	0.104	1.27156842851548E-72	Proliferating hepatocytes	GMEB2
9.20523201824452E-77	0.63653485168047	0.521	0.304	1.54261278161742E-72	Proliferating hepatocytes	STK39
9.91475051813872E-77	0.572049202468388	0.362	0.18	1.66151389182969E-72	Proliferating hepatocytes	TUBA4A
1.13697122257059E-76	0.669103468669	0.448	0.246	1.9053363747838E-72	Proliferating hepatocytes	MAEA
1.27149882182405E-76	1.13333384749772	0.128	0.037	2.13077772561274E-72	Proliferating hepatocytes	SLC25A35
1.29009354721605E-76	0.532233782166786	0.406	0.212	2.16193876642465E-72	Proliferating hepatocytes	SFN
1.64528563965829E-76	0.802522288954465	0.178	0.063	2.75716967493937E-72	Proliferating hepatocytes	RBM8A
1.77177383274165E-76	0.561273226652516	0.458	0.252	2.96913858890846E-72	Proliferating hepatocytes	STRN4
2.25576329967439E-76	0.564791934661105	0.657	0.444	3.78020813759435E-72	Proliferating hepatocytes	HNRNPU
2.52306259761737E-76	0.620149977085582	0.562	0.343	4.22814830108719E-72	Proliferating hepatocytes	CCT6A
2.6033079688037E-76	0.619659460037245	0.395	0.205	4.36262349412124E-72	Proliferating hepatocytes	CBFB
2.60558710146602E-76	0.693199121148656	0.476	0.267	4.36644286463676E-72	Proliferating hepatocytes	CDC42SE2
3.44596612550961E-76	0.559771254911285	0.383	0.195	5.77475003312901E-72	Proliferating hepatocytes	PHKG2
3.55118148729846E-76	0.777046518526172	0.324	0.156	5.95106993641475E-72	Proliferating hepatocytes	KANSL1L
3.72629482408989E-76	0.755378002104487	0.151	0.049	6.24452486620984E-72	Proliferating hepatocytes	EXPH5
4.01387536168416E-76	0.761430973233258	0.275	0.123	6.72645233111032E-72	Proliferating hepatocytes	TMEM60
4.28448197775278E-76	0.412551653668605	0.367	0.182	7.17993489831811E-72	Proliferating hepatocytes	TENT4A
4.49193023847503E-76	0.696204790404114	0.219	0.087	7.52757669363646E-72	Proliferating hepatocytes	MRE11
4.67420907812317E-76	0.633036286732535	0.45	0.249	7.83303957311881E-72	Proliferating hepatocytes	UQCC2
6.63285262996416E-76	0.761715007177485	0.44	0.243	1.11153344372939E-71	Proliferating hepatocytes	TMEM185A
6.82751384007606E-76	0.593730809123273	0.466	0.259	1.14415476931995E-71	Proliferating hepatocytes	NUDCD2
7.88488432565832E-76	0.669047801637451	0.356	0.177	1.32134891529382E-71	Proliferating hepatocytes	NUP160
8.25859662674983E-76	0.822264104745332	0.252	0.108	1.38397562271074E-71	Proliferating hepatocytes	TXNDC16
9.33970303604232E-76	0.761986454021833	0.261	0.114	1.56514743477997E-71	Proliferating hepatocytes	FOXRED1
1.08623316468673E-75	0.81448669199943	0.291	0.134	1.82030953738201E-71	Proliferating hepatocytes	EED
1.09198076813116E-75	0.61605896149209	0.427	0.229	1.8299413712342E-71	Proliferating hepatocytes	ZC3H4
1.54455678841146E-75	0.629176327905648	0.373	0.193	2.58836826601992E-71	Proliferating hepatocytes	FZD5
1.99132418212057E-75	0.804113831884859	0.357	0.179	3.33706106439765E-71	Proliferating hepatocytes	ADD3
2.11552884661558E-75	0.556501389658068	0.407	0.217	3.5452032411584E-71	Proliferating hepatocytes	ATF5
2.12545567259341E-75	1.05210810387192	0.218	0.088	3.56183861613204E-71	Proliferating hepatocytes	PDE5A
2.32613671787904E-75	0.782779218691511	0.253	0.108	3.8981399118217E-71	Proliferating hepatocytes	ALMS1
2.49886435010365E-75	0.949166218861309	0.232	0.096	4.1875968779037E-71	Proliferating hepatocytes	FSBP
2.59285161423831E-75	0.664090016685627	0.361	0.182	4.34510073514056E-71	Proliferating hepatocytes	RUSC1
2.62982807599304E-75	0.946649958291478	0.202	0.078	4.40706588974914E-71	Proliferating hepatocytes	BMP2K
3.07441430228818E-75	0.567438706161612	0.603	0.372	5.15210348777453E-71	Proliferating hepatocytes	TMEM64
3.37727070908141E-75	0.602312042006112	0.528	0.31	5.65963025427863E-71	Proliferating hepatocytes	DYNLT1
3.62769805821966E-75	0.852539337738595	0.279	0.125	6.07929640596451E-71	Proliferating hepatocytes	KRT15
3.82518791611908E-75	0.869255751600005	0.322	0.155	6.41024990983236E-71	Proliferating hepatocytes	APAF1
4.42253803710788E-75	0.636313015352541	0.409	0.217	7.41128924258538E-71	Proliferating hepatocytes	BABAM1
4.61889804403864E-75	0.817605971691241	0.242	0.102	7.74034934219995E-71	Proliferating hepatocytes	SARS
5.13787025116234E-75	0.91137597317285	0.296	0.138	8.61004296689786E-71	Proliferating hepatocytes	RASSF8
5.93234840923369E-75	0.630716198505262	0.406	0.216	9.94142946419381E-71	Proliferating hepatocytes	NUP153
7.23168366488743E-75	0.77224041502848	0.344	0.172	1.21188554856184E-70	Proliferating hepatocytes	ZADH2
7.53885847979077E-75	0.653525420117743	0.539	0.323	1.26336190404334E-70	Proliferating hepatocytes	SEPTIN8
8.49005377835782E-75	0.553357535417477	0.618	0.397	1.4227632121772E-70	Proliferating hepatocytes	EIF2AK1
8.52011821117478E-75	0.502266684207257	0.732	0.476	1.42780140982867E-70	Proliferating hepatocytes	F2RL1
8.91623538518058E-75	0.652890544543954	0.485	0.282	1.49418272584856E-70	Proliferating hepatocytes	TPP2
9.37024563239253E-75	0.624376452211117	0.554	0.337	1.57026576307634E-70	Proliferating hepatocytes	TRAPPC4
1.10948977613494E-74	0.838666827936661	0.241	0.101	1.85928296684693E-70	Proliferating hepatocytes	TIMM21
1.17754229190216E-74	0.554098219361665	0.581	0.356	1.97332537276964E-70	Proliferating hepatocytes	PPP1CA
1.30923952776125E-74	0.429202654697817	0.908	0.709	2.19402360062231E-70	Proliferating hepatocytes	ASPH
1.39332467427612E-74	0.64142097745047	0.392	0.205	2.33493348915192E-70	Proliferating hepatocytes	TADA3
1.46070761556817E-74	0.508069880475664	0.768	0.534	2.44785382216913E-70	Proliferating hepatocytes	TPD52
1.62591284032742E-74	0.792543220014098	0.416	0.228	2.72470473782069E-70	Proliferating hepatocytes	DCAF16
1.65843880043863E-74	0.602089111755386	0.397	0.207	2.77921174177505E-70	Proliferating hepatocytes	TRAPPC10

**1.87825839324193E-74**	0.553317236484835	0.587	0.36	3.14758541539483E-70	Proliferating hepatocytes	RAB8A
**2.46547151492348E-74**	0.676022535369855	0.365	0.185	4.13163716470876E-70	Proliferating hepatocytes	VEZF1
**2.78186533781861E-74**	0.837134119937623	0.317	0.153	4.66184993311642E-70	Proliferating hepatocytes	TCP11L1
**2.96032401168781E-74**	0.755138839818737	0.257	0.111	4.96091097878644E-70	Proliferating hepatocytes	VKORC1L1
**3.21809784173425E-74**	0.693890472198932	0.386	0.201	5.39288836317825E-70	Proliferating hepatocytes	ACOT7
**3.79888602348371E-74**	0.481151086366591	0.706	0.457	6.36617319815401E-70	Proliferating hepatocytes	LAGE3
**4.45828368353928E-74**	0.534026317984316	0.559	0.342	7.47119179687512E-70	Proliferating hepatocytes	TBL1XR1
**4.7442483095863E-74**	0.871588879283994	0.248	0.107	7.95041131720472E-70	Proliferating hepatocytes	KMT5C
**5.17723960034795E-74**	0.780787365074562	0.288	0.134	8.6760181222631E-70	Proliferating hepatocytes	SP2
**5.25640504386611E-74**	0.981783448066099	0.198	0.077	8.80868357251084E-70	Proliferating hepatocytes	SLC25A19
**6.10722491006323E-74**	0.654654828136821	0.383	0.201	1.0234487504284E-69	Proliferating hepatocytes	TFF3
**6.84081104033526E-74**	0.54826844376381	0.41	0.217	1.14638311413938E-69	Proliferating hepatocytes	NELFB
**7.83357013505113E-74**	0.770122760051544	0.375	0.192	1.31274968323187E-69	Proliferating hepatocytes	CCNQ
**8.24669833222471E-74**	0.680297942470882	0.407	0.22	1.38198170651422E-69	Proliferating hepatocytes	RNF19B
**8.30471937526096E-74**	0.542489420960676	0.65	0.417	1.39170487290623E-69	Proliferating hepatocytes	RAMP1
**8.55312906526024E-74**	0.932461866876249	0.213	0.085	1.43333336875631E-69	Proliferating hepatocytes	CEP68
**8.62953257514805E-74**	0.631079326506672	0.405	0.214	1.44613706894331E-69	Proliferating hepatocytes	CUL3
**8.92970292785619E-74**	0.597170516274685	0.38	0.196	1.49643961665014E-69	Proliferating hepatocytes	APBB2
**8.95778680781999E-74**	0.770964233888751	0.229	0.094	1.50114591325447E-69	Proliferating hepatocytes	PPM1D
**9.84495647112776E-74**	0.966980834464741	0.126	0.037	1.64981780543159E-69	Proliferating hepatocytes	ATRIP
**1.18920035266761E-73**	0.602735015354361	0.447	0.25	1.99286195100038E-69	Proliferating hepatocytes	TEAD1
**1.21329170360097E-73**	0.575685059124855	0.665	0.45	2.03323423689451E-69	Proliferating hepatocytes	NOTCH2NLE
**1.4315971221024E-73**	0.90290129258977	0.217	0.088	2.3990704572192E-69	Proliferating hepatocytes	IMMP1L
**1.45964992915065E-73**	0.670005705767836	0.397	0.21	2.44608135127066E-69	Proliferating hepatocytes	VCPIP1
**1.50392188498563E-73**	0.76735650239994	0.4	0.215	2.52027229485891E-69	Proliferating hepatocytes	S100PBP
**1.59054771546202E-73**	0.682532990179665	0.307	0.146	2.66543986157125E-69	Proliferating hepatocytes	MALT1
**1.80630798658763E-73**	0.713086590297552	0.346	0.172	3.02701092392354E-69	Proliferating hepatocytes	RALBP1
**1.92222018693749E-73**	0.522966784198379	0.65	0.415	3.22125658926984E-69	Proliferating hepatocytes	DDIT4
**1.99497037875794E-73**	0.970519143337895	0.251	0.111	3.35075713755056E-69	Proliferating hepatocytes	B3GLCT
**2.03076826776923E-73**	0.772957257852406	0.349	0.176	3.40316146312768E-69	Proliferating hepatocytes	SLC39A3
**2.45581590924286E-73**	0.903069493555275	0.218	0.089	4.11545630070919E-69	Proliferating hepatocytes	LRRC26
**2.61435973957643E-73**	0.736704773634617	0.249	0.107	4.38114405158218E-69	Proliferating hepatocytes	GPN3
**2.65058600021824E-73**	0.708107172124299	0.425	0.232	4.44185201916572E-69	Proliferating hepatocytes	SCAMP4
**2.65467287175796E-73**	1.02688507032683	0.204	0.08	4.44870079849198E-69	Proliferating hepatocytes	KIAA1841
**2.78380121644425E-73**	0.767876471030949	0.312	0.149	4.66509407851728E-69	Proliferating hepatocytes	RELL1
**2.86230538890545E-73**	0.697580964460754	0.314	0.15	4.79665137072775E-69	Proliferating hepatocytes	COQ6
**3.11917334312389E-73**	0.617646458155575	0.393	0.208	5.22711068840702E-69	Proliferating hepatocytes	HMGN4
**3.33122985703801E-73**	0.669884294474231	0.36	0.182	5.58247499442429E-69	Proliferating hepatocytes	PPP5C
**3.61470598557223E-73**	0.699599244485528	0.386	0.202	6.05752429062195E-69	Proliferating hepatocytes	NIN
**3.84223986698001E-73**	0.501446222168153	0.452	0.249	6.4388255690851E-69	Proliferating hepatocytes	BAZ1B
**3.86301847672231E-73**	0.846203976717287	0.258	0.114	6.47364636329124E-69	Proliferating hepatocytes	MED21
**4.53455802132917E-73**	0.694365634982718	0.329	0.162	7.59901233214342E-69	Proliferating hepatocytes	ANAPC7
**5.03177030897924E-73**	0.840924476585246	0.335	0.167	8.43224068378741E-69	Proliferating hepatocytes	LAS1L
**6.48979366860767E-73**	0.656241418384923	0.392	0.206	1.08755962298527E-68	Proliferating hepatocytes	SFPQ
**7.98980773732676E-73**	0.509454504558696	0.366	0.187	1.33893198062122E-68	Proliferating hepatocytes	C1orf198
**8.88653789605915E-73**	0.62883291358387	0.555	0.343	1.48920602062159E-68	Proliferating hepatocytes	KLF3
**8.98544486479358E-73**	0.640470966797474	0.36	0.184	1.50578085044211E-68	Proliferating hepatocytes	LY6E
**1.09788049709989E-72**	0.741407735189295	0.374	0.195	1.83982813703999E-68	Proliferating hepatocytes	PWWP2A
**1.13834415918587E-72**	0.639860069920149	0.501	0.297	1.90763714196368E-68	Proliferating hepatocytes	MICALL2
**1.69756036498439E-72**	0.662887799194665	0.433	0.238	2.84477165964085E-68	Proliferating hepatocytes	MTM1
**1.77518654641972E-72**	0.752850349658961	0.332	0.165	2.97485761449017E-68	Proliferating hepatocytes	CCND3
**1.822118481884E-72**	1.01255741167601	0.325	0.165	3.05350615194121E-68	Proliferating hepatocytes	POLA1
**2.22740989767361E-72**	0.66293599650615	0.442	0.247	3.73269350652144E-68	Proliferating hepatocytes	KHSRP
**2.24732752538022E-72**	0.479587690592754	0.465	0.26	3.76607146703217E-68	Proliferating hepatocytes	SGTA
**3.24311885468057E-72**	0.533027799485576	0.544	0.326	5.43481857667371E-68	Proliferating hepatocytes	COPS6
**3.61182370756372E-72**	0.502351577932456	0.543	0.325	6.05269416913529E-68	Proliferating hepatocytes	PKDCC
**3.68283292650082E-72**	0.698017375787507	0.395	0.21	6.17169141823007E-68	Proliferating hepatocytes	AKIP1
**4.14182209705726E-72**	0.580612358505147	0.577	0.361	6.94086547024855E-68	Proliferating hepatocytes	MICOS10
**4.23549940024369E-72**	0.484755572950347	0.742	0.508	7.09784989492838E-68	Proliferating hepatocytes	KCTD3
**4.24970524787152E-72**	1.1491634861386	0.104	0.028	7.12165605438309E-68	Proliferating hepatocytes	RPGRIP1L

**4.29562752924419E-72**	0.814724574533602	0.258	0.115	7.19861261350741E-68	Proliferating hepatocytes	GIT1
**4.64546284998538E-72**	0.583589652324783	0.353	0.177	7.7848666440055E-68	Proliferating hepatocytes	CDC23
**5.22777025376815E-72**	0.659808008181617	0.468	0.266	8.76069739126467E-68	Proliferating hepatocytes	THOP1
**5.56592612492177E-72**	0.652515654022498	0.502	0.292	9.3273790001439E-68	Proliferating hepatocytes	RAB1B
**6.65000434165467E-72**	0.556565877668002	0.588	0.365	1.11440772757449E-67	Proliferating hepatocytes	FAM199X
**6.83669260494022E-72**	0.644107276380557	0.491	0.288	1.14569294673588E-67	Proliferating hepatocytes	NOTCH2
**8.15650153558892E-72**	0.692269265670542	0.286	0.133	1.36686652733399E-67	Proliferating hepatocytes	SSNA1
**8.66028298676333E-72**	0.886760438474435	0.222	0.093	1.4512902229218E-67	Proliferating hepatocytes	TRIM11
**9.92835660294282E-72**	0.945909484568726	0.258	0.115	1.66379399952116E-67	Proliferating hepatocytes	RIOX1
**1.00795175017926E-71**	0.758929227048275	0.158	0.054	1.6891255429504E-67	Proliferating hepatocytes	MMACHC
**1.01895269153674E-71**	0.897214682433824	0.208	0.084	1.70756092047726E-67	Proliferating hepatocytes	MEX3C
**1.14587848947607E-71**	1.28282287535143	0.122	0.036	1.920263172664E-67	Proliferating hepatocytes	GEMIN2
**1.3127613577572E-71**	0.926758842767603	0.281	0.131	2.19992548332951E-67	Proliferating hepatocytes	ASF1A
**1.39459882852662E-71**	0.728899489586924	0.406	0.22	2.33706871684491E-67	Proliferating hepatocytes	C6orf62
**1.449618498221E-71**	0.512938479530948	0.437	0.238	2.42927067931876E-67	Proliferating hepatocytes	ABHD12
**1.49485059698684E-71**	0.873648780072284	0.213	0.087	2.50507063043054E-67	Proliferating hepatocytes	ELP6
**1.69244488184314E-71**	0.604083207613061	0.35	0.176	2.83619913299274E-67	Proliferating hepatocytes	SNRNP40
**1.69814590055571E-71**	0.591421883695629	0.399	0.212	2.84575290015125E-67	Proliferating hepatocytes	SF3B4
**1.75948116450411E-71**	0.594462635549661	0.432	0.238	2.94853853547598E-67	Proliferating hepatocytes	MAPK9
**1.83607969745583E-71**	0.616559123187729	0.49	0.284	3.07690235699648E-67	Proliferating hepatocytes	PAFAH1B1
**1.97814288342919E-71**	0.610189258161317	0.428	0.235	3.31497184405063E-67	Proliferating hepatocytes	RUNX1
**2.43512581207737E-71**	0.592243409314342	0.354	0.181	4.08078383587925E-67	Proliferating hepatocytes	VPS37A
**3.44723145416323E-71**	0.589777051631628	0.54	0.326	5.77687047088674E-67	Proliferating hepatocytes	SEL1L3
**3.50060801164282E-71**	0.812810147908198	0.229	0.096	5.86631890591104E-67	Proliferating hepatocytes	SNX16
**3.78440670567792E-71**	0.860581247131101	0.158	0.054	6.34190875737506E-67	Proliferating hepatocytes	LYAR
**5.71916420894925E-71**	0.914131063340953	0.221	0.092	9.58417538135716E-67	Proliferating hepatocytes	MYB
**7.00405700081213E-71**	0.489207960045368	0.74	0.512	1.1737398721961E-66	Proliferating hepatocytes	SAP18
**7.60506017170174E-71**	0.669851068592673	0.262	0.117	1.27445598357378E-66	Proliferating hepatocytes	AKNA
**7.83706000415798E-71**	0.672857074862421	0.279	0.128	1.3133345154968E-66	Proliferating hepatocytes	DGCR8
**9.59491489871792E-71**	0.656517254396754	0.458	0.259	1.60791583872715E-66	Proliferating hepatocytes	PLEKHJ1
**9.70104415568186E-71**	1.1414401549429	0.106	0.029	1.62570097960917E-66	Proliferating hepatocytes	ANKRD50
**1.05565681619634E-70**	0.60594732839665	0.409	0.22	1.76906969258182E-66	Proliferating hepatocytes	SP1
**1.08638394512065E-70**	0.602035051898002	0.319	0.156	1.82056221523319E-66	Proliferating hepatocytes	SCMH1
**1.22400640035038E-70**	0.578621711099062	0.434	0.242	2.05118992570716E-66	Proliferating hepatocytes	SET
**1.25906933145286E-70**	1.51292769727998	0.146	0.049	2.1099483856487E-66	Proliferating hepatocytes	LTBP2
**1.26758425201108E-70**	0.550939972710276	0.458	0.26	2.12421768952017E-66	Proliferating hepatocytes	TNRC18
**1.44378963215419E-70**	0.647316369593079	0.461	0.268	2.41950266556399E-66	Proliferating hepatocytes	ERCC5
**1.54972741046687E-70**	0.781678404871663	0.228	0.096	2.59703319446039E-66	Proliferating hepatocytes	MYCBP
**1.58144845630851E-70**	0.546582950562157	0.396	0.21	2.6501913230818E-66	Proliferating hepatocytes	LSM 8.00
**1.67456000032082E-70**	0.796159753634148	0.294	0.14	2.80622764853764E-66	Proliferating hepatocytes	ZNF700
**1.6979887779091E-70**	0.869843719612057	0.189	0.072	2.84548959402007E-66	Proliferating hepatocytes	PRIMPOL
**1.70060442020521E-70**	0.76472329640629	0.348	0.177	2.84987288737989E-66	Proliferating hepatocytes	BRCC3
**2.05393892924606E-70**	0.579156259129398	0.449	0.251	3.44199085763055E-66	Proliferating hepatocytes	SF3B3
**2.08139246948304E-70**	1.39601255608321	0.122	0.037	3.48799750035967E-66	Proliferating hepatocytes	NPHP4
**2.58115529182904E-70**	0.583464506979318	0.575	0.36	4.32550003804711E-66	Proliferating hepatocytes	SDCBP
**3.17686077035224E-70**	0.75765415234436	0.32	0.158	5.32378327895629E-66	Proliferating hepatocytes	FAM126A
**3.51679473751674E-70**	0.654987407679899	0.405	0.219	5.89344462113055E-66	Proliferating hepatocytes	ELF4
**3.73289433994668E-70**	0.78922392357186	0.251	0.11	6.25558433488265E-66	Proliferating hepatocytes	PPOX
**3.93455793017296E-70**	0.725205233176516	0.423	0.238	6.59353217938384E-66	Proliferating hepatocytes	COX11
**3.99712867985928E-70**	0.800995096431989	0.289	0.137	6.69838824170818E-66	Proliferating hepatocytes	FADD
**5.34905545444916E-70**	0.552626907852978	0.498	0.292	8.9639471305659E-66	Proliferating hepatocytes	RNPS1
**7.80994696399419E-70**	0.847613301307369	0.236	0.102	1.30879091222615E-65	Proliferating hepatocytes	PHF13
**7.84987125762874E-70**	0.718767944529882	0.221	0.092	1.31548142535342E-65	Proliferating hepatocytes	NRF1
**8.63386708962221E-70**	0.517572284782433	0.61	0.387	1.44686344687889E-65	Proliferating hepatocytes	CDKN1A
**1.17662682986503E-69**	0.594699584365865	0.511	0.303	1.97179124148781E-65	Proliferating hepatocytes	TDP2
**1.2239223061432E-69**	0.499072434515719	0.616	0.392	2.05104900063477E-65	Proliferating hepatocytes	TALDO1
**1.24386735377415E-69**	0.515194081620375	0.477	0.276	2.08447291145473E-65	Proliferating hepatocytes	GLRX5
**1.75895248232299E-69**	0.575753892777826	0.418	0.226	2.94765256987687E-65	Proliferating hepatocytes	HOXA13
**1.78657006663429E-69**	0.512501546065253	0.421	0.231	2.99393411766574E-65	Proliferating hepatocytes	TNPO3
**1.81335054837893E-69**	0.840232644712174	0.324	0.163	3.0388128489734E-65	Proliferating hepatocytes	KHDRBS3

**2.67172644200317E-69**	0.690039846098751	0.371	0.196	4.47727917150891E-65	Proliferating hepatocytes	ZCCHC2
**2.82625433601215E-69**	0.844137428029522	0.204	0.082	4.73623701628916E-65	Proliferating hepatocytes	NUP37
**2.85480806607286E-69**	0.664700255368226	0.494	0.293	4.78408735712489E-65	Proliferating hepatocytes	PRKAB1
**3.07918235499952E-69**	0.495920419082714	0.751	0.528	5.16009379050819E-65	Proliferating hepatocytes	FAM50A
**3.19724680220473E-69**	1.30687791063585	0.121	0.036	5.35794619113469E-65	Proliferating hepatocytes	IQCC
**3.20793601005551E-69**	0.59831260088571	0.541	0.335	5.37585916565103E-65	Proliferating hepatocytes	HECTD1
**3.79659015383718E-69**	0.826357767439507	0.192	0.075	6.36232577980035E-65	Proliferating hepatocytes	PLEK2
**3.82782082618683E-69**	0.715336841878383	0.307	0.149	6.4146621405239E-65	Proliferating hepatocytes	UBE2M
**3.86277403689734E-69**	0.5714558285136	0.358	0.184	6.47323673103256E-65	Proliferating hepatocytes	LSM14A
**4.12278438771056E-69**	0.542298361068261	0.336	0.168	6.90896207692536E-65	Proliferating hepatocytes	NR2C2
**4.34482549592241E-69**	0.787080529055122	0.318	0.157	7.28105856606678E-65	Proliferating hepatocytes	ENOX2
**4.64821960034812E-69**	0.673746651411491	0.342	0.173	7.78948640626339E-65	Proliferating hepatocytes	C2CD5
**5.04127110307322E-69**	0.765200063846968	0.33	0.166	8.4481621145301E-65	Proliferating hepatocytes	RALY
**5.66980946088996E-69**	0.957642879614576	0.275	0.129	9.5014666945594E-65	Proliferating hepatocytes	RBL1
**5.92967436189702E-69**	0.675642820756587	0.306	0.148	9.93694829566703E-65	Proliferating hepatocytes	METTL9
**5.93066317464207E-69**	0.566760602290369	0.535	0.326	9.93860534806517E-65	Proliferating hepatocytes	PYGO2
**6.07821344459739E-69**	0.481513306311244	0.913	0.735	1.01858700904563E-64	Proliferating hepatocytes	MT-ND3
**7.21771627047874E-69**	0.632918130967784	0.415	0.229	1.20954489260683E-64	Proliferating hepatocytes	ITGA6
**7.59706744501981E-69**	0.552771571008457	0.608	0.392	1.27311656243642E-64	Proliferating hepatocytes	SLC44A1
**7.87671659394297E-69**	0.541621184199007	0.6	0.385	1.31998016681296E-64	Proliferating hepatocytes	SNRPC
**8.44597022903441E-69**	0.389554810466788	0.778	0.521	1.41537569098159E-64	Proliferating hepatocytes	G6PD
**9.07462281088192E-69**	0.500953260196284	0.414	0.225	1.52072529064759E-64	Proliferating hepatocytes	NAGK
**1.06146674235396E-68**	0.611440136251345	0.441	0.25	1.77880596683677E-64	Proliferating hepatocytes	FRYL
**1.10345035858557E-68**	0.661464238705192	0.241	0.105	1.8491621109177E-64	Proliferating hepatocytes	SNTA1
**1.12752462587803E-68**	1.06727092374997	0.204	0.083	1.88950576804641E-64	Proliferating hepatocytes	TFCP2L1
**1.15990637912655E-68**	0.497075590836701	0.714	0.485	1.94377111014028E-64	Proliferating hepatocytes	IDH3G
**1.53790664166581E-68**	0.68185738344646	0.456	0.268	2.57722395010357E-64	Proliferating hepatocytes	RBM12B
**1.82725057221322E-68**	0.494878591895854	0.722	0.492	3.06210650891492E-64	Proliferating hepatocytes	AIFM1
**1.91350751580693E-68**	0.480530295858146	0.853	0.638	3.20665589498925E-64	Proliferating hepatocytes	MT-ND5
**1.92516894375962E-68**	0.821236563667924	0.31	0.153	3.22619811595237E-64	Proliferating hepatocytes	VRK3
**2.33302503632133E-68**	0.96019300556599	0.228	0.098	3.90968335586728E-64	Proliferating hepatocytes	ARSJ
**2.45622847748862E-68**	0.525067574436994	0.426	0.234	4.11614768257542E-64	Proliferating hepatocytes	GATD1
**2.50544875098024E-68**	0.600897132687353	0.576	0.356	4.19863101689269E-64	Proliferating hepatocytes	MCTS1
**2.52495687142611E-68**	0.68087814003456	0.292	0.14	4.23132272513588E-64	Proliferating hepatocytes	USP5
**2.54290787918503E-68**	0.563833284349756	0.431	0.239	4.26140502393827E-64	Proliferating hepatocytes	TLN1
**2.64793565381649E-68**	0.534627689576872	0.426	0.235	4.43741056866567E-64	Proliferating hepatocytes	NSL1
**2.69046944078466E-68**	0.941162235479054	0.229	0.099	4.50868868886694E-64	Proliferating hepatocytes	RAB23
**2.76705675916903E-68**	0.546168639850747	0.571	0.355	4.63703371701546E-64	Proliferating hepatocytes	POGK
**2.97625496753088E-68**	0.480715516520175	0.562	0.346	4.98760807458825E-64	Proliferating hepatocytes	SMARCA4
**3.17451717179662E-68**	0.642280292777809	0.433	0.243	5.31985587649677E-64	Proliferating hepatocytes	BROX
**3.7830694099767E-68**	0.606447106846097	0.439	0.25	6.33966771723896E-64	Proliferating hepatocytes	ATF4
**4.06170217554141E-68**	0.580536025741881	0.528	0.317	6.8066005057723E-64	Proliferating hepatocytes	SHROOM3
**4.12043743400492E-68**	0.775827279570278	0.294	0.142	6.90502905190545E-64	Proliferating hepatocytes	SBNO1
**4.3835714368171E-68**	0.591738553352286	0.341	0.172	7.3459890138181E-64	Proliferating hepatocytes	NUP54
**5.66621664927519E-68**	0.747343491820334	0.235	0.102	9.49544586085537E-64	Proliferating hepatocytes	ASH2L
**6.37332749002545E-68**	0.508768412496637	0.651	0.431	1.06804222077846E-63	Proliferating hepatocytes	ZNF706
**6.84250571127241E-68**	1.02967807402723	0.153	0.054	1.14666710709503E-63	Proliferating hepatocytes	SAPCD1
**7.05109885874388E-68**	0.783001030270101	0.25	0.112	1.1816231467483E-63	Proliferating hepatocytes	PDIK1L
**7.32632522882125E-68**	0.552800916355859	0.391	0.209	1.22774558184587E-63	Proliferating hepatocytes	CHERP
**8.72259772586444E-68**	0.602273057749663	0.338	0.17	1.46173292690036E-63	Proliferating hepatocytes	AGAP1
**8.95348247722978E-68**	0.933645999982889	0.261	0.121	1.50042459353417E-63	Proliferating hepatocytes	HCN3
**9.23967190920257E-68**	0.854093471979597	0.302	0.147	1.54838421854417E-63	Proliferating hepatocytes	AMMECR1
**9.37637677241767E-68**	0.630317162786204	0.36	0.187	1.57129321952175E-63	Proliferating hepatocytes	SLC4A4
**1.00475994828021E-67**	0.628242884012431	0.392	0.209	1.68377672132798E-63	Proliferating hepatocytes	EXOC5
**1.05514633578228E-67**	0.635372113851811	0.391	0.212	1.76821422950395E-63	Proliferating hepatocytes	UGGT2
**1.12979953006871E-67**	0.631717314592589	0.382	0.205	1.89331805248915E-63	Proliferating hepatocytes	SMCHD1
**1.29257981837147E-67**	0.468843913165704	0.637	0.426	2.16610525962692E-63	Proliferating hepatocytes	SPTAN1
**1.29904694547601E-67**	1.00070013911274	0.208	0.086	2.1769428712287E-63	Proliferating hepatocytes	RBBP8
**1.38855537761459E-67**	0.673916979062128	0.392	0.213	2.32694110180652E-63	Proliferating hepatocytes	GMFB
**1.40650783924615E-67**	0.604672838776772	0.449	0.256	2.35702583700869E-63	Proliferating hepatocytes	PNP

**1.509297038331E-67**	0.778714968617289	0.273	0.127	2.52927997683509E-63	Proliferating hepatocytes	UBE2D2
**1.82029008069274E-67**	0.482562796510881	0.547	0.337	3.0504421172249E-63	Proliferating hepatocytes	SRSF2
**1.92674554115672E-67**	0.612721586011991	0.38	0.202	3.22884017787044E-63	Proliferating hepatocytes	NFYB
**2.24660615034549E-67**	0.57401993527714	0.296	0.142	3.76486258674897E-63	Proliferating hepatocytes	MCOLN1
**2.33415862202412E-67**	0.71688397205341	0.326	0.166	3.91158301878803E-63	Proliferating hepatocytes	IRF2BP1
**2.94851516681238E-67**	0.551789271291324	0.503	0.298	4.94112171654419E-63	Proliferating hepatocytes	UBE2Z
**3.06708332893205E-67**	0.503489658898862	0.621	0.401	5.13981824262433E-63	Proliferating hepatocytes	RNPEP
**3.23535767471059E-67**	0.538823957640619	0.461	0.264	5.42181239128001E-63	Proliferating hepatocytes	ARPC1A
**3.7937574179249E-67**	0.538966016590657	0.636	0.421	6.35757868095854E-63	Proliferating hepatocytes	PDZD8
**4.45562988016379E-67**	0.953625245684082	0.169	0.063	7.46674455317849E-63	Proliferating hepatocytes	CDKN2B
**5.01383656710164E-67**	0.607322278429021	0.394	0.212	8.40218731914892E-63	Proliferating hepatocytes	SGPP1
**5.2282346433132E-67**	0.54187610859304	0.462	0.264	8.76147561526425E-63	Proliferating hepatocytes	SNX6
**6.412759073162E-67**	0.640825098450669	0.449	0.259	1.07465016548049E-62	Proliferating hepatocytes	GBA
**7.01355112793606E-67**	0.639389163156069	0.407	0.225	1.17533089801952E-62	Proliferating hepatocytes	POLR2B
**7.70578071010431E-67**	0.686769451406765	0.391	0.212	1.29133473139928E-62	Proliferating hepatocytes	CHIC2
**8.66949193591877E-67**	0.692054254996333	0.438	0.251	1.45283345862127E-62	Proliferating hepatocytes	DPY19L4
**8.86103379541326E-67**	1.33359418013355	0.164	0.061	1.48493204343535E-62	Proliferating hepatocytes	CTBP2
**9.25112653306858E-67**	0.524641505338971	0.672	0.457	1.55030378441163E-62	Proliferating hepatocytes	CIAO2A
**9.40828476033952E-67**	0.618111263962751	0.471	0.276	1.5766403601377E-62	Proliferating hepatocytes	SNX5
**9.46553796412116E-67**	0.656361660555244	0.396	0.214	1.58623485202742E-62	Proliferating hepatocytes	SYPL1
**9.68346559813147E-67**	0.900943728579121	0.196	0.079	1.62275516493487E-62	Proliferating hepatocytes	TMEM143
**1.02946861100104E-66**	1.00593165678895	0.191	0.076	1.72518349831554E-62	Proliferating hepatocytes	ALPK1
**1.0734976126709E-66**	0.637477162589417	0.449	0.258	1.79896729931389E-62	Proliferating hepatocytes	LRRC58
**1.11395535268976E-66**	0.601641414312773	0.304	0.15	1.8667663800375E-62	Proliferating hepatocytes	H3F3B
**1.14799197205936E-66**	0.777260013555544	0.364	0.195	1.92380494677708E-62	Proliferating hepatocytes	PIGX
**1.27765987507208E-66**	0.58759295559983	0.269	0.124	2.1411024186458E-62	Proliferating hepatocytes	RHOQ
**1.33924718490136E-66**	0.622339427473661	0.418	0.234	2.24431043245771E-62	Proliferating hepatocytes	IRF2BPL
**1.39138429066976E-66**	1.50072545772964	0.114	0.034	2.33168179430438E-62	Proliferating hepatocytes	SCML2
**1.43661863876166E-66**	0.688617450942943	0.327	0.166	2.40748551483679E-62	Proliferating hepatocytes	EXOC8
**1.52687768687773E-66**	0.483922294665671	0.52	0.307	2.5587416276697E-62	Proliferating hepatocytes	PKN1
**1.64647728664752E-66**	0.530348039961844	0.444	0.251	2.75916663696392E-62	Proliferating hepatocytes	TCP1
**1.68373035839757E-66**	0.564207159978814	0.574	0.372	2.82159533460265E-62	Proliferating hepatocytes	STT3B
**1.69475678719003E-66**	1.1763902065409	0.103	0.028	2.84007342397305E-62	Proliferating hepatocytes	XRCC4
**1.77381480279631E-66**	0.911733280842836	0.208	0.087	2.97255884652605E-62	Proliferating hepatocytes	DTD1
**1.79418337712381E-66**	0.863536609017803	0.298	0.146	3.00669250338407E-62	Proliferating hepatocytes	CHML
**1.90823538257644E-66**	0.56632283373834	0.541	0.338	3.19782085412159E-62	Proliferating hepatocytes	PHF10
**1.94972010594206E-66**	0.712237463824548	0.417	0.235	3.26734095353771E-62	Proliferating hepatocytes	ERH
**2.0190943914914E-66**	0.580949422400197	0.449	0.259	3.38359838126129E-62	Proliferating hepatocytes	ZNF652
**2.03118375680453E-66**	0.579291193712507	0.458	0.267	3.40385773965303E-62	Proliferating hepatocytes	CLN3
**2.40374424697111E-66**	0.575536678843482	0.422	0.234	4.02819460907419E-62	Proliferating hepatocytes	PAFAH1B2
**2.58092361665612E-66**	0.560287947028785	0.569	0.358	4.32511179679233E-62	Proliferating hepatocytes	PIR
**2.62387830908345E-66**	0.539940548833039	0.412	0.23	4.39709527036205E-62	Proliferating hepatocytes	DYRK1A
**2.66223126417856E-66**	0.718060058361186	0.492	0.303	4.46136715251042E-62	Proliferating hepatocytes	MYO19
**2.66721825836476E-66**	0.633041393504283	0.295	0.143	4.46972435736766E-62	Proliferating hepatocytes	CCDC82
**2.84288865599378E-66**	0.541242000683231	0.282	0.132	4.76411280971438E-62	Proliferating hepatocytes	STX2
**3.96668422760029E-66**	0.596149489955366	0.281	0.133	6.64736942861256E-62	Proliferating hepatocytes	ITM2C
**4.32952859987751E-66**	0.47370223989475	0.511	0.306	7.25542402767473E-62	Proliferating hepatocytes	UBE2I
**4.34844518048627E-66**	0.521644827579932	0.454	0.262	7.28712443345889E-62	Proliferating hepatocytes	TCERG1
**4.40202374608107E-66**	0.681213449265902	0.311	0.155	7.37691139368266E-62	Proliferating hepatocytes	RIF1
**4.52992664744955E-66**	1.22338541857554	0.18	0.07	7.59125107579595E-62	Proliferating hepatocytes	MCOLN3
**6.20542998551466E-66**	0.659578967058171	0.317	0.159	1.03990595697255E-61	Proliferating hepatocytes	PDE7A
**6.93129604304903E-66**	0.77680301530331	0.337	0.175	1.16154659089416E-61	Proliferating hepatocytes	SSTR1
**7.04444497659165E-66**	0.608058956499247	0.398	0.216	1.18050808917723E-61	Proliferating hepatocytes	ELF2
**7.37909920488066E-66**	0.691384269059658	0.362	0.191	1.2365894447539E-61	Proliferating hepatocytes	PRMT2
**7.8932775278068E-66**	0.540327285623276	0.524	0.321	1.32275544810986E-61	Proliferating hepatocytes	APH1A
**7.99746762472196E-66**	0.920262215711091	0.175	0.067	1.34021562455091E-61	Proliferating hepatocytes	WBP11
**8.30407709808892E-66**	0.677419756741596	0.393	0.213	1.39159724009774E-61	Proliferating hepatocytes	OSGEP
**8.3430155034052E-66**	0.682901336338499	0.301	0.148	1.39812253806064E-61	Proliferating hepatocytes	CLOCK
**8.74533177904087E-66**	0.583429740309207	0.238	0.105	1.46554269953167E-61	Proliferating hepatocytes	TRAM2
**9.74791143607266E-66**	0.802573365140395	0.201	0.083	1.63355499845706E-61	Proliferating hepatocytes	CMTM7

1.02658585316716E-65	1.04547734867173	0.191	0.076	1.72035257273753E-61	Proliferating hepatocytes	USP18
1.05575242671725E-65	0.629224089381529	0.34	0.176	1.76922991669277E-61	Proliferating hepatocytes	UNK
1.05993670196283E-65	0.900912205746062	0.205	0.084	1.77624192514932E-61	Proliferating hepatocytes	FBXO45
1.10032711883915E-65	0.812497052359727	0.244	0.109	1.84392818575066E-61	Proliferating hepatocytes	DEPRECATEI
1.20593530387722E-65	0.777200320453647	0.401	0.222	2.02090638223744E-61	Proliferating hepatocytes	FGF13
1.48147593716175E-65	0.495241074685525	0.408	0.225	2.48265737549566E-61	Proliferating hepatocytes	GBE1
1.55968117632847E-65	0.521092621241265	0.418	0.23	2.61371371529126E-61	Proliferating hepatocytes	BDP1
1.5741925501951E-65	0.939355741309049	0.156	0.056	2.63803187561695E-61	Proliferating hepatocytes	USP11
1.80561005443746E-65	1.07750369315789	0.176	0.068	3.0258413292263E-61	Proliferating hepatocytes	PASK
2.34295722949036E-65	0.784626877144772	0.196	0.079	3.92632772517995E-61	Proliferating hepatocytes	R3HDM1
2.3749587123023E-65	0.793961180664307	0.284	0.137	3.9799558100762E-61	Proliferating hepatocytes	CCDC85C
2.38695660795658E-65	0.565644675210015	0.364	0.191	4.00006188361364E-61	Proliferating hepatocytes	HECA
2.43525667629335E-65	0.566300955688974	0.358	0.189	4.08100313813239E-61	Proliferating hepatocytes	STRBP
2.98263820739603E-65	0.665644840195129	0.388	0.211	4.99830510795427E-61	Proliferating hepatocytes	LCLAT1
3.06941093019915E-65	0.726423579297392	0.296	0.145	5.14371883682774E-61	Proliferating hepatocytes	DDA1
3.09325838631644E-65	0.744403804573007	0.323	0.164	5.18368240378909E-61	Proliferating hepatocytes	ARPP19
3.14819408807575E-65	0.53418743884679	0.627	0.412	5.27574365279735E-61	Proliferating hepatocytes	NENF
3.23632089124478E-65	0.427365988393485	0.687	0.45	5.4234265495448E-61	Proliferating hepatocytes	OCRL
3.4165473709172E-65	0.552350497392516	0.386	0.209	5.72545008418305E-61	Proliferating hepatocytes	STK4
3.88291424304704E-65	0.473274308923577	0.4	0.217	6.50698768849823E-61	Proliferating hepatocytes	ZC3H18
4.8917861905076E-65	0.621863203062596	0.382	0.207	8.19765529805263E-61	Proliferating hepatocytes	CASP6
5.06156025881043E-65	1.0540898590424	0.176	0.068	8.48216268171452E-61	Proliferating hepatocytes	ARHGAP4
5.29932886284454E-65	0.778578750410238	0.202	0.083	8.88061530835488E-61	Proliferating hepatocytes	DGKZ
5.49280176381881E-65	0.509515883220001	0.497	0.298	9.20483719580755E-61	Proliferating hepatocytes	AGO2
5.66231407835742E-65	0.809679532893244	0.283	0.137	9.48890593251136E-61	Proliferating hepatocytes	PRUNE1
6.29953454479494E-65	0.61551807085659	0.294	0.143	1.05567599901674E-60	Proliferating hepatocytes	TNPO1
7.031312034864E-65	0.796895938376642	0.269	0.126	1.17830727080251E-60	Proliferating hepatocytes	METTL27
7.48671042326867E-65	1.19201296537258	0.182	0.072	1.25462293273136E-60	Proliferating hepatocytes	GNG4
7.66739840199339E-65	0.536416710192408	0.444	0.252	1.28490262420605E-60	Proliferating hepatocytes	MTMR1
8.06657646886879E-65	0.647171143604004	0.35	0.183	1.35179688465303E-60	Proliferating hepatocytes	CLIC4
8.35468917816718E-65	1.08769144088729	0.128	0.042	1.40007881247726E-60	Proliferating hepatocytes	ZC2HC1A
9.13316625734646E-65	0.494263180829771	0.461	0.268	1.53053600140612E-60	Proliferating hepatocytes	RBM14
1.03854414810985E-64	0.541597866363252	0.26	0.12	1.74039228340249E-60	Proliferating hepatocytes	ENKD1
1.10402914949703E-64	0.60114217674319	0.347	0.181	1.85013204872713E-60	Proliferating hepatocytes	ABCB8
1.11898372536526E-64	0.434173550718888	0.449	0.255	1.8751929269671E-60	Proliferating hepatocytes	TXLNA
1.17034287477735E-64	0.582490444550362	0.342	0.176	1.96126058955187E-60	Proliferating hepatocytes	ITGB1BP1
1.19045329345596E-64	1.04580859935443	0.205	0.087	1.99496162917351E-60	Proliferating hepatocytes	RMI2
1.4787794563996E-64	0.968063497557662	0.182	0.072	2.47813861303444E-60	Proliferating hepatocytes	GTF2E1
1.50153561193122E-64	0.54032100715632	0.324	0.164	2.51627337847434E-60	Proliferating hepatocytes	DHX8
1.64502631902686E-64	0.561556390932695	0.537	0.331	2.75673510542522E-60	Proliferating hepatocytes	TAF7
1.79342295296109E-64	0.545570051442747	0.441	0.251	3.00541818457219E-60	Proliferating hepatocytes	HMGXB4
2.14393480341841E-64	0.438452075288806	0.678	0.444	3.59280594356856E-60	Proliferating hepatocytes	ETHE1
2.16773408166688E-64	0.567360935453923	0.253	0.115	3.63268877405736E-60	Proliferating hepatocytes	IFT80
2.65980625797361E-64	0.752806723845693	0.249	0.114	4.45730332711218E-60	Proliferating hepatocytes	CPSF4
2.83289363432011E-64	0.52995753375389	0.599	0.396	4.74736315239364E-60	Proliferating hepatocytes	PLEKHF2
2.85158637419705E-64	0.768805112011463	0.257	0.12	4.77868844587941E-60	Proliferating hepatocytes	FSTL1
2.92312527988888E-64	0.541564941881297	0.582	0.366	4.89857334403778E-60	Proliferating hepatocytes	AGPAT1
3.13650943097014E-64	0.954038395579408	0.138	0.047	5.25616250441976E-60	Proliferating hepatocytes	TCF7
3.59662498460952E-64	0.560755605923159	0.458	0.268	6.02722414920863E-60	Proliferating hepatocytes	GSK3A
3.6326539088969E-64	0.659494955008047	0.359	0.192	6.08760142052943E-60	Proliferating hepatocytes	CD9
3.96446191446643E-64	0.494795575687657	0.402	0.221	6.64364527626284E-60	Proliferating hepatocytes	COPRS
4.29753662713485E-64	0.369961960903947	0.956	0.854	7.20181187975257E-60	Proliferating hepatocytes	DEPRECATEI
4.93146702332072E-64	0.497876665263585	0.588	0.37	8.26415243768086E-60	Proliferating hepatocytes	SYBU
4.95457860934205E-64	0.704793601458091	0.258	0.121	8.30288283353541E-60	Proliferating hepatocytes	CD320
4.98210587302767E-64	0.776060081242214	0.247	0.113	8.34901302201977E-60	Proliferating hepatocytes	ARL17B
5.2163481271638E-64	0.698023467919495	0.373	0.201	8.7415561915011E-60	Proliferating hepatocytes	MAP4K5
5.30453340224271E-64	0.90317516086521	0.194	0.08	8.88933707547834E-60	Proliferating hepatocytes	MDM1
5.49388613652593E-64	0.526502266366767	0.721	0.518	9.20665438759015E-60	Proliferating hepatocytes	EIF1
5.53352013618619E-64	0.504832341105922	0.372	0.198	9.27307304422082E-60	Proliferating hepatocytes	DNAJC13
5.59827477370223E-64	0.565615715594234	0.376	0.203	9.38158886577019E-60	Proliferating hepatocytes	TSN

5.59904423372179E-64	0.707876468163671	0.446	0.267	9.38287832687098E-60	Proliferating hepatocytes	ARL17A
5.66189411384426E-64	0.692249099054438	0.287	0.139	9.48820215598022E-60	Proliferating hepatocytes	CAPRIN1
5.71437043211207E-64	0.426292415601819	0.454	0.259	9.57614197013341E-60	Proliferating hepatocytes	TRA2B
5.89373339239234E-64	0.535508239870034	0.406	0.225	9.87671841897108E-60	Proliferating hepatocytes	PSMG3
6.1648961353294E-64	1.77105329527303	0.123	0.04	1.0331132943585E-59	Proliferating hepatocytes	PLPPR4
7.00600373938443E-64	0.714803265816577	0.273	0.13	1.17406610664604E-59	Proliferating hepatocytes	MCRIP1
7.09731751256126E-64	0.591663598140818	0.459	0.27	1.18936846875502E-59	Proliferating hepatocytes	DEPRECATEI
8.81942349572626E-64	0.668453489122272	0.349	0.185	1.47795898941381E-59	Proliferating hepatocytes	CNOT10
9.18136854361821E-64	0.717859276253588	0.256	0.119	1.53861374053954E-59	Proliferating hepatocytes	AARS
9.5773880580496E-64	0.709786918371092	0.275	0.131	1.60497869076795E-59	Proliferating hepatocytes	ABR
9.83788313745213E-64	0.471500267343422	0.294	0.142	1.64863245617423E-59	Proliferating hepatocytes	CPSF3
1.10698783969076E-63	0.531088047136417	0.517	0.316	1.85509022175378E-59	Proliferating hepatocytes	PHF20L1
1.14216650629097E-63	0.537501663313433	0.48	0.287	1.91404263124241E-59	Proliferating hepatocytes	ARHGEF40
1.1835031580828E-63	0.709359712718368	0.294	0.145	1.98331459231515E-59	Proliferating hepatocytes	GAR1
1.25700973357558E-63	0.545320067492838	0.548	0.345	2.10649691152596E-59	Proliferating hepatocytes	TMEM109
1.37981437381928E-63	0.614866132668033	0.35	0.184	2.31229292764636E-59	Proliferating hepatocytes	NAA38
1.5445455251049E-63	0.747977118481152	0.244	0.111	2.58834939097079E-59	Proliferating hepatocytes	APOO
1.66090524749608E-63	0.565858447762269	0.299	0.147	2.78334501375392E-59	Proliferating hepatocytes	CNOT6
1.87667118317684E-63	0.510552040942637	0.678	0.474	3.14492556876775E-59	Proliferating hepatocytes	BSG
1.91644293619887E-63	0.600591118672004	0.263	0.123	3.21157507248206E-59	Proliferating hepatocytes	PPP1R13L
2.10712188388021E-63	0.622464758336256	0.389	0.214	3.53111485300646E-59	Proliferating hepatocytes	LARP7
2.48416955859762E-63	0.61393658343376	0.409	0.228	4.1629713462979E-59	Proliferating hepatocytes	CCDC117
2.63198637035461E-63	0.543424111639708	0.379	0.207	4.41068275944026E-59	Proliferating hepatocytes	NOTCH1
2.96084246554711E-63	0.642695468509441	0.322	0.164	4.96177980376385E-59	Proliferating hepatocytes	MAP1S
3.00284806850389E-63	0.995586379768268	0.185	0.075	5.03217279319881E-59	Proliferating hepatocytes	OSBPL3
3.0755844101701E-63	0.42583415961121	0.459	0.265	5.15406435456305E-59	Proliferating hepatocytes	RUFY1
3.14375215920975E-63	0.441738947518758	0.737	0.53	5.26829986840369E-59	Proliferating hepatocytes	PUF60
3.14519775872407E-63	0.70414909133094	0.226	0.099	5.2707224040698E-59	Proliferating hepatocytes	CBX7
3.55428396679803E-63	0.554866977130498	0.387	0.212	5.95626907156015E-59	Proliferating hepatocytes	CDC42BPA
3.60677282610581E-63	0.583337901448285	0.287	0.139	6.04422990198812E-59	Proliferating hepatocytes	BTG2
3.93936566793781E-63	0.764788721667337	0.235	0.105	6.60158898633018E-59	Proliferating hepatocytes	DEPRECATEI
4.25825889425962E-63	0.576077690932825	0.399	0.221	7.13599025500028E-59	Proliferating hepatocytes	DEPRECATEI
4.82349486588936E-63	0.589685912445679	0.299	0.148	8.08321269625739E-59	Proliferating hepatocytes	COG4
5.36810863690359E-63	0.371500086708183	0.966	0.858	8.99587645372304E-59	Proliferating hepatocytes	ACTB
5.40933961767232E-63	0.669658151063846	0.343	0.18	9.06497133129528E-59	Proliferating hepatocytes	PHLPP1
5.43483451918453E-63	0.656428177985116	0.391	0.215	9.10769568724944E-59	Proliferating hepatocytes	GLO1
6.547305104327 06E-63	0.741207015711435	0.364	0.198	1.09719738938313E-58	Proliferating hepatocytes	ALG6
6.98297462354013E-63	0.425437079584844	0.746	0.504	1.17020688741285E-58	Proliferating hepatocytes	CLDN4
7.04468419092472E-63	0.743784865480901	0.231	0.103	1.18054817671517E-58	Proliferating hepatocytes	NOCT
7.21263410121664E-63	1.00328007179171	0.17	0.066	1.20869322268188E-58	Proliferating hepatocytes	PIDD1
8.62192657415958E-63	0.632720006724288	0.401	0.223	1.44486245529766E-58	Proliferating hepatocytes	RNF6
8.9318128305443E-63	0.715530476320794	0.27	0.128	1.49679319414261E-58	Proliferating hepatocytes	TTC39B
9.45328220051992E-63	0.510096439503132	0.424	0.239	1.58418103116313E-58	Proliferating hepatocytes	NUDT21
1.12143338720301E-62	0.395793305713803	0.825	0.591	1.8792980702748E-58	Proliferating hepatocytes	NDUFA1
1.18703888989086E-62	0.789332438553174	0.199	0.083	1.9892397716791E-58	Proliferating hepatocytes	CERS5
1.27522953897867E-62	0.509839237076996	0.523	0.323	2.13702966142046E-58	Proliferating hepatocytes	FNTA
1.48043479309365E-62	0.54054841542404	0.458	0.267	2.48091262626633E-58	Proliferating hepatocytes	SMARCD2
1.49512858919647E-62	1.12021663593273	0.187	0.076	2.50553648977545E-58	Proliferating hepatocytes	LLGL1
1.53326291626127E-62	0.50367034061233	0.334	0.171	2.56944199507063E-58	Proliferating hepatocytes	SEPTIN7
1.53793540864169E-62	1.1530187644328	0.106	0.031	2.57727215780174E-58	Proliferating hepatocytes	DEPRECATEI
1.57965108026073E-62	0.607162191015466	0.375	0.205	2.64717928030093E-58	Proliferating hepatocytes	CENPX
1.65661806327248E-62	0.498879759597073	0.735	0.524	2.77616055043202E-58	Proliferating hepatocytes	TSPAN6
1.67878676689862E-62	0.71038269379116	0.243	0.111	2.8133108639687E-58	Proliferating hepatocytes	RLIM
1.82337642274754E-62	0.704648723448138	0.213	0.092	3.05561420924033E-58	Proliferating hepatocytes	DGKH
1.84341851490759E-62	0.538587356577703	0.483	0.289	3.08920074728214E-58	Proliferating hepatocytes	NRP1
1.87546264746275E-62	0.516602227694216	0.567	0.368	3.14290030461808E-58	Proliferating hepatocytes	DHX15
1.92402111494373E-62	0.421825163583792	0.279	0.133	3.2242745844227E-58	Proliferating hepatocytes	INIP
2.01981581981236E-62	0.736464717377031	0.244	0.112	3.38480735084156E-58	Proliferating hepatocytes	ZFYVE1
2.54892776577294E-62	0.541106244153561	0.528	0.333	4.27149314988229E-58	Proliferating hepatocytes	PAXX
2.82459292159727E-62	1.09182169343338	0.151	0.056	4.73345281801271E-58	Proliferating hepatocytes	AKAP12

**2.87774007390573E-62**	0.568156911375965	0.257	0.12	4.82251681585123E-58	Proliferating hepatocytes	GNAI1
**3.03607531820485E-62**	0.520280323634746	0.653	0.454	5.08785501824769E-58	Proliferating hepatocytes	PICALM
**3.07825715952794E-62**	0.524264012980386	0.632	0.424	5.15854334793693E-58	Proliferating hepatocytes	KRTCAP2
**3.70248933909202E-62**	0.456010121305124	0.311	0.156	6.20463163445041E-58	Proliferating hepatocytes	UBAC1
**3.70548760738999E-62**	0.513504657623282	0.597	0.391	6.20965613246414E-58	Proliferating hepatocytes	COPS5
**3.757208202568E-62**	0.601752071306379	0.309	0.156	6.29632950586345E-58	Proliferating hepatocytes	CCDC61
**4.00403121218114E-62**	0.497200659143203	0.647	0.438	6.70995550537316E-58	Proliferating hepatocytes	STT3A
**4.24546648983764E-62**	0.832288773550433	0.184	0.074	7.11455274366992E-58	Proliferating hepatocytes	CLDN11
**4.35576718746312E-62**	0.477854254618059	0.455	0.263	7.2993946527507E-58	Proliferating hepatocytes	AP3B1
**4.39441508473654E-62**	0.546901121996899	0.577	0.365	7.3641607990015E-58	Proliferating hepatocytes	RNF187
**4.87938630815683E-62**	0.49407147266941	0.513	0.318	8.17687557520922E-58	Proliferating hepatocytes	CGGBP1
**5.13019500216162E-62**	0.677239213427774	0.305	0.153	8.59718078462245E-58	Proliferating hepatocytes	MKKS
**5.3380385497258E-62**	0.58808326721015	0.429	0.247	8.9454850016305E-58	Proliferating hepatocytes	COA6
**5.62735325364621E-62**	0.735632227041012	0.239	0.109	9.43031858246031E-58	Proliferating hepatocytes	COQ7
**5.77683861026593E-62**	0.785072186768944	0.275	0.132	9.68082614308365E-58	Proliferating hepatocytes	UBXN2A
**5.87572741205954E-62**	0.576123163021015	0.514	0.318	9.84654399712938E-58	Proliferating hepatocytes	CMTM6
**6.99913860436698E-62**	0.753136736108524	0.327	0.173	1.17291564731982E-57	Proliferating hepatocytes	RFC2
**7.9153945232398E-62**	0.836626891451396	0.223	0.099	1.32646181420453E-57	Proliferating hepatocytes	NCOA5
**8.40338721879902E-62**	0.456903313858329	0.303	0.15	1.40823963012634E-57	Proliferating hepatocytes	RNF169
**8.549904727924E-62**	0.436206180891634	0.395	0.215	1.4327930343055E-57	Proliferating hepatocytes	ROCK1
**9.88645253831347E-62**	0.783293490848201	0.26	0.123	1.65677171637057E-57	Proliferating hepatocytes	POT1
**1.01010443713192E-61**	0.52270940598604	0.583	0.382	1.69273301574568E-57	Proliferating hepatocytes	PA2G4
**1.07950058779027E-61**	0.516268146211527	0.59	0.385	1.80902708501893E-57	Proliferating hepatocytes	SRXN1
**1.23125583339557E-61**	0.620943025957393	0.288	0.142	2.0633385256043E-57	Proliferating hepatocytes	EIF4EBP1
**1.25520226582066E-61**	0.585187739842913	0.335	0.174	2.10346795706226E-57	Proliferating hepatocytes	RBBP6
**1.28883865178072E-61**	0.72991312640178	0.191	0.079	2.15983581265414E-57	Proliferating hepatocytes	ZCCHC17
**1.36675939256003E-61**	0.556162082607066	0.564	0.356	2.2904153900521E-57	Proliferating hepatocytes	TMEM38B
**1.47001586887833E-61**	0.526374643757517	0.459	0.269	2.46345259306631E-57	Proliferating hepatocytes	CAMK1
**1.56003287394739E-61**	0.724523800850211	0.257	0.122	2.61430309016103E-57	Proliferating hepatocytes	NIPSNAP3A
**1.86774469574916E-61**	0.546225284039453	0.418	0.238	3.12996656113645E-57	Proliferating hepatocytes	TAB2
**1.92695547663187E-61**	0.758213669167404	0.748	0.589	3.22919198773969E-57	Proliferating hepatocytes	H1F0
**1.9848878322095E-61**	0.672776827958183	0.21	0.09	3.32627502921668E-57	Proliferating hepatocytes	MYH10
**2.03103115047232E-61**	0.492248113012005	0.413	0.23	3.40360200196151E-57	Proliferating hepatocytes	MTR
**2.18773705910464E-61**	0.574243324430826	0.413	0.235	3.66620976364755E-57	Proliferating hepatocytes	CLDND1
**2.21368797774485E-61**	0.590276886996533	0.493	0.302	3.70969831310482E-57	Proliferating hepatocytes	NCOR1
**2.35958948745905E-61**	0.469232810455595	0.435	0.248	3.95420006308388E-57	Proliferating hepatocytes	CENPB
**2.4500570834877E-61**	0.971782407794277	0.21	0.091	4.10580566050869E-57	Proliferating hepatocytes	POGLUT3
**2.58396343346399E-61**	0.601056735726664	0.45	0.261	4.33020592179896E-57	Proliferating hepatocytes	PALLD
**2.60800146243943E-61**	0.591102239244709	0.227	0.101	4.370488850756E-57	Proliferating hepatocytes	C19orf47
**2.62317752609001E-61**	0.503984668459963	0.31	0.157	4.39592089822164E-57	Proliferating hepatocytes	TBC1D10B
**2.64389578672686E-61**	0.485308260940068	0.575	0.37	4.43064055939687E-57	Proliferating hepatocytes	GOT1
**2.88895705955788E-61**	0.557070255041164	0.409	0.229	4.8413142404071E-57	Proliferating hepatocytes	CASK
**2.91245660625006E-61**	0.549299587356026	0.353	0.189	4.88069478075385E-57	Proliferating hepatocytes	GTF2F1
**3.09014110282491E-61**	0.500706221062267	0.683	0.483	5.17845846011398E-57	Proliferating hepatocytes	HBS1L
**3.38878287483692E-61**	0.579483417886916	0.352	0.187	5.67892234165171E-57	Proliferating hepatocytes	ERAL1
**3.4020418974579E-61**	0.511014022934718	0.416	0.233	5.70114181175994E-57	Proliferating hepatocytes	ZNF219
**3.5537363112914E-61**	0.489547648265407	0.552	0.351	5.95535131046214E-57	Proliferating hepatocytes	KAT6A
**3.67979112262169E-61**	0.853058030862084	0.174	0.069	6.16659396328943E-57	Proliferating hepatocytes	DYNLL1
**3.69295235430325E-61**	0.957303617681343	0.146	0.053	6.18864955534138E-57	Proliferating hepatocytes	NEMP2
**3.86948709370067E-61**	0.68770418858557	0.299	0.15	6.48448647162358E-57	Proliferating hepatocytes	SNRPG
**4.01754625164023E-61**	0.741661571952487	0.228	0.103	6.7326040084987E-57	Proliferating hepatocytes	PRKCI
**4.30875988466466E-61**	0.555578158720684	0.293	0.146	7.22061981472104E-57	Proliferating hepatocytes	C1orf21
**4.52151580075686E-61**	0.545159774261534	0.251	0.118	7.57715617890834E-57	Proliferating hepatocytes	STK35
**5.65309341563289E-61**	0.524143689576246	0.329	0.172	9.47345394591759E-57	Proliferating hepatocytes	DNM1L
**5.8182808694404E-61**	0.750293626785614	0.311	0.16	9.75027508100822E-57	Proliferating hepatocytes	COMMD2
**5.87126451550077E-61**	0.634132348305589	0.197	0.082	9.8390650750762E-57	Proliferating hepatocytes	CFAP36
**6.14325769494832E-61**	0.60581850389772	0.371	0.203	1.02948712451944E-56	Proliferating hepatocytes	ICK
**6.38800160435568E-61**	0.623372655140895	0.606	0.414	1.07050130885793E-56	Proliferating hepatocytes	SLBP
**6.88102030067073E-61**	0.603452630178514	0.385	0.214	1.1531213819864E-56	Proliferating hepatocytes	PSMA4
**8.12417525167959E-61**	0.56525713474862	0.514	0.325	1.36144928867647E-56	Proliferating hepatocytes	CLDN15

8.54614158265615E-61	0.710126437833376	0.271	0.131	1.43216240642152E-56	Proliferating hepatocytes	SLC52A2
8.57810691162132E-61	0.726343661681144	0.361	0.196	1.4375191562495E-56	Proliferating hepatocytes	PPP2CA
8.59940840071476E-61	0.644956449173195	0.362	0.196	1.44108885979178E-56	Proliferating hepatocytes	ARHGEF18
8.87640963132028E-61	0.888447668015445	0.219	0.097	1.48750872601665E-56	Proliferating hepatocytes	PDK3
9.812235094439E-61	0.37929786304915	0.504	0.303	1.64433435712609E-56	Proliferating hepatocytes	NCOR2
1.01869316323802E-60	0.519232806862578	0.319	0.163	1.70712600295428E-56	Proliferating hepatocytes	DMWD
1.05893191602882E-60	0.439988123838387	0.556	0.353	1.77455810488109E-56	Proliferating hepatocytes	RAD23A
1.15010829702574E-60	0.621972406596597	0.363	0.196	1.92735148415574E-56	Proliferating hepatocytes	EIF2AK2
1.18515327790978E-60	0.5146913048235	0.436	0.252	1.98607986312121E-56	Proliferating hepatocytes	GSS
1.19324946458028E-60	0.630758801636711	0.271	0.132	1.99964745274363E-56	Proliferating hepatocytes	XRCC6
1.2678232488833E-60	0.539865210320812	0.373	0.204	2.12461820047863E-56	Proliferating hepatocytes	SNRPD2
1.44179795663844E-60	0.547721710664128	0.326	0.168	2.4161650157347E-56	Proliferating hepatocytes	ZBTB44
1.50604349045956E-60	0.467548208584427	0.42	0.238	2.52382768131212E-56	Proliferating hepatocytes	SZRD1
1.59291545215247E-60	0.559822806376158	0.338	0.178	2.66940771471711E-56	Proliferating hepatocytes	RETREG2
1.62354679195096E-60	0.473423850069161	0.46	0.27	2.72073971395142E-56	Proliferating hepatocytes	RAC1
1.73273812707857E-60	0.627135335280567	0.307	0.156	2.90372255335827E-56	Proliferating hepatocytes	SPTLC2
1.73708161722814E-60	0.696881985787764	0.378	0.209	2.91100137415091E-56	Proliferating hepatocytes	TEX261
1.80169558011116E-60	0.500125485631478	0.464	0.272	3.01928145315029E-56	Proliferating hepatocytes	DYNC1I2
2.00980850145408E-60	0.636731124905577	0.339	0.179	3.36803708673675E-56	Proliferating hepatocytes	CCNK
2.18348590518598E-60	0.470307620535417	0.539	0.341	3.65908567991067E-56	Proliferating hepatocytes	STAT1
2.39847048147663E-60	0.447492935045327	0.507	0.308	4.01935683285853E-56	Proliferating hepatocytes	HNRNPM
2.45821248120644E-60	0.848845996880152	0.176	0.071	4.11947247600575E-56	Proliferating hepatocytes	TOP3A
2.6183301050002E-60	0.564787494798746	0.376	0.203	4.38779758995933E-56	Proliferating hepatocytes	MICU2
3.11127725726516E-60	0.438750893661519	0.851	0.643	5.21387842772496E-56	Proliferating hepatocytes	MT-ND2
3.28571450608387E-60	0.591201072452897	0.273	0.133	5.50620036929534E-56	Proliferating hepatocytes	NSD3
3.32364226425421E-60	0.69017393266268	0.328	0.171	5.5697597064372E-56	Proliferating hepatocytes	PFN2
3.36394466551735E-60	0.940689435660167	0.144	0.052	5.63729847047397E-56	Proliferating hepatocytes	CBL
3.36777476896308E-60	0.610468185148636	0.429	0.251	5.64371695782834E-56	Proliferating hepatocytes	ABHD10
3.7317549500037E-60	0.655880953353947	0.357	0.194	6.2536749452162E-56	Proliferating hepatocytes	PPIP5K2
3.84971024932672E-60	0.414367738188298	0.378	0.206	6.45134443582172E-56	Proliferating hepatocytes	EDC4
3.88462084008746E-60	0.496166534434756	0.381	0.21	6.50984760381857E-56	Proliferating hepatocytes	KLHL2
3.98631983740388E-60	0.492762880815771	0.562	0.364	6.68027478352143E-56	Proliferating hepatocytes	SP3
4.25158526601338E-60	1.12451099906336	0.135	0.047	7.12480658878523E-56	Proliferating hepatocytes	OSBPL6
4.29712739029737E-60	0.655875894412933	0.269	0.13	7.20112608066033E-56	Proliferating hepatocytes	POLR2D
4.40730230223108E-60	0.500457999483337	0.57	0.365	7.38575719807885E-56	Proliferating hepatocytes	ADD1
4.75322749995359E-60	0.552992244698633	0.319	0.164	7.96545864442222E-56	Proliferating hepatocytes	IPO5
4.88271045503364E-60	0.624176811510402	0.393	0.22	8.18244618054538E-56	Proliferating hepatocytes	TM2D2
5.01012746079558E-60	0.631400311656674	0.366	0.2	8.39597159880124E-56	Proliferating hepatocytes	GPD1L
5.31280921964245E-60	0.44769199868436	0.71	0.478	8.90320569027681E-56	Proliferating hepatocytes	LAPTM4B
5.6118313282551E-60	0.592943186942886	0.478	0.294	9.4043069398899E-56	Proliferating hepatocytes	ABCB6
5.75089523370234E-60	0.545863056757952	0.411	0.233	9.63735023263838E-56	Proliferating hepatocytes	CTNNB1
5.91018585769775E-60	0.533969860137163	0.344	0.182	9.90428946032988E-56	Proliferating hepatocytes	SMARCC2
6.06860936729454E-60	0.644620158750297	0.376	0.21	1.01697755777122E-55	Proliferating hepatocytes	FAF2
6.65596327398778E-60	0.499245595593334	0.375	0.205	1.11540632545487E-55	Proliferating hepatocytes	CARM1
6.88448335742181E-60	0.512455098223906	0.431	0.252	1.15370172103675E-55	Proliferating hepatocytes	SKI
7.0102054941411E-60	0.5605848032626	0.385	0.214	1.17477023670817E-55	Proliferating hepatocytes	CCDC28A
7.18800652377988E-60	0.556864573771111	0.385	0.213	1.20456613325503E-55	Proliferating hepatocytes	NAA50
7.30979886539942E-60	1.02639058808294	0.122	0.04	1.22497609386363E-55	Proliferating hepatocytes	NUDT15
8.4720861700517E-60	0.737211860728977	0.171	0.068	1.41975220037726E-55	Proliferating hepatocytes	IFT22
8.48539348483122E-60	0.685895432405724	0.226	0.101	1.42198224018802E-55	Proliferating hepatocytes	SENP1
8.89279003352485E-60	0.49779322767889	0.484	0.291	1.49025375381809E-55	Proliferating hepatocytes	ENOSF1
9.24158666114244E-60	1.04959889295498	0.164	0.064	1.54870509267425E-55	Proliferating hepatocytes	ADCY3
9.86126791537141E-60	0.594117306794404	0.351	0.189	1.65255127725794E-55	Proliferating hepatocytes	VEGFB
1.0405537301911E-59	0.429320728897053	0.441	0.252	1.74375994105424E-55	Proliferating hepatocytes	TERF1
1.12297458169902E-59	0.502449205482663	0.357	0.192	1.88188080401122E-55	Proliferating hepatocytes	NFATC2
1.12442139122279E-59	0.508238241640546	0.465	0.277	1.88430536741115E-55	Proliferating hepatocytes	PTBP3
1.24898964742052E-59	0.510890002865412	0.363	0.195	2.09305685114731E-55	Proliferating hepatocytes	UBQLN4
1.28428625071768E-59	0.544271062587762	0.432	0.249	2.15220689895269E-55	Proliferating hepatocytes	HAX1
1.30892517843902E-59	0.525068003674475	0.427	0.246	2.19349681402811E-55	Proliferating hepatocytes	BTBD7
1.31721171651854E-59	0.630212514224882	0.368	0.202	2.20738339454176E-55	Proliferating hepatocytes	PBX3

1.33403683398092E-59	0.575259626375406	0.309	0.157	2.23557892638522E-55	Proliferating hepatocytes	ZDHHC20
1.36665561203592E-59	0.665091540475021	0.276	0.136	2.2902414746498E-55	Proliferating hepatocytes	TAF5L
1.46224665364145E-59	0.435605798983389	0.455	0.268	2.45043294217234E-55	Proliferating hepatocytes	MCUR1
1.50291550251484E-59	0.791825322394638	0.308	0.158	2.51858579911437E-55	Proliferating hepatocytes	P4HTM
1.53674290876588E-59	0.477919941655167	0.463	0.274	2.57527376650986E-55	Proliferating hepatocytes	SNRPA
1.54814107264706E-59	0.545890277646631	0.54	0.339	2.59437480954194E-55	Proliferating hepatocytes	CUL4B
1.63588479120452E-59	0.576106532365791	0.41	0.236	2.74141573310053E-55	Proliferating hepatocytes	ELMOD2
1.7051758391297E-59	0.532056425325379	0.549	0.352	2.85753367121356E-55	Proliferating hepatocytes	ISOC2
1.76727818408446E-59	0.337473060417072	0.303	0.152	2.96160478088874E-55	Proliferating hepatocytes	CDR2
1.78227224329967E-59	0.57814440352149	0.45	0.266	2.98673182532159E-55	Proliferating hepatocytes	ASAP1
1.96252672632216E-59	0.722710379767116	0.195	0.083	3.28880228797068E-55	Proliferating hepatocytes	CABLES2
2.04263452128908E-59	0.46870552499054	0.675	0.474	3.42304693077624E-55	Proliferating hepatocytes	ACBD5
2.0955397554632E-59	0.752136127371692	0.202	0.086	3.51170552220524E-55	Proliferating hepatocytes	GMEB1
2.10588059100955E-59	0.769658119733395	0.254	0.121	3.5290346944138E-55	Proliferating hepatocytes	SPAG1
2.15529800741992E-59	0.821672769287655	0.225	0.102	3.6118484008343E-55	Proliferating hepatocytes	PAICS
2.21400818987412E-59	0.610312209172018	0.4	0.227	3.71023492459105E-55	Proliferating hepatocytes	ANAPC4
2.24152108364289E-59	0.511627533052631	0.466	0.275	3.75634103196875E-55	Proliferating hepatocytes	ELOVL2
2.2484184133166E-59	0.668528162285884	0.288	0.144	3.76789957703596E-55	Proliferating hepatocytes	SNUPN
2.33993669381024E-59	0.581996814623434	0.397	0.223	3.92126591148719E-55	Proliferating hepatocytes	PTDSS1
2.50027728310281E-59	0.483364277787434	0.568	0.363	4.18996467102369E-55	Proliferating hepatocytes	ENSA
2.77099579344476E-59	0.514280163579982	0.395	0.223	4.64363475065473E-55	Proliferating hepatocytes	PLCB1
2.78905876254412E-59	1.05347164299111	0.16	0.061	4.67390467427144E-55	Proliferating hepatocytes	PTPDC1
2.80916243560006E-59	0.493236181746889	0.263	0.125	4.70759440957858E-55	Proliferating hepatocytes	WAPL
2.93267616831391E-59	0.651548658750145	0.373	0.207	4.91457872286045E-55	Proliferating hepatocytes	IPMK
3.02241245749194E-59	0.929958549466849	0.219	0.098	5.06495879626499E-55	Proliferating hepatocytes	MAP7D3
3.61301126074536E-59	0.832850281955377	0.206	0.09	6.05468427075708E-55	Proliferating hepatocytes	LYPD6
3.61389549475441E-59	0.502333608426604	0.454	0.268	6.05616607010944E-55	Proliferating hepatocytes	UBP1
3.82407755176105E-59	0.488912931071623	0.54	0.34	6.40838916124117E-55	Proliferating hepatocytes	GOLGA7
3.85243813561041E-59	0.653719399827652	0.245	0.115	6.45591582765593E-55	Proliferating hepatocytes	CEP170
3.96310298503879E-59	0.424082154133137	0.724	0.504	6.64136798232801E-55	Proliferating hepatocytes	PLS3
4.44188053990496E-59	0.481948907540634	0.553	0.35	7.44370340877273E-55	Proliferating hepatocytes	NBPF26
4.73887729754749E-59	0.409046597947995	0.635	0.422	7.94141057523008E-55	Proliferating hepatocytes	HPGD
4.95119159536221E-59	0.659049280019436	0.253	0.121	8.29720687550799E-55	Proliferating hepatocytes	RPA2
5.36337541138346E-59	0.443325153874735	0.436	0.253	8.9879445143964E-55	Proliferating hepatocytes	POLDIP3
5.77568003754981E-59	0.771575188264128	0.166	0.065	9.67888460692598E-55	Proliferating hepatocytes	SORT1
5.9884423443307E-59	0.571634040251023	0.321	0.166	1.00354316806294E-54	Proliferating hepatocytes	MED1
6.16673852553717E-59	0.715923253575387	0.273	0.134	1.03342204210952E-54	Proliferating hepatocytes	ABCG1
6.57215531108478E-59	1.42695905906309	0.123	0.041	1.10136178703159E-54	Proliferating hepatocytes	TERC
8.09448745775746E-59	0.607846972377384	0.245	0.115	1.35647420817099E-54	Proliferating hepatocytes	TIFA
9.05581919918158E-59	0.467483851561502	0.477	0.283	1.51757418139885E-54	Proliferating hepatocytes	SDHA
1.03528347072104E-58	0.53173094847781	0.337	0.178	1.73492804023432E-54	Proliferating hepatocytes	NANS
1.10014955431651E-58	0.43529278680223	0.471	0.281	1.84363062312361E-54	Proliferating hepatocytes	HDAC1
1.14864334458067E-58	0.442538025670755	0.547	0.341	1.92489651684829E-54	Proliferating hepatocytes	TM9SF1
1.16119404602531E-58	0.597324677692393	0.409	0.233	1.94592898232921E-54	Proliferating hepatocytes	LEMD2
1.18283878418728E-58	0.460382595506408	0.476	0.284	1.98220123454104E-54	Proliferating hepatocytes	VAPA
1.20441832325288E-58	0.565296319301513	0.443	0.262	2.01836422610717E-54	Proliferating hepatocytes	ZBTB10
1.22328776101435E-58	0.528543216979959	0.541	0.342	2.04998562990785E-54	Proliferating hepatocytes	DDX6
1.3792212895784E-58	0.380116788295365	0.496	0.299	2.31129903707549E-54	Proliferating hepatocytes	PRPF19
1.45148964590818E-58	0.532665676393196	0.42	0.24	2.43240634861293E-54	Proliferating hepatocytes	ALKBH5
1.57972205717955E-58	0.421321314466998	0.614	0.396	2.64729822342149E-54	Proliferating hepatocytes	MAP2K2
1.58238486317464E-58	0.92421802179161	0.343	0.193	2.65176055370806E-54	Proliferating hepatocytes	ATG16L2
1.92741514545435E-58	0.547550751663556	0.456	0.27	3.2299623007524E-54	Proliferating hepatocytes	SLC25A11
2.01817360491317E-58	0.458199987118617	0.643	0.435	3.38205532711349E-54	Proliferating hepatocytes	UBE2Q1
2.06636779303121E-58	0.424051756892383	0.562	0.36	3.46281914756171E-54	Proliferating hepatocytes	ANXA5
2.26689795426827E-58	0.443593708486957	0.413	0.236	3.79886759176277E-54	Proliferating hepatocytes	PLRG1
2.52078718310699E-58	0.548215113825123	0.379	0.21	4.2243351614507E-54	Proliferating hepatocytes	PSMD2
2.95622681916787E-58	0.508728853266727	0.323	0.168	4.95404490356151E-54	Proliferating hepatocytes	ZHX3
3.17382451129463E-58	0.515858495269978	0.384	0.214	5.31869511602754E-54	Proliferating hepatocytes	MBD6
3.37785711997125E-58	0.85444564071138	0.221	0.1	5.66061296164782E-54	Proliferating hepatocytes	PDSS1
3.41705696649183E-58	0.549705243284871	0.575	0.379	5.72630406444701E-54	Proliferating hepatocytes	SYTL4

**3.68477135953832E-58**	0.510738141866274	0.385	0.214	6.17493984431431E-54	Proliferating hepatocytes	BANF1
**3.97477173177808E-58**	0.701570757787316	0.289	0.146	6.6609224681137E-54	Proliferating hepatocytes	LRRC57
**4.17558340415423E-58**	0.607005612554351	0.186	0.077	6.99744266868166E-54	Proliferating hepatocytes	SCAPER
**4.36851503027128E-58**	0.532047222931671	0.409	0.235	7.32075748772862E-54	Proliferating hepatocytes	KPNA1
**4.44578720198165E-58**	0.582505463764893	0.427	0.248	7.45025019308085E-54	Proliferating hepatocytes	DEPRECATEI
**4.55865502970179E-58**	0.484326174331008	0.287	0.143	7.63939409877426E-54	Proliferating hepatocytes	ANKFY1
**5.67924897083573E-58**	0.474978039884814	0.639	0.434	9.51728542532652E-54	Proliferating hepatocytes	RNF5
**5.98707226690302E-58**	0.382924971046378	0.463	0.274	1.00331357048761E-53	Proliferating hepatocytes	DEPRECATEI
**7.26245836934366E-58**	0.455765196935413	0.542	0.34	1.21704277353461E-53	Proliferating hepatocytes	SLC25A33
**8.29715495663413E-58**	0.498512653763238	0.536	0.336	1.39043722763275E-53	Proliferating hepatocytes	C1GALT1C1
**8.84918971254474E-58**	0.628048694673671	0.347	0.188	1.48294721202825E-53	Proliferating hepatocytes	SHKBP1
**9.08584703636864E-58**	0.422012574128229	0.471	0.279	1.52260624635466E-53	Proliferating hepatocytes	USP14
**9.35253632141832E-58**	0.385389947619983	0.582	0.375	1.56729803674328E-53	Proliferating hepatocytes	DIAPH1
**9.53035186629258E-58**	0.513791901326023	0.527	0.331	1.59709636575331E-53	Proliferating hepatocytes	CETN2
**9.76387620560993E-58**	0.423434420273204	0.529	0.33	1.63623037453611E-53	Proliferating hepatocytes	PPP3R1
**1.02886770201363E-57**	0.337320764434938	0.877	0.635	1.72417649503444E-53	Proliferating hepatocytes	ACSL4
**1.05246513115903E-57**	0.587398256511654	0.301	0.153	1.76372106679631E-53	Proliferating hepatocytes	AC253572.1
**1.06065382641344E-57**	0.319337226917393	0.393	0.22	1.77744368230364E-53	Proliferating hepatocytes	HDAC6
**1.16022528527234E-57**	0.49482988060867	0.564	0.363	1.94430553305938E-53	Proliferating hepatocytes	DYNLRB1
**1.53120414275219E-57**	0.683955131671111	0.182	0.075	2.56599190242412E-53	Proliferating hepatocytes	GABPB1
**1.60688798053836E-57**	0.60678608708224	0.321	0.17	2.69282287778618E-53	Proliferating hepatocytes	PCIF1
**1.74089719140768E-57**	0.504005814922539	0.251	0.12	2.917395513361E-53	Proliferating hepatocytes	ZCCHC8
**2.19279035179857E-57**	0.539598408536411	0.408	0.233	3.67467807154404E-53	Proliferating hepatocytes	ADNP
**2.2101434144973E-57**	0.764100687579073	0.229	0.105	3.70375833401457E-53	Proliferating hepatocytes	SLX4IP
**2.2780768770693E-57**	0.832365166092849	0.172	0.069	3.81760123059273E-53	Proliferating hepatocytes	MED7
**2.35755985487326E-57**	0.704664085307809	0.241	0.113	3.9507988047966E-53	Proliferating hepatocytes	CREB1
**2.38175958509867E-57**	0.509037216890022	0.44	0.26	3.99135271270835E-53	Proliferating hepatocytes	ZNF75D
**2.43184687932223E-57**	0.901471067446242	0.155	0.06	4.07528900036819E-53	Proliferating hepatocytes	ZNF101
**2.46457962682274E-57**	0.520393234141667	0.303	0.155	4.13014253862955E-53	Proliferating hepatocytes	PIGN
**2.751728284253E-57**	0.85762367964443	0.231	0.108	4.61134625875118E-53	Proliferating hepatocytes	SDC3
**2.75703732862545E-57**	0.395115052366675	0.737	0.518	4.62024315531053E-53	Proliferating hepatocytes	PRDX1
**3.11415204772849E-57**	0.48400341228065	0.306	0.157	5.2186960015834E-53	Proliferating hepatocytes	MARK2
**3.16786131713353E-57**	0.578271175109104	0.301	0.155	5.30870199525238E-53	Proliferating hepatocytes	ARL4D
**3.24724690834976E-57**	0.504852002896615	0.541	0.34	5.44173636901254E-53	Proliferating hepatocytes	CISD2
**3.48071816436827E-57**	0.274850819281275	0.437	0.251	5.83298749984835E-53	Proliferating hepatocytes	CIC
**3.71134815370736E-57**	0.699490697695419	0.279	0.14	6.21947723598279E-53	Proliferating hepatocytes	DEPRECATEI
**3.84349060021819E-57**	0.695868272926179	0.449	0.279	6.44092154784564E-53	Proliferating hepatocytes	NUP210
**3.87980031776039E-57**	0.954074722792676	0.108	0.034	6.50176937250287E-53	Proliferating hepatocytes	UBXN10
**3.90011151257465E-57**	0.655828002781665	0.211	0.094	6.5358068727726E-53	Proliferating hepatocytes	MYCL
**3.97294776196005E-57**	0.396795749011277	0.724	0.532	6.65786585949266E-53	Proliferating hepatocytes	IDH2
**4.16341107818522E-57**	0.470699384038482	0.43	0.251	6.97704428482279E-53	Proliferating hepatocytes	SAMD4B
**4.20278195580424E-57**	0.464290031651656	0.5	0.306	7.04302200153675E-53	Proliferating hepatocytes	PSMB5
**4.20533366978468E-57**	0.528697345846562	0.429	0.252	7.04729816382517E-53	Proliferating hepatocytes	EPS8L3
**4.20730817530631E-57**	0.480989191986361	0.424	0.247	7.05060704017831E-53	Proliferating hepatocytes	TAP2
**4.41901280203501E-57**	0.470733847534733	0.409	0.236	7.40538165365027E-53	Proliferating hepatocytes	PLEKHB2
**4.47993709489404E-57**	0.653533923457401	0.294	0.151	7.50747858362343E-53	Proliferating hepatocytes	DIP2C
**4.55631927797874E-57**	0.46999936625411	0.566	0.361	7.63547984603678E-53	Proliferating hepatocytes	G3BP2
**4.61158310623007E-57**	0.458464015687098	0.344	0.184	7.72809096942036E-53	Proliferating hepatocytes	MIB1
**4.70855370118252E-57**	0.547298439604041	0.482	0.295	7.89059429244166E-53	Proliferating hepatocytes	NDUFB1
**4.71574235011042E-57**	0.654506637976632	0.296	0.15	7.90264103031505E-53	Proliferating hepatocytes	WDR41
**5.0582903567634E-57**	0.437247862521397	0.694	0.491	8.4766829798641E-53	Proliferating hepatocytes	RAB2A
**5.18582818591783E-57**	0.579568826220943	0.551	0.364	8.69041087396109E-53	Proliferating hepatocytes	PRKDC
**5.40902799385729E-57**	0.468826894341023	0.539	0.342	9.06444911210605E-53	Proliferating hepatocytes	AP1M1
**5.91510187691224E-57**	0.812451432634229	0.174	0.07	9.91252772532954E-53	Proliferating hepatocytes	CARD8
**5.99268432265706E-57**	0.893828746023083	0.2	0.088	1.00425403879087E-52	Proliferating hepatocytes	KCTD1
**6.10646766966291E-57**	0.501024427785345	0.214	0.095	1.02332185208211E-52	Proliferating hepatocytes	CABIN1
**6.21590966302413E-57**	0.727521632597809	0.303	0.156	1.04166214132958E-52	Proliferating hepatocytes	MPP6
**6.33641343162781E-57**	0.440518327340038	0.36	0.197	1.06185616287219E-52	Proliferating hepatocytes	RESF1
**6.33730726518727E-57**	0.362480458605842	0.301	0.153	1.06200595150008E-52	Proliferating hepatocytes	PITHD1
**6.53632433764204E-57**	0.514079887006578	0.369	0.204	1.09535723250205E-52	Proliferating hepatocytes	VCP

7.62982616470458E-57	0.438248020492473	0.255	0.122	1.27860626868119E-52	Proliferating hepatocytes	MTF2
7.6969162102861E-57	0.47442153626306	0.6	0.397	1.28984921851974E-52	Proliferating hepatocytes	EFR3A
8.17445396401196E-57	0.702466701569364	0.215	0.096	1.36987499528912E-52	Proliferating hepatocytes	NELFE
8.26327488651448E-57	0.714898104000183	0.216	0.097	1.3847596054821E-52	Proliferating hepatocytes	ANAPC15
8.96820891245752E-57	0.613043451054034	0.252	0.121	1.50289244954963E-52	Proliferating hepatocytes	TGFBR1
9.11105432669624E-57	0.644283628036077	0.342	0.186	1.52683048406776E-52	Proliferating hepatocytes	CPSF2
1.03410757986006E-56	0.51711695255655	0.276	0.137	1.73295748232948E-52	Proliferating hepatocytes	EIF1AD
1.06251660415993E-56	0.435182138420297	0.472	0.286	1.78056532525121E-52	Proliferating hepatocytes	AATF
1.07944474581497E-56	0.559755984279496	0.486	0.295	1.80893350503673E-52	Proliferating hepatocytes	GNB1
1.09495790761102E-56	0.581883724229873	0.291	0.149	1.83493046157454E-52	Proliferating hepatocytes	UBN1
1.10198024177153E-56	0.409536906971619	0.435	0.256	1.84669848916074E-52	Proliferating hepatocytes	SUMO3
1.12436488047086E-56	0.426909435708544	0.505	0.311	1.88421066669307E-52	Proliferating hepatocytes	SETD3
1.14131718113927E-56	0.523011165650258	0.521	0.33	1.9126193321532E-52	Proliferating hepatocytes	TMEM9
1.24949961989044E-56	0.797462928251997	0.211	0.094	2.0939114630124E-52	Proliferating hepatocytes	PPP2R3C
1.26373080426021E-56	0.570438850350831	0.44	0.261	2.11776008177926E-52	Proliferating hepatocytes	MBOAT1
1.2857373775589E-56	0.477271718800201	0.48	0.291	2.1546386973132E-52	Proliferating hepatocytes	GRB2
1.45539002127146E-56	1.4182297826736	0.105	0.033	2.43894259764672E-52	Proliferating hepatocytes	CPLX2
1.48693817673133E-56	0.41222927849281	0.928	0.783	2.49181099656637E-52	Proliferating hepatocytes	MT-ND4L
1.49872863224455E-56	0.336349114836803	0.828	0.553	2.51156944191541E-52	Proliferating hepatocytes	TSPAN3
1.59331394728527E-56	0.754782843283776	0.194	0.084	2.67007551286065E-52	Proliferating hepatocytes	DEPRECATEI
1.62112531534548E-56	0.489509796169314	0.445	0.262	2.71668180345596E-52	Proliferating hepatocytes	NSMCE2
1.62187934216724E-56	0.450567210013898	0.352	0.191	2.71794540160386E-52	Proliferating hepatocytes	FAM193A
1.63203274153245E-56	0.466141710439991	0.459	0.274	2.73496046826008E-52	Proliferating hepatocytes	DNAJC8
1.90539804432972E-56	0.745960812642941	0.274	0.137	3.19306604268774E-52	Proliferating hepatocytes	CDK8
1.90816491794589E-56	0.638287239339842	0.35	0.193	3.19770276949372E-52	Proliferating hepatocytes	HHAT
2.02386308099583E-56	0.838433419775646	0.156	0.061	3.39158975113281E-52	Proliferating hepatocytes	MTURN
2.02529660221241E-56	0.746651186680868	0.139	0.051	3.39399204598756E-52	Proliferating hepatocytes	ZBTB3
2.1050379688308E-56	0.522622094097331	0.49	0.306	3.52762262816665E-52	Proliferating hepatocytes	RAB33B
2.29487392342902E-56	0.788900053943114	0.195	0.085	3.84574972088236E-52	Proliferating hepatocytes	BCORL1
2.3039299812866E-56	0.824527051683208	0.228	0.107	3.86092586264009E-52	Proliferating hepatocytes	ARNTL2
2.30554848767021E-56	0.441007974620547	0.337	0.179	3.86363815563774E-52	Proliferating hepatocytes	ANKLE2
2.35110122517566E-56	0.760582815146067	0.175	0.073	3.93997543314938E-52	Proliferating hepatocytes	LAPTM5
2.38389573939703E-56	0.738479963453185	0.208	0.093	3.99493248008154E-52	Proliferating hepatocytes	MED26
2.40104816509681E-56	0.524364809427075	0.255	0.123	4.02367651506923E-52	Proliferating hepatocytes	TTC9
2.66781558857384E-56	0.516770684475563	0.275	0.137	4.47072536333204E-52	Proliferating hepatocytes	CCNYL1
2.69677716677622E-56	0.480413849328672	0.697	0.495	4.5192591760836E-52	Proliferating hepatocytes	UBB
2.88134766502446E-56	0.518413153373195	0.341	0.185	4.82856241704799E-52	Proliferating hepatocytes	IFT88
3.04648066475616E-56	0.618190824655721	0.292	0.15	5.10529229799838E-52	Proliferating hepatocytes	MAP7D1
3.37673084397373E-56	0.369316548022431	0.765	0.532	5.65872554833118E-52	Proliferating hepatocytes	CAPN2
3.56989120139554E-56	0.639328095224887	0.317	0.166	5.98242367529864E-52	Proliferating hepatocytes	NVL
3.76224058898182E-56	0.575863519942496	0.419	0.243	6.30476277901573E-52	Proliferating hepatocytes	PGLS
3.76666541874698E-56	0.359350134765417	0.847	0.648	6.31217790873619E-52	Proliferating hepatocytes	CFL1
4.06815331079976E-56	0.864248114511651	0.183	0.077	6.81741131823825E-52	Proliferating hepatocytes	RAD1
4.16461181993239E-56	0.507069813013891	0.34	0.184	6.9790564878427E-52	Proliferating hepatocytes	LRIG3
4.57672053217341E-56	0.466904405579196	0.46	0.274	7.66966826781619E-52	Proliferating hepatocytes	MMP14
4.93099943406169E-56	0.458394267732548	0.412	0.238	8.26336885160058E-52	Proliferating hepatocytes	RRP1B
5.07130574923495E-56	0.632986773089458	0.191	0.082	8.49849417456794E-52	Proliferating hepatocytes	KLHL15
5.30750522672135E-56	0.615569093706983	0.307	0.16	8.89431725893963E-52	Proliferating hepatocytes	ZDHHC6
5.72425972076263E-56	0.383245727052587	0.393	0.221	9.59271444005401E-52	Proliferating hepatocytes	TRAF2
6.27675904289748E-56	0.355313726880926	0.656	0.44	1.05185928040876E-51	Proliferating hepatocytes	SLC25A39
6.72342684668746E-56	0.463183656985711	0.496	0.305	1.12671187096788E-51	Proliferating hepatocytes	ESD
6.82519334411417E-56	0.553235969024547	0.39	0.222	1.14376590060665E-51	Proliferating hepatocytes	FIBP
7.05086281300128E-56	0.621370344639226	0.243	0.116	1.18158359020275E-51	Proliferating hepatocytes	KPTN
7.08110987785263E-56	0.528398524368074	0.372	0.207	1.18665239333054E-51	Proliferating hepatocytes	EFTUD2
7.46845043830777E-56	0.60437143937676	0.328	0.177	1.25156292445162E-51	Proliferating hepatocytes	ADCK2
7.55547434538326E-56	0.395639408491681	0.638	0.446	1.26614639079933E-51	Proliferating hepatocytes	EIF5
7.76507185351425E-56	0.556873329486594	0.283	0.143	1.30127074121192E-51	Proliferating hepatocytes	DSG2
8.25932047020494E-56	0.614865199496211	0.367	0.207	1.38409692439694E-51	Proliferating hepatocytes	CCDC198
8.69532081823653E-56	0.623927275495333	0.345	0.189	1.45716186272008E-51	Proliferating hepatocytes	ETAA1
8.88776599438634E-56	0.451852845912151	0.458	0.273	1.48941182533926E-51	Proliferating hepatocytes	SENP2

9.1866212381867E-56	0.712360073257177	0.2	0.088	1.53949398709533E-51	Proliferating hepatocytes	PKP4
9.62157665780468E-56	0.645035775244978	0.262	0.128	1.61238381631491E-51	Proliferating hepatocytes	WRAP73
9.76090568131069E-56	0.427849942712333	0.551	0.351	1.63573257407404E-51	Proliferating hepatocytes	RAB13
1.0957188040528E-55	0.47405404802047	0.514	0.326	1.83620557183167E-51	Proliferating hepatocytes	LRRC8A
1.12279711466329E-55	0.398175915720757	0.439	0.258	1.88158340475274E-51	Proliferating hepatocytes	USP24
1.14362184565312E-55	0.634376579695323	0.309	0.163	1.9164814889455E-51	Proliferating hepatocytes	ZFP62
1.14903614787406E-55	0.691678859205487	0.298	0.156	1.92555477660736E-51	Proliferating hepatocytes	KLHL42
1.1835019185888E-55	0.488451875814949	0.444	0.263	1.98331251517112E-51	Proliferating hepatocytes	LMBRD2
1.21652229962913E-55	0.504533342224596	0.499	0.313	2.0386480697185E-51	Proliferating hepatocytes	SGK3
1.25367302741528E-55	0.571149713479204	0.257	0.125	2.10090525934253E-51	Proliferating hepatocytes	PRKRA
1.27422584903578E-55	0.63694846764755	0.23	0.108	2.13534767781416E-51	Proliferating hepatocytes	TSPAN12
1.34909652975365E-55	0.698086055087874	0.239	0.113	2.26081596456116E-51	Proliferating hepatocytes	HLX
1.38156217498342E-55	0.760325983718255	0.148	0.056	2.31522189283721E-51	Proliferating hepatocytes	ARL13B
1.52569556068415E-55	0.621993178321092	0.456	0.279	2.5567606205945E-51	Proliferating hepatocytes	PPP1R10
1.54853332498486E-55	0.422106839350357	0.479	0.293	2.59503214600963E-51	Proliferating hepatocytes	RBM23
1.65229335345678E-55	0.592094734758127	0.309	0.162	2.76891320172287E-51	Proliferating hepatocytes	CHCHD3
1.73943318909636E-55	0.660177290763378	0.273	0.137	2.91494213828768E-51	Proliferating hepatocytes	FSD1L
1.79076437317545E-55	0.867597625651465	0.202	0.09	3.00096293656742E-51	Proliferating hepatocytes	SPSB2
1.79841542736402E-55	0.72024126726662	0.306	0.161	3.01378457317663E-51	Proliferating hepatocytes	GTPBP3
1.88352468098808E-55	0.449475444070362	0.586	0.392	3.15641066039983E-51	Proliferating hepatocytes	FAT1
1.88449128316443E-55	0.643775421959638	0.265	0.131	3.15803049232695E-51	Proliferating hepatocytes	ARHGAP21
1.89265888311527E-55	0.674896677556051	0.327	0.176	3.17171775632457E-51	Proliferating hepatocytes	RBMX2
1.95111275406515E-55	0.73366021726597	0.24	0.115	3.26967475326238E-51	Proliferating hepatocytes	NUDCD1
2.03230795105242E-55	0.663312330703584	0.334	0.18	3.40574166437365E-51	Proliferating hepatocytes	TIMM9
2.31609922828915E-55	0.466047606226331	0.365	0.202	3.88131908676695E-51	Proliferating hepatocytes	AP2M1
2.54945206578902E-55	0.634509065249739	0.34	0.185	4.27237177184924E-51	Proliferating hepatocytes	ANKS1B
2.56712447456349E-55	0.510054284611769	0.437	0.258	4.3019871944735E-51	Proliferating hepatocytes	DPYSL2
2.60383688468777E-55	0.863578408529258	0.225	0.105	4.36350985135976E-51	Proliferating hepatocytes	FUNDC2
2.82966228217112E-55	0.709492456218446	0.274	0.137	4.74194805246237E-51	Proliferating hepatocytes	ARV1
3.13079336163697E-55	0.504336419380101	0.451	0.271	5.24658351543124E-51	Proliferating hepatocytes	SLC35B2
3.20235476697216E-55	0.448700549606376	0.444	0.262	5.36650611849195E-51	Proliferating hepatocytes	CLPP
3.38681252710575E-55	0.503646786222413	0.271	0.134	5.67562043292382E-51	Proliferating hepatocytes	CD58
3.57325762589778E-55	0.601531152041557	0.275	0.139	5.9880651294795E-51	Proliferating hepatocytes	PCBP2
3.59921551905743E-55	0.364291905385788	0.387	0.219	6.03156536683644E-51	Proliferating hepatocytes	PPP1R35
3.73852820853692E-55	0.506059835943653	0.233	0.109	6.26502557186618E-51	Proliferating hepatocytes	PRKD3
4.09444942682068E-55	0.590593484113379	0.306	0.161	6.86147834946609E-51	Proliferating hepatocytes	HCFC1R1
4.2341978892244E-55	0.811772605757338	0.153	0.06	7.09566882276225E-51	Proliferating hepatocytes	WDR53
4.30353517578733E-55	0.49399946120505	0.432	0.257	7.21186424758441E-51	Proliferating hepatocytes	PSENEN
4.33028721537577E-55	0.829912064958055	0.204	0.092	7.25669531552671E-51	Proliferating hepatocytes	BUD13
5.35021607300355E-55	0.486428347780576	0.574	0.378	8.96589209513935E-51	Proliferating hepatocytes	HNRNPC
5.43075051481659E-55	0.539456264230846	0.447	0.265	9.10085171272964E-51	Proliferating hepatocytes	RC3H2
5.60669475392076E-55	0.49376550475106	0.293	0.15	9.39569906862041E-51	Proliferating hepatocytes	MTOR
5.8006857857131E-55	0.433745464120725	0.468	0.279	9.72078923969801E-51	Proliferating hepatocytes	SLC10A3
5.8846137193494E-55	0.380220272210235	0.579	0.378	9.86143567088572E-51	Proliferating hepatocytes	HSF1
5.91809788323926E-55	0.422582490832215	0.476	0.289	9.91754843273235E-51	Proliferating hepatocytes	EWSR1
6.20073453421007E-55	0.650400027983874	0.256	0.125	1.03911909324292E-50	Proliferating hepatocytes	XPOT
6.38647978652821E-55	0.494822829008607	0.386	0.22	1.0702462826264E-50	Proliferating hepatocytes	UBTD2
6.76205868574458E-55	0.859514892411513	0.223	0.104	1.13318579455708E-50	Proliferating hepatocytes	SAYSD1
7.72155568275703E-55	0.41295310410214	0.627	0.423	1.29397830131642E-50	Proliferating hepatocytes	CDC37
8.10048979285936E-55	0.406443129454083	0.533	0.33	1.35748007948737E-50	Proliferating hepatocytes	LRRC31
8.14286272110152E-55	0.465321311076213	0.328	0.174	1.36458093480219E-50	Proliferating hepatocytes	PHF2
8.52522512997707E-55	0.868161471318922	0.221	0.102	1.42865722728156E-50	Proliferating hepatocytes	HSPA14.1
8.64914215419795E-55	0.347169782489566	0.337	0.181	1.44942324220049E-50	Proliferating hepatocytes	CD82
8.73275754702981E-55	0.47640531015715	0.434	0.259	1.46343550973125E-50	Proliferating hepatocytes	SPPL2B
8.78035579399633E-55	0.384726059315293	0.753	0.583	1.47141202395791E-50	Proliferating hepatocytes	SDC1
8.87861257781771E-55	0.419767901515072	0.567	0.379	1.48787789579069E-50	Proliferating hepatocytes	SMARCA2
8.9591861046582E-55	0.624227629831855	0.187	0.08	1.50138040741862E-50	Proliferating hepatocytes	RFX1
9.68648673741633E-55	0.738453864060145	0.29	0.149	1.62326144745623E-50	Proliferating hepatocytes	BTBD3
1.00585817251275E-54	0.679984850764402	0.196	0.086	1.68561712549687E-50	Proliferating hepatocytes	RAP2B
1.10472288900402E-54	0.849233216278538	0.192	0.084	1.85129461739294E-50	Proliferating hepatocytes	FUT8

**1.14023523283923E-54**	0.882128001726803	0.222	0.104	1.91080620319198E-50	Proliferating hepatocytes	ZDHHC23
**1.15468487932022E-54**	0.465950143255362	0.422	0.247	1.93502092076483E-50	Proliferating hepatocytes	MID1
**1.20465154101573E-54**	0.51596722835545	0.236	0.111	2.01875505243416E-50	Proliferating hepatocytes	BAP1
**1.32413795985757E-54**	0.718854413435233	0.263	0.131	2.21899039312932E-50	Proliferating hepatocytes	RAD51D
**1.33695708163934E-54**	1.16443095729231	0.114	0.038	2.2404726774112E-50	Proliferating hepatocytes	BLMH
**1.3666554712604E-54**	0.393769358492076	0.696	0.481	2.29024123873818E-50	Proliferating hepatocytes	ENO1
**1.39114277828927E-54**	1.02050956953934	0.125	0.044	2.33127706785716E-50	Proliferating hepatocytes	C3orf52
**1.40168256658082E-54**	0.435254566003403	0.56	0.365	2.34893964507613E-50	Proliferating hepatocytes	HUWE1
**1.5013058480238E-54**	0.337661925542614	0.392	0.221	2.51588834011829E-50	Proliferating hepatocytes	RANBP3
**1.5307259338188E-54**	0.516805875328903	0.271	0.136	2.56519051989355E-50	Proliferating hepatocytes	PLAA
**1.57209970687382E-54**	0.523954177645444	0.412	0.239	2.63452468877915E-50	Proliferating hepatocytes	GMPS
**1.73405732024936E-54**	0.392951368533302	0.765	0.561	2.90593325727387E-50	Proliferating hepatocytes	HNRNPF
**1.89137548719803E-54**	0.412684039847043	0.523	0.331	3.16956704144646E-50	Proliferating hepatocytes	CYB5B
**2.14537740136246E-54**	0.661072814731481	0.291	0.151	3.59522344920322E-50	Proliferating hepatocytes	CMPK2
**2.37877773252833E-54**	0.707406919097296	0.274	0.139	3.98635572417097E-50	Proliferating hepatocytes	PAPSS1
**2.50132291693363E-54**	0.823293567341957	0.195	0.086	4.19171694419738E-50	Proliferating hepatocytes	ZNF250
**2.80607851646361E-54**	0.395312319621663	0.429	0.256	4.70242637788972E-50	Proliferating hepatocytes	NOP56
**2.87150133172759E-54**	0.521532577697722	0.395	0.228	4.8120619317091E-50	Proliferating hepatocytes	AIP
**3.01896508024277E-54**	0.441042486603334	0.302	0.156	5.05918168147084E-50	Proliferating hepatocytes	PIAS4
**3.07270172856391E-54**	0.428686174157178	0.375	0.211	5.1492335567274E-50	Proliferating hepatocytes	CNPPD1
**3.10472171633664E-54**	0.536185005494632	0.459	0.282	5.20289265223694E-50	Proliferating hepatocytes	RBM41
**3.53823358195293E-54**	0.505063729697252	0.374	0.211	5.92937183663671E-50	Proliferating hepatocytes	MAP4K4
**3.71057203967046E-54**	0.439915531006204	0.495	0.303	6.21817662407976E-50	Proliferating hepatocytes	MBOAT7
**3.80171590655835E-54**	0.505498008022415	0.223	0.104	6.37091551621048E-50	Proliferating hepatocytes	TBCC
**3.88532522052475E-54**	0.952053706706544	0.122	0.043	6.51102800455537E-50	Proliferating hepatocytes	CASTOR2
**3.94141715142264E-54**	0.39237599313996	0.831	0.639	6.60502686235407E-50	Proliferating hepatocytes	MPC2
**3.95855621631374E-54**	0.730535728319948	0.241	0.116	6.63374850729856E-50	Proliferating hepatocytes	CMSS1
**4.57365061427723E-54**	0.517891891428509	0.346	0.19	7.66452369940578E-50	Proliferating hepatocytes	MAGOH
**4.68877119990943E-54**	0.38747413721693	0.383	0.219	7.85744277680822E-50	Proliferating hepatocytes	CSTF3
**4.85482703858113E-54**	0.546128900915948	0.504	0.317	8.13571915125425E-50	Proliferating hepatocytes	ELOVL1
**5.14742271842112E-54**	0.442579569336325	0.416	0.243	8.62605099153011E-50	Proliferating hepatocytes	USP48
**5.22672524036241E-54**	0.528914428065302	0.434	0.255	8.75894615779933E-50	Proliferating hepatocytes	SKAP2
**5.30657860270602E-54**	0.503298587953981	0.435	0.26	8.89276442241474E-50	Proliferating hepatocytes	CLTA
**5.49667131795271E-54**	0.450746087955913	0.394	0.226	9.21132179462516E-50	Proliferating hepatocytes	SEPTIN2
**5.53851115821441E-54**	0.573233964180083	0.345	0.192	9.28143699893571E-50	Proliferating hepatocytes	GGCT
**6.13578995783601E-54**	0.396605136312455	0.494	0.305	1.02823568113416E-49	Proliferating hepatocytes	RB1CC1
**6.28920263624158E-54**	0.530597221926448	0.275	0.139	1.05394457778136E-49	Proliferating hepatocytes	SAP130
**6.44358660466067E-54**	0.439921355999598	0.514	0.328	1.07981624320904E-49	Proliferating hepatocytes	DDX46
**6.46775659206001E-54**	0.598541301443169	0.281	0.144	1.08386664969742E-49	Proliferating hepatocytes	SRSF1
**6.8988994898828E-54**	1.13351830525128	0.113	0.038	1.15611757651456E-49	Proliferating hepatocytes	SP5
**7.24306573766691E-54**	0.724155515374532	0.199	0.088	1.21379295631822E-49	Proliferating hepatocytes	BRPF1
**7.26336339254681E-54**	0.465874477418089	0.423	0.251	1.21719443732299E-49	Proliferating hepatocytes	H2AFJ
**7.59182502348523E-54**	0.664289468661103	0.243	0.117	1.27223803743566E-49	Proliferating hepatocytes	TRAPPC13
**8.49129594910312E-54**	0.518488719517882	0.49	0.307	1.4229713751507E-49	Proliferating hepatocytes	CRAT
**8.85654327325809E-54**	0.550748975267295	0.315	0.168	1.48417952173259E-49	Proliferating hepatocytes	ABL1
**9.32667382736697E-54**	0.376936682925968	0.343	0.187	1.56296399999016E-49	Proliferating hepatocytes	GMPPB
**1.02355225668686E-53**	0.414119423317432	0.56	0.364	1.71526887175584E-49	Proliferating hepatocytes	SFT2D2
**1.08524763258582E-53**	1.11183642125114	0.153	0.061	1.81865798268732E-49	Proliferating hepatocytes	CBX6
**1.10476881811417E-53**	0.419722972598166	0.49	0.301	1.85137158539572E-49	Proliferating hepatocytes	DCTN2
**1.15089525659639E-53**	0.944576953810008	0.159	0.064	1.92867027100424E-49	Proliferating hepatocytes	SLC25A14
**1.18301974118358E-53**	0.674968825012592	0.214	0.1	1.98250448227544E-49	Proliferating hepatocytes	GINS2
**1.37210400863619E-53**	0.948615316786525	0.187	0.081	2.29937189767253E-49	Proliferating hepatocytes	ARSF
**1.45622982733947E-53**	0.537417881890247	0.38	0.216	2.44034994465549E-49	Proliferating hepatocytes	CNP
**1.46389426840352E-53**	0.599097428138885	0.201	0.09	2.45319401499062E-49	Proliferating hepatocytes	TDRKH
**1.46586927047114E-53**	0.654820228638886	0.301	0.158	2.45650372345553E-49	Proliferating hepatocytes	WASF2
**1.50109855972978E-53**	0.765593712754715	0.228	0.108	2.51554096639516E-49	Proliferating hepatocytes	SLC30A6
**1.5368304333676E-53**	0.753201404588735	0.209	0.096	2.57542044023742E-49	Proliferating hepatocytes	ZDHHC2
**1.62381028167712E-53**	0.439229873460953	0.526	0.332	2.72118127003452E-49	Proliferating hepatocytes	UPF1
**1.63696412221239E-53**	0.310470710922927	0.613	0.419	2.74322447600353E-49	Proliferating hepatocytes	KLF6
**1.66451152599935E-53**	0.510693216855758	0.399	0.23	2.78938841526971E-49	Proliferating hepatocytes	CASP3

1.73358887452582E-53	0.773326897031328	0.122	0.043	2.90514823593037E-49	Proliferating hepatocytes	TTF1
1.90494047476711E-53	0.477599457386591	0.223	0.104	3.19229924761472E-49	Proliferating hepatocytes	AGPAT5
1.97919728050264E-53	0.454758926474496	0.575	0.373	3.31673880266633E-49	Proliferating hepatocytes	AMFR
2.06379462236517E-53	0.382852550869785	0.368	0.205	3.45850702815955E-49	Proliferating hepatocytes	FNIP1
2.11651245154685E-53	0.506584865247864	0.282	0.144	3.54685156630221E-49	Proliferating hepatocytes	SOCS4
2.1673655926145E-53	0.505440404552595	0.329	0.177	3.63207126010338E-49	Proliferating hepatocytes	PIGM
2.34314141646883E-53	0.552349146981513	0.223	0.105	3.92663638571846E-49	Proliferating hepatocytes	MAGI3
2.53654810643646E-53	0.635402498542234	0.254	0.125	4.25074731676622E-49	Proliferating hepatocytes	THAP12
2.64720870743443E-53	0.633742325151533	0.329	0.179	4.43619235191862E-49	Proliferating hepatocytes	TMX1
2.66875983394203E-53	0.450713920757308	0.421	0.245	4.47230772972005E-49	Proliferating hepatocytes	KCMF1
3.22868181943728E-53	0.647596462365614	0.239	0.116	5.41062499301299E-49	Proliferating hepatocytes	DTNBP1
3.62272615464957E-53	0.476924301641078	0.347	0.191	6.07096448996176E-49	Proliferating hepatocytes	WAC
3.62437825166754E-53	0.544256520492269	0.369	0.207	6.07373307414447E-49	Proliferating hepatocytes	MVB12A
3.67952062066767E-53	0.53693968320613	0.626	0.453	6.16614065611488E-49	Proliferating hepatocytes	CFLAR
3.70165420174363E-53	0.525056147967537	0.32	0.172	6.20323211128198E-49	Proliferating hepatocytes	FAM76B
3.83059781750747E-53	0.426617972508166	0.77	0.59	6.41931582257903E-49	Proliferating hepatocytes	TXNRD1
3.88089088826581E-53	0.55135053935253	0.225	0.106	6.50359695055584E-49	Proliferating hepatocytes	ENC1
4.24677891689898E-53	0.51749250574303	0.374	0.211	7.11675210893931E-49	Proliferating hepatocytes	FKBP3
4.27277912508967E-53	0.472262485325952	0.351	0.193	7.16032325782526E-49	Proliferating hepatocytes	PCNX1
4.48473183860159E-53	0.662812052014816	0.179	0.077	7.51551361512854E-49	Proliferating hepatocytes	IQGAP1
4.54689416428791E-53	0.548201003603157	0.3	0.158	7.61968524051368E-49	Proliferating hepatocytes	HINT3
4.72492774721795E-53	0.434478201397258	0.278	0.141	7.91803391878784E-49	Proliferating hepatocytes	NR1D2
4.9516600351149E-53	0.6603368941069	0.24	0.116	8.29799188684556E-49	Proliferating hepatocytes	BLOC1S5
5.00520484718437E-53	0.317911713424766	0.717	0.493	8.38772228291156E-49	Proliferating hepatocytes	TFRC
6.13015422931047E-53	0.64638004977187	0.286	0.148	1.02729124574785E-48	Proliferating hepatocytes	PLCB3
6.16179195263992E-53	0.455909302408423	0.346	0.189	1.0325930954234E-48	Proliferating hepatocytes	BOD1L1
6.29996198521486E-53	0.682359234192421	0.207	0.094	1.05574762948231E-48	Proliferating hepatocytes	UBAP2
6.73664746681946E-53	0.594113484894112	0.315	0.17	1.12892738248961E-48	Proliferating hepatocytes	GATA4
6.96339800854982E-53	0.511891424953183	0.333	0.182	1.16692623827278E-48	Proliferating hepatocytes	KLHDC10
7.06241712356131E-53	0.54342063155634	0.339	0.185	1.1835198615664E-48	Proliferating hepatocytes	CDC123
7.086923885465E-53	0.561271578331265	0.389	0.224	1.18762670472622E-48	Proliferating hepatocytes	TSR2
7.12453983239624E-53	0.423720493860368	0.552	0.362	1.19393038511296E-48	Proliferating hepatocytes	GNB2
7.22907691887083E-53	0.644599079958349	0.317	0.17	1.21144871006437E-48	Proliferating hepatocytes	GMPR2
7.62616005740903E-53	0.517775561835422	0.233	0.111	1.2779919024206E-48	Proliferating hepatocytes	DRG1
7.63005753611552E-53	0.912783652383529	0.198	0.089	1.27864504190224E-48	Proliferating hepatocytes	EPHB6
8.42955525053742E-53	0.458136242527216	0.446	0.268	1.41262486888506E-48	Proliferating hepatocytes	PSMD8
8.69271741064163E-53	0.530511278212598	0.455	0.274	1.45672558367532E-48	Proliferating hepatocytes	AKR7A2
8.90835511658755E-53	0.484567860105472	0.502	0.316	1.49286215043774E-48	Proliferating hepatocytes	TRMT2B
9.6594216378431E-53	0.436272390737104	0.366	0.205	1.61872587806975E-48	Proliferating hepatocytes	DLST
1.00236333902471E-52	0.651678940373691	0.249	0.123	1.67976048353761E-48	Proliferating hepatocytes	PAXIP1
1.00950268566969E-52	0.406953994876448	0.791	0.619	1.69172460064526E-48	Proliferating hepatocytes	RARRES2
1.01392984305741E-52	0.468464206661462	0.472	0.29	1.6991436309956E-48	Proliferating hepatocytes	EIF4E2
1.01899106245909E-52	0.37695929283152	0.37	0.209	1.70762522246894E-48	Proliferating hepatocytes	STIP1
1.03472860673092E-52	0.508529397525045	0.191	0.084	1.73399819915968E-48	Proliferating hepatocytes	SPICE1
1.12206944235223E-52	0.345783908456294	0.646	0.455	1.88036397149386E-48	Proliferating hepatocytes	LUC7L2
1.34095933572025E-52	0.432326385420524	0.491	0.309	2.2471796548E-48	Proliferating hepatocytes	KMT2B
1.35001005712285E-52	0.429081056384881	0.365	0.203	2.26234685372648E-48	Proliferating hepatocytes	BORCS8
1.36517401221678E-52	0.500080569917725	0.481	0.303	2.28775860967288E-48	Proliferating hepatocytes	RBM26
1.36534638136107E-52	0.460910559502955	0.338	0.185	2.28804746588488E-48	Proliferating hepatocytes	ZNF75A
1.37097439232034E-52	0.618195075144934	0.303	0.16	2.29747888665043E-48	Proliferating hepatocytes	NAA30
1.40139161194421E-52	0.592808938262243	0.312	0.167	2.34845206329611E-48	Proliferating hepatocytes	TMTC4
1.48758239386732E-52	0.882025354670732	0.156	0.063	2.49289057564285E-48	Proliferating hepatocytes	HAUS1
1.53740937358898E-52	0.358864822094263	0.976	0.878	2.57639062826041E-48	Proliferating hepatocytes	MT-ND4
1.55115865485793E-52	0.487305579372152	0.519	0.334	2.59943167381093E-48	Proliferating hepatocytes	DEPRECATEI
1.72321072741887E-52	0.548649089491054	0.416	0.247	2.88775653700854E-48	Proliferating hepatocytes	FAM32A
1.80455656159564E-52	0.990281650197479	0.141	0.054	3.02407588592197E-48	Proliferating hepatocytes	PIGA
1.93908747911175E-52	0.460070538690036	0.379	0.215	3.24952279749546E-48	Proliferating hepatocytes	TOMM5
2.02408633995739E-52	0.543855197553182	0.691	0.511	3.3919638885006E-48	Proliferating hepatocytes	SLPI
2.20478058264194E-52	0.647221269098567	0.21	0.097	3.69477130039136E-48	Proliferating hepatocytes	BTBD6
2.21097264842752E-52	0.56709940463221	0.328	0.179	3.70514796423484E-48	Proliferating hepatocytes	CSPP1

2.28067764957975E-52	0.84919235162056	0.19	0.085	3.82195960516575E-48	Proliferating hepatocytes	MDGA1
2.30640754472256E-52	0.45496340843673	0.55	0.356	3.86507776344606E-48	Proliferating hepatocytes	NCOA2
2.55980370405847E-52	0.614205804140587	0.267	0.135	4.28971904726119E-48	Proliferating hepatocytes	SPATA5L1
3.01076019978581E-52	0.446348269812287	0.383	0.218	5.04543194280106E-48	Proliferating hepatocytes	C2orf68
3.02276430452484E-52	0.467850261229754	0.461	0.283	5.06554842152272E-48	Proliferating hepatocytes	POLR2K
3.18188826978678E-52	0.473580994147219	0.433	0.261	5.33220836250869E-48	Proliferating hepatocytes	GET1
3.3070081532094E-52	0.466312222993184	0.492	0.307	5.54188426314831E-48	Proliferating hepatocytes	DCTN1
3.41851285363164E-52	0.949547235148664	0.116	0.04	5.7287438401159E-48	Proliferating hepatocytes	HES6
3.79706521047602E-52	0.488357360637629	0.518	0.335	6.36312187971571E-48	Proliferating hepatocytes	KIAA0232
3.94515877296832E-52	0.56848236994573	0.21	0.097	6.61129707174032E-48	Proliferating hepatocytes	KCTD2
4.02347906362813E-52	0.79955750660718	0.219	0.103	6.74254621482801E-48	Proliferating hepatocytes	PTPRU
4.12354761922447E-52	0.49358071526335	0.413	0.241	6.91024110029636E-48	Proliferating hepatocytes	MSL2
4.14111921255372E-52	0.732292313507997	0.265	0.135	6.93968757639752E-48	Proliferating hepatocytes	UBE2K
4.43277036027274E-52	0.372812278977428	0.353	0.194	7.42843656974506E-48	Proliferating hepatocytes	KMT2A
4.67540383485425E-52	0.478890670521454	0.37	0.211	7.83504174644875E-48	Proliferating hepatocytes	DIP2A
4.94673705356279E-52	0.585503535477702	0.305	0.162	8.28974195436053E-48	Proliferating hepatocytes	NOL11
4.96441078329812E-52	0.397095928547906	0.315	0.168	8.31935959065099E-48	Proliferating hepatocytes	ACAP2
5.03865854930276E-52	0.378206129425305	0.983	0.901	8.44378399692156E-48	Proliferating hepatocytes	MT-ATP6
5.26053343630301E-52	0.399488852122781	0.326	0.176	8.81560193255658E-48	Proliferating hepatocytes	MRTFA
5.41603236304812E-52	0.407619090859708	0.365	0.205	9.07618703399604E-48	Proliferating hepatocytes	TSR3
5.45306783982064E-52	0.823940377616918	0.126	0.046	9.13825108597143E-48	Proliferating hepatocytes	SPINDOC
5.54642512046913E-52	0.394884601681957	0.351	0.195	9.29469921688216E-48	Proliferating hepatocytes	GANAB
5.59140007216102E-52	0.318735682496986	0.394	0.227	9.37006824092743E-48	Proliferating hepatocytes	TRIM8
5.6528604789009E-52	1.07200284362116	0.131	0.048	9.47306359054212E-48	Proliferating hepatocytes	LSM 3.00
5.76004112773699E-52	0.511283240526808	0.383	0.22	9.65267692186164E-48	Proliferating hepatocytes	TAF8
5.82791131512962E-52	0.397515098717043	0.635	0.425	9.76641378189421E-48	Proliferating hepatocytes	CYSTM1
6.38119398147243E-52	0.42170503799085	0.27	0.136	1.06936048741515E-47	Proliferating hepatocytes	PIGQ
6.5928798262939E-52	0.532021226154326	0.179	0.077	1.10483480129033E-47	Proliferating hepatocytes	ZNF182
6.82334085680149E-52	0.50534892063487	0.425	0.252	1.14345546078279E-47	Proliferating hepatocytes	ACTR10
6.83807607821768E-52	0.410272397716611	0.696	0.503	1.14592478918772E-47	Proliferating hepatocytes	MAT2A
6.87803342029755E-52	0.919586181671653	0.121	0.043	1.15262084057346E-47	Proliferating hepatocytes	ATP23
6.95401012248075E-52	0.954556363270985	0.209	0.098	1.16535301632532E-47	Proliferating hepatocytes	GPC3
7.98496277136204E-52	0.741990421698984	0.141	0.054	1.33812006122485E-47	Proliferating hepatocytes	GLCCI1
8.08476523610785E-52	0.722183522344484	0.217	0.103	1.35484495826695E-47	Proliferating hepatocytes	PKM
8.16454523789745E-52	0.385600241039722	0.364	0.206	1.36821449096685E-47	Proliferating hepatocytes	SRCAP
8.67284309667816E-52	0.50982790173549	0.244	0.119	1.45339504614133E-47	Proliferating hepatocytes	ZNF236
8.80742114616281E-52	0.490513809491064	0.412	0.244	1.47594763567396E-47	Proliferating hepatocytes	GPATCH2L
9.01618514546428E-52	0.53283009639562	0.268	0.136	1.5109323066769E-47	Proliferating hepatocytes	DENND6A
9.09405514215623E-52	1.11837637700292	0.106	0.035	1.52398176072254E-47	Proliferating hepatocytes	ANKRD36
9.16431030819091E-52	0.427837532834244	0.324	0.175	1.53575512144663E-47	Proliferating hepatocytes	MARCH8
9.29268551293036E-52	0.478907227214835	0.481	0.298	1.55726823825687E-47	Proliferating hepatocytes	ZFAND5
1.04128110380782E-51	0.380131644075384	0.58	0.376	1.74497887376114E-47	Proliferating hepatocytes	TLE5
1.07167491852225E-51	0.508798204330309	0.374	0.215	1.79591282845958E-47	Proliferating hepatocytes	RNF113A
1.07553745711904E-51	0.567813099373677	0.3	0.159	1.80238567064009E-47	Proliferating hepatocytes	LMAN2L
1.13558082423101E-51	0.471460592855395	0.415	0.246	1.90300634524633E-47	Proliferating hepatocytes	FAF1
1.15345564565479E-51	0.405733262095197	0.542	0.349	1.93296097098829E-47	Proliferating hepatocytes	ENAH
1.17376949900475E-51	0.474606735227524	0.338	0.184	1.96700292643216E-47	Proliferating hepatocytes	UQCRFS1
1.18287773929696E-51	0.427855808393354	0.274	0.14	1.98226651551385E-47	Proliferating hepatocytes	PATL1
1.22358914381177E-51	0.574915296565095	0.258	0.129	2.05049068719976E-47	Proliferating hepatocytes	RB1
1.2582352239822E-51	0.587976564848861	0.186	0.082	2.10855058834936E-47	Proliferating hepatocytes	AKAP7
1.31751410651047E-51	0.5288479557597	0.209	0.096	2.20789013969025E-47	Proliferating hepatocytes	ZC3H6
1.32423673127615E-51	0.470712931041405	0.345	0.192	2.21915591427257E-47	Proliferating hepatocytes	PTPN12
1.40318550458813E-51	0.479134784097533	0.503	0.314	2.35145826858878E-47	Proliferating hepatocytes	PKP2
1.40643166043546E-51	0.5361519058053	0.275	0.141	2.35689817655774E-47	Proliferating hepatocytes	PIEZO1
1.44670133114752E-51	0.385131159025648	0.436	0.26	2.42438209073702E-47	Proliferating hepatocytes	RBM17
1.50035845767786E-51	0.398319991201755	0.531	0.339	2.51430070337656E-47	Proliferating hepatocytes	LONP1
1.62832889690576E-51	0.797712637809138	0.157	0.064	2.72875356543467E-47	Proliferating hepatocytes	YEATS4
1.73648702062542E-51	0.543950971177234	0.369	0.211	2.91000494916408E-47	Proliferating hepatocytes	MAP3K21
1.86746622589105E-51	1.1163830120639	0.118	0.042	3.12949990134822E-47	Proliferating hepatocytes	DEPRECATEI
1.87958637485804E-51	0.565308422580943	0.326	0.178	3.14981084698711E-47	Proliferating hepatocytes	ADSL

**1.90948913776965E-51**	0.519027753841316	0.43	0.258	3.19992189707438E-47	Proliferating hepatocytes	ENOPH1
**2.05255905210583E-51**	0.451611853941469	0.594	0.402	3.43967845951894E-47	Proliferating hepatocytes	HNRNPD
**2.11141577003573E-51**	0.437979146488186	0.636	0.431	3.53831054742587E-47	Proliferating hepatocytes	HACD3
**2.21765283022837E-51**	0.36282287520843	0.553	0.357	3.7163426128967E-47	Proliferating hepatocytes	DVL3
**2.23039740726597E-51**	0.637986525112741	0.219	0.103	3.73769997509631E-47	Proliferating hepatocytes	EFNA5
**2.2519648710965E-51**	0.44082779424879	0.345	0.191	3.77384273098351E-47	Proliferating hepatocytes	PAGR1
**2.35881783501336E-51**	0.43318861624422	0.416	0.246	3.95290692791539E-47	Proliferating hepatocytes	CTDSPL
**2.48255902488857E-51**	0.379104453509967	0.335	0.183	4.16027241390826E-47	Proliferating hepatocytes	MTMR12
**2.74749440348705E-51**	0.507257123036317	0.377	0.217	4.6042511213636E-47	Proliferating hepatocytes	DAXX
**3.00367029192636E-51**	0.451407001346753	0.318	0.173	5.0335506752102E-47	Proliferating hepatocytes	RUVBL1
**3.23383388767894E-51**	0.726814766344139	0.204	0.094	5.41925882897237E-47	Proliferating hepatocytes	SYNE3
**3.31027536778859E-51**	0.905179441343845	0.121	0.044	5.54735946134012E-47	Proliferating hepatocytes	ANKRD54
**3.73623428286936E-51**	0.546328758767805	0.246	0.122	6.26118141123248E-47	Proliferating hepatocytes	PPP4R1
**3.73753703513876E-51**	0.56950106468719	0.218	0.103	6.26336456348554E-47	Proliferating hepatocytes	SLC38A9
**3.95100232089064E-51**	0.522920658673423	0.183	0.08	6.62108968934853E-47	Proliferating hepatocytes	FRAT2
**4.01636229637995E-51**	0.688806099636225	0.179	0.078	6.73061993627352E-47	Proliferating hepatocytes	TNRC6C
**4.14156073169706E-51**	0.777871032704786	0.221	0.105	6.94042747417794E-47	Proliferating hepatocytes	DLG3
**4.40019770249445E-51**	0.542061540919368	0.231	0.11	7.3738513098402E-47	Proliferating hepatocytes	CBLL1
**4.51963561529991E-51**	0.457253192413675	0.556	0.365	7.57400536411959E-47	Proliferating hepatocytes	CRTAP
**4.55698956934614E-51**	0.585037130605447	0.263	0.134	7.63660312031026E-47	Proliferating hepatocytes	UBL7
**4.75817937657285E-51**	0.431292479134034	0.475	0.295	7.97375699926079E-47	Proliferating hepatocytes	HMGN3
**5.02763542480556E-51**	0.422033471195401	0.367	0.207	8.42531144488917E-47	Proliferating hepatocytes	TOR1A
**5.20769888354566E-51**	0.359366034106294	0.719	0.502	8.72706178904582E-47	Proliferating hepatocytes	DAP
**5.46079103687521E-51**	0.59017380314229	0.26	0.131	9.15119361959547E-47	Proliferating hepatocytes	TMEM209
**6.60450657832381E-51**	0.483068598894855	0.4	0.236	1.1067832123955E-46	Proliferating hepatocytes	CALM1
**6.66608605374812E-51**	0.47865682316219	0.323	0.175	1.11710270088711E-46	Proliferating hepatocytes	TCF20
**6.96133769960926E-51**	0.519420492902773	0.275	0.141	1.16658097170052E-46	Proliferating hepatocytes	EXOSC6
**7.06068298988818E-51**	0.504074205474558	0.412	0.245	1.18322925544546E-46	Proliferating hepatocytes	YWHAB
**8.39693922251838E-51**	0.470266943925646	0.426	0.253	1.40715907490963E-46	Proliferating hepatocytes	CAND1
**8.4210464222565E-51**	0.506287767977746	0.477	0.296	1.41119895944174E-46	Proliferating hepatocytes	TRIQK
**8.67618599208735E-51**	0.461296264615483	0.298	0.158	1.453955248554E-46	Proliferating hepatocytes	GSPT1
**8.70344109637964E-51**	0.39913752021119	0.403	0.235	1.4585226589313E-46	Proliferating hepatocytes	IMMT
**9.99928244453723E-51**	0.957286315996364	0.152	0.062	1.67567975205555E-46	Proliferating hepatocytes	SASH1
**1.0110449452631E-50**	0.548384475019452	0.205	0.094	1.6943091192719E-46	Proliferating hepatocytes	ZNF142
**1.04496416173356E-50**	0.43855115244975	0.43	0.257	1.75115094223309E-46	Proliferating hepatocytes	ATPAF1
**1.04695962292822E-50**	0.480888123895806	0.397	0.234	1.75449493610311E-46	Proliferating hepatocytes	WDR44
**1.04708182158269E-50**	0.588558362029676	0.371	0.213	1.75469971660827E-46	Proliferating hepatocytes	KCTD5
**1.0966127872998E-50**	0.504396857905955	0.284	0.148	1.83770370895701E-46	Proliferating hepatocytes	MFHAS1
**1.15077060419716E-50**	0.450735894888025	0.448	0.275	1.9284613785136E-46	Proliferating hepatocytes	EPRS
**1.21932159015837E-50**	0.904593697619361	0.128	0.048	2.0433391207874E-46	Proliferating hepatocytes	DEPRECATEI
**1.27288111506736E-50**	0.714926053754189	0.208	0.097	2.13309417262989E-46	Proliferating hepatocytes	RNF145
**1.28575368561952E-50**	0.542118995460967	0.35	0.197	2.15466602636119E-46	Proliferating hepatocytes	ZNF768
**1.34639268362479E-50**	0.391087215959626	0.414	0.244	2.25628485921842E-46	Proliferating hepatocytes	BRF1
**1.34818996167075E-50**	0.424589067766685	0.533	0.344	2.25929673776785E-46	Proliferating hepatocytes	JTB
**1.42206356350134E-50**	0.592650557412626	0.233	0.114	2.38309411971555E-46	Proliferating hepatocytes	TMEM47
**1.45800080575428E-50**	0.926364651507684	0.138	0.053	2.44331775028303E-46	Proliferating hepatocytes	PRDM5
**1.51511859393442E-50**	0.469320329881591	0.526	0.335	2.5390357397153E-46	Proliferating hepatocytes	VASP
**1.54804585833888E-50**	0.328731759747638	0.25	0.124	2.5942152494043E-46	Proliferating hepatocytes	NPC1
**1.63743802949975E-50**	0.513808156260245	0.433	0.259	2.74401864983568E-46	Proliferating hepatocytes	PCGF2
**1.66589482418637E-50**	0.446378629201083	0.52	0.332	2.79170654637152E-46	Proliferating hepatocytes	CCNI
**1.69252895497867E-50**	0.316976830451826	0.932	0.792	2.83634002275325E-46	Proliferating hepatocytes	OAZ1
**1.77515386833382E-50**	0.736314454921139	0.163	0.068	2.97480285255382E-46	Proliferating hepatocytes	CHD3
**1.80466624134991E-50**	0.380394351862739	0.381	0.22	3.02425968725417E-46	Proliferating hepatocytes	PPP6R1
**1.95512804646741E-50**	0.382268046687329	0.281	0.146	3.27640358027009E-46	Proliferating hepatocytes	PARD6B
**2.31503602068714E-50**	0.743191285691485	0.294	0.159	3.87953736346751E-46	Proliferating hepatocytes	TMEM44
**2.41006824361189E-50**	0.47507201852279	0.377	0.217	4.0387923626448E-46	Proliferating hepatocytes	ABCB10
**2.41820979582863E-50**	0.579818815570951	0.36	0.205	4.05243597584962E-46	Proliferating hepatocytes	UBAC2
**2.54853419012854E-50**	0.780637117262243	0.198	0.091	4.2708335958174E-46	Proliferating hepatocytes	STS
**2.81326562736538E-50**	0.832117083512204	0.103	0.034	4.7144705383389E-46	Proliferating hepatocytes	CBX2
**3.23947367545745E-50**	0.42507621638681	0.471	0.29	5.4287099853316E-46	Proliferating hepatocytes	LGMN

3.31243831482983E-50	0.72512049092738	0.206	0.095	5.55098412799182E-46	Proliferating hepatocytes	MAP10
3.39914635650589E-50	0.338617618088707	0.353	0.196	5.69628946423256E-46	Proliferating hepatocytes	PPP6R3
3.44624879854284E-50	0.420876685682539	0.319	0.173	5.77522373659809E-46	Proliferating hepatocytes	OXSR1
3.55524505998444E-50	0.58891538745931	0.228	0.111	5.95787967152192E-46	Proliferating hepatocytes	FBXO30
3.59604087403603E-50	0.374498834457881	0.275	0.141	6.02624529670958E-46	Proliferating hepatocytes	RBBP5
3.69365063624799E-50	0.584944429176523	0.224	0.108	6.18981973622438E-46	Proliferating hepatocytes	AHCTF1
3.72100118970964E-50	0.36407615513752	0.43	0.26	6.23565379371541E-46	Proliferating hepatocytes	SEMA4B
3.75129618445374E-50	0.399755801626982	0.291	0.154	6.28642214590757E-46	Proliferating hepatocytes	DCAKD
3.82323330445503E-50	0.710852589213751	0.215	0.103	6.40697437160575E-46	Proliferating hepatocytes	ATP1B3
3.9620591083241E-50	0.77164617174226	0.204	0.095	6.63961865372952E-46	Proliferating hepatocytes	CCDC90B
4.26168445031176E-50	0.421279508280953	0.363	0.206	7.14173080183245E-46	Proliferating hepatocytes	EHMT2
4.3303599403147E-50	0.457013672916053	0.487	0.305	7.25681718797937E-46	Proliferating hepatocytes	PDX1
4.49831262038702E-50	0.429371401163023	0.305	0.163	7.53827228924456E-46	Proliferating hepatocytes	KDM4A
4.55842101786961E-50	0.582715185351228	0.319	0.173	7.63900194174589E-46	Proliferating hepatocytes	TNFRSF11A
4.62458503867123E-50	0.690413313047768	0.252	0.127	7.74987960780524E-46	Proliferating hepatocytes	C17orf58
4.71125226164896E-50	0.474715682512101	0.292	0.154	7.89511654007132E-46	Proliferating hepatocytes	CLSTN1
4.89128347895821E-50	0.623305589513698	0.166	0.071	8.19681285403817E-46	Proliferating hepatocytes	PFAS
5.05590555770556E-50	0.285997913149253	0.413	0.244	8.47268653360298E-46	Proliferating hepatocytes	ZFP36L2
5.08300333105498E-50	0.533640780178943	0.18	0.079	8.51809698218194E-46	Proliferating hepatocytes	FAS
5.10057645926069E-50	0.814653776912109	0.185	0.083	8.54754603042907E-46	Proliferating hepatocytes	HSDL1
5.28729535319288E-50	0.391885187977686	0.603	0.399	8.86044955288062E-46	Proliferating hepatocytes	CAPN1
5.5506818224962E-50	0.430924970968402	0.313	0.169	9.30183259813913E-46	Proliferating hepatocytes	TMEM170A
5.58506328605445E-50	0.269459178721702	0.955	0.721	9.35944905477004E-46	Proliferating hepatocytes	ANXA4
5.7076013313063E-50	0.463828149861976	0.412	0.245	9.5647983110031E-46	Proliferating hepatocytes	SCAF4
6.83631470559763E-50	0.461197342550954	0.467	0.287	1.14562961836405E-45	Proliferating hepatocytes	NTPCR
7.50448933158279E-50	0.456463608321799	0.329	0.181	1.25760232218664E-45	Proliferating hepatocytes	ARPC5L
7.67007373869547E-50	0.566097558658329	0.35	0.198	1.28535095713059E-45	Proliferating hepatocytes	PDLIM1
8.40159675446999E-50	0.381620261869377	0.445	0.269	1.40793958411408E-45	Proliferating hepatocytes	SCAF1
8.5767688848107E-50	0.689035398870498	0.204	0.095	1.43729492971658E-45	Proliferating hepatocytes	MOB1A
8.68687354316946E-50	0.645163295306032	0.239	0.118	1.45574626836434E-45	Proliferating hepatocytes	PPM1L
9.03977370818248E-50	0.677013869538344	0.228	0.11	1.51488527801722E-45	Proliferating hepatocytes	TUBGCP5
9.10456213404523E-50	0.386896499504047	0.463	0.284	1.5257425224233E-45	Proliferating hepatocytes	SH3GLB2
9.23118503748442E-50	0.963068132709382	0.136	0.053	1.54696198858164E-45	Proliferating hepatocytes	TYRO3
9.56007714993105E-50	0.555924614519775	0.273	0.141	1.60207772878545E-45	Proliferating hepatocytes	C1orf35
9.73121945030395E-50	0.560931194138337	0.274	0.142	1.63075775548194E-45	Proliferating hepatocytes	SLC2A8
1.05544174698431E-49	0.681135273619915	0.274	0.143	1.7687092795963E-45	Proliferating hepatocytes	RAP1GDS1
1.05848281476217E-49	0.381088867182012	0.559	0.368	1.77380550097844E-45	Proliferating hepatocytes	PSMC3
1.06312177774559E-49	0.271253760080612	0.378	0.215	1.78157947514605E-45	Proliferating hepatocytes	PLEKHG3
1.1134781813619E-49	0.506952556889907	0.439	0.267	1.86596673632627E-45	Proliferating hepatocytes	PPP1R7
1.157745980505E-49	0.578846868387974	0.219	0.105	1.94015071413028E-45	Proliferating hepatocytes	HERC5
1.20367849458942E-49	0.475379902433997	0.383	0.222	2.01712442123295E-45	Proliferating hepatocytes	S100P
1.22187659846402E-49	0.38787020362083	0.41	0.242	2.04762080370601E-45	Proliferating hepatocytes	CREBBP
1.23324269675933E-49	0.732560400955882	0.182	0.081	2.06666811122929E-45	Proliferating hepatocytes	NAIF1
1.2529414735896E-49	0.386483180313096	0.533	0.343	2.09967932144146E-45	Proliferating hepatocytes	RNF115
1.28753747088701E-49	0.763480106964341	0.223	0.108	2.15765529371244E-45	Proliferating hepatocytes	PIP4K2A
1.29577026537347E-49	0.448083107401464	0.468	0.287	2.17145181071286E-45	Proliferating hepatocytes	NCKAP1
1.31654159362977E-49	0.5491749325805	0.25	0.126	2.20626040260478E-45	Proliferating hepatocytes	AIDA
1.43553539726174E-49	0.461608099445587	0.352	0.198	2.40567021873122E-45	Proliferating hepatocytes	ZNF687
1.51583504517724E-49	0.523946924568772	0.352	0.198	2.54023636870801E-45	Proliferating hepatocytes	RNF139
1.51620609111939E-49	0.46719398809331	0.537	0.357	2.54085816749787E-45	Proliferating hepatocytes	NUFIP2
1.69795015667548E-49	0.321914283768926	0.478	0.298	2.84542487255677E-45	Proliferating hepatocytes	CKAP4
1.78734117424983E-49	0.406029327877447	0.294	0.155	2.99522633980787E-45	Proliferating hepatocytes	XPO5
1.81458289593959E-49	0.3587195828958	0.412	0.245	3.04087801701556E-45	Proliferating hepatocytes	SPAG9
1.83696890403027E-49	0.399028375493128	0.36	0.203	3.07839248937393E-45	Proliferating hepatocytes	TMOD3
1.88065309030716E-49	0.813704304016312	0.176	0.077	3.15159844873674E-45	Proliferating hepatocytes	UNC119B
1.9617283423302E-49	0.603015785515045	0.206	0.096	3.28746435607695E-45	Proliferating hepatocytes	TCHP
1.97071232734274E-49	0.373562000001058	0.765	0.577	3.30251971816096E-45	Proliferating hepatocytes	TM9SF2
2.06554245324699E-49	0.735386247251078	0.206	0.096	3.46143604315131E-45	Proliferating hepatocytes	CIART
2.53019167353356E-49	0.744158367522203	0.148	0.06	4.24009520650754E-45	Proliferating hepatocytes	ZKSCAN5
2.57883890683703E-49	0.656099637603564	0.235	0.116	4.3216182400775E-45	Proliferating hepatocytes	ANKMY1

2.58127866921987E-49	0.638581828354368	0.26	0.133	4.32570679387865E-45	Proliferating hepatocytes	SNX7
2.6929859007996E-49	0.400514946089527	0.414	0.246	4.51290577255996E-45	Proliferating hepatocytes	DEPRECATEI
2.71569311078558E-49	0.465453554018872	0.28	0.147	4.55095851505447E-45	Proliferating hepatocytes	SNRNP48
2.76099430539851E-49	0.523432386928598	0.324	0.177	4.62687425698683E-45	Proliferating hepatocytes	INO80C
2.85752623963787E-49	0.412670334199607	0.387	0.224	4.78864247238515E-45	Proliferating hepatocytes	HINT2
3.02422352218467E-49	0.367287661358599	0.43	0.258	5.06799377847708E-45	Proliferating hepatocytes	ZMYND8
3.04347759530613E-49	0.721688426378266	0.145	0.057	5.10025975421401E-45	Proliferating hepatocytes	IGFLR1
3.08113808225649E-49	0.462103708691461	0.331	0.182	5.16337119824543E-45	Proliferating hepatocytes	SLC37A3
3.17137975537725E-49	0.418073747882521	0.279	0.145	5.3145981940612E-45	Proliferating hepatocytes	ZNF266
3.26419295233288E-49	0.425567873860002	0.47	0.292	5.47013454951945E-45	Proliferating hepatocytes	ERAP1
3.31802564202426E-49	0.379858476899739	0.498	0.312	5.56034737090426E-45	Proliferating hepatocytes	RPN2
3.53352042236882E-49	0.410308403519426	0.634	0.436	5.92147352380567E-45	Proliferating hepatocytes	CPQ
3.62768266816836E-49	0.796402635943097	0.203	0.094	6.07927061531655E-45	Proliferating hepatocytes	FHOD3
3.65760946429921E-49	0.323540979847304	0.569	0.376	6.12942194027262E-45	Proliferating hepatocytes	PTPA
3.75452750291513E-49	0.76653984652646	0.193	0.088	6.29183718938518E-45	Proliferating hepatocytes	SLC4A7
3.78808386752518E-49	0.34324809921487	0.366	0.208	6.3480709451987E-45	Proliferating hepatocytes	YY1AP1
3.82793817648214E-49	0.617666806708458	0.288	0.153	6.41485879614877E-45	Proliferating hepatocytes	CAPZA1
3.94970154630278E-49	0.483033772920829	0.36	0.205	6.6189098512942E-45	Proliferating hepatocytes	JOSD1
4.07304110826705E-49	1.02928065013212	0.129	0.049	6.82560228923392E-45	Proliferating hepatocytes	RASEF
4.07742274850841E-49	0.430494488247876	0.291	0.155	6.83294504195039E-45	Proliferating hepatocytes	DPY19L1
4.13009575601496E-49	0.327534892056112	0.252	0.126	6.92121446792987E-45	Proliferating hepatocytes	ILK
4.27257019936347E-49	0.342906715043359	0.195	0.088	7.1599731400933E-45	Proliferating hepatocytes	MYO9A
4.34331110368426E-49	0.897029445919234	0.153	0.063	7.27852074755408E-45	Proliferating hepatocytes	CARMIL1
4.48906037455161E-49	0.48119413005087	0.367	0.211	7.52276737567359E-45	Proliferating hepatocytes	VAPB
4.64619486439383E-49	0.411988249305089	0.456	0.279	7.78609335375118E-45	Proliferating hepatocytes	CNOT6L
4.75226552341115E-49	0.840409871643184	0.17	0.074	7.9638465641324E-45	Proliferating hepatocytes	SVBP
4.78249724152802E-49	0.4460086097295	0.365	0.21	8.01450887735266E-45	Proliferating hepatocytes	TIMM10
4.79249763902112E-49	0.416128728632487	0.374	0.215	8.0312675434716E-45	Proliferating hepatocytes	CNPY3
5.03845134043378E-49	0.614469443599691	0.269	0.14	8.44343675629893E-45	Proliferating hepatocytes	PDE6D
5.22786929365263E-49	0.460425679334149	0.468	0.292	8.76086336230308E-45	Proliferating hepatocytes	PIEZO2
5.97697677124098E-49	0.503202546818901	0.368	0.213	1.00162176732456E-44	Proliferating hepatocytes	C6orf89
6.21350294196192E-49	0.588513867179463	0.323	0.179	1.04125882301398E-44	Proliferating hepatocytes	C6orf136
6.38355464877042E-49	0.41294967769674	0.601	0.415	1.06975608804095E-44	Proliferating hepatocytes	FAM107B
6.51117814801771E-49	0.524920706971689	0.347	0.196	1.09114323404481E-44	Proliferating hepatocytes	LHFPL2
6.52723855669654E-49	0.418121266506084	0.534	0.35	1.09383463733121E-44	Proliferating hepatocytes	RHOA
6.80490745223608E-49	0.44315832676074	0.282	0.149	1.14036639084572E-44	Proliferating hepatocytes	TAF9
7.2387516101654E-49	0.359125467642478	0.486	0.302	1.21306999483152E-44	Proliferating hepatocytes	RASSF3
7.2752218023963E-49	0.429287318605784	0.314	0.172	1.21918166964557E-44	Proliferating hepatocytes	FOXK2
7.53510661793086E-49	0.540636777891574	0.31	0.168	1.26273316703285E-44	Proliferating hepatocytes	RAVER2
7.60597854031404E-49	0.590684778820271	0.24	0.12	1.27460988378583E-44	Proliferating hepatocytes	PAIP1
8.2775159528101E-49	0.559610121900002	0.325	0.181	1.38714612337192E-44	Proliferating hepatocytes	STXBP6
8.41056907755145E-49	0.631575022103589	0.193	0.089	1.40944316601607E-44	Proliferating hepatocytes	ZBED4
8.46704653766304E-49	0.467751678046743	0.415	0.251	1.41890765878157E-44	Proliferating hepatocytes	WIPF2
8.8062416745758E-49	0.403908420078317	0.557	0.363	1.47574997982541E-44	Proliferating hepatocytes	TXNL4A
9.14662496111699E-49	0.40620292791543	0.313	0.17	1.53279141098399E-44	Proliferating hepatocytes	GNPTAB
1.0247168166406E-48	0.413347289102822	0.334	0.186	1.71722044132632E-44	Proliferating hepatocytes	FLVCR1
1.03180894982338E-48	0.339120231059034	0.834	0.592	1.72910543811402E-44	Proliferating hepatocytes	RNF128
1.04384417521278E-48	0.318798837514861	0.487	0.308	1.74927406882158E-44	Proliferating hepatocytes	ATN1
1.08459166510859E-48	0.596521940210433	0.244	0.123	1.81755871238897E-44	Proliferating hepatocytes	JDP2
1.11883712821374E-48	0.522916250414371	0.385	0.225	1.87494725946059E-44	Proliferating hepatocytes	NDUFB4
1.1577069870755E-48	0.867193564897546	0.227	0.112	1.94008536894112E-44	Proliferating hepatocytes	PPM1H
1.19262854710245E-48	0.364341685829552	0.237	0.116	1.99860691923429E-44	Proliferating hepatocytes	RAPGEF6
1.27143852805745E-48	0.475196137703381	0.259	0.132	2.13067668531868E-44	Proliferating hepatocytes	PPME1
1.32790042092406E-48	0.609735748322794	0.24	0.12	2.22529552538453E-44	Proliferating hepatocytes	PIGU
1.55744763889698E-48	0.515202181826518	0.268	0.14	2.60997075326357E-44	Proliferating hepatocytes	ZNF672
1.5977912583949E-48	0.449347801934807	0.413	0.247	2.67757859081818E-44	Proliferating hepatocytes	GSKIP
1.67808774398361E-48	0.91420090092922	0.161	0.068	2.81213944136773E-44	Proliferating hepatocytes	MFSD14C
1.73021536618151E-48	0.49171206885381	0.286	0.152	2.89949491064697E-44	Proliferating hepatocytes	CDYL
1.76365580006834E-48	0.382118031036366	0.265	0.136	2.95553438975453E-44	Proliferating hepatocytes	PDCL
1.78295930869925E-48	0.907419378959369	0.193	0.09	2.9878832095182E-44	Proliferating hepatocytes	TRIM45

**1.91777930550148E-48**	0.408714309667786	0.49	0.305	3.21381456015937E-44	Proliferating hepatocytes	NET1
**2.07712666677854E-48**	0.458296528564718	0.487	0.305	3.48084886818747E-44	Proliferating hepatocytes	SOS 2
**2.12195749596948E-48**	0.410060907154032	0.589	0.393	3.55597637174566E-44	Proliferating hepatocytes	AKT1
**2.15124873037846E-48**	0.485095627129805	0.266	0.138	3.60506262236822E-44	Proliferating hepatocytes	TFDP2
**2.1514197356778E-48**	0.454644100643651	0.382	0.224	3.60534919304886E-44	Proliferating hepatocytes	NSF
**2.19753944092295E-48**	0.416403890715977	0.305	0.164	3.68263659509867E-44	Proliferating hepatocytes	SACM1L
**2.24248278303968E-48**	0.54838623507862	0.329	0.183	3.7579526478179E-44	Proliferating hepatocytes	CPSF1
**2.33058074523274E-48**	0.368261167144456	0.275	0.143	3.90558721286102E-44	Proliferating hepatocytes	FOXK1
**2.34933286706441E-48**	0.625745280325007	0.154	0.064	3.93701201862654E-44	Proliferating hepatocytes	NDOR1
**2.51669316301756E-48**	0.364362470852021	0.571	0.376	4.21747440258482E-44	Proliferating hepatocytes	MTCH1
**2.53493035816589E-48**	0.484985257557964	0.41	0.244	4.24803629421439E-44	Proliferating hepatocytes	TMEM258
**2.56815927903615E-48**	0.414749020444536	0.347	0.197	4.30372131980879E-44	Proliferating hepatocytes	UFD1
**2.58812758887632E-48**	0.35712065487666	0.515	0.328	4.33718421343894E-44	Proliferating hepatocytes	CD2AP
**2.59210241918415E-48**	0.521490789672031	0.204	0.095	4.3438452340688E-44	Proliferating hepatocytes	SNRNP27
**2.76335906610942E-48**	0.553235214205122	0.323	0.18	4.63083712298617E-44	Proliferating hepatocytes	ACAP3
**3.04052546298152E-48**	0.540976133968265	0.297	0.16	5.09531257086443E-44	Proliferating hepatocytes	DNTTIP1
**3.05374974295038E-48**	0.438001483238868	0.352	0.201	5.11747381923625E-44	Proliferating hepatocytes	INTS7
**3.08501966635911E-48**	0.556334940099177	0.196	0.09	5.1698759568846E-44	Proliferating hepatocytes	FP565260.6
**3.24959337091687E-48**	1.1305684265991	0.114	0.041	5.4456685709825E-44	Proliferating hepatocytes	ARHGEF2
**3.29654600892752E-48**	0.382195269575701	0.347	0.196	5.52435180176074E-44	Proliferating hepatocytes	STIM2
**3.30475383776897E-48**	0.58992825994506	0.279	0.149	5.53810648133324E-44	Proliferating hepatocytes	TMEM18
**3.31262969525415E-48**	0.707324902532692	0.194	0.09	5.55130484330691E-44	Proliferating hepatocytes	TMEM243
**3.38048801787166E-48**	0.681598481382839	0.204	0.096	5.66502182034933E-44	Proliferating hepatocytes	RSBN1
**3.43067426292453E-48**	0.41937516957354	0.344	0.195	5.74912392980892E-44	Proliferating hepatocytes	EMC8
**3.4622205953606E-48**	0.450512886320745	0.242	0.121	5.80198927370529E-44	Proliferating hepatocytes	SRSF10
**3.52659581670142E-48**	0.363414536300738	0.238	0.118	5.90986926962824E-44	Proliferating hepatocytes	P3H1
**3.62779415825531E-48**	0.55224767940076	0.292	0.156	6.07945745040425E-44	Proliferating hepatocytes	PRR12
**3.6351595833603E-48**	0.390740164774264	0.308	0.167	6.0918004297952E-44	Proliferating hepatocytes	DPAGT1
**3.86304030042154E-48**	0.4837053295823	0.19	0.086	6.47368293544642E-44	Proliferating hepatocytes	VPS29
**4.11162947912026E-48**	0.678732485608292	0.209	0.1	6.89026868110973E-44	Proliferating hepatocytes	OBI1
**4.1339983818222E-48**	0.609955238401226	0.133	0.052	6.92775448825765E-44	Proliferating hepatocytes	TCF7L1
**4.24633760167517E-48**	0.516581860620691	0.422	0.255	7.11601255288724E-44	Proliferating hepatocytes	ARPC4
**4.37563879595549E-48**	0.614900156876533	0.224	0.11	7.33269549426221E-44	Proliferating hepatocytes	RAB11A
**4.42754870630704E-48**	0.736962680259295	0.175	0.077	7.41968612202934E-44	Proliferating hepatocytes	FAM185A
**5.35515651826166E-48**	0.474099476522707	0.399	0.239	8.97417129330289E-44	Proliferating hepatocytes	DPY30
**5.36453533132727E-48**	0.456152992861068	0.332	0.187	8.98988830823824E-44	Proliferating hepatocytes	OARD1
**5.42562276206469E-48**	0.3848656748187	0.328	0.183	9.092258624668E-44	Proliferating hepatocytes	ISG20L2
**5.55275368310706E-48**	0.380013444187986	0.364	0.208	9.30530462215081E-44	Proliferating hepatocytes	ZNF384
**6.10275782752266E-48**	0.433252740407686	0.604	0.417	1.02270015673625E-43	Proliferating hepatocytes	FH
**6.17207836693875E-48**	0.474515371819634	0.306	0.167	1.0343168927316E-43	Proliferating hepatocytes	BOD1
**6.55422557466814E-48**	0.459061456063421	0.41	0.246	1.09835712180289E-43	Proliferating hepatocytes	RNF167
**6.64164489292501E-48**	0.405462597429179	0.441	0.268	1.11300685115637E-43	Proliferating hepatocytes	TTC1
**6.94675882578514E-48**	0.390770408872983	0.524	0.335	1.16413784402507E-43	Proliferating hepatocytes	HIGD2A
**7.09495733233272E-48**	0.402153163398456	0.307	0.166	1.18897294975232E-43	Proliferating hepatocytes	TOR3A
**7.22292795897285E-48**	0.520327837447812	0.291	0.157	1.21041826736467E-43	Proliferating hepatocytes	RAPGEF1
**7.35236205155031E-48**	0.584393756921924	0.232	0.115	1.2321088325988E-43	Proliferating hepatocytes	PITX1
**7.68056527734041E-48**	0.467375716847112	0.438	0.269	1.28710912917671E-43	Proliferating hepatocytes	AHCY
**7.70657379374076E-48**	0.388238406265854	0.421	0.252	1.29146763635508E-43	Proliferating hepatocytes	AZIN1
**7.93138483215037E-48**	0.400632528961881	0.394	0.232	1.32914147017176E-43	Proliferating hepatocytes	IPO8
**8.25822187892799E-48**	1.14482152643606	0.139	0.056	1.38391282247075E-43	Proliferating hepatocytes	ZFP3
**8.2666480598151E-48**	0.909360109750255	0.158	0.067	1.38532488186381E-43	Proliferating hepatocytes	SNAPC1
**8.81626195197641E-48**	0.352066202119998	0.34	0.191	1.47742917791221E-43	Proliferating hepatocytes	AAK1
**9.25759858474597E-48**	0.419386841419195	0.448	0.277	1.55138837083173E-43	Proliferating hepatocytes	TMX2
**9.73002020927453E-48**	0.427499188445546	0.293	0.157	1.63055678667023E-43	Proliferating hepatocytes	SOCS7
**9.78850042628235E-48**	0.370391984958484	0.562	0.363	1.6403569014364E-43	Proliferating hepatocytes	DEPRECATEI
**1.0074362780587E-47**	0.46203750516269	0.392	0.232	1.68826171477076E-43	Proliferating hepatocytes	AGTRAP
**1.03721590143167E-47**	0.427667851667855	0.493	0.316	1.7381664076192E-43	Proliferating hepatocytes	NUDT5
**1.07163891065712E-47**	0.342243623008068	0.307	0.167	1.79585248647921E-43	Proliferating hepatocytes	PSMD9
**1.1135289780652E-47**	0.742217699856629	0.249	0.128	1.86605186144166E-43	Proliferating hepatocytes	TSPOAP1
**1.15032926874726E-47**	0.351142278936464	0.661	0.477	1.92772178856665E-43	Proliferating hepatocytes	HADHA

1.15450640844922E-47	0.38188524545939	0.598	0.4	1.9347218392792E-43	Proliferating hepatocytes	RNF103
1.17049633722231E-47	0.453997901664515	0.299	0.162	1.96151776191715E-43	Proliferating hepatocytes	TK2
1.19667498872348E-47	0.692946960742978	0.258	0.133	2.00538794610282E-43	Proliferating hepatocytes	TBC1D7
1.26090604892843E-47	0.360849507838828	0.357	0.203	2.11302635679426E-43	Proliferating hepatocytes	RAB11FIP4
1.26639196001929E-47	1.08133576145111	0.133	0.052	2.12221964660032E-43	Proliferating hepatocytes	ZBTB34
1.2949375494376E-47	0.416280559179618	0.274	0.143	2.17005634534753E-43	Proliferating hepatocytes	TAOK2
1.30418624198453E-47	0.42760481494757	0.311	0.171	2.18555530431767E-43	Proliferating hepatocytes	RABL6
1.3270822497959E-47	0.362347188574497	0.557	0.368	2.22392443420797E-43	Proliferating hepatocytes	QKI
1.34867606762453E-47	0.364843339799082	0.353	0.201	2.26011135412519E-43	Proliferating hepatocytes	OTUD4
1.35074134863843E-47	0.402283563391004	0.273	0.142	2.26357235204828E-43	Proliferating hepatocytes	TSG101
1.36653526371998E-47	0.43290182523591	0.373	0.217	2.29003979494194E-43	Proliferating hepatocytes	TAPT1
1.42378331154684E-47	0.388010000389501	0.408	0.244	2.3859760734902E-43	Proliferating hepatocytes	PAN3
1.47347177962211E-47	0.386145546586509	0.39	0.229	2.46924400829074E-43	Proliferating hepatocytes	CNOT9
1.4785059617097E-47	0.57137982006935	0.285	0.153	2.47768029063311E-43	Proliferating hepatocytes	DYNC1H1
1.54411382172122E-47	0.549524478017333	0.276	0.146	2.58762594244043E-43	Proliferating hepatocytes	MRM2
1.65801621346931E-47	0.453133116565018	0.453	0.279	2.77850357053187E-43	Proliferating hepatocytes	GDI2
1.68643098058801E-47	0.530426773910829	0.43	0.264	2.82612103726939E-43	Proliferating hepatocytes	ARHGAP5
1.70226740956364E-47	0.374906097839733	0.369	0.214	2.85265972494675E-43	Proliferating hepatocytes	BRD7
1.76235661848696E-47	0.453732795414594	0.307	0.167	2.95335722126044E-43	Proliferating hepatocytes	ITPKA
1.80588753711131E-47	0.434365395077577	0.381	0.222	3.02630633469114E-43	Proliferating hepatocytes	LARP4B
1.82811280401523E-47	0.388993728736188	0.755	0.572	3.06355143696873E-43	Proliferating hepatocytes	LSR
1.83199357987883E-47	0.559602748852236	0.265	0.139	3.07005484116094E-43	Proliferating hepatocytes	CSGALNACT
1.85072786861806E-47	0.372495373227369	0.374	0.216	3.10144976223015E-43	Proliferating hepatocytes	PURB
1.86192785905765E-47	0.791629071221941	0.235	0.118	3.12021870620882E-43	Proliferating hepatocytes	TARDBP
1.95977449963199E-47	0.377165963107795	0.522	0.331	3.28419010648329E-43	Proliferating hepatocytes	ILVBL
1.96773755434118E-47	0.555583111352925	0.235	0.118	3.29753459356495E-43	Proliferating hepatocytes	RIC8A
2.11975393474833E-47	0.327834143846874	0.788	0.602	3.55228364385125E-43	Proliferating hepatocytes	PRDX2
2.3429428261281E-47	0.683427393233179	0.236	0.119	3.92630358802548E-43	Proliferating hepatocytes	TRIM2
2.4742479094544E-47	0.524411116724818	0.259	0.135	4.14634464666369E-43	Proliferating hepatocytes	ZDHHC5
2.50735614506129E-47	0.497129752520462	0.445	0.275	4.2018274278937E-43	Proliferating hepatocytes	SLC39A1
2.72873322252883E-47	0.548820517567451	0.242	0.123	4.57281113431382E-43	Proliferating hepatocytes	UGT2B7
2.86600754363676E-47	0.382282233042011	0.47	0.294	4.80285544162648E-43	Proliferating hepatocytes	CDIPT
2.92285006498588E-47	0.603641400461999	0.255	0.133	4.89811213890334E-43	Proliferating hepatocytes	STARD3NL
3.00725432588649E-47	0.315184211432349	0.69	0.494	5.03955679932059E-43	Proliferating hepatocytes	NFIC
3.071528995056E-47	0.502757770627533	0.41	0.245	5.14726828991484E-43	Proliferating hepatocytes	MGAT4A
3.11460396171591E-47	0.372180533609086	0.522	0.34	5.21945331904352E-43	Proliferating hepatocytes	SAFB
3.12422014802277E-47	0.623383851009603	0.265	0.14	5.23556812405655E-43	Proliferating hepatocytes	NXT1
3.36256353264755E-47	0.31593873236307	0.408	0.243	5.63498396801077E-43	Proliferating hepatocytes	ZC3HAV1
3.47347456520463E-47	0.560983073767184	0.301	0.166	5.82084867636992E-43	Proliferating hepatocytes	PKD2
3.53778428126613E-47	0.640139238544216	0.228	0.113	5.92861889854579E-43	Proliferating hepatocytes	JKAMP
3.81107712200109E-47	0.522147421422107	0.315	0.176	6.38660304104943E-43	Proliferating hepatocytes	MSH2
3.83712843609013E-47	0.484567820489928	0.43	0.264	6.43025983319984E-43	Proliferating hepatocytes	PID1
3.86501587273317E-47	0.979920174644308	0.167	0.074	6.47699359952625E-43	Proliferating hepatocytes	ISCA2
3.8827081658249E-47	0.577638051569578	0.292	0.158	6.50664234428937E-43	Proliferating hepatocytes	DYNC1LI1
4.13161318344429E-47	0.439365090946396	0.574	0.388	6.92375737281595E-43	Proliferating hepatocytes	IARS2
4.17310794223531E-47	0.727845954182425	0.168	0.074	6.99329428959793E-43	Proliferating hepatocytes	TARBP2
4.34585389319157E-47	0.467525828854833	0.298	0.162	7.28278195421044E-43	Proliferating hepatocytes	GLYR1
4.53743214560759E-47	0.344413141710372	0.588	0.403	7.60382878960921E-43	Proliferating hepatocytes	BIRC3
4.8583767026569E-47	0.498203624986871	0.298	0.162	8.14166767831244E-43	Proliferating hepatocytes	MOB4
5.05625648301834E-47	0.453454806188893	0.377	0.222	8.47327461424213E-43	Proliferating hepatocytes	METAP2
5.28848079370309E-47	0.633178702679157	0.189	0.087	8.86243611408763E-43	Proliferating hepatocytes	PSMD14
5.78437426628994E-47	0.601101911250958	0.255	0.133	9.69345439544869E-43	Proliferating hepatocytes	HSPG2
6.1611291977561E-47	0.762164514806777	0.239	0.122	1.03248203095997E-42	Proliferating hepatocytes	ADGRG2
6.48401322857431E-47	0.448501157957626	0.317	0.175	1.08659093684448E-42	Proliferating hepatocytes	RPP21
6.55049901138814E-47	0.474549633533278	0.349	0.202	1.09773262432842E-42	Proliferating hepatocytes	PLEKHA5
6.68327217927666E-47	0.549507057947984	0.299	0.164	1.11998275180318E-42	Proliferating hepatocytes	DCUN1D2
6.80686420351841E-47	0.298546687439658	0.346	0.196	1.14069430322562E-42	Proliferating hepatocytes	ILKAP
6.81683038239396E-47	0.475569929780853	0.333	0.188	1.14236443548158E-42	Proliferating hepatocytes	PDZD11
6.84571758820324E-47	0.706794391703399	0.214	0.105	1.1472053534311E-42	Proliferating hepatocytes	FOXJ2
7.36263214044035E-47	0.4627001508302	0.279	0.149	1.23382989409499E-42	Proliferating hepatocytes	PPP1R37

**7.82371675830064E-47**	0.3902824958024	0.516	0.335	1.31109845435602E-42	Proliferating hepatocytes	USP12
**7.89874768545767E-47**	0.448908449616136	0.485	0.307	1.323672137129E-42	Proliferating hepatocytes	MEA1
**8.13387366493286E-47**	0.492424801253049	0.287	0.153	1.36307454876945E-42	Proliferating hepatocytes	BRIX1
**8.5980399086149E-47**	0.409050841602023	0.41	0.246	1.44085952788569E-42	Proliferating hepatocytes	SLAIN2
**8.947835988269E-47**	0.541128476721156	0.25	0.128	1.49947835491412E-42	Proliferating hepatocytes	SNW1
**9.01911736087545E-47**	0.472805271740085	0.388	0.229	1.51142368733551E-42	Proliferating hepatocytes	EIF2S3
**9.45570083805992E-47**	0.591139585657208	0.294	0.161	1.58458634644208E-42	Proliferating hepatocytes	SPRY2
**9.46438363375418E-47**	0.510907632891699	0.247	0.126	1.58604140934453E-42	Proliferating hepatocytes	TTI1
**9.79990820994556E-47**	0.397171065522857	0.426	0.257	1.64226861782268E-42	Proliferating hepatocytes	PSMD7
**9.92203883326907E-47**	0.413470492559335	0.429	0.261	1.66273526767923E-42	Proliferating hepatocytes	FKBP1A
**1.02350086050655E-46**	0.863590546292458	0.176	0.08	1.71518274203688E-42	Proliferating hepatocytes	MAPK7
**1.05024464670953E-46**	0.420335092624605	0.477	0.301	1.75999997895583E-42	Proliferating hepatocytes	SH3BP4
**1.18170117670828E-46**	0.422030665310956	0.502	0.313	1.98029483192773E-42	Proliferating hepatocytes	UGT2B11
**1.18829132636385E-46**	0.592848729154192	0.309	0.172	1.99133860472054E-42	Proliferating hepatocytes	RPUSD3
**1.2838292721913E-46**	0.3973376223103	0.374	0.217	2.15144109433817E-42	Proliferating hepatocytes	WASHC5
**1.3006723696247E-46**	0.500632621439714	0.253	0.13	2.17966675701707E-42	Proliferating hepatocytes	COPS3
**1.3037692125741E-46**	0.349297386224222	0.534	0.346	2.18485644643168E-42	Proliferating hepatocytes	POLR1D
**1.44621451251429E-46**	0.45018145950064	0.24	0.121	2.42356628007144E-42	Proliferating hepatocytes	GTF3C2
**1.45677383916809E-46**	0.433903363325786	0.236	0.118	2.44126159967788E-42	Proliferating hepatocytes	KCTD9
**1.49037365628482E-46**	0.453188246487988	0.261	0.135	2.4975681732021E-42	Proliferating hepatocytes	SS18L2
**1.50482938464583E-46**	0.326853322350259	0.423	0.255	2.52179308278948E-42	Proliferating hepatocytes	GPR107
**1.55304994770201E-46**	0.53209828880435	0.253	0.13	2.60260110235902E-42	Proliferating hepatocytes	AQR
**1.58266349857805E-46**	0.357121799902187	0.428	0.26	2.6522274909171E-42	Proliferating hepatocytes	TAF2
**1.62403692395596E-46**	0.385615064755356	0.501	0.329	2.72156107716539E-42	Proliferating hepatocytes	TMEM245
**1.66041192042578E-46**	0.453074877470098	0.432	0.267	2.78251829624951E-42	Proliferating hepatocytes	ATP2C1
**1.75607476209593E-46**	0.463568722804214	0.429	0.264	2.94283008632036E-42	Proliferating hepatocytes	IRF2
**1.82303002986215E-46**	0.320586886880789	0.788	0.631	3.055033724043E-42	Proliferating hepatocytes	HSPA8
**1.89240760288767E-46**	0.327742082576067	0.449	0.278	3.17129666091916E-42	Proliferating hepatocytes	STAG1
**1.9204696942533E-46**	0.270109836095372	0.509	0.326	3.21832311362968E-42	Proliferating hepatocytes	FAM83H
**1.97105793597411E-46**	0.380966598578489	0.506	0.325	3.30309888910542E-42	Proliferating hepatocytes	PSMD12
**1.97743862349182E-46**	1.10312485425774	0.15	0.064	3.31379164524759E-42	Proliferating hepatocytes	NOX1
**2.08239340866822E-46**	0.448701001402641	0.399	0.238	3.4896748742462E-42	Proliferating hepatocytes	ZBTB41
**2.31222153474909E-46**	0.349695681473846	0.554	0.368	3.87482084793252E-42	Proliferating hepatocytes	AAMP
**2.42538275918523E-46**	0.480839324195046	0.304	0.166	4.0644564278426E-42	Proliferating hepatocytes	APEX2
**2.50696298658032E-46**	0.52689333943138	0.227	0.112	4.20116857291131E-42	Proliferating hepatocytes	NOA1
**2.65167579438152E-46**	0.702649968943025	0.168	0.074	4.44367829622454E-42	Proliferating hepatocytes	HTRA2
**2.72233593066538E-46**	0.34495626626235	0.521	0.334	4.56209055260904E-42	Proliferating hepatocytes	AHCYL1
**2.80648514075658E-46**	0.581934045706665	0.219	0.107	4.70310779887987E-42	Proliferating hepatocytes	CCDC7
**3.0117950313952E-46**	0.480435586381956	0.281	0.151	5.04716611361207E-42	Proliferating hepatocytes	C17orf49
**3.11814667324617E-46**	0.412665445991069	0.407	0.246	5.22539019502592E-42	Proliferating hepatocytes	AURKAIP1
**3.35616575787132E-46**	0.532161796869053	0.439	0.28	5.62426257704076E-42	Proliferating hepatocytes	MCM2
**3.37745793191879E-46**	0.751935837121395	0.21	0.102	5.65994400230951E-42	Proliferating hepatocytes	CDKN2AIPNl
**3.38851545928191E-46**	0.432395309100327	0.275	0.146	5.67847420666462E-42	Proliferating hepatocytes	DCBLD2
**3.46051768849558E-46**	0.333105362113644	0.507	0.327	5.7991355423809E-42	Proliferating hepatocytes	FUBP1
**3.60045343389282E-46**	0.335170060561782	0.431	0.263	6.03363986451758E-42	Proliferating hepatocytes	DDX24
**3.60271708421116E-46**	0.366419012437388	0.584	0.392	6.03743328972105E-42	Proliferating hepatocytes	RREB1
**3.72693258149772E-46**	0.409932558139952	0.435	0.267	6.24559362007388E-42	Proliferating hepatocytes	ARFGAP3
**4.13181849891252E-46**	0.426435974511802	0.297	0.161	6.92410144047759E-42	Proliferating hepatocytes	DGCR6L
**4.23092633674521E-46**	0.409805293057707	0.338	0.193	7.09018635511762E-42	Proliferating hepatocytes	SESN2
**4.33144788991062E-46**	0.879275125033192	0.19	0.089	7.25864037391221E-42	Proliferating hepatocytes	IFT57
**4.35901371796606E-46**	0.518669074934127	0.309	0.17	7.30483518856752E-42	Proliferating hepatocytes	MBP
**4.67470102534485E-46**	0.473075203416424	0.382	0.227	7.8338639782729E-42	Proliferating hepatocytes	PYCARD
**4.7354900592987E-46**	0.435408725644345	0.245	0.125	7.93573424137276E-42	Proliferating hepatocytes	ZNF33B
**4.88545943092391E-46**	1.04558468865923	0.11	0.04	8.18705291434228E-42	Proliferating hepatocytes	KATNBL1
**5.04592491452397E-46**	0.385931979688724	0.216	0.105	8.45596097175928E-42	Proliferating hepatocytes	IFT81
**5.53214598884543E-46**	0.468383779104092	0.353	0.206	9.27077024810716E-42	Proliferating hepatocytes	EXOSC9
**5.57307663746498E-46**	0.609079421305616	0.319	0.179	9.33936182906382E-42	Proliferating hepatocytes	DNAAF2
**5.92586913996425E-46**	0.911116583693592	0.14	0.057	9.93057150475208E-42	Proliferating hepatocytes	LMCD1
**6.10246590625559E-46**	0.756662955446199	0.134	0.054	1.02265123657031E-41	Proliferating hepatocytes	SLC22A15
**6.13176874302699E-46**	0.472537461517448	0.315	0.175	1.02756180595646E-41	Proliferating hepatocytes	VOPP1

6.18653312561747E-46	0.507303569103662	0.276	0.148	1.03673922119098E-41	Proliferating hepatocytes	DVL2
6.42110339815161E-46	0.342842693364884	0.375	0.217	1.07604850746225E-41	Proliferating hepatocytes	UBA6
6.95997644608742E-46	0.459921602441596	0.44	0.273	1.16635285283533E-41	Proliferating hepatocytes	GINM1
6.97029133740018E-46	0.258121779307653	0.36	0.207	1.16808142232152E-41	Proliferating hepatocytes	HYI
7.26699592251436E-46	0.464038886378951	0.379	0.222	1.21780317669496E-41	Proliferating hepatocytes	MAPK3
7.4814942246204E-46	0.37603554318754	0.534	0.351	1.25374880216189E-41	Proliferating hepatocytes	KTN1
7.67724319855239E-46	0.289856833396558	0.383	0.226	1.28655241521341E-41	Proliferating hepatocytes	YKT6
7.81746954605497E-46	0.318034327965862	0.427	0.258	1.31005154652789E-41	Proliferating hepatocytes	DEPRECATEI
7.93622368789242E-46	0.502116733682206	0.214	0.104	1.32995236561701E-41	Proliferating hepatocytes	VPS35
8.03874181319587E-46	0.624484096390497	0.255	0.133	1.34713235305536E-41	Proliferating hepatocytes	C9orf152
8.21498788062525E-46	0.609062164511587	0.231	0.117	1.37666766903518E-41	Proliferating hepatocytes	USP22
8.38513169730685E-46	0.398849045060867	0.22	0.108	1.40518036983468E-41	Proliferating hepatocytes	BMP2
8.44607864643859E-46	0.453998725885298	0.364	0.214	1.41539385957018E-41	Proliferating hepatocytes	NCOA6
8.50983766175531E-46	0.410930408825642	0.297	0.162	1.42607859535696E-41	Proliferating hepatocytes	ZNF106
8.62679893245808E-46	0.441759045878563	0.413	0.25	1.44567896510132E-41	Proliferating hepatocytes	PRKCA
8.63159353909031E-46	0.370405105718892	0.574	0.395	1.44648244528075E-41	Proliferating hepatocytes	LONRF3
1.02262807741838E-45	0.374806230363181	0.646	0.454	1.71372013213772E-41	Proliferating hepatocytes	PRDX4
1.08146368932398E-45	0.624556110931796	0.281	0.152	1.81231685056913E-41	Proliferating hepatocytes	ARFIP1
1.11822316272454E-45	0.645711631254412	0.243	0.125	1.87391837609379E-41	Proliferating hepatocytes	TMED8
1.13809687936138E-45	0.737415507157835	0.215	0.106	1.90722275043379E-41	Proliferating hepatocytes	ABHD17C
1.17942695005689E-45	0.422095224566546	0.634	0.45	1.97648368290533E-41	Proliferating hepatocytes	CYP4F11
1.18923200736522E-45	0.539742516607178	0.254	0.133	1.99291499794263E-41	Proliferating hepatocytes	COPG2
1.20787371964794E-45	0.289031627578457	0.202	0.095	2.02415477938603E-41	Proliferating hepatocytes	AKTIP
1.28288686203606E-45	0.683774983214842	0.195	0.093	2.14986180340003E-41	Proliferating hepatocytes	GEMIN6
1.2874260547686E-45	0.375878061085481	0.424	0.258	2.15746858258123E-41	Proliferating hepatocytes	HGH1
1.31020490404737E-45	0.425042310061451	0.304	0.167	2.19564137820258E-41	Proliferating hepatocytes	KPNA3
1.36691068665386E-45	0.349341198690391	0.265	0.138	2.29066892869454E-41	Proliferating hepatocytes	ATR
1.38207310640756E-45	0.354862288693969	0.563	0.368	2.31607811171779E-41	Proliferating hepatocytes	DUSP9
1.43266659751513E-45	0.494686692972254	0.219	0.108	2.40086268411586E-41	Proliferating hepatocytes	HRAS
1.43488562033195E-45	0.59025051224656	0.275	0.147	2.40458132255229E-41	Proliferating hepatocytes	RNF38
1.4552506355631E-45	0.408429576876878	0.462	0.288	2.43870901507664E-41	Proliferating hepatocytes	LSM 6.00
1.55347733485311E-45	0.384826231711296	0.45	0.279	2.60331731774684E-41	Proliferating hepatocytes	UBE2E3
1.73850523866688E-45	0.554351909453866	0.213	0.104	2.91338707895796E-41	Proliferating hepatocytes	INAFM1
1.74087979135384E-45	0.318428045703814	0.773	0.551	2.91736635435076E-41	Proliferating hepatocytes	RBM47
1.75908759800822E-45	0.42644154987368	0.503	0.32	2.94787899674218E-41	Proliferating hepatocytes	VDAC1
1.76958180419867E-45	0.384337722378977	0.486	0.309	2.96546518747614E-41	Proliferating hepatocytes	NDUFS6
1.79463358783817E-45	0.505822640136326	0.357	0.207	3.0074469664992E-41	Proliferating hepatocytes	RMDN1
1.87014251826229E-45	0.334015193006569	0.294	0.159	3.13398483210394E-41	Proliferating hepatocytes	POLE3
2.06197194175055E-45	0.647555433201504	0.178	0.081	3.45545257998556E-41	Proliferating hepatocytes	SP4
2.07011230053489E-45	0.3922353564874	0.32	0.179	3.46909419323637E-41	Proliferating hepatocytes	PPHLN1
2.11975513397992E-45	0.402703906637154	0.278	0.148	3.55228565352354E-41	Proliferating hepatocytes	PIP4K2C
2.14582443662932E-45	0.38703857009679	0.801	0.646	3.59597259090341E-41	Proliferating hepatocytes	DUSP1
2.1461710124612E-45	0.550926125249027	0.246	0.128	3.59655338268248E-41	Proliferating hepatocytes	ARL4C
2.39378734027335E-45	0.468753402825039	0.264	0.139	4.01150882483009E-41	Proliferating hepatocytes	NFYA
2.44003894438849E-45	0.358607930179455	0.243	0.124	4.08901726300624E-41	Proliferating hepatocytes	ARMC6
2.52291191403531E-45	0.42564551659538	0.416	0.255	4.22789578554037E-41	Proliferating hepatocytes	CDK4
2.64861102965833E-45	0.305691726728291	0.291	0.157	4.43854236350143E-41	Proliferating hepatocytes	C4orf19
2.65921770865627E-45	0.367143199218465	0.496	0.316	4.45631703616617E-41	Proliferating hepatocytes	RNF130
2.75228651706795E-45	0.318239465871762	0.286	0.153	4.61228174530247E-41	Proliferating hepatocytes	COA8
2.79864839732576E-45	0.353596277945045	0.687	0.498	4.6899749842385E-41	Proliferating hepatocytes	MTDH
2.8823171521402E-45	0.443375297369988	0.223	0.11	4.83018708355655E-41	Proliferating hepatocytes	RPP30
2.99220721811076E-45	0.367208801552538	0.511	0.329	5.01434085611001E-41	Proliferating hepatocytes	UBR5
2.99866068018587E-45	0.359038625167119	0.35	0.2	5.02515556785549E-41	Proliferating hepatocytes	VTI1B
3.11740027423434E-45	0.520158953233292	0.172	0.077	5.22413937956191E-41	Proliferating hepatocytes	CHUK
3.23705092121016E-45	0.520301233257154	0.274	0.148	5.42464993376399E-41	Proliferating hepatocytes	BAD
3.25897822432849E-45	0.382507483266825	0.432	0.266	5.46139570832969E-41	Proliferating hepatocytes	STK38
3.30234852037991E-45	0.34778312937037	0.662	0.462	5.53407565045266E-41	Proliferating hepatocytes	FRK
3.31486581685271E-45	0.317028449642897	0.472	0.297	5.55505213588178E-41	Proliferating hepatocytes	THRAP3
3.40728482779413E-45	0.364375828590194	0.708	0.507	5.7099279144174E-41	Proliferating hepatocytes	IFI30
3.45523979331539E-45	0.399755288640972	0.286	0.156	5.79029084563793E-41	Proliferating hepatocytes	RBM15B

3.61537131189096E-45	0.364804696448105	0.281	0.151	6.05863924446688E-41	Proliferating hepatocytes	DCAF12
3.76093564779871E-45	0.434524642599893	0.256	0.133	6.30257595858107E-41	Proliferating hepatocytes	DEPRECATEI
3.77571854175595E-45	0.382770521758955	0.195	0.091	6.32734913227462E-41	Proliferating hepatocytes	UACA
3.97186794081492E-45	0.391977694184498	0.423	0.257	6.65605629521764E-41	Proliferating hepatocytes	MLLT1
4.09334741691162E-45	0.272408373765306	0.408	0.246	6.8596316012605E-41	Proliferating hepatocytes	E2F4
4.18492415486883E-45	0.46135793579975	0.425	0.261	7.01309589872919E-41	Proliferating hepatocytes	UBA1
4.52361796147783E-45	0.523508480399116	0.214	0.105	7.58067897984454E-41	Proliferating hepatocytes	PDE4D
4.53176965733234E-45	0.767483202597486	0.255	0.135	7.59433959175753E-41	Proliferating hepatocytes	GPR180
4.73442301262628E-45	0.583473736056249	0.189	0.089	7.93394608455912E-41	Proliferating hepatocytes	SEC14L1
4.97571858327381E-45	0.390889094878036	0.496	0.32	8.33830920185025E-41	Proliferating hepatocytes	PAK2
5.1104618346976E-45	0.508636496202702	0.262	0.138	8.56411194258624E-41	Proliferating hepatocytes	PPIL4
5.24468142775845E-45	0.561764642578804	0.14	0.057	8.78903713663761E-41	Proliferating hepatocytes	CWF19L1
5.36485797166947E-45	0.360898878074266	0.284	0.153	8.9904289889237E-41	Proliferating hepatocytes	RIC1
5.47089085424034E-45	1.09777714897903	0.117	0.044	9.16811889353596E-41	Proliferating hepatocytes	C19orf57
5.51191950342685E-45	0.377785141708719	0.406	0.244	9.23687470384272E-41	Proliferating hepatocytes	MYH9
5.55087977731985E-45	0.61333114401172	0.163	0.071	9.3021643308326E-41	Proliferating hepatocytes	CCDC138
5.56644645868127E-45	0.509394722364668	0.31	0.173	9.32825097545807E-41	Proliferating hepatocytes	GTF2A2
6.00465215904206E-45	0.383828196224753	0.269	0.143	1.00625960881227E-40	Proliferating hepatocytes	MORF4L1
6.01119507505323E-45	0.626088261861951	0.182	0.084	1.00735607067742E-40	Proliferating hepatocytes	PARG
6.01755370908438E-45	0.401706196123736	0.476	0.305	1.00842165056836E-40	Proliferating hepatocytes	CDC16
6.54043683171764E-45	0.261031021879573	0.314	0.173	1.09604640425924E-40	Proliferating hepatocytes	SMG1
6.90585200849576E-45	0.41482580771855	0.315	0.176	1.15728267958372E-40	Proliferating hepatocytes	RIC8B
6.94535523111374E-45	0.361207274666608	0.411	0.25	1.16390262963004E-40	Proliferating hepatocytes	ABHD3
7.61535013654186E-45	0.364607737177337	0.452	0.285	1.27618037588168E-40	Proliferating hepatocytes	HECTD4
7.98774130751699E-45	0.339501037666375	0.23	0.116	1.3385856883137E-40	Proliferating hepatocytes	RALA
7.99739701447794E-45	0.309373241143174	0.358	0.208	1.34020379168621E-40	Proliferating hepatocytes	NABP2
8.42087612080415E-45	0.428935879281823	0.411	0.25	1.41117042032436E-40	Proliferating hepatocytes	VPS35L
9.01315407083057E-45	0.746799011233435	0.273	0.148	1.51042435918979E-40	Proliferating hepatocytes	KCNMB3
9.24544352676612E-45	0.713063403184624	0.181	0.084	1.54935142621547E-40	Proliferating hepatocytes	SARNP
9.53549833677076E-45	0.400735683583583	0.348	0.201	1.59795881127604E-40	Proliferating hepatocytes	EXOSC2
1.03967864094836E-44	0.680170507400419	0.194	0.092	1.74229346650127E-40	Proliferating hepatocytes	DRAM1
1.05467510203476E-44	0.38898925533528	0.525	0.35	1.76742453598984E-40	Proliferating hepatocytes	RPN1
1.20146246948885E-44	0.482499054703168	0.213	0.104	2.01341080636942E-40	Proliferating hepatocytes	CCDC97
1.20859515715436E-44	0.550058819838487	0.186	0.087	2.02536376435928E-40	Proliferating hepatocytes	DEPRECATEI
1.22289975183059E-44	0.364618572381005	0.429	0.261	2.04933540411771E-40	Proliferating hepatocytes	TPD52L2
1.22986921091447E-44	0.397308680798397	0.34	0.195	2.06101482365048E-40	Proliferating hepatocytes	ME2
1.2403054096834E-44	0.410712100908374	0.444	0.275	2.07850380554745E-40	Proliferating hepatocytes	SH3RF1
1.24077462926629E-44	0.393422141329425	0.412	0.252	2.07929012372445E-40	Proliferating hepatocytes	CCNY
1.26770687120785E-44	0.44470076216221	0.226	0.114	2.12442317477011E-40	Proliferating hepatocytes	IP6K1
1.26971618514197E-44	0.448120606276455	0.256	0.134	2.12779038306091E-40	Proliferating hepatocytes	USP28
1.27466011246919E-44	0.408655647035983	0.198	0.094	2.13607541647586E-40	Proliferating hepatocytes	DEPRECATEI
1.28594803877412E-44	0.560414234590248	0.338	0.195	2.15499172337767E-40	Proliferating hepatocytes	ZNF608
1.38127046342464E-44	0.530096442348063	0.322	0.182	2.314733042607E-40	Proliferating hepatocytes	RBM22
1.38886722432128E-44	0.349676329626927	0.213	0.105	2.3274636945176E-40	Proliferating hepatocytes	SFI1
1.39108730247235E-44	0.418553728679443	0.418	0.254	2.33118410148317E-40	Proliferating hepatocytes	ARPC1B
1.39789578750172E-44	0.466074631899771	0.246	0.127	2.34259376069539E-40	Proliferating hepatocytes	KLHL7
1.42489919516803E-44	0.554834023338136	0.193	0.091	2.38784607126259E-40	Proliferating hepatocytes	EMSY
1.54643270513616E-44	0.342024162231687	0.187	0.087	2.59151192726717E-40	Proliferating hepatocytes	DTNB
1.63358271339287E-44	0.367216375347121	0.556	0.371	2.73755791110376E-40	Proliferating hepatocytes	UQCR10
1.63455477980377E-44	0.272341187088892	0.219	0.107	2.73918689999515E-40	Proliferating hepatocytes	PPP1R18
1.65078583208907E-44	0.370142103545809	0.516	0.336	2.76638689741486E-40	Proliferating hepatocytes	PSMD1
1.66416944808787E-44	0.669272826167579	0.162	0.072	2.78881516110565E-40	Proliferating hepatocytes	ZC3H12C
1.68847358320843E-44	0.271797166392884	0.468	0.294	2.82954403074069E-40	Proliferating hepatocytes	SUN1
1.79149196655559E-44	0.505189501737085	0.301	0.167	3.00218223755385E-40	Proliferating hepatocytes	LNPEP
1.79627014079929E-44	0.26368030391637	0.294	0.16	3.01018950195145E-40	Proliferating hepatocytes	ZC3H7A
1.83093314238882E-44	0.50111628905373	0.256	0.135	3.06827776001519E-40	Proliferating hepatocytes	UBE2L3
1.9488690254064E-44	0.315574322248059	0.41	0.249	3.26591471277605E-40	Proliferating hepatocytes	PRCC
2.01189109906481E-44	0.453195947226246	0.237	0.121	3.37152710381281E-40	Proliferating hepatocytes	PELP1
2.25515076002261E-44	0.351428749639765	0.548	0.368	3.77918164364589E-40	Proliferating hepatocytes	NARS
2.25841326406414E-44	0.309470006415255	0.427	0.26	3.78464894791869E-40	Proliferating hepatocytes	GRK2

2.30229165610852E-44	0.503814992269976	0.215	0.106	3.85818035730666E-40	Proliferating hepatocytes	COA1
2.34040959749403E-44	0.312049833759768	0.626	0.409	3.9220584034805E-40	Proliferating hepatocytes	PARM1
2.40692610982089E-44	0.409014937494393	0.383	0.229	4.03352677483784E-40	Proliferating hepatocytes	LRP10
2.4074066773012E-44	0.557982032478347	0.253	0.133	4.03433210982135E-40	Proliferating hepatocytes	ZNF131
2.48650459997299E-44	0.328359883261462	0.765	0.566	4.16688440863474E-40	Proliferating hepatocytes	AKR1C2
2.49907713082335E-44	0.404717554307157	0.412	0.253	4.18795345583377E-40	Proliferating hepatocytes	EIF2S1
2.57182141187854E-44	0.507850677767911	0.233	0.119	4.30985832202605E-40	Proliferating hepatocytes	APIP
2.70071983088574E-44	0.381455609038228	0.334	0.19	4.52586629259832E-40	Proliferating hepatocytes	SF1
2.95149897750906E-44	0.420550229355545	0.51	0.329	4.94612198650969E-40	Proliferating hepatocytes	ATP5PD
3.05731086476343E-44	0.343886204686775	0.378	0.223	5.12344154717056E-40	Proliferating hepatocytes	MBTPS1
3.09714965282921E-44	0.63927499582078	0.175	0.079	5.1902033882112E-40	Proliferating hepatocytes	BRAP
3.13740227497734E-44	0.475499657860505	0.31	0.173	5.25765873240702E-40	Proliferating hepatocytes	PDS5A
3.14832638453196E-44	0.368371166043617	0.57	0.375	5.27596535519867E-40	Proliferating hepatocytes	ANXA13
3.21972627537965E-44	0.367230702854449	0.5	0.329	5.39561729228121E-40	Proliferating hepatocytes	MICAL3
3.28569093925239E-44	0.39574628718578	0.337	0.191	5.50616087599916E-40	Proliferating hepatocytes	MED6
3.3569604909954E-44	0.55936921724054	0.344	0.203	5.62559439081009E-40	Proliferating hepatocytes	SLC1A5
3.48360713206806E-44	0.690583520001001	0.157	0.069	5.83782883191965E-40	Proliferating hepatocytes	HECTD2
3.53050341638834E-44	0.608014411762311	0.211	0.104	5.91641762518359E-40	Proliferating hepatocytes	ZNF619
3.57888129496491E-44	0.442054737989265	0.416	0.256	5.9974892741022E-40	Proliferating hepatocytes	LRRC14
3.60615332677284E-44	0.584405942415707	0.313	0.178	6.04319174500593E-40	Proliferating hepatocytes	THOC7
3.61290315401204E-44	0.43241867249318	0.242	0.125	6.05450310549338E-40	Proliferating hepatocytes	BRMS1L
3.63180933433438E-44	0.41843165934168	0.275	0.149	6.08618608247755E-40	Proliferating hepatocytes	MMD
3.63494022677874E-44	0.323388330603865	0.512	0.329	6.0914328320358E-40	Proliferating hepatocytes	GUCD1
3.64042348880906E-44	0.325245974999331	0.653	0.465	6.10062168254622E-40	Proliferating hepatocytes	DSP
3.78552879713141E-44	0.480422749108062	0.426	0.261	6.34378915823282E-40	Proliferating hepatocytes	YIPF4
3.94373094491931E-44	0.450733315029017	0.412	0.254	6.60890431749579E-40	Proliferating hepatocytes	PRELID1
4.22712627153719E-44	0.398806741919972	0.505	0.323	7.08381820584203E-40	Proliferating hepatocytes	PLS1
4.27093722046373E-44	0.338269084755778	0.438	0.27	7.15723659405313E-40	Proliferating hepatocytes	C7orf50
4.29408559118758E-44	0.412928921789261	0.423	0.261	7.19602863371214E-40	Proliferating hepatocytes	KDELR1
4.3976849710168E-44	0.431876014430101	0.474	0.303	7.36964047442995E-40	Proliferating hepatocytes	MAT2B
4.53101941328554E-44	1.08417038362858	0.107	0.039	7.59308233278392E-40	Proliferating hepatocytes	USP43
4.62628154178902E-44	0.342419772957313	0.555	0.373	7.75272260773004E-40	Proliferating hepatocytes	NEDD4L
4.73477671256768E-44	0.381913142914631	0.406	0.245	7.93453881492092E-40	Proliferating hepatocytes	CENPV
4.8139198450555E-44	0.393676054199803	0.329	0.187	8.06716687634401E-40	Proliferating hepatocytes	COPS4
4.97514585121204E-44	0.268103767673967	0.479	0.301	8.33734941746114E-40	Proliferating hepatocytes	CDC42BPB
4.99729160000066E-44	0.573340522775609	0.153	0.066	8.3744612632811E-40	Proliferating hepatocytes	SH3PXD2B
5.14334199657818E-44	0.411159999500982	0.305	0.171	8.61921251786571E-40	Proliferating hepatocytes	TMEM138
5.54580565712381E-44	0.402200071957633	0.502	0.331	9.29366112020808E-40	Proliferating hepatocytes	MAN2A1
5.6912362053659E-44	0.406354860984673	0.227	0.115	9.53737363295217E-40	Proliferating hepatocytes	DEPRECATEI
6.0498469553854E-44	0.532118114423325	0.218	0.109	1.01383335278349E-39	Proliferating hepatocytes	KLC2
6.10166800782974E-44	0.356647145760064	0.501	0.323	1.02251752475211E-39	Proliferating hepatocytes	NDUFB11
6.35341169316246E-44	0.37348111861713	0.478	0.307	1.06470473154016E-39	Proliferating hepatocytes	TMUB2
6.43849000368106E-44	0.642751187165863	0.219	0.109	1.07896215481687E-39	Proliferating hepatocytes	TCFL5
6.46918191115256E-44	0.743541524087628	0.145	0.062	1.08410550467095E-39	Proliferating hepatocytes	MPV17L2
6.58131844561381E-44	0.371156082936185	0.583	0.389	1.10289734511596E-39	Proliferating hepatocytes	XIAP
6.91495254812521E-44	0.345379406630284	0.349	0.201	1.15880774801482E-39	Proliferating hepatocytes	FLII
6.98129526665826E-44	0.388049398073259	0.421	0.259	1.16992546078659E-39	Proliferating hepatocytes	IFT27
7.08481155487179E-44	0.639708030104149	0.159	0.07	1.18727272036541E-39	Proliferating hepatocytes	DNAL4
7.35224328255993E-44	0.2660920819262	0.35	0.201	1.23208892929139E-39	Proliferating hepatocytes	BRPF3
7.56061359798205E-44	0.414475141510033	0.38	0.225	1.26700762674983E-39	Proliferating hepatocytes	PSMA3
8.05800474593422E-44	0.286253555696559	0.369	0.217	1.35036043532366E-39	Proliferating hepatocytes	SLC16A1
8.3251156963119E-44	0.430855298255727	0.319	0.181	1.39512288838795E-39	Proliferating hepatocytes	RMND5B
8.54225786396835E-44	0.362017472437115	0.4	0.242	1.43151157284382E-39	Proliferating hepatocytes	SLC38A1
9.1038318939182E-44	0.389279262332387	0.241	0.124	1.52562014878281E-39	Proliferating hepatocytes	CCNH
9.22806833557955E-44	0.368112141161022	0.607	0.407	1.54643969167642E-39	Proliferating hepatocytes	ATP6V0D1
9.35982323586407E-44	0.518362261612452	0.326	0.187	1.5685191778661E-39	Proliferating hepatocytes	MAGEH1
9.36591883561721E-44	0.422965736273624	0.406	0.247	1.56954067847273E-39	Proliferating hepatocytes	VPS26C
9.41202515108181E-44	0.75419043289521	0.181	0.084	1.57726717481829E-39	Proliferating hepatocytes	PXYLP1
9.68115549031103E-44	0.539164058567999	0.206	0.101	1.62236803706632E-39	Proliferating hepatocytes	MRFAP1
9.7023203954642E-44	0.334109603303485	0.304	0.168	1.62591485187189E-39	Proliferating hepatocytes	MLXIP

9.73431651305496E-44	0.349896037765766	0.317	0.179	1.63127676125775E-39	Proliferating hepatocytes	AK1
9.98004496603411E-44	0.263991134155026	0.373	0.219	1.672455935408E-39	Proliferating hepatocytes	M6PR
1.04816076758515E-43	0.31497503284993	0.412	0.253	1.75650781431919E-39	Proliferating hepatocytes	SMC6
1.05626576061299E-43	0.465819382997382	0.293	0.161	1.77009016163525E-39	Proliferating hepatocytes	PRR14L
1.08007185003685E-43	0.38001429099294	0.283	0.154	1.80998440629175E-39	Proliferating hepatocytes	POP4
1.10428474006228E-43	0.397085418208429	0.598	0.407	1.85056036739637E-39	Proliferating hepatocytes	RAB10
1.14583053305575E-43	0.421654568071615	0.202	0.098	1.92018280729483E-39	Proliferating hepatocytes	RGMB
1.18012000517392E-43	0.337868175621749	0.19	0.09	1.97764510467046E-39	Proliferating hepatocytes	UHRF2
1.22057743144309E-43	0.457065469872292	0.45	0.28	2.04544365961233E-39	Proliferating hepatocytes	RNASEK
1.23209920377744E-43	0.376535206473789	0.359	0.209	2.06475184569023E-39	Proliferating hepatocytes	ARFGEF1
1.23546116344653E-43	0.345396911186201	0.734	0.544	2.07038581770369E-39	Proliferating hepatocytes	CNN3
1.25006976219364E-43	0.410991376628662	0.29	0.159	2.09486690748411E-39	Proliferating hepatocytes	HNRNPLL
1.2684409936701E-43	0.471644150835513	0.216	0.108	2.12565341719236E-39	Proliferating hepatocytes	NIPSNAP2
1.31381314146102E-43	0.66936983962635	0.212	0.105	2.20168806246038E-39	Proliferating hepatocytes	PPT1
1.31915882156546E-43	0.411958335301343	0.388	0.233	2.21064635317939E-39	Proliferating hepatocytes	PIP4P1
1.32061875935337E-43	0.375014662799734	0.429	0.264	2.21309291692437E-39	Proliferating hepatocytes	CFDP1
1.44372779278637E-43	0.455259150572319	0.368	0.223	2.4193990351514E-39	Proliferating hepatocytes	RNF44
1.55521662620885E-43	0.816136597338757	0.117	0.045	2.60623202220079E-39	Proliferating hepatocytes	C2CD3
1.57776430229695E-43	0.455218842496171	0.306	0.171	2.64401741778923E-39	Proliferating hepatocytes	CREB3
1.62884997139704E-43	0.39227830016253	0.536	0.363	2.72962678206716E-39	Proliferating hepatocytes	RDH10
1.64542929848343E-43	0.647347438833878	0.229	0.117	2.75741041839853E-39	Proliferating hepatocytes	TMEM30B
1.79006132661629E-43	0.461802260188845	0.221	0.11	2.99978477114358E-39	Proliferating hepatocytes	LRP12
1.79918497226594E-43	0.29190613258668	0.273	0.147	3.01507417652326E-39	Proliferating hepatocytes	INPP5E
1.83620367401135E-43	0.890505220319982	0.137	0.057	3.07711011690822E-39	Proliferating hepatocytes	STON2
1.85519878040581E-43	0.365777756292456	0.467	0.296	3.10894211620405E-39	Proliferating hepatocytes	DDB1
1.94936086920287E-43	0.303729515783542	0.431	0.265	3.26673894461017E-39	Proliferating hepatocytes	KIF1C
2.05121514544034E-43	0.75478734546332	0.159	0.07	3.43742634072892E-39	Proliferating hepatocytes	SNRPD1
2.09989840136831E-43	0.31048833339735	0.343	0.196	3.51900974101301E-39	Proliferating hepatocytes	TASOR
2.11204529958631E-43	0.475794467055538	0.196	0.094	3.53936551304673E-39	Proliferating hepatocytes	ABCC5
2.12571169424176E-43	0.326056277140189	0.368	0.216	3.56226765721035E-39	Proliferating hepatocytes	EPS8
2.13507359665005E-43	0.335724522625462	0.483	0.312	3.57795633326615E-39	Proliferating hepatocytes	TCIRG1
2.14563952899427E-43	0.556752344285268	0.22	0.11	3.5956627226886E-39	Proliferating hepatocytes	DSTYK
2.18831071066234E-43	0.402560552157667	0.486	0.31	3.66717108892796E-39	Proliferating hepatocytes	KCNJ3
2.19653574384456E-43	0.381436022259593	0.518	0.342	3.68095459953472E-39	Proliferating hepatocytes	ISOC1
2.20924323343124E-43	0.316391957990992	0.58	0.392	3.70224981058408E-39	Proliferating hepatocytes	DERL1
2.2682084600971E-43	0.3753550082035	0.368	0.218	3.80106373743072E-39	Proliferating hepatocytes	USP10
2.41194900721942E-43	0.350263516431073	0.292	0.159	4.0419441462983E-39	Proliferating hepatocytes	NUBP2
2.50236343102967E-43	0.424405640787516	0.337	0.194	4.19346063771953E-39	Proliferating hepatocytes	TRADD
2.55059579606387E-43	0.32578714154829	0.399	0.239	4.27428843504384E-39	Proliferating hepatocytes	LSM 7.00
2.65715223520855E-43	0.447979908627462	0.302	0.168	4.45285571576248E-39	Proliferating hepatocytes	BSCL2
2.8326659495923E-43	0.423447791530712	0.289	0.159	4.74698159832677E-39	Proliferating hepatocytes	HLCS
2.88116391044395E-43	0.396960196958982	0.463	0.293	4.82825448112197E-39	Proliferating hepatocytes	CLPTM1L
2.9424617814203E-43	0.448637464607967	0.366	0.217	4.93097745330414E-39	Proliferating hepatocytes	ELP5
2.98601974539034E-43	0.79555833264536	0.143	0.061	5.00397188932513E-39	Proliferating hepatocytes	PNMA1
2.99453109624716E-43	0.511778197781663	0.25	0.132	5.01823521109099E-39	Proliferating hepatocytes	TTC9C
3.08182198947085E-43	0.352194092556773	0.624	0.439	5.16451728995526E-39	Proliferating hepatocytes	CHCHD2
3.23409361699607E-43	0.693562243221805	0.211	0.105	5.41969408336202E-39	Proliferating hepatocytes	PLGRKT
3.45245210515068E-43	0.474233017214483	0.237	0.123	5.78561923781151E-39	Proliferating hepatocytes	FCF1
3.49340033984295E-43	0.335928505612742	0.446	0.278	5.85424028950882E-39	Proliferating hepatocytes	FLNB
3.72380288455634E-43	0.409645145152186	0.281	0.153	6.24034887393951E-39	Proliferating hepatocytes	GPR137
3.77780215212604E-43	0.313257274345659	0.435	0.27	6.33084084653282E-39	Proliferating hepatocytes	SORBS1
4.12423439011264E-43	0.377844179055887	0.353	0.205	6.91139199095077E-39	Proliferating hepatocytes	SNX24
4.34517165401362E-43	0.326608250534445	0.342	0.197	7.28163865779602E-39	Proliferating hepatocytes	THUMPD1
4.39229271298444E-43	0.393873144438221	0.57	0.39	7.36060412841932E-39	Proliferating hepatocytes	DAZAP2
4.67307499949528E-43	0.470445302789792	0.168	0.076	7.83113908415419E-39	Proliferating hepatocytes	CSNK1G1
4.92068878179065E-43	0.335811315928978	0.279	0.151	8.24609026052477E-39	Proliferating hepatocytes	DIPK2A
4.93247789052484E-43	0.396404250703947	0.296	0.164	8.26584644894152E-39	Proliferating hepatocytes	U2AF2
5.03022790481929E-43	0.372585848254157	0.575	0.397	8.42965592289616E-39	Proliferating hepatocytes	COX8A
5.03456623975088E-43	0.37615907621375	0.563	0.376	8.43692610457453E-39	Proliferating hepatocytes	ACMSD
5.04381909285267E-43	0.459168143485786	0.388	0.234	8.4524320358025E-39	Proliferating hepatocytes	VIRMA

5.39582345562806E-43	0.322434586450116	0.519	0.341	9.0423209469415E-39	Proliferating hepatocytes	BRD4
5.39944618503524E-43	0.401674180297193	0.274	0.149	9.04839191688205E-39	Proliferating hepatocytes	PSME2
5.40759183162618E-43	0.491510589906316	0.284	0.156	9.06204239143915E-39	Proliferating hepatocytes	XRCC5
5.51170714830414E-43	0.508056933126143	0.204	0.1	9.23651883912807E-39	Proliferating hepatocytes	THOC5
5.51837110535066E-43	0.288732147001163	0.395	0.239	9.24768629834663E-39	Proliferating hepatocytes	ZC3H3
5.73286528571546E-43	0.278932308670681	0.446	0.279	9.60713564580196E-39	Proliferating hepatocytes	CDC34
5.76324907552318E-43	0.346179185823925	0.32	0.182	9.65805280076175E-39	Proliferating hepatocytes	TMEM8A
6.16102909719896E-43	0.350393294346991	0.354	0.207	1.0324652561086E-38	Proliferating hepatocytes	ATG3
6.40403558455492E-43	0.582939328878716	0.164	0.074	1.07318828325971E-38	Proliferating hepatocytes	C1orf109
6.73012811383935E-43	0.362855854399158	0.181	0.085	1.1278348693172E-38	Proliferating hepatocytes	DNAJB6
6.91143537347367E-43	0.398053471371711	0.321	0.182	1.15821833988672E-38	Proliferating hepatocytes	CMTR1
7.10121248973406E-43	0.399541035583996	0.276	0.15	1.19002118902963E-38	Proliferating hepatocytes	UQCC3
7.16028292251496E-43	0.370476151639692	0.535	0.356	1.19992021215506E-38	Proliferating hepatocytes	INTS8
7.43282217870291E-43	0.303213265852804	0.367	0.215	1.24559234070703E-38	Proliferating hepatocytes	SLC25A43
8.0132433396E-43	0.389160384684249	0.399	0.243	1.34285931885017E-38	Proliferating hepatocytes	CNOT11
8.02281563196352E-43	0.539072882594335	0.27	0.147	1.34446344360445E-38	Proliferating hepatocytes	MED12
8.64719163454967E-43	0.407654690760654	0.271	0.146	1.44909637411783E-38	Proliferating hepatocytes	FBXW11
9.08924680707262E-43	0.293363896280032	0.289	0.159	1.52317597992923E-38	Proliferating hepatocytes	KIF21A
9.18266037967635E-43	0.3360302791878	0.342	0.2	1.53883022642616E-38	Proliferating hepatocytes	TELO2
9.33147235829615E-43	0.271989423184819	0.475	0.301	1.56376813780327E-38	Proliferating hepatocytes	PCBP1
9.55073324222088E-43	0.384049081022902	0.369	0.218	1.60051187673137E-38	Proliferating hepatocytes	GTF2H2
9.88680549855548E-43	0.500061339204719	0.245	0.128	1.65683086544793E-38	Proliferating hepatocytes	DEPRECATEI
1.03285205654828E-42	0.700117533318407	0.147	0.063	1.7308534763636E-38	Proliferating hepatocytes	SLC31A2
1.03779445539757E-42	0.303000561651549	0.347	0.202	1.73913594835526E-38	Proliferating hepatocytes	MAP3K4
1.03945234809059E-42	0.394653504796174	0.416	0.256	1.74191424493021E-38	Proliferating hepatocytes	POMP
1.12046303589639E-42	0.456764069113828	0.216	0.108	1.87767195555518E-38	Proliferating hepatocytes	TADA2A
1.14339317641246E-42	0.304504143849785	0.721	0.511	1.91609828503201E-38	Proliferating hepatocytes	COX6C
1.30115840843961E-42	0.329512721033944	0.552	0.369	2.18048126086309E-38	Proliferating hepatocytes	BAG6
1.38495322079257E-42	0.435100402271845	0.258	0.137	2.32090460740418E-38	Proliferating hepatocytes	ZNF654
1.38913432827363E-42	0.431949048939979	0.259	0.138	2.32791130732096E-38	Proliferating hepatocytes	PPARG
1.45966924136616E-42	0.42300346580593	0.255	0.136	2.44611371468141E-38	Proliferating hepatocytes	NAA40
1.46854864579347E-42	0.372835886528337	0.442	0.279	2.46099382062069E-38	Proliferating hepatocytes	BMI1
1.48438128217036E-42	0.660391701193642	0.21	0.105	2.48752615266109E-38	Proliferating hepatocytes	LCMT1
1.52334554664921E-42	0.483748523750075	0.397	0.243	2.55282246707474E-38	Proliferating hepatocytes	EIF3H
1.55633130054832E-42	0.556525860783343	0.265	0.143	2.60809999345887E-38	Proliferating hepatocytes	CYCS
1.5937292325284E-42	0.516267247984899	0.247	0.131	2.67077144787108E-38	Proliferating hepatocytes	GRK3
1.65432960322336E-42	0.43832162159668	0.284	0.155	2.7723255490817E-38	Proliferating hepatocytes	MRGBP
1.67589420317972E-42	0.270638871962479	0.382	0.227	2.80846350568858E-38	Proliferating hepatocytes	SRRM1
1.71282435458627E-42	0.810646601388662	0.167	0.077	2.87035105341567E-38	Proliferating hepatocytes	C17orf80
1.75492101309764E-42	0.381228787406181	0.544	0.366	2.94089663374903E-38	Proliferating hepatocytes	CPNE1
1.8471232569453E-42	0.575281106143521	0.221	0.112	3.09540915398893E-38	Proliferating hepatocytes	CCT2
1.90244605377464E-42	0.827410418523687	0.145	0.062	3.18811909691555E-38	Proliferating hepatocytes	KIF1BP
2.00105896842191E-42	0.504850847703137	0.262	0.141	3.35337461928144E-38	Proliferating hepatocytes	PSMG1
2.01784060320946E-42	0.326596098455031	0.287	0.158	3.38149728285842E-38	Proliferating hepatocytes	YES1
2.02830598230676E-42	0.501586291985541	0.258	0.139	3.39903516514966E-38	Proliferating hepatocytes	L2HGDH
2.18344220155649E-42	0.462381819268855	0.266	0.143	3.65901244136837E-38	Proliferating hepatocytes	MAD2L1BP
2.19577523634419E-42	0.256083026306044	0.423	0.259	3.67968014106559E-38	Proliferating hepatocytes	ANAPC5
2.21084835824727E-42	0.478020831287604	0.288	0.16	3.70493967875078E-38	Proliferating hepatocytes	NOSIP
2.29428164959867E-42	0.313672839707584	0.263	0.141	3.84475718839745E-38	Proliferating hepatocytes	GPKOW
2.40799268731268E-42	0.766536686511089	0.102	0.037	4.03531414539859E-38	Proliferating hepatocytes	ZNF232
2.43784825480052E-42	0.332168704228057	0.678	0.482	4.08534610539471E-38	Proliferating hepatocytes	ALG13
2.48859624259587E-42	0.659838519255592	0.215	0.109	4.17038958334216E-38	Proliferating hepatocytes	ARHGEF38
2.51676734513275E-42	0.456926039644086	0.343	0.202	4.21759871697346E-38	Proliferating hepatocytes	ANO8
2.56039575499654E-42	0.371383711569775	0.69	0.512	4.2907112062232E-38	Proliferating hepatocytes	SUMF2
2.58459931969463E-42	0.380433911348327	0.483	0.312	4.33127153994426E-38	Proliferating hepatocytes	PRRC2C
2.59581106297156E-42	0.364242091863703	0.272	0.147	4.35006017932774E-38	Proliferating hepatocytes	TRABD
2.61232988824119E-42	0.335718274393375	0.447	0.279	4.37774242671459E-38	Proliferating hepatocytes	MARVELD2
2.6801277514035E-42	0.29311116619363	0.438	0.272	4.49135808580199E-38	Proliferating hepatocytes	TRIM24
2.73683071060667E-42	0.428687122037519	0.42	0.261	4.58638090483466E-38	Proliferating hepatocytes	TJP3
2.75482220413948E-42	0.371867597097052	0.447	0.283	4.61653104969695E-38	Proliferating hepatocytes	ESYT1

2.82179933998551E-42	0.488449520422958	0.195	0.095	4.72877133394772E-38	Proliferating hepatocytes	RAD17
2.99744942650838E-42	0.606945662675538	0.214	0.108	5.02312574894275E-38	Proliferating hepatocytes	RFK
3.11840149706254E-42	0.476757794850981	0.291	0.163	5.2258172287774E-38	Proliferating hepatocytes	KLHL8
3.28099740699919E-42	0.352720680031431	0.509	0.335	5.49829545464925E-38	Proliferating hepatocytes	STK3
3.38620136410111E-42	0.374355957238813	0.533	0.359	5.67459624596065E-38	Proliferating hepatocytes	GCLM
3.42309496389348E-42	0.439158458513162	0.893	0.789	5.7364225404927E-38	Proliferating hepatocytes	HSPA1B
3.49982470003305E-42	0.324630283937252	0.314	0.177	5.86500623231539E-38	Proliferating hepatocytes	KLHL12
3.52970650086711E-42	0.462977487245187	0.324	0.186	5.9150821541531E-38	Proliferating hepatocytes	UBL4A
3.65216937327481E-42	0.662533174896074	0.236	0.124	6.12030543573393E-38	Proliferating hepatocytes	TP53I11
3.68287902692941E-42	0.708387875792818	0.221	0.113	6.1717686733283E-38	Proliferating hepatocytes	MECOM
3.89060370532419E-42	0.256673713567916	0.477	0.303	6.51987368938228E-38	Proliferating hepatocytes	HIPK3
3.92335664221717E-42	0.866013664114522	0.16	0.072	6.57476106102753E-38	Proliferating hepatocytes	DHRS4
3.9253314422017E-42	0.350136420424692	0.544	0.362	6.57807043084161E-38	Proliferating hepatocytes	DTX3L
4.05789321839045E-42	0.294541433817126	0.296	0.164	6.80021745537871E-38	Proliferating hepatocytes	ITGA1
4.13051442119849E-42	0.536631064078392	0.281	0.156	6.92191606704444E-38	Proliferating hepatocytes	COPS8
4.18263945017476E-42	0.330791826977516	0.31	0.175	7.00926719060286E-38	Proliferating hepatocytes	TRIM33
4.24318557275585E-42	0.377273728522661	0.27	0.146	7.11073038282425E-38	Proliferating hepatocytes	ANKRD52
4.26540447632171E-42	0.415257678422654	0.406	0.25	7.14796482141993E-38	Proliferating hepatocytes	WWC2
4.44738318260828E-42	0.547246703239654	0.342	0.205	7.45292473741496E-38	Proliferating hepatocytes	NR3C2
4.46329768566638E-42	0.545405750207822	0.235	0.122	7.47959426163972E-38	Proliferating hepatocytes	MTMR14
4.46712892119813E-42	0.326280748853058	0.41	0.249	7.48601464614383E-38	Proliferating hepatocytes	LARP1
4.69024139299783E-42	0.403752903481958	0.525	0.349	7.85990652638577E-38	Proliferating hepatocytes	NEDD8
4.75394003067617E-42	0.524241335173266	0.284	0.157	7.96665270340712E-38	Proliferating hepatocytes	C17orf75
4.92202481784782E-42	0.332830780931281	0.273	0.148	8.24832918974938E-38	Proliferating hepatocytes	ATG13
5.00443423382982E-42	0.692996615792326	0.162	0.073	8.38643088905202E-38	Proliferating hepatocytes	ZNF280C
5.03709276194375E-42	0.393711816394503	0.406	0.249	8.44116005046533E-38	Proliferating hepatocytes	ATP6V1C1
5.87001064190871E-42	0.505411526727899	0.262	0.14	9.83696383371061E-38	Proliferating hepatocytes	STAM2
5.89003395086552E-42	0.417033343527977	0.427	0.268	9.87051889486043E-38	Proliferating hepatocytes	NDUFA7
6.09873938634674E-42	0.513566002060767	0.184	0.087	1.02202674636399E-37	Proliferating hepatocytes	INPP5F
6.1869876354642E-42	0.591481243816316	0.297	0.169	1.03681538795109E-37	Proliferating hepatocytes	PDP1
6.20018253074848E-42	0.314523669254643	0.408	0.25	1.03902658850283E-37	Proliferating hepatocytes	FAAH
6.30329972577773E-42	0.390947767358735	0.241	0.126	1.05630696804583E-37	Proliferating hepatocytes	ZDHHC18
6.35806115532095E-42	0.310086411765274	0.391	0.234	1.06548388840868E-37	Proliferating hepatocytes	PAPOLA
6.47007715519027E-42	0.526541722168521	0.165	0.075	1.08425552966678E-37	Proliferating hepatocytes	PHACTR2
6.78412376312953E-42	0.316403768543783	0.268	0.145	1.13688346022525E-37	Proliferating hepatocytes	CNTLN
6.83362539551414E-42	0.488147714963835	0.202	0.099	1.14517894378026E-37	Proliferating hepatocytes	IBA57
7.31432353606799E-42	0.334418619475484	0.41	0.251	1.22573433817427E-37	Proliferating hepatocytes	AFTPH
7.3886429610185E-42	0.387796990299546	0.456	0.287	1.23818878740748E-37	Proliferating hepatocytes	SMIM7
7.53940654470686E-42	0.454136145758881	0.353	0.209	1.26345374876197E-37	Proliferating hepatocytes	CASC4
8.02854965070564E-42	0.287220278003841	0.374	0.221	1.34542435046525E-37	Proliferating hepatocytes	TOR1B
8.14933240730327E-42	0.365060621319784	0.345	0.203	1.36566512481588E-37	Proliferating hepatocytes	YME1L1
9.42733148020233E-42	0.675731446300307	0.23	0.12	1.57983220945231E-37	Proliferating hepatocytes	DEPRECATEI
9.54768531704294E-42	0.330957686168818	0.53	0.35	1.60000110543006E-37	Proliferating hepatocytes	NDUFS1
9.6322161647946E-42	0.388680122931681	0.321	0.184	1.61416678489628E-37	Proliferating hepatocytes	CYTH3
9.71019419442212E-42	0.509867734829232	0.379	0.232	1.62723434310126E-37	Proliferating hepatocytes	IRF9
9.7276199294222E-42	0.329164466699632	0.229	0.118	1.63015454777257E-37	Proliferating hepatocytes	GPRIN3
9.73960083024215E-42	0.379683160610067	0.642	0.454	1.63216230713198E-37	Proliferating hepatocytes	RER1
1.00060613201524E-41	0.435190822491826	0.259	0.139	1.67681575603114E-37	Proliferating hepatocytes	SPPL3
1.01125752104731E-41	0.322236587749726	0.241	0.126	1.69466535377108E-37	Proliferating hepatocytes	FAM53B
1.04865022117829E-41	0.323963984763882	0.664	0.48	1.75732804065058E-37	Proliferating hepatocytes	EBP
1.05518171302416E-41	0.345810333931512	0.251	0.134	1.76827351468589E-37	Proliferating hepatocytes	UBE3B
1.06291574473096E-41	0.356357494290181	0.375	0.225	1.78123420502014E-37	Proliferating hepatocytes	CHD4
1.10420072287189E-41	0.477600817700496	0.231	0.12	1.85041957138871E-37	Proliferating hepatocytes	SMIM13
1.16956538359198E-41	0.257777687073874	0.32	0.182	1.95995766982343E-37	Proliferating hepatocytes	PKN2
1.19055279236286E-41	0.418257155537754	0.495	0.327	1.99512836944168E-37	Proliferating hepatocytes	TMEM50A
1.24811027827132E-41	0.369653655656497	0.247	0.13	2.09158320432708E-37	Proliferating hepatocytes	INO80D
1.28074188500026E-41	0.335102606881407	0.567	0.371	2.14626725088343E-37	Proliferating hepatocytes	DEPRECATEI
1.29440763622418E-41	0.48595973256139	0.287	0.16	2.16916831678448E-37	Proliferating hepatocytes	STOX2
1.35635202232169E-41	0.896580258993225	0.152	0.068	2.27297471900668E-37	Proliferating hepatocytes	ANK2
1.38363961566678E-41	0.327678836694748	0.683	0.477	2.31870326793439E-37	Proliferating hepatocytes	DHRS7

1.44045272104844E-41	0.336269883533442	0.279	0.152	2.41391066993298E-37	Proliferating hepatocytes	PRR14
1.47399208507318E-41	0.432691977152348	0.388	0.236	2.47011593616563E-37	Proliferating hepatocytes	ZMYND11
1.48287960171162E-41	0.528680146639794	0.185	0.089	2.48500963654832E-37	Proliferating hepatocytes	DEPRECATEI
1.56834813390609E-41	0.62570893161833	0.103	0.038	2.62823780279982E-37	Proliferating hepatocytes	PGAP1
1.74671181844711E-41	0.260335484982715	0.319	0.183	2.92713966535367E-37	Proliferating hepatocytes	ACTR1A
1.77075494956526E-41	0.421956482045013	0.16	0.072	2.96743114448147E-37	Proliferating hepatocytes	ING3
1.80168476387605E-41	0.294636069961349	0.237	0.123	3.01926332730348E-37	Proliferating hepatocytes	DAAM1
1.83461747687062E-41	0.322325204295624	0.138	0.058	3.07445196773979E-37	Proliferating hepatocytes	ZC2HC1C
1.91502959364949E-41	0.370377826968315	0.323	0.186	3.20920659303782E-37	Proliferating hepatocytes	TIMM17B
1.95996439578138E-41	0.291764788192783	0.344	0.2	3.28450833445043E-37	Proliferating hepatocytes	ZDHHC3
2.05751543566384E-41	0.41570767497116	0.215	0.109	3.44798436708546E-37	Proliferating hepatocytes	DAGLB
2.06437942208302E-41	0.328782257998471	0.494	0.32	3.45948703552673E-37	Proliferating hepatocytes	ASB13
2.09077924488732E-41	0.855743865768056	0.181	0.087	3.50372785858217E-37	Proliferating hepatocytes	LYPD6B
2.10830100605655E-41	0.312560885817257	0.46	0.297	3.53309082594957E-37	Proliferating hepatocytes	MARCH7
2.19176339545249E-41	0.334112469952238	0.279	0.152	3.67295709809928E-37	Proliferating hepatocytes	ZNF282
2.21543621446859E-41	0.456313502632769	0.363	0.218	3.71262800820646E-37	Proliferating hepatocytes	DDX23
2.23433189770952E-41	0.445176771957573	0.274	0.151	3.74429339418161E-37	Proliferating hepatocytes	ACTR2
2.30283828723418E-41	0.30571455719576	0.215	0.109	3.85909640174705E-37	Proliferating hepatocytes	MPZL2
2.36319722274921E-41	0.562719812251688	0.184	0.088	3.96024590588312E-37	Proliferating hepatocytes	EFHC1
2.40229503617037E-41	0.41913942191423	0.391	0.238	4.0257660216143E-37	Proliferating hepatocytes	PSMA2
2.45540430577255E-41	0.449712851498263	0.333	0.195	4.11476653561364E-37	Proliferating hepatocytes	PSMC1
2.53635672726139E-41	0.297183880565535	0.394	0.238	4.25042660354464E-37	Proliferating hepatocytes	STK25
2.55178358132257E-41	0.379899575586434	0.25	0.133	4.27627892558036E-37	Proliferating hepatocytes	ADPGK
2.58579757643976E-41	0.377047222731573	0.275	0.152	4.33327957859776E-37	Proliferating hepatocytes	NCBP1
2.59496936855748E-41	0.397848914641187	0.328	0.189	4.34864966782863E-37	Proliferating hepatocytes	RAB28
2.6378651385938E-41	0.403344918934856	0.383	0.232	4.42053439925548E-37	Proliferating hepatocytes	TIMM50
2.70213701009125E-41	0.505819315478346	0.323	0.19	4.52824120151092E-37	Proliferating hepatocytes	AKR1C1
2.70762916126527E-41	0.337440401183979	0.324	0.187	4.53744494844834E-37	Proliferating hepatocytes	CCT4
2.79671696187626E-41	0.362480330224817	0.456	0.294	4.68673828471224E-37	Proliferating hepatocytes	YWHAE
3.04936778126953E-41	0.312477437157607	0.573	0.389	5.11013052785148E-37	Proliferating hepatocytes	NMT1
3.20360218228992E-41	0.461677461656288	0.243	0.129	5.36859653708146E-37	Proliferating hepatocytes	FLVCR2
3.20824937128214E-41	0.337315457011885	0.453	0.287	5.37638429639461E-37	Proliferating hepatocytes	MTIF3
3.50128675582323E-41	0.340024111891697	0.644	0.467	5.86745634540858E-37	Proliferating hepatocytes	SCARB2
3.51716053298777E-41	0.324350353320978	0.511	0.336	5.8940576211809E-37	Proliferating hepatocytes	RNF114
3.77557068074019E-41	0.411302408815526	0.294	0.164	6.32710134678441E-37	Proliferating hepatocytes	RAB22A
3.78253005042889E-41	0.381426183200455	0.386	0.236	6.33876385850873E-37	Proliferating hepatocytes	FBL
3.81099395903332E-41	0.291276533110363	0.655	0.463	6.38646367654804E-37	Proliferating hepatocytes	SLC25A1
4.09251021024878E-41	0.463818172106608	0.265	0.145	6.85822861033491E-37	Proliferating hepatocytes	TARS2
4.2045756714048E-41	0.634693259062615	0.21	0.107	7.04602791014016E-37	Proliferating hepatocytes	MDH1
4.54014972563159E-41	0.274376074334078	0.305	0.172	7.60838291021342E-37	Proliferating hepatocytes	DEPRECATEI
4.56185585484293E-41	0.369881904952391	0.388	0.237	7.64475804154578E-37	Proliferating hepatocytes	ZNF623
4.56890144622458E-41	0.997122601842348	0.125	0.051	7.65656504358315E-37	Proliferating hepatocytes	DEPRECATEI
4.77512922809066E-41	0.649431145204757	0.146	0.064	8.00216156043433E-37	Proliferating hepatocytes	BORCS6
4.83925453749462E-41	0.388622969052656	0.344	0.203	8.10962275393349E-37	Proliferating hepatocytes	CMAS
4.92096950065184E-41	0.574353635810382	0.242	0.128	8.24656068919235E-37	Proliferating hepatocytes	ATP2C2
4.93260073896415E-41	0.253983532775547	0.439	0.273	8.26605231835613E-37	Proliferating hepatocytes	SYMPK
5.09200751395579E-41	0.380083309394312	0.29	0.161	8.53318619188711E-37	Proliferating hepatocytes	PRDX3
5.12388742309639E-41	0.385604982759011	0.494	0.326	8.58661054362492E-37	Proliferating hepatocytes	ATP5F1B
5.1624236303639E-41	0.315561519035787	0.294	0.165	8.65118951976383E-37	Proliferating hepatocytes	UTP6
5.23440704792923E-41	0.332096631025697	0.606	0.42	8.7718193309198E-37	Proliferating hepatocytes	ACTN1
5.30278216410103E-41	0.511702534782105	0.253	0.136	8.8864023506005E-37	Proliferating hepatocytes	RTRAF
5.32287680092486E-41	0.410040929286785	0.343	0.202	8.92007694298988E-37	Proliferating hepatocytes	ATPSCKMT
5.36242293798239E-41	0.366540761850745	0.343	0.202	8.98634835947089E-37	Proliferating hepatocytes	MESD
5.69171542584065E-41	0.342118640785037	0.393	0.239	9.53817671062376E-37	Proliferating hepatocytes	DICER1
5.94477569217737E-41	0.524448626676441	0.294	0.166	9.96225510495084E-37	Proliferating hepatocytes	XPO4
5.9783887235601E-41	0.25237728500211	0.296	0.167	1.0018583822942E-36	Proliferating hepatocytes	SALL1
6.24178675707507E-41	0.37081525217056	0.437	0.276	1.04599862475064E-36	Proliferating hepatocytes	MAPK1IP1L
6.24506520523916E-41	0.388097739514374	0.287	0.161	1.04654802709398E-36	Proliferating hepatocytes	CAB39
6.4356229708447E-41	0.425071423183322	0.263	0.143	1.07848169745416E-36	Proliferating hepatocytes	TAF12
6.58037034290444E-41	0.311519855651168	0.469	0.299	1.10273846206393E-36	Proliferating hepatocytes	DPP9

6.77387406245414E-41	0.380522672625579	0.27	0.148	1.13516581538607E-36	Proliferating hepatocytes	ST3GAL2
6.85697749123466E-41	0.28996240016674	0.324	0.185	1.1490922879811E-36	Proliferating hepatocytes	ZNF507
6.89167991967509E-41	0.922545940549949	0.129	0.054	1.15490772093915E-36	Proliferating hepatocytes	MAATS1
7.04999919780844E-41	0.321692836950186	0.46	0.291	1.18143886556874E-36	Proliferating hepatocytes	MTFR1
7.13884996216466E-41	0.609096896416002	0.155	0.07	1.19632847665955E-36	Proliferating hepatocytes	C19orf54
7.1409119475511E-41	0.529794077025742	0.272	0.151	1.19667402417061E-36	Proliferating hepatocytes	NOL9
8.16283302808924E-41	0.493813020245549	0.315	0.182	1.36792755884719E-36	Proliferating hepatocytes	CDC42EP5
8.3846690677235E-41	0.681841363952182	0.182	0.087	1.4051028423691E-36	Proliferating hepatocytes	INTS2
8.49053076692467E-41	0.39933773411814	0.248	0.131	1.42284314592124E-36	Proliferating hepatocytes	RPE
8.49749763838001E-41	0.398871191985559	0.415	0.258	1.42401065423972E-36	Proliferating hepatocytes	TIMMDC1
8.53719976316898E-41	0.363711926145176	0.348	0.207	1.43066393631186E-36	Proliferating hepatocytes	RAB15
8.53982184241492E-41	0.584709928504409	0.233	0.122	1.43110334435189E-36	Proliferating hepatocytes	ANKRA2
8.94334185793128E-41	0.294559649172641	0.367	0.218	1.49872522855212E-36	Proliferating hepatocytes	TERF2
9.08043873870041E-41	0.366244205051568	0.492	0.32	1.52169992383142E-36	Proliferating hepatocytes	RBM10
9.28392484050279E-41	0.411485482176615	0.312	0.18	1.55580012477146E-36	Proliferating hepatocytes	SAMD1
9.48930942286829E-41	0.327441361180089	0.478	0.307	1.59021847308427E-36	Proliferating hepatocytes	PPP4C
9.56667212619059E-41	0.331831163538787	0.536	0.356	1.60318291490702E-36	Proliferating hepatocytes	MBNL1
9.68159924866952E-41	0.343032743697314	0.574	0.393	1.62244240209204E-36	Proliferating hepatocytes	ACSL3
9.68971368315335E-41	0.317699888666922	0.268	0.147	1.62380221902284E-36	Proliferating hepatocytes	RNF31
1.03594652977228E-40	0.398817883980825	0.373	0.225	1.73603919459238E-36	Proliferating hepatocytes	SNX27
1.07178169871851E-40	0.412662830311579	0.202	0.1	1.79609177071248E-36	Proliferating hepatocytes	CWC27
1.10012013090807E-40	0.94150324064123	0.112	0.043	1.84358131537575E-36	Proliferating hepatocytes	SYK
1.11103901472755E-40	0.322794763463903	0.206	0.103	1.86187918088043E-36	Proliferating hepatocytes	FAM53C
1.13048995970759E-40	0.452323597837265	0.398	0.245	1.89447507447798E-36	Proliferating hepatocytes	POF1B
1.13349486477817E-40	1.09746564176558	0.11	0.043	1.89951069439525E-36	Proliferating hepatocytes	NRARP
1.20292463423694E-40	0.461777493941395	0.362	0.22	2.01586110205426E-36	Proliferating hepatocytes	KLHL22
1.22455597943193E-40	0.551561730985014	0.253	0.137	2.05211091033202E-36	Proliferating hepatocytes	XPO6
1.22770637952266E-40	0.51271662646837	0.214	0.108	2.05739035080407E-36	Proliferating hepatocytes	TMEM116
1.32094549978005E-40	0.784243508342556	0.167	0.078	2.2136404685314E-36	Proliferating hepatocytes	PLAGL2
1.33553881570971E-40	0.388323840998144	0.255	0.137	2.23809594736633E-36	Proliferating hepatocytes	SIMC1
1.36823759096017E-40	0.426169033170135	0.21	0.105	2.29289255493106E-36	Proliferating hepatocytes	PPIL3
1.39616852820843E-40	0.2693098952732	0.369	0.22	2.33969921957168E-36	Proliferating hepatocytes	FIP1L1
1.42905324275533E-40	0.352731978918616	0.534	0.355	2.39480742420939E-36	Proliferating hepatocytes	PEX2
1.46902168868805E-40	0.266823311571508	0.789	0.559	2.46178654590343E-36	Proliferating hepatocytes	SSR4
1.5766176975755E-40	0.411008032919207	0.268	0.147	2.64209593759703E-36	Proliferating hepatocytes	ZNF449
1.59264211639874E-40	0.400262623037262	0.407	0.253	2.66894965866101E-36	Proliferating hepatocytes	AKAP8
1.59514203066482E-40	0.292432574371621	0.231	0.121	2.6731390149881E-36	Proliferating hepatocytes	FOXA2
1.64819053182047E-40	0.491080788408899	0.222	0.115	2.76203769322475E-36	Proliferating hepatocytes	TOMM34
1.65652639210638E-40	0.305638283511041	0.757	0.576	2.77600692789187E-36	Proliferating hepatocytes	TSTA3
1.66557766837779E-40	0.618905368887542	0.175	0.083	2.7911750566675E-36	Proliferating hepatocytes	KCTD10
1.68377428766695E-40	0.36886682454781	0.562	0.385	2.82166895127227E-36	Proliferating hepatocytes	KDELR2
1.72745771741388E-40	0.543096944676403	0.199	0.099	2.89487364284218E-36	Proliferating hepatocytes	CGRRF1
1.74831968376451E-40	0.423439278657926	0.371	0.225	2.92983412605257E-36	Proliferating hepatocytes	COPS7A
1.82221968223051E-40	0.385939029794711	0.385	0.234	3.05367574348189E-36	Proliferating hepatocytes	CC2D1A
1.85915791922216E-40	0.317987705250653	0.401	0.248	3.1155768410325E-36	Proliferating hepatocytes	GNL1
1.910500739652E-40	0.509802701497028	0.356	0.216	3.20161713950883E-36	Proliferating hepatocytes	SRD5A3
1.95698102975304E-40	0.379382517078363	0.37	0.223	3.27950880966014E-36	Proliferating hepatocytes	SRP68
2.08347451302604E-40	0.353354323818777	0.441	0.279	3.49148658892904E-36	Proliferating hepatocytes	DPM1
2.11273086622498E-40	0.364591745827392	0.399	0.246	3.54051438561982E-36	Proliferating hepatocytes	LYST
2.15395212448326E-40	0.432120067261913	0.269	0.148	3.60959297020904E-36	Proliferating hepatocytes	BLVRA
2.20608744173601E-40	0.802319632254634	0.187	0.091	3.69696133486121E-36	Proliferating hepatocytes	FBXO32
2.22203541147984E-40	0.395541848187491	0.396	0.244	3.72368694255791E-36	Proliferating hepatocytes	NFKB1
2.24039867768E-40	0.349148420550119	0.289	0.162	3.75446010405615E-36	Proliferating hepatocytes	PARP1
2.26061311548807E-40	0.724010070632696	0.192	0.095	3.78833545893491E-36	Proliferating hepatocytes	GNG5
2.28355855434783E-40	0.674193668823977	0.203	0.102	3.8267874253761E-36	Proliferating hepatocytes	H2AFY2
2.28548455627899E-40	0.504575406992102	0.202	0.101	3.83001501941234E-36	Proliferating hepatocytes	DEPRECATEI
2.30184186099016E-40	0.646074173170374	0.132	0.056	3.8574265906473E-36	Proliferating hepatocytes	GPATCH11
2.30276062107974E-40	0.536717250763785	0.247	0.132	3.85896624880543E-36	Proliferating hepatocytes	AP3S1
2.33710074507128E-40	0.484618437310497	0.247	0.132	3.91651342859045E-36	Proliferating hepatocytes	HFE
2.37009699859053E-40	0.419181451715893	0.243	0.128	3.97180855023801E-36	Proliferating hepatocytes	LIN52

2.3961338542861E-40	0.342019589555353	0.314	0.181	4.01544111301264E-36	Proliferating hepatocytes	EIF4G2
2.42668280238026E-40	0.255387130084971	0.211	0.106	4.06663504022884E-36	Proliferating hepatocytes	COIL
2.46753510348556E-40	0.403494709500317	0.326	0.189	4.1350953264211E-36	Proliferating hepatocytes	VSIG10
2.4976102884438E-40	0.320928699986324	0.436	0.276	4.18549532137411E-36	Proliferating hepatocytes	WWOX
2.53130660718707E-40	0.402172368705714	0.324	0.188	4.24196361232409E-36	Proliferating hepatocytes	MOSPD2
2.5535135505474E-40	0.597775049897511	0.208	0.106	4.27917800800733E-36	Proliferating hepatocytes	FAM189B
2.66674115344497E-40	0.403620936371949	0.452	0.285	4.46892482494308E-36	Proliferating hepatocytes	ZNF410
2.68820059259372E-40	0.476996645553739	0.382	0.241	4.50488655306855E-36	Proliferating hepatocytes	E2F1
2.80692123689146E-40	0.459494523187263	0.296	0.168	4.7038386087827E-36	Proliferating hepatocytes	CDC42EP4
2.81238724960959E-40	0.346871583143835	0.33	0.193	4.71299855289575E-36	Proliferating hepatocytes	ERMP1
2.95329459514731E-40	0.34814812863902	0.243	0.129	4.94913108254786E-36	Proliferating hepatocytes	FAM241A
2.95784401462761E-40	0.374992929271194	0.28	0.156	4.95675499971295E-36	Proliferating hepatocytes	APPL1
2.96624987637472E-40	0.279041292410039	0.343	0.201	4.97084154282875E-36	Proliferating hepatocytes	LSG1
3.19111833476164E-40	0.707666487937026	0.162	0.075	5.34767610539356E-36	Proliferating hepatocytes	NPAT
3.24847311381377E-40	0.589064286464636	0.228	0.119	5.44379124412912E-36	Proliferating hepatocytes	TCTEX1D2
3.29436973785435E-40	0.398580852649903	0.188	0.091	5.52070480669632E-36	Proliferating hepatocytes	TTBK2
3.34937492364053E-40	0.704823069376787	0.211	0.108	5.61288249703679E-36	Proliferating hepatocytes	ZXDB
3.51708562405164E-40	0.785496996761964	0.114	0.045	5.89393208878575E-36	Proliferating hepatocytes	GRK4
3.588147883195E-40	0.33405884210901	0.552	0.368	6.01301822265817E-36	Proliferating hepatocytes	SMIM19
3.60122999111096E-40	0.375244288445364	0.593	0.423	6.03494121910375E-36	Proliferating hepatocytes	STAU2
3.617991279045E-40	0.409715892641274	0.309	0.179	6.0630297854236E-36	Proliferating hepatocytes	SMARCB1
3.62005714118546E-40	0.341009798114877	0.292	0.164	6.0664917571986E-36	Proliferating hepatocytes	ADNP2
3.95008952462562E-40	0.278976304321286	0.782	0.597	6.61956002536762E-36	Proliferating hepatocytes	CYC1
3.99791514473296E-40	0.33857698009861	0.451	0.288	6.69970619954349E-36	Proliferating hepatocytes	CUL4A
4.17480452038245E-40	0.386840469655206	0.241	0.127	6.99613741525691E-36	Proliferating hepatocytes	MGA
4.23542087826427E-40	0.325292273033011	0.517	0.336	7.09771830779527E-36	Proliferating hepatocytes	TMEM164
4.29634922399999E-40	0.937616089953352	0.123	0.051	7.19982202957918E-36	Proliferating hepatocytes	TCEANC2
4.34554613310057E-40	0.323003002009636	0.359	0.214	7.28226620984994E-36	Proliferating hepatocytes	TAX1BP3
4.47564495596275E-40	0.320043548572113	0.287	0.161	7.50028581720238E-36	Proliferating hepatocytes	BCL7B
4.52986442533867E-40	0.43190180934236	0.332	0.196	7.59114680398254E-36	Proliferating hepatocytes	TNIP2
4.65199383065952E-40	0.345373383220874	0.725	0.516	7.79581126141923E-36	Proliferating hepatocytes	ATP6AP1
4.71691557592087E-40	0.257000410821562	0.334	0.194	7.90460712212819E-36	Proliferating hepatocytes	NRIP1
4.74974932371416E-40	0.306438659804521	0.358	0.212	7.9596299166802E-36	Proliferating hepatocytes	FNIP2
4.79820655468754E-40	0.381155911340958	0.287	0.161	8.04083454434538E-36	Proliferating hepatocytes	TCF3
4.91423537093807E-40	0.476121740460064	0.163	0.075	8.23527563461802E-36	Proliferating hepatocytes	NSUN5
4.93153103564679E-40	0.325580665993786	0.595	0.411	8.2642597095369E-36	Proliferating hepatocytes	EIF4H
5.04210944555594E-40	0.3744432562951	0.359	0.215	8.44956700886265E-36	Proliferating hepatocytes	RALGAPB
5.23510449604934E-40	0.365793167611044	0.382	0.234	8.77298811447948E-36	Proliferating hepatocytes	STXBP5
5.3192476614628E-40	0.260031101968962	0.329	0.191	8.91399523107936E-36	Proliferating hepatocytes	TGS1
5.35468492406094E-40	0.321895896595021	0.674	0.495	8.97338099574133E-36	Proliferating hepatocytes	AKR1A1
5.42328410948726E-40	0.887238389900116	0.16	0.074	9.08833951067876E-36	Proliferating hepatocytes	DNAJC14
5.45093939566824E-40	0.380368582484626	0.494	0.325	9.13468423926083E-36	Proliferating hepatocytes	TMEM68
5.56760436432469E-40	0.408575425135604	0.27	0.149	9.33019139373532E-36	Proliferating hepatocytes	SOWAHB
5.59539963403193E-40	0.517404912049746	0.276	0.155	9.37677070671071E-36	Proliferating hepatocytes	PMS1
5.71329747006868E-40	0.472723504810632	0.205	0.103	9.5743439003411E-36	Proliferating hepatocytes	UBE2N
5.76777609392756E-40	0.617926911193768	0.189	0.093	9.6656391782038E-36	Proliferating hepatocytes	POGLUT2
5.81221976938355E-40	0.40420384815214	0.322	0.188	9.74011788953296E-36	Proliferating hepatocytes	CLCN3
5.96191781388346E-40	0.404677293946614	0.412	0.26	9.99098187250591E-36	Proliferating hepatocytes	TMEM14C
5.9736039501558E-40	0.35237616419884	0.368	0.224	1.00105654996711E-35	Proliferating hepatocytes	SIPA1L1
6.08739220920847E-40	0.739218202193502	0.135	0.058	1.02012518641916E-35	Proliferating hepatocytes	MME
6.1950269765243E-40	0.727353415059063	0.139	0.06	1.03816262072594E-35	Proliferating hepatocytes	TMEM51
6.37124606252908E-40	0.842895917577628	0.119	0.048	1.06769341515862E-35	Proliferating hepatocytes	YDJC
6.38139319199728E-40	0.305542983186819	0.475	0.304	1.0693938711149E-35	Proliferating hepatocytes	CARS2
6.78547027580798E-40	0.36610449816544	0.371	0.224	1.1371091088199E-35	Proliferating hepatocytes	PPA2
6.87423580794736E-40	0.403303292694989	0.287	0.161	1.15198443669582E-35	Proliferating hepatocytes	AGA
7.08196751002577E-40	0.297818908947706	0.465	0.301	1.18679611533012E-35	Proliferating hepatocytes	RAF1
7.10073200955348E-40	0.367061222024271	0.643	0.469	1.18994067016097E-35	Proliferating hepatocytes	LAMTOR4
7.24724400532067E-40	0.311767850379749	0.519	0.341	1.21449315041164E-35	Proliferating hepatocytes	GMDS
7.44114115878751E-40	0.404585991241285	0.364	0.219	1.24698643538961E-35	Proliferating hepatocytes	C14orf119
7.47569999775989E-40	0.34016772362043	0.298	0.169	1.2527778056246E-35	Proliferating hepatocytes	TAF4

7.77136993441666E-40	0.290549391322495	0.52	0.346	1.30232617360954E-35	Proliferating hepatocytes	IMPA1
7.97156730250359E-40	0.611527574815979	0.201	0.101	1.33587524855355E-35	Proliferating hepatocytes	SGMS2
8.22906925660526E-40	0.310482359120142	0.458	0.29	1.37902742602191E-35	Proliferating hepatocytes	BTBD2
8.84557095960433E-40	0.254026630965095	0.363	0.216	1.48234078141049E-35	Proliferating hepatocytes	HTT
8.8535964884572E-40	0.254916372654272	0.382	0.232	1.48368569953566E-35	Proliferating hepatocytes	TYK2
8.94295078041642E-40	0.472370484475841	0.226	0.119	1.49865969178218E-35	Proliferating hepatocytes	BBX
9.03663991612301E-40	0.484595601947562	0.227	0.118	1.51436011714389E-35	Proliferating hepatocytes	WASHC3
9.40665457471141E-40	0.380833379813692	0.389	0.24	1.57636717363014E-35	Proliferating hepatocytes	RPRD1A
9.75413543573983E-40	0.36118221156109	0.3	0.17	1.63459801632128E-35	Proliferating hepatocytes	RBPJ
9.89932714267494E-40	0.498068520297754	0.306	0.177	1.65892924256947E-35	Proliferating hepatocytes	PTPRA
9.95981818758331E-40	0.443685717597005	0.253	0.138	1.66906633187521E-35	Proliferating hepatocytes	RHOG
1.00083965334419E-39	0.260343696872651	0.658	0.456	1.6772070910742E-35	Proliferating hepatocytes	AFF1
1.01588819216011E-39	0.306582118686142	0.329	0.194	1.70242543242191E-35	Proliferating hepatocytes	ZFYVE16
1.02195446570434E-39	0.483824033484474	0.269	0.149	1.71259129362733E-35	Proliferating hepatocytes	TDRD7
1.04031836794376E-39	0.376879768014658	0.183	0.088	1.74336552100015E-35	Proliferating hepatocytes	LRP4
1.09979431848683E-39	0.585948000638724	0.164	0.077	1.84303531892023E-35	Proliferating hepatocytes	TRIM37
1.10283176146013E-39	0.348777887865553	0.388	0.236	1.84812546585489E-35	Proliferating hepatocytes	DOCK9
1.11217845670318E-39	0.36278460461539	0.292	0.165	1.86378865774319E-35	Proliferating hepatocytes	ACTR3
1.15571171886465E-39	0.266563228885723	0.373	0.225	1.93674169847338E-35	Proliferating hepatocytes	MYO9B
1.18661671994067E-39	0.378145646531705	0.347	0.207	1.98853229927658E-35	Proliferating hepatocytes	CEBPZOS
1.20006478773089E-39	0.373001387394558	0.383	0.235	2.01106857127943E-35	Proliferating hepatocytes	FAM168B
1.22161543067407E-39	0.462617115566008	0.271	0.149	2.0471831387236E-35	Proliferating hepatocytes	SLC36A1
1.23528368240913E-39	0.589057744212949	0.188	0.093	2.07008839498121E-35	Proliferating hepatocytes	BMPR1A
1.25700801388786E-39	0.376484892360402	0.363	0.22	2.10649402967328E-35	Proliferating hepatocytes	CHCHD7
1.2637769851426E-39	0.32550276154872	0.381	0.231	2.11783747170196E-35	Proliferating hepatocytes	PHF12
1.29577446195241E-39	0.306260581604155	0.44	0.279	2.17145884333985E-35	Proliferating hepatocytes	TMUB1
1.37955732612493E-39	0.566995286379058	0.158	0.072	2.31186216712015E-35	Proliferating hepatocytes	ORC3
1.38680241548839E-39	0.32150442850974	0.289	0.162	2.32400348787544E-35	Proliferating hepatocytes	PLEKHA3
1.38931144375011E-39	0.272573817841852	0.316	0.182	2.32820811743643E-35	Proliferating hepatocytes	USPL1
1.40641103364746E-39	0.405665242056122	0.45	0.292	2.35686361018642E-35	Proliferating hepatocytes	TRIO
1.46723426072312E-39	0.365078186055306	0.562	0.385	2.4587911741198E-35	Proliferating hepatocytes	HNRNPK
1.48899881256097E-39	0.267372318114632	0.423	0.263	2.49526421008967E-35	Proliferating hepatocytes	VHL
1.50431906190321E-39	0.381458245478305	0.451	0.29	2.5209378839374E-35	Proliferating hepatocytes	MT-ND6
1.53499015009516E-39	0.530260651905418	0.131	0.055	2.57233649352946E-35	Proliferating hepatocytes	ZNF143
1.5365134898162E-39	0.349602134592729	0.347	0.205	2.57488930623399E-35	Proliferating hepatocytes	LYSMD3
1.56876643667826E-39	0.741060076745988	0.151	0.068	2.62893879458542E-35	Proliferating hepatocytes	KIF21B
1.64818216614542E-39	0.565356014909923	0.149	0.067	2.7620236740265E-35	Proliferating hepatocytes	BTBD10
1.67093738819569E-39	0.343435664687506	0.406	0.251	2.80015687513834E-35	Proliferating hepatocytes	GSK3B
2.06677210870834E-39	0.287370167521724	0.452	0.286	3.46349669977344E-35	Proliferating hepatocytes	PDLIM5
2.06908629595169E-39	0.354140360789822	0.475	0.308	3.46737481475585E-35	Proliferating hepatocytes	DHX9
2.08572223486194E-39	0.711913935671943	0.143	0.063	3.49525332118164E-35	Proliferating hepatocytes	ZNF620
2.34684372553385E-39	0.363528108264874	0.448	0.288	3.93284071524962E-35	Proliferating hepatocytes	COQ8A
2.40099419043622E-39	0.421596670478749	0.288	0.163	4.02358606433301E-35	Proliferating hepatocytes	NOM1
2.46664754260758E-39	0.862351464426114	0.121	0.05	4.13360795190178E-35	Proliferating hepatocytes	PRSS12
2.50945368630297E-39	0.38466140168726	0.305	0.174	4.20534248750652E-35	Proliferating hepatocytes	PM20D2
2.59035600426939E-39	0.41933549099762	0.184	0.089	4.34091859195465E-35	Proliferating hepatocytes	TRAF3IP1
2.59104483803659E-39	0.55767698442162	0.132	0.056	4.34207293958171E-35	Proliferating hepatocytes	NTAN1
2.61776180708925E-39	0.329710418290335	0.3	0.171	4.38684523632017E-35	Proliferating hepatocytes	DEPRECATEI
2.61909905646265E-39	0.356827246626641	0.436	0.275	4.38908619882011E-35	Proliferating hepatocytes	FAM13A
2.81927389666998E-39	0.81049968888991	0.148	0.067	4.72453919603955E-35	Proliferating hepatocytes	AKAP6
2.82106690690898E-39	0.419871847180881	0.295	0.169	4.72754392259806E-35	Proliferating hepatocytes	NRAS
2.84134480739573E-39	0.383712009624481	0.208	0.106	4.76152562823376E-35	Proliferating hepatocytes	AMD 1.00
2.85231011855149E-39	0.335117352099265	0.345	0.205	4.77990129666858E-35	Proliferating hepatocytes	CSNK2B
3.01446790207198E-39	0.355649143719082	0.368	0.224	5.05164531029223E-35	Proliferating hepatocytes	MED29
3.16381040752115E-39	0.429319125692735	0.359	0.218	5.30191348092394E-35	Proliferating hepatocytes	ZCRB1
3.17282963780757E-39	0.292740316083249	0.477	0.309	5.31702790703792E-35	Proliferating hepatocytes	YBX1
3.23329835692155E-39	0.452931074242374	0.306	0.177	5.41836138652913E-35	Proliferating hepatocytes	RBX1
3.42243388240903E-39	0.297875477968753	0.41	0.254	5.73531470014105E-35	Proliferating hepatocytes	PI4KA
3.58249242072892E-39	0.34871066475216	0.424	0.268	6.00354079865752E-35	Proliferating hepatocytes	RFC1
3.61654446248661E-39	0.30500912086372	0.447	0.286	6.06060521023506E-35	Proliferating hepatocytes	FARSA

3.80039076328922E-39	0.407759456632169	0.321	0.187	6.36869484112008E-35	Proliferating hepatocytes	STXBP3
3.80626666439657E-39	0.558172930088514	0.174	0.084	6.37854167619577E-35	Proliferating hepatocytes	ZNF574
3.81514791817712E-39	0.397216636851361	0.235	0.125	6.39342488128122E-35	Proliferating hepatocytes	TMEM185B
3.95765605187606E-39	0.329547761401604	0.322	0.187	6.6322400117339E-35	Proliferating hepatocytes	DHRSX
4.00042991385346E-39	0.302565255065145	0.721	0.528	6.70392044963563E-35	Proliferating hepatocytes	PRKACA
4.23914594174201E-39	0.389231987180113	0.242	0.131	7.10396076917126E-35	Proliferating hepatocytes	CDK2AP1
4.25991690507168E-39	0.360043850893874	0.403	0.251	7.13876874951912E-35	Proliferating hepatocytes	GNAI3
4.40935598235161E-39	0.25628624417767	0.237	0.126	7.38919875522482E-35	Proliferating hepatocytes	GCA
4.53427880769486E-39	0.27209127389962	0.381	0.233	7.59854442593505E-35	Proliferating hepatocytes	NELFA
4.90341713165641E-39	0.333161732442092	0.476	0.308	8.21714642922981E-35	Proliferating hepatocytes	PPP4R3B
4.92485462700838E-39	0.669444639392131	0.207	0.106	8.25307138394065E-35	Proliferating hepatocytes	NIPAL3
4.98901436127312E-39	0.319777111634627	0.305	0.176	8.3605902666215E-35	Proliferating hepatocytes	LEO1
5.15479635173084E-39	0.41921823095576	0.372	0.226	8.63840772623054E-35	Proliferating hepatocytes	DGUOK
5.20147158464614E-39	0.28853822488663	0.315	0.182	8.71662608155E-35	Proliferating hepatocytes	HDAC3
5.31685267404708E-39	0.317533826680775	0.256	0.14	8.9099817111681E-35	Proliferating hepatocytes	UHRF1BP1L
5.36836377772774E-39	0.808462538982293	0.166	0.078	8.99630401871614E-35	Proliferating hepatocytes	B3GNT5
5.58263504399206E-39	0.291353449697489	0.481	0.312	9.35537980672189E-35	Proliferating hepatocytes	NUDCD3
5.58790125445842E-39	0.411537408080155	0.312	0.181	9.36420492222142E-35	Proliferating hepatocytes	UCK2
5.8325116732694E-39	0.70881830542547	0.162	0.076	9.77412306206486E-35	Proliferating hepatocytes	ACER3
5.87361886146656E-39	0.271230277852673	0.509	0.334	9.84301048804567E-35	Proliferating hepatocytes	FAM177A1
6.03874396684464E-39	0.40795996524605	0.389	0.241	1.01197271396382E-34	Proliferating hepatocytes	NBN
6.08571605933862E-39	0.403172542195128	0.321	0.188	1.01984429722397E-34	Proliferating hepatocytes	SMARCE1
6.16016807222802E-39	0.452114370418897	0.242	0.13	1.03232096554397E-34	Proliferating hepatocytes	CSNK2A2
6.23570241969443E-39	0.516597493030035	0.313	0.182	1.04497901149239E-34	Proliferating hepatocytes	FAM98B
6.24213891718361E-39	0.575348354677164	0.173	0.083	1.04605763974163E-34	Proliferating hepatocytes	TRAK2
6.58365752063313E-39	0.473463958222174	0.228	0.121	1.1032893273077E-34	Proliferating hepatocytes	ARL 2.00
6.93235254748736E-39	0.356529805545182	0.367	0.224	1.16172363990793E-34	Proliferating hepatocytes	MCRS1
7.21670380739589E-39	0.370798241090841	0.478	0.31	1.2093752240434E-34	Proliferating hepatocytes	FBXL17
7.48396378810077E-39	0.449686934824536	0.328	0.193	1.25416265160993E-34	Proliferating hepatocytes	TMEM98
7.67580391299711E-39	0.269274687211692	0.41	0.254	1.28631121974006E-34	Proliferating hepatocytes	RCC1L
7.99588905192984E-39	0.377539274430065	0.409	0.255	1.3399510873224E-34	Proliferating hepatocytes	GLB1
8.08846254472228E-39	0.311841708919162	0.548	0.374	1.35546455324456E-34	Proliferating hepatocytes	PER1
8.48687565132386E-39	0.266984221733741	0.279	0.156	1.42223062164885E-34	Proliferating hepatocytes	REST
8.49839073880435E-39	0.540537491342254	0.241	0.13	1.42416032000883E-34	Proliferating hepatocytes	SLC39A8
8.88441369792067E-39	0.419845926650807	0.35	0.21	1.48885004749755E-34	Proliferating hepatocytes	CHMP5
9.14680873734899E-39	0.534450033355668	0.186	0.092	1.53282220820494E-34	Proliferating hepatocytes	WDR1
9.28330021283636E-39	0.522143023520606	0.187	0.092	1.55569544966712E-34	Proliferating hepatocytes	MANSC1
9.43981618166861E-39	0.277672366290286	0.389	0.238	1.58192439572403E-34	Proliferating hepatocytes	COLGALT1
9.95058187080566E-39	0.307931172435276	0.235	0.125	1.66751850990961E-34	Proliferating hepatocytes	SHOC2
1.02241675298873E-38	0.284596362052062	0.379	0.233	1.71336599465852E-34	Proliferating hepatocytes	KIAA2013
1.04463658083595E-38	0.337616082441019	0.177	0.085	1.75060198216489E-34	Proliferating hepatocytes	ABHD17B
1.18715950116906E-38	0.275660639218182	0.203	0.102	1.9894418920591E-34	Proliferating hepatocytes	JAK2
1.28284554811498E-38	0.278752509495784	0.341	0.202	2.14979256953108E-34	Proliferating hepatocytes	SYF2
1.28297800251842E-38	0.359323191167774	0.301	0.174	2.15001453662037E-34	Proliferating hepatocytes	KDM5A
1.35394427892499E-38	0.285506793910736	0.35	0.208	2.26893982262249E-34	Proliferating hepatocytes	DEPRECATEI
1.39334156530931E-38	0.406509869760315	0.207	0.106	2.33496179514534E-34	Proliferating hepatocytes	RRAGC
1.40247861197617E-38	0.476002257513636	0.201	0.102	2.35027365794966E-34	Proliferating hepatocytes	RALB
1.50265553593545E-38	0.384452307004324	0.326	0.193	2.51815014712063E-34	Proliferating hepatocytes	ANKRD9
1.58170280048292E-38	0.398058872932376	0.289	0.165	2.65061755304929E-34	Proliferating hepatocytes	SIRPA
1.59470183937878E-38	0.367935274650592	0.439	0.283	2.67240134243097E-34	Proliferating hepatocytes	RRP36
1.5971420840225E-38	0.349861732992378	0.428	0.273	2.67649070440491E-34	Proliferating hepatocytes	FBXO42
1.69589100443678E-38	0.31773751014428	0.516	0.346	2.84197414523516E-34	Proliferating hepatocytes	TMEM97
1.77632077427285E-38	0.306137660158491	0.467	0.301	2.97675835352645E-34	Proliferating hepatocytes	PPP4R3A
1.80721197399484E-38	0.406891100378933	0.369	0.224	3.02852582602055E-34	Proliferating hepatocytes	PHLPP2
1.87962975286312E-38	0.388356242245131	0.288	0.163	3.14988353984801E-34	Proliferating hepatocytes	SUDS3
1.88249875379623E-38	0.402028500092906	0.268	0.151	3.15469141161172E-34	Proliferating hepatocytes	GPC1
1.88978068099354E-38	0.33661044660678	0.382	0.234	3.16689446520897E-34	Proliferating hepatocytes	CHMP2B
1.91347295749523E-38	0.338366855576447	0.444	0.282	3.20659798217051E-34	Proliferating hepatocytes	PSMB2
1.9691825043731E-38	0.451472263056042	0.311	0.182	3.29995604082845E-34	Proliferating hepatocytes	SMC5
2.00547298400036E-38	0.463032319393958	0.3	0.174	3.36077162658781E-34	Proliferating hepatocytes	ASAP2

2.07321173017783E-38	0.274106478625672	0.344	0.205	3.474288217432E-34	Proliferating hepatocytes	SNRNP25
2.10668878854365E-38	0.405386691973077	0.354	0.215	3.53038907184144E-34	Proliferating hepatocytes	REEP3
2.10992158959405E-38	0.317742616680124	0.278	0.156	3.53580659984171E-34	Proliferating hepatocytes	EPHB4
2.12092909558475E-38	1.06774442980337	0.121	0.051	3.55425297838092E-34	Proliferating hepatocytes	TAF9B
2.19946621958619E-38	0.537865285642689	0.279	0.158	3.68586549078254E-34	Proliferating hepatocytes	ZFX
2.29558928757504E-38	0.371077589431202	0.268	0.149	3.84694852811826E-34	Proliferating hepatocytes	FAM200B
2.398102660418E-38	0.264847191624841	0.47	0.304	4.01874043832849E-34	Proliferating hepatocytes	ZFR
2.48826116059308E-38	0.353460832703771	0.386	0.238	4.16982805292188E-34	Proliferating hepatocytes	AP1G1
2.50321350725507E-38	0.388873715650021	0.211	0.109	4.19488519545804E-34	Proliferating hepatocytes	PFDN1
2.59647824772185E-38	0.55162726421781	0.251	0.138	4.35117824753228E-34	Proliferating hepatocytes	SDHAF3
2.59805774504814E-38	0.322098711094759	0.372	0.226	4.35382516915168E-34	Proliferating hepatocytes	C8orf33
2.61193052191498E-38	0.300027687154258	0.711	0.523	4.37707316862513E-34	Proliferating hepatocytes	ARF1
3.15459506814323E-38	0.252760831675883	0.308	0.177	5.28647041519442E-34	Proliferating hepatocytes	CAMTA1
3.17205524117256E-38	0.252048658028784	0.372	0.228	5.31573017315697E-34	Proliferating hepatocytes	CD177
3.7350917588804E-38	0.479621855022927	0.32	0.189	6.25926676953177E-34	Proliferating hepatocytes	SNRPB2
3.75901078331064E-38	0.553801840441214	0.198	0.1	6.29935027067197E-34	Proliferating hepatocytes	DOLK
3.79349168983254E-38	0.446106878499539	0.355	0.217	6.35713337382138E-34	Proliferating hepatocytes	AIMP2
3.90437319678144E-38	0.420978118308204	0.353	0.214	6.54294860316635E-34	Proliferating hepatocytes	ARL8B
3.90807771026684E-38	0.377599598450029	0.443	0.285	6.54915662686517E-34	Proliferating hepatocytes	INAVA
4.00650497217865E-38	0.371594126195533	0.403	0.251	6.71410103237698E-34	Proliferating hepatocytes	DCTD
4.15849389607467E-38	0.91235075377839	0.125	0.053	6.96880407104193E-34	Proliferating hepatocytes	SNX11
4.18201562260295E-38	0.386576126061182	0.305	0.175	7.00822178035803E-34	Proliferating hepatocytes	PSMG4
4.18764036116081E-38	0.28908918050843	0.647	0.465	7.01764771723329E-34	Proliferating hepatocytes	GSDMD
4.26791785921265E-38	0.318721608890226	0.562	0.382	7.15217674846856E-34	Proliferating hepatocytes	PSMD3
4.35396424518264E-38	0.812835826512112	0.1	0.038	7.29637328207706E-34	Proliferating hepatocytes	CEP57L1
4.57115881152295E-38	0.273953959009385	0.525	0.352	7.66034793635016E-34	Proliferating hepatocytes	TTC39C
4.91273918041125E-38	0.37272126464494	0.257	0.142	8.23276831853317E-34	Proliferating hepatocytes	NUBP1
4.95097691906076E-38	0.517204581447821	0.149	0.068	8.29684712096202E-34	Proliferating hepatocytes	EXO5
4.980698002105E-38	0.343694851363936	0.424	0.27	8.34665371192755E-34	Proliferating hepatocytes	SAMHD1
5.02425168160021E-38	0.298287210783193	0.529	0.356	8.41964096802563E-34	Proliferating hepatocytes	DLG1
5.30889890295416E-38	0.324718222624853	0.633	0.446	8.89665278157058E-34	Proliferating hepatocytes	DSTN
5.45390262996201E-38	0.389252162224729	0.229	0.122	9.13965002729034E-34	Proliferating hepatocytes	ZBED3
5.5400248886265E-38	0.280782402712928	0.342	0.204	9.2839737083603E-34	Proliferating hepatocytes	FAM168A
5.57082284603189E-38	0.360700173368904	0.326	0.193	9.33558492538024E-34	Proliferating hepatocytes	TRIM56
5.60867218106427E-38	0.302180010211798	0.415	0.261	9.3990128410275E-34	Proliferating hepatocytes	ABHD16A
5.61795457827477E-38	0.463949502937998	0.225	0.118	9.41456828227286E-34	Proliferating hepatocytes	ACTR6
5.76664984148679E-38	0.329457256868277	0.502	0.334	9.66375180436356E-34	Proliferating hepatocytes	CD164
5.91398482430836E-38	0.401255018674975	0.24	0.13	9.91065576857595E-34	Proliferating hepatocytes	ATXN1L
6.15954267473335E-38	0.431855496479335	0.362	0.22	1.03221616143182E-33	Proliferating hepatocytes	MED16
6.39773096065763E-38	0.298792766896273	0.289	0.165	1.07213175438701E-33	Proliferating hepatocytes	DEPRECATEI
6.47589531915096E-38	0.376131838257085	0.243	0.131	1.08523053758332E-33	Proliferating hepatocytes	HEATR1
7.02119785072656E-38	0.461162267595808	0.338	0.203	1.17661233582476E-33	Proliferating hepatocytes	RTF1
7.58585344975658E-38	0.372494038521179	0.529	0.358	1.27123732111021E-33	Proliferating hepatocytes	SLC35F5
7.67752144253968E-38	0.478022597264393	0.235	0.127	1.2865990433408E-33	Proliferating hepatocytes	ETNPPL
7.73976621597065E-38	0.58388776592292	0.165	0.079	1.29703002247236E-33	Proliferating hepatocytes	BOLA1
7.79022280345536E-38	0.297517258344444	0.318	0.185	1.30548553740305E-33	Proliferating hepatocytes	CADPS2
7.95904147626084E-38	0.317601130956254	0.384	0.234	1.33377617059179E-33	Proliferating hepatocytes	RETREG3
8.01919876805555E-38	0.440222674001544	0.314	0.186	1.34385732955075E-33	Proliferating hepatocytes	NIPA1
8.261361338669E-38	0.271023641173979	0.589	0.401	1.38443893313415E-33	Proliferating hepatocytes	DCAF11
8.38956842076797E-38	0.305838884784822	0.388	0.24	1.4059238759523E-33	Proliferating hepatocytes	RC3H1
8.46963604697501E-38	0.359346528302506	0.344	0.206	1.41934160875207E-33	Proliferating hepatocytes	KIAA1191
8.58474365833439E-38	0.463876985476115	0.225	0.12	1.43863134226368E-33	Proliferating hepatocytes	DEPRECATEI
8.79295129736634E-38	0.257446992298187	0.826	0.648	1.47352277841265E-33	Proliferating hepatocytes	NFKBIA
8.83724641209269E-38	0.285430581845744	0.357	0.216	1.48094575373849E-33	Proliferating hepatocytes	TCAIM
9.23301950094577E-38	0.335202363204933	0.229	0.121	1.54726940796849E-33	Proliferating hepatocytes	GKAP1
9.28153401741188E-38	0.346055178968064	0.258	0.142	1.55539947063788E-33	Proliferating hepatocytes	AP5S1
9.39087703968875E-38	0.488149918327376	0.179	0.088	1.57372317431104E-33	Proliferating hepatocytes	TTC32
9.53339015709826E-38	0.388635916557145	0.431	0.276	1.59760552252653E-33	Proliferating hepatocytes	LAPTM4A
9.54960005797741E-38	0.312339964673767	0.352	0.212	1.60032197771585E-33	Proliferating hepatocytes	UPF3A
9.78753277330199E-38	0.275498416303695	0.346	0.206	1.64019474214995E-33	Proliferating hepatocytes	SART1

**9.81808691015902E-38**	0.625478831640527	0.173	0.084	1.64531500440445E-33	Proliferating hepatocytes	ETV1
**9.8318743012714E-38**	0.425858490833799	0.308	0.18	1.64762549540706E-33	Proliferating hepatocytes	CSK
**9.98171748593268E-38**	0.303035456455411	0.738	0.575	1.6727362162926E-33	Proliferating hepatocytes	CREG1
**1.02830374559816E-37**	0.347598378231588	0.397	0.247	1.7232314168734E-33	Proliferating hepatocytes	USP54
**1.02845271532713E-37**	0.288995364218047	0.37	0.224	1.7234810603452E-33	Proliferating hepatocytes	UTP23
**1.10046664922089E-37**	0.528904582509703	0.22	0.116	1.84416201076437E-33	Proliferating hepatocytes	PPCDC
**1.14895092660696E-37**	0.294134085222144	0.369	0.227	1.92541196280794E-33	Proliferating hepatocytes	FBXO21
**1.22088801332981E-37**	0.276237000935399	0.442	0.282	2.04596413273809E-33	Proliferating hepatocytes	ZBTB7A
**1.25664450944642E-37**	0.283605200455615	0.381	0.235	2.1058848689303E-33	Proliferating hepatocytes	REV1
**1.26350435717193E-37**	0.319307908475761	0.664	0.5	2.11738060174873E-33	Proliferating hepatocytes	HSPH1
**1.27147164625592E-37**	0.68717738174432	0.192	0.098	2.13073218479568E-33	Proliferating hepatocytes	CDK5
**1.27903783490248E-37**	0.258951591286136	0.291	0.167	2.14341160372957E-33	Proliferating hepatocytes	SS18
**1.38687771105008E-37**	0.454943651991782	0.37	0.227	2.32412966817772E-33	Proliferating hepatocytes	EAPP
**1.43343353325601E-37**	0.311198798405148	0.375	0.231	2.40214791503042E-33	Proliferating hepatocytes	ATP13A1
**1.46042848182772E-37**	0.251196839473895	0.243	0.13	2.4473860498469E-33	Proliferating hepatocytes	ORC2
**1.46724042927602E-37**	0.376440496361287	0.254	0.14	2.45880151138076E-33	Proliferating hepatocytes	ZDHHC16
**1.50894899556958E-37**	0.422259673008289	0.299	0.175	2.52869672677551E-33	Proliferating hepatocytes	ARRB2
**1.51977683212384E-37**	0.79929101091338	0.191	0.097	2.54684201527312E-33	Proliferating hepatocytes	PPM1K
**1.54511777299771E-37**	0.281424913268679	0.534	0.364	2.58930836398956E-33	Proliferating hepatocytes	RHPN2
**1.59837578609235E-37**	0.316649662628125	0.625	0.442	2.67855814233356E-33	Proliferating hepatocytes	RAB5C
**1.60139140568211E-37**	0.692828616336421	0.169	0.082	2.68361171764208E-33	Proliferating hepatocytes	AREL1
**1.61069087864234E-37**	0.363718639984166	0.247	0.134	2.69919577442883E-33	Proliferating hepatocytes	VTA1
**1.63191120026382E-37**	0.485159696083239	0.222	0.117	2.73475678940211E-33	Proliferating hepatocytes	RPAP3
**1.64764434923696E-37**	0.30861122680971	0.444	0.283	2.7611224004513E-33	Proliferating hepatocytes	PLA2G12A
**1.72786670943756E-37**	0.565754228971261	0.177	0.087	2.89555903167546E-33	Proliferating hepatocytes	EIF2B2
**1.77772573582366E-37**	0.515779979351467	0.198	0.101	2.97911278809329E-33	Proliferating hepatocytes	SERTAD3
**1.80246289923293E-37**	0.323083720373741	0.427	0.268	3.02056732653454E-33	Proliferating hepatocytes	ZFYVE21
**1.85189226008159E-37**	0.353500189563352	0.488	0.317	3.10340104944473E-33	Proliferating hepatocytes	DEPRECATEI
**1.85955713315606E-37**	0.35344754680717	0.303	0.176	3.11624584374293E-33	Proliferating hepatocytes	MON1B
**1.86007409051605E-37**	0.420357793171882	0.208	0.107	3.1171121608868E-33	Proliferating hepatocytes	SLC25A17
**1.87199278025636E-37**	0.333320829142959	0.544	0.366	3.13708550115361E-33	Proliferating hepatocytes	WDR83OS
**1.88314734195304E-37**	0.297234194565869	0.338	0.203	3.1557783156449E-33	Proliferating hepatocytes	BAG2
**1.89235372440158E-37**	0.587559989467284	0.146	0.066	3.17120637135217E-33	Proliferating hepatocytes	ZNF653
**1.9049682819471E-37**	0.323084385620419	0.468	0.307	3.19234584688695E-33	Proliferating hepatocytes	STOML2
**1.96910743820963E-37**	0.467239910899614	0.324	0.194	3.29983024495169E-33	Proliferating hepatocytes	SMIM20
**2.03583219465188E-37**	0.261987801503628	0.334	0.2	3.41164759179762E-33	Proliferating hepatocytes	PTPN1
**2.05402585244855E-37**	0.350762996598609	0.246	0.134	3.44213652353328E-33	Proliferating hepatocytes	TWF1
**2.08059983965811E-37**	0.281318381152315	0.538	0.355	3.48666921129906E-33	Proliferating hepatocytes	KCNK1
**2.13131504126244E-37**	0.486761009653944	0.253	0.14	3.57165774614759E-33	Proliferating hepatocytes	NRXN3
**2.15677205582861E-37**	0.393822351812254	0.288	0.166	3.61431861115758E-33	Proliferating hepatocytes	HSBP1
**2.24008135979455E-37**	0.350888764433459	0.448	0.287	3.7539283427437E-33	Proliferating hepatocytes	TMEM129
**2.34977293423852E-37**	0.470296235199757	0.249	0.138	3.9377494831969E-33	Proliferating hepatocytes	GLTP
**2.51005880953152E-37**	0.395239687632782	0.234	0.125	4.20635655301292E-33	Proliferating hepatocytes	IL17RA
**2.52381125479692E-37**	0.337090335628902	0.214	0.111	4.22940290078868E-33	Proliferating hepatocytes	SLC39A6
**2.6324077088242E-37**	0.373410878841076	0.387	0.243	4.4113888384476E-33	Proliferating hepatocytes	SPIDR
**2.79931042756407E-37**	0.400988728681648	0.207	0.107	4.69108441451186E-33	Proliferating hepatocytes	FAM210A
**2.84011078554731E-37**	0.326640382351989	0.546	0.373	4.75945765442018E-33	Proliferating hepatocytes	MAGED2
**2.93836802881487E-37**	0.262162468161946	0.565	0.393	4.92411714268796E-33	Proliferating hepatocytes	KANK1
**3.01218443037561E-37**	0.403622990353488	0.266	0.148	5.04781866842344E-33	Proliferating hepatocytes	IGBP1
**3.03268835615487E-37**	0.450863525617721	0.22	0.117	5.08217914724434E-33	Proliferating hepatocytes	ATG4D
**3.0660414520463E-37**	0.331714165934096	0.334	0.201	5.13807226533919E-33	Proliferating hepatocytes	ATP6V1E1
**3.14770067369217E-37**	0.298244335452223	0.615	0.438	5.27491678897334E-33	Proliferating hepatocytes	SF3B5
**3.25139941735875E-37**	0.626690764481698	0.185	0.092	5.4486951436098E-33	Proliferating hepatocytes	TRPV4
**3.25346621412307E-37**	0.69166216731319	0.17	0.082	5.45215868162744E-33	Proliferating hepatocytes	C5orf30
**3.31416154384339E-37**	0.369311521189269	0.239	0.13	5.55387191517275E-33	Proliferating hepatocytes	ELAC2
**3.46859665939288E-37**	0.376875708775946	0.399	0.252	5.81267428181059E-33	Proliferating hepatocytes	AFG3L2
**3.51658588404848E-37**	0.325397509499973	0.298	0.172	5.89309462448843E-33	Proliferating hepatocytes	TP53I13
**3.5482546496265E-37**	0.324961018890616	0.219	0.115	5.94616514184409E-33	Proliferating hepatocytes	BRD9
**3.57755071273468E-37**	0.35013725956786	0.444	0.288	5.99525948440078E-33	Proliferating hepatocytes	ME1
**3.7891240040223E-37**	0.7313723268479	0.186	0.094	6.34981400594057E-33	Proliferating hepatocytes	GUCY2C

3.83334102047012E-37	0.315772818887026	0.281	0.161	6.42391288210383E-33	Proliferating hepatocytes	SH3BP5L
3.91861334675384E-37	0.311930481453416	0.373	0.23	6.56681224649009E-33	Proliferating hepatocytes	PRRC2B
4.01619100184318E-37	0.267693547011185	0.448	0.289	6.7303328808888E-33	Proliferating hepatocytes	IFI6
4.05795116098686E-37	0.548702231829468	0.195	0.099	6.80031455558178E-33	Proliferating hepatocytes	DEPRECATEI
4.27105136701357E-37	0.68671878254265	0.102	0.04	7.15742788084134E-33	Proliferating hepatocytes	ZNF138
4.35661542585682E-37	0.34024898037774	0.26	0.145	7.30081613065085E-33	Proliferating hepatocytes	ATG7
4.35887820937716E-37	0.413550157328519	0.25	0.137	7.30460810327424E-33	Proliferating hepatocytes	DEPRECATEI
4.40282355142275E-37	0.29088647646272	0.529	0.349	7.37825170747425E-33	Proliferating hepatocytes	GNAQ
4.42000584554002E-37	0.517168002045635	0.224	0.12	7.40704579595596E-33	Proliferating hepatocytes	CLASP2
4.42679259048586E-37	0.500783961089559	0.216	0.114	7.41841902313621E-33	Proliferating hepatocytes	ERAP2
4.59536446175977E-37	0.396640747557393	0.24	0.131	7.70091176501702E-33	Proliferating hepatocytes	L3MBTL2
4.63441259573584E-37	0.405602358632673	0.125	0.053	7.76634862793412E-33	Proliferating hepatocytes	DCLRE1A
4.87332850814055E-37	0.261684842298286	0.334	0.199	8.16672391394194E-33	Proliferating hepatocytes	OSTF1
5.21322837128321E-37	0.602573363450283	0.176	0.087	8.73632810459641E-33	Proliferating hepatocytes	RHPN1
5.29216145339507E-37	0.297886211845874	0.625	0.445	8.86860416359946E-33	Proliferating hepatocytes	PERP
5.32036768823598E-37	0.384478040811879	0.529	0.362	8.91587217194585E-33	Proliferating hepatocytes	UBE2R2
5.37661856383439E-37	0.337904000543075	0.55	0.374	9.01013738927368E-33	Proliferating hepatocytes	NDFIP2
5.46569532597818E-37	0.318052272884813	0.34	0.204	9.15941222727424E-33	Proliferating hepatocytes	SMAP1
5.56311111542872E-37	0.325664775717343	0.473	0.307	9.32266160723545E-33	Proliferating hepatocytes	SMYD2
5.94554505743274E-37	0.436577054879014	0.263	0.147	9.96354440724579E-33	Proliferating hepatocytes	TRAPPC11
6.41596270168187E-37	0.388027830653466	0.31	0.183	1.07518702954785E-32	Proliferating hepatocytes	ZNF275
6.41731716971295E-37	0.303531933994898	0.423	0.27	1.0754140113005E-32	Proliferating hepatocytes	RAB3IP
6.52497667670606E-37	0.347859192187581	0.456	0.297	1.0934555914824E-32	Proliferating hepatocytes	EMC3
6.70377361903221E-37	0.748418464160135	0.108	0.043	1.12341838307742E-32	Proliferating hepatocytes	ZNF789
6.72620368574556E-37	0.490612934124846	0.145	0.066	1.12717721365724E-32	Proliferating hepatocytes	PEX3
6.82439187489378E-37	0.498192453543478	0.224	0.12	1.1436315903947E-32	Proliferating hepatocytes	MTCL1
6.86375599415155E-37	0.409606866175704	0.212	0.111	1.15022822949992E-32	Proliferating hepatocytes	TRIM32
6.96975185676689E-37	0.595340703896897	0.218	0.115	1.167991016157E-32	Proliferating hepatocytes	LPCAT4
7.1486504197063E-37	0.53751831487972	0.192	0.097	1.19797083733438E-32	Proliferating hepatocytes	REXO1
7.28805725599148E-37	0.390950217286972	0.267	0.15	1.22133263495905E-32	Proliferating hepatocytes	PIAS1
7.33506671032007E-37	0.497071193683478	0.185	0.092	1.22921047931544E-32	Proliferating hepatocytes	DEPRECATEI
7.7019082020711E-37	0.341512697077402	0.347	0.21	1.29068577650308E-32	Proliferating hepatocytes	PLPP2
7.73359606021051E-37	0.459710648226987	0.252	0.14	1.29599602777008E-32	Proliferating hepatocytes	ZNF398
8.20112426949378E-37	0.544849701247753	0.262	0.149	1.37434440508177E-32	Proliferating hepatocytes	AP1AR
8.23802855723431E-37	0.408346079695134	0.229	0.123	1.38052882562133E-32	Proliferating hepatocytes	KAT5
8.50900487873981E-37	0.281378325342094	0.341	0.204	1.42593903757922E-32	Proliferating hepatocytes	ANKRD40
8.55332529534236E-37	0.422027840583581	0.192	0.098	1.43336625299347E-32	Proliferating hepatocytes	ZNF441
8.71728592640069E-37	0.383362408144098	0.353	0.214	1.46084277554623E-32	Proliferating hepatocytes	DGCR2
8.97336632923786E-37	0.31618721628825	0.327	0.196	1.50375672945368E-32	Proliferating hepatocytes	NUP214
9.10633313688123E-37	0.325498122859649	0.343	0.206	1.52603930707856E-32	Proliferating hepatocytes	RRAGA
9.3045092467318E-37	0.262912559231261	0.38	0.234	1.55924965956732E-32	Proliferating hepatocytes	ELMO1
9.51690836289594E-37	0.285248492067578	0.359	0.219	1.5948435034541E-32	Proliferating hepatocytes	PRPF38B
9.53008129217091E-37	0.286326928231027	0.187	0.093	1.597051022942E-32	Proliferating hepatocytes	CDK17
9.53821976161794E-37	0.323949437755979	0.454	0.292	1.59841486765193E-32	Proliferating hepatocytes	PDE4DIP
9.87474125394743E-37	0.266199066215373	0.427	0.27	1.65480913933651E-32	Proliferating hepatocytes	NCKIPSD
1.01009697342514E-36	0.253443336714181	0.476	0.309	1.69272050806584E-32	Proliferating hepatocytes	PPM1A
1.08358772534324E-36	0.402779208823209	0.262	0.147	1.8158763101302E-32	Proliferating hepatocytes	DEPRECATEI
1.09746658238295E-36	0.397346169602248	0.351	0.214	1.83913449875735E-32	Proliferating hepatocytes	COMMD6
1.10481199398563E-36	0.688083036810041	0.151	0.071	1.85144393952111E-32	Proliferating hepatocytes	DEPRECATEI
1.12012601353694E-36	0.370661796514267	0.552	0.383	1.87710717348521E-32	Proliferating hepatocytes	SYAP1
1.13659905405941E-36	0.332122277110111	0.273	0.154	1.90471269479276E-32	Proliferating hepatocytes	UBE2G1
1.15602366454906E-36	0.301525301419023	0.334	0.199	1.93726445705132E-32	Proliferating hepatocytes	RCAN1
1.17909119663493E-36	0.378178591690951	0.353	0.215	1.97592102732081E-32	Proliferating hepatocytes	C8orf76
1.18665229023868E-36	0.316738266319729	0.425	0.271	1.98859190798198E-32	Proliferating hepatocytes	SLC35A3
1.18708821508112E-36	0.480601229891122	0.151	0.07	1.98932243083294E-32	Proliferating hepatocytes	KIAA1586
1.19258443029777E-36	0.251986982161732	0.402	0.252	1.998532988293E-32	Proliferating hepatocytes	AKT2
1.19608142199673E-36	0.28424602425563	0.419	0.27	2.00439324698212E-32	Proliferating hepatocytes	KMT2D
1.22041956105331E-36	0.446987332976841	0.265	0.151	2.04517910041314E-32	Proliferating hepatocytes	MAGEF1
1.26543621300419E-36	0.456732429579441	0.21	0.11	2.12061800575242E-32	Proliferating hepatocytes	DBR1
1.27086087379842E-36	0.325658401359558	0.5	0.337	2.1297086523114E-32	Proliferating hepatocytes	NFIA

1.29856085314973E-36	0.298348185630931	0.269	0.152	2.17612827770832E-32	Proliferating hepatocytes	RNF20
1.32231502147582E-36	0.327586742926251	0.417	0.267	2.21593551298918E-32	Proliferating hepatocytes	C9orf78
1.33408392282651E-36	0.283513313728193	0.297	0.171	2.23565783787267E-32	Proliferating hepatocytes	PHAX
1.35814102624866E-36	0.492458403850486	0.172	0.084	2.27597273178751E-32	Proliferating hepatocytes	DEPRECATEI
1.42377067312512E-36	0.593571477509291	0.164	0.079	2.38595489402307E-32	Proliferating hepatocytes	CHST14
1.47322419447262E-36	0.372395414613669	0.206	0.107	2.46882910509721E-32	Proliferating hepatocytes	CACTIN
1.54182355473587E-36	0.374936093828315	0.172	0.084	2.58378791302638E-32	Proliferating hepatocytes	MTHFD1L
1.55656415449076E-36	0.713860686211673	0.121	0.052	2.60849021009561E-32	Proliferating hepatocytes	AEN
1.57224168538808E-36	0.259484428198893	0.455	0.294	2.63476261637335E-32	Proliferating hepatocytes	PIK3R2
1.62250547134951E-36	0.798532062896275	0.107	0.043	2.71899466888751E-32	Proliferating hepatocytes	BCL2
1.7198713736699E-36	0.390092893142963	0.42	0.27	2.88216044799602E-32	Proliferating hepatocytes	CWC15
1.7956325849097E-36	0.332337840057513	0.387	0.243	3.00912108579167E-32	Proliferating hepatocytes	MCMBP
1.83985592787072E-36	0.272134787734476	0.421	0.267	3.08323056392575E-32	Proliferating hepatocytes	DYM
1.87498655772775E-36	0.315925578126392	0.445	0.289	3.14210247344016E-32	Proliferating hepatocytes	ARCN1
1.91963258039305E-36	0.457198452238567	0.219	0.116	3.21692027822267E-32	Proliferating hepatocytes	ZNF576
1.9568716657926E-36	0.277884552823267	0.468	0.307	3.27932553753525E-32	Proliferating hepatocytes	ITPRID2
1.97270381026944E-36	0.326445893464719	0.263	0.147	3.30585704524953E-32	Proliferating hepatocytes	POM121
2.02109767899393E-36	0.432549920763082	0.177	0.088	3.38695549045802E-32	Proliferating hepatocytes	GLIS3
2.17054255001719E-36	0.331283572010786	0.469	0.309	3.6373952053188E-32	Proliferating hepatocytes	DNASE2
2.20697148257267E-36	0.438502498239264	0.288	0.166	3.69844281049528E-32	Proliferating hepatocytes	FRY
2.26448542167936E-36	0.368528577136425	0.281	0.161	3.79482466965027E-32	Proliferating hepatocytes	EHD4
2.31732062270863E-36	0.335269819528895	0.326	0.193	3.88336589953512E-32	Proliferating hepatocytes	MANBAL
2.36317414890442E-36	0.301592140315165	0.394	0.247	3.96020723873402E-32	Proliferating hepatocytes	CDC5L
2.37019295526494E-36	0.300403474012492	0.336	0.201	3.97196935443299E-32	Proliferating hepatocytes	ANKRD27
2.41936868093074E-36	0.30594922117829	0.413	0.259	4.05437803550374E-32	Proliferating hepatocytes	SNF8
2.54216890347203E-36	1.04032067169324	0.115	0.048	4.26016664843843E-32	Proliferating hepatocytes	SERPINE2
2.5747692947166E-36	0.450134524050184	0.252	0.14	4.31479838408607E-32	Proliferating hepatocytes	TMEM30A
2.60694200185955E-36	0.292749199558573	0.571	0.395	4.36871340671623E-32	Proliferating hepatocytes	PMVK
2.62157531852536E-36	0.254839963974741	0.231	0.124	4.3932359187848E-32	Proliferating hepatocytes	TESK1
2.77403230218509E-36	0.462946031004726	0.24	0.131	4.64872333200177E-32	Proliferating hepatocytes	PRKACB
2.80275071710223E-36	0.252966589019941	0.435	0.276	4.69684965171991E-32	Proliferating hepatocytes	NME1
2.99174362370184E-36	0.34165469662562	0.167	0.08	5.01356396459954E-32	Proliferating hepatocytes	LIMS1
3.4262498761221E-36	0.298016651180395	0.409	0.258	5.74170954240542E-32	Proliferating hepatocytes	FAM49B
3.69817409893698E-36	0.419312641732256	0.238	0.13	6.19740015499859E-32	Proliferating hepatocytes	POLE4
3.7060109998187E-36	0.352656590407954	0.339	0.206	6.21053323349617E-32	Proliferating hepatocytes	MAML1
3.75035460995962E-36	0.451177020427255	0.228	0.124	6.28484425537033E-32	Proliferating hepatocytes	ACD
3.79270011507788E-36	0.27394448859735	0.435	0.277	6.35580685284751E-32	Proliferating hepatocytes	VPS4A
3.8185200601815E-36	0.296949343273462	0.145	0.066	6.39907591685216E-32	Proliferating hepatocytes	CYP3A43
3.9411885570309E-36	0.316905480276395	0.201	0.104	6.60464378387239E-32	Proliferating hepatocytes	RASA2
4.18393646560784E-36	0.402174434781882	0.283	0.164	7.01144072906561E-32	Proliferating hepatocytes	CIZ1
4.2962132352207E-36	0.296758908972193	0.547	0.371	7.19959413958284E-32	Proliferating hepatocytes	DGAT1
4.49036961725207E-36	0.552648274759763	0.224	0.121	7.52496140459101E-32	Proliferating hepatocytes	VARS2
4.8398057047468E-36	0.500700712973622	0.19	0.097	8.11054640001469E-32	Proliferating hepatocytes	INTS12
4.88415621015797E-36	0.30907098555371	0.238	0.13	8.18486897698273E-32	Proliferating hepatocytes	OTUD3
5.49039782144305E-36	0.376406955415798	0.279	0.161	9.20080866917426E-32	Proliferating hepatocytes	DNAJB12
5.62358605925043E-36	0.356033158209445	0.183	0.091	9.42400551809187E-32	Proliferating hepatocytes	FASTKD5
5.69849609254539E-36	0.312864973731874	0.4	0.251	9.54953975188757E-32	Proliferating hepatocytes	PET100
5.81038362929935E-36	0.315780728052342	0.503	0.34	9.73704088597985E-32	Proliferating hepatocytes	DIAPH2
5.94709101830367E-36	0.472811847201205	0.316	0.19	9.96613512847329E-32	Proliferating hepatocytes	UTRN
6.26669182450776E-36	0.261773487881797	0.605	0.421	1.05017221595101E-31	Proliferating hepatocytes	LASP1
6.34721163244833E-36	0.298306039000798	0.452	0.292	1.06366572536569E-31	Proliferating hepatocytes	CHID1
6.35735779077612E-36	0.643578701562941	0.184	0.093	1.06536601857826E-31	Proliferating hepatocytes	ZBED8
6.77041735855318E-36	0.326115247790314	0.388	0.24	1.13458654094634E-31	Proliferating hepatocytes	SLC25A32
6.86478388004015E-36	0.274788997933131	0.404	0.254	1.15040048261713E-31	Proliferating hepatocytes	CERS6
6.87828872784582E-36	0.31977401872673	0.528	0.358	1.1526636250124E-31	Proliferating hepatocytes	COX16
7.34593952202447E-36	0.272648128597088	0.581	0.412	1.23103254510086E-31	Proliferating hepatocytes	SERINC3
7.62331711616805E-36	0.317127942864177	0.795	0.632	1.27751548232744E-31	Proliferating hepatocytes	GOLIM4
8.64234796660634E-36	0.254567014344213	0.464	0.304	1.44828467224389E-31	Proliferating hepatocytes	MAX
8.69328531245635E-36	0.420311277009015	0.279	0.162	1.45682075266144E-31	Proliferating hepatocytes	CHD8
8.80249968774396E-36	0.292551411221801	0.598	0.426	1.47512289767213E-31	Proliferating hepatocytes	DEPRECATEI

9.3003892387788E-36	0.272559727965453	0.604	0.424	1.55855922863455E-31	Proliferating hepatocytes	IL1R1
9.32602588846438E-36	0.441154554953507	0.197	0.102	1.56285541838886E-31	Proliferating hepatocytes	FAM126B
9.52090938663011E-36	0.400184388043047	0.184	0.093	1.59551399501147E-31	Proliferating hepatocytes	USP39
9.7603065059211E-36	0.458687027212514	0.229	0.125	1.63563216426226E-31	Proliferating hepatocytes	TSHZ1
1.03105318560243E-35	0.435017216249699	0.248	0.139	1.72783892843256E-31	Proliferating hepatocytes	GDE1
1.10337224783596E-35	0.266048087443148	0.435	0.276	1.84903121292351E-31	Proliferating hepatocytes	TOB2
1.11097768850292E-35	0.317657164857403	0.233	0.127	1.86177641039319E-31	Proliferating hepatocytes	NCBP3
1.1382161922618E-35	0.630514064991365	0.188	0.096	1.90742269499233E-31	Proliferating hepatocytes	ZNF689
1.15732121826573E-35	0.383011012437823	0.284	0.164	1.9394388975697E-31	Proliferating hepatocytes	FZD8
1.17423455962287E-35	0.477959385250287	0.215	0.114	1.96778227501601E-31	Proliferating hepatocytes	NEDD9
1.18578926881999E-35	0.383845999693774	0.2	0.104	1.98714565668853E-31	Proliferating hepatocytes	STK17A
1.21024912687868E-35	0.632537674777216	0.157	0.075	2.0281354868233E-31	Proliferating hepatocytes	RNF144A
1.24243526798696E-35	0.317047540034198	0.614	0.441	2.08207302209255E-31	Proliferating hepatocytes	TMEM33
1.28419319836209E-35	0.298689061209814	0.506	0.343	2.15205096181519E-31	Proliferating hepatocytes	MAPK6
1.33456493235699E-35	0.55020791726842	0.159	0.076	2.23646391364385E-31	Proliferating hepatocytes	TMEM80
1.37137490739625E-35	0.255602427545215	0.324	0.193	2.29815006981464E-31	Proliferating hepatocytes	PRPF38A
1.41203627666528E-35	0.390030266058484	0.238	0.131	2.36629039243567E-31	Proliferating hepatocytes	PCNX4
1.4822831765662E-35	0.489784688725883	0.239	0.132	2.48401014728964E-31	Proliferating hepatocytes	C4orf33
1.53243936233004E-35	0.42697803871054	0.179	0.09	2.56806188339267E-31	Proliferating hepatocytes	GFOD1
1.54817545895522E-35	0.384872454583914	0.206	0.108	2.59443243411716E-31	Proliferating hepatocytes	B3GALNT2
1.60147355925366E-35	0.34260039579027	0.235	0.129	2.68374939059728E-31	Proliferating hepatocytes	POLR3C
1.61758349172913E-35	0.569543025689693	0.2	0.105	2.71074641543967E-31	Proliferating hepatocytes	POP1
1.63175701910088E-35	0.329070548108213	0.369	0.228	2.73449841260926E-31	Proliferating hepatocytes	KIF3B
1.66766819359212E-35	0.370203705245227	0.27	0.154	2.79467835882168E-31	Proliferating hepatocytes	TBC1D13
1.68004789829764E-35	0.516214829938882	0.221	0.12	2.81542426796718E-31	Proliferating hepatocytes	SYT1
1.6853400662158E-35	0.293024446925356	0.435	0.282	2.82429288296443E-31	Proliferating hepatocytes	PARP14
1.73872482234892E-35	0.382634966766331	0.257	0.145	2.91375505729232E-31	Proliferating hepatocytes	ADPRHL2
1.82334340843975E-35	0.567576501508725	0.183	0.092	3.05555888386333E-31	Proliferating hepatocytes	MUL1
1.95545340783645E-35	0.333816879266225	0.254	0.142	3.27694882085233E-31	Proliferating hepatocytes	MFSD11
2.09936037430052E-35	0.308118034017982	0.424	0.27	3.5181081152528E-31	Proliferating hepatocytes	PYGL
2.13173302673888E-35	0.688666943664462	0.114	0.048	3.57235820620902E-31	Proliferating hepatocytes	ASTN2
2.13216386765802E-35	0.282477506326782	0.457	0.3	3.57308020942131E-31	Proliferating hepatocytes	GPR108
2.1794714613794E-35	0.417598099161569	0.148	0.069	3.6523582749796E-31	Proliferating hepatocytes	ASCC1
2.18766319587304E-35	0.291485607845218	0.247	0.137	3.66608598364404E-31	Proliferating hepatocytes	BLZF1
2.19438671635467E-35	0.466175770166285	0.259	0.146	3.67735325926716E-31	Proliferating hepatocytes	MTMR6
2.25651976325772E-35	0.266718499040997	0.454	0.293	3.78147581926729E-31	Proliferating hepatocytes	POLR2I
2.27673204315975E-35	0.29100909857969	0.421	0.267	3.81534755792712E-31	Proliferating hepatocytes	EPPK1
2.43175830451116E-35	0.572863121915921	0.149	0.07	4.0751405666998E-31	Proliferating hepatocytes	PPAT
2.49728067342846E-35	0.29931101803842	0.25	0.14	4.18494295253141E-31	Proliferating hepatocytes	SLC27A4
2.51710061433619E-35	0.250511182744986	0.274	0.156	4.21815720950458E-31	Proliferating hepatocytes	OGG1
2.54383196932849E-35	0.567793571074709	0.12	0.051	4.26295361420068E-31	Proliferating hepatocytes	RCE1
2.58466170846773E-35	0.278187364218795	0.355	0.217	4.33137609105022E-31	Proliferating hepatocytes	PIK3CB
2.60572867059591E-35	0.310242713342352	0.448	0.291	4.36668010618463E-31	Proliferating hepatocytes	TOR1AIP1
2.63671068000127E-35	0.279825586329047	0.319	0.191	4.41859975754612E-31	Proliferating hepatocytes	RPRD1B
2.72108262810057E-35	0.347078598384043	0.36	0.223	4.55999026817094E-31	Proliferating hepatocytes	VAMP3
2.92462209435334E-35	0.796113222482778	0.109	0.045	4.90108170571733E-31	Proliferating hepatocytes	CEP41
2.92956106660015E-35	0.392772991644334	0.299	0.176	4.90935843540853E-31	Proliferating hepatocytes	GUCY1A1
3.04012015481752E-35	0.338246513973941	0.516	0.348	5.0946333554432E-31	Proliferating hepatocytes	UBL3
3.17544490614727E-35	0.509259664822984	0.149	0.07	5.32141057372159E-31	Proliferating hepatocytes	ARHGEF28
3.26670428334997E-35	0.437440701562708	0.147	0.069	5.47434303803788E-31	Proliferating hepatocytes	KCTD7
3.32297219915451E-35	0.256382557317396	0.493	0.334	5.56863681134313E-31	Proliferating hepatocytes	UXT
3.32881253696841E-35	0.613806940238798	0.203	0.108	5.57842404945166E-31	Proliferating hepatocytes	F8
3.43576703662552E-35	0.376337745662863	0.218	0.117	5.75765839997705E-31	Proliferating hepatocytes	PRELID3B
3.48673621777032E-35	0.322568781710156	0.217	0.116	5.8430725537395E-31	Proliferating hepatocytes	PIP5K1C
3.53511599174707E-35	0.367634467040544	0.402	0.255	5.92414737896973E-31	Proliferating hepatocytes	LANCL1
3.57148096209562E-35	0.563214265778144	0.179	0.09	5.98508779627984E-31	Proliferating hepatocytes	KATNB1
3.68868312791943E-35	0.339751625849025	0.272	0.157	6.18149518576738E-31	Proliferating hepatocytes	YEATS2
3.98051744442291E-35	0.445676090797994	0.163	0.079	6.67055113336391E-31	Proliferating hepatocytes	JADE3
4.05106970240939E-35	0.294735033859411	0.188	0.096	6.78878260729765E-31	Proliferating hepatocytes	ZNF823
4.34503378788982E-35	0.545254980986687	0.171	0.085	7.28140762174576E-31	Proliferating hepatocytes	RRP7A

**4.3949953008831E-35**	0.455409173735857	0.223	0.122	7.3651331252199E-31	Proliferating hepatocytes	OSBPL11
**4.4510540367561E-35**	0.274034708053671	0.428	0.275	7.45907635479586E-31	Proliferating hepatocytes	CHMP4B
**4.47963071288895E-35**	0.45463266533423	0.227	0.124	7.5069651486593E-31	Proliferating hepatocytes	UIMC1
**4.53103300929489E-35**	0.693172741427194	0.162	0.08	7.59310511697637E-31	Proliferating hepatocytes	HERC6
**4.57360415814377E-35**	0.293190088729742	0.513	0.339	7.66444584821734E-31	Proliferating hepatocytes	ATP6V1A
**4.62960050539319E-35**	0.277644374393675	0.35	0.214	7.7582845269379E-31	Proliferating hepatocytes	COX7B
**4.68559525102418E-35**	0.267759497392398	0.461	0.302	7.85212052166632E-31	Proliferating hepatocytes	STXBP2
**4.69066771560716E-35**	0.315712183305692	0.406	0.258	7.86062095781449E-31	Proliferating hepatocytes	PSEN1
**4.76278745046658E-35**	0.878345232737226	0.141	0.066	7.9814792094919E-31	Proliferating hepatocytes	TMEM246
**4.90411463537667E-35**	0.442860466174993	0.251	0.142	8.21831530596423E-31	Proliferating hepatocytes	ZNF76
**4.93470315336368E-35**	0.386116684479794	0.285	0.165	8.26957554440685E-31	Proliferating hepatocytes	MNAT1
**4.95828186912031E-35**	0.286664411551058	0.327	0.198	8.30908875627181E-31	Proliferating hepatocytes	EIF5B
**5.46794159860633E-35**	0.591697582338298	0.111	0.046	9.16317653094449E-31	Proliferating hepatocytes	ZFAND4
**5.50508451180368E-35**	0.521133608617097	0.29	0.172	9.22542062488061E-31	Proliferating hepatocytes	THEM6
**5.74335341692785E-35**	0.25927984738713	0.319	0.189	9.62471165608769E-31	Proliferating hepatocytes	HSPA4
**5.76330597555677E-35**	0.346781600336276	0.275	0.159	9.65814815383803E-31	Proliferating hepatocytes	SYNJ2
**5.81721459697039E-35**	0.320328633168933	0.277	0.16	9.74848822160298E-31	Proliferating hepatocytes	YIPF2
**5.85862246356143E-35**	0.303718461902716	0.239	0.133	9.81787952443625E-31	Proliferating hepatocytes	NECAP2
**5.95402847579004E-35**	0.288127535566115	0.362	0.223	9.97776091972894E-31	Proliferating hepatocytes	ZYG11B
**6.29049382778696E-35**	0.301423287600499	0.503	0.338	1.05416095566054E-30	Proliferating hepatocytes	SPPL2A
**6.33701146126259E-35**	0.43801407297551	0.231	0.126	1.06195638067838E-30	Proliferating hepatocytes	GABPA
**6.550686735604E-35**	0.27039124879986	0.181	0.091	1.09776408315252E-30	Proliferating hepatocytes	MAP3K1
**6.79092576523661E-35**	0.364241881539616	0.253	0.143	1.13802333973835E-30	Proliferating hepatocytes	C3orf38
**7.4865794162177E-35**	0.300245964404261	0.176	0.088	1.25460097856976E-30	Proliferating hepatocytes	EIPR1
**7.49410030866013E-35**	1.09221457826821	0.103	0.042	1.25586132972527E-30	Proliferating hepatocytes	F2R
**7.55677457842515E-35**	0.29998919496903	0.36	0.223	1.26636428385249E-30	Proliferating hepatocytes	ANO6
**7.6501872007262E-35**	0.304843129684527	0.198	0.103	1.2820183710977E-30	Proliferating hepatocytes	RAB11FIP2
**7.67867189684278E-35**	0.294946851520279	0.348	0.213	1.28679183647291E-30	Proliferating hepatocytes	ISY1
**7.88978058549586E-35**	0.461025946213207	0.291	0.171	1.3221694305174E-30	Proliferating hepatocytes	BAK1
**7.93800910736306E-35**	0.296122878760984	0.232	0.127	1.3302515662119E-30	Proliferating hepatocytes	CLASP1
**8.12328371761392E-35**	0.367932498756465	0.322	0.193	1.36129988539774E-30	Proliferating hepatocytes	TMEM230
**8.43932814253109E-35**	0.259716183674006	0.33	0.198	1.41426261012536E-30	Proliferating hepatocytes	SLC30A5
**8.48641980589958E-35**	0.255948195439253	0.409	0.259	1.42215423107265E-30	Proliferating hepatocytes	SRP72
**8.55404284762048E-35**	0.299476856768168	0.398	0.253	1.43348650040424E-30	Proliferating hepatocytes	MRFAP1L1
**8.8208944756667E-35**	0.329877787109029	0.327	0.197	1.47820549623222E-30	Proliferating hepatocytes	STAT5B
**8.99164887952782E-35**	0.370603426547062	0.278	0.161	1.50682051923127E-30	Proliferating hepatocytes	NOP9
**9.14390984219559E-35**	0.300772325243236	0.392	0.248	1.53233641135514E-30	Proliferating hepatocytes	SLC37A1
**9.39798551244167E-35**	0.286778197887137	0.47	0.313	1.57491441217498E-30	Proliferating hepatocytes	BPTF
**9.49941433417953E-35**	0.294906464103687	0.317	0.19	1.59191185412181E-30	Proliferating hepatocytes	CPPED1
**9.57265660762873E-35**	0.328663255214122	0.287	0.168	1.60418579430642E-30	Proliferating hepatocytes	POLR2G
**9.6457051369792E-35**	0.379819929276771	0.148	0.07	1.61642726685497E-30	Proliferating hepatocytes	BTN2A1
**9.87521261887728E-35**	0.413471630245326	0.252	0.142	1.65488813067146E-30	Proliferating hepatocytes	MED22
**9.99320369225316E-35**	0.276206408178873	0.152	0.072	1.67466107474778E-30	Proliferating hepatocytes	DHX40
**1.00954250969277E-34**	0.528282648449228	0.166	0.081	1.69179133774314E-30	Proliferating hepatocytes	TIMM29
**1.01541997132389E-34**	0.295356767534165	0.358	0.221	1.70164078794457E-30	Proliferating hepatocytes	GOSR1
**1.02605575866947E-34**	0.899746217909077	0.159	0.078	1.71946424037831E-30	Proliferating hepatocytes	SMIM31
**1.03643475514013E-34**	0.381257244842311	0.288	0.169	1.73685736266384E-30	Proliferating hepatocytes	POU5F1
**1.04465419700849E-34**	0.469451122575878	0.256	0.146	1.75063150334683E-30	Proliferating hepatocytes	MOB3B
**1.06463130933212E-34**	1.10462257073303	0.103	0.042	1.78410914817877E-30	Proliferating hepatocytes	IL20RA
**1.06621061177965E-34**	0.557616661529952	0.164	0.081	1.78675574322034E-30	Proliferating hepatocytes	CRIP3
**1.11568129633122E-34**	0.25277745728032	0.423	0.272	1.86965871639186E-30	Proliferating hepatocytes	TAX1BP1
**1.11910472527631E-34**	0.319874451352605	0.248	0.139	1.87539569861803E-30	Proliferating hepatocytes	SMIM10L1
**1.13673534242527E-34**	0.338235822233872	0.363	0.227	1.90494108683626E-30	Proliferating hepatocytes	ATXN1
**1.17630736209929E-34**	0.274875225835521	0.354	0.217	1.97125587740598E-30	Proliferating hepatocytes	TBCE.1
**1.20429214376607E-34**	0.272620258902948	0.324	0.194	2.01815277452319E-30	Proliferating hepatocytes	MPZL1
**1.22899148937526E-34**	0.261270759486251	0.412	0.263	2.05954393789505E-30	Proliferating hepatocytes	KCTD20
**1.24387395068102E-34**	0.251528389703572	0.465	0.305	2.08448396655125E-30	Proliferating hepatocytes	PCID2
**1.25838008614527E-34**	0.265990878391894	0.559	0.389	2.10879334836224E-30	Proliferating hepatocytes	CCDC47
**1.29346489071632E-34**	0.552219932406024	0.144	0.067	2.16758846386241E-30	Proliferating hepatocytes	SENP3
**1.32264098997035E-34**	0.33911790777058	0.324	0.195	2.21648177099231E-30	Proliferating hepatocytes	ELOA

1.39573730504727E-34	0.606225867240916	0.176	0.088	2.33897657579822E-30	Proliferating hepatocytes	RUNDC1
1.39913470690166E-34	0.365567746800601	0.285	0.168	2.3446699418258E-30	Proliferating hepatocytes	ATP6V1D
1.41153257458111E-34	0.258754792484149	0.69	0.502	2.36544628848303E-30	Proliferating hepatocytes	WNK1
1.50804821176039E-34	0.305915787927227	0.393	0.249	2.52718719326806E-30	Proliferating hepatocytes	PURA
1.5130876394598E-34	0.580168194643402	0.108	0.044	2.53563226620674E-30	Proliferating hepatocytes	CFAP44
1.5260849261504E-34	0.414551299300549	0.382	0.243	2.55741311924283E-30	Proliferating hepatocytes	EBAG9
1.54208666665433E-34	0.81808381684184	0.129	0.058	2.58422883597932E-30	Proliferating hepatocytes	ZFAT
1.54840341776692E-34	0.250634440674497	0.339	0.207	2.59481444749381E-30	Proliferating hepatocytes	EPS15L1
1.56708216698636E-34	0.516018803597273	0.208	0.111	2.62611629543574E-30	Proliferating hepatocytes	MED9
1.61994599731685E-34	0.316507912074993	0.258	0.146	2.71470550230357E-30	Proliferating hepatocytes	THOC1
1.66169054677333E-34	0.469184679417595	0.133	0.06	2.78466101828274E-30	Proliferating hepatocytes	BTN3A2
1.6746977293391E-34	0.352977616780654	0.329	0.199	2.80645845482647E-30	Proliferating hepatocytes	FBXO28
1.70760472981159E-34	0.251014715675602	0.647	0.474	2.86160400621827E-30	Proliferating hepatocytes	ADAR
1.75583619106701E-34	0.266087720549036	0.634	0.467	2.94243028899009E-30	Proliferating hepatocytes	SHMT1
1.79409286666293E-34	0.490470911657343	0.25	0.141	3.00654082595374E-30	Proliferating hepatocytes	ZNF346
1.89903793190078E-34	0.340460830623758	0.29	0.171	3.18240776627934E-30	Proliferating hepatocytes	NF2
1.99544312860514E-34	0.270328579405999	0.577	0.404	3.3439635949165E-30	Proliferating hepatocytes	DBI
1.99981091712146E-34	0.367336261333574	0.145	0.068	3.35128313491215E-30	Proliferating hepatocytes	ZBTB40
2.11690622414267E-34	0.347468012532447	0.275	0.159	3.54751145041828E-30	Proliferating hepatocytes	TIGD5
2.12549225141385E-34	0.34377039381937	0.482	0.324	3.56189991491934E-30	Proliferating hepatocytes	CSTB
2.15693189294994E-34	0.334795798516382	0.268	0.154	3.61458646620551E-30	Proliferating hepatocytes	ZNF445
2.27040184967157E-34	0.305220526915342	0.291	0.17	3.80473941967962E-30	Proliferating hepatocytes	RAB35
2.36196243445307E-34	0.758909442649831	0.164	0.081	3.95817664765646E-30	Proliferating hepatocytes	PSME3
2.44270662122285E-34	0.380252336655512	0.355	0.22	4.09348775584525E-30	Proliferating hepatocytes	SLC25A38
2.44420547210133E-34	0.742022434934169	0.169	0.085	4.0959995301474E-30	Proliferating hepatocytes	PLA2G1B
2.46142342027868E-34	0.349752457674093	0.285	0.167	4.12485336770302E-30	Proliferating hepatocytes	TMEM251
2.46569319554553E-34	0.281668027682397	0.416	0.266	4.1320086570952E-30	Proliferating hepatocytes	APOOL
2.59293423224633E-34	0.252665107668183	0.426	0.276	4.3452391863984E-30	Proliferating hepatocytes	TMEM183A
2.59834504159342E-34	0.602358127530099	0.184	0.095	4.35430662070226E-30	Proliferating hepatocytes	ELOVL7
2.63807342581212E-34	0.445125244818583	0.193	0.1	4.42088344697596E-30	Proliferating hepatocytes	JRK
2.66452633690347E-34	0.283737558006734	0.351	0.216	4.46521323538284E-30	Proliferating hepatocytes	DEPRECATEI
2.69648086788535E-34	0.349648997533052	0.221	0.12	4.51876263840228E-30	Proliferating hepatocytes	TBCCD1
2.70855318918711E-34	0.476792846178785	0.194	0.101	4.53899343443976E-30	Proliferating hepatocytes	DEPRECATEI
2.86068724493312E-34	0.355536857950903	0.399	0.258	4.79393968505891E-30	Proliferating hepatocytes	DERA
2.89299357375391E-34	0.30051596060725	0.27	0.155	4.8480786308968E-30	Proliferating hepatocytes	VPS4B
2.92666472768803E-34	0.607285587538827	0.226	0.125	4.9045047506596E-30	Proliferating hepatocytes	APC
2.95457462763375E-34	0.296333618383123	0.467	0.313	4.95127616098864E-30	Proliferating hepatocytes	EMC7
2.96352659461031E-34	0.914900529501887	0.108	0.045	4.96627786724795E-30	Proliferating hepatocytes	SLAIN1
3.10400156682605E-34	0.364255370450263	0.367	0.229	5.2016858256871E-30	Proliferating hepatocytes	MARCKSL1
3.32767112986584E-34	0.381202717264954	0.261	0.149	5.57651127942918E-30	Proliferating hepatocytes	AP5M1
3.45347637189506E-34	0.250375999446757	0.341	0.209	5.78733570402174E-30	Proliferating hepatocytes	PAWR
3.50884015195845E-34	0.279213614156754	0.345	0.212	5.88011432665197E-30	Proliferating hepatocytes	PBDC1
3.55567341340947E-34	0.277551465931052	0.493	0.329	5.9585975061916E-30	Proliferating hepatocytes	PARP4
3.94497428676608E-34	0.389990284272685	0.225	0.124	6.6109879097626E-30	Proliferating hepatocytes	TOMM40
4.04190921021521E-34	0.555175254685188	0.118	0.051	6.77343145447865E-30	Proliferating hepatocytes	PANK2
4.066957909661E-34	0.261237674102631	0.188	0.096	6.8154080650099E-30	Proliferating hepatocytes	CCDC59
4.22096889228939E-34	0.501242080531482	0.245	0.138	7.07349966969856E-30	Proliferating hepatocytes	AP3M2
4.2559524745768E-34	0.274790421752057	0.483	0.325	7.1321251568958E-30	Proliferating hepatocytes	TOP 1.00
4.32768871201589E-34	0.359211079471216	0.297	0.174	7.25234074359624E-30	Proliferating hepatocytes	FNBP1L
4.46494279331458E-34	0.253362375664139	0.381	0.24	7.48235113303657E-30	Proliferating hepatocytes	ARID4B
4.73805301243637E-34	0.276404184975495	0.249	0.14	7.94002923824087E-30	Proliferating hepatocytes	SMURF1
4.98759391917652E-34	0.31030485526799	0.705	0.537	8.35820988975601E-30	Proliferating hepatocytes	NDUFA2
5.01389447201676E-34	0.314812518865809	0.274	0.159	8.40228435620568E-30	Proliferating hepatocytes	PLCG1
5.22824955735805E-34	0.597725939216709	0.143	0.067	8.76150060822062E-30	Proliferating hepatocytes	MIER2
5.25692205268559E-34	0.288098773903808	0.206	0.11	8.80954997589051E-30	Proliferating hepatocytes	CNOT3
5.43816858440471E-34	0.300957371773098	0.374	0.234	9.11328291374541E-30	Proliferating hepatocytes	ATP5PF
5.50431832532181E-34	0.847836130353669	0.12	0.053	9.2241366495743E-30	Proliferating hepatocytes	DEPRECATEI
5.59589012478456E-34	0.31213070012513	0.5	0.336	9.37759267111396E-30	Proliferating hepatocytes	CAPNS1
5.61526743544401E-34	0.537301897149293	0.206	0.11	9.41006516831708E-30	Proliferating hepatocytes	IQCB1
5.86476125209323E-34	0.283496714414242	0.489	0.328	9.82816690625783E-30	Proliferating hepatocytes	SLC35A4

6.19614793874395E-34	0.666376389427426	0.162	0.08	1.03835047157471E-29	Proliferating hepatocytes	CDKN2AIP
6.27279944308533E-34	0.326450455949578	0.435	0.284	1.05119573067224E-29	Proliferating hepatocytes	SRA1
6.5659823545496E-34	0.591880044530835	0.179	0.092	1.10032732297542E-29	Proliferating hepatocytes	R3HCC1
6.65019418824149E-34	0.270943077042259	0.687	0.503	1.11443954206551E-29	Proliferating hepatocytes	NDUFB9
6.78727111845695E-34	0.305163982898291	0.209	0.112	1.13741089403102E-29	Proliferating hepatocytes	FXYD2
6.83924952412613E-34	0.330597996496889	0.252	0.142	1.14612143525306E-29	Proliferating hepatocytes	DDX52
6.97984326906175E-34	0.759780049541223	0.146	0.07	1.16968213502937E-29	Proliferating hepatocytes	PLCE1
7.06773326939489E-34	0.257400136247502	0.455	0.3	1.1844107412852E-29	Proliferating hepatocytes	NUB1
7.2293083946428E-34	0.254497195617527	0.297	0.175	1.21148750077424E-29	Proliferating hepatocytes	EIF3K
7.35642303003501E-34	0.430060996770986	0.184	0.094	1.23278937137327E-29	Proliferating hepatocytes	ZNF707
7.37278818872023E-34	0.296546485312664	0.307	0.183	1.23553184466574E-29	Proliferating hepatocytes	ARF5
7.44894844122725E-34	0.289742352112778	0.383	0.245	1.24829477978086E-29	Proliferating hepatocytes	HAO2
7.51800130252818E-34	0.305255642939898	0.226	0.124	1.25986665827767E-29	Proliferating hepatocytes	TBP
7.72797118033484E-34	0.838618711045913	0.11	0.046	1.29505341040051E-29	Proliferating hepatocytes	MMP10
7.88161475145791E-34	0.474958273117474	0.158	0.077	1.32080100004932E-29	Proliferating hepatocytes	ZSWIM1
7.93059605690538E-34	0.264443068554252	0.486	0.326	1.3290092872162E-29	Proliferating hepatocytes	LRRC59
8.00628063377654E-34	0.502738192379823	0.241	0.135	1.34169250860827E-29	Proliferating hepatocytes	CTNNBL1
8.20743433094685E-34	0.446911668857744	0.299	0.181	1.37540184518007E-29	Proliferating hepatocytes	PIK3C2G
8.63021967140588E-34	0.35516990824718	0.345	0.213	1.4462522125342E-29	Proliferating hepatocytes	BAMBI
8.74053339897694E-34	0.429648756603593	0.248	0.14	1.46473858700056E-29	Proliferating hepatocytes	RWDD1
8.82072553995382E-34	0.358778979703917	0.265	0.153	1.47817718598546E-29	Proliferating hepatocytes	CAD
9.85655737377675E-34	0.297882592347929	0.414	0.269	1.65176188469751E-29	Proliferating hepatocytes	PPP6C
1.02688641285723E-33	0.263952469793046	0.429	0.28	1.72085625066615E-29	Proliferating hepatocytes	TOMM70
1.03791408926277E-33	0.28806553740997	0.341	0.209	1.73933643078655E-29	Proliferating hepatocytes	TADA2B
1.04679466880067E-33	0.59778786473426	0.135	0.062	1.75421850597617E-29	Proliferating hepatocytes	ZNF532
1.12001720518639E-33	0.298157396246099	0.391	0.248	1.87692483245134E-29	Proliferating hepatocytes	DEPRECATEI
1.13563782731509E-33	0.317242759920084	0.266	0.155	1.90310187101462E-29	Proliferating hepatocytes	FCHO2
1.17578729443229E-33	0.347540081119051	0.205	0.109	1.97038434800964E-29	Proliferating hepatocytes	KANSL2
1.21394587397092E-33	0.308830353914522	0.411	0.267	2.03433049560047E-29	Proliferating hepatocytes	TMEM175
1.21898262145767E-33	0.293151982325845	0.396	0.252	2.04277107703876E-29	Proliferating hepatocytes	RNF40
1.23251545251027E-33	0.451798185517174	0.197	0.104	2.06544939531671E-29	Proliferating hepatocytes	LARGE1
1.26304033282933E-33	0.397066689460717	0.207	0.112	2.11660298975538E-29	Proliferating hepatocytes	ARL 3.00
1.2842416674703E-33	0.376796911979289	0.346	0.214	2.15213218634672E-29	Proliferating hepatocytes	PGK 1.00
1.29306154931418E-33	0.348626677072528	0.263	0.151	2.1669125443407E-29	Proliferating hepatocytes	DPF2
1.35528638369776E-33	0.313292499477883	0.324	0.197	2.2711889218007E-29	Proliferating hepatocytes	DEPRECATEI
1.35744527251592E-33	0.336536591029822	0.33	0.201	2.27480678768218E-29	Proliferating hepatocytes	OTUD6B
1.37336199069637E-33	0.251708077733684	0.698	0.513	2.30148002400897E-29	Proliferating hepatocytes	ELL2
1.52605964438005E-33	0.261502569999494	0.435	0.284	2.55737075205208E-29	Proliferating hepatocytes	CASC3
1.53145573759508E-33	0.303843816066358	0.246	0.138	2.56641352506183E-29	Proliferating hepatocytes	KRT10
1.56182461211264E-33	0.433718774371126	0.207	0.112	2.61730568497835E-29	Proliferating hepatocytes	MIF4GD
1.60326859583674E-33	0.286585556139039	0.271	0.157	2.68675751290321E-29	Proliferating hepatocytes	PCGF5
1.63217636100184E-33	0.308304550097734	0.467	0.31	2.73520114576689E-29	Proliferating hepatocytes	IL10RB
1.63880003661165E-33	0.323272653920615	0.267	0.155	2.7463011013538E-29	Proliferating hepatocytes	PRPF8
1.66501793871626E-33	0.463010523637381	0.174	0.088	2.7902370617007E-29	Proliferating hepatocytes	ZUP1
1.70722492328666E-33	0.333633843580307	0.276	0.161	2.86096752644379E-29	Proliferating hepatocytes	STYX
1.7165903258539E-33	0.762086742043983	0.137	0.064	2.87666206806596E-29	Proliferating hepatocytes	PRR36
1.73100089213324E-33	0.359744894347504	0.268	0.156	2.90081129503688E-29	Proliferating hepatocytes	ATG4A
1.76622403971821E-33	0.442068473231305	0.26	0.15	2.95983824575977E-29	Proliferating hepatocytes	ABHD15
1.89840714185297E-33	0.417676462133051	0.21	0.114	3.18135068831721E-29	Proliferating hepatocytes	PPP1R1B
1.97304994430367E-33	0.390278545748576	0.173	0.087	3.30643709666408E-29	Proliferating hepatocytes	SRBD1
1.9962687658432E-33	0.303204910338868	0.283	0.167	3.34534719780004E-29	Proliferating hepatocytes	SLC41A3
2.08293934609328E-33	0.452808430178185	0.162	0.081	3.49058975618313E-29	Proliferating hepatocytes	BICD2
2.27685184651323E-33	0.600199800721777	0.278	0.164	3.81554832438686E-29	Proliferating hepatocytes	DEPRECATEI
2.29066139676537E-33	0.26205829858863	0.344	0.211	3.8386903686994E-29	Proliferating hepatocytes	ELF1
2.32328373394136E-33	0.330578961890403	0.436	0.284	3.89335888133894E-29	Proliferating hepatocytes	TMEM214
2.35119985043186E-33	0.262552893784138	0.404	0.259	3.94014070935371E-29	Proliferating hepatocytes	NSD1
2.41999314835085E-33	0.338681036502206	0.271	0.157	4.05542451800636E-29	Proliferating hepatocytes	BCL10
2.46147292317038E-33	0.319002409460029	0.456	0.303	4.12493632464892E-29	Proliferating hepatocytes	ACACB
2.49804050126563E-33	0.286691028294444	0.339	0.21	4.18621627202095E-29	Proliferating hepatocytes	SNAP47
2.52691154585129E-33	0.520639462495649	0.206	0.11	4.23459836853759E-29	Proliferating hepatocytes	C15orf41

2.56849506004254E-33	0.274890913311426	0.368	0.232	4.3042840216193E-29	Proliferating hepatocytes	CNNM3
2.69092528379793E-33	0.295996105480574	0.342	0.21	4.50945259058858E-29	Proliferating hepatocytes	NSMAF
2.70981146641165E-33	0.523116999152441	0.164	0.082	4.54110205541263E-29	Proliferating hepatocytes	N4BP2
2.74145944799938E-33	0.286843056084702	0.39	0.247	4.59413774295736E-29	Proliferating hepatocytes	COMMD3
2.79077066816829E-33	0.260817177629465	0.538	0.366	4.67677348571641E-29	Proliferating hepatocytes	SUB1
2.81736781792552E-33	0.329537955238639	0.304	0.182	4.72134498927958E-29	Proliferating hepatocytes	RDH14
2.8925543138649E-33	0.530451595862274	0.204	0.109	4.8473425191748E-29	Proliferating hepatocytes	WASF3
3.12901986907108E-33	0.366424470434865	0.344	0.214	5.24361149658931E-29	Proliferating hepatocytes	PIH1D1
3.16135188672382E-33	0.325004465258556	0.242	0.136	5.29779349177178E-29	Proliferating hepatocytes	GPR137B
3.25828141024475E-33	0.664959270321832	0.165	0.083	5.46022798728815E-29	Proliferating hepatocytes	MAP3K6
3.32815760525948E-33	0.328181480618152	0.269	0.156	5.57732651489384E-29	Proliferating hepatocytes	NGDN
3.42300002990991E-33	0.262293430444999	0.309	0.185	5.73626345012302E-29	Proliferating hepatocytes	MAP2K7
3.46685749243947E-33	0.357069423288529	0.259	0.148	5.80975978583007E-29	Proliferating hepatocytes	URM1
3.77383810744804E-33	0.617578580123834	0.107	0.045	6.32419790046143E-29	Proliferating hepatocytes	DDX20
3.85343287324628E-33	0.263490675855901	0.47	0.319	6.45758280898612E-29	Proliferating hepatocytes	IL17RB
3.86927078499833E-33	0.28615164888992	0.463	0.308	6.4841239815002E-29	Proliferating hepatocytes	SMARCA5
3.96419875690263E-33	0.300978779048876	0.367	0.23	6.64320427681743E-29	Proliferating hepatocytes	SNX2
4.16000491393199E-33	0.35993201742245	0.17	0.085	6.97133623476723E-29	Proliferating hepatocytes	AC093323.1
4.18278215279361E-33	0.294208227527007	0.293	0.173	7.00950633165152E-29	Proliferating hepatocytes	NCEH1
4.34789483092049E-33	0.28854363219765	0.453	0.297	7.28620215765655E-29	Proliferating hepatocytes	PYURF
4.53819818343691E-33	0.382952804734706	0.169	0.085	7.60511251580357E-29	Proliferating hepatocytes	SH3BP2
4.68291507524735E-33	0.250942087435456	0.342	0.209	7.84762908309951E-29	Proliferating hepatocytes	TMEM220
4.68790233920085E-33	0.261520178793557	0.494	0.334	7.85598674003279E-29	Proliferating hepatocytes	ECPAS
5.09427013506829E-33	0.262942071559164	0.322	0.195	8.53697789234744E-29	Proliferating hepatocytes	DRAM2
5.40435434431029E-33	0.316514253440627	0.196	0.103	9.05661701019518E-29	Proliferating hepatocytes	AGO4
5.47597999215295E-33	0.849836957901716	0.14	0.066	9.17664727084992E-29	Proliferating hepatocytes	ZNF212
5.61450782392087E-33	0.362332689324525	0.231	0.128	9.40879221132659E-29	Proliferating hepatocytes	NEO1
5.79707466573047E-33	0.331094612908198	0.225	0.124	9.71473772483112E-29	Proliferating hepatocytes	BAZ1A
6.20572537008348E-33	0.348132716087307	0.163	0.081	1.03995545751859E-28	Proliferating hepatocytes	TULP3
6.26170997978027E-33	0.283099765134088	0.333	0.205	1.04933735841158E-28	Proliferating hepatocytes	PRKD2
6.41577602684232E-33	0.265644342604896	0.229	0.127	1.07515574657824E-28	Proliferating hepatocytes	AAGAB
6.64858309214287E-33	0.459063262124469	0.202	0.108	1.1141695545813E-28	Proliferating hepatocytes	GPR89A
6.89120534945002E-33	0.575465669925347	0.189	0.099	1.15482819246083E-28	Proliferating hepatocytes	GGPS1
6.95991105938158E-33	0.276196999485739	0.384	0.245	1.16634189533117E-28	Proliferating hepatocytes	MIER1
6.97818041326218E-33	0.329776828613428	0.338	0.21	1.16940347365448E-28	Proliferating hepatocytes	C19orf53
7.13395211518989E-33	0.32632293125851	0.361	0.228	1.19550769546352E-28	Proliferating hepatocytes	ATRAID
7.31208561663283E-33	0.349260638853041	0.376	0.239	1.22535930763533E-28	Proliferating hepatocytes	PSMC6
7.52928687665941E-33	0.369774145787163	0.215	0.117	1.26175789479058E-28	Proliferating hepatocytes	SMARCD1
7.68155987725833E-33	0.480180542256336	0.242	0.138	1.28727580423095E-28	Proliferating hepatocytes	MTX1
7.75759519984065E-33	0.253833520927407	0.226	0.124	1.3000178035893E-28	Proliferating hepatocytes	LIAS
8.07261947588579E-33	0.263368512278511	0.441	0.292	1.35280957176894E-28	Proliferating hepatocytes	NDUFAF3
8.64030518420492E-33	0.345269249534222	0.304	0.182	1.44794234276906E-28	Proliferating hepatocytes	SLC35B4
8.66541440096721E-33	0.337012199048295	0.263	0.152	1.45215014531408E-28	Proliferating hepatocytes	DEPRECATEI
8.73311860138884E-33	0.457433792282205	0.161	0.08	1.46349601522074E-28	Proliferating hepatocytes	SMARCAL1
8.76797823968118E-33	0.338886576400039	0.281	0.166	1.46933779340577E-28	Proliferating hepatocytes	UCHL3
8.83456991207E-33	0.334785735467217	0.276	0.162	1.48049722586469E-28	Proliferating hepatocytes	GPATCH8
9.34254483803451E-33	0.636714387605316	0.143	0.068	1.56562366395782E-28	Proliferating hepatocytes	SCAI
1.01065322025079E-32	0.366478308976945	0.235	0.132	1.69365266649627E-28	Proliferating hepatocytes	FEM1A
1.07781780374389E-32	0.350811149577865	0.234	0.131	1.80620707551401E-28	Proliferating hepatocytes	EXOC1
1.11715549961795E-32	0.396258200328675	0.303	0.181	1.87212918625977E-28	Proliferating hepatocytes	USP38
1.12584758240249E-32	0.389001534104064	0.186	0.097	1.8866953785901E-28	Proliferating hepatocytes	TTLL6
1.13806671373301E-32	0.332358809202906	0.396	0.259	1.90717219887378E-28	Proliferating hepatocytes	ZMAT1
1.15097403732298E-32	0.267795628706616	0.184	0.096	1.92880229174584E-28	Proliferating hepatocytes	RAC3
1.15583222695423E-32	0.25928311749086	0.289	0.171	1.93694364592989E-28	Proliferating hepatocytes	DEPRECATEI
1.1581539877707E-32	0.258516789251879	0.166	0.083	1.94083445270613E-28	Proliferating hepatocytes	DEPRECATEI
1.16939891268269E-32	0.379023186923561	0.454	0.303	1.95967869787365E-28	Proliferating hepatocytes	EEF2K
1.2046607216116E-32	0.373379594178651	0.327	0.199	2.01877043727671E-28	Proliferating hepatocytes	USP53
1.21940908622924E-32	0.382194084220894	0.197	0.105	2.04348574670296E-28	Proliferating hepatocytes	DPM2
1.23651926105686E-32	0.336799588185096	0.298	0.178	2.07215897767908E-28	Proliferating hepatocytes	MBIP
1.26223506226472E-32	0.31716672775827	0.387	0.247	2.11525351734322E-28	Proliferating hepatocytes	SLC16A2

**1.27237785057913E-32**	0.332510595833777	0.483	0.325	2.1322508020005E-28	Proliferating hepatocytes	DNPH1
**1.31388941678509E-32**	0.312115230822906	0.452	0.301	2.20181588464845E-28	Proliferating hepatocytes	PDCD6
**1.32114868242046E-32**	0.306545509055323	0.453	0.298	2.2139809620002E-28	Proliferating hepatocytes	GIPC1
**1.34261863617755E-32**	0.347425470035118	0.236	0.132	2.24996031050634E-28	Proliferating hepatocytes	ORAI1
**1.37480472514269E-32**	0.253603436750232	0.622	0.448	2.30389775839412E-28	Proliferating hepatocytes	ADIPOR1
**1.44224094538126E-32**	0.252835659218625	0.22	0.121	2.41690737626991E-28	Proliferating hepatocytes	IMPDH2
**1.48290965388583E-32**	0.470215931574164	0.132	0.061	2.48505999798188E-28	Proliferating hepatocytes	DHRS7B
**1.48775321422076E-32**	0.615446755872098	0.143	0.068	2.49317683639114E-28	Proliferating hepatocytes	HYKK
**1.51582077928562E-32**	0.650821929172452	0.128	0.059	2.54021246192684E-28	Proliferating hepatocytes	PYCR3
**1.53724142245159E-32**	0.283311639734594	0.294	0.176	2.57610917574438E-28	Proliferating hepatocytes	WDR82
**1.56807388583354E-32**	0.255548717454142	0.506	0.344	2.62777821787984E-28	Proliferating hepatocytes	UHMK1
**1.70599091119382E-32**	0.293389631564885	0.651	0.478	2.8588995689786E-28	Proliferating hepatocytes	SSR2
**1.70932693476772E-32**	0.392648535333796	0.27	0.158	2.86449007728375E-28	Proliferating hepatocytes	PLCL2
**1.71569646039492E-32**	0.605963808072818	0.156	0.077	2.8751641283298E-28	Proliferating hepatocytes	SLC25A12
**1.94765846720591E-32**	0.266661447430768	0.429	0.278	3.26388605934367E-28	Proliferating hepatocytes	CAMK2D
**1.97197308746885E-32**	0.25016480324641	0.326	0.199	3.3046324999803E-28	Proliferating hepatocytes	CREBL2
**1.98018873366302E-32**	0.255804787287509	0.273	0.16	3.31840027987249E-28	Proliferating hepatocytes	PHF5A
**2.22398941034603E-32**	0.384494260725485	0.206	0.112	3.72696145385788E-28	Proliferating hepatocytes	DEPRECATEI
**2.31259755129235E-32**	0.389637354616764	0.234	0.131	3.87545097645573E-28	Proliferating hepatocytes	CUEDC1
**2.32694110802861E-32**	0.673718630456692	0.172	0.088	3.89948790883434E-28	Proliferating hepatocytes	EIF3E
**2.36045911656736E-32**	0.354948749150204	0.136	0.064	3.95565738754358E-28	Proliferating hepatocytes	PEAK1
**2.45206114214872E-32**	0.548033580811449	0.191	0.101	4.10916406201282E-28	Proliferating hepatocytes	ATF1
**2.70267421813443E-32**	0.346285260227029	0.643	0.49	4.52914145474968E-28	Proliferating hepatocytes	ANKRD10
**2.78457642623173E-32**	0.250961132623633	0.651	0.477	4.66639317507913E-28	Proliferating hepatocytes	GRN
**2.79435352633994E-32**	0.369045371410617	0.337	0.209	4.68277763944048E-28	Proliferating hepatocytes	LIMA1
**2.93186464201089E-32**	0.281786414924152	0.273	0.159	4.91321876708184E-28	Proliferating hepatocytes	MLST8
**3.00810997483348E-32**	0.326970687614508	0.271	0.16	5.04099069582595E-28	Proliferating hepatocytes	IPP
**3.02282656207187E-32**	0.691059064035229	0.13	0.06	5.06565275272004E-28	Proliferating hepatocytes	TOR2A
**3.0507500774448E-32**	0.517550127933796	0.139	0.066	5.11244697978199E-28	Proliferating hepatocytes	DDX41
**3.2089780672425E-32**	0.261806811325096	0.21	0.114	5.37760544508498E-28	Proliferating hepatocytes	SCYL2
**3.25838151675917E-32**	0.257646663439976	0.513	0.349	5.46039574578502E-28	Proliferating hepatocytes	GPAT4
**3.26796031175053E-32**	0.413351323983773	0.18	0.093	5.47644789043154E-28	Proliferating hepatocytes	CDK5RAP1
**3.28610107755313E-32**	0.318412787095976	0.189	0.099	5.50684818576353E-28	Proliferating hepatocytes	ZEB1
**3.39094854135003E-32**	0.505534985396182	0.195	0.105	5.68255156559438E-28	Proliferating hepatocytes	TMEM54
**3.39749500366302E-32**	0.334625506412677	0.35	0.218	5.69352212713849E-28	Proliferating hepatocytes	SHOX2
**3.72017937310635E-32**	0.311172117865693	0.301	0.181	6.23427659345162E-28	Proliferating hepatocytes	TMEM165
**3.80164767892506E-32**	0.342503740991046	0.255	0.146	6.37080118034261E-28	Proliferating hepatocytes	DHX29
**3.8117177697128E-32**	0.395618323000776	0.13	0.06	6.38767663848471E-28	Proliferating hepatocytes	NIF3L1
**3.85137261850647E-32**	0.290213483604849	0.4	0.259	6.45413023409314E-28	Proliferating hepatocytes	ATP6V0C
**4.0690250288425E-32**	0.388629952883672	0.353	0.222	6.81887214333426E-28	Proliferating hepatocytes	TULP4
**4.20008320450794E-32**	0.324630097220742	0.152	0.075	7.0384994341144E-28	Proliferating hepatocytes	ANKRD44
**4.58678258121286E-32**	0.281757548124522	0.438	0.29	7.68653024959651E-28	Proliferating hepatocytes	STMP1
**4.59197548625228E-32**	0.282273658713791	0.26	0.15	7.69523251986157E-28	Proliferating hepatocytes	NSA2
**4.74245403133032E-32**	0.473975703464713	0.182	0.095	7.94740446570334E-28	Proliferating hepatocytes	C15orf39
**4.78919237673811E-32**	0.537555173127759	0.193	0.103	8.02572858493773E-28	Proliferating hepatocytes	AP2B1
**4.96495962734398E-32**	0.342282649059478	0.231	0.129	8.32027934350304E-28	Proliferating hepatocytes	FAM120AOS
**5.12677883689327E-32**	0.298668784586302	0.505	0.343	8.59145597486574E-28	Proliferating hepatocytes	CLTB
**5.19283325466904E-32**	0.253794495701527	0.204	0.11	8.70214996817438E-28	Proliferating hepatocytes	HIP1
**5.25655946332753E-32**	0.639190004382723	0.101	0.042	8.80894234864428E-28	Proliferating hepatocytes	KANTR
**5.55147766250841E-32**	0.262147422011903	0.148	0.072	9.3031662668316E-28	Proliferating hepatocytes	ZFYVE26
**5.87749492759065E-32**	0.31079523612582	0.257	0.148	9.84950599965641E-28	Proliferating hepatocytes	DCTN6
**6.14396086579268E-32**	0.274573952098192	0.156	0.077	1.02960496188954E-27	Proliferating hepatocytes	CCDC71L
**6.42952841619851E-32**	0.363934896828727	0.214	0.118	1.07746037198655E-27	Proliferating hepatocytes	RNF214
**6.50225754675224E-32**	0.254383413872372	0.5	0.34	1.08964831968474E-27	Proliferating hepatocytes	ONECUT2
**6.54206557365188E-32**	0.361703573027485	0.191	0.102	1.09631934883258E-27	Proliferating hepatocytes	DMAC2
**6.58291071918062E-32**	0.260753828845833	0.399	0.256	1.10316417832029E-27	Proliferating hepatocytes	GOPC
**6.65183355816743E-32**	0.543249300081068	0.136	0.065	1.1147142676777E-27	Proliferating hepatocytes	WWC3
**6.93597594345265E-32**	0.452124233497443	0.236	0.134	1.16233084860379E-27	Proliferating hepatocytes	SLC49A4
**7.06647420083339E-32**	0.500942691757293	0.196	0.105	1.18419974657566E-27	Proliferating hepatocytes	TFB1M
**7.1707297951662E-32**	0.263787188406461	0.555	0.384	1.20167089907395E-27	Proliferating hepatocytes	SETD7

7.39649770470652E-32	0.465539171594431	0.174	0.089	1.23950508535472E-27	Proliferating hepatocytes	IARS
7.40861238077668E-32	0.307627585161665	0.229	0.128	1.24153526277056E-27	Proliferating hepatocytes	VCPKMT
7.58908687509176E-32	0.386939860751984	0.251	0.144	1.27177917852788E-27	Proliferating hepatocytes	CUEDC2
7.59740695339059E-32	0.328788507834656	0.194	0.104	1.2731734572492E-27	Proliferating hepatocytes	CTDNEP1
7.60850862310722E-32	0.509571730876818	0.123	0.056	1.27503387506031E-27	Proliferating hepatocytes	TNFAIP8
7.71504951692718E-32	0.289793259835112	0.3	0.18	1.29288799804666E-27	Proliferating hepatocytes	LIG3
7.74502145045466E-32	0.317318943710118	0.324	0.199	1.29791069466719E-27	Proliferating hepatocytes	NAA15
7.85637797935886E-32	0.256231239745654	0.53	0.358	1.31657182178096E-27	Proliferating hepatocytes	ZDHHC9
7.92414910329431E-32	0.64589340663466	0.197	0.106	1.32792890673006E-27	Proliferating hepatocytes	EID2
7.94271485449109E-32	0.284087521151817	0.269	0.158	1.33104015531562E-27	Proliferating hepatocytes	UQCRH
8.21644487483987E-32	0.452590796598196	0.147	0.072	1.37691183212567E-27	Proliferating hepatocytes	TSEN15
8.29051748526018E-32	0.304983983551453	0.278	0.164	1.3893249201799E-27	Proliferating hepatocytes	SEC22A
9.15603562046993E-32	0.266895940351454	0.472	0.318	1.53436844927835E-27	Proliferating hepatocytes	LAP3
9.30612805054447E-32	0.398434489138117	0.164	0.083	1.55952093871024E-27	Proliferating hepatocytes	BICC1
9.51907158379543E-32	0.262189414774353	0.718	0.54	1.59520601601244E-27	Proliferating hepatocytes	NDUFC2
1.05664570731387E-31	0.540588907530969	0.205	0.112	1.77072687631658E-27	Proliferating hepatocytes	DOCK10
1.06629850845219E-31	0.431731453470859	0.332	0.208	1.78690304046418E-27	Proliferating hepatocytes	MTX2
1.06953302753536E-31	0.371746543902653	0.222	0.123	1.79232344754375E-27	Proliferating hepatocytes	ELP4
1.08109202751394E-31	0.300833212306186	0.402	0.261	1.81169401970786E-27	Proliferating hepatocytes	GABARAP
1.08686881305451E-31	0.377647190946901	0.153	0.076	1.82137475691674E-27	Proliferating hepatocytes	DEPRECATEI
1.1095369970183E-31	0.304205882956047	0.283	0.171	1.85936209960328E-27	Proliferating hepatocytes	BRMS1
1.11401075844622E-31	0.264647687995122	0.225	0.126	1.86685922900417E-27	Proliferating hepatocytes	ACLY
1.12071300357941E-31	0.337508336799938	0.326	0.203	1.87809085139837E-27	Proliferating hepatocytes	TBL2
1.13393343668799E-31	0.266163377883058	0.352	0.221	1.90024565320173E-27	Proliferating hepatocytes	GTF2H1
1.19767507860409E-31	0.341105333172258	0.202	0.109	2.00706389672473E-27	Proliferating hepatocytes	SPAST
1.20723651911262E-31	0.461989080863026	0.172	0.089	2.02308695872893E-27	Proliferating hepatocytes	POMT1
1.2186185186418E-31	0.471802244018787	0.17	0.087	2.04216091353993E-27	Proliferating hepatocytes	WNT3
1.31146742269206E-31	0.417595716119618	0.307	0.188	2.19775710694736E-27	Proliferating hepatocytes	SLC35E1
1.32807100077603E-31	0.333372584559793	0.485	0.327	2.22558138310047E-27	Proliferating hepatocytes	RABAC1
1.40044433056765E-31	0.299078559756786	0.288	0.173	2.34686460916527E-27	Proliferating hepatocytes	SLC25A28
1.45012429177551E-31	0.550607298684551	0.13	0.06	2.43011828815739E-27	Proliferating hepatocytes	FAM118A
1.49726791961644E-31	0.289213336271237	0.219	0.121	2.50912157969323E-27	Proliferating hepatocytes	CYP20A1
1.52766747683274E-31	0.254123406992581	0.295	0.177	2.5600651576763E-27	Proliferating hepatocytes	SIK3
1.58473409017615E-31	0.77790124998607	0.123	0.056	2.65569738831719E-27	Proliferating hepatocytes	HPF1
1.59140370005345E-31	0.304666276680259	0.252	0.146	2.66687432054957E-27	Proliferating hepatocytes	HERC2
1.64933377403057E-31	0.364083846561022	0.272	0.16	2.76395353852043E-27	Proliferating hepatocytes	PRPF18
1.66512291297544E-31	0.268403561947421	0.36	0.227	2.79041297756424E-27	Proliferating hepatocytes	PPDPF
1.67840781163201E-31	0.392506754046725	0.191	0.103	2.81267581073293E-27	Proliferating hepatocytes	NOL7
1.70310017127946E-31	0.622809895943162	0.136	0.065	2.85405526703012E-27	Proliferating hepatocytes	UBE2V2
1.74632641346079E-31	0.262856415183493	0.362	0.23	2.9264938036776E-27	Proliferating hepatocytes	TIMM23
1.76723177699269E-31	0.526291123292105	0.223	0.126	2.96152701188436E-27	Proliferating hepatocytes	TWSG1
1.80345156120566E-31	0.427840818855679	0.186	0.099	3.02222412626844E-27	Proliferating hepatocytes	ZNF524
1.85446053739578E-31	0.563788308831355	0.181	0.095	3.10770496856785E-27	Proliferating hepatocytes	MOB3A
2.06954216281566E-31	0.364374828548871	0.15	0.073	3.46813875644648E-27	Proliferating hepatocytes	TRMT10A
2.23932003355242E-31	0.292950853467918	0.171	0.088	3.75265251222715E-27	Proliferating hepatocytes	DHX33
2.29683278950204E-31	0.458934245454368	0.206	0.113	3.84903238864752E-27	Proliferating hepatocytes	DEPRECATEI
2.32899767770838E-31	0.284176095176667	0.27	0.159	3.9029343083037E-27	Proliferating hepatocytes	SDE2
2.32952671503446E-31	0.353387299175535	0.181	0.095	3.90382086905475E-27	Proliferating hepatocytes	ARPC3
2.33067330665277E-31	0.305063492048118	0.469	0.315	3.90574232728872E-27	Proliferating hepatocytes	CLMN
2.39488859539579E-31	0.527543685824734	0.215	0.12	4.01335430816426E-27	Proliferating hepatocytes	LPAR1
2.40400833980758E-31	0.330552624035339	0.26	0.152	4.02863717584954E-27	Proliferating hepatocytes	EHD1
2.41473014718659E-31	0.520939221556717	0.218	0.122	4.04660478065529E-27	Proliferating hepatocytes	DRGX
2.47829646027156E-31	0.58581802109917	0.162	0.082	4.15312920812308E-27	Proliferating hepatocytes	KYAT3
2.49108905305488E-31	0.264594054832638	0.175	0.091	4.17456703510937E-27	Proliferating hepatocytes	NOC4L
2.63814703557304E-31	0.270208811440129	0.459	0.304	4.4210068022133E-27	Proliferating hepatocytes	FOXO3
2.687432094463E-31	0.256976494025965	0.436	0.289	4.50359870390109E-27	Proliferating hepatocytes	RHOBTB3
2.7014452248685E-31	0.335715234984423	0.304	0.185	4.52708190783463E-27	Proliferating hepatocytes	NDRG3
2.94007428042894E-31	0.305266599688104	0.39	0.25	4.92697647914282E-27	Proliferating hepatocytes	HERC3
2.97971609135294E-31	0.758485833805523	0.118	0.053	4.99340822588925E-27	Proliferating hepatocytes	SLC39A10
3.20739112245642E-31	0.338612823521601	0.216	0.119	5.37494604301247E-27	Proliferating hepatocytes	C7orf25

3.41471258008126E-31	0.403974601841331	0.18	0.095	5.72237534170018E-27	Proliferating hepatocytes	AP4B1
3.60015109771732E-31	0.322168812259295	0.32	0.197	6.03313320955468E-27	Proliferating hepatocytes	NMI
3.6944244335735E-31	0.424796875328048	0.232	0.132	6.19111646578248E-27	Proliferating hepatocytes	EPB41L4A
3.69498808467752E-31	0.313798688202899	0.154	0.077	6.19206103230259E-27	Proliferating hepatocytes	POMT2
3.99248622785852E-31	0.388536015886558	0.219	0.122	6.69060842064531E-27	Proliferating hepatocytes	STARD7
4.33306276793557E-31	0.264935868593019	0.666	0.489	7.26134658650643E-27	Proliferating hepatocytes	CTSH
4.46721606932773E-31	0.425830941032195	0.255	0.149	7.48616068897942E-27	Proliferating hepatocytes	DPY19L3
4.49084518447225E-31	0.28787651778964	0.401	0.264	7.52575836013859E-27	Proliferating hepatocytes	MSH6
4.52687570383967E-31	0.303723615095013	0.238	0.136	7.58613830449453E-27	Proliferating hepatocytes	SCNM1
4.6446193481008E-31	0.404058490110267	0.211	0.116	7.78345310354732E-27	Proliferating hepatocytes	DIS3L
4.90109380138562E-31	0.487806197012282	0.185	0.098	8.21325299236203E-27	Proliferating hepatocytes	SPATA2
5.20965005317357E-31	0.338652982867538	0.266	0.157	8.73033155910826E-27	Proliferating hepatocytes	FBXO4
5.4259937012908E-31	0.670311506543644	0.129	0.06	9.09288024462312E-27	Proliferating hepatocytes	DEPRECATEI
5.473736549133E-31	0.474459976725174	0.204	0.112	9.17288770903708E-27	Proliferating hepatocytes	SF3B6
5.65878281435759E-31	0.277463907693334	0.409	0.271	9.48298824030045E-27	Proliferating hepatocytes	POLD2
6.07990511005638E-31	0.33028775136716	0.189	0.1	1.01887049834325E-26	Proliferating hepatocytes	DUSP12
6.3066239311189E-31	0.336900594222742	0.232	0.132	1.0568640383769E-26	Proliferating hepatocytes	CD2BP2
6.60464905178448E-31	0.45345667233218	0.183	0.098	1.10680708809804E-26	Proliferating hepatocytes	MTFP1
6.77915980846778E-31	0.626232799347881	0.151	0.075	1.13605160070303E-26	Proliferating hepatocytes	MOSPD1
6.81783425954754E-31	0.763419959730003	0.128	0.06	1.14253266521498E-26	Proliferating hepatocytes	WDR89
6.95010392572963E-31	0.312415124209695	0.317	0.194	1.16469841587377E-26	Proliferating hepatocytes	KHNYN
6.95226670618454E-31	0.840544590130161	0.118	0.053	1.16506085462241E-26	Proliferating hepatocytes	EML1
6.97940240477328E-31	0.258618149102172	0.228	0.128	1.16960825499191E-26	Proliferating hepatocytes	CASP7
6.98717113663646E-31	0.409282319200143	0.233	0.132	1.17091013907754E-26	Proliferating hepatocytes	DEPRECATEI
7.07209987869881E-31	0.253560713012212	0.641	0.476	1.18514249767235E-26	Proliferating hepatocytes	AHSA1
7.1680732617663E-31	0.419113830433658	0.128	0.06	1.2012257172068E-26	Proliferating hepatocytes	WDR47
7.37553487685978E-31	0.46451292577059	0.16	0.081	1.23599213466416E-26	Proliferating hepatocytes	DYRK3
7.67883986085857E-31	0.360466572182488	0.238	0.136	1.28681998388268E-26	Proliferating hepatocytes	MYCBP2
8.01447031055283E-31	0.254866149364405	0.52	0.359	1.34306493464244E-26	Proliferating hepatocytes	PAPSS2
8.18588212373966E-31	0.25266341788865	0.324	0.199	1.37179012629629E-26	Proliferating hepatocytes	SUCLG2
8.28784593165888E-31	0.328783570331015	0.279	0.168	1.3888772212274E-26	Proliferating hepatocytes	PEF1
8.67882060289453E-31	0.273447299463637	0.449	0.299	1.45439675663306E-26	Proliferating hepatocytes	TCEAL1
8.70285967020379E-31	0.253030145991432	0.151	0.075	1.45842522353275E-26	Proliferating hepatocytes	REV3L
8.77513949446865E-31	0.528526059179529	0.176	0.092	1.47053787648306E-26	Proliferating hepatocytes	ABHD5
8.80332714724383E-31	0.477597820506816	0.198	0.108	1.47526156333512E-26	Proliferating hepatocytes	PIK3CA
9.21654017402681E-31	0.356088643398896	0.224	0.126	1.54450780236341E-26	Proliferating hepatocytes	ZSWIM9
9.48040538055494E-31	0.315876872403155	0.34	0.215	1.5887263336734E-26	Proliferating hepatocytes	NDUFS8
9.53076812010598E-31	0.401904896831823	0.195	0.106	1.59716612156736E-26	Proliferating hepatocytes	TMEM218
9.97322514546433E-31	0.398406551809594	0.297	0.182	1.67131306987691E-26	Proliferating hepatocytes	VPS72
1.05461066157807E-30	0.418114584484547	0.206	0.113	1.76731654667253E-26	Proliferating hepatocytes	UFSP2
1.0564903485856E-30	0.350837897406796	0.234	0.132	1.77046652615974E-26	Proliferating hepatocytes	SMYD3
1.06726932593301E-30	0.257474139854807	0.338	0.211	1.78852993639853E-26	Proliferating hepatocytes	NAE1
1.1113842413602E-30	0.404639930697077	0.229	0.13	1.86245771167142E-26	Proliferating hepatocytes	SLC44A3
1.12098447155713E-30	0.660610727243458	0.156	0.079	1.87854577743544E-26	Proliferating hepatocytes	ARL6IP6
1.13545260742872E-30	0.602469952947105	0.215	0.121	1.90279147952905E-26	Proliferating hepatocytes	FUNDC1
1.19368905312736E-30	0.412647242291302	0.233	0.133	2.00038411523082E-26	Proliferating hepatocytes	RABL3
1.23294471733561E-30	0.421660456739991	0.242	0.139	2.06616875731102E-26	Proliferating hepatocytes	ZNF606
1.25028858540298E-30	0.287111736769334	0.358	0.227	2.09523361141832E-26	Proliferating hepatocytes	WDR20
1.28610912096827E-30	0.349927018295866	0.193	0.104	2.15526166491862E-26	Proliferating hepatocytes	XPO7
1.3052737338141E-30	0.301178456209898	0.343	0.216	2.18737772312567E-26	Proliferating hepatocytes	SNX9
1.37389159011892E-30	0.470069217384437	0.212	0.118	2.30236752672128E-26	Proliferating hepatocytes	MTX3
1.37617841701781E-30	0.437205211090974	0.275	0.163	2.30619979123845E-26	Proliferating hepatocytes	CHST5
1.42394561149902E-30	0.276177459991615	0.186	0.099	2.38624805575005E-26	Proliferating hepatocytes	GZF1
1.42914562872317E-30	0.275634177523417	0.166	0.085	2.39496224461429E-26	Proliferating hepatocytes	BLOC1S3
1.53129512618223E-30	0.470560139125559	0.202	0.111	2.56614437245619E-26	Proliferating hepatocytes	C6orf141
1.57847264961539E-30	0.449767673709187	0.193	0.105	2.64520446622548E-26	Proliferating hepatocytes	SLC35A5
1.72692187845385E-30	0.407352701020214	0.223	0.126	2.89397568391297E-26	Proliferating hepatocytes	RABGGTA
1.81463633726257E-30	0.3721669701624	0.218	0.122	3.04096757398461E-26	Proliferating hepatocytes	STXBP4
1.89811291256024E-30	0.326329745501284	0.258	0.151	3.18085761886845E-26	Proliferating hepatocytes	PIGF
1.93095058956643E-30	0.300264917182259	0.864	0.733	3.23588699799542E-26	Proliferating hepatocytes	SERPINA6

1.95889877766333E-30	0.298106467531938	0.217	0.121	3.28272257160821E-26	Proliferating hepatocytes	KLF2
1.99403010766815E-30	0.273340870990789	0.335	0.21	3.34159565443028E-26	Proliferating hepatocytes	ZBTB25
2.08116734527781E-30	0.26266034336979	0.416	0.274	3.48762023721656E-26	Proliferating hepatocytes	CCT8
2.08300395454547E-30	0.268292527071987	0.16	0.081	3.49069802702729E-26	Proliferating hepatocytes	WDR48
2.15385397667581E-30	0.35274913760581	0.295	0.179	3.60942849411332E-26	Proliferating hepatocytes	CUL2
2.15505417807685E-30	0.399397830366212	0.137	0.066	3.61143979162119E-26	Proliferating hepatocytes	ASB6
2.17486374281526E-30	0.282992592293044	0.269	0.16	3.64463666020982E-26	Proliferating hepatocytes	NDUFB3
2.21273639174068E-30	0.397410339947011	0.154	0.077	3.70810364527902E-26	Proliferating hepatocytes	SLC38A6
2.25569157837867E-30	0.374833871650291	0.251	0.146	3.78008794704697E-26	Proliferating hepatocytes	PRKCD
2.29387784136655E-30	0.29564669573533	0.346	0.219	3.84408048656206E-26	Proliferating hepatocytes	UGGT1
2.53789276943661E-30	0.251998763898086	0.507	0.345	4.25300070302187E-26	Proliferating hepatocytes	ETNK1
2.53921645287125E-30	0.337853836209672	0.279	0.168	4.25521893172164E-26	Proliferating hepatocytes	UPF2
2.56588787122411E-30	0.253989850457709	0.227	0.129	4.29991489459736E-26	Proliferating hepatocytes	NFKBIL1
2.64823213508385E-30	0.286246868030454	0.293	0.178	4.43790741197352E-26	Proliferating hepatocytes	ZSWIM5
2.66117117129759E-30	0.471673075135904	0.42	0.293	4.4595906488605E-26	Proliferating hepatocytes	MCM5
2.66947827119548E-30	0.25838966998523	0.272	0.162	4.47351168686938E-26	Proliferating hepatocytes	GPS2
2.72014715376478E-30	0.297788673852216	0.172	0.09	4.55842260027903E-26	Proliferating hepatocytes	DHX58
2.7564076705802E-30	0.474357275390829	0.159	0.081	4.6191879743583E-26	Proliferating hepatocytes	DEPRECATEI
2.91274642517809E-30	0.293984897566617	0.17	0.088	4.88118045931345E-26	Proliferating hepatocytes	MCU
2.9546847597738E-30	0.301084978037706	0.343	0.216	4.95146072042894E-26	Proliferating hepatocytes	RBM24
2.95837916132204E-30	0.56665289558717	0.116	0.052	4.95765179854347E-26	Proliferating hepatocytes	APLF
2.9892920220917E-30	0.276123824440928	0.34	0.214	5.00945557062128E-26	Proliferating hepatocytes	TRMT1L
3.19186953654098E-30	0.749249793496967	0.142	0.07	5.34893496933538E-26	Proliferating hepatocytes	GPR160
3.1961382075157E-30	0.508729364587699	0.135	0.065	5.35608840815481E-26	Proliferating hepatocytes	EXTL3
3.23906591307993E-30	0.25774049084295	0.379	0.245	5.42802665713935E-26	Proliferating hepatocytes	ARAF
3.34432750742529E-30	0.278865033597978	0.271	0.162	5.6044240369433E-26	Proliferating hepatocytes	TMEM184B
3.36678137845344E-30	0.340084072885725	0.393	0.256	5.64205223401228E-26	Proliferating hepatocytes	CORO2A
3.46158645314641E-30	0.487072597650391	0.166	0.086	5.80092657818276E-26	Proliferating hepatocytes	TRIM35
3.52278652693603E-30	0.426474694946828	0.262	0.156	5.9034856618394E-26	Proliferating hepatocytes	UNC93B1
3.78432570092547E-30	0.256587986850125	0.362	0.23	6.3417730096109E-26	Proliferating hepatocytes	WIZ
3.79244248826573E-30	0.353889670990349	0.192	0.104	6.35537512183571E-26	Proliferating hepatocytes	RAB8B
3.80661489871364E-30	0.314681541348373	0.332	0.208	6.37912524726433E-26	Proliferating hepatocytes	DGKE
3.83840708921067E-30	0.567015639666832	0.132	0.063	6.43240260009923E-26	Proliferating hepatocytes	CRLF3
3.85581422566923E-30	0.573146746730548	0.247	0.145	6.4615734793765E-26	Proliferating hepatocytes	ATP5F1C
4.0216906677688E-30	0.397493367175168	0.241	0.139	6.73954922104695E-26	Proliferating hepatocytes	STRIP1
4.02827600815235E-30	0.41996521139698	0.223	0.127	6.7505849344617E-26	Proliferating hepatocytes	ST13
4.09985222770574E-30	0.284047107429321	0.237	0.138	6.87053236318928E-26	Proliferating hepatocytes	NR2C2AP
4.27443061501185E-30	0.309221681130679	0.229	0.13	7.16309082463686E-26	Proliferating hepatocytes	DEPDC5
4.55939123589963E-30	0.466188344889978	0.121	0.055	7.6406278331206E-26	Proliferating hepatocytes	PPIP5K1
4.57796041002878E-30	0.302195549733966	0.287	0.173	7.67174605512623E-26	Proliferating hepatocytes	PPP1R13B
4.77134023996357E-30	0.298576747787565	0.211	0.118	7.99581197413096E-26	Proliferating hepatocytes	SNX8
4.78488044876906E-30	0.354795603344732	0.193	0.105	8.01850265604718E-26	Proliferating hepatocytes	SLC4A1AP
4.8516065357944E-30	0.257613526454534	0.217	0.121	8.13032223268426E-26	Proliferating hepatocytes	PPP1R8
5.1229635010955E-30	0.593313886249614	0.129	0.061	8.58506223513584E-26	Proliferating hepatocytes	DEPRECATEI
5.6098517721329E-30	0.40478338813992	0.147	0.073	9.40098959974031E-26	Proliferating hepatocytes	NEK1
5.6650206355276E-30	0.627637179926767	0.112	0.05	9.49344158101715E-26	Proliferating hepatocytes	PRMT5
5.67587897746879E-30	0.253472106933498	0.364	0.232	9.5116379904422E-26	Proliferating hepatocytes	MED13
5.70477841949055E-30	0.264578912035017	0.249	0.146	9.56006767538227E-26	Proliferating hepatocytes	SVIL
5.95342379355203E-30	0.726696020536553	0.158	0.081	9.9767475932345E-26	Proliferating hepatocytes	KLHL28
6.28867996834527E-30	0.34693018995468	0.299	0.183	1.0538569890953E-25	Proliferating hepatocytes	STX12
6.51837900946394E-30	0.283408499292307	0.187	0.101	1.09234995440597E-25	Proliferating hepatocytes	MYL12A
6.70922587462578E-30	0.369243739960018	0.171	0.089	1.12433207206979E-25	Proliferating hepatocytes	QTRT2
6.73134417394943E-30	0.561170722529327	0.169	0.088	1.12803865667044E-25	Proliferating hepatocytes	FAM104B
7.086616432737E-30	0.660788012600392	0.156	0.08	1.18757518179807E-25	Proliferating hepatocytes	BICD1
7.2730295252436E-30	0.294356890634199	0.43	0.285	1.21881428784032E-25	Proliferating hepatocytes	ARRB1
7.52089731665533E-30	0.314500371088899	0.226	0.128	1.2603519723251E-25	Proliferating hepatocytes	COMMD7
7.66071301323464E-30	0.267753210539081	0.394	0.255	1.28378228675786E-25	Proliferating hepatocytes	DNASE1L1
7.84442856942106E-30	0.750126633895814	0.1	0.043	1.31456933966358E-25	Proliferating hepatocytes	MOK
8.07241292912065E-30	0.285875212674027	0.348	0.22	1.35277495866204E-25	Proliferating hepatocytes	TMEM127
9.03968236407056E-30	0.29495759755615	0.21	0.117	1.51486997057094E-25	Proliferating hepatocytes	TLK2

9.20765732241268E-30	0.266995351626944	0.348	0.222	1.54301921408992E-25	Proliferating hepatocytes	ZFYVE28
9.49913226312053E-30	0.474203757400418	0.177	0.094	1.59186458465374E-25	Proliferating hepatocytes	TMEM62
9.88960243387439E-30	0.342406115692914	0.198	0.108	1.65729957586867E-25	Proliferating hepatocytes	HCCS
1.01130264133461E-29	0.252460894166266	0.291	0.177	1.69474096634855E-25	Proliferating hepatocytes	PER3
1.01163888827222E-29	0.446679470662953	0.105	0.046	1.69530444896658E-25	Proliferating hepatocytes	DHX57
1.06548948324265E-29	0.377774882023294	0.155	0.078	1.78554727601803E-25	Proliferating hepatocytes	VPS50
1.11042730209755E-29	0.342674805682229	0.154	0.078	1.86085407285507E-25	Proliferating hepatocytes	GLMN
1.34442356262501E-29	0.367185667602707	0.193	0.105	2.25298500624699E-25	Proliferating hepatocytes	WBP1L
1.38210316610222E-29	0.257287327931222	0.375	0.241	2.31612848575411E-25	Proliferating hepatocytes	TOMM6
1.46469501932222E-29	0.365694035211229	0.187	0.101	2.45453591338018E-25	Proliferating hepatocytes	QPCTL
1.47995038509701E-29	0.253931676718979	0.288	0.174	2.48010085534556E-25	Proliferating hepatocytes	TBC1D22B
1.5461207573544E-29	0.621913035276321	0.103	0.045	2.5909891651745E-25	Proliferating hepatocytes	SNRPA1
1.62779831258806E-29	0.268123384313537	0.249	0.146	2.72786441223507E-25	Proliferating hepatocytes	EXD3
1.76394687807518E-29	0.363786736685241	0.203	0.113	2.95602217827838E-25	Proliferating hepatocytes	CXorf40B
1.76935266067169E-29	0.29871156532909	0.437	0.292	2.96508118875362E-25	Proliferating hepatocytes	STX7
1.86947851344436E-29	0.285868094623613	0.585	0.419	3.13287209283006E-25	Proliferating hepatocytes	DNAJC3
1.94092133385028E-29	0.272617710720759	0.249	0.146	3.25259597126629E-25	Proliferating hepatocytes	TINF2
2.01561406574736E-29	0.38539792295851	0.278	0.168	3.37776605137943E-25	Proliferating hepatocytes	ATP6V1F
2.10920176334564E-29	0.275607705971729	0.229	0.131	3.53460031501462E-25	Proliferating hepatocytes	SMURF2
2.12592247956201E-29	0.59952730251318	0.132	0.063	3.56262089125001E-25	Proliferating hepatocytes	PREX1
2.12621333631574E-29	0.265289167592428	0.435	0.292	3.56310830899791E-25	Proliferating hepatocytes	SELENON
2.20680450155699E-29	0.364977617450898	0.211	0.118	3.6981629837092E-25	Proliferating hepatocytes	CHMP4C
2.40009780564021E-29	0.430122339605451	0.176	0.093	4.02208390269187E-25	Proliferating hepatocytes	ATXN3
2.56731788830741E-29	0.259184565730047	0.245	0.143	4.30231131722556E-25	Proliferating hepatocytes	TTC13
2.62595285981662E-29	0.32404433011987	0.233	0.134	4.4005718024807E-25	Proliferating hepatocytes	CSNK1G3
2.67984536753159E-29	0.308858950878711	0.234	0.135	4.49088486690944E-25	Proliferating hepatocytes	PACS2
2.86166207193215E-29	0.263082414497075	0.225	0.128	4.79557330014389E-25	Proliferating hepatocytes	ASXL2
2.92201534394414E-29	0.306716446180271	0.224	0.127	4.89671331338159E-25	Proliferating hepatocytes	USP47
2.98928364067287E-29	0.664649936199308	0.14	0.069	5.0094415250396E-25	Proliferating hepatocytes	EVA1C
3.3234328455361E-29	0.265191850549212	0.198	0.108	5.5694087625494E-25	Proliferating hepatocytes	AP3M1
3.45437612307687E-29	0.264584989907027	0.632	0.461	5.78884350705222E-25	Proliferating hepatocytes	ALDH9A1
3.78576659721671E-29	0.295022514282713	0.275	0.166	6.34418766361576E-25	Proliferating hepatocytes	SDF2
3.80198560701099E-29	0.421137463008918	0.209	0.118	6.37136748022901E-25	Proliferating hepatocytes	TRAPPC1
3.89405222712112E-29	0.441856178411798	0.199	0.111	6.52565272220957E-25	Proliferating hepatocytes	RRAS
4.2043297755219E-29	0.351196361879907	0.2	0.111	7.0456158378196E-25	Proliferating hepatocytes	ATP5MPL
4.26630412003297E-29	0.726932548033418	0.132	0.064	7.14947244435125E-25	Proliferating hepatocytes	ZNF213
4.63305681179045E-29	0.286971257286297	0.307	0.19	7.76407660519844E-25	Proliferating hepatocytes	GTF3C6
4.63768475207045E-29	0.278160879973186	0.109	0.049	7.77183210751966E-25	Proliferating hepatocytes	HEY2
4.66008965275198E-29	0.417835252133411	0.208	0.117	7.80937824008177E-25	Proliferating hepatocytes	OSGIN2
4.93141802094706E-29	0.385877218507636	0.262	0.156	8.26407031950309E-25	Proliferating hepatocytes	RAI14
5.1512314647993E-29	0.262620519816484	0.196	0.107	8.63243368871067E-25	Proliferating hepatocytes	ATP2B1
5.65291258331805E-29	0.581289072007569	0.11	0.05	9.4731509071244E-25	Proliferating hepatocytes	GAS2L1
5.68228086318397E-29	0.284909549168115	0.196	0.108	9.52236627052369E-25	Proliferating hepatocytes	KARS
6.14089169829403E-29	0.351659432994374	0.238	0.139	1.02909063080011E-24	Proliferating hepatocytes	DYNLT3
6.31568842988467E-29	0.30798539196989	0.253	0.15	1.05838306708007E-24	Proliferating hepatocytes	SLC2A1
6.51128032899006E-29	0.331648721890728	0.214	0.122	1.09116035753215E-24	Proliferating hepatocytes	PTGFRN
6.55601084721914E-29	0.259187932898646	0.469	0.32	1.09865629777698E-24	Proliferating hepatocytes	C1orf43
6.68981282097251E-29	0.263247966524672	0.698	0.534	1.12107883253857E-24	Proliferating hepatocytes	TOR1AIP2
6.73290716190668E-29	0.269670551749573	0.371	0.241	1.12830058219232E-24	Proliferating hepatocytes	ATP5MF
6.85575931745182E-29	0.502447164535586	0.17	0.091	1.14888814641858E-24	Proliferating hepatocytes	WHAMM
7.12588939500195E-29	0.267811032577918	0.144	0.071	1.19415654481443E-24	Proliferating hepatocytes	CDK2AP2
7.17425528611421E-29	0.763672145558879	0.112	0.051	1.20226170084702E-24	Proliferating hepatocytes	POLR3F
7.41968884136425E-29	0.358929423265574	0.225	0.129	1.24339145603582E-24	Proliferating hepatocytes	SMARCAD1
7.69793674109907E-29	0.512933308844803	0.191	0.105	1.29002023907338E-24	Proliferating hepatocytes	PTPN9
8.74451032610906E-29	0.604888402098847	0.206	0.117	1.46540504044936E-24	Proliferating hepatocytes	ZNF57
9.0548239176866E-29	0.302024782297651	0.629	0.474	1.51740739212592E-24	Proliferating hepatocytes	ZBTB16
9.20730402565897E-29	0.278298606660344	0.406	0.269	1.54296000861993E-24	Proliferating hepatocytes	CCT3
9.66381336660778E-29	0.916998805262915	0.1	0.044	1.61946184397613E-24	Proliferating hepatocytes	C3orf14
9.81481134080397E-29	0.361908197101519	0.109	0.049	1.64476608449193E-24	Proliferating hepatocytes	ZNF646
9.92888560548947E-29	0.339224719467494	0.189	0.102	1.66388264976793E-24	Proliferating hepatocytes	TTPAL

1.01666869942627E-28	0.409908079881908	0.179	0.097	1.70373340649855E-24	Proliferating hepatocytes	EMC6
1.12988804226788E-28	0.493406061118306	0.155	0.08	1.89346638123251E-24	Proliferating hepatocytes	PARP16
1.13414231680668E-28	0.250123199822366	0.211	0.12	1.90059569450464E-24	Proliferating hepatocytes	NCOA1
1.13425931054813E-28	0.279828354113692	0.227	0.131	1.90079175261655E-24	Proliferating hepatocytes	ZNF133
1.15292469704936E-28	0.471649065329021	0.103	0.046	1.93207120731532E-24	Proliferating hepatocytes	TIAM1
1.19809252638417E-28	0.279957307848013	0.308	0.192	2.00776345571459E-24	Proliferating hepatocytes	SH3GLB1
1.21055588489327E-28	0.409293866403903	0.196	0.109	2.02864955190415E-24	Proliferating hepatocytes	CCAR2
1.22804995852855E-28	0.260705428606292	0.428	0.286	2.05796612050214E-24	Proliferating hepatocytes	ZNF664
1.30537412118579E-28	0.474694325470561	0.161	0.084	2.18754595228314E-24	Proliferating hepatocytes	ZFYVE19
1.350903377015E-28	0.344447287923135	0.194	0.107	2.26384387920173E-24	Proliferating hepatocytes	OPN3
1.38733002507681E-28	0.775712901796081	0.101	0.044	2.32488765602371E-24	Proliferating hepatocytes	CABYR
1.67802983959371E-28	0.627694302897172	0.145	0.073	2.81204240519113E-24	Proliferating hepatocytes	RCN2
1.68679044818743E-28	0.260977415936881	0.318	0.199	2.82672343307249E-24	Proliferating hepatocytes	LAMB1
1.77602976990741E-28	0.443628419795581	0.108	0.049	2.97627068841084E-24	Proliferating hepatocytes	DYRK1B
1.82672094489709E-28	0.796272299930132	0.103	0.046	3.06121895945854E-24	Proliferating hepatocytes	TLCD3A
1.89364381605175E-28	0.36199327277277	0.233	0.135	3.17336830693952E-24	Proliferating hepatocytes	HCFC2
1.93638799635782E-28	0.334948407293815	0.202	0.113	3.24499900429643E-24	Proliferating hepatocytes	CNEP1R1
1.94290624672633E-28	0.305663444854411	0.232	0.135	3.25592228826399E-24	Proliferating hepatocytes	MED18
1.94812328749518E-28	0.327495600785602	0.293	0.18	3.26466500518442E-24	Proliferating hepatocytes	LAMTOR3
2.03840582870422E-28	0.375230879847094	0.135	0.066	3.41596048774253E-24	Proliferating hepatocytes	TXNDC9
2.0492057369933E-28	0.250512606030091	0.593	0.424	3.43405897405337E-24	Proliferating hepatocytes	STBD1
2.05204896592672E-28	0.385886435768568	0.227	0.131	3.43882365709999E-24	Proliferating hepatocytes	ATXN7L1
2.13034185275315E-28	0.565148583300827	0.127	0.061	3.57002687684372E-24	Proliferating hepatocytes	DEPRECATEI
2.30025350043826E-28	0.455140115035965	0.123	0.059	3.85476481603443E-24	Proliferating hepatocytes	SLC26A2
2.41179314158013E-28	0.402665478062233	0.186	0.102	4.04168294665998E-24	Proliferating hepatocytes	RAB32
2.59050096789512E-28	0.266860643651096	0.296	0.184	4.34116152199865E-24	Proliferating hepatocytes	HEXIM1
2.6173531996877E-28	0.382871062890476	0.222	0.128	4.38616049203664E-24	Proliferating hepatocytes	TMEM184C
2.68379704520877E-28	0.316904082968517	0.223	0.129	4.49750708836087E-24	Proliferating hepatocytes	DEPRECATEI
2.73685289595837E-28	0.377065865910225	0.161	0.084	4.58641808304703E-24	Proliferating hepatocytes	CBY1
2.79595886719393E-28	0.387545348908161	0.251	0.149	4.6854678696436E-24	Proliferating hepatocytes	PAM
2.93680751163513E-28	0.281651264990494	0.289	0.176	4.92150202799815E-24	Proliferating hepatocytes	IREB2
3.01581462236791E-28	0.408554483272916	0.197	0.109	5.05390214416415E-24	Proliferating hepatocytes	RBIS
3.05964438225691E-28	0.399747843570917	0.231	0.134	5.12735205578613E-24	Proliferating hepatocytes	DHRS4L2
3.29334104101205E-28	0.561720240822706	0.162	0.085	5.518980916528E-24	Proliferating hepatocytes	STX18
3.32225459934418E-28	0.300762564961368	0.289	0.179	5.56743425758098E-24	Proliferating hepatocytes	SNRPE
3.55381166358274E-28	0.32252447478373	0.353	0.229	5.95547758583195E-24	Proliferating hepatocytes	SNX12
3.68390101853557E-28	0.255516109545221	0.251	0.149	6.1734813268619E-24	Proliferating hepatocytes	NT5C3A
3.7336155547216E-28	0.460395894686141	0.212	0.121	6.25679294660245E-24	Proliferating hepatocytes	NDUFA12
3.95894386370068E-28	0.372240988890568	0.149	0.076	6.6343981267896E-24	Proliferating hepatocytes	IPPK
4.19205031727576E-28	0.314457056765131	0.178	0.096	7.02503792169072E-24	Proliferating hepatocytes	TSR1
4.39494699461586E-28	0.314523426016336	0.276	0.168	7.36505217357726E-24	Proliferating hepatocytes	GABPB2
4.71353556526735E-28	0.265293173681846	0.528	0.368	7.89894290027503E-24	Proliferating hepatocytes	OCIAD1
4.7820413696727E-28	0.388586545891518	0.26	0.156	8.0137449272975E-24	Proliferating hepatocytes	TGDS
4.9300561979183E-28	0.359840103160621	0.249	0.148	8.26178817647149E-24	Proliferating hepatocytes	SSR1
5.08355863668038E-28	0.787236550259831	0.109	0.05	8.51902756334899E-24	Proliferating hepatocytes	ARSK
5.11348091477886E-28	0.419152397823116	0.27	0.164	8.56917131698641E-24	Proliferating hepatocytes	MFSD10
5.12522183842188E-28	0.298162116799933	0.303	0.189	8.58884675682739E-24	Proliferating hepatocytes	LYPLA1
5.57021697704602E-28	0.574687289684503	0.128	0.063	9.33456961013372E-24	Proliferating hepatocytes	ALDH1A2
5.72219727348798E-28	0.297469115942755	0.199	0.111	9.58925819091116E-24	Proliferating hepatocytes	FAM120C
6.22451167336795E-28	0.260982974412724	0.286	0.175	1.043103666223E-23	Proliferating hepatocytes	UBE2W
6.28843157711389E-28	0.467866238907176	0.166	0.089	1.05381536369275E-23	Proliferating hepatocytes	PAPLN
6.90366516912806E-28	0.403620520141408	0.2	0.111	1.15691620904248E-23	Proliferating hepatocytes	SENP7
7.19333228022254E-28	0.271576535288991	0.228	0.133	1.20545862351969E-23	Proliferating hepatocytes	REPS2
7.69640102696876E-28	0.35268909872171	0.216	0.124	1.28976288409943E-23	Proliferating hepatocytes	SSBP1
7.7670804547399E-28	0.41047891292604	0.181	0.099	1.30160734260531E-23	Proliferating hepatocytes	CNNM4
7.85848048801561E-28	0.625423085141207	0.14	0.071	1.31692416018166E-23	Proliferating hepatocytes	PROKR1
7.96132862655248E-28	0.272858476374144	0.148	0.076	1.33415945123766E-23	Proliferating hepatocytes	TCTN1
8.29628645190429E-28	0.393421855928155	0.186	0.103	1.39029168361012E-23	Proliferating hepatocytes	ERP29
8.36732907970582E-28	0.264316006910667	0.246	0.145	1.4021970071771E-23	Proliferating hepatocytes	C1GALT1
8.49265644579981E-28	0.254688741652108	0.25	0.15	1.42319936718713E-23	Proliferating hepatocytes	LSM14B

9.52788936253248E-28	0.389922473178354	0.11	0.05	1.59668369937319E-23	Proliferating hepatocytes	TAF1A
9.8737765624074E-28	0.269741737893785	0.303	0.189	1.65464747632823E-23	Proliferating hepatocytes	POLB
1.04967532797969E-27	0.610844811652357	0.109	0.05	1.75904591462836E-23	Proliferating hepatocytes	EXTL2
1.07236499862814E-27	0.444564453701896	0.105	0.048	1.79706926470104E-23	Proliferating hepatocytes	ZNF473
1.17044345014807E-27	0.261321036147226	0.287	0.178	1.96142913375814E-23	Proliferating hepatocytes	FAM110B
1.17058672774525E-27	0.409115926283976	0.201	0.113	1.96166923835548E-23	Proliferating hepatocytes	KIAA1211
1.23362552313823E-27	0.296159047665579	0.165	0.088	2.06730965167504E-23	Proliferating hepatocytes	FASTKD3
1.27933010568888E-27	0.288497970606905	0.164	0.087	2.14390139111343E-23	Proliferating hepatocytes	CAMSAP1
1.43124876530883E-27	0.581179198001605	0.108	0.049	2.39848668090454E-23	Proliferating hepatocytes	CAV1
1.4645381249013E-27	0.403963464875402	0.173	0.093	2.4542729897096E-23	Proliferating hepatocytes	NECTIN1
1.46461637111308E-27	0.449076073056842	0.186	0.102	2.4544041147113E-23	Proliferating hepatocytes	THAP6
1.57706057064365E-27	0.705618981790797	0.116	0.055	2.64283810428463E-23	Proliferating hepatocytes	FANCE
1.59609189157086E-27	0.362589681488039	0.211	0.121	2.67473079189445E-23	Proliferating hepatocytes	ARMC10
1.84000235309856E-27	0.289850861855995	0.386	0.256	3.08347594332257E-23	Proliferating hepatocytes	CLDN14
1.92130296076354E-27	0.404237755529023	0.161	0.085	3.21971950164754E-23	Proliferating hepatocytes	ARF3
1.94338091041003E-27	0.442838672106998	0.131	0.065	3.25671772966513E-23	Proliferating hepatocytes	FOCAD
2.02872713306725E-27	0.71452209583925	0.109	0.05	3.3997409295941E-23	Proliferating hepatocytes	DEPRECATEI
2.04096173119929E-27	0.50735009820946	0.173	0.094	3.42024366914377E-23	Proliferating hepatocytes	ITGAM
2.10781952125095E-27	0.354545168435055	0.192	0.108	3.53228395371234E-23	Proliferating hepatocytes	ZFP90
2.20117582878302E-27	0.344701979333307	0.198	0.111	3.68873045387458E-23	Proliferating hepatocytes	ZNF322
2.37126043250008E-27	0.392755370262699	0.122	0.059	3.97375823278364E-23	Proliferating hepatocytes	MINDY4
2.40167503556166E-27	0.298628804190703	0.291	0.178	4.02472702459422E-23	Proliferating hepatocytes	TMEM168
2.55155878815006E-27	0.384029688509632	0.272	0.167	4.27590221718187E-23	Proliferating hepatocytes	SDHAF4
2.57793370400613E-27	0.306086433473862	0.172	0.093	4.32010130117348E-23	Proliferating hepatocytes	ABHD8
2.6613332434E-27	0.287652480928461	0.165	0.088	4.45986224928973E-23	Proliferating hepatocytes	HSF2
2.72103193943244E-27	0.349534939930196	0.184	0.101	4.55990532410088E-23	Proliferating hepatocytes	TFAM
2.73659565520495E-27	0.350299359346776	0.164	0.087	4.58598699899246E-23	Proliferating hepatocytes	AGTPBP1
2.75382887121237E-27	0.287859252165234	0.304	0.19	4.61486642237769E-23	Proliferating hepatocytes	DFFA
2.82477711759315E-27	0.461251450141457	0.178	0.097	4.7337614936626E-23	Proliferating hepatocytes	UHRF1BP1
2.88891768937325E-27	0.402251583693521	0.101	0.045	4.84124826385169E-23	Proliferating hepatocytes	VPS33A
2.95074574947653E-27	0.412946698231605	0.199	0.113	4.94485972697277E-23	Proliferating hepatocytes	CSRP2
3.13449482843831E-27	0.28825937204083	0.219	0.127	5.25278643349692E-23	Proliferating hepatocytes	TMEM260
3.33841822110849E-27	0.393488415196705	0.11	0.051	5.5945212549336E-23	Proliferating hepatocytes	RASAL2
3.85543985615309E-27	0.259560821028674	0.755	0.589	6.46094611094134E-23	Proliferating hepatocytes	TMCO1
3.85674857519147E-27	0.364162788721396	0.147	0.076	6.46313926230587E-23	Proliferating hepatocytes	INPP5K
3.9663334512173E-27	0.362648750663808	0.214	0.124	6.64678159754995E-23	Proliferating hepatocytes	ZNF414
4.02763386677373E-27	0.432500721480618	0.137	0.069	6.74950883393942E-23	Proliferating hepatocytes	TIGD7
4.08004866425478E-27	0.531622658949348	0.147	0.076	6.83734555155817E-23	Proliferating hepatocytes	BAHCC1
4.40812430401948E-27	0.294250402602822	0.289	0.181	7.38713470867584E-23	Proliferating hepatocytes	NUDT2
4.59314476015461E-27	0.265432060347748	0.18	0.098	7.6971919890671E-23	Proliferating hepatocytes	ZNF782
4.72051752702403E-27	0.271050168035657	0.235	0.139	7.91064327178686E-23	Proliferating hepatocytes	RSF1
5.11772218694533E-27	0.260953963795765	0.192	0.107	8.57627884088298E-23	Proliferating hepatocytes	PTPN11
5.22423454247404E-27	0.364076290409373	0.168	0.09	8.75477224627799E-23	Proliferating hepatocytes	STARD13
5.58293458122237E-27	0.348646521079153	0.148	0.077	9.35588177121245E-23	Proliferating hepatocytes	PEX10
5.69467496440897E-27	0.436836959073249	0.157	0.083	9.54313630535656E-23	Proliferating hepatocytes	FAAP100
5.6982356072286E-27	0.288933692055894	0.173	0.094	9.54910323059369E-23	Proliferating hepatocytes	HOMEZ
5.8073527621903E-27	0.277976513511996	0.349	0.224	9.7319617588785E-23	Proliferating hepatocytes	TMLHE
5.85682469851615E-27	0.557233720148254	0.147	0.076	9.81486682977337E-23	Proliferating hepatocytes	RNF2
6.6416718810117E-27	0.443925897281374	0.146	0.075	1.11301137381994E-22	Proliferating hepatocytes	MCAT
6.66046745676819E-27	0.353140921520933	0.144	0.074	1.11616113640521E-22	Proliferating hepatocytes	PGAM1
6.80890469606978E-27	0.520373413156491	0.145	0.075	1.14103624896737E-22	Proliferating hepatocytes	CASP8AP2
7.00699842407049E-27	0.635931434495737	0.117	0.055	1.17423279590573E-22	Proliferating hepatocytes	AP1S2
9.01484890687954E-27	0.611145860417897	0.115	0.055	1.51070837981487E-22	Proliferating hepatocytes	BOLA3
9.16069424140797E-27	0.487854549203557	0.185	0.104	1.53514914097515E-22	Proliferating hepatocytes	HELQ
1.06133365304142E-26	0.352233510723219	0.134	0.067	1.77858293576681E-22	Proliferating hepatocytes	GMCL1
1.09679831457449E-26	0.263408291915655	0.193	0.109	1.83801461556393E-22	Proliferating hepatocytes	CRAMP1
1.10386457261801E-26	0.280919089135785	0.252	0.152	1.84985625079326E-22	Proliferating hepatocytes	TANGO2
1.13974380738049E-26	0.396820772547682	0.21	0.122	1.90998267240823E-22	Proliferating hepatocytes	NAIP
1.1745574177131E-26	0.349715677699885	0.326	0.21	1.96832332060361E-22	Proliferating hepatocytes	MID2
1.2874338672426E-26	0.333532461485923	0.26	0.159	2.15748167472515E-22	Proliferating hepatocytes	SFT2D1

1.2940308104052E-26	0.312249089306835	0.247	0.147	2.16853683207703E-22	Proliferating hepatocytes	NFU1
1.30940631831634E-26	0.346445470317434	0.147	0.075	2.19430310823453E-22	Proliferating hepatocytes	ANKRD65
1.33195718636865E-26	0.436886250135641	0.152	0.08	2.23209385291659E-22	Proliferating hepatocytes	TICAM2
1.5719948576761E-26	0.25609179481692	0.812	0.693	2.63434898249361E-22	Proliferating hepatocytes	DNAJB1
1.58933841913705E-26	0.426384373752261	0.171	0.093	2.66341332278988E-22	Proliferating hepatocytes	CASP10
1.60239860274778E-26	0.271332273125216	0.201	0.115	2.68529957848472E-22	Proliferating hepatocytes	TRAFD1
1.62102812606332E-26	0.279508758240431	0.256	0.158	2.71651893365691E-22	Proliferating hepatocytes	PPP1R9B
1.62685570302274E-26	0.619962761821962	0.108	0.05	2.72628478712551E-22	Proliferating hepatocytes	RCAN3
1.65717623792874E-26	0.476834189567774	0.156	0.083	2.77709593952099E-22	Proliferating hepatocytes	BCL11A
1.82582219604923E-26	0.264888755366968	0.23	0.135	3.0597128361393E-22	Proliferating hepatocytes	ATP5MD
1.82866474702397E-26	0.363505918697655	0.233	0.138	3.06447638306277E-22	Proliferating hepatocytes	MFSD6
1.88755712391691E-26	0.621634688617991	0.138	0.071	3.16316822825996E-22	Proliferating hepatocytes	ICAM3
1.90732099764466E-26	0.412348267299886	0.237	0.143	3.19628852785292E-22	Proliferating hepatocytes	LANCL2
2.75126992896741E-26	0.413930934504367	0.164	0.089	4.61057814696358E-22	Proliferating hepatocytes	CCDC127
2.77654009463798E-26	0.437397578192625	0.203	0.117	4.65292589059433E-22	Proliferating hepatocytes	SCOC
2.84130514882537E-26	0.29675697910242	0.267	0.165	4.76145916840155E-22	Proliferating hepatocytes	TMEM128
2.93424068642231E-26	0.578753129803341	0.115	0.055	4.91720054230651E-22	Proliferating hepatocytes	HSPA14
2.96472725800198E-26	0.250766461434745	0.257	0.157	4.96828993895972E-22	Proliferating hepatocytes	LAMTOR5
3.02399980932766E-26	0.287864102414196	0.203	0.116	5.06761888047129E-22	Proliferating hepatocytes	STK16
3.13779318411322E-26	0.50498302222687	0.141	0.073	5.25831381793694E-22	Proliferating hepatocytes	RTN4R
3.16020600705029E-26	0.347707159230881	0.184	0.103	5.29587322661487E-22	Proliferating hepatocytes	DOLPP1
3.16321721495284E-26	0.266236490202431	0.216	0.125	5.30091940881797E-22	Proliferating hepatocytes	PAIP2
3.24517058876149E-26	0.349136004787248	0.162	0.087	5.4382568726465E-22	Proliferating hepatocytes	VIPAS39
3.27956979217916E-26	0.70813930669963	0.104	0.048	5.49590305773384E-22	Proliferating hepatocytes	DEPRECATEI
3.29931002435941E-26	0.501656013133889	0.102	0.046	5.5289837388215E-22	Proliferating hepatocytes	AP4E1
3.32002314302855E-26	0.323130729035727	0.124	0.061	5.56369478308725E-22	Proliferating hepatocytes	FRG1
3.39887792481465E-26	0.30824182071183	0.296	0.187	5.6958396264044E-22	Proliferating hepatocytes	BMPR2
3.46142206979727E-26	0.470342578150522	0.153	0.081	5.80065110456626E-22	Proliferating hepatocytes	THAP1
3.65122423962206E-26	0.254979766563769	0.221	0.129	6.11872158075865E-22	Proliferating hepatocytes	VCL
3.71969548595916E-26	0.296930727852756	0.167	0.09	6.23346569537036E-22	Proliferating hepatocytes	NAA35
4.21572982295328E-26	0.293525922716405	0.209	0.12	7.0647200373051E-22	Proliferating hepatocytes	CEP95
4.25325505175803E-26	0.333238500643854	0.215	0.126	7.12760481573611E-22	Proliferating hepatocytes	TES
4.59108869532857E-26	0.257372380796952	0.137	0.07	7.69374643563162E-22	Proliferating hepatocytes	ZNF227
4.59843323566579E-26	0.298309281811682	0.267	0.165	7.70605441632873E-22	Proliferating hepatocytes	TRAF3
4.8071086912631E-26	0.381840291638024	0.108	0.051	8.05575274481871E-22	Proliferating hepatocytes	ZNF267
5.0766862734524E-26	0.338486688481514	0.165	0.089	8.50751085705154E-22	Proliferating hepatocytes	PDE12
5.08248407142879E-26	0.288356977683955	0.187	0.104	8.51722680690037E-22	Proliferating hepatocytes	VGLL4
5.22462356121401E-26	0.479673975349301	0.167	0.091	8.75542416388244E-22	Proliferating hepatocytes	DOP1B
5.33785877839392E-26	0.60900356524815	0.104	0.048	8.94518374083254E-22	Proliferating hepatocytes	MED19
5.49324600708646E-26	0.283078762289848	0.177	0.098	9.20558165867549E-22	Proliferating hepatocytes	DEPRECATEI
5.70132533716667E-26	0.52188968299533	0.151	0.081	9.55428100002391E-22	Proliferating hepatocytes	EML5
5.90585941579097E-26	0.267020753750971	0.193	0.109	9.89703920898251E-22	Proliferating hepatocytes	MEN1
6.176731747229E-26	0.516888299536138	0.1	0.045	1.03509670620064E-21	Proliferating hepatocytes	CTSK
6.5256727502677E-26	0.344432965344072	0.199	0.113	1.09357223948986E-21	Proliferating hepatocytes	TOM1L2
6.90459767454415E-26	0.330404610912312	0.132	0.066	1.15707247830011E-21	Proliferating hepatocytes	SETMAR
8.49688469987178E-26	0.259384027503024	0.223	0.132	1.42390793800451E-21	Proliferating hepatocytes	SHMT2
8.88011170878546E-26	0.41200089910375	0.218	0.128	1.48812912015827E-21	Proliferating hepatocytes	WDFY3
9.12647472898181E-26	0.254595057878356	0.24	0.145	1.52941463508277E-21	Proliferating hepatocytes	SIX5
1.03162797839213E-25	0.278313664396114	0.355	0.234	1.72880216618953E-21	Proliferating hepatocytes	GADD45GIP
1.1089424938325E-25	0.31017717102977	0.172	0.095	1.8583658311645E-21	Proliferating hepatocytes	POLG
1.17628114522952E-25	0.254798324382402	0.228	0.135	1.97121194317563E-21	Proliferating hepatocytes	PDRG1
1.22510111125361E-25	0.252268527948797	0.301	0.19	2.05302444223879E-21	Proliferating hepatocytes	NCK2
1.41178836416147E-25	0.250139417397772	0.125	0.062	2.3658749406618E-21	Proliferating hepatocytes	ELK1
1.66500727338929E-25	0.494210147063996	0.132	0.067	2.79021918874578E-21	Proliferating hepatocytes	ENHO
1.71667426312894E-25	0.419379884306625	0.206	0.119	2.87680273015147E-21	Proliferating hepatocytes	DPP8
1.72165644592672E-25	0.302110234844993	0.991	0.951	2.88515187208399E-21	Proliferating hepatocytes	MT-CO1
1.88766021186458E-25	0.423963132818301	0.119	0.058	3.16334098304266E-21	Proliferating hepatocytes	DEPRECATEI
2.08073943999572E-25	0.327874903421177	0.171	0.094	3.48690315354482E-21	Proliferating hepatocytes	TMED1
2.33205019100729E-25	0.250375508598881	0.193	0.11	3.90804971009002E-21	Proliferating hepatocytes	TSPAN17
2.50082737825735E-25	0.353583644784661	0.255	0.157	4.19088652048367E-21	Proliferating hepatocytes	MXRA7

**2.54216207782891E-25**	0.347947691090841	0.128	0.064	4.26015521002569E-21	Proliferating hepatocytes	TAF1B
**2.6965170903314E-25**	0.294724274850899	0.185	0.104	4.51882333997736E-21	Proliferating hepatocytes	TRMT61B
**2.71918665730642E-25**	0.411707683486864	0.144	0.076	4.55681300031409E-21	Proliferating hepatocytes	PPP4R4
**2.98368286689207E-25**	0.268911042724625	0.313	0.2	5.00005574833772E-21	Proliferating hepatocytes	SNX19
**3.0184521331778E-25**	0.50537001664674	0.146	0.077	5.05832208477936E-21	Proliferating hepatocytes	DEPRECATEI
**3.0554896702013E-25**	0.306955807995305	0.193	0.11	5.12038958932334E-21	Proliferating hepatocytes	STRADB
**3.19396002523151E-25**	0.283186455705787	0.373	0.249	5.35243821028297E-21	Proliferating hepatocytes	RASL11A
**3.55369359825737E-25**	0.330523884972743	0.225	0.135	5.95527973195971E-21	Proliferating hepatocytes	BLOC1S4
**3.5932522122286E-25**	0.26379502984206	0.34	0.223	6.02157205725268E-21	Proliferating hepatocytes	ZNF358
**3.987594632609E-25**	0.322127172190079	0.2	0.115	6.68241108532617E-21	Proliferating hepatocytes	G6PC3
**4.08321093453449E-25**	0.254940273801519	0.222	0.132	6.8426448840929E-21	Proliferating hepatocytes	NAAA
**4.39477979318014E-25**	0.303761976220001	0.271	0.168	7.36477197741127E-21	Proliferating hepatocytes	TBCE
**5.30940799054995E-25**	0.464076944207946	0.101	0.047	8.8975059105636E-21	Proliferating hepatocytes	IHH
**5.36204153809265E-25**	0.26912773018625	0.242	0.146	8.98570920953567E-21	Proliferating hepatocytes	MBTPS2
**5.63982114990075E-25**	0.277099236332102	0.14	0.073	9.45121228300368E-21	Proliferating hepatocytes	MAP2
**5.70040841619344E-25**	0.369733865889696	0.237	0.144	9.55274442385696E-21	Proliferating hepatocytes	ZFP64
**5.81652650620436E-25**	0.334748725303611	0.205	0.119	9.74733511909726E-21	Proliferating hepatocytes	FXR2
**5.86213153907679E-25**	0.383956105910969	0.132	0.067	9.82376003318488E-21	Proliferating hepatocytes	VPS16
**6.04633502871192E-25**	0.296669692276896	0.872	0.754	1.01324482411154E-20	Proliferating hepatocytes	NUPR1
**6.78607294712495E-25**	0.307617998941673	0.165	0.09	1.1372101044792E-20	Proliferating hepatocytes	CRYZL1
**7.83056075766626E-25**	0.327708351261932	0.174	0.097	1.31224537176971E-20	Proliferating hepatocytes	TGFBRAP1
**8.39266067233359E-25**	0.451153965968326	0.121	0.06	1.40644207546966E-20	Proliferating hepatocytes	DEPRECATEI
**8.45806534890634E-25**	0.306469531357572	0.207	0.12	1.41740259116972E-20	Proliferating hepatocytes	B4GALT6
**9.12234138993271E-25**	0.339826450755678	0.175	0.098	1.52872197012492E-20	Proliferating hepatocytes	FAM118B
**9.18196597586673E-25**	0.260678754788425	0.169	0.094	1.53871385823575E-20	Proliferating hepatocytes	ELMO2
**9.47189952259506E-25**	0.400634443625675	0.153	0.082	1.58730092199648E-20	Proliferating hepatocytes	ZBTB8OS
**1.04846741002353E-24**	0.333587879947501	0.15	0.08	1.75702168571744E-20	Proliferating hepatocytes	FAM222B
**1.06398501584511E-24**	0.316701598247275	0.194	0.111	1.78302608955324E-20	Proliferating hepatocytes	RWDD4
**1.06624352564735E-24**	0.392110272485874	0.183	0.104	1.78681090027983E-20	Proliferating hepatocytes	MSH3
**1.16483674109804E-24**	0.329290520854169	0.184	0.105	1.95203341073209E-20	Proliferating hepatocytes	NOMO1
**1.31565037533271E-24**	0.446692284198693	0.103	0.048	2.20476689898255E-20	Proliferating hepatocytes	METTL6
**1.37218499902909E-24**	0.3884881756155	0.218	0.13	2.29950762137295E-20	Proliferating hepatocytes	ALG1
**1.43445821732805E-24**	0.34561494751305	0.225	0.137	2.40386508059835E-20	Proliferating hepatocytes	COMTD1
**1.67245864603242E-24**	0.435170019119068	0.122	0.061	2.80270619902114E-20	Proliferating hepatocytes	HEATR6
**1.67273991282101E-24**	0.340904107532302	0.173	0.097	2.80317754590546E-20	Proliferating hepatocytes	C11orf49
**1.74351829094651E-24**	0.444440879743746	0.145	0.077	2.92178795196817E-20	Proliferating hepatocytes	DEPRECATEI
**1.93159473381391E-24**	0.268803643649169	0.229	0.138	3.23696645492536E-20	Proliferating hepatocytes	PSMB6
**1.9457075086337E-24**	0.42881984529555	0.169	0.095	3.26061664296835E-20	Proliferating hepatocytes	CCDC3
**2.3088440889434E-24**	0.341625644074369	0.127	0.065	3.86916092425135E-20	Proliferating hepatocytes	TMEM39B
**2.36855376942979E-24**	0.31955243959518	0.292	0.186	3.96922240681044E-20	Proliferating hepatocytes	ITPRIPL2
**2.38779270001269E-24**	0.290294451437156	0.307	0.198	4.00146300668126E-20	Proliferating hepatocytes	CXorf40A
**2.45698828343038E-24**	0.336826027292233	0.175	0.098	4.11742096537262E-20	Proliferating hepatocytes	LLPH
**2.56310770090909E-24**	0.490840816067263	0.115	0.056	4.29525588518345E-20	Proliferating hepatocytes	FBXO33
**2.79583061825519E-24**	0.261369255467596	0.174	0.098	4.68525295007205E-20	Proliferating hepatocytes	TRAF5
**2.92471960532729E-24**	0.268433526672444	0.235	0.142	4.90124511460748E-20	Proliferating hepatocytes	CLTC
**3.4156615216543E-24**	0.528091603079326	0.132	0.069	5.72396557798827E-20	Proliferating hepatocytes	USP46
**3.47162861148741E-24**	0.578163715722878	0.118	0.059	5.8177552271306E-20	Proliferating hepatocytes	B3GNT9
**3.6757050713771E-24**	0.266478330685837	0.254	0.157	6.15974655861375E-20	Proliferating hepatocytes	ARHGAP26
**3.70764455589435E-24**	0.535036899757734	0.175	0.099	6.21327074676776E-20	Proliferating hepatocytes	CTSE
**3.78915257487215E-24**	0.267067489743379	0.183	0.104	6.34986188497075E-20	Proliferating hepatocytes	PHF23
**3.81602662732018E-24**	0.674308332129189	0.101	0.047	6.39489742206316E-20	Proliferating hepatocytes	C1orf74
**4.63657795176936E-24**	0.252193407824234	0.14	0.074	7.7699773315751E-20	Proliferating hepatocytes	DPH3
**4.72813301569028E-24**	0.471131131268828	0.117	0.058	7.92340530769376E-20	Proliferating hepatocytes	CLUAP1
**5.05077546490938E-24**	0.578042130611984	0.121	0.061	8.46408952409514E-20	Proliferating hepatocytes	SSBP2
**5.19014295480327E-24**	0.276961889190515	0.197	0.115	8.69764156365932E-20	Proliferating hepatocytes	SNRNP35
**6.05810792221003E-24**	0.369513944480022	0.156	0.085	1.01521772560396E-19	Proliferating hepatocytes	ING4
**6.17342013589678E-24**	0.446407307751598	0.132	0.068	1.03454174637358E-19	Proliferating hepatocytes	MIPEP
**6.29374333080453E-24**	0.315646507645059	0.264	0.165	1.05470550737622E-19	Proliferating hepatocytes	GTF2IRD1
**6.90699548736563E-24**	0.257061188330508	0.273	0.172	1.15747430377273E-19	Proliferating hepatocytes	TBPL1
**7.1504387721959E-24**	0.518830555892623	0.115	0.057	1.19827052944459E-19	Proliferating hepatocytes	ARFGEF3

7.42862790892857E-24	0.283792045737416	0.182	0.104	1.24488946497825E-19	Proliferating hepatocytes	BEND7
7.58433746496695E-24	0.545044949350155	0.12	0.061	1.27098327237916E-19	Proliferating hepatocytes	ALKBH1
9.85718219728984E-24	0.324824716846886	0.156	0.085	1.65186659262183E-19	Proliferating hepatocytes	NKAPD1
1.12107944987308E-23	0.264112487147256	0.271	0.172	1.87870494209731E-19	Proliferating hepatocytes	PYM1
1.18975378246571E-23	0.326889738488016	0.162	0.09	1.99378938865604E-19	Proliferating hepatocytes	WDR77
1.36439345040031E-23	0.425889955445911	0.119	0.06	2.28645054418084E-19	Proliferating hepatocytes	C2orf92
1.37826874233019E-23	0.41835317586541	0.198	0.117	2.30970275839693E-19	Proliferating hepatocytes	NDUFA6
1.43666425023126E-23	0.262181380836289	0.172	0.096	2.40756195053754E-19	Proliferating hepatocytes	ICE2
1.45720716781413E-23	0.376167960561078	0.187	0.109	2.44198777182292E-19	Proliferating hepatocytes	ZBTB45
1.54151229265713E-23	0.297604914078607	0.237	0.145	2.58326630003481E-19	Proliferating hepatocytes	PET117
1.56733864077093E-23	0.395274751229677	0.124	0.063	2.62654609420392E-19	Proliferating hepatocytes	HACE1
1.61033768474279E-23	0.437388296583197	0.16	0.089	2.69860389209196E-19	Proliferating hepatocytes	ZNF324
1.61701573998398E-23	0.274770231616068	0.15	0.081	2.70979497706516E-19	Proliferating hepatocytes	ZNF496
1.80224092010144E-23	0.626780151667683	0.162	0.091	3.020195333906E-19	Proliferating hepatocytes	PAQR3
1.92221167041115E-23	0.336203913189941	0.162	0.091	3.221242317275E-19	Proliferating hepatocytes	DCAF13
1.96167013126228E-23	0.405180035527512	0.109	0.053	3.28736680596932E-19	Proliferating hepatocytes	FIG4
2.06743981710414E-23	0.268275459154772	0.12	0.061	3.46461564550311E-19	Proliferating hepatocytes	C19orf44
2.07660861340424E-23	0.388662596128215	0.184	0.107	3.47998071434283E-19	Proliferating hepatocytes	PDE3A
2.1665082541214E-23	0.268260472029431	0.206	0.122	3.63063453225664E-19	Proliferating hepatocytes	REXO4
2.32618763212774E-23	0.592154510561591	0.129	0.067	3.89822523391967E-19	Proliferating hepatocytes	ARHGEF35
2.45975623248573E-23	0.307768233905416	0.229	0.14	4.12205949439958E-19	Proliferating hepatocytes	B4GAT1
2.67324210107456E-23	0.516125502808177	0.12	0.061	4.47981911298074E-19	Proliferating hepatocytes	MESP1
2.70584641236781E-23	0.302889893705351	0.137	0.072	4.53445741784597E-19	Proliferating hepatocytes	KIF3A
2.95094562312434E-23	0.413553583774465	0.136	0.072	4.94519467523176E-19	Proliferating hepatocytes	SERAC1
3.06001144290067E-23	0.501966816656537	0.129	0.067	5.12796717601294E-19	Proliferating hepatocytes	PEX7
3.10653037721056E-23	0.253782848202121	0.107	0.052	5.20592360612946E-19	Proliferating hepatocytes	CDKL5
3.1769722761239E-23	0.434818217291443	0.106	0.052	5.32397014032843E-19	Proliferating hepatocytes	STARD8
3.27621421073647E-23	0.310624837989393	0.145	0.078	5.49027977435218E-19	Proliferating hepatocytes	HHLA3
3.35974241249645E-23	0.650854160676813	0.16	0.09	5.63025633486155E-19	Proliferating hepatocytes	DEPRECATEI
3.47631579298817E-23	0.267638557347829	0.19	0.11	5.82561000588958E-19	Proliferating hepatocytes	NAPEPLD
3.54831285198989E-23	0.391694427997004	0.106	0.051	5.94626267736465E-19	Proliferating hepatocytes	WDR83
3.80612840646061E-23	0.392809670145429	0.138	0.073	6.37830998354669E-19	Proliferating hepatocytes	EFL1
3.83840057741933E-23	0.646108556362477	0.137	0.073	6.43239168763931E-19	Proliferating hepatocytes	ARMC7
4.11531166987312E-23	0.339898261508336	0.171	0.097	6.89643929637337E-19	Proliferating hepatocytes	PGBD2
4.32664969618878E-23	0.317626750176178	0.221	0.135	7.25059956087316E-19	Proliferating hepatocytes	ABCB1
4.73066872444889E-23	0.288020238634496	0.157	0.087	7.92765464843144E-19	Proliferating hepatocytes	B4GALT2
4.97910591436498E-23	0.282984977120103	0.223	0.136	8.34398569129283E-19	Proliferating hepatocytes	PPP3CA
5.03382068615317E-23	0.506455426497743	0.175	0.101	8.43567670585548E-19	Proliferating hepatocytes	FMO1
5.18857448545516E-23	0.66026550450825	0.111	0.056	8.69501312272576E-19	Proliferating hepatocytes	USP49
5.35511020223202E-23	0.290098663478662	0.143	0.077	8.97409367690042E-19	Proliferating hepatocytes	PI4K2A
6.22454028248347E-23	0.309662981602434	0.265	0.168	1.04310846053858E-18	Proliferating hepatocytes	JAGN1
7.09461166497719E-23	0.250847153448825	0.33	0.218	1.18891502281688E-18	Proliferating hepatocytes	CBR4
7.16602649015949E-23	0.318587954338831	0.168	0.095	1.20088271922093E-18	Proliferating hepatocytes	NMNAT1
7.59194942381018E-23	0.255537424045031	0.194	0.114	1.27225888444211E-18	Proliferating hepatocytes	TMA7
8.01238105343197E-23	0.420540665813438	0.133	0.071	1.34271481693413E-18	Proliferating hepatocytes	SVIP
9.34613390589568E-23	0.526808401537377	0.103	0.05	1.56622511995E-18	Proliferating hepatocytes	FOXD2
9.59795055086799E-23	0.460955153550097	0.136	0.073	1.60842455331446E-18	Proliferating hepatocytes	IFT52
9.77137645574716E-23	0.378554610805362	0.195	0.116	1.63748726645411E-18	Proliferating hepatocytes	RPP38
9.7932851385873E-23	0.3562909217679	0.299	0.197	1.64115872352446E-18	Proliferating hepatocytes	FANCL
1.12279950173068E-22	0.358957585439527	0.128	0.067	1.88158740500027E-18	Proliferating hepatocytes	FTSJ1
1.3223830170647E-22	0.330751844318883	0.179	0.103	2.21604945999702E-18	Proliferating hepatocytes	DDHD1
1.40052076503273E-22	0.271911543457362	0.164	0.093	2.34699269804185E-18	Proliferating hepatocytes	TMEM41A
1.57705518997426E-22	0.294896755429103	0.198	0.118	2.64282908735887E-18	Proliferating hepatocytes	SLC48A1
1.70182350395323E-22	0.255877210481973	0.192	0.113	2.85191582792482E-18	Proliferating hepatocytes	TRANK1
1.8740857926658E-22	0.273036854626079	0.162	0.091	3.14059297134934E-18	Proliferating hepatocytes	ZBTB14
1.90616869215315E-22	0.402508665382227	0.192	0.114	3.19435749431025E-18	Proliferating hepatocytes	C6orf47
2.21274320187572E-22	0.38470668982118	0.118	0.06	3.70811505770334E-18	Proliferating hepatocytes	FAM135A
2.29387862426951E-22	0.338109306299387	0.109	0.054	3.84408179855084E-18	Proliferating hepatocytes	SLC12A6
2.49030774524783E-22	0.293127127125892	0.117	0.06	4.17325771948631E-18	Proliferating hepatocytes	MORN2
2.61681378737351E-22	0.569322207996965	0.113	0.057	4.38525654488053E-18	Proliferating hepatocytes	MIS12

**2.71434840963553E-22**	0.525673505311944	0.1	0.048	4.54870506486721E-18	Proliferating hepatocytes	DNAL1
**2.72261792223746E-22**	0.446374517996979	0.141	0.077	4.56256311408553E-18	Proliferating hepatocytes	ERCC8
**2.7384805521275E-22**	0.308010731463702	0.21	0.127	4.58914570925527E-18	Proliferating hepatocytes	HELZ2
**3.17831352112052E-22**	0.364769753201455	0.117	0.059	5.32621779869377E-18	Proliferating hepatocytes	ZSCAN25
**3.4727721415463E-22**	0.261293811010852	0.203	0.121	5.81967155480329E-18	Proliferating hepatocytes	COX15
**3.52560671813134E-22**	0.434868987708464	0.118	0.06	5.90821173824449E-18	Proliferating hepatocytes	C12orf43
**3.54351202886854E-22**	0.390669485900115	0.132	0.071	5.9382174579779E-18	Proliferating hepatocytes	GNAZ
**3.58548399064024E-22**	0.520539208376944	0.148	0.083	6.00855407151492E-18	Proliferating hepatocytes	C8orf37
**3.63034116607177E-22**	0.43690693299076	0.134	0.072	6.08372572610307E-18	Proliferating hepatocytes	DNAH11
**3.91436574999152E-22**	0.314320352410623	0.191	0.113	6.55969412383578E-18	Proliferating hepatocytes	ELAC1
**4.06825817383185E-22**	0.266342249267997	0.147	0.081	6.81758704770741E-18	Proliferating hepatocytes	KIAA1143
**4.19108974192372E-22**	0.590442838567927	0.102	0.05	7.02342818951578E-18	Proliferating hepatocytes	BTBD8
**4.66371949423738E-22**	0.437330120357059	0.163	0.093	7.81546112844301E-18	Proliferating hepatocytes	NCR3LG1
**5.80152734852585E-22**	0.378650548155762	0.147	0.082	9.72219953065961E-18	Proliferating hepatocytes	STK10
**6.42485886690033E-22**	0.423763132361024	0.116	0.06	1.07667784891516E-17	Proliferating hepatocytes	MYL12B
**6.43191769031949E-22**	0.33038446985856	0.152	0.085	1.07786076654374E-17	Proliferating hepatocytes	E2F6
**6.94185111204993E-22**	0.276809684792327	0.2	0.119	1.16331540935733E-17	Proliferating hepatocytes	VAMP4
**7.26327847175359E-22**	0.263823782124727	0.184	0.108	1.21718020629647E-17	Proliferating hepatocytes	NUDT8
**1.2283880133726E-21**	0.308778291068707	0.183	0.108	2.05853263280981E-17	Proliferating hepatocytes	EXD2
**1.41724331663019E-21**	0.28686103087339	0.185	0.11	2.37501635000887E-17	Proliferating hepatocytes	NHEJ1
**1.45137717879417E-21**	0.359817247443394	0.129	0.069	2.43221787622326E-17	Proliferating hepatocytes	FKRP
**1.46989560628314E-21**	0.29003148990677	0.144	0.079	2.46325105700928E-17	Proliferating hepatocytes	B3GNT2
**1.49598276609E-21**	0.337369533280452	0.103	0.051	2.50696791941362E-17	Proliferating hepatocytes	DUS4L
**1.59381374315067E-21**	0.278460569342177	0.145	0.08	2.6709130707719E-17	Proliferating hepatocytes	TLE3
**1.59952969540296E-21**	0.458500912131261	0.126	0.066	2.68049186355628E-17	Proliferating hepatocytes	ZNF302
**1.64331014181068E-21**	0.312426288417307	0.18	0.106	2.75385913564634E-17	Proliferating hepatocytes	TRIM16
**1.71473614690159E-21**	0.335433946678505	0.191	0.115	2.87355483497769E-17	Proliferating hepatocytes	RCHY1
**1.72528862757663E-21**	0.267475417971661	0.163	0.093	2.89123868209291E-17	Proliferating hepatocytes	WDR61
**1.86085588259376E-21**	0.469112302582084	0.101	0.05	3.11842228805062E-17	Proliferating hepatocytes	KLHDC9
**1.88941436150208E-21**	0.402975443428728	0.136	0.074	3.16628058700518E-17	Proliferating hepatocytes	GLOD4
**1.89590844922101E-21**	0.258427438814162	0.219	0.135	3.17716337920457E-17	Proliferating hepatocytes	FKBP9
**1.96751169580211E-21**	0.280120465540471	0.228	0.143	3.29715609982518E-17	Proliferating hepatocytes	CCDC167
**2.04223470831651E-21**	0.29743687739538	0.197	0.119	3.42237692419681E-17	Proliferating hepatocytes	TAB1
**2.17914789063139E-21**	0.494387885430881	0.108	0.055	3.65181603512009E-17	Proliferating hepatocytes	CHSY1
**2.20964089884506E-21**	0.670161204252016	0.105	0.053	3.70291621828456E-17	Proliferating hepatocytes	RASSF6
**2.37049950123998E-21**	0.305510878528762	0.111	0.057	3.97248306417795E-17	Proliferating hepatocytes	MAD1L1
**3.07738855289408E-21**	0.334227749683667	0.14	0.077	5.1570877369399E-17	Proliferating hepatocytes	TTC12
**3.29027561414264E-21**	0.321452354050817	0.129	0.069	5.51384387418024E-17	Proliferating hepatocytes	GCC1
**3.48887736411252E-21**	0.45963972271898	0.11	0.056	5.84666068677976E-17	Proliferating hepatocytes	URB2
**3.95124106196885E-21**	0.35613846705865	0.189	0.113	6.62148977164739E-17	Proliferating hepatocytes	SMUG1
**4.32480677672361E-21**	0.376537607787534	0.121	0.064	7.24751119643342E-17	Proliferating hepatocytes	TIPIN
**4.76359755130927E-21**	0.264288188468105	0.186	0.11	7.98283677648407E-17	Proliferating hepatocytes	FBXO8
**5.06101121506087E-21**	0.255375366237729	0.487	0.372	8.48124259419901E-17	Proliferating hepatocytes	PILRB
**6.00098148271513E-21**	0.257277365823656	0.149	0.083	1.0056444768734E-16	Proliferating hepatocytes	NOL10
**6.0155921740408E-21**	0.5261695874278	0.133	0.073	1.00809293652576E-16	Proliferating hepatocytes	SPATC1L
**6.19705206494265E-21**	0.459530766735793	0.127	0.068	1.03850198504309E-16	Proliferating hepatocytes	ANKS6
**6.24075813791227E-21**	0.458738175049679	0.134	0.073	1.04582624875134E-16	Proliferating hepatocytes	MTHFSD
**6.4843592694781E-21**	0.425937354674153	0.141	0.078	1.08664892637914E-16	Proliferating hepatocytes	LPCAT2
**6.53490010921308E-21**	0.331717482869528	0.196	0.118	1.09511856030193E-16	Proliferating hepatocytes	ATG10
**6.59435173534965E-21**	0.279767994744825	0.204	0.125	1.1050814638099E-16	Proliferating hepatocytes	FAM89B
**7.2753674330874E-21**	0.459721699652737	0.131	0.071	1.21920607443679E-16	Proliferating hepatocytes	MYPOP
**8.93907679578203E-21**	0.258825059239043	0.143	0.08	1.49801048943715E-16	Proliferating hepatocytes	DOHH
**8.95344550817641E-21**	0.377819931489948	0.123	0.065	1.5004183982602E-16	Proliferating hepatocytes	CTTNBP2NL
**1.09202969038873E-20**	0.281086046606195	0.209	0.129	1.83002335515343E-16	Proliferating hepatocytes	ATMIN
**1.14879211817665E-20**	0.280992202784093	0.244	0.156	1.92514583164042E-16	Proliferating hepatocytes	LATS2
**1.27517935875522E-20**	0.458959328342626	0.118	0.062	2.13694556940199E-16	Proliferating hepatocytes	MTMR2
**1.31575843032379E-20**	0.391516729454287	0.104	0.053	2.2049479775366E-16	Proliferating hepatocytes	ZCCHC10
**1.38377963149104E-20**	0.324546033283988	0.17	0.1	2.31893790645268E-16	Proliferating hepatocytes	CINP
**1.57579796364368E-20**	0.257767645167144	0.159	0.091	2.64072222747408E-16	Proliferating hepatocytes	ENTPD2
**1.62165633354042E-20**	0.285544967247981	0.162	0.095	2.71757168374704E-16	Proliferating hepatocytes	TUSC1

1.78562670573814E-20	0.424393143514121	0.152	0.087	2.99235323347598E-16	Proliferating hepatocytes	SLC38A11
1.96217966686788E-20	0.269246073992969	0.217	0.136	3.2882206857372E-16	Proliferating hepatocytes	MX1
2.06559104616649E-20	0.417028820367603	0.143	0.08	3.4615174751658E-16	Proliferating hepatocytes	TIMM22
2.0836057390507E-20	0.521926337868744	0.137	0.077	3.49170649750116E-16	Proliferating hepatocytes	DEPRECATEI
2.44098783726446E-20	0.409734856372435	0.107	0.055	4.09060741768778E-16	Proliferating hepatocytes	CDC42BPG
2.46202767440254E-20	0.348160246405141	0.189	0.114	4.12586597676378E-16	Proliferating hepatocytes	GALNT10
2.47102037388118E-20	0.258080948996763	0.208	0.129	4.14093594255008E-16	Proliferating hepatocytes	UTP3
2.53213998287976E-20	0.327814434143103	0.133	0.073	4.24336018330991E-16	Proliferating hepatocytes	TECPR2
2.6247832191292E-20	0.432724159161319	0.14	0.078	4.39861171861672E-16	Proliferating hepatocytes	PCNX2
2.92846640739571E-20	0.290998016254861	0.235	0.149	4.90752400551372E-16	Proliferating hepatocytes	BECN1
2.94568313484808E-20	0.436280464627594	0.108	0.056	4.93637579737842E-16	Proliferating hepatocytes	DISP1
2.96515465619192E-20	0.277827835506643	0.117	0.062	4.96900617284643E-16	Proliferating hepatocytes	ABRAXAS1
2.99344184060907E-20	0.332456147643817	0.139	0.077	5.01640983649269E-16	Proliferating hepatocytes	TRIM39
3.03995658406747E-20	0.260192932127339	0.111	0.058	5.09435924358027E-16	Proliferating hepatocytes	CAP1
3.69391790096548E-20	0.331761332728202	0.151	0.087	6.19026761843795E-16	Proliferating hepatocytes	GPANK1
3.6992454572285E-20	0.354265071991539	0.111	0.058	6.19919553722351E-16	Proliferating hepatocytes	MOB1B
3.83981224805198E-20	0.402804338311444	0.126	0.068	6.43475736528552E-16	Proliferating hepatocytes	TFEB
3.93350161888534E-20	0.292369682069871	0.245	0.157	6.59176201292805E-16	Proliferating hepatocytes	CDHR2
4.07593890967317E-20	0.269792426072819	0.259	0.169	6.83045842483029E-16	Proliferating hepatocytes	ANKRD46
4.37800023453489E-20	0.254038761672761	0.127	0.069	7.33665279303357E-16	Proliferating hepatocytes	SLC41A1
5.09552898388085E-20	0.25286199145237	0.125	0.067	8.53908747118752E-16	Proliferating hepatocytes	ZNF668
5.10704888009965E-20	0.35246769073402	0.149	0.086	8.558392513271E-16	Proliferating hepatocytes	DEPRECATEI
5.20288533044492E-20	0.418678622670454	0.237	0.152	8.7189952367596E-16	Proliferating hepatocytes	MEST
5.34925611915069E-20	0.392614232220347	0.14	0.079	8.96428340447273E-16	Proliferating hepatocytes	ZBTB42
5.6943766133054E-20	0.33686261187913	0.103	0.052	9.54263632857718E-16	Proliferating hepatocytes	NME6
6.23265567088385E-20	0.260042692408471	0.281	0.185	1.04446843732672E-15	Proliferating hepatocytes	EDARADD
7.19472037314708E-20	0.409792960693324	0.126	0.069	1.20569124013199E-15	Proliferating hepatocytes	LONRF1
7.62797638810882E-20	0.442014032418486	0.125	0.068	1.27829628311928E-15	Proliferating hepatocytes	KCTD18
8.30801647153867E-20	0.293568162595225	0.107	0.055	1.39225740030045E-15	Proliferating hepatocytes	EFCAB7
8.88775102141366E-20	0.627336779786695	0.111	0.058	1.4894093161685E-15	Proliferating hepatocytes	BTC
9.60665307676277E-20	0.280547720936893	0.184	0.11	1.60988292260391E-15	Proliferating hepatocytes	IGSF3
9.88418457472477E-20	0.417625618878994	0.12	0.065	1.65639165103238E-15	Proliferating hepatocytes	WRN
9.94101514280307E-20	0.28076738378638	0.143	0.081	1.66591531763094E-15	Proliferating hepatocytes	ATP5PO
1.03915253243498E-19	0.293002637848599	0.101	0.051	1.74141181385454E-15	Proliferating hepatocytes	SURF2
1.2568601696937E-19	0.254412385862504	0.2	0.124	2.10624627237271E-15	Proliferating hepatocytes	EVI5L
1.42810765861963E-19	0.308326112515837	0.192	0.118	2.39322281431478E-15	Proliferating hepatocytes	PFKFB2
1.44036762131781E-19	0.375371317812418	0.102	0.052	2.41376805980439E-15	Proliferating hepatocytes	TGFBR3
1.47597925322832E-19	0.276597165456798	0.127	0.07	2.47344603256001E-15	Proliferating hepatocytes	COMMD8
1.6719934880313E-19	0.505698418079271	0.108	0.057	2.80192668724286E-15	Proliferating hepatocytes	NLE1
1.72493251324609E-19	0.538389591755621	0.109	0.057	2.89064190569779E-15	Proliferating hepatocytes	TRMT12
1.90271736868942E-19	0.253534652423299	0.11	0.058	3.18857376644973E-15	Proliferating hepatocytes	GP2
2.44984193017297E-19	0.514320471952763	0.158	0.093	4.10544510658386E-15	Proliferating hepatocytes	CCL25
2.54678608188704E-19	0.305705222173076	0.257	0.169	4.2679041160263E-15	Proliferating hepatocytes	PIAS3
2.8742626196206E-19	0.337310024690558	0.128	0.07	4.8166892979602E-15	Proliferating hepatocytes	DEPRECATEI
3.01153699171814E-19	0.608581495451508	0.11	0.058	5.04673369072125E-15	Proliferating hepatocytes	KLHL9
3.70610835130604E-19	0.408423076924569	0.121	0.066	6.21069637511866E-15	Proliferating hepatocytes	AMMECR1L
3.85311424926764E-19	0.356001409817804	0.162	0.096	6.45704885892272E-15	Proliferating hepatocytes	GPRASP2
3.89562399667023E-19	0.496004876821524	0.123	0.067	6.52828669361997E-15	Proliferating hepatocytes	SLC35A2
4.26226812972204E-19	0.45826613915064	0.103	0.054	7.1427089317882E-15	Proliferating hepatocytes	TIGD3
4.52133954429187E-19	0.3078426733794	0.107	0.056	7.57686080832432E-15	Proliferating hepatocytes	NEURL4
4.82906585725401E-19	0.295033088327112	0.163	0.096	8.09254856358627E-15	Proliferating hepatocytes	DEPRECATEI
5.27608851934524E-19	0.312095245163953	0.137	0.077	8.84166914071876E-15	Proliferating hepatocytes	ARHGAP18
6.00415973682612E-19	0.420060724935161	0.117	0.063	1.00617708869732E-14	Proliferating hepatocytes	TTC4
6.19731139429167E-19	0.338671884799678	0.123	0.067	1.0385454434554E-14	Proliferating hepatocytes	ZNF517
6.44046610123375E-19	0.273387801524475	0.112	0.06	1.07929330924475E-14	Proliferating hepatocytes	DEPRECATEI
6.81393411411731E-19	0.509066237204178	0.167	0.101	1.14187907884378E-14	Proliferating hepatocytes	MDP1
7.31228997741961E-19	0.372931698431182	0.161	0.095	1.22539355441598E-14	Proliferating hepatocytes	AMZ2
8.86005486516267E-19	0.294909948340162	0.174	0.105	1.48476799430396E-14	Proliferating hepatocytes	ARAP2
9.14590500366191E-19	0.665032653387769	0.111	0.06	1.53267076051366E-14	Proliferating hepatocytes	CDYL2
1.02585921563937E-18	0.429404674365282	0.158	0.094	1.71913487356846E-14	Proliferating hepatocytes	TRMT10C

**1.03563794399398E-18**	0.279771505599529	0.265	0.177	1.73552206654511E-14	Proliferating hepatocytes	TEF
**1.08405241212684E-18**	0.315877652356837	0.137	0.078	1.81665503224216E-14	Proliferating hepatocytes	NOXA1
**1.2704695717222E-18**	0.3342293354705	0.222	0.143	2.12905290829206E-14	Proliferating hepatocytes	PLAAT2
**1.28511330352311E-18**	0.395846040859553	0.1	0.052	2.15359287404403E-14	Proliferating hepatocytes	CDPF1
**1.84641464256783E-18**	0.26494131641005	0.131	0.073	3.09422165801517E-14	Proliferating hepatocytes	PANX1
**2.20081481388938E-18**	0.262485895212183	0.265	0.175	3.68812546511582E-14	Proliferating hepatocytes	CTNNBIP1
**2.36642793158109E-18**	0.291536704937461	0.155	0.092	3.96565992774359E-14	Proliferating hepatocytes	SYNM
**2.93678571384105E-18**	0.354467203546932	0.15	0.088	4.92146549925483E-14	Proliferating hepatocytes	FAM3D
**3.86535695160684E-18**	0.306860912755455	0.107	0.057	6.47756517950274E-14	Proliferating hepatocytes	LSM 12.00
**4.11434974221588E-18**	0.337055072694941	0.108	0.058	6.89482729800537E-14	Proliferating hepatocytes	GAREM1
**4.49633194976758E-18**	0.254450160490304	0.147	0.086	7.53495308142052E-14	Proliferating hepatocytes	KAT14
**5.04718118785956E-18**	0.437649781756677	0.123	0.069	8.45806623461505E-14	Proliferating hepatocytes	SIX4
**5.19403414389337E-18**	0.308249373301537	0.171	0.104	8.70416241833651E-14	Proliferating hepatocytes	CHFR
**5.21442655880089E-18**	0.271444372764535	0.128	0.072	8.73833602723853E-14	Proliferating hepatocytes	FAM207A
**6.60556796902005E-18**	0.276605790987808	0.117	0.065	1.10696108024838E-13	Proliferating hepatocytes	SERTAD1
**6.62569582346675E-18**	0.416902273766313	0.126	0.072	1.11033410609656E-13	Proliferating hepatocytes	SIX1
**7.55908780240825E-18**	0.468815129402177	0.11	0.059	1.26675193392758E-13	Proliferating hepatocytes	DEPRECATEI
**9.00349815493394E-18**	0.273907932887977	0.106	0.057	1.50880622080383E-13	Proliferating hepatocytes	LRRC1
**9.04846815766041E-18**	0.280457262416491	0.117	0.065	1.51634229386073E-13	Proliferating hepatocytes	INTS5
**9.21545482901335E-18**	0.396523014906617	0.146	0.086	1.54432592024606E-13	Proliferating hepatocytes	TTC5
**1.15236440067457E-17**	0.290509049307009	0.242	0.16	1.93113226265044E-13	Proliferating hepatocytes	TMCC3
**1.18071541476922E-17**	0.381654298029422	0.132	0.076	1.97864289207026E-13	Proliferating hepatocytes	UTP14A
**1.34186071904236E-17**	0.295359737827037	0.105	0.056	2.24869019297119E-13	Proliferating hepatocytes	HIC2
**1.48262206449917E-17**	0.343291146482553	0.126	0.072	2.48457805568771E-13	Proliferating hepatocytes	SEMA5A
**1.55751089755368E-17**	0.636325874101632	0.103	0.056	2.61007676212045E-13	Proliferating hepatocytes	CDX2
**1.61011402026848E-17**	0.303436346239016	0.182	0.114	2.69822907516592E-13	Proliferating hepatocytes	CHCHD4
**1.61830655070386E-17**	0.424450918834689	0.109	0.06	2.71195811766953E-13	Proliferating hepatocytes	C1orf226
**1.69297823317055E-17**	0.39389220199826	0.125	0.071	2.83709292314721E-13	Proliferating hepatocytes	YTHDF1
**1.91210277082431E-17**	0.326167249111185	0.121	0.068	3.20430182334738E-13	Proliferating hepatocytes	ZNF354B
**1.97299038891098E-17**	0.344713726657943	0.109	0.059	3.30633729373702E-13	Proliferating hepatocytes	BIN3
**1.99638556452061E-17**	0.265374789066692	0.203	0.13	3.34554292902363E-13	Proliferating hepatocytes	PJA1
**2.21075451508638E-17**	0.263232022218862	0.179	0.111	3.70478241638175E-13	Proliferating hepatocytes	BAHD1
**2.25236022846478E-17**	0.285250482672224	0.118	0.065	3.77450527086127E-13	Proliferating hepatocytes	SALL4
**2.64118174214336E-17**	0.343131461015016	0.121	0.068	4.42609236348384E-13	Proliferating hepatocytes	PAIP2B
**2.77485983412267E-17**	0.296123872925764	0.13	0.074	4.65011011002276E-13	Proliferating hepatocytes	WDR4
**2.93645294851611E-17**	0.299999170993592	0.135	0.078	4.92090785112329E-13	Proliferating hepatocytes	DEPRECATEI
**2.93920507318639E-17**	0.339191768337552	0.216	0.14	4.92551986164576E-13	Proliferating hepatocytes	LRRC8E
**3.69159907905124E-17**	0.438742507329351	0.127	0.073	6.18638173667407E-13	Proliferating hepatocytes	SLC45A4
**3.81399353377475E-17**	0.419943813731984	0.127	0.074	6.39149036389973E-13	Proliferating hepatocytes	CCNE1
**4.30665110374786E-17**	0.255002018137985	0.118	0.066	7.21708591966066E-13	Proliferating hepatocytes	IQCG
**4.58322512294547E-17**	0.313776163472099	0.125	0.071	7.68056866103202E-13	Proliferating hepatocytes	DCUN1D5
**4.93939353243233E-17**	0.443824153087915	0.112	0.062	8.2774356816501E-13	Proliferating hepatocytes	SHLD1
**5.93509447793636E-17**	0.390972141704616	0.101	0.054	9.94603132612576E-13	Proliferating hepatocytes	LENG9
**6.0808118025932E-17**	0.264274870832227	0.132	0.076	1.01902244187857E-12	Proliferating hepatocytes	FDX2
**6.3420089073413E-17**	0.466773021850519	0.102	0.055	1.06279385269226E-12	Proliferating hepatocytes	CYB561D1
**6.69215726639248E-17**	0.389079784378175	0.101	0.054	1.12147171470205E-12	Proliferating hepatocytes	STN 1.00
**7.17007819231958E-17**	0.514552774075241	0.11	0.061	1.20156170346892E-12	Proliferating hepatocytes	RTN4RL1
**7.22187088447738E-17**	0.306374463165606	0.185	0.117	1.21024112282072E-12	Proliferating hepatocytes	SYDE2
**7.43646930312894E-17**	0.305163684853764	0.187	0.118	1.24620352581835E-12	Proliferating hepatocytes	TRMT61A
**7.62755787207607E-17**	0.294744244195786	0.13	0.075	1.27822614820251E-12	Proliferating hepatocytes	RTL8A
**7.81501941768447E-17**	0.319145573452951	0.162	0.099	1.30964095401556E-12	Proliferating hepatocytes	SLC44A4
**9.06973202125443E-17**	0.250381839534849	0.105	0.057	1.51990569212182E-12	Proliferating hepatocytes	MEAK7
**1.13846110986116E-16**	0.356083079968676	0.127	0.073	1.90783312790533E-12	Proliferating hepatocytes	ZNF446
**1.52478899827637E-16**	0.34693164961898	0.197	0.127	2.55524140331154E-12	Proliferating hepatocytes	WNT4
**1.61205135776233E-16**	0.36564166002787	0.1	0.054	2.70147566533811E-12	Proliferating hepatocytes	SLC30A4
**1.77134115326638E-16**	0.293131921982484	0.125	0.072	2.96841350464381E-12	Proliferating hepatocytes	NOD1
**2.25920688942085E-16**	0.337241680627678	0.118	0.067	3.78597890529146E-12	Proliferating hepatocytes	GPR135
**3.05440634909126E-16**	0.256895178488966	0.153	0.093	5.11857415980713E-12	Proliferating hepatocytes	PPP2R1A
**3.36209907916003E-16**	0.291127832204034	0.1	0.054	5.63420563685639E-12	Proliferating hepatocytes	ADPRM
**3.56262091006895E-16**	0.251976016518829	0.177	0.112	5.97024012109355E-12	Proliferating hepatocytes	AAR2

3.65663322687629E-16	0.512599286969192	0.105	0.058	6.12778596159928E-12	Proliferating hepatocytes	LACC1
4.25143212227994E-16	0.329122742271291	0.146	0.088	7.12454995051673E-12	Proliferating hepatocytes	CTNND2
5.47025878353006E-16	0.442060972964231	0.154	0.094	9.16705966943968E-12	Proliferating hepatocytes	LUZP1
9.50750730435193E-16	0.455172119577737	0.114	0.065	1.5932680740633E-11	Proliferating hepatocytes	PIGW
1.03415459165639E-15	0.371656767477365	0.101	0.056	1.73303626469778E-11	Proliferating hepatocytes	PHGR1
1.18756196039498E-15	0.405748219141489	0.1	0.055	1.99011633322991E-11	Proliferating hepatocytes	ZNF181
1.28818315127849E-15	0.28989189632098	0.109	0.062	2.15873732491249E-11	Proliferating hepatocytes	PIGB
1.4087257265135E-15	0.460460452216576	0.139	0.084	2.36074257249133E-11	Proliferating hepatocytes	ZNF16
1.607264098269E-15	0.358745568402699	0.1	0.055	2.69345317587919E-11	Proliferating hepatocytes	BCL9
2.1866188464923E-15	0.258901176007608	0.137	0.082	3.6643358629518E-11	Proliferating hepatocytes	MAP2K6
2.40970432665342E-15	0.411558028555509	0.176	0.113	4.03818251060581E-11	Proliferating hepatocytes	PPP1R3E
2.85973532231042E-15	0.275263758718213	0.122	0.071	4.7923444531278E-11	Proliferating hepatocytes	RPUSD1
3.15777034217076E-15	0.328149320164667	0.132	0.079	5.29179153940976E-11	Proliferating hepatocytes	DEPRECATEI
4.60889451525128E-15	0.282644307173489	0.103	0.057	7.7235854286581E-11	Proliferating hepatocytes	ZBTB26
6.3704784119668E-15	0.297317710283085	0.133	0.08	1.0675647722774E-10	Proliferating hepatocytes	TLR3
1.05215812600861E-14	0.257172061260794	0.108	0.062	1.76320658756522E-10	Proliferating hepatocytes	WDR73
1.15640064901795E-14	0.32861431532821	0.124	0.074	1.93789620762428E-10	Proliferating hepatocytes	SH3BP1
3.48808493491325E-14	0.295933554599083	0.125	0.076	5.84533273392762E-10	Proliferating hepatocytes	PTRHD1
3.71743500060725E-14	0.276060824570998	0.103	0.058	6.22967757401762E-10	Proliferating hepatocytes	ORAI3
6.89121226156658E-14	0.338597080615798	0.138	0.086	1.15482935079333E-09	Proliferating hepatocytes	SOWAHA
7.1541265329192E-14	0.459792336607115	0.102	0.058	1.1988885243866E-09	Proliferating hepatocytes	DEPRECATEI
7.87401579635667E-14	0.308944425370018	0.114	0.068	1.31952756715345E-09	Proliferating hepatocytes	PRDM10
8.05334218529199E-14	0.252445531900858	0.111	0.065	1.34957908341123E-09	Proliferating hepatocytes	SNX25
1.94434775850234E-13	0.267863079751136	0.114	0.068	3.25833797369823E-09	Proliferating hepatocytes	CABLES1
2.04679389124279E-13	0.273031690853443	0.135	0.084	3.43001720294467E-09	Proliferating hepatocytes	TMEM267
2.21228789841808E-13	0.268119131064164	0.109	0.064	3.70735206016902E-09	Proliferating hepatocytes	MFSD5
2.79359677868716E-13	0.382777051031361	0.105	0.062	4.68150948172395E-09	Proliferating hepatocytes	MTRF1L
5.27886277136331E-13	0.309553092962299	0.133	0.084	8.84631823225064E-09	Proliferating hepatocytes	PRMT9
6.60977346867076E-13	0.319791272822207	0.127	0.079	1.10766583787985E-08	Proliferating hepatocytes	PNMT
6.39595894093828E-12	0.288890731644778	0.146	0.095	1.07183479932244E-07	Proliferating hepatocytes	TMEM42
1.39137217832093E-11	0.250942840744538	0.104	0.063	2.33166149643021E-07	Proliferating hepatocytes	ZNF205
1.87904722963456E-11	0.354336388407478	0.125	0.081	3.14890734742159E-07	Proliferating hepatocytes	RCAN2
1.07428184684394E-10	0.306647306259046	0.132	0.087	1.80028151894108E-06	Proliferating hepatocytes	ISX
8.29450677961028E-10	0.327837360014505	0.107	0.069	1.38999344612709E-05	Proliferating hepatocytes	XPNPEP2
6.73442634410026E-09	0.349328205991668	0.1	0.065	0.000112855516674432	Proliferating hepatocytes	KCNJ11
0	2.18391197202857	0.907	0.644	0	Oxidative stress hepatocytes	STARD10
0	1.51056715267861	0.976	0.902	0	Oxidative stress hepatocytes	SQSTM1
0	2.68472211703936	1	0.971	0	Oxidative stress hepatocytes	FTL
2.78218761175433E-281	2.22678144616225	0.764	0.468	4.66238999977791E-277	Oxidative stress hepatocytes	IRAK1
9.78288196138583E-279	2.49139332099535	0.716	0.385	1.63941535908904E-274	Oxidative stress hepatocytes	SRXN1
2.48947663072108E-273	1.51200940094516	0.904	0.728	4.17186493776238E-269	Oxidative stress hepatocytes	GPX2
1.04702363878913E-249	1.21553700413395	0.922	0.666	1.75460221388282E-245	Oxidative stress hepatocytes	NQO1
5.93942301615125E-247	1.08275098330242	0.951	0.832	9.95328509046626E-243	Oxidative stress hepatocytes	PTGR1
9.24091051662393E-245	1.39309259651046	0.916	0.81	1.54859178437584E-240	Oxidative stress hepatocytes	FTH1
1.60200948121236E-240	1.12939396065778	0.921	0.798	2.68464748861567E-236	Oxidative stress hepatocytes	OAZ1
9.96594215605543E-236	1.44197311900334	0.881	0.704	1.67009258651177E-231	Oxidative stress hepatocytes	EEF2
7.10956887601419E-214	0.912051034261221	0.956	0.85	1.19142155224246E-209	Oxidative stress hepatocytes	ALDH1A1
1.47540302449565E-210	2.1313126562104	0.709	0.453	2.47248038844982E-206	Oxidative stress hepatocytes	SLC2A4RG
4.87129122358347E-194	1.45698326894456	0.784	0.531	8.16330983248117E-190	Oxidative stress hepatocytes	G6PD
8.96850042205986E-190	1.09795151458713	0.901	0.712	1.50294130072879E-185	Oxidative stress hepatocytes	AKR1B10
2.12595596276657E-178	1.22951970141169	0.835	0.704	3.56267700240422E-174	Oxidative stress hepatocytes	GPX4
3.17445502981588E-167	1.17396040272822	0.839	0.634	5.31975173896545E-163	Oxidative stress hepatocytes	DEPRECATEI
1.65477512442945E-155	0.905895278636826	0.909	0.743	2.77307215351888E-151	Oxidative stress hepatocytes	LGALS4
2.45323793248276E-155	1.926913727551	0.622	0.381	4.11113612725461E-151	Oxidative stress hepatocytes	TLE5
8.52407133611691E-154	2.27761084693401	0.488	0.219	1.42846387450647E-149	Oxidative stress hepatocytes	ETNK2
6.33297138692057E-153	1.81030566487035	0.66	0.448	1.06127934502015E-148	Oxidative stress hepatocytes	SLC25A39
1.29835833861362E-148	1.52922595589683	0.73	0.563	2.1757889038487E-144	Oxidative stress hepatocytes	MGAT4B
1.41943550249062E-141	0.77898001644125	0.97	0.945	2.37869001507378E-137	Oxidative stress hepatocytes	ALDOB
4.38006647121754E-139	1.17591604389166	0.804	0.67	7.34011539246635E-135	Oxidative stress hepatocytes	KRT8
5.44098435907658E-139	1.1394167741046	0.847	0.76	9.11800158894054E-135	Oxidative stress hepatocytes	NUPR1

**1.73753016402418E-138**	1.01341023924408	0.847	0.705	2.91175304887173E-134	Oxidative stress hepatocytes	GSTA1
**3.46096098937964E-134**	2.13385619079379	0.545	0.314	5.7998784260024E-130	Oxidative stress hepatocytes	PKN1
**2.44401729333577E-130**	0.848654643686901	0.922	0.735	4.09568418017208E-126	Oxidative stress hepatocytes	S100A10
**2.68537556094954E-127**	2.05926095585556	0.55	0.331	4.50015236503923E-123	Oxidative stress hepatocytes	GUK1
**1.08112703349705E-125**	1.77112707242198	0.6	0.405	1.81175268273436E-121	Oxidative stress hepatocytes	MAP2K2
**3.64106370630432E-124**	1.60356292113444	0.666	0.486	6.10169455902478E-120	Oxidative stress hepatocytes	MAPKAPK2
**9.47648123935184E-120**	1.82355446183635	0.589	0.39	1.58806872609058E-115	Oxidative stress hepatocytes	TMEM219
**5.38342814871482E-118**	0.723955636868152	0.903	0.782	9.02154889161629E-114	Oxidative stress hepatocytes	UGDH
**1.96688987455793E-116**	2.34608912821701	0.473	0.253	3.29611405178418E-112	Oxidative stress hepatocytes	CENPB
**2.75661400789163E-116**	1.37891552486647	0.658	0.469	4.61953375442479E-112	Oxidative stress hepatocytes	LAGE3
**3.11209312172494E-116**	1.17437013090746	0.748	0.605	5.21524565338665E-112	Oxidative stress hepatocytes	S100A16
**3.13129529612847E-114**	2.26580838478652	0.492	0.28	5.24742465725209E-110	Oxidative stress hepatocytes	LRP3
**5.89560567616247E-113**	1.33312227668812	0.675	0.513	9.87985599211307E-109	Oxidative stress hepatocytes	DAP
**7.0094213617479E-111**	1.30858665083039	0.673	0.513	1.17463883180171E-106	Oxidative stress hepatocytes	ATP5F1D
**2.85008163793289E-110**	2.08386247668076	0.517	0.311	4.77616680884794E-106	Oxidative stress hepatocytes	RNPEPL1
**3.23649754555709E-110**	1.7736254731556	0.548	0.345	5.42372258684457E-106	Oxidative stress hepatocytes	MAF 1.00
**4.12547958836218E-109**	2.38278077997713	0.432	0.224	6.91347869417734E-105	Oxidative stress hepatocytes	SLC25A23
**2.8212532007083E-104**	1.2561337682811	0.678	0.523	4.72785611374696E-100	Oxidative stress hepatocytes	CBR1
**1.21360711847463E-99**	1.56353401727743	0.599	0.429	2.03376280913979E-95	Oxidative stress hepatocytes	LASP1
**2.46591965727387E-99**	1.93335512486287	0.493	0.298	4.13238816165955E-95	Oxidative stress hepatocytes	PIK3R2
**3.0465835229904E-93**	1.6633130807332	0.568	0.402	5.10546466782731E-89	Oxidative stress hepatocytes	AKT1
**6.50420517066021E-93**	1.59424962714524	0.568	0.402	1.08997470249924E-88	Oxidative stress hepatocytes	PMVK
**3.67510733211663E-92**	2.07582674393387	0.429	0.234	6.15874486716105E-88	Oxidative stress hepatocytes	NME4
**8.52894116585186E-92**	0.674685489362532	0.869	0.738	1.42927996057345E-87	Oxidative stress hepatocytes	AKR1C3
**8.89263572947222E-90**	1.089552425511	0.708	0.514	1.49022789554496E-85	Oxidative stress hepatocytes	PEG10
**2.88635644080069E-88**	1.55094748820656	0.545	0.371	4.8369561234938E-84	Oxidative stress hepatocytes	LAMTOR1
**2.2790573591269E-87**	1.56080138311284	0.575	0.423	3.81924432242486E-83	Oxidative stress hepatocytes	SEPTIN9
**1.74387012118829E-86**	1.05873896050237	0.727	0.608	2.92237754908734E-82	Oxidative stress hepatocytes	BEX3
**2.75833087244316E-85**	1.57157360450818	0.561	0.408	4.62241087604026E-81	Oxidative stress hepatocytes	SHARPIN
**7.77809789612704E-85**	2.12324739223294	0.45	0.27	1.30345364543297E-80	Oxidative stress hepatocytes	KIF1C
**6.49591840559582E-84**	2.0039154446809	0.453	0.276	1.08858600640975E-79	Oxidative stress hepatocytes	C7orf50
**3.91125488840368E-83**	2.2984317817666	0.415	0.24	6.55448094198689E-79	Oxidative stress hepatocytes	ZNF219
**6.54675412783115E-83**	0.58318851186005	0.912	0.732	1.09710505674194E-78	Oxidative stress hepatocytes	ANXA4
**4.68046158918564E-81**	1.87427229798862	0.453	0.275	7.84351753115729E-77	Oxidative stress hepatocytes	NOP53
**9.20813474812066E-81**	0.859212629896548	0.741	0.605	1.54309922109006E-76	Oxidative stress hepatocytes	NDUFA1
**1.21357995563836E-80**	1.61405870680589	0.559	0.414	2.03371728965876E-76	Oxidative stress hepatocytes	NR2F6
**1.72500260330396E-80**	1.67411399051009	0.644	0.459	2.89075936261677E-76	Oxidative stress hepatocytes	SLC6A8
**4.15313754229601E-80**	1.07347374112262	0.724	0.643	6.95982789337965E-76	Oxidative stress hepatocytes	BRI3
**4.79530023316498E-78**	1.37117992930208	0.582	0.435	8.03596413073787E-74	Oxidative stress hepatocytes	EFHD2
**1.18425013746815E-77**	0.981141456709816	0.705	0.601	1.98456638036913E-73	Oxidative stress hepatocytes	TXNRD1
**4.51103496956866E-77**	1.23811969329768	0.661	0.555	7.55959240200316E-73	Oxidative stress hepatocytes	CAMK2N1
**1.2042666293296E-76**	2.11887776730813	0.396	0.224	2.01811001743054E-72	Oxidative stress hepatocytes	ZNF358
**9.67229875234173E-76**	2.34001940482393	0.363	0.194	1.62088382491743E-71	Oxidative stress hepatocytes	VEGFB
**1.39362985946037E-73**	0.735650376043119	0.787	0.699	2.33544491848368E-69	Oxidative stress hepatocytes	LMNA
**1.94950709709422E-73**	1.30692631614801	0.595	0.467	3.26698399331049E-69	Oxidative stress hepatocytes	ARL6IP4
**5.84306224156178E-73**	0.88081170764899	0.731	0.625	9.79180370440923E-69	Oxidative stress hepatocytes	CRYL1
**7.24478712704556E-73**	0.942797862386356	0.669	0.529	1.21408142675029E-68	Oxidative stress hepatocytes	CALM3
**4.08273368729307E-72**	1.12905580525452	0.637	0.522	6.84184511316572E-68	Oxidative stress hepatocytes	POLR2L
**6.99447599495867E-72**	0.912589244602799	0.72	0.644	1.17213428723517E-67	Oxidative stress hepatocytes	SLC9A3R1
**4.08613965668603E-70**	1.60571091188315	0.488	0.334	6.84755283667444E-66	Oxidative stress hepatocytes	SLC35A4
**3.85699588320693E-69**	0.654164059964716	0.796	0.611	6.46355370107817E-65	Oxidative stress hepatocytes	MPP1
**3.24216422535239E-68**	0.902313990509954	0.709	0.609	5.43321880884553E-64	Oxidative stress hepatocytes	CYC1
**4.15059416912253E-68**	1.65559764504965	0.492	0.346	6.95556570861554E-64	Oxidative stress hepatocytes	UBXN6
**2.55893508562972E-67**	1.46935490309924	0.553	0.427	4.28826341649829E-63	Oxidative stress hepatocytes	TAF10
**1.12235697508639E-66**	1.21539045279968	0.575	0.446	1.88084581884976E-62	Oxidative stress hepatocytes	DEPRECATEI
**2.4558489985258E-65**	1.1645906068506	0.632	0.533	4.11551175172954E-61	Oxidative stress hepatocytes	RXRA
**1.18557954572626E-64**	1.27950917442219	0.576	0.452	1.98679420272806E-60	Oxidative stress hepatocytes	AMOTL1
**2.78531336213264E-64**	1.64693073754284	0.489	0.352	4.66762813226187E-60	Oxidative stress hepatocytes	DUS1L
**7.22362620849882E-64**	1.44827877766051	0.539	0.417	1.21053528002023E-59	Oxidative stress hepatocytes	KIAA1522
**9.91012574861605E-64**	1.79405456140885	0.455	0.312	1.66073887295308E-59	Oxidative stress hepatocytes	WSB2

**7.27140586195565E-63**	1.14134493263868	0.615	0.506	1.21854219434653E-58	Oxidative stress hepatocytes	NFIC
**3.68503808490091E-62**	0.78634414874965	0.776	0.597	6.17538682267695E-58	Oxidative stress hepatocytes	NDRG1
**5.61925000941409E-62**	1.15863689210168	0.595	0.485	9.41673916577613E-58	Oxidative stress hepatocytes	EGLN1
**1.2589136643706E-61**	1.43200141505079	0.521	0.388	2.10968751875224E-57	Oxidative stress hepatocytes	IMPA2
**5.53470128538116E-61**	1.43110938562319	0.502	0.363	9.27505241404175E-57	Oxidative stress hepatocytes	TNIP1
**2.24906667226659E-60**	1.7268045211875	0.439	0.296	3.76898592938435E-56	Oxidative stress hepatocytes	FKBP8
**1.86509012281735E-58**	1.7206866567422	0.452	0.315	3.12551802781731E-54	Oxidative stress hepatocytes	DNAJC4
**6.97363947443234E-58**	0.542847557960012	0.852	0.627	1.16864250312537E-53	Oxidative stress hepatocytes	TSC22D3
**1.600438728252E-57**	1.73160559917458	0.459	0.322	2.68201522080471E-53	Oxidative stress hepatocytes	C1orf115
**3.99794606358866E-57**	1.08072675509542	0.613	0.497	6.69975801336187E-53	Oxidative stress hepatocytes	CMC4
**1.43161295768431E-56**	0.6377795235	0.798	0.746	2.39909699448737E-52	Oxidative stress hepatocytes	ECH1
**3.7905641344511E-56**	1.78081077649253	0.402	0.261	6.35222737651315E-52	Oxidative stress hepatocytes	PRELID1
**6.0537684574013E-56**	1.73104616569957	0.431	0.298	1.01449051809131E-51	Oxidative stress hepatocytes	BTBD2
**1.33106519845793E-55**	0.72158536149866	0.733	0.663	2.23059905957581E-51	Oxidative stress hepatocytes	CFL1
**3.00060870888938E-55**	1.4448476190379	0.495	0.372	5.02842007435682E-51	Oxidative stress hepatocytes	TIMM13
**6.73258332996135E-55**	1.35260029582131	0.509	0.386	1.12824631443492E-50	Oxidative stress hepatocytes	BIN1
**3.14100768636226E-54**	1.42585097250372	0.516	0.395	5.26370068080588E-50	Oxidative stress hepatocytes	GCHFR
**2.02736465947074E-53**	0.935382699391899	0.646	0.545	3.39745769634106E-49	Oxidative stress hepatocytes	ANXA2
**1.7670272353E-52**	0.847426735726719	0.645	0.543	2.96118424091573E-48	Oxidative stress hepatocytes	FAM50A
**2.94133441692424E-52**	0.728844536950991	0.728	0.653	4.92908821588164E-48	Oxidative stress hepatocytes	H3F3A
**9.7583516349848E-52**	1.60753295525493	0.45	0.329	1.63530456699075E-47	Oxidative stress hepatocytes	ASB13
**1.28188442582386E-51**	1.80862907225667	0.379	0.242	2.14818192079563E-47	Oxidative stress hepatocytes	SYNPO
**2.79708487610817E-51**	1.23649284986639	0.523	0.401	4.68735483538208E-47	Oxidative stress hepatocytes	CDKN1A
**2.86368581687518E-51**	1.43523502003969	0.455	0.324	4.79896469191943E-47	Oxidative stress hepatocytes	FBXW5
**4.89142639513831E-51**	1.99719849958264	0.326	0.191	8.19705235297278E-47	Oxidative stress hepatocytes	UBL4A
**6.7650906051815E-51**	1.13697364272643	0.589	0.496	1.13369388361632E-46	Oxidative stress hepatocytes	ENO1
**8.11959897993868E-51**	1.48609695501513	0.468	0.35	1.36068239705812E-46	Oxidative stress hepatocytes	KEAP1
**1.93527874697053E-50**	0.975143520760688	0.636	0.559	3.24314012417322E-46	Oxidative stress hepatocytes	JUND
**2.27706413206398E-50**	1.17946464091834	0.547	0.439	3.81590407251281E-46	Oxidative stress hepatocytes	GSTA2
**3.41548811862093E-50**	1.60697226932234	0.449	0.329	5.72367498918495E-46	Oxidative stress hepatocytes	EPN1
**3.54414828856693E-50**	1.84677637422326	0.388	0.26	5.93928370198045E-46	Oxidative stress hepatocytes	KANK2
**3.85401793218235E-50**	0.813654218255793	0.656	0.566	6.45856325075118E-46	Oxidative stress hepatocytes	RBM47
**4.02089002541163E-50**	0.919166470393184	0.646	0.576	6.73820750458481E-46	Oxidative stress hepatocytes	BLVRB
**1.62901884663677E-49**	1.9327687649003	0.38	0.246	2.7299097831939E-45	Oxidative stress hepatocytes	E2F1
**1.80107880240896E-49**	0.859583486433558	0.634	0.533	3.01824785707693E-45	Oxidative stress hepatocytes	PRDX1
**4.15436436031599E-49**	2.59478650191032	0.171	0.067	6.96188379501754E-45	Oxidative stress hepatocytes	ENHO
**6.28908665817917E-49**	1.48351386240497	0.469	0.353	1.05392514217767E-44	Oxidative stress hepatocytes	NAA60
**6.87648979966914E-48**	1.78544673274444	0.398	0.275	1.15236216062855E-43	Oxidative stress hepatocytes	ARPC1A
**7.20829925653428E-48**	1.03867347161493	0.597	0.503	1.20796678941002E-43	Oxidative stress hepatocytes	MIDN
**8.96781852091679E-48**	0.393144672752038	0.904	0.866	1.50282702773524E-43	Oxidative stress hepatocytes	IL32
**9.61030153634587E-48**	1.25393642599321	0.519	0.421	1.61049433146084E-43	Oxidative stress hepatocytes	ILRUN
**1.14768212373618E-47**	0.778842336611894	0.67	0.588	1.92328570295709E-43	Oxidative stress hepatocytes	PFN1
**2.00522520466929E-47**	1.09447953312572	0.561	0.429	3.36035639798479E-43	Oxidative stress hepatocytes	NBL1
**2.99502032298662E-47**	0.634711533969553	0.745	0.67	5.01905505726098E-43	Oxidative stress hepatocytes	PTMS
**8.84360573290235E-47**	0.570814096056893	0.819	0.725	1.48201144871978E-42	Oxidative stress hepatocytes	IFI27
**1.03126884599148E-46**	1.24887586759519	0.529	0.437	1.72820033211252E-42	Oxidative stress hepatocytes	DYNLL2
**1.08100844760712E-46**	1.82390170409799	0.43	0.313	1.81155395650001E-42	Oxidative stress hepatocytes	RASSF3
**2.10960570246986E-46**	0.817993780832478	0.658	0.579	3.535277236199E-42	Oxidative stress hepatocytes	ACTG1
**2.64516453296765E-46**	1.88461207408727	0.341	0.214	4.43276672434719E-42	Oxidative stress hepatocytes	PRSS3
**5.88369079061951E-45**	2.1815196254098	0.246	0.127	9.85988902692018E-41	Oxidative stress hepatocytes	METRN
**6.87511224897624E-45**	1.12482145174309	0.541	0.449	1.15213131068344E-40	Oxidative stress hepatocytes	SF3B5
**7.95390988265909E-45**	1.10512275972619	0.533	0.437	1.33291621813601E-40	Oxidative stress hepatocytes	ITPK1
**2.0591849957771E-44**	0.902738500664975	0.609	0.535	3.45078221592326E-40	Oxidative stress hepatocytes	SEPHS2
**1.37007070824502E-43**	0.906300944676505	0.615	0.529	2.295964492877E-39	Oxidative stress hepatocytes	SAP18
**1.72814964345479E-43**	1.49960475877952	0.44	0.331	2.89603317250154E-39	Oxidative stress hepatocytes	SHROOM3
**3.48269404943586E-43**	1.78148796470924	0.339	0.216	5.83629868804461E-39	Oxidative stress hepatocytes	CSNK1G2
**4.61056401638046E-43**	0.652800906817976	0.718	0.653	7.72638317865037E-39	Oxidative stress hepatocytes	MPC2
**5.74255506300484E-43**	0.844624366639111	0.661	0.577	9.6233737745835E-39	Oxidative stress hepatocytes	TMEM106C
**8.93494463904915E-43**	1.34233061187005	0.451	0.34	1.49731802261186E-38	Oxidative stress hepatocytes	GUCD1
**9.89150175889407E-43**	1.30869361586224	0.48	0.378	1.65761786475547E-38	Oxidative stress hepatocytes	USF2

1.12154146570169E-42	0.818549914214938	0.634	0.562	1.87947918822289E-38	Oxidative stress hepatocytes	EMD
4.20648302940351E-42	1.45955385512085	0.431	0.323	7.04922426067441E-38	Oxidative stress hepatocytes	NIBAN2
7.01658508966405E-42	0.816836266052674	0.653	0.589	1.1758393293259E-37	Oxidative stress hepatocytes	TSTA3
8.02116116937183E-42	0.73392603059918	0.673	0.616	1.34418618876333E-37	Oxidative stress hepatocytes	PRDX2
9.292011080251E-41	1.20804648391313	0.501	0.402	1.55715521682846E-36	Oxidative stress hepatocytes	FAM3A
2.68386526984444E-40	1.3625232218361	0.471	0.376	4.49762141920531E-36	Oxidative stress hepatocytes	HIPK2
9.80097871658066E-40	1.40448410929483	0.418	0.31	1.64244801332459E-35	Oxidative stress hepatocytes	VAT1
1.09430029205354E-39	1.2457626540163	0.481	0.389	1.83382842942332E-35	Oxidative stress hepatocytes	PTPA
1.49169364938428E-39	0.666323072012833	0.706	0.676	2.49978021763817E-35	Oxidative stress hepatocytes	ETFB
1.69004355622563E-39	1.32208618034615	0.434	0.321	2.83217499152291E-35	Oxidative stress hepatocytes	CHP2
4.39526317495603E-39	1.69166274156027	0.363	0.251	7.36558202859132E-35	Oxidative stress hepatocytes	ALKBH5
4.84623300782472E-39	1.49750777770992	0.415	0.31	8.12131727451266E-35	Oxidative stress hepatocytes	NACC1
7.72156343447359E-39	0.939642931909851	0.597	0.529	1.29397960034908E-34	Oxidative stress hepatocytes	AHNAK
1.37161443042668E-38	1.28549683188157	0.471	0.38	2.29855146250903E-34	Oxidative stress hepatocytes	RNF187
7.32417271715237E-38	1.34002426870664	0.483	0.398	1.22738486394039E-33	Oxidative stress hepatocytes	NBR1
8.98646142785497E-38	1.75973820591865	0.308	0.194	1.50595120607994E-33	Oxidative stress hepatocytes	HDAC5
2.66794082025531E-37	1.04170620280843	0.526	0.45	4.47093522658385E-33	Oxidative stress hepatocytes	UBE2Q1
3.3410466911969E-37	1.48079118278331	0.415	0.316	5.59892604510776E-33	Oxidative stress hepatocytes	NCOR2
5.34277785380705E-36	1.41797970423518	0.443	0.35	8.95342712740985E-32	Oxidative stress hepatocytes	CTDSP1
6.72843736163935E-36	1.07170945330319	0.518	0.448	1.12755153306352E-31	Oxidative stress hepatocytes	PDXK
1.66360313319516E-35	1.02684065453349	0.535	0.432	2.78786613060844E-31	Oxidative stress hepatocytes	DDIT4
1.9861382939484E-35	1.85869889238644	0.322	0.217	3.32837055299874E-31	Oxidative stress hepatocytes	TADA3
4.85085520973148E-35	0.613489855352015	0.716	0.694	8.12906316046801E-31	Oxidative stress hepatocytes	TMSB4X
6.86530145632476E-35	1.1776649884464	0.472	0.385	1.1504872180509E-30	Oxidative stress hepatocytes	SYBU
1.35321027134486E-34	1.01748798083349	0.543	0.477	2.26770977271972E-30	Oxidative stress hepatocytes	SLC25A1
3.0569621508867E-34	0.810462577940485	0.613	0.549	5.12285717245593E-30	Oxidative stress hepatocytes	NDUFA2
8.46341722257686E-34	1.37764875439497	0.409	0.313	1.41829945815943E-29	Oxidative stress hepatocytes	TMEM141
3.98681753879209E-33	1.65630763004921	0.349	0.249	6.68110883150779E-29	Oxidative stress hepatocytes	SZRD1
4.13200752837723E-33	1.23785054399047	0.442	0.354	6.92441821605455E-29	Oxidative stress hepatocytes	CLTB
5.31829241190997E-33	1.40834264239572	0.402	0.306	8.91239442387872E-29	Oxidative stress hepatocytes	NFIX
6.03558104437228E-33	0.977772884690484	0.502	0.42	1.01144267141591E-28	Oxidative stress hepatocytes	HNRNPUL1
6.17624537671343E-33	0.913978953218998	0.561	0.494	1.03501520022964E-28	Oxidative stress hepatocytes	CMBL
1.31274943487492E-32	1.14304092934956	0.476	0.39	2.19990550296339E-28	Oxidative stress hepatocytes	RHOC
1.6938087636265E-32	1.04250313232319	0.538	0.479	2.83848472608529E-28	Oxidative stress hepatocytes	RACK1
2.20614569340214E-32	2.0890401816676	0.174	0.085	3.6970589530033E-28	Oxidative stress hepatocytes	CCDC85B
4.57134527120722E-32	1.0617367581489	0.554	0.484	7.66066040548906E-28	Oxidative stress hepatocytes	NUDT16
6.38209875880464E-32	1.05576942551023	0.499	0.417	1.06951211000048E-27	Oxidative stress hepatocytes	FOSL2
7.05949979533069E-32	1.15105994761355	0.481	0.402	1.18303097570152E-27	Oxidative stress hepatocytes	MMP24OS
7.57258030990591E-32	1.0270490303635	0.543	0.479	1.26901300833403E-27	Oxidative stress hepatocytes	DSP
7.73530096068041E-32	1.50585455098348	0.357	0.258	1.29628173499082E-27	Oxidative stress hepatocytes	TRAF7
1.96543903891613E-31	0.493086319528291	0.752	0.652	3.29368274141566E-27	Oxidative stress hepatocytes	ACSL4
2.37782868661343E-31	1.94843975807137	0.278	0.179	3.98476531302679E-27	Oxidative stress hepatocytes	C19orf33
8.06620002794294E-31	0.556141574951168	0.703	0.663	1.35173380068268E-26	Oxidative stress hepatocytes	NFKBIA
1.85414972496897E-30	0.512318054177333	0.679	0.579	3.107184109103E-26	Oxidative stress hepatocytes	AKR1C2
3.2183365245608E-30	0.524459439463386	0.749	0.653	5.39328834785899E-26	Oxidative stress hepatocytes	SELENBP1
7.56841929753304E-30	1.04271019809226	0.492	0.419	1.26831570588059E-25	Oxidative stress hepatocytes	ANAPC11
1.06285264718559E-29	0.736886679075194	0.6	0.523	1.78112846615361E-25	Oxidative stress hepatocytes	PNKD
1.24471843849871E-29	2.30035596262568	0.211	0.121	2.08589915923614E-25	Oxidative stress hepatocytes	PITX1
2.17076291815208E-29	1.39005717985115	0.368	0.274	3.63776449823926E-25	Oxidative stress hepatocytes	PALLD
3.20763139921007E-29	1.77044141505483	0.321	0.227	5.37534869879624E-25	Oxidative stress hepatocytes	DOK4
4.49399914805533E-29	1.12812943549561	0.45	0.374	7.53104377231113E-25	Oxidative stress hepatocytes	PIR
6.17061562866897E-29	0.92620746420782	0.571	0.521	1.03407176705235E-24	Oxidative stress hepatocytes	MIF
6.35976523312673E-29	1.42337932013574	0.415	0.339	1.06576945776738E-24	Oxidative stress hepatocytes	FAM83H
1.68727263243603E-28	0.836443264839565	0.565	0.523	2.8275314774363E-24	Oxidative stress hepatocytes	NDUFB7
2.07969699032657E-28	0.738688686849868	0.547	0.461	3.48515621638926E-24	Oxidative stress hepatocytes	ETHE1
2.42458251431139E-28	0.873090529414547	0.546	0.478	4.06311537748302E-24	Oxidative stress hepatocytes	HTATSF1
2.86600309356308E-28	1.31746184449477	0.408	0.331	4.80284798419301E-24	Oxidative stress hepatocytes	ACTR1B
3.32031659006771E-28	0.811140161082772	0.556	0.5	5.56418654163547E-24	Oxidative stress hepatocytes	PSMB8
4.97707345617155E-28	0.670025326581116	0.641	0.582	8.34057969785228E-24	Oxidative stress hepatocytes	SMIM14
5.67060506146637E-28	1.02525366465581	0.496	0.437	9.50279996200534E-24	Oxidative stress hepatocytes	SERF2

**1.05355547086337E-27**	1.76316513017044	0.306	0.214	1.76554825807284E-23	Oxidative stress hepatocytes	TSR3
**1.3993760899965E-27**	1.34488773426432	0.391	0.308	2.34507445161613E-23	Oxidative stress hepatocytes	GIPC1
**3.1439996240328E-27**	0.381652021392495	0.912	0.88	5.26871456995417E-23	Oxidative stress hepatocytes	MT-CO2
**4.11623622179276E-27**	1.11136347782282	0.453	0.382	6.8979886604803E-23	Oxidative stress hepatocytes	AGPAT1
**1.65742770314519E-26**	0.897210275388248	0.518	0.459	2.7775173449307E-22	Oxidative stress hepatocytes	FAM114A1
**2.05666513989117E-26**	1.13186016773886	0.446	0.375	3.44655944142962E-22	Oxidative stress hepatocytes	POLR2E
**3.19008688107157E-26**	1.18311295855942	0.439	0.368	5.34594759529974E-22	Oxidative stress hepatocytes	RAD23A
**4.57472977872839E-26**	1.10999921553717	0.45	0.384	7.66633216319303E-22	Oxidative stress hepatocytes	NAA10
**9.64473979486886E-26**	1.54548872979675	0.347	0.265	1.61626549482412E-21	Oxidative stress hepatocytes	ARPC1B
**1.23955390550851E-25**	1.53644722063954	0.356	0.275	2.07724443485116E-21	Oxidative stress hepatocytes	ESRRA
**1.47798824045508E-25**	1.92135566543642	0.16	0.082	2.47681269335462E-21	Oxidative stress hepatocytes	AKR1B15
**1.80201642684136E-25**	1.42323750590747	0.353	0.271	3.01981912810074E-21	Oxidative stress hepatocytes	PCGF2
**2.36342876296133E-25**	0.543927392445227	0.665	0.652	3.96063392097059E-21	Oxidative stress hepatocytes	SOD1
**2.70321123810534E-25**	1.7624504618057	0.293	0.207	4.53004139281693E-21	Oxidative stress hepatocytes	MBD3
**6.03033358512419E-25**	0.313050335756987	0.893	0.867	1.01056330219511E-20	Oxidative stress hepatocytes	ACTB
**9.67179030211656E-25**	0.948130380751321	0.507	0.462	1.62079861882869E-20	Oxidative stress hepatocytes	ATOX1
**1.2399174960193E-24**	2.02100303120946	0.141	0.07	2.07785373982915E-20	Oxidative stress hepatocytes	DMTN
**1.36986484890169E-24**	1.83096442674062	0.319	0.239	2.29561951378946E-20	Oxidative stress hepatocytes	C19orf48
**2.63489319073259E-24**	0.798655084185589	0.519	0.469	4.41555400902968E-20	Oxidative stress hepatocytes	STK24
**3.31800426978675E-24**	1.39443551791788	0.333	0.25	5.56031155530864E-20	Oxidative stress hepatocytes	DEPRECATEI
**6.27402958165583E-24**	0.756118773198531	0.552	0.497	1.05140187729388E-19	Oxidative stress hepatocytes	UBE2A
**8.99544703028565E-24**	2.17467434986034	0.213	0.132	1.50745701333527E-19	Oxidative stress hepatocytes	CAMK2N2
**9.03358547875304E-24**	1.14978856687923	0.416	0.346	1.51384825452943E-19	Oxidative stress hepatocytes	LSM 4.00
**1.21097868552374E-23**	0.88054354777995	0.521	0.481	2.02935808120069E-19	Oxidative stress hepatocytes	ATP5MC2
**1.62977778447946E-23**	0.658013491204974	0.636	0.602	2.73118161123068E-19	Oxidative stress hepatocytes	H1F0
**1.7870043506353E-23**	1.13484420011646	0.446	0.386	2.99466189079463E-19	Oxidative stress hepatocytes	FGGY
**2.03158475837231E-23**	2.09584624969281	0.203	0.123	3.40452973808031E-19	Oxidative stress hepatocytes	SLC25A6
**2.93032246702585E-23**	1.21897659816405	0.405	0.34	4.91063439024191E-19	Oxidative stress hepatocytes	SNX17
**3.5249268523E-23**	0.848357771447237	0.505	0.44	5.90707241908434E-19	Oxidative stress hepatocytes	CITED2
**5.50046636435133E-23**	1.28146491961282	0.387	0.318	9.21768153337996E-19	Oxidative stress hepatocytes	MECP2
**7.13365661877494E-23**	1.46421405214248	0.324	0.245	1.1954581761743E-18	Oxidative stress hepatocytes	SLC25A10
**9.8990092432989E-23**	1.24448963033254	0.377	0.304	1.65887596899203E-18	Oxidative stress hepatocytes	GRB2
**1.01762625830104E-22**	1.27599446327969	0.429	0.376	1.70533808366089E-18	Oxidative stress hepatocytes	CISD3
**1.05223967698185E-22**	1.54549243354132	0.292	0.211	1.76334325068619E-18	Oxidative stress hepatocytes	MAP3K11
**2.04789267141563E-22**	0.45156324171877	0.661	0.576	3.43185853875832E-18	Oxidative stress hepatocytes	SSR4
**6.64119689885306E-22**	1.00003019088085	0.464	0.416	1.11293177630979E-17	Oxidative stress hepatocytes	DCAF11
**6.95357229891237E-22**	1.77283952932747	0.273	0.194	1.16527964585174E-17	Oxidative stress hepatocytes	JCAD
**7.75937177914034E-22**	0.603238455440834	0.606	0.567	1.30031552274834E-17	Oxidative stress hepatocytes	CIRBP
**9.8443590696791E-22**	1.67288916019455	0.25	0.169	1.64971769289682E-17	Oxidative stress hepatocytes	DGCR6L
**1.01741266576294E-21**	1.01044657699009	0.435	0.371	1.70498014528553E-17	Oxidative stress hepatocytes	GCLM
**1.1467266612247E-21**	1.21831230368081	0.417	0.357	1.92168453888035E-17	Oxidative stress hepatocytes	MKNK2
**1.26535267725119E-21**	1.36977934068363	0.374	0.307	2.12047801653754E-17	Oxidative stress hepatocytes	MLLT6
**1.34133347923879E-21**	0.780854556041908	0.566	0.545	2.24780664450837E-17	Oxidative stress hepatocytes	PRKACA
**1.46427255869605E-21**	0.819893913759165	0.521	0.474	2.45382795386283E-17	Oxidative stress hepatocytes	HNRNPA0
**1.70997005016652E-21**	1.12870740679017	0.401	0.335	2.86556781006905E-17	Oxidative stress hepatocytes	CSTB
**2.95360447862313E-21**	0.729017593565373	0.562	0.529	4.94965038527663E-17	Oxidative stress hepatocytes	COX6C
**3.53614200022623E-21**	1.30191934171946	0.378	0.313	5.92586676397912E-17	Oxidative stress hepatocytes	DNAJB2
**3.55791666649051E-21**	0.858795205504532	0.516	0.475	5.9623567497048E-17	Oxidative stress hepatocytes	CIAO2A
**3.63398600783467E-21**	1.95650760346797	0.198	0.123	6.08983375192934E-17	Oxidative stress hepatocytes	SNX8
**6.30278389817153E-21**	0.528184267172061	0.648	0.561	1.05622052565558E-16	Oxidative stress hepatocytes	ERICH5
**6.4585432977955E-21**	1.02367415583422	0.454	0.41	1.08232268584457E-16	Oxidative stress hepatocytes	COA3
**6.92405187926033E-21**	1.89270901705042	0.188	0.114	1.16033261392645E-16	Oxidative stress hepatocytes	SNTA1
**7.15584574047366E-21**	0.988284483771244	0.484	0.445	1.19917662918858E-16	Oxidative stress hepatocytes	LAMTOR2
**7.7895333839044E-21**	1.14984219485712	0.402	0.343	1.3053700044747E-16	Oxidative stress hepatocytes	PYGO2
**1.28237440004699E-20**	0.688885311819755	0.543	0.504	2.14900301959875E-16	Oxidative stress hepatocytes	YWHAZ
**1.86072820906535E-20**	1.04606223677857	0.443	0.39	3.11820833275172E-16	Oxidative stress hepatocytes	UQCR11
**2.15820917045513E-20**	1.15225653869823	0.393	0.335	3.61672692784871E-16	Oxidative stress hepatocytes	DUSP3
**2.84460292700986E-20**	0.464888150456827	0.675	0.651	4.76698558508312E-16	Oxidative stress hepatocytes	PSME1
**3.5074934452136E-20**	1.53193418540851	0.325	0.258	5.87785751548895E-16	Oxidative stress hepatocytes	CTDSPL
**3.62068451295615E-20**	0.843886122152101	0.491	0.45	6.06754310681191E-16	Oxidative stress hepatocytes	ZNF706

**4.64351766155227E-20**	0.93368016488388	0.47	0.422	7.7816068972293E-16	Oxidative stress hepatocytes	SPSB1
**4.74235344016562E-20**	1.37960401970941	0.338	0.27	7.94723589502955E-16	Oxidative stress hepatocytes	STUB1
**4.98614276005846E-20**	0.921246061412456	0.476	0.439	8.35577803730596E-16	Oxidative stress hepatocytes	ATP5ME
**5.48540913777045E-20**	1.04007556568441	0.421	0.365	9.19244863307573E-16	Oxidative stress hepatocytes	TTC39C
**6.27827352910898E-20**	1.22518327626087	0.366	0.304	1.05211307800808E-15	Oxidative stress hepatocytes	PREB
**7.37973817533226E-20**	0.9370686234656	0.461	0.417	1.23669652342218E-15	Oxidative stress hepatocytes	RAB5IF
**8.0357653034917E-20**	1.82053164174402	0.175	0.104	1.34663354955914E-15	Oxidative stress hepatocytes	DEPRECATEI
**8.84956074646502E-20**	1.68415890401314	0.27	0.197	1.48300938989261E-15	Oxidative stress hepatocytes	NCK2
**1.33138117016966E-19**	1.31522785465944	0.335	0.263	2.23112856497031E-15	Oxidative stress hepatocytes	MISP
**1.5217867391724E-19**	1.0598753158051	0.439	0.392	2.55021021750511E-15	Oxidative stress hepatocytes	MTCH1
**1.57836286629904E-19**	1.40284689628021	0.321	0.252	2.64502049134393E-15	Oxidative stress hepatocytes	KLHDC3
**1.88102207469996E-19**	1.0906332825845	0.404	0.347	3.15221679278219E-15	Oxidative stress hepatocytes	RNF10
**3.07648436278262E-19**	1.90024846161376	0.224	0.152	5.15557249515111E-15	Oxidative stress hepatocytes	TOMM40L
**3.9384890082148E-19**	0.716927601843495	0.496	0.447	6.60011987996637E-15	Oxidative stress hepatocytes	VBP1
**4.77668468392597E-19**	1.47605503133118	0.305	0.236	8.00476819332313E-15	Oxidative stress hepatocytes	C8orf33
**6.09358053972771E-19**	1.71169743940727	0.248	0.176	1.02116222684757E-14	Oxidative stress hepatocytes	ITPKA
**1.25524567243673E-18**	0.459765026405837	0.658	0.59	2.10354069786948E-14	Oxidative stress hepatocytes	DNM2
**1.47888101249797E-18**	1.7921220827498	0.227	0.154	2.47830880074409E-14	Oxidative stress hepatocytes	TRIB3
**1.60560308255311E-18**	2.70495921949228	0.127	0.067	2.69066964574249E-14	Oxidative stress hepatocytes	NDUFA4L2
**1.94781515476604E-18**	1.63379309263732	0.299	0.234	3.26414863635693E-14	Oxidative stress hepatocytes	DEAF1
**2.19646454177089E-18**	1.05477491149642	0.413	0.361	3.68083527909965E-14	Oxidative stress hepatocytes	PIK3AP1
**2.3090390245762E-18**	0.708658437291434	0.548	0.519	3.86948759738479E-14	Oxidative stress hepatocytes	NDUFB9
**2.51234545755293E-18**	1.53631693716193	0.24	0.166	4.21018851776721E-14	Oxidative stress hepatocytes	STK40
**2.66504445967142E-18**	0.82965032015538	0.477	0.425	4.46608150551736E-14	Oxidative stress hepatocytes	PGD
**3.94750124446361E-18**	0.476954537895075	0.645	0.611	6.61522258547212E-14	Oxidative stress hepatocytes	TMEM14A
**4.05224124986195E-18**	1.00924566990309	0.425	0.374	6.79074588651865E-14	Oxidative stress hepatocytes	PPP1CA
**4.25661996915818E-18**	1.57961056136577	0.333	0.275	7.13324374431527E-14	Oxidative stress hepatocytes	DEXI
**6.06910016132589E-18**	0.856699120977198	0.459	0.414	1.01705980503499E-13	Oxidative stress hepatocytes	NEK6
**6.5220142584846E-18**	1.0367993487841	0.408	0.359	1.09295914943685E-13	Oxidative stress hepatocytes	JTB
**7.50202247014885E-18**	0.665235739479358	0.54	0.496	1.25718892554754E-13	Oxidative stress hepatocytes	KRT18
**7.71364569324811E-18**	0.645341234210685	0.528	0.476	1.29265274527452E-13	Oxidative stress hepatocytes	PYGB
**7.99336500474558E-18**	1.44054159726246	0.301	0.236	1.33952810749526E-13	Oxidative stress hepatocytes	ATXN1
**8.08641291241198E-18**	0.46292644029534	0.701	0.669	1.355121075862E-13	Oxidative stress hepatocytes	PROX1
**9.94591167045289E-18**	0.971123780772937	0.431	0.387	1.6667358777345E-13	Oxidative stress hepatocytes	PIM3
**1.01767935341114E-17**	0.907265250355225	0.482	0.457	1.70542706044639E-13	Oxidative stress hepatocytes	POLDIP2
**1.25139552610786E-17**	1.38262103002247	0.318	0.256	2.09708862265155E-13	Oxidative stress hepatocytes	BCL2L1
**2.68647776380122E-17**	1.69203666746369	0.23	0.161	4.50199943657808E-13	Oxidative stress hepatocytes	DEPRECATEI
**2.73825605631936E-17**	0.875493728531885	0.461	0.419	4.58876949917999E-13	Oxidative stress hepatocytes	MAF
**2.7894661774515E-17**	0.471127743409026	0.649	0.56	4.67458742017322E-13	Oxidative stress hepatocytes	ADA
**3.06328592882622E-17**	0.588617071880649	0.602	0.579	5.13345455952698E-13	Oxidative stress hepatocytes	HNRNPF
**3.06431203712977E-17**	1.09122142826169	0.389	0.337	5.13517411182207E-13	Oxidative stress hepatocytes	CDK16
**3.61454003773836E-17**	1.32689961956689	0.32	0.256	6.05724619524194E-13	Oxidative stress hepatocytes	PGLS
**3.72514459155388E-17**	0.890357457934281	0.5	0.483	6.24259730652599E-13	Oxidative stress hepatocytes	EEF1G
**3.93230149624301E-17**	0.729695474413519	0.537	0.498	6.58975084740403E-13	Oxidative stress hepatocytes	ZBTB7B
**4.91730905239296E-17**	1.17887310940746	0.359	0.302	8.24042651000012E-13	Oxidative stress hepatocytes	MZT2B
**8.07179295624969E-17**	1.24965219850218	0.356	0.304	1.35267106360832E-12	Oxidative stress hepatocytes	RAB20
**8.17872390305771E-17**	0.728026667548421	0.528	0.511	1.37059055167441E-12	Oxidative stress hepatocytes	AKR1A1
**1.04862702843088E-16**	1.03036757795618	0.403	0.353	1.75728917424447E-12	Oxidative stress hepatocytes	KXD1
**1.09680148147066E-16**	1.25459086932972	0.346	0.288	1.83801992264854E-12	Oxidative stress hepatocytes	STK11
**1.20860599625192E-16**	2.19079574797118	0.141	0.081	2.02538192851897E-12	Oxidative stress hepatocytes	NRTN
**1.20894777712484E-16**	0.630821635449751	0.563	0.549	2.0259546849058E-12	Oxidative stress hepatocytes	PUF60
**1.49831170846494E-16**	1.59954176411081	0.232	0.163	2.51087076104554E-12	Oxidative stress hepatocytes	PPP1R9B
**2.36664495256265E-16**	1.37092075999288	0.278	0.213	3.96602361150448E-12	Oxidative stress hepatocytes	GTPBP6
**2.48822340049681E-16**	0.55408018667383	0.613	0.616	4.16976477455256E-12	Oxidative stress hepatocytes	ADI1
**2.78942874429955E-16**	0.553990928609589	0.583	0.554	4.67452468969718E-12	Oxidative stress hepatocytes	TPD52
**2.84147389283748E-16**	1.39898873779094	0.324	0.27	4.76174194961706E-12	Oxidative stress hepatocytes	HGH1
**3.02927288217896E-16**	1.28168451660048	0.353	0.303	5.0764554959555E-12	Oxidative stress hepatocytes	EGLN2
**3.17888592244912E-16**	1.06075143102153	0.411	0.368	5.32717702884023E-12	Oxidative stress hepatocytes	ISOC2
**3.83106330294024E-16**	1.06167686654516	0.395	0.35	6.42009588306725E-12	Oxidative stress hepatocytes	HIGD2A
**4.09490702487979E-16**	0.545346692488053	0.582	0.55	6.86224519229354E-12	Oxidative stress hepatocytes	SPTSSA

6.05643029162224E-16	1.37603283171575	0.3	0.238	1.01493658827006E-11	Oxidative stress hepatocytes	PYCARD
6.25335041642247E-16	0.787929129435525	0.483	0.442	1.04793646278408E-11	Oxidative stress hepatocytes	CYSTM1
6.38484180470333E-16	1.61713184221138	0.212	0.147	1.06997178963218E-11	Oxidative stress hepatocytes	CCDC85C
6.81737246859158E-16	0.603462980293001	0.547	0.507	1.14245527828658E-11	Oxidative stress hepatocytes	GPAA1
7.68375230048956E-16	1.44594723085568	0.237	0.169	1.28764321051604E-11	Oxidative stress hepatocytes	ZCCHC24
1.14952241280658E-15	0.808549502968347	0.456	0.427	1.92636965938127E-11	Oxidative stress hepatocytes	EIF4H
1.54500307864044E-15	1.50309121038141	0.266	0.205	2.58911615918565E-11	Oxidative stress hepatocytes	TNIP2
1.71540365796845E-15	1.34184091106381	0.304	0.245	2.87467345002352E-11	Oxidative stress hepatocytes	FAM168B
2.1857822382449E-15	0.830477401200373	0.493	0.48	3.66293387485081E-11	Oxidative stress hepatocytes	ZFAND3
2.2208613599464E-15	0.673271198295783	0.525	0.506	3.72171946699818E-11	Oxidative stress hepatocytes	IDH3G
2.24894508461307E-15	1.63935649908375	0.242	0.18	3.76878217279458E-11	Oxidative stress hepatocytes	CTNNBIP1
2.32682975196248E-15	1.23970812184578	0.365	0.32	3.89930129833873E-11	Oxidative stress hepatocytes	FOXP4
3.6262472382743E-15	1.29702796282495	0.331	0.278	6.07686512190007E-11	Oxidative stress hepatocytes	FASTK
3.99340444703391E-15	1.27179113592972	0.315	0.26	6.69214717233942E-11	Oxidative stress hepatocytes	FAM32A
5.23671722054098E-15	0.618256044027326	0.555	0.532	8.77569071818257E-11	Oxidative stress hepatocytes	PCK2
5.37815238203829E-15	0.761334415195996	0.504	0.483	9.01270776181977E-11	Oxidative stress hepatocytes	TBC1D9B
5.79676856493179E-15	0.955679594921441	0.407	0.368	9.71422476111269E-11	Oxidative stress hepatocytes	NDUFA13
5.8228347900429E-15	1.15440358436858	0.363	0.316	9.75790654115389E-11	Oxidative stress hepatocytes	NME2
5.96920670785084E-15	1.77696235623587	0.224	0.162	1.00031966010164E-10	Oxidative stress hepatocytes	GPR137
1.0896115954042E-14	0.873002019399179	0.456	0.431	1.82597111157835E-10	Oxidative stress hepatocytes	NENF
1.4274942787793E-14	0.721721888545253	0.481	0.458	2.39219491237836E-10	Oxidative stress hepatocytes	RAB5C
1.59671307139859E-14	1.20077820005274	0.356	0.31	2.67577176504976E-10	Oxidative stress hepatocytes	PHC2
1.61685502632521E-14	0.781494706795579	0.464	0.435	2.70952565311579E-10	Oxidative stress hepatocytes	EDF1
1.72774734298401E-14	0.937044253375878	0.499	0.485	2.8953589973726E-10	Oxidative stress hepatocytes	LAMTOR4
2.26223938480875E-14	1.47247492951815	0.262	0.204	3.7910607610625E-10	Oxidative stress hepatocytes	MPND
3.01930782446521E-14	1.28001949558973	0.325	0.273	5.0597560522388E-10	Oxidative stress hepatocytes	DEPRECATEI
3.35054047637716E-14	0.435854850551143	0.691	0.661	5.61483573031284E-10	Oxidative stress hepatocytes	MT-ND2
3.37207259669465E-14	0.574014257209559	0.547	0.537	5.6509192575409E-10	Oxidative stress hepatocytes	EIF1
3.5060169959881E-14	0.864017418476672	0.468	0.443	5.87538328187687E-10	Oxidative stress hepatocytes	SIVA1
4.08640985942512E-14	1.23756472117346	0.33	0.283	6.84800564242461E-10	Oxidative stress hepatocytes	GSK3A
4.58579129668602E-14	1.47509628526708	0.293	0.241	7.68486905498643E-10	Oxidative stress hepatocytes	TMEM250
5.26551923817674E-14	1.35086512902891	0.308	0.254	8.82395713933658E-10	Oxidative stress hepatocytes	HPCAL1
6.3648796212729E-14	0.503457239127812	0.611	0.607	1.06662652693291E-09	Oxidative stress hepatocytes	ACAA2
6.63865696057281E-14	1.72456600845325	0.203	0.143	1.11250613345279E-09	Oxidative stress hepatocytes	MEX3D
8.80370482827575E-14	0.408706025701657	0.682	0.676	1.47532485512245E-09	Oxidative stress hepatocytes	IDH1
1.2523345138768E-13	1.33980786479651	0.289	0.234	2.09866217835475E-09	Oxidative stress hepatocytes	FIBP
1.36523393245625E-13	1.52783973234842	0.193	0.132	2.28785902401018E-09	Oxidative stress hepatocytes	TTC9
1.60338106235425E-13	0.77672448554962	0.476	0.456	2.68694598429325E-09	Oxidative stress hepatocytes	TCEAL4
1.69586861549719E-13	0.421503857137584	0.764	0.751	2.84193662585019E-09	Oxidative stress hepatocytes	MT-ND3
1.82738993528554E-13	1.22356619420486	0.313	0.263	3.06234005355151E-09	Oxidative stress hepatocytes	FAM102A
1.85997815623952E-13	1.26262714776863	0.332	0.287	3.11695139422618E-09	Oxidative stress hepatocytes	GNA11
1.96343777470443E-13	0.936550254292817	0.405	0.371	3.29032902284968E-09	Oxidative stress hepatocytes	SIAH2
2.11597013606502E-13	1.24504244899	0.332	0.285	3.54594275401776E-09	Oxidative stress hepatocytes	SLC25A11
2.32535820388662E-13	0.359301866195814	0.746	0.766	3.89683527807319E-09	Oxidative stress hepatocytes	CEBPD
2.66007977996456E-13	1.13658792466377	0.197	0.135	4.45776169526461E-09	Oxidative stress hepatocytes	DEPRECATEI
2.66258372500572E-13	0.290248871423173	0.106	0.203	4.46195780636458E-09	Oxidative stress hepatocytes	GEMIN7
2.8021213883508E-13	1.25658247093386	0.313	0.264	4.69579502259827E-09	Oxidative stress hepatocytes	BOK
2.86461806077308E-13	0.610758044017075	0.498	0.464	4.80052694624352E-09	Oxidative stress hepatocytes	EZR
4.2882818359486E-13	0.93663154762313	0.422	0.401	7.18630270068267E-09	Oxidative stress hepatocytes	SMG5
4.28965117716456E-13	0.883286428137578	0.433	0.409	7.18859744269237E-09	Oxidative stress hepatocytes	ROMO1
4.33632736177867E-13	0.622153812685963	0.543	0.543	7.2668173928687E-09	Oxidative stress hepatocytes	UQCRQ
4.35249449820281E-13	0.821976447452589	0.44	0.416	7.29391028008827E-09	Oxidative stress hepatocytes	ADRM1
4.59300847489783E-13	0.838257829813744	0.45	0.43	7.69696360223379E-09	Oxidative stress hepatocytes	VPS28
4.93365939634539E-13	0.78273763584532	0.439	0.412	8.26782641639561E-09	Oxidative stress hepatocytes	TALDO1
5.47377885256097E-13	0.668947187443885	0.489	0.473	9.17295860112168E-09	Oxidative stress hepatocytes	GSR
6.00421495887501E-13	0.735838615107244	0.456	0.425	1.00618634280827E-08	Oxidative stress hepatocytes	ATP6V0D1
6.06337285110274E-13	1.2040673768678	0.314	0.264	1.0161000223878E-08	Oxidative stress hepatocytes	SRF
6.18334080343726E-13	1.34605015382472	0.273	0.219	1.03620425184002E-08	Oxidative stress hepatocytes	NABP2
6.72731811295757E-13	0.963694513055452	0.417	0.396	1.12736396936943E-08	Oxidative stress hepatocytes	HSF1
7.23167393307136E-13	1.71308876982776	0.151	0.097	1.2118839177041E-08	Oxidative stress hepatocytes	ABHD8

7.34978740890281E-13	2.07799243422802	0.143	0.091	1.23167737398393E-08	Oxidative stress hepatocytes	B4GALT2
7.36384364559525E-13	1.06992196914408	0.345	0.301	1.23403291812885E-08	Oxidative stress hepatocytes	PXDC1
8.69463608823069E-13	1.05673255316772	0.352	0.303	1.4570471156657E-08	Oxidative stress hepatocytes	DEPRECATEI
1.04190769465386E-12	1.32511259334689	0.274	0.222	1.74602891470094E-08	Oxidative stress hepatocytes	DBP
1.24215350800095E-12	1.00121766914975	0.389	0.36	2.08160084870799E-08	Oxidative stress hepatocytes	CTTN
1.26994704756959E-12	1.41469495921422	0.269	0.217	2.12817726231712E-08	Oxidative stress hepatocytes	RAB15
1.39585848255891E-12	1.53390992424368	0.222	0.167	2.33917964507222E-08	Oxidative stress hepatocytes	MLST8
1.6017134445328E-12	1.05776574554591	0.304	0.247	2.68415139034806E-08	Oxidative stress hepatocytes	CSRP1
1.90007579150255E-12	0.960801993955252	0.369	0.329	3.18414701139998E-08	Oxidative stress hepatocytes	FAM162A
2.54111361425188E-12	1.09130766344383	0.226	0.164	4.25839819476331E-08	Oxidative stress hepatocytes	ALDH3A1
3.23843502171989E-12	1.57547624493662	0.224	0.169	5.4269694093982E-08	Oxidative stress hepatocytes	BCL7C
3.31099721681693E-12	1.5523920979725	0.207	0.151	5.54856913594181E-08	Oxidative stress hepatocytes	KIAA0930
3.68884636848397E-12	1.19718885009727	0.333	0.294	6.18176874430544E-08	Oxidative stress hepatocytes	HMG20B
4.88119709022971E-12	0.817580512552565	0.389	0.349	8.17991008380695E-08	Oxidative stress hepatocytes	CAPNS1
4.8857858367637E-12	1.47825079648224	0.184	0.129	8.18759990524861E-08	Oxidative stress hepatocytes	IFFO2
5.66474457762715E-12	1.39578971153353	0.238	0.185	9.49297896318757E-08	Oxidative stress hepatocytes	FAM110B
5.69992181670588E-12	0.889649089935805	0.413	0.387	9.55192898043572E-08	Oxidative stress hepatocytes	UQCR10
5.77175417009027E-12	0.887655190391316	0.399	0.374	9.67230563823727E-08	Oxidative stress hepatocytes	HSD3B7
5.84763400612201E-12	1.87072340294212	0.192	0.14	9.79946506745927E-08	Oxidative stress hepatocytes	SCAMP5
7.99737774427941E-12	0.69637832595376	0.484	0.475	1.34020056238634E-07	Oxidative stress hepatocytes	EIF6
9.49401407904672E-12	1.34070196218994	0.297	0.254	1.59100687936665E-07	Oxidative stress hepatocytes	TSC22D4
1.04095083355788E-11	1.23619191889535	0.314	0.273	1.74442540687629E-07	Oxidative stress hepatocytes	JPT2
1.15493230618628E-11	1.19733850091893	0.317	0.277	1.93543555870696E-07	Oxidative stress hepatocytes	SGTA
1.21953436252192E-11	0.521257390981285	0.571	0.57	2.04369568471423E-07	Oxidative stress hepatocytes	OAF
1.29862039516324E-11	1.31567790032922	0.283	0.235	2.17622805821455E-07	Oxidative stress hepatocytes	DEPRECATEI
1.60431698546599E-11	1.53469799504419	0.162	0.11	2.68851440424391E-07	Oxidative stress hepatocytes	TRIM16
1.76859645177251E-11	1.29654420737609	0.307	0.267	2.96381393388037E-07	Oxidative stress hepatocytes	DNAJC5
2.00521500876289E-11	0.991798664138095	0.391	0.366	3.36033931168485E-07	Oxidative stress hepatocytes	AP3D1
2.13646614618637E-11	1.42683978302125	0.102	0.058	3.58028996777912E-07	Oxidative stress hepatocytes	TRIM16L
2.18106688401222E-11	1.40688066943108	0.242	0.19	3.65503188422768E-07	Oxidative stress hepatocytes	SPHK1
2.20868374882839E-11	0.932551373128577	0.358	0.322	3.70131222628661E-07	Oxidative stress hepatocytes	MEA1
2.22820306897995E-11	0.698965633886454	0.495	0.493	3.7340227029966E-07	Oxidative stress hepatocytes	SHC1
2.38576114600289E-11	0.751319919890496	0.441	0.414	3.99805852847164E-07	Oxidative stress hepatocytes	MYH14
3.064819055887E-11	0.894898015599526	0.424	0.396	5.13602377385543E-07	Oxidative stress hepatocytes	LSS
3.0781191056568E-11	0.816555826672918	0.446	0.437	5.15831199725967E-07	Oxidative stress hepatocytes	NDUFS7
3.19107263961243E-11	1.09190071800006	0.342	0.306	5.34759952946251E-07	Oxidative stress hepatocytes	POLR2I
3.22431257860884E-11	1.38956678145113	0.255	0.209	5.4033030192327E-07	Oxidative stress hepatocytes	ARHGEF18
3.26448634960437E-11	0.785915955465459	0.424	0.385	5.470626224667E-07	Oxidative stress hepatocytes	DUSP9
3.26728526926271E-11	1.45183019030657	0.195	0.143	5.47531665423045E-07	Oxidative stress hepatocytes	CDIP1
3.4382677693449E-11	0.832735241774367	0.438	0.42	5.76184900068183E-07	Oxidative stress hepatocytes	SELENOH
3.49772409077801E-11	1.02674247261526	0.356	0.322	5.86148603132579E-07	Oxidative stress hepatocytes	CRAT
4.20457922830786E-11	0.26276446103844	0.099	0.181	7.04603387079831E-07	Oxidative stress hepatocytes	ADNP2
4.70225829001556E-11	1.17437320665183	0.342	0.31	7.88004444240807E-07	Oxidative stress hepatocytes	RAB1B
4.80915557634747E-11	1.02879307377448	0.366	0.338	8.05918291484309E-07	Oxidative stress hepatocytes	DNPH1
5.11508516186229E-11	1.28183247376489	0.221	0.168	8.57185971424883E-07	Oxidative stress hepatocytes	RHOD
6.51045692418283E-11	1.77561244121504	0.147	0.099	1.09102237135456E-06	Oxidative stress hepatocytes	NT5DC2
6.97996217508862E-11	0.969408181884658	0.348	0.31	1.16970206130135E-06	Oxidative stress hepatocytes	PLAAT3
7.19777533813664E-11	0.865187288370801	0.382	0.352	1.20620319116494E-06	Oxidative stress hepatocytes	VASP
7.37497171126451E-11	1.38388665714175	0.304	0.266	1.23589775937371E-06	Oxidative stress hepatocytes	DNASE1L1
8.72191529333061E-11	1.33634381176076	0.278	0.235	1.46161856485634E-06	Oxidative stress hepatocytes	MED29
9.69321920810336E-11	1.39250559215776	0.239	0.192	1.62438967489396E-06	Oxidative stress hepatocytes	TMEM8A
1.12055194545876E-10	0.842598431933234	0.441	0.428	1.87782095019979E-06	Oxidative stress hepatocytes	SERP1
1.20299804770728E-10	0.764640560458826	0.434	0.418	2.01598412834786E-06	Oxidative stress hepatocytes	PHYH
1.26487253225404E-10	1.67445013607375	0.192	0.143	2.11967338955133E-06	Oxidative stress hepatocytes	KLF16
1.27327988967524E-10	0.282585079985052	0.127	0.216	2.13376243911776E-06	Oxidative stress hepatocytes	PCGF3
1.37096590269511E-10	0.892055840116681	0.401	0.381	2.29746465973647E-06	Oxidative stress hepatocytes	ENSA
1.454213995456E-10	0.817724315987407	0.417	0.389	2.43697181358517E-06	Oxidative stress hepatocytes	CNKSR3
1.48490980808356E-10	1.54731245177452	0.195	0.145	2.48841185638644E-06	Oxidative stress hepatocytes	RAP1GAP
1.4895194554076E-10	1.25427657228123	0.299	0.259	2.49613670337206E-06	Oxidative stress hepatocytes	E2F4
1.63971095198345E-10	0.264670816319884	0.717	0.702	2.74782761333387E-06	Oxidative stress hepatocytes	BST2

1.87767368194764E-10	1.41242711131324	0.257	0.214	3.14660555620786E-06	Oxidative stress hepatocytes	PPP1R12C
1.9823220950546E-10	0.643780522185304	0.467	0.456	3.3219753668925E-06	Oxidative stress hepatocytes	CHCHD2
2.01804144276867E-10	0.401622718845201	0.658	0.658	3.38183384979173E-06	Oxidative stress hepatocytes	MT-ND5
2.40668146579816E-10	1.23409035741454	0.287	0.244	4.03311680038457E-06	Oxidative stress hepatocytes	CPTP
2.85853983286171E-10	0.251496639297726	0.103	0.181	4.79034105190965E-06	Oxidative stress hepatocytes	PWP1
2.97982352929673E-10	0.758066093325622	0.439	0.43	4.99358827039546E-06	Oxidative stress hepatocytes	NDUFB10
3.22605597663141E-10	1.37242222114919	0.217	0.169	5.40622460563891E-06	Oxidative stress hepatocytes	DVL1
3.5249663750848E-10	1.04987481824327	0.343	0.313	5.9071386513671E-06	Oxidative stress hepatocytes	IDS
4.05292169041077E-10	1.92817463692785	0.147	0.101	6.79188616879036E-06	Oxidative stress hepatocytes	COX7A1
4.92759903908849E-10	0.805650270237549	0.416	0.405	8.2576704697045E-06	Oxidative stress hepatocytes	NMT1
4.95351488886716E-10	0.903716427006767	0.396	0.381	8.30110025076358E-06	Oxidative stress hepatocytes	TXNL4A
5.04396215686247E-10	1.1237460170543	0.325	0.292	8.45267178247013E-06	Oxidative stress hepatocytes	FAM174C
6.27546582574707E-10	2.02512869778123	0.117	0.075	1.05164256307869E-05	Oxidative stress hepatocytes	CAVIN1
6.60267913679057E-10	1.00880463937034	0.318	0.282	1.10647696974336E-05	Oxidative stress hepatocytes	POLD2
6.98284390644155E-10	0.311736508134659	0.125	0.211	1.17018498184147E-05	Oxidative stress hepatocytes	ADCK5
7.21204377798606E-10	0.281515912737825	0.134	0.224	1.2085942963149E-05	Oxidative stress hepatocytes	BORCS8
7.4345398789053E-10	0.324175286229278	0.121	0.205	1.24588019290695E-05	Oxidative stress hepatocytes	VPS13D
8.56925474138686E-10	1.33216838468519	0.297	0.265	1.43603570956161E-05	Oxidative stress hepatocytes	MXD4
8.6933629320524E-10	0.339970813260727	0.62	0.582	1.45683376015334E-05	Oxidative stress hepatocytes	MAOA
9.79632238650098E-10	1.2242841658879	0.259	0.217	1.64166770552983E-05	Oxidative stress hepatocytes	JOSD1
1.08528962785099E-09	1.7893799358665	0.141	0.097	1.81872835835268E-05	Oxidative stress hepatocytes	PRXL2B
1.14263943019086E-09	0.399434137822068	0.1	0.177	1.91483515711385E-05	Oxidative stress hepatocytes	NOP9
1.64183181110849E-09	0.748228655094529	0.441	0.435	2.75138174905561E-05	Oxidative stress hepatocytes	PCYT2
1.81576078385863E-09	0.76149117795951	0.443	0.438	3.04285192159029E-05	Oxidative stress hepatocytes	COX7C
1.82156601183112E-09	1.15643310347237	0.279	0.24	3.0525803226266E-05	Oxidative stress hepatocytes	UBTF
2.01156749458067E-09	1.58077528667838	0.198	0.153	3.37098480741829E-05	Oxidative stress hepatocytes	THAP7
2.19597143222411E-09	1.19606852896171	0.269	0.23	3.68000892612116E-05	Oxidative stress hepatocytes	DAXX
2.21118268130094E-09	0.369775509005041	0.102	0.178	3.70549993732412E-05	Oxidative stress hepatocytes	PEX6
2.29252540783001E-09	1.19239863253077	0.262	0.223	3.84181407844153E-05	Oxidative stress hepatocytes	CERK
2.62236121057447E-09	1.34745712701386	0.239	0.192	4.3945529166807E-05	Oxidative stress hepatocytes	CDC42EP5
2.86473540965462E-09	1.62886933327144	0.182	0.138	4.80072359949921E-05	Oxidative stress hepatocytes	CUEDC1
3.07890879226757E-09	1.08735733849485	0.301	0.263	5.159635354082E-05	Oxidative stress hepatocytes	ODC1
3.24447372910389E-09	0.261855233541671	0.163	0.257	5.4370890752323E-05	Oxidative stress hepatocytes	PSMC6
3.25720611051864E-09	0.734497896455322	0.406	0.386	5.45842600000713E-05	Oxidative stress hepatocytes	GALK1
3.41694191771758E-09	1.39654759687222	0.202	0.157	5.72611126571112E-05	Oxidative stress hepatocytes	BAD
3.43344722783889E-09	0.608200855974957	0.504	0.505	5.75377086441241E-05	Oxidative stress hepatocytes	CUTA
3.55470207778352E-09	0.365807259543849	0.127	0.209	5.95696974194962E-05	Oxidative stress hepatocytes	BOD1L1
3.74984469030293E-09	1.31848061942938	0.258	0.216	6.28398973200965E-05	Oxidative stress hepatocytes	SNRNP25
3.76635047645412E-09	0.387532713773417	0.047	0.101	6.31165012844182E-05	Oxidative stress hepatocytes	RMI2
3.77435768727914E-09	1.38329330823896	0.209	0.165	6.32506861234238E-05	Oxidative stress hepatocytes	OGG1
3.83732088076751E-09	0.250502296749731	0.068	0.129	6.4305823319902E-05	Oxidative stress hepatocytes	SRCIN1
3.83882281789696E-09	0.992840033960083	0.332	0.299	6.43309927823173E-05	Oxidative stress hepatocytes	KLHL21
3.98777825146882E-09	1.15317583422307	0.282	0.244	6.68271879381146E-05	Oxidative stress hepatocytes	GADD45GIP1
4.43399469057494E-09	1.41345671604374	0.257	0.22	7.43048830246548E-05	Oxidative stress hepatocytes	SNAP47
4.53155850477146E-09	0.275496926596258	0.151	0.238	7.59398574229601E-05	Oxidative stress hepatocytes	CHCHD7
4.62300206588912E-09	0.61725035719929	0.509	0.529	7.74722686201699E-05	Oxidative stress hepatocytes	NAPA
5.46370837662888E-09	1.44529871780018	0.188	0.144	9.15608249755467E-05	Oxidative stress hepatocytes	TP53INP2
5.48111669694269E-09	0.289215508876387	0.076	0.139	9.18525536073657E-05	Oxidative stress hepatocytes	NIPSNAP3A
6.08186495363469E-09	0.267267254404952	0.143	0.23	0.00010191989289301	Oxidative stress hepatocytes	CAMSAP2
6.14211607714442E-09	0.254410717878453	0.138	0.224	0.000102929581220786	Oxidative stress hepatocytes	AK6
6.67612822440624E-09	0.4610269429562	0.1	0.173	0.0001118785567846	Oxidative stress hepatocytes	TCP11L1
7.06800817196876E-09	0.288954443091897	0.106	0.178	0.000118445680945852	Oxidative stress hepatocytes	C7orf26
7.07077318640892E-09	0.344408457335968	0.093	0.163	0.000118492017057841	Oxidative stress hepatocytes	GIGYF2
7.95312455958993E-09	0.271328744726306	0.107	0.181	0.000133278461369608	Oxidative stress hepatocytes	PIK3C2B
8.82772132554219E-09	0.261495781364084	0.132	0.214	0.000147934953973436	Oxidative stress hepatocytes	ZNF608
1.01199537562942E-08	1.14304923128039	0.311	0.281	0.000169590185047978	Oxidative stress hepatocytes	CCDC124
1.05662543762566E-08	1.07512726890115	0.339	0.317	0.000177069290837309	Oxidative stress hepatocytes	CDC42BPB
1.0685316970519E-08	0.27418889667387	0.189	0.289	0.000179064541791958	Oxidative stress hepatocytes	SNX6
1.10067102538007E-08	1.35888941246359	0.272	0.237	0.000184450450433193	Oxidative stress hepatocytes	CHMP1A
1.12587552791148E-08	0.425489565266232	0.143	0.229	0.000188674220967405	Oxidative stress hepatocytes	ZNF7

**1.14405915709691E-08**	1.02901915916279	0.316	0.285	0.0001917214335463	Oxidative stress hepatocytes	MIEN1
**1.15770634802514E-08**	0.916032067902302	0.356	0.333	0.000194008429802053	Oxidative stress hepatocytes	ARHGDIA
**1.15782044049399E-08**	0.296776365325403	0.092	0.158	0.000194027549417983	Oxidative stress hepatocytes	SPAG16
**1.1593254777926E-08**	1.48373981066327	0.243	0.207	0.000194279763568484	Oxidative stress hepatocytes	CCNQ
**1.17881849942708E-08**	0.806362418734568	0.401	0.389	0.000197546404133989	Oxidative stress hepatocytes	ARF6
**1.19579593781296E-08**	1.53390466495858	0.181	0.138	0.000200391483258696	Oxidative stress hepatocytes	MAP3K14
**1.21618512696124E-08**	0.91028890576154	0.335	0.308	0.000203808303576164	Oxidative stress hepatocytes	FLOT1
**1.26348712182883E-08**	0.286741684100555	0.099	0.17	0.000211735171876075	Oxidative stress hepatocytes	TGDS
**1.28582863414423E-08**	0.336725749015772	0.109	0.183	0.000215479162509889	Oxidative stress hepatocytes	GABPB2
**1.29076709635743E-08**	0.250669593484508	0.127	0.207	0.000216306750007577	Oxidative stress hepatocytes	MCM3AP
**1.36686024902401E-08**	0.281986541758488	0.059	0.114	0.000229058440531444	Oxidative stress hepatocytes	NCOA5
**1.46196751065202E-08**	0.73899215106074	0.392	0.373	0.000244996515435066	Oxidative stress hepatocytes	PAPSS2
**1.66025014825222E-08**	0.271783334332186	0.14	0.222	0.000278224719844107	Oxidative stress hepatocytes	CASP2
**1.68530104288448E-08**	1.82641239148705	0.128	0.088	0.000282422748766581	Oxidative stress hepatocytes	LIMK1
**1.70008731939213E-08**	0.297573070765449	0.18	0.277	0.000284900632983733	Oxidative stress hepatocytes	LRRC14
**1.76400907930485E-08**	0.332312424230921	0.109	0.182	0.000295612641509906	Oxidative stress hepatocytes	TBCE
**1.78001801809544E-08**	0.452498757165612	0.543	0.521	0.000298295419472434	Oxidative stress hepatocytes	C2orf72
**1.84413420818578E-08**	0.279561818823562	0.116	0.192	0.000309040010607772	Oxidative stress hepatocytes	TMEM8B
**1.90327525607968E-08**	0.250642149314702	0.158	0.246	0.000318950867413833	Oxidative stress hepatocytes	EAPP
**2.12872224261305E-08**	0.284932456915941	0.163	0.254	0.000356731273417096	Oxidative stress hepatocytes	ACBD6
**2.23852704537709E-08**	0.676902297007443	0.407	0.392	0.000375132362264293	Oxidative stress hepatocytes	SMIM15
**2.31158861704794E-08**	0.339454873357898	0.065	0.122	0.000387376020444894	Oxidative stress hepatocytes	FAM76A
**2.51329682132079E-08**	0.276066463632628	0.102	0.172	0.000421178281316938	Oxidative stress hepatocytes	XRCC5
**2.68182339075198E-08**	0.324711594147383	0.071	0.129	0.000449419963822217	Oxidative stress hepatocytes	TLK2
**2.70309192983296E-08**	0.411694694122992	0.134	0.213	0.000452984145601407	Oxidative stress hepatocytes	RNF14
**2.97974326883125E-08**	0.317901634552811	0.136	0.216	0.00049934537699074	Oxidative stress hepatocytes	MAPKAPK5
**3.04123672646522E-08**	0.268381318809896	0.163	0.253	0.000509650450621041	Oxidative stress hepatocytes	PIP4P1
**3.05292642179227E-08**	0.303253940909204	0.083	0.145	0.000511609409763949	Oxidative stress hepatocytes	DHRS11
**3.07475999338818E-08**	1.26636005268374	0.303	0.278	0.00051526827969199	Oxidative stress hepatocytes	SAP30L
**3.09465416934868E-08**	1.52212584684976	0.207	0.167	0.000518602145699452	Oxidative stress hepatocytes	RAPGEF1
**3.13949637921491E-08**	0.383330380963826	0.123	0.198	0.000526116803228835	Oxidative stress hepatocytes	RETREG2
**3.38107949490199E-08**	0.744428011313253	0.412	0.388	0.000566601301755676	Oxidative stress hepatocytes	SIPA1L2
**3.54409796959272E-08**	0.809681403733282	0.332	0.301	0.000593919937744347	Oxidative stress hepatocytes	ME1
**3.70491999315023E-08**	1.3373534153558	0.265	0.233	0.000620870492452115	Oxidative stress hepatocytes	PPP1R16A
**3.72127642173755E-08**	1.74078750127125	0.144	0.105	0.000623611502754778	Oxidative stress hepatocytes	THRA
**3.83704522456493E-08**	0.312327572173826	0.076	0.135	0.00064301203873259	Oxidative stress hepatocytes	PAIP1
**3.88178435647547E-08**	0.333191590824072	0.054	0.105	0.00065050942245816	Oxidative stress hepatocytes	ELMOD3
**3.88426314627218E-08**	0.332206083789948	0.099	0.167	0.000650924818052292	Oxidative stress hepatocytes	METTL9
**4.11394980810337E-08**	0.889091567267017	0.375	0.363	0.000689415708841963	Oxidative stress hepatocytes	POLR1D
**4.20344795658538E-08**	1.25495428422212	0.269	0.237	0.000704413808564577	Oxidative stress hepatocytes	NR2F1
**4.2333472461493E-08**	0.509122138464662	0.509	0.516	0.0007094243315097	Oxidative stress hepatocytes	MORF4L2
**4.34981688350399E-08**	0.835327862058825	0.368	0.349	0.000728942313337598	Oxidative stress hepatocytes	CETN2
**4.35490402257736E-08**	0.265539126742885	0.156	0.243	0.000729794816103514	Oxidative stress hepatocytes	PPA2
**4.60946367962967E-08**	0.863567453147853	0.366	0.351	0.000772453923432341	Oxidative stress hepatocytes	MLX
**4.87982485343602E-08**	0.819371430752207	0.401	0.393	0.000817761048938808	Oxidative stress hepatocytes	C4orf3
**4.95283203545174E-08**	1.19344475413994	0.295	0.267	0.000829995592501003	Oxidative stress hepatocytes	SKI
**5.08794071148937E-08**	1.33035113036242	0.219	0.18	0.000852637104431388	Oxidative stress hepatocytes	BAK1
**5.10641743008574E-08**	1.46865667625856	0.197	0.158	0.000855733432933769	Oxidative stress hepatocytes	TLNRD1
**5.25137832124954E-08**	1.00018464257293	0.341	0.321	0.000880025979074999	Oxidative stress hepatocytes	IFI35
**5.25471921390027E-08**	0.824114896000973	0.407	0.4	0.000880585845865406	Oxidative stress hepatocytes	SETD7
**5.4450806149027E-08**	0.814506539294267	0.4	0.396	0.000912486609445394	Oxidative stress hepatocytes	RTF2
**5.55412673455143E-08**	0.331817521811516	0.069	0.125	0.000930760558176128	Oxidative stress hepatocytes	CNOT1
**5.63507914045435E-08**	0.442933240729034	0.544	0.54	0.000944326562357339	Oxidative stress hepatocytes	HNRNPH2
**5.86273261893747E-08**	0.261019962471295	0.121	0.194	0.000982476732281541	Oxidative stress hepatocytes	TOM1
**5.87282435053123E-08**	0.373460893451934	0.116	0.188	0.000984167904662023	Oxidative stress hepatocytes	DCAF10
**5.90900605340819E-08**	1.04414085920606	0.331	0.311	0.000990231234430145	Oxidative stress hepatocytes	RNPS1
**5.91288161148535E-08**	0.9179399167131	0.386	0.379	0.000990880700452714	Oxidative stress hepatocytes	ECI1
**5.95466004664476E-08**	1.10997679713627	0.244	0.205	0.000997881930616729	Oxidative stress hepatocytes	IFRD2
**6.02999229177678E-08**	0.369624034131845	0.619	0.645	0.00101050610825595	Oxidative stress hepatocytes	PEBP1
**6.18849573329905E-08**	1.33514688084873	0.232	0.195	0.00103706811498626	Oxidative stress hepatocytes	CPLX1

6.39205583799556E-08	0.326362639417581	0.087	0.149	0.0010711807173313	Oxidative stress hepatocytes	ASF1A
7.04161912305746E-08	1.07756129606103	0.274	0.24	0.00118003453264197	Oxidative stress hepatocytes	CITED4
7.16253957486684E-08	0.72364549344532	0.434	0.432	0.00120029838195619	Oxidative stress hepatocytes	NHSL1
7.24734402585729E-08	0.878923209571039	0.349	0.325	0.00121450991185316	Oxidative stress hepatocytes	NDUFS6
7.26985094107164E-08	0.793888934081464	0.394	0.383	0.00121828162070478	Oxidative stress hepatocytes	ARRDC2
7.4698689082785E-08	0.935017145004899	0.345	0.325	0.00125180063164931	Oxidative stress hepatocytes	POLD4
8.18636999571398E-08	1.11715702428782	0.269	0.235	0.00137187188388175	Oxidative stress hepatocytes	RNF44
8.49193747650938E-08	1.54994916399283	0.143	0.104	0.00142307888231344	Oxidative stress hepatocytes	MAZ
8.65336398335914E-08	0.260295520810014	0.084	0.143	0.00145013073633133	Oxidative stress hepatocytes	FAM219A
8.75850369761935E-08	1.50863568813435	0.168	0.128	0.00146775004964705	Oxidative stress hepatocytes	DEPRECATEI
9.15291021611827E-08	1.1061863232718	0.291	0.26	0.0015338446940171	Oxidative stress hepatocytes	AURKAIP1
9.98220559613756E-08	1.00940034001938	0.335	0.314	0.00167281801380073	Oxidative stress hepatocytes	ZFAND5
1.00490948705404E-07	0.656831622742891	0.466	0.475	0.00168402731840517	Oxidative stress hepatocytes	TRIR
1.07548471837931E-07	0.27220941156751	0.078	0.135	0.00180229729106005	Oxidative stress hepatocytes	TPMT
1.11503150670003E-07	0.308990199772963	0.148	0.228	0.00186856979892792	Oxidative stress hepatocytes	CASP6
1.21751368848355E-07	0.657893925155791	0.432	0.431	0.00204030943916073	Oxidative stress hepatocytes	CHMP2A
1.22479845726554E-07	0.268153787833684	0.152	0.234	0.00205251725468559	Oxidative stress hepatocytes	ATL3
1.23822326840487E-07	0.34280694378722	0.12	0.191	0.00207501455319289	Oxidative stress hepatocytes	TNFRSF11A
1.26421040587402E-07	0.286520755020943	0.138	0.216	0.00211856379816368	Oxidative stress hepatocytes	THUMPD1
1.30412923428677E-07	0.41128302262956	0.106	0.171	0.00218545977081776	Oxidative stress hepatocytes	FAM210B
1.30484625979376E-07	1.5200060913541	0.163	0.124	0.00218666136216239	Oxidative stress hepatocytes	ATG4D
1.30636131646537E-07	0.669763610162569	0.435	0.432	0.00218920029413266	Oxidative stress hepatocytes	AK4
1.31078466353738E-07	0.419918662544344	0.163	0.249	0.00219661293915594	Oxidative stress hepatocytes	MED13
1.35082227357767E-07	0.280002834449751	0.153	0.235	0.00226370796606145	Oxidative stress hepatocytes	HTT
1.37034344408856E-07	0.797092678418404	0.409	0.407	0.00229642154360362	Oxidative stress hepatocytes	FAM120A
1.39754734845804E-07	1.48000063883304	0.186	0.148	0.00234200984654599	Oxidative stress hepatocytes	PLAAT2
1.4084206899462E-07	0.379798827456244	0.058	0.109	0.00236023139221185	Oxidative stress hepatocytes	TTLL6
1.43274566780337E-07	0.322944006295073	0.2	0.302	0.00240099519010489	Oxidative stress hepatocytes	KIF13A
1.44872956244358E-07	0.264564366768131	0.153	0.235	0.00242778100074295	Oxidative stress hepatocytes	DEPRECATEI
1.44930269726534E-07	0.973653581524801	0.333	0.315	0.00242874146007726	Oxidative stress hepatocytes	SIPA1L3
1.54709934314235E-07	0.610168744184944	0.491	0.507	0.00259262907923795	Oxidative stress hepatocytes	HNF4A
1.55434095787455E-07	1.15296630230388	0.266	0.232	0.00260476457720617	Oxidative stress hepatocytes	TMEM134
1.56271344619907E-07	0.962441876706657	0.273	0.232	0.0026187951931404	Oxidative stress hepatocytes	ATF5
1.57872225309584E-07	1.68953835400033	0.104	0.069	0.002645622751738	Oxidative stress hepatocytes	WWC3
1.59003850046179E-07	0.367225068639565	0.054	0.103	0.00266458651907386	Oxidative stress hepatocytes	IMMP1L
1.61328489933279E-07	0.305041034043728	0.119	0.189	0.00270354283430189	Oxidative stress hepatocytes	XPC
1.63939766678852E-07	1.54201854718252	0.199	0.163	0.0027473026100042	Oxidative stress hepatocytes	TBC1D13
1.65189832891648E-07	0.319782114007735	0.068	0.121	0.00276825121959824	Oxidative stress hepatocytes	STRADB
1.65232394762609E-07	0.31413974945264	0.063	0.114	0.0027689644714318	Oxidative stress hepatocytes	RAB23
1.69629196864806E-07	0.67034097362116	0.456	0.464	0.00284264608106043	Oxidative stress hepatocytes	RAB4A
1.70010357354214E-07	0.312592151038193	0.091	0.151	0.00284903356854192	Oxidative stress hepatocytes	LRRC8E
1.75372235569998E-07	0.62721565110524	0.45	0.454	0.00293888792368203	Oxidative stress hepatocytes	RNF5
1.81785037290888E-07	1.22872587293502	0.244	0.21	0.00304635365492071	Oxidative stress hepatocytes	TBC1D16
1.92974365421833E-07	0.74736605487591	0.384	0.37	0.00323386441573908	Oxidative stress hepatocytes	PSMD10
1.99100409552796E-07	0.46276594703209	0.524	0.541	0.00333652466328576	Oxidative stress hepatocytes	ARF1
2.09464729417524E-07	0.267202035379073	0.113	0.18	0.00351020993557886	Oxidative stress hepatocytes	CSNK1A1
2.10541048363747E-07	1.27275896675791	0.222	0.186	0.00352824688847967	Oxidative stress hepatocytes	EIF3F
2.13090661056584E-07	0.316651841481971	0.109	0.175	0.00357097329798624	Oxidative stress hepatocytes	PHF5A
2.20190606854794E-07	0.480404848239283	0.141	0.219	0.00368995418967264	Oxidative stress hepatocytes	ZNF580
2.223547148184E-07	1.86059450905408	0.165	0.129	0.00372622031092674	Oxidative stress hepatocytes	EVI5L
2.28767591939674E-07	0.319373330319298	0.131	0.206	0.00383368730572506	Oxidative stress hepatocytes	UTRN
2.29116445403701E-07	0.703318591430383	0.45	0.464	0.00383953339207522	Oxidative stress hepatocytes	BLOC1S1
2.37843859077457E-07	0.282590659456735	0.227	0.331	0.00398578739042002	Oxidative stress hepatocytes	PPM1A
2.3821676153409E-07	0.345195358260908	0.086	0.145	0.00399203648978827	Oxidative stress hepatocytes	GRK3
2.40781771968962E-07	0.30527326869196	0.116	0.183	0.00403502093465586	Oxidative stress hepatocytes	METTL5
2.44218783128241E-07	0.404305397254843	0.155	0.235	0.00409261836766306	Oxidative stress hepatocytes	DNMT3A
2.55690338690388E-07	0.550616278209493	0.448	0.423	0.00428485869577352	Oxidative stress hepatocytes	MCM3
2.60676809318003E-07	1.68920841503587	0.166	0.13	0.0043684219705511	Oxidative stress hepatocytes	FAM89B
2.68038139085883E-07	0.783064176054756	0.391	0.386	0.00449178313480123	Oxidative stress hepatocytes	PSMC3
2.75033160746351E-07	0.317401993321964	0.103	0.167	0.00460900570778735	Oxidative stress hepatocytes	N4BP1

2.82442753387005E-07	0.298409830701511	0.115	0.182	0.00473317566125943	Oxidative stress hepatocytes	SLC41A3
2.88022801047894E-07	1.14243473432064	0.286	0.254	0.0048266860999606	Oxidative stress hepatocytes	TCF19
2.92573462197768E-07	0.390529422283515	0.12	0.19	0.00490294607951019	Oxidative stress hepatocytes	CASZ1
2.97967652733285E-07	0.280788212970135	0.096	0.157	0.00499334192450439	Oxidative stress hepatocytes	KATNAL1
3.19743025644048E-07	0.25108269130774	0.186	0.275	0.00535825362374296	Oxidative stress hepatocytes	CNPY2
3.60228726949731E-07	0.276638611392408	0.092	0.152	0.0060367130062236	Oxidative stress hepatocytes	EDC3
3.69411130237247E-07	0.763482344770994	0.402	0.404	0.00619059172051579	Oxidative stress hepatocytes	PSMB4
3.76245047251724E-07	0.429381736649563	0.081	0.136	0.00630511450184439	Oxidative stress hepatocytes	BRWD3
3.91630691489769E-07	0.31882870028317	0.178	0.266	0.00656294712798555	Oxidative stress hepatocytes	KDM3B
4.01870877084889E-07	1.37202638053604	0.216	0.183	0.00673455215818856	Oxidative stress hepatocytes	TEF
4.06703027646372E-07	0.286934582323802	0.122	0.189	0.00681552933729789	Oxidative stress hepatocytes	EIF2B1
4.07383347502342E-07	0.261004023666521	0.099	0.159	0.00682693013744424	Oxidative stress hepatocytes	SVIL
4.09355420492994E-07	0.34835412898224	0.118	0.185	0.0068599781366216	Oxidative stress hepatocytes	PDP1
4.11856120882933E-07	0.301531428221154	0.089	0.147	0.0069018848737562	Oxidative stress hepatocytes	AP3S1
4.13145843485021E-07	0.277270784363289	0.078	0.132	0.00692349804512198	Oxidative stress hepatocytes	BBX
4.1324025879231E-07	0.298013752694365	0.07	0.122	0.00692508025684152	Oxidative stress hepatocytes	AHCTF1
4.13788721964933E-07	0.251647594066358	0.198	0.291	0.00693427140268835	Oxidative stress hepatocytes	AGGF1
4.18497108229637E-07	0.273909955569877	0.112	0.176	0.00701317453971225	Oxidative stress hepatocytes	ANKS4B
4.4143285499557E-07	0.320457626247853	0.079	0.133	0.00739753178401575	Oxidative stress hepatocytes	OGFOD1
4.44593510224272E-07	0.306993199997156	0.082	0.137	0.00745049804433835	Oxidative stress hepatocytes	IFFO1
4.46378641222253E-07	0.347867380528711	0.064	0.114	0.00748041326960251	Oxidative stress hepatocytes	ZNF697
4.52988429126364E-07	0.380570078232379	0.144	0.219	0.00759118009529961	Oxidative stress hepatocytes	IRS1
4.54954797913208E-07	1.17755225640027	0.309	0.293	0.00762413250342954	Oxidative stress hepatocytes	TMUB1
4.63422009380427E-07	0.689795247736466	0.415	0.416	0.0077660260331972	Oxidative stress hepatocytes	RNH1
4.65675203014076E-07	0.415312654442289	0.116	0.182	0.00780378505210989	Oxidative stress hepatocytes	PIAS3
4.72364674670256E-07	0.342402299727912	0.113	0.179	0.00791588721812415	Oxidative stress hepatocytes	KLHL8
4.84199843781524E-07	0.35917775247594	0.058	0.105	0.00811422098209078	Oxidative stress hepatocytes	GEMIN6
4.99882056415724E-07	0.34673052084986	0.086	0.142	0.00837702350141471	Oxidative stress hepatocytes	NDUFS5
5.00444136903346E-07	0.473793632403474	0.102	0.163	0.00838644284622627	Oxidative stress hepatocytes	DVL2
5.26874364155439E-07	0.425770536835466	0.078	0.131	0.00882936059451685	Oxidative stress hepatocytes	KPTN
5.4184219852078E-07	0.633076164546782	0.482	0.5	0.00908019156281124	Oxidative stress hepatocytes	BRD2
5.45141452246207E-07	0.289799647345526	0.212	0.306	0.00913548045674194	Oxidative stress hepatocytes	EFCAB14
5.47301008181811E-07	0.324318467674958	0.09	0.148	0.00917167029511079	Oxidative stress hepatocytes	SPRYD7
5.81148246728963E-07	0.283296944434336	0.11	0.175	0.00973888231868396	Oxidative stress hepatocytes	KIF21A
5.92849001037454E-07	0.612925926589187	0.446	0.453	0.00993496355938565	Oxidative stress hepatocytes	COX4I1
5.99507581038115E-07	1.52201049602999	0.175	0.139	0.0100465480430367	Oxidative stress hepatocytes	FEM1A
6.06545953771171E-07	0.9934717907313	0.29	0.262	0.0101644970932973	Oxidative stress hepatocytes	TOLLIP
6.09935857455477E-07	1.64977002021276	0.131	0.096	0.0102213050992389	Oxidative stress hepatocytes	TSSC4
6.40648309577088E-07	0.762911468631617	0.371	0.364	0.0107359843718928	Oxidative stress hepatocytes	UBL3
6.44493673718946E-07	0.290979275594657	0.118	0.183	0.0108004249841821	Oxidative stress hepatocytes	SAP30BP
6.53952071895497E-07	0.432107555294166	0.618	0.648	0.0109589288208247	Oxidative stress hepatocytes	XBP1
6.6599904726209E-07	0.29645723443193	0.068	0.119	0.0111608120340181	Oxidative stress hepatocytes	AMD 1.00
7.14049794917461E-07	0.325056155285022	0.163	0.243	0.0119660464632268	Oxidative stress hepatocytes	SNX27
7.35129081087475E-07	0.892617831797311	0.355	0.349	0.0123192931408639	Oxidative stress hepatocytes	NAXE
7.39313303895988E-07	1.36378748373934	0.224	0.191	0.012389412346689	Oxidative stress hepatocytes	ZC3H7B
7.6380647467923E-07	0.259308913605592	0.117	0.181	0.0127998689026745	Oxidative stress hepatocytes	INO80B
7.65405391605353E-07	0.339888217179059	0.062	0.11	0.0128266635525225	Oxidative stress hepatocytes	PPM1D
7.99864149459948E-07	0.816542363858741	0.373	0.366	0.0134041234166498	Oxidative stress hepatocytes	NEDD8
8.42867210117616E-07	1.36405673507777	0.166	0.129	0.014124768707151	Oxidative stress hepatocytes	RITA1
8.50291936763785E-07	0.256795900176388	0.111	0.174	0.0142491922762875	Oxidative stress hepatocytes	C4orf19
8.826693930297E-07	0.264715354619793	0.129	0.198	0.0147917736883917	Oxidative stress hepatocytes	MED30
8.88217259752268E-07	1.19656991036092	0.29	0.27	0.0148847448389285	Oxidative stress hepatocytes	STRN4
8.92374270085409E-07	0.314513444695987	0.103	0.164	0.0149544080180913	Oxidative stress hepatocytes	MIB2
9.14701558251943E-07	0.454116277301274	0.085	0.14	0.0153285687131861	Oxidative stress hepatocytes	WDFY3
9.59891409452019E-07	0.559032779251267	0.075	0.126	0.0160858602395969	Oxidative stress hepatocytes	ALDH16A1
9.84030978183997E-07	0.42321330793226	0.146	0.22	0.0164903911324074	Oxidative stress hepatocytes	CDK14
9.8778478541151E-07	1.31980935013091	0.223	0.192	0.0165532974339261	Oxidative stress hepatocytes	RMND5B
1.02522491806363E-06	0.435251552329933	0.544	0.541	0.0171807191769103	Oxidative stress hepatocytes	GDI1
1.03134551328156E-06	0.355411138501227	0.085	0.14	0.0172832881115723	Oxidative stress hepatocytes	UTP3
1.07396718204836E-06	0.290679290588028	0.057	0.103	0.0179975420367665	Oxidative stress hepatocytes	TMEM42

1.08201220393935E-06	0.87200966044832	0.352	0.343	0.0181323605136157	Oxidative stress hepatocytes	PACSIN2
1.12522226948193E-06	0.380227862788057	0.148	0.223	0.0188564747919781	Oxidative stress hepatocytes	MAML1
1.1283433635035E-06	0.72309007056703	0.387	0.383	0.0189087780855916	Oxidative stress hepatocytes	SUB1
1.14275112146981E-06	0.380909015451638	0.151	0.227	0.0191502232935911	Oxidative stress hepatocytes	OTUD7B
1.15295403882848E-06	0.911936692921358	0.357	0.35	0.0193212037826876	Oxidative stress hepatocytes	CCNI
1.15873486204091E-06	0.3076540959178	0.077	0.128	0.0194180788180816	Oxidative stress hepatocytes	DEPRECATEI
1.16310711811627E-06	1.58499334726239	0.172	0.137	0.0194913490853925	Oxidative stress hepatocytes	FAM120AOS
1.19947419490723E-06	0.955995388517416	0.308	0.289	0.0201007885582554	Oxidative stress hepatocytes	NADK
1.2605210805115E-06	0.25383397627763	0.152	0.227	0.0211238122672118	Oxidative stress hepatocytes	EFTUD2
1.26864624085695E-06	0.698841053352714	0.079	0.133	0.0212599737042808	Oxidative stress hepatocytes	TCTEX1D2
1.33552144692163E-06	0.295431255476844	0.12	0.186	0.0223806684075127	Oxidative stress hepatocytes	GLRX2
1.34913471610457E-06	0.907148762979945	0.314	0.293	0.0226087995724804	Oxidative stress hepatocytes	CDC34
1.3502333519414E-06	0.668335177064234	0.38	0.37	0.022627210511834	Oxidative stress hepatocytes	TXNDC17
1.37520688346104E-06	0.449552421442414	0.105	0.166	0.0230457169530401	Oxidative stress hepatocytes	PIGS
1.38005267522432E-06	0.85131262899463	0.311	0.29	0.0231269227314092	Oxidative stress hepatocytes	NME1
1.41391867700381E-06	0.289847417535652	0.66	0.713	0.0236944491892298	Oxidative stress hepatocytes	HSP90AB1
1.41709742931859E-06	0.287724949730093	0.139	0.208	0.023747718720521	Oxidative stress hepatocytes	MAFG
1.44683830790572E-06	0.946463435913056	0.281	0.251	0.024246116363884	Oxidative stress hepatocytes	LMNB2
1.47086561943218E-06	0.434397478842651	0.17	0.252	0.0246487660504445	Oxidative stress hepatocytes	NELFA
1.48102386272984E-06	0.979183893180334	0.291	0.269	0.0248189978916266	Oxidative stress hepatocytes	POLDIP3
1.50019898023317E-06	0.250269417579144	0.11	0.172	0.0251403345107474	Oxidative stress hepatocytes	MTMR11
1.50582794863766E-06	0.288845910105797	0.079	0.131	0.0252346647632699	Oxidative stress hepatocytes	DTNBP1
1.52998750397924E-06	0.285651050919289	0.068	0.117	0.0256395305916841	Oxidative stress hepatocytes	PAICS
1.53491443372775E-06	0.72752655798437	0.412	0.418	0.0257220960804097	Oxidative stress hepatocytes	OPTN
1.6109151410431E-06	0.345807266164777	0.092	0.147	0.0269957159336003	Oxidative stress hepatocytes	ARHGAP21
1.61791604737851E-06	1.0559768463251	0.279	0.252	0.027113037121969	Oxidative stress hepatocytes	HMOX2
1.64381970338504E-06	0.403708521481238	0.092	0.149	0.0275471305893264	Oxidative stress hepatocytes	MYCBP2
1.64433771621596E-06	0.27577930625264	0.121	0.186	0.0275558114483471	Oxidative stress hepatocytes	MINDY1
1.65316927734454E-06	0.702151977198726	0.436	0.439	0.0277038107497399	Oxidative stress hepatocytes	PLAAT4
1.65591287587526E-06	0.298262321304574	0.068	0.115	0.0277497879739177	Oxidative stress hepatocytes	DHRS13
1.6792928917529E-06	0.666954735498037	0.417	0.414	0.028141590279995	Oxidative stress hepatocytes	COX8A
1.71106524321618E-06	0.254508109686058	0.186	0.269	0.0286740313458167	Oxidative stress hepatocytes	NR3C1
1.72978731016272E-06	0.271353480750721	0.257	0.363	0.0289877757437068	Oxidative stress hepatocytes	TJP1
1.75662811616807E-06	0.273227223449893	0.189	0.275	0.0294375739707445	Oxidative stress hepatocytes	MTMR1
1.76202876933547E-06	0.454632747488976	0.09	0.145	0.0295280781165238	Oxidative stress hepatocytes	RPAP1
1.81910935432954E-06	0.274288993758499	0.103	0.162	0.0304846345598545	Oxidative stress hepatocytes	SPOUT1
1.83352755174874E-06	1.33342704635697	0.207	0.175	0.0307262547122054	Oxidative stress hepatocytes	CCM2
1.8543422119333E-06	0.393882894328476	0.068	0.116	0.0310750667875783	Oxidative stress hepatocytes	SENP1
1.91209166714068E-06	0.590367664952142	0.469	0.485	0.0320428321579435	Oxidative stress hepatocytes	EIF4EBP2
1.96356900690979E-06	1.20361969760334	0.256	0.229	0.0329054894177943	Oxidative stress hepatocytes	ELP5
2.03733961856264E-06	0.323954866200887	0.139	0.209	0.0341417373278728	Oxidative stress hepatocytes	MAGOH
2.06668066129502E-06	1.03830829361049	0.251	0.223	0.0346334345219819	Oxidative stress hepatocytes	DDX49
2.18394248428689E-06	0.298487773599006	0.066	0.113	0.0365985081516798	Oxidative stress hepatocytes	THOC5
2.18567347172706E-06	0.81390280991199	0.385	0.386	0.0366275160392022	Oxidative stress hepatocytes	SMIM19
2.2315202698346E-06	0.421719148998122	0.089	0.143	0.0373958166818882	Oxidative stress hepatocytes	SNW1
2.24612103666947E-06	0.372095353713068	0.095	0.151	0.0376404963325071	Oxidative stress hepatocytes	BACE1
2.31445726059363E-06	0.253604346721049	0.065	0.111	0.038785674773028	Oxidative stress hepatocytes	ZNF554
2.38250673711276E-06	0.850249731815902	0.348	0.336	0.0399260479005357	Oxidative stress hepatocytes	SPR
2.39991653761345E-06	0.27519322321923	0.122	0.185	0.0402178013373262	Oxidative stress hepatocytes	DENND4B
2.4105569913263E-06	0.352566218822401	0.174	0.256	0.0403961140606461	Oxidative stress hepatocytes	RASSF4
2.48920922927608E-06	1.39350682236987	0.218	0.188	0.0417141682642086	Oxidative stress hepatocytes	FAAP20
2.51163281455092E-06	0.410228374386182	0.075	0.125	0.0420899427062443	Oxidative stress hepatocytes	SAMM50
2.54994677033702E-06	0.366947387362545	0.095	0.15	0.0427320079773077	Oxidative stress hepatocytes	RAB29
2.60321388420509E-06	0.296343323357322	0.196	0.278	0.0436246582715089	Oxidative stress hepatocytes	PSENEN
2.60351471964071E-06	1.13511590438188	0.286	0.267	0.0436296996717391	Oxidative stress hepatocytes	TBC1D14
2.64536208532215E-06	0.260045363900725	0.101	0.158	0.0443309778258286	Oxidative stress hepatocytes	MORF4L1
2.66246740605966E-06	1.19542001303152	0.238	0.207	0.0446176287907478	Oxidative stress hepatocytes	C9orf16
2.77779652211195E-06	1.16023431125428	0.257	0.232	0.0465503141175521	Oxidative stress hepatocytes	SIRT2
2.93722097452259E-06	0.31181870829982	0.185	0.268	0.0492219490910496	Oxidative stress hepatocytes	BTBD7
2.96711499851117E-06	0.635934164529742	0.399	0.391	0.0497229131450502	Oxidative stress hepatocytes	NSDHL
