# Supplementary material for: Spatiotemporal dynamics of the tumor microenvironment in hepatocellular carcinoma during combination immunotherapy
Source: Hepatol Commun. 2026 Aug 7;10(9):e1014. doi: 10.1097/HC9.0000000000001014 (PMC13456720; doi:10.1097/HC9.0000000000001014)
Supplement: Supplementary file 5 [file hc9-10-e1014-s007.pdf]

SupTable5E_C1_TNK_SeuratCluster

p_val	avg_log2FC	pct.1	pct.2	p_val_adj	cluster	gene
5.12359322472582E-28	2.90647723153029	0.284	0.089	8.58611752599553E-24	0	SATB1
4.32166599435439E-113	2.86028361248747	0.773	0.349	7.24224787333908E-109	0	IL7R
1.63330634683215E-27	2.7369596104962	0.258	0.068	2.73709477602131E-23	0	TCF7
1.18363079274935E-30	2.70094451875799	0.3	0.089	1.98352848248936E-26	0	KLF2
4.64416195561114E-17	2.4598508903508	0.3	0.153	7.78268660521315E-13	0	PER1
7.48980736918317E-15	2.15577673809306	0.283	0.145	1.25514191892772E-10	0	ERN 1.00
8.46165178501907E-12	2.12529363731236	0.254	0.14	1.4180036061335E-07	0	TNFRSF25
1.05189814266322E-44	1.94576660590926	0.546	0.258	1.76277090747502E-40	0	SERPINA1
5.19827380916495E-07	1.86662822161153	0.26	0.182	0.00871126724939862	0	ADAM19
4.62886206355584E-11	1.85476607720812	0.258	0.146	7.75704704610687E-07	0	TRAT1
1.64510130054104E-13	1.69757857720376	0.496	0.426	2.75686075944668E-09	0	TNFAIP3
3.20605453365477E-08	1.59381212450122	0.387	0.317	0.000537270618749866	0	JUNB
3.11175937426444E-07	1.48587071734892	0.455	0.414	0.00521468635939235	0	EML4
2.52990527280812E-92	1.47001823922716	0.968	0.898	4.23961525617184E-88	0	CXCR4
2.65177909197895E-08	1.34834864124595	0.393	0.315	0.000444385140233832	0	CD69
2.23446173601218E-22	1.3175472234212	0.693	0.703	3.74451097720921E-18	0	TSC22D3
2.70764738482454E-06	1.27398982656547	0.343	0.274	0.0453747548748896	0	PPP2R5C
4.75688013699405E-26	1.26973088582186	0.739	0.664	7.97157973357464E-22	0	ZFP36L2
6.06631461517196E-07	1.24266275213568	0.42	0.367	0.0101659300321052	0	CYTH1
5.20805234371236E-08	1.03661903958519	0.517	0.489	0.000872765411759317	0	AHNAK
3.36325431119975E-07	0.431082704512915	0.22	0.36	0.00563614157470854	0	PAFAH1B1
3.16798924412546E-09	0.414391445848266	0.375	0.628	5.30891637530545E-05	0	ALB
2.8672668862905E-07	0.406387196946126	0.213	0.35	0.00480496584804561	0	USF2
2.23413195818561E-07	0.368538020639829	0.227	0.372	0.00374395833552744	0	CDC37
3.62006431351464E-07	0.364367704056306	0.181	0.305	0.00606650377658783	0	ARPC2
3.13247260962383E-07	0.353473554698521	0.187	0.313	0.00524939759920761	0	SP140
2.28848687747626E-06	0.345239569589277	0.151	0.255	0.0383504630927472	0	SMARCA4
1.03464527517193E-08	0.342522560448666	0.205	0.356	0.000173385855213311	0	IRF3
2.2484938035738E-06	0.339723146530705	0.238	0.365	0.0376802591602897	0	HNRNPUL1
9.13293566738534E-08	0.338311343707304	0.176	0.304	0.00153049735914044	0	SLTM
9.18299891836903E-07	0.335986511186537	0.166	0.28	0.0153888695874028	0	DHX15
1.24079566839967E-06	0.329116239562823	0.174	0.288	0.0207932538110417	0	ZNF638
4.61170830895033E-07	0.323549746711131	0.311	0.486	0.00772830078413896	0	CLK1
3.28268236575232E-08	0.320454762541681	0.179	0.316	0.000550111910852773	0	TERF2IP
2.4004399674402E-06	0.314722420822341	0.186	0.3	0.0402265729743629	0	BRD4
4.2759033929425E-08	0.307666009751476	0.182	0.315	0.000716555890589304	0	SLC20A1
1.65538979016431E-06	0.303460192342771	0.228	0.362	0.0277410221035735	0	IL16
2.4562257497719E-06	0.301160212477765	0.179	0.292	0.0411614311146774	0	DCAF8
1.81165315565352E-07	0.271852143395037	0.228	0.376	0.00303596835824417	0	NUMA1
8.83833562784978E-07	0.271690123522103	0.174	0.288	0.0148112828451507	0	STAG2
1.93566333344527E-06	0.270974341245441	0.186	0.299	0.0324378461418758	0	ERO1A
3.81096616956635E-07	0.269937068341455	0.216	0.352	0.00638641710695929	0	HNRNPLL
9.65023162178077E-07	0.26182127755809	0.296	0.456	0.0161718581517802	0	IL10RA
4.8127047117473E-07	0.253241384639982	0.17	0.288	0.00806513055594612	0	RAB11B
4.86561448977562E-07	0.2513538143746	0.149	0.26	0.00815379676196598	0	DNAJA2
4.15835360898251E-175	4.75245463614005	0.716	0.057	6.9685689779329E-171	1	FOXP3
2.30954609600168E-81	4.14709215325405	0.356	0.024	3.87033734767962E-77	1	CCR8
6.40937984191932E-84	3.81697643783094	0.461	0.058	1.07408387390884E-79	1	IL2RA

6.54134896782515E-84	3.58665144121618	0.405	0.036	1.09619926002814E-79	1	NTNG2
5.35318015165034E-47	3.47153949954751	0.31	0.049	8.97085929813564E-43	1	GADD45A
2.24747195111076E-82	3.470876287838	0.487	0.073	3.76631349567141E-78	1	IL1R1
5.26523742107018E-113	3.32076454679003	0.641	0.093	8.82348487022942E-109	1	CTLA4
1.91575038334509E-79	3.30524512135872	0.431	0.049	3.2104144924097E-75	1	TNFRSF4
1.43727600346674E-42	3.26119028237035	0.275	0.041	2.40858712660956E-38	1	LTA
6.08477440078526E-41	3.18045620926024	0.261	0.037	1.01968649408359E-36	1	LAIR2
1.2572581613745E-44	3.14089723887756	0.261	0.033	2.10691322683138E-40	1	VDR
9.83646857354943E-42	3.06859961716438	0.333	0.07	1.64839540355541E-37	1	SDC4
1.96170950672687E-112	3.03094398601998	0.693	0.118	3.28743279137289E-108	1	TNFRSF18
8.8742691076628E-72	2.97085869212215	0.477	0.08	1.48715001706213E-67	1	TBC1D4
2.5990761854505E-64	2.83744547932658	0.487	0.1	4.35553187157795E-60	1	MAST4
7.41024093835123E-67	2.75035638817227	0.461	0.079	1.2418081764489E-62	1	LINC02694
1.1148675911016E-37	2.5655795110867	0.255	0.038	1.86829510916806E-33	1	LAYN
1.77938664228423E-62	2.51249503908658	0.487	0.097	2.98189613513992E-58	1	TNFRSF9
3.08441470279185E-82	2.44345012987096	0.81	0.349	5.16886215893858E-78	1	CTSC
2.7134897445223E-42	2.40574262487613	0.369	0.081	4.54726611387048E-38	1	ACP5
2.58905492394917E-64	2.40027000669694	0.464	0.082	4.33873824155401E-60	1	IKZF2
1.44542449581495E-24	2.34680814370687	0.265	0.071	2.4222423700867E-20	1	INPP5F
3.55825773800626E-103	2.31255686128419	0.833	0.225	5.96292831735088E-99	1	CD4
8.23285005115504E-27	2.25551956137748	0.297	0.085	1.37966101157256E-22	1	TYMP
4.04056269716942E-27	2.24136655368221	0.288	0.078	6.77117496791652E-23	1	LAPTM4B
1.64781970988257E-26	2.23374696160313	0.268	0.068	2.76141626982121E-22	1	MAGEH1
2.00253659253637E-34	2.18210080812307	0.412	0.128	3.35585082177244E-30	1	CSF1
9.35325205681859E-40	2.17184379057413	0.435	0.124	1.56741797968166E-35	1	PHLDA1
3.99839870625808E-36	2.10434733684417	0.294	0.058	6.70051655194729E-32	1	PELI1
2.92598946353064E-30	2.08975517460827	0.359	0.109	4.90337314298464E-26	1	GLRX
2.1889783406575E-41	2.06885931982081	0.428	0.116	3.66828990327384E-37	1	BATF
2.46407291189254E-66	2.06210816838807	0.673	0.205	4.12929338574953E-62	1	CD27
1.00440704949561E-53	2.04940725370104	0.464	0.103	1.68318533354475E-49	1	IL18R1
3.94279221683716E-78	2.03472413150485	0.752	0.221	6.6073311969757E-74	1	TIGIT
1.46097047913938E-36	2.01486201186872	0.389	0.103	2.44829432894178E-32	1	WHRN
2.54650694975309E-27	1.98742268705185	0.291	0.077	4.26743634639623E-23	1	ERI1
3.14973115821895E-31	1.98450220006672	0.369	0.114	5.27831947494331E-27	1	DPYSL2
2.35386214101626E-54	1.97712760449822	0.444	0.09	3.94460217591504E-50	1	STAM
2.70357287113971E-39	1.92079932850629	0.34	0.072	4.53064741745593E-35	1	IL12RB2
1.94043692870013E-32	1.90046677226986	0.458	0.161	3.25178420511568E-28	1	CISH
7.01568130870175E-28	1.89936066700128	0.275	0.068	1.17568787371224E-23	1	ABI2
5.9128444640263E-45	1.86563499496478	0.572	0.201	9.90874475281527E-41	1	PKM
2.34493572070041E-35	1.82378262341872	0.425	0.128	3.92964328074974E-31	1	DUSP4
9.04486871810428E-20	1.75841859882514	0.284	0.096	1.51573909977992E-15	1	MAN1A2
1.73616165159077E-31	1.73665180043801	0.366	0.105	2.90945969573581E-27	1	SLC12A6
1.11008701201667E-22	1.72025031551256	0.291	0.088	1.86028381473753E-18	1	PAM
6.99167454030334E-20	1.7134862557227	0.265	0.084	1.17166481946403E-15	1	INF2
2.2501583697885E-55	1.71326612251182	0.598	0.176	3.77081539609156E-51	1	ICOS
1.66168497593859E-41	1.6908122197913	0.575	0.213	2.78465168267788E-37	1	PHTF2
8.85476702961285E-41	1.68827435013323	0.438	0.122	1.48388185882252E-36	1	PRNP
5.7046555909358E-24	1.68792375317903	0.376	0.136	9.55986183929021E-20	1	THADA
3.77094881441974E-30	1.67673855334551	0.392	0.126	6.3193560232046E-26	1	CXCR3
2.86392332872998E-26	1.66511509696209	0.438	0.172	4.7993627142857E-22	1	FAM53B

4.01297169775273E-23	1.66285722138401	0.265	0.072	6.72493797109403E-19	1	ITGAM
1.89324782490398E-47	1.65752851995136	0.572	0.185	3.1727047049741E-43	1	SLAMF1
1.52769305066665E-30	1.64840495724998	0.366	0.109	2.56010801430717E-26	1	SGPP1
1.24017330999537E-54	1.62787594817189	0.673	0.219	2.07828243289023E-50	1	PIM2
1.50939192645228E-21	1.57283981065891	0.34	0.125	2.52943899034874E-17	1	NPTN
3.29803050125191E-25	1.54849774556103	0.392	0.141	5.52683951399795E-21	1	MAPKAPK3
8.14445619241313E-51	1.5455666100119	0.755	0.337	1.36484796872459E-46	1	TNFRSF1B
2.45913266717009E-29	1.53763097825011	0.454	0.168	4.12101452364364E-25	1	SIRPG
2.24353395148497E-15	1.51623998063146	0.252	0.09	3.75971419589851E-11	1	TRAF3
2.63652528894172E-26	1.50346115488296	0.314	0.089	4.41828907920853E-22	1	ADAT2
1.138509400577E-16	1.49070821577817	0.252	0.087	1.90791405348694E-12	1	GRINA
4.77226483734954E-23	1.48600345372746	0.382	0.146	7.99736141443036E-19	1	PTPRJ
9.81683111723084E-18	1.48549913299378	0.33	0.134	1.64510455862554E-13	1	RHOC
8.25221024385165E-19	1.4853883049784	0.268	0.087	1.38290539266466E-14	1	KLHL2
6.09893213875419E-21	1.46367391267408	0.343	0.127	1.02205904781243E-16	1	TGFBR1
1.20409538537024E-23	1.46359848896951	0.33	0.107	2.01782304680345E-19	1	PHACTR2
1.89429600913989E-41	1.45985339200697	0.598	0.232	3.17446125211663E-37	1	GBP2
4.42390704470766E-40	1.44866486203503	0.67	0.297	7.4135834255211E-36	1	CCND2
1.24859471483389E-54	1.44311221396166	0.801	0.347	2.09239502311864E-50	1	MAF
9.45717836545599E-14	1.43803181754566	0.261	0.106	1.58483395048312E-09	1	CREB3L2
2.49108357248968E-20	1.43356735428053	0.386	0.16	4.1745578507782E-16	1	BCL2L1
2.93748015228924E-12	1.42097560789213	0.255	0.109	4.9226292392063E-08	1	UGP2
2.25182226292838E-12	1.40831995138799	0.252	0.104	3.77360374821538E-08	1	NUDT5
4.0698583583492E-18	1.40423765563438	0.271	0.092	6.82026863692159E-14	1	SNX9
7.68713779603557E-29	1.40173025394489	0.487	0.194	1.28821055185964E-24	1	CD82
1.32969634136171E-49	1.38499930505846	0.866	0.532	2.22830512885396E-45	1	IL32
2.94207433586835E-13	1.38431769747958	0.258	0.104	4.93032817204818E-09	1	ZNRF1
1.45057425500738E-18	1.37711382423748	0.337	0.136	2.43087233654137E-14	1	DYNC1I2
3.10749555984321E-22	1.36421884719347	0.408	0.166	5.20754105918526E-18	1	CASP1
3.20573715196832E-34	1.35817877660434	0.67	0.323	5.37217431926851E-30	1	UCP2
3.8326000214748E-19	1.34913002327935	0.356	0.143	6.42267111598746E-15	1	GLCCI1
6.95328632558266E-18	1.33847137286946	0.284	0.1	1.16523172244114E-13	1	TRPS1
5.20410825391692E-42	1.33703385312762	0.771	0.359	8.72104461191398E-38	1	LTB
7.91765242725655E-26	1.33322029614736	0.516	0.232	1.32684019375965E-21	1	ENO1
4.56107952549036E-32	1.32534540922512	0.431	0.134	7.64345706881674E-28	1	IL21R
1.94972166685353E-10	1.32172065021965	0.278	0.137	3.26734356931314E-06	1	IFI6
4.04960288782112E-16	1.31999528074912	0.284	0.109	6.78632451941063E-12	1	HS3ST3B1
7.80356634275356E-13	1.31737063664022	0.265	0.112	1.30772164771864E-08	1	ATP2A2
1.09968209940845E-16	1.31629420838046	0.34	0.144	1.84284726218868E-12	1	NEDD9
7.52102739235708E-26	1.30530729349194	0.451	0.184	1.2603737704112E-21	1	CTSZ
3.65784517496977E-20	1.30178173393188	0.373	0.147	6.12981694421435E-16	1	ORMDL3
1.85058868351265E-35	1.30088115693971	0.353	0.081	3.10121651583049E-31	1	SELL
4.42821396900662E-21	1.26310782110647	0.474	0.218	7.42080096926129E-17	1	SYNGR2
2.64517502620728E-36	1.25775736969727	0.719	0.356	4.43278430891817E-32	1	S100A4
3.07426304867615E-19	1.25529752465009	0.405	0.18	5.1518500169715E-15	1	PPM1G
1.02870916552652E-17	1.2505444934618	0.304	0.112	1.72391081958934E-13	1	IGFLR1
1.86108757975268E-25	1.24092233090295	0.32	0.094	3.11881056614954E-21	1	HAPLN3
1.24550091997318E-17	1.2380571828603	0.255	0.083	2.08721044169106E-13	1	TULP4
3.05736805342333E-21	1.23452923116997	0.438	0.192	5.12353738392681E-17	1	NDFIP1
1.44227800230979E-29	1.22896828017063	0.526	0.215	2.41696947627075E-25	1	CTSB

1.16184707359196E-11	1.22717369320107	0.258	0.112	1.9470233259254E-07	1	SYT11
1.17064292081435E-11	1.22629117882426	0.297	0.146	1.96176340670068E-07	1	LMAN1
4.21750348237537E-14	1.22481133005272	0.288	0.119	7.06769233576464E-10	1	PTMS
9.33416119612195E-50	1.22004627340461	0.935	0.62	1.56421873324612E-45	1	TRAC
9.65815976234166E-19	1.21444826069156	0.363	0.146	1.61851441297322E-14	1	CAB39
3.15125151794526E-20	1.20962731041992	0.458	0.213	5.28086729377267E-16	1	CTSA
1.10536978971837E-10	1.20095076036492	0.255	0.117	1.85237869361005E-06	1	NDUFA4
7.62818996144178E-16	1.19000439515328	0.327	0.139	1.27833207373841E-11	1	SH3BGRL
7.44747935360993E-17	1.18617415215033	0.399	0.184	1.24804859007795E-12	1	SIT 1.00
8.31448650182678E-41	1.18021497488506	0.605	0.219	1.39334164797613E-36	1	CD28
6.77544952274026E-17	1.17760348123821	0.366	0.157	1.13542983102081E-12	1	ADCY3
1.17322276092018E-11	1.1727395295084	0.255	0.109	1.96608670275004E-07	1	SEC11C
2.07940354609799E-25	1.1721883124374	0.585	0.294	3.48466446255102E-21	1	ANKRD10
2.84604699269616E-26	1.17161263452547	0.435	0.163	4.76940555036022E-22	1	NCOA3
5.58685133864317E-15	1.16860887091997	0.32	0.137	9.36244547329823E-11	1	AGFG1
1.27037408204743E-13	1.16841477655557	0.278	0.114	2.12889288669508E-09	1	USP12
2.34316109230805E-13	1.1671934593415	0.337	0.161	3.92666935848984E-09	1	HADHA
3.84825810806371E-14	1.14804123085263	0.294	0.124	6.44891093749316E-10	1	GOLGA8B
1.32621600537933E-16	1.13810793103666	0.477	0.249	2.22247278181468E-12	1	TMEM173
6.04857398021071E-19	1.13067259347451	0.399	0.169	1.01362002760371E-14	1	SLC7A5
1.31473626345073E-12	1.13008304327888	0.255	0.104	2.20323503029073E-08	1	STN 1.00
8.3495263758221E-10	1.11412772592264	0.258	0.12	1.39921363006027E-05	1	IL10RB
1.89148385689821E-12	1.10501042272146	0.324	0.154	3.16974864739002E-08	1	TMED5
5.29323751209088E-19	1.10284961818786	0.382	0.162	8.8704074227619E-15	1	CD58
2.99635291610081E-10	1.09993585828164	0.297	0.149	5.02128821680174E-06	1	COX6C
3.48378662686864E-12	1.09442209630305	0.271	0.115	5.83812962930647E-08	1	AGTRAP
4.72391674652052E-21	1.07436475784889	0.451	0.194	7.91633968381908E-17	1	PCED1B
3.64945452721761E-19	1.07429281690559	0.265	0.082	6.11575589671126E-15	1	ZC3H12D
4.08651228209369E-13	1.07331942517416	0.294	0.129	6.8481772823326E-09	1	AFTPH
1.73636456067004E-14	1.06921547990403	0.474	0.262	2.90979973077085E-10	1	TFRC
2.56179042759344E-22	1.06443027315698	0.395	0.148	4.29304839856109E-18	1	MKNK2
1.98186761517303E-12	1.05924423677645	0.307	0.142	3.32121374950696E-08	1	UBASH3B
3.19480889523905E-19	1.05785767744324	0.533	0.274	5.35386074664159E-15	1	ECH1
1.50080253330019E-12	1.0534959262156	0.268	0.114	2.51504488530446E-08	1	MFHAS1
1.62011861156711E-31	1.04892727961403	0.739	0.403	2.71499476926416E-27	1	IL2RB
1.06293403648456E-08	1.0485279187219	0.278	0.146	0.000178126485834082	1	FKBP1A
1.71295549375224E-10	1.04796746588256	0.278	0.134	2.87057081643E-06	1	SOD1
4.02000223439789E-17	1.04425329138371	0.275	0.095	6.73671974440398E-13	1	UXS1
5.34963235762231E-12	1.04024533950694	0.294	0.132	8.96491390490347E-08	1	FNDC3B
3.95401187938395E-25	1.03549274720636	0.595	0.289	6.62613310747162E-21	1	CBFB
4.30614892008574E-28	1.03439476985691	0.539	0.22	7.21624436027968E-24	1	LTBP3
4.6355848399113E-10	1.02829253534367	0.271	0.132	7.76831307472336E-06	1	ATP13A3
7.38858019235296E-08	1.02379337048375	0.252	0.134	0.00123817826863451	1	COPG1
7.96905350235257E-18	1.01936985674211	0.405	0.183	1.33545398592424E-13	1	NFAT5
2.18998653524278E-09	1.0161958774761	0.284	0.149	3.66997943575986E-05	1	PPIB
9.6335542236764E-17	1.01464788654871	0.418	0.2	1.61439101680369E-12	1	MGAT1
1.25937736512335E-28	1.01198711150445	0.627	0.299	2.11046458847371E-24	1	KAT2B
4.98706716888935E-26	1.00591711858487	0.529	0.225	8.35732716162478E-22	1	FOXO1
1.06858574017652E-17	1.00079528158422	0.477	0.24	1.79073598338782E-13	1	SOCS1
5.56789010367791E-10	0.997773345122215	0.301	0.156	9.33067023574345E-06	1	SNX5

3.1837357044042E-14	0.993066470363543	0.317	0.137	5.33530429344057E-10	1	EMC4
3.42952172556754E-24	0.988009029194637	0.477	0.203	5.74719250770608E-20	1	VMP1
1.72304569511412E-13	0.976573682034685	0.297	0.128	2.88747997587224E-09	1	RNGTT
3.75886326648078E-07	0.975133439298407	0.265	0.149	0.0062991030619685	1	CHCHD2
5.62344508296358E-25	0.972473728416833	0.601	0.309	9.42376927003037E-21	1	EFHD2
1.1633574723376E-16	0.971212230613046	0.471	0.237	1.94955445214335E-12	1	LGALS1
3.23718304983121E-19	0.97097226696039	0.474	0.223	5.42487135490715E-15	1	TM9SF2
1.55874498091954E-16	0.964383445937633	0.467	0.236	2.61214483902497E-12	1	OS9
7.47831364833637E-12	0.964214874162628	0.379	0.203	1.25321580118821E-07	1	PSAP
9.86600411506986E-07	0.961818704331209	0.268	0.154	0.0165334496960341	1	SPCS1
5.64065813853838E-13	0.956069299194324	0.278	0.117	9.45261490856261E-09	1	STIMATE
2.67328965336421E-22	0.950148370094513	0.68	0.382	4.47989880110774E-18	1	ZFP36L1
5.263917899241E-13	0.939958891899145	0.284	0.121	8.82127361554807E-09	1	CCNG2
1.45774436936796E-17	0.938417369428541	0.487	0.247	2.44288801418682E-13	1	LBH
1.22189866728626E-08	0.933330232474086	0.268	0.136	0.000204765778663832	1	CLIP1
4.30999792919782E-18	0.929543005126011	0.395	0.168	7.22269452974971E-14	1	RAB11FIP1
2.0182912631623E-08	0.927799462666763	0.268	0.139	0.000338225249880738	1	HM13
4.86229526583978E-09	0.917871035009515	0.252	0.122	8.1482344064943E-05	1	ZNF292
7.14327969377545E-11	0.910956499186933	0.261	0.119	1.19707081108289E-06	1	REXO2
2.59116301867588E-10	0.902725352939289	0.327	0.171	4.34227098669704E-06	1	CMTM6
2.75403981713468E-24	0.896925553169387	0.66	0.343	4.61521992555429E-20	1	CD7
5.27658580874953E-18	0.896180002661366	0.621	0.364	8.84250249830246E-14	1	LCP2
4.39143943885434E-12	0.893878304668632	0.366	0.187	7.3591742116321E-08	1	RAB10
8.3875342824324E-22	0.890439949955078	0.824	0.559	1.40558299505002E-17	1	RNF213
1.81263255798418E-14	0.88664626414088	0.516	0.29	3.03760964066988E-10	1	GBP5
5.45967663714998E-07	0.886015903051726	0.261	0.145	0.00914932610853594	1	COX6A1
6.06531412236528E-08	0.881726014000006	0.265	0.139	0.00101642534062597	1	ERGIC3
2.24174604274391E-23	0.881383899512459	0.605	0.296	3.75671801843024E-19	1	ARL6IP5
1.99418127557809E-17	0.875445412472076	0.598	0.327	3.34184898161376E-13	1	LAMP1
1.4496222078308E-09	0.867559961810205	0.327	0.176	2.42927689588285E-05	1	ZBTB38
1.40167870104337E-09	0.860810771303347	0.255	0.117	2.34893316720848E-05	1	MAP3K5
3.4494514439806E-11	0.860699352554128	0.268	0.12	5.78059072982269E-07	1	MKNK1
4.17278597070738E-21	0.859236502835675	0.376	0.146	6.99275472971142E-17	1	ZBTB7B
9.61843686276787E-27	0.852412041080732	0.663	0.335	1.61185764946264E-22	1	TPP1
1.56089312988108E-08	0.848894137094891	0.265	0.134	0.000261574470705471	1	CHURC1
4.53585504316585E-26	0.847160357355526	0.641	0.316	7.60118588133734E-22	1	CD3D
5.95269820490144E-09	0.846628651985306	0.343	0.192	9.97553165177384E-05	1	ATP5F1E
1.40046344392125E-09	0.84438583915531	0.297	0.151	2.34689663932322E-05	1	MFSD6
2.95542428254043E-12	0.841510957655357	0.271	0.114	4.95270001268125E-08	1	COMMD7
2.79050606561439E-15	0.841083282180106	0.425	0.206	4.67633006475659E-11	1	MSI2
4.05956101977631E-09	0.83616984834089	0.275	0.139	6.80301235694115E-05	1	RFX5
7.22203721963961E-16	0.834343493954406	0.431	0.211	1.21026899726721E-11	1	IGF2R
2.31075715156308E-08	0.833848225606506	0.275	0.146	0.000387236683458941	1	MPV17
7.34236746004192E-17	0.833285354252581	0.592	0.329	1.23043393895382E-12	1	SAMHD1
4.88616126422337E-11	0.831638201376278	0.297	0.142	8.18822904658553E-07	1	ITPR1
6.07863590853971E-10	0.828279519987313	0.275	0.131	1.01865780555308E-05	1	GSK3B
1.50816186891087E-06	0.820069725606565	0.307	0.189	0.0252737765992084	1	HINT1
7.48534182934195E-10	0.818152636765729	0.34	0.184	1.25439358376112E-05	1	TLK1
7.42528679818587E-29	0.81752276212663	0.869	0.509	1.24432956163999E-24	1	SPOCK2
4.95930598198843E-14	0.812502808258484	0.497	0.267	8.31080496461621E-10	1	LMAN2

1.93107903831694E-06	0.80919082263347	0.255	0.144	0.0323610225241153	1	LAMTOR1
7.33666797337174E-07	0.801665004035993	0.252	0.136	0.0122947881897764	1	MYDGF
9.12471455526564E-10	0.795866767642519	0.294	0.145	1.52911966517142E-05	1	GALM
4.3138159449477E-22	0.79546589683455	0.503	0.232	7.22909276054335E-18	1	MOB3A
9.55071101638024E-09	0.793746245668673	0.255	0.124	0.0001600508152125	1	TRAPPC3
1.5639646248009E-19	0.793636869764959	0.431	0.185	2.62089191824134E-15	1	ISCU
2.18727445138E-09	0.793363922064092	0.363	0.202	3.66543452562261E-05	1	EID1
9.58173739620896E-07	0.790316362101795	0.304	0.183	0.016057075528567	1	NFE2L1
1.89008669900741E-07	0.787152304358081	0.252	0.133	0.00316740729019662	1	NAB1
3.26382506064294E-12	0.783557331119895	0.477	0.275	5.46951803662544E-08	1	LRBA
1.76414535168516E-06	0.779072219978412	0.275	0.156	0.0295635478035399	1	KLF9
1.08858325205873E-11	0.778904222890392	0.281	0.125	1.82424781380002E-07	1	UBA1
2.41010298755E-10	0.777967548528445	0.343	0.189	4.0388505865363E-06	1	RNF187
1.66193773047829E-19	0.764027697505708	0.663	0.36	2.78507524873553E-15	1	CHST11
4.92690855420319E-14	0.760834996782579	0.801	0.625	8.2565133551337E-10	1	TRBC2
1.20786658653085E-11	0.76027315017469	0.268	0.116	2.02414282570839E-07	1	RYBP
4.37208403704042E-07	0.753966086494845	0.278	0.154	0.00732673842927233	1	DECR1
3.98234398113888E-13	0.752273176489422	0.363	0.171	6.67361204359253E-09	1	MXD4
6.56831131817426E-07	0.750515738352664	0.265	0.149	0.0110071761069964	1	COPB2
1.27733887978134E-08	0.747546594075428	0.258	0.126	0.000214056449473758	1	AHR
7.68183214968987E-16	0.74410055536352	0.451	0.226	1.28732143164503E-11	1	RAP1A
7.55577551890653E-11	0.738045396433927	0.382	0.208	1.26619686145836E-06	1	ARPC1B
6.82213474050187E-10	0.737936896417024	0.376	0.208	1.1432533398133E-05	1	TRAM1
1.74926526195449E-13	0.734120296475638	0.307	0.131	2.93141872598333E-09	1	KDM5B
2.08148666357387E-06	0.724415136988298	0.32	0.2	0.0348815535081709	1	JUN
1.09670877877808E-15	0.722104459617765	0.601	0.34	1.8378645714763E-11	1	MT-ND5
1.13694284729939E-22	0.720554289610388	0.752	0.434	1.90528882350431E-18	1	CD3E
8.80290682880112E-15	0.717891965286404	0.431	0.214	1.47519112637049E-10	1	GNPTAB
1.9076743493197E-11	0.710384166899383	0.5	0.287	3.19688067458996E-07	1	TRAF3IP3
7.25971726303803E-12	0.693426916551976	0.451	0.255	1.21658341893991E-07	1	CKLF
5.17759700283351E-09	0.691270642188683	0.258	0.127	8.67661705734839E-05	1	TRAPPC4
1.37004224858088E-08	0.688897268182448	0.461	0.299	0.000229591680017184	1	NUCB1
6.25528219020883E-11	0.68881624646216	0.314	0.155	1.0482601894352E-06	1	DNMT3A
2.25753691864232E-12	0.686642626374162	0.291	0.127	3.7831803682608E-08	1	FAS
7.40049324514195E-13	0.6817810080657	0.592	0.35	1.24017465802089E-08	1	MT-ND2
7.33210793006819E-09	0.671445489172444	0.265	0.133	0.000122871464692083	1	MAN2B1
5.94282988760225E-09	0.670960598860077	0.356	0.198	9.95899432564385E-05	1	CTSS
1.07083832044243E-06	0.66406523204686	0.271	0.151	0.0179451085739743	1	BLOC1S1
1.73243228819701E-08	0.663652668006084	0.333	0.193	0.000290321002856054	1	ATP6V0E1
4.4269542914023E-10	0.663278785167648	0.33	0.171	7.41869000153197E-06	1	SYNE3
5.92234501270297E-16	0.660392545882867	0.569	0.318	9.92466577228763E-12	1	PTPN7
3.27843142756446E-15	0.659308238165104	0.686	0.409	5.49399538631251E-11	1	MT-CYB
5.92494640181062E-07	0.658634820415035	0.268	0.146	0.00992902518015424	1	SPPL2A
1.88241137288913E-06	0.650544115848219	0.304	0.183	0.031545449786876	1	USP48
3.95506324735459E-16	0.647461656353679	0.732	0.466	6.62789498991683E-12	1	CD2
2.12344533157504E-22	0.645771362929357	0.908	0.706	3.55846968665346E-18	1	IL2RG
3.5662025661901E-14	0.643501354253966	0.513	0.279	5.97624226042137E-10	1	CD3G
1.81078458777764E-06	0.642139266782032	0.291	0.169	0.0303451281219776	1	UBE2V1
2.98588360945819E-15	0.640276763487222	0.735	0.484	5.00374375273003E-11	1	SLA
8.99078477506147E-09	0.636050480169048	0.265	0.132	0.00015066757126048	1	ECE1

2.7500639606911E-21	0.634095337682851	0.948	0.729	4.60855718532615E-17	1	ACTB
2.66349173661949E-12	0.633864109263702	0.359	0.171	4.46347945222694E-08	1	NABP1
1.21906981902795E-07	0.633650710858318	0.395	0.236	0.00204291720272703	1	TMCO1
7.00750427870881E-13	0.631212914059441	0.343	0.16	1.17431756702602E-08	1	OTUD5
1.08229146536721E-25	0.631055036345261	0.546	0.226	1.81370403766237E-21	1	DUSP16
6.83993551396786E-12	0.630718552220244	0.454	0.254	1.14623639343073E-07	1	ADAM10
1.53350601555464E-06	0.629513007425748	0.288	0.172	0.0256984938086647	1	APOBEC3C
3.33608459820779E-21	0.6269086165564	0.725	0.409	5.59061056967661E-17	1	FTL
5.39539908715646E-09	0.626368998156898	0.373	0.214	9.04160979025679E-05	1	MAT2B
5.08335676339287E-14	0.625383010043973	0.353	0.16	8.51868926409377E-10	1	RFTN1
3.16726118931904E-09	0.623454968284873	0.451	0.277	5.30769630106084E-05	1	RCSD1
4.16567990224261E-14	0.622695414229404	0.422	0.214	6.98084638017816E-10	1	CASK
6.991635022086E-09	0.622455545065121	0.379	0.22	0.000117165819700117	1	ERBIN
9.6454042770625E-14	0.622420524103138	0.454	0.235	1.61637684875013E-09	1	BIRC3
1.08801316402322E-16	0.622078200574488	0.49	0.243	1.82329246027011E-12	1	LAT
5.21643375123328E-07	0.619711632189293	0.278	0.156	0.00874169968031674	1	PYCR2
2.4059916202155E-07	0.619023312014138	0.284	0.157	0.00403196075715713	1	PPP4R3B
1.69499664977851E-14	0.605646738054989	0.379	0.17	2.84047538569883E-10	1	CCR4
2.75552339091629E-07	0.603574184895873	0.307	0.179	0.00461770609849752	1	SEC11A
1.70638396113097E-06	0.603486351479854	0.363	0.231	0.0285955824206328	1	EIF4EBP2
2.12555697655921E-14	0.599795503174978	0.395	0.183	3.56200838131793E-10	1	HAVCR2
8.31014101495088E-07	0.595304673345565	0.359	0.224	0.0139261343128547	1	PPP1CA
1.03408707048681E-09	0.594734691692291	0.304	0.151	1.73292311272179E-05	1	EPSTI1
1.49594217802358E-11	0.593796500137269	0.379	0.197	2.50689990193191E-07	1	SURF4
1.30043621193823E-10	0.593287057826532	0.284	0.132	2.17927100396608E-06	1	NPDC1
2.13076016180191E-07	0.592787128148356	0.307	0.176	0.00357072787914764	1	PAK2
2.08687031193869E-09	0.587289028764713	0.461	0.275	3.49717726874686E-05	1	BST2
6.0491412279258E-11	0.587178930066438	0.258	0.114	1.01371508697581E-06	1	FOXN3
1.24952476011766E-06	0.582527299002259	0.288	0.171	0.0209395359300517	1	SUB1
1.6711107151129E-08	0.582472650576232	0.278	0.143	0.00028004473363862	1	PRKACA
1.8354670429842E-07	0.575521422574598	0.34	0.2	0.00307587567063292	1	PSMF1
1.86254832886541E-06	0.574234110683708	0.34	0.211	0.0312125848951265	1	FAM49B
1.69690472345304E-13	0.572458955882403	0.549	0.315	2.8436729355626E-09	1	BCL2
4.03710613476258E-08	0.572042512653445	0.369	0.218	0.000676538246063513	1	HNRNPL
2.25683086006746E-11	0.567209562742742	0.395	0.208	3.78199715530106E-07	1	SAT1
4.42602990096471E-11	0.566521900873697	0.484	0.284	7.41714090803667E-07	1	TSPAN14
1.23669068490667E-07	0.566236694472316	0.291	0.16	0.0020724462497666	1	MRTFA
3.28670816308919E-11	0.561783679326699	0.288	0.133	5.50786553970486E-07	1	IL12RB1
2.24250145252109E-13	0.559241126175342	0.431	0.225	3.75798393413485E-09	1	TAB2
4.01005763313375E-09	0.558545717032039	0.386	0.219	6.72005458160554E-05	1	ICAM3
5.10155684276376E-07	0.557217575147245	0.389	0.247	0.00854918895710352	1	SP3
2.28934708234924E-06	0.553760993719131	0.363	0.228	0.0383648784060086	1	HNRNPD
4.14221527592845E-08	0.549123689346778	0.288	0.153	0.000694152435940089	1	TXNDC15
1.06083519667289E-10	0.542443764731394	0.441	0.251	1.77774762258444E-06	1	IPCEF1
9.52690395241162E-13	0.542276696637384	0.725	0.453	1.59651856434514E-08	1	ACTG1
1.11990747689582E-09	0.541937697737433	0.487	0.29	1.87674094978201E-05	1	CD52
1.12283499427305E-06	0.541380105197106	0.291	0.17	0.0188164688340278	1	SFT2D2
1.04185364130921E-13	0.540555454435263	0.356	0.161	1.74593833210597E-09	1	N4BP1
6.40791700148569E-16	0.537025415202621	0.807	0.505	1.07383873110897E-11	1	MT-ATP6
4.89490686805255E-07	0.533307269230972	0.258	0.139	0.00820288492948247	1	ATP6AP1

5.60994226466671E-11	0.528134746455107	0.559	0.346	9.40114124712847E-07	1	H3F3A
1.82403155596662E-13	0.527489429102517	0.395	0.19	3.05671208148886E-09	1	HDAC7
4.29086770786689E-07	0.527294843856965	0.275	0.152	0.00719063610484333	1	PFKP
2.75245516917792E-14	0.525031723211087	0.435	0.213	4.61256437250837E-10	1	SERPINB9
5.73293943130805E-11	0.521262468697774	0.48	0.284	9.60725989898602E-07	1	ANXA11
1.33303133728775E-11	0.520927530957729	0.477	0.277	2.23389391502681E-07	1	HBS1L
8.23312485953304E-15	0.520213955763147	0.592	0.323	1.37970706396055E-10	1	SELPLG
4.7192689693352E-10	0.510697540633712	0.386	0.209	7.90855093881193E-06	1	ANAPC16
1.42502123306251E-09	0.509829343112305	0.268	0.127	2.38805058236616E-05	1	ZDHHC18
5.84594115918662E-07	0.509147729344019	0.343	0.206	0.00979662819456493	1	PSMB1
2.18756588251679E-08	0.508614589992055	0.422	0.257	0.000366592290592163	1	DOCK10
1.63129131396417E-08	0.505726261692849	0.435	0.266	0.000273371798394115	1	LASP1
1.57047800752729E-09	0.505718311819812	0.359	0.193	2.63180704501423E-05	1	SLC44A2
2.40988608646176E-10	0.502719716351901	0.634	0.407	4.03848710369262E-06	1	MT-ND3
6.3237019986352E-08	0.500717497165725	0.363	0.215	0.00105972598093129	1	SLC3A2
1.85340469651913E-12	0.500097476892148	0.392	0.195	3.10593559042676E-08	1	ZNRF2
2.13136681956782E-06	0.496474880661981	0.301	0.176	0.0357174451623176	1	RER1
2.35178850068452E-07	0.494321298970434	0.324	0.181	0.00394112716944712	1	DDX3X
1.85010608787489E-09	0.489789531724441	0.48	0.289	3.10040778206074E-05	1	CCR5
1.46469234347387E-09	0.487944172178953	0.422	0.243	2.45453142919351E-05	1	SCAMP2
2.48658824236217E-10	0.485151139383105	0.454	0.259	4.16702457655052E-06	1	CALM3
7.95971058606728E-09	0.483155269420218	0.275	0.136	0.000133388830001316	1	SLC6A6
9.55264281480069E-14	0.478167213446964	0.673	0.421	1.6008318829043E-09	1	PRDM1
2.51074549749337E-17	0.47775040686492	0.788	0.495	4.20750730469939E-13	1	STK17B
3.52785395664009E-15	0.474594655126084	0.49	0.249	5.91197766053747E-11	1	EMC10
3.45593486593075E-09	0.471547092041489	0.353	0.196	5.79145564832675E-05	1	IFNAR2
5.12635560455205E-12	0.470692392968795	0.595	0.356	8.59074672210832E-08	1	IKBKB
9.19920348647951E-09	0.470551533428788	0.376	0.217	0.000154160252026424	1	SLC38A10
2.7658284841626E-07	0.461933769319056	0.275	0.15	0.00463497537375968	1	SZRD1
9.35878262199721E-12	0.460247979336814	0.493	0.279	1.56834479179429E-07	1	CHD3
7.37682346192916E-10	0.460089601014421	0.807	0.574	1.23620807575009E-05	1	PFN1
5.41471232680259E-08	0.458750282278414	0.48	0.312	0.000907397491725578	1	ARHGAP30
7.83399624908776E-09	0.45471083562491	0.32	0.172	0.000131282109142213	1	OGDH
3.29169836808246E-07	0.447671297747627	0.618	0.434	0.00551622812523259	1	DDIT4
2.58201285479341E-08	0.44683455455558	0.35	0.198	0.000432693714206279	1	TMEM248
1.56009436966805E-07	0.444573774427825	0.392	0.239	0.00261440614468971	1	SEL1L3
2.74167574136874E-06	0.444202844126325	0.441	0.291	0.0459450020738573	1	SLC9A3R1
5.5093184489271E-12	0.441282642922256	0.644	0.399	9.23251585671203E-08	1	SPN
2.24277308868262E-08	0.431195839538279	0.294	0.151	0.000375843914201434	1	LPAR2
8.34786907454047E-12	0.430020418896634	0.765	0.503	1.39893589951149E-07	1	CFLAR
5.44978130601557E-08	0.429634056445347	0.503	0.326	0.00091327435126209	1	ADAR
9.55001688395675E-08	0.426969373357844	0.301	0.165	0.00160039182941347	1	TBCB
5.39101189192671E-08	0.424042352759285	0.265	0.138	0.000903425772849077	1	NCSTN
2.53926411511211E-06	0.421166748669837	0.405	0.265	0.0425529880410487	1	TRIP12
2.52413586958942E-10	0.419168072623145	0.356	0.188	4.22994689025795E-06	1	SQSTM1
1.07404572264644E-08	0.414908829695118	0.925	0.715	0.000179988582201091	1	CD74
1.45853723405148E-10	0.414244627141811	0.788	0.51	2.44421669682348E-06	1	MT-ND4
1.79369132535766E-08	0.413371092015268	0.333	0.181	0.000300586792303436	1	TNIP1
4.05146094323018E-08	0.411477422313551	0.415	0.262	0.000678943824866514	1	APBB1IP
2.61398915928825E-07	0.402001535422635	0.412	0.253	0.00438052303313526	1	EIF1

1.85932257286345E-09	0.400932920770339	0.33	0.171	3.11585276760457E-05	1	PITPNM1
1.4939829620975E-09	0.400016400316922	0.451	0.267	2.50361664788299E-05	1	SERINC3
3.83230669484115E-08	0.398999835157783	0.343	0.195	0.00064221795592148	1	LPP
5.92510084460968E-10	0.397550166095808	0.291	0.14	9.92928399539691E-06	1	TXNDC11
4.9286694705413E-07	0.392457484351254	0.477	0.32	0.0082594642987331	1	CSK
6.45682162960185E-08	0.388850945116449	0.474	0.299	0.00108203416868868	1	CMTM3
5.17701528420096E-08	0.385491647109383	0.261	0.135	0.000867564221326397	1	TPM4
8.79062273585688E-07	0.377739396707562	0.458	0.306	0.014731325580749	1	EHBP1L1
3.89375096221337E-07	0.369075991564836	0.454	0.299	0.00652514786247716	1	CCNI
2.01946049675384E-06	0.368832228908519	0.353	0.217	0.0338421190046009	1	GNAI2
6.98661859924573E-09	0.367546667679899	0.523	0.335	0.00011708175448616	1	PICALM
3.76260772867811E-09	0.3643599831328	0.503	0.31	6.30537803171878E-05	1	DOK2
3.42584401899587E-08	0.362453530796938	0.32	0.178	0.000574102940703327	1	ZMIZ2
4.28258470347654E-10	0.36127283982029	0.408	0.229	7.17675544608598E-06	1	PTPN1
1.85269639966448E-07	0.359127646319132	0.415	0.258	0.00310474862655774	1	CYBC1
3.80265794777805E-07	0.356648897537181	0.255	0.134	0.00637249418888646	1	ARIH2
2.78344730985924E-07	0.355896257568479	0.301	0.167	0.00466450100186212	1	ANKRD17
1.07009646599534E-06	0.353081863672402	0.291	0.171	0.0179326765771499	1	TMBIM1
1.11979941427875E-09	0.353008576229003	0.608	0.392	1.87655985844832E-05	1	HCLS1
6.67864972285993E-12	0.352384643212857	0.768	0.47	1.11920812055687E-07	1	MT-CO3
3.54694008740755E-07	0.352084768635973	0.301	0.168	0.00594396219847758	1	ZMYND8
1.14827896650328E-07	0.349487486832154	0.343	0.195	0.0019242858920662	1	LDB1
2.23521496936139E-07	0.347751106039002	0.317	0.18	0.00374577324565582	1	ARHGEF6
1.79860743118124E-08	0.347725593273044	0.68	0.472	0.000301410633317353	1	TMSB10
3.55929124560093E-07	0.34224798772299	0.353	0.212	0.00596466026937803	1	MAT2A
5.84866541147192E-07	0.341183612569985	0.33	0.195	0.00980119349654464	1	EFCAB14
1.02978314669148E-07	0.338877489824127	0.536	0.357	0.00172571059722558	1	ARF6
9.11832002395051E-10	0.336765318883422	0.85	0.595	1.52804806961363E-05	1	MT-CO1
2.90239574918395E-07	0.330810834138172	0.304	0.173	0.00486383479648247	1	LRP10
5.28164098075187E-07	0.325071598388615	0.373	0.222	0.00885097395554399	1	SRPRA
3.83055547015915E-16	0.323361582666077	0.641	0.353	6.41924485689271E-12	1	TRAF1
4.38841862531952E-07	0.318871599412628	0.399	0.248	0.00735411193231046	1	FCMR
2.31739102601445E-09	0.317954833105743	0.735	0.492	3.88348388139501E-05	1	CD53
1.13212392835983E-11	0.315734899182138	0.663	0.415	1.8972132791454E-07	1	FXYD5
2.1522273962299E-07	0.306542378112542	0.386	0.234	0.00360670267060206	1	SKAP1
2.01622934302373E-08	0.30539416210026	0.693	0.483	0.000337879713303916	1	RAC2
5.3972214902255E-07	0.30532291517474	0.575	0.378	0.00904466377331989	1	SH3BGRL3
2.1590936551008E-07	0.302512224084664	0.386	0.228	0.00361820914721792	1	ACADVL
9.9197638848507E-09	0.299838727988601	0.291	0.149	0.000166235403182328	1	GMIP
2.42838862458564E-12	0.297872977557027	0.977	0.913	4.06949365708061E-08	1	B2M
1.13386585788496E-11	0.296167832272026	0.392	0.203	1.90013240464362E-07	1	SH2D2A
2.73352562562105E-07	0.293791076891475	0.314	0.176	0.00458084224341575	1	IST1
1.51174048639023E-06	0.290481828620745	0.261	0.146	0.0253337470709275	1	RPA1
4.74225642890673E-09	0.289753402346977	0.784	0.512	7.9470733235619E-05	1	MT-CO2
3.06266070195636E-08	0.288724657465942	0.317	0.173	0.000513240680433847	1	CPSF6
3.35506289405043E-10	0.287646545686436	0.454	0.259	5.62241439784972E-06	1	SDF4
1.38328961262914E-06	0.286004587282749	0.356	0.217	0.0231811673284392	1	HERC4
1.42122588154283E-07	0.284051901424468	0.333	0.195	0.00238169033228948	1	SFXN1
1.77127905818113E-06	0.281915490536659	0.441	0.296	0.0296830944569994	1	NBEAL2
2.93030193942797E-10	0.281352631652152	0.359	0.188	4.91059999009338E-06	1	ARF1

5.5263407660296E-07	0.276876487886612	0.343	0.209	0.00926104185571241	1	GRB2
4.37391536981342E-07	0.271168421459201	0.546	0.372	0.00732980737673333	1	ACTN4
2.09550249194955E-06	0.270676326631307	0.464	0.309	0.0351164307600905	1	TSC22D4
2.05154034701803E-08	0.269628428495696	0.382	0.218	0.000343797131353281	1	LRPAP1
1.57695190377246E-07	0.26163123500403	0.533	0.357	0.00264265600034189	1	PSMB8
6.63588330679429E-07	0.261573219557386	0.307	0.176	0.0111204132455259	1	TOP 1.00
6.51010546747175E-08	0.25992676701645	0.418	0.252	0.00109096347423892	1	HNRNPLL
2.06297227589282E-08	0.258854649039754	0.412	0.243	0.000345712893994119	1	DAZAP2
2.22756491603793E-07	0.255992629021694	0.598	0.404	0.00373295328629636	1	IVNS1ABP
4.21853674117494E-07	0.253623556370863	0.281	0.153	0.00706942387086096	1	KDM2B
2.46487577567836E-07	0.252904221566085	0.448	0.282	0.00413063882488179	1	S100A6
2.13694201184613E-06	0.252396508640273	0.399	0.254	0.0358108742345175	1	STK24
1.8123537038827E-171	3.04283342512652	0.888	0.111	3.03714233696663E-167	2	CD8A
6.79058967871878E-43	2.58532921079974	0.301	0.048	1.13796701835969E-38	2	DTHD1
5.93653696860891E-27	2.44985611120911	0.35	0.118	9.94844865199481E-23	2	FAM3C
8.87775410019301E-41	2.36687971358821	0.364	0.081	1.48773403211035E-36	2	GFOD1
4.55990754821915E-33	2.35104103176815	0.329	0.081	7.64149306930565E-29	2	MS4A6A
1.54339478177315E-90	2.14676430182738	0.86	0.278	2.58642097529545E-86	2	CCL5
1.56647875009249E-49	2.14672701965526	0.479	0.118	2.625105089405E-45	2	CCL4
1.17680938205359E-43	2.05968015889362	0.476	0.144	1.97209716244541E-39	2	PDCD1
1.57801214760757E-23	2.03437961589451	0.346	0.125	2.64443275696077E-19	2	SNAP47
9.60808903413872E-21	2.03406166421545	0.276	0.086	1.61012356034097E-16	2	CRIM1
1.20541486116552E-83	2.0340341944832	0.727	0.165	2.02003422434118E-79	2	KLRK1
2.30752090061618E-35	2.01170521542337	0.304	0.061	3.86694352525259E-31	2	TRGC2
1.65833570209742E-48	1.9740755003012	0.626	0.236	2.77903896957486E-44	2	LYST
2.20771879939758E-97	1.96617964779221	0.839	0.211	3.69969516403046E-93	2	NKG7
9.66451372717843E-31	1.95215240995374	0.325	0.081	1.61957921040056E-26	2	ADGRG1
3.43131868477271E-65	1.79296453609692	0.734	0.228	5.75020385194211E-61	2	GZMA
1.75561309924301E-30	1.76094642529943	0.437	0.155	2.94205643171144E-26	2	TOX
2.53087918564641E-33	1.746625273697	0.371	0.098	4.24124733930626E-29	2	EOMES
5.81096885831952E-25	1.68871088478222	0.392	0.144	9.73802161277185E-21	2	ID2
7.38081477848471E-22	1.62570122306383	0.262	0.072	1.23687694057847E-17	2	FASLG
1.79866074193004E-47	1.61753972694756	0.549	0.159	3.01419567132636E-43	2	LAG3
2.48618613111127E-55	1.61359208501977	0.636	0.186	4.16635071851627E-51	2	CTSW
2.97249662752099E-36	1.60357937940045	0.406	0.104	4.98130984839967E-32	2	KLRC4
8.05662801195888E-17	1.5995873494157	0.392	0.187	1.35012972224407E-12	2	HAVCR2
2.02871481535435E-24	1.56768976797286	0.392	0.144	3.39972028757082E-20	2	BCL2L11
1.60353169904052E-19	1.53694917220599	0.381	0.166	2.6871984212521E-15	2	APOBEC3G
9.07180755431386E-25	1.52186315790396	0.542	0.279	1.52025350995192E-20	2	CCR5
6.82642275551344E-37	1.44569858348285	0.661	0.307	1.14397192536894E-32	2	SLAMF6
7.83950588750372E-12	1.4396933327011	0.262	0.116	1.31374439662787E-07	2	MAPK13
2.01570681415204E-46	1.31089431276181	0.815	0.418	3.377921479156E-42	2	IKZF3
1.88069990013249E-18	1.26626289634311	0.395	0.165	3.15167689264202E-14	2	GZMK
1.67093127915748E-14	1.26145754730984	0.252	0.09	2.8001466376121E-10	2	GZMH
1.82773703124005E-23	1.24610031524017	0.549	0.271	3.06292171695207E-19	2	SLFN5
2.67721557535382E-35	1.23460893293903	0.706	0.355	4.48647786117794E-31	2	ITM2A
4.16959396839033E-23	1.21028225789329	0.364	0.117	6.98740557222851E-19	2	KLRD1
2.24483312193689E-10	1.18912251364984	0.259	0.121	3.76189134574184E-06	2	TRIM8
5.16607526820817E-22	1.18580742884921	0.521	0.242	8.65730893446325E-18	2	CD27
1.50188393665988E-10	1.16785109821392	0.273	0.132	2.51685710105462E-06	2	RASA1

4.18769031440363E-10	1.15268698562092	0.259	0.125	7.01773142887761E-06	2	DGKH
3.5272465087156E-07	1.14998148662075	0.266	0.153	0.0059109596993056	2	UBASH3B
2.44642392685427E-14	1.13942385248017	0.367	0.179	4.09971721662239E-10	2	SLC7A5
6.62198255611868E-08	1.13858072826524	0.269	0.146	0.00110971183675437	2	TNFRSF9
2.23461300394553E-15	1.13341086326596	0.402	0.199	3.74476447201192E-11	2	SNTB2
4.20384026082346E-20	1.12275637489121	0.381	0.15	7.04479550908795E-16	2	SFMBT2
1.13094933391679E-17	1.10665548651163	0.42	0.2	1.89524489377776E-13	2	ATXN1
9.05857270691344E-19	1.09010473027359	0.364	0.152	1.51803561422455E-14	2	MAPRE2
9.41333531136392E-20	1.08920910956868	0.451	0.21	1.57748673147837E-15	2	HCST
1.24830191806881E-32	1.07618385879274	0.549	0.204	2.09190435429971E-28	2	THEMIS
1.38655518670308E-11	1.07594083105936	0.287	0.131	2.32358918187703E-07	2	CCDC69
1.31557364387602E-27	1.05004711978903	0.78	0.453	2.20463831240744E-23	2	RGS1
1.049669157087E-11	1.04792190904779	0.36	0.191	1.75903557344639E-07	2	SIRPG
1.5579214912386E-12	1.043489185866	0.371	0.191	2.61076483501765E-08	2	RBPJ
9.90250847686352E-13	1.02074490147617	0.364	0.186	1.65946237055279E-08	2	PTPN6
1.74816602970693E-21	1.02069764549731	0.507	0.244	2.92957663258287E-17	2	CXCR6
8.07052772362135E-27	1.01572335432345	0.661	0.336	1.35245903592447E-22	2	CST7
2.00395193970203E-07	0.966473660555988	0.304	0.182	0.00335822266055266	2	ZBTB38
4.69858667590494E-15	0.947165890596716	0.388	0.189	7.8738915514815E-11	2	S100PBP
2.01268965494708E-08	0.942146493096525	0.43	0.295	0.000337286532376031	2	SLC9A3R1
2.63160373728678E-12	0.938638218902398	0.465	0.276	4.41004154294519E-08	2	IDH2
9.08940909105676E-08	0.920923146997996	0.294	0.168	0.00152320317547929	2	CD84
7.63771230526498E-19	0.899754269505256	0.49	0.242	1.27992782811631E-14	2	TRAF5
1.81113490090792E-14	0.899508222948985	0.402	0.191	3.0350998669415E-10	2	ITGB7
3.18959953787159E-14	0.879128210391067	0.371	0.177	5.34513090556521E-10	2	ARAP2
3.6093407219786E-10	0.875291399351959	0.357	0.201	6.04853318189173E-06	2	SPATA13
1.40345349592312E-13	0.855459670644629	0.318	0.139	2.35190736846796E-09	2	ATP8A1
4.50300557274158E-08	0.848839722056222	0.269	0.146	0.000754613673880034	2	SP4
1.50166432411812E-25	0.848426716582893	0.654	0.331	2.51648907435714E-21	2	PRF1
2.1262511594116E-14	0.847006555280041	0.451	0.238	3.56317169294196E-10	2	CEMIP2
1.68925421738E-11	0.83121473043315	0.437	0.254	2.83085221748541E-07	2	IPCEF1
1.27472858792776E-13	0.830461166644768	0.374	0.188	2.13619016764934E-09	2	ARHGEF3
2.46310506448599E-19	0.828585987385273	0.675	0.402	4.12767146706563E-15	2	RESF1
1.00191170369326E-08	0.824860319988002	0.308	0.17	0.000167900363304917	2	APOBEC3C
1.31409439665056E-11	0.823063031826581	0.374	0.193	2.20215938990701E-07	2	ITM2C
9.39414677999887E-08	0.821563167903595	0.374	0.233	0.00157427111739221	2	ST8SIA4
3.68297972053276E-07	0.818965608345299	0.367	0.244	0.00617193741566879	2	TMCO1
5.68883985477984E-13	0.801822101388096	0.367	0.178	9.53335782864006E-09	2	RIN3
4.05207999082623E-24	0.795601316777803	0.78	0.49	6.7904756486266E-20	2	WNK1
1.50930016033114E-08	0.790600030134998	0.332	0.193	0.000252928520868292	2	GIMAP6
9.72277398523714E-07	0.78211107667226	0.266	0.157	0.0162934246444604	2	ARHGAP35
8.10808189233706E-08	0.774664798843243	0.276	0.153	0.00135875236351784	2	DAXX
6.39147266011893E-15	0.766710621213191	0.434	0.226	1.07108298838273E-10	2	PIP4K2A
9.58419065566199E-12	0.760327884349279	0.315	0.147	1.60611867007584E-07	2	CHST12
8.61119201054742E-09	0.758540042732821	0.423	0.27	0.000144306355712754	2	LASP1
9.62615471462156E-18	0.756749967152115	0.497	0.257	1.61315100707628E-13	2	PYHIN1
1.14627984828059E-06	0.75588725443481	0.364	0.245	0.0192093576974861	2	M6PR
2.51590906465827E-08	0.754882989519623	0.287	0.154	0.000421616041055433	2	TBC1D1
4.2137704499046E-20	0.748259223946909	0.636	0.344	7.06143651995013E-16	2	CBLB
1.74974986365134E-17	0.738475611665109	0.759	0.483	2.93223082150692E-13	2	SLA

5.14131534065897E-14	0.733002856423286	0.371	0.169	8.61581624787631E-10	2	ALOX5AP
1.29134690468069E-14	0.730594664367965	0.49	0.268	2.16403914286391E-10	2	IL6ST
6.20014589756008E-14	0.727838598894471	0.483	0.276	1.03902044951312E-09	2	RAB27A
4.0006323507104E-11	0.719357604908123	0.427	0.252	6.70425969332048E-07	2	TAF7
1.32140271339998E-16	0.709061111430741	0.51	0.275	2.21440666711568E-12	2	PRKCH
1.97438096657836E-07	0.706562340442481	0.329	0.205	0.00330866762379201	2	ASXL2
4.50104476158261E-10	0.70140091976336	0.479	0.283	7.54285081146014E-06	2	TIGIT
2.85182347058639E-06	0.696608888019424	0.374	0.259	0.0477908577200866	2	VPS13B
7.10445466956308E-08	0.694061251140095	0.311	0.181	0.00119056451352538	2	KLF13
6.93170551634529E-07	0.685534603835886	0.332	0.211	0.0116161521042914	2	COTL1
2.96873220309341E-06	0.681023930673934	0.262	0.153	0.0497500142594394	2	GALM
2.50152872288715E-08	0.678191676860562	0.266	0.138	0.000419206183381429	2	CHIC2
3.33803166879174E-08	0.675955079879345	0.381	0.24	0.00055938734705612	2	SSR2
1.20535653066119E-09	0.675673709814616	0.395	0.226	2.01993647408203E-05	2	CASP8
4.43204731408171E-07	0.672757866718125	0.315	0.191	0.00742722488893813	2	NDUFA3
4.49014263051847E-09	0.670560904357838	0.493	0.331	7.52458102022285E-05	2	CAPZB
1.03063279291536E-09	0.660401101285202	0.322	0.168	1.72713443436756E-05	2	KIF13B
1.26463128554359E-09	0.649705123085554	0.42	0.259	2.11926910831395E-05	2	CD99
1.95534736084332E-09	0.64095926358358	0.315	0.168	3.27677110730124E-05	2	NIN
2.32662289342975E-06	0.640426394326281	0.318	0.199	0.0389895464480957	2	ATP5F1E
1.9189088879127E-09	0.636075431509551	0.367	0.21	3.2157075143641E-05	2	APMAP
1.32934146014606E-08	0.631651519823745	0.311	0.176	0.000222771041891277	2	USP24
4.56177272048504E-11	0.626117968075453	0.458	0.278	7.64461872498882E-07	2	MAP4
2.74655352377626E-06	0.623224553400206	0.283	0.164	0.0460267439514425	2	BICDL1
1.27622637443718E-11	0.612286787260737	0.371	0.194	2.13870015828182E-07	2	GPR174
3.69273871621128E-11	0.606932199337828	0.336	0.167	6.18829154062687E-07	2	SYTL3
5.73444977223746E-10	0.600868671214699	0.49	0.312	9.60979092831553E-06	2	LCK
3.27386732722522E-12	0.598190490601787	0.556	0.337	5.48634686696402E-08	2	CD3D
3.12004447253771E-07	0.59714484142196	0.304	0.182	0.00522857052707869	2	ARPC5L
3.41955372653386E-15	0.596695345828499	0.797	0.522	5.73048813492544E-11	2	ADGRE5
1.26625791695843E-08	0.595438801459544	0.584	0.406	0.000212199501723893	2	ZFP36L1
1.58535739155225E-08	0.594886147112987	0.343	0.2	0.000265674191676326	2	RAPGEF1
8.4646374681741E-10	0.593293917900781	0.483	0.306	1.41850394691662E-05	2	TBC1D10C
3.80379231657052E-09	0.58767944614255	0.437	0.267	6.37439516410887E-05	2	ZFP36
1.55865423332995E-19	0.585401139383807	0.601	0.306	2.61199276421433E-15	2	DUSP2
1.46120242544239E-15	0.584670496624796	0.783	0.531	2.44868302455636E-11	2	LSP1
3.65482973709506E-18	0.583945305626581	0.874	0.627	6.1247636734239E-14	2	ETS1
7.24295563304439E-16	0.58192211888149	0.584	0.339	1.21377450498558E-11	2	TGFBR2
9.35141432272246E-10	0.577286724415802	0.287	0.142	1.56711001220183E-05	2	PRKACB
5.91803047865155E-07	0.577145730241928	0.329	0.2	0.00991743547612428	2	FAM102A
3.26499815736531E-07	0.563849544541293	0.444	0.296	0.00547148391211279	2	CD3G
2.07447455054536E-06	0.561702571770493	0.28	0.166	0.0347640445180391	2	ARHGAP25
2.20472212800668E-08	0.558021697700535	0.5	0.335	0.000369467334211359	2	PTPN7
2.14887002375417E-09	0.553808340775276	0.371	0.209	3.60107638580724E-05	2	MSL2
1.19908746232413E-06	0.54838394369856	0.406	0.279	0.0200943076936278	2	SUGP2
1.88357253095932E-10	0.544872268985939	0.434	0.252	3.15649084738162E-06	2	NFATC2
2.65437122594444E-08	0.543536391913472	0.458	0.298	0.00044481953004377	2	MBD2
9.35291266116924E-08	0.540072102485976	0.287	0.158	0.00156736110375874	2	SACM1L
1.83578004489175E-09	0.538708715106142	0.388	0.23	3.0764001992296E-05	2	TBL1XR1
1.59180249924651E-06	0.537069524598079	0.315	0.196	0.0266754262823731	2	RAB1B

**8.9594165815601E-07**	0.53560179624954	0.486	0.338	0.0150141903073784	2	SASH3
**5.9394831462422E-08**	0.530949281886153	0.259	0.132	0.000995338585647269	2	ADA2
**2.42771523838261E-13**	0.524759981109638	0.773	0.538	4.06836519648158E-09	2	MSN
**2.9953686570565E-12**	0.521688251594895	0.409	0.211	5.01963879549528E-08	2	GIMAP7
**1.51639004503605E-06**	0.520514090813837	0.423	0.285	0.025411664374714	2	RCSD1
**1.31401138603396E-09**	0.509089867018942	0.301	0.15	2.20202028071571E-05	2	SAMD3
**7.38749113330027E-11**	0.502496943048323	0.678	0.453	1.23799576411846E-06	2	CD3E
**2.86909085995534E-10**	0.501868998449431	0.462	0.276	4.80802246311315E-06	2	PLAAT4
**8.62423845011422E-10**	0.498759965928255	0.392	0.217	1.44524987947014E-05	2	EVI2B
**9.68586586699687E-08**	0.498262875701176	0.423	0.275	0.00162315740199134	2	DIDO1
**6.2165712648172E-08**	0.496366385967292	0.322	0.183	0.00104177301255807	2	PHF10
**3.84842414544627E-12**	0.495774534421817	0.49	0.289	6.44918918293886E-08	2	ATP11B
**9.13022174412295E-09**	0.491582554123238	0.699	0.52	0.000153004255988012	2	CFLAR
**8.04953536922508E-07**	0.486771519597286	0.294	0.171	0.0134894113717474	2	OAS2
**4.92439537511593E-11**	0.481766493125194	0.51	0.31	8.25230176961927E-07	2	RNF19A
**1.36696924875207E-06**	0.480672735678821	0.29	0.174	0.0229076706705872	2	RNF115
**6.73255632469503E-08**	0.480012716814029	0.332	0.191	0.00112824178889239	2	GGA2
**7.16283314573121E-07**	0.476584418609653	0.297	0.175	0.0120034757856164	2	CEP85L
**1.8427674103642E-06**	0.476511770997679	0.374	0.244	0.0308810962628832	2	KANSL1
**7.26917802984488E-07**	0.474118594555133	0.448	0.302	0.012181688542414	2	TLN1
**2.69767498840956E-08**	0.467997049775229	0.57	0.388	0.000452076374557674	2	PSME1
**9.83139478782138E-07**	0.467891774532915	0.336	0.205	0.0164754513854311	2	SLC4A7
**8.83402277709555E-08**	0.46516640853164	0.605	0.416	0.00148040553698567	2	MT-ND3
**1.97606440404611E-06**	0.462819253138966	0.458	0.326	0.0331148872830047	2	DOCK8
**3.54407067525883E-07**	0.461903008136835	0.472	0.323	0.00593915363759874	2	CSK
**5.93872220786125E-08**	0.460372740480148	0.552	0.381	0.000995211067593388	2	LCP2
**7.80105752425421E-12**	0.457536347109848	0.818	0.547	1.30730121991452E-07	2	IL32
**1.10272579478375E-07**	0.455570944207736	0.427	0.274	0.0018479478868986	2	MAPK1
**1.13829628305472E-10**	0.45529860194247	0.668	0.441	1.9075569111431E-06	2	VTN
**1.13974647318747E-07**	0.448843328584566	0.734	0.523	0.00190998713976756	2	MT-ATP6
**6.96815430823267E-07**	0.445704523734245	0.517	0.368	0.0116772329897363	2	MT-ND2
**1.42798035211254E-10**	0.444479632314356	0.556	0.349	2.39300947407019E-06	2	STK10
**5.50883333208063E-11**	0.442314818079496	0.832	0.612	9.23170289790072E-07	2	CELF2
**8.18618608559358E-09**	0.438478289853921	0.594	0.413	0.000137184106422377	2	ANXA6
**2.80093817456231E-07**	0.427349436517797	0.552	0.385	0.00469381219293151	2	SH3BGRL3
**8.62319057388623E-08**	0.426333813492576	0.441	0.291	0.00144507427637185	2	PSMB9
**2.46205964609864E-06**	0.413333402862701	0.399	0.268	0.041259195549321	2	DENND2D
**1.43936454840078E-06**	0.412267082987287	0.297	0.177	0.0241208711021002	2	CLSTN3
**4.38873238618884E-07**	0.410013799896593	0.448	0.298	0.00735463773277526	2	PLCG1
**1.44351309126148E-06**	0.408633467157682	0.259	0.142	0.0241903923833599	2	PTPN4
**5.60573178383769E-11**	0.402805332877716	0.322	0.153	9.3940853233552E-07	2	JAML
**4.71037022129203E-10**	0.401377475690288	0.612	0.404	7.89363841684118E-06	2	AKAP13
**1.16854734500475E-09**	0.398688373941657	0.685	0.479	1.95825164075897E-05	2	CD2
**1.03612972421063E-06**	0.390603539977463	0.304	0.181	0.0173634619183218	2	TSPYL1
**2.14590668503963E-07**	0.3857402624818	0.479	0.322	0.0035961104227894	2	CNN2
**1.65207408782801E-06**	0.379482803515705	0.266	0.155	0.0276854575638218	2	WDR33
**1.24182892588549E-06**	0.379283411872615	0.759	0.555	0.020810569139989	2	HSPA1B
**4.61200006267726E-07**	0.366332334454611	0.35	0.213	0.00772878970503456	2	PLCB2
**1.90157876504013E-08**	0.365315975643156	0.416	0.252	0.000318666569445424	2	PTPN22
**6.33875385728841E-07**	0.357871732312924	0.811	0.625	0.0106224837140439	2	TRBC2

3.8468136266258E-09	0.353197451686108	0.72	0.492	6.44649027549951E-05	2	CFL1
9.70840992497414E-07	0.348010831306711	0.462	0.316	0.0162693533522717	2	BRD2
4.50021019406898E-07	0.339689338816022	0.353	0.22	0.00754145224322079	2	ERAP2
4.27398847280961E-09	0.33373913025732	0.794	0.595	7.16234988273434E-05	2	AKNA
4.66706794319756E-09	0.321033849650975	0.374	0.206	7.82107245921046E-05	2	CLEC2B
4.78190517883693E-11	0.314406960839007	0.259	0.104	8.01351669869493E-07	2	MATK
3.59836848488859E-08	0.311954546241541	0.874	0.728	0.000603014590697629	2	CD74
2.35199639253098E-06	0.310246113968576	0.612	0.453	0.0394147555460341	2	DNAJB1
4.9400315356511E-07	0.293505145031802	0.713	0.522	0.0082785048474441	2	ITGB2
2.43160940743841E-06	0.286925715279428	0.308	0.185	0.0407489104498529	2	IQGAP2
4.84572725526191E-07	0.284997792033686	0.584	0.423	0.00812046973436791	2	CD96
1.09972048784762E-08	0.282358598457773	0.699	0.487	0.000184291159353504	2	ITGAL
2.11763828230851E-07	0.278171797182924	0.752	0.522	0.00354873823349261	2	MT-CO2
1.40582586853563E-06	0.271981469401934	0.612	0.45	0.02355882990492	2	TAP1
1.94249641479021E-06	0.27171283851553	0.542	0.371	0.0325523549190543	2	CD7
1.09116079223667E-07	0.270351425775587	0.392	0.243	0.0018285672556302	2	FGD3
2.96586244374395E-10	0.257038297794418	0.451	0.262	4.97019228322612E-06	2	IL16
4.07937604980402E-07	0.255095934244903	0.769	0.559	0.00683621838426158	2	CORO1A
7.7588898122926E-85	6.07134343756398	0.273	0.003	1.30023475474399E-80	3	KLRF1
3.42717812025655E-70	5.23653759561777	0.292	0.012	5.74326509392592E-66	3	NCAM1
4.90015498571021E-86	5.12237178696414	0.364	0.015	8.21167972505317E-82	3	FGR
2.46563293175223E-60	4.91425108895557	0.312	0.021	4.13190766703039E-56	3	KLRC1
7.34370344877315E-107	4.55991396380967	0.649	0.069	1.23065782394541E-102	3	GNLY
4.99032420016422E-53	4.18243720898007	0.403	0.054	8.3627852946352E-49	3	ITGAX
9.01994236615412E-52	3.98470099572333	0.331	0.033	1.51156194172011E-47	3	CLIC3
6.58910418704142E-57	3.80981306893385	0.351	0.032	1.1042020796644E-52	3	PLCG2
9.72503000216163E-42	3.7196749489208	0.286	0.031	1.62972052776225E-37	3	FCGR3A
2.58493082625851E-67	3.59640132546	0.513	0.069	4.33182707864402E-63	3	KLRC3
1.96529170442927E-38	3.42021667772297	0.299	0.038	3.29343583828257E-34	3	CD300A
3.7424266086316E-85	3.35637310504675	0.682	0.106	6.27155851074484E-81	3	KLRD1
1.42867417882316E-46	3.31534599508622	0.403	0.061	2.39417218887185E-42	3	NCR1
1.88553142517164E-60	3.27978502209247	0.539	0.09	3.15977356230263E-56	3	MATK
4.13253355364379E-18	3.14038250398071	0.253	0.063	6.92529972919626E-14	3	GZMB
8.01727978285282E-57	2.94945313013025	0.578	0.112	1.34353574601048E-52	3	KLRC4
1.07270098364392E-41	2.85629936416239	0.403	0.068	1.79763230839048E-37	3	AOAH
6.28471746147026E-30	2.74614335100982	0.364	0.078	1.05319295219319E-25	3	TXK
6.31404836566301E-28	2.70871850944067	0.344	0.075	1.05810822511781E-23	3	PTGDR
6.92030206673213E-22	2.62070019751095	0.351	0.099	1.15970422034297E-17	3	ADGRG1
2.09341885685812E-32	2.56359404772698	0.403	0.09	3.50815132032283E-28	3	MCTP2
1.16635681400055E-57	2.51930660286803	0.812	0.267	1.95458074890213E-53	3	NKG7
7.69414751637798E-34	2.49260255509994	0.721	0.352	1.28938524079462E-29	3	PRF1
1.62739802405928E-15	2.47287632896378	0.286	0.088	2.72719360871855E-11	3	ZEB2
2.50242221678065E-15	2.28615938686159	0.26	0.073	4.19355915088101E-11	3	SLAMF7
1.02316297081983E-29	2.16495412068072	0.597	0.228	1.71461650649988E-25	3	CTSW
9.46630732673684E-20	2.1299318075738	0.461	0.185	1.58636378181456E-15	3	RIN3
3.5392447091138E-17	2.10189279811164	0.383	0.144	5.9310662835329E-13	3	ZBTB16
5.39071594944438E-13	2.0781513118465	0.286	0.101	9.03376178807889E-09	3	SYNE1
1.43005085498694E-09	2.02458477829362	0.344	0.162	2.39647922278711E-05	3	CCL4
4.84898133381251E-14	2.00138139564882	0.357	0.142	8.125922919203E-10	3	PTPN4
1.2840848601776E-10	2.0008752188744	0.312	0.137	2.15186940868563E-06	3	APOBR

5.15824727117485E-41	2.0005229472041	0.682	0.217	8.64419077703481E-37	3	KLRK1
1.73550706027849E-16	1.93261649242604	0.396	0.153	2.90836273161469E-12	3	CHST12
5.21619853118544E-18	1.85949119201139	0.357	0.114	8.74130549856056E-14	3	KLRB1
1.08667552072277E-18	1.80187367563455	0.669	0.443	1.82105083762722E-14	3	IL2RB
4.09533183472124E-08	1.79360249187527	0.26	0.115	0.000686295708862585	3	GFOD1
2.03810074882523E-12	1.78910137585009	0.325	0.126	3.41544923488133E-08	3	EOMES
9.66716362237344E-11	1.71445822854125	0.416	0.218	1.62002327983734E-06	3	APMAP
5.86863324691895E-14	1.65906890093566	0.506	0.279	9.83465559518677E-10	3	MAPK1
5.88527932109635E-09	1.59668377445849	0.344	0.17	9.86255108629326E-05	3	METRNL
1.83969474983123E-07	1.57224820654153	0.266	0.126	0.00308296046176717	3	LPCAT1
6.40061137582228E-07	1.55123392531859	0.253	0.12	0.010726144543603	3	TNFRSF1A
5.86308013840255E-12	1.53966541710761	0.403	0.187	9.825349695935E-08	3	ABCA2
7.09730877591258E-23	1.38163103082506	0.714	0.342	1.18936700466743E-18	3	CCL5
2.67969208427422E-10	1.37020547535767	0.429	0.215	4.49062799482674E-06	3	CLEC2B
9.04607230151827E-07	1.33758801786217	0.429	0.284	0.0151594079628843	3	PYHIN1
2.10527491923311E-07	1.29214560848411	0.455	0.303	0.00352801970965084	3	CD247
3.62368359305836E-08	1.29161373385638	0.299	0.14	0.000607256896524721	3	RASSF1
8.05211308421985E-09	1.19074477330658	0.338	0.159	0.000134937311065356	3	SAMD3
1.73003822551919E-07	1.18075344877211	0.299	0.148	0.00289919805832506	3	ADAM8
1.63556513814781E-08	0.999161906110176	0.565	0.383	0.000274088005850811	3	CD7
4.27934993859324E-07	0.988186917030771	0.422	0.25	0.00717133462709454	3	PITPNC1
1.23256818506649E-06	0.975842100276055	0.532	0.38	0.0206553776453443	3	LITAF
2.40067672865866E-07	0.928042904880115	0.526	0.339	0.00402305406188618	3	DUSP2
2.5932497707711E-06	0.688499994159342	0.669	0.558	0.0434576796585821	3	ADGRE5
3.31446916504265E-101	8.33903979244411	0.289	0.001	5.55438742677848E-97	4	FBXO43
9.97094730974564E-240	7.85588894332182	0.722	0.004	1.67093135016717E-235	4	HJURP
6.42011240585024E-225	7.78654045968509	0.7	0.006	1.07588243697238E-220	4	ASPM
5.03957526655979E-142	7.6945634242201	0.422	0.002	8.44532023170089E-138	4	BUB1B
1.50455580465075E-204	7.64604496691898	0.611	0.003	2.52133461743373E-200	4	KIF18B
1.31758350271303E-179	7.20780387430249	0.544	0.003	2.20800643384649E-175	4	CDCA8
2.60518274245809E-184	7.20652605249091	0.567	0.004	4.36576523981127E-180	4	SPC25
1.87311144720213E-183	7.20480609490517	0.922	0.047	3.13896016322133E-179	4	HIST1H1B
5.44971173123591E-164	7.08505481465424	0.511	0.004	9.13262691920514E-160	4	UBE2C
3.85585065495628E-110	7.04775886034291	0.344	0.002	6.46163452757574E-106	4	PLK4
4.41323068442956E-214	6.92675834931233	0.667	0.005	7.39569198096706E-210	4	KIF2C
7.93452590585435E-177	6.88678841739147	0.556	0.004	1.32966785130307E-172	4	DLGAP5
1.59335638041236E-97	6.86807687966671	0.3	0.002	2.67014662229503E-93	4	PSRC1
6.91178357138537E-210	6.86270505242521	0.644	0.004	1.15827669089276E-205	4	ESPL1
1.48820428142831E-162	6.79144908163442	0.533	0.006	2.49393273481756E-158	4	CDCA3
3.66208280262423E-106	6.74595965107145	0.333	0.002	6.13691836063768E-102	4	E2F7
8.03076405670315E-186	6.6877545507765	0.622	0.007	1.34579544062231E-181	4	KIF23
1.72770548849993E-97	6.65034224230066	0.3	0.002	2.89528885762819E-93	4	CDC25C
4.53664569893096E-179	6.63215235502515	0.611	0.008	7.60251086226849E-175	4	FOXM1
1.6813639137508E-246	6.58116072382307	0.867	0.014	2.81762964666358E-242	4	RRM2
2.28891770227785E-188	6.57997038203756	0.622	0.007	3.83576828547722E-184	4	GTSE1
1.38737651401487E-204	6.57810772032825	0.667	0.007	2.32496556218612E-200	4	CIT
1.20534849718692E-106	6.48687573980882	0.344	0.003	2.01992301158584E-102	4	E2F8
5.35951547590356E-92	6.46341448946825	0.311	0.004	8.98147603451919E-88	4	CDC20
2.54201629655899E-166	6.44644695808129	0.544	0.006	4.25991090977356E-162	4	PRR11
2.1042877543196E-180	6.41073133465415	0.6	0.007	3.52636541868879E-176	4	ANLN

9.18630394554913E-95	6.37270207563812	0.311	0.003	1.53944081519512E-90	4	GPSM2
4.00824523573385E-137	6.34930263002091	0.456	0.005	6.71701736604278E-133	4	KIF14
3.3331898306248E-108	6.28738111584873	0.389	0.006	5.58575951816104E-104	4	TROAP
5.88981609996664E-180	6.23967107450342	0.622	0.009	9.8701538203241E-176	4	BIRC5
6.07707648623326E-165	6.20329449405301	0.533	0.005	1.01839647756297E-160	4	CDCA5
8.33525059401023E-200	6.19761584398282	0.811	0.022	1.39682129454423E-195	4	TOP2A
4.75880914869009E-83	6.19688459000693	0.278	0.003	7.97481237137485E-79	4	PBK
3.52679395248084E-90	6.18643704991385	0.322	0.005	5.91020130556739E-86	4	IQGAP3
1.86957672251372E-130	6.1857547382456	0.411	0.003	3.13303667158849E-126	4	NEIL3
2.26087551575284E-117	6.16194534203823	0.4	0.005	3.78877518929861E-113	4	MND1
7.4491242442933E-122	6.11745128232637	0.378	0.002	1.24832424085867E-117	4	ESCO2
4.54682826325914E-171	5.97672321755655	0.633	0.012	7.61957480356967E-167	4	SPC24
1.67464016767166E-224	5.9673283319629	0.867	0.02	2.80636199298416E-220	4	KIFC1
2.06062539439177E-188	5.95074383812709	0.622	0.007	3.45319603592172E-184	4	AURKB
1.22002182935108E-133	5.92579120301093	0.478	0.007	2.04451258162654E-129	4	PKMYT1
1.6421649360774E-217	5.7983872005002	0.978	0.038	2.75193999987851E-213	4	MKI67
1.00252451778392E-113	5.78536154237711	0.344	0.002	1.6800305869023E-109	4	FAM72C
9.66904315733797E-193	5.76748194102169	0.856	0.03	1.6203382523067E-188	4	TYMS
3.29102080824384E-155	5.74155568361326	0.556	0.009	5.51509267045503E-151	4	DEPDC1B
1.93930663345299E-227	5.70471025368938	0.767	0.01	3.24989005634053E-223	4	NCAPG
2.19454169055761E-180	5.65693040148176	0.689	0.014	3.67761296503645E-176	4	MYBL2
3.46980090807356E-119	5.61462109880113	0.389	0.004	5.81469236174967E-115	4	KIF20A
7.15603037317312E-136	5.55060984168439	0.444	0.004	1.19920756993635E-131	4	HIST1H2BB
7.46817880291986E-111	5.51386939818375	0.433	0.009	1.25151740379331E-106	4	NUF2
6.65314694775496E-85	5.49293664869938	0.3	0.004	1.11493436550478E-80	4	TICRR
3.23403644560534E-101	5.48742126291735	0.422	0.011	5.41959827554542E-97	4	CCNB2
1.19363244698701E-107	5.48083575784252	0.389	0.006	2.00028925466084E-103	4	FAM83D
1.11314366975258E-107	5.47962574168936	0.389	0.006	1.86540616177137E-103	4	POLQ
9.40898251874969E-99	5.46994163511435	0.378	0.007	1.57675729049207E-94	4	CCNB1
3.50140069942504E-123	5.43934086062353	0.433	0.006	5.86764729209648E-119	4	CENPA
8.93670844545052E-174	5.41200513587784	0.644	0.012	1.4976136012886E-169	4	SPAG5
4.22129609447953E-119	5.40916367701448	0.444	0.008	7.07404799512879E-115	4	CCNF
4.9225142025057E-145	5.40239314130498	0.556	0.011	8.24914930055904E-141	4	KIF15
2.52106057194692E-118	5.37928690578007	0.5	0.014	4.22479330646865E-114	4	HMMR
6.02028494104814E-59	5.36555700641335	0.256	0.007	1.00887935042085E-54	4	CLSPN
2.83550393615938E-128	5.36014020224455	0.511	0.012	4.75173749621589E-124	4	UBE2T
6.42849464650696E-116	5.34046907449705	0.456	0.01	1.07728713286164E-111	4	CKAP2L
1.57750109247273E-163	5.33401193516457	0.711	0.022	2.64357633076581E-159	4	TCF19
8.39274378440469E-156	5.32158494754297	0.6	0.012	1.40645600339054E-151	4	MELK
3.8281190093953E-116	5.31348323510921	0.422	0.007	6.41516183594465E-112	4	TTK
3.01886978540403E-81	5.31033621035302	0.289	0.004	5.05902198638008E-77	4	MCM10
8.70440287017553E-213	5.26853110085375	0.8	0.016	1.45868383298402E-208	4	HIST1H2AH
6.31316260738458E-152	5.22988323407752	0.567	0.01	1.05795978974551E-147	4	CENPI
2.04858093807557E-85	5.21854163161409	0.344	0.008	3.43301193602704E-81	4	HASPIN
5.51255103207145E-164	5.21447960576444	0.778	0.031	9.23793301954534E-160	4	TPX2
1.18539312952101E-170	5.20794072042098	0.733	0.022	1.98648180645131E-166	4	ZWINT
1.73278865091846E-102	5.20667801808362	0.389	0.007	2.90380722120915E-98	4	KNSTRN
6.65864010693083E-126	5.18957339906279	0.478	0.009	1.11585490911947E-121	4	CDCA2
1.61753265694802E-91	5.16843889168866	0.356	0.007	2.71066122651349E-87	4	CDC45
2.19176979913125E-114	5.14869362461836	0.444	0.009	3.67296782938415E-110	4	E2F2

1.35213286089989E-145	5.13606631857975	0.511	0.007	2.26590424829603E-141	4	PRC1
1.58764069707684E-171	5.11950415191402	0.756	0.025	2.66056828016137E-167	4	CDK1
4.49379407527721E-94	5.08878129846469	0.3	0.002	7.53070011134955E-90	4	DEPDC1
1.46943269167437E-97	5.08250401360146	0.3	0.002	2.46247530470791E-93	4	HIST1H2BN
1.71061755311269E-144	5.0778165455042	0.678	0.025	2.86665289550624E-140	4	CDT1
5.93515866412577E-110	5.04829754858107	0.411	0.007	9.94613888934197E-106	4	DIAPH3
2.76568392143515E-106	5.04168348756865	0.378	0.006	4.63473311554103E-102	4	NEK2
6.6805052984233E-126	5.0399638869297	0.511	0.012	1.11951907790978E-121	4	PLK1
2.73131868659709E-86	4.97590177400283	0.311	0.005	4.5771438549994E-82	4	RAD54L
1.14566934605306E-153	4.88410623168244	0.811	0.039	1.91991269011572E-149	4	HIST1H2BH
2.10005332173518E-145	4.88270853953362	0.667	0.023	3.51926935656381E-141	4	INCENP
3.69143760717858E-165	4.85045277248869	0.678	0.017	6.18611114210987E-161	4	ASF1B
1.19210515185582E-89	4.84007462035823	0.389	0.011	1.99772981347999E-85	4	WDR34
2.50630602458622E-96	4.8380928279029	0.378	0.008	4.2000676360016E-92	4	AURKA
4.22160581532933E-80	4.82269293617044	0.322	0.007	7.0745670253289E-76	4	SGO2
9.74814717163066E-144	4.79125959640523	0.622	0.018	1.63359450302187E-139	4	BUB1
2.99820352986525E-118	4.78766247662707	0.567	0.022	5.02438947534819E-114	4	TK1
1.7657057206986E-88	4.7629939696295	0.367	0.009	2.95896964674671E-84	4	EXO1
3.9063180340807E-89	4.72124562804195	0.267	0.001	6.54620776151244E-85	4	HIST1H3I
3.55377903742745E-142	4.71461266572092	0.633	0.02	5.95542291092092E-138	4	STMN1
2.4497640115727E-154	4.71248943305594	0.744	0.029	4.10531453059352E-150	4	H2AFX
9.7278452040094E-120	4.69768793557565	0.6	0.025	1.6301922992879E-115	4	PCLAF
2.51367428137474E-160	4.68401521260734	0.6	0.011	4.21241536072779E-156	4	KNL1
5.45628656001658E-106	4.64456436360645	0.433	0.01	9.14364501727578E-102	4	SHCBP1
1.60462162579403E-124	4.62795247562364	0.6	0.023	2.68902492050564E-120	4	UHRF1
3.55653669423842E-93	4.60357677176598	0.456	0.017	5.96004419220474E-89	4	ZNF367
1.27261807074334E-114	4.59652279270557	0.367	0.003	2.13265336295169E-110	4	KIF4A
1.40658552994466E-94	4.59585843781977	0.422	0.013	2.35715603108126E-90	4	DTL
3.44147744786039E-65	4.5503998478644	0.3	0.01	5.76722790712445E-61	4	BRCA1
8.08916109131623E-162	4.54848262176643	0.711	0.022	1.35558161568277E-157	4	KIF11
5.45221958025922E-93	4.52963831490942	0.422	0.014	9.1368295725984E-89	4	UBE2S
3.23892901643709E-103	4.47432136716996	0.467	0.015	5.42779724574528E-99	4	CCNA2
1.84712390016311E-128	4.46747911819572	0.678	0.032	3.09541023189334E-124	4	CENPF
7.27142316388728E-110	4.45320103241275	0.411	0.007	1.21854509380423E-105	4	CENPW
5.57046591712071E-56	4.40509120582084	0.289	0.012	9.33498678391088E-52	4	FAM111B
3.39150346699193E-143	4.38883593356948	0.522	0.009	5.68348150998507E-139	4	MXD3
2.91437625216815E-91	4.38374167025887	0.389	0.01	4.88391172338339E-87	4	STIL
1.22118603142135E-106	4.35913912584436	0.533	0.022	2.0464635514559E-102	4	CENPE
6.30877400563539E-76	4.34491945709274	0.322	0.009	1.05722434786438E-71	4	ORC1
2.9245871341502E-84	4.34176638199111	0.356	0.009	4.9010231194089E-80	4	SKA1
5.67193327501576E-72	4.3322028321758	0.278	0.006	9.50502578227141E-68	4	RACGAP1
1.21857869935487E-67	4.32108581026705	0.278	0.007	2.0420941843789E-63	4	POLE2
9.49475564207481E-114	4.29642373880836	0.4	0.006	1.5911311504989E-109	4	HIST1H2BM
2.81259838209782E-131	4.28255792332955	0.756	0.044	4.71335236871953E-127	4	HIST1H4C
7.22649719984248E-78	4.26513626827837	0.322	0.008	1.2110164007496E-73	4	RECQL4
2.82889381908684E-155	4.247090374935	0.756	0.03	4.74066026202572E-151	4	CENPM
3.71642617016874E-148	4.19724885019403	0.7	0.025	6.22798697596878E-144	4	CEP55
3.30876430881817E-118	4.19301781771857	0.478	0.011	5.54482722871749E-114	4	FBXO5
1.29097651479363E-53	4.16709618075714	0.267	0.01	2.16341844349116E-49	4	POC1A
1.06504243108471E-68	4.13715609350746	0.3	0.009	1.78479810601175E-64	4	CIP2A

**2.19698978062012E-82**	4.13017599461909	0.4	0.015	3.6817154743632E-78	4	C21orf58
**8.74771004540885E-57**	4.08417177732141	0.289	0.012	1.46594124940962E-52	4	ORC6
**7.0318722305452E-91**	4.0727709081241	0.422	0.014	1.17840114839476E-86	4	ATAD5
**1.33740008109214E-56**	3.93088211953643	0.267	0.009	2.24121505589421E-52	4	BRIP1
**1.11938532411002E-75**	3.92520541086328	0.922	0.187	1.87586592614357E-71	4	HIST1H1C
**6.05741262243983E-106**	3.89865384959443	0.622	0.035	1.01510120726847E-101	4	CKS1B
**1.14965718964298E-93**	3.8655719726496	0.844	0.11	1.9265955184037E-89	4	TUBB
**1.62733561824706E-146**	3.86145712628077	0.844	0.046	2.72708902905842E-142	4	FANCI
**1.0934205542251E-105**	3.86009124397711	0.556	0.025	1.83235416477043E-101	4	ECT2
**1.17802342577984E-107**	3.85775959826134	0.544	0.022	1.97413165692185E-103	4	ZGRF1
**2.60962629991409E-126**	3.85759708577054	0.856	0.067	4.37321175339603E-122	4	NUSAP1
**7.07284059727384E-63**	3.85161291477256	0.3	0.01	1.18526662729115E-58	4	KIF18A
**3.35183634464461E-82**	3.81204811872412	0.5	0.028	5.61700734635543E-78	4	TUBA1B
**1.78790810226887E-70**	3.76641043920631	0.433	0.025	2.99617639778217E-66	4	DTYMK
**5.43016558296559E-80**	3.74791367518869	0.5	0.03	9.09987148393374E-76	4	CENPU
**1.04807336980775E-93**	3.73796828322852	0.456	0.017	1.75636135312382E-89	4	CENPJ
**5.5338346343688E-78**	3.71075531822301	0.511	0.033	9.27360008027524E-74	4	ARHGAP11A
**8.42324925246039E-57**	3.70168655467242	0.289	0.012	1.41156810972731E-52	4	CHEK1
**3.15086081847547E-82**	3.69973212878803	0.411	0.016	5.2802125596012E-78	4	SUV39H1
**7.96447758245913E-145**	3.69815249457306	0.756	0.035	1.3346871532685E-140	4	NCAPG2
**8.82218889429367E-62**	3.69518572071695	0.344	0.017	1.47842241490573E-57	4	CDCA4
**3.33117150397929E-67**	3.68736587200901	0.4	0.022	5.5823772063685E-63	4	FANCG
**6.01812033551191E-73**	3.67471051660219	0.389	0.017	1.00851660582509E-68	4	ARHGAP33
**4.7079633245108E-49**	3.67285997812431	0.289	0.015	7.8896049392152E-45	4	SUV39H2
**3.24730965402023E-56**	3.66797362782461	0.389	0.027	5.44184151820711E-52	4	E2F1
**4.87646447533882E-47**	3.57146387590801	0.289	0.017	8.17197916777279E-43	4	DSN1
**4.95314882517139E-82**	3.56342914565044	0.422	0.017	8.30048680122221E-78	4	CENPO
**2.47830934519351E-80**	3.55607546887974	0.411	0.017	4.15315080067528E-76	4	GEN1
**1.13555915502531E-64**	3.53260689773933	0.467	0.035	1.90297003199142E-60	4	FEN1
**4.87816805232098E-66**	3.52480931488944	0.367	0.017	8.1748340220795E-62	4	CCDC18
**5.11232265848507E-97**	3.52465763716127	0.522	0.023	8.56723031108927E-93	4	C1orf112
**1.69699414681755E-50**	3.50496752071372	0.267	0.012	2.84382279123685E-46	4	WDR62
**1.8480624915636E-102**	3.4769817156662	0.756	0.063	3.09698312336228E-98	4	HIST1H1E
**4.8350407044442E-60**	3.4614410405387	0.4	0.026	8.10256121250758E-56	4	MAD2L1
**3.82022257138985E-80**	3.45368752935395	0.467	0.024	6.40192898513511E-76	4	WEE1
**1.45698191115407E-90**	3.42418332234729	0.578	0.036	2.441610286712E-86	4	TUBB4B
**5.99573787988432E-49**	3.40956984874399	0.289	0.015	1.00476575391101E-44	4	SLC37A4
**8.77896555729629E-90**	3.38362714384208	0.556	0.033	1.47117904809171E-85	4	ATAD2
**7.10566024064177E-65**	3.357612529103	0.433	0.028	1.19076654312675E-60	4	RNF26
**2.6934353247975E-95**	3.35285656496862	0.756	0.07	4.51365891729565E-91	4	TMEM106C
**1.03653221864008E-63**	3.32803058143163	0.456	0.034	1.73702069199705E-59	4	SLC43A3
**1.10949440929015E-93**	3.30895230058036	0.411	0.012	1.85929073108844E-89	4	NCAPH
**3.97862437950771E-40**	3.28119065945927	0.256	0.015	6.66737873517901E-36	4	GINS1
**1.72746930033306E-44**	3.25144073528434	0.267	0.015	2.89489305349814E-40	4	RMI2
**1.73226145083273E-36**	3.24153528580105	0.278	0.022	2.90292373930549E-32	4	IFI27L1
**5.59864666144042E-56**	3.22453127482723	0.389	0.026	9.38221207524185E-52	4	TUBG1
**1.03496535474081E-43**	3.21126086790561	0.3	0.02	1.73439494147465E-39	4	C19orf48
**1.8126214694967E-46**	3.20924114619126	0.333	0.024	3.03759105858256E-42	4	ZWILCH
**1.31317575241493E-71**	3.17805936090324	0.833	0.135	2.20061992589695E-67	4	HIST1H1D
**1.86426150899443E-56**	3.14965946501399	0.544	0.06	3.12412943677287E-52	4	MCM4

1.93026952096264E-68	3.14293685894349	0.422	0.024	3.2347456632292E-64	4	RCCD1
9.60565473342572E-46	3.13843617865654	0.278	0.015	1.60971562022748E-41	4	MIS18A
3.07806153299994E-58	3.12257185292065	0.456	0.037	5.1582155170013E-54	4	ACOT7
3.73721565943051E-56	3.11608480466939	0.333	0.018	6.26282600207365E-52	4	TEDC1
5.59985015757794E-59	3.103151156944	0.511	0.047	9.38422889406911E-55	4	DHFR
5.16218331818308E-50	3.08189391755713	0.344	0.023	8.6507868046112E-46	4	SLC1A4
1.16955839216791E-97	3.0760284885278	0.933	0.12	1.95994595359498E-93	4	LMNB1
2.48210938597278E-109	3.06860447891519	0.7	0.044	4.15951890901318E-105	4	SMC2
1.74925668158723E-54	3.05474396947289	0.4	0.03	2.93140434700389E-50	4	NDC1
2.76960674125698E-97	3.04225173458943	0.856	0.094	4.64130697699844E-93	4	TACC3
8.01417021265602E-85	3.03654989999268	0.511	0.028	1.3430146442369E-80	4	FANCA
3.43945243830377E-53	3.02517873460436	0.322	0.018	5.76383439610945E-49	4	NRM
1.29512546945127E-37	3.02365065237114	0.267	0.019	2.17037126170644E-33	4	RFC4
1.8844692414158E-67	3.00887634228011	0.456	0.03	3.1579935547646E-63	4	AC091057.6
5.75988097618433E-103	3.00850417171166	0.756	0.06	9.6524085398897E-99	4	EZH2
2.52609637020152E-52	2.95569687776763	0.311	0.017	4.2332322971837E-48	4	BCL2L12
7.34624337757504E-38	2.92485898114405	0.289	0.023	1.23108346521403E-33	4	HIRIP3
6.6339066977851E-34	2.89213951006993	0.267	0.022	1.11171008441483E-29	4	EMC9
4.76462919031091E-75	2.8881194150032	0.722	0.084	7.98456559712303E-71	4	NCAPD2
3.43648216826596E-26	2.8873561984111	0.267	0.031	5.75885681758009E-22	4	FADS2
8.73121275524826E-68	2.87104584114223	0.533	0.044	1.4631766335245E-63	4	RFWD3
5.95456911148459E-52	2.87013965770853	0.278	0.012	9.97866691702587E-48	4	TMEM201
7.76493600987845E-57	2.86050025293226	0.433	0.034	1.30124797653543E-52	4	TIMELESS
1.08299630808178E-35	2.8553827043188	0.267	0.021	1.81488521308344E-31	4	SLC29A1
6.80742436423041E-70	2.8516319409648	0.467	0.03	1.14078817495773E-65	4	CENPP
3.34996414284359E-29	2.84720622068046	0.256	0.024	5.61386991057729E-25	4	GNG5
5.70035598080298E-34	2.84383321050717	0.256	0.02	9.55265655262964E-30	4	KLF5
9.63672038240102E-60	2.83569081415005	0.689	0.098	1.61492160168276E-55	4	HELLS
1.15337557625996E-48	2.83445400708405	0.389	0.032	1.93282679069644E-44	4	ZDHHC12
1.99839434727366E-105	2.834095928267	0.944	0.103	3.34890924716119E-101	4	KIF22
3.67119236033623E-45	2.80780437000589	0.522	0.072	6.15218415745145E-41	4	MCM2
9.42653688804722E-40	2.78569338969261	0.278	0.02	1.57969905169895E-35	4	DONSON
4.1330525962863E-60	2.77813244578841	0.478	0.04	6.92616954085659E-56	4	TUBA1C
1.236819250105E-38	2.77035176749609	0.267	0.018	2.07266169932596E-34	4	CHTF18
3.26555461417301E-31	2.76985122608563	0.278	0.027	5.47241642243114E-27	4	POLD3
1.37675903252988E-65	2.75683892052639	0.444	0.03	2.30717278671357E-61	4	CHAF1A
1.51635569834288E-32	2.7544527119209	0.289	0.028	2.541108879283E-28	4	DARS2
6.39889638821614E-55	2.73818661921942	0.422	0.035	1.07232705673726E-50	4	KPNA2
7.44758707007943E-30	2.73080218758104	0.278	0.028	1.24806664120391E-25	4	GMNN
1.49902879841975E-36	2.72209529154478	0.3	0.026	2.51207246039182E-32	4	DHCR24
2.91672575554372E-75	2.67909984444232	0.389	0.016	4.88784902114016E-71	4	HIST1H2AE
5.03624536722794E-91	2.67792885638375	0.767	0.071	8.43973998640059E-87	4	LMNB2
1.53100024625928E-75	2.67656547732301	0.433	0.022	2.5656502126813E-71	4	ARHGAP19
9.82094310474891E-68	2.6743008766251	0.6	0.057	1.64579364549382E-63	4	KNTC1
1.67904464783355E-63	2.64923021583222	0.367	0.018	2.81374302083947E-59	4	CNTROB
1.14405433486007E-33	2.63843549670209	0.267	0.022	1.91720625435851E-29	4	C1GALT1C1
8.67217289278409E-30	2.62511934086609	0.256	0.023	1.45328273337276E-25	4	PXMP2
5.75028386373406E-69	2.61001770793973	0.456	0.029	9.63632569884554E-65	4	CENPN
1.57938601250944E-34	2.60687841567466	0.333	0.035	2.64673507976331E-30	4	MZT1
1.47677806918829E-111	2.60471085245318	0.633	0.031	2.47478468834573E-107	4	NDC80

**6.53521536864306E-77**	2.58339754450692	0.678	0.065	1.0951713914772E-72	4	LIG1
**7.4995660978359E-37**	2.58288229986324	0.311	0.028	1.25677728667534E-32	4	FH
**3.92262931389112E-39**	2.56909118334001	0.333	0.03	6.57354220421875E-35	4	ST3GAL3
**2.01042410484583E-61**	2.55678434055993	0.544	0.052	3.36906871490065E-57	4	KIF20B
**5.9532635282186E-59**	2.53560267837295	0.5	0.046	9.97647902058873E-55	4	PCNA
**6.92073092047234E-59**	2.51541093553237	0.422	0.03	1.15977608765275E-54	4	DNA2
**7.43859579713022E-48**	2.50255235354148	0.711	0.129	1.24655988368308E-43	4	MCM3
**2.01240750581938E-55**	2.50008990241696	0.478	0.043	3.37239249825213E-51	4	NUDT1
**4.60076812408933E-53**	2.49744373367147	0.567	0.068	7.7099672223489E-49	4	GUSB
**3.15873643929308E-93**	2.49715639740347	0.8	0.076	5.29341052496735E-89	4	NCAPD3
**1.0861798986104E-30**	2.49354986844155	0.289	0.03	1.82022027409131E-26	4	ISOC1
**1.62680162185644E-59**	2.48204732681386	0.789	0.13	2.72619415790702E-55	4	NUCKS1
**1.09418348424662E-37**	2.48192549684713	0.567	0.098	1.83363268290048E-33	4	ARL6IP1
**2.7370143970442E-29**	2.47413731747206	0.267	0.026	4.58668872656668E-25	4	BLVRB
**8.01245014251028E-42**	2.46880353453709	0.522	0.075	1.34272639488187E-37	4	CKS2
**4.64757753435744E-49**	2.4568306458372	0.478	0.051	7.78841043207619E-45	4	MBNL3
**2.66750830246909E-53**	2.45409542428061	0.644	0.09	4.4702104132777E-49	4	CDKN2C
**6.16396609674538E-44**	2.44619331954501	0.344	0.028	1.03295743849259E-39	4	RFC3
**5.75782161595733E-49**	2.4384419915362	0.356	0.026	9.64895746402129E-45	4	RAD18
**4.23260031014265E-30**	2.43698466883866	0.322	0.038	7.09299159973705E-26	4	MT2A
**6.75878980089164E-35**	2.42395796574565	0.256	0.019	1.13263799483342E-30	4	CENPH
**1.23834126634304E-69**	2.39577525674509	0.933	0.176	2.07521229413767E-65	4	HMGB2
**8.75850619802719E-63**	2.38340327050424	0.833	0.137	1.4677504686654E-58	4	SMC4
**1.18144387596203E-38**	2.35600022941733	0.433	0.053	1.97986364733717E-34	4	SCCPDH
**1.86125336594926E-40**	2.35350049672918	0.378	0.038	3.11908839065777E-36	4	BARD1
**6.83433156300007E-27**	2.35034767234667	0.3	0.038	1.14529728332755E-22	4	SEPTIN8
**1.81514857919698E-53**	2.34752259726463	0.667	0.097	3.0418259890183E-49	4	H2AFZ
**2.25917889755234E-45**	2.33863101961802	0.322	0.023	3.78593199651821E-41	4	CHEK2
**3.02117536466796E-29**	2.32559388262479	0.256	0.024	5.06288567611056E-25	4	SLC44A1
**5.8592647641975E-36**	2.32482628011638	0.256	0.018	9.81895589184217E-32	4	RCC1
**3.75874163381196E-23**	2.31409650282219	0.267	0.035	6.29889922994209E-19	4	PDLIM7
**6.53987810205797E-53**	2.29563705418228	0.556	0.063	1.09595277234288E-48	4	TTF2
**2.41569355566311E-47**	2.27416494159839	0.422	0.04	4.04821926058024E-43	4	CDK2
**1.86175676163711E-27**	2.2663123666297	0.311	0.039	3.11993198115147E-23	4	CLN6
**7.6164237937196E-38**	2.25415082412611	0.433	0.054	1.27636029935153E-33	4	HADH
**4.12170325305673E-45**	2.24781040388274	0.589	0.091	6.90715031147247E-41	4	PTTG1
**2.26904789655542E-23**	2.24195008442034	0.267	0.035	3.80247046504758E-19	4	BORA
**3.07911600517477E-42**	2.24094407949547	0.5	0.063	5.15998260147188E-38	4	TALDO1
**9.0955345133708E-56**	2.24047318189545	0.489	0.046	1.52422967375068E-51	4	POLD1
**2.05799329567185E-32**	2.24036553859819	0.322	0.035	3.44878516488689E-28	4	SHMT1
**4.04138574239428E-28**	2.23711862656164	0.256	0.025	6.77255422710433E-24	4	MFGE8
**1.1587430623997E-54**	2.23482081961315	0.622	0.078	1.94182162396942E-50	4	LSM 4.00
**4.30144760209441E-29**	2.23017333944112	0.3	0.034	7.20836589158981E-25	4	VANGL1
**4.1718803235536E-48**	2.22952102758138	0.611	0.087	6.99123704621113E-44	4	HDGF
**3.82046667902152E-28**	2.22557619275644	0.311	0.038	6.40233806070426E-24	4	CHAMP1
**2.25111876500312E-41**	2.21988744679513	0.589	0.092	3.77242482639224E-37	4	CERS2
**9.53362286531586E-31**	2.21682722991742	0.322	0.037	1.59764451976963E-26	4	GLRX5
**1.15002562439092E-34**	2.21172844995835	0.489	0.076	1.92721294135431E-30	4	MAD2L2
**1.21753000257391E-38**	2.21018037399575	0.422	0.051	2.04033677831336E-34	4	MGST3
**4.97772905441974E-46**	2.19725476080255	0.467	0.051	8.3416783493966E-42	4	CIAO2A

1.8160791044142E-25	2.19250531867832	0.256	0.028	3.04338536317731E-21	4	RMI1
7.38849079830057E-50	2.19075589466937	0.489	0.053	1.23816328797921E-45	4	WDR76
1.99041823835665E-64	2.1900119140123	0.478	0.035	3.33554288383807E-60	4	CEP85
2.86332069155738E-52	2.17846915200115	0.856	0.189	4.79835281491185E-48	4	TMPO
2.25600063529502E-26	2.14490838498303	0.278	0.033	3.7806058646274E-22	4	ACYP1
1.03015186196417E-45	2.14459246336626	0.389	0.035	1.72632849027956E-41	4	SCMH1
9.58750290320807E-39	2.13748132326451	0.333	0.03	1.60667373651961E-34	4	CENPL
3.69374476993888E-37	2.13732489076426	0.3	0.025	6.18997748546357E-33	4	YEATS4
2.50143864311072E-38	2.13654876914019	0.4	0.045	4.19191087812494E-34	4	BCAP29
3.05923801094278E-43	2.13060399738923	0.689	0.129	5.12667105873791E-39	4	MCM7
1.80130800335155E-28	2.12335812501778	0.267	0.027	3.01863195201652E-24	4	EMC6
7.378290691741E-56	2.11808005561725	0.6	0.07	1.23645395412196E-51	4	NSD2
4.49339812658464E-46	2.11254053893011	0.311	0.02	7.53003658053055E-42	4	CEP152
8.56081537402625E-25	2.10977230593248	0.411	0.076	1.43462144037932E-20	4	DUT
2.93074178452884E-59	2.10900338915648	0.711	0.101	4.91133708251342E-55	4	RRM1
3.5337210684403E-21	2.10444103401769	0.267	0.038	5.92180976649225E-17	4	HSDL2
3.94374136251728E-27	2.101116882369	0.356	0.051	6.60892177530646E-23	4	P4HB
1.96357917932305E-44	2.10001720093823	0.3	0.02	3.29056598870957E-40	4	C9orf40
2.06520917860492E-39	2.09799561378716	0.4	0.045	3.46087754150613E-35	4	MASTL
8.83186601220722E-33	2.08413841887044	0.433	0.063	1.48004410632569E-28	4	RTN3
2.33284529352955E-28	2.08051383874267	0.311	0.038	3.90938214289682E-24	4	AP2S1
5.84826716276066E-46	2.07304804460249	0.4	0.036	9.80052611135432E-42	4	CEP72
3.90629411254524E-29	2.06670644508039	0.344	0.044	6.54616767380332E-25	4	TMEM238
8.15797765953876E-23	2.0622064372127	0.333	0.054	1.36711389618551E-18	4	NABP2
1.79927678830398E-27	2.06054811271491	0.433	0.076	3.0152280418398E-23	4	DCTN3
9.02678826829449E-28	2.0456701232541	0.289	0.033	1.51270917800079E-23	4	ILVBL
2.11215867596366E-29	2.04221845935041	0.344	0.045	3.5395555091799E-25	4	BRI3BP
1.01403002643305E-29	2.04106356641942	0.267	0.026	1.69931151829651E-25	4	PCYT2
8.89061700452677E-29	2.03269423637345	0.356	0.048	1.4898895976186E-24	4	TMCO3
1.14499331229196E-32	2.02924691030198	0.356	0.042	1.91877979273887E-28	4	TMEM160
7.54043764616614E-32	2.02238505240552	0.356	0.044	1.26362654074452E-27	4	PSMD10
6.11216982114551E-34	2.01875465199704	0.367	0.043	1.02427741862756E-29	4	ACSL3
1.74384242112922E-25	2.01722731530132	0.256	0.028	2.92233112932834E-21	4	PRPS2
1.06092266087624E-18	2.01119412436614	0.278	0.047	1.77789419509639E-14	4	PRDX4
5.40987390254362E-25	2.00415930357931	0.333	0.049	9.06586668588259E-21	4	ACAD9
9.92240366493299E-33	2.00052866977265	0.544	0.098	1.66279640616947E-28	4	CARHSP1
1.66494895688765E-44	1.98899194344698	0.456	0.052	2.79012146195232E-40	4	CKAP2
6.26347247498676E-20	1.98429134115728	0.267	0.04	1.04963271735828E-15	4	NENF
1.34435550237108E-27	1.97915463533166	0.5	0.1	2.25287095087345E-23	4	NUCB2
6.12717845208837E-43	1.95777707225977	0.444	0.049	1.02679256500097E-38	4	LIN54
2.38638006114786E-17	1.95474603544964	0.3	0.058	3.99909570647158E-13	4	RNPEP
3.14022758386739E-31	1.93868527712781	0.433	0.066	5.26239338504498E-27	4	ERH
1.40134202301528E-31	1.92565046265618	0.378	0.049	2.348368962169E-27	4	BANF1
2.98865632918108E-35	1.91781388896135	0.489	0.075	5.00839027644166E-31	4	TFDP1
1.77514049551456E-28	1.90847452320122	0.344	0.046	2.9747804423833E-24	4	SLC7A1
1.87877631075101E-33	1.908332653994	0.589	0.111	3.14845334155655E-29	4	EBP
3.75131006527802E-42	1.90699555226348	0.389	0.038	6.28644540739291E-38	4	ANP32B
1.43543147752386E-29	1.90469860541415	0.5	0.091	2.40549607003449E-25	4	PMVK
3.50338467755449E-33	1.90135608233561	0.478	0.075	5.87097204264581E-29	4	ANAPC11
1.31088794735032E-21	1.90060552878072	0.356	0.065	2.19678602216966E-17	4	SPTBN1

1.57432119783475E-23	1.89282454332379	0.311	0.045	2.63824746333147E-19	4	COA3
1.45356154619112E-47	1.87876658222731	0.478	0.052	2.43587843910707E-43	4	POLH
3.71108900163115E-41	1.87837198600659	0.578	0.09	6.21904294893348E-37	4	HNRNPAB
6.37725446877344E-26	1.87806122036047	0.489	0.099	1.06870030387705E-21	4	UQCRC1
9.57348284904292E-38	1.8757820236363	0.622	0.11	1.60432425584261E-33	4	PSMC3
1.82836787057967E-17	1.8746577371267	0.3	0.057	3.06397887751741E-13	4	DAP
2.84866932325075E-20	1.86845610814488	0.278	0.043	4.77380005190361E-16	4	RFC2
4.59226507108073E-14	1.86419879935589	0.267	0.055	7.69571780611709E-10	4	SIGMAR1
4.28515768496007E-23	1.86195328313256	0.367	0.063	7.18106724845609E-19	4	CENPB
2.86804141935705E-25	1.85954417528363	0.4	0.068	4.80626381055854E-21	4	RPN1
3.43618533309647E-32	1.8567421052875	0.456	0.069	5.75835938120307E-28	4	TMEM14A
1.20371444872575E-32	1.8465975604285	0.556	0.1	2.01718467317462E-28	4	NDUFC2
4.31842267667422E-29	1.84113841126972	0.344	0.045	7.23681272157066E-25	4	VRK1
1.22206570391789E-19	1.83280761179897	0.3	0.051	2.0479377066256E-15	4	RAD23B
2.54904753539805E-24	1.83071241811038	0.344	0.054	4.27169385982005E-20	4	TLNRD1
6.67291042928684E-35	1.82795061520107	0.278	0.023	1.11824632973989E-30	4	PIDD1
4.40577515232747E-21	1.82624592942245	0.256	0.035	7.38319800027038E-17	4	FAH
5.68644547732516E-21	1.82609591697872	0.3	0.047	9.52934533090151E-17	4	RARRES2
3.65813040103554E-46	1.8221237920288	0.678	0.115	6.13029492605536E-42	4	CDC25B
1.41520596076094E-19	1.8195266235839	0.344	0.066	2.37160214904319E-15	4	ACAT2
3.14400240064705E-28	1.80877950812559	0.433	0.074	5.26871922300432E-24	4	GOLIM4
2.35533459139909E-36	1.79370316941528	0.444	0.059	3.9470697082666E-32	4	JPT2
8.51717551319804E-20	1.79238043177683	0.278	0.043	1.42730827250173E-15	4	LAGE3
2.04883445480355E-24	1.77693146694851	0.3	0.041	3.43343677935978E-20	4	SLC25A1
1.79167831085065E-31	1.7766556403459	0.511	0.093	3.00249451332352E-27	4	CKAP5
7.9969320788702E-30	1.77291992813095	0.622	0.144	1.34012587777707E-25	4	MCM5
6.38422980725102E-33	1.75491305626787	0.389	0.051	1.06986923109913E-28	4	ALYREF
4.96668297174967E-23	1.75460245122542	0.356	0.059	8.3231673240581E-19	4	ADI1
7.91052069635158E-41	1.75238737120182	0.478	0.06	1.3256450582946E-36	4	KMT5A
2.38144455321214E-35	1.74326294000227	0.344	0.036	3.9908247822729E-31	4	MCM8
3.26500918149964E-25	1.7416481873219	0.433	0.079	5.4715023863571E-21	4	PRDX6
1.37117666471251E-24	1.73558949746219	0.478	0.099	2.29781785472522E-20	4	ETFB
3.59355201851459E-23	1.73247997156429	0.433	0.086	6.02207447262675E-19	4	RAB5IF
3.68554205087096E-39	1.73180534114587	0.722	0.156	6.17623136884956E-35	4	DNMT1
1.16127978402107E-42	1.72837585473344	0.867	0.201	1.94607266206251E-38	4	FAM111A
3.11479547408158E-55	1.72824738900427	0.867	0.165	5.21977425546592E-51	4	PHF19
2.45363233895137E-25	1.72579229214497	0.478	0.094	4.11179707361471E-21	4	ACAA2
1.0401546646989E-17	1.7215944714179	0.289	0.052	1.74309118710241E-13	4	RHEB
1.99858923867767E-24	1.71793211255341	0.311	0.045	3.34923584617604E-20	4	POLA2
7.49791366848881E-31	1.71681179159683	0.422	0.063	1.25650037256536E-26	4	MGME1
8.81725574425085E-24	1.71517158045534	0.367	0.06	1.47759571762156E-19	4	CMC4
6.72640368377256E-21	1.71061604786339	0.278	0.041	1.12721072932661E-16	4	SLC25A40
1.19117906732142E-21	1.70180576086998	0.322	0.052	1.99617788101723E-17	4	SPTSSA
1.53448581620648E-49	1.70002399396351	0.433	0.039	2.57149133079881E-45	4	CEP128
2.79072873332726E-20	1.69423504576502	0.311	0.052	4.67670321130982E-16	4	AKIP1
5.62242610742781E-18	1.6933826657339	0.267	0.044	9.42206167082753E-14	4	MGST2
5.9916330688724E-35	1.69312210402545	0.422	0.055	1.00407786968164E-30	4	TOMM5
1.15415136751295E-22	1.69097222083523	0.322	0.051	1.9341268616782E-18	4	VBP1
7.91740784368312E-27	1.68757881888885	0.356	0.052	1.32679920644442E-22	4	CDC7
9.88642436653779E-18	1.68175179099662	0.333	0.067	1.6567669953444E-13	4	CD151

**3.73074274482778E-19**	1.68037435923794	0.256	0.038	6.2519786917824E-15	4	CIDEB
**4.46563544604295E-32**	1.67454733892118	0.767	0.213	7.48351188047878E-28	4	SMC1A
**1.49591120024706E-26**	1.67387511486305	0.389	0.061	2.50684798937403E-22	4	LRRC59
**9.36219435733296E-27**	1.66932918190391	0.333	0.046	1.56891653040186E-22	4	HACD3
**9.27843881971558E-28**	1.66515116577162	0.333	0.044	1.55488077740794E-23	4	MPDU1
**1.1345484637995E-32**	1.66465580761548	0.878	0.292	1.90127631563521E-28	4	CRIP1
**1.34352661012351E-30**	1.66230400948804	0.3	0.032	2.25148189324498E-26	4	LRRC45
**1.63571632667373E-24**	1.6443395154686	0.411	0.073	2.74113342023984E-20	4	MEA1
**3.2766138194658E-22**	1.64284490785483	0.367	0.065	5.49094943866078E-18	4	PGD
**6.89618940183142E-34**	1.6393918510948	0.867	0.262	1.15566341995891E-29	4	CALM3
**3.36453024264684E-26**	1.63916883331021	0.433	0.078	5.63827978062758E-22	4	H2AFY
**1.56977732754057E-28**	1.63454790387112	0.467	0.081	2.63063284549249E-24	4	TSTA3
**7.84447723779915E-30**	1.62612434113491	0.856	0.277	1.31457749551038E-25	4	IDH2
**5.80030474599142E-22**	1.62505492901654	0.411	0.081	9.72015069333243E-18	4	CANX
**6.20150011686712E-20**	1.62175021478566	0.356	0.068	1.03924738958459E-15	4	H2AFJ
**3.79582669505114E-27**	1.61468032935089	0.422	0.07	6.36104637556671E-23	4	TOPBP1
**7.8592702637589E-24**	1.61195744609832	0.4	0.071	1.31705651080072E-19	4	SSR3
**1.37258785874869E-43**	1.60966383664206	0.6	0.093	2.30018273369105E-39	4	NCAPH2
**4.47009924376813E-16**	1.60940884303195	0.256	0.046	7.49099231270663E-12	4	CPT1A
**1.25194247096928E-17**	1.60348249460009	0.278	0.048	2.09800519285032E-13	4	NME1
**2.91130446964555E-31**	1.60337985646416	0.589	0.113	4.87876403023202E-27	4	NDUFA1
**5.59764603748944E-35**	1.60053919387515	0.467	0.067	9.3805352296248E-31	4	RBL1
**8.18389235749512E-23**	1.59175628369142	0.278	0.038	1.37145668126903E-18	4	SLX4
**1.12727108316069E-31**	1.5907896630967	0.467	0.073	1.88908088116068E-27	4	HSD17B11
**1.00760502182974E-33**	1.5764238395137	0.533	0.091	1.68854449558228E-29	4	STT3A
**2.57820448841584E-28**	1.57580938109708	0.344	0.045	4.32055508168726E-24	4	RUVBL2
**1.63214003330548E-16**	1.57539354331607	0.278	0.051	2.73514026781333E-12	4	ZNF428
**5.69810149479393E-25**	1.57404231589843	0.689	0.187	9.54887848497566E-21	4	PRDX2
**1.68089655502022E-23**	1.57321448706192	0.378	0.065	2.81684644690288E-19	4	MPC2
**1.16970318699273E-18**	1.56899931215895	0.333	0.063	1.96018860076242E-14	4	ECI1
**2.31908844022764E-28**	1.56883107309932	0.422	0.067	3.88632840813348E-24	4	PRPSAP1
**2.84754297284076E-30**	1.5662827047778	0.511	0.094	4.77191251388654E-26	4	SAE1
**5.3726403325953E-16**	1.54857549752724	0.267	0.049	9.00347066936321E-12	4	SMIM19
**9.17194408708534E-26**	1.54695521270835	0.367	0.057	1.53703439011376E-21	4	POLA1
**2.23339193325607E-18**	1.54669467945801	0.322	0.06	3.74271820175053E-14	4	ILF2
**4.1835969597407E-33**	1.54111097453223	0.533	0.095	7.01087178513346E-29	4	SIVA1
**5.02712846946267E-29**	1.54019264480992	0.422	0.065	8.42446188912554E-25	4	CBX3
**4.64227389184884E-29**	1.53704909886472	0.344	0.046	7.77952258796029E-25	4	CDKN2D
**3.69239984206277E-30**	1.53352822056694	0.389	0.054	6.18772365532879E-26	4	MORF4L2
**2.6892959639195E-15**	1.53237545442842	0.278	0.054	4.5067221763363E-11	4	HLTF
**7.73448586450828E-22**	1.52859397843748	0.422	0.086	1.2961451411743E-17	4	TP53
**1.84953030363242E-53**	1.52243128920144	0.722	0.108	3.09944288282721E-49	4	ANP32E
**3.12841634995579E-19**	1.52148795613197	0.3	0.051	5.24260011925591E-15	4	TMEM19
**8.98908148244697E-20**	1.50527772101291	0.444	0.1	1.50639027482846E-15	4	COX8A
**6.23204775840664E-18**	1.49687139367234	0.256	0.041	1.04436656335378E-13	4	MYB
**6.23927181892203E-22**	1.49610424424732	0.578	0.147	1.04557717141495E-17	4	CHCHD2
**1.75196201392764E-46**	1.49336135130343	0.544	0.069	2.93593794293994E-42	4	NEMP1
**4.52854390916175E-22**	1.49333272332787	0.333	0.055	7.58893388297326E-18	4	SLC25A25
**5.09846559297561E-26**	1.4897092943969	0.4	0.065	8.54400864070853E-22	4	TPD52
**7.78218831332487E-22**	1.47876700376567	0.322	0.051	1.30413911754698E-17	4	ICMT

9.51449615946929E-30	1.47798031413819	0.433	0.067	1.59443926640386E-25	4	TTL
3.82502508895243E-20	1.47418978692332	0.3	0.049	6.40997704406648E-16	4	MID1IP1
2.95553064786561E-21	1.47190075691226	0.344	0.061	4.95287825969319E-17	4	FDXR
1.93297287388143E-33	1.4698374571505	0.478	0.073	3.2392759420505E-29	4	CCDC14
1.71948800669676E-18	1.46876168325624	0.333	0.065	2.88151800162243E-14	4	POLE3
2.46816429276628E-13	1.46736887214028	0.256	0.052	4.13614972181773E-09	4	SLC25A5
4.22858832607683E-21	1.46696462353669	0.444	0.094	7.08626831683955E-17	4	PDIA6
5.22288137405911E-26	1.46379729293379	0.411	0.069	8.75250460664826E-22	4	CDK4
1.17752601118773E-13	1.46297309501928	0.289	0.064	1.9732980895484E-09	4	TXNDC17
3.63757268385973E-28	1.45889358495035	0.344	0.046	6.09584430361214E-24	4	PRIM2
2.80116622223175E-20	1.45848953808611	0.278	0.043	4.69419435521597E-16	4	JPT1
1.47183040990823E-32	1.45791926286552	0.367	0.045	2.46649340092422E-28	4	GPAA1
1.02601613015376E-15	1.45028286716501	0.256	0.046	1.71939783091168E-11	4	UQCRH
7.38845441079831E-22	1.448714907156	0.411	0.079	1.23815719016158E-17	4	STOML2
1.32786995622087E-20	1.44457406225866	0.411	0.084	2.22524447263493E-16	4	AKR1A1
1.61940110177819E-14	1.44370303060234	0.378	0.098	2.71379236635988E-10	4	ADIPOR1
1.91551605878962E-23	1.44278271988647	0.589	0.143	3.21002181131965E-19	4	BSG
1.37965279437138E-20	1.44086183345124	0.267	0.038	2.31202215280755E-16	4	ZW10
1.18199716016338E-12	1.44007567581074	0.278	0.065	1.98079084100179E-08	4	SRM
1.65513915112935E-22	1.4387886084226	0.278	0.038	2.77368218946257E-18	4	NDUFB6
1.417541572978E-21	1.43231065167799	0.256	0.034	2.37551616799654E-17	4	HAUS5
9.80366047927539E-31	1.43128603603457	0.378	0.05	1.64289742311697E-26	4	TRIM28
3.63526190900718E-29	1.429365345507	0.278	0.028	6.09197190711423E-25	4	IMPA1
4.3716723353916E-29	1.4280662856444	0.456	0.076	7.32604849964924E-25	4	TBC1D10B
5.42833655280586E-24	1.42635528542299	0.422	0.078	9.09680639519206E-20	4	TPGS2
2.21833260638345E-25	1.42464205829506	0.689	0.198	3.71748178177739E-21	4	CALM2
4.12158236654333E-11	1.42461750947094	0.344	0.104	6.90694772985332E-07	4	INF2
9.4088871259456E-11	1.42255105725157	0.267	0.068	1.57674130456596E-06	4	PMM1
2.10269694837159E-10	1.4213724949243	0.6	0.261	3.52369954608111E-06	4	LGALS1
6.47320119197938E-23	1.41970560519896	0.433	0.087	1.0847790557519E-18	4	CBX5
1.85918149699547E-18	1.41654727677439	0.389	0.084	3.11561635266501E-14	4	HAGH
1.76552672139845E-28	1.41591731301888	0.478	0.084	2.95866967971952E-24	4	MLF 2.00
9.77413702734077E-28	1.41080326033853	0.256	0.025	1.63794988304177E-23	4	ZNF318
9.39576485246704E-20	1.40921840288586	0.333	0.062	1.57454227397643E-15	4	VMA21
5.82219297457722E-22	1.40472399093472	0.444	0.091	9.7568309867965E-18	4	SRP9
4.92935911920023E-18	1.40368716806144	0.333	0.065	8.26062001195575E-14	4	LNPK
5.66572200110141E-22	1.39296002318934	0.289	0.042	9.49461692944574E-18	4	CCDC167
6.27089548583216E-19	1.39176283126407	0.344	0.066	1.05087666551575E-14	4	DCXR
1.14304857442884E-20	1.39023652015956	0.444	0.097	1.91552080102786E-16	4	RNF5
7.78744281884336E-24	1.38941364974385	0.444	0.084	1.30501966758177E-19	4	MANF
1.26654048112339E-32	1.3877013166529	0.567	0.101	2.12246853826657E-28	4	SUMO3
1.59964889053571E-22	1.38664547397862	0.356	0.061	2.68069161075974E-18	4	HAUS4
9.82872193391594E-22	1.38516380507648	0.389	0.073	1.64709722168563E-17	4	MPP1
4.26905135695393E-26	1.3844215604459	0.522	0.112	7.1540762639834E-22	4	PLP2
2.30462228437325E-18	1.37962946284809	0.4	0.09	3.86208602415269E-14	4	TRIOBP
1.44593118668725E-25	1.37676684136106	0.311	0.041	2.42309148265049E-21	4	ANAPC15
6.44338147130218E-27	1.3766029092012	0.3	0.036	1.07978186696082E-22	4	F11R
2.30711115946192E-28	1.37606807580901	0.478	0.085	3.86625688102628E-24	4	SCLT1
1.12746351427825E-22	1.37350255690097	0.367	0.063	1.88940335722749E-18	4	MAZ
2.00802496670162E-21	1.37284520916364	0.433	0.089	3.36504823919858E-17	4	GUCD1

1.18100237071818E-25	1.37281638684737	0.533	0.112	1.97912377284952E-21	4	PGRMC1
1.38426675315777E-13	1.37273090854184	0.4	0.112	2.3197542249418E-09	4	TM7SF3
3.41835500920248E-11	1.37137182557488	0.278	0.071	5.72847932442151E-07	4	TXNDC5
1.68087707502188E-18	1.36817033917206	0.289	0.049	2.81681380232167E-14	4	CEBPG
4.1216410904674E-19	1.3668222718775	0.267	0.041	6.90704613940527E-15	4	RHNO1
1.77680185537485E-19	1.36575579076206	0.456	0.107	2.97756454923718E-15	4	HTATSF1
6.2910289029065E-20	1.36259307918024	0.378	0.074	1.05425062354907E-15	4	SLC16A1
9.14665415982905E-13	1.36248656386366	0.278	0.063	1.53279630410415E-08	4	DBI
2.30982511772305E-19	1.36012835556557	0.267	0.041	3.87080493228029E-15	4	CEP97
2.24963490893386E-36	1.3589875671656	0.878	0.235	3.76993818039137E-32	4	RBM15
3.67501770285149E-21	1.35403660607738	0.322	0.054	6.15859466643853E-17	4	HMGXB4
2.2170138358482E-17	1.3525316320259	0.289	0.052	3.71527178611442E-13	4	MCFD2
8.9636951261859E-17	1.35126524086701	0.444	0.113	1.50213602924623E-12	4	MTHFD1
3.90703413168429E-17	1.35035755554024	0.278	0.05	6.54740779787654E-13	4	IGFBP4
9.46262963739934E-23	1.34688642154046	0.478	0.1	1.58574747463538E-18	4	PPIA
8.65424662024346E-15	1.33946265672347	0.333	0.078	1.4502786486204E-10	4	MPC1
4.03069735726526E-33	1.33925262821332	0.633	0.124	6.75464263130512E-29	4	HSPB1
1.30906255313104E-20	1.33754432292833	0.322	0.055	2.193727026537E-16	4	GMPS
1.63113511044381E-15	1.33594697877084	0.289	0.057	2.73345621808173E-11	4	XRCC1
1.88089912532116E-15	1.33537051825609	0.289	0.057	3.1520107542132E-11	4	UQCRQ
1.27889265939534E-26	1.33107485742652	0.589	0.131	2.14316831861471E-22	4	SSRP1
2.14572402864061E-16	1.33017216317127	0.256	0.044	3.59580432719594E-12	4	PRIM1
3.29970188469775E-20	1.32922328069975	0.344	0.064	5.52964041837649E-16	4	SAMD1
7.18229082694407E-19	1.32547579317379	0.944	0.577	1.20360829677929E-14	4	VIM
4.68341870447876E-22	1.32352732093944	0.333	0.054	7.84847306496551E-18	4	HSD17B4
2.261787261529E-20	1.32234919623804	0.422	0.088	3.79030309287029E-16	4	SCD
1.47160440366432E-22	1.31694837913346	0.444	0.091	2.46611465966067E-18	4	CYC1
3.68982261249616E-18	1.31506542303685	0.256	0.04	6.18340473402106E-14	4	ORC3
1.85222659141913E-16	1.31166003553929	0.278	0.051	3.10396132190018E-12	4	IER3IP1
3.13334154086314E-11	1.30353357296338	0.3	0.08	5.25085375417844E-07	4	DDOST
1.15427593940635E-24	1.30248940646571	0.389	0.065	1.93433561925716E-20	4	SMARCB1
1.17505646452767E-13	1.30172278789566	0.256	0.051	1.96915962325546E-09	4	NAA20
3.4320928125802E-19	1.2997358224063	0.444	0.101	5.7515011353219E-15	4	HSD17B10
1.49564744673199E-17	1.29868020053187	0.311	0.059	2.50640599123348E-13	4	AIFM1
2.2910136039368E-22	1.29717988680091	0.289	0.041	3.83928059747728E-18	4	UBAC1
5.56035554646275E-22	1.29570382751924	0.489	0.107	9.31804382476227E-18	4	RAB2A
6.76348184518514E-17	1.29251307316138	0.378	0.086	1.13342428761613E-12	4	SCAF1
8.61010875481781E-23	1.2905612336383	0.578	0.139	1.44288202513237E-18	4	CYB5R3
8.3083059297887E-29	1.28649148060455	0.578	0.118	1.39230590771399E-24	4	BCAP31
8.30007578472468E-20	1.28475793501289	0.267	0.039	1.39092670000416E-15	4	ALG6
8.23396121819234E-23	1.28337511115812	0.578	0.138	1.37984722094467E-18	4	ERGIC3
3.78689877329887E-21	1.28255802195804	0.456	0.097	6.34608496429424E-17	4	APEH
5.92499455592143E-26	1.2821937801972	0.5	0.102	9.92910587681314E-22	4	USP1
1.796988350054E-15	1.28215306479626	0.322	0.07	3.01139307702049E-11	4	HPRT1
1.8080671566896E-24	1.27821682535495	0.444	0.083	3.02995894118043E-20	4	SLF2
4.0559537121857E-19	1.26879882682996	0.344	0.065	6.7969672308808E-15	4	MEPCE
4.28938833620653E-16	1.26312936686273	0.411	0.102	7.18815697381491E-12	4	LIMK2
2.46593418717953E-24	1.26291287808417	0.4	0.069	4.13241251087546E-20	4	IFI27
6.53809529959143E-23	1.26238438820872	0.589	0.146	1.09565401030553E-18	4	SCP2
1.71870535361973E-18	1.26150943920992	0.311	0.057	2.88020643159594E-14	4	CYB561A3

**5.08782379016821E-22**	1.25952318081102	0.711	0.214	8.52617510756389E-18	4	ANXA5
**9.23017145554356E-22**	1.25625768710915	0.322	0.052	1.54679213251999E-17	4	RBBP7
**1.48358920472968E-19**	1.25551867604255	0.544	0.14	2.48619878928599E-15	4	NDUFA2
**1.28215208146555E-27**	1.25534299915484	0.633	0.146	2.14863045811998E-23	4	DCK
**1.31100312204923E-18**	1.25018376305823	0.344	0.066	2.1969790319301E-14	4	CNP
**4.86404217265817E-20**	1.24981957207972	0.267	0.039	8.15116187294057E-16	4	APP
**1.19590181264824E-20**	1.24911628693749	0.456	0.099	2.00409225763593E-16	4	TXNRD1
**2.93274774891453E-38**	1.24859400185855	0.689	0.126	4.91469867763097E-34	4	PCNT
**3.73808704365689E-16**	1.24570122261065	0.467	0.129	6.26428626776021E-12	4	CSRP1
**2.3789107409146E-17**	1.24382560919909	0.5	0.134	3.98657861962469E-13	4	GHITM
**7.26953512407917E-19**	1.24219517817911	0.656	0.199	1.21822869609319E-14	4	ANXA2
**4.38290287996754E-23**	1.24197605505494	0.567	0.132	7.3448686462496E-19	4	COPG1
**8.18095155768824E-26**	1.23651579294655	0.578	0.128	1.3709638620374E-21	4	OAS3
**4.34041429691483E-13**	1.23636138881373	0.311	0.075	7.27366627876987E-09	4	NHP2
**1.92648463742339E-37**	1.23454133363445	0.367	0.038	3.22840295539412E-33	4	ODF2
**4.76811971697237E-14**	1.22984540892634	0.378	0.096	7.9904150217023E-10	4	COX16
**3.77588150202475E-16**	1.22808699826979	0.389	0.092	6.32762222109307E-12	4	ATP5PD
**7.55333241383192E-37**	1.22680972264906	0.622	0.112	1.26578744590995E-32	4	CDK5RAP2
**1.03648544031751E-16**	1.22635336952476	0.456	0.118	1.73694230088408E-12	4	NDUFB7
**1.85962684021478E-19**	1.22575355876052	0.467	0.108	3.11636265883192E-15	4	RBM42
**1.77030723022948E-16**	1.22494704480753	0.444	0.113	2.96668085641857E-12	4	NUDT5
**3.6023319139964E-18**	1.22280388515519	0.367	0.076	6.03678782147518E-14	4	BRI3
**2.97918360609186E-30**	1.22147767767337	0.6	0.12	4.99251588708874E-26	4	SNRPB
**3.82300426050844E-17**	1.21515539679431	0.344	0.071	6.40659053976005E-13	4	SDF2L1
**4.61228680546518E-27**	1.21483861267927	0.889	0.281	7.72927022859854E-23	4	SELENOP
**4.51788172375401E-15**	1.21149730466156	0.4	0.1	7.57106619266698E-11	4	LAMTOR2
**9.32930461649873E-23**	1.21128020867143	0.256	0.032	1.56340486763286E-18	4	NCKIPSD
**1.60125667256874E-30**	1.21051378352837	0.689	0.146	2.68338593189069E-26	4	LAMP2
**1.07505951190643E-19**	1.21016738210917	0.4	0.081	1.80158473005279E-15	4	PSMB2
**1.03901679239347E-33**	1.20936329925042	0.667	0.136	1.74118434069298E-29	4	RAD21
**5.6903211390763E-15**	1.20804957163089	0.344	0.079	9.53584016486407E-11	4	PPDPF
**5.67018551271608E-23**	1.20595521692713	0.567	0.136	9.5020968822096E-19	4	GPX4
**7.68888705517419E-16**	1.2053830023548	0.344	0.076	1.28850369270609E-11	4	CDKN2A
**2.47741124869926E-17**	1.20522396920752	0.267	0.045	4.15164577057023E-13	4	MAPK7
**5.52947933146915E-10**	1.20495314658507	0.278	0.076	9.266301463676E-06	4	PSMA2
**5.13540521244451E-14**	1.20386482599106	0.356	0.089	8.60591205501451E-10	4	FIBP
**4.62528593055265E-17**	1.20236894084752	0.289	0.054	7.75105416242014E-13	4	FAM20B
**1.58387387997757E-16**	1.19977952132254	0.322	0.065	2.65425584806641E-12	4	NDUFB1
**9.68582597287686E-18**	1.19713107836328	0.422	0.097	1.6231507165347E-13	4	AGPAT1
**6.38922140014655E-24**	1.19640684879128	0.989	0.755	1.07070572223656E-19	4	ACTB
**9.74520066028164E-12**	1.19419993845476	0.289	0.072	1.63310072665E-07	4	FANCL
**1.49469911657288E-22**	1.1860398726502	0.389	0.069	2.50481677955283E-18	4	GTF2A2
**1.12138719185886E-14**	1.18576407543048	0.356	0.086	1.87922065611707E-10	4	PGRMC2
**1.73496587887211E-16**	1.18346472960359	0.278	0.051	2.90745581981388E-12	4	PSMD2
**8.63415271628898E-17**	1.18266083587653	0.267	0.047	1.44691131219571E-12	4	C11orf80
**2.19086169277842E-26**	1.18198244835478	0.656	0.158	3.67144602475808E-22	4	AP1M1
**1.90003704680042E-19**	1.18195095347657	0.4	0.083	3.18408208302814E-15	4	AKR1C3
**4.28381042761778E-19**	1.17755181887084	0.467	0.109	7.17880951460188E-15	4	SNTB1
**1.44855314941539E-18**	1.1726404519971	0.4	0.086	2.42748536779032E-14	4	CRELD2
**4.51348016014317E-24**	1.16859016536656	0.567	0.124	7.56369005236792E-20	4	ACSL4

**4.32691625667662E-13**	1.16855205289714	0.267	0.057	7.25104626293869E-09	4	NDUFA7
**4.54194623133149E-17**	1.16818253213539	0.289	0.053	7.6113934944653E-13	4	THOP1
**2.53289340236218E-30**	1.166654646156	0.5	0.086	4.24462276367854E-26	4	CEP57
**4.18777845434563E-18**	1.16451199492721	0.444	0.105	7.01787913379241E-14	4	PSMD1
**1.1211020909073E-12**	1.16402790511619	0.311	0.076	1.87874288394245E-08	4	PFDN6
**6.73546786489363E-19**	1.16372740918935	0.4	0.086	1.12872970479887E-14	4	CEP192
**7.38338144602529E-23**	1.16355548786091	0.322	0.049	1.23730706272492E-18	4	RSRC1
**4.90420292889611E-20**	1.16323432549359	0.467	0.105	8.2184632682441E-16	4	MZT2B
**7.63075017029089E-21**	1.16295079585361	0.344	0.062	1.27876111353735E-16	4	CCNG1
**2.52774035765374E-18**	1.16235935288619	0.256	0.039	4.23598729135613E-14	4	PGAM1
**6.23460636128087E-15**	1.16147243719209	0.256	0.048	1.04479533402345E-10	4	ADPRHL2
**5.5012456767115E-26**	1.15624878412934	0.844	0.274	9.21898750503312E-22	4	S100A10
**1.60466023709494E-15**	1.15328148181381	0.344	0.077	2.6890896253237E-11	4	MZT2A
**7.67154206553703E-21**	1.15149672193409	0.511	0.125	1.28559701934269E-16	4	C17orf49
**1.91921360990911E-29**	1.14987619856883	0.322	0.038	3.2162181674857E-25	4	DBF4
**5.61459435780123E-19**	1.14933233846459	0.767	0.256	9.4089372248033E-15	4	ENO1
**9.91501360168082E-17**	1.14658663939479	0.3	0.057	1.66155797936967E-12	4	JMJD8
**6.54147542575192E-19**	1.14409174959725	0.556	0.152	1.09622045184751E-14	4	BLOC1S1
**7.59539263657373E-14**	1.14250683094731	0.3	0.068	1.27283589803703E-09	4	R3HCC1L
**7.89249449110437E-18**	1.13984270276674	0.289	0.051	1.32262422681927E-13	4	RAD54L2
**1.366847537622E-16**	1.13827645109175	0.433	0.107	2.29056310354694E-12	4	NDUFB9
**4.16694139156086E-25**	1.13818834911218	0.444	0.081	6.98296038397769E-21	4	KHSRP
**2.36974083437466E-16**	1.1371389884856	0.256	0.044	3.97121169024505E-12	4	SLC39A3
**5.22665211757685E-20**	1.13636124325727	0.944	0.478	8.75882361863528E-16	4	ACTG1
**1.08623304384589E-14**	1.13577733620628	0.3	0.063	1.82030933487695E-10	4	SLC35F6
**2.42746388212304E-12**	1.12931207792054	0.267	0.061	4.06794397366178E-08	4	USP5
**8.46807191508305E-21**	1.12894789271161	0.711	0.222	1.41907949152962E-16	4	PPP1CA
**3.29403590753011E-17**	1.1270770148541	0.322	0.063	5.52014537383896E-13	4	LGALS4
**7.35570101170707E-13**	1.12602535998218	0.278	0.063	1.23266837554187E-08	4	MUC3A
**1.33967446525582E-11**	1.11965902045525	0.256	0.059	2.2450264688757E-07	4	COA6
**4.85319171183139E-16**	1.11746193928449	0.589	0.183	8.13297867068704E-12	4	PDIA3
**6.81427872963806E-16**	1.11534976253821	0.367	0.084	1.14193682951275E-11	4	TSR3
**4.3843640402215E-10**	1.1142383126811	0.256	0.065	7.34731725860319E-06	4	ACTL6A
**7.88067963420527E-31**	1.11373449718958	0.556	0.102	1.32064429310012E-26	4	RAVER1
**1.18988633957899E-19**	1.11305809881499	0.433	0.094	1.99401152786648E-15	4	ABHD2
**1.62680005329137E-25**	1.10971943010406	0.544	0.116	2.72619152930567E-21	4	NUDT21
**1.91213880883924E-15**	1.10735321692507	0.456	0.124	3.2043622158528E-11	4	NDUFA4
**3.24185151766394E-09**	1.1070704748965	0.311	0.097	5.43269477330123E-05	4	POLDIP2
**3.6149421352115E-11**	1.10615067081641	0.256	0.06	6.05792003018742E-07	4	NDUFS6
**1.71312888166279E-17**	1.10235793982665	0.278	0.049	2.87086137989051E-13	4	CDC27
**2.36402088017961E-14**	1.10104284888581	0.3	0.065	3.96162619100498E-10	4	DDX50
**1.3039481783072E-43**	1.098888674652	0.667	0.107	2.1851563572072E-39	4	DDX39A
**4.15432294690758E-17**	1.09740124385407	0.456	0.113	6.96181439442772E-13	4	PCMT1
**2.06447328717236E-21**	1.09531939873252	0.378	0.07	3.45964433464345E-17	4	PEA15
**2.14935593761998E-14**	1.09488824236361	0.311	0.07	3.60189068026356E-10	4	NUDCD2
**7.30468963520762E-34**	1.09390773941217	0.489	0.075	1.22411988906809E-29	4	POLE
**9.84959908328343E-12**	1.09052801564167	0.267	0.062	1.65059581437664E-07	4	ATP5MF
**1.23738072584109E-13**	1.08284580727332	0.356	0.089	2.0736026203645E-09	4	CLTA
**1.08337318272303E-16**	1.0824233353035	0.578	0.171	1.81551677960726E-12	4	HADHA
**1.63446959436718E-14**	1.08177014182859	0.3	0.064	2.73904414624052E-10	4	SPCS2

**3.93990181701382E-14**	1.07995313515886	0.322	0.074	6.60248746495175E-10	4	ACAT1
**3.59488585089021E-32**	1.07954192964155	0.556	0.099	6.02430970892182E-28	4	MIS18BP1
**2.48967289174227E-18**	1.07870284697686	0.389	0.084	4.1721938319817E-14	4	CRTAP
**9.51602264144253E-29**	1.07762030879244	0.633	0.139	1.59469507425294E-24	4	MPHOSPH9
**3.86168058094789E-16**	1.07535148758551	0.322	0.067	6.47140431755248E-12	4	NAGA
**5.53293299681973E-20**	1.07473689454153	0.433	0.094	9.2720891160705E-16	4	HPS3
**2.29203048145442E-15**	1.07320475797454	0.267	0.051	3.84098468082132E-11	4	USP37
**1.92849413554027E-11**	1.07202977655996	0.256	0.06	3.23177047233839E-07	4	KCNK5
**4.22715023902784E-24**	1.06939956762479	0.256	0.03	7.08385837056285E-20	4	PASK
**1.32884239104885E-16**	1.06885050797147	0.278	0.05	2.22687407891966E-12	4	ELP5
**5.9437828130927E-18**	1.06846606500612	0.267	0.044	9.96059123818075E-14	4	PALM2-AKAP2
**2.57014170027031E-17**	1.06751870671023	0.389	0.089	4.30704346131298E-13	4	NUP58
**1.45254943661175E-15**	1.06714308105667	0.444	0.118	2.43418234587397E-11	4	KRTCAP2
**2.24819544281547E-18**	1.06620845678344	0.444	0.105	3.76752592307016E-14	4	YWHAE
**3.94088282174388E-17**	1.06604099825745	0.544	0.155	6.6041314326784E-13	4	GSDMD
**5.46331482960222E-16**	1.06570055254001	0.267	0.048	9.1554229914474E-12	4	SCAMP4
**1.62435444232073E-28**	1.06079382105535	0.622	0.134	2.72209317444108E-24	4	PRRC2A
**9.85962294136849E-16**	1.05961790327535	0.289	0.056	1.65227561251453E-11	4	FADD
**1.76345750558763E-09**	1.05659911172358	0.289	0.083	2.95520208786375E-05	4	PARK7
**2.88426609568047E-15**	1.05328255156673	0.256	0.047	4.83345312314134E-11	4	FAM162A
**3.56323059582685E-08**	1.0519888546156	0.356	0.128	0.000597126183248663	4	ECHS1
**3.32931440171089E-25**	1.05030373415638	0.4	0.067	5.57926507438712E-21	4	RCC2
**1.83788559155974E-15**	1.04895569326815	0.3	0.06	3.07992867433581E-11	4	COX7A2
**2.68532737453897E-13**	1.0480687129483	0.4	0.11	4.5000716142524E-09	4	SPATS2L
**3.35984358547224E-13**	1.04700929170564	0.267	0.057	5.63042588053438E-09	4	LRP8
**1.20749325404957E-18**	1.04507032446088	0.989	0.595	2.02351719513627E-14	4	PFN1
**4.4913180572867E-20**	1.04122765950927	0.567	0.145	7.52655080040106E-16	4	PRKACA
**5.30024102401344E-15**	1.04094777813525	0.267	0.051	8.88214390804173E-11	4	SSR1
**5.76755273914666E-18**	1.03930745748249	0.3	0.054	9.66526488026198E-14	4	PACSIN2
**5.29254522533481E-15**	1.03830085049515	0.311	0.067	8.86924728861608E-11	4	SNF8
**4.36129331287874E-09**	1.03809313048629	0.256	0.073	7.3086553337222E-05	4	IRF4
**1.70995671232659E-18**	1.0344990593571	0.533	0.144	2.86554545851691E-14	4	ATP5MG
**8.99703537979605E-17**	1.03449590963812	0.533	0.149	1.50772318894622E-12	4	EMC4
**2.50420592646599E-18**	1.03355656334429	0.644	0.189	4.19654829157171E-14	4	UBB
**8.30648349026694E-20**	1.03333673338283	0.733	0.242	1.39200050329893E-15	4	PKM
**1.98112382382328E-19**	1.03296997356249	0.756	0.277	3.31996730396306E-15	4	HNRNPF
**4.40593195236099E-17**	1.03295708054347	0.3	0.056	7.38346076576655E-13	4	SLC25A24
**2.43119301165781E-16**	1.02932929782578	0.333	0.07	4.07419324893616E-12	4	BICD1
**1.77342173351465E-11**	1.02917497950521	0.256	0.059	2.97190014102385E-07	4	MOCS2
**8.05135121544329E-13**	1.02394760318569	0.378	0.102	1.34924543668399E-08	4	PEBP1
**6.3315584977813E-18**	1.02293033729375	0.367	0.076	1.06104257305819E-13	4	METTL26
**1.45754773834118E-19**	1.01956738974925	0.467	0.105	2.44255849991216E-15	4	NDUFS1
**3.23557518081934E-13**	1.01560335777523	0.378	0.099	5.42217688801704E-09	4	PSMB4
**6.93909308442591E-17**	1.01152978804909	0.433	0.105	1.16285321908809E-12	4	HDGFL2
**1.07814287545504E-17**	1.01055939367193	0.267	0.044	1.80675183068756E-13	4	HAUS6
**3.87168675139321E-16**	1.01011167485755	0.467	0.122	6.48817265798474E-12	4	MLEC
**1.95077806939266E-16**	1.00995770360276	0.433	0.11	3.26911388868822E-12	4	SMS
**1.0535481232349E-17**	1.00845552739329	0.811	0.301	1.76553594491705E-13	4	NUCB1
**9.96065241985936E-20**	1.00793509688273	0.4	0.084	1.66920613252003E-15	4	KPNB1
**2.26820874936574E-19**	1.00712914940296	0.489	0.116	3.8010642221871E-15	4	MGAT4B

2.6297891788838E-12	1.00613800651909	0.5	0.17	4.40700070597347E-08	4	MIF
1.04514156626512E-09	1.00604288353592	0.267	0.073	1.75144823674708E-05	4	URM1
1.39761975889364E-27	1.00590705122995	0.333	0.044	2.34213119195397E-23	4	G2E3
2.15168064317587E-10	1.00586603949464	0.267	0.068	3.60578642183412E-06	4	PDZD11
1.42520718036975E-12	1.00525733354465	0.267	0.06	2.38836219286363E-08	4	NUP155
1.64139991252629E-22	1.00446567540074	0.467	0.098	2.75065797341155E-18	4	TUBGCP3
3.70218159728414E-12	1.0015735435431	0.256	0.056	6.20411592072876E-08	4	MGMT
6.72625654413771E-19	0.996815244919746	0.4	0.086	1.1271860716666E-14	4	PGP
1.42331215133608E-26	0.995509228187561	0.589	0.129	2.385186503209E-22	4	UBE2A
4.68915760739789E-14	0.993108659800355	0.344	0.084	7.85809031847739E-10	4	TIPRL
1.63790143819912E-16	0.99293193513706	0.411	0.101	2.74479523013409E-12	4	NOP56
2.24889716169746E-14	0.992342481100494	0.367	0.091	3.7687018635726E-10	4	TMX1
2.29864823197564E-14	0.991653728372996	0.322	0.072	3.85207470714478E-10	4	SUCLG1
1.42440790076541E-30	0.99072535614642	0.267	0.025	2.38702276010267E-26	4	CEP131
2.49139696885829E-15	0.989516863575785	0.256	0.046	4.17508304041272E-11	4	ZSWIM9
1.46986682679252E-15	0.985804836927679	0.444	0.124	2.46320282833891E-11	4	MCM6
5.10932870352826E-16	0.985732854438082	0.467	0.119	8.56221304137265E-12	4	RAB7A
2.25724415005303E-12	0.985436879963675	0.333	0.087	3.78268974665886E-08	4	CTNNA1
1.89698494402355E-20	0.984671451645137	0.544	0.135	3.17896736919467E-16	4	NUDC
5.3789892927349E-22	0.9816371474616	0.378	0.068	9.01411025676514E-18	4	NPC2
8.76197871278258E-15	0.980683675963231	0.356	0.083	1.4683323926881E-10	4	COMT
6.74058793328891E-22	0.979109034497672	0.522	0.12	1.12958772586056E-17	4	NNT
5.25621608308138E-19	0.978752387845214	0.311	0.055	8.80836691202778E-15	4	HAUS2
2.15934201955443E-13	0.97806798814253	0.489	0.15	3.61862535636932E-09	4	GSR
4.74630695122612E-11	0.975347181831686	0.267	0.065	7.95386118886473E-07	4	USB1
2.14852599382834E-13	0.97421549445789	0.322	0.076	3.60049986045754E-09	4	LSM 6.00
2.40983114083559E-06	0.97186167667334	0.289	0.113	0.0403839502581228	4	MS4A6A
2.09398024327079E-10	0.971570260796486	0.278	0.073	3.50909209167318E-06	4	SMIM15
1.66000758362776E-11	0.970456264092318	0.322	0.086	2.7818407086434E-07	4	CD59
1.10153190491465E-12	0.9684820525106	0.278	0.063	1.84594716625597E-08	4	UBR7
7.41388899714195E-20	0.96718116907421	0.589	0.15	1.24241951814105E-15	4	PPIB
2.11212080328794E-09	0.963673416404516	0.322	0.1	3.53949204214993E-05	4	UQCR11
2.32008278275169E-14	0.962493145976657	0.333	0.076	3.88799472733528E-10	4	GRHPR
2.70095668212649E-15	0.962381932355415	0.756	0.283	4.52626320790757E-11	4	LMAN2
1.4599341656671E-17	0.96124631268598	0.356	0.073	2.44655767482492E-13	4	LSM 2.00
3.6920909903882E-21	0.960382057198627	0.456	0.099	6.18720608169255E-17	4	HP1BP3
2.3628494870668E-10	0.956736436871536	0.256	0.063	3.95966317042654E-06	4	MCTS1
1.22370568937266E-26	0.956346334507824	0.467	0.086	2.05068599425071E-22	4	SAP30
1.72914742007859E-31	0.953786073633274	0.5	0.083	2.8977052465677E-27	4	YWHAH
8.14243428149253E-11	0.953565461792666	0.322	0.089	1.36450913689252E-06	4	PRDX1
9.34096734786993E-12	0.953562490274952	0.289	0.071	1.56535930815604E-07	4	KPNA3
1.87193509808729E-16	0.95269790113464	0.522	0.142	3.13698883737469E-12	4	PTPA
6.0639800740757E-13	0.950308537083944	0.311	0.075	1.01620178081361E-08	4	LRRC8A
4.32021051386148E-31	0.946532555064022	0.678	0.142	7.23980877912907E-27	4	DEK
3.92956961862758E-15	0.946472594979604	0.667	0.232	6.58517276689609E-11	4	EIF4EBP2
9.3826555727953E-13	0.945296805080833	0.3	0.071	1.57234542088904E-08	4	LDLR
1.39045395628428E-15	0.944025829290725	0.433	0.114	2.3301227399412E-11	4	FABP1
2.30484088012002E-10	0.942552852449867	0.3	0.083	3.86245234690514E-06	4	FASTK
2.21373669953088E-11	0.941917730205545	0.478	0.159	3.70977996107384E-07	4	COX6C
8.51710424999597E-14	0.941123142908313	0.378	0.099	1.42729633021432E-09	4	ARPC5

**4.10732967471704E-20**	0.940722545162711	0.5	0.116	6.88306306889082E-16	4	CTCF
**1.23307132031455E-19**	0.940638885296068	0.767	0.243	2.06638091858313E-15	4	CTSB
**6.96479622472737E-15**	0.939085897933834	0.489	0.141	1.16716055133981E-10	4	SOD1
**1.16914244549022E-11**	0.938000628453873	0.289	0.072	1.9592489101525E-07	4	DNAJC15
**2.39822187860941E-15**	0.936969775307963	0.422	0.106	4.01894022417365E-11	4	SEM1
**1.73334251720032E-18**	0.936819044667331	0.344	0.067	2.9047353903243E-14	4	SMPD4
**2.03560421092953E-10**	0.936561298890979	0.289	0.076	3.4112655366757E-06	4	TOMM70
**3.64242625370598E-26**	0.93505415667211	0.522	0.105	6.10397791596048E-22	4	DOT1L
**7.69061445829823E-20**	0.934573723512749	0.411	0.087	1.28879317092162E-15	4	TRIM59
**4.3231290026356E-20**	0.934044653472948	0.289	0.046	7.24469958261673E-16	4	FASN
**2.79210055487035E-23**	0.933127207965669	0.589	0.143	4.67900210985172E-19	4	RPA1
**8.07546952281165E-17**	0.931083971055124	0.4	0.093	1.35328718263278E-12	4	ARPP19
**7.61664253518014E-25**	0.930569393524647	0.322	0.046	1.27639695604549E-20	4	TECR
**1.69015797537258E-15**	0.929371941418112	0.544	0.158	2.83236673512938E-11	4	SEC61A1
**6.14563022105186E-14**	0.928554663649208	0.333	0.078	1.02988471244387E-09	4	ESD
**4.46027733406762E-12**	0.922988495895378	0.367	0.103	7.47453275643051E-08	4	SDC4
**2.46636222056591E-11**	0.922391283376915	0.289	0.072	4.13312980922435E-07	4	AFG3L2
**1.16501546338482E-20**	0.921903237279909	0.322	0.054	1.95233291354029E-16	4	HSPA13
**5.93734604467267E-34**	0.921043114686433	0.811	0.192	9.94980450166247E-30	4	CNTRL
**1.1604266231603E-09**	0.920700600458993	0.256	0.067	1.94464293509203E-05	4	C12orf75
**8.77909282143109E-13**	0.919958148854608	0.489	0.157	1.47120037501542E-08	4	NAPA
**1.77392711526436E-12**	0.918778775952038	0.444	0.135	2.97274705976001E-08	4	ITPK1
**1.05000901429003E-28**	0.917763446979033	0.411	0.063	1.75960510614723E-24	4	CEP135
**6.43562903323947E-11**	0.915667406616725	0.267	0.065	1.07848271339027E-06	4	PRELID1
**3.80484798161893E-12**	0.915276900730371	0.478	0.152	6.37616424759701E-08	4	ALDH3A2
**2.52319116227656E-13**	0.91378805007442	0.433	0.121	4.22836374974307E-09	4	NDUFB10
**3.01745860468076E-14**	0.913338406473348	0.356	0.086	5.05665712972402E-10	4	APLP2
**4.8156846418074E-12**	0.912315380422056	0.3	0.073	8.07012432274085E-08	4	TMEM258
**2.55621718370085E-16**	0.908445768878215	0.444	0.11	4.28370875644588E-12	4	ATP5MC3
**1.00245615941756E-13**	0.908042235950609	0.356	0.088	1.67991603195195E-09	4	EHMT2
**7.25113536215034E-17**	0.907069240596619	0.367	0.079	1.21514526398915E-12	4	SNX3
**1.49170688641625E-12**	0.90598790353028	0.289	0.067	2.49980240025635E-08	4	COX6B1
**1.20978315491084E-11**	0.90472625799178	0.311	0.079	2.02735461099958E-07	4	CCDC124
**1.85959363300281E-15**	0.904493261158152	0.456	0.129	3.1163070101861E-11	4	GLUL
**3.08795856973607E-11**	0.90352778571565	0.344	0.097	5.17480097116371E-07	4	PSMC2
**3.52316817097795E-14**	0.903131738138637	0.478	0.137	5.90412522092485E-10	4	CALR
**3.53827701798447E-15**	0.903100009786542	0.322	0.069	5.92944462673837E-11	4	CHID1
**1.00541078414997E-24**	0.90277448273626	0.5	0.102	1.68486739207852E-20	4	ADA
**9.42433379477802E-14**	0.901947474138266	0.378	0.097	1.5793298573289E-09	4	TMBIM6
**8.0032983102144E-15**	0.899571565198459	0.522	0.152	1.34119273082573E-10	4	POLR2L
**4.6464967560582E-09**	0.899137809180648	0.267	0.076	7.78659926380233E-05	4	ABHD10
**5.90928819115699E-09**	0.898996512076332	0.289	0.087	9.90278515074088E-05	4	LMNA
**2.98579752601584E-17**	0.898519271966301	0.578	0.173	5.00359949409735E-13	4	ADCY3
**1.56521970854412E-16**	0.89790470341201	0.578	0.169	2.62299518757823E-12	4	SF3B5
**7.67032445045518E-20**	0.897780019654304	0.678	0.206	1.28539297140728E-15	4	COTL1
**9.19728282879316E-12**	0.897354413840339	0.3	0.075	1.54128065644916E-07	4	PSMG3
**1.9246616259392E-13**	0.896499612604118	0.456	0.131	3.2253479527489E-09	4	DNAJB11
**1.47505149007796E-11**	0.895573796173595	0.4	0.121	2.47189128707265E-07	4	SEC11C
**7.53402429830221E-17**	0.893706218524332	0.311	0.06	1.26255179190948E-12	4	MTHFD2
**2.74211568763681E-15**	0.892678248562437	0.3	0.062	4.59523746934176E-11	4	SAP130

7.83565772547279E-11	0.892331025692276	0.367	0.112	1.31309952163473E-06	4	C9orf16
8.62160104233288E-28	0.891117326664291	0.656	0.15	1.44480790267414E-23	4	SMC3
1.64199079846729E-15	0.889990601037865	0.256	0.046	2.75164818007148E-11	4	TOR3A
2.22737027146759E-09	0.887164520569761	0.256	0.068	3.7326271009254E-05	4	IDH1
1.05499963429986E-10	0.887092967692921	0.256	0.063	1.76796838715971E-06	4	NDRG3
5.25328908536373E-20	0.886443853609142	0.611	0.168	8.80346184925254E-16	4	RAB8A
1.87620172989306E-19	0.884341393843177	0.422	0.091	3.14413885895479E-15	4	ENY2
1.26067231337753E-12	0.884272524536327	0.311	0.076	2.11263466275806E-08	4	MYO19
2.72338441505163E-19	0.882776095864233	0.289	0.047	4.56384760274351E-15	4	NEMP2
1.52197046569312E-08	0.881521531009366	0.278	0.084	0.000255051810640853	4	ERG28
1.93566099098701E-16	0.879081714359701	0.522	0.15	3.24378068869604E-12	4	PA2G4
5.70253274309364E-18	0.878796019608142	0.467	0.115	9.55630437087633E-14	4	TEX264
1.27651301842818E-09	0.876719469314661	0.4	0.136	2.13918051628195E-05	4	PPP1R7
5.34177544961251E-10	0.875951070955783	0.4	0.131	8.95174729846064E-06	4	COPE
1.44219385165841E-10	0.873585226800691	0.4	0.128	2.41682845660916E-06	4	HYOU1
4.18103960522982E-19	0.873103322786199	0.789	0.278	7.00658617044413E-15	4	NUP210
4.62224301822768E-22	0.872686143363115	0.511	0.113	7.74595484994594E-18	4	YWHAQ
7.94095882029758E-24	0.870750014256706	0.578	0.132	1.33074587910547E-19	4	MCMBP
1.55126405877974E-14	0.870682746371316	0.278	0.056	2.59960830970309E-10	4	ACP1
6.80475640865184E-17	0.869048311996653	0.378	0.084	1.14034107896187E-12	4	C2CD5
1.43479727098649E-15	0.865763252995521	0.344	0.077	2.40443326671916E-11	4	GTPBP2
4.23018274051608E-16	0.864682569801877	0.767	0.283	7.08894023655684E-12	4	RCSD1
9.27925307550365E-11	0.863919519367704	0.433	0.142	1.5550172303929E-06	4	MYDGF
1.49209322300573E-12	0.863865963475671	0.422	0.123	2.500449823113E-08	4	VKORC1
4.9889216325037E-16	0.86314425388882	0.467	0.121	8.36043487174969E-12	4	GNAI3
1.64817377654623E-09	0.861559343439531	0.322	0.097	2.76200961473618E-05	4	RPN2
3.52611681666989E-12	0.861392891194342	0.4	0.114	5.90906656137541E-08	4	HSPA5
4.14767211016274E-18	0.859977205431012	0.533	0.143	6.95066892221072E-14	4	SUPT16H
2.21941532469672E-20	0.855761562936786	0.556	0.14	3.71929620112676E-16	4	NUP62
8.16422924207547E-21	0.853182581214813	0.467	0.103	1.36816153638701E-16	4	PPP2R5D
4.74404221281223E-14	0.852712958047949	0.322	0.074	7.95006594023073E-10	4	PDS5B
9.4871748739564E-21	0.851725857738182	0.467	0.102	1.58986076537761E-16	4	RABL6
9.7855736421891E-17	0.850687350652795	0.556	0.158	1.63986643095805E-12	4	RNH1
4.27834610685113E-10	0.849398735533157	0.256	0.065	7.16965240586112E-06	4	MAPRE1
8.43921001019523E-15	0.847227451158685	0.6	0.189	1.41424281350852E-10	4	ATP5MC2
5.48253910547364E-11	0.846151483337772	0.333	0.096	9.18763903295273E-07	4	GSTP1
1.34132338987121E-15	0.845354042371254	0.378	0.092	2.24778973674618E-11	4	DCAF15
5.65506735343104E-14	0.845123336777409	0.4	0.108	9.47676187087974E-10	4	GYPC
1.91138803116837E-14	0.843614217052569	0.389	0.096	3.20310406263195E-10	4	NDUFB11
1.06234882953546E-11	0.842826048805299	0.322	0.085	1.78028416853553E-07	4	GDI2
4.57736690772924E-13	0.842742833118084	0.344	0.087	7.67075146397266E-09	4	SMIM12
1.53954417584008E-27	0.84162988729946	0.689	0.164	2.57996812987281E-23	4	ANKRD36C
8.45298099604109E-12	0.841468266907843	0.311	0.079	1.41655055531657E-07	4	XRCC5
3.27245991952161E-22	0.839742789090452	0.611	0.152	5.48398833313432E-18	4	PYGO2
1.57270459228049E-09	0.837178648991841	0.3	0.087	2.63553835574365E-05	4	FDPS
9.11611754559976E-09	0.836187793903281	0.256	0.072	0.000152767897829161	4	PGM1
3.21450384285898E-11	0.833556580275951	0.344	0.099	5.38686553986308E-07	4	KATNAL1
1.63094421918243E-16	0.832763173437458	0.267	0.047	2.73313632250592E-12	4	ARL6IP6
7.02037769275233E-24	0.832554605310601	0.456	0.087	1.17647489375144E-19	4	SPATA5
3.87067678121614E-09	0.831887359010664	0.256	0.07	6.486480149962E-05	4	NUTF2

3.70652902355617E-16	0.83152090553358	0.489	0.133	6.21140133767543E-12	4	RTN4
1.90597062851013E-09	0.827425218144546	0.344	0.108	3.19402557925728E-05	4	GPI
3.00005804916102E-29	0.82698006864555	0.511	0.091	5.02749727878403E-25	4	CANT1
7.4566642510014E-13	0.826414012449492	0.344	0.087	1.24958779518281E-08	4	MFF
1.45886630304702E-10	0.824563750640837	0.267	0.068	2.44476815064619E-06	4	PAGR1
9.58811516864325E-10	0.823601093118314	0.289	0.08	1.60677633996124E-05	4	BEX3
4.05977887254926E-13	0.821462043243607	0.3	0.068	6.80337743461805E-09	4	COMMD4
2.23469177996488E-10	0.818462126315745	0.478	0.168	3.74489648486515E-06	4	TMED5
1.59615971124267E-11	0.816818986699818	0.322	0.085	2.67484444410047E-07	4	MESD
1.04438467729952E-25	0.816186762810683	0.444	0.08	1.75017984221854E-21	4	ETHE1
1.0916773781859E-14	0.815678444737158	0.389	0.1	1.82943295036393E-10	4	AP2A1
1.20186195511106E-22	0.8155710337067	0.722	0.2	2.01408026437512E-18	4	NONO
4.8114576523829E-16	0.814040865809011	0.622	0.198	8.06304073386327E-12	4	PPM1G
8.15463480911388E-18	0.812379684549152	0.4	0.089	1.3665537013113E-13	4	RALY
3.42201056401325E-25	0.811351140022231	0.511	0.104	5.73460530317341E-21	4	SELENOK
2.20324568810047E-13	0.811277409866391	0.289	0.064	3.69219912411876E-09	4	TRAP1
1.02623003614834E-14	0.810612385983496	0.578	0.184	1.71975629457739E-10	4	MTDH
2.55825700212055E-06	0.809586737093326	0.267	0.096	0.0428712708415362	4	ATOX1
5.34172176592892E-19	0.808012707298928	0.467	0.108	8.95165733534368E-15	4	RBMX
7.02277348931922E-11	0.807748042506939	0.411	0.127	1.17687638134012E-06	4	EDF1
2.87989650610311E-19	0.8063369538351	0.533	0.134	4.82613056492759E-15	4	PRPF19
7.56855538833803E-14	0.806105523085973	0.411	0.112	1.26833851197769E-09	4	SMC6
1.51427417066518E-10	0.802931375851046	0.356	0.104	2.5376206552007E-06	4	JTB
9.00901712596399E-23	0.801386797851111	0.444	0.088	1.50973108996905E-18	4	NUP85
6.53229414400792E-14	0.801306082174762	0.278	0.058	1.09468185265285E-09	4	EIPR1
2.09174425592827E-10	0.800040853292887	0.267	0.068	3.50534502408459E-06	4	TOR1A
1.1937173512144E-22	0.79992167083682	0.611	0.155	2.00043153716509E-18	4	EIF4A3
3.46307483258458E-09	0.799191027671413	0.256	0.069	5.80342080444524E-05	4	EPN1
1.07561327418887E-12	0.797412411957289	0.289	0.067	1.80251272488572E-08	4	PPP5C
2.81270788697409E-17	0.796830825654549	0.311	0.06	4.71353587699118E-13	4	GMPPB
6.29665003120331E-11	0.796494049614692	0.433	0.139	1.05519261222905E-06	4	SNRPC
6.97662224889243E-19	0.796383471564745	0.811	0.301	1.16914235646939E-14	4	H1FX
4.72076791063538E-17	0.795524514551643	0.333	0.068	7.91106286464277E-13	4	NUP93
4.59864804922463E-15	0.795328095684315	0.278	0.055	7.70641440089064E-11	4	TAF4
2.98423592867262E-22	0.791347800668299	0.789	0.243	5.00098256926957E-18	4	LBR
2.02959851436498E-08	0.791283824300224	0.267	0.081	0.000340120119037284	4	MSH6
1.1050053560721E-10	0.790522734470803	0.267	0.067	1.85176797570562E-06	4	TXN2
5.31907630393298E-10	0.788896592792384	0.444	0.158	8.91370807013088E-06	4	CXCR3
1.276565505178E-13	0.78687022323688	0.589	0.189	2.13926847357729E-09	4	HINT1
5.20052857359428E-11	0.78573466500314	0.411	0.126	8.71504578362929E-07	4	DDT
1.68340292262154E-13	0.785414335307092	0.333	0.079	2.82104661772919E-09	4	GTF3C6
4.20513836080144E-13	0.785381091858327	0.433	0.121	7.04697086503106E-09	4	CHMP2A
6.62583041164267E-18	0.784659318078544	0.533	0.14	1.11035666038308E-13	4	GC
7.49839508782063E-27	0.781159040684771	0.478	0.087	1.25658104881698E-22	4	TAF15
1.1150935823733E-17	0.777852249971717	0.278	0.047	1.86867382534118E-13	4	PGM2
4.26264387708127E-18	0.775940513129032	0.478	0.117	7.14333860921279E-14	4	MED4
2.03359711099018E-09	0.774565719361072	0.278	0.078	3.40790203859735E-05	4	MLLT1
8.74162489742579E-13	0.773844713883905	0.267	0.059	1.46492150031061E-08	4	NCBP1
6.44528635135943E-11	0.773242108189188	0.411	0.13	1.08010108676081E-06	4	CD63
9.06312726630076E-10	0.771973458626001	0.389	0.13	1.51879886728668E-05	4	PSMD3

1.1348226145927E-21	0.771783368252765	0.667	0.176	1.90173573753445E-17	4	HNRNPUL2
1.19330315824816E-19	0.770788546925276	0.678	0.193	1.99973743259226E-15	4	RNF167
4.08505346835904E-18	0.769346585417407	0.311	0.057	6.84573260227608E-14	4	LRRFIP2
9.5830385958131E-10	0.768955521869879	0.311	0.09	1.60592560788636E-05	4	STMP1
5.14242110147765E-19	0.767839196967605	0.722	0.226	8.61766928185625E-15	4	HNRNPD
5.38905793754153E-09	0.767434884493475	0.311	0.095	9.0309832917321E-05	4	CSTB
8.13253389292967E-19	0.767084168741955	0.267	0.042	1.36285002977715E-14	4	PMAIP1
1.13654422231576E-10	0.762474222658759	0.256	0.062	1.90462080775675E-06	4	UPF3A
1.76430086403947E-11	0.762278768550071	0.333	0.091	2.95661538795735E-07	4	RTCB
1.47907795908119E-08	0.762084106563654	0.356	0.122	0.000247863884382825	4	VPS28
6.20657471652116E-11	0.758376405596992	0.289	0.074	1.04009779099462E-06	4	ABHD12
9.16244953754883E-15	0.758334353862171	0.611	0.193	1.53544329350243E-10	4	RAB1B
9.67608154526368E-11	0.757219817772208	0.267	0.067	1.62151774535529E-06	4	SUPT4H1
1.68798238247519E-10	0.756178129148863	0.322	0.09	2.82872087655192E-06	4	NIT1
4.67875953693438E-07	0.755848084360264	0.256	0.084	0.00784066523199463	4	P2RY11
1.65908269241576E-23	0.755537791378869	0.322	0.048	2.78029077595033E-19	4	EXOSC8
2.17822985233153E-12	0.754467211363857	0.311	0.077	3.65027758653718E-08	4	TMEM189
1.59456271731776E-24	0.751153269139037	0.278	0.035	2.6721682016811E-20	4	SARNP
1.03459894377482E-14	0.751149425740564	0.3	0.063	1.73378090997784E-10	4	CDK11B
4.35700144005939E-12	0.749585295643166	0.3	0.074	7.30146301325152E-08	4	GIGYF2
4.67759805644591E-12	0.749132786751154	0.311	0.077	7.83871882299206E-08	4	PRCC
1.88664944104875E-20	0.747553297017644	0.578	0.148	3.1616471333095E-16	4	HNRNPM
3.9746935383897E-13	0.747168465370077	0.4	0.109	6.66079143163345E-09	4	COPS6
3.50913106440353E-12	0.745969394537262	0.422	0.124	5.88060183772743E-08	4	SLC25A11
6.26825394628121E-17	0.7454991653262	0.556	0.164	1.0504339963178E-12	4	FZR1
2.16090028115775E-10	0.745004570749148	0.411	0.132	3.62123669116416E-06	4	EIF6
5.138763831694E-10	0.744602951823001	0.622	0.245	8.6115404291528E-06	4	TMCO1
1.06379942627976E-13	0.742424079204466	0.589	0.19	1.78271507855962E-09	4	ST6GAL1
1.04369594256569E-11	0.739380092574618	0.322	0.085	1.74902566055158E-07	4	TRAPPC1
4.7454444623083E-11	0.739289567480976	0.267	0.065	7.95241582993624E-07	4	PSME2
2.7745268154841E-14	0.738276861048242	0.278	0.057	4.64955203738826E-10	4	TMEM128
6.23997410252511E-13	0.733675583846286	0.278	0.061	1.04569486010116E-08	4	CCT5
2.23837104182048E-14	0.73254511268446	0.378	0.094	3.75106219188276E-10	4	PHACTR4
6.78602597882536E-18	0.732424691728167	0.5	0.129	1.13720223353155E-13	4	C12orf57
1.86172892128632E-12	0.731608327849034	0.3	0.072	3.11988532629162E-08	4	CAP1
5.94413141290941E-14	0.731578640101286	0.967	0.506	9.9611754217536E-10	4	CFL1
2.60163247940933E-11	0.731010264995832	0.289	0.074	4.35981570899416E-07	4	TOX2
1.80627861147661E-20	0.730699955689929	0.389	0.075	3.0269616971125E-16	4	NUP107
6.39062815061915E-13	0.730523676856965	0.467	0.141	1.07094146548076E-08	4	SLC1A5
1.71933550919392E-19	0.728921533640854	0.578	0.151	2.88126244630716E-15	4	ZFP91
2.00374205343459E-15	0.727607189721712	0.578	0.18	3.35787093314568E-11	4	ELAVL1
4.38563161241629E-09	0.726746345628763	0.278	0.079	7.34944145608722E-05	4	CAMK1
9.54018831379575E-11	0.726714065072071	0.289	0.076	1.59874475762589E-06	4	UBXN7
4.34690914155322E-12	0.724449160667977	0.322	0.084	7.28455033941488E-08	4	ATL3
3.28357662873834E-14	0.724225740974762	0.289	0.062	5.50261771443971E-10	4	DKC1
1.67432515743021E-14	0.722982952884986	0.3	0.064	2.80583409882154E-10	4	CCT4
2.05220046319282E-16	0.719674853218299	0.6	0.181	3.43907753621853E-12	4	SLC38A2
3.02527686180886E-15	0.71911919272921	0.256	0.047	5.06975896501929E-11	4	CXorf56
2.8487760807475E-15	0.718295823479476	0.411	0.105	4.77397895611666E-11	4	ZNF384
1.90774747807196E-14	0.717814291753477	0.478	0.136	3.197003223753E-10	4	SLC25A39

1.01696744141072E-13	0.716610313752856	0.522	0.16	1.70423403831608E-09	4	SERF2
5.23390022505821E-11	0.714667255349732	0.3	0.078	8.77096999715254E-07	4	SLC39A9
6.82157687074131E-17	0.712690102582501	0.511	0.136	1.14315985199883E-12	4	DPP9
2.40714834435098E-08	0.711846574682466	0.322	0.105	0.000403389919546338	4	PLEKHF1
5.33105799520596E-13	0.710573114754865	0.5	0.151	8.93378698836615E-09	4	COPB2
3.9355218856563E-22	0.709001255745784	0.544	0.125	6.59514757598283E-18	4	KHDRBS1
2.02554777483842E-13	0.708461329197283	0.633	0.216	3.39441296107423E-09	4	TRAM1
1.12454851857805E-18	0.70775446079874	0.478	0.113	1.88451840743309E-14	4	RAD23A
4.55767448764608E-16	0.707362462953449	0.611	0.193	7.6377509063973E-12	4	NFATC2IP
1.29467203324695E-14	0.707123945610628	0.344	0.081	2.16961139331524E-10	4	THOC2
3.72521917928643E-16	0.706782583550554	0.5	0.14	6.24272230064819E-12	4	SRPK1
3.65662190606422E-11	0.706362544100554	0.389	0.116	6.12776699018242E-07	4	LPGAT1
1.31625475975326E-09	0.705758936362597	0.256	0.067	2.20577972639452E-05	4	PSMD8
6.96473915466669E-13	0.704413971890262	0.667	0.246	1.16715098753904E-08	4	UBA52
1.48943360067135E-20	0.704020320376699	0.544	0.134	2.49599282800505E-16	4	SF3A2
6.18430783434718E-13	0.7037460666219	0.311	0.075	1.0363663068799E-08	4	RUVBL1
5.83714581487325E-12	0.701346367120414	0.311	0.079	9.7818889565646E-08	4	PIH1D1
1.31753173071629E-10	0.700358833280243	0.367	0.111	2.20791967433436E-06	4	SHC1
2.39372838103585E-14	0.69984738924192	0.533	0.158	4.01141002093988E-10	4	NDUFS2
7.82527056300034E-22	0.699441659509131	0.378	0.069	1.3113588409476E-17	4	ATP1A1
5.64398033903817E-17	0.699261797252215	0.467	0.119	9.45818225216017E-13	4	FAR1
1.63342442076707E-11	0.697600434788008	0.322	0.084	2.73729264432146E-07	4	PSMC4
1.47467063825924E-17	0.697374749597007	0.722	0.238	2.47125305559483E-13	4	LDHB
6.83961234414372E-28	0.695893541784605	0.5	0.091	1.1461822366316E-23	4	CENPT
1.86265450038773E-21	0.69546136724783	0.311	0.049	3.12143641174976E-17	4	RANGRF
7.01679429766189E-10	0.695073614060143	0.278	0.075	1.17587438840218E-05	4	TIMM23
1.24042263627032E-15	0.69410718180209	0.3	0.06	2.07870025386181E-11	4	TCP1
2.51864474627895E-10	0.693985998065285	0.256	0.064	4.22074486581427E-06	4	WRAP73
1.2723302269231E-14	0.692958245630616	0.467	0.132	2.13217099427772E-10	4	TXLNA
5.5858228664702E-13	0.692719381533107	0.256	0.053	9.36072195963076E-09	4	KLHL15
1.27250886720912E-07	0.692270380015464	0.267	0.084	0.00213247035966904	4	GOT2
1.47157795764498E-19	0.692190109175212	0.411	0.088	2.46607034142146E-15	4	RNF168
8.30021390503456E-13	0.69131729422186	0.3	0.07	1.39094984620569E-08	4	PYURF
1.9665005280265E-16	0.690926488683335	0.367	0.081	3.2954615848668E-12	4	TTLL5
6.65384708840713E-08	0.689392215909562	0.489	0.195	0.00111505169507527	4	CUTA
5.72714568738837E-09	0.685365098984438	0.289	0.086	9.59755074292542E-05	4	ACTR10
1.20521522317088E-14	0.685072697829877	0.356	0.085	2.01969967098976E-10	4	SSR4
1.43878206768369E-20	0.68446148553935	0.5	0.116	2.41111098902433E-16	4	NDUFAF3
5.33647994752438E-09	0.684413370457999	0.367	0.121	8.94287309606136E-05	4	NCOA4
9.21751548073924E-10	0.682410991449084	0.322	0.096	1.54467124426228E-05	4	MBOAT1
2.02724008546073E-16	0.682084839984243	0.389	0.089	3.3972489352151E-12	4	HSPB11
1.76440602292231E-15	0.681608058375007	0.311	0.065	2.9567916132132E-11	4	ATP6V0B
3.18406099122947E-15	0.680854487604873	0.344	0.078	5.33584940910235E-11	4	PSMD7
2.5059968040171E-12	0.679728663690011	0.289	0.068	4.19954944417186E-08	4	LDAH
1.50430659340601E-18	0.677245941707901	0.578	0.152	2.5209169892298E-14	4	PSMC5
3.65304552657437E-17	0.677060162760482	0.433	0.103	6.12177369343333E-13	4	PTPRA
4.26501024188528E-14	0.676924754639235	0.756	0.288	7.14730416335136E-10	4	PPP1R18
7.01005702658749E-19	0.676364430144705	0.478	0.115	1.17474535651553E-14	4	IRAK1
3.79001655555671E-17	0.676213327949314	0.5	0.132	6.35130974380193E-13	4	PTBP1
2.84541906521313E-13	0.675839561490678	0.289	0.064	4.76835326948416E-09	4	GABPB2

4.18460038219647E-18	0.673729467936756	0.689	0.212	7.01255332048484E-14	4	MAT2A
9.09033502754578E-10	0.673633786888538	0.5	0.181	1.52335834391612E-05	4	PUF60
1.39983160837611E-19	0.672814049432232	0.522	0.129	2.34583780931668E-15	4	CCNG2
1.90076212566751E-12	0.672315173322106	0.256	0.055	3.18529717019361E-08	4	CEP44
6.17627968908703E-13	0.67199798255581	0.744	0.284	1.0350209502972E-08	4	BST2
5.51939607805362E-10	0.671490371204049	0.333	0.1	9.24940394760225E-06	4	AP1B1
1.95194520596228E-11	0.671356315047186	0.322	0.086	3.27106977615158E-07	4	LARP7
9.10845864818288E-14	0.670197168050372	0.356	0.089	1.52639550026249E-09	4	CCDC47
4.18464287499324E-10	0.670076592618831	0.278	0.074	7.01262452991366E-06	4	PSMD9
2.79005895003673E-07	0.670051207101521	0.3	0.102	0.00467558078847155	4	HTATIP2
1.1232150406765E-12	0.668994525245281	0.278	0.062	1.88228376516567E-08	4	CNOT10
9.83727649527622E-10	0.668374302935933	0.311	0.09	1.64853079507839E-05	4	XBP1
1.18331177287118E-14	0.668293793838931	0.311	0.068	1.98299386897753E-10	4	ADK
2.44099527300756E-09	0.665074121414028	0.267	0.074	4.09061987850608E-05	4	GSS
4.85762388560156E-11	0.664056441514503	0.378	0.11	8.1404061074911E-07	4	OCIAD2
4.91987597697689E-14	0.663558855752447	0.4	0.105	8.24472816221787E-10	4	RBM15B
7.67656426700912E-16	0.663555959144382	0.311	0.063	1.28643863986539E-11	4	GIT1
2.5840550433459E-21	0.662041582341163	0.322	0.052	4.33035944163905E-17	4	ATP5PB
4.99323190095037E-19	0.660971269142451	0.789	0.27	8.36765801961263E-15	4	HNRNPA3
6.19233130297023E-10	0.660806737521287	0.344	0.107	1.03771087975175E-05	4	SUSD3
4.58269043113773E-19	0.660163275111595	0.411	0.087	7.67967262450061E-15	4	SFPQ
1.74497433498168E-09	0.656838966117692	0.256	0.068	2.92422799056229E-05	4	LEO1
1.14088888347093E-10	0.656655867418195	0.3	0.079	1.91190159092058E-06	4	YWHAG
2.60263973627826E-10	0.655773417179016	0.378	0.117	4.3615036700551E-06	4	CNPPD1
2.30515004561245E-10	0.654789195655417	0.289	0.078	3.86297044643735E-06	4	E2F3
3.34201677409909E-12	0.654425385462334	0.422	0.12	5.60055171003526E-08	4	ATP5IF1
3.23942778313212E-17	0.654419344917903	0.567	0.162	5.4286330789728E-13	4	SET
7.39817922983494E-07	0.653405137136228	0.267	0.089	0.0123978687533574	4	DNPH1
3.37231416467448E-16	0.653217417233354	0.467	0.12	5.65132407716149E-12	4	CAND1
1.18067541822002E-16	0.649319439436089	0.367	0.081	1.97857586585311E-12	4	MLH1
4.99215391616694E-13	0.64765746091511	0.878	0.418	8.36585153271256E-09	4	SPN
2.27488822480157E-14	0.646868429204254	0.378	0.094	3.81225768712247E-10	4	SNRNP40
3.13830972530374E-13	0.646830287668608	0.278	0.06	5.25917943766401E-09	4	INTS6L
1.95490481763923E-10	0.645820995725648	0.289	0.078	3.27602949339983E-06	4	ARID3B
9.07561585676882E-14	0.645510167654438	0.478	0.142	1.52089170527732E-09	4	IMMT
2.50585158801197E-08	0.644165665225122	0.311	0.1	0.000419930609119046	4	WWP1
1.54742079107009E-11	0.64376103552604	0.267	0.062	2.59316776167526E-07	4	MAGOH
6.93947461615522E-18	0.643288315931143	0.5	0.127	1.16291715617529E-13	4	CHD4
2.16616256519513E-16	0.642613075721065	0.322	0.066	3.63005522675399E-12	4	EXOSC2
5.04727655527224E-17	0.642062581295151	0.8	0.289	8.45822605132522E-13	4	PSMB9
4.71637299264116E-15	0.640906105844561	0.267	0.051	7.90369786106805E-11	4	THOC6
1.53307923452604E-11	0.640903787525515	0.256	0.059	2.56913418121875E-07	4	STK35
7.9686564566434E-10	0.64034759302702	0.356	0.11	1.3353874490043E-05	4	RCC1L
1.301593299662E-12	0.63985798183187	0.422	0.121	2.18121005157358E-08	4	UGDH
2.97732206188824E-11	0.638322049375571	0.311	0.081	4.98939631131232E-07	4	BRMS1
2.10535003234039E-17	0.638113721412285	0.389	0.084	3.52814558419602E-13	4	GTF2A1
3.99921768434798E-12	0.637833718864178	0.3	0.073	6.70188899543035E-08	4	FAF2
2.86703811406198E-09	0.63738184073679	0.367	0.116	4.80458247154506E-05	4	ALDH9A1
4.24137453020794E-13	0.637123979019816	0.611	0.218	7.10769543772247E-09	4	SLBP
2.88753889893642E-10	0.636569531026242	0.389	0.127	4.83893768683765E-06	4	GBP1

1.6958411323268E-10	0.635430772390719	0.278	0.071	2.84189056955325E-06	4	YIF1B
1.68061637021642E-17	0.633296195229443	0.678	0.221	2.81637691320868E-13	4	TMEM109
1.83507439303945E-11	0.632976808357964	0.389	0.114	3.07521766785551E-07	4	KEAP1
1.06299903844466E-17	0.630642166592759	0.367	0.078	1.78137378862556E-13	4	BRD8
3.46324344651438E-14	0.630327798226583	0.533	0.161	5.80370336766879E-10	4	CLIC1
3.14258031925572E-09	0.629339196638272	0.311	0.092	5.26633609900873E-05	4	CASC4
5.0599625648951E-13	0.629265161402321	0.578	0.197	8.47948526625122E-09	4	PRKDC
1.58619490708156E-09	0.628742157135114	0.267	0.071	2.65814542528728E-05	4	TMEM147
5.06426369141947E-21	0.627763632654572	0.489	0.107	8.48669309408075E-17	4	TDP1
6.50215758593251E-15	0.627528608498005	0.289	0.059	1.08963156825057E-10	4	PHF6
1.85170800665885E-11	0.626297958370905	0.367	0.104	3.10309227755889E-07	4	TMEM250
1.3242352945254E-10	0.626044611334061	0.367	0.11	2.21915350656566E-06	4	PIN1
3.77061470279582E-11	0.625436652603689	0.322	0.087	6.31879611894523E-07	4	S100A11
5.30936373390395E-08	0.624665484892456	0.433	0.162	0.000889743174527624	4	DECR1
1.71120917535456E-07	0.623334256938492	0.278	0.091	0.00286764433605917	4	MCRIP1
6.62374726832169E-19	0.622558493934597	0.311	0.054	1.11000756722535E-14	4	SPPL2B
1.14208533296675E-11	0.618433363087198	0.378	0.11	1.91390660098569E-07	4	YEATS2
1.93088312939304E-13	0.61829677125033	0.267	0.055	3.23577394823686E-09	4	EMC1
1.58453251414339E-15	0.618007551334899	0.867	0.357	2.6553595872015E-11	4	H3F3A
1.42416157940097E-16	0.615384599124613	0.356	0.077	2.38660997476015E-12	4	TAOK2
4.36221504154434E-15	0.61490486619828	0.511	0.144	7.31019996662E-11	4	UBA2
1.63500700216197E-09	0.613338718645687	0.322	0.098	2.73994473422303E-05	4	ANXA4
1.99737785496957E-13	0.612789928121698	0.278	0.059	3.347205809358E-09	4	ELOB
2.68886279723448E-11	0.612420054517063	0.656	0.246	4.50599627560553E-07	4	TM9SF2
1.09703744639075E-10	0.610227634726487	0.678	0.278	1.83841535266162E-06	4	TFRC
6.83184542810119E-11	0.610196183069	0.356	0.102	1.1448806568412E-06	4	GPS1
7.22627065777982E-13	0.609442751291186	0.267	0.058	1.21097843683074E-08	4	BICD2
2.65101711020504E-12	0.609206402619679	0.4	0.116	4.44257447328161E-08	4	MED14
4.88317790827387E-10	0.609056895123357	0.956	0.574	8.18322953868536E-06	4	CORO1A
2.80823800723146E-12	0.608949946862058	0.344	0.09	4.70604525251848E-08	4	AZIN1
1.67121356941375E-12	0.608676471936328	0.778	0.292	2.80061969962356E-08	4	SLC9A3R1
5.33126139705279E-08	0.608659751283899	0.333	0.112	0.000893412784918106	4	DDRGK1
1.68290337272613E-07	0.607831977252364	0.333	0.117	0.00282020947201445	4	CDIPT
1.75738557265952E-09	0.60758966388815	0.444	0.15	2.94502674266283E-05	4	COX6A1
1.15261357803523E-09	0.607239684002716	0.278	0.076	1.93154983407145E-05	4	MIEN1
1.05491472323018E-08	0.606831334407175	0.944	0.63	0.000176782609318914	4	LCP1
5.18117355750736E-09	0.606378513055082	0.411	0.143	8.68261064767084E-05	4	FAM3C
7.04902276526785E-11	0.603647314534192	0.433	0.138	1.18127523500359E-06	4	POLR3GL
3.47418610392097E-08	0.601866983453107	0.356	0.123	0.000582204107295076	4	FDFT1
1.25071718413635E-13	0.601758538645264	0.478	0.14	2.0959518571757E-09	4	TPM4
1.95560812953793E-19	0.599478173889725	0.544	0.14	3.27720810347966E-15	4	NDE1
4.16959495142378E-11	0.598476978657104	0.333	0.092	6.98740721959598E-07	4	GRN
3.16539131950362E-13	0.598154747412121	0.344	0.086	5.30456277322416E-09	4	ELAC2
5.06275737552917E-18	0.596744854204577	0.722	0.23	8.48416880991179E-14	4	RHOA
6.22196697659627E-12	0.595745785563149	0.267	0.062	1.042677225938E-07	4	U2AF2
1.31578139712175E-18	0.595477451172657	0.489	0.121	2.20498646529664E-14	4	NUP205
2.44904282160574E-07	0.594744059860427	0.333	0.118	0.0041041059604469	4	TNFSF10
2.77545290414425E-09	0.594270214075775	0.444	0.159	4.65110397676493E-05	4	NACC1
1.57854003057094E-10	0.594007590202948	0.533	0.186	2.64531738323078E-06	4	NFE2L1
2.02708750600307E-19	0.593821046797503	0.444	0.1	3.39699324255995E-15	4	SH3GLB2

3.652455968727E-10	0.593097057592258	0.344	0.102	6.12078571239271E-06	4	GOPC
1.23542816060422E-13	0.591133506758651	0.8	0.319	2.07033051154056E-09	4	CBFB
7.56229112317736E-10	0.591004241796709	0.256	0.066	1.26728874642206E-05	4	TMEM123
2.17282451564798E-12	0.589651589384939	0.356	0.094	3.64121932332289E-08	4	G6PD
2.12448264023954E-17	0.589534248106183	0.444	0.11	3.56020800851342E-13	4	PI4K2B
3.38769970381636E-19	0.589464646453354	0.633	0.177	5.67710716365546E-15	4	RNF4
3.31760048695952E-20	0.589047847748533	0.656	0.177	5.55963489604676E-16	4	SEC11A
3.31219730079227E-13	0.58858617281206	0.4	0.109	5.55058023666769E-09	4	LEMD2
9.44750141327318E-13	0.588018135167364	0.589	0.201	1.58321228683632E-08	4	TWF2
1.33701408166334E-19	0.587783206992239	0.5	0.115	2.24056819805143E-15	4	SP1
5.89958157821391E-08	0.586668293046881	0.311	0.1	0.000988651880877087	4	UQCR10
3.61387957177E-12	0.585157041520108	0.333	0.087	6.05613938637216E-08	4	NAA38
5.55784111595174E-17	0.584785828231275	0.689	0.223	9.31383014211193E-13	4	POLR2A
1.2331431667128E-09	0.584478119897171	0.322	0.096	2.06650131877732E-05	4	HAT1
3.26550625579046E-14	0.582994586625523	0.622	0.218	5.47233538345366E-10	4	DCAF7
3.87194286594505E-07	0.58225006449344	0.256	0.083	0.00648860185475071	4	CALU
3.69670876619602E-11	0.580010578815397	0.644	0.242	6.19494455039129E-07	4	SSR2
3.36894641396469E-15	0.579632417773708	0.656	0.219	5.64568040052203E-11	4	GNAI2
3.25608801955079E-08	0.579096123975077	0.389	0.136	0.000545655230316321	4	MICOS10
7.2570151673523E-25	0.578027692785201	0.489	0.097	1.2161306017449E-20	4	RANGAP1
1.25206522355082E-19	0.577692069680912	0.589	0.155	2.09821090162647E-15	4	CNPY3
1.82295964428226E-10	0.577635967390145	0.356	0.103	3.05491577188821E-06	4	DNAJC3
1.02386704554315E-15	0.57761502922172	0.311	0.064	1.71579639492121E-11	4	BCL7B
8.58803754982727E-14	0.577534943776232	0.256	0.051	1.43918333260005E-09	4	SFMBT1
1.02758057441703E-09	0.577388206487351	0.333	0.099	1.72201952660806E-05	4	GSTA2
7.87398258186178E-09	0.577056471930874	0.311	0.098	0.00013195220010684	4	AGT
8.58242426480557E-12	0.576707684929934	0.3	0.075	1.43824265829612E-07	4	RNF220
4.55242700724791E-09	0.57649969252552	0.389	0.129	7.62895717874605E-05	4	CLPTM1
1.54427671739231E-07	0.57571426812949	0.267	0.084	0.00258789892300603	4	IFI35
1.75746316772465E-14	0.574635967060994	0.933	0.474	2.94515677647297E-10	4	ALB
4.31278563259237E-13	0.573334319461046	0.289	0.065	7.2273661630983E-09	4	CAPRIN1
1.17229720380468E-09	0.571624613254975	0.4	0.129	1.96453565413588E-05	4	TMED9
2.62672840295109E-11	0.571281260282393	0.344	0.095	4.40187145766544E-07	4	UNC50
6.81121955374035E-16	0.570975425820818	0.711	0.253	1.14142417281581E-11	4	ATP2A3
4.38366128471504E-10	0.570911749838101	0.344	0.1	7.34613958092547E-06	4	ROMO1
4.32809188026992E-10	0.56959849649873	0.3	0.084	7.25301637295633E-06	4	DHX8
6.89140967695862E-09	0.567605788812671	0.3	0.091	0.000115486243366473	4	DRG1
1.95452951970903E-15	0.567459760004003	0.589	0.177	3.27540056912839E-11	4	TOR1AIP1
1.81913091100963E-14	0.566198797185513	0.556	0.169	3.04849958066993E-10	4	ADRM1
1.50197885631496E-18	0.564983293776723	0.311	0.055	2.51701616741261E-14	4	CEP78
1.2817703232622E-16	0.564972866242668	0.544	0.152	2.1479907077228E-12	4	DHX9
7.47302830775436E-08	0.564790641878381	0.322	0.108	0.00125233008381348	4	SRPRB
3.08814777053396E-15	0.563841797223231	0.389	0.096	5.17511803386081E-11	4	PARP1
1.56891289217862E-11	0.563390576934916	0.378	0.11	2.62918422471293E-07	4	PBRM1
6.32910054330981E-10	0.563292879750442	0.389	0.121	1.06063066904786E-05	4	GSTO1
6.36747344811334E-11	0.562374063646814	0.589	0.211	1.06706120043483E-06	4	PSMB1
6.66902364009416E-13	0.5617910024212	0.267	0.058	1.11759498160698E-08	4	AARS2
1.97994556013846E-09	0.561542061989209	0.267	0.073	3.31799276968003E-05	4	SPIDR
2.69728260906972E-15	0.561510989659428	0.4	0.1	4.52010619627903E-11	4	EXOSC9
1.3884321326098E-10	0.561472920240355	0.333	0.094	2.32673456782751E-06	4	INPP1

1.22079261685281E-10	0.560411981330308	0.389	0.121	2.04580426732194E-06	4	ALDH1A1
2.25017341329656E-08	0.556925483333959	0.411	0.151	0.000377084060600238	4	ATP5F1D
3.92829225345718E-15	0.556819655302394	0.378	0.089	6.58303215834355E-11	4	BCOR
1.59686003809269E-11	0.556486050241244	0.356	0.099	2.67601805183573E-07	4	ANAPC7
1.19628339911729E-15	0.555499777674508	0.511	0.144	2.00473172024075E-11	4	SF3B3
4.36156934876009E-13	0.554925880075651	0.333	0.081	7.30911791465217E-09	4	ADNP
1.81487937616326E-12	0.554703524644263	0.322	0.08	3.04137485857439E-08	4	BUD31
3.16891254882655E-09	0.55455010146986	0.333	0.105	5.31046364932353E-05	4	TRMT112
1.10416026310226E-11	0.553352348406043	0.489	0.153	1.85035176890677E-07	4	EIF2AK1
2.07658143738724E-11	0.551404141612826	0.289	0.073	3.47993517277354E-07	4	SLFN11
8.51053694795366E-09	0.551116432842063	0.967	0.538	0.000142619578173807	4	MT-CO2
8.22754001945141E-15	0.550656952185615	0.344	0.078	1.37877115645967E-10	4	SMC5
3.39291113808283E-14	0.55064242692342	0.322	0.073	5.6858404851992E-10	4	SGTA
1.15991085852951E-18	0.550546726649784	0.6	0.164	1.94377861672375E-14	4	HMGN1
4.41809331979393E-10	0.54947836034105	0.467	0.158	7.40384078531067E-06	4	SPCS1
4.22921208884893E-11	0.547850675262309	0.544	0.192	7.08731361849304E-07	4	ARL6IP4
2.9834630291721E-12	0.547401460364805	0.278	0.064	4.9996873442866E-08	4	UBE2D2
1.64817757533263E-13	0.545511242955069	0.322	0.076	2.76201598074242E-09	4	MTOR
4.22686890174351E-20	0.545297584220827	0.6	0.155	7.08338690554177E-16	4	AKT1
1.1142674895299E-10	0.545284548063623	0.4	0.121	1.86728945895421E-06	4	YIPF3
3.14396389568896E-11	0.545280103181835	0.311	0.083	5.26865469639556E-07	4	NUP214
4.2850171255805E-15	0.544520485948933	0.4	0.099	7.18083169904781E-11	4	C6orf89
4.39922182140687E-08	0.543770249808154	0.422	0.156	0.000737221592831364	4	CTSD
3.01454700885654E-16	0.543686308544156	0.456	0.116	5.0517778774418E-12	4	PNRC2
4.74133243631599E-18	0.543450398985026	0.344	0.068	7.94552489677834E-14	4	EIF4E
8.65447278962604E-18	0.543250622735108	0.611	0.176	1.45031655008553E-13	4	UBE2I
6.52625815954498E-18	0.541177348260068	0.411	0.091	1.09367034237655E-13	4	SF1
1.6759955096771E-23	0.541083987289676	0.644	0.163	2.80863327511689E-19	4	TUBA4A
2.80994387542099E-07	0.540962300965201	0.256	0.08	0.00470890394643049	4	SIAH2
6.85689857515132E-07	0.538574210758359	0.378	0.149	0.0114907906322386	4	NARS
7.28681601101873E-09	0.538567559208837	0.833	0.443	0.000122112462712652	4	HSPA1A
1.70098313088917E-08	0.538072246436508	0.311	0.097	0.000285050753074408	4	ETFA
4.49971924465162E-15	0.53784530128918	0.6	0.188	7.54062951018718E-11	4	ARPC4
7.15859875314293E-09	0.533974547140935	0.344	0.111	0.000119963797905169	4	PPP1R10
4.99452253291053E-11	0.533604345935303	0.356	0.102	8.36982086065146E-07	4	UBTF
1.39216438346874E-11	0.533512058107309	0.667	0.269	2.33298907381691E-07	4	LUC7L2
2.3990164899269E-14	0.532842234739244	0.544	0.165	4.0202718338195E-10	4	UHMK1
1.72566033824386E-16	0.53066849061387	0.556	0.157	2.89186159482906E-12	4	NMT1
5.39887234311748E-19	0.529657494672723	0.433	0.096	9.04743027259626E-15	4	TRA2B
3.74183935326185E-18	0.528339251049001	0.467	0.111	6.2705743881962E-14	4	HNRNPR
1.2858043792881E-09	0.528198513198491	0.867	0.458	2.154750978811E-05	4	DNAJB1
2.82346070775201E-08	0.527049906767203	0.344	0.116	0.000473155545405081	4	SLC35A4
9.20464774739623E-19	0.52601350496697	0.456	0.103	1.54251486950866E-14	4	PLEKHJ1
1.08241845654036E-11	0.525437253642326	0.333	0.09	1.81391684947033E-07	4	CLU
2.74711527737473E-10	0.525370519094771	0.889	0.5	4.60361578182457E-06	4	RAC2
4.9788407127161E-07	0.524927083818986	0.278	0.094	0.00834354126636965	4	ORMDL2
1.63232247362878E-15	0.522877694543363	0.722	0.253	2.73544600130711E-11	4	TACC1
1.78838863180472E-07	0.521636925524038	0.311	0.105	0.00299698166917835	4	LAPTM4B
2.2338309736191E-09	0.521492083609104	0.256	0.068	3.7434539455909E-05	4	NDUFS3
5.12395826341915E-13	0.521309851485452	0.467	0.138	8.58672925783781E-09	4	DGKZ

8.04972135464144E-12	0.521008858009605	0.433	0.129	1.34897230461081E-07	4	PARP14
7.32466183837769E-14	0.521003358685611	0.9	0.407	1.22746683087533E-09	4	SUN2
3.35385811409939E-12	0.520755373224052	0.256	0.057	5.62039542760776E-08	4	PSMB6
1.65928064699068E-08	0.520114494050236	0.3	0.092	0.000278062250822697	4	CPNE3
3.08108939614089E-09	0.519438938920758	0.344	0.114	5.16328961005291E-05	4	IER5
6.16153027203903E-12	0.519278974461226	0.344	0.092	1.0325492429883E-07	4	SNX19
5.45916455371254E-08	0.518609847924583	0.311	0.104	0.000914846795911148	4	PPT1
5.27765239362695E-13	0.517904821328101	0.611	0.208	8.84428988124004E-09	4	ATF6B
2.05342897616903E-10	0.517889488453243	0.289	0.076	3.44113627826407E-06	4	DNAJA1
5.05012645175481E-08	0.5174548845443	0.4	0.144	0.000846300190785071	4	ATP13A3
4.7083148617578E-09	0.517099099967675	0.378	0.124	7.89019404533371E-05	4	SEC13
1.23638998016827E-17	0.515914200089456	0.356	0.072	2.07194232876599E-13	4	EEF1AKMT2
1.47489486654338E-13	0.515097972760319	0.556	0.179	2.4716288173534E-09	4	NCL
3.63991629147877E-11	0.513165702333883	0.278	0.069	6.09977172126012E-07	4	ERCC3
1.03626951567112E-11	0.512284237896446	0.378	0.107	1.73658045436166E-07	4	PRR12
5.12806772608497E-17	0.510618566428977	0.533	0.144	8.5936158953732E-13	4	DAZAP1
8.25808987020133E-09	0.510528748507152	0.4	0.136	0.000138389070044834	4	ARHGAP1
2.94647528736196E-09	0.510015713205323	0.3	0.087	4.93770328656117E-05	4	PSMA4
1.82256611145172E-13	0.508375325011607	0.378	0.099	3.05425628957079E-09	4	API5
1.15983652508279E-07	0.507118038386625	0.422	0.159	0.00194365404873374	4	LMAN1
1.24986834401904E-14	0.505934004035301	0.489	0.142	2.09452937090711E-10	4	HCFC1
2.14708470697462E-10	0.504720003351719	0.267	0.067	3.59808455194808E-06	4	AGGF1
1.61798492754585E-19	0.503986613555166	0.622	0.166	2.71141914158134E-15	4	CYB5B
7.28469488990186E-10	0.503729270421841	0.533	0.199	1.22076916964975E-05	4	RNF187
7.05362120070984E-09	0.503075608628815	0.378	0.126	0.000118204584081495	4	PIGT
4.7219233492999E-10	0.502890167254839	0.922	0.433	7.91299914875677E-06	4	MT-CYB
2.0395131512617E-16	0.501406393458719	0.622	0.187	3.41781613888435E-12	4	ERAP1
5.20140644508818E-09	0.501314801467784	0.544	0.213	8.71651692067877E-05	4	EID1
3.96791443622846E-12	0.499377328180876	0.311	0.076	6.64943101223165E-08	4	NDUFA9
5.76295661616986E-08	0.497039177413681	0.311	0.102	0.000965756269737745	4	NQO1
1.0713280022997E-12	0.496863457665784	0.256	0.054	1.79533146625384E-08	4	CTDSPL2
2.67376362507318E-19	0.496303447297915	0.556	0.139	4.48069308289763E-15	4	ARID1A
4.8236332933831E-08	0.495001406205136	0.4	0.146	0.000808344467305139	4	MYO18A
2.60174513546391E-15	0.494133880777689	0.644	0.208	4.36000449801041E-11	4	MTA2
6.55669621440488E-11	0.493430990448242	0.333	0.092	1.09877115160997E-06	4	EIF2S3
7.8459045435883E-11	0.493242488041856	0.378	0.113	1.31481668341453E-06	4	VPS26B
8.02493601279094E-10	0.492793543300225	0.278	0.076	1.34481877702351E-05	4	GATD1
3.12874382265694E-08	0.492650569581802	0.267	0.078	0.00052431488980085	4	SURF1
7.57747929213287E-09	0.492202040051004	0.267	0.075	0.000126983397977563	4	DCAF6
1.39629433482447E-12	0.49213951793926	0.389	0.11	2.33991004629885E-08	4	REV1
8.10546531709974E-07	0.489990248623096	0.278	0.096	0.0135831387783957	4	SIPA1L1
8.6520201839749E-08	0.487039044965013	0.256	0.078	0.00144990554243051	4	CDIP1
3.3042560787326E-07	0.486929009483743	0.256	0.082	0.00553727233674009	4	FLOT1
5.58511074645111E-15	0.485454506470203	0.367	0.087	9.35952858890277E-11	4	PLEKHF2
3.68238896896023E-16	0.48526940240521	0.444	0.111	6.17094743418355E-12	4	AGPS
1.6598766877666E-08	0.485123358414233	0.378	0.129	0.000278162135335927	4	IARS2
1.24776223397419E-07	0.48444176119641	0.289	0.093	0.00209099995169395	4	NME2
5.1496165187538E-12	0.48408999266251	0.278	0.066	8.62972736212762E-08	4	ZFAND5
2.76887292685101E-08	0.484063292893828	0.778	0.393	0.000464007725081692	4	SH3BGRL3
1.28553637471222E-14	0.48294446415444	0.322	0.071	2.15430185674273E-10	4	GNPAT

1.11523607821288E-14	0.482807751300352	0.511	0.15	1.86891261986915E-10	4	HDAC1
5.99307571353805E-08	0.481532421853469	0.289	0.094	0.00100431962807471	4	PHC1
7.77661091849419E-12	0.481047178465931	0.389	0.113	1.30320445772126E-07	4	RBM3
4.3879576804714E-14	0.480982129227839	0.478	0.139	7.35333948093398E-10	4	TRIM26
2.62465478503265E-14	0.480430896809269	0.756	0.275	4.39839648875771E-10	4	SUGP2
6.82717668459331E-14	0.480231150053425	0.367	0.092	1.14409826880415E-09	4	MUS81
1.04017996812294E-07	0.47819578483772	0.367	0.129	0.00174313359058043	4	NDUFA13
5.31710582888048E-12	0.47796466861591	0.311	0.078	8.91040594803791E-08	4	TRNAU1AP
4.82545942874923E-07	0.476981830077542	0.422	0.17	0.00808650491069796	4	IFITM2
6.91672740487342E-08	0.476004609495011	0.389	0.142	0.00115910517850869	4	ZMAT2
3.17867947882071E-13	0.474278385245953	0.544	0.172	5.32683107060775E-09	4	SUB1
2.13552582217918E-15	0.47396603511417	0.533	0.154	3.57871417280787E-11	4	PFKP
3.84209278328472E-10	0.47385899225984	0.289	0.078	6.43857908622853E-06	4	PAPSS1
4.64990482059543E-09	0.473306781969237	0.522	0.206	7.79231049835381E-05	4	SIT 1.00
2.09261252751682E-14	0.472627307998466	0.567	0.172	3.50680007361269E-10	4	APOBEC3C
4.40870151972945E-08	0.472573105866005	0.944	0.569	0.000738810200676262	4	HSPA1B
1.70881023728724E-10	0.47256829159202	0.3	0.079	2.86362419564595E-06	4	ATXN10
2.78185569267491E-20	0.472384157327476	0.478	0.104	4.66183376978461E-16	4	SLC39A7
2.69277268290489E-07	0.471214065230405	0.789	0.364	0.00451254846201201	4	MT-ND5
8.05688793872908E-08	0.470605018890629	0.322	0.107	0.00135017328077222	4	PLD3
1.26561988024197E-10	0.470547995362105	0.356	0.104	2.1209257953095E-06	4	APH1A
4.94599738482944E-14	0.469718881509359	0.456	0.128	8.28850241749717E-10	4	SMARCD2
2.91658950487995E-10	0.469298546109555	0.456	0.152	4.88762069227781E-06	4	SAP18
1.46335862781936E-10	0.468883637188917	0.378	0.117	2.45229638849969E-06	4	ANKRD36B
2.4794948642204E-14	0.468730282114525	0.622	0.208	4.15513749346055E-10	4	DYNLL2
3.60426681992822E-17	0.468291036792895	0.667	0.205	6.04003033683571E-13	4	BUB3
5.28040551732743E-10	0.467831731109898	0.433	0.144	8.84890356593731E-06	4	ELOVL1
6.95470790678954E-13	0.466833351826116	0.333	0.083	1.16546995101979E-08	4	NDUFAF8
2.56886214457351E-16	0.466485532468261	0.567	0.165	4.30489918187629E-12	4	BAZ1B
4.43874331344772E-08	0.465785204478567	0.256	0.076	0.000743844604467568	4	OGG1
4.54050051695501E-20	0.465545014668371	0.411	0.084	7.60897076631321E-16	4	ABCB8
1.00294104739219E-10	0.464832266496158	0.367	0.109	1.68072860721983E-06	4	WWP2
2.21081593901348E-12	0.464138940194253	0.344	0.089	3.70488535059879E-08	4	CTDNEP1
1.03539008050649E-14	0.463834668867416	0.378	0.093	1.73510669691277E-10	4	CGAS
1.82690470374456E-15	0.462849430854954	0.478	0.129	3.06152690253514E-11	4	MEAF6
1.61126590374034E-09	0.461903864789158	0.367	0.118	2.70015940148807E-05	4	ZNRF1
2.43023371823622E-12	0.461217206562149	0.378	0.105	4.07258566502026E-08	4	NUP160
7.55868200406395E-14	0.460093636560892	0.344	0.083	1.26668393024104E-09	4	TNPO2
1.09509610486769E-12	0.459567594077419	0.556	0.183	1.83516205253728E-08	4	THRAP3
1.31359485172426E-17	0.458434106933821	0.489	0.122	2.20132225251951E-13	4	TXNL4A
2.57247835369174E-11	0.458277596855545	0.533	0.184	4.31095922511661E-07	4	APOBEC3G
2.36525106805084E-17	0.457625285304475	0.611	0.178	3.9636877398396E-13	4	STAG1
1.83799353543918E-14	0.457618955172866	0.489	0.138	3.08010956668897E-10	4	CDKAL1
1.1079136974067E-08	0.457521250316509	0.378	0.128	0.000185664177411414	4	MAPK13
1.96504564698015E-10	0.456501648029218	0.333	0.095	3.29302349520933E-06	4	MYD88
8.1473687304243E-10	0.455998174193212	0.433	0.142	1.3653360518445E-05	4	TUFM
2.28708676521811E-11	0.45415631518832	0.467	0.147	3.83270000115251E-07	4	SEPTIN11
7.47150124668301E-10	0.453497650826386	0.267	0.07	1.25207417891914E-05	4	EMC8
1.03150635663652E-16	0.452940730735693	0.411	0.095	1.72859835245147E-12	4	NUP188
5.7490988279599E-07	0.451712437968729	0.267	0.089	0.0096343398158952	4	NSF

**5.21767851948043E-12**	0.451465532317914	0.489	0.156	8.74378566294531E-08	4	STK11
**3.96151741872818E-10**	0.451267110592812	0.622	0.237	6.63871089030468E-06	4	CTSA
**7.24952740615303E-07**	0.450289728912858	0.356	0.131	0.0121487580272312	4	RDH11
**1.42629588591852E-08**	0.448302451216463	0.956	0.541	0.000239018664562225	4	ARHGDIB
**5.92660323740648E-10**	0.447131453140572	0.278	0.073	9.93180170524577E-06	4	C1orf43
**1.76229456482225E-13**	0.446906157517672	0.367	0.093	2.95325323172913E-09	4	FAF1
**6.8354416587519E-13**	0.446269736316444	0.311	0.075	1.14548331317364E-08	4	TGS1
**9.33547999224016E-10**	0.445078041169826	0.333	0.102	1.56443973709961E-05	4	CDKN1A
**4.72587394712039E-07**	0.444989949827016	0.267	0.087	0.00791961956058435	4	HMG20B
**2.20877442690009E-12**	0.444961158036779	0.611	0.221	3.70146418459917E-08	4	ERO1A
**1.42308130689214E-06**	0.444443251105375	0.267	0.093	0.0238479965408985	4	TMX2
**6.77295888470252E-10**	0.444279022678512	0.456	0.153	1.13501244989845E-05	4	FKBP1A
**1.20914289567608E-14**	0.443348860613964	0.822	0.328	2.02628166457398E-10	4	HNRNPA0
**3.30138575673474E-09**	0.441855679719976	0.344	0.109	5.53246225113609E-05	4	SOCS4
**5.75738058083727E-14**	0.440102539920268	0.433	0.12	9.64821837736709E-10	4	SNRPA
**9.57148312993609E-13**	0.439983636551993	0.367	0.097	1.60398914291469E-08	4	C2orf68
**6.53400583160716E-07**	0.43902068024639	0.267	0.089	0.0109496869726073	4	PRKAB1
**5.28129570706361E-09**	0.435499550681416	0.356	0.116	8.85039534589719E-05	4	RO60
**1.1692409153737E-12**	0.434280471565422	0.544	0.178	1.95941392598324E-08	4	FLOT2
**1.89642114250266E-15**	0.43364686267398	0.3	0.06	3.17802255060596E-11	4	USP7
**9.61316322506958E-10**	0.432704122801312	0.411	0.137	1.61097389325716E-05	4	QARS
**4.14524664904723E-12**	0.43235680017645	0.667	0.259	6.94660433447334E-08	4	GNG2
**9.76950176967822E-08**	0.432248716766531	0.367	0.132	0.00163717310656268	4	IL10RB
**5.92558405181105E-09**	0.431766767185773	0.544	0.216	9.93009375402495E-05	4	SNTB2
**1.53694773740317E-08**	0.431091230467605	0.5	0.185	0.000257561701834024	4	CNDP2
**1.39478506379603E-11**	0.431022393745165	0.256	0.059	2.33738080990939E-07	4	ZCCHC8
**8.45548160586855E-07**	0.431005853804674	0.278	0.096	0.0141696960751145	4	TMEM64
**2.18363415946746E-12**	0.430857368518235	0.456	0.136	3.65933412443558E-08	4	PSMA7
**9.1081562060248E-11**	0.430689950409825	0.489	0.166	1.52634481700564E-06	4	MRTFA
**5.96467017021879E-12**	0.430633617579973	0.344	0.09	9.99559427125265E-08	4	AP2B1
**1.63080336924464E-12**	0.430427221572587	0.267	0.059	2.73290028618017E-08	4	MOB1A
**4.68873264602E-09**	0.430335546075809	0.922	0.495	7.85737816820031E-05	4	HSPA8
**7.47068455861103E-10**	0.428123201451158	0.3	0.085	1.25193731833204E-05	4	BCCIP
**9.86257441163887E-11**	0.42806137725127	0.289	0.075	1.65277021990244E-06	4	SH2D3C
**2.74611367578651E-08**	0.427828141956609	0.344	0.114	0.000460193729788304	4	TMED7
**2.42460455225346E-13**	0.426525710795105	0.378	0.097	4.06315230866634E-09	4	CASP2
**3.36131983769273E-08**	0.426334414723593	0.489	0.189	0.000563289978400547	4	REEP5
**1.42243304319976E-07**	0.425855469769368	0.322	0.113	0.00238371329379416	4	CALHM2
**1.62183011648039E-14**	0.425544147662134	0.556	0.167	2.71786290919783E-10	4	KIF2A
**1.29628123158805E-08**	0.423063352903483	0.311	0.096	0.000217230808789525	4	CDK6
**1.27061962949217E-13**	0.421927390700308	0.467	0.132	2.12930437510299E-09	4	DNAJC1
**4.46546157273924E-08**	0.421165533190547	0.478	0.183	0.000748322050359642	4	COX4I1
**2.283870675388E-07**	0.418996966081386	0.922	0.501	0.00382731047781522	4	MT-CO3
**2.87233569751803E-14**	0.418586250908653	0.522	0.158	4.81346016190072E-10	4	SRSF3
**5.89899344120392E-12**	0.418014340080141	0.333	0.087	9.88553320876954E-08	4	POLR2G
**3.42850379558452E-16**	0.417692626873562	0.411	0.097	5.74548666064054E-12	4	UBE2J2
**1.13290808093915E-14**	0.417568730773364	0.489	0.137	1.89852736203784E-10	4	KBTBD2
**1.32481239951816E-07**	0.41719525270076	0.289	0.096	0.00222012061911253	4	INPP5F
**4.77842893745334E-08**	0.414295885321264	0.322	0.108	0.000800769121338431	4	KLHL2
**8.23074208142535E-13**	0.413924681498257	0.578	0.195	1.37930775800526E-08	4	NPLOC4

2.2306594745303E-08	0.413497037097348	0.3	0.092	0.000373813914741788	4	KIAA2013
1.02429726656551E-14	0.412943643267302	0.267	0.051	1.71651735931048E-10	4	DTD1
3.34862915002172E-11	0.412848771029415	0.433	0.137	5.6116327296064E-07	4	EIF3B
5.48512054458885E-07	0.411636446321568	0.456	0.184	0.00919196500862	4	RER1
2.17769277003003E-07	0.411430890018837	0.456	0.181	0.00364937754401633	4	RTF2
1.31415077319519E-16	0.411402196511444	0.333	0.069	2.20225386572049E-12	4	ILK
3.98879874762177E-16	0.410963680157127	0.467	0.12	6.68442894126457E-12	4	SUZ12
2.01054978012118E-11	0.410930150937823	0.467	0.147	3.36927932152707E-07	4	DNAJC8
1.57813519996009E-09	0.410686553133645	0.389	0.127	2.64463896809311E-05	4	POLR1D
4.12250379323581E-08	0.409984599550102	0.311	0.1	0.000690849185670458	4	MAPK6
7.5193958903446E-13	0.408862515770295	0.344	0.088	1.26010036330395E-08	4	PAF1
8.88910904117333E-08	0.408458191459973	0.3	0.1	0.00148963689311983	4	NCLN
5.69479089321984E-10	0.407831283487387	0.489	0.17	9.54333057885781E-06	4	MGAT2
5.74841661261423E-23	0.407716590433415	0.878	0.268	9.63319655941893E-19	4	HNRNPUL1
1.16109546904664E-08	0.407449411892473	0.433	0.153	0.000194576378702837	4	SPPL2A
1.56997853759018E-13	0.406373394233154	0.478	0.14	2.63097003329363E-09	4	NOTCH1
1.1928996805413E-17	0.405791656099093	0.344	0.069	1.99906128465111E-13	4	ATL2
2.15503529756602E-09	0.40578431055126	0.322	0.098	3.61140815166113E-05	4	MTA1
2.41675068097001E-06	0.405504465199661	0.356	0.138	0.0404999079116955	4	PTMS
6.23548522442938E-12	0.405459449903307	0.3	0.074	1.04494261390988E-07	4	NIPAL3
1.38522566686619E-09	0.403384242274342	0.278	0.078	2.32136117253436E-05	4	TELO2
2.83662515549295E-16	0.402085881958062	0.389	0.089	4.75361643557508E-12	4	EFTUD2
2.22180825926889E-17	0.400934426322199	0.533	0.141	3.72330628088281E-13	4	DDX23
4.0516015295741E-10	0.400742942334913	0.356	0.108	6.78967384326028E-06	4	OPA1
7.39478508203539E-12	0.399111200370271	0.344	0.093	1.23921808404749E-07	4	ILKAP
4.33235306472839E-10	0.398836614201237	0.278	0.073	7.26015726587183E-06	4	GET3
1.32868779971215E-11	0.39822937738949	0.311	0.079	2.22661501475762E-07	4	SP2
2.19161832944048E-07	0.397957238940499	0.767	0.354	0.00367271399647636	4	LAMP1
2.86394234694396E-11	0.397616666129348	0.378	0.11	4.79939458500869E-07	4	AMFR
7.47598521890718E-08	0.397607486442919	0.889	0.488	0.00125282560298446	4	TMSB10
5.52726555301644E-13	0.396840230848313	0.444	0.126	9.26259161374495E-09	4	INTS1
1.16840874066437E-18	0.395871772960868	0.589	0.156	1.95801936760535E-14	4	PPP4C
1.32513136556266E-08	0.395807216311685	0.267	0.076	0.000222065514240991	4	HDHD2
8.236164629042E-09	0.395129447873123	0.533	0.221	0.000138021646853486	4	MAP4K1
3.85825435472255E-07	0.395021999294507	0.278	0.091	0.00646566264764404	4	SMPD1
5.37357117223072E-07	0.394178595332231	0.267	0.088	0.00900503057042423	4	RAP1GDS1
1.2901262724749E-14	0.393335582643429	0.567	0.178	2.16199360741344E-10	4	SRSF2
4.73213103729441E-09	0.391755714365516	0.667	0.278	7.93010519229798E-05	4	ITM2B
1.61855453944437E-08	0.391338810720438	0.433	0.158	0.000271237369720088	4	DYNC1I2
2.93636054407582E-11	0.390098090512311	0.478	0.154	4.92075299976226E-07	4	ZC3H4
7.37979495735487E-18	0.389260935536384	0.511	0.128	1.23670603895353E-13	4	PMF1
1.84393894381041E-14	0.38881119564656	0.411	0.105	3.09007288203748E-10	4	METTL23
1.82223409034936E-13	0.388539512090438	0.5	0.148	3.05369988860745E-09	4	CNOT9
9.55599652803692E-11	0.387973058621165	0.356	0.104	1.60139389816843E-06	4	CBL
7.27635479266875E-15	0.387278316045149	0.322	0.07	1.21937153615543E-10	4	GRK5
7.39340992461453E-16	0.387265532791795	0.511	0.141	1.2389876351669E-11	4	SART1
1.85059064235205E-15	0.386879248228465	0.478	0.129	3.10121979845357E-11	4	TPR
3.24283121101439E-15	0.386859121233422	0.5	0.137	5.43433654341791E-11	4	PPP3CB
1.31528284439947E-14	0.386452870135093	0.611	0.2	2.20415099064463E-10	4	DHX15
1.15552964821594E-10	0.385930911852467	0.933	0.439	1.93643658448027E-06	4	FTL

**1.76451331292565E-06**	0.384713680881342	0.278	0.096	0.029569714098008	4	FAM174C
**8.50328644638378E-16**	0.383086948305223	0.511	0.142	1.42498074268499E-11	4	RBM14
**3.41472268320759E-15**	0.381858538021281	0.478	0.132	5.72239227251928E-11	4	YBX1
**2.93781873265368E-09**	0.381419341382595	0.256	0.068	4.92319663218104E-05	4	ZFP62
**2.95763850315181E-10**	0.380499096609756	0.356	0.105	4.95641060358181E-06	4	PHTF1
**2.12259195640145E-13**	0.380241457374697	0.278	0.059	3.55703960053755E-09	4	PPP1R8
**3.75603516285268E-15**	0.379560995956658	0.456	0.123	6.29436372590852E-11	4	ADSL
**4.37565324491047E-10**	0.37954008103866	0.378	0.118	7.33271970782097E-06	4	ZMIZ1
**1.86352262687186E-13**	0.377122617451904	0.789	0.299	3.12289121811186E-09	4	MBD2
**2.09355585762127E-10**	0.375499083546032	0.478	0.166	3.50838090620172E-06	4	CRKL
**5.17839802457058E-07**	0.374486117245373	0.356	0.141	0.00867795940957537	4	CAPG
**6.67037124703889E-12**	0.374092578598436	0.856	0.362	1.11782081357878E-07	4	PSMB8
**1.03916824888004E-08**	0.373865228108553	0.633	0.258	0.000174143815147318	4	OS9
**3.21281124733989E-07**	0.372431471043959	0.289	0.099	0.00538402908829219	4	GNS
**1.38522313715367E-12**	0.371621578180311	0.489	0.149	2.32135693324211E-08	4	LRRFIP1
**1.90932052333528E-09**	0.371470269540969	0.389	0.126	3.19963933300527E-05	4	ATP2A2
**3.80919386146048E-12**	0.370915625687932	0.333	0.086	6.38344707303547E-08	4	PDS5A
**4.9720383272032E-11**	0.370545359129949	0.667	0.265	8.33214182872712E-07	4	CD99
**1.25375400693885E-09**	0.368771275106834	0.278	0.076	2.10104096482812E-05	4	COPS3
**9.35653778899224E-08**	0.368682668368576	0.489	0.197	0.00156796860267932	4	CMPK1
**2.20766677101585E-15**	0.36814835297908	0.5	0.139	3.69960797486836E-11	4	RECQL
**4.31171255967208E-10**	0.36807717045753	0.367	0.113	7.22556790749847E-06	4	TXNDC12
**6.22889291715406E-08**	0.367766586616094	0.289	0.091	0.00104383787505668	4	STRN
**5.68292759503079E-14**	0.366917430579665	0.356	0.086	9.5234500637526E-10	4	MOB4
**3.00823330790811E-07**	0.36643731612677	0.5	0.203	0.00504119737739241	4	ATP5F1E
**1.25451782277669E-08**	0.366300613803417	0.278	0.083	0.000210232096740917	4	EXOC8
**1.9846657386822E-08**	0.365685859048096	0.478	0.185	0.000332590284488363	4	UBC
**2.98981198032497E-17**	0.36559350743114	0.511	0.131	5.01032691662859E-13	4	PSMD13
**6.44984267284393E-08**	0.365179173678232	0.267	0.081	0.00108086463511519	4	DDX1
**8.66321188324398E-11**	0.36466782663364	0.289	0.075	1.45178104739403E-06	4	MFN2
**2.11609211771196E-06**	0.363843019091561	0.333	0.124	0.0354614717086171	4	HADHB
**1.45848732367384E-06**	0.363146787833748	0.756	0.395	0.0244413305701262	4	ITM2A
**9.06188660598232E-11**	0.362974715186275	0.467	0.155	1.51859095743052E-06	4	GALM
**7.37200552132716E-12**	0.36204967037945	0.911	0.421	1.23540068526401E-07	4	IFI16
**7.16680041038827E-09**	0.361499688274668	0.344	0.109	0.000120101241277287	4	RAB6A
**5.58137303620046E-14**	0.361441604322188	0.644	0.225	9.35326493406472E-10	4	EMP3
**3.14117628849629E-11**	0.361152729479114	0.378	0.111	5.26398322426208E-07	4	DCTD
**1.70393472551244E-10**	0.361148169701961	0.4	0.123	2.85545381301375E-06	4	USP14
**1.15736779920863E-09**	0.359848717503163	0.267	0.071	1.93951695791382E-05	4	CEBPD
**4.44627956664536E-12**	0.358845308903885	0.589	0.205	7.4510752977843E-08	4	HNRNPK
**1.82260670035844E-07**	0.357876768661116	0.489	0.206	0.00305432430846067	4	HAVCR2
**7.07023588395983E-11**	0.357428492730028	0.411	0.126	1.18483012943399E-06	4	UGGT1
**1.51317521336669E-08**	0.356551846411579	0.378	0.129	0.00025357790225599	4	PFDN2
**2.01075481445096E-06**	0.355874884048915	0.256	0.089	0.0336962291805692	4	ARL8B
**5.01208931867274E-14**	0.355694061020859	0.322	0.073	8.39925928023178E-10	4	RB1
**5.06311694855225E-07**	0.355121131458152	0.289	0.097	0.00848477138238385	4	FPGS
**1.73570210273458E-08**	0.354921825428917	0.578	0.228	0.000290868958376261	4	PSMB10
**8.51164604708772E-13**	0.35483280960065	0.433	0.124	1.42638164457096E-08	4	SEC22B
**9.91531117183498E-15**	0.353192575286131	0.578	0.174	1.66160784617611E-10	4	SRI
**3.24940120636487E-11**	0.352209807249057	0.322	0.086	5.44534654162625E-07	4	PRPS1

**9.65054858608819E-12**	0.350377927907982	0.467	0.145	1.61723893205666E-07	4	MARK2
**2.71937598213449E-08**	0.349515513784472	0.344	0.114	0.000455713027086098	4	GLYCTK
**9.7083846580891E-09**	0.349101019521958	0.256	0.073	0.000162693110100257	4	UBE2O
**3.17001835052401E-09**	0.348687721016306	0.356	0.114	5.31231675180814E-05	4	LRRC8C
**1.07611149903115E-14**	0.34847547254102	0.4	0.099	1.80334765007641E-10	4	ZADH2
**1.39931750902775E-09**	0.347734516815627	0.311	0.091	2.3449762816287E-05	4	GBF1
**5.21483091525328E-10**	0.347045712970649	0.278	0.075	8.73901364778144E-06	4	MDC 1.00
**3.42923939893099E-11**	0.345816968510061	0.311	0.083	5.74671938472855E-07	4	TAGLN2
**1.78951192937599E-06**	0.345764427574381	0.256	0.087	0.0299886409124828	4	LRRC41
**8.78062887315213E-12**	0.345755788966796	0.4	0.116	1.47145778656283E-07	4	IPO9
**1.67272066878969E-09**	0.345748399885402	0.256	0.068	2.80314529675776E-05	4	LSM 1.00
**1.11853665637552E-09**	0.345075489360342	0.3	0.086	1.8744437287541E-05	4	RBM27
**2.25629642127833E-06**	0.343882333091875	0.333	0.126	0.0378110154277823	4	EMC7
**4.03740757456502E-09**	0.343353909921381	0.344	0.107	6.76588761345606E-05	4	MIA2
**1.12239458035419E-06**	0.343059980701224	0.267	0.091	0.0188090883775755	4	NAA10
**7.08267814851467E-10**	0.342728737548883	0.311	0.09	1.18691520412809E-05	4	SEC63
**2.28065416838057E-09**	0.341987368838664	0.3	0.087	3.82192025537217E-05	4	FBL
**1.18039417826296E-11**	0.341808779442213	0.644	0.235	1.97810456393307E-07	4	ACADVL
**1.11476975647816E-10**	0.340533220168761	0.7	0.287	1.8681311579061E-06	4	DRAP1
**4.66630503318253E-08**	0.340050789372189	0.667	0.306	0.000781979397460729	4	CD52
**2.13551165301501E-11**	0.340036351677348	0.389	0.113	3.57869042812256E-07	4	NDUFS7
**1.63762768132869E-09**	0.33983484873197	0.456	0.158	2.74433646837061E-05	4	RAB5C
**1.65442402148912E-12**	0.339767671894164	0.344	0.092	2.77248377521147E-08	4	SRRT
**3.07733980522209E-13**	0.339476280096412	0.456	0.133	5.15700604559117E-09	4	NUP153
**1.01233523929756E-06**	0.3379044619641	0.344	0.131	0.0169647139401485	4	MFHAS1
**2.19904323911834E-14**	0.336230667311328	0.289	0.061	3.68515666011452E-10	4	MYADM
**4.72737192252195E-15**	0.335925962498498	0.389	0.094	7.92212986776228E-11	4	ZCCHC3
**1.6805743396815E-07**	0.33581002128456	0.311	0.104	0.00281630647843825	4	CDV3
**9.19953458860774E-11**	0.33520058496542	0.3	0.079	1.54165800635889E-06	4	ZNF830
**2.23993586511927E-11**	0.335131138294068	0.667	0.244	3.75368452276687E-07	4	SEL1L3
**5.79215750813467E-09**	0.33345245054362	0.489	0.187	9.70649755213208E-05	4	ARPC5L
**9.24434142160969E-09**	0.332233721318505	0.256	0.071	0.000154916673543335	4	CUL4B
**1.01001897657319E-07**	0.332232112830041	0.278	0.087	0.00169258980094136	4	PRPF31
**3.39072118185948E-10**	0.331846928413257	0.3	0.082	5.68217055656012E-06	4	CHFR
**2.17949801079651E-08**	0.331810686243778	0.411	0.148	0.000365240276649279	4	AGFG2
**1.8051282398238E-07**	0.331326347195314	0.267	0.084	0.00302503390429672	4	SLC50A1
**2.29219961389297E-12**	0.329442769420006	0.478	0.145	3.84126811296184E-08	4	TNPO3
**1.23096247303564E-11**	0.328443717707362	0.722	0.281	2.06284691231313E-07	4	TRIR
**1.01870100639087E-10**	0.326176732627354	0.267	0.067	1.70713914650982E-06	4	TMEM154
**2.68235063667387E-13**	0.325536871905571	0.344	0.085	4.49508319693807E-09	4	SSU72
**1.26632408611075E-08**	0.325233863281328	0.289	0.087	0.00021221059035044	4	PPP2CA
**3.39375058315937E-10**	0.325037005698971	0.278	0.074	5.68724722725847E-06	4	EMSY
**1.26290122119914E-12**	0.324293570584415	0.5	0.154	2.11636986648553E-08	4	SZRD1
**6.36569470037967E-13**	0.324201161364504	0.9	0.415	1.06676311788962E-08	4	HNRNPA2B1
**1.98113942965825E-16**	0.322790152708525	0.511	0.14	3.31999345622129E-12	4	FAM50A
**2.30425818064938E-10**	0.322309939991358	0.256	0.063	3.86147585913223E-06	4	KDM1A
**1.52122775694708E-15**	0.322135453566993	0.656	0.206	2.54927347509191E-11	4	TMEM50A
**1.16206791364396E-08**	0.322022458080063	0.489	0.193	0.000194739340968454	4	EIF4A1
**3.60103450939961E-09**	0.321647780865658	0.3	0.091	6.03461363085186E-05	4	VAMP1
**1.16748240339817E-08**	0.321295889256935	0.311	0.097	0.000195646701161466	4	ELF4

2.82760441062064E-18	0.319165790292775	0.356	0.07	4.73849947131806E-14	4	NAA40
1.73003474101527E-11	0.318911377069381	0.3	0.075	2.89919221899338E-07	4	GLE1
5.89507014057781E-09	0.318805588517116	0.289	0.085	9.8789585415803E-05	4	GALK2
1.17053014239763E-09	0.318785064849766	0.278	0.076	1.96157441262995E-05	4	MFSD14B
8.94330807163891E-07	0.317340577420558	0.289	0.101	0.0149871956664525	4	KXD1
6.6863353321705E-12	0.31549510352171	0.478	0.149	1.12049607496513E-07	4	DCTN2
2.39587828384874E-10	0.315238813460893	0.478	0.158	4.01501282807372E-06	4	CD164
1.00023448505305E-10	0.314775322083872	0.544	0.197	1.67619295005191E-06	4	RUNX1
1.26151288406755E-07	0.314228378849304	0.311	0.102	0.00211404329112041	4	POLR2H
8.21076526864499E-08	0.313610233291143	0.322	0.107	0.00137596004371953	4	SCAMP3
2.39266625818414E-08	0.312091622540683	0.522	0.202	0.000400963011546498	4	RAB10
8.38477782770426E-15	0.311278094684446	0.378	0.092	1.40512106836668E-10	4	ENO2
6.58176421267502E-07	0.309249468284315	0.311	0.109	0.0110297204676008	4	C6orf62
9.45767971054963E-09	0.308932002244632	0.389	0.134	0.000158491796589391	4	DGKH
1.85199723540064E-09	0.308561334263744	0.533	0.194	3.10357696708439E-05	4	NDUFA3
1.82933406895921E-09	0.307025902426281	0.322	0.096	3.06559803276185E-05	4	ZNF148
1.01476827346054E-07	0.306442550269816	0.278	0.089	0.00170054867266517	4	CNOT6
1.49873438369902E-18	0.306029823457172	0.444	0.1	2.51157908020282E-14	4	GMFG
1.54173433702944E-15	0.303437793725425	0.422	0.105	2.58363840199394E-11	4	PET100
2.57536811495637E-07	0.303412366223127	0.311	0.107	0.00431580188704389	4	EIF2AK2
8.01044806788028E-14	0.303150827329125	0.411	0.112	1.34239088721538E-09	4	CMC2
3.35601171753668E-09	0.302930242380869	0.344	0.109	5.62400443624797E-05	4	MBOAT7
4.26100998577783E-08	0.302770628406078	0.289	0.091	0.000714060053416649	4	LARP1
1.69296218577983E-09	0.302487101034299	0.422	0.14	2.83706603092983E-05	4	ATP6AP2
1.29406533076198E-11	0.302186529775553	0.522	0.174	2.16859468129093E-07	4	DR1
2.16461620895782E-06	0.302153519498323	0.344	0.132	0.0362746384297151	4	ANXA7
1.9666770951844E-11	0.302138449719703	0.511	0.163	3.29575747611002E-07	4	SNX5
1.58095415448419E-13	0.300202373083953	0.556	0.172	2.6493629720846E-09	4	ACTR1B
2.18218610333553E-07	0.299485180909507	0.278	0.089	0.00365690747196968	4	PHC2
1.68937014051491E-10	0.299344900326104	0.267	0.067	2.83104648147489E-06	4	DROSHA
2.28956963767204E-08	0.295813232533711	0.333	0.111	0.00038368607988108	4	PIK3CG
2.2373932506439E-11	0.295273560340239	0.611	0.226	3.74942360942905E-07	4	EVI2B
2.20623023403646E-15	0.293031645685207	0.778	0.274	3.6972006261983E-11	4	NUMA1
3.25052172303081E-09	0.29189135925727	0.267	0.075	5.44722430345502E-05	4	COX17
9.01072953848195E-07	0.291278576432997	0.489	0.211	0.0151001805605881	4	PSMF1
2.43750044453147E-10	0.290433033515729	0.467	0.156	4.08476324494584E-06	4	ASCC3
1.03055653803788E-06	0.287508939447422	0.278	0.096	0.0172700664644389	4	PHKB
2.00524595695901E-06	0.286640349320213	0.267	0.092	0.0336039117467191	4	C21orf91
3.16312743322898E-14	0.285305062337355	0.267	0.053	5.30076895260512E-10	4	OTULINL
8.3484883755924E-08	0.284321395183113	0.3	0.099	0.00139903968198178	4	ANP32A
1.4373708509852E-13	0.284251891500383	0.511	0.155	2.408746072081E-09	4	CALM1
9.22522382022507E-10	0.283385948979062	0.556	0.211	1.54596300779332E-05	4	SURF4
3.85534325724552E-10	0.282292180099561	0.389	0.123	6.46078423049205E-06	4	PRKCSH
1.31031337221242E-16	0.28182204220272	0.489	0.126	2.19582314915357E-12	4	CIC
4.29987355017233E-13	0.280188440575042	0.422	0.119	7.20572809537879E-09	4	LPCAT4
7.31617706390055E-14	0.279172028675349	0.422	0.114	1.22604495236845E-09	4	KHDC4
1.42053806017836E-08	0.278290611746532	0.356	0.121	0.000238053768124689	4	CHMP4A
1.207107900374E-06	0.275565263720908	0.533	0.222	0.0202287141944675	4	MT-ND1
2.29004997253741E-12	0.275352241621828	0.289	0.068	3.83766574397819E-08	4	FGFR1OP
4.80941604209468E-15	0.274388942846561	0.422	0.107	8.05961940334226E-11	4	SMNDC1

**3.0837572239717E-08**	0.273722935895613	0.3	0.094	0.000516776035593177	4	SELENON
**2.94129403753511E-09**	0.273466771480248	0.389	0.128	4.92902054810134E-05	4	PPM1M
**1.04365711416256E-09**	0.273423531410957	0.344	0.105	1.74896059191361E-05	4	PNP
**6.43176708429655E-07**	0.272553586489381	0.789	0.4	0.0107783552798642	4	S100A4
**8.18094436438294E-10**	0.272332276779273	0.589	0.22	1.37096265658329E-05	4	ARPC1B
**1.8375255376328E-09**	0.271541005702891	0.567	0.214	3.07932529596505E-05	4	IQGAP1
**6.6123051329955E-08**	0.271063626996447	0.378	0.134	0.00110809009418739	4	PEX2
**1.27225277175289E-11**	0.270978997256052	0.478	0.15	2.13204119490349E-07	4	TMOD3
**5.2287665896165E-14**	0.270654000374608	0.578	0.181	8.76236705087932E-10	4	SMARCA4
**7.57100791552635E-15**	0.27063545018772	0.344	0.078	1.26874950648391E-10	4	ARL17B
**5.29053266085188E-09**	0.269385231764496	0.356	0.115	8.86587463305558E-05	4	ITFG1
**2.31298740957991E-11**	0.269115076670103	0.544	0.193	3.87610430097401E-07	4	P2RY10
**9.62815612309267E-13**	0.268998479675639	0.422	0.12	1.61348640310787E-08	4	ZNF83
**1.43660315185702E-12**	0.268825290436617	0.622	0.211	2.407459561882E-08	4	GRB2
**9.16257849829256E-09**	0.267526052013097	0.356	0.118	0.000153546490474387	4	N4BP2
**6.48996104935809E-13**	0.26669369921093	0.4	0.109	1.08758767265143E-08	4	EPRS
**4.92867924688022E-08**	0.265796833026729	0.289	0.091	0.000825948068192187	4	SRP68
**6.55351570566745E-09**	0.265292988827673	0.389	0.129	0.000109823816195575	4	EIF4G1
**3.37132102171989E-12**	0.264928596965633	0.689	0.258	5.64965976819819E-08	4	STK24
**9.11512715697063E-13**	0.262938176671647	0.567	0.187	1.52751300896514E-08	4	UBE2R2
**2.75996720195972E-11**	0.262551885895856	0.822	0.337	4.6251530370441E-07	4	PTPN7
**7.24350895945227E-07**	0.262358130767638	0.478	0.197	0.0121386723142501	4	CAPN1
**9.34951766998649E-12**	0.262212231828338	0.322	0.083	1.56679217113634E-07	4	IFNAR1
**1.45704390326503E-10**	0.262056370103182	0.256	0.062	2.44171417309153E-06	4	ACLY
**1.19002914821201E-07**	0.26081156833858	0.278	0.089	0.00199425084657369	4	FN1
**1.62039478913512E-10**	0.260592584098342	0.511	0.18	2.71545758763264E-06	4	SLC2A4RG
**1.76060421227616E-09**	0.259332830084363	0.333	0.101	2.95042053893239E-05	4	NF2
**2.02429495038594E-13**	0.259092006635107	0.444	0.124	3.39231347785675E-09	4	PPP1R35
**1.02644971054833E-06**	0.258169629173013	0.267	0.091	0.0172012442493689	4	COLGALT1
**1.18073794909124E-13**	0.25760238440815	0.444	0.126	1.97868065508711E-09	4	LTB4R
**8.87045188625395E-14**	0.257488667946243	0.511	0.151	1.48651032709844E-09	4	PURB
**2.55423156360771E-09**	0.255554144389814	0.389	0.127	4.2803812542938E-05	4	URI1
**2.48249536852745E-14**	0.255059092046312	0.378	0.094	4.16016573857831E-10	4	PSIP1
**1.5714158644503E-09**	0.253555212019059	0.833	0.373	2.63337870564581E-05	4	MYO1G
**1.88071271179183E-06**	0.252669416419562	0.8	0.37	0.0315169836242074	4	MT-ND2
**5.67875939510014E-13**	0.251826568242362	0.356	0.09	9.51646499430881E-09	4	TBC1D5
**9.34643619390933E-07**	0.251102709648162	0.322	0.116	0.0156627577737533	4	DNASE2
**7.00492093324368E-16**	0.250971218510444	0.756	0.246	1.17388464999298E-11	4	SP3

SupTable5F_C1_TNK_TNKSubtype

p_val	avg_log2FC	pct.1	pct.2	p_val_adj	cluster	gene
4.32166599435439E-113	2.86028361248747	0.773	0.349	7.24224787333908E-109	Naive/Memory T cell	IL7R
2.52990527280812E-92	1.47001823922716	0.968	0.898	4.23961525617184E-88	Naive/Memory T cell	CXCR4
1.05189814266322E-44	1.94576660590926	0.546	0.258	1.76277090747502E-40	Naive/Memory T cell	SERPINA1
1.18363079274935E-30	2.70094451875799	0.3	0.089	1.98352848248936E-26	Naive/Memory T cell	KLF2
5.12359322472582E-28	2.90647723153029	0.284	0.089	8.58611752599553E-24	Naive/Memory T cell	SATB1
1.63330634683215E-27	2.7369596104962	0.258	0.068	2.73709477602131E-23	Naive/Memory T cell	TCF7
4.75688013699405E-26	1.26973088582186	0.739	0.664	7.97157973357464E-22	Naive/Memory T cell	ZFP36L2
2.23446173601218E-22	1.3175472234212	0.693	0.703	3.74451097720921E-18	Naive/Memory T cell	TSC22D3
5.14561975177021E-21	3.19448284488245	0.206	0.057	8.62302958001652E-17	Naive/Memory T cell	CD40LG
4.64416195561114E-17	2.4598508903508	0.3	0.153	7.78268660521315E-13	Naive/Memory T cell	PER1
7.48980736918317E-15	2.15577673809306	0.283	0.145	1.25514191892772E-10	Naive/Memory T cell	ERN 1.00
1.64510130054104E-13	1.69757857720376	0.496	0.426	2.75686075944668E-09	Naive/Memory T cell	TNFAIP3
8.46165178501907E-12	2.12529363731236	0.254	0.14	1.4180036061335E-07	Naive/Memory T cell	TNFRSF25
1.1865006849376E-11	2.13516828329432	0.181	0.075	1.98833784781843E-07	Naive/Memory T cell	RASGRP2
1.21943719872971E-11	2.42453915883153	0.104	0.024	2.04353285763125E-07	Naive/Memory T cell	IGHG1
2.19148788492809E-11	2.24796098543778	0.22	0.109	3.6724953975625E-07	Naive/Memory T cell	CDC14A
2.21392343868217E-11	2.38741879543308	0.151	0.055	3.71009289854358E-07	Naive/Memory T cell	ITGA5
4.62886206355584E-11	1.85476607720812	0.258	0.146	7.75704704610687E-07	Naive/Memory T cell	TRAT1
3.16798924412546E-09	0.414391445848266	0.375	0.628	5.30891637530545E-05	Naive/Memory T cell	ALB
5.13154443924502E-09	1.55898479741799	0.231	0.132	8.5994421712868E-05	Naive/Memory T cell	MGAT4A
1.03464527517193E-08	0.342522560448666	0.205	0.356	0.000173385855213311	Naive/Memory T cell	IRF3
1.70689937598432E-08	3.21972386861126	0.1	0.033	0.000286042197427452	Naive/Memory T cell	RBP4
2.65177909197895E-08	1.34834864124595	0.393	0.315	0.000444385140233832	Naive/Memory T cell	CD69
2.7303764243139E-08	0.315890904097282	0.074	0.167	0.000457556481186524	Naive/Memory T cell	GPS2
3.20605453365477E-08	1.59381212450122	0.387	0.317	0.000537270618749866	Naive/Memory T cell	JUNB
3.28268236575232E-08	0.320454762541681	0.179	0.316	0.000550111910852773	Naive/Memory T cell	TERF2IP
4.2759033929425E-08	0.307666009751476	0.182	0.315	0.000716555890589304	Naive/Memory T cell	SLC20A1
4.37318626929782E-08	2.09010242241866	0.192	0.108	0.00073285855500893	Naive/Memory T cell	USP36
4.69554389857043E-08	0.271781210265906	0.107	0.215	0.000786879246522432	Naive/Memory T cell	RBM23
5.20805234371236E-08	1.03661903958519	0.517	0.489	0.000872765411759317	Naive/Memory T cell	AHNAK
6.79506878354304E-08	0.336022848407473	0.114	0.222	0.00113871762674614	Naive/Memory T cell	SLC5A3
7.97187667829817E-08	0.330248444396965	0.088	0.183	0.00133592709374921	Naive/Memory T cell	DEPRECATED-ENSG00000205937
9.13293566738534E-08	0.338311343707304	0.176	0.304	0.00153049735914044	Naive/Memory T cell	SLTM
1.24720816839307E-07	0.34207921529749	0.095	0.189	0.0020900714485931	Naive/Memory T cell	ANP32E
1.627447299502E-07	0.371372501929194	0.13	0.24	0.00272727618450545	Naive/Memory T cell	STIM2
1.76836335789198E-07	0.324631154543394	0.043	0.114	0.00296342331515539	Naive/Memory T cell	DEPRECATED-ENSG00000084463
1.81165315565352E-07	0.271852143395037	0.228	0.376	0.00303596835824417	Naive/Memory T cell	NUMA1
2.23413195818561E-07	0.368538020639829	0.227	0.372	0.00374395833552744	Naive/Memory T cell	CDC37
2.8672668862905E-07	0.406387196946126	0.213	0.35	0.00480496584804561	Naive/Memory T cell	USF2
3.11175937426444E-07	1.48587071734892	0.455	0.414	0.00521468635939235	Naive/Memory T cell	EML4
3.13247260962383E-07	0.353473554698521	0.187	0.313	0.00524939759920761	Naive/Memory T cell	SP140
3.36325431119975E-07	0.431082704512915	0.22	0.36	0.00563614157470854	Naive/Memory T cell	PAFAH1B1
3.62006431351464E-07	0.364367704056306	0.181	0.305	0.00606650377658783	Naive/Memory T cell	ARPC2
3.81096616956635E-07	0.269937068341455	0.216	0.352	0.00638641710695929	Naive/Memory T cell	HNRNPLL
3.96676354935565E-07	2.13638807008462	0.119	0.053	0.0066475023560102	Naive/Memory T cell	GPR183
4.61170830895033E-07	0.323549746711131	0.311	0.486	0.00772830078413896	Naive/Memory T cell	CLK1
4.8127047117473E-07	0.253241384639982	0.17	0.288	0.00806513055594612	Naive/Memory T cell	RAB11B
4.86561448977562E-07	0.2513538143746	0.149	0.26	0.00815379676196598	Naive/Memory T cell	DNAJA2
5.19827380916495E-07	1.86662822161153	0.26	0.182	0.00871126724939862	Naive/Memory T cell	ADAM19
5.34406545378386E-07	0.253449784167462	0.14	0.248	0.00895558488745099	Naive/Memory T cell	NIN
5.78240139661486E-07	0.434742540657048	0.113	0.212	0.00969014826044719	Naive/Memory T cell	RBM14
5.82787942137688E-07	1.81816298306078	0.219	0.139	0.00976636033434337	Naive/Memory T cell	ANXA1
6.06631461517196E-07	1.24266275213568	0.42	0.367	0.0101659300321052	Naive/Memory T cell	CYTH1
8.83833562784978E-07	0.271690123522103	0.174	0.288	0.0148112828451507	Naive/Memory T cell	STAG2
9.18299891836903E-07	0.335986511186537	0.166	0.28	0.0153888695874028	Naive/Memory T cell	DHX15
9.65023162178077E-07	0.26182127755809	0.296	0.456	0.0161718581517802	Naive/Memory T cell	IL10RA
1.16737279812451E-06	0.286785523706647	0.116	0.213	0.0195628333509706	Naive/Memory T cell	RABGGTB
1.24079566839967E-06	0.329116239562823	0.174	0.288	0.0207932538110417	Naive/Memory T cell	ZNF638
1.65308396116302E-06	0.386880838766983	0.137	0.239	0.0277023810211698	Naive/Memory T cell	RNPS1
1.65538979016431E-06	0.303460192342771	0.228	0.362	0.0277410221035735	Naive/Memory T cell	IL16
1.6734992244982E-06	0.25354736167946	0.089	0.173	0.0280445000041409	Naive/Memory T cell	KHDC4
1.91258393958103E-06	0.424075730909074	0.143	0.245	0.032051081659499	Naive/Memory T cell	YWHAB
1.93566333344527E-06	0.270974341245441	0.186	0.299	0.0324378461418758	Naive/Memory T cell	ERO1A
2.0938024899932E-06	0.390613618580065	0.14	0.242	0.0350879421273061	Naive/Memory T cell	FBRS

2.2484938035738E-06	0.339723146530705	0.238	0.365	0.0376802591602897	Naive/Memory T cell	HNRNPUL1
2.28848687747626E-06	0.345239569589277	0.151	0.255	0.0383504630927472	Naive/Memory T cell	SMARCA4
2.31744675317312E-06	1.8083276403715	0.158	0.087	0.0388357726896751	Naive/Memory T cell	CTSH
2.4004399674402E-06	0.314722420822341	0.186	0.3	0.0402265729743629	Naive/Memory T cell	BRD4
2.4562257497719E-06	0.301160212477765	0.179	0.292	0.0411614311146774	Naive/Memory T cell	DCAF8
2.70764738482454E-06	1.27398982656547	0.343	0.274	0.0453747548748896	Naive/Memory T cell	PPP2R5C
2.752069818938E-06	0.307305037699802	0.106	0.194	0.046119186025763	Naive/Memory T cell	GUK1
4.15835360898251E-175	4.75245463614005	0.716	0.057	6.9685689779329E-171	Treg	FOXP3
5.26523742107018E-113	3.32076454679003	0.641	0.093	8.82348487022942E-109	Treg	CTLA4
1.96170950672687E-112	3.03094398601998	0.693	0.118	3.28743279137289E-108	Treg	TNFRSF18
3.55825773800626E-103	2.31255686128419	0.833	0.225	5.96292831735088E-99	Treg	CD4
6.40937984191932E-84	3.81697643783094	0.461	0.058	1.07408387390884E-79	Treg	IL2RA
6.54134896782515E-84	3.58665144121618	0.405	0.036	1.09619926002814E-79	Treg	NTNG2
2.24747195111076E-82	3.470876287838	0.487	0.073	3.76631349567141E-78	Treg	IL1R1
3.08441470279185E-82	2.44345012987096	0.81	0.349	5.16886215893858E-78	Treg	CTSC
2.30954609600168E-81	4.14709215325405	0.356	0.024	3.87033734767962E-77	Treg	CCR8
1.91575038334509E-79	3.30524512135872	0.431	0.049	3.2104144924097E-75	Treg	TNFRSF4
3.94279221683716E-78	2.03472413150485	0.752	0.221	6.6073311969757E-74	Treg	TIGIT
8.8742691076628E-72	2.97085869212215	0.477	0.08	1.48715001706213E-67	Treg	TBC1D4
7.41024093835123E-67	2.75035638817227	0.461	0.079	1.2418081764489E-62	Treg	LINC02694
2.46407291189254E-66	2.06210816838807	0.673	0.205	4.12929338574953E-62	Treg	CD27
2.58905492394917E-64	2.40027000669694	0.464	0.082	4.33873824155401E-60	Treg	IKZF2
2.5990761854505E-64	2.83744547932658	0.487	0.1	4.35553187157795E-60	Treg	MAST4
1.77938664228423E-62	2.51249503908658	0.487	0.097	2.98189613513992E-58	Treg	TNFRSF9
2.2501583697885E-55	1.71326612251182	0.598	0.176	3.77081539609156E-51	Treg	ICOS
1.24017330999537E-54	1.62787594817189	0.673	0.219	2.07828243289023E-50	Treg	PIM2
1.24859471483389E-54	1.44311221396166	0.801	0.347	2.09239502311864E-50	Treg	MAF
2.35386214101626E-54	1.97712760449822	0.444	0.09	3.94460217591504E-50	Treg	STAM
1.00440704949561E-53	2.04940725370104	0.464	0.103	1.68318533354475E-49	Treg	IL18R1
8.14445619241313E-51	1.5455666100119	0.755	0.337	1.36484796872459E-46	Treg	TNFRSF1B
3.14734838965942E-50	4.48654163297958	0.222	0.015	5.27432643139125E-46	Treg	IL1R2
9.33416119612195E-50	1.22004627340461	0.935	0.62	1.56421873324612E-45	Treg	TRAC
1.32969634136171E-49	1.38499930505846	0.866	0.532	2.22830512885396E-45	Treg	IL32
8.24021780711412E-48	4.75564747216933	0.196	0.01	1.38089570011618E-43	Treg	CLEC7A
1.89324782490398E-47	1.65752851995136	0.572	0.185	3.1727047049741E-43	Treg	SLAMF1
5.35318015165034E-47	3.47153949954751	0.31	0.049	8.97085929813564E-43	Treg	GADD45A
5.9128444640263E-45	1.86563499496478	0.572	0.201	9.90874475281527E-41	Treg	PKM
1.2572581613745E-44	3.14089723887756	0.261	0.033	2.10691322683138E-40	Treg	VDR
6.62161702699155E-44	3.62980605488068	0.239	0.026	1.10965058138324E-39	Treg	CES4A
1.43727600346674E-42	3.26119028237035	0.275	0.041	2.40858712660956E-38	Treg	LTA
2.7134897445223E-42	2.40574262487613	0.369	0.081	4.54726611387048E-38	Treg	ACP5
5.20410825391692E-42	1.33703385312762	0.771	0.359	8.72104461191398E-38	Treg	LTB
9.83646857354943E-42	3.06859961716438	0.333	0.07	1.64839540355541E-37	Treg	SDC4
1.66168497593859E-41	1.6908122197913	0.575	0.213	2.78465168267788E-37	Treg	PHTF2
1.89429600913989E-41	1.45985339200697	0.598	0.232	3.17446125211663E-37	Treg	GBP2
2.1889783406575E-41	2.06885931982081	0.428	0.116	3.66828990327384E-37	Treg	BATF
6.08477440078526E-41	3.18045620926024	0.261	0.037	1.01968649408359E-36	Treg	LAIR2
8.31448650182678E-41	1.18021497488506	0.605	0.219	1.39334164797613E-36	Treg	CD28
8.85476702961285E-41	1.68827435013323	0.438	0.122	1.48388185882252E-36	Treg	PRNP
4.42390704470766E-40	1.44866486203503	0.67	0.297	7.4135834255211E-36	Treg	CCND2
9.35325205681859E-40	2.17184379057413	0.435	0.124	1.56741797968166E-35	Treg	PHLDA1
2.70357287113971E-39	1.92079932850629	0.34	0.072	4.53064741745593E-35	Treg	IL12RB2
1.07341541836612E-38	3.50925541983306	0.216	0.024	1.79882955809795E-34	Treg	IKZF4
1.1148675911016E-37	2.5655795110867	0.255	0.038	1.86829510916806E-33	Treg	LAYN
1.46097047913938E-36	2.01486201186872	0.389	0.103	2.44829432894178E-32	Treg	WHRN
2.64517502620728E-36	1.25775736969727	0.719	0.356	4.43278430891817E-32	Treg	S100A4
3.99839870625808E-36	2.10434733684417	0.294	0.058	6.70051655194729E-32	Treg	PELI1
6.25449174694571E-36	2.6901798788555	0.203	0.023	1.04812772695316E-31	Treg	SLC9A7
1.85058868351265E-35	1.30088115693971	0.353	0.081	3.10121651583049E-31	Treg	SELL
2.34493572070041E-35	1.82378262341872	0.425	0.128	3.92964328074974E-31	Treg	DUSP4
2.00253659253637E-34	2.18210080812307	0.412	0.128	3.35585082177244E-30	Treg	CSF1
3.20573715196832E-34	1.35817877660434	0.67	0.323	5.37217431926851E-30	Treg	UCP2
2.27074899698138E-33	3.02278419668199	0.163	0.014	3.8053211691414E-29	Treg	COL5A1
6.46504687210487E-33	3.8312961750537	0.15	0.011	1.08341255482733E-28	Treg	IL1RL1
1.86077185819567E-32	3.34581110749805	0.209	0.03	3.1182814799643E-28	Treg	ZBED2
1.94043692870013E-32	1.90046677226986	0.458	0.161	3.25178420511568E-28	Treg	CISH

4.56107952549036E-32	1.32534540922512	0.431	0.134	7.64345706881674E-28	Treg	IL21R
6.24693374990985E-32	4.26895146580433	0.144	0.01	1.04686115780989E-27	Treg	DEPRECATED-ENSG00000179934
1.62011861156711E-31	1.04892727961403	0.739	0.403	2.71499476926416E-27	Treg	IL2RB
1.73616165159077E-31	1.73665180043801	0.366	0.105	2.90945969573581E-27	Treg	SLC12A6
2.27578592450737E-31	2.51274633435501	0.216	0.033	3.81376205228944E-27	Treg	TNS3
3.01494422249611E-31	3.12540314411764	0.16	0.015	5.05244352805898E-27	Treg	CSF2RB
3.14973115821895E-31	1.98450220006672	0.369	0.114	5.27831947494331E-27	Treg	DPYSL2
1.52769305066665E-30	1.64840495724998	0.366	0.109	2.56010801430717E-26	Treg	SGPP1
1.84650971802373E-30	2.72262720166163	0.219	0.036	3.09438098546417E-26	Treg	ENTPD1
2.3180462436841E-30	2.9547770099959	0.216	0.036	3.88458189516582E-26	Treg	TBC1D8
2.92598946353064E-30	2.08975517460827	0.359	0.109	4.90337314298464E-26	Treg	GLRX
3.77094881441974E-30	1.67673855334551	0.392	0.126	6.3193560232046E-26	Treg	CXCR3
1.44227800230979E-29	1.22896828017063	0.526	0.215	2.41696947627075E-25	Treg	CTSB
2.45913266717009E-29	1.53763097825011	0.454	0.168	4.12101452364364E-25	Treg	SIRPG
7.42528679818587E-29	0.81752276212663	0.869	0.509	1.24432956163999E-24	Treg	SPOCK2
7.68713779603557E-29	1.40173025394489	0.487	0.194	1.28821055185964E-24	Treg	CD82
1.25937736512335E-28	1.01198711150445	0.627	0.299	2.11046458847371E-24	Treg	KAT2B
2.41386545559356E-28	2.24519380648105	0.245	0.051	4.04515573048369E-24	Treg	ARHGEF12
4.30614892008574E-28	1.03439476985691	0.539	0.22	7.21624436027968E-24	Treg	LTBP3
7.01568130870175E-28	1.89936066700128	0.275	0.068	1.17568787371224E-23	Treg	ABI2
2.54650694975309E-27	1.98742268705185	0.291	0.077	4.26743634639623E-23	Treg	ERI1
2.78443157170688E-27	2.27832198569663	0.232	0.048	4.6661504278664E-23	Treg	SLC35F2
4.04056269716942E-27	2.24136655368221	0.288	0.078	6.77117496791652E-23	Treg	LAPTM4B
4.95447294177529E-27	2.56217639962695	0.245	0.056	8.30270575582704E-23	Treg	SLC16A1
8.23285005115504E-27	2.25551956137748	0.297	0.085	1.37966101157256E-22	Treg	TYMP
9.61843686276787E-27	0.852412041080732	0.663	0.335	1.61185764946264E-22	Treg	TPP1
1.29240797745832E-26	2.76918592223613	0.163	0.021	2.16581728862465E-22	Treg	MCAM
1.64781970988257E-26	2.23374696160313	0.268	0.068	2.76141626982121E-22	Treg	MAGEH1
2.63652528894172E-26	1.50346115488296	0.314	0.089	4.41828907920853E-22	Treg	ADAT2
2.84604699269616E-26	1.17161263452547	0.435	0.163	4.76940555036022E-22	Treg	NCOA3
2.86392332872998E-26	1.66511509696209	0.438	0.172	4.7993627142857E-22	Treg	FAM53B
4.53585504316585E-26	0.847160357355526	0.641	0.316	7.60118588133734E-22	Treg	CD3D
4.98706716888935E-26	1.00591711858487	0.529	0.225	8.35732716162478E-22	Treg	FOXO1
7.52102739235708E-26	1.30530729349194	0.451	0.184	1.2603737704112E-21	Treg	CTSZ
7.91765242725655E-26	1.33322029614736	0.516	0.232	1.32684019375965E-21	Treg	ENO1
1.08229146536721E-25	0.631055036345261	0.546	0.226	1.81370403766237E-21	Treg	DUSP16
1.86108757975268E-25	1.24092233090295	0.32	0.094	3.11881056614954E-21	Treg	HAPLN3
2.07940354609799E-25	1.1721883124374	0.585	0.294	3.48466446255102E-21	Treg	ANKRD10
3.29803050125191E-25	1.54849774556103	0.392	0.141	5.52683951399795E-21	Treg	MAPKAPK3
3.95401187938395E-25	1.03549274720636	0.595	0.289	6.62613310747162E-21	Treg	CBFB
5.62344508296358E-25	0.972473728416833	0.601	0.309	9.42376927003037E-21	Treg	EFHD2
1.40587862578386E-24	2.24823883271681	0.176	0.029	2.3559714010886E-20	Treg	COL9A2
1.44542449581495E-24	2.34680814370687	0.265	0.071	2.4222423700867E-20	Treg	INPP5F
2.75403981713468E-24	0.896925553169387	0.66	0.343	4.61521992555429E-20	Treg	CD7
2.88026179997566E-24	2.33523787791615	0.209	0.043	4.82674272439921E-20	Treg	EPHX2
3.42952172556754E-24	0.988009029194637	0.477	0.203	5.74719250770608E-20	Treg	VMP1
5.7046555909358E-24	1.68792375317903	0.376	0.136	9.55986183929021E-20	Treg	THADA
1.20409538537024E-23	1.46359848896951	0.33	0.107	2.01782304680345E-19	Treg	PHACTR2
1.51084803866044E-23	2.69463592283459	0.163	0.026	2.53187914318716E-19	Treg	CD80
2.24174604274391E-23	0.881383899512459	0.605	0.296	3.75671801843024E-19	Treg	ARL6IP5
3.22933634510313E-23	2.1561219446946	0.248	0.066	5.41172184712382E-19	Treg	DNPH1
4.01297169775273E-23	1.66285722138401	0.265	0.072	6.72493797109403E-19	Treg	ITGAM
4.77226483734954E-23	1.48600345372746	0.382	0.146	7.99736141443036E-19	Treg	PTPRJ
9.3338720389263E-23	3.78659053068393	0.105	0.008	1.56417027628327E-18	Treg	TNFRSF8
1.11008701201667E-22	1.72025031551256	0.291	0.088	1.86028381473753E-18	Treg	PAM
1.13694284729939E-22	0.720554289610388	0.752	0.434	1.90528882350431E-18	Treg	CD3E
2.12344533157504E-22	0.645771362929357	0.908	0.706	3.55846968665346E-18	Treg	IL2RG
2.56179042759344E-22	1.06443027315698	0.395	0.148	4.29304839856109E-18	Treg	MKNK2
2.67328965336421E-22	0.950148370094513	0.68	0.382	4.47989880110774E-18	Treg	ZFP36L1
3.10749555984321E-22	1.36421884719347	0.408	0.166	5.20754105918526E-18	Treg	CASP1
4.3138159449477E-22	0.79546589683455	0.503	0.232	7.22909276054335E-18	Treg	MOB3A
4.45374959759882E-22	2.32063350752508	0.225	0.057	7.4635935756561E-18	Treg	CDIP1
4.81527865871036E-22	2.4823135589753	0.229	0.06	8.06944397626683E-18	Treg	CAMK1
8.3875342824324E-22	0.890439949955078	0.824	0.559	1.40558299505002E-17	Treg	RNF213
8.66922389230411E-22	3.04701794659046	0.121	0.014	1.45278853987232E-17	Treg	CTTNBP2NL
1.27365210843351E-21	3.39809965756538	0.124	0.015	2.13438620331288E-17	Treg	ZC2HC1A

1.3996375137349E-21	2.64625310207896	0.163	0.029	2.34551254551695E-17	Treg	TRIB1
1.50939192645228E-21	1.57283981065891	0.34	0.125	2.52943899034874E-17	Treg	NPTN
1.58077635080096E-21	2.95058306905429	0.124	0.015	2.64906500867225E-17	Treg	IL7
2.00552087646435E-21	2.49757886143953	0.16	0.028	3.36085188477895E-17	Treg	PLAGL1
2.02571205604842E-21	2.6541348955928	0.18	0.036	3.39468826352594E-17	Treg	KIAA1324
2.7500639606911E-21	0.634095337682851	0.948	0.729	4.60855718532615E-17	Treg	ACTB
3.05736805342333E-21	1.23452923116997	0.438	0.192	5.12353738392681E-17	Treg	NDFIP1
3.33608459820779E-21	0.6269086165564	0.725	0.409	5.59061056967661E-17	Treg	FTL
4.17278597070738E-21	0.859236502835675	0.376	0.146	6.99275472971142E-17	Treg	ZBTB7B
4.42821396900662E-21	1.26310782110647	0.474	0.218	7.42080096926129E-17	Treg	SYNGR2
4.72391674652052E-21	1.07436475784889	0.451	0.194	7.91633968381908E-17	Treg	PCED1B
4.74670275745389E-21	2.55318398515065	0.137	0.02	7.95452448094122E-17	Treg	HDAC9
4.82530249284077E-21	1.93957988998884	0.245	0.068	8.08624191750256E-17	Treg	B3GAT1
6.09893213875419E-21	1.46367391267408	0.343	0.127	1.02205904781243E-16	Treg	TGFBR1
8.41832784894731E-21	2.00591385358433	0.199	0.046	1.41074338092659E-16	Treg	SERPINE2
8.94165811181839E-21	1.84975532646866	0.186	0.04	1.49844306637853E-16	Treg	RTKN2
2.49108357248968E-20	1.43356735428053	0.386	0.16	4.1745578507782E-16	Treg	BCL2L1
3.15125151794526E-20	1.20962731041992	0.458	0.213	5.28086729377267E-16	Treg	CTSA
3.65784517496977E-20	1.30178173393188	0.373	0.147	6.12981694421435E-16	Treg	ORMDL3
5.36325416276405E-20	3.18232608066599	0.134	0.021	8.98774132595999E-16	Treg	MRC1
6.8801892816528E-20	2.28149430064349	0.124	0.017	1.15298211981938E-15	Treg	PARD6G
6.99167454030334E-20	1.7134862557227	0.265	0.084	1.17166481946403E-15	Treg	INF2
9.04486871810428E-20	1.75841859882514	0.284	0.096	1.51573909977992E-15	Treg	MAN1A2
1.5639646248009E-19	0.793636869764959	0.431	0.185	2.62089191824134E-15	Treg	ISCU
1.66193773047829E-19	0.764027697505708	0.663	0.36	2.78507524873553E-15	Treg	CHST11
2.26624264017349E-19	1.76548405903656	0.219	0.06	3.79776941640274E-15	Treg	NAMPT
3.07426304867615E-19	1.25529752465009	0.405	0.18	5.1518500169715E-15	Treg	PPM1G
3.19480889523905E-19	1.05785767744324	0.533	0.274	5.35386074664159E-15	Treg	ECH1
3.23718304983121E-19	0.97097226696039	0.474	0.223	5.42487135490715E-15	Treg	TM9SF2
3.64945452721761E-19	1.07429281690559	0.265	0.082	6.11575589671126E-15	Treg	ZC3H12D
3.8326000214748E-19	1.34913002327935	0.356	0.143	6.42267111598746E-15	Treg	GLCCI1
4.05633858843119E-19	1.8501471875822	0.245	0.076	6.79761220649299E-15	Treg	TIMP1
4.83609767656359E-19	2.23305499129625	0.118	0.016	8.10433248638527E-15	Treg	WNT10A
5.29323751209088E-19	1.10284961818786	0.382	0.162	8.8704074227619E-15	Treg	CD58
6.04857398021071E-19	1.13067259347451	0.399	0.169	1.01362002760371E-14	Treg	SLC7A5
7.19612615707466E-19	3.08313932775755	0.144	0.026	1.20592682140257E-14	Treg	GADD45G
8.25221024385165E-19	1.4853883049784	0.268	0.087	1.38290539266466E-14	Treg	KLHL2
9.65815976234166E-19	1.21444826069156	0.363	0.146	1.61851441297322E-14	Treg	CAB39
1.45057425500738E-18	1.37711382423748	0.337	0.136	2.43087233654137E-14	Treg	DYNC1I2
3.00731141814383E-18	1.80898977349357	0.154	0.031	5.03965247452543E-14	Treg	GNA15
4.0698583583492E-18	1.40423765563438	0.271	0.092	6.82026863692159E-14	Treg	SNX9
4.30999792919782E-18	0.929543005126011	0.395	0.168	7.22269452974971E-14	Treg	RAB11FIP1
5.27658580874953E-18	0.896180002661366	0.621	0.364	8.84250249830246E-14	Treg	LCP2
5.75621771162427E-18	2.1782420117574	0.131	0.022	9.64626964113995E-14	Treg	MINDY1
6.95328632558266E-18	1.33847137286946	0.284	0.1	1.16523172244114E-13	Treg	TRPS1
7.96905350235257E-18	1.01936985674211	0.405	0.183	1.33545398592424E-13	Treg	NFAT5
9.81683111723084E-18	1.48549913299378	0.33	0.134	1.64510455862554E-13	Treg	RHOC
1.02870916552652E-17	1.2505444934618	0.304	0.112	1.72391081958934E-13	Treg	IGFLR1
1.06858574017652E-17	1.00079528158422	0.477	0.24	1.79073598338782E-13	Treg	SOCS1
1.24550091997318E-17	1.2380571828603	0.255	0.083	2.08721044169106E-13	Treg	TULP4
1.31871123849505E-17	2.39161782990388	0.131	0.023	2.20989629347001E-13	Treg	TSPAN13
1.33834513398607E-17	2.21057730719495	0.17	0.04	2.24279877553385E-13	Treg	SLC41A1
1.45774436936796E-17	0.938417369428541	0.487	0.247	2.44288801418682E-13	Treg	LBH
1.99418127557809E-17	0.875445412472076	0.598	0.327	3.34184898161376E-13	Treg	LAMP1
2.27121230553615E-17	1.92829228745277	0.245	0.081	3.80609758161748E-13	Treg	LDLRAD4
2.36600028227626E-17	1.86858956203481	0.183	0.046	3.96494327303856E-13	Treg	C3AR1
2.51074549749337E-17	0.47775040686492	0.788	0.495	4.20750730469939E-13	Treg	STK17B
2.57122957919902E-17	2.39550802031614	0.124	0.021	4.30886652882171E-13	Treg	SMPD3
2.6537345592031E-17	1.55388761018717	0.137	0.026	4.44712837431256E-13	Treg	PTP4A3
4.02000223439789E-17	1.04425329138371	0.275	0.095	6.73671974440398E-13	Treg	UXS1
6.77544952274026E-17	1.17760348123821	0.366	0.157	1.13542983102081E-12	Treg	ADCY3
7.34236746004192E-17	0.833285354252581	0.592	0.329	1.23043393895382E-12	Treg	SAMHD1
7.44747935360993E-17	1.18617415215033	0.399	0.184	1.24804859007795E-12	Treg	SIT 1.00
8.54146126749633E-17	1.2074838223406	0.232	0.073	1.43137807920704E-12	Treg	LRRC8B
9.45194666895665E-17	1.48749727508573	0.222	0.07	1.58395722278376E-12	Treg	MICAL2
9.4964619270009E-17	2.33716322175174	0.137	0.027	1.59141708972681E-12	Treg	KBTBD8

9.6335542236764E-17	1.01464788654871	0.418	0.2	1.61439101680369E-12	Treg	MGAT1
1.08801316402322E-16	0.622078200574488	0.49	0.243	1.82329246027011E-12	Treg	LAT
1.09968209940845E-16	1.31629420838046	0.34	0.144	1.84284726218868E-12	Treg	NEDD9
1.138509400577E-16	1.49070821577817	0.252	0.087	1.90791405348694E-12	Treg	GRINA
1.1633574723376E-16	0.971212230613046	0.471	0.237	1.94955445214335E-12	Treg	LGALS1
1.32621600537933E-16	1.13810793103666	0.477	0.249	2.22247278181468E-12	Treg	TMEM173
1.55874498091954E-16	0.964383445937633	0.467	0.236	2.61214483902497E-12	Treg	OS9
3.37218807497095E-16	0.86991470364418	0.239	0.077	5.65111277603632E-12	Treg	KSR1
3.40570971784817E-16	3.06758725038855	0.101	0.014	5.70728834516996E-12	Treg	LMCD1
3.83055547015915E-16	0.323361582666077	0.641	0.353	6.41924485689271E-12	Treg	TRAF1
3.83721970427439E-16	1.23339709632468	0.19	0.052	6.43041278042302E-12	Treg	SNAP23
3.95506324735459E-16	0.647461656353679	0.732	0.466	6.62789498991683E-12	Treg	CD2
4.04960288782112E-16	1.31999528074912	0.284	0.109	6.78632451941063E-12	Treg	HS3ST3B1
5.91284263404529E-16	2.2048559183033	0.101	0.014	9.9087416861331E-12	Treg	GPAT3
5.92234501270297E-16	0.660392545882867	0.569	0.318	9.92466577228763E-12	Treg	PTPN7
6.12980471046441E-16	1.43405700829617	0.19	0.053	1.02723267337963E-11	Treg	SLC26A11
6.40791700148569E-16	0.537025415202621	0.807	0.505	1.07383873110897E-11	Treg	MT-ATP6
7.22203721963961E-16	0.834343493954406	0.431	0.211	1.21026899726721E-11	Treg	IGF2R
7.62818996144178E-16	1.19000439515328	0.327	0.139	1.27833207373841E-11	Treg	SH3BGRL
7.68183214968987E-16	0.74410055536352	0.451	0.226	1.28732143164503E-11	Treg	RAP1A
1.09670877877808E-15	0.722104459617765	0.601	0.34	1.8378645714763E-11	Treg	MT-ND5
2.24353395148497E-15	1.51623998063146	0.252	0.09	3.75971419589851E-11	Treg	TRAF3
2.79050606561439E-15	0.841083282180106	0.425	0.206	4.67633006475659E-11	Treg	MSI2
2.98588360945819E-15	0.640276763487222	0.735	0.484	5.00374375273003E-11	Treg	SLA
3.27843142756446E-15	0.659308238165104	0.686	0.409	5.49399538631251E-11	Treg	MT-CYB
3.52250287427967E-15	0.645055417800961	0.222	0.071	5.90301031671787E-11	Treg	SGSH
3.52785395664009E-15	0.474594655126084	0.49	0.249	5.91197766053747E-11	Treg	EMC10
3.88419373789213E-15	1.32579385046373	0.242	0.085	6.50913186595963E-11	Treg	PTPN9
4.91024627886567E-15	1.19998405318377	0.235	0.084	8.22859071412308E-11	Treg	PCYOX1L
5.58685133864317E-15	1.16860887091997	0.32	0.137	9.36244547329823E-11	Treg	AGFG1
7.32386794595948E-15	1.2726507847052	0.186	0.054	1.22733379038389E-10	Treg	ZNF589
8.23312485953304E-15	0.520213955763147	0.592	0.323	1.37970706396055E-10	Treg	SELPLG
8.80290682880112E-15	0.717891965286404	0.431	0.214	1.47519112637049E-10	Treg	GNPTAB
8.88874604368937E-15	1.86425698164178	0.203	0.067	1.48957606200146E-10	Treg	BEX3
9.11250901349302E-15	1.64464304730589	0.216	0.073	1.52707426048116E-10	Treg	CD59
1.34321993596546E-14	1.69970326584407	0.147	0.036	2.25096796869093E-10	Treg	FAM184A
1.37041281895457E-14	1.84367450177259	0.173	0.049	2.29653780200407E-10	Treg	RCL1
1.68413141708457E-14	2.26000229834335	0.111	0.021	2.82226742875033E-10	Treg	EVC
1.69499664977851E-14	0.605646738054989	0.379	0.17	2.84047538569883E-10	Treg	CCR4
1.73636456067004E-14	1.06921547990403	0.474	0.262	2.90979973077085E-10	Treg	TFRC
1.81263255798418E-14	0.88664626414088	0.516	0.29	3.03760964066988E-10	Treg	GBP5
2.12555697655921E-14	0.599795503174978	0.395	0.183	3.56200838131793E-10	Treg	HAVCR2
2.75245516917792E-14	0.525031723211087	0.435	0.213	4.61256437250837E-10	Treg	SERPINB9
3.1837357044042E-14	0.993066470363543	0.317	0.137	5.33530429344057E-10	Treg	EMC4
3.54008765328439E-14	1.1219881047779	0.248	0.094	5.93247888937398E-10	Treg	MBOAT7
3.5662025661901E-14	0.643501354253966	0.513	0.279	5.97624226042137E-10	Treg	CD3G
3.84825810806371E-14	1.14804123085263	0.294	0.124	6.44891093749316E-10	Treg	GOLGA8B
4.16567990224261E-14	0.622695414229404	0.422	0.214	6.98084638017816E-10	Treg	CASK
4.21750348237537E-14	1.22481133005272	0.288	0.119	7.06769233576464E-10	Treg	PTMS
4.59414746888692E-14	2.11409770455735	0.141	0.034	7.6988723283607E-10	Treg	TMPRSS3
4.92690855420319E-14	0.760834996782579	0.801	0.625	8.2565133551337E-10	Treg	TRBC2
4.95930598198843E-14	0.812502808258484	0.497	0.267	8.31080496461621E-10	Treg	LMAN2
4.99014222951094E-14	1.46956512226968	0.229	0.083	8.36248034821444E-10	Treg	CD79B
5.08335676339287E-14	0.625383010043973	0.353	0.16	8.51868926409377E-10	Treg	RFTN1
5.60025758486041E-14	1.70496466540059	0.144	0.036	9.38491166070907E-10	Treg	GADD45B
8.1187626554403E-14	1.71231834209789	0.199	0.065	1.36054224579869E-09	Treg	NCALD
8.75342935802178E-14	1.34697748878421	0.199	0.065	1.46689969181729E-09	Treg	GCNT1
9.45717836545599E-14	1.43803181754566	0.261	0.106	1.58483395048312E-09	Treg	CREB3L2
9.55264281480069E-14	0.478167213446964	0.673	0.421	1.6008318829043E-09	Treg	PRDM1
9.6454042770625E-14	0.622420524103138	0.454	0.235	1.61637684875013E-09	Treg	BIRC3
1.04185364130921E-13	0.540555454435263	0.356	0.161	1.74593833210597E-09	Treg	N4BP1
1.14066514000266E-13	2.6370378955079	0.105	0.019	1.91152664161645E-09	Treg	KLHL11
1.27037408204743E-13	1.16841477655557	0.278	0.114	2.12889288669508E-09	Treg	USP12
1.47307404213646E-13	1.42016876437549	0.199	0.065	2.46857747981228E-09	Treg	CDKN2B
1.69690472345304E-13	0.572458955882403	0.549	0.315	2.8436729355626E-09	Treg	BCL2
1.72304569511412E-13	0.976573682034685	0.297	0.128	2.88747997587224E-09	Treg	RNGTT

1.74926526195449E-13	0.734120296475638	0.307	0.131	2.93141872598333E-09	Treg	KDM5B
1.82403155596662E-13	0.527489429102517	0.395	0.19	3.05671208148886E-09	Treg	HDAC7
2.14951066671226E-13	1.87902550111647	0.124	0.028	3.60214997527641E-09	Treg	SIRPB1
2.24250145252109E-13	0.559241126175342	0.431	0.225	3.75798393413485E-09	Treg	TAB2
2.34316109230805E-13	1.1671934593415	0.337	0.161	3.92666935848984E-09	Treg	HADHA
2.94207433586835E-13	1.38431769747958	0.258	0.104	4.93032817204818E-09	Treg	ZNRF1
3.98234398113888E-13	0.752273176489422	0.363	0.171	6.67361204359253E-09	Treg	MXD4
4.08651228209369E-13	1.07331942517416	0.294	0.129	6.8481772823326E-09	Treg	AFTPH
5.263917899241E-13	0.939958891899145	0.284	0.121	8.82127361554807E-09	Treg	CCNG2
5.64065813853838E-13	0.956069299194324	0.278	0.117	9.45261490856261E-09	Treg	STIMATE
7.00750427870881E-13	0.631212914059441	0.343	0.16	1.17431756702602E-08	Treg	OTUD5
7.40049324514195E-13	0.6817810080657	0.592	0.35	1.24017465802089E-08	Treg	MT-ND2
7.80356634275356E-13	1.31737063664022	0.265	0.112	1.30772164771864E-08	Treg	ATP2A2
9.52690395241162E-13	0.542276696637384	0.725	0.453	1.59651856434514E-08	Treg	ACTG1
1.31473626345073E-12	1.13008304327888	0.255	0.104	2.20323503029073E-08	Treg	STN 1.00
1.47366290907327E-12	1.46804914061665	0.216	0.08	2.46956430302499E-08	Treg	VPS54
1.50080253330019E-12	1.0534959262156	0.268	0.114	2.51504488530446E-08	Treg	MFHAS1
1.85340469651913E-12	0.500097476892148	0.392	0.195	3.10593559042676E-08	Treg	ZNRF2
1.89148385689821E-12	1.10501042272146	0.324	0.154	3.16974864739002E-08	Treg	TMED5
1.94097406385067E-12	1.580025464751	0.232	0.094	3.25268433620096E-08	Treg	PLD3
1.98186761517303E-12	1.05924423677645	0.307	0.142	3.32121374950696E-08	Treg	UBASH3B
1.99230742539953E-12	0.795953333506966	0.154	0.044	3.33870878348452E-08	Treg	GDPD5
2.25182226292838E-12	1.40831995138799	0.252	0.104	3.77360374821538E-08	Treg	NUDT5
2.25753691864232E-12	0.686642626374162	0.291	0.127	3.7831803682608E-08	Treg	FAS
2.33495853589533E-12	1.44795482351288	0.203	0.072	3.91292351445339E-08	Treg	SGMS1
2.42838862458564E-12	0.297872977557027	0.977	0.913	4.06949365708061E-08	Treg	B2M
2.57637871586904E-12	1.35678236846808	0.242	0.1	4.31749545205334E-08	Treg	SPATS2L
2.66349173661949E-12	0.633864109263702	0.359	0.171	4.46347945222694E-08	Treg	NABP1
2.86951468895066E-12	1.45813433472648	0.216	0.082	4.80873271574352E-08	Treg	INPP1
2.93748015228924E-12	1.42097560789213	0.255	0.109	4.9226292392063E-08	Treg	UGP2
2.95542428254043E-12	0.841510957655357	0.271	0.114	4.95270001268125E-08	Treg	COMMD7
3.24246356081219E-12	1.1801580852919	0.199	0.072	5.43372043520906E-08	Treg	TIAM1
3.26382506064294E-12	0.783557331119895	0.477	0.275	5.46951803662544E-08	Treg	LRBA
3.48378662686864E-12	1.09442209630305	0.271	0.115	5.83812962930647E-08	Treg	AGTRAP
3.48481047913359E-12	1.8583538763409	0.105	0.022	5.83984540093206E-08	Treg	NFE2L3
3.51835226505818E-12	1.42805324226808	0.219	0.084	5.8960547257845E-08	Treg	DGCR2
3.61763039544643E-12	1.70238650234513	0.141	0.039	6.06242501668913E-08	Treg	TMEM273
4.10408284484192E-12	1.55052563202873	0.127	0.033	6.87762203138609E-08	Treg	PPP1R3F
4.39143943885434E-12	0.893878304668632	0.366	0.187	7.3591742116321E-08	Treg	RAB10
5.12635560455205E-12	0.470692392968795	0.595	0.356	8.59074672210832E-08	Treg	IKBKB
5.16218543064124E-12	1.81368160179629	0.183	0.064	8.65079034466859E-08	Treg	TOX2
5.34963235762231E-12	1.04024533950694	0.294	0.132	8.96491390490347E-08	Treg	FNDC3B
5.36453893017325E-12	1.76736750385987	0.147	0.043	8.98989433918434E-08	Treg	SETBP1
5.5093184489271E-12	0.441282642922256	0.644	0.399	9.23251585671203E-08	Treg	SPN
6.67864972285993E-12	0.352384643212857	0.768	0.47	1.11920812055687E-07	Treg	MT-CO3
6.83993551396786E-12	0.630718552220244	0.454	0.254	1.14623639343073E-07	Treg	ADAM10
7.25971726303803E-12	0.693426916551976	0.451	0.255	1.21658341893991E-07	Treg	CKLF
7.47831364833637E-12	0.964214874162628	0.379	0.203	1.25321580118821E-07	Treg	PSAP
8.34786907454047E-12	0.430020418896634	0.765	0.503	1.39893589951149E-07	Treg	CFLAR
9.24033808157039E-12	0.795763976182243	0.248	0.102	1.54849585570957E-07	Treg	BACH1
9.35878262199721E-12	0.460247979336814	0.493	0.279	1.56834479179429E-07	Treg	CHD3
9.44539448749507E-12	1.44404613986881	0.193	0.069	1.58285920821442E-07	Treg	CLNK
1.08858325205873E-11	0.778904222890392	0.281	0.125	1.82424781380002E-07	Treg	UBA1
1.13212392835983E-11	0.315734899182138	0.663	0.415	1.8972132791454E-07	Treg	FXYD5
1.13386585788496E-11	0.296167832272026	0.392	0.203	1.90013240464362E-07	Treg	SH2D2A
1.16184707359196E-11	1.22717369320107	0.258	0.112	1.9470233259254E-07	Treg	SYT11
1.17064292081435E-11	1.22629117882426	0.297	0.146	1.96176340670068E-07	Treg	LMAN1
1.17322276092018E-11	1.1727395295084	0.255	0.109	1.96608670275004E-07	Treg	SEC11C
1.20786658653085E-11	0.76027315017469	0.268	0.116	2.02414282570839E-07	Treg	RYBP
1.33303133728775E-11	0.520927530957729	0.477	0.277	2.23389391502681E-07	Treg	HBS1L
1.49594217802358E-11	0.593796500137269	0.379	0.197	2.50689990193191E-07	Treg	SURF4
1.9076743493197E-11	0.710384166899383	0.5	0.287	3.19688067458996E-07	Treg	TRAF3IP3
2.25683086006746E-11	0.567209562742742	0.395	0.208	3.78199715530106E-07	Treg	SAT1
2.26186800558456E-11	0.894729402602588	0.242	0.101	3.79043840375861E-07	Treg	IER5
2.49787186052337E-11	0.691220238599481	0.235	0.095	4.18593366386507E-07	Treg	GALNT10
3.28670816308919E-11	0.561783679326699	0.288	0.133	5.50786553970486E-07	Treg	IL12RB1

3.4494514439806E-11	0.860699352554128	0.268	0.12	5.78059072982269E-07	Treg	MKNK1
4.32753305814797E-11	1.10986283572073	0.144	0.043	7.25207989884436E-07	Treg	SMYD3
4.42602990096471E-11	0.566521900873697	0.484	0.284	7.41714090803667E-07	Treg	TSPAN14
4.88616126422337E-11	0.831638201376278	0.297	0.142	8.18822904658553E-07	Treg	ITPR1
5.30608229075413E-11	1.68958967631613	0.147	0.046	8.89193270284578E-07	Treg	KCNN4
5.60994226466671E-11	0.528134746455107	0.559	0.346	9.40114124712847E-07	Treg	H3F3A
5.73293943130805E-11	0.521262468697774	0.48	0.284	9.60725989898602E-07	Treg	ANXA11
6.0491412279258E-11	0.587178930066438	0.258	0.114	1.01371508697581E-06	Treg	FOXN3
6.25528219020883E-11	0.68881624646216	0.314	0.155	1.0482601894352E-06	Treg	DNMT3A
6.4617297072049E-11	2.08457682453697	0.108	0.026	1.0828566643334E-06	Treg	ADPRH
7.14327969377545E-11	0.910956499186933	0.261	0.119	1.19707081108289E-06	Treg	REXO2
7.55577551890653E-11	0.738045396433927	0.382	0.208	1.26619686145836E-06	Treg	ARPC1B
7.96171064805295E-11	1.10427386831047	0.248	0.111	1.33422347040071E-06	Treg	GSTO1
8.67042175534221E-11	1.7245492203684	0.167	0.059	1.45298927776025E-06	Treg	OAS1
8.909391424026E-11	0.873197910588955	0.19	0.071	1.49303581483828E-06	Treg	FYCO1
9.87993160084174E-11	1.19739699431099	0.216	0.087	1.65567893766906E-06	Treg	ITPA
1.06083519667289E-10	0.542443764731394	0.441	0.251	1.77774762258444E-06	Treg	IPCEF1
1.10536978971837E-10	1.20095076036492	0.255	0.117	1.85237869361005E-06	Treg	NDUFA4
1.12535400494892E-10	1.23250634255153	0.19	0.071	1.88586824149339E-06	Treg	VAV3
1.30043621193823E-10	0.593287057826532	0.284	0.132	2.17927100396608E-06	Treg	NPDC1
1.38429840852814E-10	1.88514756936276	0.15	0.05	2.31980727301146E-06	Treg	HLF
1.41737839986073E-10	1.50189696901003	0.114	0.031	2.37524272248662E-06	Treg	PRKCI
1.45853723405148E-10	0.414244627141811	0.788	0.51	2.44421669682348E-06	Treg	MT-ND4
1.49602496793699E-10	1.15301042348827	0.206	0.083	2.5070386412688E-06	Treg	GRSF1
1.50613417429198E-10	1.25491528024208	0.16	0.055	2.5239796492785E-06	Treg	SKAP2
1.62912848027911E-10	0.954613322299241	0.173	0.062	2.73009350725173E-06	Treg	NCF4
1.71295549375224E-10	1.04796746588256	0.278	0.134	2.87057081643E-06	Treg	SOD1
1.86952194049218E-10	1.58478298709533	0.167	0.06	3.1329448678768E-06	Treg	CDK14
1.94972166685353E-10	1.32172065021965	0.278	0.137	3.26734356931314E-06	Treg	IFI6
2.3475903336087E-10	1.21753049020524	0.209	0.087	3.93409188106146E-06	Treg	ARPP19
2.36881301548338E-10	1.57342295826887	0.173	0.064	3.96965685134704E-06	Treg	FANCL
2.40988608646176E-10	0.502719716351901	0.634	0.407	4.03848710369262E-06	Treg	MT-ND3
2.41010298755E-10	0.777967548528445	0.343	0.189	4.0388505865363E-06	Treg	RNF187
2.48658824236217E-10	0.485151139383105	0.454	0.259	4.16702457655052E-06	Treg	CALM3
2.50648876459828E-10	0.684944029908604	0.183	0.068	4.2003738717138E-06	Treg	CLCC1
2.52413586958942E-10	0.419168072623145	0.356	0.188	4.22994689025795E-06	Treg	SQSTM1
2.59116301867588E-10	0.902725352939289	0.327	0.171	4.34227098669704E-06	Treg	CMTM6
2.60452570240048E-10	1.7470353650405	0.147	0.049	4.36466417208273E-06	Treg	TSPAN33
2.93030193942797E-10	0.281352631652152	0.359	0.188	4.91059999009338E-06	Treg	ARF1
2.99635291610081E-10	1.09993585828164	0.297	0.149	5.02128821680174E-06	Treg	COX6C
3.02949563148208E-10	1.25870052744088	0.17	0.061	5.07682877923768E-06	Treg	PGAP3
3.3413216562137E-10	1.96863381127272	0.118	0.033	5.59938683148291E-06	Treg	SLC41A2
3.35506289405043E-10	0.287646545686436	0.454	0.259	5.62241439784972E-06	Treg	SDF4
3.51642287986883E-10	1.16776454184438	0.245	0.111	5.89282146208418E-06	Treg	HADHB
3.86376495117773E-10	2.31176421672189	0.101	0.026	6.47489730518363E-06	Treg	SGPP2
3.99243387390644E-10	0.812215289113305	0.225	0.096	6.69052068589241E-06	Treg	C14orf119
4.28258470347654E-10	0.36127283982029	0.408	0.229	7.17675544608598E-06	Treg	PTPN1
4.4269542914023E-10	0.663278785167648	0.33	0.171	7.41869000153197E-06	Treg	SYNE3
4.6355848399113E-10	1.02829253534367	0.271	0.132	7.76831307472336E-06	Treg	ATP13A3
4.7192689693352E-10	0.510697540633712	0.386	0.209	7.90855093881193E-06	Treg	ANAPC16
5.51269685557266E-10	1.3763655860152	0.167	0.061	9.23817739056867E-06	Treg	DESI1
5.55800101956734E-10	1.72648205194221	0.147	0.05	9.31409810859095E-06	Treg	COPS7A
5.56789010367791E-10	0.997773345122215	0.301	0.156	9.33067023574345E-06	Treg	SNX5
5.62527610304772E-10	1.30525808973545	0.212	0.09	9.42683769348737E-06	Treg	HTATIP2
5.84832142239388E-10	1.12782554911421	0.242	0.111	9.80061703964766E-06	Treg	NCOA4
5.92510084460968E-10	0.397550166095808	0.291	0.14	9.92928399539691E-06	Treg	TXNDC11
6.07863590853971E-10	0.828279519987313	0.275	0.131	1.01865780555308E-05	Treg	GSK3B
6.44611828969371E-10	1.52669840382382	0.209	0.092	1.08024050298687E-05	Treg	ROMO1
6.82213474050187E-10	0.737936896417024	0.376	0.208	1.1432533398133E-05	Treg	TRAM1
6.94958088178088E-10	0.73668653805485	0.219	0.095	1.16461076416884E-05	Treg	LFNG
7.30749572015425E-10	1.75986536914461	0.101	0.026	1.22459013278345E-05	Treg	DEPRECATED-ENSG00000196743
7.37682346192916E-10	0.460089601014421	0.807	0.574	1.23620807575009E-05	Treg	PFN1
7.48534182934195E-10	0.818152636765729	0.34	0.184	1.25439358376112E-05	Treg	TLK1
8.08871593010657E-10	1.07828998448662	0.167	0.061	1.35550701556726E-05	Treg	MIIP
8.3495263758221E-10	1.11412772592264	0.258	0.12	1.39921363006027E-05	Treg	IL10RB
8.47045348796228E-10	0.846959156562699	0.219	0.092	1.41947859551272E-05	Treg	AKAP5

8.52384347529135E-10	1.15971002241136	0.222	0.097	1.42842568958932E-05	Treg	SAR1B
9.11832002395051E-10	0.336765318883422	0.85	0.595	1.52804806961363E-05	Treg	MT-CO1
9.12471455526564E-10	0.795866767642519	0.294	0.145	1.52911966517142E-05	Treg	GALM
1.03408707048681E-09	0.594734691692291	0.304	0.151	1.73292311272179E-05	Treg	EPSTI1
1.03798313826246E-09	1.34078924989249	0.199	0.084	1.73945214310023E-05	Treg	ATOX1
1.11979941427875E-09	0.353008576229003	0.608	0.392	1.87655985844832E-05	Treg	HCLS1
1.11990747689582E-09	0.541937697737433	0.487	0.29	1.87674094978201E-05	Treg	CD52
1.16186681784366E-09	1.85372093518454	0.108	0.03	1.9470564133424E-05	Treg	GRK3
1.33887282029467E-09	1.13009079566025	0.235	0.108	2.2436830722498E-05	Treg	CCDC141
1.40046344392125E-09	0.84438583915531	0.297	0.151	2.34689663932322E-05	Treg	MFSD6
1.40167870104337E-09	0.860810771303347	0.255	0.117	2.34893316720848E-05	Treg	MAP3K5
1.42502123306251E-09	0.509829343112305	0.268	0.127	2.38805058236616E-05	Treg	ZDHHC18
1.4496222078308E-09	0.867559961810205	0.327	0.176	2.42927689588285E-05	Treg	ZBTB38
1.46469234347387E-09	0.487944172178953	0.422	0.243	2.45453142919351E-05	Treg	SCAMP2
1.4939829620975E-09	0.400016400316922	0.451	0.267	2.50361664788299E-05	Treg	SERINC3
1.57047800752729E-09	0.505718311819812	0.359	0.193	2.63180704501423E-05	Treg	SLC44A2
1.69709713767363E-09	1.10586138127421	0.18	0.07	2.84399538331348E-05	Treg	ADORA2A
1.81781821180012E-09	1.30076971816184	0.18	0.071	3.04629975933464E-05	Treg	PSMB5
1.83429056684057E-09	0.909800456882461	0.229	0.104	3.07390413191142E-05	Treg	EED
1.85010608787489E-09	0.489789531724441	0.48	0.289	3.10040778206074E-05	Treg	CCR5
1.85932257286345E-09	0.400932920770339	0.33	0.171	3.11585276760457E-05	Treg	PITPNM1
1.90368555001114E-09	0.971321991391843	0.232	0.107	3.19019624470867E-05	Treg	HSP90B1
1.96162425617063E-09	1.32172183667199	0.176	0.07	3.28728992849074E-05	Treg	STAMBPL1
2.08687031193869E-09	0.587289028764713	0.461	0.275	3.49717726874686E-05	Treg	BST2
2.11566144061413E-09	1.68411860984597	0.114	0.034	3.54542544218116E-05	Treg	AP1S2
2.18727445138E-09	0.793363922064092	0.363	0.202	3.66543452562261E-05	Treg	EID1
2.18998653524278E-09	1.0161958774761	0.284	0.149	3.66997943575986E-05	Treg	PPIB
2.31739102601445E-09	0.317954833105743	0.735	0.492	3.88348388139501E-05	Treg	CD53
2.55660712526226E-09	1.24596899135517	0.209	0.092	4.28436222051449E-05	Treg	LIPA
2.70929184865427E-09	2.16962375247095	0.105	0.029	4.54023127997483E-05	Treg	PHLPP1
2.93868891792616E-09	0.74578106741839	0.108	0.031	4.92465488866066E-05	Treg	POU2F2
3.03928088954626E-09	1.2354804993077	0.173	0.068	5.09322691470162E-05	Treg	IKBKE
3.16726118931904E-09	0.623454968284873	0.451	0.277	5.30769630106084E-05	Treg	RCSD1
3.41412843272339E-09	1.08562986749919	0.248	0.121	5.72139642755785E-05	Treg	ANXA7
3.45593486593075E-09	0.471547092041489	0.353	0.196	5.79145564832675E-05	Treg	IFNAR2
3.51084130078753E-09	1.18859023837818	0.15	0.054	5.88346785185974E-05	Treg	ZDHHC21
3.76260772867811E-09	0.3643599831328	0.503	0.31	6.30537803171878E-05	Treg	DOK2
4.01005763313375E-09	0.558545717032039	0.386	0.219	6.72005458160554E-05	Treg	ICAM3
4.01785787915898E-09	0.887955921882922	0.193	0.082	6.73312623389461E-05	Treg	C21orf91
4.05956101977631E-09	0.83616984834089	0.275	0.139	6.80301235694115E-05	Treg	RFX5
4.53128131691845E-09	1.58067820210096	0.118	0.036	7.59352123089194E-05	Treg	GOLGA5
4.74225642890673E-09	0.289753402346977	0.784	0.512	7.9470733235619E-05	Treg	MT-CO2
4.86229526583978E-09	0.917871035009515	0.252	0.122	8.1482344064943E-05	Treg	ZNF292
5.17759700283351E-09	0.691270642188683	0.258	0.127	8.67661705734839E-05	Treg	TRAPPC4
5.37013585802213E-09	1.40454440891899	0.157	0.06	8.99927367087348E-05	Treg	TMEM154
5.39539908715646E-09	0.626368998156898	0.373	0.214	9.04160979025679E-05	Treg	MAT2B
5.43795708966141E-09	1.65364850527126	0.118	0.037	9.11292849085459E-05	Treg	TDRD3
5.94282988760225E-09	0.670960598860077	0.356	0.198	9.95899432564385E-05	Treg	CTSS
5.95269820490144E-09	0.846628651985306	0.343	0.192	9.97553165177384E-05	Treg	ATP5F1E
5.98622358717908E-09	0.683359628311175	0.209	0.092	0.000100317134873947	Treg	P4HA1
6.18568542459725E-09	2.14413488084514	0.118	0.038	0.000103659716345401	Treg	MYB
6.26029331447163E-09	1.32923241043454	0.203	0.086	0.000104909995363916	Treg	TTN
6.98661859924573E-09	0.367546667679899	0.523	0.335	0.00011708175448616	Treg	PICALM
6.991635022086E-09	0.622455545065121	0.379	0.22	0.000117165819700117	Treg	ERBIN
7.04272829798647E-09	1.5338882653156	0.147	0.055	0.000118022040817657	Treg	TKFC
7.14900695815962E-09	0.983561730855159	0.108	0.032	0.000119803058604839	Treg	MOB3C
7.14990471431572E-09	1.22044299244042	0.186	0.079	0.000119818103202503	Treg	NSF
7.33210793006819E-09	0.671445489172444	0.265	0.133	0.000122871464692083	Treg	MAN2B1
7.73444619865332E-09	1.18784330856366	0.176	0.073	0.000129613849397032	Treg	CALU
7.83399624908776E-09	0.45471083562491	0.32	0.172	0.000131282109142213	Treg	OGDH
7.95971058606728E-09	0.483155269420218	0.275	0.136	0.000133388830001316	Treg	SLC6A6
8.24985721290257E-09	1.13020050932847	0.186	0.079	0.000138251107173821	Treg	CPEB4
8.72309227709044E-09	1.69502292373105	0.121	0.039	0.000146181580379482	Treg	TNIP3
8.8454127962258E-09	0.751724793945833	0.216	0.098	0.000148231427639152	Treg	SUSD3
8.99078477506147E-09	0.636050480169048	0.265	0.132	0.00015066757126048	Treg	ECE1
9.19920348647951E-09	0.470551533428788	0.376	0.217	0.000154160252026424	Treg	SLC38A10

9.55071101638024E-09	0.793746245668673	0.255	0.124	0.0001600508152125	Treg	TRAPPC3
9.70516266724785E-09	1.07428052892723	0.176	0.073	0.00016263911597774	Treg	PSMD7
9.9197638848507E-09	0.299838727988601	0.291	0.149	0.000166235403182328	Treg	GMIP
9.96651675115402E-09	1.25643503003732	0.193	0.085	0.000167018887715839	Treg	NDFIP2
1.06293403648456E-08	1.0485279187219	0.278	0.146	0.000178126485834082	Treg	FKBP1A
1.07404572264644E-08	0.414908829695118	0.925	0.715	0.000179988582201091	Treg	CD74
1.12352426741366E-08	0.806890423422575	0.219	0.1	0.00018828019673318	Treg	ANTXR2
1.18258525327919E-08	1.03766552220354	0.186	0.081	0.000198177636744527	Treg	CTNNA1
1.22189866728626E-08	0.933330232474086	0.268	0.136	0.000204765778663832	Treg	CLIP1
1.27733887978134E-08	0.747546594075428	0.258	0.126	0.000214056449473758	Treg	AHR
1.32203562582807E-08	1.41706166462666	0.173	0.071	0.000221546730176268	Treg	E2F3
1.34324879545237E-08	1.26157696027499	0.17	0.07	0.000225101633141908	Treg	ATP6V1E1
1.37004224858088E-08	0.688897268182448	0.461	0.299	0.000229591680017184	Treg	NUCB1
1.37637285671001E-08	0.851422029418418	0.167	0.065	0.000230652563327463	Treg	PITPNM2
1.4443868078362E-08	1.14122074029153	0.163	0.065	0.000242050341257191	Treg	IRF4
1.56089312988108E-08	0.848894137094891	0.265	0.134	0.000261574470705471	Treg	CHURC1
1.59138585203533E-08	0.834651988248849	0.173	0.07	0.00026668444108408	Treg	PGK 1.00
1.63129131396417E-08	0.505726261692849	0.435	0.266	0.000273371798394115	Treg	LASP1
1.63182962991134E-08	1.01630359747126	0.147	0.055	0.000273462009380542	Treg	TRMT1L
1.66528940916284E-08	0.717130719062105	0.239	0.117	0.000279069199187509	Treg	MAP2K3
1.6711107151129E-08	0.582472650576232	0.278	0.143	0.00028004473363862	Treg	PRKACA
1.73243228819701E-08	0.663652668006084	0.333	0.193	0.000290321002856054	Treg	ATP6V0E1
1.79242663620598E-08	1.35432649335036	0.118	0.038	0.000300374855695399	Treg	ZBED6CL
1.79369132535766E-08	0.413371092015268	0.333	0.181	0.000300586792303436	Treg	TNIP1
1.79860743118124E-08	0.347725593273044	0.68	0.472	0.000301410633317353	Treg	TMSB10
1.81273699832117E-08	1.266993443461	0.163	0.066	0.000303778466178662	Treg	DNAJC15
1.98979208631946E-08	1.45758280782227	0.118	0.039	0.000333449357825416	Treg	KIF1B
2.01622934302373E-08	0.30539416210026	0.693	0.483	0.000337879713303916	Treg	RAC2
2.0182912631623E-08	0.927799462666763	0.268	0.139	0.000338225249880738	Treg	HM13
2.05154034701803E-08	0.269628428495696	0.382	0.218	0.000343797131353281	Treg	LRPAP1
2.06297227589282E-08	0.258854649039754	0.412	0.243	0.000345712893994119	Treg	DAZAP2
2.12859192012719E-08	1.10669907249328	0.176	0.075	0.000356709433974915	Treg	GTF3C6
2.18756588251679E-08	0.508614589992055	0.422	0.257	0.000366592290592163	Treg	DOCK10
2.24277308868262E-08	0.431195839538279	0.294	0.151	0.000375843914201434	Treg	LPAR2
2.29776645760691E-08	1.00761395923838	0.141	0.052	0.000385059702965766	Treg	RCBTB1
2.31075715156308E-08	0.833848225606506	0.275	0.146	0.000387236683458941	Treg	MPV17
2.32947181531313E-08	1.55596028059923	0.15	0.059	0.000390372886810175	Treg	LEPROT
2.48456950020433E-08	1.19685749401788	0.167	0.07	0.000416364156844242	Treg	CLTB
2.58201285479341E-08	0.44683455455558	0.35	0.198	0.000432693714206279	Treg	TMEM248
2.66218368762539E-08	1.16752148119659	0.212	0.1	0.000446128742372263	Treg	GPI
2.74995591885379E-08	1.19988501822477	0.17	0.071	0.000460837612881517	Treg	PAPSS1
2.87018354673664E-08	0.586005682408537	0.206	0.093	0.000480985358762127	Treg	RHBDD2
2.91377081145728E-08	1.32779962697332	0.154	0.061	0.000488289712584011	Treg	ECI1
2.96286214950613E-08	1.18065859772899	0.167	0.069	0.000496516439014236	Treg	KLC2
2.99231382580192E-08	1.365723853973	0.199	0.092	0.000501451950927886	Treg	MAPK6
3.06266070195636E-08	0.288724657465942	0.317	0.173	0.000513240680433847	Treg	CPSF6
3.2761731374292E-08	0.951247730398655	0.199	0.092	0.000549021094370385	Treg	VOPP1
3.32362618175029E-08	1.05663661432847	0.216	0.101	0.000556973275537714	Treg	ZKSCAN1
3.42584401899587E-08	0.362453530796938	0.32	0.178	0.000574102940703327	Treg	ZMIZ2
3.52563214225535E-08	0.752524585846273	0.18	0.078	0.000590825434399151	Treg	NIPA1
3.60971432573311E-08	0.812158350698318	0.239	0.118	0.000604915926706354	Treg	PIGT
3.83230669484115E-08	0.398999835157783	0.343	0.195	0.00064221795592148	Treg	LPP
4.03710613476258E-08	0.572042512653445	0.369	0.218	0.000676538246063513	Treg	HNRNPL
4.05146094323018E-08	0.411477422313551	0.415	0.262	0.000678943824866514	Treg	APBB1IP
4.14221527592845E-08	0.549123689346778	0.288	0.153	0.000694152435940089	Treg	TXNDC15
4.43030388969775E-08	1.19304902689261	0.167	0.07	0.00074243032583555	Treg	LAX1
4.44986041894385E-08	1.16072647331775	0.167	0.07	0.00074570760900661	Treg	ATP6V1B2
4.87380059458838E-08	0.399273633424839	0.212	0.097	0.000816751503641121	Treg	PRPF39
4.97814375690733E-08	0.312735929549887	0.141	0.053	0.000834237330782531	Treg	CCDC50
5.08929631070594E-08	0.276786331082323	0.242	0.119	0.000852864275748101	Treg	CNST
5.12767427301916E-08	0.735286553848952	0.196	0.09	0.000859295654672551	Treg	GABPB1
5.17701528420096E-08	0.385491647109383	0.261	0.135	0.000867564221326397	Treg	TPM4
5.2147437410296E-08	1.11185554094923	0.183	0.082	0.000873886756121741	Treg	APLP2
5.39101189192671E-08	0.424042352759285	0.265	0.138	0.000903425772849077	Treg	NCSTN
5.41471232680259E-08	0.458750282278414	0.48	0.312	0.000907397491725578	Treg	ARHGAP30
5.44978130601557E-08	0.429634056445347	0.503	0.326	0.00091327435126209	Treg	ADAR

5.58434533928587E-08	0.852608140236383	0.183	0.081	0.000935824591957526	Treg	LIMS1
5.86580520393018E-08	1.62532345805134	0.118	0.041	0.00098299163607462	Treg	CEACAM1
6.06531412236528E-08	0.881726014000006	0.265	0.139	0.00101642534062597	Treg	ERGIC3
6.16553162615431E-08	1.09639985267499	0.203	0.095	0.00103321978991094	Treg	CDKN1A
6.3237019986352E-08	0.500717497165725	0.363	0.215	0.00105972598093129	Treg	SLC3A2
6.37990651832589E-08	0.464776129390184	0.242	0.119	0.00106914473434105	Treg	TAOK3
6.45682162960185E-08	0.388850945116449	0.474	0.299	0.00108203416868868	Treg	CMTM3
6.51010546747175E-08	0.25992676701645	0.418	0.252	0.00109096347423892	Treg	HNRNPLL
6.61176866103257E-08	1.09463173979949	0.154	0.063	0.00110800019221584	Treg	LDAH
6.71452712544631E-08	0.723642272813002	0.147	0.058	0.00112522045568229	Treg	ACVR1
6.82571156599586E-08	0.721435842729233	0.114	0.038	0.00114385274422959	Treg	DEPRECATED-ENSG00000072803
6.89717989453181E-08	0.778668243100325	0.108	0.035	0.00115582940672564	Treg	PRDM8
7.00227491177599E-08	1.21812066157805	0.17	0.073	0.00117344122971542	Treg	YWHAG
7.04426676220433E-08	0.858674681075613	0.206	0.097	0.0011804782240102	Treg	BTG3
7.06282848398434E-08	1.19905499434746	0.114	0.038	0.0011835887973461	Treg	UHRF1BP1L
7.13698767294285E-08	0.93457442279226	0.239	0.123	0.00119601639423176	Treg	COX7A2L
7.25860615014453E-08	0.500036734874809	0.199	0.091	0.00121639721864122	Treg	RAB3GAP1
7.38858019235296E-08	1.02379337048375	0.252	0.134	0.00123817826863451	Treg	COPG1
8.1179098743836E-08	1.25070949277972	0.154	0.063	0.0013603993367492	Treg	PMM1
8.4550941616129E-08	1.28779150381887	0.176	0.08	0.00141690467960309	Treg	COMT
8.80330346457722E-08	1.29940811725094	0.16	0.068	0.00147525759459385	Treg	AAAS
9.55001688395675E-08	0.426969373357844	0.301	0.165	0.00160039182941347	Treg	TBCB
9.82434393415737E-08	0.607815199523193	0.196	0.09	0.00164636355648609	Treg	SNX10
1.0240652313153E-07	0.758899685175467	0.196	0.092	0.00171612851463818	Treg	PITPNA
1.02524591440697E-07	0.485145229693594	0.219	0.106	0.00171810710336321	Treg	TNIP2
1.02978314669148E-07	0.338877489824127	0.536	0.357	0.00172571059722558	Treg	ARF6
1.14827896650328E-07	0.349487486832154	0.343	0.195	0.0019242858920662	Treg	LDB1
1.16922711651374E-07	0.444938091837937	0.245	0.127	0.00195939080185372	Treg	FMNL3
1.21906981902795E-07	0.633650710858318	0.395	0.236	0.00204291720272703	Treg	TMCO1
1.23669068490667E-07	0.566236694472316	0.291	0.16	0.0020724462497666	Treg	MRTFA
1.29717832421592E-07	0.709201675825263	0.206	0.098	0.00217381143572104	Treg	STIP1
1.33323601181738E-07	0.858579643525259	0.193	0.09	0.00223423690860357	Treg	GNS
1.33766602840044E-07	1.19691685920753	0.105	0.034	0.00224166073039345	Treg	PLCXD1
1.36701825257558E-07	0.884636440901005	0.19	0.087	0.00229084918766615	Treg	SIPA1L1
1.40881764257898E-07	1.33195251431308	0.147	0.06	0.00236089660543386	Treg	HDAC8
1.42122588154283E-07	0.284051901424468	0.333	0.195	0.00238169033228948	Treg	SFXN1
1.45670110707363E-07	0.840987678478049	0.108	0.036	0.002441139715234	Treg	C20orf194
1.56009436966805E-07	0.444573774427825	0.392	0.239	0.00261440614468971	Treg	SEL1L3
1.57695190377246E-07	0.26163123500403	0.533	0.357	0.00264265600034189	Treg	PSMB8
1.58945172983549E-07	0.460761027684959	0.206	0.097	0.00266360320885832	Treg	ST3GAL5
1.61287453291718E-07	0.328486453703676	0.245	0.124	0.00270285514226261	Treg	WDR83OS
1.65708013296819E-07	0.814468146160193	0.242	0.124	0.0027769348868281	Treg	YIPF4
1.65713370427524E-07	0.754935128208379	0.212	0.104	0.00277702466162445	Treg	IL27RA
1.67848873156506E-07	1.14052757241659	0.134	0.052	0.00281281141635672	Treg	VPS72
1.68836127122679E-07	0.665416083565314	0.232	0.117	0.00282935581832186	Treg	RNF145
1.72920783352715E-07	0.984690709500412	0.203	0.096	0.00289780648742479	Treg	PPT1
1.7711606344091E-07	1.44074397083165	0.124	0.047	0.00296811099114277	Treg	FAM110A
1.82801560948231E-07	0.741407196399381	0.235	0.12	0.00306338855837045	Treg	MBD6
1.8354670429842E-07	0.575521422574598	0.34	0.2	0.00307587567063292	Treg	PSMF1
1.85269639966448E-07	0.359127646319132	0.415	0.258	0.00310474862655774	Treg	CYBC1
1.89008669900741E-07	0.787152304358081	0.252	0.133	0.00316740729019662	Treg	NAB1
1.89541673270856E-07	1.33594501926523	0.108	0.037	0.00317633936067301	Treg	MCF2L2
1.89674822439931E-07	1.29021365341864	0.118	0.042	0.00317857067444836	Treg	TM2D2
1.91273557547397E-07	0.451358207132798	0.137	0.053	0.00320536227737928	Treg	TGIF2
1.97000351699131E-07	1.22879550023535	0.111	0.038	0.00330133189377404	Treg	DEPRECATED-ENSG00000204397
2.02459776951892E-07	0.986734530404035	0.206	0.1	0.0033928209421598	Treg	NFYC
2.02811891984188E-07	0.94827514657969	0.186	0.087	0.00339872168587102	Treg	NUP58
2.03799734236166E-07	0.915194850541513	0.239	0.123	0.00341527594632966	Treg	TRIM8
2.06008376658808E-07	0.55242369723553	0.216	0.107	0.0034522883760483	Treg	TXNDC12
2.07810708937926E-07	1.45692873806004	0.137	0.055	0.00348249186038177	Treg	HIVEP3
2.08194328345543E-07	0.759528375633659	0.225	0.112	0.0034889205544146	Treg	SESN3
2.13076016180191E-07	0.592787128148356	0.307	0.176	0.00357072787914764	Treg	PAK2
2.1522273962299E-07	0.306542378112542	0.386	0.234	0.00360670267060206	Treg	SKAP1
2.1590936551008E-07	0.302512224084664	0.386	0.228	0.00361820914721792	Treg	ACADVL
2.20107039434172E-07	1.24862453742731	0.17	0.077	0.00368855376683786	Treg	SNX3
2.22756491603793E-07	0.255992629021694	0.598	0.404	0.00373295328629636	Treg	IVNS1ABP

2.23521496936139E-07	0.347751106039002	0.317	0.18	0.00374577324565582	Treg	ARHGEF6
2.25036599675156E-07	1.40858655597151	0.137	0.055	0.00377116333735626	Treg	UEVLD
2.28258999378671E-07	1.32559204870506	0.134	0.053	0.00382516431158778	Treg	BMPR2
2.35178850068452E-07	0.494321298970434	0.324	0.181	0.00394112716944712	Treg	DDX3X
2.36584359396829E-07	0.648760146227302	0.154	0.064	0.00396468069477206	Treg	DNAJB4
2.4059916202155E-07	0.619023312014138	0.284	0.157	0.00403196075715713	Treg	PPP4R3B
2.41913879552756E-07	1.58568307193177	0.114	0.041	0.00405399279354508	Treg	MCCC2
2.42991647928462E-07	0.992681700474862	0.203	0.1	0.00407205403598517	Treg	FAM126A
2.46487577567836E-07	0.252904221566085	0.448	0.282	0.00413063882488179	Treg	S100A6
2.55571432691175E-07	0.412924647878655	0.203	0.096	0.00428286606903871	Treg	WDR6
2.61398915928825E-07	0.402001535422635	0.412	0.253	0.00438052303313526	Treg	EIF1
2.73352562562105E-07	0.293791076891475	0.314	0.176	0.00458084224341575	Treg	IST1
2.75552339091629E-07	0.603574184895873	0.307	0.179	0.00461770609849752	Treg	SEC11A
2.7658284841626E-07	0.461933769319056	0.275	0.15	0.00463497537375968	Treg	SZRD1
2.78344730985924E-07	0.355896257568479	0.301	0.167	0.00466450100186212	Treg	ANKRD17
2.87244964739379E-07	1.30822559626223	0.154	0.065	0.0048136511191025	Treg	FAM136A
2.90239574918395E-07	0.330810834138172	0.304	0.173	0.00486383479648247	Treg	LRP10
2.93062680554017E-07	1.29593941952236	0.118	0.043	0.00491114440072421	Treg	NINJ2
2.97649249719804E-07	0.681988079728911	0.219	0.108	0.00498800612680447	Treg	HECTD1
3.0069710062899E-07	0.871024539055879	0.245	0.131	0.00503908201234062	Treg	OAS3
3.04402495743529E-07	1.21299358422799	0.141	0.058	0.00510117702367006	Treg	CD320
3.10971349938942E-07	0.851652492032875	0.212	0.108	0.00521125788227679	Treg	SMS
3.16850273906744E-07	0.67184150021819	0.118	0.043	0.00530977689012922	Treg	SUMO4
3.18496899665001E-07	1.49966726080158	0.124	0.048	0.00533737104458608	Treg	APOL1
3.29169836808246E-07	0.447671297747627	0.618	0.434	0.00551622812523259	Treg	DDIT4
3.49873988104976E-07	0.337752265843592	0.167	0.072	0.00586318829266318	Treg	PLPP5
3.54694008740755E-07	0.352084768635973	0.301	0.168	0.00594396219847758	Treg	ZMYND8
3.55929124560093E-07	0.34224798772299	0.353	0.212	0.00596466026937803	Treg	MAT2A
3.70584937181576E-07	0.680882473930213	0.131	0.05	0.00621026237728886	Treg	TCP11L2
3.74291965876387E-07	1.08712257553605	0.196	0.095	0.00627238476415649	Treg	NF2
3.75886326648078E-07	0.975133439298407	0.265	0.149	0.0062991030619685	Treg	CHCHD2
3.80265794777805E-07	0.356648897537181	0.255	0.134	0.00637249418888646	Treg	ARIH2
3.80754320417225E-07	0.798763492669453	0.212	0.106	0.00638068090155185	Treg	C9orf16
3.89375096221337E-07	0.369075991564836	0.454	0.299	0.00652514786247716	Treg	CCNI
3.94480619692713E-07	0.471035450816273	0.203	0.099	0.00661070622481049	Treg	JAK2
4.02723830547593E-07	0.860522520827302	0.239	0.125	0.00674884595231656	Treg	C16orf58
4.11702220437728E-07	0.400564196877104	0.248	0.131	0.00689930581009545	Treg	GATA3
4.11714579226707E-07	1.82603953009973	0.101	0.035	0.00689951291868115	Treg	NDUFB3
4.21853674117494E-07	0.253623556370863	0.281	0.153	0.00706942387086096	Treg	KDM2B
4.29086770786689E-07	0.527294843856965	0.275	0.152	0.00719063610484333	Treg	PFKP
4.32945832785849E-07	1.08993773268673	0.154	0.066	0.00725530626582525	Treg	GALNT2
4.37208403704042E-07	0.753966086494845	0.278	0.154	0.00732673842927233	Treg	DECR1
4.37391536981342E-07	0.271168421459201	0.546	0.372	0.00732980737673333	Treg	ACTN4
4.38841862531952E-07	0.318871599412628	0.399	0.248	0.00735411193231046	Treg	FCMR
4.58182008335644E-07	1.09342170483433	0.144	0.06	0.00767821409568873	Treg	TMED8
4.64160095057625E-07	0.344590880658778	0.219	0.111	0.00777839487297568	Treg	EPM2AIP1
4.6801549376087E-07	1.32221661783387	0.141	0.059	0.00784300364444466	Treg	LGALS3BP
4.73471206122284E-07	1.03452838542517	0.193	0.094	0.00793443047219723	Treg	SCFD1
4.76002960777115E-07	1.19515347176575	0.141	0.059	0.00797685761670289	Treg	MTHFD2
4.80382530799329E-07	0.817563556425435	0.219	0.112	0.00805025045113516	Treg	ZMIZ1
4.8889810641339E-07	1.18373333847321	0.17	0.077	0.0081929544672756	Treg	PARK7
4.89490686805255E-07	0.533307269230972	0.258	0.139	0.00820288492948247	Treg	ATP6AP1
4.9178045840838E-07	0.900451379348487	0.163	0.072	0.00824125692200762	Treg	MFSD1
4.9286694705413E-07	0.392457484351254	0.477	0.32	0.0082594642987331	Treg	CSK
5.10155684276376E-07	0.557217575147245	0.389	0.247	0.00854918895710352	Treg	SP3
5.21643375123328E-07	0.619711632189293	0.278	0.156	0.00874169968031674	Treg	PYCR2
5.28164098075187E-07	0.325071598388615	0.373	0.222	0.00885097395554399	Treg	SRPRA
5.2866466269314E-07	0.72540698388255	0.206	0.105	0.00885936241741163	Treg	SLC39A7
5.3972214902255E-07	0.30532291517474	0.575	0.378	0.00904466377331989	Treg	SH3BGRL3
5.45967663714998E-07	0.886015903051726	0.261	0.145	0.00914932610853594	Treg	COX6A1
5.5263407660296E-07	0.276876487886612	0.343	0.209	0.00926104185571241	Treg	GRB2
5.6484831592762E-07	1.10857642169656	0.134	0.054	0.00946572807831506	Treg	SLC35D1
5.81653291047022E-07	0.780628165958511	0.203	0.102	0.00974734585136599	Treg	AQP3
5.84594115918662E-07	0.509147729344019	0.343	0.206	0.00979662819456493	Treg	PSMB1
5.84866541147192E-07	0.341183612569985	0.33	0.195	0.00980119349654464	Treg	EFCAB14
5.92494640181062E-07	0.658634820415035	0.268	0.146	0.00992902518015424	Treg	SPPL2A

5.93730833410453E-07	0.714832337351368	0.16	0.071	0.00994974130629237	Treg	TRIM25
6.29840346164435E-07	1.25525758536475	0.134	0.055	0.0105548645210236	Treg	NDUFA8
6.44586564961542E-07	0.942343526610022	0.108	0.038	0.0108019816556255	Treg	TMED3
6.56831131817426E-07	0.750515738352664	0.265	0.149	0.0110071761069964	Treg	COPB2
6.63588330679429E-07	0.261573219557386	0.307	0.176	0.0111204132455259	Treg	TOP 1.00
7.33666797337174E-07	0.801665004035993	0.252	0.136	0.0122947881897764	Treg	MYDGF
7.97618104446778E-07	1.09764852285941	0.176	0.084	0.0133664841943191	Treg	SMPD1
8.31014101495088E-07	0.595304673345565	0.359	0.224	0.0139261343128547	Treg	PPP1CA
8.59467047354454E-07	0.360424528797445	0.225	0.114	0.0144029487795659	Treg	FIP1L1
8.65994657638846E-07	1.08385840534912	0.124	0.049	0.0145123384727118	Treg	DIP2B
8.79062273585688E-07	0.377739396707562	0.458	0.306	0.014731325580749	Treg	EHBP1L1
8.94386105430781E-07	0.887747489047397	0.212	0.11	0.014988122354809	Treg	PABPC1L
9.23205556308695E-07	0.794380307293217	0.225	0.121	0.0154710787126211	Treg	ATP5ME
9.24302611640823E-07	1.15002159173467	0.19	0.095	0.0154894631658769	Treg	PMVK
9.58173739620896E-07	0.790316362101795	0.304	0.183	0.016057075528567	Treg	NFE2L1
9.86600411506986E-07	0.961818704331209	0.268	0.154	0.0165334496960341	Treg	SPCS1
1.03736964763296E-06	1.23069645667817	0.134	0.056	0.0173842405550332	Treg	VDAC2
1.07009646599534E-06	0.353081863672402	0.291	0.171	0.0179326765771499	Treg	TMBIM1
1.07083832044243E-06	0.66406523204686	0.271	0.151	0.0179451085739743	Treg	BLOC1S1
1.10589959191751E-06	0.373981120374755	0.248	0.134	0.0185326653613537	Treg	TMED10
1.12283499427305E-06	0.541380105197106	0.291	0.17	0.0188164688340278	Treg	SFT2D2
1.17206096392509E-06	0.58020768253068	0.15	0.066	0.0196413976334566	Treg	NCR3
1.18521645483551E-06	0.693230928337197	0.144	0.062	0.0198618573501334	Treg	TOM1
1.21605280710873E-06	0.915923650705917	0.157	0.071	0.0203786129415281	Treg	ABHD10
1.24952476011766E-06	0.582527299002259	0.288	0.171	0.0209395359300517	Treg	SUB1
1.25582183883308E-06	1.05403498797663	0.144	0.063	0.0210450623751648	Treg	IFI27L2
1.30181166946679E-06	0.826758171770938	0.124	0.05	0.0218157599569245	Treg	GPHN
1.37607059202486E-06	0.795119117835114	0.167	0.079	0.0230601909811526	Treg	GSDMB
1.38328961262914E-06	0.286004587282749	0.356	0.217	0.0231811673284392	Treg	HERC4
1.40713386671342E-06	0.815486001554768	0.239	0.129	0.0235807493383836	Treg	DEPRECATED-ENSG00000141524
1.49692461021758E-06	0.868915749962339	0.225	0.122	0.0250854626180262	Treg	ECHS1
1.50816186891087E-06	0.820069725606565	0.307	0.189	0.0252737765992084	Treg	HINT1
1.51174048639023E-06	0.290481828620745	0.261	0.146	0.0253337470709275	Treg	RPA1
1.53350601555464E-06	0.629513007425748	0.288	0.172	0.0256984938086647	Treg	APOBEC3C
1.54006347192075E-06	1.08315712648769	0.114	0.045	0.025808383662448	Treg	ZDHHC16
1.5790654993793E-06	1.01860965473644	0.176	0.086	0.0264619796385984	Treg	LGALS3
1.57946865182649E-06	1.12366295142277	0.18	0.088	0.0264687356673083	Treg	USO1
1.59617020948007E-06	0.7007076470067	0.239	0.13	0.026748620370467	Treg	MICOS10
1.65246733338362E-06	0.99456309002773	0.108	0.041	0.0276920475728427	Treg	HOXB2
1.69463089823261E-06	1.00826815568106	0.163	0.077	0.028398624592582	Treg	SMARCE1
1.69848600550074E-06	1.38804393319204	0.101	0.037	0.0284632284801815	Treg	DCP1B
1.70638396113097E-06	0.603486351479854	0.363	0.231	0.0285955824206328	Treg	EIF4EBP2
1.74343626637198E-06	0.833923327044895	0.134	0.056	0.0292165049518617	Treg	TTC19
1.76414535168516E-06	0.779072219978412	0.275	0.156	0.0295635478035399	Treg	KLF9
1.77127905818113E-06	0.281915490536659	0.441	0.296	0.0296830944569994	Treg	NBEAL2
1.81078458777764E-06	0.642139266782032	0.291	0.169	0.0303451281219776	Treg	UBE2V1
1.85859170298482E-06	1.08037756457261	0.15	0.069	0.0311462797586196	Treg	NMD3
1.86254832886541E-06	0.574234110683708	0.34	0.211	0.0312125848951265	Treg	FAM49B
1.86390261395026E-06	1.00231646403395	0.157	0.072	0.0312352800045784	Treg	LY75
1.88241137288913E-06	0.650544115848219	0.304	0.183	0.031545449786876	Treg	USP48
1.90214174664183E-06	1.15180702558831	0.127	0.053	0.0318760913902238	Treg	NT5C
1.92722700180212E-06	1.01949920509761	0.147	0.066	0.0322964700961999	Treg	NAGA
1.93107903831694E-06	0.80919082263347	0.255	0.144	0.0323610225241153	Treg	LAMTOR1
2.00353573818205E-06	0.924027436233248	0.225	0.126	0.0335752519004547	Treg	TMED9
2.01946049675384E-06	0.368832228908519	0.353	0.217	0.0338421190046009	Treg	GNAI2
2.08148666357387E-06	0.724415136988298	0.32	0.2	0.0348815535081709	Treg	JUN
2.08407091927937E-06	0.927912690767922	0.111	0.043	0.0349248604652838	Treg	CNNM4
2.09550249194955E-06	0.270676326631307	0.464	0.309	0.0351164307600905	Treg	TSC22D4
2.10374671392145E-06	0.301065442788796	0.193	0.095	0.0352545874318956	Treg	EIF4G3
2.13136681956782E-06	0.496474880661981	0.301	0.176	0.0357174451623176	Treg	RER1
2.13694201184613E-06	0.252396508640273	0.399	0.254	0.0358108742345175	Treg	STK24
2.15189090134542E-06	1.23054727064094	0.137	0.06	0.0360613877247465	Treg	EHD4
2.18392924045275E-06	0.625808278070928	0.147	0.065	0.0365982862115072	Treg	ARL 15.00
2.20981601900513E-06	0.787217208611997	0.141	0.06	0.0370320968464879	Treg	BCL3
2.21951388216506E-06	1.08778995514422	0.186	0.095	0.037194613637322	Treg	UQCR10
2.2383416367822E-06	0.762675662022979	0.16	0.073	0.0375101291491962	Treg	UTS2

2.28934708234924E-06	0.553760993719131	0.363	0.228	0.0383648784060086	Treg	HNRNPD
2.34651421589402E-06	0.870704408707709	0.219	0.119	0.0393228852299519	Treg	RAB7A
2.35977397102127E-06	0.666495680653919	0.17	0.081	0.0395450922063745	Treg	ZMYM5
2.44554237360999E-06	0.580035844040335	0.209	0.109	0.0409823990969562	Treg	GOLGB1
2.53926411511211E-06	0.421166748669837	0.405	0.265	0.0425529880410487	Treg	TRIP12
2.57200455155849E-06	1.12435877974659	0.144	0.065	0.0431016522750172	Treg	EPN1
2.63066020771662E-06	0.855929068893736	0.127	0.053	0.044084603760915	Treg	ZCCHC2
2.74167574136874E-06	0.444202844126325	0.441	0.291	0.0459450020738573	Treg	SLC9A3R1
2.8891501539899E-06	1.13336940061443	0.127	0.053	0.0484163782805627	Treg	FOPNL
2.91482675745759E-06	0.5424694075334	0.163	0.077	0.0488466668014743	Treg	TFE3
1.8123537038827E-171	3.04283342512652	0.888	0.111	3.03714233696663E-167	CD8+ Teff/Tex	CD8A
2.20771879939758E-97	1.96617964779221	0.839	0.211	3.69969516403046E-93	CD8+ Teff/Tex	NKG7
1.54339478177315E-90	2.14676430182738	0.86	0.278	2.58642097529545E-86	CD8+ Teff/Tex	CCL5
1.20541486116552E-83	2.0340341944832	0.727	0.165	2.02003422434118E-79	CD8+ Teff/Tex	KLRK1
3.43131868477271E-65	1.79296453609692	0.734	0.228	5.75020385194211E-61	CD8+ Teff/Tex	GZMA
2.48618613111127E-55	1.61359208501977	0.636	0.186	4.16635071851627E-51	CD8+ Teff/Tex	CTSW
1.56647875009249E-49	2.14672701965526	0.479	0.118	2.625105089405E-45	CD8+ Teff/Tex	CCL4
1.65833570209742E-48	1.9740755003012	0.626	0.236	2.77903896957486E-44	CD8+ Teff/Tex	LYST
2.05720340828149E-48	3.22629922818114	0.248	0.023	3.44746147159813E-44	CD8+ Teff/Tex	CD8B
1.79866074193004E-47	1.61753972694756	0.549	0.159	3.01419567132636E-43	CD8+ Teff/Tex	LAG3
2.01570681415204E-46	1.31089431276181	0.815	0.418	3.377921479156E-42	CD8+ Teff/Tex	IKZF3
1.17680938205359E-43	2.05968015889362	0.476	0.144	1.97209716244541E-39	CD8+ Teff/Tex	PDCD1
6.79058967871878E-43	2.58532921079974	0.301	0.048	1.13796701835969E-38	CD8+ Teff/Tex	DTHD1
8.87775410019301E-41	2.36687971358821	0.364	0.081	1.48773403211035E-36	CD8+ Teff/Tex	GFOD1
6.82642275551344E-37	1.44569858348285	0.661	0.307	1.14397192536894E-32	CD8+ Teff/Tex	SLAMF6
2.97249662752099E-36	1.60357937940045	0.406	0.104	4.98130984839967E-32	CD8+ Teff/Tex	KLRC4
2.30752090061618E-35	2.01170521542337	0.304	0.061	3.86694352525259E-31	CD8+ Teff/Tex	TRGC2
2.67721557535382E-35	1.23460893293903	0.706	0.355	4.48647786117794E-31	CD8+ Teff/Tex	ITM2A
8.504343612841E-35	3.89960801214461	0.185	0.019	1.42515790263989E-30	CD8+ Teff/Tex	VCAM1
2.53087918564641E-33	1.746625273697	0.371	0.098	4.24124733930626E-29	CD8+ Teff/Tex	EOMES
4.55990754821915E-33	2.35104103176815	0.329	0.081	7.64149306930565E-29	CD8+ Teff/Tex	MS4A6A
1.24830191806881E-32	1.07618385879274	0.549	0.204	2.09190435429971E-28	CD8+ Teff/Tex	THEMIS
9.66451372717843E-31	1.95215240995374	0.325	0.081	1.61957921040056E-26	CD8+ Teff/Tex	ADGRG1
1.67388449712475E-30	2.9938518292136	0.227	0.039	2.80509564028166E-26	CD8+ Teff/Tex	CCL4L2
1.75561309924301E-30	1.76094642529943	0.437	0.155	2.94205643171144E-26	CD8+ Teff/Tex	TOX
8.62215341074418E-28	2.46186459263625	0.234	0.047	1.44490046857251E-23	CD8+ Teff/Tex	PECAM1
1.31557364387602E-27	1.05004711978903	0.78	0.453	2.20463831240744E-23	CD8+ Teff/Tex	RGS1
5.93653696860891E-27	2.44985611120911	0.35	0.118	9.94844865199481E-23	CD8+ Teff/Tex	FAM3C
8.07052772362135E-27	1.01572335432345	0.661	0.336	1.35245903592447E-22	CD8+ Teff/Tex	CST7
1.31619583986762E-26	2.5572643815816	0.178	0.026	2.20568098845015E-22	CD8+ Teff/Tex	CRTAM
1.50166432411812E-25	0.848426716582893	0.654	0.331	2.51648907435714E-21	CD8+ Teff/Tex	PRF1
5.81096885831952E-25	1.68871088478222	0.392	0.144	9.73802161277185E-21	CD8+ Teff/Tex	ID2
9.07180755431386E-25	1.52186315790396	0.542	0.279	1.52025350995192E-20	CD8+ Teff/Tex	CCR5
2.02871481535435E-24	1.56768976797286	0.392	0.144	3.39972028757082E-20	CD8+ Teff/Tex	BCL2L11
4.05207999082623E-24	0.795601316777803	0.78	0.49	6.7904756486266E-20	CD8+ Teff/Tex	WNK1
1.57801214760757E-23	2.03437961589451	0.346	0.125	2.64443275696077E-19	CD8+ Teff/Tex	SNAP47
1.82773703124005E-23	1.24610031524017	0.549	0.271	3.06292171695207E-19	CD8+ Teff/Tex	SLFN5
4.16959396839033E-23	1.21028225789329	0.364	0.117	6.98740557222851E-19	CD8+ Teff/Tex	KLRD1
3.47714145276103E-22	3.08122718851571	0.108	0.009	5.82699364653694E-18	CD8+ Teff/Tex	CLECL1
5.16607526820817E-22	1.18580742884921	0.521	0.242	8.65730893446325E-18	CD8+ Teff/Tex	CD27
7.38081477848471E-22	1.62570122306383	0.262	0.072	1.23687694057847E-17	CD8+ Teff/Tex	FASLG
1.74816602970693E-21	1.02069764549731	0.507	0.244	2.92957663258287E-17	CD8+ Teff/Tex	CXCR6
9.60808903413872E-21	2.03406166421545	0.276	0.086	1.61012356034097E-16	CD8+ Teff/Tex	CRIM1
1.26451916328753E-20	2.08361681397253	0.192	0.041	2.11908121383724E-16	CD8+ Teff/Tex	CCL3
4.20384026082346E-20	1.12275637489121	0.381	0.15	7.04479550908795E-16	CD8+ Teff/Tex	SFMBT2
4.2137704499046E-20	0.748259223946909	0.636	0.344	7.06143651995013E-16	CD8+ Teff/Tex	CBLB
9.41333531136392E-20	1.08920910956868	0.451	0.21	1.57748673147837E-15	CD8+ Teff/Tex	HCST
1.17557021214295E-19	1.35572131063749	0.231	0.062	1.97002056150916E-15	CD8+ Teff/Tex	SLAMF7
1.55865423332995E-19	0.585401139383807	0.601	0.306	2.61199276421433E-15	CD8+ Teff/Tex	DUSP2
1.60353169904052E-19	1.53694917220599	0.381	0.166	2.6871984212521E-15	CD8+ Teff/Tex	APOBEC3G
2.46310506448599E-19	0.828585987385273	0.675	0.402	4.12767146706563E-15	CD8+ Teff/Tex	RESF1
7.63771230526498E-19	0.899754269505256	0.49	0.242	1.27992782811631E-14	CD8+ Teff/Tex	TRAF5
9.05857270691344E-19	1.09010473027359	0.364	0.152	1.51803561422455E-14	CD8+ Teff/Tex	MAPRE2
1.18108470464202E-18	3.68700504090077	0.119	0.017	1.9792617480391E-14	CD8+ Teff/Tex	ETV1
1.88069990013249E-18	1.26626289634311	0.395	0.165	3.15167689264202E-14	CD8+ Teff/Tex	GZMK
2.20614465831201E-18	1.34922446418015	0.371	0.157	3.69705721839927E-14	CD8+ Teff/Tex	DEPRECATED-ENSG00000115738

3.65482973709506E-18	0.583945305626581	0.874	0.627	6.1247636734239E-14	CD8+ Teff/Tex	ETS1
4.56053163580419E-18	2.62650390900256	0.143	0.027	7.64253891528067E-14	CD8+ Teff/Tex	AFAP1L2
9.62615471462156E-18	0.756749967152115	0.497	0.257	1.61315100707628E-13	CD8+ Teff/Tex	PYHIN1
1.13094933391679E-17	1.10665548651163	0.42	0.2	1.89524489377776E-13	CD8+ Teff/Tex	ATXN1
1.74974986365134E-17	0.738475611665109	0.759	0.483	2.93223082150692E-13	CD8+ Teff/Tex	SLA
2.66339316394719E-17	2.33472622076208	0.22	0.067	4.4633142641427E-13	CD8+ Teff/Tex	SARDH
7.51953479736056E-17	1.56954222569241	0.22	0.066	1.26012364134168E-12	CD8+ Teff/Tex	MCOLN2
8.05662801195888E-17	1.5995873494157	0.392	0.187	1.35012972224407E-12	CD8+ Teff/Tex	HAVCR2
1.32140271339998E-16	0.709061111430741	0.51	0.275	2.21440666711568E-12	CD8+ Teff/Tex	PRKCH
5.13232131600633E-16	1.15624385571754	0.248	0.081	8.60074406136341E-12	CD8+ Teff/Tex	KLRC3
7.24295563304439E-16	0.58192211888149	0.584	0.339	1.21377450498558E-11	CD8+ Teff/Tex	TGFBR2
1.46120242544239E-15	0.584670496624796	0.783	0.531	2.44868302455636E-11	CD8+ Teff/Tex	LSP1
2.21320198256734E-15	1.23238045532403	0.234	0.078	3.70888388238635E-11	CD8+ Teff/Tex	NSMF
2.23461300394553E-15	1.13341086326596	0.402	0.199	3.74476447201192E-11	CD8+ Teff/Tex	SNTB2
3.19487138250317E-15	2.60230309338842	0.175	0.049	5.35396546279882E-11	CD8+ Teff/Tex	KCNK5
3.41955372653386E-15	0.596695345828499	0.797	0.522	5.73048813492544E-11	CD8+ Teff/Tex	ADGRE5
4.69858667590494E-15	0.947165890596716	0.388	0.189	7.8738915514815E-11	CD8+ Teff/Tex	S100PBP
6.39147266011893E-15	0.766710621213191	0.434	0.226	1.07108298838273E-10	CD8+ Teff/Tex	PIP4K2A
1.27202882485065E-14	2.84567560199845	0.122	0.025	2.13166590468471E-10	CD8+ Teff/Tex	HECTD2
1.29134690468069E-14	0.730594664367965	0.49	0.268	2.16403914286391E-10	CD8+ Teff/Tex	IL6ST
1.67093127915748E-14	1.26145754730984	0.252	0.09	2.8001466376121E-10	CD8+ Teff/Tex	GZMH
1.81113490090792E-14	0.899508222948985	0.402	0.191	3.0350998669415E-10	CD8+ Teff/Tex	ITGB7
2.1262511594116E-14	0.847006555280041	0.451	0.238	3.56317169294196E-10	CD8+ Teff/Tex	CEMIP2
2.44642392685427E-14	1.13942385248017	0.367	0.179	4.09971721662239E-10	CD8+ Teff/Tex	SLC7A5
3.18959953787159E-14	0.879128210391067	0.371	0.177	5.34513090556521E-10	CD8+ Teff/Tex	ARAP2
5.14131534065897E-14	0.733002856423286	0.371	0.169	8.61581624787631E-10	CD8+ Teff/Tex	ALOX5AP
6.20014589756008E-14	0.727838598894471	0.483	0.276	1.03902044951312E-09	CD8+ Teff/Tex	RAB27A
8.22292521839186E-14	1.87297614987201	0.178	0.054	1.37799780809811E-09	CD8+ Teff/Tex	NFIL3
9.81266250148208E-14	1.47786569712547	0.154	0.041	1.64440598199837E-09	CD8+ Teff/Tex	ITGA1
1.27472858792776E-13	0.830461166644768	0.374	0.188	2.13619016764934E-09	CD8+ Teff/Tex	ARHGEF3
1.40345349592312E-13	0.855459670644629	0.318	0.139	2.35190736846796E-09	CD8+ Teff/Tex	ATP8A1
2.42771523838261E-13	0.524759981109638	0.773	0.538	4.06836519648158E-09	CD8+ Teff/Tex	MSN
5.68883985477984E-13	0.801822101388096	0.367	0.178	9.53335782864006E-09	CD8+ Teff/Tex	RIN3
6.66626091926155E-13	1.8466037310792	0.199	0.069	1.11713200484985E-08	CD8+ Teff/Tex	KIAA1671
6.70965820753344E-13	2.37404080466064	0.122	0.028	1.12440452241845E-08	CD8+ Teff/Tex	SEMA7A
9.90250847686352E-13	1.02074490147617	0.364	0.186	1.65946237055279E-08	CD8+ Teff/Tex	PTPN6
1.53734843056575E-12	1.75124658015545	0.119	0.027	2.57628849994208E-08	CD8+ Teff/Tex	ZNF683
1.5579214912386E-12	1.043489185866	0.371	0.191	2.61076483501765E-08	CD8+ Teff/Tex	RBPJ
2.23229733472815E-12	1.24309598212297	0.224	0.085	3.74088387353743E-08	CD8+ Teff/Tex	PIK3AP1
2.63160373728678E-12	0.938638218902398	0.465	0.276	4.41004154294519E-08	CD8+ Teff/Tex	IDH2
2.9953686570565E-12	0.521688251594895	0.409	0.211	5.01963879549528E-08	CD8+ Teff/Tex	GIMAP7
3.0530648644435E-12	1.51113445545004	0.126	0.031	5.11632609983441E-08	CD8+ Teff/Tex	ADGRG5
3.27386732722522E-12	0.598190490601787	0.556	0.337	5.48634686696402E-08	CD8+ Teff/Tex	CD3D
3.84842414544627E-12	0.495774534421817	0.49	0.289	6.44918918293886E-08	CD8+ Teff/Tex	ATP11B
5.53023678792267E-12	1.42941727251826	0.192	0.067	9.26757080920081E-08	CD8+ Teff/Tex	DEPRECATED-ENSG00000100450
5.55637754576651E-12	1.38373422954238	0.199	0.07	9.31137749119551E-08	CD8+ Teff/Tex	NCR1
7.67100537293682E-12	0.952592824056067	0.336	0.168	1.28550708039675E-07	CD8+ Teff/Tex	DEPRECATED-ENSG00000150867
7.80105752425421E-12	0.457536347109848	0.818	0.547	1.30730121991452E-07	CD8+ Teff/Tex	IL32
7.83950588750372E-12	1.4396933327011	0.262	0.116	1.31374439662787E-07	CD8+ Teff/Tex	MAPK13
9.58419065566199E-12	0.760327884349279	0.315	0.147	1.60611867007584E-07	CD8+ Teff/Tex	CHST12
1.049669157087E-11	1.04792190904779	0.36	0.191	1.75903557344639E-07	CD8+ Teff/Tex	SIRPG
1.27622637443718E-11	0.612286787260737	0.371	0.194	2.13870015828182E-07	CD8+ Teff/Tex	GPR174
1.31409439665056E-11	0.823063031826581	0.374	0.193	2.20215938990701E-07	CD8+ Teff/Tex	ITM2C
1.38655518670308E-11	1.07594083105936	0.287	0.131	2.32358918187703E-07	CD8+ Teff/Tex	CCDC69
1.68925421738E-11	0.83121473043315	0.437	0.254	2.83085221748541E-07	CD8+ Teff/Tex	IPCEF1
1.99173943672473E-11	2.09044397408362	0.112	0.027	3.33775694806331E-07	CD8+ Teff/Tex	BMF
3.6116292106151E-11	2.15799688800079	0.133	0.038	6.05236823114879E-07	CD8+ Teff/Tex	TNIP3
3.69273871621128E-11	0.606932199337828	0.336	0.167	6.18829154062687E-07	CD8+ Teff/Tex	SYTL3
3.88408503640101E-11	1.00280430932839	0.178	0.06	6.50894970400082E-07	CD8+ Teff/Tex	GZMB
4.0006323507104E-11	0.719357604908123	0.427	0.252	6.70425969332048E-07	CD8+ Teff/Tex	TAF7
4.56177272048504E-11	0.626117968075453	0.458	0.278	7.64461872498882E-07	CD8+ Teff/Tex	MAP4
4.78190517883693E-11	0.314406960839007	0.259	0.104	8.01351669869493E-07	CD8+ Teff/Tex	MATK
4.92439537511593E-11	0.481766493125194	0.51	0.31	8.25230176961927E-07	CD8+ Teff/Tex	RNF19A
5.50883333208063E-11	0.442314818079496	0.832	0.612	9.23170289790072E-07	CD8+ Teff/Tex	CELF2
5.60573178383769E-11	0.402805332877716	0.322	0.153	9.3940853233552E-07	CD8+ Teff/Tex	JAML
5.80538906199739E-11	1.56599053036547	0.245	0.108	9.72867099009523E-07	CD8+ Teff/Tex	CCDC141

6.52874569136192E-11	1.08492471772007	0.21	0.083	1.09408720295843E-06	CD8+ Teff/Tex	PRR5L
7.38749113330027E-11	0.502496943048323	0.678	0.453	1.23799576411846E-06	CD8+ Teff/Tex	CD3E
1.01038887542618E-10	1.39705470799146	0.199	0.078	1.69320967743919E-06	CD8+ Teff/Tex	ABI3
1.12888203870822E-10	1.2095873199681	0.192	0.073	1.89178052046723E-06	CD8+ Teff/Tex	HOPX
1.13829628305472E-10	0.45529860194247	0.668	0.441	1.9075569111431E-06	CD8+ Teff/Tex	VTN
1.23957434946243E-10	2.55334835976608	0.101	0.024	2.07727869482914E-06	CD8+ Teff/Tex	SEMA4A
1.42798035211254E-10	0.444479632314356	0.556	0.349	2.39300947407019E-06	CD8+ Teff/Tex	STK10
1.50188393665988E-10	1.16785109821392	0.273	0.132	2.51685710105462E-06	CD8+ Teff/Tex	RASA1
1.59078184800653E-10	1.07222348199825	0.217	0.09	2.66583222088935E-06	CD8+ Teff/Tex	ABHD17B
1.8340457229825E-10	0.923439852755271	0.213	0.084	3.07349382257407E-06	CD8+ Teff/Tex	ZEB2
1.88357253095932E-10	0.544872268985939	0.434	0.252	3.15649084738162E-06	CD8+ Teff/Tex	NFATC2
2.24483312193689E-10	1.18912251364984	0.259	0.121	3.76189134574184E-06	CD8+ Teff/Tex	TRIM8
2.62617096148994E-10	1.94545534958142	0.112	0.029	4.40093729726485E-06	CD8+ Teff/Tex	GIMAP8
2.71797107570191E-10	0.397047596521324	0.182	0.067	4.55477592866127E-06	CD8+ Teff/Tex	SYTL2
2.86909085995534E-10	0.501868998449431	0.462	0.276	4.80802246311315E-06	CD8+ Teff/Tex	PLAAT4
2.96586244374395E-10	0.257038297794418	0.451	0.262	4.97019228322612E-06	CD8+ Teff/Tex	IL16
3.6093407219786E-10	0.875291399351959	0.357	0.201	6.04853318189173E-06	CD8+ Teff/Tex	SPATA13
4.18769031440363E-10	1.15268698562092	0.259	0.125	7.01773142887761E-06	CD8+ Teff/Tex	DGKH
4.50104476158261E-10	0.70140091976336	0.479	0.283	7.54285081146014E-06	CD8+ Teff/Tex	TIGIT
4.71037022129203E-10	0.401377475690288	0.612	0.404	7.89363841684118E-06	CD8+ Teff/Tex	AKAP13
4.9043046463684E-10	2.03410155779099	0.161	0.059	8.21863372638416E-06	CD8+ Teff/Tex	GPD2
5.73444977223746E-10	0.600868671214699	0.49	0.312	9.60979092831553E-06	CD8+ Teff/Tex	LCK
6.08642798679535E-10	1.97240242489569	0.168	0.063	1.01996360202716E-05	CD8+ Teff/Tex	HMOX1
8.33158594271403E-10	0.665163077129981	0.224	0.095	1.39620717228002E-05	CD8+ Teff/Tex	ARHGAP26
8.4646374681741E-10	0.593293917900781	0.483	0.306	1.41850394691662E-05	CD8+ Teff/Tex	TBC1D10C
8.50670642718562E-10	1.7545202161903	0.105	0.027	1.42555386306777E-05	CD8+ Teff/Tex	IFNG
8.62423845011422E-10	0.498759965928255	0.392	0.217	1.44524987947014E-05	CD8+ Teff/Tex	EVI2B
9.35141432272246E-10	0.577286724415802	0.287	0.142	1.56711001220183E-05	CD8+ Teff/Tex	PRKACB
1.03063279291536E-09	0.660401101285202	0.322	0.168	1.72713443436756E-05	CD8+ Teff/Tex	KIF13B
1.16854734500475E-09	0.398688373941657	0.685	0.479	1.95825164075897E-05	CD8+ Teff/Tex	CD2
1.20535653066119E-09	0.675673709814616	0.395	0.226	2.01993647408203E-05	CD8+ Teff/Tex	CASP8
1.26463128554359E-09	0.649705123085554	0.42	0.259	2.11926910831395E-05	CD8+ Teff/Tex	CD99
1.31401138603396E-09	0.509089867018942	0.301	0.15	2.20202028071571E-05	CD8+ Teff/Tex	SAMD3
1.59919397188102E-09	1.10437936348341	0.136	0.043	2.67992925807821E-05	CD8+ Teff/Tex	FCRL6
1.83578004489175E-09	0.538708715106142	0.388	0.23	3.0764001992296E-05	CD8+ Teff/Tex	TBL1XR1
1.9189088879127E-09	0.636075431509551	0.367	0.21	3.2157075143641E-05	CD8+ Teff/Tex	APMAP
1.95534736084332E-09	0.64095926358358	0.315	0.168	3.27677110730124E-05	CD8+ Teff/Tex	NIN
2.14887002375417E-09	0.553808340775276	0.371	0.209	3.60107638580724E-05	CD8+ Teff/Tex	MSL2
3.80379231657052E-09	0.58767944614255	0.437	0.267	6.37439516410887E-05	CD8+ Teff/Tex	ZFP36
3.8468136266258E-09	0.353197451686108	0.72	0.492	6.44649027549951E-05	CD8+ Teff/Tex	CFL1
4.14098165773926E-09	0.861927899595066	0.189	0.078	6.93945706203945E-05	CD8+ Teff/Tex	SLF1
4.27398847280961E-09	0.33373913025732	0.794	0.595	7.16234988273434E-05	CD8+ Teff/Tex	AKNA
4.49014263051847E-09	0.670560904357838	0.493	0.331	7.52458102022285E-05	CD8+ Teff/Tex	CAPZB
4.66706794319756E-09	0.321033849650975	0.374	0.206	7.82107245921046E-05	CD8+ Teff/Tex	CLEC2B
6.9061521704745E-09	1.23642493728117	0.21	0.096	0.000115733298072812	CD8+ Teff/Tex	AKAP5
7.79469281142511E-09	0.625631332727675	0.164	0.062	0.000130623462133862	CD8+ Teff/Tex	RGS2
8.18618608559358E-09	0.438478289853921	0.594	0.413	0.000137184106422377	CD8+ Teff/Tex	ANXA6
8.61119201054742E-09	0.758540042732821	0.423	0.27	0.000144306355712754	CD8+ Teff/Tex	LASP1
9.13022174412295E-09	0.491582554123238	0.699	0.52	0.000153004255988012	CD8+ Teff/Tex	CFLAR
9.24817091529071E-09	1.3315010661629	0.126	0.041	0.000154980848198442	CD8+ Teff/Tex	RASGEF1A
1.00191170369326E-08	0.824860319988002	0.308	0.17	0.000167900363304917	CD8+ Teff/Tex	APOBEC3C
1.09972048784762E-08	0.282358598457773	0.699	0.487	0.000184291159353504	CD8+ Teff/Tex	ITGAL
1.15201861787101E-08	1.52393129513351	0.196	0.088	0.000193055279982824	CD8+ Teff/Tex	GALNT1
1.26625791695843E-08	0.595438801459544	0.584	0.406	0.000212199501723893	CD8+ Teff/Tex	ZFP36L1
1.32934146014606E-08	0.631651519823745	0.311	0.176	0.000222771041891277	CD8+ Teff/Tex	USP24
1.50930016033114E-08	0.790600030134998	0.332	0.193	0.000252928520868292	CD8+ Teff/Tex	GIMAP6
1.53039410301675E-08	1.40503735573417	0.119	0.038	0.000256463443783547	CD8+ Teff/Tex	ATP8B4
1.58535739155225E-08	0.594886147112987	0.343	0.2	0.000265674191676326	CD8+ Teff/Tex	RAPGEF1
1.90157876504013E-08	0.365315975643156	0.416	0.252	0.000318666569445424	CD8+ Teff/Tex	PTPN22
2.01268965494708E-08	0.942146493096525	0.43	0.295	0.000337286532376031	CD8+ Teff/Tex	SLC9A3R1
2.14008804898916E-08	1.31806631416052	0.196	0.09	0.000358635955249603	CD8+ Teff/Tex	FUT8
2.20472212800668E-08	0.558021697700535	0.5	0.335	0.000369467334211359	CD8+ Teff/Tex	PTPN7
2.22635787005819E-08	1.07471896854587	0.196	0.085	0.000373093051864351	CD8+ Teff/Tex	TXK
2.50152872288715E-08	0.678191676860562	0.266	0.138	0.000419206183381429	CD8+ Teff/Tex	CHIC2
2.51590906465827E-08	0.754882989519623	0.287	0.154	0.000421616041055433	CD8+ Teff/Tex	TBC1D1
2.65437122594444E-08	0.543536391913472	0.458	0.298	0.00044481953004377	CD8+ Teff/Tex	MBD2

2.68376467740403E-08	0.702014961965193	0.227	0.11	0.000449745284639367	CD8+ Teff/Tex	SBNO1
2.69767498840956E-08	0.467997049775229	0.57	0.388	0.000452076374557674	CD8+ Teff/Tex	PSME1
3.33803166879174E-08	0.675955079879345	0.381	0.24	0.00055938734705612	CD8+ Teff/Tex	SSR2
3.59836848488859E-08	0.311954546241541	0.874	0.728	0.000603014590697629	CD8+ Teff/Tex	CD74
3.69422713880956E-08	1.26349310995944	0.175	0.075	0.000619078583921707	CD8+ Teff/Tex	USP28
4.50300557274158E-08	0.848839722056222	0.269	0.146	0.000754613673880034	CD8+ Teff/Tex	SP4
5.93872220786125E-08	0.460372740480148	0.552	0.381	0.000995211067593388	CD8+ Teff/Tex	LCP2
5.9394831462422E-08	0.530949281886153	0.259	0.132	0.000995338585647269	CD8+ Teff/Tex	ADA2
6.2165712648172E-08	0.496366385967292	0.322	0.183	0.00104177301255807	CD8+ Teff/Tex	PHF10
6.62198255611868E-08	1.13858072826524	0.269	0.146	0.00110971183675437	CD8+ Teff/Tex	TNFRSF9
6.73255632469503E-08	0.480012716814029	0.332	0.191	0.00112824178889239	CD8+ Teff/Tex	GGA2
7.10445466956308E-08	0.694061251140095	0.311	0.181	0.00119056451352538	CD8+ Teff/Tex	KLF13
8.10808189233706E-08	0.774664798843243	0.276	0.153	0.00135875236351784	CD8+ Teff/Tex	DAXX
8.62319057388623E-08	0.426333813492576	0.441	0.291	0.00144507427637185	CD8+ Teff/Tex	PSMB9
8.83402277709555E-08	0.46516640853164	0.605	0.416	0.00148040553698567	CD8+ Teff/Tex	MT-ND3
9.08940909105676E-08	0.920923146997996	0.294	0.168	0.00152320317547929	CD8+ Teff/Tex	CD84
9.35291266116924E-08	0.540072102485976	0.287	0.158	0.00156736110375874	CD8+ Teff/Tex	SACM1L
9.39414677999887E-08	0.821563167903595	0.374	0.233	0.00157427111739221	CD8+ Teff/Tex	ST8SIA4
9.68586586699687E-08	0.498262875701176	0.423	0.275	0.00162315740199134	CD8+ Teff/Tex	DIDO1
9.72276517442135E-08	1.40733351406233	0.189	0.089	0.00162934098792953	CD8+ Teff/Tex	FAM32A
1.01178025171746E-07	1.13521310590865	0.175	0.077	0.00169554134582812	CD8+ Teff/Tex	P2RY11
1.09116079223667E-07	0.270351425775587	0.392	0.243	0.0018285672556302	CD8+ Teff/Tex	FGD3
1.10272579478375E-07	0.455570944207736	0.427	0.274	0.0018479478868986	CD8+ Teff/Tex	MAPK1
1.13974647318747E-07	0.448843328584566	0.734	0.523	0.00190998713976756	CD8+ Teff/Tex	MT-ATP6
1.32573538731818E-07	1.19942259577644	0.15	0.061	0.0022216673620678	CD8+ Teff/Tex	SIDT1
1.32945642305257E-07	0.904722540591759	0.178	0.08	0.00222790307375149	CD8+ Teff/Tex	TARBP1
1.34689641199625E-07	0.918044501721254	0.185	0.085	0.00225712900722331	CD8+ Teff/Tex	CD2BP2
1.97438096657836E-07	0.706562340442481	0.329	0.205	0.00330866762379201	CD8+ Teff/Tex	ASXL2
2.00395193970203E-07	0.966473660555988	0.304	0.182	0.00335822266055266	CD8+ Teff/Tex	ZBTB38
2.11763828230851E-07	0.278171797182924	0.752	0.522	0.00354873823349261	CD8+ Teff/Tex	MT-CO2
2.14590668503963E-07	0.3857402624818	0.479	0.322	0.0035961104227894	CD8+ Teff/Tex	CNN2
2.61838254361589E-07	1.80684946806407	0.115	0.042	0.00438788546659151	CD8+ Teff/Tex	EOGT
2.80093817456231E-07	0.427349436517797	0.552	0.385	0.00469381219293151	CD8+ Teff/Tex	SH3BGRL3
3.12004447253771E-07	0.59714484142196	0.304	0.182	0.00522857052707869	CD8+ Teff/Tex	ARPC5L
3.26499815736531E-07	0.563849544541293	0.444	0.296	0.00547148391211279	CD8+ Teff/Tex	CD3G
3.5272465087156E-07	1.14998148662075	0.266	0.153	0.0059109596993056	CD8+ Teff/Tex	UBASH3B
3.54407067525883E-07	0.461903008136835	0.472	0.323	0.00593915363759874	CD8+ Teff/Tex	CSK
3.68297972053276E-07	0.818965608345299	0.367	0.244	0.00617193741566879	CD8+ Teff/Tex	TMCO1
4.07937604980402E-07	0.255095934244903	0.769	0.559	0.00683621838426158	CD8+ Teff/Tex	CORO1A
4.32726391733037E-07	1.26322685657232	0.115	0.042	0.00725162887266224	CD8+ Teff/Tex	SCPEP1
4.35739871255447E-07	1.15484238522846	0.14	0.057	0.00730212876249879	CD8+ Teff/Tex	SAMD9
4.38873238618884E-07	0.410013799896593	0.448	0.298	0.00735463773277526	CD8+ Teff/Tex	PLCG1
4.43204731408171E-07	0.672757866718125	0.315	0.191	0.00742722488893813	CD8+ Teff/Tex	NDUFA3
4.50021019406898E-07	0.339689338816022	0.353	0.22	0.00754145224322079	CD8+ Teff/Tex	ERAP2
4.61200006267726E-07	0.366332334454611	0.35	0.213	0.00772878970503456	CD8+ Teff/Tex	PLCB2
4.72013242355955E-07	0.79107344268963	0.164	0.073	0.0079099979154011	CD8+ Teff/Tex	ZBED4
4.84572725526191E-07	0.284997792033686	0.584	0.423	0.00812046973436791	CD8+ Teff/Tex	CD96
4.9400315356511E-07	0.293505145031802	0.713	0.522	0.0082785048474441	CD8+ Teff/Tex	ITGB2
5.74083409015303E-07	1.30822368244457	0.154	0.069	0.00962048976827845	CD8+ Teff/Tex	ZNF75A
5.91803047865155E-07	0.577145730241928	0.329	0.2	0.00991743547612428	CD8+ Teff/Tex	FAM102A
6.07721120446026E-07	0.952548869664368	0.126	0.048	0.0101841905364345	CD8+ Teff/Tex	CWF19L1
6.33875385728841E-07	0.357871732312924	0.811	0.625	0.0106224837140439	CD8+ Teff/Tex	TRBC2
6.57171193886896E-07	1.07478896813421	0.136	0.056	0.0110128748671566	CD8+ Teff/Tex	ST8SIA1
6.93170551634529E-07	0.685534603835886	0.332	0.211	0.0116161521042914	CD8+ Teff/Tex	COTL1
6.96815430823267E-07	0.445704523734245	0.517	0.368	0.0116772329897363	CD8+ Teff/Tex	MT-ND2
7.16283314573121E-07	0.476584418609653	0.297	0.175	0.0120034757856164	CD8+ Teff/Tex	CEP85L
7.26917802984488E-07	0.474118594555133	0.448	0.302	0.012181688542414	CD8+ Teff/Tex	TLN1
7.90560066159069E-07	0.402514465308868	0.231	0.12	0.0132482055886937	CD8+ Teff/Tex	LPCAT1
8.04953536922508E-07	0.486771519597286	0.294	0.171	0.0134894113717474	CD8+ Teff/Tex	OAS2
8.14773107054211E-07	0.681884957481365	0.171	0.078	0.0136539677280145	CD8+ Teff/Tex	GIMAP1
8.76536023829513E-07	0.952859024030231	0.21	0.11	0.014688990687335	CD8+ Teff/Tex	ZNF831
8.9594165815601E-07	0.53560179624954	0.486	0.338	0.0150141903073784	CD8+ Teff/Tex	SASH3
9.20043278923559E-07	0.830396290690262	0.241	0.134	0.015418085268201	CD8+ Teff/Tex	PCNX2
9.70840992497414E-07	0.348010831306711	0.462	0.316	0.0162693533522717	CD8+ Teff/Tex	BRD2
9.72277398523714E-07	0.78211107667226	0.266	0.157	0.0162934246444604	CD8+ Teff/Tex	ARHGAP35
9.83139478782138E-07	0.467891774532915	0.336	0.205	0.0164754513854311	CD8+ Teff/Tex	SLC4A7

1.0116975471126E-06	0.939142435026851	0.136	0.057	0.0169540274945129	CD8+ Teff/Tex	MLLT3
1.03612972421063E-06	0.390603539977463	0.304	0.181	0.0173634619183218	CD8+ Teff/Tex	TSPYL1
1.08015700540009E-06	1.22497973025861	0.147	0.064	0.0181012710964947	CD8+ Teff/Tex	DEPRECATED-ENSG00000168214
1.14627984828059E-06	0.75588725443481	0.364	0.245	0.0192093576974861	CD8+ Teff/Tex	M6PR
1.19908746232413E-06	0.54838394369856	0.406	0.279	0.0200943076936278	CD8+ Teff/Tex	SUGP2
1.24182892588549E-06	0.379283411872615	0.759	0.555	0.020810569139989	CD8+ Teff/Tex	HSPA1B
1.29583445198781E-06	0.783002809017293	0.199	0.101	0.0217155937464117	CD8+ Teff/Tex	FAM78A
1.36696924875207E-06	0.480672735678821	0.29	0.174	0.0229076706705872	CD8+ Teff/Tex	RNF115
1.4028547588067E-06	1.31637685572582	0.147	0.066	0.0235090400480827	CD8+ Teff/Tex	LZIC
1.40582586853563E-06	0.271981469401934	0.612	0.45	0.02355882990492	CD8+ Teff/Tex	TAP1
1.43936454840078E-06	0.412267082987287	0.297	0.177	0.0241208711021002	CD8+ Teff/Tex	CLSTN3
1.44351309126148E-06	0.408633467157682	0.259	0.142	0.0241903923833599	CD8+ Teff/Tex	PTPN4
1.51639004503605E-06	0.520514090813837	0.423	0.285	0.025411664374714	CD8+ Teff/Tex	RCSD1
1.51657294915265E-06	1.72841417326614	0.108	0.041	0.0254147294819002	CD8+ Teff/Tex	MAP4K3
1.55107295075328E-06	1.45653740888902	0.147	0.065	0.0259928805087234	CD8+ Teff/Tex	DEPRECATED-ENSG00000244509
1.59180249924651E-06	0.537069524598079	0.315	0.196	0.0266754262823731	CD8+ Teff/Tex	RAB1B
1.65207408782801E-06	0.379482803515705	0.266	0.155	0.0276854575638218	CD8+ Teff/Tex	WDR33
1.8427674103642E-06	0.476511770997679	0.374	0.244	0.0308810962628832	CD8+ Teff/Tex	KANSL1
1.94249641479021E-06	0.27171283851553	0.542	0.371	0.0325523549190543	CD8+ Teff/Tex	CD7
1.97606440404611E-06	0.462819253138966	0.458	0.326	0.0331148872830047	CD8+ Teff/Tex	DOCK8
2.07447455054536E-06	0.561702571770493	0.28	0.166	0.0347640445180391	CD8+ Teff/Tex	ARHGAP25
2.24975332356809E-06	1.01787703471067	0.112	0.043	0.037701366196354	CD8+ Teff/Tex	MXRA7
2.32662289342975E-06	0.640426394326281	0.318	0.199	0.0389895464480957	CD8+ Teff/Tex	ATP5F1E
2.35199639253098E-06	0.310246113968576	0.612	0.453	0.0394147555460341	CD8+ Teff/Tex	DNAJB1
2.43160940743841E-06	0.286925715279428	0.308	0.185	0.0407489104498529	CD8+ Teff/Tex	IQGAP2
2.46205964609864E-06	0.413333402862701	0.399	0.268	0.041259195549321	CD8+ Teff/Tex	DENND2D
2.55232463660253E-06	1.05629448727264	0.196	0.103	0.0427718562601851	CD8+ Teff/Tex	FOXN2
2.7132593133017E-06	0.53266654434376	0.245	0.137	0.0454687995723099	CD8+ Teff/Tex	RASSF1
2.73402324331261E-06	0.578446206922715	0.248	0.137	0.0458167615114328	CD8+ Teff/Tex	TRERF1
2.74655352377626E-06	0.623224553400206	0.283	0.164	0.0460267439514425	CD8+ Teff/Tex	BICDL1
2.77037033861071E-06	0.39367962167285	0.22	0.118	0.0464258661344382	CD8+ Teff/Tex	TTC39B
2.80629434747541E-06	0.932840744586393	0.21	0.114	0.0470278806749929	CD8+ Teff/Tex	MFSD10
2.85182347058639E-06	0.696608888019424	0.374	0.259	0.0477908577200866	CD8+ Teff/Tex	VPS13B
2.96873220309341E-06	0.681023930673934	0.262	0.153	0.0497500142594394	CD8+ Teff/Tex	GALM
7.34370344877315E-107	4.55991396380967	0.649	0.069	1.23065782394541E-102	NK cell	GNLY
4.90015498571021E-86	5.12237178696414	0.364	0.015	8.21167972505317E-82	NK cell	FGR
3.7424266086316E-85	3.35637310504675	0.682	0.106	6.27155851074484E-81	NK cell	KLRD1
7.7588898122926E-85	6.07134343756398	0.273	0.003	1.30023475474399E-80	NK cell	KLRF1
3.42717812025655E-70	5.23653759561777	0.292	0.012	5.74326509392592E-66	NK cell	NCAM1
2.58493082625851E-67	3.59640132546	0.513	0.069	4.33182707864402E-63	NK cell	KLRC3
4.92961569790504E-62	7.42522601726823	0.195	0.002	8.26104998654926E-58	NK cell	FGFBP2
1.88553142517164E-60	3.27978502209247	0.539	0.09	3.15977356230263E-56	NK cell	MATK
2.46563293175223E-60	4.91425108895557	0.312	0.021	4.13190766703039E-56	NK cell	KLRC1
1.16635681400055E-57	2.51930660286803	0.812	0.267	1.95458074890213E-53	NK cell	NKG7
6.58910418704142E-57	3.80981306893385	0.351	0.032	1.1042020796644E-52	NK cell	PLCG2
8.01727978285282E-57	2.94945313013025	0.578	0.112	1.34353574601048E-52	NK cell	KLRC4
4.99032420016422E-53	4.18243720898007	0.403	0.054	8.3627852946352E-49	NK cell	ITGAX
9.01994236615412E-52	3.98470099572333	0.331	0.033	1.51156194172011E-47	NK cell	CLIC3
1.42867417882316E-46	3.31534599508622	0.403	0.061	2.39417218887185E-42	NK cell	NCR1
2.64154323715591E-46	4.58717169893955	0.234	0.015	4.42669815682587E-42	NK cell	SH2D1B
9.72503000216163E-42	3.7196749489208	0.286	0.031	1.62972052776225E-37	NK cell	FCGR3A
1.07270098364392E-41	2.85629936416239	0.403	0.068	1.79763230839048E-37	NK cell	AOAH
5.15824727117485E-41	2.0005229472041	0.682	0.217	8.64419077703481E-37	NK cell	KLRK1
1.96529170442927E-38	3.42021667772297	0.299	0.038	3.29343583828257E-34	NK cell	CD300A
3.44808560954977E-35	3.96195075429264	0.201	0.016	5.7783018644835E-31	NK cell	KLRC2
3.74123363789965E-35	3.14275548962341	0.234	0.023	6.26955933039223E-31	NK cell	TRDC
7.69414751637798E-34	2.49260255509994	0.721	0.352	1.28938524079462E-29	NK cell	PRF1
2.09341885685812E-32	2.56359404772698	0.403	0.09	3.50815132032283E-28	NK cell	MCTP2
8.6024101758269E-31	7.38119600085607	0.104	0.002	1.44159189726507E-26	NK cell	OSBPL5
6.28471746147026E-30	2.74614335100982	0.364	0.078	1.05319295219319E-25	NK cell	TXK
1.02316297081983E-29	2.16495412068072	0.597	0.228	1.71461650649988E-25	NK cell	CTSW
1.3695168585716E-29	4.72272876259401	0.143	0.008	2.29503635159429E-25	NK cell	FSD1
6.31404836566301E-28	2.70871850944067	0.344	0.075	1.05810822511781E-23	NK cell	PTGDR
5.71139403893354E-26	5.10454606577752	0.123	0.007	9.57115413044483E-22	NK cell	CD160
1.01826733194129E-25	4.20594209099216	0.136	0.01	1.70641239486721E-21	NK cell	S1PR5
4.10707696430708E-25	3.47933894733945	0.188	0.023	6.8826395767858E-21	NK cell	IGFBP2

2.59020419159592E-24	5.45604293546889	0.136	0.011	4.34066418427644E-20	NK cell	MYOM2
9.87706933288146E-24	3.7083923531833	0.149	0.014	1.65519927880428E-19	NK cell	C1orf21
7.09730877591258E-23	1.38163103082506	0.714	0.342	1.18936700466743E-18	NK cell	CCL5
5.78437277223813E-22	3.34900263312202	0.117	0.008	9.69345189171665E-18	NK cell	SYNGR1
6.92030206673213E-22	2.62070019751095	0.351	0.099	1.15970422034297E-17	NK cell	ADGRG1
1.0392777135378E-21	4.29157055585168	0.13	0.012	1.74162159234665E-17	NK cell	NMUR1
7.07670678251127E-20	3.2232820843297	0.221	0.043	1.18591452261324E-15	NK cell	FCRL6
7.70652627731641E-20	3.72265181923006	0.156	0.021	1.29145967355268E-15	NK cell	CD244
9.46630732673684E-20	2.1299318075738	0.461	0.185	1.58636378181456E-15	NK cell	RIN3
1.08667552072277E-18	1.80187367563455	0.669	0.443	1.82105083762722E-14	NK cell	IL2RB
4.13253355364379E-18	3.14038250398071	0.253	0.063	6.92529972919626E-14	NK cell	GZMB
5.21619853118544E-18	1.85949119201139	0.357	0.114	8.74130549856056E-14	NK cell	KLRB1
6.2213221697447E-18	2.29245187262708	0.214	0.044	1.04256916920582E-13	NK cell	TRGC1
1.42224348832271E-17	2.3405993981927	0.201	0.04	2.38339563773119E-13	NK cell	IRF8
3.5392447091138E-17	2.10189279811164	0.383	0.144	5.9310662835329E-13	NK cell	ZBTB16
4.05048450223187E-17	2.50948390276885	0.201	0.041	6.78780192884017E-13	NK cell	SPRY2
7.49814329679348E-17	3.15614122260779	0.221	0.051	1.25653885367665E-12	NK cell	SLC15A4
1.73550706027849E-16	1.93261649242604	0.396	0.153	2.90836273161469E-12	NK cell	CHST12
4.1339934555382E-16	3.92152772588099	0.11	0.012	6.92774623279092E-12	NK cell	CTBP2
4.18833385925823E-16	1.73624528788058	0.221	0.051	7.01880988134494E-12	NK cell	ABCB1
1.36134139259867E-15	2.53957545211193	0.234	0.06	2.28133590571685E-11	NK cell	LAT2
1.62739802405928E-15	2.47287632896378	0.286	0.088	2.72719360871855E-11	NK cell	ZEB2
2.50242221678065E-15	2.28615938686159	0.26	0.073	4.19355915088101E-11	NK cell	SLAMF7
3.91789395229915E-15	2.23173131892876	0.156	0.028	6.56560668526292E-11	NK cell	TYROBP
6.46860162522092E-15	2.63036011587527	0.182	0.038	1.08400826035452E-10	NK cell	ATP8B4
4.84898133381251E-14	2.00138139564882	0.357	0.142	8.125922919203E-10	NK cell	PTPN4
5.86863324691895E-14	1.65906890093566	0.506	0.279	9.83465559518677E-10	NK cell	MAPK1
8.65273838817823E-14	2.21707164762269	0.247	0.072	1.45002589909091E-09	NK cell	DGKD
1.96634128058725E-13	2.13440492193776	0.156	0.031	3.29519471800812E-09	NK cell	DEPRECATED-ENSG00000145287
3.00942581632782E-13	1.63701902641705	0.221	0.06	5.04319578300216E-09	NK cell	AUTS2
5.39071594944438E-13	2.0781513118465	0.286	0.101	9.03376178807889E-09	NK cell	SYNE1
1.17554308481638E-12	3.15822991159979	0.104	0.015	1.9699751015353E-08	NK cell	CDHR1
2.03810074882523E-12	1.78910137585009	0.325	0.126	3.41544923488133E-08	NK cell	EOMES
5.86308013840255E-12	1.53966541710761	0.403	0.187	9.825349695935E-08	NK cell	ABCA2
6.61803471804629E-11	1.89804970418979	0.143	0.032	1.1090502580502E-06	NK cell	ZNF683
9.66716362237344E-11	1.71445822854125	0.416	0.218	1.62002327983734E-06	NK cell	APMAP
1.21612022286159E-10	1.6259831473429	0.234	0.079	2.03797426947145E-06	NK cell	HOPX
1.2840848601776E-10	2.0008752188744	0.312	0.137	2.15186940868563E-06	NK cell	APOBR
1.87123541425513E-10	2.87974867907502	0.143	0.035	3.13581630720875E-06	NK cell	CMC1
2.67969208427422E-10	1.37020547535767	0.429	0.215	4.49062799482674E-06	NK cell	CLEC2B
6.28298943231594E-10	2.1835575318179	0.195	0.063	1.05290336906751E-05	NK cell	YPEL1
6.36098011150855E-10	2.23106064456231	0.247	0.093	1.0659730470866E-05	NK cell	ITGAM
9.63523128069174E-10	2.88184482331124	0.123	0.028	1.61467205801832E-05	NK cell	GAS7
1.0643455006203E-09	1.9139868325823	0.247	0.094	1.78363018993949E-05	NK cell	GSTP1
1.43005085498694E-09	2.02458477829362	0.344	0.162	2.39647922278711E-05	NK cell	CCL4
1.86950474418194E-09	1.95857934086798	0.156	0.044	3.1329160503001E-05	NK cell	CD38
5.88527932109635E-09	1.59668377445849	0.344	0.17	9.86255108629326E-05	NK cell	METRNL
7.11145519256735E-09	1.72916873616864	0.24	0.096	0.000119173766117044	NK cell	PIK3AP1
7.80521002855596E-09	2.16688820018728	0.175	0.057	0.000130799709658541	NK cell	PTPN12
8.05211308421985E-09	1.19074477330658	0.338	0.159	0.000134937311065356	NK cell	SAMD3
1.31327049925805E-08	1.72926941278606	0.234	0.091	0.000220077870265664	NK cell	FASLG
1.36691229706249E-08	2.12455339525018	0.247	0.105	0.000229067162741732	NK cell	GZMH
1.63556513814781E-08	0.999161906110176	0.565	0.383	0.000274088005850811	NK cell	CD7
3.62368359305836E-08	1.29161373385638	0.299	0.14	0.000607256896524721	NK cell	RASSF1
4.09533183472124E-08	1.79360249187527	0.26	0.115	0.000686295708862585	NK cell	GFOD1
1.04413119180095E-07	1.22907107131154	0.149	0.046	0.00174975505122003	NK cell	MAP3K8
1.12224333331395E-07	1.38011533452635	0.136	0.04	0.00188065537796752	NK cell	PLAC8
1.17728272220212E-07	1.74332624635048	0.175	0.062	0.00197289038586632	NK cell	HSH2D
1.73003822551919E-07	1.18075344877211	0.299	0.148	0.00289919805832506	NK cell	ADAM8
1.7799606147868E-07	1.47481835814418	0.221	0.093	0.00298285799825973	NK cell	TSPAN32
1.83969474983123E-07	1.57224820654153	0.266	0.126	0.00308296046176717	NK cell	LPCAT1
2.10527491923311E-07	1.29214560848411	0.455	0.303	0.00352801970965084	NK cell	CD247
2.40067672865866E-07	0.928042904880115	0.526	0.339	0.00402305406188618	NK cell	DUSP2
3.01973532862927E-07	1.90200085524665	0.201	0.081	0.00506047246371692	NK cell	USP28
4.27934993859324E-07	0.988186917030771	0.422	0.25	0.00717133462709454	NK cell	PITPNC1
5.28801265283337E-07	1.60743076778145	0.143	0.047	0.00886165160361816	NK cell	PDE4A

6.12047291612403E-07	1.80236747790096	0.169	0.064	0.0102566885128407	NK cell	ABHD15
6.40061137582228E-07	1.55123392531859	0.253	0.12	0.010726144543603	NK cell	TNFRSF1A
9.04607230151827E-07	1.33758801786217	0.429	0.284	0.0151594079628843	NK cell	PYHIN1
9.60092369548839E-07	1.68612205924323	0.221	0.1	0.0160892279288994	NK cell	TSPOAP1
1.11488524268114E-06	1.37824838403295	0.175	0.068	0.0186832468968506	NK cell	NOL4L
1.23256818506649E-06	0.975842100276055	0.532	0.38	0.0206553776453443	NK cell	LITAF
1.26145554553363E-06	2.29061161010238	0.195	0.082	0.0211394720320526	NK cell	PLEK
1.66598218582252E-06	1.44568891862184	0.214	0.096	0.0279185294700137	NK cell	SLC16A3
1.7033991047782E-06	1.97724425837314	0.156	0.058	0.0285455621978731	NK cell	CCL3
2.12548419518235E-06	1.34279579446218	0.136	0.046	0.0356188641428659	NK cell	NUGGC
2.46630830866016E-06	2.40370008202971	0.123	0.04	0.041330394636527	NK cell	LRMP
2.5932497707711E-06	0.688499994159342	0.669	0.558	0.0434576796585821	NK cell	ADGRE5
1.6813639137508E-246	6.58116072382307	0.867	0.014	2.81762964666358E-242	Proliferating T/NK	RRM2
9.97094730974564E-240	7.85588894332182	0.722	0.004	1.67093135016717E-235	Proliferating T/NK	HJURP
1.93930663345299E-227	5.70471025368938	0.767	0.01	3.24989005634053E-223	Proliferating T/NK	NCAPG
6.42011240585024E-225	7.78654045968509	0.7	0.006	1.07588243697238E-220	Proliferating T/NK	ASPM
1.67464016767166E-224	5.9673283319629	0.867	0.02	2.80636199298416E-220	Proliferating T/NK	KIFC1
1.6421649360774E-217	5.7983872005002	0.978	0.038	2.75193999987851E-213	Proliferating T/NK	MKI67
4.41323068442956E-214	6.92675834931233	0.667	0.005	7.39569198096706E-210	Proliferating T/NK	KIF2C
8.70440287017553E-213	5.26853110085375	0.8	0.016	1.45868383298402E-208	Proliferating T/NK	HIST1H2AH
6.91178357138537E-210	6.86270505242521	0.644	0.004	1.15827669089276E-205	Proliferating T/NK	ESPL1
1.38737651401487E-204	6.57810772032825	0.667	0.007	2.32496556218612E-200	Proliferating T/NK	CIT
1.50455580465075E-204	7.64604496691898	0.611	0.003	2.52133461743373E-200	Proliferating T/NK	KIF18B
3.19547060207763E-204	6.37154298825847	0.744	0.014	5.3549696349617E-200	Proliferating T/NK	DEPRECATED-ENSG00000188486
8.33525059401023E-200	6.19761584398282	0.811	0.022	1.39682129454423E-195	Proliferating T/NK	TOP2A
9.66904315733797E-193	5.76748194102169	0.856	0.03	1.6203382523067E-188	Proliferating T/NK	TYMS
2.06062539439177E-188	5.95074383812709	0.622	0.007	3.45319603592172E-184	Proliferating T/NK	AURKB
2.28891770227785E-188	6.57997038203756	0.622	0.007	3.83576828547722E-184	Proliferating T/NK	GTSE1
8.03076405670315E-186	6.6877545507765	0.622	0.007	1.34579544062231E-181	Proliferating T/NK	KIF23
2.60518274245809E-184	7.20652605249091	0.567	0.004	4.36576523981127E-180	Proliferating T/NK	SPC25
1.87311144720213E-183	7.20480609490517	0.922	0.047	3.13896016322133E-179	Proliferating T/NK	HIST1H1B
2.1042877543196E-180	6.41073133465415	0.6	0.007	3.52636541868879E-176	Proliferating T/NK	ANLN
2.19454169055761E-180	5.65693040148176	0.689	0.014	3.67761296503645E-176	Proliferating T/NK	MYBL2
5.88981609996664E-180	6.23967107450342	0.622	0.009	9.8701538203241E-176	Proliferating T/NK	BIRC5
1.31758350271303E-179	7.20780387430249	0.544	0.003	2.20800643384649E-175	Proliferating T/NK	CDCA8
4.53664569893096E-179	6.63215235502515	0.611	0.008	7.60251086226849E-175	Proliferating T/NK	FOXM1
7.93452590585435E-177	6.88678841739147	0.556	0.004	1.32966785130307E-172	Proliferating T/NK	DLGAP5
8.93670844545052E-174	5.41200513587784	0.644	0.012	1.4976136012886E-169	Proliferating T/NK	SPAG5
1.58764069707684E-171	5.11950415191402	0.756	0.025	2.66056828016137E-167	Proliferating T/NK	CDK1
4.54682826325914E-171	5.97672321755655	0.633	0.012	7.61957480356967E-167	Proliferating T/NK	SPC24
1.18539312952101E-170	5.20794072042098	0.733	0.022	1.98648180645131E-166	Proliferating T/NK	ZWINT
2.54201629655899E-166	6.44644695808129	0.544	0.006	4.25991090977356E-162	Proliferating T/NK	PRR11
3.69143760717858E-165	4.85045277248869	0.678	0.017	6.18611114210987E-161	Proliferating T/NK	ASF1B
6.07707648623326E-165	6.20329449405301	0.533	0.005	1.01839647756297E-160	Proliferating T/NK	CDCA5
5.44971173123591E-164	7.08505481465424	0.511	0.004	9.13262691920514E-160	Proliferating T/NK	UBE2C
5.51255103207145E-164	5.21447960576444	0.778	0.031	9.23793301954534E-160	Proliferating T/NK	TPX2
1.57750109247273E-163	5.33401193516457	0.711	0.022	2.64357633076581E-159	Proliferating T/NK	TCF19
1.48820428142831E-162	6.79144908163442	0.533	0.006	2.49393273481756E-158	Proliferating T/NK	CDCA3
8.08916109131623E-162	4.54848262176643	0.711	0.022	1.35558161568277E-157	Proliferating T/NK	KIF11
2.51367428137474E-160	4.68401521260734	0.6	0.011	4.21241536072779E-156	Proliferating T/NK	KNL1
8.39274378440469E-156	5.32158494754297	0.6	0.012	1.40645600339054E-151	Proliferating T/NK	MELK
2.82889381908684E-155	4.247090374935	0.756	0.03	4.74066026202572E-151	Proliferating T/NK	CENPM
3.29102080824384E-155	5.74155568361326	0.556	0.009	5.51509267045503E-151	Proliferating T/NK	DEPDC1B
2.4497640115727E-154	4.71248943305594	0.744	0.029	4.10531453059352E-150	Proliferating T/NK	H2AFX
1.14566934605306E-153	4.88410623168244	0.811	0.039	1.91991269011572E-149	Proliferating T/NK	HIST1H2BH
6.31316260738458E-152	5.22988323407752	0.567	0.01	1.05795978974551E-147	Proliferating T/NK	CENPI
3.71642617016874E-148	4.19724885019403	0.7	0.025	6.22798697596878E-144	Proliferating T/NK	CEP55
1.62733561824706E-146	3.86145712628077	0.844	0.046	2.72708902905842E-142	Proliferating T/NK	FANCI
1.35213286089989E-145	5.13606631857975	0.511	0.007	2.26590424829603E-141	Proliferating T/NK	PRC1
2.10005332173518E-145	4.88270853953362	0.667	0.023	3.51926935656381E-141	Proliferating T/NK	INCENP
4.9225142025057E-145	5.40239314130498	0.556	0.011	8.24914930055904E-141	Proliferating T/NK	KIF15
7.96447758245913E-145	3.69815249457306	0.756	0.035	1.3346871532685E-140	Proliferating T/NK	NCAPG2
1.71061755311269E-144	5.0778165455042	0.678	0.025	2.86665289550624E-140	Proliferating T/NK	CDT1
9.74814717163066E-144	4.79125959640523	0.622	0.018	1.63359450302187E-139	Proliferating T/NK	BUB1
3.39150346699193E-143	4.38883593356948	0.522	0.009	5.68348150998507E-139	Proliferating T/NK	MXD3
3.55377903742745E-142	4.71461266572092	0.633	0.02	5.95542291092092E-138	Proliferating T/NK	STMN1

5.03957526655979E-142	7.6945634242201	0.422	0.002	8.44532023170089E-138	Proliferating T/NK	BUB1B
4.00824523573385E-137	6.34930263002091	0.456	0.005	6.71701736604278E-133	Proliferating T/NK	KIF14
7.15603037317312E-136	5.55060984168439	0.444	0.004	1.19920756993635E-131	Proliferating T/NK	HIST1H2BB
1.22002182935108E-133	5.92579120301093	0.478	0.007	2.04451258162654E-129	Proliferating T/NK	PKMYT1
2.81259838209782E-131	4.28255792332955	0.756	0.044	4.71335236871953E-127	Proliferating T/NK	HIST1H4C
1.86957672251372E-130	6.1857547382456	0.411	0.003	3.13303667158849E-126	Proliferating T/NK	NEIL3
1.84712390016311E-128	4.46747911819572	0.678	0.032	3.09541023189334E-124	Proliferating T/NK	CENPF
2.83550393615938E-128	5.36014020224455	0.511	0.012	4.75173749621589E-124	Proliferating T/NK	UBE2T
2.60962629991409E-126	3.85759708577054	0.856	0.067	4.37321175339603E-122	Proliferating T/NK	NUSAP1
6.65864010693083E-126	5.18957339906279	0.478	0.009	1.11585490911947E-121	Proliferating T/NK	CDCA2
6.6805052984233E-126	5.0399638869297	0.511	0.012	1.11951907790978E-121	Proliferating T/NK	PLK1
1.60462162579403E-124	4.62795247562364	0.6	0.023	2.68902492050564E-120	Proliferating T/NK	UHRF1
3.50140069942504E-123	5.43934086062353	0.433	0.006	5.86764729209648E-119	Proliferating T/NK	CENPA
7.4491242442933E-122	6.11745128232637	0.378	0.002	1.24832424085867E-117	Proliferating T/NK	ESCO2
9.7278452040094E-120	4.69768793557565	0.6	0.025	1.6301922992879E-115	Proliferating T/NK	PCLAF
3.46980090807356E-119	5.61462109880113	0.389	0.004	5.81469236174967E-115	Proliferating T/NK	KIF20A
4.22129609447953E-119	5.40916367701448	0.444	0.008	7.07404799512879E-115	Proliferating T/NK	CCNF
2.52106057194692E-118	5.37928690578007	0.5	0.014	4.22479330646865E-114	Proliferating T/NK	HMMR
2.99820352986525E-118	4.78766247662707	0.567	0.022	5.02438947534819E-114	Proliferating T/NK	TK1
3.30876430881817E-118	4.19301781771857	0.478	0.011	5.54482722871749E-114	Proliferating T/NK	FBXO5
2.26087551575284E-117	6.16194534203823	0.4	0.005	3.78877518929861E-113	Proliferating T/NK	MND1
3.8281190093953E-116	5.31348323510921	0.422	0.007	6.41516183594465E-112	Proliferating T/NK	TTK
6.42849464650696E-116	5.34046907449705	0.456	0.01	1.07728713286164E-111	Proliferating T/NK	CKAP2L
1.27261807074334E-114	4.59652279270557	0.367	0.003	2.13265336295169E-110	Proliferating T/NK	KIF4A
2.19176979913125E-114	5.14869362461836	0.444	0.009	3.67296782938415E-110	Proliferating T/NK	E2F2
9.49475564207481E-114	4.29642373880836	0.4	0.006	1.5911311504989E-109	Proliferating T/NK	HIST1H2BM
1.00252451778392E-113	5.78536154237711	0.344	0.002	1.6800305869023E-109	Proliferating T/NK	FAM72C
1.47677806918829E-111	2.60471085245318	0.633	0.031	2.47478468834573E-107	Proliferating T/NK	NDC80
7.46817880291986E-111	5.51386939818375	0.433	0.009	1.25151740379331E-106	Proliferating T/NK	NUF2
3.85585065495628E-110	7.04775886034291	0.344	0.002	6.46163452757574E-106	Proliferating T/NK	PLK4
5.93515866412577E-110	5.04829754858107	0.411	0.007	9.94613888934197E-106	Proliferating T/NK	DIAPH3
7.27142316388728E-110	4.45320103241275	0.411	0.007	1.21854509380423E-105	Proliferating T/NK	CENPW
2.48210938597278E-109	3.06860447891519	0.7	0.044	4.15951890901318E-105	Proliferating T/NK	SMC2
3.3331898306248E-108	6.28738111584873	0.389	0.006	5.58575951816104E-104	Proliferating T/NK	TROAP
1.11314366975258E-107	5.47962574168936	0.389	0.006	1.86540616177137E-103	Proliferating T/NK	POLQ
1.17802342577984E-107	3.85775959826134	0.544	0.022	1.97413165692185E-103	Proliferating T/NK	ZGRF1
1.19363244698701E-107	5.48083575784252	0.389	0.006	2.00028925466084E-103	Proliferating T/NK	FAM83D
1.20534849718692E-106	6.48687573980882	0.344	0.003	2.01992301158584E-102	Proliferating T/NK	E2F8
1.22118603142135E-106	4.35913912584436	0.533	0.022	2.0464635514559E-102	Proliferating T/NK	CENPE
2.76568392143515E-106	5.04168348756865	0.378	0.006	4.63473311554103E-102	Proliferating T/NK	NEK2
3.66208280262423E-106	6.74595965107145	0.333	0.002	6.13691836063768E-102	Proliferating T/NK	E2F7
5.45628656001658E-106	4.64456436360645	0.433	0.01	9.14364501727578E-102	Proliferating T/NK	SHCBP1
6.05741262243983E-106	3.89865384959443	0.622	0.035	1.01510120726847E-101	Proliferating T/NK	CKS1B
1.0934205542251E-105	3.86009124397711	0.556	0.025	1.83235416477043E-101	Proliferating T/NK	ECT2
1.99839434727366E-105	2.834095928267	0.944	0.103	3.34890924716119E-101	Proliferating T/NK	KIF22
3.23892901643709E-103	4.47432136716996	0.467	0.015	5.42779724574528E-99	Proliferating T/NK	CCNA2
5.75988097618433E-103	3.00850417171166	0.756	0.06	9.6524085398897E-99	Proliferating T/NK	EZH2
1.73278865091846E-102	5.20667801808362	0.389	0.007	2.90380722120915E-98	Proliferating T/NK	KNSTRN
1.8480624915636E-102	3.4769817156662	0.756	0.063	3.09698312336228E-98	Proliferating T/NK	HIST1H1E
3.23403644560534E-101	5.48742126291735	0.422	0.011	5.41959827554542E-97	Proliferating T/NK	CCNB2
3.31446916504265E-101	8.33903979244411	0.289	0.001	5.55438742677848E-97	Proliferating T/NK	FBXO43
9.40898251874969E-99	5.46994163511435	0.378	0.007	1.57675729049207E-94	Proliferating T/NK	CCNB1
1.16955839216791E-97	3.0760284885278	0.933	0.12	1.95994595359498E-93	Proliferating T/NK	LMNB1
1.46943269167437E-97	5.08250401360146	0.3	0.002	2.46247530470791E-93	Proliferating T/NK	HIST1H2BN
1.59335638041236E-97	6.86807687966671	0.3	0.002	2.67014662229503E-93	Proliferating T/NK	PSRC1
1.72770548849993E-97	6.65034224230066	0.3	0.002	2.89528885762819E-93	Proliferating T/NK	CDC25C
2.76960674125698E-97	3.04225173458943	0.856	0.094	4.64130697699844E-93	Proliferating T/NK	TACC3
5.11232265848507E-97	3.52465763716127	0.522	0.023	8.56723031108927E-93	Proliferating T/NK	C1orf112
2.50630602458622E-96	4.8380928279029	0.378	0.008	4.2000676360016E-92	Proliferating T/NK	AURKA
2.6934353247975E-95	3.35285656496862	0.756	0.07	4.51365891729565E-91	Proliferating T/NK	TMEM106C
9.18630394554913E-95	6.37270207563812	0.311	0.003	1.53944081519512E-90	Proliferating T/NK	GPSM2
1.40658552994466E-94	4.59585843781977	0.422	0.013	2.35715603108126E-90	Proliferating T/NK	DTL
4.49379407527721E-94	5.08878129846469	0.3	0.002	7.53070011134955E-90	Proliferating T/NK	DEPDC1
1.04807336980775E-93	3.73796828322852	0.456	0.017	1.75636135312382E-89	Proliferating T/NK	CENPJ
1.10949440929015E-93	3.30895230058036	0.411	0.012	1.85929073108844E-89	Proliferating T/NK	NCAPH
1.14965718964298E-93	3.8655719726496	0.844	0.11	1.9265955184037E-89	Proliferating T/NK	TUBB

3.15873643929308E-93	2.49715639740347	0.8	0.076	5.29341052496735E-89	Proliferating T/NK	NCAPD3
3.55653669423842E-93	4.60357677176598	0.456	0.017	5.96004419220474E-89	Proliferating T/NK	ZNF367
5.45221958025922E-93	4.52963831490942	0.422	0.014	9.1368295725984E-89	Proliferating T/NK	UBE2S
5.35951547590356E-92	6.46341448946825	0.311	0.004	8.98147603451919E-88	Proliferating T/NK	CDC20
1.61753265694802E-91	5.16843889168866	0.356	0.007	2.71066122651349E-87	Proliferating T/NK	CDC45
2.91437625216815E-91	4.38374167025887	0.389	0.01	4.88391172338339E-87	Proliferating T/NK	STIL
5.03624536722794E-91	2.67792885638375	0.767	0.071	8.43973998640059E-87	Proliferating T/NK	LMNB2
7.0318722305452E-91	4.0727709081241	0.422	0.014	1.17840114839476E-86	Proliferating T/NK	ATAD5
1.45698191115407E-90	3.42418332234729	0.578	0.036	2.441610286712E-86	Proliferating T/NK	TUBB4B
3.52679395248084E-90	6.18643704991385	0.322	0.005	5.91020130556739E-86	Proliferating T/NK	IQGAP3
8.77896555729629E-90	3.38362714384208	0.556	0.033	1.47117904809171E-85	Proliferating T/NK	ATAD2
1.19210515185582E-89	4.84007462035823	0.389	0.011	1.99772981347999E-85	Proliferating T/NK	WDR34
3.9063180340807E-89	4.72124562804195	0.267	0.001	6.54620776151244E-85	Proliferating T/NK	HIST1H3I
1.7657057206986E-88	4.7629939696295	0.367	0.009	2.95896964674671E-84	Proliferating T/NK	EXO1
2.73131868659709E-86	4.97590177400283	0.311	0.005	4.5771438549994E-82	Proliferating T/NK	RAD54L
2.04858093807557E-85	5.21854163161409	0.344	0.008	3.43301193602704E-81	Proliferating T/NK	HASPIN
6.65314694775496E-85	5.49293664869938	0.3	0.004	1.11493436550478E-80	Proliferating T/NK	TICRR
8.01417021265602E-85	3.03654989999268	0.511	0.028	1.3430146442369E-80	Proliferating T/NK	FANCA
2.34030616012466E-84	3.34246805793408	0.622	0.049	3.92188506313691E-80	Proliferating T/NK	DEPRECATED-ENSG00000132646
2.9245871341502E-84	4.34176638199111	0.356	0.009	4.9010231194089E-80	Proliferating T/NK	SKA1
4.75880914869009E-83	6.19688459000693	0.278	0.003	7.97481237137485E-79	Proliferating T/NK	PBK
2.19698978062012E-82	4.13017599461909	0.4	0.015	3.6817154743632E-78	Proliferating T/NK	C21orf58
3.15086081847547E-82	3.69973212878803	0.411	0.016	5.2802125596012E-78	Proliferating T/NK	SUV39H1
3.35183634464461E-82	3.81204811872412	0.5	0.028	5.61700734635543E-78	Proliferating T/NK	TUBA1B
4.95314882517139E-82	3.56342914565044	0.422	0.017	8.30048680122221E-78	Proliferating T/NK	CENPO
3.01886978540403E-81	5.31033621035302	0.289	0.004	5.05902198638008E-77	Proliferating T/NK	MCM10
2.47830934519351E-80	3.55607546887974	0.411	0.017	4.15315080067528E-76	Proliferating T/NK	GEN1
3.82022257138985E-80	3.45368752935395	0.467	0.024	6.40192898513511E-76	Proliferating T/NK	WEE1
4.22160581532933E-80	4.82269293617044	0.322	0.007	7.0745670253289E-76	Proliferating T/NK	SGO2
5.43016558296559E-80	3.74791367518869	0.5	0.03	9.09987148393374E-76	Proliferating T/NK	CENPU
5.5338346343688E-78	3.71075531822301	0.511	0.033	9.27360008027524E-74	Proliferating T/NK	ARHGAP11A
7.22649719984248E-78	4.26513626827837	0.322	0.008	1.2110164007496E-73	Proliferating T/NK	RECQL4
3.54699127199855E-77	6.36979588923518	0.233	0.001	5.94404797361518E-73	Proliferating T/NK	SAPCD2
6.53521536864306E-77	2.58339754450692	0.678	0.065	1.0951713914772E-72	Proliferating T/NK	LIG1
6.30877400563539E-76	4.34491945709274	0.322	0.009	1.05722434786438E-71	Proliferating T/NK	ORC1
1.11938532411002E-75	3.92520541086328	0.922	0.187	1.87586592614357E-71	Proliferating T/NK	HIST1H1C
1.53100024625928E-75	2.67656547732301	0.433	0.022	2.5656502126813E-71	Proliferating T/NK	ARHGAP19
2.91672575554372E-75	2.67909984444232	0.389	0.016	4.88784902114016E-71	Proliferating T/NK	HIST1H2AE
4.76462919031091E-75	2.8881194150032	0.722	0.084	7.98456559712303E-71	Proliferating T/NK	NCAPD2
2.39208074827757E-74	5.6431110157098	0.244	0.002	4.00864891796354E-70	Proliferating T/NK	TRIP13
3.00136956945061E-74	5.57267896292643	0.244	0.002	5.02969512448533E-70	Proliferating T/NK	KIF24
9.09734504704875E-74	5.13633712722775	0.289	0.006	1.52453308298443E-69	Proliferating T/NK	DEPRECATED-ENSG00000117399
6.01812033551191E-73	3.67471051660219	0.389	0.017	1.00851660582509E-68	Proliferating T/NK	ARHGAP33
5.67193327501576E-72	4.3322028321758	0.278	0.006	9.50502578227141E-68	Proliferating T/NK	RACGAP1
1.31317575241493E-71	3.17805936090324	0.833	0.135	2.20061992589695E-67	Proliferating T/NK	HIST1H1D
1.78790810226887E-70	3.76641043920631	0.433	0.025	2.99617639778217E-66	Proliferating T/NK	DTYMK
6.80742436423041E-70	2.8516319409648	0.467	0.03	1.14078817495773E-65	Proliferating T/NK	CENPP
1.23834126634304E-69	2.39577525674509	0.933	0.176	2.07521229413767E-65	Proliferating T/NK	HMGB2
4.26023201223789E-69	7.71219287128833	0.2	0.001	7.13929680610826E-65	Proliferating T/NK	MTFR2
5.75028386373406E-69	2.61001770793973	0.456	0.029	9.63632569884554E-65	Proliferating T/NK	CENPN
1.06504243108471E-68	4.13715609350746	0.3	0.009	1.78479810601175E-64	Proliferating T/NK	CIP2A
1.93026952096264E-68	3.14293685894349	0.422	0.024	3.2347456632292E-64	Proliferating T/NK	RCCD1
8.73121275524826E-68	2.87104584114223	0.533	0.044	1.4631766335245E-63	Proliferating T/NK	RFWD3
9.82094310474891E-68	2.6743008766251	0.6	0.057	1.64579364549382E-63	Proliferating T/NK	KNTC1
1.21857869935487E-67	4.32108581026705	0.278	0.007	2.0420941843789E-63	Proliferating T/NK	POLE2
1.8844692414158E-67	3.00887634228011	0.456	0.03	3.1579935547646E-63	Proliferating T/NK	AC091057.6
3.33117150397929E-67	3.68736587200901	0.4	0.022	5.5823772063685E-63	Proliferating T/NK	FANCG
4.87816805232098E-66	3.52480931488944	0.367	0.017	8.1748340220795E-62	Proliferating T/NK	CCDC18
5.95429136928454E-66	6.80393430708254	0.211	0.002	9.97820147664704E-62	Proliferating T/NK	NRGN
1.37675903252988E-65	2.75683892052639	0.444	0.03	2.30717278671357E-61	Proliferating T/NK	CHAF1A
3.44147744786039E-65	4.5503998478644	0.3	0.01	5.76722790712445E-61	Proliferating T/NK	BRCA1
7.10566024064177E-65	3.357612529103	0.433	0.028	1.19076654312675E-60	Proliferating T/NK	RNF26
1.13555915502531E-64	3.53260689773933	0.467	0.035	1.90297003199142E-60	Proliferating T/NK	FEN1
1.22434477402648E-64	2.90637857344713	0.456	0.032	2.05175697231357E-60	Proliferating T/NK	DEPRECATED-ENSG00000120802
1.99041823835665E-64	2.1900119140123	0.478	0.035	3.33554288383807E-60	Proliferating T/NK	CEP85
1.03653221864008E-63	3.32803058143163	0.456	0.034	1.73702069199705E-59	Proliferating T/NK	SLC43A3

1.67904464783355E-63	2.64923021583222	0.367	0.018	2.81374302083947E-59	Proliferating T/NK	CNTROB
7.07284059727384E-63	3.85161291477256	0.3	0.01	1.18526662729115E-58	Proliferating T/NK	KIF18A
8.38039058752104E-63	5.56012850591141	0.211	0.002	1.40438585465678E-58	Proliferating T/NK	TRAIP
8.75850619802719E-63	2.38340327050424	0.833	0.137	1.4677504686654E-58	Proliferating T/NK	SMC4
8.82218889429367E-62	3.69518572071695	0.344	0.017	1.47842241490573E-57	Proliferating T/NK	CDCA4
2.01042410484583E-61	2.55678434055993	0.544	0.052	3.36906871490065E-57	Proliferating T/NK	KIF20B
4.13093115398519E-61	4.72458925933439	0.244	0.006	6.92261442784838E-57	Proliferating T/NK	RAD51
4.1330525962863E-60	2.77813244578841	0.478	0.04	6.92616954085659E-56	Proliferating T/NK	TUBA1C
4.8350407044442E-60	3.4614410405387	0.4	0.026	8.10256121250758E-56	Proliferating T/NK	MAD2L1
9.63672038240102E-60	2.83569081415005	0.689	0.098	1.61492160168276E-55	Proliferating T/NK	HELLS
9.88343628636906E-60	5.05979836278832	0.233	0.005	1.65626625286973E-55	Proliferating T/NK	CCDC34
1.62680162185644E-59	2.48204732681386	0.789	0.13	2.72619415790702E-55	Proliferating T/NK	NUCKS1
2.93074178452884E-59	2.10900338915648	0.711	0.101	4.91133708251342E-55	Proliferating T/NK	RRM1
5.59985015757794E-59	3.103151156944	0.511	0.047	9.38422889406911E-55	Proliferating T/NK	DHFR
5.9532635282186E-59	2.53560267837295	0.5	0.046	9.97647902058873E-55	Proliferating T/NK	PCNA
6.02028494104814E-59	5.36555700641335	0.256	0.007	1.00887935042085E-54	Proliferating T/NK	CLSPN
6.18394827505028E-59	4.64542917525208	0.244	0.006	1.03630605193293E-54	Proliferating T/NK	RAD51AP1
6.92073092047234E-59	2.51541093553237	0.422	0.03	1.15977608765275E-54	Proliferating T/NK	DNA2
2.20043512797084E-58	5.27007545724543	0.222	0.004	3.68748918745353E-54	Proliferating T/NK	DEPRECATED-ENSG00000090889
3.07806153299994E-58	3.12257185292065	0.456	0.037	5.1582155170013E-54	Proliferating T/NK	ACOT7
6.15502434019889E-58	4.41807684472297	0.189	0.002	1.03145897893053E-53	Proliferating T/NK	ERCC6L
5.55888585037981E-57	4.84272720767338	0.244	0.007	9.31558090806648E-53	Proliferating T/NK	GGH
7.76493600987845E-57	2.86050025293226	0.433	0.034	1.30124797653543E-52	Proliferating T/NK	TIMELESS
8.42324925246039E-57	3.70168655467242	0.289	0.012	1.41156810972731E-52	Proliferating T/NK	CHEK1
8.74771004540885E-57	4.08417177732141	0.289	0.012	1.46594124940962E-52	Proliferating T/NK	ORC6
1.33740008109214E-56	3.93088211953643	0.267	0.009	2.24121505589421E-52	Proliferating T/NK	BRIP1
1.86426150899443E-56	3.14965946501399	0.544	0.06	3.12412943677287E-52	Proliferating T/NK	MCM4
3.24730965402023E-56	3.66797362782461	0.389	0.027	5.44184151820711E-52	Proliferating T/NK	E2F1
3.73721565943051E-56	3.11608480466939	0.333	0.018	6.26282600207365E-52	Proliferating T/NK	TEDC1
5.57046591712071E-56	4.40509120582084	0.289	0.012	9.33498678391088E-52	Proliferating T/NK	FAM111B
5.59864666144042E-56	3.22453127482723	0.389	0.026	9.38221207524185E-52	Proliferating T/NK	TUBG1
7.378290691741E-56	2.11808005561725	0.6	0.07	1.23645395412196E-51	Proliferating T/NK	NSD2
9.0955345133708E-56	2.24047318189545	0.489	0.046	1.52422967375068E-51	Proliferating T/NK	POLD1
2.01240750581938E-55	2.50008990241696	0.478	0.043	3.37239249825213E-51	Proliferating T/NK	NUDT1
3.11479547408158E-55	1.72824738900427	0.867	0.165	5.21977425546592E-51	Proliferating T/NK	PHF19
5.02441189747578E-55	4.09867129059959	0.244	0.007	8.41990945778991E-51	Proliferating T/NK	RAD54B
6.39889638821614E-55	2.73818661921942	0.422	0.035	1.07232705673726E-50	Proliferating T/NK	KPNA2
6.5814071317475E-55	3.21095587776005	0.244	0.007	1.10291220713825E-50	Proliferating T/NK	DBF4B
1.1587430623997E-54	2.23482081961315	0.622	0.078	1.94182162396942E-50	Proliferating T/NK	LSM 4.00
1.74925668158723E-54	3.05474396947289	0.4	0.03	2.93140434700389E-50	Proliferating T/NK	NDC1
4.16093833311917E-54	5.47694617500744	0.178	0.002	6.97290045864111E-50	Proliferating T/NK	C17orf53
1.29097651479363E-53	4.16709618075714	0.267	0.01	2.16341844349116E-49	Proliferating T/NK	POC1A
1.6475958548327E-53	4.72873988292156	0.233	0.007	2.76104113352865E-49	Proliferating T/NK	SGO1
1.70706261398806E-53	6.70451612391985	0.167	0.001	2.86069552852119E-49	Proliferating T/NK	PIMREG
1.81106331600788E-53	3.54241052051406	0.167	0.001	3.034979904966E-49	Proliferating T/NK	HIST1H2AK
1.81514857919698E-53	2.34752259726463	0.667	0.097	3.0418259890183E-49	Proliferating T/NK	H2AFZ
1.84953030363242E-53	1.52243128920144	0.722	0.108	3.09944288282721E-49	Proliferating T/NK	ANP32E
2.66750830246909E-53	2.45409542428061	0.644	0.09	4.4702104132777E-49	Proliferating T/NK	CDKN2C
3.43945243830377E-53	3.02517873460436	0.322	0.018	5.76383439610945E-49	Proliferating T/NK	NRM
4.60076812408933E-53	2.49744373367147	0.567	0.068	7.7099672223489E-49	Proliferating T/NK	GUSB
6.53987810205797E-53	2.29563705418228	0.556	0.063	1.09595277234288E-48	Proliferating T/NK	TTF2
2.52609637020152E-52	2.95569687776763	0.311	0.017	4.2332322971837E-48	Proliferating T/NK	BCL2L12
2.86332069155738E-52	2.17846915200115	0.856	0.189	4.79835281491185E-48	Proliferating T/NK	TMPO
5.95456911148459E-52	2.87013965770853	0.278	0.012	9.97866691702587E-48	Proliferating T/NK	TMEM201
1.69699414681755E-50	3.50496752071372	0.267	0.012	2.84382279123685E-46	Proliferating T/NK	WDR62
2.86077817587298E-50	3.06175509474512	0.211	0.006	4.79409206712794E-46	Proliferating T/NK	ATP8B3
5.16218331818308E-50	3.08189391755713	0.344	0.023	8.6507868046112E-46	Proliferating T/NK	SLC1A4
7.38849079830057E-50	2.19075589466937	0.489	0.053	1.23816328797921E-45	Proliferating T/NK	WDR76
1.0607392821097E-49	3.9313270738932	0.233	0.008	1.77758688895943E-45	Proliferating T/NK	SMTN
1.53448581620648E-49	1.70002399396351	0.433	0.039	2.57149133079881E-45	Proliferating T/NK	CEP128
1.61039612858305E-49	6.296881017226	0.156	0.001	2.69870183227947E-45	Proliferating T/NK	ARHGEF39
2.76728858389126E-49	9.49026178756386	0.133	0	4.63742220888497E-45	Proliferating T/NK	FAM72D
4.64757753435744E-49	2.4568306458372	0.478	0.051	7.78841043207619E-45	Proliferating T/NK	MBNL3
4.7079633245108E-49	3.67285997812431	0.289	0.015	7.8896049392152E-45	Proliferating T/NK	SUV39H2
5.75782161595733E-49	2.4384419915362	0.356	0.026	9.64895746402129E-45	Proliferating T/NK	RAD18
5.99573787988432E-49	3.40956984874399	0.289	0.015	1.00476575391101E-44	Proliferating T/NK	SLC37A4

1.15337557625996E-48	2.83445400708405	0.389	0.032	1.93282679069644E-44	Proliferating T/NK	ZDHHC12
1.38908751214946E-48	5.7223275802344	0.178	0.003	2.32783285286006E-44	Proliferating T/NK	DSCC1
2.33348504413553E-48	4.82072950076878	0.222	0.007	3.91045423696232E-44	Proliferating T/NK	SKA3
2.3953722958601E-48	4.33234841528907	0.211	0.006	4.01416489340235E-44	Proliferating T/NK	PIF1
2.72626136361762E-48	4.37329693409838	0.211	0.006	4.56866879315041E-44	Proliferating T/NK	OIP5
4.1718803235536E-48	2.22952102758138	0.611	0.087	6.99123704621113E-44	Proliferating T/NK	HDGF
7.43859579713022E-48	2.50255235354148	0.711	0.129	1.24655988368308E-43	Proliferating T/NK	MCM3
1.45356154619112E-47	1.87876658222731	0.478	0.052	2.43587843910707E-43	Proliferating T/NK	POLH
2.41569355566311E-47	2.27416494159839	0.422	0.04	4.04821926058024E-43	Proliferating T/NK	CDK2
4.87646447533882E-47	3.57146387590801	0.289	0.017	8.17197916777279E-43	Proliferating T/NK	DSN1
1.75196201392764E-46	1.49336135130343	0.544	0.069	2.93593794293994E-42	Proliferating T/NK	NEMP1
1.8126214694967E-46	3.20924114619126	0.333	0.024	3.03759105858256E-42	Proliferating T/NK	ZWILCH
3.65813040103554E-46	1.8221237920288	0.678	0.115	6.13029492605536E-42	Proliferating T/NK	CDC25B
4.49339812658464E-46	2.11254053893011	0.311	0.02	7.53003658053055E-42	Proliferating T/NK	CEP152
4.97772905441974E-46	2.19725476080255	0.467	0.051	8.3416783493966E-42	Proliferating T/NK	CIAO2A
5.84826716276066E-46	2.07304804460249	0.4	0.036	9.80052611135432E-42	Proliferating T/NK	CEP72
9.60565473342572E-46	3.13843617865654	0.278	0.015	1.60971562022748E-41	Proliferating T/NK	MIS18A
1.03015186196417E-45	2.14459246336626	0.389	0.035	1.72632849027956E-41	Proliferating T/NK	SCMH1
2.25917889755234E-45	2.33863101961802	0.322	0.023	3.78593199651821E-41	Proliferating T/NK	CHEK2
3.67119236033623E-45	2.80780437000589	0.522	0.072	6.15218415745145E-41	Proliferating T/NK	MCM2
4.12170325305673E-45	2.24781040388274	0.589	0.091	6.90715031147247E-41	Proliferating T/NK	PTTG1
1.66494895688765E-44	1.98899194344698	0.456	0.052	2.79012146195232E-40	Proliferating T/NK	CKAP2
1.72746930033306E-44	3.25144073528434	0.267	0.015	2.89489305349814E-40	Proliferating T/NK	RMI2
1.96357917932305E-44	2.10001720093823	0.3	0.02	3.29056598870957E-40	Proliferating T/NK	C9orf40
6.16396609674538E-44	2.44619331954501	0.344	0.028	1.03295743849259E-39	Proliferating T/NK	RFC3
1.03496535474081E-43	3.21126086790561	0.3	0.02	1.73439494147465E-39	Proliferating T/NK	C19orf48
1.3039481783072E-43	1.098888674652	0.667	0.107	2.1851563572072E-39	Proliferating T/NK	DDX39A
1.37258785874869E-43	1.60966383664206	0.6	0.093	2.30018273369105E-39	Proliferating T/NK	NCAPH2
3.05923801094278E-43	2.13060399738923	0.689	0.129	5.12667105873791E-39	Proliferating T/NK	MCM7
3.45373517829979E-43	3.45881965728356	0.211	0.008	5.7877694117948E-39	Proliferating T/NK	DDX11
6.12717845208837E-43	1.95777707225977	0.444	0.049	1.02679256500097E-38	Proliferating T/NK	LIN54
1.16127978402107E-42	1.72837585473344	0.867	0.201	1.94607266206251E-38	Proliferating T/NK	FAM111A
3.07911600517477E-42	2.24094407949547	0.5	0.063	5.15998260147188E-38	Proliferating T/NK	TALDO1
3.75131006527802E-42	1.90699555226348	0.389	0.038	6.28644540739291E-38	Proliferating T/NK	ANP32B
7.59865655919353E-42	3.65080673460284	0.244	0.013	1.27338286618965E-37	Proliferating T/NK	LMO7
8.01245014251028E-42	2.46880353453709	0.522	0.075	1.34272639488187E-37	Proliferating T/NK	CKS2
9.95078824757304E-42	4.51148824969301	0.178	0.005	1.66755309452829E-37	Proliferating T/NK	DDIAS
2.25111876500312E-41	2.21988744679513	0.589	0.092	3.77242482639224E-37	Proliferating T/NK	CERS2
2.9235743621562E-41	6.76346556072959	0.122	0.001	4.89932591610136E-37	Proliferating T/NK	EME1
3.71108900163115E-41	1.87837198600659	0.578	0.09	6.21904294893348E-37	Proliferating T/NK	HNRNPAB
4.20833852101977E-41	4.34714728963313	0.222	0.01	7.05233369352494E-37	Proliferating T/NK	CDC6
4.63329032242556E-41	5.21968716183763	0.156	0.003	7.76446792232075E-37	Proliferating T/NK	XRCC2
7.91052069635158E-41	1.75238737120182	0.478	0.06	1.3256450582946E-36	Proliferating T/NK	KMT5A
1.86125336594926E-40	2.35350049672918	0.378	0.038	3.11908839065777E-36	Proliferating T/NK	BARD1
3.97862437950771E-40	3.28119065945927	0.256	0.015	6.66737873517901E-36	Proliferating T/NK	GINS1
9.42653688804722E-40	2.78569338969261	0.278	0.02	1.57969905169895E-35	Proliferating T/NK	DONSON
1.0902770870058E-39	2.88086769360582	0.178	0.006	1.82708634240431E-35	Proliferating T/NK	CEP76
2.06520917860492E-39	2.09799561378716	0.4	0.045	3.46087754150613E-35	Proliferating T/NK	MASTL
3.68554205087096E-39	1.73180534114587	0.722	0.156	6.17623136884956E-35	Proliferating T/NK	DNMT1
3.92262931389112E-39	2.56909118334001	0.333	0.03	6.57354220421875E-35	Proliferating T/NK	ST3GAL3
5.18749057522213E-39	3.61139733963246	0.233	0.013	8.69319670595725E-35	Proliferating T/NK	WDR90
9.58750290320807E-39	2.13748132326451	0.333	0.03	1.60667373651961E-34	Proliferating T/NK	CENPL
1.18144387596203E-38	2.35600022941733	0.433	0.053	1.97986364733717E-34	Proliferating T/NK	SCCPDH
1.21753000257391E-38	2.21018037399575	0.422	0.051	2.04033677831336E-34	Proliferating T/NK	MGST3
1.236819250105E-38	2.77035176749609	0.267	0.018	2.07266169932596E-34	Proliferating T/NK	CHTF18
2.50143864311072E-38	2.13654876914019	0.4	0.045	4.19191087812494E-34	Proliferating T/NK	BCAP29
2.93274774891453E-38	1.24859400185855	0.689	0.126	4.91469867763097E-34	Proliferating T/NK	PCNT
7.34624337757504E-38	2.92485898114405	0.289	0.023	1.23108346521403E-33	Proliferating T/NK	HIRIP3
7.6164237937196E-38	2.25415082412611	0.433	0.054	1.27636029935153E-33	Proliferating T/NK	HADH
9.57348284904292E-38	1.8757820236363	0.622	0.11	1.60432425584261E-33	Proliferating T/NK	PSMC3
9.60306764179767E-38	3.37300765519014	0.211	0.01	1.60928207541245E-33	Proliferating T/NK	GINS4
1.09418348424662E-37	2.48192549684713	0.567	0.098	1.83363268290048E-33	Proliferating T/NK	ARL6IP1
1.13900740995087E-37	3.79067807182697	0.211	0.01	1.90874861759567E-33	Proliferating T/NK	CEP70
1.29512546945127E-37	3.02365065237114	0.267	0.019	2.17037126170644E-33	Proliferating T/NK	RFC4
1.92648463742339E-37	1.23454133363445	0.367	0.038	3.22840295539412E-33	Proliferating T/NK	ODF2
2.45406162559769E-37	8.78532635317217	0.1	0	4.11251647217662E-33	Proliferating T/NK	CCDC150

3.69374476993888E-37	2.13732489076426	0.3	0.025	6.18997748546357E-33	Proliferating T/NK	YEATS4
7.4995660978359E-37	2.58288229986324	0.311	0.028	1.25677728667534E-32	Proliferating T/NK	FH
7.55333241383192E-37	1.22680972264906	0.622	0.112	1.26578744590995E-32	Proliferating T/NK	CDK5RAP2
1.49902879841975E-36	2.72209529154478	0.3	0.026	2.51207246039182E-32	Proliferating T/NK	DHCR24
1.6525729987964E-36	3.7515968396752	0.178	0.007	2.769381831383E-32	Proliferating T/NK	HIST1H4J
1.73226145083273E-36	3.24153528580105	0.278	0.022	2.90292373930549E-32	Proliferating T/NK	IFI27L1
2.24963490893386E-36	1.3589875671656	0.878	0.235	3.76993818039137E-32	Proliferating T/NK	RBM15
2.35533459139909E-36	1.79370316941528	0.444	0.059	3.9470697082666E-32	Proliferating T/NK	JPT2
4.356096517347E-36	5.75669591606579	0.133	0.002	7.2999465437701E-32	Proliferating T/NK	DMC1
5.8592647641975E-36	2.32482628011638	0.256	0.018	9.81895589184217E-32	Proliferating T/NK	RCC1
1.08299630808178E-35	2.8553827043188	0.267	0.021	1.81488521308344E-31	Proliferating T/NK	SLC29A1
2.15990187735667E-35	3.30278177671887	0.189	0.009	3.6195635660743E-31	Proliferating T/NK	CDKN3
2.30051120900549E-35	2.84416012332385	0.211	0.012	3.8551966840514E-31	Proliferating T/NK	PARPBP
2.38144455321214E-35	1.74326294000227	0.344	0.036	3.9908247822729E-31	Proliferating T/NK	MCM8
2.98865632918108E-35	1.91781388896135	0.489	0.075	5.00839027644166E-31	Proliferating T/NK	TFDP1
5.59764603748944E-35	1.60053919387515	0.467	0.067	9.3805352296248E-31	Proliferating T/NK	RBL1
5.9916330688724E-35	1.69312210402545	0.422	0.055	1.00407786968164E-30	Proliferating T/NK	TOMM5
6.36845541080411E-35	4.39623409146412	0.156	0.005	1.06722575774255E-30	Proliferating T/NK	CCDC15
6.67291042928684E-35	1.82795061520107	0.278	0.023	1.11824632973989E-30	Proliferating T/NK	PIDD1
6.75878980089164E-35	2.42395796574565	0.256	0.019	1.13263799483342E-30	Proliferating T/NK	CENPH
6.95483583231402E-35	4.96874724873568	0.122	0.002	1.16549138877918E-30	Proliferating T/NK	ARHGAP11B
1.15002562439092E-34	2.21172844995835	0.489	0.076	1.92721294135431E-30	Proliferating T/NK	MAD2L2
1.57938601250944E-34	2.60687841567466	0.333	0.035	2.64673507976331E-30	Proliferating T/NK	MZT1
4.45024032891648E-34	3.17961063247202	0.244	0.018	7.45771274319823E-30	Proliferating T/NK	TMEM9
5.10148722723353E-34	6.58481569905181	0.111	0.001	8.54907229539795E-30	Proliferating T/NK	RDM1
5.13699312233495E-34	2.21264226124331	0.222	0.014	8.6085730744089E-30	Proliferating T/NK	LRR1
5.70035598080298E-34	2.84383321050717	0.256	0.02	9.55265655262964E-30	Proliferating T/NK	KLF5
5.92861574119153E-34	2.08703779832813	0.222	0.014	9.93517425908876E-30	Proliferating T/NK	MAST2
5.93734604467267E-34	0.921043114686433	0.811	0.192	9.94980450166247E-30	Proliferating T/NK	CNTRL
6.11216982114551E-34	2.01875465199704	0.367	0.043	1.02427741862756E-29	Proliferating T/NK	ACSL3
6.6339066977851E-34	2.89213951006993	0.267	0.022	1.11171008441483E-29	Proliferating T/NK	EMC9
6.89618940183142E-34	1.6393918510948	0.867	0.262	1.15566341995891E-29	Proliferating T/NK	CALM3
7.30468963520762E-34	1.09390773941217	0.489	0.075	1.22411988906809E-29	Proliferating T/NK	POLE
1.00760502182974E-33	1.5764238395137	0.533	0.091	1.68854449558228E-29	Proliferating T/NK	STT3A
1.03901679239347E-33	1.20936329925042	0.667	0.136	1.74118434069298E-29	Proliferating T/NK	RAD21
1.14405433486007E-33	2.63843549670209	0.267	0.022	1.91720625435851E-29	Proliferating T/NK	C1GALT1C1
1.87877631075101E-33	1.908332653994	0.589	0.111	3.14845334155655E-29	Proliferating T/NK	EBP
1.93297287388143E-33	1.4698374571505	0.478	0.073	3.2392759420505E-29	Proliferating T/NK	CCDC14
2.26642567833284E-33	4.03816259795685	0.167	0.007	3.79807615175018E-29	Proliferating T/NK	CHAF1B
3.50338467755449E-33	1.90135608233561	0.478	0.075	5.87097204264581E-29	Proliferating T/NK	ANAPC11
3.75708318380755E-33	2.45425885641937	0.144	0.004	6.29611999942469E-29	Proliferating T/NK	ZNF850
4.03069735726526E-33	1.33925262821332	0.633	0.124	6.75464263130512E-29	Proliferating T/NK	HSPB1
4.04445485428766E-33	3.06533224674897	0.211	0.013	6.77769744481526E-29	Proliferating T/NK	NEURL1B
4.1835969597407E-33	1.54111097453223	0.533	0.095	7.01087178513346E-29	Proliferating T/NK	SIVA1
6.38422980725102E-33	1.75491305626787	0.389	0.051	1.06986923109913E-28	Proliferating T/NK	ALYREF
8.83186601220722E-33	2.08413841887044	0.433	0.063	1.48004410632569E-28	Proliferating T/NK	RTN3
9.92240366493299E-33	2.00052866977265	0.544	0.098	1.66279640616947E-28	Proliferating T/NK	CARHSP1
1.1345484637995E-32	1.66465580761548	0.878	0.292	1.90127631563521E-28	Proliferating T/NK	CRIP1
1.14499331229196E-32	2.02924691030198	0.356	0.042	1.91877979273887E-28	Proliferating T/NK	TMEM160
1.20371444872575E-32	1.8465975604285	0.556	0.1	2.01718467317462E-28	Proliferating T/NK	NDUFC2
1.26654048112339E-32	1.3877013166529	0.567	0.101	2.12246853826657E-28	Proliferating T/NK	SUMO3
1.47183040990823E-32	1.45791926286552	0.367	0.045	2.46649340092422E-28	Proliferating T/NK	GPAA1
1.51635569834288E-32	2.7544527119209	0.289	0.028	2.541108879283E-28	Proliferating T/NK	DARS2
2.05799329567185E-32	2.24036553859819	0.322	0.035	3.44878516488689E-28	Proliferating T/NK	SHMT1
2.89151940936633E-32	3.24228813744165	0.178	0.009	4.84560822621609E-28	Proliferating T/NK	SLC25A10
3.43618533309647E-32	1.8567421052875	0.456	0.069	5.75835938120307E-28	Proliferating T/NK	TMEM14A
3.59488585089021E-32	1.07954192964155	0.556	0.099	6.02430970892182E-28	Proliferating T/NK	MIS18BP1
4.46563544604295E-32	1.67454733892118	0.767	0.213	7.48351188047878E-28	Proliferating T/NK	SMC1A
7.54043764616614E-32	2.02238505240552	0.356	0.044	1.26362654074452E-27	Proliferating T/NK	PSMD10
1.12727108316069E-31	1.5907896630967	0.467	0.073	1.88908088116068E-27	Proliferating T/NK	HSD17B11
1.40134202301528E-31	1.92565046265618	0.378	0.049	2.348368962169E-27	Proliferating T/NK	BANF1
1.72914742007859E-31	0.953786073633274	0.5	0.083	2.8977052465677E-27	Proliferating T/NK	YWHAH
1.79167831085065E-31	1.7766556403459	0.511	0.093	3.00249451332352E-27	Proliferating T/NK	CKAP5
2.91130446964555E-31	1.60337985646416	0.589	0.113	4.87876403023202E-27	Proliferating T/NK	NDUFA1
3.14022758386739E-31	1.93868527712781	0.433	0.066	5.26239338504498E-27	Proliferating T/NK	ERH
3.26555461417301E-31	2.76985122608563	0.278	0.027	5.47241642243114E-27	Proliferating T/NK	POLD3

4.32021051386148E-31	0.946532555064022	0.678	0.142	7.23980877912907E-27	Proliferating T/NK	DEK
7.49791366848881E-31	1.71681179159683	0.422	0.063	1.25650037256536E-26	Proliferating T/NK	MGME1
7.88067963420527E-31	1.11373449718958	0.556	0.102	1.32064429310012E-26	Proliferating T/NK	RAVER1
9.53362286531586E-31	2.21682722991742	0.322	0.037	1.59764451976963E-26	Proliferating T/NK	GLRX5
9.80366047927539E-31	1.43128603603457	0.378	0.05	1.64289742311697E-26	Proliferating T/NK	TRIM28
1.0861798986104E-30	2.49354986844155	0.289	0.03	1.82022027409131E-26	Proliferating T/NK	ISOC1
1.34352661012351E-30	1.66230400948804	0.3	0.032	2.25148189324498E-26	Proliferating T/NK	LRRC45
1.42440790076541E-30	0.99072535614642	0.267	0.025	2.38702276010267E-26	Proliferating T/NK	CEP131
1.60125667256874E-30	1.21051378352837	0.689	0.146	2.68338593189069E-26	Proliferating T/NK	LAMP2
2.53289340236218E-30	1.166654646156	0.5	0.086	4.24462276367854E-26	Proliferating T/NK	CEP57
2.84754297284076E-30	1.5662827047778	0.511	0.094	4.77191251388654E-26	Proliferating T/NK	SAE1
2.97918360609186E-30	1.22147767767337	0.6	0.12	4.99251588708874E-26	Proliferating T/NK	SNRPB
3.07757242421797E-30	4.29464104251038	0.156	0.007	5.15739586850447E-26	Proliferating T/NK	TEX30
3.69239984206277E-30	1.53352822056694	0.389	0.054	6.18772365532879E-26	Proliferating T/NK	MORF4L2
4.23260031014265E-30	2.43698466883866	0.322	0.038	7.09299159973705E-26	Proliferating T/NK	MT2A
4.26560284340466E-30	4.25250410269018	0.122	0.003	7.14829724497752E-26	Proliferating T/NK	EFCAB11
4.33045882587252E-30	3.72773732110796	0.122	0.003	7.25698290039718E-26	Proliferating T/NK	ZNF257
7.44758707007943E-30	2.73080218758104	0.278	0.028	1.24806664120391E-25	Proliferating T/NK	GMNN
7.84447723779915E-30	1.62612434113491	0.856	0.277	1.31457749551038E-25	Proliferating T/NK	IDH2
7.9969320788702E-30	1.77291992813095	0.622	0.144	1.34012587777707E-25	Proliferating T/NK	MCM5
8.67217289278409E-30	2.62511934086609	0.256	0.023	1.45328273337276E-25	Proliferating T/NK	PXMP2
9.51449615946929E-30	1.47798031413819	0.433	0.067	1.59443926640386E-25	Proliferating T/NK	TTL
1.01403002643305E-29	2.04106356641942	0.267	0.026	1.69931151829651E-25	Proliferating T/NK	PCYT2
1.43543147752386E-29	1.90469860541415	0.5	0.091	2.40549607003449E-25	Proliferating T/NK	PMVK
1.91921360990911E-29	1.14987619856883	0.322	0.038	3.2162181674857E-25	Proliferating T/NK	DBF4
2.11215867596366E-29	2.04221845935041	0.344	0.045	3.5395555091799E-25	Proliferating T/NK	BRI3BP
2.7370143970442E-29	2.47413731747206	0.267	0.026	4.58668872656668E-25	Proliferating T/NK	BLVRB
3.00005804916102E-29	0.82698006864555	0.511	0.091	5.02749727878403E-25	Proliferating T/NK	CANT1
3.02117536466796E-29	2.32559388262479	0.256	0.024	5.06288567611056E-25	Proliferating T/NK	SLC44A1
3.34996414284359E-29	2.84720622068046	0.256	0.024	5.61386991057729E-25	Proliferating T/NK	GNG5
3.63526190900718E-29	1.429365345507	0.278	0.028	6.09197190711423E-25	Proliferating T/NK	IMPA1
3.90629411254524E-29	2.06670644508039	0.344	0.044	6.54616767380332E-25	Proliferating T/NK	TMEM238
4.30144760209441E-29	2.23017333944112	0.3	0.034	7.20836589158981E-25	Proliferating T/NK	VANGL1
4.31842267667422E-29	1.84113841126972	0.344	0.045	7.23681272157066E-25	Proliferating T/NK	VRK1
4.3716723353916E-29	1.4280662856444	0.456	0.076	7.32604849964924E-25	Proliferating T/NK	TBC1D10B
4.64227389184884E-29	1.53704909886472	0.344	0.046	7.77952258796029E-25	Proliferating T/NK	CDKN2D
5.02712846946267E-29	1.54019264480992	0.422	0.065	8.42446188912554E-25	Proliferating T/NK	CBX3
8.3083059297887E-29	1.28649148060455	0.578	0.118	1.39230590771399E-24	Proliferating T/NK	BCAP31
8.89061700452677E-29	2.03269423637345	0.356	0.048	1.4898895976186E-24	Proliferating T/NK	TMCO3
9.51602264144253E-29	1.07762030879244	0.633	0.139	1.59469507425294E-24	Proliferating T/NK	MPHOSPH9
1.05000901429003E-28	0.917763446979033	0.411	0.063	1.75960510614723E-24	Proliferating T/NK	CEP135
1.56977732754057E-28	1.63454790387112	0.467	0.081	2.63063284549249E-24	Proliferating T/NK	TSTA3
1.62435444232073E-28	1.06079382105535	0.622	0.134	2.72209317444108E-24	Proliferating T/NK	PRRC2A
1.76552672139845E-28	1.41591731301888	0.478	0.084	2.95866967971952E-24	Proliferating T/NK	MLF 2.00
1.77514049551456E-28	1.90847452320122	0.344	0.046	2.9747804423833E-24	Proliferating T/NK	SLC7A1
1.80130800335155E-28	2.12335812501778	0.267	0.027	3.01863195201652E-24	Proliferating T/NK	EMC6
2.30711115946192E-28	1.37606807580901	0.478	0.085	3.86625688102628E-24	Proliferating T/NK	SCLT1
2.31908844022764E-28	1.56883107309932	0.422	0.067	3.88632840813348E-24	Proliferating T/NK	PRPSAP1
2.33284529352955E-28	2.08051383874267	0.311	0.038	3.90938214289682E-24	Proliferating T/NK	AP2S1
2.57820448841584E-28	1.57580938109708	0.344	0.045	4.32055508168726E-24	Proliferating T/NK	RUVBL2
3.14400240064705E-28	1.80877950812559	0.433	0.074	5.26871922300432E-24	Proliferating T/NK	GOLIM4
3.41810247277209E-28	3.25042247218795	0.167	0.009	5.72805612387146E-24	Proliferating T/NK	LIN9
3.63757268385973E-28	1.45889358495035	0.344	0.046	6.09584430361214E-24	Proliferating T/NK	PRIM2
3.82046667902152E-28	2.22557619275644	0.311	0.038	6.40233806070426E-24	Proliferating T/NK	CHAMP1
4.04138574239428E-28	2.23711862656164	0.256	0.025	6.77255422710433E-24	Proliferating T/NK	MFGE8
4.3006630150704E-28	4.28303528131884	0.133	0.005	7.20705108065497E-24	Proliferating T/NK	RIBC2
6.83961234414372E-28	0.695893541784605	0.5	0.091	1.1461822366316E-23	Proliferating T/NK	CENPT
8.20758507687919E-28	3.42327848061055	0.2	0.015	1.37542710718341E-23	Proliferating T/NK	TONSL
8.62160104233288E-28	0.891117326664291	0.656	0.15	1.44480790267414E-23	Proliferating T/NK	SMC3
9.02678826829449E-28	2.0456701232541	0.289	0.033	1.51270917800079E-23	Proliferating T/NK	ILVBL
9.27843881971558E-28	1.66515116577162	0.333	0.044	1.55488077740794E-23	Proliferating T/NK	MPDU1
9.77413702734077E-28	1.41080326033853	0.256	0.025	1.63794988304177E-23	Proliferating T/NK	ZNF318
1.00045800346916E-27	2.19514039232656	0.2	0.015	1.67656752221362E-23	Proliferating T/NK	BLM
1.28215208146555E-27	1.25534299915484	0.633	0.146	2.14863045811998E-23	Proliferating T/NK	DCK
1.34435550237108E-27	1.97915463533166	0.5	0.1	2.25287095087345E-23	Proliferating T/NK	NUCB2
1.39761975889364E-27	1.00590705122995	0.333	0.044	2.34213119195397E-23	Proliferating T/NK	G2E3

1.48474469821853E-27	3.21322901090328	0.156	0.008	2.48813516527461E-23	Proliferating T/NK	SSX2IP
1.53954417584008E-27	0.84162988729946	0.689	0.164	2.57996812987281E-23	Proliferating T/NK	ANKRD36C
1.55624202377355E-27	2.20410826052346	0.244	0.023	2.60795038343971E-23	Proliferating T/NK	TM9SF1
1.79927678830398E-27	2.06054811271491	0.433	0.076	3.0152280418398E-23	Proliferating T/NK	DCTN3
1.86175676163711E-27	2.2663123666297	0.311	0.039	3.11993198115147E-23	Proliferating T/NK	CLN6
2.16184731211455E-27	4.32425953096043	0.1	0.002	3.62282372564157E-23	Proliferating T/NK	FAM72B
3.28163828278615E-27	2.75578753386642	0.211	0.017	5.49936943429303E-23	Proliferating T/NK	PERP
3.79582669505114E-27	1.61468032935089	0.422	0.07	6.36104637556671E-23	Proliferating T/NK	TOPBP1
3.94374136251728E-27	2.101116882369	0.356	0.051	6.60892177530646E-23	Proliferating T/NK	P4HB
4.61228680546518E-27	1.21483861267927	0.889	0.281	7.72927022859854E-23	Proliferating T/NK	SELENOP
4.80927381768262E-27	3.05679557674817	0.167	0.01	8.05938106367253E-23	Proliferating T/NK	SAC3D1
6.44338147130218E-27	1.3766029092012	0.3	0.036	1.07978186696082E-22	Proliferating T/NK	F11R
6.83433156300007E-27	2.35034767234667	0.3	0.038	1.14529728332755E-22	Proliferating T/NK	SEPTIN8
7.49839508782063E-27	0.781159040684771	0.478	0.087	1.25658104881698E-22	Proliferating T/NK	TAF15
7.91740784368312E-27	1.68757881888885	0.356	0.052	1.32679920644442E-22	Proliferating T/NK	CDC7
9.36219435733296E-27	1.66932918190391	0.333	0.046	1.56891653040186E-22	Proliferating T/NK	HACD3
1.21897420558147E-26	4.06035493119973	0.111	0.003	2.04275697371342E-22	Proliferating T/NK	PRRG4
1.22370568937266E-26	0.956346334507824	0.467	0.086	2.05068599425071E-22	Proliferating T/NK	SAP30
1.27889265939534E-26	1.33107485742652	0.589	0.131	2.14316831861471E-22	Proliferating T/NK	SSRP1
1.42331215133608E-26	0.995509228187561	0.589	0.129	2.385186503209E-22	Proliferating T/NK	UBE2A
1.49591120024706E-26	1.67387511486305	0.389	0.061	2.50684798937403E-22	Proliferating T/NK	LRRC59
2.19086169277842E-26	1.18198244835478	0.656	0.158	3.67144602475808E-22	Proliferating T/NK	AP1M1
2.24660225679068E-26	2.26025436479035	0.244	0.025	3.76485606192983E-22	Proliferating T/NK	LPCAT3
2.25600063529502E-26	2.14490838498303	0.278	0.033	3.7806058646274E-22	Proliferating T/NK	ACYP1
3.36453024264684E-26	1.63916883331021	0.433	0.078	5.63827978062758E-22	Proliferating T/NK	H2AFY
3.43648216826596E-26	2.8873561984111	0.267	0.031	5.75885681758009E-22	Proliferating T/NK	FADS2
3.64242625370598E-26	0.93505415667211	0.522	0.105	6.10397791596048E-22	Proliferating T/NK	DOT1L
4.26905135695393E-26	1.3844215604459	0.522	0.112	7.1540762639834E-22	Proliferating T/NK	PLP2
5.09846559297561E-26	1.4897092943969	0.4	0.065	8.54400864070853E-22	Proliferating T/NK	TPD52
5.22288137405911E-26	1.46379729293379	0.411	0.069	8.75250460664826E-22	Proliferating T/NK	CDK4
5.5012456767115E-26	1.15624878412934	0.844	0.274	9.21898750503312E-22	Proliferating T/NK	S100A10
5.56621033043034E-26	1.88841575386224	0.189	0.014	9.32785527173516E-22	Proliferating T/NK	GLA
5.92499455592143E-26	1.2821937801972	0.5	0.102	9.92910587681314E-22	Proliferating T/NK	USP1
6.37725446877344E-26	1.87806122036047	0.489	0.099	1.06870030387705E-21	Proliferating T/NK	UQCRC1
8.18095155768824E-26	1.23651579294655	0.578	0.128	1.3709638620374E-21	Proliferating T/NK	OAS3
9.17194408708534E-26	1.54695521270835	0.367	0.057	1.53703439011376E-21	Proliferating T/NK	POLA1
1.04438467729952E-25	0.816186762810683	0.444	0.08	1.75017984221854E-21	Proliferating T/NK	ETHE1
1.18100237071818E-25	1.37281638684737	0.533	0.112	1.97912377284952E-21	Proliferating T/NK	PGRMC1
1.32144942748802E-25	2.27934747819728	0.233	0.023	2.21448495058443E-21	Proliferating T/NK	CENPK
1.44593118668725E-25	1.37676684136106	0.311	0.041	2.42309148265049E-21	Proliferating T/NK	ANAPC15
1.62680005329137E-25	1.10971943010406	0.544	0.116	2.72619152930567E-21	Proliferating T/NK	NUDT21
1.74384242112922E-25	2.01722731530132	0.256	0.028	2.92233112932834E-21	Proliferating T/NK	PRPS2
1.8160791044142E-25	2.19250531867832	0.256	0.028	3.04338536317731E-21	Proliferating T/NK	RMI1
2.03927040117278E-25	1.99309105413492	0.2	0.017	3.41740933828535E-21	Proliferating T/NK	HSPA14
2.21833260638345E-25	1.42464205829506	0.689	0.198	3.71748178177739E-21	Proliferating T/NK	CALM2
2.45363233895137E-25	1.72579229214497	0.478	0.094	4.11179707361471E-21	Proliferating T/NK	ACAA2
2.86804141935705E-25	1.85954417528363	0.4	0.068	4.80626381055854E-21	Proliferating T/NK	RPN1
3.26500918149964E-25	1.7416481873219	0.433	0.079	5.4715023863571E-21	Proliferating T/NK	PRDX6
3.31746464036229E-25	3.06442976079707	0.167	0.011	5.55940724431912E-21	Proliferating T/NK	HYI
3.32931440171089E-25	1.05030373415638	0.4	0.067	5.57926507438712E-21	Proliferating T/NK	RCC2
3.42201056401325E-25	0.811351140022231	0.511	0.104	5.73460530317341E-21	Proliferating T/NK	SELENOK
3.53300193869564E-25	3.55043554952065	0.133	0.006	5.92060464886615E-21	Proliferating T/NK	CBR3
4.16694139156086E-25	1.13818834911218	0.444	0.081	6.98296038397769E-21	Proliferating T/NK	KHSRP
5.40987390254362E-25	2.00415930357931	0.333	0.049	9.06586668588259E-21	Proliferating T/NK	ACAD9
5.69810149479393E-25	1.57404231589843	0.689	0.187	9.54887848497566E-21	Proliferating T/NK	PRDX2
6.57661412348106E-25	3.21128979724345	0.122	0.005	1.10210899481296E-20	Proliferating T/NK	LRRCC1
6.89937751261742E-25	2.15408055097696	0.222	0.022	1.15619768356443E-20	Proliferating T/NK	FOXRED1
7.2570151673523E-25	0.578027692785201	0.489	0.097	1.2161306017449E-20	Proliferating T/NK	RANGAP1
7.61664253518014E-25	0.930569393524647	0.322	0.046	1.27639695604549E-20	Proliferating T/NK	TECR
8.12779599317043E-25	2.04101964447224	0.222	0.022	1.3620560525355E-20	Proliferating T/NK	CEP89
8.56081537402625E-25	2.10977230593248	0.411	0.076	1.43462144037932E-20	Proliferating T/NK	DUT
8.90792917422855E-25	3.63447381462125	0.111	0.004	1.49279077101722E-20	Proliferating T/NK	ATP23
9.63413432374444E-25	1.60350083606606	0.389	0.067	1.61448822997309E-20	Proliferating T/NK	DEPRECATED-ENSG00000102007
1.00541078414997E-24	0.90277448273626	0.5	0.102	1.68486739207852E-20	Proliferating T/NK	ADA
1.15427593940635E-24	1.30248940646571	0.389	0.065	1.93433561925716E-20	Proliferating T/NK	SMARCB1
1.37117666471251E-24	1.73558949746219	0.478	0.099	2.29781785472522E-20	Proliferating T/NK	ETFB

1.59456271731776E-24	0.751153269139037	0.278	0.035	2.6721682016811E-20	Proliferating T/NK	SARNP
1.63571632667373E-24	1.6443395154686	0.411	0.073	2.74113342023984E-20	Proliferating T/NK	MEA1
1.67113367666116E-24	2.11515961538203	0.189	0.015	2.80048581534877E-20	Proliferating T/NK	GSTCD
1.8080671566896E-24	1.27821682535495	0.444	0.083	3.02995894118043E-20	Proliferating T/NK	SLF2
1.87622574651192E-24	1.09619353772755	0.7	0.187	3.14417910600467E-20	Proliferating T/NK	DEPRECATED-ENSG00000089248
1.95599756609182E-24	2.25791521151726	0.178	0.014	3.27786072125667E-20	Proliferating T/NK	TMEM107
1.96918442757474E-24	3.46729753702895	0.156	0.01	3.29995926372975E-20	Proliferating T/NK	HMGB3
1.99858923867767E-24	1.71793211255341	0.311	0.045	3.34923584617604E-20	Proliferating T/NK	POLA2
2.04883445480355E-24	1.77693146694851	0.3	0.041	3.43343677935978E-20	Proliferating T/NK	SLC25A1
2.46593418717953E-24	1.26291287808417	0.4	0.069	4.13241251087546E-20	Proliferating T/NK	IFI27
2.54904753539805E-24	1.83071241811038	0.344	0.054	4.27169385982005E-20	Proliferating T/NK	TLNRD1
3.02940324801004E-24	2.85683753172758	0.2	0.018	5.07667396301522E-20	Proliferating T/NK	IPO5
3.49414055686571E-24	1.98527340550671	0.2	0.018	5.85548074519556E-20	Proliferating T/NK	SASS6
4.12242990056649E-24	1.99657802225313	0.167	0.012	6.90836802736932E-20	Proliferating T/NK	COQ2
4.22715023902784E-24	1.06939956762479	0.256	0.03	7.08385837056285E-20	Proliferating T/NK	PASK
4.51348016014317E-24	1.16859016536656	0.567	0.124	7.56369005236792E-20	Proliferating T/NK	ACSL4
5.42833655280586E-24	1.42635528542299	0.422	0.078	9.09680639519206E-20	Proliferating T/NK	TPGS2
6.06742849806296E-24	3.21634909692212	0.133	0.007	1.01677966770539E-19	Proliferating T/NK	PARP2
6.38922140014655E-24	1.19640684879128	0.989	0.755	1.07070572223656E-19	Proliferating T/NK	ACTB
7.02037769275233E-24	0.832554605310601	0.456	0.087	1.17647489375144E-19	Proliferating T/NK	SPATA5
7.78744281884336E-24	1.38941364974385	0.444	0.084	1.30501966758177E-19	Proliferating T/NK	MANF
7.83032599191666E-24	1.66231124198102	0.189	0.016	1.31220602972539E-19	Proliferating T/NK	REEP4
7.8592702637589E-24	1.61195744609832	0.4	0.071	1.31705651080072E-19	Proliferating T/NK	SSR3
7.94095882029758E-24	0.870750014256706	0.578	0.132	1.33074587910547E-19	Proliferating T/NK	MCMBP
8.81725574425085E-24	1.71517158045534	0.367	0.06	1.47759571762156E-19	Proliferating T/NK	CMC4
9.93287070820493E-24	1.69678546551665	0.189	0.016	1.66455047328098E-19	Proliferating T/NK	DEPRECATED-ENSG00000006634
1.01784918658518E-23	2.67486964369764	0.222	0.023	1.70571166687944E-19	Proliferating T/NK	TP73
1.24364746376873E-23	2.21319931476176	0.178	0.014	2.08410441978364E-19	Proliferating T/NK	HAUS8
1.26515386885797E-23	2.39528870716734	0.178	0.014	2.12014485343219E-19	Proliferating T/NK	CDCA7
1.57432119783475E-23	1.89282454332379	0.311	0.045	2.63824746333147E-19	Proliferating T/NK	COA3
1.65908269241576E-23	0.755537791378869	0.322	0.048	2.78029077595033E-19	Proliferating T/NK	EXOSC8
1.6759955096771E-23	0.541083987289676	0.644	0.163	2.80863327511689E-19	Proliferating T/NK	TUBA4A
1.68089655502022E-23	1.57321448706192	0.378	0.065	2.81684644690288E-19	Proliferating T/NK	MPC2
1.91551605878962E-23	1.44278271988647	0.589	0.143	3.21002181131965E-19	Proliferating T/NK	BSG
2.067265571388E-23	1.29246671572274	0.233	0.026	3.46432364453201E-19	Proliferating T/NK	TMEM214
2.26904789655542E-23	2.24195008442034	0.267	0.035	3.80247046504758E-19	Proliferating T/NK	BORA
2.79210055487035E-23	0.933127207965669	0.589	0.143	4.67900210985172E-19	Proliferating T/NK	RPA1
3.19897136873953E-23	3.47769068266641	0.111	0.004	5.3608362197337E-19	Proliferating T/NK	BRCA2
3.24549415076922E-23	4.08158826656115	0.1	0.003	5.43879909785905E-19	Proliferating T/NK	CDC25A
3.59355201851459E-23	1.73247997156429	0.433	0.086	6.02207447262675E-19	Proliferating T/NK	RAB5IF
3.75874163381196E-23	2.31409650282219	0.267	0.035	6.29889922994209E-19	Proliferating T/NK	PDLIM7
3.76346941951541E-23	2.53217999953998	0.189	0.017	6.30682205322392E-19	Proliferating T/NK	SEPHS1
3.78840062950355E-23	2.41308292812232	0.1	0.003	6.34860177492206E-19	Proliferating T/NK	RNFT2
4.28515768496007E-23	1.86195328313256	0.367	0.063	7.18106724845609E-19	Proliferating T/NK	CENPB
4.38290287996754E-23	1.24197605505494	0.567	0.132	7.3448686462496E-19	Proliferating T/NK	COPG1
4.96668297174967E-23	1.75460245122542	0.356	0.059	8.3231673240581E-19	Proliferating T/NK	ADI1
5.67018551271608E-23	1.20595521692713	0.567	0.136	9.5020968822096E-19	Proliferating T/NK	GPX4
5.74841661261423E-23	0.407716590433415	0.878	0.268	9.63319655941893E-19	Proliferating T/NK	HNRNPUL1
6.47320119197938E-23	1.41970560519896	0.433	0.087	1.0847790557519E-18	Proliferating T/NK	CBX5
6.53809529959143E-23	1.26238438820872	0.589	0.146	1.09565401030553E-18	Proliferating T/NK	SCP2
7.38338144602529E-23	1.16355548786091	0.322	0.049	1.23730706272492E-18	Proliferating T/NK	RSRC1
8.15797765953876E-23	2.0622064372127	0.333	0.054	1.36711389618551E-18	Proliferating T/NK	NABP2
8.18389235749512E-23	1.59175628369142	0.278	0.038	1.37145668126903E-18	Proliferating T/NK	SLX4
8.23396121819234E-23	1.28337511115812	0.578	0.138	1.37984722094467E-18	Proliferating T/NK	ERGIC3
8.34639629292805E-23	1.72321803346829	0.233	0.026	1.39868909076888E-18	Proliferating T/NK	KDELR2
8.61010875481781E-23	1.2905612336383	0.578	0.139	1.44288202513237E-18	Proliferating T/NK	CYB5R3
8.68904642148404E-23	2.26873398520732	0.211	0.022	1.4561103993123E-18	Proliferating T/NK	HEBP1
9.00901712596399E-23	0.801386797851111	0.444	0.088	1.50973108996905E-18	Proliferating T/NK	NUP85
9.32930461649873E-23	1.21128020867143	0.256	0.032	1.56340486763286E-18	Proliferating T/NK	NCKIPSD
9.46262963739934E-23	1.34688642154046	0.478	0.1	1.58574747463538E-18	Proliferating T/NK	PPIA
1.12746351427825E-22	1.37350255690097	0.367	0.063	1.88940335722749E-18	Proliferating T/NK	MAZ
1.15415136751295E-22	1.69097222083523	0.322	0.051	1.9341268616782E-18	Proliferating T/NK	VBP1
1.1937173512144E-22	0.79992167083682	0.611	0.155	2.00043153716509E-18	Proliferating T/NK	EIF4A3
1.20186195511106E-22	0.8155710337067	0.722	0.2	2.01408026437512E-18	Proliferating T/NK	NONO
1.47160440366432E-22	1.31694837913346	0.444	0.091	2.46611465966067E-18	Proliferating T/NK	CYC1
1.49469911657288E-22	1.1860398726502	0.389	0.069	2.50481677955283E-18	Proliferating T/NK	GTF2A2

1.59964889053571E-22	1.38664547397862	0.356	0.061	2.68069161075974E-18	Proliferating T/NK	HAUS4
1.64139991252629E-22	1.00446567540074	0.467	0.098	2.75065797341155E-18	Proliferating T/NK	TUBGCP3
1.65513915112935E-22	1.4387886084226	0.278	0.038	2.77368218946257E-18	Proliferating T/NK	NDUFB6
2.2910136039368E-22	1.29717988680091	0.289	0.041	3.83928059747728E-18	Proliferating T/NK	UBAC1
2.84487870099846E-22	1.29636613277508	0.244	0.03	4.76744772713322E-18	Proliferating T/NK	IKBIP
2.98423592867262E-22	0.791347800668299	0.789	0.243	5.00098256926957E-18	Proliferating T/NK	LBR
3.25288994385496E-22	1.025395583727	0.367	0.064	5.45119296791214E-18	Proliferating T/NK	DEPRECATED-ENSG00000054118
3.27245991952161E-22	0.839742789090452	0.611	0.152	5.48398833313432E-18	Proliferating T/NK	PYGO2
3.2766138194658E-22	1.64284490785483	0.367	0.065	5.49094943866078E-18	Proliferating T/NK	PGD
3.9355218856563E-22	0.709001255745784	0.544	0.125	6.59514757598283E-18	Proliferating T/NK	KHDRBS1
4.17023590420129E-22	1.91479819286217	0.211	0.023	6.98848132826053E-18	Proliferating T/NK	DERA
4.52854390916175E-22	1.49333272332787	0.333	0.055	7.58893388297326E-18	Proliferating T/NK	SLC25A25
4.62224301822768E-22	0.872686143363115	0.511	0.113	7.74595484994594E-18	Proliferating T/NK	YWHAQ
4.68341870447876E-22	1.32352732093944	0.333	0.054	7.84847306496551E-18	Proliferating T/NK	HSD17B4
5.08782379016821E-22	1.25952318081102	0.711	0.214	8.52617510756389E-18	Proliferating T/NK	ANXA5
5.3789892927349E-22	0.9816371474616	0.378	0.068	9.01411025676514E-18	Proliferating T/NK	NPC2
5.56035554646275E-22	1.29570382751924	0.489	0.107	9.31804382476227E-18	Proliferating T/NK	RAB2A
5.66572200110141E-22	1.39296002318934	0.289	0.042	9.49461692944574E-18	Proliferating T/NK	CCDC167
5.80030474599142E-22	1.62505492901654	0.411	0.081	9.72015069333243E-18	Proliferating T/NK	CANX
5.82219297457722E-22	1.40472399093472	0.444	0.091	9.7568309867965E-18	Proliferating T/NK	SRP9
5.82878980251809E-22	2.36258469890647	0.244	0.031	9.76788595105982E-18	Proliferating T/NK	GAMT
6.23927181892203E-22	1.49610424424732	0.578	0.147	1.04557717141495E-17	Proliferating T/NK	CHCHD2
6.74058793328891E-22	0.979109034497672	0.522	0.12	1.12958772586056E-17	Proliferating T/NK	NNT
7.38845441079831E-22	1.448714907156	0.411	0.079	1.23815719016158E-17	Proliferating T/NK	STOML2
7.73448586450828E-22	1.52859397843748	0.422	0.086	1.2961451411743E-17	Proliferating T/NK	TP53
7.78218831332487E-22	1.47876700376567	0.322	0.051	1.30413911754698E-17	Proliferating T/NK	ICMT
7.82527056300034E-22	0.699441659509131	0.378	0.069	1.3113588409476E-17	Proliferating T/NK	ATP1A1
9.23017145554356E-22	1.25625768710915	0.322	0.052	1.54679213251999E-17	Proliferating T/NK	RBBP7
9.81765926882924E-22	3.00209821255364	0.111	0.005	1.6452433402704E-17	Proliferating T/NK	KLF11
9.82872193391594E-22	1.38516380507648	0.389	0.073	1.64709722168563E-17	Proliferating T/NK	MPP1
1.1348226145927E-21	0.771783368252765	0.667	0.176	1.90173573753445E-17	Proliferating T/NK	HNRNPUL2
1.19117906732142E-21	1.70180576086998	0.322	0.052	1.99617788101723E-17	Proliferating T/NK	SPTSSA
1.31088794735032E-21	1.90060552878072	0.356	0.065	2.19678602216966E-17	Proliferating T/NK	SPTBN1
1.417541572978E-21	1.43231065167799	0.256	0.034	2.37551616799654E-17	Proliferating T/NK	HAUS5
1.52797722511042E-21	1.92257037384354	0.233	0.028	2.56058423384004E-17	Proliferating T/NK	CCHCR1
1.86265450038773E-21	0.69546136724783	0.311	0.049	3.12143641174976E-17	Proliferating T/NK	RANGRF
1.9558341234243E-21	2.12623122523983	0.211	0.023	3.27758682403444E-17	Proliferating T/NK	WDHD1
2.00802496670162E-21	1.37284520916364	0.433	0.089	3.36504823919858E-17	Proliferating T/NK	GUCD1
2.06447328717236E-21	1.09531939873252	0.378	0.07	3.45964433464345E-17	Proliferating T/NK	PEA15
2.5840550433459E-21	0.662041582341163	0.322	0.052	4.33035944163905E-17	Proliferating T/NK	ATP5PB
2.80714558030889E-21	2.15798371645942	0.2	0.021	4.70421456348164E-17	Proliferating T/NK	DHCR7
2.95553064786561E-21	1.47190075691226	0.344	0.061	4.95287825969319E-17	Proliferating T/NK	FDXR
3.5337210684403E-21	2.10444103401769	0.267	0.038	5.92180976649225E-17	Proliferating T/NK	HSDL2
3.67501770285149E-21	1.35403660607738	0.322	0.054	6.15859466643853E-17	Proliferating T/NK	HMGXB4
3.6920909903882E-21	0.960382057198627	0.456	0.099	6.18720608169255E-17	Proliferating T/NK	HP1BP3
3.78689877329887E-21	1.28255802195804	0.456	0.097	6.34608496429424E-17	Proliferating T/NK	APEH
3.91047955527808E-21	2.64069217755659	0.167	0.014	6.553181638735E-17	Proliferating T/NK	LACTB2
4.22858832607683E-21	1.46696462353669	0.444	0.094	7.08626831683955E-17	Proliferating T/NK	PDIA6
4.40577515232747E-21	1.82624592942245	0.256	0.035	7.38319800027038E-17	Proliferating T/NK	FAH
4.60301625976478E-21	1.03484667657491	0.233	0.029	7.71373464811382E-17	Proliferating T/NK	MYEF2
5.06426369141947E-21	0.627763632654572	0.489	0.107	8.48669309408075E-17	Proliferating T/NK	TDP1
5.68644547732516E-21	1.82609591697872	0.3	0.047	9.52934533090151E-17	Proliferating T/NK	RARRES2
6.72640368377256E-21	1.71061604786339	0.278	0.041	1.12721072932661E-16	Proliferating T/NK	SLC25A40
6.84510419831767E-21	2.0495038804319	0.2	0.022	1.14710256155407E-16	Proliferating T/NK	IMPA2
7.28477692693527E-21	1.85596464044001	0.2	0.022	1.22078291741581E-16	Proliferating T/NK	WRAP53
7.36071888330644E-21	2.34619156895263	0.2	0.022	1.23350927046449E-16	Proliferating T/NK	MEP1A
7.63075017029089E-21	1.16295079585361	0.344	0.062	1.27876111353735E-16	Proliferating T/NK	CCNG1
7.67154206553703E-21	1.15149672193409	0.511	0.125	1.28559701934269E-16	Proliferating T/NK	C17orf49
8.16422924207547E-21	0.853182581214813	0.467	0.103	1.36816153638701E-16	Proliferating T/NK	PPP2R5D
8.38959249266813E-21	1.69705079588035	0.189	0.019	1.40592790992133E-16	Proliferating T/NK	PPP2R3B
8.46807191508305E-21	1.12894789271161	0.711	0.222	1.41907949152962E-16	Proliferating T/NK	PPP1CA
8.77836444698563E-21	2.54217994447941	0.2	0.022	1.47107831402585E-16	Proliferating T/NK	CENPX
9.12704593122267E-21	2.11409415981014	0.211	0.024	1.52951035715429E-16	Proliferating T/NK	GLO1
9.48717487395640E-21	0.851725857738182	0.467	0.102	1.58986076537761E-16	Proliferating T/NK	RABL6
1.08207932109256E-20	2.50009434114635	0.144	0.01	1.81334852628691E-16	Proliferating T/NK	WDYHV1
1.14304857442884E-20	1.39023652015956	0.444	0.097	1.91552080102786E-16	Proliferating T/NK	RNF5

1.16501546338482E-20	0.921903237279909	0.322	0.054	1.95233291354029E-16	Proliferating T/NK	HSPA13
1.19590181264824E-20	1.24911628693749	0.456	0.099	2.00409225763593E-16	Proliferating T/NK	TXNRD1
1.30906255313104E-20	1.33754432292833	0.322	0.055	2.193727026537E-16	Proliferating T/NK	GMPS
1.32786995622087E-20	1.44457406225866	0.411	0.084	2.22524447263493E-16	Proliferating T/NK	AKR1A1
1.37965279437138E-20	1.44086183345124	0.267	0.038	2.31202215280755E-16	Proliferating T/NK	ZW10
1.43878206768369E-20	0.68446148553935	0.5	0.116	2.41111098902433E-16	Proliferating T/NK	NDUFAF3
1.48943360067135E-20	0.704020320376699	0.544	0.134	2.49599282800505E-16	Proliferating T/NK	SF3A2
1.55325313291596E-20	2.08369527773837	0.167	0.015	2.60294160014056E-16	Proliferating T/NK	OCLN
1.78537840987336E-20	2.35912882211425	0.233	0.03	2.99193713926578E-16	Proliferating T/NK	CD99L2
1.80627861147661E-20	0.730699955689929	0.389	0.075	3.0269616971125E-16	Proliferating T/NK	NUP107
1.81065297301906E-20	3.66964647484292	0.111	0.006	3.03429225218535E-16	Proliferating T/NK	RNASEH2A
1.85343631672657E-20	2.00020212920203	0.222	0.027	3.10598857957039E-16	Proliferating T/NK	RRBP1
1.88664944104875E-20	0.747553297017644	0.578	0.148	3.1616471333095E-16	Proliferating T/NK	HNRNPM
1.89698494402355E-20	0.984671451645137	0.544	0.135	3.17896736919467E-16	Proliferating T/NK	NUDC
2.21941532469672E-20	0.855761562936786	0.556	0.14	3.71929620112676E-16	Proliferating T/NK	NUP62
2.261787261529E-20	1.32234919623804	0.422	0.088	3.79030309287029E-16	Proliferating T/NK	SCD
2.78185569267491E-20	0.472384157327476	0.478	0.104	4.66183376978461E-16	Proliferating T/NK	SLC39A7
2.79072873332726E-20	1.69423504576502	0.311	0.052	4.67670321130982E-16	Proliferating T/NK	AKIP1
2.80116622223175E-20	1.45848953808611	0.278	0.043	4.69419435521597E-16	Proliferating T/NK	JPT1
2.84866932325075E-20	1.86845610814488	0.278	0.043	4.77380005190361E-16	Proliferating T/NK	RFC2
3.29970188469775E-20	1.32922328069975	0.344	0.064	5.52964041837649E-16	Proliferating T/NK	SAMD1
3.31760048695952E-20	0.589047847748533	0.656	0.177	5.55963489604676E-16	Proliferating T/NK	SEC11A
3.59230126740632E-20	2.01580069591793	0.189	0.02	6.01997846391951E-16	Proliferating T/NK	ZNF519
3.82502508895243E-20	1.47418978692332	0.3	0.049	6.40997704406648E-16	Proliferating T/NK	MID1IP1
4.10732967471704E-20	0.940722545162711	0.5	0.116	6.88306306889082E-16	Proliferating T/NK	CTCF
4.22686890174351E-20	0.545297584220827	0.6	0.155	7.08338690554177E-16	Proliferating T/NK	AKT1
4.29755256855791E-20	2.96837278721899	0.178	0.018	7.20183859438934E-16	Proliferating T/NK	GINS2
4.3231290026356E-20	0.934044653472948	0.289	0.046	7.24469958261673E-16	Proliferating T/NK	FASN
4.4913180572867E-20	1.04122765950927	0.567	0.145	7.52655080040106E-16	Proliferating T/NK	PRKACA
4.54050051695501E-20	0.465545014668371	0.411	0.084	7.60897076631321E-16	Proliferating T/NK	ABCB8
4.86404217265817E-20	1.24981957207972	0.267	0.039	8.15116187294057E-16	Proliferating T/NK	APP
4.90420292889611E-20	1.16323432549359	0.467	0.105	8.2184632682441E-16	Proliferating T/NK	MZT2B
5.22665211757685E-20	1.13636124325727	0.944	0.478	8.75882361863528E-16	Proliferating T/NK	ACTG1
5.25328908536373E-20	0.886443853609142	0.611	0.168	8.80346184925254E-16	Proliferating T/NK	RAB8A
5.53293299681973E-20	1.07473689454153	0.433	0.094	9.2720891160705E-16	Proliferating T/NK	HPS3
5.83286910923971E-20	2.50947055324251	0.1	0.004	9.7747220532639E-16	Proliferating T/NK	ERI2
5.97956926340169E-20	2.91333444424043	0.122	0.007	1.00205621716086E-15	Proliferating T/NK	GGCX
6.20150011686712E-20	1.62175021478566	0.356	0.068	1.03924738958459E-15	Proliferating T/NK	H2AFJ
6.26347247498676E-20	1.98429134115728	0.267	0.04	1.04963271735828E-15	Proliferating T/NK	NENF
6.2910289029065E-20	1.36259307918024	0.378	0.074	1.05425062354907E-15	Proliferating T/NK	SLC16A1
7.41388899714195E-20	0.96718116907421	0.589	0.15	1.24241951814105E-15	Proliferating T/NK	PPIB
7.67032445045518E-20	0.897780019654304	0.678	0.206	1.28539297140728E-15	Proliferating T/NK	COTL1
7.69061445829823E-20	0.934573723512749	0.411	0.087	1.28879317092162E-15	Proliferating T/NK	TRIM59
7.72550844564741E-20	1.88837387699039	0.167	0.015	1.29464070532159E-15	Proliferating T/NK	CABLES2
7.78537645549114E-20	2.48468136669532	0.167	0.015	1.30467338641121E-15	Proliferating T/NK	IFT122
8.30007578472468E-20	1.28475793501289	0.267	0.039	1.39092670000416E-15	Proliferating T/NK	ALG6
8.30648349026694E-20	1.03333673338283	0.733	0.242	1.39200050329893E-15	Proliferating T/NK	PKM
8.51717551319804E-20	1.79238043177683	0.278	0.043	1.42730827250173E-15	Proliferating T/NK	LAGE3
8.98908148244697E-20	1.50527772101291	0.444	0.1	1.50639027482846E-15	Proliferating T/NK	COX8A
9.39576485246704E-20	1.40921840288586	0.333	0.062	1.57454227397643E-15	Proliferating T/NK	VMA21
9.96065241985936E-20	1.00793509688273	0.4	0.084	1.66920613252003E-15	Proliferating T/NK	KPNB1
9.99900432549943E-20	1.06500536369009	0.233	0.031	1.6756331448672E-15	Proliferating T/NK	CSE1L
1.07505951190643E-19	1.21016738210917	0.4	0.081	1.80158473005279E-15	Proliferating T/NK	PSMB2
1.18988633957899E-19	1.11305809881499	0.433	0.094	1.99401152786648E-15	Proliferating T/NK	ABHD2
1.19330315824816E-19	0.770788546925276	0.678	0.193	1.99973743259226E-15	Proliferating T/NK	RNF167
1.22206570391789E-19	1.83280761179897	0.3	0.051	2.0479377066256E-15	Proliferating T/NK	RAD23B
1.23307132031455E-19	0.940638885296068	0.767	0.243	2.06638091858313E-15	Proliferating T/NK	CTSB
1.25206522355082E-19	0.577692069680912	0.589	0.155	2.09821090162647E-15	Proliferating T/NK	CNPY3
1.33701408166334E-19	0.587783206992239	0.5	0.115	2.24056819805143E-15	Proliferating T/NK	SP1
1.39983160837611E-19	0.672814049432232	0.522	0.129	2.34583780931668E-15	Proliferating T/NK	CCNG2
1.41520596076094E-19	1.8195266235839	0.344	0.066	2.37160214904319E-15	Proliferating T/NK	ACAT2
1.45754773834118E-19	1.01956738974925	0.467	0.105	2.44255849991216E-15	Proliferating T/NK	NDUFS1
1.47157795764498E-19	0.692190109175212	0.411	0.088	2.46607034142146E-15	Proliferating T/NK	RNF168
1.48358920472968E-19	1.25551867604255	0.544	0.14	2.48619789928599E-15	Proliferating T/NK	NDUFA2
1.51750737578423E-19	1.62079295293809	0.244	0.035	2.54303886033921E-15	Proliferating T/NK	SNX8
1.61798492754585E-19	0.503986613555166	0.622	0.166	2.71141914158134E-15	Proliferating T/NK	CYB5B

1.71933550919392E-19	0.728921533640854	0.578	0.151	2.88126244630716E-15	Proliferating T/NK	ZFP91
1.77680185537485E-19	1.36575579076206	0.456	0.107	2.97756454923718E-15	Proliferating T/NK	HTATSF1
1.85962684021478E-19	1.22575355876052	0.467	0.108	3.11636265883192E-15	Proliferating T/NK	RBM42
1.87620172989306E-19	0.884341393843177	0.422	0.091	3.14413885895479E-15	Proliferating T/NK	ENY2
1.90003704680042E-19	1.18195095347657	0.4	0.083	3.18408208302814E-15	Proliferating T/NK	AKR1C3
1.95560812953793E-19	0.599478173889725	0.544	0.14	3.27720810347966E-15	Proliferating T/NK	NDE1
1.98112382382328E-19	1.03296997356249	0.756	0.277	3.31996730396306E-15	Proliferating T/NK	HNRNPF
2.01826877600275E-19	0.952202938179965	0.244	0.035	3.3822148148254E-15	Proliferating T/NK	ZNF184
2.02708750600307E-19	0.593821046797503	0.444	0.1	3.39699324255995E-15	Proliferating T/NK	SH3GLB2
2.26820874936574E-19	1.00712914940296	0.489	0.116	3.8010642221871E-15	Proliferating T/NK	MGAT4B
2.28137772225394E-19	1.60678164480591	0.222	0.029	3.82313278695316E-15	Proliferating T/NK	ZFP30
2.30982511772305E-19	1.36012835556557	0.267	0.041	3.87080493228029E-15	Proliferating T/NK	CEP97
2.55202405335906E-19	2.4690685490483	0.178	0.018	4.27668190861911E-15	Proliferating T/NK	AAMDC
2.60704186776394E-19	2.43749123403119	0.178	0.018	4.36888076199881E-15	Proliferating T/NK	TPM1
2.67376362507318E-19	0.496303447297915	0.556	0.139	4.48069308289763E-15	Proliferating T/NK	ARID1A
2.72338441505163E-19	0.882776095864233	0.289	0.047	4.56384760274351E-15	Proliferating T/NK	NEMP2
2.87989650610311E-19	0.8063369538351	0.533	0.134	4.82613056492759E-15	Proliferating T/NK	PRPF19
3.12841634995579E-19	1.52148795613197	0.3	0.051	5.24260011925591E-15	Proliferating T/NK	TMEM19
3.18046625824689E-19	1.88451194396361	0.178	0.018	5.32982535557014E-15	Proliferating T/NK	SPIN4
3.2120933143803E-19	2.7213236231412	0.144	0.012	5.3828259762385E-15	Proliferating T/NK	FGGY
3.21660389441456E-19	1.4324195192205	0.167	0.016	5.39038480625992E-15	Proliferating T/NK	TNFAIP8L1
3.38769970381636E-19	0.589464646453354	0.633	0.177	5.67710716365546E-15	Proliferating T/NK	RNF4
3.4320928125802E-19	1.2997358224063	0.444	0.101	5.7515011353219E-15	Proliferating T/NK	HSD17B10
3.73074274482778E-19	1.68037435923794	0.256	0.038	6.2519786917824E-15	Proliferating T/NK	CIDEB
3.92031755440538E-19	3.52372564309297	0.122	0.008	6.56966815767254E-15	Proliferating T/NK	GINS3
4.0559537121857E-19	1.26879882682996	0.344	0.065	6.7969672308808E-15	Proliferating T/NK	MEPCE
4.1216410904674E-19	1.3668222718775	0.267	0.041	6.90704613940527E-15	Proliferating T/NK	RHNO1
4.18103960522982E-19	0.873103322786199	0.789	0.278	7.00658617044413E-15	Proliferating T/NK	NUP210
4.28381042761778E-19	1.17755181887084	0.467	0.109	7.17880951460188E-15	Proliferating T/NK	SNTB1
4.58269043113773E-19	0.660163275111595	0.411	0.087	7.67967262450061E-15	Proliferating T/NK	SFPQ
4.99323190095037E-19	0.660971269142451	0.789	0.27	8.36765801961263E-15	Proliferating T/NK	HNRNPA3
5.14242110147765E-19	0.767839196967605	0.722	0.226	8.61766928185625E-15	Proliferating T/NK	HNRNPD
5.25621608308138E-19	0.978752387845214	0.311	0.055	8.80836691202778E-15	Proliferating T/NK	HAUS2
5.33212178017946E-19	1.91438159198298	0.233	0.033	8.93556967922474E-15	Proliferating T/NK	TSPAN3
5.33756474304312E-19	2.35530145926456	0.156	0.014	8.94469099639167E-15	Proliferating T/NK	LAPTM4A
5.34172176592892E-19	0.808012707298928	0.467	0.108	8.95165733534368E-15	Proliferating T/NK	RBMX
5.39887234311748E-19	0.529657494672723	0.433	0.096	9.04743027259626E-15	Proliferating T/NK	TRA2B
5.61459435780123E-19	1.14933233846459	0.767	0.256	9.4089372248033E-15	Proliferating T/NK	ENO1
6.27089548583216E-19	1.39176283126407	0.344	0.066	1.05087666551575E-14	Proliferating T/NK	DCXR
6.54147542575192E-19	1.14409174959725	0.556	0.152	1.09622045184751E-14	Proliferating T/NK	BLOC1S1
6.62374726832169E-19	0.622558493934597	0.311	0.054	1.11000756722535E-14	Proliferating T/NK	SPPL2B
6.72625654413771E-19	0.996815244919746	0.4	0.086	1.12718607166666E-14	Proliferating T/NK	PGP
6.73546786489363E-19	1.16372740918935	0.4	0.086	1.12872970479887E-14	Proliferating T/NK	CEP192
6.97662224889243E-19	0.796383471564745	0.811	0.301	1.16914235646939E-14	Proliferating T/NK	H1FX
7.01005702658749E-19	0.676364430144705	0.478	0.115	1.17474535651553E-14	Proliferating T/NK	IRAK1
7.18229082694407E-19	1.32547579317379	0.944	0.577	1.20360829677929E-14	Proliferating T/NK	VIM
7.26953512407917E-19	1.24219517817911	0.656	0.199	1.21822869609319E-14	Proliferating T/NK	ANXA2
8.13253389292967E-19	0.767084168741955	0.267	0.042	1.36285002977715E-14	Proliferating T/NK	PMAIP1
9.20464774739623E-19	0.52601350496697	0.456	0.103	1.54251486950866E-14	Proliferating T/NK	PLEKHJ1
1.06092266087624E-18	2.01119412436614	0.278	0.047	1.77789419509639E-14	Proliferating T/NK	PRDX4
1.06173983244318E-18	3.78885314502222	0.1	0.005	1.77926361120829E-14	Proliferating T/NK	KANK1
1.0723098387766E-18	1.77315361952542	0.167	0.017	1.79697682782182E-14	Proliferating T/NK	ATRIP
1.12454851857805E-18	0.70775446079874	0.478	0.113	1.88451840743309E-14	Proliferating T/NK	RAD23A
1.15991085852951E-18	0.550546726649784	0.6	0.164	1.94377861672375E-14	Proliferating T/NK	HMGN1
1.16840874066437E-18	0.395871772960868	0.589	0.156	1.95801936760535E-14	Proliferating T/NK	PPP4C
1.16970318699273E-18	1.56899931215895	0.333	0.063	1.96018860076242E-14	Proliferating T/NK	ECI1
1.20749325404957E-18	1.04507032446088	0.989	0.595	2.02351719513627E-14	Proliferating T/NK	PFN1
1.31100312204923E-18	1.25018376305823	0.344	0.066	2.1969790319301E-14	Proliferating T/NK	CNP
1.31578139712175E-18	0.595477451172657	0.489	0.121	2.20498646529664E-14	Proliferating T/NK	NUP205
1.44855314941539E-18	1.1726404519971	0.4	0.086	2.42748536779032E-14	Proliferating T/NK	CRELD2
1.49873438369902E-18	0.306029823457172	0.444	0.1	2.51157908020282E-14	Proliferating T/NK	GMFG
1.50197885631496E-18	0.564983293776723	0.311	0.055	2.51701616741261E-14	Proliferating T/NK	CEP78
1.50430659340601E-18	0.677245941707901	0.578	0.152	2.5209169892298E-14	Proliferating T/NK	PSMC5
1.53664815859264E-18	2.72857829268456	0.144	0.012	2.57511498416955E-14	Proliferating T/NK	L2HGDH
1.68087707502188E-18	1.36817033917206	0.289	0.049	2.81681380232167E-14	Proliferating T/NK	CEBPG
1.69507366797889E-18	2.41649557251584	0.144	0.012	2.84060445279902E-14	Proliferating T/NK	MCOLN1

1.70995671232659E-18	1.0344990593571	0.533	0.144	2.86554545851691E-14	Proliferating T/NK	ATP5MG
1.71870535361973E-18	1.26150943920992	0.311	0.057	2.88020643159594E-14	Proliferating T/NK	CYB561A3
1.71948800669676E-18	1.46876168325624	0.333	0.065	2.88151800162243E-14	Proliferating T/NK	POLE3
1.73334251720032E-18	0.936819044667331	0.344	0.067	2.9047353903243E-14	Proliferating T/NK	SMPD4
1.85918149699547E-18	1.41654727677439	0.389	0.084	3.11561635266501E-14	Proliferating T/NK	HAGH
2.23339193325607E-18	1.54669467945801	0.322	0.06	3.74271820175053E-14	Proliferating T/NK	ILF2
2.24819544281547E-18	1.06620845678344	0.444	0.105	3.76752592307016E-14	Proliferating T/NK	YWHAE
2.30462228437325E-18	1.37962946284809	0.4	0.09	3.86208602415269E-14	Proliferating T/NK	TRIOBP
2.48967289174227E-18	1.07870284697686	0.389	0.084	4.1721938319817E-14	Proliferating T/NK	CRTAP
2.50164664489094E-18	1.22179638652189	0.244	0.036	4.19225944750824E-14	Proliferating T/NK	SYPL1
2.50420592646599E-18	1.03355656334429	0.644	0.189	4.19654829157171E-14	Proliferating T/NK	UBB
2.52774035765374E-18	1.16235935288619	0.256	0.039	4.23598729135613E-14	Proliferating T/NK	PGAM1
2.82760441062064E-18	0.319165790292775	0.356	0.07	4.73849947131806E-14	Proliferating T/NK	NAA40
3.17854038729634E-18	1.88786463256268	0.111	0.007	5.32659798103121E-14	Proliferating T/NK	REXO5
3.39066693324373E-18	2.38250786766094	0.178	0.02	5.68207964672984E-14	Proliferating T/NK	RFC5
3.58768619317506E-18	1.95136029540542	0.222	0.031	6.01224452252277E-14	Proliferating T/NK	ADAM15
3.6023319139964E-18	1.22280388515519	0.367	0.076	6.03678782147518E-14	Proliferating T/NK	BRI3
3.68982261249616E-18	1.31506542303685	0.256	0.04	6.18340473402106E-14	Proliferating T/NK	ORC3
3.74183935326185E-18	0.528339251049001	0.467	0.111	6.2705743881962E-14	Proliferating T/NK	HNRNPR
4.08505346835904E-18	0.769346585417407	0.311	0.057	6.84573260227608E-14	Proliferating T/NK	LRRFIP2
4.08829979587728E-18	2.29855408822578	0.122	0.009	6.85117279793114E-14	Proliferating T/NK	CDH1
4.14767211016274E-18	0.859977205431012	0.533	0.143	6.95066892221072E-14	Proliferating T/NK	SUPT16H
4.18460038219647E-18	0.673729467936756	0.689	0.212	7.01255332048484E-14	Proliferating T/NK	MAT2A
4.18777845434563E-18	1.16451199492721	0.444	0.105	7.01787913379241E-14	Proliferating T/NK	PSMD1
4.26264387708127E-18	0.775940513129032	0.478	0.117	7.14333860921279E-14	Proliferating T/NK	MED4
4.74133243631599E-18	0.543450398985026	0.344	0.068	7.94552489677834E-14	Proliferating T/NK	EIF4E
4.92935911920023E-18	1.40368716806144	0.333	0.065	8.26062001195575E-14	Proliferating T/NK	LNPK
4.95879962726862E-18	0.46937827490703	0.167	0.017	8.30995641537675E-14	Proliferating T/NK	CTPS1
5.06275737552917E-18	0.596744854204577	0.722	0.23	8.48416880991179E-14	Proliferating T/NK	RHOA
5.44925013028724E-18	0.525254023620907	0.456	0.107	9.13185336833535E-14	Proliferating T/NK	DEPRECATED-ENSG00000265241
5.62242610742781E-18	1.6933826657339	0.267	0.044	9.42206167082753E-14	Proliferating T/NK	MGST2
5.70253274309364E-18	0.878796019608142	0.467	0.115	9.55630437087633E-14	Proliferating T/NK	TEX264
5.76755273914666E-18	1.03930745748249	0.3	0.054	9.66526488026198E-14	Proliferating T/NK	PACSIN2
5.9437828130927E-18	1.06846606500612	0.267	0.044	9.96059123818075E-14	Proliferating T/NK	PALM2-AKAP2
6.23204775840664E-18	1.49687139367234	0.256	0.041	1.04436656335378E-13	Proliferating T/NK	MYB
6.3315584977813E-18	1.02293033729375	0.367	0.076	1.06104257305819E-13	Proliferating T/NK	METTL26
6.52625815954498E-18	0.541177348260068	0.411	0.091	1.09367034237655E-13	Proliferating T/NK	SF1
6.62583041164267E-18	0.784659318078544	0.533	0.14	1.11035666038308E-13	Proliferating T/NK	GC
6.78602597882536E-18	0.732424691728167	0.5	0.129	1.13720223353155E-13	Proliferating T/NK	C12orf57
6.93947461615522E-18	0.643288315931143	0.5	0.127	1.16291715617529E-13	Proliferating T/NK	CHD4
7.1406919591856E-18	1.80045693741414	0.211	0.028	1.19663715852032E-13	Proliferating T/NK	DNAJC9
7.37979495735487E-18	0.389260935536384	0.511	0.128	1.23670603895353E-13	Proliferating T/NK	PMF1
7.89249449110437E-18	1.13984270276674	0.289	0.051	1.32262422681927E-13	Proliferating T/NK	RAD54L2
8.15463480911388E-18	0.812379684549152	0.4	0.089	1.3665537013113E-13	Proliferating T/NK	RALY
8.65447278962604E-18	0.543250622735108	0.611	0.176	1.45031655008553E-13	Proliferating T/NK	UBE2I
9.44353479091726E-18	1.73555922743322	0.222	0.031	1.58254756026191E-13	Proliferating T/NK	HPSE
9.68582597287686E-18	1.19713107836328	0.422	0.097	1.6231507165347E-13	Proliferating T/NK	AGPAT1
9.88642436653779E-18	1.68175179099662	0.333	0.067	1.6567669953444E-13	Proliferating T/NK	CD151
1.0401546646989E-17	1.7215944714179	0.289	0.052	1.74309118710241E-13	Proliferating T/NK	RHEB
1.0535481232349E-17	1.00845552739329	0.811	0.301	1.76553594491705E-13	Proliferating T/NK	NUCB1
1.06299903844466E-17	0.630642166592759	0.367	0.078	1.78137378862556E-13	Proliferating T/NK	BRD8
1.07814287545504E-17	1.01055939367193	0.267	0.044	1.80675183068756E-13	Proliferating T/NK	HAUS6
1.07985837814626E-17	0.974401995585113	0.333	0.065	1.8096266700975E-13	Proliferating T/NK	DEPRECATED-ENSG00000147872
1.1150935823733E-17	0.777852249971717	0.278	0.047	1.86867382534118E-13	Proliferating T/NK	PGM2
1.1928996805413E-17	0.405791656099093	0.344	0.069	1.99906128465111E-13	Proliferating T/NK	ATL2
1.23638998016827E-17	0.515914200089456	0.356	0.072	2.07194232876599E-13	Proliferating T/NK	EEF1AKMT2
1.25194247096928E-17	1.60348249460009	0.278	0.048	2.09800519285032E-13	Proliferating T/NK	NME1
1.31359485172426E-17	0.458434106933821	0.489	0.122	2.20132225251951E-13	Proliferating T/NK	TXNL4A
1.4599341656671E-17	0.96124631268598	0.356	0.073	2.44655767482492E-13	Proliferating T/NK	LSM 2.00
1.47467063825924E-17	0.697374749597007	0.722	0.238	2.47125305559483E-13	Proliferating T/NK	LDHB
1.49564744673199E-17	1.29868020053187	0.311	0.059	2.50640599123348E-13	Proliferating T/NK	AIFM1
1.63230169548235E-17	1.38793321772648	0.244	0.038	2.73541118128932E-13	Proliferating T/NK	DIAPH2
1.66326388295683E-17	2.110184624807	0.189	0.023	2.78729761505906E-13	Proliferating T/NK	MSH5
1.68061637021642E-17	0.633296195229443	0.678	0.221	2.81637691320868E-13	Proliferating T/NK	TMEM109
1.71312888166279E-17	1.10235793982665	0.278	0.049	2.87086137989051E-13	Proliferating T/NK	CDC27
1.74282392087497E-17	1.66467617438913	0.244	0.039	2.92062432660227E-13	Proliferating T/NK	MMS22L

1.78019577304272E-17	1.41949765681238	0.211	0.029	2.98325207646499E-13	Proliferating T/NK	AIDA
1.82836787057967E-17	1.8746577371267	0.3	0.057	3.06397887751741E-13	Proliferating T/NK	DAP
1.83021611285339E-17	3.51600675472379	0.1	0.006	3.06707616191971E-13	Proliferating T/NK	IGF2BP3
2.10535003234039E-17	0.638113721412285	0.389	0.084	3.52814558419602E-13	Proliferating T/NK	GTF2A1
2.12448264023954E-17	0.589534248106183	0.444	0.11	3.56020800851342E-13	Proliferating T/NK	PI4K2B
2.2170138358482E-17	1.3525316320259	0.289	0.052	3.71527178611442E-13	Proliferating T/NK	MCFD2
2.21982873642216E-17	1.41486395552427	0.244	0.038	3.71998899649625E-13	Proliferating T/NK	CRYL1
2.22180825926889E-17	0.400934426322199	0.533	0.141	3.72330628088281E-13	Proliferating T/NK	DDX23
2.36525106805084E-17	0.457625285304475	0.611	0.178	3.9636877398396E-13	Proliferating T/NK	STAG1
2.3789107409146E-17	1.24382560919909	0.5	0.134	3.98657861962469E-13	Proliferating T/NK	GHITM
2.38638006114786E-17	1.95474603544964	0.3	0.058	3.99909570647158E-13	Proliferating T/NK	RNPEP
2.47741124869926E-17	1.20522396920752	0.267	0.045	4.15164577057023E-13	Proliferating T/NK	MAPK7
2.52135257562821E-17	3.04299295919111	0.111	0.007	4.22528264623776E-13	Proliferating T/NK	RCAN1
2.57014170027031E-17	1.06751870671023	0.389	0.089	4.30704346131298E-13	Proliferating T/NK	NUP58
2.57673960652062E-17	2.84809166346059	0.122	0.009	4.31810023260725E-13	Proliferating T/NK	SDC2
2.59969407918052E-17	2.82023483886869	0.122	0.009	4.35656733789071E-13	Proliferating T/NK	HAUS7
2.80299821267466E-17	2.41658708019401	0.111	0.007	4.6972644048002E-13	Proliferating T/NK	ING2
2.81270788697409E-17	0.796830825654549	0.311	0.06	4.71353587699118E-13	Proliferating T/NK	GMPPB
2.98579752601584E-17	0.898519271966301	0.578	0.173	5.00359949409735E-13	Proliferating T/NK	ADCY3
2.98981198032497E-17	0.36559350743114	0.511	0.131	5.01032691662859E-13	Proliferating T/NK	PSMD13
2.99916947550842E-17	2.59186781218975	0.156	0.016	5.02600820705701E-13	Proliferating T/NK	CTNNAL1
3.06285161148514E-17	1.84234463578903	0.233	0.036	5.13272673052679E-13	Proliferating T/NK	VDAC1
3.23942778313212E-17	0.654419344917903	0.567	0.162	5.4286330789728E-13	Proliferating T/NK	SET
3.29403590753011E-17	1.1270770148541	0.322	0.063	5.52014537383896E-13	Proliferating T/NK	LGALS4
3.29632770140593E-17	1.7426195358466	0.167	0.018	5.52398596201605E-13	Proliferating T/NK	SLC19A1
3.4037611291777E-17	2.52544621382924	0.144	0.014	5.704022900276E-13	Proliferating T/NK	UBL4A
3.60426681992822E-17	0.468291036792895	0.667	0.205	6.04003033683571E-13	Proliferating T/NK	BUB3
3.65304552657437E-17	0.677060162760482	0.433	0.103	6.12177369343333E-13	Proliferating T/NK	PTPRA
3.75423408120402E-17	1.87568397765693	0.211	0.03	6.2913454732817E-13	Proliferating T/NK	QDPR
3.79001655555671E-17	0.676213327949314	0.5	0.132	6.35130974380193E-13	Proliferating T/NK	PTBP1
3.82300426050844E-17	1.21515539679431	0.344	0.071	6.40659053976005E-13	Proliferating T/NK	SDF2L1
3.84994876999698E-17	2.2925426784325	0.167	0.018	6.45174414876094E-13	Proliferating T/NK	RAD51C
3.90703413168429E-17	1.35035755554024	0.278	0.05	6.54740779787654E-13	Proliferating T/NK	IGFBP4
3.93585766063291E-17	1.93709681652933	0.156	0.016	6.59571026768864E-13	Proliferating T/NK	FANCD2
3.94088282174388E-17	1.06604099825745	0.544	0.155	6.6041314326784E-13	Proliferating T/NK	GSDMD
4.15432294690758E-17	1.09740124385407	0.456	0.113	6.96181439442772E-13	Proliferating T/NK	PCMT1
4.40593195236099E-17	1.03295708054347	0.3	0.056	7.38346076576655E-13	Proliferating T/NK	SLC25A24
4.54194623133149E-17	1.16818253213539	0.289	0.053	7.6113934944653E-13	Proliferating T/NK	THOP1
4.62528593055265E-17	1.20236894084752	0.289	0.054	7.75105416242014E-13	Proliferating T/NK	FAM20B
4.72076791063538E-17	0.795524514551643	0.333	0.068	7.91106286464277E-13	Proliferating T/NK	NUP93
4.72325696582924E-17	1.8176466268167	0.233	0.036	7.91523402333663E-13	Proliferating T/NK	ZFAND2A
4.84119269236505E-17	1.90458499013043	0.189	0.024	8.11287071386535E-13	Proliferating T/NK	CDH17
5.04727655527224E-17	0.642062581295151	0.8	0.289	8.45822605132522E-13	Proliferating T/NK	PSMB9
5.12806772608497E-17	0.510618566428977	0.533	0.144	8.5936158953732E-13	Proliferating T/NK	DAZAP1
5.33282669484489E-17	0.550479703041754	0.3	0.057	8.93675097522106E-13	Proliferating T/NK	DEPRECATED-ENSG00000137575
5.55784111595174E-17	0.584785828231275	0.689	0.223	9.31383014211193E-13	Proliferating T/NK	POLR2A
5.64398033903817E-17	0.699261797252215	0.467	0.119	9.45818225216017E-13	Proliferating T/NK	FAR1
6.26825394628121E-17	0.7454991653262	0.556	0.164	1.0504339963178E-12	Proliferating T/NK	FZR1
6.76348184518514E-17	1.29251307316138	0.378	0.086	1.13342428761613E-12	Proliferating T/NK	SCAF1
6.80475640865184E-17	0.869048311996653	0.378	0.084	1.14034107896187E-12	Proliferating T/NK	C2CD5
6.82157687074131E-17	0.712690102582501	0.511	0.136	1.14315985199883E-12	Proliferating T/NK	DPP9
6.93909308442591E-17	1.01152978804909	0.433	0.105	1.16285321908809E-12	Proliferating T/NK	HDGFL2
7.25113536215034E-17	0.907069240596619	0.367	0.079	1.21514526398915E-12	Proliferating T/NK	SNX3
7.53402429830221E-17	0.893706218524332	0.311	0.06	1.26255179190948E-12	Proliferating T/NK	MTHFD2
8.07546952281165E-17	0.931083971055124	0.4	0.093	1.35328718263278E-12	Proliferating T/NK	ARPP19
8.63415271628898E-17	1.18266083587653	0.267	0.047	1.44691131219571E-12	Proliferating T/NK	C11orf80
8.9636951261859E-17	1.35126524086701	0.444	0.113	1.50213602924623E-12	Proliferating T/NK	MTHFD1
8.99703537979605E-17	1.03449590963812	0.533	0.149	1.50772318894622E-12	Proliferating T/NK	EMC4
9.7855736421891E-17	0.850687350652795	0.556	0.158	1.63986643095805E-12	Proliferating T/NK	RNH1
9.91501360168082E-17	1.14658663939479	0.3	0.057	1.66155797936967E-12	Proliferating T/NK	JMJD8
1.03150635663652E-16	0.452940730735693	0.411	0.095	1.72859835245147E-12	Proliferating T/NK	NUP188
1.03648544031751E-16	1.22635336952476	0.456	0.118	1.73694230088408E-12	Proliferating T/NK	NDUFB7
1.08337318272303E-16	1.0824233353035	0.578	0.171	1.81551677960726E-12	Proliferating T/NK	HADHA
1.18067541822002E-16	0.649319439436089	0.367	0.081	1.97857586585311E-12	Proliferating T/NK	MLH1
1.21818381107957E-16	2.130988645872	0.167	0.019	2.04143243060714E-12	Proliferating T/NK	GSTM4
1.2817703232622E-16	0.564972866242668	0.544	0.152	2.1479907077228E-12	Proliferating T/NK	DHX9

1.31031337221242E-16	0.28182204220272	0.489	0.126	2.19582314915357E-12	Proliferating T/NK	CIC
1.31415077319519E-16	0.411402196511444	0.333	0.069	2.20225386572049E-12	Proliferating T/NK	ILK
1.32884239104885E-16	1.06885050797147	0.278	0.05	2.22687407891966E-12	Proliferating T/NK	ELP5
1.366847537622E-16	1.13827645109175	0.433	0.107	2.29056310354694E-12	Proliferating T/NK	NDUFB9
1.37540478204949E-16	2.09356325868056	0.156	0.017	2.30490333375853E-12	Proliferating T/NK	PTK2
1.42416157940097E-16	0.615384599124613	0.356	0.077	2.38660997476015E-12	Proliferating T/NK	TAOK2
1.44052840991164E-16	2.20403211314516	0.122	0.01	2.41403750932993E-12	Proliferating T/NK	FIGNL1
1.44377696596551E-16	2.10582252877965	0.156	0.017	2.419481439565E-12	Proliferating T/NK	CENPS
1.53382712890934E-16	1.97133495014827	0.189	0.025	2.57038750262627E-12	Proliferating T/NK	ATP5MPL
1.56521970854412E-16	0.89790470341201	0.578	0.169	2.62299518757823E-12	Proliferating T/NK	SF3B5
1.58387387997757E-16	1.19977952132254	0.322	0.065	2.65425584806641E-12	Proliferating T/NK	NDUFB1
1.59088511016844E-16	1.59405830471103	0.222	0.034	2.66600526762021E-12	Proliferating T/NK	TUBGCP4
1.63094421918243E-16	0.832763173437458	0.267	0.047	2.73313632250592E-12	Proliferating T/NK	ARL6IP6
1.63214003330548E-16	1.57539354331607	0.278	0.051	2.73514026781333E-12	Proliferating T/NK	ZNF428
1.63630317685226E-16	2.54590235806612	0.122	0.01	2.74211686376902E-12	Proliferating T/NK	BRCC3
1.63790143819912E-16	0.99293193513706	0.411	0.101	2.74479523013409E-12	Proliferating T/NK	NOP56
1.67621664179743E-16	1.07703244357469	0.2	0.028	2.80900384832413E-12	Proliferating T/NK	TMEM164
1.72566033824386E-16	0.53066849061387	0.556	0.157	2.89186159482906E-12	Proliferating T/NK	NMT1
1.73496587887211E-16	1.18346472960359	0.278	0.051	2.90745581981388E-12	Proliferating T/NK	PSMD2
1.76525431288137E-16	3.06279631817688	0.111	0.008	2.9582131775266E-12	Proliferating T/NK	CCDC171
1.77030723022948E-16	1.22494704480753	0.444	0.113	2.96668085641857E-12	Proliferating T/NK	NUDT5
1.85222659141913E-16	1.31166003553929	0.278	0.051	3.10396132190018E-12	Proliferating T/NK	IER3IP1
1.87193509808729E-16	0.95269790113464	0.522	0.142	3.13698883737469E-12	Proliferating T/NK	PTPA
1.87204330119937E-16	1.19787068017293	0.211	0.031	3.1371701641499E-12	Proliferating T/NK	WDR5
1.93566099098701E-16	0.879081714359701	0.522	0.15	3.24378068869604E-12	Proliferating T/NK	PA2G4
1.95077806939266E-16	1.00995770360276	0.433	0.11	3.26911388868822E-12	Proliferating T/NK	SMS
1.9665005280265E-16	0.690926488683335	0.367	0.081	3.2954615848668E-12	Proliferating T/NK	TTLL5
1.98113942965825E-16	0.322790152708525	0.511	0.14	3.31999345622129E-12	Proliferating T/NK	FAM50A
2.02724008546073E-16	0.682084839984243	0.389	0.089	3.3972489352151E-12	Proliferating T/NK	HSPB11
2.0395131512617E-16	0.501406393458719	0.622	0.187	3.41781613888435E-12	Proliferating T/NK	ERAP1
2.05220046319282E-16	0.719674853218299	0.6	0.181	3.43907753621853E-12	Proliferating T/NK	SLC38A2
2.11216171266582E-16	1.85793756945478	0.189	0.025	3.53956059808537E-12	Proliferating T/NK	NQO2
2.14572402864061E-16	1.33017216317127	0.256	0.044	3.59580432719594E-12	Proliferating T/NK	PRIM1
2.16616256519513E-16	0.642613075721065	0.322	0.066	3.63005522675399E-12	Proliferating T/NK	EXOSC2
2.36974083437466E-16	1.1371389884856	0.256	0.044	3.97121169024505E-12	Proliferating T/NK	SLC39A3
2.37591735461948E-16	1.16195894076875	0.211	0.031	3.98156230287132E-12	Proliferating T/NK	SLC40A1
2.43119301165781E-16	1.02932929782578	0.333	0.07	4.07419324893616E-12	Proliferating T/NK	BICD1
2.490027836469E-16	1.94973965803512	0.211	0.031	4.17278864835475E-12	Proliferating T/NK	PAFAH1B3
2.55621718370085E-16	0.908445768878215	0.444	0.11	4.28370875644588E-12	Proliferating T/NK	ATP5MC3
2.56886214457351E-16	0.466485532468261	0.567	0.165	4.30489918187629E-12	Proliferating T/NK	BAZ1B
2.62672570718152E-16	2.02792925563608	0.189	0.025	4.40186694009479E-12	Proliferating T/NK	TMEM70
2.6519310202735E-16	2.98290239438524	0.144	0.015	4.44410600377433E-12	Proliferating T/NK	LRRC20
2.83662515549295E-16	0.402085881958062	0.389	0.089	4.75361643557508E-12	Proliferating T/NK	EFTUD2
2.84219938838731E-16	2.13935525577651	0.133	0.012	4.76295773505946E-12	Proliferating T/NK	B3GLCT
3.01454700885654E-16	0.543686308544156	0.456	0.116	5.0517778774418E-12	Proliferating T/NK	PNRC2
3.11889477577326E-16	2.42890333648669	0.133	0.012	5.22664386524083E-12	Proliferating T/NK	DEPRECATED-ENSG00000176485
3.37231416467448E-16	0.653217417233354	0.467	0.12	5.65132407716149E-12	Proliferating T/NK	CAND1
3.42850379558452E-16	0.417692626873562	0.411	0.097	5.74548666064054E-12	Proliferating T/NK	UBE2J2
3.68238896896023E-16	0.48526940240521	0.444	0.111	6.17094743418355E-12	Proliferating T/NK	AGPS
3.70652902355617E-16	0.83152090553358	0.489	0.133	6.21140133767543E-12	Proliferating T/NK	RTN4
3.72521917928643E-16	0.706782583550554	0.5	0.14	6.24272230064819E-12	Proliferating T/NK	SRPK1
3.73808704365689E-16	1.24570122261065	0.467	0.129	6.26428626776021E-12	Proliferating T/NK	CSRP1
3.77588150202475E-16	1.22808699826979	0.389	0.092	6.32762222109307E-12	Proliferating T/NK	ATP5PD
3.86168058094789E-16	1.07535148758551	0.322	0.067	6.47140431755248E-12	Proliferating T/NK	NAGA
3.87168675139321E-16	1.01011167485755	0.467	0.122	6.48817265798474E-12	Proliferating T/NK	MLEC
3.98879874762177E-16	0.410963680157127	0.467	0.12	6.68442894126457E-12	Proliferating T/NK	SUZ12
4.0254306206676E-16	1.16823578971287	0.244	0.041	6.74581663411476E-12	Proliferating T/NK	KATNA1
4.10330667974993E-16	1.39282920540676	0.222	0.035	6.87632133392493E-12	Proliferating T/NK	BLOC1S5
4.23018274051608E-16	0.864682569801877	0.767	0.283	7.08894023655684E-12	Proliferating T/NK	RCSD1
4.28938833620653E-16	1.26312936686273	0.411	0.102	7.18815697381491E-12	Proliferating T/NK	LIMK2
4.39970696443464E-16	0.921691210126144	0.2	0.028	7.37302893099957E-12	Proliferating T/NK	IMPDH2
4.47009924376813E-16	1.60940884303195	0.256	0.046	7.49099231270663E-12	Proliferating T/NK	CPT1A
4.55767448764608E-16	0.707362462953449	0.611	0.193	7.6377509063973E-12	Proliferating T/NK	NFATC2IP
4.8114576523829E-16	0.814040865809011	0.622	0.198	8.06304073386327E-12	Proliferating T/NK	PPM1G
4.85319171183139E-16	1.11746193928449	0.589	0.183	8.13297867068704E-12	Proliferating T/NK	PDIA3
4.88652026273658E-16	1.95051573473865	0.144	0.015	8.18883065629395E-12	Proliferating T/NK	MPND

4.9889216325037E-16	0.86314425388882	0.467	0.121	8.36043487174969E-12	Proliferating T/NK	GNAI3
5.10932870352826E-16	0.985732854438082	0.467	0.119	8.56221304137265E-12	Proliferating T/NK	RAB7A
5.3726403325953E-16	1.54857549752724	0.267	0.049	9.00347066936321E-12	Proliferating T/NK	SMIM19
5.46331482960222E-16	1.06570055254001	0.267	0.048	9.1554229914474E-12	Proliferating T/NK	SCAMP4
5.61180996660177E-16	1.63727373109857	0.211	0.032	9.40427114203125E-12	Proliferating T/NK	PRAP1
6.44829050477456E-16	1.87953347825697	0.2	0.029	1.08060452279012E-11	Proliferating T/NK	CCP110
6.48281344461155E-16	1.8268887490307	0.178	0.023	1.086389877048E-11	Proliferating T/NK	CPT2
6.81121955374035E-16	0.570975425820818	0.711	0.253	1.14142417281581E-11	Proliferating T/NK	ATP2A3
6.81427872963806E-16	1.11534976253821	0.367	0.084	1.14193682951275E-11	Proliferating T/NK	TSR3
7.00492093324368E-16	0.250971218510444	0.756	0.246	1.17388464999298E-11	Proliferating T/NK	SP3
7.39340992461453E-16	0.387265532791795	0.511	0.141	1.2389876351669E-11	Proliferating T/NK	SART1
7.60946829006158E-16	1.16241125793244	0.244	0.042	1.27519469604852E-11	Proliferating T/NK	NHLRC3
7.67656426700912E-16	0.663555959144382	0.311	0.063	1.28643863986539E-11	Proliferating T/NK	GIT1
7.68888705517419E-16	1.2053830023548	0.344	0.076	1.28850369270609E-11	Proliferating T/NK	CDKN2A
7.78565370965308E-16	1.10339403238992	0.211	0.032	1.30471984866366E-11	Proliferating T/NK	CDK19
7.92174892085243E-16	2.02619213401001	0.2	0.029	1.32752668415645E-11	Proliferating T/NK	FAM200B
8.07332076761008E-16	1.69851931832735	0.211	0.032	1.3529270942361E-11	Proliferating T/NK	CFAP20
8.13521536331476E-16	0.526536973300927	0.178	0.023	1.36329939058429E-11	Proliferating T/NK	DEPRECATED-ENSG00000153487
8.4675610469434E-16	0.958413408288505	0.356	0.08	1.41899388024677E-11	Proliferating T/NK	DEPRECATED-ENSG00000174903
8.50328644638378E-16	0.383086948305223	0.511	0.142	1.42498074268499E-11	Proliferating T/NK	RBM14
8.67094663617676E-16	1.1801559518832	0.167	0.02	1.4530772372905E-11	Proliferating T/NK	CNIH1
8.70466901220955E-16	0.687625304712661	0.244	0.041	1.45872843306608E-11	Proliferating T/NK	MDM1
9.85962294136849E-16	1.05961790327535	0.289	0.056	1.65227561251453E-11	Proliferating T/NK	FADD
1.02386704554315E-15	0.57761502922172	0.311	0.064	1.71579639492121E-11	Proliferating T/NK	BCL7B
1.02601613015376E-15	1.45028286716501	0.256	0.046	1.71939783091168E-11	Proliferating T/NK	UQCRH
1.03363014269248E-15	1.22508774633235	0.244	0.043	1.73215739312406E-11	Proliferating T/NK	EXOSC3
1.03387035969415E-15	1.81705765415671	0.2	0.03	1.73255994877545E-11	Proliferating T/NK	CDCA7L
1.16894561095138E-15	1.23288292543735	0.222	0.036	1.95891905483232E-11	Proliferating T/NK	CCDC134
1.17713884655348E-15	1.31539086991428	0.167	0.02	1.97264927905432E-11	Proliferating T/NK	RDX
1.19486347225007E-15	1.23112722516438	0.222	0.035	2.00235220679667E-11	Proliferating T/NK	TOR1AIP2
1.19628339911729E-15	0.555499777674508	0.511	0.144	2.00473172024075E-11	Proliferating T/NK	SF3B3
1.24042263627032E-15	0.69410718180209	0.3	0.06	2.07870025386181E-11	Proliferating T/NK	TCP1
1.26921800278746E-15	2.06571555234485	0.133	0.013	2.12695552907123E-11	Proliferating T/NK	SRD5A3
1.29346493339189E-15	0.304407077174797	0.2	0.029	2.16758853537813E-11	Proliferating T/NK	TPRKB
1.32527515035164E-15	1.74973319282351	0.222	0.036	2.22089609695928E-11	Proliferating T/NK	RPP21
1.34132338987121E-15	0.845354042371254	0.378	0.092	2.24778973674618E-11	Proliferating T/NK	DCAF15
1.39045395628428E-15	0.944025829290725	0.433	0.114	2.3301227399412E-11	Proliferating T/NK	FABP1
1.43479727098649E-15	0.865763252995521	0.344	0.077	2.40443326671916E-11	Proliferating T/NK	GTPBP2
1.45254943661175E-15	1.06714308105667	0.444	0.118	2.43418234587397E-11	Proliferating T/NK	KRTCAP2
1.46986682679252E-15	0.985804836927679	0.444	0.124	2.46320282833891E-11	Proliferating T/NK	MCM6
1.52122775694708E-15	0.322135453566993	0.656	0.206	2.54927347509191E-11	Proliferating T/NK	TMEM50A
1.54173433702944E-15	0.303437793725425	0.422	0.105	2.58363840199394E-11	Proliferating T/NK	PET100
1.58453251414339E-15	0.618007551334899	0.867	0.357	2.6553595872015E-11	Proliferating T/NK	H3F3A
1.60466023709494E-15	1.15328148181381	0.344	0.077	2.68908962532237E-11	Proliferating T/NK	MZT2A
1.63113511044381E-15	1.33594697877084	0.289	0.057	2.73345621808173E-11	Proliferating T/NK	XRCC1
1.63232247362878E-15	0.522877694543363	0.722	0.253	2.73544600130711E-11	Proliferating T/NK	TACC1
1.64199079846729E-15	0.889990601037865	0.256	0.046	2.75164818007148E-11	Proliferating T/NK	TOR3A
1.66112796198182E-15	1.9416228917537	0.189	0.026	2.78371823868913E-11	Proliferating T/NK	CRLS1
1.69015797537258E-15	0.929371941418112	0.544	0.158	2.83236673512938E-11	Proliferating T/NK	SEC61A1
1.70793082650301E-15	1.02943266371597	0.189	0.026	2.86215047905374E-11	Proliferating T/NK	WDR54
1.72791138038151E-15	2.63783227237109	0.1	0.007	2.89563389124334E-11	Proliferating T/NK	C17orf58
1.76440602292231E-15	0.681608058375007	0.311	0.065	2.9567916132132E-11	Proliferating T/NK	ATP6V0B
1.796988350054E-15	1.28215306479626	0.322	0.07	3.01139307702049E-11	Proliferating T/NK	HPRT1
1.82690470374456E-15	0.462849430854954	0.478	0.129	3.06152690253514E-11	Proliferating T/NK	MEAF6
1.83788559155974E-15	1.04895569326815	0.3	0.06	3.07992867433581E-11	Proliferating T/NK	COX7A2
1.85059064235205E-15	0.386879248228465	0.478	0.129	3.10121979845357E-11	Proliferating T/NK	TPR
1.85959363300281E-15	0.904493261158152	0.456	0.129	3.1163070101861E-11	Proliferating T/NK	GLUL
1.88089912532116E-15	1.33537051825609	0.289	0.057	3.1520107542132E-11	Proliferating T/NK	UQCRQ
1.89642114250266E-15	0.43364686267398	0.3	0.06	3.17802255060596E-11	Proliferating T/NK	USP7
1.90848305692208E-15	1.20109700410262	0.189	0.026	3.19823590679003E-11	Proliferating T/NK	ITPKC
1.91213880883924E-15	1.10735321692507	0.456	0.124	3.2043622158528E-11	Proliferating T/NK	NDUFA4
1.92180865511468E-15	0.965337133777342	0.233	0.039	3.22056694424118E-11	Proliferating T/NK	CDC37L1
1.95452951970903E-15	0.567459760004003	0.589	0.177	3.27540056912839E-11	Proliferating T/NK	TOR1AIP1
2.00374205343459E-15	0.727607189721712	0.578	0.18	3.35787093314568E-11	Proliferating T/NK	ELAVL1
2.06007247776527E-15	0.417333510166403	0.222	0.036	3.45226945823903E-11	Proliferating T/NK	DEPRECATED-ENSG00000145241
2.12075456529996E-15	1.52068614073879	0.233	0.039	3.55396050052968E-11	Proliferating T/NK	CES2

2.13552582217918E-15	0.47396603511417	0.533	0.154	3.57871417280787E-11	Proliferating T/NK	PFKP
2.19203408241031E-15	1.13823205056995	0.189	0.026	3.6734107153032E-11	Proliferating T/NK	GMFB
2.20623023403646E-15	0.293031645685207	0.778	0.274	3.6972006261983E-11	Proliferating T/NK	NUMA1
2.20766677101585E-15	0.36814835297908	0.5	0.139	3.69960797486836E-11	Proliferating T/NK	RECQL
2.25071096916513E-15	1.31739565306986	0.211	0.033	3.77174144212692E-11	Proliferating T/NK	CBX1
2.29203048145442E-15	1.07320475797454	0.267	0.051	3.84098468082132E-11	Proliferating T/NK	USP37
2.31114785368642E-15	2.11081863919568	0.178	0.024	3.8730215732077E-11	Proliferating T/NK	PAQR4
2.39822187860941E-15	0.936969775307963	0.422	0.106	4.01894022417365E-11	Proliferating T/NK	SEM1
2.49139696885829E-15	0.989516863575785	0.256	0.046	4.17508304041272E-11	Proliferating T/NK	ZSWIM9
2.60174513546391E-15	0.494133880777689	0.644	0.208	4.36000449801041E-11	Proliferating T/NK	MTA2
2.6892959639195E-15	1.53237545442842	0.278	0.054	4.5067221763363E-11	Proliferating T/NK	HLTF
2.69728260906972E-15	0.561510989659428	0.4	0.1	4.52010619627903E-11	Proliferating T/NK	EXOSC9
2.70095668212649E-15	0.962381932355415	0.756	0.283	4.52626320790757E-11	Proliferating T/NK	LMAN2
2.74211568763681E-15	0.892678248562437	0.3	0.062	4.59523746934176E-11	Proliferating T/NK	SAP130
2.8487760807475E-15	0.718295823479476	0.411	0.105	4.77397895611666E-11	Proliferating T/NK	ZNF384
2.88426609568047E-15	1.05328255156673	0.256	0.047	4.83345312314134E-11	Proliferating T/NK	FAM162A
2.97916834611783E-15	1.33696882380288	0.167	0.021	4.99249031442426E-11	Proliferating T/NK	TMEM39B
3.02527686180886E-15	0.71911919272921	0.256	0.047	5.06975896501929E-11	Proliferating T/NK	CXorf56
3.06156366367187E-15	1.51456112905853	0.244	0.044	5.13056838758131E-11	Proliferating T/NK	EOGT
3.08814777053396E-15	0.563841797223231	0.389	0.096	5.17511803386081E-11	Proliferating T/NK	PARP1
3.1163574494345E-15	1.7145483815226	0.2	0.03	5.22239181376234E-11	Proliferating T/NK	ABCD3
3.18406099122947E-15	0.680854487604873	0.344	0.078	5.33584940910235E-11	Proliferating T/NK	PSMD7
3.24283121101439E-15	0.386859121233422	0.5	0.137	5.43433654341791E-11	Proliferating T/NK	PPP3CB
3.36894641396469E-15	0.579632417773708	0.656	0.219	5.64568040052203E-11	Proliferating T/NK	GNAI2
3.41472268320759E-15	0.381858538021281	0.478	0.132	5.72239227251928E-11	Proliferating T/NK	YBX1
3.47638858964597E-15	1.43059149316374	0.178	0.024	5.82573199852872E-11	Proliferating T/NK	ANO8
3.53827701798447E-15	0.903100009786542	0.322	0.069	5.92944462673837E-11	Proliferating T/NK	CHID1
3.75603516285268E-15	0.379560995956658	0.456	0.123	6.29436372590852E-11	Proliferating T/NK	ADSL
3.81458724023165E-15	1.58328623625756	0.156	0.018	6.39248529718019E-11	Proliferating T/NK	ZNF75D
3.92829225345718E-15	0.556819655302394	0.378	0.089	6.58303215834355E-11	Proliferating T/NK	BCOR
3.92956961862758E-15	0.946472594979604	0.667	0.232	6.58517276689609E-11	Proliferating T/NK	EIF4EBP2
4.14317821267997E-15	2.36559837425946	0.144	0.016	6.9431380488091E-11	Proliferating T/NK	GOT1
4.2850171255805E-15	0.544520485948933	0.4	0.099	7.18083169904781E-11	Proliferating T/NK	C6orf89
4.29562162779545E-15	1.2034955769047	0.211	0.033	7.19860272385962E-11	Proliferating T/NK	HDDC3
4.31089742863234E-15	1.0657605771746	0.189	0.027	7.22420191090208E-11	Proliferating T/NK	GTPBP3
4.36221504154434E-15	0.61490486619828	0.511	0.144	7.31019996662E-11	Proliferating T/NK	UBA2
4.44048839461143E-15	2.24234281314146	0.189	0.028	7.44137045168984E-11	Proliferating T/NK	NR2C2AP
4.49971924465162E-15	0.53784530128918	0.6	0.188	7.54062951018718E-11	Proliferating T/NK	ARPC4
4.51788172375401E-15	1.21149730466156	0.4	0.1	7.57106619266698E-11	Proliferating T/NK	LAMTOR2
4.59864804922463E-15	0.795328095684315	0.278	0.055	7.70641440089064E-11	Proliferating T/NK	TAF4
4.61243229756028E-15	1.70105943958147	0.211	0.034	7.72951404425151E-11	Proliferating T/NK	TTLL12
4.67673156769983E-15	1.04488251201149	0.2	0.03	7.83726676115138E-11	Proliferating T/NK	FOCAD
4.71637299264116E-15	0.640906105844561	0.267	0.051	7.90369786106805E-11	Proliferating T/NK	THOC6
4.72737192252195E-15	0.335925962498498	0.389	0.094	7.92212986776228E-11	Proliferating T/NK	ZCCHC3
4.80941604209468E-15	0.274388942846561	0.422	0.107	8.05961940334226E-11	Proliferating T/NK	SMNDC1
4.90452835180315E-15	1.56725719764055	0.144	0.016	8.21900861195171E-11	Proliferating T/NK	SMG7
5.02931182601041E-15	1.47808998987652	0.211	0.034	8.42812075802824E-11	Proliferating T/NK	TOP3A
5.29254522533481E-15	1.03830085049515	0.311	0.067	8.86924728861608E-11	Proliferating T/NK	SNF8
5.30024102401344E-15	1.04094777813525	0.267	0.051	8.88214390804173E-11	Proliferating T/NK	SSR1
5.58511074645111E-15	0.485454506470203	0.367	0.087	9.35952858890277E-11	Proliferating T/NK	PLEKHF2
5.6903211390763E-15	1.20804957163089	0.344	0.079	9.53584016486407E-11	Proliferating T/NK	PPDPF
6.23460636128087E-15	1.16147243719209	0.256	0.048	1.04479533402345E-10	Proliferating T/NK	ADPRHL2
6.50215758593251E-15	0.627528608498005	0.289	0.059	1.08963156825057E-10	Proliferating T/NK	PHF6
6.57358678999875E-15	1.81276820279893	0.244	0.045	1.10160167426799E-10	Proliferating T/NK	TMEM205
6.82711301373503E-15	1.474240624263	0.211	0.034	1.14408759884172E-10	Proliferating T/NK	PLS3
6.84670807708234E-15	2.665397993476	0.111	0.009	1.14737133955746E-10	Proliferating T/NK	C18orf54
6.96479622472737E-15	0.939085897933834	0.489	0.141	1.16716055133981E-10	Proliferating T/NK	SOD1
7.2597257998283E-15	1.50617308545249	0.211	0.034	1.21658484953523E-10	Proliferating T/NK	GFPT1
7.27635479266875E-15	0.387278316045149	0.322	0.07	1.21937153615543E-10	Proliferating T/NK	GRK5
7.57012218282223E-15	0.933766219355227	0.244	0.044	1.26860107539735E-10	Proliferating T/NK	BAP1
7.57100791552635E-15	0.27063545018772	0.344	0.078	1.26874950648391E-10	Proliferating T/NK	ARL17B
7.70036347676375E-15	1.17865933373807	0.211	0.034	1.29042691143607E-10	Proliferating T/NK	FKBP2
7.95411189661333E-15	2.15114051807723	0.111	0.009	1.33295007163446E-10	Proliferating T/NK	CEP57L1
7.96121883295111E-15	0.753875079786871	0.244	0.044	1.33414105202595E-10	Proliferating T/NK	POU2F1
8.0032983102144E-15	0.899571565198459	0.522	0.152	1.34119273082573E-10	Proliferating T/NK	POLR2L
8.22754001945141E-15	0.550656952185615	0.344	0.078	1.37877115645967E-10	Proliferating T/NK	SMC5

8.38477782770426E-15	0.311278094684446	0.378	0.092	1.40512106836668E-10	Proliferating T/NK	ENO2
8.43921001019523E-15	0.847227451158685	0.6	0.189	1.41424281350852E-10	Proliferating T/NK	ATP5MC2
8.51864917093983E-15	0.918017229484874	0.189	0.028	1.4275552280661E-10	Proliferating T/NK	TAF5
8.57801966211297E-15	2.01092782144469	0.156	0.019	1.43750453497689E-10	Proliferating T/NK	CEP250
8.65424662024346E-15	1.33946265672347	0.333	0.078	1.4502786486204E-10	Proliferating T/NK	MPC1
8.76197871278258E-15	0.980683675963231	0.356	0.083	1.4683323926881E-10	Proliferating T/NK	COMT
8.87644802620992E-15	2.10232664110805	0.211	0.035	1.48751516023226E-10	Proliferating T/NK	ACTN1
9.16244953754883E-15	0.758334353862171	0.611	0.193	1.53544329350243E-10	Proliferating T/NK	RAB1B
9.24346037666934E-15	1.97173312373328	0.156	0.019	1.54901908992225E-10	Proliferating T/NK	NDUFAF5
9.91531117183498E-15	0.353192575286131	0.578	0.174	1.66160784617611E-10	Proliferating T/NK	SRI
1.02429726656551E-14	0.412943643267302	0.267	0.051	1.71651735931048E-10	Proliferating T/NK	DTD1
1.02623003614834E-14	0.810612385983496	0.578	0.184	1.71975629457739E-10	Proliferating T/NK	MTDH
1.03459894377482E-14	0.751149425740564	0.3	0.063	1.73378090997784E-10	Proliferating T/NK	CDK11B
1.03539008050649E-14	0.463834668867416	0.378	0.093	1.73510669691277E-10	Proliferating T/NK	CGAS
1.07611149903115E-14	0.34847547254102	0.4	0.099	1.80334765007641E-10	Proliferating T/NK	ZADH2
1.08623304384589E-14	1.13577733620628	0.3	0.063	1.82030933487695E-10	Proliferating T/NK	SLC35F6
1.0916773781859E-14	0.815678444737158	0.389	0.1	1.82943295036393E-10	Proliferating T/NK	AP2A1
1.11523607821288E-14	0.482807751300352	0.511	0.15	1.86891261986915E-10	Proliferating T/NK	HDAC1
1.12138719185886E-14	1.18576407543048	0.356	0.086	1.87922065611707E-10	Proliferating T/NK	PGRMC2
1.13290808093915E-14	0.417568730773364	0.489	0.137	1.89852736203784E-10	Proliferating T/NK	KBTBD2
1.18331177287118E-14	0.668293793838931	0.311	0.068	1.98299386897753E-10	Proliferating T/NK	ADK
1.20521522317088E-14	0.685072697829877	0.356	0.085	2.01969967098976E-10	Proliferating T/NK	SSR4
1.20914289567608E-14	0.443348860613964	0.822	0.328	2.02628166457398E-10	Proliferating T/NK	HNRNPA0
1.24986834401904E-14	0.505934004035301	0.489	0.142	2.09452937090711E-10	Proliferating T/NK	HCFC1
1.2723302269231E-14	0.692958245630616	0.467	0.132	2.13217099427772E-10	Proliferating T/NK	TXLNA
1.28553637471222E-14	0.48294446415444	0.322	0.071	2.15430185674273E-10	Proliferating T/NK	GNPAT
1.2901262724749E-14	0.393335582643429	0.567	0.178	2.16199360741344E-10	Proliferating T/NK	SRSF2
1.29467203324695E-14	0.707123945610628	0.344	0.081	2.16961139331524E-10	Proliferating T/NK	THOC2
1.31528284439947E-14	0.386452870135093	0.611	0.2	2.20415099064463E-10	Proliferating T/NK	DHX15
1.37871594315946E-14	2.72298265537581	0.122	0.012	2.31045217754662E-10	Proliferating T/NK	PLOD1
1.47108701506019E-14	1.88391808196859	0.144	0.017	2.46524761983786E-10	Proliferating T/NK	ALG10
1.55126405877974E-14	0.870682746371316	0.278	0.056	2.59960830970309E-10	Proliferating T/NK	ACP1
1.55658365064991E-14	1.78547684280998	0.167	0.022	2.60852288175912E-10	Proliferating T/NK	VNN 1.00
1.61300821892169E-14	2.06789274482272	0.133	0.014	2.70307917326897E-10	Proliferating T/NK	ATP9A
1.61940110177819E-14	1.44370303060234	0.378	0.098	2.71379236635988E-10	Proliferating T/NK	ADIPOR1
1.62183011648039E-14	0.425544147662134	0.556	0.167	2.71786290919783E-10	Proliferating T/NK	KIF2A
1.63446959436718E-14	1.08177014182859	0.3	0.064	2.73904414624052E-10	Proliferating T/NK	SPCS2
1.63541243573719E-14	1.73772181509308	0.211	0.035	2.74062415980839E-10	Proliferating T/NK	SNRNP25
1.66844620892941E-14	1.5840772419151	0.222	0.038	2.7959821569239E-10	Proliferating T/NK	ZNF7
1.67432515743021E-14	0.722982952884986	0.3	0.064	2.80583409882154E-10	Proliferating T/NK	CCT4
1.75367313797321E-14	1.85211771127851	0.133	0.014	2.93880544461551E-10	Proliferating T/NK	NADK2
1.75746316772465E-14	0.574635967060994	0.933	0.474	2.94515677647297E-10	Proliferating T/NK	ALB
1.81913091100963E-14	0.566198797185513	0.556	0.169	3.04849958066993E-10	Proliferating T/NK	ADRM1
1.83799353543918E-14	0.457618955172866	0.489	0.138	3.08010956668897E-10	Proliferating T/NK	CDKAL1
1.84393894381041E-14	0.38881119564656	0.411	0.105	3.09007288203748E-10	Proliferating T/NK	METTL23
1.90774747807196E-14	0.717814291753477	0.478	0.136	3.197003223753E-10	Proliferating T/NK	SLC25A39
1.91138803116837E-14	0.843614217052569	0.389	0.096	3.20310406263195E-10	Proliferating T/NK	NDUFB11
1.97381582821521E-14	1.82827695839026	0.133	0.014	3.30772056492305E-10	Proliferating T/NK	NTAN1
2.00015563883284E-14	1.54392162935255	0.2	0.031	3.35186081955607E-10	Proliferating T/NK	SGCB
2.06229092744341E-14	0.728814491881661	0.189	0.028	3.45598713620967E-10	Proliferating T/NK	PSMA5
2.09261252751682E-14	0.472627307998466	0.567	0.172	3.50680007361269E-10	Proliferating T/NK	APOBEC3C
2.12337928844184E-14	0.326236572832191	0.233	0.041	3.55835901157084E-10	Proliferating T/NK	PPM1D
2.14935593761998E-14	1.09488824236361	0.311	0.07	3.60189068026356E-10	Proliferating T/NK	NUDCD2
2.19904323911834E-14	0.336230667311328	0.289	0.061	3.68515666011452E-10	Proliferating T/NK	MYADM
2.23837104182048E-14	0.73254511268446	0.378	0.094	3.75106219188276E-10	Proliferating T/NK	PHACTR4
2.24889716169746E-14	0.992342481100494	0.367	0.091	3.7687018635726E-10	Proliferating T/NK	TMX1
2.27488822480157E-14	0.646868429204254	0.378	0.094	3.81225768712247E-10	Proliferating T/NK	SNRNP40
2.29864823197564E-14	0.991653728372996	0.322	0.072	3.85207470714478E-10	Proliferating T/NK	SUCLG1
2.32008278275169E-14	0.962493145976657	0.333	0.076	3.88799472733528E-10	Proliferating T/NK	GRHPR
2.34098606124161E-14	2.32618326324985	0.178	0.026	3.92302444142868E-10	Proliferating T/NK	CETN2
2.36402088017961E-14	1.10104284888581	0.3	0.065	3.96162619100498E-10	Proliferating T/NK	DDX50
2.39372838103585E-14	0.69984738924192	0.533	0.158	4.01141002093988E-10	Proliferating T/NK	NDUFS2
2.3990164899269E-14	0.532842234739244	0.544	0.165	4.0202718338195E-10	Proliferating T/NK	UHMK1
2.46764282769956E-14	0.919202924085117	0.178	0.025	4.13527585065893E-10	Proliferating T/NK	ZNF653
2.4794948642204E-14	0.468730282114525	0.622	0.208	4.15513749346055E-10	Proliferating T/NK	DYNLL2
2.48249536852745E-14	0.255059092046312	0.378	0.094	4.16016573857831E-10	Proliferating T/NK	PSIP1

2.62465478503265E-14	0.480430896809269	0.756	0.275	4.39839648875771E-10	Proliferating T/NK	SUGP2
2.7745268154841E-14	0.738276861048242	0.278	0.057	4.64955203738826E-10	Proliferating T/NK	TMEM128
2.84108140445872E-14	1.33735041094866	0.2	0.032	4.76108421759192E-10	Proliferating T/NK	TMEM38B
2.87233569751803E-14	0.418586250908653	0.522	0.158	4.81346016190072E-10	Proliferating T/NK	SRSF3
3.01745860468076E-14	0.913338406473348	0.356	0.086	5.05665712972402E-10	Proliferating T/NK	APLP2
3.05958784590501E-14	2.07349657340661	0.178	0.026	5.12725731216762E-10	Proliferating T/NK	PAXIP1
3.16312743322898E-14	0.285305062337355	0.267	0.053	5.30076895260512E-10	Proliferating T/NK	OTULINL
3.24483794174769E-14	1.41170902226377	0.211	0.036	5.43769942278077E-10	Proliferating T/NK	SMIM14
3.26550625579046E-14	0.582994586625523	0.622	0.218	5.47233538345366E-10	Proliferating T/NK	DCAF7
3.28357662873834E-14	0.724225740974762	0.289	0.062	5.50261771443971E-10	Proliferating T/NK	DKC1
3.36343315801111E-14	2.09011661678055	0.111	0.01	5.63644128619502E-10	Proliferating T/NK	EPB41L2
3.37218964072224E-14	0.673165643507335	0.244	0.046	5.65111539992233E-10	Proliferating T/NK	PATL1
3.37930457004772E-14	1.20866580333409	0.244	0.046	5.66303859848597E-10	Proliferating T/NK	MED8
3.39035129460004E-14	2.47407422791749	0.111	0.01	5.68155069949074E-10	Proliferating T/NK	CENPQ
3.39291113808283E-14	0.55064242692342	0.322	0.073	5.6858404851992E-10	Proliferating T/NK	SGTA
3.42044609008913E-14	1.68255529235656	0.233	0.044	5.73198355777136E-10	Proliferating T/NK	CD38
3.46324344651438E-14	0.630327798226583	0.533	0.161	5.80370336766879E-10	Proliferating T/NK	CLIC1
3.52316817097795E-14	0.903131738138637	0.478	0.137	5.90412522092485E-10	Proliferating T/NK	CALR
3.63009462712082E-14	1.63516588452469	0.167	0.023	6.08331257612906E-10	Proliferating T/NK	SLC2A8
3.67145801542865E-14	1.44481004222646	0.111	0.01	6.15262934225533E-10	Proliferating T/NK	ANKRD13B
3.86755817051559E-14	0.349116073606028	0.622	0.203	6.48125398215002E-10	Proliferating T/NK	DEPRECATED-ENSG00000124831
3.93990181701382E-14	1.07995313515886	0.322	0.074	6.60248746495175E-10	Proliferating T/NK	ACAT1
4.26501024188528E-14	0.676924754639235	0.756	0.288	7.14730416335136E-10	Proliferating T/NK	PPP1R18
4.3879576804714E-14	0.480982129227839	0.478	0.139	7.35333948093398E-10	Proliferating T/NK	TRIM26
4.59226507108073E-14	1.86419879935589	0.267	0.055	7.69571780611709E-10	Proliferating T/NK	SIGMAR1
4.68915760739789E-14	0.993108659800355	0.344	0.084	7.85809031847739E-10	Proliferating T/NK	TIPRL
4.74404221281223E-14	0.852712958047949	0.322	0.074	7.95006594023073E-10	Proliferating T/NK	PDS5B
4.76811971697237E-14	1.22984540892634	0.378	0.096	7.9904150217023E-10	Proliferating T/NK	COX16
4.91987597697689E-14	0.663558855752447	0.4	0.105	8.24472816221787E-10	Proliferating T/NK	RBM15B
4.94599738482944E-14	0.469718881509359	0.456	0.128	8.28850241749717E-10	Proliferating T/NK	SMARCD2
5.01208931867274E-14	0.355694061020859	0.322	0.073	8.39925928023178E-10	Proliferating T/NK	RB1
5.03882030068841E-14	0.841495258172673	0.222	0.039	8.44405505989364E-10	Proliferating T/NK	PROSER3
5.13540521244451E-14	1.20386482599106	0.356	0.089	8.60591205501451E-10	Proliferating T/NK	FIBP
5.18497910605888E-14	1.01607978371794	0.211	0.036	8.68898798593347E-10	Proliferating T/NK	ASH2L
5.2287665896165E-14	0.270654000374608	0.578	0.181	8.76236705087932E-10	Proliferating T/NK	SMARCA4
5.46361227332788E-14	1.35424373277803	0.233	0.043	9.15592144764286E-10	Proliferating T/NK	INSIG1
5.58137303620046E-14	0.361441604322188	0.644	0.225	9.35326493406472E-10	Proliferating T/NK	EMP3
5.6458143666755E-14	1.8584149709313	0.222	0.04	9.46125571567481E-10	Proliferating T/NK	NAXE
5.6532718495739E-14	2.11415733780195	0.156	0.02	9.47375296551593E-10	Proliferating T/NK	ACOT13
5.65506735343104E-14	0.845123336777409	0.4	0.108	9.47676187087974E-10	Proliferating T/NK	GYPC
5.6569124289413E-14	1.67142902847552	0.133	0.015	9.47985384841983E-10	Proliferating T/NK	AIMP2
5.68292759503079E-14	0.366917430579665	0.356	0.086	9.5234500637526E-10	Proliferating T/NK	MOB4
5.75738058083727E-14	0.440102539920268	0.433	0.12	9.64821837736709E-10	Proliferating T/NK	SNRPA
5.7905915961577E-14	1.81250525784056	0.189	0.03	9.70387339684106E-10	Proliferating T/NK	RAB33B
5.80336019908866E-14	1.20021286642629	0.3	0.067	9.72527102163277E-10	Proliferating T/NK	DEPRECATED-ENSG00000244509
5.92992342883455E-14	1.00614599922267	0.133	0.015	9.93736568204093E-10	Proliferating T/NK	DEPRECATED-ENSG00000104897
5.94413141290941E-14	0.731578640101286	0.967	0.506	9.9611754217536E-10	Proliferating T/NK	CFL1
6.14563022105186E-14	0.928554663649208	0.333	0.078	1.02988471244387E-09	Proliferating T/NK	ESD
6.53229414400792E-14	0.801306082174762	0.278	0.058	1.09468185265285E-09	Proliferating T/NK	EIPR1
6.82717668459331E-14	0.480231150053425	0.367	0.092	1.14409826880415E-09	Proliferating T/NK	MUS81
6.86863787707873E-14	1.35989069906128	0.267	0.055	1.15104633544085E-09	Proliferating T/NK	DEPRECATED-ENSG00000167797
6.87568462843032E-14	2.11883457707277	0.1	0.008	1.15222723003235E-09	Proliferating T/NK	DCLRE1B
7.24361636654328E-14	1.39057247534036	0.156	0.02	1.21388523070532E-09	Proliferating T/NK	SAPCD1
7.31617706390055E-14	0.279172028675349	0.422	0.114	1.22604495236845E-09	Proliferating T/NK	KHDC4
7.32466183837769E-14	0.521003358685611	0.9	0.407	1.22746683087533E-09	Proliferating T/NK	SUN2
7.55868200406395E-14	0.460093636560892	0.344	0.083	1.26668393024104E-09	Proliferating T/NK	TNPO2
7.56855538833803E-14	0.806105523085973	0.411	0.112	1.26833851197769E-09	Proliferating T/NK	SMC6
7.59539263657373E-14	1.14250683094731	0.3	0.068	1.27283589803703E-09	Proliferating T/NK	R3HCC1L
8.01044806788028E-14	0.303150827329125	0.411	0.112	1.34239088721538E-09	Proliferating T/NK	CMC2
8.51710424999597E-14	0.941123142908313	0.378	0.099	1.42729633021432E-09	Proliferating T/NK	ARPC5
8.58803754982727E-14	0.577534943776232	0.256	0.051	1.43918333260005E-09	Proliferating T/NK	SFMBT1
8.87045188625395E-14	0.257488667946243	0.511	0.151	1.48651032709844E-09	Proliferating T/NK	PURB
8.89296603359737E-14	0.879860975567578	0.211	0.036	1.48966822931025E-09	Proliferating T/NK	PPP1R37
9.07561585676882E-14	0.645510167654438	0.478	0.142	1.52089170527732E-09	Proliferating T/NK	IMMT
9.10845864818288E-14	0.670197168050372	0.356	0.089	1.52639550026249E-09	Proliferating T/NK	CCDC47
9.25736593807703E-14	2.23290432950319	0.144	0.018	1.55134938390295E-09	Proliferating T/NK	HSD17B8

9.42433379477802E-14	0.901947474138266	0.378	0.097	1.5793298573289E-09	Proliferating T/NK	TMBIM6
9.51520608733345E-14	1.35275682659386	0.167	0.023	1.59455823611534E-09	Proliferating T/NK	PRIMPOL
9.81971981863283E-14	3.09110409055712	0.122	0.013	1.64558864720649E-09	Proliferating T/NK	GPC1
1.00245615941756E-13	0.908042235950609	0.356	0.088	1.67991603195195E-09	Proliferating T/NK	EHMT2
1.01696744141072E-13	0.716610313752856	0.522	0.16	1.70423403831608E-09	Proliferating T/NK	SERF2
1.06379942627976E-13	0.742424079204466	0.589	0.19	1.78271507855962E-09	Proliferating T/NK	ST6GAL1
1.12488901146134E-13	1.33001719991729	0.2	0.033	1.88508900540691E-09	Proliferating T/NK	PAAF1
1.13105772587211E-13	1.0666540713615	0.211	0.036	1.89542653701648E-09	Proliferating T/NK	SLC27A4
1.17505646452767E-13	1.30172278789566	0.256	0.051	1.96915962325546E-09	Proliferating T/NK	NAA20
1.17752601118773E-13	1.46297309501928	0.289	0.064	1.9732980895484E-09	Proliferating T/NK	TXNDC17
1.17784183021731E-13	1.28545263313743	0.322	0.077	1.97382733907817E-09	Proliferating T/NK	DEPRECATED-ENSG00000163463
1.18073794909124E-13	0.25760238440815	0.444	0.126	1.97868065508711E-09	Proliferating T/NK	LTB4R
1.23542816060422E-13	0.591133506758651	0.8	0.319	2.07033051154056E-09	Proliferating T/NK	CBFB
1.23738072584109E-13	1.08284580727332	0.356	0.089	2.0736026203645E-09	Proliferating T/NK	CLTA
1.25071718413635E-13	0.601758538645264	0.478	0.14	2.0959518571757E-09	Proliferating T/NK	TPM4
1.26084477387482E-13	0.884509343436354	0.244	0.047	2.11292367205942E-09	Proliferating T/NK	HPS4
1.27061962949217E-13	0.421927390700308	0.467	0.132	2.12930437510299E-09	Proliferating T/NK	DNAJC1
1.276565505178E-13	0.78687022323688	0.589	0.189	2.13926847357729E-09	Proliferating T/NK	HINT1
1.29264953186789E-13	1.17294265486544	0.189	0.03	2.16622208550422E-09	Proliferating T/NK	BLVRA
1.34618888279973E-13	1.05334500487049	0.222	0.041	2.25594332979579E-09	Proliferating T/NK	NFYB
1.37169624959858E-13	1.58879839128012	0.189	0.03	2.2986885750773E-09	Proliferating T/NK	NTPCR
1.38426675315777E-13	1.37273090854184	0.4	0.112	2.3197542249418E-09	Proliferating T/NK	TM7SF3
1.39155110865922E-13	1.12620527813619	0.211	0.037	2.33196134789113E-09	Proliferating T/NK	TMEM14C
1.43534411519551E-13	1.73220946284604	0.167	0.024	2.40534966824464E-09	Proliferating T/NK	LSR
1.4373708509852E-13	0.284251891500383	0.511	0.155	2.408746072081E-09	Proliferating T/NK	CALM1
1.47489486654338E-13	0.515097972760319	0.556	0.179	2.4716288173534E-09	Proliferating T/NK	NCL
1.47720913900436E-13	1.01576218682652	0.189	0.03	2.47550707514351E-09	Proliferating T/NK	DUS3L
1.52798612391419E-13	0.974854170466401	0.167	0.024	2.5605991464554E-09	Proliferating T/NK	LYN
1.56769187358691E-13	1.46544354352638	0.222	0.041	2.62713804175694E-09	Proliferating T/NK	QSOX1
1.56997853759018E-13	0.406373394233154	0.478	0.14	2.63097003329363E-09	Proliferating T/NK	NOTCH1
1.58095415448419E-13	0.300202373083953	0.556	0.172	2.6493629720846E-09	Proliferating T/NK	ACTR1B
1.58689765492465E-13	1.25527989505698	0.211	0.037	2.65932309012273E-09	Proliferating T/NK	GON7
1.64440071767312E-13	1.05514224643596	0.111	0.01	2.75568672267662E-09	Proliferating T/NK	DEPRECATED-ENSG00000104442
1.64817757533263E-13	0.545511242955069	0.322	0.076	2.76201598074242E-09	Proliferating T/NK	MTOR
1.68340292262154E-13	0.785414335307092	0.333	0.079	2.82104661772919E-09	Proliferating T/NK	GTF3C6
1.75515962726619E-13	1.42272046845655	0.233	0.044	2.94129650337268E-09	Proliferating T/NK	PSENEN
1.76229456482225E-13	0.446906157517672	0.367	0.093	2.95325323172913E-09	Proliferating T/NK	FAF1
1.78849426823062E-13	1.59999498311629	0.167	0.024	2.99715869470088E-09	Proliferating T/NK	DEPRECATED-ENSG00000148834
1.79345805107637E-13	1.68436231293314	0.2	0.034	3.00547700199378E-09	Proliferating T/NK	PRDX3
1.82223409034936E-13	0.388539512090438	0.5	0.148	3.05369988860745E-09	Proliferating T/NK	CNOT9
1.82256611145172E-13	0.508375325011607	0.378	0.099	3.05425628957079E-09	Proliferating T/NK	API5
1.86352262687186E-13	0.377122617451904	0.789	0.299	3.12289121811186E-09	Proliferating T/NK	MBD2
1.9223002647217E-13	0.367825093727481	0.222	0.041	3.22139078362063E-09	Proliferating T/NK	ING5
1.9246616259392E-13	0.896499612604118	0.456	0.131	3.2253479527489E-09	Proliferating T/NK	DNAJB11
1.93088312939304E-13	0.61829677125033	0.267	0.055	3.23577394823686E-09	Proliferating T/NK	EMC1
1.99737785496957E-13	0.612789928121698	0.278	0.059	3.347205809358E-09	Proliferating T/NK	ELOB
2.02429495038594E-13	0.259092006635107	0.444	0.124	3.39231347785675E-09	Proliferating T/NK	PPP1R35
2.02554777483842E-13	0.708461329197283	0.633	0.216	3.39441296107423E-09	Proliferating T/NK	TRAM1
2.12259195640145E-13	0.380241457374697	0.278	0.059	3.55703960053755E-09	Proliferating T/NK	PPP1R8
2.14852599382834E-13	0.97421549445789	0.322	0.076	3.60049986045754E-09	Proliferating T/NK	LSM 6.00
2.15934201955443E-13	0.97806798814253	0.489	0.15	3.61862535636932E-09	Proliferating T/NK	GSR
2.20324568810047E-13	0.811277409866391	0.289	0.064	3.69219912411876E-09	Proliferating T/NK	TRAP1
2.42460455225346E-13	0.426525710795105	0.378	0.097	4.06315230866634E-09	Proliferating T/NK	CASP2
2.46816429276628E-13	1.46736887214028	0.256	0.052	4.13614972181773E-09	Proliferating T/NK	SLC25A5
2.52319116227656E-13	0.91378805007442	0.433	0.121	4.22836374974307E-09	Proliferating T/NK	NDUFB10
2.52958409128631E-13	1.14046314014258	0.222	0.041	4.2390770201776E-09	Proliferating T/NK	FARSB
2.56213057471629E-13	2.15678727475495	0.144	0.018	4.29361841710956E-09	Proliferating T/NK	TMEM97
2.68235063667387E-13	0.325536871905571	0.344	0.085	4.49508319693807E-09	Proliferating T/NK	SSU72
2.68532737453897E-13	1.0480687129483	0.4	0.11	4.5000716142524E-09	Proliferating T/NK	SPATS2L
2.79576627942676E-13	1.06062075859721	0.233	0.045	4.68514513106336E-09	Proliferating T/NK	R3HDM1
2.84541906521313E-13	0.675839561490678	0.289	0.064	4.76835326948416E-09	Proliferating T/NK	GABPB2
2.94789761770936E-13	1.58106500453734	0.233	0.046	4.94008682775735E-09	Proliferating T/NK	IFITM3
2.95181119618177E-13	1.5375923428557	0.189	0.031	4.94664520256141E-09	Proliferating T/NK	NET1
3.00760496695696E-13	1.06161652749489	0.256	0.053	5.04014440362648E-09	Proliferating T/NK	DEPRECATED-ENSG00000198074
3.07733980522209E-13	0.339476280096412	0.456	0.133	5.15700604559117E-09	Proliferating T/NK	NUP153
3.13830972530374E-13	0.646830287668608	0.278	0.06	5.25917943766401E-09	Proliferating T/NK	INTS6L

3.16539131950362E-13	0.598154747412121	0.344	0.086	5.30456277322416E-09	Proliferating T/NK	ELAC2
3.17867947882071E-13	0.474278385245953	0.544	0.172	5.32683107060775E-09	Proliferating T/NK	SUB1
3.17998049221918E-13	1.92576958041032	0.1	0.009	5.3290113088609E-09	Proliferating T/NK	PKN3
3.18173086634265E-13	0.955998321319828	0.189	0.031	5.33194458581702E-09	Proliferating T/NK	MED26
3.23557518081934E-13	1.01560335777523	0.378	0.099	5.42217688801704E-09	Proliferating T/NK	PSMB4
3.31219730079227E-13	0.58858617281206	0.4	0.109	5.55058023666769E-09	Proliferating T/NK	LEMD2
3.35984358547224E-13	1.04700929170564	0.267	0.057	5.63042588053438E-09	Proliferating T/NK	LRP8
3.4778934769212E-13	2.26294342508056	0.1	0.009	5.82825388862455E-09	Proliferating T/NK	DEPRECATED-ENSG00000165948
3.88061025904995E-13	1.63965075184804	0.2	0.035	6.50312667211591E-09	Proliferating T/NK	PIGU
3.90815437723294E-13	2.09454285719779	0.133	0.016	6.54928510536696E-09	Proliferating T/NK	NTHL1
3.93627126175722E-13	0.869552505263229	0.244	0.049	6.59640338045276E-09	Proliferating T/NK	NUDT2
3.9746935383897E-13	0.747168465370077	0.4	0.109	6.66079143163345E-09	Proliferating T/NK	COPS6
4.05977887254926E-13	0.821462043243607	0.3	0.068	6.80337743461805E-09	Proliferating T/NK	COMMD4
4.11560245327047E-13	0.918426267364782	0.278	0.062	6.89692659119066E-09	Proliferating T/NK	DEPRECATED-ENSG00000078668
4.19624726706242E-13	1.59121098021771	0.189	0.031	7.0320711701432E-09	Proliferating T/NK	UCK2
4.20513836080144E-13	0.785381091858327	0.433	0.121	7.04697086503106E-09	Proliferating T/NK	CHMP2A
4.24137453020794E-13	0.637123979019816	0.611	0.218	7.10769543772247E-09	Proliferating T/NK	SLBP
4.29987355017233E-13	0.280188440575042	0.422	0.119	7.20572809537879E-09	Proliferating T/NK	LPCAT4
4.31278563259237E-13	0.573334319461046	0.289	0.065	7.2273661630983E-09	Proliferating T/NK	CAPRIN1
4.32691625667662E-13	1.16855205289714	0.267	0.057	7.25104626293869E-09	Proliferating T/NK	NDUFA7
4.34041429691483E-13	1.23636138881373	0.311	0.075	7.27366627876987E-09	Proliferating T/NK	NHP2
4.36156934876009E-13	0.554925880075651	0.333	0.081	7.30911791465217E-09	Proliferating T/NK	ADNP
4.57736690772924E-13	0.842742833118084	0.344	0.087	7.67075146397266E-09	Proliferating T/NK	SMIM12
4.58966837357594E-13	2.25000390137893	0.144	0.019	7.69136626043856E-09	Proliferating T/NK	STK3
4.89410656826007E-13	2.45008242704324	0.111	0.011	8.20154378709022E-09	Proliferating T/NK	NLRX1
4.92157052880521E-13	2.09927478357289	0.122	0.014	8.24756789217177E-09	Proliferating T/NK	TRIB3
4.99215391616694E-13	0.64765746091511	0.878	0.418	8.36585153271256E-09	Proliferating T/NK	SPN
5.02122476046222E-13	1.18381320680858	0.233	0.046	8.41456845358259E-09	Proliferating T/NK	VPS25
5.05741633431554E-13	1.4438316870897	0.178	0.028	8.47521829304597E-09	Proliferating T/NK	RHOB
5.0599625648951E-13	0.629265161402321	0.578	0.197	8.47948526625122E-09	Proliferating T/NK	PRKDC
5.12395826341915E-13	0.521309851485452	0.467	0.138	8.58672925783781E-09	Proliferating T/NK	DGKZ
5.22542197841379E-13	1.04720775671473	0.189	0.031	8.75676215142582E-09	Proliferating T/NK	KIAA1328
5.25034745450951E-13	0.497845536596964	0.189	0.031	8.79853226426704E-09	Proliferating T/NK	TIFA
5.27765239362695E-13	0.517904821328101	0.611	0.208	8.84428988124004E-09	Proliferating T/NK	ATF6B
5.33105799520596E-13	0.710573114754865	0.5	0.151	8.93378698836615E-09	Proliferating T/NK	COPB2
5.4839102359407E-13	2.1268848425127	0.122	0.014	9.18993677338943E-09	Proliferating T/NK	MACROD1
5.50140543557334E-13	1.80565857480885	0.189	0.032	9.2192552289338E-09	Proliferating T/NK	CARM1
5.50312262220116E-13	1.91679629509334	0.111	0.011	9.2221328902847E-09	Proliferating T/NK	SLCO4A1
5.52726555301644E-13	0.396840230848313	0.444	0.126	9.26259161374495E-09	Proliferating T/NK	INTS1
5.5858228664702E-13	0.692719381533107	0.256	0.053	9.36072195963076E-09	Proliferating T/NK	KLHL15
5.67875939510014E-13	0.251826568242362	0.356	0.09	9.51646499430881E-09	Proliferating T/NK	TBC1D5
5.84827312502421E-13	1.20462015887441	0.2	0.035	9.80053610291557E-09	Proliferating T/NK	HACE1
5.87483200841433E-13	1.26350931345532	0.2	0.035	9.84504347970073E-09	Proliferating T/NK	C2CD3
6.0639800740757E-13	0.950308537083944	0.311	0.075	1.01620178081361E-08	Proliferating T/NK	LRRC8A
6.14133633948328E-13	0.950385549653974	0.111	0.011	1.02916514377061E-08	Proliferating T/NK	MIER2
6.17627968908703E-13	0.67199798255581	0.744	0.284	1.0350209502972E-08	Proliferating T/NK	BST2
6.18430783434718E-13	0.7037460666219	0.311	0.075	1.03636663068799E-08	Proliferating T/NK	RUVBL1
6.23997410252511E-13	0.733675583846286	0.278	0.061	1.04569486010116E-08	Proliferating T/NK	CCT5
6.36569470037967E-13	0.324201161364504	0.9	0.415	1.06676311788962E-08	Proliferating T/NK	HNRNPA2B1
6.39062815061915E-13	0.730523676856965	0.467	0.141	1.07094146548076E-08	Proliferating T/NK	SLC1A5
6.48996104935809E-13	0.26669369921093	0.4	0.109	1.08758767265143E-08	Proliferating T/NK	EPRS
6.54902560919647E-13	1.20131764182129	0.2	0.035	1.09748571158915E-08	Proliferating T/NK	VAMP7
6.66902364009416E-13	0.5617910024212	0.267	0.058	1.11759498160698E-08	Proliferating T/NK	AARS2
6.67207772222963E-13	1.35307386442107	0.156	0.022	1.11810678469124E-08	Proliferating T/NK	NFIC
6.76697481150232E-13	1.34433453405243	0.211	0.039	1.13400963891156E-08	Proliferating T/NK	HEXB
6.8354416587519E-13	0.446269736316444	0.311	0.075	1.14548331317364E-08	Proliferating T/NK	TGS1
6.83977067268742E-13	1.55925615480894	0.144	0.019	1.14620876932896E-08	Proliferating T/NK	SKP2
6.95470790678954E-13	0.466833351826116	0.333	0.083	1.16546995101979E-08	Proliferating T/NK	NDUFAF8
6.96473915466669E-13	0.704413971890262	0.667	0.246	1.16715098753904E-08	Proliferating T/NK	UBA52
7.22627065777982E-13	0.609442751291186	0.267	0.058	1.21097843683074E-08	Proliferating T/NK	BICD2
7.35570101170707E-13	1.12602535998218	0.278	0.063	1.23266837554187E-08	Proliferating T/NK	MUC3A
7.4566642510014E-13	0.826414012449492	0.344	0.087	1.24958779518281E-08	Proliferating T/NK	MFF
7.5193958903446E-13	0.408862515770295	0.344	0.088	1.26010036330395E-08	Proliferating T/NK	PAF1
8.05135121544329E-13	1.02394760318569	0.378	0.102	1.34924543668399E-08	Proliferating T/NK	PEBP1
8.1706792139485E-13	0.973369093082884	0.244	0.05	1.36924242267349E-08	Proliferating T/NK	TMEM134
8.23074208142535E-13	0.413924681498257	0.578	0.195	1.37930775800526E-08	Proliferating T/NK	NPLOC4

8.30021390503456E-13	0.69131729422186	0.3	0.07	1.39094984620569E-08	Proliferating T/NK	PYURF
8.51164604708772E-13	0.35483280960065	0.433	0.124	1.42638164457096E-08	Proliferating T/NK	SEC22B
8.66000958155901E-13	0.769044373562374	0.189	0.032	1.45124440567766E-08	Proliferating T/NK	B3GNTL1
8.74162489742579E-13	0.773844713883905	0.267	0.059	1.46492150031061E-08	Proliferating T/NK	NCBP1
8.77909282143109E-13	0.919958148854608	0.489	0.157	1.47120037501542E-08	Proliferating T/NK	NAPA
8.8266457232433E-13	1.07948338794704	0.233	0.046	1.47916929030111E-08	Proliferating T/NK	PGAM5
9.11512715697063E-13	0.262938176671647	0.567	0.187	1.52751300896514E-08	Proliferating T/NK	UBE2R2
9.14665415982905E-13	1.36248656386366	0.278	0.063	1.53279630410415E-08	Proliferating T/NK	DBI
9.29753722084189E-13	0.669011692001269	0.233	0.046	1.55808128746868E-08	Proliferating T/NK	ENDOD1
9.3826555727953E-13	0.945296805080833	0.3	0.071	1.57234542088904E-08	Proliferating T/NK	LDLR
9.44750141327318E-13	0.588018135167364	0.589	0.201	1.58321228683632E-08	Proliferating T/NK	TWF2
9.57148312993609E-13	0.439983636551993	0.367	0.097	1.60398914291469E-08	Proliferating T/NK	C2orf68
9.62815612309267E-13	0.268998479675639	0.422	0.12	1.61348640310787E-08	Proliferating T/NK	ZNF83
9.95331885487488E-13	1.0853627804046	0.233	0.047	1.66797717369993E-08	Proliferating T/NK	NUP54
1.05037935805607E-12	1.43832984975073	0.222	0.043	1.76022572823036E-08	Proliferating T/NK	RASSF7
1.0713280022997E-12	0.496863457665784	0.256	0.054	1.79533146625384E-08	Proliferating T/NK	CTDSPL2
1.07561327418887E-12	0.797412411957289	0.289	0.067	1.80251272488572E-08	Proliferating T/NK	PPP5C
1.09509610486769E-12	0.459567594077419	0.556	0.183	1.83516205253728E-08	Proliferating T/NK	THRAP3
1.10153190491465E-12	0.9684820525106	0.278	0.063	1.84594716625597E-08	Proliferating T/NK	UBR7
1.11119897617493E-12	0.450345519869401	0.222	0.043	1.86214724427394E-08	Proliferating T/NK	PSMA3
1.1211020909073E-12	1.16402790511619	0.311	0.076	1.87874288394245E-08	Proliferating T/NK	PFDN6
1.12321504067650E-12	0.668994525245281	0.278	0.062	1.88228376516567E-08	Proliferating T/NK	CNOT10
1.141454089969E-12	1.3308435490995	0.211	0.039	1.91284876397004E-08	Proliferating T/NK	ALG3
1.15391615343316E-12	1.45169106839593	0.244	0.052	1.93373268992329E-08	Proliferating T/NK	POLD2
1.1692409153737E-12	0.434280471565422	0.544	0.178	1.95941392598324E-08	Proliferating T/NK	FLOT2
1.18199716016338E-12	1.44007567581074	0.278	0.065	1.98079084100179E-08	Proliferating T/NK	SRM
1.26067231337753E-12	0.884272524536327	0.311	0.076	2.11263466275806E-08	Proliferating T/NK	MYO19
1.26290122119914E-12	0.324293570584415	0.5	0.154	2.11636986648553E-08	Proliferating T/NK	SZRD1
1.2971793403063E-12	1.12144900504348	0.233	0.047	2.17381313848529E-08	Proliferating T/NK	SRGAP2B
1.301593299662E-12	0.63985798183187	0.422	0.121	2.18121005157358E-08	Proliferating T/NK	UGDH
1.33107593264753E-12	1.29832343039474	0.2	0.036	2.23061704793074E-08	Proliferating T/NK	ADCK5
1.38282716442735E-12	2.12993519646413	0.122	0.014	2.31734176214736E-08	Proliferating T/NK	NAT14
1.38522313715367E-12	0.371621578180311	0.489	0.149	2.32135693324211E-08	Proliferating T/NK	LRRFIP1
1.39629433482447E-12	0.49213951793926	0.389	0.11	2.33991004629885E-08	Proliferating T/NK	REV1
1.42520718036975E-12	1.00525733354465	0.267	0.06	2.38836219286363E-08	Proliferating T/NK	NUP155
1.43660315185702E-12	0.268825290436617	0.622	0.211	2.407459561882E-08	Proliferating T/NK	GRB2
1.47628798174106E-12	2.43324515422374	0.122	0.014	2.47396339980166E-08	Proliferating T/NK	TUBB2A
1.49170688641625E-12	0.90598790353028	0.289	0.067	2.49980240025635E-08	Proliferating T/NK	COX6B1
1.49209322300573E-12	0.863865963475671	0.422	0.123	2.500449823113E-08	Proliferating T/NK	VKORC1
1.52677684191839E-12	1.11284241976647	0.233	0.047	2.55857263168684E-08	Proliferating T/NK	OGFOD3
1.53267563036968E-12	1.44317082958239	0.167	0.026	2.56845782137351E-08	Proliferating T/NK	NDUFA6
1.59059017419665E-12	1.35011027068396	0.211	0.04	2.66551101391875E-08	Proliferating T/NK	PPIL3
1.63080336924464E-12	0.430427221572587	0.267	0.059	2.73290028618017E-08	Proliferating T/NK	MOB1A
1.63146404428828E-12	0.636928304456904	0.233	0.047	2.7340074454183E-08	Proliferating T/NK	DEPRECATED-ENSG00000176101
1.65442402148912E-12	0.339767671894164	0.344	0.092	2.77248377521147E-08	Proliferating T/NK	SRRT
1.67073167012222E-12	0.969406329159154	0.233	0.047	2.79981213279082E-08	Proliferating T/NK	BRPF1
1.67121356941375E-12	0.608676471936328	0.778	0.292	2.80061969962356E-08	Proliferating T/NK	SLC9A3R1
1.73740286876171E-12	1.10423298076813	0.156	0.023	2.91153972747087E-08	Proliferating T/NK	ZNF736
1.77392711526436E-12	0.918778775952038	0.444	0.135	2.97274705976001E-08	Proliferating T/NK	ITPK1
1.79609657170784E-12	0.337154552699695	0.5	0.163	3.009898634868E-08	Proliferating T/NK	DEPRECATED-ENSG00000135976
1.80558446361054E-12	1.24721093416491	0.111	0.012	3.02579844411855E-08	Proliferating T/NK	MORC4
1.81487937616326E-12	0.554703524644263	0.322	0.08	3.04137485857439E-08	Proliferating T/NK	BUD31
1.86172892128632E-12	0.731608327849034	0.3	0.072	3.11988532629162E-08	Proliferating T/NK	CAP1
1.88026713748339E-12	1.7974031192625	0.167	0.026	3.15095166899467E-08	Proliferating T/NK	KRT8
1.90076212566751E-12	0.672315173322106	0.256	0.055	3.18529717019361E-08	Proliferating T/NK	CEP44
2.03485554933793E-12	1.26299326987213	0.111	0.012	3.4100109295805E-08	Proliferating T/NK	FANCB
2.06934285494888E-12	0.351801160243569	0.389	0.107	3.46780475632334E-08	Proliferating T/NK	DEPRECATED-ENSG00000116489
2.10178041926664E-12	1.52661431900778	0.178	0.03	3.52216362660704E-08	Proliferating T/NK	SHMT2
2.13209493860662E-12	1.11469180324777	0.178	0.03	3.57296469811698E-08	Proliferating T/NK	C19orf47
2.1618527376991E-12	0.504939533665553	0.233	0.047	3.62283281783615E-08	Proliferating T/NK	RAD51D
2.17282451564798E-12	0.589651589384939	0.356	0.094	3.64121932332289E-08	Proliferating T/NK	G6PD
2.17822985233153E-12	0.754467211363857	0.311	0.077	3.65027758653718E-08	Proliferating T/NK	TMEM189
2.18363415946746E-12	0.430857368518235	0.456	0.136	3.65933412443558E-08	Proliferating T/NK	PSMA7
2.20877442690009E-12	0.444961158036779	0.611	0.221	3.70146418459917E-08	Proliferating T/NK	ERO1A
2.21081593901348E-12	0.464138940194253	0.344	0.089	3.70488535059879E-08	Proliferating T/NK	CTDNEP1
2.25724415005303E-12	0.985436879963675	0.333	0.087	3.78268974665886E-08	Proliferating T/NK	CTNNA1

2.2716189036692E-12	2.08986372165576	0.2	0.038	3.80677895876885E-08	Proliferating T/NK	DEPRECATED-ENSG00000134049
2.29004997253741E-12	0.275352241621828	0.289	0.068	3.83766574397819E-08	Proliferating T/NK	FGFR1OP
2.29219961389297E-12	0.329442769420006	0.478	0.145	3.84126811296184E-08	Proliferating T/NK	TNPO3
2.39055453901739E-12	0.579130886871852	0.178	0.03	4.00609129648535E-08	Proliferating T/NK	RP2
2.42212256403251E-12	0.744668386366665	0.222	0.044	4.05899299280568E-08	Proliferating T/NK	NOM1
2.42746388212304E-12	1.12931207792054	0.267	0.061	4.06794397366178E-08	Proliferating T/NK	USP5
2.43023371823622E-12	0.461217206562149	0.378	0.105	4.07258566502026E-08	Proliferating T/NK	NUP160
2.5059968040171E-12	0.679728663690011	0.289	0.068	4.19954944417186E-08	Proliferating T/NK	LDAH
2.6297891788838E-12	1.00613800651909	0.5	0.17	4.40700070597347E-08	Proliferating T/NK	MIF
2.65101711020504E-12	0.609206402619679	0.4	0.116	4.44257447328161E-08	Proliferating T/NK	MED14
2.65432562065208E-12	1.0575846309031	0.178	0.03	4.44811887508876E-08	Proliferating T/NK	MIS12
2.74786984754305E-12	1.11278547992157	0.2	0.037	4.60488029051265E-08	Proliferating T/NK	INIP
2.80823800723146E-12	0.608949946862058	0.344	0.09	4.70604525251848E-08	Proliferating T/NK	AZIN1
2.9834630291721E-12	0.547401460364805	0.278	0.064	4.99968734428666E-08	Proliferating T/NK	UBE2D2
3.12095741434305E-12	0.892052748176584	0.167	0.026	5.23010043495609E-08	Proliferating T/NK	PPIH
3.13659003228217E-12	0.685631188578921	0.233	0.048	5.25629757609846E-08	Proliferating T/NK	CORO1C
3.29769466067861E-12	1.394408009338	0.156	0.023	5.52627671236522E-08	Proliferating T/NK	MDK
3.34201677409909E-12	0.654425385462334	0.422	0.12	5.60055171003526E-08	Proliferating T/NK	ATP5IF1
3.35385811409939E-12	0.520755373224052	0.256	0.057	5.62039542760776E-08	Proliferating T/NK	PSMB6
3.37132102171989E-12	0.264928596965633	0.689	0.258	5.64965976819819E-08	Proliferating T/NK	STK24
3.47638101044264E-12	1.36728518161893	0.189	0.033	5.82571929729978E-08	Proliferating T/NK	P3H1
3.50913106440353E-12	0.745969394537262	0.422	0.124	5.88060183772743E-08	Proliferating T/NK	SLC25A11
3.52611681666989E-12	0.861392891194342	0.4	0.114	5.90906656137541E-08	Proliferating T/NK	HSPA5
3.57145732405545E-12	1.24985469887214	0.189	0.033	5.98504818365212E-08	Proliferating T/NK	PSMD11
3.61387957177E-12	0.585157041520108	0.333	0.087	6.05613938637216E-08	Proliferating T/NK	NAA38
3.66107309256828E-12	1.54139868987389	0.156	0.023	6.13522628852592E-08	Proliferating T/NK	NUP37
3.70218159728414E-12	1.0015735435431	0.256	0.056	6.20411592072876E-08	Proliferating T/NK	MGMT
3.80006223290519E-12	1.39406123492267	0.144	0.02	6.36814428990252E-08	Proliferating T/NK	IBA57
3.80484798161893E-12	0.915276900730371	0.478	0.152	6.37616424759701E-08	Proliferating T/NK	ALDH3A2
3.80919386146048E-12	0.370915625687932	0.333	0.086	6.38344707303547E-08	Proliferating T/NK	PDS5A
3.8695305064195E-12	1.12023432526589	0.2	0.037	6.48455922265779E-08	Proliferating T/NK	ALMS1
3.90288304942184E-12	0.728702042900049	0.2	0.037	6.54045141422111E-08	Proliferating T/NK	NUDT19
3.93421052788362E-12	0.605250567919803	0.233	0.048	6.59295000262737E-08	Proliferating T/NK	ATXN7L3
3.96791443622846E-12	0.499377328180876	0.311	0.076	6.64943101223165E-08	Proliferating T/NK	NDUFA9
3.99921768434798E-12	0.637833718864178	0.3	0.073	6.70188899543035E-08	Proliferating T/NK	FAF2
4.1289106567634E-12	1.6040014874098	0.144	0.02	6.9192284786041E-08	Proliferating T/NK	GLTP
4.14524664904723E-12	0.43235680017645	0.667	0.259	6.94660433447334E-08	Proliferating T/NK	GNG2
4.15506541896637E-12	1.38628896000049	0.122	0.015	6.96305862910384E-08	Proliferating T/NK	NT5DC3
4.18295821889634E-12	0.859885366780732	0.244	0.053	7.00980138322649E-08	Proliferating T/NK	MFAP1
4.20788526107268E-12	1.73406786377559	0.122	0.015	7.05157412050559E-08	Proliferating T/NK	PDE4DIP
4.31863108802797E-12	1.553741684329	0.2	0.038	7.23716197731727E-08	Proliferating T/NK	FUCA1
4.34690914155322E-12	0.724449160667977	0.322	0.084	7.28455033941488E-08	Proliferating T/NK	ATL3
4.35700144005939E-12	0.749585295643166	0.3	0.074	7.30146301325152E-08	Proliferating T/NK	GIGYF2
4.43993453804304E-12	1.35778138218073	0.233	0.049	7.44044229885252E-08	Proliferating T/NK	CCT3
4.44627956664536E-12	0.358845308903885	0.589	0.205	7.4510752977843E-08	Proliferating T/NK	HNRNPK
4.46027733406762E-12	0.922988495895378	0.367	0.103	7.47453275643051E-08	Proliferating T/NK	SDC4
4.49680465852154E-12	0.843205409071994	0.222	0.044	7.53574524675039E-08	Proliferating T/NK	HS2ST1
4.67759805644591E-12	0.749132786751154	0.311	0.077	7.83871882299206E-08	Proliferating T/NK	PRCC
4.71386202793556E-12	0.537879878337853	0.122	0.015	7.89948998641441E-08	Proliferating T/NK	CTSL
4.8156846418074E-12	0.912315380422056	0.3	0.073	8.07012432274085E-08	Proliferating T/NK	TMEM258
5.14819761864202E-12	2.01538369912927	0.122	0.015	8.62734956932029E-08	Proliferating T/NK	CRAT
5.1496165187538E-12	0.48408999266251	0.278	0.066	8.62972736212762E-08	Proliferating T/NK	ZFAND5
5.21767851948043E-12	0.451465532317914	0.489	0.156	8.74378566294531E-08	Proliferating T/NK	STK11
5.24709598010198E-12	1.07484807154196	0.333	0.091	8.7930834434549E-08	Proliferating T/NK	DEPRECATED-ENSG00000163191
5.2600748157926E-12	0.612988791295112	0.222	0.046	8.81483337630524E-08	Proliferating T/NK	DEPRECATED-ENSG00000188529
5.31710582888048E-12	0.47796466861591	0.311	0.078	8.91040594803791E-08	Proliferating T/NK	TRNAU1AP
5.83714581487325E-12	0.701346367120414	0.311	0.079	9.7818889565646E-08	Proliferating T/NK	PIH1D1
5.89899341120392E-12	0.418014340080141	0.333	0.087	9.88553320876954E-08	Proliferating T/NK	POLR2G
5.96467017021879E-12	0.430633617579973	0.344	0.09	9.99559427125265E-08	Proliferating T/NK	AP2B1
6.06015056311076E-12	1.85550886308778	0.1	0.01	1.0155600313661E-07	Proliferating T/NK	PPP1R13L
6.13713494098251E-12	2.28359798866938	0.144	0.021	1.02846107340985E-07	Proliferating T/NK	ARNTL2
6.16153027203903E-12	0.519278974461226	0.344	0.092	1.0325492429883E-07	Proliferating T/NK	SNX19
6.2093777615236E-12	0.623998019663649	0.222	0.045	1.04056752527612E-07	Proliferating T/NK	FOXJ3
6.22196697659627E-12	0.595745785563149	0.267	0.062	1.042677225938E-07	Proliferating T/NK	U2AF2
6.23548522442938E-12	0.405459449903307	0.3	0.074	1.04494261390988E-07	Proliferating T/NK	NIPAL3
6.47929562955726E-12	2.0193017318956	0.144	0.021	1.08580036160121E-07	Proliferating T/NK	PEMT

6.52213156532285E-12	1.43726968690786	0.133	0.018	1.0929788077168E-07	Proliferating T/NK	MSMO1
6.570320542542E-12	0.850724288697639	0.2	0.038	1.10105431651919E-07	Proliferating T/NK	CLK2
6.67037124703889E-12	0.374092578598436	0.856	0.362	1.11782081357878E-07	Proliferating T/NK	PSMB8
6.6863353321705E-12	0.31549510352171	0.478	0.149	1.12049607496513E-07	Proliferating T/NK	DCTN2
6.83863848895715E-12	1.81960428031203	0.111	0.012	1.14601903797944E-07	Proliferating T/NK	ALKBH4
7.07315796761552E-12	1.17561361190816	0.111	0.012	1.18531981221301E-07	Proliferating T/NK	LIPE
7.11386458703681E-12	1.78675626637102	0.133	0.018	1.19214142749563E-07	Proliferating T/NK	KIAA1522
7.14117745086565E-12	0.886672025841144	0.233	0.049	1.19671851721607E-07	Proliferating T/NK	DPAGT1
7.28933159263976E-12	1.04568976760608	0.244	0.053	1.22154618829457E-07	Proliferating T/NK	CNPY2
7.37200552132716E-12	0.36204967037945	0.911	0.421	1.23540068526401E-07	Proliferating T/NK	IFI16
7.37858075994468E-12	1.73540603855661	0.144	0.021	1.23650256375153E-07	Proliferating T/NK	SVBP
7.39478508203539E-12	0.399111200370271	0.344	0.093	1.23921808404749E-07	Proliferating T/NK	ILKAP
7.59261006702232E-12	1.59855474853276	0.2	0.038	1.2723695950316E-07	Proliferating T/NK	SLC27A3
7.77661091849419E-12	0.481047178465931	0.389	0.113	1.30320445772126E-07	Proliferating T/NK	RBM3
8.04972135464144E-12	0.521008858009605	0.433	0.129	1.34897230461081E-07	Proliferating T/NK	PARP14
8.45298099604109E-12	0.841468266907843	0.311	0.079	1.41655055531657E-07	Proliferating T/NK	XRCC5
8.58242426480557E-12	0.576707684929934	0.3	0.075	1.43824265829612E-07	Proliferating T/NK	RNF220
8.78062887315213E-12	0.345755788966796	0.4	0.116	1.47145778656283E-07	Proliferating T/NK	IPO9
8.91149807826266E-12	1.52889448955618	0.156	0.024	1.49338884795526E-07	Proliferating T/NK	AK4
9.19728282879316E-12	0.897354413840339	0.3	0.075	1.54128065644916E-07	Proliferating T/NK	PSMG3
9.23218245726231E-12	1.2761520551757	0.178	0.031	1.54712913618802E-07	Proliferating T/NK	GTF2H3
9.34096734786993E-12	0.953562490274952	0.289	0.071	1.56535930815604E-07	Proliferating T/NK	KPNA3
9.34951766998649E-12	0.262212231828338	0.322	0.083	1.56679217113634E-07	Proliferating T/NK	IFNAR1
9.51872741831683E-12	0.7513978674397	0.244	0.054	1.59514834076153E-07	Proliferating T/NK	XPOT
9.65054858608819E-12	0.350377927907982	0.467	0.145	1.61723893205666E-07	Proliferating T/NK	MARK2
9.74520066028164E-12	1.19419993845476	0.289	0.072	1.63310072665E-07	Proliferating T/NK	FANCL
9.78206123248174E-12	1.08071113248928	0.233	0.049	1.63927782133929E-07	Proliferating T/NK	PRDX5
9.84959908328343E-12	1.09052801564167	0.267	0.062	1.65059581437664E-07	Proliferating T/NK	ATP5MF
1.02702689714077E-11	1.72335920569111	0.167	0.028	1.7210916742285E-07	Proliferating T/NK	BDH1
1.03626951567112E-11	0.512284237896446	0.378	0.107	1.73658045436166E-07	Proliferating T/NK	PRR12
1.04369594256569E-11	0.739380092574618	0.322	0.085	1.74902566055158E-07	Proliferating T/NK	TRAPPC1
1.06234882953546E-11	0.842826048805299	0.322	0.085	1.78028416853553E-07	Proliferating T/NK	GDI2
1.0782813484843E-11	1.72393255046385	0.178	0.031	1.80698388379E-07	Proliferating T/NK	MPST
1.08241845654036E-11	0.525437253642326	0.333	0.09	1.81391684947033E-07	Proliferating T/NK	CLU
1.10416026310226E-11	0.553352348406043	0.489	0.153	1.85035176890677E-07	Proliferating T/NK	EIF2AK1
1.14208533296675E-11	0.618433363087198	0.378	0.11	1.91390660098569E-07	Proliferating T/NK	YEATS2
1.16914244549022E-11	0.938000628453873	0.289	0.072	1.9592489101525E-07	Proliferating T/NK	DNAJC15
1.18039417826296E-11	0.341808779442213	0.644	0.235	1.97810456393307E-07	Proliferating T/NK	ACADVL
1.18697357960754E-11	2.12831317495117	0.122	0.015	1.98913032470632E-07	Proliferating T/NK	HIBADH
1.20978315491084E-11	0.90472625799178	0.311	0.079	2.02735461099958E-07	Proliferating T/NK	CCDC124
1.21743945130202E-11	1.04949885237501	0.189	0.035	2.04018503249193E-07	Proliferating T/NK	GCC1
1.23096247303564E-11	0.328443717707362	0.722	0.281	2.06284691231313E-07	Proliferating T/NK	TRIR
1.25175369153619E-11	1.37241334623524	0.2	0.039	2.09768883627635E-07	Proliferating T/NK	ATPSCKMT
1.25538341387044E-11	0.536874195142076	0.233	0.05	2.10377152496408E-07	Proliferating T/NK	SRSF1
1.27225277175289E-11	0.270978997256052	0.478	0.15	2.13204119490349E-07	Proliferating T/NK	TMOD3
1.29406533076198E-11	0.302186529775553	0.522	0.174	2.16859468129093E-07	Proliferating T/NK	DR1
1.32868779971215E-11	0.39822937738949	0.311	0.079	2.22661501475762E-07	Proliferating T/NK	SP2
1.33967446525582E-11	1.11965902045525	0.256	0.059	2.2450264688757E-07	Proliferating T/NK	COA6
1.34549835709585E-11	1.20841412109073	0.244	0.055	2.25478614682123E-07	Proliferating T/NK	MSH2
1.36013870574684E-11	0.731373834400253	0.222	0.047	2.27932044309055E-07	Proliferating T/NK	LRRC57
1.39216438346874E-11	0.533512058107309	0.667	0.269	2.33298907381691E-07	Proliferating T/NK	LUC7L2
1.39478506379603E-11	0.431022393745165	0.256	0.059	2.33738080990939E-07	Proliferating T/NK	ZCCHC8
1.42632703727247E-11	0.442959447226861	0.256	0.059	2.3902388490612E-07	Proliferating T/NK	DEPRECATED-ENSG00000093000
1.4383235407384E-11	2.12895142530206	0.133	0.018	2.41034258956942E-07	Proliferating T/NK	RAB13
1.45152997294962E-11	1.69699766474265	0.178	0.032	2.43247392866897E-07	Proliferating T/NK	CRACR2B
1.47505149007796E-11	0.895573796173595	0.4	0.121	2.47189128707265E-07	Proliferating T/NK	SEC11C
1.5227855347435E-11	1.7453599628581	0.111	0.013	2.55188399912315E-07	Proliferating T/NK	ZNF343
1.53116407842032E-11	1.8006354678171	0.167	0.028	2.56592476261678E-07	Proliferating T/NK	OTC
1.53307923452604E-11	0.640903787525515	0.256	0.059	2.56913418121875E-07	Proliferating T/NK	STK35
1.54742079107009E-11	0.64376103552604	0.267	0.062	2.59316776167526E-07	Proliferating T/NK	MAGOH
1.56891289217862E-11	0.563390576934916	0.378	0.11	2.62918422471293E-07	Proliferating T/NK	PBRM1
1.57391558240368E-11	1.96144933308715	0.133	0.018	2.63756773299208E-07	Proliferating T/NK	SPSB1
1.59615971124267E-11	0.816818986699818	0.322	0.085	2.67484444410047E-07	Proliferating T/NK	MESD
1.59686003809269E-11	0.556486050241244	0.356	0.099	2.67601805183573E-07	Proliferating T/NK	ANAPC7
1.6155368506001E-11	1.03563591619753	0.144	0.022	2.70731665423564E-07	Proliferating T/NK	SNAPC5
1.63342442076707E-11	0.697600434788008	0.322	0.084	2.73729264432146E-07	Proliferating T/NK	PSMC4

**1.66000758362776E-11**	0.970456264092318	0.322	0.086	2.7818407086434E-07	Proliferating T/NK	CD59
**1.69394394222487E-11**	1.12198295718254	0.144	0.022	2.83871125838044E-07	Proliferating T/NK	GPANK1
**1.73003474101527E-11**	0.318911377069381	0.3	0.075	2.89919221899338E-07	Proliferating T/NK	GLE1
**1.75749323659501E-11**	1.12099523698979	0.144	0.022	2.94520716588591E-07	Proliferating T/NK	DEPRECATED-ENSG00000139343
**1.76430086403947E-11**	0.762278768550071	0.333	0.091	2.95661538795735E-07	Proliferating T/NK	RTCB
**1.77342173351465E-11**	1.02917497950521	0.256	0.059	2.97190014102385E-07	Proliferating T/NK	MOCS2
**1.78074456798219E-11**	1.95360654215696	0.111	0.013	2.98417174702456E-07	Proliferating T/NK	LSM 5.00
**1.78589470393822E-11**	0.488401942176641	0.156	0.025	2.99280234485967E-07	Proliferating T/NK	DDX20
**1.82337540939367E-11**	1.34581479145141	0.189	0.036	3.05561251106192E-07	Proliferating T/NK	MED7
**1.83507439303945E-11**	0.632976808357964	0.389	0.114	3.07521766785551E-07	Proliferating T/NK	KEAP1
**1.84693526378578E-11**	1.09702591143561	0.167	0.028	3.09509411505221E-07	Proliferating T/NK	CIITA
**1.85170800665885E-11**	0.626297958370905	0.367	0.104	3.10309227755889E-07	Proliferating T/NK	TMEM250
**1.92849413554027E-11**	1.07202977655996	0.256	0.06	3.23177047233839E-07	Proliferating T/NK	KCNK5
**1.95194520596228E-11**	0.671356315047186	0.322	0.086	3.27106977615158E-07	Proliferating T/NK	LARP7
**1.9666770951844E-11**	0.302138449719703	0.511	0.163	3.29575747611002E-07	Proliferating T/NK	SNX5
**1.97600359222567E-11**	2.0041746767776	0.111	0.013	3.31138681985178E-07	Proliferating T/NK	DEPRECATED-ENSG00000087087
**2.00183880174489E-11**	1.56958396771759	0.111	0.013	3.35468146396408E-07	Proliferating T/NK	SYBU
**2.01054978012118E-11**	0.410930150937823	0.467	0.147	3.36927932152707E-07	Proliferating T/NK	DNAJC8
**2.0746193713369E-11**	1.60483974485568	0.2	0.04	3.47664714248637E-07	Proliferating T/NK	SERPINA6
**2.07658143738724E-11**	0.551404141612826	0.289	0.073	3.47993517277354E-07	Proliferating T/NK	SLFN11
**2.13551165301501E-11**	0.340036351677348	0.389	0.113	3.57869042812256E-07	Proliferating T/NK	NDUFS7
**2.1498995112477E-11**	1.8325457659433	0.1	0.01	3.6028016009489E-07	Proliferating T/NK	BTBD11
**2.15602590954912E-11**	0.789800276528679	0.233	0.051	3.61306821922242E-07	Proliferating T/NK	COMTD1
**2.18093384368862E-11**	0.875916756871433	0.244	0.055	3.65480893524934E-07	Proliferating T/NK	CPSF3
**2.21373669953088E-11**	0.941917730205545	0.478	0.159	3.70977996107384E-07	Proliferating T/NK	COX6C
**2.2373932506439E-11**	0.295273560340239	0.611	0.226	3.74942360942905E-07	Proliferating T/NK	EVI2B
**2.23993586511927E-11**	0.335131138294068	0.667	0.244	3.75368452276687E-07	Proliferating T/NK	SEL1L3
**2.24301802341097E-11**	1.96995666639315	0.1	0.01	3.7588496036321E-07	Proliferating T/NK	NCS1
**2.28708676521811E-11**	0.45415631518832	0.467	0.147	3.83270000115251E-07	Proliferating T/NK	SEPTIN11
**2.31298740957991E-11**	0.269115076670103	0.544	0.193	3.87610430097401E-07	Proliferating T/NK	P2RY10
**2.35005578440759E-11**	0.973280170509234	0.2	0.039	3.93822348351024E-07	Proliferating T/NK	COX15
**2.40768810060091E-11**	0.676627018025689	0.178	0.032	4.034803718987E-07	Proliferating T/NK	MMGT1
**2.46636222056591E-11**	0.922391283376915	0.289	0.072	4.13312980922435E-07	Proliferating T/NK	AFG3L2
**2.49407088991394E-11**	0.74628705969366	0.2	0.039	4.17956399731779E-07	Proliferating T/NK	ZUP1
**2.5052566417309E-11**	1.41509274866311	0.178	0.032	4.19830908021264E-07	Proliferating T/NK	AP1S1
**2.57247835369174E-11**	0.458277596855545	0.533	0.184	4.31095922511661E-07	Proliferating T/NK	APOBEC3G
**2.60163247940933E-11**	0.731010264995832	0.289	0.074	4.35981570899416E-07	Proliferating T/NK	TOX2
**2.62672840295109E-11**	0.571281260282393	0.344	0.095	4.40187145766544E-07	Proliferating T/NK	UNC50
**2.68886279723448E-11**	0.612420054517063	0.656	0.246	4.50599627560553E-07	Proliferating T/NK	TM9SF2
**2.75996720195972E-11**	0.262551885895856	0.822	0.337	4.6251530370441E-07	Proliferating T/NK	PTPN7
**2.86394234694396E-11**	0.397616666129348	0.378	0.11	4.79939458500869E-07	Proliferating T/NK	AMFR
**2.93636054407582E-11**	0.390098090512311	0.478	0.154	4.92075299976226E-07	Proliferating T/NK	ZC3H4
**2.97732206188824E-11**	0.638322049375571	0.311	0.081	4.98939631131232E-07	Proliferating T/NK	BRMS1
**2.98769872188967E-11**	0.722384983362714	0.233	0.051	5.0067855181427E-07	Proliferating T/NK	RPAP3
**2.99241345584482E-11**	1.79162568472506	0.167	0.029	5.01468646930476E-07	Proliferating T/NK	DEPRECATED-ENSG00000180817
**3.08795856973607E-11**	0.90352778571565	0.344	0.097	5.17480097116371E-07	Proliferating T/NK	PSMC2
**3.10693551633961E-11**	1.96336475765931	0.133	0.019	5.20660253828192E-07	Proliferating T/NK	MSANTD3
**3.13334154086314E-11**	1.30353357296338	0.3	0.08	5.25085375417844E-07	Proliferating T/NK	DDOST
**3.13599024502168E-11**	1.49495044255705	0.144	0.022	5.25529245260734E-07	Proliferating T/NK	DPF3
**3.14117628849629E-11**	0.361152729479114	0.378	0.111	5.26398322426208E-07	Proliferating T/NK	DCTD
**3.14171939815081E-11**	0.677561877603455	0.233	0.051	5.26489336742112E-07	Proliferating T/NK	NOL9
**3.14396389568896E-11**	0.545280103181835	0.311	0.083	5.26865469639556E-07	Proliferating T/NK	NUP214
**3.21450384285898E-11**	0.833556580275951	0.344	0.099	5.38686553986308E-07	Proliferating T/NK	KATNAL1
**3.24940120636487E-11**	0.352209807249057	0.322	0.086	5.44534654162625E-07	Proliferating T/NK	PRPS1
**3.33626988164551E-11**	1.22849621135435	0.133	0.019	5.59092106766154E-07	Proliferating T/NK	MDP1
**3.34862915002172E-11**	0.412848771029415	0.433	0.137	5.6116327296064E-07	Proliferating T/NK	EIF3B
**3.4079123310453E-11**	1.73306770541388	0.178	0.033	5.71097948436571E-07	Proliferating T/NK	CTNNBIP1
**3.41835500920248E-11**	1.37137182557488	0.278	0.071	5.72847932442151E-07	Proliferating T/NK	TXNDC5
**3.42923939893099E-11**	0.345816968510061	0.311	0.083	5.74671938472855E-07	Proliferating T/NK	TAGLN2
**3.47355968591324E-11**	1.57837957877351	0.167	0.029	5.82099132165341E-07	Proliferating T/NK	GALK1
**3.6149421352115E-11**	1.10615067081641	0.256	0.06	6.05792003018742E-07	Proliferating T/NK	NDUFS6
**3.63991629147877E-11**	0.513165702333883	0.278	0.069	6.09977172126012E-07	Proliferating T/NK	ERCC3
**3.65662190606422E-11**	0.706362544100554	0.389	0.116	6.12776699018242E-07	Proliferating T/NK	LPGAT1
**3.67712579425229E-11**	1.57554079038517	0.144	0.022	6.16212740600798E-07	Proliferating T/NK	MDH1
**3.6960232596134E-11**	0.999442299424213	0.144	0.022	6.19379577846014E-07	Proliferating T/NK	DEPRECATED-ENSG00000099797
**3.69670876619602E-11**	0.580010578815397	0.644	0.242	6.19494455039129E-07	Proliferating T/NK	SSR2

**3.70450427868694E-11**	1.1149370390965	0.244	0.056	6.20800827022357E-07	Proliferating T/NK	PSMD4
**3.71501456133258E-11**	1.44867310472352	0.144	0.022	6.22562140188114E-07	Proliferating T/NK	SLC2A13
**3.77061470279582E-11**	0.625436652603689	0.322	0.087	6.31879611894523E-07	Proliferating T/NK	S100A11
**4.12085933190273E-11**	0.920239642914352	0.244	0.056	6.90573606840259E-07	Proliferating T/NK	SLC41A3
**4.12158236654333E-11**	1.42461750947094	0.344	0.104	6.90694772985332E-07	Proliferating T/NK	INF2
**4.16959495142378E-11**	0.598476978657104	0.333	0.092	6.98740721959598E-07	Proliferating T/NK	GRN
**4.1962765789078E-11**	1.21276377103549	0.133	0.019	7.03212029093369E-07	Proliferating T/NK	EIF3E
**4.22921208884893E-11**	0.547850675262309	0.544	0.192	7.08731361849304E-07	Proliferating T/NK	ARL6IP4
**4.25659785239459E-11**	1.25748637280284	0.211	0.044	7.13320668104285E-07	Proliferating T/NK	HIGD1A
**4.27117180049935E-11**	1.2148174027053	0.178	0.033	7.15762970327681E-07	Proliferating T/NK	RMDN1
**4.28165807138116E-11**	1.15288134682592	0.211	0.044	7.17520259602054E-07	Proliferating T/NK	UCK1
**4.28666110739981E-11**	1.84903227511762	0.111	0.014	7.1835866837806E-07	Proliferating T/NK	VAV2
**4.7454444623083E-11**	0.739289567480976	0.267	0.065	7.95241582993624E-07	Proliferating T/NK	PSME2
**4.74630695122612E-11**	0.975347181831686	0.267	0.065	7.95386118886473E-07	Proliferating T/NK	USB1
**4.85762388560156E-11**	0.664056441514503	0.378	0.11	8.1404061074911E-07	Proliferating T/NK	OCIAD2
**4.9720383272032E-11**	0.370545359129949	0.667	0.265	8.33214182872712E-07	Proliferating T/NK	CD99
**4.99452253291053E-11**	0.533604345935303	0.356	0.102	8.36982086065146E-07	Proliferating T/NK	UBTF
**5.00474559656532E-11**	0.414611872212025	0.222	0.048	8.38695267072415E-07	Proliferating T/NK	SLC9A9
**5.04696611347531E-11**	1.67460205704736	0.111	0.014	8.45770581296193E-07	Proliferating T/NK	FXYD3
**5.07870209897615E-11**	1.49577310431049	0.111	0.014	8.51088897746423E-07	Proliferating T/NK	IFT88
**5.20052857359428E-11**	0.78573466500314	0.411	0.126	8.71504578362929E-07	Proliferating T/NK	DDT
**5.23390022505821E-11**	0.714667255349732	0.3	0.078	8.77096999715254E-07	Proliferating T/NK	SLC39A9
**5.31038598451905E-11**	1.04145682805769	0.211	0.045	8.89914483285702E-07	Proliferating T/NK	NUBP1
**5.3732520239528E-11**	1.46553977046372	0.111	0.014	9.00449574174011E-07	Proliferating T/NK	ALCAM
**5.44142020713861E-11**	1.42985635319329	0.167	0.03	9.11873198312288E-07	Proliferating T/NK	RNF128
**5.48253910547364E-11**	0.846151483337772	0.333	0.096	9.18763903295273E-07	Proliferating T/NK	GSTP1
**5.54026436468367E-11**	1.55595020686733	0.167	0.03	9.2843750223369E-07	Proliferating T/NK	ZHX1
**5.69894710410825E-11**	2.6930903559962	0.1	0.011	9.55029555706461E-07	Proliferating T/NK	GAS2L3
**5.75022622680396E-11**	1.1316540349037	0.144	0.023	9.63622911087808E-07	Proliferating T/NK	FANCC
**6.06426786990702E-11**	1.56056255142231	0.122	0.017	1.01625000963902E-06	Proliferating T/NK	EPS8L2
**6.09917363954784E-11**	2.31926515679677	0.122	0.017	1.02209951851543E-06	Proliferating T/NK	TRIM47
**6.20657471652116E-11**	0.758376405596992	0.289	0.074	1.04009779099462E-06	Proliferating T/NK	ABHD12
**6.29665003120331E-11**	0.796494049614692	0.433	0.139	1.05519261222905E-06	Proliferating T/NK	SNRPC
**6.36747344811334E-11**	0.562374063646814	0.589	0.211	1.06706120043483E-06	Proliferating T/NK	PSMB1
**6.43562903323947E-11**	0.915667406616725	0.267	0.065	1.07848271339027E-06	Proliferating T/NK	PRELID1
**6.44528635135943E-11**	0.773242108189188	0.411	0.13	1.08010108676081E-06	Proliferating T/NK	CD63
**6.55196648189336E-11**	1.39436865896326	0.144	0.023	1.09797854303569E-06	Proliferating T/NK	HAUS1
**6.55669621440488E-11**	0.493430990448242	0.333	0.092	1.09877115160997E-06	Proliferating T/NK	EIF2S3
**6.57474496494487E-11**	0.58777618278754	0.244	0.057	1.10179576122546E-06	Proliferating T/NK	DEPRECATED-ENSG00000055070
**6.63605739076907E-11**	0.954997174793801	0.2	0.041	1.11207049754508E-06	Proliferating T/NK	NFKBIB
**6.70430630580522E-11**	0.996473538173605	0.122	0.017	1.12350765072684E-06	Proliferating T/NK	TRIP6
**6.83184542810119E-11**	0.610196183069	0.356	0.102	1.1448806568412E-06	Proliferating T/NK	GPS1
**6.86854992209861E-11**	1.73398188339721	0.133	0.02	1.15103159594528E-06	Proliferating T/NK	F2RL1
**6.95544664816268E-11**	1.16188100590675	0.211	0.045	1.1655937492991E-06	Proliferating T/NK	WASL
**7.02277348931922E-11**	0.807748042506939	0.411	0.127	1.17687638134012E-06	Proliferating T/NK	EDF1
**7.04902276526785E-11**	0.603647314534192	0.433	0.138	1.18127523500359E-06	Proliferating T/NK	POLR3GL
**7.07023588395983E-11**	0.357428492730028	0.411	0.126	1.18483012943399E-06	Proliferating T/NK	UGGT1
**7.12949607897l9E-11**	1.44645564301348	0.133	0.02	1.19476095291411E-06	Proliferating T/NK	PAK4
**7.1573766651546E-11**	1.1557290839258	0.156	0.026	1.19943318154661E-06	Proliferating T/NK	PRKCA
**7.22499815142937E-11**	1.43344022896087	0.1	0.011	1.21076519021653E-06	Proliferating T/NK	ATG4C
**7.30308964623164E-11**	0.820653025376639	0.222	0.049	1.2238517629155E-06	Proliferating T/NK	TESC
**7.44739408137071E-11**	1.10971796672934	0.178	0.033	1.2480343001561E-06	Proliferating T/NK	UBTD2
**7.65402270312433E-11**	0.572359970864154	0.244	0.057	1.28266112458958E-06	Proliferating T/NK	STAMBP
**7.6705353055161E-11**	0.53366871054451	0.189	0.037	1.28542830649839E-06	Proliferating T/NK	ZNF92
**7.80755757225846E-11**	1.25252060037056	0.222	0.05	1.30839049795907E-06	Proliferating T/NK	CISD2
**7.83565772547279E-11**	0.892331025692276	0.367	0.112	1.31309952163473E-06	Proliferating T/NK	C9orf16
**7.8459045435883E-11**	0.493242488041856	0.378	0.113	1.31481668341453E-06	Proliferating T/NK	VPS26B
**7.96339481838782E-11**	0.48962581540595	0.144	0.023	1.33450570366543E-06	Proliferating T/NK	FCGRT
**8.01008423064217E-11**	1.45849894101233	0.156	0.026	1.34232991537102E-06	Proliferating T/NK	RHOU
**8.14243428149253E-11**	0.953565461792666	0.322	0.089	1.36450913689252E-06	Proliferating T/NK	PRDX1
**8.49640564647521E-11**	0.762469694558346	0.244	0.057	1.42382765823632E-06	Proliferating T/NK	ACOX1
**8.66321188324398E-11**	0.36466782663364	0.289	0.075	1.45178104739403E-06	Proliferating T/NK	MFN2
**8.83390860022928E-11**	1.38366763128603	0.2	0.041	1.48038640322642E-06	Proliferating T/NK	PCBD1
**8.84499363426583E-11**	1.17156061472306	0.189	0.038	1.48224403323027E-06	Proliferating T/NK	DCTN5
**9.06188660598232E-11**	0.362974715186275	0.467	0.155	1.51859095743052E-06	Proliferating T/NK	GALM
**9.1081562060248E-11**	0.430689950409825	0.489	0.166	1.52634481700564E-06	Proliferating T/NK	MRTFA

9.12778478797057E-11	1.08042318940654	0.222	0.049	1.52963417476811E-06	Proliferating T/NK	COX7B
9.19953458860774E-11	0.33520058496542	0.3	0.079	1.54165800635889E-06	Proliferating T/NK	ZNF830
9.27925307550365E-11	0.863919519367704	0.433	0.142	1.5550172303929E-06	Proliferating T/NK	MYDGF
9.34639717338838E-11	1.29207895064634	0.167	0.03	1.56626923831642E-06	Proliferating T/NK	TRIAP1
9.4088871259456E-11	1.42255105725157	0.267	0.068	1.57674130456596E-06	Proliferating T/NK	PMM1
9.54018831379575E-11	0.726714065072071	0.289	0.076	1.59874475762589E-06	Proliferating T/NK	UBXN7
9.55599652803692E-11	0.387973058621165	0.356	0.104	1.60139389816843E-06	Proliferating T/NK	CBL
9.67608154526368E-11	0.757219817772208	0.267	0.067	1.62151774535529E-06	Proliferating T/NK	SUPT4H1
9.86257441163887E-11	0.42806137725127	0.289	0.075	1.65277021990244E-06	Proliferating T/NK	SH2D3C
9.93814657215643E-11	1.51031596009158	0.178	0.034	1.66543460256197E-06	Proliferating T/NK	PPIL1
1.00023448505305E-10	0.314775322083872	0.544	0.197	1.67619295005191E-06	Proliferating T/NK	RUNX1
1.00272373947846E-10	0.89816983908518	0.233	0.053	1.680364442618E-06	Proliferating T/NK	LYPLAL1
1.00294104739219E-10	0.464832266496158	0.367	0.109	1.68072860721983E-06	Proliferating T/NK	WWP2
1.01870100639087E-10	0.326176732627354	0.267	0.067	1.70713914650982E-06	Proliferating T/NK	TMEM154
1.05050625158482E-10	0.620104993789141	0.233	0.054	1.76043837640585E-06	Proliferating T/NK	RAE1
1.05499963429986E-10	0.887092967692921	0.256	0.063	1.76796838715971E-06	Proliferating T/NK	NDRG3
1.08962469806064E-10	1.22197909388212	0.189	0.038	1.82599306901002E-06	Proliferating T/NK	ALG8
1.09399342879797E-10	0.258178483182686	0.189	0.038	1.83331418797964E-06	Proliferating T/NK	TIMM9
1.09703744639075E-10	0.610227634726487	0.678	0.278	1.83841535266162E-06	Proliferating T/NK	TFRC
1.10438940086637E-10	1.27136694468785	0.2	0.041	1.85073575797185E-06	Proliferating T/NK	GCLC
1.10500053560721E-10	0.790522734470803	0.267	0.067	1.85176797570562E-06	Proliferating T/NK	TXN2
1.1142674895299E-10	0.545284548063623	0.4	0.121	1.86728945895421E-06	Proliferating T/NK	YIPF3
1.11476975647816E-10	0.340533220168761	0.7	0.287	1.8681311579061E-06	Proliferating T/NK	DRAP1
1.13654422231576E-10	0.762474222658759	0.256	0.062	1.90462080775675E-06	Proliferating T/NK	UPF3A
1.13953063786577E-10	0.929795398460894	0.167	0.03	1.90962544293545E-06	Proliferating T/NK	GLOD4
1.14088888347093E-10	0.656655867418195	0.3	0.079	1.91190159092058E-06	Proliferating T/NK	YWHAG
1.15552964821594E-10	0.385930911852467	0.933	0.439	1.93643658448027E-06	Proliferating T/NK	FTL
1.2110489616757E-10	1.42620794039833	0.178	0.035	2.02947584997613E-06	Proliferating T/NK	RNASEH2C
1.22008400621275E-10	0.798573038243034	0.211	0.046	2.04461677761133E-06	Proliferating T/NK	SMIM26
1.22079261685281E-10	0.560411981330308	0.389	0.121	2.04580426732194E-06	Proliferating T/NK	ALDH1A1
1.24236826698741E-10	0.47681741554729	0.244	0.059	2.08196074181749E-06	Proliferating T/NK	WWOX
1.26561988024197E-10	0.470547995362105	0.356	0.104	2.1209257953095E-06	Proliferating T/NK	APH1A
1.27034605760342E-10	0.793848617328999	0.211	0.046	2.12884592333181E-06	Proliferating T/NK	EIF2AK3
1.28579728704711E-10	2.1513446047434	0.111	0.014	2.15473909363355E-06	Proliferating T/NK	TMEM186
1.30963899681331E-10	1.0775398769985	0.178	0.034	2.19469303085975E-06	Proliferating T/NK	MMUT
1.31753173071629E-10	0.700358833280243	0.367	0.111	2.20791967433436E-06	Proliferating T/NK	SHC1
1.32020747582361E-10	1.51711747889624	0.189	0.038	2.2124036879852E-06	Proliferating T/NK	AKR1C2
1.3242352945254E-10	0.626044611334061	0.367	0.11	2.21915350656566E-06	Proliferating T/NK	PIN1
1.35044111228321E-10	1.01802348506254	0.222	0.051	2.26306921596421E-06	Proliferating T/NK	ELP6
1.36119770120446E-10	0.696613491465556	0.144	0.023	2.28109510767844E-06	Proliferating T/NK	CIAPIN1
1.37137914762335E-10	0.660848191672154	0.233	0.054	2.29815717558721E-06	Proliferating T/NK	TIMM50
1.38205547172432E-10	0.995827821307123	0.167	0.03	2.31604855951562E-06	Proliferating T/NK	TCTEX1D2
1.38828279496534E-10	0.775171612003337	0.178	0.034	2.32648430780292E-06	Proliferating T/NK	CCDC137
1.3884321326098E-10	0.561472920240355	0.333	0.094	2.32673456782751E-06	Proliferating T/NK	INPP1
1.3948432853661E-10	0.784447199166455	0.144	0.023	2.3374783776165E-06	Proliferating T/NK	CNPY4
1.42834931012999E-10	0.894787651951274	0.211	0.046	2.39362777391584E-06	Proliferating T/NK	SAMM50
1.44219385165841E-10	0.873585226800691	0.4	0.128	2.41682845660916E-06	Proliferating T/NK	HYOU1
1.45704390326503E-10	0.262056370103182	0.256	0.062	2.44171417309153E-06	Proliferating T/NK	ACLY
1.45886630304702E-10	0.824563750640837	0.267	0.068	2.44476815064619E-06	Proliferating T/NK	PAGR1
1.46335862781936E-10	0.468883637188917	0.378	0.117	2.45229638849969E-06	Proliferating T/NK	ANKRD36B
1.47826095118767E-10	0.964014874727007	0.156	0.027	2.47726970200029E-06	Proliferating T/NK	SPICE1
1.4863145520397E-10	0.597075592498051	0.111	0.014	2.49076592630813E-06	Proliferating T/NK	MICB
1.51427417066518E-10	0.802931375851046	0.356	0.104	2.5376206552007E-06	Proliferating T/NK	JTB
1.53420132935229E-10	1.68064261496407	0.133	0.02	2.57101458772857E-06	Proliferating T/NK	FUT4
1.5342136337291E-10	0.717898990737133	0.189	0.038	2.57103520740322E-06	Proliferating T/NK	NEDD1
1.54216393349713E-10	1.63622223140784	0.133	0.02	2.58435831975449E-06	Proliferating T/NK	POLE4
1.57854003057094E-10	0.594007590202948	0.533	0.186	2.64531738323078E-06	Proliferating T/NK	NFE2L1
1.62039478913512E-10	0.260592584098342	0.511	0.18	2.71545758763264E-06	Proliferating T/NK	SLC2A4RG
1.65503450626188E-10	1.41483805970782	0.167	0.031	2.77350682559367E-06	Proliferating T/NK	CPQ
1.68798238247519E-10	0.756178129148863	0.322	0.09	2.82872087655192E-06	Proliferating T/NK	NIT1
1.68937014051491E-10	0.299344900326104	0.267	0.067	2.83104648147489E-06	Proliferating T/NK	DROSHA
1.6958411323268E-10	0.635430772390719	0.278	0.071	2.84189056955325E-06	Proliferating T/NK	YIF1B
1.70393472551244E-10	0.361148169701961	0.4	0.123	2.85545381301375E-06	Proliferating T/NK	USP14
1.70881023728724E-10	0.47256829159202	0.3	0.079	2.86362419564595E-06	Proliferating T/NK	ATXN10
1.72105886930874E-10	0.605224586531444	0.233	0.054	2.88415045318758E-06	Proliferating T/NK	GINM1
1.72695113669131E-10	0.931805302363984	0.156	0.027	2.8940247148673E-06	Proliferating T/NK	BUD13

**1.72747723716561E-10**	1.40477177245198	0.222	0.051	2.89490635404213E-06	Proliferating T/NK	THYN1
**1.737171146912E-10**	1.64912035655805	0.122	0.017	2.91115140799513E-06	Proliferating T/NK	PRKAR1B
**1.73729820944051E-10**	2.4573407645382	0.1	0.012	2.9113643393804E-06	Proliferating T/NK	ABCB6
**1.76353238033312E-10**	1.31222959331397	0.178	0.035	2.95532756296224E-06	Proliferating T/NK	POGLUT3
**1.78447833218273E-10**	0.483345147541822	0.211	0.046	2.99042878907181E-06	Proliferating T/NK	METTL15
**1.79166501795317E-10**	1.33233052987472	0.244	0.059	3.00247223708593E-06	Proliferating T/NK	MDH2
**1.80648691010643E-10**	2.5361056036079	0.1	0.012	3.02731076395635E-06	Proliferating T/NK	PXMP4
**1.81583526465312E-10**	0.880129522824254	0.156	0.027	3.0429767365057E-06	Proliferating T/NK	ING1
**1.81827715571505E-10**	1.57845363268489	0.1	0.012	3.04706885754728E-06	Proliferating T/NK	ATAD3A
**1.82295964428226E-10**	0.577635967390145	0.356	0.103	3.05491577188821E-06	Proliferating T/NK	DNAJC3
**1.8758852074527E-10**	1.31212717482569	0.167	0.031	3.14360843064924E-06	Proliferating T/NK	PIGX
**1.95490481763923E-10**	0.645820995725648	0.289	0.078	3.27602949339983E-06	Proliferating T/NK	ARID3B
**1.96504564698015E-10**	0.456501648029218	0.333	0.095	3.29302349520933E-06	Proliferating T/NK	MYD88
**1.97856392954593E-10**	1.04397989866612	0.1	0.012	3.31567743313306E-06	Proliferating T/NK	CDK2AP1
**2.03560421092953E-10**	0.936561298890979	0.289	0.076	3.4112655366757E-06	Proliferating T/NK	TOMM70
**2.05342897616903E-10**	0.517889488453243	0.289	0.076	3.44113627826407E-06	Proliferating T/NK	DNAJA1
**2.07050694515957E-10**	1.09452488666043	0.1	0.012	3.4697555386984E-06	Proliferating T/NK	LGMN
**2.09174425592827E-10**	0.800040853292887	0.267	0.068	3.50534502408459E-06	Proliferating T/NK	TOR1A
**2.09355585762127E-10**	0.375499083546032	0.478	0.166	3.50838090620172E-06	Proliferating T/NK	CRKL
**2.09398024327079E-10**	0.971570260796486	0.278	0.073	3.50909209167318E-06	Proliferating T/NK	SMIM15
**2.09753426847079E-10**	1.75133567136947	0.1	0.012	3.51504792710334E-06	Proliferating T/NK	PARM1
**2.10269694837159E-10**	1.4213724949243	0.6	0.261	3.52369954608111E-06	Proliferating T/NK	LGALS1
**2.11818264660738E-10**	0.514784090535295	0.211	0.046	3.54965047918466E-06	Proliferating T/NK	ZNF398
**2.14708470697462E-10**	0.504720003351719	0.267	0.067	3.59808455194808E-06	Proliferating T/NK	AGGF1
**2.15168064317587E-10**	1.00586603949464	0.267	0.068	3.60578642183412E-06	Proliferating T/NK	PDZD11
**2.16090028115775E-10**	0.745004570749148	0.411	0.132	3.62123669116416E-06	Proliferating T/NK	EIF6
**2.16952062035731E-10**	0.972906690504534	0.233	0.055	3.63568265559478E-06	Proliferating T/NK	NUBP2
**2.18070065049928E-10**	0.946895363600508	0.1	0.012	3.65441815010669E-06	Proliferating T/NK	EIF4EBP3
**2.18070065049928E-10**	1.49570704909263	0.1	0.012	3.65441815010669E-06	Proliferating T/NK	ST7
**2.2111678063708E-10**	1.01777738252415	0.189	0.038	3.70547500991619E-06	Proliferating T/NK	STARD10
**2.21486538887789E-10**	1.04830398987561	0.144	0.024	3.71167141868157E-06	Proliferating T/NK	TXNDC16
**2.23469177996488E-10**	0.818462126315745	0.478	0.168	3.74489648486515E-06	Proliferating T/NK	TMED5
**2.25854273919539E-10**	0.628999296488017	0.222	0.051	3.78486592234364E-06	Proliferating T/NK	RNF8
**2.27097855652443E-10**	2.37456641724769	0.111	0.015	3.80570586502363E-06	Proliferating T/NK	ALDH5A1
**2.2924754402115E-10**	1.54333559511392	0.2	0.043	3.84173034270642E-06	Proliferating T/NK	PCK2
**2.30425818064938E-10**	0.322309939991358	0.256	0.063	3.86147585913223E-06	Proliferating T/NK	KDM1A
**2.30484088012002E-10**	0.942552852449867	0.3	0.083	3.86245234690514E-06	Proliferating T/NK	FASTK
**2.30515004561245E-10**	0.654789195655417	0.289	0.078	3.86297044643735E-06	Proliferating T/NK	E2F3
**2.31091023944013E-10**	1.2380308712254	0.2	0.043	3.87262337925377E-06	Proliferating T/NK	RNF181
**2.3628494870668E-10**	0.956736436871536	0.256	0.063	3.95966317042654E-06	Proliferating T/NK	MCTS1
**2.38801017155199E-10**	0.293609140241753	0.667	0.281	4.00182744548683E-06	Proliferating T/NK	DEPRECATED-ENSG00000132002
**2.39587828384874E-10**	0.315238813460893	0.478	0.158	4.01501282807372E-06	Proliferating T/NK	CD164
**2.40685560772002E-10**	1.41321831255183	0.189	0.039	4.03340862741721E-06	Proliferating T/NK	NDUFB3
**2.43750044453147E-10**	0.290433033515729	0.467	0.156	4.08476324494584E-06	Proliferating T/NK	ASCC3
**2.51864474627895E-10**	0.693985998065285	0.256	0.064	4.22074486581427E-06	Proliferating T/NK	WRAP73
**2.54626062175568E-10**	0.954948827974082	0.2	0.043	4.26702354993816E-06	Proliferating T/NK	C17orf75
**2.60263973627826E-10**	0.655773417179016	0.378	0.117	4.3615036700551E-06	Proliferating T/NK	CNPPD1
**2.64006680474229E-10**	1.2232192234632	0.233	0.055	4.42422395138713E-06	Proliferating T/NK	ELOC
**2.69252403858081E-10**	0.794578845557679	0.2	0.043	4.51213178385372E-06	Proliferating T/NK	COA8
**2.74711527737473E-10**	0.525370519094771	0.889	0.5	4.60361578182457E-06	Proliferating T/NK	RAC2
**2.85492854291223E-10**	2.03134927087112	0.111	0.015	4.78428925221232E-06	Proliferating T/NK	MEX3D
**2.88753889893642E-10**	0.636569531026242	0.389	0.127	4.83893768683765E-06	Proliferating T/NK	GBP1
**2.91658950487995E-10**	0.469298546109555	0.456	0.152	4.88762069227781E-06	Proliferating T/NK	SAP18
**2.9225253916807E-10**	1.18028288908496	0.111	0.015	4.89756805137852E-06	Proliferating T/NK	AIF1
**2.95763850315181E-10**	0.380499096609756	0.356	0.105	4.95641060358181E-06	Proliferating T/NK	PHTF1
**3.03394772794256E-10**	1.04474446037671	0.144	0.024	5.08428960248615E-06	Proliferating T/NK	VPS29
**3.06244045718748E-10**	1.95962690352713	0.111	0.015	5.13203771815478E-06	Proliferating T/NK	ENTPD5
**3.21585384272316E-10**	1.35504413773932	0.2	0.044	5.38912786963548E-06	Proliferating T/NK	HRAS
**3.25468635255523E-10**	1.24878324199238	0.189	0.039	5.45420338961205E-06	Proliferating T/NK	TMEM141
**3.26553966636903E-10**	1.17717966807557	0.211	0.047	5.47239137290122E-06	Proliferating T/NK	PIGQ
**3.3147798130873E-10**	1.08083087800987	0.189	0.039	5.55490801077169E-06	Proliferating T/NK	SH3PXD2A
**3.3542578552149E-10**	1.17151072002178	0.222	0.052	5.62106531376913E-06	Proliferating T/NK	BTBD6
**3.3659552325508E-10**	1.23932711846701	0.189	0.039	5.64066777870863E-06	Proliferating T/NK	MANEA
**3.39072118185948E-10**	0.331846928413257	0.3	0.082	5.68217055656012E-06	Proliferating T/NK	CHFR
**3.39375058315937E-10**	0.325037005698971	0.278	0.074	5.68724722725847E-06	Proliferating T/NK	EMSY
**3.43004439453479E-10**	1.15997865567122	0.178	0.035	5.7480683963614E-06	Proliferating T/NK	HSD17B6

3.5306124480493E-10	0.848187903967356	0.2	0.044	5.91660034044101E-06	Proliferating T/NK	FBF1
3.53566903753677E-10	1.05038315257347	0.189	0.039	5.92507417310412E-06	Proliferating T/NK	DEPRECATED-ENSG00000128228
3.65180936933796E-10	0.901514469673276	0.222	0.051	6.11970214113656E-06	Proliferating T/NK	USP39
3.652455968727E-10	0.593097057592258	0.344	0.102	6.12078571239271E-06	Proliferating T/NK	GOPC
3.6548271061607E-10	1.51228689770958	0.122	0.018	6.12475926450411E-06	Proliferating T/NK	CLDN15
3.84209278328472E-10	0.47385899225984	0.289	0.078	6.43857908622853E-06	Proliferating T/NK	PAPSS1
3.85534325724552E-10	0.282292180099561	0.389	0.123	6.46078423049205E-06	Proliferating T/NK	PRKCSH
3.8564521146184E-10	1.83450032207628	0.122	0.018	6.46264245367752E-06	Proliferating T/NK	ARMT1
3.87720340195833E-10	1.27845614619606	0.122	0.018	6.49741746100177E-06	Proliferating T/NK	PLEKHG4
3.89806095239762E-10	0.761009567798299	0.122	0.018	6.53237054402794E-06	Proliferating T/NK	ZNF626
3.93399351369911E-10	0.991711535972936	0.244	0.06	6.59258633025698E-06	Proliferating T/NK	NRAS
3.94126594981456E-10	1.67565793881538	0.144	0.025	6.60477347869924E-06	Proliferating T/NK	POP7
3.96151741872818E-10	0.451267110592812	0.622	0.237	6.63871089030468E-06	Proliferating T/NK	CTSA
4.0516015295741E-10	0.400742942334913	0.356	0.108	6.78967384326028E-06	Proliferating T/NK	OPA1
4.10656860610751E-10	0.631243629518949	0.2	0.043	6.88178767011497E-06	Proliferating T/NK	CAMKK2
4.18464287499324E-10	0.670076592618831	0.278	0.074	7.01262452991366E-06	Proliferating T/NK	PSMD9
4.27834610685113E-10	0.849398735533157	0.256	0.065	7.16965240586112E-06	Proliferating T/NK	MAPRE1
4.31171255967208E-10	0.36807717045753	0.367	0.113	7.22556790749847E-06	Proliferating T/NK	TXNDC12
4.32809188026992E-10	0.56959849649873	0.3	0.084	7.25301637295633E-06	Proliferating T/NK	DHX8
4.33235306472839E-10	0.398836614201237	0.278	0.073	7.26015726587183E-06	Proliferating T/NK	GET3
4.34426509210035E-10	0.607448631077603	0.222	0.051	7.28011944134176E-06	Proliferating T/NK	OARD1
4.37565324491047E-10	0.37954008103866	0.378	0.118	7.33271970782097E-06	Proliferating T/NK	ZMIZ1
4.38366128471504E-10	0.570911749838101	0.344	0.1	7.34613958092547E-06	Proliferating T/NK	ROMO1
4.3843640402215E-10	1.1142383126811	0.256	0.065	7.34731725860319E-06	Proliferating T/NK	ACTL6A
4.41809331979393E-10	0.54947836034105	0.467	0.158	7.40384078531067E-06	Proliferating T/NK	SPCS1
4.48144922084965E-10	0.50861230859957	0.189	0.039	7.51001260429985E-06	Proliferating T/NK	LCORL
4.7219233492999E-10	0.502890167254839	0.922	0.433	7.91299914875677E-06	Proliferating T/NK	MT-CYB
4.80987623577916E-10	0.780158701163383	0.244	0.061	8.06039059591872E-06	Proliferating T/NK	BRD3
4.81262786323143E-10	0.779239519557278	0.2	0.044	8.06500177320323E-06	Proliferating T/NK	RAD51B
4.88317790827387E-10	0.609056895123357	0.956	0.574	8.18322953868536E-06	Proliferating T/NK	CORO1A
4.91023245109726E-10	0.930267508726454	0.222	0.052	8.22856754154879E-06	Proliferating T/NK	UQCC2
4.98456223768725E-10	0.561183736199	0.2	0.043	8.35312939791629E-06	Proliferating T/NK	PPCDC
4.99480212108401E-10	0.501312124113058	0.244	0.06	8.37028939451259E-06	Proliferating T/NK	PLEKHG2
5.138763831694E-10	0.744602951823001	0.622	0.245	8.6115404291528E-06	Proliferating T/NK	TMCO1
5.21483091525328E-10	0.347045712970649	0.278	0.075	8.73901364778144E-06	Proliferating T/NK	MDC 1.00
5.21812323093142E-10	0.84432357928888	0.178	0.036	8.74453091039488E-06	Proliferating T/NK	SNX15
5.25437297560256E-10	0.847324954669572	0.222	0.052	8.80527823251478E-06	Proliferating T/NK	ERAL1
5.28040551732743E-10	0.467831731109898	0.433	0.144	8.84890356593731E-06	Proliferating T/NK	ELOVL1
5.30775876667007E-10	1.02069730681097	0.189	0.04	8.8947421411857E-06	Proliferating T/NK	INTS2
5.31907630393298E-10	0.788896592792384	0.444	0.158	8.91370807013088E-06	Proliferating T/NK	CXCR3
5.34177544961251E-10	0.875951070955783	0.4	0.131	8.95174729846064E-06	Proliferating T/NK	COPE
5.4127896565529E-10	2.12245522889949	0.1	0.012	9.07075290645135E-06	Proliferating T/NK	LRP11
5.4127896565529E-10	1.80976116844076	0.1	0.012	9.07075290645135E-06	Proliferating T/NK	ST3GAL4
5.51939607805362E-10	0.671490371204049	0.333	0.1	9.24940394760225E-06	Proliferating T/NK	AP1B1
5.52947933146915E-10	1.20495314658507	0.278	0.076	9.266301463676E-06	Proliferating T/NK	PSMA2
5.53028735724857E-10	0.770533254453836	0.156	0.028	9.26765555327716E-06	Proliferating T/NK	ROGDI
5.62963285973741E-10	0.755969499219385	0.244	0.06	9.43413874634795E-06	Proliferating T/NK	TEX261
5.69479089321984E-10	0.407831283487387	0.489	0.17	9.54333057885781E-06	Proliferating T/NK	MGAT2
5.76382635954388E-10	1.17222921908531	0.178	0.036	9.65902021332363E-06	Proliferating T/NK	BAG3
5.79649690734068E-10	1.69940007590835	0.1	0.012	9.71376951732152E-06	Proliferating T/NK	KIF1BP
5.83266731231709E-10	1.1606217492935	0.1	0.012	9.77438388198098E-06	Proliferating T/NK	TMLHE
5.92660323740648E-10	0.447131453140572	0.278	0.073	9.93180170524577E-06	Proliferating T/NK	C1orf43
5.97754334661946E-10	0.626767220173501	0.189	0.039	1.00171671402648E-05	Proliferating T/NK	NOL12
5.98649200728026E-10	0.840646320106727	0.189	0.04	1.00321633058003E-05	Proliferating T/NK	PDCD5
6.14588257855847E-10	1.01183965925886	0.189	0.04	1.02992700251483E-05	Proliferating T/NK	MAPK8
6.17268063589488E-10	0.766562814455688	0.211	0.049	1.03441782096326E-05	Proliferating T/NK	POLR3K
6.17398215915737E-10	1.57220108113564	0.133	0.022	1.03463593023159E-05	Proliferating T/NK	SPRTN
6.19233130297023E-10	0.660806737521287	0.344	0.107	1.03771087975175E-05	Proliferating T/NK	SUSD3
6.2368659219219E-10	0.972419642680977	0.211	0.049	1.04517399119567E-05	Proliferating T/NK	TRIM24
6.31337470736312E-10	0.517750222734454	0.2	0.044	1.05799533345991E-05	Proliferating T/NK	RTRAF
6.32700016834154E-10	1.05958413592709	0.2	0.044	1.06027868821068E-05	Proliferating T/NK	MED21
6.32910054330981E-10	0.563292879750442	0.389	0.121	1.06063066904786E-05	Proliferating T/NK	GSTO1
6.44899693490954E-10	0.356549527603947	0.222	0.052	1.08072290635214E-05	Proliferating T/NK	FAM110A
6.77295888470252E-10	0.444279022678512	0.456	0.153	1.13501244989845E-05	Proliferating T/NK	FKBP1A
6.7823520069002E-10	2.12754591190695	0.111	0.015	1.13658654931633E-05	Proliferating T/NK	PUSL1
6.89829036569452E-10	1.92139831423242	0.111	0.015	1.15601549948309E-05	Proliferating T/NK	STAP2

7.01240208089406E-10	1.23761819004683	0.133	0.022	1.17513834071623E-05	Proliferating T/NK	TOR2A
7.01679429766189E-10	0.695073614060143	0.278	0.075	1.17587438840218E-05	Proliferating T/NK	TIMM23
7.07103590612409E-10	0.684916399585136	0.222	0.053	1.18496419714827E-05	Proliferating T/NK	RAB4A
7.08131077027051E-10	0.933038621551059	0.133	0.022	1.18668605888193E-05	Proliferating T/NK	RNF215
7.08267814851467E-10	0.342728737548883	0.311	0.09	1.18691520412809E-05	Proliferating T/NK	SEC63
7.14576396675218E-10	1.65593008950102	0.122	0.018	1.19748712554833E-05	Proliferating T/NK	ACSF2
7.22080298944645E-10	1.18614024859403	0.122	0.018	1.21006216497144E-05	Proliferating T/NK	FABP5
7.25452111932824E-10	0.262698251683893	0.211	0.048	1.21571264917703E-05	Proliferating T/NK	TFAM
7.2826468791135E-10	1.06615147811651	0.144	0.025	1.22042596400184E-05	Proliferating T/NK	APIP
7.28469488990186E-10	0.503729270421841	0.533	0.199	1.22076916964975E-05	Proliferating T/NK	RNF187
7.45055930999938E-10	1.72591714071241	0.122	0.018	1.2485647291697E-05	Proliferating T/NK	TAF6L
7.47068455861103E-10	0.428123201451158	0.3	0.085	1.25193731833204E-05	Proliferating T/NK	BCCIP
7.47150124668301E-10	0.453497650826386	0.267	0.07	1.25207417891914E-05	Proliferating T/NK	EMC8
7.56229112317736E-10	0.591004241796709	0.256	0.066	1.26728874642206E-05	Proliferating T/NK	TMEM123
7.59273370993488E-10	1.8267990235165	0.111	0.015	1.27239031511089E-05	Proliferating T/NK	DHRS7B
7.62438546584267E-10	1.123546345245	0.189	0.041	1.27769451636591E-05	Proliferating T/NK	TADA2A
7.63563811466267E-10	1.81959584672812	0.111	0.015	1.27958023525517E-05	Proliferating T/NK	SRD5A1
7.76802839034893E-10	1.24009353165429	0.122	0.018	1.30176619765467E-05	Proliferating T/NK	DEPRECATED-ENSG00000053372
7.96704396968308E-10	1.15502604682375	0.167	0.033	1.33511722843949E-05	Proliferating T/NK	PARVB
7.9686564566434E-10	0.64034759302702	0.356	0.11	1.3353874490043E-05	Proliferating T/NK	RCC1L
7.97114893657373E-10	1.1135411334321	0.178	0.036	1.33580513879103E-05	Proliferating T/NK	AAR2
7.98821586501788E-10	0.462940903500803	0.211	0.049	1.3386652146597E-05	Proliferating T/NK	C12orf65
8.02493601279094E-10	0.492793543300225	0.278	0.076	1.34481877702351E-05	Proliferating T/NK	GATD1
8.03259963324367E-10	1.43872387675819	0.111	0.015	1.34610304653897E-05	Proliferating T/NK	WDCP
8.12350928449083E-10	0.402950597317199	0.111	0.015	1.36133768589497E-05	Proliferating T/NK	CCDC88A
8.1473687304243E-10	0.455998174193212	0.433	0.142	1.3653360518445E-05	Proliferating T/NK	TUFM
8.18094436438294E-10	0.272332276779273	0.589	0.22	1.37096265658329E-05	Proliferating T/NK	ARPC1B
8.35519945512393E-10	0.258968922461046	0.111	0.015	1.40016432468967E-05	Proliferating T/NK	ADAM9
8.43477631350159E-10	0.790322343472699	0.167	0.033	1.4134998146166E-05	Proliferating T/NK	CORO2A
8.85463306526881E-10	1.24277200509861	0.156	0.029	1.48385940907775E-05	Proliferating T/NK	ERP29
8.85691999061627E-10	0.671398141444384	0.167	0.033	1.48424265202747E-05	Proliferating T/NK	AP002495.1
8.8866321145768E-10	1.46074371069726	0.178	0.037	1.48922180976078E-05	Proliferating T/NK	FUOM
9.06312726630076E-10	0.771973458626001	0.389	0.13	1.51879886728668E-05	Proliferating T/NK	PSMD3
9.09033502754578E-10	0.673633786888538	0.5	0.181	1.52335834391612E-05	Proliferating T/NK	PUF60
9.21751548073924E-10	0.682410991449084	0.322	0.096	1.54467124426228E-05	Proliferating T/NK	MBOAT1
9.22522382022507E-10	0.283385948979062	0.556	0.211	1.54596300779332E-05	Proliferating T/NK	SURF4
9.25541890317505E-10	0.948808934630307	0.233	0.058	1.55102309979407E-05	Proliferating T/NK	NDUFS8
9.33547999224016E-10	0.445078041169826	0.333	0.102	1.56443973709961E-05	Proliferating T/NK	CDKN1A
9.52642590941457E-10	0.745414808478661	0.144	0.025	1.59643845389969E-05	Proliferating T/NK	C12orf4
9.5830385958131E-10	0.768955521869879	0.311	0.09	1.60592560788636E-05	Proliferating T/NK	STMP1
9.58811516864325E-10	0.823601093118314	0.289	0.08	1.60677633996124E-05	Proliferating T/NK	BEX3
9.61316322506958E-10	0.432704122801312	0.411	0.137	1.61097389325716E-05	Proliferating T/NK	QARS
9.7309513973263E-10	1.25593046158901	0.133	0.022	1.63071283516394E-05	Proliferating T/NK	ATG10
9.8235195763637E-10	1.31809411214528	0.167	0.033	1.64622541060703E-05	Proliferating T/NK	COX5B
9.83727649527622E-10	0.668374302935933	0.311	0.09	1.64853079507839E-05	Proliferating T/NK	XBP1
1.00860750486545E-09	0.929208460154364	0.2	0.046	1.69022445665352E-05	Proliferating T/NK	MBD3
1.02758057441703E-09	0.577388206487351	0.333	0.099	1.72201952660806E-05	Proliferating T/NK	GSTA2
1.03758899354826E-09	0.765495406391281	0.222	0.054	1.73879163538817E-05	Proliferating T/NK	GCH1
1.04365711416256E-09	0.273423531410957	0.344	0.105	1.74896059191361E-05	Proliferating T/NK	PNP
1.04514156626512E-09	1.00604288353592	0.267	0.073	1.75144823674708E-05	Proliferating T/NK	URM1
1.06647809766599E-09	0.862238011876135	0.2	0.045	1.78720399606867E-05	Proliferating T/NK	MRE11
1.08577423329287E-09	0.805126518524111	0.233	0.058	1.81954046015219E-05	Proliferating T/NK	CDC23
1.09379108754976E-09	0.791806827671204	0.178	0.037	1.83297510451588E-05	Proliferating T/NK	WBP11
1.09671542368671E-09	1.37082271348929	0.167	0.033	1.83787570701419E-05	Proliferating T/NK	DBNDD2
1.11853665637552E-09	0.345075489360342	0.3	0.086	1.8744437287541E-05	Proliferating T/NK	RBM27
1.15261357803523E-09	0.607239684002716	0.278	0.076	1.93154983407145E-05	Proliferating T/NK	MIEN1
1.15736779920863E-09	0.359848717503163	0.267	0.071	1.93951695791382E-05	Proliferating T/NK	CEBPD
1.1604266231603E-09	0.920700600458993	0.256	0.067	1.94464293509203E-05	Proliferating T/NK	C12orf75
1.17053014239763E-09	0.318785064849766	0.278	0.076	1.96157441262995E-05	Proliferating T/NK	MFSD14B
1.17229720380468E-09	0.571624613254975	0.4	0.129	1.96453565413588E-05	Proliferating T/NK	TMED9
1.19452716427641E-09	0.810422658645492	0.211	0.049	2.00178862189441E-05	Proliferating T/NK	CHMP3
1.21686539777772E-09	0.945214289306342	0.2	0.045	2.0392230335959E-05	Proliferating T/NK	ARF4
1.21865964301943E-09	0.74699960008466	0.133	0.022	2.04222982977196E-05	Proliferating T/NK	GMCL1
1.2331431667128E-09	0.584478119897171	0.322	0.096	2.06650131877732E-05	Proliferating T/NK	HAT1
1.24182102206371E-09	0.365751309302527	0.233	0.057	2.08104366877436E-05	Proliferating T/NK	XPO4
1.24933270242339E-09	0.645913162031925	0.2	0.045	2.09363174272112E-05	Proliferating T/NK	GMPR2

1.25375400693885E-09	0.368771275106834	0.278	0.076	2.10104096482812E-05	Proliferating T/NK	COPS3
1.27651301842818E-09	0.876719469314661	0.4	0.136	2.13918051628195E-05	Proliferating T/NK	PPP1R7
1.2858043792881E-09	0.528198513198491	0.867	0.458	2.154750978811E-05	Proliferating T/NK	DNAJB1
1.31625475975326E-09	0.705758936362597	0.256	0.067	2.20577972639452E-05	Proliferating T/NK	PSMD8
1.34427854773357E-09	1.5568061348398	0.167	0.034	2.25274199029192E-05	Proliferating T/NK	HECTD2
1.37527953165797E-09	1.55174100442628	0.1	0.013	2.30469343915243E-05	Proliferating T/NK	LYL1
1.37688437219486E-09	0.256107679738856	0.233	0.058	2.30738283092415E-05	Proliferating T/NK	XRCC6
1.38522566686619E-09	0.403384242274342	0.278	0.078	2.32136117253436E-05	Proliferating T/NK	TELO2
1.38650064508937E-09	1.17065724248389	0.178	0.037	2.32349778104077E-05	Proliferating T/NK	PRKAB2
1.39929102882406E-09	0.632259681579342	0.133	0.022	2.34493190610337E-05	Proliferating T/NK	DEPRECATED-ENSG00000132570
1.39931750902775E-09	0.347734516815627	0.311	0.091	2.3449762816287E-05	Proliferating T/NK	GBF1
1.4188427862947E-09	0.586036601972312	0.244	0.063	2.37769674127265E-05	Proliferating T/NK	CD320
1.41940280637241E-09	0.274119603813229	0.133	0.022	2.37863522291888E-05	Proliferating T/NK	OSBPL11
1.44265572135842E-09	0.96021510188095	0.189	0.041	2.41760245785244E-05	Proliferating T/NK	MMD
1.45527446555097E-09	1.4877684389147	0.178	0.038	2.43874894937031E-05	Proliferating T/NK	ACADS
1.46885063589047E-09	1.16000370113303	0.1	0.013	2.46149989562526E-05	Proliferating T/NK	XRCC4
1.46890337712868E-09	1.24905877010962	0.167	0.033	2.46158827939224E-05	Proliferating T/NK	COA5
1.47870562274104E-09	0.878097347191128	0.178	0.038	2.47801488258943E-05	Proliferating T/NK	NBL1
1.48990276474493E-09	1.14073498007572	0.122	0.019	2.49677905315955E-05	Proliferating T/NK	ELF3
1.5714158644503E-09	0.253555212019059	0.833	0.373	2.63337870564581E-05	Proliferating T/NK	MYO1G
1.57270459228049E-09	0.837178648991841	0.3	0.087	2.63553835574365E-05	Proliferating T/NK	FDPS
1.57813519996009E-09	0.410686553133645	0.389	0.127	2.64463896809311E-05	Proliferating T/NK	POLR1D
1.58619490708156E-09	0.628742157135114	0.267	0.071	2.65814542528728E-05	Proliferating T/NK	TMEM147
1.61126590374034E-09	0.461903864789158	0.367	0.118	2.70015940148807E-05	Proliferating T/NK	ZNRF1
1.62901256896251E-09	1.19653892672864	0.111	0.016	2.72989926306737E-05	Proliferating T/NK	ENOX2
1.63500700216197E-09	0.613338718645687	0.322	0.098	2.73994473422303E-05	Proliferating T/NK	ANXA4
1.63762768132869E-09	0.33983484873197	0.456	0.158	2.74433646837061E-05	Proliferating T/NK	RAB5C
1.64817377654623E-09	0.861559343439531	0.322	0.097	2.76200961473618E-05	Proliferating T/NK	RPN2
1.67272066878969E-09	0.345748399885402	0.256	0.068	2.80314529675776E-05	Proliferating T/NK	LSM 1.00
1.69296218577983E-09	0.302487101034299	0.422	0.14	2.83706603092983E-05	Proliferating T/NK	ATP6AP2
1.73521135756454E-09	0.546676278061392	0.178	0.037	2.90786719300665E-05	Proliferating T/NK	GOLPH3L
1.74497433498168E-09	0.656838966117692	0.256	0.068	2.92422799056229E-05	Proliferating T/NK	LEO1
1.74857720832059E-09	0.92876924556673	0.111	0.016	2.93026568570364E-05	Proliferating T/NK	CCDC138
1.75738557265952E-09	0.60758966388815	0.444	0.15	2.94502674266283E-05	Proliferating T/NK	COX6A1
1.76060421227616E-09	0.259332830084363	0.333	0.101	2.95042053893239E-05	Proliferating T/NK	NF2
1.76318898788042E-09	1.02909093643068	0.244	0.063	2.95475210589001E-05	Proliferating T/NK	TIMM17A
1.76345750558763E-09	1.05659911172358	0.289	0.083	2.95520208786375E-05	Proliferating T/NK	PARK7
1.77396352844098E-09	0.873999573946284	0.2	0.046	2.9728080809614E-05	Proliferating T/NK	CPPED1
1.78705678822836E-09	0.291123528477517	0.111	0.016	2.99474976571309E-05	Proliferating T/NK	C6orf136
1.82636101129467E-09	0.635413964918681	0.111	0.016	3.06061578272761E-05	Proliferating T/NK	GPATCH11
1.82933406895921E-09	0.307025902426281	0.322	0.096	3.06559803276185E-05	Proliferating T/NK	ZNF148
1.8375255376328E-09	0.271541005702891	0.567	0.214	3.07932529596505E-05	Proliferating T/NK	IQGAP1
1.85199723540064E-09	0.308561334263744	0.533	0.194	3.10357696708439E-05	Proliferating T/NK	NDUFA3
1.90411762544429E-09	0.563280238430508	0.244	0.064	3.19092031671954E-05	Proliferating T/NK	ALAS1
1.90597062851013E-09	0.827425218144546	0.344	0.108	3.19402557925728E-05	Proliferating T/NK	GPI
1.90932052333528E-09	0.371470269540969	0.389	0.126	3.19963933300527E-05	Proliferating T/NK	ATP2A2
1.93101491311255E-09	0.404101744507177	0.178	0.038	3.235994791394E-05	Proliferating T/NK	CEP95
1.93574162592773E-09	1.0103759951011	0.211	0.051	3.24391581672968E-05	Proliferating T/NK	ARPC3
1.97994556013846E-09	0.561542061989209	0.267	0.073	3.31799276968003E-05	Proliferating T/NK	SPIDR
1.98526471815441E-09	1.21827563022301	0.133	0.023	3.32690661468317E-05	Proliferating T/NK	PAK1
2.01326060776125E-09	1.42598196744412	0.189	0.043	3.3738221264863E-05	Proliferating T/NK	BTN3A2
2.03359711099018E-09	0.774565719361072	0.278	0.078	3.40790203859735E-05	Proliferating T/NK	MLLT1
2.06428446659976E-09	1.14785324944977	0.156	0.03	3.45932790912788E-05	Proliferating T/NK	PDIK1L
2.11212080328794E-09	0.963673416404516	0.322	0.1	3.53949204214993E-05	Proliferating T/NK	UQCR11
2.14144944219518E-09	0.876487712907405	0.167	0.034	3.58864097523069E-05	Proliferating T/NK	C2orf69
2.15503529756602E-09	0.40578431055126	0.322	0.098	3.61140815166113E-05	Proliferating T/NK	MTA1
2.16414310835615E-09	0.833324962497032	0.244	0.064	3.62667102098324E-05	Proliferating T/NK	SMARCAD1
2.18687930827487E-09	0.8608681021107	0.189	0.042	3.66477234480702E-05	Proliferating T/NK	PELO
2.22737027146759E-09	0.887164520569761	0.256	0.068	3.7326271009254E-05	Proliferating T/NK	IDH1
2.2338309736191E-09	0.521492083609104	0.256	0.068	3.7434539455909E-05	Proliferating T/NK	NDUFS3
2.24646397640089E-09	1.15583991018424	0.156	0.03	3.76462433165262E-05	Proliferating T/NK	TBCE
2.25220456731741E-09	1.45184171261207	0.144	0.026	3.77424441391051E-05	Proliferating T/NK	DEPRECATED-ENSG00000117691
2.26381346743414E-09	0.950517827111736	0.222	0.055	3.79369860872613E-05	Proliferating T/NK	HMGCR
2.28065416838057E-09	0.341987368838664	0.3	0.087	3.82192025537217E-05	Proliferating T/NK	FBL
2.38137887670126E-09	1.17331955904873	0.178	0.038	3.99071472157598E-05	Proliferating T/NK	NVL
2.40697890883757E-09	0.414220660566745	0.211	0.051	4.03361525543E-05	Proliferating T/NK	DDHD2

2.44099527300756E-09	0.665074121414028	0.267	0.074	4.09061987850608E-05	Proliferating T/NK	GSS
2.49385953669512E-09	0.846551966680813	0.133	0.023	4.17920981159368E-05	Proliferating T/NK	SCO1
2.50435638977519E-09	0.545573098049835	0.189	0.042	4.19680043798526E-05	Proliferating T/NK	CCT2
2.55423156360771E-09	0.255554144389814	0.389	0.127	4.2803812542938E-05	Proliferating T/NK	URI1
2.69211099041015E-09	0.851118729130865	0.144	0.026	4.51143959772932E-05	Proliferating T/NK	BCS1L
2.70382354388036E-09	0.572891728761307	0.144	0.026	4.53106749483471E-05	Proliferating T/NK	FAR2
2.75332749741096E-09	0.452798632864218	0.156	0.03	4.61402622016128E-05	Proliferating T/NK	RNF130
2.77545290414425E-09	0.594270214075775	0.444	0.159	4.65110397676493E-05	Proliferating T/NK	NACC1
2.8248330649633E-09	0.641932317759688	0.244	0.065	4.7338552502655E-05	Proliferating T/NK	FKBP3
2.83598186809029E-09	1.29007826247644	0.144	0.026	4.7525384145457E-05	Proliferating T/NK	DPP3
2.86703811406198E-09	0.63738184073679	0.367	0.116	4.80458247154506E-05	Proliferating T/NK	ALDH9A1
2.90752357023671E-09	0.392402649762277	0.189	0.042	4.87242799900267E-05	Proliferating T/NK	ALDH18A1
2.91225911260535E-09	1.13835104859306	0.2	0.047	4.88036382090404E-05	Proliferating T/NK	PGAP2
2.93781873265368E-09	0.381419341382595	0.256	0.068	4.92319663218104E-05	Proliferating T/NK	ZFP62
2.94129403753511E-09	0.273466771480248	0.389	0.128	4.92902054810134E-05	Proliferating T/NK	PPM1M
2.94647528736196E-09	0.510015713205323	0.3	0.087	4.93770328656117E-05	Proliferating T/NK	PSMA4
3.08108939614089E-09	0.519438938920758	0.344	0.114	5.16328961005291E-05	Proliferating T/NK	IER5
3.12152929475471E-09	1.66225152028895	0.1	0.014	5.23105879214994E-05	Proliferating T/NK	TCFL5
3.14258031925572E-09	0.629339196638272	0.311	0.092	5.26633609900873E-05	Proliferating T/NK	CASC4
3.1430199939368E-09	1.90111720182957	0.133	0.023	5.26707290583928E-05	Proliferating T/NK	CD109
3.16891254882655E-09	0.55455010146986	0.333	0.105	5.31046364932353E-05	Proliferating T/NK	TRMT112
3.17001835052401E-09	0.348687721016306	0.356	0.114	5.31231675180814E-05	Proliferating T/NK	LRRC8C
3.24185151766394E-09	1.1070704748965	0.311	0.097	5.43269477330123E-05	Proliferating T/NK	POLDIP2
3.25052172303081E-09	0.29189135925727	0.267	0.075	5.44722430345502E-05	Proliferating T/NK	COX17
3.28779432913848E-09	1.72986104738954	0.1	0.014	5.50968573677026E-05	Proliferating T/NK	TENT5A
3.30138575673474E-09	0.441855679719976	0.344	0.109	5.53246225113609E-05	Proliferating T/NK	SOCS4
3.35601171753668E-09	0.302930242380869	0.344	0.109	5.62400443624797E-05	Proliferating T/NK	MBOAT7
3.36571564631645E-09	1.68125676654242	0.133	0.023	5.64026628009711E-05	Proliferating T/NK	MVK
3.46307483258458E-09	0.799191027671413	0.256	0.069	5.80342080444524E-05	Proliferating T/NK	EPN1
3.47079451060684E-09	0.474304639722979	0.2	0.047	5.81635744087495E-05	Proliferating T/NK	NINJ1
3.53380161948618E-09	1.47223407047337	0.111	0.017	5.92194475393494E-05	Proliferating T/NK	ALDH2
3.60103450939961E-09	0.321647780865658	0.3	0.091	6.03461363085186E-05	Proliferating T/NK	VAMP1
3.6859859137907E-09	1.39177378642646	0.111	0.017	6.17697519433045E-05	Proliferating T/NK	UBD
3.70545180224403E-09	1.6553606626322	0.111	0.017	6.20959613020054E-05	Proliferating T/NK	NXT2
3.74061959150467E-09	0.60951857805721	0.222	0.056	6.26853031144353E-05	Proliferating T/NK	SSNA1
3.75434452774831E-09	1.5038733115421	0.133	0.023	6.29153055960062E-05	Proliferating T/NK	CYSTM1
3.81586652460398E-09	1.28954517924726	0.178	0.039	6.39462912193136E-05	Proliferating T/NK	HINT2
3.84618484667498E-09	0.948508411569813	0.167	0.035	6.44543656605794E-05	Proliferating T/NK	POT1
3.87067678121614E-09	0.831887359010664	0.256	0.07	6.486480149962E-05	Proliferating T/NK	NUTF2
3.90992353333671E-09	0.306457517028823	0.189	0.043	6.55224985716566E-05	Proliferating T/NK	DAD1
3.9192462435125E-09	1.21703308855974	0.167	0.035	6.56787285487824E-05	Proliferating T/NK	SLC25A13
4.03740757456502E-09	0.343353909921381	0.344	0.107	6.76588761345606E-05	Proliferating T/NK	MIA2
4.07383999743545E-09	0.81940641600925	0.167	0.035	6.82694106770233E-05	Proliferating T/NK	COPS9
4.36129331287874E-09	1.03809313048629	0.256	0.073	7.3086553337222E-05	Proliferating T/NK	IRF4
4.38563161241629E-09	0.726746345628763	0.278	0.079	7.34944145608722E-05	Proliferating T/NK	CAMK1
4.39783612164716E-09	1.12366061032103	0.178	0.039	7.36989377265631E-05	Proliferating T/NK	DEPRECATED-ENSG00000196757
4.5241189882354E-09	0.459474385837508	0.144	0.027	7.58151860048488E-05	Proliferating T/NK	MAP3K9
4.54662787834902E-09	1.38781617875611	0.2	0.048	7.61923899853728E-05	Proliferating T/NK	NINJ2
4.55242700724791E-09	0.57649969252552	0.389	0.129	7.62895717874605E-05	Proliferating T/NK	CLPTM1
4.6464967560582E-09	0.899137809180648	0.267	0.076	7.78659926380233E-05	Proliferating T/NK	ABHD10
4.64990482059543E-09	0.473306781969237	0.522	0.206	7.79231049835381E-05	Proliferating T/NK	SIT 1.00
4.67697302635288E-09	1.22535518876238	0.178	0.039	7.83767139756216E-05	Proliferating T/NK	UBIAD1
4.68873264602E-09	0.430335546075809	0.922	0.495	7.85737816820031E-05	Proliferating T/NK	HSPA8
4.70502983371769E-09	0.704821913641299	0.233	0.06	7.8846889953441E-05	Proliferating T/NK	GPX2
4.7083148617578E-09	0.517099099967675	0.378	0.124	7.89019404533371E-05	Proliferating T/NK	SEC13
4.73213103729441E-09	0.391755714365516	0.667	0.278	7.93010519229798E-05	Proliferating T/NK	ITM2B
4.91958186540539E-09	0.599404490449966	0.244	0.065	8.24423529004635E-05	Proliferating T/NK	RBX1
5.01050091336668E-09	1.70009642058292	0.122	0.02	8.39659743061988E-05	Proliferating T/NK	NELFE
5.120880859172E-09	0.399427931495254	0.222	0.056	8.58157214380044E-05	Proliferating T/NK	CSTF1
5.18117355750736E-09	0.606378513055082	0.411	0.143	8.68261064767084E-05	Proliferating T/NK	FAM3C
5.20140644508818E-09	0.501314801467784	0.544	0.213	8.71651692067877E-05	Proliferating T/NK	EID1
5.28129570706361E-09	0.435499550681416	0.356	0.116	8.85039534589719E-05	Proliferating T/NK	RO60
5.29053266085188E-09	0.269385231764496	0.356	0.115	8.86587463305558E-05	Proliferating T/NK	ITFG1
5.29291759966377E-09	0.441383285848863	0.222	0.057	8.86987131351654E-05	Proliferating T/NK	TMEM11
5.31894154539419E-09	1.23380853328654	0.167	0.035	8.91348224177159E-05	Proliferating T/NK	NRIP1
5.33647994752438E-09	0.684413370457999	0.367	0.121	8.94287309606136E-05	Proliferating T/NK	NCOA4

5.38905793754153E-09	0.767434884493475	0.311	0.095	9.0309832917321E-05	Proliferating T/NK	CSTB
5.4543016872686E-09	1.98290037667487	0.189	0.045	9.14031876752472E-05	Proliferating T/NK	SF3B4
5.71374239008676E-09	1.58921368240105	0.133	0.024	9.57508949730739E-05	Proliferating T/NK	NANS
5.72714568738837E-09	0.685365098984438	0.289	0.086	9.59755074292542E-05	Proliferating T/NK	ACTR10
5.79215750813467E-09	0.33345245054362	0.489	0.187	9.70649755213208E-05	Proliferating T/NK	ARPC5L
5.8377278208149E-09	0.863711885602184	0.222	0.057	9.78286428212162E-05	Proliferating T/NK	DEPRECATED-ENSG00000106538
5.89454714339829E-09	1.56846403315799	0.133	0.024	9.87808210290685E-05	Proliferating T/NK	GATB
5.89507014057781E-09	0.318805588517116	0.289	0.085	9.8789585415803E-05	Proliferating T/NK	GALK2
5.90082384554463E-09	1.27355001812782	0.189	0.044	9.88860060036369E-05	Proliferating T/NK	ZCRB1
5.90928819115699E-09	0.898996512076332	0.289	0.087	9.90278515074088E-05	Proliferating T/NK	LMNA
5.92499194554721E-09	0.500820827228732	0.122	0.02	9.92910150234801E-05	Proliferating T/NK	ZFP1
5.92558405181105E-09	0.431766767185773	0.544	0.216	9.93009375402495E-05	Proliferating T/NK	SNTB2
5.97372965559099E-09	0.878868468681357	0.133	0.024	0.000100107761568394	Proliferating T/NK	ZNF260
6.00976080798279E-09	1.09191301928321	0.144	0.028	0.000100711571620176	Proliferating T/NK	JAG2
6.11538969081667E-09	1.77568696249545	0.111	0.017	0.000102481700438706	Proliferating T/NK	CREG1
6.22450616855209E-09	0.358266905568168	0.222	0.057	0.000104310274372596	Proliferating T/NK	TMEM209
6.24211034300131E-09	1.55013887435972	0.111	0.017	0.000104605285128016	Proliferating T/NK	RBFA
6.29030246344336E-09	0.913561294351003	0.167	0.035	0.000105412888682384	Proliferating T/NK	ZFP64
6.32554763734392E-09	1.98924341643624	0.133	0.025	0.000106003527306609	Proliferating T/NK	SFXN5
6.3345769547024E-09	1.28204815916091	0.122	0.02	0.000106154840606903	Proliferating T/NK	UCHL3
6.39733046952881E-09	0.388273100344158	0.244	0.067	0.000107206464008364	Proliferating T/NK	CNOT4
6.41018670322751E-09	0.499182307110415	0.244	0.065	0.000107421908772687	Proliferating T/NK	NFIL3
6.47864754829117E-09	0.7187641736088	0.144	0.028	0.000108569175614263	Proliferating T/NK	SPINDOC
6.52867674566876E-09	1.31969793742239	0.133	0.024	0.000109407564903917	Proliferating T/NK	USP31
6.55351570566745E-09	0.265292988827673	0.389	0.129	0.000109823816195575	Proliferating T/NK	EIF4G1
6.77502320620439E-09	1.21768740906713	0.111	0.017	0.000113535838889573	Proliferating T/NK	NECTIN3
6.84642486015843E-09	0.453937164302518	0.189	0.044	0.000114732387806535	Proliferating T/NK	TIMM22
6.89140967695862E-09	0.567605788812671	0.3	0.091	0.000115486243366473	Proliferating T/NK	DRG1
7.02371564819234E-09	1.03137822123445	0.189	0.044	0.000117703426832407	Proliferating T/NK	DIS3L
7.03953740902487E-09	1.50312982590155	0.133	0.024	0.000117968567900439	Proliferating T/NK	GBE1
7.04034230449478E-09	0.48853443796681	0.167	0.035	0.000117982056338724	Proliferating T/NK	SPATA5L1
7.05362120070984E-09	0.503075608628815	0.378	0.126	0.000118204584081495	Proliferating T/NK	PIGT
7.11351616821762E-09	0.552640058292181	0.244	0.066	0.000119208303946991	Proliferating T/NK	CHSY1
7.15859875314293E-09	0.533974547140935	0.344	0.111	0.000119963797905169	Proliferating T/NK	PPP1R10
7.16680041038827E-09	0.361499688274668	0.344	0.109	0.000120101241277287	Proliferating T/NK	RAB6A
7.2626280419522E-09	1.1663516001653	0.178	0.04	0.000121707120727035	Proliferating T/NK	ATP6V1C1
7.28681601101873E-09	0.538567559208837	0.833	0.443	0.000122112462712652	Proliferating T/NK	HSPA1A
7.42333931897004E-09	0.914359638832706	0.133	0.024	0.0001244003203073	Proliferating T/NK	YIPF2
7.56321194749782E-09	0.360728367643909	0.233	0.061	0.000126744305816169	Proliferating T/NK	CYREN
7.57747929213287E-09	0.492202040051004	0.267	0.075	0.000126983397977563	Proliferating T/NK	DCAF6
7.63829761365319E-09	0.601899634627384	0.156	0.031	0.0001280025914096	Proliferating T/NK	PFAS
7.71531659533784E-09	0.958338869325472	0.144	0.028	0.000129293275504671	Proliferating T/NK	SLC35A5
7.8582798670189E-09	0.776673369997706	0.189	0.044	0.000131689054011503	Proliferating T/NK	IRF2BP1
7.87398258186178E-09	0.577056471930874	0.311	0.098	0.00013195220010684	Proliferating T/NK	AGT
7.88208031343369E-09	0.891715987285637	0.156	0.031	0.000132087901892522	Proliferating T/NK	COQ5
8.236164629042E-09	0.395129447873123	0.533	0.221	0.000138021646853486	Proliferating T/NK	MAP4K1
8.25808987020133E-09	0.510528748507152	0.4	0.136	0.000138389070044834	Proliferating T/NK	ARHGAP1
8.26341681387014E-09	1.37626686876599	0.1	0.014	0.000138478338966836	Proliferating T/NK	KATNBL1
8.27738372943487E-09	0.692546172648609	0.178	0.04	0.00013871239653787	Proliferating T/NK	ZNF562
8.42491005837957E-09	0.657353747825515	0.189	0.044	0.000141184642758325	Proliferating T/NK	SMG9
8.42698279366823E-09	0.840519341890934	0.167	0.036	0.000141219377656292	Proliferating T/NK	MLST8
8.51053694795366E-09	0.551116432842063	0.967	0.538	0.000142619578173807	Proliferating T/NK	MT-CO2
8.58958090624453E-09	1.42153254128751	0.1	0.014	0.000143944196826846	Proliferating T/NK	HKDC1
8.77741379103499E-09	0.944152098118079	0.167	0.036	0.000147091900310164	Proliferating T/NK	PIGC
8.9699316079648E-09	0.857027202592915	0.2	0.049	0.000150318113886274	Proliferating T/NK	MALSU1
9.11611754559976E-09	0.836187793903281	0.256	0.072	0.000152767897829161	Proliferating T/NK	PGM1
9.16257849829256E-09	0.267526052013097	0.356	0.118	0.000153546490474387	Proliferating T/NK	N4BP2
9.24434142160969E-09	0.332233721318505	0.256	0.071	0.000154916673543335	Proliferating T/NK	CUL4B
9.44569202127598E-09	0.43196253873858	0.2	0.049	0.000158290906892543	Proliferating T/NK	FAM102B
9.45767971054963E-09	0.308932002244632	0.389	0.134	0.000158491796589391	Proliferating T/NK	DGKH
9.54214456357285E-09	1.18337836887707	0.133	0.025	0.000159907258596354	Proliferating T/NK	CEP295
9.54533382151543E-09	0.727483330545841	0.156	0.032	0.000159960704180956	Proliferating T/NK	UBXN2B
9.7083846580891E-09	0.349101019521958	0.256	0.073	0.000162693110100257	Proliferating T/NK	UBE2O
9.77442144623699E-09	1.86202530508057	0.122	0.021	0.00016379975459604	Proliferating T/NK	RWDD4
9.95638638188406E-09	0.412758474841624	0.244	0.068	0.000166849122987613	Proliferating T/NK	IFI27L2
1.03916824888004E-08	0.373865228108553	0.633	0.258	0.000174143815147318	Proliferating T/NK	OS9

1.05310942540841E-08	0.758038476350595	0.233	0.062	0.000176480077509941	Proliferating T/NK	SNAP29
1.05491472323018E-08	0.606831334407175	0.944	0.63	0.000176782609318914	Proliferating T/NK	LCP1
1.06290073342824E-08	0.667739076216767	0.122	0.021	0.000178120904907904	Proliferating T/NK	ETS2
1.06290101169263E-08	1.49112320964172	0.122	0.021	0.000178120951539451	Proliferating T/NK	IPO11
1.08834523819111E-08	0.312665550929948	0.233	0.062	0.000182384895016065	Proliferating T/NK	DRG2
1.0956248254691E-08	0.402416571488619	0.222	0.058	0.000183604808252112	Proliferating T/NK	SS18L2
1.1079136974067E-08	0.457521250316509	0.378	0.128	0.000185664177411414	Proliferating T/NK	MAPK13
1.11714169485912E-08	0.430801759705112	0.189	0.044	0.000187210605224491	Proliferating T/NK	KAT5
1.12655979044209E-08	0.94366012707552	0.211	0.054	0.000188788889682286	Proliferating T/NK	TMED2
1.16109546904664E-08	0.407449411892473	0.433	0.153	0.000194576378702837	Proliferating T/NK	SPPL2A
1.16206791364396E-08	0.322022458080063	0.489	0.193	0.000194739340968454	Proliferating T/NK	EIF4A1
1.16748240339817E-08	0.321295889256935	0.311	0.097	0.000195646701161466	Proliferating T/NK	ELF4
1.17980391373963E-08	0.363763405193466	0.211	0.054	0.000197711539864487	Proliferating T/NK	ERC1
1.2139137990777E-08	0.450502542700505	0.2	0.049	0.000203427674449442	Proliferating T/NK	SEC22A
1.25215099598876E-08	0.76129581835122	0.178	0.041	0.000209835463907796	Proliferating T/NK	SLC5A6
1.25451782277669E-08	0.366300613803417	0.278	0.083	0.000210232096740917	Proliferating T/NK	EXOC8
1.25593737201738E-08	0.983627243740832	0.211	0.054	0.000210469984802672	Proliferating T/NK	RAC1
1.26632408611075E-08	0.325233863281328	0.289	0.087	0.00021221059035044	Proliferating T/NK	PPP2CA
1.28394293181254E-08	0.987316340646636	0.156	0.033	0.000215163156513145	Proliferating T/NK	NDOR1
1.29628123158805E-08	0.423063352903483	0.311	0.096	0.000217230808789525	Proliferating T/NK	CDK6
1.32513136556266E-08	0.395807216311685	0.267	0.076	0.000222065514240991	Proliferating T/NK	HDHD2
1.34895611648836E-08	0.516572890088342	0.178	0.041	0.000226058066001119	Proliferating T/NK	ATRN
1.35048147784543E-08	0.714071718391024	0.211	0.054	0.000226313686057338	Proliferating T/NK	DDX59
1.367310304344E-08	1.45081602445624	0.156	0.033	0.000229133860801967	Proliferating T/NK	ADH5
1.37324123809302E-08	1.76966644578597	0.111	0.018	0.000230127766679628	Proliferating T/NK	MSRB1
1.38920067817712E-08	1.09296697543888	0.178	0.041	0.000232802249648922	Proliferating T/NK	ALAD
1.39044857539474E-08	0.891868039588509	0.222	0.06	0.000233011372264651	Proliferating T/NK	ASPH
1.39411911521142E-08	0.600968933768631	0.2	0.049	0.00023362648132713	Proliferating T/NK	PLOD3
1.42053806017836E-08	0.278290611746532	0.356	0.121	0.000238053768124689	Proliferating T/NK	CHMP4A
1.42629588591852E-08	0.448302451216463	0.956	0.541	0.000239018664562225	Proliferating T/NK	ARHGDIB
1.4357774156717E-08	0.82756424023861	0.111	0.018	0.000240607579318263	Proliferating T/NK	FANCM
1.44800814162419E-08	0.502310865893689	0.178	0.041	0.000242657204373381	Proliferating T/NK	ACOT8
1.45010641849695E-08	0.46911439028091	0.233	0.063	0.000243008833611719	Proliferating T/NK	SENP1
1.4565428110845E-08	0.508847878635184	0.222	0.059	0.00024408744428154	Proliferating T/NK	RAB3IP
1.47899549068099E-08	1.25145582840648	0.111	0.018	0.00024785006432832	Proliferating T/NK	ZNF689
1.47907795908119E-08	0.762084106563654	0.356	0.122	0.000247863884382825	Proliferating T/NK	VPS28
1.48631993485339E-08	1.68071734363433	0.111	0.018	0.000249077494682732	Proliferating T/NK	SNX24
1.51317521336669E-08	0.356551846411579	0.378	0.129	0.00025357790225599	Proliferating T/NK	PFDN2
1.52197046569312E-08	0.881521531009366	0.278	0.084	0.000255051810640853	Proliferating T/NK	ERG28
1.5360560491543E-08	1.53898462646064	0.144	0.029	0.000257412272717278	Proliferating T/NK	AKR7A2
1.53694773740317E-08	0.431091230467605	0.5	0.185	0.000257561701834024	Proliferating T/NK	CNDP2
1.54840390022414E-08	1.54769985581697	0.144	0.029	0.000259481525599562	Proliferating T/NK	SF3B6
1.5530815650263E-08	0.339564076313829	0.244	0.067	0.000260265408667107	Proliferating T/NK	ANKH
1.56755574621935E-08	0.829754527285401	0.178	0.041	0.00026269099195144	Proliferating T/NK	DEPRECATED-ENSG00000147123
1.61730178632459E-08	0.594450520969385	0.211	0.054	0.000271027433352274	Proliferating T/NK	SNUPN
1.61855453944437E-08	0.391338810720438	0.433	0.158	0.000271237369720088	Proliferating T/NK	DYNC1I2
1.62318231594391E-08	0.79581113411591	0.222	0.059	0.000272012892505881	Proliferating T/NK	BRAT1
1.64893050871719E-08	0.659254516043301	0.244	0.068	0.000276327774650826	Proliferating T/NK	RRAGA
1.65928064699068E-08	0.520114494050236	0.3	0.092	0.000278062250822697	Proliferating T/NK	CPNE3
1.6598766877666E-08	0.485123358414233	0.378	0.129	0.000278162135335927	Proliferating T/NK	IARS2
1.69358607005005E-08	1.44973670584654	0.133	0.025	0.000283811153618987	Proliferating T/NK	KCTD12
1.70010344695603E-08	0.387489786465344	0.167	0.037	0.000284903335640892	Proliferating T/NK	DEPRECATED-ENSG00000122912
1.70098313088917E-08	0.538072246436508	0.311	0.097	0.000285050753074408	Proliferating T/NK	ETFA
1.71521099284288E-08	0.619981618515527	0.233	0.063	0.00028743505818061	Proliferating T/NK	METAP2
1.73570210273458E-08	0.354921825428917	0.578	0.228	0.000290868958376261	Proliferating T/NK	PSMB10
1.88436089422636E-08	0.573177230438343	0.189	0.046	0.000315781198654454	Proliferating T/NK	RBM28
1.96709422058439E-08	0.680085622628645	0.156	0.033	0.000329645649485531	Proliferating T/NK	SPATA33
1.97868451962833E-08	0.929419991849716	0.178	0.041	0.000331587951799316	Proliferating T/NK	SEC24D
1.9846657386822E-08	0.365685859048096	0.478	0.185	0.000332590284488363	Proliferating T/NK	UBC
1.99863142844467E-08	0.785555482765356	0.144	0.029	0.000334930654778757	Proliferating T/NK	MTCH2
1.99956581261035E-08	0.562408757540931	0.244	0.068	0.000335087238877243	Proliferating T/NK	DEPRECATED-ENSG00000168214
2.00425902118225E-08	0.688334433592159	0.167	0.037	0.000335873726769721	Proliferating T/NK	ITGAV
2.02959851436498E-08	0.791283824300224	0.267	0.081	0.000340120119037284	Proliferating T/NK	MSH6
2.03125744569342E-08	0.541424048009122	0.133	0.025	0.000340398122749303	Proliferating T/NK	CD68
2.03877735614293E-08	0.396540586726115	0.233	0.063	0.000341658309342433	Proliferating T/NK	CAPZA2
2.14438889127729E-08	0.814655212443625	0.211	0.055	0.000359356690400249	Proliferating T/NK	NADK

2.17949801079651E-08	0.331810686243778	0.411	0.148	0.000365240276649279	Proliferating T/NK	AGFG2
2.23036729027458E-08	1.24835530802188	0.178	0.042	0.000373764950504215	Proliferating T/NK	H6PD
2.2306594745303E-08	0.413497037097348	0.3	0.092	0.000373813914741788	Proliferating T/NK	KIAA2013
2.25017341329656E-08	0.556925483333959	0.411	0.151	0.000377084060600238	Proliferating T/NK	ATP5F1D
2.25780965034498E-08	0.398816188979712	0.233	0.064	0.000378363741204812	Proliferating T/NK	DYNC1LI1
2.27578023549941E-08	0.385439673806	0.133	0.025	0.000381375251864992	Proliferating T/NK	NAIF1
2.28956963767204E-08	0.295813232533711	0.333	0.111	0.00038368607988108	Proliferating T/NK	PIK3CG
2.29498835674929E-08	0.281083791373778	0.133	0.025	0.000384594148824046	Proliferating T/NK	ACVR1B
2.39266625818414E-08	0.312091622540683	0.522	0.202	0.000400963011546498	Proliferating T/NK	RAB10
2.40714834435098E-08	0.711846574682466	0.322	0.105	0.000403389919546338	Proliferating T/NK	PLEKHF1
2.50585158801197E-08	0.644165665225122	0.311	0.1	0.000419930609119046	Proliferating T/NK	WWP1
2.59827960028229E-08	0.395729645093524	0.233	0.065	0.000435419695415305	Proliferating T/NK	HDAC6
2.67581165674421E-08	0.528712077220877	0.844	0.489	0.000448412517437195	Proliferating T/NK	DEPRECATED-ENSG00000204389
2.71282900940578E-08	0.580692582556287	0.211	0.055	0.00045461588539622	Proliferating T/NK	DDX19A
2.71937598213449E-08	0.349515513784472	0.344	0.114	0.000455713027086098	Proliferating T/NK	GLYCTK
2.73790156080312E-08	0.696697738771158	0.178	0.042	0.000458817543559387	Proliferating T/NK	POP5
2.74611367578651E-08	0.427828141956609	0.344	0.114	0.000460193729788304	Proliferating T/NK	TMED7
2.76530318447869E-08	1.0447018204423	0.244	0.071	0.00046340950765494	Proliferating T/NK	MGST1
2.76887292685101E-08	0.484063292893828	0.778	0.393	0.000464007725081692	Proliferating T/NK	SH3BGRL3
2.82346070775201E-08	0.527049906767203	0.344	0.116	0.000473155545405081	Proliferating T/NK	SLC35A4
2.85709159425819E-08	1.13862950602371	0.156	0.034	0.000478791409365788	Proliferating T/NK	ASL
2.87062858307821E-08	0.363401388529458	0.222	0.06	0.000481059937952247	Proliferating T/NK	PIAS4
2.88419981896744E-08	0.816914812045391	0.222	0.061	0.000483334205662563	Proliferating T/NK	APPL1
2.88923298107394E-08	0.692928140866582	0.233	0.064	0.000484177662968371	Proliferating T/NK	GANAB
2.89472926782865E-08	0.251571971657336	0.233	0.065	0.000485098730702725	Proliferating T/NK	EXOSC7
2.91517764283142E-08	0.484915431863517	0.244	0.068	0.000488525469385689	Proliferating T/NK	SIK2
2.9471288902079E-08	1.04851508463956	0.178	0.042	0.00049387985942104	Proliferating T/NK	LRIF1
2.96776682233145E-08	0.855834809341623	0.111	0.018	0.000497338364086305	Proliferating T/NK	ZNF569
2.97146191511684E-08	1.15969174950423	0.244	0.07	0.00049795758773528	Proliferating T/NK	EEF1AKNMT
2.98965225510563E-08	0.48015281705912	0.189	0.046	0.000501005924910602	Proliferating T/NK	EFL1
3.01694444998914E-08	0.893806891208443	0.222	0.06	0.00050557955092918	Proliferating T/NK	POMP
3.0343459411849E-08	0.831007027807978	0.244	0.07	0.000508495692823766	Proliferating T/NK	TSN
3.05410957518862E-08	0.432511289442659	0.111	0.018	0.00051180768261011	Proliferating T/NK	SAAL1
3.0837572239717E-08	0.273722935895613	0.3	0.094	0.000516776035593177	Proliferating T/NK	SELENON
3.117418847507E-08	1.19860353527828	0.189	0.047	0.000522417050465223	Proliferating T/NK	CISD3
3.12636833427605E-08	1.00158998275261	0.167	0.038	0.000523916805457981	Proliferating T/NK	PSMB3
3.12874382265694E-08	0.492650569581802	0.267	0.078	0.00052431488980085	Proliferating T/NK	SURF1
3.13904437424974E-08	1.83643738839808	0.1	0.015	0.000526041056236771	Proliferating T/NK	PDLIM5
3.15353669946899E-08	0.302059631349484	0.233	0.065	0.000528469680097014	Proliferating T/NK	GADD45GIP1
3.20359037995987E-08	1.26517751542518	0.2	0.052	0.000536857675873675	Proliferating T/NK	SPAST
3.21086417096349E-08	0.7180472627796	0.211	0.055	0.000538076617770062	Proliferating T/NK	CUEDC2
3.22637616616752E-08	0.876569654161168	0.122	0.022	0.000540676117926353	Proliferating T/NK	SORBS3
3.24133927004064E-08	0.339248596491487	0.222	0.059	0.00054318363487341	Proliferating T/NK	TMEM30A
3.25608801955079E-08	0.579096123975077	0.389	0.136	0.000545655230316321	Proliferating T/NK	MICOS10
3.3434253885719E-08	0.836328463306469	0.144	0.03	0.00056029122661688	Proliferating T/NK	DEPRECATED-ENSG00000119900
3.36131983769273E-08	0.426334414723593	0.489	0.189	0.000563289978400547	Proliferating T/NK	REEP5
3.47418610392097E-08	0.601866983453107	0.356	0.123	0.000582204107295076	Proliferating T/NK	FDFT1
3.4932664426093E-08	0.517276777228458	0.167	0.038	0.000585401590452466	Proliferating T/NK	TMPPE
3.55654687288971E-08	0.827414589975658	0.2	0.052	0.000596006124958858	Proliferating T/NK	ZNF672
3.56323059582685E-08	1.0519888546156	0.356	0.128	0.000597126183248663	Proliferating T/NK	ECHS1
3.61890637350483E-08	0.494290673390053	0.189	0.047	0.00060645633007194	Proliferating T/NK	PREB
3.66558343778024E-08	0.974911411178999	0.122	0.022	0.000614278472503212	Proliferating T/NK	ZNF41
3.68637805371387E-08	0.785662760168603	0.133	0.026	0.000617763234241371	Proliferating T/NK	IQCE
3.75681542928735E-08	0.496016988758961	0.167	0.038	0.000629567129639974	Proliferating T/NK	RRP7A
3.91365867120907E-08	1.05989391354257	0.156	0.034	0.000655850920121216	Proliferating T/NK	HSPBP1
3.91726774218968E-08	0.532324688479499	0.244	0.07	0.000656455728236146	Proliferating T/NK	SPPL3
3.92387263197296E-08	0.490015729280444	0.244	0.071	0.000657562575666028	Proliferating T/NK	RGS10
3.92419089941176E-08	1.9314482645045	0.122	0.023	0.000657615910923422	Proliferating T/NK	PIK3R2
4.01573558824571E-08	0.355826375639088	0.2	0.051	0.000672956969878216	Proliferating T/NK	ZNF236
4.03391082926297E-08	1.01799901580838	0.2	0.052	0.000676002776767888	Proliferating T/NK	MTLN
4.12250379323581E-08	0.409984599550102	0.311	0.1	0.000690849185670458	Proliferating T/NK	MAPK6
4.26100998577783E-08	0.302770628406078	0.289	0.091	0.000714060053416649	Proliferating T/NK	LARP1
4.39922182140687E-08	0.543770249808154	0.422	0.156	0.000737221592831364	Proliferating T/NK	CTSD
4.40870151972945E-08	0.472573105866005	0.944	0.569	0.000738810200676262	Proliferating T/NK	HSPA1B
4.4171333685268E-08	1.26027474060502	0.167	0.039	0.000740223209897721	Proliferating T/NK	NUDT16
4.43874331344772E-08	0.465785204478567	0.256	0.076	0.000743844604467568	Proliferating T/NK	OGG1

4.44457733448736E-08	0.343581770949591	0.156	0.034	0.000744822269713392	Proliferating T/NK	CEP164
4.44911253230883E-08	0.615645013241524	0.233	0.065	0.000745582278164313	Proliferating T/NK	ATP6V0E2
4.46546157273924E-08	0.421165533190547	0.478	0.183	0.000748322050359642	Proliferating T/NK	COX4I1
4.52782988867783E-08	0.461044685535089	0.189	0.047	0.000758773732744631	Proliferating T/NK	CHMP5
4.62510984347743E-08	0.358887178410097	0.222	0.06	0.000775075907569948	Proliferating T/NK	DNAJC11
4.62719288358654E-08	1.1654282706923	0.2	0.052	0.000775424983431432	Proliferating T/NK	UBE2E3
4.64002258486268E-08	0.913002352387717	0.144	0.03	0.000777574984771288	Proliferating T/NK	TMEM80
4.66630503318253E-08	0.340050789372189	0.667	0.306	0.000781979397460729	Proliferating T/NK	CD52
4.72401949906305E-08	1.03700848718284	0.156	0.035	0.000791651187652985	Proliferating T/NK	CCNQ
4.72852792515606E-08	0.566970921649027	0.211	0.055	0.000792406709697653	Proliferating T/NK	RFXANK
4.72903223731472E-08	1.44685753229826	0.111	0.019	0.000792491222329201	Proliferating T/NK	EXO5
4.73151815284346E-08	0.2659002846178	0.189	0.047	0.000792907812053507	Proliferating T/NK	SRBD1
4.73275557092252E-08	0.794085023952104	0.2	0.052	0.000793115178575196	Proliferating T/NK	DGCR8
4.77842893745334E-08	0.414295885321264	0.322	0.108	0.000800769121338431	Proliferating T/NK	KLHL2
4.8236332933831E-08	0.495001406205136	0.4	0.146	0.000808344467305139	Proliferating T/NK	MYO18A
4.83576625620245E-08	0.917283101683838	0.167	0.039	0.000810377709214406	Proliferating T/NK	CRADD
4.86712120217135E-08	0.40909317367506	0.189	0.047	0.000815632171059875	Proliferating T/NK	TEX10
4.88332116283506E-08	1.09491552839839	0.2	0.052	0.0008183469604679	Proliferating T/NK	POFUT1
4.92867924688022E-08	0.265796833026729	0.289	0.091	0.000825948068192187	Proliferating T/NK	SRP68
5.05012645175481E-08	0.5174548845443	0.4	0.144	0.000846300190785071	Proliferating T/NK	ATP13A3
5.19740640552744E-08	0.678985607048713	0.189	0.047	0.000870981365438289	Proliferating T/NK	YARS2
5.26293885812837E-08	1.08037593708278	0.111	0.019	0.000881963293845153	Proliferating T/NK	GTF2E1
5.30936373390395E-08	0.624665484892456	0.433	0.162	0.000889743174527624	Proliferating T/NK	DECR1
5.32515804768847E-08	1.17075083046799	0.133	0.026	0.000892389985631633	Proliferating T/NK	CETN3
5.33126139705279E-08	0.608659751283899	0.333	0.112	0.000893412784918106	Proliferating T/NK	DDRGK1
5.41565794781119E-08	0.402530873965027	0.211	0.057	0.000907555958894199	Proliferating T/NK	FXR2
5.45916455371254E-08	0.518609847924583	0.311	0.104	0.000914846795911148	Proliferating T/NK	PPT1
5.47553562348805E-08	0.433163881254122	0.244	0.071	0.000917590259784127	Proliferating T/NK	KLHL22
5.53850263945505E-08	0.2879611559695	0.111	0.019	0.000928142272319878	Proliferating T/NK	YDJC
5.56498029038742E-08	0.650951523393937	0.2	0.052	0.000932579397063124	Proliferating T/NK	PTEN
5.59142024251233E-08	0.345085995670075	0.156	0.035	0.000937010204240216	Proliferating T/NK	ODC1
5.59142024251233E-08	0.353154505256805	0.156	0.035	0.000937010204240216	Proliferating T/NK	NR2C1
5.63343804590518E-08	0.405245021431514	0.233	0.067	0.00094405154773279	Proliferating T/NK	GNB5
5.71151609994187E-08	0.658824959150592	0.122	0.023	0.000957135868028259	Proliferating T/NK	AARS
5.73606541079195E-08	0.896074853676329	0.122	0.023	0.000961249841540514	Proliferating T/NK	NPM3
5.76295661616986E-08	0.497039177413681	0.311	0.102	0.000965756269737745	Proliferating T/NK	NQO1
5.76935182920485E-08	0.630311356238109	0.211	0.057	0.000966827979538149	Proliferating T/NK	CMAS
5.81401951535873E-08	0.439969556905748	0.244	0.071	0.000974313390383815	Proliferating T/NK	CCDC92
5.89958157821391E-08	0.586668293046881	0.311	0.1	0.000988651880877087	Proliferating T/NK	UQCR10
5.96166083743037E-08	0.612791211567671	0.122	0.023	0.000999055123136581	Proliferating T/NK	WBP4
5.96292870585718E-08	0.369840343169935	0.156	0.035	0.000999267592527547	Proliferating T/NK	BTBD10
5.98554102700809E-08	0.598344779882313	0.189	0.048	0.00100305696530602	Proliferating T/NK	AHI1
5.99307571353805E-08	0.481532421853469	0.289	0.094	0.00100431962807471	Proliferating T/NK	PHC1
6.03972153182861E-08	0.917976254134499	0.233	0.067	0.00101213653430384	Proliferating T/NK	ABHD14B
6.0906812059463E-08	1.22785515835322	0.122	0.023	0.00102067635649248	Proliferating T/NK	BAG2
6.22889291715406E-08	0.367766586616094	0.289	0.091	0.00104383787505668	Proliferating T/NK	STRN
6.2542298375606E-08	1.03424554139519	0.244	0.073	0.00104808383617841	Proliferating T/NK	UBL5
6.40834414775219E-08	0.603252924389288	0.222	0.063	0.00107391031228031	Proliferating T/NK	DEPRECATED-ENSG00000134250
6.41388085182335E-08	0.641620437738454	0.144	0.031	0.00107483815314856	Proliferating T/NK	ATP10A
6.4436604443203E-08	0.760650317491799	0.167	0.039	0.0010798286172592	Proliferating T/NK	PDRG1
6.44984267284393E-08	0.365179173678232	0.267	0.081	0.00108086463511519	Proliferating T/NK	DDX1
6.54565855441086E-08	1.72012970922017	0.1	0.016	0.00109692146054817	Proliferating T/NK	RPA3
6.54949087328263E-08	0.529871131551509	0.222	0.062	0.0010975636805447	Proliferating T/NK	VDAC2
6.6123051329955E-08	0.271063626996447	0.378	0.134	0.00110809009418739	Proliferating T/NK	PEX2
6.65384708840713E-08	0.689392215909562	0.489	0.195	0.00111505169507527	Proliferating T/NK	CUTA
6.68359264748963E-08	0.922482466059021	0.144	0.031	0.00112003645586631	Proliferating T/NK	C9orf85
6.68359377656405E-08	0.30952000718497	0.144	0.031	0.0011200366450766	Proliferating T/NK	EEA1
6.73518405336344E-08	0.806237091253516	0.222	0.063	0.00112868214366265	Proliferating T/NK	MEGF9
6.7778660080098E-08	1.37499721102561	0.1	0.016	0.00113583478562228	Proliferating T/NK	PLOD2
6.91672740487342E-08	0.476004609495011	0.389	0.142	0.00115910517850869	Proliferating T/NK	ZMAT2
7.05302163481582E-08	1.13069857505033	0.1	0.016	0.00118194536556243	Proliferating T/NK	B3GNT9
7.12769578431114E-08	1.32419385571941	0.133	0.027	0.00119445925953486	Proliferating T/NK	RNF141
7.24436800418892E-08	0.468734056749285	0.211	0.057	0.00121401119014198	Proliferating T/NK	RNF31
7.42151723380962E-08	0.385665074538344	0.211	0.057	0.00124369785804182	Proliferating T/NK	MCCC1
7.43537909395375E-08	0.748263262830452	0.2	0.052	0.00124602082856477	Proliferating T/NK	PLPBP
7.47302830775436E-08	0.564790641878381	0.322	0.108	0.00125233008381348	Proliferating T/NK	SRPRB

7.47598521890718E-08	0.397607486442919	0.889	0.488	0.00125282560298446	Proliferating T/NK	TMSB10
7.58549832759249E-08	1.1479330744506	0.211	0.059	0.00127117780973795	Proliferating T/NK	FOPNL
7.713513677416E-08	0.859551692128336	0.2	0.053	0.00129263062206137	Proliferating T/NK	POP4
7.74915427825149E-08	1.53289310131713	0.111	0.02	0.00129860327394939	Proliferating T/NK	CDK2AP2
7.76899883299047E-08	1.09742285249013	0.211	0.059	0.00130192882443254	Proliferating T/NK	TIMM10
7.89093111322934E-08	1.50479538941948	0.111	0.02	0.00132236223595497	Proliferating T/NK	CLDN1
8.05688793872908E-08	0.470605018890629	0.322	0.107	0.00135017328077222	Proliferating T/NK	PLD3
8.21076526864499E-08	0.313610233291143	0.322	0.107	0.00137596004371953	Proliferating T/NK	SCAMP3
8.27579965116791E-08	0.759392326273011	0.156	0.035	0.00138685850554272	Proliferating T/NK	MYCBP
8.3484883755924E-08	0.284321395183113	0.3	0.099	0.00139903968198178	Proliferating T/NK	ANP32A
8.55725537489908E-08	0.59082322032345	0.167	0.04	0.00143402485572559	Proliferating T/NK	PLCXD1
8.6520201839749E-08	0.487039044965013	0.256	0.078	0.00144990554243051	Proliferating T/NK	CDIP1
8.65394699731146E-08	1.18598731956874	0.156	0.036	0.00145022843780945	Proliferating T/NK	ID3
8.68478401724852E-08	0.410437096918522	0.211	0.058	0.00145539610561051	Proliferating T/NK	COPS8
8.75620642596332E-08	0.644774906609468	0.111	0.02	0.00146736507286293	Proliferating T/NK	CEBPB
8.87850255049532E-08	0.587003523482953	0.167	0.039	0.00148785945741201	Proliferating T/NK	FAM210A
8.88910904117333E-08	0.408458191459973	0.3	0.1	0.00148963689311983	Proliferating T/NK	NCLN
9.07817495851533E-08	1.49834049106792	0.111	0.02	0.001521320559548	Proliferating T/NK	FNTB
9.09712040359732E-08	0.82609555401344	0.156	0.035	0.00152449543723484	Proliferating T/NK	ERLIN2
9.13339484345596E-08	0.66235001280817	0.244	0.073	0.00153057430786635	Proliferating T/NK	EMC2
9.18092500205503E-08	0.398866672294544	0.2	0.053	0.00153853941184438	Proliferating T/NK	COX20
9.35653778899224E-08	0.368682668368576	0.489	0.197	0.00156796860267932	Proliferating T/NK	CMPK1
9.38866226951349E-08	0.28185250370027	0.133	0.027	0.00157335202312507	Proliferating T/NK	VPS16
9.40704852898632E-08	0.458216384521563	0.156	0.036	0.00157643319248753	Proliferating T/NK	ARFIP2
9.66261367943983E-08	0.860370925763797	0.122	0.023	0.00161926080040053	Proliferating T/NK	CEP162
9.68983542056587E-08	0.435969246618323	0.211	0.058	0.00162382261977843	Proliferating T/NK	TM9SF4
9.76950176967822E-08	0.432248716766531	0.367	0.132	0.00163717310656268	Proliferating T/NK	IL10RB
9.84166853381835E-08	0.850597838186178	0.167	0.039	0.00164926681289728	Proliferating T/NK	CNIH4
9.9077324300574E-08	0.887651812606368	0.122	0.023	0.00166033780062902	Proliferating T/NK	GCA
1.01001897657319E-07	0.332232112830041	0.278	0.087	0.00169258980094136	Proliferating T/NK	PRPF31
1.0115636668019E-07	0.260546739225154	0.2	0.054	0.00169517839282663	Proliferating T/NK	DMAC1
1.01476827346054E-07	0.306442550269816	0.278	0.089	0.00170054867266517	Proliferating T/NK	CNOT6
1.0220133600993E-07	0.281499592982094	0.222	0.063	0.0017126899888544	Proliferating T/NK	HIST3H2BB
1.03677034305943E-07	0.634066435076653	0.2	0.054	0.00173741974089898	Proliferating T/NK	CACUL1
1.04017996812294E-07	0.47819578483772	0.367	0.129	0.00174313359058043	Proliferating T/NK	NDUFA13
1.06344096025034E-07	0.262266390684271	0.233	0.068	0.00178211436118753	Proliferating T/NK	DEPRECATED-ENSG00000196313
1.06641295885931E-07	0.832152671236989	0.222	0.064	0.00178709483645643	Proliferating T/NK	IMP4
1.07010608042951E-07	0.666989787689255	0.144	0.031	0.00179328376958377	Proliferating T/NK	TCTN3
1.08059826911952E-07	0.825889846835113	0.189	0.049	0.00181086657939049	Proliferating T/NK	ALG1
1.10709975127001E-07	1.18507270609609	0.133	0.028	0.00185527776317828	Proliferating T/NK	AGAP3
1.12510216878842E-07	0.66569997597357	0.167	0.04	0.00188544621445563	Proliferating T/NK	SLC39A1
1.15983652508279E-07	0.507118038386625	0.422	0.159	0.00194365404873374	Proliferating T/NK	LMAN1
1.16242330042147E-07	0.907999493516698	0.189	0.049	0.00194798896684629	Proliferating T/NK	CHCHD1
1.18655988298477E-07	1.50286596019889	0.122	0.024	0.00198843705190587	Proliferating T/NK	PLAAT3
1.19002914821201E-07	0.26081156833858	0.278	0.089	0.00199425084657369	Proliferating T/NK	FN1
1.24776223397419E-07	0.48444176119641	0.289	0.093	0.00209099995169395	Proliferating T/NK	NME2
1.26151288406755E-07	0.314228378849304	0.311	0.102	0.00211404329112041	Proliferating T/NK	POLR2H
1.27250886720912E-07	0.692270380015464	0.267	0.084	0.00213247035966904	Proliferating T/NK	GOT2
1.27772052044245E-07	1.26421039190482	0.167	0.041	0.00214120404815745	Proliferating T/NK	MSRA
1.31171154461492E-07	0.917522856484096	0.133	0.028	0.00219816620646568	Proliferating T/NK	NOMO1
1.31676251462137E-07	0.60137249519271	0.133	0.028	0.0022066306220025	Proliferating T/NK	RBAK
1.32374271447319E-07	0.533841372940031	0.233	0.068	0.00221832804091417	Proliferating T/NK	ZNF654
1.32481239951816E-07	0.41719525270076	0.289	0.096	0.00222012061911253	Proliferating T/NK	INPP5F
1.32743117394346E-07	1.58130300055999	0.1	0.017	0.00222450916129445	Proliferating T/NK	GSTM1
1.34372273147402E-07	0.742897499540948	0.211	0.06	0.00225181055340416	Proliferating T/NK	LRRC40
1.35023650019311E-07	0.858727056233334	0.167	0.041	0.00226272632702362	Proliferating T/NK	CLOCK
1.35318222117116E-07	1.08578748162946	0.1	0.017	0.00226766276623863	Proliferating T/NK	GPN3
1.36095173424244E-07	0.672080634864404	0.233	0.069	0.00228068291624348	Proliferating T/NK	C19orf53
1.39436372314217E-07	0.97782974846513	0.144	0.032	0.00233667472724164	Proliferating T/NK	COMMD2
1.40950480519967E-07	0.89287935970968	0.144	0.032	0.0023620481525536	Proliferating T/NK	RABIF
1.41520782881651E-07	0.716711202067478	0.222	0.065	0.0023716052795307	Proliferating T/NK	ATP6V1F
1.42243304319976E-07	0.425855469769368	0.322	0.113	0.00238371329379416	Proliferating T/NK	CALHM2
1.4255772804987E-07	0.281068185692933	0.244	0.075	0.00238898240665973	Proliferating T/NK	XPR1
1.46784309245437E-07	0.832834001135327	0.222	0.064	0.00245981145433503	Proliferating T/NK	ELOVL6
1.47735886704294E-07	0.3804869825271	0.133	0.028	0.00247575798939055	Proliferating T/NK	SRGAP2C
1.54427671739231E-07	0.57571426812949	0.267	0.084	0.00258789892300603	Proliferating T/NK	IFI35

1.55307566676812E-07	0.632868775963647	0.111	0.02	0.00260264420237002	Proliferating T/NK	LNX2
1.56674729722939E-07	1.12932194673376	0.111	0.02	0.00262555512069701	Proliferating T/NK	RFXAP
1.5798626009143E-07	0.715030291031291	0.122	0.024	0.00264753374661219	Proliferating T/NK	CMPK2
1.59926978792673E-07	0.601352855124952	0.122	0.024	0.00268005631060762	Proliferating T/NK	FANCE
1.6377944285702E-07	0.680744429217709	0.211	0.059	0.00274461590339794	Proliferating T/NK	SEPHS2
1.65888453210103E-07	0.273441768617424	0.122	0.024	0.00277995869889491	Proliferating T/NK	ZNF441
1.6805743396815E-07	0.33581002128456	0.311	0.104	0.00281630647843825	Proliferating T/NK	CDV3
1.68290337272613E-07	0.607831977252364	0.333	0.117	0.00282020947201445	Proliferating T/NK	CDIPT
1.70285739316403E-07	0.484758629399675	0.244	0.074	0.00285364841946429	Proliferating T/NK	TRAF7
1.71120917535456E-07	0.623334256938492	0.278	0.091	0.00286764433605917	Proliferating T/NK	MCRIP1
1.75311998663072E-07	0.475897078647222	0.178	0.045	0.00293787847359576	Proliferating T/NK	PHAX
1.78571066752197E-07	0.973459603680093	0.2	0.055	0.00299249393663332	Proliferating T/NK	ALG5
1.78838863180472E-07	0.521636925524038	0.311	0.105	0.00299698166917835	Proliferating T/NK	LAPTM4B
1.79631951892372E-07	0.254164905336193	0.222	0.064	0.00301027224981237	Proliferating T/NK	FKBP15
1.8051282398238E-07	0.331326347195314	0.267	0.084	0.00302503390429672	Proliferating T/NK	SLC50A1
1.82260670035844E-07	0.357876768661116	0.489	0.206	0.00305432430846067	Proliferating T/NK	HAVCR2
1.87649821390205E-07	0.564839804988766	0.167	0.041	0.00314463570685706	Proliferating T/NK	SRGAP2
1.91296854560605E-07	0.713649938095656	0.167	0.041	0.00320575268872662	Proliferating T/NK	DEPRECATED-ENSG00000109536
1.94854479259105E-07	0.786072851867234	0.144	0.033	0.00326537136342408	Proliferating T/NK	ZMYM1
1.95543111788279E-07	0.5213546043157	0.2	0.054	0.00327691146734798	Proliferating T/NK	OSBPL9
1.97318307526428E-07	0.527085433099395	0.2	0.055	0.00330666019752787	Proliferating T/NK	ZNF544
2.05001192642112E-07	0.367140108639191	0.167	0.041	0.00343540998629651	Proliferating T/NK	LRMP
2.06182183775366E-07	1.34378329449305	0.144	0.033	0.00345520103570758	Proliferating T/NK	PCCB
2.08951279120205E-07	0.286459266973865	0.2	0.055	0.00350160553549639	Proliferating T/NK	RLF
2.10186433393119E-07	0.337628861091426	0.244	0.076	0.00352230425080189	Proliferating T/NK	CD70
2.12761856909744E-07	0.574174319571606	0.189	0.05	0.00356546319809349	Proliferating T/NK	DCLRE1C
2.13759906157879E-07	0.722416501557606	0.233	0.07	0.00358218850739374	Proliferating T/NK	DEPRECATED-ENSG00000163931
2.15012131834559E-07	0.886410270232494	0.167	0.041	0.00360317330528355	Proliferating T/NK	CAAP1
2.15767586426036E-07	1.66183855633156	0.1	0.017	0.00361583321332751	Proliferating T/NK	AADAC
2.17769277003003E-07	0.411430890018837	0.456	0.181	0.00364937754401633	Proliferating T/NK	RTF2
2.18218610333553E-07	0.299485180909507	0.278	0.089	0.00365690747196968	Proliferating T/NK	PHC2
2.19161832944048E-07	0.397957238940499	0.767	0.354	0.00367271399647636	Proliferating T/NK	LAMP1
2.26590724177119E-07	1.58588125051603	0.1	0.017	0.00379720735576017	Proliferating T/NK	GPX3
2.27523758165665E-07	1.15959335860407	0.178	0.046	0.00381284313934021	Proliferating T/NK	CRYZ
2.27554618510017E-07	0.493621448947451	0.144	0.033	0.00381336029699086	Proliferating T/NK	SC5D
2.28372361058222E-07	0.671296748442184	0.167	0.041	0.00382706402661369	Proliferating T/NK	C1orf122
2.283870675388E-07	0.418996966081386	0.922	0.501	0.00382731047781522	Proliferating T/NK	MT-CO3
2.28898321335817E-07	0.410929609019565	0.189	0.051	0.00383587806894562	Proliferating T/NK	CEP63
2.28973241004604E-07	0.314326393716206	0.178	0.046	0.00383713357275515	Proliferating T/NK	EXT2
2.29423126012031E-07	0.483220684275939	0.189	0.051	0.00384467274570962	Proliferating T/NK	TMEM18
2.30313710292159E-07	1.72927820531191	0.1	0.017	0.003859597157076	Proliferating T/NK	MAOA
2.329214475008E-07	1.02896209687861	0.122	0.025	0.0039032976172184	Proliferating T/NK	TICAM1
2.35718425067294E-07	0.870587405514319	0.122	0.025	0.00395016936727771	Proliferating T/NK	TM4SF4
2.35894766230037E-07	1.53514756646826	0.133	0.029	0.00395312449248297	Proliferating T/NK	TRAPPC2L
2.38190640961951E-07	0.995252968195168	0.144	0.033	0.00399159876124038	Proliferating T/NK	TBCE.1
2.39598039870438E-07	0.262472361799904	0.211	0.06	0.0040151839521488	Proliferating T/NK	HACD4
2.4045184637282E-07	0.550003389582725	0.189	0.051	0.00402949204151572	Proliferating T/NK	TIMM8B
2.42532947190123E-07	0.434473286742155	0.167	0.041	0.00406436712901208	Proliferating T/NK	SHLD1
2.44305427805071E-07	0.584531319119804	0.122	0.025	0.00409407035915738	Proliferating T/NK	EID3
2.44904282160574E-07	0.594744059860427	0.333	0.118	0.0041041059604469	Proliferating T/NK	TNFSF10
2.54759437033827E-07	1.13274363753002	0.111	0.021	0.00426925864581287	Proliferating T/NK	ARHGAP21
2.57536811495637E-07	0.303412366223127	0.311	0.107	0.00431580188704389	Proliferating T/NK	EIF2AK2
2.58678538685899E-07	0.484152609633057	0.1	0.017	0.0043349349512983	Proliferating T/NK	PPIP5K1
2.60245634699583E-07	1.18243485093246	0.111	0.021	0.00436119634629561	Proliferating T/NK	DEPRECATED-ENSG00000071894
2.63352945413559E-07	0.375833151697411	0.311	0.104	0.00441326865924043	Proliferating T/NK	DEPRECATED-ENSG00000067064
2.65827115022632E-07	0.911044470824257	0.144	0.033	0.00445473079354927	Proliferating T/NK	FAM122C
2.69277268290489E-07	0.471214065230405	0.789	0.364	0.00451254846201201	Proliferating T/NK	MT-ND5
2.71562412032178E-07	1.36149241943487	0.111	0.021	0.00455084290083524	Proliferating T/NK	LRFN3
2.73312739527661E-07	0.737014342337035	0.144	0.033	0.00458017488900454	Proliferating T/NK	PHF13
2.79005895003673E-07	0.670051207101521	0.3	0.102	0.00467558078847155	Proliferating T/NK	HTATIP2
2.80994387542099E-07	0.540962300965201	0.256	0.08	0.00470890394643049	Proliferating T/NK	SIAH2
2.82185941265047E-07	0.408519412837453	0.189	0.051	0.00472887200371966	Proliferating T/NK	COX10
2.84559746559811E-07	0.460160545436314	0.111	0.021	0.00476865223284931	Proliferating T/NK	TTI2
2.84862008910087E-07	0.675922879650951	0.2	0.057	0.00477371754531524	Proliferating T/NK	C16orf87
2.86354775514803E-07	0.775397523772708	0.211	0.062	0.00479873332807706	Proliferating T/NK	HIVEP3
2.87145853601981E-07	1.63801980216055	0.122	0.025	0.004811990214662	Proliferating T/NK	AHCY

2.94067259450604E-07	0.978531043187623	0.2	0.056	0.00492797913387321	Proliferating T/NK	ACADM
2.95092910680464E-07	0.291355006702021	0.189	0.051	0.00494516699718322	Proliferating T/NK	TAF12
2.99073455848987E-07	0.383297449610325	0.144	0.033	0.00501187297311732	Proliferating T/NK	ZNF524
2.99657838956075E-07	0.803957381557008	0.244	0.076	0.0050216660652259	Proliferating T/NK	LIMA1
2.99813638533821E-07	0.799684520664178	0.211	0.061	0.00502427695454978	Proliferating T/NK	DDX55
3.00823330790811E-07	0.36643731612677	0.5	0.203	0.00504119737739241	Proliferating T/NK	ATP5F1E
3.03918568382978E-07	0.552891338563659	0.2	0.056	0.00509306736896194	Proliferating T/NK	PPM1F
3.1281780376861E-07	0.925125137364013	0.144	0.033	0.00524220075555436	Proliferating T/NK	TMEM200A
3.21281124733989E-07	0.372431471043959	0.289	0.099	0.00538402908829219	Proliferating T/NK	GNS
3.2604483358875E-07	0.883685278411013	0.144	0.033	0.00546385932128028	Proliferating T/NK	GM2A
3.3042560787326E-07	0.486929009483743	0.256	0.082	0.00553727233674009	Proliferating T/NK	FLOT1
3.44945668246777E-07	1.39600954844574	0.122	0.025	0.0057805995084795	Proliferating T/NK	YIPF6
3.5292149938408E-07	0.443746683016606	0.144	0.033	0.00591425848667842	Proliferating T/NK	STXBP4
3.62161280970951E-07	1.09079892904068	0.178	0.047	0.0060690987465112	Proliferating T/NK	MCCC2
3.692597704642E-07	1.27816415270908	0.144	0.034	0.00618805523343906	Proliferating T/NK	HPGD
3.77580274222141E-07	0.384507706986816	0.156	0.038	0.00632749023541463	Proliferating T/NK	TUBGCP5
3.79119163419611E-07	0.847981648963555	0.178	0.047	0.00635327894058585	Proliferating T/NK	ARL 1.00
3.79445366171389E-07	1.68352240669948	0.1	0.018	0.00635874544630014	Proliferating T/NK	MAP3K20
3.80111900923579E-07	0.947354024408705	0.122	0.025	0.00636991523567734	Proliferating T/NK	PLA2G12A
3.85538875879994E-07	1.29417086585778	0.133	0.03	0.00646086048199694	Proliferating T/NK	ZC3HC1
3.85825435472255E-07	0.395021999294507	0.278	0.091	0.00646566264764404	Proliferating T/NK	SMPD1
3.87194286594505E-07	0.58225006449344	0.256	0.083	0.00648860185475071	Proliferating T/NK	CALU
3.88108163175935E-07	1.79827018670508	0.1	0.018	0.00650391659850232	Proliferating T/NK	PRADC1
3.89137739794143E-07	0.568652814963376	0.211	0.061	0.00652117024347025	Proliferating T/NK	HACD2
3.9212143393066E-07	1.25760502353056	0.156	0.038	0.00657117098981	Proliferating T/NK	TSFM
3.95291862585748E-07	1.36340322131626	0.111	0.022	0.00662430103321196	Proliferating T/NK	PIN4
4.005572140132E-07	0.718726253152873	0.1	0.018	0.00671253779243321	Proliferating T/NK	MLXIPL
4.08012685659032E-07	0.647516871263765	0.222	0.067	0.00683747658627406	Proliferating T/NK	PYM1
4.09214199979053E-07	0.338802567524673	0.189	0.052	0.00685761156324897	Proliferating T/NK	DEPRECATED-ENSG00000238083
4.10711890061027E-07	1.06348045302538	0.122	0.025	0.00688270985364269	Proliferating T/NK	ANKRD54
4.13029800177306E-07	0.572594964324509	0.189	0.052	0.0069215533913713	Proliferating T/NK	LCMT1
4.13391142923359E-07	0.629472064606036	0.1	0.018	0.00692760877310965	Proliferating T/NK	NETO2
4.15803617771031E-07	0.498754893978509	0.2	0.057	0.00696803702660693	Proliferating T/NK	PIGK
4.2023510444197E-07	0.664251577463212	0.211	0.062	0.00704229988023853	Proliferating T/NK	TEX2
4.24266387803222E-07	0.993422383277963	0.178	0.047	0.00710985612680639	Proliferating T/NK	SRXN1
4.32257319165847E-07	0.356098068127098	0.233	0.071	0.00724376815458126	Proliferating T/NK	B3GAT3
4.32338638512521E-07	1.19994495516235	0.167	0.043	0.00724513090419283	Proliferating T/NK	RUSC1
4.32415684597452E-07	0.907724098175569	0.1	0.018	0.00724642204248409	Proliferating T/NK	HDAC2
4.38892551114167E-07	0.965690220847504	0.156	0.038	0.00735496137157121	Proliferating T/NK	RCN1
4.40586739341704E-07	0.696636887509551	0.133	0.03	0.00738335257788828	Proliferating T/NK	PRDM10
4.50257768909408E-07	1.04875122758128	0.1	0.018	0.00754541969138386	Proliferating T/NK	DDX41
4.50257768909408E-07	1.00072415754059	0.1	0.018	0.00754541969138386	Proliferating T/NK	IFT46
4.56720411759094E-07	0.911774722757764	0.133	0.03	0.00765372066025889	Proliferating T/NK	C1orf35
4.56964769611374E-07	1.0203611139071	0.111	0.022	0.00765781560914741	Proliferating T/NK	PSME3
4.5905422680168E-07	0.458382174583672	0.178	0.047	0.00769283073274255	Proliferating T/NK	KIAA0930
4.60474741634215E-07	0.870702303767797	0.1	0.018	0.00771663572030617	Proliferating T/NK	MTX1
4.60755899001197E-07	1.41141855730325	0.111	0.022	0.00772134735546206	Proliferating T/NK	PIGP
4.66143042446905E-07	0.46259733527993	0.244	0.079	0.00781162510532524	Proliferating T/NK	ATF6
4.67875953693438E-07	0.755848084360264	0.256	0.084	0.00784066523199463	Proliferating T/NK	P2RY11
4.71249925119325E-07	0.600855058078003	0.178	0.047	0.00789720624514965	Proliferating T/NK	MXRA7
4.72587394712039E-07	0.444989949827016	0.267	0.087	0.00791961956058435	Proliferating T/NK	HMG20B
4.7709626993831E-07	0.950833461886213	0.211	0.065	0.0079951792916262	Proliferating T/NK	FHOD1
4.82545942874923E-07	0.476981830077542	0.422	0.17	0.00808650491069796	Proliferating T/NK	IFITM2
4.83757888496037E-07	0.468422680949816	0.178	0.047	0.0081068146954166	Proliferating T/NK	THUMPD3
4.84743601297467E-07	0.268986935059063	0.178	0.048	0.00812333327054295	Proliferating T/NK	MIER3
4.91010371836628E-07	0.919541687838376	0.144	0.034	0.0082283518112382	Proliferating T/NK	DHTKD1
4.97764956409024E-07	0.433133346151992	0.222	0.066	0.00834154513950242	Proliferating T/NK	ATRAID
4.9788407127161E-07	0.524927083818986	0.278	0.094	0.00834354126636965	Proliferating T/NK	ORMDL2
5.00905376056906E-07	0.503333272344662	0.222	0.067	0.00839417229196164	Proliferating T/NK	TFG
5.0274506186287E-07	0.668768058967742	0.144	0.034	0.00842500174669798	Proliferating T/NK	WDR61
5.03853247912166E-07	0.522455258099041	0.178	0.047	0.00844357272851208	Proliferating T/NK	WDSUB1
5.06311694855225E-07	0.355121131458152	0.289	0.097	0.00848477138238385	Proliferating T/NK	FPGS
5.17839802457058E-07	0.374486117245373	0.356	0.141	0.00867795940957537	Proliferating T/NK	CAPG
5.21773210007694E-07	0.306267295196262	0.156	0.038	0.00874387545330894	Proliferating T/NK	HENMT1
5.24740192667669E-07	1.27734602478133	0.122	0.026	0.00879359614872479	Proliferating T/NK	RTL8C
5.33563573001862E-07	0.287861370682722	0.156	0.038	0.0089414583563652	Proliferating T/NK	RBBP5

5.37357117223072E-07	0.394178595332231	0.267	0.088	0.00900503057042423	Proliferating T/NK	RAP1GDS1
5.40973088541175E-07	0.561992086088761	0.178	0.047	0.00906562701777301	Proliferating T/NK	CCNC
5.48512054458885E-07	0.411636446321568	0.456	0.184	0.009191965008622	Proliferating T/NK	RER1
5.55465698463096E-07	1.00501662698619	0.122	0.026	0.00930849417484456	Proliferating T/NK	BCKDHB
5.56172194670473E-07	0.670040644450177	0.144	0.034	0.00932033363828779	Proliferating T/NK	TTC5
5.63947727998325E-07	0.425417497729364	0.122	0.026	0.00945063602579593	Proliferating T/NK	FAAP100
5.74764230146716E-07	1.23341546088002	0.144	0.035	0.00963189896879866	Proliferating T/NK	DGCR6L
5.7490988279599E-07	0.451712437968729	0.267	0.089	0.0096343398158952	Proliferating T/NK	NSF
5.79269335338281E-07	0.743296474459879	0.189	0.053	0.00970739552159891	Proliferating T/NK	PEX19
5.87679753279852E-07	0.291043563201949	0.222	0.068	0.00984833730546375	Proliferating T/NK	AQR
5.88921011746225E-07	0.677926029847631	0.189	0.053	0.00986913831484324	Proliferating T/NK	TTI1
5.92385395673289E-07	0.922600113812035	0.122	0.026	0.00992719446069297	Proliferating T/NK	DMAC2
5.94719158217322E-07	0.484393287043075	0.189	0.052	0.00996630365340588	Proliferating T/NK	SMARCAL1
5.9748500191195E-07	1.37473158228949	0.133	0.03	0.0100126536620405	Proliferating T/NK	NSDHL
6.09250267333756E-07	0.993235930833605	0.167	0.044	0.0102098159799791	Proliferating T/NK	MCUR1
6.20431834705546E-07	0.346599503162019	0.211	0.063	0.0103971966859955	Proliferating T/NK	ATG5
6.21455118581887E-07	1.00795018167417	0.111	0.022	0.0104143448771953	Proliferating T/NK	CTNND1
6.2464120974698E-07	0.28043510664517	0.178	0.048	0.0104677373929399	Proliferating T/NK	RIC8A
6.26662198077568E-07	0.633192701137622	0.244	0.079	0.0105016051153839	Proliferating T/NK	CLTB
6.26916599260666E-07	0.550619600917692	0.122	0.026	0.0105058683704102	Proliferating T/NK	DNAJC14
6.37797975648767E-07	0.605806406638561	0.189	0.053	0.010688218475922	Proliferating T/NK	CFAP298
6.3902074058177E-07	0.253731488983743	0.222	0.068	0.0107087095706693	Proliferating T/NK	ASF1A
6.43176708429655E-07	0.272553586489381	0.789	0.4	0.0107783552798642	Proliferating T/NK	S100A4
6.5137639728286E-07	0.686500837219616	0.156	0.039	0.0109157656656662	Proliferating T/NK	CTNNBL1
6.53400583160716E-07	0.43902068024639	0.267	0.089	0.0109496869726073	Proliferating T/NK	PRKAB1
6.58176421267502E-07	0.309249468284315	0.311	0.109	0.0110297204676008	Proliferating T/NK	C6orf62
6.58848638182174E-07	1.11807937820622	0.144	0.035	0.0110409854786569	Proliferating T/NK	PLXNB2
6.59300487652331E-07	1.19177699397099	0.1	0.018	0.0110485575720778	Proliferating T/NK	TIGD7
6.596295732909E-07	0.790310042351734	0.156	0.039	0.0110540723892089	Proliferating T/NK	COQ6
6.78288972050491E-07	0.48303112398781	0.133	0.03	0.0113667665936221	Proliferating T/NK	ERLIN1
6.85689857515132E-07	0.538574210758359	0.378	0.149	0.0114907906322386	Proliferating T/NK	NARS
6.89986609587324E-07	0.57224374634862	0.144	0.035	0.0115627956034644	Proliferating T/NK	NSRP1
6.92987175710824E-07	1.25953406461423	0.111	0.022	0.011613079090562	Proliferating T/NK	DEPRECATED-ENSG00000181061
7.00079050384935E-07	1.04402773995261	0.133	0.03	0.0117319247263507	Proliferating T/NK	ACTA2
7.13540266241494E-07	0.765054974666856	0.156	0.039	0.011957507781675	Proliferating T/NK	COPG2
7.19993594414922E-07	1.19621608359817	0.111	0.022	0.0120656526552053	Proliferating T/NK	SYNJ2BP
7.22595462259219E-07	1.19445692759006	0.1	0.018	0.01210925475654	Proliferating T/NK	INTS5
7.24350895945227E-07	0.262358130767638	0.478	0.197	0.0121386723142501	Proliferating T/NK	CAPN1
7.24952740615303E-07	0.450289728912858	0.356	0.131	0.0121487580272312	Proliferating T/NK	RDH11
7.25751294100889E-07	0.402706702108038	0.1	0.018	0.0121621401865427	Proliferating T/NK	FGFR1
7.39817922983494E-07	0.653405137136228	0.267	0.089	0.0123978687533574	Proliferating T/NK	DNPH1
7.48215684898562E-07	1.28983823697093	0.1	0.018	0.0125385984475301	Proliferating T/NK	PHYH
7.50286390564201E-07	0.402155811816605	0.2	0.057	0.0125732993330749	Proliferating T/NK	DDX49
7.64612637327268E-07	0.531057371500023	0.156	0.039	0.0128133785763304	Proliferating T/NK	CDAN1
7.67226537419349E-07	0.658953532165089	0.178	0.049	0.0128571823140734	Proliferating T/NK	RTL10
7.70223958728324E-07	0.943668413288516	0.211	0.063	0.0129074131003693	Proliferating T/NK	TKFC
7.80885634219626E-07	0.543700424048299	0.189	0.053	0.0130860814582525	Proliferating T/NK	DEPRECATED-ENSG00000155115
7.96282948697823E-07	0.303522688790517	0.211	0.062	0.0133441096542781	Proliferating T/NK	UEVLD
7.987855201106E-07	1.732589082205	0.111	0.023	0.0133860477460134	Proliferating T/NK	UQCC3
8.06196866536162E-07	0.669342451194444	0.122	0.026	0.013510247089413	Proliferating T/NK	FARP2
8.10522643890893E-07	0.850420384207855	0.189	0.054	0.0135827384663236	Proliferating T/NK	NIPSNAP1
8.10546531709974E-07	0.489990248623096	0.278	0.096	0.0135831387783957	Proliferating T/NK	SIPA1L1
8.1272136196109E-07	0.511574110811413	0.189	0.054	0.013619584583744	Proliferating T/NK	APEX2
8.1823051175316E-07	1.21543583309807	0.122	0.026	0.0137119069159594	Proliferating T/NK	DPH3
8.23498917997396E-07	0.480536729868865	0.2	0.059	0.0138001948678004	Proliferating T/NK	MYO1C
8.30608009985496E-07	0.78481487517157	0.133	0.031	0.0139193290313369	Proliferating T/NK	MTHFD1L
8.31197772295432E-07	0.397013549437883	0.167	0.044	0.0139292122681268	Proliferating T/NK	CIAO3
8.33488355261071E-07	1.38277732336093	0.133	0.031	0.013967597857465	Proliferating T/NK	RAB15
8.45548160586855E-07	0.431005853804674	0.278	0.096	0.0141696960751145	Proliferating T/NK	TMEM64
8.47230136151532E-07	0.383345094624296	0.189	0.054	0.0141978826216274	Proliferating T/NK	RIOK1
8.47673477894717E-07	1.33401885427998	0.111	0.023	0.0142053121425597	Proliferating T/NK	TTC38
8.52377453093944E-07	0.630125172085647	0.156	0.039	0.0142841413589483	Proliferating T/NK	CASP8AP2
8.6974387547241E-07	0.263697828628316	0.2	0.058	0.0145751678651666	Proliferating T/NK	CEBPZOS
8.94330807163891E-07	0.317340577420558	0.289	0.101	0.0149871956664525	Proliferating T/NK	KXD1
9.01072953848195E-07	0.291278576432997	0.489	0.211	0.0151001805605881	Proliferating T/NK	PSMF1
9.06563976633436E-07	0.867652549938812	0.111	0.023	0.0151921991204231	Proliferating T/NK	MAP3K13

9.20923940028532E-07	0.846942572795252	0.156	0.039	0.0154328433869981	Proliferating T/NK	UROD
9.25660965424672E-07	0.599246695119635	0.2	0.059	0.0155122264585866	Proliferating T/NK	TCAF1
9.25982849555152E-07	0.717513687595947	0.244	0.081	0.0155176205928452	Proliferating T/NK	NCALD
9.34643619390933E-07	0.251102709648162	0.322	0.116	0.0156627577737533	Proliferating T/NK	DNASE2
9.61597088361588E-07	0.41536408621221	0.156	0.039	0.0161144440067635	Proliferating T/NK	ATP6V1D
9.6546626371588E-07	0.35561312409999	0.167	0.044	0.0161792836473507	Proliferating T/NK	UBE2Q2
9.70157233168502E-07	1.50743709240015	0.133	0.031	0.0162578949134378	Proliferating T/NK	DCTPP1
9.73838152049041E-07	0.421493173293228	0.278	0.096	0.0163195797520378	Proliferating T/NK	DEPRECATED-ENSG00000179010
9.9313624436188E-07	0.426733856359577	0.178	0.049	0.0166429771830164	Proliferating T/NK	INTS9
9.97938217970615E-07	0.331928881202553	0.222	0.07	0.0167234486567516	Proliferating T/NK	CNOT1
1.01233523929756E-06	0.3379044619641	0.344	0.131	0.0169647139401485	Proliferating T/NK	MFHAS1
1.02022884000175E-06	1.35753432661568	0.111	0.023	0.0170969949007493	Proliferating T/NK	PPAT
1.02644971054833E-06	0.258169629173013	0.267	0.091	0.0172012442493689	Proliferating T/NK	COLGALT1
1.02849125096324E-06	0.34919194332508	0.222	0.069	0.017235456383642	Proliferating T/NK	SLC25A46
1.03055653803788E-06	0.287508939447422	0.278	0.096	0.0172700664644389	Proliferating T/NK	PHKB
1.03060129066135E-06	0.658356502606192	0.211	0.064	0.0172708164289028	Proliferating T/NK	AURKAIP1
1.05278105415169E-06	0.901098456400936	0.189	0.054	0.017642504905474	Proliferating T/NK	OST4
1.06012564393627E-06	0.309302661658455	0.167	0.044	0.017765585541084	Proliferating T/NK	SLC38A5
1.06032029846387E-06	1.30049774122187	0.156	0.041	0.0177688475616575	Proliferating T/NK	MAGED1
1.06842171479351E-06	0.58634433843021	0.2	0.059	0.0179046110965097	Proliferating T/NK	VPS72
1.08288165272748E-06	0.822148014088377	0.211	0.065	0.018146930736407	Proliferating T/NK	POLR2I
1.08516174904063E-06	0.320998876257103	0.222	0.07	0.0181851405904229	Proliferating T/NK	MIOS
1.12239458035419E-06	0.343059980701224	0.267	0.091	0.0188090883775755	Proliferating T/NK	NAA10
1.12373495735227E-06	0.305459637007305	0.2	0.06	0.0188315504153093	Proliferating T/NK	TMEM94
1.12387193945004E-06	0.40480005916937	0.178	0.05	0.0188338459613038	Proliferating T/NK	POLR2D
1.13415802945728E-06	0.86460553431383	0.111	0.023	0.0190062202576451	Proliferating T/NK	SMIM20
1.15864341281269E-06	0.745250984939222	0.167	0.046	0.0194165463119151	Proliferating T/NK	DBP
1.16576278096239E-06	0.531700646516907	0.2	0.059	0.0195358526833677	Proliferating T/NK	SLC25A20
1.18801388934881E-06	0.993127363139388	0.122	0.027	0.0199087367577073	Proliferating T/NK	BCKDK
1.19815711288255E-06	0.550819711971578	0.189	0.055	0.0200787168976857	Proliferating T/NK	GMEB1
1.207107900374E-06	0.275565263720908	0.533	0.222	0.0202287141944675	Proliferating T/NK	MT-ND1
1.21458837215101E-06	0.49641301974582	0.211	0.065	0.0203540719405067	Proliferating T/NK	TPRA1
1.21461023027676E-06	0.270289621617047	0.2	0.06	0.0203544382389779	Proliferating T/NK	PLXNC1
1.21646798540745E-06	1.17869379503305	0.144	0.036	0.020385570499458	Proliferating T/NK	SAYSD1
1.22113800335777E-06	0.36229632435345	0.222	0.07	0.0204638306602695	Proliferating T/NK	TCP11L1
1.23925660968567E-06	1.17997917408267	0.133	0.031	0.0207674622651125	Proliferating T/NK	CHCHD5
1.24970449826437E-06	1.00350876065326	0.122	0.027	0.0209425479819143	Proliferating T/NK	LHPP
1.29762303352843E-06	0.594492620565411	0.2	0.06	0.0217455667958695	Proliferating T/NK	ROCK2
1.31509641443901E-06	0.457402397768439	0.167	0.045	0.0220383857131689	Proliferating T/NK	BAG1
1.31632262681067E-06	0.537174471383428	0.189	0.054	0.0220589345800932	Proliferating T/NK	C7orf50
1.32518670823417E-06	0.421161523420644	0.156	0.04	0.0222074788565882	Proliferating T/NK	STK16
1.33515938418018E-06	0.822189597229862	0.189	0.055	0.0223746009600915	Proliferating T/NK	KARS
1.35631056035984E-06	0.306247475844448	0.233	0.075	0.0227290523705102	Proliferating T/NK	DHX38
1.36055744478465E-06	0.51033371311629	0.156	0.041	0.0228002233355011	Proliferating T/NK	ZDHHC13
1.392937505434E-06	0.80408391564632	0.167	0.045	0.0233428467160629	Proliferating T/NK	CHRAC1
1.39842786941785E-06	0.660051333622618	0.144	0.036	0.0234348542357044	Proliferating T/NK	VPS35
1.4191259102555E-06	0.967385457626368	0.178	0.051	0.0237817120040618	Proliferating T/NK	CHST14
1.42308130689214E-06	0.444443251105375	0.267	0.093	0.0238479965408985	Proliferating T/NK	TMX2
1.42971696387281E-06	0.285614176693988	0.156	0.041	0.0239591968805806	Proliferating T/NK	PEAK1
1.45848732367384E-06	0.363146787833748	0.756	0.395	0.0244413305701262	Proliferating T/NK	ITM2A
1.46956889804587E-06	0.710607868259374	0.122	0.027	0.0246270355934527	Proliferating T/NK	HSD17B12
1.49109775914783E-06	0.595718977785423	0.222	0.071	0.0249878162477993	Proliferating T/NK	GRAMD4
1.5297653922337E-06	1.42416044977725	0.111	0.023	0.0256358084430523	Proliferating T/NK	MAOB
1.53822557238188E-06	0.348137519258267	0.189	0.054	0.0257775841419755	Proliferating T/NK	ZMPSTE24
1.58952097921662E-06	0.481394423183797	0.222	0.071	0.0266371925697121	Proliferating T/NK	SPATA20
1.59137311018021E-06	0.433807411868349	0.189	0.055	0.0266682305803999	Proliferating T/NK	GOLGA1
1.61340054871018E-06	0.996477719517584	0.133	0.032	0.0270373663952852	Proliferating T/NK	LANCL2
1.6201026826796E-06	0.908559195509204	0.111	0.023	0.0271496807563447	Proliferating T/NK	GTPBP8
1.63647836959603E-06	0.525129690478009	0.122	0.028	0.0274241045176903	Proliferating T/NK	GEMIN4
1.65066614461891E-06	0.536050024908999	0.156	0.041	0.0276618632515237	Proliferating T/NK	GTF3C5
1.67514920420967E-06	0.578733006901558	0.178	0.051	0.0280721503641456	Proliferating T/NK	MYO1E
1.70941213700786E-06	0.913432349895609	0.189	0.056	0.0286463285919778	Proliferating T/NK	DUSP23
1.73631354151502E-06	1.61965196755765	0.1	0.02	0.0290971423287087	Proliferating T/NK	ORC5
1.76451331292565E-06	0.384713680881342	0.278	0.096	0.029569714098008	Proliferating T/NK	FAM174C
1.78951192937599E-06	0.345764427574381	0.256	0.087	0.0299886409124828	Proliferating T/NK	LRRC41
1.79783254716656E-06	0.28826994438542	0.233	0.076	0.0301280778254173	Proliferating T/NK	XPO1

1.81582138603947E-06	0.266488560291932	0.156	0.041	0.0304295347872494	Proliferating T/NK	DESI2
1.81729380822243E-06	0.402300215823885	0.211	0.066	0.0304542096381915	Proliferating T/NK	CERS5
1.83370480110457E-06	1.85861982335777	0.122	0.028	0.0307292250569103	Proliferating T/NK	SCLY
1.85126460365434E-06	0.590369910248153	0.111	0.023	0.0310234922280394	Proliferating T/NK	DEPRECATED-ENSG00000108064
1.85126524364663E-06	0.511928594892	0.111	0.023	0.0310235029530302	Proliferating T/NK	KIF3C
1.85430731193507E-06	1.085309123926	0.1	0.02	0.031074481933408	Proliferating T/NK	LYRM4
1.8791958614135E-06	0.442295171250751	0.133	0.032	0.0314915642455675	Proliferating T/NK	PRMT3
1.88071271179183E-06	0.252669416419562	0.8	0.37	0.0315169836242074	Proliferating T/NK	MT-ND2
1.88249878615687E-06	0.738188639562993	0.144	0.037	0.0315469146584169	Proliferating T/NK	CLPB
1.90552985739761E-06	1.74820901118417	0.111	0.024	0.0319328693502692	Proliferating T/NK	CD200
1.93361986407826E-06	1.03118446516009	0.156	0.041	0.0324036016822235	Proliferating T/NK	KDELR1
1.95882516929918E-06	0.757444434496083	0.178	0.051	0.0328259921871157	Proliferating T/NK	ZNF277
1.96609329257305E-06	0.576287920911109	0.2	0.061	0.0329477913969391	Proliferating T/NK	IFT27
1.97330574893755E-06	0.913037757124902	0.122	0.028	0.0330686577406954	Proliferating T/NK	OSGIN2
2.00524595695901E-06	0.286640349320213	0.267	0.092	0.0336039117467191	Proliferating T/NK	C21orf91
2.01075481445096E-06	0.355874884048915	0.256	0.089	0.0336962291805692	Proliferating T/NK	ARL8B
2.01462077763198E-06	0.537408289936772	0.156	0.041	0.0337610149915567	Proliferating T/NK	SDHAF3
2.11609211771196E-06	0.363843019091561	0.333	0.124	0.0354614717086171	Proliferating T/NK	HADHB
2.13003446936888E-06	0.658776962814788	0.133	0.032	0.0356951176376837	Proliferating T/NK	DEPRECATED-ENSG00000254986
2.1471876966458E-06	0.367115175756265	0.167	0.046	0.0359825714203904	Proliferating T/NK	ZNF275
2.14861753233757E-06	0.34355779357645	0.1	0.02	0.0360065326069129	Proliferating T/NK	SIAH1
2.15823567558077E-06	0.735205528456704	0.133	0.032	0.0361677134513825	Proliferating T/NK	CCR1
2.16461620895782E-06	0.302153519498323	0.344	0.132	0.0362746384297151	Proliferating T/NK	ANXA7
2.17045305264805E-06	0.361488613560727	0.144	0.037	0.036372452256276	Proliferating T/NK	ALDH16A1
2.17247068769637E-06	0.483729459048266	0.133	0.032	0.0364062637844158	Proliferating T/NK	FLNB
2.18101196760531E-06	0.868613281368343	0.133	0.033	0.0365493985531299	Proliferating T/NK	TBP
2.18797350749179E-06	0.525087673196587	0.156	0.041	0.0366660600385474	Proliferating T/NK	MPHOSPH10
2.18902608732268E-06	1.67910066334523	0.111	0.024	0.0366836991713535	Proliferating T/NK	TMEM251
2.25629642127833E-06	0.343882333091875	0.333	0.126	0.0378110154277823	Proliferating T/NK	EMC7
2.28018557957686E-06	0.912481362197142	0.144	0.037	0.0382113499425491	Proliferating T/NK	POLR1B
2.28510826248418E-06	0.298546014704692	0.244	0.083	0.03829384426271	Proliferating T/NK	AP2M1
2.30492679761312E-06	0.635313294898015	0.244	0.083	0.0386259632744006	Proliferating T/NK	ATPAF1
2.30737788962443E-06	0.56933555881667	0.222	0.071	0.0386670386743262	Proliferating T/NK	CERS6
2.3924208900253E-06	0.402093533132522	0.244	0.081	0.0400921892750439	Proliferating T/NK	GLUD1
2.40348090409908E-06	0.623261365970463	0.211	0.066	0.0402775329908923	Proliferating T/NK	LGALS3BP
2.40940627223584E-06	0.6167133980311	0.233	0.078	0.0403768303101282	Proliferating T/NK	FBXL6
2.40983114083559E-06	0.97186167667334	0.289	0.113	0.0403839502581228	Proliferating T/NK	MS4A6A
2.41675068097001E-06	0.405504465199661	0.356	0.138	0.0404999079116955	Proliferating T/NK	PTMS
2.43063238413857E-06	1.26237653446297	0.122	0.028	0.0407325374933941	Proliferating T/NK	NME4
2.49417462822195E-06	0.621537267654038	0.111	0.024	0.0417973784197435	Proliferating T/NK	GPX7
2.52323733030724E-06	0.321072054628953	0.244	0.083	0.0422844111812888	Proliferating T/NK	PYCARD
2.54919896588068E-06	0.846376689199537	0.2	0.062	0.0427194762702284	Proliferating T/NK	TRIM11
2.55643912300749E-06	0.418537673971583	0.167	0.046	0.0428408068233595	Proliferating T/NK	CWC22
2.55825700212055E-06	0.809586737093326	0.267	0.096	0.0428712708415362	Proliferating T/NK	ATOX1
2.57086838439084E-06	0.381645907794859	0.167	0.047	0.0430826123856217	Proliferating T/NK	ARMC6
2.58679345864652E-06	0.542328899706424	0.167	0.047	0.0433494847799983	Proliferating T/NK	ERI3
2.67535685344008E-06	0.282109595837667	0.178	0.051	0.0448336301499488	Proliferating T/NK	LGALS9
2.67629196443466E-06	0.839153201454137	0.133	0.033	0.044849300739996	Proliferating T/NK	SSBP3
2.69589333427773E-06	0.998995310597293	0.122	0.028	0.0451777804958261	Proliferating T/NK	C1D
2.7649465431226E-06	0.846093261246217	0.156	0.042	0.0463349741696485	Proliferating T/NK	CPLANE1
2.78700732556714E-06	0.366106052648798	0.222	0.071	0.0467046687618541	Proliferating T/NK	DCAF12
2.79994671614203E-06	0.586057129848375	0.122	0.028	0.0469215070691082	Proliferating T/NK	MCU
2.80007844724529E-06	0.398676243954624	0.167	0.047	0.0469237146189365	Proliferating T/NK	LARS2
2.81831395970951E-06	0.313923133426658	0.133	0.033	0.0472293053368119	Proliferating T/NK	BTRC
2.86469072229446E-06	0.76379999819748	0.189	0.057	0.0480064871242106	Proliferating T/NK	SMARCA1
2.88793725766559E-06	0.733326689814332	0.122	0.028	0.0483960525639599	Proliferating T/NK	GEMIN6
2.8978761763567E-06	0.933298849555554	0.122	0.028	0.0485626089633855	Proliferating T/NK	NRBF2

SupTable5G_C1_Mye_SeuratCluster

p_val	avg_log2FC	pct.1	pct.2	p_val_adj	cluster	gene
1.484316333928E-86	4.01636528627424	0.383	0.024	2.48741731239654E-82	0	CCL13
2.48200479527063E-182	3.02489403705744	0.904	0.278	4.15934363591453E-178	0	RNASE1
1.01465728335921E-97	3.01173317128813	0.639	0.156	1.70036267545337E-93	0	SPP1
6.68718485829659E-113	2.97815746661727	0.556	0.065	1.12063843855334E-108	0	COLEC12
2.54046054255807E-48	2.95588726048739	0.3	0.047	4.25730377721881E-44	0	AKR1C2
1.54346840610957E-122	2.86802382913649	0.597	0.069	2.58654435495842E-118	0	IL2RA
1.29600056782545E-78	2.80816230508524	0.376	0.029	2.1718377515619E-74	0	KCNJ5
5.37819938626212E-160	2.50938047485849	0.9	0.333	9.01278653149806E-156	0	F13A1
2.50497154348304E-159	2.48976885140257	0.874	0.265	4.19783131256888E-155	0	EPHX1
7.34178534839057E-172	2.48666116394563	0.891	0.25	1.23033638868329E-167	0	FOLR2
3.11568913800893E-152	2.41464878548331	0.855	0.212	5.22127185747536E-148	0	CD209
1.89021147791214E-129	2.37518076130208	0.731	0.141	3.16761639468516E-125	0	GPR34
3.83468989898648E-56	2.33277148491585	0.417	0.09	6.42617333272155E-52	0	MUC3A
4.03773318167278E-63	2.28164043694906	0.469	0.11	6.76643326584725E-59	0	AKR1B10
1.32659689031318E-78	2.2703656794949	0.498	0.092	2.22311106878683E-74	0	ACE
1.66177240710908E-132	2.26667779410582	0.814	0.233	2.78479819983339E-128	0	ALDH1A1
8.71003557047396E-49	2.23975900434654	0.396	0.102	1.45962776090003E-44	0	CBR1
5.28104854246265E-93	2.21427078702173	0.525	0.077	8.84998114745891E-89	0	WLS
5.12638517608336E-44	2.20945581247001	0.331	0.072	8.59079627808049E-40	0	ST5
1.27939855435959E-61	2.17344774269114	0.386	0.064	2.1440160973958E-57	0	NAGLU
6.42990471285571E-64	2.16612953914827	0.475	0.11	1.07752343178036E-59	0	GSTA1
1.79243315372065E-104	2.13648799002428	0.721	0.207	3.00375947900507E-100	0	GATM
1.1854483423227E-68	2.13334567357674	0.48	0.101	1.98657433206437E-64	0	ADORA3
2.89975936607504E-60	2.11380054842892	0.53	0.152	4.85941674566855E-56	0	MMP9
9.2038600186551E-35	2.0962885914969	0.269	0.057	1.54238286192622E-30	0	BAAT
2.13299726548659E-52	2.08817927150437	0.364	0.071	3.57447681750243E-48	0	HYAL2
1.65693717153869E-49	2.06169808158137	0.376	0.083	2.77669531206454E-45	0	NBL1
1.51367782983623E-30	2.0542181784569	0.251	0.058	2.53662130723955E-26	0	RNASE4
7.16893979521575E-31	2.02315481659946	0.258	0.062	1.20137093088226E-26	0	TMEM9
4.412441789764E-34	2.00316322764962	0.257	0.052	7.39436995128651E-30	0	SULT1C2
1.12348274139758E-110	1.98688032462686	0.771	0.236	1.88273237803407E-106	0	CPM
9.10448182363907E-35	1.96823418421235	0.266	0.055	1.52572906400544E-30	0	LRP3
1.71737475692689E-152	1.96315803831575	0.939	0.381	2.87797661765809E-148	0	DAB2
4.63381823369252E-61	1.95126219031306	0.348	0.044	7.76535259602193E-57	0	FAM174B
2.20580964314629E-33	1.94223560974351	0.266	0.06	3.69649579998455E-29	0	SLPI
2.73074394551976E-106	1.92340625287418	0.649	0.132	4.57618070390202E-102	0	PMP22
4.68609541712443E-124	1.91678281584684	0.9	0.375	7.85295870001712E-120	0	CTSD
1.51241989468376E-106	1.91400920295631	0.771	0.26	2.53451325951104E-102	0	IDH1
1.31494337083446E-43	1.90154720266104	0.287	0.047	2.2035821008444E-39	0	HPGDS
3.30036251619753E-33	1.9000202434763	0.264	0.059	5.53074750464382E-29	0	TMEM236
6.289546719661E-54	1.89780647512756	0.379	0.075	1.05400223928079E-49	0	SLC2A8
8.28664216677963E-61	1.88767129008311	0.554	0.172	1.38867549430893E-56	0	CES1
2.34342555755618E-61	1.87801369457524	0.427	0.085	3.92711254935264E-57	0	TREM2
5.60705136857597E-125	1.8642659771119	0.874	0.309	9.3962966834596E-121	0	A2M
9.46100636430192E-29	1.85939600355956	0.263	0.07	1.58547544652972E-24	0	MUC13
1.7326566153813E-34	1.85685635831803	0.315	0.084	2.90358595605599E-30	0	RHOBTB3
1.14661540506578E-85	1.84956814373307	0.668	0.189	1.92149809580923E-81	0	PTGR1

3.86285817698133E-49	1.84736044921838	0.293	0.04	6.47337773298532E-45	0	CD28
2.16576289577062E-37	1.8216718426589	0.345	0.096	3.62938546073241E-33	0	MCFD2
3.5809103076936E-72	1.81824169621841	0.569	0.149	6.00088949363294E-68	0	PDK4
6.61966791899596E-41	1.81607017806533	0.373	0.105	1.10932394986534E-36	0	PCK2
2.67569873548255E-66	1.80931916847982	0.38	0.049	4.48393594092167E-62	0	SLC7A8
1.16287712335517E-58	1.80612003537358	0.504	0.138	1.94874948331859E-54	0	GPX2
7.57277679352901E-106	1.80424234737263	0.887	0.4	1.26904593505959E-101	0	APOE
1.60781567175416E-53	1.79692057154504	0.48	0.137	2.69437750272562E-49	0	H1F0
1.10135702597072E-62	1.79441042293159	0.522	0.147	1.84565410412173E-58	0	BEX3
2.36520902680146E-35	1.79267018321627	0.322	0.086	3.96361728711389E-31	0	POR
1.38205360108689E-27	1.7921152149963	0.258	0.069	2.3160454247014E-23	0	FAH
4.25554445841778E-116	1.78036125840542	0.893	0.434	7.13144140341652E-112	0	PLD3
5.41845463492416E-118	1.77195745716579	0.843	0.257	9.08024627720591E-114	0	NRP1
1.67422806546604E-32	1.77068038313102	0.257	0.056	2.80567139210799E-28	0	S100A16
1.34318890964835E-30	1.76545869285999	0.273	0.07	2.2509159747887E-26	0	PEG10
1.47786023786115E-38	1.76178487575359	0.385	0.117	2.47659818660772E-34	0	DTNA
8.155532428001E-97	1.76134094579321	0.642	0.148	1.36670412428441E-92	0	VAT1
8.86527863396396E-67	1.75774024597656	0.547	0.153	1.48564339347968E-62	0	NUPR1
2.54755706810918E-42	1.75431060874777	0.322	0.068	4.26919613473737E-38	0	HSD3B7
9.36071785495843E-71	1.74953663891164	0.62	0.193	1.56866909813393E-66	0	UGDH
2.4908530344694E-94	1.74587101155563	0.644	0.156	4.17417151516382E-90	0	CD59
9.20958143297644E-24	1.7442658596541	0.263	0.084	1.54334165653819E-19	0	HSD17B6
2.61985201114472E-36	1.74148540874345	0.269	0.054	4.39034800027631E-32	0	CTDSPL
7.05056616632208E-28	1.72804468502036	0.254	0.066	1.18153387815225E-23	0	CMBL
7.11596562091479E-119	1.72695421413303	0.906	0.421	1.1924935187529E-114	0	MRC1
4.08973559957979E-56	1.72579148900852	0.54	0.175	6.85357891777581E-52	0	CERS2
2.2760006099862E-22	1.71615062555093	0.27	0.095	3.81412182221487E-18	0	SERPINA6
3.90859283503573E-31	1.70922038808784	0.35	0.115	6.55001987295288E-27	0	UGT2B4
8.23574582096053E-27	1.70752208281006	0.269	0.078	1.38014628467656E-22	0	CCNG1
9.28515403207119E-39	1.70212954698978	0.26	0.043	1.55600611269449E-34	0	GAL3ST4
1.18524568514426E-92	1.6955697040051	0.724	0.22	1.98623471916475E-88	0	ENPP2
4.48917066365686E-32	1.68547500980697	0.34	0.106	7.52295219815616E-28	0	RARRES2
5.95960354934684E-29	1.67544720770718	0.255	0.065	9.98710362799544E-25	0	RPP25
1.01249250742974E-105	1.67448696742331	0.914	0.391	1.69673494395075E-101	0	STAB1
2.15330189287571E-43	1.67185590542297	0.454	0.151	3.60850331208112E-39	0	LGALS4
6.70324880540741E-39	1.67103575339977	0.345	0.089	1.12333043481017E-34	0	NAPRT
9.95183185081483E-40	1.67033106464673	0.293	0.058	1.66772798155955E-35	0	SCN9A
1.63712361766104E-81	1.66945145710909	0.691	0.22	2.74349175847637E-77	0	ALDOB
8.20999853317896E-29	1.66456994686536	0.253	0.064	1.37583155419013E-24	0	CLTC
3.7067298280091E-39	1.66440911810062	0.361	0.102	6.21173784577765E-35	0	PNPO
6.24964053394076E-44	1.65584131610654	0.438	0.138	1.04731476067779E-39	0	MGST1
1.53226651879605E-119	1.65461005526275	0.894	0.358	2.56777223219843E-115	0	SLCO2B1
2.22143361892897E-30	1.6472728891026	0.293	0.083	3.72267845860117E-26	0	QDPR
8.3503891487341E-38	1.64545351256813	0.357	0.102	1.39935821354486E-33	0	ERRFI1
9.45347609002261E-120	1.64517008308394	0.917	0.52	1.58421352316599E-115	0	CTSC
2.10737884954891E-41	1.64210049389674	0.258	0.037	3.53154547607406E-37	0	ANTXR1
3.35965970841976E-60	1.64195721809516	0.498	0.131	5.63011773936984E-56	0	IGFBP4
4.57283404728907E-54	1.63727274867066	0.431	0.106	7.66315529644702E-50	0	CD276
3.17739954652981E-90	1.63690624932167	0.765	0.258	5.32468616007466E-86	0	ANXA4
2.63367709568268E-60	1.63547755964022	0.557	0.176	4.41351607694503E-56	0	MPC2

**1.88571976365614E-46**	1.63212265171807	0.453	0.142	3.16008917993496E-42	0	AIFM1
**2.93967102021264E-34**	1.63152922266368	0.351	0.106	4.92630069567234E-30	0	ADH1C
**6.61404780573485E-85**	1.62621263055565	0.833	0.346	1.10838213128505E-80	0	GPNMB
**2.30749359048767E-54**	1.62407247600069	0.421	0.097	3.86689775893924E-50	0	GNPDA1
**9.81806273444935E-26**	1.61869756836745	0.251	0.07	1.64531095303902E-21	0	GAMT
**5.74795302260516E-102**	1.61638463826893	0.772	0.231	9.63241967528173E-98	0	MMP14
**3.20791268186098E-27**	1.61351899600641	0.257	0.068	5.37582007226263E-23	0	CISD3
**1.39694554974803E-36**	1.61105606889775	0.348	0.099	2.34100135226775E-32	0	ITPKC
**1.16939584385217E-79**	1.60365657239387	0.48	0.072	1.95967355512747E-75	0	IGF1
**2.63353963027232E-99**	1.60274970408541	0.705	0.172	4.41328571241035E-95	0	TSPAN4
**5.55555554739514E-38**	1.59325457422403	0.478	0.187	9.30999998632478E-34	0	AGT
**8.22330314688427E-112**	1.59038707265275	0.917	0.44	1.37806114135487E-107	0	SLC40A1
**3.44927288207615E-32**	1.58797720449242	0.38	0.136	5.78029149578321E-28	0	SOD1
**9.76265548018822E-34**	1.58402916599802	0.409	0.15	1.63602580536994E-29	0	TDO2
**7.84343644611513E-42**	1.58057537067278	0.456	0.157	1.31440307963997E-37	0	PRDX2
**6.50091949246941E-102**	1.58009227278584	0.758	0.215	1.08942408854802E-97	0	PIK3IP1
**8.32789467456627E-94**	1.57387330301891	0.668	0.154	1.39558858956382E-89	0	CMKLR1
**3.02533225179682E-85**	1.57229110218215	0.578	0.119	5.06985178756111E-81	0	TCN2
**1.22373869864158E-94**	1.57107379609417	0.792	0.265	2.05074131118356E-90	0	FUCA1
**1.43870173298058E-67**	1.56716621844259	0.612	0.188	2.41097636412885E-63	0	IFI27
**3.29798034395502E-79**	1.56632555718114	0.601	0.146	5.52675546039982E-75	0	DHRS3
**6.06164038455356E-107**	1.56410335690027	0.848	0.271	1.01580969564349E-102	0	MS4A4A
**6.06615720918721E-79**	1.55814311663183	0.739	0.257	1.01656662511559E-74	0	SPINK1
**2.40647476368269E-34**	1.54848102441402	0.337	0.096	4.03277040897945E-30	0	SLC9A3R1
**6.29191202570197E-72**	1.54845192554733	0.682	0.228	1.05439861726714E-67	0	PGRMC1
**2.87068322175606E-90**	1.54540251335293	0.61	0.127	4.81069094301881E-86	0	PLA2G7
**6.20701284262828E-93**	1.54271498075871	0.671	0.155	1.04017121216765E-88	0	NPL
**4.11351443068595E-30**	1.53650915440106	0.292	0.083	6.89342748294352E-26	0	TEX261
**3.83569041622725E-45**	1.53387093570879	0.454	0.144	6.42784999951363E-41	0	TALDO1
**1.02893766077054E-91**	1.53115970914719	0.698	0.189	1.72429373191927E-87	0	PDGFC
**1.1095720328249E-68**	1.52234072168936	0.422	0.065	1.85942081260797E-64	0	GYPC
**4.16704859678553E-34**	1.51275672261654	0.354	0.109	6.98314003849319E-30	0	TMEM14A
**2.27774973266739E-24**	1.51103993019857	0.283	0.094	3.81705300200401E-20	0	DUSP23
**1.95981303852628E-62**	1.51022574724483	0.448	0.095	3.28425468996234E-58	0	RELL1
**2.99834515418785E-69**	1.50998644661768	0.541	0.138	5.024626809388E-65	0	TNFSF12
**1.52537908275095E-42**	1.50438983504281	0.327	0.068	2.55623026687404E-38	0	GBGT1
**4.75665087225893E-41**	1.4973188318907	0.456	0.153	7.97119553173151E-37	0	ETFB
**1.23115247536577E-29**	1.49282811295389	0.312	0.099	2.06316531821795E-25	0	LACTB2
**3.68116476077738E-91**	1.49014714139092	0.84	0.278	6.16889590611074E-87	0	PLTP
**5.86846887938482E-68**	1.48698854547718	0.695	0.25	9.83438014807307E-64	0	GCLC
**1.7622334076827E-75**	1.48253703942478	0.794	0.329	2.95315074459467E-71	0	CD93
**3.23637505293867E-79**	1.48189659823135	0.685	0.211	5.42351731371462E-75	0	C2
**3.6470721363754E-48**	1.48067754760011	0.446	0.128	6.1117634861379E-44	0	PNKD
**1.34313891430903E-25**	1.46972375910143	0.27	0.082	2.25083219259908E-21	0	ORM2
**3.99161858052105E-25**	1.46646821191446	0.303	0.104	6.68915441723717E-21	0	HACD3
**6.30569280024881E-52**	1.46524161946844	0.601	0.227	1.0567079994657E-47	0	AKR1C3
**1.65895494369258E-28**	1.46456260037326	0.277	0.078	2.78007669464002E-24	0	MGMT
**1.49858818022659E-32**	1.4612571975615	0.322	0.094	2.51133407242371E-28	0	SULT2A1
**2.18903760027991E-73**	1.45757932331336	0.682	0.23	3.66838921054907E-69	0	ACP5
**1.91249325648028E-81**	1.44861767185588	0.681	0.214	3.20495619920965E-77	0	IQGAP2

1.14808962478635E-19	1.44587411370499	0.257	0.093	1.92396859321696E-15	0	PGM1
7.16281970130696E-40	1.44275844296799	0.422	0.135	1.20034532554502E-35	0	SCCPDH
8.89483719405967E-42	1.43998267690823	0.478	0.166	1.49059681698052E-37	0	AMBP
1.03891281464402E-21	1.42663945019115	0.27	0.095	1.74101009478045E-17	0	TSPAN8
1.0332085965383E-131	1.4265598959663	0.975	0.843	1.73145096607889E-127	0	CTSB
1.15223187176304E-72	1.42384169615935	0.66	0.216	1.9309101707005E-68	0	TMEM176A
2.52246633881648E-41	1.42329265001677	0.367	0.094	4.22714909058865E-37	0	GIMAP1
4.24683013969181E-26	1.42231064706682	0.313	0.106	7.11683794809554E-22	0	PLS3
2.71905918420047E-79	1.42216857343396	0.652	0.18	4.55659938088315E-75	0	ITM2C
6.66859403709897E-45	1.42131783380841	0.403	0.11	1.11752298873704E-40	0	PPM1M
8.17656063203316E-53	1.41381239846506	0.475	0.139	1.37022803071612E-48	0	RCAN1
3.53923055461984E-23	1.41166339935312	0.321	0.123	5.93104256343193E-19	0	HMGCS2
1.42266927273222E-59	1.40514927106925	0.419	0.083	2.38410916724466E-55	0	SGMS1
8.62817911779275E-107	1.40323662492964	0.926	0.526	1.44591025655971E-102	0	LAMP1
5.95411297727005E-89	1.40100373328104	0.848	0.355	9.97790252730915E-85	0	CREG1
5.27217176239604E-62	1.39184570934807	0.586	0.181	8.83510543942328E-58	0	SH3PXD2B
1.51599028550788E-32	1.38822075579348	0.264	0.061	2.54049652045411E-28	0	FECH
3.85137635412992E-52	1.38699649766459	0.501	0.152	6.45413649425092E-48	0	FUOM
1.64894771627182E-30	1.38519022267198	0.308	0.089	2.76330658292832E-26	0	ACTR10
5.97520202181339E-36	1.38476664841453	0.46	0.174	1.00132435481549E-31	0	SMARCA1
1.2865522904266E-86	1.38304484785384	0.936	0.632	2.15600432829689E-82	0	LGMN
3.65757028939118E-26	1.37994872630443	0.343	0.127	6.12935629096174E-22	0	GSTA2
3.3583515568642E-22	1.37993229839253	0.279	0.097	5.62792553899303E-18	0	HMGCS1
7.60351254608209E-91	1.37881654904811	0.836	0.328	1.27419663247244E-86	0	TNFSF13
2.80446718066617E-30	1.37582296112709	0.296	0.083	4.69972610136037E-26	0	RETSAT
1.14219450191559E-68	1.3722205938363	0.451	0.08	1.91408954631014E-64	0	GLMP
1.17980173130435E-92	1.37149548695834	0.938	0.491	1.97711174131983E-88	0	CD14
6.68288730663691E-75	1.36985812168918	0.579	0.13	1.11991825484621E-70	0	IGSF21
3.66174584410907E-30	1.36910706362539	0.374	0.133	6.13635368555797E-26	0	ZHX1
1.41721038987814E-81	1.36904745649282	0.833	0.308	2.37496117135779E-77	0	VTN
1.28766153786898E-42	1.36762502396967	0.403	0.113	2.15786320516083E-38	0	EMP2
3.88503395479708E-17	1.36527493493007	0.29	0.128	6.51053990144894E-13	0	CXCL9
1.85245957962405E-72	1.36332701130214	0.758	0.308	3.10435176353398E-68	0	IGSF6
1.18110810278549E-21	1.35978604327738	0.258	0.087	1.97930095864793E-17	0	RIDA
2.66709315372825E-25	1.35899692249609	0.382	0.157	4.4695147070178E-21	0	PIGR
2.47561118595813E-34	1.34892928034577	0.328	0.09	4.14862922542864E-30	0	UROD
5.45145454710384E-122	1.34856682547573	0.964	0.647	9.13554753003661E-118	0	C1QC
1.86747221432637E-20	1.33954462449516	0.277	0.098	3.12950993676813E-16	0	RBP4
1.41984051127763E-91	1.33910997372925	0.657	0.142	2.37936872879905E-87	0	CD163L1
4.52341240141431E-40	1.33701127946378	0.476	0.168	7.5803345022901E-36	0	CFH
3.19421620637473E-29	1.33635588544188	0.337	0.111	5.35286751864277E-25	0	CLDN7
3.5167523078568E-41	1.33268792461327	0.313	0.066	5.89337351750643E-37	0	GHDC
7.59462978876502E-48	1.33217843840288	0.392	0.097	1.27270806000124E-43	0	BAG5
2.3386565047627E-94	1.33195082004011	0.907	0.451	3.91912057068134E-90	0	TMEM176B
3.71407214352395E-24	1.32880642182497	0.279	0.091	6.22404209811743E-20	0	COA3
4.93015207966271E-36	1.32825683098383	0.412	0.139	8.26194885509877E-32	0	SDHB
1.42731422766038E-28	1.32543585242221	0.334	0.113	2.39189318271327E-24	0	HMGCL
7.42291054575584E-81	1.32536264793969	0.594	0.134	1.24393134925776E-76	0	TNFRSF21
1.90857014834601E-58	1.31908179521352	0.721	0.311	3.19838185459825E-54	0	CAPG
1.26132046924537E-61	1.31901528326653	0.525	0.144	2.11372084236139E-57	0	CD99

1.49614653781282E-55	1.3174730579185	0.576	0.192	2.50724236806672E-51	0	IL32
1.91524250659097E-46	1.31691523879392	0.541	0.193	3.20956339254514E-42	0	CD151
1.0031131805929E-36	1.31155098618399	0.454	0.166	1.68101706803758E-32	0	NQO1
3.75196740855572E-50	1.31041543390711	0.443	0.118	6.28754698325768E-46	0	BCAT1
2.4604140149465E-40	1.30865435667331	0.361	0.097	4.12316180624735E-36	0	GPX3
1.85361252063196E-28	1.30761039325242	0.327	0.108	3.10628386207503E-24	0	TP53INP1
4.36395232498568E-36	1.30550848321926	0.382	0.118	7.313111306211E-32	0	PRKAB1
2.50184039367524E-37	1.30499297266534	0.273	0.053	4.19258413172096E-33	0	EVA1B
6.00614054446518E-87	1.30468866056867	0.724	0.216	1.00650903244148E-82	0	SLC1A3
1.06481798628104E-27	1.30451831142152	0.261	0.07	1.78442198140977E-23	0	CPT2
8.17585207551409E-36	1.30292666050104	0.412	0.142	1.37010929081465E-31	0	TPD52
1.49010201604859E-19	1.30193929005709	0.251	0.089	2.49711295849423E-15	0	FAM114A1
1.5638582005383E-68	1.29883717545317	0.434	0.07	2.62071357246209E-64	0	SLC18B1
4.71403962588713E-45	1.29880900720283	0.399	0.103	7.89978760506166E-41	0	APOC1
3.67775633905804E-44	1.2976684152716	0.462	0.143	6.16318407299346E-40	0	SLC37A4
4.73504283164613E-65	1.29631201328638	0.491	0.113	7.93498477727259E-61	0	TMEM86A
3.16561196132588E-36	1.29542036431874	0.393	0.124	5.30493252478991E-32	0	CFB
1.61473893419774E-23	1.28776689864837	0.271	0.088	2.70597950592857E-19	0	MDK
1.31051740825704E-39	1.28671527653902	0.409	0.127	2.19616507275715E-35	0	CMC4
3.72848325744418E-21	1.28579715333845	0.251	0.083	6.24819224282495E-17	0	TMEM141
6.50512978007861E-46	1.28448439638751	0.541	0.192	1.09012964854557E-41	0	ECHS1
2.51984324465088E-110	1.28295900542255	0.978	0.706	4.22275330938594E-106	0	SELENOP
1.0738958462476E-73	1.28284021822086	0.826	0.34	1.79963465914172E-69	0	FMN1
4.90463711171296E-35	1.28053352864788	0.408	0.139	8.21919087180858E-31	0	ACAA2
2.34889456593838E-38	1.27587253093493	0.52	0.203	3.93627751359955E-34	0	TF
8.58950597213229E-66	1.27436886706686	0.727	0.279	1.43942941080993E-61	0	SCP2
1.17974905601527E-23	1.27263736639368	0.293	0.101	1.97702346807038E-19	0	HSDL2
2.88934054726565E-26	1.2701972770243	0.382	0.153	4.84195688910777E-22	0	SERPINA7
6.04146180117898E-67	1.26970325198151	0.496	0.108	1.01242816864157E-62	0	AMDHD2
1.27660650351313E-19	1.26885478316238	0.264	0.097	2.1393371785873E-15	0	FDPS
7.34276908743286E-31	1.26803827920519	0.382	0.136	1.230501243672E-26	0	POLDIP2
7.38535467814832E-40	1.26423731248603	0.456	0.156	1.2376377369641E-35	0	ACOX1
1.55799418136233E-29	1.26255868407229	0.286	0.078	2.610886649127E-25	0	CYP4V2
2.42647744996461E-35	1.26101461715902	0.385	0.124	4.0662909106507E-31	0	ATF5
1.01021612418694E-48	1.25975012022134	0.538	0.185	1.69292018091247E-44	0	RRBP1
1.11875266081592E-62	1.25811433955021	0.711	0.271	1.87480570899531E-58	0	ST6GAL1
2.45563136176353E-78	1.25567098369575	0.656	0.189	4.11514703604332E-74	0	CHID1
5.42380617290612E-31	1.25386895544016	0.411	0.157	9.08921438455608E-27	0	ALDH3A2
1.31460561444952E-28	1.25129084615441	0.379	0.141	2.2030160886945E-24	0	TSTA3
3.56543271136652E-41	1.25048190792787	0.46	0.15	5.97495213770802E-37	0	FN1
2.09388882467011E-79	1.24558240458976	0.782	0.29	3.50893889238217E-75	0	C3AR1
7.15674613301821E-47	1.2448655394649	0.386	0.092	1.19932751697119E-42	0	PINK1
2.58213777084932E-34	1.2437493266966	0.324	0.088	4.32714647638928E-30	0	TMEM129
1.02725338444524E-22	1.24310082256002	0.29	0.105	1.72147122165333E-18	0	CISH
1.07724927633245E-62	1.24053777385339	0.566	0.17	1.80525433727793E-58	0	SLC35F6
1.28515720127503E-46	1.23935203595502	0.451	0.134	2.1536664378967E-42	0	ZNF189
6.98253176017984E-20	1.2374445817218	0.3	0.12	1.17013267237094E-15	0	HACD2
7.24431206763148E-27	1.23742185917182	0.363	0.138	1.21400181629368E-22	0	ZKSCAN1
5.41350651509316E-34	1.23719391480102	0.399	0.138	9.07195421799312E-30	0	PCBD1
3.02371770361745E-65	1.2355876806322	0.448	0.083	5.06714612772213E-61	0	HOMER3

4.72050936986907E-59	1.23509269303692	0.615	0.206	7.91062960202659E-55	0	ASPH
1.83201379990301E-37	1.23506603155792	0.428	0.145	3.07008872587746E-33	0	IARS2
2.01398162275279E-44	1.23296084311205	0.502	0.172	3.37503040340912E-40	0	MLEC
2.01469504842475E-27	1.22991180793906	0.263	0.069	3.3762259621502E-23	0	AIG1
5.9997025738779E-25	1.22859098913623	0.299	0.099	1.00543015733046E-20	0	GIPC1
1.62960881404263E-24	1.22758659858546	0.343	0.133	2.73089845057264E-20	0	ERICH5
6.30839826136641E-31	1.22631633115419	0.354	0.117	1.05716138063978E-26	0	SLC35D2
6.05532928056647E-61	1.22391532897055	0.612	0.209	1.01475208083733E-56	0	PRDX1
2.51537079928125E-59	1.22389838624059	0.598	0.197	4.21525838543552E-55	0	GAS6
1.86930844137057E-74	1.22328325223716	0.845	0.393	3.1325870860488E-70	0	CTSA
1.05922427793557E-35	1.21992194723467	0.379	0.12	1.77504804496443E-31	0	SLC22A18
2.7410219737347E-26	1.21968935178279	0.34	0.122	4.59340462358462E-22	0	SRP9
2.47918046832854E-27	1.20619767967744	0.341	0.12	4.15461062882497E-23	0	STARD10
2.86048195330888E-51	1.20562414676626	0.588	0.212	4.79359565735503E-47	0	KLF9
1.38812807040835E-60	1.19204606558315	0.685	0.261	2.32622502039031E-56	0	PTMS
1.97144210062263E-25	1.18923220106274	0.35	0.135	3.30374267222341E-21	0	VNN 1.00
1.39123276638078E-49	1.18671332110586	0.524	0.18	2.33142786990091E-45	0	CYB5A
9.84397043982422E-101	1.18667057429816	0.959	0.683	1.64965256630574E-96	0	C1QA
3.7618492702639E-37	1.18369233456724	0.263	0.047	6.30410700710825E-33	0	TGFA
4.61076642715587E-41	1.18240131482533	0.511	0.19	7.72672237862781E-37	0	COX6C
3.84368300955085E-25	1.18187682677698	0.303	0.102	6.44124398740531E-21	0	PAQR8
2.84515982753685E-54	1.18126317765387	0.718	0.299	4.76791883898626E-50	0	APOC3
1.49056349060067E-23	1.18109116306542	0.282	0.094	2.4978862975486E-19	0	PSMD10
1.30037660978774E-42	1.1789874700276	0.531	0.196	2.1791711226823E-38	0	APOH
1.88261347337692E-52	1.17730911083967	0.478	0.128	3.15488365868504E-48	0	BLVRA
2.76319689649114E-24	1.17614508002671	0.295	0.1	4.63056535913985E-20	0	SLC2A4RG
1.21925667169999E-64	1.17205970893667	0.659	0.237	2.04323033043484E-60	0	SCD
2.3984324199662E-51	1.17051551453406	0.578	0.203	4.01929304937935E-47	0	TMCO1
5.92728920195146E-39	1.16375320006884	0.3	0.063	9.93295124463026E-35	0	SLC45A4
3.80319508558402E-36	1.16271033848443	0.472	0.183	6.37339432442171E-32	0	CLU
1.59536851558803E-60	1.16146014406286	0.627	0.219	2.67351855842241E-56	0	CRYL1
4.68877123780704E-77	1.15641106588096	0.903	0.451	7.85744284031704E-73	0	MS4A7
5.07503587682139E-73	1.15610202314868	0.832	0.373	8.50474512237728E-69	0	NCOA4
7.41417689972369E-27	1.15481196191047	0.331	0.114	1.2424677648557E-22	0	GRHPR
3.07295475509585E-33	1.15398184427103	0.444	0.165	5.14965757858963E-29	0	DDT
2.0214315460407E-73	1.15103067520436	0.798	0.325	3.38751498485501E-69	0	LAMP2
3.34431627308879E-32	1.15001826589378	0.334	0.1	5.60440521044219E-28	0	C9orf16
4.79728750196251E-42	1.1499907840141	0.376	0.097	8.03929439578878E-38	0	ANTXR2
1.52170919638945E-39	1.14949006207809	0.44	0.143	2.55008027130944E-35	0	GIMAP6
1.62364537244628E-36	1.14821637186066	0.457	0.167	2.72090491514547E-32	0	P4HB
6.83323546032195E-28	1.1466140309289	0.348	0.12	1.14511359844075E-23	0	VBP1
2.22542838142054E-37	1.14620276491226	0.472	0.176	3.72937288158453E-33	0	NDUFC2
1.10081333546355E-83	1.14514915073395	0.932	0.608	1.84474298756982E-79	0	CD68
1.27197494161484E-73	1.14193638730538	0.859	0.372	2.13157560715815E-69	0	LAIR1
3.63759934334042E-69	1.14034008006754	0.698	0.249	6.09588897956987E-65	0	SCPEP1
2.51855765879737E-37	1.1399970229098	0.28	0.054	4.22059892461263E-33	0	TNS1
7.5314554718826E-20	1.13597563276962	0.305	0.125	1.26212130797809E-15	0	DSTN
1.12086649900988E-26	1.13497423309514	0.377	0.144	1.87834807904076E-22	0	LPGAT1
5.01457999300016E-85	1.1339843297404	0.923	0.441	8.40343315226967E-81	0	MAF
9.08240652702367E-72	1.12607972683116	0.742	0.269	1.52202968579863E-67	0	BLVRB

1.83535413243712E-78	1.12486264620452	0.592	0.134	3.07568645513813E-74	0	NRP2
1.37920817667484E-28	1.12077086107747	0.388	0.145	2.31127706247169E-24	0	UQCRQ
8.21838384203347E-34	1.12045040690704	0.29	0.068	1.37723676424797E-29	0	NMRK1
2.09067311328666E-61	1.11938539026239	0.541	0.151	3.50355000324578E-57	0	MGAT5
7.42077323302479E-49	1.11769762808218	0.37	0.076	1.24357317839029E-44	0	HPSE
1.2031804901334E-65	1.11686412884131	0.53	0.127	2.01628986536555E-61	0	PDGFB
1.50595011981178E-52	1.115982110847	0.536	0.174	2.52367121078058E-48	0	G6PD
3.72698787414706E-72	1.11470634356898	0.866	0.426	6.24568627949565E-68	0	TIMP2
3.61038851237992E-57	1.1144361003308	0.731	0.297	6.05028906904627E-53	0	GC
6.75398669897017E-64	1.11057020468521	0.782	0.32	1.13183309101342E-59	0	SLAMF8
3.38315181896624E-61	1.10989557118772	0.527	0.132	5.66948581822362E-57	0	GFRA2
5.30818735468878E-17	1.10858171442231	0.258	0.102	8.89546036898746E-13	0	PSMB2
8.75804393545679E-60	1.10846671444741	0.464	0.103	1.46767300270385E-55	0	FARP1
7.80209326556166E-61	1.10695801864438	0.588	0.185	1.30747478944282E-56	0	MYO1E
1.18577135144385E-69	1.10638493002062	0.839	0.38	1.9871156307496E-65	0	LIPA
4.71608421259449E-78	1.10599852330358	0.785	0.285	7.90321392346585E-74	0	AP2A2
2.13488837650203E-37	1.10579266760828	0.303	0.067	3.5776459413421E-33	0	APH1B
1.72393584301213E-72	1.105789458288	0.888	0.522	2.88897168571972E-68	0	ITM2B
1.42896824235632E-19	1.10500811008207	0.26	0.096	2.39466498054071E-15	0	VDAC1
2.98744199347475E-48	1.10362203573927	0.491	0.154	5.00635529266498E-44	0	MGAT4B
8.25298870169673E-22	1.10190026010772	0.313	0.12	1.38303584663034E-17	0	FH
1.15687837721806E-33	1.09950436118343	0.379	0.123	1.93869678454202E-29	0	PGRMC2
5.47874613235579E-62	1.09886701670578	0.687	0.258	9.18128276860184E-58	0	UNC93B1
4.3759765543356E-28	1.09446634239924	0.26	0.067	7.33326150975559E-24	0	HS2ST1
6.21024860409886E-43	1.09329507856576	0.414	0.119	1.04071346107489E-38	0	LY96
1.61215580520051E-33	1.0913348694278	0.392	0.132	2.70165069835502E-29	0	NDUFB9
4.900190059423E-46	1.08946431653264	0.556	0.205	8.21173850158107E-42	0	MGST2
1.51463336283985E-23	1.08566226816959	0.276	0.092	2.53822258944702E-19	0	SMIM19
8.20244718209022E-58	1.0822493279041	0.538	0.149	1.37456609877468E-53	0	DNASE2
8.3503635863227E-95	1.0791644371878	0.971	0.687	1.39935392979596E-90	0	C1QB
9.04880811093998E-43	1.07865389055354	0.541	0.198	1.51639926323132E-38	0	PEBP1
4.43830452695142E-59	1.07810502796452	0.682	0.254	7.43771072626519E-55	0	SERPING1
9.24014957346906E-21	1.07381072914174	0.253	0.086	1.54846426552194E-16	0	TMCO3
1.73844422986937E-30	1.07290140644913	0.389	0.138	2.91328484041509E-26	0	PRDX4
1.17999278507497E-16	1.07265303392435	0.273	0.113	1.97743190922864E-12	0	CEBPG
1.91627070191447E-70	1.07186842328843	0.585	0.15	3.21128644226827E-66	0	GIMAP5
3.17771908813235E-47	1.07120635007546	0.618	0.254	5.32522164789219E-43	0	XBP1
2.69626195028237E-25	1.06984766357364	0.282	0.089	4.5183957762832E-21	0	SMYD2
2.13332910961259E-65	1.06920878914357	0.723	0.276	3.57503292188878E-61	0	FERMT3
4.04800137947335E-39	1.06508280622132	0.287	0.053	6.78364071172144E-35	0	DPEP3
4.46294388316959E-50	1.06150566668689	0.637	0.24	7.4790013594156E-46	0	APOC2
1.39696599272976E-41	1.06025829487266	0.38	0.102	2.34103561061654E-37	0	ZC3H12D
2.18801434925317E-72	1.0588901808028	0.736	0.264	3.66667444647846E-68	0	CYFIP1
1.16784417201945E-33	1.05765320653032	0.376	0.124	1.95707326347019E-29	0	CYB561A3
3.64178614011688E-31	1.05627673825519	0.386	0.14	6.10290521360787E-27	0	RDH11
1.08614693469564E-17	1.05346492034528	0.26	0.101	1.82016503316295E-13	0	CPD
2.9050793894147E-20	1.05215958828598	0.3	0.12	4.86833204078116E-16	0	TMEM205
1.18056623931837E-37	1.04922157780953	0.338	0.083	1.97839290384973E-33	0	PDCD1LG2
8.39461465101249E-59	1.04728823295443	0.687	0.256	1.40676952321667E-54	0	BCAP31
5.55249862510253E-50	1.04542571931857	0.536	0.185	9.30487719594682E-46	0	PEPD

2.06412434917989E-24	1.04288471741203	0.356	0.14	3.45905958435566E-20	0	ACAT1
3.32711910135935E-22	1.04232276145473	0.264	0.087	5.575586190058E-18	0	C6orf89
8.45959575740904E-40	1.04101758666292	0.547	0.215	1.41765905702661E-35	0	TXNRD1
4.18844116858723E-55	1.03991390837109	0.599	0.206	7.01898971031848E-51	0	ABHD12
3.68094660747067E-81	1.03794429386447	0.923	0.609	6.16853032479936E-77	0	GRN
3.54362885885909E-41	1.03418218599931	0.406	0.117	5.93841324167607E-37	0	CLN6
9.77628652622849E-26	1.0323754961306	0.341	0.122	1.63831009606537E-21	0	HADH
2.06826015871663E-19	1.03063626770199	0.263	0.097	3.46599037397733E-15	0	GLUD1
4.52438068542187E-30	1.0293221094248	0.364	0.126	7.58195715262997E-26	0	KCTD3
1.46311940474651E-31	1.02855772675914	0.303	0.081	2.45189549847421E-27	0	FOXRED2
1.4260798517165E-44	1.02854840208311	0.424	0.117	2.38982461550652E-40	0	HECTD3
8.24144605177073E-30	1.02829531922043	0.348	0.116	1.38110152935574E-25	0	LAGE3
1.69665073480313E-25	1.0274322489252	0.322	0.113	2.84324730138308E-21	0	SLC25A1
1.17780972029473E-52	1.0253678621174	0.611	0.219	1.9737735292699E-48	0	RTN3
2.32526119470581E-31	1.02253423408392	0.303	0.083	3.89667271008799E-27	0	TBC1D16
1.82287937456576E-59	1.02214619549307	0.646	0.223	3.05478125589731E-55	0	FABP1
2.25818009178504E-15	1.02052240990979	0.258	0.109	3.78425819781337E-11	0	NUCB2
5.83105857873823E-52	1.02044404758239	0.546	0.177	9.77168796624953E-48	0	WASF2
4.19749288880781E-28	1.01936727380676	0.309	0.093	7.03415858306413E-24	0	NLN
6.58529972944696E-48	1.019105884538	0.428	0.109	1.10356452866072E-43	0	PXN
3.93520146655531E-34	1.01749060655639	0.379	0.12	6.5946106176534E-30	0	NAGPA
6.58622491358345E-66	1.017222722403	0.701	0.251	1.10371957101831E-61	0	GM2A
1.04223695103E-18	1.01329533316332	0.26	0.096	1.74658068253607E-14	0	ACADM
7.45292934190073E-74	1.01123327361974	0.628	0.159	1.24896189911572E-69	0	TMEM37
8.53663418762192E-19	1.00983566121926	0.263	0.098	1.43056915716168E-14	0	MTMR4
6.30982756326408E-35	1.00944699848075	0.528	0.215	1.0574009030518E-30	0	AFDN
2.6912722254366E-16	1.00837072892461	0.257	0.104	4.51003399538665E-12	0	EBP
9.44257368577857E-23	1.00816560847177	0.287	0.099	1.58238649826277E-18	0	HPRT1
4.83709389456714E-39	1.00759893960656	0.511	0.192	8.10600194851562E-35	0	CAT
8.67251977272025E-23	1.00743785282143	0.271	0.09	1.45334086351246E-18	0	KDELR2
4.2590930148518E-27	1.00732388107464	0.277	0.08	7.13738807428864E-23	0	IL12RB1
6.01093531679375E-25	1.00614897498348	0.327	0.119	1.0073125403883E-20	0	SAR1B
8.78611973963717E-32	1.00603817262623	0.356	0.111	1.4723779459684E-27	0	APOL1
4.62842581512503E-35	1.00325149949462	0.389	0.124	7.75631598098652E-31	0	DNPH1
8.13855125950687E-70	1.00321749899515	0.794	0.337	1.36385842006816E-65	0	AP1B1
3.04309177789388E-27	0.99729203731244	0.341	0.119	5.09961320139456E-23	0	RABL6
3.28488123958538E-42	0.995077960126595	0.453	0.142	5.50480398129717E-38	0	MBNL3
2.933976089119E-26	0.989465107181754	0.302	0.099	4.91675713014562E-22	0	KCNQ1
2.2394696399714E-24	0.989257858540306	0.364	0.142	3.75290322266408E-20	0	AGPAT2
5.32111706791608E-19	0.98067895981865	0.327	0.139	8.91712798241376E-15	0	ETFA
2.70459530012012E-38	0.980079579620305	0.328	0.079	4.53236080394129E-34	0	BAIAP2
2.61402273674413E-33	0.976698814648869	0.401	0.139	4.38057930223581E-29	0	NACC1
7.9270472765256E-30	0.975509266414943	0.414	0.159	1.32841458260016E-25	0	CUTA
2.05048808599137E-27	0.974855419619948	0.28	0.079	3.43620793450433E-23	0	PER3
8.76662042008277E-29	0.974234847869784	0.351	0.121	1.46911024999747E-24	0	SLC25A24
2.19506164980789E-22	0.974082649238507	0.277	0.091	3.67848431274806E-18	0	IAH1
5.78831074889913E-41	0.969253099911457	0.533	0.202	9.70005115300517E-37	0	C3
1.24297687384401E-24	0.968537195931008	0.299	0.101	2.08298064518778E-20	0	PIGS
2.03666487124788E-32	0.965314958151382	0.389	0.131	3.41304299123719E-28	0	SLC31A1
4.98216979026019E-72	0.962866897462878	0.938	0.572	8.34912013451803E-68	0	MPEG1

**1.52837176663196E-26**	0.956989903199516	0.358	0.133	2.56124540652183E-22	0	IFI35
**5.6208074775519E-22**	0.956852698849096	0.277	0.096	9.41934917088148E-18	0	TMED2
**1.40691962253507E-30**	0.95564175242369	0.29	0.077	2.35771590344427E-26	0	PLEKHA1
**6.01396602247259E-20**	0.953149435909336	0.273	0.101	1.00782042604596E-15	0	SNX4
**3.81329036858445E-16**	0.952638321562877	0.257	0.103	6.39031199967382E-12	0	STOML2
**7.17436237180195E-34**	0.952606309601777	0.412	0.14	1.20227964626657E-29	0	WSB2
**1.12023862771124E-56**	0.952115612363913	0.64	0.218	1.87729589231849E-52	0	VSIG4
**5.7098821818231E-42**	0.95021738323093	0.388	0.101	9.56862056029915E-38	0	TRIM47
**3.32366230967081E-16**	0.94962610814881	0.284	0.123	5.56979329854634E-12	0	MARVELD1
**1.55569211839201E-42**	0.947814895199599	0.447	0.139	2.60702885200134E-38	0	AGTRAP
**1.26860881449264E-32**	0.94521508854712	0.437	0.166	2.12593465132677E-28	0	TPM1
**1.46049657354653E-20**	0.943666471839875	0.293	0.111	2.44750015794927E-16	0	MCTS1
**3.3602180598123E-50**	0.943534106422576	0.682	0.28	5.63105342463346E-46	0	NAGA
**2.825949047877E-45**	0.939954371315704	0.493	0.165	4.73572541443228E-41	0	CLTA
**3.11027943848882E-15**	0.938005922185297	0.257	0.107	5.21220628301956E-11	0	GALK1
**8.96799703563948E-17**	0.937904521497683	0.293	0.125	1.50285694323246E-12	0	MAP3K13
**8.4411708348429E-48**	0.936319819771459	0.554	0.199	1.41457140850297E-43	0	MXD4
**4.44951336508902E-25**	0.935427332911823	0.38	0.149	7.45649449721618E-21	0	COX6A1
**1.43887674717797E-62**	0.935102763068368	0.682	0.245	2.41126965292085E-58	0	TMBIM1
**3.26656530137132E-22**	0.934875008230304	0.302	0.113	5.47411013203806E-18	0	AFG3L2
**9.5422603667802E-41**	0.929668416721788	0.343	0.079	1.59909199226503E-36	0	SLC38A6
**2.43927155832192E-33**	0.925437110453446	0.392	0.131	4.08773127743587E-29	0	TMEM51
**6.83280011537956E-19**	0.924809620193645	0.292	0.118	1.14504064333531E-14	0	ROMO1
**1.11345640688658E-30**	0.923460908423597	0.374	0.132	1.86593024666053E-26	0	DCAF6
**3.14722115704979E-25**	0.922530410878998	0.322	0.114	5.27411321498404E-21	0	C1orf43
**1.40705840965136E-19**	0.921096589283095	0.299	0.116	2.35794848289374E-15	0	TXNDC17
**2.16173650984953E-47**	0.920083164162221	0.579	0.211	3.62263804320584E-43	0	MGST3
**1.13237915049611E-59**	0.918355894817244	0.835	0.407	1.89764098040138E-55	0	CD63
**3.61945572558896E-72**	0.916186873913013	0.766	0.246	6.06548390494198E-68	0	MERTK
**3.9294822544647E-55**	0.915614095866679	0.653	0.243	6.58502636203194E-51	0	SESN1
**6.16346776690381E-37**	0.913186611621686	0.464	0.166	1.03287392837774E-32	0	ATRN
**1.00594205066355E-26**	0.909537908829715	0.376	0.141	1.68575768850197E-22	0	HTATIP2
**5.54849069610355E-23**	0.908717952535959	0.289	0.101	9.29816070853033E-19	0	ARHGAP35
**6.09412313945298E-26**	0.907588183090496	0.364	0.141	1.02125315570953E-21	0	LMAN1
**5.91227288154567E-31**	0.905975254767365	0.377	0.132	9.90778689489423E-27	0	GPAA1
**4.36348720296426E-63**	0.905490276402563	0.762	0.305	7.3123318547275E-59	0	LGALS3BP
**3.27498382921973E-42**	0.903490626058407	0.598	0.241	5.48821790100642E-38	0	ECH1
**2.14229481334717E-21**	0.903339982768485	0.347	0.144	3.59005764820718E-17	0	FADS2
**2.63571056624125E-45**	0.90193980308377	0.44	0.124	4.41692376690708E-41	0	EBI3
**2.21101758440352E-46**	0.900668433994034	0.517	0.17	3.70522326794341E-42	0	EHD4
**1.12932670541732E-18**	0.900586975809643	0.266	0.102	1.89252569293835E-14	0	POMP
**2.14215857197624E-46**	0.89835881347236	0.467	0.144	3.58982933491778E-42	0	EDEM2
**1.34189767793381E-40**	0.898339583586758	0.495	0.172	2.24875212868148E-36	0	IL4I1
**1.18026477654567E-71**	0.897066611940021	0.653	0.189	1.97788771253523E-67	0	RENBP
**3.15147215303129E-21**	0.896320182018736	0.335	0.137	5.28123703404983E-17	0	CDC123
**1.26539402024628E-65**	0.894282923862865	0.541	0.123	2.12054729912872E-61	0	RAB3IL1
**4.27135017873937E-57**	0.891709509718414	0.949	0.802	7.15792862953143E-53	0	CTSZ
**3.96961750058534E-33**	0.890343315244002	0.351	0.105	6.65228500748091E-29	0	RAB13
**3.65034862910345E-17**	0.889183496174771	0.271	0.11	6.11725423265156E-13	0	SLC25A5
**1.34115299213855E-35**	0.887142372655249	0.538	0.216	2.24750418422578E-31	0	DECR1

**3.85187274872293E-39**	0.887080375466043	0.518	0.197	6.45496835230989E-35	0	CAPN1
**3.73483874190922E-32**	0.886247357209188	0.356	0.115	6.25884276369147E-28	0	VPS26C
**1.07214928821624E-28**	0.884244984670436	0.403	0.156	1.79670777719278E-24	0	SURF4
**6.67779868716791E-46**	0.883969027170874	0.669	0.283	1.1190655039956E-41	0	HSD17B4
**6.16866177309851E-41**	0.88241212864928	0.444	0.138	1.03374433993585E-36	0	OAS1
**2.25210056898507E-27**	0.881530445629038	0.421	0.169	3.77407013350517E-23	0	SPTSSA
**1.19816952136175E-54**	0.881115074739637	0.472	0.116	2.00789248389801E-50	0	SLC36A1
**2.55029812308989E-34**	0.880962492053586	0.534	0.224	4.27378959467403E-30	0	PPIB
**4.76838463676348E-40**	0.879868367522598	0.463	0.155	7.99085897428824E-36	0	SHMT1
**3.73765681870582E-28**	0.879114243049951	0.308	0.095	6.26356529678721E-24	0	TMEM115
**4.31807720345988E-27**	0.87640498869476	0.286	0.084	7.23623377755807E-23	0	CTNS
**4.22153692464095E-27**	0.87459580266612	0.299	0.094	7.0744515783133E-23	0	EMB
**4.95567954311118E-59**	0.872016133738156	0.646	0.223	8.30472777834572E-55	0	PLBD2
**3.65169537318698E-23**	0.871213402523616	0.325	0.125	6.11951110638674E-19	0	RAB5B
**5.06102185959875E-37**	0.870910161245647	0.482	0.179	8.48126043231559E-33	0	ALDH9A1
**6.95745723538081E-27**	0.868979787159928	0.43	0.178	1.16593068350512E-22	0	MLF 2.00
**4.38687880948912E-52**	0.8685745770166	0.504	0.146	7.35153150894187E-48	0	MYO5A
**2.79617652262255E-26**	0.864917081444038	0.341	0.12	4.68583261661088E-22	0	NUDT5
**2.14020481373298E-33**	0.86244805178294	0.347	0.104	3.58655522685372E-29	0	MFSD12
**5.35193900824975E-54**	0.859392505476725	0.691	0.28	8.96877939002494E-50	0	CD81
**1.62944707700942E-35**	0.857486554763458	0.376	0.114	2.73062741165239E-31	0	PARVB
**3.70512316063979E-20**	0.852177515147742	0.325	0.135	6.20904539260015E-16	0	SRPRB
**4.49142554093616E-56**	0.849795488945018	0.673	0.244	7.52673092150081E-52	0	ARHGAP18
**6.36874114008039E-33**	0.848454843687057	0.409	0.14	1.06727364025467E-28	0	KLHDC3
**2.30022081136667E-54**	0.847734876614027	0.827	0.33	3.85471003568826E-50	0	FKBP5
**2.47376849001561E-64**	0.846083792420404	0.955	0.627	4.14554123556815E-60	0	CD163
**3.28332576892788E-38**	0.844295660539072	0.541	0.211	5.50219732356934E-34	0	ESYT1
**3.56681448214144E-17**	0.843864365378728	0.257	0.1	5.97726770917262E-13	0	ECI1
**5.88128794336083E-19**	0.840849252383646	0.283	0.109	9.85586233548408E-15	0	BCKDHA
**2.11257085740379E-26**	0.836290100640514	0.386	0.149	3.54024624283727E-22	0	SNRPC
**3.48710782769347E-70**	0.835101987757406	0.705	0.221	5.84369529764872E-66	0	ASAH1
**1.50724228062451E-25**	0.834995322929026	0.279	0.084	2.52583661387056E-21	0	P2RY11
**3.98487565347302E-20**	0.834858757633217	0.338	0.142	6.67785462009009E-16	0	SHC1
**5.55426231093913E-40**	0.834807194081177	0.493	0.176	9.30783278067179E-36	0	NLK
**1.46532019513523E-15**	0.834250461674847	0.295	0.131	2.45558358300762E-11	0	NDUFAB1
**3.40976926580049E-29**	0.833952201062504	0.377	0.132	5.71409133562845E-25	0	KIAA2013
**5.71729592339024E-25**	0.833706056642239	0.34	0.125	9.58104450841736E-21	0	PDHB
**1.44528157089899E-34**	0.833098170185799	0.351	0.101	2.42200285651253E-30	0	MSRB2
**2.19941331098838E-30**	0.832275798494751	0.412	0.155	3.68577682655432E-26	0	TM7SF3
**1.6093088595537E-59**	0.832273285886089	0.955	0.646	2.6968797868401E-55	0	FCGRT
**3.00449146909166E-23**	0.831775482581194	0.26	0.081	5.03492680390381E-19	0	FCGR1A
**2.87673154840601E-42**	0.829181779751923	0.729	0.346	4.8208267288188E-38	0	ANXA2
**3.38665002413657E-62**	0.828108699112585	0.583	0.164	5.67534811044807E-58	0	RHBDF2
**4.84834724578097E-48**	0.827793818051867	0.476	0.14	8.12486031447974E-44	0	PLA2G15
**1.31747697489586E-15**	0.826502735606999	0.274	0.12	2.20782791453048E-11	0	SCAMP3
**2.82319589593965E-53**	0.826335346784697	0.66	0.25	4.73111168241567E-49	0	SNX6
**1.42749213946377E-19**	0.825862357558438	0.295	0.115	2.39219132731338E-15	0	C4BPA
**2.95483466674564E-25**	0.825726296991728	0.293	0.095	4.95171193453235E-21	0	TECR
**3.64879604282134E-47**	0.820843299415675	0.52	0.169	6.11465240856001E-43	0	SCAMP4
**5.52444226731203E-29**	0.817875472662096	0.382	0.137	9.25786035156149E-25	0	PIP5K1C

1.43292672683471E-10	0.817357901896126	0.282	0.145	2.40129860882961E-06	0	MTHFD1
1.65871064565899E-34	0.816692192730148	0.414	0.141	2.77966729999534E-30	0	TEX264
5.85606327237382E-31	0.816668517361001	0.379	0.129	9.81359083184404E-27	0	TXNDC5
1.55939665753107E-50	0.816486594300736	0.763	0.33	2.61323691869057E-46	0	SLA
4.63078169432834E-48	0.811377632212696	0.798	0.36	7.76026396335544E-44	0	MT-ND2
7.80108461416434E-21	0.811359625371738	0.292	0.108	1.30730575964166E-16	0	PREB
1.64384186176512E-37	0.810381016087793	0.433	0.141	2.75475019194598E-33	0	WASHC2A
6.87472623365282E-65	0.810345300160253	0.803	0.339	1.15206662223554E-60	0	PLXND1
1.13928583771879E-21	0.809257829099985	0.286	0.104	1.90921520684914E-17	0	ARPC3
1.15597928461565E-50	0.807867344086152	0.543	0.182	1.93719008515891E-46	0	WWP1
7.34014684155939E-25	0.807804271898056	0.29	0.094	1.23006180770852E-20	0	TDRKH
3.66289374583865E-56	0.807779413899524	0.522	0.144	6.13827733927642E-52	0	ITGB5
1.58841310884374E-46	0.806020589460098	0.557	0.194	2.66186268780034E-42	0	GBA
2.48470081802687E-23	0.805177352788919	0.26	0.079	4.16386163084942E-19	0	IP6K1
1.16525624557009E-41	0.804574759635317	0.53	0.196	1.95273641632635E-37	0	SSR4
3.99415941288829E-50	0.804420382109668	0.554	0.187	6.69341234411819E-46	0	SLC11A2
3.93873604028263E-53	0.803766271182104	0.72	0.314	6.60053385630564E-49	0	RAB5C
2.30849054060836E-33	0.801970452791116	0.424	0.148	3.86856844795149E-29	0	CYBRD1
3.67335077477949E-33	0.801851706706829	0.385	0.124	6.15580122837546E-29	0	PXDC1
1.47300687338832E-53	0.801660933173946	0.749	0.308	2.46846491842415E-49	0	SIRPA
2.03008232818494E-22	0.801375919169727	0.315	0.119	3.40201196557233E-18	0	ACER3
8.24836644029953E-46	0.799878547389785	0.567	0.213	1.38226124806539E-41	0	RNF13
4.05588622047807E-23	0.799133886275883	0.277	0.09	6.79685412827715E-19	0	SF3A1
2.00724109126465E-58	0.798902041785735	0.565	0.163	3.3637346207413E-54	0	SLC38A7
2.27748498016532E-37	0.797856198176465	0.329	0.081	3.81660932976104E-33	0	RRAGD
8.0028364638109E-41	0.797255933101437	0.396	0.112	1.34111533460543E-36	0	NPC1
1.59222891104052E-45	0.795798698015728	0.733	0.316	2.6682572091217E-41	0	LMAN2
1.13341443148933E-46	0.7956310285962	0.684	0.276	1.89937590428981E-42	0	RCSD1
2.54099189231418E-54	0.795222953621797	0.567	0.185	4.2581942131401E-50	0	PSME4
2.03685569617469E-20	0.794841055424617	0.261	0.092	3.41336277564954E-16	0	ERLIN2
2.13155728990129E-30	0.794782488097965	0.347	0.11	3.57206370641659E-26	0	COMMD5
7.16630946963258E-28	0.79460720935204	0.344	0.119	1.20093014092103E-23	0	C6orf120
1.8456564919511E-32	0.792600194652902	0.379	0.122	3.09295114921165E-28	0	SLC39A1
1.04965708098622E-25	0.792351775360914	0.345	0.128	1.75901533631671E-21	0	RBM15B
4.47416688968809E-33	0.792298872507974	0.47	0.184	7.4978088737393E-29	0	CIAO2A
2.45199670832575E-23	0.791263929969776	0.324	0.12	4.10905608381229E-19	0	DARS
3.53600117560378E-29	0.791073531719754	0.421	0.165	5.92563077007681E-25	0	ANXA7
1.3851785844202E-41	0.79104045222596	0.556	0.214	2.32128227177137E-37	0	VOPP1
3.68678564000724E-27	0.790677805648835	0.332	0.112	6.17831537552414E-23	0	SFXN3
1.08032662979527E-40	0.790376220283792	0.585	0.234	1.81041136621092E-36	0	NDUFA1
1.41755061900469E-29	0.790132315107269	0.469	0.193	2.37553132732805E-25	0	DAP
2.5004230392767E-43	0.787087234129566	0.581	0.224	4.1902089292199E-39	0	PHC2
1.77358553908959E-18	0.786268554104099	0.261	0.098	2.97217464640633E-14	0	KDELR1
1.6236847635253E-52	0.78568975626382	0.495	0.136	2.7209709267157E-48	0	ITSN1
1.79611470117087E-49	0.785347104544564	0.501	0.148	3.00992901622215E-45	0	ACP2
3.5836819560047E-36	0.78529832588694	0.479	0.176	6.00553422187268E-32	0	RTCB
1.23851217390249E-19	0.785070375569933	0.306	0.125	2.07549870102579E-15	0	NIT1
5.29530045842268E-21	0.784400688149422	0.325	0.129	8.87386450822473E-17	0	PSMB4
1.65230255968179E-28	0.782563422233955	0.374	0.135	2.76892862951474E-24	0	SHARPIN
1.14671227286426E-26	0.781634304496897	0.37	0.142	1.92166042686593E-22	0	GALNT1

1.84340373000621E-18	0.780258253840624	0.255	0.094	3.0891759707444E-14	0	CEBPZOS
2.32250990711536E-16	0.78004050999027	0.257	0.102	3.89206210234391E-12	0	COMMD7
1.59828255927818E-29	0.7783744801175	0.335	0.107	2.67840191283838E-25	0	SNX12
1.20337764260059E-25	0.776854651781726	0.37	0.14	2.01662025347006E-21	0	PPP1R7
4.60168371018796E-57	0.774920124046593	0.702	0.275	7.71150156153299E-53	0	LRRC25
1.73813948023854E-46	0.774310717988788	0.823	0.371	2.91277414098375E-42	0	S100A10
1.39687527552202E-30	0.772749592370831	0.456	0.179	2.34088358671981E-26	0	HINT1
7.85879985394068E-21	0.772349794075129	0.38	0.168	1.31697767952338E-16	0	PRDX6
8.42583084470244E-26	0.771596915909801	0.306	0.102	1.41200073295524E-21	0	C5orf24
2.21174769451127E-19	0.769543224858704	0.287	0.11	3.70644678646198E-15	0	TMEM123
1.27260999017334E-15	0.76923676678754	0.254	0.104	2.13263982153248E-11	0	ISOC2
9.21522058783076E-50	0.769224213854601	0.927	0.522	1.54428666610868E-45	0	ALB
4.32266598802994E-33	0.768148297255791	0.36	0.111	7.24392366274057E-29	0	MAPK3
4.25943739553239E-42	0.768012805471131	0.411	0.115	7.13796518743318E-38	0	PRXL2A
1.34209423415842E-20	0.767989862760092	0.289	0.105	2.24908151760268E-16	0	TNFAIP8L2
7.27973646370605E-42	0.767261505969647	0.637	0.266	1.21993823658786E-37	0	HSD17B11
5.37469463114301E-28	0.766643988391244	0.405	0.152	9.00691326286945E-24	0	COX8A
4.37759379306598E-25	0.766076868260535	0.338	0.125	7.33597167841996E-21	0	NHLRC3
1.40048308895593E-62	0.764791528721804	0.642	0.193	2.34692956047234E-58	0	MTSS1
1.36782463429148E-21	0.764300177231259	0.295	0.108	2.29220052214566E-17	0	ENOPH1
1.60412448436868E-28	0.762654973443811	0.393	0.148	2.68819181090504E-24	0	PLOD1
2.57868184136146E-53	0.762638519411463	0.557	0.176	4.32135502975353E-49	0	BCL2L1
9.64040511649851E-48	0.762149217697234	0.527	0.173	1.61553908942282E-43	0	SNX9
7.59528436262526E-48	0.761636199798173	0.903	0.561	1.27281775348874E-43	0	NPC2
6.14508697585278E-30	0.760711335615767	0.324	0.097	1.02979367541341E-25	0	APPL2
3.87787542450406E-28	0.76007430353378	0.369	0.134	6.4985436363839E-24	0	TMEM35B
5.47207294524423E-59	0.759542500723466	0.647	0.225	9.17009984164029E-55	0	VEGFB
1.2362433258327E-34	0.758614810783178	0.626	0.28	2.07169656543044E-30	0	ACSL4
7.81721489403353E-24	0.7539822030915	0.276	0.087	1.31000887194214E-19	0	TGFBRAP1
6.71146663626595E-18	0.75280846758169	0.266	0.102	1.12470757890545E-13	0	UBD
5.18247532220663E-42	0.751404825301299	0.48	0.154	8.68479214495387E-38	0	DRAM1
1.59330531317744E-20	0.74845262100189	0.341	0.143	2.67006104382276E-16	0	GOLGA7
1.19935562257105E-28	0.74810824943062	0.258	0.064	2.00988015230457E-24	0	MEGF6
1.45212834479623E-46	0.747911876401069	0.538	0.176	2.43347668020952E-42	0	SLC43A3
9.69826593064087E-23	0.74773766463891	0.357	0.143	1.6252354046568E-18	0	SMIM14
1.42061006682674E-20	0.74740792342882	0.279	0.102	2.38065834998825E-16	0	TPGS1
9.56910688459738E-35	0.746822602777898	0.475	0.183	1.60359093172083E-30	0	CALU
5.67799650709614E-39	0.745638635946757	0.417	0.125	9.51518654659171E-35	0	KLF7
7.97409500092463E-41	0.745599196539505	0.557	0.215	1.33629884025495E-36	0	DHRS7
1.87412243259361E-25	0.744753260399993	0.315	0.105	3.14065437254037E-21	0	TSPAN15
7.2644901862977E-24	0.744640088746618	0.325	0.121	1.21738326541977E-19	0	GID8
3.28725158657677E-27	0.743992869848695	0.318	0.102	5.50877620878534E-23	0	ZFYVE21
1.7559326396353E-34	0.742366536832487	0.438	0.155	2.94259191750083E-30	0	SEL1L3
3.74264445635277E-12	0.740126402685933	0.286	0.142	6.27192357995596E-08	0	DCXR
1.97781607917459E-20	0.739228199666434	0.254	0.084	3.31442418548078E-16	0	KCNE3
9.29075139866023E-29	0.738505534152881	0.385	0.139	1.55694411938748E-24	0	NAA20
1.25258784341052E-26	0.737804038429956	0.403	0.159	2.09908670798735E-22	0	MAP2K2
1.24168692175298E-32	0.73744959121135	0.456	0.174	2.08081894347364E-28	0	CLPTM1
1.13334467838598E-25	0.736864472179099	0.308	0.102	1.89925901203922E-21	0	BCAP29
1.11764032212485E-15	0.736594036200379	0.273	0.117	1.87294165181682E-11	0	FIBP

4.76318088328763E-19	0.735062718816809	0.283	0.11	7.98213852421341E-15	0	PPP2R5A
1.54068487146446E-19	0.733236287838064	0.28	0.102	2.58187970760014E-15	0	KLHL12
7.4819435606688E-25	0.732682125141703	0.348	0.131	1.25382410189688E-20	0	FUCA2
2.76321352959036E-21	0.731198188461614	0.295	0.107	4.63059323288752E-17	0	STUB1
7.43090026709267E-36	0.730843871161557	0.453	0.163	1.24527026675939E-31	0	DNASE1L1
7.75972394909125E-48	0.730087747355801	0.637	0.256	1.30037453938871E-43	0	TWF2
1.67020610038977E-44	0.729457135286818	0.569	0.206	2.79893138303317E-40	0	PLOD3
2.29387955172855E-12	0.729191108014982	0.286	0.141	3.84408335278671E-08	0	PSMG2
4.69957302936679E-31	0.727293626955738	0.395	0.137	7.87554448261287E-27	0	C20orf194
2.06817535445103E-21	0.72726806710475	0.293	0.108	3.46584825898903E-17	0	SYVN1
2.14788220849478E-21	0.727037566549944	0.299	0.112	3.59942100499555E-17	0	TM9SF1
2.20493139390679E-16	0.726584210519337	0.298	0.131	3.695024029909E-12	0	IMMP2L
1.85837325899914E-32	0.722842042993039	0.308	0.082	3.11426190743076E-28	0	SNX24
9.25770687453484E-54	0.719706644986337	0.742	0.311	1.55140651803455E-49	0	TNS3
1.46427012322855E-34	0.719067181241614	0.421	0.144	2.45382387250641E-30	0	ACBD5
6.59591676770046E-22	0.719012355500774	0.356	0.143	1.10534373193124E-17	0	ADCK2
1.38600653702422E-39	0.718211758531856	0.528	0.194	2.32266975474519E-35	0	YIPF3
6.7476293477922E-20	0.717836245874576	0.298	0.116	1.13076772610302E-15	0	LRRC58
1.06811117108933E-57	0.716727776295623	0.419	0.08	1.7899407005115E-53	0	HSD17B14
1.39008898216807E-39	0.715588761128639	0.897	0.543	2.32951111631726E-35	0	CD4
9.38841483052059E-23	0.715302707046096	0.361	0.146	1.57331055729864E-18	0	UQCR11
1.02173839925832E-59	0.714333818382128	0.627	0.197	1.7122292094771E-55	0	LHFPL2
1.55064923854974E-41	0.714203991289192	0.549	0.201	2.59857799396166E-37	0	CYB5R1
1.48871178586708E-27	0.713854667321271	0.441	0.183	2.49478321075605E-23	0	ADA
1.21375883499778E-37	0.712931741989623	0.485	0.177	2.03401705568927E-33	0	TMEM147
1.44281423253891E-28	0.71264613735044	0.508	0.223	2.4178680908887E-24	0	SEC61A1
5.24291233318864E-21	0.712201130644377	0.325	0.13	8.78607248795752E-17	0	TRAPPC4
4.53988557891318E-50	0.711501355315143	0.595	0.21	7.60794025314271E-46	0	NFIC
3.82432832755493E-17	0.711467562908999	0.344	0.155	6.40880941131656E-13	0	HTATSF1
2.27485028766321E-17	0.710345083139883	0.267	0.103	3.81219411206601E-13	0	NMRAL1
2.81215579656186E-21	0.70987338090267	0.284	0.102	4.71261068387837E-17	0	TFG
8.58460012818645E-41	0.709825364930379	0.672	0.273	1.43860728948149E-36	0	NUDT16
4.97013731019331E-53	0.709762470239445	0.626	0.222	8.32895610442196E-49	0	SWAP70
3.59799599206724E-39	0.708977288645932	0.376	0.102	6.02952168350628E-35	0	DSC2
1.28421674965185E-44	0.708660557899293	0.639	0.258	2.15209042906656E-40	0	SDF4
3.85431891470461E-39	0.705854641747838	0.627	0.272	6.45906763726199E-35	0	OTULINL
6.34402581010315E-43	0.705584194946535	0.843	0.447	1.06313184525709E-38	0	MAN2B1
1.1811473503816E-17	0.704786029712757	0.293	0.12	1.97936672976948E-13	0	ATP2B4
1.06932392394539E-18	0.704034229894395	0.334	0.145	1.79197303174769E-14	0	SLC44A1
9.7030113490433E-41	0.703798627295179	0.493	0.17	1.62603064187268E-36	0	GIMAP8
3.43418975142693E-42	0.703487063822614	0.611	0.242	5.75501518544125E-38	0	CALM3
2.78526614721156E-15	0.703438206876148	0.263	0.11	4.66754900949713E-11	0	TMEM14C
2.63532120401066E-44	0.703346960380718	0.817	0.421	4.41627127368107E-40	0	BST2
2.38093484114003E-24	0.703189515141629	0.366	0.145	3.98997060678246E-20	0	NENF
1.55810002842273E-45	0.702749095694618	0.491	0.156	2.61106402763081E-41	0	SNX29
3.54131619755065E-32	0.701812181926082	0.531	0.224	5.93453768385538E-28	0	CYC1
8.45221439887853E-40	0.700310810872953	0.692	0.295	1.41642208896406E-35	0	RNASE6
8.56000322078981E-27	0.69903019326284	0.408	0.161	1.43448533973996E-22	0	CAMK1
3.81685839801346E-14	0.698651956367409	0.253	0.109	6.39629130339096E-10	0	LNPK
7.14368294437342E-51	0.698329202765384	0.769	0.343	1.1971383878181E-46	0	SCAMP2

4.28549155147558E-47	0.697376508792503	0.803	0.387	7.18162674196277E-43	0	ATP6AP1
5.79196770920739E-21	0.696881550145787	0.286	0.104	9.70617948708974E-17	0	CTBS
2.20466221429654E-36	0.696724253526389	0.543	0.223	3.69457293871815E-32	0	SPPL2A
1.06424887702194E-43	0.695128118484578	0.595	0.223	1.78346826811337E-39	0	PTAFR
1.09093013314032E-45	0.692481181121675	0.878	0.428	1.82818071711654E-41	0	MT-CYB
7.68568010093653E-17	0.691414837269824	0.348	0.161	1.28796627131494E-12	0	RNF5
6.26858095424195E-35	0.6909074454388	0.478	0.178	1.05048879631187E-30	0	DCTN2
1.94841544146308E-25	0.689595910207595	0.363	0.139	3.26515459680383E-21	0	APEH
7.81048554929983E-27	0.688630781890948	0.376	0.14	1.30888116835166E-22	0	COPS7A
1.09507843949021E-22	0.687877475112458	0.383	0.162	1.83513244889769E-18	0	NDUFB10
3.5016425526242E-24	0.68562508056374	0.366	0.146	5.86805258968763E-20	0	MAP3K5
1.10077507479837E-46	0.684562666024764	0.456	0.129	1.84467887034711E-42	0	SPRED1
1.24400162027753E-37	0.683321337065228	0.691	0.31	2.08469791526109E-33	0	IFITM3
4.97172666631644E-24	0.682287406734509	0.284	0.091	8.33161954741309E-20	0	TMEM185A
9.161500090698E-43	0.681434533494266	0.628	0.255	1.53528418519917E-38	0	TSPAN14
2.27696977395456E-18	0.681308581177175	0.261	0.099	3.81574594719305E-14	0	GATD3B
8.4847054363365E-26	0.677637952444313	0.319	0.108	1.42186693702127E-21	0	DPEP2
1.21462823101668E-23	0.676161403979222	0.328	0.12	2.03547398953776E-19	0	COPS6
3.40660584382968E-24	0.675615528127306	0.284	0.092	5.70879007308978E-20	0	NUDT14
4.19337743872883E-15	0.674328179900659	0.283	0.127	7.02726191182177E-11	0	GPS1
1.39118836075155E-30	0.674039531238025	0.453	0.179	2.33135345494744E-26	0	CHMP2A
7.90761676390845E-38	0.673988602037937	0.572	0.234	1.32515841729578E-33	0	KIAA0930
7.15282286294119E-17	0.672926989499873	0.283	0.118	1.19867005537168E-12	0	SLC35E3
2.37541521973952E-43	0.672880957183609	0.807	0.404	3.98072082523949E-39	0	MARCKS
1.12659924411486E-14	0.671903293362038	0.254	0.106	1.88795501328769E-10	0	ERLIN1
2.83013992409097E-18	0.671371313934204	0.302	0.125	4.74274848479165E-14	0	RETREG2
1.97619266491663E-17	0.670292716087031	0.284	0.117	3.31170366786729E-13	0	SRP68
2.99570654219485E-19	0.670084639266639	0.293	0.113	5.02020502341012E-15	0	VPS4B
2.91832174846451E-41	0.670031423468895	0.56	0.218	4.89052358607682E-37	0	ATOX1
1.86109941448488E-29	0.669911094249712	0.478	0.196	3.11883039879376E-25	0	DPP9
1.37463470241983E-27	0.669715311914557	0.332	0.111	2.30361283431515E-23	0	FKBP8
3.11669850319976E-15	0.669159121867534	0.293	0.133	5.22296335166216E-11	0	FADS1
9.36128123557625E-37	0.66877086306801	0.485	0.181	1.56876350945787E-32	0	APH1A
2.38711621971408E-54	0.668000703134754	0.759	0.317	4.00032936099685E-50	0	RBM47
4.71431834157213E-23	0.667759397328024	0.325	0.122	7.90025467680658E-19	0	TTC1
3.43802086366606E-58	0.667306659414314	0.583	0.169	5.76143536333159E-54	0	STARD13
1.70897383717631E-25	0.667023527184302	0.412	0.171	2.86389835634006E-21	0	TRPV2
4.13827736651196E-34	0.666120521445938	0.537	0.216	6.93492521080074E-30	0	ALCAM
2.5776941902599E-17	0.665823770895071	0.254	0.098	4.31969992403754E-13	0	PPDPF
1.50585077640944E-31	0.665681801815866	0.427	0.16	2.52350473110694E-27	0	PYGB
1.65041142052023E-14	0.66506858239694	0.261	0.113	2.7657594585078E-10	0	ZNF664
2.96965266998482E-34	0.663315517043044	0.396	0.131	4.97654394436057E-30	0	ITPA
1.14676125495393E-26	0.662470400489374	0.386	0.15	1.9217425110518E-22	0	NDUFAF3
2.81650040503929E-36	0.661280181978847	0.388	0.116	4.71989137876483E-32	0	PYGL
3.68860196671373E-24	0.659483470851958	0.269	0.083	6.18135917581887E-20	0	TSPAN31
1.93677691277944E-15	0.65943237961738	0.26	0.108	3.24565075043578E-11	0	NUP160
1.46208495152214E-20	0.658573653074703	0.373	0.16	2.45016196176081E-16	0	DYNLRB1
2.18116381067315E-41	0.657491049383508	0.57	0.223	3.65519431392607E-37	0	SLC31A2
1.3491718926198E-47	0.655094756020654	0.673	0.277	2.26094225765226E-43	0	TRAM1
6.17946331269263E-30	0.654522715152764	0.405	0.148	1.03555446194103E-25	0	UQCR10

**8.96714704106827E-53**	0.654437736452359	0.776	0.334	1.50271450114222E-48	0	GAA
**2.2750681808539E-25**	0.653995179373135	0.399	0.164	3.81255925747496E-21	0	HAGH
**4.12684693363397E-23**	0.652853479409956	0.356	0.141	6.9157700913838E-19	0	VMA21
**3.48088565867598E-21**	0.652347775441361	0.276	0.096	5.83326818680921E-17	0	ACO2
**1.10759975458868E-17**	0.652236593289234	0.293	0.12	1.85611566873972E-13	0	SLC50A1
**2.73625875144728E-40**	0.651245514170398	0.636	0.26	4.58542241567534E-36	0	SELPLG
**3.38017707628544E-27**	0.651161069225633	0.341	0.116	5.66450074443914E-23	0	OSTM1
**2.07310265990263E-27**	0.650820731447843	0.39	0.148	3.47410543746483E-23	0	EEF2K
**5.52457305321683E-43**	0.650211665457942	0.611	0.248	9.25807952258076E-39	0	MAN1A1
**1.08195766858508E-18**	0.649665232737994	0.305	0.125	1.81314466101487E-14	0	C18orf32
**4.82827159510674E-18**	0.648580070863798	0.26	0.099	8.09121753907987E-14	0	ZDHHC6
**4.24754695505197E-33**	0.648176069519217	0.845	0.509	7.11803918727608E-29	0	FCER1G
**2.25849340774662E-36**	0.646842490153495	0.824	0.461	3.78478325270178E-32	0	TYROBP
**6.13897372390386E-25**	0.646323386101381	0.311	0.106	1.02876921665181E-20	0	TMEM106E
**3.12870007778536E-41**	0.645784697503494	0.56	0.214	5.2430755903527E-37	0	TMED5
**1.18405012784198E-16**	0.644770061158574	0.269	0.109	1.98423120423758E-12	0	GALK2
**5.77658940800828E-21**	0.643717025058558	0.409	0.182	9.68040852994027E-17	0	SPCS1
**8.27044559663173E-21**	0.642078658001076	0.305	0.116	1.38596127308354E-16	0	AKIP1
**2.00753284210412E-56**	0.640924420026983	0.729	0.262	3.36422353679809E-52	0	SIGLEC1
**1.73432861391456E-19**	0.640799475086491	0.302	0.12	2.90638789119802E-15	0	C7orf50
**9.60891982559033E-27**	0.640497641091611	0.332	0.112	1.61026278437243E-22	0	CUL4B
**2.04763459479725E-49**	0.639213461935679	0.898	0.465	3.43142605396123E-45	0	MAFB
**5.83951657945238E-17**	0.63907745065905	0.311	0.138	9.78586188384629E-13	0	SEC11C
**1.30066164342709E-44**	0.63845069611137	0.769	0.351	2.17964878205512E-40	0	COLGALT1
**9.56901901196806E-42**	0.63810393121485	0.422	0.121	1.60357620602561E-37	0	ME1
**8.14407224426398E-18**	0.636921356066889	0.271	0.106	1.36478362669376E-13	0	NDUFS3
**3.29485898032677E-16**	0.636855970602093	0.263	0.106	5.52152467923161E-12	0	POLR3GL
**4.61695144384103E-36**	0.636786138409518	0.424	0.14	7.73708722958879E-32	0	TANGO2
**9.87212374179139E-24**	0.635825504307463	0.26	0.079	1.6543704966494E-19	0	ZNF561
**1.59645111481127E-46**	0.635608676547206	0.792	0.38	2.67533277820072E-42	0	GNS
**2.50523026192141E-40**	0.634650282115128	0.601	0.25	4.19826487292791E-36	0	ATP6V0B
**7.77089125020714E-21**	0.633769299564867	0.255	0.086	1.30224595570971E-16	0	RFFL
**4.96449637120784E-24**	0.633321554325501	0.379	0.155	8.3195030188701E-20	0	IPO8
**3.18064289329531E-27**	0.632214685230576	0.401	0.157	5.33012136058428E-23	0	RPN1
**6.01904413704702E-33**	0.632059597368472	0.522	0.216	1.00867141648634E-28	0	FGG
**3.94608616580701E-22**	0.628971951783603	0.322	0.123	6.6128511966594E-18	0	RALBP1
**1.10907283036792E-39**	0.62894101413492	0.791	0.376	1.85858424913056E-35	0	MT-ND5
**2.53783260280647E-16**	0.628330759795506	0.258	0.102	4.25289987578308E-12	0	SKP1
**1.68378851132109E-24**	0.627564930083183	0.414	0.177	2.82169278727188E-20	0	MPC1
**1.33329961131636E-23**	0.627501067049142	0.386	0.159	2.23434348864395E-19	0	TXNL4A
**7.23506504117857E-40**	0.62658373519978	0.402	0.117	1.2124521996007E-35	0	RBMS1
**2.34918374036482E-43**	0.625477867485799	0.946	0.816	3.93676211210336E-39	0	PSAP
**9.34424241706351E-30**	0.624807693567984	0.27	0.065	1.5659081442515E-25	0	SASH1
**1.64266914809997E-20**	0.624651152096588	0.258	0.089	2.75278495838593E-16	0	TMEM68
**1.66531680638522E-34**	0.62460257655391	0.601	0.267	2.79073790414035E-30	0	RBPJ
**8.92799994079246E-29**	0.623321998886606	0.386	0.14	1.496154230078E-24	0	MILR1
**3.22909893091405E-17**	0.623069423366798	0.279	0.113	5.41132398842576E-13	0	SURF1
**3.31896583464843E-36**	0.621893296002657	0.353	0.097	5.56192294570384E-32	0	ANKH
**2.25213780640978E-32**	0.621610547021681	0.321	0.089	3.77413253598151E-28	0	ZNF358
**4.90424545994175E-18**	0.621262564858353	0.276	0.106	8.21853454177038E-14	0	CANT1

1.54981457537789E-18	0.621022751268227	0.293	0.116	2.59717926541826E-14	0	NNT
7.38405223862306E-26	0.620898121787708	0.402	0.159	1.23741947414845E-21	0	KRTCAP2
2.93918401213833E-19	0.620334440625901	0.344	0.146	4.92548456754141E-15	0	PPM1G
5.96496321325144E-42	0.620240547259358	0.678	0.292	9.99608535276677E-38	0	CD164
2.14680012713404E-45	0.618916442539879	0.772	0.349	3.59760765305123E-41	0	RASSF4
2.16241094846054E-18	0.61702284892337	0.282	0.108	3.62376826743018E-14	0	EIF4EBP1
2.80979948294211E-43	0.616933919257192	0.599	0.235	4.70866197351439E-39	0	IRAK1
2.0260121066637E-19	0.61684605076561	0.34	0.144	3.39519108834703E-15	0	GIMAP7
9.88120160978535E-28	0.616618985594177	0.435	0.178	1.65589176576783E-23	0	RNF187
1.39022428339572E-24	0.616075690766951	0.444	0.194	2.32973785411455E-20	0	PNPLA2
2.67184617708788E-25	0.615279448015148	0.334	0.119	4.47747982356387E-21	0	EXOC7
2.41050626653183E-22	0.612931736668171	0.3	0.108	4.03952640145404E-18	0	NDUFS6
5.02694375695453E-40	0.612880796487931	0.569	0.222	8.4241523479044E-36	0	SPATS2L
1.80075278985653E-21	0.612310397466546	0.367	0.157	3.01770152524157E-17	0	PIEZO1
3.68452415942553E-12	0.610784212932441	0.286	0.14	6.1745255863653E-08	0	IL1RAP
1.03076800315991E-40	0.609255249417451	0.602	0.248	1.72736101969538E-36	0	HM13
1.56175643005261E-26	0.60860723969704	0.459	0.194	2.61719142548216E-22	0	MLX
2.50415514159646E-23	0.607320783581257	0.347	0.135	4.19646318628735E-19	0	XPO6
2.9874912632234E-24	0.607117325100076	0.338	0.125	5.00643785890977E-20	0	AKT1S1
2.12498557914708E-20	0.606289667153681	0.316	0.125	3.56105083353467E-16	0	GUCD1
7.85734082684159E-43	0.604335824858842	0.583	0.229	1.31673317576211E-38	0	SNX2
2.4212597201691E-23	0.604022594560238	0.406	0.174	4.05754703905937E-19	0	PYCR2
1.06385193803337E-24	0.602803588011872	0.39	0.159	1.78280307775632E-20	0	APPL1
5.62987700781179E-21	0.602489050371451	0.331	0.13	9.434547889691E-17	0	RAB5IF
1.23301127792366E-28	0.601598426838705	0.396	0.146	2.06628029954447E-24	0	ZBED1
4.29834914866421E-43	0.598737373592815	0.657	0.272	7.20317350333148E-39	0	NECAP2
1.36561835517858E-40	0.598626723542712	0.489	0.166	2.28850323960827E-36	0	CD40
4.91284513208555E-21	0.597816543782238	0.283	0.102	8.23294587234896E-17	0	CYB561D2
1.18269133140803E-17	0.59732657463545	0.287	0.12	1.98195413317357E-13	0	ATRAID
8.0315521097596E-54	0.597311679761756	0.826	0.349	1.34592750255351E-49	0	SDC3
1.0331555860087E-25	0.596417501598373	0.289	0.089	1.73136213103338E-21	0	TMEM59
2.52623566886431E-37	0.595899873279375	0.72	0.332	4.23346573388282E-33	0	MT-ND1
5.90896675180607E-16	0.595423617350721	0.261	0.107	9.90224648267661E-12	0	ZNF362
1.9155288631228E-38	0.594751148605735	0.769	0.37	3.21004326882118E-34	0	CAPZB
1.90222970837248E-29	0.594687909677723	0.435	0.171	3.1877565452906E-25	0	TOR1AIP2
1.20938615349588E-14	0.594447508470922	0.25	0.106	2.0266893160284E-10	0	ANO6
5.36706937030372E-35	0.593677154134743	0.433	0.152	8.99413485075497E-31	0	TMEM179E
3.97508004281267E-39	0.593094731107647	0.627	0.272	6.66143913574546E-35	0	PGD
1.14868052487542E-42	0.592271533436649	0.621	0.248	1.92495882358623E-38	0	SCARB2
1.8677042213749E-20	0.591637548048221	0.393	0.175	3.12989873418006E-16	0	PON2
5.65387181908905E-30	0.591396863252353	0.453	0.177	9.47475839442942E-26	0	TMEM173
5.85533194160329E-24	0.591370619275737	0.454	0.204	9.81236526773879E-20	0	NDUFB7
8.81294665150989E-18	0.591168625566704	0.322	0.142	1.47687359986003E-13	0	PSMC4
4.77433158396384E-26	0.590527301475875	0.347	0.126	8.0008248684066E-22	0	CNP
1.17970258367046E-29	0.590439369154465	0.499	0.21	1.97694558971495E-25	0	PTPRJ
1.76808820477049E-23	0.589417889248058	0.315	0.11	2.96296221355439E-19	0	EMC3
9.73018057101054E-35	0.588675612879784	0.504	0.197	1.63058366008995E-30	0	ARPIN
1.87391684577239E-31	0.588674312597387	0.576	0.257	3.14030985014538E-27	0	DCAF7
6.55825205188197E-23	0.588624370294896	0.417	0.18	1.09903187885438E-18	0	MGAT2
1.77895320470379E-42	0.586497733590232	0.617	0.251	2.98116978044261E-38	0	ATP6V0D1

5.40447579687348E-25	0.586079599659966	0.438	0.187	9.05682054040058E-21	0	DTX3L
4.80660371325223E-25	0.585995821605291	0.405	0.166	8.05490650266809E-21	0	BSDC1
6.31804510757487E-38	0.585839424564136	0.591	0.241	1.0587779991274E-33	0	LASP1
7.25376971532325E-20	0.581836484220037	0.287	0.108	1.21558672889387E-15	0	TSR3
4.55373659378759E-43	0.580550964004029	0.512	0.173	7.63115178386925E-39	0	TRPM2
3.72845223560014E-35	0.579736338499963	0.546	0.22	6.24814025641871E-31	0	SAP30
1.46829110398985E-53	0.579629373882576	0.573	0.176	2.46056223206619E-49	0	TLR4
1.80555834551141E-22	0.579418551441476	0.325	0.124	3.02575467540802E-18	0	GALNT2
3.02876435495997E-41	0.579000166941073	0.695	0.307	5.07560330604191E-37	0	GOLIM4
4.16714313377986E-21	0.578990627830269	0.318	0.122	6.98329846358829E-17	0	VPS52
1.54567885570441E-19	0.578610420198493	0.388	0.176	2.59024862638945E-15	0	SSR3
1.38163458750452E-37	0.578584344316752	0.765	0.356	2.31534324174007E-33	0	FPR3
2.76649305946762E-38	0.578581471601954	0.424	0.129	4.63608906905584E-34	0	ZDHHC14
5.38738318621729E-46	0.577932932948423	0.774	0.351	9.02817674346293E-42	0	RNF130
3.78893068811161E-36	0.577153735571453	0.668	0.302	6.34949004713744E-32	0	PRKACA
3.57285973865793E-41	0.576471041228556	0.836	0.441	5.98739835004297E-37	0	MGAT1
8.32959145988203E-18	0.576313415946712	0.253	0.093	1.39587293684703E-13	0	SCRIB
8.72913662745754E-20	0.575161770994702	0.271	0.1	1.46282871602934E-15	0	GCNT1
5.17824917716173E-39	0.575122371605143	0.46	0.153	8.67770997108764E-35	0	TMEM273
1.77440127379131E-24	0.573697951689151	0.374	0.15	2.97354165461948E-20	0	GATAD1
7.84693871096249E-41	0.573507268457355	0.406	0.111	1.31498998918309E-36	0	OLFML2B
1.31722463540384E-42	0.573141281205303	0.671	0.291	2.20740504400975E-38	0	CD84
1.57367134386245E-20	0.573098963401784	0.345	0.142	2.63715843804469E-16	0	ESD
1.88289882320843E-13	0.572440450995592	0.282	0.131	3.15536184793268E-09	0	COX14
7.8631213901276E-18	0.572241189247616	0.277	0.11	1.31770188255758E-13	0	GINM1
8.77268296116683E-24	0.571509814269297	0.287	0.094	1.47012621063234E-19	0	SAP30L
1.70802810092227E-42	0.56868050866702	0.554	0.204	2.86231349152555E-38	0	DENND4C
2.91092036987223E-34	0.568433850106738	0.55	0.228	4.87812035583188E-30	0	REEP5
1.09613026289258E-39	0.5681019167663	0.676	0.294	1.83689509455539E-35	0	PTTG1IP
3.584398570369E-22	0.567923269417274	0.254	0.081	6.00673512422438E-18	0	NRAS
2.03444274392197E-23	0.56753529795507	0.334	0.125	3.40931915026444E-19	0	HEBP1
6.62676530230022E-45	0.566828721044531	0.644	0.265	1.11051332935947E-40	0	TBC1D14
3.79513573454281E-30	0.566814803378029	0.438	0.168	6.35988846394684E-26	0	HADHB
1.10032520533975E-34	0.565754102796105	0.649	0.297	1.84392497910836E-30	0	LGALS9
1.76435406825703E-25	0.564785238527804	0.303	0.097	2.95670454758513E-21	0	ILVBL
1.79556186468656E-18	0.564755935090774	0.255	0.093	3.00900257284174E-14	0	GATD1
4.12672777136225E-22	0.564188722169055	0.325	0.121	6.91557039924886E-18	0	RAB32
9.24931108185038E-20	0.564169406272764	0.276	0.102	1.54999955109649E-15	0	ZMPSTE24
1.16126269110926E-42	0.56353348044803	0.475	0.149	1.94604401776089E-38	0	CCR1
7.87885659199825E-48	0.562699160329591	0.792	0.364	1.32033878768707E-43	0	SLC15A3
1.186313270909E-28	0.562608355198429	0.369	0.131	1.98802377938929E-24	0	MCRIP1
9.88955654646299E-22	0.562543994232263	0.287	0.101	1.65729188605627E-17	0	DOK3
2.081688031053E-27	0.562339268958063	0.428	0.172	3.48849280243861E-23	0	P2RY13
2.5523353870885E-27	0.56203982453638	0.395	0.151	4.2772036416829E-23	0	SLC35F5
2.17754980992687E-11	0.560789996775275	0.263	0.127	3.64913797147545E-07	0	DEPTOR
9.05047930721311E-43	0.560541580273409	0.467	0.147	1.51667932230277E-38	0	SIAH2
6.90961482689255E-32	0.560055152353779	0.392	0.133	1.15791325269065E-27	0	CHMP3
1.32583174647275E-19	0.55943796040639	0.366	0.161	2.22182884073903E-15	0	RPN2
1.37837640205741E-21	0.559053861727203	0.286	0.101	2.30988317456781E-17	0	UBE2W
3.26608423157222E-24	0.559003706186232	0.292	0.097	5.47330395526873E-20	0	SLC15A4

1.13935791594335E-17	0.558853954054819	0.257	0.098	1.90933599553787E-13	0	C11orf68
2.92884159645465E-25	0.557936814175502	0.331	0.117	4.90815274733871E-21	0	FAM13A
1.65674809249548E-41	0.557758381122193	0.585	0.234	2.77637845340392E-37	0	CSTB
2.09446970413092E-34	0.556955815385137	0.784	0.406	3.5099123301826E-30	0	RNH1
3.48931156591222E-40	0.556617696544706	0.729	0.33	5.8473883221557E-36	0	TM9SF2
1.79611660620718E-33	0.556595668340415	0.401	0.135	3.00993220868199E-29	0	DBP
1.79378210965183E-33	0.556336325802378	0.563	0.233	3.00602005935454E-29	0	RNPEP
4.0562711058814E-41	0.556078051794203	0.418	0.115	6.79749911923605E-37	0	FAM20A
7.13281070400988E-29	0.555951601869736	0.382	0.137	1.19531641777798E-24	0	PAPSS2
4.10172084160593E-34	0.555617394697544	0.567	0.246	6.87366378636322E-30	0	TMEM219
9.90902293086463E-27	0.555376008421797	0.366	0.139	1.6605540627543E-22	0	ALKBH5
7.45656410589525E-22	0.555058815464804	0.376	0.161	1.24957101286593E-17	0	CLPTM1L
9.65574681374362E-13	0.554716748242574	0.254	0.117	1.61811005104716E-08	0	TCP11L1
5.81641475373527E-23	0.554676097099495	0.401	0.173	9.74714784430956E-19	0	FGA
8.3653751849623E-34	0.554624496812213	0.43	0.149	1.40186957349598E-29	0	SLC49A4
6.94439030599239E-47	0.55325826785829	0.56	0.188	1.16374092747821E-42	0	NCF4
2.44813750765105E-18	0.553210866887416	0.29	0.116	4.10258883532163E-14	0	NELFB
4.10028976679019E-33	0.552755682158129	0.938	0.672	6.87126559118701E-29	0	CSF1R
3.24717796836072E-31	0.549238104417742	0.894	0.538	5.44162083937889E-27	0	CYBA
2.04287813093857E-42	0.548294942904735	0.808	0.399	3.42345517182686E-38	0	APLP2
2.10545926796528E-38	0.547255051717861	0.595	0.235	3.52832864125622E-34	0	GIMAP4
3.75970464077282E-18	0.546893770805647	0.27	0.103	6.3005130370071E-14	0	MED10
4.19394400453526E-31	0.54620058056946	0.396	0.138	7.02821136280018E-27	0	PRKACB
1.01249327203722E-17	0.545770663689006	0.293	0.121	1.69673622527998E-13	0	CCDC124
1.35654335949998E-22	0.544470216209135	0.299	0.107	2.27329536185006E-18	0	ELAC2
1.98836340953421E-16	0.544024837630447	0.25	0.096	3.33209940169744E-12	0	PDLIM2
5.81022278515147E-20	0.543570670555455	0.328	0.137	9.73677134335683E-16	0	HDGFL2
1.55962520342782E-55	0.543138821807633	0.605	0.201	2.61361991590434E-51	0	TPD52L2
9.9568603406451E-16	0.542768541162661	0.311	0.139	1.66857065588531E-11	0	GPI
2.49113446880369E-26	0.542720986871152	0.344	0.121	4.17464314282123E-22	0	EMC7
2.77506374749969E-36	0.542529603801126	0.595	0.259	4.65045182805998E-32	0	MYDGF
1.50007645846904E-19	0.542045061287599	0.279	0.105	2.51382812910242E-15	0	ATP6V1H
1.52380687592183E-38	0.54166768879955	0.454	0.147	2.55359556266981E-34	0	PILRA
1.39411447575647E-20	0.541593382248365	0.312	0.122	2.33625703847269E-16	0	MCM3AP
9.0312298552403E-39	0.54141965218477	0.668	0.309	1.51345349914117E-34	0	ATP6AP2
1.12462931520991E-20	0.541200938147533	0.36	0.154	1.88465380642877E-16	0	ARHGAP1
8.95749928011895E-20	0.540718283195778	0.283	0.105	1.50109772936233E-15	0	GSK3A
1.76620143671114E-43	0.539391391898578	0.647	0.265	2.95980036764052E-39	0	PECAM1
5.75506410157816E-26	0.539130615610056	0.43	0.179	9.64433642142468E-22	0	ORMDL1
2.09821688290797E-34	0.53828323811082	0.316	0.076	3.51619185237717E-30	0	SCN1B
3.22403864222056E-28	0.537150981214169	0.358	0.124	5.40284395663322E-24	0	KEAP1
2.63086205533124E-31	0.537061320970981	0.566	0.256	4.4087986323241E-27	0	COPG1
3.3732918992862E-25	0.535326502534897	0.393	0.157	5.65296256482382E-21	0	PSMD1
3.3192333446399E-18	0.535180603524866	0.253	0.091	5.56237123894755E-14	0	LEO1
1.42582709420515E-44	0.534120313564789	0.434	0.115	2.38940104446899E-40	0	SLC46A1
3.20652994803417E-28	0.533579428176688	0.93	0.625	5.37350288691567E-24	0	MT-ATP6
9.94791532096464E-19	0.533294326484969	0.327	0.136	1.66707164948725E-14	0	RECQL
5.38313620556506E-21	0.532303049140419	0.327	0.131	9.02105965328592E-17	0	PPP4R3B
4.1280943165068E-25	0.532086921361473	0.443	0.185	6.91786045560209E-21	0	EIF6
1.01140991379551E-14	0.531987578615276	0.269	0.119	1.69492073353851E-10	0	FLVCR2

3.81690258999159E-22	0.531914815551635	0.337	0.134	6.3963653603079E-18	0	CDV3
3.32313246984334E-42	0.531881732683768	0.527	0.189	5.56890539296347E-38	0	WASHC4
3.80353993827741E-27	0.531380508882857	0.399	0.154	6.37397222856529E-23	0	PSMC2
2.88763362764344E-18	0.531204007017229	0.26	0.097	4.83909643320488E-14	0	SLC25A45
1.58702007714546E-29	0.53112352704173	0.418	0.159	2.65952824528036E-25	0	ORM1
3.26011187206729E-35	0.530384782934684	0.425	0.147	5.46329547521037E-31	0	ATP6V0A1
9.70135818757928E-22	0.530365710497262	0.366	0.156	1.62575360507454E-17	0	NDUFS1
2.07472155292385E-25	0.530335684260055	0.459	0.203	3.47681837838979E-21	0	COPB2
4.32034517048253E-29	0.530091501372864	0.401	0.149	7.24003443669462E-25	0	LAPTM4A
1.18001779497634E-32	0.530081493519348	0.524	0.213	1.97747382082135E-28	0	WDR81
2.65539986066313E-31	0.529648944682568	0.604	0.278	4.44991908649927E-27	0	ATP6V0E1
2.749223563192E-20	0.529129992858376	0.367	0.158	4.60714884719715E-16	0	DUS1L
1.87878276536445E-36	0.527127502891286	0.583	0.241	3.14846415819774E-32	0	TSPO
5.92950049856495E-29	0.525993266660065	0.493	0.209	9.93665693549515E-25	0	ARHGDIA
2.77702085979945E-19	0.523307073071132	0.27	0.1	4.65373155685192E-15	0	DOK1
2.72832433486666E-20	0.523132129471858	0.313	0.122	4.57212592036955E-16	0	SLC39A11
5.92958187610707E-26	0.522790048756292	0.361	0.136	9.93679330798023E-22	0	TMBIM4
3.94717930207828E-16	0.522101223576731	0.25	0.1	6.61468307442278E-12	0	PRKCA
1.16157536049939E-40	0.521179998053444	0.553	0.209	1.94656798912488E-36	0	BSG
4.57548921380928E-27	0.520958906861181	0.424	0.17	7.6676048245016E-23	0	KIF16B
3.0948216312421E-26	0.520930112629302	0.453	0.19	5.1863020896355E-22	0	AP2S1
1.39895340308671E-17	0.520835236030127	0.287	0.119	2.34436611289271E-13	0	ZDHHC3
6.33329375942213E-19	0.519798880051373	0.305	0.12	1.06133336820396E-14	0	MAPK1IP1L
1.99053847579471E-30	0.519246356382058	0.343	0.107	3.33574437773677E-26	0	MORF4L2
2.60546135641094E-14	0.517840329858798	0.284	0.132	4.36623214107345E-10	0	NME4
1.43903202690171E-36	0.516923885215321	0.774	0.388	2.41152987068188E-32	0	M6PR
3.70849407685323E-15	0.515286142671004	0.26	0.108	6.21469437399064E-11	0	DCUN1D1
9.14361363202726E-14	0.514327158125454	0.255	0.112	1.53228677245513E-09	0	GANAB
6.67115747526883E-14	0.51302952541274	0.253	0.11	1.11795256970555E-09	0	ERCC6L2
1.93421235408654E-28	0.512557765836648	0.488	0.21	3.24135306297823E-24	0	PUF60
2.60007001197392E-13	0.511778866817742	0.279	0.13	4.3571973260659E-09	0	NMI
3.43725998759416E-51	0.511458891854821	0.605	0.212	5.7601602872103E-47	0	MGAT4A
1.077325012024E-30	0.511206634345148	0.443	0.17	1.80538125514982E-26	0	C16orf58
3.67181313921993E-31	0.510480251618361	0.504	0.207	6.15322445870475E-27	0	SAP18
1.68754629295343E-28	0.509611681949002	0.341	0.113	2.82799007773136E-24	0	CYSTM1
2.09194960813878E-26	0.509120616547972	0.374	0.139	3.50568915331896E-22	0	IFITM2
1.13506520241991E-31	0.509050538064509	0.453	0.172	1.90214226621528E-27	0	SEPTIN11
6.13449747237761E-30	0.507054494000329	0.478	0.195	1.02801908642104E-25	0	POLR2L
5.17264667162939E-20	0.506827801093597	0.358	0.153	8.66832129231653E-16	0	CCT6A
8.10340524438797E-35	0.506335438607857	0.502	0.202	1.35796865085454E-30	0	RNPEPL1
1.38010404965556E-36	0.505675624819823	0.505	0.19	2.31277836641278E-32	0	DDRGK1
3.61348405876859E-31	0.504965481819264	0.525	0.222	6.0554765856844E-27	0	STAU1
5.82745211621984E-51	0.504664965442867	0.697	0.269	9.76564425636121E-47	0	AKR1B1
6.64421371529381E-42	0.504531445718696	0.672	0.285	1.11343733440894E-37	0	IL18BP
3.74279833158147E-17	0.503442995312315	0.319	0.139	6.27218144406423E-13	0	LRRC47
5.2810913785681E-26	0.50331039157455	0.344	0.122	8.85005293220441E-22	0	NSL1
5.17068260096469E-31	0.503245732492656	0.512	0.216	8.66502990269663E-27	0	RAB2A
4.37433359844907E-38	0.502812901504391	0.689	0.326	7.33050824428095E-34	0	AKR1A1
7.51383071899847E-20	0.502234375612444	0.35	0.149	1.25916775188976E-15	0	UNC50
5.76734102089292E-24	0.502122745295179	0.279	0.088	9.66491008281236E-20	0	FAM234A

4.00429059277723E-33	0.501918715623574	0.399	0.131	6.71039017537608E-29	0	LRP10
3.37234150217578E-28	0.501458654003666	0.382	0.135	5.65136988934617E-24	0	CD302
9.0129932612558E-28	0.501181515759764	0.427	0.168	1.51039741072125E-23	0	ANKFY1
1.83377584113774E-25	0.499933148229013	0.325	0.112	3.07304155457862E-21	0	RAPH1
3.9680439143533E-39	0.49982956765526	0.511	0.185	6.64964799167326E-35	0	BIN1
4.46368597917828E-43	0.499204836755956	0.61	0.243	7.48024496390696E-39	0	ATP6V0C
5.24800783389894E-13	0.498516261269264	0.264	0.122	8.79461152804785E-09	0	RBM10
1.1154511529869E-14	0.498224893505322	0.267	0.115	1.86927304217544E-10	0	RCC1L
6.49780165504198E-25	0.497730318457822	0.324	0.111	1.08890160135193E-20	0	SLC30A7
3.94122946806689E-18	0.496854353542456	0.271	0.103	6.6047123425865E-14	0	PARP1
1.16768337803226E-22	0.494628206165336	0.367	0.15	1.95680380490646E-18	0	VPS4A
9.63810736715277E-24	0.494026724200341	0.315	0.113	1.61515403258746E-19	0	SLC35B2
1.88753528633549E-21	0.493917661516416	0.351	0.14	3.16313163284101E-17	0	PSMC3
1.12616264353211E-43	0.492697782234213	0.583	0.205	1.88722335803111E-39	0	VCAM1
6.749359861151E-13	0.492510655261525	0.263	0.122	1.13105772553168E-08	0	GTPBP2
2.19069373179248E-28	0.491898002577193	0.434	0.172	3.67116455573783E-24	0	FIS1
2.38538483412818E-15	0.490970792527171	0.271	0.115	3.99742790503201E-11	0	MEAF6
3.14714745811451E-29	0.490912675841169	0.43	0.172	5.2739897103083E-25	0	TRAPPC5
6.27159884645387E-28	0.489663053947193	0.332	0.108	1.05099453468874E-23	0	SLC29A3
9.96883922430108E-20	0.489298325082084	0.338	0.144	1.67057807720837E-15	0	SOS 2
1.29621312923719E-33	0.488243754431495	0.443	0.157	2.17219396197568E-29	0	FAM20C
2.67315612069485E-25	0.486338894164177	0.388	0.152	4.47967502706043E-21	0	UBAC1
1.05106337679584E-22	0.485770421928175	0.315	0.115	1.76137200683447E-18	0	LEPROT
4.16981455808244E-15	0.48451573412983	0.267	0.115	6.98777523643455E-11	0	HLX
6.91547981719638E-13	0.484445384871176	0.254	0.114	1.15889610776577E-08	0	RALY
4.28747938060287E-27	0.483926672985171	0.412	0.158	7.18495794601428E-23	0	UBL3
4.75828508656727E-18	0.483333190840729	0.328	0.145	7.97393414806943E-14	0	ACTR1B
8.75849422848476E-21	0.482884763904941	0.364	0.153	1.46774846280948E-16	0	CCSAP
2.82281006135868E-43	0.482671493788926	0.771	0.362	4.73046510082487E-39	0	DPP7
8.41483931098908E-23	0.482126877335666	0.303	0.107	1.41015877173555E-18	0	KLHL21
1.20460826713464E-17	0.48198356066373	0.271	0.106	2.01868253406423E-13	0	CENPB
2.25516237990791E-12	0.481949853280282	0.277	0.131	3.77920111624968E-08	0	HSD17B10
3.95335528791055E-15	0.481625460580064	0.367	0.177	6.62503279148051E-11	0	CHPT1
1.47788623673064E-36	0.48131489796225	0.524	0.194	2.47664175551321E-32	0	ETV5
7.68838018784158E-21	0.480656889127208	0.871	0.556	1.28841875187849E-16	0	CYBB
9.26634031478152E-24	0.480306306213953	0.37	0.147	1.55285330995109E-19	0	SEPHS2
4.45879714782918E-21	0.479264865990599	0.385	0.167	7.47205226033214E-17	0	PPP1R10
3.4966130634008E-31	0.479134305941866	0.515	0.211	5.85962417164705E-27	0	ELOVL1
9.32163301445243E-23	0.478892365179667	0.337	0.129	1.56211926056194E-18	0	FNDC3A
1.65094008633358E-35	0.47887964558555	0.431	0.147	2.76664539667782E-31	0	ATP6V1C1
1.0618972587535E-24	0.478342942874293	0.448	0.193	1.77952742621911E-20	0	ARPC5
2.76590807350771E-21	0.478231834838187	0.27	0.093	4.63510874958422E-17	0	TDP2
1.024236851641E-30	0.47763642923856	0.296	0.076	1.71641611597999E-26	0	LAMB2
5.89252864570267E-30	0.476665822769789	0.37	0.123	9.87469950446854E-26	0	PEAK1
3.53251206420366E-22	0.476261364113425	0.312	0.115	5.91978371719249E-18	0	SEM1
2.70540933985542E-19	0.4749469503563	0.28	0.107	4.53372497172972E-15	0	CLCC1
3.3101931967478E-33	0.474817885139172	0.414	0.143	5.54722175910997E-29	0	ATP6V1F
4.77871024495957E-30	0.474147590858459	0.354	0.114	8.00816262850324E-26	0	ENTR1
8.32113675859201E-21	0.473999011200824	0.318	0.123	1.39445609800485E-16	0	YKT6
2.48820400069128E-32	0.473352407791055	0.491	0.195	4.16973226435844E-28	0	PIGT

4.60715941064077E-19	0.471284682420047	0.303	0.121	7.72067774035181E-15	0	DCAF12
1.01691590466579E-26	0.470976818239552	0.851	0.475	1.70414767303893E-22	0	TSC22D3
9.55825953109646E-34	0.470947685942059	0.675	0.309	1.60177313222114E-29	0	SYK
4.7616593682852E-47	0.470561847773755	0.806	0.341	7.97958876937234E-43	0	LRP1
1.35273575142835E-14	0.469076115900853	0.309	0.14	2.26691457224364E-10	0	MIA2
9.82100895458084E-29	0.468514360010972	0.393	0.144	1.64580468060866E-24	0	GTF2F1
1.65413887306272E-35	0.468049096973897	0.498	0.19	2.77200592347851E-31	0	TBC1D15
3.25977060398703E-21	0.467683022841621	0.289	0.102	5.46272357816146E-17	0	RTL8C
1.82698368168885E-31	0.467352341818515	0.414	0.147	3.06165925377417E-27	0	CAB39
7.85892278131153E-29	0.467215322394627	0.412	0.157	1.31699827969219E-24	0	CDIPT
4.2283371716459E-30	0.466957763065141	0.325	0.096	7.08584743224419E-26	0	B3GNT2
4.00808830422256E-34	0.466157362556557	0.331	0.088	6.71675438021616E-30	0	HNMT
1.32449454549458E-30	0.465488696975058	0.605	0.27	2.21958795933981E-26	0	GNB2
1.24532112625519E-19	0.46456663095931	0.353	0.155	2.08690914337844E-15	0	UBA1
8.23170628939687E-22	0.464303111303692	0.415	0.179	1.37946933997713E-17	0	PPM1B
1.93124907858567E-24	0.464072319819195	0.406	0.17	3.23638720589386E-20	0	TMEM9B
6.43186221143581E-37	0.462375689629516	0.642	0.289	1.07785146939241E-32	0	SEC14L1
4.72041917775229E-23	0.461614374349753	0.345	0.135	7.91047845807728E-19	0	MLXIP
9.86111302531372E-22	0.461228841871132	0.668	0.374	1.65252532078207E-17	0	LGALS1
2.30403768693538E-18	0.460533033578149	0.295	0.12	3.86110635576631E-14	0	MESD
2.02196629502049E-27	0.460145701911212	0.424	0.173	3.38841111719533E-23	0	DDB1
1.33142968205126E-24	0.458628842521484	0.399	0.162	2.2312098611815E-20	0	MOGS
5.15315055272901E-19	0.458004855470652	0.27	0.099	8.63564969626327E-15	0	VCP
2.40706586417353E-27	0.457858431264085	0.377	0.135	4.03376097518201E-23	0	ABHD16A
1.4631935755149E-16	0.45642837426745	0.305	0.134	2.45201979384788E-12	0	MYO1C
2.45866494658415E-19	0.456250315973927	0.345	0.147	4.12023071748573E-15	0	SART1
3.46244091458244E-22	0.454827943546923	0.315	0.117	5.80235848465725E-18	0	REXO2
3.61588736773317E-36	0.454573547394274	0.66	0.295	6.05950405084725E-32	0	SUSD6
9.08601076747042E-21	0.454296910190317	0.296	0.11	1.52263368441269E-16	0	CTSO
4.13487644532684E-14	0.453516601808233	0.287	0.133	6.92922594707873E-10	0	PYURF
1.5426402504259E-19	0.453261128041625	0.337	0.143	2.58515653166372E-15	0	C14orf119
7.39064005745462E-30	0.453006411268663	0.462	0.186	1.23852346082824E-25	0	SELENON
9.6098300480421E-22	0.452798577769561	0.377	0.156	1.61041531945089E-17	0	ANAPC11
1.08093725356702E-26	0.452093678176976	0.46	0.193	1.81143464952761E-22	0	HUWE1
2.21709372733582E-16	0.452078747482351	0.261	0.106	3.71540566826937E-12	0	CCDC28A
2.21803561587343E-14	0.451693495075795	0.258	0.108	3.7169840850807E-10	0	TEX2
8.8168901701058E-22	0.449913044182698	0.293	0.105	1.47753445470633E-17	0	ENOSF1
1.69523151471955E-33	0.449289444781606	0.576	0.25	2.84086897236701E-29	0	CRTAP
3.0187111994526E-28	0.449058662706967	0.403	0.152	5.05875622804267E-24	0	TCF12
3.83592212710183E-26	0.448719049479324	0.351	0.127	6.42823830059725E-22	0	DOCK7
1.92770963887154E-18	0.448499986255758	0.369	0.167	3.23045581282093E-14	0	NUP43
1.48260892504141E-15	0.448402191111131	0.347	0.169	2.4845560365844E-11	0	EIF2AK1
9.88606908503455E-17	0.447965535241492	0.318	0.139	1.65670745727009E-12	0	SUPT16H
1.13255658256677E-36	0.447520519272713	0.761	0.363	1.89793832106539E-32	0	ACSL1
1.06966171204332E-36	0.446981424080004	0.762	0.37	1.7925390970422E-32	0	TNFRSF1A
1.17087930108994E-23	0.446897591348006	0.415	0.177	1.96215953276652E-19	0	YWHAB
1.39637333147549E-19	0.445500083563607	0.341	0.143	2.34004242888662E-15	0	SNX27
7.65119029449859E-22	0.445471408484	0.328	0.127	1.28218646955207E-17	0	BTF3L4
1.12802343413682E-17	0.44499142413666	0.29	0.12	1.89034167092648E-13	0	PSMB5
2.926759149714E-37	0.44356746475787	0.684	0.31	4.90466298309073E-33	0	IFNGR2

1.63258926577982E-14	0.443443340898289	0.309	0.143	2.73589309159382E-10	0	VDAC3
5.7815637397555E-31	0.443190650206237	0.618	0.287	9.68874451508227E-27	0	TLN1
2.90807550730447E-29	0.443151620203328	0.507	0.22	4.87335293514083E-25	0	CD46
1.3965323162705E-21	0.44298601116328	0.415	0.189	2.34030885560611E-17	0	CTDSP1
6.85069662145997E-20	0.441786981594785	0.335	0.135	1.14803973982426E-15	0	MCM3
3.56558846276793E-17	0.441555991444164	0.253	0.094	5.97521314590649E-13	0	TMEM70
4.08499930493637E-25	0.441479780467843	0.324	0.112	6.84564183521237E-21	0	CREB3L2
3.34873712852458E-12	0.441342989722588	0.263	0.126	5.61181367998149E-08	0	PEX2
4.44423643709128E-27	0.440962208828346	0.504	0.22	7.44765142127757E-23	0	PTPA
2.49904006546806E-38	0.440935200028347	0.46	0.155	4.18789134171137E-34	0	EPAS1
8.21425230236476E-23	0.440866205250989	0.347	0.136	1.37654440083029E-18	0	METTL23
1.22332254362497E-21	0.440622748038116	0.396	0.173	2.05004391860673E-17	0	IFI6
6.1627545297074E-17	0.440239451790606	0.376	0.176	1.03275440408837E-12	0	FKBP1A
1.23775705383662E-23	0.437923153876308	0.509	0.245	2.07423327081941E-19	0	GOLPH3
4.35181143203206E-28	0.436738407291591	0.527	0.236	7.29276559779932E-24	0	SSR2
7.20811152481677E-31	0.436658054498367	0.504	0.213	1.2079353293288E-26	0	HEXB
6.16081369823366E-24	0.436429325899821	0.313	0.109	1.03242915955E-19	0	CDS2
9.15448431415012E-14	0.436293195811878	0.284	0.132	1.53410848136528E-09	0	STMP1
2.28267759132733E-15	0.435844805459183	0.366	0.179	3.82531110754633E-11	0	LONP2
2.96685115947015E-19	0.435605496173418	0.325	0.137	4.97184917304007E-15	0	IPO9
1.55612802055787E-23	0.435481511975156	0.901	0.569	2.60775933685088E-19	0	KCTD12
1.21380381674107E-15	0.435327882071154	0.335	0.153	2.03409243609468E-11	0	ZBTB16
7.89161233398579E-19	0.433420612894516	0.39	0.176	1.32247639492934E-14	0	TMEM106C
9.76146325494457E-18	0.433380826404419	0.28	0.113	1.63582601226361E-13	0	ITPRIP
1.25947578488926E-24	0.430979894606643	0.395	0.161	2.11062952031742E-20	0	BROX
6.5597585080354E-25	0.430291573448285	0.459	0.2	1.09928433077657E-20	0	EMC4
2.41272309553807E-23	0.429816997509166	0.44	0.192	4.04324136350269E-19	0	AKT1
8.25038730428108E-14	0.429453604864215	0.254	0.111	1.38259990445142E-09	0	BABAM1
4.36974461792432E-17	0.429039902885859	0.338	0.155	7.32281803071758E-13	0	SCAF1
1.36016348814103E-25	0.428320941459278	0.357	0.129	2.27936197342674E-21	0	TADA3
1.27547001835523E-18	0.426687526307625	0.277	0.104	2.1374326567597E-14	0	PGK 1.00
2.26987264610521E-19	0.426686249651215	0.279	0.102	3.80385258034312E-15	0	LPCAT3
9.88806157198391E-23	0.425858770976028	0.47	0.219	1.65704135823306E-18	0	ADI1
3.29696073247569E-23	0.425840128373791	0.446	0.199	5.52504679548276E-19	0	TRAPPC3
1.21004540872383E-30	0.425408010692603	0.56	0.245	2.02779409593939E-26	0	HNRNPH2
8.00090019349546E-30	0.424442221277805	0.454	0.175	1.34079085442597E-25	0	VKORC1
2.50794053773492E-27	0.423938073020244	0.486	0.207	4.20280675313618E-23	0	DYNC1I2
8.95822201726605E-33	0.423868590685738	0.401	0.135	1.50121884565344E-28	0	SGPL1
3.97191161939671E-17	0.42235966321311	0.329	0.145	6.65612949178501E-13	0	GIMAP2
1.47422379118306E-21	0.421565867720268	0.408	0.183	2.47050422926458E-17	0	EGLN1
1.74378188986441E-21	0.420921281951722	0.363	0.155	2.92222969103478E-17	0	YIPF4
1.58067494523187E-36	0.420824549009712	0.668	0.301	2.64889507321956E-32	0	NFE2L1
3.09255238258362E-12	0.418877977492406	0.303	0.156	5.18249928273363E-08	0	NDRG1
1.16939848635685E-13	0.418751240459225	0.311	0.149	1.9596779834368E-09	0	PGP
6.99314578569016E-27	0.418627489016948	0.543	0.242	1.17191137076596E-22	0	RAB31
4.5834928955646E-23	0.41785596607322	0.331	0.124	7.68101739438715E-19	0	FAM199X
1.49314545033043E-25	0.417832979070087	0.472	0.208	2.50221314566374E-21	0	SRGAP2B
3.97513780306974E-23	0.417671277749656	0.433	0.189	6.66153593038427E-19	0	MAN2A1
7.00931331306396E-20	0.415902601812797	0.322	0.133	1.17462072500326E-15	0	ADGRG6
7.96164657770433E-25	0.415210167485789	0.514	0.238	1.33421273349169E-20	0	SNX17

**3.45713226815785E-21**	0.414837273142583	0.914	0.652	5.79346225497893E-17	0	TPP1
**7.63069410471261E-32**	0.414727917679178	0.56	0.24	1.27875171806774E-27	0	GSDMD
**4.92143418798067E-15**	0.414578359839785	0.27	0.116	8.247339412218E-11	0	PRPF31
**1.66242325833692E-18**	0.414023964435503	0.26	0.094	2.78588889632101E-14	0	PBDC1
**8.09834185066581E-20**	0.413327304674965	0.344	0.146	1.35712012733458E-15	0	IMMT
**5.84799901670606E-22**	0.412946660313373	0.45	0.21	9.80007675219601E-18	0	PPP1CA
**8.82517437775531E-22**	0.412247829795874	0.457	0.215	1.47892272222423E-17	0	LAMTOR2
**3.36406070529159E-41**	0.411418157357148	0.493	0.168	5.63749292992765E-37	0	UBXN6
**3.22818040631714E-31**	0.411012283287049	0.576	0.25	5.40978472490627E-27	0	CTNNA1
**1.78533023942266E-29**	0.410986979237037	0.476	0.195	2.9918564152245E-25	0	ANXA6
**4.79114497731759E-32**	0.410285976379165	0.64	0.303	8.02900075298882E-28	0	LAMTOR1
**1.17372312507596E-25**	0.409325441069387	0.377	0.146	1.9669252130023E-21	0	LRRC41
**6.15215037563346E-24**	0.408947545393563	0.377	0.151	1.03097735994865E-19	0	STX12
**2.72455656793541E-21**	0.408155071837242	0.313	0.121	4.56581189654617E-17	0	EIF2S1
**9.79574394589673E-28**	0.407529436960265	0.337	0.109	1.64157077045337E-23	0	LZTS2
**2.03048330318901E-24**	0.406945095020594	0.363	0.136	3.40268391948414E-20	0	PMVK
**6.9515312956581E-24**	0.406796746783758	0.52	0.243	1.16493761452638E-19	0	S100A11
**4.51579937818393E-28**	0.405284909282843	0.498	0.213	7.56757659796063E-24	0	ADD1
**1.02512756051261E-12**	0.405268557487952	0.255	0.116	1.71790876590703E-08	0	EIF5B
**2.04505390963212E-20**	0.404937617314254	0.357	0.151	3.4271013417615E-16	0	ZC3H4
**3.00834251959107E-34**	0.403978506411817	0.446	0.161	5.04138039433071E-30	0	PAPSS1
**3.27547239524986E-27**	0.403559761929753	0.408	0.155	5.48903663995971E-23	0	IFFO1
**6.15257323994968E-22**	0.403129650094515	0.312	0.118	1.03104822355077E-17	0	VPS26B
**1.64919152082984E-40**	0.402992228779584	0.617	0.251	2.76371515060665E-36	0	GUSB
**9.02217397718377E-28**	0.402976066088389	0.491	0.212	1.51193591509646E-23	0	SEC31A
**2.75434473761232E-35**	0.402786894890605	0.721	0.346	4.61573091129073E-31	0	AFF1
**2.51835893592471E-45**	0.401507291123853	0.554	0.176	4.22026590482263E-41	0	KCNMA1
**3.82517499518044E-27**	0.401207299454265	0.483	0.209	6.41022825692337E-23	0	PYCARD
**1.93502981656899E-20**	0.399749452768861	0.284	0.103	3.24272296660631E-16	0	GRAMD1A
**1.07949317175819E-26**	0.399306741527418	0.412	0.163	1.80901465723237E-22	0	RSU1
**8.33131827528204E-18**	0.396883403158618	0.309	0.131	1.39616231657176E-13	0	GALM
**1.07666031395673E-13**	0.396738570609336	0.273	0.121	1.80426735412868E-09	0	SMC3
**1.03741143123368E-26**	0.396407396581803	0.311	0.097	1.73849407646139E-22	0	TANC2
**2.21919265754381E-48**	0.396359369035199	0.742	0.308	3.71892305551192E-44	0	TTYH3
**1.63859555453702E-12**	0.395872578109848	0.251	0.114	2.74595843029314E-08	0	PTEN
**1.1526710806264E-16**	0.395299950276469	0.274	0.111	1.93164619691372E-12	0	SLC2A13
**1.52114514970694E-19**	0.394257094573453	0.395	0.18	2.54913504187888E-15	0	RAB1B
**2.15209772254435E-29**	0.393810115788727	0.525	0.223	3.60648536343981E-25	0	TMED9
**1.17919494868793E-26**	0.392609384337244	0.402	0.156	1.97609489501124E-22	0	ITFG1
**3.4670708369724E-17**	0.392476269922857	0.269	0.105	5.81011730859834E-13	0	MTIF3
**5.2521811450642E-17**	0.392379489510768	0.289	0.12	8.80160516289859E-13	0	ERLEC1
**2.05436767410018E-15**	0.392177649884105	0.312	0.139	3.44270934825709E-11	0	RPA1
**1.27094451137395E-14**	0.390939528458525	0.255	0.105	2.12984881216047E-10	0	YWHAQ
**5.13031410836945E-18**	0.390229491120332	0.289	0.116	8.59738038280552E-14	0	RNF103
**2.04560478233289E-38**	0.389257616472107	0.679	0.299	3.42802449423346E-34	0	ANXA1
**2.64904968217871E-34**	0.388740057282306	0.527	0.21	4.43927745739508E-30	0	ZFAND5
**1.42134068988426E-29**	0.388567504529684	0.43	0.165	2.38188272810804E-25	0	CAPZA2
**6.45434864158754E-15**	0.387837597121228	0.279	0.123	1.08161974535724E-10	0	MON1B
**9.11828876680517E-25**	0.387542579236443	0.348	0.13	1.52804283154121E-20	0	RETREG3
**3.3534549126436E-19**	0.386512518762976	0.325	0.131	5.61971974260814E-15	0	SQOR

2.10965932709064E-20	0.386315834162625	0.761	0.403	3.5353671003385E-16	0	ARHGDIB
2.73819198843876E-19	0.386124509391	0.38	0.172	4.58866213422568E-15	0	PARP10
6.97214780666026E-23	0.38579321208223	0.334	0.127	1.16839252944013E-18	0	ALDH3B1
2.63980957125886E-30	0.385758707093528	0.424	0.157	4.4237928795156E-26	0	FEZ2
4.48864508925316E-30	0.385607054473981	0.582	0.263	7.52207144057044E-26	0	MPV17
1.39362800535254E-17	0.385561627081752	0.308	0.129	2.33544181136978E-13	0	PRPF8
3.012586688546E-34	0.384610277283983	0.536	0.213	5.04849277266539E-30	0	FRMD4B
4.56133981482902E-24	0.3843691129866	0.433	0.186	7.64389326169046E-20	0	PDIA6
1.75844314009868E-18	0.383456062449223	0.337	0.145	2.94679901417737E-14	0	COX16
2.77864622836243E-13	0.382994914021208	0.271	0.122	4.65645534948976E-09	0	REEP3
6.16529378157578E-16	0.382430618020763	0.34	0.157	1.03317993191647E-11	0	PDCD6
2.58675950470171E-30	0.382295945099465	0.511	0.219	4.33489157797912E-26	0	RAB35
9.56902025195113E-26	0.382185152342304	0.598	0.275	1.60357641382197E-21	0	ENO1
2.2968675109651E-29	0.381692876913378	0.517	0.222	3.84909057487531E-25	0	CANX
3.38909177333654E-30	0.379616960672261	0.376	0.127	5.67943999375738E-26	0	NHSL1
1.03172716761675E-33	0.37914245325851	0.419	0.142	1.72896838749214E-29	0	SLC9A9
1.66547169489192E-15	0.37893141967252	0.267	0.112	2.79099746629988E-11	0	ANKRD40
6.06005697759759E-29	0.378515843156423	0.553	0.246	1.0155443483058E-24	0	INF2
4.1963987255205E-23	0.377576692481345	0.385	0.161	7.03232498422725E-19	0	TMBIM6
1.91554765261645E-17	0.377410579031349	0.354	0.159	3.21007475625464E-13	0	ATP1A1
5.02853993646246E-35	0.377043443701141	0.552	0.225	8.42682722552379E-31	0	ATP6V1A
2.55886326203399E-22	0.376775293353763	0.287	0.099	4.28814305451656E-18	0	TMX1
7.37105688913619E-23	0.376662452167091	0.515	0.237	1.23524171348144E-18	0	NUCKS1
5.69209648966083E-33	0.376266607622463	0.406	0.135	9.53881529737362E-29	0	CBLB
1.67025852870266E-42	0.375905095605326	0.708	0.31	2.79901924239992E-38	0	ARRDC2
1.57114904834509E-33	0.374958585393972	0.791	0.398	2.6329315752167E-29	0	DNM2
4.0827791494872E-15	0.374859993230601	0.261	0.109	6.84192129871065E-11	0	KDM3A
2.91797803220791E-24	0.374811293315511	0.366	0.142	4.88994758637402E-20	0	TCEAL4
1.91433864016069E-15	0.373447488346066	0.353	0.168	3.20804869318128E-11	0	RASSF1
2.86640815588216E-25	0.372705920684614	0.676	0.345	4.80352678762732E-21	0	CMTM3
1.06408103436878E-20	0.372382771878081	0.353	0.149	1.78318699739519E-16	0	DUSP22
6.95066829265639E-23	0.372289079816132	0.379	0.156	1.16479299248336E-18	0	GOLGB1
2.31640801638628E-23	0.370569783562916	0.444	0.194	3.88183655386012E-19	0	NDUFA4
1.45070660789352E-24	0.370494530069235	0.367	0.143	2.43109413350797E-20	0	LYPLAL1
1.42649917571152E-22	0.370409790747605	0.313	0.113	2.39052731865736E-18	0	SECTM1
1.41581076294778E-23	0.370264738231057	0.463	0.203	2.37261567654789E-19	0	UQCRC1
3.95279525071532E-17	0.369838536101495	0.298	0.125	6.62409428114873E-13	0	PLEKHJ1
9.9437036220377E-34	0.369364987819696	0.675	0.317	1.66636585298108E-29	0	PIK3AP1
2.34616451317412E-28	0.368833334768029	0.485	0.203	3.9317024911772E-24	0	RGS10
9.36246387328744E-15	0.36867872632178	0.251	0.104	1.56896169588551E-10	0	BMPR2
8.77784676827084E-17	0.367977183604625	0.29	0.12	1.47099156142683E-12	0	HDHD5
2.12452322928458E-17	0.366807821549758	0.337	0.15	3.56027602763509E-13	0	AP1G1
2.09638031284798E-37	0.365696212169687	0.605	0.245	3.51311412827064E-33	0	TBXAS1
3.07470486517704E-30	0.365136340112182	0.594	0.272	5.15259041306368E-26	0	APMAP
2.8531373632935E-16	0.36479964681467	0.306	0.135	4.78128759340725E-12	0	RRP36
2.89945807427288E-11	0.364225705939414	0.26	0.127	4.85891184086649E-07	0	DGUOK
4.53614420918063E-22	0.364009940405615	0.772	0.436	7.6016704657449E-18	0	ANXA5
6.36051576820388E-27	0.36171128208415	0.451	0.187	1.06589523243561E-22	0	LTBR
1.30283379607851E-16	0.36129824870871	0.257	0.102	2.18328887546837E-12	0	SNRK
3.42124903683189E-27	0.359920949342386	0.62	0.292	5.73332913592288E-23	0	TSPAN3

1.11679309529531E-30	0.358734582814802	0.562	0.244	1.87152186909588E-26	0	CREBL2
4.79300401043671E-14	0.357655216480163	0.255	0.109	8.03211612068984E-10	0	PRCC
1.62919604086521E-30	0.357322785081964	0.527	0.223	2.73020672528192E-26	0	GLB1
9.79699996762033E-21	0.35689343530601	0.328	0.131	1.64178125457381E-16	0	AP2M1
4.73829353955303E-31	0.356845731645386	0.448	0.167	7.94043231358297E-27	0	NAIP
7.31924553262089E-29	0.356743975789366	0.446	0.173	1.22655916635661E-24	0	DUSP3
2.99436643556766E-13	0.356661999268997	0.254	0.114	5.01795927272429E-09	0	FRAT2
1.88964345327367E-17	0.356626864988041	0.25	0.092	3.16666449899602E-13	0	PIK3R2
4.25184023683789E-18	0.356494212552613	0.295	0.118	7.12523386889294E-14	0	LIN7C
7.37974842401633E-19	0.356421155812591	0.251	0.088	1.23669824089666E-14	0	DGCR2
1.45506406364043E-18	0.356164296886169	0.345	0.152	2.43839635784863E-14	0	TMEM19
2.90747854949427E-36	0.35603865044899	0.636	0.273	4.87235255324249E-32	0	NDFIP1
2.06216368636349E-37	0.355996525911132	0.644	0.28	3.45577390560794E-33	0	OSTF1
7.96467939138299E-28	0.355763275014718	0.544	0.242	1.33472097240796E-23	0	KIF5B
8.89111589174464E-18	0.354931424772857	0.881	0.541	1.48997320113857E-13	0	ITGB2
3.70793639264954E-12	0.354486596454996	0.258	0.12	6.2137598068021E-08	0	KDM4A
7.70540286361775E-32	0.35355123565245	0.53	0.221	1.29127141188506E-27	0	STT3A
1.23437477315984E-26	0.353161581588114	0.357	0.127	2.06856524486125E-22	0	GDE1
9.77110413461607E-14	0.353158220301696	0.284	0.13	1.63744163087896E-09	0	MAN2B2
8.69366018621786E-33	0.352247234570406	0.518	0.214	1.45688357400639E-28	0	MAP3K3
7.46174718109127E-29	0.352107001503096	0.653	0.317	1.25043959260727E-24	0	RUNX1
4.9867483063644E-34	0.351594629177316	0.626	0.277	8.35679281180547E-30	0	COMT
1.01845447526215E-20	0.351411767979917	0.29	0.104	1.7067260096443E-16	0	GALC
8.34347456926014E-16	0.350982050249397	0.343	0.162	1.39819946831661E-11	0	SNX14
3.56694262227699E-31	0.350883458050151	0.554	0.243	5.97748244641177E-27	0	TMEM50A
8.00307209465527E-22	0.350668976504389	0.385	0.161	1.34115482162233E-17	0	IRF9
2.61335070814547E-45	0.350666359428814	0.547	0.186	4.37945311671017E-41	0	SELENOS
1.1104331537301E-16	0.350457587997797	0.309	0.137	1.86086387902091E-12	0	CDC16
2.72719995345686E-20	0.349811527206959	0.263	0.089	4.57024168200301E-16	0	RAB39A
9.17883152887104E-20	0.348795079910998	0.251	0.085	1.53818858760821E-15	0	FIG4
4.53593914012661E-29	0.348334878352321	0.617	0.277	7.60132681102417E-25	0	PRKCD
2.46812968552691E-19	0.348115468099509	0.36	0.155	4.136091727006E-15	0	TTL
4.59725306061905E-30	0.347065684438447	0.415	0.153	7.70407667898541E-26	0	GAS2L3
1.2290764983238E-25	0.347063054391182	0.788	0.411	2.05968639589102E-21	0	PICALM
7.48233848500123E-26	0.347008547983104	0.514	0.226	1.25389028331651E-21	0	ITPK1
3.80881474828747E-28	0.346935201377369	0.644	0.31	6.38281175518015E-24	0	CHKA
8.05368734345084E-25	0.34685270734228	0.837	0.49	1.34963692501549E-20	0	NUCB1
1.35547271880971E-15	0.346843694068132	0.298	0.133	2.27150118218132E-11	0	CALM1
1.28070490823389E-18	0.34620449316908	0.341	0.149	2.14620528521835E-14	0	OCIAD1
1.89469499616367E-27	0.345884545426844	0.595	0.279	3.17512987457108E-23	0	ARL6IP5
2.88862724598081E-26	0.345462927969007	0.261	0.068	4.84076153881463E-22	0	ZKSCAN4
3.85218542951301E-20	0.345192941202115	0.421	0.196	6.4554923427779E-16	0	TUFM
5.13556145237761E-20	0.345153030055904	0.89	0.577	8.6061738818944E-16	0	TXNIP
8.24197581263403E-16	0.344688024870756	0.315	0.14	1.38119030668121E-11	0	ATF7
1.86252074702244E-21	0.343679665583357	0.334	0.131	3.1212122678602E-17	0	ORMDL2
2.3534490192064E-17	0.343055850973019	0.284	0.116	3.94390986638609E-13	0	MUS81
4.29604868373191E-33	0.341321769641763	0.628	0.283	7.19931838419793E-29	0	OGFRL1
9.58530769064126E-16	0.340046212418292	0.269	0.111	1.60630586279766E-11	0	MAPRE1
2.00886275352165E-24	0.339699884999492	0.457	0.198	3.36645220235159E-20	0	RFX5
6.09729907108277E-20	0.33952052249867	0.303	0.119	1.02178537833205E-15	0	ANAPC7

6.64505908940575E-17	0.339117809450668	0.345	0.156	1.11357900220262E-12	0	SIVA1
2.21105336647929E-24	0.339031887185347	0.48	0.218	3.70528323154599E-20	0	TLE5
8.03828523203189E-16	0.336956096844127	0.318	0.144	1.3470558391839E-11	0	CCNDBP1
6.16707314272511E-19	0.336754219346928	0.377	0.169	1.03347811725787E-14	0	EDF1
5.18425090266966E-30	0.335939423097297	0.639	0.305	8.68776766269382E-26	0	CORO1B
3.32328111727559E-12	0.335606893413562	0.351	0.181	5.56915449633043E-08	0	NME2
7.82226791788224E-14	0.33418917638964	0.283	0.13	1.31085565767871E-09	0	IVD
1.98733857671989E-15	0.332104356842672	0.263	0.109	3.33038198686719E-11	0	MBTPS1
3.24581567637046E-25	0.332035329484455	0.462	0.203	5.43933791046161E-21	0	DNAJC3
4.27864477656132E-15	0.332006501731419	0.337	0.158	7.17015291656145E-11	0	NDUFB5
5.05073601133628E-21	0.331257149214795	0.395	0.175	8.46402340779734E-17	0	SRI
2.65714115143767E-21	0.331081465899622	0.379	0.16	4.45283714157925E-17	0	KDSR
1.28985707037273E-18	0.330972691040606	0.308	0.125	2.16154247853063E-14	0	LDLRAD4
1.09455611760574E-31	0.330101087103158	0.572	0.25	1.83425714188369E-27	0	TMUB2
1.22738514835661E-15	0.330070017325573	0.332	0.154	2.056852031616E-11	0	APRT
2.37332240011295E-32	0.330061133902249	0.522	0.216	3.97721367810928E-28	0	PIK3R1
5.04369266202634E-23	0.329796288073572	0.421	0.18	8.45222016302375E-19	0	OSBPL3
1.99301734625951E-28	0.329605594710372	0.424	0.163	3.33989846886169E-24	0	PPFIBP2
8.61246985326682E-24	0.329577117432714	0.283	0.091	1.44327769801045E-19	0	CLIP2
1.27395747903853E-17	0.329349544263691	0.254	0.097	2.13489794337277E-13	0	SLC39A3
4.16029116486252E-28	0.329106568807151	0.591	0.267	6.97181593407661E-24	0	SEPTIN9
2.90234968004487E-18	0.32823372988167	0.299	0.122	4.8637575938192E-14	0	LMF2
1.38097687399803E-26	0.327759613813108	0.698	0.349	2.3142410454459E-22	0	ARPC1B
2.36339533169997E-23	0.327474726578971	0.463	0.203	3.96057789686281E-19	0	HNRNPUL2
2.25998722907311E-22	0.32708445140574	0.435	0.192	3.78728659848071E-18	0	GHITM
3.99376337642476E-14	0.326699668678852	0.255	0.111	6.69274866621262E-10	0	EIF2S3
5.24530344102582E-18	0.326267428358739	0.327	0.144	8.79007950647107E-14	0	TIMM13
1.54113654134744E-32	0.326010214737555	0.585	0.25	2.58263661599005E-28	0	IGFLR1
1.88739025613432E-33	0.325534116220329	0.66	0.306	3.16288859122989E-29	0	NEK6
3.78593736972386E-16	0.325144813635781	0.276	0.117	6.34447384418325E-12	0	WDR41
4.8534566125752E-29	0.324729672238905	0.61	0.282	8.13342259135352E-25	0	KCNAB2
2.38795940248914E-33	0.324442631215929	0.533	0.208	4.0017423666913E-29	0	NADK
2.2398328741524E-23	0.323349393262615	0.403	0.169	3.75351193050459E-19	0	SLC25A11
1.52622566070705E-20	0.323161781892817	0.292	0.11	2.55764896221288E-16	0	KIF1C
4.10848206657458E-26	0.323141002651499	0.54	0.245	6.88499424716568E-22	0	UBE2L6
3.92568413275116E-30	0.322918949074695	0.569	0.244	6.5786614696644E-26	0	HADHA
5.5896778318467E-18	0.322900773867596	0.279	0.109	9.3671821106087E-14	0	NBN
5.40798276980142E-16	0.322805508990509	0.29	0.125	9.06269752563321E-12	0	XIAP
2.54207339213395E-26	0.322703803533679	0.269	0.074	4.26000659053807E-22	0	SLC17A5
1.63902550298948E-20	0.322299237927272	0.546	0.27	2.74667893790978E-16	0	PKM
4.57603303209995E-26	0.322219282263151	0.507	0.231	7.6685161551931E-22	0	NBR1
5.02450600825019E-16	0.321113748172548	0.274	0.114	8.42006716862566E-12	0	SPCS2
1.80496871637949E-36	0.320685119664591	0.475	0.164	3.02476657490875E-32	0	BMP2K
2.62106611064245E-39	0.320019900950585	0.615	0.255	4.39238258821462E-35	0	RAP2B
4.97389016619783E-23	0.319051828914189	0.409	0.171	8.33524514051432E-19	0	CHCHD2
8.66083610054719E-16	0.318934187153042	0.327	0.152	1.4513829137297E-11	0	LIX1L
1.29810561079814E-32	0.318540107834874	0.586	0.256	2.17536538257552E-28	0	TM6SF1
5.10961620679224E-25	0.318379125627875	0.842	0.488	8.56269483934244E-21	0	GRINA
6.4635976676745E-13	0.3178801161506	0.311	0.151	1.08316969714889E-08	0	CRKL
1.95031063455818E-29	0.31613248527997	0.447	0.172	3.2683305613926E-25	0	P2RX7

8.56618373996839E-12	0.315302286529869	0.276	0.138	1.4355210711439E-07	0	DNTTIP1
6.55235240698554E-34	0.314973916494662	0.569	0.239	1.09804321636264E-29	0	ABCG1
1.52223321394181E-17	0.313968736502025	0.361	0.165	2.55095841992369E-13	0	FBXW2
3.08199243210392E-23	0.313703942629669	0.269	0.084	5.16480291771975E-19	0	SDSL
4.33814702852535E-22	0.313488526353504	0.296	0.104	7.26986679040278E-18	0	SLC49A3
4.98577393368473E-15	0.313106768141915	0.316	0.146	8.35515995806887E-11	0	APOA1
8.01145500515161E-23	0.313090590447663	0.34	0.13	1.34255962976331E-18	0	DERA
1.97652039132144E-19	0.312124807220895	0.266	0.097	3.31225287177647E-15	0	PYM1
2.18542597241002E-13	0.311590591190623	0.906	0.631	3.66233684456471E-09	0	MT-ND4
1.77900902932384E-20	0.310822565261958	0.399	0.179	2.9812633313409E-16	0	PGM2
1.36479930592368E-39	0.310745844289752	0.583	0.216	2.28713067686691E-35	0	ARHGEF10
5.36978069907897E-22	0.310729182538956	0.385	0.162	8.99867849551654E-18	0	ERP44
2.30479791098822E-11	0.310048950891904	0.263	0.126	3.86238033923405E-07	0	NDUFA9
1.16628979621801E-17	0.31000392441375	0.34	0.148	1.95446844050213E-13	0	BIN2
5.81960170779607E-13	0.308830074523283	0.328	0.168	9.75248854192466E-09	0	SRP72
1.97952622342601E-15	0.307953628004277	0.27	0.113	3.31729004521731E-11	0	COMMD4
6.41864201396345E-37	0.306659190277168	0.633	0.276	1.07563602869999E-32	0	ARF1
1.05724649750896E-28	0.306647220672754	0.566	0.256	1.77173368052552E-24	0	FAM50A
4.27131865098236E-24	0.305484692318059	0.338	0.122	7.15787579531624E-20	0	ARNT
4.13077700764416E-16	0.30426359178344	0.253	0.098	6.92235610941008E-12	0	G6PC3
1.20400668740186E-20	0.304260999101243	0.372	0.156	2.01767440674803E-16	0	STIM1
2.80182487513984E-30	0.304138427752639	0.601	0.276	4.69529812575934E-26	0	NCSTN
1.92949760968895E-22	0.303533075403896	0.338	0.131	3.23345209431674E-18	0	AHCYL1
2.85645284227736E-20	0.303380543602314	0.29	0.108	4.7868436730884E-16	0	TM2D2
2.76083727959552E-15	0.303204166284692	0.277	0.12	4.62661111314618E-11	0	UBQLN2
3.88869407364695E-17	0.30259415909243	0.263	0.102	6.51667352861756E-13	0	RMDN3
2.64512886183654E-20	0.302471371993742	0.328	0.13	4.43270694666568E-16	0	UBP1
7.75958064582815E-11	0.302147849168564	0.325	0.172	1.30035052462788E-06	0	ANKRD22
1.52315310790406E-38	0.301987012327616	0.447	0.144	2.55249997822562E-34	0	SORBS3
1.87364045693993E-16	0.301886238275205	0.361	0.171	3.13984667773993E-12	0	TOR1AIP1
2.8337932907091E-23	0.301266469850759	0.395	0.162	4.74887079657031E-19	0	TAX1BP1
2.67263119214065E-31	0.300846293528818	0.431	0.159	4.4787953517893E-27	0	SUSD1
3.43490668144573E-15	0.300311971145088	0.284	0.121	5.75621661676676E-11	0	GET3
3.86343071001005E-17	0.299632512183736	0.499	0.266	6.47433718383485E-13	0	RAP1A
1.41291590446527E-22	0.299604248026414	0.396	0.166	2.3677644727029E-18	0	CLIC1
2.17825942633003E-12	0.299168049837539	0.276	0.132	3.65032714664387E-08	0	EPRS
5.07957353285893E-21	0.298847199213432	0.311	0.119	8.512349326365E-17	0	WBP2
7.00847619386997E-43	0.298434236752465	0.713	0.292	1.17448044056873E-38	0	RGL1
2.07399588539066E-16	0.298290164039396	0.293	0.125	3.47560230473766E-12	0	SMIM7
4.32814531132338E-26	0.29768388956565	0.374	0.139	7.25310591271572E-22	0	N4BP1
2.22135670738125E-21	0.297350485414974	0.525	0.251	3.7225495702295E-17	0	CYB5R3
4.17069069498571E-35	0.297299345740939	0.521	0.194	6.98924346665705E-31	0	IRS2
1.14791781233279E-16	0.297147508326417	0.35	0.16	1.92368066990729E-12	0	MAP1S
1.79535082672192E-15	0.296582919225027	0.929	0.662	3.0086489154206E-11	0	MT-CO2
3.51341781315293E-27	0.296407201774501	0.376	0.139	5.88778557128168E-23	0	SPPL3
1.22353027745386E-24	0.296313741224164	0.299	0.097	2.05039203895717E-20	0	MITF
4.48671831715273E-25	0.296305862419877	0.572	0.265	7.51884255588454E-21	0	SERPINB1
1.3209929602132E-14	0.2951312812353	0.97	0.81	2.21372000272527E-10	0	MS4A6A
1.1544226515302E-33	0.294933557779584	0.431	0.149	1.93458147943431E-29	0	EPS15
2.78495944920032E-22	0.294759214463258	0.343	0.131	4.66703504496989E-18	0	BCKDK

3.02235812252855E-29	0.294250495409587	0.579	0.264	5.06486774173335E-25	0	CORO7
9.79505983727421E-22	0.29332669904127	0.383	0.158	1.64145612753041E-17	0	DNAJB12
1.27020708894375E-29	0.293112035529009	0.508	0.214	2.12861303965194E-25	0	GNPTAB
3.02531289506336E-37	0.293039476268246	0.476	0.163	5.06981934954718E-33	0	KIF1B
1.44081475677972E-41	0.29269333624613	0.528	0.182	2.41451736941145E-37	0	SMS
1.21071765638124E-34	0.292517862464089	0.379	0.112	2.02892064856368E-30	0	RIN2
3.11783211277877E-19	0.291635100878954	0.35	0.151	5.22486305459466E-15	0	PCYT1A
3.34912008549364E-30	0.291372777034725	0.521	0.223	5.61245543927025E-26	0	DNAJC5
7.68016132820019E-13	0.291302079526514	0.269	0.126	1.28704143537979E-08	0	MYC
2.2439779417446E-32	0.290823648622711	0.657	0.307	3.7604582347756E-28	0	SUMF2
2.84550811216501E-27	0.290449258925981	0.324	0.106	4.76850249436613E-23	0	ZMYND11
1.89139324310382E-20	0.289653923994639	0.398	0.182	3.16959679679338E-16	0	DELE1
5.97736495834466E-18	0.288597674616917	0.35	0.157	1.0016868197194E-13	0	SMG5
1.16008389666254E-24	0.288016464434316	0.383	0.154	1.94406859402709E-20	0	AGFG1
1.30688396530345E-15	0.286856054936855	0.293	0.13	2.19007614905551E-11	0	DCTD
1.46972595902265E-23	0.286807924298639	0.46	0.205	2.46296676213015E-19	0	PARP4
3.02759047201309E-26	0.286115773921553	0.537	0.248	5.07363611299954E-22	0	IQGAP1
6.84856383566793E-20	0.285929440613908	0.302	0.117	1.14768232758123E-15	0	WASHC5
3.17517220566978E-22	0.285852012606174	0.61	0.298	5.32095358226141E-18	0	IDH2
3.44959708444487E-19	0.285806258968859	0.319	0.131	5.78083479411271E-15	0	STK25
1.97428179040299E-29	0.284001291035208	0.601	0.273	3.30850142435733E-25	0	RAB14
2.52325408146118E-17	0.282625096263038	0.282	0.112	4.22846918971264E-13	0	EIF3D
1.18901639118121E-16	0.282568931236575	0.329	0.147	1.99255366834147E-12	0	VAC14
5.78478358641332E-22	0.282143508084479	0.322	0.123	9.69414033411145E-18	0	TMEM8A
5.79928082120328E-25	0.282060232598344	0.343	0.123	9.71843480017246E-21	0	MTMR6
2.45089827285913E-27	0.281660287951835	0.354	0.121	4.10721532565732E-23	0	SIRT2
8.23303372298179E-26	0.281504195151815	0.447	0.189	1.37969179129729E-21	0	DCTN1
2.25092639012824E-15	0.280655525748991	0.364	0.178	3.77210244457691E-11	0	HYOU1
4.38377929882731E-26	0.280493946905955	0.563	0.261	7.34633734897481E-22	0	N4BP2L2
6.84788490182221E-25	0.280364763962711	0.704	0.354	1.14756855184737E-20	0	PPT1
4.10604838800731E-21	0.280230215414354	0.463	0.213	6.88091588862265E-17	0	SF3B5
3.73572096584125E-34	0.280032424781541	0.502	0.192	6.26032119455677E-30	0	FOXO3
2.89106816135443E-12	0.279719460387329	0.271	0.129	4.84485202479775E-08	0	LRRC59
1.42556736229452E-15	0.279169515415021	0.315	0.141	2.38896578573315E-11	0	USP14
1.35386548252379E-15	0.278611849985791	0.309	0.14	2.26880777561336E-11	0	CLIC4
1.08775987410196E-28	0.278218427416115	0.466	0.188	1.82286799702007E-24	0	RAC1
2.48128859415174E-19	0.278209537578213	0.425	0.196	4.15814342607949E-15	0	ATP2C1
1.73039127459754E-19	0.277675721885055	0.343	0.145	2.89978969797056E-15	0	GSR
2.27891542016397E-20	0.277204233685037	0.382	0.165	3.81900646111079E-16	0	AP2A1
3.16650339633656E-41	0.27695373221236	0.504	0.172	5.30642639158081E-37	0	ITGAV
3.89893765984724E-20	0.275968891251805	0.327	0.129	6.53383973037201E-16	0	NAA60
5.12026327541492E-13	0.275899669558242	0.255	0.115	8.58053719694033E-09	0	MZT2B
3.4904101917605E-14	0.27531990792917	0.279	0.125	5.84922939935224E-10	0	SEC63
1.32409397435926E-14	0.274917541976137	0.298	0.134	2.21891668223125E-10	0	MYO1D
1.4966658158804E-27	0.274650109505935	0.749	0.381	2.50811257425238E-23	0	DDIT4
3.63171219610794E-25	0.274635515421532	0.552	0.258	6.08602329823769E-21	0	ARL6IP4
1.61046506037084E-21	0.273919498534202	0.351	0.139	2.69881734816946E-17	0	MBD6
4.07047874945479E-36	0.273370479929118	0.795	0.414	6.82130828833633E-32	0	MPP1
2.91667974213956E-32	0.272637400639807	0.666	0.31	4.88777191187747E-28	0	SERINC1
7.64322638015691E-30	0.27256475537142	0.435	0.166	1.2808518767867E-25	0	DIP2B

1.89973559618512E-20	0.272201360396424	0.681	0.352	3.18357691208702E-16	0	METTL7A
2.14604487193211E-20	0.271838654802636	0.408	0.183	3.59634199638384E-16	0	TMEM165
1.88409410394821E-33	0.271387369066141	0.633	0.277	3.15736489939642E-29	0	TMEM106A
9.63213039619371E-29	0.271291458729795	0.406	0.149	1.61415241179414E-24	0	MAPK6
2.35022134582078E-19	0.271233912940838	0.422	0.2	3.93850093132647E-15	0	CTBP2
1.0108406505824E-18	0.270811811916364	0.35	0.151	1.69396676224599E-14	0	GPR108
6.04299838954366E-34	0.27028479274593	0.57	0.237	1.01268567011973E-29	0	PKN1
3.34686653948081E-10	0.269789513028382	0.261	0.134	5.60867894686194E-06	0	SNRPB
7.26191274631186E-19	0.269456779816296	0.406	0.185	1.21695133802694E-14	0	DNAJC13
1.04234424830645E-25	0.269366645658155	0.527	0.244	1.74676049131195E-21	0	DEGS1
2.86495601263629E-16	0.268628379005345	0.343	0.158	4.8010932859759E-12	0	DYNLT1
8.47451673543624E-24	0.267889791795102	0.435	0.187	1.4201595145244E-19	0	MAT2B
2.0330679742888E-30	0.267455374812235	0.537	0.228	3.40701531131317E-26	0	TMEM33
2.08012603987383E-22	0.266647681890686	0.55	0.263	3.48587521762056E-18	0	CNTRL
6.99680569241334E-17	0.2655782409159	0.877	0.562	1.17252469793463E-12	0	CTSS
6.13792851803692E-18	0.264161557328669	0.289	0.115	1.02859406105263E-13	0	AGO4
6.00112314772849E-30	0.263822027671239	0.594	0.259	1.00566821709634E-25	0	ITGAM
7.45551482550754E-14	0.263132881456794	0.251	0.106	1.24939517445855E-09	0	PLD2
5.08240177062047E-28	0.26196441124985	0.656	0.323	8.51708888720578E-24	0	LRPAP1
5.19266339656057E-13	0.261738435466575	0.302	0.149	8.7018653199562E-09	0	RNF38
1.19464408679548E-32	0.260620015121278	0.479	0.182	2.00198456065187E-28	0	ZSWIM8
1.23852659328266E-13	0.260432035846489	0.25	0.11	2.07552286502308E-09	0	NUDT9
1.03534413161488E-25	0.260207611181604	0.463	0.201	1.73502969576021E-21	0	UBE2Q1
3.64836359361554E-22	0.259160084436997	0.388	0.163	6.11392771018093E-18	0	MEF2C
3.29736409907653E-30	0.259124066061833	0.525	0.225	5.52572275723246E-26	0	PREX1
6.35437846550916E-22	0.258874638261018	0.308	0.114	1.06486674325003E-17	0	NRIP1
8.99555968459038E-19	0.258802532864055	0.832	0.487	1.50747589194366E-14	0	FCGR2A
6.96469409997286E-12	0.257956907363011	0.263	0.124	1.16714343727345E-07	0	PTPN18
5.4780301614196E-26	0.257947646326291	0.476	0.211	9.18008294450697E-22	0	IDH3G
1.74242559969822E-30	0.257822671740974	0.382	0.128	2.91995681997428E-26	0	RHOU
1.99997291233006E-15	0.257720178894068	0.267	0.11	3.35155460648272E-11	0	CARS2
4.67169829250473E-25	0.257607753857038	0.369	0.136	7.82883199857943E-21	0	STON2
1.06811480429141E-17	0.257075198241606	0.446	0.221	1.78994678903155E-13	0	ATF6B
5.40768227666929E-33	0.256960692654046	0.55	0.23	9.0621939592424E-29	0	FAM91A1
5.08172890649873E-21	0.256792048928036	0.737	0.395	8.51596130151057E-17	0	LCP2
5.28782170940982E-13	0.256767137589783	0.315	0.157	8.86133162062898E-09	0	ADAM15
5.52658465972886E-22	0.256575882925776	0.372	0.156	9.26145057277362E-18	0	TXLNA
8.1546284075455E-21	0.25570900199292	0.337	0.135	1.36655262853647E-16	0	BTBD2
2.40800411874523E-24	0.255286204824568	0.469	0.206	4.03533330219326E-20	0	CMPK1
1.0199887273067E-24	0.255121455002447	0.473	0.208	1.70929710922057E-20	0	RAP2A
2.40162812872377E-14	0.25443196427327	0.261	0.112	4.02464841811529E-10	0	CDC34
4.77806218262567E-33	0.253923738111052	0.749	0.347	8.0070766056441E-29	0	MSR1
1.14918547865942E-24	0.253806736554121	0.348	0.126	1.92580502513745E-20	0	SIGLEC9
5.77832444299705E-20	0.253449285894122	0.345	0.142	9.68331610157446E-16	0	OSBP
8.01989632269407E-18	0.252932624601748	0.372	0.173	1.34397422575707E-13	0	CNPPD1
2.12199928397511E-27	0.25288944206787	0.358	0.121	3.55604640008549E-23	0	PGPEP1
1.87244925957639E-17	0.2528384086347	0.393	0.182	3.13785046919811E-13	0	PRPF19
1.08776593867048E-13	0.252804425957167	0.277	0.127	1.82287816002399E-09	0	INTS1
3.19910688852985E-20	0.251985961864549	0.403	0.183	5.36106332379832E-16	0	ATP5ME
1.45591014569555E-29	0.251982962873844	0.446	0.175	2.4398142221566E-25	0	ZNF706

1.90098141020072E-21	0.250936081243945	0.517	0.245	3.18566464721437E-17	0	SFMBT2
3.62619213475621E-20	0.250666842395281	0.373	0.152	6.07677277942446E-16	0	TUBB
7.04242000314617E-13	0.250496949688506	0.315	0.16	1.18016874412724E-08	0	PPIA
6.0959470553388E-28	0.250008650165517	0.361	0.124	1.02155880753368E-23	0	PITHD1
4.10735112287525E-96	5.64977843049556	0.366	0.015	6.88309901171434E-92	1	CD5L
2.37991861421243E-66	5.18125308987065	0.311	0.031	3.9882676136972E-62	1	CCL4
2.4296777101094E-70	4.8717336784248	0.358	0.048	4.07165390660134E-66	1	CCL3
3.85466980497115E-65	4.1421557761179	0.433	0.107	6.45965565917065E-61	1	CCL4L2
8.56895111344972E-72	4.07323280084174	0.48	0.131	1.4359848275919E-67	1	KLF2
2.25864474344922E-108	3.80981329482099	0.665	0.206	3.78503686107221E-104	1	NR4A2
4.66651941161983E-97	3.35285614147714	0.611	0.162	7.82015322999251E-93	1	NR4A1
4.69945515579438E-22	3.06531310893114	0.383	0.228	7.87534695008023E-18	1	IFRD1
1.92377184101097E-23	2.9696509189441	0.402	0.246	3.22385685116618E-19	1	KLF4
4.18694331921203E-26	2.89238970257834	0.369	0.179	7.01647961433553E-22	1	BAG3
1.02675350973948E-08	2.86825315163663	0.282	0.206	0.000172063353162143	1	EXT1
1.58888953430651E-39	2.86517281689198	0.584	0.417	2.66266108159084E-35	1	CHMP1B
4.52819072407671E-12	2.83394005870927	0.337	0.241	7.58834201540774E-08	1	APOA2
3.96501765892472E-08	2.81110319398493	0.315	0.258	0.000664457659282605	1	SELENOK
1.75422578390956E-51	2.72073421311094	0.582	0.312	2.93973156867564E-47	1	ATF3
3.08577838427707E-14	2.67956877845578	0.379	0.278	5.17114741637152E-10	1	APBB3
1.33761752189701E-19	2.60431351703	0.294	0.137	2.24157944319502E-15	1	ID3
2.00134291738869E-06	2.4976020260076	0.263	0.203	0.0335385046095996	1	OTUD1
1.39133543798419E-09	2.47180206902954	0.325	0.246	2.33159992697391E-05	1	ZNF331
7.45470632633185E-33	2.44945496800461	0.398	0.172	1.24925968616669E-28	1	NR4A3
2.26399926100824E-99	2.44095939295829	0.851	0.653	3.79400996159761E-95	1	RHOB
3.95850745825392E-22	2.43540635477802	0.344	0.175	6.63366679854192E-18	1	CXCL12
4.76337916921473E-12	2.40875816869292	0.377	0.288	7.98247081177005E-08	1	SERPINH1
1.48147234234115E-10	2.29531553527506	0.29	0.191	2.48265135129529E-06	1	ZFAND2A
6.56412025996043E-31	2.28295236981518	0.634	0.579	1.10001527316417E-26	1	HMOX1
9.26835292386064E-24	2.27420840091578	0.466	0.319	1.55319058298057E-19	1	HSPE1
7.7690955020823E-30	2.21857671031225	0.507	0.329	1.30194502423895E-25	1	PPP1R15A
2.06808044937299E-15	2.1734588168807	0.395	0.273	3.46568921705926E-11	1	IER3
2.34864534518763E-07	2.12418510191377	0.338	0.288	0.00393585986946542	1	RASGEF1B
1.18188706120465E-34	2.09207220874945	0.646	0.546	1.98060633716676E-30	1	IER5
1.54116482512282E-29	2.0803881729642	0.493	0.294	2.58268401394082E-25	1	DNAJA4
1.36542862042489E-66	2.07310507505675	0.774	0.571	2.28818528210802E-62	1	PFKFB3
6.61652285933655E-07	1.9948051785055	0.321	0.269	0.0110879690076762	1	TAGAP
3.8536522483489E-11	1.95465800455287	0.25	0.135	6.45795043778308E-07	1	SDS
2.44557779888889E-11	1.94980552972222	0.449	0.409	4.098299275378E-07	1	GADD45B
4.17751886106432E-34	1.94612364248351	0.671	0.638	7.00068610737159E-30	1	JUN
7.16229070417072E-55	1.94226714644162	0.764	0.659	1.20025667620493E-50	1	SOD2
2.04947511886665E-57	1.87981654535704	0.754	0.578	3.43451040419673E-53	1	HSPH1
3.34810732829622E-20	1.87077740738246	0.534	0.454	5.61075826075881E-16	1	HSPD1
2.40235317493958E-09	1.86952739004171	0.354	0.275	4.02586345056375E-05	1	PHACTR1
1.04511470671577E-64	1.86052707182837	0.805	0.641	1.75140322551429E-60	1	FOS
2.46343378449902E-38	1.85534632329015	0.602	0.387	4.12822233606345E-34	1	CD83
7.62119243362241E-35	1.80519497428583	0.689	0.692	1.27715942802644E-30	1	KLF6
7.69880214422845E-14	1.80279776869536	0.497	0.458	1.2901652633298E-09	1	P4HA1
1.40501976558087E-11	1.79548587995562	0.346	0.238	2.35453212316043E-07	1	SOCS3
3.04761406734815E-07	1.78605403461988	0.443	0.445	0.00510719165406203	1	INSIG1

5.19214252289129E-21	1.77283304994822	0.58	0.553	8.70099243986122E-17	1	ARL4C
1.04511952994882E-72	1.76766554666046	0.838	0.728	1.75141130828824E-68	1	DNAJB1
1.74533138789218E-08	1.71514469668473	0.416	0.384	0.000292482633982971	1	UGCG
2.13125525188355E-101	1.68238256501183	0.952	0.849	3.57155755110646E-97	1	HSPA1B
4.09028230843328E-67	1.64773189700326	0.863	0.785	6.85449509247249E-63	1	JUNB
2.85816343424653E-13	1.62371477671972	0.524	0.51	4.78971028311034E-09	1	NAMPT
1.73849379284882E-26	1.61499238501846	0.683	0.748	2.91336789805606E-22	1	HSPB1
1.00751355113995E-48	1.58563386620352	0.797	0.785	1.68839120900033E-44	1	HSP90AA1
3.59065597860724E-82	1.57118796066507	0.913	0.792	6.01722128895001E-78	1	DUSP1
1.12227953142678E-11	1.46026663058664	0.571	0.648	1.880716038765E-07	1	WSB1
8.04239285513626E-21	1.41268949533171	0.675	0.728	1.34774419466373E-16	1	SGK1
9.40316631192933E-69	1.39952554714994	0.903	0.896	1.57578261055312E-64	1	SAT1
3.21709705441455E-20	1.37818712109282	0.662	0.67	5.3912112437879E-16	1	ZFP36
1.28153460498376E-08	1.34976079512549	0.524	0.552	0.000214759569103178	1	CTSL
9.29282546551585E-07	1.30618614072463	0.447	0.428	0.0155729169151115	1	PLAUR
1.19895305526724E-50	1.29958177638886	0.845	0.829	2.00920553001684E-46	1	HSPA1A
6.72023787149131E-07	1.25908809739442	0.559	0.684	0.0112617746250451	1	PNRC1
1.6511356965791E-09	1.18588187688665	0.563	0.624	2.76697320032726E-05	1	GNA13
3.15474971496221E-07	1.12236243035135	0.116	0.25	0.00528672957233367	1	IDI1
7.63799811186393E-10	1.09937423884729	0.615	0.685	1.27997572358616E-05	1	RGS2
1.39167758720471E-07	1.08660332869349	0.207	0.413	0.00233217330063766	1	STX4
2.47186824856158E-08	1.02192659797577	0.151	0.323	0.00041423568109395	1	SPATA20
9.13431407909199E-23	0.997289919434833	0.795	0.91	1.53072835337424E-18	1	SERPINA1
1.05534021792858E-20	0.986712932185764	0.76	0.84	1.76853913720472E-16	1	MCL1
2.43292411064777E-07	0.960086181474692	0.195	0.385	0.00407709422462353	1	CPEB4
5.7862738532983E-10	0.956813935920544	0.161	0.358	9.69663772335729E-06	1	AFF4
4.71859099017152E-07	0.955018807122081	0.17	0.338	0.00790741478132943	1	ELMSAN1
2.45374912433441E-08	0.953116762791395	0.139	0.303	0.000411199278255961	1	HEATR3
1.11956318807484E-30	0.936988205427113	0.812	0.89	1.87616399057582E-26	1	FTH1
9.59385794714188E-09	0.933300387877178	0.197	0.405	0.000160773871478204	1	MIDN
1.09750843744633E-07	0.932113861468965	0.118	0.259	0.00183920463947256	1	JOSD1
1.25454264123107E-07	0.92658801158415	0.182	0.364	0.00210236255817503	1	EXOC3
6.50622600785151E-10	0.924292262657782	0.172	0.381	1.09031335439576E-05	1	SMS
8.82134037014144E-08	0.911396419177119	0.205	0.41	0.0014782802192283	1	STOM
3.40387609651198E-08	0.895565641477339	0.132	0.289	0.000570421556253477	1	NCEH1
1.68890127299414E-06	0.888288454052737	0.213	0.404	0.0283026075328357	1	SLC37A2
6.74404087268959E-13	0.865020646781414	0.263	0.57	1.13016636944532E-08	1	TFRC
1.95121424658886E-08	0.859371060907855	0.207	0.422	0.000326984483443362	1	ELL2
2.63036857741606E-11	0.854634493764002	0.143	0.343	4.40797166203383E-07	1	USP4
2.5932866499313E-06	0.846627928432897	0.6	0.644	0.0434582976795487	1	RGS1
1.2064117774619E-06	0.844494783618957	0.182	0.353	0.0202170485667064	1	SLC38A2
7.8357084819976E-07	0.843818475040782	0.141	0.285	0.0131310802741316	1	DDX27
3.08963780434896E-08	0.841692515122381	0.135	0.295	0.000517761503252798	1	VPS26A
8.14661194530575E-09	0.836134713942836	0.143	0.314	0.000136520922979434	1	PIP4P1
1.64274816993538E-07	0.825110364374709	0.12	0.259	0.00275291738317772	1	NOTCH2
9.43262039944633E-09	0.822998979222314	0.116	0.268	0.000158071852653922	1	RABEP1
7.27298530999848E-08	0.813914152150852	0.124	0.272	0.00121880687824955	1	STON2
4.80888180611637E-09	0.811269691800474	0.195	0.409	8.05872413068981E-05	1	GPR137B
7.56598664981386E-25	0.807543465550934	0.884	0.884	1.26790804277581E-20	1	FTL
1.08481483491499E-10	0.807494177436274	0.128	0.308	1.81793270035055E-06	1	GASK1B

**1.80128294611365E-12**	0.807053148641421	0.834	0.852	3.01858996109726E-08	1	FGL2
**7.1145278490212E-08**	0.806049576351401	0.255	0.506	0.00119225257693897	1	RNASET2
**1.26846650063243E-06**	0.804517922151653	0.263	0.501	0.0212569616175983	1	ADAM9
**2.35308610135968E-06**	0.799191384321761	0.251	0.467	0.0394330168865855	1	NUFIP2
**1.39744183191516E-08**	0.797242062997709	0.141	0.309	0.000234183302192343	1	TBC1D12
**3.18008934368503E-25**	0.783135846436891	0.886	0.846	5.32919372214738E-21	1	GLUL
**7.03562786088419E-09**	0.780310517553953	0.675	0.784	0.000117903051692697	1	MFSD1
**4.38204336221352E-11**	0.780226756785291	0.145	0.347	7.34342826639742E-07	1	KIF1B
**2.72229342698214E-11**	0.77614103825422	0.112	0.291	4.56201932493668E-07	1	PHACTR4
**4.15041346219789E-08**	0.774883201482053	0.114	0.256	0.000695526287995122	1	HSD17B14
**9.33730080830143E-11**	0.768246200370367	0.103	0.268	1.56474486945515E-06	1	SLC2A9
**1.27959356486536E-07**	0.765506379641725	0.29	0.559	0.00214434289600137	1	SH3BP5
**2.88157330615293E-10**	0.760252711296724	0.162	0.362	4.82894054645108E-06	1	ITGAV
**3.12242838035464E-07**	0.757989567953541	0.195	0.381	0.00523256547979831	1	SPG7
**8.2804290069196E-15**	0.756485380608173	0.186	0.458	1.38763429297959E-10	1	ALG13
**2.43777774671128E-08**	0.752448693949419	0.13	0.287	0.000408522794793876	1	ME1
**2.50153935116273E-07**	0.751048442422055	0.147	0.302	0.00419207964467851	1	ACAP2
**4.98988056975978E-07**	0.75077165946962	0.23	0.436	0.00836204185880344	1	TFEC
**6.06457421881161E-10**	0.748531806571918	0.099	0.25	1.01630134758845E-05	1	STRN4
**4.99491297088148E-09**	0.745489211609398	0.151	0.33	8.37047515660319E-05	1	LRRK1
**3.39199103942648E-08**	0.732595624348933	0.153	0.327	0.000568429858387089	1	EPAS1
**3.14190103755763E-11**	0.726616762692754	0.128	0.314	5.26519775873908E-07	1	FGB
**1.27017287089648E-08**	0.724465105812263	0.213	0.432	0.000212855569704831	1	JDP2
**2.91730030577178E-09**	0.71979246886807	0.12	0.28	4.88881185241234E-05	1	WIPF2
**1.53588870078453E-10**	0.719441542853111	0.114	0.285	2.57384228477471E-06	1	WDFY3
**3.64159154141083E-08**	0.71892367167001	0.205	0.413	0.000610257910509627	1	CELF1
**4.60395668299395E-11**	0.714253534999276	0.145	0.342	7.71531060936127E-07	1	PCED1A
**6.9799180314587E-15**	0.713058472481069	0.135	0.366	1.16969466371185E-10	1	NEU1
**2.11336927310251E-12**	0.710044296807989	0.147	0.361	3.54158422786519E-08	1	UBE2D3
**3.35556234908534E-08**	0.709952165516973	0.147	0.313	0.000562325138459721	1	PPP1R15B
**1.01171051061916E-07**	0.708463651074177	0.114	0.251	0.00169542447369559	1	ZNF330
**4.81625403586275E-10**	0.708221893898738	0.203	0.432	8.07107851329879E-06	1	BNIP3L
**4.32346418224263E-08**	0.697561420988705	0.226	0.448	0.000724526127660219	1	DOCK4
**1.6691979403708E-08**	0.695751661198448	0.17	0.355	0.000279724190847339	1	CERT1
**8.32335273897974E-11**	0.689815915056873	0.162	0.369	1.39482745199822E-06	1	EMD
**2.06633482834775E-06**	0.688946045528886	0.149	0.29	0.0346276390534516	1	FNBP4
**3.66725385855721E-19**	0.687769327368285	0.797	0.808	6.14558401617017E-15	1	CXCR4
**2.67189085153197E-06**	0.686284132909817	0.161	0.308	0.0447755468899727	1	DNMT3A
**7.7319846592242E-08**	0.681108636632112	0.234	0.456	0.00129572598919279	1	TRA2A
**3.58809188054504E-09**	0.678861579474072	0.19	0.397	6.01292437341737E-05	1	CALCOCO2
**1.2420257941056E-08**	0.678791033053382	0.188	0.388	0.000208138682576216	1	ADAM10
**3.83036441369813E-10**	0.677390404103911	0.182	0.402	6.41892468447532E-06	1	LARP4
**1.75991119695775E-07**	0.676436797578648	0.25	0.48	0.00294925918386179	1	BRD2
**2.74403868178258E-08**	0.675663071195582	0.228	0.449	0.000459846002293125	1	EZR
**1.90449833603092E-12**	0.674522653949081	0.091	0.263	3.19155831152062E-08	1	NANS
**9.00033172788644E-08**	0.671530899444924	0.13	0.277	0.00150827559095921	1	VAV1
**1.05447422031433E-09**	0.664995815052371	0.209	0.436	1.76708789840275E-05	1	ATG7
**5.45158584319023E-08**	0.656053783526356	0.164	0.341	0.000913576755601818	1	RAB3IL1
**1.04748790701085E-10**	0.655980411894999	0.161	0.369	1.75538023456878E-06	1	GNB4
**1.57698049412512E-10**	0.64951849305685	0.188	0.41	2.64270391205487E-06	1	DENND1A

**1.62127068455229E-06**	0.649039975969056	0.232	0.427	0.0271692541317272	1	DOCK5
**5.12321980411076E-08**	0.64610986834593	0.253	0.493	0.000858549174772881	1	PLEKHO2
**7.31352291588702E-11**	0.64562344923948	0.191	0.42	1.22560017024435E-06	1	SP3
**1.76437579003931E-06**	0.64543051886404	0.255	0.471	0.0295674094894788	1	HNRNPH3
**5.03919959767532E-08**	0.642190806836116	0.188	0.38	0.000844469068578431	1	NKTR
**1.12095597619721E-09**	0.640469239103162	0.11	0.265	1.87849802491129E-05	1	TBC1D9
**7.93265706127704E-10**	0.64023057547916	0.118	0.283	1.32935467032881E-05	1	ZBTB4
**5.35355991545943E-12**	0.636688439536636	0.184	0.423	8.97149570632691E-08	1	SETD5
**1.39650171521369E-11**	0.63640566725974	0.135	0.33	2.3402575743551E-07	1	RNF10
**5.38801531017942E-13**	0.635141333871372	0.166	0.404	9.02923605679867E-09	1	TLR4
**1.26211589111402E-10**	0.633261071325569	0.19	0.414	2.11505381032888E-06	1	AKAP9
**2.68089865784032E-08**	0.633143064635571	0.197	0.401	0.000449264997080881	1	FRMD4B
**7.23405589355333E-12**	0.629487643189871	0.087	0.252	1.21228308664167E-07	1	CCNT2
**1.80270430093828E-07**	0.628976004126659	0.29	0.555	0.00302097186751237	1	EVL
**1.3966700770739E-09**	0.62860185324157	0.133	0.306	2.34053971516044E-05	1	RBM17
**4.8864791455103E-12**	0.62583950134637	0.124	0.317	8.18876175204617E-08	1	NT5C2
**4.8289849894289E-11**	0.625132912145753	0.162	0.374	8.09241304528494E-07	1	ARRB1
**3.45731416235184E-09**	0.625072049598376	0.108	0.257	5.79376707326921E-05	1	ABCD4
**9.77209681594707E-11**	0.623983406605814	0.118	0.288	1.63760798441641E-06	1	UAP1L1
**2.10957747073241E-10**	0.617750515397759	0.186	0.403	3.53522992545337E-06	1	YPEL5
**7.39295404766749E-09**	0.615448991002776	0.168	0.357	0.000123891123930812	1	CACUL1
**1.87694152637789E-06**	0.612035142315118	0.186	0.353	0.0314537860990407	1	SPAG9
**1.27574937000772E-10**	0.611885061815727	0.168	0.377	2.13790079425894E-06	1	DDAH2
**4.36235089578097E-15**	0.608340083337422	0.079	0.268	7.31042763114975E-11	1	TTC37
**1.58659349361931E-10**	0.607039464025971	0.132	0.314	2.65881337660723E-06	1	CMTM7
**4.10872238134279E-08**	0.605070282983912	0.234	0.465	0.000688539696665425	1	FYB1
**2.54185393421016E-11**	0.604149990200833	0.143	0.341	4.25963882294939E-07	1	SNRNP70
**5.98302038535724E-08**	0.601313489665104	0.279	0.534	0.00100263455617817	1	GGA2
**2.15859785052947E-07**	0.599202397535707	0.195	0.378	0.00361737827791729	1	PNISR
**5.16871207936674E-15**	0.597143522507734	0.159	0.407	8.66172770260279E-11	1	SLC25A3
**1.59105073583916E-08**	0.593651428014601	0.199	0.401	0.000266628282311927	1	HNRNPDL
**1.40475960739131E-11**	0.592770546058348	0.224	0.487	2.35409615006635E-07	1	NAPA
**2.76851042004163E-09**	0.592115481591449	0.172	0.367	4.63946976190576E-05	1	ILRUN
**6.44256817065747E-10**	0.590842931157613	0.197	0.414	1.07964557403878E-05	1	SREBF1
**1.66276402882776E-13**	0.589948073808285	0.099	0.285	2.78645995950955E-09	1	RNF19B
**9.54045858845001E-11**	0.589352612560218	0.114	0.287	1.59879005025245E-06	1	VHL
**2.05726684326654E-11**	0.58852844882899	0.172	0.395	3.44756777594608E-07	1	C16orf72
**2.48376358866552E-13**	0.585192441060612	0.079	0.251	4.16229102188569E-09	1	PRKAA1
**6.16895969136469E-07**	0.584405828453616	0.306	0.553	0.010337942650789	1	TTYH3
**1.1121697439932E-09**	0.582967005454202	0.124	0.29	1.8637740569838E-05	1	SLC46A1
**8.55210758488822E-07**	0.582204660893957	0.149	0.294	0.0143316218907557	1	TAF1D
**4.1057004230793E-07**	0.570730464415875	0.199	0.383	0.00688033276899629	1	SAFB2
**5.84948170651857E-07**	0.5671638587462	0.126	0.262	0.00980256144378381	1	PARP8
**4.09746631620316E-18**	0.565908409101132	0.83	0.896	6.86653405269325E-14	1	SRGN
**6.46265084090377E-12**	0.564215072615156	0.12	0.308	1.08301102791865E-07	1	SLC9A9
**4.50210581756691E-13**	0.563413621756567	0.12	0.318	7.54462892907862E-09	1	LRMDA
**2.1912812290062E-07**	0.560235372669288	0.126	0.264	0.00367214908356858	1	PHYKPL
**1.29359218498287E-06**	0.559789491598398	0.325	0.586	0.0216780178359429	1	CD300A
**1.13646097712282E-06**	0.559428508006474	0.238	0.444	0.0190448130546242	1	RAB20
**5.714947472582E-10**	0.558767564471257	0.25	0.507	9.57710897455291E-06	1	LMNA

4.40847535228019E-13	0.556591812446732	0.151	0.374	7.38772299535114E-09	1	HSP90B1
1.13330151065725E-10	0.55591160628054	0.124	0.3	1.89918667155942E-06	1	GUK1
1.54962530496324E-12	0.555297286262386	0.13	0.334	2.5968620860574E-08	1	ENTPD6
2.68112203608884E-12	0.554628183831997	0.155	0.373	4.49302430807769E-08	1	NDUFA10
2.41391831420048E-07	0.554037293180352	0.161	0.321	0.00404524431093717	1	GPBP1
2.7901195374454E-06	0.545269748975497	0.308	0.556	0.04675682320851	1	SLC3A2
9.20729647968843E-07	0.545034296877925	0.203	0.382	0.0154295874406619	1	MS4A4E
5.99816420906003E-07	0.544634229847114	0.267	0.5	0.0100517235815428	1	REL
5.39873689979756E-09	0.540662695292759	0.12	0.273	9.04720329668074E-05	1	EIF1B
2.22511357880541E-13	0.537212824324043	0.118	0.32	3.7288453353621E-09	1	PFDN5
1.06959386823451E-08	0.53707957868246	0.25	0.497	0.00017924254043874	1	STAT6
5.60049698622528E-12	0.535793287213528	0.091	0.256	9.38531284951632E-08	1	PCMTD2
3.94981856610894E-13	0.530517470939873	0.18	0.426	6.61910595308536E-09	1	EIF5
1.03654580556993E-11	0.530411351626814	0.112	0.293	1.73704346097409E-07	1	NR3C1
1.27567198600499E-13	0.528749175859911	0.182	0.433	2.13777111414717E-09	1	MAPKAPK2
1.1440556728481E-10	0.527826659464276	0.106	0.271	1.91720849655885E-06	1	RHOT2
3.48779114757091E-12	0.526691305588939	0.184	0.424	5.84484040509934E-08	1	PLEKHM2
6.9999428659061E-11	0.524416950695449	0.097	0.257	1.17305042546854E-06	1	YTHDC2
8.73161453300058E-11	0.523484657174316	0.143	0.334	1.46324396344024E-06	1	ITSN1
1.76369532740773E-10	0.5220679641955	0.106	0.268	2.95560062966987E-06	1	WDR11
7.91131774953894E-15	0.519304188356771	0.132	0.359	1.32577862846774E-10	1	RELCH
5.23574837968445E-08	0.517078589581789	0.114	0.255	0.00087740671346752	1	NFAM1
1.13034852223584E-12	0.516434631600619	0.126	0.328	1.89423805356282E-08	1	HERC4
4.0756627132633E-12	0.514950744776539	0.118	0.306	6.82999557488665E-08	1	NCK1
2.10524925054202E-11	0.512913298846744	0.124	0.305	3.52797669405832E-07	1	IKBKG
1.19064441079619E-09	0.512336441693225	0.104	0.258	1.99528190361225E-05	1	SRC
2.04647974921448E-09	0.510987920500606	0.159	0.345	3.42949076373362E-05	1	MVB12B
5.52228862669847E-08	0.509147928397621	0.114	0.254	0.00092542512806213	1	FBXW7
8.39162757595003E-13	0.509081766466554	0.159	0.381	1.40626894917771E-08	1	KMT2D
4.29887244030066E-10	0.509016249330008	0.155	0.347	7.20405043545584E-06	1	SOAT1
1.58781528224738E-13	0.508719428788969	0.083	0.26	2.66086084999016E-09	1	ADGRG6
3.78985303204298E-07	0.507427953369524	0.118	0.25	0.00635103571109763	1	PPP2R5C
3.01419381527119E-10	0.503210976675027	0.174	0.383	5.05118599563146E-06	1	LGALS8
7.22649521482549E-15	0.50300859786379	0.083	0.272	1.21101606810045E-10	1	H2AFJ
1.136269823095E-10	0.501562291907317	0.128	0.31	1.90416096954261E-06	1	SEC22B
4.62087578377945E-07	0.501061232707854	0.145	0.289	0.0077436636384576	1	NFKB2
2.7313468208442E-14	0.499536995887227	0.11	0.316	4.57719100237072E-10	1	ZBTB1
2.26180890655423E-12	0.499190559873113	0.159	0.38	3.79033936560358E-08	1	FOXO3
2.42158884184118E-13	0.498273236900518	0.151	0.375	4.05809858115745E-09	1	DCAF8
6.40555124212604E-13	0.497864861561446	0.147	0.366	1.07344227715548E-08	1	TIAL1
1.67906770140423E-07	0.494040738331668	0.306	0.576	0.0028137816540132	1	DAPK1
7.93560584923199E-11	0.491227727845236	0.17	0.384	1.3298488282143E-06	1	PTPRC
3.50488714952891E-11	0.489654978384014	0.095	0.256	5.87348988518056E-07	1	ZRANB2
3.37440438727471E-08	0.487422267674331	0.112	0.253	0.000565482687219496	1	SF3B1
8.1227805525006E-13	0.485163426547504	0.126	0.326	1.36121556498805E-08	1	SUSD1
1.58424581616713E-11	0.483603488041532	0.112	0.291	2.65487913873287E-07	1	PPIG
2.75570509741837E-10	0.481572792793691	0.23	0.483	4.6180106022537E-06	1	STAT2
2.39276843817756E-12	0.48118766422832	0.108	0.293	4.00980134869796E-08	1	BEX4
1.00642401136131E-16	0.480615209329762	0.085	0.289	1.68656535823928E-12	1	RHOU
1.57264435268873E-10	0.480048764628216	0.095	0.25	2.63543740623578E-06	1	DCAF5

7.08917048302753E-13	0.479486804876152	0.168	0.404	1.18800318954575E-08	1	UBE2B
2.54373838338038E-08	0.477608321725324	0.12	0.266	0.000426279678286883	1	ZNF592
8.19644553727599E-13	0.47660554561652	0.083	0.253	1.37356034313671E-08	1	SIAH1
5.22909262908308E-09	0.473294672372959	0.118	0.272	8.76291342781743E-05	1	ARHGEF7
7.08723461994512E-08	0.472863944301819	0.118	0.258	0.0011876787776104	1	SP140L
1.21410753733154E-12	0.471735684121547	0.153	0.373	2.0346014110602E-08	1	CASP1
6.39450198679392E-11	0.470668520489707	0.17	0.387	1.07159064294693E-06	1	IFNAR2
1.10200694595741E-10	0.470095822977324	0.13	0.308	1.84674324003542E-06	1	IFFO1
5.76568194546646E-12	0.46823572924581	0.159	0.374	9.6621298042127E-08	1	ABI3
1.36828089765184E-12	0.467271025218977	0.104	0.288	2.29296512828495E-08	1	MEPCE
3.60213533486087E-09	0.466658277192767	0.191	0.395	6.03645839415985E-05	1	CLN8
2.17767567052424E-10	0.462706308661044	0.23	0.479	3.64934888866451E-06	1	RBM33
2.51568863723669E-07	0.462535500124456	0.789	0.743	0.00421579101828125	1	CD163
3.74634898207805E-15	0.461263484086985	0.157	0.405	6.2781316241664E-11	1	IL18
1.52740194205842E-13	0.460160451322581	0.126	0.335	2.55962017450151E-09	1	PLA2G15
1.25234854350152E-09	0.459287302064364	0.288	0.578	2.09868568919985E-05	1	CHST11
8.42892000427928E-12	0.459146697271758	0.153	0.36	1.41251841431712E-07	1	TRIM14
4.43572076327282E-11	0.4582703354454	0.137	0.33	7.4333808550926E-07	1	DNAJC7
5.40561881687099E-12	0.456184673230773	0.104	0.279	9.05873601331241E-08	1	MAP3K2
2.11500095365103E-12	0.45416143236648	0.184	0.428	3.54431859812839E-08	1	UBAP2L
7.20702767575406E-12	0.452996938179774	0.13	0.322	1.20775369790287E-07	1	MKLN1
1.55931414253718E-08	0.45225686812246	0.11	0.253	0.000261309864006381	1	JMJD6
6.19414392428024E-10	0.4488379678491	0.147	0.332	1.03801463883088E-05	1	STARD3
1.81256293918731E-12	0.446818238593177	0.114	0.301	3.03749297349009E-08	1	ZDHHC14
8.83740215559641E-12	0.445974519676701	0.174	0.4	1.48097185323485E-07	1	RAB1A
6.419108044912E-16	0.445842539556717	0.101	0.312	1.07571412616635E-11	1	PPP4R3A
8.01480836418065E-07	0.44487427932047	0.226	0.418	0.0134312158566939	1	ARHGEF10
3.33347095708328E-11	0.444535708806704	0.133	0.322	5.58623062988016E-07	1	PPFIBP2
2.18660553572892E-08	0.443937588125976	0.161	0.333	0.000366431355677452	1	SCARB1
2.195384051655E-12	0.443780652410781	0.149	0.363	3.67902459376345E-08	1	CEPT1
1.35801746359437E-11	0.442204437660407	0.122	0.309	2.27576566549145E-07	1	FBXL3
9.993362588862E-10	0.441497510757691	0.103	0.253	1.67468770264149E-05	1	WDFY1
6.32342083613462E-14	0.439715785326852	0.11	0.31	1.05967886371944E-09	1	MAPK6
5.1425558290846E-10	0.439419642843616	0.12	0.288	8.61789505837997E-06	1	FER
8.61753398752466E-14	0.438142882422827	0.095	0.283	1.44412634562938E-09	1	KLF3
4.82356517593114E-12	0.437857177418744	0.137	0.338	8.08333052182541E-08	1	RIPOR1
5.02672867147105E-09	0.437135399985555	0.153	0.33	8.42379190765119E-05	1	ARHGAP15
2.37231182572385E-11	0.435939119444545	0.112	0.286	3.97552015754803E-07	1	MOV10
9.0394509954773E-14	0.435734442768024	0.149	0.38	1.51483119782209E-09	1	ARMH3
1.75133742711118E-10	0.435717662060337	0.191	0.414	2.93489126035292E-06	1	IQSEC1
1.03268674709894E-14	0.434998654596126	0.112	0.32	1.7305764507884E-10	1	SPRED1
2.01062648050323E-07	0.434633876527011	0.279	0.523	0.00336940785602732	1	DENND3
1.42073381111857E-10	0.434240105142968	0.132	0.313	2.38086572067249E-06	1	INPP4A
6.30178409348278E-14	0.434233090161586	0.166	0.406	1.05605297838584E-09	1	NCF4
2.14898763993605E-14	0.433842155927687	0.168	0.423	3.60127348700483E-10	1	NFATC2
2.10003627809686E-09	0.433804869673224	0.137	0.309	3.51924079483471E-05	1	GAB2
4.80788371657905E-12	0.430449321743258	0.209	0.459	8.05705153224317E-08	1	SFT2D2
1.77028675712814E-11	0.429618622598599	0.205	0.454	2.96664654759534E-07	1	EPB41L3
7.72362506893296E-07	0.429332785700266	0.232	0.428	0.0129432508905179	1	MTSS1
1.53664604351582E-11	0.429045889469881	0.097	0.263	2.57511143972382E-07	1	ABRACL

9.48891130978222E-14	0.426478816226345	0.126	0.336	1.5901517572933E-09	1	CPED1
2.87439608567665E-09	0.425006968822961	0.186	0.389	4.81691296037693E-05	1	SH2B3
4.56147417541707E-10	0.42383239405239	0.101	0.254	7.64411842316392E-06	1	TUBGCP6
2.77255964265139E-09	0.423585529022067	0.118	0.273	4.64625544915521E-05	1	TRAK1
4.43759276396557E-11	0.423167722782391	0.112	0.285	7.4365179538535E-07	1	ARL8B
9.91758114126936E-09	0.423111339067791	0.261	0.507	0.000166198824765392	1	MDM4
6.03712553094592E-17	0.421549077189674	0.12	0.353	1.01170149647592E-12	1	SNX10
9.8303993659446E-12	0.420437898242257	0.184	0.417	1.647378325745E-07	1	PABPC4
8.75816891088118E-16	0.418050174642648	0.149	0.396	1.46769394608547E-11	1	GSAP
4.80164438081654E-11	0.413130575555241	0.153	0.353	8.04659565337236E-07	1	CREBRF
1.588964084233E-18	0.411860040200019	0.132	0.388	2.66278601235765E-14	1	HELZ
5.7320910568436E-09	0.410845982758202	0.304	0.595	9.60583819305851E-05	1	MYO1F
4.20387325167263E-16	0.410269030726911	0.087	0.29	7.04485079515299E-12	1	NUDCD3
8.81356883002594E-11	0.410030321705488	0.124	0.3	1.47697786453575E-06	1	SON
2.19555932446228E-10	0.409466920920253	0.24	0.499	3.67931831593388E-06	1	TCF25
1.54820042665506E-13	0.407761402696764	0.103	0.293	2.59447427498854E-09	1	PTOV1
2.83225128899916E-12	0.407366801323793	0.095	0.265	4.7462867101048E-08	1	TNRC6B
3.60007330993696E-12	0.406894204729835	0.116	0.3	6.03300285279235E-08	1	KDM4C
1.50399465649312E-10	0.406420207138997	0.116	0.285	2.52039424535117E-06	1	EXOC4
2.35897847788926E-13	0.406140832063109	0.087	0.265	3.95317613324682E-09	1	C6orf62
3.16029919068668E-13	0.405052381867886	0.087	0.263	5.29602938375274E-09	1	PHACTR2
9.02656871696549E-15	0.40419873952206	0.191	0.457	1.51267238558908E-10	1	POLR2A
1.00529257152414E-17	0.404027399588598	0.184	0.476	1.68466929136016E-13	1	B4GALT1
3.25238909957337E-14	0.403967150107223	0.112	0.318	5.45035365306506E-10	1	SKI
5.87348596733409E-14	0.4006180117895	0.095	0.282	9.84278778405847E-10	1	KLHL5
2.58459538730582E-10	0.399068930942393	0.097	0.25	4.33126495004709E-06	1	ZNF24
1.65838184844886E-10	0.396613769548527	0.118	0.285	2.7791163016306E-06	1	VPS41
8.40818377929839E-14	0.396321784590025	0.132	0.344	1.40904343773482E-09	1	TRIM26
2.38565198591864E-07	0.394303858037145	0.315	0.573	0.00399787559800245	1	TNFRSF1B
1.24760703714175E-15	0.392128000281324	0.081	0.276	2.09073987284215E-11	1	PITHD1
1.07510581466668E-09	0.391656941966238	0.112	0.271	1.80166232421842E-05	1	SESN3
1.43537323986341E-11	0.391242431004242	0.159	0.369	2.40539847536309E-07	1	SYAP1
2.86131244074672E-12	0.38849172830543	0.099	0.272	4.79498738820335E-08	1	TRAPPC9
6.69356591927512E-15	0.388403876193017	0.215	0.503	1.12170777675213E-10	1	TMEM106A
1.0718502264554E-13	0.388065510197086	0.104	0.297	1.79620660949396E-09	1	SGPL1
3.05424150601488E-12	0.387559280612118	0.174	0.405	5.11829791577974E-08	1	ITSN2
3.5967351295605E-09	0.387482063011108	0.178	0.372	6.02740873011749E-05	1	MORC3
1.04950198761021E-09	0.386954362912196	0.203	0.42	1.7587554308372E-05	1	LILRB2
1.11704810755897E-11	0.386358731350802	0.099	0.268	1.87194921864732E-07	1	RIN2
4.09309135445787E-17	0.384589093133031	0.104	0.327	6.85920249180049E-13	1	BTAF1
8.91378456267028E-12	0.384013462499701	0.141	0.343	1.49377201701229E-07	1	SLC4A7
1.23132726220113E-06	0.381188364770555	0.313	0.574	0.0206345822599666	1	IL10RA
1.31247499400457E-09	0.380563466388146	0.11	0.264	2.19944559495286E-05	1	DVL3
8.69772930498183E-17	0.379545670478464	0.133	0.382	1.45756547692886E-12	1	MANBA
2.36116294199699E-17	0.376217037242717	0.153	0.419	3.95683685819856E-13	1	SND1
9.8940327801957E-12	0.375755897472901	0.108	0.285	1.65804201330519E-07	1	PPP1R12C
3.29048491243477E-13	0.375322446240699	0.081	0.252	5.51419461625819E-09	1	PSPC1
1.19439687351306E-12	0.37226677551904	0.091	0.264	2.00157028063319E-08	1	MAPRE2
1.63971617709835E-08	0.369921238334971	0.132	0.286	0.000274783636958142	1	PIM3
1.32019408370291E-12	0.368058461572822	0.101	0.28	2.21238124546934E-08	1	NEK7

2.88153440833214E-13	0.366064037496703	0.126	0.33	4.828875361483E-09	1	FAM20C
1.62148501431356E-20	0.364615278310677	0.159	0.458	2.71728458698667E-16	1	LYN
1.07536730683516E-17	0.363903482729125	0.215	0.531	1.80210053279436E-13	1	EIF1
5.29851815755123E-17	0.36356071352402	0.159	0.427	8.87925672842435E-13	1	INPP5D
1.43248700377786E-09	0.363457643216994	0.184	0.387	2.40056172093095E-05	1	RBM25
4.32970937521418E-14	0.363308149651214	0.11	0.312	7.25572697098393E-10	1	DCTN4
9.329087230108E-15	0.363053964734392	0.164	0.412	1.5633684380215E-10	1	PNPLA6
8.65310733851709E-18	0.362086179872486	0.199	0.505	1.45008772778869E-13	1	ZFAND3
1.15195525065532E-11	0.361786010049976	0.091	0.253	1.93044660904819E-07	1	UBE4B
3.58352903630914E-13	0.361681531577798	0.083	0.254	6.00527795904686E-09	1	CDK4
4.43057122107815E-10	0.360399899903408	0.106	0.263	7.42475125228276E-06	1	CUX1
1.55091206229692E-10	0.358345986550905	0.19	0.41	2.59901843399717E-06	1	TMEM37
3.78794739749404E-15	0.357530248326054	0.099	0.301	6.3478422487205E-11	1	ZGPAT
1.79856846658911E-11	0.356251062696022	0.188	0.417	3.01404103631003E-07	1	STK10
3.50278128534057E-10	0.35552778035009	0.159	0.355	5.86996087797374E-06	1	RALGDS
1.6613138263984E-11	0.355026132343867	0.133	0.326	2.78402971027844E-07	1	CYB5R4
8.22965509022464E-09	0.35365533121969	0.114	0.263	0.000137912560001985	1	KDM2B
8.01310054321928E-09	0.353295745227096	0.211	0.423	0.000134283538903269	1	JMJD1C
1.41361048075904E-10	0.352436554585676	0.265	0.538	2.368928443656E-06	1	PARVG
8.26646798861388E-10	0.352171965999826	0.188	0.395	1.38529470553191E-05	1	SBNO2
2.94755008209634E-10	0.349489773723447	0.135	0.314	4.93950442757705E-06	1	MEF2D
2.17688418980741E-14	0.347704580452944	0.087	0.272	3.64802252527926E-10	1	IWS1
1.61151050262405E-12	0.346951422178749	0.11	0.295	2.70056930029738E-08	1	TOB1
1.83714329458077E-19	0.346798883218141	0.151	0.435	3.07868473305846E-15	1	RREB1
1.00312668300708E-09	0.346783600598836	0.106	0.259	1.68103969538326E-05	1	TRIM47
1.02374568825259E-07	0.346447368608519	0.139	0.287	0.0017155930243737	1	ANKRD13D
7.85101857123833E-13	0.345859469727863	0.12	0.314	1.31567369216812E-08	1	SLC36A1
6.33789389268109E-15	0.34513679358298	0.077	0.263	1.0621042585355E-10	1	ABCF1
3.36240461449669E-15	0.344583027661647	0.097	0.298	5.63471765297355E-11	1	NFKB1
1.6820435787768E-11	0.341306276811306	0.162	0.372	2.81876862931416E-07	1	IL16
4.76743555575449E-16	0.341201139022824	0.19	0.476	7.98926850433337E-12	1	DRAM2
1.2958347175516E-11	0.341153200638357	0.145	0.343	2.17155981967297E-07	1	ST3GAL1
5.60769924937522E-16	0.340841228325179	0.24	0.564	9.397382402103E-12	1	PDXK
5.26554406932756E-13	0.340695365348135	0.108	0.299	8.82399875137912E-09	1	ME2
2.75677474283339E-10	0.338693918117695	0.13	0.303	4.61980311404019E-06	1	CLEC2D
1.8410792056737E-09	0.338545568242714	0.273	0.537	3.08528053286799E-05	1	RNF144B
3.70675014197169E-11	0.338137367575967	0.255	0.538	6.21177188791617E-07	1	LPAR6
3.1294093714429E-16	0.33611267850815	0.17	0.442	5.24426422466402E-12	1	BNIP2
1.64579270135372E-12	0.335425151542334	0.087	0.254	2.75801940892857E-08	1	KIF13B
1.31817634261143E-12	0.33540583808865	0.168	0.4	2.20899991494823E-08	1	AAK1
3.22245738343103E-09	0.333452648654891	0.108	0.259	5.40019408315372E-05	1	BCL2L11
1.44112363733643E-14	0.332112080905914	0.13	0.348	2.41503499144838E-10	1	WDFY2
3.2482077193491E-08	0.327565206240319	0.342	0.647	0.000544334649608521	1	ARRB2
9.50806054261517E-12	0.323817172186033	0.162	0.378	1.59336078573145E-07	1	ISCU
2.26980936864025E-11	0.322384906025586	0.29	0.604	3.80374653996734E-07	1	CIRBP
2.92211297400664E-11	0.322134979121933	0.091	0.25	4.89687692184032E-07	1	PABPC1L
1.47348867375767E-12	0.32139485728379	0.089	0.259	2.46927231948311E-08	1	YPEL2
2.94529737994907E-15	0.320026361676375	0.217	0.508	4.93572934931865E-11	1	STAT3
4.08047302024919E-18	0.31989749455335	0.124	0.374	6.83805668733359E-14	1	MAGT1
4.74646753400222E-10	0.319653839036893	0.284	0.564	7.95413029348093E-06	1	TNS3

1.03742181117718E-14	0.319184544533501	0.095	0.29	1.73851147117072E-10	1	RIOK3
7.93443475174565E-16	0.317740111375454	0.091	0.294	1.32965257569754E-11	1	PAPSS2
5.49737254440615E-09	0.317308238024556	0.203	0.406	9.21249690991582E-05	1	CD163L1
4.87410263254021E-11	0.315454486955482	0.12	0.295	8.16802119161088E-07	1	PILRB
9.44694311150835E-12	0.315219158912521	0.246	0.52	1.58311872662657E-07	1	AKR1B1
4.48652641326222E-16	0.313372170894619	0.093	0.299	7.51852096334482E-12	1	CASC3
4.25125598324066E-13	0.313132130682403	0.193	0.447	7.12425477671469E-09	1	TMEM259
7.553146664166E-12	0.31255381891361	0.126	0.316	1.26575631798094E-07	1	ADGRE2
4.15429679347645E-11	0.312401470488206	0.099	0.263	6.96177056650783E-07	1	EVI5
2.12353079163361E-15	0.311093905319455	0.12	0.339	3.5586129006196E-11	1	SMARCA4
7.21603803389389E-15	0.310351342385451	0.178	0.442	1.20926365371994E-10	1	GNPTG
2.17697540415308E-13	0.308577928305387	0.079	0.25	3.64817538227974E-09	1	UBE2E2
8.23326204135948E-11	0.308466700521121	0.112	0.281	1.37973005289102E-06	1	ATN1
1.30477597438529E-13	0.308148169095038	0.103	0.293	2.18654357787486E-09	1	ZNF410
2.30623984844114E-14	0.308098772420459	0.226	0.521	3.86479673801767E-10	1	ACADVL
6.26078528370173E-15	0.307899205011126	0.118	0.334	1.04918239784274E-10	1	SNX5
2.63079447949944E-17	0.307521885729718	0.099	0.321	4.40868538874516E-13	1	WIPI2
2.25650105605203E-13	0.306834096559331	0.168	0.402	3.78144446973199E-09	1	STARD13
4.14344683823262E-11	0.30550412790789	0.292	0.589	6.94358821151022E-07	1	CLEC7A
7.711748571433E-18	0.302419852922685	0.135	0.39	1.29233482560074E-13	1	SLBP
5.31500592389532E-12	0.302180521895157	0.097	0.266	8.90688692726378E-08	1	UPP1
4.32050579023689E-13	0.302137381972538	0.182	0.422	7.24030360327898E-09	1	STX16
2.3577482556904E-13	0.301597118528482	0.104	0.294	3.95111452688598E-09	1	HPS3
1.18381669600241E-13	0.301578537765491	0.182	0.43	1.98384001916083E-09	1	RSRC2
8.25400755633518E-14	0.299053152566108	0.13	0.342	1.38320658629065E-09	1	GNG10
1.41643006873966E-10	0.297882749054229	0.103	0.263	2.37365350919392E-06	1	PGPEP1
4.37178425683366E-15	0.297110403493325	0.13	0.353	7.32623605760185E-11	1	BMP2K
1.71272875835653E-12	0.296292837391403	0.114	0.302	2.87019085325388E-08	1	SCIMP
7.21210794103075E-12	0.295075911405369	0.104	0.28	1.20860504875793E-07	1	ZNF609
2.72049439099173E-14	0.294991268899793	0.118	0.325	4.55900450042394E-10	1	MARCH6
4.26608746940642E-17	0.29390155693615	0.106	0.33	7.14910938123128E-13	1	SORBS3
1.38571476002396E-14	0.29310435643163	0.077	0.258	2.32218079484815E-10	1	LOXL3
1.1315858984825E-16	0.291836644545202	0.072	0.265	1.89631164867698E-12	1	MOSPD2
4.32198661179084E-13	0.290707957649899	0.083	0.254	7.2427851640391E-09	1	PIP4K2B
7.94609224394367E-12	0.290666273626733	0.095	0.26	1.33160613824008E-07	1	KLHDC2
3.06256748597129E-13	0.290432919829686	0.083	0.255	5.13225059299068E-09	1	RB1
1.50412761599868E-12	0.290254215240221	0.114	0.303	2.52061705889059E-08	1	TBC1D22A
2.35095654395038E-12	0.28911765703486	0.11	0.294	3.93973297635204E-08	1	TCERG1
9.91280297480612E-16	0.288763129895677	0.072	0.257	1.66118752251801E-11	1	USO1
3.9457361916927E-18	0.288452220638899	0.126	0.374	6.61226471003863E-14	1	FUS
8.51468714444329E-10	0.288051426199412	0.317	0.626	1.42689127166581E-05	1	IKBKB
8.08220254227176E-16	0.286100177106873	0.147	0.393	1.3544155020339E-11	1	SERP1
2.44171821025879E-10	0.286097158386393	0.11	0.272	4.09183137675168E-06	1	NPEPL1
1.04902437403866E-14	0.2860658369877	0.083	0.27	1.75795504601399E-10	1	TOMM20
1.06082975961152E-18	0.285780327815335	0.17	0.46	1.77773851115698E-14	1	H2AFY
7.90387447622225E-17	0.285590457453087	0.147	0.403	1.32453128472532E-12	1	BCL2L1
1.20204389121619E-14	0.282250934959299	0.12	0.333	2.01438515290009E-10	1	SPEN
1.89960295215424E-19	0.281811290338286	0.164	0.453	3.18335462722007E-15	1	MGAT4A
1.02490559461925E-15	0.280611882184994	0.07	0.253	1.71753679546294E-11	1	ST3GAL6
1.30412232623716E-13	0.279739857160057	0.112	0.308	2.18544819430823E-09	1	THBD

3.98265644819548E-20	0.278922701851528	0.099	0.346	6.67413567588598E-16	1	DIP2B
8.22769839100643E-19	0.276971911668763	0.122	0.374	1.37879769636486E-14	1	TSPYL1
1.51880907566351E-13	0.276366295788374	0.234	0.523	2.54522024899691E-09	1	SERPINB6
3.67934632512994E-15	0.276292167834169	0.083	0.272	6.16584857165275E-11	1	NFX1
6.04904266410091E-16	0.275854328944253	0.077	0.27	1.01369856965003E-11	1	DERA
3.17947389682411E-14	0.27540195573653	0.089	0.277	5.32816235629785E-10	1	REV1
1.79166299808829E-12	0.274870241858334	0.132	0.331	3.00246885219636E-08	1	LPXN
5.47467896242942E-17	0.273742763859326	0.166	0.439	9.17446700523921E-13	1	LPP
6.96780631354979E-16	0.27332501629904	0.132	0.367	1.16766498202467E-11	1	DNAJB11
2.49597156445472E-15	0.273009003746354	0.07	0.25	4.18274914771321E-11	1	TRRAP
1.88626577670116E-14	0.272998392366864	0.12	0.33	3.16100418859581E-10	1	KANSL1
2.49441549908242E-13	0.27257770327343	0.101	0.287	4.18014149336233E-09	1	ANKRD11
6.12287004505504E-10	0.272527697174826	0.306	0.598	1.02607056215032E-05	1	SDCBP
9.23609775557313E-17	0.272182065899761	0.222	0.538	1.54778526187894E-12	1	MINK1
2.34665276094287E-19	0.271270384538967	0.132	0.401	3.93252069678807E-15	1	ZFHX3
1.12765131794385E-14	0.270504539051731	0.106	0.31	1.88971807861031E-10	1	EWSR1
2.60225176579041E-17	0.27039454402959	0.103	0.326	4.36085350911157E-13	1	KIDINS220
3.49807969621201E-10	0.269859891327256	0.155	0.344	5.8620819549121E-06	1	GFRA2
3.2769549141919E-13	0.269334430799308	0.095	0.277	5.49152104520279E-09	1	PYGL
2.90683679249703E-14	0.269059771174229	0.226	0.519	4.87127709686653E-10	1	SLC7A7
1.79961151679437E-13	0.268933082471691	0.11	0.304	3.01578897984401E-09	1	P2RX4
3.4900663884205E-11	0.267694494926914	0.178	0.397	5.84865325371507E-07	1	HECA
4.15076325479625E-16	0.26739568989041	0.101	0.312	6.95584906238756E-12	1	MAEA
1.51627175506966E-11	0.266157464627273	0.095	0.257	2.54096820714573E-07	1	ENTR1
2.99686207883326E-18	0.265929927553411	0.11	0.35	5.02214147170878E-14	1	LMBRD1
4.95753863088154E-13	0.265695242556101	0.097	0.276	8.30784323763128E-09	1	METTL17
5.44879512967837E-09	0.265298417294598	0.176	0.363	9.13109087831501E-05	1	TRIM38
7.35406820012267E-12	0.263898438824201	0.261	0.551	1.23239474897656E-07	1	CCNL2
5.48602698177839E-16	0.262896779172561	0.079	0.273	9.19348401606422E-12	1	DOCK7
4.17566984330213E-18	0.262501432879475	0.099	0.329	6.99758752340571E-14	1	ANKRD17
3.28462864390493E-14	0.26142678092929	0.095	0.285	5.50438068145589E-10	1	ZFC3H1
2.55878429155085E-13	0.261371557653212	0.168	0.4	4.28801071578092E-09	1	SLTM
4.63150530431502E-18	0.260975935475301	0.145	0.413	7.76147658897112E-14	1	TENT2
2.62092626974417E-14	0.258890031285675	0.091	0.28	4.39214824283728E-10	1	SLK
2.67296665425504E-14	0.25859362007114	0.075	0.253	4.4793575192006E-10	1	GLRX3
4.77782924537701E-12	0.254727833036906	0.13	0.325	8.00668624940279E-08	1	ASXL1
4.44360182175092E-17	0.25179993509501	0.166	0.44	7.44658793289019E-13	1	SPATS2L
1.65440206564142E-14	0.251150535835324	0.168	0.413	2.77244698160189E-10	1	ST8SIA4
1.28075584785826E-14	0.251122490202607	0.141	0.372	2.14629064984087E-10	1	POGZ
5.762648526556E-80	4.59816307337202	0.348	0.027	9.65704640080255E-76	2	CD1E
1.12856022108819E-81	3.92722544755659	0.37	0.032	1.89124121849959E-77	2	CD1C
1.30033298635118E-56	3.65037935531731	0.269	0.025	2.1790980185273E-52	2	CD1B
1.20069280536922E-100	3.32286214213311	0.686	0.227	2.01212100323773E-96	2	CLEC10A
1.78070241272744E-28	3.19304634304773	0.339	0.129	2.98410110324864E-24	2	S100B
7.63809643826715E-37	3.17759057139067	0.271	0.058	1.27999220112481E-32	2	CST7
3.48376344756414E-47	3.08056291059109	0.278	0.041	5.83809078542798E-43	2	ACAP1
1.85847231082204E-47	2.96011413927178	0.336	0.069	3.11442789847557E-43	2	LGALS2
1.62514490486657E-40	2.90368230391569	0.305	0.067	2.7234178315754E-36	2	PLD4
4.37708471818641E-46	2.67324959631229	0.476	0.179	7.33511857073679E-42	2	FCGR2B
3.26237549591719E-34	2.66770177448936	0.325	0.095	5.46708885605803E-30	2	SLC38A1

6.84116650025377E-44	2.66240744762733	0.409	0.12	1.14644268211253E-39	2	SPN
2.27254623374507E-26	2.58793874962088	0.266	0.078	3.80833297850998E-22	2	PARM1
1.17233423635052E-42	2.57235150802242	0.278	0.046	1.9645977132762E-38	2	ADAM8
1.36775723054508E-34	2.51567530376066	0.28	0.066	2.29208756694745E-30	2	CCR5
2.74931622733346E-46	2.45920842110328	0.612	0.302	4.60730413376541E-42	2	CYTIP
2.50017395003728E-38	2.42028868347944	0.503	0.246	4.18979150547248E-34	2	FGR
2.16125490903494E-17	2.38851018482475	0.309	0.145	3.62183097656076E-13	2	SATB1
2.41798917892833E-23	2.38242169340867	0.366	0.166	4.05206626604809E-19	2	IL1R1
2.81095067625521E-33	2.35916957599054	0.332	0.1	4.71059114326848E-29	2	PALM2-AK/
2.01170855336576E-58	2.35643738098081	0.691	0.443	3.37122119373033E-54	2	JAML
1.25136135428211E-31	2.35311855174588	0.37	0.14	2.09703135750597E-27	2	NDRG2
4.63828106462825E-19	2.27350777777773	0.323	0.148	7.77283140810402E-15	2	SULF2
2.98962592139617E-29	2.22144073374369	0.409	0.175	5.01001511907571E-25	2	S100A6
2.30131317351701E-19	2.18831638954463	0.293	0.121	3.85654061617981E-15	2	ITPR1
6.09510402631247E-29	2.11539024168422	0.289	0.084	1.02141753272944E-24	2	CD52
1.92166548427953E-14	2.06543746195435	0.33	0.182	3.22032701855563E-10	2	CKLF
1.01110252943889E-19	2.05960951902934	0.404	0.23	1.69440561883369E-15	2	MNDA
2.87613324110648E-31	2.05398273123825	0.526	0.3	4.81982408544623E-27	2	STK17B
2.0921215484737E-22	1.97741112191654	0.257	0.082	3.50597729093223E-18	2	CRIP1
3.71639249403585E-09	1.97167219996145	0.255	0.146	6.22793054150528E-05	2	ITGA5
9.59016121513964E-82	1.9116571613988	0.896	0.722	1.6071192164331E-77	2	LYZ
1.64079093708774E-15	1.86697545003565	0.323	0.165	2.74963745237163E-11	2	S100A4
2.26475522755051E-15	1.83842266252916	0.255	0.109	3.79527681032915E-11	2	HAPLN3
3.34393386343729E-20	1.82091904048061	0.336	0.148	5.60376436834821E-16	2	ADAM28
1.30815799026658E-19	1.80097038230435	0.454	0.278	2.19221116008874E-15	2	APOBR
5.11522167536274E-19	1.79907791526477	0.413	0.239	8.57208848357288E-15	2	PAK1
2.27035652471967E-23	1.77036891245239	0.47	0.257	3.80466346412523E-19	2	CORO1A
1.07443251887902E-19	1.72307573630527	0.352	0.165	1.80053401513747E-15	2	TES
2.90858650786338E-52	1.70471716904594	0.754	0.481	4.87420926987744E-48	2	LSP1
1.87551192033077E-11	1.7028063312355	0.341	0.205	3.14298287609031E-07	2	FPR1
5.14127162786417E-15	1.69196967149715	0.352	0.185	8.61574299397477E-11	2	SLC2A3
4.84768040295906E-17	1.65520142121846	0.393	0.218	8.1237428192788E-13	2	UVRAG
1.85639636492045E-22	1.64739066283257	0.411	0.208	3.11094902833369E-18	2	ST14
2.38564555636364E-14	1.63525838445045	0.463	0.308	3.99786482335419E-10	2	FMNL1
5.22977532098313E-17	1.63162508668564	0.436	0.275	8.76405748290353E-13	2	RTN1
1.58000132900048E-22	1.61103870913542	0.476	0.282	2.647766227139E-18	2	ANPEP
1.62542081691354E-17	1.61059582126213	0.318	0.145	2.72388020498371E-13	2	FLNA
9.91686747138036E-13	1.59131515844939	0.361	0.213	1.66186865085392E-08	2	IL17RA
3.81109739262408E-13	1.58535521655645	0.4	0.257	6.38663701055943E-09	2	ANKRD44
1.62229821786902E-37	1.58001789960469	0.767	0.582	2.7186473535049E-33	2	CSF2RA
5.85929818247079E-12	1.56697607170131	0.334	0.192	9.81901189418456E-08	2	CSF3R
1.52182243943511E-13	1.5534457929197	0.429	0.271	2.55027004400536E-09	2	CD37
2.0234915422381E-14	1.48707300021211	0.49	0.365	3.39096712648261E-10	2	COTL1
3.05227259380237E-13	1.47403587486481	0.413	0.257	5.11499841269401E-09	2	PALD1
1.23054883081777E-20	1.47095151961304	0.515	0.335	2.06215373068443E-16	2	IL2RG
7.69377298634577E-43	1.47056494705544	0.734	0.552	1.28932247705182E-38	2	CIITA
3.45673751733241E-12	1.46101558734504	0.449	0.325	5.79280073154565E-08	2	ZNF385A
4.69376939894476E-14	1.45977183184448	0.395	0.246	7.86581875875163E-10	2	MYD88
2.88135121943017E-12	1.45242916456806	0.42	0.29	4.82856837352108E-08	2	CALHM6
1.32805921335187E-12	1.44924405608704	0.318	0.181	2.22556162973506E-08	2	ARHGAP31

4.23175461364403E-09	1.43716017015481	0.372	0.258	7.09157438154466E-05	2	MBOAT7
1.0936979637537E-07	1.43515795653207	0.422	0.332	0.00183281904765845	2	FGD4
3.50784565941429E-14	1.41556258156473	0.343	0.182	5.87844775604646E-10	2	ALOX5AP
6.09245495570327E-09	1.3879165300732	0.314	0.2	0.000102097360147675	2	LRRK2
5.79834940718259E-17	1.385106190067	0.526	0.383	9.71687393655658E-13	2	AHR
3.62378024604311E-12	1.38210646754001	0.46	0.333	6.07273093631904E-08	2	SERPINB9
1.59676701921801E-11	1.3820701967893	0.339	0.202	2.67586217080554E-07	2	WARS
6.62421678833672E-18	1.38193108864307	0.467	0.282	1.11008624938947E-13	2	ALOX5
1.54140779202265E-09	1.37700438273905	0.411	0.293	2.58309117787156E-05	2	RELT
1.80655577942613E-12	1.37451237033753	0.406	0.262	3.02742617516231E-08	2	VASH1
7.88403852360333E-14	1.35763903321376	0.345	0.188	1.32120717578545E-09	2	CRYBG1
3.20544325072871E-09	1.33532740964709	0.363	0.243	5.37168179957117E-05	2	FOXN2
1.90643067637564E-07	1.32544551060691	0.323	0.221	0.00319479652747029	2	PSTPIP2
2.93677882473504E-11	1.32322799776944	0.354	0.215	4.92145395449098E-07	2	STK38L
1.81819272521419E-09	1.31322150488208	0.262	0.145	3.04692736891394E-05	2	RASSF5
1.83885653754823E-06	1.30563540976514	0.28	0.19	0.0308155578562333	2	SNX20
8.72358206646686E-10	1.30267500184804	0.415	0.302	1.46189788269852E-05	2	GLIPR1
1.28661878449151E-06	1.2926686100451	0.352	0.256	0.0215611575905087	2	EMP3
1.15040026997043E-09	1.29137658018342	0.352	0.223	1.92784077241645E-05	2	DEF6
3.39399293999277E-12	1.25501912960771	0.506	0.411	5.68765336883989E-08	2	PEA15
8.55050300606512E-09	1.24997104923242	0.474	0.392	0.000143289329375639	2	VASP
2.08672636888027E-09	1.22993980133581	0.391	0.272	3.49693604896956E-05	2	DIAPH1
6.08285740217993E-07	1.22190676716418	0.264	0.171	0.0101936524345731	2	MYOF
3.61863863151181E-08	1.21913655726399	0.4	0.292	0.000606411461868749	2	EDEM1
6.76848817963249E-08	1.21862915661387	0.264	0.159	0.00113426324914281	2	SKIL
9.0562441753856E-12	1.19881945079398	0.465	0.32	1.51764539891112E-07	2	AKNA
6.78748182741356E-17	1.18687172653798	0.628	0.571	1.13744620463796E-12	2	GSN
2.84364520716831E-09	1.1772543801036	0.481	0.373	4.76538063817265E-05	2	PLSCR1
8.05796101216814E-09	1.17244844076289	0.472	0.388	0.000135035310641914	2	IL13RA1
3.92640867956504E-31	1.17079505919941	0.758	0.607	6.5798756652151E-27	2	ITGAX
1.82096166673327E-13	1.14230035733104	0.278	0.128	3.05156756111161E-09	2	HDAC9
6.02931131318728E-07	1.12755452768655	0.402	0.312	0.0101039198986392	2	MRTFA
2.17128294694581E-29	1.12293413783526	0.716	0.628	3.63863596249179E-25	2	LCP1
9.52439269442028E-32	1.10283535404016	0.758	0.649	1.59609772773095E-27	2	CTSH
4.93224262250952E-28	1.09171916903034	0.862	0.785	8.26545218680145E-24	2	CXCR4
1.2757398401389E-12	1.09094043016742	0.562	0.52	2.13788482410476E-08	2	CPVL
2.72926005911915E-06	1.08146830480931	0.377	0.29	0.0457369400707188	2	ENTPD1
2.49561320593753E-12	1.07494053098587	0.637	0.543	4.18214861051011E-08	2	ZFP36L2
2.43857782443248E-25	1.06692260959605	0.72	0.655	4.08656871818395E-21	2	SAMHD1
3.908933465994E-35	1.06257762269444	0.826	0.658	6.55059070231275E-31	2	TXNIP
8.10224650903552E-09	1.05901163738886	0.33	0.215	0.000135777446998417	2	SAMD9L
2.5223109460591E-06	1.05550216272706	0.345	0.248	0.0422688868340583	2	APAF1
1.03036518516243E-08	1.03920591430016	0.348	0.229	0.00017266859772952	2	ADGRE2
2.25175076583464E-06	0.976485765946301	0.492	0.415	0.0377348393338569	2	KDM2A
3.14087160985335E-07	0.961044620871266	0.53	0.472	0.00526347264379224	2	GNAQ
5.98069899005077E-08	0.947474556723643	0.348	0.24	0.00100224553675271	2	LST1
8.50470708841788E-08	0.945322715181935	0.558	0.505	0.00142521881387707	2	SH3BGRL3
1.57143382160378E-08	0.935090025075521	0.621	0.55	0.000263340879824361	2	EFHD2
5.35124291296632E-08	0.929735934886794	0.458	0.355	0.000896761287354896	2	CNN2
4.68585611848133E-08	0.912523492925606	0.341	0.232	0.000785255768335101	2	LPCAT2

7.19526965843135E-09	0.895261994099733	0.521	0.492	0.000120578328935993	2	IFNGR1
1.29358950508056E-06	0.891289037556146	0.458	0.38	0.02167797292614	2	PTPN6
7.17535642965678E-07	0.875662983529234	0.433	0.345	0.0120244623048188	2	DSE
2.79231712112732E-10	0.865579189901568	0.616	0.572	4.67936503158516E-06	2	SYNGR2
6.25644305680842E-07	0.858938898562274	0.253	0.152	0.0104845472745995	2	LMNB1
1.01304926894135E-07	0.852725024667941	0.485	0.421	0.00169766796489191	2	MBNL1
4.1103282438396E-15	0.828783039607097	0.91	0.824	6.8880880710264E-11	2	IFI30
8.60368117403339E-07	0.825943559344511	0.533	0.462	0.0144180489114452	2	ITGA4
6.02860650589904E-08	0.821245891875678	0.35	0.235	0.00101027387825856	2	PBX2
3.4924748643363E-09	0.817206358528602	0.621	0.588	5.85268937765477E-05	2	SMAP2
2.4793588867181E-09	0.770881771842621	0.521	0.422	4.15490962236219E-05	2	SLC8A1
7.28748693876967E-12	0.736736658621902	0.698	0.66	1.22123706119902E-07	2	CELF2
5.21722601575055E-07	0.728458037972024	0.379	0.285	0.00874302735719477	2	DOCK10
1.55955248132772E-10	0.723626883127258	0.673	0.699	2.613498048209E-06	2	TMSB10
6.62185442729796E-24	0.703970482696215	0.851	0.816	1.10969036492659E-19	2	CST3
1.01692923075519E-17	0.673342254108876	0.795	0.736	1.70417000489954E-13	2	SPI1
2.54430045502218E-06	0.662008139594193	0.58	0.534	0.0426373870252617	2	LENG8
2.85358816910393E-10	0.659428987093785	0.842	0.793	4.78204305378436E-06	2	LITAF
6.46437933239168E-08	0.623301192388503	0.682	0.652	0.0010833006885222	2	EEF2
1.11039882835963E-10	0.600938242999717	0.709	0.664	1.86080635656507E-06	2	ITGB2
4.09814100817855E-11	0.588821030089858	0.289	0.148	6.86766470150562E-07	2	BCL6
9.14211584872429E-17	0.58822912627074	0.874	0.839	1.53203577392922E-12	2	ACTB
5.88906002119525E-08	0.544905864371529	0.679	0.642	0.0009868886783519	2	PFN1
7.46141736059677E-13	0.518585500142908	0.725	0.683	1.25038432128881E-08	2	HSPA6
8.66512041567702E-09	0.511270785817816	0.334	0.201	0.000145210087925916	2	GRASP
1.06670867695973E-31	0.501244946443066	0.991	0.987	1.78759040084911E-27	2	CD74
5.20406796826162E-09	0.355442962354786	0.84	0.832	8.72097710121282E-05	2	HSPA1A
1.69122443217242E-114	6.75287496561972	0.383	0.005	2.83415390343454E-110	3	TROAP
1.00660363373467E-111	6.50742919152023	0.383	0.005	1.68686636941256E-107	3	ASPM
4.99786273008065E-112	6.34063762913713	0.426	0.009	8.37541836306915E-108	3	KIF20A
2.27744967495408E-115	6.24881822789887	0.447	0.01	3.81655016528805E-111	3	PLK1
5.77910042781453E-127	5.79755426141556	0.681	0.038	9.68461649693159E-123	3	MKI67
1.52688615141311E-130	5.77830961467006	0.436	0.005	2.55875581253809E-126	3	HJURP
2.12268685958317E-111	5.64526207542016	0.404	0.007	3.55719863928948E-107	3	KIF18B
1.69421246621312E-109	5.57039352664034	0.436	0.011	2.83916125087995E-105	3	GTSE1
1.06838309980543E-102	5.56994487637141	0.309	0.002	1.79039639865394E-98	3	TTK
2.2978616011028E-96	5.46939266116264	0.34	0.005	3.85075647112807E-92	3	SPC25
7.64988084623167E-77	5.43639257180784	0.298	0.007	1.2819670322115E-72	3	FAM83D
1.72193019388615E-81	5.41646110167235	0.298	0.005	2.88561061891441E-77	3	PKMYT1
4.73998170302632E-141	5.38676252901897	0.574	0.016	7.94326133793151E-137	3	NCAPG
6.16498731149211E-114	5.29600263508878	0.479	0.014	1.03312857365985E-109	3	KIF2C
1.61226376418481E-97	5.26193091936855	0.457	0.018	2.7018316160209E-93	3	BIRC5
5.61793447745293E-87	5.2592755898544	0.34	0.008	9.41453459731563E-83	3	CDC20
4.4313342978512E-101	5.24607882270437	0.362	0.006	7.42603001633904E-97	3	KIF14
3.18185439190457E-122	5.17488345913167	0.521	0.016	5.33215158995367E-118	3	IQGAP3
5.59680304083276E-94	5.16389532042208	0.319	0.004	9.37912253582753E-90	3	BUB1B
2.18552971369239E-70	5.11808864075673	0.266	0.005	3.6625106942057E-66	3	OIP5
4.0144067039315E-124	5.11511127385189	0.511	0.014	6.7273427544484E-120	3	KIF23
8.06883165228424E-61	4.98265514336553	0.702	0.123	1.35217480828979E-56	3	HIST1H1B
1.79208739945721E-79	4.97230121167392	0.372	0.014	3.00318006401039E-75	3	CCNB1

2.30131288609573E-134	4.96067165525156	0.479	0.008	3.85654013451923E-130	3	AURKB
3.15549671862353E-100	4.94948265371306	0.479	0.019	5.28798140106931E-96	3	KIF11
8.17112883141048E-85	4.94402371947914	0.34	0.008	1.36931776956777E-80	3	CDCA3
5.57800625217166E-81	4.94356938416458	0.362	0.012	9.34762287738926E-77	3	CCNF
3.30674733100444E-91	4.89306341360759	0.543	0.034	5.54144717729725E-87	3	RRM2
7.93350537514507E-56	4.8701039392177	0.255	0.009	1.32949683076681E-51	3	HIST1H2BE
2.29241824184319E-105	4.83359234626727	0.511	0.021	3.84163448968081E-101	3	ANLN
4.81440637165018E-79	4.78012699436422	0.298	0.006	8.06798219761136E-75	3	E2F8
6.11239345451793E-124	4.77200617480092	0.543	0.018	1.02431489510812E-119	3	BUB1
4.1108753214567E-103	4.75252686658398	0.585	0.034	6.88900486369714E-99	3	CENPM
2.98002106802553E-110	4.71888959773895	0.553	0.025	4.99391930579719E-106	3	TPX2
9.67205183089954E-85	4.71776866514639	0.585	0.049	1.62084244582215E-80	3	CDK1
8.77536584783194E-65	4.69822984390142	0.255	0.006	1.47057580877968E-60	3	NEURL1B
8.70126336501213E-73	4.60832063916566	0.351	0.014	1.45815771470873E-68	3	CCNB2
3.29704998250029E-82	4.5862058588243	0.309	0.006	5.52519636067399E-78	3	CKAP2L
2.80020866645814E-88	4.57586420146442	0.479	0.025	4.69258968325054E-84	3	HIST1H2BH
2.9752357563851E-95	4.55452977014921	0.479	0.021	4.98590008055015E-91	3	MYBL2
1.02401464824891E-76	4.54206519291638	0.532	0.042	1.71604374753553E-72	3	TOP2A
8.6999684491534E-124	4.5219023823394	0.511	0.014	1.45794071270913E-119	3	ESPL1
6.1089652083603E-123	4.51130064310358	0.574	0.022	1.02374038961702E-118	3	KIFC1
2.15298571076632E-96	4.50707724676555	0.596	0.04	3.6079734541022E-92	3	CENPF
5.21558683471738E-64	4.50516966848886	0.298	0.011	8.74028041761939E-60	3	DLGAP5
4.28548284831515E-80	4.47879990495437	0.468	0.027	7.18161215720653E-76	3	UBE2C
2.76113770433638E-63	4.44820365043788	0.415	0.03	4.62711456492691E-59	3	HIST1H2AH
7.38324915916691E-87	4.40842783002111	0.468	0.024	1.23728489409319E-82	3	MELK
3.69205675856763E-102	4.32268459977957	0.468	0.016	6.18714871600764E-98	3	CENPI
3.69184519143702E-61	4.3136695138393	0.351	0.019	6.18679417181016E-57	3	ARHGAP11
1.51568421826244E-99	4.28350367121118	0.372	0.007	2.53998361296419E-95	3	KNL1
2.57044977102285E-99	4.27886297349018	0.511	0.024	4.3075597262801E-95	3	CEP55
1.30947697616324E-92	4.27481801248472	0.468	0.021	2.19442151665436E-88	3	NDC80
6.44506443416617E-66	4.26585950132633	0.266	0.007	1.08006389787757E-61	3	RAD54L
2.60485134792376E-51	4.24829182365722	0.266	0.012	4.36520988885064E-47	3	RAD51
1.90428273760387E-81	4.20453541583264	0.319	0.007	3.19119701167656E-77	3	CDCA5
1.54594664229295E-100	4.19798391209557	0.489	0.02	2.59069738315453E-96	3	CIT
8.13759254998617E-32	4.16396896641668	0.33	0.042	1.36369775952668E-27	3	IDO1
1.14819694288984E-88	4.15498974431395	0.617	0.051	1.9241484368948E-84	3	NUSAP1
3.41576767450066E-67	4.11554902812551	0.33	0.013	5.72414346892821E-63	3	POLQ
5.86867378780213E-63	4.10154911356208	0.319	0.014	9.83472353359881E-59	3	AURKA
5.4656405684003E-91	4.07252892189036	0.372	0.01	9.15932046452522E-87	3	CDCA8
1.15892462790629E-33	4.01537491783687	0.298	0.032	1.94212589144536E-29	3	FLT3
2.32205025489628E-51	3.97466856992519	0.489	0.061	3.89129181715519E-47	3	TYMS
2.76643232894886E-68	3.97363222732619	0.298	0.009	4.6359872968525E-64	3	DEPDC1B
1.72906730231214E-55	3.96800780690781	0.298	0.015	2.89757098521469E-51	3	CLSPN
1.36540235073103E-53	3.92793431338644	0.255	0.01	2.28814125935507E-49	3	DIAPH3
1.09905061008355E-73	3.92592379444986	0.617	0.066	1.84178901237801E-69	3	TK1
4.65801361451814E-76	3.92162839215143	0.277	0.005	7.8058992152095E-72	3	TICRR
5.67835946508625E-78	3.80405978425942	0.436	0.023	9.51579479159154E-74	3	SPC24
2.42578113130887E-71	3.77671489138913	0.319	0.01	4.06512401984741E-67	3	CDCA2
2.86985652693344E-82	3.75927756776178	0.426	0.019	4.80930556783506E-78	3	NCAPH
1.36908661787645E-67	3.7006328813377	0.415	0.026	2.29431535423736E-63	3	FOXM1

5.29475124029473E-75	3.69891321357304	0.34	0.012	8.87294412848591E-71	3	KIF15
4.02292334987926E-43	3.65339946733678	0.255	0.015	6.74161494972766E-39	3	GINS4
1.36750816585154E-35	3.6499285774833	0.255	0.02	2.29167018433401E-31	3	MAD2L1
1.1498375159264E-71	3.63694404978957	0.585	0.059	1.92689770918946E-67	3	H2AFX
6.36616766314657E-81	3.63439064705682	0.489	0.03	1.0668423769901E-76	3	SHCBP1
2.38430257329007E-46	3.63171399227954	0.521	0.076	3.9956142523195E-42	3	HIST1H1D
2.78947856947406E-57	3.62925995954007	0.277	0.011	4.67460818672462E-53	3	SGO2
5.38868674282723E-50	3.62133320271553	0.372	0.031	9.03036124362988E-46	3	CCNA2
1.82704185400294E-67	3.61208186019414	0.543	0.053	3.06175673893813E-63	3	UHRF1
1.07706848095794E-13	3.59767746195767	0.277	0.07	1.80495136038932E-09	3	RAB7B
1.07969779116763E-65	3.58521800206973	0.596	0.07	1.80935755843871E-61	3	KIF22
2.18786313508505E-41	3.53854569917231	0.319	0.028	3.66642104177552E-37	3	BARD1
7.61991765105541E-67	3.52614789674165	0.351	0.016	1.27694579996387E-62	3	CENPA
4.75821303537571E-64	3.51103757687522	0.447	0.034	7.97381340468262E-60	3	FANCI
3.4572615241176E-59	3.51080152421658	0.649	0.101	5.79367886211628E-55	3	NCAPD2
2.59593532923217E-48	3.46099588300958	0.287	0.017	4.35026842472726E-44	3	GPSM2
6.79632493689777E-65	3.43565007306108	0.436	0.032	1.13892813292533E-60	3	PRC1
4.80510169603488E-61	3.43324368220097	0.5	0.048	8.05238942221526E-57	3	FANCA
7.57273901398215E-43	3.41929373007399	0.362	0.035	1.26903960396313E-38	3	AC091057.
2.8921679499852E-52	3.40242137940708	0.266	0.012	4.84669505058519E-48	3	RACGAP1
3.32722845986028E-43	3.37532779689431	0.372	0.038	5.57576945303386E-39	3	CENPE
7.62380667612933E-51	3.33513794571037	0.394	0.035	1.27759752278575E-46	3	PTTG1
4.68335083641185E-41	3.33027644880578	0.5	0.081	7.84835933165899E-37	3	HELLS
3.2024010258941E-59	3.32354892815182	0.436	0.036	5.36658363919334E-55	3	ASF1B
2.95000790430905E-35	3.26669051192959	0.468	0.081	4.94362324604111E-31	3	HIST1H1E
7.08576253508248E-51	3.24723899055541	0.532	0.073	1.18743208562912E-46	3	TCF19
1.24342085209228E-38	3.24420477567559	0.362	0.041	2.08372466393625E-34	3	ZNF367
2.7326214470124E-22	3.19639974721751	0.33	0.06	4.57932702090337E-18	3	ZNF366
8.64119022414387E-34	3.18333684410592	0.319	0.036	1.44809065776203E-29	3	DTL
1.31324446471006E-55	3.17996316391815	0.34	0.021	2.20073507396111E-51	3	STIL
1.98969755181945E-52	3.1703715555027	0.404	0.035	3.33433515733904E-48	3	CDT1
2.26734911604827E-23	3.15304840617877	0.638	0.238	3.79962364867369E-19	3	HIST1H1C
2.06260068906248E-49	3.15078100007366	0.351	0.027	3.4565062347309E-45	3	NUF2
4.13818870856752E-22	3.14072373940655	0.266	0.039	6.93477663781746E-18	3	RMI2
6.16120991795634E-27	3.12542111215401	0.277	0.034	1.03249555805112E-22	3	SAC3D1
3.34968578512366E-61	3.09009621924034	0.766	0.144	5.61340343871022E-57	3	LMNB1
2.59844933005573E-31	3.0742040522215	0.287	0.031	4.3544813873074E-27	3	CENPH
1.02065204455315E-18	3.05580044739737	0.415	0.109	1.71040869626217E-14	3	MARCKSL1
1.71628306006535E-17	3.05022296298818	0.702	0.309	2.87614715205752E-13	3	WDFY4
7.35185887522909E-50	3.04964931557534	0.426	0.043	1.23202451031089E-45	3	HMMR
1.43021371065384E-40	3.04694295714758	0.436	0.059	2.3967521363137E-36	3	STMN1
1.37149122091691E-30	3.03452502959466	0.298	0.035	2.29834498801256E-26	3	SUV39H1
3.6430826473676E-45	3.03411242152059	0.266	0.015	6.10507790045862E-41	3	MND1
6.7324464122808E-63	2.98288041723304	0.33	0.015	1.12822336977002E-58	3	BRIP1
4.86982915972263E-46	2.97455332335687	0.574	0.095	8.16085970586319E-42	3	INCENP
1.30816608649607E-33	2.95477290487802	0.479	0.091	2.19222472775011E-29	3	HIST1H4C
2.11235519207298E-38	2.94827707580149	0.319	0.03	3.5398848308759E-34	3	BRCA1
7.32416034765472E-62	2.93807120116866	0.628	0.084	1.22738279105998E-57	3	TACC3
1.50791684383819E-45	2.93327118329695	0.255	0.013	2.52696704690404E-41	3	EXO1
6.69592641657119E-13	2.92514446025367	0.266	0.065	1.122103348889E-08	3	PLEKHM3

7.33560455248491E-50	2.88773424074203	0.574	0.089	1.22930061090542E-45	3	NCAPG2
9.0551318631469E-54	2.88723282258754	0.33	0.02	1.51745899762616E-49	3	MXD3
1.94381477825745E-18	2.840965216901	0.287	0.054	3.25744480540383E-14	3	AUTS2
3.6213302176077E-45	2.83502879300271	0.5	0.072	6.06862517866698E-41	3	ZWINT
1.28057856011557E-35	2.83061728184685	0.479	0.082	2.14599355104167E-31	3	CENPU
8.39977912948674E-32	2.83038072132563	0.66	0.215	1.40763498651939E-27	3	TUBB
3.88971376340822E-36	2.81200616998078	0.723	0.218	6.51838232471949E-32	3	HMGB2
9.68504663475646E-29	2.76451648619575	0.287	0.034	1.62302011505249E-24	3	CENPL
1.1134619118405E-41	2.74401734521964	0.606	0.121	1.86593947186231E-37	3	CDKN2C
2.33065675157826E-11	2.71975621135062	0.319	0.098	3.90571458429484E-07	3	TRAF1
2.23475378570353E-08	2.70455108664975	0.34	0.132	0.000374500039408198	3	FSCN1
5.04689011088933E-36	2.70016299434735	0.383	0.049	8.45757844782834E-32	3	KNSTRN
1.85271000480873E-72	2.6916846411298	0.511	0.039	3.10477142605847E-68	3	SPAG5
3.3305810915909E-44	2.64973360502514	0.617	0.119	5.58138779328803E-40	3	PHF19
1.54535614222512E-34	2.64198668077047	0.457	0.076	2.58970782314086E-30	3	PCLAF
5.82507668170295E-36	2.62376602730305	0.394	0.053	9.7616635031978E-32	3	ORC6
4.77727135091859E-27	2.61611439254663	0.362	0.061	8.00575132986937E-23	3	FEN1
2.19305101193766E-30	2.59922665460794	0.468	0.088	3.67511488580514E-26	3	EZH2
6.44441382761157E-34	2.58153113867308	0.287	0.028	1.07995486923115E-29	3	FBXO5
1.24984511212415E-33	2.5758024051989	0.521	0.104	2.09449043889765E-29	3	CRIP1
4.90902053171211E-41	2.50804495564616	0.287	0.021	8.22653660704315E-37	3	RECQL4
1.02719312613925E-46	2.50005768069831	0.553	0.084	1.72137024078416E-42	3	DTYMK
1.87340855865564E-42	2.48343859166529	0.638	0.13	3.13945806259512E-38	3	LMNB2
6.65133650829609E-08	2.47954524940636	0.309	0.117	0.00111463097206026	3	LY75
6.17979249389885E-20	2.47777874996683	0.277	0.047	1.03560962612757E-15	3	WDR76
4.04629671983863E-30	2.4734961928632	0.415	0.07	6.78078404310558E-26	3	SLC1A4
2.83161105105276E-32	2.46410102949238	0.33	0.041	4.74521379935422E-28	3	MCM8
7.03621224186982E-14	2.45888637055035	0.277	0.067	1.17912844749254E-09	3	ADAM19
1.28803539965349E-30	2.45398181817295	0.319	0.039	2.15848972273932E-26	3	GINS1
9.30595635314755E-47	2.43564842384938	0.511	0.069	1.55949216566047E-42	3	CHAF1A
3.1051467124103E-35	2.43450819616603	0.404	0.057	5.20360486065718E-31	3	PRR11
4.82902901586842E-44	2.43131098731896	0.34	0.029	8.09248682479229E-40	3	CENPO
4.65168026720033E-21	2.42862043559648	0.287	0.049	7.79528579177432E-17	3	LIN54
2.94487228185294E-26	2.42846832197739	0.309	0.044	4.93501696992916E-22	3	E2F1
8.27471751891372E-36	2.42284696988227	0.394	0.053	1.38667716181956E-31	3	KIF20B
6.43401172354631E-28	2.42220695178904	0.351	0.053	1.07821168463189E-23	3	RCCD1
5.49496828146791E-19	2.40269442544807	0.287	0.052	9.20846784608393E-15	3	OXCT1
2.6965451365795E-32	2.38259892205044	0.351	0.045	4.51887033987992E-28	3	UBE2T
1.46629476954848E-28	2.3650424897984	0.309	0.039	2.45721677480935E-24	3	TEDC1
3.24798584332965E-28	2.34709688886162	0.553	0.136	5.44297467625183E-24	3	MCM4
1.30360017324154E-47	2.33359218985666	0.362	0.03	2.18457317031817E-43	3	ATAD5
6.02249178722792E-25	2.33304511238958	0.277	0.037	1.00924917370365E-20	3	ZWILCH
3.99229162669937E-29	2.33292877561972	0.372	0.059	6.69028230802281E-25	3	CKAP2
3.4612787928216E-23	2.32040725042008	0.309	0.05	5.80041100101044E-19	3	TRERF1
2.56036944308074E-29	2.30403822911126	0.532	0.125	4.2906671127147E-25	3	RRM1
1.94135339842044E-08	2.29406761319001	0.426	0.183	0.000325332002507298	3	BIRC3
4.22465022409781E-28	2.28663862798684	0.532	0.129	7.07966884554311E-24	3	CKS1B
3.47140373133869E-32	2.2573103290703	0.33	0.039	5.81737837297737E-28	3	ARHGAP19
1.33476374079424E-36	2.23741025267672	0.447	0.068	2.23679707682298E-32	3	PCNA
3.87154865807303E-40	2.23558679459315	0.415	0.052	6.48794124119879E-36	3	ECT2

2.00654185668177E-24	2.20595747963727	0.266	0.035	3.36256284342731E-20	3	BORA
9.08197280421006E-19	2.19775681388466	0.383	0.093	1.52195700252952E-14	3	TUBB4B
2.84943461866864E-28	2.19705471548919	0.426	0.079	4.77508253396491E-24	3	RFWD3
1.18730177094746E-24	2.19384523975614	0.309	0.045	1.98968030775376E-20	3	WEE1
4.49525642989477E-23	2.19193144790699	0.309	0.051	7.53315072521766E-19	3	UBE2S
2.86059679216129E-37	2.19120887582212	0.702	0.181	4.7937881043039E-33	3	H2AFZ
6.26135777296671E-22	2.18334742373005	0.255	0.036	1.04927833559376E-17	3	ZGRF1
2.88143836143778E-23	2.17844299775421	0.34	0.061	4.82871440609742E-19	3	HIRIP3
2.32607129010051E-21	2.16090875420662	0.255	0.036	3.89803026795044E-17	3	NRM
1.43077529135327E-29	2.15135203016762	0.34	0.047	2.39769323324982E-25	3	NSD2
1.9720896323311E-26	2.1341529143985	0.34	0.053	3.30482780586046E-22	3	NRGN
1.73317110046686E-23	2.13316123700644	0.649	0.238	2.90444813016236E-19	3	TMEM106C
6.32059498173565E-22	2.13116032792521	0.436	0.107	1.05920530703926E-17	3	MCM2
1.57541203549323E-30	2.11702952876709	0.255	0.024	2.64007548907956E-26	3	SKA3
9.11530593907739E-10	2.11499010711989	0.255	0.073	1.52754296927059E-05	3	ST7
3.5430190833143E-14	2.10585424241621	0.436	0.138	5.9373913798181E-10	3	KIF2A
5.67473436385949E-16	2.08788306954165	0.309	0.069	9.50971984695573E-12	3	PLP2
8.73920850792624E-12	2.07951314243256	0.404	0.137	1.46451656175828E-07	3	NIBAN1
6.2916874579003E-16	2.07818639566455	0.415	0.121	1.05436098419493E-11	3	NAV1
2.67239091066468E-07	2.0678363182056	0.394	0.186	0.00447839268809186	3	CPNE3
2.76953230197055E-23	2.06476593467632	0.564	0.17	4.64118223164224E-19	3	MCM7
6.84841612257986E-32	2.06034499622162	0.447	0.076	1.14765757382193E-27	3	CENPN
1.97616686405527E-24	2.04457148589248	0.532	0.146	3.31166043078382E-20	3	TUBA1B
5.59137430986204E-23	2.04275258165308	0.426	0.092	9.3700250684668E-19	3	MASTL
1.6054313273601E-42	2.03901788337255	0.574	0.096	2.69038181839005E-38	3	NUP210
1.50753361900609E-06	2.03871604785502	0.436	0.207	0.0252632483873041	3	KDM2B
2.01996844478822E-13	2.0382495374408	0.287	0.071	3.3850631197761E-09	3	CCNE2
2.1737288509975E-28	2.03780000429045	0.372	0.059	3.64273480850162E-24	3	CDK2
1.28346760337592E-33	2.02623750345746	0.702	0.199	2.15083500973736E-29	3	TMPO
2.28312292860793E-25	2.0184827371178	0.309	0.044	3.82605740376118E-21	3	GGH
3.39867688273824E-23	2.0124645000147	0.309	0.049	5.69550272009274E-19	3	GEN1
4.70500520408516E-25	1.99843349721367	0.362	0.062	7.88464772100591E-21	3	VRK1
4.24692993062733E-21	1.99321465177142	0.309	0.055	7.11700517774528E-17	3	RBL1
7.911141762742E-22	1.99010036794787	0.489	0.133	1.3257491366003E-17	3	ATAD2
1.9880801906613E-21	1.98863866709337	0.394	0.084	3.3316247835102E-17	3	SNRNP25
3.92346479534529E-25	1.9808949125542	0.319	0.049	6.57494230403964E-21	3	NETO2
1.59216226827705E-08	1.98056561432652	0.309	0.115	0.000266814552917867	3	SLAMF7
2.05667967050051E-06	1.96001173244028	0.33	0.15	0.0344658379182475	3	DAPP1
1.43282621948967E-09	1.94890379731419	0.309	0.106	2.40113017862078E-05	3	NBEAL2
1.18428921174636E-25	1.94829883883708	0.266	0.033	1.98463186104456E-21	3	FANCG
1.18933631533365E-20	1.93512196239502	0.5	0.137	1.99308979723612E-16	3	BRI3BP
1.02495419669822E-34	1.91374240292186	0.34	0.039	1.71761824282687E-30	3	CCDC18
1.84018832726225E-27	1.89402425385565	0.298	0.038	3.08378759882608E-23	3	RFC3
6.20914910227403E-10	1.88761432863956	0.298	0.094	1.04052920655908E-05	3	FCHO1
1.25616322312032E-17	1.88676465151783	0.553	0.186	2.10507832930503E-13	3	NDE1
3.39119174141497E-13	1.88260027240802	0.489	0.18	5.68295912026321E-09	3	NLRC5
1.41668522385746E-13	1.8636427269446	0.309	0.079	2.37408109814034E-09	3	TOMM34
4.50577108341335E-14	1.86012673893484	0.309	0.077	7.55077118158409E-10	3	RAB27A
2.72487634295019E-13	1.85787243479584	0.394	0.127	4.56634777551593E-09	3	JPT2
6.4206109792713E-10	1.8522463439397	0.287	0.093	1.07596598790628E-05	3	CCSER1

**7.62934547668668E-29**	1.85144148479276	0.585	0.156	1.27852571498315E-24	3	SMC4
**3.75840028528234E-16**	1.84791825870721	0.298	0.065	6.29832719807615E-12	3	POLH
**7.6548709977584E-13**	1.83819502778577	0.351	0.103	1.28280328180435E-08	3	PRMT1
**2.97088073536526E-11**	1.83609250161182	0.277	0.076	4.97860193632511E-07	3	PTPN22
**4.35982591759099E-18**	1.83296244219605	0.319	0.065	7.30619627269897E-14	3	CENPX
**3.85012367994209E-29**	1.82622019355682	0.287	0.033	6.45203726284695E-25	3	CHTF18
**8.43003483244748E-17**	1.82320859452349	0.394	0.106	1.41270523722155E-12	3	DHFR
**7.66818463882193E-21**	1.82286495340302	0.362	0.073	1.28503438177378E-16	3	C19orf48
**3.98411326481802E-11**	1.82131437956588	0.287	0.082	6.67657700918203E-07	3	PMM1
**1.71087489316155E-22**	1.82002446725992	0.33	0.058	2.86708414596013E-18	3	RCC1
**1.52214849516894E-31**	1.81421225684172	0.415	0.065	2.5508164482041E-27	3	TIMELESS
**4.27174962577223E-25**	1.80341244417412	0.564	0.153	7.15859802286911E-21	3	RANGAP1
**5.38061557576435E-08**	1.80161503911284	0.298	0.107	0.00090168355818659	3	TMEM39A
**1.10460636978483E-07**	1.79892054121468	0.298	0.112	0.00185109935448542	3	DENND1B
**4.96024326613927E-11**	1.79736160879357	0.309	0.093	8.3123756653962E-07	3	ADAM8
**1.47438748853588E-22**	1.79423535738652	0.734	0.3	2.47077855328843E-18	3	SMC1A
**6.15467284958763E-13**	1.79050118551318	0.287	0.073	1.0314000761339E-08	3	KATNA1
**8.54344584269427E-19**	1.78489858580313	0.404	0.102	1.43171065431871E-14	3	ACOT7
**2.36081476700083E-24**	1.78407482492637	0.436	0.095	3.95625338654E-20	3	KNTC1
**2.4962093110736E-06**	1.78357264804366	0.255	0.098	0.0418314756349714	3	C11orf24
**5.88526782580457E-19**	1.76572318668616	0.266	0.044	9.8625318224833E-15	3	LRR1
**4.24831740308418E-13**	1.75185378004491	0.298	0.075	7.11933030408847E-09	3	ESS2
**1.17457745979751E-16**	1.73280220116472	0.319	0.072	1.96835690712866E-12	3	FZD2
**2.49130372126284E-23**	1.72728243191929	0.457	0.103	4.17492677609227E-19	3	MPHOSPH9
**2.09808579618082E-19**	1.72431549608337	0.319	0.062	3.51597217723981E-15	3	TUBB6
**9.27243830431088E-18**	1.7187191433776	0.277	0.05	1.55387521103642E-13	3	FAM72C
**1.95855808170616E-07**	1.70780239706273	0.277	0.103	0.00328215163332318	3	ST3GAL2
**2.22892955920923E-30**	1.70709726003455	0.447	0.079	3.73524015532283E-26	3	SMC2
**6.47408639282222E-16**	1.701707264338	0.543	0.203	1.08492739770915E-11	3	ARL6IP1
**1.41288841146755E-15**	1.69888268652497	0.255	0.049	2.36771839993731E-11	3	NEDD1
**4.96147799371294E-20**	1.69464053037615	0.617	0.221	8.31444482186414E-16	3	SUN2
**4.38260065999042E-13**	1.68102507364588	0.362	0.109	7.34436218601195E-09	3	PSMG3
**3.11062372239706E-09**	1.67493900823934	0.468	0.205	5.21278323399299E-05	3	VAC14
**1.65828439026053E-09**	1.67004891331413	0.415	0.17	2.7789529811986E-05	3	BCL6
**1.45388844941901E-14**	1.6689770047229	0.319	0.079	2.43642626353638E-10	3	INIP
**1.97958429295574E-17**	1.6637657035236	0.457	0.139	3.31738735813523E-13	3	CDC25B
**1.76728149792871E-24**	1.6626787227017	0.479	0.109	2.96161033422893E-20	3	RNF26
**1.47560529433766E-06**	1.66140895380377	0.287	0.116	0.0247281935225104	3	SRF
**1.47049613477616E-10**	1.65771138899249	0.606	0.271	2.46425742265789E-06	3	NUB1
**2.36709380910887E-14**	1.65533226760631	0.33	0.084	3.96677580530464E-10	3	DONSON
**1.15358917626399E-13**	1.65503724342776	0.351	0.098	1.9331847415832E-09	3	CST7
**2.88132219317285E-12**	1.64891641876594	0.372	0.121	4.82851973131906E-08	3	MCM6
**5.6508852584266E-13**	1.64527526219625	0.5	0.198	9.4697535160713E-09	3	MCM3
**5.78452397403174E-11**	1.64270908557796	0.255	0.067	9.69370527568238E-07	3	SUSD3
**5.37784876492335E-09**	1.63846412124216	0.309	0.102	9.01219896025855E-05	3	BCOR
**4.08566152841084E-19**	1.63074838400308	0.84	0.399	6.84675158931089E-15	3	TAP1
**9.33776848681495E-34**	1.6307270902901	0.457	0.074	1.56482324302045E-29	3	LIG1
**8.73907484935424E-16**	1.62935925589159	0.521	0.187	1.46449416325478E-11	3	TUBA1C
**1.96420200668535E-08**	1.61098416755325	0.266	0.085	0.00032916097228033	3	BZW1
**1.23614320235985E-14**	1.60233707328997	0.309	0.076	2.07152877851464E-10	3	POLE

2.84322029557441E-22	1.59986109752039	0.457	0.109	4.76466857132359E-18	3	TTF2
2.6374264312483E-07	1.59957673114427	0.298	0.115	0.0044197992134859	3	EBNA1BP2
2.10244722757592E-06	1.59760728923573	0.298	0.119	0.0352328106397172	3	DAAM1
6.18699934961907E-08	1.59552974475482	0.489	0.236	0.00103681735100916	3	SHTN1
9.39899399938528E-15	1.59365191933699	0.574	0.207	1.57508341441698E-10	3	ID2
1.52710418598987E-12	1.59292984117256	0.447	0.153	2.55912119488183E-08	3	SLC25A28
5.37080852314656E-19	1.591766194661	0.745	0.337	9.00040092308901E-15	3	H1FX
4.35147801151801E-15	1.5908235177685	0.457	0.142	7.29220685170188E-11	3	PALM2-AK/
3.34461906005893E-14	1.59078299613718	0.298	0.071	5.60491262084675E-10	3	MZT2A
8.62268475314678E-09	1.58716511317037	0.415	0.173	0.000144498951093234	3	SPAG7
2.08267372732427E-08	1.5864030547287	0.298	0.104	0.000349014463225001	3	CEP68
2.65420982122935E-18	1.58468525716564	0.489	0.14	4.44792481841614E-14	3	KMT5A
1.02416113481947E-13	1.58229134266371	0.34	0.094	1.71628922973047E-09	3	HLTF
3.91312690201691E-14	1.58183664279722	0.33	0.087	6.55761806239994E-10	3	JPT1
5.73885806648145E-22	1.57476736904164	0.585	0.173	9.61717834780961E-18	3	TP53
1.47277153554711E-08	1.57227760303105	0.33	0.121	0.000246807053926985	3	DVL2
1.18060723851433E-12	1.5722713403639	0.84	0.513	1.97846161030231E-08	3	CPVL
7.3348436948333E-24	1.57204470647724	0.755	0.286	1.22917310638016E-19	3	CORO1A
4.41047641754942E-11	1.57101286970154	0.309	0.093	7.39107638052931E-07	3	ALYREF
7.17509852054459E-18	1.56650314785877	0.404	0.104	1.20240301007286E-13	3	USP1
2.56070268691276E-06	1.55917196499218	0.362	0.162	0.0429122556272841	3	BID
5.37364455186646E-25	1.55871990569598	0.5	0.119	9.00515354001781E-21	3	NCAPD3
1.69211426697243E-13	1.55665376327524	0.255	0.055	2.8356450885924E-09	3	PXMP4
1.68661385254447E-12	1.55432686129339	0.372	0.112	2.82642749409403E-08	3	CNTLN
1.12364235040176E-16	1.54884321208629	0.298	0.062	1.88299985080327E-12	3	SPINDOC
8.93994199614479E-12	1.542580231235	0.319	0.092	1.49815547971394E-07	3	PSMB3
2.69013568967504E-22	1.53621479837938	0.734	0.306	4.50812938875743E-18	3	DNMT1
1.18805131694914E-19	1.53242478865356	0.372	0.08	1.99093639694336E-15	3	ACTL6A
2.07375348864142E-06	1.51855252154581	0.298	0.128	0.0347519609626528	3	LGALS2
1.95342926047696E-16	1.51791559135238	0.426	0.12	3.27355675470729E-12	3	ANP32E
1.01941419404767E-11	1.51571242249279	0.351	0.11	1.70833430638509E-07	3	SFXN1
1.3407576785733E-09	1.51079244678429	0.266	0.079	2.24684171775313E-05	3	ELP6
2.58027564259523E-17	1.5102455568459	0.883	0.531	4.32402592186108E-13	3	LSP1
6.1922363050026E-10	1.49957549690663	0.33	0.11	1.03769495999234E-05	3	TOPBP1
1.41024573356648E-13	1.49543396577156	0.511	0.186	2.36328980031072E-09	3	CBL
8.83668075275902E-09	1.49426903006126	0.415	0.174	0.000148085096054736	3	C1orf54
1.93292949977122E-19	1.49226982737063	0.298	0.053	3.23920325571661E-15	3	PRIM1
7.46860019252043E-07	1.48865576617241	0.564	0.318	0.0125158802026257	3	ANPEP
1.52402210304514E-13	1.48791468653549	0.394	0.121	2.55395624028304E-09	3	CD52
2.35692619038391E-14	1.48708482453868	0.287	0.065	3.94973690984535E-10	3	CEP85
1.22181855567304E-07	1.48550134036938	0.426	0.192	0.00204752353559688	3	MTPN
1.35942447832823E-15	1.48505420838593	0.351	0.087	2.27812354078245E-11	3	MZT1
2.28671547212308E-10	1.48484752287363	0.319	0.103	3.83207778818386E-06	3	PLPP1
1.07644079728522E-11	1.48358516107138	0.309	0.086	1.80389948809057E-07	3	ACD
3.04680038590754E-25	1.46856559563402	0.309	0.045	5.10582808670385E-21	3	CENPW
2.32107033601186E-26	1.46280569262581	0.479	0.102	3.88964966908867E-22	3	IMPA2
2.61343716862351E-13	1.46079178396953	0.489	0.189	4.37959800717929E-09	3	CKAP5
3.56639815141517E-13	1.45637349380837	0.447	0.154	5.97657002214154E-09	3	PARP1
1.02484750878148E-08	1.45597438713066	0.319	0.113	0.000171743945521601	3	SYPL1
4.3588477453519E-14	1.45113917649495	0.553	0.206	7.30455705166071E-10	3	PPM1G

**7.9568433579127E-07**	1.4498589085965	0.277	0.109	0.0133340780991901	3	CD1C
**1.87600753933065E-21**	1.44124775164992	0.479	0.124	3.1438134344103E-17	3	POLD1
**4.60772476656465E-09**	1.43942872412163	0.266	0.08	7.72162516380904E-05	3	POLR2D
**4.0342874833571E-09**	1.43922608775395	0.255	0.078	6.76065896460983E-05	3	FLVCR1
**3.70110915807534E-15**	1.43862350289337	0.426	0.134	6.20231872710266E-11	3	KPNA2
**5.31451986568171E-15**	1.43392636816141	0.298	0.069	8.90607239090941E-11	3	NDC1
**1.09626087814622E-14**	1.43084310432228	0.596	0.227	1.83711397959744E-10	3	DCP2
**1.45252753283785E-18**	1.42944417420475	0.798	0.4	2.43414563952966E-14	3	IDH2
**5.85587473137606E-13**	1.42808590228047	0.298	0.076	9.81327487484E-09	3	GMNN
**1.2468479733012E-12**	1.42695492332725	0.319	0.089	2.08946783365815E-08	3	DSN1
**4.22441363657429E-09**	1.42459640418074	0.362	0.138	7.07927237217119E-05	3	PSMD8
**3.16784019438466E-14**	1.42440524031444	0.755	0.343	5.30866659774981E-10	3	SERPINB9
**1.37783480741299E-18**	1.42337221422574	0.372	0.086	2.30897557026269E-14	3	POLA2
**1.28920880840274E-09**	1.41970904132403	0.309	0.101	2.16045612112132E-05	3	MCOLN2
**2.65213135555391E-11**	1.4172380599716	0.34	0.105	4.44444172563724E-07	3	HMCES
**2.16841528260177E-13**	1.41670424491073	0.521	0.205	3.63383033058405E-09	3	SSRP1
**1.03401874387386E-17**	1.4161103766192	0.287	0.055	1.73280861098382E-13	3	ODF2
**8.77012128308825E-08**	1.4105099640962	0.532	0.256	0.00146969692461993	3	RAB8B
**1.16113198208115E-10**	1.40782423176284	0.394	0.142	1.94582497557159E-06	3	ZNHIT1
**1.57608590230233E-12**	1.40679648290071	0.319	0.089	2.64120475507824E-08	3	MDC 1.00
**8.14318890192405E-19**	1.40557527200204	0.649	0.237	1.36463559618443E-14	3	LBR
**3.1888231327246E-08**	1.40533009272499	0.383	0.159	0.000534382980581989	3	ZBTB33
**3.31241933230302E-07**	1.40329766000887	0.277	0.099	0.00555095231707341	3	C5
**5.33390962422363E-14**	1.40022696906714	0.457	0.148	8.93856574827396E-10	3	SEPTIN6
**4.01615753073148E-18**	1.39695483231347	0.457	0.124	6.73027678999981E-14	3	PTPN7
**3.90036901433218E-08**	1.3911765867645	0.309	0.112	0.000653623839421788	3	BZW2
**8.13011116874423E-11**	1.39091325401066	0.479	0.196	1.36244402965816E-06	3	PRRC2A
**1.69142631495205E-06**	1.39075788141908	0.383	0.177	0.0283449221859665	3	ATP2B4
**1.04330024659946E-14**	1.39033330602538	0.468	0.153	1.74836255325137E-10	3	ATP5MF
**1.34266786928658E-09**	1.38996970971774	0.372	0.133	2.25004281535045E-05	3	PDE7A
**9.46183813903208E-15**	1.38632704883937	0.596	0.252	1.585614835339E-10	3	FAM111A
**2.56204560079225E-19**	1.38613877125299	0.766	0.32	4.29347601780765E-15	3	TAP2
**6.16799534031923E-12**	1.3837368168341	0.447	0.166	1.0336326591307E-07	3	SMC3
**3.45569670881047E-13**	1.38114243344546	0.266	0.062	5.79105654462458E-09	3	PAFAH1B3
**2.77578138432464E-11**	1.38090852458497	0.255	0.066	4.65165444385123E-07	3	EMC9
**1.04485209000354E-08**	1.37253187634491	0.33	0.116	0.000175096313242793	3	TTC9C
**7.26856255930773E-18**	1.37228461270673	0.713	0.306	1.21806571368879E-13	3	CSRP1
**5.24651125035347E-13**	1.36874578586525	0.266	0.062	8.79210355334235E-09	3	RNF8
**2.81567823756983E-08**	1.3684475147621	0.404	0.174	0.000471851359051952	3	ST3GAL5
**8.5705164374109E-15**	1.36453808022917	0.436	0.135	1.43624714458132E-10	3	ILF2
**1.54117087157878E-10**	1.3638902639441	0.67	0.342	2.58269414659172E-06	3	CYB5R3
**1.16034412607822E-14**	1.36377841270064	0.436	0.135	1.94450468648189E-10	3	CEP192
**2.85048096207626E-11**	1.36263709224926	0.362	0.117	4.77683599624739E-07	3	PRPSAP1
**2.13492425140582E-06**	1.36062782134164	0.277	0.109	0.0357770606050588	3	CREB3
**1.94625259003509E-09**	1.35691130080227	0.319	0.11	3.26153009038081E-05	3	PRPS2
**1.02687839057491E-08**	1.34614071070961	0.266	0.086	0.000172084280692543	3	CDCA7L
**1.83849792898228E-13**	1.34566175685399	0.436	0.149	3.08095482938851E-09	3	NUP205
**8.29823944481549E-14**	1.34167815963531	0.277	0.062	1.39061896616218E-09	3	ZDHHC12
**6.28308461754679E-16**	1.33663193575251	0.33	0.077	1.05291932020849E-11	3	C1orf112
**2.76448958135249E-13**	1.33568790237527	0.415	0.135	4.6327316404305E-09	3	TFDP1

3.73875825626334E-18	1.32885884850826	0.702	0.31	6.26541108584611E-14	3	DCK
3.42442360893979E-23	1.328347423003	0.5	0.122	5.73864908386131E-19	3	NEMP1
3.53318877564113E-10	1.32735202157149	0.394	0.152	5.9209177502194E-06	3	YWHAQ
2.52802558378104E-18	1.32721281841608	0.521	0.153	4.23646527330026E-14	3	DAXX
1.10715269540625E-08	1.32663970117946	0.266	0.085	0.000185536648696179	3	HINT2
1.72535387874327E-14	1.32638431229375	0.415	0.129	2.89134802999796E-10	3	SAE1
5.02392712739106E-11	1.32635482277619	0.33	0.101	8.41909708008194E-07	3	AIDA
1.72093079231776E-12	1.32502740451902	0.255	0.061	2.8839358217661E-08	3	LRRC45
8.94255724957741E-11	1.32389330347298	0.468	0.181	1.49859374388418E-06	3	MYADM
5.49937090191247E-08	1.32174049683958	0.404	0.172	0.000921584575742492	3	KAT2B
5.42524548444497E-10	1.31659557846659	0.383	0.136	9.09162638283289E-06	3	SRPK1
9.00545443855273E-08	1.31504757582349	0.585	0.334	0.00150913405481267	3	BASP1
3.74221937784832E-14	1.31444321176212	0.362	0.1	6.27121123339821E-10	3	TUBG1
2.49906328344748E-15	1.31331726484672	0.447	0.133	4.18793025040128E-11	3	ERAP2
1.09691521560484E-18	1.31093164049226	0.489	0.139	1.8382105183106E-14	3	RAVER1
2.23911243060122E-06	1.3041030794064	0.447	0.234	0.0375230461120152	3	RNF115
4.07565054413809E-15	1.30352317252634	0.426	0.123	6.82997518186661E-11	3	PSME2
3.72964411636157E-08	1.30084787312157	0.309	0.113	0.000625013761019872	3	SEH1L
1.74094904894719E-13	1.29984126105464	0.351	0.098	2.9174824162257E-09	3	NUP107
1.21276492052991E-13	1.2994180024147	0.436	0.143	2.03235145382403E-09	3	DIPK2A
6.4102495150174E-12	1.2992138977476	0.362	0.113	1.07422961372662E-07	3	GLIPR2
4.26256477560053E-27	1.29889986911324	0.372	0.06	7.14320605095136E-23	3	CENPJ
5.61700516497587E-10	1.29846756278645	0.362	0.125	9.41297725546656E-06	3	UPF2
4.44618812667471E-09	1.2980804299985	0.287	0.092	7.45092206268147E-05	3	NABP2
7.0706933408568E-11	1.2903457721985	0.5	0.203	1.18490679006078E-06	3	DAZAP1
1.59859966174463E-16	1.28150974765235	0.543	0.189	2.67893331315165E-12	3	RPA1
3.58740904557562E-11	1.28120682880467	0.426	0.162	6.01178007857563E-07	3	PIK3CG
1.88127268193483E-16	1.27739021531408	0.532	0.173	3.15263676038639E-12	3	DNTTIP1
1.46662508119556E-12	1.27728917386638	0.372	0.11	2.45777031106752E-08	3	CASP2
4.0308209127123E-07	1.27332506633933	0.287	0.11	0.00675484968552327	3	PHF6
1.68039442606573E-09	1.27259459625274	0.404	0.16	2.81600497920094E-05	3	HPRT1
1.145841696905E-08	1.27185490715662	0.309	0.104	0.00019202015156734	3	ZNF595
1.71596985567976E-09	1.26606207671995	0.351	0.123	2.87562228414814E-05	3	APEX2
6.09514450261776E-09	1.26141667013399	0.457	0.209	0.000102142431574868	3	ALOX5AP
1.1054998584171E-18	1.26041597205815	0.394	0.095	1.85259666273538E-14	3	CBX5
2.76905413528724E-10	1.2602203153697	0.266	0.075	4.64038091991436E-06	3	PLGRKT
2.30103606838808E-08	1.25762967325605	0.319	0.11	0.000385607624340474	3	CNIH1
8.39958111067311E-12	1.25231220873554	0.564	0.248	1.4076018025266E-07	3	CHCHD2
1.50636445432989E-06	1.2494211489507	0.255	0.095	0.0252436555256602	3	POLD2
9.89129755158991E-15	1.2475321988505	0.415	0.127	1.65758364369544E-10	3	PCNT
3.95825244265414E-14	1.24629975767585	0.266	0.056	6.63323944339982E-10	3	CDKN2A
3.54209199232639E-20	1.24577499716739	0.532	0.152	5.93583776074057E-16	3	RAD21
8.84426179435483E-11	1.24413986273589	0.415	0.149	1.48212139149798E-06	3	CCDC12
2.59777897116301E-16	1.24404544905966	0.266	0.05	4.35335799987497E-12	3	TSPAN6
9.32104693369265E-12	1.24249383734334	0.33	0.098	1.56202104514821E-07	3	TMEM160
2.43092569523297E-12	1.23859306115821	0.564	0.235	4.07374528007141E-08	3	PACS1
1.08713888315697E-14	1.23654460520843	0.553	0.218	1.82182734039444E-10	3	PA2G4
2.46364741064608E-09	1.23612851450455	0.266	0.08	4.1285803307607E-05	3	CBX1
1.85569486265063E-08	1.23363524472732	0.415	0.171	0.000310977345082992	3	SUZ12
1.57780745550387E-09	1.23167531587601	0.298	0.093	2.64408973393338E-05	3	NEPRO

8.23225680843817E-07	1.22635440782908	0.34	0.144	0.0137956159595807	3	THOC6
1.32779225698144E-22	1.22580143784219	0.574	0.164	2.22511426424949E-18	3	BUB3
8.70423054393864E-13	1.22508413862119	0.468	0.162	1.45865495455324E-08	3	FIBP
3.99811671869299E-24	1.22502093324696	0.989	0.84	6.70004399718572E-20	3	ACTB
3.94108467069514E-07	1.22420716649583	0.277	0.102	0.00660446969115092	3	ZNF700
1.26973296221676E-14	1.22386866632039	0.574	0.226	2.12781849808285E-10	3	MIS18BP1
1.01055072835485E-14	1.22228332705965	0.638	0.283	1.69348091057705E-10	3	MCM5
3.68764196894347E-08	1.22204831621414	0.287	0.098	0.000617975041155547	3	ISOC1
1.92073076728061E-13	1.22152115023387	0.553	0.208	3.21876061980885E-09	3	ACTR3
3.8142490555335E-10	1.21616885996529	0.415	0.155	6.39191856726304E-06	3	DUSP23
4.23123106797011E-09	1.2147468708924	0.404	0.158	7.09069702370432E-05	3	DNAJC15
7.24282881211769E-07	1.21410004081067	0.372	0.166	0.0121375325233468	3	HSDL2
5.78771825102622E-15	1.21338707147008	0.479	0.153	9.69905824506974E-11	3	PYGO2
2.48583858111355E-09	1.21299938838669	0.266	0.079	4.1657682942301E-05	3	CLECL1
5.62311009078248E-20	1.21236292125729	0.777	0.313	9.42320789013328E-16	3	CSK
5.73553355002308E-14	1.21075807750537	0.468	0.158	9.61160712312868E-10	3	RBMX
4.83455012996275E-14	1.21000046433061	0.543	0.192	8.10173910779157E-10	3	MDFIC
2.88268584041976E-15	1.20792761868335	0.734	0.359	4.83080493137543E-11	3	PKM
1.63376083496942E-09	1.20685891267333	0.277	0.084	2.73785640724175E-05	3	EED
5.25244711229541E-14	1.20402402048029	0.574	0.248	8.80205087078465E-10	3	PRPF19
6.30494353499713E-10	1.20353815922049	0.298	0.091	1.05658243759482E-05	3	CEP83
8.99160975339195E-07	1.20069667590833	0.287	0.108	0.0150681396247342	3	LAS1L
2.74548670314072E-09	1.19911034381494	0.489	0.214	4.60088661712322E-05	3	LRRK2
8.30653880188732E-15	1.19505214413527	0.372	0.105	1.39200977242028E-10	3	NUDT1
1.98237873406886E-12	1.19484915095504	0.5	0.193	3.3220702825526E-08	3	SUPT16H
5.87804844477925E-12	1.19427233883795	0.436	0.15	9.85043358376106E-08	3	TMEM11
1.90511721863089E-14	1.19419234960687	0.755	0.371	3.19259543498164E-10	3	GSTP1
1.22556165468254E-14	1.18838076890354	0.394	0.107	2.05379622091699E-10	3	TMEM97
8.05917371423749E-12	1.18836522936413	0.404	0.134	1.35055633103192E-07	3	CHD3
5.26022601497677E-08	1.18662504478518	0.372	0.147	0.000881508675589807	3	CNOT10
2.77967555898897E-09	1.18634044676273	0.319	0.107	4.65818030175372E-05	3	REPIN1
3.53180475105231E-11	1.1834012942294	0.447	0.167	5.91859840181346E-07	3	S100B
1.32951627609127E-14	1.17772774065236	0.372	0.1	2.22800337547375E-10	3	PSTPIP1
2.03819072398103E-08	1.17611441842107	0.415	0.172	0.000341560001524741	3	TRMT112
1.78864977934202E-08	1.17197678559577	0.351	0.13	0.000299741930022135	3	TRAPPC1
9.81140992522613E-17	1.17001939016702	0.585	0.22	1.6441960752694E-12	3	LSM 4.00
2.18585932000363E-07	1.16524703514449	0.319	0.124	0.00366306304846209	3	SEC61B
7.07855775625394E-17	1.16446343752042	0.436	0.122	1.18622470879303E-12	3	HNRNPR
6.68446629405563E-15	1.16286227736811	0.585	0.224	1.12018286155784E-10	3	ENSA
4.51965163887116E-14	1.1625302469365	0.543	0.201	7.5740322164203E-10	3	PAXX
4.61465968775794E-14	1.16150419798392	0.287	0.067	7.73324670474476E-10	3	DARS2
3.50558397181085E-22	1.16143996802017	0.574	0.167	5.87465761996063E-18	3	RBM15
1.66127274557468E-10	1.16004300610749	0.426	0.152	2.78396086703405E-06	3	QSER1
2.34184778063347E-10	1.15705186115324	0.33	0.109	3.92446851078557E-06	3	RFC2
2.28655169419498E-13	1.15391791919667	0.617	0.269	3.83180332913195E-09	3	RAC2
3.09552419310075E-13	1.15214680395019	0.415	0.131	5.18747944279824E-09	3	DCTN3
1.68732410354934E-12	1.15072910281075	0.372	0.111	2.82761773272798E-08	3	ALG5
8.57957403641805E-18	1.14881530513989	0.926	0.636	1.43776501702294E-13	3	PFN1
1.2958083780131E-07	1.14747685783651	0.351	0.137	0.00217151567987436	3	SOS 1
5.98185061264049E-16	1.14729700165504	0.287	0.06	1.00243852566629E-11	3	CEP128

8.45712405269794E-15	1.14308859547979	0.447	0.142	1.41724484875112E-10	3	ICMT
8.41124518773021E-11	1.14281835645461	0.415	0.156	1.40955646855983E-06	3	MZT2B
4.50225763049997E-13	1.14247183201171	0.564	0.219	7.54488333719186E-09	3	TXNDC12
3.27342216370819E-15	1.14214270280344	0.351	0.089	5.48560086194218E-11	3	THOP1
3.01836340009653E-09	1.13969428731183	0.319	0.11	5.05817338588177E-05	3	ERG28
5.79534304634384E-09	1.13349093490353	0.33	0.118	9.711835877063E-05	3	PSMD2
7.5881031664943E-12	1.13336944924867	0.319	0.095	1.27161432864111E-07	3	RPA2
3.6570222565283E-10	1.13265080331943	0.372	0.133	6.12843789749013E-06	3	TOR3A
4.41573084294361E-10	1.13135607929365	0.606	0.288	7.3998817466049E-06	3	SNX3
8.15725446869581E-09	1.12973024193726	0.394	0.156	0.000136699270386404	3	DENND5A
1.42575530293074E-16	1.12925223280969	0.628	0.241	2.38928073665134E-12	3	ITGAL
1.09145615874775E-08	1.12856151720074	0.255	0.081	0.000182906223082948	3	BATF3
2.23004596168595E-09	1.12831261512211	0.33	0.11	3.73711102259331E-05	3	ARPP19
2.07342513664825E-11	1.12697334141518	0.5	0.208	3.47464584399514E-07	3	PSMC3
2.47890252155026E-09	1.12675306934417	0.298	0.095	4.15414484561392E-05	3	PPP1CC
6.85095983509535E-10	1.12440266537549	0.606	0.279	1.14808384916528E-05	3	PLCG2
4.14580313637661E-10	1.12340645702378	0.255	0.07	6.94753689593993E-06	3	PFAS
1.1206964191825E-06	1.12007410060471	0.489	0.249	0.0187806305926604	3	SLC25A11
3.41601939757929E-11	1.11533883613143	0.298	0.082	5.72456530646337E-07	3	SMARCD1
3.22241049241888E-11	1.1147418273909	0.383	0.129	5.40011550319556E-07	3	SAMD1
9.18128841706923E-07	1.11126301395619	0.457	0.216	0.0153860031293246	3	HECTD1
7.85842410730252E-12	1.11124943667841	0.436	0.151	1.31691471190176E-07	3	HDAC9
4.50097659014272E-09	1.11123145403508	0.255	0.077	7.54273656976117E-05	3	CRAT
5.07746416181638E-07	1.10927816685406	0.298	0.115	0.00850881444237189	3	SPINT1
1.97419679603905E-07	1.10854229002228	0.277	0.098	0.00330835899080223	3	EIPR1
3.57823078556759E-16	1.10639707422892	0.521	0.175	5.99639915045416E-12	3	SPN
2.17214768397337E-12	1.10618977415848	0.691	0.342	3.64008508880258E-08	3	SLC1A5
1.6091214593139E-15	1.10513435761765	0.936	0.634	2.69656574151823E-11	3	LCP1
3.04108858757765E-14	1.10139116524881	0.34	0.087	5.09625625506263E-10	3	PFKP
3.96431594137659E-10	1.09833784429823	0.266	0.075	6.64340065455889E-06	3	SNRPE
3.31480060540501E-14	1.09356553213564	0.681	0.283	5.55494285453772E-10	3	PPP1CA
9.54527945268093E-07	1.09321904263563	0.362	0.158	0.0159959793068027	3	DOT1L
1.04706888893132E-13	1.09178154639878	0.34	0.092	1.75467804407111E-09	3	CHEK2
1.79363184950543E-06	1.09049351220555	0.298	0.119	0.0300576825340121	3	ELOF1
7.97284491373809E-08	1.08998147681239	0.33	0.122	0.00133608935064423	3	DPAGT1
2.34787854106209E-09	1.08572171762035	0.415	0.163	3.93457485911185E-05	3	UBTF
4.80453261064001E-15	1.08482927707687	0.723	0.312	8.05143574891053E-11	3	MRTFA
2.91713947750628E-10	1.08392640606464	0.383	0.13	4.88854233640502E-06	3	KLHL22
1.10212526654015E-08	1.08222082462892	0.383	0.152	0.000184694152166799	3	ERLIN1
9.46607952883896E-15	1.08087319320489	0.713	0.301	1.58632560744283E-10	3	APOBR
1.3438702499315E-12	1.07791360748051	0.383	0.119	2.25205776483521E-08	3	CCDC14
6.48337371882637E-15	1.07708042727774	0.351	0.088	1.08648376780092E-10	3	LYL1
3.81679057570871E-08	1.07655223706206	0.33	0.121	0.000639617764677265	3	PSMD4
8.08341054034681E-09	1.07644800660273	0.457	0.19	0.000135461793835132	3	TMX2
2.61737849222772E-11	1.07590470661142	0.468	0.175	4.38620287727521E-07	3	CIC
5.08341378173377E-08	1.07486093832817	0.33	0.124	0.000851878481542945	3	XRCC6
7.60528227262013E-07	1.07418203778716	0.34	0.142	0.0127449320324568	3	PPP1R35
5.25995673976766E-15	1.07291581944569	0.34	0.083	8.81463550450265E-11	3	MAP4K4
2.50417492395943E-09	1.07239563417955	0.362	0.139	4.19649633757122E-05	3	DUT
1.55422573431764E-08	1.07119703483011	0.33	0.119	0.000260457148556951	3	COX17

1.52505911003526E-11	1.06961290900429	0.532	0.216	2.5556940565971E-07	3	DNAJC8
1.31239471255197E-08	1.06828610726122	0.394	0.158	0.00021993110592946	3	EIF5B
2.65349225773837E-14	1.06732507172749	0.5	0.175	4.44672232551796E-10	3	SNRPA
6.12515374468612E-18	1.06651814552834	0.362	0.083	1.0264532645345E-13	3	PIM1
2.6860934303826E-08	1.06570825723845	0.287	0.098	0.000450135537063515	3	PGAP2
4.54243977620037E-16	1.06038074732266	0.691	0.309	7.61222057695657E-12	3	PSMB9
4.35063137986623E-08	1.05942416509307	0.585	0.29	0.000729078806637982	3	EPSTI1
8.46025508188426E-11	1.0582452235985	0.372	0.126	1.41776954662216E-06	3	MTMR1
6.1179177656469E-09	1.05711363691264	0.351	0.128	0.000102524065916711	3	HK2
7.53353441871972E-10	1.05469745623991	0.266	0.077	1.26246969788905E-05	3	SNRPG
4.31045292555969E-11	1.05423147508722	0.936	0.819	7.22345701265294E-07	3	CST3
1.12494294018863E-07	1.05409803796405	0.266	0.091	0.00188517937916811	3	SUCO
1.96119453670213E-13	1.05377840012074	0.489	0.167	3.28656980460543E-09	3	SNRPB
2.78175720602519E-09	1.05334738596845	0.277	0.084	4.66166872585701E-05	3	CTDSPL2
2.94494805893411E-12	1.05328721522989	0.426	0.139	4.93514395716179E-08	3	NSMAF
4.29614735773478E-22	1.05293420404149	0.383	0.075	7.19948374209194E-18	3	JADE1
2.48090153491806E-11	1.05214883544526	0.426	0.149	4.15749479221569E-07	3	SRSF9
2.80552536051845E-06	1.0518062777819	0.362	0.163	0.0470149939915682	3	TPRA1
3.98894231177143E-15	1.05062823412606	0.489	0.161	6.68466952606657E-11	3	TAGLN2
1.8362117676778E-06	1.04952950473007	0.479	0.241	0.0307712368027445	3	BROX
1.12968810796966E-11	1.04815806021234	0.426	0.147	1.89313133133556E-07	3	ECI1
2.55164074729163E-09	1.04573433666793	0.372	0.135	4.27603956431131E-05	3	SDF2L1
2.48599465231696E-15	1.04499103633446	0.351	0.087	4.16602983835276E-11	3	TPGS2
2.03386167742327E-16	1.03692426890929	0.787	0.375	3.40834539902592E-12	3	COTL1
1.98508431162094E-12	1.034449112098	0.277	0.069	3.32660428941437E-08	3	ANP32B
8.23153488449938E-13	1.03296411381161	0.511	0.197	1.37944061594441E-08	3	CRKL
1.76266696888331E-09	1.03130636098547	0.33	0.112	2.95387730645465E-05	3	SLC16A1
1.20729356106509E-07	1.03006086396464	0.255	0.086	0.00202318254963289	3	BRD9
2.17377996897413E-12	1.02995449801989	0.287	0.074	3.64282047200684E-08	3	CBFA2T3
1.44292341679263E-14	1.02954751365744	0.574	0.21	2.41805106186109E-10	3	SMARCD2
1.55694506409276E-12	1.02776401719085	0.713	0.378	2.60912853840665E-08	3	HNRNPUL1
1.97370696020948E-07	1.02379499670796	0.287	0.107	0.00330753812391904	3	UQCC2
4.4978318099216E-09	1.02322495021492	0.404	0.161	7.53746654706662E-05	3	LSM 6.00
6.10772492192238E-09	1.02316718423762	0.447	0.185	0.000102353254241575	3	DCXR
1.3519164520699E-08	1.02282747321064	0.319	0.111	0.000226554159037873	3	XRCC5
4.97376389577255E-13	1.02237366120879	0.66	0.329	8.33503353653565E-09	3	NUCKS1
6.62786462226636E-08	1.02233603259532	0.564	0.284	0.0011106975533994	3	TAB2
5.35185507501949E-08	1.02099124223165	0.319	0.117	0.000896863873471765	3	SIGMAR1
2.00540229042142E-10	1.01924630151563	0.436	0.17	3.36065315828822E-06	3	R3HDM4
2.18056455696716E-13	1.01540857266961	0.298	0.072	3.65419008456557E-09	3	F2RL1
1.16978915218366E-14	1.01111346052921	0.532	0.18	1.96033266122938E-10	3	PSMG2
5.74736365966365E-09	1.0098107651393	0.309	0.102	9.63143202086435E-05	3	SEC22C
2.81364304360959E-08	1.00963451280617	0.287	0.096	0.000471510301248095	3	XRCC1
1.2680974705512E-12	1.00958561796106	0.606	0.265	2.12507774114969E-08	3	PAK1
2.05611294436445E-12	1.00935477439553	0.734	0.395	3.44563407216594E-08	3	PSMB8
3.79188712338808E-09	1.00925715010407	0.319	0.108	6.35444444137374E-05	3	HCFC1R1
6.67496257217012E-10	1.00535807433115	0.543	0.243	1.11859022784427E-05	3	ELOVL5
6.04693752171597E-14	1.00298214803057	0.681	0.285	1.01334578988916E-09	3	PPP4C
4.37652193581672E-15	1.00251933553823	0.415	0.123	7.33417546004165E-11	3	MAD2L2
4.68003753106866E-11	1.00107673732591	0.617	0.288	7.84280689456486E-07	3	UQCRC1

1.42710050562836E-07	1.00080470799779	0.532	0.271	0.002391535027332	3	CLPTM1
3.1554247061027E-10	0.998497256846742	0.457	0.179	5.2878607224869E-06	3	BUD23
5.10884406727775E-14	0.998333389765736	0.489	0.163	8.56140088794405E-10	3	HDGF
1.46538226651021E-12	0.997373142077034	0.479	0.173	2.4556876022178E-08	3	LNPEP
5.36834545150129E-14	0.996501767222076	0.574	0.209	8.99627330762585E-10	3	CRYBG1
4.81169030042785E-08	0.994672536057359	0.585	0.29	0.000806343060545699	3	ANXA6
5.94142225852441E-07	0.994186171288186	0.277	0.101	0.0099566354208352	3	CHCHD1
5.87849461969043E-08	0.992545177541436	0.447	0.198	0.000985118128367722	3	RECQL
1.78947565441324E-07	0.992481807072075	0.298	0.11	0.0029988033016657	3	NT5DC3
2.55952460091654E-06	0.992051613303423	0.309	0.128	0.0428925132621593	3	ELP3
6.0527700292848E-09	0.991787308933434	0.787	0.473	0.000101432320150755	3	LGALS1
6.82053078447502E-08	0.991110749325022	0.436	0.193	0.00114298454886232	3	RABL6
7.83552536691442E-09	0.990605943548186	0.383	0.152	0.000131307734098752	3	ARL 2.00
2.84084918874107E-12	0.990026292863422	0.585	0.256	4.76069507049228E-08	3	NONO
8.05334172228701E-08	0.988562104024811	0.436	0.196	0.00134957900582086	3	RAB5IF
7.15109257274348E-08	0.988349903499961	0.372	0.153	0.00119838009334035	3	EBP
2.28422712629318E-08	0.987599820675904	0.415	0.17	0.00038279078182421	3	HACD3
7.04362077548545E-10	0.987576892818171	0.415	0.158	1.18036996955585E-05	3	ADSL
1.15848430672414E-19	0.987050518369848	0.426	0.107	1.94138800120832E-15	3	NCAPH2
5.55851352741123E-08	0.986734237264596	0.468	0.208	0.000931495696923573	3	EGLN2
1.61582580550891E-08	0.984832235629411	0.298	0.102	0.000270780088487183	3	VAV3
8.91109212983243E-13	0.983715785136408	0.436	0.15	1.49332081911732E-08	3	UBALD2
8.7289719438058E-11	0.981733618329073	0.457	0.173	1.46280111834298E-06	3	FLNA
1.4243613730856E-15	0.98083026319037	0.67	0.281	2.38694478901685E-11	3	DIAPH1
1.80413647396014E-12	0.980486632653693	0.404	0.133	3.0233719030624E-08	3	HAT1
3.5056049312599E-13	0.97655140917532	0.606	0.246	5.87469274380533E-09	3	RAB1B
2.03265254417655E-07	0.976014765797659	0.532	0.269	0.00340631913353107	3	SEPTIN11
5.35268466358142E-09	0.975541374496109	0.394	0.155	8.97002895922975E-05	3	KCTD9
3.32233928479227E-11	0.974857602348354	0.564	0.246	5.56757617345489E-07	3	AP1M1
1.05676373707841E-07	0.974216749733119	0.309	0.117	0.001770924670596	3	PLD4
1.36316720273496E-10	0.973522850779806	0.287	0.081	2.28439559834325E-06	3	CNOT3
1.06131141474667E-15	0.973401304148118	0.383	0.101	1.77854566883246E-11	3	TDP1
2.97353937575825E-07	0.972503646623611	0.266	0.096	0.00498305728589567	3	MICAL3
2.09900777645266E-13	0.969023741494395	0.67	0.312	3.51751723177937E-09	3	HNRNPD
9.39419209147299E-11	0.968066773448507	0.468	0.184	1.57427871068904E-06	3	MTHFD1
1.47272918807806E-06	0.964255513967386	0.447	0.215	0.0246799957338121	3	GATAD2B
1.2767042088272E-09	0.964176103427189	0.628	0.312	2.13950091315262E-05	3	TSPAN33
1.20976622517831E-07	0.963773326099042	0.426	0.187	0.00202732624015381	3	GRHPR
1.32858982459236E-11	0.96375579787079	0.362	0.112	2.22645082805187E-07	3	PARM1
3.71650163069951E-10	0.961560498795671	0.362	0.122	6.22811343272625E-06	3	NHP2
1.09660705352294E-12	0.960189748986959	0.436	0.143	1.83769410029375E-08	3	DHX8
9.0831157141192E-11	0.960147770819311	0.287	0.082	1.5221485313721E-06	3	MAZ
5.08436215644101E-13	0.959193266749045	0.34	0.092	8.52037410176385E-09	3	ANG
1.41796464674265E-14	0.959115316052362	0.894	0.599	2.37622515501133E-10	3	CFL1
1.30451764243888E-12	0.958843258012842	0.5	0.189	2.18611066519908E-08	3	FZR1
1.51708735242929E-09	0.958624667630072	0.362	0.128	2.542334985201E-05	3	MFSD14B
9.64561785103867E-07	0.956582404092173	0.277	0.104	0.0161641263947706	3	NUP155
8.19121392487973E-08	0.954479750839687	0.468	0.207	0.00137268362953135	3	GOLGA7
3.95696550845215E-08	0.954348531088383	0.266	0.087	0.000663108279906411	3	GNG5
3.59772916555111E-14	0.954226860292195	0.447	0.143	6.02907453563056E-10	3	POLE3

1.24562304622334E-08	0.953724728174785	0.543	0.257	0.000208741510086107	3	ETFB
1.42937826002943E-07	0.952636175056311	0.362	0.152	0.00239535208815732	3	FIP1L1
9.66402201780137E-07	0.952348353745784	0.266	0.098	0.0161949680974315	3	PEF1
2.18253046949739E-08	0.951875828876363	0.266	0.085	0.000365748456078372	3	ALG8
6.0806324632363E-07	0.951751007816839	0.415	0.201	0.0101899238818914	3	PGP
3.71418616394925E-09	0.948697829342782	0.362	0.13	6.22423317354615E-05	3	UBR7
7.81298543853391E-07	0.947751366662414	0.33	0.136	0.0130930009978951	3	HAPLN3
1.79319739535611E-06	0.945683973950185	0.309	0.128	0.0300504019513777	3	CHAMP1
6.97319882147658E-08	0.944445494980928	0.489	0.221	0.00116856865850304	3	VHL
5.91752240462557E-11	0.942137373003415	0.489	0.191	9.91658404567153E-07	3	STIM2
2.42616022286806E-06	0.941388898468421	0.479	0.237	0.0406575930148229	3	THBD
2.60112221839968E-07	0.941056787301988	0.383	0.164	0.00435896061359418	3	CEBPG
4.06834671962702E-09	0.940653554531038	0.479	0.207	6.81773543275096E-05	3	AATF
1.91427709070354E-11	0.939171594485103	0.277	0.073	3.207945548601E-07	3	ANAPC15
1.16850512922511E-11	0.938137127741329	0.394	0.129	1.95818089555544E-07	3	ARPC5L
3.98814959355772E-08	0.936641966339369	0.351	0.13	0.000668334108888403	3	SRXN1
1.28454985702886E-07	0.936518301682898	0.34	0.135	0.00215264865040897	3	AQR
1.05266786875619E-08	0.936150239527396	0.34	0.122	0.000176406081446162	3	ITGB1BP1
1.56860922515574E-10	0.934351198156616	0.564	0.257	2.62867533951599E-06	3	FOXN2
1.18818158491301E-07	0.932994437141335	0.372	0.152	0.00199115469999721	3	PSMB2
8.46494191116893E-10	0.93266024454249	0.415	0.158	1.41855496547369E-05	3	LPCAT3
3.19459416107261E-10	0.931770138691297	0.66	0.307	5.35350089512549E-06	3	CDC42SE2
3.9933675496618E-07	0.931032807649652	0.383	0.163	0.00669208533972324	3	SCAF4
1.1306889754979E-12	0.929830905613677	0.33	0.089	1.89480858513939E-08	3	RPIA
8.3578993303209E-11	0.928143009495846	0.681	0.343	1.40061676977518E-06	3	PSMB10
5.09107164012882E-08	0.927441158165092	0.277	0.092	0.000853161785452788	3	TBCC
1.13363184738477E-06	0.926514245935754	0.287	0.109	0.018997402498474	3	NUDT2
3.37943547115806E-08	0.92594766348917	0.266	0.086	0.000566325796256667	3	ZUP1
1.08398031684857E-06	0.92578613456003	0.511	0.273	0.0181653421497484	3	FBXO7
5.02306698978095E-10	0.925425436133719	0.266	0.075	8.41765566147491E-06	3	SRSF1
1.967548681684E-07	0.923660552509866	0.415	0.187	0.00329721808076605	3	ACSL5
1.00177088517131E-08	0.922856143357553	0.457	0.196	0.000167876764937008	3	USP14
1.27714109393718E-11	0.922119475657268	0.532	0.21	2.14023304521992E-07	3	SUB1
2.44144326917974E-09	0.921857009108739	0.404	0.16	4.09137063049141E-05	3	CANT1
2.20002736086554E-07	0.91920255620903	0.362	0.146	0.00368680585133847	3	C6orf89
2.99416583248707E-07	0.91854435833283	0.383	0.165	0.00501762310208183	3	MEAF6
1.03546711717901E-06	0.918190744236811	0.277	0.106	0.0173523579496858	3	DHCR24
1.76443502571382E-07	0.917406071112191	0.415	0.188	0.00295684021609122	3	YKT6
2.25200868211111E-08	0.916753974126957	0.681	0.363	0.000377391614948179	3	IL2RG
6.14847583816244E-11	0.915821281963248	0.404	0.14	1.03036158095926E-06	3	CLNS1A
1.37990962570879E-11	0.914464417273308	0.766	0.435	2.31245255076279E-07	3	HNRNPF
9.45523748345425E-12	0.91380761988358	0.532	0.21	1.58450869747726E-07	3	SCAF1
5.06334590913652E-07	0.913587165100507	0.511	0.262	0.00848515507453098	3	DDT
2.07864303748255E-09	0.913179174352736	0.564	0.251	3.48339000221326E-05	3	RAB4B
8.09839321384128E-08	0.912816185845502	0.266	0.089	0.00135712873477552	3	PDRG1
8.80373478756074E-09	0.911173267620671	0.457	0.197	0.000147532987569943	3	MED4
9.08889256375168E-11	0.907571992259117	0.489	0.195	1.52311661583351E-06	3	AIP
2.76548929763496E-14	0.906395204161785	0.255	0.053	4.63440696497667E-10	3	PPP2R3B
7.15159505500601E-13	0.901343956613496	0.394	0.126	1.19846429931791E-08	3	CARHSP1
5.16974669837647E-10	0.901021464344102	0.266	0.075	8.66346151713929E-06	3	EXOSC3

2.82375560853248E-16	0.899714958630215	0.489	0.151	4.73204964877873E-12	3	OAS3
5.9651116421238E-11	0.899132216223402	0.33	0.1	9.99633408987106E-07	3	REEP4
4.85359855970276E-12	0.899050294158522	0.415	0.139	8.13366046634988E-08	3	SLC38A1
1.73324206651877E-09	0.897668998847081	0.319	0.104	2.90456705507216E-05	3	PELP1
1.31005479463905E-09	0.897659406852859	0.34	0.119	2.19538982485613E-05	3	ERAL1
7.11305374187564E-07	0.896770486643646	0.255	0.091	0.0119200554606352	3	NSRP1
5.29410348840991E-13	0.896085074072135	0.617	0.246	8.87185862587732E-09	3	SEPTIN2
9.65511201008051E-10	0.895804050102226	0.277	0.082	1.61800367064929E-05	3	PSIP1
3.56953898384536E-07	0.894580038356876	0.34	0.136	0.00598183342912806	3	CLASP2
2.14134383242528E-11	0.893271274167254	0.33	0.096	3.58846399437829E-07	3	RUVBL1
4.23062269400317E-10	0.892674618001908	0.723	0.357	7.08967751061051E-06	3	PTPN1
2.6265041072674E-12	0.892236855488985	0.309	0.082	4.40149558295871E-08	3	ATG16L1
6.06068041947224E-09	0.892173801861419	0.372	0.141	0.000101564882469516	3	TBC1D10B
2.40626817097547E-16	0.891049593952424	0.394	0.104	4.0324242009207E-12	3	SMPD4
2.66637775643692E-13	0.890638014891077	0.5	0.175	4.46831584423698E-09	3	NUP50
3.00288829686478E-12	0.890266136625244	0.553	0.224	5.03224020788599E-08	3	DEK
2.28628702724304E-10	0.888259233528726	0.564	0.262	3.83135980025388E-06	3	ELAVL1
5.08629194759266E-09	0.8881518983716	0.372	0.146	8.52360804577578E-05	3	FAF1
1.61707338566105E-13	0.887988257757689	0.426	0.132	2.70989157969078E-09	3	ANKRD36C
6.27474365955068E-07	0.887643990559368	0.287	0.108	0.010515215424675	3	CSNK1E
8.90269247068556E-09	0.886782989391435	0.383	0.15	0.000149191320423749	3	CNOT11
4.68256203139794E-07	0.885353778110057	0.606	0.323	0.00784703745221667	3	CCNG2
7.94425224817278E-14	0.885201083394361	0.702	0.32	1.3312977917488E-09	3	RAB8A
6.30762266086871E-08	0.884735106352526	0.372	0.155	0.00105703140550838	3	PSMA4
2.71530323540587E-06	0.883963773806042	0.457	0.227	0.0455030516189316	3	ITPRID2
2.99355243242289E-08	0.883819541603535	0.532	0.263	0.000501659516625429	3	FLOT2
3.35700191647022E-07	0.883272573577271	0.298	0.11	0.00562566381162079	3	NOC2L
1.32201349025481E-11	0.882206558044577	0.543	0.226	2.21543020696901E-07	3	SNRPC
3.2056388282828E-10	0.882049941950781	0.5	0.213	5.37200954843632E-06	3	TMEM19
2.11771746729652E-10	0.881866805346442	0.543	0.235	3.54887093169552E-06	3	ATG3
3.10389568399257E-11	0.881745240615155	0.277	0.074	5.20150838723475E-07	3	BCL2A1
9.28403228226444E-09	0.880931673791139	0.266	0.084	0.000155581812986188	3	PPP1R14B
3.51777053390771E-08	0.873752520985519	0.298	0.102	0.000589507986072254	3	CPSF3
7.62478775004635E-09	0.87347232162529	0.298	0.099	0.000127776193115277	3	MYCL
3.36294256959326E-07	0.87230641898683	0.287	0.106	0.00563561915812439	3	AFF3
4.48781527739705E-08	0.871713662298669	0.394	0.158	0.000752068084186198	3	SNTB2
1.27556183261142E-16	0.869486723140364	0.457	0.132	2.13758651909021E-12	3	SNRNP40
2.30555206822989E-07	0.869210885709259	0.33	0.131	0.00386364415593965	3	NDUFB6
3.14243598186549E-09	0.86884824901645	0.298	0.099	5.26609421841018E-05	3	RUVBL2
9.25519349744924E-08	0.86779589215249	0.372	0.152	0.00155098532630254	3	RAD23B
2.35099258406899E-12	0.86749888087709	0.606	0.258	3.93979337238281E-08	3	PRKDC
2.0348575954971E-07	0.86291981953419	0.298	0.11	0.00341001435853404	3	GNL1
4.23343142367874E-11	0.862552712966458	0.372	0.123	7.09438437980083E-07	3	PPP2R5D
4.50000204415553E-09	0.86082179313834	0.628	0.314	7.54110342559583E-05	3	ELOVL1
3.47845432724964E-09	0.860484476180124	0.691	0.386	5.82919376160495E-05	3	ENO1
5.20290675235641E-09	0.859844166227596	0.479	0.21	8.71903113559888E-05	3	FADS2
3.85063039524736E-11	0.858354562748901	0.521	0.211	6.45288641635552E-07	3	SRRM1
6.9012515128852E-07	0.85809801186779	0.351	0.143	0.011565117285293	3	ATG12
2.71039131334242E-11	0.855945556859263	0.926	0.679	4.54207376289922E-07	3	TMSB10
1.16633158691014E-06	0.854559633080343	0.298	0.118	0.0195453847334401	3	TMEM250

1.63858383587896E-06	0.85145884174316	0.33	0.139	0.0274593879216596	3	TIMM17A
3.06787404492633E-09	0.850044842841694	0.745	0.411	5.14114332448755E-05	3	PTMS
2.21527122082687E-06	0.849614346100217	0.34	0.145	0.0371235151186167	3	CCDC85B
1.78046560056034E-07	0.849368420846429	0.298	0.108	0.00298370425341903	3	ATP5MC1
2.55318264214519E-09	0.848547698192669	0.5	0.213	4.27862347170691E-05	3	POLDIP3
3.48626043143273E-08	0.847619998712197	0.564	0.279	0.000584227523099497	3	PPP6R1
1.05161611783437E-07	0.846845161078519	0.351	0.136	0.00176229829026683	3	GOT2
4.18809933289411E-08	0.846649356158515	0.372	0.145	0.000701841686206395	3	MANBAL
7.13145475501131E-10	0.846120107800069	0.287	0.085	1.1950891878448E-05	3	MRE11
9.47432712183864E-09	0.844833557231586	0.777	0.547	0.000158770773907772	3	ACTG1
7.2967076735428E-09	0.844692127734806	0.298	0.098	0.00012227822719323	3	PTK2
2.72259583648961E-08	0.844620814506995	0.372	0.14	0.00045625261027893	3	MALSU1
3.25961729584253E-11	0.844605328566423	0.83	0.477	5.46246666437291E-07	3	UCP2
7.8167200309349E-09	0.839188896571555	0.468	0.217	0.000130992594278407	3	SIVA1
5.58879966684309E-12	0.838284049642655	0.67	0.313	9.36571048169566E-08	3	PTPA
3.18925564313587E-08	0.8382805132365	0.287	0.096	0.000534455460676709	3	GPR65
7.71425723235954E-10	0.836592027951796	0.309	0.096	1.29275522699881E-05	3	NUBP2
1.1982742277841E-08	0.835813386766965	0.404	0.159	0.000200806795092059	3	NAA40
5.30624647682393E-12	0.835301966397583	0.436	0.148	8.89220784586154E-08	3	SRRT
6.36110450337533E-10	0.835057692287824	0.34	0.115	1.06599389267564E-05	3	APOBEC3C
9.24299715267853E-08	0.834966993741679	0.426	0.18	0.00154894146284587	3	PLEKHJ1
4.81777027186151E-07	0.83438262777451	0.34	0.139	0.00807361942158551	3	APOL3
1.25051926278528E-08	0.834290609376656	0.255	0.078	0.000209562018057557	3	ERI2
3.52432277153424E-07	0.834230272159269	0.457	0.216	0.00590606010053708	3	HTATSF1
1.98522741967857E-08	0.833586373133673	0.436	0.194	0.000332684410989735	3	LDHB
2.91744951907162E-12	0.833295794031127	0.255	0.059	4.88906190406021E-08	3	UBAP2
6.82774224114581E-08	0.831672113777771	0.255	0.083	0.00114419304477121	3	R3HCC1
3.26911097532651E-10	0.831265195089843	0.681	0.349	5.47837617245216E-06	3	DSE
2.11448087903991E-08	0.83072947606948	0.649	0.344	0.000354344705709508	3	EIF4H
2.77862542216763E-06	0.8291160052308	0.553	0.304	0.0465642048246851	3	PYCARD
3.22256516984163E-08	0.8282685924375	0.266	0.086	0.000540037471162061	3	GPKOW
6.50816064513644E-13	0.827608741849223	0.947	0.659	1.09063756091196E-08	3	VIM
6.96802449250753E-13	0.824576945809891	0.457	0.153	1.16770154445441E-08	3	CSTF3
6.38824741459604E-12	0.82451004992896	0.479	0.176	1.070542501738E-07	3	PAF1
4.25774581245105E-07	0.824412658494911	0.33	0.133	0.00713513043250546	3	RBM12B
1.79821444196778E-07	0.823658914575995	0.33	0.129	0.0030134477618496	3	RPP25
4.14634987894025E-07	0.823180406930717	0.277	0.101	0.00694845312712808	3	GALNT10
1.24333202255564E-07	0.820236327417148	0.287	0.099	0.00208357580339874	3	UCHL5
2.91640899556446E-06	0.820034662359132	0.351	0.151	0.0488731819476692	3	KDELR2
9.54401844213905E-09	0.820017895808677	0.33	0.118	0.000159938661053366	3	PRPF38A
6.99934121817967E-12	0.819847264725334	0.521	0.203	1.17294960134255E-07	3	CKLF
8.85535571963905E-07	0.818208926537524	0.266	0.101	0.0148398051149711	3	RMND5B
1.31527703221879E-06	0.816947731063173	0.585	0.334	0.0220414125059225	3	SNX17
5.82370604873196E-09	0.816942338860537	0.298	0.097	9.75936659646502E-05	3	MICOS13
7.78541292171507E-10	0.81635818676912	0.596	0.277	1.30467949742101E-05	3	ARPC5
1.28393203431201E-10	0.816313977900306	0.277	0.076	2.15161330310007E-06	3	TMEM209
1.49834628309783E-14	0.816039204357759	0.511	0.173	2.51092870121535E-10	3	DDX39A
7.63665991041046E-10	0.814091176801104	0.404	0.153	1.27975146778658E-05	3	DCAF15
2.83762807499749E-10	0.814034396263535	0.266	0.073	4.7552971280808E-06	3	WDR61
1.24359622703945E-08	0.812817902766134	0.426	0.169	0.000208401855727272	3	ASCC2

5.95849473296371E-11	0.812358357106185	0.734	0.389	9.98524547350058E-07	3	ARF6
6.00263488393652E-09	0.811645393708404	0.319	0.108	0.000100592155385008	3	CEP63
2.6640961836614E-08	0.811548909117811	0.691	0.366	0.000446449238457977	3	DCAF7
9.69106849137079E-07	0.810306051679728	0.426	0.204	0.0162402925778392	3	H2AFJ
1.53365773857629E-06	0.809959594355373	0.511	0.266	0.0257010363830615	3	RASSF2
1.54905631634372E-09	0.809541593516311	0.362	0.126	2.59590857492881E-05	3	RLIM
1.49752699153604E-06	0.809253626629418	0.319	0.132	0.025095557324161	3	CEP57
1.41112202972023E-07	0.807171867473618	0.404	0.177	0.00236475829740516	3	CCDC124
4.04526986906165E-10	0.806660796714305	0.426	0.153	6.7790632465735E-06	3	NUCB2
3.41325745728979E-09	0.805559532911551	0.457	0.186	5.71993684692623E-05	3	LRBA
1.69769609625539E-13	0.805401154163702	0.33	0.088	2.84499911810478E-09	3	EXOSC8
9.94836057193525E-11	0.804987019710911	0.628	0.306	1.66714626464491E-06	3	NUP62
9.61647805932334E-09	0.80477579535252	0.628	0.319	0.000161152939318141	3	SEC61A1
3.30524244412837E-13	0.804723725688917	0.67	0.286	5.53892528787033E-09	3	SYMPK
1.86974700875406E-10	0.804059923593401	0.83	0.46	3.13332203727005E-06	3	ITGA4
7.06651116797461E-11	0.802297819822593	0.309	0.092	1.18420594152918E-06	3	SF3A3
4.47934342877637E-07	0.801291961868293	0.319	0.126	0.00750648371794344	3	ZNF672
1.37130822553505E-09	0.800559813137237	0.468	0.193	2.29803832435164E-05	3	PNN
1.09421704228598E-07	0.797357356344725	0.319	0.12	0.00183368891946284	3	DCAF16
4.63697443175822E-11	0.796683171708086	0.426	0.148	7.77064175274043E-07	3	TAF15
1.77594803240044E-08	0.796412984962347	0.319	0.111	0.000297613371269666	3	KLHL8
4.22877210268117E-13	0.794058400576184	0.553	0.223	7.0865762896731E-09	3	HAUS4
6.08414436273996E-10	0.790307733291733	0.287	0.087	1.01958091230796E-05	3	POLD3
1.35934831284401E-08	0.790103053866452	0.309	0.103	0.000227799590266399	3	DCAF10
2.13899792659398E-06	0.789379106520928	0.426	0.201	0.0358453272538618	3	MIER1
6.52760997209126E-09	0.789061244761349	0.777	0.456	0.000109389687912305	3	MYL6
5.35040488850074E-10	0.788647960842976	0.66	0.356	8.96620851214954E-06	3	TMEM109
1.26728067676696E-07	0.788558180533542	0.394	0.174	0.00212370895812607	3	ATL3
3.35460575751364E-08	0.788448108258911	0.543	0.25	0.000562164832844135	3	PGM2
1.23770154519529E-07	0.787611391906538	0.298	0.109	0.00207414024943827	3	CCT5
3.88177694611576E-08	0.786911204511449	0.479	0.217	0.00065050818063008	3	CLN6
3.40754120713252E-10	0.785328662550801	0.734	0.426	5.71035755491269E-06	3	CALM2
1.53819651090706E-07	0.781876842842555	0.511	0.252	0.00257770971297805	3	RBM42
1.2961103725562E-08	0.781360991551396	0.298	0.099	0.000217202176232968	3	RBBP7
2.92996352380662E-07	0.781340077667059	0.383	0.164	0.00491003287319513	3	PURB
4.31933818418129E-08	0.781251985726271	0.351	0.135	0.000723834692905101	3	SMC6
1.72927721321399E-08	0.780837265896267	0.521	0.244	0.000289792275390401	3	ST14
1.51375760349394E-06	0.780687523586711	0.309	0.122	0.0253675499193514	3	ZBTB48
3.11306713246904E-07	0.779805859084154	0.426	0.196	0.00521687790059162	3	HADH
1.92031560392145E-06	0.777188528267141	0.585	0.332	0.0321806488905157	3	CYC1
1.0145700284753E-07	0.775904164021485	0.34	0.132	0.0017002164537189	3	NAA50
7.90748811528363E-07	0.773708775468489	0.298	0.113	0.0132513685835923	3	AP3M1
1.34798175218849E-08	0.773255161370691	0.372	0.15	0.000225894782031747	3	MLH1
5.24636201140063E-08	0.773081223115219	0.287	0.097	0.000879185345870517	3	PXMP2
2.19589077390551E-08	0.772178447715988	0.394	0.155	0.000367987375891086	3	ZNF362
1.73236137724319E-06	0.771172879273182	0.287	0.112	0.0290309119598414	3	ROCK2
1.56795537658581E-06	0.769878633923756	0.468	0.233	0.026275796200825	3	YIF1B
7.14520479024914E-09	0.769291193417241	0.553	0.258	0.000119739341874995	3	YWHAE
1.05571236427612E-07	0.766522666823932	0.34	0.129	0.00176916278005391	3	ADNP
9.62548532512441E-08	0.765558507200751	0.436	0.19	0.00161303883078435	3	RNF145

1.27637048552033E-09	0.765303083839084	0.638	0.29	2.13894165963496E-05	3	DOCK10
2.09168665145134E-09	0.765234282872097	0.894	0.568	3.50524849050215E-05	3	GSN
6.93946530391472E-09	0.763441005964594	0.511	0.233	0.000116291559563003	3	RNF4
1.29069587971483E-06	0.762330975990918	0.266	0.099	0.0216294815522611	3	LSM 2.00
1.2889403626023E-06	0.759929536312165	0.277	0.106	0.0216000625964893	3	DDX19A
8.04244398325379E-09	0.759811180228441	0.468	0.195	0.000134775276271367	3	RYBP
1.18952677300043E-12	0.759495531921644	0.511	0.183	1.99340896619413E-08	3	GUCD1
8.61187345935543E-09	0.759315468165918	0.426	0.178	0.000144317775431878	3	HCFC1
4.0255946073863E-10	0.759234418457413	0.457	0.183	6.74609144305796E-06	3	RCC2
5.59542485443189E-08	0.75891196605943	0.309	0.11	0.000937681297105697	3	CNOT6
1.53560667372598E-10	0.758315481291302	0.394	0.135	2.57336966383001E-06	3	KPNB1
6.5211316208896E-07	0.75805005383574	0.372	0.16	0.0109281123702868	3	NDUFS3
5.27997199106826E-07	0.755728297770411	0.309	0.121	0.0088481770626322	3	FAF2
1.67278634331963E-08	0.755018384916918	0.319	0.112	0.000280325535413504	3	BANF1
9.1673146625847E-12	0.75435632968602	0.585	0.257	1.53625859115594E-07	3	MNDA
5.21718199600515E-07	0.753378093896138	0.394	0.169	0.00874295358890543	3	NFATC3
4.56569266855706E-07	0.753207371477715	0.436	0.195	0.00765118777396793	3	DEDD
4.43977268934406E-09	0.750882849204498	0.34	0.125	7.44017107280277E-05	3	PNP
5.12163241475046E-07	0.750664363084239	0.426	0.195	0.00858283160063881	3	TRAPPC4
1.05286535124546E-08	0.750016794964086	0.362	0.139	0.000176439175561714	3	CTCF
1.01247506846754E-15	0.749043812593036	0.33	0.076	1.69670571973791E-11	3	MSH5
7.03762547308065E-07	0.74858661903986	0.298	0.116	0.0117936527677886	3	SMARCE1
3.5002312923725E-07	0.747898528863214	0.394	0.169	0.00586568759975783	3	PRR14
2.6767471628976E-07	0.74637994040814	0.362	0.151	0.00448569289558379	3	KDELR1
5.22993504146701E-09	0.746206236080632	0.404	0.166	8.76432514249042E-05	3	ILVBL
1.38166161939121E-10	0.745926974143462	0.436	0.169	2.31538854177579E-06	3	HNRNPLL
6.93612255187715E-07	0.745018362479853	0.33	0.135	0.0116235541724357	3	SLU7
1.40304493345679E-06	0.744953178304338	0.277	0.104	0.0235122269948689	3	ATXN3
1.48020983854983E-07	0.744104399145526	0.298	0.11	0.0024805356474418	3	CENPP
6.07672628679754E-09	0.743109778140654	0.351	0.13	0.000101833779114153	3	MYO19
1.14167824283197E-11	0.741896886115076	0.532	0.204	1.91322439933782E-07	3	PPIA
1.29918323182114E-10	0.741082879441326	0.394	0.134	2.17717125988586E-06	3	FARP2
2.94832285184511E-09	0.740324148966426	0.5	0.206	4.94079943512203E-05	3	RC3H2
1.71284822381033E-07	0.740214226091456	0.543	0.267	0.00287039105346134	3	P4HB
5.42784997489482E-08	0.74019434894328	0.362	0.141	0.000909599098792874	3	RNF20
7.48875132215986E-07	0.739861063761655	0.426	0.195	0.0125496494656755	3	PTDSS1
1.15663647523784E-06	0.73744851145772	0.479	0.238	0.0193829140520357	3	EDF1
1.579502832647E-07	0.737014923254693	0.638	0.347	0.00264693084694984	3	ETV6
2.94612195319251E-06	0.736110009810492	0.277	0.108	0.0493711116916	3	VPS72
3.03113196170652E-08	0.736083675562861	0.511	0.226	0.000507957094142778	3	MSL1
1.70016144720401E-07	0.733573349128332	0.319	0.122	0.00284913055322449	3	SPOPL
2.65119174842487E-07	0.732622889777537	0.543	0.269	0.0044428671320104	3	MYD88
4.81693996605003E-11	0.732601775778249	0.255	0.065	8.07222799510664E-07	3	FAM110A
7.33068104057736E-07	0.732319703622341	0.277	0.103	0.0122847552877995	3	ERGIC1
1.39875275121876E-07	0.732217116777481	0.383	0.159	0.00234402986049239	3	CENPB
2.54648456604175E-07	0.731614271475229	0.372	0.155	0.00426739883577276	3	CLIP2
5.73135518183869E-07	0.731212688467472	0.383	0.165	0.00960460501372527	3	CASP3
5.05461442170491E-07	0.730906243390076	0.436	0.194	0.0084705228478931	3	CAPZA1
2.54185495731766E-10	0.727841456338174	0.362	0.118	4.25964053747294E-06	3	LSM 1.00
3.16433875906343E-08	0.727029551029445	0.468	0.212	0.00053027988924385	3	DIDO1

**8.37814940584675E-09**	0.725846954762618	0.479	0.205	0.00014040102774318	3	SHC1
**4.13665100870789E-07**	0.725589874545418	0.34	0.136	0.00693219976039269	3	DNAJB6
**2.81604638463776E-12**	0.724563081205077	0.404	0.127	4.71913053137595E-08	3	TAF11
**2.84528595119249E-10**	0.724425872051125	0.596	0.277	4.76813019700838E-06	3	MIF
**1.09816798130067E-09**	0.723219272012922	0.67	0.344	1.84030990306366E-05	3	ARPC4
**2.32770049564875E-08**	0.72281756764394	0.596	0.295	0.000390076049060818	3	FGR
**9.51171194772406E-08**	0.719236063708217	0.404	0.162	0.0015939726881996	3	USP8
**1.67021852556307E-07**	0.719158280802332	0.585	0.289	0.0027989522051386	3	MOB3A
**2.03623263425332E-09**	0.718615721323675	0.436	0.17	3.41231864848172E-05	3	CUL3
**2.08463141017281E-06**	0.71803606097941	0.479	0.238	0.034934253171676	3	UHMK1
**2.07463360096184E-10**	0.716579143472219	0.521	0.216	3.47667098849186E-06	3	TBCB
**2.05808255033529E-08**	0.716077740838016	0.489	0.221	0.000344893473785188	3	EEF1B2
**4.2373759473922E-10**	0.715441961623518	0.511	0.217	7.10099461263984E-06	3	TYK2
**6.88299446850283E-13**	0.715215641927449	0.564	0.218	1.1534522130317E-08	3	EFTUD2
**3.49934010739484E-09**	0.7149082910065	0.394	0.148	5.86419415197227E-05	3	MFN2
**9.55867934407268E-12**	0.71416812960413	0.564	0.227	1.6018434844797E-07	3	ARID1A
**1.43674861182909E-09**	0.714077306645121	0.447	0.178	2.40770332370319E-05	3	LMF2
**6.61376778569421E-07**	0.713204634242312	0.468	0.222	0.0110833520552664	3	TSTA3
**5.15270685926697E-14**	0.712877609001699	0.585	0.216	8.63490615475959E-10	3	NFATC2IP
**5.76665391253545E-07**	0.712663418292556	0.447	0.21	0.0096637586266269	3	ESD
**2.49178431156406E-08**	0.710449378929636	0.426	0.183	0.000417573214931905	3	FADS1
**1.11764111700475E-08**	0.709614587149607	0.383	0.148	0.000187294298387656	3	COPS3
**1.23809552343997E-06**	0.708377649349516	0.798	0.535	0.020748004781807	3	S100A10
**4.34592504321262E-07**	0.707836461753754	0.426	0.195	0.0072829011874157	3	NUDT5
**2.00642276571265E-14**	0.70652242493324	0.255	0.052	3.36236327078125E-10	3	BCL2L12
**1.28206909487063E-06**	0.706387672924595	0.309	0.124	0.021484913891842	3	KLF10
**3.50811243812416E-09**	0.703819878068248	0.457	0.192	5.87889482380846E-05	3	C17orf49
**4.77649455976245E-07**	0.702366782940805	0.287	0.107	0.00800444958324992	3	MIB1
**3.17481130538475E-09**	0.701650093497266	0.383	0.143	5.32034878556377E-05	3	SYNE3
**5.46994374508008E-12**	0.701592557249321	0.585	0.237	9.1665317280052E-08	3	CNOT8
**6.23758502747727E-07**	0.701534327096349	0.394	0.172	0.0104529449890464	3	RIPK1
**3.33972385579712E-11**	0.70087650641131	0.383	0.126	5.59670923754481E-07	3	CCAR2
**3.38847144798958E-09**	0.700677308216421	0.255	0.074	5.67840045254094E-05	3	ZPR1
**1.3179325239927E-06**	0.699740727533134	0.447	0.218	0.0220859132370697	3	AGPAT2
**2.04086433777426E-07**	0.699648290388779	0.883	0.579	0.00342008045724211	3	TAPBP
**2.4483969192098E-12**	0.699346093636811	0.638	0.29	4.10302355721179E-08	3	CBFB
**9.71395969508733E-08**	0.699338371868596	0.426	0.189	0.00162786536570274	3	RAB32
**9.07636187897841E-08**	0.695843851856175	0.34	0.13	0.0015210167236792	3	DENND4A
**1.78509500816386E-09**	0.69471743209674	0.447	0.183	2.991462214681E-05	3	LMO4
**2.10594485772301E-08**	0.694488150253842	0.319	0.109	0.000352914239257222	3	NDUFB4
**2.00356431066761E-09**	0.693255229827818	0.383	0.136	3.35757307181678E-05	3	DYNC1LI1
**2.07284430135661E-09**	0.6908442548354	0.351	0.123	3.47367248021341E-05	3	CABIN1
**8.04191461518305E-08**	0.690120747656144	0.489	0.229	0.00134766405121238	3	NDUFAF3
**3.83495431812919E-09**	0.68622093754959	0.649	0.32	6.4266164463209E-05	3	UBE2A
**1.12362068689252E-09**	0.686203417561803	0.298	0.092	1.88296354709449E-05	3	SCML1
**9.20498355746648E-10**	0.683475400747849	0.511	0.221	1.54257114456023E-05	3	ATP1A1
**5.97449785266407E-08**	0.678965333327793	0.809	0.529	0.00100120635014945	3	ARHGDIB
**8.21297405015434E-07**	0.678604515866627	0.606	0.326	0.0137633019132486	3	ESYT1
**5.99076716824746E-10**	0.678238131254318	0.532	0.224	1.00393276205491E-05	3	COX6A1
**6.75812118171577E-09**	0.676556059711726	0.511	0.227	0.000113252594763193	3	NACC1

**2.94054393957381E-07**	0.675511927252628	0.638	0.374	0.0049277635339378	3	CALM3
**3.14597174734795E-08**	0.674617807453569	0.511	0.224	0.00052720194542057	3	CDC73
**5.12910033931711E-12**	0.674178083803632	0.691	0.328	8.59534634862762E-08	3	FMNL1
**3.43967265177174E-07**	0.673830113454903	0.447	0.196	0.00576420342983909	3	SRPRB
**1.02008059781239E-06**	0.673522480745803	0.277	0.1	0.01709451065814	3	GBE1
**1.91206366170126E-08**	0.673360934513509	0.617	0.327	0.000320423628427897	3	TMED9
**8.17908093294272E-11**	0.673164198290302	0.5	0.196	1.37065038274254E-06	3	KHDRBS1
**5.61070144325659E-09**	0.67212364365001	0.309	0.102	9.4024134786094E-05	3	ERGIC2
**8.74089217357764E-09**	0.670239434306786	0.511	0.217	0.000146479871044814	3	POLDIP2
**4.51882905789816E-08**	0.669553960539763	0.638	0.335	0.000757265373522574	3	COX4I1
**1.06879551831415E-08**	0.669128914689486	0.298	0.098	0.000179108752959086	3	COX20
**2.49965789645953E-10**	0.668093728407607	0.298	0.088	4.18892670288688E-06	3	CZIB
**6.10735939149149E-11**	0.667778010494492	0.649	0.306	1.02347128682614E-06	3	SUMO3
**6.2216481064242E-07**	0.667251709637995	0.309	0.121	0.0104262378967457	3	AURKAIP1
**2.66442780566569E-07**	0.66611205559649	0.436	0.194	0.00446504811673456	3	CLIC4
**5.50936739306732E-10**	0.665578639170183	0.372	0.132	9.23259787730222E-06	3	PIN1
**1.93874408218328E-09**	0.66389434725038	0.628	0.312	3.24894733292273E-05	3	CCDC88A
**2.09340765036312E-08**	0.663855749081106	0.574	0.29	0.000350813254047852	3	HNRNPUL2
**2.43501313012108E-11**	0.663090195534956	0.787	0.42	4.08059500345691E-07	3	HNRNPA0
**1.11421252582185E-07**	0.661435058347992	0.447	0.195	0.00186719735077225	3	RAB3GAP1
**4.09977417817717E-07**	0.660607788155929	0.468	0.223	0.00687040156778931	3	EIF4G1
**8.90360831127439E-08**	0.660249611870521	0.936	0.756	0.00149206668080336	3	LYZ
**1.64389664615682E-07**	0.659840768049274	0.457	0.216	0.00275484199962959	3	SERTAD2
**7.7699677959058E-07**	0.659768585368962	0.309	0.121	0.0130209120323789	3	SCNM1
**1.05518691184693E-06**	0.659516035585014	0.33	0.133	0.0176828222687309	3	SLC25A25
**6.63447896740033E-08**	0.659426840322604	0.447	0.188	0.00111180598535695	3	GID8
**5.44974892383317E-14**	0.657695928377254	0.362	0.099	9.13268924655962E-10	3	THOC3
**1.02883466294323E-07**	0.65685685593431	0.543	0.267	0.00172412112816026	3	MICOS10
**3.91099223657343E-08**	0.65675570316719	0.468	0.209	0.000655404079004975	3	BRD1
**2.13407514991856E-08**	0.656754029235364	0.585	0.289	0.000357628313623352	3	NMT1
**2.75126221290479E-07**	0.65615307994707	0.298	0.108	0.00461056521638585	3	SRD5A3
**1.5847908037786E-06**	0.654394829811251	0.298	0.114	0.0265579242897217	3	FAM3A
**3.76892748718502E-07**	0.654353719070919	0.468	0.221	0.00631596868302465	3	CPSF6
**1.32348437984408E-09**	0.653284267190488	0.596	0.276	2.21789512374271E-05	3	LAT2
**2.30904851968999E-07**	0.652740479012786	0.436	0.2	0.00386950350929648	3	ABHD3
**1.0881815101313E-08**	0.652310285478212	0.5	0.232	0.000182357457467804	3	HMG20B
**7.83910960937726E-11**	0.651417833666623	0.362	0.119	1.31367798833944E-06	3	CDK5RAP2
**6.53266649963567E-08**	0.651340255615709	0.255	0.084	0.00109474425200895	3	N4BP2
**2.81035298785877E-07**	0.649143776211539	0.372	0.151	0.00470958953705372	3	FDPS
**3.62439957332402E-07**	0.6462639155374	0.426	0.189	0.00607376880497639	3	MYO1C
**4.98119125026979E-08**	0.643956008129108	0.532	0.257	0.000834748029720211	3	SPCS1
**4.26122492355019E-08**	0.64092557467914	0.457	0.201	0.000714096072688541	3	DYM
**7.16413887811322E-08**	0.640773948091896	0.819	0.554	0.00120056639319421	3	EFHD2
**6.17414666902221E-08**	0.640190627456426	0.426	0.175	0.00103466349879474	3	GPS1
**3.28714559210863E-07**	0.640074651155955	0.436	0.195	0.00550859858325564	3	STARD10
**6.19709277817644E-09**	0.639309608807336	0.511	0.228	0.000103850880776681	3	ANAPC11
**3.26143829167076E-07**	0.639169867847844	0.479	0.229	0.00546551828918186	3	FBXW5
**3.92921207462478E-08**	0.63913896002954	0.277	0.092	0.000658457359465621	3	CENPC
**1.50701234928412E-08**	0.636503170472475	0.489	0.226	0.000252545129493033	3	HNRNPAB
**8.70970861053735E-09**	0.636098246045903	0.436	0.18	0.000145957296895385	3	DCAF12

3.68660555429768E-12	0.636008753605636	0.287	0.073	6.17801358789205E-08	3	TOP3A
1.67171480016764E-08	0.63592258517949	0.457	0.194	0.000280145966212094	3	ZRANB2
3.36335860411514E-09	0.635234867701603	0.404	0.155	5.63631634877615E-05	3	ANP32A
1.64868379349303E-07	0.63521830921661	0.5	0.237	0.00276286430113561	3	SHISA5
2.6791500426331E-07	0.633264836105225	0.489	0.227	0.00448971964144455	3	DUS1L
4.24558739772811E-07	0.632659512734519	0.362	0.152	0.00711475536111277	3	CSNK1G2
6.79886804549412E-10	0.631910093746823	0.436	0.166	1.13935430706391E-05	3	ARAF
4.252378337347E-07	0.631286084881867	0.298	0.115	0.0071261356177261	3	NOP2
1.9908954615595E-06	0.629744198340903	0.681	0.375	0.0333634261448141	3	SPINT2
1.17085608931099E-08	0.629339186752068	0.447	0.189	0.000196212063446735	3	RRP36
2.29528356721043E-09	0.628967394953616	0.287	0.09	3.84643620193124E-05	3	ACAP1
6.51028339772657E-10	0.628641514494023	0.277	0.081	1.09099329179102E-05	3	RIOK1
9.97622895730252E-09	0.627896510095677	0.426	0.179	0.000167181644866476	3	DR1
5.54911526044552E-09	0.627409926102391	0.404	0.156	9.2992073534546E-05	3	SFPQ
1.39858441491997E-10	0.62718319960947	0.67	0.332	2.34374776252289E-06	3	TRIR
1.4079109326966E-07	0.62550015705461	0.372	0.155	0.00235937714101297	3	TNPO2
2.11915060772445E-08	0.625497580891506	0.66	0.347	0.000355127258842463	3	RNPEP
1.89997541514271E-08	0.624941607071672	0.383	0.148	0.00031839788006961 5	3	NAA15
3.66079224377159E-10	0.624242526033901	0.404	0.145	6.13475564211243E-06	3	NUP133
5.17899562337341E-07	0.6237622886254	0.426	0.193	0.00867896086564916	3	ARL17A
4.0060701854744E-10	0.6234358277635	0.617	0.284	6.71337241681799E-06	3	TMED10
2.82901053224852E-08	0.622904788136778	0.553	0.263	0.000474085584994207	3	SCAF11
5.97824009922001E-09	0.622115805813979	0.553	0.258	0.000100183347582729	3	SMARCA4
1.47423809988507E-06	0.621646494813641	0.319	0.128	0.0247052820778739	3	CBWD2
1.30493465365714E-06	0.621387795618229	0.691	0.404	0.0218680949259863	3	AHR
1.93034710976349E-07	0.620201208879818	0.532	0.263	0.00323487568654166	3	MLF 2.00
3.25645416087378E-07	0.620078472259804	0.394	0.169	0.00545716588279229	3	HEXIM1
1.19963594780574E-06	0.619114835333084	0.33	0.137	0.0201034992133285	3	GYS1
1.52837273397375E-06	0.616420499940655	0.266	0.101	0.0256124702759321	3	PPIE
1.27293605950651E-09	0.616207282135106	0.521	0.224	2.13318624852101E-05	3	SERBP1
6.85469385501458E-09	0.615780339787818	0.713	0.385	0.000114870959622334	3	GNB2
2.80096399135238E-09	0.615653495001807	0.511	0.211	4.69385545670833E-05	3	NUP98
1.10667680825087E-10	0.615559239211018	0.415	0.147	1.85456899526681E-06	3	CHERP
8.01005030301244E-08	0.614877727942606	0.617	0.358	0.00134232422977883	3	HADHA
1.10020387244123E-10	0.612924679313011	0.521	0.21	1.84372164943702E-06	3	KBTBD2
2.63709709645782E-08	0.612157698440764	0.617	0.327	0.000441924731424401	3	STT3A
9.49088458171537E-08	0.611541636472828	0.447	0.195	0.00159048243820386	3	VDAC3
7.61673416517961E-09	0.611445097681601	0.426	0.167	0.00012764123114008	3	PREB
2.13479692374672E-08	0.610667311687854	0.702	0.383	0.000357749268481476	3	PDIA3
1.82025071591604E-07	0.610380353062276	0.628	0.321	0.00305037614973209	3	GBA
1.91847192154159E-07	0.610025122349295	0.34	0.13	0.00321497524611939	3	RASA2
1.60065749771517E-06	0.609915810627135	0.426	0.193	0.0268238183467108	3	RFTN1
7.21716662037731E-11	0.609068005114471	0.691	0.334	1.20945278224283E-06	3	SUGP2
2.37208106671801E-07	0.609033115270355	0.638	0.357	0.00397513345160604	3	SNRNP200
3.74535876063918E-09	0.60889796292782	0.426	0.166	6.27647221107914E-05	3	MIIP
1.0538692178922E-08	0.608336711647074	0.287	0.092	0.000176607403534374	3	SPATA5
1.74085384660833E-08	0.607279223998778	0.66	0.344	0.000291732287614624	3	SERF2
5.33432451782365E-07	0.607137042518334	0.574	0.299	0.00893926102696887	3	TMEM248
5.98764185148078E-07	0.606759143378802	0.319	0.123	0.0100340902147115	3	ALG12
6.02829289252904E-13	0.606239043563834	0.319	0.084	1.01022132293002E-08	3	POLA1

2.94497463505598E-11	0.605682353502743	0.649	0.303	4.93518849342681E-07	3	ARHGDIA
1.27981935714622E-06	0.604921907887179	0.479	0.239	0.0214472127870564	3	COX8A
4.90979434372105E-09	0.60334200698645	0.277	0.086	8.22783336120774E-05	3	CHCHD3
1.81140762588696E-06	0.600730019086961	0.574	0.306	0.0303555689946137	3	RNPEPL1
1.47358780973851E-07	0.598253337493777	0.415	0.184	0.0024694384515598	3	CNOT9
1.33845218782953E-07	0.597244500492378	0.34	0.13	0.00224297817636472	3	MOB4
7.74174115895906E-07	0.596580893790484	0.468	0.222	0.0129736098341836	3	RNF5
9.05150431462965E-09	0.596212881514409	0.415	0.159	0.000151685109304564	3	TECR
2.7020841080721E-06	0.596032199455565	0.638	0.369	0.0452815254830722	3	TAF10
5.62326545028651E-07	0.595392828820824	0.468	0.228	0.00942346824159014	3	STIM1
7.13509702141529E-07	0.595380402594608	0.372	0.164	0.0119569955884877	3	ECHDC1
1.92862382020366E-07	0.594837299315815	0.479	0.23	0.00323198779789729	3	PILRB
6.09328293161656E-07	0.594498217891396	0.277	0.102	0.010211123536803	3	TNFRSF11A
2.46984369149908E-06	0.593719178001891	0.319	0.134	0.0413896405821416	3	MMD
7.60537863811603E-09	0.592762194816191	0.628	0.301	0.000127450935217548	3	ADI1
1.30301736296674E-07	0.59247949755065	0.287	0.1	0.00218359649685966	3	SC5D
3.71010241460857E-10	0.592346934499372	0.543	0.225	6.21738962640105E-06	3	RPN2
4.73217169127156E-10	0.59127115069052	0.521	0.212	7.93017332023288E-06	3	BAZ1B
1.01592864439802E-10	0.59086646254925	0.5	0.196	1.70249322228221E-06	3	HDGFL2
7.96568367551997E-07	0.589845719454842	0.309	0.124	0.0133488927034364	3	FN3KRP
7.48691915379242E-09	0.589645681287087	0.638	0.331	0.000125465791179253	3	NDUFV2
7.52056094765169E-10	0.589464954567592	0.628	0.306	1.26029560360747E-05	3	ACTN4
1.0453465147296E-07	0.587159531203937	0.415	0.176	0.00175179168938386	3	PSMD7
9.93035422326026E-08	0.585947373404537	0.66	0.351	0.00166412876073395	3	KIAA0930
3.02575538387877E-10	0.585782053952189	0.521	0.21	5.07056087230405E-06	3	APEH
5.34636647663076E-08	0.585774317945623	0.33	0.12	0.000895944094153783	3	HMGXB4
8.6395867834352E-10	0.582423446163268	0.468	0.185	1.44782195316807E-05	3	PTBP1
1.64793632200546E-08	0.582138069998862	0.457	0.197	0.000276161168841675	3	C9orf78
1.20589004624734E-08	0.581583146967736	0.415	0.166	0.000202083053950129	3	CCDC6
1.49607811005943E-07	0.581132882351554	0.468	0.213	0.0025071276968376	3	PRKCSH
5.98806983720384E-08	0.580606590360797	0.734	0.417	0.00100348074331862	3	MBD2
5.6151856619891E-08	0.579293078500958	0.34	0.13	0.000940992813236133	3	CTPS1
1.5580103559104E-07	0.576614918127522	0.755	0.477	0.00261091375443465	3	PPT1
1.52697786814819E-09	0.575369673772271	0.585	0.268	2.55890951144274E-05	3	TUFM
1.24393051851247E-07	0.575109294640025	0.372	0.153	0.0020845787629232	3	CRELD2
2.97119905469838E-06	0.573580879955248	0.277	0.109	0.0497913537586354	3	GGCT
2.08364544021008E-06	0.573292792786963	0.266	0.102	0.0349177302870406	3	NTPCR
6.21242042298818E-07	0.572504302843596	0.638	0.34	0.0104107741448436	3	MGST3
9.76273401519385E-09	0.572002108478482	0.319	0.11	0.000163603896626618	3	NOP16
3.14027075262026E-09	0.571864109640659	0.84	0.544	5.26246572724103E-05	3	HNRNPA2B
8.45500395942733E-08	0.571798608761609	0.521	0.241	0.00141688956352083	3	NEDD8
8.76770505226471E-13	0.571028980785307	0.628	0.261	1.46929201265852E-08	3	EMP3
5.33409702414771E-08	0.570096729403322	0.468	0.203	0.000893887979306674	3	IWS1
2.25189860891978E-09	0.567177342424038	0.543	0.243	3.77373168882776E-05	3	MTA2
2.68130327059083E-07	0.566502719999191	0.319	0.125	0.0044933280208561	3	GLE1
3.98332671317197E-07	0.565243783994562	0.394	0.176	0.00667525890593359	3	HDHD5
1.34456670878008E-06	0.564553911259642	0.319	0.132	0.0225322489057366	3	HARS
7.86583827561454E-07	0.563757824793949	0.34	0.142	0.0131815717822748	3	ADSS
7.4930265206691E-07	0.563745917451953	0.415	0.196	0.0125568138433373	3	NOP56
1.52565634368876E-08	0.563673152522442	0.553	0.263	0.000255669490075362	3	HIPK1

**8.94125315285589E-07**	0.562871563671082	0.255	0.092	0.0149837520335559	3	CDK19
**1.56138659249933E-06**	0.561730130436954	0.489	0.252	0.0261657165171038	3	SHKBP1
**1.14050393854798E-07**	0.560545010724938	0.383	0.153	0.0019112565002187	3	DNAJC1
**2.10584622621337E-07**	0.559322164490154	0.585	0.303	0.00352897710588837	3	CEP350
**5.83235959253011E-08**	0.557769642831006	0.84	0.519	0.000977386820516196	3	DBNL
**2.46050176585931E-08**	0.55759372217056	0.404	0.162	0.000412330885922703	3	COMMD4
**1.41392543470322E-06**	0.557030219903214	0.266	0.101	0.0236945624347566	3	CNOT1
**1.21331662133795E-08**	0.554747194443217	0.415	0.167	0.000203327599403814	3	NDUFA9
**5.00693913718286E-09**	0.550991657166456	0.468	0.196	8.39062860609103E-05	3	WWP2
**2.0609113011825E-07**	0.550660659584611	0.723	0.395	0.00345367515852164	3	PGD
**1.55830461320088E-13**	0.549823018387171	0.394	0.114	2.61140687080204E-09	3	RNGTT
**6.51175629541714E-07**	0.548928407788996	0.649	0.364	0.01091240119986	3	LASP1
**7.7649417259808E-08**	0.54752880046364	0.585	0.286	0.00130124893443986	3	UBXN1
**1.1066515463475E-07**	0.545457932483825	0.436	0.185	0.00185452666136915	3	CDK2AP1
**8.87342873438032E-07**	0.545266577146845	0.266	0.095	0.0148700918730745	3	SP2
**5.90517531612229E-07**	0.545231269803957	0.33	0.132	0.00989589279475774	3	PSMA2
**1.42338885261874E-07**	0.544211182159831	0.309	0.113	0.00238531503921849	3	SNRPD2
**1.32390587101035E-06**	0.543844843914998	0.543	0.283	0.0221860145863915	3	THRAP3
**1.11294269213347E-06**	0.543374919116025	0.266	0.098	0.0186506936347726	3	CPOX
**1.5080630777669E-07**	0.5416743929658	0.457	0.209	0.00252721210572176	3	PSMD13
**3.25326878592997E-07**	0.540658671410324	0.511	0.247	0.00545182783146144	3	HNRNPL
**4.60060385122877E-07**	0.540648942754092	0.319	0.122	0.00770969193388918	3	ASGR2
**2.04045269020375E-11**	0.539701905858739	0.532	0.212	3.41939061824344E-07	3	SET
**1.70086334222778E-06**	0.539394555698895	0.862	0.535	0.0285030678890531	3	PLEKHO1
**5.45398269383455E-07**	0.53921509311482	0.33	0.132	0.00913978419832794	3	FAM20B
**4.93225714952779E-07**	0.538148210004213	0.489	0.243	0.00826547653117866	3	MAP2K2
**7.48799435326901E-08**	0.536890890083657	0.319	0.115	0.00125483809372082	3	CCT8
**3.56338997974422E-07**	0.535464441131769	0.255	0.09	0.00597152892805537	3	ABI2
**2.1388975680014E-06**	0.53512264975203	0.404	0.186	0.0358436454445675	3	DCAF11
**9.74310766479683E-09**	0.534112009032542	0.617	0.318	0.000163274998246665	3	ADRM1
**9.66371582815674E-07**	0.53405560650671	0.383	0.166	0.0161944549848251	3	SDR39U1
**1.87852668920051E-06**	0.533410557546483	0.479	0.233	0.0314803502576222	3	CLPTM1L
**2.67815910783345E-06**	0.53302431013031	0.319	0.132	0.044880590329073	3	MAP3K14
**1.97675038248635E-10**	0.531485536631158	0.606	0.268	3.31263829097063E-06	3	MYH9
**1.57293640227711E-06**	0.531213081881017	0.372	0.164	0.0263592682293598	3	PHF2
**1.25714556891889E-10**	0.529947610954296	0.479	0.182	2.10672454439427E-06	3	ZNF384
**1.45654213001776E-07**	0.52932741513764	0.564	0.284	0.00244087330148376	3	AGPAT3
**5.77419174807415E-07**	0.528438132072626	0.298	0.113	0.00967639053142266	3	COPS7B
**4.73107902739597E-07**	0.528119170993264	0.543	0.28	0.00792834223411017	3	AP2S1
**2.30621166284525E-07**	0.527573027830851	0.372	0.152	0.00386474950459607	3	TRAP1
**1.68857381798227E-06**	0.527337292857251	0.511	0.26	0.0282971200417469	3	AGPS
**5.43543477318871E-07**	0.526646279410776	0.309	0.115	0.00910870159290965	3	YIPF5
**1.38615756386495E-06**	0.525465684424215	0.479	0.25	0.0232292284552488	3	PFDN2
**1.34602290273522E-06**	0.522983451006077	0.383	0.166	0.0225566518040369	3	GTPBP2
**5.65165511876895E-10**	0.522274767892517	0.298	0.09	9.47104364803301E-06	3	GABPB1
**3.14030468132553E-09**	0.521136918309756	0.33	0.113	5.26252258496532E-05	3	TAF6
**5.98962889846607E-08**	0.520876705857761	0.596	0.306	0.00100374201080494	3	DDX6
**3.21080967748628E-10**	0.520512621473877	0.532	0.231	5.3806748575315E-06	3	KDM3B
**6.45287044613171E-10**	0.520303795947751	0.436	0.173	1.08137202936275E-05	3	PNRC2
**2.34738821691365E-07**	0.520282925587853	0.372	0.156	0.00393375317390389	3	NUP93

1.06641712477184E-06	0.520117145770463	0.479	0.234	0.0178710181769264	3	SRSF3
6.92131006859329E-10	0.519405802577599	0.713	0.365	1.15987314129486E-05	3	EZR
5.6373964507265E-08	0.519304529110789	0.287	0.099	0.000944714897212747	3	TFAM
5.88875273524351E-07	0.517247042334409	0.266	0.096	0.00986837183372108	3	PSMA3
3.62248161095023E-07	0.516131491332366	0.319	0.123	0.0060705546836304	3	KDM1B
1.52568914275515E-08	0.51549027843106	0.457	0.19	0.000255674986542908	3	S100A4
4.49996840941305E-07	0.514448750501418	0.447	0.207	0.00754104706049439	3	ADAM15
1.32899225105045E-07	0.514388090045029	0.511	0.24	0.00222712521431034	3	CCND3
6.90282185404827E-09	0.514361055233731	0.415	0.162	0.000115677488630141	3	SF1
3.03498025623249E-09	0.514333959724632	0.638	0.326	5.0860199133944E-05	3	UBB
4.19835620791018E-10	0.512677564177965	0.489	0.197	7.03560533321588E-06	3	SNX20
4.6657592236938E-08	0.512043207398505	0.511	0.229	0.000781887930706607	3	XRN2
7.77304786534374E-08	0.511923032228775	0.649	0.361	0.0013026073612743	3	CNTRL
1.55781473844048E-07	0.511460999221576	0.596	0.302	0.00261058593867855	3	EVI2B
5.05260344618064E-07	0.510435739289002	0.479	0.223	0.00846715285510952	3	PRDX4
4.38992594269507E-07	0.50995340934553	0.362	0.148	0.0073566378947684	3	PPDPF
3.37016434938845E-07	0.509928173054029	0.33	0.125	0.00564772141670517	3	ZNF768
3.9163252823133E-09	0.509746830799968	0.415	0.156	6.56297790810063E-05	3	TMEM14C
2.92985124742367E-09	0.509636692667499	0.372	0.133	4.90984472043258E-05	3	TTLL5
2.2563889778141E-07	0.508428278174342	0.298	0.112	0.00378125664902086	3	TMEM30A
5.01247146862307E-10	0.50761004032701	0.5	0.204	8.39989968711855E-06	3	TRABD
1.96765884352599E-07	0.506867909932896	0.468	0.215	0.00329740268998086	3	ATP5PD
5.17589818785933E-10	0.503681891669012	0.457	0.178	8.67377018321467E-06	3	DYRK1A
1.11076587936704E-06	0.503631465144656	0.351	0.148	0.0186142146064329	3	WRNIP1
1.2728202816285E-08	0.503624748679641	0.574	0.289	0.000213299222795304	3	NCL
2.21044588996738E-07	0.501104770284781	0.33	0.124	0.00370426522240733	3	ASCC3
2.49953016890039E-09	0.500849968087677	0.691	0.363	4.18871265704327E-05	3	CNN2
4.34397228089766E-07	0.499780184443343	0.404	0.181	0.00727962874832829	3	POLR2B
5.64869782147353E-08	0.498412971903868	0.468	0.203	0.000946608780922534	3	ACTR1B
2.80484046513229E-07	0.498380869196419	0.351	0.139	0.0047003516514687	3	GTPBP4
6.64961185771069E-09	0.495306144099888	0.457	0.186	0.000111434195511516	3	VARS
2.95905137996941E-08	0.495064432752891	0.447	0.187	0.000495877830255274	3	GALM
1.24104516127617E-07	0.493544810724867	0.394	0.164	0.0020797434812666	3	NUBP1
5.79027562602265E-07	0.493522246127477	0.33	0.131	0.00970334389408876	3	JMJD8
1.58219631526587E-07	0.492911179879403	0.5	0.233	0.00265144458512254	3	MTMR10
8.74293373135346E-08	0.492751723391631	0.596	0.287	0.00146514083470021	3	CPNE1
7.83416252978339E-09	0.492749727850293	0.468	0.192	0.00013128489567411	3	SEC16A
5.130185494921E-12	0.492587202032219	0.67	0.28	8.59716485238862E-08	3	FUS
3.35125387438604E-08	0.491999112170484	0.819	0.509	0.000561603124269612	3	DPYSL2
1.16260248817336E-06	0.491686973706882	0.457	0.218	0.0194828924968092	3	CFB
6.7707940539492E-07	0.490248519189317	0.511	0.258	0.0113464966756081	3	DYNLL2
3.33146197787025E-07	0.490224808421122	0.34	0.139	0.00558286398251496	3	TIMM10
4.81741633040003E-11	0.489988224040449	0.34	0.103	8.07302628648436E-07	3	LRRC40
4.61209335923481E-07	0.489659578063927	0.777	0.463	0.00772894605140569	3	RHOA
2.85647755463683E-06	0.488817008299405	0.713	0.414	0.0478688508606039	3	LAMTOR4
1.07252953308205E-09	0.488609925218982	0.532	0.231	1.7973449915389E-05	3	CYB5B
1.49324407481598E-06	0.48725197245916	0.468	0.229	0.0250237842057662	3	TUBGCP3
1.60224783067241E-06	0.487031366838664	0.66	0.374	0.0268504691464083	3	SLC38A10
2.32819782162935E-09	0.486559977389707	0.436	0.173	3.90159390948647E-05	3	TNPO3
2.01585884153276E-11	0.485837235528013	0.447	0.155	3.3781762466406E-07	3	FAM122B

3.63650754074539E-08	0.485175887761252	0.628	0.302	0.000609405933678113	3	EDEM1
1.54820328659234E-06	0.485162336335966	0.383	0.169	0.0259447906767144	3	ELK4
9.68375497580863E-07	0.484915366657215	0.511	0.272	0.0162280365884601	3	VKORC1
2.17605976146994E-08	0.482223393413327	0.383	0.149	0.000364664094827132	3	HDAC1
1.35234060716209E-06	0.481759273395683	0.351	0.144	0.0226625238948222	3	HSD17B6
2.08858858660026E-06	0.481602697904519	0.319	0.127	0.0350005675342472	3	TCF3
1.09268168881757E-06	0.48016087868591	0.479	0.228	0.0183111597412048	3	DLD
1.78535350475622E-09	0.478898326165096	0.691	0.371	2.99189540327048E-05	3	HNRNPA3
5.00494468458061E-07	0.477644085988657	0.383	0.166	0.00838728630242019	3	LARP7
2.44517599224589E-09	0.477018809764809	0.255	0.074	4.09762592780566E-05	3	CEP131
4.03499180126468E-09	0.476745576628178	0.628	0.301	6.76183926055935E-05	3	YWHAH
1.81899148340386E-06	0.476580384029195	0.287	0.113	0.0304826592788818	3	PDS5B
3.18644740919224E-14	0.476286001883958	0.479	0.152	5.33984856832435E-10	3	TTC39C
1.2136370745754E-08	0.475581035991131	0.553	0.276	0.000203381300957346	3	UPF1
1.42763967541632E-08	0.474168050811668	0.394	0.153	0.000239243856806267	3	LNPK
5.25857976530889E-07	0.47310056373176	1	0.987	0.00881232797070464	3	CD74
4.39950121996118E-07	0.471317712871179	0.617	0.34	0.00737268414441094	3	NDUFA2
1.36584643611579E-12	0.471229199499855	0.447	0.149	2.28888545764283E-08	3	MGME1
1.78828984916669E-07	0.470321636357946	0.34	0.134	0.00299681612923354	3	CTNNBIP1
6.74197479972993E-08	0.468960789264189	0.5	0.225	0.00112982013693874	3	PIEZO1
2.37035317905876E-08	0.46884061704246	0.532	0.26	0.000397223785746667	3	ATP5MG
4.29311440508851E-09	0.467310916897398	0.383	0.141	7.19440112004733E-05	3	NUDT21
1.59002544850988E-06	0.466224854805297	0.309	0.125	0.0266456464661286	3	TCOF1
1.18314537525209E-09	0.465597437197798	0.681	0.345	1.98271501984746E-05	3	YWHAZ
7.54269227243435E-10	0.463035250717449	0.564	0.244	1.26400437101455E-05	3	NSFL1C
2.64519675312327E-07	0.462787841234813	0.511	0.248	0.00443282071888397	3	ATP5F1B
1.08354678201432E-06	0.462168110795746	0.34	0.14	0.0181580769729961	3	ATIC
2.5024484742401E-06	0.461079502511698	0.309	0.124	0.0419360315313155	3	GTF3C2
1.6317107327893E-06	0.460811191725619	0.319	0.13	0.027344208460083	3	UBL5
1.88330943286638E-07	0.460785713873355	0.468	0.217	0.00315604994759747	3	AFTPH
9.97981246996343E-07	0.459333777968745	0.415	0.185	0.0167241697371647	3	AKAP11
6.97221184731767E-08	0.458685329450169	0.5	0.247	0.0011684032613735	3	UXT
7.55131677078688E-08	0.457232178484741	0.426	0.183	0.00126544966444847	3	TXN2
1.47498373728903E-08	0.456688383811646	0.521	0.231	0.000247177774694895	3	NUP43
7.2818654219064E-08	0.456644381398324	0.457	0.194	0.00122029500740307	3	FAM32A
4.33429752926225E-08	0.45636120223343	0.383	0.15	0.000726341579953767	3	EHMT2
2.81458260952493E-07	0.456181156520228	0.33	0.126	0.00471667753704187	3	STARD7
2.08331571095699E-09	0.454882440833366	0.415	0.15	3.49122046842173E-05	3	SERPINA6
1.02441460403883E-07	0.454817627595171	0.394	0.17	0.00171671399344827	3	SOGA1
1.93192356666678E-06	0.454763152015931	0.362	0.152	0.0323751751302018	3	ANO6
5.21234832584979E-07	0.448527680714814	0.468	0.221	0.00873485332445907	3	STK11
1.78032131592022E-06	0.448153056645161	0.298	0.118	0.029834624612191	3	C19orf53
2.21259026005657E-06	0.447694884746781	0.383	0.17	0.0370785875780279	3	ENOPH1
3.62518193190802E-07	0.447633745041375	0.468	0.221	0.00607507988149146	3	S100A6
9.33202425200145E-07	0.446228956575625	0.33	0.133	0.015638606241504	3	SLC4A2
2.27147605100809E-06	0.445551093147535	0.404	0.188	0.0380653956627936	3	PRPF8
7.7189958434663E-09	0.444637307706261	0.426	0.171	0.000129354932344808	3	RNF169
1.97414801851785E-06	0.444395215995418	0.681	0.407	0.0330827724943222	3	TSPAN3
9.91817459769672E-07	0.4440189153123	0.511	0.249	0.0166208769908202	3	FGA
4.15732377342185E-07	0.443393967097779	0.404	0.174	0.00696684317950034	3	INTS1

1.4654330126008E-06	0.443391141609985	0.309	0.12	0.0245577264251642	3	SIRT7
5.433726818133E-09	0.442546704467646	0.287	0.09	9.10583940182729E-05	3	U2AF2
2.11374472067537E-06	0.441362347751705	0.415	0.193	0.0354221340290779	3	API5
1.98875094556357E-07	0.439780356361277	0.521	0.245	0.00333274883457543	3	LPCAT2
1.40555395957103E-07	0.438567575771801	0.649	0.346	0.00235542732544913	3	CALR
1.18987162744469E-06	0.437691204705723	0.66	0.358	0.0199398687327182	3	ARL6IP4
6.75719510587803E-07	0.43711489676907	0.394	0.169	0.0113237075584304	3	MTHFD2
1.82835323012724E-07	0.434197651539778	0.543	0.261	0.00306395434304723	3	PI4KA
3.09349791945377E-07	0.43286417653579	0.383	0.155	0.00518408381342063	3	GCNT1
1.06242650775851E-07	0.432355014550578	0.404	0.164	0.00178041434170171	3	BRMS1
5.09455271598682E-09	0.431391229239457	0.468	0.19	8.53745144145072E-05	3	CTDNEP1
2.43342580552865E-07	0.430455913240469	0.394	0.17	0.0040779349649049	3	CAND1
2.25721337907659E-06	0.430380543687885	0.309	0.123	0.0378263818065656	3	MED1
1.64966865435233E-09	0.429928546528415	0.33	0.109	2.76451473096363E-05	3	BRD8
9.4706237939162E-07	0.428723343105693	0.734	0.491	0.0158708713538448	3	IFI16
1.78236916200062E-07	0.428305896906265	0.298	0.109	0.00298689424168064	3	SERPINB8
1.83646988676371E-08	0.428183224057247	0.266	0.085	0.000307755623623863	3	ECSIT
9.66419573346486E-07	0.427639588144647	0.564	0.289	0.0161952592101404	3	SEC13
1.54826945331204E-06	0.427603121679975	0.362	0.156	0.0259458994986031	3	PRCC
3.31902320903733E-08	0.427276206094872	0.372	0.144	0.000556201909370476	3	PIH1D1
5.25397308233096E-13	0.426476809373293	0.351	0.098	8.80460809137023E-09	3	DEPDC5
1.4307420163472E-06	0.426081602591694	0.521	0.263	0.0239763747099464	3	PDHA1
6.17760480839509E-07	0.425865261592538	0.521	0.27	0.0103524301379085	3	PDIA6
9.05281440728406E-07	0.425738657540053	0.383	0.166	0.0151707063837266	3	EIF3H
5.98745221891887E-09	0.425100886482637	0.574	0.264	0.000100337724284642	3	TRIM26
3.91096204318715E-08	0.424617713127853	0.468	0.204	0.000655399019197302	3	MED14
5.1999238466376E-08	0.42338373437304	0.436	0.18	0.000871403238219529	3	TIMMDC1
6.73583904894037E-08	0.422871525112364	0.277	0.093	0.00112879190782143	3	FNTB
1.03958128012543E-06	0.420012415270319	0.543	0.279	0.0174213030923419	3	ANKRD44
9.83014919133209E-07	0.419816351697928	0.298	0.116	0.0164733640148343	3	CA2
1.50728527188946E-10	0.419446775640382	0.468	0.177	2.52590865863236E-06	3	SART3
2.19065593235455E-07	0.418130438009203	0.404	0.167	0.00367110121143975	3	NUDT22
1.30276576739553E-07	0.414995684256844	0.426	0.185	0.00218317487300142	3	CALM1
3.1565167994142E-07	0.414125871339339	0.585	0.292	0.00528969085245831	3	ACTR1A
1.39385692062549E-06	0.41391783047632	0.468	0.232	0.023358254275842	3	DYNLRB1
1.16872101973231E-06	0.413634149903053	0.33	0.138	0.0195854268486741	3	CXXC1
4.76888906424894E-08	0.409907009602921	0.479	0.21	0.000799170429386838	3	KHSRP
1.1512425440749E-06	0.409898982197925	0.468	0.22	0.0192925225536072	3	SLC39A7
2.6571124790935E-06	0.409805539562793	0.511	0.254	0.0445278909246489	3	CPPED1
3.67454712683489E-07	0.409600212710741	0.319	0.122	0.0061578060751499	3	NCKIPSD
4.636844975188E-07	0.409364934246105	0.543	0.269	0.00777042480942005	3	SNX10
7.24244189454961E-07	0.409118579763351	0.426	0.189	0.0121368841268862	3	AAAS
6.4533443333021E-07	0.40443433378242	0.468	0.22	0.0108145144337477	3	UBA1
1.13473279662831E-06	0.402555252489393	0.34	0.139	0.0190158522058972	3	CUEDC2
8.46157202156371E-10	0.400772638620386	0.681	0.323	1.41799023937365E-05	3	IKZF1
2.08924814762376E-06	0.40061058936093	0.681	0.397	0.0350116204578789	3	VASP
4.87949264607616E-07	0.400498486028985	0.383	0.163	0.00817705377629443	3	TELO2
2.84635374323997E-07	0.399065398753842	0.628	0.323	0.00476991960292154	3	SLC25A39
5.83712433813203E-08	0.398700750233521	0.426	0.178	0.000978185296584166	3	UBL7
2.18284401901996E-09	0.398373211134031	0.372	0.131	3.65801000707365E-05	3	LRRC57

6.54624825932146E-11	0.396560459690976	0.617	0.272	1.09702028329709E-06	3	HMGN1
3.31834885661085E-11	0.396143737447565	0.394	0.129	5.56088901390846E-07	3	COQ2
1.2000039353889E-06	0.395904146430314	0.479	0.227	0.0201096659492472	3	TOB1
3.0646191804201E-07	0.39520187763996	0.415	0.176	0.00513568882254801	3	NMI
3.64101592167892E-07	0.394621976170583	0.564	0.288	0.00610161448154954	3	NDUFB7
6.03602143933452E-07	0.393827488698789	0.585	0.309	0.0101151647280368	3	PACSIN2
6.3317752668498E-07	0.391868029106802	0.298	0.116	0.0106107889921869	3	MVD
1.48911354674568E-07	0.391489969743318	0.415	0.179	0.00249545648163641	3	SF3A2
8.82110658358543E-09	0.391220153412986	0.447	0.178	0.000147824104127725	3	NME4
1.28491136626857E-07	0.389914165686598	0.362	0.144	0.00215325446759286	3	TMUB1
2.41714680722124E-06	0.38843897939555	0.628	0.329	0.0405065461954136	3	TBL1XR1
5.80502086124368E-07	0.388070585969574	0.404	0.176	0.00972805395927216	3	JTB
6.82523301529976E-11	0.384585771501013	0.404	0.138	1.14377254870393E-06	3	NUTF2
7.7974849503466E-07	0.384446617806422	0.394	0.172	0.0130670252797908	3	MEA1
2.51761114129653E-06	0.383408159875177	0.574	0.303	0.0421901275058472	3	DYNC1I2
2.8995972622484E-06	0.380064387019424	0.606	0.324	0.0485914509207587	3	TSC22D4
6.21454243893621E-07	0.377378443811826	0.447	0.204	0.0104143302191693	3	KEAP1
1.28826396103891E-06	0.376178676725798	0.319	0.128	0.02158872745909	3	COMMD1
8.07340875354505E-10	0.375775755461366	0.362	0.122	1.35294183891908E-05	3	FBXO46
9.20986061671528E-07	0.375590167627722	0.553	0.281	0.0154338844214915	3	PSMD3
2.96038873985516E-06	0.373882283056239	0.543	0.283	0.0496101945024927	3	DNAJB11
1.72749767594981E-08	0.373640732739403	0.436	0.18	0.00028949406053567	3	HP1BP3
1.48214068812983E-09	0.37306320389675	0.436	0.164	2.48377136516797E-05	3	RBM10
9.04818226228194E-09	0.372057963659464	0.277	0.086	0.000151629438351321	3	TMEM50B
6.34362122688122E-07	0.371733547423237	0.702	0.415	0.0106306404520075	3	FAM49B
2.2202329430434E-06	0.369724019968629	0.606	0.317	0.0372066636595213	3	C3
3.78536176419751E-08	0.36867251041633	0.638	0.314	0.000634350924444218	3	CORO1C
8.68069584106322E-07	0.36759607988416	0.553	0.28	0.0145471100904538	3	SELENON
4.84287867402812E-08	0.366161558217922	0.394	0.158	0.000811569608193632	3	ELP5
1.37794320226054E-07	0.365754550027338	0.489	0.222	0.00230915721834821	3	WARS
6.85484997006686E-08	0.363595572009179	0.564	0.281	0.00114873575798381	3	ATP5F1E
9.90781388692093E-08	0.362922788503229	0.404	0.17	0.00166035145117021	3	PID1
2.22571394941198E-06	0.362113513616129	0.394	0.179	0.037298514364246	3	COX14
6.5331940884968E-08	0.361950894645851	0.5	0.224	0.00109483266535029	3	EIF2AK1
8.54642022106756E-08	0.357529260678708	0.426	0.179	0.0014322091006465	3	TMX4
7.1627936973968E-07	0.357121050444977	0.681	0.361	0.0120034096780976	3	COPG1
8.74148833168734E-07	0.356897788769263	0.34	0.141	0.0146489861462416	3	AHCY
6.5811142664098E-07	0.354857701612406	0.5	0.24	0.0110286312876495	3	ZFR
6.42153721510873E-07	0.354621809342523	0.66	0.369	0.0107612120650792	3	SFT2D2
9.88805706251322E-09	0.351940597157223	0.543	0.244	0.000165704060253596	3	CYTH2
5.24511168822665E-10	0.351679278397454	0.681	0.34	8.78975816713023E-06	3	ILF3
2.35560202565052E-06	0.344866029754486	0.596	0.313	0.0394751787458515	3	TF
7.73281930926715E-07	0.336845946746533	0.319	0.129	0.0129586585984699	3	ATL2
3.33518396364014E-07	0.336422341060957	0.511	0.238	0.00558910128626815	3	RPN1
5.46898142786989E-07	0.335772414747101	0.521	0.251	0.00916491907682436	3	PYCR2
2.21270138259553E-07	0.335083128069077	0.67	0.368	0.0037080449769536	3	FAR1
5.28185580447733E-08	0.329048866538229	0.5	0.232	0.00088513339571431	3	FARSA
1.61951014633549E-08	0.324655656765849	0.404	0.159	0.000271397510322902	3	MPPE1
1.11239490273395E-06	0.321520621038927	0.372	0.159	0.0186415137800156	3	OXSR1
1.83782827461918E-07	0.318544089955316	0.5	0.232	0.00307983262260682	3	DBNDD2

6.19471681878092E-07	0.314650423122089	0.691	0.375	0.0103811064449131	3	EIF3A
4.29800604809332E-07	0.312618409665641	0.447	0.198	0.00720259853539479	3	IPO9
2.34450753048602E-07	0.312081793770602	0.66	0.356	0.00392892571958847	3	GABARAP
3.45678031942367E-07	0.31124224891002	0.436	0.2	0.00579287245929018	3	ANKLE2
1.41278684640152E-08	0.310062515908367	0.287	0.092	0.000236754819719966	3	DDB2
1.15376479028175E-06	0.309435905522338	0.766	0.458	0.0193347903555416	3	GNAI2
3.83035516037398E-09	0.306746733038496	0.691	0.346	6.41890917775472E-05	3	BAX
2.87437145549554E-06	0.306591322151363	0.617	0.331	0.0481687168511943	3	PPIB
3.41579822146943E-07	0.304151983761998	0.5	0.23	0.00572419465953847	3	DNAJC4
2.05318271750382E-08	0.29849148869498	0.447	0.187	0.00034407235979929	3	PSPC1
7.6087430323732E-08	0.294986598386281	0.372	0.147	0.0012750731573651	3	MCUR1
1.49435375043329E-07	0.294302903121948	0.553	0.263	0.00250423801497611	3	CTDSP1
1.38907304955843E-06	0.294253692746801	0.468	0.222	0.0232780861645002	3	NUP58
2.86510865468659E-08	0.293214417304516	0.372	0.137	0.000480134908352378	3	TIMM23
1.26096139060349E-11	0.293182063958393	0.468	0.167	2.11311909837333E-07	3	CMC2
6.22203581970396E-07	0.292445950466308	0.426	0.192	0.0104268876266599	3	FMR1
7.7493122669548E-08	0.292046869470097	0.351	0.133	0.00129862974969628	3	GSPT1
6.07159937595239E-10	0.291223635004008	0.532	0.223	1.0174786234221E-05	3	STAG1
1.6823693572622E-07	0.290792831507176	0.511	0.236	0.0028193145689	3	C1orf162
1.42956995515893E-07	0.282752603912288	0.426	0.19	0.00239567333085533	3	CDK4
5.44529410091486E-07	0.281905109738564	0.351	0.14	0.00912522385431312	3	ATP5PB
2.05028615331702E-08	0.281478702157649	0.628	0.324	0.000343586953572867	3	PCBP1
2.21476662792867E-07	0.279842743855489	0.266	0.092	0.00371150591508286	3	CDC27
1.10563332582257E-09	0.277874139097358	0.574	0.243	1.85282032741346E-05	3	BTAF1
2.94269741654278E-06	0.277362695741913	0.287	0.111	0.0493137233064239	3	TRIM24
5.53994527061552E-07	0.274602835135544	0.649	0.347	0.00928384028449749	3	PSMF1
2.65763293435338E-07	0.272938083434497	0.489	0.224	0.00445366127138939	3	TRIOBP
2.65160287444656E-06	0.272064055820393	0.489	0.251	0.0444355609699755	3	PPP1R15B
1.5178427459827E-06	0.270560661974251	0.521	0.251	0.0254360087371781	3	HTT
2.61221015455117E-09	0.270020696122353	0.436	0.166	4.37754177699686E-05	3	SMARCC1
1.60181971139828E-06	0.269385741066812	0.457	0.213	0.0268432947236124	3	PPP6C
8.10800228934845E-08	0.26772939974293	0.33	0.122	0.00135873902364901	3	PSMD9
5.82263298331801E-07	0.266512779220075	0.362	0.148	0.00975756835344432	3	CXorf21
2.98041307025599E-08	0.259976856566951	0.649	0.341	0.0004994576223135	3	DOCK8
1.69224898170107E-07	0.259583861263704	0.372	0.149	0.00283587084353465	3	RSRC1
1.40110914664989E-06	0.259480450976602	0.457	0.216	0.0234797870795588	3	DNM1L
7.56792200467394E-07	0.251767383720137	0.351	0.142	0.0126823236954326	3	NUP214
3.39125369617411E-07	0.251052758331908	0.372	0.149	0.00568306294404857	3	ACIN1
4.64265801462298E-08	0.250446383132075	0.298	0.103	0.000778016630090519	3	MTOR

SupTable5H_C1_Mye_MyeloidSubtype

p_val	avg_log2FC	pct.1	pct.2	p_val_adj	cluster	gene
2.48200479527063E-182	3.02489403705744	0.904	0.278	4.15934363591453E-178	FOLR2+ resident-like macrophages	RNASE1
7.34178534839057E-172	2.48666116394563	0.891	0.25	1.23033638868329E-167	FOLR2+ resident-like macrophages	FOLR2
5.37819938626212E-160	2.50938047485849	0.9	0.333	9.01278653149806E-156	FOLR2+ resident-like macrophages	F13A1
2.50497154348304E-159	2.48976885140257	0.874	0.265	4.19783131256888E-155	FOLR2+ resident-like macrophages	EPHX1
1.71737475692689E-152	1.96315803831575	0.939	0.381	2.87797661765809E-148	FOLR2+ resident-like macrophages	DAB2
3.11568913800893E-152	2.41464878548331	0.855	0.212	5.22127185747536E-148	FOLR2+ resident-like macrophages	CD209
1.66177240710908E-132	2.26667779410582	0.814	0.233	2.78479819983339E-128	FOLR2+ resident-like macrophages	ALDH1A1
1.0332085965383E-131	1.4265598959663	0.975	0.843	1.73145096607889E-127	FOLR2+ resident-like macrophages	CTSB
1.89021147791214E-129	2.37518076130208	0.731	0.141	3.16761639468516E-125	FOLR2+ resident-like macrophages	GPR34
1.1908663249113E-128	2.07304651020633	0.774	0.168	1.99565378728635E-124	FOLR2+ resident-like macrophages	DEPRECATED-ENSG00000110079
5.60705136857597E-125	1.8642659771119	0.874	0.309	9.3962966834596E-121	FOLR2+ resident-like macrophages	A2M
4.68609541712443E-124	1.91678281584684	0.9	0.375	7.85295870001712E-120	FOLR2+ resident-like macrophages	CTSD
1.54346840610957E-122	2.86802382913649	0.597	0.069	2.58654435495842E-118	FOLR2+ resident-like macrophages	IL2RA
5.45145454710384E-122	1.34856682547573	0.964	0.647	9.13554753003661E-118	FOLR2+ resident-like macrophages	C1QC
9.45347609002261E-120	1.64517008308394	0.917	0.52	1.58421352316599E-115	FOLR2+ resident-like macrophages	CTSC
1.53226651879605E-119	1.65461005526275	0.894	0.358	2.56777223219843E-115	FOLR2+ resident-like macrophages	SLCO2B1
7.11596562091479E-119	1.72695421413303	0.906	0.421	1.19249351875529E-114	FOLR2+ resident-like macrophages	MRC1
5.41845463492416E-118	1.77195745716579	0.843	0.257	9.08024627720591E-114	FOLR2+ resident-like macrophages	NRP1
4.25554445841778E-116	1.78036125840542	0.893	0.434	7.13144140341652E-112	FOLR2+ resident-like macrophages	PLD3
1.43125156457695E-113	1.7851973222485	0.836	0.281	2.39849137191805E-109	FOLR2+ resident-like macrophages	DEPRECATED-ENSG00000175899
6.68718485829659E-113	2.97815746661727	0.556	0.065	1.12063843855334E-108	FOLR2+ resident-like macrophages	COLEC12
8.22330314688427E-112	1.59038707265275	0.917	0.44	1.37806114135487E-107	FOLR2+ resident-like macrophages	SLC40A1
1.12348274139758E-110	1.98688032462686	0.771	0.236	1.88273237803407E-106	FOLR2+ resident-like macrophages	CPM
2.51984324465088E-110	1.28295900542255	0.978	0.706	4.22275330938594E-106	FOLR2+ resident-like macrophages	SELENOP
6.06164038455356E-107	1.56410335690027	0.848	0.271	1.01580969564349E-102	FOLR2+ resident-like macrophages	MS4A4A
8.62817911779275E-107	1.40323662492964	0.926	0.526	1.44591025655971E-102	FOLR2+ resident-like macrophages	LAMP1
1.51241989468376E-106	1.91400920295631	0.771	0.26	2.53451325951104E-102	FOLR2+ resident-like macrophages	IDH1
2.73074394551976E-106	1.92340625287418	0.649	0.132	4.57618070390202E-102	FOLR2+ resident-like macrophages	PMP22
7.57277679352901E-106	1.80424234737263	0.887	0.4	1.26904593505959E-101	FOLR2+ resident-like macrophages	APOE
1.01249250742974E-105	1.67448696742331	0.914	0.391	1.69673494395075E-101	FOLR2+ resident-like macrophages	STAB1
1.79243315372065E-104	2.13648799002428	0.721	0.207	3.00375947900507E-100	FOLR2+ resident-like macrophages	GATM
5.74795302260516E-102	1.61638463826893	0.772	0.231	9.63241967528173E-98	FOLR2+ resident-like macrophages	MMP14
6.50091949246941E-102	1.58009227278584	0.758	0.215	1.08942408854802E-97	FOLR2+ resident-like macrophages	PIK3IP1
9.84397043982422E-101	1.18667057429816	0.959	0.683	1.64965256630574E-96	FOLR2+ resident-like macrophages	C1QA
2.63353963027232E-99	1.60274970408541	0.705	0.172	4.41328571241035E-95	FOLR2+ resident-like macrophages	TSPAN4
1.01465728335921E-97	3.01173317128813	0.639	0.156	1.70036267545337E-93	FOLR2+ resident-like macrophages	SPP1
8.155532428001E-97	1.76134094579321	0.642	0.148	1.36670412428441E-92	FOLR2+ resident-like macrophages	VAT1
8.3503635863227E-95	1.0791644371878	0.971	0.687	1.39935392979596E-90	FOLR2+ resident-like macrophages	C1QB
1.22373869864158E-94	1.57107379609417	0.792	0.265	2.05074131118356E-90	FOLR2+ resident-like macrophages	FUCA1
2.3386565047627E-94	1.33195082004011	0.907	0.451	3.91912057068134E-90	FOLR2+ resident-like macrophages	TMEM176B
2.4908530344694E-94	1.74587101155563	0.644	0.156	4.17417151516382E-90	FOLR2+ resident-like macrophages	CD59
8.32789467456627E-94	1.57387330301891	0.668	0.154	1.39558858956382E-89	FOLR2+ resident-like macrophages	CMKLR1
5.28104854246265E-93	2.21427078702173	0.525	0.077	8.84998114745891E-89	FOLR2+ resident-like macrophages	WLS
6.20701284262828E-93	1.54271498075871	0.671	0.155	1.04017121216765E-88	FOLR2+ resident-like macrophages	NPL
1.17980173130435E-92	1.37149548695834	0.938	0.491	1.97711174131983E-88	FOLR2+ resident-like macrophages	CD14
1.18524568514426E-92	1.6955697040051	0.724	0.22	1.98623471916475E-88	FOLR2+ resident-like macrophages	ENPP2
1.02893766077054E-91	1.53115970914719	0.698	0.189	1.72429373191927E-87	FOLR2+ resident-like macrophages	PDGFC
1.41984051127763E-91	1.33910997372925	0.657	0.142	2.37936872879905E-87	FOLR2+ resident-like macrophages	CD163L1
3.68116476077738E-91	1.49014714139092	0.84	0.278	6.16889590611074E-87	FOLR2+ resident-like macrophages	PLTP
7.60351254608209E-91	1.37881654904811	0.836	0.328	1.27419663247244E-86	FOLR2+ resident-like macrophages	TNFSF13
2.87068322175606E-90	1.54540251335293	0.61	0.127	4.81069094301881E-86	FOLR2+ resident-like macrophages	PLA2G7
3.17739954652981E-90	1.63690624932167	0.765	0.258	5.32468616007466E-86	FOLR2+ resident-like macrophages	ANXA4
5.95411297727005E-89	1.40100373328104	0.848	0.355	9.97790252730915E-85	FOLR2+ resident-like macrophages	CREG1
6.00614054446518E-87	1.30468866056867	0.724	0.216	1.00650903244148E-82	FOLR2+ resident-like macrophages	SLC1A3
1.2865522904266E-86	1.38304484785384	0.936	0.632	2.15600432829689E-82	FOLR2+ resident-like macrophages	LGMN
1.484316333928E-86	4.01636528627424	0.383	0.024	2.48741731239654E-82	FOLR2+ resident-like macrophages	CCL13
1.14661540506578E-85	1.84956814373307	0.668	0.189	1.92149809580923E-81	FOLR2+ resident-like macrophages	PTGR1
3.02533225179682E-85	1.57229110218215	0.578	0.119	5.06985178756111E-81	FOLR2+ resident-like macrophages	TCN2
5.01457999300016E-85	1.1339843297404	0.923	0.441	8.40343315226967E-81	FOLR2+ resident-like macrophages	MAF
6.61404780573485E-85	1.62621263055565	0.833	0.346	1.10838213128505E-80	FOLR2+ resident-like macrophages	GPNMB
1.10081333546355E-83	1.14514915073395	0.932	0.608	1.84474298756982E-79	FOLR2+ resident-like macrophages	CD68
1.41721038987814E-81	1.36904745649282	0.833	0.308	2.37496117135779E-77	FOLR2+ resident-like macrophages	VTN
1.63712361766104E-81	1.66945145710909	0.691	0.22	2.74349175847637E-77	FOLR2+ resident-like macrophages	ALDOB
1.91249325648028E-81	1.44861767185588	0.681	0.214	3.20495619920965E-77	FOLR2+ resident-like macrophages	IQGAP2
3.68094660747067E-81	1.03794429386447	0.923	0.609	6.16853032479936E-77	FOLR2+ resident-like macrophages	GRN
7.42291054575584E-81	1.32536264793969	0.594	0.134	1.24393134925776E-76	FOLR2+ resident-like macrophages	TNFRSF21
1.16939584385217E-79	1.60365657239387	0.48	0.072	1.95967355512747E-75	FOLR2+ resident-like macrophages	IGF1
2.09388882467011E-79	1.24558240458976	0.782	0.29	3.50893889238217E-75	FOLR2+ resident-like macrophages	C3AR1
2.71905918420047E-79	1.42216857343396	0.652	0.18	4.55659938088315E-75	FOLR2+ resident-like macrophages	ITM2C

3.23637505293867E-79	1.48189659823135	0.685	0.211	5.42351731371462E-75	FOLR2+ resident-like macrophages	C2
3.29798034395502E-79	1.56632555718114	0.601	0.146	5.52675546039982E-75	FOLR2+ resident-like macrophages	DHRS3
6.06615720918721E-79	1.55814311663183	0.739	0.257	1.01656662511559E-74	FOLR2+ resident-like macrophages	SPINK1
1.29600056782545E-78	2.80816230508524	0.376	0.029	2.1718377515619E-74	FOLR2+ resident-like macrophages	KCNJ5
1.32659689031318E-78	2.2703656794949	0.498	0.092	2.22311106878683E-74	FOLR2+ resident-like macrophages	ACE
1.83535413243712E-78	1.12486264620452	0.592	0.134	3.07568645513813E-74	FOLR2+ resident-like macrophages	NRP2
2.45563136176353E-78	1.25567098369575	0.656	0.189	4.11514703604332E-74	FOLR2+ resident-like macrophages	CHID1
4.71608421259449E-78	1.10599852330358	0.785	0.285	7.90321392346585E-74	FOLR2+ resident-like macrophages	AP2A2
4.68877123780704E-77	1.15641106588096	0.903	0.451	7.85744284031704E-73	FOLR2+ resident-like macrophages	MS4A7
1.7622334076827E-75	1.48253703942478	0.794	0.329	2.95315074459467E-71	FOLR2+ resident-like macrophages	CD93
6.68288730663691E-75	1.36985812168918	0.579	0.13	1.11991825484621E-70	FOLR2+ resident-like macrophages	IGSF21
1.86930844137057E-74	1.22328325223716	0.845	0.393	3.1325870860488E-70	FOLR2+ resident-like macrophages	CTSA
7.45292934190073E-74	1.01123327361974	0.628	0.159	1.24896189911572E-69	FOLR2+ resident-like macrophages	TMEM37
1.0738958462476E-73	1.28284021822086	0.826	0.34	1.79963465914172E-69	FOLR2+ resident-like macrophages	FMN1
1.27197494161484E-73	1.14193638730538	0.859	0.372	2.13157560715815E-69	FOLR2+ resident-like macrophages	LAIR1
2.0214315460407E-73	1.15103067520436	0.798	0.325	3.38751498485501E-69	FOLR2+ resident-like macrophages	LAMP2
2.18903760027991E-73	1.45757932331336	0.682	0.23	3.66838921054907E-69	FOLR2+ resident-like macrophages	ACP5
5.07503587682139E-73	1.15610202314868	0.832	0.373	8.50474512237728E-69	FOLR2+ resident-like macrophages	NCOA4
1.15223187176304E-72	1.42384169615935	0.66	0.216	1.9309101707005E-68	FOLR2+ resident-like macrophages	TMEM176A
1.72393584301213E-72	1.105789458288	0.888	0.522	2.88897168571972E-68	FOLR2+ resident-like macrophages	ITM2B
1.85245957962405E-72	1.36332701130214	0.758	0.308	3.10435176353398E-68	FOLR2+ resident-like macrophages	IGSF6
2.18801434925317E-72	1.0588901808028	0.736	0.264	3.66667444647846E-68	FOLR2+ resident-like macrophages	CYFIP1
3.5809103076936E-72	1.81824169621841	0.569	0.149	6.00088949363294E-68	FOLR2+ resident-like macrophages	PDK4
3.61945572558896E-72	0.91618687391301	0.766	0.246	6.06548390494198E-68	FOLR2+ resident-like macrophages	MERTK
3.72698787414706E-72	1.11470634356898	0.866	0.426	6.24568627949565E-68	FOLR2+ resident-like macrophages	TIMP2
4.98216979026019E-72	0.96286689746287	0.938	0.572	8.34912013451803E-68	FOLR2+ resident-like macrophages	MPEG1
6.29191202570197E-72	1.54845192554733	0.682	0.228	1.05439861726714E-67	FOLR2+ resident-like macrophages	PGRMC1
9.08240652702367E-72	1.12607972683116	0.742	0.269	1.52202968579863E-67	FOLR2+ resident-like macrophages	BLVRB
1.18026477654567E-71	0.89706661194002	0.653	0.189	1.97788771253523E-67	FOLR2+ resident-like macrophages	RENBP
9.36071785495843E-71	1.74953663891164	0.62	0.193	1.56866909813393E-66	FOLR2+ resident-like macrophages	UGDH
1.91627070191447E-70	1.07186842328843	0.585	0.15	3.21128644226827E-66	FOLR2+ resident-like macrophages	GIMAP5
3.48710782769347E-70	0.83510198775740	0.705	0.221	5.84369529764872E-66	FOLR2+ resident-like macrophages	ASAH1
8.13855125950687E-70	1.00321749899515	0.794	0.337	1.36385842006816E-65	FOLR2+ resident-like macrophages	AP1B1
1.18577135144385E-69	1.10638493002062	0.839	0.38	1.9871156307496E-65	FOLR2+ resident-like macrophages	LIPA
2.99834515418785E-69	1.50998644661768	0.541	0.138	5.024626809388E-65	FOLR2+ resident-like macrophages	TNFSF12
3.63759934334042E-69	1.14034008006754	0.698	0.249	6.09588897956987E-65	FOLR2+ resident-like macrophages	SCPEP1
1.1095720328249E-68	1.52234072168936	0.422	0.065	1.85942081260797E-64	FOLR2+ resident-like macrophages	GYPC
1.14219450191559E-68	1.3722205938363	0.451	0.08	1.91408954631014E-64	FOLR2+ resident-like macrophages	GLMP
1.1854483423227E-68	2.13334567357674	0.48	0.101	1.98657433206437E-64	FOLR2+ resident-like macrophages	ADORA3
1.5638582005383E-68	1.29883717545317	0.434	0.07	2.62071357246209E-64	FOLR2+ resident-like macrophages	SLC18B1
5.86846887938482E-68	1.48698854547718	0.695	0.25	9.83438014807307E-64	FOLR2+ resident-like macrophages	GCLC
1.43870173298058E-67	1.56716621844259	0.612	0.188	2.41097636412885E-63	FOLR2+ resident-like macrophages	IFI27
6.04146180117898E-67	1.26970325198151	0.496	0.108	1.01242816864157E-62	FOLR2+ resident-like macrophages	AMDHD2
6.42713397839248E-67	1.4211421700944	0.544	0.142	1.07705911209901E-62	FOLR2+ resident-like macrophages	DEPRECATED-ENSG00000079215
8.86527863396396E-67	1.75774024597656	0.547	0.153	1.48564339347968E-62	FOLR2+ resident-like macrophages	NUPR1
2.67569873548255E-66	1.80931916847982	0.38	0.049	4.48393594092167E-62	FOLR2+ resident-like macrophages	SLC7A8
6.58622491358345E-66	1.017222722403	0.701	0.251	1.10371957101831E-61	FOLR2+ resident-like macrophages	GM2A
8.58950597213229E-66	1.27436886706686	0.727	0.279	1.43942941080993E-61	FOLR2+ resident-like macrophages	SCP2
1.2031804901334E-65	1.11686412884131	0.53	0.127	2.01628986536555E-61	FOLR2+ resident-like macrophages	PDGFB
1.26539402024628E-65	0.89428292386286	0.541	0.123	2.12054729912872E-61	FOLR2+ resident-like macrophages	RAB3IL1
2.13332910961259E-65	1.06920878914357	0.723	0.276	3.57503292188878E-61	FOLR2+ resident-like macrophages	FERMT3
3.02371770361745E-65	1.2355876806322	0.448	0.083	5.06714612772213E-61	FOLR2+ resident-like macrophages	HOMER3
4.73504283164613E-65	1.29631201328638	0.491	0.113	7.93498477727259E-61	FOLR2+ resident-like macrophages	TMEM86A
6.87472623365282E-65	0.81034530016025	0.803	0.339	1.15206662223554E-60	FOLR2+ resident-like macrophages	PLXND1
1.21925667169999E-64	1.17205970893667	0.659	0.237	2.04323033043484E-60	FOLR2+ resident-like macrophages	SCD
1.81956319835248E-64	1.27609244187516	0.501	0.115	3.04922400779908E-60	FOLR2+ resident-like macrophages	DEPRECATED-ENSG00000196329
2.47376849001561E-64	0.84608379242040	0.955	0.627	4.14554123556815E-60	FOLR2+ resident-like macrophages	CD163
6.42990471285571E-64	2.16612953914827	0.475	0.11	1.07752343178036E-59	FOLR2+ resident-like macrophages	GSTA1
6.75398669897017E-64	1.11057020468521	0.782	0.32	1.13183309101342E-59	FOLR2+ resident-like macrophages	SLAMF8
4.03773318167278E-63	2.28164043694906	0.469	0.11	6.76643326584725E-59	FOLR2+ resident-like macrophages	AKR1B10
4.36348720296426E-63	0.90549027640256	0.762	0.305	7.3123318547275E-59	FOLR2+ resident-like macrophages	LGALS3BP
1.07724927633245E-62	1.24053777385339	0.566	0.17	1.80525433727793E-58	FOLR2+ resident-like macrophages	SLC35F6
1.10135702597072E-62	1.79441042293159	0.522	0.147	1.84565410412173E-58	FOLR2+ resident-like macrophages	BEX3
1.11875266081592E-62	1.25811433955021	0.711	0.271	1.87480570899531E-58	FOLR2+ resident-like macrophages	ST6GAL1
1.40048308895593E-62	0.76479152872180	0.642	0.193	2.34692956047234E-58	FOLR2+ resident-like macrophages	MTSS1
1.43887674717797E-62	0.93510276306836	0.682	0.245	2.41126965292085E-58	FOLR2+ resident-like macrophages	TMBIM1
1.95981303852628E-62	1.51022574724483	0.448	0.095	3.28425468996234E-58	FOLR2+ resident-like macrophages	RELL1
3.38665002413657E-62	0.82810869911258	0.583	0.164	5.67534811044807E-58	FOLR2+ resident-like macrophages	RHBDF2
5.27217176239604E-62	1.39184570934807	0.586	0.181	8.83510543942328E-58	FOLR2+ resident-like macrophages	SH3PXD2B
5.47874613235579E-62	1.09886701670578	0.687	0.258	9.18128276860184E-58	FOLR2+ resident-like macrophages	UNC93B1
1.26132046924537E-61	1.31901528326653	0.525	0.144	2.11372084236139E-57	FOLR2+ resident-like macrophages	CD99

1.27939855435959E-61	2.17344774269114	0.386	0.064	2.1440160973958E-57	FOLR2+ resident-like macrophages	NAGLU
2.09067311328666E-61	1.11938539026239	0.541	0.151	3.50355000324578E-57	FOLR2+ resident-like macrophages	MGAT5
2.34342555755618E-61	1.87801369457524	0.427	0.085	3.92711254935264E-57	FOLR2+ resident-like macrophages	TREM2
3.38315181896624E-61	1.10989557118772	0.527	0.132	5.66948581822362E-57	FOLR2+ resident-like macrophages	GFRA2
4.63381823369252E-61	1.95126219031306	0.348	0.044	7.76535259602193E-57	FOLR2+ resident-like macrophages	FAM174B
6.05532928056647E-61	1.22391532897055	0.612	0.209	1.01475208083733E-56	FOLR2+ resident-like macrophages	PRDX1
7.80209326556166E-61	1.10695801864438	0.588	0.185	1.30747478944282E-56	FOLR2+ resident-like macrophages	MYO1E
8.28664216677963E-61	1.88767129008311	0.554	0.172	1.38867549430893E-56	FOLR2+ resident-like macrophages	CES1
1.38812807040835E-60	1.19204606558315	0.685	0.261	2.32622502039031E-56	FOLR2+ resident-like macrophages	PTMS
1.59536851558803E-60	1.16146014406286	0.627	0.219	2.67351855842241E-56	FOLR2+ resident-like macrophages	CRYL1
2.63367709568268E-60	1.63547755964022	0.557	0.176	4.41351607694503E-56	FOLR2+ resident-like macrophages	MPC2
2.89975936607504E-60	2.11380054842892	0.53	0.152	4.85941674566855E-56	FOLR2+ resident-like macrophages	MMP9
3.35965970841976E-60	1.64195721809516	0.498	0.131	5.63011773936984E-56	FOLR2+ resident-like macrophages	IGFBP4
8.75804393545679E-60	1.10846671444741	0.464	0.103	1.46767300270385E-55	FOLR2+ resident-like macrophages	FARP1
1.02173839925832E-59	0.714333818382128	0.627	0.197	1.7122292094771E-55	FOLR2+ resident-like macrophages	LHFPL2
1.13237915049611E-59	0.918355894817244	0.835	0.407	1.89764098040138E-55	FOLR2+ resident-like macrophages	CD63
1.42266927273222E-59	1.40514927106925	0.419	0.083	2.38410916724466E-55	FOLR2+ resident-like macrophages	SGMS1
1.6093088595537E-59	0.832273285886089	0.955	0.646	2.6968797868401E-55	FOLR2+ resident-like macrophages	FCGRT
1.82287937456576E-59	1.02214619549307	0.646	0.223	3.05478125589731E-55	FOLR2+ resident-like macrophages	FABP1
2.51537079928125E-59	1.22389838624059	0.598	0.197	4.21525838543552E-55	FOLR2+ resident-like macrophages	GAS6
4.31390321150525E-59	0.948968984121505	0.607	0.197	7.22923900184051E-55	FOLR2+ resident-like macrophages	DEPRECATED-ENSG00000196743
4.43830452695142E-59	1.07810502796452	0.682	0.254	7.43771072626519E-55	FOLR2+ resident-like macrophages	SERPING1
4.72050936986907E-59	1.23509269303692	0.615	0.206	7.91062960202659E-55	FOLR2+ resident-like macrophages	ASPH
4.95567954311118E-59	0.872016133738156	0.646	0.223	8.30472777834572E-55	FOLR2+ resident-like macrophages	PLBD2
5.47207294524423E-59	0.759542500723466	0.647	0.225	9.17009984164029E-55	FOLR2+ resident-like macrophages	VEGFB
8.39461465101249E-59	1.04728823295443	0.687	0.256	1.40676952321667E-54	FOLR2+ resident-like macrophages	BCAP31
1.16287712335517E-58	1.80612003537358	0.504	0.138	1.94874948331859E-54	FOLR2+ resident-like macrophages	GPX2
1.90857014834601E-58	1.31908179521352	0.721	0.311	3.19838185459825E-54	FOLR2+ resident-like macrophages	CAPG
2.00724109126465E-58	0.798902041785735	0.565	0.163	3.3637346207413E-54	FOLR2+ resident-like macrophages	SLC38A7
3.43802086366606E-58	0.667306659414314	0.583	0.169	5.76143536333159E-54	FOLR2+ resident-like macrophages	STARD13
8.20244718209022E-58	1.0822493279041	0.538	0.149	1.37456609877468E-53	FOLR2+ resident-like macrophages	DNASE2
1.06811117108933E-57	0.716727776295623	0.419	0.08	1.7899407005115E-53	FOLR2+ resident-like macrophages	HSD17B14
3.61038851237992E-57	1.1144361003308	0.731	0.297	6.05028906904627E-53	FOLR2+ resident-like macrophages	GC
4.27135017873937E-57	0.891709509718414	0.949	0.802	7.15792862953143E-53	FOLR2+ resident-like macrophages	CTSZ
4.60168371018796E-57	0.774920124046593	0.702	0.275	7.71150156153299E-53	FOLR2+ resident-like macrophages	LRRC25
1.12023862771124E-56	0.952115612363913	0.64	0.218	1.87729589231849E-52	FOLR2+ resident-like macrophages	VSIG4
2.00753284210412E-56	0.640924420026983	0.729	0.262	3.36422353679809E-52	FOLR2+ resident-like macrophages	SIGLEC1
3.66289374583865E-56	0.807779413899524	0.522	0.144	6.13827733927642E-52	FOLR2+ resident-like macrophages	ITGB5
3.83468989898648E-56	2.33277148491585	0.417	0.09	6.42617333272155E-52	FOLR2+ resident-like macrophages	MUC3A
4.08973559957979E-56	1.72579148900852	0.54	0.175	6.85357891777581E-52	FOLR2+ resident-like macrophages	CERS2
4.49142554093616E-56	0.849795488945018	0.673	0.244	7.52673092150081E-52	FOLR2+ resident-like macrophages	ARHGAP18
1.49614653781282E-55	1.3174730579185	0.576	0.192	2.50724236806672E-51	FOLR2+ resident-like macrophages	IL32
1.55962520342782E-55	0.543138821807633	0.605	0.201	2.61361991590434E-51	FOLR2+ resident-like macrophages	TPD52L2
3.9294822544647E-55	0.915614095866679	0.653	0.243	6.58502636203194E-51	FOLR2+ resident-like macrophages	SESN1
4.18844116858723E-55	1.03991390837109	0.599	0.206	7.01898971031848E-51	FOLR2+ resident-like macrophages	ABHD12
1.19816952136175E-54	0.881115074739637	0.472	0.116	2.00789248389801E-50	FOLR2+ resident-like macrophages	SLC36A1
2.30022081136667E-54	0.847734876614027	0.827	0.33	3.85471003568826E-50	FOLR2+ resident-like macrophages	FKBP5
2.30749359048767E-54	1.62407247600069	0.421	0.097	3.86689775893924E-50	FOLR2+ resident-like macrophages	GNPDA1
2.38711621971408E-54	0.668000703134754	0.759	0.317	4.00032936099685E-50	FOLR2+ resident-like macrophages	RBM47
2.54099189231418E-54	0.795222953621797	0.567	0.185	4.2581942131401E-50	FOLR2+ resident-like macrophages	PSME4
2.84515982753685E-54	1.18126317765387	0.718	0.299	4.76791883898626E-50	FOLR2+ resident-like macrophages	APOC3
4.57283404728907E-54	1.63727274867066	0.431	0.106	7.66315529644702E-50	FOLR2+ resident-like macrophages	CD276
5.35193900824975E-54	0.859392505476725	0.691	0.28	8.96877939002494E-50	FOLR2+ resident-like macrophages	CD81
6.289546719661E-54	1.89780647512756	0.379	0.075	1.05400223928079E-49	FOLR2+ resident-like macrophages	SLC2A8
8.0315521097596E-54	0.597311679761756	0.826	0.349	1.34592750255351E-49	FOLR2+ resident-like macrophages	SDC3
9.25770687453484E-54	0.719706644986337	0.742	0.311	1.55140651803455E-49	FOLR2+ resident-like macrophages	TNS3
1.46829110398985E-53	0.579629373882576	0.573	0.176	2.46056223206619E-49	FOLR2+ resident-like macrophages	TLR4
1.47300687338832E-53	0.801660933173946	0.749	0.308	2.46846491842415E-49	FOLR2+ resident-like macrophages	SIRPA
1.60781567175416E-53	1.79692057154504	0.48	0.137	2.69437750272562E-49	FOLR2+ resident-like macrophages	H1F0
2.57868184136146E-53	0.762638519411463	0.557	0.176	4.32135502975353E-49	FOLR2+ resident-like macrophages	BCL2L1
2.82319589593965E-53	0.826335346784697	0.66	0.25	4.73111168241567E-49	FOLR2+ resident-like macrophages	SNX6
3.93873604028263E-53	0.803766271182104	0.72	0.314	6.60053385630564E-49	FOLR2+ resident-like macrophages	RAB5C
4.97013731019331E-53	0.709762470239445	0.626	0.222	8.32895610442196E-49	FOLR2+ resident-like macrophages	SWAP70
8.17656063203316E-53	1.41381239846506	0.475	0.139	1.37022803071612E-48	FOLR2+ resident-like macrophages	RCAN1
8.96714704106827E-53	0.654437736452359	0.776	0.334	1.50271450114222E-48	FOLR2+ resident-like macrophages	GAA
1.17780972029473E-52	1.0253678621174	0.611	0.219	1.9737735292699E-48	FOLR2+ resident-like macrophages	RTN3
1.50595011981178E-52	1.115982110847	0.536	0.174	2.52367121078058E-48	FOLR2+ resident-like macrophages	G6PD
1.6236847635253E-52	0.78568975626382	0.495	0.136	2.7209709267157E-48	FOLR2+ resident-like macrophages	ITSN1
1.88261347337692E-52	1.17730911083967	0.478	0.128	3.15488365868504E-48	FOLR2+ resident-like macrophages	BLVRA
2.13299726548659E-52	2.08817927150437	0.364	0.071	3.57447681750243E-48	FOLR2+ resident-like macrophages	HYAL2
3.85137635412992E-52	1.38699649766459	0.501	0.152	6.45413649425092E-48	FOLR2+ resident-like macrophages	FUOM

4.38687880948912E-52	0.8685745770166	0.504	0.146	7.35153150894187E-48	FOLR2+ resident-like macrophages	MYO5A
5.83105857873823E-52	1.02044404758239	0.546	0.177	9.77168796624953E-48	FOLR2+ resident-like macrophages	WASF2
6.30569280024881E-52	1.46524161946844	0.601	0.227	1.0567079994657E-47	FOLR2+ resident-like macrophages	AKR1C3
2.3984324199662E-51	1.17051551453406	0.578	0.203	4.01929304937935E-47	FOLR2+ resident-like macrophages	TMCO1
2.86048195330888E-51	1.20562414676626	0.588	0.212	4.79359565735503E-47	FOLR2+ resident-like macrophages	KLF9
3.43725998759416E-51	0.51145889185482	0.605	0.212	5.7601602872103E-47	FOLR2+ resident-like macrophages	MGAT4A
5.82745211621984E-51	0.50466496544286	0.697	0.269	9.76564425636121E-47	FOLR2+ resident-like macrophages	AKR1B1
6.87894276332995E-51	0.96433444032110	0.631	0.239	1.15277322827883E-46	FOLR2+ resident-like macrophages	DEPRECATED-ENSG00000119900
7.14368294437342E-51	0.69832920276538	0.769	0.343	1.1971383878181E-46	FOLR2+ resident-like macrophages	SCAMP2
1.15597928461565E-50	0.80786734408615	0.543	0.182	1.93719008515891E-46	FOLR2+ resident-like macrophages	WWP1
1.55939665753107E-50	0.81648659430073	0.763	0.33	2.61323691869057E-46	FOLR2+ resident-like macrophages	SLA
3.3602180598123E-50	0.94353410642257	0.682	0.28	5.63105342463346E-46	FOLR2+ resident-like macrophages	NAGA
3.75196740855572E-50	1.31041543390711	0.443	0.118	6.28754698325768E-46	FOLR2+ resident-like macrophages	BCAT1
3.99415941288829E-50	0.80442038210966	0.554	0.187	6.69341234411819E-46	FOLR2+ resident-like macrophages	SLC11A2
4.46294388316959E-50	1.06150566668689	0.637	0.24	7.4790013594156E-46	FOLR2+ resident-like macrophages	APOC2
4.53988557891318E-50	0.71150135531514	0.595	0.21	7.60794025314271E-46	FOLR2+ resident-like macrophages	NFIC
5.55249862510253E-50	1.04542571931857	0.536	0.185	9.30487719594682E-46	FOLR2+ resident-like macrophages	PEPD
9.21522058783076E-50	0.76922421385460	0.927	0.522	1.54428666610868E-45	FOLR2+ resident-like macrophages	ALB
1.39123276638078E-49	1.18671332110586	0.524	0.18	2.33142786990091E-45	FOLR2+ resident-like macrophages	CYB5A
1.65693717153869E-49	2.06169808158137	0.376	0.083	2.77669531206454E-45	FOLR2+ resident-like macrophages	NBL1
1.79611470117087E-49	0.78534710454456	0.501	0.148	3.00992901622215E-45	FOLR2+ resident-like macrophages	ACP2
2.04763459479725E-49	0.63921346193567	0.898	0.465	3.43142605396123E-45	FOLR2+ resident-like macrophages	MAFB
3.86285817698133E-49	1.84736044921838	0.293	0.04	6.47337773298532E-45	FOLR2+ resident-like macrophages	CD28
7.42077323302479E-49	1.11769762808218	0.37	0.076	1.24357317839029E-44	FOLR2+ resident-like macrophages	HPSE
8.71003557047396E-49	2.23975900434654	0.396	0.102	1.45962776090003E-44	FOLR2+ resident-like macrophages	CBR1
1.01021612418694E-48	1.25975012022134	0.538	0.185	1.69292018091247E-44	FOLR2+ resident-like macrophages	RRBP1
2.21919265754381E-48	0.39635936903519	0.742	0.308	3.71892305551192E-44	FOLR2+ resident-like macrophages	TTYH3
2.54046054255807E-48	2.95588726048739	0.3	0.047	4.25730377721881E-44	FOLR2+ resident-like macrophages	AKR1C2
2.98744199347475E-48	1.10362203573927	0.491	0.154	5.00635529266498E-44	FOLR2+ resident-like macrophages	MGAT4B
3.6470721363754E-48	1.48067754760011	0.446	0.128	6.1117634861379E-44	FOLR2+ resident-like macrophages	PNKD
4.63078169432834E-48	0.81137763221269	0.798	0.36	7.76026396335544E-44	FOLR2+ resident-like macrophages	MT-ND2
4.84834724578097E-48	0.82779381805186	0.476	0.14	8.12486031447974E-44	FOLR2+ resident-like macrophages	PLA2G15
6.58529972944696E-48	1.019105884538	0.428	0.109	1.10356452866072E-43	FOLR2+ resident-like macrophages	PXN
7.59462978876502E-48	1.33217843840288	0.392	0.097	1.27270806000124E-43	FOLR2+ resident-like macrophages	BAG5
7.59528436262526E-48	0.76163619979817	0.903	0.561	1.27281775348874E-43	FOLR2+ resident-like macrophages	NPC2
7.75972394909125E-48	0.73008774735580	0.637	0.256	1.30037453938871E-43	FOLR2+ resident-like macrophages	TWF2
7.87885659199825E-48	0.56269916032959	0.792	0.364	1.32033878768707E-43	FOLR2+ resident-like macrophages	SLC15A3
8.4411708348429E-48	0.93631981977145	0.554	0.199	1.41457140850297E-43	FOLR2+ resident-like macrophages	MXD4
9.64040511649851E-48	0.76214921769723	0.527	0.173	1.61553908942282E-43	FOLR2+ resident-like macrophages	SNX9
1.3491718926198E-47	0.65509475602065	0.673	0.277	2.26094225765226E-43	FOLR2+ resident-like macrophages	TRAM1
2.16173650984953E-47	0.92008316416222	0.579	0.211	3.62263804320584E-43	FOLR2+ resident-like macrophages	MGST3
3.17771908813235E-47	1.07120635007546	0.618	0.254	5.32522164789219E-43	FOLR2+ resident-like macrophages	XBP1
3.64879604282134E-47	0.82084329941567	0.52	0.169	6.11465240856001E-43	FOLR2+ resident-like macrophages	SCAMP4
4.28549155147558E-47	0.69737650879250	0.803	0.387	7.18162674196277E-43	FOLR2+ resident-like macrophages	ATP6AP1
4.7616593682852E-47	0.47056184777375	0.806	0.341	7.97958876937234E-43	FOLR2+ resident-like macrophages	LRP1
6.94439030599239E-47	0.55325826785829	0.56	0.188	1.16374092747821E-42	FOLR2+ resident-like macrophages	NCF4
7.15674613301821E-47	1.2448655394649	0.386	0.092	1.19932751697119E-42	FOLR2+ resident-like macrophages	PINK1
1.10077507479837E-46	0.68456266602476	0.456	0.129	1.84467887034711E-42	FOLR2+ resident-like macrophages	SPRED1
1.13341443148933E-46	0.7956310285962	0.684	0.276	1.89937590428981E-42	FOLR2+ resident-like macrophages	RCSD1
1.28515720127503E-46	1.23935203595502	0.451	0.134	2.1536664378967E-42	FOLR2+ resident-like macrophages	ZNF189
1.45212834479623E-46	0.74791187640106	0.538	0.176	2.43347668020952E-42	FOLR2+ resident-like macrophages	SLC43A3
1.58841310884374E-46	0.80602058946009	0.557	0.194	2.66186268780034E-42	FOLR2+ resident-like macrophages	GBA
1.59645111481127E-46	0.63560867654720	0.792	0.38	2.67533277820072E-42	FOLR2+ resident-like macrophages	GNS
1.73813948023854E-46	0.77431071798878	0.823	0.371	2.91277414098375E-42	FOLR2+ resident-like macrophages	S100A10
1.88571976365614E-46	1.63212265171807	0.453	0.142	3.16008917993496E-42	FOLR2+ resident-like macrophages	AIFM1
1.91524250659097E-46	1.31691523879392	0.541	0.193	3.20956339254514E-42	FOLR2+ resident-like macrophages	CD151
2.14215857197624E-46	0.89835881347236	0.467	0.144	3.58982933491778E-42	FOLR2+ resident-like macrophages	EDEM2
2.21101758440352E-46	0.90066843399403	0.517	0.17	3.70522326794341E-42	FOLR2+ resident-like macrophages	EHD4
4.900190059423E-46	1.08946431653264	0.556	0.205	8.21173850158107E-42	FOLR2+ resident-like macrophages	MGST2
5.38738318621729E-46	0.57793293294842	0.774	0.351	9.02817674346293E-42	FOLR2+ resident-like macrophages	RNF130
6.50512978007861E-46	1.28448439638751	0.541	0.192	1.09012964854557E-41	FOLR2+ resident-like macrophages	ECHS1
6.67779868716791E-46	0.88396902717087	0.669	0.283	1.1190655039956E-41	FOLR2+ resident-like macrophages	HSD17B4
8.24836644029953E-46	0.79987854738978	0.567	0.213	1.38226124806539E-41	FOLR2+ resident-like macrophages	RNF13
1.09093013314032E-45	0.69248118112167	0.878	0.428	1.82818071711654E-41	FOLR2+ resident-like macrophages	MT-CYB
1.55810002842273E-45	0.70274909569461	0.491	0.156	2.61106402763081E-41	FOLR2+ resident-like macrophages	SNX29
1.59222891104052E-45	0.79579869801572	0.733	0.316	2.6682572091217E-41	FOLR2+ resident-like macrophages	LMAN2
2.14680012713404E-45	0.61891644253987	0.772	0.349	3.59760765305123E-41	FOLR2+ resident-like macrophages	RASSF4
2.51835893592471E-45	0.40150729112385	0.554	0.176	4.22026590482263E-41	FOLR2+ resident-like macrophages	KCNMA1
2.61335070814547E-45	0.35066635942881	0.547	0.186	4.37945311671017E-41	FOLR2+ resident-like macrophages	SELENOS
2.63571056624125E-45	0.90193980308377	0.44	0.124	4.41692376690708E-41	FOLR2+ resident-like macrophages	EBI3
2.825949047877E-45	0.93995437131570	0.493	0.165	4.73572541443228E-41	FOLR2+ resident-like macrophages	CLTA

3.83569041622725E-45	1.53387093570879	0.454	0.144	6.42784999951363E-41	FOLR2+ resident-like macrophages	TALDO1
4.71403962588713E-45	1.29880900720283	0.399	0.103	7.89978760506166E-41	FOLR2+ resident-like macrophages	APOC1
6.62676530230022E-45	0.566828721044531	0.644	0.265	1.11051332935947E-40	FOLR2+ resident-like macrophages	TBC1D14
6.66859403709897E-45	1.42131783380841	0.403	0.11	1.11752298873704E-40	FOLR2+ resident-like macrophages	PPM1M
1.28421674965185E-44	0.70866055789929	0.639	0.258	2.15209042906656E-40	FOLR2+ resident-like macrophages	SDF4
1.30066164342709E-44	0.63845069611137	0.769	0.351	2.17964878205512E-40	FOLR2+ resident-like macrophages	COLGALT1
1.42582709420515E-44	0.53412031356478	0.434	0.115	2.38940104446899E-40	FOLR2+ resident-like macrophages	SLC46A1
1.4260798517165E-44	1.02854840208311	0.424	0.117	2.38982461550652E-40	FOLR2+ resident-like macrophages	HECTD3
1.67020610038977E-44	0.72945713528681	0.569	0.206	2.79893138303317E-40	FOLR2+ resident-like macrophages	PLOD3
2.01398162275279E-44	1.23296084311205	0.502	0.172	3.37503040340912E-40	FOLR2+ resident-like macrophages	MLEC
2.63532120401066E-44	0.70334696038071	0.817	0.421	4.41627127368107E-40	FOLR2+ resident-like macrophages	BST2
3.67775633905804E-44	1.2976684152716	0.462	0.143	6.16318407299346E-40	FOLR2+ resident-like macrophages	SLC37A4
4.19661895847E-44	2.05156071025307	0.327	0.066	7.03269405060403E-40	FOLR2+ resident-like macrophages	DEPRECATED-ENSG00000198074
5.12638517608336E-44	2.20945581247001	0.331	0.072	8.59079627808049E-40	FOLR2+ resident-like macrophages	ST5
6.24964053394076E-44	1.65584131610654	0.438	0.138	1.04731476067779E-39	FOLR2+ resident-like macrophages	MGST1
1.06424887702194E-43	0.69512811848457	0.595	0.223	1.78346826811337E-39	FOLR2+ resident-like macrophages	PTAFR
1.12616264353211E-43	0.49269778223421	0.583	0.205	1.88722335803111E-39	FOLR2+ resident-like macrophages	VCAM1
1.31494337083446E-43	1.90154720266104	0.287	0.047	2.2035821008444E-39	FOLR2+ resident-like macrophages	HPGDS
1.76620143671114E-43	0.53939139189857	0.647	0.265	2.95980036764052E-39	FOLR2+ resident-like macrophages	PECAM1
2.15330189287571E-43	1.67185590542297	0.454	0.151	3.60850331208112E-39	FOLR2+ resident-like macrophages	LGALS4
2.34918374036482E-43	0.62547786748579	0.946	0.816	3.93676211210336E-39	FOLR2+ resident-like macrophages	PSAP
2.37541521973952E-43	0.67288095718360	0.807	0.404	3.98072082523949E-39	FOLR2+ resident-like macrophages	MARCKS
2.5004230392767E-43	0.78708723412956	0.581	0.224	4.1902089292199E-39	FOLR2+ resident-like macrophages	PHC2
2.80979948294211E-43	0.61693391925719	0.599	0.235	4.70866197351439E-39	FOLR2+ resident-like macrophages	IRAK1
2.82281006135868E-43	0.48267149378892	0.771	0.362	4.73046510082487E-39	FOLR2+ resident-like macrophages	DPP7
4.29834914866421E-43	0.59873737359281	0.657	0.272	7.20317350333148E-39	FOLR2+ resident-like macrophages	NECAP2
4.46368597917828E-43	0.49920483675595	0.61	0.243	7.48024496390696E-39	FOLR2+ resident-like macrophages	ATP6V0C
4.55373659378759E-43	0.58055096400402	0.512	0.173	7.63115178386925E-39	FOLR2+ resident-like macrophages	TRPM2
5.52457305321683E-43	0.65021166545794	0.611	0.248	9.25807952258076E-39	FOLR2+ resident-like macrophages	MAN1A1
6.21024860409886E-43	1.09329507856576	0.414	0.119	1.04071346107489E-38	FOLR2+ resident-like macrophages	LY96
6.34402581010315E-43	0.70558419494653	0.843	0.447	1.06313184525709E-38	FOLR2+ resident-like macrophages	MAN2B1
7.00847619386997E-43	0.29843423675246	0.713	0.292	1.17448044056873E-38	FOLR2+ resident-like macrophages	RGL1
7.85734082684159E-43	0.60433582485884	0.583	0.229	1.31673317576211E-38	FOLR2+ resident-like macrophages	SNX2
9.04880811093998E-43	1.07865389055354	0.541	0.198	1.51639926323132E-38	FOLR2+ resident-like macrophages	PEBP1
9.05047930721311E-43	0.56054158027340	0.467	0.147	1.51667932230277E-38	FOLR2+ resident-like macrophages	SIAH2
9.161500090698E-43	0.68143453349426	0.628	0.255	1.53528418519917E-38	FOLR2+ resident-like macrophages	TSPAN14
1.14868052487542E-42	0.59227153343664	0.621	0.248	1.92495882358623E-38	FOLR2+ resident-like macrophages	SCARB2
1.16126269110926E-42	0.56353348044803	0.475	0.149	1.94604401776089E-38	FOLR2+ resident-like macrophages	CCR1
1.28766153786898E-42	1.36762502396967	0.403	0.113	2.15786320516083E-38	FOLR2+ resident-like macrophages	EMP2
1.30037660978774E-42	1.1789874700276	0.531	0.196	2.1791711226823E-38	FOLR2+ resident-like macrophages	APOH
1.31722463540384E-42	0.57314128120530	0.671	0.291	2.20740504400975E-38	FOLR2+ resident-like macrophages	CD84
1.52537908275095E-42	1.50438983504281	0.327	0.068	2.55623026687404E-38	FOLR2+ resident-like macrophages	GBGT1
1.55569211839201E-42	0.94781489519959	0.447	0.139	2.60702885200134E-38	FOLR2+ resident-like macrophages	AGTRAP
1.67025852870266E-42	0.37590509560532	0.708	0.31	2.79901924239992E-38	FOLR2+ resident-like macrophages	ARRDC2
1.70802810092227E-42	0.56868050866702	0.554	0.204	2.86231349152555E-38	FOLR2+ resident-like macrophages	DENND4C
1.77895320470379E-42	0.58649773359023	0.617	0.251	2.98116978044261E-38	FOLR2+ resident-like macrophages	ATP6V0D1
2.04287813093857E-42	0.54829494290473	0.808	0.399	3.42345517182686E-38	FOLR2+ resident-like macrophages	APLP2
2.54755706810918E-42	1.75431060874777	0.322	0.068	4.26919613473737E-38	FOLR2+ resident-like macrophages	HSD3B7
2.87673154840601E-42	0.82918177975192	0.729	0.346	4.8208267288188E-38	FOLR2+ resident-like macrophages	ANXA2
3.27498382921973E-42	0.90349062605840	0.598	0.241	5.48821790100642E-38	FOLR2+ resident-like macrophages	ECH1
3.28488123958538E-42	0.99507796012659	0.453	0.142	5.50480398129717E-38	FOLR2+ resident-like macrophages	MBNL3
3.32313246984334E-42	0.53188173268376	0.527	0.189	5.56890539296347E-38	FOLR2+ resident-like macrophages	WASHC4
3.43418975142693E-42	0.70348706382261	0.611	0.242	5.75501518544125E-38	FOLR2+ resident-like macrophages	CALM3
4.25943739553239E-42	0.76801280547113	0.411	0.115	7.13796518743318E-38	FOLR2+ resident-like macrophages	PRXL2A
4.79728750196251E-42	1.1499907840141	0.376	0.097	8.03929439578878E-38	FOLR2+ resident-like macrophages	ANTXR2
5.18247532220663E-42	0.75140482530129	0.48	0.154	8.68479214495387E-38	FOLR2+ resident-like macrophages	DRAM1
5.7098821818231E-42	0.95021738323093	0.388	0.101	9.56862056029915E-38	FOLR2+ resident-like macrophages	TRIM47
5.96496321325144E-42	0.62024054725935	0.678	0.292	9.99608535276677E-38	FOLR2+ resident-like macrophages	CD164
6.64421371529381E-42	0.50453144571869	0.672	0.285	1.11343733440894E-37	FOLR2+ resident-like macrophages	IL18BP
7.27973646370605E-42	0.76726150596964	0.637	0.266	1.21993823658786E-37	FOLR2+ resident-like macrophages	HSD17B11
7.84343644611513E-42	1.58057537067278	0.456	0.157	1.31440307963997E-37	FOLR2+ resident-like macrophages	PRDX2
8.89483719405967E-42	1.43998267690823	0.478	0.166	1.49059681698052E-37	FOLR2+ resident-like macrophages	AMBP
9.56901901196806E-42	0.63810393121485	0.422	0.121	1.60357620602561E-37	FOLR2+ resident-like macrophages	ME1
1.07713019234403E-41	3.44041492294412	0.199	0.012	1.80505477633012E-37	FOLR2+ resident-like macrophages	GSDMA
1.16525624557009E-41	0.80457475963531	0.53	0.196	1.95273641632635E-37	FOLR2+ resident-like macrophages	SSR4
1.27694993345308E-41	1.9910624546274	0.247	0.032	2.13991269848067E-37	FOLR2+ resident-like macrophages	TRIM32
1.3851785844202E-41	0.79104045222596	0.556	0.214	2.32128227177137E-37	FOLR2+ resident-like macrophages	VOPP1
1.39696599272976E-41	1.06025829487266	0.38	0.102	2.34103561061654E-37	FOLR2+ resident-like macrophages	ZC3H12D
1.44081475677972E-41	0.29269333624613	0.528	0.182	2.41451736941145E-37	FOLR2+ resident-like macrophages	SMS
1.55064923854974E-41	0.71420399128919	0.549	0.201	2.59857799396166E-37	FOLR2+ resident-like macrophages	CYB5R1
1.65674809249548E-41	0.55775838112219	0.585	0.234	2.77637845340392E-37	FOLR2+ resident-like macrophages	CSTB

2.10737884954891E-41	1.64210049389674	0.258	0.037	3.53154547607406E-37	FOLR2+ resident-like macrophages	ANTXR1
2.18116381067315E-41	0.657491049383508	0.57	0.223	3.65519431392607E-37	FOLR2+ resident-like macrophages	SLC31A2
2.52246633881648E-41	1.42329265001677	0.367	0.094	4.22714909058865E-37	FOLR2+ resident-like macrophages	GIMAP1
2.91832174846451E-41	0.670031423468895	0.56	0.218	4.89052358607682E-37	FOLR2+ resident-like macrophages	ATOX1
3.02876435495997E-41	0.579000166941073	0.695	0.307	5.07560330604191E-37	FOLR2+ resident-like macrophages	GOLIM4
3.12870007778536E-41	0.645784697503494	0.56	0.214	5.2430755903527E-37	FOLR2+ resident-like macrophages	TMED5
3.16650339633656E-41	0.27695373221236	0.504	0.172	5.30642639158081E-37	FOLR2+ resident-like macrophages	ITGAV
3.36406070529159E-41	0.411418157357148	0.493	0.168	5.63749292992765E-37	FOLR2+ resident-like macrophages	UBXN6
3.5167523078568E-41	1.33268792461327	0.313	0.066	5.89337351750643E-37	FOLR2+ resident-like macrophages	GHDC
3.54362885885909E-41	1.03418218599931	0.406	0.117	5.93841324167607E-37	FOLR2+ resident-like macrophages	CLN6
3.56543271136652E-41	1.25048190792787	0.46	0.15	5.97495213770802E-37	FOLR2+ resident-like macrophages	FN1
3.57285973865793E-41	0.576471041228556	0.836	0.441	5.98739835004297E-37	FOLR2+ resident-like macrophages	MGAT1
4.0562711058814E-41	0.556078051794203	0.418	0.115	6.79749911923605E-37	FOLR2+ resident-like macrophages	FAM20A
4.61076642715587E-41	1.18240131482533	0.511	0.19	7.72672237862781E-37	FOLR2+ resident-like macrophages	COX6C
4.75665087225893E-41	1.4973188318907	0.456	0.153	7.97119553173151E-37	FOLR2+ resident-like macrophages	ETFB
5.78831074889913E-41	0.969253099911457	0.533	0.202	9.70005115300517E-37	FOLR2+ resident-like macrophages	C3
6.16866177309851E-41	0.88241212864928	0.444	0.138	1.03374433993585E-36	FOLR2+ resident-like macrophages	OAS1
6.61966791899596E-41	1.81607017806533	0.373	0.105	1.10932394986534E-36	FOLR2+ resident-like macrophages	PCK2
7.84693871096249E-41	0.573507268457355	0.406	0.111	1.31498998918309E-36	FOLR2+ resident-like macrophages	OLFML2B
7.97409500092463E-41	0.745599196539505	0.557	0.215	1.33629884025495E-36	FOLR2+ resident-like macrophages	DHRS7
8.0028364638109E-41	0.797255933101437	0.396	0.112	1.34111533460543E-36	FOLR2+ resident-like macrophages	NPC1
8.58460012818645E-41	0.709825364930379	0.672	0.273	1.43860728948149E-36	FOLR2+ resident-like macrophages	NUDT16
9.5422603667802E-41	0.929668416721788	0.343	0.079	1.59909199226503E-36	FOLR2+ resident-like macrophages	SLC38A6
9.7030113490433E-41	0.703798627295179	0.493	0.17	1.62603064187268E-36	FOLR2+ resident-like macrophages	GIMAP8
1.03076800315991E-40	0.609255249417451	0.602	0.248	1.72736101969538E-36	FOLR2+ resident-like macrophages	HM13
1.08032662979527E-40	0.790376220283792	0.585	0.234	1.81041136621092E-36	FOLR2+ resident-like macrophages	NDUFA1
1.16157536049939E-40	0.521179998053444	0.553	0.209	1.94656798912488E-36	FOLR2+ resident-like macrophages	BSG
1.34189767793381E-40	0.898339583586758	0.495	0.172	2.24875212868148E-36	FOLR2+ resident-like macrophages	IL4I1
1.36561835517858E-40	0.598626723542712	0.489	0.166	2.28850323960827E-36	FOLR2+ resident-like macrophages	CD40
1.64919152082984E-40	0.402992228779584	0.617	0.251	2.76371515060665E-36	FOLR2+ resident-like macrophages	GUSB
2.4604140149465E-40	1.30865435667331	0.361	0.097	4.12316180624735E-36	FOLR2+ resident-like macrophages	GPX3
2.50523026192141E-40	0.634650282115128	0.601	0.25	4.19826487292791E-36	FOLR2+ resident-like macrophages	ATP6V0B
2.73625875144728E-40	0.651245514170398	0.636	0.26	4.58542241567534E-36	FOLR2+ resident-like macrophages	SELPLG
3.48931156591222E-40	0.556617696544706	0.729	0.33	5.8473883221557E-36	FOLR2+ resident-like macrophages	TM9SF2
4.52341240141431E-40	1.33701127946378	0.476	0.168	7.5803345022901E-36	FOLR2+ resident-like macrophages	CFH
4.76838463676348E-40	0.879868367522598	0.463	0.155	7.99085897428824E-36	FOLR2+ resident-like macrophages	SHMT1
5.02694375695453E-40	0.612880796487931	0.569	0.222	8.4241523479044E-36	FOLR2+ resident-like macrophages	SPATS2L
5.55426231093913E-40	0.834807194081177	0.493	0.176	9.30783278067179E-36	FOLR2+ resident-like macrophages	NLK
7.16281970130696E-40	1.44275844296799	0.422	0.135	1.20034532554502E-35	FOLR2+ resident-like macrophages	SCCPDH
7.23506504117857E-40	0.62658373519978	0.402	0.117	1.2124521996007E-35	FOLR2+ resident-like macrophages	RBMS1
7.38535467814832E-40	1.26423731248603	0.456	0.156	1.2376377369641E-35	FOLR2+ resident-like macrophages	ACOX1
8.45221439887853E-40	0.700310810872953	0.692	0.295	1.41642208896406E-35	FOLR2+ resident-like macrophages	RNASE6
8.45959575740904E-40	1.04101758666292	0.547	0.215	1.41765905702661E-35	FOLR2+ resident-like macrophages	TXNRD1
9.95183185081483E-40	1.67033106464673	0.293	0.058	1.66772798155955E-35	FOLR2+ resident-like macrophages	SCN9A
1.09613026289258E-39	0.5681019167663	0.676	0.294	1.83689509455539E-35	FOLR2+ resident-like macrophages	PTTG1IP
1.10907283036792E-39	0.62894101413492	0.791	0.376	1.85858424913056E-35	FOLR2+ resident-like macrophages	MT-ND5
1.31051740825704E-39	1.28671527653902	0.409	0.127	2.19616507275715E-35	FOLR2+ resident-like macrophages	CMC4
1.36479930592368E-39	0.310745844289752	0.583	0.216	2.28713067686691E-35	FOLR2+ resident-like macrophages	ARHGEF10L
1.38600653702422E-39	0.718211758531856	0.528	0.194	2.32266975474519E-35	FOLR2+ resident-like macrophages	YIPF3
1.39008898216807E-39	0.715588761128639	0.897	0.543	2.32951111631726E-35	FOLR2+ resident-like macrophages	CD4
1.52170919638945E-39	1.14949006207809	0.44	0.143	2.55008027130944E-35	FOLR2+ resident-like macrophages	GIMAP6
2.62106611064245E-39	0.320019900950585	0.615	0.255	4.39238258821462E-35	FOLR2+ resident-like macrophages	RAP2B
3.59799599206724E-39	0.708977288645932	0.376	0.102	6.02952168350628E-35	FOLR2+ resident-like macrophages	DSC2
3.7067298280091E-39	1.66440911810062	0.361	0.102	6.21173784577765E-35	FOLR2+ resident-like macrophages	PNPO
3.85187274872293E-39	0.887080375466043	0.518	0.197	6.45496835230989E-35	FOLR2+ resident-like macrophages	CAPN1
3.85431891470461E-39	0.705854641747838	0.627	0.272	6.45906763726199E-35	FOLR2+ resident-like macrophages	OTULINL
3.9680439143533E-39	0.49982956765526	0.511	0.185	6.64964799167326E-35	FOLR2+ resident-like macrophages	BIN1
3.97508004281267E-39	0.593094731107647	0.627	0.272	6.66143913574546E-35	FOLR2+ resident-like macrophages	PGD
4.04800137947335E-39	1.06508280622132	0.287	0.053	6.78364071172144E-35	FOLR2+ resident-like macrophages	DPEP3
4.83709389456714E-39	1.00759893960656	0.511	0.192	8.10600194851562E-35	FOLR2+ resident-like macrophages	CAT
5.17824917716173E-39	0.575122371605143	0.46	0.153	8.67770997108764E-35	FOLR2+ resident-like macrophages	TMEM273
5.67799650709614E-39	0.745638635946757	0.417	0.125	9.51518654659171E-35	FOLR2+ resident-like macrophages	KLF7
5.92728920195146E-39	1.16375320006884	0.3	0.063	9.93295124463026E-35	FOLR2+ resident-like macrophages	SLC45A4
6.70324880540741E-39	1.67103575339977	0.345	0.089	1.12333043481017E-34	FOLR2+ resident-like macrophages	NAPRT
9.0312298552403E-39	0.54141965218477	0.668	0.309	1.51345349914117E-34	FOLR2+ resident-like macrophages	ATP6AP2
9.28515403207119E-39	1.70212954698978	0.26	0.043	1.55600611269449E-34	FOLR2+ resident-like macrophages	GAL3ST4
1.47786023786115E-38	1.76178487575359	0.385	0.117	2.47659818660772E-34	FOLR2+ resident-like macrophages	DTNA
1.52315310790406E-38	0.301987012327616	0.447	0.144	2.55249997822562E-34	FOLR2+ resident-like macrophages	SORBS3
1.52380687592183E-38	0.54166768879955	0.454	0.147	2.55359556266981E-34	FOLR2+ resident-like macrophages	PILRA
1.9155288631228E-38	0.594751148605735	0.769	0.37	3.21004326882118E-34	FOLR2+ resident-like macrophages	CAPZB
2.04560478233289E-38	0.389257616472107	0.679	0.299	3.42802449423346E-34	FOLR2+ resident-like macrophages	ANXA1

2.10545926796528E-38	0.547255051717861	0.595	0.235	3.52832864125622E-34	FOLR2+ resident-like macrophages	GIMAP4
2.34889456593838E-38	1.27587253093493	0.52	0.203	3.93627751359955E-34	FOLR2+ resident-like macrophages	TF
2.49904006546806E-38	0.440935200028347	0.46	0.155	4.18789134171137E-34	FOLR2+ resident-like macrophages	EPAS1
2.70459530012012E-38	0.980079579620305	0.328	0.079	4.53236080394129E-34	FOLR2+ resident-like macrophages	BAIAP2
2.76649305946762E-38	0.578581471601954	0.424	0.129	4.63608906905584E-34	FOLR2+ resident-like macrophages	ZDHHC14
3.28332576892788E-38	0.844295660539072	0.541	0.211	5.50219732356934E-34	FOLR2+ resident-like macrophages	ESYT1
4.37433359844907E-38	0.502812901504391	0.689	0.326	7.33050824428095E-34	FOLR2+ resident-like macrophages	AKR1A1
5.55555554739514E-38	1.59325457422403	0.478	0.187	9.30999998632478E-34	FOLR2+ resident-like macrophages	AGT
6.31804510757487E-38	0.585839424564136	0.591	0.241	1.0587779991274E-33	FOLR2+ resident-like macrophages	LASP1
7.90761676390845E-38	0.673988602037937	0.572	0.234	1.32515841729578E-33	FOLR2+ resident-like macrophages	KIAA0930
8.3503891487341E-38	1.64545351256813	0.357	0.102	1.39935821354486E-33	FOLR2+ resident-like macrophages	ERRFI1
1.0906081580636E-37	1.76901670148794	0.248	0.04	1.82764115128299E-33	FOLR2+ resident-like macrophages	MAPK13
1.18056623931837E-37	1.04922157780953	0.338	0.083	1.97839290384973E-33	FOLR2+ resident-like macrophages	PDCD1LG2
1.21375883499778E-37	0.712931741989623	0.485	0.177	2.03401705568927E-33	FOLR2+ resident-like macrophages	TMEM147
1.24400162027753E-37	0.683321337065228	0.691	0.31	2.08469791526109E-33	FOLR2+ resident-like macrophages	IFITM3
1.38163458750452E-37	0.578584344316752	0.765	0.356	2.31534324174007E-33	FOLR2+ resident-like macrophages	FPR3
1.64384186176512E-37	0.810381016087793	0.433	0.141	2.75475019194598E-33	FOLR2+ resident-like macrophages	WASHC2A
1.83201379990301E-37	1.23506603155792	0.428	0.145	3.07008872587746E-33	FOLR2+ resident-like macrophages	IARS2
2.06216368636349E-37	0.355996525911132	0.644	0.28	3.45577390560794E-33	FOLR2+ resident-like macrophages	OSTF1
2.09638031284798E-37	0.365696212169687	0.605	0.245	3.51311412827064E-33	FOLR2+ resident-like macrophages	TBXAS1
2.13488837650203E-37	1.10579266760828	0.303	0.067	3.5776459413421E-33	FOLR2+ resident-like macrophages	APH1B
2.16576289577062E-37	1.8216718426589	0.345	0.096	3.62938546073241E-33	FOLR2+ resident-like macrophages	MCFD2
2.22542838142054E-37	1.14620276491226	0.472	0.176	3.72937288158453E-33	FOLR2+ resident-like macrophages	NDUFC2
2.27748498016532E-37	0.797856198176465	0.329	0.081	3.81660932976104E-33	FOLR2+ resident-like macrophages	RRAGD
2.50184039367524E-37	1.30499297266534	0.273	0.053	4.19258413172096E-33	FOLR2+ resident-like macrophages	EVA1B
2.51855765879737E-37	1.1399970229098	0.28	0.054	4.22059892461263E-33	FOLR2+ resident-like macrophages	TNS1
2.52623566886431E-37	0.595899873279375	0.72	0.332	4.23346573388282E-33	FOLR2+ resident-like macrophages	MT-ND1
2.926759149714E-37	0.44356746475787	0.684	0.31	4.90466298309073E-33	FOLR2+ resident-like macrophages	IFNGR2
3.02531289506336E-37	0.293039476268246	0.476	0.163	5.06981934954718E-33	FOLR2+ resident-like macrophages	KIF1B
3.05955450928059E-37	0.628347948315876	0.656	0.286	5.12720144665242E-33	FOLR2+ resident-like macrophages	DEPRECATED-ENSG00000089248
3.7618492702639E-37	1.18369233456724	0.263	0.047	6.30410700710825E-33	FOLR2+ resident-like macrophages	TGFA
5.06102185959875E-37	0.870910161245647	0.482	0.179	8.48126043231559E-33	FOLR2+ resident-like macrophages	ALDH9A1
6.16346776690381E-37	0.913186611621686	0.464	0.166	1.03287392837774E-32	FOLR2+ resident-like macrophages	ATRN
6.41864201396345E-37	0.306659190277168	0.633	0.276	1.07563602869999E-32	FOLR2+ resident-like macrophages	ARF1
6.43186221143581E-37	0.462375689629516	0.642	0.289	1.07785146939241E-32	FOLR2+ resident-like macrophages	SEC14L1
9.36128123557625E-37	0.66877086306801	0.485	0.181	1.56876350945787E-32	FOLR2+ resident-like macrophages	APH1A
1.0031131805929E-36	1.31155098618399	0.454	0.166	1.68101706803758E-32	FOLR2+ resident-like macrophages	NQO1
1.06966171204332E-36	0.446981424080004	0.762	0.37	1.7925390970422E-32	FOLR2+ resident-like macrophages	TNFRSF1A
1.13255658256677E-36	0.447520519272713	0.761	0.363	1.89793832106539E-32	FOLR2+ resident-like macrophages	ACSL1
1.38010404965556E-36	0.505675624819823	0.505	0.19	2.31277836641278E-32	FOLR2+ resident-like macrophages	DDRGK1
1.39694554974803E-36	1.61105606889775	0.348	0.099	2.34100135226775E-32	FOLR2+ resident-like macrophages	ITPKC
1.43903202690171E-36	0.516923885215321	0.774	0.388	2.41152987068188E-32	FOLR2+ resident-like macrophages	M6PR
1.47788623673064E-36	0.48131489796225	0.524	0.194	2.47664175551321E-32	FOLR2+ resident-like macrophages	ETV5
1.58067494523187E-36	0.420824549009712	0.668	0.301	2.64889507321956E-32	FOLR2+ resident-like macrophages	NFE2L1
1.62364537244628E-36	1.14821637186066	0.457	0.167	2.72090491514547E-32	FOLR2+ resident-like macrophages	P4HB
1.80496871637949E-36	0.320685119664591	0.475	0.164	3.02476657490875E-32	FOLR2+ resident-like macrophages	BMP2K
1.87878276536445E-36	0.527127502891286	0.583	0.241	3.14846415819774E-32	FOLR2+ resident-like macrophages	TSPO
2.20466221429654E-36	0.696724253526389	0.543	0.223	3.69457293871815E-32	FOLR2+ resident-like macrophages	SPPL2A
2.25849340774662E-36	0.646842490153495	0.824	0.461	3.78478325270178E-32	FOLR2+ resident-like macrophages	TYROBP
2.61985201114472E-36	1.74148540874345	0.269	0.054	4.39034800027631E-32	FOLR2+ resident-like macrophages	CTDSPL
2.77506374749969E-36	0.542529603801126	0.595	0.259	4.65045182805998E-32	FOLR2+ resident-like macrophages	MYDGF
2.81650040503929E-36	0.661280181978847	0.388	0.116	4.71989137876483E-32	FOLR2+ resident-like macrophages	PYGL
2.90747854949427E-36	0.35603865044899	0.636	0.273	4.87235255324249E-32	FOLR2+ resident-like macrophages	NDFIP1
3.16561196132588E-36	1.29542036431874	0.393	0.124	5.30493252478991E-32	FOLR2+ resident-like macrophages	CFB
3.31896583464843E-36	0.621893296002657	0.353	0.097	5.56192294570384E-32	FOLR2+ resident-like macrophages	ANKH
3.5836819560047E-36	0.78529832588694	0.479	0.176	6.00553422187268E-32	FOLR2+ resident-like macrophages	RTCB
3.61588736773317E-36	0.454573547394274	0.66	0.295	6.05950405084725E-32	FOLR2+ resident-like macrophages	SUSD6
3.78893068811161E-36	0.577153735571453	0.668	0.302	6.34949004713744E-32	FOLR2+ resident-like macrophages	PRKACA
3.80319508558402E-36	1.16271033848443	0.472	0.183	6.37339432442171E-32	FOLR2+ resident-like macrophages	CLU
4.07047874945479E-36	0.273370479929118	0.795	0.414	6.82130828833633E-32	FOLR2+ resident-like macrophages	MPP1
4.36395232498568E-36	1.30550848321926	0.382	0.118	7.313111306211E-32	FOLR2+ resident-like macrophages	PRKAB1
4.61695144384103E-36	0.636786138409518	0.424	0.14	7.73708722958879E-32	FOLR2+ resident-like macrophages	TANGO2
4.93015207966271E-36	1.32825683098383	0.412	0.139	8.26194885509877E-32	FOLR2+ resident-like macrophages	SDHB
5.97520202181339E-36	1.38476664841453	0.46	0.174	1.00132435481549E-31	FOLR2+ resident-like macrophages	SMARCA1
7.43090026709267E-36	0.730843871161557	0.453	0.163	1.24527026675939E-31	FOLR2+ resident-like macrophages	DNASE1L1
8.17585207551409E-36	1.30292666050104	0.412	0.142	1.37010929081465E-31	FOLR2+ resident-like macrophages	TPD52
1.05922427793557E-35	1.21992194723467	0.379	0.12	1.77504804496443E-31	FOLR2+ resident-like macrophages	SLC22A18
1.34115299213855E-35	0.887142372655249	0.538	0.216	2.24750418422578E-31	FOLR2+ resident-like macrophages	DECR1
1.62944707700942E-35	0.857486554763458	0.376	0.114	2.73062741165239E-31	FOLR2+ resident-like macrophages	PARVB
1.65094008633358E-35	0.47887964558555	0.431	0.147	2.76664539667782E-31	FOLR2+ resident-like macrophages	ATP6V1C1
1.65413887306272E-35	0.468049096973897	0.498	0.19	2.77200592347851E-31	FOLR2+ resident-like macrophages	TBC1D15

**2.36520902680146E-35**	1.79267018321627	0.322	0.086	3.96361728711389E-31	FOLR2+ resident-like macrophages	POR
**2.42647744996461E-35**	1.26101461715902	0.385	0.124	4.0662909106507E-31	FOLR2+ resident-like macrophages	ATF5
**2.75434473761232E-35**	0.402786894890605	0.721	0.346	4.61573091129073E-31	FOLR2+ resident-like macrophages	AFF1
**3.26011187206729E-35**	0.530384782934684	0.425	0.147	5.46329547521037E-31	FOLR2+ resident-like macrophages	ATP6V0A1
**3.72845223560014E-35**	0.579736338499963	0.546	0.22	6.24814025641871E-31	FOLR2+ resident-like macrophages	SAP30
**4.17069069498571E-35**	0.297299345740939	0.521	0.194	6.98924346665705E-31	FOLR2+ resident-like macrophages	IRS2
**4.62842581512503E-35**	1.00325149949462	0.389	0.124	7.75631598098652E-31	FOLR2+ resident-like macrophages	DNPH1
**4.90463711171296E-35**	1.28053352864788	0.408	0.139	8.21919087180858E-31	FOLR2+ resident-like macrophages	ACAA2
**5.02853993646246E-35**	0.377043443701141	0.552	0.225	8.42682722552379E-31	FOLR2+ resident-like macrophages	ATP6V1A
**5.36706937030372E-35**	0.593677154134743	0.433	0.152	8.99413485075497E-31	FOLR2+ resident-like macrophages	TMEM179B
**6.26858095424195E-35**	0.6909074454388	0.478	0.178	1.05048879631187E-30	FOLR2+ resident-like macrophages	DCTN2
**6.30982756326408E-35**	1.00944699848075	0.528	0.215	1.0574009030518E-30	FOLR2+ resident-like macrophages	AFDN
**8.10340524438797E-35**	0.506335438607857	0.502	0.202	1.35796865085454E-30	FOLR2+ resident-like macrophages	RNPEPL1
**9.10448182363907E-35**	1.96823418421235	0.266	0.055	1.52572906400544E-30	FOLR2+ resident-like macrophages	LRP3
**9.2038600186551E-35**	2.0962885914969	0.269	0.057	1.54238286192622E-30	FOLR2+ resident-like macrophages	BAAT
**9.56910688459738E-35**	0.746822602777898	0.475	0.183	1.60359093172083E-30	FOLR2+ resident-like macrophages	CALU
**9.73018057101054E-35**	0.588675612879784	0.504	0.197	1.63058366008995E-30	FOLR2+ resident-like macrophages	ARPIN
**1.10032520533975E-34**	0.565754102796105	0.649	0.297	1.84392497910836E-30	FOLR2+ resident-like macrophages	LGALS9
**1.21071765638124E-34**	0.292517862464089	0.379	0.112	2.02892064856368E-30	FOLR2+ resident-like macrophages	RIN2
**1.2362433258327E-34**	0.758614810783178	0.626	0.28	2.07169656543044E-30	FOLR2+ resident-like macrophages	ACSL4
**1.44528157089899E-34**	0.833098170185799	0.351	0.101	2.42200285651253E-30	FOLR2+ resident-like macrophages	MSRB2
**1.46427012322855E-34**	0.719067181241614	0.421	0.144	2.45382387250641E-30	FOLR2+ resident-like macrophages	ACBD5
**1.65871064565899E-34**	0.816692192730148	0.414	0.141	2.77966729999534E-30	FOLR2+ resident-like macrophages	TEX264
**1.66531680638522E-34**	0.62460257655391	0.601	0.267	2.79073790414035E-30	FOLR2+ resident-like macrophages	RBPJ
**1.7326566153813E-34**	1.85685635831803	0.315	0.084	2.90358595605599E-30	FOLR2+ resident-like macrophages	RHOBTB3
**1.7559326396353E-34**	0.742366536832487	0.438	0.155	2.94259191750083E-30	FOLR2+ resident-like macrophages	SEL1L3
**2.09446970413092E-34**	0.556955815385137	0.784	0.406	3.5099123301826E-30	FOLR2+ resident-like macrophages	RNH1
**2.09821688290797E-34**	0.53828323811082	0.316	0.076	3.51619185237717E-30	FOLR2+ resident-like macrophages	SCN1B
**2.173170696299E-34**	1.73074031134997	0.306	0.08	3.64179945285787E-30	FOLR2+ resident-like macrophages	DEPRECATED-ENSG00000159228
**2.40647476368269E-34**	1.54848102441402	0.337	0.096	4.03277040897945E-30	FOLR2+ resident-like macrophages	SLC9A3R1
**2.47561118595813E-34**	1.34892928034577	0.328	0.09	4.14862922542864E-30	FOLR2+ resident-like macrophages	UROD
**2.55029812308989E-34**	0.880962492053586	0.534	0.224	4.27378959467403E-30	FOLR2+ resident-like macrophages	PPIB
**2.58213777084932E-34**	1.2437493266966	0.324	0.088	4.32714647638928E-30	FOLR2+ resident-like macrophages	TMEM129
**2.64904968217871E-34**	0.388740057282306	0.527	0.21	4.43927745739508E-30	FOLR2+ resident-like macrophages	ZFAND5
**2.91092036987223E-34**	0.568433850106738	0.55	0.228	4.87812035583188E-30	FOLR2+ resident-like macrophages	REEP5
**2.93967102021264E-34**	1.63152922266368	0.351	0.106	4.92630069567234E-30	FOLR2+ resident-like macrophages	ADH1C
**2.96965266998482E-34**	0.663315517043044	0.396	0.131	4.97654394436057E-30	FOLR2+ resident-like macrophages	ITPA
**3.00834251959107E-34**	0.403978506411817	0.446	0.161	5.04138039433071E-30	FOLR2+ resident-like macrophages	PAPSS1
**3.012586688546E-34**	0.384610277283983	0.536	0.213	5.04849277266539E-30	FOLR2+ resident-like macrophages	FRMD4B
**3.73572096584125E-34**	0.280032424781541	0.502	0.192	6.26032119455677E-30	FOLR2+ resident-like macrophages	FOXO3
**3.93520146655531E-34**	1.01749060655639	0.379	0.12	6.5946106176534E-30	FOLR2+ resident-like macrophages	NAGPA
**4.00808830422256E-34**	0.466157362556557	0.331	0.088	6.71675438021616E-30	FOLR2+ resident-like macrophages	HNMT
**4.10172084160593E-34**	0.555617394697544	0.567	0.246	6.87366378636322E-30	FOLR2+ resident-like macrophages	TMEM219
**4.13827736651196E-34**	0.666120521445938	0.537	0.216	6.93492521080074E-30	FOLR2+ resident-like macrophages	ALCAM
**4.16704859678553E-34**	1.51275672261654	0.354	0.109	6.98314003849319E-30	FOLR2+ resident-like macrophages	TMEM14A
**4.412441789764E-34**	2.00316322764962	0.257	0.052	7.39436995128651E-30	FOLR2+ resident-like macrophages	SULT1C2
**4.9867483063644E-34**	0.351594629177316	0.626	0.277	8.35679281180547E-30	FOLR2+ resident-like macrophages	COMT
**5.41350651509316E-34**	1.23719391480102	0.399	0.138	9.07195421799312E-30	FOLR2+ resident-like macrophages	PCBD1
**6.04299838954366E-34**	0.27028479274593	0.57	0.237	1.01268567011973E-29	FOLR2+ resident-like macrophages	PKN1
**6.55235240698554E-34**	0.314973916494662	0.569	0.239	1.09804321636264E-29	FOLR2+ resident-like macrophages	ABCG1
**7.17436237180195E-34**	0.952606309601777	0.412	0.14	1.20227964626657E-29	FOLR2+ resident-like macrophages	WSB2
**8.21838384203347E-34**	1.12045040690704	0.29	0.068	1.37723676424797E-29	FOLR2+ resident-like macrophages	NMRK1
**8.3653751849623E-34**	0.554624496812213	0.43	0.149	1.40186957349598E-29	FOLR2+ resident-like macrophages	SLC49A4
**9.55825953109646E-34**	0.470947685942059	0.675	0.309	1.60177313222114E-29	FOLR2+ resident-like macrophages	SYK
**9.76265548018822E-34**	1.58402916599802	0.409	0.15	1.63602580536994E-29	FOLR2+ resident-like macrophages	TDO2
**9.9437036220377E-34**	0.369364987819696	0.675	0.317	1.66636585298108E-29	FOLR2+ resident-like macrophages	PIK3AP1
**1.03172716761675E-33**	0.37914245325851	0.419	0.142	1.72896838749214E-29	FOLR2+ resident-like macrophages	SLC9A9
**1.1544226515302E-33**	0.294933557779584	0.431	0.149	1.93458147943431E-29	FOLR2+ resident-like macrophages	EPS15
**1.15687837721806E-33**	1.09950436118343	0.379	0.123	1.93869678454202E-29	FOLR2+ resident-like macrophages	PGRMC2
**1.16784417201945E-33**	1.05765320653032	0.376	0.124	1.95707326347019E-29	FOLR2+ resident-like macrophages	CYB561A3
**1.29621312923719E-33**	0.488243754431495	0.443	0.157	2.17219396197568E-29	FOLR2+ resident-like macrophages	FAM20C
**1.57114904834509E-33**	0.374958585393972	0.791	0.398	2.6329315752167E-29	FOLR2+ resident-like macrophages	DNM2
**1.61215580520051E-33**	1.0913348694278	0.392	0.132	2.70165069835502E-29	FOLR2+ resident-like macrophages	NDUFB9
**1.69523151471955E-33**	0.449289444781606	0.576	0.25	2.84086897236701E-29	FOLR2+ resident-like macrophages	CRTAP
**1.79378210965183E-33**	0.556336325802378	0.563	0.233	3.00602005935454E-29	FOLR2+ resident-like macrophages	RNPEP
**1.79611660620718E-33**	0.556595668340415	0.401	0.135	3.00993220868199E-29	FOLR2+ resident-like macrophages	DBP
**1.88409410394821E-33**	0.271387369066141	0.633	0.277	3.15736489939642E-29	FOLR2+ resident-like macrophages	TMEM106A
**1.88739025613432E-33**	0.325534116220329	0.66	0.306	3.16288859122989E-29	FOLR2+ resident-like macrophages	NEK6
**2.14020481373298E-33**	0.86244805178294	0.347	0.104	3.58655522685372E-29	FOLR2+ resident-like macrophages	MFSD12
**2.20580964314629E-33**	1.94223560974351	0.266	0.06	3.69649579998455E-29	FOLR2+ resident-like macrophages	SLPI
**2.30849054060836E-33**	0.801970452791116	0.424	0.148	3.86856844795149E-29	FOLR2+ resident-like macrophages	CYBRD1

2.38795940248914E-33	0.32444263121592	0.533	0.208	4.0017423666913E-29	FOLR2+ resident-like macrophages	NADK
2.43927155832192E-33	0.92543711045344	0.392	0.131	4.08773127743587E-29	FOLR2+ resident-like macrophages	TMEM51
2.61402273674413E-33	0.97669881464886	0.401	0.139	4.38057930223581E-29	FOLR2+ resident-like macrophages	NACC1
3.07295475509585E-33	1.15398184427103	0.444	0.165	5.14965757858963E-29	FOLR2+ resident-like macrophages	DDT
3.30036251619753E-33	1.9000202434763	0.264	0.059	5.53074750464382E-29	FOLR2+ resident-like macrophages	TMEM236
3.3101931967478E-33	0.47481788513917	0.414	0.143	5.54722175910997E-29	FOLR2+ resident-like macrophages	ATP6V1F
3.67335077477949E-33	0.80185170670682	0.385	0.124	6.15580122837546E-29	FOLR2+ resident-like macrophages	PXDC1
3.96961750058534E-33	0.89034331524400	0.351	0.105	6.65228500748091E-29	FOLR2+ resident-like macrophages	RAB13
4.00429059277723E-33	0.50191871562357	0.399	0.131	6.71039017537608E-29	FOLR2+ resident-like macrophages	LRP10
4.10028976679019E-33	0.55275568215812	0.938	0.672	6.87126559118701E-29	FOLR2+ resident-like macrophages	CSF1R
4.24754695505197E-33	0.64817606951921	0.845	0.509	7.11803918727608E-29	FOLR2+ resident-like macrophages	FCER1G
4.29604868373191E-33	0.34132176964176	0.628	0.283	7.19931838419793E-29	FOLR2+ resident-like macrophages	OGFRL1
4.32266598802994E-33	0.768148297255791	0.36	0.111	7.24392366274057E-29	FOLR2+ resident-like macrophages	MAPK3
4.47416688968809E-33	0.79229887250797	0.47	0.184	7.4978088737393E-29	FOLR2+ resident-like macrophages	CIAO2A
4.77806218262567E-33	0.25392373811105	0.749	0.347	8.0070766056441E-29	FOLR2+ resident-like macrophages	MSR1
5.40768227666929E-33	0.25696069265404	0.55	0.23	9.0621939592424E-29	FOLR2+ resident-like macrophages	FAM91A1
5.69209648966083E-33	0.37626660762246	0.406	0.135	9.53881529737362E-29	FOLR2+ resident-like macrophages	CBLB
6.01904413704702E-33	0.63205959736847	0.522	0.216	1.00867141648634E-28	FOLR2+ resident-like macrophages	FGG
6.36874114008039E-33	0.84845484368705	0.409	0.14	1.06727364025467E-28	FOLR2+ resident-like macrophages	KLHDC3
8.69366018621786E-33	0.35224723457040	0.518	0.214	1.45688357400639E-28	FOLR2+ resident-like macrophages	MAP3K3
8.95822201726605E-33	0.42386859068573	0.401	0.135	1.50121884565344E-28	FOLR2+ resident-like macrophages	SGPL1
1.18001779497634E-32	0.53008149351934	0.524	0.213	1.97747382082135E-28	FOLR2+ resident-like macrophages	WDR81
1.19464408679548E-32	0.26062001512127	0.479	0.182	2.00198456065187E-28	FOLR2+ resident-like macrophages	ZSWIM8
1.24168692175298E-32	0.73744959121135	0.456	0.174	2.08081894347364E-28	FOLR2+ resident-like macrophages	CLPTM1
1.26860881449264E-32	0.94521508854712	0.437	0.166	2.12593465132677E-28	FOLR2+ resident-like macrophages	TPM1
1.29810561079814E-32	0.31854010783487	0.586	0.256	2.17536538257552E-28	FOLR2+ resident-like macrophages	TM6SF1
1.49858818022659E-32	1.4612571975615	0.322	0.094	2.51133407242371E-28	FOLR2+ resident-like macrophages	SULT2A1
1.51599028550788E-32	1.38822075579348	0.264	0.061	2.54049652045411E-28	FOLR2+ resident-like macrophages	FECH
1.54113654134744E-32	0.32601021473755	0.585	0.25	2.58263661599005E-28	FOLR2+ resident-like macrophages	IGFLR1
1.67422806546604E-32	1.77068038313102	0.257	0.056	2.80567139210799E-28	FOLR2+ resident-like macrophages	S100A16
1.8456564919511E-32	0.79260019465290	0.379	0.122	3.09295114921165E-28	FOLR2+ resident-like macrophages	SLC39A1
1.85837325899914E-32	0.72284204299303	0.308	0.082	3.11426190743076E-28	FOLR2+ resident-like macrophages	SNX24
2.03666487124788E-32	0.96531495815138	0.389	0.131	3.41304299123719E-28	FOLR2+ resident-like macrophages	SLC31A1
2.2439779417446E-32	0.29082364862271	0.657	0.307	3.7604582347756E-28	FOLR2+ resident-like macrophages	SUMF2
2.25213780640978E-32	0.62161054702168	0.321	0.089	3.77413253598151E-28	FOLR2+ resident-like macrophages	ZNF358
2.37332240011295E-32	0.33006113390224	0.522	0.216	3.97721367810928E-28	FOLR2+ resident-like macrophages	PIK3R1
2.48820400069128E-32	0.47335240779105	0.491	0.195	4.16973226435844E-28	FOLR2+ resident-like macrophages	PIGT
2.91667974213956E-32	0.27263740063980	0.666	0.31	4.88777191187747E-28	FOLR2+ resident-like macrophages	SERINC1
3.34431627308879E-32	1.15001826589378	0.334	0.1	5.60440521044219E-28	FOLR2+ resident-like macrophages	C9orf16
3.44927288207615E-32	1.58797720449242	0.38	0.136	5.78029149578321E-28	FOLR2+ resident-like macrophages	SOD1
3.54131619755065E-32	0.70181218192608	0.531	0.224	5.93453768385538E-28	FOLR2+ resident-like macrophages	CYC1
3.73483874190922E-32	0.88624735720918	0.356	0.115	6.25884276369147E-28	FOLR2+ resident-like macrophages	VPS26C
4.48917066365686E-32	1.68547500980697	0.34	0.106	7.52295219815616E-28	FOLR2+ resident-like macrophages	RARRES2
4.79114497731759E-32	0.41028597637916	0.64	0.303	8.02900075298882E-28	FOLR2+ resident-like macrophages	LAMTOR1
6.90961482689255E-32	0.56005515235377	0.392	0.133	1.15791325269065E-27	FOLR2+ resident-like macrophages	CHMP3
7.63069410471261E-32	0.41472791767917	0.56	0.24	1.27875171806774E-27	FOLR2+ resident-like macrophages	GSDMD
7.70540286361775E-32	0.35355123565245	0.53	0.221	1.29127141188506E-27	FOLR2+ resident-like macrophages	STT3A
8.78611973963717E-32	1.00603817262623	0.356	0.111	1.4723779459684E-27	FOLR2+ resident-like macrophages	APOL1
1.09455611760574E-31	0.33010108710315	0.572	0.25	1.83425714188369E-27	FOLR2+ resident-like macrophages	TMUB2
1.13506520241991E-31	0.50905053806450	0.453	0.172	1.90214226621528E-27	FOLR2+ resident-like macrophages	SEPTIN11
1.46311940474651E-31	1.02855772675914	0.303	0.081	2.45189549847421E-27	FOLR2+ resident-like macrophages	FOXRED2
1.50585077640944E-31	0.66568180181586	0.427	0.16	2.52350473110694E-27	FOLR2+ resident-like macrophages	PYGB
1.82698368168885E-31	0.46735234181851	0.414	0.147	3.06165925377417E-27	FOLR2+ resident-like macrophages	CAB39
1.87391684577239E-31	0.58867431259738	0.576	0.257	3.14030985014538E-27	FOLR2+ resident-like macrophages	DCAF7
2.32526119470581E-31	1.02253423408392	0.303	0.083	3.89667271008799E-27	FOLR2+ resident-like macrophages	TBC1D16
2.63086205533124E-31	0.53706132097098	0.566	0.256	4.4087986323241E-27	FOLR2+ resident-like macrophages	COPG1
2.65539986066313E-31	0.52964894468256	0.604	0.278	4.44991908649927E-27	FOLR2+ resident-like macrophages	ATP6V0E1
2.67263119214065E-31	0.30084629352881	0.431	0.159	4.4787953517893E-27	FOLR2+ resident-like macrophages	SUSD1
3.22818040631714E-31	0.41101228328704	0.576	0.25	5.40978472490627E-27	FOLR2+ resident-like macrophages	CTNNA1
3.24717796836072E-31	0.54923810441774	0.894	0.538	5.44162083937889E-27	FOLR2+ resident-like macrophages	CYBA
3.4966130634008E-31	0.47913430594186	0.515	0.211	5.85962417164705E-27	FOLR2+ resident-like macrophages	ELOVL1
3.56694262227699E-31	0.35088345805015	0.554	0.243	5.97748244641177E-27	FOLR2+ resident-like macrophages	TMEM50A
3.61348405876859E-31	0.50496548181926	0.525	0.222	6.0554765856844E-27	FOLR2+ resident-like macrophages	STAU1
3.64178614011688E-31	1.05627673825519	0.386	0.14	6.10290521360787E-27	FOLR2+ resident-like macrophages	RDH11
3.67181313921993E-31	0.51048025161836	0.504	0.207	6.15322445870475E-27	FOLR2+ resident-like macrophages	SAP18
3.90859283503573E-31	1.70922038808784	0.35	0.115	6.55001987295288E-27	FOLR2+ resident-like macrophages	UGT2B4
4.17985577111381E-31	1.69978728284177	0.171	0.018	7.00460230123252E-27	FOLR2+ resident-like macrophages	MAPK12
4.19394400453526E-31	0.54620058056946	0.396	0.138	7.02821136280018E-27	FOLR2+ resident-like macrophages	PRKACB
4.69957302936679E-31	0.72729362695573	0.395	0.137	7.87554448261287E-27	FOLR2+ resident-like macrophages	C20orf194
4.73829353955303E-31	0.35684573164538	0.448	0.167	7.94043231358297E-27	FOLR2+ resident-like macrophages	NAIP
5.17068260096469E-31	0.50324573249265	0.512	0.216	8.66502990269663E-27	FOLR2+ resident-like macrophages	RAB2A

5.42380617290612E-31	1.25386895544016	0.411	0.157	9.08921438455608E-27	FOLR2+ resident-like macrophages	ALDH3A2
5.7815637397555E-31	0.443190650206237	0.618	0.287	9.68874451508227E-27	FOLR2+ resident-like macrophages	TLN1
5.85606327237382E-31	0.816668517361001	0.379	0.129	9.81359083184404E-27	FOLR2+ resident-like macrophages	TXNDC5
5.91227288154567E-31	0.905975254767365	0.377	0.132	9.90778689489423E-27	FOLR2+ resident-like macrophages	GPAA1
6.30839826136641E-31	1.22631633115419	0.354	0.117	1.05716138063978E-26	FOLR2+ resident-like macrophages	SLC35D2
7.16893979521575E-31	2.02315481659946	0.258	0.062	1.20137093088226E-26	FOLR2+ resident-like macrophages	TMEM9
7.20811152481677E-31	0.436658054498367	0.504	0.213	1.2079353293288E-26	FOLR2+ resident-like macrophages	HEXB
7.34276908743286E-31	1.26803827920519	0.382	0.136	1.230501243672E-26	FOLR2+ resident-like macrophages	POLDIP2
9.76537875273838E-31	1.15573589836148	0.212	0.036	1.6364821713839E-26	FOLR2+ resident-like macrophages	PRSS36
1.024236851641E-30	0.47763642923856	0.296	0.076	1.71641611597999E-26	FOLR2+ resident-like macrophages	LAMB2
1.077325012024E-30	0.511206634345148	0.443	0.17	1.80538125514982E-26	FOLR2+ resident-like macrophages	C16orf58
1.11345640688658E-30	0.923460908423597	0.374	0.132	1.86593024666053E-26	FOLR2+ resident-like macrophages	DCAF6
1.11679309529531E-30	0.358734582814802	0.562	0.244	1.87152186909588E-26	FOLR2+ resident-like macrophages	CREBL2
1.21004540872383E-30	0.425408010692603	0.56	0.245	2.02779409593939E-26	FOLR2+ resident-like macrophages	HNRNPH2
1.32449454549458E-30	0.465488696975058	0.605	0.27	2.21958795933981E-26	FOLR2+ resident-like macrophages	GNB2
1.34318890964835E-30	1.76545869285999	0.273	0.07	2.2509159747887E-26	FOLR2+ resident-like macrophages	PEG10
1.39118836075155E-30	0.674039531238025	0.453	0.179	2.33135345494744E-26	FOLR2+ resident-like macrophages	CHMP2A
1.39687527552202E-30	0.772749592370831	0.456	0.179	2.34088358671981E-26	FOLR2+ resident-like macrophages	HINT1
1.40691962253507E-30	0.95564175242369	0.29	0.077	2.35771590344427E-26	FOLR2+ resident-like macrophages	PLEKHA1
1.4920204324483E-30	1.2344556577193	0.21	0.034	2.50032784069685E-26	FOLR2+ resident-like macrophages	SIGLEC8
1.51367782983623E-30	2.0542181784569	0.251	0.058	2.53662130723955E-26	FOLR2+ resident-like macrophages	RNASE4
1.60621046982538E-30	1.12142863618787	0.219	0.04	2.69168750533338E-26	FOLR2+ resident-like macrophages	HGF
1.62919604086521E-30	0.357322785081964	0.527	0.223	2.73020672528192E-26	FOLR2+ resident-like macrophages	GLB1
1.64894771627182E-30	1.38519022267198	0.308	0.089	2.76330658292832E-26	FOLR2+ resident-like macrophages	ACTR10
1.73844422986937E-30	1.07290140644913	0.389	0.138	2.91328484041509E-26	FOLR2+ resident-like macrophages	PRDX4
1.74242559969822E-30	0.257822671740974	0.382	0.128	2.91995681997428E-26	FOLR2+ resident-like macrophages	RHOU
1.76951084176668E-30	1.22596973656382	0.239	0.05	2.9653462686326E-26	FOLR2+ resident-like macrophages	QPRT
1.99053847579471E-30	0.519246356382058	0.343	0.107	3.33574437773677E-26	FOLR2+ resident-like macrophages	MORF4L2
2.02990164112428E-30	2.07305657251138	0.18	0.023	3.40170917019607E-26	FOLR2+ resident-like macrophages	MAMDC2
2.0330679742888E-30	0.267455374812235	0.537	0.228	3.40701531131317E-26	FOLR2+ resident-like macrophages	TMEM33
2.09252961051003E-30	0.651174868571805	0.231	0.044	3.50666112129271E-26	FOLR2+ resident-like macrophages	BCAM
2.13155728990129E-30	0.794782488097965	0.347	0.11	3.57206370641659E-26	FOLR2+ resident-like macrophages	COMMD5
2.19941331098838E-30	0.832275798494751	0.412	0.155	3.68577682655432E-26	FOLR2+ resident-like macrophages	TM7SF3
2.22143361892897E-30	1.6472728891026	0.293	0.083	3.72267845860117E-26	FOLR2+ resident-like macrophages	QDPR
2.58675950470171E-30	0.382295945099465	0.511	0.219	4.33489157797912E-26	FOLR2+ resident-like macrophages	RAB35
2.63980957125886E-30	0.385758707093528	0.424	0.157	4.4237928795156E-26	FOLR2+ resident-like macrophages	FEZ2
2.80182487513984E-30	0.304138427752639	0.601	0.276	4.69529812575934E-26	FOLR2+ resident-like macrophages	NCSTN
2.80446718066617E-30	1.37582296112709	0.296	0.083	4.69972610136037E-26	FOLR2+ resident-like macrophages	RETSAT
3.07470486517704E-30	0.365136340112182	0.594	0.272	5.15259041306368E-26	FOLR2+ resident-like macrophages	APMAP
3.29736409907653E-30	0.259124066061833	0.525	0.225	5.52572275723246E-26	FOLR2+ resident-like macrophages	PREX1
3.34912008549364E-30	0.291372777034725	0.521	0.223	5.61245543927025E-26	FOLR2+ resident-like macrophages	DNAJC5
3.38909177333654E-30	0.379616960672261	0.376	0.127	5.67943999375738E-26	FOLR2+ resident-like macrophages	NHSL1
3.66174584410907E-30	1.36910706362539	0.374	0.133	6.13635368555797E-26	FOLR2+ resident-like macrophages	ZHX1
3.79513573454281E-30	0.566814803378029	0.438	0.168	6.35988846394684E-26	FOLR2+ resident-like macrophages	HADHB
3.92568413275116E-30	0.322918949074695	0.569	0.244	6.5786614696644E-26	FOLR2+ resident-like macrophages	HADHA
4.11351443068595E-30	1.53650915440106	0.292	0.083	6.89342748294352E-26	FOLR2+ resident-like macrophages	TEX261
4.2283371716459E-30	0.466957763065141	0.325	0.096	7.08584743224419E-26	FOLR2+ resident-like macrophages	B3GNT2
4.48864508925316E-30	0.385607054473981	0.582	0.263	7.52207144057044E-26	FOLR2+ resident-like macrophages	MPV17
4.52438068542187E-30	1.0293221094248	0.364	0.126	7.58195715262997E-26	FOLR2+ resident-like macrophages	KCTD3
4.59725306061905E-30	0.347065684438447	0.415	0.153	7.70407667898541E-26	FOLR2+ resident-like macrophages	GAS2L3
4.77871024495957E-30	0.474147590858459	0.354	0.114	8.00816262850324E-26	FOLR2+ resident-like macrophages	ENTR1
5.18425090266966E-30	0.335939423097297	0.639	0.305	8.68776766269382E-26	FOLR2+ resident-like macrophages	CORO1B
5.65387181908905E-30	0.591396863252353	0.453	0.177	9.47475839442942E-26	FOLR2+ resident-like macrophages	TMEM173
5.89252864570267E-30	0.476665822769789	0.37	0.123	9.87469950446854E-26	FOLR2+ resident-like macrophages	PEAK1
6.00112314772849E-30	0.263822027671239	0.594	0.259	1.00566821709634E-25	FOLR2+ resident-like macrophages	ITGAM
6.13449747237761E-30	0.507054494000329	0.478	0.195	1.02801908642104E-25	FOLR2+ resident-like macrophages	POLR2L
6.14508697585278E-30	0.760711335615767	0.324	0.097	1.02979367541341E-25	FOLR2+ resident-like macrophages	APPL2
6.17946331269263E-30	0.654522715152764	0.405	0.148	1.03555446194103E-25	FOLR2+ resident-like macrophages	UQCR10
7.39064005745462E-30	0.453006411268663	0.462	0.186	1.23852346082824E-25	FOLR2+ resident-like macrophages	SELENON
7.64322638015691E-30	0.27256475537142	0.435	0.166	1.2808518767867E-25	FOLR2+ resident-like macrophages	DIP2B
7.9270472765256E-30	0.975509266414943	0.414	0.159	1.32841458260016E-25	FOLR2+ resident-like macrophages	CUTA
8.00090019349546E-30	0.424442221277805	0.454	0.175	1.34079085442597E-25	FOLR2+ resident-like macrophages	VKORC1
8.24144605177073E-30	1.02829531922043	0.348	0.116	1.38110152935574E-25	FOLR2+ resident-like macrophages	LAGE3
8.90291513697571E-30	0.946598577095721	0.212	0.038	1.49195051865439E-25	FOLR2+ resident-like macrophages	CNRIP1
9.34424241706351E-30	0.624807693567984	0.27	0.065	1.5659081442515E-25	FOLR2+ resident-like macrophages	SASH1
1.17970258367046E-29	0.590439369154465	0.499	0.21	1.97694558971495E-25	FOLR2+ resident-like macrophages	PTPRJ
1.23115247536577E-29	1.49282811295389	0.312	0.099	2.06316531821795E-25	FOLR2+ resident-like macrophages	LACTB2
1.27020708894375E-29	0.293112035529009	0.508	0.214	2.12861303965194E-25	FOLR2+ resident-like macrophages	GNPTAB
1.41755061900469E-29	0.790132315107269	0.469	0.193	2.37553132732805E-25	FOLR2+ resident-like macrophages	DAP
1.42134068988426E-29	0.388567504529684	0.43	0.165	2.38188272810804E-25	FOLR2+ resident-like macrophages	CAPZA2
1.45591014569555E-29	0.251982962873844	0.446	0.175	2.4398142221566E-25	FOLR2+ resident-like macrophages	ZNF706

1.55799418136233E-29	1.26255868407229	0.286	0.078	2.610886649127E-25	FOLR2+ resident-like macrophages	CYP4V2
1.58702007714546E-29	0.53112352704173	0.418	0.159	2.65952824528036E-25	FOLR2+ resident-like macrophages	ORM1
1.59828255927818E-29	0.7783744801175	0.335	0.107	2.67840191283838E-25	FOLR2+ resident-like macrophages	SNX12
1.65095272433922E-29	0.315115311431339	0.395	0.143	2.76666657544767E-25	FOLR2+ resident-like macrophages	DEPRECATED-ENSG00000118263
1.69615492041487E-29	2.00841522021988	0.218	0.044	2.84241641563123E-25	FOLR2+ resident-like macrophages	WFS1
1.78533023942266E-29	0.410986979237037	0.476	0.195	2.9918564152245E-25	FOLR2+ resident-like macrophages	ANXA6
1.79471850993185E-29	0.929005763314019	0.361	0.12	3.0075892789438E-25	FOLR2+ resident-like macrophages	DEPRECATED-ENSG00000163463
1.86109941448488E-29	0.669911094249712	0.478	0.196	3.11883039879376E-25	FOLR2+ resident-like macrophages	DPP9
1.90222970837248E-29	0.594687909677723	0.435	0.171	3.1877565452906E-25	FOLR2+ resident-like macrophages	TOR1AIP2
1.93152622458953E-29	2.5119099674322	0.155	0.014	3.23685164716714E-25	FOLR2+ resident-like macrophages	EPB41L1
1.95031063455818E-29	0.31613248527997	0.447	0.172	3.2683305613926E-25	FOLR2+ resident-like macrophages	P2RX7
1.97428179040299E-29	0.284001291035208	0.601	0.273	3.30850142435733E-25	FOLR2+ resident-like macrophages	RAB14
2.15209772254435E-29	0.393810115788727	0.525	0.223	3.60648536343981E-25	FOLR2+ resident-like macrophages	TMED9
2.2968675109651E-29	0.381692876913378	0.517	0.222	3.84909057487531E-25	FOLR2+ resident-like macrophages	CANX
2.55800499685979E-29	1.0225101892261	0.247	0.057	4.28670477373764E-25	FOLR2+ resident-like macrophages	NPDC1
2.90807550730447E-29	0.443151620203328	0.507	0.22	4.87335293514083E-25	FOLR2+ resident-like macrophages	CD46
3.02235812252855E-29	0.294250495409587	0.579	0.264	5.06486774173335E-25	FOLR2+ resident-like macrophages	CORO7
3.14714745811451E-29	0.490912675841169	0.43	0.172	5.2739897103083E-25	FOLR2+ resident-like macrophages	TRAPPC5
3.19421620637473E-29	1.33635588544188	0.337	0.111	5.35286751864277E-25	FOLR2+ resident-like macrophages	CLDN7
3.40976926580049E-29	0.833952201062504	0.377	0.132	5.71409133562845E-25	FOLR2+ resident-like macrophages	KIAA2013
3.53600117560378E-29	0.791073531719754	0.421	0.165	5.92563077007681E-25	FOLR2+ resident-like macrophages	ANXA7
4.32034517048253E-29	0.530091501372864	0.401	0.149	7.24003443669462E-25	FOLR2+ resident-like macrophages	LAPTM4A
4.38816174664951E-29	1.6112487069604	0.34	0.118	7.35368145503524E-25	FOLR2+ resident-like macrophages	DEPRECATED-ENSG00000106538
4.53593914012661E-29	0.348334878352321	0.617	0.277	7.60132681102417E-25	FOLR2+ resident-like macrophages	PRKCD
4.8534566125752E-29	0.324729672238905	0.61	0.282	8.13342259135352E-25	FOLR2+ resident-like macrophages	KCNAB2
5.17821053078218E-29	0.784392730167897	0.212	0.038	8.67764520748478E-25	FOLR2+ resident-like macrophages	HSF4
5.52444226731203E-29	0.817875472662096	0.382	0.137	9.25786035156149E-25	FOLR2+ resident-like macrophages	PIP5K1C
5.92950049856495E-29	0.525993266660065	0.493	0.209	9.93665693549515E-25	FOLR2+ resident-like macrophages	ARHGDIA
5.95960354934684E-29	1.67544720770718	0.255	0.065	9.98710362799544E-25	FOLR2+ resident-like macrophages	RPP25
6.06005697759759E-29	0.378515843156423	0.553	0.246	1.0155443483058E-24	FOLR2+ resident-like macrophages	INF2
7.13281070400988E-29	0.555951601869736	0.382	0.137	1.19531641777798E-24	FOLR2+ resident-like macrophages	PAPSS2
7.31924553262089E-29	0.356743975789366	0.446	0.173	1.22655916635661E-24	FOLR2+ resident-like macrophages	DUSP3
7.46174718109127E-29	0.352107001503096	0.653	0.317	1.25043959260727E-24	FOLR2+ resident-like macrophages	RUNX1
7.85892278131153E-29	0.467215322394627	0.412	0.157	1.31699827969219E-24	FOLR2+ resident-like macrophages	CDIPT
8.20999853317896E-29	1.66456994686536	0.253	0.064	1.37583155419013E-24	FOLR2+ resident-like macrophages	CLTC
8.76662042008277E-29	0.974234847869784	0.351	0.121	1.46911024999747E-24	FOLR2+ resident-like macrophages	SLC25A24
8.92799994079246E-29	0.623321998886606	0.386	0.14	1.496154230078E-24	FOLR2+ resident-like macrophages	MILR1
9.29075139866023E-29	0.738505534152881	0.385	0.139	1.55694411938748E-24	FOLR2+ resident-like macrophages	NAA20
9.46100636430192E-29	1.85939600355956	0.263	0.07	1.58547544652972E-24	FOLR2+ resident-like macrophages	MUC13
9.63213039619371E-29	0.271291458729795	0.406	0.149	1.61415241179414E-24	FOLR2+ resident-like macrophages	MAPK6
9.82100895458084E-29	0.468514360010972	0.393	0.144	1.64580468060866E-24	FOLR2+ resident-like macrophages	GTF2F1
1.04779602678493E-28	1.95394030581014	0.163	0.018	1.75589658168618E-24	FOLR2+ resident-like macrophages	CTSF
1.05724649750896E-28	0.306647220672754	0.566	0.256	1.77173368052552E-24	FOLR2+ resident-like macrophages	FAM50A
1.07214928821624E-28	0.884244984670436	0.403	0.156	1.79670777719278E-24	FOLR2+ resident-like macrophages	SURF4
1.08775987410196E-28	0.278218427416115	0.466	0.188	1.82286799702007E-24	FOLR2+ resident-like macrophages	RAC1
1.186313270909E-28	0.562608355198429	0.369	0.131	1.98802377938929E-24	FOLR2+ resident-like macrophages	MCRIP1
1.19935562257105E-28	0.74810824943062	0.258	0.064	2.00988015230457E-24	FOLR2+ resident-like macrophages	MEGF6
1.23301127792366E-28	0.601598426838705	0.396	0.146	2.06628029954447E-24	FOLR2+ resident-like macrophages	ZBED1
1.31460561444952E-28	1.25129084615441	0.379	0.141	2.2030160886945E-24	FOLR2+ resident-like macrophages	TSTA3
1.37920817667484E-28	1.12077086107747	0.388	0.145	2.31127706247169E-24	FOLR2+ resident-like macrophages	UQCRQ
1.42731422766038E-28	1.32543585242221	0.334	0.113	2.39189318271327E-24	FOLR2+ resident-like macrophages	HMGCL
1.44281423253891E-28	0.71264613735044	0.508	0.223	2.4178680908887E-24	FOLR2+ resident-like macrophages	SEC61A1
1.58827449139075E-28	0.87130630642633	0.21	0.04	2.66163039267262E-24	FOLR2+ resident-like macrophages	OSBPL1A
1.60412448436868E-28	0.762654973443811	0.393	0.148	2.68819181090504E-24	FOLR2+ resident-like macrophages	PLOD1
1.65230255968179E-28	0.782563422233955	0.374	0.135	2.76892862951474E-24	FOLR2+ resident-like macrophages	SHARPIN
1.65895494369258E-28	1.46456260037326	0.277	0.078	2.78007669464002E-24	FOLR2+ resident-like macrophages	MGMT
1.68754629295343E-28	0.509611681949002	0.341	0.113	2.82799007773136E-24	FOLR2+ resident-like macrophages	CYSTM1
1.85361252063196E-28	1.30761039325242	0.327	0.108	3.10628386207503E-24	FOLR2+ resident-like macrophages	TP53INP1
1.93421235408654E-28	0.512557765836648	0.488	0.21	3.24135306297823E-24	FOLR2+ resident-like macrophages	PUF60
1.99301734625951E-28	0.329605594710372	0.424	0.163	3.33989846886169E-24	FOLR2+ resident-like macrophages	PPFIBP2
2.19069373179248E-28	0.491898002577193	0.434	0.172	3.67116455573783E-24	FOLR2+ resident-like macrophages	FIS1
2.34616451317412E-28	0.368833334768029	0.485	0.203	3.9317024911772E-24	FOLR2+ resident-like macrophages	RGS10
3.0187111994526E-28	0.449058662706967	0.403	0.152	5.05875622804267E-24	FOLR2+ resident-like macrophages	TCF12
3.20652994803417E-28	0.533579428176688	0.93	0.625	5.37350288691567E-24	FOLR2+ resident-like macrophages	MT-ATP6
3.22403864222056E-28	0.537150981214169	0.358	0.124	5.40284395663322E-24	FOLR2+ resident-like macrophages	KEAP1
3.37234150217578E-28	0.501458654003666	0.382	0.135	5.65136988934617E-24	FOLR2+ resident-like macrophages	CD302
3.69288992152544E-28	1.97738372485904	0.225	0.048	6.18854493049233E-24	FOLR2+ resident-like macrophages	EPDR1
3.73765681870582E-28	0.879114243049951	0.308	0.095	6.26356529678721E-24	FOLR2+ resident-like macrophages	TMEM115
3.80881474828747E-28	0.346935201377369	0.644	0.31	6.38281175518015E-24	FOLR2+ resident-like macrophages	CHKA
3.87787542450406E-28	0.76007430353378	0.369	0.134	6.4985436363839E-24	FOLR2+ resident-like macrophages	TMEM35B
4.16029116486252E-28	0.329106568807151	0.591	0.267	6.97181593407661E-24	FOLR2+ resident-like macrophages	SEPTIN9

4.19749288880781E-28	1.01936727380676	0.309	0.093	7.03415858306413E-24	FOLR2+ resident-like macrophages	NLN
4.35181143203206E-28	0.436738407291591	0.527	0.236	7.29276559779932E-24	FOLR2+ resident-like macrophages	SSR2
4.3759765543356E-28	1.09446634239924	0.26	0.067	7.33326150975559E-24	FOLR2+ resident-like macrophages	HS2ST1
4.51579937818393E-28	0.405284909282843	0.498	0.213	7.56757659796063E-24	FOLR2+ resident-like macrophages	ADD1
5.08240177062047E-28	0.26196441124985	0.656	0.323	8.51708888720578E-24	FOLR2+ resident-like macrophages	LRPAP1
5.37469463114301E-28	0.766643988391244	0.405	0.152	9.00691326286945E-24	FOLR2+ resident-like macrophages	COX8A
5.5746982065144E-28	1.22203914626406	0.2	0.036	9.34207925447683E-24	FOLR2+ resident-like macrophages	EYA2
6.0959470553388E-28	0.250008650165517	0.361	0.124	1.02155880753368E-23	FOLR2+ resident-like macrophages	PITHD1
6.27159884645387E-28	0.489663053947193	0.332	0.108	1.05099453468874E-23	FOLR2+ resident-like macrophages	SLC29A3
6.50186485893046E-28	1.30753094225213	0.19	0.031	1.08958251305957E-23	FOLR2+ resident-like macrophages	SMIM3
6.83323546032195E-28	1.1466140309289	0.348	0.12	1.14511359844075E-23	FOLR2+ resident-like macrophages	VBP1
7.05056616632208E-28	1.72804468502036	0.254	0.066	1.18153387815225E-23	FOLR2+ resident-like macrophages	CMBL
7.16630946963258E-28	0.79460720935204	0.344	0.119	1.20093014092103E-23	FOLR2+ resident-like macrophages	C6orf120
7.96467939138299E-28	0.355763275014718	0.544	0.242	1.33472097240796E-23	FOLR2+ resident-like macrophages	KIF5B
8.00066456826535E-28	1.9459087284387	0.199	0.036	1.34075136834991E-23	FOLR2+ resident-like macrophages	RARRES1
9.0129932612558E-28	0.501181515759764	0.427	0.168	1.51039741072125E-23	FOLR2+ resident-like macrophages	ANKFY1
9.02217397718377E-28	0.402976066088389	0.491	0.212	1.51193591509646E-23	FOLR2+ resident-like macrophages	SEC31A
9.79574394589673E-28	0.407529436960265	0.337	0.109	1.64157077045337E-23	FOLR2+ resident-like macrophages	LZTS2
9.88120160978535E-28	0.616618985594177	0.435	0.178	1.65589176576783E-23	FOLR2+ resident-like macrophages	RNF187
1.06481798628104E-27	1.30451831142152	0.261	0.07	1.78442198140977E-23	FOLR2+ resident-like macrophages	CPT2
1.37463470241983E-27	0.669715311914557	0.332	0.111	2.30361283431515E-23	FOLR2+ resident-like macrophages	FKBP8
1.38205360108689E-27	1.7921152149963	0.258	0.069	2.3160454247014E-23	FOLR2+ resident-like macrophages	FAH
1.48871178586708E-27	0.713854667321271	0.441	0.183	2.49478321075605E-23	FOLR2+ resident-like macrophages	ADA
1.4966658158804E-27	0.274650109505935	0.749	0.381	2.50811257425238E-23	FOLR2+ resident-like macrophages	DDIT4
1.73745011182725E-27	0.837276861410991	0.247	0.06	2.9116188974001E-23	FOLR2+ resident-like macrophages	SLC27A1
1.89469499616367E-27	0.345884545426844	0.595	0.279	3.17512987457108E-23	FOLR2+ resident-like macrophages	ARL6IP5
2.01469504842475E-27	1.22991180793906	0.263	0.069	3.3762259621502E-23	FOLR2+ resident-like macrophages	AIG1
2.02050269060199E-27	3.50157326465279	0.125	0.006	3.38595840891082E-23	FOLR2+ resident-like macrophages	GPRC5B
2.02196629502049E-27	0.460145701911212	0.424	0.173	3.38841111719533E-23	FOLR2+ resident-like macrophages	DDB1
2.05048808599137E-27	0.974855419619948	0.28	0.079	3.43620793450433E-23	FOLR2+ resident-like macrophages	PER3
2.07310265990263E-27	0.650820731447843	0.39	0.148	3.47410543746483E-23	FOLR2+ resident-like macrophages	EEF2K
2.081688031053E-27	0.562339268958063	0.428	0.172	3.48849280243861E-23	FOLR2+ resident-like macrophages	P2RY13
2.12199928397511E-27	0.25288944206787	0.358	0.121	3.55604640008549E-23	FOLR2+ resident-like macrophages	PGPEP1
2.25210056898507E-27	0.881530445629038	0.421	0.169	3.77407013350517E-23	FOLR2+ resident-like macrophages	SPTSSA
2.40706586417353E-27	0.457858431264085	0.377	0.135	4.03376097518201E-23	FOLR2+ resident-like macrophages	ABHD16A
2.44759004393605E-27	1.23458015522719	0.193	0.034	4.10167139562804E-23	FOLR2+ resident-like macrophages	TSHZ3
2.45089827285913E-27	0.281660287951835	0.354	0.121	4.10721532565732E-23	FOLR2+ resident-like macrophages	SIRT2
2.47918046832854E-27	1.20619767967744	0.341	0.12	4.15461062882497E-23	FOLR2+ resident-like macrophages	STARD10
2.50794053773492E-27	0.423938073020244	0.486	0.207	4.20280675313618E-23	FOLR2+ resident-like macrophages	DYNC1I2
2.5523353870885E-27	0.56203982453638	0.395	0.151	4.2772036416829E-23	FOLR2+ resident-like macrophages	SLC35F5
2.56565645685484E-27	2.28704584591826	0.177	0.028	4.29952709039734E-23	FOLR2+ resident-like macrophages	PIR
2.84550811216501E-27	0.290449258925981	0.324	0.106	4.76850249436613E-23	FOLR2+ resident-like macrophages	ZMYND11
3.04309177789388E-27	0.99729203731244	0.341	0.119	5.09961320139456E-23	FOLR2+ resident-like macrophages	RABL6
3.18064289329531E-27	0.632214685230576	0.401	0.157	5.33012136058428E-23	FOLR2+ resident-like macrophages	RPN1
3.20791268186098E-27	1.61351899600641	0.257	0.068	5.37582007226263E-23	FOLR2+ resident-like macrophages	CISD3
3.27547239524986E-27	0.403559761929753	0.408	0.155	5.48903663995971E-23	FOLR2+ resident-like macrophages	IFFO1
3.28725158657677E-27	0.743992869848695	0.318	0.102	5.50877620878534E-23	FOLR2+ resident-like macrophages	ZFYVE21
3.34786024380039E-27	1.02786186079065	0.241	0.058	5.61034419656069E-23	FOLR2+ resident-like macrophages	PFKFB2
3.38017707628544E-27	0.651161069225633	0.341	0.116	5.66450074443914E-23	FOLR2+ resident-like macrophages	OSTM1
3.42124903683189E-27	0.359920949342386	0.62	0.292	5.73332913592288E-23	FOLR2+ resident-like macrophages	TSPAN3
3.51341781315293E-27	0.296407201774501	0.376	0.139	5.88778557128168E-23	FOLR2+ resident-like macrophages	SPPL3
3.68678564000724E-27	0.790677805648835	0.332	0.112	6.17831537552414E-23	FOLR2+ resident-like macrophages	SFXN3
3.80353993827741E-27	0.531380508882857	0.399	0.154	6.37397222856529E-23	FOLR2+ resident-like macrophages	PSMC2
3.82517499518044E-27	0.401207299454265	0.483	0.209	6.41022825692337E-23	FOLR2+ resident-like macrophages	PYCARD
4.22153692464095E-27	0.87459580266612	0.299	0.094	7.0744515783133E-23	FOLR2+ resident-like macrophages	EMB
4.2590930148518E-27	1.00732388107464	0.277	0.08	7.13738807428864E-23	FOLR2+ resident-like macrophages	IL12RB1
4.28747938060287E-27	0.483926672985171	0.412	0.158	7.18495794601428E-23	FOLR2+ resident-like macrophages	UBL3
4.31807720345988E-27	0.87640498869476	0.286	0.084	7.23623377755807E-23	FOLR2+ resident-like macrophages	CTNS
4.35083413555346E-27	2.33196687939391	0.179	0.028	7.29112784436049E-23	FOLR2+ resident-like macrophages	TFF1
4.44423643709128E-27	0.440962208828346	0.504	0.22	7.44765142127757E-23	FOLR2+ resident-like macrophages	PTPA
4.57548921380928E-27	0.520958906861181	0.424	0.17	7.6676048245016E-23	FOLR2+ resident-like macrophages	KIF16B
6.36051576820388E-27	0.36171128208415	0.451	0.187	1.06589523243561E-22	FOLR2+ resident-like macrophages	LTBR
6.88373211497566E-27	1.97910654671106	0.244	0.064	1.15357582782762E-22	FOLR2+ resident-like macrophages	CRYZ
6.95745723538081E-27	0.868979787159928	0.43	0.178	1.16593068350512E-22	FOLR2+ resident-like macrophages	MLF 2.00
6.99314578569016E-27	0.418627489016948	0.543	0.242	1.17191137076596E-22	FOLR2+ resident-like macrophages	RAB31
7.24431206763148E-27	1.23742185917182	0.363	0.138	1.21400181629368E-22	FOLR2+ resident-like macrophages	ZKSCAN1
7.41417689972369E-27	1.15481196191047	0.331	0.114	1.2424677648557E-22	FOLR2+ resident-like macrophages	GRHPR
7.81048554929983E-27	0.688630781890948	0.376	0.14	1.30888116835166E-22	FOLR2+ resident-like macrophages	COPS7A
7.93161065156747E-27	1.57813535174444	0.237	0.059	1.32917931298968E-22	FOLR2+ resident-like macrophages	PHYH
8.23574582096053E-27	1.70752208281006	0.269	0.078	1.38014628467656E-22	FOLR2+ resident-like macrophages	CCNG1
8.56000322078981E-27	0.69903019326284	0.408	0.161	1.43448533973996E-22	FOLR2+ resident-like macrophages	CAMK1

9.5526628104967E-27	0.519799946051934	0.183	0.029	1.60083523378304E-22	FOLR2+ resident-like macrophages	MMP2
9.60891982559033E-27	0.640497641091611	0.332	0.112	1.61026278437243E-22	FOLR2+ resident-like macrophages	CUL4B
9.90902293086463E-27	0.555376008421797	0.366	0.139	1.6605540627543E-22	FOLR2+ resident-like macrophages	ALKBH5
1.00594205066355E-26	0.909537908829715	0.376	0.141	1.68575768850197E-22	FOLR2+ resident-like macrophages	HTATIP2
1.01691590466579E-26	0.470976818239552	0.851	0.475	1.70414767303893E-22	FOLR2+ resident-like macrophages	TSC22D3
1.0306055811053E-26	2.44279194349783	0.181	0.031	1.72708883281625E-22	FOLR2+ resident-like macrophages	SPR
1.03741143123368E-26	0.396407396581803	0.311	0.097	1.73849407646139E-22	FOLR2+ resident-like macrophages	TANC2
1.07949317175819E-26	0.399306741527418	0.412	0.163	1.80901465723237E-22	FOLR2+ resident-like macrophages	RSU1
1.08093725356702E-26	0.452093678176976	0.46	0.193	1.81143464952761E-22	FOLR2+ resident-like macrophages	HUWE1
1.12086649900988E-26	1.13497423309514	0.377	0.144	1.87834807904076E-22	FOLR2+ resident-like macrophages	LPGAT1
1.14671227286426E-26	0.781634304496897	0.37	0.142	1.92166042686593E-22	FOLR2+ resident-like macrophages	GALNT1
1.14676125495393E-26	0.662470400489374	0.386	0.15	1.9217425110518E-22	FOLR2+ resident-like macrophages	NDUFAF3
1.17919494868793E-26	0.392609384337244	0.402	0.156	1.97609489501124E-22	FOLR2+ resident-like macrophages	ITFG1
1.23437477315984E-26	0.353161581588114	0.357	0.127	2.06856524486125E-22	FOLR2+ resident-like macrophages	GDE1
1.25258784341052E-26	0.737804038429956	0.403	0.159	2.09908670798735E-22	FOLR2+ resident-like macrophages	MAP2K2
1.28469263887713E-26	0.91050572835714	0.247	0.063	2.15288792423029E-22	FOLR2+ resident-like macrophages	B3GNT7
1.38097687399803E-26	0.327759613813108	0.698	0.349	2.3142410454459E-22	FOLR2+ resident-like macrophages	ARPC1B
1.38637061109663E-26	0.646402812061122	0.245	0.063	2.32327987007574E-22	FOLR2+ resident-like macrophages	SNX18
1.48889800897026E-26	2.11285530427671	0.138	0.011	2.49509528343237E-22	FOLR2+ resident-like macrophages	MPPED2
1.52837176663196E-26	0.956989903199516	0.358	0.133	2.56124540652183E-22	FOLR2+ resident-like macrophages	IFI35
1.56175643005261E-26	0.60860723969704	0.459	0.194	2.61719142548216E-22	FOLR2+ resident-like macrophages	MLX
2.09194960813878E-26	0.509120616547972	0.374	0.139	3.50568915331896E-22	FOLR2+ resident-like macrophages	IFITM2
2.11257085740379E-26	0.836290100640514	0.386	0.149	3.54024624283727E-22	FOLR2+ resident-like macrophages	SNRPC
2.49113446880369E-26	0.542720986871152	0.344	0.121	4.17464314282123E-22	FOLR2+ resident-like macrophages	EMC7
2.54207339213395E-26	0.322703803533679	0.269	0.074	4.26000659053807E-22	FOLR2+ resident-like macrophages	SLC17A5
2.7410219737347E-26	1.21968935178279	0.34	0.122	4.59340462358462E-22	FOLR2+ resident-like macrophages	SRP9
2.79617652262255E-26	0.864917081444038	0.341	0.12	4.68583261661088E-22	FOLR2+ resident-like macrophages	NUDT5
2.88862724598081E-26	0.345462927969007	0.261	0.068	4.84076153881463E-22	FOLR2+ resident-like macrophages	ZKSCAN4
2.88934054726565E-26	1.2701972770243	0.382	0.153	4.84195688910777E-22	FOLR2+ resident-like macrophages	SERPINA7
2.933976089119E-26	0.989465107181754	0.302	0.099	4.91675713014562E-22	FOLR2+ resident-like macrophages	KCNQ1
3.02759047201309E-26	0.286115773921553	0.537	0.248	5.07363611299954E-22	FOLR2+ resident-like macrophages	IQGAP1
3.0948216312421E-26	0.520930112629302	0.453	0.19	5.1863020896355E-22	FOLR2+ resident-like macrophages	AP2S1
3.65757028939118E-26	1.37994872630443	0.343	0.127	6.12935629096174E-22	FOLR2+ resident-like macrophages	GSTA2
3.83592212710183E-26	0.448719049479324	0.351	0.127	6.42823830059725E-22	FOLR2+ resident-like macrophages	DOCK7
3.85177345494715E-26	1.8417043806998	0.222	0.052	6.45480195580043E-22	FOLR2+ resident-like macrophages	DEPRECATED-ENSG00000245848
4.01801658921019E-26	0.912254025693713	0.248	0.065	6.73339220019844E-22	FOLR2+ resident-like macrophages	TPRG1L
4.10848206657458E-26	0.323141002651499	0.54	0.245	6.88499424716568E-22	FOLR2+ resident-like macrophages	UBE2L6
4.24683013969181E-26	1.42231064706682	0.313	0.106	7.11683794809554E-22	FOLR2+ resident-like macrophages	PLS3
4.32814531132338E-26	0.29768388956565	0.374	0.139	7.25310591271572E-22	FOLR2+ resident-like macrophages	N4BP1
4.38377929882731E-26	0.280493946905955	0.563	0.261	7.34633734897481E-22	FOLR2+ resident-like macrophages	N4BP2L2
4.57603303209995E-26	0.322219282263151	0.507	0.231	7.6685161551931E-22	FOLR2+ resident-like macrophages	NBR1
4.77433158396384E-26	0.590527301475875	0.347	0.126	8.0008248684066E-22	FOLR2+ resident-like macrophages	CNP
5.2810913785681E-26	0.50331039157455	0.344	0.122	8.85005293220441E-22	FOLR2+ resident-like macrophages	NSL1
5.4780301614196E-26	0.257947646326291	0.476	0.211	9.18008294450697E-22	FOLR2+ resident-like macrophages	IDH3G
5.75506410157816E-26	0.539130615610056	0.43	0.179	9.64433642142468E-22	FOLR2+ resident-like macrophages	ORMDL1
5.92958187610707E-26	0.522790048756292	0.361	0.136	9.93679330798023E-22	FOLR2+ resident-like macrophages	TMBIM4
6.09412313945298E-26	0.907588183090496	0.364	0.141	1.02125315570953E-21	FOLR2+ resident-like macrophages	LMAN1
7.38405223862306E-26	0.620898121787708	0.402	0.159	1.23741947414845E-21	FOLR2+ resident-like macrophages	KRTCAP2
7.48233848500123E-26	0.347008547983104	0.514	0.226	1.25389028331651E-21	FOLR2+ resident-like macrophages	ITPK1
8.23303372298179E-26	0.281504195151815	0.447	0.189	1.37969179129729E-21	FOLR2+ resident-like macrophages	DCTN1
8.42583084470244E-26	0.771596915909801	0.306	0.102	1.41200073295524E-21	FOLR2+ resident-like macrophages	C5orf24
8.4847054363365E-26	0.677637952444313	0.319	0.108	1.42186693702127E-21	FOLR2+ resident-like macrophages	DPEP2
9.56902025195113E-26	0.382185152342304	0.598	0.275	1.60357641382197E-21	FOLR2+ resident-like macrophages	ENO1
9.77628652622849E-26	1.0323754961306	0.341	0.122	1.63831009606537E-21	FOLR2+ resident-like macrophages	HADH
9.81806273444935E-26	1.61869756836745	0.251	0.07	1.64531095303902E-21	FOLR2+ resident-like macrophages	GAMT
1.0331555860087E-25	0.596417501598373	0.289	0.089	1.73136213103338E-21	FOLR2+ resident-like macrophages	TMEM59
1.03534413161488E-25	0.260207611181604	0.463	0.201	1.73502969576021E-21	FOLR2+ resident-like macrophages	UBE2Q1
1.04234424830645E-25	0.269366645658155	0.527	0.244	1.74676049131195E-21	FOLR2+ resident-like macrophages	DEGS1
1.04965708098622E-25	0.792351775360914	0.345	0.128	1.75901533631671E-21	FOLR2+ resident-like macrophages	RBM15B
1.13334467838598E-25	0.736864472179099	0.308	0.102	1.89925901203922E-21	FOLR2+ resident-like macrophages	BCAP29
1.17372312507596E-25	0.409325441069387	0.377	0.146	1.9669252130023E-21	FOLR2+ resident-like macrophages	LRRC41
1.20337764260059E-25	0.776854651781726	0.37	0.14	2.01662025347006E-21	FOLR2+ resident-like macrophages	PPP1R7
1.2290764983238E-25	0.347063054391182	0.788	0.411	2.05968639589102E-21	FOLR2+ resident-like macrophages	PICALM
1.34313891430903E-25	1.46972375910143	0.27	0.082	2.25083219259908E-21	FOLR2+ resident-like macrophages	ORM2
1.36016348814103E-25	0.428320941459278	0.357	0.129	2.27936197342674E-21	FOLR2+ resident-like macrophages	TADA3
1.49314545033043E-25	0.417832979070087	0.472	0.208	2.50221314566374E-21	FOLR2+ resident-like macrophages	SRGAP2B
1.50724228062451E-25	0.834995322929026	0.279	0.084	2.52583661387056E-21	FOLR2+ resident-like macrophages	P2RY11
1.69665073480313E-25	1.0274322489252	0.322	0.113	2.84324730138308E-21	FOLR2+ resident-like macrophages	SLC25A1
1.70897383717631E-25	0.667023527184302	0.412	0.171	2.86389835634006E-21	FOLR2+ resident-like macrophages	TRPV2
1.76435406825703E-25	0.564785238527804	0.303	0.097	2.95670454758513E-21	FOLR2+ resident-like macrophages	ILVBL
1.83377584113774E-25	0.499933148229013	0.325	0.112	3.07304155457862E-21	FOLR2+ resident-like macrophages	RAPH1

1.87412243259361E-25	0.744753260399993	0.315	0.105	3.14065437254037E-21	FOLR2+ resident-like macrophages	TSPAN15
1.94841544146308E-25	0.689595910207595	0.363	0.139	3.26515459680383E-21	FOLR2+ resident-like macrophages	APEH
1.97144210062263E-25	1.18923220106274	0.35	0.135	3.30374267222341E-21	FOLR2+ resident-like macrophages	VNN 1.00
2.07472155292385E-25	0.530335684260055	0.459	0.203	3.47681837838979E-21	FOLR2+ resident-like macrophages	COPB2
2.194825247519E-25	0.503739305834982	0.245	0.064	3.67808814979234E-21	FOLR2+ resident-like macrophages	LILRB4
2.2750681808539E-25	0.653995179373135	0.399	0.164	3.81255925747496E-21	FOLR2+ resident-like macrophages	HAGH
2.66709315372825E-25	1.35899692249609	0.382	0.157	4.4695147070178E-21	FOLR2+ resident-like macrophages	PIGR
2.67184617708788E-25	0.615279448015148	0.334	0.119	4.47747982356387E-21	FOLR2+ resident-like macrophages	EXOC7
2.67315612069485E-25	0.486338894164177	0.388	0.152	4.47967502706043E-21	FOLR2+ resident-like macrophages	UBAC1
2.69626195028237E-25	1.06984766357364	0.282	0.089	4.5183957762832E-21	FOLR2+ resident-like macrophages	SMYD2
2.74766347027496E-25	0.61415919511786	0.247	0.065	4.60453444348677E-21	FOLR2+ resident-like macrophages	MAP1LC3B
2.86640815588216E-25	0.372705920684614	0.676	0.345	4.80352678762732E-21	FOLR2+ resident-like macrophages	CMTM3
2.92884159645465E-25	0.557936814175502	0.331	0.117	4.90815274733871E-21	FOLR2+ resident-like macrophages	FAM13A
2.95483466674564E-25	0.825726299917288	0.293	0.095	4.95171193453235E-21	FOLR2+ resident-like macrophages	TECR
3.14722115704979E-25	0.922530410878998	0.322	0.114	5.27411321498404E-21	FOLR2+ resident-like macrophages	C1orf43
3.24581567637046E-25	0.332035329484455	0.462	0.203	5.43933791046161E-21	FOLR2+ resident-like macrophages	DNAJC3
3.3732918992862E-25	0.535326502534897	0.393	0.157	5.65296256482382E-21	FOLR2+ resident-like macrophages	PSMD1
3.46018102084253E-25	1.76807264720697	0.164	0.026	5.79857135472792E-21	FOLR2+ resident-like macrophages	ZNF552
3.57816904583455E-25	3.5427352638507	0.135	0.013	5.99629568700953E-21	FOLR2+ resident-like macrophages	ACSM5
3.63171219610794E-25	0.274635515421532	0.552	0.258	6.08602329823769E-21	FOLR2+ resident-like macrophages	ARL6IP4
3.84368300955085E-25	1.18187682677698	0.303	0.102	6.44124398740531E-21	FOLR2+ resident-like macrophages	PAQR8
3.99161858052105E-25	1.46646821191446	0.303	0.104	6.68915441723717E-21	FOLR2+ resident-like macrophages	HACD3
4.08499930493637E-25	0.441479780467843	0.324	0.112	6.84564183521237E-21	FOLR2+ resident-like macrophages	CREB3L2
4.1280943165068E-25	0.532086921361473	0.443	0.185	6.91786045560209E-21	FOLR2+ resident-like macrophages	EIF6
4.30228012309495E-25	1.46930210035016	0.199	0.043	7.20976103028252E-21	FOLR2+ resident-like macrophages	MVK
4.37759379306598E-25	0.766076868260535	0.338	0.125	7.33597167841996E-21	FOLR2+ resident-like macrophages	NHLRC3
4.44951336508902E-25	0.935427332911823	0.38	0.149	7.45649449721618E-21	FOLR2+ resident-like macrophages	COX6A1
4.48671831715273E-25	0.296305862419877	0.572	0.265	7.51884255588454E-21	FOLR2+ resident-like macrophages	SERPINB1
4.67169829250473E-25	0.257607753857038	0.369	0.136	7.82883199857943E-21	FOLR2+ resident-like macrophages	STON2
4.74776483400379E-25	0.995115338338617	0.212	0.048	7.95630430882355E-21	FOLR2+ resident-like macrophages	TFCP2L1
4.80660371325223E-25	0.585995821605291	0.405	0.166	8.05490650266809E-21	FOLR2+ resident-like macrophages	BSDC1
5.10961620679224E-25	0.318379125627875	0.842	0.488	8.56269483934244E-21	FOLR2+ resident-like macrophages	GRINA
5.40447579687348E-25	0.586079599659966	0.438	0.187	9.05682054040058E-21	FOLR2+ resident-like macrophages	DTX3L
5.71729592339024E-25	0.833706056642239	0.34	0.125	9.58104450841736E-21	FOLR2+ resident-like macrophages	PDHB
5.79928082120328E-25	0.282060232598344	0.343	0.123	9.71843480017246E-21	FOLR2+ resident-like macrophages	MTMR6
5.9997025738779E-25	1.22859098913623	0.299	0.099	1.00543015733046E-20	FOLR2+ resident-like macrophages	GIPC1
6.01093531679375E-25	1.00614897498348	0.327	0.119	1.0073125403883E-20	FOLR2+ resident-like macrophages	SAR1B
6.13897372390386E-25	0.646323386101381	0.311	0.106	1.02876921665181E-20	FOLR2+ resident-like macrophages	TMEM106B
6.49780165504198E-25	0.497730318457822	0.324	0.111	1.08890160135193E-20	FOLR2+ resident-like macrophages	SLC30A7
6.5597585080354E-25	0.430291573448285	0.459	0.2	1.09928433077657E-20	FOLR2+ resident-like macrophages	EMC4
6.84788490182221E-25	0.280364763962711	0.704	0.354	1.14756855184737E-20	FOLR2+ resident-like macrophages	PPT1
7.34014684155939E-25	0.807804271898056	0.29	0.094	1.23006180770852E-20	FOLR2+ resident-like macrophages	TDRKH
7.4819435606688E-25	0.732682125141703	0.348	0.131	1.25382410189688E-20	FOLR2+ resident-like macrophages	FUCA2
7.55422413481846E-25	0.795872482677236	0.228	0.056	1.26593688051288E-20	FOLR2+ resident-like macrophages	ICA1
7.96164657770433E-25	0.415210167485789	0.514	0.238	1.33421273349169E-20	FOLR2+ resident-like macrophages	SNX17
8.05368734345084E-25	0.34685270734228	0.837	0.49	1.34963692501549E-20	FOLR2+ resident-like macrophages	NUCB1
9.11828876680517E-25	0.387542579236443	0.348	0.13	1.52804283154121E-20	FOLR2+ resident-like macrophages	RETREG3
1.0199887273067E-24	0.255121455002447	0.473	0.208	1.70929710922057E-20	FOLR2+ resident-like macrophages	RAP2A
1.0618972587535E-24	0.478342942874293	0.448	0.193	1.77952742621911E-20	FOLR2+ resident-like macrophages	ARPC5
1.06385193803337E-24	0.602803588011872	0.39	0.159	1.78280307775632E-20	FOLR2+ resident-like macrophages	APPL1
1.14918547865942E-24	0.253806736554121	0.348	0.126	1.92580502513745E-20	FOLR2+ resident-like macrophages	SIGLEC9
1.16008389666254E-24	0.288016464434316	0.383	0.154	1.94406859402709E-20	FOLR2+ resident-like macrophages	AGFG1
1.16825606700417E-24	0.476265901664711	0.495	0.223	1.95776351708559E-20	FOLR2+ resident-like macrophages	DEPRECATED-ENSG00000163191
1.22353027745386E-24	0.296313741224164	0.299	0.097	2.05039203895717E-20	FOLR2+ resident-like macrophages	MITF
1.24297687384401E-24	0.968537195931008	0.299	0.101	2.08298064518778E-20	FOLR2+ resident-like macrophages	PIGS
1.25947578488926E-24	0.430979894606643	0.395	0.161	2.11062952031742E-20	FOLR2+ resident-like macrophages	BROX
1.33142968205126E-24	0.458628842521484	0.399	0.162	2.2312098611815E-20	FOLR2+ resident-like macrophages	MOGS
1.39022428339572E-24	0.616075690766951	0.444	0.194	2.32973785411455E-20	FOLR2+ resident-like macrophages	PNPLA2
1.45070660789352E-24	0.370494530069235	0.367	0.143	2.43109413350797E-20	FOLR2+ resident-like macrophages	LYPLAL1
1.59614464246241E-24	2.36926666152886	0.205	0.049	2.6748191918385E-20	FOLR2+ resident-like macrophages	MAOA
1.62960881404263E-24	1.22758659858546	0.343	0.133	2.73089845057264E-20	FOLR2+ resident-like macrophages	ERICH5
1.68378851132109E-24	0.627564930083183	0.414	0.177	2.82169278727188E-20	FOLR2+ resident-like macrophages	MPC1
1.77440127379131E-24	0.573697951689151	0.374	0.15	2.97354165461948E-20	FOLR2+ resident-like macrophages	GATAD1
1.93124907858567E-24	0.464072319819195	0.406	0.17	3.23638720589386E-20	FOLR2+ resident-like macrophages	TMEM9B
2.00886275352165E-24	0.339699884999492	0.457	0.198	3.36645220235159E-20	FOLR2+ resident-like macrophages	RFX5
2.03048330318901E-24	0.406945095020594	0.363	0.136	3.40268391948414E-20	FOLR2+ resident-like macrophages	PMVK
2.06412434917989E-24	1.04288471741203	0.356	0.14	3.45905958435566E-20	FOLR2+ resident-like macrophages	ACAT1
2.21105336647929E-24	0.339031887185347	0.48	0.218	3.70528323154599E-20	FOLR2+ resident-like macrophages	TLE5
2.2394696399714E-24	0.989257858540306	0.364	0.142	3.75290322266408E-20	FOLR2+ resident-like macrophages	AGPAT2
2.27774973266739E-24	1.51103993019857	0.283	0.094	3.81705300200401E-20	FOLR2+ resident-like macrophages	DUSP23
2.29543089936358E-24	2.20685460114303	0.168	0.029	3.84668310115349E-20	FOLR2+ resident-like macrophages	LDLR

2.38093484114003E-24	0.70318951514162‌9	0.366	0.145	3.98997060678246E-20	FOLR2+ resident-like macrophages	NENF
2.40800411874523E-24	0.25528620482456‌8	0.469	0.206	4.03533330219326E-20	FOLR2+ resident-like macrophages	CMPK1
2.46717147425148E-24	1.85590891732196	0.184	0.037	4.13448595655063E-20	FOLR2+ resident-like macrophages	FXYD3
2.76319689649114E-24	1.17614508002671	0.295	0.1	4.63056535913985E-20	FOLR2+ resident-like macrophages	SLC2A4RG
2.76433184950505E-24	1.77214169647911	0.244	0.071	4.63246731340056E-20	FOLR2+ resident-like macrophages	CEBPA
2.91797803220791E-24	0.37481129331551‌1	0.366	0.142	4.88994758637402E-20	FOLR2+ resident-like macrophages	TCEAL4
2.9874912632234E-24	0.60711732510007‌6	0.338	0.125	5.00643785890977E-20	FOLR2+ resident-like macrophages	AKT1S1
3.22864748051081E-24	1.80954305818112	0.216	0.055	5.41056744784002E-20	FOLR2+ resident-like macrophages	PPARA
3.26608423157222E-24	0.55900370618623‌2	0.292	0.097	5.47330395526873E-20	FOLR2+ resident-like macrophages	SLC15A4
3.40660584382968E-24	0.67561552812730‌6	0.284	0.092	5.70879007308978E-20	FOLR2+ resident-like macrophages	NUDT14
3.5016425526242E-24	0.68562508056374	0.366	0.146	5.86805258968763E-20	FOLR2+ resident-like macrophages	MAP3K5
3.68860196671373E-24	0.65948347085195‌8	0.269	0.083	6.18135917581887E-20	FOLR2+ resident-like macrophages	TSPAN31
3.71407214352395E-24	1.32880642182497	0.279	0.091	6.22404209811743E-20	FOLR2+ resident-like macrophages	COA3
4.27131865098236E-24	0.30548469231805‌9	0.338	0.122	7.15787579531624E-20	FOLR2+ resident-like macrophages	ARNT
4.46219134761656E-24	1.53923694916075	0.218	0.054	7.47774026033583E-20	FOLR2+ resident-like macrophages	PHETA1
4.56133981482902E-24	0.3843691129866	0.433	0.186	7.64389326169046E-20	FOLR2+ resident-like macrophages	PDIA6
4.96449637120784E-24	0.63332155432550‌1	0.379	0.155	8.3195030188701E-20	FOLR2+ resident-like macrophages	IPO8
4.97172666631644E-24	0.68228740673450‌9	0.284	0.091	8.33161954741309E-20	FOLR2+ resident-like macrophages	TMEM185A
5.76734102089292E-24	0.50212274529517‌9	0.279	0.088	9.66491008281236E-20	FOLR2+ resident-like macrophages	FAM234A
5.85533194160329E-24	0.59137061927573‌7	0.454	0.204	9.81236526773879E-20	FOLR2+ resident-like macrophages	NDUFB7
6.15215037563346E-24	0.40894754539356‌3	0.377	0.151	1.03097735994865E-19	FOLR2+ resident-like macrophages	STX12
6.16081369823366E-24	0.43642932589982‌1	0.313	0.109	1.03242915955E-19	FOLR2+ resident-like macrophages	CDS2
6.9211875895757E-24	1.14139807921861	0.213	0.053	1.1598526162611E-19	FOLR2+ resident-like macrophages	ARSD
6.9515312956581E-24	0.40679674678375‌8	0.52	0.243	1.16493761452638E-19	FOLR2+ resident-like macrophages	S100A11
7.21972972522765E-24	2.60093002527378	0.158	0.026	1.20988230735365E-19	FOLR2+ resident-like macrophages	CYP27A1
7.2644901862977E-24	0.74464008874661‌8	0.325	0.121	1.21738326541977E-19	FOLR2+ resident-like macrophages	GID8
7.81721489403353E-24	0.7539822030915	0.276	0.087	1.31000887194214E-19	FOLR2+ resident-like macrophages	TGFBRAP1
8.47451673543624E-24	0.26788979179510‌2	0.435	0.187	1.4201595145244E-19	FOLR2+ resident-like macrophages	MAT2B
8.5214795282842E-24	1.89585727984121	0.244	0.072	1.42802953934987E-19	FOLR2+ resident-like macrophages	SNTB1
8.61246985326682E-24	0.32957711743271‌4	0.283	0.091	1.44327769801045E-19	FOLR2+ resident-like macrophages	CLIP2
8.77268296116683E-24	0.57150981426929‌7	0.287	0.094	1.47012621063234E-19	FOLR2+ resident-like macrophages	SAP30L
8.80240067531315E-24	1.66774479893527	0.247	0.072	1.47510630516898E-19	FOLR2+ resident-like macrophages	C19orf12
9.20958143297644E-24	1.7442658596541	0.263	0.084	1.54334165653819E-19	FOLR2+ resident-like macrophages	HSD17B6
9.26634031478152E-24	0.48030630621395‌3	0.37	0.147	1.55285330995109E-19	FOLR2+ resident-like macrophages	SEPHS2
9.26925538267293E-24	0.47377581897268‌7	0.2	0.046	1.55334181702833E-19	FOLR2+ resident-like macrophages	HECTD2
9.63810736715277E-24	0.49402672420034‌1	0.315	0.113	1.61515403258746E-19	FOLR2+ resident-like macrophages	SLC35B2
9.87212374179139E-24	0.63582550430746‌3	0.26	0.079	1.6543704966494E-19	FOLR2+ resident-like macrophages	ZNF561
1.07073297560562E-23	2.10661208397423	0.167	0.03	1.79433432051989E-19	FOLR2+ resident-like macrophages	MAP7
1.17087930108994E-23	0.44689759134800‌6	0.415	0.177	1.96215953276652E-19	FOLR2+ resident-like macrophages	YWHAB
1.17974905601527E-23	1.27263736639368	0.293	0.101	1.97702346807038E-19	FOLR2+ resident-like macrophages	HSDL2
1.21462823101668E-23	0.67616140397922‌2	0.328	0.12	2.03547398953776E-19	FOLR2+ resident-like macrophages	COPS6
1.23775705383662E-23	0.43792315387630‌8	0.509	0.245	2.07423327081941E-19	FOLR2+ resident-like macrophages	GOLPH3
1.33329961131636E-23	0.62750106704914‌2	0.386	0.159	2.23434348864395E-19	FOLR2+ resident-like macrophages	TXNL4A
1.34110647917771E-23	1.2377102787792	0.245	0.072	2.24742623780601E-19	FOLR2+ resident-like macrophages	ASL
1.41581076294778E-23	0.37026473823105‌7	0.463	0.203	2.37261567654789E-19	FOLR2+ resident-like macrophages	UQCRC1
1.46972595902265E-23	0.28680792429863‌9	0.46	0.205	2.46296676213015E-19	FOLR2+ resident-like macrophages	PARP4
1.49056349060067E-23	1.18109116306542	0.282	0.094	2.4978862975486E-19	FOLR2+ resident-like macrophages	PSMD10
1.51463336283985E-23	1.08566226816959	0.276	0.092	2.53822258944702E-19	FOLR2+ resident-like macrophages	SMIM19
1.5161511517236E-23	1.47341918087567	0.177	0.034	2.5407661000584E-19	FOLR2+ resident-like macrophages	DIP2C
1.55612802055787E-23	0.43548151197515‌6	0.901	0.569	2.60775933685088E-19	FOLR2+ resident-like macrophages	KCTD12
1.61448381491441E-23	0.38909438207533‌8	0.199	0.045	2.70555197703357E-19	FOLR2+ resident-like macrophages	SFXN2
1.61473893419774E-23	1.28776689864837	0.271	0.088	2.70597950592857E-19	FOLR2+ resident-like macrophages	MDK
1.76808820477049E-23	0.58941788924805‌8	0.315	0.11	2.96296221355439E-19	FOLR2+ resident-like macrophages	EMC3
1.89481286628811E-23	1.78303291377757	0.164	0.028	3.17532740132562E-19	FOLR2+ resident-like macrophages	SCAMP5
2.03444274392197E-23	0.56753529795507	0.334	0.125	3.40931915026444E-19	FOLR2+ resident-like macrophages	HEBP1
2.2398328741524E-23	0.32334939326261‌5	0.403	0.169	3.75351193050459E-19	FOLR2+ resident-like macrophages	SLC25A11
2.31640801638628E-23	0.37056978356291‌6	0.444	0.194	3.88183655386012E-19	FOLR2+ resident-like macrophages	NDUFA4
2.36339533169997E-23	0.32747472657897‌1	0.463	0.203	3.96057789686281E-19	FOLR2+ resident-like macrophages	HNRNPUL2
2.41272309553807E-23	0.42981699750916‌6	0.44	0.192	4.04324136350269E-19	FOLR2+ resident-like macrophages	AKT1
2.4212597201691E-23	0.60402259456023‌8	0.406	0.174	4.05754703905937E-19	FOLR2+ resident-like macrophages	PYCR2
2.45199670832575E-23	0.79126392996977‌6	0.324	0.12	4.10905608381229E-19	FOLR2+ resident-like macrophages	DARS
2.48470081802687E-23	0.80517735278891‌9	0.26	0.079	4.16386163084942E-19	FOLR2+ resident-like macrophages	IP6K1
2.50415514159646E-23	0.60732078358125‌7	0.347	0.135	4.19646318628735E-19	FOLR2+ resident-like macrophages	XPO6
2.5479695373895E-23	1.60030028863195	0.244	0.072	4.26988735075733E-19	FOLR2+ resident-like macrophages	GLYCTK
2.60371238232908E-23	1.32460198468356	0.163	0.028	4.36330121030707E-19	FOLR2+ resident-like macrophages	WNT5B
2.62710558870526E-23	2.51069814437149	0.173	0.034	4.40250354555228E-19	FOLR2+ resident-like macrophages	FRK
2.8337932907091E-23	0.30126646985075‌9	0.395	0.162	4.74887079657031E-19	FOLR2+ resident-like macrophages	TAX1BP1
2.90715096529855E-23	0.85777438771902‌3	0.242	0.07	4.8718035876473E-19	FOLR2+ resident-like macrophages	ZBTB38
3.00449146909166E-23	0.83177548258119‌4	0.26	0.081	5.03492680390381E-19	FOLR2+ resident-like macrophages	FCGR1A
3.08199243210392E-23	0.31370394262966‌9	0.269	0.084	5.16480291771975E-19	FOLR2+ resident-like macrophages	SDSL
3.29696073247569E-23	0.42584012837379‌1	0.446	0.199	5.52504679548276E-19	FOLR2+ resident-like macrophages	TRAPPC3

3.3126348492257E-23	1.59081899600998	0.239	0.071	5.55131348033244E-19	FOLR2+ resident-like macrophages	PEX19
3.53923055461984E-23	1.41166339935312	0.321	0.123	5.93104256343193E-19	FOLR2+ resident-like macrophages	HMGCS2
3.65169537318698E-23	0.87121340252361€	0.325	0.125	6.11951110638674E-19	FOLR2+ resident-like macrophages	RAB5B
3.97513780306974E-23	0.41767127774965€	0.433	0.189	6.66153593038427E-19	FOLR2+ resident-like macrophages	MAN2A1
4.05588622047807E-23	0.79913388627588З	0.277	0.09	6.79685412827715E-19	FOLR2+ resident-like macrophages	SF3A1
4.06579438471765E-23	1.09125688966901	0.199	0.046	6.81345822990983E-19	FOLR2+ resident-like macrophages	ACOT13
4.12684693363397E-23	0.65285347940995€	0.356	0.141	6.9157700913838E-19	FOLR2+ resident-like macrophages	VMA21
4.1963987255205E-23	0.37757669248134€	0.385	0.161	7.03232498422725E-19	FOLR2+ resident-like macrophages	TMBIM6
4.35848841420705E-23	0.78618958988448€	0.244	0.072	7.30395488452818E-19	FOLR2+ resident-like macrophages	BLNK
4.5834928955646E-23	0.41785596607322	0.331	0.124	7.68101739438715E-19	FOLR2+ resident-like macrophages	FAM199X
4.71431834157213E-23	0.667759397328024	0.325	0.122	7.90025467680658E-19	FOLR2+ resident-like macrophages	TTC1
4.72041917775229E-23	0.46161437434975З	0.345	0.135	7.91047845807728E-19	FOLR2+ resident-like macrophages	MLXIP
4.97389016619783E-23	0.319051828914189	0.409	0.171	8.33524514051432E-19	FOLR2+ resident-like macrophages	CHCHD2
5.04369266202634E-23	0.329796288073572	0.421	0.18	8.45222016302375E-19	FOLR2+ resident-like macrophages	OSBPL3
5.07330496316048E-23	1.7840011421807	0.197	0.047	8.50184445726433E-19	FOLR2+ resident-like macrophages	PROX1
5.54849069610355E-23	0.908717952535959	0.289	0.101	9.29816070853033E-19	FOLR2+ resident-like macrophages	ARHGAP35
5.67561117386262E-23	2.49571509435605	0.161	0.029	9.51118920515897E-19	FOLR2+ resident-like macrophages	EHHADH
5.81641475373527E-23	0.554676097099495	0.401	0.173	9.74714784430956E-19	FOLR2+ resident-like macrophages	FGA
6.18406311078895E-23	0.29291463993166€	0.242	0.069	1.03632529610601E-18	FOLR2+ resident-like macrophages	RHOBTB1
6.55825205188197E-23	0.58862437029489€	0.417	0.18	1.09903187885438E-18	FOLR2+ resident-like macrophages	MGAT2
6.66177030894537E-23	1.69325732101087	0.205	0.051	1.11637946837307E-18	FOLR2+ resident-like macrophages	APOB
6.95066829265639E-23	0.372289079816132	0.379	0.156	1.16479299248336E-18	FOLR2+ resident-like macrophages	GOLGB1
6.97214780666026E-23	0.38579321208223	0.334	0.127	1.16839252944013E-18	FOLR2+ resident-like macrophages	ALDH3B1
7.10588433151025E-23	2.26355369096078	0.168	0.032	1.19080409627449E-18	FOLR2+ resident-like macrophages	FN3K
7.37105688913619E-23	0.376662452167091	0.515	0.237	1.23524171348144E-18	FOLR2+ resident-like macrophages	NUCKS1
7.43785535393107E-23	2.50878366781115	0.131	0.015	1.24643580021177E-18	FOLR2+ resident-like macrophages	SOCS2
8.01145500515161E-23	0.31309059044766З	0.34	0.13	1.34255962976331E-18	FOLR2+ resident-like macrophages	DERA
8.03827805764964E-23	1.35738719914286	0.222	0.062	1.34705463690093E-18	FOLR2+ resident-like macrophages	DEPRECATED-ENSG00000099797
8.21425230236476E-23	0.440866205250989	0.347	0.136	1.37654440083029E-18	FOLR2+ resident-like macrophages	METTL23
8.41483931098908E-23	0.48212687733566€	0.303	0.107	1.41015877173555E-18	FOLR2+ resident-like macrophages	KLHL21
8.67251977272025E-23	1.00743785282143	0.271	0.09	1.45334086351246E-18	FOLR2+ resident-like macrophages	KDELR2
9.13012119822567E-23	1.09994498371747	0.247	0.075	1.53002571039866E-18	FOLR2+ resident-like macrophages	HEBP2
9.32163301445243E-23	0.478892365179667	0.337	0.129	1.56211926056194E-18	FOLR2+ resident-like macrophages	FNDC3A
9.38841483052059E-23	0.71530270704609€	0.361	0.146	1.57331055729864E-18	FOLR2+ resident-like macrophages	UQCR11
9.39364701689383E-23	1.84540885557401	0.222	0.061	1.57418736709107E-18	FOLR2+ resident-like macrophages	PCTP
9.44257368577857E-23	1.00816560847177	0.287	0.099	1.58238649826277E-18	FOLR2+ resident-like macrophages	HPRT1
9.69826593064087E-23	0.74773766463891	0.357	0.143	1.6252354046568E-18	FOLR2+ resident-like macrophages	SMIM14
9.88806157198391E-23	0.425858770976028	0.47	0.219	1.65704135823306E-18	FOLR2+ resident-like macrophages	ADI1
1.02725338444524E-22	1.24310082256002	0.29	0.105	1.72147122165333E-18	FOLR2+ resident-like macrophages	CISH
1.05106337679584E-22	0.485770421928175	0.315	0.115	1.76137200683447E-18	FOLR2+ resident-like macrophages	LEPROT
1.09507843949021E-22	0.687877475112458	0.383	0.162	1.83513244889769E-18	FOLR2+ resident-like macrophages	NDUFB10
1.16768337803226E-22	0.49462820616533€	0.367	0.15	1.95680380490646E-18	FOLR2+ resident-like macrophages	VPS4A
1.27021131087237E-22	1.45234129006156	0.225	0.064	2.12862011475991E-18	FOLR2+ resident-like macrophages	AAMDC
1.3427038455687E-22	1.31670273988531	0.247	0.075	2.25010310440403E-18	FOLR2+ resident-like macrophages	ECI2
1.35654335949998E-22	0.544470216209135	0.299	0.107	2.27329536185006E-18	FOLR2+ resident-like macrophages	ELAC2
1.41291590446527E-22	0.299604248026414	0.396	0.166	2.3677644727029E-18	FOLR2+ resident-like macrophages	CLIC1
1.42649917571152E-22	0.370409790747605	0.313	0.113	2.39052731865736E-18	FOLR2+ resident-like macrophages	SECTM1
1.70647480966684E-22	1.88952104288907	0.218	0.06	2.85971048603969E-18	FOLR2+ resident-like macrophages	TM4SF20
1.80555834551141E-22	0.57941855144147€	0.325	0.124	3.02575467540802E-18	FOLR2+ resident-like macrophages	GALNT2
1.81199411980426E-22	0.42984681912386	0.221	0.059	3.03653974596797E-18	FOLR2+ resident-like macrophages	FAXDC2
1.92949760968895E-22	0.30353307540389€	0.338	0.131	3.23345209431674E-18	FOLR2+ resident-like macrophages	AHCYL1
1.9744592886085E-22	0.634337151339222	0.17	0.032	3.30879887585012E-18	FOLR2+ resident-like macrophages	KLHDC8B
2.03008232818494E-22	0.801375919169727	0.315	0.119	3.40201196557233E-18	FOLR2+ resident-like macrophages	ACER3
2.08012603987383E-22	0.26664768189068€	0.55	0.263	3.48587521762056E-18	FOLR2+ resident-like macrophages	CNTRL
2.10182972173362E-22	0.498300989132098	0.242	0.072	3.5222462476812E-18	FOLR2+ resident-like macrophages	ACOT11
2.19506164980789E-22	0.974082649238507	0.277	0.091	3.67848431274806E-18	FOLR2+ resident-like macrophages	IAH1
2.24192812693524E-22	0.890407123741881	0.229	0.065	3.75702315511808E-18	FOLR2+ resident-like macrophages	TMEM263
2.25998722907311E-22	0.32708445140574	0.435	0.192	3.78728659848071E-18	FOLR2+ resident-like macrophages	GHITM
2.2616073147147E-22	0.986302285664848	0.216	0.058	3.79000153799889E-18	FOLR2+ resident-like macrophages	BACE1
2.2760006099862E-22	1.71615062555093	0.27	0.095	3.81412182221487E-18	FOLR2+ resident-like macrophages	SERPINA6
2.41050626653183E-22	0.612931736668171	0.3	0.108	4.03952640145404E-18	FOLR2+ resident-like macrophages	NDUFS6
2.55886326203399E-22	0.37677529335376З	0.287	0.099	4.28814305451656E-18	FOLR2+ resident-like macrophages	TMX1
2.78495944920032E-22	0.294759214463258	0.343	0.131	4.66703504496989E-18	FOLR2+ resident-like macrophages	BCKDK
3.17517220566978E-22	0.285852012606174	0.61	0.298	5.32095358226141E-18	FOLR2+ resident-like macrophages	IDH2
3.26656530137132E-22	0.934875008230304	0.302	0.113	5.47411013203806E-18	FOLR2+ resident-like macrophages	AFG3L2
3.32711910135935E-22	1.04232276145473	0.264	0.087	5.575586190058E-18	FOLR2+ resident-like macrophages	C6orf89
3.3583515568642E-22	1.37993229839253	0.279	0.097	5.62792553899303E-18	FOLR2+ resident-like macrophages	HMGCS1
3.46244091458244E-22	0.45482794354692З	0.315	0.117	5.80235848465725E-18	FOLR2+ resident-like macrophages	REXO2
3.53251206420366E-22	0.476261364113425	0.312	0.115	5.91978371719249E-18	FOLR2+ resident-like macrophages	SEM1
3.584398570369E-22	0.567923269417274	0.254	0.081	6.00673512422438E-18	FOLR2+ resident-like macrophages	NRAS
3.64836359361554E-22	0.259160084436997	0.388	0.163	6.11392771018093E-18	FOLR2+ resident-like macrophages	MEF2C

**3.81690258999159E-22**	0.531914815551635	0.337	0.134	6.3963653603079E-18	FOLR2+ resident-like macrophages	CDV3
**3.94608616580701E-22**	0.628971951783603	0.322	0.123	6.6128511966594E-18	FOLR2+ resident-like macrophages	RALBP1
**4.12672777136225E-22**	0.564188722169055	0.325	0.121	6.91557039924886E-18	FOLR2+ resident-like macrophages	RAB32
**4.33814702852535E-22**	0.313488526353504	0.296	0.104	7.26986679040278E-18	FOLR2+ resident-like macrophages	SLC49A3
**4.51938339741115E-22**	1.1830988493071	0.194	0.046	7.57358269738161E-18	FOLR2+ resident-like macrophages	C1S
**4.53614420918063E-22**	0.364009940405615	0.772	0.436	7.6016704657449E-18	FOLR2+ resident-like macrophages	ANXA5
**4.71386692611869E-22**	1.32601471318982	0.2	0.05	7.8994981947897E-18	FOLR2+ resident-like macrophages	SIDT2
**5.36978069907897E-22**	0.310729182538956	0.385	0.162	8.99867849551654E-18	FOLR2+ resident-like macrophages	ERP44
**5.52658465972886E-22**	0.256575882925776	0.372	0.156	9.26145057277362E-18	FOLR2+ resident-like macrophages	TXLNA
**5.6208074775519E-22**	0.956852698849096	0.277	0.096	9.41934917088148E-18	FOLR2+ resident-like macrophages	TMED2
**5.78478358641332E-22**	0.282143508084479	0.322	0.123	9.69414033411145E-18	FOLR2+ resident-like macrophages	TMEM8A
**5.84799901670606E-22**	0.412946660313373	0.45	0.21	9.80007675219601E-18	FOLR2+ resident-like macrophages	PPP1CA
**6.15257323994968E-22**	0.403129650094515	0.312	0.118	1.03104822355077E-17	FOLR2+ resident-like macrophages	VPS26B
**6.35437846550916E-22**	0.258874638261018	0.308	0.114	1.06486674325003E-17	FOLR2+ resident-like macrophages	NRIP1
**6.59591676770046E-22**	0.719012355500774	0.356	0.143	1.10534373193124E-17	FOLR2+ resident-like macrophages	ADCK2
**7.45656410589525E-22**	0.555058815464804	0.376	0.161	1.24957101286593E-17	FOLR2+ resident-like macrophages	CLPTM1L
**7.65119029449859E-22**	0.445471408484	0.328	0.127	1.28218646955207E-17	FOLR2+ resident-like macrophages	BTF3L4
**7.86231896095805E-22**	2.03878785096857	0.183	0.043	1.31756741147735E-17	FOLR2+ resident-like macrophages	HNF4G
**8.00307209465527E-22**	0.350668976504389	0.385	0.161	1.34115482162233E-17	FOLR2+ resident-like macrophages	IRF9
**8.23170628939687E-22**	0.464303111303692	0.415	0.179	1.37946933997713E-17	FOLR2+ resident-like macrophages	PPM1B
**8.25298870169673E-22**	1.10190026010772	0.313	0.12	1.38303584663034E-17	FOLR2+ resident-like macrophages	FH
**8.8168901701058E-22**	0.449913044182698	0.293	0.105	1.47753445470633E-17	FOLR2+ resident-like macrophages	ENOSF1
**8.82517437775531E-22**	0.412247829795874	0.457	0.215	1.47892272222423E-17	FOLR2+ resident-like macrophages	LAMTOR2
**8.8325551268678E-22**	0.694365261562839	0.238	0.07	1.48015958816051E-17	FOLR2+ resident-like macrophages	TPST2
**9.6098300480421E-22**	0.452798577769561	0.377	0.156	1.61041531945089E-17	FOLR2+ resident-like macrophages	ANAPC11
**9.70135818757928E-22**	0.530365710497262	0.366	0.156	1.62575360507454E-17	FOLR2+ resident-like macrophages	NDUFS1
**9.79505983727421E-22**	0.29332669904127	0.383	0.158	1.64145612753041E-17	FOLR2+ resident-like macrophages	DNAJB12
**9.86111302531372E-22**	0.461228841871132	0.668	0.374	1.65252532078207E-17	FOLR2+ resident-like macrophages	LGALS1
**9.88955654646299E-22**	0.562543994232263	0.287	0.101	1.65729188605627E-17	FOLR2+ resident-like macrophages	DOK3
**1.03891281464402E-21**	1.42663945019115	0.27	0.095	1.74101009478045E-17	FOLR2+ resident-like macrophages	TSPAN8
**1.06554721571522E-21**	1.67729957080838	0.209	0.057	1.78564402409557E-17	FOLR2+ resident-like macrophages	HIBADH
**1.13127276405257E-21**	1.81571283690629	0.228	0.069	1.89578689799929E-17	FOLR2+ resident-like macrophages	OTC
**1.13928583771879E-21**	0.809257829099985	0.286	0.104	1.90921520684914E-17	FOLR2+ resident-like macrophages	ARPC3
**1.18075761355774E-21**	1.25759469203261	0.186	0.045	1.97871360880007E-17	FOLR2+ resident-like macrophages	RDH10
**1.18110810278549E-21**	1.35978604327738	0.258	0.087	1.97930095864793E-17	FOLR2+ resident-like macrophages	RIDA
**1.21676847715679E-21**	0.41764028286146	0.206	0.051	2.03906061401935E-17	FOLR2+ resident-like macrophages	PLXDC1
**1.22332254362497E-21**	0.440622748038116	0.396	0.173	2.05004391860673E-17	FOLR2+ resident-like macrophages	IFI6
**1.36782463429148E-21**	0.764300177231259	0.295	0.108	2.29220052214566E-17	FOLR2+ resident-like macrophages	ENOPH1
**1.37837640205741E-21**	0.559053861727203	0.286	0.101	2.30988317456781E-17	FOLR2+ resident-like macrophages	UBE2W
**1.3965323162705E-21**	0.44298601116328	0.415	0.189	2.34030885560611E-17	FOLR2+ resident-like macrophages	CTDSP1
**1.3992341068658E-21**	3.38291308212094	0.125	0.015	2.34483651628571E-17	FOLR2+ resident-like macrophages	METTL7B
**1.41298366882301E-21**	0.520103486318514	0.237	0.071	2.3678780322136E-17	FOLR2+ resident-like macrophages	SMPD1
**1.47422379118306E-21**	0.421565867720268	0.408	0.183	2.47050422926458E-17	FOLR2+ resident-like macrophages	EGLN1
**1.61046506037084E-21**	0.273919498534202	0.351	0.139	2.69881734816946E-17	FOLR2+ resident-like macrophages	MBD6
**1.61850864537187E-21**	1.24437325170078	0.239	0.075	2.71229678791419E-17	FOLR2+ resident-like macrophages	HMOX2
**1.69777224910113E-21**	0.827749734489947	0.226	0.066	2.84512673504367E-17	FOLR2+ resident-like macrophages	CHMP6
**1.74378188986441E-21**	0.420921281951722	0.363	0.155	2.92222969103478E-17	FOLR2+ resident-like macrophages	YIPF4
**1.7923206698576E-21**	1.51194996855015	0.216	0.062	3.00357097854736E-17	FOLR2+ resident-like macrophages	KRT8
**1.80075278985653E-21**	0.612310397466546	0.367	0.157	3.01770152524157E-17	FOLR2+ resident-like macrophages	PIEZO1
**1.86252074702244E-21**	0.343679665583357	0.334	0.131	3.1212122678602E-17	FOLR2+ resident-like macrophages	ORMDL2
**1.88753528633549E-21**	0.493917661516416	0.351	0.14	3.16313163284101E-17	FOLR2+ resident-like macrophages	PSMC3
**1.90098141020072E-21**	0.250936081243945	0.517	0.245	3.18566464721437E-17	FOLR2+ resident-like macrophages	SFMBT2
**1.90452782963381E-21**	0.835687408912663	0.244	0.076	3.19160773690034E-17	FOLR2+ resident-like macrophages	GMFB
**1.95869186007369E-21**	0.29521160917412	0.226	0.065	3.28237581911148E-17	FOLR2+ resident-like macrophages	MMADHC
**2.04646121798915E-21**	0.916779879194614	0.164	0.033	3.42945970910621E-17	FOLR2+ resident-like macrophages	ZNF219
**2.06817535445103E-21**	0.72726806710475	0.293	0.108	3.46584825898903E-17	FOLR2+ resident-like macrophages	SYVN1
**2.14229481334717E-21**	0.903339982768485	0.347	0.144	3.59005764820718E-17	FOLR2+ resident-like macrophages	FADS2
**2.14788220849478E-21**	0.727037566549944	0.299	0.112	3.59942100499555E-17	FOLR2+ resident-like macrophages	TM9SF1
**2.22135670738125E-21**	0.297350485414974	0.525	0.251	3.7225495702295E-17	FOLR2+ resident-like macrophages	CYB5R3
**2.27061721242399E-21**	1.00711637023982	0.226	0.065	3.80510032458013E-17	FOLR2+ resident-like macrophages	SMIM30
**2.65714115143767E-21**	0.331081465899622	0.379	0.16	4.45283714157925E-17	FOLR2+ resident-like macrophages	KDSR
**2.72455656793541E-21**	0.408155071837242	0.313	0.121	4.56581189654617E-17	FOLR2+ resident-like macrophages	EIF2S1
**2.76321352959036E-21**	0.731198188461614	0.295	0.107	4.63059323288752E-17	FOLR2+ resident-like macrophages	STUB1
**2.76590807350771E-21**	0.478231834838187	0.27	0.093	4.63510874958422E-17	FOLR2+ resident-like macrophages	TDP2
**2.81215579656186E-21**	0.70987338090267	0.284	0.102	4.71261068387837E-17	FOLR2+ resident-like macrophages	TFG
**2.93267619093566E-21**	1.21834511141255	0.196	0.05	4.91457876076997E-17	FOLR2+ resident-like macrophages	GCLM
**2.93565653529079E-21**	1.18964049600078	0.219	0.063	4.91957322184031E-17	FOLR2+ resident-like macrophages	UBE2E1
**3.15147215303129E-21**	0.896320182018736	0.335	0.137	5.28123703404983E-17	FOLR2+ resident-like macrophages	CDC123
**3.25977060398703E-21**	0.467683022841621	0.289	0.102	5.46272357816146E-17	FOLR2+ resident-like macrophages	RTL8C
**3.45713226815785E-21**	0.414837273142583	0.914	0.652	5.79346225497893E-17	FOLR2+ resident-like macrophages	TPP1
**3.48088565867598E-21**	0.652347775441361	0.276	0.096	5.83326818680921E-17	FOLR2+ resident-like macrophages	ACO2

3.72848325744418E-21	1.28579715333845	0.251	0.083	6.24819224282495E-17	FOLR2+ resident-like macrophages	TMEM141
3.83154439919713E-21	0.470820046774193	0.149	0.027	6.42090210417455E-17	FOLR2+ resident-like macrophages	ZBED3
4.10604838800731E-21	0.280230215414354	0.463	0.213	6.88091588862265E-17	FOLR2+ resident-like macrophages	SF3B5
4.16714313377986E-21	0.578990627830269	0.318	0.122	6.98329846358829E-17	FOLR2+ resident-like macrophages	VPS52
4.45879714782918E-21	0.479264865990599	0.385	0.167	7.47205226033214E-17	FOLR2+ resident-like macrophages	PPP1R10
4.84348614399646E-21	1.5132921728056	0.241	0.078	8.11671408010927E-17	FOLR2+ resident-like macrophages	SRXN1
4.91284513208555E-21	0.597816543782238	0.283	0.102	8.23294587234896E-17	FOLR2+ resident-like macrophages	CYB561D2
5.05073601133628E-21	0.331257149214795	0.395	0.175	8.46402340779734E-17	FOLR2+ resident-like macrophages	SRI
5.07957353285893E-21	0.298847199213432	0.311	0.119	8.512349326365E-17	FOLR2+ resident-like macrophages	WBP2
5.08172890649873E-21	0.256792048928036	0.737	0.395	8.51596130151057E-17	FOLR2+ resident-like macrophages	LCP2
5.24291233318864E-21	0.712201130644377	0.325	0.13	8.78607248795752E-17	FOLR2+ resident-like macrophages	TRAPPC4
5.29527719941371E-21	1.56746504580664	0.231	0.072	8.87382553077749E-17	FOLR2+ resident-like macrophages	FMO5
5.29530045842268E-21	0.784400688149422	0.325	0.129	8.87386450822473E-17	FOLR2+ resident-like macrophages	PSMB4
5.38313620556506E-21	0.532303049140419	0.327	0.131	9.02105965328592E-17	FOLR2+ resident-like macrophages	PPP4R3B
5.62987700781179E-21	0.602489050371451	0.331	0.13	9.434547889691E-17	FOLR2+ resident-like macrophages	RAB5IF
5.67241807981019E-21	2.08340190351231	0.2	0.055	9.50583821814591E-17	FOLR2+ resident-like macrophages	CPTP
5.77658940800828E-21	0.643717025058558	0.409	0.182	9.68040852994027E-17	FOLR2+ resident-like macrophages	SPCS1
5.79196770920739E-21	0.696881550145787	0.286	0.104	9.70617948708974E-17	FOLR2+ resident-like macrophages	CTBS
5.82810083004513E-21	1.49774895283085	0.171	0.038	9.76673137098963E-17	FOLR2+ resident-like macrophages	PDE6B
7.68838018784158E-21	0.480656889127208	0.871	0.556	1.28841875187849E-16	FOLR2+ resident-like macrophages	CYBB
7.77089125020714E-21	0.633769299564867	0.255	0.086	1.30224595570971E-16	FOLR2+ resident-like macrophages	RFFL
7.80108461416434E-21	0.811359625371738	0.292	0.108	1.30730575964166E-16	FOLR2+ resident-like macrophages	PREB
7.85879985394068E-21	0.772349794075129	0.38	0.168	1.31697767952338E-16	FOLR2+ resident-like macrophages	PRDX6
7.94146444894171E-21	0.337912644008985	0.241	0.074	1.33083061235365E-16	FOLR2+ resident-like macrophages	ABTB1
8.1546284075455E-21	0.25570900199292	0.337	0.135	1.36655262853647E-16	FOLR2+ resident-like macrophages	BTBD2
8.27044559663173E-21	0.642078658001076	0.305	0.116	1.38596127308354E-16	FOLR2+ resident-like macrophages	AKIP1
8.32113675859201E-21	0.473999011200824	0.318	0.123	1.39445609800485E-16	FOLR2+ resident-like macrophages	YKT6
8.44000381032437E-21	0.373668796508221	0.286	0.102	1.41437583853416E-16	FOLR2+ resident-like macrophages	DEPRECATED-ENSG00000067064
8.75849422848476E-21	0.482884763904941	0.364	0.153	1.46774846280948E-16	FOLR2+ resident-like macrophages	CCSAP
9.08601076747042E-21	0.454296910190317	0.296	0.11	1.52263368441269E-16	FOLR2+ resident-like macrophages	CTSO
9.24014957346906E-21	1.07381072914174	0.253	0.086	1.54846426552194E-16	FOLR2+ resident-like macrophages	TMCO3
9.79699996762033E-21	0.35689343530601	0.328	0.131	1.64178125457381E-16	FOLR2+ resident-like macrophages	AP2M1
1.01845447526215E-20	0.351411767979917	0.29	0.104	1.7067260096443E-16	FOLR2+ resident-like macrophages	GALC
1.02361804002005E-20	1.18163799359003	0.2	0.054	1.71537911146559E-16	FOLR2+ resident-like macrophages	HAGHL
1.06408103436878E-20	0.372382771878081	0.353	0.149	1.78318699739519E-16	FOLR2+ resident-like macrophages	DUSP22
1.12462931520991E-20	0.541200938147533	0.36	0.154	1.88465380642877E-16	FOLR2+ resident-like macrophages	ARHGAP1
1.17719914549875E-20	1.61031123428709	0.208	0.059	1.97275032802681E-16	FOLR2+ resident-like macrophages	GGCX
1.20175896219567E-20	1.69574065096253	0.177	0.042	2.0139076688475E-16	FOLR2+ resident-like macrophages	PCYOX1
1.20400668740186E-20	0.304260999101243	0.372	0.156	2.01767440674803E-16	FOLR2+ resident-like macrophages	STIM1
1.34209423415842E-20	0.767989862760092	0.289	0.105	2.24908151760268E-16	FOLR2+ resident-like macrophages	TNFAIP8L2
1.35612148885289E-20	0.438210133296509	0.224	0.066	2.27258839101968E-16	FOLR2+ resident-like macrophages	DAAM1
1.39411447575647E-20	0.541593382248365	0.312	0.122	2.33625703847269E-16	FOLR2+ resident-like macrophages	MCM3AP
1.42061006682674E-20	0.74740792342882	0.279	0.102	2.38065834998825E-16	FOLR2+ resident-like macrophages	TPGS1
1.46049657354653E-20	0.943666471839875	0.293	0.111	2.44750015794927E-16	FOLR2+ resident-like macrophages	MCTS1
1.46208495152214E-20	0.658573653074703	0.373	0.16	2.45016196176081E-16	FOLR2+ resident-like macrophages	DYNLRB1
1.52622566070705E-20	0.323161781892817	0.292	0.11	2.55764896221288E-16	FOLR2+ resident-like macrophages	KIF1C
1.57367134386245E-20	0.573098963401784	0.345	0.142	2.63715843804469E-16	FOLR2+ resident-like macrophages	ESD
1.59330531317744E-20	0.74845262100189	0.341	0.143	2.67006104382276E-16	FOLR2+ resident-like macrophages	GOLGA7
1.62207805770175E-20	1.44578811238063	0.237	0.077	2.71827840909659E-16	FOLR2+ resident-like macrophages	RNF14
1.63902550298948E-20	0.322299237927272	0.546	0.27	2.74667893790978E-16	FOLR2+ resident-like macrophages	PKM
1.64266914809997E-20	0.624651152096588	0.258	0.089	2.75278495838593E-16	FOLR2+ resident-like macrophages	TMEM68
1.71018529469045E-20	2.84826534055539	0.129	0.019	2.86592851684226E-16	FOLR2+ resident-like macrophages	DCBLD2
1.77900902932384E-20	0.310822565261958	0.399	0.179	2.9812633313409E-16	FOLR2+ resident-like macrophages	PGM2
1.86747221432637E-20	1.33954462449516	0.277	0.098	3.12950993676813E-16	FOLR2+ resident-like macrophages	RBP4
1.8677042213749E-20	0.591637548048221	0.393	0.175	3.12989873418006E-16	FOLR2+ resident-like macrophages	PON2
1.89139324310382E-20	0.289653923994639	0.398	0.182	3.16959679679338E-16	FOLR2+ resident-like macrophages	DELE1
1.89973559618512E-20	0.272201360396424	0.681	0.352	3.18357691208702E-16	FOLR2+ resident-like macrophages	METTL7A
1.92062640650099E-20	1.41811065667454	0.148	0.028	3.21858573201435E-16	FOLR2+ resident-like macrophages	DISP1
1.93502981656899E-20	0.399749452768861	0.284	0.103	3.24272296660631E-16	FOLR2+ resident-like macrophages	GRAMD1A
1.97781607917459E-20	0.739228199666434	0.254	0.084	3.31442418548078E-16	FOLR2+ resident-like macrophages	KCNE3
2.03685569617469E-20	0.794841055424617	0.261	0.092	3.41336277564954E-16	FOLR2+ resident-like macrophages	ERLIN2
2.04505390963212E-20	0.404937617314254	0.357	0.151	3.4271013417615E-16	FOLR2+ resident-like macrophages	ZC3H4
2.04848295804421E-20	0.981363771299697	0.229	0.07	3.43284774109048E-16	FOLR2+ resident-like macrophages	CCS
2.0536667690492E-20	0.811253758661728	0.181	0.044	3.44153477157265E-16	FOLR2+ resident-like macrophages	TMEM150A
2.10965932709064E-20	0.386315834162625	0.761	0.403	3.5353671003385E-16	FOLR2+ resident-like macrophages	ARHGDIB
2.12498557914708E-20	0.606289667153681	0.316	0.125	3.56105083353467E-16	FOLR2+ resident-like macrophages	GUCD1
2.14604487193211E-20	0.271838654802636	0.408	0.183	3.59634199638384E-16	FOLR2+ resident-like macrophages	TMEM165
2.17359299476213E-20	1.54920610470397	0.196	0.053	3.64250714062238E-16	FOLR2+ resident-like macrophages	GOLGA2
2.27891542016397E-20	0.277204233685037	0.382	0.165	3.81900646111079E-16	FOLR2+ resident-like macrophages	AP2A1
2.64512886183654E-20	0.302471371993742	0.328	0.13	4.43270694666568E-16	FOLR2+ resident-like macrophages	UBP1
2.72719995345686E-20	0.349811527206959	0.263	0.089	4.57024168200301E-16	FOLR2+ resident-like macrophages	RAB39A

2.72832433486666E-20	0.52313212947185	0.313	0.122	4.57212592036955E-16	FOLR2+ resident-like macrophages	SLC39A11
2.749223563192E-20	0.52912999285837	0.367	0.158	4.60714884719715E-16	FOLR2+ resident-like macrophages	DUS1L
2.81495115084255E-20	3.17357631932712	0.11	0.011	4.71729513858195E-16	FOLR2+ resident-like macrophages	CHST13
2.85645284227736E-20	0.30338054360231	0.29	0.108	4.7868436730884E-16	FOLR2+ resident-like macrophages	TM2D2
2.9050793894147E-20	1.05215958828598	0.3	0.12	4.86833204078116E-16	FOLR2+ resident-like macrophages	TMEM205
3.19910688852985E-20	0.25198596186454	0.403	0.183	5.36106332379832E-16	FOLR2+ resident-like macrophages	ATP5ME
3.62619213475621E-20	0.25066684239528	0.373	0.152	6.07677277942446E-16	FOLR2+ resident-like macrophages	TUBB
3.70512316063979E-20	0.85217751514774	0.325	0.135	6.20904539260015E-16	FOLR2+ resident-like macrophages	SRPRB
3.77935513248937E-20	1.12521135486679	0.221	0.068	6.33344333102569E-16	FOLR2+ resident-like macrophages	TMEM208
3.85218542951301E-20	0.34519294120211	0.421	0.196	6.4554923427779E-16	FOLR2+ resident-like macrophages	TUFM
3.89893765984724E-20	0.27596889125180	0.327	0.129	6.53383973037201E-16	FOLR2+ resident-like macrophages	NAA60
3.98487565347302E-20	0.83485875763321	0.338	0.142	6.67785462009009E-16	FOLR2+ resident-like macrophages	SHC1
4.0448658365918E-20	0.94735084596736	0.202	0.056	6.77838616896054E-16	FOLR2+ resident-like macrophages	PLAAT3
4.19117843923625E-20	1.38747929657464	0.226	0.071	7.02357682847211E-16	FOLR2+ resident-like macrophages	ABCD3
5.0874954638275E-20	0.26214002220679	0.215	0.06	8.52562489828213E-16	FOLR2+ resident-like macrophages	EDA
5.13556145237761E-20	0.34515303005590	0.89	0.577	8.6061738818944E-16	FOLR2+ resident-like macrophages	TXNIP
5.17264667162939E-20	0.50682780109359	0.358	0.153	8.66832129231653E-16	FOLR2+ resident-like macrophages	CCT6A
5.50682093131088E-20	1.44310747501336	0.247	0.084	9.22833051669078E-16	FOLR2+ resident-like macrophages	CRLS1
5.77832444299705E-20	0.25344928589412	0.345	0.142	9.68331610157446E-16	FOLR2+ resident-like macrophages	OSBP
5.81022278515147E-20	0.54357067055545	0.328	0.137	9.73677134335683E-16	FOLR2+ resident-like macrophages	HDGFL2
6.01396602247259E-20	0.95314943590933	0.273	0.101	1.00782042604596E-15	FOLR2+ resident-like macrophages	SNX4
6.09729907108277E-20	0.33952052249867	0.303	0.119	1.02178537833205E-15	FOLR2+ resident-like macrophages	ANAPC7
6.61076771085509E-20	2.87838395430692	0.125	0.018	1.1078324529851E-15	FOLR2+ resident-like macrophages	MTSS2
6.7476293477922E-20	0.71783624587457	0.298	0.116	1.13076772610302E-15	FOLR2+ resident-like macrophages	LRRC58
6.84856383566793E-20	0.28592944061390	0.302	0.117	1.14768232758123E-15	FOLR2+ resident-like macrophages	WASHC5
6.85069662145997E-20	0.44178698159478	0.335	0.135	1.14803973982426E-15	FOLR2+ resident-like macrophages	MCM3
6.98253176017984E-20	1.2374445817218	0.3	0.12	1.17013267237094E-15	FOLR2+ resident-like macrophages	HACD2
7.00931331306396E-20	0.41590260181279	0.322	0.133	1.17462072500326E-15	FOLR2+ resident-like macrophages	ADGRG6
7.25376971532325E-20	0.58183648422003	0.287	0.108	1.21558672889387E-15	FOLR2+ resident-like macrophages	TSR3
7.51383071899847E-20	0.50223437561244	0.35	0.149	1.25916775188976E-15	FOLR2+ resident-like macrophages	UNC50
7.5314554718826E-20	1.13597563276962	0.305	0.125	1.26212130797809E-15	FOLR2+ resident-like macrophages	DSTN
7.57162451119342E-20	0.75457198399357	0.2	0.055	1.26885283558579E-15	FOLR2+ resident-like macrophages	BBS2
8.09834185066581E-20	0.41332730467496	0.344	0.146	1.35712012733458E-15	FOLR2+ resident-like macrophages	IMMT
8.72913662745754E-20	0.57516177099470	0.271	0.1	1.46282871602934E-15	FOLR2+ resident-like macrophages	GCNT1
8.95749928011895E-20	0.54071828319577	0.283	0.105	1.50109772936233E-15	FOLR2+ resident-like macrophages	GSK3A
9.17883152887104E-20	0.34879507991099	0.251	0.085	1.53818858760821E-15	FOLR2+ resident-like macrophages	FIG4
9.24931108185038E-20	0.56416940627276	0.276	0.102	1.54999955109649E-15	FOLR2+ resident-like macrophages	ZMPSTE24
9.27030134229677E-20	1.51737624015109	0.218	0.067	1.55351709894209E-15	FOLR2+ resident-like macrophages	C11orf54
9.96883922430108E-20	0.48929832508208	0.338	0.144	1.67057807720837E-15	FOLR2+ resident-like macrophages	SOS 2
1.05658100153623E-19	1.17431221519746	0.224	0.072	1.77061844237442E-15	FOLR2+ resident-like macrophages	CETN2
1.07012480927581E-19	0.43985588965299	0.248	0.083	1.7933151553844E-15	FOLR2+ resident-like macrophages	RRAGC
1.14808962478635E-19	1.44587411370499	0.257	0.093	1.92396859321696E-15	FOLR2+ resident-like macrophages	PGM1
1.15915548477797E-19	1.02362094813923	0.234	0.076	1.94251276139092E-15	FOLR2+ resident-like macrophages	ALG3
1.23851217390249E-19	0.78507037556993	0.306	0.125	2.07549870102579E-15	FOLR2+ resident-like macrophages	NIT1
1.24332725349698E-19	0.66550438677252	0.244	0.083	2.08356781141024E-15	FOLR2+ resident-like macrophages	FAM219B
1.24532112625519E-19	0.46456663095931	0.353	0.155	2.08690914337844E-15	FOLR2+ resident-like macrophages	UBA1
1.27660650351313E-19	1.26885478316238	0.264	0.097	2.1393371785873E-15	FOLR2+ resident-like macrophages	FDPS
1.32583174647275E-19	0.55943796040639	0.366	0.161	2.22182884073903E-15	FOLR2+ resident-like macrophages	RPN2
1.39637333147549E-19	0.44550008356360	0.341	0.143	2.34004242888662E-15	FOLR2+ resident-like macrophages	SNX27
1.40705840965136E-19	0.92109658928309	0.299	0.116	2.35794848289374E-15	FOLR2+ resident-like macrophages	TXNDC17
1.42749213946377E-19	0.82586235755843	0.295	0.115	2.39219132731338E-15	FOLR2+ resident-like macrophages	C4BPA
1.42896824235632E-19	1.10500811008207	0.26	0.096	2.39466498054071E-15	FOLR2+ resident-like macrophages	VDAC1
1.49010201604859E-19	1.30193929005709	0.251	0.089	2.49711295849423E-15	FOLR2+ resident-like macrophages	FAM114A1
1.50007645846904E-19	0.54204506128759	0.279	0.105	2.51382812910242E-15	FOLR2+ resident-like macrophages	ATP6V1H
1.52114514970694E-19	0.39425709457345	0.395	0.18	2.54913504187888E-15	FOLR2+ resident-like macrophages	RAB1B
1.54068487146446E-19	0.73323628783806	0.28	0.102	2.58187970760014E-15	FOLR2+ resident-like macrophages	KLHL12
1.5426402504259E-19	0.45326112804162	0.337	0.143	2.58515653166372E-15	FOLR2+ resident-like macrophages	C14orf119
1.54567885570441E-19	0.57861042019849	0.388	0.176	2.59024862638945E-15	FOLR2+ resident-like macrophages	SSR3
1.69188725757349E-19	0.47215891500876	0.215	0.065	2.83526466624166E-15	FOLR2+ resident-like macrophages	IL15RA
1.73039127459754E-19	0.27767572188505	0.343	0.145	2.89978969797056E-15	FOLR2+ resident-like macrophages	GSR
1.73432861391456E-19	0.64079947508649	0.302	0.12	2.90638789119802E-15	FOLR2+ resident-like macrophages	C7orf50
1.84157602263374E-19	0.97646877665912	0.203	0.06	3.08611309872963E-15	FOLR2+ resident-like macrophages	AKR7A2
1.88324580767598E-19	1.32440777735336	0.228	0.074	3.15594332450341E-15	FOLR2+ resident-like macrophages	PCCB
1.97652039132144E-19	0.31212480722089	0.266	0.097	3.31225287177647E-15	FOLR2+ resident-like macrophages	PYM1
2.00080931155866E-19	1.64853155431226	0.237	0.082	3.35295624431E-15	FOLR2+ resident-like macrophages	RNF128
2.02412535931243E-19	1.35948790739599	0.215	0.066	3.39202927713576E-15	FOLR2+ resident-like macrophages	CASC4
2.0260121066637E-19	0.61684605076561	0.34	0.144	3.39519108834703E-15	FOLR2+ resident-like macrophages	GIMAP7
2.06826015871663E-19	1.03063626770199	0.263	0.097	3.46599037397733E-15	FOLR2+ resident-like macrophages	GLUD1
2.07514329264188E-19	1.38762539393715	0.196	0.056	3.47752512980926E-15	FOLR2+ resident-like macrophages	PDK2
2.08685312200509E-19	1.42091848339785	0.205	0.06	3.49714846185613E-15	FOLR2+ resident-like macrophages	CNN3
2.16318322381762E-19	0.98235928112658	0.237	0.082	3.62506244647358E-15	FOLR2+ resident-like macrophages	KRCC1

2.21174769451127E-19	0.769543224858704	0.287	0.11	3.70644678646198E-15	FOLR2+ resident-like macrophages	TMEM123
2.26987264610521E-19	0.426686249651215	0.279	0.102	3.80385258034312E-15	FOLR2+ resident-like macrophages	LPCAT3
2.35022134582078E-19	0.271233912940838	0.422	0.2	3.93850093132647E-15	FOLR2+ resident-like macrophages	CTBP2
2.45866494658415E-19	0.456250315973927	0.345	0.147	4.12023071748573E-15	FOLR2+ resident-like macrophages	SART1
2.46812968552691E-19	0.348115468099509	0.36	0.155	4.136091727006E-15	FOLR2+ resident-like macrophages	TTL
2.48128859415174E-19	0.278209537578213	0.425	0.196	4.15814342607949E-15	FOLR2+ resident-like macrophages	ATP2C1
2.70540933985542E-19	0.4749469503563	0.28	0.107	4.53372497172972E-15	FOLR2+ resident-like macrophages	CLCC1
2.71078221939303E-19	1.58611315334909	0.237	0.082	4.54272884325883E-15	FOLR2+ resident-like macrophages	CYP51A1
2.73819198843876E-19	0.386124509391	0.38	0.172	4.58866213422568E-15	FOLR2+ resident-like macrophages	PARP10
2.77702085979945E-19	0.523307073071132	0.27	0.1	4.65373155685192E-15	FOLR2+ resident-like macrophages	DOK1
2.89044151489112E-19	2.24232895046547	0.138	0.026	4.84380189065454E-15	FOLR2+ resident-like macrophages	TOM1L1
2.93918401213833E-19	0.620334440625901	0.344	0.146	4.92548456754141E-15	FOLR2+ resident-like macrophages	PPM1G
2.96685115947015E-19	0.435605496173418	0.325	0.137	4.97184917304007E-15	FOLR2+ resident-like macrophages	IPO9
2.96753143173393E-19	1.32560243561983	0.186	0.05	4.97298917329971E-15	FOLR2+ resident-like macrophages	ADH5
2.99570654219485E-19	0.670084639266639	0.293	0.113	5.02020502341012E-15	FOLR2+ resident-like macrophages	VPS4B
3.11783211277877E-19	0.291635100878954	0.35	0.151	5.22486305459466E-15	FOLR2+ resident-like macrophages	PCYT1A
3.27405870585475E-19	1.6072274167341	0.186	0.051	5.4866675792714E-15	FOLR2+ resident-like macrophages	PCED1B
3.31196014711085E-19	0.296787902039989	0.21	0.062	5.55018281452836E-15	FOLR2+ resident-like macrophages	ARHGAP10
3.3534549126436E-19	0.386512518762976	0.325	0.131	5.61971974260814E-15	FOLR2+ resident-like macrophages	SQOR
3.44959708444487E-19	0.285806258968859	0.319	0.131	5.78083479411271E-15	FOLR2+ resident-like macrophages	STK25
3.56587756476733E-19	1.82542167734	0.168	0.042	5.97569762303709E-15	FOLR2+ resident-like macrophages	FAHD1
3.88956516425403E-19	1.35014113730223	0.226	0.074	6.5181333022569E-15	FOLR2+ resident-like macrophages	NAXE
3.89544490764299E-19	1.39501277278003	0.235	0.082	6.52798657622812E-15	FOLR2+ resident-like macrophages	ALAD
4.16237226136768E-19	1.86088269167666	0.203	0.062	6.97530343559995E-15	FOLR2+ resident-like macrophages	ABAT
4.60715941064077E-19	0.471284682420047	0.303	0.121	7.72067774035181E-15	FOLR2+ resident-like macrophages	DCAF12
4.76318088328763E-19	0.735062718816809	0.283	0.11	7.98213852421341E-15	FOLR2+ resident-like macrophages	PPP2R5A
4.82004032081606E-19	1.24297914717785	0.222	0.071	8.07742356962356E-15	FOLR2+ resident-like macrophages	OSTC
5.15135390142687E-19	1.01739847763832	0.219	0.07	8.63263886801114E-15	FOLR2+ resident-like macrophages	PDIA5
5.15315055272901E-19	0.458004855470652	0.27	0.099	8.63564969626327E-15	FOLR2+ resident-like macrophages	VCP
5.16849332566602E-19	0.40252604697003	0.181	0.046	8.66136111515111E-15	FOLR2+ resident-like macrophages	RILPL1
5.17914957356093E-19	0.96478075695194	0.176	0.045	8.6792188553734E-15	FOLR2+ resident-like macrophages	UFSP2
5.32111706791608E-19	0.98067895981865	0.327	0.139	8.91712798241376E-15	FOLR2+ resident-like macrophages	ETFA
5.88128794336083E-19	0.840849252383646	0.283	0.109	9.85586233548408E-15	FOLR2+ resident-like macrophages	BCKDHA
5.9252559255983E-19	0.87275004466424	0.218	0.07	9.92954388011762E-15	FOLR2+ resident-like macrophages	DHDDS
6.10944846968156E-19	1.77237161960995	0.158	0.036	1.02382137454924E-14	FOLR2+ resident-like macrophages	SDC2
6.16707314272511E-19	0.336754219346928	0.377	0.169	1.03347811725787E-14	FOLR2+ resident-like macrophages	EDF1
6.33329375942213E-19	0.519798880051373	0.305	0.12	1.06133336820396E-14	FOLR2+ resident-like macrophages	MAPK1IP1L
6.51753858314455E-19	2.16820437411953	0.142	0.028	1.09220911576336E-14	FOLR2+ resident-like macrophages	SLC43A1
6.83280011537956E-19	0.924809620193645	0.292	0.118	1.14504064333531E-14	FOLR2+ resident-like macrophages	ROMO1
6.84598973585803E-19	0.530667523236036	0.229	0.076	1.14725095993509E-14	FOLR2+ resident-like macrophages	ACSS1
7.1551649098424E-19	1.1216753300039	0.219	0.07	1.19906253559139E-14	FOLR2+ resident-like macrophages	ACO1
7.21323211334573E-19	0.419094741166783	0.17	0.042	1.20879343755448E-14	FOLR2+ resident-like macrophages	IPO13
7.26191274631186E-19	0.269456779816296	0.406	0.185	1.21695133802694E-14	FOLR2+ resident-like macrophages	DNAJC13
7.37974842401633E-19	0.356421155812591	0.251	0.088	1.23669824089666E-14	FOLR2+ resident-like macrophages	DGCR2
7.89161233398579E-19	0.433420612894516	0.39	0.176	1.32247639492934E-14	FOLR2+ resident-like macrophages	TMEM106C
8.09614129154716E-19	1.43952339995289	0.229	0.078	1.35675135763747E-14	FOLR2+ resident-like macrophages	LEAP2
8.53663418762192E-19	1.00983566121926	0.263	0.098	1.43056915716168E-14	FOLR2+ resident-like macrophages	MTMR4
8.95891264811686E-19	1.67379646332893	0.161	0.039	1.50133458157142E-14	FOLR2+ resident-like macrophages	GALE
8.99555968459038E-19	0.258802532864055	0.832	0.487	1.50747589194366E-14	FOLR2+ resident-like macrophages	FCGR2A
9.94791532096464E-19	0.533294326484969	0.327	0.136	1.66707164948725E-14	FOLR2+ resident-like macrophages	RECQL
9.9780855651871E-19	1.08748667863934	0.245	0.086	1.67212757901405E-14	FOLR2+ resident-like macrophages	SDC4
1.0108406505824E-18	0.270811811916364	0.35	0.151	1.69396676224599E-14	FOLR2+ resident-like macrophages	GPR108
1.01948899935481E-18	0.809380071510159	0.212	0.067	1.70845966511879E-14	FOLR2+ resident-like macrophages	TCTA
1.04223695103E-18	1.01329533316332	0.26	0.096	1.74658068253607E-14	FOLR2+ resident-like macrophages	ACADM
1.04418568687507E-18	2.07105855156323	0.12	0.018	1.74984637406525E-14	FOLR2+ resident-like macrophages	CCL18
1.06932392394539E-18	0.704034229894395	0.334	0.145	1.79197303174769E-14	FOLR2+ resident-like macrophages	SLC44A1
1.08195766858508E-18	0.649665232737994	0.305	0.125	1.81314466101487E-14	FOLR2+ resident-like macrophages	C18orf32
1.12306028560541E-18	0.675396718314419	0.142	0.028	1.88202442661754E-14	FOLR2+ resident-like macrophages	BBS5
1.12932670541732E-18	0.900586975809643	0.266	0.102	1.89252569293835E-14	FOLR2+ resident-like macrophages	POMP
1.27547001835523E-18	0.426687526307625	0.277	0.104	2.1374326567597E-14	FOLR2+ resident-like macrophages	PGK 1.00
1.28070490823389E-18	0.34620449316908	0.341	0.149	2.14620528521835E-14	FOLR2+ resident-like macrophages	OCIAD1
1.28985707037273E-18	0.330972691040606	0.308	0.125	2.16154247853063E-14	FOLR2+ resident-like macrophages	LDLRAD4
1.4316028005881E-18	2.09844101618674	0.135	0.026	2.39907997322554E-14	FOLR2+ resident-like macrophages	DEPRECATED-ENSG00000146416
1.45506406364043E-18	0.356164296886169	0.345	0.152	2.43839635784863E-14	FOLR2+ resident-like macrophages	TMEM19
1.54981457537789E-18	0.621022751268227	0.293	0.116	2.59717926541826E-14	FOLR2+ resident-like macrophages	NNT
1.57416266329812E-18	0.761398742109183	0.225	0.075	2.63798179115499E-14	FOLR2+ resident-like macrophages	NIPAL2
1.66242325833692E-18	0.414023964435503	0.26	0.094	2.78588889632101E-14	FOLR2+ resident-like macrophages	PBDC1
1.68655825894261E-18	0.436650120946355	0.242	0.084	2.82633433033603E-14	FOLR2+ resident-like macrophages	SEC23A
1.74760657048965E-18	2.09079938783208	0.171	0.045	2.92863909082655E-14	FOLR2+ resident-like macrophages	MTUS1
1.75844314009868E-18	0.383456062449223	0.337	0.145	2.94679901417737E-14	FOLR2+ resident-like macrophages	COX16
1.77358553908959E-18	0.786268554104099	0.261	0.098	2.97217464640633E-14	FOLR2+ resident-like macrophages	KDELR1

1.78878090524497E-18	1.4894313942692	0.225	0.077	2.99763904100953E-14	FOLR2+ resident-like macrophages	FASN
1.79556186468656E-18	0.564755935090774	0.255	0.093	3.00900257284174E-14	FOLR2+ resident-like macrophages	GATD1
1.80043279384514E-18	1.26593936845969	0.129	0.023	3.01716527592569E-14	FOLR2+ resident-like macrophages	HS1BP3
1.84308134216931E-18	0.775806202449445	0.244	0.085	3.08863571320732E-14	FOLR2+ resident-like macrophages	SDHC
1.84340373000621E-18	0.780258253840624	0.255	0.094	3.0891759707444E-14	FOLR2+ resident-like macrophages	CEBPZOS
1.92770963887154E-18	0.448499986255758	0.369	0.167	3.23045581282093E-14	FOLR2+ resident-like macrophages	NUP43
1.93903444071837E-18	0.3514210294898	0.183	0.048	3.24943391575585E-14	FOLR2+ resident-like macrophages	LTBP2
2.1058719036813E-18	0.652289988223244	0.206	0.064	3.52902013618912E-14	FOLR2+ resident-like macrophages	SNX11
2.1122393141697E-18	1.24191620202507	0.126	0.021	3.53969064268558E-14	FOLR2+ resident-like macrophages	EPHB2
2.16241094846054E-18	0.61702284892337	0.282	0.108	3.62376826743018E-14	FOLR2+ resident-like macrophages	EIF4EBP1
2.18423540471649E-18	0.667347997750367	0.21	0.065	3.66034169122389E-14	FOLR2+ resident-like macrophages	ZDHHC21
2.27696977395456E-18	0.681308581177175	0.261	0.099	3.81574594719305E-14	FOLR2+ resident-like macrophages	GATD3B
2.30403768693538E-18	0.460533033578149	0.295	0.12	3.86110635576631E-14	FOLR2+ resident-like macrophages	MESD
2.44489705049972E-18	0.368012278722043	0.241	0.083	4.09715847722742E-14	FOLR2+ resident-like macrophages	NOP10
2.44813750765105E-18	0.553210866887416	0.29	0.116	4.10258883532163E-14	FOLR2+ resident-like macrophages	NELFB
2.47217966791874E-18	1.0198857188048	0.248	0.09	4.14287868749822E-14	FOLR2+ resident-like macrophages	JUP
2.48861193301535E-18	0.831518075223233	0.205	0.064	4.17041587734713E-14	FOLR2+ resident-like macrophages	DCUN1D4
2.54529207022617E-18	0.701281525796517	0.193	0.056	4.26540045128501E-14	FOLR2+ resident-like macrophages	OPLAH
2.62217783670514E-18	0.722608555205532	0.218	0.07	4.39424561875048E-14	FOLR2+ resident-like macrophages	MARCH2
2.83013992409097E-18	0.671371313934204	0.302	0.125	4.74274848479165E-14	FOLR2+ resident-like macrophages	RETREG2
2.86147947329016E-18	1.34762568225787	0.192	0.057	4.79526730133966E-14	FOLR2+ resident-like macrophages	PBLD
2.88763362764344E-18	0.531204007017229	0.26	0.097	4.83909643320488E-14	FOLR2+ resident-like macrophages	SLC25A45
2.90234968004487E-18	0.32823372988167	0.299	0.122	4.8637575938192E-14	FOLR2+ resident-like macrophages	LMF2
2.9070688651639E-18	0.822584809545227	0.228	0.077	4.87166600424167E-14	FOLR2+ resident-like macrophages	TSR2
3.00023339042241E-18	0.341589854953589	0.247	0.087	5.02779111566988E-14	FOLR2+ resident-like macrophages	TMEM87B
3.3192333446399E-18	0.535180603524866	0.253	0.091	5.56237123894755E-14	FOLR2+ resident-like macrophages	LEO1
3.33128772141749E-18	0.843696235543693	0.239	0.083	5.58257196355142E-14	FOLR2+ resident-like macrophages	PIN1
3.38362046467153E-18	1.40171150864587	0.183	0.052	5.67027117469655E-14	FOLR2+ resident-like macrophages	GRAMD2B
3.43318361780511E-18	0.488230897614491	0.238	0.083	5.75332910671781E-14	FOLR2+ resident-like macrophages	MMD
3.6228016181983E-18	0.37014725165887	0.244	0.086	6.07109095177672E-14	FOLR2+ resident-like macrophages	ARHGEF40
3.75970464077282E-18	0.546893770805647	0.27	0.103	6.3005130370071E-14	FOLR2+ resident-like macrophages	MED10
3.90019960705339E-18	0.495295080070578	0.235	0.082	6.53595450150008E-14	FOLR2+ resident-like macrophages	PATZ1
3.90362242836695E-18	2.19178422561334	0.148	0.033	6.54169046545733E-14	FOLR2+ resident-like macrophages	TMEM45B
3.94122946806689E-18	0.496854353542456	0.271	0.103	6.6047123425865E-14	FOLR2+ resident-like macrophages	PARP1
4.25184023683789E-18	0.356494212552613	0.295	0.118	7.12523386889294E-14	FOLR2+ resident-like macrophages	LIN7C
4.43427880223341E-18	1.43225560398772	0.199	0.061	7.43096441678275E-14	FOLR2+ resident-like macrophages	UGT2B11
4.7138030003313E-18	0.320098540511302	0.215	0.067	7.89939106795519E-14	FOLR2+ resident-like macrophages	ALKBH7
4.75828508656727E-18	0.483333190840729	0.328	0.145	7.97393414806943E-14	FOLR2+ resident-like macrophages	ACTR1B
4.77394316588963E-18	1.20802787944995	0.113	0.016	8.00017395739784E-14	FOLR2+ resident-like macrophages	PTCRA
4.82827159510674E-18	0.648580070863798	0.26	0.099	8.09121753907987E-14	FOLR2+ resident-like macrophages	ZDHHC6
4.85230802334491E-18	1.58558078747756	0.135	0.027	8.1314977855214E-14	FOLR2+ resident-like macrophages	MARCH9
4.86436604848471E-18	1.11390071065997	0.229	0.081	8.15170462405067E-14	FOLR2+ resident-like macrophages	DEPRECATED-ENSG00000133142
4.90349816978149E-18	1.83734511387991	0.179	0.05	8.21728223291982E-14	FOLR2+ resident-like macrophages	MPI
4.90424545994175E-18	0.621262564858353	0.276	0.106	8.21853454177038E-14	FOLR2+ resident-like macrophages	CANT1
5.03572193046693E-18	0.612482149606872	0.228	0.077	8.43886281107649E-14	FOLR2+ resident-like macrophages	ARFIP1
5.13031410836945E-18	0.390229491120332	0.289	0.116	8.59738038280552E-14	FOLR2+ resident-like macrophages	RNF103
5.24530344102582E-18	0.326267428358739	0.327	0.144	8.79007950647107E-14	FOLR2+ resident-like macrophages	TIMM13
5.32560449192269E-18	0.690201909563089	0.218	0.072	8.92464800756404E-14	FOLR2+ resident-like macrophages	SLC27A4
5.37616472881449E-18	0.61187496104089	0.181	0.05	9.00937685254733E-14	FOLR2+ resident-like macrophages	TOR4A
5.38878402035391E-18	1.02616835628747	0.206	0.065	9.03052426130909E-14	FOLR2+ resident-like macrophages	COA8
5.5896778318467E-18	0.322900773867596	0.279	0.109	9.3671821106087E-14	FOLR2+ resident-like macrophages	NBN
5.97736495834466E-18	0.288597674616917	0.35	0.157	1.0016868197194E-13	FOLR2+ resident-like macrophages	SMG5
6.10595343772223E-18	1.62934974301698	0.16	0.041	1.02323567709349E-13	FOLR2+ resident-like macrophages	CCL15
6.13792851803692E-18	0.264161557328669	0.289	0.115	1.02859406105263E-13	FOLR2+ resident-like macrophages	AGO4
6.27561224061057E-18	0.888864861355878	0.218	0.072	1.05166709928152E-13	FOLR2+ resident-like macrophages	ARL 1.00
6.71146663626595E-18	0.75280846758169	0.266	0.102	1.12470757890545E-13	FOLR2+ resident-like macrophages	UBD
7.22525170683996E-18	1.41846463244773	0.157	0.038	1.21080768103224E-13	FOLR2+ resident-like macrophages	TMEM192
7.8631213901276E-18	0.572241189247616	0.277	0.11	1.31770188255758E-13	FOLR2+ resident-like macrophages	GINM1
7.99784823464111E-18	1.33721735438856	0.239	0.088	1.34027940716116E-13	FOLR2+ resident-like macrophages	GOT2
8.01989632269407E-18	0.252932624601748	0.372	0.173	1.34397422575707E-13	FOLR2+ resident-like macrophages	CNPPD1
8.04791486266512E-18	0.824276290085723	0.229	0.08	1.34866957268542E-13	FOLR2+ resident-like macrophages	RNF220
8.14407224426398E-18	0.636921356066889	0.271	0.106	1.36478362669376E-13	FOLR2+ resident-like macrophages	NDUFS3
8.32959145988203E-18	0.576313415946712	0.253	0.093	1.39587293684703E-13	FOLR2+ resident-like macrophages	SCRIB
8.33131827528204E-18	0.396883403158618	0.309	0.131	1.39616231657176E-13	FOLR2+ resident-like macrophages	GALM
8.76171113158177E-18	1.01374957499927	0.237	0.085	1.46828755143047E-13	FOLR2+ resident-like macrophages	UGP2
8.81294665150989E-18	0.591168625566704	0.322	0.142	1.47687359986003E-13	FOLR2+ resident-like macrophages	PSMC4
8.89111589174464E-18	0.354931424772857	0.881	0.541	1.48997320113857E-13	FOLR2+ resident-like macrophages	ITGB2
9.30532381175332E-18	0.442868924208503	0.248	0.09	1.55938616437362E-13	FOLR2+ resident-like macrophages	RFNG
9.52303301599676E-18	1.64597527188581	0.215	0.073	1.59586987282074E-13	FOLR2+ resident-like macrophages	MPST
9.76146325494457E-18	0.433380826404419	0.28	0.113	1.63582601226361E-13	FOLR2+ resident-like macrophages	ITPRIP
9.83677347515364E-18	2.42847993944353	0.12	0.021	1.64844649896625E-13	FOLR2+ resident-like macrophages	HAO1

1.00799752567274E-17	1.29080064481954	0.179	0.05	1.68920225352238E-13	FOLR2+ resident-like macrophages	CRNKL1
1.01249327203722E-17	0.545770663689006	0.293	0.121	1.69673622527998E-13	FOLR2+ resident-like macrophages	CCDC124
1.06463177140047E-17	1.70170429869064	0.147	0.034	1.78410992251292E-13	FOLR2+ resident-like macrophages	ACMSD
1.06811480429141E-17	0.257075198241606	0.446	0.221	1.78994678903155E-13	FOLR2+ resident-like macrophages	ATF6B
1.07333133026302E-17	1.78839986021905	0.173	0.047	1.79868864325476E-13	FOLR2+ resident-like macrophages	DEPRECATED-ENSG00000184500
1.08614693469564E-17	1.05346492034528	0.26	0.101	1.82016503316295E-13	FOLR2+ resident-like macrophages	CPD
1.10759975458868E-17	0.652236593289234	0.293	0.12	1.85611566873972E-13	FOLR2+ resident-like macrophages	SLC50A1
1.12802343413682E-17	0.44499142413666	0.29	0.12	1.89034167092648E-13	FOLR2+ resident-like macrophages	PSMB5
1.13935791594335E-17	0.558853954054819	0.257	0.098	1.90933599553787E-13	FOLR2+ resident-like macrophages	C11orf68
1.15422409047356E-17	0.268956219565511	0.219	0.073	1.93424873081558E-13	FOLR2+ resident-like macrophages	NFATC1
1.16628979621801E-17	0.31000392441375	0.34	0.148	1.95446844050213E-13	FOLR2+ resident-like macrophages	BIN2
1.1811473503816E-17	0.704786029712757	0.293	0.12	1.97936672976948E-13	FOLR2+ resident-like macrophages	ATP2B4
1.18269133140803E-17	0.59732657463545	0.287	0.12	1.98195413317357E-13	FOLR2+ resident-like macrophages	ATRAID
1.20460826713464E-17	0.48198356066373	0.271	0.106	2.01868253406423E-13	FOLR2+ resident-like macrophages	CENPB
1.22879452865351E-17	1.66252183644714	0.179	0.051	2.05921387111755E-13	FOLR2+ resident-like macrophages	DPP4
1.23140972404811E-17	2.32333249639327	0.16	0.042	2.06359641555982E-13	FOLR2+ resident-like macrophages	ALDH7A1
1.23440486758208E-17	0.63379435928248	0.241	0.087	2.06861567709405E-13	FOLR2+ resident-like macrophages	BCL2L2
1.24846179183928E-17	1.0693594960348	0.122	0.021	2.09217227076426E-13	FOLR2+ resident-like macrophages	OBSL1
1.27395747903853E-17	0.329349544263691	0.254	0.097	2.13489794337277E-13	FOLR2+ resident-like macrophages	SLC39A3
1.29053368464596E-17	2.02082031202865	0.138	0.029	2.1626763487297E-13	FOLR2+ resident-like macrophages	FAR2
1.321183314083E-17	0.313611808113359	0.231	0.08	2.2140389977403E-13	FOLR2+ resident-like macrophages	SUCLG2
1.35745346908056E-17	2.11044860378047	0.126	0.024	2.27482052348521E-13	FOLR2+ resident-like macrophages	ALDH5A1
1.39362800535254E-17	0.385561627081752	0.308	0.129	2.33544181136978E-13	FOLR2+ resident-like macrophages	PRPF8
1.39895340308671E-17	0.520835236030127	0.287	0.119	2.34436611289271E-13	FOLR2+ resident-like macrophages	ZDHHC3
1.42563336169365E-17	1.18489778629244	0.194	0.06	2.38907638752621E-13	FOLR2+ resident-like macrophages	VAPB
1.52223321394181E-17	0.313968736502025	0.361	0.165	2.55095841992369E-13	FOLR2+ resident-like macrophages	FBXW2
1.58188355880815E-17	1.75075624938596	0.135	0.028	2.6509204678507E-13	FOLR2+ resident-like macrophages	MAPRE3
1.63945984157702E-17	1.23074752286895	0.131	0.026	2.74740680251477E-13	FOLR2+ resident-like macrophages	FAIM
1.67614234132052E-17	0.554629633009692	0.179	0.05	2.80887933558493E-13	FOLR2+ resident-like macrophages	ZCCHC24
1.75423300491911E-17	1.63452156136107	0.173	0.047	2.93974366964344E-13	FOLR2+ resident-like macrophages	NECAB3
1.81230279728355E-17	1.25121310475146	0.136	0.028	3.03705702768777E-13	FOLR2+ resident-like macrophages	LAMP5
1.83485071216804E-17	1.07884201586873	0.113	0.017	3.0748428234512E-13	FOLR2+ resident-like macrophages	PRR5
1.83957804384511E-17	0.4423218658791	0.232	0.082	3.08276488587564E-13	FOLR2+ resident-like macrophages	ENPP4
1.87244925957639E-17	0.2528384086347	0.393	0.182	3.13785046919811E-13	FOLR2+ resident-like macrophages	PRPF19
1.88964345327367E-17	0.356626864988041	0.25	0.092	3.16666449899602E-13	FOLR2+ resident-like macrophages	PIK3R2
1.90392350502593E-17	0.797402523194371	0.222	0.076	3.19059500972245E-13	FOLR2+ resident-like macrophages	WASHC3
1.91554765261645E-17	0.377410579031349	0.354	0.159	3.21007475625464E-13	FOLR2+ resident-like macrophages	ATP1A1
1.97619266491663E-17	0.670292716087031	0.284	0.117	3.31170366786729E-13	FOLR2+ resident-like macrophages	SRP68
2.00802385022143E-17	0.687247256401014	0.221	0.076	3.36504636820108E-13	FOLR2+ resident-like macrophages	TMEM60
2.1083411495243E-17	1.74859923241296	0.189	0.058	3.53315809837282E-13	FOLR2+ resident-like macrophages	AK4
2.12452322928458E-17	0.366807821549758	0.337	0.15	3.56027602763509E-13	FOLR2+ resident-like macrophages	AP1G1
2.15492528044755E-17	1.0383109021944	0.173	0.047	3.611223784974E-13	FOLR2+ resident-like macrophages	POMGNT1
2.16613485454281E-17	1.3286278904089	0.234	0.085	3.63000878924284E-13	FOLR2+ resident-like macrophages	DEPRECATED-ENSG00000213759
2.1761183726104E-17	2.41537673167609	0.128	0.025	3.6467391688205E-13	FOLR2+ resident-like macrophages	HPN
2.27485028766321E-17	0.710345083139883	0.267	0.103	3.81219411206601E-13	FOLR2+ resident-like macrophages	NMRAL1
2.3534490192064E-17	0.343055850973019	0.284	0.116	3.94390986638609E-13	FOLR2+ resident-like macrophages	MUS81
2.52325408146118E-17	0.282625096263038	0.282	0.112	4.22846918971264E-13	FOLR2+ resident-like macrophages	EIF3D
2.5776941902599E-17	0.665823770895071	0.254	0.098	4.31969992403754E-13	FOLR2+ resident-like macrophages	PPDPF
2.79106427414535E-17	0.878882737673772	0.221	0.077	4.67726551061279E-13	FOLR2+ resident-like macrophages	ALG12
2.98247995944297E-17	1.19283336428302	0.163	0.043	4.99803991603452E-13	FOLR2+ resident-like macrophages	PRDX3
2.98606321651501E-17	0.464947439367578	0.237	0.086	5.00404473823586E-13	FOLR2+ resident-like macrophages	PDLIM5
3.07679463034854E-17	0.706975353374736	0.138	0.029	5.15609244153809E-13	FOLR2+ resident-like macrophages	PLCL1
3.1716282278701E-17	1.59978878566972	0.163	0.044	5.31501458426472E-13	FOLR2+ resident-like macrophages	ICK
3.22909893091405E-17	0.623069423366798	0.279	0.113	5.41132398842576E-13	FOLR2+ resident-like macrophages	SURF1
3.26327003206298E-17	0.515288595989195	0.232	0.083	5.46858791973114E-13	FOLR2+ resident-like macrophages	SMIM29
3.29834908250923E-17	0.956111655346942	0.144	0.032	5.52737339246897E-13	FOLR2+ resident-like macrophages	METTL27
3.3715672559669E-17	1.49596856872105	0.208	0.07	5.65007240754934E-13	FOLR2+ resident-like macrophages	NSDHL
3.4670708369724E-17	0.392476269922857	0.269	0.105	5.81011730859834E-13	FOLR2+ resident-like macrophages	MTIF3
3.47208615830416E-17	0.745337184177318	0.149	0.035	5.81852198408611E-13	FOLR2+ resident-like macrophages	ZNF438
3.56558846276793E-17	0.441555991444164	0.253	0.094	5.97521314590649E-13	FOLR2+ resident-like macrophages	TMEM70
3.56681448214144E-17	0.843864365378728	0.257	0.1	5.97726770917262E-13	FOLR2+ resident-like macrophages	ECI1
3.57266083371964E-17	0.537013852661682	0.245	0.091	5.98706502514738E-13	FOLR2+ resident-like macrophages	TLR6
3.65034862910345E-17	0.889183496174771	0.271	0.11	6.11725423265156E-13	FOLR2+ resident-like macrophages	SLC25A5
3.74279833158147E-17	0.503442995312315	0.319	0.139	6.27218144406423E-13	FOLR2+ resident-like macrophages	LRRC47
3.76082970865575E-17	0.343498610018678	0.231	0.081	6.3023984257653E-13	FOLR2+ resident-like macrophages	TRIM4
3.81482977300296E-17	0.481328840992282	0.219	0.076	6.39289173359836E-13	FOLR2+ resident-like macrophages	PRKAR2A
3.82432832755493E-17	0.711467562908999	0.344	0.155	6.40880941131656E-13	FOLR2+ resident-like macrophages	HTATSF1
3.83580222691612E-17	1.21650179003476	0.151	0.037	6.42803737186603E-13	FOLR2+ resident-like macrophages	FCGR1B
3.86343071001005E-17	0.299632512183736	0.499	0.266	6.47433718383485E-13	FOLR2+ resident-like macrophages	RAP1A
3.88503395479708E-17	1.36527493493007	0.29	0.128	6.51053990144894E-13	FOLR2+ resident-like macrophages	CXCL9
3.88869407364695E-17	0.30259415909243	0.263	0.102	6.51667352861756E-13	FOLR2+ resident-like macrophages	RMDN3

3.95279525071532E-17	0.369838536101495	0.298	0.125	6.62409428114873E-13	FOLR2+ resident-like macrophages	PLEKHJ1
3.97191161939671E-17	0.42235966321311	0.329	0.145	6.65612949178501E-13	FOLR2+ resident-like macrophages	GIMAP2
4.36974461792432E-17	0.429039902885859	0.338	0.155	7.32281803071758E-13	FOLR2+ resident-like macrophages	SCAF1
4.42881160779123E-17	0.268553730665509	0.234	0.083	7.42180249233655E-13	FOLR2+ resident-like macrophages	USP22
4.45611089623462E-17	0.486647204616128	0.2	0.064	7.46755063990998E-13	FOLR2+ resident-like macrophages	RBM41
4.57279587874702E-17	1.25208784015554	0.212	0.073	7.66309133360425E-13	FOLR2+ resident-like macrophages	POLR2I
4.81316821740712E-17	0.345488215040249	0.16	0.041	8.06590729873085E-13	FOLR2+ resident-like macrophages	GATD3A
4.84284766574688E-17	0.58021299258436	0.216	0.073	8.11564411825862E-13	FOLR2+ resident-like macrophages	TRIM65
5.18517661379917E-17	0.674310125654248	0.213	0.074	8.68931896940466E-13	FOLR2+ resident-like macrophages	GOLPH3L
5.2521811450642E-17	0.392379489510768	0.289	0.12	8.80160516289859E-13	FOLR2+ resident-like macrophages	ERLEC1
5.30818735468878E-17	1.10858171442231	0.258	0.102	8.89546036898746E-13	FOLR2+ resident-like macrophages	PSMB2
5.56075337453552E-17	0.82333486263965	0.221	0.077	9.31871050504662E-13	FOLR2+ resident-like macrophages	LMBRD2
5.57565098570863E-17	0.495890759071117	0.226	0.08	9.34367592185052E-13	FOLR2+ resident-like macrophages	SNRPB2
5.83951657945238E-17	0.63907745065905	0.311	0.138	9.78586188384629E-13	FOLR2+ resident-like macrophages	SEC11C
5.89904397251309E-17	1.59316040356354	0.154	0.039	9.88561788913744E-13	FOLR2+ resident-like macrophages	PLOD2
6.16045610515853E-17	1.03814334257545	0.141	0.031	1.03236923410247E-12	FOLR2+ resident-like macrophages	MYO5B
6.1627545297074E-17	0.440239451790606	0.376	0.176	1.03275440408837E-12	FOLR2+ resident-like macrophages	FKBP1A
6.27721964720643E-17	0.995404455600464	0.216	0.075	1.05193646847885E-12	FOLR2+ resident-like macrophages	AURKAIP1
6.64505908940575E-17	0.339117809450668	0.345	0.156	1.11357900220262E-12	FOLR2+ resident-like macrophages	SIVA1
6.85886860586112E-17	1.88869691111584	0.168	0.047	1.14940920097021E-12	FOLR2+ resident-like macrophages	RAMP1
6.99680569241334E-17	0.2655782409159	0.877	0.562	1.17252469793463E-12	FOLR2+ resident-like macrophages	CTSS
7.04319594284628E-17	1.14784176932365	0.226	0.082	1.18029877610218E-12	FOLR2+ resident-like macrophages	HAAO
7.15282286294119E-17	0.672926989499873	0.283	0.118	1.19867005537168E-12	FOLR2+ resident-like macrophages	SLC35E3
7.68568010093653E-17	0.691414837269824	0.348	0.161	1.28796627131494E-12	FOLR2+ resident-like macrophages	RNF5
8.34643201321852E-17	1.52792006022966	0.196	0.063	1.39869507677516E-12	FOLR2+ resident-like macrophages	DHCR24
8.77784676827084E-17	0.367977183604625	0.29	0.12	1.47099156142683E-12	FOLR2+ resident-like macrophages	HDHD5
8.96799703563948E-17	0.937904521497683	0.293	0.125	1.50285694323246E-12	FOLR2+ resident-like macrophages	MAP3K13
8.98112222896327E-17	0.470902774473561	0.193	0.061	1.50505646312966E-12	FOLR2+ resident-like macrophages	DEPRECATED-ENSG00000117691
9.20707833641287E-17	0.376749245437694	0.203	0.066	1.54292218761607E-12	FOLR2+ resident-like macrophages	UHRF1BP1
9.49605946196166E-17	0.403577955056527	0.222	0.078	1.59134964463554E-12	FOLR2+ resident-like macrophages	DERL2
9.88606908503455E-17	0.447965535241492	0.318	0.139	1.65670745727009E-12	FOLR2+ resident-like macrophages	SUPT16H
1.1104331537301E-16	0.350457587997797	0.309	0.137	1.86086387902091E-12	FOLR2+ resident-like macrophages	CDC16
1.14791781233279E-16	0.297147508326417	0.35	0.16	1.92368066990729E-12	FOLR2+ resident-like macrophages	MAP1S
1.1526710806264E-16	0.395299950276469	0.274	0.111	1.93164619691372E-12	FOLR2+ resident-like macrophages	SLC2A13
1.17225220046124E-16	0.694113140863198	0.187	0.057	1.96446023753295E-12	FOLR2+ resident-like macrophages	CCDC32
1.17999278507497E-16	1.07265303392435	0.273	0.113	1.97743190922864E-12	FOLR2+ resident-like macrophages	CEBPG
1.18252567442853E-16	0.856041901953739	0.196	0.064	1.98167652520733E-12	FOLR2+ resident-like macrophages	VEZF1
1.18405012784198E-16	0.644770061158574	0.269	0.109	1.98423120423758E-12	FOLR2+ resident-like macrophages	GALK2
1.18901639118121E-16	0.282568931236575	0.329	0.147	1.99255366834147E-12	FOLR2+ resident-like macrophages	VAC14
1.20893181172458E-16	0.902944238968946	0.248	0.099	2.02592793008805E-12	FOLR2+ resident-like macrophages	NDUFA5
1.28065502594464E-16	0.757098721309766	0.179	0.053	2.14612169247803E-12	FOLR2+ resident-like macrophages	GLCCI1
1.29581134851985E-16	0.504537227829054	0.148	0.036	2.17152065784956E-12	FOLR2+ resident-like macrophages	PTPRM
1.30283379607851E-16	0.36129824870871	0.257	0.102	2.18328887546837E-12	FOLR2+ resident-like macrophages	SNRK
1.4631935755149E-16	0.45642837426745	0.305	0.134	2.45201979384788E-12	FOLR2+ resident-like macrophages	MYO1C
1.47603669967234E-16	2.34216623944992	0.128	0.027	2.47354230131091E-12	FOLR2+ resident-like macrophages	DIO1
1.71033745841512E-16	1.53363080208047	0.171	0.05	2.86618351281206E-12	FOLR2+ resident-like macrophages	ZDHHC4
1.72152478977689E-16	0.611733784156657	0.232	0.086	2.88493124270811E-12	FOLR2+ resident-like macrophages	CCDC97
1.76632912110685E-16	0.300968556137907	0.231	0.083	2.96001434115085E-12	FOLR2+ resident-like macrophages	NICN1
1.87355866694948E-16	1.08613894517794	0.115	0.02	3.13970961407394E-12	FOLR2+ resident-like macrophages	P3H2
1.87364045693993E-16	0.301886238275205	0.361	0.171	3.13984667773993E-12	FOLR2+ resident-like macrophages	TOR1AIP1
1.96627503478142E-16	0.357805657881463	0.189	0.059	3.2950837032867E-12	FOLR2+ resident-like macrophages	MITD1
1.98836340953421E-16	0.544024837630447	0.25	0.096	3.33209940169744E-12	FOLR2+ resident-like macrophages	PDLIM2
2.00868618488164E-16	2.28580743772654	0.118	0.022	3.36615630862465E-12	FOLR2+ resident-like macrophages	SCAMP1
2.07229612739938E-16	0.253828661437974	0.235	0.086	3.47275385029588E-12	FOLR2+ resident-like macrophages	ZC3H7B
2.07399588539066E-16	0.298290164039396	0.293	0.125	3.47560230473766E-12	FOLR2+ resident-like macrophages	SMIM7
2.0896915062408E-16	0.545150493569097	0.218	0.078	3.50190502615833E-12	FOLR2+ resident-like macrophages	EAPP
2.19076951739352E-16	0.817549468703546	0.245	0.095	3.67129155724806E-12	FOLR2+ resident-like macrophages	SRM
2.20493139390679E-16	0.726584210519337	0.298	0.131	3.695024029909E-12	FOLR2+ resident-like macrophages	IMMP2L
2.20712762425635E-16	0.872480991298449	0.147	0.036	3.69870447272879E-12	FOLR2+ resident-like macrophages	RIPK3
2.21709372733582E-16	0.452078747482351	0.261	0.106	3.71540566826937E-12	FOLR2+ resident-like macrophages	CCDC28A
2.32250990711536E-16	0.78004050999027	0.257	0.102	3.89206210234391E-12	FOLR2+ resident-like macrophages	COMMD7
2.39668293413366E-16	0.35642578371689	0.173	0.05	4.01636126102118E-12	FOLR2+ resident-like macrophages	GNB5
2.41884370142096E-16	0.584054835768713	0.245	0.096	4.05349827484125E-12	FOLR2+ resident-like macrophages	RNF6
2.43668512208393E-16	0.267335914229254	0.148	0.036	4.08339692758825E-12	FOLR2+ resident-like macrophages	RAB42
2.44699660659227E-16	0.864938817525572	0.213	0.076	4.10067691332733E-12	FOLR2+ resident-like macrophages	NDRG3
2.53783260280647E-16	0.628330759795506	0.258	0.102	4.25289985578308E-12	FOLR2+ resident-like macrophages	SKP1
2.54134662294269E-16	0.616133601485008	0.239	0.092	4.25878867072737E-12	FOLR2+ resident-like macrophages	CUEDC2
2.58318092485952E-16	0.986959093662399	0.106	0.016	4.32889459387958E-12	FOLR2+ resident-like macrophages	PGBD5
2.64197343195225E-16	0.644277660149451	0.205	0.069	4.42741907726558E-12	FOLR2+ resident-like macrophages	TLR7
2.64676692660898E-16	0.58262987066192	0.244	0.094	4.43545201561133E-12	FOLR2+ resident-like macrophages	RNF20
2.68787936012251E-16	1.73202064199203	0.215	0.08	4.50434823169331E-12	FOLR2+ resident-like macrophages	ASGR2

**2.6912722254366E-16**	1.00837072892461	0.257	0.104	4.51003399538665E-12	FOLR2+ resident-like macrophages	EBP
**2.73234337564716E-16**	1.15354581674996	0.189	0.06	4.5788610289095E-12	FOLR2+ resident-like macrophages	DFFA
**2.76170182952091E-16**	0.596030542071466	0.155	0.041	4.62805992591114E-12	FOLR2+ resident-like macrophages	SLC48A1
**2.77207505921901E-16**	1.21237994139512	0.186	0.059	4.64544338423922E-12	FOLR2+ resident-like macrophages	AP1S1
**2.7883578352706E-16**	1.29042405999645	0.221	0.082	4.67273006034648E-12	FOLR2+ resident-like macrophages	COA6
**2.79649201054782E-16**	0.636208326013535	0.228	0.083	4.68636131127604E-12	FOLR2+ resident-like macrophages	UBL5
**2.8531373632935E-16**	0.36479964681467	0.306	0.135	4.78128759340725E-12	FOLR2+ resident-like macrophages	RRP36
**2.86495601263629E-16**	0.268628379005345	0.343	0.158	4.8010932859759E-12	FOLR2+ resident-like macrophages	DYNLT1
**3.02922609738888E-16**	0.566234870547366	0.244	0.093	5.07637709400429E-12	FOLR2+ resident-like macrophages	HSD17B12
**3.08126555705331E-16**	0.85266115513843	0.215	0.076	5.16358482050993E-12	FOLR2+ resident-like macrophages	SLC30A9
**3.10413518787432E-16**	2.62603581385463	0.1	0.014	5.20190974783978E-12	FOLR2+ resident-like macrophages	COBLL1
**3.10480145441115E-16**	0.908113533658897	0.194	0.065	5.20302627730221E-12	FOLR2+ resident-like macrophages	OCRL
**3.29485898032677E-16**	0.636855970602093	0.263	0.106	5.52152467923161E-12	FOLR2+ resident-like macrophages	POLR3GL
**3.32366230967081E-16**	0.94962610814881	0.284	0.123	5.56979329854634E-12	FOLR2+ resident-like macrophages	MARVELD1
**3.58354285130892E-16**	0.941586300634791	0.184	0.058	6.00530111022349E-12	FOLR2+ resident-like macrophages	ALG1
**3.63587976729847E-16**	0.462916942202254	0.193	0.063	6.09300731403877E-12	FOLR2+ resident-like macrophages	SUFU
**3.69158501736675E-16**	1.09071295859755	0.224	0.083	6.18635817210321E-12	FOLR2+ resident-like macrophages	COQ8A
**3.74382742503775E-16**	1.99480891334948	0.106	0.017	6.27390599887826E-12	FOLR2+ resident-like macrophages	SLAMF6
**3.78593736972386E-16**	0.325144813635781	0.276	0.117	6.34447384418325E-12	FOLR2+ resident-like macrophages	WDR41
**3.79355791068048E-16**	0.35784399891349	0.222	0.08	6.35724434671835E-12	FOLR2+ resident-like macrophages	SLC25A43
**3.81329036858445E-16**	0.952638321562877	0.257	0.103	6.39031199967382E-12	FOLR2+ resident-like macrophages	STOML2
**3.94717930207828E-16**	0.522101223576731	0.25	0.1	6.61468307442278E-12	FOLR2+ resident-like macrophages	PRKCA
**4.00950220176902E-16**	2.40717605800161	0.122	0.025	6.71912378972452E-12	FOLR2+ resident-like macrophages	ACACB
**4.13077700764416E-16**	0.30426359178344	0.253	0.098	6.92235610941008E-12	FOLR2+ resident-like macrophages	G6PC3
**4.19392550190057E-16**	0.395093456295901	0.226	0.084	7.02818035608497E-12	FOLR2+ resident-like macrophages	PIGO
**4.23291682034077E-16**	0.547804502873384	0.232	0.088	7.09352200752706E-12	FOLR2+ resident-like macrophages	PRDX5
**4.36336013820444E-16**	0.369874190503803	0.239	0.092	7.31211891960301E-12	FOLR2+ resident-like macrophages	CAPRIN1
**4.3937234956889E-16**	0.608831237204094	0.222	0.081	7.36300183407547E-12	FOLR2+ resident-like macrophages	OPTN
**4.41381882925123E-16**	1.1938191060924	0.189	0.062	7.39667759405921E-12	FOLR2+ resident-like macrophages	OXLD1
**4.43960572058605E-16**	1.33238831364777	0.224	0.083	7.4398912665581E-12	FOLR2+ resident-like macrophages	PPA2
**4.82877021155537E-16**	1.28405090390845	0.171	0.052	8.09205312052449E-12	FOLR2+ resident-like macrophages	NR2F6
**4.83749511618766E-16**	0.44017067650869	0.176	0.052	8.10667431570729E-12	FOLR2+ resident-like macrophages	PLB1
**4.86400288160289E-16**	1.16764399023315	0.238	0.094	8.15109602899013E-12	FOLR2+ resident-like macrophages	EPS15L1
**5.02450600825019E-16**	0.321113748172548	0.274	0.114	8.42006716862566E-12	FOLR2+ resident-like macrophages	SPCS2
**5.03808031771268E-16**	0.515065738268149	0.187	0.06	8.4428149964229E-12	FOLR2+ resident-like macrophages	TBC1D4
**5.13837508994675E-16**	1.67370408549454	0.184	0.061	8.61088897573276E-12	FOLR2+ resident-like macrophages	GBE1
**5.22931271212769E-16**	0.504165315528842	0.255	0.102	8.76328224298359E-12	FOLR2+ resident-like macrophages	DEPRECATED-ENSG00000168214
**5.40798276980142E-16**	0.322805508990509	0.29	0.125	9.06269752563321E-12	FOLR2+ resident-like macrophages	XIAP
**5.59120475816339E-16**	0.670770741910047	0.194	0.065	9.36974093373021E-12	FOLR2+ resident-like macrophages	ATP6V1D
**5.61691609258782E-16**	0.609993489017373	0.202	0.068	9.41282798795868E-12	FOLR2+ resident-like macrophages	TMOD2
**5.84931123172581E-16**	0.780279392973552	0.205	0.071	9.80227576212611E-12	FOLR2+ resident-like macrophages	ATPAF1
**5.90896675180607E-16**	0.595423617350721	0.261	0.107	9.90224648267661E-12	FOLR2+ resident-like macrophages	ZNF362
**6.16529378157578E-16**	0.382430618020763	0.34	0.157	1.03317993191647E-11	FOLR2+ resident-like macrophages	PDCD6
**6.35298848106027E-16**	0.846667461681751	0.193	0.065	1.06463380965608E-11	FOLR2+ resident-like macrophages	ASCC1
**6.39591159819874E-16**	0.566252031566054	0.235	0.092	1.07182686562614E-11	FOLR2+ resident-like macrophages	TMEM38B
**6.63845389884015E-16**	0.3209221266737	0.226	0.085	1.11247210436763E-11	FOLR2+ resident-like macrophages	STAC3
**6.76142347770516E-16**	1.19727651430107	0.142	0.035	1.13307934639383E-11	FOLR2+ resident-like macrophages	JMJD4
**6.96567561035323E-16**	1.49323406696065	0.132	0.03	1.16730791878299E-11	FOLR2+ resident-like macrophages	TMEM220
**7.03534115524246E-16**	0.894466650215777	0.151	0.04	1.17898247079553E-11	FOLR2+ resident-like macrophages	ABHD6
**7.05981831554126E-16**	1.94062940017916	0.131	0.029	1.18308433531184E-11	FOLR2+ resident-like macrophages	STAP2
**7.21327793730556E-16**	0.847141265512725	0.2	0.069	1.20880111673367E-11	FOLR2+ resident-like macrophages	SMIM20
**8.00046696733242E-16**	0.686795450634837	0.224	0.083	1.34071825438557E-11	FOLR2+ resident-like macrophages	TWSG1
**8.03828523203189E-16**	0.336956096844127	0.318	0.144	1.3470558391839E-11	FOLR2+ resident-like macrophages	CCNDBP1
**8.04444235653857E-16**	0.658645706372312	0.179	0.056	1.34808765010873E-11	FOLR2+ resident-like macrophages	LMAN2L
**8.24197581263403E-16**	0.344688024870756	0.315	0.14	1.38119030668121E-11	FOLR2+ resident-like macrophages	ATF7
**8.34347456926014E-16**	0.350982050249397	0.343	0.162	1.39819946831661E-11	FOLR2+ resident-like macrophages	SNX14
**8.36053707702879E-16**	0.352408314374899	0.237	0.09	1.40105880336848E-11	FOLR2+ resident-like macrophages	PRPS1
**8.52163661180859E-16**	0.771056367487825	0.209	0.076	1.42805586340688E-11	FOLR2+ resident-like macrophages	TMEM199
**8.66083610054719E-16**	0.318934187153042	0.327	0.152	1.4513829137297E-11	FOLR2+ resident-like macrophages	LIX1L
**8.70609969845713E-16**	1.82328860719847	0.154	0.043	1.45896818746745E-11	FOLR2+ resident-like macrophages	NAXD
**8.7174265539915E-16**	1.64922465326352	0.163	0.046	1.4608663419179E-11	FOLR2+ resident-like macrophages	PTGFRN
**9.34446470373468E-16**	1.19990430412803	0.186	0.061	1.56594539505186E-11	FOLR2+ resident-like macrophages	FAM136A
**9.58530769064126E-16**	0.340046212418292	0.269	0.111	1.60630586279766E-11	FOLR2+ resident-like macrophages	MAPRE1
**9.9568603406451E-16**	0.542768541162661	0.311	0.139	1.66857065588531E-11	FOLR2+ resident-like macrophages	GPI
**1.00636916622072E-15**	0.334582565277658	0.235	0.088	1.68647344875268E-11	FOLR2+ resident-like macrophages	TAF13
**1.03006328803203E-15**	1.37533604320327	0.152	0.042	1.72618005808408E-11	FOLR2+ resident-like macrophages	RPAP1
**1.03130913521055E-15**	1.84879364330701	0.183	0.061	1.72826784878583E-11	FOLR2+ resident-like macrophages	NOL9
**1.04015020102956E-15**	1.02737284827609	0.206	0.073	1.74308370688534E-11	FOLR2+ resident-like macrophages	NDUFA7
**1.06975399765792E-15**	1.2498877051943	0.113	0.021	1.79269374927514E-11	FOLR2+ resident-like macrophages	ATP13A2
**1.09834699784768E-15**	0.39893000889464	0.247	0.099	1.84060989899314E-11	FOLR2+ resident-like macrophages	TMEM144
**1.10135623993901E-15**	0.681040408630618	0.232	0.088	1.8456527868898E-11	FOLR2+ resident-like macrophages	KIAA0232

**1.11764032212485E-15**	0.736594036200379	0.273	0.117	1.87294165181682E-11	FOLR2+ resident-like macrophages	FIBP
**1.13037877925549E-15**	0.296724423440722	0.221	0.082	1.89428875827635E-11	FOLR2+ resident-like macrophages	TLR5
**1.13438523091602E-15**	0.383617378447188	0.238	0.092	1.90100276996906E-11	FOLR2+ resident-like macrophages	GPD1L
**1.21380381674107E-15**	0.435327882071154	0.335	0.153	2.03409243609468E-11	FOLR2+ resident-like macrophages	ZBTB16
**1.22738514835661E-15**	0.330070017325573	0.332	0.154	2.056852031616E-11	FOLR2+ resident-like macrophages	APRT
**1.2345587263318E-15**	0.754741664783325	0.197	0.067	2.06887351358683E-11	FOLR2+ resident-like macrophages	CSGALNACT1
**1.27260999017334E-15**	0.76923676678754	0.254	0.104	2.13263982153248E-11	FOLR2+ resident-like macrophages	ISOC2
**1.30688396530345E-15**	0.286856054936855	0.293	0.13	2.19007614905551E-11	FOLR2+ resident-like macrophages	DCTD
**1.31747697489586E-15**	0.826502735606999	0.274	0.12	2.20782791453048E-11	FOLR2+ resident-like macrophages	SCAMP3
**1.3188453930671E-15**	1.15437606395296	0.194	0.066	2.21012110970185E-11	FOLR2+ resident-like macrophages	GSKIP
**1.31898252848919E-15**	0.948666878100437	0.118	0.023	2.21035092124218E-11	FOLR2+ resident-like macrophages	DEPRECATED-ENSG00000275385
**1.32586423812436E-15**	1.02265203632449	0.145	0.037	2.2218832902488E-11	FOLR2+ resident-like macrophages	SEPTIN10
**1.35305330033476E-15**	1.02536130948035	0.247	0.1	2.26744672070099E-11	FOLR2+ resident-like macrophages	CLUH
**1.35386548252379E-15**	0.278611849985791	0.309	0.14	2.26880777561336E-11	FOLR2+ resident-like macrophages	CLIC4
**1.35547271880971E-15**	0.346843694068132	0.298	0.133	2.27150118218132E-11	FOLR2+ resident-like macrophages	CALM1
**1.38221613431528E-15**	1.14517327774856	0.179	0.057	2.31631779788554E-11	FOLR2+ resident-like macrophages	KLHDC10
**1.42556736229452E-15**	0.279169515415021	0.315	0.141	2.38896578573315E-11	FOLR2+ resident-like macrophages	USP14
**1.44642513426629E-15**	0.440649894038262	0.229	0.086	2.42391924000345E-11	FOLR2+ resident-like macrophages	ZNF518A
**1.46532019513523E-15**	0.834250461674847	0.295	0.131	2.45558358300762E-11	FOLR2+ resident-like macrophages	NDUFAB1
**1.48260892504141E-15**	0.448402191111131	0.347	0.169	2.4845560365844E-11	FOLR2+ resident-like macrophages	EIF2AK1
**1.53373025837619E-15**	0.655638009563522	0.228	0.085	2.57022516698682E-11	FOLR2+ resident-like macrophages	CAP1
**1.61463062830037E-15**	0.709646254948759	0.206	0.073	2.70579800690576E-11	FOLR2+ resident-like macrophages	ARL2BP
**1.64985187177823E-15**	0.887942379875854	0.218	0.083	2.76482176672596E-11	FOLR2+ resident-like macrophages	S100A13
**1.66547169489192E-15**	0.37893141967252	0.267	0.112	2.79099746629988E-11	FOLR2+ resident-like macrophages	ANKRD40
**1.74817363005401E-15**	1.56459863219779	0.138	0.034	2.92958936924451E-11	FOLR2+ resident-like macrophages	SLC6A8
**1.79535082672192E-15**	0.296582919225027	0.929	0.662	3.0086489154206E-11	FOLR2+ resident-like macrophages	MT-CO2
**1.8139547260554E-15**	0.454362632522453	0.228	0.086	3.03982532992364E-11	FOLR2+ resident-like macrophages	MORF4L1
**1.84951511844535E-15**	0.905590174030971	0.228	0.087	3.09941743549072E-11	FOLR2+ resident-like macrophages	SEC24D
**1.8691006024215E-15**	0.650746603337965	0.197	0.069	3.13223878953795E-11	FOLR2+ resident-like macrophages	BMI1
**1.91433864016069E-15**	0.373447488346066	0.353	0.168	3.20804869318128E-11	FOLR2+ resident-like macrophages	RASSF1
**1.93677691277944E-15**	0.65943237961738	0.26	0.108	3.24565075043578E-11	FOLR2+ resident-like macrophages	NUP160
**1.97952622342601E-15**	0.307953628004277	0.27	0.113	3.31729004521731E-11	FOLR2+ resident-like macrophages	COMMD4
**1.98651401307907E-15**	0.847834193695347	0.208	0.075	3.32900018311791E-11	FOLR2+ resident-like macrophages	TMEM250
**1.98733857671989E-15**	0.332104356842672	0.263	0.109	3.33038198686719E-11	FOLR2+ resident-like macrophages	MBTPS1
**1.99997291233006E-15**	0.257720178894068	0.267	0.11	3.35155460648272E-11	FOLR2+ resident-like macrophages	CARS2
**2.05436767410018E-15**	0.392177649884105	0.312	0.139	3.44270934825709E-11	FOLR2+ resident-like macrophages	RPA1
**2.1083782296007E-15**	0.482450313027508	0.235	0.091	3.53322023716485E-11	FOLR2+ resident-like macrophages	SDF2L1
**2.18904712231561E-15**	0.824734373557611	0.179	0.057	3.6684051675765E-11	FOLR2+ resident-like macrophages	MRNIP
**2.19296988341126E-15**	0.498013804801945	0.234	0.091	3.67497893062059E-11	FOLR2+ resident-like macrophages	ACBD6
**2.25092639012824E-15**	0.280655525748991	0.364	0.178	3.77210244457691E-11	FOLR2+ resident-like macrophages	HYOU1
**2.25336525685917E-15**	0.261914708637211	0.219	0.082	3.7761894974446E-11	FOLR2+ resident-like macrophages	PDPK1
**2.25818009178504E-15**	1.02052240990979	0.258	0.109	3.78425819781337E-11	FOLR2+ resident-like macrophages	NUCB2
**2.28267759132733E-15**	0.435844805459183	0.366	0.179	3.82531110754633E-11	FOLR2+ resident-like macrophages	LONP2
**2.36569316851652E-15**	2.38615092166464	0.106	0.019	3.96442861179998E-11	FOLR2+ resident-like macrophages	NAV2
**2.38538483412818E-15**	0.490970792527171	0.271	0.115	3.99742790503201E-11	FOLR2+ resident-like macrophages	MEAF6
**2.4738798093848E-15**	0.294444681851293	0.222	0.083	4.14572778456704E-11	FOLR2+ resident-like macrophages	SLC35A5
**2.50808135903212E-15**	0.933729232933533	0.17	0.052	4.20304274146602E-11	FOLR2+ resident-like macrophages	NDUFAF7
**2.66479942830679E-15**	0.612416639485706	0.244	0.097	4.46567088195651E-11	FOLR2+ resident-like macrophages	METTL26
**2.74204856750018E-15**	0.771326385198262	0.222	0.083	4.59512498941681E-11	FOLR2+ resident-like macrophages	BRAT1
**2.76083727959552E-15**	0.303204166284692	0.277	0.12	4.62661111314618E-11	FOLR2+ resident-like macrophages	UBQLN2
**2.78526614721156E-15**	0.703438206876148	0.263	0.11	4.66754900949713E-11	FOLR2+ resident-like macrophages	TMEM14C
**2.89854673592999E-15**	1.23110803744577	0.167	0.051	4.85738462007148E-11	FOLR2+ resident-like macrophages	COPRS
**2.93862701362929E-15**	0.36904868476444	0.239	0.094	4.92455114943996E-11	FOLR2+ resident-like macrophages	AHCY
**2.98593529868924E-15**	0.678470080616439	0.221	0.084	5.00383037354343E-11	FOLR2+ resident-like macrophages	ANKRD12
**3.01289167739906E-15**	1.36638519536946	0.158	0.046	5.04900387298534E-11	FOLR2+ resident-like macrophages	PROS1
**3.11027943848882E-15**	0.938005922185297	0.257	0.107	5.21220628301956E-11	FOLR2+ resident-like macrophages	GALK1
**3.11669850319976E-15**	0.669159121867534	0.293	0.133	5.22296335166216E-11	FOLR2+ resident-like macrophages	FADS1
**3.13448911810691E-15**	1.86594618766165	0.132	0.032	5.25277686412356E-11	FOLR2+ resident-like macrophages	KIF3B
**3.1399770160107E-15**	0.336350724810719	0.228	0.087	5.26197348343073E-11	FOLR2+ resident-like macrophages	PDCL
**3.15592534429194E-15**	1.28325139238356	0.199	0.071	5.28869969196443E-11	FOLR2+ resident-like macrophages	ACAT2
**3.17638582301068E-15**	0.450235225317312	0.228	0.087	5.32298736220129E-11	FOLR2+ resident-like macrophages	PLA2G2A
**3.2725749330015E-15**	0.304002213286173	0.212	0.077	5.48418107272391E-11	FOLR2+ resident-like macrophages	TCAIM
**3.378304515889E-15**	0.361034411377413	0.229	0.088	5.66136270772679E-11	FOLR2+ resident-like macrophages	CRELD1
**3.43065163174726E-15**	1.36061962648007	0.187	0.065	5.74908600448206E-11	FOLR2+ resident-like macrophages	PRAP1
**3.43490668144573E-15**	0.300311971145088	0.284	0.121	5.75621661676676E-11	FOLR2+ resident-like macrophages	GET3
**3.60683919685861E-15**	0.594178083702359	0.235	0.094	6.04434112609565E-11	FOLR2+ resident-like macrophages	SENP3
**3.70849407685323E-15**	0.515286142671004	0.26	0.108	6.21469437399064E-11	FOLR2+ resident-like macrophages	DCUN1D1
**3.76378019238583E-15**	0.962967912553653	0.235	0.094	6.30734284640018E-11	FOLR2+ resident-like macrophages	LAPTM4B
**3.76915529348385E-15**	0.55781909044488	0.222	0.083	6.31635044082024E-11	FOLR2+ resident-like macrophages	NCOA7
**3.89335928493951E-15**	1.47016643666341	0.186	0.064	6.52449148970162E-11	FOLR2+ resident-like macrophages	GCHFR
**3.93344554965188E-15**	0.425412113911359	0.248	0.1	6.59166805210662E-11	FOLR2+ resident-like macrophages	KHNYN

3.95335528791055E-15	0.48162546058006	0.367	0.177	6.62503279148051E-11	FOLR2+ resident-like macrophages	CHPT1
4.0827791494872E-15	0.37485999323060	0.261	0.109	6.84192129871065E-11	FOLR2+ resident-like macrophages	KDM3A
4.1189613912739E-15	1.19859639890691	0.104	0.018	6.90255549949679E-11	FOLR2+ resident-like macrophages	ARMC9
4.1454566807394E-15	0.31599064771187	0.164	0.049	6.94695630558308E-11	FOLR2+ resident-like macrophages	HFE
4.16981455808244E-15	0.48451573412983	0.267	0.115	6.98777523643455E-11	FOLR2+ resident-like macrophages	HLX
4.19337743872883E-15	0.67432817990065	0.283	0.127	7.02726191182177E-11	FOLR2+ resident-like macrophages	GPS1
4.27864477656132E-15	0.33200650173141	0.337	0.158	7.17015291656145E-11	FOLR2+ resident-like macrophages	NDUFB5
4.33474999172943E-15	0.75013397963647	0.17	0.052	7.26417403614018E-11	FOLR2+ resident-like macrophages	AK6
4.44657957527248E-15	0.25149134592779	0.142	0.037	7.45157805224163E-11	FOLR2+ resident-like macrophages	ENOX2
4.44700561736351E-15	0.59345557426639	0.216	0.081	7.45229201357777E-11	FOLR2+ resident-like macrophages	SEC61B
4.78392012391964E-15	0.28867981835480	0.224	0.085	8.01689334366454E-11	FOLR2+ resident-like macrophages	SEC24A
4.92143418798067E-15	0.41457835983978	0.27	0.116	8.247339412218E-11	FOLR2+ resident-like macrophages	PRPF31
4.98577393368473E-15	0.31310676814191	0.316	0.146	8.35515995806887E-11	FOLR2+ resident-like macrophages	APOA1
5.08834361947361E-15	0.60530669552213	0.247	0.102	8.52704623751388E-11	FOLR2+ resident-like macrophages	MED22
5.12059381523504E-15	0.77664819665264	0.199	0.07	8.58109111557088E-11	FOLR2+ resident-like macrophages	AGPAT5
5.57165451473878E-15	1.88878760849715	0.151	0.044	9.33697863579926E-11	FOLR2+ resident-like macrophages	OAF
5.71197608474127E-15	0.36230989985535	0.235	0.092	9.57212952280943E-11	FOLR2+ resident-like macrophages	SLC9A6
5.73651270093553E-15	0.86942899771278	0.202	0.074	9.61324798422776E-11	FOLR2+ resident-like macrophages	COQ9
5.88698433708031E-15	0.46238765743279	0.213	0.08	9.86540835207919E-11	FOLR2+ resident-like macrophages	ARFRP1
6.06185285857022E-15	1.45677945863374	0.158	0.047	1.0158453020392E-10	FOLR2+ resident-like macrophages	FMO3
6.20948214038241E-15	0.39235816958232	0.248	0.102	1.04058501708528E-10	FOLR2+ resident-like macrophages	KLF16
6.22455623502669E-15	0.72089292707273	0.122	0.027	1.04311113386577E-10	FOLR2+ resident-like macrophages	SH3D21
6.24159184235365E-15	0.46690553295866	0.235	0.094	1.04596596094162E-10	FOLR2+ resident-like macrophages	ERH
6.334697412414E-15	0.71822378162128	0.247	0.102	1.06156859237234E-10	FOLR2+ resident-like macrophages	C1GALT1C1
6.4078641807151E-15	1.82328267751885	0.136	0.035	1.07382987940424E-10	FOLR2+ resident-like macrophages	SSX2IP
6.45434864158754E-15	0.38783759712122	0.279	0.123	1.08161974535724E-10	FOLR2+ resident-like macrophages	MON1B
6.52524665456884E-15	0.39686962448933	0.239	0.096	1.09350083437265E-10	FOLR2+ resident-like macrophages	ABL1
6.69767674318056E-15	0.46622993801409	0.189	0.065	1.1223966686222E-10	FOLR2+ resident-like macrophages	RILP
6.71841801221592E-15	0.61604476614269	0.164	0.048	1.12587249048714E-10	FOLR2+ resident-like macrophages	PLEKHG5
6.9314776260356E-15	1.32580140020191	0.228	0.091	1.16157702057105E-10	FOLR2+ resident-like macrophages	KHK
7.6955074073039E-15	0.38602191369141	0.2	0.071	1.28961313131599E-10	FOLR2+ resident-like macrophages	SLC52A2
8.21417806850197E-15	0.53467164716172	0.231	0.091	1.37653196071956E-10	FOLR2+ resident-like macrophages	AQR
8.3167604558871E-15	0.42116985180054	0.128	0.029	1.39372271719756E-10	FOLR2+ resident-like macrophages	HS3ST2
8.39765453459203E-15	1.08194075653231	0.194	0.069	1.40727894690693E-10	FOLR2+ resident-like macrophages	SLC17A9
8.40841903980876E-15	1.35559535301018	0.176	0.059	1.40908286269115E-10	FOLR2+ resident-like macrophages	GOT1
8.60887173310775E-15	0.63973548336603	0.241	0.097	1.4426747250342E-10	FOLR2+ resident-like macrophages	COA4
8.63517319291157E-15	1.06207913694371	0.184	0.063	1.44708232366812E-10	FOLR2+ resident-like macrophages	L3HYPDH
8.79676865830547E-15	1.13829193683112	0.241	0.1	1.47416249175883E-10	FOLR2+ resident-like macrophages	ZNHIT1
8.84418972809677E-15	0.47339184648757	0.247	0.101	1.48210931463446E-10	FOLR2+ resident-like macrophages	NUDCD2
9.36246387328744E-15	0.36867872632178	0.251	0.104	1.56896169588551E-10	FOLR2+ resident-like macrophages	BMPR2
9.42657237846108E-15	0.70453490388453	0.226	0.088	1.57970499918251E-10	FOLR2+ resident-like macrophages	UBR7
9.59925800339588E-15	1.31268755929991	0.177	0.059	1.60864365620908E-10	FOLR2+ resident-like macrophages	COX7B
1.01140991379551E-14	0.53198757861527	0.269	0.119	1.69492073353851E-10	FOLR2+ resident-like macrophages	FLVCR2
1.04261586663975E-14	0.32300977566100	0.205	0.075	1.74721566931489E-10	FOLR2+ resident-like macrophages	NEK3
1.05731314754962E-14	1.50793438628378	0.197	0.071	1.77184537266365E-10	FOLR2+ resident-like macrophages	ERG28
1.06216745851963E-14	1.32552526235695	0.131	0.032	1.7799802269872E-10	FOLR2+ resident-like macrophages	BPHL
1.0653867498626E-14	0.2765303973017	0.167	0.051	1.78537511541974E-10	FOLR2+ resident-like macrophages	CEP104
1.06993633494079E-14	0.62888518835034	0.202	0.075	1.79299931009378E-10	FOLR2+ resident-like macrophages	ABHD15
1.0711731710009E-14	0.68087011451995	0.155	0.046	1.79507199996331E-10	FOLR2+ resident-like macrophages	FAM131A
1.09150458088006E-14	1.93542934971602	0.106	0.02	1.82914337663881E-10	FOLR2+ resident-like macrophages	PCDH12
1.09769655781087E-14	2.64463202285439	0.103	0.019	1.83951989157945E-10	FOLR2+ resident-like macrophages	SEC16B
1.1154511529869E-14	0.49822489350532	0.267	0.115	1.86927304217544E-10	FOLR2+ resident-like macrophages	RCC1L
1.12659924411486E-14	0.67190329336203	0.254	0.106	1.88795501328769E-10	FOLR2+ resident-like macrophages	ERLIN1
1.12725320534677E-14	0.31405842503670	0.21	0.078	1.88905092152012E-10	FOLR2+ resident-like macrophages	PBXIP1
1.13386872993919E-14	1.37006468465261	0.132	0.033	1.9001372176321E-10	FOLR2+ resident-like macrophages	CASP6
1.14336003381959E-14	1.04280101520919	0.202	0.074	1.91604274467486E-10	FOLR2+ resident-like macrophages	EBNA1BP2
1.19619590352668E-14	0.73104162359478	0.161	0.048	2.00458509513001E-10	FOLR2+ resident-like macrophages	CALML4
1.19838894418357E-14	1.3803110080607	0.135	0.035	2.00826019266283E-10	FOLR2+ resident-like macrophages	PAFAH2
1.20938615349588E-14	0.59444750847092	0.25	0.106	2.0266893160284E-10	FOLR2+ resident-like macrophages	ANO6
1.24261796782713E-14	0.56856608775473	0.179	0.059	2.08237919048471E-10	FOLR2+ resident-like macrophages	KIAA1191
1.26353401678736E-14	0.88507393759449	0.176	0.058	2.11743030533225E-10	FOLR2+ resident-like macrophages	NPRL3
1.27094451137395E-14	0.39093952845852	0.255	0.105	2.12984881216047E-10	FOLR2+ resident-like macrophages	YWHAQ
1.31318102116638E-14	0.28648251681872	0.21	0.078	2.20062875527062E-10	FOLR2+ resident-like macrophages	ZFYVE26
1.3209929602132E-14	0.2951312812353	0.97	0.81	2.21372000272527E-10	FOLR2+ resident-like macrophages	MS4A6A
1.32409397435926E-14	0.27491754197613	0.298	0.134	2.21891668223125E-10	FOLR2+ resident-like macrophages	MYO1D
1.35273575142835E-14	0.46907611590085	0.309	0.14	2.26691457224364E-10	FOLR2+ resident-like macrophages	MIA2
1.41107754324876E-14	1.58351175830131	0.131	0.033	2.36468374697628E-10	FOLR2+ resident-like macrophages	LLGL2
1.41431840206654E-14	0.79682272976642	0.113	0.024	2.3701147781831E-10	FOLR2+ resident-like macrophages	APBA1
1.45671219523475E-14	1.94955121928469	0.145	0.042	2.4411582967744E-10	FOLR2+ resident-like macrophages	CFI
1.55684536529388E-14	0.61928152416838	0.226	0.09	2.60896146315948E-10	FOLR2+ resident-like macrophages	PHF23
1.63258926577982E-14	0.44344334089828	0.309	0.143	2.73589309159382E-10	FOLR2+ resident-like macrophages	VDAC3

1.65041142052023E-14	0.66506858239694	0.261	0.113	2.7657594585078E-10	FOLR2+ resident-like macrophages	ZNF664
1.65151823538212E-14	0.384527384616563	0.226	0.089	2.76761425885336E-10	FOLR2+ resident-like macrophages	MTIF2
1.74112059029651E-14	1.51357819424878	0.215	0.085	2.91776988521889E-10	FOLR2+ resident-like macrophages	CES2
1.81277929579537E-14	2.01041223577734	0.103	0.019	3.03785554389388E-10	FOLR2+ resident-like macrophages	MT1X
1.90703097933358E-14	0.422342271653843	0.247	0.102	3.19580251516722E-10	FOLR2+ resident-like macrophages	DPP8
1.91730081554296E-14	0.382753829024828	0.245	0.102	3.21301270668689E-10	FOLR2+ resident-like macrophages	SPTLC1
1.9189691513019E-14	0.583170022939126	0.197	0.072	3.21580850375173E-10	FOLR2+ resident-like macrophages	MMGT1
1.98187381703704E-14	1.0288341227632	0.209	0.08	3.32122414259067E-10	FOLR2+ resident-like macrophages	FAF2
2.01650673595043E-14	0.351418249205453	0.239	0.098	3.37926198810574E-10	FOLR2+ resident-like macrophages	XPC
2.12130310785317E-14	0.383386790267137	0.206	0.076	3.55487974814034E-10	FOLR2+ resident-like macrophages	NCK2
2.18230719605413E-14	0.558021050932647	0.212	0.082	3.65711039914751E-10	FOLR2+ resident-like macrophages	DBR1
2.21803561587343E-14	0.451693495075795	0.258	0.108	3.7169840850807E-10	FOLR2+ resident-like macrophages	TEX2
2.32015273537247E-14	0.864927534658025	0.16	0.049	3.88811195393718E-10	FOLR2+ resident-like macrophages	GNA11
2.34307680919258E-14	0.510459896949467	0.234	0.096	3.92652811684493E-10	FOLR2+ resident-like macrophages	NDUFA11
2.35327152496901E-14	0.591464300268323	0.173	0.057	3.94361242154306E-10	FOLR2+ resident-like macrophages	MIGA1
2.40162812872377E-14	0.25443196427327	0.261	0.112	4.02464841811529E-10	FOLR2+ resident-like macrophages	CDC34
2.52253863193044E-14	1.29700303352565	0.197	0.073	4.22727023938903E-10	FOLR2+ resident-like macrophages	PCYT2
2.60546135641094E-14	0.517840329858798	0.284	0.132	4.36623214107345E-10	FOLR2+ resident-like macrophages	NME4
2.64806839384189E-14	1.41015957164227	0.174	0.059	4.43763301440025E-10	FOLR2+ resident-like macrophages	TRIQK
2.65921856842716E-14	0.618408224455859	0.157	0.047	4.45631847697023E-10	FOLR2+ resident-like macrophages	TBCEL
2.69843624574824E-14	0.609634388362605	0.122	0.028	4.52203946062491E-10	FOLR2+ resident-like macrophages	LGALSL
2.70230398410174E-14	0.321733986361363	0.241	0.1	4.5285210165577E-10	FOLR2+ resident-like macrophages	RSF1
2.79081475678313E-14	0.476968223327616	0.245	0.102	4.67684736941717E-10	FOLR2+ resident-like macrophages	NMD3
2.84486628457337E-14	1.0530910067552	0.155	0.047	4.76742691968805E-10	FOLR2+ resident-like macrophages	DEPRECATED-ENSG00000150337
2.89419144991976E-14	0.664575167634998	0.221	0.089	4.85008603177554E-10	FOLR2+ resident-like macrophages	SNAPIN
3.02819721609513E-14	0.388617297185182	0.184	0.064	5.07465289473222E-10	FOLR2+ resident-like macrophages	LYPLA1
3.33056204335214E-14	1.06311251647952	0.213	0.084	5.58135587224951E-10	FOLR2+ resident-like macrophages	KNG1
3.35969049962522E-14	0.439716250956032	0.184	0.064	5.63016933927194E-10	FOLR2+ resident-like macrophages	EOGT
3.36409637713394E-14	0.888711445386132	0.161	0.051	5.63755270880105E-10	FOLR2+ resident-like macrophages	ZNF581
3.41407907004714E-14	0.69945147609231	0.163	0.052	5.72131370558501E-10	FOLR2+ resident-like macrophages	JOSD2
3.48008782330293E-14	0.250325762443835	0.245	0.102	5.83193117429104E-10	FOLR2+ resident-like macrophages	TMEM65
3.4904101917605E-14	0.27531990792917	0.279	0.125	5.84922939935224E-10	FOLR2+ resident-like macrophages	SEC63
3.5120058146431E-14	0.845971513519341	0.213	0.083	5.88541934417891E-10	FOLR2+ resident-like macrophages	FN3KRP
3.54853090378547E-14	0.52561341324107	0.244	0.102	5.9466280885637E-10	FOLR2+ resident-like macrophages	SPRYD3
3.55787060389711E-14	1.3156398443316	0.139	0.039	5.96227955801077E-10	FOLR2+ resident-like macrophages	ZCCHC14
3.61503095164552E-14	0.282755940744173	0.18	0.062	6.05806886876755E-10	FOLR2+ resident-like macrophages	SPRY2
3.63493934688206E-14	1.66450015654241	0.113	0.025	6.09143135750495E-10	FOLR2+ resident-like macrophages	A1CF
3.72004711502432E-14	0.61808183852561	0.181	0.064	6.23405495535776E-10	FOLR2+ resident-like macrophages	ABHD11
3.73594124915354E-14	1.55164992925739	0.167	0.055	6.26069034533151E-10	FOLR2+ resident-like macrophages	ECHDC3
3.81685839801346E-14	0.698651956367409	0.253	0.109	6.39629130339096E-10	FOLR2+ resident-like macrophages	LNPK
3.86127607064992E-14	0.851693999330885	0.148	0.044	6.47072643919513E-10	FOLR2+ resident-like macrophages	AGA
3.99376337642476E-14	0.326699668678852	0.255	0.111	6.69274866621262E-10	FOLR2+ resident-like macrophages	EIF2S3
4.13487644532684E-14	0.453516601808233	0.287	0.133	6.92922594707873E-10	FOLR2+ resident-like macrophages	PYURF
4.47276640556523E-14	0.595405106640827	0.202	0.075	7.49546194244621E-10	FOLR2+ resident-like macrophages	NKAP
4.64595208750341E-14	0.484021159082156	0.135	0.036	7.78568650823822E-10	FOLR2+ resident-like macrophages	C3orf62
4.646081294951E-14	0.690243517281875	0.142	0.04	7.78590303407888E-10	FOLR2+ resident-like macrophages	SFXN4
4.78295921252836E-14	0.432816727402329	0.219	0.085	8.01528304835503E-10	FOLR2+ resident-like macrophages	TFPT
4.79300401043671E-14	0.357655216480163	0.255	0.109	8.03211612068984E-10	FOLR2+ resident-like macrophages	PRCC
4.87008198305268E-14	1.47492243532274	0.151	0.046	8.16128338719968E-10	FOLR2+ resident-like macrophages	MMUT
5.1286074380001E-14	1.26265296165936	0.192	0.07	8.59452034460057E-10	FOLR2+ resident-like macrophages	CBR4
5.13421136029387E-14	1.79466200556087	0.11	0.024	8.60391139758047E-10	FOLR2+ resident-like macrophages	CCDC106
5.14248890239147E-14	0.559225793408808	0.21	0.082	8.61778290262763E-10	FOLR2+ resident-like macrophages	FASTK
5.35657954301419E-14	0.922157429463785	0.134	0.036	8.97655599818317E-10	FOLR2+ resident-like macrophages	FBXO8
5.37933253138425E-14	0.472594898619643	0.115	0.026	9.01468545609373E-10	FOLR2+ resident-like macrophages	BMPR1A
5.60238921983335E-14	1.24180913855663	0.151	0.046	9.38848385459672E-10	FOLR2+ resident-like macrophages	MFSD5
5.70908440412744E-14	0.747437186317264	0.231	0.096	9.56728364443676E-10	FOLR2+ resident-like macrophages	PFDN6
6.23576539384729E-14	1.7939097023762	0.161	0.053	1.04498956470093E-09	FOLR2+ resident-like macrophages	FGGY
6.2874700209204E-14	0.348098063688502	0.184	0.065	1.05365422610584E-09	FOLR2+ resident-like macrophages	TNFRSF11A
6.64707216003919E-14	0.413326510628016	0.192	0.07	1.11391635257937E-09	FOLR2+ resident-like macrophages	DAPK3
6.67115747526883E-14	0.51302952541274	0.253	0.11	1.11795256970555E-09	FOLR2+ resident-like macrophages	ERCC6L2
6.70457718711179E-14	0.459171796942873	0.215	0.083	1.12355304501619E-09	FOLR2+ resident-like macrophages	GLE1
6.75463170851244E-14	0.590385727130079	0.193	0.071	1.13194118171251E-09	FOLR2+ resident-like macrophages	SPTY2D1
6.80540631878179E-14	0.460360530104143	0.212	0.083	1.14044999090145E-09	FOLR2+ resident-like macrophages	SEPTIN8
6.80941978115151E-14	1.51724363686968	0.183	0.066	1.14112256692537E-09	FOLR2+ resident-like macrophages	SMAD5
6.8662281734658E-14	0.328819636871096	0.165	0.054	1.1506425173094E-09	FOLR2+ resident-like macrophages	POLI
7.21047725264198E-14	0.428926276163168	0.141	0.04	1.20833177799774E-09	FOLR2+ resident-like macrophages	KIFAP3
7.22149231873167E-14	0.575796586487466	0.174	0.059	1.21017768277305E-09	FOLR2+ resident-like macrophages	GET1
7.25577229327951E-14	0.581032231715926	0.197	0.073	1.21592232090778E-09	FOLR2+ resident-like macrophages	SELENOT
7.2791276784177E-14	1.62743509337285	0.145	0.043	1.21983621634924E-09	FOLR2+ resident-like macrophages	ACY1
7.37950332103917E-14	0.308215094285879	0.218	0.086	1.23665716653974E-09	FOLR2+ resident-like macrophages	PSMB6
7.45551482550754E-14	0.263132881456794	0.251	0.106	1.24939517445855E-09	FOLR2+ resident-like macrophages	PLD2

7.57765118151944E-14	1.0548273649701	0.104	0.021	1.26986278499903E-09	FOLR2+ resident-like macrophages	THRA
7.58559904363324E-14	0.43551483782743	0.221	0.09	1.27119468773206E-09	FOLR2+ resident-like macrophages	JMJD8
7.76963029394733E-14	2.263196499255	0.116	0.028	1.30203464465969E-09	FOLR2+ resident-like macrophages	GJB1
7.82226791788224E-14	0.33418917638964	0.283	0.13	1.31085565767871E-09	FOLR2+ resident-like macrophages	IVD
8.25038730428108E-14	0.42945360486421	0.254	0.111	1.38259990445142E-09	FOLR2+ resident-like macrophages	BABAM1
8.69407329344359E-14	2.07008987992264	0.142	0.043	1.45695280251528E-09	FOLR2+ resident-like macrophages	ASB9
8.83134650876304E-14	0.55168256265000	0.155	0.047	1.47995704793851E-09	FOLR2+ resident-like macrophages	NKAPD1
8.96841630195277E-14	0.83238397623808	0.141	0.041	1.50292720388125E-09	FOLR2+ resident-like macrophages	FOXP4
9.01860845509456E-14	2.18669432237696	0.11	0.025	1.51133840490475E-09	FOLR2+ resident-like macrophages	MAL2
9.04194118370815E-14	0.86748250296311	0.165	0.054	1.51524850356581E-09	FOLR2+ resident-like macrophages	IL17RC
9.13791408111508E-14	0.79415445444941	0.205	0.08	1.53133164171326E-09	FOLR2+ resident-like macrophages	ELMOD2
9.14361363202726E-14	0.51432715812545	0.255	0.112	1.53228677245513E-09	FOLR2+ resident-like macrophages	GANAB
9.15448431415012E-14	0.43629319581187	0.284	0.132	1.53410848136528E-09	FOLR2+ resident-like macrophages	STMP1
9.48337676587757E-14	1.68062363984636	0.174	0.063	1.58922427842576E-09	FOLR2+ resident-like macrophages	AGXT
9.49804939521335E-14	0.34069670323791	0.232	0.099	1.59168311764985E-09	FOLR2+ resident-like macrophages	TINF2
9.543722543867E-14	0.41154470256202	0.21	0.083	1.59933702390123E-09	FOLR2+ resident-like macrophages	NCKIPSD
9.77110413461607E-14	0.35315822030169	0.284	0.13	1.63744163087896E-09	FOLR2+ resident-like macrophages	MAN2B2
9.91405182598054E-14	0.85423518196324	0.21	0.084	1.66139680499782E-09	FOLR2+ resident-like macrophages	RFC1
9.92914123042219E-14	0.36630972578433	0.222	0.089	1.66392548739415E-09	FOLR2+ resident-like macrophages	SCAND1
1.04944809560697E-13	1.01276888541712	0.16	0.051	1.75866511861815E-09	FOLR2+ resident-like macrophages	BSCL2
1.07666031395673E-13	0.39673857060933	0.273	0.121	1.80426735412868E-09	FOLR2+ resident-like macrophages	SMC3
1.08776593867048E-13	0.25280442595716	0.277	0.127	1.82287816002399E-09	FOLR2+ resident-like macrophages	INTS1
1.10064850842098E-13	1.13042778489127	0.248	0.11	1.84446677041188E-09	FOLR2+ resident-like macrophages	CDH17
1.12687931723964E-13	0.74399958560268	0.197	0.074	1.88842435983018E-09	FOLR2+ resident-like macrophages	GLRX5
1.13122776734441E-13	1.06489008985815	0.122	0.03	1.89571149251577E-09	FOLR2+ resident-like macrophages	NEIL2
1.16939848636585E-13	0.41875124045922	0.311	0.149	1.9596779834368E-09	FOLR2+ resident-like macrophages	PGP
1.18015091765139E-13	0.30851452681753	0.196	0.073	1.97769690780021E-09	FOLR2+ resident-like macrophages	MLST8
1.19887441649129E-13	1.00651562831386	0.17	0.058	2.0090737471561E-09	FOLR2+ resident-like macrophages	PTS
1.22266389228365E-13	1.17311948662357	0.176	0.062	2.04894015068894E-09	FOLR2+ resident-like macrophages	CASK
1.22477397915235E-13	1.10238137591526	0.12	0.029	2.05247623426351E-09	FOLR2+ resident-like macrophages	CAV2
1.23852659328266E-13	0.26043203584648	0.25	0.11	2.07552286502308E-09	FOLR2+ resident-like macrophages	NUDT9
1.28461826063905E-13	1.43075805387103	0.123	0.031	2.15276328117892E-09	FOLR2+ resident-like macrophages	SLC25A4
1.29323359805362E-13	0.37931382391048	0.206	0.081	2.16720086361825E-09	FOLR2+ resident-like macrophages	ABCB8
1.31009521651442E-13	0.68988252843545	0.234	0.1	2.19545756383487E-09	FOLR2+ resident-like macrophages	MALSU1
1.31158574022518E-13	0.74348313548225	0.226	0.094	2.19795538346935E-09	FOLR2+ resident-like macrophages	VIRMA
1.31302021130502E-13	1.68444617840163	0.125	0.032	2.20035927010495E-09	FOLR2+ resident-like macrophages	NIPA1
1.32173366155576E-13	0.91331854280834	0.142	0.042	2.21496127003515E-09	FOLR2+ resident-like macrophages	TMEM255B
1.34865919924846E-13	0.42329561363440	0.231	0.097	2.26008308610058E-09	FOLR2+ resident-like macrophages	AARS2
1.3660135123192E-13	1.01077898617814	0.187	0.069	2.28916544394451E-09	FOLR2+ resident-like macrophages	DEPRECATED-ENSG00000105701
1.42348059764056E-13	1.52521444330251	0.144	0.043	2.38546878552604E-09	FOLR2+ resident-like macrophages	DHCR7
1.4403419671223E-13	1.08351033215532	0.183	0.066	2.41372506850355E-09	FOLR2+ resident-like macrophages	DTD1
1.46049282917478E-13	0.55328223398508	0.16	0.051	2.4474938831311E-09	FOLR2+ resident-like macrophages	LAMTOR5
1.46055022437158E-13	0.29659357175962	0.202	0.077	2.44759066600189E-09	FOLR2+ resident-like macrophages	CC2D1B
1.46444104872873E-13	0.78378482031953	0.209	0.083	2.4541103094596E-09	FOLR2+ resident-like macrophages	WDR44
1.49923810716951E-13	1.13421727993716	0.174	0.062	2.51242321999466E-09	FOLR2+ resident-like macrophages	TOR1B
1.50632286798602E-13	0.59579451053982	0.171	0.059	2.52429586217097E-09	FOLR2+ resident-like macrophages	PHAX
1.52884922102636E-13	1.05028100283507	0.181	0.065	2.56204552459597E-09	FOLR2+ resident-like macrophages	LDAH
1.54303352159721E-13	1.13919446144951	0.187	0.069	2.58581557549261E-09	FOLR2+ resident-like macrophages	SLC25A30
1.57827932433584E-13	0.64239332386823	0.238	0.104	2.644880491722E-09	FOLR2+ resident-like macrophages	DUSP6
1.6204592915969E-13	1.03207295536818	0.193	0.073	2.71556568085808E-09	FOLR2+ resident-like macrophages	GOLM1
1.6273056216047E-13	0.97663178725137	0.18	0.065	2.72703876068515E-09	FOLR2+ resident-like macrophages	ZMAT5
1.6466838366431E-13	0.76336094175353	0.215	0.087	2.75951277344651E-09	FOLR2+ resident-like macrophages	TRMT1L
1.71623876828297E-13	0.76152704758173	0.148	0.046	2.8760729278886E-09	FOLR2+ resident-like macrophages	RNF214
1.71659040525112E-13	0.77490300362111	0.225	0.093	2.87666220111983E-09	FOLR2+ resident-like macrophages	TOR1A
1.74185680893735E-13	0.75041239461882	0.173	0.059	2.91900364041722E-09	FOLR2+ resident-like macrophages	MAGEH1
1.74398552660358E-13	0.34904442864055	0.235	0.1	2.92257094548228E-09	FOLR2+ resident-like macrophages	NDUFA8
1.82435157749408E-13	2.26921736247902	0.103	0.022	3.05724837356458E-09	FOLR2+ resident-like macrophages	ELOVL2
1.8386466170674E-13	0.43876739835427	0.193	0.071	3.08120400088154E-09	FOLR2+ resident-like macrophages	TRMT2B
1.84194771183949E-13	0.57783891637611	0.208	0.082	3.08673597550061E-09	FOLR2+ resident-like macrophages	HGH1
1.88289882320843E-13	0.57244045099559	0.282	0.131	3.15536184793268E-09	FOLR2+ resident-like macrophages	COX14
1.91012196131711E-13	0.31103213028051	0.229	0.096	3.20098238277521E-09	FOLR2+ resident-like macrophages	PHF14
1.93311626833545E-13	0.86448337096950	0.212	0.085	3.23951624247654E-09	FOLR2+ resident-like macrophages	SLC25A46
1.93706682880993E-13	1.12744545157078	0.176	0.063	3.24613659171967E-09	FOLR2+ resident-like macrophages	NUBP2
1.98037418815764E-13	0.8807340016122	0.113	0.027	3.31871106451457E-09	FOLR2+ resident-like macrophages	LAG3
2.01780020093394E-13	0.86141292021786	0.148	0.046	3.3814295767251E-09	FOLR2+ resident-like macrophages	TOMM40L
2.0614449683781E-13	0.88497809623643	0.136	0.039	3.45456947800803E-09	FOLR2+ resident-like macrophages	CDK18
2.07199370473352E-13	1.37672953583684	0.129	0.035	3.47224705039243E-09	FOLR2+ resident-like macrophages	TP53I3
2.09031097244075E-13	1.31995671982227	0.194	0.076	3.50294312761621E-09	FOLR2+ resident-like macrophages	TKFC
2.09722236398221E-13	0.50462045118687	0.235	0.102	3.51452523756139E-09	FOLR2+ resident-like macrophages	CHMP1A
2.16863547452375E-13	1.31907417248498	0.106	0.023	3.6341993282069E-09	FOLR2+ resident-like macrophages	ORAI3
2.18542597241002E-13	0.31159059119062	0.906	0.631	3.66233684456471E-09	FOLR2+ resident-like macrophages	MT-ND4

2.21595698847895E-13	1.57603316861944	0.138	0.04	3.71350072129303E-09	FOLR2+ resident-like macrophages	DHRS1
2.25383537337147E-13	0.76293865296699	0.18	0.065	3.7769773186959E-09	FOLR2+ resident-like macrophages	COX11
2.33293238343943E-13	1.35504676119404	0.123	0.032	3.9095280881678E-09	FOLR2+ resident-like macrophages	LGR4
2.34525371866935E-13	0.396751833974964	0.218	0.087	3.93017618174609E-09	FOLR2+ resident-like macrophages	INSIG2
2.39578494573839E-13	0.660610820790596	0.161	0.053	4.01485641206839E-09	FOLR2+ resident-like macrophages	MLH3
2.48832806820007E-13	0.316857900685865	0.181	0.065	4.16994017668967E-09	FOLR2+ resident-like macrophages	SLC4A1AP
2.56539413900992E-13	0.490286583702626	0.193	0.074	4.29908749815282E-09	FOLR2+ resident-like macrophages	DDX60
2.57499967455493E-13	0.760331595626565	0.215	0.087	4.31518445461915E-09	FOLR2+ resident-like macrophages	CHAMP1
2.59886345311103E-13	0.386888928876557	0.147	0.045	4.35517537472346E-09	FOLR2+ resident-like macrophages	YIPF6
2.60007001197392E-13	0.511778866817742	0.279	0.13	4.3571973260659E-09	FOLR2+ resident-like macrophages	NMI
2.77864622836243E-13	0.382994914021208	0.271	0.122	4.65645534948976E-09	FOLR2+ resident-like macrophages	REEP3
2.7942494018879E-13	1.07835804133236	0.129	0.035	4.68260314768374E-09	FOLR2+ resident-like macrophages	MATK
2.8019792625041E-13	1.12323163902548	0.171	0.061	4.69555684810438E-09	FOLR2+ resident-like macrophages	PECR
2.83972042406871E-13	0.304870931938108	0.203	0.079	4.75880348665434E-09	FOLR2+ resident-like macrophages	OST4
2.85418138514601E-13	0.953706592005231	0.163	0.054	4.78303716522768E-09	FOLR2+ resident-like macrophages	MUL1
2.89468538856241E-13	1.0266615214184	0.145	0.045	4.85091377415289E-09	FOLR2+ resident-like macrophages	CYSLTR1
2.92325222256789E-13	0.634638746076113	0.158	0.052	4.89878607457927E-09	FOLR2+ resident-like macrophages	MAML2
2.98379242348152E-13	1.00233281206421	0.151	0.047	5.00023934327034E-09	FOLR2+ resident-like macrophages	TBC1D13
2.99436643556766E-13	0.356661999268997	0.254	0.114	5.01795927272429E-09	FOLR2+ resident-like macrophages	FRAT2
3.24453113778333E-13	0.379812508542229	0.134	0.037	5.4371852806973E-09	FOLR2+ resident-like macrophages	FXYD6
3.27645423015089E-13	0.671518352840478	0.176	0.064	5.49068199888686E-09	FOLR2+ resident-like macrophages	GPR137
3.37226232542115E-13	0.421650473746846	0.171	0.06	5.65123720494076E-09	FOLR2+ resident-like macrophages	NFU1
3.58503997225402E-13	0.275444350201438	0.228	0.094	6.00780998550329E-09	FOLR2+ resident-like macrophages	NDUFB2
3.62415287532407E-13	0.264689665216429	0.181	0.065	6.07335538846807E-09	FOLR2+ resident-like macrophages	WWC3
3.87229255567649E-13	0.603122793660153	0.197	0.076	6.48918786480266E-09	FOLR2+ resident-like macrophages	DEPRECATED-ENSG00000174903
4.02075251944123E-13	1.61638686528965	0.115	0.028	6.73797707207962E-09	FOLR2+ resident-like macrophages	ALDH1L1
4.18934039489845E-13	0.575019382617703	0.209	0.084	7.02049663377082E-09	FOLR2+ resident-like macrophages	RCN1
4.35405089542433E-13	1.09598742892975	0.167	0.059	7.2965184905521E-09	FOLR2+ resident-like macrophages	SFXN5
4.75996909930915E-13	0.276050944392312	0.216	0.089	7.97675621662228E-09	FOLR2+ resident-like macrophages	MTM1
5.08442455532305E-13	0.39499472674799	0.244	0.107	8.52047866981036E-09	FOLR2+ resident-like macrophages	MFN2
5.12026327541492E-13	0.275899669558242	0.255	0.115	8.58053719694033E-09	FOLR2+ resident-like macrophages	MZT2B
5.19266339656057E-13	0.261738435466575	0.302	0.149	8.7018653199562E-09	FOLR2+ resident-like macrophages	RNF38
5.24800783389894E-13	0.498516261269264	0.264	0.122	8.79461152804785E-09	FOLR2+ resident-like macrophages	RBM10
5.28782170940982E-13	0.256767137589783	0.315	0.157	8.86133162062898E-09	FOLR2+ resident-like macrophages	ADAM15
5.31707044048509E-13	0.454159978167339	0.197	0.077	8.91034664416491E-09	FOLR2+ resident-like macrophages	CREB5
5.34742209354883E-13	0.877387896588436	0.209	0.085	8.96120994436913E-09	FOLR2+ resident-like macrophages	L3MBTL2
5.38542784759964E-13	1.74145874196294	0.129	0.037	9.02489998700747E-09	FOLR2+ resident-like macrophages	BHMT
5.38768951641936E-13	0.46435108552991	0.155	0.051	9.02869009161556E-09	FOLR2+ resident-like macrophages	LARS2
5.81960170779607E-13	0.308830074523283	0.328	0.168	9.75248854192466E-09	FOLR2+ resident-like macrophages	SRP72
5.95616047096401E-13	0.901411403818367	0.155	0.051	9.98133371724148E-09	FOLR2+ resident-like macrophages	UBASH3B
6.03893484164056E-13	0.504790904369058	0.225	0.097	1.01200470076213E-08	FOLR2+ resident-like macrophages	WDR36
6.24961697579603E-13	0.933384531293107	0.19	0.073	1.0473108128039E-08	FOLR2+ resident-like macrophages	ECHDC2
6.25413709490276E-13	0.703426488571834	0.17	0.06	1.04806829436381E-08	FOLR2+ resident-like macrophages	ZC3H14
6.33912869934192E-13	1.38938371532239	0.103	0.023	1.06231118743572E-08	FOLR2+ resident-like macrophages	FRAT1
6.38096937229793E-13	2.08389041339027	0.128	0.036	1.06932284740969E-08	FOLR2+ resident-like macrophages	TM7SF2
6.41952624230874E-13	0.462208308439734	0.245	0.11	1.0757842076861E-08	FOLR2+ resident-like macrophages	DDX1
6.42646678243364E-13	0.491973706009642	0.155	0.051	1.07694730340023E-08	FOLR2+ resident-like macrophages	DEPRECATED-ENSG00000122642
6.4635976676745E-13	0.3178801161506	0.311	0.151	1.08316969714889E-08	FOLR2+ resident-like macrophages	CRKL
6.47402928151837E-13	1.00678665284217	0.216	0.09	1.08491782699685E-08	FOLR2+ resident-like macrophages	TOMM70
6.4904120020916E-13	0.348577820415036	0.234	0.1	1.08766324331051E-08	FOLR2+ resident-like macrophages	WAC
6.749359861151E-13	0.492510655261525	0.263	0.122	1.13105772553168E-08	FOLR2+ resident-like macrophages	GTPBP2
6.90159288780425E-13	1.64588673200086	0.139	0.043	1.15656893613824E-08	FOLR2+ resident-like macrophages	NIPSNAP1
6.91547981719638E-13	0.484445384871176	0.254	0.114	1.15889610776577E-08	FOLR2+ resident-like macrophages	RALY
7.04242000314617E-13	0.250496949688506	0.315	0.16	1.18016874412724E-08	FOLR2+ resident-like macrophages	PPIA
7.25843388235992E-13	1.09315792663196	0.17	0.061	1.21636835000587E-08	FOLR2+ resident-like macrophages	NADK2
7.40321227275845E-13	0.280494979327547	0.224	0.095	1.24063031266886E-08	FOLR2+ resident-like macrophages	ALG2
7.44475173899726E-13	0.385545793173977	0.231	0.1	1.24759149642116E-08	FOLR2+ resident-like macrophages	COX7A2
7.50121528379498E-13	0.594012370804662	0.187	0.071	1.25705365725836E-08	FOLR2+ resident-like macrophages	BORCS6
7.68016132820019E-13	0.291302079526514	0.269	0.126	1.28704143537979E-08	FOLR2+ resident-like macrophages	MYC
7.8067050136779E-13	0.721084055092618	0.179	0.065	1.30824762619214E-08	FOLR2+ resident-like macrophages	EXT2
8.13401802178601E-13	0.773834134684778	0.234	0.104	1.3630987400909E-08	FOLR2+ resident-like macrophages	CSNK2A2
8.25854465946875E-13	0.400840864305201	0.21	0.085	1.38396691403377E-08	FOLR2+ resident-like macrophages	RSAD1
8.27673380215264E-13	0.407910153820635	0.194	0.074	1.38701505056474E-08	FOLR2+ resident-like macrophages	RNASEH1
8.34147502894674E-13	0.896421946668561	0.135	0.04	1.39786438535089E-08	FOLR2+ resident-like macrophages	ZNF639
8.41556707686593E-13	1.39324413781938	0.161	0.056	1.41028073074119E-08	FOLR2+ resident-like macrophages	ABHD8
8.59068755265232E-13	0.389128568336752	0.215	0.089	1.43962742007348E-08	FOLR2+ resident-like macrophages	ZNF337
8.59448022481554E-13	0.908860398706215	0.128	0.036	1.44026299607459E-08	FOLR2+ resident-like macrophages	DEPRECATED-ENSG00000134597
9.00033132662676E-13	0.39361686430707	0.215	0.089	1.50827552371611E-08	FOLR2+ resident-like macrophages	COMMD3
9.000365998576E-13	0.666409315877658	0.196	0.077	1.50828133404137E-08	FOLR2+ resident-like macrophages	POLR2K
9.65574681374362E-13	0.554716748242574	0.254	0.117	1.61811005104716E-08	FOLR2+ resident-like macrophages	TCP11L1
9.93462717531035E-13	0.352305849115722	0.194	0.076	1.66484482203851E-08	FOLR2+ resident-like macrophages	JADE2

1.02291520754961E-12	1.33472168655732	0.163	0.058	1.71420130481163E-08	FOLR2+ resident-like macrophages	MMAB
1.02512756051261E-12	0.405268557487952	0.255	0.116	1.71790876590703E-08	FOLR2+ resident-like macrophages	EIF5B
1.02782154844029E-12	1.24090895963572	0.151	0.05	1.72242335087624E-08	FOLR2+ resident-like macrophages	MTFR1
1.0318130856681E-12	0.491874847412399	0.208	0.085	1.72911236896259E-08	FOLR2+ resident-like macrophages	TIMM50
1.03844451727319E-12	1.48339001125829	0.116	0.03	1.74022532204641E-08	FOLR2+ resident-like macrophages	KLF5
1.06789181221865E-12	0.298519142336999	0.209	0.085	1.78957309891602E-08	FOLR2+ resident-like macrophages	TRAPPC8
1.08240998497419E-12	0.268346877338217	0.241	0.108	1.81390265281976E-08	FOLR2+ resident-like macrophages	RBM22
1.08600741310469E-12	0.844027198255528	0.149	0.048	1.81993122288084E-08	FOLR2+ resident-like macrophages	COG8
1.09507073582481E-12	1.08676528715758	0.196	0.079	1.83511953909521E-08	FOLR2+ resident-like macrophages	IGSF8
1.17193396721417E-12	0.294819875768538	0.245	0.109	1.96392694225751E-08	FOLR2+ resident-like macrophages	CNOT11
1.21670787382204E-12	0.331281695973381	0.174	0.064	2.03895905495097E-08	FOLR2+ resident-like macrophages	ADPRH
1.21841446444868E-12	1.53244767115385	0.157	0.055	2.0418189595231E-08	FOLR2+ resident-like macrophages	CAMK2N1
1.22451940842314E-12	1.7039502295004	0.132	0.039	2.0520496246355E-08	FOLR2+ resident-like macrophages	POLR3H
1.2302288165022E-12	0.497827532382236	0.194	0.077	2.06161745069439E-08	FOLR2+ resident-like macrophages	SLC16A1
1.25417310868081E-12	1.26204859574576	0.157	0.054	2.1017432955273E-08	FOLR2+ resident-like macrophages	ZCCHC9
1.27258020692725E-12	0.291475956789799	0.197	0.079	2.13258991076868E-08	FOLR2+ resident-like macrophages	C1GALT1
1.32686576481733E-12	0.852833311341387	0.216	0.091	2.22356164868088E-08	FOLR2+ resident-like macrophages	GFM1
1.33692284579387E-12	0.800882194461379	0.167	0.06	2.24041530498138E-08	FOLR2+ resident-like macrophages	CLN5
1.34213858474034E-12	0.799499800661202	0.125	0.035	2.24915584030785E-08	FOLR2+ resident-like macrophages	AKTIP
1.41644173870614E-12	1.1106260679286	0.158	0.054	2.37367306572375E-08	FOLR2+ resident-like macrophages	CDCA7L
1.41940482039298E-12	1.74486560976388	0.119	0.032	2.37863859801455E-08	FOLR2+ resident-like macrophages	FKBP9
1.42885891944904E-12	0.274194448978579	0.224	0.096	2.39448177721271E-08	FOLR2+ resident-like macrophages	TBC1D20
1.45209119976487E-12	1.771378976097	0.109	0.027	2.43341443256596E-08	FOLR2+ resident-like macrophages	PON1
1.469870249685E-12	0.31660494844285	0.186	0.071	2.46320856442213E-08	FOLR2+ resident-like macrophages	FASTKD2
1.5290903604673E-12	0.872383360894161	0.174	0.065	2.5624496260711E-08	FOLR2+ resident-like macrophages	ISOC1
1.55806126227475E-12	2.28518780341603	0.104	0.025	2.61099906332002E-08	FOLR2+ resident-like macrophages	PDZK1
1.5746585752563E-12	0.37260305999484	0.238	0.107	2.63881284041451E-08	FOLR2+ resident-like macrophages	SRP19
1.59099815881052E-12	1.50949251677386	0.112	0.028	2.66619471453467E-08	FOLR2+ resident-like macrophages	CDC42EP1
1.62486567504912E-12	1.13992062737861	0.192	0.076	2.72294989824732E-08	FOLR2+ resident-like macrophages	DHFR
1.63859555453702E-12	0.395872578109848	0.251	0.114	2.74595843029314E-08	FOLR2+ resident-like macrophages	PTEN
1.64593597845748E-12	0.904499118665669	0.109	0.027	2.75825951269904E-08	FOLR2+ resident-like macrophages	XRRA1
1.64624918822612E-12	0.428577763810582	0.186	0.071	2.75878438962933E-08	FOLR2+ resident-like macrophages	NFS1
1.65214498803202E-12	0.270053069365598	0.206	0.085	2.76866457094406E-08	FOLR2+ resident-like macrophages	DPAGT1
1.67737001265505E-12	0.322655604304625	0.179	0.066	2.81093666720733E-08	FOLR2+ resident-like macrophages	NTPCR
1.71579335427575E-12	1.69219476409293	0.103	0.024	2.87532650309531E-08	FOLR2+ resident-like macrophages	SLC47A1
1.73502179937224E-12	0.368832985435201	0.228	0.102	2.907549531388E-08	FOLR2+ resident-like macrophages	ATP5PB
1.73895289233725E-12	0.415733947431028	0.222	0.095	2.91413725697876E-08	FOLR2+ resident-like macrophages	EXOC8
1.79471308010159E-12	0.982998003018846	0.183	0.071	3.00758017963425E-08	FOLR2+ resident-like macrophages	RAB33B
1.84943133512952E-12	1.02259471537045	0.129	0.038	3.09927703141004E-08	FOLR2+ resident-like macrophages	ARMC10
1.85299263935484E-12	0.759558116191792	0.218	0.092	3.10524506503084E-08	FOLR2+ resident-like macrophages	JPT2
1.87625120446961E-12	0.985518554915037	0.241	0.109	3.14422176845017E-08	FOLR2+ resident-like macrophages	FDFT1
1.90606879644231E-12	1.53444546825374	0.12	0.033	3.19419008907803E-08	FOLR2+ resident-like macrophages	NDUFA6
1.93451779827969E-12	0.459027171790638	0.167	0.06	3.24186492635711E-08	FOLR2+ resident-like macrophages	PAQR7
1.94218107286923E-12	0.543114317826483	0.237	0.106	3.25470704191426E-08	FOLR2+ resident-like macrophages	SF3B2
1.95867012704636E-12	0.360040345345535	0.235	0.102	3.28233939890429E-08	FOLR2+ resident-like macrophages	THOC6
1.99199859784586E-12	0.870611185979666	0.2	0.082	3.3381912502701E-08	FOLR2+ resident-like macrophages	ACAD9
2.0515229963732E-12	1.50658259368066	0.141	0.046	3.43794223732221E-08	FOLR2+ resident-like macrophages	DNAAF5
2.05326045234271E-12	0.366564619765575	0.199	0.08	3.44085386603591E-08	FOLR2+ resident-like macrophages	COX18
2.06649075252191E-12	0.761428889840477	0.115	0.029	3.46302520307621E-08	FOLR2+ resident-like macrophages	RBM18
2.09107228205337E-12	0.34379705076619	0.206	0.086	3.50421893026504E-08	FOLR2+ resident-like macrophages	MRS2
2.1224379110303E-12	0.543988207823718	0.123	0.034	3.55678145130458E-08	FOLR2+ resident-like macrophages	GON7
2.1591668692542E-12	0.747652380226709	0.164	0.06	3.61833183949618E-08	FOLR2+ resident-like macrophages	VAV2
2.1665381108997E-12	0.384128822278295	0.155	0.053	3.63068456624571E-08	FOLR2+ resident-like macrophages	TTLL12
2.17825942633003E-12	0.299168049837539	0.276	0.132	3.65032714664387E-08	FOLR2+ resident-like macrophages	EPRS
2.19189080562295E-12	0.3991550595648	0.206	0.084	3.67317061206293E-08	FOLR2+ resident-like macrophages	BCL9L
2.25516237990791E-12	0.481949853280282	0.277	0.131	3.77920111624968E-08	FOLR2+ resident-like macrophages	HSD17B10
2.29387955172855E-12	0.729191108014982	0.286	0.141	3.84408335278671E-08	FOLR2+ resident-like macrophages	PSMG2
2.30401420851741E-12	0.655110742721882	0.202	0.082	3.86106701063348E-08	FOLR2+ resident-like macrophages	THUMPD3
2.40319207479929E-12	0.867633713416091	0.219	0.093	4.02726927894865E-08	FOLR2+ resident-like macrophages	AKAP1
2.43631265520552E-12	0.829788700931563	0.215	0.092	4.0827727475934E-08	FOLR2+ resident-like macrophages	PPCS
2.4405592813808E-12	1.23656573860037	0.161	0.058	4.08988924373795E-08	FOLR2+ resident-like macrophages	PIGQ
2.44106558227496E-12	0.41262629245523	0.219	0.094	4.09073770277637E-08	FOLR2+ resident-like macrophages	CFDP1
2.48165222012416E-12	0.823899656246519	0.208	0.086	4.15875279048407E-08	FOLR2+ resident-like macrophages	SHFL
2.5626285241835E-12	1.22035460280402	0.203	0.085	4.29445288082672E-08	FOLR2+ resident-like macrophages	PLPBP
2.57272706374361E-12	0.319457253872139	0.176	0.065	4.31137601342154E-08	FOLR2+ resident-like macrophages	PMPCA
2.63077199196219E-12	0.35596347227251	0.177	0.066	4.40864770413024E-08	FOLR2+ resident-like macrophages	DEPRECATED-ENSG00000134049
2.76859573781043E-12	2.18990475631149	0.112	0.029	4.63961273742271E-08	FOLR2+ resident-like macrophages	CDHR5
2.78359546775674E-12	0.604313074431047	0.189	0.075	4.66474928486675E-08	FOLR2+ resident-like macrophages	MOB2
2.78446704333272E-12	0.424841837589517	0.228	0.1	4.66620987121697E-08	FOLR2+ resident-like macrophages	GMPPB
2.86373190442527E-12	0.353124230236923	0.17	0.063	4.79904192543587E-08	FOLR2+ resident-like macrophages	TMEM63B
2.86850342049892E-12	0.649014809978776	0.177	0.068	4.80703803207209E-08	FOLR2+ resident-like macrophages	CPLANE1

2.89106816135443E-12	0.27971946038732	0.271	0.129	4.84485202479775E-08	FOLR2+ resident-like macrophages	LRRC59
2.90572727391195E-12	1.05210469471136	0.128	0.038	4.86941776562164E-08	FOLR2+ resident-like macrophages	IFT88
2.94889767528898E-12	0.385573611621568	0.231	0.102	4.94176272424927E-08	FOLR2+ resident-like macrophages	PDP1
2.97929601895483E-12	0.42248708034487	0.18	0.068	4.99270426856451E-08	FOLR2+ resident-like macrophages	SIRT6
3.09255238258362E-12	0.418877977492406	0.303	0.156	5.18249928273363E-08	FOLR2+ resident-like macrophages	NDRG1
3.12445137788046E-12	0.724208833585307	0.181	0.07	5.23595561905208E-08	FOLR2+ resident-like macrophages	NIT2
3.14636080328917E-12	0.740404891543295	0.115	0.03	5.27267143415199E-08	FOLR2+ resident-like macrophages	EIF4EBP3
3.21250992667465E-12	0.587656948316937	0.168	0.062	5.38352413512138E-08	FOLR2+ resident-like macrophages	UQCRFS1
3.28180824039951E-12	0.893986511756936	0.149	0.05	5.49965424926149E-08	FOLR2+ resident-like macrophages	ZNF398
3.29348665641809E-12	1.26486639757036	0.164	0.06	5.51922493882543E-08	FOLR2+ resident-like macrophages	ENTPD5
3.31801156094614E-12	0.628069106257796	0.157	0.055	5.56032377383354E-08	FOLR2+ resident-like macrophages	ZSCAN26
3.32328111727559E-12	0.335606893413562	0.351	0.181	5.56915449633043E-08	FOLR2+ resident-like macrophages	NME2
3.34873712852458E-12	0.441342989722588	0.263	0.126	5.61181367998149E-08	FOLR2+ resident-like macrophages	PEX2
3.44852793389808E-12	1.69215836565015	0.139	0.046	5.7790431116264E-08	FOLR2+ resident-like macrophages	CLMN
3.46543183220809E-12	0.344370511938048	0.184	0.072	5.80737066441431E-08	FOLR2+ resident-like macrophages	KCTD10
3.47027678362882E-12	0.603008615288635	0.245	0.112	5.81548983400517E-08	FOLR2+ resident-like macrophages	UBE2E3
3.47866411036893E-12	0.615068678220842	0.209	0.088	5.82954531615626E-08	FOLR2+ resident-like macrophages	NREP
3.52581374963917E-12	0.260660824916501	0.21	0.089	5.90855868164532E-08	FOLR2+ resident-like macrophages	DPY30
3.5270838047047E-12	0.679737898585674	0.193	0.079	5.91068703992413E-08	FOLR2+ resident-like macrophages	TATDN3
3.66369581276561E-12	0.873352235201569	0.131	0.04	6.13962144303261E-08	FOLR2+ resident-like macrophages	DTX2
3.68400180736356E-12	0.719456431262307	0.212	0.09	6.17365022877985E-08	FOLR2+ resident-like macrophages	NSMCE1
3.68452415942553E-12	0.610784212932441	0.286	0.14	6.1745255863653E-08	FOLR2+ resident-like macrophages	IL1RAP
3.69176713430447E-12	0.739418843148242	0.1	0.023	6.18666336366743E-08	FOLR2+ resident-like macrophages	ACVRL1
3.70793639264954E-12	0.354486596454996	0.258	0.12	6.2137598068021E-08	FOLR2+ resident-like macrophages	KDM4A
3.74264445635277E-12	0.740126402685933	0.286	0.142	6.27192357995596E-08	FOLR2+ resident-like macrophages	DCXR
3.74856347845735E-12	1.07832636464567	0.164	0.06	6.28184267719882E-08	FOLR2+ resident-like macrophages	PPP1R37
3.84964649522142E-12	0.831730279620331	0.107	0.027	6.45123759669206E-08	FOLR2+ resident-like macrophages	ABCB6
3.92418391888795E-12	0.831180293997629	0.141	0.046	6.57614741127243E-08	FOLR2+ resident-like macrophages	AAR2
3.92983335736603E-12	1.4332583159051	0.141	0.046	6.58561474027399E-08	FOLR2+ resident-like macrophages	TMEM238
3.96255760206057E-12	0.650185649915618	0.163	0.059	6.6404540295331E-08	FOLR2+ resident-like macrophages	INO80
4.08895725505084E-12	1.06819420218602	0.109	0.028	6.85227456801419E-08	FOLR2+ resident-like macrophages	RHPN1
4.25437159834444E-12	0.44221034214936	0.196	0.08	7.12947592450561E-08	FOLR2+ resident-like macrophages	TMEM138
4.70257231302326E-12	1.47976085863002	0.138	0.045	7.88057068216438E-08	FOLR2+ resident-like macrophages	PEAK3
4.70432104364766E-12	0.481082724833679	0.189	0.075	7.88350120494474E-08	FOLR2+ resident-like macrophages	ZNF26
4.76635586557132E-12	0.587411471561747	0.128	0.038	7.98745915952441E-08	FOLR2+ resident-like macrophages	TBCCD1
4.79960972480184E-12	1.07338586381322	0.152	0.053	8.04318597682293E-08	FOLR2+ resident-like macrophages	DEPRECATED-ENSG00000109536
5.07648090132477E-12	0.435813593696617	0.218	0.096	8.50716669444005E-08	FOLR2+ resident-like macrophages	ZNF580
5.13670685999232E-12	0.865288678381652	0.177	0.068	8.60809335597512E-08	FOLR2+ resident-like macrophages	MAGOH
5.25091536439386E-12	0.67823989895474	0.19	0.077	8.79948396765123E-08	FOLR2+ resident-like macrophages	MBD3
5.43854954867058E-12	0.516564042285735	0.228	0.1	9.11392133366216E-08	FOLR2+ resident-like macrophages	TFDP1
5.57243436268956E-12	1.47315864404156	0.149	0.052	9.33828550499516E-08	FOLR2+ resident-like macrophages	TUBB2A
5.59665325499412E-12	1.91278804163158	0.11	0.029	9.37887152471914E-08	FOLR2+ resident-like macrophages	HGD
5.60480711631299E-12	0.562805743607393	0.125	0.036	9.3925357655173E-08	FOLR2+ resident-like macrophages	RTN2
5.63288384766705E-12	0.406045757618758	0.202	0.083	9.43958675192045E-08	FOLR2+ resident-like macrophages	PSMD2
5.66165189523722E-12	0.440694689077703	0.2	0.083	9.48779624603853E-08	FOLR2+ resident-like macrophages	RAP1GDS1
6.17687072430074E-12	0.884822785413357	0.151	0.053	1.03511999597832E-07	FOLR2+ resident-like macrophages	VTA1
6.20695378353922E-12	1.04635972600147	0.11	0.029	1.0401613150455E-07	FOLR2+ resident-like macrophages	ZDHHC24
6.37539748449957E-12	0.424821214506193	0.176	0.068	1.06838911045244E-07	FOLR2+ resident-like macrophages	CRCP
6.39607977270606E-12	0.481459435898481	0.235	0.108	1.07185504831008E-07	FOLR2+ resident-like macrophages	ICMT
6.72323446003066E-12	0.555124831835432	0.161	0.059	1.12667963081194E-07	FOLR2+ resident-like macrophages	FCF1
6.72798242585087E-12	0.515297196485952	0.115	0.031	1.12747529492409E-07	FOLR2+ resident-like macrophages	ZC3H12C
6.78929198255476E-12	0.316258313362842	0.228	0.102	1.13774955043653E-07	FOLR2+ resident-like macrophages	PTPRA
6.81856287566704E-12	1.42673085911477	0.165	0.063	1.14265476670428E-07	FOLR2+ resident-like macrophages	ASB13
6.85065373562821E-12	1.86811645495387	0.123	0.037	1.14803255301658E-07	FOLR2+ resident-like macrophages	CNKSR3
6.96469409997286E-12	0.257956907363011	0.263	0.124	1.16714343727345E-07	FOLR2+ resident-like macrophages	PTPN18
7.2688239560386E-12	0.970866319779044	0.165	0.062	1.21810951855295E-07	FOLR2+ resident-like macrophages	NUP54
7.40885476978172E-12	1.32366987303441	0.112	0.03	1.24157588232002E-07	FOLR2+ resident-like macrophages	PRELID3A
7.57515170555547E-12	0.433898261437746	0.228	0.102	1.26944392281699E-07	FOLR2+ resident-like macrophages	TMEM64
7.5960325680761E-12	0.835814700069862	0.231	0.104	1.27294313775819E-07	FOLR2+ resident-like macrophages	EIF3K
7.80632809620667E-12	0.635687619043985	0.122	0.035	1.30818446236231E-07	FOLR2+ resident-like macrophages	TP53INP2
7.85592534792777E-12	0.495960288650635	0.176	0.067	1.31649596980574E-07	FOLR2+ resident-like macrophages	CFAP298
7.86340572532573E-12	0.414728244541778	0.229	0.103	1.31774953145009E-07	FOLR2+ resident-like macrophages	CLPP
7.98100839804599E-12	0.2768292833879	0.149	0.051	1.33745738734455E-07	FOLR2+ resident-like macrophages	DEPRECATED-ENSG00000176101
7.99378538959239E-12	0.363278571321261	0.239	0.11	1.33959855558789E-07	FOLR2+ resident-like macrophages	BRD3
8.0397738200731E-12	0.866049490833959	0.163	0.06	1.34730529676785E-07	FOLR2+ resident-like macrophages	MTCH2
8.07394837147991E-12	0.608580569834399	0.184	0.074	1.3530322680926E-07	FOLR2+ resident-like macrophages	HIGD1A
8.19842899321107E-12	0.429552817920626	0.179	0.069	1.37389273068231E-07	FOLR2+ resident-like macrophages	UBN1
8.29034507405366E-12	1.76189671064941	0.103	0.026	1.38929602750991E-07	FOLR2+ resident-like macrophages	GCDH
8.34939996856562E-12	0.478244690689452	0.149	0.051	1.39919244673223E-07	FOLR2+ resident-like macrophages	KLHL7
8.38568203338919E-12	1.46225181667922	0.142	0.048	1.40527259515536E-07	FOLR2+ resident-like macrophages	BTD
8.56618373996839E-12	0.315302286529869	0.276	0.138	1.4355210711439E-07	FOLR2+ resident-like macrophages	DNTTIP1

8.57424019124805E-12	0.808074565730082	0.125	0.037	1.43687117124935E-07	FOLR2+ resident-like macrophages	ZNF524
8.63776786695541E-12	0.771830633930096	0.142	0.047	1.44751713914439E-07	FOLR2+ resident-like macrophages	NDFIP2
8.7063854665308E-12	0.559939325981608	0.122	0.036	1.45901607648123E-07	FOLR2+ resident-like macrophages	DNAJC24
8.76642058382103E-12	0.58536480917652	0.215	0.096	1.46907676143673E-07	FOLR2+ resident-like macrophages	ZC3H13
8.90626049025492E-12	1.46900605945239	0.102	0.025	1.49251113295692E-07	FOLR2+ resident-like macrophages	ZNF185
8.92399692765189E-12	1.97044083002332	0.107	0.028	1.4954834051359E-07	FOLR2+ resident-like macrophages	ATP6V0E2
9.01499074666542E-12	0.284599993988088	0.163	0.06	1.51073214932619E-07	FOLR2+ resident-like macrophages	SLC38A9
9.24914629848816E-12	0.373506662243882	0.239	0.109	1.54997193670065E-07	FOLR2+ resident-like macrophages	FAM162A
9.4896959674469E-12	0.794105484264837	0.181	0.072	1.59028325022475E-07	FOLR2+ resident-like macrophages	TMEM230
9.86996174468844E-12	0.254524756687236	0.209	0.087	1.65400818917489E-07	FOLR2+ resident-like macrophages	GPD2
9.91152713345049E-12	0.888626620614657	0.138	0.045	1.66097371702363E-07	FOLR2+ resident-like macrophages	NRBP2
9.94438663602542E-12	0.298446413145773	0.228	0.103	1.66648031246514E-07	FOLR2+ resident-like macrophages	WDR20
1.01806403511523E-11	0.305704093075206	0.245	0.115	1.7060717100461E-07	FOLR2+ resident-like macrophages	TNPO2
1.03241887528298E-11	0.383164486782118	0.232	0.107	1.73012755119921E-07	FOLR2+ resident-like macrophages	MFSD14A
1.03744931324043E-11	0.630110442339178	0.179	0.071	1.73855755912831E-07	FOLR2+ resident-like macrophages	NIPSNAP3A
1.04179045739768E-11	0.984077209664017	0.177	0.07	1.74583244850703E-07	FOLR2+ resident-like macrophages	GALT
1.09696762104615E-11	0.451246132809141	0.152	0.053	1.83829833934914E-07	FOLR2+ resident-like macrophages	TULP4
1.10832935318198E-11	1.1320154963956	0.134	0.043	1.85733833006236E-07	FOLR2+ resident-like macrophages	IRF2BP1
1.13232497441745E-11	0.565417666020233	0.136	0.045	1.89755019212877E-07	FOLR2+ resident-like macrophages	ARRDC4
1.13790853366534E-11	0.595785887309307	0.144	0.048	1.90690712071638E-07	FOLR2+ resident-like macrophages	PDE12
1.14898578166173E-11	0.690752591610696	0.165	0.063	1.92547037290873E-07	FOLR2+ resident-like macrophages	GAB1
1.1499881214872E-11	0.446897846071918	0.167	0.063	1.92715009398825E-07	FOLR2+ resident-like macrophages	CCNC
1.16599532476408E-11	2.46172976641176	0.102	0.026	1.95397496523964E-07	FOLR2+ resident-like macrophages	STBD1
1.22140260740641E-11	0.323320572478603	0.18	0.071	2.04682648949166E-07	FOLR2+ resident-like macrophages	ATP5PF
1.22287466454251E-11	0.496714424281057	0.174	0.068	2.04929336284033E-07	FOLR2+ resident-like macrophages	TBC1D23
1.26576635257472E-11	1.03750684799982	0.179	0.071	2.12117125364471E-07	FOLR2+ resident-like macrophages	MED18
1.27566732410618E-11	0.268046759920301	0.184	0.074	2.13776330173714E-07	FOLR2+ resident-like macrophages	RIC8A
1.30504755892795E-11	1.21995014685948	0.152	0.055	2.18699869925146E-07	FOLR2+ resident-like macrophages	STAMBPL1
1.30998257786382E-11	0.453713071471379	0.209	0.092	2.19526880398418E-07	FOLR2+ resident-like macrophages	URM1
1.31205911816475E-11	0.35861872544538	0.183	0.072	2.19874867022048E-07	FOLR2+ resident-like macrophages	COQ4
1.31719651372472E-11	0.717707165330638	0.168	0.065	2.20735791769988E-07	FOLR2+ resident-like macrophages	DAD1
1.32877233439062E-11	0.577636184861595	0.144	0.048	2.2267566779718E-07	FOLR2+ resident-like macrophages	RUNDC1
1.35466006762794E-11	0.88396468021259	0.104	0.027	2.27013934133091E-07	FOLR2+ resident-like macrophages	CCDC71L
1.35476322188381E-11	0.904789968051293	0.123	0.037	2.27031220723288E-07	FOLR2+ resident-like macrophages	PRELID2
1.38256243431432E-11	0.845915872868773	0.147	0.051	2.31689812742394E-07	FOLR2+ resident-like macrophages	ZSCAN29
1.43763342509926E-11	0.660123713449116	0.205	0.088	2.40918609378133E-07	FOLR2+ resident-like macrophages	PARN
1.45756808650719E-11	0.502333910774725	0.173	0.067	2.44259259936875E-07	FOLR2+ resident-like macrophages	NUDT19
1.46616045525452E-11	0.383865312095792	0.149	0.052	2.45699169091553E-07	FOLR2+ resident-like macrophages	VIPAS39
1.48553894396683E-11	0.479739586765203	0.209	0.09	2.48946616229961E-07	FOLR2+ resident-like macrophages	TMLHE
1.48778575174535E-11	0.391696329748134	0.149	0.053	2.49323136277485E-07	FOLR2+ resident-like macrophages	CDIP1
1.4979721335169E-11	0.354189453265526	0.2	0.084	2.51030170134763E-07	FOLR2+ resident-like macrophages	CAMSAP2
1.51162478477399E-11	1.0050942127648	0.183	0.074	2.53318081432426E-07	FOLR2+ resident-like macrophages	VPS72
1.51311023894503E-11	1.78897382625351	0.125	0.039	2.53567013842409E-07	FOLR2+ resident-like macrophages	HSD17B8
1.51516167092138E-11	1.26019049856916	0.186	0.077	2.53910792813005E-07	FOLR2+ resident-like macrophages	CCT3
1.51789444031488E-11	1.04433940902974	0.149	0.054	2.54368750307968E-07	FOLR2+ resident-like macrophages	GTF2E1
1.55302578203973E-11	0.437461285021797	0.203	0.087	2.60256060554218E-07	FOLR2+ resident-like macrophages	MED1
1.55804008011818E-11	0.826495622353769	0.104	0.027	2.61096356626205E-07	FOLR2+ resident-like macrophages	TMEM242
1.59591887682643E-11	0.630402105232024	0.147	0.05	2.67444085378574E-07	FOLR2+ resident-like macrophages	MCCC2
1.59729060173619E-11	1.5826218468197	0.134	0.044	2.6767395903895E-07	FOLR2+ resident-like macrophages	DEPRECATED-ENSG00000001630
1.6346532261286E-11	0.428422207850702	0.113	0.031	2.73935187634631E-07	FOLR2+ resident-like macrophages	POMT1
1.66020052333199E-11	0.382434043732495	0.194	0.08	2.78216403699974E-07	FOLR2+ resident-like macrophages	H2AFY2
1.66923660509313E-11	0.29766587082783	0.2	0.084	2.79730670281507E-07	FOLR2+ resident-like macrophages	TMEM214
1.67438546544677E-11	0.497518490191793	0.134	0.043	2.80593516299569E-07	FOLR2+ resident-like macrophages	TUSC2
1.68480020670605E-11	0.314631320652863	0.138	0.046	2.823388186398E-07	FOLR2+ resident-like macrophages	SENP7
1.72144010068499E-11	0.289083700514354	0.237	0.109	2.8847893207279E-07	FOLR2+ resident-like macrophages	ABHD10
1.75272179414863E-11	0.25505063759081	0.186	0.076	2.93721118263427E-07	FOLR2+ resident-like macrophages	MED6
1.76258562713388E-11	1.28640094379985	0.161	0.061	2.95374099395095E-07	FOLR2+ resident-like macrophages	PDZD11
1.83933244799677E-11	0.317562816378735	0.141	0.047	3.08235331635299E-07	FOLR2+ resident-like macrophages	ZFP30
1.84606608651437E-11	0.635213387019589	0.125	0.038	3.09363754778078E-07	FOLR2+ resident-like macrophages	PPIC
1.88179820773697E-11	0.413309302886853	0.148	0.052	3.15351743652562E-07	FOLR2+ resident-like macrophages	SLC39A13
1.90270268818295E-11	0.52933624380675	0.183	0.074	3.18854916485699E-07	FOLR2+ resident-like macrophages	SLC35B4
1.90948854472491E-11	1.25086403019486	0.141	0.048	3.19992090325E-07	FOLR2+ resident-like macrophages	ARMC1
2.0494294381126E-11	0.814328302629151	0.157	0.058	3.4344338523891E-07	FOLR2+ resident-like macrophages	REXO4
2.13148927822395E-11	0.302113857964675	0.151	0.053	3.5719497324477E-07	FOLR2+ resident-like macrophages	RANBP10
2.17754980992687E-11	0.560789996775275	0.263	0.127	3.64913797147545E-07	FOLR2+ resident-like macrophages	DEPTOR
2.30479791098822E-11	0.310048950891904	0.263	0.126	3.86238033923405E-07	FOLR2+ resident-like macrophages	NDUFA9
2.32629694067856E-11	0.70151076703159	0.197	0.083	3.89840841318913E-07	FOLR2+ resident-like macrophages	TMEM167A
2.37250923013301E-11	0.810174081883409	0.205	0.088	3.97585096785689E-07	FOLR2+ resident-like macrophages	AIMP2
2.40737156089087E-11	0.266843349356812	0.219	0.097	4.03427326174093E-07	FOLR2+ resident-like macrophages	EIF2AK2
2.45483686768083E-11	0.586635113014663	0.244	0.117	4.11381562285953E-07	FOLR2+ resident-like macrophages	YTHDF2
2.49925933739573E-11	0.679872294909946	0.164	0.064	4.18825879760777E-07	FOLR2+ resident-like macrophages	RNF139

2.50386362597596E-11	0.331387471335854	0.187	0.077	4.19597466441051E-07	FOLR2+ resident-like macrophages	IMPACT
2.55978956742046E-11	0.962289435896774	0.189	0.079	4.28969535708321E-07	FOLR2+ resident-like macrophages	USP5
2.61801999000292E-11	0.882797569307527	0.123	0.038	4.38727789924689E-07	FOLR2+ resident-like macrophages	ALKBH6
2.66967845137108E-11	0.535602224409046	0.187	0.079	4.47384714880766E-07	FOLR2+ resident-like macrophages	CNIH1
2.68359809704954E-11	0.307634753958448	0.202	0.086	4.49717369103561E-07	FOLR2+ resident-like macrophages	GALNT11
2.76751453736117E-11	1.55745655836131	0.1	0.026	4.63780086170985E-07	FOLR2+ resident-like macrophages	TIGD2
2.77281716281809E-11	0.397476730608052	0.193	0.082	4.64668700145056E-07	FOLR2+ resident-like macrophages	B3GAT3
2.78309044371174E-11	0.607044997542624	0.151	0.054	4.66390296557213E-07	FOLR2+ resident-like macrophages	TRAK2
2.79763616645771E-11	0.267154697741698	0.235	0.108	4.68827868774984E-07	FOLR2+ resident-like macrophages	TMX3
2.83350042978347E-11	0.360166727515559	0.199	0.085	4.74838002023113E-07	FOLR2+ resident-like macrophages	HSPB11
2.86821013599472E-11	0.852591025576123	0.141	0.048	4.80654654589994E-07	FOLR2+ resident-like macrophages	RPUSD3
2.89141392280301E-11	0.568284719290542	0.176	0.07	4.84543145183329E-07	FOLR2+ resident-like macrophages	ZNF428
2.89945807427288E-11	0.364225705939414	0.26	0.127	4.85891184086649E-07	FOLR2+ resident-like macrophages	DGUOK
3.02316108755948E-11	0.354690524318348	0.154	0.056	5.06621335053217E-07	FOLR2+ resident-like macrophages	CHST12
3.0402743369425E-11	0.657499858898415	0.165	0.064	5.09489173384824E-07	FOLR2+ resident-like macrophages	GLO1
3.06205555269319E-11	0.727205105028488	0.134	0.044	5.13139269520324E-07	FOLR2+ resident-like macrophages	CEP250
3.16455049697911E-11	0.556973229061123	0.12	0.036	5.30315372283759E-07	FOLR2+ resident-like macrophages	CARD6
3.27228708084131E-11	0.382536875309732	0.176	0.07	5.48369869007387E-07	FOLR2+ resident-like macrophages	PCBP2
3.38202832711209E-11	1.9370441774964	0.104	0.028	5.66760307057445E-07	FOLR2+ resident-like macrophages	DEPRECATED-ENSG00000176485
3.38298880161601E-11	0.356446686182073	0.206	0.091	5.6692126337481E-07	FOLR2+ resident-like macrophages	PPP2R5D
3.63419709985706E-11	0.320146259802352	0.248	0.12	6.09018749994046E-07	FOLR2+ resident-like macrophages	PYGO2
3.69994072830235E-11	0.44072946340808	0.16	0.061	6.20036067248908E-07	FOLR2+ resident-like macrophages	NFKBIL1
3.70987054460776E-11	0.714198867791512	0.171	0.067	6.21700105865368E-07	FOLR2+ resident-like macrophages	ABHD14B
3.71183075235496E-11	0.541581454147368	0.186	0.077	6.22028597479644E-07	FOLR2+ resident-like macrophages	RNF185
3.82623512094836E-11	0.990960714803093	0.152	0.056	6.41200481568527E-07	FOLR2+ resident-like macrophages	MBIP
3.88064657733326E-11	0.273294410851009	0.209	0.092	6.50318753429508E-07	FOLR2+ resident-like macrophages	OTUD6B
3.89136373302997E-11	1.10095788286743	0.128	0.042	6.52114734381162E-07	FOLR2+ resident-like macrophages	ZFP3
4.01298540474683E-11	0.463039053762941	0.177	0.072	6.72496094127473E-07	FOLR2+ resident-like macrophages	SEC22C
4.0498002274127E-11	0.345835601342215	0.174	0.07	6.7866552210982E-07	FOLR2+ resident-like macrophages	UHRF1BP1L
4.11307414753174E-11	0.335210212058271	0.189	0.078	6.89268965643369E-07	FOLR2+ resident-like macrophages	PCID2
4.12255669810702E-11	0.824450148693404	0.152	0.056	6.90858051468774E-07	FOLR2+ resident-like macrophages	YIPF2
4.28530609712199E-11	0.298697614804947	0.18	0.073	7.18131595755704E-07	FOLR2+ resident-like macrophages	TMEM126B
4.30467568981554E-11	0.532920273046121	0.1	0.026	7.21377552099287E-07	FOLR2+ resident-like macrophages	DNMBP
4.32797523013009E-11	1.0398803637678	0.123	0.039	7.25282089065201E-07	FOLR2+ resident-like macrophages	ZNF268
4.33960019588194E-11	0.317471750179919	0.154	0.057	7.27230200825896E-07	FOLR2+ resident-like macrophages	C12orf4
4.4955515364093E-11	0.572874233673916	0.12	0.037	7.5336452647147E-07	FOLR2+ resident-like macrophages	BET1
4.74004849958462E-11	1.01493970311071	0.141	0.049	7.9433732756039E-07	FOLR2+ resident-like macrophages	TIGD5
4.84421183582331E-11	0.768621729119455	0.147	0.053	8.1179301944727E-07	FOLR2+ resident-like macrophages	CBX1
4.90341097335309E-11	0.309375369203644	0.219	0.099	8.21713610914511E-07	FOLR2+ resident-like macrophages	SMC6
4.91775909954148E-11	0.263155558534543	0.173	0.069	8.24118069901162E-07	FOLR2+ resident-like macrophages	CHCHD1
5.0478328471219E-11	0.666072013982504	0.123	0.039	8.45915828520688E-07	FOLR2+ resident-like macrophages	RPF2
5.08821106347744E-11	0.294389819958261	0.168	0.066	8.52682410017549E-07	FOLR2+ resident-like macrophages	TRPT1
5.29445798348392E-11	0.488860820640519	0.155	0.058	8.87245268872236E-07	FOLR2+ resident-like macrophages	NKIRAS2
5.33867149478079E-11	0.263193938198442	0.199	0.087	8.94654569095365E-07	FOLR2+ resident-like macrophages	HMGXB4
5.47418835223897E-11	0.969287676192823	0.126	0.041	9.17364484068206E-07	FOLR2+ resident-like macrophages	FAM8A1
5.47429598915909E-11	0.520937766121875	0.17	0.067	9.17382521863281E-07	FOLR2+ resident-like macrophages	FEM1A
5.71649945178817E-11	0.489665238246262	0.16	0.061	9.57970978130662E-07	FOLR2+ resident-like macrophages	NRBF2
5.7514554886926E-11	0.436251376892395	0.18	0.074	9.63828910795105E-07	FOLR2+ resident-like macrophages	OMA1
5.8396055656414E-11	0.82935062714066	0.16	0.062	9.78601100690186E-07	FOLR2+ resident-like macrophages	UTP23
5.84403315706033E-11	0.864233833961903	0.122	0.039	9.7934307646017E-07	FOLR2+ resident-like macrophages	IFIT5
5.86161596632313E-11	0.521053603727091	0.157	0.059	9.82289603636429E-07	FOLR2+ resident-like macrophages	RASGRP4
5.88875889520281E-11	0.313961838824936	0.126	0.041	9.86838215658087E-07	FOLR2+ resident-like macrophages	KCTD2
5.9149102478969E-11	0.422807348957346	0.155	0.058	9.91220659342562E-07	FOLR2+ resident-like macrophages	ZDHHC16
5.94040598521724E-11	0.47637920112756	0.176	0.071	9.95493235002705E-07	FOLR2+ resident-like macrophages	C5orf51
5.95275628500848E-11	0.694029522158915	0.151	0.056	9.97562898241721E-07	FOLR2+ resident-like macrophages	TSR1
6.09034044121941E-11	0.283604006942422	0.151	0.055	1.02061925113955E-06	FOLR2+ resident-like macrophages	DEPRECATED-ENSG00000140365
6.18696932011834E-11	0.734388163249796	0.125	0.04	1.03681231866543E-06	FOLR2+ resident-like macrophages	ZNF740
6.27606337754419E-11	1.19604667479109	0.122	0.039	1.05174270080885E-06	FOLR2+ resident-like macrophages	AP5S1
6.27805360401984E-11	0.268145843081235	0.209	0.093	1.05207622296164E-06	FOLR2+ resident-like macrophages	ATG13
6.35076387540247E-11	0.278308332068069	0.171	0.067	1.06426101023995E-06	FOLR2+ resident-like macrophages	AAGAB
6.55110309376308E-11	0.318578877944376	0.202	0.089	1.09783385645282E-06	FOLR2+ resident-like macrophages	WDR77
6.60328573005105E-11	0.490869902428116	0.16	0.062	1.10657862264196E-06	FOLR2+ resident-like macrophages	NOA1
6.73995784676768E-11	0.33661365864134	0.122	0.038	1.12948213596133E-06	FOLR2+ resident-like macrophages	PANK2
6.81600730663624E-11	0.27012051500163	0.139	0.048	1.1422265044461E-06	FOLR2+ resident-like macrophages	FUNDC1
6.82347061405007E-11	0.682575609232045	0.197	0.087	1.14347720550251E-06	FOLR2+ resident-like macrophages	SF3B4
6.90382987580201E-11	0.470548711624812	0.131	0.044	1.1569438105869E-06	FOLR2+ resident-like macrophages	ZBTB45
6.91114591455671E-11	1.28800843092172	0.107	0.03	1.15816983236141E-06	FOLR2+ resident-like macrophages	DOHH
6.9228281532264E-11	0.354242425801077	0.194	0.083	1.16012754191768E-06	FOLR2+ resident-like macrophages	TAOK2
7.0650642382409E-11	0.682189873244917	0.186	0.079	1.18396346504441E-06	FOLR2+ resident-like macrophages	TMEM168
7.25158679964355E-11	0.527016569779895	0.104	0.028	1.21522091588427E-06	FOLR2+ resident-like macrophages	AGL
7.37440247854044E-11	0.455551300323332	0.224	0.103	1.23580236735381E-06	FOLR2+ resident-like macrophages	ATIC

7.54764100011852E-11	0.58811652849519:	0.161	0.063	1.26483367879986E-06	FOLR2+ resident-like macrophages	C9orf64
7.57503142171775E-11	1.0774920234478	0.183	0.08	1.26942376565146E-06	FOLR2+ resident-like macrophages	NDUFB4
7.59421908154119E-11	1.22865571616236	0.134	0.046	1.27263923368467E-06	FOLR2+ resident-like macrophages	MMP25
7.61512947171379E-11	0.57497882775568	0.215	0.101	1.2761433968698E-06	FOLR2+ resident-like macrophages	GSS
7.75958064582815E-11	0.30214784916856	0.325	0.172	1.30035052462788E-06	FOLR2+ resident-like macrophages	ANKRD22
7.91269119722497E-11	0.28727126315560	0.213	0.095	1.32600879083096E-06	FOLR2+ resident-like macrophages	NEDD4L
7.95813056515338E-11	0.53833830426094	0.17	0.067	1.3336235201084E-06	FOLR2+ resident-like macrophages	RCL1
7.96860504318483E-11	0.26854391589489	0.215	0.098	1.33537883313691E-06	FOLR2+ resident-like macrophages	CSNK2B
7.98515115876938E-11	0.39196897388580	0.176	0.072	1.33815163118657E-06	FOLR2+ resident-like macrophages	DEPRECATED-ENSG00000155115
7.99866966069749E-11	0.48267096540425	0.116	0.035	1.34041706173968E-06	FOLR2+ resident-like macrophages	BBS9
8.0516562760335E-11	0.60583757429482	0.151	0.056	1.34929655873769E-06	FOLR2+ resident-like macrophages	CIR1
8.12703028165354E-11	0.33260762229816	0.158	0.061	1.3619277345995E-06	FOLR2+ resident-like macrophages	TMEM181
8.33966812980997E-11	0.44147275471027	0.218	0.1	1.39756158519356E-06	FOLR2+ resident-like macrophages	DCTN3
8.4305773823983E-11	0.43424233450608	0.139	0.049	1.41279615774231E-06	FOLR2+ resident-like macrophages	DDR1
8.50164343845344E-11	0.32223312472997	0.154	0.056	1.42470540741603E-06	FOLR2+ resident-like macrophages	PNPLA7
8.50204805648148E-11	0.75368074780092	0.155	0.059	1.42477321330517E-06	FOLR2+ resident-like macrophages	ZBTB22
8.6709591316876E-11	0.29305059152726	0.221	0.102	1.45307933128821E-06	FOLR2+ resident-like macrophages	MORC2
8.8279995097401E-11	0.96474343020362	0.12	0.038	1.47939615784225E-06	FOLR2+ resident-like macrophages	HDHD3
9.38864297947006E-11	1.56866829112266	0.138	0.049	1.57334879049959E-06	FOLR2+ resident-like macrophages	GASK1A
9.43234944242051E-11	0.49523068267852	0.132	0.045	1.58067311956083E-06	FOLR2+ resident-like macrophages	GLI4
9.45585360211993E-11	0.50542228834339	0.229	0.11	1.58461194664326E-06	FOLR2+ resident-like macrophages	ELF2
9.69763000305701E-11	0.43734322457213	0.202	0.09	1.62512883591229E-06	FOLR2+ resident-like macrophages	WASL
9.73237848323066E-11	0.32907674332406	0.157	0.06	1.63095198621979E-06	FOLR2+ resident-like macrophages	RAB28
1.01623348213845E-10	0.32849163261608	0.155	0.059	1.70300406936762E-06	FOLR2+ resident-like macrophages	INTS14
1.03220388875406E-10	0.84732105673311	0.135	0.047	1.72976727677405E-06	FOLR2+ resident-like macrophages	DNAJC14
1.04095879554245E-10	1.35935296498543	0.197	0.09	1.74443874957003E-06	FOLR2+ resident-like macrophages	PSMD4
1.05073397321862E-10	0.50967303211557	0.177	0.073	1.76081999231976E-06	FOLR2+ resident-like macrophages	RNF31
1.05526660814444E-10	0.82512931539602	0.129	0.044	1.76841578192846E-06	FOLR2+ resident-like macrophages	KIF1BP
1.05812456941974E-10	1.76077453840448	0.138	0.049	1.7732051534336E-06	FOLR2+ resident-like macrophages	TMEM69
1.1304996952216E-10	0.39674429813462	0.212	0.098	1.89449138925236E-06	FOLR2+ resident-like macrophages	UBAC2
1.18184472897628E-10	1.33837263225093	0.184	0.082	1.98053539681845E-06	FOLR2+ resident-like macrophages	TMEM97
1.19948853468994E-10	1.33114418668636	0.131	0.046	2.01010288643341E-06	FOLR2+ resident-like macrophages	PIGC
1.23413981482648E-10	0.65163187596211	0.132	0.046	2.06817150168622E-06	FOLR2+ resident-like macrophages	RIOK2
1.27676583791908E-10	0.36842095335121	0.203	0.092	2.1396041911848E-06	FOLR2+ resident-like macrophages	ZNF768
1.27733542523906E-10	1.13282229553139	0.157	0.062	2.14055870561562E-06	FOLR2+ resident-like macrophages	TACO1
1.29213024465181E-10	0.65678088053781	0.116	0.036	2.16535186398751E-06	FOLR2+ resident-like macrophages	ACBD4
1.29803037454402E-10	0.27943545111184	0.138	0.049	2.17523930166087E-06	FOLR2+ resident-like macrophages	GFM2
1.29872155444731E-10	0.42595623723840	0.219	0.102	2.1763975809428E-06	FOLR2+ resident-like macrophages	AHDC1
1.30422297721686E-10	0.36965836596116	0.154	0.059	2.18561686522002E-06	FOLR2+ resident-like macrophages	FBXW11
1.31302256515564E-10	0.73063079495202	0.119	0.038	2.20036321468782E-06	FOLR2+ resident-like macrophages	MINPP1
1.31558289602195E-10	0.64361437713324	0.176	0.072	2.20465381715358E-06	FOLR2+ resident-like macrophages	ALDH6A1
1.31605294350613E-10	0.63878661071510	0.221	0.105	2.20544152272757E-06	FOLR2+ resident-like macrophages	PPAN
1.34550597082901E-10	0.32585169427791	0.218	0.101	2.25479890591526E-06	FOLR2+ resident-like macrophages	CBFA2T2
1.35330129821189E-10	0.7293936652155	0.167	0.066	2.26786231554348E-06	FOLR2+ resident-like macrophages	ORMDL3
1.37081776788278E-10	0.37154443096564	0.128	0.043	2.29721641541796E-06	FOLR2+ resident-like macrophages	GUF1
1.37221375058321E-10	1.05669208671399	0.147	0.055	2.29955580322734E-06	FOLR2+ resident-like macrophages	MCEE
1.38965877185469E-10	1.15985853639088	0.141	0.051	2.32879016987409E-06	FOLR2+ resident-like macrophages	CRAT
1.40480785237764E-10	0.53638725145933	0.113	0.034	2.35417699901444E-06	FOLR2+ resident-like macrophages	SERTAD1
1.42633986519402E-10	0.50324305360575	0.176	0.073	2.39026034609215E-06	FOLR2+ resident-like macrophages	UBA5
1.43292672683471E-10	0.81735790189612	0.282	0.145	2.40129860882961E-06	FOLR2+ resident-like macrophages	MTHFD1
1.4382881275092E-10	0.63151714618708	0.245	0.121	2.41028324407992E-06	FOLR2+ resident-like macrophages	ATP5MF
1.44967564696679E-10	0.34380630084708	0.176	0.073	2.42936644918695E-06	FOLR2+ resident-like macrophages	USP7
1.55273837792523E-10	0.62741238293911	0.193	0.085	2.60207897372711E-06	FOLR2+ resident-like macrophages	SIGMAR1
1.5607862690379E-10	0.7391221142087	0.206	0.094	2.61556562965372E-06	FOLR2+ resident-like macrophages	EEF1AKNMT
1.5775753509199E-10	0.72019706374412	0.205	0.093	2.64370077307156E-06	FOLR2+ resident-like macrophages	TAPBPL
1.60005336447212E-10	0.40521542627388	0.104	0.029	2.68136942818238E-06	FOLR2+ resident-like macrophages	PINX1
1.62943176710163E-10	0.98884122193974	0.155	0.06	2.73060175530891E-06	FOLR2+ resident-like macrophages	POP4
1.6674262043098E-10	1.60145468650205	0.106	0.031	2.79427283318237E-06	FOLR2+ resident-like macrophages	PRG4
1.68214417097511E-10	0.78267722612225	0.112	0.034	2.81893720172009E-06	FOLR2+ resident-like macrophages	LTC4S
1.68686333132826E-10	0.25202925925316	0.151	0.057	2.82684557063989E-06	FOLR2+ resident-like macrophages	PLA2G12A
1.709272476392E-10	0.94487233672134	0.118	0.038	2.86439881593772E-06	FOLR2+ resident-like macrophages	ACAD10
1.72169301640807E-10	0.32191556568673	0.151	0.058	2.88521315689665E-06	FOLR2+ resident-like macrophages	TBCE.1
1.73342173706505E-10	1.48983991603081	0.119	0.039	2.90486814697362E-06	FOLR2+ resident-like macrophages	DEPRECATED-ENSG00000114021
1.74789928174845E-10	0.36964082300688	0.131	0.045	2.92912961635405E-06	FOLR2+ resident-like macrophages	GPANK1
1.77551482186901E-10	0.54643202071221	0.142	0.052	2.97540773848809E-06	FOLR2+ resident-like macrophages	MDH1
1.78927061132879E-10	0.65681813305573	0.149	0.057	2.99845969046479E-06	FOLR2+ resident-like macrophages	C4orf48
1.79611199519209E-10	1.12342632210647	0.112	0.034	3.0099244815429E-06	FOLR2+ resident-like macrophages	MYLK
1.81411693385321E-10	0.89532382338382	0.136	0.048	3.04009715775121E-06	FOLR2+ resident-like macrophages	APOBEC3G
1.83368045647074E-10	0.45208175409785	0.222	0.104	3.07288170895367E-06	FOLR2+ resident-like macrophages	PXK
1.84758284974875E-10	1.41999078801892	0.115	0.036	3.09617933960895E-06	FOLR2+ resident-like macrophages	RABIF
1.87556826969437E-10	0.77192171177311	0.129	0.045	3.14307730635383E-06	FOLR2+ resident-like macrophages	DEPRECATED-ENSG00000186591

1.89679560927861E-10	1.59207809933703	0.106	0.031	3.17865008202909E-06	FOLR2+ resident-like macrophages	SLC25A23
1.90708649368542E-10	0.285396861771245	0.192	0.083	3.19589554611803E-06	FOLR2+ resident-like macrophages	LRRC8A
1.92561041308427E-10	0.793051763413631	0.154	0.06	3.22693793024661E-06	FOLR2+ resident-like macrophages	PFDN1
1.93072212940366E-10	0.880838138543111	0.136	0.048	3.23550414445466E-06	FOLR2+ resident-like macrophages	MAGED1
2.00388552949565E-10	0.681775010986598	0.123	0.041	3.35811137032881E-06	FOLR2+ resident-like macrophages	ATMIN
2.01097394879593E-10	0.669723211047629	0.171	0.071	3.36999014339222E-06	FOLR2+ resident-like macrophages	TMEM160
2.12515292677728E-10	0.868197010425717	0.171	0.071	3.56133127469337E-06	FOLR2+ resident-like macrophages	UQCRH
2.14372802613614E-10	0.595648341960101	0.19	0.084	3.59245942619894E-06	FOLR2+ resident-like macrophages	PES 1.00
2.37328431356055E-10	0.530854684095037	0.158	0.063	3.97714985266477E-06	FOLR2+ resident-like macrophages	BCL7C
2.43404296658217E-10	0.447414279135248	0.144	0.053	4.07896920339841E-06	FOLR2+ resident-like macrophages	DMAC1
2.47996206707741E-10	0.463755522984097	0.21	0.095	4.15592043200832E-06	FOLR2+ resident-like macrophages	CARHSP1
2.52252845463671E-10	0.936399722798144	0.174	0.074	4.22725318428021E-06	FOLR2+ resident-like macrophages	OGFOD2
2.52429936922514E-10	0.430460660792212	0.161	0.065	4.23022088294749E-06	FOLR2+ resident-like macrophages	XPA
2.57516687447679E-10	0.334984542338683	0.231	0.113	4.3154646482482E-06	FOLR2+ resident-like macrophages	GORASP1
2.58331070588741E-10	0.350116918923501	0.154	0.061	4.32911208092612E-06	FOLR2+ resident-like macrophages	DPY19L4
2.60344251864671E-10	1.53810506709655	0.145	0.056	4.36284897274815E-06	FOLR2+ resident-like macrophages	MEP1A
2.69733170401147E-10	0.286182833972988	0.187	0.083	4.52018846958243E-06	FOLR2+ resident-like macrophages	FAM3A
2.70220398310495E-10	0.3408834585434	0.219	0.102	4.52835343488727E-06	FOLR2+ resident-like macrophages	SMIM26
2.70585318040049E-10	0.297854806388607	0.171	0.071	4.53446875971514E-06	FOLR2+ resident-like macrophages	SLC10A7
2.72691388246531E-10	0.598612216671229	0.176	0.075	4.56976228423536E-06	FOLR2+ resident-like macrophages	XPO4
2.72881615092405E-10	0.334303009863983	0.158	0.063	4.57295010571853E-06	FOLR2+ resident-like macrophages	PDHX
2.73365754216276E-10	1.11244579939632	0.161	0.065	4.58106330915635E-06	FOLR2+ resident-like macrophages	POLD2
2.76626148881498E-10	0.389096077653169	0.164	0.066	4.63570100295614E-06	FOLR2+ resident-like macrophages	ZNF91
2.81763105244704E-10	0.300179087508196	0.205	0.094	4.72178611769075E-06	FOLR2+ resident-like macrophages	TCF3
2.82716543685451E-10	1.58437932176149	0.1	0.028	4.73776383908078E-06	FOLR2+ resident-like macrophages	MANEAL
2.93441513733996E-10	0.735998558512735	0.132	0.046	4.9174928871543E-06	FOLR2+ resident-like macrophages	IFI27L2
2.97072668494488E-10	0.34367331315957	0.192	0.084	4.97834377863064E-06	FOLR2+ resident-like macrophages	MVB12A
2.98197076294317E-10	0.350055476419842	0.125	0.042	4.99718660454016E-06	FOLR2+ resident-like macrophages	CFAP410
3.01091294096938E-10	0.573123442462519	0.194	0.086	5.04568790647649E-06	FOLR2+ resident-like macrophages	PRKAG1
3.02216280013462E-10	0.652990233266896	0.135	0.048	5.0645404204656E-06	FOLR2+ resident-like macrophages	SDHAF4
3.12643814438036E-10	1.32354103781731	0.11	0.034	5.23928504235261E-06	FOLR2+ resident-like macrophages	SLC35D1
3.12831694796253E-10	0.613312473460129	0.136	0.049	5.24243354139562E-06	FOLR2+ resident-like macrophages	HCFC2
3.14387262807693E-10	0.802310337029933	0.104	0.03	5.26850175013132E-06	FOLR2+ resident-like macrophages	VWA5A
3.14539530973255E-10	1.23808963641283	0.12	0.04	5.2710534600498E-06	FOLR2+ resident-like macrophages	FAM210B
3.22094152817801E-10	0.366878806383714	0.179	0.077	5.39765381292071E-06	FOLR2+ resident-like macrophages	DEPRECATED-ENSG00000078668
3.2295353239429E-10	1.77209136953683	0.116	0.038	5.41205529586351E-06	FOLR2+ resident-like macrophages	FPGT
3.34686653948081E-10	0.269789513028382	0.261	0.134	5.60867894686194E-06	FOLR2+ resident-like macrophages	SNRPB
3.35544064185014E-10	0.510413953953137	0.109	0.033	5.62304742761246E-06	FOLR2+ resident-like macrophages	BTBD10
3.3939281046279E-10	1.16229439775532	0.144	0.054	5.68754471773543E-06	FOLR2+ resident-like macrophages	ETFDH
3.53868352505286E-10	0.520502784036723	0.18	0.078	5.93012585128359E-06	FOLR2+ resident-like macrophages	CFAP97
3.53982983129894E-10	0.366244362993416	0.155	0.062	5.93204683129076E-06	FOLR2+ resident-like macrophages	TLK2
3.55955124779748E-10	0.777800301693577	0.192	0.086	5.96509598105902E-06	FOLR2+ resident-like macrophages	TCAF1
3.56248344353022E-10	1.27323760309901	0.115	0.037	5.97000975466795E-06	FOLR2+ resident-like macrophages	BLOC1S4
3.62558293572784E-10	0.601287629864543	0.189	0.084	6.07575188369272E-06	FOLR2+ resident-like macrophages	YIPF5
3.64953989227067E-10	0.258713626272	0.216	0.103	6.1158989514672E-06	FOLR2+ resident-like macrophages	TSN
3.70019890354854E-10	0.47407695889706	0.179	0.078	6.20079332256664E-06	FOLR2+ resident-like macrophages	CD2BP2
3.76922446277574E-10	0.334329424975452	0.199	0.09	6.31646635471959E-06	FOLR2+ resident-like macrophages	PSMD9
3.7765320776168E-10	0.473708888539294	0.134	0.047	6.32871245567023E-06	FOLR2+ resident-like macrophages	AP3S1
4.03177185758054E-10	0.573939016589836	0.148	0.057	6.75644327893347E-06	FOLR2+ resident-like macrophages	SMIM10L1
4.16640367862351E-10	0.744640030008367	0.181	0.08	6.98205928463728E-06	FOLR2+ resident-like macrophages	DHX58
4.17454958040463E-10	0.402050784958081	0.184	0.082	6.99571018684208E-06	FOLR2+ resident-like macrophages	KLF13
4.23695249059669E-10	0.581504682353206	0.141	0.053	7.10028498374193E-06	FOLR2+ resident-like macrophages	ERI2
4.25002496292463E-10	0.317879853540554	0.183	0.079	7.1221918328691E-06	FOLR2+ resident-like macrophages	MTPAP
4.2800508443015E-10	0.332393514214467	0.21	0.1	7.17250920488045E-06	FOLR2+ resident-like macrophages	LSM14B
4.28764054263215E-10	0.542985562027755	0.158	0.064	7.18522802134296E-06	FOLR2+ resident-like macrophages	TSG101
4.30140606200716E-10	0.944516608910818	0.179	0.077	7.2082962787116E-06	FOLR2+ resident-like macrophages	MSMO1
4.47027240960442E-10	0.54590505531723	0.208	0.097	7.49128250401508E-06	FOLR2+ resident-like macrophages	MFSD14B
4.51757926470109E-10	0.41866085671967	0.177	0.076	7.57055933178609E-06	FOLR2+ resident-like macrophages	C19orf25
4.55193125620266E-10	0.414189160805811	0.144	0.054	7.62812639914442E-06	FOLR2+ resident-like macrophages	POLR2C
4.6045776356098E-10	0.377592218087772	0.19	0.085	7.7163512017549E-06	FOLR2+ resident-like macrophages	MDH2
4.64781466994325E-10	0.603340738234529	0.152	0.061	7.78880782389089E-06	FOLR2+ resident-like macrophages	ZNF12
4.73013059752664E-10	0.754068492069725	0.138	0.051	7.92675285533515E-06	FOLR2+ resident-like macrophages	ARL 16.00
4.77558087757624E-10	0.79397105969587	0.131	0.046	8.00291843464226E-06	FOLR2+ resident-like macrophages	DHRS7B
4.85576558789083E-10	0.531736606700143	0.183	0.08	8.13729197218745E-06	FOLR2+ resident-like macrophages	RFT1
4.87085001071333E-10	0.270017126291473	0.18	0.078	8.1625704479534E-06	FOLR2+ resident-like macrophages	UXS1
4.88601793427371E-10	0.508445481548042	0.141	0.052	8.18798885425589E-06	FOLR2+ resident-like macrophages	EDNRB
4.9603421519921E-10	0.879308491527318	0.149	0.059	8.31254137830835E-06	FOLR2+ resident-like macrophages	HINT2
5.03515839736786E-10	0.637742874711243	0.167	0.07	8.43791844230907E-06	FOLR2+ resident-like macrophages	ANAPC13
5.11659842889745E-10	0.420011790078092	0.209	0.098	8.57439564714634E-06	FOLR2+ resident-like macrophages	ACADS
5.15583566379718E-10	0.896248630616556	0.149	0.058	8.64014940539132E-06	FOLR2+ resident-like macrophages	DEPRECATED-ENSG00000050130
5.17625178924912E-10	0.625992477223691	0.154	0.061	8.67436274842367E-06	FOLR2+ resident-like macrophages	EIF4ENIF1

5.19665899939554E-10	0.82776843389459	0.106	0.032	8.70856115118705E-06	FOLR2+ resident-like macrophages	ZNF345
5.20046837849473E-10	0.51488582390926	0.129	0.046	8.71494490868146E-06	FOLR2+ resident-like macrophages	NOL6
5.24179759986949E-10	0.30678782861314	0.16	0.065	8.7842044178613E-06	FOLR2+ resident-like macrophages	CYCS
5.28229677970942E-10	0.80736407207844	0.144	0.055	8.85207294343704E-06	FOLR2+ resident-like macrophages	ACOT8
5.28554775306157E-10	0.52787660400002	0.19	0.085	8.85752092458057E-06	FOLR2+ resident-like macrophages	GRPEL1
5.29536711425504E-10	0.75479575135711	0.118	0.039	8.87397621006859E-06	FOLR2+ resident-like macrophages	TCEAL3
5.39548191074841E-10	0.26156816285857	0.165	0.068	9.04174858603219E-06	FOLR2+ resident-like macrophages	PIGM
5.61301123993906E-10	0.33505061779686	0.228	0.112	9.40628423588987E-06	FOLR2+ resident-like macrophages	SYT11
5.65848859421477E-10	0.32543960358804	0.145	0.055	9.48249518618511E-06	FOLR2+ resident-like macrophages	SLC35E2A
5.69636350162847E-10	0.39381103461362	0.165	0.068	9.54596595602898E-06	FOLR2+ resident-like macrophages	TMEM161A
5.70998860161682E-10	1.00135920967856	0.141	0.053	9.56879889858946E-06	FOLR2+ resident-like macrophages	EHBP1
5.72918123658748E-10	0.82222604749832	0.144	0.056	9.6009619162733E-06	FOLR2+ resident-like macrophages	AMOTL1
5.79326774815847E-10	0.66838072932065	0.155	0.062	9.70835809236396E-06	FOLR2+ resident-like macrophages	NDUFC1
5.82315823882371E-10	0.65734090433021	0.113	0.036	9.75844857662077E-06	FOLR2+ resident-like macrophages	BLZF1
5.84189597587197E-10	1.3336691228063	0.116	0.038	9.78984927636624E-06	FOLR2+ resident-like macrophages	LRRC42
5.84365261784822E-10	0.59321617428699	0.18	0.079	9.79279305699004E-06	FOLR2+ resident-like macrophages	GFPT1
5.99600345782144E-10	0.26916951765955	0.232	0.114	1.00481025946172E-05	FOLR2+ resident-like macrophages	COPS3
6.02219676524151E-10	0.35450650318726	0.142	0.054	1.00919973391917E-05	FOLR2+ resident-like macrophages	DAG1
6.03783369245151E-10	0.69920928014905	0.179	0.079	1.01182017018102E-05	FOLR2+ resident-like macrophages	NUDT2
6.06449085311658E-10	0.56418731145181	0.179	0.079	1.01628737716528E-05	FOLR2+ resident-like macrophages	SRD5A3
6.0894439663703E-10	0.43939616286158	0.192	0.086	1.02046901988434E-05	FOLR2+ resident-like macrophages	TRNT1
6.09665876942937E-10	0.32879663858376	0.161	0.066	1.02167807658097E-05	FOLR2+ resident-like macrophages	MTRR
6.16702794844912E-10	0.39650785684073	0.18	0.079	1.0334705436011E-05	FOLR2+ resident-like macrophages	REPIN1
6.21462691621387E-10	0.49035229862013	0.18	0.079	1.04144717861912E-05	FOLR2+ resident-like macrophages	TDRD7
6.25502053246701E-10	0.70185593835450	0.125	0.044	1.04821634083082E-05	FOLR2+ resident-like macrophages	FAM114A2
6.40247061432176E-10	0.50754028168585	0.161	0.066	1.07292602554804E-05	FOLR2+ resident-like macrophages	ERP29
6.49623052371946E-10	0.43111502531757	0.179	0.079	1.08863831116491E-05	FOLR2+ resident-like macrophages	TARS2
6.5935085809682E-10	0.37688645491822	0.179	0.077	1.10494016799865E-05	FOLR2+ resident-like macrophages	UQCC2
6.88350180774347E-10	0.45580006221139	0.109	0.034	1.15353723294165E-05	FOLR2+ resident-like macrophages	TMEM91
6.89978339657148E-10	0.53217268448502	0.152	0.061	1.15626570159745E-05	FOLR2+ resident-like macrophages	CLOCK
7.07294226339328E-10	0.77909696054334	0.131	0.047	1.18528366449945E-05	FOLR2+ resident-like macrophages	LRFN4
7.15490991360966E-10	0.77752476053027	0.116	0.039	1.19901980332271E-05	FOLR2+ resident-like macrophages	EMC6
7.27960848654106E-10	1.33753749900944	0.109	0.034	1.21991679017455E-05	FOLR2+ resident-like macrophages	SORD
7.3367258622567E-10	0.67112208290107	0.148	0.059	1.22948851999698E-05	FOLR2+ resident-like macrophages	SMARCD1
7.34326122599302E-10	0.63870822513178	0.164	0.068	1.23058371625191E-05	FOLR2+ resident-like macrophages	C11orf24
7.57787922942962E-10	0.81458018860018	0.128	0.046	1.26990100126781E-05	FOLR2+ resident-like macrophages	EBPL
7.58920506430665E-10	0.55104262983708	0.171	0.074	1.27179898467651E-05	FOLR2+ resident-like macrophages	COG2
7.71132005565076E-10	0.89946767808015	0.122	0.042	1.29226301492595E-05	FOLR2+ resident-like macrophages	ZBTB14
7.98351675356539E-10	0.36509367255629	0.177	0.076	1.33787773756249E-05	FOLR2+ resident-like macrophages	MTX2
8.03518862053488E-10	0.32360061375488	0.208	0.098	1.34653690902923E-05	FOLR2+ resident-like macrophages	CTPS1
8.04393166836668E-10	1.40188819001094	0.12	0.042	1.34800206898489E-05	FOLR2+ resident-like macrophages	MAOB
8.10515156927124E-10	1.15190800298385	0.132	0.048	1.35826129997847E-05	FOLR2+ resident-like macrophages	FBXL4
8.12415961217321E-10	0.99584145102388	0.134	0.049	1.36144666780799E-05	FOLR2+ resident-like macrophages	RNF170
8.13842591697985E-10	0.76825975492469	0.145	0.056	1.36383741516748E-05	FOLR2+ resident-like macrophages	COQ5
8.14037930104305E-10	0.42916470518156	0.192	0.088	1.36416476326879E-05	FOLR2+ resident-like macrophages	RNF26
8.19071778866476E-10	1.60881581554635	0.103	0.031	1.37260048702444E-05	FOLR2+ resident-like macrophages	TBC1D24
8.34867573681691E-10	0.61219097627299	0.144	0.055	1.39907107997578E-05	FOLR2+ resident-like macrophages	PRPF18
8.63530866187264E-10	0.93352286452058	0.147	0.058	1.44710502555662E-05	FOLR2+ resident-like macrophages	COMMD2
8.71693195355698E-10	0.37478256463485	0.164	0.068	1.46078345677708E-05	FOLR2+ resident-like macrophages	DEPRECATED-ENSG00000099810
8.81214350258276E-10	0.58623278275255	0.193	0.088	1.47673900816282E-05	FOLR2+ resident-like macrophages	GMPS
9.09625166232067E-10	0.54266932662905	0.112	0.036	1.5243498535717E-05	FOLR2+ resident-like macrophages	TMEM126A
9.35764844647069E-10	0.33079902204601	0.216	0.105	1.56815472665956E-05	FOLR2+ resident-like macrophages	TIMM17A
9.36625744801515E-10	0.27435334231621	0.134	0.049	1.56959742313838E-05	FOLR2+ resident-like macrophages	CCDC90B
9.60349027100723E-10	0.99984718668413	0.126	0.046	1.60935289961539E-05	FOLR2+ resident-like macrophages	ASRGL1
9.60719607534892E-10	1.12961721008144	0.109	0.035	1.60997391830697E-05	FOLR2+ resident-like macrophages	ZNF234
9.75667439459548E-10	0.54891511468035	0.128	0.046	1.63502349504631E-05	FOLR2+ resident-like macrophages	JKAMP
9.91746236899611E-10	0.59729537022499	0.144	0.056	1.66196834379637E-05	FOLR2+ resident-like macrophages	BRK1
9.9203398882332E-10	0.25853877385721	0.245	0.124	1.66245055847012E-05	FOLR2+ resident-like macrophages	RAD21
1.02148924569267E-09	0.36810275634025	0.187	0.084	1.71181167793178E-05	FOLR2+ resident-like macrophages	PRR12
1.02249186448115E-09	1.1443199332685	0.132	0.049	1.71349186649752E-05	FOLR2+ resident-like macrophages	SLC30A5
1.03511544787482E-09	0.29272745932343	0.187	0.083	1.73464646754863E-05	FOLR2+ resident-like macrophages	DEPRECATED-ENSG00000175826
1.03709171112038E-09	0.50718834927381	0.165	0.07	1.73795828949554E-05	FOLR2+ resident-like macrophages	PGAP2
1.04257658250722E-09	0.90380708471357	0.136	0.052	1.74714983696561E-05	FOLR2+ resident-like macrophages	POFUT1
1.04795132008709E-09	0.65356874367997	0.209	0.099	1.75615682220195E-05	FOLR2+ resident-like macrophages	MYO19
1.06546234358074E-09	0.43770839473627	0.21	0.101	1.7855017953726E-05	FOLR2+ resident-like macrophages	NOP14
1.08076213106445E-09	0.31727854939103	0.142	0.055	1.81114117923781E-05	FOLR2+ resident-like macrophages	ANTKMT
1.08665534696237E-09	0.57853196487603	0.119	0.041	1.82101703043953E-05	FOLR2+ resident-like macrophages	PLCG1
1.08827217711918E-09	0.38449720429354	0.197	0.09	1.82372651441633E-05	FOLR2+ resident-like macrophages	DHX38
1.09308860622259E-09	1.17849434132662	0.103	0.031	1.83179788630782E-05	FOLR2+ resident-like macrophages	GTF2IRD2B
1.11667149269867E-09	0.70147073943386	0.148	0.059	1.87131808746443E-05	FOLR2+ resident-like macrophages	FAM221A
1.13232210222594E-09	1.62823540822125	0.123	0.045	1.89754537891023E-05	FOLR2+ resident-like macrophages	DGAT2

1.13351710541039E-09	0.322835362334732	0.128	0.046	1.89954796524673E-05	FOLR2+ resident-like macrophages	RASL11A
1.14342449883631E-09	0.477731561968997	0.149	0.06	1.91615077514989E-05	FOLR2+ resident-like macrophages	DEPRECATED-ENSG00000139318
1.18370232575138E-09	0.272993259828219	0.152	0.062	1.98364835749417E-05	FOLR2+ resident-like macrophages	PCNX3
1.20090949799251E-09	0.269560016372714	0.241	0.119	2.01248413673585E-05	FOLR2+ resident-like macrophages	TUBA1B
1.22804862640143E-09	0.662737914662932	0.145	0.058	2.05796388812351E-05	FOLR2+ resident-like macrophages	RPRD1B
1.23139606599646E-09	0.468773979655028	0.152	0.062	2.06357352739688E-05	FOLR2+ resident-like macrophages	TAF12
1.2699752714377E-09	0.304636016591874	0.139	0.053	2.12822455987531E-05	FOLR2+ resident-like macrophages	ZNF281
1.32090657429178E-09	0.633706013258238	0.144	0.057	2.21357523719817E-05	FOLR2+ resident-like macrophages	SSBP1
1.32351139194002E-09	0.464974740219933	0.126	0.046	2.21794039061308E-05	FOLR2+ resident-like macrophages	GLOD4
1.34657235834275E-09	1.0699101082258	0.177	0.08	2.25658595811077E-05	FOLR2+ resident-like macrophages	ATP5MC1
1.3715989651467E-09	0.652724205681179	0.109	0.035	2.29852554579285E-05	FOLR2+ resident-like macrophages	RAB11FIP3
1.39328180978549E-09	1.74441323025845	0.136	0.053	2.33486165683853E-05	FOLR2+ resident-like macrophages	PPP2R1B
1.4198519667473E-09	0.43479825804463	0.202	0.095	2.37938792587513E-05	FOLR2+ resident-like macrophages	USP53
1.42072933195241E-09	0.445273627581593	0.163	0.068	2.38085821448585E-05	FOLR2+ resident-like macrophages	NAT9
1.50140577855824E-09	0.860152037876483	0.148	0.061	2.51605580370789E-05	FOLR2+ resident-like macrophages	WRAP73
1.52381086379669E-09	0.509827323883816	0.171	0.075	2.5536022455505E-05	FOLR2+ resident-like macrophages	RNF113A
1.52923203564703E-09	0.789796716958512	0.144	0.057	2.56268704533728E-05	FOLR2+ resident-like macrophages	DMAC2
1.54361280043663E-09	0.528763781913509	0.184	0.084	2.5867863309717E-05	FOLR2+ resident-like macrophages	SNRPD2
1.57727910213176E-09	1.02441783392062	0.115	0.04	2.6432043193524E-05	FOLR2+ resident-like macrophages	SMUG1
1.61733435698941E-09	0.752994976757082	0.142	0.056	2.71032891544285E-05	FOLR2+ resident-like macrophages	LRP11
1.63738682887203E-09	1.18938917054787	0.135	0.052	2.74393284782375E-05	FOLR2+ resident-like macrophages	MPLKIP
1.64127166627889E-09	0.628632696595528	0.145	0.058	2.75044305835016E-05	FOLR2+ resident-like macrophages	VRK3
1.65249335034708E-09	0.650526499381352	0.151	0.063	2.76924835651164E-05	FOLR2+ resident-like macrophages	PIGK
1.70789128804589E-09	0.588581131834716	0.122	0.044	2.8620842205073E-05	FOLR2+ resident-like macrophages	HIBCH
1.80896058076337E-09	0.27297053880515	0.139	0.054	3.03145614124325E-05	FOLR2+ resident-like macrophages	BRIX1
1.81102277883936E-09	0.888727605570823	0.135	0.052	3.03491197277899E-05	FOLR2+ resident-like macrophages	METTL5
1.84012199085293E-09	0.519727330471086	0.141	0.055	3.08367643227135E-05	FOLR2+ resident-like macrophages	RGP1
1.88136106765112E-09	1.52494794048438	0.112	0.038	3.15278487716975E-05	FOLR2+ resident-like macrophages	WDR7
1.9296264657209E-09	1.13537188439707	0.136	0.053	3.23366803125508E-05	FOLR2+ resident-like macrophages	ETAA1
2.0195276166651E-09	0.465314587856509	0.154	0.065	3.38432438000737E-05	FOLR2+ resident-like macrophages	RUSC1
2.07786007664858E-09	0.439374455513401	0.104	0.033	3.48207791644769E-05	FOLR2+ resident-like macrophages	INTS12
2.14733283322112E-09	0.565089911917745	0.174	0.077	3.59850036191195E-05	FOLR2+ resident-like macrophages	TMCC3
2.15074583219031E-09	0.416980875163117	0.123	0.045	3.60421986558452E-05	FOLR2+ resident-like macrophages	MTERF3
2.15332727090964E-09	0.252968358218665	0.155	0.065	3.60854584059037E-05	FOLR2+ resident-like macrophages	DEPRECATED-ENSG00000181061
2.17625195878959E-09	0.799879092758224	0.103	0.032	3.6469630325396E-05	FOLR2+ resident-like macrophages	VGLL4
2.18098464674228E-09	0.731896523180647	0.136	0.052	3.65489407101071E-05	FOLR2+ resident-like macrophages	HYI
2.24593610613592E-09	1.28174571024263	0.109	0.036	3.76373972666258E-05	FOLR2+ resident-like macrophages	SMAD1
2.28389704583116E-09	1.31599962802129	0.155	0.065	3.82735466940385E-05	FOLR2+ resident-like macrophages	DSN1
2.35258575019845E-09	0.450650467042356	0.167	0.073	3.94246320018256E-05	FOLR2+ resident-like macrophages	TICAM1
2.43983837281214E-09	0.793776582322784	0.145	0.059	4.08868114515858E-05	FOLR2+ resident-like macrophages	IPMK
2.53833699688746E-09	0.518189312669578	0.148	0.061	4.253745139384E-05	FOLR2+ resident-like macrophages	GPKOW
2.59242312668775E-09	0.517206909404993	0.158	0.067	4.34438267570334E-05	FOLR2+ resident-like macrophages	COX15
2.71682970195138E-09	0.539857757453422	0.177	0.079	4.55286321453013E-05	FOLR2+ resident-like macrophages	GGCT
2.73355709664455E-09	1.41622699160406	0.132	0.052	4.58089498255694E-05	FOLR2+ resident-like macrophages	F2RL1
2.73790017471785E-09	0.703769099821203	0.131	0.049	4.58817311279218E-05	FOLR2+ resident-like macrophages	NEURL4
2.749572897582E-09	0.359026920563987	0.242	0.126	4.60773426176792E-05	FOLR2+ resident-like macrophages	NAA40
2.82096519744629E-09	0.431994770448226	0.17	0.076	4.72737347788049E-05	FOLR2+ resident-like macrophages	ERGIC2
2.92536614530501E-09	0.649113712930087	0.179	0.082	4.90232858630214E-05	FOLR2+ resident-like macrophages	ERI3
2.94277741889841E-09	0.805071063222167	0.183	0.083	4.93150639858995E-05	FOLR2+ resident-like macrophages	SFXN1
2.96649009545552E-09	0.556459379128817	0.119	0.043	4.97124410196435E-05	FOLR2+ resident-like macrophages	SYNJ2BP
2.97088784896886E-09	0.29335516752792	0.194	0.091	4.97861385730201E-05	FOLR2+ resident-like macrophages	DEPRECATED-ENSG00000196313
3.09943080932023E-09	0.278204986091536	0.179	0.082	5.19402615025885E-05	FOLR2+ resident-like macrophages	STK32C
3.11357223680992E-09	1.20904376175986	0.106	0.035	5.21772435444607E-05	FOLR2+ resident-like macrophages	WDR24
3.2793298354179E-09	0.767023763746306	0.149	0.063	5.49550093819332E-05	FOLR2+ resident-like macrophages	THYN1
3.38449448594958E-09	0.437549385841784	0.206	0.102	5.6717358595543E-05	FOLR2+ resident-like macrophages	DHTKD1
3.47106575262634E-09	0.618394744816652	0.168	0.075	5.81681198825121E-05	FOLR2+ resident-like macrophages	SAP130
3.49282539050685E-09	0.671578368919602	0.151	0.063	5.85327678941138E-05	FOLR2+ resident-like macrophages	MPG
3.49356361794635E-09	0.444647205724655	0.167	0.073	5.85451391095449E-05	FOLR2+ resident-like macrophages	UBN2
3.58587601598932E-09	0.847429287216255	0.131	0.05	6.0092110275949E-05	FOLR2+ resident-like macrophages	LSR
3.60206822590178E-09	0.623202157824403	0.128	0.048	6.0363459329662E-05	FOLR2+ resident-like macrophages	LLPH
3.63097159992982E-09	0.592087512457781	0.103	0.033	6.08478220716239E-05	FOLR2+ resident-like macrophages	DPM2
3.71734457968086E-09	0.640288825063112	0.12	0.044	6.22952604662919E-05	FOLR2+ resident-like macrophages	RIC8B
3.77727792290251E-09	0.266786298389807	0.177	0.082	6.32996234320003E-05	FOLR2+ resident-like macrophages	ABCB10
3.83240148759863E-09	0.292250200254879	0.192	0.09	6.42233841291778E-05	FOLR2+ resident-like macrophages	COMMD6
3.98304081047209E-09	0.391696302742037	0.208	0.101	6.67477979018913E-05	FOLR2+ resident-like macrophages	COQ2
3.98729565936131E-09	0.337292298591241	0.128	0.047	6.68191006595769E-05	FOLR2+ resident-like macrophages	RNASEH2C
4.14386251739278E-09	1.16825948774144	0.134	0.052	6.94428480664682E-05	FOLR2+ resident-like macrophages	ZNF7
4.18171547825976E-09	0.926580191303328	0.1	0.031	7.00771879846771E-05	FOLR2+ resident-like macrophages	TCP11L2
4.26504028545856E-09	0.660356724383271	0.148	0.062	7.14735451037145E-05	FOLR2+ resident-like macrophages	DYRK1B
4.32686614881122E-09	0.595024460911331	0.141	0.057	7.25096229217784E-05	FOLR2+ resident-like macrophages	COQ6
4.37472175561811E-09	0.628314673130919	0.141	0.056	7.33115871806483E-05	FOLR2+ resident-like macrophages	SLC35C1

4.41168436044418E-09	0.25453643888128€	0.192	0.09	7.39310065123236E-05	FOLR2+ resident-like macrophages	CIAO2B
4.42143347340057E-09	0.89451761758397	0.126	0.047	7.40943821472467E-05	FOLR2+ resident-like macrophages	ARL 11.00
4.78102546001958E-09	0.50149898999805	0.141	0.056	8.01204246590082E-05	FOLR2+ resident-like macrophages	PIP4K2C
4.83693480358242E-09	0.29017289136977€	0.194	0.095	8.10573534384342E-05	FOLR2+ resident-like macrophages	GTF3C2
4.94467571453794E-09	0.63906927254644	0.176	0.08	8.28628756242268E-05	FOLR2+ resident-like macrophages	MTERF4
4.9856120425239E-09	0.67394537401397	0.109	0.037	8.35488866086155E-05	FOLR2+ resident-like macrophages	MANEA
5.01885402913826E-09	0.46968341485042	0.181	0.083	8.4105955820299E-05	FOLR2+ resident-like macrophages	TOPBP1
5.25059470880487E-09	0.86172917444703	0.112	0.039	8.79894661301519E-05	FOLR2+ resident-like macrophages	AARS
5.27479403024302E-09	1.23306633515134	0.104	0.035	8.83949983588125E-05	FOLR2+ resident-like macrophages	DOLK
5.27948005906272E-09	1.16380760134072	0.161	0.072	8.84735268297731E-05	FOLR2+ resident-like macrophages	PXMP2
5.3137038815338E-09	1.14134002477825	0.132	0.052	8.90470496467434E-05	FOLR2+ resident-like macrophages	TMEM184C
5.40097843724574E-09	0.35707045655734	0.218	0.11	9.05095966513641E-05	FOLR2+ resident-like macrophages	SLC39A9
5.42435342813706E-09	0.55011381055897	0.151	0.064	9.09013147487209E-05	FOLR2+ resident-like macrophages	NAE1
5.45012009772818E-09	0.34114659461732	0.128	0.048	9.13331125977288E-05	FOLR2+ resident-like macrophages	PPP2R5B
5.48019172300923E-09	0.70755556879933	0.168	0.076	9.18370528941887E-05	FOLR2+ resident-like macrophages	VPS25
5.57389690844526E-09	0.93470211747951	0.139	0.057	9.34073643917256E-05	FOLR2+ resident-like macrophages	GPALPP1
5.66480447413339E-09	0.66004523085617	0.184	0.086	9.49307933775273E-05	FOLR2+ resident-like macrophages	PLEKHA7
5.73145461851024E-09	0.63431943825229	0.189	0.089	9.60477164969945E-05	FOLR2+ resident-like macrophages	YIF1A
5.75227284188389E-09	0.25574740933515	0.118	0.043	9.63965882842902E-05	FOLR2+ resident-like macrophages	DDX28
5.77281955970384E-09	0.50936776027318	0.138	0.055	9.6740910181517E-05	FOLR2+ resident-like macrophages	AGFG2
5.77840131243403E-09	0.38543015015374	0.112	0.039	9.68344491937695E-05	FOLR2+ resident-like macrophages	MRFAP1
5.79089705779484E-09	0.96088519186685	0.116	0.042	9.7043852894526E-05	FOLR2+ resident-like macrophages	RPP38
5.86240959656802E-09	0.43181981875288	0.131	0.05	9.8242260019287E-05	FOLR2+ resident-like macrophages	SPHK1
6.25825159779134E-09	0.295634684359091	0.17	0.077	0.000104875780275787	FOLR2+ resident-like macrophages	GTF2F2
6.30583362803088E-09	0.41633419839194	0.187	0.088	0.000105673159938541	FOLR2+ resident-like macrophages	TMEM170A
6.8480285407363E-09	0.28602718520043	0.21	0.106	0.000114759262285659	FOLR2+ resident-like macrophages	HAT1
7.06482814591161E-09	0.33441669127258	0.151	0.064	0.000118392390069187	FOLR2+ resident-like macrophages	SNAP47
7.10647294512651E-09	1.3399236215341	0.107	0.037	0.00011909027361443	FOLR2+ resident-like macrophages	ALDOC
7.28054818041045E-09	0.49207421322999	0.119	0.044	0.000122007426407318	FOLR2+ resident-like macrophages	HRAS
7.38995199812799E-09	0.69213789331843	0.131	0.051	0.000123840815584629	FOLR2+ resident-like macrophages	IMPDH1
7.55618939984729E-09	0.59459043339817	0.109	0.038	0.000126626621962641	FOLR2+ resident-like macrophages	C15orf40
7.62409927445401E-09	0.46386119039686	0.184	0.086	0.0001277646556413	FOLR2+ resident-like macrophages	MCM2
7.70040028568092E-09	0.44868391271728	0.135	0.054	0.000129043307987441	FOLR2+ resident-like macrophages	FP565260.6
7.74278961719199E-09	0.78497658743032	0.131	0.051	0.000129753668404903	FOLR2+ resident-like macrophages	TRAPPC13
8.16701661264053E-09	0.26999886055426	0.167	0.075	0.00013686286439463	FOLR2+ resident-like macrophages	EIF2B1
8.21751690069382E-09	0.31615431170269	0.147	0.061	0.000137709148221827	FOLR2+ resident-like macrophages	PRXL2C
8.34499864129109E-09	0.38583479690408	0.171	0.078	0.000139845487230756	FOLR2+ resident-like macrophages	DDX19A
8.47208182894137E-09	1.25504883913628	0.125	0.047	0.000141975147289399	FOLR2+ resident-like macrophages	TTC38
8.58278329298328E-09	0.44938503955977	0.186	0.089	0.000143830282423814	FOLR2+ resident-like macrophages	TMEM164
8.64589204257229E-09	0.56262473271079	0.142	0.059	0.000144887858849426	FOLR2+ resident-like macrophages	FGL1
8.73224982289215E-09	0.32360344789943	0.131	0.051	0.000146335042532027	FOLR2+ resident-like macrophages	C1D
8.79627111601825E-09	0.26746959890022€	0.104	0.035	0.000147407911362234	FOLR2+ resident-like macrophages	GABPA
8.83282719971036E-09	0.84153444740632	0.115	0.042	0.000148020518212746	FOLR2+ resident-like macrophages	C6orf47
9.03526656472011E-09	0.71003976855810	0.102	0.033	0.00015141299709158	FOLR2+ resident-like macrophages	RFK
9.18363235766656E-09	1.18252444317367	0.125	0.048	0.000153899311049776	FOLR2+ resident-like macrophages	CCNB1IP1
9.24290421257299E-09	1.29408686120437	0.128	0.05	0.000154892588794298	FOLR2+ resident-like macrophages	ATP5F1C
9.27207642202285E-09	0.52659957200983	0.144	0.061	0.000155381456680259	FOLR2+ resident-like macrophages	YIPF1
9.27258027568972E-09	0.47062683174289	0.181	0.085	0.000155389900260008	FOLR2+ resident-like macrophages	LRIG2
9.28612602311779E-09	0.46919774335223	0.11	0.039	0.000155616899895408	FOLR2+ resident-like macrophages	ARMC7
9.36184818760373E-09	0.83585158822785	0.141	0.059	0.000156885851927863	FOLR2+ resident-like macrophages	NSUN3
9.64140114343904E-09	1.3858886117356	0.1	0.033	0.000161570600361751	FOLR2+ resident-like macrophages	WDYHV1
9.69883675875512E-09	0.42303883807181	0.183	0.087	0.000162533106403218	FOLR2+ resident-like macrophages	ALG5
1.00043012464678E-08	1.34559200570693	0.116	0.043	0.000167652080288307	FOLR2+ resident-like macrophages	CLYBL
1.00341824823262E-08	1.49688965688333	0.103	0.035	0.000168152830038822	FOLR2+ resident-like macrophages	C19orf33
1.02555354097077E-08	0.48430002995641	0.11	0.039	0.000171862262395882	FOLR2+ resident-like macrophages	MDP1
1.03720633970792E-08	0.38889430960195	0.103	0.034	0.000173815038408254	FOLR2+ resident-like macrophages	PAM
1.08236808738027E-08	0.27155929673779	0.189	0.09	0.000181383244083186	FOLR2+ resident-like macrophages	PAM16
1.11515506105043E-08	0.86672205373184	0.163	0.073	0.000186877685130832	FOLR2+ resident-like macrophages	TUBB4B
1.176683268021E-08	0.43792686550990	0.115	0.042	0.000197188582054959	FOLR2+ resident-like macrophages	ZNF708
1.19869351559322E-08	0.47614505701663	0.194	0.095	0.000200877059343113	FOLR2+ resident-like macrophages	LRRC8D
1.21984252035368E-08	0.88512416677633	0.106	0.037	0.00020442120956087	FOLR2+ resident-like macrophages	RBIS
1.29748629516459E-08	0.42496875018665	0.19	0.092	0.000217432753343682	FOLR2+ resident-like macrophages	SCNM1
1.31568059602038E-08	0.36134294496972€	0.145	0.062	0.000220481754281095	FOLR2+ resident-like macrophages	LCMT1
1.3179831521269E-08	1.14082352680738	0.109	0.039	0.000220867616633426	FOLR2+ resident-like macrophages	LCLAT1
1.3382353521154E-08	0.51994900984459	0.206	0.103	0.000224261480307499	FOLR2+ resident-like macrophages	RBM12B
1.34836788304459E-08	0.92693736079630	0.113	0.042	0.000225959489840612	FOLR2+ resident-like macrophages	RAB3IP
1.35079515720902E-08	0.46821531918003	0.145	0.062	0.000226366252445087	FOLR2+ resident-like macrophages	BRD9
1.37509803557754E-08	0.57453040182247	0.142	0.06	0.000230438928802084	FOLR2+ resident-like macrophages	R3HCC1
1.3805677864314E-08	1.47409952508196	0.104	0.037	0.000231355549650174	FOLR2+ resident-like macrophages	C4BPB
1.43096234369808E-08	0.2926858763148	0.145	0.062	0.000239800669556925	FOLR2+ resident-like macrophages	HEMK1
1.43181584520413E-08	0.30409755248710	0.125	0.048	0.000239943699339308	FOLR2+ resident-like macrophages	GFER

1.43203145927697E-08	0.290879396816786	0.12	0.046	0.000239979831945635	FOLR2+ resident-like macrophages	CSNK1G3
1.47258725325568E-08	0.398413366462803	0.141	0.059	0.000246776171900587	FOLR2+ resident-like macrophages	TMEM120A
1.49061682949073E-08	0.656925077893822	0.189	0.093	0.000249797568286057	FOLR2+ resident-like macrophages	DCAF16
1.5206184307863E-08	1.19181575202152	0.134	0.055	0.000254825236631169	FOLR2+ resident-like macrophages	PSPH
1.57488271768846E-08	0.425971557172099	0.104	0.036	0.000263918845830232	FOLR2+ resident-like macrophages	DEPRECATED-ENSG00000185619
1.58508727043731E-08	1.06061748361167	0.132	0.054	0.000265628924779885	FOLR2+ resident-like macrophages	BIVM
1.60208107579123E-08	0.51065140661719	0.1	0.033	0.000268476746681094	FOLR2+ resident-like macrophages	GADD45A
1.67770304960828E-08	1.07357044061894	0.148	0.065	0.000281149477053356	FOLR2+ resident-like macrophages	SERPINF2
1.68739126835355E-08	1.02079754020936	0.11	0.04	0.000282773028750688	FOLR2+ resident-like macrophages	PRKAB2
1.70921580027388E-08	0.298176108803128	0.163	0.074	0.000286430383809897	FOLR2+ resident-like macrophages	PSMC1
1.73350977201963E-08	0.377027774315292	0.193	0.095	0.00029050156759505	FOLR2+ resident-like macrophages	GEMIN7
1.78130294219366E-08	0.568645995807523	0.138	0.057	0.000298510747052814	FOLR2+ resident-like macrophages	COG1
1.88911290607828E-08	1.02300984285485	0.12	0.046	0.000316577540800598	FOLR2+ resident-like macrophages	H6PD
1.9648435912757E-08	0.620132686345468	0.164	0.075	0.000329268489025982	FOLR2+ resident-like macrophages	TERF1
1.96887143332159E-08	1.69646563804425	0.106	0.038	0.000329943474796031	FOLR2+ resident-like macrophages	ACADSB
2.01135806048929E-08	0.328071599628609	0.164	0.074	0.000337063383776795	FOLR2+ resident-like macrophages	RNF123
2.03114829507114E-08	0.256671676928436	0.148	0.065	0.000340379831288021	FOLR2+ resident-like macrophages	POLD3
2.03292477462278E-08	0.76543306936185	0.144	0.063	0.000340677533731286	FOLR2+ resident-like macrophages	ERCC2
2.05415562220217E-08	0.312503547558699	0.128	0.05	0.00034423539916864	FOLR2+ resident-like macrophages	DEPRECATED-ENSG00000115325
2.08262333732957E-08	0.513677593791649	0.205	0.103	0.000349006018869689	FOLR2+ resident-like macrophages	NME1
2.09576361886899E-08	0.646182103368536	0.123	0.048	0.000351208067250066	FOLR2+ resident-like macrophages	RANGRF
2.10468088851916E-08	0.374884617890562	0.168	0.079	0.000352702423298041	FOLR2+ resident-like macrophages	GMPPA
2.24136855329648E-08	1.30858367340546	0.109	0.04	0.000375608542161424	FOLR2+ resident-like macrophages	IQCE
2.24172043834126E-08	0.422869927271104	0.113	0.042	0.000375667511057229	FOLR2+ resident-like macrophages	CDR2
2.24212157176021E-08	1.20758020025021	0.132	0.055	0.000375734732995576	FOLR2+ resident-like macrophages	F5
2.32926407802605E-08	0.369756007690788	0.231	0.122	0.000390338074195606	FOLR2+ resident-like macrophages	SMARCC2
2.32948736431004E-08	0.580687209173	0.103	0.036	0.000390375492511076	FOLR2+ resident-like macrophages	COPS9
2.35194077791832E-08	0.520466512120566	0.151	0.066	0.000394138235563552	FOLR2+ resident-like macrophages	MTHFS
2.47911306268341E-08	0.797652865413067	0.129	0.053	0.000415449767044486	FOLR2+ resident-like macrophages	ZNF573
2.49643648633449E-08	1.0412858312266	0.109	0.04	0.000418352826379933	FOLR2+ resident-like macrophages	ZCCHC4
2.50445299487464E-08	0.626759790546525	0.168	0.078	0.000419696232881093	FOLR2+ resident-like macrophages	OGFOD1
2.56494874531515E-08	0.410712694381486	0.17	0.08	0.000429834110739913	FOLR2+ resident-like macrophages	TOMM5
2.59566138620054E-08	0.406291615232828	0.119	0.046	0.000434980935099487	FOLR2+ resident-like macrophages	ING4
2.59926707745448E-08	1.19581825343968	0.102	0.035	0.000435585176839822	FOLR2+ resident-like macrophages	ADH1A
2.60344578568394E-08	0.296048572804282	0.151	0.067	0.000436285444764915	FOLR2+ resident-like macrophages	CYP2U1
2.66728838966141E-08	0.282365644087372	0.136	0.057	0.000446984188339459	FOLR2+ resident-like macrophages	FOPNL
2.67908980885193E-08	1.01250967033041	0.161	0.075	0.000448961870167406	FOLR2+ resident-like macrophages	C5
2.75419797210016E-08	1.10879346771051	0.116	0.045	0.000461548496164545	FOLR2+ resident-like macrophages	HELQ
2.85671493369295E-08	0.480870676979371	0.129	0.052	0.000478728288588265	FOLR2+ resident-like macrophages	NMNAT1
2.89066415221337E-08	1.15345951316764	0.142	0.062	0.000484417498627916	FOLR2+ resident-like macrophages	PMM1
2.92209369637519E-08	0.597326692464516	0.112	0.042	0.000489684461638554	FOLR2+ resident-like macrophages	SSH3
2.92738995494626E-08	0.571492248996164	0.171	0.082	0.000490572008649894	FOLR2+ resident-like macrophages	CHST14
2.95456837666314E-08	0.318609083874924	0.168	0.08	0.00049512656856121	FOLR2+ resident-like macrophages	SDHAF2
3.01943518969829E-08	0.730315004076629	0.126	0.052	0.000505996949089639	FOLR2+ resident-like macrophages	APOL4
3.16168739632673E-08	0.323163865463802	0.126	0.051	0.000529835573876434	FOLR2+ resident-like macrophages	FBXO3
3.28755373913233E-08	0.469095303050269	0.102	0.036	0.000550928255603797	FOLR2+ resident-like macrophages	B3GALT6
3.35314897390701E-08	0.639826784972127	0.12	0.048	0.000561920705047337	FOLR2+ resident-like macrophages	TBC1D31
3.37270809715754E-08	0.578781554353731	0.144	0.064	0.000565198422921661	FOLR2+ resident-like macrophages	HDDC3
3.44699233447748E-08	0.458746138389541	0.116	0.045	0.000577646975411736	FOLR2+ resident-like macrophages	TSPAN17
3.53210312674815E-08	0.695625292493415	0.131	0.054	0.000591909841980455	FOLR2+ resident-like macrophages	METTL15
3.54157227538886E-08	0.583927932574684	0.132	0.055	0.000593496681909665	FOLR2+ resident-like macrophages	TCHP
3.55941396997223E-08	0.601283059549928	0.158	0.072	0.000596486593087946	FOLR2+ resident-like macrophages	PPP1CC
3.57151030985786E-08	0.338811044963324	0.149	0.066	0.00059851369772598	FOLR2+ resident-like macrophages	RBMS2
3.72569976214875E-08	0.600259695465711	0.144	0.063	0.000624352766140887	FOLR2+ resident-like macrophages	NOC3L
3.8481120658426E-08	1.2080344994863	0.104	0.038	0.000644866619993903	FOLR2+ resident-like macrophages	MTX1
3.89747444096424E-08	0.386140638481627	0.138	0.059	0.000653138766816787	FOLR2+ resident-like macrophages	GLRX2
3.9194741472943E-08	0.492159868699144	0.161	0.074	0.000656825477603579	FOLR2+ resident-like macrophages	PPCDC
4.01415678393741E-08	0.448880552891011	0.135	0.057	0.000672692393852231	FOLR2+ resident-like macrophages	KNOP1
4.06400016664524E-08	0.461143274135759	0.139	0.061	0.000681045147926409	FOLR2+ resident-like macrophages	ZNF282
4.13859229526489E-08	0.846839717175639	0.103	0.037	0.000693545296840491	FOLR2+ resident-like macrophages	DGKQ
4.14502276457317E-08	0.66937946143254	0.116	0.045	0.000694622914887171	FOLR2+ resident-like macrophages	EXD3
4.17450656487273E-08	0.430507104120469	0.11	0.042	0.000699563810141372	FOLR2+ resident-like macrophages	TRUB1
4.25410356768208E-08	0.448977972522449	0.102	0.036	0.000712902675872163	FOLR2+ resident-like macrophages	FOXO4
4.28489624892664E-08	0.739725956039128	0.116	0.046	0.000718062913395125	FOLR2+ resident-like macrophages	POT1
4.3034368246408E-08	0.643951430952795	0.141	0.061	0.000721169943073306	FOLR2+ resident-like macrophages	ALOX15B
4.36132454818974E-08	1.22063540042476	0.12	0.048	0.000730870767785637	FOLR2+ resident-like macrophages	BDH1
4.3680227853466E-08	0.34961936552094	0.165	0.079	0.000731993258368383	FOLR2+ resident-like macrophages	NUP155
4.43209268807364E-08	0.488714907032154	0.12	0.047	0.00074273009266738	FOLR2+ resident-like macrophages	ZNF32
4.44034223913877E-08	0.491296809451008	0.135	0.057	0.000744112552434876	FOLR2+ resident-like macrophages	TRIM11
4.44847272133029E-08	0.473715238168873	0.152	0.069	0.00074547505864053	FOLR2+ resident-like macrophages	BMS1
4.49619523198114E-08	0.596449705286214	0.131	0.054	0.0007534723969754	FOLR2+ resident-like macrophages	OCIAD2

4.56606814662331E-08	0.376570827150447	0.17	0.081	0.000765181700011135	FOLR2+ resident-like macrophages	AP5M1
4.63407733596434E-08	0.473821515410781	0.208	0.108	0.000776578679960904	FOLR2+ resident-like macrophages	ANKRD36C
4.89499390550113E-08	0.78485300564972	0.113	0.044	0.00082030307868388	FOLR2+ resident-like macrophages	ZNF839
4.97931847538179E-08	0.336618116992605	0.177	0.085	0.00083443419010448	FOLR2+ resident-like macrophages	PRPS2
5.05988660965273E-08	0.435562419177213	0.155	0.071	0.000847935798045605	FOLR2+ resident-like macrophages	PKD2
5.07159126090752E-08	0.831323697590305	0.136	0.059	0.000849897263502882	FOLR2+ resident-like macrophages	ELP6
5.42780671713093E-08	0.743020239532819	0.123	0.05	0.000909591849656801	FOLR2+ resident-like macrophages	MSH2
5.83704465598771E-08	0.613336729220644	0.107	0.04	0.00097817194345042	FOLR2+ resident-like macrophages	OGG1
5.88158163829852E-08	1.46946798431878	0.1	0.036	0.000985635450946065	FOLR2+ resident-like macrophages	TRMT11
5.90509306595712E-08	0.730775419858737	0.116	0.046	0.000989575495993095	FOLR2+ resident-like macrophages	TYW3
5.96623866057981E-08	1.65248388800082	0.134	0.059	0.000999822274739964	FOLR2+ resident-like macrophages	CYP3A5
6.00469228466924E-08	0.328584629997873	0.12	0.048	0.00100626633306487	FOLR2+ resident-like macrophages	TBCE
6.08140200090476E-08	0.435270989001199	0.125	0.051	0.00101912134731162	FOLR2+ resident-like macrophages	FABP5
6.37020501573205E-08	1.2715288185118	0.103	0.038	0.00106751895653638	FOLR2+ resident-like macrophages	LIG4
6.53828207995006E-08	0.406551238225372	0.173	0.083	0.00109568531095803	FOLR2+ resident-like macrophages	POLR2F
6.54997705211305E-08	0.934157817850499	0.134	0.057	0.00109764515439311	FOLR2+ resident-like macrophages	ANXA13
6.58515262610783E-08	0.503504373050685	0.11	0.042	0.00110353987708315	FOLR2+ resident-like macrophages	ALG6
6.69094893759067E-08	0.492484861609364	0.145	0.065	0.00112126922296144	FOLR2+ resident-like macrophages	MYL12A
6.77412246831756E-08	0.739395156987271	0.12	0.048	0.00113520744324066	FOLR2+ resident-like macrophages	ASNSD1
6.87563506435768E-08	1.15643309935012	0.118	0.047	0.00115221892408506	FOLR2+ resident-like macrophages	HPS6
7.15107991717094E-08	0.437069336138171	0.135	0.059	0.00119837797251951	FOLR2+ resident-like macrophages	ZNF780A
7.18153341589681E-08	0.509782894305578	0.112	0.043	0.00120348136983599	FOLR2+ resident-like macrophages	CCDC71
7.48141121561641E-08	0.291155239286945	0.199	0.102	0.001253734891513	FOLR2+ resident-like macrophages	TOMM22
7.5021154262181E-08	0.980310549830872	0.115	0.046	0.00125720450312563	FOLR2+ resident-like macrophages	URGCP
7.74791898651031E-08	0.312714999574288	0.131	0.056	0.0012983962637594	FOLR2+ resident-like macrophages	MOSPD3
7.79680581086804E-08	0.328726344021382	0.122	0.05	0.00130658871778527	FOLR2+ resident-like macrophages	PIGV
8.07046687503924E-08	0.300964691046176	0.192	0.098	0.00135244883891908	FOLR2+ resident-like macrophages	MCM6
8.22634687877889E-08	0.437786887220798	0.158	0.074	0.00137857120994577	FOLR2+ resident-like macrophages	HLTF
8.3479530758523E-08	0.534145437056555	0.147	0.065	0.00139894997645133	FOLR2+ resident-like macrophages	SPATA13
8.61786652226942E-08	1.00986459362547	0.104	0.039	0.00144418207180191	FOLR2+ resident-like macrophages	STEAP3
8.7231722476045E-08	0.857669840828223	0.115	0.046	0.00146182920525356	FOLR2+ resident-like macrophages	SHMT2
8.86866055339685E-08	0.423630909565236	0.106	0.04	0.00148621013553824	FOLR2+ resident-like macrophages	CACTIN
9.09202168314597E-08	0.417753556557393	0.148	0.068	0.0015236409936616	FOLR2+ resident-like macrophages	NSRP1
9.13194396798466E-08	0.787003063019182	0.113	0.045	0.00153033117015487	FOLR2+ resident-like macrophages	PAIP1
9.57083915176282E-08	0.44044601555809	0.165	0.081	0.00160388122505241	FOLR2+ resident-like macrophages	DCAF10
9.63310325777717E-08	1.19927608075307	0.158	0.077	0.0016143154439383	FOLR2+ resident-like macrophages	TM4SF4
9.67307506863858E-08	0.302218201993148	0.134	0.058	0.00162101392000245	FOLR2+ resident-like macrophages	PRMT7
9.98413847738815E-08	0.538358685049098	0.145	0.066	0.00167314192604071	FOLR2+ resident-like macrophages	SPOUT1
9.98475282731004E-08	0.32115475550672	0.11	0.043	0.00167324487880062	FOLR2+ resident-like macrophages	ZSCAN30
1.0144888277332E-07	0.251781420873453	0.179	0.089	0.00170008037751529	FOLR2+ resident-like macrophages	SOCS5
1.04223061745344E-07	0.472071771833249	0.122	0.05	0.00174657006872848	FOLR2+ resident-like macrophages	NAIF1
1.05164679507715E-07	0.96071949303921	0.123	0.052	0.00176234969919029	FOLR2+ resident-like macrophages	TOR2A
1.06308424700398E-07	0.520873326338033	0.158	0.075	0.00178151658112928	FOLR2+ resident-like macrophages	LSM 2.00
1.14592879302313E-07	0.784926243076504	0.102	0.038	0.00192034747134816	FOLR2+ resident-like macrophages	ZNF175
1.15913525441451E-07	0.681877987418253	0.136	0.061	0.00194247885934783	FOLR2+ resident-like macrophages	C1orf112
1.20976252546752E-07	0.35434920656246	0.171	0.084	0.00202732004017847	FOLR2+ resident-like macrophages	CCT5
1.22179860076753E-07	0.587046344230729	0.131	0.057	0.00204749009516622	FOLR2+ resident-like macrophages	SHPK
1.27086000686347E-07	1.17791647231849	0.104	0.04	0.0021297071995018	FOLR2+ resident-like macrophages	SIRT5
1.27857904012107E-07	0.722002326980665	0.134	0.059	0.00214264275543489	FOLR2+ resident-like macrophages	FARSB
1.3116752591933E-07	0.837663872624955	0.104	0.04	0.00219810539935613	FOLR2+ resident-like macrophages	MACROD1
1.37750493994497E-07	0.520271917266468	0.129	0.056	0.00230842277835978	FOLR2+ resident-like macrophages	FBXO22
1.37895036995334E-07	0.445650296726064	0.161	0.078	0.00231084502996781	FOLR2+ resident-like macrophages	RECQL5
1.38720879729718E-07	0.620081727891445	0.119	0.049	0.00232468450251061	FOLR2+ resident-like macrophages	CUL9
1.38932764184824E-07	0.787012920326147	0.163	0.079	0.00232823526220927	FOLR2+ resident-like macrophages	AADAC
1.40870801134742E-07	0.312033002134196	0.167	0.083	0.002360712885416	FOLR2+ resident-like macrophages	XPO7
1.41138158387984E-07	0.832667773660131	0.106	0.041	0.00236519325826583	FOLR2+ resident-like macrophages	FRA10AC1
1.44955936984686E-07	0.690001151564775	0.134	0.059	0.00242917159198936	FOLR2+ resident-like macrophages	GMNN
1.4532379917257E-07	0.368787674333194	0.132	0.058	0.00243533622653393	FOLR2+ resident-like macrophages	ZNF75D
1.47258925395467E-07	0.351336642151591	0.11	0.044	0.00246776507177723	FOLR2+ resident-like macrophages	CLK2
1.48533157875564E-07	0.558926038428546	0.126	0.054	0.00248911865967871	FOLR2+ resident-like macrophages	GTPBP3
1.48555916846619E-07	0.612966614662452	0.102	0.038	0.00248950005451563	FOLR2+ resident-like macrophages	SOCS7
1.48746307513956E-07	0.679590895649798	0.109	0.043	0.00249269062131888	FOLR2+ resident-like macrophages	ZNF671
1.49331842908434E-07	0.746163465398653	0.113	0.046	0.00250250302345954	FOLR2+ resident-like macrophages	PPP5C
1.50824219004231E-07	0.516052520930786	0.173	0.086	0.0025275122620729	FOLR2+ resident-like macrophages	TRIM24
1.56288495675467E-07	0.770038256736898	0.139	0.064	0.00261908261052948	FOLR2+ resident-like macrophages	SLC26A2
1.57608120602813E-07	1.65855891499571	0.1	0.038	0.00264119688506194	FOLR2+ resident-like macrophages	PERP
1.61917035551329E-07	0.364314839669256	0.126	0.054	0.00271340568176918	FOLR2+ resident-like macrophages	RPP21
1.62331977105426E-07	0.332600738627953	0.176	0.088	0.00272035927233272	FOLR2+ resident-like macrophages	BANF1
1.62527483334205E-07	0.340961940023022	0.118	0.047	0.00272363556571461	FOLR2+ resident-like macrophages	SLC25A20
1.62702721722999E-07	0.56985886676313	0.163	0.079	0.00272657221063402	FOLR2+ resident-like macrophages	ZNF700
1.64129650479577E-07	0.532847974806495	0.104	0.041	0.00275048468273675	FOLR2+ resident-like macrophages	ZNF805

1.70771316555807E-07	0.909103813825022	0.12	0.05	0.00286178572284222	FOLR2+ resident-like macrophages	BRCC3
1.71414575676838E-07	0.255093323532348	0.134	0.06	0.00287256545919246	FOLR2+ resident-like macrophages	ANKRD52
1.7148284829517E-07	0.362484183391395	0.17	0.083	0.00287370957173046	FOLR2+ resident-like macrophages	HMCES
1.74434405323573E-07	0.595096261034815	0.11	0.044	0.00292317176441244	FOLR2+ resident-like macrophages	ASF1A
1.77050949927044E-07	0.252780898139604	0.109	0.043	0.0029670198188774	FOLR2+ resident-like macrophages	SETD4
1.82218629351648E-07	1.29122851193703	0.104	0.041	0.00305361979067491	FOLR2+ resident-like macrophages	TCEA3
1.82315122075594E-07	0.494453822987102	0.145	0.067	0.00305523681574281	FOLR2+ resident-like macrophages	DDX52
1.92996698728418E-07	0.713323181073053	0.136	0.062	0.00323423867729083	FOLR2+ resident-like macrophages	PDCD5
2.01106374730806E-07	0.438368749481282	0.113	0.046	0.00337014062773884	FOLR2+ resident-like macrophages	CDK5
2.0792218004819E-07	0.718168875824062	0.122	0.052	0.00348435989324757	FOLR2+ resident-like macrophages	SPRED2
2.08379393706481E-07	0.477346459064402	0.119	0.049	0.00349202187973322	FOLR2+ resident-like macrophages	BCLAF3
2.17062019176059E-07	0.452362602990411	0.164	0.081	0.00363752531735239	FOLR2+ resident-like macrophages	POM121
2.20260631729118E-07	0.341726196833885	0.157	0.076	0.00369112766651656	FOLR2+ resident-like macrophages	MICOS13
2.34255471216405E-07	0.534475388353798	0.131	0.059	0.00392565318664451	FOLR2+ resident-like macrophages	TADA2A
2.56045041728753E-07	0.688505213824624	0.158	0.077	0.00429080280929045	FOLR2+ resident-like macrophages	SLC5A6
2.66743389058427E-07	0.321433930494323	0.147	0.068	0.00447008571384112	FOLR2+ resident-like macrophages	CDK6
2.78139683624397E-07	0.847903771981184	0.116	0.048	0.00466106481817764	FOLR2+ resident-like macrophages	RIOX2
2.8140692921997E-07	0.349384846881387	0.106	0.042	0.00471581731986826	FOLR2+ resident-like macrophages	FBF1
2.83116386722982E-07	0.606198897211931	0.11	0.045	0.00474446440870374	FOLR2+ resident-like macrophages	NAPG
2.85872997715585E-07	0.62778813568438	0.129	0.057	0.00479065969571777	FOLR2+ resident-like macrophages	CHCHD5
2.85978945440613E-07	0.283068551763413	0.131	0.058	0.00479243516769379	FOLR2+ resident-like macrophages	TIMM8A
2.86022693176536E-07	0.34113960714159	0.145	0.068	0.00479316829225239	FOLR2+ resident-like macrophages	SNAPC3
2.92143843780961E-07	0.387087639319208	0.164	0.081	0.00489574653408135	FOLR2+ resident-like macrophages	POLR1A
3.00559480039888E-07	0.300245395218144	0.129	0.057	0.00503677576650844	FOLR2+ resident-like macrophages	GSTM4
3.08644019932388E-07	1.20530426708015	0.106	0.043	0.00517225648602696	FOLR2+ resident-like macrophages	F2
3.10869379860113E-07	0.562081364102549	0.115	0.047	0.00520954906769578	FOLR2+ resident-like macrophages	ZNF512B
3.25592144526255E-07	0.534362710542541	0.148	0.07	0.00545627315797098	FOLR2+ resident-like macrophages	PAOX
3.30762949568011E-07	0.492523932495068	0.144	0.067	0.00554292550886072	FOLR2+ resident-like macrophages	ANO10
3.42929175705341E-07	0.256272780452348	0.135	0.06	0.0057468071264701	FOLR2+ resident-like macrophages	CPT1A
3.56510260972213E-07	0.577011456242234	0.1	0.039	0.00597439895337234	FOLR2+ resident-like macrophages	MIS18A
3.61546808355722E-07	0.50447963440343	0.163	0.082	0.00605880141442518	FOLR2+ resident-like macrophages	AIDA
3.66210948806854E-07	0.503042005943615	0.11	0.046	0.00613696308010525	FOLR2+ resident-like macrophages	ZNF689
3.78861507815684E-07	0.712373966570775	0.115	0.048	0.00634896114797523	FOLR2+ resident-like macrophages	ZC3H11A
3.83375554490948E-07	0.287134099741545	0.144	0.067	0.00642460754215931	FOLR2+ resident-like macrophages	PDK1
3.87642801235727E-07	0.66879561133707	0.174	0.09	0.00649611806310831	FOLR2+ resident-like macrophages	SEH1L
4.12556860963068E-07	0.550818314060576	0.113	0.047	0.00691362787601909	FOLR2+ resident-like macrophages	TMEM107
4.13509278402619E-07	0.69625010449229	0.147	0.07	0.0069295884874711	FOLR2+ resident-like macrophages	TRMU
4.3673767884764E-07	0.3233326795953	0.104	0.042	0.00731885002212875	FOLR2+ resident-like macrophages	GYG1
4.40502710477344E-07	0.556074754711575	0.11	0.046	0.00738194442217933	FOLR2+ resident-like macrophages	LRRC45
4.74618261764989E-07	0.422835950231118	0.142	0.067	0.00795365283065769	FOLR2+ resident-like macrophages	EIF2B4
4.7923949503876E-07	0.4173161417103	0.158	0.079	0.00803109545785954	FOLR2+ resident-like macrophages	SC5D
4.79666286780482E-07	0.401009902882004	0.147	0.071	0.00803824763386731	FOLR2+ resident-like macrophages	SSNA1
4.94188776069906E-07	0.285913200550955	0.123	0.054	0.00828161550937948	FOLR2+ resident-like macrophages	PEX1
5.13589927814753E-07	0.97305556379369	0.102	0.041	0.00860674001031963	FOLR2+ resident-like macrophages	SCYL3
5.26667370072098E-07	0.362774275990253	0.125	0.056	0.00882589178766823	FOLR2+ resident-like macrophages	GABPB2
5.31638256271697E-07	0.260175534175904	0.103	0.041	0.0089091938986011	FOLR2+ resident-like macrophages	NFXL1
5.33275002531195E-07	0.505606974545534	0.134	0.062	0.00893662249241777	FOLR2+ resident-like macrophages	SAMM50
5.3800448518408E-07	0.554639757814907	0.103	0.042	0.00901587916271481	FOLR2+ resident-like macrophages	FAM120C
5.43500108410191E-07	0.546741887209073	0.12	0.053	0.00910797481673798	FOLR2+ resident-like macrophages	PSMA5
6.09245797186324E-07	0.3968000488635	0.183	0.097	0.0102097410692484	FOLR2+ resident-like macrophages	FAM118B
6.49243515733436E-07	0.484964379513657	0.107	0.045	0.0108800228366609	FOLR2+ resident-like macrophages	ZNF557
6.60053326763855E-07	0.371740227283334	0.154	0.075	0.0110611736499087	FOLR2+ resident-like macrophages	TLR8
6.6354070065653E-07	0.64219294206493	0.12	0.053	0.0111196150616021	FOLR2+ resident-like macrophages	VAMP4
6.87117550567348E-07	0.467780882494238	0.179	0.095	0.0115147159124076	FOLR2+ resident-like macrophages	TTC9C
6.91369176882905E-07	0.268209646842951	0.129	0.059	0.0115859646662037	FOLR2+ resident-like macrophages	NDUFB3
7.03387287087103E-07	0.765560165985487	0.134	0.062	0.0117873641570057	FOLR2+ resident-like macrophages	NXT2
7.1427956505456E-07	0.489297928127129	0.1	0.04	0.0119698969511843	FOLR2+ resident-like macrophages	C9orf85
7.20992948292543E-07	0.426735908915383	0.125	0.057	0.0120823998274864	FOLR2+ resident-like macrophages	RWDD1
7.24421278215073E-07	0.375937057146293	0.148	0.072	0.0121398517803282	FOLR2+ resident-like macrophages	SNRNP35
7.38763983483935E-07	0.970946051305239	0.1	0.04	0.0123802068352238	FOLR2+ resident-like macrophages	GTF2IRD1
7.50789400918396E-07	0.275942945167378	0.176	0.093	0.0125817287805905	FOLR2+ resident-like macrophages	CNTLN
7.51958710929839E-07	1.49125571374866	0.115	0.051	0.0126013240777622	FOLR2+ resident-like macrophages	ATPSCKMT
7.54003041867922E-07	0.403723289360036	0.116	0.05	0.0126355829756226	FOLR2+ resident-like macrophages	EMC9
7.62369453622591E-07	0.44289772402482	0.102	0.041	0.0127757873038074	FOLR2+ resident-like macrophages	ATG9A
8.3179899128299E-07	0.391441301655856	0.119	0.053	0.0139392874959203	FOLR2+ resident-like macrophages	TMEM101
8.35583314003591E-07	0.412805483468154	0.11	0.047	0.0140027051760722	FOLR2+ resident-like macrophages	PUS7L
8.3781041876917E-07	0.709585160312703	0.171	0.09	0.0140400269977338	FOLR2+ resident-like macrophages	XRCC5
8.74965404365529E-07	0.375892208704689	0.151	0.075	0.0146626702463575	FOLR2+ resident-like macrophages	PSKH1
1.00849973123871E-06	0.268288843059506	0.112	0.047	0.0169004384960982	FOLR2+ resident-like macrophages	MINDY1
1.07001605604839E-06	0.994859569110582	0.107	0.046	0.0179313290672589	FOLR2+ resident-like macrophages	UBIAD1
1.07054575013628E-06	0.589830714865089	0.126	0.059	0.0179402056807837	FOLR2+ resident-like macrophages	C6orf136

1.10638713158308E-06	0.411788345736965	0.139	0.067	0.0185408355510692	FOLR2+ resident-like macrophages	RHNO1
1.12004757614512E-06	0.333615557063869	0.107	0.046	0.01876975728104	FOLR2+ resident-like macrophages	GLCE
1.20312264634681E-06	0.615634413271622	0.139	0.067	0.0201619293074798	FOLR2+ resident-like macrophages	CCNQ
1.20598651299782E-06	0.514055498041132	0.136	0.065	0.0202099219848174	FOLR2+ resident-like macrophages	GPATCH4
1.22010112700249E-06	0.484658970267518	0.142	0.069	0.0204464546863077	FOLR2+ resident-like macrophages	ZNF224
1.2728680719205E-06	0.93060239238605	0.115	0.05	0.0213307231492437	FOLR2+ resident-like macrophages	CYP2R1
1.30543792918685E-06	0.321823578832389	0.104	0.044	0.0218765288173132	FOLR2+ resident-like macrophages	FAM102A
1.34279562123632E-06	0.780127096642598	0.154	0.08	0.0225025690206782	FOLR2+ resident-like macrophages	UCHL5
1.35740552305708E-06	0.732758204837364	0.173	0.091	0.0227474017553906	FOLR2+ resident-like macrophages	HMGCR
1.40798150132764E-06	0.645061234697318	0.11	0.048	0.0235949539992487	FOLR2+ resident-like macrophages	CNIH4
1.42880187805866E-06	0.709590123901151	0.107	0.046	0.023943861872507	FOLR2+ resident-like macrophages	GTF2H5
1.42987037156088E-06	0.580291188879559	0.135	0.064	0.0239617676866172	FOLR2+ resident-like macrophages	HOMEZ
1.46558543225197E-06	0.288031041401108	0.109	0.046	0.0245602806736785	FOLR2+ resident-like macrophages	EVI5L
1.50879784283605E-06	0.319192850213654	0.116	0.051	0.0252844342502466	FOLR2+ resident-like macrophages	HEATR6
1.53205719833658E-06	0.74104580872592	0.119	0.054	0.0256742145297244	FOLR2+ resident-like macrophages	EIF3M
1.57753965138293E-06	0.785464630960676	0.118	0.053	0.0264364094778751	FOLR2+ resident-like macrophages	HMBS
1.5923697622061E-06	0.517037064405137	0.12	0.055	0.0266849324750498	FOLR2+ resident-like macrophages	ATXN7L3
1.65340940769461E-06	0.252575112251572	0.129	0.061	0.0277078348541463	FOLR2+ resident-like macrophages	ERMARD
1.66098179072701E-06	0.401591375252371	0.106	0.045	0.0278347328490033	FOLR2+ resident-like macrophages	PLSCR3
1.67038917487561E-06	0.352591067858015	0.115	0.051	0.0279923817925655	FOLR2+ resident-like macrophages	PLG
1.74615622405051E-06	1.01300117496577	0.106	0.046	0.0292620860026384	FOLR2+ resident-like macrophages	UQCC3
1.81423400605565E-06	0.899412326151078	0.103	0.044	0.0304029334734806	FOLR2+ resident-like macrophages	FTCDNL1
1.84601794003931E-06	0.995030475751226	0.125	0.059	0.0309355686391787	FOLR2+ resident-like macrophages	SRSF1
1.85215371290915E-06	0.780760216446397	0.1	0.042	0.0310383919209316	FOLR2+ resident-like macrophages	RBM45
1.8833637330821E-06	0.434256033336382	0.116	0.053	0.0315614094389899	FOLR2+ resident-like macrophages	SKP2
1.8942854551929E-06	0.45916353512853	0.12	0.055	0.0317444356581226	FOLR2+ resident-like macrophages	ANO8
1.93105583829415E-06	0.46119047303326	0.115	0.051	0.0323606337381333	FOLR2+ resident-like macrophages	TRIM23
1.94911430163584E-06	0.330259018675273	0.136	0.066	0.0326632574668135	FOLR2+ resident-like macrophages	DEPRECATED-ENSG00000147123
1.968032801922E-06	0.584799641995465	0.132	0.064	0.0329802936946088	FOLR2+ resident-like macrophages	WDR1
1.97903941224908E-06	0.97781753079408	0.131	0.062	0.03316474247047	FOLR2+ resident-like macrophages	CXCL10
1.9835496181242E-06	0.371466055182257	0.177	0.095	0.0332403245005254	FOLR2+ resident-like macrophages	MAPK9
1.98562642937093E-06	0.611718739847289	0.116	0.052	0.033275127703398	FOLR2+ resident-like macrophages	DCLRE1B
2.02281510085835E-06	0.49581091001171	0.1	0.042	0.0338983354601843	FOLR2+ resident-like macrophages	THAP6
2.0667674946712E-06	0.354843855751072	0.206	0.115	0.0346348896757	FOLR2+ resident-like macrophages	CKS1B
2.08000500634388E-06	0.71863183774807	0.104	0.045	0.0348567238963107	FOLR2+ resident-like macrophages	DEPRECATED-ENSG00000254986
2.18562267606515E-06	0.79906649579609	0.119	0.055	0.0366266648054998	FOLR2+ resident-like macrophages	PFAS
2.24987010448787E-06	1.07168371604317	0.102	0.044	0.0377033232110077	FOLR2+ resident-like macrophages	FLYWCH2
2.43544836836158E-06	0.562982672489693	0.129	0.063	0.0408132437570034	FOLR2+ resident-like macrophages	BCS1L
2.74396333514199E-06	0.536652514471316	0.139	0.07	0.0459833375703094	FOLR2+ resident-like macrophages	DONSON
2.76796498484341E-06	0.625942264981069	0.123	0.059	0.0463855572160059	FOLR2+ resident-like macrophages	ARMC6
2.78969726181894E-06	0.951080555205909	0.138	0.069	0.0467497467135619	FOLR2+ resident-like macrophages	GNG5
2.89752630327618E-06	1.06095872287051	0.139	0.07	0.0485567457903022	FOLR2+ resident-like macrophages	LRRC14
2.25864474344922E-108	3.80981329482099	0.665	0.206	3.78503686107221E-104	Inflammatory macrophages	NR4A2
2.13125525188355E-101	1.68238256501183	0.952	0.849	3.57155755110646E-97	Inflammatory macrophages	HSPA1B
2.26399926100824E-99	2.44095939295829	0.851	0.653	3.79400996159761E-95	Inflammatory macrophages	RHOB
2.3877745419596E-98	1.60832635521975	0.948	0.892	4.0014325774159E-94	Inflammatory macrophages	DEPRECATED-ENSG00000204389
4.66651941161983E-97	3.35285614147714	0.611	0.162	7.82015322999251E-93	Inflammatory macrophages	NR4A1
4.10735112287525E-96	5.64977843049556	0.366	0.015	6.88309901171434E-92	Inflammatory macrophages	CD5L
3.59065597860724E-82	1.57118796066507	0.913	0.792	6.01722128895001E-78	Inflammatory macrophages	DUSP1
1.04511952994882E-72	1.76766554666046	0.838	0.728	1.75141130828824E-68	Inflammatory macrophages	DNAJB1
8.56895111344972E-72	4.07323280084174	0.48	0.131	1.4359848275919E-67	Inflammatory macrophages	KLF2
2.4296777101094E-70	4.8717336784248	0.358	0.048	4.07165390660134E-66	Inflammatory macrophages	CCL3
9.40316631192933E-69	1.39952554714994	0.903	0.896	1.57578261055312E-64	Inflammatory macrophages	SAT1
4.09028230843328E-67	1.64773189700326	0.863	0.785	6.85449509247249E-63	Inflammatory macrophages	JUNB
1.36542862042489E-66	2.07310507505675	0.774	0.571	2.28818528210802E-62	Inflammatory macrophages	PFKFB3
2.37991861421243E-66	5.18125308987065	0.311	0.031	3.9882676136972E-62	Inflammatory macrophages	CCL4
3.85466980497115E-65	4.1421557761179	0.433	0.107	6.45965565917065E-61	Inflammatory macrophages	CCL4L2
1.04511470671577E-64	1.86052707182837	0.805	0.641	1.75140322551429E-60	Inflammatory macrophages	FOS
2.04947511886665E-57	1.87981654535704	0.754	0.578	3.43451040419673E-53	Inflammatory macrophages	HSPH1
7.16229070417072E-55	1.94226714644162	0.764	0.659	1.20025667620493E-50	Inflammatory macrophages	SOD2
1.75422578390956E-51	2.72073421311094	0.582	0.312	2.93973156867564E-47	Inflammatory macrophages	ATF3
1.19895305526724E-50	1.29958177638886	0.845	0.829	2.00920553001684E-46	Inflammatory macrophages	HSPA1A
1.00751355113995E-48	1.58563386620352	0.797	0.785	1.68839120900033E-44	Inflammatory macrophages	HSP90AA1
1.7367502468149E-42	5.27904004205812	0.217	0.024	2.91044606361241E-38	Inflammatory macrophages	MARCO
1.58888953430651E-39	2.86517281689198	0.584	0.417	2.66266108159084E-35	Inflammatory macrophages	CHMP1B
2.46343378449902E-38	1.85534632329015	0.602	0.387	4.12822233606345E-34	Inflammatory macrophages	CD83
4.68202928730916E-35	4.10683719465194	0.199	0.028	7.84614467967269E-31	Inflammatory macrophages	CXCL8
7.62119243362241E-35	1.80519497428583	0.689	0.692	1.27715942802644E-30	Inflammatory macrophages	KLF6
1.18188706120465E-34	2.09207220874945	0.646	0.546	1.98060633716676E-30	Inflammatory macrophages	IER5
4.17751886106432E-34	1.94612364248351	0.671	0.638	7.00068610737159E-30	Inflammatory macrophages	JUN
7.45470632633185E-33	2.44945496800461	0.398	0.172	1.24925968616669E-28	Inflammatory macrophages	NR4A3

**1.37406696143581E-32**	1.8664432350849	0.617	0.489	2.30266141397412E-28	Inflammatory macrophages	DEPRECATED-ENSG00000132002
**6.56412025996043E-31**	2.28295236981518	0.634	0.579	1.10001527316417E-26	Inflammatory macrophages	HMOX1
**1.11956318807484E-30**	0.936988205427113	0.812	0.89	1.87616399057582E-26	Inflammatory macrophages	FTH1
**7.7690955020823E-30**	2.21857671031225	0.507	0.329	1.30194502423895E-25	Inflammatory macrophages	PPP1R15A
**1.54116482512282E-29**	2.0803881729642	0.493	0.294	2.58268401394082E-25	Inflammatory macrophages	DNAJA4
**1.73849379284882E-26**	1.61499238501846	0.683	0.748	2.91336789805606E-22	Inflammatory macrophages	HSPB1
**4.18694331921203E-26**	2.89238970257834	0.369	0.179	7.01647961433553E-22	Inflammatory macrophages	BAG3
**3.18008934368503E-25**	0.783135846436891	0.886	0.846	5.32919372214738E-21	Inflammatory macrophages	GLUL
**3.34865075293697E-25**	3.77262760163216	0.217	0.06	5.61166893177178E-21	Inflammatory macrophages	PLPP3
**7.56598664981386E-25**	0.807543465550934	0.884	0.884	1.26790804277581E-20	Inflammatory macrophages	FTL
**9.26835292386064E-24**	2.27420840091578	0.466	0.319	1.55319058298057E-19	Inflammatory macrophages	HSPE1
**1.20265147788613E-23**	3.51192808067129	0.219	0.064	2.01540334664157E-19	Inflammatory macrophages	FOSB
**1.92377184101097E-23**	2.9696509189441	0.402	0.246	3.22385685116618E-19	Inflammatory macrophages	KLF4
**4.47254325857894E-23**	3.8386858834032	0.17	0.037	7.49508799272658E-19	Inflammatory macrophages	DLL1
**9.13431407909199E-23**	0.997289919434833	0.795	0.91	1.53072835337424E-18	Inflammatory macrophages	SERPINA1
**9.51216698130905E-23**	4.28664150328694	0.195	0.053	1.59404894272777E-18	Inflammatory macrophages	DEPRECATED-ENSG00000276085
**2.90760222768852E-22**	4.87115121204554	0.12	0.015	4.87255981316042E-18	Inflammatory macrophages	HES4
**3.95850745825392E-22**	2.43540635477802	0.344	0.175	6.63366679854192E-18	Inflammatory macrophages	CXCL12
**4.69945515579438E-22**	3.06531310893114	0.383	0.228	7.87534695008023E-18	Inflammatory macrophages	IFRD1
**9.07139756949808E-22**	2.73483281275982	0.246	0.087	1.52018480469649E-17	Inflammatory macrophages	HBEGF
**5.19214252289129E-21**	1.77283304994822	0.58	0.553	8.70099243986122E-17	Inflammatory macrophages	ARL4C
**8.04239285513626E-21**	1.41268949533171	0.675	0.728	1.34774419466373E-16	Inflammatory macrophages	SGK1
**1.05534021792858E-20**	0.986712932185764	0.76	0.84	1.76853913720472E-16	Inflammatory macrophages	MCL1
**1.62148501431356E-20**	0.364615278310677	0.159	0.458	2.71728458698667E-16	Inflammatory macrophages	LYN
**3.21709705441455E-20**	1.37818712109282	0.662	0.67	5.3912112437879E-16	Inflammatory macrophages	ZFP36
**3.34810732829622E-20**	1.87077740738246	0.534	0.454	5.61075826075881E-16	Inflammatory macrophages	HSPD1
**3.98265644819548E-20**	0.278922701851528	0.099	0.346	6.67413567588598E-16	Inflammatory macrophages	DIP2B
**1.33761752189701E-19**	2.60431351703	0.294	0.137	2.24157944319502E-15	Inflammatory macrophages	ID3
**1.83714329458077E-19**	0.346798883218141	0.151	0.435	3.07868473305846E-15	Inflammatory macrophages	RREB1
**1.89960295215424E-19**	0.281811290338286	0.164	0.453	3.18335462722007E-15	Inflammatory macrophages	MGAT4A
**2.34665276094287E-19**	0.271270384538967	0.132	0.401	3.93252069678807E-15	Inflammatory macrophages	ZFHX3
**3.66725385855721E-19**	0.687769327368285	0.797	0.808	6.14558401617017E-15	Inflammatory macrophages	CXCR4
**8.22769839100643E-19**	0.276971911668763	0.122	0.374	1.37879769636486E-14	Inflammatory macrophages	TSPYL1
**1.06082975961152E-18**	0.285780327815335	0.17	0.46	1.77773851115698E-14	Inflammatory macrophages	H2AFY
**1.588964084233E-18**	0.411860040200019	0.132	0.388	2.66278601235765E-14	Inflammatory macrophages	HELZ
**2.99686207883326E-18**	0.265929927553411	0.11	0.35	5.02214147170878E-14	Inflammatory macrophages	LMBRD1
**3.9457361916927E-18**	0.288452220638899	0.126	0.374	6.61226471003863E-14	Inflammatory macrophages	FUS
**4.08047302024919E-18**	0.31989749455335	0.124	0.374	6.83805668733359E-14	Inflammatory macrophages	MAGT1
**4.09746631620316E-18**	0.565908409101132	0.83	0.896	6.86653405269325E-14	Inflammatory macrophages	SRGN
**4.17566984330213E-18**	0.262501432879475	0.099	0.329	6.99758752340571E-14	Inflammatory macrophages	ANKRD17
**4.63150530431502E-18**	0.260975935475301	0.145	0.413	7.76147658897112E-14	Inflammatory macrophages	TENT2
**7.711748571433E-18**	0.302419852922685	0.135	0.39	1.29233482560074E-13	Inflammatory macrophages	SLBP
**8.65310733851709E-18**	0.362086179872486	0.199	0.505	1.45008772778869E-13	Inflammatory macrophages	ZFAND3
**1.00529257152414E-17**	0.404027399588598	0.184	0.476	1.68466929136016E-13	Inflammatory macrophages	B4GALT1
**1.07536730683516E-17**	0.363903482729125	0.215	0.531	1.80210053279436E-13	Inflammatory macrophages	EIF1
**2.19228345034523E-17**	0.301604361586315	0.054	0.238	3.67382860608854E-13	Inflammatory macrophages	CMC2
**2.36116294199699E-17**	0.376217037242717	0.153	0.419	3.95683685819856E-13	Inflammatory macrophages	SND1
**2.60225176579041E-17**	0.27039454402959	0.103	0.326	4.36085350911157E-13	Inflammatory macrophages	KIDINS220
**2.63079447949944E-17**	0.307521885729718	0.099	0.321	4.40868538874516E-13	Inflammatory macrophages	WIPI2
**4.09309135445787E-17**	0.384589093133031	0.104	0.327	6.85920249180049E-13	Inflammatory macrophages	BTAF1
**4.26608746940642E-17**	0.29390155693615	0.106	0.33	7.14910938123128E-13	Inflammatory macrophages	SORBS3
**4.44360182175092E-17**	0.25179993509501	0.166	0.44	7.44658793289019E-13	Inflammatory macrophages	SPATS2L
**5.29851815755123E-17**	0.36356071352402	0.159	0.427	8.87925672842435E-13	Inflammatory macrophages	INPP5D
**5.47467896242942E-17**	0.273742763859326	0.166	0.439	9.17446700523921E-13	Inflammatory macrophages	LPP
**6.03712553094592E-17**	0.421549077189674	0.12	0.353	1.01170149647592E-12	Inflammatory macrophages	SNX10
**7.90387447622225E-17**	0.285590457453087	0.147	0.403	1.32453128472532E-12	Inflammatory macrophages	BCL2L1
**8.69772930498183E-17**	0.379545670478464	0.133	0.382	1.45756547692886E-12	Inflammatory macrophages	MANBA
**9.23609775557313E-17**	0.272182065899761	0.222	0.538	1.54778526187894E-12	Inflammatory macrophages	MINK1
**1.00642401136131E-16**	0.480615209329762	0.085	0.289	1.68656535823928E-12	Inflammatory macrophages	RHOU
**1.1315858984825E-16**	0.291836644545202	0.072	0.265	1.89631164867698E-12	Inflammatory macrophages	MOSPD2
**3.1294093714429E-16**	0.33611267850815	0.17	0.442	5.24426422466402E-12	Inflammatory macrophages	BNIP2
**4.15076325479625E-16**	0.26739568989041	0.101	0.312	6.95584906238756E-12	Inflammatory macrophages	MAEA
**4.20387325167263E-16**	0.410269030726911	0.087	0.29	7.04485079515299E-12	Inflammatory macrophages	NUDCD3
**4.48652641326222E-16**	0.313372170894619	0.093	0.299	7.51852096334482E-12	Inflammatory macrophages	CASC3
**4.76743555575449E-16**	0.341201139022824	0.19	0.476	7.98926850433337E-12	Inflammatory macrophages	DRAM2
**5.48602698177839E-16**	0.262896779172561	0.079	0.273	9.19348401606422E-12	Inflammatory macrophages	DOCK7
**5.60769924937522E-16**	0.340841228325179	0.24	0.564	9.397382402103E-12	Inflammatory macrophages	PDXK
**6.04904266410091E-16**	0.275854328944253	0.077	0.27	1.01369856965003E-11	Inflammatory macrophages	DERA
**6.419108044912E-16**	0.445842539556717	0.101	0.312	1.07571412616635E-11	Inflammatory macrophages	PPP4R3A
**6.96780631354979E-16**	0.27332501629904	0.132	0.367	1.16766498202467E-11	Inflammatory macrophages	DNAJB11
**7.93443475174565E-16**	0.317740111375454	0.091	0.294	1.32965257569754E-11	Inflammatory macrophages	PAPSS2

8.08220254227176E-16	0.286100177106873	0.147	0.393	1.3544155020339E-11	Inflammatory macrophages	SERP1
8.75816891088118E-16	0.418050174642648	0.149	0.396	1.46769394608547E-11	Inflammatory macrophages	GSAP
9.91280297480612E-16	0.288763129895677	0.072	0.257	1.66118752251801E-11	Inflammatory macrophages	USO1
1.02490559461925E-15	0.280611882184994	0.07	0.253	1.71753679546294E-11	Inflammatory macrophages	ST3GAL6
1.18878048824855E-15	0.448434750559966	0.054	0.226	1.99215834220693E-11	Inflammatory macrophages	SUGT1
1.24760703714175E-15	0.392128000281324	0.081	0.276	2.09073987284215E-11	Inflammatory macrophages	PITHD1
2.06808044937299E-15	2.1734588168807	0.395	0.273	3.46568921705926E-11	Inflammatory macrophages	IER3
2.12353079163361E-15	0.311093905319455	0.12	0.339	3.5586129006196E-11	Inflammatory macrophages	SMARCA4
2.49597156445472E-15	0.273009003746354	0.07	0.25	4.18274914771321E-11	Inflammatory macrophages	TRRAP
2.94529737994907E-15	0.320026361676375	0.217	0.508	4.93572934931865E-11	Inflammatory macrophages	STAT3
3.36240461449669E-15	0.344583027661647	0.097	0.298	5.63471765297355E-11	Inflammatory macrophages	NFKB1
3.67934632512994E-15	0.276292167834169	0.083	0.272	6.16584857165275E-11	Inflammatory macrophages	NFX1
3.74634898207805E-15	0.461263484086985	0.157	0.405	6.2781316241664E-11	Inflammatory macrophages	IL18
3.78794739749404E-15	0.357530248326054	0.099	0.301	6.3478422487205E-11	Inflammatory macrophages	ZGPAT
4.22214674640381E-15	4.1661665935452	0.114	0.025	7.07547351762351E-11	Inflammatory macrophages	LYVE1
4.36235089578097E-15	0.608340083337422	0.079	0.268	7.31042763114975E-11	Inflammatory macrophages	TTC37
4.37178425683366E-15	0.297110403493325	0.13	0.353	7.32623605760185E-11	Inflammatory macrophages	BMP2K
5.16871207936674E-15	0.597143522507734	0.159	0.407	8.66172770260279E-11	Inflammatory macrophages	SLC25A3
6.26078528370173E-15	0.307899205011126	0.118	0.334	1.04918239784274E-10	Inflammatory macrophages	SNX5
6.33789389268109E-15	0.34513679358298	0.077	0.263	1.0621042585355E-10	Inflammatory macrophages	ABCF1
6.69356591927512E-15	0.388403876193017	0.215	0.503	1.12170777675213E-10	Inflammatory macrophages	TMEM106A
6.9799180314587E-15	0.713058472481069	0.135	0.366	1.16969466371185E-10	Inflammatory macrophages	NEU1
7.21603803389389E-15	0.310351342385451	0.178	0.442	1.20926365371994E-10	Inflammatory macrophages	GNPTG
7.22649521482549E-15	0.50300859786379	0.083	0.272	1.21101606810045E-10	Inflammatory macrophages	H2AFJ
7.91131774953894E-15	0.519304188356771	0.132	0.359	1.32577862846774E-10	Inflammatory macrophages	RELCH
8.2804290069196E-15	0.756485380608173	0.186	0.458	1.38763429297959E-10	Inflammatory macrophages	ALG13
9.02656871696549E-15	0.40419873952206	0.191	0.457	1.51267238558908E-10	Inflammatory macrophages	POLR2A
9.329087230108E-15	0.363053964734392	0.164	0.412	1.5633684380215E-10	Inflammatory macrophages	PNPLA6
9.94245722320228E-15	2.41888039679601	0.246	0.116	1.66615698146424E-10	Inflammatory macrophages	DUSP2
1.03268674709894E-14	0.434998654596126	0.112	0.32	1.7305764507884E-10	Inflammatory macrophages	SPRED1
1.03742181117718E-14	0.319184544533501	0.095	0.29	1.73851147117072E-10	Inflammatory macrophages	RIOK3
1.04902437403866E-14	0.2860658369877	0.083	0.27	1.75795504601399E-10	Inflammatory macrophages	TOMM20
1.06111977444833E-14	0.33577823514596	0.066	0.239	1.77822451802052E-10	Inflammatory macrophages	PPP3CA
1.12765131794385E-14	0.270504539051731	0.106	0.31	1.88971807861031E-10	Inflammatory macrophages	EWSR1
1.20204389121619E-14	0.282250934959299	0.12	0.333	2.01438515290009E-10	Inflammatory macrophages	SPEN
1.23345157747698E-14	0.301173776686363	0.041	0.192	2.06701815353592E-10	Inflammatory macrophages	TIMM8B
1.28075584785826E-14	0.251122490202607	0.141	0.372	2.14629064984087E-10	Inflammatory macrophages	POGZ
1.36217515119052E-14	0.333900722751633	0.072	0.248	2.28273311836508E-10	Inflammatory macrophages	BRWD1
1.38571476002396E-14	0.29310435643163	0.077	0.258	2.32218079484815E-10	Inflammatory macrophages	LOXL3
1.44112363733643E-14	0.332112080905914	0.13	0.348	2.41503499144838E-10	Inflammatory macrophages	WDFY2
1.65440206564142E-14	0.251150535835324	0.168	0.413	2.77244698160189E-10	Inflammatory macrophages	ST8SIA4
1.88626577670116E-14	0.272998392366864	0.12	0.33	3.16100418859581E-10	Inflammatory macrophages	KANSL1
2.14898763993605E-14	0.433842155927687	0.168	0.423	3.60127348700483E-10	Inflammatory macrophages	NFATC2
2.17688418980741E-14	0.347704580452944	0.087	0.272	3.64802252527926E-10	Inflammatory macrophages	IWS1
2.30623984844114E-14	0.308098772420459	0.226	0.521	3.86479673801767E-10	Inflammatory macrophages	ACADVL
2.62092626974417E-14	0.258890031285675	0.091	0.28	4.39214824283728E-10	Inflammatory macrophages	SLK
2.67296665425504E-14	0.25859362007114	0.075	0.253	4.4793575192006E-10	Inflammatory macrophages	GLRX3
2.72049439099173E-14	0.294991268899793	0.118	0.325	4.55900450042394E-10	Inflammatory macrophages	MARCH6
2.7313468208442E-14	0.499536995887227	0.11	0.316	4.57719100237072E-10	Inflammatory macrophages	ZBTB1
2.76984342085216E-14	0.292982712767127	0.062	0.227	4.64170360466405E-10	Inflammatory macrophages	TMEM41B
2.90683679249703E-14	0.269059771174229	0.226	0.519	4.87127709686653E-10	Inflammatory macrophages	SLC7A7
3.08577838427707E-14	2.67956877845578	0.379	0.278	5.17114741637152E-10	Inflammatory macrophages	APBB3
3.17947389682411E-14	0.27540195573653	0.089	0.277	5.32816235629785E-10	Inflammatory macrophages	REV1
3.25238909957337E-14	0.403967150107223	0.112	0.318	5.45035365306506E-10	Inflammatory macrophages	SKI
3.28462864390493E-14	0.26142678092929	0.095	0.285	5.50438068145589E-10	Inflammatory macrophages	ZFC3H1
4.32970937521418E-14	0.363308149651214	0.11	0.312	7.25572697098393E-10	Inflammatory macrophages	DCTN4
5.87348596733409E-14	0.4006180117895	0.095	0.282	9.84278778405847E-10	Inflammatory macrophages	KLHL5
6.30178409348278E-14	0.434233090161586	0.166	0.406	1.05605297838584E-09	Inflammatory macrophages	NCF4
6.32342083613462E-14	0.439715785326852	0.11	0.31	1.05967886371944E-09	Inflammatory macrophages	MAPK6
7.69880214422845E-14	1.80279776869536	0.497	0.458	1.2901652633298E-09	Inflammatory macrophages	P4HA1
8.25400755633518E-14	0.299053152566108	0.13	0.342	1.38320658629065E-09	Inflammatory macrophages	GNG10
8.40818377929839E-14	0.396321784590025	0.132	0.344	1.40904343773482E-09	Inflammatory macrophages	TRIM26
8.51203192218455E-14	0.380133001342726	0.07	0.237	1.42644630951969E-09	Inflammatory macrophages	TNPO3
8.61753398752466E-14	0.438142882422827	0.095	0.283	1.44412634562938E-09	Inflammatory macrophages	KLF3
9.0394509954773E-14	0.435734442768024	0.149	0.38	1.51483119782209E-09	Inflammatory macrophages	ARMH3
9.48891130978222E-14	0.426478816226345	0.126	0.336	1.5901517572933E-09	Inflammatory macrophages	CPED1
1.07103674395194E-13	0.254354385784279	0.066	0.228	1.79484337551465E-09	Inflammatory macrophages	TM2D2
1.0718502264554E-13	0.388065510197086	0.104	0.297	1.79620660949396E-09	Inflammatory macrophages	SGPL1
1.18381669600241E-13	0.301578537765491	0.182	0.43	1.98384001916083E-09	Inflammatory macrophages	RSRC2
1.1997756137072E-13	3.95511816483302	0.112	0.028	2.01058397345052E-09	Inflammatory macrophages	CCL3L1
1.27567198600499E-13	0.528749175859911	0.182	0.433	2.13777111414717E-09	Inflammatory macrophages	MAPKAPK2

1.30412232623716E-13	0.279739857160057	0.112	0.308	2.18544819430823E-09	Inflammatory macrophages	THBD
1.30477597438529E-13	0.308148169095038	0.103	0.293	2.18654357787486E-09	Inflammatory macrophages	ZNF410
1.51880907566351E-13	0.276366295788374	0.234	0.523	2.54522024899691E-09	Inflammatory macrophages	SERPINB6
1.52740194205842E-13	0.460160451322581	0.126	0.335	2.55962017450151E-09	Inflammatory macrophages	PLA2G15
1.54820042665506E-13	0.407761402696764	0.103	0.293	2.59447427498854E-09	Inflammatory macrophages	PTOV1
1.58781528224738E-13	0.508719428788969	0.083	0.26	2.66086084999016E-09	Inflammatory macrophages	ADGRG6
1.61320823359668E-13	0.30211203634772	0.052	0.203	2.70341435786131E-09	Inflammatory macrophages	UEVLD
1.66276402882776E-13	0.589948073808285	0.099	0.285	2.78645995950955E-09	Inflammatory macrophages	RNF19B
1.71268194435233E-13	0.328123192426831	0.074	0.241	2.87011240234564E-09	Inflammatory macrophages	STX3
1.79961151679437E-13	0.268933082471691	0.11	0.304	3.01578897984401E-09	Inflammatory macrophages	P2RX4
2.17697540415308E-13	0.308577928305387	0.079	0.25	3.64817538227974E-09	Inflammatory macrophages	UBE2E2
2.22511357880541E-13	0.537218243240430	0.118	0.32	3.7288453353621E-09	Inflammatory macrophages	PFDN5
2.25650105605203E-13	0.306834096559331	0.168	0.402	3.78144446973199E-09	Inflammatory macrophages	STARD13
2.33393860478981E-13	0.310257886819321	0.064	0.223	3.91121431390676E-09	Inflammatory macrophages	NBN
2.3577482556904E-13	0.301597118528482	0.104	0.294	3.95111452688598E-09	Inflammatory macrophages	HPS3
2.35897847788926E-13	0.406140832063109	0.087	0.265	3.95317613324682E-09	Inflammatory macrophages	C6orf62
2.42158884184118E-13	0.498273236900518	0.151	0.375	4.05809858115745E-09	Inflammatory macrophages	DCAF8
2.48376358866552E-13	0.585192441060612	0.079	0.251	4.16229102188569E-09	Inflammatory macrophages	PRKAA1
2.49441549908242E-13	0.27257770327343	0.101	0.287	4.18014149336233E-09	Inflammatory macrophages	ANKRD11
2.55878429155085E-13	0.261371557653212	0.168	0.4	4.28801071578092E-09	Inflammatory macrophages	SLTM
2.85816343424653E-13	1.62371477671972	0.524	0.51	4.78971028311034E-09	Inflammatory macrophages	NAMPT
2.88153440833214E-13	0.366064037496703	0.126	0.33	4.828875361483E-09	Inflammatory macrophages	FAM20C
2.98849857576446E-13	0.327927885099965	0.07	0.23	5.00812591326608E-09	Inflammatory macrophages	MYC
3.06256748597129E-13	0.290432919829686	0.083	0.255	5.13225059299068E-09	Inflammatory macrophages	RB1
3.16029919068668E-13	0.405052381867886	0.087	0.263	5.29602938375274E-09	Inflammatory macrophages	PHACTR2
3.2769549141919E-13	0.269334430799308	0.095	0.277	5.49152104520279E-09	Inflammatory macrophages	PYGL
3.29048491243477E-13	0.375322446240699	0.081	0.252	5.51419461625819E-09	Inflammatory macrophages	PSPC1
3.36015120652553E-13	0.263501761047351	0.058	0.211	5.63094139189548E-09	Inflammatory macrophages	HMG20A
3.36911652136166E-13	0.579534511383025	0.114	0.312	5.64596546649788E-09	Inflammatory macrophages	DEPRECATED-ENSG00000068305
3.37145674456134E-13	0.351170541739312	0.068	0.228	5.64988721253589E-09	Inflammatory macrophages	SLC33A1
3.58352903630914E-13	0.361681531577798	0.083	0.254	6.00527795904686E-09	Inflammatory macrophages	CDK4
3.94981856610894E-13	0.530517470939873	0.18	0.426	6.61910595308536E-09	Inflammatory macrophages	EIF5
4.25125598324066E-13	0.313132130682403	0.193	0.447	7.12425477671469E-09	Inflammatory macrophages	TMEM259
4.32050579023689E-13	0.302137381972538	0.182	0.422	7.24030360327898E-09	Inflammatory macrophages	STX16
4.32198661179084E-13	0.290707957649899	0.083	0.254	7.2427851640391E-09	Inflammatory macrophages	PIP4K2B
4.37007298362782E-13	0.37068387832226	0.11	0.303	7.32336830596351E-09	Inflammatory macrophages	DEPRECATED-ENSG00000163931
4.40847535228019E-13	0.556591812446732	0.151	0.374	7.38772299535114E-09	Inflammatory macrophages	HSP90B1
4.48147296750296E-13	0.349002027141827	0.054	0.204	7.51005239894146E-09	Inflammatory macrophages	MCUR1
4.50210581756691E-13	0.563413621756567	0.12	0.318	7.54462892907862E-09	Inflammatory macrophages	LRMDA
4.54316675892464E-13	0.267508638460973	0.079	0.248	7.61343885460592E-09	Inflammatory macrophages	GGA1
4.95753863088154E-13	0.265695242556101	0.097	0.276	8.30784323763128E-09	Inflammatory macrophages	METTL17
5.03097796418202E-13	0.495132635779663	0.056	0.206	8.43091287237623E-09	Inflammatory macrophages	MLH1
5.26554406932756E-13	0.340695365348135	0.108	0.299	8.82399875137912E-09	Inflammatory macrophages	ME2
5.38801531017942E-13	0.635141333871372	0.166	0.404	9.02923605679867E-09	Inflammatory macrophages	TLR4
6.16083711398392E-13	0.502707157950308	0.074	0.237	1.03243308356142E-08	Inflammatory macrophages	SETD7
6.35062229003013E-13	0.262244769168104	0.058	0.21	1.06423728336325E-08	Inflammatory macrophages	UBLCP1
6.40555124212604E-13	0.497864861561446	0.147	0.366	1.07344227715548E-08	Inflammatory macrophages	TIAL1
6.74404087268959E-13	0.865020646781414	0.263	0.57	1.13016636944532E-08	Inflammatory macrophages	TFRC
7.01995072819227E-13	0.329988848419912	0.075	0.24	1.17640334303046E-08	Inflammatory macrophages	MON2
7.08917048302753E-13	0.479486804876152	0.168	0.404	1.18800318954575E-08	Inflammatory macrophages	UBE2B
7.10751730398505E-13	0.339143654193448	0.044	0.184	1.19107774980182E-08	Inflammatory macrophages	GTF2H2C
7.85101857123833E-13	0.345859469727863	0.12	0.314	1.31567369216812E-08	Inflammatory macrophages	SLC36A1
8.1227805525006E-13	0.485163426547504	0.126	0.326	1.36121556498805E-08	Inflammatory macrophages	SUSD1
8.19644553727599E-13	0.47660554561652	0.083	0.253	1.37356034313671E-08	Inflammatory macrophages	SIAH1
8.39162757595003E-13	0.509081766466554	0.159	0.381	1.40626894917771E-08	Inflammatory macrophages	KMT2D
9.03174638626979E-13	0.264757997985897	0.064	0.219	1.51354005941109E-08	Inflammatory macrophages	TNFAIP8
9.11078224864037E-13	0.260913711618339	0.05	0.193	1.52678488922715E-08	Inflammatory macrophages	MGA
1.02312076771846E-12	0.623610668199121	0.068	0.224	1.71454578254259E-08	Inflammatory macrophages	FUT11
1.02395319417389E-12	0.39136036755924	0.081	0.249	1.71594076279661E-08	Inflammatory macrophages	FAM168A
1.05587010255078E-12	0.293456351246471	0.054	0.2	1.76942711785459E-08	Inflammatory macrophages	TXNL1
1.13034852223584E-12	0.516434631600619	0.126	0.328	1.89423805356282E-08	Inflammatory macrophages	HERC4
1.13991737417071E-12	0.312203490437649	0.064	0.216	1.91027353563527E-08	Inflammatory macrophages	MIOS
1.19439687351306E-12	0.37226677551904	0.091	0.264	2.00157028063319E-08	Inflammatory macrophages	MAPRE2
1.20673286528666E-12	0.27920914912464	0.064	0.216	2.02224293564738E-08	Inflammatory macrophages	CD80
1.21410753733154E-12	0.471735684121547	0.153	0.373	2.0346014110602E-08	Inflammatory macrophages	CASP1
1.27890375025823E-12	0.625896632384747	0.072	0.231	2.14318690468274E-08	Inflammatory macrophages	ABCF3
1.31817634261143E-12	0.33540583808865	0.168	0.4	2.20899991494823E-08	Inflammatory macrophages	AAK1
1.32019408370291E-12	0.368058461572822	0.101	0.28	2.21238124546934E-08	Inflammatory macrophages	NEK7
1.36828089765184E-12	0.467271025218977	0.104	0.288	2.29296512828495E-08	Inflammatory macrophages	MEPCE
1.47348867375767E-12	0.32139485728379	0.089	0.259	2.46927231948311E-08	Inflammatory macrophages	YPEL2
1.47618195274919E-12	0.36085556005616	0.054	0.198	2.47378571641709E-08	Inflammatory macrophages	YME1L1

1.49631351643377E-12	0.30855321790206	0.07	0.226	2.50752219083971E-08	Inflammatory macrophages	ENOSF1
1.50412761599868E-12	0.29025421524022	0.114	0.303	2.52061705889059E-08	Inflammatory macrophages	TBC1D22A
1.54962530496324E-12	0.55529728626238	0.13	0.334	2.5968620860574E-08	Inflammatory macrophages	ENTPD6
1.55294000148956E-12	0.43386204365846	0.077	0.241	2.6024168544962E-08	Inflammatory macrophages	TRPM7
1.61151050262405E-12	0.34695142217874	0.11	0.295	2.70056930029738E-08	Inflammatory macrophages	TOB1
1.64579270135372E-12	0.33542515154233	0.087	0.254	2.75801940892857E-08	Inflammatory macrophages	KIF13B
1.71272875835653E-12	0.29629283739140	0.114	0.302	2.87019085325388E-08	Inflammatory macrophages	SCIMP
1.79166299808829E-12	0.27487024185833	0.132	0.331	3.00246885219636E-08	Inflammatory macrophages	LPXN
1.80128294611365E-12	0.80705314864142	0.834	0.852	3.01858996109726E-08	Inflammatory macrophages	FGL2
1.81256293918731E-12	0.44681823859317	0.114	0.301	3.03749297349009E-08	Inflammatory macrophages	ZDHHC14
1.87154128987447E-12	0.49731406427664	0.077	0.238	3.13632889357163E-08	Inflammatory macrophages	PHC3
1.90449833603092E-12	0.67452265394908	0.091	0.263	3.19155831152062E-08	Inflammatory macrophages	NANS
2.10575316436529E-12	0.58700946425567	0.112	0.298	3.52882115284336E-08	Inflammatory macrophages	DEPRECATED-ENSG00000118263
2.11336927310251E-12	0.71004429680798	0.147	0.361	3.54158422786519E-08	Inflammatory macrophages	UBE2D3
2.11500095365103E-12	0.45416143236648	0.184	0.428	3.54431859812839E-08	Inflammatory macrophages	UBAP2L
2.18264590075494E-12	0.297201579725	0.056	0.201	3.65767800048513E-08	Inflammatory macrophages	FAM126A
2.195384051655E-12	0.44378065241078	0.149	0.363	3.67902459376345E-08	Inflammatory macrophages	CEPT1
2.26017610644635E-12	0.37651125347806	0.052	0.193	3.78760311918279E-08	Inflammatory macrophages	RMC1
2.26180890655423E-12	0.49919055987311	0.159	0.38	3.79033936560358E-08	Inflammatory macrophages	FOXO3
2.31055563512336E-12	0.28852964409741	0.077	0.237	3.87202913333972E-08	Inflammatory macrophages	ARFGAP3
2.35095654395038E-12	0.28911765703486	0.11	0.294	3.93973297635204E-08	Inflammatory macrophages	TCERG1
2.39276843817756E-12	0.48118766422832	0.108	0.293	4.00980134869796E-08	Inflammatory macrophages	BEX4
2.47442942266384E-12	0.37352244349461	0.066	0.218	4.14664882650006E-08	Inflammatory macrophages	TNFSF10
2.53183294943452E-12	0.49344012820728	0.074	0.232	4.24284565666236E-08	Inflammatory macrophages	RSBN1L
2.68112203608884E-12	0.55462818383199	0.155	0.373	4.49302430807769E-08	Inflammatory macrophages	NDUFA10
2.83225128899916E-12	0.40736680132379	0.095	0.265	4.7462867101048E-08	Inflammatory macrophages	TNRC6B
2.86131244074672E-12	0.38849172830543	0.099	0.272	4.79498738820335E-08	Inflammatory macrophages	TRAPPC9
3.05424150601488E-12	0.38755928061211	0.174	0.405	5.11829791577974E-08	Inflammatory macrophages	ITSN2
3.07334405960058E-12	0.45784499380929	0.081	0.246	5.15030997507864E-08	Inflammatory macrophages	EIF4G2
3.07793154612927E-12	0.44292940681607	0.075	0.234	5.15799768500344E-08	Inflammatory macrophages	CD2AP
3.2237190938502E-12	0.33121668121081	0.07	0.223	5.40230845747417E-08	Inflammatory macrophages	NISCH
3.37264858027132E-12	0.36984855044912	0.058	0.201	5.65188449081869E-08	Inflammatory macrophages	LSS
3.38895884948683E-12	0.29829566417914	0.085	0.249	5.67921723997003E-08	Inflammatory macrophages	KPNA6
3.48779114757091E-12	0.52669130558893	0.184	0.424	5.84484040509934E-08	Inflammatory macrophages	PLEKHM2
3.60007330993696E-12	0.40689420472983	0.116	0.3	6.03300285279235E-08	Inflammatory macrophages	KDM4C
3.72018999855465E-12	0.61082091363994	0.079	0.241	6.23429439957788E-08	Inflammatory macrophages	PAF1
4.0756627132633E-12	0.51495074477653	0.118	0.306	6.82999557488665E-08	Inflammatory macrophages	NCK1
4.09476776661877E-12	2.31071796519586	0.19	0.083	6.86201182329973E-08	Inflammatory macrophages	CFP
4.5103627672873E-12	0.31616333976945	0.052	0.19	7.55846592542006E-08	Inflammatory macrophages	RHEB
4.52819072407671E-12	2.83394005870927	0.337	0.241	7.58834201540774E-08	Inflammatory macrophages	APOA2
4.63305863621484E-12	0.39184685916355	0.081	0.242	7.76407966256883E-08	Inflammatory macrophages	TBRG1
4.76337916921473E-12	2.40875816869292	0.377	0.288	7.98247081177005E-08	Inflammatory macrophages	SERPINH1
4.77782924537701E-12	0.25472783303690	0.13	0.325	8.00668624940279E-08	Inflammatory macrophages	ASXL1
4.80788371657905E-12	0.43044932174325	0.209	0.459	8.05705153224317E-08	Inflammatory macrophages	SFT2D2
4.82356517593114E-12	0.43785717741874	0.137	0.338	8.08333052182541E-08	Inflammatory macrophages	RIPOR1
4.8864791455103E-12	0.62583950134637	0.124	0.317	8.18876175204617E-08	Inflammatory macrophages	NT5C2
4.90638903377166E-12	0.40568511860191	0.043	0.174	8.22212674279455E-08	Inflammatory macrophages	ARF4
5.31500592389532E-12	0.30218052189515	0.097	0.266	8.90688692726378E-08	Inflammatory macrophages	UPP1
5.35355991545943E-12	0.63668843953663	0.184	0.423	8.97149570632691E-08	Inflammatory macrophages	SETD5
5.40561881687099E-12	0.45618467323077	0.104	0.279	9.05873601331241E-08	Inflammatory macrophages	MAP3K2
5.51084420863308E-12	0.25415299219402	0.079	0.237	9.23507272482731E-08	Inflammatory macrophages	ARIH2
5.60049698622528E-12	0.53579328721352	0.091	0.256	9.38531284951632E-08	Inflammatory macrophages	PCMTD2
5.76568194546646E-12	0.46823572924581	0.159	0.374	9.6621298042127E-08	Inflammatory macrophages	ABI3
5.96423721902948E-12	0.36739636669069	0.017	0.124	9.9948687316496E-08	Inflammatory macrophages	IPO13
6.15969688771232E-12	0.49005646479577	0.044	0.175	1.03224200444283E-07	Inflammatory macrophages	PNP
6.34757775228926E-12	2.15620445245858	0.335	0.232	1.06372707972863E-07	Inflammatory macrophages	DEPRECATED-ENSG00000115738
6.3664322322498E-12	0.41361521695844	0.07	0.219	1.06688671348042E-07	Inflammatory macrophages	UBXN2B
6.39213417274212E-12	0.65193673729750	0.074	0.228	1.07119384466812E-07	Inflammatory macrophages	DNAJC11
6.46265084090377E-12	0.56421507261515	0.12	0.308	1.08301102791865E-07	Inflammatory macrophages	SLC9A9
6.96212904343243E-12	0.44933452780969	0.07	0.22	1.16671358509841E-07	Inflammatory macrophages	FBXW4
7.20702767575406E-12	0.45299693817977	0.13	0.322	1.20775369790287E-07	Inflammatory macrophages	MKLN1
7.21210794103075E-12	0.29507591140536	0.104	0.28	1.20860504875793E-07	Inflammatory macrophages	ZNF609
7.23405589355333E-12	0.62948764318987	0.087	0.252	1.21228308664167E-07	Inflammatory macrophages	CCNT2
7.35406820012267E-12	0.26389843882420	0.261	0.551	1.23239474897656E-07	Inflammatory macrophages	CCNL2
7.553146664166E-12	0.31255381891361	0.126	0.316	1.26575631798094E-07	Inflammatory macrophages	ADGRE2
7.5972741579772E-12	0.37999898343114	0.07	0.219	1.27315120339382E-07	Inflammatory macrophages	WDR37
7.64139043630942E-12	0.48408721293870	0.048	0.182	1.28054420931673E-07	Inflammatory macrophages	RNF11
7.94609224394367E-12	0.29066627362673	0.095	0.26	1.33160613824008E-07	Inflammatory macrophages	KLHDC2
8.42892000427928E-12	0.45914669727175	0.153	0.36	1.41251841431712E-07	Inflammatory macrophages	TRIM14
8.83740215559641E-12	0.44597451967670	0.174	0.4	1.48097185323485E-07	Inflammatory macrophages	RAB1A
8.91378456267028E-12	0.38401346249970	0.141	0.343	1.49377201701229E-07	Inflammatory macrophages	SLC4A7

9.44694311150835E-12	0.315219158912521	0.246	0.52	1.58311872662657E-07	Inflammatory macrophages	AKR1B1
9.50806054261517E-12	0.323817172186033	0.162	0.378	1.59336078573145E-07	Inflammatory macrophages	ISCU
9.8303993659446E-12	0.420437898242257	0.184	0.417	1.647378325745E-07	Inflammatory macrophages	PABPC4
9.8940327801957E-12	0.375755897472901	0.108	0.285	1.65804201330519E-07	Inflammatory macrophages	PPP1R12C
1.02981426448003E-11	0.482549878377299	0.074	0.224	1.72576274441564E-07	Inflammatory macrophages	MIIP
1.03654580556993E-11	0.530411351626814	0.112	0.293	1.73704346097409E-07	Inflammatory macrophages	NR3C1
1.11704810755897E-11	0.386358731350802	0.099	0.268	1.87194921864732E-07	Inflammatory macrophages	RIN2
1.12227953142678E-11	1.46026663058664	0.571	0.648	1.880716038765E-07	Inflammatory macrophages	WSB1
1.12976912469607E-11	0.6085436847138	0.083	0.241	1.89326709916568E-07	Inflammatory macrophages	HP1BP3
1.15195525065532E-11	0.361786010049976	0.091	0.253	1.93044660904819E-07	Inflammatory macrophages	UBE4B
1.26278001657472E-11	3.39626506250961	0.205	0.102	2.11616675177591E-07	Inflammatory macrophages	CXCL2
1.2958347175516E-11	0.341153200638357	0.145	0.343	2.17155981967297E-07	Inflammatory macrophages	ST3GAL1
1.35801746359437E-11	0.442204437660407	0.122	0.309	2.27576566549145E-07	Inflammatory macrophages	FBXL3
1.36145354527885E-11	0.501008965150954	0.064	0.208	2.2815238511783E-07	Inflammatory macrophages	SUMF1
1.39650171521369E-11	0.63640566725974	0.135	0.33	2.3402575743551E-07	Inflammatory macrophages	RNF10
1.40475960739131E-11	0.592770546058348	0.224	0.487	2.35409615006635E-07	Inflammatory macrophages	NAPA
1.40501976558087E-11	1.79548587995562	0.346	0.238	2.35453212316043E-07	Inflammatory macrophages	SOCS3
1.40971027853627E-11	0.346398860744294	0.048	0.179	2.36239248477109E-07	Inflammatory macrophages	CCDC91
1.43537323986341E-11	0.391242431004242	0.159	0.369	2.40539847536309E-07	Inflammatory macrophages	SYAP1
1.47371052405356E-11	0.29545457254436	0.077	0.23	2.46964409620896E-07	Inflammatory macrophages	ZBED6
1.50942338338234E-11	0.651379651562526	0.041	0.166	2.52949170587213E-07	Inflammatory macrophages	RNF181
1.51627175506966E-11	0.266157464627273	0.095	0.257	2.54096820714573E-07	Inflammatory macrophages	ENTR1
1.53664604351582E-11	0.429045889469881	0.097	0.263	2.57511143972382E-07	Inflammatory macrophages	ABRACL
1.56122848615406E-11	0.428222210586583	0.056	0.192	2.61630669709698E-07	Inflammatory macrophages	RFXANK
1.56404497955051E-11	0.627740326850625	0.075	0.228	2.62102657673074E-07	Inflammatory macrophages	SLC25A36
1.58403491157897E-11	0.68532189110047	0.085	0.246	2.65452570482404E-07	Inflammatory macrophages	ANKH
1.58424581616713E-11	0.483603488041532	0.112	0.291	2.65487913873287E-07	Inflammatory macrophages	PPIG
1.6613138263984E-11	0.355026132343867	0.133	0.326	2.78402971027844E-07	Inflammatory macrophages	CYB5R4
1.6820435787768E-11	0.341306276811306	0.162	0.372	2.81876862931416E-07	Inflammatory macrophages	IL16
1.69521167982136E-11	0.390482683913287	0.062	0.203	2.84083573304463E-07	Inflammatory macrophages	NIPSNAP2
1.77028675712814E-11	0.429618622598599	0.205	0.454	2.96664654759534E-07	Inflammatory macrophages	EPB41L3
1.79856846658911E-11	0.356251062696022	0.188	0.417	3.01404103631003E-07	Inflammatory macrophages	STK10
2.05726684326654E-11	0.58852844882899	0.172	0.395	3.44756777594608E-07	Inflammatory macrophages	C16orf72
2.10524925054202E-11	0.512913298846744	0.124	0.305	3.52797669405832E-07	Inflammatory macrophages	IKBKG
2.26980936864025E-11	0.322384906025586	0.29	0.604	3.80374653996734E-07	Inflammatory macrophages	CIRBP
2.37231182572385E-11	0.435939119444545	0.112	0.286	3.97552015754803E-07	Inflammatory macrophages	MOV10
2.44557779888889E-11	1.94980552972222	0.449	0.409	4.098299275378E-07	Inflammatory macrophages	GADD45B
2.54185393421016E-11	0.604149990200833	0.143	0.341	4.25963882294939E-07	Inflammatory macrophages	SNRNP70
2.61952450444969E-11	2.90695350842196	0.159	0.065	4.38979916455679E-07	Inflammatory macrophages	HES1
2.63036857741606E-11	0.854634493764002	0.143	0.343	4.40797166203383E-07	Inflammatory macrophages	USP4
2.72229342698214E-11	0.77614103825422	0.112	0.291	4.56201932493668E-07	Inflammatory macrophages	PHACTR4
2.7419209154008E-11	0.422724325035105	0.075	0.223	4.59491107002867E-07	Inflammatory macrophages	ZBTB25
2.80540959504117E-11	0.253315849620924	0.083	0.237	4.70130539936999E-07	Inflammatory macrophages	ATXN7
2.87316116572652E-11	0.408089030799321	0.05	0.179	4.8148434815245E-07	Inflammatory macrophages	ATR
2.88161583950461E-11	0.350865111220421	0.06	0.197	4.82901182384183E-07	Inflammatory macrophages	C3orf38
2.92211297400664E-11	0.322134979121933	0.091	0.25	4.89687692184032E-07	Inflammatory macrophages	PABPC1L
2.93858833371834E-11	0.745994611823913	0.079	0.232	4.92448632964519E-07	Inflammatory macrophages	RASA1
2.99020002498703E-11	0.390537638182944	0.083	0.237	5.01097720187326E-07	Inflammatory macrophages	KIAA1109
3.14190103755763E-11	0.726617676292754	0.128	0.314	5.26519775873908E-07	Inflammatory macrophages	FGB
3.19058989800655E-11	0.263703209400424	0.081	0.232	5.34679055107938E-07	Inflammatory macrophages	KIAA2026
3.26696133747515E-11	0.61380999424993	0.064	0.204	5.47477380934086E-07	Inflammatory macrophages	MYCBP2
3.32873938562575E-11	0.300737291458846	0.074	0.218	5.57830146243164E-07	Inflammatory macrophages	USP8
3.33347095708328E-11	0.444535708806704	0.133	0.322	5.58623062988016E-07	Inflammatory macrophages	PPFIBP2
3.38784856256751E-11	0.379047146034111	0.054	0.186	5.67735662115064E-07	Inflammatory macrophages	VPS54
3.4900663884205E-11	0.267694494926914	0.178	0.397	5.84865325371507E-07	Inflammatory macrophages	HECA
3.50488714952891E-11	0.489654978384014	0.095	0.256	5.87348988518056E-07	Inflammatory macrophages	ZRANB2
3.70675014197169E-11	0.338137367575967	0.255	0.538	6.21177188791617E-07	Inflammatory macrophages	LPAR6
3.73802893914094E-11	0.557194835927471	0.068	0.21	6.26418889621239E-07	Inflammatory macrophages	OTUD4
3.76712621671903E-11	0.364211467262431	0.058	0.192	6.31295011397775E-07	Inflammatory macrophages	VPS13D
3.8536522483489E-11	1.95465800455287	0.25	0.135	6.45795043778308E-07	Inflammatory macrophages	SDS
3.87368082780285E-11	0.68562101617876	0.07	0.214	6.49151433123201E-07	Inflammatory macrophages	SNX24
4.0850831574938E-11	0.266305682563768	0.043	0.165	6.84578235532811E-07	Inflammatory macrophages	MMADHC
4.14344683823262E-11	0.30550412790789	0.292	0.589	6.94358821151022E-07	Inflammatory macrophages	CLEC7A
4.15429679347645E-11	0.312401470488206	0.099	0.263	6.96177056650783E-07	Inflammatory macrophages	EVI5
4.29261136035847E-11	0.419820994406895	0.079	0.229	7.19355811768872E-07	Inflammatory macrophages	HVCN1
4.33159222548202E-11	0.333978021858775	0.037	0.154	7.25888225146276E-07	Inflammatory macrophages	ABCB10
4.38204336221352E-11	0.780226756785291	0.145	0.347	7.34342826639742E-07	Inflammatory macrophages	KIF1B
4.43572076327282E-11	0.4582703354454	0.137	0.33	7.4333808550926E-07	Inflammatory macrophages	DNAJC7
4.43759276396557E-11	0.423167722782391	0.112	0.285	7.4365179538535E-07	Inflammatory macrophages	ARL8B
4.54426083148985E-11	0.326890475780634	0.054	0.184	7.61527230141068E-07	Inflammatory macrophages	CDYL
4.58808803313631E-11	0.330337695693967	0.068	0.208	7.68871792592982E-07	Inflammatory macrophages	KLHDC4

4.60395668299395E-11	0.714253534999276	0.145	0.342	7.71531060936127E-07	Inflammatory macrophages	PCED1A
4.80164438081654E-11	0.413130575555241	0.153	0.353	8.04659565337236E-07	Inflammatory macrophages	CREBRF
4.8289849894289E-11	0.625132912145753	0.162	0.374	8.09241304528494E-07	Inflammatory macrophages	ARRB1
4.87410263254021E-11	0.315454486955482	0.12	0.295	8.16802119161088E-07	Inflammatory macrophages	PILRB
5.03312427998601E-11	0.533412099906551	0.081	0.232	8.43450966840055E-07	Inflammatory macrophages	YY1
5.05775476455473E-11	0.414717417766884	0.058	0.192	8.47578543444082E-07	Inflammatory macrophages	MAP2K7
5.87917499483928E-11	0.37433602605416	0.068	0.208	9.85232145635167E-07	Inflammatory macrophages	BCL10
6.39450198679392E-11	0.470668520489707	0.17	0.387	1.07159064294693E-06	Inflammatory macrophages	IFNAR2
6.4718941196585E-11	0.423309794594083	0.064	0.2	1.08456001657237E-06	Inflammatory macrophages	DYRK2
6.83379709998227E-11	0.688917914214454	0.07	0.211	1.14520771801503E-06	Inflammatory macrophages	ATXN2
6.9999428659061E-11	0.524416950695449	0.097	0.257	1.17305042546854E-06	Inflammatory macrophages	YTHDC2
7.03157287916126E-11	0.460088851134918	0.085	0.237	1.17835098308984E-06	Inflammatory macrophages	NUP50
7.06618688566091E-11	0.563470261527318	0.058	0.19	1.18415159829906E-06	Inflammatory macrophages	FIG4
7.11593208769319E-11	0.256424717075672	0.07	0.21	1.19248789925562E-06	Inflammatory macrophages	DOT1L
7.13524928169135E-11	0.280149729681378	0.044	0.166	1.19572507462584E-06	Inflammatory macrophages	TOB2
7.23980215018834E-11	0.525407696900538	0.054	0.183	1.21324604432856E-06	Inflammatory macrophages	PACS2
7.2409891635096E-11	0.331481183038807	0.081	0.23	1.21344496402094E-06	Inflammatory macrophages	GTPBP6
7.31352291588702E-11	0.64562344923948	0.191	0.42	1.22560017024435E-06	Inflammatory macrophages	SP3
7.35902201078923E-11	0.620789352701223	0.074	0.218	1.23322490856806E-06	Inflammatory macrophages	LIMK2
7.93560584923199E-11	0.491227727845236	0.17	0.384	1.3298488282143E-06	Inflammatory macrophages	PTPRC
8.14110912419331E-11	0.376219290907553	0.041	0.159	1.36428706703232E-06	Inflammatory macrophages	EPS8
8.16533554273986E-11	0.480982147083209	0.075	0.219	1.36834693025234E-06	Inflammatory macrophages	C15orf48
8.23326204135948E-11	0.308466700521121	0.112	0.281	1.37973005289102E-06	Inflammatory macrophages	ATN1
8.32335273897974E-11	0.689815915056873	0.162	0.369	1.39482745199822E-06	Inflammatory macrophages	EMD
8.73161453300058E-11	0.523484657174316	0.143	0.334	1.46324396344024E-06	Inflammatory macrophages	ITSN1
8.81356883002594E-11	0.410030321705488	0.124	0.3	1.47697786453575E-06	Inflammatory macrophages	SON
9.0152253956356E-11	0.312086075626162	0.037	0.152	1.51077147180061E-06	Inflammatory macrophages	IRF5
9.33730080830143E-11	0.768246200370367	0.103	0.268	1.56474486945515E-06	Inflammatory macrophages	SLC2A9
9.40655358958664E-11	0.466171153339	0.081	0.23	1.57635025054293E-06	Inflammatory macrophages	TRAF6
9.54045858845001E-11	0.589352612560218	0.114	0.287	1.59879005025245E-06	Inflammatory macrophages	VHL
9.77209681594707E-11	0.623983406605814	0.118	0.288	1.63760798441641E-06	Inflammatory macrophages	UAP1L1
9.94365020057973E-11	0.502327191001348	0.06	0.192	1.66635690061315E-06	Inflammatory macrophages	PHF20
1.04695544926538E-10	0.311132154609991	0.056	0.185	1.75448794187893E-06	Inflammatory macrophages	CLASP2
1.04748790701085E-10	0.655980411894999	0.161	0.369	1.75538023456878E-06	Inflammatory macrophages	GNB4
1.08481483491499E-10	0.807494177436274	0.128	0.308	1.81793270035055E-06	Inflammatory macrophages	GASK1B
1.10200694595741E-10	0.470095822977324	0.13	0.308	1.84674324003542E-06	Inflammatory macrophages	IFFO1
1.10231581471164E-10	0.3028366048287	0.079	0.225	1.84726084229376E-06	Inflammatory macrophages	SOGA1
1.12601162854053E-10	0.701985224195231	0.118	0.294	1.88697028710822E-06	Inflammatory macrophages	DEPRECATED-ENSG00000166136
1.13330151065725E-10	0.55591160628054	0.124	0.3	1.89918667155942E-06	Inflammatory macrophages	GUK1
1.136269823095E-10	0.501562291907317	0.128	0.31	1.90416096954261E-06	Inflammatory macrophages	SEC22B
1.1440556728481E-10	0.527826659464276	0.106	0.271	1.91720849655885E-06	Inflammatory macrophages	RHOT2
1.22390695612972E-10	0.487765185507622	0.058	0.188	2.05102327708219E-06	Inflammatory macrophages	TOLLIP
1.26211589111402E-10	0.633261071325569	0.19	0.414	2.11505381032888E-06	Inflammatory macrophages	AKAP9
1.27210467286624E-10	0.407657581225887	0.05	0.174	2.13179301078924E-06	Inflammatory macrophages	GDAP2
1.27574937000772E-10	0.611885061815727	0.168	0.377	2.13790079425894E-06	Inflammatory macrophages	DDAH2
1.31155899822292E-10	0.463159955310614	0.075	0.218	2.19791056922197E-06	Inflammatory macrophages	ZXDC
1.3616982687695E-10	0.286117013122009	0.087	0.236	2.28193395880392E-06	Inflammatory macrophages	TTC14
1.39435078223266E-10	0.327165101589869	0.083	0.23	2.33665304086549E-06	Inflammatory macrophages	CDKAL1
1.40468067930986E-10	0.321298206878847	0.044	0.163	2.35396388238746E-06	Inflammatory macrophages	IMPA1
1.41361048075904E-10	0.352436554585676	0.265	0.538	2.368928443656E-06	Inflammatory macrophages	PARVG
1.41643006873966E-10	0.297882749054229	0.103	0.263	2.37365350919392E-06	Inflammatory macrophages	PGPEP1
1.42073381111857E-10	0.434240105142968	0.132	0.313	2.38086572067249E-06	Inflammatory macrophages	INPP4A
1.48147234234115E-10	2.29531553527506	0.29	0.191	2.48265135129529E-06	Inflammatory macrophages	ZFAND2A
1.50399465649312E-10	0.406420207138997	0.116	0.285	2.52039424535117E-06	Inflammatory macrophages	EXOC4
1.53588870078453E-10	0.719441542853111	0.114	0.285	2.57384228477471E-06	Inflammatory macrophages	WDFY3
1.55091206229692E-10	0.358345986550905	0.19	0.41	2.59901843399717E-06	Inflammatory macrophages	TMEM37
1.57264435268873E-10	0.480048764628216	0.095	0.25	2.63543740623578E-06	Inflammatory macrophages	DCAF5
1.57698049412512E-10	0.64951849305685	0.188	0.41	2.64270391205487E-06	Inflammatory macrophages	DENND1A
1.57840821876348E-10	0.61556125188519	0.079	0.223	2.64509649300384E-06	Inflammatory macrophages	HEXIM1
1.58659349361931E-10	0.607039464025971	0.132	0.314	2.65881337660723E-06	Inflammatory macrophages	CMTM7
1.65838184844886E-10	0.396613769548527	0.118	0.285	2.7791163016306E-06	Inflammatory macrophages	VPS41
1.67770919964875E-10	0.557478746282448	0.054	0.179	2.81150507677137E-06	Inflammatory macrophages	NEMP1
1.75133742711118E-10	0.435717662060337	0.191	0.414	2.93489126035292E-06	Inflammatory macrophages	IQSEC1
1.76369532740773E-10	0.5220679641955	0.106	0.268	2.95560062966987E-06	Inflammatory macrophages	WDR11
1.76562085598189E-10	0.258053131377007	0.048	0.17	2.95882743045445E-06	Inflammatory macrophages	KDM1B
1.92393555468099E-10	0.650997893784576	0.077	0.22	3.2241312025344E-06	Inflammatory macrophages	SLC25A44
1.9574262582106E-10	0.443891655540732	0.068	0.202	3.28025492350932E-06	Inflammatory macrophages	HSPA9
2.10957747073241E-10	0.617750515397759	0.186	0.403	3.53522992545337E-06	Inflammatory macrophages	YPEL5
2.15786639447244E-10	0.274495041866997	0.087	0.234	3.61615250385692E-06	Inflammatory macrophages	WASHC1
2.17767567052424E-10	0.462706308661044	0.23	0.479	3.64934888866451E-06	Inflammatory macrophages	RBM33
2.18923434333289E-10	0.271234050063583	0.041	0.154	3.66871891255726E-06	Inflammatory macrophages	UHRF1BP1

2.19555932446228E-10	0.40946692092025	0.24	0.499	3.67931831593388E-06	Inflammatory macrophages	TCF25
2.38952039575799E-10	0.44994879479879	0.066	0.199	4.00435827921125E-06	Inflammatory macrophages	TIMM44
2.39703695123806E-10	0.33333797602446	0.058	0.185	4.01695452288474E-06	Inflammatory macrophages	MED24
2.44171821025879E-10	0.28609715838639	0.11	0.272	4.09183137675168E-06	Inflammatory macrophages	NPEPL1
2.54933873626623E-10	0.28239517454597	0.056	0.181	4.27218185423495E-06	Inflammatory macrophages	MMD
2.55256893027594E-10	0.28445063110478	0.089	0.237	4.27759501335641E-06	Inflammatory macrophages	PWWP2B
2.58101312235977E-10	0.54218440433289	0.052	0.175	4.3252617904505E-06	Inflammatory macrophages	CXorf38
2.58459538730582E-10	0.39906893094239	0.097	0.25	4.33126495004709E-06	Inflammatory macrophages	ZNF24
2.61719214403197E-10	0.52607268038928	0.037	0.148	4.38589059496878E-06	Inflammatory macrophages	RICTOR
2.65000923976946E-10	0.53866544152767	0.048	0.168	4.44088548400565E-06	Inflammatory macrophages	BORCS8
2.7231074082324E-10	0.41222407241516	0.048	0.167	4.56338339471585E-06	Inflammatory macrophages	CDK5RAP2
2.75570509741837E-10	0.48157279279369	0.23	0.483	4.6180106022537E-06	Inflammatory macrophages	STAT2
2.75677474283339E-10	0.33869391811769	0.13	0.303	4.61980311404019E-06	Inflammatory macrophages	CLEC2D
2.79393734931886E-10	0.59282672265807	0.044	0.161	4.68208020998855E-06	Inflammatory macrophages	SHLD2
2.83078001227649E-10	0.27903211833444	0.066	0.197	4.74382114457294E-06	Inflammatory macrophages	DIPK2A
2.83817780183528E-10	0.34239443909163	0.058	0.184	4.75621836031557E-06	Inflammatory macrophages	KLHL36
2.84781931164086E-10	0.48574424017157	0.046	0.164	4.77237560244775E-06	Inflammatory macrophages	NGDN
2.87559744938759E-10	0.46018311627426	0.074	0.21	4.81892620568372E-06	Inflammatory macrophages	PATL1
2.88157330615293E-10	0.76025271129672	0.162	0.362	4.82894054645108E-06	Inflammatory macrophages	ITGAV
2.93041255906927E-10	0.60289802256811	0.075	0.214	4.91078536648829E-06	Inflammatory macrophages	SIK2
2.94755008209634E-10	0.34948977372344	0.135	0.314	4.93950442757705E-06	Inflammatory macrophages	MEF2D
3.01419381527119E-10	0.50321097667502	0.174	0.383	5.05118599563146E-06	Inflammatory macrophages	LGALS8
3.40673395457245E-10	0.27335238142656	0.05	0.17	5.70900476107252E-06	Inflammatory macrophages	ASPSCR1
3.46712924303693E-10	0.52138866496953	0.058	0.183	5.81021518548128E-06	Inflammatory macrophages	NOP10
3.49807969621201E-10	0.26985989132725	0.155	0.344	5.8620819549121E-06	Inflammatory macrophages	GFRA2
3.50278128534057E-10	0.35552778035009	0.159	0.355	5.86996087797374E-06	Inflammatory macrophages	RALGDS
3.79557255596349E-10	0.46845486813387	0.033	0.139	6.36062048928362E-06	Inflammatory macrophages	BICRAL
3.83036441369813E-10	0.67739040410391	0.182	0.402	6.41892468447532E-06	Inflammatory macrophages	LARP4
3.88639000614453E-10	0.33616701487521	0.027	0.128	6.512812372297E-06	Inflammatory macrophages	POLI
4.26391686912718E-10	0.50271139808207	0.068	0.2	7.14547188928333E-06	Inflammatory macrophages	ATF7IP
4.29887244030066E-10	0.50901624933000	0.155	0.347	7.20405043545584E-06	Inflammatory macrophages	SOAT1
4.36312328841277E-10	0.57060020597549	0.056	0.179	7.31172200672213E-06	Inflammatory macrophages	RARS
4.41808926605148E-10	0.30804920283149	0.037	0.145	7.40383399204907E-06	Inflammatory macrophages	GTF2H2
4.42908706067276E-10	0.32346758644901	0.114	0.276	7.42226409627541E-06	Inflammatory macrophages	DEPRECATED-ENSG00000161547
4.43057122107815E-10	0.36039989990340	0.106	0.263	7.42475125228276E-06	Inflammatory macrophages	CUX1
4.50329778942277E-10	0.56258038708122	0.062	0.19	7.54662643551468E-06	Inflammatory macrophages	METTL9
4.51371519886569E-10	0.31727208761987	0.035	0.142	7.56408393025912E-06	Inflammatory macrophages	MITD1
4.53557015854389E-10	0.69797490768974	0.033	0.138	7.60070847168786E-06	Inflammatory macrophages	HECTD2
4.56115828920581E-10	0.40228384320077	0.07	0.202	7.6435890610511E-06	Inflammatory macrophages	LAMB2
4.56147417541707E-10	0.42383239405239	0.101	0.254	7.64411842316392E-06	Inflammatory macrophages	TUBGCP6
4.74646753400222E-10	0.31965383903689	0.284	0.564	7.95413029348093E-06	Inflammatory macrophages	TNS3
4.81625403586275E-10	0.70822189389873	0.203	0.432	8.07107851329879E-06	Inflammatory macrophages	BNIP3L
4.97692813830277E-10	0.41295006743804	0.075	0.213	8.34033617416778E-06	Inflammatory macrophages	EEA1
5.11829946027571E-10	0.35882273729609	0.064	0.191	8.57724623553004E-06	Inflammatory macrophages	FXR1
5.1425558290846E-10	0.43941964284361	0.12	0.288	8.61789505837997E-06	Inflammatory macrophages	FER
5.1587305212738E-10	0.25036205365505	0.046	0.162	8.64500060755064E-06	Inflammatory macrophages	SCAP
5.1959447605308E-10	0.41903144158242	0.079	0.218	8.70736422969752E-06	Inflammatory macrophages	MITF
5.55107362568082E-10	0.30958475071683	0.095	0.242	9.30248918191592E-06	Inflammatory macrophages	CBX4
5.714947472582E-10	0.55876756447125	0.25	0.507	9.57710897455291E-06	Inflammatory macrophages	LMNA
5.7862738532983E-10	0.95681393592054	0.161	0.358	9.69663772335729E-06	Inflammatory macrophages	AFF4
5.82016467373356E-10	0.42839233543873	0.095	0.246	9.7534319602427E-06	Inflammatory macrophages	SDCCAG8
6.0030933270362E-10	0.59571500466638	0.043	0.155	1.00599837974473E-05	Inflammatory macrophages	DDX18
6.06457421881161E-10	0.74853180657191	0.099	0.25	1.01630134758845E-05	Inflammatory macrophages	STRN4
6.10994445858306E-10	0.43351428023660	0.068	0.198	1.02390449236935E-05	Inflammatory macrophages	MAPKAPK5
6.12287004505504E-10	0.27252769717482	0.306	0.598	1.02607056215032E-05	Inflammatory macrophages	SDCBP
6.19414392428024E-10	0.4488379678491	0.147	0.332	1.03801463883088E-05	Inflammatory macrophages	STARD3
6.21755561329357E-10	0.37490843979220	0.066	0.195	1.04193796967574E-05	Inflammatory macrophages	CHCHD10
6.24686815125369E-10	0.37422043924893	0.085	0.227	1.04685016478709E-05	Inflammatory macrophages	TNRC6A
6.34419851311786E-10	0.44083292422776	0.077	0.214	1.06316078682829E-05	Inflammatory macrophages	TPRA1
6.44256817065747E-10	0.59084293115761	0.197	0.414	1.07964557403878E-05	Inflammatory macrophages	SREBF1
6.50622600785151E-10	0.92429226265778	0.172	0.381	1.09031335439576E-05	Inflammatory macrophages	SMS
6.60941343597625E-10	0.63235883263913	0.079	0.215	1.1076055036009E-05	Inflammatory macrophages	AP5Z1
6.81472527713477E-10	0.69031270156256	0.07	0.201	1.14201166194225E-05	Inflammatory macrophages	AVL9
6.84655338417951E-10	0.26879186677113	0.089	0.231	1.1473454161208E-05	Inflammatory macrophages	RBM15
6.88909788063898E-10	0.42393106120613	0.068	0.197	1.15447502283748E-05	Inflammatory macrophages	KMT5A
6.90635927590972E-10	0.76679824536758	0.085	0.229	1.15736768745695E-05	Inflammatory macrophages	C2CD5
7.13124247856971E-10	0.44491977385223	0.079	0.217	1.19505361455871E-05	Inflammatory macrophages	PCCA
7.63799811186393E-10	1.09937423884729	0.615	0.685	1.27997572358616E-05	Inflammatory macrophages	RGS2
7.78432666316165E-10	0.42602288112733	0.043	0.153	1.30449746221263E-05	Inflammatory macrophages	SPAST
7.93265706127704E-10	0.64023057547916	0.118	0.283	1.32935467032881E-05	Inflammatory macrophages	ZBTB4
7.98691366327792E-10	0.47799769078662	0.056	0.176	1.33844699169211E-05	Inflammatory macrophages	GAPVD1

8.26646798861388E-10	0.35217196599982	0.188	0.395	1.38529470553191E-05	Inflammatory macrophages	SBNO2
8.51468714444329E-10	0.28805142619941	0.317	0.626	1.42689127166581E-05	Inflammatory macrophages	IKBKB
8.95152154023578E-10	0.42210916511171	0.085	0.225	1.50009597971271E-05	Inflammatory macrophages	CCNYL1
9.12362671951891E-10	0.46708939905771	0.083	0.222	1.52893736565698E-05	Inflammatory macrophages	PRR14
9.60992555572604E-10	0.36382680758556	0.048	0.163	1.61043132462857E-05	Inflammatory macrophages	COG5
9.993362588862E-10	0.44149751075769	0.103	0.253	1.67468770264149E-05	Inflammatory macrophages	WDFY1
1.00312668300708E-09	0.34678360059883	0.106	0.259	1.68103969538326E-05	Inflammatory macrophages	TRIM47
1.00451328422209E-09	0.45356947367087	0.058	0.179	1.68336336169937E-05	Inflammatory macrophages	PLA2G2A
1.00517807675497E-09	0.53100658204864	0.085	0.226	1.68447742102597E-05	Inflammatory macrophages	NUP42
1.04950198761021E-09	0.38695436291219	0.203	0.42	1.7587554308372E-05	Inflammatory macrophages	LILRB2
1.05447422031433E-09	0.66499581505237	0.209	0.436	1.76708789840275E-05	Inflammatory macrophages	ATG7
1.07510581466668E-09	0.39165694196623	0.112	0.271	1.80166232421842E-05	Inflammatory macrophages	SESN3
1.0850764930694E-09	0.77154367709271	0.06	0.183	1.8183711870857E-05	Inflammatory macrophages	GTF2A1
1.1121697439932E-09	0.58296700545420	0.124	0.29	1.8637740569838E-05	Inflammatory macrophages	SLC46A1
1.12095597619721E-09	0.64046923910316	0.11	0.265	1.87849802491129E-05	Inflammatory macrophages	TBC1D9
1.16625990121867E-09	0.63501411852250	0.099	0.248	1.95441834246225E-05	Inflammatory macrophages	PHF11
1.17032443304416E-09	0.56527388262835	0.087	0.228	1.9612296848954E-05	Inflammatory macrophages	PLAGL1
1.19064441079619E-09	0.51233644169322	0.104	0.258	1.99528190361225E-05	Inflammatory macrophages	SRC
1.19685758382175E-09	0.47306094334020	0.075	0.209	2.0056939389685E-05	Inflammatory macrophages	TTC39C
1.21286665966641E-09	0.46806361426524	0.05	0.165	2.03252194826896E-05	Inflammatory macrophages	ARAP2
1.21989422159576E-09	0.30374781673438	0.062	0.185	2.04429873655017E-05	Inflammatory macrophages	CAMKK2
1.25234854350152E-09	0.45928730206436	0.288	0.578	2.09868568919985E-05	Inflammatory macrophages	CHST11
1.31247499400457E-09	0.38056346638814	0.11	0.264	2.19944559495286E-05	Inflammatory macrophages	DVL3
1.32023854346833E-09	0.35427191820957	0.064	0.188	2.21245575114424E-05	Inflammatory macrophages	ZMYM6
1.32779326264941E-09	0.51800618019838	0.068	0.194	2.22511594954788E-05	Inflammatory macrophages	ERCC3
1.36988358590487E-09	0.8757439306368	0.052	0.169	2.29565091325938E-05	Inflammatory macrophages	ELMO2
1.39133543798419E-09	2.47180206902954	0.325	0.246	2.33159992697391E-05	Inflammatory macrophages	ZNF331
1.3966700770739E-09	0.62860185324157	0.133	0.306	2.34053971516044E-05	Inflammatory macrophages	RBM17
1.40871233941172E-09	0.44216634507321	0.031	0.131	2.36072013838616E-05	Inflammatory macrophages	ZCCHC24
1.43248700377786E-09	0.36345764321699	0.184	0.387	2.40056172093095E-05	Inflammatory macrophages	RBM25
1.47210684876741E-09	0.84627197856491	0.093	0.237	2.46695665716442E-05	Inflammatory macrophages	POLR2H
1.47424934166961E-09	0.39664288110310	0.052	0.167	2.47054704676993E-05	Inflammatory macrophages	LSM14A
1.48448225531776E-09	0.26179750995268	0.033	0.134	2.4876953634615E-05	Inflammatory macrophages	BOD1
1.49063538082426E-09	0.26291802807859	0.039	0.144	2.4980067711853E-05	Inflammatory macrophages	BBS2
1.61431204738311E-09	0.47350517214641	0.043	0.151	2.70526412900462E-05	Inflammatory macrophages	RAB3GAP2
1.61464172680613E-09	0.39819137364288	0.07	0.196	2.70581660578171E-05	Inflammatory macrophages	C16orf70
1.6511356965791E-09	1.18588187688665	0.563	0.624	2.76697320032726E-05	Inflammatory macrophages	GNA13
1.6737683179482E-09	0.49166401667420	0.062	0.184	2.80490094721759E-05	Inflammatory macrophages	MPND
1.69577544001033E-09	0.42556532063376	0.056	0.174	2.84178048236931E-05	Inflammatory macrophages	RAB11FIP2
1.7082226559457E-09	0.27799368484405	0.089	0.228	2.8626395268338E-05	Inflammatory macrophages	APPL2
1.77986375848718E-09	0.29930755565393	0.054	0.17	2.98269568647282E-05	Inflammatory macrophages	PITPNM1
1.8410792056737E-09	0.33854556824271	0.273	0.537	3.08528053286799E-05	Inflammatory macrophages	RNF144B
2.04647974921448E-09	0.51098792050060	0.159	0.345	3.42949076373362E-05	Inflammatory macrophages	MVB12B
2.05373050217729E-09	0.29360035622882	0.081	0.214	3.44164157554871E-05	Inflammatory macrophages	URI1
2.08260760763744E-09	0.65663122357133	0.054	0.17	3.49003382887882E-05	Inflammatory macrophages	ASCC3
2.08885831374248E-09	0.32685375054023	0.081	0.214	3.50050876216965E-05	Inflammatory macrophages	FLCN
2.09952635665916E-09	0.48862438479722	0.046	0.157	3.51838626848942E-05	Inflammatory macrophages	HSPA13
2.10003627809686E-09	0.43380486967322	0.137	0.309	3.51924079483471E-05	Inflammatory macrophages	GAB2
2.10057499754911E-09	0.44889029723150	0.072	0.2	3.52014358089281E-05	Inflammatory macrophages	COX6B1
2.20803863160173E-09	0.52737344980190	0.068	0.192	3.70023113883818E-05	Inflammatory macrophages	PDE8A
2.29768667165442E-09	0.48636157742522	0.081	0.215	3.85046332435847E-05	Inflammatory macrophages	ABL2
2.39751650728639E-09	0.36861949680394	0.035	0.135	4.01775816291054E-05	Inflammatory macrophages	UBQLN1
2.40020556069217E-09	0.52993593321136	0.048	0.16	4.02226447860793E-05	Inflammatory macrophages	SESTD1
2.40235317493958E-09	1.86952739004171	0.354	0.275	4.02586345056375E-05	Inflammatory macrophages	PHACTR1
2.54088250624575E-09	0.66239843727096	0.046	0.156	4.25801090396662E-05	Inflammatory macrophages	IMP3
2.73386187272591E-09	0.38227877012168	0.066	0.188	4.58140572631409E-05	Inflammatory macrophages	AKT2
2.76851042004163E-09	0.59211548159144	0.172	0.367	4.63946976190576E-05	Inflammatory macrophages	ILRUN
2.77255964265139E-09	0.42358552902206	0.118	0.273	4.64625544915521E-05	Inflammatory macrophages	TRAK1
2.77436105550343E-09	0.71018775942741	0.056	0.173	4.64927425681265E-05	Inflammatory macrophages	COMMD1
2.79163219108704E-09	0.54683842603543	0.066	0.189	4.67821722582366E-05	Inflammatory macrophages	ABL1
2.87439608567665E-09	0.42500696882296	0.186	0.389	4.81691296037693E-05	Inflammatory macrophages	SH2B3
2.91730030577178E-09	0.71979246886807	0.12	0.28	4.88881185241234E-05	Inflammatory macrophages	WIPF2
2.9684604476784E-09	0.39877851593360	0.033	0.131	4.97454601821946E-05	Inflammatory macrophages	CNNM3
2.972708351706E-09	0.48429540091278	0.06	0.178	4.98166465578892E-05	Inflammatory macrophages	NDUFB1
2.97439140512718E-09	0.67059963842819	0.081	0.215	4.98448511671213E-05	Inflammatory macrophages	DEPRECATED-ENSG00000067064
3.04074936148206E-09	0.67622662910805	0.093	0.233	5.09568777997163E-05	Inflammatory macrophages	EIF2AK4
3.08166387019037E-09	0.85972325285922	0.074	0.201	5.16425231366502E-05	Inflammatory macrophages	WDR6
3.12816227713982E-09	0.45232842914054	0.05	0.162	5.24217434403092E-05	Inflammatory macrophages	MAPK7
3.22245738343103E-09	0.33345264865489	0.108	0.259	5.40019408315372E-05	Inflammatory macrophages	BCL2L11
3.34695996149385E-09	0.31526594832852	0.035	0.134	5.60883550347139E-05	Inflammatory macrophages	B4GALT3
3.39486654410419E-09	0.30186641901232	0.06	0.178	5.6891173546098E-05	Inflammatory macrophages	ZNF394

3.42431818968255E-09	0.25170453493244	0.023	0.113	5.73847242227002E-05	Inflammatory macrophages	CTPS2
3.45731416235184E-09	0.625072049598376	0.108	0.257	5.79376707326921E-05	Inflammatory macrophages	ABCD4
3.50018183140523E-09	0.325620982916856	0.072	0.197	5.86560471306888E-05	Inflammatory macrophages	RIT1
3.58809188054504E-09	0.678861579474072	0.19	0.397	6.01292437341737E-05	Inflammatory macrophages	CALCOCO2
3.5967351295605E-09	0.387482063011108	0.178	0.372	6.02740873011749E-05	Inflammatory macrophages	MORC3
3.60213533486087E-09	0.466658277192767	0.191	0.395	6.03645839415985E-05	Inflammatory macrophages	CLN8
3.70799980456443E-09	0.558547398392485	0.048	0.158	6.21386607248908E-05	Inflammatory macrophages	PDS5A
3.75265538770911E-09	0.321680012788904	0.023	0.113	6.28869989872293E-05	Inflammatory macrophages	BLMH
3.83774216546444E-09	0.450379133833427	0.039	0.14	6.4312883208853E-05	Inflammatory macrophages	ZDHHC17
3.87542993751576E-09	0.335690818781428	0.037	0.137	6.49444548928891E-05	Inflammatory macrophages	NLRC4
3.93207512299454E-09	0.507247399789713	0.077	0.206	6.58937149111425E-05	Inflammatory macrophages	SH3GLB2
3.96921984115816E-09	0.325765311746088	0.031	0.126	6.65161860981285E-05	Inflammatory macrophages	TIMM9
4.03209590997098E-09	2.8909934472241	0.197	0.109	6.75698632592937E-05	Inflammatory macrophages	SNAPC1
4.19464889935003E-09	0.661303571831961	0.033	0.13	7.02939262553078E-05	Inflammatory macrophages	ACTR8
4.26128840819185E-09	0.303029617363081	0.068	0.188	7.1410671144479E-05	Inflammatory macrophages	RPP30
4.32973900526296E-09	0.623146894331417	0.044	0.15	7.25577662501967E-05	Inflammatory macrophages	WDR18
4.49733004658195E-09	0.337982211467047	0.046	0.153	7.53662569206203E-05	Inflammatory macrophages	STX17
4.50224876175256E-09	0.464361005233816	0.037	0.136	7.54486847494494E-05	Inflammatory macrophages	CNRIP1
4.75559624706076E-09	0.288702347732033	0.056	0.17	7.96942819082443E-05	Inflammatory macrophages	ZCCHC2
4.80888180611637E-09	0.811269691800474	0.195	0.409	8.05872413068981E-05	Inflammatory macrophages	GPR137B
4.86726088263367E-09	0.589110963016201	0.052	0.162	8.1565557871175E-05	Inflammatory macrophages	FUT4
4.93058948762648E-09	0.651999451933827	0.029	0.122	8.26268186336446E-05	Inflammatory macrophages	HFE
4.99491297088148E-09	0.745489211609398	0.151	0.33	8.37047515660319E-05	Inflammatory macrophages	LRRK1
5.02672867147105E-09	0.437135399985555	0.153	0.33	8.42379190765119E-05	Inflammatory macrophages	ARHGAP15
5.03799627999905E-09	0.803140201501229	0.072	0.197	8.4426741660224E-05	Inflammatory macrophages	COMMD10
5.16356794599452E-09	0.392179882218085	0.054	0.166	8.65310716389762E-05	Inflammatory macrophages	SLC7A6
5.21085366511093E-09	0.264679041344915	0.041	0.142	8.7323485719929E-05	Inflammatory macrophages	PIGF
5.22909262908308E-09	0.473294672372959	0.118	0.272	8.76291342781743E-05	Inflammatory macrophages	ARHGEF7
5.24508716754859E-09	0.291631243680948	0.033	0.129	8.78971707537793E-05	Inflammatory macrophages	ECD
5.25239927746486E-09	0.294002890607279	0.081	0.211	8.80197070917561E-05	Inflammatory macrophages	BCLAF1
5.26275644295525E-09	0.665897435704615	0.048	0.156	8.8193272471044E-05	Inflammatory macrophages	IL15RA
5.30233355346574E-09	0.413707016813077	0.284	0.555	8.88565056889788E-05	Inflammatory macrophages	DEPRECATED-ENSG00000026297
5.30643137595401E-09	0.34629128184737	0.039	0.139	8.89251769982373E-05	Inflammatory macrophages	MRRF
5.39873689979756E-09	0.540662695292759	0.12	0.273	9.04720329668074E-05	Inflammatory macrophages	EIF1B
5.44879512967837E-09	0.265298417294598	0.176	0.363	9.13109087831501E-05	Inflammatory macrophages	TRIM38
5.49257323858288E-09	0.503078001576932	0.037	0.135	9.20445423321719E-05	Inflammatory macrophages	PPIL4
5.49737254440615E-09	0.317308238024556	0.203	0.406	9.21249690991582E-05	Inflammatory macrophages	CD163L1
5.71731035294494E-09	0.3641805821244	0.064	0.182	9.58106868946512E-05	Inflammatory macrophages	DMAP1
5.7320910568436E-09	0.410845982758202	0.304	0.595	9.60583819305851E-05	Inflammatory macrophages	MYO1F
5.75730869310952E-09	0.425310234170273	0.035	0.131	9.64809790791293E-05	Inflammatory macrophages	POGLUT1
5.79430832534038E-09	0.350778955690638	0.041	0.142	9.71010189160541E-05	Inflammatory macrophages	TNFRSF11A
5.97950965293034E-09	0.62120046386757	0.041	0.141	0.000100204622763807	Inflammatory macrophages	DEPDC5
6.04831558724082E-09	0.794706082056776	0.056	0.168	0.000101357672610982	Inflammatory macrophages	MSH6
6.21449398973897E-09	0.503895130460702	0.06	0.175	0.000104142490280046	Inflammatory macrophages	DEPRECATED-ENSG00000176788
6.25008054699547E-09	0.479862840329524	0.066	0.185	0.00010473884980655	Inflammatory macrophages	KPNA2
6.27883726170332E-09	0.607653826347051	0.033	0.128	0.000105220754831624	Inflammatory macrophages	FKBP3
6.36992992820401E-09	0.600320047253663	0.07	0.192	0.000106747285736843	Inflammatory macrophages	ARHGEF18
6.60090075472034E-09	0.738351918481086	0.104	0.248	0.000110617894847603	Inflammatory macrophages	DGLUCY
6.62915497025044E-09	0.275126892280534	0.019	0.103	0.000111091378991457	Inflammatory macrophages	PAK1IP1
6.83130267515072E-09	0.379216118326568	0.027	0.117	0.000114478970230176	Inflammatory macrophages	BRPF3
6.99582843068474E-09	0.491806612749238	0.072	0.194	0.000117236092841415	Inflammatory macrophages	PDK3
7.01266737594765E-09	0.372894718294397	0.029	0.121	0.000117518279886131	Inflammatory macrophages	CHM
7.03562786088419E-09	0.780310517553953	0.675	0.784	0.000117903051692697	Inflammatory macrophages	MFSD1
7.0899144256299E-09	0.678303880456108	0.046	0.152	0.000118812785944706	Inflammatory macrophages	OPN3
7.39295404766749E-09	0.615448991002776	0.168	0.357	0.000123891123930812	Inflammatory macrophages	CACUL1
7.39687004904807E-09	0.47089994769715	0.035	0.131	0.000123956748281948	Inflammatory macrophages	PREPL
7.89115649426045E-09	0.423147374523716	0.093	0.228	0.000132240000530817	Inflammatory macrophages	UBE2H
8.01310054321928E-09	0.353295745227096	0.211	0.423	0.000134283538903269	Inflammatory macrophages	JMJD1C
8.14661194530575E-09	0.836134713942836	0.143	0.314	0.000136520922979434	Inflammatory macrophages	PIP4P1
8.22965509022464E-09	0.35365533121969	0.114	0.263	0.000137912560001985	Inflammatory macrophages	KDM2B
8.24305439690387E-09	0.395777062019861	0.07	0.189	0.000138137105583315	Inflammatory macrophages	DEPRECATED-ENSG00000196821
8.28381681471275E-09	0.359400685224118	0.039	0.137	0.000138820202180956	Inflammatory macrophages	AP2B1
8.35221687449696E-09	0.551047975785921	0.05	0.157	0.00013996645038282	Inflammatory macrophages	MVD
8.71724243851359E-09	0.417614268481827	0.039	0.137	0.000146083548784611	Inflammatory macrophages	ZNF830
8.75760349240163E-09	0.368764236335793	0.064	0.18	0.000146759919325667	Inflammatory macrophages	DPEP3
9.43262039944633E-09	0.822998979222314	0.116	0.268	0.000158071852653922	Inflammatory macrophages	RABEP1
9.59385794714188E-09	0.933300387877178	0.197	0.405	0.000160773871478204	Inflammatory macrophages	MIDN
9.71873866146347E-09	0.390822793128928	0.06	0.173	0.000162866622488805	Inflammatory macrophages	ACOT11
9.91758114126936E-09	0.423111339067791	0.261	0.507	0.000166198824765392	Inflammatory macrophages	MDM4
9.94642433815069E-09	0.496821402306558	0.039	0.136	0.000166682179058729	Inflammatory macrophages	SLC25A19
1.02675350973948E-08	2.86825315163663	0.282	0.206	0.000172063353162143	Inflammatory macrophages	EXT1

1.06959386823451E-08	0.53707957868246	0.25	0.497	0.00017924254043874	Inflammatory macrophages	STAT6
1.07131205813654E-08	0.657128941463145	0.085	0.215	0.000179530474702521	Inflammatory macrophages	AGO3
1.08099884452755E-08	0.399201179903466	0.062	0.175	0.000181153786365927	Inflammatory macrophages	DENND4A
1.103490333137E-08	0.415284442267261	0.064	0.179	0.000184922910027098	Inflammatory macrophages	GMDS
1.12292374697891E-08	0.364558663068254	0.046	0.149	0.000188179561518725	Inflammatory macrophages	ADK
1.13549116749849E-08	0.370205690131586	0.033	0.126	0.000190285609849396	Inflammatory macrophages	RSL1D1
1.17094179585055E-08	0.565633856182669	0.06	0.173	0.000196226426148636	Inflammatory macrophages	EPC2
1.17676552333218E-08	0.604197998546798	0.046	0.15	0.000197202366400008	Inflammatory macrophages	ACVR1
1.21215218668966E-08	0.614514854513519	0.089	0.222	0.000203132463445453	Inflammatory macrophages	CEMIP2
1.21433574920202E-08	0.889320798462821	0.072	0.193	0.000203498384851275	Inflammatory macrophages	TCP1
1.22765961642166E-08	0.474396091938545	0.039	0.135	0.000205731198519941	Inflammatory macrophages	IFT20
1.2420257941056E-08	0.678791033053382	0.188	0.388	0.000208138682576216	Inflammatory macrophages	ADAM10
1.26575624251706E-08	0.295591355830324	0.043	0.142	0.000212115431121009	Inflammatory macrophages	C5orf51
1.27017287089648E-08	0.724465105812263	0.213	0.432	0.000212855569704831	Inflammatory macrophages	JDP2
1.28078540625578E-08	0.517311569019373	0.077	0.201	0.000214634018380344	Inflammatory macrophages	MAN1B1
1.28153460498376E-08	1.34976079512549	0.524	0.552	0.000214759569103178	Inflammatory macrophages	CTSL
1.34856205895761E-08	0.633635738543135	0.072	0.19	0.000225992029840116	Inflammatory macrophages	ACYP2
1.36204660065278E-08	0.415930464614867	0.023	0.108	0.000228251769337394	Inflammatory macrophages	C1D
1.39744183191516E-08	0.797242062997709	0.141	0.309	0.000234183302192343	Inflammatory macrophages	TBC1D12
1.4082718594149E-08	0.785184184402289	0.099	0.237	0.000235998198200749	Inflammatory macrophages	CNEP1R1
1.53028497912892E-08	0.562132922990419	0.085	0.212	0.000256445156802424	Inflammatory macrophages	SPIDR
1.55931414253718E-08	0.45225686812246	0.11	0.253	0.000261309864006381	Inflammatory macrophages	JMJD6
1.59105073583916E-08	0.593651428014601	0.199	0.401	0.000266628282311927	Inflammatory macrophages	HNRNPDL
1.59769046483171E-08	0.490748383298436	0.06	0.171	0.000267740968096498	Inflammatory macrophages	ARHGEF12
1.61075995628172E-08	0.725326865175472	0.05	0.155	0.00026993115347369	Inflammatory macrophages	ODR4
1.63971617709835E-08	0.369921238334971	0.132	0.286	0.000274783636958142	Inflammatory macrophages	PIM3
1.6691979403708E-08	0.695751661198448	0.17	0.355	0.000279724190847339	Inflammatory macrophages	CERT1
1.67492228433831E-08	0.62766372117758	0.052	0.158	0.000280683476409414	Inflammatory macrophages	SLC19A1
1.68244794256596E-08	0.530462674509526	0.079	0.202	0.000281944626215204	Inflammatory macrophages	PWWP3A
1.69676669327922E-08	0.289569323331394	0.029	0.117	0.000284344162459732	Inflammatory macrophages	TMEM120A
1.70093879681677E-08	0.557957120303581	0.041	0.138	0.000285043323570554	Inflammatory macrophages	ZCCHC8
1.74533138789218E-08	1.71514469668473	0.416	0.384	0.000292482633982971	Inflammatory macrophages	UGCG
1.74876903340783E-08	0.407985551416202	0.048	0.151	0.000293058714618484	Inflammatory macrophages	CENPP
1.79114163834717E-08	0.489867694396069	0.033	0.124	0.000300159515754219	Inflammatory macrophages	SLC25A26
1.82236238868415E-08	0.710997698057361	0.054	0.161	0.000305391489095689	Inflammatory macrophages	RNGTT
1.83911695173539E-08	0.402615376046157	0.021	0.103	0.000308199218771816	Inflammatory macrophages	DMAC2L
1.86255392635064E-08	0.431351687850382	0.048	0.151	0.00031212678697784	Inflammatory macrophages	ARHGAP10
1.86752588305001E-08	1.00669524458764	0.072	0.191	0.00031295998748152	Inflammatory macrophages	TMUB1
1.90663820818632E-08	0.360709963206681	0.035	0.126	0.000319514430927864	Inflammatory macrophages	HCCS
1.95121424658886E-08	0.859371060907855	0.207	0.422	0.000326984483443362	Inflammatory macrophages	ELL2
1.99314438258025E-08	0.421949053121985	0.029	0.117	0.000334011135632799	Inflammatory macrophages	TBCEL
2.01926030766852E-08	0.352996717365828	0.025	0.109	0.00033838764235909	Inflammatory macrophages	NOL11
2.14540627674605E-08	0.323878829083952	0.039	0.133	0.000359527183857103	Inflammatory macrophages	HARS2
2.18660553572892E-08	0.443937588125976	0.161	0.333	0.000366431355677452	Inflammatory macrophages	SCARB1
2.19245098269868E-08	0.785812846975435	0.06	0.17	0.000367410935680645	Inflammatory macrophages	RHOBTB1
2.24016118249943E-08	0.71607974892088	0.075	0.197	0.000375406210963255	Inflammatory macrophages	TLNRD1
2.40234224932946E-08	0.790359659603816	0.041	0.137	0.000402584514142631	Inflammatory macrophages	SPRY2
2.40929817273005E-08	0.615640256376891	0.056	0.163	0.000403750187786102	Inflammatory macrophages	ZMAT3
2.43357545474271E-08	0.428795339222677	0.048	0.149	0.000407818574705784	Inflammatory macrophages	TOGARAM1
2.43777774671128E-08	0.752448693949419	0.13	0.287	0.000408522794793876	Inflammatory macrophages	ME1
2.45374912433441E-08	0.953116762791395	0.139	0.303	0.000411199278255961	Inflammatory macrophages	HEATR3
2.47186824856158E-08	1.02192659797577	0.151	0.323	0.00041423568109395	Inflammatory macrophages	SPATA20
2.54373838338038E-08	0.477608321725324	0.12	0.266	0.000426279678286883	Inflammatory macrophages	ZNF592
2.54692086607972E-08	0.859905687512677	0.029	0.116	0.000426812998737639	Inflammatory macrophages	SVBP
2.61486896587313E-08	0.282596599620654	0.077	0.197	0.000438199741301019	Inflammatory macrophages	GNL2
2.68089865784032E-08	0.633143064635571	0.197	0.401	0.000449264997080881	Inflammatory macrophages	FRMD4B
2.73844918730332E-08	0.581975910821088	0.103	0.24	0.00045890931480829	Inflammatory macrophages	MTMR3
2.74403868178258E-08	0.675663071195582	0.228	0.449	0.000459846002293125	Inflammatory macrophages	EZR
2.85284253861847E-08	0.334881116269823	0.068	0.181	0.000478079352621684	Inflammatory macrophages	RPTOR
2.89948529002894E-08	0.677453470548638	0.062	0.171	0.00048589574490305	Inflammatory macrophages	FAM172A
2.91083436341908E-08	0.355080457274922	0.056	0.162	0.000487797622621769	Inflammatory macrophages	ZFYVE26
3.06814763799755E-08	0.591666320777408	0.044	0.142	0.000514160181175629	Inflammatory macrophages	ATXN3
3.07761580676055E-08	0.308651626647867	0.037	0.128	0.000515746856896934	Inflammatory macrophages	TOM1L2
3.08963780434896E-08	0.841692515122381	0.135	0.295	0.000517761503252798	Inflammatory macrophages	VPS26A
3.24718062014906E-08	0.288531648416049	0.035	0.125	0.000544162528324579	Inflammatory macrophages	PSEN2
3.2482077193491E-08	0.327565206240319	0.342	0.647	0.000544334649608521	Inflammatory macrophages	ARRB2
3.35133942000165E-08	0.678910875999461	0.06	0.168	0.000561617460003877	Inflammatory macrophages	PCGF3
3.35556234908534E-08	0.709952165516973	0.147	0.313	0.000562325138459721	Inflammatory macrophages	PPP1R15B
3.36047676892286E-08	0.855387577317676	0.087	0.214	0.000563148696936094	Inflammatory macrophages	RNF141
3.36653490303148E-08	0.536802080091843	0.037	0.128	0.000564163919050016	Inflammatory macrophages	DENND10
3.37440438727471E-08	0.487422267674331	0.112	0.253	0.000565482687219496	Inflammatory macrophages	SF3B1

3.39199103942648E-08	0.732595624348933	0.153	0.327	0.000568429858387089	Inflammatory macrophages	EPAS1
3.40387609651198E-08	0.895565641477339	0.132	0.289	0.000570421556253477	Inflammatory macrophages	NCEH1
3.55893259032425E-08	0.324777099030987	0.048	0.147	0.000596405923486537	Inflammatory macrophages	PDSS2
3.5889020956957E-08	0.642579031964015	0.087	0.212	0.000601428213196685	Inflammatory macrophages	INO80E
3.64159154141083E-08	0.71892367167001	0.205	0.413	0.000610257910509627	Inflammatory macrophages	CELF1
3.71511510139735E-08	0.878740994580934	0.048	0.148	0.000622578988692168	Inflammatory macrophages	FBXL20
3.82376975397667E-08	0.518060011528337	0.046	0.144	0.00064078733537141	Inflammatory macrophages	KAT5
3.82782574292813E-08	0.303124342241843	0.05	0.151	0.000641467037999896	Inflammatory macrophages	NFYA
3.84379291142961E-08	0.665948934784911	0.103	0.238	0.000644142816097374	Inflammatory macrophages	SUMO2
3.93163875119861E-08	0.628992526957907	0.023	0.104	0.000658864021925863	Inflammatory macrophages	KIFAP3
3.96501765892472E-08	2.81110319398493	0.315	0.258	0.000664457659282605	Inflammatory macrophages	SELENOK
3.96911103680685E-08	0.403182570224443	0.079	0.198	0.000665143627548092	Inflammatory macrophages	CARD8
4.02765110753666E-08	0.325621967021363	0.021	0.1	0.000674953772600993	Inflammatory macrophages	THOC5
4.09780557751724E-08	0.340127731159035	0.037	0.127	0.000686710258680339	Inflammatory macrophages	ZNF449
4.10872238134279E-08	0.605070282983912	0.234	0.465	0.000688539696665425	Inflammatory macrophages	FYB1
4.15041346219789E-08	0.774883201482053	0.114	0.256	0.000695526287995122	Inflammatory macrophages	HSD17B14
4.32346418224263E-08	0.697561420988705	0.226	0.448	0.000724526127660219	Inflammatory macrophages	DOCK4
4.41162640279319E-08	0.641341111320361	0.06	0.167	0.000739300352580082	Inflammatory macrophages	LPAR5
4.51521050262881E-08	0.476597282446774	0.044	0.14	0.000756658976030536	Inflammatory macrophages	B4GALT7
4.56539529385755E-08	0.483259778487307	0.066	0.176	0.000765068943344649	Inflammatory macrophages	FEM1C
4.70811586952839E-08	3.02193291466079	0.141	0.067	0.000788986057415568	Inflammatory macrophages	PTGS2
4.76245034158666E-08	0.630951462439067	0.054	0.157	0.000798091428243092	Inflammatory macrophages	MAP3K4
4.81468709010996E-08	0.994939380885269	0.085	0.208	0.000806845262560627	Inflammatory macrophages	PELI1
4.94047828987071E-08	0.397135222105576	0.025	0.106	0.000827925351816534	Inflammatory macrophages	TMEM39B
5.03919959767532E-08	0.642190806836116	0.188	0.38	0.000844469068578431	Inflammatory macrophages	NKTR
5.08957978913993E-08	0.554936134815878	0.062	0.17	0.00085291178106407	Inflammatory macrophages	SS18
5.12321980411076E-08	0.64610986834593	0.253	0.493	0.000858549174772881	Inflammatory macrophages	PLEKHO2
5.15657822491264E-08	0.386467155740953	0.089	0.212	0.00086413937893086	Inflammatory macrophages	ZNF516
5.23574837968445E-08	0.517078589581789	0.114	0.255	0.00087740671346752	Inflammatory macrophages	NFAM1
5.36835439138276E-08	0.376423519431234	0.054	0.156	0.000899628828907922	Inflammatory macrophages	DIS3L2
5.45158584319023E-08	0.656053783526356	0.164	0.341	0.000913576755601818	Inflammatory macrophages	RAB3IL1
5.5028178691197E-08	2.48353888603524	0.199	0.116	0.000922162218507079	Inflammatory macrophages	IL1RN
5.52228862669847E-08	0.509147928397621	0.114	0.254	0.00092542512806213	Inflammatory macrophages	FBXW7
5.54196805807943E-08	0.570547763441813	0.035	0.123	0.000928723007172951	Inflammatory macrophages	C4orf33
5.73895554654312E-08	0.408805791546148	0.046	0.143	0.000961734170489697	Inflammatory macrophages	SLC37A1
5.96526519488152E-08	0.657763427998443	0.054	0.156	0.000999659141358246	Inflammatory macrophages	COG4
5.98302038535724E-08	0.601313489665104	0.279	0.534	0.00100263455617817	Inflammatory macrophages	GGA2
6.249615069083E-08	0.489623889766259	0.081	0.198	0.00104731049327693	Inflammatory macrophages	ATP2B1
6.33398651161637E-08	0.255260742662046	0.037	0.126	0.00106144945961667	Inflammatory macrophages	CCDC174
6.33475857930501E-08	0.392553174425257	0.041	0.131	0.00106157884271993	Inflammatory macrophages	TRIP11
6.3618408981101E-08	0.481275496549241	0.07	0.18	0.00106611729770529	Inflammatory macrophages	CEBPZ
6.41073948306411E-08	0.659126500467336	0.031	0.116	0.00107431172257188	Inflammatory macrophages	HINT3
6.49638212475653E-08	0.455212604787049	0.079	0.196	0.0010886637164667	Inflammatory macrophages	ZC3H3
6.5646440218826E-08	0.260596772003738	0.027	0.108	0.00110010304518709	Inflammatory macrophages	TOP3A
7.06074503915621E-08	0.770856372132648	0.052	0.153	0.0011832396536618	Inflammatory macrophages	FAXDC2
7.08723461994512E-08	0.472863944301819	0.118	0.258	0.0011876787776104	Inflammatory macrophages	SP140L
7.10367047185357E-08	0.317641318748284	0.043	0.135	0.00119043309767322	Inflammatory macrophages	FAM192A
7.1145278490212E-08	0.806049576351401	0.255	0.506	0.00119225257693897	Inflammatory macrophages	RNASET2
7.27298530999848E-08	0.813914152150852	0.124	0.272	0.00121880687824955	Inflammatory macrophages	STON2
7.32986712718489E-08	0.355457935500285	0.06	0.163	0.00122833913317364	Inflammatory macrophages	ACSF2
7.47331803794653E-08	0.826248988272192	0.108	0.244	0.00125237863679908	Inflammatory macrophages	TPCN1
7.4889386569433E-08	0.456301532454757	0.039	0.128	0.00125499634013056	Inflammatory macrophages	DDX55
7.61717790302775E-08	0.866667575729392	0.099	0.23	0.00127648667298939	Inflammatory macrophages	TMC6
7.7319846592242E-08	0.681108636632112	0.234	0.456	0.00129572598919279	Inflammatory macrophages	TRA2A
7.7821176641105E-08	0.310449227381732	0.054	0.154	0.00130412727815164	Inflammatory macrophages	ADAT2
7.78528228424902E-08	0.477334052932779	0.058	0.16	0.00130465760519445	Inflammatory macrophages	ATG14
7.91549600186741E-08	0.289109263474421	0.035	0.122	0.00132647881999294	Inflammatory macrophages	WHAMM
8.02294688020304E-08	0.292690255456054	0.043	0.135	0.00134448543818442	Inflammatory macrophages	ULK1
8.13852733310827E-08	0.839662082831123	0.072	0.184	0.00136385441048228	Inflammatory macrophages	CREB1
8.19381556488903E-08	0.71858561167885	0.074	0.187	0.0013731196123641	Inflammatory macrophages	LTA4H
8.64988078349869E-08	0.966515101394132	0.083	0.203	0.00144954702169871	Inflammatory macrophages	NSMCE3
8.82134037014144E-08	0.911396419177119	0.205	0.41	0.0014782802192283	Inflammatory macrophages	STOM
8.92644597225662E-08	1.18160029088665	0.054	0.155	0.00149589381603076	Inflammatory macrophages	FOXK2
9.00033172788644E-08	0.671530899444924	0.13	0.277	0.00150827559095921	Inflammatory macrophages	VAV1
9.1363294660451E-08	0.374700510740858	0.023	0.1	0.00153106609191984	Inflammatory macrophages	CCDC112
9.15298083553192E-08	0.259352480562493	0.035	0.121	0.00153385652841844	Inflammatory macrophages	ZSCAN26
9.32603502466615E-08	0.57728768289122	0.031	0.114	0.00156285694943355	Inflammatory macrophages	DPY19L3
9.66003796267587E-08	0.52792923919936	0.048	0.144	0.00161882916178522	Inflammatory macrophages	TLE4
9.76714972710569E-08	0.831650644140501	0.079	0.195	0.00163677895126837	Inflammatory macrophages	ACIN1
9.85530149570182E-08	0.731669975125802	0.087	0.207	0.00165155142464971	Inflammatory macrophages	ZNF326
1.00048656643399E-07	0.418677021052412	0.043	0.134	0.00167661538803008	Inflammatory macrophages	CD180

1.01171051061916E-07	0.708463651074177	0.114	0.251	0.00169542447369559	Inflammatory macrophages	ZNF330
1.01766737188364E-07	0.447209747334602	0.052	0.15	0.0017054069818026	Inflammatory macrophages	TRNAU1AP
1.02374568825259E-07	0.346447368608519	0.139	0.287	0.0017155930243737	Inflammatory macrophages	ANKRD13D
1.04793765880636E-07	0.437568412057078	0.054	0.153	0.0017561339286277	Inflammatory macrophages	CCDC92
1.05035819123758E-07	0.291146658801801	0.025	0.103	0.00176019025687593	Inflammatory macrophages	ANP32B
1.06338879909302E-07	0.273398384139712	0.048	0.144	0.00178202694952008	Inflammatory macrophages	ZNHIT6
1.07389236750659E-07	0.423351049667284	0.089	0.21	0.00179962882946754	Inflammatory macrophages	EI24
1.09512365437223E-07	0.676124618073045	0.039	0.127	0.00183520821999699	Inflammatory macrophages	PLA2G4A
1.09750843744633E-07	0.932113861468965	0.118	0.259	0.00183920463947256	Inflammatory macrophages	JOSD1
1.22051103459438E-07	0.802076494431786	0.025	0.103	0.00204533239177325	Inflammatory macrophages	NOL12
1.24653161074132E-07	0.310947470274133	0.031	0.113	0.0020889376732803	Inflammatory macrophages	FOPNL
1.25454264123107E-07	0.92658801158415	0.182	0.364	0.00210236255817503	Inflammatory macrophages	EXOC3
1.27959356486536E-07	0.765506379641725	0.29	0.559	0.00214434289600137	Inflammatory macrophages	SH3BP5
1.34823500800417E-07	0.266704512923279	0.041	0.129	0.00225937222641339	Inflammatory macrophages	XPA
1.35773793472864E-07	0.541439572245472	0.035	0.119	0.00227529723101826	Inflammatory macrophages	BCAS3
1.36501574816204E-07	0.304462645279475	0.033	0.116	0.00228749339076995	Inflammatory macrophages	IDE
1.38818409855165E-07	0.935724441189696	0.091	0.214	0.00232631891235286	Inflammatory macrophages	MBOAT1
1.39167758720471E-07	1.08660332869349	0.207	0.413	0.00233217330063766	Inflammatory macrophages	STX4
1.39956052985068E-07	0.269901379351086	0.033	0.116	0.00234538353592377	Inflammatory macrophages	SIRPB1
1.41271591018345E-07	0.471878412713682	0.066	0.17	0.00236742932228543	Inflammatory macrophages	PLXNA3
1.44292186463747E-07	0.442146879119607	0.095	0.217	0.00241804846075947	Inflammatory macrophages	USP25
1.44477021684401E-07	0.65404341277643	0.072	0.181	0.00242114592938719	Inflammatory macrophages	CPEB2
1.44715171703218E-07	0.593943321207414	0.06	0.161	0.00242513684740253	Inflammatory macrophages	HMGXB3
1.45110188344376E-07	0.427294245412738	0.037	0.122	0.00243175653627504	Inflammatory macrophages	DIS3L
1.45241245096584E-07	0.597955149159283	0.106	0.237	0.00243395278532856	Inflammatory macrophages	DNAJB2
1.49219492746358E-07	0.608515752353493	0.031	0.113	0.00250062025944347	Inflammatory macrophages	OARD1
1.49260305756594E-07	0.311015597685278	0.06	0.161	0.002501304203869	Inflammatory macrophages	GIGYF2
1.5510250723203E-07	0.387355971339793	0.048	0.141	0.00259920781619435	Inflammatory macrophages	GTF2F2
1.60674818011464E-07	0.275551620463723	0.05	0.144	0.00269258860023611	Inflammatory macrophages	DGKZ
1.62803157411662E-07	0.547926153803738	0.091	0.211	0.00272825531190463	Inflammatory macrophages	ARHGAP25
1.64274816993538E-07	0.825110364374709	0.12	0.259	0.00275291738317772	Inflammatory macrophages	NOTCH2
1.64800831560438E-07	0.797531224215508	0.043	0.131	0.00276173233528982	Inflammatory macrophages	MICAL3
1.65921836877577E-07	0.342214174640404	0.029	0.108	0.00278051814239444	Inflammatory macrophages	IKZF5
1.67187839246999E-07	0.360015401459209	0.046	0.137	0.00280173381010121	Inflammatory macrophages	OPLAH
1.67906770140423E-07	0.494040738331668	0.306	0.576	0.0028137816540132	Inflammatory macrophages	DAPK1
1.68457902504036E-07	0.548789590128778	0.077	0.189	0.00282301753016263	Inflammatory macrophages	ZC3H7A
1.69873607860302E-07	0.518597994896194	0.06	0.16	0.00284674192052294	Inflammatory macrophages	SIK3
1.74085153823686E-07	0.837542583706095	0.064	0.167	0.00291731900777732	Inflammatory macrophages	HERC2
1.75614064670682E-07	0.452191919823073	0.043	0.131	0.00294294049575129	Inflammatory macrophages	TMEM63B
1.75991119695775E-07	0.676436797578648	0.25	0.48	0.00294925918386179	Inflammatory macrophages	BRD2
1.76264213526064E-07	0.69277758955019	0.072	0.18	0.00295383569026978	Inflammatory macrophages	FARP2
1.76887045698545E-07	0.566212070725871	0.048	0.14	0.00296427311181622	Inflammatory macrophages	ZRANB1
1.77324305521804E-07	0.92092611365413	0.052	0.148	0.00297160071193439	Inflammatory macrophages	TMEM251
1.80270430093828E-07	0.628976004126659	0.29	0.555	0.00302097186751237	Inflammatory macrophages	EVL
1.82016598445646E-07	0.384427415586583	0.039	0.125	0.00305023415675213	Inflammatory macrophages	CDK6
1.86412435468806E-07	0.797901136081397	0.075	0.187	0.00312389959358626	Inflammatory macrophages	CALCOCO1
1.87315911131891E-07	0.405777090243024	0.101	0.226	0.00313904003874824	Inflammatory macrophages	SLC23A2
1.90824393764519E-07	0.658145477766869	0.041	0.128	0.00319783519070581	Inflammatory macrophages	ARL 15.00
1.91749730390398E-07	0.292478024246954	0.039	0.124	0.00321334198188229	Inflammatory macrophages	HELB
1.9489782910162E-07	0.930574094714382	0.052	0.148	0.00326609782008494	Inflammatory macrophages	SUN1
1.94924818390851E-07	0.357520659952033	0.048	0.14	0.00326655010659387	Inflammatory macrophages	TFCP2L1
2.01062648050323E-07	0.434633876527011	0.279	0.523	0.00336940785602732	Inflammatory macrophages	DENND3
2.08220933985081E-07	0.313658497940326	0.037	0.121	0.00348936641172199	Inflammatory macrophages	CHUK
2.08685550296059E-07	0.663819191343978	0.054	0.15	0.00349715245186136	Inflammatory macrophages	MPP5
2.09235734441992E-07	0.596308641598388	0.054	0.151	0.0035063724377789	Inflammatory macrophages	USP6NL
2.09366299615627E-07	0.261036866236039	0.05	0.144	0.00350856044895867	Inflammatory macrophages	SAMD8
2.10619313878416E-07	0.458763083448677	0.083	0.197	0.00352955846197449	Inflammatory macrophages	EZH1
2.15729957774113E-07	0.587081908448558	0.079	0.191	0.00361520263237859	Inflammatory macrophages	SSH1
2.15859785052947E-07	0.599202397535707	0.195	0.378	0.00361737827791729	Inflammatory macrophages	PNISR
2.1912812290062E-07	0.560235372669288	0.126	0.264	0.00367214908356858	Inflammatory macrophages	PHYKPL
2.29860913719484E-07	0.980528581440712	0.048	0.14	0.00385200919211111	Inflammatory macrophages	CPNE2
2.31842752363357E-07	0.642003616479218	0.029	0.107	0.00388522084410513	Inflammatory macrophages	PSMD11
2.34864534518763E-07	2.12418510191377	0.338	0.288	0.00393585986946542	Inflammatory macrophages	RASGEF1B
2.38565198591864E-07	0.394303858037145	0.315	0.573	0.00399787559800245	Inflammatory macrophages	TNFRSF1B
2.41391831420048E-07	0.554037293180352	0.161	0.321	0.00404524431093717	Inflammatory macrophages	GPBP1
2.43292411064777E-07	0.960086181474692	0.195	0.385	0.00407709422462353	Inflammatory macrophages	CPEB4
2.43755107661215E-07	0.450358031926276	0.05	0.143	0.00408484809418663	Inflammatory macrophages	RCHY1
2.45488766739948E-07	0.856831975753261	0.077	0.188	0.00411390075302804	Inflammatory macrophages	UVSSA
2.48486963553024E-07	0.410122599007428	0.041	0.126	0.00416414453522157	Inflammatory macrophages	INAFM2
2.50153935116273E-07	0.751048442422055	0.147	0.302	0.00419207964467851	Inflammatory macrophages	ACAP2
2.51568863723669E-07	0.462535500124456	0.789	0.743	0.00421579101828125	Inflammatory macrophages	CD163

2.54934167619164E-07	0.41304937060939	0.079	0.189	0.00427218678096195	Inflammatory macrophages	NOP58
2.63291408204311E-07	0.85089209891194	0.099	0.223	0.00441223741868785	Inflammatory macrophages	B3GNT2
2.64979111025099E-07	0.25301045048905	0.027	0.103	0.0044405199425586	Inflammatory macrophages	ZNF438
2.67317561965844E-07	0.58340340388819	0.112	0.244	0.00447970770342361	Inflammatory macrophages	TATDN2
2.73407712537811E-07	0.38985606861992	0.025	0.1	0.00458176644670864	Inflammatory macrophages	POLR3A
2.73916742512706E-07	0.59733532431271	0.06	0.158	0.00459029677102794	Inflammatory macrophages	MAFG
2.77185897091603E-07	0.38681338483352	0.058	0.155	0.00464508126346108	Inflammatory macrophages	MAFK
2.86216652077485E-07	0.77051093862503	0.093	0.212	0.00479641865551449	Inflammatory macrophages	H3F3B
2.88693036446645E-07	0.31113038648344	0.031	0.109	0.00483791790477287	Inflammatory macrophages	KLF12
2.97041154723361E-07	0.29478758839387	0.087	0.2	0.00497781567085409	Inflammatory macrophages	HDAC9
3.00679041114721E-07	0.56233776673612	0.048	0.138	0.0050387793710005	Inflammatory macrophages	EOGT
3.02952015865969E-07	0.77691832251543	0.039	0.123	0.0050768698818819	Inflammatory macrophages	CNOT4
3.04761406734815E-07	1.78605403461988	0.443	0.445	0.00510719165406203	Inflammatory macrophages	INSIG1
3.06621464044898E-07	0.95540590683513	0.035	0.117	0.00513836249446439	Inflammatory macrophages	ING3
3.12242838035464E-07	0.75798956795354	0.195	0.381	0.00523256547979831	Inflammatory macrophages	SPG7
3.15474971496221E-07	1.12236243035135	0.116	0.25	0.00528672957233367	Inflammatory macrophages	IDI1
3.31153055846597E-07	0.28414318286651	0.029	0.105	0.00554946290987727	Inflammatory macrophages	CCDC90B
3.3469339844081E-07	0.72549859299688	0.089	0.206	0.0056087919710711	Inflammatory macrophages	OXSR1
3.61849917738492E-07	0.77558093993703	0.037	0.118	0.00606388092146164	Inflammatory macrophages	PVR
3.70006115628035E-07	0.44024279183108	0.033	0.112	0.00620056248569462	Inflammatory macrophages	WDR27
3.73475687299104E-07	0.46481399283323	0.031	0.108	0.00625870556775838	Inflammatory macrophages	CAAP1
3.77068884689837E-07	0.80090302385331	0.058	0.155	0.00631892036963229	Inflammatory macrophages	SUCNR1
3.78985303204298E-07	0.50742795336952	0.118	0.25	0.00635103571109763	Inflammatory macrophages	PPP2R5C
3.82163323955951E-07	0.31467846290991	0.081	0.191	0.00640429298285382	Inflammatory macrophages	DENND6A
3.85856063033276E-07	1.00434253418339	0.116	0.249	0.00646617590431164	Inflammatory macrophages	LRCH1
3.94408355907537E-07	0.43538964549650	0.054	0.147	0.0066094952282985	Inflammatory macrophages	CRTC3
3.98708686186227E-07	0.81553519808455	0.103	0.227	0.0066815601631088	Inflammatory macrophages	TRIB1
4.03177741713328E-07	0.37949263684523	0.041	0.125	0.00675645259563194	Inflammatory macrophages	PDE3B
4.03210536532644E-07	0.31203707259919	0.046	0.134	0.00675700217121405	Inflammatory macrophages	GTDC1
4.1057004230793E-07	0.57073046441587	0.199	0.383	0.00688033276899629	Inflammatory macrophages	SAFB2
4.31520236528513E-07	1.08531674757633	0.101	0.223	0.00723141612374481	Inflammatory macrophages	ZZEF1
4.48728789326167E-07	0.78979223419942	0.072	0.175	0.00751979705152791	Inflammatory macrophages	ZKSCAN4
4.55056793582166E-07	0.41740443555910	0.06	0.156	0.00762584174684994	Inflammatory macrophages	KANSL3
4.62087578377945E-07	0.50106123270785	0.145	0.289	0.0077436636384576	Inflammatory macrophages	NFKB2
4.62401714156435E-07	0.62655400231204	0.058	0.153	0.00774892792583355	Inflammatory macrophages	PHF8
4.71859099017152E-07	0.95501880712208	0.17	0.338	0.00790741478132943	Inflammatory macrophages	ELMSAN1
4.83964487474998E-07	0.34686123875392	0.054	0.146	0.00811027688110602	Inflammatory macrophages	EEFSEC
4.94246346710205E-07	0.43628729239866	0.066	0.165	0.00828258027816962	Inflammatory macrophages	LILRB4
4.95216664852181E-07	0.78155971620312	0.041	0.124	0.00829884086959285	Inflammatory macrophages	AC118549.1
4.98988056975978E-07	0.75077165946962	0.23	0.436	0.00836204185880344	Inflammatory macrophages	TFEC
5.03649745463093E-07	0.58756350625403	0.083	0.193	0.00844016243447051	Inflammatory macrophages	WWOX
5.20133449769884E-07	0.99125054181725	0.087	0.2	0.00871639635124372	Inflammatory macrophages	GALNS
5.31061526229534E-07	0.99746057761333	0.05	0.14	0.00889952905655454	Inflammatory macrophages	FZD1
5.6348567938679E-07	0.55987420629306	0.046	0.133	0.00944289301516383	Inflammatory macrophages	BIRC2
5.7080832921256E-07	0.54684426551429	0.052	0.143	0.00956560598094408	Inflammatory macrophages	ARMCX1
5.78680035254031E-07	0.83808076123181	0.079	0.187	0.00969752003078706	Inflammatory macrophages	CLK4
5.79017973218022E-07	0.75845484302627	0.056	0.149	0.00970318319518762	Inflammatory macrophages	ARMCX3
5.84948170651857E-07	0.5671638587462	0.126	0.262	0.00980256144378381	Inflammatory macrophages	PARP8
5.94605176912738E-07	0.26276639657892	0.035	0.113	0.00996439355470366	Inflammatory macrophages	YARS2
5.99816420906003E-07	0.54463422984711	0.267	0.5	0.0100517235815428	Inflammatory macrophages	REL
6.06095201298023E-07	0.32404641130311	0.039	0.119	0.0101569433833523	Inflammatory macrophages	STK11IP
6.16895969136469E-07	0.58440582845361	0.306	0.553	0.010337942650789	Inflammatory macrophages	TTYH3
6.18736508899143E-07	0.38191191474708	0.048	0.135	0.0103687864161318	Inflammatory macrophages	METTL21A
6.24496937712864E-07	0.32192574979425	0.046	0.132	0.0104653196821922	Inflammatory macrophages	CERK
6.61652285933655E-07	1.9948051785055	0.321	0.269	0.0110879690076762	Inflammatory macrophages	TAGAP
6.67933602965874E-07	0.88253284028339	0.091	0.205	0.0111932313185021	Inflammatory macrophages	PHIP
6.72023787149131E-07	1.25908809739442	0.559	0.684	0.0112617746250451	Inflammatory macrophages	PNRC1
6.77131850508199E-07	0.62056488741266	0.048	0.135	0.0113473755508164	Inflammatory macrophages	NT5DC1
6.85097594856247E-07	0.66897386120098	0.043	0.126	0.011480865494601	Inflammatory macrophages	CDC27
7.01562773505655E-07	0.50713146872463	0.046	0.132	0.0117567889584078	Inflammatory macrophages	VPS37A
7.08058265478403E-07	0.98248139714511	0.087	0.198	0.0118656404128871	Inflammatory macrophages	MBTD1
7.09186091183523E-07	1.09678819796571	0.114	0.241	0.0118845405160535	Inflammatory macrophages	SLC29A1
7.15724710644846E-07	0.60952188779180	0.095	0.209	0.0119941147009863	Inflammatory macrophages	KDM7A
7.20199639257957E-07	0.68210135576717	0.116	0.243	0.0120691055546849	Inflammatory macrophages	VPS8
7.65152833121339E-07	0.52226433116535	0.039	0.119	0.0128224311774474	Inflammatory macrophages	C12orf4
7.72362506893296E-07	0.42933278570026	0.232	0.428	0.0129432508905179	Inflammatory macrophages	MTSS1
7.8357084819976E-07	0.84381847504078	0.141	0.285	0.0131310802741316	Inflammatory macrophages	DDX27
7.97415399583802E-07	0.93044868094938	0.056	0.148	0.0133630872662254	Inflammatory macrophages	BRD8
8.01480836418065E-07	0.44487427932047	0.226	0.418	0.0134312158566939	Inflammatory macrophages	ARHGEF10L
8.12551176653137E-07	0.38920467287770	0.068	0.165	0.0136167326183533	Inflammatory macrophages	PPP2R2D
8.47402143716078E-07	0.66210442461314	0.046	0.131	0.014200765124394	Inflammatory macrophages	ZNF621

8.49693575185578E-07	0.289300609307447	0.039	0.118	0.0142391649329599	Inflammatory macrophages	ECSIT
8.55210758488822E-07	0.582204660893957	0.149	0.294	0.0143316218907557	Inflammatory macrophages	TAF1D
8.55799246247823E-07	0.348156512652447	0.043	0.125	0.01434148376862	Inflammatory macrophages	SRBD1
8.56236260093559E-07	0.613055737338177	0.043	0.125	0.0143488072466479	Inflammatory macrophages	SYNJ1
8.57750547851589E-07	0.38127351081503	0.039	0.118	0.0143741836808969	Inflammatory macrophages	RNFT1
8.6187506551217E-07	0.47373431569902	0.046	0.131	0.01444330234785	Inflammatory macrophages	GPR65
9.05199336669721E-07	0.353059044423471	0.054	0.144	0.0151693304839112	Inflammatory macrophages	NUDT4
9.20729647968843E-07	0.545034296877925	0.203	0.382	0.0154295874406619	Inflammatory macrophages	MS4A4E
9.26684801683783E-07	0.303853549773601	0.058	0.148	0.0155293839066168	Inflammatory macrophages	SLC35E2B
9.29282546551585E-07	1.30618614072463	0.447	0.428	0.0155729169151115	Inflammatory macrophages	PLAUR
9.57883595951662E-07	0.368336139985157	0.089	0.197	0.016052213300958	Inflammatory macrophages	PPIF
9.6127378237721E-07	0.607816964389232	0.072	0.171	0.0161090260450773	Inflammatory macrophages	AKAP8L
9.7784915396541E-07	0.274513762218374	0.033	0.108	0.0163867961221523	Inflammatory macrophages	GSTM4
9.82145683183262E-07	0.422301350822844	0.031	0.104	0.0164587973587851	Inflammatory macrophages	TOP2B
1.02384785626978E-06	0.97547850666285	0.064	0.16	0.017157642375369	Inflammatory macrophages	ZNF407
1.02497459916421E-06	0.548334647230844	0.043	0.124	0.0171765243327938	Inflammatory macrophages	POU2F1
1.03548621531504E-06	0.258730158592237	0.05	0.136	0.0173526779962494	Inflammatory macrophages	ZFAND1
1.0852663357154E-06	0.804902306086084	0.039	0.117	0.0181868932539187	Inflammatory macrophages	ATG16L1
1.09734736386564E-06	0.816091879312319	0.106	0.226	0.0183893471236604	Inflammatory macrophages	ELL
1.11095525184166E-06	0.47587780248039	0.074	0.172	0.0186173881103626	Inflammatory macrophages	NOTCH1
1.12975182946999E-06	0.654509197775385	0.095	0.208	0.0189323811582581	Inflammatory macrophages	CHORDC1
1.13646097712282E-06	0.559428508006474	0.238	0.444	0.0190448130546242	Inflammatory macrophages	RAB20
1.16128321397139E-06	0.853708263159197	0.077	0.181	0.0194607840997326	Inflammatory macrophages	TRAF5
1.18043531185184E-06	0.919260066933314	0.058	0.149	0.0197817349560132	Inflammatory macrophages	SUPT7L
1.2064117774619E-06	0.844494783618957	0.182	0.353	0.0202170485667064	Inflammatory macrophages	SLC38A2
1.23132726220113E-06	0.381188364770555	0.313	0.574	0.0206345822599666	Inflammatory macrophages	IL10RA
1.23740747189192E-06	1.1754601374301	0.116	0.246	0.0207364744139648	Inflammatory macrophages	ZSWIM6
1.26728500572499E-06	0.704549266996998	0.06	0.152	0.0212371621259394	Inflammatory macrophages	CPEB3
1.26846650063243E-06	0.804517922151653	0.263	0.501	0.0212569616175983	Inflammatory macrophages	ADAM9
1.27247245868776E-06	0.701086064025426	0.054	0.143	0.0213240934626894	Inflammatory macrophages	RASAL2
1.29359218498287E-06	0.559789491598398	0.325	0.586	0.0216780178359429	Inflammatory macrophages	CD300A
1.30792972870507E-06	0.943462881939775	0.037	0.113	0.0219182863936396	Inflammatory macrophages	FAM120AOS
1.37187127611173E-06	0.725751935078221	0.046	0.13	0.0229898188450803	Inflammatory macrophages	ZNF496
1.37606942288077E-06	0.365098205264768	0.044	0.125	0.0230601713886359	Inflammatory macrophages	EYA2
1.38076120859666E-06	0.491808842790855	0.054	0.141	0.0231387963336628	Inflammatory macrophages	CLCN6
1.40784313077308E-06	0.520301597236047	0.07	0.166	0.0235926351854953	Inflammatory macrophages	VAMP2
1.49471511753644E-06	0.634525200285268	0.041	0.119	0.0250484359396756	Inflammatory macrophages	PARP6
1.50368965341135E-06	0.685394408806701	0.037	0.113	0.0251988312118674	Inflammatory macrophages	ZNF84
1.50561026677374E-06	1.10009641177181	0.081	0.185	0.0252310168505944	Inflammatory macrophages	BLOC1S6
1.55857850363638E-06	0.297972242679872	0.046	0.128	0.0261186585639385	Inflammatory macrophages	PCGF5
1.56502048628831E-06	0.69572259740713	0.079	0.182	0.0262266133092195	Inflammatory macrophages	PHF1
1.56611856180734E-06	0.763480042430217	0.103	0.219	0.0262450148587674	Inflammatory macrophages	TANK
1.59764625967227E-06	0.397576057217943	0.044	0.126	0.0267733560195879	Inflammatory macrophages	ATP10D
1.6149607335056E-06	0.808158049454398	0.106	0.224	0.0270635119720868	Inflammatory macrophages	DUSP16
1.62127068455229E-06	0.649039975969056	0.232	0.427	0.0271692541317272	Inflammatory macrophages	DOCK5
1.62856351901751E-06	0.727511634766426	0.043	0.122	0.0272914674516955	Inflammatory macrophages	ATE1
1.63491687788953E-06	0.63805773953215	0.05	0.135	0.0273979370396728	Inflammatory macrophages	RUVBL2
1.65977644096926E-06	0.710851770812254	0.072	0.169	0.0278145335977629	Inflammatory macrophages	ABTB1
1.66688681280778E-06	0.654539634252597	0.095	0.206	0.0279336892090328	Inflammatory macrophages	PI4K2B
1.68546282829678E-06	0.367825073261329	0.044	0.125	0.0282449860765974	Inflammatory macrophages	RABGEF1
1.68743022428683E-06	0.57404299368785	0.039	0.115	0.0282779556985986	Inflammatory macrophages	DEPRECATED-ENSG00000122642
1.68890127299414E-06	0.888288454052737	0.213	0.404	0.0283026075328357	Inflammatory macrophages	SLC37A2
1.70286269764355E-06	0.762558086601381	0.041	0.119	0.0285365730871107	Inflammatory macrophages	INTS13
1.73247716941974E-06	0.385511812002208	0.043	0.122	0.029032852405136	Inflammatory macrophages	INVS
1.76202316580406E-06	0.638105295551516	0.033	0.105	0.0295279842125445	Inflammatory macrophages	ZNF133
1.76437579003931E-06	0.64543051886404	0.255	0.471	0.0295674094894788	Inflammatory macrophages	HNRNPH3
1.79340533714988E-06	0.803551595377441	0.099	0.211	0.0300538866399577	Inflammatory macrophages	OSER1
1.81942271958893E-06	1.01809424228965	0.089	0.197	0.0304898859348712	Inflammatory macrophages	GCN1
1.87662322325262E-06	0.423425037104926	0.033	0.105	0.0314484519752675	Inflammatory macrophages	THAP12
1.87694152637789E-06	0.612035142315118	0.186	0.353	0.0314537860990407	Inflammatory macrophages	SPAG9
1.92398777388713E-06	0.784310453026121	0.103	0.217	0.0322421871148005	Inflammatory macrophages	ARHGEF11
1.95109681645236E-06	0.44047297760299	0.039	0.114	0.0326964804501086	Inflammatory macrophages	WDR45
1.98276871202075E-06	0.441353224645468	0.044	0.124	0.0332272380760437	Inflammatory macrophages	BTBD9
2.00134291738869E-06	2.4976020260076	0.263	0.203	0.0335385046095996	Inflammatory macrophages	OTUD1
2.04835093155035E-06	0.520314519325053	0.054	0.139	0.0343262649109208	Inflammatory macrophages	ZNF276
2.06112943768273E-06	0.906680891207726	0.033	0.104	0.0345404071166873	Inflammatory macrophages	RAB2B
2.06633482834775E-06	0.688946045528886	0.149	0.29	0.0346276390534516	Inflammatory macrophages	FNBP4
2.07293898925142E-06	0.268186745907278	0.037	0.111	0.0347383115818753	Inflammatory macrophages	PLEKHG3
2.16366250596119E-06	0.450342502393449	0.043	0.121	0.0362586562748977	Inflammatory macrophages	CIDEB
2.1851402073459E-06	0.463228868238038	0.093	0.201	0.0366185795947026	Inflammatory macrophages	CENPT
2.19724825371725E-06	0.466541194972078	0.07	0.165	0.0368214862357937	Inflammatory macrophages	GNG2

2.3135569159746E-06	0.424902305538802	0.041	0.117	0.0387705867979023	Inflammatory macrophages	KBTBD8
2.31635367232706E-06	0.321017040302656	0.039	0.113	0.0388174548408568	Inflammatory macrophages	ANAPC2
2.35308610135968E-06	0.799191384321761	0.251	0.467	0.0394330168865855	Inflammatory macrophages	NUFIP2
2.41321839192407E-06	0.482574761619732	0.052	0.136	0.0404407138118636	Inflammatory macrophages	WWC3
2.41770423223341E-06	0.638015783676189	0.039	0.114	0.0405158875237675	Inflammatory macrophages	TMED8
2.4319905832696E-06	0.667590226290706	0.046	0.126	0.0407552981944319	Inflammatory macrophages	ZNF277
2.48448370320123E-06	0.66407134974014	0.046	0.126	0.0416349778982463	Inflammatory macrophages	CRAMP1
2.52917022592705E-06	0.272499960747533	0.064	0.153	0.0423838346460855	Inflammatory macrophages	CMPK2
2.55875988861899E-06	0.555274697742068	0.079	0.176	0.0428796982134771	Inflammatory macrophages	EIF4G3
2.56677603751627E-06	0.473408369950659	0.062	0.151	0.0430140328366977	Inflammatory macrophages	AC253572.1
2.5932866499313E-06	0.846627928432897	0.6	0.644	0.0434582976795487	Inflammatory macrophages	RGS1
2.63079393105226E-06	0.358053173958502	0.037	0.11	0.0440868446965738	Inflammatory macrophages	INPP5E
2.67189085153197E-06	0.686284132909817	0.161	0.308	0.0447755468899727	Inflammatory macrophages	DNMT3A
2.67725044069591E-06	0.955223867831615	0.039	0.113	0.0448653628851821	Inflammatory macrophages	BRAF
2.73001338566086E-06	0.602721606574325	0.033	0.104	0.0457495643169046	Inflammatory macrophages	BRAP
2.74953342318633E-06	0.914143892986685	0.048	0.13	0.0460766811057566	Inflammatory macrophages	SFXN2
2.76339180975685E-06	0.636348101425301	0.031	0.1	0.0463089199479053	Inflammatory macrophages	KCNK13
2.7901195374454E-06	0.545269748975497	0.308	0.556	0.04675682320851	Inflammatory macrophages	SLC3A2
2.82495126150284E-06	0.482996500864542	0.044	0.122	0.0473405332402646	Inflammatory macrophages	RBM38
2.83428851688614E-06	0.502283841798937	0.035	0.107	0.0474970069659779	Inflammatory macrophages	SLC30A4
2.87825367415735E-06	1.07199291908053	0.089	0.196	0.0482337750715289	Inflammatory macrophages	ADRB2
2.90131315352511E-06	1.01416243923631	0.068	0.162	0.0486202058267737	Inflammatory macrophages	LACC1
2.90155436841097E-06	0.343726340649184	0.039	0.113	0.0486242481058311	Inflammatory macrophages	PTPN2
1.20069280536922E-100	3.32286214213311	0.686	0.227	2.01212100323773E-96	cDC2	CLEC10A
9.59016121513964E-82	1.9116571613988	0.896	0.722	1.6071192164331E-77	cDC2	LYZ
1.12856022108819E-81	3.92722544755659	0.37	0.032	1.89124121849959E-77	cDC2	CD1C
5.762648526556E-80	4.59816307337202	0.348	0.027	9.65704640080255E-76	cDC2	CD1E
2.01170855336576E-58	2.35643738098081	0.691	0.443	3.37122119373033E-54	cDC2	JAML
1.30033298635118E-56	3.65037935531731	0.269	0.025	2.1790980185273E-52	cDC2	CD1B
2.90858650786338E-52	1.70471716904594	0.754	0.481	4.87420926987744E-48	cDC2	LSP1
1.06127313725713E-48	6.66987047907856	0.181	0.005	1.77848152341549E-44	cDC2	CLEC4F
1.85847231082204E-47	2.96011413927178	0.336	0.069	3.11442789847557E-43	cDC2	LGALS2
3.48376344756414E-47	3.08056291059109	0.278	0.041	5.83809078542798E-43	cDC2	ACAP1
7.6910676736159E-47	2.78537076112071	0.454	0.157	1.28886912074455E-42	cDC2	DEPRECATED-ENSG00000072694
2.74931622733346E-46	2.45920842110328	0.612	0.302	4.60730413376541E-42	cDC2	CYTIP
4.37708471818641E-46	2.67324959631229	0.476	0.179	7.33511857073679E-42	cDC2	FCGR2B
6.84116650025377E-44	2.66240744762733	0.409	0.12	1.14644268211253E-39	cDC2	SPN
7.69377298634577E-43	1.47056494705544	0.734	0.552	1.28932247705182E-38	cDC2	CIITA
1.17233423635052E-42	2.57235150802242	0.278	0.046	1.9645977132762E-38	cDC2	ADAM8
1.62514490486657E-40	2.90368230391569	0.305	0.067	2.7234178315754E-36	cDC2	PLD4
6.28816035404729E-40	4.16094331942429	0.199	0.02	1.05376991213124E-35	cDC2	CD1A
4.6476854880137E-39	5.28151668533691	0.16	0.008	7.78859134081336E-35	cDC2	FCER1A
2.50017395003728E-38	2.42028868347944	0.503	0.246	4.18979150547248E-34	cDC2	FGR
6.83107860318198E-38	3.81958591183213	0.172	0.013	1.14475215232124E-33	cDC2	CCR6
1.62229821786902E-37	1.58001789960469	0.767	0.582	2.7186473535049E-33	cDC2	CSF2RA
7.63809643826715E-37	3.17759057139067	0.271	0.058	1.27999220112481E-32	cDC2	CST7
2.05646279352391E-36	3.31541597234648	0.219	0.033	3.44622034938737E-32	cDC2	CLEC5A
3.908933465994E-35	1.06257762269444	0.826	0.658	6.55059070231275E-31	cDC2	TXNIP
1.36775723054508E-34	2.51567530376066	0.28	0.066	2.29208756694745E-30	cDC2	CCR5
1.48723678382263E-34	3.44577265987924	0.237	0.045	2.49231140232996E-30	cDC2	TRPC6
2.97632357175131E-34	2.567983106161	0.212	0.032	4.98772304154085E-30	cDC2	ADAM19
3.26237549591719E-34	2.66770177448936	0.325	0.095	5.46708885605803E-30	cDC2	SLC38A1
2.81095067625521E-33	2.35916957599054	0.332	0.1	4.71059114326848E-29	cDC2	PALM2-AKAP2
4.45589304471121E-33	4.70657652472873	0.172	0.019	7.46718556432705E-29	cDC2	CCR2
2.18798106264829E-32	3.0789984580402	0.237	0.049	3.666618664786E-28	cDC2	P2RY14
9.52439269442028E-32	1.10283535404016	0.758	0.649	1.59609772773095E-27	cDC2	CTSH
1.06670867695973E-31	0.501244946443066	0.991	0.987	1.78759040084911E-27	cDC2	CD74
1.25136135428211E-31	2.35311855174588	0.37	0.14	2.09703135750597E-27	cDC2	NDRG2
2.87613324110648E-31	2.05398273123825	0.526	0.3	4.81982408544623E-27	cDC2	STK17B
3.92640867956504E-31	1.17079505919941	0.758	0.607	6.5798756652151E-27	cDC2	ITGAX
1.37256203024863E-29	2.51238295166613	0.203	0.037	2.30013945029065E-25	cDC2	RUFY4
2.17128294694581E-29	1.12293413783526	0.716	0.628	3.63863596249179E-25	cDC2	LCP1
2.98962592139617E-29	2.22144073374369	0.409	0.175	5.01001511907571E-25	cDC2	S100A6
6.09510402631247E-29	2.11539024168422	0.289	0.084	1.02141753272944E-24	cDC2	CD52
1.41868505630086E-28	3.27714797428799	0.242	0.06	2.37743241734898E-24	cDC2	GPAT3
1.78070241272744E-28	3.19304634304773	0.339	0.129	2.98410110324864E-24	cDC2	S100B
3.02344009732006E-28	2.90931699158754	0.19	0.033	5.06668091508896E-24	cDC2	CD300LB
4.93224262250952E-28	1.09171916903034	0.862	0.785	8.26545218680145E-24	cDC2	CXCR4
2.27254623374507E-26	2.58793874962088	0.266	0.078	3.80833297850998E-22	cDC2	PARM1
2.88235360222995E-26	4.82838310331383	0.147	0.019	4.83024816661695E-22	cDC2	CD300E
7.46073679303343E-26	2.97714288778807	0.158	0.024	1.25027027177654E-21	cDC2	QPCT

2.43857782443248E-25	1.06692260959605	0.72	0.655	4.08656871818395E-21	cDC2	SAMHD1
2.59275034097974E-24	2.64157842414907	0.228	0.063	4.34493102141384E-20	cDC2	CEACAM4
6.57347137679146E-24	4.7210554105192	0.117	0.012	1.10158233332271E-19	cDC2	CLEC4D
6.62185442729796E-24	0.70397048269621!	0.851	0.816	1.10969036492659E-19	cDC2	CST3
7.01158483358112E-24	3.66077334764186	0.12	0.012	1.17500138641152E-19	cDC2	CLEC6A
2.22451377891504E-23	2.0166810155776	0.181	0.038	3.72784019070582E-19	cDC2	ZNF366
2.27035652471967E-23	1.77036891245239	0.47	0.257	3.80466346412523E-19	cDC2	CORO1A
2.41798917892833E-23	2.38242169340867	0.366	0.166	4.05206626604809E-19	cDC2	IL1R1
1.58000132900048E-22	1.61103870913542	0.476	0.282	2.647766227139E-18	cDC2	ANPEP
1.85639636492045E-22	1.64739066283257	0.411	0.208	3.11094902833369E-18	cDC2	ST14
1.96123173583615E-22	2.10906639903882	0.172	0.035	3.28663214291421E-18	cDC2	PKIB
2.0921215484737E-22	1.97741112191654	0.257	0.082	3.50597729093223E-18	cDC2	CRIP1
4.17675805132788E-22	3.10539735020396	0.174	0.038	6.99941114241525E-18	cDC2	IL1R2
5.45476392787062E-22	2.80742586968706	0.167	0.035	9.14109339032559E-18	cDC2	ACPP
7.43097998468296E-22	3.13668358663863	0.142	0.024	1.24528362583317E-17	cDC2	PADI2
1.03789976734449E-21	2.94909033620324	0.192	0.048	1.7393124301159E-17	cDC2	CCND2
2.32143727625494E-21	2.58506243068129	0.233	0.075	3.89026458754802E-17	cDC2	IL27RA
3.61497285333795E-21	1.49522573647607	0.149	0.027	6.05797150762374E-17	cDC2	IDO1
7.31264227165715E-21	2.34625414401993	0.165	0.035	1.2254525918843E-16	cDC2	CD48
1.23054883081777E-20	1.47095151961304	0.515	0.335	2.06215373068443E-16	cDC2	IL2RG
1.62841469034236E-20	2.89207719063096	0.126	0.019	2.72889733807572E-16	cDC2	AREG
2.87734817571774E-20	2.67769568860687	0.192	0.052	4.82186007286779E-16	cDC2	LTB
3.34393386343729E-20	1.82091904048061	0.336	0.148	5.60376436834821E-16	cDC2	ADAM28
1.01110252943889E-19	2.05960951902934	0.404	0.23	1.69440561883369E-15	cDC2	MNDA
1.07443251887902E-19	1.72307573630527	0.352	0.165	1.80053401513747E-15	cDC2	TES
1.30815799026658E-19	1.80097038230435	0.454	0.278	2.19221116008874E-15	cDC2	APOBR
1.59260831673971E-19	2.73822482347877	0.174	0.044	2.6688930171924E-15	cDC2	MEFV
2.30131317351701E-19	2.18831638954463	0.293	0.121	3.85654061617981E-15	cDC2	ITPR1
2.74214054520779E-19	2.75019123609652	0.12	0.018	4.59527912565922E-15	cDC2	GABBR1
3.48324986636189E-19	1.72624151164189	0.214	0.065	5.83723012604925E-15	cDC2	CCSER1
3.81092602358915E-19	3.41208914138398	0.126	0.022	6.3863498303307E-15	cDC2	IL18R1
4.63828106462825E-19	2.27350777777773	0.323	0.148	7.77283140810402E-15	cDC2	SULF2
5.11522167536274E-19	1.79907791526477	0.413	0.239	8.57208848357288E-15	cDC2	PAK1
1.01872996093201E-18	2.07195400559531	0.239	0.086	1.70718766852986E-14	cDC2	SPINT1
1.87625048964134E-18	3.05618097120154	0.129	0.024	3.14422057054097E-14	cDC2	PRAM1
2.82254271197557E-18	2.22940019687277	0.106	0.015	4.73001707672866E-14	cDC2	GGT5
4.60112212489741E-18	2.90418031340855	0.102	0.013	7.71056045690308E-14	cDC2	TRABD2A
6.62421678833672E-18	1.38193108864307	0.467	0.282	1.11008624938947E-13	cDC2	ALOX5
1.01692923075519E-17	0.67334225410887(	0.795	0.736	1.70417000489954E-13	cDC2	SPI1
1.62542081691354E-17	1.61059582126213	0.318	0.145	2.72388020498371E-13	cDC2	FLNA
2.16125490903494E-17	2.38851018482475	0.309	0.145	3.62183097656076E-13	cDC2	SATB1
3.29598284202326E-17	2.88028440864621	0.12	0.022	5.52340804666258E-13	cDC2	TSPAN32
3.44692193595987E-17	2.44017685001742	0.217	0.076	5.77635178028155E-13	cDC2	MCOLN2
4.84768040295906E-17	1.65520142121846	0.393	0.218	8.1237428192788E-13	cDC2	UVRAG
5.22977532098313E-17	1.63162508668564	0.436	0.275	8.76405748290353E-13	cDC2	RTN1
5.79834940718259E-17	1.385106190067	0.526	0.383	9.71687393655658E-13	cDC2	AHR
6.78748182741356E-17	1.18687172653798	0.628	0.571	1.13744620463796E-12	cDC2	GSN
7.30930262123665E-17	3.3076834488318	0.113	0.02	1.22489293326684E-12	cDC2	RUNX2
9.14211584872429E-17	0.58822912627074	0.874	0.839	1.53203577392922E-12	cDC2	ACTB
1.86539227662573E-16	1.6920463557413	0.246	0.098	3.12602437716939E-12	cDC2	TMEM131L
1.95501103690982E-16	2.23040601062282	0.108	0.018	3.27620749565348E-12	cDC2	PELI2
2.76937530435611E-16	1.8860423175426	0.167	0.048	4.64091913503997E-12	cDC2	CLEC12A
3.82288350051185E-16	2.27483070710224	0.108	0.019	6.40638817015776E-12	cDC2	ATP2A3
6.86772754226478E-16	2.32106869391785	0.14	0.035	1.15089378153273E-11	cDC2	FHL3
1.64079093708774E-15	1.86697545003565	0.323	0.165	2.74963745237163E-11	cDC2	S100A4
1.83403993606224E-15	2.74384911860823	0.117	0.025	3.0734841248531E-11	cDC2	CD101
2.26475522755051E-15	1.83842266252916	0.255	0.109	3.79527681032915E-11	cDC2	HAPLN3
4.1103282438396E-15	0.82878303960709?	0.91	0.824	6.8880880710264E-11	cDC2	IFI30
5.14127162786417E-15	1.69196967149715	0.352	0.185	8.61574299397477E-11	cDC2	SLC2A3
5.35080658214491E-15	1.9419476384441	0.172	0.055	8.96688167035843E-11	cDC2	KCNMB1
7.32202886753474E-15	3.63276323360186	0.122	0.028	1.22702559762147E-10	cDC2	CYSLTR2
8.69106848404163E-15	2.31776810306243	0.131	0.032	1.4564492565557E-10	cDC2	MBOAT2
1.58586966506609E-14	2.45451924448545	0.221	0.091	2.65760038471775E-10	cDC2	CYP2S1
1.92166548427953E-14	2.06543746195435	0.33	0.182	3.22032701855563E-10	cDC2	CKLF
2.0234915422381E-14	1.48707300021211	0.49	0.365	3.39096712648261E-10	cDC2	COTL1
2.38564555636364E-14	1.63525838445045	0.463	0.308	3.99786482335419E-10	cDC2	FMNL1
3.32824932292311E-14	2.55168056936556	0.129	0.032	5.57748021535454E-10	cDC2	ADAMTS10
3.50784565941429E-14	1.41556258156473	0.343	0.182	5.87844775604646E-10	cDC2	ALOX5AP
4.27606512294852E-14	2.02130111898826	0.129	0.033	7.16582993303713E-10	cDC2	CX3CR1
4.69376939894476E-14	1.45977183184448	0.395	0.246	7.86581875875163E-10	cDC2	MYD88
5.97563180093518E-14	1.62823645104982	0.111	0.024	1.00139637720072E-09	cDC2	FLT3

7.28611105554659E-14	2.33088264359067	0.208	0.083	1.2210064906885E-09	cDC2	PSTPIP1
7.88403852360333E-14	1.35763903321376	0.345	0.188	1.32120717578545E-09	cDC2	CRYBG1
8.55939380091857E-14	1.87871380384704	0.158	0.051	1.43438321315793E-09	cDC2	VDR
1.52182243943511E-13	1.5534457929197	0.429	0.271	2.55027004400536E-09	cDC2	CD37
1.82096166673327E-13	1.14230035733104	0.278	0.128	3.05156756111161E-09	cDC2	HDAC9
2.33342340070607E-13	2.70350552975744	0.149	0.047	3.91035093490324E-09	cDC2	MYO1G
2.48840364014084E-13	2.40185278979046	0.201	0.081	4.17006682014801E-09	cDC2	MAP4K1
3.05227259380237E-13	1.47403587486481	0.413	0.257	5.11499841269401E-09	cDC2	PALD1
3.81109739262408E-13	1.58535521655645	0.4	0.257	6.38663701055943E-09	cDC2	ANKRD44
7.46141736059677E-13	0.51858550014290	0.725	0.683	1.25038432128881E-08	cDC2	HSPA6
9.91686747138036E-13	1.59131515844939	0.361	0.213	1.66186865085392E-08	cDC2	IL17RA
1.2757398401389E-12	1.09094043016742	0.562	0.52	2.13788482410476E-08	cDC2	CPVL
1.32805921335187E-12	1.44924405608704	0.318	0.181	2.22556162973506E-08	cDC2	ARHGAP31
1.80655577942613E-12	1.37451237033753	0.406	0.262	3.02742617516231E-08	cDC2	VASH1
2.25417802063315E-12	1.98760727460245	0.147	0.048	3.77755152697704E-08	cDC2	CD1D
2.49519534349696E-12	1.90567986778387	0.217	0.096	4.18144835663221E-08	cDC2	GLIPR2
2.49561320593753E-12	1.07494053098587	0.637	0.543	4.18214861051011E-08	cDC2	ZFP36L2
2.88135121943017E-12	1.45242916456806	0.42	0.29	4.82856837352108E-08	cDC2	CALHM6
3.18527800867518E-12	1.77304943247947	0.16	0.056	5.33788888693786E-08	cDC2	PCNX2
3.39399293999277E-12	1.25501912960771	0.506	0.411	5.68765336883989E-08	cDC2	PEA15
3.45673751733241E-12	1.46101558734504	0.449	0.325	5.79280073154565E-08	cDC2	ZNF385A
3.62378024604311E-12	1.38210646754001	0.46	0.333	6.07273093631904E-08	cDC2	SERPINB9
5.82940169903816E-12	1.78525889692298	0.208	0.091	9.76891136724815E-08	cDC2	NT5DC3
5.85929818247079E-12	1.56697607170131	0.334	0.192	9.81901189418456E-08	cDC2	CSF3R
7.28748693876967E-12	0.73673665862190	0.698	0.66	1.22123706119902E-07	cDC2	CELF2
7.84233101370278E-12	1.71096811999301	0.233	0.108	1.31421783127631E-07	cDC2	HK2
9.0562441753856E-12	1.19881945079398	0.465	0.32	1.51764539891112E-07	cDC2	AKNA
1.30164434648172E-11	1.07069685185749	0.214	0.095	2.18129559583406E-07	cDC2	MARCKSL1
1.59676701921801E-11	1.3820701967893	0.339	0.202	2.67586217080554E-07	cDC2	WARS
1.709639707342E-11	1.76507812055783	0.104	0.026	2.86501422156372E-07	cDC2	OLR1
1.87551192033077E-11	1.7028063312355	0.341	0.205	3.14298287609031E-07	cDC2	FPR1
2.11927317812971E-11	2.32152240834298	0.165	0.064	3.55147799190977E-07	cDC2	SVIL
2.43686190530757E-11	1.88249456626659	0.223	0.107	4.08369318091443E-07	cDC2	MOB3B
2.49569918247886E-11	2.86070162551067	0.19	0.083	4.18229268999807E-07	cDC2	MYCL
2.93677882473504E-11	1.32322799776944	0.354	0.215	4.92145395449098E-07	cDC2	STK38L
3.9082268581851E-11	2.11298898096098	0.106	0.028	6.54940656894659E-07	cDC2	RIPOR2
4.09814100817855E-11	0.58882103008985	0.289	0.148	6.86766470150562E-07	cDC2	BCL6
1.11039882835963E-10	0.60093824299971	0.709	0.664	1.86080635656507E-06	cDC2	ITGB2
1.18628997074368E-10	1.73029522330243	0.183	0.079	1.98798473297226E-06	cDC2	DEPRECATED-ENSG00000057294
1.27776603850836E-10	1.81508786774743	0.158	0.062	2.14128032733231E-06	cDC2	PKP2
1.3938910256825E-10	2.1957744944449	0.126	0.042	2.33588258083873E-06	cDC2	RGS14
1.55955248132772E-10	0.72362688312725	0.673	0.699	2.613498048209E-06	cDC2	TMSB10
2.66748402419807E-10	2.25392359407873	0.158	0.064	4.47016972775113E-06	cDC2	NOD2
2.77111153972025E-10	1.10845206780575	0.183	0.078	4.6438287182632E-06	cDC2	FCHO1
2.79231712112732E-10	0.86557918990156	0.616	0.572	4.67936503158516E-06	cDC2	SYNGR2
2.85358816910393E-10	0.65942898709378	0.842	0.793	4.78204305378436E-06	cDC2	LITAF
2.90580741590393E-10	1.7985860107221	0.19	0.085	4.86955206757181E-06	cDC2	SPECC1
6.81999086104188E-10	1.63271634039263	0.208	0.098	1.14289406849 34E-05	cDC2	SLAMF7
7.84920974952479E-10	1.46636987012244	0.212	0.104	1.31537056982536E-05	cDC2	SLC9A7
8.72358206646686E-10	1.30267500184804	0.415	0.302	1.46189788269852E-05	cDC2	GLIPR1
9.81280423086927E-10	1.55830873658472	0.19	0.086	1.64442973300907E-05	cDC2	MX2
1.15040026997043E-09	1.29137658018342	0.352	0.223	1.92784077241645E-05	cDC2	DEF6
1.50488998450957E-09	1.36944066076878	0.102	0.031	2.52189463604114E-05	cDC2	RHOH
1.54140779202265E-09	1.37700438273905	0.411	0.293	2.58309117787156E-05	cDC2	RELT
1.68027397494473E-09	1.73082028882796	0.172	0.076	2.81580312721238E-05	cDC2	PFKP
1.81819272521419E-09	1.31322150488208	0.262	0.145	3.04692736891394E-05	cDC2	RASSF5
2.08672636888027E-09	1.22993980133581	0.391	0.272	3.49693604896956E-05	cDC2	DIAPH1
2.4793588867181E-09	0.77088177184262	0.521	0.422	4.15490962236219E-05	cDC2	SLC8A1
2.69574401667031E-09	1.53746877341682	0.217	0.112	4.51752782313611E-05	cDC2	SMCO4
2.84364520716831E-09	1.1772543801036	0.481	0.373	4.76538063817265E-05	cDC2	PLSCR1
3.12817188739265E-09	1.53683922667941	0.242	0.132	5.24219044889261E-05	cDC2	DAPP1
3.20544325072871E-09	1.33532740964709	0.363	0.243	5.37168179957117E-05	cDC2	FOXN2
3.4924748643363E-09	0.81720635852860	0.621	0.588	5.85268937765477E-05	cDC2	SMAP2
3.51332012812651E-09	2.35007684451317	0.192	0.095	5.88762187071441E-05	cDC2	TET2
3.71639249403585E-09	1.97167219996145	0.255	0.146	6.22793054150528E-05	cDC2	ITGA5
4.23175461364403E-09	1.43716017015481	0.372	0.258	7.09157438154466E-05	cDC2	MBOAT7
4.34122759463469E-09	1.41728813638656	0.124	0.045	7.27502920308882E-05	cDC2	SNAI3
4.6226092694355E-09	1.53645039041683	0.201	0.1	7.74656861372E-05	cDC2	PCYOX1L
5.20406796826162E-09	0.35544296235478	0.84	0.832	8.72097710121282E-05	cDC2	HSPA1A
6.09245495570327E-09	1.3879165300732	0.314	0.2	0.000102097360147675	cDC2	LRRK2
6.27990367858734E-09	1.64413794777272	0.167	0.075	0.000105238625845767	cDC2	CLEC4E

6.79160331113002E-09	2.02956265943428	0.169	0.076	0.000113813688287917	cDC2	SLC25A37
6.87767915389268E-09	1.92904394499771	0.176	0.082	0.000115256147260934	cDC2	SMAD3
7.19526965843135E-09	0.89526199409973	0.521	0.492	0.000120578328935993	cDC2	IFNGR1
8.05796101216814E-09	1.17244844076289	0.472	0.388	0.000135035310641914	cDC2	IL13RA1
8.10224650903552E-09	1.05901163738886	0.33	0.215	0.000135777446998417	cDC2	SAMD9L
8.15809043748073E-09	1.77579829689836	0.142	0.058	0.000136713279551302	cDC2	AQP3
8.29412498037213E-09	0.90222906491186	0.217	0.109	0.000138992946421076	cDC2	NAV1
8.55050300606512E-09	1.24997104923242	0.474	0.392	0.000143289329375639	cDC2	VASP
8.56454996250916E-09	1.67999480314395	0.244	0.138	0.000143524728271729	cDC2	RARA
8.66512041567702E-09	0.51127078581781	0.334	0.201	0.000145210087925916	cDC2	GRASP
9.00951790176636E-09	1.42057115309868	0.321	0.205	0.000150981500997801	cDC2	DEPRECATED-ENSG00000059804
9.43072288020096E-09	1.92124431332542	0.126	0.048	0.000158040054026408	cDC2	NRGN
1.03036518516243E-08	1.03920591430016	0.348	0.229	0.00017266859772952	cDC2	ADGRE2
1.57143382160378E-08	0.93509002507552	0.621	0.55	0.000263340879824361	cDC2	EFHD2
1.61766025581675E-08	2.5786741709262	0.104	0.035	0.000271087505669771	cDC2	DEPRECATED-ENSG00000128487
1.75004900625709E-08	1.94423816594373	0.104	0.035	0.000293273212468563	cDC2	SP140
2.00283682518221E-08	1.66053041398704	0.129	0.052	0.000335635395164035	cDC2	DEPRECATED-ENSG00000180817
2.90083389955334E-08	1.52688473978145	0.167	0.079	0.000486121744887148	cDC2	ITPRIPL1
3.3903100166695E-08	1.68955568500674	0.248	0.145	0.000568148152593475	cDC2	CD55
3.61863863151181E-08	1.21913655726399	0.4	0.292	0.000606411461868749	cDC2	EDEM1
3.86934159623528E-08	1.56911777000041	0.104	0.036	0.000648424264697108	cDC2	BCL11A
3.93480782709742E-08	1.28836883765371	0.305	0.195	0.000659395095664986	cDC2	DEPRECATED-ENSG00000188313
4.31615962919559E-08	1.10751904095162	0.178	0.087	0.000723302030660597	cDC2	TRAF1
4.68585611848133E-08	0.91252349292560	0.341	0.232	0.000785255768335101	cDC2	LPCAT2
5.34436555612157E-08	1.26505636197793	0.172	0.082	0.000895608779894853	cDC2	CAPN3
5.35124291296632E-08	0.92973593488679	0.458	0.355	0.000896761287354896	cDC2	CNN2
5.88906002119525E-08	0.54490586437152	0.679	0.642	0.0009868886783519	cDC2	PFN1
5.98069899005077E-08	0.94747455672364	0.348	0.24	0.00100224553675271	cDC2	LST1
6.02860650589904E-08	0.82124589187567	0.35	0.235	0.00101027387825856	cDC2	PBX2
6.46437933239168E-08	0.62330119238850	0.682	0.652	0.0010833006885222	cDC2	EEF2
6.76848817963249E-08	1.21862915661387	0.264	0.159	0.00113426324914281	cDC2	SKIL
7.51402314295036E-08	1.62715360533534	0.199	0.105	0.00125919999829562	cDC2	DGKA
8.50470708841788E-08	0.94532271518193	0.558	0.505	0.00142521881387707	cDC2	SH3BGRL3
1.01304926894135E-07	0.85272502466794	0.485	0.421	0.00169766796489191	cDC2	MBNL1
1.0936979637537E-07	1.43515795653207	0.422	0.332	0.00183281904765845	cDC2	FGD4
1.11345470029564E-07	1.53909867710531	0.111	0.042	0.00186592738675543	cDC2	MAP3K20
1.24858443205303E-07	1.5148214753958	0.12	0.048	0.00209237779123446	cDC2	PPA1
1.38187753930543E-07	1.70831158270182	0.12	0.048	0.00231575038036805	cDC2	LY9
1.61323845659606E-07	1.97223761620922	0.167	0.084	0.00270346500556368	cDC2	MAML3
1.90643067637564E-07	1.32544551060691	0.323	0.221	0.00319479652747029	cDC2	PSTPIP2
2.55539167053681E-07	0.82846057275443	0.149	0.07	0.00428232536148559	cDC2	P2RY12
3.05337141463525E-07	1.34082537504231	0.12	0.049	0.00511683981664575	cDC2	RASGRP2
3.14087160985335E-07	0.96104462087126	0.53	0.472	0.00526347264379224	cDC2	GNAQ
3.62401106872769E-07	2.08297910915682	0.151	0.074	0.00607311774897386	cDC2	N4BP2
4.76182757773524E-07	1.08547717294197	0.239	0.146	0.00797987065476872	cDC2	SOCS1
5.21722601575055E-07	0.72845803797202	0.379	0.285	0.00874302735719477	cDC2	DOCK10
6.02931131318728E-07	1.12755452768655	0.402	0.312	0.0101039198986392	cDC2	MRTFA
6.08285740217993E-07	1.22190676716418	0.264	0.171	0.0101936524345731	cDC2	MYOF
6.25644305680842E-07	0.85893889856227	0.253	0.152	0.0104845472745995	cDC2	LMNB1
6.59326534278194E-07	1.77650555571917	0.174	0.09	0.011048994061434	cDC2	HIST2H2BE
6.76468300135235E-07	0.66692478304729	0.135	0.062	0.0113362557736663	cDC2	ARHGEF3
7.17535642965678E-07	0.87566298352923	0.433	0.345	0.0120244623048188	cDC2	DSE
7.35432378363493E-07	1.62313206770134	0.244	0.153	0.0123243757966154	cDC2	LYST
8.25990670815706E-07	1.1935789969456	0.217	0.129	0.0138419516615296	cDC2	NIBAN1
8.60368117403339E-07	0.82594355934451	0.533	0.462	0.0144180489114452	cDC2	ITGA4
8.85894155085479E-07	1.92991268160002	0.126	0.057	0.0148458142509225	cDC2	SH2B2
1.28661878449151E-06	1.2926686100451	0.352	0.256	0.0215611575905087	cDC2	EMP3
1.29358950508056E-06	0.89128903755614	0.458	0.38	0.02167797292614	cDC2	PTPN6
1.83885653754823E-06	1.30563540976514	0.28	0.19	0.0308155578562333	cDC2	SNX20
2.00599705026203E-06	0.38406360126509	0.05	0.138	0.033616498568291	cDC2	CHST15
2.06379510068867E-06	1.30968896958518	0.149	0.076	0.0345850782973407	cDC2	C12orf45
2.25175076583464E-06	0.97648576594630	0.492	0.415	0.0377348393338569	cDC2	KDM2A
2.5223109460591E-06	1.05550216272706	0.345	0.248	0.0422688868340583	cDC2	APAF1
2.54430045502218E-06	0.66200813959419	0.58	0.534	0.0426373870252617	cDC2	LENG8
2.72926005911915E-06	1.08146830480931	0.377	0.29	0.0457369400707188	cDC2	ENTPD1
2.87239087290325E-06	1.29945659916873	0.221	0.138	0.0481355262481127	cDC2	TBC1D1
4.73998170302632E-141	5.38676252901897	0.574	0.016	7.94326133793151E-137	Proliferating macrophages	NCAPG
2.30131288609573E-134	4.96067165525156	0.479	0.008	3.85654013451923E-130	Proliferating macrophages	AURKB
1.52688615141311E-130	5.77830961467006	0.436	0.005	2.55875581253809E-126	Proliferating macrophages	HJURP
5.77910042781453E-127	5.79755426141556	0.681	0.038	9.68461649693159E-123	Proliferating macrophages	MKI67
4.0144067039315E-124	5.11511127385189	0.511	0.014	6.7273427544484E-120	Proliferating macrophages	KIF23

6.11239345451793E-124	4.77200617480092	0.543	0.018	1.02431489510812E-119	Proliferating macrophages	BUB1
8.6999684491534E-124	4.5219023823394	0.511	0.014	1.45794071270913E-119	Proliferating macrophages	ESPL1
6.1089652083603E-123	4.51130064310358	0.574	0.022	1.02374038961702E-118	Proliferating macrophages	KIFC1
3.18185439190457E-122	5.17488345913167	0.521	0.016	5.33215158995367E-118	Proliferating macrophages	IQGAP3
2.27744967495408E-115	6.24881822789887	0.447	0.01	3.81655016528805E-111	Proliferating macrophages	PLK1
1.69122443217242E-114	6.75287496561972	0.383	0.005	2.83415390343454E-110	Proliferating macrophages	TROAP
6.16498731149211E-114	5.29600263508878	0.479	0.014	1.03312857365985E-109	Proliferating macrophages	KIF2C
4.99786273008065E-112	6.34063762913713	0.426	0.009	8.37541836306915E-108	Proliferating macrophages	KIF20A
1.00660363373467E-111	6.50742919152023	0.383	0.005	1.68686636941256E-107	Proliferating macrophages	ASPM
2.12268685958317E-111	5.64526207542016	0.404	0.007	3.55719863928948E-107	Proliferating macrophages	KIF18B
2.98002106802553E-110	4.71888959773895	0.553	0.025	4.99391930579719E-106	Proliferating macrophages	TPX2
1.69421246621312E-109	5.57039352664034	0.436	0.011	2.83916125087995E-105	Proliferating macrophages	GTSE1
2.29241824184319E-105	4.83359234626727	0.511	0.021	3.84163448968081E-101	Proliferating macrophages	ANLN
4.1108753214567E-103	4.75252686658398	0.585	0.034	6.88900486369714E-99	Proliferating macrophages	CENPM
1.02636677006749E-102	5.98289916791961	0.351	0.005	1.71998543327911E-98	Proliferating macrophages	DEPRECATED-ENSG00000117399
1.06838309980543E-102	5.56994487637141	0.309	0.002	1.79039639865394E-98	Proliferating macrophages	TTK
3.69205675856763E-102	4.32268459977957	0.468	0.016	6.18714871600764E-98	Proliferating macrophages	CENPI
4.4313342978512E-101	5.24607882270437	0.362	0.006	7.42603001633904E-97	Proliferating macrophages	KIF14
1.54594664229295E-100	4.19798391209557	0.489	0.02	2.59069738315453E-96	Proliferating macrophages	CIT
3.15549671862353E-100	4.94948265371306	0.479	0.019	5.28798140106931E-96	Proliferating macrophages	KIF11
1.51568421826244E-99	4.28350367121118	0.372	0.007	2.53998361296419E-95	Proliferating macrophages	KNL1
2.57044977102285E-99	4.27886297349018	0.511	0.024	4.3075597262801E-95	Proliferating macrophages	CEP55
1.61226376418481E-97	5.26193091936855	0.457	0.018	2.7018316160209E-93	Proliferating macrophages	BIRC5
2.15298571076632E-96	4.50707724676555	0.596	0.04	3.6079734541022E-92	Proliferating macrophages	CENPF
2.2978616011028E-96	5.46939266116264	0.34	0.005	3.85075647112807E-92	Proliferating macrophages	SPC25
2.9752357563851E-95	4.55452977014921	0.479	0.021	4.98590008055015E-91	Proliferating macrophages	MYBL2
5.59680304083276E-94	5.16389532042208	0.319	0.004	9.37912253582753E-90	Proliferating macrophages	BUB1B
1.30947697616324E-92	4.27481801248472	0.468	0.021	2.19442151665436E-88	Proliferating macrophages	NDC80
3.30674733100444E-91	4.89306341360759	0.543	0.034	5.54144717729725E-87	Proliferating macrophages	RRM2
5.4656405684003E-91	4.07252892189036	0.372	0.01	9.15932046452522E-87	Proliferating macrophages	CDCA8
1.14819694288984E-88	4.15498974431395	0.617	0.051	1.9241484368948E-84	Proliferating macrophages	NUSAP1
2.80020866645814E-88	4.57586420146442	0.479	0.025	4.69258968325054E-84	Proliferating macrophages	HIST1H2BH
5.61793447745293E-87	5.2592755898544	0.34	0.008	9.41453459731563E-83	Proliferating macrophages	CDC20
7.38324915916691E-87	4.40842783002111	0.468	0.024	1.23728489409319E-82	Proliferating macrophages	MELK
4.99755990490896E-86	3.97661212026933	0.489	0.028	8.37491088864643E-82	Proliferating macrophages	DEPRECATED-ENSG00000188486
8.17112883141048E-85	4.94402371947914	0.34	0.008	1.36931776956777E-80	Proliferating macrophages	CDCA3
9.67205183089954E-85	4.71776866514639	0.585	0.049	1.62084244582215E-80	Proliferating macrophages	CDK1
2.86985652693344E-82	3.75927756776178	0.426	0.019	4.80930556783506E-78	Proliferating macrophages	NCAPH
3.29704998250029E-82	4.5862058588243	0.309	0.006	5.52519636067399E-78	Proliferating macrophages	CKAP2L
1.72193019388615E-81	5.41646110167235	0.298	0.005	2.88561061891441E-77	Proliferating macrophages	PKMYT1
1.90428273760387E-81	4.20453541583264	0.319	0.007	3.19119701167656E-77	Proliferating macrophages	CDCA5
5.57800625217166E-81	4.94356938416458	0.362	0.012	9.34762287738926E-77	Proliferating macrophages	CCNF
6.36616766314657E-81	3.63439064705682	0.489	0.03	1.0668423769901E-76	Proliferating macrophages	SHCBP1
4.28548284831515E-80	4.47879990495437	0.468	0.027	7.18161215720653E-76	Proliferating macrophages	UBE2C
1.79208739945721E-79	4.97230121167392	0.372	0.014	3.00318006401039E-75	Proliferating macrophages	CCNB1
4.81440637165018E-79	4.78012699436422	0.298	0.006	8.06798219761136E-75	Proliferating macrophages	E2F8
5.67835946508625E-78	3.80405978425942	0.436	0.023	9.51579479159154E-74	Proliferating macrophages	SPC24
7.64988084623167E-77	5.43639257180784	0.298	0.007	1.2819670322115E-72	Proliferating macrophages	FAM83D
1.02401464824891E-76	4.54206519291638	0.532	0.042	1.71604374753553E-72	Proliferating macrophages	TOP2A
2.04055764350997E-76	7.04146903995819	0.245	0.002	3.41956649899401E-72	Proliferating macrophages	NEK2
4.65801361451814E-76	3.92162839215143	0.277	0.005	7.8058992152095E-72	Proliferating macrophages	TICRR
5.29475124029473E-75	3.69891321357304	0.34	0.012	8.87294412848591E-71	Proliferating macrophages	KIF15
1.09905061008355E-73	3.92592379444986	0.617	0.066	1.84178901237801E-69	Proliferating macrophages	TK1
8.70126336501213E-73	4.60832063916566	0.351	0.014	1.45815771470873E-68	Proliferating macrophages	CCNB2
1.85271000480873E-72	2.6916846411298	0.511	0.039	3.10477142605847E-68	Proliferating macrophages	SPAG5
1.1498375159264E-71	3.63694404978957	0.585	0.059	1.92689770918946E-67	Proliferating macrophages	H2AFX
2.42578113130887E-71	3.77671489138913	0.319	0.01	4.06512401984741E-67	Proliferating macrophages	CDCA2
2.18552971369239E-70	5.11808864075673	0.266	0.005	3.6625106942057E-66	Proliferating macrophages	OIP5
2.76643232894886E-68	3.97363222732619	0.298	0.009	4.6359872968525E-64	Proliferating macrophages	DEPDC1B
1.35539745163479E-67	8.35525696891514	0.202	0.001	2.27137504944958E-63	Proliferating macrophages	XCR1
1.36908661787645E-67	3.7006328813377	0.415	0.026	2.29431535423736E-63	Proliferating macrophages	FOXM1
1.82704185400294E-67	3.61208186019414	0.543	0.053	3.06175673893813E-63	Proliferating macrophages	UHRF1
3.41576767450066E-67	4.11554902812551	0.33	0.013	5.72414346892821E-63	Proliferating macrophages	POLQ
7.61991765105541E-67	3.52614789674165	0.351	0.016	1.27694579996387E-62	Proliferating macrophages	CENPA
6.44506443416617E-66	4.26585950132633	0.266	0.007	1.08006389787757E-61	Proliferating macrophages	RAD54L
1.07969779116763E-65	3.58521800206973	0.596	0.07	1.80935755843871E-61	Proliferating macrophages	KIF22
6.79632493689777E-65	3.43565007306108	0.436	0.032	1.13892813292533E-60	Proliferating macrophages	PRC1
8.77536584783194E-65	4.69822984390142	0.255	0.006	1.47057580877968E-60	Proliferating macrophages	NEURL1B
1.21369429912665E-64	3.58383771920386	0.213	0.002	2.03390890647643E-60	Proliferating macrophages	KIF18A
4.75821303537571E-64	3.51103757687522	0.447	0.034	7.97381340468262E-60	Proliferating macrophages	FANCI
5.21558683471738E-64	4.50516966848886	0.298	0.011	8.74028041761939E-60	Proliferating macrophages	DLGAP5

2.76113770433638E-63	4.44820365043788	0.415	0.03	4.62711456492691E-59	Proliferating macrophages	HIST1H2AH
2.9946521668906E-63	4.74927455702066	0.245	0.005	5.01843810127526E-59	Proliferating macrophages	NEIL3
5.86867378780213E-63	4.10154911356208	0.319	0.014	9.83472353359881E-59	Proliferating macrophages	AURKA
6.7324464122808E-63	2.98288041723304	0.33	0.015	1.12822336977002E-58	Proliferating macrophages	BRIP1
7.32416034765472E-62	2.93807120116866	0.628	0.084	1.22738279105998E-57	Proliferating macrophages	TACC3
2.81882083227887E-61	7.2555220438845	0.202	0.002	4.72377995073293E-57	Proliferating macrophages	CLEC9A
3.34968578512366E-61	3.09009621924034	0.766	0.144	5.61340343871022E-57	Proliferating macrophages	LMNB1
3.69184519143702E-61	4.3136695138393	0.351	0.019	6.18679417181016E-57	Proliferating macrophages	ARHGAP11A
4.80510169603488E-61	3.43324368220097	0.5	0.048	8.05238942221526E-57	Proliferating macrophages	FANCA
8.06883165228424E-61	4.98265514336553	0.702	0.123	1.35217480828979E-56	Proliferating macrophages	HIST1H1B
9.37447927174402E-60	6.69474655788587	0.181	0.001	1.57097523635886E-55	Proliferating macrophages	PIMREG
3.2024010258941E-59	3.32354892815182	0.436	0.036	5.36658363919334E-55	Proliferating macrophages	ASF1B
3.4572615241176E-59	3.51080152421658	0.649	0.101	5.79367886211628E-55	Proliferating macrophages	NCAPD2
1.98063099038683E-58	5.70743505925829	0.223	0.005	3.31914141369025E-54	Proliferating macrophages	E2F7
2.78947856947406E-57	3.62925995954007	0.277	0.011	4.67460818672462E-53	Proliferating macrophages	SGO2
3.28809125817958E-56	4.2131869485926	0.223	0.005	5.51018333045734E-52	Proliferating macrophages	HIST1H2BM
7.68441218902224E-56	5.61322797193613	0.234	0.007	1.28775379463635E-51	Proliferating macrophages	SKA1
7.93350537514507E-56	4.8701039392177	0.255	0.009	1.32949683076681E-51	Proliferating macrophages	HIST1H2BB
1.31324446471006E-55	3.17996316391815	0.34	0.021	2.20073507396111E-51	Proliferating macrophages	STIL
1.72906730231214E-55	3.96800780690781	0.298	0.015	2.89757098521469E-51	Proliferating macrophages	CLSPN
3.66027459863211E-55	3.85010828406737	0.234	0.007	6.13388817238769E-51	Proliferating macrophages	DEPDC1
9.0551318631469E-54	2.88723282258754	0.33	0.02	1.51745899762616E-49	Proliferating macrophages	MXD3
1.36540235073103E-53	3.92793431338644	0.255	0.01	2.28814125935507E-49	Proliferating macrophages	DIAPH3
1.98969755181945E-52	3.1703715555027	0.404	0.035	3.33433515733904E-48	Proliferating macrophages	CDT1
2.8921679499852E-52	3.40242137940708	0.266	0.012	4.84669505058519E-48	Proliferating macrophages	RACGAP1
8.0230867345695E-52	3.89795842233964	0.191	0.004	1.34450887497916E-47	Proliferating macrophages	FBXO43
1.0274373057509E-51	3.73903268678248	0.245	0.009	1.72177943697736E-47	Proliferating macrophages	HASPIN
2.32205025489628E-51	3.97466856992519	0.489	0.061	3.89129181715519E-47	Proliferating macrophages	TYMS
2.60485134792376E-51	4.24829182365722	0.266	0.012	4.36520988885064E-47	Proliferating macrophages	RAD51
7.08576253508248E-51	3.24723899055541	0.532	0.073	1.18743208562912E-46	Proliferating macrophages	TCF19
7.62380667612933E-51	3.33513794571037	0.394	0.035	1.27759752278575E-46	Proliferating macrophages	PTTG1
5.38868674282723E-50	3.62133320271553	0.372	0.031	9.03036124362988E-46	Proliferating macrophages	CCNA2
5.92822301303934E-50	6.19943620977152	0.17	0.002	9.93451612525133E-46	Proliferating macrophages	SGO1
7.33560455248491E-50	2.88773424074203	0.574	0.089	1.22930061090542E-45	Proliferating macrophages	NCAPG2
7.35185887522909E-50	3.04964931557534	0.426	0.043	1.23202451031089E-45	Proliferating macrophages	HMMR
1.09423942935506E-49	5.25108225487703	0.17	0.002	1.83372643571321E-45	Proliferating macrophages	SLFN13
2.06260068906248E-49	3.15078100007366	0.351	0.027	3.4565062347309E-45	Proliferating macrophages	NUF2
2.59593532923217E-48	3.46099588300958	0.287	0.017	4.35026842472726E-44	Proliferating macrophages	GPSM2
1.30360017324154E-47	2.33359218985666	0.362	0.03	2.18457317031817E-43	Proliferating macrophages	ATAD5
1.85808096178846E-47	3.23193657089958	0.245	0.011	3.1137720757651E-43	Proliferating macrophages	KIF4A
9.30595635314755E-47	2.43564842384938	0.511	0.069	1.55949216566047E-42	Proliferating macrophages	CHAF1A
1.02719312613925E-46	2.50005768069831	0.553	0.084	1.72137024078416E-42	Proliferating macrophages	DTYMK
2.38430257329007E-46	3.63171399227954	0.521	0.076	3.9956142523195E-42	Proliferating macrophages	HIST1H1D
4.86982915972263E-46	2.97455332335687	0.574	0.095	8.16085970586319E-42	Proliferating macrophages	INCENP
1.50791684383819E-45	2.93327118329695	0.255	0.013	2.52696704690404E-41	Proliferating macrophages	EXO1
2.37733482486247E-45	3.23886975840569	0.223	0.009	3.98393769950452E-41	Proliferating macrophages	ARHGEF39
3.6213302176077E-45	2.83502879300271	0.5	0.072	6.06862517866698E-41	Proliferating macrophages	ZWINT
3.6430826473676E-45	3.03411242152059	0.266	0.015	6.10507790045862E-41	Proliferating macrophages	MND1
3.3305810915909E-44	2.64973360502514	0.617	0.119	5.58138779328803E-40	Proliferating macrophages	PHF19
4.25870512954989E-44	4.54195739667792	0.181	0.005	7.1367380560997E-40	Proliferating macrophages	CDC25C
4.82902901586842E-44	2.43131098731896	0.34	0.029	8.09248682479229E-40	Proliferating macrophages	CENPO
2.17275236188415E-43	4.92280334141542	0.223	0.01	3.64109840804545E-39	Proliferating macrophages	HIST1H2AE
3.32722845986028E-43	3.37532779689431	0.372	0.038	5.57576945303386E-39	Proliferating macrophages	CENPE
4.02292334987926E-43	3.65339946733678	0.255	0.015	6.74161494972766E-39	Proliferating macrophages	GINS4
7.57273901398215E-43	3.41929373007399	0.362	0.035	1.26903960396313E-38	Proliferating macrophages	AC091057.6
1.6054313273601E-42	2.03901788337255	0.574	0.096	2.69038181839005E-38	Proliferating macrophages	NUP210
1.87340855865564E-42	2.48343859166529	0.638	0.13	3.13945806259512E-38	Proliferating macrophages	LMNB2
8.64724608367233E-42	3.0067523174996	0.234	0.012	1.44910549870181E-37	Proliferating macrophages	WDR62
1.1134619118405E-41	2.74401734521964	0.606	0.121	1.86593947186231E-37	Proliferating macrophages	CDKN2C
2.18786313508505E-41	3.53854569917231	0.319	0.028	3.66642104177552E-37	Proliferating macrophages	BARD1
4.68335083641185E-41	3.33027644880578	0.5	0.081	7.84835933165899E-37	Proliferating macrophages	HELLS
4.90902053171211E-41	2.50804495564616	0.287	0.021	8.22653660704315E-37	Proliferating macrophages	RECQL4
6.48995958258699E-41	3.50697390836763	0.245	0.015	1.08758742684993E-36	Proliferating macrophages	CDC45
1.43021371065384E-40	3.04694295714758	0.436	0.059	2.3967521363137E-36	Proliferating macrophages	STMN1
3.87154865807303E-40	2.23558679459315	0.415	0.052	6.48794124119879E-36	Proliferating macrophages	ECT2
2.81774798758865E-39	2.23977981335882	0.447	0.061	4.72198207760105E-35	Proliferating macrophages	DEPRECATED-ENSG00000120802
3.96513379643755E-39	5.28570674935248	0.181	0.007	6.64477121607004E-35	Proliferating macrophages	CLNK
9.00003852243085E-39	5.63512237999042	0.138	0.002	1.50822645558896E-34	Proliferating macrophages	SAPCD2
1.24342085209228E-38	3.24420477567559	0.362	0.041	2.08372466393625E-34	Proliferating macrophages	ZNF367
2.11235519207298E-38	2.94827707580149	0.319	0.03	3.5398848308759E-34	Proliferating macrophages	BRCA1
5.30886946477469E-38	3.637547808239	0.234	0.015	8.89660344906942E-34	Proliferating macrophages	ARHGAP33

1.94141861470661E-37	3.69703828680572	0.213	0.012	3.25342931452534E-33	Proliferating macrophages	CENPK
2.86059679216129E-37	2.19120887582212	0.702	0.181	4.7937881043039E-33	Proliferating macrophages	H2AFZ
1.33476374079424E-36	2.23741025267672	0.447	0.068	2.23679707682298E-32	Proliferating macrophages	PCNA
2.40335579718792E-36	4.73106762588987	0.138	0.003	4.02754364492752E-32	Proliferating macrophages	APOBEC3B
3.88971376340822E-36	2.81200616998078	0.723	0.218	6.51838232471949E-32	Proliferating macrophages	HMGB2
5.04689011088933E-36	2.70016299434735	0.383	0.049	8.45757844782834E-32	Proliferating macrophages	KNSTRN
5.82507668170295E-36	2.62376602730305	0.394	0.053	9.7616635031978E-32	Proliferating macrophages	ORC6
8.27471751891372E-36	2.42284696988227	0.394	0.053	1.38667716181956E-31	Proliferating macrophages	KIF20B
1.28057856011557E-35	2.83061728184685	0.479	0.082	2.14599355104167E-31	Proliferating macrophages	CENPU
1.36750816585154E-35	3.6499285774833	0.255	0.02	2.29167018433401E-31	Proliferating macrophages	MAD2L1
2.95000790430905E-35	3.26669051192959	0.468	0.081	4.94362324604111E-31	Proliferating macrophages	HIST1H1E
3.04965486473281E-35	3.20034223794618	0.213	0.013	5.11061162231925E-31	Proliferating macrophages	RAD54B
3.1051467124103E-35	2.43450819616603	0.404	0.057	5.20360486065718E-31	Proliferating macrophages	PRR11
4.10850086089641E-35	4.09185278969453	0.128	0.002	6.88502574269021E-31	Proliferating macrophages	HIST1H3I
1.02495419669822E-34	1.91374240292186	0.34	0.039	1.71761824282687E-30	Proliferating macrophages	CCDC18
1.54535614222512E-34	2.64198668077047	0.457	0.076	2.58970782314086E-30	Proliferating macrophages	PCLAF
2.24416851503312E-34	3.16529190758929	0.202	0.012	3.7607775974925E-30	Proliferating macrophages	BLM
6.44441382761157E-34	2.58153113867308	0.287	0.028	1.07995486923115E-29	Proliferating macrophages	FBXO5
8.64119022414387E-34	3.18333684410592	0.319	0.036	1.44809065776203E-29	Proliferating macrophages	DTL
9.33776848681495E-34	1.6307270902901	0.457	0.074	1.56482324302045E-29	Proliferating macrophages	LIG1
1.15892462790629E-33	4.01537491783687	0.298	0.032	1.94212589144536E-29	Proliferating macrophages	FLT3
1.24984511212415E-33	2.5758024051989	0.521	0.104	2.09449043889765E-29	Proliferating macrophages	CRIP1
1.28346760337592E-33	2.02623750345746	0.702	0.199	2.15083500973736E-29	Proliferating macrophages	TMPO
1.30816608649607E-33	2.95477290487802	0.479	0.091	2.19222472775011E-29	Proliferating macrophages	HIST1H4C
2.56170765641331E-33	3.33651051703749	0.213	0.014	4.29290969061742E-29	Proliferating macrophages	C21orf58
3.79810042809299E-33	8.00679649179206	0.106	0.001	6.36485669739822E-29	Proliferating macrophages	SLCO5A1
1.14022308701288E-32	3.77580850977557	0.213	0.015	1.91078584921618E-28	Proliferating macrophages	PARPBP
2.27005549596459E-32	4.3780647324198	0.138	0.004	3.80415900013746E-28	Proliferating macrophages	E2F2
2.6965451365795E-32	2.38259892205044	0.351	0.045	4.51887033987992E-28	Proliferating macrophages	UBE2T
2.70836065029602E-32	4.1420234691412	0.181	0.01	4.53867077776607E-28	Proliferating macrophages	MCM10
2.83161105105276E-32	2.46410102949238	0.33	0.041	4.74521379935422E-28	Proliferating macrophages	MCM8
3.47140373133869E-32	2.2573103290703	0.33	0.039	5.81737837297737E-28	Proliferating macrophages	ARHGAP19
4.52738196475321E-32	3.39493772330223	0.234	0.019	7.58698669653343E-28	Proliferating macrophages	FAM111B
6.84841612257986E-32	2.06034499622162	0.447	0.076	1.14765757382193E-27	Proliferating macrophages	CENPN
7.36694908362619E-32	2.97891052233292	0.234	0.019	1.23455332743408E-27	Proliferating macrophages	POC1A
8.13759254998617E-32	4.16396896641668	0.33	0.042	1.36369775952668E-27	Proliferating macrophages	IDO1
8.39977912948674E-32	2.83038072132563	0.66	0.215	1.40763498651939E-27	Proliferating macrophages	TUBB
1.39125006030944E-31	2.69342616286041	0.191	0.012	2.33145685106656E-27	Proliferating macrophages	MTFR2
1.52214849516894E-31	1.81421225684172	0.415	0.065	2.5508164482041E-27	Proliferating macrophages	TIMELESS
2.59844933005573E-31	3.0742040522215	0.287	0.031	4.3544813873074E-27	Proliferating macrophages	CENPH
5.36218638650114E-31	2.67849337415337	0.181	0.01	8.9859519464986E-27	Proliferating macrophages	PIF1
1.28803539965349E-30	2.45398181817295	0.319	0.039	2.15848972273932E-26	Proliferating macrophages	GINS1
1.37149122091691E-30	3.03452502959466	0.298	0.035	2.29834498801256E-26	Proliferating macrophages	SUV39H1
1.57541203549323E-30	2.11702952876709	0.255	0.024	2.64007548907956E-26	Proliferating macrophages	SKA3
1.82189423975495E-30	6.38825508246398	0.106	0.002	3.05313036698134E-26	Proliferating macrophages	CYYR1
2.19305101193766E-30	2.59922665460794	0.468	0.088	3.67511488580514E-26	Proliferating macrophages	EZH2
2.22892955920923E-30	1.70709726003455	0.447	0.079	3.73524015532283E-26	Proliferating macrophages	SMC2
4.04629671983863E-30	2.4734961928632	0.415	0.07	6.78078404310558E-26	Proliferating macrophages	SLC1A4
5.99244844582046E-30	3.09530918355616	0.181	0.011	1.00421451055059E-25	Proliferating macrophages	REXO5
8.20933625067973E-30	2.95049953441106	0.191	0.013	1.37572056888891E-25	Proliferating macrophages	ORC1
1.43077529135327E-29	2.15135203016762	0.34	0.047	2.39769323324982E-25	Proliferating macrophages	NSD2
2.56036944308074E-29	2.30403822911126	0.532	0.125	4.2906671127147E-25	Proliferating macrophages	RRM1
3.38326885491964E-29	2.97194592238718	0.17	0.01	5.66968194707434E-25	Proliferating macrophages	ESCO2
3.85012367994209E-29	1.82622019355682	0.287	0.033	6.45203726284695E-25	Proliferating macrophages	CHTF18
3.99229162669937E-29	2.33292877561972	0.372	0.059	6.69028230802281E-25	Proliferating macrophages	CKAP2
6.8619756833423E-29	3.80768574982713	0.213	0.018	1.1499298850145E-24	Proliferating macrophages	PSRC1
7.62934547668668E-29	1.85144148479276	0.585	0.156	1.27852571498315E-24	Proliferating macrophages	SMC4
9.68504663475646E-29	2.76451648619575	0.287	0.034	1.62302011505249E-24	Proliferating macrophages	CENPL
1.46629476954848E-28	2.3650424897984	0.309	0.039	2.45721677480935E-24	Proliferating macrophages	TEDC1
2.1737288509975E-28	2.03780000429045	0.372	0.059	3.64273480850162E-24	Proliferating macrophages	CDK2
2.84943461866864E-28	2.19705471548919	0.426	0.079	4.77508253396491E-24	Proliferating macrophages	RFWD3
3.24798584332965E-28	2.34709688886162	0.553	0.136	5.44297467625183E-24	Proliferating macrophages	MCM4
4.22465022409781E-28	2.28663862798684	0.532	0.129	7.07966884554311E-24	Proliferating macrophages	CKS1B
6.43401172354631E-28	2.42220695178904	0.351	0.053	1.07821168463189E-23	Proliferating macrophages	RCCD1
6.68408791894904E-28	2.68406833440486	0.191	0.014	1.12011945345748E-23	Proliferating macrophages	ZNF724
1.16030913023972E-27	4.17877922696054	0.138	0.006	1.94444604045572E-23	Proliferating macrophages	TEDC2
1.80541678406278E-27	2.84497174743508	0.202	0.016	3.02551744673241E-23	Proliferating macrophages	RNFT2
1.84018832726225E-27	1.89402425385565	0.298	0.038	3.08378759882608E-23	Proliferating macrophages	RFC3
4.26256477560053E-27	1.29889986911324	0.372	0.06	7.14320605095136E-23	Proliferating macrophages	CENPJ
4.77727135091859E-27	2.61611439254663	0.362	0.061	8.00575132986937E-23	Proliferating macrophages	FEN1
6.16120991795634E-27	3.12542111215401	0.277	0.034	1.03249555805112E-22	Proliferating macrophages	SAC3D1

1.96106459910493E-26	4.12974286160798	0.138	0.007	3.28635205518005E-22	Proliferating macrophages	CDC25A
1.9720896323311E-26	2.1341529143985	0.34	0.053	3.30482780586046E-22	Proliferating macrophages	NRGN
2.32107033601186E-26	1.46280569262581	0.479	0.102	3.88964966908867E-22	Proliferating macrophages	IMPA2
2.94487228185294E-26	2.42846832197739	0.309	0.044	4.93501696992916E-22	Proliferating macrophages	E2F1
4.54085151861659E-26	2.57227516227928	0.234	0.024	7.60955897489769E-22	Proliferating macrophages	CHEK1
6.55683466539636E-26	2.91221684613973	0.213	0.02	1.09879435322712E-21	Proliferating macrophages	CDC6
1.05926436687994E-25	2.14592700141175	0.234	0.025	1.7751152260174E-21	Proliferating macrophages	CCDC15
1.18428921174636E-25	1.94829883883708	0.266	0.033	1.98463186104456E-21	Proliferating macrophages	FANCG
2.28312292860793E-25	2.0184827371178	0.309	0.044	3.82605740376118E-21	Proliferating macrophages	GGH
3.04680038590754E-25	1.46856559563402	0.309	0.045	5.10582808670385E-21	Proliferating macrophages	CENPW
3.92346479534529E-25	1.9808949125542	0.319	0.049	6.57494230403964E-21	Proliferating macrophages	NETO2
4.27174962577223E-25	1.80341244417412	0.564	0.153	7.15859802286911E-21	Proliferating macrophages	RANGAP1
4.70500520408516E-25	1.99843349721367	0.362	0.062	7.88464772100591E-21	Proliferating macrophages	VRK1
5.37364455186646E-25	1.55871990569598	0.5	0.119	9.00515354001781E-21	Proliferating macrophages	NCAPD3
6.02249178722792E-25	2.33304511238958	0.277	0.037	1.00924917370365E-20	Proliferating macrophages	ZWILCH
6.56344847799607E-25	3.713033750189	0.213	0.021	1.09990269594258E-20	Proliferating macrophages	CDCA4
1.18730177094746E-24	2.19384523975614	0.309	0.045	1.98968030775376E-20	Proliferating macrophages	WEE1
1.30684317118783E-24	2.58936543315634	0.245	0.029	2.19000778627656E-20	Proliferating macrophages	DNA2
1.76728149792871E-24	1.6626787227017	0.479	0.109	2.96161033422893E-20	Proliferating macrophages	RNF26
1.78379460826101E-24	3.01050502882639	0.245	0.029	2.9892830045238E-20	Proliferating macrophages	C9orf40
1.97616686405527E-24	2.04457148589248	0.532	0.146	3.31166043078382E-20	Proliferating macrophages	TUBA1B
2.00654185668177E-24	2.20595747963727	0.266	0.035	3.36256284342731E-20	Proliferating macrophages	BORA
2.36081476700083E-24	1.78407482492637	0.436	0.095	3.95625338654E-20	Proliferating macrophages	KNTC1
3.06578600170166E-24	3.61740502854154	0.181	0.015	5.13764418165165E-20	Proliferating macrophages	TRIM59
3.99811671869299E-24	1.22502093324696	0.989	0.84	6.70004399718572E-20	Proliferating macrophages	ACTB
6.12968040368613E-24	3.43375542019449	0.191	0.018	1.02721184204972E-19	Proliferating macrophages	PKN3
7.3348436948333E-24	1.57204470647724	0.755	0.286	1.22917310638016E-19	Proliferating macrophages	CORO1A
1.0054302424554E-23	2.89539149215407	0.149	0.01	1.68490000030675E-19	Proliferating macrophages	HIST1H2BN
1.28362653443162E-23	3.24594620471262	0.181	0.016	2.1511013464005E-19	Proliferating macrophages	HSH2D
1.73317110046686E-23	2.13316123700644	0.649	0.238	2.90444813016236E-19	Proliferating macrophages	TMEM106C
2.26734911604827E-23	3.15304840617877	0.638	0.238	3.79962364867369E-19	Proliferating macrophages	HIST1H1C
2.49130372126284E-23	1.72728243191929	0.457	0.103	4.17492677609227E-19	Proliferating macrophages	MPHOSPH9
2.76953230197055E-23	2.06476593467632	0.564	0.17	4.64118223164224E-19	Proliferating macrophages	MCM7
2.88143836143778E-23	2.17844299775421	0.34	0.061	4.82871440609742E-19	Proliferating macrophages	HIRIP3
3.30286832410166E-23	1.97962496883357	0.191	0.018	5.53494673752955E-19	Proliferating macrophages	RAD51AP1
3.39867688273824E-23	2.0124645000147	0.309	0.049	5.69550272009274E-19	Proliferating macrophages	GEN1
3.42442360893979E-23	1.328347423003	0.5	0.122	5.73864908386131E-19	Proliferating macrophages	NEMP1
3.4612787928216E-23	2.32040725042008	0.309	0.05	5.80041100101044E-19	Proliferating macrophages	TRERF1
4.49525642989477E-23	2.19193144790699	0.309	0.051	7.53315072521766E-19	Proliferating macrophages	UBE2S
5.59137430986204E-23	2.04275258165308	0.426	0.092	9.3700250684668E-19	Proliferating macrophages	MASTL
1.32779225698144E-22	1.22580143784219	0.574	0.164	2.22511426424949E-18	Proliferating macrophages	BUB3
1.3893824573771E-22	3.25671482543197	0.106	0.004	2.32832712207254E-18	Proliferating macrophages	EME1
1.47438748853588E-22	1.79423535738652	0.734	0.3	2.47077855328843E-18	Proliferating macrophages	SMC1A
1.71087489316155E-22	1.82002446725992	0.33	0.058	2.86708414596013E-18	Proliferating macrophages	RCC1
2.69013568967504E-22	1.53621479837938	0.734	0.306	4.50812938875743E-18	Proliferating macrophages	DNMT1
2.7326214470124E-22	3.19639974721751	0.33	0.06	4.57932702090337E-18	Proliferating macrophages	ZNF366
2.84322029557441E-22	1.59986109752039	0.457	0.109	4.76466857132359E-18	Proliferating macrophages	TTF2
3.50558397181085E-22	1.16143996802017	0.574	0.167	5.87465761996063E-18	Proliferating macrophages	RBM15
3.70699564172876E-22	2.94960061447661	0.213	0.024	6.21218329640905E-18	Proliferating macrophages	CIP2A
3.72583152856397E-22	2.18726083751711	0.447	0.114	6.24437484755675E-18	Proliferating macrophages	DEPRECATED-ENSG00000132646
4.13818870856752E-22	3.14072373940655	0.266	0.039	6.93477663781746E-18	Proliferating macrophages	RMI2
4.29614735773478E-22	1.05293420404149	0.383	0.075	7.19948374209194E-18	Proliferating macrophages	JADE1
5.73885806648145E-22	1.57476736904164	0.585	0.173	9.61717834780961E-18	Proliferating macrophages	TP53
6.26135777296671E-22	2.18334742373005	0.255	0.036	1.04927833559376E-17	Proliferating macrophages	ZGRF1
6.32059498173565E-22	2.13116032792521	0.436	0.107	1.05920530703926E-17	Proliferating macrophages	MCM2
7.911141762742E-22	1.99010036794787	0.489	0.133	1.3257491366003E-17	Proliferating macrophages	ATAD2
9.76323213075072E-22	5.07716726831705	0.117	0.006	1.6361224404712E-17	Proliferating macrophages	MELTF
1.35639927389957E-21	2.71435484501384	0.309	0.055	2.2730539032009E-17	Proliferating macrophages	DEPRECATED-ENSG00000176014
1.87600753933065E-21	1.44124775164992	0.479	0.124	3.1438134344103E-17	Proliferating macrophages	POLD1
1.9880801906613E-21	1.98863866709337	0.394	0.084	3.3316247835102E-17	Proliferating macrophages	SNRNP25
2.32607129010051E-21	2.16090875420662	0.255	0.036	3.89803026795044E-17	Proliferating macrophages	NRM
4.24692993062733E-21	1.99321465177142	0.309	0.055	7.11700517774528E-17	Proliferating macrophages	RBL1
4.65168026720033E-21	2.42862043559648	0.287	0.049	7.79528579177432E-17	Proliferating macrophages	LIN54
6.82928655907809E-21	2.55806855573562	0.223	0.029	1.14445184157031E-16	Proliferating macrophages	WDR34
7.66818463882193E-21	1.82286495340302	0.362	0.073	1.28503438177378E-16	Proliferating macrophages	C19orf48
9.7799358500776E-21	4.55239916198965	0.117	0.007	1.638921649756E-16	Proliferating macrophages	FBXO27
1.18933631533365E-20	1.93512196239502	0.5	0.137	1.99308979723612E-16	Proliferating macrophages	BRI3BP
1.65291745979491E-20	2.56562981049052	0.17	0.016	2.76995907912431E-16	Proliferating macrophages	CDKN3
3.54209199232639E-20	1.24577499716739	0.532	0.152	5.93583776074057E-16	Proliferating macrophages	RAD21
4.96147799371294E-20	1.69464053037615	0.617	0.221	8.31444482186414E-16	Proliferating macrophages	SUN2
5.62311009078248E-20	1.21236292125729	0.777	0.313	9.42320789013328E-16	Proliferating macrophages	CSK

6.17979249389885E-20	2.47777874996683	0.277	0.047	1.03560962612757E-15	Proliferating macrophages	WDR76
6.57567687405836E-20	3.7050922764143	0.106	0.005	1.1019519305547E-15	Proliferating macrophages	RIBC2
6.86473569003295E-20	2.13101259011168	0.213	0.027	1.15039240693572E-15	Proliferating macrophages	CEP72
1.15848430672414E-19	0.987050518369848	0.426	0.107	1.94138800120832E-15	Proliferating macrophages	NCAPH2
1.18805131694914E-19	1.53242478865356	0.372	0.08	1.99093639694336E-15	Proliferating macrophages	ACTL6A
1.93292949977122E-19	1.49226982737063	0.298	0.053	3.23920325571661E-15	Proliferating macrophages	PRIM1
2.09808579618082E-19	1.72431549608337	0.319	0.062	3.51597217723981E-15	Proliferating macrophages	TUBB6
2.13620241778646E-19	2.66045804665564	0.128	0.009	3.57984801172654E-15	Proliferating macrophages	FANCB
2.56204560079225E-19	1.38613877125299	0.766	0.32	4.29347601780765E-15	Proliferating macrophages	TAP2
3.19813930118131E-19	1.62810857575742	0.213	0.028	5.35944184091964E-15	Proliferating macrophages	HAUS8
4.08566152841084E-19	1.63074838400308	0.84	0.399	6.84675158931089E-15	Proliferating macrophages	TAP1
5.37080852314656E-19	1.591766194661	0.745	0.337	9.00040092308901E-15	Proliferating macrophages	H1FX
5.49496828146791E-19	2.40269442544807	0.287	0.052	9.20846784608393E-15	Proliferating macrophages	OXCT1
5.88526782580457E-19	1.76572318668616	0.266	0.044	9.8625318224833E-15	Proliferating macrophages	LRR1
8.14318890192405E-19	1.40557527200204	0.649	0.237	1.36463559618443E-14	Proliferating macrophages	LBR
8.54344584269427E-19	1.78489858580313	0.404	0.102	1.43171065431871E-14	Proliferating macrophages	ACOT7
8.94227626063333E-19	2.09011160689756	0.17	0.018	1.49854665575693E-14	Proliferating macrophages	CDKN2D
9.04629318506463E-19	2.0804890220208	0.106	0.006	1.51597781195313E-14	Proliferating macrophages	HIST1H4J
9.08197280421006E-19	2.19775681388466	0.383	0.093	1.52195700252952E-14	Proliferating macrophages	TUBB4B
1.02065204455315E-18	3.05580044739737	0.415	0.109	1.71040869626217E-14	Proliferating macrophages	MARCKSL1
1.09691521560484E-18	1.31093164049226	0.489	0.139	1.8382105183106E-14	Proliferating macrophages	RAVER1
1.1054998584171E-18	1.26041597205815	0.394	0.095	1.85259666273538E-14	Proliferating macrophages	CBX5
1.37783480741299E-18	1.42337221422574	0.372	0.086	2.30897557026269E-14	Proliferating macrophages	POLA2
1.45252753283785E-18	1.42944417420475	0.798	0.4	2.43414563952966E-14	Proliferating macrophages	IDH2
1.81194553083402E-18	2.66063098369935	0.202	0.027	3.03645832057165E-14	Proliferating macrophages	TONSL
1.94381477825745E-18	2.840965216901	0.287	0.054	3.25744480540383E-14	Proliferating macrophages	AUTS2
2.23313613914933E-18	2.59714846534083	0.202	0.027	3.74228954198645E-14	Proliferating macrophages	LIN9
2.24649488259444E-18	2.21787039958272	0.16	0.016	3.76467612425176E-14	Proliferating macrophages	PSMC3IP
2.52802558378104E-18	1.32721281841608	0.521	0.153	4.23646527330026E-14	Proliferating macrophages	DAXX
2.65420982122935E-18	1.58468525716564	0.489	0.14	4.44792481841614E-14	Proliferating macrophages	KMT5A
3.01569077217692E-18	2.09752696149579	0.223	0.033	5.05369459601408E-14	Proliferating macrophages	BRCA2
3.73875825626334E-18	1.32885884850826	0.702	0.31	6.26541108584611E-14	Proliferating macrophages	DCK
4.01615753073148E-18	1.39695483231347	0.457	0.124	6.73027678999981E-14	Proliferating macrophages	PTPN7
4.14512739657563E-18	2.34751303556394	0.245	0.04	6.94640449118144E-14	Proliferating macrophages	SLC25A10
4.35982591759099E-18	1.83296244219605	0.319	0.065	7.30619627269897E-14	Proliferating macrophages	CENPX
4.38376229368727E-18	3.00195397037294	0.149	0.015	7.34630885176112E-14	Proliferating macrophages	MYB
5.05417226586045E-18	2.1335174063552	0.191	0.025	8.46978188312895E-14	Proliferating macrophages	FYN
6.12515374468612E-18	1.06651814552834	0.362	0.083	1.0264532645345E-13	Proliferating macrophages	PIM1
7.17509852054459E-18	1.56650314785877	0.404	0.104	1.20240301007286E-13	Proliferating macrophages	USP1
7.26856255930773E-18	1.37228461270673	0.713	0.306	1.21806571368879E-13	Proliferating macrophages	CSRP1
8.13357300365851E-18	3.20099193820385	0.106	0.007	1.36302416395309E-13	Proliferating macrophages	DEPRECATED-ENSG00000090889
8.57957403641805E-18	1.14881530513989	0.926	0.636	1.43776501702294E-13	Proliferating macrophages	PFN1
8.69155607794812E-18	2.1032977593566	0.149	0.015	1.45653096754255E-13	Proliferating macrophages	DDX11
9.27243830431088E-18	1.7187191433776	0.277	0.05	1.55387521103642E-13	Proliferating macrophages	FAM72C
1.03401874387386E-17	1.4161103766192	0.287	0.055	1.73280861098382E-13	Proliferating macrophages	ODF2
1.25616322312032E-17	1.88676465151783	0.553	0.186	2.10507832930503E-13	Proliferating macrophages	NDE1
1.59192014053098E-17	2.41973560371975	0.138	0.013	2.66773977150181E-13	Proliferating macrophages	TRIP13
1.71628306006535E-17	3.05022296298818	0.702	0.309	2.87614715205752E-13	Proliferating macrophages	WDFY4
1.72907187028361E-17	1.41281898717124	0.245	0.04	2.89757864022128E-13	Proliferating macrophages	DEPRECATED-ENSG00000102007
1.97958429295574E-17	1.6637657035236	0.457	0.139	3.31738735813523E-13	Proliferating macrophages	CDC25B
2.58027564259523E-17	1.5102455568459	0.883	0.531	4.32402592186108E-13	Proliferating macrophages	LSP1
2.88035334165063E-17	2.21688066278704	0.202	0.029	4.82689612993813E-13	Proliferating macrophages	CDC7
4.00638814647342E-17	1.8322315262185	0.181	0.023	6.71390525586016E-13	Proliferating macrophages	MYBL1
7.07855775625394E-17	1.16446343752042	0.436	0.122	1.18622470879303E-12	Proliferating macrophages	HNRNPR
8.11042723479363E-17	2.68523830033889	0.16	0.018	1.35914539600672E-12	Proliferating macrophages	CEP97
8.43003483244748E-17	1.82320859452349	0.394	0.106	1.41270523722155E-12	Proliferating macrophages	DHFR
9.81140992522613E-17	1.17001939016702	0.585	0.22	1.6441960752694E-12	Proliferating macrophages	LSM 4.00
1.11401541021797E-16	1.89542384790676	0.191	0.027	1.86686702444328E-12	Proliferating macrophages	ITGB7
1.12364235040176E-16	1.54884321208629	0.298	0.062	1.88299985080327E-12	Proliferating macrophages	SPINDOC
1.17457745979751E-16	1.73280220116472	0.319	0.072	1.96835690712866E-12	Proliferating macrophages	FZD2
1.27556183261142E-16	0.869486723140364	0.457	0.132	2.13758651909021E-12	Proliferating macrophages	SNRNP40
1.42575530293074E-16	1.12925223280969	0.628	0.241	2.38928073665134E-12	Proliferating macrophages	ITGAL
1.5849710700319E-16	2.51385265160012	0.128	0.012	2.65609451915946E-12	Proliferating macrophages	P2RY10
1.59859966174463E-16	1.28150974765235	0.543	0.189	2.67893331315165E-12	Proliferating macrophages	RPA1
1.70230233987702E-16	1.21019151997576	0.468	0.143	2.85271826116591E-12	Proliferating macrophages	DEPRECATED-ENSG00000179051
1.82788383466507E-16	2.99007392746117	0.149	0.016	3.06316773013173E-12	Proliferating macrophages	DSCC1
1.87879587294095E-16	2.19225783285886	0.191	0.027	3.14848612387444E-12	Proliferating macrophages	NEMP2
1.88127268193483E-16	1.27739021531408	0.532	0.173	3.15263676038639E-12	Proliferating macrophages	DNTTIP1
1.95342926047696E-16	1.51791559135238	0.426	0.12	3.27355675470729E-12	Proliferating macrophages	ANP32E
2.03386167742327E-16	1.03692426890929	0.787	0.375	3.40834539902592E-12	Proliferating macrophages	COTL1
2.27842171502062E-16	2.3196407601833	0.16	0.019	3.81817911003156E-12	Proliferating macrophages	RFC5

2.40626817097547E-16	0.891049593952424	0.394	0.104	4.0324242009207E-12	Proliferating macrophages	SMPD4
2.59777897116301E-16	1.24404544905966	0.266	0.05	4.35335799987497E-12	Proliferating macrophages	TSPAN6
2.82375560853248E-16	0.899714958630215	0.489	0.151	4.73204964877873E-12	Proliferating macrophages	OAS3
2.98955114644352E-16	1.99403216132699	0.213	0.034	5.00988981121005E-12	Proliferating macrophages	CEP152
3.50857858986685E-16	1.39085132875702	0.223	0.037	5.87967600089886E-12	Proliferating macrophages	DBF4B
3.57823078556759E-16	1.10639707422892	0.521	0.175	5.99639915045416E-12	Proliferating macrophages	SPN
3.75840028528234E-16	1.84791825870721	0.298	0.065	6.29832719807615E-12	Proliferating macrophages	POLH
4.54243977620037E-16	1.06038074732266	0.691	0.309	7.61222057695657E-12	Proliferating macrophages	PSMB9
5.67473436385949E-16	2.08788306954165	0.309	0.069	9.50971984695573E-12	Proliferating macrophages	PLP2
5.98185061264049E-16	1.14729700165504	0.287	0.06	1.00243852566629E-11	Proliferating macrophages	CEP128
6.28308461754679E-16	1.33663193575251	0.33	0.077	1.05291932020849E-11	Proliferating macrophages	C1orf112
6.2916874579003E-16	2.07818639566455	0.415	0.121	1.05436098419493E-11	Proliferating macrophages	NAV1
6.47408639282222E-16	1.701707264338	0.543	0.203	1.08492739770915E-11	Proliferating macrophages	ARL6IP1
6.68549902181862E-16	2.43063111564866	0.128	0.012	1.12035592607636E-11	Proliferating macrophages	DDIAS
8.73907484935424E-16	1.62935925589159	0.521	0.187	1.46449416325478E-11	Proliferating macrophages	TUBA1C
1.01247506846754E-15	0.749043812593036	0.33	0.076	1.69670571973791E-11	Proliferating macrophages	MSH5
1.06131141474667E-15	0.973401304148118	0.383	0.101	1.77854566883246E-11	Proliferating macrophages	TDP1
1.17493502652392E-15	2.48129165484192	0.202	0.033	1.96895611744878E-11	Proliferating macrophages	ICAM3
1.34516840096219E-15	4.12805531314032	0.138	0.015	2.25423320633244E-11	Proliferating macrophages	RAB30
1.35942447832823E-15	1.48505420838593	0.351	0.087	2.27812354078245E-11	Proliferating macrophages	MZT1
1.37208031561968E-15	1.49472646838236	0.191	0.029	2.29933219291546E-11	Proliferating macrophages	GINS3
1.41288841146755E-15	1.69888268652497	0.255	0.049	2.36771839993731E-11	Proliferating macrophages	NEDD1
1.4243613730856E-15	0.98083026319037	0.67	0.281	2.38694478901685E-11	Proliferating macrophages	DIAPH1
1.6091214593139E-15	1.10513435761765	0.936	0.634	2.69656574151823E-11	Proliferating macrophages	LCP1
1.67938582790896E-15	1.75905651608542	0.202	0.032	2.81431477040984E-11	Proliferating macrophages	POLE2
2.48599465231696E-15	1.04499103633446	0.351	0.087	4.16602983835276E-11	Proliferating macrophages	TPGS2
2.49906328344748E-15	1.31331726484672	0.447	0.133	4.18793025040128E-11	Proliferating macrophages	ERAP2
2.65068233565518E-15	1.67957824413525	0.245	0.047	4.44201345809095E-11	Proliferating macrophages	MMS22L
2.82330566851117E-15	1.99525932728519	0.181	0.026	4.73129563929103E-11	Proliferating macrophages	FANCD2
2.88268584041976E-15	1.20792761868335	0.734	0.359	4.83080493137543E-11	Proliferating macrophages	PKM
3.27342216370819E-15	1.14214270280344	0.351	0.089	5.48560086194218E-11	Proliferating macrophages	THOP1
3.30598212567844E-15	2.06426481699702	0.138	0.015	5.54016484621193E-11	Proliferating macrophages	PLK4
3.70110915807534E-15	1.43862350289337	0.426	0.134	6.20231872710266E-11	Proliferating macrophages	KPNA2
3.92029604789327E-15	0.992674267233036	0.213	0.036	6.56963211705954E-11	Proliferating macrophages	SCMH1
3.98894231177143E-15	1.05062823412606	0.489	0.161	6.68466952606657E-11	Proliferating macrophages	TAGLN2
4.07565054413809E-15	1.30352317252634	0.426	0.123	6.82997518186661E-11	Proliferating macrophages	PSME2
4.35147801151801E-15	1.5908235177685	0.457	0.142	7.29220685170188E-11	Proliferating macrophages	PALM2-AKAP2
4.37652193581672E-15	1.00251933553823	0.415	0.123	7.33417546004165E-11	Proliferating macrophages	MAD2L2
4.80453261064001E-15	1.08482927707687	0.723	0.312	8.05143574891053E-11	Proliferating macrophages	MRTFA
5.25995673976766E-15	1.07291581944569	0.34	0.083	8.81463550450265E-11	Proliferating macrophages	MAP4K4
5.31451986568171E-15	1.43392636816141	0.298	0.069	8.90607239090941E-11	Proliferating macrophages	NDC1
5.78771825102622E-15	1.21338707147008	0.479	0.153	9.69905824506974E-11	Proliferating macrophages	PYGO2
6.48337371882637E-15	1.07708042727774	0.351	0.088	1.08648376780092E-10	Proliferating macrophages	LYL1
6.68446629405563E-15	1.16286227736811	0.585	0.224	1.12018286155784E-10	Proliferating macrophages	ENSA
7.16261183466482E-15	1.73239233084417	0.117	0.011	1.20031049125313E-10	Proliferating macrophages	HIST1H3H
7.84312083559745E-15	1.53267232176128	0.245	0.048	1.31435018962942E-10	Proliferating macrophages	SPIN4
8.30653880188732E-15	1.19505214413527	0.372	0.105	1.39200977242028E-10	Proliferating macrophages	NUDT1
8.45712405269794E-15	1.14308859547979	0.447	0.142	1.41724484875112E-10	Proliferating macrophages	ICMT
8.5705164374109E-15	1.36453808022917	0.436	0.135	1.43624714458132E-10	Proliferating macrophages	ILF2
9.39899399938528E-15	1.59365191933699	0.574	0.207	1.57508341441698E-10	Proliferating macrophages	ID2
9.46183813903208E-15	1.38632704883937	0.596	0.252	1.585614835339E-10	Proliferating macrophages	FAM111A
9.46350901529581E-15	2.88524944464794	0.16	0.022	1.58589484078327E-10	Proliferating macrophages	TSPAN13
9.46607952883896E-15	1.08087319320489	0.713	0.301	1.58632560744283E-10	Proliferating macrophages	APOBR
9.89129755158991E-15	1.2475321988505	0.415	0.127	1.65758364369544E-10	Proliferating macrophages	PCNT
1.01055072835485E-14	1.22228332705965	0.638	0.283	1.69348091057705E-10	Proliferating macrophages	MCM5
1.04330024659946E-14	1.39033330602538	0.468	0.153	1.74836255325137E-10	Proliferating macrophages	ATP5MF
1.05471961854667E-14	3.35879328895882	0.106	0.009	1.76749913676051E-10	Proliferating macrophages	SLC24A4
1.0804594185848E-14	4.68909788426938	0.106	0.009	1.81063389366441E-10	Proliferating macrophages	GCSAM
1.08713888315697E-14	1.23654460520843	0.553	0.218	1.82182734039444E-10	Proliferating macrophages	PA2G4
1.09626087814622E-14	1.43084310432228	0.596	0.227	1.83711397959744E-10	Proliferating macrophages	DCP2
1.16034412607822E-14	1.36377841270064	0.436	0.135	1.94450468648189E-10	Proliferating macrophages	CEP192
1.16978915218366E-14	1.01111346052921	0.532	0.18	1.96033266122938E-10	Proliferating macrophages	PSMG2
1.22556165468254E-14	1.18838076890354	0.394	0.107	2.05379622091699E-10	Proliferating macrophages	TMEM97
1.23614320235985E-14	1.60233707328997	0.309	0.076	2.07152877851464E-10	Proliferating macrophages	POLE
1.26973296221676E-14	1.22386866632039	0.574	0.226	2.12781849808285E-10	Proliferating macrophages	MIS18BP1
1.32951627609127E-14	1.17772774065236	0.372	0.1	2.22800337547375E-10	Proliferating macrophages	PSTPIP1
1.41796464674265E-14	0.959115316052362	0.894	0.599	2.37622515501133E-10	Proliferating macrophages	CFL1
1.44292341679263E-14	1.02954751365744	0.574	0.21	2.41805106186109E-10	Proliferating macrophages	SMARCD2
1.45388844941901E-14	1.6689770047229	0.319	0.079	2.43642626353638E-10	Proliferating macrophages	INIP
1.49834628309783E-14	0.816039204357759	0.511	0.173	2.51092870121535E-10	Proliferating macrophages	DDX39A
1.53718711586854E-14	2.85189765203721	0.106	0.009	2.5760181687725E-10	Proliferating macrophages	MNS1

1.72535387874327E-14	1.32638431229375	0.415	0.129	2.89134802999796E-10	Proliferating macrophages	SAE1
1.90511721863089E-14	1.19419234960687	0.755	0.371	3.19259543498164E-10	Proliferating macrophages	GSTP1
1.98710513169419E-14	2.18625124866699	0.17	0.025	3.32999077969312E-10	Proliferating macrophages	ZNF90
2.00642276571265E-14	0.70652242493324	0.255	0.052	3.36236327078125E-10	Proliferating macrophages	BCL2L12
2.35692619038391E-14	1.48708482453868	0.287	0.065	3.94973690984535E-10	Proliferating macrophages	CEP85
2.36709380910887E-14	1.65533226760631	0.33	0.084	3.96677580530464E-10	Proliferating macrophages	DONSON
2.46669453231268E-14	2.68760268160867	0.138	0.016	4.13368669724958E-10	Proliferating macrophages	CCDC34
2.65349225773837E-14	1.06732507172749	0.5	0.175	4.44672232551796E-10	Proliferating macrophages	SNRPA
2.76548929763496E-14	0.906395204161785	0.255	0.053	4.63440696497667E-10	Proliferating macrophages	PPP2R3B
2.81033592789001E-14	1.31299857873672	0.213	0.038	4.70956094795807E-10	Proliferating macrophages	TMEM71
2.915390542065E-14	2.07321618260371	0.117	0.012	4.88561147039253E-10	Proliferating macrophages	DEPRECATED-ENSG00000136943
2.95990081224048E-14	4.49199074485488	0.138	0.017	4.96020178115259E-10	Proliferating macrophages	DUSP4
3.04108858757765E-14	1.10139116524881	0.34	0.087	5.09625625506263E-10	Proliferating macrophages	PFKP
3.16784019438466E-14	1.42440524031444	0.755	0.343	5.30866659774981E-10	Proliferating macrophages	SERPINB9
3.18644740919224E-14	0.47628600188395	0.479	0.152	5.33984856832435E-10	Proliferating macrophages	TTC39C
3.31480060540501E-14	1.09356553213564	0.681	0.283	5.55494285453772E-10	Proliferating macrophages	PPP1CA
3.34461906005893E-14	1.59078299613718	0.298	0.071	5.60491262084675E-10	Proliferating macrophages	MZT2A
3.5430190833143E-14	2.10585424241621	0.436	0.138	5.9373913798181E-10	Proliferating macrophages	KIF2A
3.59772916555111E-14	0.95422686029219	0.447	0.143	6.02907453563056E-10	Proliferating macrophages	POLE3
3.74221937784832E-14	1.31444321176212	0.362	0.1	6.27121123339821E-10	Proliferating macrophages	TUBG1
3.91312690201691E-14	1.58183664279722	0.33	0.087	6.55761806239994E-10	Proliferating macrophages	JPT1
3.91509244325665E-14	1.89262763441786	0.319	0.082	6.56091191640949E-10	Proliferating macrophages	DEPRECATED-ENSG00000167797
3.95825244265414E-14	1.24629975767585	0.266	0.056	6.63323944339982E-10	Proliferating macrophages	CDKN2A
4.3588477453519E-14	1.45113917649495	0.553	0.206	7.30455705166071E-10	Proliferating macrophages	PPM1G
4.50577108341335E-14	1.86012673893484	0.309	0.077	7.55077118158409E-10	Proliferating macrophages	RAB27A
4.51965163887116E-14	1.1625302469365	0.543	0.201	7.5740322164203E-10	Proliferating macrophages	PAXX
4.61465968775794E-14	1.16150419798392	0.287	0.067	7.73324670474476E-10	Proliferating macrophages	DARS2
4.81314110053414E-14	1.2721914112488	0.255	0.055	8.06586185627511E-10	Proliferating macrophages	DEPRECATED-ENSG00000188529
4.83455012996275E-14	1.21000046433061	0.543	0.192	8.10173910779157E-10	Proliferating macrophages	MDFIC
5.10884406727775E-14	0.99833338976573	0.489	0.163	8.56140088794405E-10	Proliferating macrophages	HDGF
5.15270685926697E-14	0.71287760900169	0.585	0.216	8.63490615475959E-10	Proliferating macrophages	NFATC2IP
5.25244711229541E-14	1.20402402048029	0.574	0.248	8.80205087078465E-10	Proliferating macrophages	PRPF19
5.33390962422363E-14	1.40022696906714	0.457	0.148	8.93856574827396E-10	Proliferating macrophages	SEPTIN6
5.36834545150129E-14	0.99650176722207	0.574	0.209	8.99627330762585E-10	Proliferating macrophages	CRYBG1
5.44974892383317E-14	0.65769592837725	0.362	0.099	9.13268924655962E-10	Proliferating macrophages	THOC3
5.73553355002308E-14	1.21075807750537	0.468	0.158	9.61160712312868E-10	Proliferating macrophages	RBMX
6.04693752171597E-14	1.00298214803057	0.681	0.285	1.01334578988916E-09	Proliferating macrophages	PPP4C
6.09391318330009E-14	1.7184071655152	0.234	0.047	1.02121797125743E-09	Proliferating macrophages	PAQR4
6.54977452560469E-14	1.6282885810976	0.234	0.047	1.09761121500083E-09	Proliferating macrophages	LMO7
6.70714063495927E-14	1.18932323222796	0.223	0.042	1.12398262760647E-09	Proliferating macrophages	FANCC
7.03621224186982E-14	2.45888637055035	0.277	0.067	1.17912844749254E-09	Proliferating macrophages	ADAM19
7.94425224817278E-14	0.88520108339436	0.702	0.32	1.3312977917488E-09	Proliferating macrophages	RAB8A
8.29823944481549E-14	1.34167815963531	0.277	0.062	1.39061896616218E-09	Proliferating macrophages	ZDHHC12
8.51222853871468E-14	1.43223229073117	0.617	0.243	1.42647925851781E-09	Proliferating macrophages	DEPRECATED-ENSG00000115738
1.00469146282978E-13	2.23043733722507	0.17	0.026	1.68366195341015E-09	Proliferating macrophages	CHAF1B
1.02416113481947E-13	1.58229134266371	0.34	0.094	1.71628922973047E-09	Proliferating macrophages	HLTF
1.04706888893132E-13	1.09178154639878	0.34	0.092	1.75467804407111E-09	Proliferating macrophages	CHEK2
1.07706848095794E-13	3.59767746195767	0.277	0.07	1.80495136038932E-09	Proliferating macrophages	RAB7B
1.12863477308608E-13	1.90539424905099	0.191	0.033	1.89136615273765E-09	Proliferating macrophages	CDAN1
1.15358917626399E-13	1.65503724342776	0.351	0.098	1.9331847415832E-09	Proliferating macrophages	CST7
1.21276492052991E-13	1.2994180024147	0.436	0.143	2.03235145382403E-09	Proliferating macrophages	DIPK2A
1.24672806692164E-13	1.95350620260157	0.202	0.036	2.08926689454728E-09	Proliferating macrophages	CBR3
1.41024573356648E-13	1.49543396577156	0.511	0.186	2.36328980031072E-09	Proliferating macrophages	CBL
1.41668522385746E-13	1.8636427269446	0.309	0.079	2.37408109814034E-09	Proliferating macrophages	TOMM34
1.52402210304514E-13	1.48791468653549	0.394	0.121	2.55395624028304E-09	Proliferating macrophages	CD52
1.55830461320088E-13	0.54982301838717	0.394	0.114	2.61140687080204E-09	Proliferating macrophages	RNGTT
1.61707338566105E-13	0.88798825775768	0.426	0.132	2.70989157969078E-09	Proliferating macrophages	ANKRD36C
1.69211426697243E-13	1.55665376327524	0.255	0.055	2.8356450885924E-09	Proliferating macrophages	PXMP4
1.69769609625539E-13	0.80540115416370	0.33	0.088	2.84499911810478E-09	Proliferating macrophages	EXOSC8
1.74094904894719E-13	1.29984126105464	0.351	0.098	2.9174824162257E-09	Proliferating macrophages	NUP107
1.80556819214168E-13	2.11719593266697	0.202	0.037	3.02577117639102E-09	Proliferating macrophages	WRAP53
1.83849792898228E-13	1.34566175685399	0.436	0.149	3.08095482938851E-09	Proliferating macrophages	NUP205
1.92073076728061E-13	1.22152115023387	0.553	0.208	3.21876061980885E-09	Proliferating macrophages	ACTR3
1.96119453670213E-13	1.05377840012074	0.489	0.167	3.28656980460543E-09	Proliferating macrophages	SNRPB
2.01996844478822E-13	2.0382495374408	0.287	0.071	3.3850631197761E-09	Proliferating macrophages	CCNE2
2.09900777645266E-13	0.96902374149439	0.67	0.312	3.51751723177937E-09	Proliferating macrophages	HNRNPD
2.1042969940207E-13	2.25702594143048	0.181	0.03	3.52638090257989E-09	Proliferating macrophages	DNAJC9
2.16841528260177E-13	1.41670424491073	0.521	0.205	3.63383033058405E-09	Proliferating macrophages	SSRP1
2.18056455696716E-13	1.01540857266961	0.298	0.072	3.65419008456557E-09	Proliferating macrophages	F2RL1
2.28655169419498E-13	1.15391791919667	0.617	0.269	3.83180332913195E-09	Proliferating macrophages	RAC2
2.61343716862351E-13	1.46079178396953	0.489	0.189	4.37959800717929E-09	Proliferating macrophages	CKAP5

2.66637775643692E-13	0.89063801489107	0.5	0.175	4.46831584423698E-09	Proliferating macrophages	NUP50
2.72487634295019E-13	1.85787243479584	0.394	0.127	4.56634777551593E-09	Proliferating macrophages	JPT2
2.76448958135249E-13	1.33568790237527	0.415	0.135	4.6327316404305E-09	Proliferating macrophages	TFDP1
2.88589148702621E-13	1.88214685296669	0.181	0.03	4.83617695395852E-09	Proliferating macrophages	ZEB1
3.09552419310075E-13	1.15214680395019	0.415	0.131	5.18747944279824E-09	Proliferating macrophages	DCTN3
3.30524244412837E-13	0.80472372568891	0.67	0.286	5.53892528787033E-09	Proliferating macrophages	SYMPK
3.39119174141497E-13	1.88260027240802	0.489	0.18	5.68295912026321E-09	Proliferating macrophages	NLRC5
3.40558150125167E-13	0.81356952477353	0.16	0.024	5.70707347979755E-09	Proliferating macrophages	BAHCC1
3.45569670881047E-13	1.38114243344546	0.266	0.062	5.79105654462458E-09	Proliferating macrophages	PAFAH1B3
3.5056049312599E-13	0.97655140917532	0.606	0.246	5.87469274380533E-09	Proliferating macrophages	RAB1B
3.56639815141517E-13	1.45637349380837	0.447	0.154	5.97657002214154E-09	Proliferating macrophages	PARP1
4.22877210268117E-13	0.79405840057618	0.553	0.223	7.0865762896731E-09	Proliferating macrophages	HAUS4
4.24831740308418E-13	1.75185378004491	0.298	0.075	7.11933030408847E-09	Proliferating macrophages	ESS2
4.38260065999042E-13	1.68102507364588	0.362	0.109	7.34436218601195E-09	Proliferating macrophages	PSMG3
4.50225763049997E-13	1.14247183201171	0.564	0.219	7.54488333719186E-09	Proliferating macrophages	TXNDC12
4.97376389577255E-13	1.02237366120879	0.66	0.329	8.33503353653565E-09	Proliferating macrophages	NUCKS1
5.00012916251039E-13	2.48191811792107	0.149	0.021	8.37921645053491E-09	Proliferating macrophages	FLNB
5.01454603496158E-13	1.3804493674751	0.245	0.053	8.40337624538861E-09	Proliferating macrophages	CEP78
5.08436215644101E-13	0.95919326674904	0.34	0.092	8.52037410176385E-09	Proliferating macrophages	ANG
5.24651125035347E-13	1.36874578586525	0.266	0.062	8.79210355334235E-09	Proliferating macrophages	RNF8
5.25397308233096E-13	0.42647680937329	0.351	0.098	8.80460809137023E-09	Proliferating macrophages	DEPDC5
5.29410348840991E-13	0.89608507407213	0.617	0.246	8.87185862587732E-09	Proliferating macrophages	SEPTIN2
5.6508852584266E-13	1.64527526219625	0.5	0.198	9.4697535160713E-09	Proliferating macrophages	MCM3
5.74780327133529E-13	1.72642933505101	0.245	0.054	9.63216872210367E-09	Proliferating macrophages	CETN3
5.85587473137606E-13	1.42808590228047	0.298	0.076	9.81327487484E-09	Proliferating macrophages	GMNN
6.02829289252904E-13	0.60623904356383	0.319	0.084	1.01022132293002E-08	Proliferating macrophages	POLA1
6.07419082623428E-13	1.51412474481276	0.149	0.021	1.01791289866034E-08	Proliferating macrophages	NUDT15
6.15467284958763E-13	1.79050118551318	0.287	0.073	1.0314000761339E-08	Proliferating macrophages	KATNA1
6.45102583672153E-13	0.88260531718308	0.213	0.041	1.08106290971779E-08	Proliferating macrophages	DBF4
6.50816064513644E-13	0.82760874184922	0.947	0.659	1.09063756091196E-08	Proliferating macrophages	VIM
6.55777425085493E-13	0.95449465333308	0.223	0.045	1.09895180895827E-08	Proliferating macrophages	HMGB3
6.69592641657119E-13	2.92514446025367	0.266	0.065	1.122103348889E-08	Proliferating macrophages	PLEKHM3
6.88299446850283E-13	0.71521564192744	0.564	0.218	1.1534522130317E-08	Proliferating macrophages	EFTUD2
6.96802449250753E-13	0.82457694580989	0.457	0.153	1.16770154445441E-08	Proliferating macrophages	CSTF3
6.97288188808271E-13	1.96701813671342	0.191	0.035	1.1685155468049E-08	Proliferating macrophages	ATAD3A
7.15159505500601E-13	0.90134395661349	0.394	0.126	1.19846429931791E-08	Proliferating macrophages	CARHSP1
7.6548709977584E-13	1.83819502778577	0.351	0.103	1.28280328180435E-08	Proliferating macrophages	PRMT1
8.23153488449938E-13	1.03296411381161	0.511	0.197	1.37944061594441E-08	Proliferating macrophages	CRKL
8.70423054393864E-13	1.22508413862119	0.468	0.162	1.45865495455324E-08	Proliferating macrophages	FIBP
8.76770505226471E-13	0.57102898078530	0.628	0.261	1.46929201265852E-08	Proliferating macrophages	EMP3
8.91109212983243E-13	0.98371578513640	0.436	0.15	1.49332081911732E-08	Proliferating macrophages	UBALD2
1.09378848711916E-12	1.94328302491191	0.234	0.052	1.83297074671428E-08	Proliferating macrophages	LRRCC1
1.09660705352294E-12	0.96018974898695	0.436	0.143	1.83769410029375E-08	Proliferating macrophages	DHX8
1.09681352066722E-12	1.84396490736257	0.245	0.055	1.83804009793413E-08	Proliferating macrophages	RMI1
1.1306889754979E-12	0.92983090561367	0.33	0.089	1.89480858513939E-08	Proliferating macrophages	RPIA
1.18060723851433E-12	1.5722713403639	0.84	0.513	1.97846161030231E-08	Proliferating macrophages	CPVL
1.18952677300043E-12	0.75949553192164	0.511	0.183	1.99340896619413E-08	Proliferating macrophages	GUCD1
1.2468479733012E-12	1.42695492332725	0.319	0.089	2.08946783365815E-08	Proliferating macrophages	DSN1
1.2680974705512E-12	1.00958561796106	0.606	0.265	2.12507774114969E-08	Proliferating macrophages	PAK1
1.30451764243888E-12	0.95884325801284	0.5	0.189	2.18611066519908E-08	Proliferating macrophages	FZR1
1.3438702499315E-12	1.07791360748051	0.383	0.119	2.25205776483521E-08	Proliferating macrophages	CCDC14
1.36584643611579E-12	0.47122919949985	0.447	0.149	2.28888545764283E-08	Proliferating macrophages	MGME1
1.36993257116732E-12	1.81752224262846	0.17	0.029	2.2957330027622E-08	Proliferating macrophages	RAVER2
1.43607795805248E-12	1.96944985751664	0.245	0.056	2.40657944210435E-08	Proliferating macrophages	FGFR1OP
1.46538226651021E-12	0.99737314207703	0.479	0.173	2.4556876022178E-08	Proliferating macrophages	LNPEP
1.46662508119556E-12	1.27728917386638	0.372	0.11	2.45777031106752E-08	Proliferating macrophages	CASP2
1.52710418598987E-12	1.59292984117256	0.447	0.153	2.55912119488183E-08	Proliferating macrophages	SLC25A28
1.55694506409276E-12	1.02776401719085	0.713	0.378	2.60912853840665E-08	Proliferating macrophages	HNRNPUL1
1.57608590230233E-12	1.40679648290071	0.319	0.089	2.64120475507824E-08	Proliferating macrophages	MDC 1.00
1.6429832371236E-12	2.00894243963453	0.223	0.049	2.75331130877173E-08	Proliferating macrophages	ECE1
1.68661385254447E-12	1.55432686129339	0.372	0.112	2.82642749409403E-08	Proliferating macrophages	CNTLN
1.68732410354934E-12	1.15072910281075	0.372	0.111	2.82761773272798E-08	Proliferating macrophages	ALG5
1.72093079231776E-12	1.32502740451902	0.255	0.061	2.8839358217661E-08	Proliferating macrophages	LRRC45
1.80413647396014E-12	0.98048663265369	0.404	0.133	3.0233719030624E-08	Proliferating macrophages	HAT1
1.98237873406886E-12	1.19484915095504	0.5	0.193	3.3220702825526E-08	Proliferating macrophages	SUPT16H
1.98508431162094E-12	1.034449112098	0.277	0.069	3.32660428941437E-08	Proliferating macrophages	ANP32B
2.05611294436445E-12	1.00935477439553	0.734	0.395	3.44563407216594E-08	Proliferating macrophages	PSMB8
2.17214768397337E-12	1.10618977415848	0.691	0.342	3.64008508880258E-08	Proliferating macrophages	SLC1A5
2.17377996897413E-12	1.02995449801989	0.287	0.074	3.64282047200684E-08	Proliferating macrophages	CBFA2T3
2.32682779142077E-12	2.8184762072528	0.223	0.05	3.89929801286293E-08	Proliferating macrophages	CADM1
2.35099258406899E-12	0.86749888087709	0.606	0.258	3.93979337238281E-08	Proliferating macrophages	PRKDC

2.43092569523297E-12	1.23859306115821	0.564	0.235	4.07374528007141E-08	Proliferating macrophages	PACS1
2.4483969192098E-12	0.699346093636811	0.638	0.29	4.10302355721179E-08	Proliferating macrophages	CBFB
2.6265041072674E-12	0.892236855488985	0.309	0.082	4.40149558295871E-08	Proliferating macrophages	ATG16L1
2.81604638463776E-12	0.724563081205077	0.404	0.127	4.71913053137595E-08	Proliferating macrophages	TAF11
2.84084918874107E-12	0.990026292863422	0.585	0.256	4.76069507049228E-08	Proliferating macrophages	NONO
2.88132219317285E-12	1.64891641876594	0.372	0.121	4.82851973131906E-08	Proliferating macrophages	MCM6
2.91744951907162E-12	0.833295794031127	0.255	0.059	4.88906190406021E-08	Proliferating macrophages	UBAP2
2.94494805893411E-12	1.05328721522989	0.426	0.139	4.93514395716179E-08	Proliferating macrophages	NSMAF
3.00288829686478E-12	0.890266136625244	0.553	0.224	5.03224020788599E-08	Proliferating macrophages	DEK
3.12804904360139E-12	2.61045083815718	0.106	0.012	5.2419845872672E-08	Proliferating macrophages	C4orf46
3.21118667540724E-12	2.11462080326689	0.202	0.04	5.38130663064744E-08	Proliferating macrophages	PARP2
3.68660555429768E-12	0.636008753605636	0.287	0.073	6.17801358789205E-08	Proliferating macrophages	TOP3A
4.61998877374362E-12	2.18037913256829	0.16	0.026	7.74217718703956E-08	Proliferating macrophages	PATJ
4.85359855970276E-12	0.899050294158522	0.415	0.139	8.13366046634988E-08	Proliferating macrophages	SLC38A1
5.12910033931711E-12	0.674178083803632	0.691	0.328	8.59534634862762E-08	Proliferating macrophages	FMNL1
5.130185494921E-12	0.492587202032219	0.67	0.28	8.59716485238862E-08	Proliferating macrophages	FUS
5.17758864964326E-12	1.75579394666384	0.213	0.045	8.67660305907218E-08	Proliferating macrophages	NT5DC2
5.30624647682393E-12	0.835301966397583	0.436	0.148	8.89220784586154E-08	Proliferating macrophages	SRRT
5.37827252765891E-12	1.8697014667904	0.16	0.027	9.0129091018508E-08	Proliferating macrophages	WDHD1
5.46994374508008E-12	0.701592557249321	0.585	0.237	9.1665317280052E-08	Proliferating macrophages	CNOT8
5.58879966684309E-12	0.838284049642655	0.67	0.313	9.36571048169566E-08	Proliferating macrophages	PTPA
5.87804844477925E-12	1.19427233883795	0.436	0.15	9.85043358376106E-08	Proliferating macrophages	TMEM11
6.16799534031923E-12	1.3837368168341	0.447	0.166	1.0336326591307E-07	Proliferating macrophages	SMC3
6.38824741459604E-12	0.82451004992896	0.479	0.176	1.070542501738E-07	Proliferating macrophages	PAF1
6.4102495150174E-12	1.2992138977476	0.362	0.113	1.07422961372662E-07	Proliferating macrophages	GLIPR2
6.4804017554964E-12	1.39920149126617	0.234	0.053	1.08598572618609E-07	Proliferating macrophages	NUP37
6.51339615686127E-12	1.82019769010469	0.17	0.03	1.09151492796681E-07	Proliferating macrophages	CENPQ
6.99934121817967E-12	0.819847264725334	0.521	0.203	1.17294960134255E-07	Proliferating macrophages	CKLF
7.5881031664943E-12	1.13336944924867	0.319	0.095	1.27161432864111E-07	Proliferating macrophages	RPA2
7.85842410730252E-12	1.11124943667841	0.436	0.151	1.31691471190176E-07	Proliferating macrophages	HDAC9
8.05917371423749E-12	1.18836522936413	0.404	0.134	1.35055633103192E-07	Proliferating macrophages	CHD3
8.39958111067311E-12	1.25231220873554	0.564	0.248	1.4076018025266E-07	Proliferating macrophages	CHCHD2
8.73920850792624E-12	2.07951314243256	0.404	0.137	1.46451656175828E-07	Proliferating macrophages	NIBAN1
8.93994199614479E-12	1.542580231235	0.319	0.092	1.49815547971394E-07	Proliferating macrophages	PSMB3
9.1673146625847E-12	0.75435632968602	0.585	0.257	1.53625859115594E-07	Proliferating macrophages	MNDA
9.32104693369265E-12	1.24249383734334	0.33	0.098	1.56202104514821E-07	Proliferating macrophages	TMEM160
9.41182138265814E-12	2.0195344588379	0.234	0.055	1.57723302730585E-07	Proliferating macrophages	METTL4
9.45523748345425E-12	0.91380761988358	0.532	0.21	1.58450869747726E-07	Proliferating macrophages	SCAF1
9.45541087647832E-12	2.63656727053353	0.106	0.012	1.58453775468024E-07	Proliferating macrophages	RGS3
9.55867934407268E-12	0.71416812960413	0.564	0.227	1.6018434844797E-07	Proliferating macrophages	ARID1A
1.01941419404767E-11	1.51571242249279	0.351	0.11	1.70833430638509E-07	Proliferating macrophages	SFXN1
1.07644079728522E-11	1.48358516107138	0.309	0.086	1.80389948809057E-07	Proliferating macrophages	ACD
1.12968810796966E-11	1.04815806021234	0.426	0.147	1.89313133133556E-07	Proliferating macrophages	ECI1
1.14167824283197E-11	0.741896886115076	0.532	0.204	1.91322439933782E-07	Proliferating macrophages	PPIA
1.16850512922511E-11	0.938137127741329	0.394	0.129	1.95818089555544E-07	Proliferating macrophages	ARPC5L
1.17598457470136E-11	1.39637370918036	0.16	0.027	1.97071495028453E-07	Proliferating macrophages	ENC1
1.22777204100256E-11	1.22972385492198	0.223	0.05	2.05750038631208E-07	Proliferating macrophages	CCDC167
1.23667866263589E-11	1.59889496203889	0.213	0.046	2.07242610284522E-07	Proliferating macrophages	RAD18
1.26096139060349E-11	0.293182063958393	0.468	0.167	2.11311909837333E-07	Proliferating macrophages	CMC2
1.27714109393718E-11	0.922119475657268	0.532	0.21	2.14023304521992E-07	Proliferating macrophages	SUB1
1.32201349025481E-11	0.882206558044577	0.543	0.226	2.21543020696901E-07	Proliferating macrophages	SNRPC
1.32858982459236E-11	0.96375579787079	0.362	0.112	2.22645082805187E-07	Proliferating macrophages	PARM1
1.37990962570879E-11	0.914464417273308	0.766	0.435	2.31245255076279E-07	Proliferating macrophages	HNRNPF
1.41794260002117E-11	2.04388698721122	0.191	0.039	2.37618820911548E-07	Proliferating macrophages	SAPCD1
1.43380653725822E-11	1.06645718878708	0.16	0.027	2.40277299513732E-07	Proliferating macrophages	ZNF714
1.52505911003526E-11	1.06961290900429	0.532	0.216	2.5556940565971E-07	Proliferating macrophages	DNAJC8
1.64409513427982E-11	2.38578795828906	0.245	0.062	2.75517462602612E-07	Proliferating macrophages	CCND1
1.91427709070354E-11	0.939171594485103	0.277	0.073	3.207945548601E-07	Proliferating macrophages	ANAPC15
2.01585884153276E-11	0.485837235528013	0.447	0.155	3.3781762466406E-07	Proliferating macrophages	FAM122B
2.04045269020375E-11	0.539701905858739	0.532	0.212	3.41939061824344E-07	Proliferating macrophages	SET
2.07342513664825E-11	1.12697334141518	0.5	0.208	3.47464584399514E-07	Proliferating macrophages	PSMC3
2.14134383242528E-11	0.893271274167254	0.33	0.096	3.58846399437829E-07	Proliferating macrophages	RUVBL1
2.33065675157826E-11	2.71975621135062	0.319	0.098	3.90571458429484E-07	Proliferating macrophages	TRAF1
2.36732539844459E-11	1.43345224999103	0.149	0.024	3.96716390271344E-07	Proliferating macrophages	LRP8
2.43501313012108E-11	0.663090195534956	0.787	0.42	4.08059500345691E-07	Proliferating macrophages	HNRNPA0
2.48090153491806E-11	1.05214883544526	0.426	0.149	4.15749479221569E-07	Proliferating macrophages	SRSF9
2.61737849222772E-11	1.07590470661142	0.468	0.175	4.38620287727521E-07	Proliferating macrophages	CIC
2.65213135555391E-11	1.4172380599716	0.34	0.105	4.44444172563724E-07	Proliferating macrophages	HMCES
2.71039131334242E-11	0.855945556859263	0.926	0.679	4.54207376289922E-07	Proliferating macrophages	TMSB10
2.77578138432464E-11	1.38090852458497	0.255	0.066	4.65165444385123E-07	Proliferating macrophages	EMC9
2.83749770852216E-11	1.49804499756618	0.245	0.059	4.75507865994143E-07	Proliferating macrophages	MFSD6

2.85048096207626E-11	1.36263709224926	0.362	0.117	4.77683599624739E-07	Proliferating macrophages	PRPSAP1
2.94497463505598E-11	0.605682353502743	0.649	0.303	4.93518849342681E-07	Proliferating macrophages	ARHGDIA
2.97088073536526E-11	1.83609250161182	0.277	0.076	4.97860193632511E-07	Proliferating macrophages	PTPN22
2.99403765407324E-11	1.87373520838201	0.16	0.028	5.01740830069594E-07	Proliferating macrophages	GINS2
3.10389568399257E-11	0.881745240615155	0.277	0.074	5.20150838723475E-07	Proliferating macrophages	BCL2A1
3.22241049241888E-11	1.1147418273909	0.383	0.129	5.40011550319556E-07	Proliferating macrophages	SAMD1
3.25961729584253E-11	0.844605328566423	0.83	0.477	5.46246666437291E-07	Proliferating macrophages	UCP2
3.29605224625087E-11	1.63879381922399	0.149	0.025	5.5235243542672E-07	Proliferating macrophages	LPL
3.31834885661085E-11	0.396143737447565	0.394	0.129	5.56088901390846E-07	Proliferating macrophages	COQ2
3.32233928479227E-11	0.974857602348354	0.564	0.246	5.56757617345489E-07	Proliferating macrophages	AP1M1
3.33972385579712E-11	0.70087650641131	0.383	0.126	5.59670923754481E-07	Proliferating macrophages	CCAR2
3.41601939757929E-11	1.11533883613143	0.298	0.082	5.72456530646337E-07	Proliferating macrophages	SMARCD1
3.53180475105231E-11	1.1834012942294	0.447	0.167	5.91859840181346E-07	Proliferating macrophages	S100B
3.58740904557562E-11	1.28120682880467	0.426	0.162	6.01178007857563E-07	Proliferating macrophages	PIK3CG
3.85063039524736E-11	0.858354562748901	0.521	0.211	6.45288641635552E-07	Proliferating macrophages	SRRM1
3.98411326481802E-11	1.82131437956588	0.287	0.082	6.67657700918203E-07	Proliferating macrophages	PMM1
4.22942123120373E-11	1.60638107805645	0.138	0.022	7.08766409925121E-07	Proliferating macrophages	TMEM121B
4.23343142367874E-11	0.862552712966458	0.372	0.123	7.09438437980083E-07	Proliferating macrophages	PPP2R5D
4.29281796126618E-11	2.41056835911325	0.138	0.022	7.19390433948987E-07	Proliferating macrophages	GNAO1
4.31045292555969E-11	1.05423147508722	0.936	0.819	7.22345701265294E-07	Proliferating macrophages	CST3
4.41047641754942E-11	1.57101286970154	0.309	0.093	7.39107638052931E-07	Proliferating macrophages	ALYREF
4.63697443175822E-11	0.796683171708086	0.426	0.148	7.77064175274043E-07	Proliferating macrophages	TAF15
4.68003753106866E-11	1.00107673732591	0.617	0.288	7.84280689456486E-07	Proliferating macrophages	UQCRC1
4.77537494970181E-11	2.43588235743728	0.191	0.041	8.0025733407103E-07	Proliferating macrophages	DEPRECATED-ENSG00000196757
4.81693996605003E-11	0.732601775778249	0.255	0.065	8.07222799510664E-07	Proliferating macrophages	FAM110A
4.81741633040003E-11	0.489988224040449	0.34	0.103	8.07302628648436E-07	Proliferating macrophages	LRRC40
4.83320983885121E-11	2.14069400381833	0.117	0.016	8.09949304794685E-07	Proliferating macrophages	CEP76
4.96024326613927E-11	1.79736160879357	0.309	0.093	8.3123756653962E-07	Proliferating macrophages	ADAM8
5.02392712739106E-11	1.32635482277619	0.33	0.101	8.41909708008194E-07	Proliferating macrophages	AIDA
5.12826115294749E-11	1.06184899449654	0.181	0.036	8.59394004010941E-07	Proliferating macrophages	SMTN
5.28523551968245E-11	3.66307067708787	0.17	0.034	8.85699768388385E-07	Proliferating macrophages	GPR157
5.6758166804889E-11	2.09115392170039	0.17	0.032	9.5115335931633E-07	Proliferating macrophages	POGLUT3
5.78452397403174E-11	1.64270908557796	0.255	0.067	9.69370527568238E-07	Proliferating macrophages	SUSD3
5.91752240462557E-11	0.942137373003415	0.489	0.191	9.91658404567153E-07	Proliferating macrophages	STIM2
5.95849473296371E-11	0.812358357106185	0.734	0.389	9.98524547350058E-07	Proliferating macrophages	ARF6
5.9651116421238E-11	0.899132216223402	0.33	0.1	9.99633408987106E-07	Proliferating macrophages	REEP4
6.10735939149149E-11	0.667778010494492	0.649	0.306	1.02347128682614E-06	Proliferating macrophages	SUMO3
6.14847583816244E-11	0.915821281963248	0.404	0.14	1.03036158095926E-06	Proliferating macrophages	CLNS1A
6.54624825932146E-11	0.396560459690976	0.617	0.272	1.09702028329709E-06	Proliferating macrophages	HMGN1
6.59615730266044E-11	2.08172581468255	0.245	0.063	1.10538404077984E-06	Proliferating macrophages	PIM2
6.62725974786099E-11	2.60294103796358	0.138	0.022	1.11059618854654E-06	Proliferating macrophages	CYFIP2
6.82523301529976E-11	0.384585771501013	0.404	0.138	1.14377254870393E-06	Proliferating macrophages	NUTF2
7.06651116797461E-11	0.802297819822593	0.309	0.092	1.18420594152918E-06	Proliferating macrophages	SF3A3
7.0706933408568E-11	1.2903457721985	0.5	0.203	1.18490679006078E-06	Proliferating macrophages	DAZAP1
7.21716662037731E-11	0.609068005114471	0.691	0.334	1.20945278224283E-06	Proliferating macrophages	SUGP2
7.83910960937726E-11	0.651417833666623	0.362	0.119	1.31367798833944E-06	Proliferating macrophages	CDK5RAP2
8.13011116874423E-11	1.39091325401066	0.479	0.196	1.36244402965816E-06	Proliferating macrophages	PRRC2A
8.17908093294272E-11	0.673164198290302	0.5	0.196	1.37065038274254E-06	Proliferating macrophages	KHDRBS1
8.22401130253839E-11	3.40197186856964	0.128	0.019	1.37817981407938E-06	Proliferating macrophages	IDO2
8.3578993303209E-11	0.928143009495846	0.681	0.343	1.40061676977518E-06	Proliferating macrophages	PSMB10
8.41124518773021E-11	1.14281835645461	0.415	0.156	1.40955646855983E-06	Proliferating macrophages	MZT2B
8.46025508188426E-11	1.0582452235985	0.372	0.126	1.41776954662216E-06	Proliferating macrophages	MTMR1
8.7289719438058E-11	0.981733618329073	0.457	0.173	1.46280111834298E-06	Proliferating macrophages	FLNA
8.83130840799709E-11	1.03432362045683	0.213	0.049	1.47995066301215E-06	Proliferating macrophages	CX3CR1
8.84426179435483E-11	1.24413986273589	0.415	0.149	1.48212139149798E-06	Proliferating macrophages	CCDC12
8.94255724957741E-11	1.32389330347298	0.468	0.181	1.49859374388418E-06	Proliferating macrophages	MYADM
9.0831157141192E-11	0.960147770819311	0.287	0.082	1.5221485313721E-06	Proliferating macrophages	MAZ
9.08889256375168E-11	0.907571992259117	0.489	0.195	1.52311661583351E-06	Proliferating macrophages	AIP
9.30254734314443E-11	1.64170187820781	0.213	0.049	1.55892088376414E-06	Proliferating macrophages	ZNF92
9.39419209147299E-11	0.968066773448507	0.468	0.184	1.57427871068904E-06	Proliferating macrophages	MTHFD1
9.57889733566365E-11	0.848503637015173	0.223	0.054	1.60523161551051E-06	Proliferating macrophages	CD109
9.94836057193525E-11	0.804987019710911	0.628	0.306	1.66714626464491E-06	Proliferating macrophages	NUP62
1.00432641440806E-10	1.6183672982197	0.202	0.045	1.68305020526502E-06	Proliferating macrophages	GNAI1
1.01592864439802E-10	0.59086646254925	0.5	0.196	1.70249322228221E-06	Proliferating macrophages	HDGFL2
1.04462692281285E-10	1.64048037603645	0.202	0.045	1.75058579724977E-06	Proliferating macrophages	SLC7A1
1.0489302610191E-10	1.41613188406978	0.106	0.013	1.7577973314158E-06	Proliferating macrophages	PBK
1.10020387244123E-10	0.612924679313011	0.521	0.21	1.84372164943702E-06	Proliferating macrophages	KBTBD2
1.10667680825087E-10	0.615559239211018	0.415	0.147	1.85456899526681E-06	Proliferating macrophages	CHERP
1.16113198208115E-10	1.40782423176284	0.394	0.142	1.94582497557159E-06	Proliferating macrophages	ZNHIT1
1.23061278262586E-10	1.00821443908952	0.223	0.053	2.06226090112441E-06	Proliferating macrophages	DGCR8
1.25714556891889E-10	0.529947610954296	0.479	0.182	2.10672454439427E-06	Proliferating macrophages	ZNF384

1.26268872489234E-10	0.99547109965900§	0.234	0.059	2.11601376517458E-06	Proliferating macrophages	NPM3
1.28393203431201E-10	0.81631397790030€	0.277	0.076	2.15161330310007E-06	Proliferating macrophages	TMEM209
1.29918323182114E-10	0.74108287944132€	0.394	0.134	2.17717125988586E-06	Proliferating macrophages	FARP2
1.36316720273496E-10	0.97352285077980€	0.287	0.081	2.28439559834325E-06	Proliferating macrophages	CNOT3
1.38166161939121E-10	0.74592697414346Ź	0.436	0.169	2.31538854177579E-06	Proliferating macrophages	HNRNPLL
1.39858441491997E-10	0.62718319960947	0.67	0.332	2.34374776252289E-06	Proliferating macrophages	TRIR
1.42714470132164E-10	1.11248669503547	0.202	0.045	2.3916090904748E-06	Proliferating macrophages	RAB11FIP4
1.47049613477616E-10	1.65771138899249	0.606	0.271	2.46425742265789E-06	Proliferating macrophages	NUB1
1.4826245584244E-10	0.76609072400684∠	0.223	0.052	2.48458223500761E-06	Proliferating macrophages	CPB2
1.50728527188946E-10	0.41944677564038Ź	0.468	0.177	2.52590865863236E-06	Proliferating macrophages	SART3
1.52012508195118E-10	1.14197658060043	0.181	0.038	2.54742561233378E-06	Proliferating macrophages	SLX4
1.53560667372598E-10	0.75831548129130Ź	0.394	0.135	2.57336966383001E-06	Proliferating macrophages	KPNB1
1.54117087157878E-10	1.3638902639441	0.67	0.342	2.58269414659172E-06	Proliferating macrophages	CYB5R3
1.56860922515574E-10	0.93435119815661€	0.564	0.257	2.62867533951599E-06	Proliferating macrophages	FOXN2
1.62460054565686E-10	3.38350509223853	0.149	0.027	2.72250559441177E-06	Proliferating macrophages	TOX
1.63119607831334E-10	1.46023136693997	0.149	0.026	2.73355838803749E-06	Proliferating macrophages	C18orf54
1.66127274557468E-10	1.16004300610749	0.426	0.152	2.78396086703405E-06	Proliferating macrophages	QSER1
1.721135281567E-10	1.00680471769976	0.191	0.042	2.88427850484998E-06	Proliferating macrophages	TBC1D10C
1.73654691117059E-10	1.22771412685243	0.149	0.026	2.91010531373967E-06	Proliferating macrophages	EPS8L2
1.80637564514139E-10	1.65101195796582	0.213	0.051	3.02712430612794E-06	Proliferating macrophages	PDLIM7
1.86974700875406E-10	0.80405992359340	0.83	0.46	3.13332203727005E-06	Proliferating macrophages	ITGA4
1.97675038248635E-10	0.53148553663115€	0.606	0.268	3.31263829097063E-06	Proliferating macrophages	MYH9
2.00540229042142E-10	1.01924630151563	0.436	0.17	3.36065315828822E-06	Proliferating macrophages	R3HDM4
2.07463360096184E-10	0.71657914347221§	0.521	0.216	3.47667098849186E-06	Proliferating macrophages	TBCB
2.11771746729652E-10	0.88186680534644Ź	0.543	0.235	3.54887093169552E-06	Proliferating macrophages	ATG3
2.28628702724304E-10	0.88825923352872€	0.564	0.262	3.83135980025388E-06	Proliferating macrophages	ELAVL1
2.28671547212308E-10	1.48484752287363	0.319	0.103	3.83207778818386E-06	Proliferating macrophages	PLPP1
2.34184778063347E-10	1.15705186115324	0.33	0.109	3.92446851078557E-06	Proliferating macrophages	RFC2
2.4658275184427E-10	0.39372732802402ἓ	0.5	0.189	4.13223375540628E-06	Proliferating macrophages	DEPRECATED-ENSG00000106538
2.49965789645953E-10	0.66809372840760ï	0.298	0.088	4.18892670288688E-06	Proliferating macrophages	CZIB
2.52079418930098E-10	1.00353298655235	0.245	0.063	4.22434690243059E-06	Proliferating macrophages	IFI27L1
2.54185495731766E-10	0.72784145633817∠	0.362	0.118	4.25964053747294E-06	Proliferating macrophages	LSM 1.00
2.74582541746122E-10	2.25305159508318	0.149	0.027	4.6014542345815E-06	Proliferating macrophages	LDLRAD3
2.76905413528724E-10	1.2602203153697	0.266	0.075	4.64038091991436E-06	Proliferating macrophages	PLGRKT
2.83762807499749E-10	0.81403439626353Ś	0.266	0.073	4.7552971280808E-06	Proliferating macrophages	WDR61
2.84528595119249E-10	0.72442587205112Ś	0.596	0.277	4.76813019700838E-06	Proliferating macrophages	MIF
2.91713947750628E-10	1.08392640606464	0.383	0.13	4.88854233640502E-06	Proliferating macrophages	KLHL22
3.02575538387877E-10	0.58578205395218§	0.521	0.21	5.07056087230405E-06	Proliferating macrophages	APEH
3.1554247061027E-10	0.99849725684674Ź	0.457	0.179	5.2878607224869E-06	Proliferating macrophages	BUD23
3.19459416107261E-10	0.93177013869129ï	0.66	0.307	5.35350089512549E-06	Proliferating macrophages	CDC42SE2
3.2056388282828E-10	0.88204994195078	0.5	0.213	5.37200954843632E-06	Proliferating macrophages	TMEM19
3.21080967748628E-10	0.52051262147387ï	0.532	0.231	5.3806748575315E-06	Proliferating macrophages	KDM3B
3.26911097532651E-10	0.83126519508984Ś	0.681	0.349	5.47837617245216E-06	Proliferating macrophages	DSE
3.33443368821458E-10	1.27678488007117	0.202	0.047	5.58784397471E-06	Proliferating macrophages	CXXC5
3.40754120713252E-10	0.78532866255080	0.734	0.426	5.71035755491269E-06	Proliferating macrophages	CALM2
3.53318877564113E-10	1.32735202157149	0.394	0.152	5.9209177502194E-06	Proliferating macrophages	YWHAQ
3.6570222565283E-10	1.13265080331943	0.372	0.133	6.12843789749013E-06	Proliferating macrophages	TOR3A
3.66079224377159E-10	0.62424252603390	0.404	0.145	6.13475564211243E-06	Proliferating macrophages	NUP133
3.71010241460857E-10	0.59234693449937Ź	0.543	0.225	6.21738962640105E-06	Proliferating macrophages	RPN2
3.71650163069951E-10	0.96156049879567	0.362	0.122	6.22811343272625E-06	Proliferating macrophages	NHP2
3.8142490555335E-10	1.21616885996529	0.415	0.155	6.39191856726304E-06	Proliferating macrophages	DUSP23
3.96431594137659E-10	1.09833784429823	0.266	0.075	6.64340065455889E-06	Proliferating macrophages	SNRPE
4.0060701854744E-10	0.6234358277635	0.617	0.284	6.71337241681799E-06	Proliferating macrophages	TMED10
4.0255946073863E-10	0.75923441845741Ś	0.457	0.183	6.74609144305796E-06	Proliferating macrophages	RCC2
4.02943318854235E-10	2.75042421460047	0.106	0.015	6.75252413735926E-06	Proliferating macrophages	PTTG2
4.04526986906165E-10	0.80666079671430Ś	0.426	0.153	6.7790632465735E-06	Proliferating macrophages	NUCB2
4.14145218471002E-10	2.20092930165638	0.149	0.027	6.94024557113704E-06	Proliferating macrophages	ZNF320
4.14580313637661E-10	1.12340645702378	0.255	0.07	6.94753689593993E-06	Proliferating macrophages	PFAS
4.19835620791018E-10	0.51267756417796Ś	0.489	0.197	7.03560533321588E-06	Proliferating macrophages	SNX20
4.21958967957136E-10	0.62928023479252	0.17	0.035	7.07118838502568E-06	Proliferating macrophages	ATP2A3
4.23062269400317E-10	0.89267461800190ἓ	0.723	0.357	7.08967751061051E-06	Proliferating macrophages	PTPN1
4.2373759473922E-10	0.71544196162351ἓ	0.511	0.217	7.10099461263984E-06	Proliferating macrophages	TYK2
4.2926623945567E-10	1.80903440197106	0.16	0.031	7.19364364079811E-06	Proliferating macrophages	PAK4
4.41573084294361E-10	1.13135607929365	0.606	0.288	7.3998817466049E-06	Proliferating macrophages	SNX3
4.5544831397224E-10	0.77436213597962ἓ	0.234	0.06	7.63240284554679E-06	Proliferating macrophages	POLR3K
4.73217169127156E-10	0.59127115069052	0.521	0.212	7.93017332023288E-06	Proliferating macrophages	BAZ1B
4.80855306140208E-10	1.16555543943626	0.223	0.056	8.05817322029761E-06	Proliferating macrophages	FANCL
5.01247146862307E-10	0.50761004032701	0.5	0.204	8.39989968711855E-06	Proliferating macrophages	TRABD
5.02306698978095E-10	0.92542543613371§	0.266	0.075	8.41765566147491E-06	Proliferating macrophages	SRSF1
5.16974669837647E-10	0.90102146434410Ź	0.266	0.075	8.66346151713929E-06	Proliferating macrophages	EXOSC3
5.17589818785933E-10	0.50368189166901Ź	0.457	0.178	8.67377018321467E-06	Proliferating macrophages	DYRK1A

5.24511168822665E-10	0.351679278397454	0.681	0.34	8.78975816713023E-06	Proliferating macrophages	ILF3
5.35040488850074E-10	0.788647960842976	0.66	0.356	8.96620851214954E-06	Proliferating macrophages	TMEM109
5.37254143993977E-10	2.27793436148704	0.117	0.018	9.00330494505107E-06	Proliferating macrophages	RPA3
5.39520711319356E-10	0.499439666351778	0.245	0.065	9.04128808028977E-06	Proliferating macrophages	SRSF10
5.42524548444497E-10	1.31659557846659	0.383	0.136	9.09162638283289E-06	Proliferating macrophages	SRPK1
5.50936739306732E-10	0.665578639170183	0.372	0.132	9.23259787730222E-06	Proliferating macrophages	PIN1
5.61700516497587E-10	1.29846756278645	0.362	0.125	9.41297725546656E-06	Proliferating macrophages	UPF2
5.65165511876895E-10	0.522274767892517	0.298	0.09	9.47104364803301E-06	Proliferating macrophages	GABPB1
5.84923701404697E-10	0.865080342365461	0.202	0.047	9.80215138813992E-06	Proliferating macrophages	DEPRECATED-ENSG00000258366
5.99076716824746E-10	0.678238131254318	0.532	0.224	1.00393276205491E-05	Proliferating macrophages	COX6A1
6.07159937595239E-10	0.291223635004008	0.532	0.223	1.0174786234221E-05	Proliferating macrophages	STAG1
6.08414436273996E-10	0.790307733291733	0.287	0.087	1.01958091230796E-05	Proliferating macrophages	POLD3
6.1922363050026E-10	1.49957549690663	0.33	0.11	1.03769495999234E-05	Proliferating macrophages	TOPBP1
6.20914910227403E-10	1.88761432863956	0.298	0.094	1.04052920655908E-05	Proliferating macrophages	FCHO1
6.23263010750093E-10	1.34613577631364	0.16	0.032	1.04446415341501E-05	Proliferating macrophages	HIST3H2BB
6.30494353499713E-10	1.20353815922049	0.298	0.091	1.05658243759482E-05	Proliferating macrophages	CEP83
6.36110450337533E-10	0.835057692287824	0.34	0.115	1.06599389267564E-05	Proliferating macrophages	APOBEC3C
6.4206109792713E-10	1.8522463439397	0.287	0.093	1.07596598790628E-05	Proliferating macrophages	CCSER1
6.45287044613171E-10	0.520303795947751	0.436	0.173	1.08137202936275E-05	Proliferating macrophages	PNRC2
6.51028339772657E-10	0.628641514494023	0.277	0.081	1.09099329179102E-05	Proliferating macrophages	RIOK1
6.67496257217012E-10	1.00535807433115	0.543	0.243	1.11859022784427E-05	Proliferating macrophages	ELOVL5
6.79886804549412E-10	0.631910093746823	0.436	0.166	1.13935430706391E-05	Proliferating macrophages	ARAF
6.85095983509535E-10	1.12440266537549	0.606	0.279	1.14808384916528E-05	Proliferating macrophages	PLCG2
6.92131006859329E-10	0.519405802577599	0.713	0.365	1.15987314129486E-05	Proliferating macrophages	EZR
7.04362077548545E-10	0.987576892818171	0.415	0.158	1.18036996555585E-05	Proliferating macrophages	ADSL
7.13145475501131E-10	0.846120107800069	0.287	0.085	1.1950891878448E-05	Proliferating macrophages	MRE11
7.28470088156228E-10	1.38947413394695	0.223	0.058	1.22077017373221E-05	Proliferating macrophages	FBF1
7.52056094765169E-10	0.589464954567592	0.628	0.306	1.26029560360747E-05	Proliferating macrophages	ACTN4
7.53353441871972E-10	1.05469745623991	0.266	0.077	1.26246969788905E-05	Proliferating macrophages	SNRPG
7.54269227243435E-10	0.463035250717449	0.564	0.244	1.26400437101455E-05	Proliferating macrophages	NSFL1C
7.63665991041046E-10	0.814091176801104	0.404	0.153	1.27975146778658E-05	Proliferating macrophages	DCAF15
7.71425723235954E-10	0.836592027951796	0.309	0.096	1.29275522699881E-05	Proliferating macrophages	NUBP2
7.78541292171507E-10	0.81635818676912	0.596	0.277	1.30467949742101E-05	Proliferating macrophages	ARPC5
8.07340875354505E-10	0.375775755461366	0.362	0.122	1.35294183891908E-05	Proliferating macrophages	FBXO46
8.46157202156371E-10	0.400772638620386	0.681	0.323	1.41799023937365E-05	Proliferating macrophages	IKZF1
8.46494191116893E-10	0.93266024454249	0.415	0.158	1.41855496547369E-05	Proliferating macrophages	LPCAT3
8.6395867834352E-10	0.582423446163268	0.468	0.185	1.44782195316807E-05	Proliferating macrophages	PTBP1
8.65983150490615E-10	1.20680715786948	0.223	0.058	1.45121456359217E-05	Proliferating macrophages	IQCE
8.84907257681482E-10	1.38212522402132	0.17	0.036	1.48292758242263E-05	Proliferating macrophages	PPAT
9.11530593907739E-10	2.11499010711989	0.255	0.073	1.52754296927059E-05	Proliferating macrophages	ST7
9.20498355746648E-10	0.683475400747849	0.511	0.221	1.54257114456023E-05	Proliferating macrophages	ATP1A1
9.45135470144344E-10	1.5540601420138	0.138	0.025	1.58385802086789E-05	Proliferating macrophages	RBBP8
9.65511201008051E-10	0.895804050102226	0.277	0.082	1.61800367064929E-05	Proliferating macrophages	PSIP1
9.94459714850581E-10	1.26805284236464	0.17	0.036	1.6665155901466E-05	Proliferating macrophages	CDK2AP2
1.07252953308205E-09	0.488609925218982	0.532	0.231	1.7973449915389E-05	Proliferating macrophages	CYB5B
1.09816798130067E-09	0.723219272012922	0.67	0.344	1.84030990306366E-05	Proliferating macrophages	ARPC4
1.10563332582257E-09	0.277874139097358	0.574	0.243	1.85282032741346E-05	Proliferating macrophages	BTAF1
1.12362068689252E-09	0.686203417561803	0.298	0.092	1.88296354709449E-05	Proliferating macrophages	SCML1
1.16466545121704E-09	0.731783945200351	0.426	0.163	1.95174636314952E-05	Proliferating macrophages	DEPRECATED-ENSG00000073578
1.18314537525209E-09	0.465597437197798	0.681	0.345	1.98271501984746E-05	Proliferating macrophages	YWHAZ
1.24063812250353E-09	2.02515117188543	0.191	0.045	2.07906136569142E-05	Proliferating macrophages	BCL11A
1.24810921845997E-09	0.526710569359289	0.213	0.054	2.09158142829523E-05	Proliferating macrophages	HIVEP3
1.25497564925226E-09	1.30995637227201	0.16	0.032	2.10308819301693E-05	Proliferating macrophages	SGMS2
1.27293605950651E-09	0.616207282135106	0.521	0.224	2.13318624852101E-05	Proliferating macrophages	SERBP1
1.27637048552033E-09	0.765303083839084	0.638	0.29	2.13894165963496E-05	Proliferating macrophages	DOCK10
1.2767042088272E-09	0.964176103427189	0.628	0.312	2.13950091315262E-05	Proliferating macrophages	TSPAN33
1.28920880840274E-09	1.41970904132403	0.309	0.101	2.16045612112132E-05	Proliferating macrophages	MCOLN2
1.31005479463905E-09	0.897659406852859	0.34	0.119	2.19538982485613E-05	Proliferating macrophages	ERAL1
1.32348437984408E-09	0.653284267190488	0.596	0.276	2.21789512374271E-05	Proliferating macrophages	LAT2
1.3407576785733E-09	1.51079244678429	0.266	0.079	2.24684171775313E-05	Proliferating macrophages	ELP6
1.34266786928658E-09	1.38996970971774	0.372	0.133	2.25004281535045E-05	Proliferating macrophages	PDE7A
1.37130822553505E-09	0.800559813137237	0.468	0.193	2.29803832435164E-05	Proliferating macrophages	PNN
1.43282621948967E-09	1.94890379731419	0.309	0.106	2.40113017862078E-05	Proliferating macrophages	NBEAL2
1.43516632260927E-09	1.38031032020836	0.17	0.036	2.40505172342861E-05	Proliferating macrophages	POMGNT2
1.43674861182909E-09	0.714077306645121	0.447	0.178	2.40770332370319E-05	Proliferating macrophages	LMF2
1.45252490155158E-09	1.19298508421714	0.245	0.07	2.43414123002013E-05	Proliferating macrophages	RGCC
1.48214068812983E-09	0.37306320389675	0.436	0.164	2.48377136516797E-05	Proliferating macrophages	RBM10
1.50990501434705E-09	1.50215123545817	0.138	0.025	2.53029882304279E-05	Proliferating macrophages	PCDH1
1.51708735242929E-09	0.958624667630072	0.362	0.128	2.542334985201E-05	Proliferating macrophages	MFSD14B
1.52697786814819E-09	0.575369673772271	0.585	0.268	2.55890951144274E-05	Proliferating macrophages	TUFM
1.54905631634372E-09	0.809541593516311	0.362	0.126	2.59590857492881E-05	Proliferating macrophages	RLIM

1.57780745550387E-09	1.23167531587601	0.298	0.093	2.64408973393338E-05	Proliferating macrophages	NEPRO
1.60173295371948E-09	1.22656661180354	0.213	0.054	2.68418408384311E-05	Proliferating macrophages	RAI1
1.62573725813447E-09	1.37268057929689	0.234	0.064	2.72441049718175E-05	Proliferating macrophages	MYO1G
1.63376083496942E-09	1.20685891267333	0.277	0.084	2.73785640724175E-05	Proliferating macrophages	EED
1.64966865435233E-09	0.42992854652841	0.33	0.109	2.76451473096363E-05	Proliferating macrophages	BRD8
1.65828439026053E-09	1.67004891331413	0.415	0.17	2.7789529811986E-05	Proliferating macrophages	BCL6
1.65980438891533E-09	1.16988002060999	0.372	0.138	2.78150019494431E-05	Proliferating macrophages	DEPRECATED-ENSG00000205937
1.68039442606573E-09	1.27259459625274	0.404	0.16	2.81600497920094E-05	Proliferating macrophages	HPRT1
1.68134983259177E-09	1.05080931152757	0.181	0.041	2.81760604945729E-05	Proliferating macrophages	ASAP2
1.71596985567976E-09	1.26606207671995	0.351	0.123	2.87562228414814E-05	Proliferating macrophages	APEX2
1.73324206651877E-09	0.89766899884708	0.319	0.104	2.90456705507216E-05	Proliferating macrophages	PELP1
1.76266696888331E-09	1.03130636098547	0.33	0.112	2.95387730645465E-05	Proliferating macrophages	SLC16A1
1.78509500816386E-09	0.69471743209674	0.447	0.183	2.991462214681E-05	Proliferating macrophages	LMO4
1.78535350475622E-09	0.47889832616509	0.691	0.371	2.99189540327048E-05	Proliferating macrophages	HNRNPA3
1.79331492515395E-09	1.86378146652434	0.149	0.029	3.00523715157299E-05	Proliferating macrophages	BHMT2
1.91440775308931E-09	1.11896694872317	0.191	0.045	3.20816451262706E-05	Proliferating macrophages	MIS12
1.93874408218328E-09	0.66389434725038	0.628	0.312	3.24894733292273E-05	Proliferating macrophages	CCDC88A
1.94625259003509E-09	1.35691130080227	0.319	0.11	3.26153009038081E-05	Proliferating macrophages	PRPS2
2.00356431066761E-09	0.69325522982781	0.383	0.136	3.35757307181678E-05	Proliferating macrophages	DYNC1LI1
2.03623263425332E-09	0.71861572132367	0.436	0.17	3.41231864848172E-05	Proliferating macrophages	CUL3
2.07284430135661E-09	0.6908442548354	0.351	0.123	3.47367248021341E-05	Proliferating macrophages	CABIN1
2.07864303748255E-09	0.91317917435273	0.564	0.251	3.48339000221326E-05	Proliferating macrophages	RAB4B
2.08331571095699E-09	0.45488244083336	0.415	0.15	3.49122046842173E-05	Proliferating macrophages	SERPINA6
2.09168665145134E-09	0.76523428287209	0.894	0.568	3.50524849050215E-05	Proliferating macrophages	GSN
2.18284401901996E-09	0.39837321113403	0.372	0.131	3.65801000707365E-05	Proliferating macrophages	LRRC57
2.23004596168595E-09	1.12831261512211	0.33	0.11	3.73711102259331E-05	Proliferating macrophages	ARPP19
2.25189860891978E-09	0.56717734242403	0.543	0.243	3.77373168882776E-05	Proliferating macrophages	MTA2
2.25822797613311E-09	1.18027978399058	0.213	0.055	3.78433844240386E-05	Proliferating macrophages	MIS18A
2.29528356721043E-09	0.62896739495361	0.287	0.09	3.84643620193124E-05	Proliferating macrophages	ACAP1
2.314644284342E-09	1.30357707608027	0.351	0.129	3.87888089170032E-05	Proliferating macrophages	DEPRECATED-ENSG00000176788
2.32819782162935E-09	0.48655997738970	0.436	0.173	3.90159390948647E-05	Proliferating macrophages	TNPO3
2.34787854106209E-09	1.08572171762035	0.415	0.163	3.93457485911185E-05	Proliferating macrophages	UBTF
2.39426197493018E-09	2.31142338019641	0.202	0.052	4.012304217588E-05	Proliferating macrophages	AP3M2
2.40168432789232E-09	0.92194424505727	0.213	0.054	4.02474259668195E-05	Proliferating macrophages	GXYLT1
2.44144326917974E-09	0.92185700910873	0.404	0.16	4.09137063049141E-05	Proliferating macrophages	CANT1
2.44517599224589E-09	0.47701880976480	0.255	0.074	4.09762592780566E-05	Proliferating macrophages	CEP131
2.46364741064608E-09	1.23612851450455	0.266	0.08	4.1285803307607E-05	Proliferating macrophages	CBX1
2.47890252155026E-09	1.12675306934417	0.298	0.095	4.15414484561392E-05	Proliferating macrophages	PPP1CC
2.48583858111355E-09	1.21299938838669	0.266	0.079	4.1657682942301E-05	Proliferating macrophages	CLECL1
2.49953016890039E-09	0.50084996808767	0.691	0.363	4.18871265704327E-05	Proliferating macrophages	CNN2
2.50417492395943E-09	1.07239563417955	0.362	0.139	4.19649633757122E-05	Proliferating macrophages	DUT
2.55164074729163E-09	1.04573433666793	0.372	0.135	4.27603956431131E-05	Proliferating macrophages	SDF2L1
2.55318264214519E-09	0.84854769819266	0.5	0.213	4.27862347170691E-05	Proliferating macrophages	POLDIP3
2.5713245424142E-09	1.34372414564358	0.16	0.034	4.30902566817772E-05	Proliferating macrophages	TOE1
2.61221015455117E-09	0.27002069612235	0.436	0.166	4.37754177699686E-05	Proliferating macrophages	SMARCC1
2.69979531264589E-09	1.60463800232652	0.138	0.026	4.52431698493198E-05	Proliferating macrophages	PIDD1
2.74548670314072E-09	1.19911034381494	0.489	0.214	4.60088661712322E-05	Proliferating macrophages	LRRK2
2.77967555898897E-09	1.18634044676273	0.319	0.107	4.65818030175372E-05	Proliferating macrophages	REPIN1
2.78175720602519E-09	1.05334738596845	0.277	0.084	4.66166872585701E-05	Proliferating macrophages	CTDSPL2
2.80096399135238E-09	0.61565349500180	0.511	0.211	4.69385545670833E-05	Proliferating macrophages	NUP98
2.92313316243925E-09	0.27727679543578	0.223	0.059	4.8985865536157E-05	Proliferating macrophages	DEPRECATED-ENSG00000204516
2.92985124742367E-09	0.50963669266749	0.372	0.133	4.90984472043258E-05	Proliferating macrophages	TTLL5
2.94832285184511E-09	0.74032414896642	0.5	0.206	4.94079943512203E-05	Proliferating macrophages	RC3H2
3.01087576580213E-09	1.55491140151604	0.128	0.022	5.04562560833122E-05	Proliferating macrophages	LRIG1
3.01836340009653E-09	1.13969428731183	0.319	0.11	5.05817338588177E-05	Proliferating macrophages	ERG28
3.03498025623249E-09	0.51433395972463	0.638	0.326	5.0860199133944E-05	Proliferating macrophages	UBB
3.06787404492633E-09	0.85004484284169	0.745	0.411	5.14114332448755E-05	Proliferating macrophages	PTMS
3.11062372239706E-09	1.67493900823934	0.468	0.205	5.21278323399299E-05	Proliferating macrophages	VAC14
3.14027075262026E-09	0.57186410964065	0.84	0.544	5.26246572724103E-05	Proliferating macrophages	HNRNPA2B1
3.14030468132553E-09	0.52113691830975	0.33	0.113	5.26252258496532E-05	Proliferating macrophages	TAF6
3.14243598186549E-09	0.86884824901645	0.298	0.099	5.26609421841018E-05	Proliferating macrophages	RUVBL2
3.16815846607103E-09	1.42525323271662	0.202	0.051	5.30919995744183E-05	Proliferating macrophages	RFC4
3.17481130538475E-09	0.70165009349726	0.383	0.143	5.32034878556377E-05	Proliferating macrophages	SYNE3
3.23818248207744E-09	0.89316839525462	0.202	0.05	5.42654620346538E-05	Proliferating macrophages	MORC4
3.36335860411514E-09	0.63523486770160	0.404	0.155	5.63631634877615E-05	Proliferating macrophages	ANP32A
3.38847144798958E-09	0.70067730821642	0.255	0.074	5.67840045254094E-05	Proliferating macrophages	ZPR1
3.4082299687313E-09	0.80857041030610	0.702	0.417	5.71151178159992E-05	Proliferating macrophages	DEPRECATED-ENSG00000089248
3.41325745728979E-09	0.80555953291155	0.457	0.186	5.71993684692623E-05	Proliferating macrophages	LRBA
3.47845432724964E-09	0.86048447618012	0.691	0.386	5.82919376160495E-05	Proliferating macrophages	ENO1
3.49934010739484E-09	0.7149082910065	0.394	0.148	5.86419415197227E-05	Proliferating macrophages	MFN2
3.50811243812416E-09	0.70381987806824	0.457	0.192	5.87889482380846E-05	Proliferating macrophages	C17orf49

3.60129230450557E-09	1.66172943310622	0.149	0.03	6.03504564389043E-05	Proliferating macrophages	DEPRECATED-ENSG00000171234
3.60138806889774E-09	1.31249315661122	0.213	0.055	6.03520612585883E-05	Proliferating macrophages	RGS14
3.71418616394925E-09	0.948697829342782	0.362	0.13	6.22423317354615E-05	Proliferating macrophages	UBR7
3.74535876063918E-09	0.60889796292782	0.426	0.166	6.27647221107914E-05	Proliferating macrophages	MIIP
3.79188712338808E-09	1.00925715010407	0.319	0.108	6.35444444137374E-05	Proliferating macrophages	HCFC1R1
3.83035516037398E-09	0.306746733038496	0.691	0.346	6.41890917775472E-05	Proliferating macrophages	BAX
3.83495431812919E-09	0.68622093754959	0.649	0.32	6.4266164463209E-05	Proliferating macrophages	UBE2A
3.89450971358437E-09	1.23304993390571	0.202	0.051	6.52641937802469E-05	Proliferating macrophages	KIAA1522
3.9163252823133E-09	0.509746830799968	0.415	0.156	6.56297790810063E-05	Proliferating macrophages	TMEM14C
4.0342874833571E-09	1.43922608775395	0.255	0.078	6.76065896460983E-05	Proliferating macrophages	FLVCR1
4.03499180126468E-09	0.476745576628178	0.628	0.301	6.76183926055935E-05	Proliferating macrophages	YWHAH
4.06834671962702E-09	0.940653554531038	0.479	0.207	6.81773543275096E-05	Proliferating macrophages	AATF
4.09132076892267E-09	1.6123711128407	0.181	0.042	6.85623534456061E-05	Proliferating macrophages	DEPRECATED-ENSG00000109084
4.22441363657429E-09	1.42459640418074	0.362	0.138	7.07927237217119E-05	Proliferating macrophages	PSMD8
4.23123106797011E-09	1.2147468708924	0.404	0.158	7.09069702370432E-05	Proliferating macrophages	DNAJC15
4.29311440508851E-09	0.467310916897398	0.383	0.141	7.19440112004733E-05	Proliferating macrophages	NUDT21
4.43977268934406E-09	0.750882849204498	0.34	0.125	7.44017107280277E-05	Proliferating macrophages	PNP
4.44618812667471E-09	1.2980804299985	0.287	0.092	7.45092206268147E-05	Proliferating macrophages	NABP2
4.4978318099216E-09	1.02322495021492	0.404	0.161	7.53746654706662E-05	Proliferating macrophages	LSM 6.00
4.50000204415553E-09	0.86082179313834	0.628	0.314	7.54110342559583E-05	Proliferating macrophages	ELOVL1
4.50097659014272E-09	1.11123145403508	0.255	0.077	7.54273656976117E-05	Proliferating macrophages	CRAT
4.60772476656465E-09	1.43942872412163	0.266	0.08	7.72162516380904E-05	Proliferating macrophages	POLR2D
4.79450504201896E-09	0.968237769131112	0.245	0.071	8.03463154941538E-05	Proliferating macrophages	CCP110
4.81391695275198E-09	0.837992856593252	0.202	0.052	8.06716202942176E-05	Proliferating macrophages	RAD51C
4.90979434372105E-09	0.60334200698645	0.277	0.086	8.22783336120774E-05	Proliferating macrophages	CHCHD3
5.00693913718286E-09	0.550991657166456	0.468	0.196	8.39062860609103E-05	Proliferating macrophages	WWP2
5.08629194759266E-09	0.8881518983716	0.372	0.146	8.52360804575578E-05	Proliferating macrophages	FAF1
5.09455271598682E-09	0.431391229239457	0.468	0.19	8.53745144145072E-05	Proliferating macrophages	CTDNEP1
5.18051354907286E-09	0.954885791141551	0.213	0.057	8.68150460553629E-05	Proliferating macrophages	DEPRECATED-ENSG00000071564
5.18773592579562E-09	0.737459543834169	0.128	0.023	8.6936078644483E-05	Proliferating macrophages	KLHL25
5.20290675235641E-09	0.859844166227596	0.479	0.21	8.71903113559888E-05	Proliferating macrophages	FADS2
5.22993504146701E-09	0.746206236080632	0.404	0.166	8.76432514249042E-05	Proliferating macrophages	ILVBL
5.35268466358142E-09	0.975541374496109	0.394	0.155	8.97002895922975E-05	Proliferating macrophages	KCTD9
5.37784876492335E-09	1.63846412124216	0.309	0.102	9.01219896025855E-05	Proliferating macrophages	BCOR
5.433726818133E-09	0.442546704467646	0.287	0.09	9.10583940182729E-05	Proliferating macrophages	U2AF2
5.54911526044552E-09	0.627409926102391	0.404	0.156	9.2992073534546E-05	Proliferating macrophages	SFPQ
5.61070144325659E-09	0.67212364365001	0.309	0.102	9.4024134786094E-05	Proliferating macrophages	ERGIC2
5.74736365966365E-09	1.0098107651393	0.309	0.102	9.63143202086435E-05	Proliferating macrophages	SEC22C
5.79534304634384E-09	1.13349093490353	0.33	0.118	9.711835877063E-05	Proliferating macrophages	PSMD2
5.82370604873196E-09	0.816942338860537	0.298	0.097	9.75936659646502E-05	Proliferating macrophages	MICOS13
5.97322823849685E-09	1.47359088603	0.149	0.03	0.00010009935882073	Proliferating macrophages	IL17RB
5.97824009922001E-09	0.622115805813979	0.553	0.258	0.000100183347582729	Proliferating macrophages	SMARCA4
5.98745221891887E-09	0.425100886482637	0.574	0.264	0.000100337724284642	Proliferating macrophages	TRIM26
6.00263488393652E-09	0.811645393708404	0.319	0.108	0.000100592155385008	Proliferating macrophages	CEP63
6.0527700292848E-09	0.991787308933434	0.787	0.473	0.000101432320150755	Proliferating macrophages	LGALS1
6.06068041947224E-09	0.892173801861419	0.372	0.141	0.000101564882469516	Proliferating macrophages	TBC1D10B
6.07672628679754E-09	0.743109778140654	0.351	0.13	0.000101833779114153	Proliferating macrophages	MYO19
6.09514450261776E-09	1.26141667013399	0.457	0.209	0.000102142431574868	Proliferating macrophages	ALOX5AP
6.10772492192238E-09	1.02316718423762	0.447	0.185	0.000102353254241575	Proliferating macrophages	DCXR
6.1179177656469E-09	1.05711363691264	0.351	0.128	0.000102524065916711	Proliferating macrophages	HK2
6.19709277817644E-09	0.639309608807336	0.511	0.228	0.000103850880776681	Proliferating macrophages	ANAPC11
6.52760997209126E-09	0.789061244761349	0.777	0.456	0.000109389687912305	Proliferating macrophages	MYL6
6.63569743993937E-09	1.83382379186	0.213	0.058	0.000111201017698504	Proliferating macrophages	HEATR5A
6.64961185771069E-09	0.495306144099888	0.457	0.186	0.000111434195511516	Proliferating macrophages	VARS
6.65025034137436E-09	2.80158376455335	0.149	0.032	0.000111444895220752	Proliferating macrophages	ENPP1
6.65579093238031E-09	2.38511287932903	0.117	0.02	0.000111537744444829	Proliferating macrophages	PTGER3
6.75812118171577E-09	0.676556059711726	0.511	0.227	0.000113252594763193	Proliferating macrophages	NACC1
6.85469385501458E-09	0.615780339787818	0.713	0.385	0.000114870959622334	Proliferating macrophages	GNB2
6.87501583206407E-09	0.525432048866466	0.245	0.07	0.00011521151531373	Proliferating macrophages	CPSF1
6.90282185404827E-09	0.514361055233731	0.415	0.162	0.000115677488630141	Proliferating macrophages	SF1
6.93946530391472E-09	0.763441005964594	0.511	0.233	0.000116291559563003	Proliferating macrophages	RNF4
7.14520479024914E-09	0.769291193417241	0.553	0.258	0.000119739341874995	Proliferating macrophages	YWHAE
7.2967076735428E-09	0.844692127734806	0.298	0.098	0.00012227822719323	Proliferating macrophages	PTK2
7.37201554819323E-09	0.427960200862575	0.245	0.069	0.000123540236556622	Proliferating macrophages	SKP2
7.48691915379242E-09	0.589645681287087	0.638	0.331	0.000125465791179253	Proliferating macrophages	NDUFV2
7.57247258090405E-09	0.695374749479252	0.245	0.071	0.00012689949551079	Proliferating macrophages	NUP88
7.60537863811603E-09	0.592762194816191	0.628	0.301	0.000127450935217548	Proliferating macrophages	ADI1
7.61673416517961E-09	0.611445097681601	0.426	0.167	0.00012764123114008	Proliferating macrophages	PREB
7.62478775004635E-09	0.87347232162529	0.298	0.099	0.000127776193115277	Proliferating macrophages	MYCL
7.7189958434663E-09	0.444637307706261	0.426	0.171	0.000129354932344808	Proliferating macrophages	RNF169
7.75882576877571E-09	1.78059180714479	0.128	0.024	0.000130022402233143	Proliferating macrophages	CCNE1

7.8167200309349E-09	0.839188896571555	0.468	0.217	0.000130992594278407	Proliferating macrophages	SIVA1
7.83416252978339E-09	0.492749727850293	0.468	0.192	0.00013128489567411	Proliferating macrophages	SEC16A
7.83552536691442E-09	0.990605943548186	0.383	0.152	0.000131307734098752	Proliferating macrophages	ARL 2.00
8.04244398325379E-09	0.759811180228441	0.468	0.195	0.000134775276271367	Proliferating macrophages	RYBP
8.08341054034681E-09	1.07644800660273	0.457	0.19	0.000135461793835132	Proliferating macrophages	TMX2
8.15725446869581E-09	1.12973024193726	0.394	0.156	0.000136699270386404	Proliferating macrophages	DENND5A
8.37814940584675E-09	0.725846954762618	0.479	0.205	0.00014040102774318	Proliferating macrophages	SHC1
8.53812201014324E-09	0.57114849082067	0.245	0.073	0.00014308184864598	Proliferating macrophages	SPHK1
8.57531977851396E-09	1.46167420416576	0.117	0.02	0.000143705208848337	Proliferating macrophages	HDGFL3
8.61187345935543E-09	0.759315468165918	0.426	0.178	0.000144317775431878	Proliferating macrophages	HCFC1
8.62268475314678E-09	1.58716511317037	0.415	0.173	0.000144498951093234	Proliferating macrophages	SPAG7
8.70970861053735E-09	0.636098246045903	0.436	0.18	0.000145957296895385	Proliferating macrophages	DCAF12
8.72239548924959E-09	1.56637687099649	0.213	0.058	0.000146169903608845	Proliferating macrophages	SH3BP4
8.74089217357764E-09	0.670239434306786	0.511	0.217	0.000146479871044814	Proliferating macrophages	POLDIP2
8.80373478756074E-09	0.911173267620671	0.457	0.197	0.000147532987569943	Proliferating macrophages	MED4
8.82110658358543E-09	0.391220153412986	0.447	0.178	0.000147824104127725	Proliferating macrophages	NME4
8.83668075275902E-09	1.49426903006126	0.415	0.174	0.000148085096054736	Proliferating macrophages	C1orf54
8.90269247068556E-09	0.886782989391435	0.383	0.15	0.000149191320423749	Proliferating macrophages	CNOT11
9.04818226228194E-09	0.372057963659464	0.277	0.086	0.000151629438351321	Proliferating macrophages	TMEM50B
9.05150431462965E-09	0.596212881514409	0.415	0.159	0.000151685109304564	Proliferating macrophages	TECR
9.06908189719922E-09	0.763214821590424	0.223	0.062	0.000151979674433265	Proliferating macrophages	PRRG4
9.20614290366268E-09	1.03011287953612	0.191	0.048	0.000154276542779579	Proliferating macrophages	CSE1L
9.28403228226444E-09	0.880931673791139	0.266	0.084	0.000155581812986188	Proliferating macrophages	PPP1R14B
9.47432712183864E-09	0.844833557231586	0.777	0.547	0.000158770773907772	Proliferating macrophages	ACTG1
9.54401844213905E-09	0.820017895808677	0.33	0.118	0.000159938661053366	Proliferating macrophages	PRPF38A
9.61647805932334E-09	0.80477579535252	0.628	0.319	0.000161152939318141	Proliferating macrophages	SEC61A1
9.74310766479683E-09	0.534112009032542	0.617	0.318	0.000163274998246665	Proliferating macrophages	ADRM1
9.75763016515575E-09	1.56372544602428	0.138	0.027	0.00016351836630768	Proliferating macrophages	XRCC3
9.76273401519385E-09	0.572002108478482	0.319	0.11	0.000163603896626618	Proliferating macrophages	NOP16
9.77655335008744E-09	1.52955016541261	0.149	0.032	0.000163835481040765	Proliferating macrophages	CCDC126
9.88805706251322E-09	0.351940597157223	0.543	0.244	0.000165704060253596	Proliferating macrophages	CYTH2
9.91499937840393E-09	0.530659955785328	0.138	0.027	0.000166155559583293	Proliferating macrophages	PASK
9.97622895730252E-09	0.627896510095677	0.426	0.179	0.000167181644866476	Proliferating macrophages	DR1
1.00177088517131E-08	0.922856143357553	0.457	0.196	0.000167876764937008	Proliferating macrophages	USP14
1.02484750878148E-08	1.45597438713066	0.319	0.113	0.000171743945521601	Proliferating macrophages	SYPL1
1.02687839057491E-08	1.34614071070961	0.266	0.086	0.000172084280692543	Proliferating macrophages	CDCA7L
1.04485209000354E-08	1.37253187634491	0.33	0.116	0.000175096313242793	Proliferating macrophages	TTC9C
1.05266786875619E-08	0.936150239527396	0.34	0.122	0.000176406081446162	Proliferating macrophages	ITGB1BP1
1.05286535124546E-08	0.750016794964086	0.362	0.139	0.000176439175561714	Proliferating macrophages	CTCF
1.0538692178922E-08	0.608336711647074	0.287	0.092	0.000176607403534374	Proliferating macrophages	SPATA5
1.06879551831415E-08	0.669128914689486	0.298	0.098	0.000179108752959086	Proliferating macrophages	COX20
1.0881815101313E-08	0.652310285478212	0.5	0.232	0.000182357457467804	Proliferating macrophages	HMG20B
1.09145615874775E-08	1.12856151720074	0.255	0.081	0.000182906223082948	Proliferating macrophages	BATF3
1.09250402044668E-08	0.33834282983979	0.298	0.097	0.000183081823746454	Proliferating macrophages	DEPRECATED-ENSG00000054118
1.10212526654015E-08	1.08222082462892	0.383	0.152	0.000184694152166799	Proliferating macrophages	ERLIN1
1.10715269540625E-08	1.32663970117946	0.266	0.085	0.000185536648696179	Proliferating macrophages	HINT2
1.11764111700475E-08	0.709614587149607	0.383	0.148	0.000187294298387656	Proliferating macrophages	COPS3
1.1301139788572E-08	1.6336349596404	0.202	0.054	0.000189384500576889	Proliferating macrophages	COMTD1
1.145841696905E-08	1.27185490715662	0.309	0.104	0.00019202015156734	Proliferating macrophages	ZNF595
1.17085608931099E-08	0.629339186752068	0.447	0.189	0.000196212063446735	Proliferating macrophages	RRP36
1.1838451251646E-08	1.98741866372443	0.234	0.069	0.000198388766075084	Proliferating macrophages	VDR
1.1982742277841E-08	0.835813386766965	0.404	0.159	0.000200806795092059	Proliferating macrophages	NAA40
1.20589004624734E-08	0.581583146967736	0.415	0.166	0.000202083053950129	Proliferating macrophages	CCDC6
1.21331662133795E-08	0.554747194443217	0.415	0.167	0.000203327599403814	Proliferating macrophages	NDUFA9
1.2136370745754E-08	0.475581035991131	0.553	0.276	0.000203381300957346	Proliferating macrophages	UPF1
1.23855177992314E-08	0.670409430993381	0.191	0.049	0.000207556507279519	Proliferating macrophages	FAM135A
1.24359622703945E-08	0.812817902766134	0.426	0.169	0.000208401855727272	Proliferating macrophages	ASCC2
1.24562304622334E-08	0.953724728174785	0.543	0.257	0.000208741510086107	Proliferating macrophages	ETFB
1.25051926278528E-08	0.834290609376656	0.255	0.078	0.000209562018057557	Proliferating macrophages	ERI2
1.2728202816285E-08	0.503624748679641	0.574	0.289	0.000213299222795304	Proliferating macrophages	NCL
1.2961103725562E-08	0.781360991551396	0.298	0.099	0.000217202176232968	Proliferating macrophages	RBBP7
1.30162042283506E-08	1.88448852778059	0.191	0.05	0.000218125550458699	Proliferating macrophages	QPCT
1.31239471255197E-08	1.06828610726122	0.394	0.158	0.00021993110592946	Proliferating macrophages	EIF5B
1.34569774922037E-08	0.779605825407713	0.223	0.065	0.00022551202881435	Proliferating macrophages	HERC5
1.34798175218849E-08	0.773255161370691	0.372	0.15	0.000225894782031747	Proliferating macrophages	MLH1
1.3519164520699E-08	1.02282747321064	0.319	0.111	0.000226554159037873	Proliferating macrophages	XRCC5
1.3572012274064E-08	1.41181721676346	0.234	0.069	0.000227439781688764	Proliferating macrophages	ASRGL1
1.35934831284401E-08	0.790103053866452	0.309	0.103	0.000227799590266399	Proliferating macrophages	DCAF10
1.39461741078402E-08	1.1395142419248	0.245	0.073	0.000233709985699186	Proliferating macrophages	RNF170
1.40040628014465E-08	1.38323507553889	0.213	0.059	0.00023468008442664	Proliferating macrophages	IFT80
1.41278684640152E-08	0.310062515908367	0.287	0.092	0.000236754819719966	Proliferating macrophages	DDB2

1.42763967541632E-08	0.474168050811668	0.394	0.153	0.000239243856806267	Proliferating macrophages	LNPK
1.44806207431498E-08	0.935906430307154	0.234	0.069	0.000242666242413705	Proliferating macrophages	CLEC12A
1.456712518364E-08	1.28678094828344	0.128	0.024	0.000244115883827439	Proliferating macrophages	DEPRECATED-ENSG00000087087
1.47277153554711E-08	1.57227760303105	0.33	0.121	0.000246807053926985	Proliferating macrophages	DVL2
1.47498373728903E-08	0.456688383811646	0.521	0.231	0.000247177774694895	Proliferating macrophages	NUP43
1.50701234928412E-08	0.636503170472475	0.489	0.226	0.000252545129493033	Proliferating macrophages	HNRNPAB
1.52565634368876E-08	0.563673152522442	0.553	0.263	0.000255669490075362	Proliferating macrophages	HIPK1
1.52568914275515E-08	0.51549027843106	0.457	0.19	0.000255674986542908	Proliferating macrophages	S100A4
1.55422573431764E-08	1.07119703483011	0.33	0.119	0.000260457148556951	Proliferating macrophages	COX17
1.55595846082315E-08	1.69018201618697	0.181	0.045	0.000260747518864744	Proliferating macrophages	EXO5
1.57992745183414E-08	2.30751854093645	0.234	0.07	0.000264764242378365	Proliferating macrophages	ATP8A1
1.58365460385883E-08	0.588325997130722	0.223	0.064	0.000265388838514663	Proliferating macrophages	PPIH
1.59216226827705E-08	1.98056561432652	0.309	0.115	0.000266814552917867	Proliferating macrophages	SLAMF7
1.61582580550891E-08	0.984832235629411	0.298	0.102	0.000270780088487183	Proliferating macrophages	VAV3
1.61951014633549E-08	0.324655656765849	0.404	0.159	0.000271397510322902	Proliferating macrophages	MPPE1
1.64793632200546E-08	0.582138069998862	0.457	0.197	0.000276161168841675	Proliferating macrophages	C9orf78
1.66483613497472E-08	0.778104058224166	0.213	0.059	0.000278993239499064	Proliferating macrophages	FAM102A
1.67171480016764E-08	0.63592258517949	0.457	0.194	0.000280145966212094	Proliferating macrophages	ZRANB2
1.67278634331963E-08	0.755018384916918	0.319	0.112	0.000280325535413504	Proliferating macrophages	BANF1
1.6946361107697E-08	1.24877684437731	0.181	0.045	0.000283987119442787	Proliferating macrophages	DEPRECATED-ENSG00000145241
1.7229166521672E-08	1.9874406588665	0.16	0.038	0.000288726372570179	Proliferating macrophages	AIM2
1.72749767594981E-08	0.373640732739403	0.436	0.18	0.00028949406053567	Proliferating macrophages	HP1BP3
1.72927721321399E-08	0.780837265896267	0.521	0.244	0.000289792275390401	Proliferating macrophages	ST14
1.74085384660833E-08	0.607279223998778	0.66	0.344	0.000291732287614624	Proliferating macrophages	SERF2
1.75653317292025E-08	1.09411201828446	0.128	0.024	0.000294359829117976	Proliferating macrophages	EPB41L5
1.77594803240044E-08	0.796412984962347	0.319	0.111	0.000297613371269666	Proliferating macrophages	KLHL8
1.78864977934202E-08	1.17197678559577	0.351	0.13	0.000299741930022135	Proliferating macrophages	TRAPPC1
1.83646988676371E-08	0.428183224057247	0.266	0.085	0.000307755623623863	Proliferating macrophages	ECSIT
1.85569486265063E-08	1.23363524472732	0.415	0.171	0.000310977345082992	Proliferating macrophages	SUZ12
1.89997541514271E-08	0.624941607071672	0.383	0.148	0.000318397880069615	Proliferating macrophages	NAA15
1.91206366170126E-08	0.673360934513509	0.617	0.327	0.000320423628427897	Proliferating macrophages	TMED9
1.94135339842044E-08	2.29406761319001	0.426	0.183	0.000325332002507298	Proliferating macrophages	BIRC3
1.96420200668535E-08	1.61098416755325	0.266	0.085	0.00032916097228033	Proliferating macrophages	BZW1
1.96862578373462E-08	1.39959492778677	0.245	0.077	0.000329902308838247	Proliferating macrophages	GTF3C5
1.98522741967857E-08	0.833586373133673	0.436	0.194	0.000332684410989735	Proliferating macrophages	LDHB
2.03819072398103E-08	1.17611441842107	0.415	0.172	0.000341560001524741	Proliferating macrophages	TRMT112
2.05028615331702E-08	0.281478702157649	0.628	0.324	0.000343586953572867	Proliferating macrophages	PCBP1
2.05318271750382E-08	0.29849148869498	0.447	0.187	0.00034407235979929	Proliferating macrophages	PSPC1
2.05808255033529E-08	0.716077740838016	0.489	0.221	0.000344893473785188	Proliferating macrophages	EEF1B2
2.08267372732427E-08	1.5864030547287	0.298	0.104	0.000349014463225001	Proliferating macrophages	CEP68
2.08841846171814E-08	3.40096668303976	0.149	0.034	0.000349977165814726	Proliferating macrophages	LAMP3
2.09340765036312E-08	0.663855749081106	0.574	0.29	0.000350813254047852	Proliferating macrophages	HNRNPUL2
2.10594485772301E-08	0.694488150253842	0.319	0.109	0.000352914239257222	Proliferating macrophages	NDUFB4
2.11448087903991E-08	0.83072947606948	0.649	0.344	0.000354344705709508	Proliferating macrophages	EIF4H
2.11915060772445E-08	0.625497580891506	0.66	0.347	0.000355127258842463	Proliferating macrophages	RNPEP
2.13407514991856E-08	0.656754029235364	0.585	0.289	0.000357628313623352	Proliferating macrophages	NMT1
2.13479692374672E-08	0.610667311687854	0.702	0.383	0.000357749268481476	Proliferating macrophages	PDIA3
2.17605976146994E-08	0.482223393413327	0.383	0.149	0.000364664094827132	Proliferating macrophages	HDAC1
2.17799488654551E-08	1.24338372737318	0.223	0.065	0.000364988383087296	Proliferating macrophages	TMEM107
2.18253046949739E-08	0.951875828876363	0.266	0.085	0.000365748456078372	Proliferating macrophages	ALG8
2.19589077390551E-08	0.772178447715988	0.394	0.155	0.000367987375891086	Proliferating macrophages	ZNF362
2.23475378570353E-08	2.70455108664975	0.34	0.132	0.000374500039408198	Proliferating macrophages	FSCN1
2.25200868211111E-08	0.916753974126957	0.681	0.363	0.000377391614948179	Proliferating macrophages	IL2RG
2.28422712629318E-08	0.987599820675904	0.415	0.17	0.00038279078182421	Proliferating macrophages	HACD3
2.30103606838808E-08	1.25762967325605	0.319	0.11	0.000385607624340474	Proliferating macrophages	CNIH1
2.32770049564875E-08	0.72281756764394	0.596	0.295	0.000390076049060818	Proliferating macrophages	FGR
2.33498107378154E-08	0.979098805799388	0.234	0.07	0.00039129612834311	Proliferating macrophages	THAP4
2.35018120899852E-08	0.863985086866923	0.245	0.073	0.000393843367003972	Proliferating macrophages	XPOT
2.37035317905876E-08	0.46884061704246	0.532	0.26	0.000397223785746667	Proliferating macrophages	ATP5MG
2.44581511827989E-08	3.04698224952797	0.106	0.018	0.000409869697521344	Proliferating macrophages	MYEF2
2.44926090160557E-08	1.37704533011305	0.17	0.042	0.000410447141891062	Proliferating macrophages	G2E3
2.44926090160557E-08	1.69196758453994	0.17	0.042	0.000410447141891062	Proliferating macrophages	STARD9
2.46050176585931E-08	0.55759372217056	0.404	0.162	0.000412330885922703	Proliferating macrophages	COMMD4
2.49178431156406E-08	0.710449378929636	0.426	0.183	0.000417573214931905	Proliferating macrophages	FADS1
2.52699602668353E-08	1.74151229277683	0.17	0.041	0.000423473994151626	Proliferating macrophages	POLR1E
2.63709709645782E-08	0.612157698440764	0.617	0.327	0.000441924731424401	Proliferating macrophages	STT3A
2.6640961836614E-08	0.811548909117811	0.691	0.366	0.000446449238457977	Proliferating macrophages	DCAF7
2.6860934303826E-08	1.06570825723845	0.287	0.098	0.000450135537063515	Proliferating macrophages	PGAP2
2.72259583648961E-08	0.844620814506995	0.372	0.14	0.00045625261027893	Proliferating macrophages	MALSU1
2.81364304360959E-08	1.00963451280617	0.287	0.096	0.000471510301248095	Proliferating macrophages	XRCC1
2.81567823756983E-08	1.3684475147621	0.404	0.174	0.000471851359051952	Proliferating macrophages	ST3GAL5

2.82901053224852E-08	0.62290478813677	0.553	0.263	0.000474085584994207	Proliferating macrophages	SCAF11
2.86510865468659E-08	0.29321441730451	0.372	0.137	0.000480134908352378	Proliferating macrophages	TIMM23
2.95905137996941E-08	0.49506443275289	0.447	0.187	0.000495877830255274	Proliferating macrophages	GALM
2.98041307025599E-08	0.25997685656695	0.649	0.341	0.0004994576223135	Proliferating macrophages	DOCK8
2.99355243242289E-08	0.88381954160353	0.532	0.263	0.000501659516625429	Proliferating macrophages	FLOT2
3.00185389124035E-08	1.33208767057332	0.223	0.065	0.000503050675094057	Proliferating macrophages	DCPS
3.03113196170652E-08	0.73608367556286	0.511	0.226	0.000507957094142778	Proliferating macrophages	MSL1
3.05687270899436E-08	2.06879933999788	0.138	0.03	0.000512270728573276	Proliferating macrophages	TRABD2A
3.08801039242077E-08	0.30627455212896	0.138	0.029	0.000517488781561872	Proliferating macrophages	SASS6
3.14597174734795E-08	0.67461780745356	0.511	0.224	0.00052720194542057	Proliferating macrophages	CDC73
3.16433875906343E-08	0.72702955102944	0.468	0.212	0.00053027988924385	Proliferating macrophages	DIDO1
3.1888231327246E-08	1.40533009272499	0.383	0.159	0.000534382980581989	Proliferating macrophages	ZBTB33
3.18925564313587E-08	0.8382805132365	0.287	0.096	0.000534455460676709	Proliferating macrophages	GPR65
3.22256516984163E-08	0.8282685924375	0.266	0.086	0.00054003747116206	Proliferating macrophages	GPKOW
3.31902320903733E-08	0.42727620609487	0.372	0.144	0.000556201909370476	Proliferating macrophages	PIH1D1
3.33498871143831E-08	1.34662245094408	0.149	0.033	0.000558877408262832	Proliferating macrophages	ZBTB26
3.35125387438604E-08	0.49199911217048	0.819	0.509	0.000561603124269612	Proliferating macrophages	DPYSL2
3.35460575751364E-08	0.78844810825891	0.543	0.25	0.000562164832844135	Proliferating macrophages	PGM2
3.37943547115806E-08	0.92594766348917	0.266	0.086	0.000566325796256667	Proliferating macrophages	ZUP1
3.41008368824148E-08	1.19415091636775	0.245	0.075	0.000571461824475508	Proliferating macrophages	ARMC6
3.4621592435582E-08	1.38477251524528	0.191	0.052	0.000580188646035483	Proliferating macrophages	MAP3K20
3.48626043143273E-08	0.84761999871219	0.564	0.279	0.000584227523099497	Proliferating macrophages	PPP6R1
3.50479990326707E-08	0.77617575068521	0.191	0.05	0.000587334367789495	Proliferating macrophages	YEATS4
3.51745320089395E-08	1.43069811275691	0.234	0.072	0.000589454807405808	Proliferating macrophages	CLEC5A
3.51777053390771E-08	0.87375252098551	0.298	0.102	0.000589507986072254	Proliferating macrophages	CPSF3
3.63650754074539E-08	0.48517588776125	0.628	0.302	0.000609405933678113	Proliferating macrophages	EDEM1
3.6429031872717E-08	2.09313594408882	0.234	0.073	0.000610477716122992	Proliferating macrophages	C1orf21
3.66826174602139E-08	0.85801809434341	0.213	0.06	0.000614727303398265	Proliferating macrophages	ZMYM1
3.68764196894347E-08	1.22204831621414	0.287	0.098	0.000617975041155547	Proliferating macrophages	ISOC1
3.72964411636157E-08	1.30084787312157	0.309	0.113	0.000625013761019872	Proliferating macrophages	SEH1L
3.7501612285377E-08	1.73307246468622	0.16	0.038	0.000628452018678347	Proliferating macrophages	DOLPP1
3.78536176419751E-08	0.36867251041633	0.638	0.314	0.000634350924444218	Proliferating macrophages	CORO1C
3.81679057570871E-08	1.07655223706206	0.33	0.121	0.000639617764677265	Proliferating macrophages	PSMD4
3.88177694611576E-08	0.78691120451144	0.479	0.217	0.00065050818063008	Proliferating macrophages	CLN6
3.90036901433218E-08	1.3911765867645	0.309	0.112	0.000653623839421788	Proliferating macrophages	BZW2
3.91096204318715E-08	0.42461771312785	0.468	0.204	0.000655399019197302	Proliferating macrophages	MED14
3.91099223657343E-08	0.65675570316719	0.468	0.209	0.000655404079004975	Proliferating macrophages	BRD1
3.92921207462478E-08	0.63913896002954	0.277	0.092	0.000658457359465621	Proliferating macrophages	CENPC
3.95696550845215E-08	0.95434853108838	0.266	0.087	0.00066310827990641	Proliferating macrophages	GNG5
3.98814959355772E-08	0.93664196633936	0.351	0.13	0.000668334108888403	Proliferating macrophages	SRXN1
4.18809933289411E-08	0.84664935615851	0.372	0.145	0.000701841686206395	Proliferating macrophages	MANBAL
4.21710777878882E-08	1.55352907065311	0.223	0.065	0.00070670292156943	Proliferating macrophages	PKNOX1
4.26122492355019E-08	0.64092557467914	0.457	0.201	0.000714096072688541	Proliferating macrophages	DYM
4.26226148006719E-08	0.35762482709043	0.234	0.07	0.00071426977882966	Proliferating macrophages	MAST2
4.31933818418129E-08	0.78125198572627	0.351	0.135	0.000723834692905101	Proliferating macrophages	SMC6
4.33429752926225E-08	0.45636120223343	0.383	0.15	0.000726341579953767	Proliferating macrophages	EHMT2
4.35063137986623E-08	1.05942416509307	0.585	0.29	0.000729078806637982	Proliferating macrophages	EPSTI1
4.37348991042973E-08	1.00942126049199	0.234	0.072	0.000732909439189815	Proliferating macrophages	C1orf35
4.48781527739705E-08	0.87171366229866	0.394	0.158	0.000752068084186198	Proliferating macrophages	SNTB2
4.51882905789816E-08	0.66955396053976	0.638	0.335	0.000757265373522574	Proliferating macrophages	COX4I1
4.60944513394357E-08	1.601420967761	0.234	0.074	0.000772450815546263	Proliferating macrophages	SELENOI
4.64265801462298E-08	0.25044638313207	0.298	0.103	0.000778016630090519	Proliferating macrophages	MTOR
4.6657592236938E-08	0.51204320739850	0.511	0.229	0.000781887930706607	Proliferating macrophages	XRN2
4.69643839550268E-08	0.73208294827066	0.213	0.061	0.000787029146318339	Proliferating macrophages	PPIL1
4.76888906424894E-08	0.40990700960292	0.479	0.21	0.000799170429386838	Proliferating macrophages	KHSRP
4.81169030042785E-08	0.99467253605735	0.585	0.29	0.000806343060545699	Proliferating macrophages	ANXA6
4.84287867402812E-08	0.36616155821792	0.394	0.158	0.000811569608193632	Proliferating macrophages	ELP5
4.98119125026979E-08	0.64395600812910	0.532	0.257	0.000834748029720211	Proliferating macrophages	SPCS1
5.05793668927465E-08	0.60997373270408	0.181	0.046	0.000847609030388646	Proliferating macrophages	ITGB3BP
5.08341378173377E-08	1.07486093832817	0.33	0.124	0.000851878481542945	Proliferating macrophages	XRCC6
5.09107164012882E-08	0.92744115816509	0.277	0.092	0.000853161785452788	Proliferating macrophages	TBCC
5.10723567797725E-08	1.40102293579757	0.138	0.03	0.000855870554915428	Proliferating macrophages	DEPRECATED-ENSG00000139343
5.11226437842063E-08	1.43354676133011	0.128	0.025	0.000856713264535728	Proliferating macrophages	RNF157
5.18316682057312E-08	1.94440390828241	0.138	0.03	0.000868595095791643	Proliferating macrophages	PDSS1
5.1999238466376E-08	0.42338373437304	0.436	0.18	0.000871403238219529	Proliferating macrophages	TIMMDC1
5.24636201140063E-08	0.77308122311521	0.287	0.097	0.000879185345870517	Proliferating macrophages	PXMP2
5.26022601497677E-08	1.18662504478518	0.372	0.147	0.000881508675589807	Proliferating macrophages	CNOT10
5.26660587251926E-08	1.623077573162	0.202	0.058	0.000882577812116777	Proliferating macrophages	MTFP1
5.28185580447733E-08	0.32904886653822	0.5	0.232	0.00088513339571431	Proliferating macrophages	FARSA
5.33409702414771E-08	0.57009672940332	0.468	0.203	0.000893887979306674	Proliferating macrophages	IWS1
5.34636647663076E-08	0.58577431794562	0.33	0.12	0.000895944094153783	Proliferating macrophages	HMGXB4

5.35185507501949E-08	1.02099124223165	0.319	0.117	0.000896863873471765	Proliferating macrophages	SIGMAR1
5.38061557576435E-08	1.80161503911284	0.298	0.107	0.00090168355818659	Proliferating macrophages	TMEM39A
5.42784997489482E-08	0.74019434894328	0.362	0.141	0.000909599098792874	Proliferating macrophages	RNF20
5.47391751180318E-08	1.05432836824053	0.191	0.052	0.000917319096627977	Proliferating macrophages	KIAA0586
5.49937090191247E-08	1.32174049683958	0.404	0.172	0.000921584575742492	Proliferating macrophages	KAT2B
5.55851352741123E-08	0.986734237264596	0.468	0.208	0.000931495696923573	Proliferating macrophages	EGLN2
5.59542485443189E-08	0.75891196605943	0.309	0.11	0.000937681297105697	Proliferating macrophages	CNOT6
5.6151856619891E-08	0.579293078500958	0.34	0.13	0.000940992813236133	Proliferating macrophages	CTPS1
5.6373964507265E-08	0.519304529110789	0.287	0.099	0.000944714897212747	Proliferating macrophages	TFAM
5.64869782147353E-08	0.498412971903868	0.468	0.203	0.000946608780922534	Proliferating macrophages	ACTR1B
5.83235959253011E-08	0.557769642831006	0.84	0.519	0.000977386820516196	Proliferating macrophages	DBNL
5.83712433813203E-08	0.398700750233521	0.426	0.178	0.000978185296584166	Proliferating macrophages	UBL7
5.87849461969043E-08	0.992545177541436	0.447	0.198	0.000985118128367722	Proliferating macrophages	RECQL
5.97449785266407E-08	0.678965333327793	0.809	0.529	0.00100120635014945	Proliferating macrophages	ARHGDIB
5.98806983720384E-08	0.580606590360797	0.734	0.417	0.00100348074331862	Proliferating macrophages	MBD2
5.98962889846607E-08	0.520876705857761	0.596	0.306	0.00100374201080494	Proliferating macrophages	DDX6
6.00226365365729E-08	0.650558495216432	0.191	0.051	0.00100585934307989	Proliferating macrophages	CEP295
6.17414666902221E-08	0.640190627456426	0.426	0.175	0.00103466349879474	Proliferating macrophages	GPS1
6.18699934961907E-08	1.59552974475482	0.489	0.236	0.00103681735100916	Proliferating macrophages	SHTN1
6.21270139069071E-08	1.32210957366404	0.202	0.058	0.00104112449905195	Proliferating macrophages	CD1A
6.30762266086871E-08	0.884735106352526	0.372	0.155	0.00105703140550838	Proliferating macrophages	PSMA4
6.38384697301121E-08	1.97798867823781	0.213	0.066	0.00106980507573722	Proliferating macrophages	UNG
6.38934685880987E-08	1.1758581909213	0.149	0.034	0.00107072674659936	Proliferating macrophages	STK26
6.39041453048066E-08	0.513855945139256	0.181	0.047	0.00107090566701795	Proliferating macrophages	FTSJ1
6.53266649963567E-08	0.651340255615709	0.255	0.084	0.00109474425200895	Proliferating macrophages	N4BP2
6.5331940884968E-08	0.361950894645851	0.5	0.224	0.00109483266535029	Proliferating macrophages	EIF2AK1
6.62786462226636E-08	1.02233603259532	0.564	0.284	0.0011106975533994	Proliferating macrophages	TAB2
6.63447896740033E-08	0.659426840322604	0.447	0.188	0.00111180598535695	Proliferating macrophages	GID8
6.65133650829609E-08	2.47954524940636	0.309	0.117	0.00111463097206026	Proliferating macrophages	LY75
6.73583904894037E-08	0.422871525112364	0.277	0.093	0.00112879190782143	Proliferating macrophages	FNTB
6.74197479972993E-08	0.468960789264189	0.5	0.225	0.00112982013693874	Proliferating macrophages	PIEZO1
6.82053078447502E-08	0.991110749325022	0.436	0.193	0.00114298454886232	Proliferating macrophages	RABL6
6.82774224114581E-08	0.831672113777771	0.255	0.083	0.00114419304477121	Proliferating macrophages	R3HCC1
6.85484997006686E-08	0.363595572009179	0.564	0.281	0.00114873575798381	Proliferating macrophages	ATP5F1E
6.97221184731767E-08	0.458685329450169	0.5	0.247	0.0011684032613735	Proliferating macrophages	UXT
6.97319882147658E-08	0.944445494980928	0.489	0.221	0.00116856865850304	Proliferating macrophages	VHL
7.12485646567385E-08	1.19075617936856	0.191	0.052	0.00119398344651762	Proliferating macrophages	BYSL
7.15109257274348E-08	0.988349903499961	0.372	0.153	0.00119838009334035	Proliferating macrophages	EBP
7.16413887811322E-08	0.640773948091896	0.819	0.554	0.00120056639319421	Proliferating macrophages	EFHD2
7.2818654219064E-08	0.456644381398324	0.457	0.194	0.00122029500740307	Proliferating macrophages	FAM32A
7.32547286753868E-08	0.941039354232589	0.245	0.077	0.00122760274314213	Proliferating macrophages	PPP2R1B
7.4232824288686E-08	0.684883294187332	0.138	0.03	0.0012439936694298	Proliferating macrophages	C19orf38
7.48799435326901E-08	0.536890890083657	0.319	0.115	0.00125483809372082	Proliferating macrophages	CCT8
7.55131677078688E-08	0.457232178484741	0.426	0.183	0.00126544966444847	Proliferating macrophages	TXN2
7.6087430323732E-08	0.294986598386281	0.372	0.147	0.0012750731573651	Proliferating macrophages	MCUR1
7.72462394197112E-08	1.68831764206801	0.138	0.03	0.00129449248019552	Proliferating macrophages	TMEM154
7.7493122669548E-08	0.292046869470097	0.351	0.133	0.00129862974969628	Proliferating macrophages	GSPT1
7.7649417259808E-08	0.54752880046364	0.585	0.286	0.00130124893443986	Proliferating macrophages	UBXN1
7.77304786534374E-08	0.511923032228775	0.649	0.361	0.0013026073612743	Proliferating macrophages	CNTRL
7.97284491373809E-08	1.08998147681239	0.33	0.122	0.00133608935064423	Proliferating macrophages	DPAGT1
8.01005030301244E-08	0.614877727942606	0.617	0.358	0.00134232422977883	Proliferating macrophages	HADHA
8.04191461518305E-08	0.690120747656144	0.489	0.229	0.00134766405121238	Proliferating macrophages	NDUFAF3
8.05334172228701E-08	0.988562104024811	0.436	0.196	0.00134957900582086	Proliferating macrophages	RAB5IF
8.09839321384128E-08	0.912816185845502	0.266	0.089	0.00135712873477552	Proliferating macrophages	PDRG1
8.10800228934845E-08	0.26772939974293	0.33	0.122	0.00135873902364901	Proliferating macrophages	PSMD9
8.19121392487973E-08	0.954479750839687	0.468	0.207	0.00137268362953135	Proliferating macrophages	GOLGA7
8.3498993473698E-08	1.30655024449871	0.234	0.074	0.00139927613263223	Proliferating macrophages	POP7
8.45500395942733E-08	0.571798608761609	0.521	0.241	0.00141688956352083	Proliferating macrophages	NEDD8
8.54642022106756E-08	0.357529260678708	0.426	0.179	0.0014322091006465	Proliferating macrophages	TMX4
8.55662217763479E-08	2.00060375895578	0.213	0.065	0.00143391874452804	Proliferating macrophages	DEPRECATED-ENSG00000168924
8.74293373135346E-08	0.492751723391631	0.596	0.287	0.00146514083470021	Proliferating macrophages	CPNE1
8.77012128308825E-08	1.4105099640962	0.532	0.256	0.00146969692461993	Proliferating macrophages	RAB8B
8.806903148924E-08	0.777582645092532	0.245	0.078	0.00147586082969668	Proliferating macrophages	LTB
8.90360831127439E-08	0.660249611870521	0.936	0.756	0.00149206668080336	Proliferating macrophages	LYZ
8.9593002364674E-08	0.938996269131532	0.191	0.053	0.00150139953362721	Proliferating macrophages	TUBGCP4
9.00545443855273E-08	1.31504757582349	0.585	0.334	0.00150913405481267	Proliferating macrophages	BASP1
9.07636187897841E-08	0.695843851856175	0.34	0.13	0.0015210167236792	Proliferating macrophages	DENND4A
9.11894020601009E-08	2.09737877207489	0.117	0.023	0.00152815199972317	Proliferating macrophages	CTNNAL1
9.19584101536293E-08	0.495442339357819	0.17	0.043	0.00154103903735452	Proliferating macrophages	PIGU
9.20411708657844E-08	0.491171927973866	0.202	0.058	0.00154242594136882	Proliferating macrophages	BUD13
9.24299715267853E-08	0.834966993741679	0.426	0.18	0.00154894146284587	Proliferating macrophages	PLEKHJ1

9.25519349744924E-08	0.86779589215249	0.372	0.152	0.00155098532630254	Proliferating macrophages	RAD23B
9.29204049730351E-08	1.03709466732851	0.245	0.079	0.00155716014653812	Proliferating macrophages	MDH1
9.36025201321347E-08	1.23561009043625	0.191	0.052	0.00156859103237431	Proliferating macrophages	CSTF2
9.41801211911629E-08	0.95835355270350	0.138	0.03	0.00157827047092151	Proliferating macrophages	TMEM143
9.49088458171537E-08	0.61154163647282	0.447	0.195	0.00159048243820386	Proliferating macrophages	VDAC3
9.51171194772406E-08	0.71923606370821	0.404	0.162	0.0015939726881996	Proliferating macrophages	USP8
9.62548532512441E-08	0.76555850720075	0.436	0.19	0.00161303883078435	Proliferating macrophages	RNF145
9.71395969508733E-08	0.69933837186859	0.426	0.189	0.00162786536570274	Proliferating macrophages	RAB32
9.80860387153131E-08	1.33144953865438	0.128	0.027	0.00164372583679122	Proliferating macrophages	SH2D3C
9.90781388692093E-08	0.36292278850322	0.404	0.17	0.00166035145117021	Proliferating macrophages	PID1
9.93035422326026E-08	0.58594737340453	0.66	0.351	0.00166412876073395	Proliferating macrophages	KIAA0930
1.01128239508124E-07	0.84850491742633	0.245	0.079	0.00169470703767713	Proliferating macrophages	SARNP
1.0145700284753E-07	0.77590416402148	0.34	0.132	0.0017002164537189	Proliferating macrophages	NAA50
1.02441460403883E-07	0.45481762759517	0.394	0.17	0.00171671399344827	Proliferating macrophages	SOGA1
1.02883466294323E-07	0.65685685593431	0.543	0.267	0.00172412112816026	Proliferating macrophages	MICOS10
1.0453465147296E-07	0.58715953120393	0.415	0.176	0.00175179168938386	Proliferating macrophages	PSMD7
1.05161611783437E-07	0.84684516107851	0.351	0.136	0.00176229829026683	Proliferating macrophages	GOT2
1.05571236427612E-07	0.76652266682393	0.34	0.129	0.00176916278005391	Proliferating macrophages	ADNP
1.05676373707841E-07	0.97421674973311	0.309	0.117	0.001770924670596	Proliferating macrophages	PLD4
1.05925451529932E-07	0.84141827797733	0.17	0.043	0.0017750987167386	Proliferating macrophages	SDC1
1.06242650775851E-07	0.43235501455057	0.404	0.164	0.00178041434170171	Proliferating macrophages	BRMS1
1.09071235072751E-07	0.66851933930473	0.128	0.027	0.00182781575734917	Proliferating macrophages	BBS7
1.09421704228598E-07	0.79735735634472	0.319	0.12	0.00183368891946284	Proliferating macrophages	DCAF16
1.10016707786887E-07	0.98093787458778	0.149	0.035	0.00184365998909265	Proliferating macrophages	CABLES2
1.10460636978483E-07	1.79892054121468	0.298	0.112	0.00185109935448542	Proliferating macrophages	DENND1B
1.1066515463475E-07	0.54545793248382	0.436	0.185	0.00185452666136915	Proliferating macrophages	CDK2AP1
1.11421252582185E-07	0.66143505834799	0.447	0.195	0.00186719735077225	Proliferating macrophages	RAB3GAP1
1.12494294018863E-07	1.05409803796405	0.266	0.091	0.00188517937916811	Proliferating macrophages	SUCO
1.14050393854798E-07	0.56054501072493	0.383	0.153	0.0019112565002187	Proliferating macrophages	DNAJC1
1.18818158491301E-07	0.93299443714133	0.372	0.152	0.00199115469999721	Proliferating macrophages	PSMB2
1.20729356106509E-07	1.03006086396464	0.255	0.086	0.00202318254963289	Proliferating macrophages	BRD9
1.20976622517831E-07	0.96377332609904	0.426	0.187	0.00202732624015381	Proliferating macrophages	GRHPR
1.21876273584264E-07	1.13492983801091	0.245	0.079	0.0020424025927251	Proliferating macrophages	ALDH7A1
1.22077144277667E-07	1.06854528274862	0.16	0.039	0.00204576878380515	Proliferating macrophages	MARVELD2
1.22181855567304E-07	1.48550134036938	0.426	0.192	0.00204752353559688	Proliferating macrophages	MTPN
1.22862744967219E-07	1.4500258163507	0.149	0.035	0.00205893388016065	Proliferating macrophages	MAP3K21
1.23401549073014E-07	0.96395073235150	0.106	0.019	0.00206796315936556	Proliferating macrophages	HS3ST1
1.23770154519529E-07	0.78761139190653	0.298	0.109	0.00207414024943827	Proliferating macrophages	CCT5
1.24104516127617E-07	0.49354481072486	0.394	0.164	0.0020797434812666	Proliferating macrophages	NUBP1
1.24333202255564E-07	0.82023632741714	0.287	0.099	0.00208357580339874	Proliferating macrophages	UCHL5
1.24393051851247E-07	0.57510929464002	0.372	0.153	0.0020845787629232	Proliferating macrophages	CRELD2
1.26439017661494E-07	0.52259057296714	0.213	0.063	0.00211886505797132	Proliferating macrophages	SEMA4B
1.26728067676696E-07	0.78855818053354	0.394	0.174	0.00212370895812607	Proliferating macrophages	ATL3
1.28454985702886E-07	0.93651830168289	0.34	0.135	0.00215264865040897	Proliferating macrophages	AQR
1.28491136626857E-07	0.38991416568659	0.362	0.144	0.00215325446759286	Proliferating macrophages	TMUB1
1.2958083780131E-07	1.14747685783651	0.351	0.137	0.00217151567987436	Proliferating macrophages	SOS 1
1.30276576739553E-07	0.41499568425684	0.426	0.185	0.00218317487300142	Proliferating macrophages	CALM1
1.30278912538485E-07	1.27233299892412	0.181	0.049	0.00218321401631994	Proliferating macrophages	TMTC4
1.30301736296674E-07	0.59247949755065	0.287	0.1	0.00218359649685966	Proliferating macrophages	SC5D
1.32899225105045E-07	0.51438809004502	0.511	0.24	0.00222712521431034	Proliferating macrophages	CCND3
1.33845218782953E-07	0.59724450049237	0.34	0.13	0.00224297817636472	Proliferating macrophages	MOB4
1.37501641849637E-07	0.68425850573835	0.106	0.019	0.00230425251411621	Proliferating macrophages	DCLK2
1.37543361987166E-07	1.53041916838559	0.202	0.059	0.00230495166018093	Proliferating macrophages	RCAN3
1.37794320226054E-07	0.36575455002733	0.489	0.222	0.00230915721834821	Proliferating macrophages	WARS
1.39454339341379E-07	1.07169095795367	0.213	0.065	0.00233697581868282	Proliferating macrophages	BOP 1.00
1.39875275121876E-07	0.73221711677748	0.383	0.159	0.00234402986049239	Proliferating macrophages	CENPB
1.40555395957103E-07	0.43856757577180	0.649	0.346	0.00235542732544913	Proliferating macrophages	CALR
1.4079109326966E-07	0.62550015705461	0.372	0.155	0.00235937714101297	Proliferating macrophages	TNPO2
1.41112202972023E-07	0.80717186747361	0.404	0.177	0.00236475829740516	Proliferating macrophages	CCDC124
1.42338885261874E-07	0.54421118215983	0.309	0.113	0.00238531503921849	Proliferating macrophages	SNRPD2
1.42710050562836E-07	1.00080470799779	0.532	0.271	0.002391535027332	Proliferating macrophages	CLPTM1
1.42937826002943E-07	0.95263617505631	0.362	0.152	0.00239535208815732	Proliferating macrophages	FIP1L1
1.42956995515893E-07	0.28275260391228	0.426	0.19	0.00239567333085533	Proliferating macrophages	CDK4
1.45654213001776E-07	0.52932741513764	0.564	0.284	0.00244087330148376	Proliferating macrophages	AGPAT3
1.47358780973851E-07	0.59825333749377	0.415	0.184	0.0024694384515598	Proliferating macrophages	CNOT9
1.48020983854983E-07	0.74410439914552	0.298	0.11	0.0024805356474418	Proliferating macrophages	CENPP
1.48911354674568E-07	0.39148996974331	0.415	0.179	0.00249545648163641	Proliferating macrophages	SF3A2
1.49435375043329E-07	0.29430290312194	0.553	0.263	0.00250423801497611	Proliferating macrophages	CTDSP1
1.49607811005943E-07	0.58113288235155	0.468	0.213	0.0025071276968376	Proliferating macrophages	PRKCSH
1.5080630777669E-07	0.5416743929658	0.457	0.209	0.00252721210572176	Proliferating macrophages	PSMD13
1.53819651090706E-07	0.78187684284255	0.511	0.252	0.00257770971297805	Proliferating macrophages	RBM42

1.54774566597963E-07	1.56804680015042	0.213	0.064	0.00259371218704867	Proliferating macrophages	ASB6
1.55467866507831E-07	0.81317216035105	0.202	0.059	0.00260533050693823	Proliferating macrophages	OPA3
1.55781473844048E-07	0.51146099922157	0.596	0.302	0.00261058593867855	Proliferating macrophages	EVI2B
1.5580103559104E-07	0.57661491812752	0.755	0.477	0.00261091375443465	Proliferating macrophages	PPT1
1.579502832647E-07	0.73701492325469	0.638	0.347	0.00264693084694984	Proliferating macrophages	ETV6
1.58219631526587E-07	0.49291117987940	0.5	0.233	0.00265144458512254	Proliferating macrophages	MTMR10
1.60014463087403E-07	1.93275736321794	0.117	0.024	0.0026815223724187	Proliferating macrophages	SUV39H2
1.64389664615682E-07	0.65984076804927	0.457	0.216	0.00275484199962959	Proliferating macrophages	SERTAD2
1.64868379349303E-07	0.63521830921661	0.5	0.237	0.00276286430113561	Proliferating macrophages	SHISA5
1.65282279148574E-07	2.93823579793508	0.16	0.041	0.00276980043397181	Proliferating macrophages	TNFAIP8L1
1.67021852556307E-07	0.71915828080233	0.585	0.289	0.0027989522051386	Proliferating macrophages	MOB3A
1.6823693572622E-07	0.29079283150717	0.511	0.236	0.0028193145689	Proliferating macrophages	C1orf162
1.69224898170107E-07	0.25958386126370	0.372	0.149	0.00283587084353465	Proliferating macrophages	RSRC1
1.70016144720401E-07	0.73357334912833	0.319	0.122	0.00284913055322449	Proliferating macrophages	SPOPL
1.71284822381033E-07	0.74021422609145	0.543	0.267	0.00287039105346134	Proliferating macrophages	P4HB
1.76252969876386E-07	0.44416079916132	0.234	0.076	0.00295364726918847	Proliferating macrophages	IDH3A
1.76443502571382E-07	0.91740607111219	0.415	0.188	0.00295684021609122	Proliferating macrophages	YKT6
1.78046560056034E-07	0.84936842084642	0.298	0.108	0.00298370425341903	Proliferating macrophages	ATP5MC1
1.78236916200062E-07	0.42830589690626	0.298	0.109	0.00298689424168064	Proliferating macrophages	SERPINB8
1.78828984916669E-07	0.47032163635794	0.34	0.134	0.00299681612923354	Proliferating macrophages	CTNNBIP1
1.78947565441324E-07	0.99248180707207	0.298	0.11	0.0029988033016657	Proliferating macrophages	NT5DC3
1.79821444196778E-07	0.82365891457599	0.33	0.129	0.0030134477618496	Proliferating macrophages	RPP25
1.80035575441194E-07	1.18114078577236	0.223	0.071	0.00301703617324354	Proliferating macrophages	DCTPP1
1.80989282892989E-07	0.57717927567698	0.223	0.069	0.0030330184027207	Proliferating macrophages	TBC1D31
1.82025071591604E-07	0.61038035306227	0.628	0.321	0.00305037614973209	Proliferating macrophages	GBA
1.82835323012724E-07	0.43419765153977	0.543	0.261	0.00306395434304723	Proliferating macrophages	PI4KA
1.83782827461918E-07	0.31854408995531	0.5	0.232	0.00307983262260682	Proliferating macrophages	DBNDD2
1.87182222847697E-07	0.93282890378003	0.234	0.076	0.0031367996904817	Proliferating macrophages	BAP1
1.87562801691698E-07	0.87611028120805	0.149	0.036	0.00314317743074947	Proliferating macrophages	ZDHHC13
1.87614694953979E-07	0.83811444937906	0.245	0.081	0.00314404705803878	Proliferating macrophages	NXT2
1.88330943286638E-07	0.46078571387335	0.468	0.217	0.00315604994759747	Proliferating macrophages	AFTPH
1.91847192154159E-07	0.61002512234929	0.34	0.13	0.00321497524611939	Proliferating macrophages	RASA2
1.92862382020366E-07	0.59483729931581	0.479	0.23	0.00323198779789729	Proliferating macrophages	PILRB
1.93034710976349E-07	0.62020120887981	0.532	0.263	0.00323487568654166	Proliferating macrophages	MLF 2.00
1.95855808170616E-07	1.70780239706273	0.277	0.103	0.00328215163332318	Proliferating macrophages	ST3GAL2
1.967548681684E-07	0.92366055250986	0.415	0.187	0.00329721808076605	Proliferating macrophages	ACSL5
1.96765884352599E-07	0.50686790993289	0.468	0.215	0.00329740268998086	Proliferating macrophages	ATP5PD
1.96979387765567E-07	1.83112763299682	0.106	0.02	0.00330098058017537	Proliferating macrophages	SMIM27
1.97370696020948E-07	1.02379499670796	0.287	0.107	0.00330753812391904	Proliferating macrophages	UQCC2
1.97419679603905E-07	1.10854229002228	0.277	0.098	0.00330835899080223	Proliferating macrophages	EIPR1
1.97544461492467E-07	0.87541844460266	0.191	0.054	0.00331045008569077	Proliferating macrophages	ALMS1
1.98875094556357E-07	0.43978035636127	0.521	0.245	0.00333274883457543	Proliferating macrophages	LPCAT2
2.01610001882685E-07	1.97134439670438	0.128	0.028	0.00337858041155003	Proliferating macrophages	TJP2
2.03265254417655E-07	0.97601476579765	0.532	0.269	0.00340631913353107	Proliferating macrophages	SEPTIN11
2.0348575954971E-07	0.86291981953419	0.298	0.11	0.00341001435853404	Proliferating macrophages	GNL1
2.04086433777426E-07	0.69964829038877	0.883	0.579	0.00342008045724211	Proliferating macrophages	TAPBP
2.043873326748E-07	0.80532376179811	0.16	0.04	0.0034251229209643	Proliferating macrophages	DEPRECATED-ENSG00000101444
2.0609113011825E-07	0.55066065958461	0.723	0.395	0.00345367515852164	Proliferating macrophages	PGD
2.10584622621337E-07	0.55932216449015	0.585	0.303	0.00352897710588837	Proliferating macrophages	CEP350
2.14064090952887E-07	0.48509609512809	0.234	0.075	0.00358728603618848	Proliferating macrophages	THAP12
2.15734248843838E-07	0.86973044828323	0.181	0.05	0.00361527454212504	Proliferating macrophages	TRMT10C
2.16112939985275E-07	1.7072025070799	0.106	0.02	0.00362162064827323	Proliferating macrophages	MMACHC
2.18585932000363E-07	1.16524703514449	0.319	0.124	0.00366306304846209	Proliferating macrophages	SEC61B
2.19065593235455E-07	0.41813043800920	0.404	0.167	0.00367110121143975	Proliferating macrophages	NUDT22
2.20002736086554E-07	0.91920255620903	0.362	0.146	0.00368680585133847	Proliferating macrophages	C6orf89
2.21044588996738E-07	0.50110477028478	0.33	0.124	0.00370426522240733	Proliferating macrophages	ASCC3
2.21270138259553E-07	0.33508312806907	0.67	0.368	0.0037080449769536	Proliferating macrophages	FAR1
2.21476662792867E-07	0.27984274385548	0.266	0.092	0.00371150591508286	Proliferating macrophages	CDC27
2.2563889778141E-07	0.50842827817434	0.298	0.112	0.00378125664902086	Proliferating macrophages	TMEM30A
2.28250578425068E-07	1.59815597276638	0.106	0.02	0.0038250231932473	Proliferating macrophages	COPZ2
2.30555206822989E-07	0.86921088570925	0.33	0.131	0.00386364415593965	Proliferating macrophages	NDUFB6
2.30621166284525E-07	0.52757302783085	0.372	0.152	0.00386474950459607	Proliferating macrophages	TRAP1
2.30904851968999E-07	0.65274047901278	0.436	0.2	0.00386950350929648	Proliferating macrophages	ABHD3
2.31143714079257E-07	1.70420078488844	0.106	0.02	0.00387350636054018	Proliferating macrophages	CLGN
2.34450753048602E-07	0.31208179377060	0.66	0.356	0.00392892571958847	Proliferating macrophages	GABARAP
2.34738821691365E-07	0.52028292558785	0.372	0.156	0.00393375317390389	Proliferating macrophages	NUP93
2.37208106671801E-07	0.60903311527035	0.638	0.357	0.00397513345160604	Proliferating macrophages	SNRNP200
2.42441342598713E-07	0.52814614970361	0.234	0.075	0.00406283201926923	Proliferating macrophages	INPP1
2.43342580552865E-07	0.43045591324046	0.394	0.17	0.0040779349649049	Proliferating macrophages	CAND1
2.4455572748952E-07	0.43624089929070	0.245	0.08	0.00409826488126937	Proliferating macrophages	BCS1L
2.50518217064016E-07	0.80738410471189	0.138	0.032	0.00419818428155878	Proliferating macrophages	ATP8B3

2.54648456604175E-07	0.731614271475229	0.372	0.155	0.00426739883577276	Proliferating macrophages	CLIP2
2.60112221839968E-07	0.941056787301988	0.383	0.164	0.00435896061359418	Proliferating macrophages	CEBPG
2.61826357269433E-07	0.455483220716264	0.202	0.061	0.00438768609512115	Proliferating macrophages	CNTROB
2.63447450113367E-07	1.32835672013685	0.16	0.041	0.0044148523689998	Proliferating macrophages	DEPRECATED-ENSG00000053372
2.6374264312483E-07	1.59957673114427	0.298	0.115	0.0044197992134859	Proliferating macrophages	EBNA1BP2
2.64519675312327E-07	0.462787841234813	0.511	0.248	0.00443282071888397	Proliferating macrophages	ATP5F1B
2.65119174842487E-07	0.732622889777537	0.543	0.269	0.0044428671320104	Proliferating macrophages	MYD88
2.65763293435338E-07	0.272938083434497	0.489	0.224	0.00445366127138939	Proliferating macrophages	TRIOBP
2.66442780566569E-07	0.66611205559649	0.436	0.194	0.00446504811673456	Proliferating macrophages	CLIC4
2.67239091066468E-07	2.0678363182056	0.394	0.186	0.00447839268809186	Proliferating macrophages	CPNE3
2.6767471628976E-07	0.74637994040814	0.362	0.151	0.00448569289558379	Proliferating macrophages	KDELR1
2.6791500426331E-07	0.633264836105225	0.489	0.227	0.00448971964144455	Proliferating macrophages	DUS1L
2.68130327059083E-07	0.566502719999191	0.319	0.125	0.00449332802085611	Proliferating macrophages	GLE1
2.75126221290479E-07	0.65615307994707	0.298	0.108	0.00461056521638585	Proliferating macrophages	SRD5A3
2.75741735174072E-07	0.870977817130926	0.16	0.041	0.0046208799980471	Proliferating macrophages	ZSWIM1
2.80484046513229E-07	0.498380869196419	0.351	0.139	0.0047003516514687	Proliferating macrophages	GTPBP4
2.81035298785877E-07	0.649143776211539	0.372	0.151	0.00470958953705372	Proliferating macrophages	FDPS
2.81458260952493E-07	0.456181156520228	0.33	0.126	0.00471667753704187	Proliferating macrophages	STARD7
2.84635374323997E-07	0.399065398753842	0.628	0.323	0.00476991960292154	Proliferating macrophages	SLC25A39
2.86317769853573E-07	0.815954151875728	0.234	0.077	0.00479811318720618	Proliferating macrophages	GALNT7
2.90605193393166E-07	2.55459205710172	0.16	0.042	0.00486996183088267	Proliferating macrophages	DUSP5
2.92996352380662E-07	0.781340077667059	0.383	0.164	0.00491003287319513	Proliferating macrophages	PURB
2.94054393957381E-07	0.675511927252628	0.638	0.374	0.0049277635339378	Proliferating macrophages	CALM3
2.97353937575825E-07	0.972503646623611	0.266	0.096	0.00498305728589567	Proliferating macrophages	MICAL3
2.99416583248707E-07	0.91854435833283	0.383	0.165	0.00501762310208183	Proliferating macrophages	MEAF6
3.00930444850034E-07	0.403361343499923	0.223	0.072	0.00504299239479686	Proliferating macrophages	NOL12
3.0646191804201E-07	0.39520187763996	0.415	0.176	0.00513568882254801	Proliferating macrophages	NMI
3.09349791945377E-07	0.43286417653579	0.383	0.155	0.00518408381342063	Proliferating macrophages	GCNT1
3.11306713246904E-07	0.779805859084154	0.426	0.196	0.00521687790059162	Proliferating macrophages	HADH
3.15354526347658E-07	1.53295763726785	0.191	0.056	0.00528471115253405	Proliferating macrophages	RRP9
3.1565167994142E-07	0.414125871339339	0.585	0.292	0.00528969085245831	Proliferating macrophages	ACTR1A
3.17028032307913E-07	1.03263679628617	0.191	0.055	0.00531275576541601	Proliferating macrophages	TP53I11
3.18294638662011E-07	0.813892744414651	0.234	0.078	0.00533398155469798	Proliferating macrophages	RRP7A
3.25326878592997E-07	0.540658671410324	0.511	0.247	0.00545182783146144	Proliferating macrophages	HNRNPL
3.25645416087378E-07	0.620078472259804	0.394	0.169	0.00545716588279229	Proliferating macrophages	HEXIM1
3.26143829167076E-07	0.639169867847844	0.479	0.229	0.00546551828918186	Proliferating macrophages	FBXW5
3.28714559210863E-07	0.640074651155955	0.436	0.195	0.00550859858325564	Proliferating macrophages	STARD10
3.31241933230302E-07	1.40329766000887	0.277	0.099	0.00555095231707341	Proliferating macrophages	C5
3.32761168205283E-07	1.71143219921006	0.106	0.021	0.00557641165678413	Proliferating macrophages	ZNF519
3.33146197787025E-07	0.490224808421122	0.34	0.139	0.00558286398251496	Proliferating macrophages	TIMM10
3.33518396364014E-07	0.336422341060957	0.511	0.238	0.00558910128626815	Proliferating macrophages	RPN1
3.35700191647022E-07	0.883272573577271	0.298	0.11	0.00562566381162079	Proliferating macrophages	NOC2L
3.36294256959326E-07	0.87230641898683	0.287	0.106	0.00563561915812439	Proliferating macrophages	AFF3
3.37016434938845E-07	0.509928173054029	0.33	0.125	0.00564772141670517	Proliferating macrophages	ZNF768
3.39125369617411E-07	0.251052758331908	0.372	0.149	0.00568306294404857	Proliferating macrophages	ACIN1
3.41579822146943E-07	0.304151983761998	0.5	0.23	0.00572419465953847	Proliferating macrophages	DNAJC4
3.43150665696158E-07	0.365047165047362	0.372	0.151	0.00575051885573621	Proliferating macrophages	DEPRECATED-ENSG00000265241
3.43967265177174E-07	0.673830113454903	0.447	0.196	0.00576420342983909	Proliferating macrophages	SRPRB
3.45043638617472E-07	1.23823297581694	0.213	0.067	0.0057822412959516	Proliferating macrophages	ING2
3.45678031942367E-07	0.31124224891002	0.436	0.2	0.00579287245929018	Proliferating macrophages	ANKLE2
3.5002312923725E-07	0.747898528863214	0.394	0.169	0.00586568759975783	Proliferating macrophages	PRR14
3.52432277153424E-07	0.834230272159269	0.457	0.216	0.00590606010053708	Proliferating macrophages	HTATSF1
3.56338997974422E-07	0.535464441131769	0.255	0.09	0.00597152892805537	Proliferating macrophages	ABI2
3.56953898384536E-07	0.894580038356876	0.34	0.136	0.00598183342912806	Proliferating macrophages	CLASP2
3.57664353579082E-07	0.444688076052	0.16	0.041	0.00599373923727825	Proliferating macrophages	ZNF480
3.62248161095023E-07	0.516131491332366	0.319	0.123	0.0060705546836304	Proliferating macrophages	KDM1B
3.62439957332402E-07	0.6462639155374	0.426	0.189	0.00607376880497639	Proliferating macrophages	MYO1C
3.62518193190802E-07	0.447633745041375	0.468	0.221	0.00607507988149146	Proliferating macrophages	S100A6
3.64101592167892E-07	0.394621976170583	0.564	0.288	0.00610161448154954	Proliferating macrophages	NDUFB7
3.67454712683489E-07	0.409600212710741	0.319	0.122	0.0061578060751499	Proliferating macrophages	NCKIPSD
3.76892748718502E-07	0.654353719070919	0.468	0.221	0.00631596868302465	Proliferating macrophages	CPSF6
3.77451336698768E-07	0.509001072695815	0.181	0.05	0.00632532950039796	Proliferating macrophages	PSMD14
3.82798094857743E-07	0.441179872135306	0.213	0.065	0.00641493047362606	Proliferating macrophages	NCBP2AS2
3.84256755517817E-07	1.41825525210534	0.16	0.043	0.00643937470896757	Proliferating macrophages	METTL2B
3.8592214662107E-07	0.575231055593156	0.16	0.042	0.00646728333307589	Proliferating macrophages	RIPOR2
3.94108467069514E-07	1.22420716649583	0.277	0.102	0.00660446969115092	Proliferating macrophages	ZNF700
3.98332671317197E-07	0.565243783994562	0.394	0.176	0.00667525890593359	Proliferating macrophages	HDHD5
3.98545469921047E-07	2.5636091924657	0.117	0.025	0.0066788249849369	Proliferating macrophages	NUDT8
3.9933675496618E-07	0.931032807649652	0.383	0.163	0.00669208533972324	Proliferating macrophages	SCAF4
4.0308209127123E-07	1.27332506633933	0.287	0.11	0.00675484968552327	Proliferating macrophages	PHF6
4.0311263286529E-07	1.4121090605496	0.17	0.046	0.00675536150155653	Proliferating macrophages	ENKD1

4.0419503800916E-07	0.289759589095852	0.202	0.06	0.00677350044695751	Proliferating macrophages	ZNF101
4.06986018408106E-07	1.08589804416467	0.128	0.029	0.00682027169648305	Proliferating macrophages	STOML1
4.09977417817717E-07	0.660607788155929	0.468	0.223	0.00687040156778931	Proliferating macrophages	EIF4G1
4.10622185255925E-07	1.05826126893082	0.223	0.074	0.00688120658051878	Proliferating macrophages	PRIM2
4.11626611399383E-07	0.830658678934553	0.245	0.084	0.00689803875383086	Proliferating macrophages	DEPRECATED-ENSG00000140365
4.13665100870789E-07	0.725589874545418	0.34	0.136	0.00693219976039269	Proliferating macrophages	DNAJB6
4.14634987894025E-07	0.823180406930717	0.277	0.101	0.00694845312712808	Proliferating macrophages	GALNT10
4.15732377342185E-07	0.443393967097779	0.404	0.174	0.00696684317950034	Proliferating macrophages	INTS1
4.23092124137285E-07	0.803524843474708	0.128	0.029	0.00709017781629263	Proliferating macrophages	ECM1
4.24558739772811E-07	0.632659512734519	0.362	0.152	0.00711475536111277	Proliferating macrophages	CSNK1G2
4.252378337347E-07	0.631286084881867	0.298	0.115	0.0071261356177261	Proliferating macrophages	NOP2
4.25774581245105E-07	0.824412658494911	0.33	0.133	0.00713513043250546	Proliferating macrophages	RBM12B
4.29800604809332E-07	0.312618409665641	0.447	0.198	0.00720259853539479	Proliferating macrophages	IPO9
4.31147425091158E-07	1.11628613081101	0.149	0.038	0.00722516854967763	Proliferating macrophages	IMMP1L
4.34397228089766E-07	0.499780184443343	0.404	0.181	0.00727962874832829	Proliferating macrophages	POLR2B
4.34592504321262E-07	0.707836461753754	0.426	0.195	0.0072829011874157	Proliferating macrophages	NUDT5
4.38992594269507E-07	0.50995340934553	0.362	0.148	0.0073566378947684	Proliferating macrophages	PPDPF
4.39950121996118E-07	0.471317712871179	0.617	0.34	0.00737268414441094	Proliferating macrophages	NDUFA2
4.43821273358178E-07	0.854828207727406	0.245	0.083	0.00743755689893635	Proliferating macrophages	CAD
4.47934342877637E-07	0.801291961868293	0.319	0.126	0.00750648371794344	Proliferating macrophages	ZNF672
4.49996840941305E-07	0.514448750501418	0.447	0.207	0.00754104706049439	Proliferating macrophages	ADAM15
4.56569266855706E-07	0.753207371477715	0.436	0.195	0.00765118777396793	Proliferating macrophages	DEDD
4.59723890742399E-07	2.08143508757015	0.245	0.083	0.00770405296106113	Proliferating macrophages	ZNF282
4.60060385122877E-07	0.540648942754092	0.319	0.122	0.00770969193388918	Proliferating macrophages	ASGR2
4.61209335923481E-07	0.489659578063927	0.777	0.463	0.00772894605140569	Proliferating macrophages	RHOA
4.636844975188E-07	0.409364934246105	0.543	0.269	0.00777042480942005	Proliferating macrophages	SNX10
4.68256203139794E-07	0.885353778110057	0.606	0.323	0.00784703745221667	Proliferating macrophages	CCNG2
4.73107902739597E-07	0.528119170993264	0.543	0.28	0.00792834223411017	Proliferating macrophages	AP2S1
4.7670013330104E-07	1.37140170906831	0.298	0.115	0.00798854083385883	Proliferating macrophages	DEPRECATED-ENSG00000099797
4.77649455976245E-07	0.702366782940805	0.287	0.107	0.00800444958324992	Proliferating macrophages	MIB1
4.7810481049653E-07	0.717644635770071	0.16	0.042	0.00801208041430085	Proliferating macrophages	GPR89A
4.81777027186151E-07	0.83438262777451	0.34	0.139	0.00807361942158551	Proliferating macrophages	APOL3
4.87949264607616E-07	0.400498486028985	0.383	0.163	0.00817705377629443	Proliferating macrophages	TELO2
4.90003036673068E-07	0.930746752938634	0.213	0.067	0.00821147088856727	Proliferating macrophages	CCT2
4.91719678410848E-07	2.3253452695929	0.16	0.042	0.00824023837080899	Proliferating macrophages	SLC12A2
4.93225714952779E-07	0.538148210004213	0.489	0.243	0.00826547653117866	Proliferating macrophages	MAP2K2
5.00494468458061E-07	0.477644085988657	0.383	0.166	0.00838728630242019	Proliferating macrophages	LARP7
5.05260344618064E-07	0.510435739289002	0.479	0.223	0.00846715285510952	Proliferating macrophages	PRDX4
5.05461442170491E-07	0.730906243390076	0.436	0.194	0.0084705228478931	Proliferating macrophages	CAPZA1
5.06334590913652E-07	0.913587165100507	0.511	0.262	0.00848515507453098	Proliferating macrophages	DDT
5.07746416181638E-07	1.10927816685406	0.298	0.115	0.00850881444237189	Proliferating macrophages	SPINT1
5.07895405371413E-07	1.41727461956144	0.16	0.042	0.00851131120321415	Proliferating macrophages	ZNF439
5.12163241475046E-07	0.750664363084239	0.426	0.195	0.00858283160063881	Proliferating macrophages	TRAPPC4
5.15568997901858E-07	1.35946825560001	0.181	0.052	0.00863990526683934	Proliferating macrophages	MEX3D
5.15746672318276E-07	0.303590520070733	0.234	0.077	0.00864288273470967	Proliferating macrophages	DEPRECATED-ENSG00000093000
5.17899562337341E-07	0.6237622886254	0.426	0.193	0.00867896086564916	Proliferating macrophages	ARL17A
5.21234832584979E-07	0.448527680714814	0.468	0.221	0.00873485332445907	Proliferating macrophages	STK11
5.21718199600515E-07	0.753378093896138	0.394	0.169	0.00874295358890543	Proliferating macrophages	NFATC3
5.25857976530889E-07	0.47310056373176	1	0.987	0.00881232797070464	Proliferating macrophages	CD74
5.27997199106826E-07	0.755728297770411	0.309	0.121	0.0088481770626322	Proliferating macrophages	FAF2
5.33432451782365E-07	0.607137042518334	0.574	0.299	0.00893926102696887	Proliferating macrophages	TMEM248
5.34220818424762E-07	0.76048821234415	0.213	0.066	0.00895247247516217	Proliferating macrophages	ZNF318
5.37385836155983E-07	0.860526221603174	0.117	0.025	0.00900551184230197	Proliferating macrophages	ZNF569
5.37432377480563E-07	0.931395852535559	0.17	0.047	0.00900629178181928	Proliferating macrophages	DEPRECATED-ENSG00000104442
5.43543477318871E-07	0.526646279410776	0.309	0.115	0.00910870159290965	Proliferating macrophages	YIPF5
5.44529410091486E-07	0.281905109738564	0.351	0.14	0.00912522385431312	Proliferating macrophages	ATP5PB
5.45398269383455E-07	0.53921509311482	0.33	0.132	0.00913978419832794	Proliferating macrophages	FAM20B
5.46796878525913E-07	1.02136962534335	0.245	0.084	0.00916322209033724	Proliferating macrophages	RPRD1B
5.46898142786989E-07	0.335772414747101	0.521	0.251	0.00916491907682436	Proliferating macrophages	PYCR2
5.53994527061552E-07	0.274602835135544	0.649	0.347	0.00928384028449749	Proliferating macrophages	PSMF1
5.61535863768385E-07	0.687417248589802	0.202	0.061	0.0094102180050306	Proliferating macrophages	ACPP
5.62326545028651E-07	0.595392828820824	0.468	0.228	0.00942346824159014	Proliferating macrophages	STIM1
5.73135518183869E-07	0.731212688467472	0.383	0.165	0.00960460501372527	Proliferating macrophages	CASP3
5.76665391253545E-07	0.712663418292556	0.447	0.21	0.0096637586266269	Proliferating macrophages	ESD
5.77419174807415E-07	0.528438132072626	0.298	0.113	0.00967639053142266	Proliferating macrophages	COPS7B
5.79027562602265E-07	0.493522246127477	0.33	0.131	0.00970334389408876	Proliferating macrophages	JMJD8
5.80502086124368E-07	0.388070585969574	0.404	0.176	0.00972805395927216	Proliferating macrophages	JTB
5.82263298331801E-07	0.266512779220075	0.362	0.148	0.00975756835344432	Proliferating macrophages	CXorf21
5.85380547360072E-07	1.00592165139884	0.191	0.057	0.00980980721266008	Proliferating macrophages	MRTO4
5.88875273524351E-07	0.517247042334409	0.266	0.096	0.00986837183372108	Proliferating macrophages	PSMA3
5.90517531612229E-07	0.545231269803957	0.33	0.132	0.00989589279475774	Proliferating macrophages	PSMA2

5.94142225852441E-07	0.994186171288186	0.277	0.101	0.0099566354208352	Proliferating macrophages	CHCHD1
5.98764185148078E-07	0.606759143378802	0.319	0.123	0.0100340902147115	Proliferating macrophages	ALG12
6.00595782112133E-07	0.594781896182629	0.234	0.078	0.0100647841166351	Proliferating macrophages	NDUFB3
6.00832855406973E-07	0.497098092381189	0.149	0.038	0.0100687569909101	Proliferating macrophages	RGMB
6.02929573762157E-07	0.952633302564141	0.138	0.033	0.0101038937971062	Proliferating macrophages	MATN2
6.03602143933452E-07	0.393827488698789	0.585	0.309	0.0101151647280368	Proliferating macrophages	PACSIN2
6.0806324632363E-07	0.951751007816839	0.415	0.201	0.0101899238818914	Proliferating macrophages	PGP
6.08685070947579E-07	1.58976654775173	0.213	0.069	0.0102003444189395	Proliferating macrophages	BDH1
6.09328293161656E-07	0.594498217891396	0.277	0.102	0.010211123536803	Proliferating macrophages	TNFRSF11A
6.17760480839509E-07	0.425865261592538	0.521	0.27	0.0103524301379085	Proliferating macrophages	PDIA6
6.19471681878092E-07	0.314650423122089	0.691	0.375	0.0103811064449131	Proliferating macrophages	EIF3A
6.21242042298818E-07	0.572504302843596	0.638	0.34	0.0104107741448436	Proliferating macrophages	MGST3
6.21454243893621E-07	0.377378443811826	0.447	0.204	0.0104143302191693	Proliferating macrophages	KEAP1
6.2216481064242E-07	0.667251709637995	0.309	0.121	0.0104262378967457	Proliferating macrophages	AURKAIP1
6.22203581970396E-07	0.292445950466308	0.426	0.192	0.0104268876266599	Proliferating macrophages	FMR1
6.23758502747727E-07	0.701534327096349	0.394	0.172	0.0104529449890464	Proliferating macrophages	RIPK1
6.27474365955068E-07	0.887643990559368	0.287	0.108	0.010515215424675	Proliferating macrophages	CSNK1E
6.3317752668498E-07	0.391868029106802	0.298	0.116	0.0106107889921869	Proliferating macrophages	MVD
6.34362122688122E-07	0.371733547423237	0.702	0.415	0.0106306404520075	Proliferating macrophages	FAM49B
6.42153721510873E-07	0.354621809342523	0.66	0.369	0.0107612120650792	Proliferating macrophages	SFT2D2
6.4533443333021E-07	0.40443433378242	0.468	0.22	0.0108145144337477	Proliferating macrophages	UBA1
6.45372506761321E-07	0.437699836738686	0.181	0.052	0.0108151524683062	Proliferating macrophages	ST3GAL4
6.51175629541714E-07	0.548928407788996	0.649	0.364	0.01091240119986	Proliferating macrophages	LASP1
6.5211316208896E-07	0.75805005383574	0.372	0.16	0.0109281123702868	Proliferating macrophages	NDUFS3
6.5811142664098E-07	0.354857701612406	0.5	0.24	0.0110286312876495	Proliferating macrophages	ZFR
6.61376778569421E-07	0.713204634242312	0.468	0.222	0.0110833520552664	Proliferating macrophages	TSTA3
6.63333533114055E-07	1.84513924662777	0.234	0.082	0.0111161433479253	Proliferating macrophages	GPN3
6.75719510587803E-07	0.43711489676907	0.394	0.169	0.0113237075584304	Proliferating macrophages	MTHFD2
6.7707940539492E-07	0.490248519189317	0.511	0.258	0.0113464966756081	Proliferating macrophages	DYNLL2
6.9012515128852E-07	0.85809801186779	0.351	0.143	0.011565117285293	Proliferating macrophages	ATG12
6.93612255187715E-07	0.745018362479853	0.33	0.135	0.0116235541724357	Proliferating macrophages	SLU7
6.98560980469835E-07	1.56302114723711	0.149	0.039	0.0117064849107135	Proliferating macrophages	USP31
7.03762547308065E-07	0.74858661903986	0.298	0.116	0.0117936527677886	Proliferating macrophages	SMARCE1
7.11305374187564E-07	0.896770486643646	0.255	0.091	0.0119200554606352	Proliferating macrophages	NSRP1
7.13509702141529E-07	0.595380402594608	0.372	0.164	0.0119569955884877	Proliferating macrophages	ECHDC1
7.1627936973968E-07	0.357121050444977	0.681	0.361	0.0120034096780976	Proliferating macrophages	COPG1
7.24244189454961E-07	0.409118579763351	0.426	0.189	0.0121368841268862	Proliferating macrophages	AAAS
7.24282881211769E-07	1.21410004081067	0.372	0.166	0.0121375325233468	Proliferating macrophages	HSDL2
7.25699099733368E-07	0.659897581446907	0.234	0.079	0.0121612655133318	Proliferating macrophages	IMPDH2
7.33068104057736E-07	0.732319703622341	0.277	0.103	0.0122847552877995	Proliferating macrophages	ERGIC1
7.43115669277257E-07	1.03887715576597	0.128	0.03	0.0124531323857483	Proliferating macrophages	TAF5
7.46860019252043E-07	1.48865576617241	0.564	0.318	0.0125158802026257	Proliferating macrophages	ANPEP
7.48875132215986E-07	0.739861063761655	0.426	0.195	0.0125496494656755	Proliferating macrophages	PTDSS1
7.4930265206691E-07	0.563745917451953	0.415	0.196	0.0125568138433373	Proliferating macrophages	NOP56
7.49634807209209E-07	1.26299484228404	0.149	0.038	0.0125623800992119	Proliferating macrophages	URB2
7.52801317930279E-07	0.514435667001785	0.213	0.067	0.0126154444858756	Proliferating macrophages	CDK14
7.56792200467394E-07	0.251767383720137	0.351	0.142	0.0126823236954326	Proliferating macrophages	NUP214
7.60528227262013E-07	1.07418203778716	0.34	0.142	0.0127449320324568	Proliferating macrophages	PPP1R35
7.65459991096132E-07	0.657136254286891	0.234	0.081	0.012827578530789	Proliferating macrophages	ORAI2
7.73281930926715E-07	0.336845946746533	0.319	0.129	0.0129586585984699	Proliferating macrophages	ATL2
7.74174115895906E-07	0.596580893790484	0.468	0.222	0.0129736098341836	Proliferating macrophages	RNF5
7.7699677959058E-07	0.659768585368962	0.309	0.121	0.0130209120323789	Proliferating macrophages	SCNM1
7.7974849503466E-07	0.384446617806422	0.394	0.172	0.0130670252797908	Proliferating macrophages	MEA1
7.81298543853391E-07	0.947751366662414	0.33	0.136	0.0130930009978951	Proliferating macrophages	HAPLN3
7.86583827561454E-07	0.563757824793949	0.34	0.142	0.0131815717822748	Proliferating macrophages	ADSS
7.90748811528363E-07	0.773708775468489	0.298	0.113	0.0132513685835923	Proliferating macrophages	AP3M1
7.9568433579127E-07	1.4498589085965	0.277	0.109	0.0133340780991901	Proliferating macrophages	CD1C
7.96568367551997E-07	0.589845719454842	0.309	0.124	0.0133488927034364	Proliferating macrophages	FN3KRP
8.14954325037545E-07	0.65381455306643	0.181	0.052	0.0136570045789792	Proliferating macrophages	ARMT1
8.21297405015434E-07	0.678604515866627	0.606	0.326	0.0137633019132486	Proliferating macrophages	ESYT1
8.23225680843817E-07	1.22635440782908	0.34	0.144	0.0137956159595807	Proliferating macrophages	THOC6
8.25131606090283E-07	1.79025835879957	0.106	0.022	0.013827555454861	Proliferating macrophages	MTRF1L
8.44850962926267E-07	0.539436945107304	0.149	0.039	0.0141580124367184	Proliferating macrophages	EPHB6
8.45714327638038E-07	1.43265509952423	0.245	0.089	0.0141724807025582	Proliferating macrophages	P2RY14
8.51455104703757E-07	1.27079787228308	0.138	0.035	0.0142686846446256	Proliferating macrophages	PIN4
8.57344352619973E-07	1.53552605426454	0.181	0.055	0.0143673766612055	Proliferating macrophages	MYCBP
8.68069584106322E-07	0.36759607988416	0.553	0.28	0.0145471100904538	Proliferating macrophages	SELENON
8.71004585641955E-07	0.956091658391273	0.191	0.059	0.0145962948461879	Proliferating macrophages	KATNAL1
8.74148833168734E-07	0.356897788769263	0.34	0.141	0.0146489861462416	Proliferating macrophages	AHCY
8.85535571963905E-07	0.818208926537524	0.266	0.101	0.0148398051149711	Proliferating macrophages	RMND5B
8.87342873438032E-07	0.545266577146845	0.266	0.095	0.0148700918730745	Proliferating macrophages	SP2

8.94125315285589E-07	0.562871563671082	0.255	0.092	0.0149837520335559	Proliferating macrophages	CDK19
8.99160975339195E-07	1.20069667590833	0.287	0.108	0.0150681396247342	Proliferating macrophages	LAS1L
9.05281440728406E-07	0.425738657540053	0.383	0.166	0.0151707063837266	Proliferating macrophages	EIF3H
9.18128841706923E-07	1.11126301395619	0.457	0.216	0.0153860031293246	Proliferating macrophages	HECTD1
9.20986061671528E-07	0.375590167627722	0.553	0.281	0.0154338844214915	Proliferating macrophages	PSMD3
9.25637594776391E-07	0.782663090548158	0.213	0.069	0.0155118348132628	Proliferating macrophages	SACS
9.33202425200145E-07	0.446228956575625	0.33	0.133	0.015638606241504	Proliferating macrophages	SLC4A2
9.4706237939162E-07	0.428723343105693	0.734	0.491	0.0158708713538448	Proliferating macrophages	IFI16
9.50049452402699E-07	1.13780591979832	0.223	0.074	0.0159209287233644	Proliferating macrophages	RNF121
9.53960965807034E-07	1.90070988507485	0.117	0.026	0.0159864778649943	Proliferating macrophages	ZNF296
9.54527945268093E-07	1.09321904263563	0.362	0.158	0.0159959793068027	Proliferating macrophages	DOT1L
9.57220653114165E-07	1.3745444250411	0.17	0.049	0.0160411037048872	Proliferating macrophages	CMTM8
9.64561785103867E-07	0.956582404092173	0.277	0.104	0.0161641263947706	Proliferating macrophages	NUP155
9.66371582815674E-07	0.53405560650671	0.383	0.166	0.0161944549848251	Proliferating macrophages	SDR39U1
9.66402201780137E-07	0.952348353745784	0.266	0.098	0.0161949680974315	Proliferating macrophages	PEF1
9.66419573346486E-07	0.427639588144647	0.564	0.289	0.0161952592101404	Proliferating macrophages	SEC13
9.68375497580863E-07	0.484915366657215	0.511	0.272	0.0162280365884601	Proliferating macrophages	VKORC1
9.69106849137079E-07	0.810306051679728	0.426	0.204	0.0162402925778392	Proliferating macrophages	H2AFJ
9.80798745929882E-07	0.837385661204465	0.149	0.039	0.016436225384293	Proliferating macrophages	KIAA1328
9.83014919133209E-07	0.419816351697928	0.298	0.116	0.0164733640148343	Proliferating macrophages	CA2
9.8894612601538E-07	0.873314289998465	0.223	0.075	0.0165727591797657	Proliferating macrophages	IMPDH1
9.91817459769672E-07	0.4440189153123	0.511	0.249	0.0166208769908202	Proliferating macrophages	FGA
9.97981246996343E-07	0.459333777968745	0.415	0.185	0.0167241697371647	Proliferating macrophages	AKAP11
1.01212986875061E-06	2.09927448636533	0.17	0.05	0.0169612723405227	Proliferating macrophages	VANGL1
1.02008059781239E-06	0.673522480745803	0.277	0.1	0.01709451065814	Proliferating macrophages	GBE1
1.03546711717901E-06	0.918190744236811	0.277	0.106	0.0173523579496858	Proliferating macrophages	DHCR24
1.03958128012543E-06	0.420012415270319	0.543	0.279	0.0174213030923419	Proliferating macrophages	ANKRD44
1.05518691184693E-06	0.659516035585014	0.33	0.133	0.0176828222687309	Proliferating macrophages	SLC25A25
1.05876346354855E-06	1.43962279035145	0.234	0.081	0.0177427581221467	Proliferating macrophages	FADD
1.06641712477184E-06	0.520117145770463	0.479	0.234	0.0178710181769264	Proliferating macrophages	SRSF3
1.08354678201432E-06	0.462168110795746	0.34	0.14	0.0181580769729961	Proliferating macrophages	ATIC
1.08398031684857E-06	0.92578613456003	0.511	0.273	0.0181653421497484	Proliferating macrophages	FBXO7
1.09268168881757E-06	0.48016087868591	0.479	0.228	0.0183111597412048	Proliferating macrophages	DLD
1.11076587936704E-06	0.503631465144656	0.351	0.148	0.0186142146064329	Proliferating macrophages	WRNIP1
1.11239490273395E-06	0.321520621038927	0.372	0.159	0.0186415137800156	Proliferating macrophages	OXSR1
1.11294269213347E-06	0.543374919116025	0.266	0.098	0.0186506936347726	Proliferating macrophages	CPOX
1.1206964191825E-06	1.12007410060471	0.489	0.249	0.0187806305926604	Proliferating macrophages	SLC25A11
1.12603250305775E-06	0.358786684257046	0.234	0.08	0.0188700526862417	Proliferating macrophages	ASH2L
1.13363184738477E-06	0.926514245935754	0.287	0.109	0.018997402498474	Proliferating macrophages	NUDT2
1.13473279662831E-06	0.402555252489393	0.34	0.139	0.0190158522058972	Proliferating macrophages	CUEDC2
1.14679885867689E-06	1.75405696252195	0.181	0.055	0.0192180552737073	Proliferating macrophages	KIAA0040
1.1512425440749E-06	0.409898982197925	0.468	0.22	0.0192925225536072	Proliferating macrophages	SLC39A7
1.15376479028175E-06	0.309435905522338	0.766	0.458	0.0193347903555416	Proliferating macrophages	GNAI2
1.15406569117389E-06	0.875063991348418	0.245	0.089	0.019339832852692	Proliferating macrophages	MYL12A
1.15663647523784E-06	0.73744851145772	0.479	0.238	0.0193829140520357	Proliferating macrophages	EDF1
1.16260248817336E-06	0.491686973706882	0.457	0.218	0.0194828924968092	Proliferating macrophages	CFB
1.16633158691014E-06	0.854559633080343	0.298	0.118	0.0195453847334401	Proliferating macrophages	TMEM250
1.16872101973231E-06	0.413634149903053	0.33	0.138	0.0195854268486741	Proliferating macrophages	CXXC1
1.18315374633628E-06	1.36164704866836	0.117	0.026	0.0198272904811034	Proliferating macrophages	RHPN2
1.18987162744469E-06	0.437691204705723	0.66	0.358	0.0199398687327182	Proliferating macrophages	ARL6IP4
1.19963594780574E-06	0.619114835333084	0.33	0.137	0.0201034992133285	Proliferating macrophages	GYS1
1.2000039353889E-06	0.395904146430314	0.479	0.227	0.0201096659492472	Proliferating macrophages	TOB1
1.20766880248015E-06	0.950823808762103	0.191	0.059	0.0202381137919624	Proliferating macrophages	LLGL1
1.21193440623757E-06	0.330686750153416	0.234	0.08	0.0203095967797292	Proliferating macrophages	FOPNL
1.22040077623145E-06	1.16881091392334	0.213	0.07	0.0204514762080867	Proliferating macrophages	GLOD4
1.23219032663973E-06	1.77850633780352	0.191	0.062	0.0206490454938286	Proliferating macrophages	LRRC42
1.23809552343997E-06	0.708377649349516	0.798	0.535	0.020748004781807	Proliferating macrophages	S100A10
1.27981935714622E-06	0.604921907887179	0.479	0.239	0.0214472127870564	Proliferating macrophages	COX8A
1.28051499168707E-06	0.890316845926091	0.138	0.035	0.021458870230692	Proliferating macrophages	MTRF1
1.28206909487063E-06	0.706387672924595	0.309	0.124	0.021484913891842	Proliferating macrophages	KLF10
1.28826396103891E-06	0.376178676725798	0.319	0.128	0.02158872745909	Proliferating macrophages	COMMD1
1.2889403626023E-06	0.759929536312165	0.277	0.106	0.0216000625964893	Proliferating macrophages	DDX19A
1.29069587971483E-06	0.762330975990918	0.266	0.099	0.0216294815522611	Proliferating macrophages	LSM 2.00
1.30493465365714E-06	0.621387795618229	0.691	0.404	0.0218680949259863	Proliferating macrophages	AHR
1.31527703221879E-06	0.816947731063173	0.585	0.334	0.0220414125059225	Proliferating macrophages	SNX17
1.3179325239927E-06	0.699740727533134	0.447	0.218	0.0220859132370697	Proliferating macrophages	AGPAT2
1.32334516672782E-06	1.35072187127541	0.17	0.05	0.0221766183040248	Proliferating macrophages	NUAK2
1.32390587101035E-06	0.543844843914998	0.543	0.283	0.0221860145863915	Proliferating macrophages	THRAP3
1.32882204918613E-06	0.872183216021269	0.213	0.07	0.0222683999002612	Proliferating macrophages	RAD51D
1.33491724012817E-06	1.0159588368539	0.213	0.071	0.0223705431100679	Proliferating macrophages	UBE2N
1.33856281802474E-06	0.840958129433008	0.277	0.107	0.0224316357044586	Proliferating macrophages	DEPRECATED-ENSG00000105701

1.34069135640141E-06	2.01012767351467	0.128	0.031	0.0224673057505748	Proliferating macrophages	EGLN3
1.34456670878008E-06	0.564553911259642	0.319	0.132	0.0225322489057366	Proliferating macrophages	HARS
1.34602290273522E-06	0.522983451006077	0.383	0.166	0.0225566518040369	Proliferating macrophages	GTPBP2
1.35234060716209E-06	0.481759273395683	0.351	0.144	0.0226625238948222	Proliferating macrophages	HSD17B6
1.38615756386495E-06	0.525465684424215	0.479	0.25	0.0232292284552488	Proliferating macrophages	PFDN2
1.38907304955843E-06	0.294253692746801	0.468	0.222	0.0232780861645002	Proliferating macrophages	NUP58
1.39095515471578E-06	0.498832308641598	0.277	0.104	0.0233096264827271	Proliferating macrophages	DEPRECATED-ENSG00000155115
1.39385692062549E-06	0.41391783047632	0.468	0.232	0.023358254275842	Proliferating macrophages	DYNLRB1
1.39520026464294E-06	0.812655796776563	0.17	0.049	0.0233807660348863	Proliferating macrophages	ZC3HC1
1.3974118648537E-06	1.50762130267615	0.16	0.045	0.0234178280312183	Proliferating macrophages	SPATA33
1.40110914664989E-06	0.259480450976602	0.457	0.216	0.0234797870795588	Proliferating macrophages	DNM1L
1.40304493345679E-06	0.744953178304338	0.277	0.104	0.0235122269948689	Proliferating macrophages	ATXN3
1.41392543470322E-06	0.557030219903214	0.266	0.101	0.0236945624347566	Proliferating macrophages	CNOT1
1.42092005719741E-06	0.681222017326726	0.245	0.087	0.0238117783185142	Proliferating macrophages	DEPRECATED-ENSG00000128228
1.42238983398641E-06	0.908177070668829	0.213	0.071	0.0238364088379442	Proliferating macrophages	HMBS
1.4307420163472E-06	0.426081602591694	0.521	0.263	0.0239763747099464	Proliferating macrophages	PDHA1
1.4654330126008E-06	0.443391141609985	0.309	0.12	0.0245577264251642	Proliferating macrophages	SIRT7
1.47272918807806E-06	0.964255513967386	0.447	0.215	0.0246799957338121	Proliferating macrophages	GATAD2B
1.47423809988507E-06	0.621646494813641	0.319	0.128	0.0247052820778739	Proliferating macrophages	CBWD2
1.47560529433766E-06	1.66140895380377	0.287	0.116	0.0247281935225104	Proliferating macrophages	SRF
1.49324407481598E-06	0.48725197245916	0.468	0.229	0.0250237842057662	Proliferating macrophages	TUBGCP3
1.49431033671053E-06	0.67960176347842	0.138	0.035	0.0250416526225951	Proliferating macrophages	DEPRECATED-ENSG00000020922
1.49752699153604E-06	0.809253626629418	0.319	0.132	0.025095557324161	Proliferating macrophages	CEP57
1.49891589464803E-06	0.595596132434732	0.17	0.05	0.0251188325625116	Proliferating macrophages	RNASEH2B
1.50636445432989E-06	1.2494211489507	0.255	0.095	0.0252436555256602	Proliferating macrophages	POLD2
1.50753361900609E-06	2.03871604785502	0.436	0.207	0.0252632483873041	Proliferating macrophages	KDM2B
1.51295438439526E-06	1.30181044147152	0.128	0.031	0.0253540895736957	Proliferating macrophages	TLE1
1.51375760349394E-06	0.780687523586711	0.309	0.122	0.0253675499193514	Proliferating macrophages	ZBTB48
1.5178427459827E-06	0.270560661974251	0.521	0.251	0.0254360087371781	Proliferating macrophages	HTT
1.52837273397375E-06	0.616420499940655	0.266	0.101	0.0256124702759321	Proliferating macrophages	PPIE
1.53365773857629E-06	0.809959594355373	0.511	0.266	0.0257010363830615	Proliferating macrophages	RASSF2
1.54820328659234E-06	0.485162336335966	0.383	0.169	0.0259447906767144	Proliferating macrophages	ELK4
1.54826945331204E-06	0.427603121679975	0.362	0.156	0.0259458994986031	Proliferating macrophages	PRCC
1.55774585432702E-06	1.39673055687605	0.106	0.022	0.0261047050268122	Proliferating macrophages	DLG5
1.56138659249933E-06	0.561730130436954	0.489	0.252	0.0261657165171038	Proliferating macrophages	SHKBP1
1.56795537658581E-06	0.769878633923756	0.468	0.233	0.026275796200825	Proliferating macrophages	YIF1B
1.57293640227711E-06	0.531213081881017	0.372	0.164	0.0263592682293598	Proliferating macrophages	PHF2
1.5847908037786E-06	0.654394829811251	0.298	0.114	0.0265579242897217	Proliferating macrophages	FAM3A
1.59002544850988E-06	0.466224854805297	0.309	0.125	0.0266456464661286	Proliferating macrophages	TCOF1
1.60065749771517E-06	0.609915810627135	0.426	0.193	0.0268238183467108	Proliferating macrophages	RFTN1
1.60181971139828E-06	0.269385741066812	0.457	0.213	0.0268432947236124	Proliferating macrophages	PPP6C
1.60224783067241E-06	0.487031366838664	0.66	0.374	0.0268504691464083	Proliferating macrophages	SLC38A10
1.61661389684405E-06	0.268804658081927	0.245	0.086	0.0270912156833125	Proliferating macrophages	UBE2D2
1.61983350538726E-06	0.513490821868333	0.213	0.072	0.0271451698832798	Proliferating macrophages	MIEF1
1.62004443220714E-06	0.615914420930469	0.202	0.065	0.0271487045949272	Proliferating macrophages	ZNF736
1.6317107327893E-06	0.460811191725619	0.319	0.13	0.027344208460083	Proliferating macrophages	UBL5
1.63858383587896E-06	0.85145884174316	0.33	0.139	0.0274593879216596	Proliferating macrophages	TIMM17A
1.65390745625908E-06	1.09968977449878	0.16	0.045	0.0277161811519896	Proliferating macrophages	UBXN2A
1.67151060292237E-06	1.03779984957037	0.223	0.076	0.0280111746837731	Proliferating macrophages	GASK1A
1.67179865676288E-06	1.86635142706239	0.117	0.027	0.0280160018900323	Proliferating macrophages	PUS3
1.68315642271437E-06	0.718232781921099	0.181	0.054	0.0282063353318475	Proliferating macrophages	SLC6A19
1.68639110092296E-06	1.62425851985268	0.149	0.041	0.0282605420692669	Proliferating macrophages	C17orf80
1.68857381798227E-06	0.527337292857251	0.511	0.26	0.0282971200417469	Proliferating macrophages	AGPS
1.69142631495205E-06	1.39075788141908	0.383	0.177	0.0283449221859665	Proliferating macrophages	ATP2B4
1.69830926250488E-06	0.693078166822821	0.191	0.059	0.0284602666210567	Proliferating macrophages	DSP
1.69932219885653E-06	0.811607025876514	0.383	0.171	0.0284772414084377	Proliferating macrophages	DEPRECATED-ENSG00000150867
1.70086334222778E-06	0.539394555698895	0.862	0.535	0.0285030678890531	Proliferating macrophages	PLEKHO1
1.73236137724319E-06	0.771172879273182	0.287	0.112	0.0290309119598414	Proliferating macrophages	ROCK2
1.74914167852942E-06	0.694353773961014	0.234	0.083	0.029312116248796	Proliferating macrophages	DMAC2
1.77667673100578E-06	0.398495822822326	0.202	0.066	0.0297735486581949	Proliferating macrophages	GRK6
1.78032131592022E-06	0.448153056645161	0.298	0.118	0.029834624612191	Proliferating macrophages	C19orf53
1.79319739535611E-06	0.945683973950185	0.309	0.128	0.0300504019513777	Proliferating macrophages	CHAMP1
1.79363184950543E-06	1.09049351220555	0.298	0.119	0.0300576825340121	Proliferating macrophages	ELOF1
1.80290424883037E-06	1.84294757202715	0.117	0.027	0.0302130694018994	Proliferating macrophages	FIGNL1
1.80882428623167E-06	0.861398871589376	0.234	0.083	0.0303122773886703	Proliferating macrophages	MAGEF1
1.81140762588696E-06	0.600730019086961	0.574	0.306	0.0303555689946137	Proliferating macrophages	RNPEPL1
1.81899148340386E-06	0.476580384029195	0.287	0.113	0.0304826592788818	Proliferating macrophages	PDS5B
1.8362117676778E-06	1.04952950473007	0.479	0.241	0.0307712368027445	Proliferating macrophages	BROX
1.84385300319528E-06	0.543236416300521	0.266	0.101	0.0308992886275465	Proliferating macrophages	DEPRECATED-ENSG00000134049
1.87852668920051E-06	0.533410557546483	0.479	0.233	0.0314803502576222	Proliferating macrophages	CLPTM1L
1.90114871801389E-06	0.678934917357013	0.138	0.036	0.0318594502164768	Proliferating macrophages	DHRS13

1.92031560392145E-06	0.777188528267141	0.585	0.332	0.0321806488905157	Proliferating macrophages	CYC1
1.93192356666678E-06	0.454763152015931	0.362	0.152	0.0323751751302018	Proliferating macrophages	ANO6
1.95024696605422E-06	1.12788908875211	0.245	0.09	0.0326822386571367	Proliferating macrophages	UBE3B
1.97155754571229E-06	0.646930227912829	0.191	0.06	0.0330393613510465	Proliferating macrophages	ZNF544
1.97414801851785E-06	0.444395215995418	0.681	0.407	0.0330827724943222	Proliferating macrophages	TSPAN3
1.9908954615595E-06	0.629744198340903	0.681	0.375	0.0333634261448141	Proliferating macrophages	SPINT2
2.02559296782484E-06	1.08662272235121	0.213	0.072	0.0339448869548087	Proliferating macrophages	MSH2
2.02671806566951E-06	0.486833771837805	0.202	0.066	0.0339637413444896	Proliferating macrophages	USP28
2.05667967050051E-06	1.96001173244028	0.33	0.15	0.0344658379182475	Proliferating macrophages	DAPP1
2.05668228290839E-06	1.34341533336972	0.138	0.036	0.0344658816969789	Proliferating macrophages	FAM83H
2.07375348864142E-06	1.51855252154581	0.298	0.128	0.0347519609626528	Proliferating macrophages	LGALS2
2.08364544021008E-06	0.573292792786963	0.266	0.102	0.0349177302870406	Proliferating macrophages	NTPCR
2.08463141017281E-06	0.71803606097941	0.479	0.238	0.034934253171676	Proliferating macrophages	UHMK1
2.08858858660026E-06	0.481602697904519	0.319	0.127	0.0350005675342472	Proliferating macrophages	TCF3
2.08924814762376E-06	0.40061058936093	0.681	0.397	0.0350116204578789	Proliferating macrophages	VASP
2.09660005328464E-06	0.933735678542754	0.245	0.091	0.0351348236929439	Proliferating macrophages	HCCS
2.10244722757592E-06	1.59760728923573	0.298	0.119	0.0352328106397172	Proliferating macrophages	DAAM1
2.11374472067537E-06	0.441362347751705	0.415	0.193	0.0354221340290779	Proliferating macrophages	API5
2.13118966775515E-06	1.3772463596387	0.245	0.09	0.0357144764525759	Proliferating macrophages	TLK2
2.13492425140582E-06	1.36062782134164	0.277	0.109	0.0357770606050588	Proliferating macrophages	CREB3
2.1388975680014E-06	0.53512264975203	0.404	0.186	0.0358436454445675	Proliferating macrophages	DCAF11
2.13899792659398E-06	0.789379106520928	0.426	0.201	0.0358453272538618	Proliferating macrophages	MIER1
2.16965504324371E-06	1.09653663852041	0.17	0.05	0.0363590792146781	Proliferating macrophages	TIMM21
2.19624762056049E-06	0.621630898792405	0.17	0.049	0.0368047176253527	Proliferating macrophages	FMC1
2.21259026005657E-06	0.447694884746781	0.383	0.17	0.0370785875780279	Proliferating macrophages	ENOPH1
2.21527122082687E-06	0.849614346100217	0.34	0.145	0.0371235151186167	Proliferating macrophages	CCDC85B
2.21703304662155E-06	1.91904564867747	0.128	0.032	0.0371530397952839	Proliferating macrophages	ATRIP
2.2202329430434E-06	0.369724019968629	0.606	0.317	0.0372066636595213	Proliferating macrophages	C3
2.22571394941198E-06	0.362113513616129	0.394	0.179	0.037298514364246	Proliferating macrophages	COX14
2.23911243060122E-06	1.3041030794064	0.447	0.234	0.0375230461120152	Proliferating macrophages	RNF115
2.25721337907659E-06	0.430380543687885	0.309	0.123	0.0378263818065656	Proliferating macrophages	MED1
2.27147605100809E-06	0.445551093147535	0.404	0.188	0.0380653956627936	Proliferating macrophages	PRPF8
2.28487267440886E-06	0.643547642247425	0.234	0.083	0.0382898962777436	Proliferating macrophages	ARL17B
2.28823534229734E-06	1.30060892655384	0.128	0.032	0.0383462478662188	Proliferating macrophages	PARP16
2.35560202565052E-06	0.344866029754486	0.596	0.313	0.0394751787458515	Proliferating macrophages	TF
2.38853405816747E-06	0.886889895329947	0.138	0.036	0.0400270537467704	Proliferating macrophages	FAM207A
2.39511092815647E-06	0.522334571107013	0.245	0.089	0.0401372689340461	Proliferating macrophages	THYN1
2.41714680722124E-06	0.38843897939555	0.628	0.329	0.0405065461954136	Proliferating macrophages	TBL1XR1
2.42616022286806E-06	0.941388898468421	0.479	0.237	0.0406575930148229	Proliferating macrophages	THBD
2.46984369149908E-06	0.593719178001891	0.319	0.134	0.0413896405821416	Proliferating macrophages	MMD
2.4962093110736E-06	1.78357264804366	0.255	0.098	0.0418314756349714	Proliferating macrophages	C11orf24
2.5024484742401E-06	0.461079502511698	0.309	0.124	0.0419360315313155	Proliferating macrophages	GTF3C2
2.51696646619142E-06	2.00689946947437	0.149	0.042	0.0421793240404359	Proliferating macrophages	CD101
2.51761114129653E-06	0.383408159875177	0.574	0.303	0.0421901275058472	Proliferating macrophages	DYNC1I2
2.53113933714878E-06	1.0504832286373	0.128	0.032	0.0424168330119393	Proliferating macrophages	CNPY4
2.5553414181298E-06	0.585560374509397	0.234	0.083	0.0428224114850192	Proliferating macrophages	GPN1
2.55952460091654E-06	0.992051613303423	0.309	0.128	0.0428925132621593	Proliferating macrophages	ELP3
2.56070268691276E-06	1.55917196499218	0.362	0.162	0.0429122556272841	Proliferating macrophages	BID
2.56916157829662E-06	0.787263912768068	0.191	0.061	0.0430540097290948	Proliferating macrophages	TMEM201
2.58083634037726E-06	0.748783975038035	0.138	0.036	0.0432496553920422	Proliferating macrophages	MDM1
2.6428094530248E-06	0.355671592635683	0.202	0.065	0.0442882008137896	Proliferating macrophages	ARL6IP6
2.65160287444656E-06	0.272064055820393	0.489	0.251	0.0444355609699755	Proliferating macrophages	PPP1R15B
2.6571124790935E-06	0.409805539562793	0.511	0.254	0.0445278909246489	Proliferating macrophages	CPPED1
2.67463044977167E-06	1.05303641370546	0.117	0.027	0.0448214570772736	Proliferating macrophages	PAWR
2.67815910783345E-06	0.53302431013031	0.319	0.132	0.044880590329073	Proliferating macrophages	MAP3K14
2.7020841080721E-06	0.596032199455565	0.638	0.369	0.0452815254830722	Proliferating macrophages	TAF10
2.71530323540587E-06	0.883963773806042	0.457	0.227	0.0455030516189316	Proliferating macrophages	ITPRID2
2.735096656966E-06	0.750841334883084	0.223	0.077	0.0458347497774362	Proliferating macrophages	FBXO22
2.74984769070268E-06	1.51854157160082	0.181	0.057	0.0460819476007956	Proliferating macrophages	P2RY1
2.77862542216763E-06	0.8291160052308	0.553	0.304	0.0465642048246851	Proliferating macrophages	PYCARD
2.80552536051845E-06	1.0518062777819	0.362	0.163	0.0470149939915682	Proliferating macrophages	TPRA1
2.82658834661257E-06	0.640816151727668	0.181	0.056	0.0473679675125334	Proliferating macrophages	PERP
2.85647755463683E-06	0.488817008299405	0.713	0.414	0.0478688508606039	Proliferating macrophages	LAMTOR4
2.87250540447349E-06	0.881050540891025	0.223	0.078	0.0481374455681667	Proliferating macrophages	SLC26A6
2.87437145549554E-06	0.306591322151363	0.617	0.331	0.0481687168511943	Proliferating macrophages	PPIB
2.8995972622484E-06	0.380064387019424	0.606	0.324	0.0485914509207587	Proliferating macrophages	TSC22D4
2.91496003677721E-06	1.12827430021571	0.128	0.032	0.0488489002963125	Proliferating macrophages	TRMT6
2.91640899556446E-06	0.820034662359132	0.351	0.151	0.0488731819476692	Proliferating macrophages	KDELR2
2.94269741654278E-06	0.277362695741913	0.287	0.111	0.0493137233064239	Proliferating macrophages	TRIM24
2.94612195319251E-06	0.736110009810492	0.277	0.108	0.0493711116916	Proliferating macrophages	VPS72
2.96038873985516E-06	0.373882283056239	0.543	0.283	0.0496101945024927	Proliferating macrophages	DNAJB11

**2.97119905469838E-06**	0.57358087995524{	0.277	0.109	0.0497913537586354	Proliferating macrophages	GGCT

SupTable5I_C2_Total_SeuratCluster

p_val	avg_log2FC	pct.1	pct.2	p_val_adj	cluster	gene
0	5.295858709791	0.306	0.017	0	0	KLRG1
0	5.22265215247238	0.596	0.039	0	0	IL7R
0	4.0687879592297	0.284	0.024	0	0	TC2N
0	3.72523112835436	0.26	0.029	0	0	GZMK
0	3.20261256329908	0.375	0.06	0	0	LTB
0	2.91790736484385	0.386	0.077	0	0	CD69
0	2.7480670547152	0.27	0.051	0	0	CD96
0	2.74401871051212	0.749	0.361	0	0	CXCR4
0	2.74076871639292	0.299	0.062	0	0	CD6
2.30624075164681E-137	2.70734199973095	0.289	0.172	3.76170929001112E-133	0	SATB1
5.9404591940936E-215	2.70534198048936	0.25	0.099	9.68948299148607E-211	0	PARP8
0	2.61972101650324	0.374	0.105	0	0	STAT4
6.60984993589908E-139	2.58728330266107	0.297	0.179	1.0781326230445E-134	0	PIK3R1
0	2.41009173907094	0.264	0.06	0	0	BCL11B
3.67319957912086E-223	2.34825090504226	0.272	0.109	5.99135583350404E-219	0	STK17A
0	2.3393608128972	0.487	0.261	0	0	SYNE2
0	2.30053747574373	0.4	0.096	0	0	SPOCK2
7.93719258994984E-287	2.25064917675936	0.467	0.292	1.29463548334672E-282	0	BTG1
0	2.25052782669522	0.449	0.123	0	0	ACAP1
7.37668199615107E-286	2.25025217300615	0.281	0.095	1.2032106003922E-281	0	DGKA
0	2.2381905136162	0.452	0.197	0	0	DUSP2
7.96261882118532E-150	2.23236617662143	0.375	0.263	1.29878275592354E-145	0	PDCD4
1.80637116900242E-151	2.22997460679255	0.323	0.195	2.94637201375985E-147	0	RBL2
2.63640337253447E-296	2.22611900231032	0.312	0.114	4.30023754094097E-292	0	TBC1D10C
0	2.1759112160338	0.414	0.167	0	0	IKZF1
0	2.1474288892834	0.305	0.091	0	0	SEPTIN1
1.04881685694919E-220	2.10004542400876	0.272	0.109	1.71072517536982E-216	0	DEF6
0	2.07820477589926	0.564	0.388	0	0	ZFP36L2
0	2.03602002265937	0.347	0.102	0	0	ZAP70
1.03503835330132E-91	2.01635846732156	0.294	0.205	1.68825105806978E-87	0	ATM
8.15661670169917E-119	2.00273465625145	0.265	0.151	1.33042575021415E-114	0	CDC42SE2
0	1.99135795379874	0.304	0.091	0	0	ITK
2.53068334478974E-237	1.97134588522395	0.271	0.1	4.12779760368655E-233	0	RASAL3
2.7308912890511E-49	1.9437959360988	0.257	0.2	4.45435678157124E-45	0	KLHDC4
2.90883742372086E-285	1.93233210632596	0.27	0.083	4.74460472183109E-281	0	GZMA
3.36596386344421E-96	1.91844439210013	0.277	0.178	5.49022365766385E-92	0	PPP2R5C
5.17342781709701E-273	1.89313709292906	0.278	0.092	8.43837811246694E-269	0	PYHIN1
2.62168920856015E-37	1.87811319379027	0.26	0.221	4.27623726808247E-33	0	HECA
0	1.87123553233545	0.444	0.199	0	0	TMC8
6.09406019539585E-308	1.84330792229866	0.271	0.077	9.94002158471017E-304	0	TRBC1
1.30963184082932E-179	1.83075186672481	0.354	0.198	2.13614049557671E-175	0	TNFAIP3
3.5047980150315E-106	1.82670972078885	0.257	0.147	5.71667604231787E-102	0	SLC38A1
1.17031770901616E-137	1.76701451918827	0.286	0.157	1.90890521517626E-133	0	NLRP1
0	1.76003290421453	0.611	0.305	0	0	WIPF1
0	1.75593428932564	0.591	0.296	0	0	AKNA
3.36634492198168E-110	1.74710574687293	0.314	0.205	5.49084520224432E-106	0	MLLT6
4.93202534374806E-113	1.72605756797389	0.254	0.141	8.04462653818746E-109	0	ARHGAP45
0	1.72552096088279	0.462	0.216	0	0	CYTIP

3.53206563808625E-143	1.70718368129668	0.276	0.142	5.76115226228249E-139	0	JAK3
4.60474033582438E-99	1.70169067732074	0.428	0.392	7.51079196176314E-95	0	AHNAK
5.40950461634266E-195	1.70133174237878	0.38	0.219	8.82344297971651E-191	0	CD44
3.51306206676658E-235	1.69000543153301	0.258	0.087	5.73015553710297E-231	0	CD2
1.98238611494876E-126	1.68703693055388	0.265	0.142	3.23346999209292E-122	0	PIK3CD
5.57069672248543E-178	1.68665858291647	0.404	0.26	9.08636342404598E-174	0	STK4
6.41830153411029E-244	1.67900719257922	0.497	0.341	1.04688916322873E-239	0	NLRC5
2.29867858908893E-26	1.64990030213724	0.274	0.254	3.74937464666296E-22	0	PCSK7
4.07858510177998E-153	1.64619562565788	0.329	0.189	6.65258015951333E-149	0	LIMD2
0	1.64335013161313	0.531	0.228	0	0	CORO1A
4.46163683366512E-21	1.64293091210612	0.282	0.276	7.27737583939118E-17	0	EML4
0	1.6369604747031	0.537	0.332	0	0	ARHGEF1
3.28358825975467E-56	1.63614757110107	0.265	0.198	5.35586081048584E-52	0	SIPA1
0	1.62782034309625	0.393	0.138	0	0	TRAC
5.76845414863298E-37	1.62052320160758	0.256	0.212	9.40892556183526E-33	0	MBNL1
1.5054338659277E-82	1.61602835092695	0.383	0.324	2.45551317871468E-78	0	SUN2
1.76551941344042E-123	1.58560806608007	0.269	0.147	2.87973871526267E-119	0	ARHGAP15
6.62113640484029E-103	1.58198208123028	0.297	0.191	1.0799735589935E-98	0	ARHGDIB
0	1.57875575834356	0.584	0.385	0	0	EVL
5.53736251888656E-140	1.57640127259688	0.273	0.139	9.03199200455587E-136	0	STK17B
7.67600043206533E-261	1.54995717408897	0.426	0.22	1.25203243047418E-256	0	IL2RG
9.47905471629839E-141	1.54544915598303	0.269	0.134	1.54612861477543E-136	0	TRAF1
6.31121067153903E-26	1.47659881373453	0.35	0.37	1.02942157263473E-21	0	CDC42SE1
2.03347084425689E-109	1.46354992055065	0.267	0.151	3.31679429406741E-105	0	PTPRC
2.70441082586323E-139	1.43586784334462	0.357	0.221	4.41116449806551E-135	0	ITGAL
1.99366008311807E-193	1.42007606368212	0.355	0.179	3.25185896157389E-189	0	ETS1
2.25406437835133E-208	1.41313422596799	0.304	0.126	3.67660440752886E-204	0	CCL5
3.63779274827588E-107	1.41020220099748	0.27	0.152	5.93360375171279E-103	0	CNOT6L
1.80235780571694E-136	1.3833592255129	0.374	0.246	2.93982581690491E-132	0	ARHGAP4
4.22830142611598E-257	1.35911313567932	0.397	0.183	6.89678245613777E-253	0	CLEC2D
2.50759790395356E-129	1.33911373655413	0.472	0.388	4.09014294113865E-125	0	CDKN1B
2.68253211506895E-53	1.32960605753518	0.373	0.337	4.37547813288896E-49	0	OGT
1.54569802255138E-129	1.30741324029572	0.378	0.251	2.52118804458356E-125	0	CELF2
8.44787026809302E-67	1.30240048125503	0.29	0.209	1.37793211942865E-62	0	ADGRE5
1.52553999833914E-49	1.29371456614861	0.33	0.28	2.48830829129097E-45	0	TRIM22
0	1.29134483490769	0.435	0.165	0	0	TRBC2
2.73656954257519E-25	1.28941695963904	0.347	0.357	4.46361858089439E-21	0	CYTH1
7.40584705130627E-98	1.28352680154682	0.373	0.276	1.20796771253857E-93	0	LCP1
4.03737828095621E-134	1.27535558478444	0.271	0.131	6.58536771406768E-130	0	NKG7
1.7400508298814E-24	1.24048561444816	0.352	0.362	2.83819690861955E-20	0	OGA
3.40159668390648E-57	1.23829406921512	0.255	0.176	5.54834435111986E-53	0	GBP5
1.75117770506748E-24	1.13081825603389	0.337	0.336	2.85634595473557E-20	0	TXNIP
5.48138628220168E-62	1.08724392386789	0.473	0.483	8.94068916489916E-58	0	RNF213
2.46711859417466E-09	1.07805983277917	0.353	0.41	4.02411713895829E-05	0	LUC7L3
5.50943179144019E-77	1.067304678421	0.255	0.153	8.98643419501809E-73	0	ARHGAP9
1.39297714149415E-28	1.05837964406434	0.271	0.231	2.27208501549111E-24	0	IL10RA
5.78592273178341E-09	1.05386105587369	0.201	0.291	9.43741856781191E-05	0	N4BP2L2
2.63426274995023E-21	1.04921125547733	0.178	0.288	4.29674597144383E-17	0	HSP90AA1
1.1535712684854E-14	1.03728212389596	0.174	0.264	1.88159009602654E-10	0	MPRIP
5.39724595428321E-07	1.03051925283299	0.208	0.292	0.00880344787603134	0	IRF3

1.16307348629128E-44	1.03032951470693	0.31	0.249	1.8970891634897E-40	0	ARL4C
3.12619825469926E-11	1.02919510521163	0.181	0.266	5.09914197323996E-07	0	DDX39B
4.53405884468946E-12	0.982908488508084	0.186	0.277	7.39550338157298E-08	0	UBA52
7.75270923065516E-14	0.978421195655858	0.195	0.296	1.26454440261216E-09	0	UBR2
2.07032345169802E-69	0.945471905288422	0.316	0.219	3.37690458206465E-65	0	ITGA4
6.53749071945532E-23	0.93896725660641	0.165	0.271	1.06633011125036E-18	0	CAPN2
8.44994481689774E-08	0.938463713361053	0.203	0.287	0.00137827049908419	0	FAM193B
9.36447074895186E-70	0.934813282809463	0.363	0.275	1.52743882386154E-65	0	ITPKB
4.11310832905176E-89	0.932777260716252	0.571	0.602	6.70889099551633E-85	0	LENG8
2.20366940499433E-18	0.895906263243219	0.169	0.265	3.59440516648625E-14	0	ILF3
4.4000760916207E-09	0.89562673116618	0.193	0.276	7.17696411304253E-05	0	PSMB9
8.37417373956888E-15	0.888263493714099	0.166	0.251	1.36591147866108E-10	0	VPS13C
4.81321729655205E-14	0.887951930156876	0.192	0.29	7.85083873240606E-10	0	CHD2
1.34717056476185E-07	0.885670832665784	0.352	0.401	0.00219736990818305	0	TAP1
2.93232380032329E-17	0.858841832806755	0.168	0.26	4.78291335070732E-13	0	KMT2D
1.80769167146998E-13	0.845213897123197	0.177	0.263	2.94852588533468E-09	0	TRA2A
2.68497701701623E-11	0.834891914788091	0.199	0.292	4.37946601245517E-07	0	TAP2
8.30802222739386E-24	0.831339097650406	0.178	0.292	1.35512150551021E-19	0	LAMTOR4
1.75570044087832E-14	0.822959286599857	0.201	0.304	2.86372298911662E-10	0	SMAP2
1.2023367191175E-33	0.809173030225361	0.16	0.282	1.96113142255255E-29	0	MAPKAPK2
2.42920878202465E-07	0.805333733555122	0.192	0.266	0.00396228244436041	0	PNISR
6.31103697839859E-22	0.80371684421214	0.179	0.287	1.02939324154659E-17	0	CLK1
5.76211043196754E-19	0.796182971290894	0.16	0.251	9.39857832558225E-15	0	NFKB2
3.92941709845908E-36	0.783336819822729	0.166	0.297	6.4092722292966E-32	0	SNRNP70
3.15482741053897E-28	0.781177979924912	0.181	0.305	5.14583898933012E-24	0	HNRNPH3
4.5504030068819E-28	0.774906886366316	0.148	0.252	7.42216234452507E-24	0	SLTM
7.3134027421567E-20	0.773279922901944	0.182	0.287	1.19288912127318E-15	0	LUC7L
6.13393281937234E-22	0.76248316799412	0.204	0.328	1.00050578216782E-17	0	DNM2
1.03824803564092E-07	0.760347340351768	0.252	0.358	0.00169348637093391	0	TUT7
1.89194781763029E-23	0.758736414743861	0.2	0.324	3.08595608533677E-19	0	PSME1
3.33011419897564E-47	0.754828074223087	0.128	0.251	5.43174926994916E-43	0	PLEC
3.82209409834484E-31	0.754284754484096	0.167	0.288	6.23421768381026E-27	0	OPTN
8.94481818391904E-22	0.750231298859237	0.194	0.309	1.45898929397903E-17	0	EFHD2
4.14262109199144E-31	0.749650388550676	0.175	0.301	6.75702926314724E-27	0	TRIR
3.04753713731001E-17	0.748849640912665	0.218	0.339	4.97083782466635E-13	0	LTBP3
4.1291278767819E-10	0.7469641003077	0.253	0.369	6.73502047981895E-06	0	IKBKB
1.18539078050987E-113	0.745424159887021	0.324	0.173	1.93349090208965E-109	0	CD8A
2.06892107503364E-20	0.737972638492057	0.165	0.263	3.37461716548737E-16	0	MED15
3.40038855692844E-11	0.737727386382173	0.208	0.301	5.54637377520597E-07	0	AKAP13
6.08373632512798E-10	0.716771321572913	0.257	0.375	9.92318231991625E-06	0	RSRP1
2.0852192919221E-200	0.714780425628136	0.777	0.723	3.40120118705413E-196	0	TMSB4X
2.66487584819318E-37	0.709672504127928	0.164	0.294	4.3466789959879E-33	0	RBM39
4.93309621538525E-20	0.707752232037633	0.214	0.338	8.04637323691488E-16	0	HNRNPA0
6.96726726023346E-58	0.705920208769397	0.122	0.256	1.13643096281668E-53	0	SUPT5H
5.96228174598334E-41	0.70393855647936	0.553	0.662	9.72507775587343E-37	0	TAPBP
3.5142669572691E-41	0.696631956940315	0.156	0.288	5.73212083400164E-37	0	ASH1L
2.68691766314663E-49	0.691589021217614	0.129	0.256	4.38263140035848E-45	0	B4GALT1
5.50694548069762E-19	0.687908467443712	0.177	0.277	8.98237877356589E-15	0	MSN
2.98973247062392E-38	0.687463694635116	0.157	0.284	4.87655263283467E-34	0	PSMB10
6.24789185757395E-156	0.683440533526864	0.743	0.773	1.01909364088889E-151	0	B2M

4.16344775241165E-36	0.671674872639069	0.178	0.314	6.79099962895864E-32	0	TBC1D2B
3.3647004084835E-39	0.667618606434282	0.207	0.368	5.48816283627743E-35	0	TCF25
2.42710041484762E-33	0.656115300790472	0.207	0.358	3.95884348665795E-29	0	HSPA8
7.42642513591546E-13	0.647640788876733	0.266	0.399	1.21132420391917E-08	0	WNK1
8.99206611991574E-35	0.644151506074677	0.183	0.319	1.46669590481946E-30	0	PSMB8
1.54964445678872E-55	0.639217467017719	0.134	0.273	2.52762507346808E-51	0	SETD5
1.69877569525725E-35	0.63769003517674	0.206	0.36	2.77087303653409E-31	0	DPP7
3.39875418375184E-51	0.634056702690983	0.147	0.288	5.54370794911763E-47	0	FBXO7
2.88165928759414E-22	0.631872897615453	0.176	0.28	4.7002744639948E-18	0	SPTAN1
5.95563335011534E-63	0.627031253017796	0.124	0.265	9.71423355737313E-59	0	CSNK1D
7.78856331453783E-63	0.623812266560009	0.122	0.262	1.27039256223426E-58	0	CELF1
1.74182898001022E-41	0.621911914826043	0.196	0.354	2.84109724929466E-37	0	DBNL
1.6514009257243E-16	0.620627329155466	0.298	0.474	2.6936000499489E-12	0	TGOLN2
3.58151886283027E-35	0.617933548850621	0.2	0.346	5.84181541716245E-31	0	TCIRG1
6.48893757440492E-54	0.615650657550669	0.38	0.292	1.05841060776119E-49	0	LSP1
4.14968593760194E-56	0.615184640794003	0.122	0.253	6.76855273282253E-52	0	ZBTB7A
5.86516116781906E-50	0.614538722414142	0.131	0.259	9.56666438082967E-46	0	CDC37
1.14310049037749E-56	0.611873577904421	0.158	0.314	1.86451120985472E-52	0	DUS1L
4.56361482077916E-40	0.611456277801014	0.214	0.383	7.44371213417289E-36	0	EMC10
1.22103889350025E-22	0.6079068170193	0.253	0.41	1.99163653918825E-18	0	MDM4
9.74949815261049E-40	0.60579996902041	0.156	0.284	1.5902406436723E-35	0	POLR2A
2.27877737406998E-50	0.604655465665201	0.176	0.334	3.71691377484554E-46	0	BRD2
8.20863025232657E-53	0.602755928851399	0.157	0.308	1.33890968045699E-48	0	PER1
3.22325440326545E-30	0.60203602083232	0.192	0.324	5.25745025716628E-26	0	RBM6
4.57652596004767E-50	0.601503157693375	0.187	0.351	7.46477149343376E-46	0	TMEM259
1.38833689407578E-44	0.589100419682731	0.153	0.286	2.26451630792701E-40	0	SRSF5
2.04390613025809E-52	0.586648893508119	0.133	0.266	3.33381528906397E-48	0	RERE
1.33669688608685E-49	0.583836913277107	0.174	0.329	2.18028629089627E-45	0	SPG7
2.3373053540761E-36	0.578976727722866	0.166	0.293	3.81237876303352E-32	0	LPCAT1
2.18437573472678E-23	0.574518022513014	0.212	0.34	3.56293526091285E-19	0	ACTG1
1.97436982316132E-47	0.550153278477115	0.208	0.383	3.22039461855844E-43	0	HNRNPA2B1
1.39159912889943E-62	0.547418837873856	0.117	0.253	2.26983733914786E-58	0	APOL2
3.24095985573022E-57	0.546168982811484	0.119	0.25	5.28632962068156E-53	0	SUGP2
3.09472013943009E-54	0.545346160153837	0.153	0.301	5.04779801942443E-50	0	LUC7L2
2.43821187194564E-68	0.543401191921089	0.121	0.266	3.97696738433053E-64	0	USF2
7.95844689429694E-61	0.53852122691708	0.167	0.334	1.29810227292877E-56	0	ADAR
2.93488501606126E-73	0.534567145405423	0.132	0.291	4.78709094969753E-69	0	C11orf58
4.69164326061277E-50	0.532266174201824	0.196	0.366	7.6525393223855E-46	0	IQSEC1
6.75464474474332E-73	0.510777170645349	0.113	0.258	1.10175010431508E-68	0	HBS1L
2.29080066613E-48	0.505026667518299	0.199	0.368	3.73652496652465E-44	0	CCNL1
1.13308406563499E-64	0.503908948485726	0.145	0.302	1.84817341945723E-60	0	HNRNPF
3.08194648918781E-74	0.502326202165574	0.119	0.271	5.02696291851423E-70	0	CNBP
1.27557711717088E-47	0.495166572177978	0.167	0.313	2.08059383581743E-43	0	CYBC1
1.82655480696179E-12	0.489320959306137	0.309	0.46	2.97929354563537E-08	0	CFLAR
2.72483774219223E-78	0.483516900302956	0.105	0.251	4.44448284128975E-74	0	ARSA
8.84443164227214E-08	0.474174499546492	0.295	0.287	0.00144261524517101	0	ITGB2
5.31659645804735E-77	0.463596760743182	0.121	0.276	8.67190048272104E-73	0	SERINC1
2.88718018446224E-66	0.455568947739138	0.162	0.333	4.70927959887635E-62	0	MINK1
1.74253799218162E-69	0.45526394215751	0.148	0.313	2.84225371904743E-65	0	SRSF4
1.67036569400658E-67	0.444038595140603	0.111	0.25	2.72453348349414E-63	0	DENND4B

2.30515246228862E-67	0.440604203981818	0.114	0.254	3.75993418123897E-63	0	SYMPK
3.54488512490211E-66	0.438818548045815	0.114	0.252	5.78206212722783E-62	0	SRRM2
7.5142933349482E-08	0.437483451456423	0.349	0.505	0.0012256563858634	0	CCNL2
7.3398640581993E-15	0.435761599052971	0.348	0.538	1.19720522653289E-10	0	JUND
3.92257440252075E-77	0.431150081175322	0.118	0.272	6.39811110795159E-73	0	INTS11
3.66999686607498E-78	0.430294522383518	0.142	0.313	5.98613188825489E-74	0	SELENOF
6.0401008709415E-51	0.426094106223396	0.189	0.352	9.85200853059269E-47	0	IER2
7.26361712934641E-45	0.425536260727144	0.184	0.334	1.18476858996769E-40	0	LY6E
1.44295631365503E-61	0.416919543688314	0.153	0.309	2.35360604320273E-57	0	PPP1R9B
1.58449542900891E-85	0.406460956558996	0.112	0.271	2.58447049425643E-81	0	LGALS8
6.53215051838577E-38	0.40181445646039	0.57	0.556	1.0654590710539E-33	0	IL32
1.8548363712759E-12	0.401039728359801	0.274	0.39	3.02542360518812E-08	0	MAF
4.65739439114205E-58	0.398844411447431	0.151	0.301	7.59667599139179E-54	0	TLN1
3.17116984472023E-95	0.395169656552753	0.096	0.255	5.17249513372318E-91	0	SND1
1.72439463379848E-06	0.394589163985401	0.322	0.439	0.028126600871887	0	TMSB10
1.12431819842923E-29	0.392539319413325	0.294	0.485	1.83387541345792E-25	0	LITAF
8.99010307758398E-60	0.392215964665437	0.178	0.348	1.46637571298472E-55	0	DRAP1
3.66985112558771E-87	0.387190362187808	0.103	0.258	5.98589417094611E-83	0	RAB11B
3.03978927577373E-91	0.386886876383745	0.119	0.289	4.95820028771453E-87	0	BSDC1
3.13789919186449E-90	0.386662554501037	0.145	0.333	5.11822737185017E-86	0	SUMF2
1.01964965536454E-91	0.382871532332661	0.116	0.284	1.66315055286511E-87	0	PPP6R2
1.09288922628425E-98	0.381675782512989	0.099	0.263	1.78261161699225E-94	0	LPP
9.1982701176596E-79	0.376017396824659	0.109	0.258	1.50032983889146E-74	0	OGFR
1.912530013574E-81	0.364310960287727	0.113	0.267	3.11952770514055E-77	0	MAP4
1.62251132975762E-89	0.363402091638672	0.143	0.329	2.64647822996766E-85	0	MTCH1
4.19917862539394E-45	0.358831377947715	0.225	0.401	6.84928025588005E-41	0	CFL1
6.41971094866049E-81	0.351682942812014	0.135	0.304	1.04711905283601E-76	0	APMAP
9.94158299211106E-91	0.350981479259977	0.1	0.255	1.62157160184324E-86	0	UBXN4
7.0178927272805E-71	0.350446119771009	0.12	0.267	1.14468848274672E-66	0	PICALM
7.98623230608458E-107	0.347820557157134	0.09	0.254	1.30263435144546E-102	0	C16orf58
1.4088890291573E-93	0.341751371804479	0.12	0.294	2.29803889545847E-89	0	SRPRA
5.00331063000073E-96	0.337430736192375	0.147	0.344	8.16089996859418E-92	0	STAT6
2.79670380395725E-54	0.33584077538469	0.221	0.412	4.56170357463467E-50	0	CDK5RAP3
2.17267015661578E-95	0.329572959280577	0.153	0.353	3.54384229245599E-91	0	SNX1
1.43825348563264E-68	0.328289743340662	0.178	0.359	2.3459352604154E-64	0	TNFRSF14
1.17143732429674E-64	0.324393266255705	0.117	0.254	1.91073141966042E-60	0	GGA2
1.13772826910189E-77	0.320670018043868	0.109	0.257	1.85574857973209E-73	0	CALM3
1.96521037625668E-64	0.319339847167694	0.174	0.346	3.20545464471227E-60	0	MBD2
4.53239831376617E-89	0.318708200793104	0.113	0.275	7.392794889584E-85	0	STAT3
4.33469910388401E-86	0.311935281689459	0.109	0.265	7.0703277083452E-82	0	TRPC4AP
2.16528259012137E-98	0.305573857914682	0.103	0.269	3.53179243274697E-94	0	UBR4
3.39926704844613E-95	0.301775980068285	0.123	0.3	5.54454448272049E-91	0	ARL6IP4
8.12342207254712E-101	0.290358900030317	0.113	0.289	1.32501137425316E-96	0	POGZ
1.873537263184E-97	0.287231249199831	0.107	0.275	3.05592662997943E-93	0	STXBP2
4.09175617301549E-97	0.287084358391281	0.112	0.282	6.67406349380557E-93	0	CALCOCO2
5.88132541420911E-98	0.283454944589622	0.092	0.25	9.59302988311648E-94	0	ZSWIM8
4.03863904873426E-102	0.269609900933086	0.139	0.336	6.58742415239045E-98	0	DPYD
2.65758092599395E-96	0.266803406004091	0.158	0.363	4.33478024838874E-92	0	EIF3A
2.52668484715529E-106	0.260222402472496	0.092	0.259	4.12127565419499E-102	0	TRIM27
1.32962165490822E-79	0.257673394773284	0.158	0.338	2.16874588132079E-75	0	ACTN4

1.81995738310279E-115	0.256494243843156	0.091	0.265	2.96853248757896E-111	0	NARS
1.6981117199997E-96	0.251659527105785	0.099	0.26	2.76979002649151E-92	0	TXNDC11
0	5.72637097479363	0.911	0.053	0	1	ITGAX
0	5.42324560245664	0.463	0.015	0	1	PLA2G7
0	5.26651085425185	0.593	0.024	0	1	EMILIN2
0	5.24043023800258	0.336	0.011	0	1	FPR3
0	5.05797617722743	0.297	0.012	0	1	CD300LF
0	4.77423908475672	0.299	0.024	0	1	MMP19
0	4.69609199094342	0.408	0.02	0	1	ADGRE2
0	4.67913432954191	0.441	0.033	0	1	HSPA6
0	4.67669008383387	0.461	0.028	0	1	KCNMA1
0	4.63077924325642	0.567	0.031	0	1	CLEC7A
0	4.61473647244168	0.589	0.036	0	1	C5AR1
0	4.57133199263765	0.362	0.045	0	1	IL1RN
0	4.55154314580126	0.367	0.017	0	1	TFEC
0	4.54785425159386	0.577	0.04	0	1	GPNMB
0	4.50856961756906	0.618	0.036	0	1	LILRB2
0	4.48035007312011	0.632	0.048	0	1	PLAUR
0	4.47429148285548	0.315	0.022	0	1	NCF2
0	4.46592565878741	0.715	0.044	0	1	MS4A7
0	4.40995622967738	0.513	0.022	0	1	CSF2RA
0	4.40467437294109	0.296	0.025	0	1	RAB7B
0	4.39839556676674	0.263	0.015	0	1	IL18
0	4.1920042032472	0.38	0.024	0	1	CSF3R
0	4.18438198968876	0.406	0.05	0	1	TLR2
0	4.14464846759018	0.62	0.038	0	1	MSR1
0	4.12284871755702	0.404	0.028	0	1	FAM49A
0	4.09731968531108	0.32	0.022	0	1	EPB41L3
0	4.06013655473868	0.811	0.068	0	1	FGL2
0	4.04715134673232	0.284	0.038	0	1	MERTK
0	4.02302573979714	0.492	0.038	0	1	SLC8A1
0	4.00109215062521	0.267	0.02	0	1	IGSF6
0	3.98712219689738	0.589	0.091	0	1	DMXL2
0	3.95082355420975	0.295	0.024	0	1	FCGR2A
0	3.91665826502966	0.415	0.037	0	1	PLXDC2
0	3.90651522675842	0.422	0.02	0	1	GPR183
0	3.89824422168947	0.332	0.025	0	1	HCK
0	3.86488451857835	0.836	0.175	0	1	TNFAIP2
0	3.76632376099352	0.25	0.016	0	1	SLAMF8
0	3.73738775097637	0.73	0.052	0	1	CSF1R
0	3.7336266858168	0.783	0.082	0	1	SPI1
0	3.73170261718356	0.325	0.022	0	1	CAPG
0	3.72917017615444	0.401	0.057	0	1	SLC43A2
0	3.69802090465828	0.315	0.037	0	1	HBEGF
0	3.67929479430286	0.315	0.04	0	1	LHFPL2
0	3.67338776769807	0.298	0.039	0	1	SLC37A2
0	3.66523861377491	0.718	0.097	0	1	SGK1
0	3.65568497000108	0.267	0.034	0	1	TNFSF13
0	3.63833565223335	0.295	0.046	0	1	GLA
0	3.63631870674841	0.431	0.069	0	1	ASAH1

0	3.61845337974816	0.293	0.027	0	1	LRRC25
0	3.61504330101712	0.559	0.067	0	1	SLC15A3
0	3.61429348307158	0.25	0.018	0	1	C3AR1
0	3.59982698065079	0.542	0.104	0	1	MPP1
0	3.58993186189317	0.768	0.205	0	1	LGMN
0	3.58514752183029	0.423	0.089	0	1	SCO2
0	3.58316704756661	0.5	0.088	0	1	ATP6V1B2
0	3.57722540435424	0.967	0.322	0	1	SAT1
0	3.55958265260986	0.375	0.04	0	1	LGALS3
0	3.5542139870329	0.692	0.119	0	1	CD68
0	3.55061585300646	0.9	0.264	0	1	IFI30
0	3.54007189315667	0.371	0.034	0	1	NR4A3
0	3.53937492755819	0.413	0.053	0	1	FMNL2
0	3.50472458935566	0.626	0.055	0	1	TYROBP
0	3.49455078222586	0.275	0.023	0	1	CALHM6
0	3.49231061593699	0.64	0.058	0	1	CYBB
0	3.49226045947858	0.451	0.049	0	1	AIF1
0	3.47439442527207	0.493	0.041	0	1	FGD2
0	3.46913044694516	0.599	0.06	0	1	KCTD12
0	3.46569476082396	0.287	0.026	0	1	MS4A4A
0	3.46544742295897	0.384	0.038	0	1	SEMA4A
0	3.45098761641325	0.57	0.056	0	1	RGS2
0	3.43062238188231	0.451	0.036	0	1	CD72
0	3.40067621496021	0.392	0.08	0	1	FCHO2
0	3.39719206199657	0.616	0.063	0	1	CD83
0	3.37088533265166	0.929	0.329	0	1	PSAP
0	3.36440755424232	0.382	0.035	0	1	LILRB1
0	3.34674855017249	0.3	0.065	0	1	RNF144B
0	3.3458486639258	0.46	0.035	0	1	LAIR1
0	3.3277036756613	0.294	0.044	0	1	ZNF385A
0	3.3237928115157	0.41	0.082	0	1	GM2A
0	3.32026417451407	0.306	0.076	0	1	AVPI1
0	3.31969019575973	0.63	0.077	0	1	FCER1G
0	3.31704155837706	0.741	0.065	0	1	MPEG1
0	3.30827767169569	0.708	0.149	0	1	LIPA
0	3.30628924179702	0.514	0.048	0	1	CPVL
0	3.26949438070342	0.428	0.036	0	1	SIGLEC1
0	3.25668108599997	0.691	0.073	0	1	THEMIS2
0	3.24749211328972	0.412	0.058	0	1	ADAP2
0	3.2298637026116	0.339	0.067	0	1	SIRPA
0	3.19972530590278	0.748	0.117	0	1	CIITA
0	3.18319675388724	0.82	0.239	0	1	CTSZ
0	3.18017382182072	0.282	0.022	0	1	IL4I1
0	3.16516933485299	0.262	0.033	0	1	SLC7A7
0	3.15915215747862	0.283	0.029	0	1	SCIMP
0	3.1457000507136	0.38	0.109	0	1	KYNU
0	3.14276232982644	0.459	0.124	0	1	APBB3
0	3.13600761650744	0.427	0.062	0	1	IL18BP
0	3.13095309911055	0.346	0.077	0	1	DAPK1
0	3.11469513455763	0.586	0.171	0	1	TGFBI

0	3.11420501324152	0.624	0.16	0	1	MFSD1
0	3.11079302228265	0.746	0.1	0	1	C1QA
0	3.09580479471883	0.459	0.128	0	1	INSIG1
0	3.07131333793981	0.864	0.277	0	1	LYZ
0	3.06141857199088	0.538	0.112	0	1	MAFB
0	3.04937353535789	0.766	0.089	0	1	C1QC
0	3.04709236922575	0.271	0.049	0	1	DEPRECATE
0	3.04509878925187	0.32	0.076	0	1	RASSF4
0	3.0240103980954	0.734	0.111	0	1	C1QB
0	3.0179275160712	0.41	0.118	0	1	DOCK5
4.81182375332454E-271	3.01568038848712	0.307	0.101	7.84856572404765E-267	1	FGD4
0	3.01238758202854	0.348	0.097	0	1	SRC
0	2.9702472405784	0.474	0.134	0	1	ABCA1
0	2.96510580306914	0.386	0.039	0	1	FOLR2
0	2.96291504599848	0.415	0.037	0	1	HK3
0	2.95822484075705	0.758	0.079	0	1	CD163
0	2.94829052741544	0.358	0.083	0	1	PDE4DIP
0	2.93338966069378	0.59	0.168	0	1	NAMPT
0	2.91729651031109	0.337	0.06	0	1	LYN
0	2.88229534545943	0.797	0.279	0	1	GLUL
0	2.8753498898872	0.328	0.073	0	1	C9orf72
0	2.87345257301234	0.319	0.042	0	1	MRC2
0	2.86864785724459	0.297	0.029	0	1	FGR
0	2.86247125550619	0.348	0.092	0	1	RNF130
0	2.83853738829912	0.26	0.041	0	1	TNFSF13B
0	2.83800452717145	0.83	0.275	0	1	TYMP
0	2.82386817251547	0.507	0.069	0	1	PLEK
0	2.82029761293523	0.284	0.036	0	1	GRK3
0	2.81847891073936	0.262	0.057	0	1	CCDC88A
0	2.81543403853602	0.54	0.122	0	1	FOS
0	2.80482527698284	0.433	0.146	0	1	SLCO2B1
0	2.78507207773991	0.626	0.157	0	1	TTYH3
0	2.78481338771448	0.645	0.086	0	1	MS4A6A
0	2.77072262060269	0.802	0.267	0	1	GRN
0	2.76952608226049	0.454	0.062	0	1	NFKBID
0	2.73816966423267	0.39	0.106	0	1	RAB20
0	2.73641189443675	0.369	0.09	0	1	RASGEF1B
0	2.73302079681158	0.601	0.154	0	1	HMOX1
0	2.73144033636575	0.318	0.04	0	1	BASP1
0	2.72517233627226	0.48	0.141	0	1	CTSH
0	2.72166947942498	0.711	0.211	0	1	CTSS
0	2.7175364104247	0.52	0.142	0	1	NPC2
0	2.71748650646335	0.25	0.032	0	1	CSF2RB
0	2.71540935697997	0.78	0.155	0	1	PFKFB3
0	2.70671997716956	0.361	0.109	0	1	MCOLN1
0	2.6932712952031	0.656	0.226	0	1	PLD3
0	2.67231331163445	0.691	0.133	0	1	SDC3
7.44094390457674E-219	2.67198640043808	0.31	0.12	1.21369236027551E-214	1	ELL2
0	2.67071966976505	0.447	0.156	0	1	ODF3B
0	2.6664801427189	0.682	0.243	0	1	MAT2A

0	2.6652365052814	0.622	0.191	0	1	CTSL
0	2.65979788359395	0.286	0.063	0	1	RGL1
9.67893220477452E-295	2.65644229148852	0.285	0.078	1.57873063192077E-290	1	IFNGR2
0	2.65185109870141	0.312	0.077	0	1	RENBP
0	2.64382953200273	0.401	0.118	0	1	ACP5
9.1226199678767E-242	2.64094101213518	0.341	0.134	1.48799054296037E-237	1	NR1H3
0	2.63756642257907	0.373	0.048	0	1	CFD
0	2.63055186205355	0.609	0.082	0	1	ADA2
0	2.59777577066574	0.275	0.061	0	1	ADAM9
0	2.58308580055025	0.357	0.099	0	1	CLN8
0	2.58129606727991	0.693	0.197	0	1	MARCKS
0	2.55867215341107	0.371	0.109	0	1	NPL
0	2.54615344932164	0.268	0.05	0	1	CD9
0	2.54311879114505	0.296	0.075	0	1	DEPRECATE
0	2.52991175755651	0.622	0.153	0	1	NAGK
0	2.52062065675465	0.334	0.061	0	1	PTPRE
0	2.50804578316361	0.41	0.117	0	1	PLSCR1
0	2.5027049455051	0.506	0.082	0	1	TIMP2
0	2.50249045463049	0.282	0.032	0	1	GAS7
0	2.49102063224896	0.364	0.112	0	1	SCPEP1
6.95557656964007E-221	2.4886658497623	0.358	0.152	1.13452409427399E-216	1	TFRC
0	2.48620041212109	0.279	0.039	0	1	NRROS
0	2.46419460488044	0.508	0.076	0	1	FCGR3A
0	2.46132426237794	0.304	0.049	0	1	VASH1
0	2.456277930754	0.372	0.052	0	1	SLC16A3
0	2.43098327144205	0.379	0.081	0	1	HPS3
0	2.4294863804711	0.354	0.1	0	1	ZFYVE16
0	2.4242301709214	0.919	0.372	0	1	CTSB
0	2.4221445717497	0.346	0.074	0	1	UBXN11
4.58697214839291E-240	2.41342414589506	0.278	0.086	7.48181027124368E-236	1	DAB2
0	2.40675776386121	0.354	0.088	0	1	GSAP
9.03120064301414E-260	2.39112962371133	0.335	0.121	1.47307913688204E-255	1	CYFIP1
2.92826164884048E-295	2.38023219614862	0.283	0.075	4.77628757542371E-291	1	HEATR3
0	2.37754298790879	0.722	0.228	0	1	FCGRT
0	2.37249306162373	0.399	0.129	0	1	NINJ1
0	2.37178343428188	0.274	0.049	0	1	DSE
0	2.36874142571581	0.465	0.159	0	1	GNS
0	2.36289505592336	0.488	0.071	0	1	KLHL6
0	2.36285733316935	0.35	0.045	0	1	AXL
0	2.35742864670258	0.603	0.128	0	1	GNA13
0	2.35316730392409	0.985	0.773	0	1	CD74
0	2.35101372929636	0.454	0.149	0	1	TSPAN4
0	2.35041251375082	0.564	0.199	0	1	GAA
0	2.33459379150752	0.483	0.175	0	1	CXCL16
4.76591924859909E-245	2.33347808913633	0.292	0.094	7.77369088638998E-241	1	DOCK4
0	2.32024453921999	0.531	0.173	0	1	QKI
0	2.28785623688918	0.474	0.121	0	1	SH3TC1
0	2.27114824984445	0.837	0.299	0	1	ARAP1
0	2.25991540198735	0.467	0.059	0	1	AOAH
0	2.25712479801201	0.483	0.099	0	1	GSN

2.27746969256123E-241	2.24828906347927	0.309	0.106	3.71478081553662E-237	1	JDP2
1.66425697585836E-279	2.23505570383303	0.337	0.112	2.71456955332257E-275	1	IER3
0	2.23493847615011	0.403	0.118	0	1	WARS
0	2.22639912481071	0.486	0.079	0	1	ZNF331
2.84276996757517E-299	2.21983744394637	0.409	0.16	4.63684209411186E-295	1	TNS3
0	2.21274112427578	0.472	0.141	0	1	STX7
0	2.20024548350739	0.587	0.108	0	1	ZEB2
0	2.19809253868294	0.823	0.319	0	1	CST3
0	2.19794375662824	0.38	0.085	0	1	ATG7
2.34567363900859E-162	2.19647388856885	0.291	0.127	3.82602827258691E-158	1	TBC1D8
0	2.19560517004729	0.642	0.149	0	1	PLEKHO1
0	2.19538976496629	0.34	0.065	0	1	TMEM106A
0	2.19230479862155	0.455	0.136	0	1	SDS
1.3997309987097E-95	2.18796220230713	0.255	0.135	2.28310123199538E-91	1	FAM20A
3.18665157888312E-193	2.18618592645745	0.252	0.084	5.19774739031625E-189	1	C15orf39
0	2.15571097790271	0.891	0.411	0	1	FTH1
3.99714463389034E-220	2.10415842891677	0.33	0.13	6.51974261233853E-216	1	OAZ2
3.51366678849138E-110	2.09136122600611	0.266	0.135	5.7311418987083E-106	1	FAM20C
0	2.08996714807395	0.47	0.169	0	1	STX4
0	2.08892478167968	0.498	0.11	0	1	PLEKHO2
0	2.0879480518729	0.403	0.105	0	1	IFNGR1
2.58689065195807E-171	2.07073106637256	0.283	0.114	4.21947734240881E-167	1	AMPD2
0	2.06530243965328	0.712	0.291	0	1	CEBPD
0	2.05933583890761	0.68	0.253	0	1	MGAT1
6.84549723545348E-294	2.04758660082224	0.267	0.063	1.11656905407482E-289	1	MRC1
4.46064299417984E-225	2.03511613861706	0.264	0.081	7.27575478780673E-221	1	SMS
0	2.0324317478879	0.306	0.055	0	1	ABR
6.47627164252181E-227	2.02409400130191	0.285	0.093	1.05634466761173E-222	1	SLC26A11
0	2.00984924016681	0.411	0.074	0	1	RELT
0	1.99990895117971	0.689	0.269	0	1	LRP1
1.41471393403006E-231	1.99203033599789	0.262	0.076	2.30753989779643E-227	1	RCN3
0	1.99171534977462	0.373	0.099	0	1	SH2B3
0	1.98727473310963	0.468	0.159	0	1	MAN2B1
2.1643103258054E-263	1.97888793458263	0.411	0.173	3.53020657242119E-259	1	LAP3
4.77101785999922E-293	1.9754699200807	0.45	0.187	7.78200723144472E-289	1	CHMP1B
3.78383697373187E-213	1.97351583847791	0.297	0.106	6.17181648785405E-209	1	YTHDF3
0	1.97258023076247	0.375	0.075	0	1	DENND3
1.37782963710598E-186	1.97101479611714	0.34	0.153	2.24737792108356E-182	1	CORO1B
0	1.96771385521182	0.583	0.134	0	1	NABP1
0	1.93813859968131	0.823	0.351	0	1	DUSP1
2.70965034630664E-137	1.9377323974223	0.282	0.128	4.41971067986075E-133	1	SRA1
0	1.92982014400767	0.427	0.123	0	1	RAP2B
1.04736178831772E-180	1.92938067275462	0.273	0.101	1.70835181292504E-176	1	DDAH2
9.00405240450034E-126	1.9276656604445	0.253	0.113	1.46865098769805E-121	1	PGD
0	1.92283151167253	0.705	0.283	0	1	CD14
9.03270593902902E-294	1.91867708160468	0.441	0.183	1.47332466571502E-289	1	HERPUD1
0	1.9079260248909	0.347	0.077	0	1	RILPL2
6.57935938188279E-294	1.89968888197909	0.408	0.153	1.0731593087789E-289	1	ATF3
0	1.88898704535539	0.311	0.08	0	1	LGALS9
0	1.8805657884941	0.627	0.129	0	1	CD4

9.40272386024934E-282	1.86471890401988	0.402	0.155	1.53367828884527E-277	1	ANXA5
1.29300305814698E-146	1.85253659177809	0.325	0.162	2.10901728814354E-142	1	RBM47
0	1.83951755351583	0.59	0.2	0	1	ARRB2
9.0125165680017E-130	1.83528811008268	0.254	0.111	1.47003157740676E-125	1	ACP2
4.22081557632496E-165	1.8308209632153	0.384	0.198	6.88457228654364E-161	1	ACSL1
0	1.8273451021914	0.539	0.245	0	1	CEBPB
3.08877805938214E-143	1.80792144804047	0.266	0.113	5.03810589265821E-139	1	KIAA0930
0	1.80421865221879	0.584	0.299	0	1	RHOB
0	1.80058582113714	0.407	0.143	0	1	ATP6AP1
3.92735558350082E-210	1.78992671580839	0.279	0.094	6.40590969224819E-206	1	H2AFY
0	1.77874713820115	0.588	0.176	0	1	DEPRECATE
0	1.7694202997872	0.912	0.371	0	1	SRGN
2.48976448422103E-282	1.76664797124875	0.398	0.149	4.06105485021291E-278	1	GNAQ
2.82858940226871E-300	1.76276396381124	0.303	0.08	4.61371217404049E-296	1	STX11
0	1.75745227464872	0.405	0.137	0	1	COLGALT1
0	1.75329645142757	0.42	0.122	0	1	RNASET2
0	1.75250939916948	0.697	0.297	0	1	SOD2
0	1.7504502051812	0.712	0.333	0	1	CD63
0	1.74350396420835	0.413	0.138	0	1	SH3BP5
1.36744131526601E-155	1.73793832144684	0.326	0.156	2.23043352933039E-151	1	PLBD2
0	1.72760244238606	0.76	0.214	0	1	LAPTM5
0	1.71776030661219	0.647	0.201	0	1	SAMHD1
8.20260255142293E-115	1.71610216022064	0.263	0.125	1.33792650216259E-110	1	ITPRIPL2
4.37063917024231E-226	1.714904918537	0.423	0.204	7.12894955058223E-222	1	CREG1
0	1.71416532473072	0.319	0.056	0	1	TRPS1
0	1.70819397210607	0.387	0.112	0	1	PTPN6
1.51884188091917E-257	1.70593405689488	0.277	0.078	2.47738299196727E-253	1	RHBDF2
0	1.70022062623141	0.495	0.143	0	1	IRF8
1.01365353085072E-269	1.69870873905193	0.308	0.091	1.65337027417061E-265	1	JAK2
1.33527743608669E-171	1.69419159612429	0.25	0.089	2.17797102600099E-167	1	SDCBP
0	1.69360491533226	0.366	0.094	0	1	ATG16L2
9.58257079642481E-98	1.68144015361173	0.271	0.144	1.56301312260485E-93	1	SLC35F6
0	1.67813212243535	0.744	0.326	0	1	VMP1
0	1.67162313578988	0.554	0.166	0	1	CTSC
5.59798737673271E-166	1.67026892188082	0.341	0.16	9.13087721018873E-162	1	RUFY3
5.18602970549284E-227	1.66789755307997	0.26	0.076	8.45893305262937E-223	1	MKNK1
0	1.66562249738464	0.26	0.051	0	1	NRP2
1.04612155804395E-110	1.64818095474379	0.264	0.127	1.70632887332549E-106	1	ZMIZ1
1.22377045671701E-123	1.64673256329227	0.25	0.109	1.99609199195112E-119	1	CACUL1
0	1.64339969822366	0.357	0.069	0	1	CYTH4
5.13853161087346E-88	1.62917132730324	0.284	0.163	8.38145891049571E-84	1	ATP1B1
0	1.62496211595036	0.774	0.372	0	1	TPP1
2.41992353802955E-220	1.61554359304381	0.446	0.224	3.94713728288E-216	1	BLVRB
2.13777316140848E-184	1.60977941246729	0.399	0.201	3.48692180357337E-180	1	AKR1A1
6.12664253226198E-193	1.6043588185169	0.292	0.108	9.99316663437252E-189	1	TGIF1
0	1.5904760298304	0.265	0.053	0	1	CFP
0	1.59010155259858	0.299	0.032	0	1	WDFY4
0	1.56830063324127	0.39	0.069	0	1	CD300A
1.70856554113851E-229	1.56274671137995	0.272	0.08	2.78684125415102E-225	1	DPYSL2
0	1.54100161851163	0.581	0.255	0	1	NOTCH2NL(

0	1.54060449072778	0.486	0.127	0	1	REL
0	1.53626100177282	0.438	0.15	0	1	GRB2
0	1.52417630082042	0.331	0.087	0	1	VCAM1
1.86249180565427E-167	1.5105822238337	0.316	0.136	3.03791038420268E-163	1	TPD52L2
0	1.50498611953992	0.711	0.217	0	1	ITGB2
0	1.5036536206684	0.425	0.077	0	1	CD5L
1.86056232422996E-241	1.49286026808395	0.501	0.258	3.03476320705148E-237	1	GRINA
0	1.49220032544601	0.478	0.184	0	1	SYNGR2
6.6991318207322E-241	1.48166826775946	0.315	0.101	1.09269539127963E-236	1	ETV6
8.86349527203539E-283	1.4734313038541	0.272	0.064	1.44572471382169E-278	1	LPAR6
1.0987482964907E-258	1.47212527933031	0.279	0.075	1.79216834640599E-254	1	GSTP1
5.42502085967405E-175	1.47145139096443	0.258	0.088	8.84875152421434E-171	1	SCARF1
6.09476730569104E-87	1.47064286868136	0.289	0.167	9.94117495231265E-83	1	NCOA4
0	1.46938581644261	0.881	0.411	0	1	FTL
1.21299776970002E-107	1.46480917180594	0.343	0.199	1.9785206621577E-103	1	GADD45B
7.41483900388214E-73	1.46021674038714	0.297	0.188	1.20943438992322E-68	1	SCARB1
0	1.45957938361725	0.743	0.351	0	1	WSB1
2.18272344529152E-160	1.45303155665739	0.293	0.12	3.560240211615E-156	1	DENND1A
4.54378697604212E-187	1.44304106645552	0.261	0.085	7.41137093662231E-183	1	DYSF
0	1.43379466286684	0.465	0.098	0	1	PARVG
0	1.43092765167877	0.602	0.301	0	1	PLXNB2
0	1.42897241977309	0.345	0.08	0	1	ST8SIA4
0	1.42055472060552	0.36	0.082	0	1	APOBR
1.76212107553611E-147	1.41884633525634	0.286	0.122	2.87419568630695E-143	1	AP1B1
0	1.41785327778092	0.53	0.197	0	1	FOSL2
2.79382594582017E-174	1.40311502856799	0.387	0.187	4.55700950022728E-170	1	ICAM1
1.64899441630771E-184	1.39995902272406	0.388	0.181	2.68967479243951E-180	1	MYO15B
0	1.39931543035532	0.301	0.071	0	1	FAR1
1.0334146822293E-54	1.3971456953748	0.255	0.163	1.68560268818421E-50	1	MGLL
0	1.39230788738041	0.566	0.204	0	1	MYO9B
2.06964717889435E-270	1.39192860561072	0.49	0.224	3.37580151349458E-266	1	LGALS3BP
0	1.388242101152	0.697	0.301	0	1	RNF149
3.14577045977484E-197	1.38712068135475	0.454	0.238	5.13106619693874E-193	1	APLP2
0	1.37823802879126	0.521	0.219	0	1	LPCAT1
1.28831264311297E-139	1.37104776870123	0.293	0.13	2.10136675218157E-135	1	MYD88
1.18033275761971E-172	1.36214900147046	0.266	0.095	1.92524076095351E-168	1	CREBL2
2.36946081627276E-237	1.36007221564907	0.52	0.273	3.86482753742249E-233	1	RNH1
1.020750324982E-195	1.3590306406921	0.48	0.251	1.66494585507815E-191	1	HSPA1A
4.88505750013827E-251	1.35706865351203	0.591	0.318	7.96801728847554E-247	1	CTSD
1.07075778792837E-249	1.35036505163266	0.269	0.07	1.74651302788997E-245	1	LFNG
3.56931028006514E-94	1.35017783997744	0.382	0.243	5.82190199781425E-90	1	SLC40A1
0	1.34851824918771	0.371	0.08	0	1	FERMT3
2.78006424856523E-205	1.34296123997183	0.325	0.121	4.53456279583475E-201	1	ACOT9
1.06678926206107E-238	1.34160363763345	0.517	0.268	1.74003996534781E-234	1	BRI3
7.01225782594397E-286	1.33129126756213	0.532	0.249	1.14376937398972E-281	1	DPYD
1.70711705834078E-92	1.32804664764814	0.341	0.208	2.78447863385964E-88	1	RXRA
1.19298043715782E-100	1.3250900330164	0.282	0.143	1.94587039104813E-96	1	SERPINB6
1.04741072642429E-151	1.3243488757915	0.348	0.167	1.70843163587066E-147	1	RTN4
1.48598398385689E-131	1.31547729202086	0.326	0.159	2.42378847606898E-127	1	LGALS1
1.80703805804659E-236	1.31497209328402	0.466	0.22	2.9474597764798E-232	1	CLCN7

4.3498887759054E-81	1.31144594444743	0.252	0.135	7.0951035823793E-77	1	WDR81
7.83143184458606E-86	1.30624924034549	0.363	0.229	1.27738484817043E-81	1	EXT1
2.95867120975449E-132	1.30406054843027	0.35	0.185	4.82588861023055E-128	1	HEXA
0	1.3015945414195	0.723	0.315	0	1	EHBP1L1
2.37357776973203E-123	1.29108583908287	0.296	0.143	3.87154270020991E-119	1	NOTCH2NLE
0	1.28309804680219	0.723	0.392	0	1	LITAF
3.64329087536754E-217	1.27220205757037	0.462	0.226	5.942571746812E-213	1	NFE2L2
2.77529439980952E-124	1.2618383941937	0.387	0.215	4.5267826955293E-120	1	DEPRECATE
1.04502253290072E-122	1.25866391272076	0.314	0.157	1.70453625341436E-118	1	PIK3AP1
1.57036470820787E-113	1.25074844344421	0.25	0.11	2.56142187555786E-109	1	PRNP
2.5302117524422E-120	1.23414897247346	0.29	0.139	4.12702838940846E-116	1	SFT2D2
0	1.223965409951	0.483	0.122	0	1	PLCB2
0	1.22248385500431	0.767	0.286	0	1	CYBA
1.43630889062502E-73	1.20614729153667	0.313	0.197	2.34276343149847E-69	1	NADK
3.88667628140079E-108	1.2012766015505	0.41	0.24	6.33955768259282E-104	1	GBP1
5.00681651108387E-110	1.18405800920726	0.388	0.229	8.1666184112289E-106	1	CDKN1A
2.26620576687129E-293	1.17410847025551	0.577	0.272	3.69640822634376E-289	1	TNFRSF14
3.24101584000948E-112	1.16924829961651	0.304	0.151	5.28642093663947E-108	1	KDM6B
7.08287500996358E-166	1.16777696968138	0.411	0.21	1.15528774287516E-161	1	VAMP8
6.89309624074659E-116	1.16474623354403	0.258	0.113	1.12433292782818E-111	1	AFF1
8.19965749020365E-189	1.162223416342	0.304	0.11	1.33744613322712E-184	1	MAP3K8
7.58228773662544E-281	1.1584909319863	0.535	0.239	1.23674695272098E-276	1	EFHD2
3.89671293249945E-216	1.15763107227965	0.532	0.279	6.35592846419986E-212	1	NFKBIA
0	1.15477841597379	0.646	0.199	0	1	ARL4C
9.00529658673104E-250	1.14534334069687	0.289	0.079	1.4688539262617E-245	1	ABI3
1.84093013057421E-112	1.13092589378296	0.264	0.117	3.00274113597959E-108	1	NRP1
0	1.11918843911717	0.696	0.288	0	1	VIM
0	1.11556044882459	0.666	0.211	0	1	HCLS1
5.44766311764215E-99	1.11245036050656	0.28	0.143	8.88568331118612E-95	1	VEGFB
1.46217731359529E-170	1.1117325292093	0.305	0.119	2.38495741620528E-166	1	GPCPD1
1.29951741364661E-213	1.11133247139095	0.526	0.274	2.11964285339898E-209	1	ZYX
4.5200132594458E-183	1.10984469334863	0.354	0.146	7.37259362748204E-179	1	IFNAR2
1.35180269058577E-131	1.10813547038756	0.314	0.144	2.20492536861445E-127	1	UGCG
2.72570494178276E-254	1.10714870449181	0.298	0.084	4.44589733054185E-250	1	NR4A1
4.73666353801199E-115	1.10400486880158	0.388	0.224	7.72597189685136E-111	1	MT-ND3
7.1444345886545E-103	1.10376533590007	0.378	0.23	1.16532872575544E-98	1	CTSA
6.97072292533243E-131	1.10363785791381	0.273	0.116	1.13699461635097E-126	1	PNPLA6
1.1319013263075E-169	1.10223326941177	0.46	0.243	1.84624425334017E-165	1	TMEM176B
4.94728111374392E-132	1.09521547940769	0.549	0.341	8.06951022462771E-128	1	SELENOP
9.85692998860617E-231	1.09405771893958	0.33	0.108	1.60776385044155E-226	1	ARHGEF2
2.16312923751332E-72	1.09315409753929	0.302	0.181	3.52828009930798E-68	1	IER5
1.66855650510532E-127	1.08979149981539	0.288	0.128	2.72158251547729E-123	1	SNX6
5.49517499473785E-169	1.08176129804961	0.368	0.165	8.96317993391691E-165	1	PTPN1
3.47026719175255E-234	1.08135314834672	0.344	0.114	5.66035281646758E-230	1	RUNX1
2.62706913490436E-157	1.07954393462719	0.439	0.239	4.2850124659425E-153	1	STAT2
6.22401182455791E-35	1.05874874351576	0.269	0.196	1.01519856870364E-30	1	IRS2
9.00134223073652E-230	1.05116808977782	0.429	0.178	1.46820893125543E-225	1	M6PR
1.20730198227712E-188	1.04082344735389	0.471	0.238	1.96923026329222E-184	1	PER1
0	1.02471632901322	0.71	0.218	0	1	METRNL
9.96403797320714E-131	1.02356356486073	0.378	0.199	1.62523423380982E-126	1	PARP14

0	1.01662913863358	0.449	0.124	0	1	DOK2
5.45718716111738E-249	1.01610003597738	0.667	0.369	8.90121797849856E-245	1	TMSB10
0	1.01426412753971	0.452	0.15	0	1	MICAL1
2.73967294823296E-116	1.0100364573199	0.267	0.119	4.46868054586278E-112	1	CERT1
0	1.00980753502374	0.822	0.483	0	1	JUNB
2.18435569066342E-134	1.00763386501113	0.342	0.168	3.56290256704111E-130	1	PLEKHB2
0	0.999273162320429	0.447	0.128	0	1	UCP2
7.54501903424991E-73	0.995782804862117	0.326	0.203	1.2306680546765E-68	1	BACH1
1.90800623846303E-292	0.991616463446043	0.721	0.402	3.11214897555705E-288	1	ZFP36
0	0.987984921372813	0.579	0.198	0	1	TNFRSF1B
3.62709372397163E-177	0.98525158775698	0.454	0.228	5.91615257317013E-173	1	SLC3A2
8.35860325723789E-47	0.974150997221733	0.304	0.212	1.36337177728807E-42	1	NUDC
5.83200732592654E-119	0.963470551561418	0.356	0.189	9.51258714931878E-115	1	ARRDC2
6.86505609639097E-91	0.96055084501972	0.282	0.147	1.11975929988233E-86	1	ARMH3
1.18661755770032E-90	0.956470252253577	0.273	0.14	1.93549189836498E-86	1	PLEKHM2
4.24013184731016E-71	0.95485922877513	0.268	0.152	6.9160790561476E-67	1	FBXL5
2.47742018503951E-130	0.938954573711265	0.37	0.191	4.04092006381794E-126	1	DENND4B
2.57737814370715E-271	0.93432799322509	0.346	0.1	4.20396149020073E-267	1	ADCY7
0	0.929661594156349	0.412	0.079	0	1	STAB1
3.75660840730868E-90	0.922938723299798	0.305	0.167	6.12740397316119E-86	1	DNAJC5
8.40055118188219E-220	0.919818832087832	0.278	0.082	1.3702139032768E-215	1	CXCL12
2.67314485782574E-178	0.904460765318959	0.297	0.102	4.36016657759956E-174	1	COTL1
0	0.90386958961525	0.414	0.113	0	1	NCKAP1L
1.82193374073843E-87	0.902233745601518	0.347	0.206	2.97175612451846E-83	1	ITPR2
8.57876277162655E-37	0.900018470394538	0.272	0.195	1.39928199568001E-32	1	ADIPOR1
0	0.89540044464671	0.582	0.183	0	1	IL10RA
2.77102158692149E-59	0.888330664572715	0.314	0.206	4.51981331042764E-55	1	PDXK
0	0.885949712978872	0.493	0.149	0	1	MYO1F
3.23632070185433E-34	0.884597763273439	0.255	0.182	5.2787626967946E-30	1	RREB1
6.8248572051986E-85	0.877819000554256	0.285	0.153	1.11320245873994E-80	1	SP3
1.39314239903387E-66	0.866028233642988	0.25	0.14	2.27235456706415E-62	1	TMEM127
1.23550701038437E-184	0.862994373690521	0.421	0.188	2.01523548463795E-180	1	RIN3
3.91398345803269E-118	0.854991781972989	0.28	0.125	6.38409841839712E-114	1	SGSH
4.33529729353249E-137	0.851996409953028	0.463	0.264	7.07130341548084E-133	1	MINK1
6.0923717827189E-117	0.844830065663797	0.406	0.234	9.9372676147928E-113	1	EIF4A1
1.20606639897657E-64	0.842933060121692	0.31	0.194	1.96721490337069E-60	1	INPPL1
9.40749655427417E-40	0.841307840400919	0.286	0.204	1.53445676296766E-35	1	LAMP2
5.36004703731928E-202	0.841049148323899	0.699	0.413	8.74277272257147E-198	1	MT-CO3
2.11043409069067E-61	0.830012874730084	0.307	0.196	3.44232904532555E-57	1	YBX1
2.73951847417695E-97	0.823648995396918	0.365	0.213	4.46842858323002E-93	1	STXBP2
1.97698019749057E-117	0.82243604605896	0.268	0.115	3.22465240012687E-113	1	SLC6A6
4.17685072177775E-123	0.820660104546708	0.385	0.206	6.81286121229168E-119	1	ARPC1B
4.24952152435752E-155	0.82043114772023	0.583	0.343	6.93139455837956E-151	1	MT-ATP6
4.09710134972426E-65	0.814947439364553	0.267	0.156	6.68278201153525E-61	1	TNRC18
7.31885279860692E-259	0.812626509259885	0.884	0.616	1.19377807998078E-254	1	MCL1
1.70638087280827E-118	0.807411062649804	0.353	0.177	2.78327784163758E-114	1	SH3BGRL3
1.33453738906766E-104	0.802262556987477	0.365	0.206	2.17676393530826E-100	1	IGF2R
6.61607812200273E-133	0.800896882779538	0.46	0.264	1.07914850247987E-128	1	H3F3A
8.96444242964759E-49	0.796286209253907	0.252	0.158	1.46219020469982E-44	1	TMUB2
4.99847179482748E-41	0.794390369770413	0.259	0.174	8.1530073445431E-37	1	GUSB

1.53612184871981E-207	0.789201968033632	0.833	0.541	2.50556834744689E-203	1	MT-CO1
8.79958403395014E-101	0.789169161394574	0.357	0.2	1.43530015177761E-96	1	CNPY3
2.35156575735771E-41	0.788158180067956	0.254	0.169	3.83563890682616E-37	1	SLC23A2
1.18448582951209E-162	0.78063004591873	0.398	0.176	1.93201483651717E-158	1	SOCS3
8.40903110178327E-43	0.775116198203199	0.302	0.214	1.37159706301187E-38	1	NAPA
8.43945195560285E-235	0.771652087659171	0.382	0.13	1.37655900847838E-230	1	NR4A2
6.35637919766253E-240	0.769851516697404	0.388	0.128	1.03678901093074E-235	1	LCP2
1.21816365879403E-252	0.762331780235008	0.306	0.083	1.98694674385894E-248	1	CD84
3.59627644553852E-85	0.756499621148603	0.298	0.163	5.86588651031787E-81	1	TWF2
3.96525944469768E-51	0.755445931567172	0.348	0.243	6.46773468024639E-47	1	GPX4
1.14754152994526E-156	0.755268365771278	0.527	0.294	1.87175498949371E-152	1	HSPA1B
5.73912351753592E-70	0.75253772379536	0.327	0.199	9.36108436945285E-66	1	BTG2
2.46712514241214E-36	0.750940731865945	0.253	0.175	4.02412781978844E-32	1	RAB1A
2.36940556673221E-17	0.750089937943343	0.271	0.226	3.8647374198969E-13	1	CTNND1
6.73998813352716E-33	0.745420045608735	0.276	0.202	1.09935946445961E-28	1	RAB7A
6.37008106294275E-250	0.742847618226601	0.343	0.101	1.03902392217659E-245	1	SAMSN1
1.96696673142202E-51	0.730360961622164	0.259	0.158	3.20831943562246E-47	1	VASP
1.72441138003221E-71	0.72559153679455	0.267	0.145	2.81268740197054E-67	1	TTLL3
7.05088515492292E-73	0.725385369574199	0.409	0.268	1.15006987761948E-68	1	SLC12A7
3.11651211599504E-44	0.72508393443018	0.333	0.241	5.08334291239952E-40	1	GNPTG
2.48823871564878E-184	0.714557009383237	0.304	0.103	4.05856616909472E-180	1	CMTM3
3.77233248924464E-116	0.70884389699816	0.321	0.155	6.15305152320694E-112	1	SUSD6
8.13024915780134E-254	0.707672770317409	0.912	0.705	1.32612494012898E-249	1	TMSB4X
1.37030662808924E-59	0.705669401928563	0.255	0.148	2.23510714107636E-55	1	TENT2
1.14577738983998E-121	0.688139991997891	0.344	0.165	1.86887750056798E-117	1	PLXND1
4.0904273446713E-96	0.686521134928618	0.318	0.163	6.67189604189335E-92	1	SRSF7
2.65767104126098E-54	0.664053038836994	0.308	0.192	4.33492723540079E-50	1	SLC20A1
3.28434449244032E-114	0.662848132028531	0.351	0.175	5.3570943016194E-110	1	TACC1
1.3021077735413E-109	0.661628284997364	0.253	0.107	2.12386798942321E-105	1	PKM
2.94551859846742E-106	0.660302176095141	0.484	0.309	4.80443538596022E-102	1	TBC1D9B
6.47024798650641E-157	0.658135972857557	0.266	0.09	1.05536214907906E-152	1	TAGAP
4.92462599834446E-110	0.65810230275996	0.633	0.42	8.03255746589965E-106	1	ZFP36L1
3.37895470728051E-65	0.657764307799216	0.314	0.191	5.51141302304524E-61	1	EDEM1
1.32311293295456E-29	0.655125727346157	0.285	0.212	2.15812950494218E-25	1	MT-CYB
7.91501289286843E-86	0.651828244845051	0.414	0.263	1.29101775295577E-81	1	MTCH1
4.03186076899278E-48	0.649476468452693	0.288	0.187	6.57636810030412E-44	1	SREBF1
1.55751063958813E-24	0.644059859808416	0.282	0.22	2.54045560423221E-20	1	EPN1
4.38619642215466E-50	0.643711346344112	0.407	0.292	7.15432498417647E-46	1	MIDN
6.49552138224213E-221	0.633115769813478	0.411	0.147	1.05948449265751E-216	1	CHST11
2.0739003102082E-63	0.631411928890819	0.304	0.184	3.38273879598059E-59	1	ZMIZ2
1.13074743824949E-64	0.629992532531168	0.366	0.243	1.84436214652875E-60	1	CNDP2
4.43736364151131E-08	0.629900748096478	0.25	0.224	0.000723778383566909	1	ETS2
6.49403792250633E-118	0.624178374198456	0.436	0.245	1.05924252554001E-113	1	PPP1R9B
7.61861493442281E-99	0.624013442324446	0.25	0.109	1.2426722819537E-94	1	UTRN
6.99670748791039E-40	0.616409898675736	0.286	0.198	1.14123295835306E-35	1	IRAK1
4.97304641351101E-284	0.616372448888633	0.48	0.156	8.11153600507781E-280	1	DOCK2
8.84809004077182E-141	0.615143200039044	0.64	0.389	1.44321196655029E-136	1	MT-CO2
1.16012131981005E-57	0.614940251065449	0.36	0.238	1.89227388474217E-53	1	IFI6
9.04730786486592E-61	0.614442091760011	0.277	0.163	1.47570638583828E-56	1	PPP1CB
6.96540610539058E-68	0.611817462132023	0.345	0.213	1.13612738985026E-63	1	PICALM

1.40934278515112E-65	0.611005891788283	0.335	0.207	2.29877901685999E-61	1	AKIRIN2
2.36525598623065E-104	0.587696486249526	0.35	0.18	3.85796903914081E-100	1	MAP7D1
4.45751936331344E-118	0.587199249382883	0.594	0.387	7.27065983350056E-114	1	STAT1
2.03570941014094E-61	0.587028184969603	0.275	0.158	3.32044561888089E-57	1	KIAA0100
9.42432456879335E-101	0.581644478898604	0.463	0.285	1.53720158041588E-96	1	ANKRD10
4.16869447204896E-123	0.579221190009827	0.495	0.294	6.79955755335906E-119	1	DPP7
9.58661420953106E-18	0.575335577822214	0.303	0.244	1.56367264371661E-13	1	VEGFA
3.07696249103259E-59	0.574762377418393	0.4	0.28	5.01883351912326E-55	1	LRPAP1
3.06648379311662E-154	0.572408270382781	0.646	0.387	5.00174171495252E-150	1	CFLAR
3.82195704958238E-54	0.570557288659305	0.42	0.297	6.23399414357382E-50	1	MT-ND4L
6.72125380914132E-146	0.5683830926056	0.53	0.256	1.09630370880904E-141	1	ITPKB
1.04938331575026E-173	0.564999993045024	0.389	0.158	1.71164912632026E-169	1	FNBP1
1.78238040715158E-102	0.562784766397524	0.332	0.165	2.90724068210494E-98	1	ANKRD13D
2.58334753691873E-19	0.559277060321587	0.275	0.222	4.21369816746815E-15	1	PRKACA
3.73829949083129E-106	0.559058764776567	0.5	0.305	6.09754029949492E-102	1	TUT7
2.40271459949869E-30	0.555592056315636	0.311	0.235	3.91906778324231E-26	1	TNFRSF1A
6.0402863426184E-48	0.553992558085856	0.365	0.256	9.85231105344488E-44	1	PIM3
3.00085949196755E-94	0.551845493210924	0.529	0.333	4.89470191734827E-90	1	MT-ND4
1.21705693135446E-26	0.548128567787018	0.27	0.203	1.98514156073225E-22	1	RAB5C
7.52898750791288E-84	0.543138213668354	0.462	0.302	1.22805315241567E-79	1	ITM2B
1.1292194812529E-46	0.536523594083077	0.31	0.208	1.84186989587161E-42	1	OGFR
2.63756655409097E-41	0.533210576535387	0.273	0.18	4.30213480637778E-37	1	RHOA
5.25153564909432E-95	0.531705738824006	0.759	0.6	8.56577979723775E-91	1	ACTB
2.15954156171596E-32	0.523335091965754	0.254	0.177	3.5224282413149E-28	1	RAB10
1.93069627166174E-197	0.522162175975138	0.572	0.252	3.14915868870747E-193	1	LCP1
1.96409043497191E-105	0.521459032217251	0.271	0.12	3.20362790848269E-101	1	RAPGEF1
1.30068930461925E-83	0.5180277020932	0.591	0.394	2.12155432476445E-79	1	LAMP1
2.87329224562486E-39	0.517914302424404	0.255	0.166	4.68662698183871E-35	1	ADAM10
1.21133813862511E-277	0.5175697596106	0.604	0.22	1.97581363791141E-273	1	ARHGAP4
5.32177693079442E-31	0.513457260413166	0.255	0.18	8.68035035181878E-27	1	PTTG1IP
2.67744285034829E-178	0.512719547245365	0.426	0.182	4.3671770332031E-174	1	KCNAB2
8.83778288327518E-162	0.503711781718053	0.275	0.093	1.44153076609101E-157	1	PRKCB
2.65322640197263E-21	0.497773494933443	0.26	0.202	4.32767758425755E-17	1	HM13
8.16516225449833E-87	0.496773691733594	0.458	0.293	1.33181961533122E-82	1	DBNL
6.32103869658763E-57	0.496101003390586	0.351	0.228	1.03102462180041E-52	1	NCOR2
1.12044013792607E-42	0.49170356958493	0.283	0.187	1.82754990897122E-38	1	GPATCH2L
1.13343080397467E-104	0.491027240545387	0.471	0.285	1.84873898436308E-100	1	TCIRG1
1.71010106986179E-93	0.490299539078833	0.433	0.257	2.78934585505157E-89	1	LRCH4
4.74677660378328E-118	0.489979247616868	0.326	0.149	7.74246731843091E-114	1	CORO7
7.94625439275393E-48	0.481660181950161	0.316	0.211	1.29611355400209E-43	1	B4GALT1
1.39730969844096E-72	0.48047996521807	0.452	0.305	2.27915184912705E-68	1	IQSEC1
1.04735373892304E-49	0.479927747097733	0.28	0.175	1.70833868355737E-45	1	ST3GAL1
1.09367195009842E-46	0.477033685822629	0.285	0.176	1.78388831780553E-42	1	SERPINB9
4.59094087276041E-75	0.473344484376658	0.394	0.244	7.48828365755951E-71	1	TLN1
3.95861421590854E-211	0.471898877324145	0.423	0.154	6.45689564756842E-207	1	CD53
3.62570941050519E-37	0.471197986194594	0.255	0.166	5.91389461947501E-33	1	MRTFA
1.80335991478804E-89	0.4696000479385	0.625	0.41	2.94146035701077E-85	1	OS9
3.15027885293661E-13	0.459286883359168	0.29	0.249	5.1384198370249E-09	1	FNDC3B
1.87804583695679E-43	0.448228346936611	0.299	0.199	3.06328056466022E-39	1	TRIM38
1.14451409231797E-128	0.445507323924994	0.379	0.18	1.86681693597984E-124	1	CREM

2.55499532020127E-88	0.437533280787879	0.33	0.174	4.16745286678028E-84	1	INPP4A
1.42831877167125E-55	0.43536810924766	0.402	0.28	2.32973074847298E-51	1	STAT6
3.0501895370022E-06	0.429437411456986	0.308	0.281	0.0497516415380429	1	ISG15
1.06303344974287E-129	0.423721381090755	0.538	0.298	1.73391385987559E-125	1	GRK2
1.1717188268256E-12	0.420809174133719	0.271	0.224	1.91119057843523E-08	1	SLC30A1
2.37097493562334E-23	0.414209234449673	0.261	0.196	3.86729721749523E-19	1	AAMP
4.04471157086848E-45	0.403016434658909	0.468	0.34	6.59732904324358E-41	1	KLF6
2.11359678171263E-60	0.396625013840024	0.406	0.268	3.44748771065147E-56	1	BST2
8.69278569688582E-70	0.389739470895265	0.405	0.259	1.41788027501905E-65	1	SMAP2
1.68092167530298E-08	0.382427025573741	0.279	0.249	0.000274175134458669	1	INF2
2.21340022157812E-12	0.38224662366611	0.269	0.225	3.61027710141608E-08	1	METTL7A
1.88837529534947E-51	0.380580341789464	0.385	0.264	3.08012894424452E-47	1	TBC1D2B
2.25037705834846E-34	0.367488091882662	0.259	0.172	3.67059001987217E-30	1	KMT2C
5.41073382816097E-84	0.364090599457634	0.86	0.75	8.82544794711336E-80	1	B2M
3.25226849331289E-134	0.362061104794678	0.506	0.294	5.30477513944266E-130	1	IVNS1ABP
6.22080333488404E-07	0.361451145660611	0.25	0.226	0.0101467523195294	1	IRF7
1.44495434661984E-60	0.352214839013371	0.33	0.203	2.35686503477162E-56	1	GGA2
3.14282737047628E-35	0.351930062838351	0.278	0.188	5.12626572398385E-31	1	PPP1R12C
2.82854403219263E-40	0.34950298669071	0.36	0.26	4.61363817090939E-36	1	ANXA11
3.57451567736745E-43	0.348901743386378	0.26	0.163	5.83039252135405E-39	1	NUFIP2
4.76223101573457E-16	0.348432138524136	0.328	0.267	7.76767500976466E-12	1	ACAA1
3.85462933199755E-47	0.34162580275577	0.434	0.311	6.2872859034212E-43	1	CCNL1
4.49074763374664E-151	0.323840168100714	0.375	0.148	7.32485846540414E-147	1	FMNL1
1.51168886591524E-105	0.32377194710541	0.274	0.116	2.46571570919436E-101	1	PTK2B
2.09046021132107E-145	0.320180181252388	0.374	0.152	3.40974965068579E-141	1	JAML
2.46257478530348E-58	0.312075135363141	0.391	0.259	4.0167057323085E-54	1	CYBC1
1.29850003856112E-07	0.28800933199226	0.253	0.221	0.00211798341289704	1	FUOM
6.43486884718038E-15	0.281901176473822	0.255	0.206	1.04959145766359E-10	1	ZSWIM8
5.57874017383676E-98	0.281624379380946	0.25	0.102	9.09948309754515E-94	1	SFMBT2
3.39611674812313E-09	0.26926228434381	0.251	0.217	5.53940602786364E-05	1	TRIM8
8.67647340014922E-07	0.267326132697068	0.252	0.227	0.0141521957629834	1	MYDGF
1.56104064434746E-59	0.261096429661622	0.479	0.325	2.54621339499514E-55	1	RSRP1
1.21047670177591E-35	0.252078741776871	0.519	0.393	1.97440854826669E-31	1	NUCB1
5.75456550225896E-227	3.5373673934406	0.288	0.097	9.38627179073458E-223	2	HSD11B1
0	3.28589968351273	0.973	0.213	0	2	TTR
0	3.26800279407846	0.555	0.148	0	2	PRAP1
0	3.18765723250305	0.954	0.207	0	2	HP
0	3.13352657145027	0.969	0.206	0	2	CES1
0	3.12603982673692	0.534	0.149	0	2	HPR
8.41464424609483E-136	3.11963771561695	0.253	0.107	1.37251262298053E-131	2	APOA5
0	3.09495272914034	0.987	0.222	0	2	RBP4
0	3.09389229019325	0.827	0.197	0	2	FABP1
0	3.09213858468894	0.989	0.217	0	2	APOA1
0	3.06150477846526	0.849	0.174	0	2	AMBP
0	3.00302241093311	0.4	0.139	0	2	STARD10
0	2.9616695678877	0.98	0.258	0	2	APOC3
2.09653628480745E-300	2.9568480314992	0.379	0.134	3.41966033414944E-296	2	MBL2
0	2.94866358746166	0.578	0.162	0	2	SPINK1
0	2.94782132522616	0.998	0.387	0	2	APOA2
0	2.9439016460451	0.498	0.146	0	2	SERPIND1

2.46098307351967E-110	2.9375978618727	0.252	0.12	4.01410949121794E-106	2	ANGPTL3
0	2.91930401915594	0.417	0.157	0	2	DEPRECATE
0	2.91708290570627	0.909	0.197	0	2	AGT
0	2.91614210019265	0.65	0.16	0	2	ORM2
0	2.91348011918448	0.957	0.261	0	2	EPHX1
8.22985283454464E-295	2.90980640634312	0.378	0.136	1.34237129584258E-290	2	AADAC
0	2.90867549572725	0.631	0.167	0	2	GSTA1
0	2.90527829650658	0.917	0.225	0	2	GC
0	2.90020948888286	0.721	0.17	0	2	F2
0	2.8951940314395	0.71	0.165	0	2	SERPINC1
0	2.89174843011497	0.582	0.164	0	2	DHCR24
8.74720451050562E-249	2.88502148953879	0.365	0.144	1.42675652770857E-244	2	MYH14
1.57492109624859E-188	2.86454626777526	0.31	0.125	2.56885380009107E-184	2	ANG
3.9060552362142E-123	2.86022649270753	0.259	0.119	6.37116669578898E-119	2	OTC
0	2.85911626480315	0.805	0.182	0	2	AHSG
0	2.85783124702898	0.941	0.212	0	2	CLU
2.06981675730608E-199	2.85103024891723	0.312	0.123	3.37607811284195E-195	2	ADH1A
1.13755144901942E-133	2.83001049093902	0.27	0.12	1.85546016849558E-129	2	UGT2B4
0	2.82338244462842	0.514	0.182	0	2	CEBPA
0	2.81549449956638	0.656	0.175	0	2	HMGCS2
0	2.81262554618323	0.99	0.234	0	2	TF
8.61837902713137E-287	2.80270908174032	0.374	0.135	1.4057438031154E-282	2	SERPINA7
0	2.80073258503474	0.83	0.19	0	2	ORM1
8.44763070912367E-165	2.79154851464428	0.303	0.132	1.37789304496516E-160	2	MASP2
0	2.77592793480591	0.997	0.36	0	2	ALB
1.89507378227552E-140	2.77230185055121	0.281	0.126	3.0910548462696E-136	2	SERPINA10
0	2.77215298631952	0.498	0.158	0	2	ADH1C
0	2.76172046052021	0.676	0.168	0	2	LRG1
0	2.75970373854928	0.524	0.167	0	2	CES2
0	2.75798749665319	0.997	0.25	0	2	FGG
3.50368833085715E-147	2.75711602894176	0.287	0.127	5.7148660364611E-143	2	GJB1
0	2.75233926744226	0.405	0.145	0	2	SDC1
1.25275427687164E-227	2.7471357662547	0.341	0.132	2.04336750100533E-223	2	HSD17B6
0	2.73886933752231	0.519	0.155	0	2	PLG
0	2.73543863058051	0.584	0.17	0	2	KRT8
0	2.73369650951969	0.992	0.26	0	2	VTN
0	2.72812465206292	0.643	0.209	0	2	NUPR1
4.17846889058732E-142	2.71748904682223	0.289	0.132	6.81550060743697E-138	2	HAO1
0	2.71731729963349	0.998	0.375	0	2	SERPINA1
0	2.69945019176284	0.994	0.273	0	2	FGB
0	2.69431822114493	0.634	0.172	0	2	ASGR1
0	2.69222342993303	0.809	0.184	0	2	ITIH2
0	2.69204588098789	0.65	0.201	0	2	CYP 20.00
6.85310757799782E-271	2.6908541150088	0.379	0.144	1.11781037704722E-266	2	MGST1
3.62282303902808E-129	2.69032797793896	0.272	0.124	5.90918665895871E-125	2	SERPINA5
0	2.68018441668797	0.522	0.164	0	2	TFR2
0	2.67539074501188	0.502	0.165	0	2	KRT18
0	2.67508602246139	0.915	0.276	0	2	MT1G
0	2.66755514086086	0.995	0.265	0	2	FGA
2.70013220871548E-152	2.66015801942984	0.32	0.152	4.40418564563583E-148	2	LRP3

0	2.65994340299627	0.498	0.16	0	2	IGFBP2
0	2.64662975129497	0.926	0.208	0	2	PLA2G2A
0	2.63767425325191	0.489	0.161	0	2	AZGP1
0	2.6318767689109	0.489	0.198	0	2	ECHS1
0	2.63003735811396	0.72	0.203	0	2	ALDOB
0	2.62915585005559	0.694	0.176	0	2	ASGR2
0	2.61907437920128	0.684	0.173	0	2	SERPINF2
0	2.60861007663331	0.712	0.188	0	2	APOH
0	2.60736122076291	0.805	0.195	0	2	APOB
1.07689704002277E-93	2.60147525687374	0.252	0.129	1.75652676198114E-89	2	HSD17B2
0	2.59202109320704	0.516	0.155	0	2	TM4SF4
0	2.58152944668772	0.984	0.271	0	2	C3
3.54871818824567E-251	2.57371709905461	0.402	0.172	5.7883142368475E-247	2	PGRMC1
1.52419935033323E-113	2.56975285835906	0.273	0.133	2.48612156032852E-109	2	SULT2A1
6.26899868769157E-83	2.56848385536482	0.252	0.136	1.02253637594937E-78	2	DSP
0	2.56788360769831	0.477	0.145	0	2	HRG
0	2.56430120015768	0.566	0.163	0	2	ITIH1
0	2.55706795175508	0.57	0.163	0	2	APCS
5.91255630573901E-308	2.55690233996824	0.412	0.153	9.6439705902909E-304	2	SERINC2
0	2.54819499905375	0.78	0.2	0	2	FGL1
0	2.54590716356014	0.424	0.157	0	2	AOX1
1.70206324136671E-207	2.54013282346931	0.348	0.145	2.77623535299325E-203	2	HNF4A
2.86053311361358E-179	2.52896562255105	0.347	0.16	4.66581556161511E-175	2	QPRT
5.84066972674674E-190	2.52214948447865	0.341	0.148	9.52671639129661E-186	2	PROC
0	2.51948688167384	0.559	0.166	0	2	TM4SF5
1.32256491707464E-142	2.51706727703977	0.289	0.129	2.15723563624044E-138	2	GPX2
6.99915717273392E-272	2.51235422927137	0.393	0.151	1.14163252644463E-267	2	CFHR2
0	2.51159688579271	0.627	0.164	0	2	SERPINA3
4.54171898677961E-240	2.51019126275776	0.372	0.149	7.40799783933622E-236	2	GAMT
0	2.5092191746992	0.817	0.192	0	2	KNG1
0	2.50869180055828	0.753	0.265	0	2	MT1E
0	2.50860172920692	0.889	0.198	0	2	HPX
0	2.50182351618332	0.916	0.289	0	2	MT1F
0	2.49132358342572	0.73	0.261	0	2	APOC1
0	2.47452317419205	0.947	0.281	0	2	MT1B
0	2.47423401544069	0.665	0.261	0	2	ALDH2
1.02888176330887E-102	2.47342813806695	0.264	0.132	1.67820904413309E-98	2	RGN
7.60304253847698E-253	2.47012763070898	0.379	0.148	1.24013226845098E-248	2	HABP2
2.77338175913025E-204	2.46365947752062	0.363	0.16	4.52366298731735E-200	2	FAH
0	2.46118871260134	0.53	0.212	0	2	GATM
1.73431391584182E-210	2.45817194044158	0.359	0.151	2.8288394281296E-206	2	CLDN1
0	2.45514296747495	0.646	0.188	0	2	C4BPA
0	2.42662526847407	0.922	0.236	0	2	APOC2
5.16660042657132E-138	2.42132470094368	0.306	0.146	8.42724195578048E-134	2	SOD1
1.60101339798644E-121	2.41604300502808	0.286	0.139	2.61141295345568E-117	2	LSR
4.63777751638284E-167	2.41462367653883	0.34	0.16	7.56467890697205E-163	2	MPST
2.73988278719852E-188	2.40836004494366	0.347	0.152	4.4690228141995E-184	2	DEPRECATE
0	2.3989534041895	0.999	0.437	0	2	APOE
0	2.39523955368815	0.906	0.248	0	2	FN1
4.67765710410483E-162	2.38096385263556	0.324	0.147	7.62972650250538E-158	2	ADH1B

0	2.37119239391888	0.516	0.183	0	2	SDC4
9.77587233470766E-128	2.37111118458517	0.296	0.143	1.59454253651417E-123	2	PON1
0	2.34248526160881	0.768	0.189	0	2	IGFBP1
0	2.33907182103179	0.474	0.191	0	2	CYB5A
1.00889705816118E-113	2.33664605232608	0.284	0.141	1.6456119915667E-109	2	F7
4.41115921327361E-100	2.3333481341069	0.256	0.126	7.19504179277058E-96	2	HPD
0	2.32566308992696	0.583	0.217	0	2	CERS2
0	2.32325474195546	0.431	0.156	0	2	LBP
0	2.31482735896307	0.515	0.168	0	2	TDO2
6.46467177732929E-281	2.3106094986182	0.412	0.16	1.05445261360018E-276	2	DEPRECATE
3.38800879306279E-137	2.30880000724117	0.334	0.172	5.52618114236472E-133	2	ACAT1
0	2.30645340958834	0.446	0.173	0	2	SPON2
2.54961326267888E-126	2.290676542548	0.322	0.168	4.15867419275551E-122	2	PCK2
0	2.28171286863136	0.963	0.235	0	2	CFB
0	2.27960378822874	0.467	0.173	0	2	SERPINF1
2.23603162610477E-195	2.27487025859348	0.39	0.187	3.64719118533948E-191	2	ADI1
4.65821723225995E-152	2.2545444534404	0.344	0.169	7.59801812753921E-148	2	DCXR
9.21707947923386E-77	2.24875448901845	0.264	0.15	1.50339783385784E-72	2	NADK2
2.20609224621564E-267	2.23774116928949	0.406	0.158	3.59835706280233E-263	2	BDH1
0	2.2369790119665	0.987	0.397	0	2	MT2A
1.09628803725141E-134	2.23641171607252	0.332	0.172	1.78815541756078E-130	2	SEPHS2
3.46424991178374E-66	2.2342397482627	0.253	0.149	5.65053803111045E-62	2	PGM1
0	2.23386941208154	0.515	0.221	0	2	ETFB
4.55426995559846E-271	2.21127293073262	0.476	0.225	7.42846972457665E-267	2	HYOU1
0	2.20929268468214	0.495	0.195	0	2	OAF
5.75138758100212E-139	2.20831102799962	0.336	0.17	9.38108828337256E-135	2	TFPI
2.08027109022363E-170	2.19791448358379	0.374	0.186	3.39313017526376E-166	2	FURIN
1.89805683737607E-100	2.19261274707913	0.296	0.16	3.09592050744411E-96	2	TCEA3
7.19502136127591E-173	2.18448376217507	0.39	0.201	1.17357993423771E-168	2	FUOM
1.64849529396527E-162	2.15712912519856	0.374	0.19	2.68886067398675E-158	2	HDGF
2.89954173587918E-148	2.15211463963421	0.343	0.171	4.72944252539252E-144	2	S100A16
0	2.14957875684531	0.556	0.268	0	2	RRBP1
0	2.14625703012347	0.736	0.212	0	2	C1S
6.30157329776013E-181	2.12896758723191	0.364	0.164	1.02784962059765E-176	2	UGDH
0	2.11776448064543	0.541	0.255	0	2	SCP2
0	2.09429122708877	0.491	0.193	0	2	GDF15
2.5674214044941E-160	2.089202418051	0.389	0.203	4.18772105287032E-156	2	MGAT4B
6.0324172551821E-85	2.08761445751013	0.272	0.15	9.83947578492752E-81	2	C5
3.48915964393091E-93	2.08748892375989	0.307	0.176	5.69116829521571E-89	2	CLUH
2.97016007811688E-80	2.08719651583531	0.252	0.135	4.84462810341644E-76	2	ARG1
0	2.08489903666747	0.655	0.183	0	2	CP
4.36397474720565E-173	2.06735580679587	0.374	0.181	7.11807921016713E-169	2	SLC9A3R2
1.13264520839533E-104	2.05992447794352	0.325	0.184	1.84745759941362E-100	2	NDUFC2
0	2.02849742734048	0.644	0.288	0	2	SERPING1
0	2.0193629139767	0.561	0.281	0	2	ECH1
0	2.00154897017811	0.67	0.292	0	2	LMNA
1.57137996404688E-89	1.99648683473523	0.294	0.166	2.56307785935686E-85	2	PRDX2
5.29480669561252E-152	1.98766401952876	0.319	0.142	8.63635920121358E-148	2	AKR1B10
4.28132488645517E-202	1.97950682046753	0.401	0.187	6.98326902229703E-198	2	GPX3
0	1.97863127031995	0.542	0.177	0	2	MAT1A

3.24285842796607E-244	1.97432764032515	0.463	0.221	5.28942638185546E-240	2	XBP1
0	1.9739413042593	0.91	0.407	0	2	SQSTM1
1.10453971420028E-95	1.96636325241623	0.265	0.132	1.80161472783207E-91	2	NNMT
3.62634581340106E-173	1.96210636532303	0.397	0.201	5.91493265623848E-169	2	ANPEP
4.27544132506638E-143	1.95979970486833	0.387	0.215	6.97367234531578E-139	2	P4HB
0	1.95873118290162	0.84	0.296	0	2	MT1X
1.14950271508047E-101	1.94926269679817	0.315	0.174	1.87495387856776E-97	2	PDIA5
5.54675764737185E-54	1.94658042013075	0.25	0.156	9.04731639862822E-50	2	SELENBP1
3.53461225731608E-233	1.93161414362219	0.393	0.156	5.76530605290825E-229	2	TAT
6.49063290814145E-113	1.93158709203534	0.303	0.153	1.05868713364695E-108	2	OSGIN1
2.0410989683321E-289	1.93063121351472	0.455	0.18	3.32923652724649E-285	2	BAAT
1.46167273871393E-79	1.92994193698886	0.251	0.132	2.3841344041163E-75	2	FMO3
0	1.92899786892247	0.721	0.237	0	2	COL18A1
0	1.92638919024813	0.601	0.203	0	2	C1R
1.79196097423381E-274	1.92518378706732	0.436	0.17	2.92286754507277E-270	2	FMO5
5.16777113872912E-302	1.92160238650917	0.46	0.174	8.42915150438107E-298	2	HPN
2.62360229093934E-107	1.91289823717886	0.334	0.187	4.27935769675116E-103	2	ASL
3.57236359643901E-161	1.90653845972085	0.344	0.156	5.82688226215167E-157	2	SLC38A3
6.24060307103275E-68	1.89681497139332	0.274	0.164	1.01790476691615E-63	2	GGCX
3.2320009304226E-112	1.89623439510785	0.33	0.178	5.2717167176123E-108	2	MTSS2
5.18071958890741E-86	1.88703602626964	0.314	0.188	8.45027172146688E-82	2	PRDX6
1.64412533692434E-53	1.87195538277204	0.264	0.169	2.6817328370573E-49	2	SPTSSA
2.70209766746311E-60	1.8603075959987	0.273	0.173	4.40739150539907E-56	2	ZNHIT1
0	1.85642640735369	0.647	0.179	0	2	AGXT
9.24840731275901E-211	1.84122518572467	0.445	0.217	1.50850771678412E-206	2	SHMT1
7.73989853704992E-212	1.84118876128373	0.343	0.131	1.26245485037821E-207	2	CHI3L1
6.35808752389777E-108	1.83545058490306	0.359	0.213	1.03706765602297E-103	2	METTL7A
2.20406649697794E-273	1.83246527011794	0.536	0.275	3.59505286322071E-269	2	IFITM3
0	1.80440967600195	0.719	0.336	0	2	A2M
0	1.79754393898829	0.671	0.298	0	2	C2
2.64842164436051E-107	1.79667131475367	0.269	0.128	4.31984054411642E-103	2	G0S2
4.37130479172871E-61	1.77743732667104	0.267	0.163	7.13003524578871E-57	2	CFI
9.13017890394162E-74	1.77716503353453	0.283	0.167	1.48922348102192E-69	2	DHRS7
5.38978161532695E-73	1.77075691737864	0.305	0.187	8.79127279275979E-69	2	ALDH1A1
0	1.7419962387381	0.651	0.315	0	2	CTSD
8.7063620807028E-65	1.7394401427061	0.294	0.186	1.42009471898343E-60	2	ACOX1
5.13586533318585E-92	1.73829920993162	0.331	0.199	8.37710994495944E-88	2	TXNDC5
3.48406508828945E-212	1.73217832038898	0.46	0.227	5.68285856550892E-208	2	DEPRECATE
2.72754446362756E-253	1.7294330372553	0.54	0.279	4.44889777462292E-249	2	DEPRECATE
2.64241359638158E-77	1.72356079811054	0.327	0.207	4.310040817058E-73	2	UQCRC1
3.92047525792486E-69	1.71645595705581	0.296	0.182	6.39468719320123E-65	2	IFI27
2.03017712280546E-104	1.69600585982338	0.269	0.126	3.31142190500798E-100	2	PCK1
1.83786536509818E-55	1.66470516376982	0.295	0.196	2.99774219701165E-51	2	PDIA6
0	1.65383952368785	0.607	0.247	0	2	MT1H
2.06029936646142E-94	1.64298765323101	0.336	0.193	3.36055429663521E-90	2	F11R
5.3700710976366E-61	1.63853723096659	0.259	0.153	8.75912296735506E-57	2	GPT
7.48769109591682E-136	1.63261199436651	0.427	0.254	1.22131729465499E-131	2	NDUFS2
0	1.63259121463989	0.822	0.215	0	2	CFH
1.58445467458872E-85	1.6180394613004	0.346	0.213	2.58440401972166E-81	2	AGRN
1.56119993829823E-68	1.61566287426105	0.278	0.163	2.54647321935824E-64	2	EPS8L2

2.61322961925498E-39	1.5676774110119	0.275	0.196	4.2624388319668E-35	2	IRS2
5.99436124202069E-47	1.56730588207314	0.273	0.182	9.77740262185995E-43	2	HAGH
4.91905684586006E-261	1.56232677287522	0.585	0.326	8.02347362128235E-257	2	PTMS
4.56534512152897E-47	1.5577016034897	0.276	0.186	7.4465344277259E-43	2	CALR
2.34792491403192E-50	1.53021291784434	0.29	0.197	3.82970032727747E-46	2	DDRGK1
2.23068148258979E-239	1.51240995705161	0.494	0.226	3.63846456625221E-235	2	MT1M
8.53960788130968E-188	1.47681258779262	0.43	0.203	1.39289544152042E-183	2	MT1A
1.4428393633006E-92	1.47458201027992	0.38	0.237	2.35341528547961E-88	2	ST6GAL1
5.52579981531253E-43	1.46582146006069	0.291	0.206	9.01313207875627E-39	2	CYC1
3.0389022374995E-283	1.44357127153267	0.649	0.389	4.95675343958544E-279	2	LAMP1
4.70549015340145E-297	1.43124028741195	0.626	0.334	7.6751249892131E-293	2	SELENOP
1.29918732794932E-32	1.43103688607048	0.286	0.218	2.11910445061814E-28	2	CD151
2.71175331296427E-26	1.42263087730683	0.279	0.22	4.42314082877602E-22	2	NECTIN2
4.22090901704671E-57	1.41949974958386	0.346	0.246	6.8847246977049E-53	2	GPX4
1.20043907340909E-45	1.41879944369594	0.311	0.221	1.95803617263756E-41	2	RHOC
4.5673323764109E-39	1.41674985121204	0.298	0.22	7.44977583916382E-35	2	MYDGF
1.44950910482164E-48	1.40508605536341	0.315	0.223	2.36429430087457E-44	2	DHRS3
1.62047122448905E-34	1.38729702714719	0.275	0.2	2.6431506142641E-30	2	DDT
1.66373812655236E-93	1.38255058039701	0.402	0.255	2.71372325821956E-89	2	TMEM176B
3.29615856103324E-88	1.38152110279285	0.394	0.259	5.37636422890132E-84	2	ACAA1
2.67830995539223E-25	1.38083279843596	0.274	0.217	4.36859136824027E-21	2	NFIC
1.33695939919083E-24	1.35129420330383	0.257	0.198	2.18071447602016E-20	2	MPC1
0	1.31807548141011	0.559	0.185	0	2	ITIH3
2.71669537338927E-46	1.31048723365807	0.257	0.163	4.43120182353524E-42	2	ITIH4
2.94492809246778E-37	1.30166173836874	0.304	0.227	4.8034722116242E-33	2	PPIB
0	1.29609102036771	0.872	0.422	0	2	FTL
7.68449270636427E-235	1.29140445044049	0.538	0.272	1.25341760533508E-230	2	IGFBP4
1.16464692564935E-150	1.28274349518369	0.279	0.11	1.89965560042666E-146	2	CRP
9.34946744414355E-67	1.28146777457279	0.4	0.29	1.52499163481425E-62	2	BRI3
8.56410795753128E-135	1.27951699717267	0.515	0.339	1.39689164895293E-130	2	MT-ND4
5.6094744771415E-29	1.27164202084717	0.277	0.21	9.1496138196655E-25	2	PDIA3
9.31502329357695E-44	1.27087757328512	0.316	0.227	1.51937344941534E-39	2	RNF187
7.64328549542904E-30	1.2673166183858	0.269	0.199	1.24669629715943E-25	2	ACSL4
1.07911717496674E-34	1.26267574652531	0.29	0.213	1.76014802408826E-30	2	ZBTB7B
4.93211566688833E-108	1.26088636318072	0.336	0.171	8.04477386426156E-104	2	UPB1
2.86083167102067E-47	1.26025243938505	0.348	0.258	4.66630253860182E-43	2	APLP2
3.75258307052527E-50	1.25464520109346	0.34	0.244	6.12083824633376E-46	2	BLVRB
1.24347090135045E-119	1.23481598381717	0.504	0.344	2.02822538719272E-115	2	SDF4
1.33669902563368E-159	1.21528868420278	0.591	0.401	2.18028978071109E-155	2	MT-CO2
6.14076863855262E-22	1.21508003744545	0.269	0.216	1.00162077263432E-17	2	MT-CYB
1.12088776427431E-72	1.21052365318594	0.394	0.268	1.82828003230783E-68	2	ISG15
6.17504617541117E-52	1.19740933623679	0.269	0.162	1.00721178167132E-47	2	HGD
1.9451867102485E-13	1.14349043116552	0.267	0.231	3.17279404308632E-09	2	CREG1
5.35736653933194E-28	1.13392959172115	0.291	0.223	8.73840056230433E-24	2	HSD17B4
1.97812269213648E-27	1.12664561094683	0.304	0.24	3.2265159231438E-23	2	SLC25A39
3.42037699298537E-25	1.12068862062382	0.286	0.222	5.57897691325844E-21	2	SLC30A1
1.18574097277584E-36	1.11332641316488	0.331	0.252	1.93406210069468E-32	2	CYB5R3
5.68783240627417E-93	1.11032280683505	0.325	0.18	9.2774234378738E-89	2	DEPP1
8.82689749491254E-37	1.10132899438702	0.269	0.188	1.43975525039518E-32	2	CALD1
3.40378374831547E-17	1.09268828323316	0.272	0.227	5.55191167187737E-13	2	BSG

3.33956250716487E-120	1.08014618311641	0.38	0.203	5.44716040543663E-116	2	THBS1
2.30908008446657E-86	1.07941043973867	0.337	0.187	3.76634052577343E-82	2	ERRFI1
9.29190614243986E-114	1.07485694081537	0.344	0.171	1.51560281089337E-109	2	ELF3
2.22873078221556E-131	1.07259524994934	0.488	0.3	3.63528277887181E-127	2	HSPB1
4.66039847943155E-113	1.06903258123121	0.546	0.391	7.6015759598008E-109	2	NUCB1
7.58590387260851E-74	1.06096929446494	0.449	0.328	1.23733678066117E-69	2	LMAN2
2.04987348366281E-108	1.0505287462641	0.52	0.357	3.34354863920241E-104	2	MT-ATP6
6.73941587758075E-22	1.03056962411729	0.256	0.197	1.0992661237922E-17	2	FIS1
4.80115927358954E-111	1.02055962972306	0.57	0.422	7.8311708911519E-107	2	OS9
1.84187335545431E-43	1.01117554998828	0.33	0.231	3.00427963008153E-39	2	SAT2
8.38135274364485E-67	0.987342821674301	0.399	0.273	1.36708244601591E-62	2	SLC2A4RG
1.13330061951369E-148	0.947168711718456	0.398	0.179	1.84852664048879E-144	2	CYP3A5
2.34280227739627E-137	0.939852517177082	0.554	0.339	3.82134479466105E-133	2	ACADVL
1.94431842291175E-93	0.938629850227453	0.339	0.175	3.17137777961135E-89	2	C4BPB
2.7201581776459E-36	0.923372019238299	0.365	0.283	4.43685000355823E-32	2	IDH2
7.16318941133998E-26	0.92047612757611	0.347	0.285	1.16838782488366E-21	2	GRINA
1.07079207997155E-23	0.900969729000076	0.36	0.308	1.7465689616416E-19	2	MT-ND4L
3.51689572709922E-34	0.877404173744608	0.311	0.223	5.73640862047154E-30	2	ECHDC2
1.70927531937257E-34	0.859978829737344	0.412	0.335	2.78799897342859E-30	2	PLXNB2
5.23931512213376E-18	0.834540974254474	0.31	0.259	8.54584689571237E-14	2	CSRP1
2.27185336279027E-76	0.809853271259772	0.474	0.325	3.7056200200472E-72	2	CD14
2.04499208346363E-15	0.79203245818375	0.292	0.246	3.33558658733753E-11	2	CDKN1A
2.76119296155634E-06	0.748597823862311	0.263	0.246	0.0450378183959455	2	MT-ND3
2.83591987282628E-76	0.744055346102882	0.572	0.437	4.62566890456694E-72	2	MT-CO3
3.31108006709938E-96	0.730881195952424	0.355	0.179	5.4007026974458E-92	2	PAH
1.74650057422633E-59	0.715994033371633	0.47	0.338	2.84871708662057E-55	2	SOD2
2.87523094864324E-19	0.712775648477178	0.35	0.297	4.68978920033199E-15	2	HSP90AB1
9.48973536449516E-132	0.703982526432206	0.756	0.558	1.54787073530281E-127	2	MT-CO1
1.11824286857078E-39	0.611650156784738	0.483	0.39	1.8239659429258E-35	2	APOL6
2.99222042282903E-13	0.578085582220501	0.301	0.258	4.88061073167643E-09	2	SLC40A1
1.3548898761756E-61	0.577021490246275	0.605	0.492	2.20996087703002E-57	2	EEF2
4.28218108198308E-127	0.568609987967408	0.686	0.45	6.98466556282261E-123	2	FTH1
2.41646633881839E-16	0.538054575900237	0.298	0.236	3.94149824524668E-12	2	GLYCTK
3.5847970967892E-08	0.534873977403461	0.313	0.288	0.000584716254457286	2	LASP1
3.43230847449106E-42	0.53472862365533	0.48	0.375	5.59843835274237E-38	2	CD63
4.22366982732671E-168	0.522161730750741	0.474	0.207	6.88922785535259E-164	2	EFNA1
2.93436026111922E-08	0.495423570184797	0.289	0.26	0.000478623502191156	2	APMAP
1.87461614781203E-45	0.440998113290172	0.68	0.541	3.0576863986962E-41	2	IL32
2.24265411802706E-13	0.34456675692042	0.399	0.347	3.65799313191394E-09	2	JUN
1.10544246841768E-07	0.296475214530453	0.378	0.348	0.00180308721023609	2	CEBPD
1.90753147830298E-83	0.27973758865194	0.525	0.338	3.11137459425999E-79	2	LYZ
6.43724637237397E-218	0.27232376011736	0.781	0.403	1.04997925579792E-213	2	CTSB
0	4.22876495662278	0.378	0.033	0	3	PDCD1
0	4.1291332532618	0.273	0.025	0	3	TNFRSF9
0	3.85464276733552	0.512	0.069	0	3	LAG3
0	3.80528353927022	0.376	0.035	0	3	KLRC4
0	3.72321044221975	0.621	0.14	0	3	HAVCR2
0	3.44201435890243	0.807	0.126	0	3	CD8A
0	3.41582591603307	0.813	0.198	0	3	RGS1
1.86324934762582E-281	3.34140720395776	0.359	0.126	3.03914601091247E-277	3	ADGRG1

2.9170462216606E-229	3.20350493976377	0.299	0.1	4.75799409215061E-225	3	WHRN
0	3.13334270380095	0.293	0.044	0	3	CXCR6
0	3.11696726788129	0.493	0.069	0	3	TIGIT
0	2.965559434797	0.53	0.079	0	3	KLRK1
0	2.91271607259641	0.551	0.111	0	3	NKG7
0	2.88031381766105	0.421	0.126	0	3	LYST
0	2.83053551774298	0.52	0.12	0	3	CCL5
0	2.82641902089184	0.292	0.051	0	3	CD7
0	2.74201583043252	0.35	0.07	0	3	PTPN22
2.06861294912843E-261	2.68167292422655	0.285	0.078	3.37411458132339E-257	3	BICDL1
0	2.58693550844556	0.406	0.08	0	3	PRF1
0	2.54226377264749	0.29	0.064	0	3	ITM2A
0	2.50176725149668	0.39	0.09	0	3	IKZF3
2.61486899466234E-254	2.46585696365336	0.25	0.06	4.26511281719375E-250	3	SYTL3
0	2.45384278208546	0.657	0.17	0	3	TRBC2
3.23680876162347E-291	2.43458267920834	0.364	0.118	5.27955877108403E-287	3	PRDM1
0	2.3947833684532	0.478	0.136	0	3	ARHGAP9
0	2.30884322024455	0.558	0.252	0	3	METRNL
0	2.30493689061221	0.37	0.093	0	3	GZMA
0	2.30324849426578	0.426	0.146	0	3	CNOT6L
0	2.25795733843211	0.606	0.182	0	3	CLEC2D
2.17804216645927E-145	2.24227883106745	0.255	0.097	3.55260457771171E-141	3	VPS37B
1.14559937236764E-178	2.23144764767991	0.337	0.143	1.86858713626886E-174	3	NR4A2
3.61551651373635E-215	2.21689851106788	0.285	0.088	5.89726898555536E-211	3	CCND2
5.12516767412693E-124	2.21532340388301	0.281	0.128	8.35966099326843E-120	3	RAB27A
1.95349088713229E-257	2.18892905991654	0.425	0.176	3.18633898600148E-253	3	SLA
2.96328845639767E-75	2.17520028620675	0.291	0.177	4.83341980123025E-71	3	ABCA2
1.60908178827407E-78	2.17201172023654	0.271	0.152	2.62457330485384E-74	3	PGGHG
3.79459549635406E-159	2.16730187905727	0.401	0.22	6.1893647141031E-155	3	CBLB
0	2.14037819150076	0.546	0.2	0	3	ITGA4
2.60832830302057E-85	2.1244470657682	0.281	0.158	4.25444429505685E-81	3	CEMIP2
7.93084883315647E-264	2.10811410230771	0.405	0.154	1.29360075317615E-259	3	JAML
1.34603569934436E-104	2.10323459065294	0.252	0.116	2.19551882920058E-100	3	TRAF5
1.04582990986512E-301	2.08457061729714	0.322	0.083	1.705853165981E-297	3	CST7
3.40952595970023E-217	2.0700500131447	0.305	0.097	5.56127779286705E-213	3	TRBC1
5.36203633462438E-216	2.04786940216768	0.262	0.073	8.74601746540583E-212	3	SH2D2A
2.15291575811163E-281	2.01359237530183	0.353	0.105	3.51162089305589E-277	3	PYHIN1
1.18445398591777E-91	1.99760687580756	0.323	0.193	1.93196289643048E-87	3	CREM
2.63707762243159E-104	1.99074789602302	0.382	0.246	4.30133730994817E-100	3	RNF19A
0	1.9286413504052	0.524	0.153	0	3	TRAC
0	1.91029504944411	0.499	0.182	0	3	ETS1
0	1.9071960873791	0.422	0.124	0	3	ZAP70
1.44554339152694E-270	1.88016443709551	0.339	0.098	2.35782582591959E-266	3	CD2
8.14646504141733E-252	1.87428828705102	0.48	0.222	1.32876991290558E-247	3	ITGAL
4.01288695975263E-292	1.84925278293099	0.336	0.092	6.54541992005251E-288	3	IL2RB
1.15395483875733E-194	1.83874323207057	0.272	0.084	1.88221573749708E-190	3	CD3E
6.24897463766457E-249	1.82028369615198	0.351	0.112	1.01927025314947E-244	3	ITK
2.11208806972462E-90	1.7440711008184	0.294	0.156	3.44502685052783E-86	3	SLC38A1
2.69338003764272E-185	1.73575236655554	0.318	0.116	4.39317217939904E-181	3	RASAL3
1.82561715498409E-164	1.7277651756173	0.352	0.155	2.97776414149456E-160	3	PTPRC

5.41991134529666E-168	1.68926677567111	0.294	0.108	8.84041739531339E-164	3	FYN
5.69075301405769E-135	1.60825236248171	0.323	0.147	9.28218724122949E-131	3	CCDC88B
2.16176329069691E-244	1.56478556414452	0.544	0.281	3.52605210345573E-240	3	LSP1
7.78152845716451E-176	1.54331288531835	0.319	0.117	1.2692451066481E-171	3	SEPTIN1
2.35056681725784E-53	1.54022567946285	0.264	0.164	3.83400953562926E-49	3	HNRNPLL
8.29966027553416E-24	1.53172767211518	0.263	0.216	1.35375758754238E-19	3	RIN3
9.65996670285129E-260	1.52018642793194	0.645	0.403	1.57563716890207E-255	3	EVL
3.20659924840578E-248	1.51708067734633	0.441	0.167	5.23028403407467E-244	3	ACAP1
0	1.50378645629281	0.646	0.327	0	3	AKNA
8.38923599492879E-265	1.49937463077931	0.511	0.222	1.36836828313284E-260	3	DUSP2
7.42376474257194E-212	1.46707435173287	0.581	0.353	1.21089026716091E-207	3	ARHGEF1
0	1.46319433577852	0.649	0.34	0	3	WIPF1
2.27112534916897E-112	1.45517531578855	0.313	0.151	3.7044325570295E-108	3	STK17B
2.76518462818904E-281	1.44557154183072	0.573	0.262	4.51029264703914E-277	3	CORO1A
2.63095080914885E-87	1.44356697245374	0.349	0.211	4.29134386480269E-83	3	ADGRE5
7.43797337076952E-48	1.43616311849551	0.409	0.362	1.21320783650622E-43	3	WNK1
5.79459961330213E-195	1.43019663528536	0.481	0.239	9.45157142925711E-191	3	IL2RG
1.87282085181107E-51	1.40299326168031	0.332	0.242	3.05475809138903E-47	3	TNFRSF1B
1.00701015153827E-143	1.40106808386936	0.45	0.257	1.64253425817407E-139	3	CELF2
2.20501399097389E-207	1.40067236437777	0.497	0.244	3.59659832067752E-203	3	CYTIP
1.0619596263459E-88	1.39585506096683	0.32	0.177	1.7321623465328E-84	3	GBP5
8.5288070264987E-26	1.36688282975083	0.315	0.274	1.3911337140922E-21	3	AKAP13
8.8761616650321E-15	1.35906948014813	0.277	0.253	1.44779072918339E-10	3	SPTAN1
1.19988378495905E-83	1.34608515366766	0.295	0.157	1.95713044164671E-79	3	JAK3
8.48739399078071E-18	1.34220429971199	0.306	0.283	1.38437883383624E-13	3	PLAAT4
1.22852419791088E-70	1.32492475778453	0.444	0.351	2.00384581921243E-66	3	MAF
1.86300728141938E-32	1.31615773141149	0.295	0.229	3.03875117672314E-28	3	IFI16
1.71035309130833E-70	1.31520795112339	0.252	0.132	2.78975692723302E-66	3	RAC2
5.93616523712804E-124	1.2998674980903	0.534	0.391	9.68247911827954E-120	3	CDKN1B
2.13053144511905E-169	1.29804793648528	0.33	0.125	3.47510984013367E-165	3	CD69
8.02510324164999E-56	1.29737637725684	0.263	0.157	1.30897458974553E-51	3	FYB1
2.58268037080449E-27	1.27018905793477	0.258	0.193	4.2126099528192E-23	3	PPP2R5C
5.76617733773781E-64	1.26974901058888	0.274	0.157	9.40521185558414E-60	3	PIK3CD
1.54400076416426E-33	1.24833844329842	0.252	0.176	2.51841964642833E-29	3	CHST11
1.62684009643352E-107	1.24097590242842	0.382	0.215	2.65353888129272E-103	3	TNFAIP3
1.43048955914691E-60	1.23749423450914	0.422	0.335	2.33327151992452E-56	3	OGT
2.86932330353538E-09	1.23534025755799	0.261	0.252	4.68015324039656E-05	3	MSN
9.59985792851112E-80	1.22679827866498	0.286	0.15	1.56583282671945E-75	3	TRAF1
4.01522118853135E-68	1.22568300546746	0.27	0.146	6.54922728061349E-64	3	TBC1D10C
7.04550361907942E-72	1.21849608729893	0.401	0.279	1.14919209530804E-67	3	STK4
3.18762735205525E-26	1.20603174032684	0.343	0.301	5.19933897393732E-22	3	RABGAP1L
1.42627079907991E-111	1.16618809549238	0.415	0.235	2.32639030037925E-107	3	TMC8
4.5007970323838E-44	1.16114451700807	0.252	0.158	7.34125003952122E-40	3	ARHGAP30
7.55540441297803E-33	1.14764859158549	0.381	0.332	1.23236201380085E-28	3	SUN2
7.83929572579977E-21	1.14275636667177	0.32	0.281	1.2786675258352E-16	3	FAM118A
2.89187187900583E-47	1.11210261871569	0.264	0.164	4.71693222184641E-43	3	ARHGAP15
1.97569246563731E-58	1.10070698944409	0.324	0.208	3.22255198070101E-54	3	LIMD2
7.70494055661832E-32	1.09482597589003	0.414	0.386	1.25675285419001E-27	3	TAP1
1.2705298229318E-29	1.07831927697078	0.343	0.285	2.07236119418406E-25	3	TRIM22
2.80195087464142E-24	1.06020940629016	0.284	0.223	4.57026207162762E-20	3	MLLT6

**5.95219238878468E-54**	1.05360470857818	0.511	0.477	9.70862100534669E-50	3	RNF213
**1.1965550277078E-78**	1.04359426619258	0.484	0.363	1.95170090569419E-74	3	NLRC5
**7.71850410687749E-29**	0.977810874229314	0.253	0.179	1.25896520487279E-24	3	NLRP1
**7.27842525760306E-12**	0.956771100497396	0.356	0.36	1.18718394376764E-07	3	OGA
**6.05856745204717E-87**	0.933763125353734	0.304	0.149	9.88212937103415E-83	3	SPOCK2
**1.97703575468907E-44**	0.920768075216626	0.258	0.156	3.22474301947335E-40	3	STAT4
**1.96716707604863E-15**	0.86630477821495	0.449	0.499	3.20864621774292E-11	3	JUND
**6.53487899169859E-08**	0.858355392439013	0.327	0.334	0.00106590411233596	3	GRK2
**3.18932579426475E-26**	0.847912604123725	0.501	0.549	5.20210930302524E-22	3	OAZ1
**1.91508163620998E-06**	0.84211180272305	0.185	0.265	0.0312368965682209	3	SRSF5
**9.14094111809547E-42**	0.827718413236608	0.53	0.512	1.49097890577255E-37	3	ARGLU1
**6.50759379264308E-111**	0.82605340158643	0.617	0.427	1.06145362351801E-106	3	SRGN
**3.20425212294199E-41**	0.819799916674625	0.374	0.277	5.22645563773068E-37	3	ITGB2
**7.14936914137007E-22**	0.766002251038475	0.327	0.269	1.16613360064887E-17	3	ARHGAP4
**6.16916266552305E-07**	0.7353992967813	0.183	0.263	0.0100625212237346	3	POLR2A
**1.20241204806534E-41**	0.725397901620312	0.584	0.596	1.96125429159937E-37	3	LENG8
**1.95409110830758E-11**	0.720426709353452	0.183	0.285	3.18731800676049E-07	3	MEF2D
**2.79252367663417E-33**	0.720173379814459	0.306	0.214	4.55488536895799E-29	3	IKZF1
**4.21830457683414E-07**	0.708673893941458	0.196	0.282	0.00688047659527417	3	UBR2
**8.99911082878941E-07**	0.707883543215816	0.213	0.304	0.0146784496728384	3	RBM6
**4.08859175221521E-09**	0.66774263483013	0.171	0.256	6.66890200703823E-05	3	NCOR2
**1.85720113009409E-08**	0.657814795890792	0.262	0.237	0.000302928076329648	3	IL10RA
**3.66615336790955E-13**	0.652271219889096	0.309	0.273	5.97986275839727E-09	3	HCLS1
**1.36196003672204E-16**	0.651373893883047	0.307	0.257	2.22149301589732E-12	3	ARL4C
**1.41169296547482E-07**	0.630248308030431	0.206	0.298	0.00230261239598597	3	PSMB8
**1.72984152716717E-15**	0.612055788153135	0.186	0.305	2.82154451496238E-11	3	LASP1
**4.36772378966336E-11**	0.603671104686453	0.18	0.275	7.12419427331991E-07	3	RBM39
**8.20603429997311E-12**	0.599558530028001	0.425	0.43	1.33848625466861E-07	3	ZFP36L2
**1.58536770498098E-06**	0.588017701210277	0.195	0.273	0.0258589326359448	3	PKN1
**2.00125115105314E-16**	0.583344043222084	0.34	0.289	3.26424075248278E-12	3	ITPKB
**1.70296647024817E-13**	0.578425888422406	0.169	0.269	2.7777086096218E-09	3	ASH1L
**6.03702868685675E-48**	0.577266389297361	0.61	0.552	9.84699749113205E-44	3	IL32
**8.74241363528899E-07**	0.574483918973312	0.226	0.321	0.0142597508805199	3	ACTG1
**3.12434396684811E-10**	0.57303341313944	0.183	0.277	5.09611744432595E-06	3	TLN1
**4.13176837360279E-21**	0.572609498807592	0.169	0.297	6.73932739418352E-17	3	TMEM140
**2.814868911592E-08**	0.568954982158712	0.281	0.254	0.000459133268169772	3	CD44
**1.36142779985931E-13**	0.565856727154512	0.167	0.266	2.22062488435052E-09	3	PSMB10
**8.67656966502922E-11**	0.562128330003287	0.169	0.257	1.41523527806292E-06	3	DDX39B
**9.85400566128015E-12**	0.559186021539516	0.224	0.345	1.6072868634114E-07	3	TCF25
**8.35978509680176E-11**	0.549466509843889	0.183	0.279	1.36356454713933E-06	3	CHD2
**2.04049501530542E-07**	0.54381148747629	0.205	0.293	0.00332825141946466	3	LRCH4
**1.74587713227886E-17**	0.534614565435952	0.188	0.312	2.84770019046005E-13	3	BRD2
**7.66989657197105E-13**	0.525507773935375	0.208	0.323	1.2510368298542E-08	3	HNRNPA0
**1.12949052277526E-20**	0.515301574598428	0.41	0.349	1.84231199169873E-16	3	CYBA
**1.11078540075889E-17**	0.511677941970979	0.565	0.631	1.81180206717783E-13	3	ACTB
**8.73641274344247E-16**	0.511255860232774	0.174	0.283	1.4249962825829E-11	3	N4BP2L2
**1.12065806253271E-19**	0.509367726849465	0.154	0.267	1.82790536579711E-15	3	MAPKAPK2
**3.02525557154831E-23**	0.501428676979794	0.154	0.279	4.93449436275244E-19	3	APMAP
**5.36300091048578E-14**	0.496755681284999	0.19	0.302	8.74759078509336E-10	3	SLC2A4RG
**7.15977836825726E-15**	0.48748444013846	0.204	0.326	1.16783144964644E-10	3	ANKRD10

1.42758097223982E-17	0.486847555727868	0.159	0.268	2.32852732382036E-13	3	FBXO7
2.72096206588001E-19	0.483011152492169	0.168	0.285	4.43816122565688E-15	3	TRIR
2.73333579042259E-26	0.475845194553087	0.136	0.259	4.45834400775829E-22	3	PPP6R2
1.25806712689329E-52	0.474820355257292	0.744	0.734	2.05203329067564E-48	3	TMSB4X
6.89499592714854E-54	0.470233592025506	0.745	0.769	1.1246427856772E-49	3	B2M
1.90923026911055E-25	0.467764584777973	0.144	0.268	3.11414549194622E-21	3	C11orf58
1.23166148948668E-07	0.456636064261886	0.285	0.418	0.00200896305550173	3	APOL6
1.85259824846933E-22	0.455800350462417	0.164	0.29	3.02177300307832E-18	3	SRSF4
1.28011837065646E-19	0.454610973676747	0.195	0.329	2.08800107437775E-15	3	TMEM259
1.04158666020984E-14	0.452381316298419	0.194	0.309	1.69893200146828E-10	3	PSME1
1.63538795966109E-21	0.444091999142438	0.178	0.308	2.66748130100321E-17	3	SPG7
8.55229387438101E-24	0.440926533526502	0.155	0.28	1.39496465385029E-19	3	HNRNPF
1.09532807178034E-26	0.43880283422005	0.135	0.257	1.78658961788091E-22	3	FLII
1.61163981045955E-12	0.437214884852625	0.19	0.29	2.62874569484057E-08	3	EML4
3.13648829317472E-25	0.429054208399379	0.165	0.299	5.11592605499728E-21	3	GSDMD
1.7335450410685E-17	0.428936893719281	0.208	0.339	2.82758531648683E-13	3	DPP7
4.12389218881082E-24	0.426174065890419	0.161	0.29	6.72648054916933E-20	3	ANXA11
5.58580290578756E-20	0.423449552169729	0.159	0.273	9.11100311963008E-16	3	OPTN
2.3423806628492E-24	0.409749888963953	0.142	0.26	3.82065709917332E-20	3	CAPN2
2.1338469487401E-13	0.405256874910682	0.251	0.39	3.48051775808998E-09	3	MDM4
4.13409520424665E-15	0.398497140596258	0.17	0.271	6.74312268764671E-11	3	PILRB
4.50079951187493E-16	0.393839645450838	0.223	0.358	7.34125408381919E-12	3	HNRNPA2B1
4.90873883216023E-20	0.386666404303899	0.232	0.39	8.00664390913655E-16	3	NDUFV1
1.77364690841488E-14	0.38603590865778	0.18	0.282	2.89299547231551E-10	3	NOP53
5.02027677208262E-12	0.385555730077455	0.292	0.452	8.18857344294397E-08	3	TGOLN2
1.73759161407297E-11	0.384060228549606	0.221	0.332	2.83418568171442E-07	3	PNRC1
1.07738956628736E-19	0.38125612252669	0.216	0.361	1.75733012157131E-15	3	EMC10
2.01321445859561E-32	0.371167838906643	0.172	0.331	3.2837541034153E-28	3	SLC38A10
1.35381204144991E-30	0.367756449134773	0.128	0.255	2.20820282080895E-26	3	SERINC1
8.5865165165625E-07	0.358235445583498	0.267	0.371	0.0140054670901651	3	RNF149
1.14264750389624E-19	0.357612906465896	0.158	0.269	1.86377234360516E-15	3	UBA52
4.69833649277252E-33	0.348228516081229	0.125	0.256	7.66345665336126E-29	3	SETD5
4.89130938212383E-20	0.347913051720707	0.206	0.345	7.97821473318218E-16	3	CCNL1
2.03563724154884E-35	0.343878983404087	0.13	0.27	3.32032790469032E-31	3	SRPRA
7.75893512200152E-32	0.332960004074575	0.124	0.251	1.26555990774967E-27	3	STXBP2
3.836218470895E-07	0.325355706345725	0.577	0.666	0.00625725594787684	3	MCL1
1.40944986960473E-14	0.324529963524324	0.246	0.382	2.29895368231227E-10	3	CDC42SE1
2.16697162076769E-31	0.321880015539149	0.164	0.313	3.53454741063417E-27	3	ADAR
5.70627051530527E-12	0.314983558455814	0.244	0.375	9.30749783751443E-08	3	PTMS
1.54556706671358E-25	0.297200642369256	0.189	0.334	2.52097444251652E-21	3	TNFRSF14
1.51714517925498E-39	0.294645567357889	0.118	0.26	2.47461550188279E-35	3	EIF4H
5.42295329150582E-23	0.267542602234215	0.227	0.386	8.84537911377514E-19	3	CDK5RAP3
6.7141956946628E-31	0.254849252528872	0.147	0.282	1.09515245975645E-26	3	LAMTOR4
1.00678119463176E-38	0.252765957408998	0.128	0.275	1.64216080656386E-34	3	NDUFV2
1.14993579474195E-34	0.252514444509756	0.141	0.283	1.87566027480359E-30	3	SNRNP70
0	3.04096145945158	0.253	0.012	0	4	C9
0	2.91325337512873	0.7	0.079	0	4	CRP
0	2.83749316394584	0.524	0.023	0	4	TSPAN8
0	2.81298566994562	0.459	0.017	0	4	APOF
0	2.79204652432862	0.378	0.03	0	4	CPS1

0	2.76161262130146	0.951	0.117	0	4	HRG
0	2.73825021274754	0.99	0.152	0	4	SERPINA3
0	2.66138689703297	0.771	0.055	0	4	SERPINA6
0	2.61974832163297	0.627	0.027	0	4	PGLYRP2
0	2.59374159408197	1	0.299	0	4	FGA
0	2.58450877490393	0.674	0.039	0	4	CYP4A11
0	2.57733773447664	0.87	0.057	0	4	RNASE4
0	2.5749406042801	0.498	0.021	0	4	SERPINA11
0	2.56472075390671	0.282	0.015	0	4	LCN2
0	2.55886389120201	0.305	0.015	0	4	HGFAC
0	2.54019893263082	0.251	0.013	0	4	CFHR3
0	2.53287900128944	0.735	0.058	0	4	CYP2C9
0	2.53079628848439	0.996	0.196	0	4	ITIH2
0	2.52715344524426	0.509	0.024	0	4	CCL16
0	2.51593668490905	0.713	0.041	0	4	SLC22A1
0	2.51407171666113	1	0.293	0	4	VTN
0	2.50606190296392	0.701	0.038	0	4	TST
0	2.50522042364414	1	0.306	0	4	FGB
0	2.50478223467593	0.767	0.043	0	4	APOM
0	2.50469036003306	0.292	0.014	0	4	AL135905.2
0	2.50052542941909	0.983	0.129	0	4	TM4SF4
0	2.49923575582578	0.999	0.235	0	4	PLA2G2A
0	2.4985091189714	1	0.269	0	4	TF
0	2.49488196990818	0.566	0.029	0	4	RIDA
0	2.49269441777636	0.868	0.062	0	4	C8B
0	2.49221546682375	0.754	0.046	0	4	CFHR4
0	2.49149156530457	0.972	0.104	0	4	HABP2
0	2.48649003687596	0.998	0.167	0	4	SERPINF2
0	2.4762933833115	1	0.404	0	4	SERPINA1
0	2.47279470215683	0.806	0.049	0	4	REEP6
0	2.47208967324236	0.968	0.119	0	4	LBP
0	2.47190527455375	0.6	0.039	0	4	PIGR
0	2.47137262512366	0.738	0.052	0	4	CYP2C8
0	2.46325655350052	0.998	0.162	0	4	LRG1
0	2.46046741643704	0.892	0.081	0	4	GPX2
0	2.45966950328451	0.797	0.083	0	4	PCK1
0	2.45480439005049	0.555	0.033	0	4	GLS2
0	2.44790405839271	0.754	0.042	0	4	DEPRECATE
0	2.44685800159048	0.568	0.029	0	4	SLC51A
0	2.44337434582148	0.572	0.027	0	4	PPP1R1A
0	2.4368182414848	0.95	0.089	0	4	PON1
0	2.43419013848152	0.921	0.076	0	4	GJB1
0	2.43239532885295	0.997	0.204	0	4	KNG1
0	2.43006439002657	0.903	0.072	0	4	SERPINA5
0	2.42626132300326	0.923	0.087	0	4	HSD17B6
0	2.42572769418126	0.986	0.129	0	4	PLG
0	2.42221051276827	0.999	0.253	0	4	APOA1
0	2.42109586448027	0.505	0.029	0	4	FETUB
0	2.41681732997324	0.716	0.097	0	4	NNMT
0	2.41325534597766	0.854	0.073	0	4	LEPR

0	2.413042878554	0.464	0.039	0	4	SAA1
0	2.41041105971747	1	0.285	0	4	FGG
0	2.4099156697322	0.987	0.143	0	4	ITIH1
0	2.40947471976008	0.992	0.164	0	4	SERPINC1
0	2.40829556969578	0.987	0.152	0	4	ORM2
0	2.40718513327338	0.98	0.118	0	4	SERPIND1
0	2.40689264801742	0.428	0.023	0	4	SLC6A11
0	2.40492085013324	1	0.303	0	4	C3
0	2.39681520445543	1	0.22	0	4	HPX
0	2.39676858897657	0.277	0.013	0	4	SLC15A1
0	2.39614062382417	0.712	0.046	0	4	SLC17A4
0	2.39344195693906	1	0.241	0	4	CLU
0	2.38798594631211	0.997	0.174	0	4	CP
0	2.38566802254234	0.48	0.023	0	4	TTPA
0	2.38444337787795	0.959	0.105	0	4	GAMT
0	2.38100017084179	0.928	0.077	0	4	ANG
0	2.3799428820476	0.258	0.01	0	4	UGT1A4
0	2.37946974680382	0.747	0.063	0	4	G6PC
0	2.37897823736014	0.468	0.02	0	4	CCL15
0	2.37779607655833	0.601	0.037	0	4	TMEM45A
0	2.37662285479389	0.553	0.026	0	4	MYORG
0	2.37645292302345	0.752	0.048	0	4	DNAJC22
0	2.37505119221486	0.837	0.063	0	4	PDZK1IP1
0	2.37222623744451	0.998	0.192	0	4	AMBP
0	2.37069089769417	0.96	0.107	0	4	SLC38A3
0	2.37024119184704	0.6	0.029	0	4	DEPRECATE
0	2.36854197477095	1	0.227	0	4	CFH
0	2.3667635510482	1	0.39	0	4	ALB
0	2.3640006658276	0.488	0.024	0	4	PLA2G12B
0	2.3608538927946	0.998	0.204	0	4	ORM1
0	2.3596941087139	0.959	0.104	0	4	DEPRECATE
0	2.3541342944769	0.675	0.047	0	4	GNMT
0	2.35349167558952	0.401	0.018	0	4	AGXT2
0	2.33981812839439	0.856	0.072	0	4	DPYS
0	2.3365698461655	0.297	0.017	0	4	SAA4
0	2.3347555240462	0.42	0.02	0	4	PRODH2
0	2.33227933898669	0.838	0.06	0	4	APOC4
0	2.33132768790897	1	0.265	0	4	CFB
0	2.33101266078469	0.596	0.029	0	4	UGT2B15
0	2.32878295584028	0.941	0.094	0	4	SERPINA7
0	2.32864326737648	0.592	0.026	0	4	RNF128
0	2.32803127633347	0.701	0.043	0	4	DEPRECATE
0	2.32602729396263	0.789	0.05	0	4	AMN
0	2.32534834138613	0.378	0.018	0	4	SMLR1
0	2.32360985712322	0.945	0.116	0	4	TAT
0	2.3235124507906	0.65	0.038	0	4	CYP4F11
0	2.32169074594815	0.819	0.053	0	4	SERPINA4
0	2.3172038924102	0.984	0.145	0	4	TM4SF5
0	2.31683230819063	0.961	0.101	0	4	MGST1
0	2.31507398995901	0.743	0.05	0	4	AFM

0	2.31484807741774	0.984	0.152	0	4	KRT8
0	2.31419227652041	0.613	0.049	0	4	CYP2B6
0	2.3139785561509	0.401	0.018	0	4	MAL2
0	2.31371006407712	0.884	0.079	0	4	ADH1A
0	2.31177160382471	0.651	0.046	0	4	CDO1
0	2.31161491468806	0.457	0.018	0	4	CLDN3
0	2.31105637579351	0.254	0.014	0	4	PIK3C2G
0	2.31046467892588	0.832	0.064	0	4	SLC2A2
0	2.31013863944229	0.836	0.064	0	4	C6
0	2.31001230352687	0.561	0.047	0	4	BHMT
0	2.308051882452	0.964	0.106	0	4	SDC1
0	2.30699382349294	0.338	0.015	0	4	LECT2
0	2.30633740333054	0.414	0.02	0	4	AGMO
0	2.30614933504592	0.618	0.034	0	4	SLCO1B1
0	2.30532537102328	0.879	0.075	0	4	C8A
0	2.30520476996291	0.895	0.075	0	4	PON3
0	2.30473037055384	0.893	0.075	0	4	HJV
0	2.29677123084289	0.933	0.082	0	4	MASP2
0	2.29635169527933	0.898	0.075	0	4	TMEM45B
0	2.29502471444614	0.319	0.012	0	4	GJB2
0	2.29326157085583	0.49	0.021	0	4	UGT2B10
0	2.29275433587744	0.683	0.042	0	4	LGALS4
0	2.29254468975082	0.543	0.029	0	4	ASPDH
0	2.28944106112435	0.867	0.066	0	4	CPB2
0	2.28892542209204	0.425	0.021	0	4	F12
0	2.28863902398482	0.972	0.145	0	4	APCS
0	2.2871323646536	1	0.261	0	4	APOC2
0	2.28649760500712	0.993	0.194	0	4	AHSG
0	2.28397075788445	0.997	0.172	0	4	ASGR2
0	2.28089400710003	0.931	0.088	0	4	F7
0	2.28020833247384	0.999	0.222	0	4	AGT
0	2.27741262910679	0.581	0.037	0	4	SLC27A5
0	2.27724788582863	0.997	0.177	0	4	C4BPA
0	2.27442976853698	0.952	0.134	0	4	IGFBP2
0	2.27055246611964	0.903	0.096	0	4	AKR1B10
0	2.2698989372745	0.757	0.044	0	4	PERP
0	2.26895141239924	0.999	0.29	0	4	APOC3
0	2.26888681486942	0.569	0.035	0	4	DIO1
0	2.26380960299885	0.999	0.205	0	4	APOB
0	2.26376976098504	0.93	0.086	0	4	LSR
0	2.26181306476715	0.716	0.049	0	4	TM7SF2
0	2.26162258481317	0.922	0.087	0	4	F5
0	2.26138628257412	0.953	0.105	0	4	CLDN1
0	2.26020475157899	1	0.25	0	4	GC
0	2.25825728189009	0.964	0.118	0	4	BDH1
0	2.257440232023	0.257	0.014	0	4	PROZ
0	2.25662490421321	0.716	0.048	0	4	PIPOX
0	2.25481186867654	0.68	0.043	0	4	SEMA4G
0	2.25426935754756	0.33	0.015	0	4	CYP4F2
0	2.25293019467394	0.986	0.156	0	4	GSTA1

0	2.25233821836537	0.947	0.095		0	4	AADAC
0	2.25221711000274	0.983	0.13		0	4	AZGP1
0	2.25102751338593	0.38	0.026		0	4	MUC13
0	2.24856917736851	0.738	0.043		0	4	CMBL
0	2.24751381467419	0.292	0.014		0	4	KLB
0	2.2439541732365	0.468	0.028		0	4	DEPRECATE
0	2.24179049237989	0.581	0.036		0	4	ABCG5
0	2.24114292077062	0.931	0.094		0	4	MBL2
0	2.23956807806068	0.994	0.211		0	4	FABP1
0	2.23851392484176	0.995	0.17		0	4	F2
0	2.23593745464384	0.506	0.04		0	4	ADH4
0	2.23487191169057	0.639	0.041		0	4	PDZK1
0	2.23449079707525	0.881	0.076		0	4	HSD17B2
0	2.23397181759877	0.906	0.076		0	4	C8G
0	2.23195785495027	0.494	0.028		0	4	IRF6
0	2.23135510857874	0.436	0.027		0	4	PTPRD
0	2.23080646482843	0.98	0.129		0	4	PAH
0	2.22914857398272	0.884	0.088		0	4	SLC22A7
0	2.22858576377252	0.305	0.013		0	4	UGT1A9
0	2.22602958203908	0.955	0.1		0	4	PROC
0	2.22552208992605	0.695	0.037		0	4	MAOB
0	2.2250491506811	0.979	0.144		0	4	SPINK1
0	2.22418493346022	0.775	0.07		0	4	HAL
0	2.22353399378098	0.736	0.046		0	4	GLTPD2
0	2.22324978002132	0.393	0.021		0	4	CTH
0	2.22264662878939	0.884	0.071		0	4	ABCC2
0	2.22252018846605	0.338	0.019		0	4	NR1I3
0	2.22238890282715	0.623	0.041		0	4	ABCC6
0	2.22042412235284	0.987	0.146		0	4	DHCR24
0	2.2201385928669	0.766	0.061		0	4	DEPRECATE
0	2.21947137298628	0.861	0.069		0	4	OTC
0	2.21907015158869	0.841	0.081		0	4	LEAP2
0	2.2162473166347	0.378	0.019		0	4	TM6SF2
0	2.21424756625863	0.996	0.186		0	4	APOH
0	2.21382825917726	0.797	0.057		0	4	F13B
0	2.21325989034277	0.383	0.017		0	4	ERICH5
0	2.21263719746936	0.953	0.104		0	4	HGD
0	2.21209886443015	0.664	0.042		0	4	CDH1
0	2.2072929308745	0.979	0.141		0	4	CES2
0	2.20577201706132	0.491	0.027		0	4	ADGRV1
0	2.20495106653067	0.943	0.1		0	4	HNF4A
0	2.20382494266177	0.707	0.051		0	4	SLPI
0	2.20340582637971	0.874	0.083		0	4	SLC38A4
0	2.20330253372153	0.677	0.037		0	4	CPN2
0	2.20301240628773	0.652	0.038		0	4	F10
0	2.20178476128086	0.88	0.076		0	4	OCLN
0	2.200753399142	0.778	0.055		0	4	SHBG
0	2.20072342451934	0.809	0.061		0	4	EHHADH
0	2.20005114251712	0.865	0.067		0	4	TMPRSS6
0	2.19853646886124	0.882	0.082		0	4	ARG1

0	2.19823891423659	0.767	0.056	0	4	ACSM1
0	2.19570729462469	0.777	0.054	0	4	DEPRECATE
0	2.19487101313041	0.893	0.079	0	4	RGN
0	2.19367465236096	0.329	0.019	0	4	DEPDC7
0	2.19300269713758	0.999	0.257	0	4	RBP4
0	2.19043434873065	0.752	0.053	0	4	FGGY
0	2.18934562332673	0.567	0.033	0	4	CLDN4
0	2.18883835038908	0.987	0.196	0	4	IGFBP1
0	2.18763463425854	0.296	0.016	0	4	NIPAL1
0	2.18716958042605	0.684	0.045	0	4	RARRES2
0	2.18701209048614	0.679	0.044	0	4	EVA1A
0	2.18555323788494	0.381	0.031	0	4	DEFB1
0	2.18216914341824	0.922	0.096	0	4	GPT
0	2.18139728168796	0.391	0.018	0	4	SLC16A2
0	2.17952826376489	0.721	0.047	0	4	UGT1A1
0	2.17813819161394	0.747	0.052	0	4	PROS1
0	2.17796782933343	0.812	0.059	0	4	CDHR5
0	2.17783061380417	0.529	0.03	0	4	PLGLB1
0	2.17770742798819	0.62	0.04	0	4	PRG4
0	2.17721561731881	0.855	0.073	0	4	CREB3L3
0	2.17571431640289	0.967	0.119	0	4	AOX1
0	2.17237236576647	0.622	0.041	0	4	SLC22A25
0	2.17148711247489	0.522	0.037	0	4	LOXL4
0	2.17103256138026	0.87	0.077	0	4	METTL7B
0	2.17031944710588	0.402	0.022	0	4	PLGLB2
0	2.17006090730436	0.494	0.025	0	4	CFHR5
0	2.16923228969265	0.997	0.207	0	4	FGL1
0	2.16743285334151	0.98	0.141	0	4	TDO2
0	2.16688021151265	0.815	0.069	0	4	CLDN7
0	2.16626897958539	0.876	0.078	0	4	SERPINA10
0	2.16568357100043	0.31	0.015	0	4	ENPP7
0	2.16532318492543	0.927	0.095	0	4	C5
0	2.164375915624	0.386	0.028	0	4	MUC3A
0	2.16349597553014	0.314	0.016	0	4	SPTBN2
0	2.16270913646977	0.98	0.123	0	4	C4BPB
0	2.16018375538091	0.501	0.03	0	4	ASPG
0	2.16000988148534	0.945	0.112	0	4	CFHR2
0	2.1593314325321	0.967	0.13	0	4	ADH1C
0	2.15675725213649	0.655	0.041	0	4	CYP2D6
0	2.15434812327053	0.519	0.03	0	4	GPLD1
0	2.1542454742622	0.655	0.039	0	4	GOLT1A
0	2.15288982408579	0.293	0.014	0	4	FOXA1
0	2.15287823870517	0.807	0.072	0	4	ALDH1L1
0	2.15183867621048	0.25	0.012	0	4	RTP3
0	2.14998706029767	1	0.246	0	4	TTR
0	2.14771727142655	0.621	0.034	0	4	GCAT
0	2.14680864688499	0.8	0.065	0	4	SLC6A1
0	2.14634260316435	0.816	0.064	0	4	BHMT2
0	2.14525135497459	0.733	0.049	0	4	ALDH4A1
0	2.14391897860433	0.804	0.058	0	4	AKR1C8P

0	2.14376762508993	0.817	0.08	0	4	HPD
0	2.14302821540757	0.865	0.071	0	4	ERBB3
0	2.14131830231332	0.593	0.038	0	4	KNDC1
0	2.14084251082843	0.999	0.238	0	4	HP
0	2.14061091079222	1	0.289	0	4	EPHX1
0	2.13880410338213	0.283	0.014	0	4	ACMSD
0	2.13784893348297	0.987	0.137	0	4	TFR2
0	2.13756097632664	0.442	0.021	0	4	SMOC1
0	2.12966022735704	0.976	0.133	0	4	FMO5
0	2.12921545543807	0.454	0.02	0	4	DEPRECATE
0	2.1281696138281	0.99	0.169	0	4	AGXT
0	2.12673818378206	0.644	0.042	0	4	RAB17
0	2.12497049232875	0.974	0.137	0	4	KRT18
0	2.12409127044371	0.319	0.017	0	4	DEPRECATE
0	2.1208802159107	0.4	0.018	0	4	ARSE
0	2.12078684091393	0.848	0.074	0	4	UGT2B4
0	2.11917732285374	0.285	0.016	0	4	GRB14
0	2.11843584466756	0.987	0.153	0	4	MAT1A
0	2.11589214745792	0.344	0.018	0	4	RIPK4
0	2.1135758846562	0.908	0.082	0	4	HAO1
0	2.11253321075449	0.936	0.108	0	4	GGCX
0	2.11230589830648	0.8	0.063	0	4	MYOM1
0	2.11158753221189	0.449	0.02	0	4	C1orf56
0	2.11140518998469	0.822	0.065	0	4	KLKB1
0	2.10974662114409	0.958	0.115	0	4	SERINC2
0	2.10768579727554	0.805	0.07	0	4	MDK
0	2.10729576604168	0.871	0.075	0	4	ACSM2A
0	2.10629808651357	0.983	0.155	0	4	SDC4
0	2.10376515067695	0.999	0.27	0	4	FN1
0	2.10275957430816	0.999	0.308	0	4	MT1B
0	2.10238927337654	0.826	0.067	0	4	A1CF
0	2.10157186519742	0.859	0.071	0	4	LDHD
0	2.10033622318167	0.97	0.14	0	4	SERPINF1
0	2.09699746873369	0.512	0.028	0	4	RBP5
0	2.09647798823033	1	0.415	0	4	APOA2
0	2.0963767279696	0.823	0.069	0	4	KHK
0	2.09301148660872	0.815	0.067	0	4	AKR1C3
0	2.09038319387362	0.293	0.013	0	4	AKR1C4
0	2.08951062892679	0.999	0.212	0	4	C1S
0	2.08927731207374	0.345	0.016	0	4	MAOA
0	2.08783716201941	0.989	0.138	0	4	HPN
0	2.08739915962325	0.711	0.055	0	4	STEAP3
0	2.08701604871644	0.347	0.019	0	4	TMEM92
0	2.0850131812425	0.914	0.101	0	4	ADH1B
0	2.08501005267208	0.382	0.019	0	4	FOXA2
0	2.08443946395067	0.294	0.018	0	4	CES3
0	2.08419783750382	0.708	0.045	0	4	C2orf72
0	2.08378722120308	0.519	0.033	0	4	TRIM55
0	2.08368471282222	0.966	0.129	0	4	PRAP1
0	2.08306566931034	0.397	0.02	0	4	F9

0	2.08281578070225	0.661	0.048	0	4	ACOT12
0	2.08218142149001	0.676	0.039	0	4	AGMAT
0	2.08128063577161	0.58	0.032	0	4	ALDH8A1
0	2.08063429045458	0.532	0.034	0	4	EPB41L5
0	2.07768373859393	0.7	0.051	0	4	PALM3
0	2.07595518886525	0.536	0.034	0	4	GHR
0	2.07475105918214	0.402	0.021	0	4	RTN4RL2
0	2.07435552937792	0.811	0.062	0	4	APOA5
0	2.07327857861352	0.967	0.127	0	4	HPR
0	2.06993340813954	0.797	0.087	0	4	FMO3
0	2.06969672862171	0.99	0.166	0	4	HMGCS2
0	2.06946164984843	0.58	0.039	0	4	LGR4
0	2.06920583569896	0.959	0.102	0	4	ITIH4
0	2.06845929603487	0.287	0.014	0	4	ANKS4B
0	2.06756998522588	0.382	0.022	0	4	MOGAT3
0	2.06656848282	0.751	0.052	0	4	ALDH6A1
0	2.06583912292985	0.953	0.115	0	4	FAH
0	2.06564302615723	0.264	0.015	0	4	SUGCT
0	2.06410397538736	0.324	0.018	0	4	ETV4
0	2.0629740860511	0.699	0.055	0	4	AJUBA
0	2.06107184971065	0.966	0.121	0	4	DEPRECATE
0	2.06083901663983	0.75	0.053	0	4	FOXA3
0	2.06082374468906	0.885	0.078	0	4	ESRP2
0	2.0593812543769	0.875	0.079	0	4	MPC2
0	2.05936404437935	0.579	0.038	0	4	HOOK1
0	2.05602205754089	0.501	0.028	0	4	CYP2J2
0	2.05496269901024	0.929	0.107	0	4	CFI
0	2.05464544822065	0.47	0.025	0	4	PKLR
0	2.05384666313146	0.842	0.072	0	4	KLF15
0	2.05222937729823	0.465	0.022	0	4	GGH
0	2.05184557995797	0.655	0.039	0	4	DGAT2
0	2.05119798956992	0.577	0.032	0	4	PACSIN3
0	2.05074712839457	0.31	0.015	0	4	SALL1
0	2.05070002487771	0.706	0.054	0	4	SLC30A10
0	2.04926334464668	0.499	0.031	0	4	IL17RB
0	2.04717728161725	0.277	0.011	0	4	SEZ6L2
0	2.04659810155514	0.756	0.06	0	4	AMDHD1
0	2.04612233892321	0.28	0.012	0	4	SOWAHA
0	2.04598807139516	1	0.239	0	4	CES1
0	2.04560117900675	0.792	0.07	0	4	AKR1C2
0	2.04526564529675	0.315	0.016	0	4	ELOVL2
0	2.04513140368192	1	0.298	0	4	MT1G
0	2.04401244023006	0.287	0.019	0	4	PTPN3
0	2.04288769334842	0.416	0.028	0	4	RDH16
0	2.03992428468722	0.76	0.069	0	4	ANGPTL4
0	2.03716896335432	0.998	0.328	0	4	A2M
0	2.03600904537478	0.518	0.03	0	4	ACSM2B
0	2.03384179230731	0.75	0.056	0	4	GCKR
0	2.03275141424025	0.968	0.141	0	4	F11R
0	2.03180011697048	0.483	0.032	0	4	ZNF648

**0**	2.03082586092702	0.526	0.032		0	4	GAGE1
**0**	2.02821866931905	0.932	0.102		0	4	PON2
**0**	2.02792588703507	0.763	0.06		0	4	CHDH
**0**	2.02571827881022	0.914	0.099		0	4	SELENBP1
**0**	2.02516851092892	0.9	0.092		0	4	ENTPD5
**0**	2.0239860143584	0.753	0.051		0	4	GPRC5C
**0**	2.02322968191086	0.275	0.018		0	4	CSMD1
**0**	2.02221054611324	0.392	0.023		0	4	AR
**0**	2.02091105879337	0.704	0.051		0	4	FTCD
**0**	2.01961709155017	0.562	0.035		0	4	SLC12A8
**0**	2.01860744808217	0.72	0.053		0	4	F11
**0**	2.01848288149341	0.9	0.083		0	4	ONECUT1
**0**	2.01843560546394	0.375	0.017		0	4	ANXA9
**0**	2.01838670074833	0.279	0.019		0	4	CECR2
**0**	2.01681866150052	0.3	0.016		0	4	SLC7A9
**0**	2.01576792109041	0.757	0.06		0	4	GPT2
**0**	2.01557547068522	0.601	0.041		0	4	GRTP1
**0**	2.01512373319027	0.589	0.036		0	4	PC
**0**	2.0128748985095	0.796	0.059		0	4	CDH2
**0**	2.01280432156209	0.707	0.051		0	4	CYP2C18
**0**	2.0111959787405	0.944	0.106		0	4	TCEA3
**0**	2.00864433684265	0.734	0.05		0	4	PCDH1
**0**	2.00838002013519	0.87	0.08		0	4	SLC7A2
**0**	2.00799617929614	0.531	0.033		0	4	GBP7
**0**	2.00607091226032	0.748	0.057		0	4	SLC39A14
**0**	2.00468377219052	0.989	0.161		0	4	ASGR1
**0**	1.99959816664331	0.418	0.025		0	4	FRK
**0**	1.99694136364	0.333	0.017		0	4	GSTA2
**0**	1.99691048954844	0.927	0.102		0	4	STARD10
**0**	1.99577157931468	0.601	0.033		0	4	TSPAN6
**0**	1.99505063675462	0.36	0.023		0	4	FYB2
**0**	1.99432205023949	0.38	0.019		0	4	DBNDD1
**0**	1.99047464555992	0.706	0.049		0	4	DECR2
**0**	1.98917026064413	0.993	0.202		0	4	ALDOB
**0**	1.9865565767129	0.971	0.146		0	4	BAAT
**0**	1.9864572269138	0.859	0.076		0	4	DEPRECATE
**0**	1.98566315014007	0.763	0.059		0	4	ACOX2
**0**	1.98511270064837	0.428	0.028		0	4	ABCB11
**0**	1.98455064449381	0.752	0.055		0	4	TUBB2A
**0**	1.98212014940322	0.602	0.041		0	4	ABCB4
**0**	1.98150328776557	0.683	0.046		0	4	CAMSAP3
**0**	1.98104796018438	0.569	0.036		0	4	GPD1
**0**	1.97704315579327	0.992	0.163		0	4	ITIH3
**0**	1.97526480027379	0.49	0.031		0	4	MARVELD2
**0**	1.97493339256717	0.993	0.196		0	4	CERS2
**0**	1.97434197645451	0.951	0.194		0	4	CYP 20.00
**0**	1.97429583749222	0.58	0.035		0	4	LAD1
**0**	1.97225181332197	0.546	0.026		0	4	OLFM2
**0**	1.97047453138598	0.989	0.249		0	4	DEPRECATE
**0**	1.96885542314361	0.788	0.062		0	4	CBS

0	1.96764148194806	0.252	0.01		0	4	DEPRECATE
0	1.96555586057875	0.977	0.157		0	4	CYB5A
0	1.96476313953414	0.589	0.036		0	4	TMEM184A
0	1.96436142979848	0.955	0.132		0	4	PGRMC1
0	1.96376159685052	0.755	0.056		0	4	FNDC4
0	1.96370905682469	0.725	0.056		0	4	PTPRH
0	1.96326535149584	0.504	0.033		0	4	UNC5CL
0	1.96152092848628	0.84	0.065		0	4	NIPSNAP1
0	1.96143543461709	0.805	0.063		0	4	CAMK2N1
0	1.9613365294936	0.909	0.08		0	4	DSP
0	1.96095757171271	0.871	0.078		0	4	OGDHL
0	1.95882673370714	0.687	0.044		0	4	C1orf115
0	1.95786633516178	0.479	0.03		0	4	MET
0	1.95759981204739	0.344	0.019		0	4	PKP2
0	1.95739750683366	0.998	0.311		0	4	MT1F
0	1.95587633732816	0.748	0.057		0	4	EGFR
0	1.955016509501	0.85	0.076		0	4	PCCB
0	1.9546666906836	0.759	0.06		0	4	NR1I2
0	1.95258477723697	0.272	0.019		0	4	TBX3
0	1.94733750099766	0.96	0.122		0	4	ELF3
0	1.94703942012061	0.43	0.026		0	4	FGFR4
0	1.94650261735498	0.377	0.023		0	4	GAS2
0	1.9455910406682	0.702	0.05		0	4	PROX1
0	1.94271841168793	0.912	0.098		0	4	SOD1
0	1.94179511246687	0.683	0.047		0	4	ATP2B2
0	1.94177769428675	0.973	0.136		0	4	CYP3A5
0	1.94106656667956	0.422	0.027		0	4	TMPRSS2
0	1.93952049698622	0.443	0.025		0	4	CBLC
0	1.93884362791628	0.37	0.021		0	4	SLC26A1
0	1.93705387264634	0.357	0.023		0	4	RNF165
0	1.93694219112956	0.9	0.105		0	4	OSGIN1
0	1.93675846817914	0.604	0.041		0	4	SLC25A18
0	1.93663047457788	0.942	0.112		0	4	MPST
0	1.93574185650915	0.296	0.022		0	4	PPP1R9A
0	1.93549898553593	0.277	0.011		0	4	NAT8
0	1.93418990423324	0.99	0.172		0	4	EFNA1
0	1.93310955691075	0.471	0.028		0	4	PBLD
0	1.9324271600556	0.842	0.072		0	4	ANGPTL3
0	1.93006966907024	0.487	0.032		0	4	GDA
0	1.92941074356416	0.362	0.024		0	4	RAB3B
0	1.92776687040894	0.933	0.109		0	4	PCSK6
0	1.92734884091207	0.718	0.051		0	4	GOT1
0	1.92691006184641	0.503	0.034		0	4	MOCOS
0	1.92469821243362	0.718	0.054		0	4	ALDH1B1
0	1.92179663102339	0.488	0.031		0	4	TJP3
0	1.9213366491084	0.279	0.019		0	4	CCDC187
0	1.92034256462199	0.936	0.122		0	4	TFPI
0	1.9168200221797	0.944	0.123		0	4	ACAT1
0	1.91654755110995	0.946	0.1		0	4	MYH14
0	1.91648015598959	0.373	0.024		0	4	DEPRECATE

0	1.91637586969272	0.995	0.307	0	4	MT1X
0	1.91346419260474	0.31	0.022	0	4	GREB1L
0	1.91209078300139	0.891	0.086	0	4	MYO1B
0	1.9117347879513	0.292	0.014	0	4	TMEM82
0	1.91101796350937	0.314	0.017	0	4	PSAT1
0	1.91052830281912	0.279	0.013	0	4	PANX2
0	1.9101699054286	0.779	0.066	0	4	CYP21A2
0	1.90984027223177	0.493	0.027	0	4	SPR
0	1.90956162358953	0.443	0.027	0	4	GLYATL1
0	1.90885849955966	0.521	0.031	0	4	SHMT2
0	1.90725842493133	0.846	0.087	0	4	SULT2A1
0	1.90626205429885	1	0.423	0	4	MT2A
0	1.90597361667837	0.618	0.044	0	4	HNF1A
0	1.90397999281423	0.571	0.04	0	4	XYLB
0	1.90345665077226	0.582	0.036	0	4	SIAE
0	1.90151522217312	0.974	0.186	0	4	GATM
0	1.89957363691821	0.772	0.066	0	4	PLEKHA6
0	1.89945916889863	0.993	0.259	0	4	APOC1
0	1.89914660371143	0.32	0.017	0	4	ETNK2
0	1.89693252722578	0.733	0.056	0	4	CITED4
0	1.89636079773262	0.74	0.057	0	4	BPHL
0	1.8912745824349	0.527	0.032	0	4	STBD1
0	1.89076939515264	0.897	0.097	0	4	NADK2
0	1.8891005268777	0.948	0.122	0	4	PDIA5
0	1.88445435295531	0.389	0.047	0	4	HAMP
0	1.88334968172634	0.83	0.078	0	4	MST1
0	1.88234617725202	0.468	0.031	0	4	CENPV
0	1.88172942420633	0.923	0.123	0	4	DCXR
0	1.87968980607918	0.869	0.079	0	4	KIAA1522
0	1.87867835317404	0.519	0.033	0	4	COBL
0	1.87516065227981	0.293	0.022	0	4	SLC28A1
0	1.87373250413109	0.918	0.116	0	4	SLC37A4
0	1.87170363219646	0.45	0.028	0	4	NUDT7
0	1.8708287320893	0.705	0.049	0	4	PHYH
0	1.86614426677826	0.921	0.136	0	4	ALDH1A1
0	1.86500476897865	0.438	0.027	0	4	FUT6
0	1.86363314404783	0.959	0.108	0	4	MLXIPL
0	1.86179647951224	0.926	0.1	0	4	SEC16B
0	1.86082305839575	0.953	0.144	0	4	ADI1
0	1.8605634229022	0.952	0.137	0	4	ERRFI1
0	1.86014366042416	0.889	0.083	0	4	PTPRF
0	1.85979937744548	0.59	0.046	0	4	FST
0	1.85915110986278	0.629	0.047	0	4	WWC1
0	1.85753416581259	0.665	0.042	0	4	RAMP1
0	1.85596405454905	0.937	0.121	0	4	UGDH
0	1.85243323089445	0.31	0.021	0	4	EPDR1
0	1.85052656164514	0.296	0.018	0	4	THPO
0	1.84971878670333	0.297	0.019	0	4	CYP4F12
0	1.8493988133666	0.376	0.021	0	4	VIL1
0	1.84802723835978	0.553	0.034	0	4	RHOD

	0	1.84790137775881	0.731	0.056	0	4	BAIAP2L1
	0	1.84706532424432	0.914	0.12	0	4	PCK2
	0	1.84166442422155	0.524	0.035	0	4	FAM110C
	0	1.84141168769474	0.43	0.027	0	4	C4orf19
	0	1.83940107756419	0.588	0.045	0	4	RND1
	0	1.83877720309568	0.742	0.06	0	4	PLA1A
	0	1.8386401161066	0.794	0.076	0	4	MMP15
	0	1.83845772816777	0.362	0.023	0	4	NR0B2
	0	1.83632364742024	0.332	0.02	0	4	EPB41L4B
	0	1.83325741015497	0.616	0.048	0	4	PPARGC1A
	0	1.8331963546599	0.766	0.067	0	4	SAMD5
	0	1.83179412604424	0.598	0.044	0	4	NAGS
	0	1.82999808205588	0.387	0.021	0	4	PROSER2
	0	1.82880406777071	0.809	0.081	0	4	TSKU
	0	1.82613612434847	0.636	0.045	0	4	ECHDC3
	0	1.82437991948238	0.725	0.077	0	4	TMEM176A
	0	1.82430710681551	0.398	0.026	0	4	CLDN12
	0	1.82368729028614	0.549	0.037	0	4	SORD
	0	1.82206434963747	0.553	0.039	0	4	SHANK2
	0	1.82202708240077	0.747	0.072	0	4	TNFRSF12A
	0	1.82060065718785	0.392	0.025	0	4	AQP7
	0	1.8203580146531	0.752	0.065	0	4	KANK1
	0	1.81806792382561	0.937	0.114	0	4	QPRT
	0	1.81760021006192	0.379	0.026	0	4	DNAH14
	0	1.81749562907474	0.78	0.113	0	4	UBD
	0	1.81699945381072	0.348	0.022	0	4	MPV17L
	0	1.81318412242817	0.899	0.094	0	4	PGM1
	0	1.81303527687778	0.826	0.075	0	4	ABAT
	0	1.81277584756591	0.652	0.051	0	4	CYP4F3
	0	1.81168298560015	0.943	0.122	0	4	SEPHS2
	0	1.81126210070739	0.269	0.017	0	4	STXBP6
	0	1.81050359908847	0.587	0.045	0	4	SLC30A2
	0	1.80814438455475	0.513	0.04	0	4	PFKFB1
	0	1.80739292849924	0.638	0.048	0	4	SGK2
	0	1.80704340208159	0.983	0.184	0	4	XBP1
	0	1.80641662421622	0.945	0.106	0	4	EPS8L2
	0	1.80633705841917	0.675	0.053	0	4	COBLL1
	0	1.80518047279158	0.697	0.052	0	4	DAG1
	0	1.80517753962879	0.994	0.272	0	4	SERPING1
	0	1.80258065311592	0.997	0.234	0	4	COL18A1
	0	1.80079002575617	0.459	0.033	0	4	ERBB2
	0	1.80025199724314	0.758	0.066	0	4	MARC2
	0	1.80004162641853	0.311	0.021	0	4	SEC14L4
	0	1.79966244151467	0.859	0.08	0	4	PLIN5
	0	1.79435250489065	0.555	0.04	0	4	RHPN2
	0	1.79251347480996	0.943	0.122	0	4	UPB1
	0	1.79242369650152	0.877	0.09	0	4	CGN
	0	1.78603316426034	0.516	0.038	0	4	PLXNB1
	0	1.78516318880317	0.495	0.032	0	4	MAP7
	0	1.78254801889963	0.504	0.034	0	4	RAP1GAP

0	1.78100141741704	0.583	0.036		0	4	ASS1
0	1.778125609766	0.995	0.265		0	4	MT1E
0	1.77663529377232	0.707	0.057		0	4	RTKN
0	1.77233115057772	0.715	0.053		0	4	PXMP2
0	1.77199539223014	0.841	0.082		0	4	DUSP23
0	1.77169728264983	0.808	0.086		0	4	NEDD4L
0	1.77147168764342	0.812	0.069		0	4	TSPAN13
0	1.77018730152932	0.735	0.057		0	4	XDH
0	1.76783593812384	0.65	0.043		0	4	CADM1
0	1.76695381204349	0.406	0.022		0	4	COPZ2
0	1.76647524807849	0.354	0.02		0	4	ITPKA
0	1.76591880551315	0.677	0.048		0	4	ALDH7A1
0	1.76525206610891	0.881	0.094		0	4	PCYT2
0	1.76350489073671	0.691	0.059		0	4	HLF
0	1.76300672369478	0.444	0.03		0	4	TMEM220
0	1.76167165821655	0.684	0.049		0	4	ALDH5A1
0	1.76010226569137	0.63	0.043		0	4	GPC3
0	1.75895904388859	0.794	0.073		0	4	ACY1
0	1.75838342469472	0.395	0.028		0	4	ADGRA3
0	1.75823034540513	0.616	0.042		0	4	ACOT13
0	1.75795866999703	0.992	0.185		0	4	C1R
0	1.75762722049627	0.286	0.019		0	4	FBXO27
0	1.75450830598415	0.688	0.057		0	4	HAAO
0	1.75137695755591	0.284	0.021		0	4	SCN8A
0	1.75135934729355	0.333	0.021		0	4	THRB
0	1.75048858294499	0.83	0.076		0	4	NR2F6
0	1.75039232385999	0.527	0.036		0	4	STAP2
0	1.74841473725089	0.591	0.047		0	4	SOX5
0	1.74811485715892	0.25	0.016		0	4	VNN 1.00
0	1.74502701844566	0.598	0.046		0	4	DHODH
0	1.744871679795	0.681	0.052		0	4	SMIM14
0	1.74330937038792	1	0.463		0	4	APOE
0	1.74312545942831	0.997	0.285		0	4	C2
0	1.74042217770472	0.429	0.034		0	4	ZDHHC11
0	1.7400064048671	0.473	0.032		0	4	BTNL8
0	1.73971009191342	0.991	0.249		0	4	ALDH2
0	1.73970067971031	0.324	0.018		0	4	TLCD2
0	1.73780749454385	0.485	0.035		0	4	CACNA1H
0	1.73753096508542	0.974	0.166		0	4	ECHS1
0	1.73607253495273	0.292	0.019		0	4	ABCA10
0	1.73506468344428	0.652	0.047		0	4	DEPRECATE
0	1.73363935780299	0.856	0.096		0	4	IDH1
0	1.7326289961236	0.413	0.027		0	4	AS3MT
0	1.73009085470495	0.295	0.022		0	4	FMO4
0	1.72975860261863	0.531	0.04		0	4	TUFT1
0	1.72745570748198	0.959	0.178		0	4	GLYCTK
0	1.72666713816613	0.682	0.055		0	4	AMACR
0	1.72653557033234	0.601	0.047		0	4	ACSM5
0	1.72648873886506	0.855	0.081		0	4	PECR
0	1.72507176052147	0.94	0.136		0	4	PRDX6

0	1.72243372015613	0.411	0.034	0	4	VNN 3.00
0	1.72168944023684	0.272	0.016	0	4	MYO5B
0	1.7208295734938	0.582	0.036	0	4	DDAH1
0	1.72012113421602	0.468	0.035	0	4	FRRS1
0	1.71872003159359	0.869	0.093	0	4	ACAA2
0	1.71834436935066	0.672	0.051	0	4	SDC2
0	1.71818105882483	0.975	0.192	0	4	ETFB
0	1.71525837090804	0.413	0.022	0	4	KDELR3
0	1.71446631467252	0.443	0.033	0	4	CAP2
0	1.71397151918625	0.393	0.028	0	4	ASAP3
0	1.7104930672217	0.712	0.062	0	4	SPSB1
0	1.71003732882152	0.462	0.035	0	4	CLMN
0	1.70957303709862	0.434	0.029	0	4	MARC1
0	1.70918708640685	0.611	0.048	0	4	PCBD1
0	1.7083269816037	0.991	0.228	0	4	MT1H
0	1.70812055159898	0.851	0.101	0	4	PTGR1
0	1.70752603268308	0.744	0.063	0	4	SHROOM1
0	1.70606744823678	0.306	0.02	0	4	SH2D4A
0	1.70580360555423	0.726	0.056	0	4	TXNDC17
0	1.70474745028462	0.298	0.015	0	4	C1orf53
0	1.70332924827828	0.367	0.023	0	4	SLC2A10
0	1.70307756572396	0.516	0.044	0	4	ANGPTL8
0	1.7024918727116	0.675	0.051	0	4	FAM83H
0	1.70157021620772	0.714	0.058	0	4	HACD3
0	1.69891401766829	0.735	0.064	0	4	SLC35C1
0	1.69823055600517	0.972	0.178	0	4	SHMT1
0	1.69697071125121	0.918	0.168	0	4	GDF15
0	1.69629875695915	0.976	0.164	0	4	OAF
0	1.69543789691974	0.579	0.041	0	4	FZD5
0	1.69483962024994	0.472	0.036	0	4	SLC6A12
0	1.69482112843484	0.969	0.21	0	4	TMEM176B
0	1.69436051920462	0.355	0.024	0	4	CYP4F22
0	1.69432513309208	0.592	0.046	0	4	SORBS2
0	1.6896941536451	0.942	0.123	0	4	S100A16
0	1.68897712876175	0.71	0.057	0	4	GALK1
0	1.6882087768949	0.251	0.02	0	4	IGSF9
0	1.6881973049099	0.689	0.059	0	4	ACO1
0	1.68673501641608	0.369	0.022	0	4	ZDHHC9
0	1.68598688349305	0.364	0.023	0	4	PLEKHA4
0	1.68447048362029	0.371	0.022	0	4	DEPRECATE
0	1.68347334735334	0.423	0.036	0	4	EEF1A2
0	1.68255790372035	0.259	0.014	0	4	TMEM98
0	1.6819434984084	0.953	0.136	0	4	ASL
0	1.68137889227085	0.266	0.014	0	4	PLEK2
0	1.67910373779749	0.981	0.193	0	4	MT1M
0	1.67790595551014	0.477	0.03	0	4	MSRB1
0	1.67788789129018	0.962	0.138	0	4	SPON2
0	1.67748111357815	0.732	0.059	0	4	MCFD2
0	1.67694366744592	0.59	0.045	0	4	P4HA2
0	1.67640164214678	0.786	0.071	0	4	FBXO17

0	1.67456023545398	0.658	0.05		0	4	CTSF
0	1.67332009921701	0.93	0.167		0	4	MT1A
0	1.67303460677301	0.587	0.049		0	4	AQP9
0	1.6725842883253	0.392	0.028		0	4	MYRF
0	1.67019700129755	0.567	0.043		0	4	CAPN5
0	1.66895046886484	0.283	0.016		0	4	MMACHC
0	1.66789313013051	0.93	0.124		0	4	ALDH3A2
0	1.66784418154961	0.441	0.031		0	4	PLEKHG6
0	1.66618555105002	0.334	0.02		0	4	LRRC20
0	1.66329771998052	0.473	0.04		0	4	CYP2C19
0	1.66163348293501	0.798	0.072		0	4	NR5A2
0	1.65999430218099	0.324	0.022		0	4	ACSS3
0	1.65713212933794	0.474	0.033		0	4	FAT1
0	1.65534461471756	0.3	0.018		0	4	NTN1
0	1.6550106491794	0.918	0.123		0	4	DEPRECATE
0	1.65325143724088	0.45	0.031		0	4	SOWAHC
0	1.65295161095614	0.816	0.076		0	4	CGNL1
0	1.65130359972819	0.661	0.062		0	4	CD24
0	1.65092965760776	0.661	0.046		0	4	EBPL
0	1.64872925327456	0.465	0.031		0	4	CXADR
0	1.6470606643149	0.583	0.052		0	4	GPAM
0	1.64395699582352	0.254	0.021		0	4	SLC6A15
0	1.64268517247718	0.895	0.125		0	4	SLC27A3
0	1.64174077606648	0.27	0.018		0	4	ACADL
0	1.63939946767744	0.585	0.039		0	4	HOMER2
0	1.63869434406939	0.314	0.015		0	4	CADM4
0	1.63756029573273	0.384	0.029		0	4	INHBE
0	1.63284237459792	0.663	0.057		0	4	GALE
0	1.63057395285245	0.49	0.038		0	4	HKDC1
0	1.63045851150846	0.797	0.078		0	4	PLIN4
0	1.62995579460462	0.932	0.132		0	4	ACOX1
0	1.62864610031967	0.502	0.035		0	4	DEPRECATE
0	1.6277977179825	0.948	0.128		0	4	MTSS2
0	1.6271182432305	0.768	0.068		0	4	CRLS1
0	1.62659665112879	0.78	0.072		0	4	CEP72
0	1.62300373334271	0.517	0.039		0	4	TOM1L1
0	1.62164075274684	0.721	0.064		0	4	PTPRK
0	1.62156748051315	0.535	0.045		0	4	DHRS2
0	1.61904955360598	0.636	0.051		0	4	FN3K
0	1.61776823886163	0.641	0.049		0	4	CPT2
0	1.61504592153	0.504	0.033		0	4	CYP7B1
0	1.61377000184874	0.546	0.041		0	4	ONECUT2
0	1.61124682036937	0.71	0.053		0	4	FAM50B
0	1.61044441973554	0.487	0.035		0	4	TRIB3
0	1.61041331114737	0.983	0.198		0	4	NUPR1
0	1.60571769525669	0.982	0.227		0	4	SCP2
0	1.6052243583757	0.99	0.245		0	4	IFITM3
0	1.60267688565921	0.804	0.084		0	4	SCCPDH
0	1.60204378212496	0.701	0.064		0	4	DTNA
0	1.60017529340531	0.362	0.031		0	4	SLC17A2

0	1.59941088599326	0.536	0.04		0	4	ENPP1
0	1.59895204705314	0.296	0.017		0	4	TPD52L1
0	1.59871515297886	0.693	0.056		0	4	RORC
0	1.59747632464491	0.96	0.156		0	4	CEBPA
0	1.59528097972895	0.999	0.422		0	4	SQSTM1
0	1.59290061280703	0.549	0.044		0	4	EXOC3L4
0	1.59245755014391	0.447	0.041		0	4	PPP1R3C
0	1.59233304244752	0.72	0.065		0	4	PRKAB2
0	1.59130881709713	0.301	0.02		0	4	PTPRU
0	1.59092160502874	0.908	0.105		0	4	LRP3
0	1.58817889087377	0.884	0.126		0	4	GCLC
0	1.58695693327875	0.605	0.044		0	4	SLC25A10
0	1.58677086968355	0.867	0.114		0	4	ANXA4
0	1.58605011375454	0.449	0.033		0	4	IGF2BP3
0	1.58565708203731	0.716	0.067		0	4	SLC41A2
0	1.58562566175186	0.4	0.028		0	4	RETREG1
0	1.58325484135318	0.637	0.051		0	4	MTHFS
0	1.58227397530841	0.443	0.03		0	4	NT5E
0	1.58119423347125	0.339	0.025		0	4	SEMA6C
0	1.57630685921435	0.288	0.019		0	4	PANK1
0	1.57520074936968	0.572	0.044		0	4	CCDC85C
0	1.57487149871377	0.609	0.054		0	4	COL5A3
0	1.57445576772861	0.958	0.158		0	4	ANPEP
0	1.5742548950425	0.952	0.164		0	4	SLC30A1
0	1.57249885605218	0.723	0.064		0	4	FGFR3
0	1.57230881076754	0.953	0.141		0	4	FURIN
0	1.56769883139348	0.352	0.025		0	4	NPAS2
0	1.56747642142484	0.978	0.242		0	4	RRBP1
0	1.56642621607461	0.992	0.28		0	4	LMNA
0	1.5643431988985	0.704	0.057		0	4	NAXE
0	1.56374456252208	0.403	0.025		0	4	TLCD4
0	1.56365940822877	0.456	0.035		0	4	MLPH
0	1.56362567176224	0.267	0.016		0	4	ACKR2
0	1.56156371065859	0.87	0.117		0	4	SPTSSA
0	1.55932218269564	0.453	0.033		0	4	PLOD2
0	1.55723313969798	0.864	0.096		0	4	BEX3
0	1.55646132372677	0.755	0.076		0	4	DTX4
0	1.5563602777429	0.945	0.166		0	4	HSD17B4
0	1.5560290890566	0.502	0.039		0	4	LRRC75B
0	1.55133638767443	0.479	0.033		0	4	HYAL1
0	1.54779974564129	0.587	0.06		0	4	SNCG
0	1.5452208782172	0.564	0.053		0	4	CHRD
0	1.54334907663917	0.406	0.032		0	4	OXER1
0	1.54331140724886	0.315	0.018		0	4	CHST13
0	1.54212536903855	0.657	0.047		0	4	HSD17B8
0	1.53402238183172	0.73	0.066		0	4	MACROD1
0	1.53122323889658	0.449	0.034		0	4	FAM83G
0	1.52997940322843	0.428	0.032		0	4	SMO
0	1.52897734750955	0.912	0.123		0	4	ABCC3
0	1.52595806916384	0.603	0.05		0	4	CEP170B

0	1.52593747820019	0.747	0.065		0	4	SDHC
0	1.52219079496501	0.465	0.036		0	4	NOL3
0	1.52194390103221	0.727	0.067		0	4	LAMC1
0	1.52056739730073	0.635	0.048		0	4	MFSD3
0	1.52043084829137	0.717	0.062		0	4	CEACAM1
0	1.52015349640736	0.336	0.029		0	4	RASD1
0	1.51863882615537	0.829	0.094		0	4	TOR1AIP2
0	1.51639229836147	0.643	0.051		0	4	UQCRQ
0	1.51608275711087	0.338	0.029		0	4	PNPLA3
0	1.51219957522292	0.362	0.026		0	4	ATP7B
0	1.5088926913402	0.95	0.165		0	4	METTL7A
0	1.50698259848322	0.272	0.029		0	4	DEPRECATE
0	1.5068375675637	0.644	0.055		0	4	GCLM
0	1.5063328319639	0.484	0.039		0	4	FAAH
0	1.5063007928909	0.924	0.125		0	4	CLUH
0	1.50615852935525	0.524	0.041		0	4	FAM174B
0	1.50428071570551	0.357	0.029		0	4	TEAD3
0	1.50392827359652	0.929	0.134		0	4	NDUFC2
0	1.50137314806218	0.526	0.035		0	4	SLC25A4
0	1.49585531352738	0.271	0.018		0	4	IRS1
0	1.49584035441844	0.968	0.176		0	4	SAT2
0	1.49178299361202	0.96	0.17		0	4	P4HB
0	1.49070428772634	0.937	0.142		0	4	DDT
0	1.48935414833423	0.715	0.067		0	4	TTC38
0	1.48875851264445	0.944	0.137		0	4	SLC9A3R2
0	1.48828971186709	0.763	0.072		0	4	LRP5
0	1.4854888889735	0.589	0.044		0	4	TMEM9
0	1.48418631539141	0.732	0.066		0	4	GOT2
0	1.48255005739695	0.496	0.036		0	4	BOK
0	1.48182369753504	0.427	0.03		0	4	PIR
0	1.48038453902044	0.379	0.027		0	4	ARHGEF26
0	1.4796947488125	0.481	0.032		0	4	PTGFRN
0	1.47931387238127	0.587	0.07		0	4	FGF21
0	1.47865194888897	0.966	0.19		0	4	ST6GAL1
0	1.47833543814632	0.773	0.071		0	4	SIGMAR1
0	1.47648353830898	0.475	0.037		0	4	LIPG
0	1.47448238189685	0.883	0.109		0	4	H6PD
0	1.47354042841503	0.559	0.046		0	4	HYI
0	1.47351674132244	0.38	0.027		0	4	KLHL23
0	1.47310378849117	0.293	0.016		0	4	GPC6
0	1.47151183750794	0.887	0.145		0	4	ACSL4
0	1.4685164829891	0.441	0.036		0	4	RAPGEFL1
0	1.46668862806559	0.742	0.073		0	4	OPLAH
0	1.46360285705271	0.485	0.032		0	4	HINT2
0	1.4608510803332	0.276	0.018		0	4	WIPF3
0	1.46038426123028	0.939	0.143		0	4	NIT1
0	1.45948764729779	0.95	0.163		0	4	AGRN
0	1.45909220168695	0.353	0.028		0	4	VSIG10
0	1.45825689969845	0.348	0.027		0	4	SPIRE2
0	1.45555202863656	0.583	0.057		0	4	PPP1R3B

0	1.45465414063169	0.724	0.063		0	4	ACADSB
0	1.45415006749858	0.755	0.103		0	4	CHI3L1
0	1.4533243696974	0.294	0.02		0	4	TADA1
0	1.45301224326913	0.482	0.039		0	4	PAWR
0	1.45252879389278	0.954	0.159		0	4	MGAT4B
0	1.45225405585831	0.413	0.035		0	4	SLC25A33
0	1.44465219928985	0.493	0.038		0	4	TLE1
0	1.43894725405135	0.286	0.018		0	4	B9D1
0	1.43709789834591	0.251	0.018		0	4	ARL4D
0	1.43507924930009	0.943	0.146		0	4	GPX3
0	1.43208247726805	0.729	0.073		0	4	AIG1
0	1.4320399344276	0.481	0.038		0	4	MCRIP2
0	1.4283779444201	0.479	0.035		0	4	DOK4
0	1.42790366762469	0.401	0.034		0	4	KCNT2
0	1.42346295618584	0.255	0.017		0	4	SPTLC3
0	1.41934722599509	0.953	0.168		0	4	DHRS3
0	1.41928640559339	0.564	0.046		0	4	CAV2
0	1.41876703036303	0.555	0.045		0	4	AIMP2
0	1.41756241139356	0.387	0.026		0	4	GLT1D1
0	1.41655082921978	0.804	0.082		0	4	PEX19
0	1.41479623298419	0.865	0.2		0	4	DEPRECATE
0	1.41475670863984	0.422	0.027		0	4	SLC25A15
0	1.41406025526475	0.686	0.061		0	4	DNAJB9
0	1.41273647776966	0.412	0.032		0	4	CLDN23
0	1.41266232683347	0.596	0.053		0	4	DEPRECATE
0	1.41258146232195	0.322	0.021		0	4	ARHGEF5
0	1.41252300997467	0.662	0.071		0	4	HSD11B1
0	1.41066517765311	0.778	0.088		0	4	RILP
0	1.41007577215845	0.881	0.121		0	4	ZNHIT1
0	1.40852212467297	0.547	0.041		0	4	CEP70
0	1.40668062460501	0.732	0.063		0	4	TMEM14A
0	1.40594576472351	0.883	0.122		0	4	CAT
0	1.40571465534284	0.502	0.036		0	4	AK4
0	1.40444347124442	0.914	0.115		0	4	PRDX2
0	1.4030018174096	0.824	0.092		0	4	PEBP1
0	1.40214614473759	0.622	0.056		0	4	PPARA
0	1.39989776009899	0.874	0.111		0	4	HTATIP2
0	1.39745291094079	0.909	0.141		0	4	MPC1
0	1.39585066962813	0.937	0.159		0	4	FUOM
0	1.39542857818088	0.602	0.049		0	4	AP1S1
0	1.39520722508148	0.492	0.035		0	4	LMO7
0	1.39362509729058	0.334	0.021		0	4	C8orf82
0	1.39239928598678	0.747	0.071		0	4	OTUD7B
0	1.39032385252126	0.457	0.03		0	4	NTHL1
0	1.38969407321863	0.883	0.128		0	4	HSP90B1
0	1.38912644415862	0.481	0.034		0	4	RNF152
0	1.38905554894709	0.67	0.062		0	4	FAM162A
0	1.38411409220093	0.676	0.065		0	4	FH
0	1.38158446659855	0.549	0.052		0	4	SRXN1
0	1.3813424195263	0.921	0.147		0	4	HDGF

0	1.38023842653211	0.744	0.073		0	4	PRDX4
0	1.37875584126911	0.89	0.112		0	4	AFDN
0	1.37393575727603	0.394	0.03		0	4	PARD3
0	1.3732489114132	0.498	0.037		0	4	GLDC
0	1.37294761565299	0.726	0.074		0	4	UAP1
0	1.37002926215469	0.497	0.054		0	4	PLIN2
0	1.36992111065036	0.809	0.096		0	4	GRHPR
0	1.36822099848302	0.777	0.076		0	4	C1RL
0	1.365937795983	0.726	0.065		0	4	MMUT
0	1.3620619115472	0.776	0.084		0	4	QDPR
0	1.36187362016891	0.795	0.085		0	4	PINK1
0	1.36026595942588	0.501	0.039		0	4	GNG12
0	1.35610676754541	0.689	0.06		0	4	SLC43A1
0	1.3545634947128	0.847	0.11		0	4	MGST3
0	1.35383710272056	0.414	0.03		0	4	VASN
0	1.35343154419914	0.773	0.078		0	4	HMGCL
0	1.35020152806252	0.789	0.083		0	4	RETSAT
0	1.3475867182683	0.96	0.192		0	4	HYOU1
0	1.34526969412787	0.964	0.166		0	4	ECHDC2
0	1.34481282847813	0.622	0.052		0	4	PCYOX1
0	1.34429214946614	0.752	0.079		0	4	COQ8A
0	1.34350339833286	0.51	0.043		0	4	ABCA6
0	1.34285691804153	0.643	0.054		0	4	HTRA1
0	1.34237335155731	0.871	0.126		0	4	WDR13
0	1.34212361312713	0.577	0.051		0	4	ENAH
0	1.34082555854431	0.264	0.017		0	4	SHISA4
0	1.33913126317716	0.479	0.037		0	4	NFIX
0	1.33848845321271	0.267	0.022		0	4	CCDC24
0	1.33818711746615	0.584	0.051		0	4	ISOC2
0	1.33811107184855	0.524	0.05		0	4	BCAT2
0	1.33755161625237	0.547	0.049		0	4	DLG5
0	1.3367567236536	0.92	0.125		0	4	INSR
0	1.33585848612969	0.311	0.025		0	4	NUDT12
0	1.33579858572409	0.876	0.113		0	4	CXXC5
0	1.3351390194763	0.551	0.044		0	4	CHPF
0	1.33504928740228	0.379	0.032		0	4	SH3D19
0	1.33407752496436	0.865	0.108		0	4	CYP27A1
0	1.33192572870747	0.281	0.02		0	4	SOX13
0	1.33045065465668	0.509	0.046		0	4	DEPRECATE
0	1.32984253824999	0.579	0.045		0	4	SEC14L2
0	1.3294484497205	0.434	0.032		0	4	PPIC
0	1.32802193810913	0.282	0.017		0	4	DEPRECATE
0	1.32411662406154	0.329	0.024		0	4	CACFD1
0	1.32247121165253	0.744	0.081		0	4	PXDC1
0	1.32237134699702	0.837	0.103		0	4	CHCHD10
0	1.32050245438567	0.85	0.136		0	4	IFI27
0	1.31835899389308	0.658	0.057		0	4	ACADS
0	1.31728010181086	0.37	0.026		0	4	DEPRECATE
0	1.31613533896723	0.519	0.046		0	4	DDR1
0	1.31240511148119	0.306	0.022		0	4	SOX12

0	1.31142207499821	0.519	0.048	0	4	SLC47A1
0	1.30994716358208	0.704	0.108	0	4	P4HA1
0	1.30901592658193	0.668	0.06	0	4	ABCD3
0	1.30779076235614	0.603	0.061	0	4	SDSL
0	1.30767821573866	0.283	0.019	0	4	BOLA1
0	1.30753032367638	0.255	0.019	0	4	GRHL1
0	1.30725629805416	0.618	0.058	0	4	FGFR2
0	1.3069197826857	0.989	0.308	0	4	ACADVL
0	1.30622342386965	0.391	0.025	0	4	PDIA4
0	1.30613824967154	0.437	0.039	0	4	AASS
0	1.30449671160038	0.419	0.033	0	4	YAP1
0	1.3013871754386	0.561	0.047	0	4	FKBP4
0	1.29019524323705	0.811	0.099	0	4	PAPSS2
0	1.28908152797888	0.583	0.059	0	4	MAP3K13
0	1.28871435618507	0.592	0.144	0	4	SDS
0	1.28818056588981	0.848	0.112	0	4	IL6R
0	1.28787307050199	0.443	0.034	0	4	ZNF768
0	1.28576595803098	0.892	0.164	0	4	THBS1
0	1.28498137963079	0.299	0.02	0	4	GNPNAT1
0	1.28497096441623	0.837	0.112	0	4	SPTBN1
0	1.28250462790212	0.863	0.109	0	4	ARHGEF10L
0	1.28250096508543	0.473	0.039	0	4	THNSL2
0	1.28198749325862	0.885	0.138	0	4	POR
0	1.28071636492234	0.402	0.033	0	4	TACC2
0	1.28067608686589	0.429	0.036	0	4	CBR1
0	1.28007559613569	0.887	0.133	0	4	CALR
0	1.2793943481728	0.44	0.035	0	4	MTFR1
0	1.27919962232809	0.626	0.065	0	4	ADGRG6
0	1.27912576482627	0.391	0.025	0	4	INHBB
0	1.27526220534162	0.362	0.026	0	4	LIN7A
0	1.27186493428595	0.691	0.064	0	4	PSMD4
0	1.26940460358719	0.782	0.092	0	4	SHB
0	1.26913939587916	0.796	0.102	0	4	PRXL2A
0	1.26855133772157	0.644	0.061	0	4	MGST2
0	1.26219137757198	0.713	0.072	0	4	PNPO
0	1.26200421975599	0.514	0.05	0	4	ZNF595
0	1.26197072560811	0.748	0.079	0	4	CHP1
0	1.26052690509353	0.371	0.03	0	4	ZKSCAN3
0	1.26049486713103	0.331	0.03	0	4	BCO2
0	1.25982531106928	0.476	0.033	0	4	ENPEP
0	1.25690721962267	0.505	0.038	0	4	GPC1
0	1.25533786151962	0.979	0.255	0	4	ECH1
0	1.25358979802629	0.28	0.017	0	4	RTN4R
0	1.25344028636617	0.837	0.094	0	4	NDUFS6
0	1.25192355328467	0.771	0.091	0	4	GSTO1
0	1.25151256883143	0.671	0.064	0	4	SLC25A1
0	1.25006368796361	0.605	0.043	0	4	TMEM238
0	1.24892886817441	0.286	0.019	0	4	MSMO1
0	1.24582357058366	0.54	0.042	0	4	SH3BGRL2
0	1.24510851539933	0.942	0.203	0	4	CSRP1

0	1.24427715985844	0.557	0.046	0	4	AAMDC
0	1.24316246177841	0.667	0.061	0	4	SELENOM
0	1.24311317256501	0.315	0.023	0	4	CDC42EP4
0	1.2428333477984	0.559	0.051	0	4	CUL7
0	1.24280647749459	0.254	0.018	0	4	PALMD
0	1.24079579835551	0.835	0.104	0	4	POLDIP2
0	1.23996696423374	0.888	0.116	0	4	DHRS7
0	1.23987318911746	0.432	0.034	0	4	ASB13
0	1.23912182095181	0.36	0.028	0	4	OPN3
0	1.2351627453462	0.93	0.162	0	4	NECTIN2
0	1.23472012783577	0.413	0.035	0	4	FAXDC2
0	1.23023550978042	0.634	0.066	0	4	RIOX2
0	1.2292453800829	0.436	0.036	0	4	ADAMTSL3
0	1.22767092007167	0.364	0.024	0	4	ZNF593
0	1.22687695530335	0.569	0.045	0	4	CISD3
0	1.22598023539795	0.551	0.043	0	4	ELMO3
0	1.22560273256837	0.829	0.104	0	4	SLC25A13
0	1.22545005765393	0.325	0.031	0	4	LPIN3
0	1.22224647196255	0.312	0.021	0	4	VDAC1
0	1.21965132295627	0.812	0.096	0	4	KIF1C
0	1.21922625054326	0.6	0.054	0	4	CPTP
0	1.2191153149274	0.482	0.071	0	4	SCD
0	1.21842894705912	0.757	0.081	0	4	NME4
0	1.2179669473707	0.42	0.032	0	4	B4GALT2
0	1.21557004971516	0.719	0.071	0	4	CRYZ
0	1.21526593943008	0.766	0.082	0	4	ECI1
0	1.21466851557629	0.324	0.022	0	4	MANEAL
0	1.21190599854721	0.923	0.162	0	4	CD151
0	1.21066165991992	0.378	0.031	0	4	SULT1A1
0	1.2089998247372	0.255	0.015	0	4	B4GAT1
0	1.20549622441147	0.931	0.149	0	4	TXNDC5
0	1.20348410769609	0.348	0.026	0	4	FAHD1
0	1.20195352876966	0.511	0.047	0	4	ZNF219
0	1.19945409569366	0.296	0.022	0	4	EMP2
0	1.19895578748292	0.708	0.073	0	4	MSRB2
0	1.19676678953379	0.823	0.105	0	4	TOB1
0	1.19401049666895	0.639	0.059	0	4	TMEM205
0	1.19389780056315	0.834	0.107	0	4	SIL1
0	1.18908340786091	0.678	0.063	0	4	PTPRS
0	1.18888931248992	0.314	0.024	0	4	SIX5
0	1.18814425362595	0.363	0.029	0	4	CCDC125
0	1.18786006933856	0.551	0.046	0	4	LACTB2
0	1.18597344436377	0.335	0.021	0	4	SLC27A2
0	1.18580744647727	0.616	0.062	0	4	KCTD3
0	1.18473994427119	0.566	0.045	0	4	PLS3
0	1.18399529756931	0.289	0.021	0	4	ACOT2
0	1.18174189502727	0.439	0.033	0	4	ADCY9
0	1.18125947461027	0.627	0.067	0	4	ALS2CL
0	1.17530362035962	0.782	0.099	0	4	CD59
0	1.17358523672043	0.807	0.1	0	4	ATP5MC3

0	1.17284627295723	0.279	0.024	0	4	TXNRD3
0	1.1724405093478	0.575	0.051	0	4	WFS1
0	1.17030494582772	0.661	0.066	0	4	FLNB
0	1.1698861622755	0.508	0.034	0	4	SLC25A23
0	1.16782062805092	0.743	0.072	0	4	LHPP
0	1.16678216275539	0.289	0.022	0	4	LARGE1
0	1.16554551990305	0.524	0.047	0	4	NLN
0	1.1638946029392	0.79	0.094	0	4	RAB13
0	1.16380358762073	0.58	0.061	0	4	NR1D1
0	1.16291862919987	0.363	0.028	0	4	IGF1
0	1.16176043594009	0.311	0.024	0	4	PTGR2
0	1.16058279681385	0.472	0.039	0	4	AHCY
0	1.15880885205351	0.993	0.314	0	4	SELENOP
0	1.15872782769288	0.361	0.029	0	4	GBE1
0	1.15802224791018	0.614	0.064	0	4	ABLIM3
0	1.15597800486343	0.29	0.017	0	4	RDH5
0	1.15583015481795	0.555	0.053	0	4	HHEX
0	1.15543210568947	0.857	0.105	0	4	SEC11C
0	1.15322710434347	0.907	0.158	0	4	UQCRC1
0	1.15098671300811	0.915	0.154	0	4	PDIA3
0	1.15030054459426	0.899	0.14	0	4	LPGAT1
0	1.14697262469824	0.802	0.091	0	4	RNF5
0	1.14668136420494	0.426	0.04	0	4	TEAD1
0	1.14462598315497	0.7	0.066	0	4	ACADM
0	1.14326017857768	0.792	0.103	0	4	SLC29A1
0	1.14308095234531	0.542	0.039	0	4	CCT3
0	1.14198440850467	0.533	0.049	0	4	MPND
0	1.14193604630817	0.318	0.022	0	4	PDXP
0	1.14062766282688	0.878	0.13	0	4	HAGH
0	1.13876910745068	0.757	0.099	0	4	H1F0
0	1.13634916293474	0.578	0.054	0	4	COL27A1
0	1.12961873411173	0.585	0.058	0	4	FAM114A1
0	1.12812947812029	0.558	0.047	0	4	COA3
0	1.12785457266552	0.696	0.064	0	4	KIAA1671
0	1.12678749804966	0.567	0.055	0	4	TOMM40L
0	1.12658646302455	0.871	0.096	0	4	NR1H4
0	1.12651861451822	0.579	0.053	0	4	HIBADH
0	1.12413607266799	0.424	0.04	0	4	SMARCA1
0	1.12404944632131	0.319	0.025	0	4	JADE3
0	1.12390748155922	0.611	0.055	0	4	CKAP4
0	1.12382805504825	0.263	0.021	0	4	MROH6
0	1.12186728739901	0.764	0.093	0	4	NAPRT
0	1.12163767245041	0.366	0.042	0	4	ROBO1
0	1.11949465524307	0.333	0.028	0	4	CDC42BPA
0	1.11927788247044	0.458	0.044	0	4	MTUS1
0	1.11815065695322	0.284	0.023	0	4	GLCE
0	1.11800441234004	0.755	0.081	0	4	FOXP4
0	1.11742280178031	0.391	0.034	0	4	IL1RAP
0	1.11472074679299	0.416	0.03	0	4	CMTM8
0	1.1145907831983	0.642	0.051	0	4	TSTD1

0	1.11417419065039	0.736	0.073	0	4	AIFM1
0	1.11404385340795	0.282	0.023	0	4	NT5DC3
0	1.1130809063366	0.854	0.126	0	4	ITGB5
0	1.11043283533029	0.765	0.08	0	4	HSD17B10
0	1.10960626892965	0.79	0.088	0	4	PSMB4
0	1.10937619705473	0.848	0.121	0	4	SPATS2L
0	1.1073058206269	0.787	0.094	0	4	CIAO2A
0	1.10362496386959	0.598	0.054	0	4	NME1
0	1.10341277380238	0.889	0.135	0	4	DEPP1
0	1.10308136760694	0.589	0.059	0	4	GLMP
0	1.10253394533265	0.381	0.025	0	4	FDX1
0	1.10138301929567	0.468	0.04	0	4	WLS
0	1.10036584419541	0.882	0.144	0	4	IRS2
0	1.09714816056336	0.91	0.144	0	4	DDRGK1
0	1.0958912347576	0.343	0.022	0	4	ATP5PF
0	1.09535698391626	0.551	0.047	0	4	KDELR2
0	1.09524263149953	0.316	0.028	0	4	CTDSPL
0	1.09508346125588	0.984	0.301	0	4	PTMS
0	1.09477511488793	0.589	0.051	0	4	ATP9A
0	1.09383222760197	0.315	0.023	0	4	CRAT
0	1.09088282267218	0.453	0.038	0	4	CX3CL1
0	1.08866331062544	0.746	0.084	0	4	ATRN
0	1.08858974034834	0.62	0.056	0	4	AGPAT2
0	1.08457758906402	0.699	0.069	0	4	UGP2
0	1.08436977325195	0.926	0.168	0	4	RHOC
0	1.08339911050039	0.288	0.02	0	4	C1QBP
0	1.08274726814668	0.332	0.026	0	4	SPRYD7
0	1.08119375378395	0.626	0.06	0	4	CHPT1
0	1.08095497143218	0.956	0.213	0	4	ACAA1
0	1.07884520297998	0.344	0.03	0	4	CRACR2B
0	1.07765737391144	0.8	0.101	0	4	GALNT2
0	1.07562802056462	0.752	0.085	0	4	NDUFB9
0	1.07513821954118	0.338	0.025	0	4	GNE
0	1.07513815864596	0.697	0.067	0	4	NDUFB6
0	1.07423439321904	0.887	0.143	0	4	ADAM15
0	1.07375898954952	0.424	0.043	0	4	MAP3K20
0	1.07189094683404	0.496	0.047	0	4	ZFAND2A
0	1.07089534645246	0.844	0.11	0	4	DEPRECATE
0	1.06887598754885	0.344	0.032	0	4	RBFOX2
0	1.06828509937695	0.516	0.055	0	4	EEPD1
0	1.06630576792029	0.526	0.045	0	4	POLD2
0	1.06592428945327	0.38	0.03	0	4	ZFYVE9
0	1.0614266519774	0.888	0.146	0	4	PDIA6
0	1.06063764707075	0.882	0.133	0	4	ETFA
0	1.05889939572031	0.919	0.158	0	4	ZBTB7B
0	1.05846823746794	0.423	0.039	0	4	MAGI1
0	1.05593618675221	0.534	0.046	0	4	ALG3
0	1.05273853145931	0.54	0.048	0	4	PLSCR4
0	1.05262858940731	0.555	0.044	0	4	SDF2L1
0	1.05200019706075	0.294	0.027	0	4	PHETA1

	0	1.0512379335908	0.502	0.051	0	4	MPDZ
	0	1.05093776741768	0.436	0.038	0	4	COPRS
	0	1.0501964019083	0.769	0.088	0	4	C16orf70
	0	1.04856796001165	0.657	0.072	0	4	SLC17A9
	0	1.047363102426	0.398	0.042	0	4	SIPA1L2
	0	1.04646382307523	0.734	0.076	0	4	JUP
	0	1.04630831073631	0.282	0.017	0	4	RYR1
	0	1.04351839679327	0.607	0.065	0	4	IL17RC
	0	1.04331893064712	0.787	0.12	0	4	ASPH
	0	1.04328986355185	0.285	0.024	0	4	HFE
	0	1.04320195995578	0.854	0.095	0	4	MASP1
	0	1.04269164108512	0.788	0.089	0	4	CARHSP1
	0	1.04150608343081	0.33	0.026	0	4	CYP51A1
	0	1.04143009536472	0.533	0.062	0	4	RHOBTB3
	0	1.04074608833849	0.904	0.154	0	4	CYC1
	0	1.04060003509783	0.605	0.058	0	4	COMTD1
	0	1.0362619142764	0.511	0.049	0	4	GATA4
	0	1.03563057884976	0.305	0.029	0	4	ITGA7
	0	1.03251884426601	0.854	0.118	0	4	YIPF3
	0	1.03172680342949	0.437	0.039	0	4	CKS1B
	0	1.03142488991108	0.714	0.075	0	4	GOLM1
	0	1.02928852643112	0.847	0.141	0	4	TXNRD1
	0	1.02831590419866	0.311	0.028	0	4	FAM89A
	0	1.02798333929873	0.298	0.02	0	4	MPP6
	0	1.02785036124102	0.617	0.068	0	4	ALAS1
	0	1.02551866979286	0.375	0.031	0	4	TMEM14C
	0	1.02324070115686	0.467	0.044	0	4	SLC16A1
	0	1.02158790627739	0.648	0.067	0	4	CRYL1
	0	1.02116762577173	0.476	0.045	0	4	HIGD1A
	0	1.01982563559025	0.608	0.053	0	4	RASSF7
	0	1.0192208454956	0.412	0.033	0	4	PDK2
	0	1.01788015291488	0.621	0.058	0	4	TBC1D24
	0	1.01727452217695	0.435	0.035	0	4	CFL2
	0	1.01575780060073	0.309	0.028	0	4	NEDD4
	0	1.0140885776139	0.885	0.154	0	4	LAMP2
	0	1.01378689919476	0.407	0.039	0	4	NAV2
	0	1.01366993460871	0.713	0.064	0	4	VWA1
	0	1.01133495118633	0.502	0.041	0	4	DEPRECATE
	0	1.0112329443823	0.934	0.196	0	4	GPX4
	0	1.00821612005948	0.699	0.077	0	4	EBNA1BP2
	0	1.00812592427618	0.722	0.073	0	4	MIF
	0	1.00788534672517	0.729	0.09	0	4	GBA
	0	1.00780384609478	0.747	0.097	0	4	GUCD1
	0	1.00743534770817	0.727	0.081	0	4	DCAF6
	0	1.00567180978102	0.665	0.073	0	4	SLC22A18
	0	1.00477099017448	0.907	0.161	0	4	NFIC
	0	1.00397800979624	0.282	0.027	0	4	IFIT1
	0	1.00303561906206	0.545	0.047	0	4	TACO1
	0	1.00135138686267	0.961	0.187	0	4	VEGFA
	0	1.00104696378619	0.252	0.021	0	4	ZNF205

0	0.997243874963092	0.943	0.207	0	4	APLP2
0	0.996088587287348	0.465	0.032	0	4	GCHFR
0	0.994959712969933	0.557	0.049	0	4	KLC4
0	0.994509284374735	0.254	0.019	0	4	SH3BP4
0	0.988350873658837	0.453	0.042	0	4	CYSTM1
0	0.987759524538222	0.373	0.038	0	4	ABHD11
0	0.987370129482747	0.48	0.046	0	4	MAPRE3
0	0.986423461638925	0.957	0.213	0	4	NDUFS2
0	0.98515933400936	0.698	0.076	0	4	SLC50A1
0	0.983930976587237	0.778	0.103	0	4	ATOX1
0	0.983181420843549	0.27	0.027	0	4	SLC45A3
0	0.980800728958454	0.372	0.036	0	4	WWC2
0	0.975797612018922	0.898	0.168	0	4	MYDGF
0	0.973301565402216	0.827	0.112	0	4	DNPH1
0	0.972182462542272	0.497	0.041	0	4	LNX2
0	0.969198215522371	0.99	0.299	0	4	CTSD
0	0.966164130934806	0.543	0.055	0	4	PDGFA
0	0.9653700633082	0.587	0.049	0	4	SPRYD4
0	0.965283316079943	0.346	0.025	0	4	DSTN
0	0.963157293195848	0.691	0.067	0	4	NDUFAB1
0	0.962927301172242	0.268	0.019	0	4	TMEM254
0	0.962856644419276	0.617	0.07	0	4	NHSL1
0	0.962645634120658	0.974	0.264	0	4	HSPB1
0	0.961647790185576	0.423	0.049	0	4	PDK4
0	0.958325859288307	0.752	0.083	0	4	TSTA3
0	0.957083218230651	0.569	0.066	0	4	PVR
0	0.954047226512569	0.638	0.053	0	4	EPHX2
0	0.952075514518934	0.751	0.084	0	4	NDUFA4
0	0.951179009720707	0.674	0.082	0	4	CEBPG
0	0.950876546321096	0.361	0.03	0	4	OBSL1
0	0.950007473911366	0.68	0.078	0	4	TOLLIP
0	0.948732325467171	0.864	0.142	0	4	SCARB1
0	0.948620771518976	0.577	0.054	0	4	RPN1
0	0.948009692971394	0.926	0.173	0	4	PPIB
0	0.947917698421717	0.317	0.023	0	4	HYPK
0	0.946245561560388	0.39	0.035	0	4	MPZL1
0	0.946034041671513	0.69	0.083	0	4	PLOD1
0	0.94541831659537	0.638	0.084	0	4	MID1IP1
0	0.944902516495329	0.684	0.079	0	4	SHC2
0	0.944846215692398	0.564	0.107	0	4	G0S2
0	0.944344518720336	0.779	0.104	0	4	SLC22A23
0	0.944027230114886	0.526	0.045	0	4	YIPF2
0	0.941586365604187	0.724	0.075	0	4	IGSF8
0	0.9365133149963	0.394	0.031	0	4	SNAPIN
0	0.936374285571403	0.62	0.071	0	4	NFIB
0	0.935517261827122	0.348	0.026	0	4	AKR7A2
0	0.934414081544055	0.659	0.063	0	4	PFDN6
0	0.933071270442715	0.733	0.092	0	4	IFRD2
0	0.927015447927829	0.652	0.071	0	4	TNKS1BP1
0	0.926411846405494	0.722	0.076	0	4	SPCS1

0	0.9253746968685	0.543	0.046	0	4	GRPEL1
0	0.923046066189849	0.697	0.087	0	4	PNKD
0	0.921269205653638	0.286	0.02	0	4	PAIP2B
0	0.920836696120662	0.892	0.166	0	4	ETS2
0	0.919090950852907	0.91	0.17	0	4	SHC1
0	0.917013363107236	0.883	0.136	0	4	SNTB1
0	0.914575953237509	0.715	0.081	0	4	SDHB
0	0.914086143536276	0.626	0.069	0	4	SYNPO
0	0.913009698913299	0.787	0.091	0	4	ALDH9A1
0	0.910569854966038	0.746	0.098	0	4	CYB5R1
0	0.910180881583016	0.562	0.059	0	4	DCUN1D4
0	0.907677284128229	0.424	0.046	0	4	TNFRSF21
0	0.9074755031659	0.52	0.042	0	4	TMED2
0	0.906996867801167	0.811	0.112	0	4	FKBP2
0	0.906970228734051	0.548	0.056	0	4	ALAD
0	0.906811313589376	0.726	0.106	0	4	TRIP6
0	0.90572206280275	0.717	0.079	0	4	AURKAIP1
0	0.904711460583708	0.294	0.027	0	4	C1QTNF3
0	0.904191387219483	0.586	0.053	0	4	COX6C
0	0.900752471294543	0.303	0.022	0	4	COX5B
0	0.899742193027885	0.274	0.024	0	4	GAS8
0	0.8992662091927	0.255	0.026	0	4	IGF2BP2
0	0.899139099579546	0.615	0.066	0	4	TIMM17A
0	0.898638219994038	0.648	0.067	0	4	PSMC4
0	0.89811804974225	0.771	0.092	0	4	ROMO1
0	0.896938386102625	0.665	0.074	0	4	ZCCHC24
0	0.895946466249954	0.33	0.028	0	4	CD302
0	0.895412552898421	0.275	0.025	0	4	ATP8B1
0	0.894257366528592	0.311	0.021	0	4	GTF2H5
0	0.89383397198166	0.371	0.029	0	4	TSHZ2
0	0.893254944159591	0.376	0.032	0	4	IMPA2
0	0.891552421756711	0.79	0.125	0	4	PLOD3
0	0.887811127960363	0.465	0.055	0	4	TRIB1
0	0.886672075550669	0.653	0.063	0	4	HAX1
0	0.885896291308536	0.712	0.08	0	4	NDUFA1
0	0.88453304044761	0.65	0.061	0	4	NUCB2
0	0.884478046415752	0.526	0.048	0	4	ACAT2
0	0.884017694661238	0.342	0.03	0	4	TUBA1C
0	0.883695497285802	0.259	0.024	0	4	TUSC3
0	0.882262742353574	0.339	0.036	0	4	SH3RF1
0	0.882097607774783	0.557	0.067	0	4	RHOU
0	0.882047349670394	0.588	0.055	0	4	MAGED1
0	0.881788646164464	0.857	0.144	0	4	MTSS1
0	0.880441947391666	0.99	0.37	0	4	LAMP1
0	0.880023787028204	0.573	0.054	0	4	UQCC2
0	0.87863965690594	0.422	0.032	0	4	MAIP1
0	0.878511654571966	0.9	0.149	0	4	TMCO1
0	0.878335653344403	0.65	0.069	0	4	LAMTOR2
0	0.874288343085984	0.534	0.055	0	4	NEU1
0	0.873700451535237	0.314	0.023	0	4	NPR 2.00

	0	0.873161017991068	0.607	0.064	0	4	LRRC59
	0	0.872294656043986	0.569	0.054	0	4	DEPRECATE
	0	0.870685796176724	0.628	0.076	0	4	KIFC3
	0	0.870297785934692	0.542	0.058	0	4	NDRG2
	0	0.870102048706772	0.266	0.024	0	4	SORT1
	0	0.869337650045038	0.365	0.033	0	4	PCTP
	0	0.867600168572174	0.347	0.032	0	4	CARD10
	0	0.867457870424603	0.601	0.063	0	4	NAA20
	0	0.867364264408664	0.626	0.072	0	4	KIAA1217
	0	0.86651958681888	0.454	0.043	0	4	TCEAL4
	0	0.864062171735566	0.256	0.018	0	4	ZNHIT2
	0	0.86338487388023	0.314	0.032	0	4	CNKSR3
	0	0.860947782088518	0.528	0.049	0	4	UMPS
	0	0.859993917595566	0.648	0.08	0	4	CDC42EP1
	0	0.858128336601519	0.399	0.032	0	4	ZNF787
	0	0.856883225337969	0.793	0.139	0	4	QSOX1
	0	0.856076740006112	0.433	0.051	0	4	KLF11
	0	0.855217663972488	0.626	0.074	0	4	S100A13
	0	0.85439753643148	0.771	0.086	0	4	SRPRB
	0	0.852714620947906	0.771	0.093	0	4	VARS
	0	0.851454657480307	0.318	0.03	0	4	ENTPD7
	0	0.847804451206066	0.259	0.023	0	4	ZBED3
	0	0.846122461426573	0.315	0.035	0	4	DCBLD1
	0	0.845131188251864	0.636	0.072	0	4	UROD
	0	0.843963567728231	0.595	0.067	0	4	DUSP3
	0	0.842624807861663	0.943	0.199	0	4	CYB5R3
	0	0.842549193694588	0.545	0.048	0	4	CERS4
	0	0.842271494124168	0.972	0.305	0	4	MT-ND4
	0	0.841776886743096	0.796	0.124	0	4	ATP1B1
	0	0.84159069137042	0.631	0.068	0	4	ESD
	0	0.840854093007149	0.721	0.091	0	4	BCAR1
	0	0.839900806164589	0.62	0.06	0	4	TMEM208
	0	0.839403650832742	0.624	0.067	0	4	PRPSAP1
	0	0.839360714884304	0.384	0.029	0	4	NECTIN3
	0	0.836565216414278	0.402	0.043	0	4	ADCY6
	0	0.835964853502447	0.553	0.06	0	4	TALDO1
	0	0.835146319700735	0.759	0.11	0	4	LTBR
	0	0.834023117772374	0.599	0.059	0	4	YIF1A
	0	0.832951065383407	0.45	0.055	0	4	PLEKHH3
	0	0.827025761836896	0.316	0.023	0	4	SEC61G
	0	0.826761641515979	0.937	0.193	0	4	BLVRB
	0	0.825390080346627	0.432	0.042	0	4	NCKAP1
	0	0.824615153488199	0.543	0.063	0	4	ACSS2
	0	0.823828216114109	0.388	0.035	0	4	GMNN
	0	0.823524425923607	0.284	0.023	0	4	MTX1
	0	0.823317773511943	0.344	0.038	0	4	CDK18
	0	0.821246882651297	0.39	0.037	0	4	FUCA2
	0	0.816623886739234	0.448	0.039	0	4	CPT1A
	0	0.816120113447952	0.682	0.075	0	4	NIT2
	0	0.812098920997357	0.463	0.04	0	4	MTCH2

0	0.811918455737775	0.762	0.094	0	4	SYVN1
0	0.811178073947695	0.869	0.132	0	4	POLD4
0	0.810310120848268	0.51	0.05	0	4	C1orf198
0	0.809175772831414	0.773	0.099	0	4	MGMT
0	0.808877776137434	0.554	0.054	0	4	FAM174A
0	0.806044783829802	0.542	0.057	0	4	BAIAP2
0	0.80553320251372	0.419	0.046	0	4	ALCAM
0	0.805130604902196	0.8	0.117	0	4	NDST1
0	0.80499121518338	0.296	0.023	0	4	SNRPE
0	0.802614449277825	0.564	0.064	0	4	PRDX1
0	0.801987322086867	0.87	0.165	0	4	ACSL1
0	0.799243758653218	0.441	0.049	0	4	HNMT
0	0.798915695057379	0.579	0.06	0	4	ZCCHC14
0	0.798781519749477	0.383	0.029	0	4	DEPRECATE
0	0.797992842833936	0.576	0.053	0	4	TARS2
0	0.79750995866802	0.291	0.022	0	4	DEPRECATE
0	0.795971044290085	0.452	0.046	0	4	BLNK
0	0.794851000213962	0.45	0.041	0	4	SLC20A2
0	0.793661126381524	0.488	0.052	0	4	SFXN5
0	0.792730576583519	0.28	0.022	0	4	PUS3
0	0.791706620089575	0.815	0.099	0	4	HEBP2
0	0.790448158681756	0.542	0.061	0	4	HSF4
0	0.788515638122961	0.31	0.03	0	4	TMEM150A
0	0.788172447082045	0.31	0.03	0	4	NKIRAS1
0	0.78701117691044	0.855	0.127	0	4	PMVK
0	0.786033625226022	0.626	0.069	0	4	MYO6
0	0.785192555126665	0.325	0.033	0	4	RPH3AL
0	0.784709300135869	0.534	0.056	0	4	TSPAN9
0	0.777304964564065	0.782	0.11	0	4	HSPD1
0	0.777288810047534	0.568	0.059	0	4	SRM
0	0.776221428274826	0.95	0.232	0	4	GRINA
0	0.77600708389504	0.3	0.03	0	4	SPRED2
0	0.774544031939737	0.978	0.285	0	4	LMAN2
0	0.772105506134366	0.492	0.046	0	4	PPA1
0	0.771880666523673	0.26	0.028	0	4	SDCBP2
0	0.770816714263445	0.337	0.031	0	4	AMOTL2
0	0.76886953442718	0.938	0.228	0	4	SLC12A7
0	0.767836106171078	0.497	0.056	0	4	ARVCF
0	0.767741537381217	0.638	0.076	0	4	DAP
0	0.767623583566812	0.618	0.087	0	4	DDIT3
0	0.7671437304085	0.726	0.114	0	4	DST
0	0.766734838344247	0.365	0.043	0	4	GADD45G
0	0.766587587263727	0.333	0.023	0	4	DEPRECATE
0	0.761768372034549	0.326	0.03	0	4	RAB11FIP5
0	0.761551846135749	0.502	0.049	0	4	TCEAL8
0	0.761185805870956	0.419	0.036	0	4	HACD2
0	0.760844121252851	0.459	0.044	0	4	LAPTM4A
0	0.760454480284119	0.888	0.172	0	4	CTNND1
0	0.760381938751459	0.656	0.074	0	4	NUDT16
0	0.759473943753628	0.277	0.029	0	4	CADPS2

0	0.754901757287457	0.382	0.042	0	4	ABTB2
0	0.753413734859524	0.323	0.031	0	4	TMEM70
0	0.753244873906471	0.279	0.026	0	4	WDR19
0	0.752554703862564	0.38	0.037	0	4	TICAM1
0	0.749513657264143	0.89	0.149	0	4	ZKSCAN1
0	0.748792458108128	0.78	0.106	0	4	EIF4G1
0	0.747096446554059	0.431	0.047	0	4	BCAM
0	0.745758053206788	0.551	0.049	0	4	COX6A1
0	0.745333088245747	0.45	0.037	0	4	AIFM2
0	0.745029232630053	0.265	0.026	0	4	ZNF697
0	0.744159016069645	0.369	0.04	0	4	FERMT2
0	0.743862725432486	0.496	0.04	0	4	DMTN
0	0.74366389001613	0.543	0.068	0	4	ARHGEF40
0	0.743514269541417	0.958	0.243	0	4	BRI3
0	0.741944910477123	0.416	0.039	0	4	ERGIC1
0	0.740556128540621	0.715	0.086	0	4	KRTCAP2
0	0.73950669979366	0.465	0.038	0	4	RALGPS2
0	0.73920579471283	0.733	0.098	0	4	MSRA
0	0.738899950631294	0.704	0.092	0	4	AHR
0	0.737902585449626	0.602	0.064	0	4	CANX
0	0.737659236834936	0.357	0.038	0	4	CCDC9B
0	0.73723629216283	0.412	0.041	0	4	SOCS6
0	0.73610700238513	0.897	0.133	0	4	CALD1
0	0.736018899689526	0.729	0.098	0	4	SLC39A7
0	0.735809282951455	0.539	0.058	0	4	CTSO
0	0.735363724592791	0.863	0.125	0	4	MIA2
0	0.733746919862538	0.337	0.033	0	4	ERMAP
0	0.732496731735573	0.293	0.03	0	4	CBX8
0	0.730031654899367	0.973	0.323	0	4	MT-ATP6
0	0.729204062449126	0.254	0.026	0	4	GATA6
0	0.728551290332545	0.786	0.127	0	4	CPEB4
0	0.726897749576062	0.853	0.164	0	4	MT-CYB
0	0.726710765072377	0.502	0.068	0	4	MYC
0	0.724070160390667	0.404	0.049	0	4	SLC19A2
0	0.723323695278697	0.957	0.232	0	4	IDH2
0	0.722204113334014	0.618	0.085	0	4	TRIO
0	0.721800633320005	0.285	0.027	0	4	HOOK2
0	0.719528503074819	0.31	0.026	0	4	PHLPP1
0	0.718082481817628	0.286	0.024	0	4	AARS
0	0.715616821301739	0.695	0.082	0	4	ZNF622
0	0.715061097782541	0.542	0.066	0	4	SLC46A1
0	0.714660539103843	0.734	0.102	0	4	FUCA1
0	0.713681500801722	0.626	0.073	0	4	HSPA5
0	0.713034710241	0.741	0.091	0	4	SNRPC
0	0.712978878439063	0.516	0.048	0	4	TPD52
0	0.711591604961451	0.259	0.025	0	4	TPMT
0	0.709208937787383	0.377	0.051	0	4	KLF10
0	0.707841776665256	0.258	0.023	0	4	SEMA5A
0	0.707449284110345	0.577	0.059	0	4	RNF181
0	0.706850852643577	0.576	0.061	0	4	GLUD1

0	0.706376933124383	0.525	0.052	0	4	SF3B4
0	0.705476057389518	0.261	0.029	0	4	TTC8
0	0.705180083659627	0.551	0.067	0	4	ST5
0	0.704961092650791	0.402	0.041	0	4	TP53INP2
0	0.704936216592606	0.402	0.044	0	4	NFIA
0	0.703721752482995	0.871	0.148	0	4	MLF 2.00
0	0.70329900811813	0.936	0.198	0	4	MYO18A
0	0.702367459579343	0.487	0.048	0	4	TOP1MT
0	0.702130388432112	0.417	0.042	0	4	RCN1
0	0.701534620238587	0.633	0.069	0	4	HDHD3
0	0.700602246005148	0.544	0.058	0	4	LPCAT3
0	0.699722299959384	0.311	0.036	0	4	OCRL
0	0.698578447703083	0.732	0.104	0	4	SIK2
0	0.698570302836714	0.755	0.123	0	4	MGLL
0	0.695463624908565	0.51	0.059	0	4	NQO2
0	0.694604198330074	0.383	0.034	0	4	PEMT
0	0.691965242094485	0.441	0.046	0	4	C11orf24
0	0.690940162244424	0.365	0.032	0	4	UQCC3
0	0.68927687699716	0.319	0.024	0	4	PHB
0	0.687942860175654	0.489	0.05	0	4	NPIPB15
0	0.686722180617214	0.564	0.063	0	4	ACSF2
0	0.68504487002775	0.43	0.038	0	4	MOSPD3
0	0.684813554133975	0.554	0.051	0	4	ELOB
0	0.68385563590797	0.392	0.037	0	4	RBFA
0	0.683549803835079	0.62	0.069	0	4	TMEM106C
0	0.683174107846123	0.956	0.299	0	4	SOD2
0	0.682517078948454	0.345	0.026	0	4	RPP25L
0	0.681968014470426	0.431	0.043	0	4	PPP1R16A
0	0.680786341392817	0.274	0.027	0	4	SLC66A1
0	0.680344015950922	0.912	0.187	0	4	SLC25A39
0	0.676840504157275	0.885	0.175	0	4	CREG1
0	0.676663375104303	0.516	0.039	0	4	OCIAD2
0	0.676526450554407	0.306	0.027	0	4	LRRC42
0	0.67601378425572	0.731	0.09	0	4	IMP4
0	0.674963301743205	0.356	0.031	0	4	POLR2I
0	0.674652207549652	0.506	0.054	0	4	HSDL2
0	0.672029151797036	0.999	0.431	0	4	FTL
0	0.671697633225318	0.777	0.108	0	4	PSMC3
0	0.67164307070146	0.268	0.029	0	4	FBLIM1
0	0.667881221813307	0.792	0.13	0	4	RBM47
0	0.667607130212957	0.852	0.14	0	4	MAN1A1
0	0.665232417819725	0.752	0.091	0	4	PLEKHG3
0	0.662763311119283	0.35	0.03	0	4	HSPBP1
0	0.661692372630699	0.36	0.031	0	4	NDUFC1
0	0.661350368977426	0.416	0.048	0	4	ZNF358
0	0.659553715899434	0.771	0.098	0	4	AKAP1
0	0.65935457357794	0.809	0.114	0	4	SAR1B
0	0.658492501071206	0.38	0.031	0	4	RITA1
0	0.657639420732813	0.441	0.031	0	4	EPHA1
0	0.655605478665134	0.718	0.093	0	4	COX8A

0	0.654479101526873	0.468	0.051	0	4	MCUR1
0	0.653921706850786	0.252	0.018	0	4	CPE
0	0.653372144659681	0.805	0.118	0	4	EIF6
0	0.653142780218381	0.274	0.03	0	4	ROBO3
0	0.652878349530598	0.577	0.077	0	4	SLC25A29
0	0.651870551070244	0.975	0.244	0	4	IGFBP4
0	0.651191402451937	0.261	0.02	0	4	UQCRFS1
0	0.651035566779724	0.848	0.138	0	4	HADHA
0	0.650395616146646	0.65	0.089	0	4	HSD3B7
0	0.650257881282996	0.684	0.084	0	4	MANF
0	0.650069845916983	0.801	0.117	0	4	GPAA1
0	0.648414273819937	0.29	0.03	0	4	RRAGD
0	0.648405919871247	0.463	0.047	0	4	C1orf43
0	0.644914614927282	0.591	0.064	0	4	SLC25A16
0	0.643666021241055	0.309	0.031	0	4	PEX10
0	0.641160918793485	0.438	0.038	0	4	NOMO1
0	0.640652116071891	0.474	0.049	0	4	DEPRECATE
0	0.640305394062664	0.282	0.025	0	4	DUSP8
0	0.639907890517413	0.494	0.044	0	4	FGFRL1
0	0.639893547653654	0.71	0.101	0	4	HEXB
0	0.638550947912004	0.565	0.074	0	4	GRAMD4
0	0.637994016071542	0.338	0.027	0	4	POP7
0	0.637855670495455	0.387	0.034	0	4	HADH
0	0.635077437406379	0.356	0.036	0	4	SUOX
7.62680569342451E-288	0.633815818215854	0.987	0.391	1.24400827665447E-283	4	OS9
0	0.632788001437651	0.712	0.093	0	4	RAB5IF
0	0.632084518674783	0.28	0.025	0	4	FDX2
0	0.631821411428501	0.492	0.045	0	4	ALKBH7
0	0.630580783431647	0.291	0.033	0	4	PSD3
0	0.630523367255614	0.557	0.053	0	4	ZNRD2
0	0.629975507239051	0.66	0.083	0	4	HDHD5
0	0.627837590106209	0.555	0.051	0	4	DPY30
0	0.627284654854276	0.377	0.036	0	4	AFF3
0	0.626608667151018	0.398	0.037	0	4	PSMB2
0	0.62524009422603	0.508	0.053	0	4	GLRX5
0	0.625081910490127	0.428	0.039	0	4	ETFDH
0	0.62479828867823	0.976	0.308	0	4	SDF4
0	0.623208451023163	0.787	0.122	0	4	SLC66A2
0	0.619689808149333	0.491	0.045	0	4	DAD1
0	0.618808290313159	0.611	0.064	0	4	TMBIM6
0	0.617661351823216	0.57	0.054	0	4	POLR2F
0	0.616532915797606	0.339	0.029	0	4	GLRX2
0	0.616346916637463	0.563	0.06	0	4	DHCR7
0	0.616182199810207	0.632	0.064	0	4	ADHFE1
0	0.614734316335017	0.817	0.119	0	4	ACBD5
0	0.614624344985997	0.54	0.064	0	4	EHBP1
0	0.614272299427979	0.434	0.037	0	4	PRDX5
0	0.612945238204738	0.755	0.1	0	4	TMED10
0	0.611889986557339	0.563	0.055	0	4	SLC25A20
0	0.611124604149806	0.357	0.033	0	4	ZSCAN2

0	0.610404057311927	0.563	0.067	0	4	IL13RA1
0	0.610394650798157	0.266	0.022	0	4	ALG8
0	0.609369323107324	0.841	0.146	0	4	TMED9
0	0.608374153563063	0.877	0.173	0	4	BSG
0	0.604114943971379	0.464	0.063	0	4	RAPH1
0	0.603819460805054	0.397	0.036	0	4	BANF1
0	0.603061260830655	0.723	0.088	0	4	TSR3
0	0.602597696909588	0.301	0.035	0	4	MICU3
0	0.6015688526262	0.693	0.103	0	4	FAM20A
0	0.599977968729832	0.927	0.202	0	4	PNPLA2
0	0.597940038287541	0.691	0.08	0	4	ZNF444
0	0.593216453058282	0.259	0.022	0	4	SLIRP
0	0.592670816304593	0.707	0.092	0	4	PCCA
0	0.591419450474306	0.386	0.044	0	4	TCEAL9
0	0.590103181753021	0.36	0.042	0	4	EID3
0	0.589521281769129	0.76	0.127	0	4	MT-ND5
0	0.589455194038371	0.578	0.076	0	4	DEPRECATE
0	0.589433515220188	0.968	0.287	0	4	PLXNB2
0	0.589054234698914	0.716	0.101	0	4	FAM20C
0	0.588120666432506	0.315	0.035	0	4	ZNF704
0	0.586322101621483	0.812	0.112	0	4	ECI2
0	0.585617901953198	0.486	0.056	0	4	TJP1
0	0.584408867615794	0.483	0.054	0	4	TEX2
0	0.579453123355584	0.658	0.069	0	4	C11orf54
0	0.57915111344953	0.864	0.171	0	4	PRKACA
0	0.578512306532772	0.35	0.038	0	4	FBP1
0	0.575996149231822	0.261	0.02	0	4	TRMT10C
0	0.575320972462597	0.333	0.036	0	4	SMCO4
0	0.574625027401488	0.695	0.096	0	4	RTN3
0	0.574502855974376	0.84	0.149	0	4	NFE2L1
0	0.57446671468801	0.581	0.067	0	4	PSMB5
0	0.573359043122257	0.819	0.135	0	4	DGAT1
0	0.572093596837501	0.535	0.069	0	4	CTIF
0	0.571146358278119	0.536	0.059	0	4	NECAB3
0	0.570872202530618	0.612	0.091	0	4	HS6ST1
0	0.569368659836566	0.931	0.224	0	4	ISG15
0	0.568025597289503	0.509	0.047	0	4	MEA1
0	0.566209492054619	0.827	0.136	0	4	NDUFB7
0	0.564412869225016	0.819	0.13	0	4	EGLN1
0	0.564408754226687	0.41	0.037	0	4	FAM8A1
0	0.56366810890594	0.675	0.079	0	4	EI24
0	0.563278994697279	0.508	0.053	0	4	GPANK1
0	0.562250432853606	0.318	0.027	0	4	PSMB6
0	0.56074981580933	0.886	0.159	0	4	COPB2
0	0.560581363295071	0.581	0.072	0	4	CTTN
0	0.559534442575136	0.383	0.04	0	4	MYLK
0	0.557906134969242	0.648	0.093	0	4	MT-ND1
0	0.555792932664829	0.529	0.054	0	4	ATP5PD
0	0.554470511974507	0.532	0.059	0	4	LAMA5
0	0.552917234580343	0.305	0.035	0	4	TBC1D16

0	0.551587413874008	0.272	0.028	0	4	CCNB1IP1
0	0.550887942604424	0.926	0.21	0	4	GOLPH3
0	0.549840302932113	0.66	0.088	0	4	MYO1C
0	0.549211081660347	0.747	0.093	0	4	IFITM2
0	0.54909238735474	0.373	0.038	0	4	LARP1B
0	0.548679848799746	0.326	0.035	0	4	ALS2
0	0.547396865149939	0.411	0.045	0	4	EFNB1
0	0.545760222036103	0.487	0.053	0	4	EXOSC4
0	0.544611756944717	0.548	0.061	0	4	RFNG
0	0.544456347456963	0.529	0.052	0	4	ABHD14B
0	0.543024915770869	0.425	0.044	0	4	NME2
0	0.542347427172438	0.805	0.125	0	4	STT3A
0	0.542222203549845	0.32	0.03	0	4	SSBP3
0	0.541582960908059	0.788	0.123	0	4	DNAJB11
0	0.54068079056355	0.565	0.12	0	4	EGR1
0	0.539948099375579	0.57	0.074	0	4	NAGLU
0	0.539537518187798	0.4	0.039	0	4	PRRC1
0	0.539320103890063	0.494	0.051	0	4	HGH1
0	0.538448377108404	0.263	0.021	0	4	MAGEF1
0	0.537200590817113	0.251	0.024	0	4	ATAD3A
0	0.537044830987455	0.335	0.032	0	4	GRAMD2B
0	0.535047779851464	0.906	0.194	0	4	GNPTG
0	0.532002223536635	0.73	0.089	0	4	PGRMC2
0	0.531915309132609	0.516	0.049	0	4	MBNL3
0	0.531402617567792	0.674	0.09	0	4	MTHFD1
0	0.531148131352661	0.48	0.047	0	4	PDCD5
0	0.530150230843561	0.383	0.034	0	4	TMEM14B
0	0.527944493228201	0.573	0.071	0	4	TNS2
0	0.52694296161267	0.68	0.084	0	4	PMM1
0	0.524305488089324	0.335	0.029	0	4	USE1
0	0.52348256469348	0.268	0.029	0	4	LRP6
0	0.522635871529328	0.782	0.117	0	4	NDUFB5
0	0.520792299299935	0.527	0.053	0	4	GPATCH4
0	0.519805515479312	0.472	0.046	0	4	COX6B1
0	0.519588737370868	0.55	0.07	0	4	BLVRA
0	0.518841462134458	0.289	0.03	0	4	LRRC28
0	0.518557756816529	0.362	0.053	0	4	IFIT2
0	0.51851224582838	0.265	0.031	0	4	MCC
0	0.518251998242498	0.793	0.115	0	4	TRAP1
0	0.517198737117548	0.58	0.075	0	4	ITPKC
0	0.514972187641738	0.256	0.026	0	4	NET1
0	0.512775850330883	0.817	0.13	0	4	PFDN2
0	0.510376705873831	0.638	0.07	0	4	SMPD1
0	0.510271803448793	0.719	0.093	0	4	SPCS2
0	0.510226709218037	0.255	0.022	0	4	MECR
0	0.509611193226634	0.83	0.148	0	4	ADIPOR1
0	0.509599018054526	0.584	0.065	0	4	BCKDHA
0	0.509318187794143	0.495	0.049	0	4	TSFM
0	0.509087725063033	0.379	0.039	0	4	RNF26
1.03461712149922E-284	0.508658328615694	0.987	0.373	1.68756398687738E-280	4	MT-CO2

0	0.508526655984205	0.844	0.154	0	4	RAB7A
1.0973625959337E-159	0.50829856876985	0.99	0.519	1.78990813022745E-155	4	IL32
0	0.503316148255833	0.315	0.032	0	4	ST7
0	0.502388483248405	0.547	0.075	0	4	CD276
0	0.500688089989929	0.548	0.076	0	4	KIF13A
0	0.499327899461606	0.551	0.056	0	4	ARL 1.00
0	0.499210697990151	0.286	0.028	0	4	MUL1
0	0.494510323092634	0.695	0.09	0	4	SLC4A2
0	0.494469376547561	0.507	0.062	0	4	DCPS
0	0.494421072671708	0.289	0.026	0	4	HSD17B12
6.53538461340032E-288	0.493855911979424	0.988	0.357	1.06598658429173E-283	4	NUCB1
0	0.493170066427757	0.492	0.056	0	4	NFKBIL1
0	0.492270959907117	0.311	0.034	0	4	CPNE8
0	0.49170863170143	0.84	0.132	0	4	SELENOO
0	0.491101341456531	0.346	0.039	0	4	GNA11
0	0.491048825305783	0.286	0.024	0	4	CNIH1
0	0.491016359477775	0.639	0.084	0	4	GADD45A
0	0.49032525167466	0.539	0.058	0	4	TOMM70
0	0.489919574656999	0.504	0.054	0	4	DHTKD1
0	0.489520651955411	0.322	0.041	0	4	CAMSAP2
0	0.488671897806797	0.411	0.05	0	4	ADAMTS10
0	0.487862905644217	0.655	0.098	0	4	HES1
0	0.487351667381331	0.66	0.084	0	4	HMGCS1
0	0.487283460307686	0.831	0.14	0	4	POLR2L
0	0.485660470867615	0.944	0.245	0	4	HSP90AB1
0	0.484612672282543	0.272	0.022	0	4	COX7B
0	0.483632463726174	0.298	0.029	0	4	DEPTOR
0	0.481764494505016	0.408	0.052	0	4	CCND1
0	0.47921528523326	0.513	0.054	0	4	MPLKIP
0	0.479198378635342	0.373	0.046	0	4	TEAD2
0	0.474518393087483	0.429	0.041	0	4	DEPRECATE
0	0.473576778611434	0.451	0.044	0	4	VPS25
0	0.47258750369582	0.425	0.04	0	4	ATP5MC1
0	0.470473455652063	0.315	0.037	0	4	SNX21
0	0.468898403801792	0.25	0.02	0	4	MCAT
0	0.468683497432319	0.316	0.029	0	4	BAD
0	0.468300235900987	0.408	0.038	0	4	PHB2
0	0.465610855008184	0.553	0.069	0	4	TMEM147
0	0.465305814366549	0.766	0.124	0	4	TMED7
0	0.461529148330667	0.284	0.027	0	4	ABCB6
0	0.46148060135572	0.488	0.046	0	4	MDH2
0	0.461337838131021	0.725	0.099	0	4	NDUFA2
0	0.460261247763119	0.715	0.093	0	4	SEM1
0	0.458636253366901	0.541	0.063	0	4	DVL1
0	0.458571178846636	0.263	0.036	0	4	TENT5A
0	0.456375026714643	0.859	0.141	0	4	OBSCN
0	0.456319550969744	0.777	0.119	0	4	TMED5
0	0.453880306254978	0.57	0.068	0	4	SSR3
0	0.453397697779719	0.88	0.166	0	4	TUFM
0	0.453144289552126	0.333	0.031	0	4	FAM136A

0	0.452909960074483	0.699	0.085	0	4	TFG
0	0.45239590583286	0.414	0.055	0	4	RGL3
0	0.451954408095407	0.394	0.035	0	4	COPZ1
0	0.449543736818895	0.932	0.231	0	4	LASP1
0	0.448965801838845	0.272	0.026	0	4	EXOSC5
0	0.448591946272102	0.49	0.046	0	4	SLC35D1
0	0.448259832696632	0.285	0.023	0	4	PSMB3
0	0.447768753452854	0.445	0.048	0	4	GPR137
0	0.444765387249327	0.258	0.022	0	4	SMIM13
0	0.444586211233903	0.668	0.119	0	4	LIMK2
0	0.443341246636417	0.394	0.044	0	4	PIK3R2
0	0.441471997667079	0.423	0.038	0	4	PLAAT3
0	0.440850566199702	0.449	0.036	0	4	AQP3
0	0.4399850961016	0.962	0.259	0	4	DCAF8
0	0.439737901560062	0.771	0.116	0	4	MAGED2
0	0.438444776886765	0.618	0.083	0	4	NRBP2
0	0.438301151210139	0.818	0.123	0	4	CUTA
0	0.437276195310695	0.74	0.11	0	4	PPP2R1B
0	0.437147027485866	0.461	0.052	0	4	MORF4L2
0	0.43606272297024	0.273	0.026	0	4	RAB43
0	0.435087763504614	0.27	0.03	0	4	PKDCC
0	0.43333641780727	0.43	0.052	0	4	DEPRECATE
0	0.43277710253908	0.31	0.026	0	4	TMEM123
0	0.432421177262727	0.652	0.077	0	4	COX17
0	0.430689858830348	0.508	0.05	0	4	MCCC2
0	0.427361932840235	0.758	0.102	0	4	NNT
0	0.425377772388254	0.621	0.079	0	4	ANAPC11
0	0.424757148872701	0.341	0.037	0	4	BCKDK
0	0.424059771349561	0.526	0.054	0	4	FAM20B
0	0.423052547634084	0.846	0.145	0	4	FIS1
0	0.422954565301254	0.738	0.107	0	4	EDEM3
0	0.422924860808154	0.516	0.066	0	4	PYGL
0	0.422187073535626	0.608	0.1	0	4	TGM2
0	0.420770800223162	0.344	0.03	0	4	NDUFAF1
0	0.420696768253401	0.572	0.064	0	4	EBP
0	0.419184414754397	0.464	0.057	0	4	IFT43
0	0.419151956623978	0.565	0.064	0	4	TMEM161A
0	0.417451323559194	0.932	0.257	0	4	MT-ND4L
0	0.41611364654352	0.348	0.046	0	4	SCML1
0	0.415033393515419	0.301	0.035	0	4	TRPV1
0	0.413505590037472	0.63	0.074	0	4	COX7C
0	0.412011567960207	0.774	0.127	0	4	CALM2
0	0.411409069953927	0.462	0.048	0	4	TMEM63B
0	0.41137259350943	0.662	0.081	0	4	ATP5F1A
0	0.410051704201506	0.958	0.271	0	4	LRP1
0	0.409455114963523	0.929	0.202	0	4	APMAP
0	0.407605797676489	0.268	0.028	0	4	RHBDD3
0	0.406670068897846	0.308	0.029	0	4	FAHD2A
0	0.405451846628437	0.768	0.129	0	4	FASN
0	0.405254011916616	0.328	0.04	0	4	NREP

	0	0.404935993684507	0.763	0.131	0	4	NCOA4
	0	0.404268452748114	0.611	0.071	0	4	RNF11
	0	0.403100425177887	0.713	0.107	0	4	C7orf50
	0	0.402911638479717	0.855	0.148	0	4	EPAS1
	0	0.402825104882444	0.334	0.033	0	4	SIRT5
	0	0.402556240824476	0.677	0.087	0	4	DCAF11
	0	0.402374237203198	0.35	0.035	0	4	LANCL2
	0	0.402056598205016	0.467	0.046	0	4	BCKDHB
	0	0.40158858041952	0.849	0.152	0	4	ERGIC3
	0	0.401360217946974	0.748	0.102	0	4	GHITM
	0	0.401091735813077	0.313	0.031	0	4	TOMM6
	0	0.40020485042434	0.342	0.032	0	4	NT5C3B
	0	0.398886347546364	0.453	0.042	0	4	POLR2K
	0	0.398457599823385	0.859	0.147	0	4	LMAN1
	0	0.397767529315547	0.349	0.035	0	4	PEX5
	0	0.397289524409853	0.923	0.195	0	4	NDUFV2
	0	0.396654825748097	0.442	0.045	0	4	ACOT7
	0	0.39346180493894	0.367	0.047	0	4	L3MBTL4
	0	0.391911371028517	0.653	0.09	0	4	HADHB
	0	0.390424261232054	0.441	0.044	0	4	GFER
	0	0.390021549084848	0.31	0.029	0	4	AVEN
	0	0.388779348278383	0.733	0.108	0	4	GOLIM4
	0	0.388474514881316	0.395	0.042	0	4	DEPRECATE
	0	0.388322573247596	0.406	0.041	0	4	UQCR10
	0	0.388242006303343	0.886	0.17	0	4	CMPK1
	0	0.387480097855494	0.723	0.105	0	4	NMD3
	0	0.387304301258767	0.846	0.193	0	4	MT-ND3
	0	0.387187660909615	0.825	0.199	0	4	CDKN1A
	0	0.386820834705257	0.736	0.115	0	4	NSFL1C
	0	0.38622998507672	0.302	0.025	0	4	SNTA1
	0	0.386198440543551	0.369	0.056	0	4	MAFF
	0	0.384227387735661	0.364	0.053	0	4	TRIM47
	0	0.383924832048703	0.279	0.035	0	4	TDRD10
	0	0.383576629505223	0.398	0.04	0	4	TTLL12
	0	0.382291651669647	0.727	0.107	0	4	POLR2H
	0	0.382260689313191	0.796	0.166	0	4	GADD45B
	0	0.37857465590382	0.509	0.058	0	4	PTS
	0	0.375429156816221	0.839	0.145	0	4	DTX3L
	0	0.37458912505252	0.49	0.046	0	4	GCDH
	0	0.372214804860276	0.339	0.033	0	4	CCT5
	0	0.370448978334832	0.419	0.044	0	4	VARS2
	0	0.369548786776974	0.669	0.11	0	4	MT-ND2
	0	0.36637091800494	0.367	0.039	0	4	MAD2L1BP
	0	0.364558172533386	0.39	0.037	0	4	ZCRB1
	0	0.364110588906173	0.919	0.31	0	4	LYZ
	0	0.36392196020474	0.631	0.087	0	4	ILVBL
	0	0.363235955764959	0.705	0.09	0	4	PRKCSH
	0	0.362155627222593	0.296	0.029	0	4	IMPDH2
	0	0.360537127459512	0.656	0.09	0	4	NSUN6
	0	0.360209331125883	0.296	0.039	0	4	MAPK12

0	0.359991172653417	0.459	0.054	0	4	NENF
0	0.359647537066063	0.366	0.037	0	4	C8orf33
0	0.359000414258502	0.373	0.048	0	4	SERPINE1
0	0.358570116496011	0.427	0.044	0	4	WASL
0	0.358383541906264	0.747	0.101	0	4	SUB1
0	0.357373888120003	0.579	0.083	0	4	SLC25A25
0	0.357090593896768	0.742	0.11	0	4	SCYL1
0	0.356299712804019	0.267	0.025	0	4	APOOL
0	0.356163419520147	0.341	0.037	0	4	IER3IP1
0	0.354844625318107	0.668	0.094	0	4	MEAF6
0	0.354268234289486	0.901	0.204	0	4	SLC40A1
0	0.353762342152758	0.253	0.026	0	4	SMIM20
0	0.351452410447356	0.379	0.065	0	4	CXCL2
0	0.350933136149226	0.712	0.102	0	4	KEAP1
0	0.3497004147653	0.559	0.066	0	4	SNAP29
0	0.348650662405139	0.794	0.136	0	4	LONP1
0	0.346263865470743	0.348	0.042	0	4	TAGLN2
0	0.345670539288161	0.466	0.051	0	4	WDR18
0	0.345329890478701	0.31	0.033	0	4	RPF2
0	0.344435968833915	0.456	0.054	0	4	DEPRECATE
0	0.344176935172212	0.468	0.07	0	4	ARHGEF11
0	0.341642956706346	0.946	0.228	0	4	SLC2A4RG
0	0.340458714279339	0.526	0.067	0	4	MUC20
0	0.340455821948562	0.279	0.024	0	4	DEPRECATE
0	0.338290493036542	0.707	0.11	0	4	RNPEP
0	0.338196880535024	0.397	0.039	0	4	DPAGT1
0	0.33797096453883	0.378	0.038	0	4	RRS1
0	0.337522230441274	0.919	0.197	0	4	BAG6
0	0.33678126330469	0.43	0.054	0	4	RRP12
0	0.336425101704825	0.677	0.109	0	4	CTNNA1
0	0.335558383542233	0.365	0.036	0	4	ATP5MF
0	0.335363019276284	0.682	0.093	0	4	COMMD4
0	0.334890835333569	0.406	0.046	0	4	ARF4
0	0.33450336282998	0.593	0.069	0	4	SAP18
0	0.334284389926231	0.656	0.084	0	4	NDUFS4
0	0.332971681008426	0.579	0.069	0	4	GADD45GIP
0	0.331553774344186	0.543	0.061	0	4	COX7A2
0	0.33120606890169	0.765	0.11	0	4	APEH
0	0.329787950635483	0.565	0.069	0	4	PHKA2
0	0.329678989136084	0.636	0.084	0	4	ZNF512B
0	0.329053116028392	0.562	0.067	0	4	COQ9
0	0.328096335866479	0.793	0.134	0	4	UBXN6
0	0.327750961956747	0.663	0.091	0	4	ATP5ME
0	0.327631604057035	0.311	0.029	0	4	TMEM8B
0	0.325592299391643	0.283	0.036	0	4	TTC28
0	0.32468277412357	0.348	0.033	0	4	MED18
0	0.323482745723615	0.434	0.06	0	4	PPARG
0	0.32326477642998	0.66	0.086	0	4	NDUFA13
0	0.323197857982599	0.825	0.142	0	4	ILRUN
0	0.323067130351177	0.506	0.056	0	4	FBXO31

0	0.322593571118133	0.507	0.078	0	4	SHTN1
0	0.322204279331674	0.308	0.028	0	4	HIBCH
0	0.321875673851113	0.786	0.127	0	4	MAGT1
0	0.321759274907837	0.625	0.107	0	4	TMEM37
0	0.32168042424692	0.449	0.062	0	4	ATP6V0A1
0	0.320061852872965	0.815	0.132	0	4	NUCKS1
0	0.318468418564805	0.317	0.034	0	4	MMAB
0	0.316746973910637	0.343	0.037	0	4	FOXRED1
0	0.315848793770283	0.549	0.058	0	4	ACBD4
0	0.311499788496833	0.269	0.025	0	4	HMBS
3.97450866553891E-128	0.310791957646691	0.994	0.546	6.48282108436051E-124	4	MT-CO1
0	0.31071467483282	0.396	0.035	0	4	ZNF275
0	0.310456750625267	0.377	0.054	0	4	SESTD1
0	0.310212553660788	0.282	0.024	0	4	LRATD2
0	0.309048408506414	0.252	0.034	0	4	IFIT3
0	0.308132674765582	0.553	0.062	0	4	NDUFB11
0	0.307241753327779	0.295	0.028	0	4	SSR1
0	0.307187974211809	0.448	0.061	0	4	LRMDA
0	0.306875866514288	0.63	0.078	0	4	GIPC1
0	0.306623716979012	0.323	0.03	0	4	RAB15
0	0.305268798536611	0.271	0.026	0	4	LAMTOR5
0	0.305044744741882	0.371	0.042	0	4	SEMA4B
0	0.304951987502837	0.267	0.028	0	4	BAG3
0	0.304389121041696	0.792	0.131	0	4	GALT
0	0.302947402949081	0.601	0.076	0	4	DEPRECATE
0	0.302360249532498	0.82	0.138	0	4	REXO2
0	0.298526287784337	0.659	0.09	0	4	WSB2
0	0.297309651545233	0.69	0.098	0	4	APH1A
0	0.296461651120268	0.345	0.04	0	4	GORAB
0	0.296080731563588	0.405	0.04	0	4	RUVBL2
0	0.295418639606599	0.573	0.067	0	4	SSR4
0	0.295275418450634	0.593	0.074	0	4	DENND10
0	0.292933962582049	0.407	0.058	0	4	CLCN5
0	0.292715227675766	0.648	0.083	0	4	UBA5
0	0.291365144660292	0.833	0.174	0	4	AKR1A1
0	0.291249216999808	0.408	0.052	0	4	EPHA2
0	0.290861942534016	0.621	0.085	0	4	APEX1
0	0.290590567206632	0.692	0.113	0	4	ABHD2
0	0.287431602897175	0.747	0.118	0	4	MLEC
0	0.286865216478551	0.845	0.148	0	4	DAP3
0	0.286585235782538	0.455	0.053	0	4	TM9SF1
0	0.286098217044089	0.338	0.032	0	4	BSCL2
0	0.285935962477773	0.267	0.023	0	4	TMEM256
0	0.284831735127444	0.663	0.089	0	4	ZBTB20
0	0.283575083160111	0.257	0.024	0	4	FDXR
0	0.282956410635766	0.629	0.098	0	4	LAMB2
0	0.282774537658641	0.31	0.033	0	4	DXO
0	0.281993384673548	0.551	0.06	0	4	KLHDC3
0	0.279620704643423	0.69	0.109	0	4	ESRRA
0	0.278992867166898	0.277	0.025	0	4	OXSM

**0**	0.278839902647493	0.454	0.058	0	4	DEPRECATE
**0**	0.277792770747274	0.781	0.126	0	4	LPIN2
**0**	0.276839065225198	0.585	0.07	0	4	EIF2D
**0**	0.276529621796134	0.755	0.122	0	4	SEC61A1
**0**	0.276244373632742	0.367	0.049	0	4	EDA
**0**	0.276220535186311	0.439	0.061	0	4	CPPED1
**0**	0.274029438663778	0.349	0.036	0	4	NDUFB2
**0**	0.273707611610171	0.629	0.084	0	4	YWHAE
**0**	0.273571009359445	0.803	0.14	0	4	SEL1L
**0**	0.272790331488909	0.819	0.149	0	4	CRELD2
**0**	0.271962572399471	0.503	0.056	0	4	CDIP1
**0**	0.271023595883389	0.629	0.081	0	4	DEXI
**0**	0.270912923883941	0.601	0.08	0	4	NUDT14
**0**	0.269858405295144	0.619	0.079	0	4	NDUFB10
**0**	0.268740759073853	0.467	0.052	0	4	MED8
**0**	0.268357995508765	0.514	0.078	0	4	PPFIBP2
**0**	0.266959614939237	0.593	0.065	0	4	PPP1R11
**0**	0.266205027126606	0.331	0.042	0	4	USP6NL
**4.57880478889471E-192**	0.266033197607769	0.973	0.406	7.46848849116617E-188	4	MT-CO3
**0**	0.26361509589562	0.366	0.05	0	4	ST3GAL6
**0**	0.263123326205833	0.533	0.062	0	4	WDR46
**0**	0.26281333352473	0.54	0.061	0	4	TKFC
**0**	0.26254818228022	0.794	0.123	0	4	TMEM8A
**0**	0.260733841015803	0.561	0.078	0	4	TFIP11
**0**	0.260491601205835	0.465	0.057	0	4	PCBP4
**0**	0.260078889906665	0.845	0.155	0	4	LONP2
**0**	0.257022458438015	0.283	0.026	0	4	ALPL
**0**	0.256557518103334	0.527	0.058	0	4	TMEM120A
**0**	0.255310405934176	0.387	0.038	0	4	UBL5
**0**	0.254899855199353	0.83	0.169	0	4	NUDC
**0**	0.254676281676087	0.793	0.132	0	4	IMMP2L
**0**	0.254188809097176	0.43	0.052	0	4	CAMK1
**0**	0.251363368742676	0.581	0.069	0	4	SNX4
**0**	0.251027949898119	0.435	0.046	0	4	EMC3
**0**	0.250908334500993	0.298	0.028	0	4	S1PR2
**0**	0.25078885439843	0.332	0.034	0	4	NELFE
**0**	0.250374677667554	0.846	0.172	0	4	EPN1
**0**	4.39289287703229	0.451	0.048	0	5	CYP2C19
**0**	4.25674204515211	0.436	0.052	0	5	PFKFB1
**0**	4.17686900226422	0.461	0.062	0	5	SLC22A25
**0**	4.17125532008334	0.636	0.082	0	5	AKR1C8P
**0**	4.1086062695994	0.512	0.064	0	5	PPARGC1A
**0**	4.01462568894307	0.34	0.045	0	5	VNN 3.00
**0**	3.94153742756879	0.662	0.092	0	5	CEP72
**0**	3.93651118650288	0.256	0.036	0	5	FYB2
**0**	3.87536350987838	0.578	0.079	0	5	GCKR
**0**	3.86344917984747	0.388	0.059	0	5	ABCG5
**0**	3.84001985518845	0.275	0.039	0	5	DNAH14
**0**	3.82569534374475	0.343	0.05	0	5	GDA
**0**	3.7974806162445	0.35	0.055	0	5	HKDC1

0	3.76619874311713	0.627	0.087	0	5	MYOM1
0	3.76544488507045	0.882	0.126	0	5	MLXIPL
0	3.76329415727534	0.496	0.077	0	5	FTCD
0	3.7586554587719	0.594	0.083	0	5	NR1I2
0	3.73525997830103	0.68	0.099	0	5	ABCC2
0	3.72049091739169	0.959	0.186	0	5	EFNA1
0	3.70737937152222	0.495	0.07	0	5	ACOT12
0	3.7002188915753	0.525	0.077	0	5	F11
0	3.68410816102294	0.424	0.065	0	5	ABCC6
0	3.66612535087544	0.479	0.074	0	5	SLC17A4
0	3.64092151214456	0.574	0.086	0	5	SHROOM1
0	3.62692733807429	0.336	0.05	0	5	ZNF648
0	3.62509706781018	0.303	0.04	0	5	SLC17A2
0	3.61899301375626	0.514	0.078	0	5	SLC30A10
0	3.61352591614078	0.702	0.104	0	5	PLIN5
0	3.60233302135558	0.923	0.152	0	5	CYP3A5
0	3.57588332277506	0.413	0.067	0	5	PDZK1
0	3.55771344607732	0.658	0.099	0	5	ERBB3
0	3.55311273993833	0.311	0.052	0	5	IL17RB
0	3.53145421604592	0.599	0.09	0	5	CYP21A2
0	3.52357958370693	0.475	0.073	0	5	CYP4F3
0	3.51968960310782	0.57	0.084	0	5	DTNA
2.16149015770223E-292	3.51459250279904	0.353	0.077	3.5256065962281E-288	5	CYP2B6
0	3.51376726210705	0.909	0.147	0	5	PAH
0	3.46153646049379	0.367	0.063	0	5	KNDC1
0	3.46152604663474	0.391	0.058	0	5	TUFT1
0	3.4592674430088	0.845	0.143	0	5	ELF3
0	3.44084842706885	0.891	0.143	0	5	C4BPB
0	3.42510017911307	0.336	0.051	0	5	MLPH
0	3.42430715200107	0.435	0.067	0	5	SOX5
0	3.42297645046889	0.793	0.122	0	5	SEC16B
0	3.41812874241289	0.328	0.058	0	5	GLS2
0	3.4119632170033	0.85	0.142	0	5	UPB1
0	3.39678781630397	0.452	0.07	0	5	GPAM
0	3.39309698833207	0.482	0.078	0	5	CYP2C18
1.68918473877035E-293	3.39054844724326	0.278	0.049	2.75522922740832E-289	5	DEPRECATE
0	3.38134397943105	0.337	0.053	0	5	CACNA1H
0	3.38092773824397	0.417	0.068	0	5	RAB17
0	3.36799935655501	0.55	0.087	0	5	CHDH
0	3.32848319467939	0.382	0.07	0	5	FST
0	3.32610612562435	0.556	0.098	0	5	AKR1C2
0	3.32523007016641	0.704	0.11	0	5	ONECUT1
3.86353312489717E-270	3.32484528542101	0.256	0.043	6.30180888001977E-266	5	MUC3A
0	3.32463844970991	0.669	0.116	0	5	SLC22A7
8.54202110173636E-279	3.32115068936017	0.265	0.046	1.39328906190422E-274	5	PTPRD
0	3.31470059552916	0.672	0.107	0	5	SLC7A2
0	3.2990601979107	0.326	0.048	0	5	ZDHHC11
0	3.29503521319273	0.396	0.065	0	5	GRTP1
0	3.29024076670007	0.594	0.092	0	5	SLC6A1
0	3.28334958129213	0.948	0.178	0	5	ITIH3

0	3.27729750875711	0.425	0.071	0	5	CHRD
0	3.27659060489158	0.339	0.064	0	5	TMEM184A
0	3.27607359001202	0.438	0.065	0	5	DLG5
0	3.26994330879018	0.468	0.081	0	5	AFM
4.3616690272931E-259	3.26993208136104	0.256	0.046	7.11431835041777E-255	5	C4orf19
2.85297192041995E-263	3.26884400568642	0.252	0.044	4.65348249939698E-259	5	ADGRA3
0	3.26719350722333	0.541	0.082	0	5	ALS2CL
0	3.25626218099424	0.559	0.097	0	5	HAL
0	3.25609130533091	0.31	0.049	0	5	CAP2
0	3.25376743035406	0.435	0.072	0	5	SGK2
5.84744704219272E-298	3.24640988523485	0.272	0.046	9.53777087052054E-294	5	ABCB11
0	3.24596367505161	0.391	0.068	0	5	NAGS
0	3.2454693830031	0.792	0.129	0	5	HGD
1.95002951279077E-290	3.24452215654522	0.263	0.043	3.18069313831303E-286	5	ASAP3
0	3.24379134734058	0.36	0.057	0	5	PLXNB1
0	3.23045955898057	0.521	0.081	0	5	HLF
0	3.22435926466294	0.351	0.059	0	5	TOM1L1
0	3.20649301779273	0.646	0.104	0	5	ACSM2A
0	3.20515802762759	0.264	0.039	0	5	BCO2
0	3.19718812347829	0.596	0.096	0	5	FBXO17
0	3.18807335283447	0.495	0.083	0	5	RTKN
0	3.17991149833869	0.27	0.04	0	5	LPIN3
0	3.17033519874003	0.472	0.078	0	5	PALM3
0	3.17015321163227	0.772	0.129	0	5	ITIH4
0	3.16877506111018	0.596	0.107	0	5	MST1
0	3.16769804261302	0.799	0.13	0	5	EPS8L2
0	3.15951481681344	0.412	0.073	0	5	SEMA4G
2.14357375668405E-268	3.14911565415433	0.258	0.045	3.49638315452735E-264	5	TMPRSS2
0	3.13765720292223	0.319	0.055	0	5	MOCOS
0	3.13009108339715	0.601	0.098	0	5	NR5A2
0	3.09975533952091	0.808	0.16	0	5	ERRFI1
0	3.09598180734119	0.437	0.075	0	5	ATP2B2
0	3.09597723601658	0.314	0.058	0	5	FAM110C
1.81746749833411E-299	3.08263096566395	0.304	0.057	2.96447123653277E-295	5	TRIM55
0	3.0454305915747	0.567	0.1	0	5	DTX4
0	3.04115838746092	0.353	0.062	0	5	SHANK2
0	3.03975413170215	0.628	0.107	0	5	OCLN
0	3.03849662365506	0.344	0.064	0	5	HOOK1
0	3.03648872083448	0.361	0.071	0	5	RND1
0	3.03536323540835	0.344	0.065	0	5	LGR4
0	3.0339611310306	0.503	0.071	0	5	HSF4
0	3.03051758496498	0.307	0.053	0	5	CLMN
0	3.01576697230271	0.592	0.105	0	5	LEPR
0	3.01375568701448	0.364	0.065	0	5	EXOC3L4
0	3.00530782588764	0.488	0.085	0	5	BAIAP2L1
0	2.99860696720406	0.693	0.135	0	5	IL6R
0	2.99027401931661	0.472	0.087	0	5	XDH
0	2.98775215289576	0.647	0.109	0	5	ESRP2
0	2.98745273480465	0.527	0.089	0	5	FGFR3
0	2.98375508969634	0.891	0.203	0	5	VEGFA

7.95562103447272E-279	2.97991189777711	0.288	0.055	1.29764134693284E-274	5	FRRS1
0	2.97488014258542	0.583	0.126	0	5	CHI3L1
0	2.9718851887834	0.288	0.048	0	5	KCNT2
0	2.97139277571427	0.565	0.098	0	5	A1CF
0	2.96840858045699	0.34	0.055	0	5	GLDC
0	2.96726157884576	0.654	0.113	0	5	PTPRF
1.26571395637422E-267	2.96529834654827	0.288	0.056	2.06450603424199E-263	5	UNC5CL
0	2.96032437980878	0.74	0.148	0	5	ABCC3
0	2.95988298591819	0.35	0.069	0	5	SLC25A18
6.01687131058658E-253	2.94637579830281	0.271	0.053	9.81411879469777E-249	5	MET
7.00306098295592E-306	2.93681194151002	0.317	0.061	1.14226927692994E-301	5	SLC12A8
0	2.93202745310053	0.727	0.136	0	5	AFDN
5.49730461623062E-266	2.91160239755337	0.29	0.058	8.96665355953376E-262	5	COBL
0	2.90874466787024	0.612	0.115	0	5	SLC38A4
0	2.90262485118397	0.355	0.062	0	5	RHPN2
1.56780215012554E-232	2.90201496688203	0.266	0.055	2.55724208706977E-228	5	ASPG
0	2.90061259031835	0.526	0.096	0	5	SAMD5
0	2.8952204409901	0.486	0.087	0	5	CYP2C9
0	2.89335548613487	0.518	0.09	0	5	PRKAB2
2.35082334460581E-266	2.88372537464245	0.295	0.06	3.83442795738654E-262	5	GHR
3.34056141956493E-223	2.88225002545155	0.266	0.057	5.44878973145235E-219	5	ACSM2B
0	2.87970882200109	0.514	0.092	0	5	SLC41A2
0	2.87680456338308	0.318	0.05	0	5	EID3
0	2.87437357652931	0.477	0.092	0	5	GPT2
0	2.86983152914641	0.733	0.136	0	5	PCSK6
0	2.8641398338809	0.666	0.117	0	5	CGN
0	2.86360832877044	0.519	0.083	0	5	FGF21
0	2.85659588909905	0.426	0.082	0	5	COBLL1
0	2.85648427734633	0.688	0.133	0	5	ARHGEF10L
0	2.8560892515813	0.539	0.095	0	5	PLEKHA6
2.80384506031936E-257	2.85570996975274	0.272	0.052	4.5733516778869E-253	5	CENPV
0	2.84824766314723	0.403	0.077	0	5	CAMSAP3
0	2.83409212091822	0.712	0.124	0	5	GPT
0	2.82798733451159	0.39	0.074	0	5	WWC1
0	2.82311110801299	0.498	0.096	0	5	MARC2
0	2.81948353941959	0.482	0.091	0	5	ACOX2
0	2.80728025525164	0.426	0.084	0	5	DNAJC22
0	2.80686211349977	0.366	0.065	0	5	DHRS2
0	2.80455979604016	0.53	0.097	0	5	BHMT2
0	2.80270754501552	0.509	0.132	0	5	P4HA1
0	2.79935190684672	0.352	0.062	0	5	MAPRE3
0	2.79509919144229	0.374	0.071	0	5	SORBS2
0	2.79424296777336	0.465	0.085	0	5	PTPRH
0	2.79157476412464	0.52	0.091	0	5	SLC17A9
0	2.78609779922717	0.567	0.113	0	5	LEAP2
0	2.77593366250918	0.5	0.094	0	5	KANK1
3.11788432602862E-289	2.77557875226163	0.308	0.06	5.08558112418528E-285	5	LRRC75B
2.04471740673023E-291	2.7735169366883	0.291	0.053	3.33513856211768E-287	5	RAPGEFL1
0	2.76780263538346	0.712	0.131	0	5	PON2
0	2.76696863190777	0.395	0.089	0	5	CYP2C8

0	2.7619119175335	0.45	0.085	0	5	ABLIM3
9.47545766148124E-296	2.76184559850377	0.337	0.071	1.54554189916421E-291	5	CYP4F11
1.69212339673044E-259	2.7609126743871	0.291	0.06	2.76002247240702E-255	5	EPB41L5
4.45370577814333E-256	2.75960615508276	0.27	0.051	7.26443949472959E-252	5	SLC25A33
8.12898843809182E-289	2.75841467406983	0.343	0.075	1.32591930413716E-284	5	ACSM5
9.10413830754445E-197	2.73465890790827	0.254	0.058	1.48497599934357E-192	5	PLGLB1
0	2.73367365608663	0.896	0.188	0	5	AGXT
0	2.73025538592539	0.458	0.095	0	5	G6PC
0	2.72604288775878	0.365	0.077	0	5	CDO1
0	2.72208642679231	0.602	0.131	0	5	PTGR1
3.66438816115197E-218	2.71886150880559	0.257	0.054	5.97698352965498E-214	5	IGF2BP3
0	2.71845470003711	0.83	0.198	0	5	SAT2
0	2.71575620433762	0.693	0.121	0	5	NR1H4
4.56886371641707E-307	2.71467781535718	0.324	0.063	7.45227360784789E-303	5	ENPP1
0	2.71417057690344	0.581	0.114	0	5	NEDD4L
7.16383255840335E-293	2.7086614382346	0.307	0.059	1.16849272860117E-288	5	PAWR
0	2.70689621540768	0.542	0.108	0	5	PLIN4
0	2.69919843190469	0.885	0.212	0	5	MT1M
0	2.68569724845565	0.867	0.16	0	5	HPN
6.39968246912832E-292	2.68446993183956	0.321	0.064	1.04385220753952E-287	5	ABCA6
1.03137294412054E-269	2.68041264275667	0.293	0.058	1.68227240915501E-265	5	LIPG
0	2.67919628506521	0.817	0.2	0	5	GLYCTK
1.9466099490311E-228	2.67537790482403	0.263	0.055	3.17511548786462E-224	5	TJP3
0	2.66673694698916	0.804	0.188	0	5	MT1A
1.43945331502796E-227	2.66392082625498	0.274	0.059	2.3478923021421E-223	5	RAP1GAP
0	2.65759951656862	0.366	0.072	0	5	HNF1A
0	2.64949764078924	0.439	0.088	0	5	ADGRG6
1.70945458597564E-299	2.64580124971691	0.348	0.075	2.78829137518486E-295	5	CDH1
0	2.6383905581115	0.532	0.107	0	5	KLF15
1.55307219754656E-256	2.63783653262151	0.292	0.059	2.53321606141819E-252	5	PPP1R3C
0	2.62490547168926	0.466	0.096	0	5	TTC38
6.67571512680188E-209	2.62478713608865	0.255	0.055	1.08887589433265E-204	5	BTNL8
1.09842757768085E-237	2.62198816489851	0.27	0.056	1.79164522195524E-233	5	FAT1
0	2.61989479086076	0.66	0.135	0	5	OSGIN1
0	2.61756919048675	0.465	0.083	0	5	RORC
0	2.61670529797308	0.311	0.056	0	5	ADCY6
7.01989206924061E-221	2.61510074901734	0.261	0.055	1.14501459541384E-216	5	ERBB2
0	2.61164879004022	0.593	0.111	0	5	OGDHL
2.90748200649195E-298	2.60672914323536	0.304	0.057	4.74239390078903E-294	5	DEPRECATE
0	2.60623816596654	0.361	0.068	0	5	ZNF595
4.23238271498259E-226	2.60253732204265	0.256	0.052	6.9034394464081E-222	5	YAP1
2.91956560433403E-233	2.60122242238793	0.273	0.057	4.76210345722923E-229	5	SLC6A12
0	2.59404401207967	0.498	0.102	0	5	OPLAH
0	2.58183237598975	0.815	0.158	0	5	FMO5
0	2.57907382591046	0.85	0.187	0	5	ECHDC2
0	2.57191906921542	0.674	0.153	0	5	GCLC
0	2.56754638778745	0.576	0.114	0	5	KIAA1522
0	2.56529824962835	0.431	0.091	0	5	SLC39A14
0	2.56312819030627	0.385	0.079	0	5	COL5A3
5.05560993302844E-287	2.56080103496136	0.337	0.072	8.24620536176269E-283	5	SLC30A2

5.93002683583256E-192	2.55745982019209	0.259	0.061	9.67246677192649E-188	5	GBP7
0	2.55256828060551	0.387	0.083	0	5	DECR2
0	2.55137303190184	0.859	0.175	0	5	MAT1A
1.86776906976154E-234	2.55054398155131	0.256	0.05	3.04651812968804E-230	5	TACC2
0	2.54696513194676	0.808	0.17	0	5	BAAT
0	2.54301969583904	0.519	0.115	0	5	PCK1
0	2.54108841383233	0.501	0.104	0	5	CLDN7
0	2.53929076298023	0.598	0.128	0	5	SLC29A1
0	2.53481684662322	0.43	0.09	0	5	BPHL
4.86948630597713E-231	2.53233411005509	0.307	0.072	7.94261911367929E-227	5	ABCB4
0	2.51562094367695	0.434	0.097	0	5	DEPRECATE
2.32281126463218E-260	2.51457171850385	0.288	0.057	3.78873745374155E-256	5	AASS
0	2.50605577571309	0.489	0.1	0	5	ANGPTL4
0	2.50033675860721	0.365	0.076	0	5	CEP170B
0	2.50031812906744	0.366	0.074	0	5	ENAH
2.03361664451286E-260	2.49691456850609	0.312	0.068	3.31703210886492E-256	5	XYLB
0	2.49553149062048	0.551	0.114	0	5	HJV
0	2.48906722119009	0.408	0.081	0	5	NR1D1
0	2.48842503631906	0.408	0.093	0	5	EGFR
0	2.4883497036533	0.453	0.094	0	5	PTPRK
0	2.48657510575424	0.964	0.241	0	5	CFH
0	2.48566226800784	0.375	0.072	0	5	EEPD1
0	2.48514203497093	0.41	0.075	0	5	COL27A1
0	2.48193527829195	0.396	0.081	0	5	MAP3K13
0	2.47802931944307	0.479	0.102	0	5	KLKB1
5.19086407700436E-284	2.47367564599756	0.324	0.067	8.46681839600181E-280	5	DEPRECATE
6.2417821643731E-246	2.46832161355168	0.27	0.053	1.0180970888309E-241	5	FAM83G
0	2.4671203161403	0.435	0.095	0	5	AMDHD1
0	2.46651337519907	0.927	0.323	0	5	ACADVL
0	2.46500403144262	0.377	0.081	0	5	PPP1R3B
2.5413869647318E-211	2.46060232664281	0.305	0.074	4.14525627817404E-207	5	BHMT
0	2.45693966514685	0.352	0.068	0	5	PLEKHH3
1.52501754341382E-221	2.45664598251676	0.252	0.051	2.48745611506228E-217	5	PLEKHG6
5.73671365487593E-253	2.45624747347041	0.27	0.051	9.35715364246813E-249	5	ABHD11
2.01154501298521E-281	2.43981931637568	0.302	0.059	3.28103107068018E-277	5	FAAH
0	2.4393153139595	0.727	0.149	0	5	UGDH
0	2.43905462744867	0.422	0.086	0	5	AJUBA
4.0747492116174E-236	2.436958761359	0.281	0.059	6.64632343906914E-232	5	MAP3K20
0	2.43622099022529	0.532	0.109	0	5	DPYS
0	2.43370699727704	0.436	0.096	0	5	F13B
5.4382124074941E-238	2.43259550076956	0.275	0.057	8.87026825786363E-234	5	NOL3
5.1650945309342E-214	2.42877266312386	0.28	0.065	8.42478568940677E-210	5	DIO1
0	2.42856350897518	0.507	0.099	0	5	NRBP2
0	2.42740412062267	0.699	0.14	0	5	SLC38A3
1.85553614237488E-302	2.42558017609999	0.347	0.072	3.02656500182766E-298	5	PLIN2
0	2.41505293907162	0.412	0.073	0	5	NQO2
9.18960551021478E-291	2.41501473714918	0.261	0.042	1.49891655477113E-286	5	MICU3
0	2.41288966913608	0.752	0.17	0	5	NIT1
0	2.41278467355699	0.544	0.113	0	5	C8A
0	2.4114165056844	0.707	0.147	0	5	TAT

0	2.41052364003924	0.606	0.132	0	5	DST
0	2.3994878331098	0.55	0.116	0	5	PECR
0	2.39384819260658	0.365	0.074	0	5	CUL7
0	2.38981864654509	0.732	0.157	0	5	MTSS2
6.20607450340211E-255	2.38975546067314	0.416	0.11	1.01227281224992E-250	5	CRP
0	2.38792100527774	0.581	0.132	0	5	AKR1B10
1.32872707273369E-205	2.38492501708363	0.282	0.068	2.16728672833592E-201	5	SLCO1B1
0	2.38240545987069	0.555	0.114	0	5	TMEM45B
0	2.37999062262331	0.35	0.069	0	5	MPDZ
0	2.37911708085151	0.376	0.081	0	5	RHOBTB3
2.93370977300078E-232	2.37771749368071	0.257	0.05	4.78517401074158E-228	5	CCDC9B
0	2.37608008069367	0.392	0.083	0	5	PPARA
0	2.36969898781939	0.494	0.101	0	5	UAP1
0	2.35946871605792	0.45	0.104	0	5	AIG1
1.41378933463988E-231	2.35324437740921	0.297	0.068	2.3060317837311E-227	5	ONECUT2
0	2.35108152847113	0.551	0.115	0	5	C16orf70
0	2.34245388485371	0.53	0.109	0	5	CGNL1
2.24846486480321E-251	2.34113983944676	0.325	0.075	3.66747104098051E-247	5	CYP2D6
1.2850353678158E-230	2.33718536582897	0.274	0.058	2.09602118844435E-226	5	TEAD1
2.29291869667912E-226	2.32897846592937	0.301	0.071	3.73997968615332E-222	5	CAPN5
4.6341444886857E-302	2.32727488775309	0.381	0.087	7.55875307549524E-298	5	GALE
1.39768921518414E-267	2.3190506992607	0.36	0.086	2.27977087888685E-263	5	PROX1
2.95531401748536E-292	2.30925880878037	0.316	0.062	4.82041269392038E-288	5	BLNK
1.7249361413303E-286	2.30782899542245	0.259	0.042	2.81354334012385E-282	5	TRPV1
1.15381568131451E-208	2.30748934607516	0.259	0.056	1.88198875779209E-204	5	NAV2
0	2.30293342413396	0.654	0.154	0	5	WDR13
2.38273093265753E-207	2.30176540871213	0.304	0.077	3.88647242425769E-203	5	DHODH
0	2.29618201232664	0.484	0.107	0	5	ALDH1L1
0	2.29552017347965	0.529	0.121	0	5	ARG1
0	2.29482334367534	0.421	0.095	0	5	PLA1A
1.75236348639205E-278	2.28168558105189	0.318	0.066	2.85828008265407E-274	5	CEP70
0	2.27926775014095	0.591	0.128	0	5	PCYT2
0	2.26696099506765	0.743	0.15	0	5	DEPRECATE
0	2.26207818196293	0.615	0.141	0	5	H6PD
0	2.25797298223226	0.348	0.069	0	5	SFXN5
7.13232252745793E-238	2.25135812357054	0.273	0.056	1.16335312745366E-233	5	MAGI1
1.13693822546068E-283	2.24442059226224	0.327	0.067	1.85445993954891E-279	5	RGL3
1.96901847156876E-182	2.24207441274396	0.26	0.064	3.21166602897581E-178	5	STAP2
0	2.23358571734395	0.362	0.073	0	5	ARVCF
1.0041110681156E-252	2.22243412178494	0.371	0.095	1.63780556320336E-248	5	TUBB2A
0	2.21209490506296	0.448	0.108	0	5	DDIT3
0	2.21136391766165	0.392	0.079	0	5	DCUN1D4
0	2.20900977712094	0.339	0.061	0	5	ADAMTS10
0	2.20111716465695	0.81	0.203	0	5	SHMT1
0	2.19901566637846	0.435	0.095	0	5	KIAA1217
2.33521225804328E-187	2.19836394773282	0.253	0.059	3.8089647140944E-183	5	SMARCA1
0	2.19489095443394	0.735	0.172	0	5	ZKSCAN1
0	2.19234502533843	0.748	0.17	0	5	F11R
0	2.18856803270355	0.403	0.095	0	5	NHSL1
5.5218147301427E-291	2.18750371238732	0.395	0.095	9.00663200633575E-287	5	SPSB1

1.1485022086084E-243	2.18572759089767	0.339	0.083	1.87332195246117E-239	5	C2orf72
0	2.18364248680683	0.904	0.246	0	5	MT1H
1.07704355768942E-240	2.18113276513314	0.295	0.063	1.75676574694721E-236	5	SLC19A2
1.91181850979195E-270	2.17987920156348	0.355	0.084	3.11836717132164E-266	5	ALDH5A1
0	2.17464943974967	0.42	0.101	0	5	CBS
0	2.17367871495067	0.734	0.192	0	5	SLC30A1
0	2.17104236081592	0.67	0.156	0	5	ALDH3A2
4.58061823494116E-283	2.16972185511463	0.381	0.092	7.47144640301253E-279	5	FOXA3
0	2.16859816155766	0.712	0.149	0	5	BDH1
1.18410235477959E-242	2.16513167796706	0.293	0.063	1.93138935088099E-238	5	MTUS1
0	2.16300224859929	0.811	0.189	0	5	OAF
0	2.16034055649668	0.713	0.222	0	5	DEPRECATE
0	2.15656116176257	0.578	0.126	0	5	F5
0	2.15071226497786	0.884	0.195	0	5	CP
0	2.1501488419905	0.728	0.194	0	5	HSD17B4
0	2.15012232240009	0.415	0.091	0	5	SLC43A1
0	2.14156242252667	0.395	0.087	0	5	CTIF
0	2.14054855079648	0.562	0.13	0	5	SLC22A23
0	2.1369013946125	0.488	0.121	0	5	FMO3
6.36239591326766E-266	2.12987467120744	0.383	0.096	1.03777039741309E-261	5	ACSM1
1.30014816772314E-184	2.12564843917665	0.259	0.062	2.12067167637321E-180	5	THNSL2
1.22228128808907E-206	2.12268397779996	0.27	0.062	1.99366300900208E-202	5	TLE1
0	2.12136696902613	0.434	0.099	0	5	KIFC3
1.83946502924092E-148	2.11420804686174	0.256	0.071	3.00035140919487E-144	5	TMEM45A
0	2.11401134803392	0.75	0.16	0	5	OBSCN
1.60811195104928E-206	2.10035625621119	0.3	0.074	2.62299140335648E-202	5	BCAT2
5.11325568538042E-184	2.09938830918178	0.317	0.09	8.340231348424E-180	5	CFHR4
0	2.09920890768002	0.61	0.14	0	5	SPTBN1
0	2.09494333019462	0.426	0.097	0	5	TNKS1BP1
0	2.09394966087559	0.923	0.251	0	5	COL18A1
1.64400870498151E-278	2.09235292878126	0.417	0.108	2.68154259869534E-274	5	C6
0	2.09098020992466	0.401	0.082	0	5	EHBP1
0	2.08624273991645	0.711	0.167	0	5	ASL
1.04352756542739E-192	2.07893173009011	0.289	0.074	1.70209781196861E-188	5	HYI
0	2.07723604196538	0.604	0.17	0	5	TXNRD1
2.82014047980556E-290	2.07642343400654	0.373	0.085	4.59993113661085E-286	5	FGFR2
0	2.07535912920168	0.635	0.142	0	5	TCEA3
4.97900513620445E-257	2.07400268324036	0.372	0.093	8.12125527766308E-253	5	CD24
0	2.06441033585473	0.466	0.107	0	5	CEBPG
0	2.0596112847928	0.567	0.134	0	5	C5
0	2.05960327364116	0.648	0.171	0	5	MTSS1
0	2.05728584171564	0.762	0.17	0	5	TDO2
2.50055232449124E-196	2.05662565994251	0.334	0.093	4.07865089647766E-192	5	STEAP3
2.55616406922443E-284	2.05652510993459	0.388	0.094	4.16935921331198E-280	5	FNDC4
3.17892969950851E-171	2.05216867332907	0.261	0.067	5.18515223286832E-167	5	LAD1
0	2.04950100420816	0.557	0.136	0	5	SLC25A13
0	2.04694629658209	0.441	0.105	0	5	TNFRSF12A
0	2.04136639049778	0.422	0.096	0	5	SLC25A29
4.82847812249336E-191	2.03110845768652	0.252	0.057	7.87573066559891E-187	5	ABTB2
0	2.03096421971578	0.478	0.112	0	5	LDHD

3.98496993342917E-157	2.03065806201904	0.272	0.077	6.49988445841632E-153	5	GOLT1A
0	2.02956733480725	0.336	0.066	0	5	DEPRECATE
0	2.02750564238203	0.458	0.104	0	5	OTUD7B
0	2.02697674162182	0.404	0.095	0	5	FLNB
0	2.02182326627748	0.868	0.217	0	5	IGFBP1
0	2.01865669573578	0.739	0.191	0	5	AGRN
0	2.015848211632	0.605	0.151	0	5	SLC37A4
2.26431301937052E-255	2.01551340348525	0.355	0.086	3.69332096589525E-251	5	GCLM
0	2.01375460829848	0.624	0.144	0	5	GGCX
0	2.01178023577975	0.789	0.25	0	5	SLC12A7
0	2.00322453550983	0.719	0.194	0	5	GDF15
4.80080447018577E-207	2.00233382544428	0.262	0.057	7.83059217132001E-203	5	SCML1
4.11086265804622E-274	2.00096576315793	0.274	0.048	6.70522808153919E-270	5	TUBG2
2.60251447405108E-244	1.99805705161601	0.357	0.09	4.24496135862472E-240	5	KCTD3
0	1.97616913103939	0.588	0.136	0	5	SELENBP1
9.26554663564663E-273	1.97572709712266	0.382	0.094	1.51130331174032E-268	5	RIOX2
5.76368569644125E-254	1.97500656340897	0.388	0.102	9.40114773946533E-250	5	MACROD1
2.7605307528736E-239	1.97387749894919	0.314	0.073	4.50270171101212E-235	5	UMPS
5.6354338644967E-281	1.97174624228558	0.41	0.106	9.19195617638057E-277	5	CRLS1
2.84587530610302E-207	1.9582240066107	0.334	0.091	4.64190721178464E-203	5	AMACR
2.92883386473837E-303	1.95798554411575	0.404	0.095	4.77722091677476E-299	5	NFIB
1.45684448198058E-171	1.95643439045407	0.259	0.065	2.37625903455853E-167	5	KLF10
0	1.95486159117756	0.41	0.094	0	5	KIF13A
0	1.95463660766794	0.507	0.127	0	5	MYO1B
8.01525139658841E-155	1.9544762040889	0.276	0.079	1.30736765529754E-150	5	CYP4A11
5.35597168204534E-255	1.95364681793109	0.367	0.092	8.73612541058415E-251	5	IL17RC
0	1.95200839667417	0.466	0.101	0	5	NIT2
1.16734183313362E-289	1.95164445890075	0.424	0.106	1.90405126402425E-285	5	SLC2A2
0	1.95087476889501	0.756	0.173	0	5	PABPC1L
0	1.94500842538972	0.548	0.107	0	5	NSUN6
0	1.94309979850238	0.561	0.129	0	5	F7
5.41072311139753E-304	1.93640079049131	0.365	0.079	8.82543046700051E-300	5	LAMA5
0	1.93235454545592	0.67	0.156	0	5	INSR
0	1.92874988254252	0.522	0.122	0	5	DSP
0	1.92542863088056	0.504	0.119	0	5	C8G
0	1.92396511611647	0.409	0.074	0	5	DAPK2
0	1.92333586557265	0.36	0.071	0	5	ZSCAN26
0	1.92180357235597	0.649	0.165	0	5	ACOX1
5.4357702280682E-224	1.9211128194391	0.291	0.065	8.86628481900203E-220	5	SESTD1
2.00137932953226E-179	1.91620737796868	0.255	0.061	3.26444982440007E-175	5	NCKAP1
0	1.91372853673643	0.445	0.087	0	5	ADHFE1
0	1.9017664331686	0.981	0.278	0	5	CFB
0	1.89994393369165	0.793	0.22	0	5	MYO18A
0	1.89975580132547	0.597	0.16	0	5	SLC27A3
9.35525426183488E-247	1.89328204407807	0.266	0.051	1.52593552264789E-242	5	GORAB
7.52145828810945E-294	1.89228179023996	0.454	0.122	1.22682506137353E-289	5	SCCPDH
3.02186576732157E-273	1.8917488390751	0.346	0.078	4.92896525307821E-269	5	DEPRECATE
8.31517307576228E-299	1.89056892840027	0.45	0.115	1.35628788038759E-294	5	CREB3L3
1.99922570801747E-153	1.89039405574541	0.262	0.073	3.2609370523473E-149	5	ELMO3
5.88798615455595E-154	1.89025050359525	0.266	0.074	9.6038942166962E-150	5	SLC47A1

1.00729015831617E-184	1.88853754810679	0.277	0.068	1.6429909772295E-180	5	MAFF
5.9221618239593E-302	1.88583194132844	0.433	0.107	9.65963815106001E-298	5	AKR1C3
0	1.88574774099204	0.558	0.135	0	5	NADK2
0	1.88268266097894	0.419	0.085	0	5	SLC25A16
2.09983679281813E-141	1.87380024476651	0.278	0.085	3.42504379276565E-137	5	DEPRECATE
0	1.87312971007279	0.656	0.175	0	5	DDT
0	1.87308474027358	0.39	0.084	0	5	MUC20
2.82872134046791E-224	1.8705794552637	0.347	0.09	4.61392737843721E-220	5	PVR
1.31102148675342E-254	1.86714297946335	0.327	0.074	2.1384071470435E-250	5	KLC4
0	1.86595432473898	0.633	0.148	0	5	CFHR2
0	1.86413659926859	0.945	0.321	0	5	MT1X
0	1.86267456744792	0.353	0.071	0	5	USP35
0	1.86139417547206	0.574	0.127	0	5	MASP1
0	1.85411843462168	0.597	0.144	0	5	CFI
0	1.85236243060526	0.449	0.11	0	5	TMPRSS6
7.96726378363008E-248	1.85214365178081	0.359	0.089	1.2995403957479E-243	5	ARHGEF40
3.89770982469289E-214	1.84843274970609	0.348	0.095	6.35755449505657E-210	5	ACO1
1.01904251110186E-274	1.847836127219	0.387	0.096	1.66216023985825E-270	5	MYO6
1.17702703869942E-202	1.83784283747072	0.326	0.087	1.91984880282262E-198	5	ACSS2
5.07071422429194E-169	1.8377793919351	0.308	0.09	8.27084197124259E-165	5	TM7SF2
1.37818869847431E-227	1.83751196007961	0.372	0.102	2.24796358608145E-223	5	CDH2
0	1.83407252937647	0.452	0.111	0	5	C1RL
4.77170561943281E-287	1.82796412876502	0.451	0.119	7.78312903585686E-283	5	TSKU
1.85204693724941E-230	1.82437267255959	0.342	0.085	3.02087375934751E-226	5	ARHGEF11
0	1.81497686748493	0.53	0.132	0	5	ENTPD5
6.2714044141066E-177	1.81282298244406	0.26	0.064	1.02292877398493E-172	5	ALCAM
3.2380017171719E-178	1.81280656335073	0.282	0.074	5.28150460087908E-174	5	CCDC85C
2.76829739281606E-190	1.81230233187744	0.283	0.069	4.51536987742227E-186	5	PLA2G4C
0	1.8084928493185	0.473	0.121	0	5	RILP
3.74742032401569E-233	1.80529815226712	0.335	0.082	6.11241729050199E-229	5	DCPS
1.76053306945891E-188	1.80306222317558	0.27	0.065	2.87160548959443E-184	5	C11orf24
0	1.79976707079262	0.973	0.433	0	5	SQSTM1
6.19343220649961E-208	1.79088333007789	0.271	0.06	1.01021072720215E-203	5	SERPINE1
0	1.78782976592169	0.658	0.15	0	5	SERINC2
3.38746718926351E-264	1.78663134311898	0.347	0.08	5.52529773240772E-260	5	NECAB3
2.12211622212109E-286	1.7855472754339	0.482	0.136	3.46138376990171E-282	5	IDH1
2.11017870267199E-224	1.78362586343847	0.351	0.093	3.44191248192829E-220	5	ABCD3
1.98434565811697E-295	1.78312747465843	0.459	0.12	3.23666620295458E-291	5	PON3
4.99324413254994E-294	1.78200469282573	0.426	0.108	8.1444805046022E-290	5	LRP5
0	1.77059518586745	0.547	0.155	0	5	CPEB4
0	1.76295690294028	0.348	0.069	0	5	GABRE
0	1.76020696480573	0.617	0.174	0	5	ADAM15
3.02490074377652E-193	1.7593191695438	0.311	0.083	4.93391560317388E-189	5	NDRG2
0	1.75117507635268	0.592	0.139	0	5	HNF4A
0	1.75003026984199	0.58	0.122	0	5	PLA2G6
0	1.74818956209831	0.633	0.159	0	5	PDIA5
0	1.74738557630143	0.522	0.118	0	5	PLEKHG3
1.47469391762283E-302	1.74668571758531	0.445	0.114	2.40537324903461E-298	5	FOXP4
1.8903421888757E-168	1.74320264314673	0.263	0.068	3.08333714427515E-164	5	HIGD1A
4.82961866260476E-156	1.74310185909258	0.263	0.072	7.87759100057462E-152	5	ZFAND2A

0	1.74111402037975	0.744	0.17	0	5	SERPINF1
3.55252326412581E-186	1.74058272657246	0.294	0.076	5.79452069611562E-182	5	PPARG
0	1.73978174371274	0.563	0.132	0	5	PON1
0	1.73945320178579	0.617	0.139	0	5	PROC
1.00577306321416E-276	1.7387453322866	0.472	0.135	1.64051644340862E-272	5	PAPSS2
8.6619994460389E-153	1.73713037690711	0.302	0.093	1.4128587296434E-148	5	GOT1
2.68301677354799E-155	1.73541127937595	0.291	0.085	4.37626865933412E-151	5	GNMT
9.11499230306377E-168	1.7351111486595	0.268	0.07	1.48674639455273E-163	5	DEPRECATE
1.17117410818544E-241	1.73458944236678	0.305	0.067	1.91030208786127E-237	5	SHPK
1.74790365163699E-245	1.73349228506335	0.404	0.109	2.85100564618509E-241	5	C8B
0	1.73233488697954	0.665	0.154	0	5	AOX1
0	1.73120264495853	0.467	0.12	0	5	BCAR1
0	1.72845466616174	0.822	0.186	0	5	ASGR1
1.31577814021297E-151	1.71076729716408	0.261	0.073	2.14616572450138E-147	5	ZNF219
6.489884199344E-288	1.70998388381302	0.473	0.132	1.058565011755E-283	5	TOR1AIP2
1.43016540330267E-170	1.70082864295508	0.301	0.086	2.33274278932698E-166	5	FN3K
0	1.69945612987251	0.695	0.177	0	5	TP53INP1
0	1.69400104270801	0.771	0.174	0	5	TM4SF5
0	1.69398791629707	0.546	0.153	0	5	SPTSSA
1.40848917092455E-246	1.69088329076255	0.371	0.096	2.29738668669503E-242	5	PTPRS
2.01713550804351E-245	1.68938073066824	0.346	0.083	3.29014972716977E-241	5	YPEL2
0	1.68880018841231	0.733	0.188	0	5	CYB5A
0	1.68361191621963	0.482	0.126	0	5	SULT2A1
0	1.68268152533506	0.683	0.171	0	5	SPON2
7.08815102244277E-184	1.6813727622332	0.332	0.095	1.15614831327064E-179	5	FGGY
9.92942847006515E-299	1.67894444395583	0.439	0.112	1.61958907775233E-294	5	DCAF6
2.76288946530155E-283	1.67623711453347	0.44	0.118	4.50654900685335E-279	5	ATRN
2.49146062915482E-207	1.66362002194512	0.351	0.096	4.06382143221443E-203	5	SHTN1
0	1.65702106187298	0.954	0.275	0	5	APOC2
2.55285345640137E-127	1.65658592738785	0.265	0.083	4.16395927273627E-123	5	HHEX
0	1.65612961389112	0.601	0.151	0	5	MPST
2.0076775282379E-227	1.65405046204945	0.305	0.071	3.27472281630884E-223	5	NPIPB15
0	1.65193301107423	0.786	0.217	0	5	CYP 20.00
6.16552978391035E-226	1.64708366638813	0.419	0.121	1.00565956305362E-221	5	HPD
1.4071806528454E-168	1.64687904106243	0.293	0.082	2.29525236285612E-164	5	PDGFA
2.03679769871426E-154	1.64406442821405	0.291	0.087	3.32222072637283E-150	5	MTHFS
0	1.63539600268248	0.738	0.186	0	5	SDC4
1.03337854770645E-194	1.63442925503096	0.263	0.06	1.68554374916399E-190	5	L3MBTL4
0	1.63261207973596	0.674	0.188	0	5	ZBTB7B
3.93919676541899E-190	1.63064770975808	0.35	0.104	6.42522384407491E-186	5	MMUT
1.03842847630796E-231	1.62477762744105	0.41	0.118	1.69378068770591E-227	5	ABAT
2.52863342530039E-232	1.62174128554101	0.403	0.112	4.12445398000747E-228	5	KHK
4.28936362913051E-209	1.62173369715537	0.319	0.083	6.99638101547478E-205	5	RASSF7
0	1.62067336981801	0.678	0.192	0	5	MGAT4B
2.84549451184482E-293	1.61995373229856	0.498	0.14	4.64128609827009E-289	5	LIMK2
3.08986904046421E-207	1.61909402574361	0.362	0.103	5.03988539190117E-203	5	SLC35C1
8.90166803934399E-234	1.61784116887706	0.436	0.131	1.4519510738974E-229	5	FAM20A
0	1.61737332555667	0.544	0.147	0	5	NDST1
3.64917788978431E-159	1.61527799402957	0.266	0.071	5.9521740560272E-155	5	STK3
1.17210587470418E-228	1.61495315306672	0.323	0.079	1.91182189222999E-224	5	ATP9A

**2.2333030458972E-280**	1.61465935195073	0.438	0.117	3.64274059816292E-276	5	NR2F6
**0**	1.60971636450456	0.638	0.157	0	5	LBP
**0**	1.607363053231	0.718	0.176	0	5	APCS
**6.35407064754E-213**	1.60338861988925	0.353	0.096	1.03641246332025E-208	5	TNS2
**0**	1.60022038634466	0.873	0.229	0	5	FGL1
**9.56759806256094E-177**	1.59938553651548	0.291	0.078	1.56057091998431E-172	5	TJP1
**5.87895275438641E-186**	1.59644096454069	0.339	0.099	9.58915983767968E-182	5	SYNPO
**1.67714664040825E-152**	1.59452053761429	0.316	0.099	2.73559388516989E-148	5	ALAS1
**0**	1.59420882846736	0.449	0.106	0	5	ZNF512B
**2.06372367580716E-278**	1.59225824789132	0.463	0.124	3.36613968760905E-274	5	RGN
**5.6365419499604E-160**	1.58469861262863	0.29	0.082	9.19376357458041E-156	5	RAPH1
**0**	1.58372136042304	0.825	0.202	0	5	C4BPA
**2.58945929437815E-174**	1.5829124055072	0.334	0.1	4.2236670550602E-170	5	FH
**0**	1.58034028830859	0.623	0.198	0	5	ETS2
**1.69046449560229E-258**	1.58027269287434	0.409	0.109	2.7573166387769E-254	5	SHC2
**0**	1.57545835816721	0.546	0.134	0	5	PPP2R1B
**0**	1.57291554676321	0.593	0.176	0	5	MPC1
**9.74660956836729E-143**	1.57255841971393	0.289	0.09	1.58976948669639E-138	5	FAM83H
**0**	1.57068552093765	0.712	0.213	0	5	EXT1
**3.41135477618018E-218**	1.57018105264299	0.269	0.057	5.56426077542749E-214	5	ZBTB41
**0**	1.56850624585949	0.475	0.117	0	5	MEAF6
**0**	1.56693709980393	0.532	0.131	0	5	POLR2H
**1.37593521966671E-206**	1.56592574941778	0.338	0.091	2.24428793679838E-202	5	ST5
**1.51036397806976E-235**	1.56486348462426	0.541	0.183	2.46355468462959E-231	5	ACSL4
**0**	1.56409770245366	0.58	0.163	0	5	CLUH
**1.75090286641405E-123**	1.56014814451508	0.259	0.081	2.85589766540796E-119	5	SRXN1
**4.08629100772509E-303**	1.55650953615431	0.459	0.12	6.66514926270039E-299	5	PCCA
**0**	1.5560831958261	0.673	0.199	0	5	SHC1
**0**	1.55516821300549	0.59	0.146	0	5	CLDN1
**1.50707780069015E-162**	1.55119305370492	0.312	0.092	2.4581946007057E-158	5	TCF7L2
**1.52541796140027E-150**	1.54975612682118	0.27	0.077	2.48810923683997E-146	5	SEC14L2
**9.76985098447226E-100**	1.54834756533155	0.254	0.09	1.59356039407727E-95	5	ALDH7A1
**0**	1.54587870868432	0.799	0.224	0	5	NUPR1
**0**	1.54148840357933	0.601	0.174	0	5	PRDX6
**5.40154447361453E-284**	1.54055169339789	0.568	0.174	8.81045919091265E-280	5	ALDH1A1
**0**	1.53618563338384	0.955	0.321	0	5	MT1B
**0**	1.53118603057707	0.721	0.17	0	5	TFR2
**0**	1.52657006600432	0.544	0.155	0	5	SPATS2L
**1.99895626128305E-167**	1.52656311869364	0.307	0.09	3.26049755777878E-163	5	TBC1D24
**5.2418703800493E-135**	1.52623993482805	0.284	0.09	8.55001477689841E-131	5	UGT1A1
**1.00036877433352E-217**	1.52312155119359	0.398	0.114	1.63170150781541E-213	5	CPB2
**0**	1.51804699160341	0.693	0.233	0	5	CSRP1
**0**	1.51684675757141	0.606	0.155	0	5	GALT
**0**	1.51490811297942	0.427	0.085	0	5	NFRKB
**0**	1.51296344716921	0.583	0.175	0	5	LPGAT1
**3.42819963245652E-176**	1.51261074230525	0.372	0.119	5.59173642049983E-172	5	PXDC1
**0**	1.51219033301903	0.736	0.228	0	5	PNPLA2
**0**	1.51176555246538	0.527	0.142	0	5	ADH1B
**1.61516790484286E-199**	1.50436769753691	0.366	0.106	2.63450036958919E-195	5	EHHADH
**0**	1.50239521046722	0.833	0.226	0	5	ALDOB

1.85058243515229E-204	1.50093944861634	0.367	0.107	3.01848500997689E-200	5	CRYZ
0	1.50060474950052	0.598	0.154	0	5	FAH
1.70405900570394E-123	1.50001806673686	0.278	0.091	2.77949064420369E-119	5	PIPOX
1.02670674424007E-142	1.4978651440575	0.256	0.071	1.67466137052998E-138	5	ANGPTL8
3.29124599964404E-168	1.49649117361336	0.308	0.09	5.3683513500194E-164	5	ACSF2
0	1.4963313546251	0.822	0.28	0	5	DCAF8
0	1.49367816635747	0.847	0.209	0	5	APOH
0	1.49307183085879	0.906	0.224	0	5	APOB
0	1.49245218423692	0.916	0.238	0	5	HPX
0	1.49184772841077	0.787	0.223	0	5	CERS2
1.53950977246449E-182	1.49122120897131	0.253	0.059	2.51109438986683E-178	5	SFXN4
5.91431021226807E-145	1.49054062338522	0.288	0.088	9.64683138723045E-141	5	FAM114A1
9.43133941686089E-211	1.48959482191689	0.362	0.1	1.53834577228418E-206	5	TFIP11
1.02302013033938E-128	1.48857100899543	0.27	0.085	1.66864813459657E-124	5	EVA1A
0	1.48673607624798	0.746	0.174	0	5	ITIH1
1.71433755326847E-194	1.48220668719121	0.38	0.115	2.79625598313621E-190	5	ACY1
2.1521683273503E-213	1.47747393041616	0.372	0.105	3.51040175874108E-209	5	ZCCHC24
1.59336114409706E-288	1.4716934970726	0.381	0.088	2.59893136213672E-284	5	KLHL20
1.06434351930267E-206	1.47121282764648	0.348	0.096	1.73605071433458E-202	5	PRPSAP1
3.17522291865518E-152	1.46750988683387	0.286	0.084	5.17910610261847E-148	5	TOMM40L
2.39264241779781E-116	1.46711985728115	0.259	0.084	3.90263904767001E-112	5	LGALS4
0	1.46657730541121	0.894	0.224	0	5	KNG1
0	1.46429873479348	0.839	0.197	0	5	ASGR2
1.59876536409617E-172	1.46342789752488	0.337	0.099	2.60774618537726E-168	5	SERPINA6
1.43220561864874E-231	1.46078928933787	0.324	0.077	2.33607058457796E-227	5	RALGAPA2
0	1.45930622256057	0.588	0.146	0	5	HABP2
1.25243328348672E-171	1.45522009147437	0.342	0.106	2.04284392869518E-167	5	LAMC1
0	1.45491619823249	0.576	0.163	0	5	ACAT1
6.42225544552414E-161	1.45378893312975	0.253	0.065	1.04753408571944E-156	5	PICK1
6.52463687675741E-151	1.45358246904115	0.328	0.105	1.0642335209679E-146	5	S100A13
2.59257880132545E-166	1.44869290674819	0.315	0.093	4.22875528284194E-162	5	RHOU
0	1.44659433656611	0.453	0.114	0	5	ZBTB20
1.50003958251435E-237	1.44631683072023	0.427	0.125	2.44671456303916E-233	5	IFRD2
7.04576253093293E-234	1.4461822560072	0.446	0.135	1.14923432642047E-229	5	SIK2
9.28340194643857E-207	1.44478364479012	0.417	0.13	1.5142156914836E-202	5	SHB
2.36401062806846E-118	1.44377225247651	0.285	0.097	3.85593773544246E-114	5	HAAO
3.00388031760663E-193	1.44372344141025	0.447	0.149	4.89962918604817E-189	5	UBD
3.68242364351542E-162	1.44122874237701	0.328	0.101	6.006401204938E-158	5	CEACAM1
4.23806806381682E-230	1.43944030765528	0.415	0.118	6.91271281889162E-226	5	UGT2B4
0	1.4391731682445	0.565	0.142	0	5	MYH14
5.68284706951856E-158	1.43818358385052	0.283	0.079	9.26929185509172E-154	5	LRMDA
2.27877728132476E-268	1.43730339975616	0.455	0.122	3.71691362356882E-264	5	SERPINA10
6.03246995728194E-175	1.43488714095349	0.311	0.089	9.83956174732258E-171	5	ZCCHC14
7.73329073278691E-202	1.43062012996698	0.337	0.09	1.26137705142487E-197	5	FLCN
1.36505526433622E-179	1.42736285376029	0.286	0.075	2.22654164165881E-175	5	CERS4
0	1.42640663442463	0.596	0.168	0	5	DEPP1
0	1.42571727819409	0.52	0.131	0	5	QTRT1
7.82447154189178E-144	1.42465289752613	0.263	0.074	1.27624955319797E-139	5	CLCN5
3.47808973522258E-268	1.42342614521062	0.551	0.175	5.67311216712155E-264	5	POR
4.09523530236256E-181	1.42339362667446	0.286	0.073	6.67973830168358E-177	5	SORBS1

2.4263116987771E-149	1.4132146213202	0.443	0.171	3.95755701187533E-145	5	ATF3
5.98704452819337E-175	1.41112340442845	0.288	0.077	9.76546832993621E-171	5	IFT43
2.47587053685891E-206	1.40710912921911	0.433	0.138	4.03839243267058E-202	5	TRIP6
1.29591521141952E-264	1.40677287357207	0.374	0.091	2.11376730134639E-260	5	PHKA2
0	1.39950306365512	0.426	0.096	0	5	PAN2
0	1.39895842898894	0.678	0.189	0	5	CEBPA
8.05861595588151E-290	1.39647552236376	0.476	0.131	1.31444084856383E-285	5	SPATA20
0	1.39456715863784	0.932	0.326	0	5	MT1F
3.24703932246297E-220	1.39304395176909	0.318	0.077	5.29624583886936E-216	5	THBS3
1.17956560904061E-183	1.39256351461245	0.3	0.078	1.92398946490614E-179	5	STX3
0	1.3904648356001	0.624	0.182	0	5	GPX3
0	1.38626266404243	0.777	0.195	0	5	HMGCS2
2.35074126980857E-228	1.38579551539584	0.321	0.077	3.83429408518477E-224	5	FBXO31
2.73295762110058E-104	1.38556460616901	0.256	0.087	4.45772717577715E-100	5	RARRES2
9.21975717553289E-127	1.38459854176443	0.296	0.099	1.50383459290117E-122	5	AMN
5.8463852612828E-190	1.38292656556836	0.325	0.088	9.53603899967838E-186	5	SNCG
1.56558694984348E-194	1.38056866634257	0.374	0.112	2.5536288738897E-190	5	GADD45A
5.47936779761416E-121	1.3792439788877	0.251	0.079	8.93739681468845E-117	5	P4HA2
1.10949782929823E-269	1.37774414563642	0.486	0.143	1.80970190936834E-265	5	NSFL1C
3.96685025152035E-276	1.37563393482917	0.594	0.197	6.47032944525484E-272	5	ACSL1
2.62480960434351E-127	1.37320776185957	0.291	0.097	4.2813269456447E-123	5	GPRC5C
0	1.37166304378351	0.87	0.234	0	5	C1S
1.33938400608782E-272	1.37009143479698	0.562	0.179	2.18466925232984E-268	5	IRS2
4.12488160203802E-177	1.36864426290727	0.334	0.098	6.72809438108422E-173	5	CTTN
5.88472910581018E-160	1.36617811663247	0.272	0.074	9.59858164448698E-156	5	SMAD5
9.01780539315558E-205	1.36179676151774	0.403	0.123	1.47089423767761E-200	5	PEX19
6.22002361555775E-163	1.35639242278161	0.272	0.073	1.01454805193362E-158	5	ZNF281
3.41455738337174E-159	1.34837290752488	0.329	0.103	5.56948454801765E-155	5	ACADSB
8.40504193386178E-264	1.3465295581164	0.425	0.112	1.3709463898322E-259	5	GTPBP2
0	1.34374144685882	0.688	0.236	0	5	NFKBIZ
8.02464118626107E-116	1.33760557957804	0.254	0.08	1.30889922389104E-111	5	CXCL2
5.09710586871925E-197	1.32890734632827	0.459	0.155	8.31388938246797E-193	5	ASPH
0	1.3283514106096	0.551	0.145	0	5	MGST1
5.68558789237947E-175	1.32650300962481	0.374	0.118	9.27376241126015E-171	5	MMP15
5.64982100763274E-153	1.32497397714165	0.32	0.1	9.21542304554977E-149	5	PPFIBP2
2.73516314854433E-125	1.32334109953748	0.286	0.095	4.46132461159065E-121	5	PCDH1
0	1.31937941001546	0.654	0.168	0	5	AZGP1
4.07519531871543E-234	1.31669407592357	0.445	0.129	6.64705108435674E-230	5	HAO1
4.94910541142139E-201	1.31416240559909	0.404	0.124	8.07248583656943E-197	5	AHR
0	1.31401476858084	0.554	0.149	0	5	GAMT
2.83889121789265E-248	1.31244380074385	0.407	0.109	4.63051546550471E-244	5	FOXN2
6.26259191050538E-261	1.31210747790255	0.353	0.083	1.02149136652253E-256	5	SLC25A42
3.27508231723512E-148	1.3105722738317	0.251	0.068	5.34198676764221E-144	5	BCKDHB
3.34743511121858E-102	1.30581106746095	0.263	0.093	5.46000140990862E-98	5	GLTPD2
1.84012961578542E-213	1.30153816947823	0.433	0.135	3.00143541630759E-209	5	KIF1C
1.58676431934909E-158	1.30051569566531	0.279	0.077	2.5881712812903E-154	5	PCBP4
0	1.29822006657136	0.565	0.164	0	5	SELENOO
0	1.29607337782419	0.657	0.167	0	5	TM4SF4
0	1.29102763475374	0.568	0.158	0	5	MIA2
1.62264401344684E-164	1.29087345413274	0.654	0.312	2.64669465033314E-160	5	TXNIP

2.77316372935208E-172	1.29077507658076	0.36	0.113	4.52330735894618E-168	5	TRIO
2.5673187194356E-291	1.28803922291963	0.631	0.204	4.18755356327141E-287	5	DHRS3
3.18878930729401E-209	1.28801984885039	0.266	0.057	5.20123423912726E-205	5	NBPF12
3.92685004150789E-216	1.2855871629394	0.705	0.295	6.40508510270352E-212	5	HSPB1
7.05556611080808E-140	1.28270619403408	0.257	0.073	1.15083338833391E-135	5	RRP12
1.29971149302272E-200	1.28264067698284	0.279	0.065	2.11995941626936E-196	5	SEPSECS
1.68570709525607E-216	1.2825913023732	0.308	0.073	2.74955684307217E-212	5	ZFAND1
4.91145848376804E-235	1.28195701896272	0.432	0.122	8.01107993287405E-231	5	HSD17B2
8.36539822367921E-234	1.28154426186626	0.314	0.07	1.36448010426432E-229	5	GABBR1
3.42380509481769E-205	1.2807341767668	0.313	0.079	5.58456849015713E-201	5	GTF2H4
6.71353877814531E-296	1.27707529084401	0.703	0.243	1.09504531010328E-291	5	ACAA1
1.67016842397531E-169	1.27526677000684	0.293	0.081	2.72421171634612E-165	5	PTS
3.06738195887762E-162	1.2747484944412	0.338	0.106	5.00320671312528E-158	5	GOT2
1.36087095102415E-204	1.27139452729869	0.33	0.087	2.21971660821548E-200	5	RANBP10
0	1.26836049939652	0.639	0.194	0	5	THBS1
2.53187035221494E-199	1.26698454567233	0.355	0.1	4.12973373149778E-195	5	ITPKC
2.53460887081917E-197	1.26222291417471	0.316	0.081	4.13420052919315E-193	5	KAT2A
6.81344868172378E-205	1.26092433196608	0.473	0.159	1.11134161447597E-200	5	PLOD3
7.0274063017882E-272	1.25828519255915	0.448	0.12	1.14624024188467E-267	5	ZC3H12A
4.97699840311136E-128	1.25721681917615	0.296	0.099	8.11798209531494E-124	5	TIMM17A
5.86036234530181E-165	1.2537145259853	0.413	0.146	9.55883702142179E-161	5	LTBR
1.00152258675987E-268	1.25169989418813	0.606	0.204	1.63358349126403E-264	5	CTNND1
6.54413656878578E-96	1.25148042490372	0.262	0.095	1.06741411573465E-91	5	ALDH4A1
1.56658857594149E-151	1.25101691023104	0.345	0.112	2.55526262621816E-147	5	PDZK1IP1
0	1.24986883640509	0.619	0.169	0	5	ADH1C
2.97072850015084E-212	1.24554320940814	0.337	0.087	4.84555525659603E-208	5	ARHGAP12
3.73056792105794E-250	1.24313470789073	0.419	0.116	6.08492933603761E-246	5	TFG
0	1.2418105460639	0.81	0.194	0	5	SERPINF2
7.87531360503023E-249	1.24025848895177	0.518	0.159	1.28454240211648E-244	5	QPRT
2.43191820536923E-157	1.23852535403222	0.346	0.113	3.96670178477775E-153	5	SLC50A1
3.81366622253179E-232	1.23594713089513	0.366	0.095	6.22047097557161E-228	5	SLC5A6
5.62672450686501E-250	1.22347734284657	0.413	0.111	9.17775034314752E-246	5	CRY2
2.61677533771238E-221	1.22182827624867	0.427	0.129	4.26822225334266E-217	5	SYVN1
2.34964320499316E-189	1.22043388596972	0.307	0.08	3.83250303166434E-185	5	PHLDB2
4.43343692773278E-289	1.21838463502644	0.377	0.085	7.23137897282493E-285	5	MCF2L
8.42215063643504E-116	1.21519045109391	0.252	0.081	1.37373699030892E-111	5	NFKBIL1
0	1.21176618196106	0.981	0.433	0	5	MT2A
4.50051141661908E-125	1.21165165284222	0.302	0.102	7.34078417164737E-121	5	GRAMD4
1.37006794551298E-233	1.20998630336465	0.461	0.134	2.23471782592622E-229	5	LSR
1.46073099872162E-206	1.20737289982582	0.514	0.18	2.38259833201484E-202	5	SCARB1
5.91856903639614E-188	1.2060086037082	0.266	0.063	9.65377795526575E-184	5	TMEM39A
9.21087981333208E-302	1.20454211962557	0.539	0.157	1.5023866063526E-297	5	SMG5
3.0308312242457E-105	1.2040989814049	0.28	0.101	4.94358880986716E-101	5	CITED4
1.86530359152175E-232	1.2036776343807	0.518	0.167	3.04249668813113E-228	5	HSP90B1
1.72179190128478E-123	1.20252293622126	0.252	0.077	2.8084147701856E-119	5	C1orf198
5.65579595081985E-146	1.2000109740367	0.344	0.116	9.22516877538225E-142	5	TSPAN13
2.83931144115619E-207	1.19974040171345	0.417	0.125	4.63120089166986E-203	5	G0S2
8.96190395182958E-188	1.19885871304885	0.35	0.102	1.46177615358292E-183	5	PLCXD1
1.86345294284912E-256	1.19552074512956	0.411	0.109	3.03947809508119E-252	5	FAM160A2
1.74866570700167E-225	1.19509673831089	0.449	0.131	2.85224863469042E-221	5	MASP2

6.04517435751714E-163	1.19320170863966	0.255	0.064	9.86028389454621E-159	5	ARFIP2
7.3180320196062E-292	1.19207143934402	0.53	0.155	1.19364420271797E-287	5	LPIN2
1.39653394077131E-258	1.19104565169015	0.534	0.164	2.27788651079208E-254	5	DEPRECATE
2.48397907239521E-234	1.1907662069659	0.337	0.081	4.05161826498383E-230	5	GPHN
7.63732012331489E-281	1.19020343667261	0.664	0.22	1.24572328531389E-276	5	XBP1
1.35675351265136E-176	1.18898009089661	0.367	0.113	2.21300065448564E-172	5	CHKA
3.88226773881567E-233	1.18783836221265	0.371	0.098	6.33236690878224E-229	5	HDHD3
7.9275712089393E-294	1.18751162247682	0.739	0.257	1.29306613989009E-289	5	SCP2
2.10930367969455E-172	1.18681507906236	0.308	0.087	3.44048523194978E-168	5	TP53BP2
4.95201815214157E-244	1.18587162672396	0.401	0.108	8.07723680795812E-240	5	MORC2
1.91504382980919E-124	1.18090672400624	0.322	0.115	3.12362799080177E-120	5	EBNA1BP2
1.09475237720847E-186	1.1802637775701	0.383	0.118	1.78565060246474E-182	5	MTHFR
0	1.1800256135606	0.926	0.287	0	5	FN1
1.09592565567379E-296	1.17865414941746	0.549	0.15	1.78756433696952E-292	5	SDC1
0	1.17722839588063	0.551	0.126	0	5	PATJ
1.06633891428422E-149	1.17679210147222	0.328	0.106	1.739305403089E-145	5	UGP2
0	1.17600831917408	0.842	0.307	0	5	C2
6.94592664259155E-147	1.17509072041889	0.323	0.104	1.13295009467311E-142	5	TMEM14A
4.81219657695999E-206	1.17455249083709	0.427	0.128	7.84917383667944E-202	5	GPX2
2.18317792071644E-164	1.17017770481016	0.347	0.109	3.56098150648058E-160	5	CAMK2N1
9.32099735190465E-154	1.16838354425052	0.372	0.125	1.52034787806917E-149	5	DEPRECATE
1.9488375171292E-239	1.16637794034278	0.312	0.07	3.17874887418943E-235	5	ABCA5
0	1.16402195865547	0.624	0.18	0	5	ATP2B4
1.88265612580987E-132	1.16251181958606	0.319	0.108	3.07080040680848E-128	5	CDHR5
2.73281744782639E-235	1.16205667758268	0.466	0.135	4.45749853914963E-231	5	HSD17B6
1.84190295519508E-118	1.16185566685319	0.302	0.104	3.0043279102187E-114	5	SERPINA4
8.9686444978692E-281	1.15921574362558	0.592	0.192	1.46287560404744E-276	5	COPB2
0	1.15899949815268	0.569	0.155	0	5	CHTOP
6.51878624669897E-204	1.15861765428263	0.251	0.052	1.06327922469907E-199	5	TRMT11
5.90550107318363E-234	1.15700418326007	0.385	0.103	9.63246280046982E-230	5	LSS
1.41633453733261E-265	1.15481279553971	0.548	0.173	2.31018326384322E-261	5	SNTB1
2.84739407122981E-183	1.15349257979701	0.455	0.157	4.64438446958295E-179	5	CXXC5
6.13365481705945E-214	1.15131621767666	0.452	0.14	1.00046043721057E-209	5	PGM1
0	1.15019601863825	0.819	0.29	0	5	MT1E
3.41625076049749E-106	1.14873320465298	0.286	0.102	5.57224661544746E-102	5	DEPRECATE
6.14926438697212E-282	1.14395503878119	0.456	0.122	1.00300651415902E-277	5	RB1CC1
3.51044396625057E-147	1.14115670379879	0.324	0.104	5.7258851533513E-143	5	LYPLAL1
8.68622313403893E-284	1.14108517867743	0.419	0.104	1.41680985539309E-279	5	SETDB1
1.53688538339658E-89	1.13872776829031	0.271	0.104	2.50681374885816E-85	5	CRYL1
3.31526992636236E-159	1.13745503179947	0.305	0.089	5.40753677688964E-155	5	MAFG
1.14316584623715E-149	1.13733277242408	0.318	0.099	1.86461781179742E-145	5	CDC42BPB
3.30807276081246E-117	1.13683812737031	0.293	0.102	5.3957974801612E-113	5	SLC25A1
4.69832531158559E-145	1.13629420040645	0.404	0.149	7.66343841572725E-141	5	HSPD1
2.49863568251214E-272	1.13471009160843	0.432	0.113	4.07552466174555E-268	5	KDM5B
8.30458562096667E-227	1.13425534550506	0.566	0.198	1.35456096063587E-222	5	NFIC
2.48020546917876E-167	1.1337439529945	0.304	0.088	4.04546314077748E-163	5	EPHX2
0	1.13339155116905	0.563	0.16	0	5	IMMP2L
9.7664837313492E-147	1.13267923010476	0.318	0.102	1.59301116142037E-142	5	KIAA1671
7.95848557727757E-75	1.13234609933514	0.36	0.169	1.29810858250974E-70	5	SDS
3.77863601040344E-172	1.13115150845594	0.399	0.133	6.16333319656905E-168	5	MSRA

2.67282482023838E-241	1.13099587501671	0.413	0.113	4.35964456429082E-237	5	ULK1
1.85207049790808E-157	1.12874224111854	0.342	0.109	3.02091218913786E-153	5	SLC25A25
3.19114911162235E-105	1.12735540763553	0.271	0.096	5.20508331596722E-101	5	CHPT1
7.76546934875349E-112	1.12686988636556	0.325	0.122	1.26662570547518E-107	5	HMGCL
1.04293917221022E-190	1.12641829366099	0.413	0.132	1.70113808379209E-186	5	GUCD1
3.62540998891814E-177	1.12617265850011	0.309	0.085	5.91340623292438E-173	5	PIEZO2
0	1.12384314028786	0.827	0.332	0	5	CDK5RAP3
8.22121338255774E-157	1.11867602222571	0.353	0.114	1.34096211482899E-152	5	SLC38A7
8.30009782618406E-265	1.11792438822294	0.443	0.119	1.35382895642888E-260	5	CSAD
6.77894427607394E-218	1.11628518398502	0.566	0.2	1.10571360087042E-213	5	CD151
1.2994917609585E-160	1.1146659142583	0.388	0.132	2.11960101129941E-156	5	NAPRT
1.99518451890995E-235	1.11453690754813	0.527	0.171	3.25434546879401E-231	5	ETFA
1.19028329105399E-287	1.11272801031287	0.686	0.239	1.94147107603817E-283	5	XAF1
0	1.10893919764806	0.981	0.315	0	5	C3
3.60191480931391E-215	1.10774886873591	0.277	0.061	5.87508324547191E-211	5	PLCH2
4.391817817948E-204	1.10744265123103	0.737	0.33	7.16349404285498E-200	5	KLF6
5.6200319039484E-201	1.10633282757431	0.297	0.071	9.16683403853023E-197	5	ZKSCAN8
1.34097887925172E-202	1.10582993746588	0.355	0.099	2.18727064994749E-198	5	ODR4
3.42942280567315E-169	1.10541659423042	0.308	0.088	5.59373153833348E-165	5	DVL1
1.27903455173986E-276	1.10505287527811	0.557	0.163	2.08623325734289E-272	5	TFPI
3.91014636595006E-178	1.10430009861684	0.446	0.156	6.37783973750114E-174	5	MGLL
2.80284146732164E-213	1.10426901130962	0.341	0.088	4.57171471734833E-209	5	KIAA0232
1.37350219723728E-184	1.10376521337215	0.312	0.084	2.24031943391373E-180	5	CTPS1
0	1.09972280506382	0.657	0.178	0	5	CES2
2.55279402164654E-162	1.09884943176441	0.331	0.103	4.16386232870768E-158	5	C11orf54
8.34185453942185E-244	1.09663820478073	0.36	0.088	1.3606398939251E-239	5	MSTO1
5.17641798292328E-293	1.08841998490846	0.612	0.177	8.44325537194616E-289	5	KRT18
3.46096796837113E-86	1.08830373507496	0.256	0.098	5.64518485321015E-82	5	PXMP2
1.46751723843838E-145	1.08741374587185	0.435	0.167	2.39366736761685E-141	5	RBM47
4.03631962101859E-130	1.08714700772209	0.288	0.091	6.58364093384343E-126	5	MYC
0	1.08573165157384	0.778	0.199	0	5	F2
3.51848932562454E-135	1.084008517738	0.33	0.113	5.7390079390262E-131	5	CDC42EP1
1.77978335664845E-202	1.08207128633168	0.5	0.168	2.90300463302929E-198	5	SEPHS2
2.25533048835531E-145	1.0808574360356	0.352	0.12	3.67866955955634E-141	5	COQ8A
9.2382124789376E-160	1.07912407996467	0.699	0.329	1.50684483743951E-155	5	SOD2
1.38993819890203E-213	1.07909860470189	0.469	0.142	2.26712819622911E-209	5	SERPINA7
0	1.07722049932796	0.633	0.168	0	5	PLG
0	1.07398028277221	0.599	0.179	0	5	MOV10
1.7744380474403E-241	1.07311042848581	0.405	0.111	2.89428589917988E-237	5	TRAF4
6.86066044335333E-305	1.07134133744126	0.487	0.127	1.11904232491536E-300	5	IP6K2
2.11866852198582E-226	1.06663363484659	0.443	0.133	3.45576022621107E-222	5	SLC25A44
1.13482250953189E-294	1.06400511139494	0.481	0.129	1.85100899529746E-290	5	TIMM44
2.45687677177897E-201	1.06199767660002	0.425	0.128	4.00741170244867E-197	5	NNMT
4.98688352351303E-103	1.06014479683695	0.274	0.096	8.1341057152021E-99	5	FNIP2
7.6210790412201E-201	1.05995075696482	0.346	0.096	1.24307420241341E-196	5	EIF2D
2.05446656720065E-154	1.05919170720738	0.363	0.12	3.35104041776099E-150	5	ANGPTL3
1.78679115079502E-260	1.05770721541139	0.452	0.127	2.91443504606176E-256	5	GSE1
0	1.05694530175815	0.615	0.183	0	5	SHFL
3.15237293231642E-166	1.05446328177156	0.387	0.124	5.14183548990131E-162	5	SERPINA5
5.47736280522852E-277	1.04628339259943	0.563	0.177	8.93412647160824E-273	5	MPV17

2.19587102358327E-208	1.0462735386678	0.397	0.118	3.58168522656668E-204	5	PI4K2B
3.06047882718482E-135	1.04296466617194	0.344	0.12	4.99194701502115E-131	5	MYO1C
6.12838483290056E-233	1.04234914018385	0.492	0.148	9.9960085009441E-229	5	STARD10
3.51967316309353E-187	1.04045752093543	0.374	0.113	5.74093889632185E-183	5	PWWP3A
1.75657115566455E-141	1.0401988982264	0.424	0.159	2.86514321200446E-137	5	ANXA4
1.22310912674094E-208	1.03929078086228	0.344	0.091	1.99501329662714E-204	5	FBXO9
7.41618701103511E-128	1.03858377236932	0.302	0.101	1.20965426336994E-123	5	TMEM106C
1.76513701929413E-186	1.03285101805094	0.438	0.145	2.87911499217066E-182	5	TOB1
2.70333648136932E-277	1.03121415370245	0.444	0.117	4.40941213476149E-273	5	MICAL3
6.06421106758007E-161	1.02644982815494	0.317	0.095	9.89133467232986E-157	5	OSBPL9
2.48446571951237E-216	1.02473299842723	0.439	0.137	4.05241203509662E-212	5	NNT
3.54947910232139E-90	1.02457503105103	0.283	0.111	5.78955536379643E-86	5	SDHC
1.89923062530683E-176	1.02367866473216	0.466	0.166	3.09783507293797E-172	5	ITGB5
4.12783802792802E-192	1.02246345637648	0.362	0.106	6.73291660735339E-188	5	ZBED6
9.44979179325347E-165	1.02143192814083	0.288	0.079	1.54135553939757E-160	5	FEM1C
3.3770097300467E-138	1.01993370276812	0.334	0.113	5.50824057067917E-134	5	TOLLIP
1.26377774673865E-190	1.01952161697056	0.402	0.125	2.06134788270541E-186	5	HES1
1.17645329635801E-84	1.0189112149443	0.261	0.1	1.91891297168956E-80	5	PROS1
2.33764112540394E-185	1.01727634081572	0.374	0.115	3.81292643964637E-181	5	HMGCS1
1.71006096337571E-226	1.01704208123199	0.401	0.112	2.78928043736212E-222	5	GBA2
2.05339725611633E-155	1.01495721996971	0.35	0.115	3.34929626445135E-151	5	HDHD5
1.15069485685089E-284	1.01201121893969	0.525	0.153	1.87689838100948E-280	5	PI4KB
6.23138646519705E-179	1.01082330652903	0.447	0.153	1.01640144633829E-174	5	LRP3
0	1.0105657443619	0.681	0.182	0	5	DHCR24
3.52023415335875E-211	1.00948898092316	0.515	0.168	5.74185392754345E-207	5	S100A16
1.53070138612008E-75	1.00884415339138	0.27	0.112	2.49672703090047E-71	5	SLC22A18
5.43846892274902E-269	1.00578908348068	0.637	0.22	8.87068665989593E-265	5	ZFAND3
1.57331202982903E-145	1.00547575585298	0.376	0.129	2.56622925185413E-141	5	MPC2
3.50726371046394E-302	1.00494900341678	0.573	0.169	5.72069783813773E-298	5	CALD1
0	1.00266474745985	0.921	0.252	0	5	PLA2G2A
0	1.00148874152201	0.658	0.212	0	5	PPP6R2
4.6929469792016E-211	0.995153477676699	0.44	0.139	7.65466581777572E-207	5	EDEM3
0	0.993189311487296	0.814	0.22	0	5	AHSG
5.11900335296394E-225	0.992371404800693	0.502	0.152	8.34960636901948E-221	5	DEPRECATE
8.16737330036843E-207	0.989211079275275	0.431	0.133	1.3321802590231E-202	5	TRIM25
3.0778662329003E-172	0.984598750122529	0.403	0.128	5.02030761248367E-168	5	GJB1
6.65740001239059E-197	0.983492497774205	0.588	0.228	1.08588851602103E-192	5	INF2
1.04220723715479E-245	0.977661925799621	0.486	0.146	1.69994422452317E-241	5	INTS3
2.95571259274912E-239	0.974995595821308	0.479	0.147	4.8210628100331E-235	5	TMEM263
7.71750854412642E-223	0.974186851522176	0.373	0.1	1.25880281863246E-218	5	ACBD6
1.9982880085741E-173	0.973854996569328	0.359	0.112	3.25940757078521E-169	5	IGSF8
9.32257900760919E-94	0.972387986570089	0.301	0.118	1.52060586193113E-89	5	MID1IP1
1.31491151176579E-188	0.972143024654999	0.335	0.094	2.14475216684119E-184	5	MTMR4
4.16828580556749E-88	0.970012639598695	0.267	0.102	6.79889097746114E-84	5	HACD3
1.01643240335058E-172	0.965817956239031	0.307	0.085	1.65790289310513E-168	5	FNIP1
1.66031489638337E-141	0.965724960847609	0.356	0.12	2.70813962749092E-137	5	OTC
9.64050228702575E-110	0.9648843391144	0.277	0.096	1.57246232803677E-105	5	ARHGEF12
8.35855888907143E-152	0.96435673212481	0.345	0.114	1.36336454039644E-147	5	APEX1
3.37441692389628E-206	0.964236749015656	0.436	0.137	5.50401144456721E-202	5	ESRRA
8.80410018041742E-231	0.964119767803867	0.474	0.148	1.43603678042788E-226	5	ECI2

7.34834164879339E-102	0.961664887289061	0.316	0.122	1.19858800633469E-97	5	CHP1
1.83334789027017E-188	0.960190889393287	0.296	0.075	2.99037374381967E-184	5	ZNF44
1.90148405643613E-180	0.959221823222905	0.285	0.073	3.10151064445298E-176	5	IMPA1
0	0.958537170611738	0.758	0.215	0	5	C1R
5.93616488866658E-163	0.954360497629542	0.439	0.159	9.68247854990405E-159	5	SLC66A2
3.99398659678901E-192	0.951264406178408	0.419	0.135	6.51459153802255E-188	5	AKAP1
4.04938992095887E-176	0.949211825894374	0.559	0.222	6.60495990007602E-172	5	TNFRSF1A
3.09205894907109E-146	0.948660259482255	0.268	0.075	5.04345735182986E-142	5	NAT9
1.49494137178424E-111	0.948101891663294	0.344	0.131	2.43839887151728E-107	5	DUSP23
9.24853785133898E-181	0.946820240932821	0.367	0.113	1.5085290089319E-176	5	UBA5
8.76900468000695E-146	0.946639518358473	0.381	0.129	1.43031235335593E-141	5	ADH1A
1.49790705093527E-101	0.94656102166809	0.263	0.092	2.44323619078053E-97	5	COMTD1
1.83931558816856E-134	0.944049653672753	0.261	0.075	3.00010765586173E-130	5	NBPF26
1.22435947318711E-278	0.94321061572007	0.625	0.208	1.9970527367155E-274	5	UBAP2L
8.4578479240674E-199	0.9422286065839	0.308	0.077	1.37955957489463E-194	5	PKD1
8.20853613509913E-120	0.939518969625056	0.266	0.086	1.33889432899602E-115	5	TOMM70
1.46408774920088E-203	0.937940428346781	0.443	0.143	2.38807352772155E-199	5	DCTN2
1.10090208444427E-212	0.937391755071158	0.549	0.194	1.79568138993705E-208	5	BCL3
7.30694875813837E-94	0.936387062791159	0.296	0.113	1.19183641193995E-89	5	APOC4
3.7260632347839E-110	0.936266240955245	0.266	0.09	6.07758174225602E-106	5	RFNG
1.37769090420188E-121	0.934828609152171	0.378	0.144	2.24715163384369E-117	5	PRXL2A
4.99252276398131E-195	0.934644177942753	0.556	0.2	8.14330388032992E-191	5	FUOM
1.41467341711971E-209	0.934644128032991	0.555	0.186	2.30747381066396E-205	5	ADI1
2.44887550396431E-136	0.93431680289783	0.318	0.106	3.99436083451619E-132	5	DEPRECATE
2.94848277938557E-183	0.932801734661424	0.396	0.126	4.8092702614558E-179	5	HECTD3
1.18642465298169E-144	0.932637738223225	0.27	0.076	1.93517725147843E-140	5	RECQL5
8.07309388368738E-214	0.931841732087019	0.439	0.137	1.31680234336825E-209	5	IBTK
1.34545531827597E-178	0.930143206823301	0.312	0.086	2.19457216963993E-174	5	ACAD11
3.27880407271446E-209	0.928357472373212	0.408	0.121	5.34805732300456E-205	5	SUN1
0	0.928214942172676	0.843	0.228	0	5	ORM1
3.96885997088525E-111	0.927839660500091	0.331	0.125	6.47360749851094E-107	5	ECI1
2.02224103101941E-134	0.926905405195132	0.257	0.075	3.29847734569576E-130	5	SUCO
5.83115791823836E-181	0.925455262843028	0.375	0.117	9.51120168043858E-177	5	PMM1
3.39315936398264E-214	0.924442037002737	0.487	0.161	5.53458223859208E-210	5	KLHL21
1.31800426170289E-128	0.923870294617808	0.25	0.073	2.14979675126359E-124	5	SMURF1
2.8576382883968E-186	0.922606771173625	0.518	0.191	4.66109381220402E-182	5	NADK
3.63651306095985E-158	0.921389792153013	0.351	0.112	5.9315164537316E-154	5	KIF1B
6.80308724366285E-148	0.912188507419119	0.259	0.071	1.10965156031385E-143	5	CPOX
1.25631330662528E-150	0.91157959892186	0.337	0.108	2.0491726344365E-146	5	DUSP10
4.00041112962293E-218	0.910672132606405	0.327	0.08	6.52507059352795E-214	5	POLE
1.47200061479669E-297	0.909725307852715	0.518	0.145	2.40098020279488E-293	5	TNKS
3.48796812932838E-267	0.90842641513471	0.635	0.218	5.68922481574753E-263	5	POGZ
2.21040529439701E-107	0.908152129675085	0.255	0.086	3.60539207569097E-103	5	SLC25A20
1.41394392607045E-90	0.908021790428144	0.279	0.108	2.30628393781351E-86	5	UROD
0	0.907940311075624	0.983	0.297	0	5	FGG
1.02941444872986E-175	0.907627339487826	0.42	0.143	1.67907790732327E-171	5	PGPEP1
4.35044613425953E-104	0.904907645610114	0.339	0.13	7.09601268959072E-100	5	METTL7B
8.10879123891008E-111	0.903754394132396	0.31	0.112	1.32262493897862E-106	5	APOA5
1.28513826393769E-126	0.903452003389226	0.275	0.088	2.09618902230877E-122	5	TSTD1
2.24056605641521E-244	0.900418132805195	0.346	0.08	3.65458729461885E-240	5	KIFC2

2.91157307189959E-179	0.899209949262785	0.27	0.065	4.74906683757543E-175	5	TBC1D22B
8.67621113286711E-152	0.898478458498939	0.337	0.108	1.41517679788195E-147	5	DLGAP4
7.04813701233811E-173	0.896878007874891	0.323	0.093	1.14962162808247E-168	5	DNAJC10
1.88203749908185E-220	0.896196784005487	0.456	0.142	3.0697913647524E-216	5	GNL3
1.56477547772954E-167	0.896116776665607	0.33	0.1	2.55230528172465E-163	5	BRPF3
6.07324552684258E-304	0.895715347807883	0.432	0.102	9.90607077883293E-300	5	GSDMB
4.85701836414447E-281	0.894025100958508	0.532	0.157	7.92228265375605E-277	5	VPS45
1.85786434968422E-105	0.893564650266496	0.313	0.117	3.03036254076993E-101	5	NIPSNAP1
5.93468489701385E-258	0.893478655064703	0.442	0.123	9.68006453551929E-254	5	LLGL2
1.73888292253867E-235	0.89232361013983	0.668	0.252	2.83629193495283E-231	5	TMEM140
3.93344412617799E-203	0.891932990794778	0.48	0.16	6.41584071420892E-199	5	FASN
3.01611833415308E-190	0.891767762499416	0.456	0.145	4.91959061483708E-186	5	AADAC
4.63906212840521E-212	0.891091151410003	0.409	0.122	7.56677423764174E-208	5	ARNT
1.15123923984438E-184	0.889598309213723	0.346	0.099	1.87778632411017E-180	5	DDI2
0	0.887122872459663	0.902	0.241	0	5	AGT
7.23177107159486E-142	0.886967396229123	0.392	0.14	1.17957417948784E-137	5	EGR1
6.78916240039593E-196	0.886621818285629	0.341	0.095	1.10738027912858E-191	5	SLC35D2
7.66590145516674E-139	0.885114990328768	0.379	0.131	1.25038518635225E-134	5	ANG
6.24127526800173E-186	0.884105844435189	0.525	0.186	1.01801440896376E-181	5	FURIN
1.12854005630413E-152	0.882961809459955	0.288	0.083	1.84076168583767E-148	5	BBC3
1.50891478832797E-224	0.882422098931595	0.615	0.227	2.46119091124175E-220	5	FNDC3B
0	0.882058535345758	0.719	0.195	0	5	LRG1
4.358254577902E-124	0.881872593557386	0.255	0.078	7.10874904201596E-120	5	NFS1
0	0.87761845179477	0.841	0.22	0	5	ITIH2
7.43469971265023E-231	0.877519461160228	0.442	0.131	1.21267387013038E-226	5	KPNA1
1.31597575197317E-134	0.876617442278135	0.454	0.182	2.14648804904344E-130	5	SREBF1
0	0.8754151603626	0.694	0.187	0	5	SERPINA3
0	0.874701428056206	0.913	0.315	0	5	MT1G
2.16100065635281E-271	0.874606158092276	0.55	0.169	3.52480817057707E-267	5	MTG1
9.88200299575471E-94	0.874467976717638	0.282	0.106	1.61185350863755E-89	5	NFIL3
1.3219715480941E-92	0.87404484934756	0.295	0.116	2.15626779209629E-88	5	LHPP
4.62926249261826E-104	0.873617507739827	0.269	0.094	7.55079005170965E-100	5	ACVR1B
9.13586174271535E-242	0.867171403886304	0.438	0.123	1.4901504088543E-237	5	TENT4A
1.06203354223666E-182	0.866260729123718	0.354	0.103	1.73228291074221E-178	5	ENOSF1
2.92661144975288E-122	0.866234542838366	0.377	0.144	4.77359593569193E-118	5	NAB2
7.17410852698217E-142	0.863096490967242	0.345	0.115	1.17016884183606E-137	5	FGD4
2.41336895094716E-202	0.862835184308859	0.628	0.243	3.93644609588992E-198	5	GOLPH3
2.39100975574545E-182	0.862702097184052	0.461	0.16	3.89997601259641E-178	5	SLC23A2
7.65315334044721E-226	0.86233888382468	0.548	0.188	1.24830584136034E-221	5	LONP2
1.58017712624335E-232	0.859776854840393	0.443	0.131	2.57742691061553E-228	5	RGS14
8.03415807023834E-194	0.857466861816801	0.456	0.143	1.31045152283658E-189	5	MBL2
0	0.857140091102985	0.955	0.253	0	5	CES1
2.398316485411E-130	0.854578486548748	0.354	0.127	3.91189401935388E-126	5	LAMB2
2.84718296090463E-101	0.853522116009803	0.304	0.112	4.64404012753154E-97	5	RNASE4
0	0.851588236946152	0.683	0.192	0	5	GSTA1
2.06108954037346E-218	0.8491487432825	0.875	0.437	3.36184314930315E-214	5	CCNL2
1.36529103161903E-163	0.846423431230733	0.331	0.101	2.22692620167379E-159	5	DENND10
3.70935677382855E-171	0.841159099789685	0.401	0.131	6.05033183379175E-167	5	TBC1D8
5.93593264222598E-227	0.841132130632795	0.338	0.083	9.6820997327348E-223	5	HIP1R
2.29188325543903E-259	0.839901450972545	0.629	0.192	3.7382907779466E-255	5	KRT8

6.13966139456494E-143	0.838519581538583	0.26	0.072	1.00144017006749E-138	5	CCDC14
4.51746151009686E-232	0.836093447307796	0.519	0.17	7.368431469119E-228	5	NFX1
7.52742537889005E-107	0.83293496798025	0.333	0.128	1.22779835355076E-102	5	RETSAT
1.5390175937708E-119	0.830946422769922	0.354	0.133	2.51029159719955E-115	5	CARHSP1
1.10026952305792E-200	0.830376439683117	0.446	0.145	1.79464961905978E-196	5	LARP4
3.8867173406428E-182	0.828864210790364	0.334	0.094	6.33962465432247E-178	5	RXRB
1.40639391753577E-218	0.828791232447067	0.356	0.094	2.2939691188926E-214	5	VWA8
6.72346891920118E-170	0.818849193109426	0.474	0.166	1.0966650154109E-165	5	PCK2
1.14489140822211E-153	0.818236494527306	0.308	0.092	1.86743237595109E-149	5	ZNF146
3.3715094167135E-119	0.818134867673721	0.366	0.14	5.4992690096014E-115	5	PHC2
2.03422023410849E-219	0.816150273018503	0.342	0.086	3.31801662385436E-215	5	PITPNM2
4.06314113633526E-83	0.814399958372822	0.296	0.12	6.62738950747644E-79	5	MDK
1.01408351561552E-159	0.814003393527172	0.297	0.085	1.65407162232048E-155	5	XPO5
2.47952115724533E-170	0.812802875308998	0.831	0.422	4.04434695958286E-166	5	ZFP36L1
2.05071938466247E-243	0.804167592908802	0.642	0.233	3.34492838832295E-239	5	NPLOC4
4.85438723156244E-245	0.801396082908831	0.564	0.17	7.9179910134015E-241	5	HPR
4.48056528846587E-161	0.7995481094096	0.401	0.139	7.30825004201668E-157	5	NMD3
9.23804333374152E-138	0.796842706985463	0.44	0.167	1.50681724816658E-133	5	CAT
2.85779132719475E-118	0.796155123340535	0.388	0.152	4.66134343378735E-114	5	SIL1
6.83786687831873E-218	0.794858414885558	0.642	0.223	1.11532446652257E-213	5	GATM
5.06872528319285E-238	0.792009274154963	0.489	0.152	8.26759780941585E-234	5	KLHDC2
3.29951550190208E-181	0.789976398721482	0.346	0.101	5.38183973515249E-177	5	RAF1
2.08318821262964E-135	0.789083217782644	0.278	0.084	3.3978882936202E-131	5	SEC24A
6.70830210468201E-227	0.788905969564686	0.473	0.147	1.09419115629468E-222	5	TNRC6A
5.53439336694228E-194	0.788698615782453	0.695	0.275	9.02714902081955E-190	5	RRBP1
8.66726551234504E-251	0.788143349868571	0.792	0.285	1.4137176777186E-246	5	APOC1
1.02787724492026E-183	0.786716445956883	0.486	0.172	1.67657057418943E-179	5	POLD4
8.03772320049414E-232	0.786581177815233	0.636	0.233	1.3110330312326E-227	5	LIMD1
3.93617091474521E-201	0.783044748217077	0.815	0.352	6.42028837904091E-197	5	A2M
2.58824469938019E-196	0.781031186286812	0.385	0.112	4.22168592915903E-192	5	UVSSA
6.80304869114851E-178	0.780645826181124	0.694	0.283	1.10964527201323E-173	5	DEPRECATE
5.39795762434893E-100	0.779193686756007	0.35	0.14	8.80460868107554E-96	5	H1F0
2.90226680277079E-96	0.7743488549957	0.385	0.165	4.73388738199944E-92	5	ATP1B1
1.05028068849411E-163	0.774038702869404	0.341	0.105	1.71311283100274E-159	5	CCDC159
1.40794582934651E-76	0.770122587835461	0.297	0.127	2.2965004422471E-72	5	NME4
7.77055254574074E-219	0.768636758470322	0.447	0.136	1.26745482573577E-214	5	COG3
1.14667571217715E-105	0.767403835483397	0.258	0.088	1.87034275413215E-101	5	ACBD4
1.63851474617351E-196	0.764975544236355	0.563	0.208	2.67258140248361E-192	5	LGALS8
1.87687919584253E-163	0.764353642441061	0.293	0.079	3.06137765633875E-159	5	ZNF621
4.61016603513682E-160	0.762938221480442	0.351	0.113	7.51964181991166E-156	5	FBXL6
5.13731235601787E-145	0.762600578062771	0.33	0.107	8.37947018390075E-141	5	PANK3
8.54156515867237E-77	0.76168541382516	0.264	0.105	1.39321469303105E-72	5	SHBG
6.30513755237783E-77	0.76133781967315	0.304	0.13	1.02843098616835E-72	5	QDPR
0	0.751707869115783	0.709	0.198	0	5	SERPINC1
1.78505729736911E-106	0.751468379740393	0.275	0.097	2.91160695773875E-102	5	COQ9
4.21226803195069E-150	0.750060815757317	0.355	0.118	6.87063038691477E-146	5	ATF6
6.75309121252382E-195	0.749324560893779	0.765	0.308	1.10149670767476E-190	5	LMNA
9.20413910787821E-185	0.749180920291893	0.496	0.177	1.50128712988602E-180	5	ILRUN
3.48708152969874E-104	0.74909819162977	0.362	0.143	5.68777868309161E-100	5	ACAA2
5.1735776011412E-97	0.748382922183144	0.279	0.101	8.43862242522141E-93	5	NCOA7

9.14183520439507E-100	0.748150681760058	0.275	0.1	1.49112474018888E-95	5	SNX4
3.402546663565E-162	0.747901636210227	0.436	0.156	5.54989386294088E-158	5	ATP13A3
2.63630355304E-217	0.746925012799812	0.408	0.118	4.30007472536354E-213	5	DLG1
8.43153744532905E-183	0.744051096845817	0.384	0.12	1.37526807270762E-178	5	ASPSCR1
1.9061393892877E-128	0.741942328592282	0.257	0.076	3.10910395786716E-124	5	ARFGEF2
7.10091016711864E-162	0.74114771729949	0.348	0.109	1.15822945735872E-157	5	CD2AP
3.60256687281535E-173	0.740479898740416	0.511	0.182	5.87614682624911E-169	5	SLC9A3R2
1.55667635283636E-142	0.740399412741899	0.4	0.142	2.53909479911139E-138	5	DOCK5
1.09285858683571E-101	0.740354875494681	0.266	0.093	1.78256164098773E-97	5	MAPK6
3.11918220046483E-119	0.740090003808877	0.383	0.148	5.08769808717819E-115	5	CHCHD10
1.16600932727381E-165	0.739827264522768	0.364	0.116	1.90187781371631E-161	5	DHX16
0	0.739268208636473	0.973	0.318	0	5	FGB
2.44056063778605E-117	0.739125761413048	0.303	0.106	3.98079845629283E-113	5	SLC35B1
2.68566446864288E-55	0.735941501702567	0.265	0.121	4.38058731480339E-51	5	TMEM176A
4.45328957280543E-205	0.733231333349145	0.598	0.207	7.26376062220293E-201	5	ECHS1
2.12249268040546E-114	0.73215336384693	0.334	0.124	3.46199781100934E-110	5	MTHFD1
0	0.732121230357166	0.99	0.425	0	5	APOA2
1.42240026763978E-137	0.731646406798486	0.51	0.206	2.32007707654725E-133	5	NECTIN2
1.22201243880583E-209	0.731408908717896	0.455	0.143	1.99322448893619E-205	5	PIP5K1A
3.03546996243978E-257	0.730895398531544	0.61	0.185	4.95115505573553E-253	5	SPINK1
2.40564444938027E-194	0.730292167117893	0.732	0.299	3.92384666138416E-190	5	LRP1
0	0.729474465157807	0.976	0.311	0	5	FGA
3.8289316740462E-145	0.724222054253202	0.498	0.198	6.24537045353676E-141	5	GADD45B
0	0.723069062516355	0.896	0.308	0	5	EPHX1
3.89982178021219E-241	0.721454182549415	0.536	0.161	6.36099930570411E-237	5	HRG
1.0536700226817E-229	0.71816920831311	0.443	0.131	1.71864117399612E-225	5	ZBTB16
3.34399550096801E-116	0.712391538150412	0.265	0.084	5.45439106162892E-112	5	HOOK3
2.29317454970805E-153	0.709602889150519	0.381	0.13	3.7403970080288E-149	5	CCDC50
0	0.708975778829001	0.669	0.188	0	5	ORM2
0	0.704101413511896	0.879	0.27	0	5	GC
1.13715948729593E-235	0.70181208202245	0.557	0.163	1.85482083972838E-231	5	SERPIND1
4.36827274869786E-78	0.698949075349244	0.286	0.119	7.12508968040108E-74	5	SIGMAR1
4.52708658087541E-99	0.697610242128695	0.269	0.095	7.38413092206589E-95	5	LRRFIP2
3.16582218391193E-215	0.69356580980299	0.49	0.16	5.16377256417875E-211	5	MAN2A2
1.40455976589204E-100	0.691103833525984	0.362	0.146	2.2909774341465E-96	5	BEX3
3.55510825696161E-178	0.69010690230457	0.461	0.161	5.79873707793008E-174	5	IKBKG
2.8356275788254E-115	0.687474205003356	0.275	0.09	4.62519214382211E-111	5	MIB2
4.83212200376437E-75	0.684352214220769	0.258	0.105	7.88167420034006E-71	5	ESD
5.0740614386308E-102	0.682829833226605	0.334	0.132	8.27630161255069E-98	5	FLOT1
5.33194095113897E-87	0.680440747807018	0.299	0.119	8.69692888540278E-83	5	SLC43A3
2.31816961601996E-120	0.678963382717773	0.267	0.085	3.78116646069015E-116	5	TRIM24
3.23860416102855E-160	0.67879310047469	0.414	0.143	5.28248724705367E-156	5	NUMB
1.04749748049032E-118	0.676110189428887	0.406	0.158	1.70857314042776E-114	5	HTATIP2
4.70109329670716E-170	0.675769207012854	0.496	0.185	7.66795327625904E-166	5	DAP3
4.44388176800241E-78	0.67446735129012	0.302	0.129	7.24841555178873E-74	5	SLC4A2
9.8921937936374E-142	0.673395071911415	0.305	0.095	1.6135157296802E-137	5	DNAJC11
2.17115172722323E-91	0.673190895523431	0.258	0.095	3.5413655822738E-87	5	TMEM161A
0	0.67172410559336	0.962	0.282	0	5	TF
0	0.669329819358959	0.965	0.306	0	5	VTN
7.26959426747032E-177	0.668169415408451	0.399	0.129	1.18574352096708E-172	5	AGFG2

1.117655266567E-141	0.668168074290684	0.355	0.121	1.82300750529744E-137	5	ATP11A
5.37962847873801E-101	0.667696279513159	0.257	0.089	8.77471201166957E-97	5	CDC5L
7.15835590032164E-173	0.667558668133306	0.314	0.088	1.16759943090146E-168	5	WDR59
7.09568572441325E-209	0.665111811732856	0.442	0.135	1.15737729850904E-204	5	PPP1R12B
8.30860484003748E-168	0.661182248406934	0.355	0.11	1.35521653545851E-163	5	CBWD2
0	0.660503195033261	0.893	0.26	0	5	CLU
1.3634642921102E-111	0.652792867808426	0.375	0.149	2.22394660686095E-107	5	TNFSF10
2.1371688793225E-113	0.652729708628089	0.276	0.093	3.48593615906293E-109	5	DHRS1
3.00555481598908E-124	0.652708637097108	0.312	0.107	4.90236046035978E-120	5	VDAC3
6.70937119597646E-176	0.651346973790522	0.554	0.2	1.09436553577572E-171	5	ANPEP
9.7357656366149E-135	0.649484368179304	0.291	0.09	1.58800073298826E-130	5	ATXN2
1.54195899870533E-101	0.649293555459556	0.502	0.233	2.51508932278826E-97	5	CDKN1A
2.13640297134575E-136	0.647309277283003	0.438	0.167	3.48468688656205E-132	5	RUFY3
3.13680723514531E-129	0.647056543955953	0.389	0.146	5.11644628124551E-125	5	EIF4G1
5.08874920950519E-134	0.64607897748718	0.346	0.121	8.30025883562391E-130	5	DCAF11
2.47364772414287E-190	0.644874495999713	0.671	0.278	4.03476680284944E-186	5	SNX1
2.04391038552788E-95	0.642553020909398	0.344	0.139	3.33382222983453E-91	5	PEBP1
6.17863384426817E-115	0.642022445019856	0.448	0.189	1.00779696633858E-110	5	NFE2L1
3.4307911253971E-96	0.641867194582561	0.364	0.151	5.59596340463521E-92	5	POLDIP2
1.74592280297571E-187	0.639898773393178	0.426	0.137	2.84777468393368E-183	5	NPEPPS
2.41733990172498E-130	0.639713873236818	0.261	0.077	3.94292311370361E-126	5	ZNF76
1.34552019446059E-108	0.639604384154033	0.274	0.092	2.19467798918467E-104	5	PACSIN2
3.0006580760707E-126	0.638154293439693	0.261	0.079	4.89437338787893E-122	5	STRN3
2.55242162638986E-102	0.635238663765713	0.343	0.137	4.1632549148045E-98	5	SLC39A7
7.75081081746677E-183	0.634916723356921	0.382	0.116	1.264234752437E-178	5	IDUA
4.26194264572506E-139	0.632687594341346	0.384	0.139	6.95165464944215E-135	5	AP1M1
1.08501986475913E-97	0.630007764908981	0.252	0.087	1.76977590140862E-93	5	TSC2
2.35731493615449E-238	0.628792609206276	0.54	0.175	3.84501639236159E-234	5	ORMDL3
8.8549317437666E-68	0.623279668717129	0.289	0.127	1.44432791672577E-63	5	PNKD
2.53169918768963E-134	0.621926046636471	0.284	0.086	4.12945454504055E-130	5	POFUT2
5.9233603671411E-201	0.621087771884216	0.738	0.28	9.66159309484386E-197	5	ALDH2
6.55198811939339E-100	0.619656090306499	0.263	0.092	1.06869478215426E-95	5	GFOD2
1.43912040127535E-92	0.618015332915767	0.35	0.146	2.34734928652022E-88	5	GALNT2
1.8604040402454E-209	0.617779685881681	0.475	0.154	3.03450503004427E-205	5	RNF115
9.39135331202747E-84	0.617246227025021	0.323	0.135	1.5318236387248E-79	5	KYNU
1.9615746939263E-147	0.614018219184642	0.358	0.121	3.19952448326319E-143	5	ARHGAP1
1.93847354743035E-235	0.613467382188539	0.737	0.273	3.16184420321365E-231	5	IGFBP4
1.0302846164812E-101	0.613230490577908	0.299	0.109	1.68049723794248E-97	5	WDFY2
8.10361843927225E-101	0.612525631050375	0.406	0.168	1.3217812036297E-96	5	ZNHIT1
1.52885247713961E-217	0.612314282682722	0.593	0.213	2.49371127546242E-213	5	SETD5
1.41054002451365E-146	0.611480000530226	0.707	0.317	2.30073183398422E-142	5	PLXNB2
1.13277818837932E-128	0.61031821731497	0.619	0.28	1.84767450306551E-124	5	HSP90AB1
8.53492988102852E-122	0.608957238943246	0.438	0.175	1.39213241289456E-117	5	HAGH
2.66601978077744E-127	0.608023195210036	0.445	0.172	4.34854486442608E-123	5	DCXR
1.13711360802151E-65	0.607472027210732	0.271	0.118	1.85474600604389E-61	5	AIFM1
4.03672911218891E-139	0.607427945421327	0.493	0.194	6.58430885489133E-135	5	TXNDC5
1.39851597397146E-169	0.60672326190023	0.424	0.144	2.28111940514485E-165	5	SCRIB
8.87921560911758E-161	0.606050956226524	0.342	0.105	1.44828885800317E-156	5	NPHP3
6.8954951347696E-207	0.605411097520623	0.394	0.115	1.12472421143227E-202	5	CLSTN3
2.01295037353545E-144	0.60507448817603	0.476	0.187	3.28332335427367E-140	5	EPAS1

1.8239969339104E-164	0.601338702342167	0.388	0.126	2.97512139890126E-160	5	USF1
7.28553633258885E-132	0.601248552912918	0.325	0.111	1.18834383120857E-127	5	RNF103
1.73715893989923E-160	0.5998502480727	0.695	0.279	2.83347994686963E-156	5	IFITM3
8.48696316126997E-65	0.599795429909426	0.251	0.107	1.38430856123474E-60	5	PSMD4
4.94442448289213E-80	0.597800665031675	0.325	0.14	8.06485077404535E-76	5	CYB5R1
3.36246247942907E-77	0.59757960785058	0.316	0.138	5.48451255019676E-73	5	VARS
1.1719479493986E-121	0.591826545354094	0.549	0.236	1.91156430026406E-117	5	GPX4
1.70453332410258E-172	0.591081245551181	0.346	0.101	2.78026430494371E-168	5	RNPC3
6.17592871613438E-112	0.590677024306315	0.314	0.114	1.00735573288868E-107	5	PEX13
3.91425655435905E-173	0.59043285844603	0.636	0.263	6.38454386581505E-169	5	DPYD
4.38343586917911E-143	0.590292848907255	0.328	0.105	7.14982224621804E-139	5	ACAP3
2.40865774032427E-183	0.58990622049202	0.377	0.115	3.92876164024292E-179	5	SMAD4
4.27284779963338E-135	0.588873790251645	0.477	0.18	6.96944204598201E-131	5	PGRMC1
0	0.588327755356315	0.958	0.271	0	5	RBP4
3.73929749408835E-172	0.587792561741914	0.4	0.127	6.09916814260751E-168	5	IFRD1
1.31137184898453E-119	0.586387064537527	0.312	0.107	2.13897862287866E-115	5	HSD11B1
1.20379001718531E-139	0.585610031712856	0.599	0.251	1.96350189703095E-135	5	NDUFS2
3.22989338354505E-104	0.580490807781153	0.278	0.098	5.26827909790032E-100	5	TMCO3
1.41720761234599E-173	0.579191206950311	0.368	0.113	2.31160733649754E-169	5	RBCK1
1.27722131969507E-73	0.576991966571493	0.325	0.145	2.08327569455463E-69	5	CD59
2.15174818238828E-137	0.574328622272369	0.295	0.09	3.50971646029352E-133	5	PIAS2
6.81079067533256E-83	0.573403663991481	0.327	0.139	1.11090806705349E-78	5	CIAO2A
6.26272480699243E-131	0.573129790647809	0.613	0.268	1.02151304326853E-126	5	GRINA
5.8412437880701E-131	0.572982711965299	0.518	0.211	9.52765274272114E-127	5	RHOC
1.5737811312827E-130	0.571471314538387	0.368	0.134	2.56699440323521E-126	5	HGSNAT
5.90624570388975E-186	0.56936945605417	0.39	0.119	9.63367736761457E-182	5	ZFYVE27
3.14482282259231E-141	0.569135585247303	0.55	0.225	5.12952050593032E-137	5	SLC25A39
8.5462244502595E-149	0.568491602979016	0.389	0.136	1.39397467008183E-144	5	POGK
7.7537666259068E-171	0.566960543621649	0.399	0.131	1.26471687435166E-166	5	PRKAA1
8.43632823772257E-148	0.56506360146099	0.316	0.097	1.37604949885493E-143	5	CBWD1
3.05928990540594E-121	0.563904898553818	0.528	0.23	4.99000776470762E-117	5	CTSA
2.4398635290853E-182	0.562856053856825	0.536	0.2	3.97966140229103E-178	5	LPP
1.05539742881306E-192	0.561415747924396	0.536	0.178	1.72145874613699E-188	5	IGFBP2
1.16227448240489E-77	0.56091864051064	0.625	0.341	1.89578590825061E-73	5	MT-ND4
7.08357363738484E-152	0.559147583638427	0.399	0.14	1.15540169599384E-147	5	RSAD1
3.64634209845133E-73	0.558306525662798	0.304	0.13	5.94754859678396E-69	5	PCCB
2.29807481950477E-98	0.556524698001789	0.592	0.293	3.74838983809424E-94	5	MT-ND4L
1.77231508320045E-115	0.556335423701312	0.379	0.148	2.89082313220825E-111	5	NR1H3
5.72466964317705E-99	0.555869330491072	0.265	0.093	9.33750865498608E-95	5	GNL2
5.18571960339753E-182	0.555809607680646	0.521	0.189	8.45842724510171E-178	5	ZSWIM8
1.11433631350193E-133	0.555705801268063	0.6	0.249	1.817593960953E-129	5	TMEM176B
1.06484588525029E-140	0.55437126438595	0.374	0.132	1.73687012343174E-136	5	GOLGA3
1.40846780778169E-130	0.554010135260717	0.544	0.232	2.29735184127272E-126	5	GNPTG
5.62777156462371E-133	0.552080580865824	0.39	0.145	9.17945819905773E-129	5	CDC34
2.68779262661918E-178	0.550809432531391	0.525	0.196	4.38405855327855E-174	5	TRIM27
1.30134643490528E-68	0.54928989011808	0.353	0.167	2.122626169974E-64	5	MT-ND5
9.35180584097308E-143	0.549057995345159	0.497	0.199	1.52537305072112E-138	5	APOL2
4.89173172927922E-137	0.549027525289273	0.445	0.173	7.97890362362734E-133	5	RREB1
4.29500845240773E-159	0.546805756350436	0.35	0.109	7.00558828672225E-155	5	GTF3C3
1.36480049582898E-135	0.54619312153938	0.366	0.131	2.22612608874666E-131	5	MPPE1

5.89415182648216E-143	0.542133065277959	0.291	0.086	9.61395104417506E-139	5	MBD4
1.88802334195846E-138	0.539196750492347	0.349	0.12	3.07955487306844E-134	5	CARS2
0	0.538959839593914	0.917	0.307	0	5	APOC3
3.88376559584982E-78	0.537304155480861	0.258	0.101	6.33481006339064E-74	5	ITPRID2
2.26365943653832E-128	0.536634587246796	0.441	0.174	3.69225490693766E-124	5	DGAT1
8.94795972852041E-144	0.53643221150012	0.376	0.131	1.45950171131896E-139	5	DEDD
1.1457146841286E-118	0.535526773586838	0.422	0.17	1.86877522128216E-114	5	EGLN1
8.52745734200591E-118	0.533904488730588	0.399	0.158	1.39091356705458E-113	5	GPAA1
3.6388442540366E-97	0.531040542166366	0.271	0.099	5.93531886275909E-93	5	PITRM1
2.31684942103763E-106	0.530179300867462	0.294	0.105	3.77901309065448E-102	5	GOLGB1
1.28971373981856E-143	0.52970940673767	0.331	0.107	2.10365208101806E-139	5	SLC35A3
1.73206403229414E-226	0.528952099709198	0.632	0.228	2.82516964307497E-222	5	ASH1L
9.53096088677132E-120	0.528651810523271	0.25	0.075	1.55459503024127E-115	5	GTF2H2C
6.68607152029213E-150	0.527871833566045	0.269	0.073	1.09056512567485E-145	5	FBXO44
4.25298450975242E-148	0.527756140798881	0.504	0.199	6.93704303385717E-144	5	BACH1
2.5052794515873E-64	0.527561671151769	0.274	0.121	4.08636131348404E-60	5	JUP
2.50314156371183E-115	0.526486848552872	0.323	0.117	4.08287420457037E-111	5	NAA60
1.65064547936514E-142	0.526430301338153	0.571	0.24	2.69236784139248E-138	5	SLC40A1
5.68821643272241E-133	0.525762334566394	0.252	0.071	9.27804982341352E-129	5	MED24
1.83658304134521E-175	0.525631005480805	0.466	0.165	2.99565059873818E-171	5	CLINT1
4.10597883120176E-116	0.525505040052448	0.273	0.089	6.69726207157319E-112	5	MTMR12
8.55996034839293E-167	0.5245340140529	0.374	0.119	1.39621513242637E-162	5	ZNF33A
1.29892367979421E-189	0.524386071849313	0.449	0.148	2.11867441411233E-185	5	ZFR
1.82220123376698E-164	0.524262510545726	0.447	0.158	2.97219243239732E-160	5	USP12
1.22862400148947E-68	0.521726255564439	0.31	0.139	2.00400860882947E-64	5	TMEM37
6.56412657930622E-98	0.521358132590599	0.272	0.098	1.07067468635064E-93	5	SLC33A1
8.65877114264745E-180	0.51668295685104	0.502	0.181	1.41233216107723E-175	5	MROH1
2.40157299614026E-95	0.514463382975669	0.344	0.141	3.91720571400438E-91	5	ARL8A
3.75258382443788E-111	0.512463529856974	0.394	0.159	6.12083947604062E-107	5	EIF6
6.27447054418072E-152	0.511709343527975	0.363	0.12	1.02342889046132E-147	5	HECTD1
6.93884629036188E-107	0.509481803489304	0.353	0.139	1.13179521842093E-102	5	RING1
6.13547975108982E-102	0.509126682189506	0.285	0.102	1.00075810220026E-97	5	PLPP1
4.20011346667604E-89	0.508457153233189	0.369	0.157	6.85080507549528E-85	5	CYP27A1
3.3054703851736E-119	0.506483219360407	0.264	0.082	5.39155274525666E-115	5	HPS4
3.12012670800705E-130	0.505054517990904	0.319	0.106	5.08923867343029E-126	5	ZXDC
8.36033192793671E-126	0.505029459097797	0.312	0.105	1.36365374076576E-121	5	UBR3
4.9649722587102E-80	0.502615669033146	0.295	0.122	8.09836625118221E-76	5	MANF
1.66994128195406E-121	0.502558838151698	0.296	0.099	2.72384122499527E-117	5	PMM2
3.98795284577668E-85	0.50154706774884	0.258	0.097	6.50474988674635E-81	5	ZBTB18
1.11653398440319E-121	0.500936690161719	0.464	0.189	1.82117858196005E-117	5	MLF 2.00
4.07146657276549E-129	0.500138349258573	0.405	0.154	6.64096912683779E-125	5	TRAP1
2.50071318880501E-140	0.499640065992715	0.392	0.141	4.07891328225985E-136	5	MAFK
1.57133853839999E-86	0.49949444843244	0.315	0.13	2.56301028998422E-82	5	COMMD4
3.46829755240253E-55	0.49786495894204	0.282	0.134	5.65714013772377E-51	5	GBA
3.13232669311856E-107	0.496545633284991	0.31	0.114	5.10913806914568E-103	5	MFN2
2.67564300582732E-151	0.495206063291927	0.502	0.196	4.36424130680495E-147	5	TRIM26
7.60544853808654E-79	0.494877570045502	0.273	0.108	1.2405247110473E-74	5	ZNF516
6.7083302209446E-102	0.494111992828429	0.29	0.103	1.09419574233827E-97	5	PDE8A
2.85775151166426E-108	0.494094577030117	0.309	0.111	4.66127849067557E-104	5	ID1
6.35408943488892E-55	0.493440890254066	0.255	0.117	1.03641552772473E-50	5	PNPO

6.03162915924996E-92	0.49255195476918	0.369	0.156	9.83819032165261E-88	5	FKBP2
5.03553714224564E-150	0.491577740434478	0.315	0.094	8.21346463271686E-146	5	RNF138
1.1192990206282E-94	0.488620365737838	0.41	0.178	1.82568863254666E-90	5	QSOX1
1.5246034559421E-150	0.488061548092286	0.376	0.129	2.48678069698715E-146	5	PPP2R5A
4.1819437922192E-96	0.487693529245092	0.383	0.163	6.82116851948874E-92	5	ACBD5
1.17525852089748E-94	0.487635584139065	0.304	0.119	1.91696417343588E-90	5	ZNF444
9.45622671519354E-168	0.486819446563283	0.695	0.3	1.54240513951522E-163	5	CCNL1
1.49019791040063E-113	0.485300695742106	0.281	0.094	2.43066181165448E-109	5	ZNF680
4.16917466423065E-93	0.484512277962582	0.463	0.212	6.80034079482661E-89	5	AKR1A1
5.60531802735925E-148	0.48422179242615	0.519	0.207	9.14283423442566E-144	5	COPG1
5.67267683290865E-47	0.484108316641598	0.259	0.127	9.2527031821573E-43	5	HSD3B7
1.01002005877951E-49	0.480504523471632	0.268	0.13	1.64744371787526E-45	5	MT-ND1
5.416846064497E-170	0.479280308759955	0.438	0.151	8.83541761580106E-166	5	RIOK3
2.20612807502449E-74	0.478477586419841	0.323	0.143	3.59841550317245E-70	5	MGMT
1.93861505708898E-65	0.473962074684894	0.309	0.142	3.16207501961783E-61	5	FAM20C
4.91501046377064E-98	0.473325862291147	0.283	0.105	8.01687356745629E-94	5	SCAP
3.11103940750884E-117	0.472632896052354	0.432	0.176	5.07441637758767E-113	5	SLC38A2
5.8187948299103E-163	0.471409144305552	0.475	0.174	9.49103624706669E-159	5	OXA1L
1.3511816787919E-122	0.471398076286313	0.329	0.116	2.20391243627746E-118	5	ZNF316
1.65890778491939E-143	0.469906948306839	0.281	0.082	2.70584448798202E-139	5	DMAP1
5.16768229751873E-134	0.467454933103926	0.331	0.113	8.42900659548281E-130	5	TPM1
1.11178661861723E-142	0.46612334487399	0.353	0.118	1.81343515362657E-138	5	FNDC3A
8.27899220965741E-133	0.465543240777255	0.322	0.107	1.35038641931722E-128	5	AARS2
5.02416121056227E-193	0.46515979248486	0.395	0.117	8.19490935054811E-189	5	DENND4A
1.70513094868799E-124	0.461918562999391	0.296	0.097	2.78123909040497E-120	5	SEC24B
6.18689798716577E-138	0.46139258912849	0.467	0.184	1.00914493068661E-133	5	DTX3L
1.09557994645858E-155	0.458644353014736	0.413	0.144	1.78700045066859E-151	5	MKLN1
5.23828715090907E-144	0.458541435686759	0.461	0.178	8.54417017184779E-140	5	DPP9
6.98434067476958E-182	0.455112651995345	0.508	0.182	1.13921580746167E-177	5	DYNC1LI2
4.92201530039601E-104	0.453043627740113	0.286	0.102	8.02829915647593E-100	5	ZNF687
4.2400275594794E-110	0.447816894308039	0.374	0.149	6.91590895226684E-106	5	RMDN3
5.04668789275483E-100	0.447006923184976	0.29	0.107	8.2316526218724E-96	5	MRS2
4.68363967817819E-94	0.444987344670691	0.28	0.103	7.63948467907645E-90	5	ZMAT3
7.46019111080747E-120	0.444007180534949	0.328	0.117	1.21683177208381E-115	5	CCS
7.06036588506368E-78	0.443749560821008	0.251	0.096	1.15161627951274E-73	5	DAPK3
5.61282372167366E-125	0.443192563323772	0.335	0.118	9.1550767724219E-121	5	SORL1
9.66120748551638E-74	0.442004984510732	0.327	0.143	1.57583955296258E-69	5	GRHPR
5.06281328702824E-185	0.440193461341492	0.489	0.171	8.25795475247177E-181	5	NSUN2
1.24436825206993E-59	0.439590400641429	0.404	0.209	2.02968905595127E-55	5	MT-CYB
1.08226099651592E-114	0.437708123004933	0.335	0.122	1.76527591141711E-110	5	USP32
8.46796637279492E-72	0.435693044845688	0.351	0.159	1.38120999506658E-67	5	MGST3
1.31656838163598E-140	0.434772260446934	0.773	0.341	2.14745468728644E-136	5	SELENOP
2.63782889982261E-95	0.433420475969949	0.452	0.199	4.30256271850066E-91	5	CYC1
0	0.433413812816667	0.881	0.258	0	5	HP
1.9496985192891E-128	0.432918928964216	0.31	0.103	3.18015325481246E-124	5	PUS1
3.72611356686424E-156	0.43060623714805	0.35	0.11	6.07766383891227E-152	5	PCID2
4.7045092652141E-94	0.429591991220433	0.412	0.18	7.67352506249072E-90	5	TNS3
2.89455846657017E-124	0.426734472862744	0.699	0.305	4.7213143148226E-120	5	SERPING1
3.95207462347072E-80	0.426144014057864	0.273	0.108	6.44622891834309E-76	5	ARFGAP3
2.90280653827477E-103	0.425954953261089	0.365	0.148	4.73476774457998E-99	5	SCYL1

3.27244323504153E-71	0.425666486226029	0.279	0.117	5.33768216067625E-67	5	GLRX
6.14045387900609E-118	0.424911538885573	0.569	0.235	1.00156943220468E-113	5	ETFB
1.17991022729832E-115	0.422228775471581	0.266	0.085	1.92455157174629E-111	5	TTC31
3.36718338966237E-90	0.421978335698901	0.25	0.089	5.49221282687828E-86	5	ZBTB48
6.2685713488281E-128	0.421018323089763	0.382	0.142	1.02246667270735E-123	5	PARP9
1.48785954184144E-72	0.42029594402392	0.318	0.141	2.42684769869757E-68	5	KEAP1
6.65496009668916E-116	0.419810078516944	0.321	0.113	1.08549054137097E-111	5	TUBGCP3
5.60613678906752E-95	0.417990081433133	0.269	0.097	9.14416971664803E-91	5	ZNF326
3.2868814250469E-103	0.417804095548934	0.318	0.121	5.361232292394E-99	5	GMPPB
2.085404270654E-181	0.415833729014712	0.538	0.195	3.40150290586374E-177	5	SRRM2
2.7863627991231E-150	0.415184486036901	0.571	0.233	4.54483636164969E-146	5	NDUFV2
1.63075032161462E-104	0.414210284955551	0.387	0.16	2.6599168495856E-100	5	SEC61A1
5.10326300049255E-283	0.412164337639808	0.773	0.221	8.32393228010339E-279	5	AMBP
2.12942996246656E-48	0.407042781192534	0.377	0.204	3.47331321177921E-44	5	ICAM1
1.17481343145763E-127	0.406231321350995	0.38	0.14	1.91623818805054E-123	5	CLIP1
1.27693068511613E-58	0.405785193590934	0.348	0.172	2.08280164049292E-54	5	NCOA4
5.63410737787997E-106	0.403915530497671	0.415	0.174	9.18979254406002E-102	5	LONP1
3.98335653206999E-64	0.402329531298445	0.3	0.136	6.49725283945936E-60	5	ELL2
1.65464766577453E-74	0.401241597854265	0.297	0.127	2.69889580764484E-70	5	KRTCAP2
9.62301911432367E-173	0.400864109037821	0.574	0.224	1.56961064773733E-168	5	BSDC1
1.96008833972766E-156	0.399402750370673	0.513	0.199	3.19710009092978E-152	5	RAB11B
3.96418750868432E-93	0.399057927123617	0.282	0.106	6.465986245415E-89	5	ACBD3
1.30069300137286E-110	0.398206698543059	0.302	0.107	2.12156035453927E-106	5	SPPL2B
5.63315723859544E-128	0.396667065489099	0.551	0.236	9.18824277187303E-124	5	BAG6
9.48088456692143E-111	0.396530907096843	0.288	0.099	1.54642708171055E-106	5	PIGO
8.54653912833679E-135	0.396099635667509	0.485	0.197	1.39402599722301E-130	5	SND1
1.56882963852921E-55	0.390443330785858	0.285	0.134	2.55891802340499E-51	5	PINK1
3.24209101947688E-77	0.390373379024633	0.275	0.112	5.28817466186873E-73	5	NUDT14
3.12924847972237E-75	0.390275040828179	0.252	0.1	5.10411719527516E-71	5	COA4
1.54517056339068E-164	0.389920332476677	0.414	0.137	2.52032770594654E-160	5	CHKB
4.81857585041377E-293	0.388094452213228	0.973	0.415	7.8595790696099E-289	5	SERPINA1
5.36167643146145E-156	0.386282794521468	0.588	0.242	8.74543042735677E-152	5	SNRNP70
8.81489840157382E-138	0.385924496356531	0.288	0.086	1.43779807828071E-133	5	PLXNA3
1.89017370200056E-104	0.385922530713853	0.258	0.086	3.08306232533312E-100	5	TTC37
9.23854759375313E-46	0.385628842966735	0.597	0.361	1.50689949801707E-41	5	MT-ATP6
6.66481979645639E-117	0.385493205851947	0.296	0.101	1.087098757E-112	5	ZBTB37
5.75201493569362E-110	0.385203973452273	0.324	0.12	9.38211156160986E-106	5	HDAC5
8.94865227280797E-115	0.384540731284375	0.268	0.086	1.45961467221771E-110	5	ATG2B
5.69233385311998E-81	0.383718523210447	0.431	0.199	9.284765747824E-77	5	LAMP2
2.06906361434012E-102	0.383113186552628	0.298	0.11	3.37484966135018E-98	5	SEC63
4.98435739116782E-114	0.3823115904441	0.394	0.156	8.12998534073383E-110	5	SAR1B
2.36147842213838E-130	0.382168076843669	0.261	0.076	3.85180745434991E-126	5	ZNF91
5.76898380281741E-116	0.3815597391236	0.33	0.118	9.40978948077548E-112	5	AKAP8L
5.55573281185297E-183	0.381038309886209	0.592	0.227	9.06195578941337E-179	5	MAPKAPK2
3.72483122334536E-124	0.379259211211845	0.331	0.116	6.07557220839861E-120	5	CUL4A
2.16922531806106E-105	0.379087464877535	0.273	0.094	3.53822341628939E-101	5	ARFGAP2
1.16291219481978E-113	0.378424827630465	0.266	0.086	1.89682608097054E-109	5	SMYD2
5.36651343354377E-107	0.377705109936988	0.351	0.135	8.75332006145325E-103	5	MDM2
1.07851143599415E-120	0.376990363922194	0.337	0.118	1.75916000325005E-116	5	WWOX
2.92276098499944E-121	0.375447156203084	0.422	0.168	4.76731544263259E-117	5	RAB1A

9.75354376845195E-93	0.375184725570163	0.592	0.282	1.5909005240722E-88	5	BRI3
4.56399670077412E-74	0.374668150199406	0.295	0.126	7.44433501863267E-70	5	WSB2
6.3013489850856E-104	0.373887166245757	0.369	0.15	1.02781303295731E-99	5	HSPE1
1.88130811686924E-154	0.372596672299038	0.592	0.239	3.06860166942541E-150	5	APMAP
6.88061257459495E-83	0.372467979187613	0.273	0.107	1.12229671704218E-78	5	TIPRL
1.43631320072485E-163	0.371599432206303	0.544	0.212	2.3427704617023E-159	5	INTS11
2.16531356054667E-114	0.370995532378745	0.42	0.17	3.53184294860767E-110	5	TSC22D2
1.20556097079783E-98	0.37009373515977	0.303	0.114	1.96639049946834E-94	5	USP14
3.04203272719637E-83	0.369636565170417	0.258	0.098	4.96185958133E-79	5	RRP1
1.29489799262658E-77	0.367920543126746	0.418	0.193	2.11210811577322E-73	5	DDRGK1
2.17465601538714E-71	0.367563379472358	0.369	0.173	3.54708142669796E-67	5	SLCO2B1
1.4776263273476E-172	0.367312252858764	0.377	0.115	2.41015630253666E-168	5	WASHC1
5.81300884621051E-94	0.365931992731983	0.286	0.107	9.48159872905396E-90	5	UBE2J2
1.31915887088467E-133	0.365927435219258	0.337	0.112	2.15168003429999E-129	5	GATD1
6.0324217440877E-109	0.365750159608405	0.414	0.172	9.83948310678145E-105	5	NCSTN
2.14388557116499E-72	0.365142311896848	0.305	0.134	3.49689175512721E-68	5	SNRPC
1.89631575109877E-87	0.363107639691323	0.259	0.094	3.0930806216172E-83	5	DHX34
2.85317961758902E-114	0.361526864526507	0.328	0.119	4.65382127424946E-110	5	TRAPPC4
2.96722163377859E-83	0.360261832337416	0.3	0.123	4.83983520685626E-79	5	NCBP2
5.6247199034848E-61	0.359598615058839	0.274	0.124	9.17448063457406E-57	5	ZNF622
7.26531127883493E-98	0.357716191095693	0.351	0.143	1.18504492269077E-93	5	ITPA
1.85842531640972E-118	0.356678060185353	0.515	0.224	3.03127753359589E-114	5	COPA
4.8618398788769E-118	0.355865715528427	0.452	0.187	7.93014702643611E-114	5	CRELD2
1.1451992881812E-51	0.354889542783429	0.266	0.125	1.86793455895236E-47	5	HS6ST1
4.54262406674301E-87	0.353075337882491	0.257	0.094	7.40947411526452E-83	5	TRMT1
1.10481894955938E-104	0.353058023563805	0.388	0.159	1.80207018862631E-100	5	SPG21
3.13605330768646E-85	0.351942477003695	0.278	0.108	5.11521655016738E-81	5	SCRN2
5.02742333747839E-135	0.350711830723557	0.377	0.133	8.20023020576101E-131	5	CLASRP
4.10313092920342E-107	0.349745303569007	0.301	0.107	6.6926168586237E-103	5	NOP56
1.86112753849438E-103	0.349688700442112	0.394	0.164	3.03568512803819E-99	5	IRF2BP2
7.64678846148824E-56	0.349476672250867	0.295	0.14	1.24726766595335E-51	5	RNF5
7.50105737133934E-143	0.347844149782159	0.315	0.097	1.22349746783916E-138	5	FAM160B2
3.59819471080905E-113	0.347790178846984	0.304	0.106	5.86901539280063E-109	5	IPO9
7.53882631981994E-155	0.346674642769851	0.597	0.247	1.22965796102583E-150	5	MEF2D
4.39846449881256E-69	0.346656492134168	0.346	0.153	7.17433544401317E-65	5	SOD1
2.42137244396403E-104	0.346192878854252	0.282	0.099	3.94950059334973E-100	5	GBF1
8.58236283147007E-140	0.346188791658707	0.619	0.272	1.39986920144108E-135	5	BRD2
1.05145966530038E-115	0.345477178379215	0.323	0.116	1.71503586007145E-111	5	SERINC5
2.47421303558193E-77	0.344796653476487	0.461	0.214	4.03568888233768E-73	5	METTL7A
4.97075068257214E-42	0.344720950149486	0.27	0.135	8.10779143834342E-38	5	IER3
5.5660011439467E-62	0.344313963619625	0.284	0.129	9.07870446589146E-58	5	TSTA3
7.20484818713509E-122	0.342271042082898	0.358	0.132	1.1751827878036E-117	5	GNL1
3.96337782987867E-180	0.342220357064873	0.523	0.192	6.4646655783151E-176	5	MARCH6
1.61180463893876E-87	0.341299543614736	0.433	0.197	2.629014546573E-83	5	ARRDC2
5.6804177926862E-89	0.340611143777723	0.367	0.157	9.26532946165045E-85	5	MAGED2
3.94100716591359E-76	0.339437058910095	0.271	0.109	6.42817678832165E-72	5	CNN3
3.1835340758239E-69	0.338958385183641	0.289	0.126	5.19266243107636E-65	5	HADHB
2.71278929325701E-97	0.33845334451032	0.447	0.194	4.42483061623151E-93	5	TMCO1
5.0629135980735E-113	0.338442212905003	0.322	0.116	8.25811836981768E-109	5	GSK3B
2.02060077240585E-116	0.336571344015851	0.452	0.188	3.29580191987119E-112	5	ARRDC3

3.99560498305753E-64	0.335410385753877	0.319	0.149	6.51723128786514E-60	5	RNPEP
1.77513226393564E-152	0.335308741523124	0.599	0.249	2.89541823570543E-148	5	SRSF4
1.0350732952764E-119	0.335182817445766	0.415	0.166	1.68830805192533E-115	5	PDHA1
8.41455467961138E-277	0.335041082639725	0.964	0.402	1.37249801379141E-272	5	ALB
2.2613689434243E-113	0.334637998458433	0.43	0.178	3.68851888361938E-109	5	REXO2
2.94450725876905E-102	0.334580150834543	0.259	0.087	4.8027857897782E-98	5	E4F1
4.42855982735501E-82	0.331624578065246	0.407	0.18	7.22342393439876E-78	5	IFI27
1.98834043073487E-137	0.331497423812171	0.412	0.154	3.24318207657165E-133	5	TMEM41B
2.4195144089566E-53	0.331122120847706	0.3	0.146	3.94646995244911E-49	5	MT-ND2
1.94998069004449E-88	0.330674536036784	0.53	0.259	3.18061350353156E-84	5	BHLHE40
1.68656834454653E-69	0.330558098782267	0.254	0.104	2.75096162678985E-65	5	ACO2
8.80321274684231E-101	0.330337641629763	0.327	0.128	1.43589203113745E-96	5	UHMK1
1.37620024440711E-114	0.330038871869611	0.41	0.165	2.24472021865243E-110	5	DVL3
1.05742269725898E-97	0.328440541730223	0.531	0.234	1.72476216149912E-93	5	ST6GAL1
1.4126793484031E-28	0.328057640868539	0.885	0.637	2.30422128518029E-24	5	MCL1
2.51032838558379E-58	0.326316895028393	0.286	0.134	4.09459662972572E-54	5	SRPRB
3.52536630607809E-112	0.323571673917595	0.481	0.207	5.75022498184397E-108	5	ITPR2
6.59106036431644E-118	0.32340932458249	0.369	0.14	1.07506785602365E-113	5	PITPNB
4.68760509272343E-124	0.32326992156502	0.308	0.103	7.64595266674118E-120	5	NCK1
1.76459791762083E-137	0.32260813303274	0.513	0.209	2.87823566343133E-133	5	MAP4
2.71076811713312E-98	0.321060049331491	0.53	0.237	4.42153387585583E-94	5	HYOU1
2.93425255544081E-97	0.320611683496407	0.534	0.241	4.7860593431795E-93	5	CYB5R3
3.5934652772356E-121	0.320291168668254	0.333	0.117	5.86130121369898E-117	5	TPR
5.2933111150321E-100	0.319511671182558	0.349	0.141	8.63391975972886E-96	5	PTOV1
1.00725165049727E-148	0.316425676555017	0.521	0.207	1.6429281671261E-144	5	CELF1
4.33034589855751E-142	0.316416735223836	0.495	0.197	7.06322719513716E-138	5	UBXN4
1.51100483867897E-117	0.316348254007608	0.325	0.116	2.46459999236927E-113	5	MAP4K4
2.94154838853254E-82	0.316235889430943	0.34	0.147	4.79795957653543E-78	5	GOLIM4
7.37879415232547E-65	0.315265499788885	0.261	0.112	1.20355511418581E-60	5	PSMD7
1.70907237037561E-94	0.314139020414299	0.304	0.116	2.78766794331966E-90	5	RIPK1
1.72984712679105E-98	0.312763875028816	0.295	0.109	2.82155364850888E-94	5	GPAT4
8.54188200635351E-104	0.31103509780088	0.296	0.107	1.39326637405632E-99	5	ABCF3
5.14231028343176E-64	0.308357121969791	0.304	0.139	8.38762230330555E-60	5	ALDH9A1
1.4919019348699E-150	0.308274234314888	0.75	0.344	2.43344124596629E-146	5	MDM4
1.90637009089925E-103	0.307886512785543	0.286	0.1	3.10948025526577E-99	5	RYBP
1.2055900431554E-101	0.307292738059084	0.327	0.125	1.96643791939078E-97	5	ENTPD6
1.99637700176453E-121	0.30723110737666	0.314	0.106	3.25629052757813E-117	5	TRPM7
7.38279276105898E-131	0.305520294769428	0.613	0.273	1.20420732725633E-126	5	STAT6
8.54999448906935E-41	0.305487871671591	0.652	0.408	1.3945896011121E-36	5	MT-CO2
4.49100736323427E-73	0.304970424268338	0.263	0.108	7.32528211017142E-69	5	VWA1
1.0011394662585E-104	0.304565153846981	0.365	0.147	1.63295858341425E-100	5	OSBP
3.53464041647159E-124	0.303079121592128	0.284	0.09	5.76535198330682E-120	5	BRD9
5.27886150293712E-101	0.302796205292404	0.342	0.134	8.61035099744073E-97	5	MAVS
2.00435962250643E-94	0.302513779168947	0.254	0.087	3.26931098027024E-90	5	ERC1
4.4962415957956E-131	0.299879116734192	0.311	0.101	7.33381966690221E-127	5	SPATA13
2.45230271270731E-83	0.298656009652428	0.288	0.115	3.9999509546969E-79	5	SUCLG1
2.26787411374313E-141	0.293649756259553	0.337	0.11	3.69912946692642E-137	5	RAPGEF5
5.34187040331252E-100	0.292509164154921	0.437	0.191	8.71312481484305E-96	5	HEXA
2.4706810622514E-156	0.292073083140436	0.478	0.177	4.02992788063826E-152	5	ZNF644
5.17231776164504E-160	0.290475827830792	0.39	0.127	8.43656750101922E-156	5	AP1G2

1.1495944890567E-89	0.290121787434648	0.394	0.166	1.87510357110038E-85	5	PRDX2
7.3445159573106E-64	0.28992080640933	0.309	0.141	1.19796399779693E-59	5	RAB13
1.36279467477295E-84	0.289867320803251	0.251	0.09	2.22285439402215E-80	5	FER
1.74959665928696E-71	0.288094675604845	0.362	0.168	2.85376711096297E-67	5	CALM2
3.26161889919746E-53	0.288092401971789	0.307	0.151	5.32002658648098E-49	5	ABHD2
1.06313494129663E-107	0.288049816724594	0.377	0.15	1.73407940274894E-103	5	AFF4
4.87274297036019E-58	0.287491161640868	0.326	0.158	7.94793105895451E-54	5	CALU
4.45389784581014E-133	0.285221968804942	0.341	0.116	7.26475277630092E-129	5	CDC16
7.99361531336254E-115	0.284072353230043	0.296	0.099	1.30383859376256E-110	5	MTR
1.01794987228248E-105	0.283203192191787	0.255	0.082	1.66037803667996E-101	5	TRMU
1.65134703122573E-124	0.280730857291862	0.392	0.148	2.69351214263228E-120	5	EIF5
1.49943751546566E-54	0.280604874046529	0.261	0.121	2.44573253147604E-50	5	TIMM13
1.02821251498025E-86	0.28042554422192	0.407	0.182	1.67711743318428E-82	5	ODF3B
1.42127709101433E-83	0.278569081025908	0.409	0.184	2.31824506315347E-79	5	MAN1A1
2.05759049059515E-97	0.277591598769346	0.289	0.107	3.35613584920975E-93	5	CLCC1
1.58505503445479E-152	0.276310701105215	0.472	0.177	2.5853832666992E-148	5	STAU2
1.70394322234525E-99	0.276249943810612	0.348	0.139	2.77930178996734E-95	5	AUP1
1.78536082816209E-116	0.275015315185032	0.362	0.136	2.91210204681519E-112	5	CPSF7
9.09434787256224E-64	0.275011621316114	0.282	0.126	1.48337908149363E-59	5	IARS2
1.75884386823164E-101	0.273673876132511	0.389	0.162	2.86885023347263E-97	5	ATF4
2.69818069224707E-77	0.273467579577374	0.334	0.146	4.4010025271242E-73	5	HEBP2
4.07293010887311E-40	0.273088145546209	0.272	0.14	6.64335630058293E-36	5	GSTO1
9.58650392789724E-45	0.271791982692575	0.289	0.147	1.56365465567932E-40	5	NDUFS6
1.8930229893998E-130	0.27060576644174	0.463	0.186	3.08770979801002E-126	5	HGS
5.8598816568687E-143	0.269510166303339	0.639	0.276	9.55805297051854E-139	5	NOTCH2NLC
1.07402008620986E-72	0.268526402074195	0.265	0.109	1.7518341626169E-68	5	JTB
7.52787068091665E-126	0.268146950914116	0.301	0.1	1.22787098676432E-121	5	SFXN1
9.69911397321133E-152	0.266026138392433	0.408	0.141	1.5820224801705E-147	5	GON4L
1.1452398089561E-141	0.264629954098091	0.974	0.473	1.86800065238829E-137	5	APOE
5.41280459330794E-82	0.264159555094948	0.256	0.095	8.82882557214458E-78	5	GATAD1
2.24664581656368E-84	0.262706415584866	0.51	0.237	3.66450399139701E-80	5	BLVRB
1.34116744071342E-107	0.262121286600708	0.413	0.172	2.18757821254767E-103	5	PA2G4
6.61221224941169E-44	0.26114714705863	0.251	0.124	1.07851794000154E-39	5	ILVBL
8.37552632263843E-93	0.260866205094831	0.254	0.089	1.36613209848555E-88	5	MTOR
5.2162394672842E-143	0.260405195778856	0.342	0.11	8.50820819508725E-139	5	CCNT2
2.00419534778502E-76	0.259895706480334	0.38	0.174	3.26904303177215E-72	5	PFDN2
1.59581222675299E-143	0.258326383203523	0.381	0.132	2.6029293230568E-139	5	TAF11
9.15988641355271E-131	0.258092597836216	0.376	0.135	1.49406907291458E-126	5	VPS53
1.22414517448949E-105	0.257757369401259	0.433	0.185	1.9967031941098E-101	5	DEPRECATE
4.3484435334252E-97	0.257489290626413	0.286	0.103	7.09274624736985E-93	5	TARBP1
4.65017966666622E-82	0.257134431205614	0.254	0.095	7.58490805429928E-78	5	YKT6
2.25905478015583E-91	0.256392604923489	0.521	0.248	3.68474425191218E-87	5	STAT2
3.23331868117467E-68	0.256181803489543	0.273	0.117	5.27386610086401E-64	5	PGAP3
8.31481812936578E-108	0.255533805463502	0.284	0.097	1.35622998508085E-103	5	ZDHHC8
1.39456131698232E-81	0.25533253742582	0.27	0.103	2.27466896412987E-77	5	TFE3
7.67421066082446E-270	0.255081779051694	0.756	0.24	1.25174050088708E-265	5	FABP1
4.12259581509968E-37	0.254380785316511	0.265	0.139	6.7243660340091E-33	5	ANXA2
7.6347497039321E-153	0.254159304364789	0.42	0.15	1.24530402420836E-148	5	ELP2
1.74660204366068E-51	0.253277856655065	0.275	0.133	2.84888259341494E-47	5	PGRMC2
4.00601961993252E-77	0.252634287031348	0.666	0.336	6.53421860207193E-73	5	PTMS

4.35694335421151E-128	0.251339777289469	0.391	0.146	7.1066103050544E-124	5	U2SURP
5.41710366458723E-67	0.250282536372108	0.344	0.16	8.83583778730823E-63	5	DEPRECATE
0	9.61951392079353	0.367	0.002	0	6	CLEC4M
0	9.39797504129931	0.436	0.003	0	6	STAB2
0	9.10245597737304	0.575	0.003	0	6	PTPRB
0	9.06854693055544	0.654	0.004	0	6	EFNB2
0	9.01050330314084	0.279	0.001	0	6	DEPRECATE
0	8.85640352372396	0.572	0.003	0	6	PCDH17
0	8.7346640319069	0.314	0.001	0	6	NOVA2
0	8.72447345947902	0.256	0.002	0	6	STC1
0	8.6512297702329	0.494	0.004	0	6	VWF
0	8.59751482628861	0.398	0.002	0	6	KDR
0	8.53759341379706	0.309	0.001	0	6	ARHGEF15
0	8.51788559920429	0.494	0.005	0	6	FCN2
0	8.49838809818728	0.431	0.002	0	6	NOSTRIN
0	8.36018607186848	0.514	0.006	0	6	OIT3
0	8.303523285716	0.695	0.006	0	6	FLT1
0	8.20261510754433	0.455	0.004	0	6	RASIP1
0	8.12426238989049	0.292	0.003	0	6	DLL4
0	8.05577047058289	0.303	0.002	0	6	TEK
0	8.05434106999171	0.502	0.008	0	6	PLVAP
0	7.98140476975005	0.363	0.003	0	6	PCDH12
0	7.97792591995352	0.516	0.004	0	6	ROBO4
0	7.87488353394872	0.373	0.005	0	6	CLEC4G
0	7.85536466302178	0.567	0.005	0	6	MMRN2
0	7.84043317304123	0.35	0.002	0	6	TIE1
0	7.74552518897372	0.268	0.001	0	6	SOX18
0	7.61978502303256	0.273	0.001	0	6	ECSCR
0	7.61976725915754	0.534	0.004	0	6	RAMP3
0	7.54352354782696	0.359	0.002	0	6	ADGRL4
0	7.50631786935024	0.504	0.014	0	6	SHANK3
0	7.50098940335234	0.463	0.005	0	6	EXOC3L2
0	7.42645364444587	0.698	0.02	0	6	FCN3
0	7.37785551490906	0.52	0.007	0	6	PDE2A
0	7.34798092995119	0.329	0.003	0	6	NPR 1.00
0	7.34729553214517	0.317	0.011	0	6	STC2
0	7.32654104881496	0.51	0.009	0	6	CRHBP
0	7.31535198117163	0.336	0.003	0	6	CLEC14A
0	7.27868084640609	0.391	0.004	0	6	CLDN5
0	7.27487477469971	0.781	0.019	0	6	DNASE1L3
0	7.21058717138763	0.52	0.015	0	6	SEMA3F
0	7.19800369189142	0.929	0.092	0	6	EGFL7
0	7.16184692563454	0.581	0.01	0	6	AFAP1L1
0	6.99007937962436	0.463	0.006	0	6	ESAM
0	6.92300220036324	0.417	0.006	0	6	HECW2
0	6.78524992238964	0.585	0.007	0	6	LDB2
0	6.72046548108535	0.397	0.005	0	6	KANK3
0	6.69100066701582	0.442	0.008	0	6	TMEM255B
0	6.67597460673075	0.265	0.004	0	6	LYVE1
0	6.63778379293753	0.311	0.007	0	6	ITGB3

0	6.52827047639902	0.311	0.004	0	6	LIMCH1
0	6.49694809934007	0.413	0.022	0	6	PLK2
0	6.40329038415898	0.775	0.033	0	6	HSPG2
0	6.37409463396232	0.325	0.004	0	6	ADGRF5
0	6.36182471307301	0.378	0.011	0	6	AKAP12
0	6.32408754881065	0.431	0.021	0	6	CCL14
0	6.32407151400087	0.273	0.014	0	6	SEMA6A
0	6.2717658639674	0.654	0.009	0	6	FLT4
0	6.1680141611557	0.275	0.008	0	6	APOLD1
0	6.15371844877324	0.275	0.006	0	6	CAVIN2
0	6.06687732624267	0.4	0.027	0	6	ADM
0	6.02066003420531	0.506	0.038	0	6	FZD4
0	5.99528069180278	0.261	0.006	0	6	CALCRL
0	5.91394848095899	0.784	0.032	0	6	CDH5
0	5.90513840283956	0.311	0.008	0	6	APLNR
0	5.88266918057601	0.414	0.011	0	6	RNASE1
0	5.87074265383864	0.581	0.023	0	6	SMAD6
0	5.8680926234907	0.267	0.005	0	6	ARAP3
0	5.86443760066236	0.381	0.009	0	6	ARHGAP23
0	5.83463131325892	0.5	0.011	0	6	ADAMTS4
0	5.72736325697225	0.251	0.024	0	6	PODXL
0	5.70994741676957	0.317	0.01	0	6	GNG11
0	5.69484032019954	0.283	0.01	0	6	LAMA4
0	5.68609191874467	0.495	0.039	0	6	PPFIBP1
0	5.59827888203182	0.449	0.047	0	6	ARHGAP29
0	5.583244737832	0.377	0.015	0	6	EMP1
0	5.50231085986802	0.64	0.041	0	6	KALRN
0	5.4726280169739	0.598	0.068	0	6	HYAL2
0	5.42749810664024	0.357	0.027	0	6	SPRY4
1.42477360773706E-302	5.38239135797777	0.256	0.036	2.32394823157991E-298	6	CA2
0	5.36264359850204	0.462	0.01	0	6	MCAM
0	5.35247527892338	0.265	0.006	0	6	ADCY4
0	5.34547119858874	0.337	0.03	0	6	SPRY1
0	5.28418522977808	0.312	0.031	0	6	WWTR1
0	5.27887306714403	0.851	0.081	0	6	STAB1
0	5.25534773558046	0.325	0.039	0	6	TM4SF1
0	5.23134204175607	0.577	0.022	0	6	TCIM
0	5.20573325972477	0.268	0.022	0	6	KCNE3
0	5.20172138245199	0.29	0.032	0	6	PLXNA2
0	5.17054388658915	0.686	0.09	0	6	RAPGEF5
0	5.153281885077	0.416	0.061	0	6	GRB10
0	5.14630028740239	0.4	0.039	0	6	DOCK6
0	5.06559903347756	0.452	0.023	0	6	TINAGL1
0	5.04291493931439	0.268	0.019	0	6	EPB41L4A
0	5.00270330647736	0.884	0.196	0	6	PLPP3
0	4.98927948640287	0.324	0.014	0	6	CD36
0	4.9660255576683	0.51	0.074	0	6	LIMS2
0	4.93690191231988	0.273	0.006	0	6	ST6GALNAC
0	4.92970807546716	0.305	0.014	0	6	PALD1
0	4.8637243528921	0.832	0.083	0	6	ENG

0	4.79487352730396	0.463	0.083	0	6	MCF2L
5.80886577361193E-289	4.7831856633286	0.253	0.037	9.47484096333841E-285	6	PTPRG
0	4.78258227050724	0.265	0.012	0	6	PLEKHG1
0	4.77004873905073	0.451	0.057	0	6	SHE
0	4.73696821366625	0.365	0.071	0	6	GABRE
0	4.72616753956207	0.393	0.018	0	6	DCHS1
0	4.72253739526713	0.311	0.04	0	6	MID1
0	4.67333725745954	0.474	0.077	0	6	PIEZO2
0	4.67221399139903	0.867	0.053	0	6	NPDC1
0	4.62364707407527	0.507	0.032	0	6	FXYD6
0	4.61520244959288	0.295	0.022	0	6	ADAMTS9
0	4.59163545672536	0.397	0.023	0	6	TP53I11
0	4.5718854466838	0.342	0.028	0	6	DLL1
0	4.56695815597299	0.573	0.096	0	6	ID1
0	4.56530728153202	0.357	0.032	0	6	TMEM44
0	4.37799161907145	0.329	0.029	0	6	RASGRP3
0	4.35763887689884	0.654	0.056	0	6	CD93
0	4.29448655705649	0.434	0.083	0	6	EPHB4
0	4.26764280462591	0.321	0.046	0	6	PDGFB
0	4.21186331872725	0.524	0.053	0	6	NRP2
0	4.21060230040869	0.468	0.055	0	6	SMTN
0	4.19370500733859	0.831	0.054	0	6	COL4A1
0	4.17029634743963	0.288	0.016	0	6	PXDN
3.28874207415E-265	4.15095848425	0.257	0.041	5.36426719714607E-261	6	PEAR1
0	4.05148494680693	0.492	0.041	0	6	TCF4
0	4.0265937019528	0.704	0.149	0	6	APP
0	3.92917948313353	0.324	0.023	0	6	FSCN1
0	3.90989456889712	0.831	0.062	0	6	SPARC
0	3.81350500642227	0.413	0.096	0	6	PLPP1
0	3.80626013715626	0.844	0.045	0	6	COL4A2
8.71299582528951E-207	3.78402689050909	0.25	0.05	1.42117674906297E-202	6	DEPRECATE
0	3.76441361422366	0.256	0.033	0	6	PIK3R3
0	3.75174277881825	0.274	0.035	0	6	CAVIN1
4.70476199389428E-115	3.71622317591831	0.29	0.108	7.67393728824096E-111	6	VWA1
6.47760565653753E-284	3.70180449255781	0.345	0.073	1.05656225863784E-279	6	DLC1
0	3.7016921375215	0.332	0.038	0	6	PHLDB1
0	3.68551171400516	0.255	0.022	0	6	FGFR1
0	3.68376085671109	0.441	0.1	0	6	CNN3
0	3.68175723253506	0.437	0.032	0	6	LRRC32
0	3.67020012364997	0.265	0.016	0	6	TMEM204
0	3.66931476075005	0.434	0.076	0	6	PRCP
0	3.65179306945516	0.541	0.095	0	6	NID1
3.39585061624284E-266	3.62825547048406	0.275	0.047	5.53897194015369E-262	6	LIFR
4.3922381021109E-191	3.62347594353319	0.284	0.069	7.1641795683531E-187	6	EPHA2
0	3.59470090878982	0.522	0.064	0	6	GRASP
1.9675668259425E-244	3.59223326137024	0.317	0.07	3.20929824979482E-240	6	TRPM4
0	3.57870210667266	0.409	0.071	0	6	NAV1
0	3.57280764976297	0.252	0.03	0	6	FAM43A
0	3.56723267706978	0.396	0.051	0	6	ID3
0	3.56109451461036	0.472	0.122	0	6	TGM2

0	3.53678536134903	0.54	0.088	0	6	NR4A1
2.16685973100626E-199	3.53561199434059	0.392	0.129	3.53436490724431E-195	6	HES1
0	3.52198603420989	0.537	0.085	0	6	SCARF1
0	3.51932239425624	0.305	0.037	0	6	ZBTB46
9.62801152526823E-180	3.51220022900442	0.296	0.08	1.5704249598865E-175	6	TJP1
0	3.5118487279348	0.532	0.084	0	6	DYSF
0	3.50716266384462	0.426	0.067	0	6	SERPINH1
0	3.50610845024956	0.5	0.07	0	6	TIMP3
0	3.46567884477206	0.306	0.045	0	6	RGCC
0	3.44956654040174	0.39	0.056	0	6	MARCKSL1
0	3.42355533726626	0.283	0.035	0	6	LMO2
2.57104124936977E-183	3.40164400878228	0.341	0.105	4.19362538184703E-179	6	KIAA1217
0	3.36798250795445	0.265	0.029	0	6	LAMB1
0	3.36677290104205	0.564	0.111	0	6	NRP1
0	3.35287534360433	0.304	0.037	0	6	GDPD5
2.70547994971017E-154	3.34415962227849	0.287	0.085	4.41290834597225E-150	6	TSPAN9
0	3.3418921234915	0.289	0.041	0	6	PEAK1
0	3.34050427243536	0.467	0.05	0	6	ITPRIP
0	3.33209415041981	0.258	0.027	0	6	MEF2C
7.5169187859359E-160	3.2530596683512	0.281	0.078	1.226084623174E-155	6	IL1R1
0	3.24084673949468	0.48	0.07	0	6	ITGA9
0	3.21756277544164	0.694	0.301	0	6	IVNS1ABP
1.37770526189034E-188	3.20149560921221	0.339	0.1	2.24717505266934E-184	6	TNS2
2.1376860824751E-140	3.18657663649065	0.308	0.104	3.48677976912514E-136	6	MYO6
0	3.18402147054639	0.362	0.064	0	6	CD9
0	3.16353442595151	0.47	0.075	0	6	PECAM1
7.07789479423779E-291	3.15257998585368	0.359	0.075	1.15447541988813E-286	6	PALM2-AKA
3.18053654161711E-101	3.14876267983933	0.253	0.089	5.18777315303167E-97	6	LAMA5
6.32178964817957E-124	3.14594672326893	0.253	0.078	1.03114710951457E-119	6	SORBS1
1.57857104305399E-142	3.1397327043153	0.311	0.105	2.57480722832537E-138	6	NFIB
0	3.13630385090528	0.265	0.035	0	6	HIC1
0	3.084826068856	0.57	0.184	0	6	EPAS1
3.03930115985169E-146	3.07466526557127	0.311	0.103	4.9574041218341E-142	6	CTTN
2.28424948832278E-222	3.06795055238282	0.26	0.049	3.72583934040328E-218	6	RBPMS
0	3.06228440674012	0.349	0.061	0	6	UPP1
3.04658201065265E-165	3.06147251974363	0.287	0.078	4.96927991757554E-161	6	TNS1
1.36673266854359E-282	3.03073797806972	0.259	0.038	2.22927765566145E-278	6	DEPRECATE
1.63029147421124E-119	3.00648815379358	0.314	0.121	2.65916842358596E-115	6	JUP
1.11970438554055E-133	2.9877065193977	0.262	0.078	1.82634982325519E-129	6	PTK2
0	2.96902378766785	0.466	0.087	0	6	SEC14L1
1.065387587092E-128	2.92075619590531	0.264	0.081	1.73775369330577E-124	6	RILPL1
0	2.91836859015917	0.279	0.03	0	6	S1PR1
1.3630762531981E-144	2.89902509193238	0.282	0.084	2.22331367659142E-140	6	PHLDB2
7.49984011528781E-308	2.88187576071314	0.389	0.083	1.22329892120459E-303	6	PPM1F
0	2.88143862289243	0.363	0.049	0	6	IFI44L
0	2.85189764467609	0.308	0.035	0	6	NEDD9
5.06837222883981E-146	2.83987200687929	0.265	0.073	8.26702194246061E-142	6	STARD8
6.28075733772084E-217	2.83609337051991	0.403	0.126	1.02445432935565E-212	6	LAMB2
3.29936462108738E-118	2.8348585462321	0.29	0.103	5.38159363345562E-114	6	CDC42BPB
0	2.82398513255068	0.619	0.185	0	6	MYO15B

1.64211900971696E-251	2.81476335551914	0.315	0.064	2.67846031674934E-247	6	DGKE
2.09007818828211E-134	2.81450149257246	0.289	0.092	3.40912653290695E-130	6	EDNRB
7.50131862414721E-199	2.80658467942975	0.255	0.052	1.22354008078465E-194	6	FKBP1A
4.47153642996475E-156	2.80532967898359	0.363	0.131	7.2935230709155E-152	6	BCAR1
1.01898463294466E-133	2.78378084504385	0.324	0.118	1.66206583479604E-129	6	SHC2
1.71319339705764E-223	2.77504120002168	0.477	0.175	2.79438974994072E-219	6	INSR
0	2.75752830524313	0.458	0.074	0	6	ABCG1
1.39176329915687E-179	2.75038174380818	0.421	0.159	2.27010511725478E-175	6	NDST1
9.10542805941257E-258	2.73426833191645	0.525	0.186	1.48518637077078E-253	6	ARRDC3
0	2.69899699610161	0.64	0.162	0	6	PLXND1
1.84249827128963E-259	2.69070326656224	0.318	0.063	3.00529893030052E-255	6	SLC25A37
5.65770281005377E-156	2.68711955378929	0.259	0.066	9.22827905347871E-152	6	MAST4
1.44706560910467E-151	2.62976115384976	0.333	0.109	2.36030871501063E-147	6	DOCK4
0	2.61954765010233	0.686	0.252	0	6	BHLHE40
2.54179458446842E-257	2.60877214265189	0.35	0.076	4.14592114672644E-253	6	MRC1
0	2.58216185974831	0.505	0.092	0	6	DUSP5
7.11015792315666E-107	2.54138676451057	0.284	0.103	1.15973785884608E-102	6	NCOA7
1.89906076989765E-233	2.53620978441451	0.38	0.101	3.09755802178006E-229	6	PIK3C2B
4.61549951405995E-233	2.49478881453817	0.348	0.084	7.52834125738318E-229	6	MICALL2
0	2.46126908017801	0.589	0.184	0	6	SOCS3
1.09198372561109E-182	2.45420002488509	0.412	0.138	1.78113465484425E-178	6	GSN
1.92058019668563E-274	2.44957751032331	0.501	0.157	3.13265835881394E-270	6	ITGA5
6.23081915934902E-304	2.43757091816875	0.477	0.125	1.01630891308142E-299	6	IFI44
0	2.40398112026354	0.756	0.278	0	6	IGFBP4
5.4446089545297E-119	2.3843826757733	0.323	0.122	8.8807016657334E-115	6	MMP14
0	2.37838842667036	0.713	0.056	0	6	IGFBP7
2.18556573750485E-239	2.37506482433221	0.464	0.147	3.56487627444416E-235	6	ARHGEF7
0	2.3674791847008	0.499	0.129	0	6	PXN
1.46259949032832E-80	2.36099740238943	0.32	0.148	2.38564602867453E-76	6	EGR1
2.26316936047118E-264	2.36049108509122	0.324	0.062	3.69145554386454E-260	6	KSR1
1.26952330005386E-123	2.35933141957417	0.3	0.102	2.07071945471785E-119	6	DAB2
5.52063096855575E-96	2.35145082406275	0.282	0.109	9.00470117281128E-92	6	NDRG1
0	2.34747075105895	0.622	0.152	0	6	DEPRECATE
6.9252233082996E-63	2.34638154413468	0.27	0.131	1.12957317381675E-58	6	PLIN4
1.8820012670735E-302	2.31869356793619	0.402	0.085	3.06973226672359E-298	6	SNRK
0	2.31569883270635	0.275	0.033	0	6	SH2D3C
3.11786443322475E-140	2.31094793875535	0.265	0.073	5.08554867703289E-136	6	SEMA4C
1.56597027436913E-184	2.29019562726193	0.304	0.076	2.55425411452349E-180	6	TAOK2
4.42118764180326E-130	2.28455025430254	0.272	0.082	7.2113991625453E-126	6	TMEM184B
0	2.27469833764477	0.593	0.141	0	6	SLC2A3
0	2.2677446973095	0.544	0.162	0	6	STOM
1.21398509250638E-102	2.25859957421058	0.285	0.106	1.98013108438715E-98	6	PDE8A
1.31475545549094E-205	2.24172614244227	0.266	0.052	2.14449762345126E-201	6	DISC1
2.3522319590117E-166	2.22372068283515	0.322	0.093	3.83672554834399E-162	6	NLK
7.64049807842789E-60	2.21741990486873	0.287	0.149	1.24624164157237E-55	6	CD59
3.46067422673672E-175	2.20989511664118	0.271	0.063	5.64470573123027E-171	6	DOCK9
0	2.20951591398434	0.859	0.391	0	6	DUSP1
0	2.19841644002258	0.52	0.121	0	6	NOTCH1
2.88596830427699E-160	2.19558634154317	0.364	0.122	4.7073029011062E-156	6	HIPK2
5.01133874156621E-194	2.19530150441069	0.336	0.088	8.17399462136865E-190	6	RCN3

**4.93266640911742E-175**	2.17907480873875	0.535	0.235	8.04567217991143E-171	6	CTSL
**2.53424520630344E-157**	2.16790703774368	0.439	0.181	4.13360735600154E-153	6	IFI27
**3.20652090821415E-125**	2.11782669771074	0.423	0.192	5.2301562533881E-121	6	SLC9A3R2
**2.37176834722131E-46**	2.0950630111066	0.274	0.154	3.86859135115267E-42	6	SLC22A23
**2.19942998941232E-239**	2.09164334755518	0.351	0.078	3.58749025573043E-235	6	LPAR6
**3.22472384037642E-128**	2.08234386172987	0.389	0.153	5.25984705603798E-124	6	MS4A6A
**2.09724813506182E-229**	2.04995648522583	0.265	0.048	3.42082143309934E-225	6	C11orf96
**3.9072744519922E-215**	2.04629478298528	0.474	0.159	6.37315535864448E-211	6	SH3BP5
**1.14187696190567E-93**	2.02598138863284	0.356	0.167	1.86251551256433E-89	6	AFDN
**1.07812629755344E-117**	2.02527682890368	0.441	0.219	1.75853180393941E-113	6	SHC1
**1.228599520495E-156**	2.02403332858281	0.402	0.144	2.00396867787939E-152	6	WARS
**3.12141277043723E-167**	2.02286651389618	0.296	0.077	5.09133636986016E-163	6	RGS3
**1.57967876581571E-163**	2.01052964025706	0.314	0.086	2.576614034922E-159	6	SMAD7
**8.78216968026135E-93**	1.99762050743995	0.315	0.134	1.43245969654743E-88	6	SELENON
**4.55053873663601E-202**	1.971525336895	0.395	0.114	7.42238373332699E-198	6	HMBOX1
**6.7617230654915E-40**	1.95914902788772	0.252	0.14	1.10290464921232E-35	6	NPL
**4.96922843989407E-107**	1.93533171416364	0.356	0.151	8.10530850831122E-103	6	SERPINB6
**3.94692900498439E-202**	1.92885094159965	0.29	0.063	6.43783590003004E-198	6	CDK17
**1.38264971679056E-46**	1.92723068204317	0.272	0.149	2.25523995305708E-42	6	CTNNA1
**7.74449916059234E-118**	1.92215676760935	0.456	0.214	1.26320525808422E-113	6	DDIT4
**4.18328711625797E-219**	1.91676719960162	0.505	0.167	6.82335961532837E-215	6	SGK1
**4.82273400626709E-52**	1.9140406316074	0.279	0.148	7.86636143762225E-48	6	PHC2
**1.46925550907792E-104**	1.90007808639392	0.267	0.09	2.39650266085699E-100	6	TTLL5
**3.68747363890551E-208**	1.86908166305292	0.462	0.147	6.01463825241878E-204	6	PLEKHO2
**6.92157448409772E-208**	1.86601978311736	0.443	0.138	1.12897801410118E-203	6	PGS1
**2.0218920595708E-218**	1.86408804686974	0.442	0.139	3.29790813836593E-214	6	TIMP1
**4.98530415405979E-123**	1.86277479215694	0.385	0.155	8.13152960568693E-119	6	UGCG
**1.48412786634456E-53**	1.85612998228862	0.293	0.157	2.42076096279462E-49	6	LIMK2
**2.76222271520251E-228**	1.85516521569732	0.545	0.196	4.50546147076681E-224	6	TGFBR2
**3.96100393291422E-96**	1.83074825021401	0.415	0.216	6.46079351497638E-92	6	ETS2
**6.19111181668694E-60**	1.82621058057477	0.283	0.14	1.00983224841981E-55	6	AGFG2
**2.26226669310579E-142**	1.82475424056449	0.275	0.074	3.68998320312486E-138	6	VASH1
**3.44802880767976E-178**	1.81838275487393	0.363	0.103	5.62407978820645E-174	6	DENND3
**1.55924471687625E-93**	1.81515874654846	0.416	0.221	2.54328405769686E-89	6	CTNND1
**6.31907856311705E-227**	1.80788912667115	0.385	0.095	1.03070490443002E-222	6	ARHGAP26
**2.69363958886103E-294**	1.78580394474243	0.858	0.492	4.39359553339123E-290	6	ARGLU1
**1.30984899059335E-97**	1.77769461174332	0.382	0.17	2.13649468855682E-93	6	FOS
**1.83511572999275E-72**	1.76676324979679	0.285	0.127	2.99325726719118E-68	6	SLC12A4
**2.28260194602627E-185**	1.73306032330584	0.256	0.052	3.72315203416344E-181	6	ICAM2
**6.12541876273663E-44**	1.7245967588675	0.321	0.199	9.99117054389971E-40	6	ADAM15
**3.69669994108838E-135**	1.71671367711814	0.294	0.088	6.02968727390925E-131	6	DMWD
**7.07579237026931E-101**	1.70978481463975	0.308	0.118	1.15413249351463E-96	6	TNFRSF10B
**4.96120867513435E-110**	1.70804535696476	0.383	0.167	8.09222747001164E-106	6	KLHL5
**7.1307034077106E-86**	1.70183422732497	0.256	0.094	1.16308903283168E-81	6	ASAP1
**1.16857866561979E-51**	1.6848433619472	0.301	0.166	1.90606866149243E-47	6	SPTBN1
**3.21377278473078E-264**	1.68416905169718	0.304	0.051	5.24198478917437E-260	6	SYNE1
**1.66047194852114E-184**	1.68368361392069	0.647	0.335	2.70839579523283E-180	6	JUN
**9.52076605739934E-167**	1.68080902830303	0.4	0.127	1.55293215162241E-162	6	TIMP2
**1.54282147542162E-127**	1.67826309787488	0.41	0.168	2.5164961085602E-123	6	CCDC85B
**1.25063214450718E-82**	1.6722017258705	0.299	0.127	2.03990609090565E-78	6	ECE1

1.26424808939484E-38	1.65363241145034	0.278	0.164	2.06211505861192E-34	6	ACTN1
8.26916959774656E-48	1.64806667922826	0.267	0.14	1.34878425308844E-43	6	CCDC50
4.95549371704563E-67	1.64023049471529	0.345	0.183	8.08290580187312E-63	6	S100A16
2.47911385451296E-86	1.6241240963075	0.297	0.121	4.04368260809609E-82	6	RGL2
3.61401879235748E-108	1.62199096381353	0.394	0.176	5.89482605221428E-104	6	CYTH2
1.37756538427971E-64	1.61329889960544	0.405	0.242	2.24694689829864E-60	6	CDKN1A
7.11178133968083E-172	1.60973797039628	0.56	0.253	1.16000265431534E-167	6	XAF1
7.63204417190005E-69	1.59762163060202	0.333	0.168	1.24486272487862E-64	6	TRIOBP
2.81477473900333E-119	1.57749835327661	0.321	0.109	4.59117907678832E-115	6	GBP4
1.00606389179865E-32	1.54767012504329	0.264	0.161	1.64099081391278E-28	6	APOL1
1.68339567522049E-74	1.5328744369305	0.362	0.184	2.74578668585214E-70	6	SLC38A2
1.89650887178022E-49	1.52454038970335	0.252	0.124	3.09339562076072E-45	6	KLF9
7.62215800359968E-129	1.51689307692739	0.533	0.273	1.24325019196714E-124	6	BST2
7.44315157953936E-86	1.51136819123993	0.339	0.15	1.21405245413867E-81	6	PML
4.34569011961277E-103	1.50620211816105	0.295	0.105	7.08825515410038E-99	6	CNST
4.04240657792482E-77	1.46615720070712	0.253	0.096	6.59356936925318E-73	6	OAS3
6.58587255047892E-35	1.4643411131975	0.299	0.183	1.07422167170862E-30	6	ATF3
2.03636334327263E-92	1.46117089482715	0.534	0.311	3.32151224921199E-88	6	HSPB1
2.48821281552024E-18	1.45080213396048	0.271	0.194	4.05852392339506E-14	6	DEPP1
4.25539491566361E-190	1.44346063343453	0.624	0.279	6.94097464693891E-186	6	FCGRT
2.6345662124815E-156	1.44125472997976	0.434	0.156	4.29724094917857E-152	6	CEMIP2
1.14475842421676E-26	1.38776828642577	0.256	0.164	1.86721546573996E-22	6	CALU
2.21806021845072E-141	1.38732592991524	0.548	0.251	3.61787802231497E-137	6	MARCKS
1.3989585745899E-56	1.37827974911675	0.374	0.224	2.28184133101359E-52	6	RHOC
4.71898463318038E-116	1.37198415813261	0.554	0.298	7.69713583518052E-112	6	IER2
1.11202521533591E-46	1.3641380193716	0.289	0.157	1.8138243287344E-42	6	MED10
8.29898145406119E-90	1.34522982609734	0.311	0.122	1.35364686497192E-85	6	TSPAN14
1.06205657124157E-46	1.34058682666108	0.256	0.127	1.73232047335212E-42	6	STRN4
9.59580136138767E-67	1.32809320250732	0.265	0.112	1.56517116005594E-62	6	HDAC1
1.44883448668431E-112	1.30969786482858	0.345	0.124	2.36319393123078E-108	6	CHST12
4.09721512442472E-40	1.30894805164285	0.339	0.22	6.68296758944917E-36	6	NECTIN2
6.28190014448913E-63	1.30032797733187	0.321	0.159	1.02464073256762E-58	6	TBCD
1.76418065074786E-157	1.29838189541484	0.453	0.163	2.87755505943483E-153	6	HDAC7
3.6342586706571E-57	1.29095617363021	0.32	0.162	5.92783931770879E-53	6	SH3TC1
5.81590608766446E-41	1.28187612746731	0.37	0.246	9.4863244195895E-37	6	INF2
1.11272594146507E-43	1.25868932443146	0.281	0.151	1.81496728312368E-39	6	PLSCR1
9.04215969442264E-59	1.23863935021791	0.4	0.239	1.47486666775728E-54	6	BSDC1
1.0603147507416E-184	1.23208401375805	0.664	0.272	1.72947938993463E-180	6	ITPKB
1.21644719094097E-86	1.22997185669159	0.425	0.216	1.98414701314381E-82	6	CDC37
3.68151226287732E-42	1.22202182341157	0.255	0.133	6.0049146519792E-38	6	ATN1
6.80350010458777E-19	1.18783813278325	0.253	0.177	1.10971890205931E-14	6	SLC23A2
7.03264887318217E-87	1.17865180873108	0.264	0.093	1.14709535770474E-82	6	CRIM1
1.57771517637841E-48	1.15632665417713	0.425	0.275	2.57341122419082E-44	6	HSPA1A
4.0103252276306E-122	1.15242935487459	0.322	0.102	6.54124147878827E-118	6	PLCG1
6.09408706402193E-16	1.14360625057672	0.259	0.195	9.94006541012617E-12	6	SNTB1
1.4471836636332E-42	1.14059144792125	0.354	0.217	2.36050127375211E-38	6	PARP14
3.75883891107676E-15	1.1381051028995	0.263	0.201	6.1310421478573E-11	6	MTSS1
2.23079304997668E-78	1.13242419901542	0.505	0.296	3.63864654381697E-74	6	IFITM3
4.85963770359762E-42	1.12267593602427	0.309	0.177	7.92655505833808E-38	6	GNAQ
6.16339753846949E-99	1.07624274462522	0.257	0.08	1.00531177249976E-94	6	DEPRECATE

1.55137778516509E-43	1.07054665891811	0.269	0.14	2.53045230538277E-39	6	CMIP
1.12012107033782E-18	1.06279114020668	0.284	0.211	1.82702947782801E-14	6	TXNDC5
3.89656747678293E-142	1.05284017874941	0.758	0.45	6.35569121138063E-138	6	CCNL2
6.55945989848087E-44	1.05253010285868	0.36	0.217	1.06991350404121E-39	6	QKI
1.47548694860224E-65	1.05135824922296	0.607	0.438	2.40666676186511E-61	6	ZFP36
1.03605102205218E-130	1.05080936623889	0.36	0.116	1.68990282206932E-126	6	SLC44A2
8.38062713408863E-46	1.04951645332438	0.321	0.18	1.3669640918412E-41	6	JMJD1C
0	1.04740403281539	0.37	0.048	0	6	COL6A2
1.92042319892909E-60	1.04374982540899	0.499	0.317	3.13240227977324E-56	6	HSPA1B
2.2682465196736E-33	1.04365268195139	0.271	0.158	3.69973689823961E-29	6	SIN3B
8.41196284554731E-29	1.03717906416424	0.265	0.164	1.37207525973722E-24	6	INTS3
3.37515111203166E-99	0.988686518421474	0.551	0.295	5.50520897883483E-95	6	MACF1
6.1909237043419E-56	0.984019807605046	0.255	0.112	1.00980156541521E-51	6	PRKD2
6.83648874171678E-17	0.983158893239151	0.26	0.186	1.11509967866142E-12	6	TFPI
1.72955889752454E-36	0.980045065740619	0.256	0.14	2.82108351775227E-32	6	TBC1D15
1.70957971014302E-57	0.972542275457774	0.453	0.282	2.78849546521428E-53	6	SPG7
1.61788081931076E-34	0.949078090936379	0.302	0.184	2.63892540437779E-30	6	IL6ST
2.44212385842656E-18	0.92991715268783	0.421	0.339	3.98334822547957E-14	6	CD14
3.46480919584225E-41	0.928708613557292	0.403	0.263	5.65145027933829E-37	6	PRKAR1A
1.09866392350969E-29	0.897345767672853	0.428	0.309	1.79203072563666E-25	6	NFKBIA
1.35636261670511E-39	0.8909074095768	0.435	0.302	2.21236306410771E-35	6	MYL6
6.39706778535294E-47	0.881681096044135	0.459	0.299	1.04342572646892E-42	6	MIDN
3.38656064850023E-110	0.879567589880788	0.557	0.269	5.52381907376872E-106	6	FAM118A
1.12170776074579E-27	0.87927238724094	0.25	0.147	1.82961752855247E-23	6	CENPT
1.96072337946059E-35	0.878835324306934	0.324	0.193	3.19813590423817E-31	6	TACC1
4.48576525020075E-32	0.86733757492588	0.27	0.156	7.31673169960244E-28	6	TTLL3
6.99371443905536E-37	0.856709123531126	0.467	0.332	1.14074476215432E-32	6	RHOB
2.1567825267148E-09	0.856351027125958	0.266	0.224	3.51792797932452E-05	6	CD151
1.23074876904459E-41	0.85031135074239	0.368	0.223	2.00747431718864E-37	6	PICALM
3.03454584680614E-17	0.835029021802629	0.296	0.221	4.94964773072549E-13	6	ADRM1
8.36218739662951E-47	0.83164314383016	0.421	0.265	1.36395638626424E-42	6	SRSF4
2.67982678659494E-27	0.828580070709504	0.321	0.209	4.37106547161501E-23	6	SBNO2
1.28693922091163E-36	0.812170239654546	0.311	0.182	2.09912656322896E-32	6	AKAP17A
5.35584467446686E-15	0.80805346519735	0.297	0.226	8.73591824852289E-11	6	AGRN
4.3061471743033E-13	0.792521072680171	0.301	0.236	7.02375665600611E-09	6	DEPRECATE
8.19244293721568E-20	0.785421231285242	0.354	0.267	1.33626936748925E-15	6	PIM3
1.7091765347479E-27	0.785314933451924	0.331	0.224	2.7878378458273E-23	6	UBR4
1.13315197621676E-17	0.78069815357359	0.266	0.189	1.84828418840715E-13	6	CRK
2.00278152377131E-19	0.768383685865682	0.284	0.2	3.26673694342339E-15	6	DYNC1LI2
1.0469024423824E-80	0.762685076918036	0.335	0.143	1.70760257376993E-76	6	ADGRG1
1.33961019861025E-56	0.747533428198826	0.426	0.234	2.18503819495318E-52	6	FOSL2
6.93500531468601E-13	0.727215974468844	0.281	0.218	1.13116871687844E-08	6	TRIM8
1.18785096490437E-10	0.693823002804485	0.273	0.217	1.93750370885552E-06	6	BACH1
9.33520839933809E-34	0.683653761340789	0.366	0.235	1.52266584201604E-29	6	MPRIP
3.37086484227123E-15	0.678779205530321	0.357	0.284	5.4982176442286E-11	6	SLC12A7
9.93165382722038E-09	0.674738100999315	0.265	0.216	0.000161995205575792	6	BCL3
3.23120282231533E-43	0.67239266070733	0.41	0.253	5.27041492347853E-39	6	LUC7L
6.96955386316098E-31	0.664643108609241	0.491	0.331	1.13680393062019E-26	6	LMNA
1.1886096042496E-17	0.663897291787765	0.345	0.266	1.93874112549153E-13	6	GOLPH3
2.97244419129967E-36	0.652975735759662	0.339	0.203	4.8483537204289E-32	6	UBA7

**5.21508858891298E-71**	0.643676885526978	0.29	0.113	8.50633099737597E-67	6	SFMBT2
**7.57819493168575E-08**	0.629758488063503	0.298	0.259	0.00123607937530726	6	CYB5R3
**1.38753280563276E-30**	0.619190715727221	0.315	0.193	2.26320475926759E-26	6	ASXL1
**5.04570517427417E-18**	0.615094724007245	0.321	0.237	8.23004970975859E-14	6	CALCOCO2
**1.13291030869538E-18**	0.607882759001499	0.342	0.257	1.84789000451303E-14	6	NPLOC4
**2.61005176392498E-47**	0.597412459313986	0.428	0.257	4.25725543213804E-43	6	FAM193B
**8.46155444255352E-66**	0.591935614821973	0.606	0.384	1.3801641451249E-61	6	LUC7L3
**6.34160840164662E-10**	0.587900222984782	0.268	0.216	1.03437974639258E-05	6	TRIM27
**4.66680294991345E-14**	0.577261059955498	0.299	0.226	7.61202229160383E-10	6	MAP4
**5.97629633033809E-27**	0.57641534088504	0.321	0.204	9.74793694441446E-23	6	KLC1
**8.95699578818451E-08**	0.561683899091859	0.293	0.253	0.00146097558301078	6	FNDC3B
**2.13321247550416E-12**	0.561552363996618	0.342	0.278	3.47948286879483E-08	6	TMEM140
**5.31553280201494E-10**	0.558940158463536	0.25	0.194	8.67016555336656E-06	6	HSF1
**6.41189202636734E-53**	0.555505368148254	0.32	0.15	1.04584370842078E-48	6	PITPNC1
**1.11694700376808E-08**	0.536220251382721	0.256	0.206	0.000182185225784611	6	MOV10
**4.47079232683535E-17**	0.531214728453017	0.319	0.235	7.29230936430114E-13	6	SETD5
**3.61003952613584E-14**	0.521483165627085	0.383	0.306	5.88833547108016E-10	6	ZYX
**2.75356820807347E-31**	0.519230049254986	0.42	0.285	4.49134510418864E-27	6	RBM6
**9.14437866296403E-40**	0.510821400828632	0.359	0.191	1.49153960371606E-35	6	CD4
**1.34310097517239E-30**	0.492989143671826	0.275	0.153	2.19073200060369E-26	6	ABCC1
**3.09945162802944E-13**	0.491587652088308	0.302	0.23	5.05551555047882E-09	6	RERE
**1.00837254944224E-19**	0.489786695803402	0.384	0.287	1.64475646539523E-15	6	MINK1
**1.60170112561066E-58**	0.474019849975949	0.55	0.283	2.61253470598355E-54	6	COL18A1
**2.97780373619621E-41**	0.467116815342083	0.551	0.382	4.85709567410964E-37	6	DDX5
**9.60337689645734E-07**	0.466711009830322	0.299	0.259	0.0156640680558116	6	MYO18A
**1.00618355622267E-08**	0.466368901249593	0.307	0.252	0.00016411859985548	6	ST6GAL1
**2.96854187294684E-27**	0.459849420256967	0.398	0.271	4.8419886489636E-23	6	JAK1
**4.94462403833789E-07**	0.452677810091791	0.308	0.263	0.00806517626893294	6	CSRP1
**1.53220606320671E-11**	0.44744653652818	0.262	0.197	2.49918130969646E-07	6	GPATCH2L
**8.58057859940433E-10**	0.437362901809158	0.25	0.195	1.39957817534884E-05	6	STAU2
**4.39066430561973E-12**	0.432541676114639	0.275	0.207	7.16161254889633E-08	6	FUS
**8.41482569448677E-11**	0.42855672153999	0.262	0.197	1.37254221902774E-06	6	PPP1R12C
**6.27772250781312E-11**	0.41745829724507	0.34	0.279	1.0239593182494E-06	6	GSDMD
**3.17187772387652E-46**	0.416041263549307	0.287	0.133	5.17364975541499E-42	6	CLEC2B
**9.89067773989345E-52**	0.414086599582075	0.772	0.583	1.61326844615402E-47	6	LENG8
**3.17515266368564E-07**	0.402695542334141	0.344	0.301	0.00517899150973764	6	HSP90AB1
**2.10344673345263E-20**	0.402374706937006	0.408	0.305	3.43093196693458E-16	6	ANKRD10
**4.98698055801121E-09**	0.397611488925732	0.315	0.257	8.13426398817209E-05	6	SLC3A2
**1.11840305785037E-06**	0.388279168644271	0.285	0.245	0.0182422722765974	6	POGZ
**5.14212115080815E-10**	0.381012822763603	0.25	0.19	8.38731380908317E-06	6	PNN
**1.69343142605297E-14**	0.362615919200616	0.266	0.185	2.76215599903499E-10	6	EHMT1
**1.77286983183839E-08**	0.3560148621603	0.284	0.233	0.000289172798271161	6	INTS11
**1.43793336616463E-39**	0.350154186306691	0.537	0.351	2.34541311355113E-35	6	MAF
**1.92493541170886E-06**	0.349900831580869	0.417	0.343	0.0313976215003832	6	VIM
**2.31586613983499E-08**	0.344260115240004	0.304	0.252	0.000377740926068485	6	FBXO7
**4.65160024109582E-12**	0.340147493941159	0.263	0.192	7.58722515325139E-08	6	TIA1
**2.81720848994237E-08**	0.323737267136952	0.415	0.355	0.0004595148767945	6	KLF6
**9.61578383808899E-10**	0.31885343939853	0.331	0.268	1.5684305018307E-05	6	PER1
**9.76786095090341E-14**	0.317609177413481	0.333	0.251	1.59323579970186E-09	6	SPTAN1
**1.01260750726134E-23**	0.316218157682544	0.548	0.416	1.65166410509397E-19	6	CFLAR

6.73192548362782E-94	0.316130028151586	0.505	0.202	1.09804436563453E-89	6	ETS1
2.12671333022436E-11	0.314207541867212	0.394	0.307	3.46888211292896E-07	6	DEPRECATE
8.79326077214523E-10	0.307117804593088	0.27	0.208	1.43426876454461E-05	6	ALG13
3.78085649252622E-07	0.307019989430849	0.37	0.322	0.00616695502495951	6	ITM2B
1.22793612856378E-09	0.292790483200065	0.281	0.218	2.00288661930038E-05	6	MARCH7
3.71706372268681E-11	0.286740124123903	0.273	0.204	6.06290263807446E-07	6	MAX
1.2770768072136E-56	0.272851646708667	0.379	0.176	2.0830399802461E-52	6	NLRP1
5.20931011190458E-13	0.269533023037139	0.321	0.241	8.49690572352755E-09	6	DDX39B
0	7.19454844910539	0.832	0.057	0	7	MARCO
0	5.37391165231946	0.903	0.095	0	7	CD5L
0	4.79773084787917	0.549	0.032	0	7	VSIG4
0	4.71593452689126	0.725	0.057	0	7	CFP
0	4.33957459221137	0.253	0.011	0	7	TMEM26
0	4.32717227250774	0.733	0.063	0	7	AXL
0	4.24748403216191	0.339	0.016	0	7	SIGLEC11
0	4.11932965866933	0.405	0.014	0	7	TIMD4
0	4.11088450533573	0.684	0.07	0	7	CFD
0	4.07684793951515	0.299	0.015	0	7	TRPV4
0	4.03975800537487	0.595	0.041	0	7	LILRB5
0	3.98103688469348	0.695	0.067	0	7	HK3
0	3.95442038068908	0.929	0.147	0	7	CD163
0	3.94253566791007	0.817	0.11	0	7	FCGR3A
0	3.92124463546723	0.708	0.064	0	7	FOLR2
0	3.88697782189194	0.92	0.185	0	7	SDC3
0	3.87271547431918	0.977	0.154	0	7	C1QC
0	3.78791597141743	0.96	0.17	0	7	C1QB
0	3.73721452580112	0.689	0.068	0	7	SIGLEC1
0	3.70749667660922	0.539	0.053	0	7	PLTP
0	3.69813530446309	0.955	0.162	0	7	C1QA
0	3.63573257845811	0.263	0.018	0	7	CETP
0	3.53850338279361	0.341	0.026	0	7	VMO1
0	3.37781525037955	0.818	0.194	0	7	HMOX1
5.50946051771216E-273	3.28659411002031	0.409	0.076	8.9864810504403E-269	7	PLA2G15
0	3.20986313264564	0.402	0.046	0	7	CALHM6
0	3.20580675693166	0.768	0.132	0	7	FCER1G
0	3.19731667564001	0.889	0.203	0	7	LIPA
0	3.16154311760026	0.725	0.114	0	7	TYROBP
0	3.15959020852954	0.454	0.049	0	7	LRRC25
0	3.15634651561641	0.587	0.089	0	7	ADAP2
0	3.14522310526341	0.801	0.123	0	7	CSF1R
0	3.13794030365024	0.418	0.049	0	7	MS4A4A
7.71792223888336E-233	3.13368834926376	0.305	0.047	1.25887029638426E-228	7	NCEH1
4.16157899637135E-247	3.12813849373434	0.41	0.082	6.78795150098131E-243	7	TCN2
0	3.11600388739057	0.889	0.264	0	7	PLD3
0	3.10894972109938	0.444	0.051	0	7	SCIMP
1.78693837108113E-287	3.10149686844824	0.251	0.024	2.91467517707043E-283	7	CMKLR1
3.76102272465355E-217	3.10128678112142	0.405	0.092	6.1346041661824E-213	7	PYGL
0	3.09700376185534	0.591	0.127	0	7	MCOLN1
4.89906583410277E-210	3.02222604512337	0.29	0.047	7.99086628200502E-206	7	RAB3IL1
0	3.0076237341085	0.549	0.074	0	7	MRC1

1.28983456027512E-239	2.92735018640648	0.259	0.032	2.10384915126474E-235	7	CCR1
1.41755823730959E-238	2.9066231563941	0.255	0.031	2.31217924087567E-234	7	DEPRECATE
0	2.88822258951142	0.609	0.095	0	7	CPVL
0	2.88651518631011	0.555	0.105	0	7	PDE4DIP
0	2.88540480366244	0.749	0.117	0	7	CYBB
6.70972645453921E-294	2.87334620509215	0.369	0.054	1.09442348199989E-289	7	SLC7A7
0	2.8713685289666	0.561	0.089	0	7	AIF1
0	2.87066240884584	0.806	0.177	0	7	CD68
2.70381786348833E-292	2.86694976361659	0.311	0.038	4.41019731713582E-288	7	FPR1
1.65358430628794E-287	2.85281159395542	0.524	0.12	2.69716136198625E-283	7	JDP2
0	2.85125192878141	0.67	0.153	0	7	MAFB
0	2.84736699813858	0.686	0.087	0	7	CXCL12
0	2.83568540374278	0.729	0.244	0	7	SLC40A1
0	2.83234093576571	0.475	0.064	0	7	BASP1
5.43243057758761E-283	2.81532717368695	0.333	0.045	8.86083751510315E-279	7	SIGLEC9
0	2.81329452735316	0.495	0.089	0	7	EPB41L2
0	2.80992439316361	0.654	0.101	0	7	VCAM1
0	2.78508424417008	0.84	0.277	0	7	FCGRT
0	2.78470044268583	0.499	0.079	0	7	RGL1
6.85464277453304E-263	2.78182932128214	0.661	0.232	1.11806078295408E-258	7	EXT1
2.38741399449246E-299	2.77441285291317	0.359	0.05	3.89411096641664E-295	7	RNF135
0	2.77367321749284	0.334	0.04	0	7	KMO
0	2.75457027053742	0.52	0.067	0	7	LILRB1
0	2.75182141094778	0.69	0.099	0	7	MSR1
6.9298846611021E-266	2.6897393520996	0.383	0.064	1.13033348707236E-261	7	SLC37A2
1.17525277456723E-172	2.67513809476418	0.27	0.047	1.91695480059661E-168	7	IGSF6
1.09779096681617E-166	2.66558065422231	0.322	0.071	1.79060684597385E-162	7	SMPDL3A
5.924314616081E-127	2.65119803764902	0.336	0.097	9.66314957028972E-123	7	SLC46A1
0	2.6301344109249	0.897	0.261	0	7	LGMN
2.03478423514644E-223	2.61930644337227	0.462	0.112	3.31893656594735E-219	7	DDAH2
0	2.61792292460803	0.944	0.369	0	7	CST3
3.78731430776479E-199	2.60562933716031	0.277	0.043	6.17748836739515E-195	7	SLC31A2
0	2.60012216113608	0.898	0.322	0	7	GRN
3.61617991585459E-275	2.59456945419965	0.601	0.166	5.89835106075042E-271	7	ABCA1
0	2.59086853381064	0.387	0.052	0	7	EPB41L3
0	2.57548291139188	0.436	0.039	0	7	ADAMTS2
1.35574372731929E-135	2.57041549128563	0.321	0.082	2.21135359363049E-131	7	SCARB2
3.79185162116211E-282	2.56741842554286	0.623	0.175	6.18488917927751E-278	7	TSPAN4
0	2.56547715505851	0.785	0.137	0	7	MPEG1
0	2.54170592887997	0.447	0.061	0	7	CSF3R
0	2.53745051730595	0.985	0.43	0	7	CTSB
0	2.51806817765605	0.653	0.117	0	7	KCTD12
3.76513931389715E-139	2.51594496441757	0.255	0.051	6.14131873489764E-135	7	SECTM1
7.08376450228853E-227	2.50070364586867	0.292	0.042	1.15543282796828E-222	7	C3AR1
2.47880739375661E-286	2.49866361249502	0.36	0.051	4.04318273995641E-282	7	FRMD4A
7.5867776358374E-237	2.48780146620788	0.597	0.18	1.23747930018144E-232	7	NPC2
0	2.47139279707828	0.598	0.12	0	7	SLC15A3
1.17236049974243E-279	2.47120196527953	0.694	0.234	1.91223721112988E-275	7	GAA
0	2.46051468556054	0.74	0.243	0	7	LGALS3BP
0	2.45882883776289	0.806	0.262	0	7	CTSS

3.17081504698883E-153	2.45225524398128	0.456	0.152	5.17191642314348E-149	7	NR1H3
5.76700787142236E-114	2.45137103200029	0.259	0.063	9.40656653907702E-110	7	SCN1B
0	2.45020249593653	0.792	0.15	0	7	FGL2
0	2.44990513395732	0.321	0.037	0	7	CCL3
1.27234579775364E-215	2.44915757841535	0.269	0.037	2.07532323071597E-211	7	NFAM1
1.44063159683832E-255	2.43925293905103	0.564	0.152	2.34981419760299E-251	7	NINJ1
0	2.43872581352921	0.882	0.3	0	7	CTSZ
1.56687613680679E-122	2.42068317093509	0.41	0.145	2.55573166674555E-118	7	FUCA1
1.03420273285646E-224	2.4206048250446	0.289	0.042	1.68688807756217E-220	7	IL18
0	2.40969864840483	0.964	0.393	0	7	PSAP
1.2455357471059E-180	2.40915741560455	0.326	0.066	2.03159335710443E-176	7	UNC93B1
5.89944361307128E-273	2.4044171072577	0.372	0.057	9.62258247728057E-269	7	HCK
0	2.39831032646964	0.692	0.119	0	7	MS4A7
4.41736472365881E-201	2.38922818696993	0.485	0.133	7.20516360075989E-197	7	RAB20
0	2.38382737759751	0.517	0.094	0	7	DYSF
0	2.37696354828729	0.784	0.159	0	7	SPI1
0	2.37138159664693	0.599	0.101	0	7	LILRB2
0	2.35597485369614	0.73	0.144	0	7	MS4A6A
3.8195622047612E-131	2.34937013480275	0.298	0.073	6.230087912186E-127	7	SNX10
7.87335124412908E-195	2.34665399030717	0.277	0.044	1.28422232142989E-190	7	LST1
0	2.34113168131504	0.936	0.333	0	7	IFI30
1.85305147110164E-112	2.323279711914	0.317	0.093	3.02251225451388E-108	7	ADAMTSL4
1.45164444244283E-142	2.30698653153183	0.297	0.068	2.3677772500685E-138	7	ME1
1.03867990361887E-160	2.2757283130452	0.3	0.061	1.69419079079274E-156	7	DRAM1
7.53542104833597E-147	2.27417644169156	0.272	0.055	1.22910252719408E-142	7	ACE
1.96371793892168E-243	2.25635092802473	0.392	0.07	3.20302033017515E-239	7	MRC2
4.79860750895162E-250	2.25604187582542	0.452	0.091	7.82700870785099E-246	7	FMNL2
0	2.25579695150752	0.818	0.335	0	7	GLUL
1.09144973389869E-255	2.24221246337074	0.318	0.045	1.78026366096215E-251	7	CCL4L2
0	2.22625189020955	0.964	0.46	0	7	FTL
1.51105745721698E-134	2.22416859171432	0.302	0.071	2.46468581846662E-130	7	ZNF385A
0	2.22154230214431	0.77	0.232	0	7	CTSL
0	2.21592242214256	0.599	0.113	0	7	PLEK
0	2.19193570792431	0.603	0.098	0	7	GPNMB
4.21534730171033E-177	2.1884829029547	0.525	0.173	6.87565298381972E-173	7	SLCO2B1
2.53786007629262E-216	2.18775260071763	0.323	0.053	4.13950357044089E-212	7	CSF2RB
0	2.17766843246143	0.867	0.368	0	7	CD63
1.30029200868471E-181	2.17765501919059	0.323	0.063	2.12090629536563E-177	7	MERTK
5.89084462823932E-179	2.17079388242891	0.567	0.201	9.60855667312116E-175	7	ICAM1
0	2.16851065923085	0.515	0.09	0	7	FGD2
4.06158902888E-121	2.16186221417305	0.407	0.14	6.62485786500616E-117	7	TMEM37
3.03366930631801E-135	2.16128488755625	0.399	0.118	4.94821800553531E-131	7	GM2A
0	2.15884561199606	0.566	0.098	0	7	C5AR1
7.69321969338337E-173	2.15001572126088	0.467	0.138	1.25484106418776E-168	7	COMT
0	2.14570808911205	0.721	0.18	0	7	CD4
2.56358078956761E-173	2.14031361166648	0.254	0.042	4.18145662586373E-169	7	S100A9
0	2.1389703370504	0.999	0.796	0	7	CD74
1.06479415985664E-122	2.1371144074393	0.298	0.074	1.73678575414216E-118	7	PPT1
2.50515329250598E-134	2.13004441104639	0.467	0.168	4.0861555354065E-130	7	CORO1B
0	2.12373855467298	0.493	0.087	0	7	SLC8A1

3.47589166489019E-110	2.10663039543326	0.401	0.142	5.66952689460239E-106	7	CYFIP1
6.43357630494244E-110	2.10538517960265	0.314	0.09	1.04938063109916E-105	7	RNF144B
2.12027404007869E-227	2.09683795088249	0.551	0.152	3.45837898677235E-223	7	MPP1
1.92235179086659E-85	2.09405478894868	0.298	0.098	3.13554800608249E-81	7	SCD
3.71028325319602E-154	2.08649703515831	0.38	0.1	6.05184301428803E-150	7	RASSF4
9.13326890245794E-224	2.08261865872286	0.633	0.211	1.48972749067991E-219	7	MFSD1
7.54070955320717E-121	2.04319279890166	0.297	0.075	1.22996513522362E-116	7	KLF4
2.43287877144895E-161	2.040522457368	0.555	0.205	3.96826856411038E-157	7	CXCL16
1.33030001404848E-278	2.0385806559742	0.863	0.413	2.16985235291448E-274	7	TPP1
4.8009248464131E-148	2.03820551782502	0.7	0.396	7.8307885169844E-144	7	WSB1
8.47701521987679E-207	2.02995163374436	0.62	0.215	1.3826859525141E-202	7	TGFBI
1.02025129301765E-185	2.02615708002995	0.392	0.089	1.66413188404108E-181	7	TLR2
2.36496937060961E-252	2.02388215543053	0.769	0.311	3.85750154040134E-248	7	LRP1
5.14741468248304E-229	2.02110362554561	0.406	0.078	8.39594808859809E-225	7	KCNMA1
0	2.01924099270947	0.494	0.06	0	7	CDH5
4.62364713783884E-156	2.00313641799997	0.534	0.19	7.54163084652893E-152	7	MAN2B1
0	1.99585372570858	0.662	0.142	0	7	THEMIS2
1.23525843534518E-174	1.98964628690609	0.437	0.117	2.01483003389152E-170	7	PRNP
5.25027585007702E-133	1.98405585959139	0.503	0.191	8.56372493906063E-129	7	GNS
5.96465537231036E-222	1.97243130060577	0.545	0.148	9.72894937777543E-218	7	DMXL2
5.48503572161525E-113	1.97222170356084	0.285	0.072	8.94664176552663E-109	7	LHFPL2
4.73489861673456E-141	1.95852924758373	0.38	0.105	7.72309313375573E-137	7	DAPK1
9.85291196480529E-130	1.95594643481007	0.255	0.053	1.60710847057939E-125	7	OTUD1
2.2816392278258E-96	1.95507518449016	0.354	0.123	3.72158174450667E-92	7	ACP2
5.02828205674451E-52	1.94914607331686	0.264	0.109	8.20163086275597E-48	7	CD276
3.31040623430028E-247	1.94695453275051	0.434	0.083	5.39960360876718E-243	7	CD72
9.03946717288802E-178	1.94662738318772	0.333	0.067	1.47442749056976E-173	7	HBEGF
0	1.94631614944079	0.59	0.125	0	7	CD83
7.146820089992E-160	1.93526432694815	0.425	0.118	1.16571782487859E-155	7	RASGEF1B
2.13534303401914E-174	1.91904946884833	0.648	0.272	3.48295802278862E-170	7	CEBPB
8.07872517408045E-242	1.91361561740684	0.79	0.326	1.31772086314426E-237	7	CD14
5.75053370419529E-151	1.90771144363561	0.38	0.096	9.37969552491294E-147	7	DPYSL2
9.89136657586188E-188	1.90650294371212	0.711	0.325	1.61338080218883E-183	7	RHOB
6.89602997404695E-264	1.90077661884736	0.482	0.094	1.1248114490668E-259	7	CLEC7A
1.55234889091875E-111	1.89404909090971	0.34	0.101	2.53203627597757E-107	7	RENBP
3.5576890800439E-119	1.88992687187102	0.522	0.224	5.80294665845961E-115	7	CREG1
1.18486656487593E-173	1.87220799027418	0.413	0.101	1.93263585396914E-169	7	LGALS9
1.13688647825429E-268	1.87122519055539	0.631	0.176	1.85437553468058E-264	7	IRF8
3.47458123017912E-210	1.85870914041514	0.699	0.251	5.66738944454516E-206	7	MARCKS
4.86492111674898E-138	1.85854750278108	0.291	0.064	7.93517283352926E-134	7	TNFSF13B
1.54401716678879E-185	1.85779985019399	0.493	0.129	2.51844640074919E-181	7	TIMP2
1.08933716985663E-124	1.84793821415658	0.482	0.179	1.77681785775315E-120	7	ANXA5
7.03165709511699E-151	1.84607106504383	0.264	0.049	1.14693358878453E-146	7	LRRK1
1.75844766058192E-134	1.83698789258991	0.389	0.111	2.86820397917516E-130	7	ASAH1
4.90488586376786E-129	1.83267674658404	0.403	0.126	8.00035933239176E-125	7	ZFYVE16
1.43878812408692E-132	1.82972444817411	0.53	0.215	2.34680730919818E-128	7	RXRA
1.07309532939093E-123	1.82440088701154	0.289	0.067	1.75032579176954E-119	7	ADGRE2
3.66158101744354E-189	1.81537130251402	0.395	0.084	5.97240479755215E-185	7	LAIR1
1.54788740579824E-57	1.81418071073842	0.267	0.1	2.52475914759751E-53	7	SIRPA
2.87664549112525E-299	1.8089388235171	0.563	0.115	4.69209646057439E-295	7	PLAUR

8.12167037654718E-81	1.80892831497396	0.4	0.172	1.32472565511861E-76	7	ATP1B1
1.85253417448839E-196	1.80886942666829	0.431	0.1	3.02166849200801E-192	7	UBXN11
4.77283547063294E-286	1.8058469239926	0.46	0.08	7.78497193614939E-282	7	ITGA9
3.52688715567125E-130	1.7997508265428	0.568	0.243	5.75270563961537E-126	7	BLVRB
2.96662778990942E-115	1.77742407574363	0.253	0.058	4.83886658812125E-111	7	FRMD4B
7.11746152447472E-226	1.77538372228182	0.782	0.338	1.16092914925707E-221	7	TYMP
6.5854999363585E-119	1.77388851121185	0.291	0.072	1.07416089461943E-114	7	MXD1
1.04979770422763E-296	1.77374185708166	0.522	0.099	1.71232503536569E-292	7	CD300A
1.59784782477402E-108	1.76899108586192	0.301	0.082	2.60624958698891E-104	7	IL1RN
4.11226860266883E-147	1.76620867108128	0.31	0.07	6.70752131781312E-143	7	MYO9A
7.52759214015377E-148	1.76312606283104	0.36	0.089	1.22782555398048E-143	7	LYN
1.26591603404483E-90	1.75986475497111	0.395	0.155	2.06483564313052E-86	7	TNFSF10
8.20244187441861E-181	1.75768491486539	0.416	0.1	1.33790029413642E-176	7	STX11
2.48129988000595E-46	1.75649574851553	0.271	0.119	4.04724823427771E-42	7	DNASE2
5.83393301427263E-96	1.74934516129723	0.36	0.124	9.51572813958008E-92	7	SRC
4.69668627249342E-63	1.74683737287857	0.307	0.122	7.66076497906402E-59	7	RNF130
3.21692037571652E-193	1.74107549458256	0.617	0.205	5.24711882483121E-189	7	NAGK
6.46754777335615E-168	1.73312838931558	0.516	0.162	1.05492171731212E-163	7	COLGALT1
1.02053532811458E-73	1.71800322300415	0.266	0.085	1.66459517368769E-69	7	ADAM9
1.73939402994677E-58	1.71218380288532	0.33	0.141	2.83712560224617E-54	7	SCPEP1
3.33440917017462E-109	1.70150608860911	0.4	0.136	5.43875479747182E-105	7	NPL
1.57757667273516E-117	1.69662521563346	0.338	0.096	2.57318531089832E-113	7	HEATR3
4.70588386150186E-178	1.68705997894024	0.299	0.054	7.67576716649568E-174	7	MMP19
5.61599405748748E-152	1.68210018611153	0.375	0.092	9.16024790716784E-148	7	RCN3
2.03649848744414E-104	1.66820984565862	0.331	0.098	3.32173268287014E-100	7	SLC43A2
1.42209111615223E-256	1.66397994300646	0.864	0.335	2.3195728195559E-252	7	CYBA
7.22162610020942E-140	1.65006317380797	0.594	0.242	1.17791943320516E-135	7	IFI6
3.27507453282124E-228	1.64482251198044	0.578	0.142	5.34197407048473E-224	7	ADA2
5.16491948335124E-112	1.64139277181224	0.49	0.196	8.4245001692942E-108	7	LAP3
1.35430000231677E-163	1.64087785457715	0.836	0.439	2.20899873377889E-159	7	MT-CO3
4.03578752795672E-189	1.63614522639607	0.637	0.219	6.58277303685021E-185	7	DEPRECATE
4.18790492421918E-182	1.6317965778103	0.745	0.336	6.83089172189391E-178	7	CEBPD
1.16946317641066E-180	1.6313452853634	0.505	0.14	1.90751138704343E-176	7	GSN
3.75873324819635E-68	1.62414027618093	0.337	0.136	6.13086980113306E-64	7	VAMP5
2.38822970586138E-67	1.62282722800239	0.439	0.214	3.89544147323049E-63	7	MT-CYB
6.69606744725794E-67	1.61405957745874	0.357	0.154	1.09219556132224E-62	7	SLC35F6
5.96522850314524E-119	1.60893018954106	0.379	0.115	9.7298842114802E-115	7	FCHO2
9.85287835006864E-61	1.60586076840662	0.333	0.145	1.6071029876797E-56	7	FAM20A
2.68416840125445E-122	1.6039048377572	0.463	0.168	4.37814707928614E-118	7	PIK3AP1
8.57990458777598E-108	1.59551473399686	0.423	0.149	1.39946823731214E-103	7	WARS
4.6737784466664E-192	1.58954870437617	0.677	0.254	7.62340002435756E-188	7	TNFAIP2
2.64141006817359E-89	1.58785035610353	0.405	0.162	4.30840396219794E-85	7	ACTN1
3.65233552004465E-87	1.5850196519067	0.321	0.106	5.95732446674482E-83	7	DAB2
3.75730801485028E-89	1.58319321195697	0.284	0.081	6.12854510302229E-85	7	LGALS3
3.26760463245127E-63	1.58273998683208	0.278	0.101	5.32978991599126E-59	7	IFNGR2
7.06793561728785E-145	1.57902758812723	0.324	0.072	1.15285097853582E-140	7	FAM49A
2.73049784252314E-223	1.57824353456677	0.786	0.273	4.4537150309395E-219	7	LAPTM5
2.22584706826408E-143	1.57096633955835	0.478	0.153	3.63057915304554E-139	7	RNASET2
3.78475409433904E-130	1.56137745780144	0.415	0.127	6.17331240327641E-126	7	PNPLA6
1.94290294196431E-170	1.53637551450318	0.602	0.209	3.16906898863799E-166	7	TTYH3

1.0959622285377E-55	1.53575452196959	0.364	0.173	1.78762399096785E-51	7	PLBD2
5.26711392277586E-124	1.53479525898513	0.343	0.093	8.59118951943971E-120	7	MKNK1
7.92889670204517E-50	1.53452343020996	0.256	0.103	1.29328234107059E-45	7	ATP6V0C
1.42780079777354E-118	1.53252440835213	0.406	0.127	2.32888588124842E-114	7	NRP1
3.82273607024841E-160	1.53203478130185	0.694	0.299	6.23526480418218E-156	7	MGAT1
1.03263898240073E-47	1.52783404602508	0.353	0.179	1.68433744419384E-43	7	RBM47
6.98589066495919E-125	1.52261069410339	0.356	0.1	1.13946862636149E-120	7	C9orf72
4.23998743786566E-96	1.52234690817043	0.369	0.127	6.91584350990268E-92	7	CLN8
2.95328204779625E-88	1.51258486667396	0.399	0.154	4.81709834816047E-84	7	SERPINB6
3.07370681635782E-92	1.50839458865094	0.484	0.218	5.01352318816123E-88	7	EPN1
3.0822978185295E-198	1.5038056082474	0.81	0.399	5.02753597180346E-194	7	SAT1
4.02647225317676E-90	1.49994317487464	0.282	0.082	6.56757889215662E-86	7	ME2
1.49326150596384E-160	1.49816851305913	0.389	0.093	2.43565884237763E-156	7	TMEM106A
7.07513301546175E-103	1.49648851663841	0.284	0.073	1.15402494615197E-98	7	DSE
1.05064199167215E-63	1.49486031548123	0.263	0.091	1.71370215261644E-59	7	PRCP
1.75672260576094E-61	1.49438647176107	0.309	0.122	2.86539024225667E-57	7	CD81
4.89603501134944E-134	1.48830282261944	0.315	0.073	7.98592270701208E-130	7	NR4A3
3.11684155599446E-91	1.48070390721005	0.515	0.237	5.08388026198256E-87	7	MT-ND3
4.84841690521577E-80	1.47838502078848	0.347	0.13	7.90825281409744E-76	7	AMPD2
1.77621975574849E-204	1.47716755230785	0.693	0.228	2.89719204360137E-200	7	PFKFB3
1.37441402735048E-36	1.46979291750725	0.267	0.128	2.24180672001136E-32	7	PGD
6.68675283541287E-158	1.46247618581776	0.259	0.045	1.09067625498419E-153	7	CCL4
5.80771383857223E-175	1.44859977828934	0.952	0.568	9.47296204209517E-171	7	MT-CO1
2.17244110359422E-180	1.44837596120415	0.799	0.329	3.54346868407253E-176	7	VIM
1.71871162084973E-55	1.43870374249557	0.251	0.093	2.80339052476799E-51	7	SGPP1
4.07244211665536E-162	1.42558900074326	0.425	0.106	6.64256033647655E-158	7	DENND3
1.11959316640277E-71	1.42281609857012	0.368	0.152	1.82616841371955E-67	7	DOCK5
4.0803535093261E-80	1.4153050159896	0.276	0.087	6.65546460906181E-76	7	GPR137B
6.94102245970852E-116	1.40996034661886	0.726	0.363	1.13215017340306E-111	7	MT-ATP6
7.41453703412799E-104	1.40811602783179	0.522	0.227	1.20938513563662E-99	7	VAMP8
2.69172599289284E-133	1.40260298900703	0.744	0.356	4.39047426700751E-129	7	SELENOP
1.76929951785993E-56	1.39517079910513	0.297	0.12	2.88590444358134E-52	7	ATP6AP2
2.59245990905909E-34	1.38505790037957	0.267	0.13	4.22856135766629E-30	7	KIAA0930
1.82524530982234E-114	1.38411360844624	0.264	0.06	2.97715762485122E-110	7	TFEC
5.65942709292514E-220	1.37629033903857	0.905	0.431	9.2310915312702E-216	7	SRGN
8.6940956579587E-60	1.36909481461063	0.433	0.222	1.41809394276964E-55	7	AKR1A1
1.03965907209015E-121	1.36732288993348	0.34	0.088	1.69578791248625E-117	7	SLC16A3
5.15945110902403E-203	1.3671680201342	0.582	0.154	8.4155807039291E-199	7	DOK2
1.28839568126051E-90	1.36575670646016	0.39	0.144	2.10150219570403E-86	7	MYD88
2.56542574826904E-76	1.35508844121674	0.461	0.216	4.18446593800164E-72	7	ACSL1
3.38502805619972E-98	1.34812934590064	0.397	0.137	5.52131926246736E-94	7	ATP6V1B2
5.02902559669471E-62	1.34776221456062	0.409	0.197	8.20284365076873E-58	7	MTSS1
5.22119928327079E-63	1.34238340460417	0.468	0.243	8.51629815094298E-59	7	CTSA
1.01306866590663E-145	1.34141275511864	0.787	0.396	1.6524163009603E-141	7	TMSB10
2.5165074260035E-78	1.34089738278658	0.549	0.282	4.1046752625543E-74	7	GRINA
8.22842015539892E-143	1.33537994494983	0.59	0.207	1.34213761154712E-138	7	CTSC
1.53006062055339E-51	1.33387987842489	0.259	0.1	2.49568187818464E-47	7	SWAP70
2.18900093506472E-74	1.33248608339485	0.355	0.14	3.57047942518406E-70	7	KYNU
5.87660025120978E-38	1.3318836906421	0.334	0.178	9.58532266974827E-34	7	NCOA4
3.92781202827389E-29	1.32532734852843	0.267	0.142	6.40665419931754E-25	7	RTN3

4.58777250994791E-61	1.3185203235927	0.493	0.26	7.48311574097604E-57	7	APLP2
8.95079432545578E-117	1.31845153903669	0.717	0.343	1.45996406242509E-112	7	CTSD
2.31624335788715E-139	1.31236904343932	0.738	0.329	3.77802454104974E-135	7	C2
2.60218145891887E-102	1.31115219675344	0.513	0.213	4.24441817764256E-98	7	QKI
1.47119781613385E-54	1.2973865725777	0.372	0.174	2.39967075789592E-50	7	ATP6AP1
2.56193226428452E-97	1.29651887112697	0.385	0.134	4.17876771627448E-93	7	CD82
1.00026110389992E-182	1.29281682775719	0.871	0.464	1.63152588657116E-178	7	FTH1
5.24089691806107E-104	1.27844696200612	0.695	0.348	8.54842696304942E-100	7	MT-ND4
3.45396994011839E-120	1.27652857166298	0.802	0.41	5.63377036932711E-116	7	MT-CO2
1.45350718779568E-73	1.27231788750192	0.251	0.073	2.37081557401353E-69	7	PLA2G7
2.44933538935677E-92	1.27034589568907	0.594	0.297	3.99511095357983E-88	7	RNH1
7.5717978760588E-88	1.26876154864556	0.254	0.067	1.23503595156395E-83	7	NRROS
9.66056096472371E-33	1.25992381154365	0.255	0.124	1.57573409895608E-28	7	SORBS3
7.43849907757768E-104	1.24637293411525	0.355	0.107	1.2132935845437E-99	7	RILPL2
2.15679622421523E-76	1.24324214650875	0.329	0.118	3.51795032131746E-72	7	GSAP
4.34740670800349E-96	1.24189852083129	0.507	0.218	7.09105508142449E-92	7	NAMPT
5.44702317373638E-60	1.23787109789849	0.385	0.178	8.88463949868141E-56	7	RUFY3
9.31828228730959E-37	1.23030856399165	0.266	0.125	1.51990502388307E-32	7	FGD4
1.19675796584448E-67	1.22701965323378	0.26	0.084	1.95203191808894E-63	7	ARRB1
4.27090533965377E-128	1.22499573619557	0.482	0.153	6.96627369950926E-124	7	PLEKHO2
3.37494502753233E-94	1.22319868341096	0.51	0.22	5.50487283440798E-90	7	ARPC1B
2.12944005457275E-72	1.22162716046084	0.347	0.13	3.47332967301361E-68	7	SH2B3
1.90784330699268E-100	1.21558975006693	0.469	0.181	3.11188321803576E-96	7	GRB2
4.23538284137328E-70	1.21200668175534	0.551	0.294	6.90833295256395E-66	7	BRI3
2.45965246297077E-94	1.21034511860713	0.298	0.084	4.01193913235163E-90	7	GRASP
9.60725174823191E-52	1.20856866800546	0.402	0.206	1.56703883265411E-47	7	NADK
2.89789314744059E-103	1.19840798820151	0.306	0.083	4.72675351279035E-99	7	HSPA6
8.1803967110179E-68	1.19712607013594	0.3	0.105	1.33430450753413E-63	7	SCARF1
1.70960492354416E-42	1.19565786711306	0.305	0.146	2.78853659079287E-38	7	WDR81
7.60728379768691E-52	1.19342404530763	0.263	0.104	1.24082406024071E-47	7	C6orf62
7.73696472480898E-173	1.17712378440306	0.972	0.726	1.26197631626359E-168	7	TMSB4X
2.01192114687416E-86	1.17389065958848	0.613	0.313	3.28164458266644E-82	7	ITM2B
2.04195384747215E-95	1.17240414305963	0.721	0.398	3.33063092061182E-91	7	NUCB1
9.17297549972816E-69	1.16628215004695	0.348	0.137	1.49620403376066E-64	7	DENND1A
1.09124057042931E-98	1.15146658144959	0.251	0.06	1.77992249442724E-94	7	IFI44L
4.5793016201159E-21	1.14744568042446	0.259	0.153	7.46929887257105E-17	7	CTNNA1
3.00100160086173E-58	1.14709680156719	0.33	0.138	4.89493371116557E-54	7	AP1B1
1.33214688408451E-81	1.14503808190093	0.557	0.277	2.17286478263024E-77	7	SLC12A7
2.34384604838479E-131	1.13714493511077	0.509	0.164	3.82304728952044E-127	7	ZEB2
4.46872055746676E-155	1.12687833757523	0.714	0.259	7.28893010128403E-151	7	HCLS1
4.00594898874217E-75	1.12679338113161	0.617	0.333	6.53410339553736E-71	7	PLXNB2
1.98762629843577E-37	1.12131461706619	0.455	0.277	3.24201725537858E-33	7	ISG15
5.51151040545548E-27	1.1161935147538	0.287	0.161	8.98982462233844E-23	7	DST
3.25946015559727E-97	1.11549320584292	0.513	0.215	5.31650545979471E-93	7	SYNGR2
5.20476325241996E-127	1.11396020142486	0.71	0.271	8.4894893410222E-123	7	ITGB2
6.63896106389412E-44	1.11055948878884	0.368	0.186	1.08288093913177E-39	7	RTN4
8.47276464839841E-27	1.10636057788653	0.324	0.191	1.38199264180026E-22	7	IFI27
3.37156319299525E-128	1.10623433486362	0.638	0.25	5.49935672409456E-124	7	SAMHD1
1.31947884016208E-176	1.09849066452083	0.498	0.143	2.15220193618836E-172	7	TIMP1
3.91990917665268E-68	1.08952364002702	0.262	0.085	6.39376385803819E-64	7	ARHGAP27

3.54690218655479E-27	1.08386344816238	0.331	0.198	5.78535215648952E-23	7	SCARB1
3.59301950139857E-148	1.08324066960486	0.689	0.246	5.8605741087312E-144	7	ARL4C
4.89922685234954E-147	1.08225512047597	0.532	0.16	7.99112891886733E-143	7	UCP2
3.79298101635282E-30	1.08133847780136	0.326	0.187	6.18673133577308E-26	7	RREB1
2.29163673452864E-96	1.08116762777198	0.67	0.342	3.73788867768967E-92	7	SOD2
2.80452918252003E-41	1.07862719379187	0.293	0.137	4.57446754960841E-37	7	TGM2
4.69715303303255E-26	1.07213394076812	0.34	0.207	7.66152631217939E-22	7	RAB7A
4.99322176149278E-124	1.07121443950093	0.37	0.098	8.14444401517088E-120	7	ABI3
3.07640938492731E-42	1.0704568056846	0.313	0.149	5.01793134775493E-38	7	TMEM127
1.87623354145097E-66	1.06702258495865	0.532	0.278	3.06032452946067E-62	7	BST2
2.68225244300647E-83	1.06655485056052	0.338	0.113	4.37502195978785E-79	7	JAK2
1.64939036857247E-13	1.06642420202558	0.263	0.177	2.69032063017856E-09	7	MT-ND5
3.05740334368367E-54	1.06381331997262	0.305	0.124	4.98693059388243E-50	7	TLNRD1
1.61433014296999E-77	1.0551114342726	0.525	0.25	2.63313389619834E-73	7	SLC3A2
8.21923427168145E-30	1.05382624904377	0.256	0.129	1.34063930205396E-25	7	TMBIM1
9.40698310077096E-100	1.04547628030542	0.693	0.364	1.53437301356675E-95	7	ARAP1
1.07558826615808E-76	1.042920462607	0.274	0.085	1.75439202093044E-72	7	MVB12B
6.17764190485728E-19	1.04034660941073	0.297	0.186	1.00763517110127E-14	7	CTSH
4.96863870005294E-106	1.03449135363486	0.709	0.36	8.10434658365635E-102	7	EHBP1L1
5.75847506005585E-29	1.03150737626542	0.27	0.143	9.3926486704571E-25	7	ELL2
5.71548088502611E-69	1.02807632493608	0.499	0.251	9.32252087156609E-65	7	CNDP2
3.01624718521116E-102	1.01686525525537	0.361	0.108	4.91980078379793E-98	7	NFKBID
9.69794607011264E-52	1.00917664247959	0.339	0.154	1.58183198349607E-47	7	SFT2D2
5.73271178592888E-104	1.00886983675844	0.731	0.407	9.35062619402859E-100	7	DUSP1
1.13626771621588E-76	1.00598877378774	0.409	0.162	1.85336627191973E-72	7	SH3TC1
4.48967366993358E-33	1.00537873931758	0.261	0.127	7.32310672302866E-29	7	SPNS1
5.66365251520612E-31	1.00333655538533	0.364	0.214	9.2379836175527E-27	7	GADD45B
1.49867401085651E-17	0.999108941215109	0.255	0.155	2.44448717910806E-13	7	OAZ2
3.18375163987024E-50	0.99884858149899	0.453	0.245	5.19301729979235E-46	7	INF2
2.44153829303388E-51	0.998132993303279	0.308	0.13	3.98239310976757E-47	7	SCO2
8.22938949463294E-58	0.99799118262253	0.295	0.113	1.34229572046958E-53	7	SLC26A11
3.78866714254554E-22	0.996162538020397	0.302	0.178	6.17969497620603E-18	7	LGALS1
1.70720512349922E-48	0.992478966695136	0.371	0.183	2.78462227693958E-44	7	PTTG1IP
1.23080623043249E-55	0.990103361363261	0.351	0.151	2.00756804245844E-51	7	PLSCR1
1.84374686427606E-35	0.98687370578329	0.279	0.136	3.00733551032068E-31	7	GNAI3
1.58328961295081E-59	0.982074469904806	0.372	0.159	2.58250368768406E-55	7	RAP2B
2.551747637059E-118	0.97943795676789	0.37	0.1	4.16215557080693E-114	7	CYTH4
1.37310367796921E-38	0.978793063975526	0.262	0.117	2.23966940913557E-34	7	RIPOR1
6.27117914953632E-94	0.973960106978153	0.592	0.269	1.02289203108087E-89	7	EFHD2
1.23015539505883E-17	0.964857540332287	0.285	0.183	2.00650646488045E-13	7	ITGB5
2.14255640246056E-26	0.964444067650936	0.366	0.225	3.49472374805343E-22	7	PRKACA
4.28296386341397E-46	0.96178131067078	0.413	0.215	6.98594235761453E-42	7	CNPY3
2.27879114545559E-59	0.960912203319676	0.355	0.151	3.71693623735261E-55	7	PLEKHM2
1.71441148575857E-31	0.95954341394693	0.251	0.121	2.79637657442081E-27	7	OAS1
7.47061592882645E-56	0.95565564736289	0.27	0.099	1.21853216415088E-51	7	OAS3
1.6251883496977E-18	0.954571407326049	0.257	0.156	2.65084471719191E-14	7	GABARAP
1.80005281544608E-67	0.951614865577825	0.36	0.143	2.9360661472741E-63	7	SNX6
4.69733654477401E-40	0.951521713291989	0.328	0.164	7.66182563818089E-36	7	ATP2A2
2.28719546356541E-40	0.95026244671799	0.366	0.192	3.73064452062155E-36	7	IER5
9.68023907270104E-55	0.948213067069432	0.371	0.171	1.57894379514827E-50	7	ADAM10

5.19814456365431E-63	0.946138817902827	0.501	0.258	8.47869359777655E-59	7	STAT2
4.44673243811626E-22	0.945515407128812	0.252	0.14	7.25306527981143E-18	7	ITPRIPL2
4.12583727108831E-40	0.943829154825876	0.515	0.306	6.72965317287215E-36	7	MT-ND4L
9.27561636845861E-72	0.938518580787615	0.27	0.084	1.51294578585928E-67	7	ABR
1.61793847078582E-16	0.932922566289998	0.251	0.157	2.63901943969875E-12	7	RNPEP
7.7481178246699E-43	0.931383917311794	0.407	0.218	1.26379549838191E-38	7	CHMP1B
1.5867368385539E-85	0.929546908566075	0.394	0.142	2.58812645736527E-81	7	PTPN6
3.93309950703757E-61	0.929092777147768	0.392	0.169	6.41527860592898E-57	7	SH3BP5
2.80306156590832E-68	0.924482354089422	0.629	0.321	4.57207372015306E-64	7	SERPING1
3.24805496990456E-59	0.923789002920234	0.344	0.14	5.29790246141133E-55	7	IFNGR1
4.21239439946477E-37	0.922150436895108	0.341	0.18	6.87083650496699E-33	7	GUSB
9.87830133769437E-116	0.921235922987202	0.326	0.083	1.61124973119133E-111	7	PTGER4
3.53038709633282E-55	0.919016307055949	0.283	0.108	5.75841439282846E-51	7	NECAP2
6.52496747986704E-19	0.910814999770671	0.255	0.153	1.06428744564111E-14	7	ABHD16A
1.1592897563526E-66	0.909821390248075	0.516	0.255	1.89091752158672E-62	7	TLN1
5.05765981501887E-109	0.905808709536485	0.614	0.242	8.24954892427729E-105	7	CD44
6.38137969449382E-71	0.904761479013615	0.416	0.176	1.04086684196889E-66	7	STOM
2.84219522625837E-20	0.902849940309173	0.278	0.169	4.63590463355002E-16	7	CLTA
8.92669111028003E-20	0.899207815099106	0.268	0.159	1.45603258699778E-15	7	VEGFB
2.26833210051327E-35	0.896328985895799	0.351	0.187	3.69987648914719E-31	7	RHOA
5.20317268295525E-72	0.896174396005015	0.329	0.116	8.48689496316832E-68	7	HPS3
6.12708041466218E-68	0.894600942887098	0.728	0.41	9.99388086435548E-64	7	LAMP1
2.50379222306798E-115	0.8928287403786	0.529	0.186	4.08393549504619E-111	7	NABP1
4.85484284515498E-109	0.89167785592166	0.383	0.112	7.91873416473228E-105	7	RELT
1.7178036905126E-20	0.891108035652814	0.32	0.201	2.80190959959509E-16	7	ADAM15
7.99743883362852E-48	0.889071689472129	0.29	0.121	1.30446224815315E-43	7	AP2A2
5.86078103051394E-32	0.888143601858626	0.36	0.206	9.55951993887129E-28	7	YBX1
1.55722910548713E-86	0.887645310121948	0.302	0.089	2.53999639396006E-82	7	ABCG1
1.35335250745626E-49	0.887328395232821	0.479	0.256	2.2074532749119E-45	7	GBP1
9.57847924137337E-53	0.883827161225258	0.255	0.092	1.56234574906041E-48	7	IQGAP1
1.97665972101994E-16	0.882588374712754	0.256	0.155	3.22412967095562E-12	7	ACP5
1.62853498510916E-56	0.880790192115144	0.543	0.301	2.65630341421155E-52	7	ZYX
2.0918122809521E-80	0.864267553883811	0.655	0.346	3.41195501146097E-76	7	RNF149
5.55333788345817E-29	0.862713422482224	0.315	0.173	9.05804942170863E-25	7	TRIOBP
1.86380224862822E-44	0.855323694398406	0.331	0.155	3.04004784773749E-40	7	TPD52L2
2.08307820626019E-28	0.854579935472594	0.438	0.267	3.39770886223099E-24	7	TMEM176B
1.19807179041938E-19	0.847006546678823	0.256	0.151	1.95417489735306E-15	7	LAMTOR1
1.0617887036108E-81	0.84624266431921	0.346	0.113	1.73188355445958E-77	7	FERMT3
1.88665643911714E-103	0.846154209549292	0.487	0.175	3.07732531784396E-99	7	SGK1
2.67678390442207E-98	0.844879906964755	0.315	0.087	4.36610222650283E-94	7	CD38
1.26473276864885E-81	0.841958051375639	0.433	0.172	2.06290561894314E-77	7	FOS
3.65820411557457E-45	0.837160373871544	0.276	0.114	5.96689673291369E-41	7	RHOG
5.25146351184091E-72	0.832586947822553	0.518	0.246	8.5656621341637E-68	7	ARRB2
3.90164967374495E-37	0.829878200983457	0.356	0.19	6.36398078284539E-33	7	TNS3
9.77926720651794E-43	0.826055857715281	0.351	0.169	1.59509627405514E-38	7	INSIG1
1.99933171453664E-36	0.825879614922873	0.328	0.166	3.26110995958071E-32	7	VASP
5.84101463081986E-54	0.825626195247057	0.436	0.217	9.52727896433027E-50	7	AKIRIN2
7.62787517664983E-109	0.82424059552972	0.375	0.108	1.24418272006335E-104	7	AOAH
2.20311752385195E-54	0.815910209830273	0.277	0.1	3.59350499315492E-50	7	EMILIN2
4.73919439143906E-44	0.811823382263035	0.382	0.194	7.73009997187625E-40	7	SREBF1

2.49360925019663E-65	0.806623943348377	0.399	0.167	4.06732604799573E-61	7	IFNAR2
2.72873997904354E-116	0.8030899893856	0.614	0.225	4.45084777981792E-112	7	IL10RA
6.49189589514329E-43	0.797698245905426	0.343	0.166	1.05889313945682E-38	7	APBB3
2.07834261855957E-74	0.796400270516284	0.292	0.091	3.38998464513252E-70	7	PECAM1
9.18622702167828E-44	0.785811740599474	0.42	0.226	1.49836548950594E-39	7	LGALS8
1.05602789117284E-84	0.784763885426919	0.641	0.285	1.72248709329203E-80	7	LCP1
4.06138530945235E-31	0.780967419148321	0.33	0.177	6.62452557824772E-27	7	TWF2
5.86824992409878E-70	0.780155584664742	0.849	0.614	9.57170245119752E-66	7	ACTB
2.30321041734785E-22	0.777129663049773	0.266	0.152	3.75676651173608E-18	7	BCL2L1
3.58330167156661E-77	0.772767713178674	0.936	0.759	5.84472335649229E-73	7	B2M
2.2300953272086E-80	0.769153502216923	0.516	0.208	3.63750848820994E-76	7	PLEKHO1
6.3721667328981E-22	0.766103607966781	0.255	0.144	1.03936411580301E-17	7	AMDHD2
2.35059205966514E-10	0.763706572631735	0.292	0.213	3.83405070851981E-06	7	LAMP2
6.55585002138053E-20	0.75837986748579	0.325	0.208	1.06932469698738E-15	7	RAB5C
3.79050290786064E-51	0.757186378801193	0.361	0.161	6.18268929301148E-47	7	UGCG
2.49987591590902E-37	0.755830094929328	0.357	0.186	4.0775476064392E-33	7	PLEKHB2
4.52067886604304E-37	0.751974843259331	0.293	0.14	7.37367929840281E-33	7	SH3BP2
3.87451366737042E-57	0.751896139232803	0.437	0.205	6.3197192428479E-53	7	M6PR
7.02470067230635E-15	0.751870716751178	0.301	0.204	1.14579892665989E-10	7	NFE2L1
1.09216461369323E-59	0.746539772171077	0.561	0.306	1.78142970139502E-55	7	NFKBIA
1.81775334225532E-62	0.74062843236434	0.524	0.262	2.96493747655265E-58	7	PPP1R9B
2.23241542509335E-16	0.740306497136984	0.325	0.22	3.64129279986977E-12	7	NFIC
1.70576861430985E-31	0.732676763273548	0.332	0.182	2.7822791868008E-27	7	RAB10
2.91707067135276E-14	0.730692221929362	0.325	0.224	4.75803397204349E-10	7	NECTIN2
2.5360863311665E-36	0.72917290674485	0.483	0.289	4.13661041476567E-32	7	LRPAP1
3.58805312196287E-52	0.726501001893447	0.313	0.126	5.85247344723364E-48	7	TSPAN14
2.60851026470071E-34	0.72249075611627	0.271	0.128	4.25474109275333E-30	7	YTHDF3
4.89144876091547E-63	0.720588385122042	0.369	0.151	7.97844207392923E-59	7	EHD1
1.27624710970283E-118	0.71882221610822	0.549	0.185	2.08168666063629E-114	7	MYO1F
1.30312838919843E-53	0.718691125558791	0.322	0.131	2.12553271562157E-49	7	MAP3K8
2.23873450818225E-106	0.715674316340799	0.485	0.162	3.65159985629606E-102	7	PLCB2
2.27142047805795E-61	0.711104906855876	0.511	0.253	3.70491394176032E-57	7	LPCAT1
8.50293141342163E-20	0.708538638235193	0.34	0.22	1.3869131428432E-15	7	NUDC
3.56457193491959E-12	0.708052960036828	0.318	0.223	5.81417328304735E-08	7	CD151
8.71675922776235E-20	0.706682108459019	0.376	0.249	1.42179059764032E-15	7	GNPTG
3.68244575006908E-29	0.70531939675683	0.292	0.153	6.00643726293767E-25	7	P4HA1
2.35102407009411E-42	0.702037993809876	0.391	0.195	3.83475536073051E-38	7	SH3BGRL3
4.98791828548047E-70	0.692761727048914	0.348	0.125	8.13579351544719E-66	7	ATG16L2
3.11474716336056E-51	0.686600044612131	0.434	0.216	5.08046409815741E-47	7	PARP14
1.25702944598147E-79	0.686484466594955	0.43	0.164	2.05034072934038E-75	7	CORO7
1.6728045131178E-53	0.686221105658172	0.524	0.283	2.72851144134645E-49	7	MINK1
3.76161567034672E-43	0.684449869892443	0.507	0.29	6.13557131990253E-39	7	LY6E
1.39481474417299E-86	0.683241236854042	0.348	0.109	2.27508232922057E-82	7	ST8SIA4
1.73330733077097E-32	0.682424272250384	0.266	0.122	2.82719758722054E-28	7	PKM
3.50764035858051E-55	0.680972960294127	0.587	0.301	5.72131218888067E-51	7	ALDH2
3.79357219204087E-15	0.671463463034641	0.337	0.227	6.18769560243786E-11	7	METTL7A
1.22426650051643E-87	0.660697956321482	0.733	0.347	1.99690108899235E-83	7	LYZ
1.82666688368662E-105	0.660500083148613	0.452	0.144	2.97947635398125E-101	7	NCKAP1L
2.48407933987912E-42	0.660079096845035	0.356	0.172	4.05178181127683E-38	7	SUSD6
4.07554834311084E-42	0.655339333131079	0.429	0.232	6.64762690244809E-38	7	DEPRECATE

**4.73140500958083E-85**	0.653532434679956	0.37	0.121	7.7173947111273E-81	7	RGS2
**5.72408243723455E-65**	0.65343407790874	0.47	0.211	9.33655086337328E-61	7	RIN3
**6.07970756961569E-66**	0.652679525187134	0.26	0.08	9.91661101680016E-62	7	GIMAP6
**2.01795053433141E-73**	0.650688836055255	0.355	0.123	3.29147911654796E-69	7	KLHL6
**1.32933085761823E-47**	0.64953357863314	0.322	0.141	2.16827156186109E-43	7	SGSH
**4.5624271940284E-63**	0.648162872026083	0.457	0.212	7.44177499617972E-59	7	GGA2
**5.58637712883231E-08**	0.641652046267841	0.287	0.216	0.000911193973483837	7	PDIA3
**1.71809818863471E-45**	0.639937954104148	0.499	0.281	2.80238995548207E-41	7	DPYD
**1.66505602107442E-40**	0.637068164417593	0.68	0.432	2.71587287597449E-36	7	OS9
**7.64219769290497E-19**	0.634050435062915	0.289	0.177	1.24651886568973E-14	7	IFI35
**1.72089359275504E-34**	0.632374174058711	0.517	0.327	2.80694953914275E-30	7	TBC1D9B
**3.05884812708709E-22**	0.620937780268999	0.32	0.193	4.98928718009175E-18	7	ODF3B
**9.76167609130039E-09**	0.620265027653381	0.301	0.227	0.000159222698725201	7	MYDGF
**1.21679021461926E-42**	0.61200547582837	0.369	0.182	1.98470651906547E-38	7	STX7
**4.47404563180272E-60**	0.604843629210244	0.32	0.121	7.29761583003341E-56	7	ETNK1
**9.0438783946527E-42**	0.599221699209575	0.68	0.441	1.4751470049518E-37	7	ZFP36L1
**3.5146524784377E-21**	0.598756263512036	0.297	0.176	5.73274965757973E-17	7	SLC23A2
**3.61071115413784E-34**	0.598646528500137	0.379	0.206	5.88943096351423E-30	7	STX4
**1.09623079981103E-18**	0.59678009281845	0.251	0.141	1.78806205757177E-14	7	GNAI2
**7.18399916916225E-98**	0.596365018914272	0.402	0.125	1.17178210448205E-93	7	SAMSN1
**8.78502836354384E-70**	0.593599809627525	0.455	0.186	1.43292597637764E-65	7	GNA13
**6.45406412435141E-43**	0.589834772506793	0.309	0.133	1.05272239932296E-38	7	ARHGEF2
**2.50373840254973E-12**	0.580113894808656	0.28	0.194	4.08384770839886E-08	7	DEPRECATE
**1.33090784675289E-31**	0.571651953745491	0.251	0.116	2.17084378883864E-27	7	IFFO1
**3.98012376455791E-27**	0.568657181151487	0.302	0.166	6.4919798723704E-23	7	TMUB2
**2.1086672683072E-112**	0.5648062117903	0.271	0.063	3.43944718133588E-108	7	WDFY4
**2.49258268998601E-08**	0.552660308342828	0.252	0.186	0.000406565162563617	7	PMVK
**5.51456352970835E-11**	0.552416200647401	0.272	0.191	8.99480457330728E-07	7	TMEM219
**2.89184974299571E-29**	0.546468070932331	0.433	0.265	4.7168961158003E-25	7	PRKAR1A
**3.73530002906851E-52**	0.54603161250127	0.271	0.097	6.09264787741364E-48	7	FAR1
**4.62192596588423E-32**	0.534711079425166	0.459	0.281	7.53882344295377E-28	7	SUMF2
**2.8178399648512E-77**	0.532751071922744	0.685	0.349	4.5961787666688E-73	7	MAF
**2.67777194351532E-51**	0.529249279183805	0.251	0.088	4.36771381706783E-47	7	TRPS1
**2.0487207458188E-98**	0.523021024163681	0.991	0.489	3.34166840850504E-94	7	APOE
**5.15542544320388E-66**	0.52232975600814	0.27	0.086	8.40901444040985E-62	7	CSF2RA
**9.37618958647718E-13**	0.518755335025437	0.351	0.248	1.52935028345029E-08	7	CDKN1A
**5.07171593442544E-33**	0.518464608502628	0.394	0.222	8.27247586064134E-29	7	IGF2R
**1.62241962176081E-10**	0.518426769403071	0.316	0.23	2.64632864505406E-06	7	BSG
**1.05921594587307E-90**	0.511109437042704	0.359	0.109	1.72768712931357E-86	7	PRKCB
**1.94157308294656E-18**	0.506158760112475	0.259	0.15	3.16689985559413E-14	7	ARHGDIA
**7.22035293892403E-08**	0.505009816040367	0.275	0.206	0.0011777117678679	7	PDIA6
**2.94041781988622E-33**	0.502739869820024	0.476	0.29	4.79611550601642E-29	7	STAT6
**4.29086974038379E-13**	0.502490463469552	0.279	0.188	6.99883763354001E-09	7	ARF1
**1.03183488630583E-26**	0.501732228114966	0.251	0.123	1.68302588305343E-22	7	ATG7
**2.29272085152862E-37**	0.501501604829656	0.602	0.378	3.73965698092834E-33	7	VMP1
**3.84075130823147E-31**	0.499156879437826	0.385	0.22	6.26464945885635E-27	7	ITPR2
**4.53384143226034E-35**	0.495936504701846	0.49	0.294	7.39514876015984E-31	7	NOTCH2NLC
**5.02899054564497E-11**	0.49204431552874	0.286	0.203	8.20278647900151E-07	7	ADIPOR1
**4.38017030048767E-23**	0.489596784692839	0.352	0.215	7.14449577712544E-19	7	HERPUD1
**4.07444089394396E-07**	0.485749323019901	0.256	0.196	0.006645820542112	7	POLR2L

1.34380859979477E-42	0.481584979049297	0.402	0.196	2.19188620712524E-38	7	MAP7D1
2.88049910302459E-12	0.474394712837466	0.333	0.23	4.69838208694342E-08	7	DHRS3
3.21985434587203E-27	0.466370177071971	0.357	0.205	5.25190442355187E-23	7	MYO15B
1.01813420759321E-53	0.462347100173102	0.324	0.129	1.66067870600528E-49	7	SEL1L3
7.56890107368775E-47	0.459318191158226	0.485	0.247	1.23456345412921E-42	7	MYO9B
6.90851881286882E-29	0.456278724060925	0.416	0.249	1.12684850356703E-24	7	CLCN7
1.12867621316245E-35	0.45417784388236	0.39	0.21	1.84098377128927E-31	7	DENND4B
2.64146439138315E-61	0.451566849922488	0.351	0.129	4.30849256878505E-57	7	ZNF331
3.65567722978123E-29	0.45041718083482	0.376	0.217	5.96277512949617E-25	7	OGFR
5.37057099098862E-22	0.445840138730359	0.425	0.278	8.75993834340153E-18	7	HSPA1A
2.22847697812427E-08	0.44329517153628	0.34	0.255	0.000363486879901849	7	GPX4
3.57926549190635E-72	0.442001915302366	0.394	0.141	5.83813994384845E-68	7	PARVG
1.1894311507016E-54	0.440183845909221	0.347	0.139	1.94008114990937E-50	7	RUNX1
9.67610472423192E-34	0.43938980360869	0.263	0.123	1.57826944156947E-29	7	GALNS
1.39543975230201E-19	0.43804759877761	0.323	0.201	2.27610177997982E-15	7	GNB2
2.70744210374191E-27	0.437795254515098	0.403	0.239	4.41610881541342E-23	7	NCOR2
3.7247147433321E-39	0.436208840228271	0.668	0.439	6.07538221784899E-35	7	ZFP36
1.06083095353925E-21	0.435691925670431	0.395	0.253	1.73032136831788E-17	7	EIF4A1
1.05607581464538E-35	0.435225405889838	0.513	0.309	1.72256526126808E-31	7	DBNL
6.4528956920342E-36	0.424571089904203	0.501	0.297	1.0525318163277E-31	7	MBD2
9.05622120466497E-26	0.420072634895601	0.263	0.127	1.4771602406929E-21	7	CMTM3
2.73195514034742E-16	0.419368466718185	0.284	0.178	4.45609202942068E-12	7	BNIP2
2.30603731207595E-25	0.41397785471862	0.252	0.128	3.76137745972708E-21	7	NXF1
1.02992550522257E-29	0.410896205289459	0.374	0.211	1.67991149156853E-25	7	BTG2
1.12180683575965E-08	0.410165860757125	0.278	0.206	0.000182977912980756	7	HEXA
1.76272650479765E-06	0.410103774674016	0.277	0.215	0.0287518320197544	7	CYC1
2.599641878159E-26	0.40846594405861	0.422	0.259	4.24027586746515E-22	7	LAMTOR4
2.42066961691224E-16	0.400531759552613	0.311	0.201	3.94835421214555E-12	7	AAMP
3.38620687540941E-13	0.400446189751856	0.306	0.211	5.52324203448029E-09	7	SEC22B
4.5690867615138E-62	0.40024599346765	0.308	0.109	7.45263741670515E-58	7	KLF2
2.64689717246142E-67	0.396001682367308	0.355	0.126	4.31735397800183E-63	7	ADCY7
1.30241084745167E-28	0.393434264878254	0.501	0.316	2.12436233327842E-24	7	DPP7
1.40071594165536E-80	0.391100480424112	0.643	0.274	2.28470777243406E-76	7	METRNL
4.17507320155252E-61	0.391065379369927	0.436	0.182	6.80996189905231E-57	7	FNBP1
8.695679418832E-68	0.390205484899343	0.603	0.262	1.41835227000569E-63	7	ARHGAP4
1.60812910317631E-35	0.389404985403019	0.464	0.264	2.62301938019087E-31	7	PER1
2.21944320928666E-16	0.38887290732192	0.292	0.181	3.62013381866747E-12	7	GNAQ
2.88358200227111E-21	0.383356891133286	0.489	0.317	4.70341060390441E-17	7	HSPB1
7.84718460423102E-23	0.379612475808023	0.525	0.352	1.27995428079612E-18	7	CFL1
2.69883857036904E-10	0.378722273681257	0.271	0.193	4.40207559212895E-06	7	SNX17
7.07697502238017E-30	0.376590248826867	0.482	0.299	1.15432539590043E-25	7	MAT2A
1.17235059693269E-07	0.372647435387988	0.282	0.212	0.00191222105865692	7	MTDH
8.12540328617214E-18	0.369750073609703	0.738	0.546	1.32533453000754E-13	7	MT-ND6
1.45895519922754E-47	0.366001405959453	0.268	0.103	2.37970182546005E-43	7	TRIM14
2.75889311607836E-11	0.364022862024003	0.309	0.218	4.50003056163542E-07	7	TRIM8
1.83147049828398E-14	0.360727035580932	0.27	0.172	2.987311529751E-10	7	EFR3A
2.7516935715127E-17	0.360675457947002	0.295	0.182	4.48828738449437E-13	7	DNAJC5
2.16995304858743E-14	0.357937893291211	0.509	0.358	3.53941041755096E-10	7	SDF4
1.40617545788557E-11	0.357808262030437	0.338	0.242	2.29361278935716E-07	7	TNFRSF1A
5.99672964603265E-11	0.349768999157425	0.309	0.223	9.78126572564385E-07	7	NAPA

1.30946181139658E-11	0.344684005667263	0.276	0.189	2.13586316056896E-07	7	RNF114
7.91506332172538E-10	0.342370753689028	0.29	0.208	1.29102597840663E-05	7	IRAK1
1.55093079070733E-25	0.340513010176083	0.33	0.187	2.52972321272273E-21	7	ITSN2
2.37749220997231E-28	0.334704426406054	0.314	0.167	3.87792754368584E-24	7	ZNF609
1.01120386119935E-23	0.333452075845614	0.502	0.337	1.64937461800226E-19	7	EMC10
4.5409670145337E-30	0.330371056756066	0.274	0.124	7.40677129740591E-26	7	COTL1
1.09250310370134E-13	0.328611520482106	0.323	0.218	1.78198181244726E-09	7	CALM3
8.25409920134635E-34	0.327451054121223	0.39	0.201	1.3463261207316E-29	7	SOCS3
3.55710954049689E-103	0.326171996619239	0.53	0.184	5.80200137150447E-99	7	HAVCR2
3.58822582916522E-15	0.32522222802948	0.26	0.162	5.85275514995139E-11	7	NOTCH2NLE
2.5522281120552E-11	0.320914947325275	0.347	0.251	4.16293927357324E-07	7	FNDC3B
2.00966437698593E-57	0.313781593133896	0.303	0.108	3.27796356530175E-53	7	CD84
4.18588144900156E-16	0.302431716808349	0.372	0.249	6.82759123146644E-12	7	PSMB10
5.98389704852052E-16	0.301618128266848	0.346	0.231	9.76033447584183E-12	7	STXBP2
3.78440562231486E-16	0.298097616627258	0.507	0.353	6.17274401055777E-12	7	KLF6
6.25513256510835E-37	0.294228899549285	0.37	0.182	1.02027467269482E-32	7	ANKRD13D
1.42858978112467E-19	0.293071445946018	0.421	0.28	2.33017279199244E-15	7	MTCH1
2.18003574788126E-06	0.2897751181081	0.302	0.24	0.0355585630836913	7	SERF2
1.11647970580858E-14	0.284748381968277	0.41	0.287	1.82109004814438E-10	7	H3F3A
5.46210836316006E-11	0.281861927751517	0.316	0.226	8.90924495115037E-07	7	IRF7
2.35517947163669E-41	0.280293725416176	0.269	0.106	3.84153323618661E-37	7	RCSD1
5.73003122098951E-19	0.273984766600655	0.821	0.629	9.34625392455599E-15	7	TAPBP
2.80067298434047E-48	0.266994736829114	0.43	0.194	4.56817770475774E-44	7	STK10
5.58397722550358E-11	0.264367665056915	0.432	0.299	9.10802525251888E-07	7	RRBP1
8.68906361857492E-62	0.261426439839589	0.369	0.14	1.41727316682575E-57	7	TMC6
1.26384932508801E-09	0.255157529314445	0.321	0.236	2.06146463415106E-05	7	SERINC1
4.93924602484168E-15	0.2541808735899	0.374	0.255	8.05640419111927E-11	7	NFE2L2
2.07464428519395E-23	0.253353790231721	0.403	0.247	3.38395229357986E-19	7	POLR2A
0	9.65859791971707	0.259	0.002	0	8	PAPPA
0	9.57562125647862	0.616	0.003	0	8	EFEMP1
0	9.45116208885606	0.903	0.022	0	8	COL1A1
0	9.35416981137559	0.736	0.006	0	8	THBS2
0	9.26925170049953	0.325	0.002	0	8	TNC
0	9.22200247367718	0.399	0.002	0	8	LAMC3
0	9.17075724997958	0.372	0.001	0	8	PTH1R
0	9.17049628655996	0.469	0.002	0	8	LUM
0	9.17007332462631	0.858	0.012	0	8	COL1A2
0	9.1587305911463	0.925	0.019	0	8	COL3A1
0	9.15523267521946	0.311	0.002	0	8	ALDH1A3
0	9.13517515934498	0.369	0.001	0	8	ID4
0	8.95756455529533	0.787	0.009	0	8	DCN
0	8.83276641127174	0.404	0.003	0	8	COL16A1
0	8.76407044391341	0.406	0.002	0	8	SVEP1
0	8.71363539422	0.335	0.001	0	8	MXRA8
0	8.69974346967723	0.259	0.003	0	8	ADRA1B
0	8.66985351945702	0.369	0.001	0	8	COL14A1
0	8.6346776556345	0.264	0.001	0	8	FAM163B
0	8.62327881395	0.391	0.001	0	8	MFAP4
0	8.54779806739749	0.296	0.001	0	8	PDGFRA
0	8.35738179434354	0.252	0.001	0	8	FAM180A

0	8.3473361101944	0.258	0.002	0	8	HHIP
0	8.34078853025633	0.265	0.005	0	8	HEYL
0	8.24398221721909	0.816	0.017	0	8	BGN
0	8.16580584852335	0.518	0.003	0	8	PODN
0	8.10582063293103	0.268	0.002	0	8	ITGBL1
0	8.06667492633014	0.62	0.006	0	8	GGT5
0	8.05625960485932	0.452	0.005	0	8	COLEC10
0	8.04783270120623	0.358	0.002	0	8	NGFR
0	7.98642423410805	0.581	0.005	0	8	MYL9
0	7.8156375091194	0.319	0.002	0	8	PRKG1
0	7.77459847575682	0.289	0.002	0	8	PLA2G5
0	7.73388715701681	0.406	0.003	0	8	FBLN1
0	7.72123232674577	0.283	0.002	0	8	STEAP4
0	7.5902843842791	0.594	0.007	0	8	CRISPLD2
0	7.51517708923477	0.297	0.003	0	8	FHL2
0	7.50962964814776	0.713	0.011	0	8	AEBP1
0	7.50907494867531	0.725	0.012	0	8	COL5A1
0	7.50163386074706	0.317	0.003	0	8	CCDC3
0	7.44243202428609	0.504	0.031	0	8	CCN2
0	7.39313574051327	0.512	0.024	0	8	PDGFRB
0	7.38369501760132	0.304	0.002	0	8	LAMA2
0	7.38326803195448	0.323	0.004	0	8	OLFML3
0	7.34027136259667	0.633	0.014	0	8	C7
0	7.33458175996992	0.648	0.044	0	8	CCN1
0	7.24156905118331	0.478	0.004	0	8	FBLN5
0	7.23212711420624	0.258	0.006	0	8	NOTCH3
0	7.22588365150943	0.874	0.089	0	8	COL6A1
0	7.22006416598186	0.712	0.018	0	8	EMILIN1
0	7.1962670262085	0.39	0.009	0	8	NFASC
0	7.1652668404283	0.385	0.003	0	8	ELN
0	7.1533044381004	0.419	0.015	0	8	ADAMTS13
0	7.03748290776865	0.669	0.088	0	8	IGFBP3
0	6.95258485245285	0.881	0.036	0	8	COL6A2
0	6.88725630381574	0.453	0.01	0	8	COL5A2
0	6.8044929097702	0.538	0.006	0	8	CCL21
0	6.78970054242759	0.663	0.014	0	8	FBN1
0	6.78813529458359	0.595	0.02	0	8	FSTL1
0	6.72646759729528	0.664	0.015	0	8	TNXB
0	6.71137100050908	0.748	0.07	0	8	COLEC11
0	6.69442467691912	0.423	0.009	0	8	HGF
0	6.61454507246805	0.473	0.016	0	8	ACTA2
0	6.58255819652422	0.318	0.003	0	8	COL4A4
0	6.58134539608171	0.599	0.037	0	8	ADAMTSL2
0	6.52582710065431	0.252	0.002	0	8	SYNPO2
0	6.50517605883328	0.469	0.006	0	8	FBLN2
0	6.47545826154834	0.364	0.01	0	8	CCL2
0	6.43970283670863	0.296	0.009	0	8	FSTL3
0	6.39897282580128	0.346	0.009	0	8	CCDC102B
0	6.38627627442516	0.321	0.013	0	8	HMCN1
0	6.3673388790301	0.397	0.007	0	8	ANGPTL2

0	6.34979687088107	0.277	0.006	0	8	SEPTIN4
0	6.25212139541361	0.286	0.005	0	8	DENND2A
0	6.19914966846667	0.761	0.057	0	8	TAGLN
0	6.11621669428883	0.37	0.012	0	8	ECM1
0	6.06395539117787	0.596	0.014	0	8	COL6A3
0	5.89127585990455	0.946	0.063	0	8	IGFBP7
0	5.88449954335063	0.385	0.011	0	8	RIPOR3
0	5.83677875649643	0.645	0.039	0	8	C11orf96
1.0027526392818E-260	5.8181205280996	0.255	0.028	1.63558982993255E-256	8	SOD3
0	5.78888999246031	0.459	0.012	0	8	INMT
0	5.77734357703288	0.311	0.032	0	8	LTBP2
0	5.6776719878428	0.348	0.021	0	8	RBP1
0	5.66183706009326	0.652	0.032	0	8	ADAMTS2
0	5.64186854174461	0.399	0.012	0	8	C1QTNF1
0	5.63868345454589	0.351	0.027	0	8	PCOLCE
0	5.62048737179585	0.321	0.023	0	8	DMD
0	5.58901671481053	0.284	0.018	0	8	VIPR1
0	5.53754132459975	0.335	0.024	0	8	RGS16
0	5.52300038551764	0.348	0.012	0	8	NEURL1B
0	5.51696635907745	0.42	0.02	0	8	LOXL2
0	5.47696904662515	0.396	0.012	0	8	PTGIR
0	5.42469205199996	0.278	0.009	0	8	KAZN
0	5.36382892233738	0.269	0.02	0	8	PTGDS
0	5.36041907814952	0.438	0.018	0	8	ADAMTS1
0	5.30228349841991	0.346	0.01	0	8	ADGRA2
0	5.30169616983194	0.252	0.01	0	8	EHD2
0	5.29086023586347	0.283	0.02	0	8	SCARF2
0	5.2483370540245	0.353	0.017	0	8	ARHGEF17
0	5.22811420495878	0.277	0.009	0	8	THY1
0	5.21690039967841	0.273	0.018	0	8	MATN2
0	5.17659620955921	0.302	0.01	0	8	MMP2
0	5.15431715030169	0.638	0.109	0	8	TPM1
0	5.1518355205895	0.277	0.009	0	8	FKBP10
0	5.13061368608978	0.335	0.022	0	8	LMCD1
0	5.11033988238604	0.352	0.012	0	8	CLEC11A
0	5.08015572022188	0.291	0.011	0	8	TBXA2R
0	5.05058161840631	0.389	0.041	0	8	MYH10
0	5.03295096871119	0.532	0.022	0	8	GEM
0	4.96924503350576	0.493	0.026	0	8	LAMB1
0	4.96412610806401	0.361	0.014	0	8	OSMR
0	4.92789403156035	0.792	0.103	0	8	LTBP4
6.55546811550664E-263	4.91119736608254	0.303	0.04	1.06926240432029E-258	8	PKDCC
0	4.80842385739806	0.419	0.026	0	8	JAG1
0	4.78133383252056	0.701	0.136	0	8	TIMP1
0	4.70628598649024	0.603	0.081	0	8	MICALL2
0	4.69360879326922	0.872	0.064	0	8	COL4A2
0	4.62805416273089	0.44	0.058	0	8	TANC1
1.6481392812686E-241	4.62548669450887	0.289	0.04	2.68827998167722E-237	8	PALLD
5.32300252356062E-219	4.61706692833707	0.259	0.035	8.68234941617973E-215	8	SEMA5A
0	4.58874732815335	0.449	0.047	0	8	RBPMS

0	4.5848624781218	0.331	0.016	0	8	RBMS3
1.19483210123965E-114	4.55330136374185	0.272	0.068	1.948890640332E-110	8	SERPINE1
1.27195257649304E-259	4.47149375476777	0.358	0.059	2.07468184751779E-255	8	MYLK
1.2249558834635E-279	4.41693624044936	0.254	0.025	1.99802554151731E-275	8	PBX1
0	4.37906533820534	0.669	0.089	0	8	CXCL12
0	4.37030161035343	0.369	0.02	0	8	PXDN
0	4.33375107430521	0.894	0.079	0	8	SPARC
1.10681706221077E-188	4.29995825487003	0.284	0.049	1.80532931017198E-184	8	AMOTL2
0	4.25424163272668	0.864	0.072	0	8	COL4A1
0	4.23649668056486	0.424	0.047	0	8	LIFR
2.69209686184907E-279	4.20166291934544	0.551	0.144	4.39107919136202E-275	8	MASP1
0	4.19724988528257	0.337	0.041	0	8	TPM2
0	4.16393605566292	0.425	0.041	0	8	PHLDB1
6.00914614695997E-199	4.08705647897265	0.366	0.079	9.8015182803064E-195	8	C1orf198
4.19319286646579E-209	4.05811250425749	0.312	0.053	6.83951688449234E-205	8	NR2F2
0	4.04914924537969	0.74	0.178	0	8	CALD1
2.1025336515655E-293	4.04392575547208	0.374	0.056	3.42944263906848E-289	8	NR2F1
1.97858387351563E-226	3.98539005532652	0.384	0.076	3.22726815609135E-222	8	IFITM10
1.96112062970188E-256	3.98242452081672	0.255	0.028	3.19878385910673E-252	8	VCL
0	3.96624064106066	0.53	0.038	0	8	LRRC32
0	3.93585306356804	0.504	0.05	0	8	MEGF6
7.04482318052487E-235	3.91779581514607	0.284	0.039	1.14908110897541E-230	8	NIBAN2
0	3.8620141268574	0.34	0.018	0	8	ANK3
1.14737373627819E-238	3.86187092026934	0.448	0.103	1.87148130124336E-234	8	PTPRS
0	3.84896846487254	0.279	0.022	0	8	PRSS23
0	3.7765537839697	0.338	0.038	0	8	CAVIN1
8.28464368449461E-129	3.72291448206419	0.327	0.09	1.35130823137792E-124	8	COL27A1
8.28733297369828E-224	3.69501025653142	0.518	0.149	1.35174688133993E-219	8	NR1H4
0	3.64396096374851	0.46	0.065	0	8	ITGA1
1.42081737428337E-306	3.63770080874247	0.462	0.082	2.3174952191936E-302	8	TIMP3
8.37108008392886E-290	3.59995147645673	0.431	0.075	1.36540687248964E-285	8	SERPINH1
2.11896252686931E-189	3.56136694312908	0.314	0.058	3.45623977757654E-185	8	UACA
5.53669514722541E-217	3.54786192597365	0.415	0.093	9.03090345463937E-213	8	EDNRB
4.92649794047827E-165	3.52415351985651	0.326	0.071	8.03561079071411E-161	8	KANK2
5.40872617124427E-106	3.51398306710289	0.297	0.086	8.82217325791654E-102	8	ARVCF
4.27223794296521E-70	3.4953090580489	0.275	0.1	6.96844730877055E-66	8	FGFR2
3.11012244459766E-303	3.49131388966264	0.682	0.208	5.07292071938324E-299	8	THBS1
1.56702165440877E-212	3.44250270559701	0.314	0.052	2.55596902050614E-208	8	DAAM1
1.16237483432657E-304	3.44080013430427	0.517	0.107	1.89594959227007E-300	8	NID1
1.93099522849434E-153	3.37135499935736	0.37	0.098	3.14964631719713E-149	8	ST5
0	3.36188302089733	0.527	0.079	0	8	ITGA9
1.50034579089169E-166	3.36113159159938	0.282	0.052	2.44721401952343E-162	8	CPED1
0	3.34343669026665	0.519	0.088	0	8	CRIM1
2.53599678103244E-129	3.33468361976275	0.312	0.08	4.13646434954201E-125	8	SORBS1
6.0712761317542E-161	3.29252386385852	0.342	0.079	9.90285849850428E-157	8	DLC1
0	3.23790129877404	0.426	0.042	0	8	TCIM
8.61810024095339E-131	3.20279241468897	0.264	0.057	1.40569833030191E-126	8	BMP1
9.94448809027129E-198	3.17295832480702	0.272	0.04	1.62204545240415E-193	8	TINAGL1
1.42089861811198E-247	3.16473603700518	0.387	0.068	2.31762773600245E-243	8	SMTN
6.90577802121525E-168	3.14478819856754	0.338	0.074	1.12640145304042E-163	8	PDLIM7

9.23521321212896E-292	3.13613096121035	0.406	0.064	1.50635562703035E-287	8	GRK5
5.34385965848505E-114	3.13298792789455	0.463	0.189	8.71636948895497E-110	8	DEPP1
0	3.13179860126873	0.796	0.233	0	8	C1R
0	3.12256069472455	0.283	0.018	0	8	IGFBP5
3.91129109546365E-277	3.11073172074022	0.472	0.094	6.37970690581076E-273	8	SARDH
2.23513944883777E-200	3.10449013864451	0.345	0.066	3.64573595499928E-196	8	LIMA1
0	3.10039038322492	0.508	0.051	0	8	ITM2C
4.38512557363877E-196	3.08603612918092	0.433	0.109	7.15257832316219E-192	8	CNN3
4.95666598104237E-188	3.06698305876805	0.506	0.159	8.08481788167821E-184	8	ACTN1
1.42220775985768E-170	3.05455953947856	0.516	0.182	2.31976307710386E-166	8	QSOX1
1.98329333591001E-71	3.04244451787763	0.254	0.084	3.23494976020282E-67	8	PLSCR4
3.42892342183534E-142	3.03697288299966	0.331	0.082	5.59291699335562E-138	8	TNS1
2.47681430199827E-104	3.02905184501722	0.323	0.101	4.03993180798937E-100	8	CTIF
4.76543986246939E-171	3.01570428974202	0.449	0.131	7.77290895967381E-167	8	LAMB2
1.24131254050364E-203	3.0096932233207	0.4	0.088	2.02470488481549E-199	8	PELO
7.56934416531867E-65	2.95908828010776	0.259	0.093	1.23463572680513E-60	8	NDRG2
4.77218391817067E-167	2.85354719809058	0.283	0.051	7.78390918892818E-163	8	SLCO3A1
4.03380504933669E-229	2.80742723019604	0.609	0.199	6.57953941597307E-225	8	GPX3
1.71712946672898E-111	2.80197455024318	0.302	0.082	2.80080987318164E-107	8	IL1R1
2.71112997239233E-173	2.73929193071841	0.481	0.145	4.42212409796914E-169	8	PPP1R12B
6.05058057680292E-153	2.73406552999997	0.372	0.096	9.86910197882324E-149	8	SEPTIN11
1.81497238533254E-207	2.72551965466731	0.346	0.062	2.96040145771591E-203	8	ID3
1.04774743674237E-172	2.72526479201349	0.342	0.072	1.70898084407049E-168	8	MXRA7
6.354600773124E-209	2.69385646206668	0.42	0.091	1.03649893210425E-204	8	RCN3
6.68022522244563E-83	2.68879175978035	0.374	0.15	1.08961153603311E-78	8	EGR1
6.27131862848338E-296	2.59801145760156	0.782	0.289	1.02291478149192E-291	8	IGFBP4
3.11482242212409E-198	2.54783551732631	0.367	0.072	5.0805868527266E-194	8	MRC2
1.672282565378E-135	2.54045788299903	0.302	0.069	2.72766009238806E-131	8	KALRN
1.2907955541011E-161	2.53816800950927	0.391	0.097	2.1054166282943E-157	8	DPYSL2
1.5612050901028E-46	2.50612018339681	0.254	0.107	2.54648162246668E-42	8	SELENOM
7.78108985235942E-194	2.50470745263057	0.541	0.169	1.26917356581835E-189	8	APP
2.46570617495041E-164	2.47631884653148	0.254	0.041	4.02181334196161E-160	8	HIC1
5.51704469514697E-132	2.45600855952048	0.501	0.197	8.99885160225422E-128	8	EPAS1
1.22415006349019E-158	2.42833327114068	0.283	0.052	1.99671116855885E-154	8	FXYD6
9.11246590799719E-75	2.42821775091947	0.255	0.078	1.48633431425342E-70	8	IER5L
1.3612523358671E-187	2.42461381003233	0.63	0.248	2.22033868503282E-183	8	LGALS3BP
7.09649689255042E-50	2.39150040174933	0.27	0.115	1.1575096081439E-45	8	TNKS1BP1
2.29019499765623E-56	2.3875763186698	0.273	0.108	3.73553706067708E-52	8	TNS2
0	2.3657704157201	0.587	0.111	0	8	ENG
1.44521779983617E-205	2.36261095720387	0.506	0.13	2.35729475331278E-201	8	TIMP2
2.18379107772872E-66	2.34016069690454	0.262	0.09	3.56198162688332E-62	8	PHLDB2
2.44969347224162E-49	2.32048586286347	0.281	0.122	3.99569502257331E-45	8	CDC42EP1
2.50351124401346E-58	2.29679414013018	0.268	0.102	4.08347719011036E-54	8	ITGAV
1.00905499672822E-103	2.28228591339024	0.258	0.063	1.64586960516341E-99	8	MYADM
7.03560969898471E-91	2.27753837982495	0.35	0.121	1.1475782980014E-86	8	CD81
9.4179949607121E-138	2.26640356991246	0.579	0.254	1.53616915804175E-133	8	CSRP1
3.20146209190513E-67	2.26382243638346	0.321	0.127	5.22190481810646E-63	8	MMP14
1.2685942091997E-106	2.20303470014077	0.38	0.127	2.06920401462563E-102	8	SLC12A4
2.30712116108765E-37	2.15119822336206	0.286	0.139	3.76314532585007E-33	8	IER3
4.98844411110342E-74	2.1396481616747	0.309	0.108	8.13665118962079E-70	8	NR4A1

3.79641373409388E-98	2.1293399447036	0.45	0.185	6.19233044168052E-94	8	SLC38A2
3.49734121735041E-86	2.09195105437284	0.254	0.069	5.70451325962025E-82	8	MARCKSL1
1.01794298725907E-48	2.05222490726878	0.352	0.181	1.66036680651826E-44	8	ITGB5
1.96786942932414E-119	2.04194388236215	0.406	0.128	3.2097918261706E-115	8	NRP1
2.84051663812459E-248	2.03112391336252	0.775	0.26	4.63316668844501E-244	8	C1S
3.270910132176E-65	2.02256683881198	0.272	0.095	5.33518151659228E-61	8	FER
1.21650027034964E-93	2.0068007036322	0.275	0.074	1.9842335909673E-89	8	DSE
3.32528195652068E-65	2.0051140086034	0.335	0.137	5.42386739928087E-61	8	LZTS2
1.83534010818502E-73	2.00496176736516	0.415	0.188	2.99362325046059E-69	8	TNS3
2.8051636887853E-88	1.96384404692913	0.369	0.132	4.5755024927777E-84	8	PHLDA1
8.11986076526527E-91	1.91313174206813	0.433	0.174	1.32443048942242E-86	8	LGALS1
3.95901555225919E-91	1.8810162205324	0.347	0.118	6.45755026728996E-87	8	RAPGEF5
1.15123655823736E-173	1.87887511945276	0.361	0.074	1.87778195014096E-169	8	SYTL2
4.94143437183946E-35	1.86921485712935	0.277	0.14	8.05997360390734E-31	8	BCAR1
6.61857891117462E-173	1.83746033666176	0.503	0.137	1.07955640620169E-168	8	FLNA
6.05269235867913E-43	1.82903676124654	0.322	0.161	9.87254650624152E-39	8	APOL1
8.99210421760926E-32	1.79586268895592	0.257	0.127	1.46670211893425E-27	8	KLF9
1.47015459245325E-62	1.78191794417118	0.414	0.2	2.39796915575049E-58	8	SPON2
9.19423906789389E-37	1.71889677458619	0.27	0.129	1.49967233436417E-32	8	CLSTN3
3.42262917149402E-25	1.70031367541048	0.27	0.155	5.5826504416239E-21	8	TRIP6
6.06817433043888E-99	1.68282800171817	0.404	0.14	9.89779915037885E-95	8	NNMT
3.84186656779823E-170	1.6180601972291	0.342	0.068	6.2664685587357E-166	8	HSPG2
5.45957087661307E-32	1.6110835286835	0.303	0.165	8.90510605684359E-28	8	CALU
2.72604510810851E-42	1.60608298878002	0.257	0.109	4.44645217583579E-38	8	PLPP1
5.68828891238136E-37	1.59040816915033	0.265	0.123	9.27816804498524E-33	8	ANKS1A
1.81123649423006E-15	1.58579817076163	0.304	0.216	2.95430784573865E-11	8	GADD45B
3.51292234715726E-41	1.53451940424236	0.288	0.133	5.7299276404482E-37	8	ITGB1
1.18527359316175E-45	1.51273650432443	0.25	0.098	1.93329975780613E-41	8	ASAP1
9.55352657434181E-18	1.50294386664183	0.258	0.163	1.55827571954089E-13	8	DST
3.55883945971819E-17	1.49771459272049	0.281	0.179	5.80482304274634E-13	8	FOS
2.19985295800631E-35	1.48074413436446	0.259	0.12	3.58818015980408E-31	8	PTPA
7.45573724728501E-38	1.46830732383582	0.293	0.14	1.21610530240466E-33	8	G0S2
1.04081950336713E-56	1.46697227003666	0.386	0.184	1.69768069194212E-52	8	IL6ST
4.63404351890559E-109	1.4587012903437	0.253	0.054	7.5585883836869E-105	8	TRIB2
3.45913050363271E-20	1.45156626118466	0.327	0.212	5.64218776447531E-16	8	ICAM1
1.23341865087965E-68	1.44484412887749	0.477	0.24	2.0118291614498E-64	8	EXT1
5.9336287875746E-39	1.43983632507222	0.287	0.135	9.67834191541293E-35	8	ATN1
1.40425732383753E-24	1.43731480054722	0.289	0.168	2.29048412091139E-20	8	NEK6
1.66085980415579E-55	1.42745652768994	0.348	0.147	2.70902842655851E-51	8	GSN
2.50811641868896E-77	1.41487883056016	0.493	0.234	4.09098869052356E-73	8	MPRIP
1.55827701296493E-64	1.40909816321094	0.579	0.331	2.5417056358471E-60	8	RHOB
2.79590409145773E-131	1.38409684068812	0.735	0.333	4.5603991635767E-127	8	VIM
8.61105483219021E-87	1.35577993421623	0.55	0.258	1.40454915367855E-82	8	MARCKS
1.9520199088202E-25	1.34702118896578	0.252	0.133	3.18393967327662E-21	8	ECE1
1.53817897895625E-88	1.29885359117319	0.273	0.072	2.50892373257554E-84	8	TMEM173
1.21180979879745E-95	1.28358741889376	0.608	0.3	1.97658296281851E-91	8	LTBP3
2.98254543306539E-42	1.25318127106647	0.338	0.162	4.86482985587296E-38	8	UGCG
1.03746580183919E-80	1.22697402981765	0.304	0.092	1.6922104693799E-76	8	RFTN1
2.66147296697545E-60	1.20868177244469	0.418	0.195	4.34112855643366E-56	8	SKI
1.29587296300792E-16	1.20825694059485	0.264	0.166	2.11369838996223E-12	8	ADH1B

1.30366163401068E-59	1.18555276653034	0.552	0.321	2.12640249123482E-55	8	LRP1
5.9561478439753E-30	1.18473594437826	0.364	0.216	9.71507274830811E-26	8	TRIM8
3.23033944361835E-29	1.16654295080666	0.479	0.318	5.26900666648589E-25	8	HSPB1
3.19660048456441E-64	1.15531810395584	0.322	0.115	5.21397505037301E-60	8	VCAM1
9.48644158896929E-136	1.13776430800401	0.795	0.315	1.54733348757678E-131	8	FN1
1.11817503618186E-42	1.12887922601098	0.54	0.345	1.82385530151622E-38	8	CEBPD
6.96910978433344E-14	1.12518855106348	0.309	0.223	1.13673149692263E-09	8	CD151
2.39944976160525E-20	1.12358165192071	0.365	0.249	3.91374250615432E-16	8	INF2
4.25621098621628E-14	1.08714352606789	0.313	0.224	6.94230573961737E-10	8	RXRA
1.48378630612209E-17	1.05368000323727	0.319	0.204	2.42020384391573E-13	8	SOCS3
1.73054617985032E-47	1.0012978392383	0.57	0.332	2.82269387395385E-43	8	LMNA
5.89293469797939E-18	0.993140846183556	0.282	0.173	9.61196578587419E-14	8	DEPRECATE
3.1958324008816E-36	0.98849737816376	0.517	0.338	5.21272222907797E-32	8	PLXNB2
1.75768090243807E-21	0.973130127494904	0.502	0.382	2.86695331996673E-17	8	VMP1
1.79161028309093E-11	0.968694761401987	0.299	0.221	2.92229553274961E-07	8	NFIC
3.29669982516653E-12	0.967561710971871	0.284	0.199	5.37724708482913E-08	8	ALDH1A1
4.50735575463535E-28	0.945016132127568	0.323	0.177	7.35194797138572E-24	8	CCDC85B
2.68611400474036E-10	0.936792490925154	0.302	0.23	4.38132055313201E-06	8	CTNND1
1.52846784992831E-33	0.922815326835746	0.462	0.289	2.49308391001806E-29	8	ACTN4
4.9822515614205E-16	0.910435927200257	0.357	0.258	8.12655052183298E-12	8	CYB5R3
1.7212733688603E-14	0.902093248373993	0.33	0.234	2.80756899194804E-10	8	PPIB
1.7706491914217E-28	0.884008120494889	0.508	0.348	2.88810589612794E-24	8	JUN
2.89665797755581E-43	0.875740724236564	0.319	0.139	4.72473882719128E-39	8	NOTCH1
3.88873469723122E-26	0.870590798000834	0.369	0.225	6.34291516465384E-22	8	MAP4
2.4233420858489E-08	0.867124163489818	0.257	0.188	0.000395271327622814	8	ANXA5
1.98183410986898E-16	0.853839887349931	0.313	0.199	3.23256961660729E-12	8	IGFBP2
2.29261848338737E-11	0.842029966601578	0.273	0.193	3.73949000825314E-07	8	REXO2
1.59231657864984E-26	0.831655994453303	0.467	0.303	2.59722757143576E-22	8	IFITM3
1.52252403181578E-41	0.829953150750874	0.59	0.381	2.48338894829471E-37	8	CD63
3.63818620868738E-07	0.790992982508245	0.273	0.214	0.00593424552498999	8	TXNDC5
1.11587584781586E-18	0.736852481145952	0.351	0.23	1.82010509537245E-14	8	RERE
9.75943158559807E-08	0.733188925173103	0.257	0.191	0.0015918608859269	8	TSPAN4
1.30794627915815E-15	0.721635143183507	0.345	0.238	2.13339117593486E-11	8	CALCOCO2
4.84730946369931E-41	0.697581621370045	0.371	0.17	7.90644646623994E-37	8	ZEB2
1.70149847884141E-10	0.692195583084506	0.376	0.285	2.77531416883823E-06	8	BST2
5.982677988845E-12	0.688368579819144	0.34	0.252	9.75834606760509E-08	8	FNDC3B
7.11759354198588E-21	0.669477511803215	0.445	0.299	1.16095068263332E-16	8	RRBP1
5.51719028052379E-28	0.65180242846726	0.509	0.291	8.99908906656236E-24	8	COL18A1
1.08936527637618E-07	0.646666143951135	0.296	0.23	0.00177686370229719	8	SHC1
2.21762739642608E-18	0.64498651955277	0.43	0.306	3.61717204631059E-14	8	ANKRD10
2.6281801821451E-09	0.626621451686768	0.293	0.221	4.28682469509687E-05	8	LPP
3.06037446217154E-35	0.620742647648725	0.361	0.179	4.991776785248E-31	8	ARID5B
1.93737630596075E-11	0.610879850328596	0.253	0.168	3.16005449265258E-07	8	ADD1
4.89380326039965E-19	0.568076527134071	0.275	0.158	7.98228249803787E-15	8	TTLL3
4.00967994256744E-12	0.55142985878365	0.283	0.187	6.54018895432175E-08	8	PLXND1
2.80727914989479E-16	0.523154941310102	0.478	0.328	4.57895302139338E-12	8	SERPING1
6.00447683970603E-20	0.521960992729862	0.258	0.133	9.7939021732445E-16	8	KCTD12
9.6992570098362E-08	0.45215626805895	0.377	0.308	0.00158204581087438	8	MYL6
1.51666276525882E-07	0.442729036716783	0.272	0.204	0.00247382863641367	8	CD99
2.14810801920377E-09	0.428848982578601	0.301	0.217	3.50377899012327E-05	8	CNN2

4.06409686383423E-19	0.421253364886728	0.623	0.463	6.62894839460002E-15	8	CCNL2
5.61650882613822E-13	0.415142058385412	0.311	0.207	9.16108754631405E-09	8	KLC1
2.59353086092305E-40	0.395127238884993	0.625	0.383	4.23030818725159E-36	8	CST3
2.39993803565781E-08	0.393658397561561	0.519	0.419	0.000391453892996145	8	LAMP1
8.86108734262467E-08	0.390917980302597	0.291	0.219	0.00144533195645551	8	PCED1A
7.12476984772653E-15	0.381572753503872	0.431	0.305	1.16212120986267E-10	8	MACF1
1.90133947310685E-13	0.366034024530634	0.267	0.168	3.10127481458459E-09	8	ANXA6
7.28788470634601E-10	0.316549856081533	0.585	0.45	1.1887268744521E-05	8	MT-CO3
3.18572863810553E-09	0.315598637129835	0.289	0.201	5.19624198161393E-05	8	MAP7D1
5.92582835339663E-10	0.307272802697964	0.255	0.171	9.66561862722524E-06	8	LDB1
4.01399760495309E-08	0.296209730528608	0.303	0.227	0.000654723149343898	8	SMAD3
1.84524341961216E-06	0.279458555512411	0.265	0.2	0.0300977654172939	8	OBSCN
2.712551729637E-60	0.27648052589094	0.662	0.279	4.4244431262109E-56	8	CFH
1.89192749016932E-12	0.272678667698423	0.401	0.282	3.08592292921518E-08	8	FAM118A
0	7.40712568393881	0.436	0.003	0	9	FOXP3
0	6.26258379583298	0.267	0.008	0	9	TNFRSF4
0	5.67052224280818	0.541	0.02	0	9	TNFRSF18
0	4.25452449393254	0.265	0.018	0	9	NTNG2
2.4507298744204E-175	3.91511289297098	0.253	0.04	3.99738549816711E-171	9	TBC1D4
0	3.63508432684061	0.316	0.031	0	9	CTLA4
6.21738577210333E-118	3.35765886624335	0.254	0.057	1.01411779328777E-113	9	STAM
0	3.33053277529216	0.391	0.046	0	9	ICOS
3.06470155263026E-175	3.23481541313985	0.275	0.046	4.99883470249521E-171	9	ZC3H12D
0	3.13670534844748	0.674	0.1	0	9	TIGIT
2.01465514290571E-203	3.07888444664935	0.332	0.058	3.28610400359351E-199	9	DUSP4
2.3375332208101E-145	2.88752530604307	0.279	0.055	3.81275043646336E-141	9	IL21R
1.69435914171314E-74	2.74770220901231	0.289	0.101	2.76366919604831E-70	9	ADAT2
4.79720142041318E-186	2.73593474159688	0.375	0.08	7.82471523683594E-182	9	PIM2
2.64282994587579E-179	2.71784844996894	0.284	0.047	4.310719924718E-175	9	TNFRSF9
2.82807332385284E-87	2.61094571407015	0.333	0.116	4.61287039853637E-83	9	WHRN
9.38524810068105E-105	2.45847022769116	0.369	0.123	1.53082781770209E-100	9	DUSP16
4.60481666885393E-81	2.37815712772004	0.279	0.088	7.51091646856764E-77	9	ENTPD1
2.98449126287984E-71	2.35503777959249	0.272	0.091	4.86800369888331E-67	9	PHTF2
4.01990013387424E-105	2.3514947257345	0.33	0.097	6.55685910836228E-101	9	TNIK
1.21193925124604E-195	2.30529979315515	0.537	0.153	1.97679411270742E-191	9	TRAF1
1.53082817974578E-106	2.28026524941337	0.516	0.244	2.49693384398335E-102	9	TNFRSF1B
4.85571758243943E-290	2.26439809830651	0.641	0.15	7.92016094871696E-286	9	SPOCK2
1.55552830395165E-163	2.17803533048594	0.697	0.35	2.53722221657553E-159	9	MAF
2.4711479780399E-33	2.17381545336179	0.25	0.123	4.03068946698089E-29	9	PKM
1.01907946138068E-203	2.08030754509693	0.502	0.121	1.66222050945803E-199	9	LTB
8.04074077217752E-161	2.04297754011598	0.445	0.116	1.31152522734987E-156	9	CD2
1.13456606155572E-178	2.03884138250247	0.449	0.109	1.85059070300353E-174	9	IL2RB
3.71061084659148E-87	2.01119942756512	0.415	0.167	6.05237735187537E-83	9	HNRNPLL
1.23265903758999E-204	2.00768626033591	0.614	0.183	2.01059015621303E-200	9	TRAC
3.59938589502972E-91	2.0051518676181	0.289	0.082	5.87095833338298E-87	9	TNFRSF25
1.35114280481494E-110	1.99959356387258	0.294	0.072	2.20384902893366E-106	9	CD7
4.76430151768888E-32	1.99561894400299	0.254	0.126	7.77105220550232E-28	9	KAT2B
5.45803242678278E-70	1.98278504143334	0.271	0.088	8.9025966913254E-66	9	S100A4
2.59874872778631E-91	1.94107669206047	0.297	0.084	4.23881904989225E-87	9	ITM2A
5.37312557420888E-29	1.93283736501466	0.284	0.16	8.7641051240921E-25	9	KDM2B

9.38054430345442E-80	1.93153462758568	0.316	0.105	1.53006058133645E-75	9	CCND2
2.72979487091393E-79	1.88357415138474	0.428	0.193	4.45256841394772E-75	9	CD4
1.0396258280712E-34	1.86800806705267	0.289	0.148	1.69573368816693E-30	9	MSI2
2.74216311712572E-63	1.84028151542316	0.258	0.084	4.47274226034376E-59	9	SKAP1
4.40095893737919E-24	1.81112434265473	0.301	0.188	7.1784041227592E-20	9	SERPINB9
3.84736620063654E-29	1.79666966431372	0.306	0.176	6.27543900985827E-25	9	HDAC7
6.58146762472333E-37	1.79573659666986	0.365	0.217	1.07350318426862E-32	9	CTSC
3.04581450081248E-54	1.78853941188862	0.369	0.179	4.96802803227524E-50	9	ARID5B
3.34804516909209E-81	1.779123218938	0.259	0.071	5.46099647530611E-77	9	CD28
2.0682781624294E-26	1.75806701073657	0.286	0.162	3.3735685107386E-22	9	PGGHG
4.70424074240785E-58	1.75332450086922	0.352	0.155	7.67308707494144E-54	9	BCL2
4.14908500289335E-22	1.73707727376308	0.285	0.177	6.76757254821935E-18	9	PBXIP1
2.11865496314832E-134	1.73476462718932	0.612	0.256	3.45573811039123E-130	9	IL2RG
2.47820801040735E-85	1.70286502438814	0.458	0.196	4.04220508577543E-81	9	SLA
9.6945748033542E-51	1.67495408553201	0.319	0.14	1.5812820961751E-46	9	PIK3IP1
1.77047972142782E-37	1.65262015704257	0.44	0.306	2.88782947362092E-33	9	LTBP3
2.03784105369743E-47	1.62591334269101	0.346	0.167	3.32392254268588E-43	9	TSC22D3
1.70910377147823E-120	1.51365749523243	0.79	0.551	2.78771916165815E-116	9	IL32
5.34454123290938E-07	1.47071730541075	0.255	0.217	0.00871748120499849	9	ZNRF2
6.45086296395145E-40	1.4439177966805	0.25	0.102	1.05220025805012E-35	9	MYO1G
2.72518426030935E-21	1.43382037811019	0.373	0.276	4.44504804699058E-17	9	SMAP2
1.85417260398923E-18	1.43375841910464	0.357	0.272	3.02434093436683E-14	9	GBP2
2.33981682441037E-99	1.42029334894567	0.541	0.217	3.81647522229575E-95	9	TRBC2
1.36747802234572E-18	1.40932518963138	0.298	0.196	2.2304934022481E-14	9	NABP1
2.66729345503424E-24	1.39068094548908	0.286	0.165	4.35062235450635E-20	9	NBEAL2
3.49425784680361E-25	1.38651150981145	0.294	0.17	5.69948397392136E-21	9	UCP2
2.36356670115057E-87	1.3820989662747	0.564	0.264	3.8552136462467E-83	9	CYTIP
2.23917563453479E-37	1.34754838937028	0.356	0.189	3.6523193774897E-33	9	GBP5
1.15849258302914E-37	1.3271615819575	0.29	0.133	1.88961725217882E-33	9	DGKA
1.14948384328505E-58	1.31159594681328	0.343	0.134	1.87492309678224E-54	9	ITK
9.16082878753599E-50	1.30648724302271	0.36	0.164	1.494222783535E-45	9	STK17B
2.41415430054886E-27	1.29515343584985	0.314	0.181	3.93772707962524E-23	9	CHST11
2.84647383188531E-27	1.28486677873886	0.364	0.225	4.64288346718813E-23	9	MLLT6
1.11079082043828E-33	1.23878324797613	0.483	0.34	1.81181090721688E-29	9	OGT
3.95574571729774E-32	1.21386815281995	0.288	0.141	6.45221683948434E-28	9	RAC2
2.58867272897755E-24	1.17857812072531	0.505	0.421	4.22238408823529E-20	9	CFLAR
1.33971761177387E-18	1.13849517512538	0.252	0.146	2.18521339656436E-14	9	FXYD5
2.80834273191302E-38	1.1376437382487	0.591	0.477	4.58068783002332E-34	9	RNF213
9.1187917866968E-31	1.13291585370062	0.326	0.176	1.48736612832811E-26	9	FMNL1
1.65748801863972E-29	1.11305311462416	0.317	0.169	2.70352870720325E-25	9	JAK3
4.55461219425037E-40	1.10783913686944	0.417	0.228	7.42902795004177E-36	9	TNFAIP3
1.35027102240589E-15	1.09361426971914	0.263	0.168	2.20242706464625E-11	9	DOK2
1.38022327948032E-09	1.09356536086016	0.277	0.212	2.25128219116036E-05	9	EZR
2.23780847453208E-21	1.08435131175659	0.452	0.356	3.65008940280927E-17	9	OGA
3.12457757431865E-27	1.08279651108489	0.342	0.198	5.09649848147114E-23	9	DOCK2
5.09628612139811E-07	1.07922988121505	0.265	0.216	0.00831255229261246	9	CSK
2.84446699157882E-10	1.06636434102024	0.324	0.26	4.63961010996421E-06	9	RNF19A
4.83145052999213E-48	1.06188613632986	0.295	0.116	7.88057895947017E-44	9	TRBC1
4.57396303273891E-17	1.02780248017461	0.343	0.237	7.46059110270044E-13	9	IL10RA
4.67773950225301E-45	1.0182395181839	0.52	0.305	7.62986090212489E-41	9	LSP1

5.16567887425392E-21	1.01001321295481	0.428	0.31	8.42573881179556E-17	9	SYNE2
1.06138369228677E-08	0.997813835929054	0.374	0.341	0.000173122294048896	9	IKBKB
7.01686313531516E-43	0.987062812202427	0.457	0.25	1.14452054600126E-38	9	TMC8
6.59428342329033E-51	0.969799340075637	0.706	0.59	1.07559356917289E-46	9	LENG8
1.34272326033321E-19	0.961411483245926	0.283	0.164	2.1901159099295E-15	9	CCDC88B
4.36385718289233E-38	0.958091930306002	0.409	0.214	7.11788745101568E-34	9	ETS1
3.2109175624592E-29	0.955555250554286	0.325	0.171	5.23732763612721E-25	9	ARHGAP9
1.8516681536774E-42	0.951974855937773	0.506	0.292	3.0202559254632E-38	9	CORO1A
3.98780689006668E-30	0.948470151187037	0.412	0.247	6.50451181838777E-26	9	ITGAL
6.92571478981502E-18	0.93956360741619	0.498	0.446	1.12965333936673E-13	9	CIRBP
9.07627129284814E-23	0.913805882489388	0.306	0.174	1.48043061057646E-18	9	PTPRC
1.10822176428414E-27	0.904867861286778	0.28	0.137	1.80762051972386E-23	9	SEPTIN1
4.15092748355349E-24	0.895151586911664	0.365	0.22	6.7705778184241E-20	9	IKZF1
1.03973977065501E-67	0.886354003518848	0.542	0.262	1.69591953991539E-63	9	RGS1
4.11255789143013E-16	0.866725204919482	0.277	0.172	6.70799317671168E-12	9	ARHGAP15
2.64472142968547E-37	0.85748868837236	0.428	0.226	4.31380512395997E-33	9	CLEC2D
5.16461180231195E-20	0.838703167960369	0.503	0.405	8.42399831075102E-16	9	CDKN1B
8.51216112417373E-19	0.836541313445404	0.275	0.157	1.38841860096398E-14	9	TBC1D10C
3.23153696450859E-30	0.818589142337086	0.317	0.154	5.27095994280996E-26	9	ZAP70
9.67267891574401E-39	0.808057609910024	0.561	0.358	1.57771065794701E-34	9	AKNA
1.9229465905391E-18	0.806890651445453	0.417	0.291	3.13651818382833E-14	9	ITPKB
2.50117777369472E-10	0.794853433641137	0.293	0.213	4.07967106667346E-06	9	ARHGDIB
1.40010879671407E-09	0.790199549820853	0.49	0.5	2.28371745832032E-05	9	PFN1
1.38473328674848E-11	0.777926591432139	0.342	0.254	2.25863846401545E-07	9	CD44
4.00919836977347E-14	0.738588470358271	0.323	0.218	6.53940346093751E-10	9	LIMD2
3.51049154813213E-10	0.732678368136995	0.396	0.331	5.72596276415832E-06	9	BTG1
3.94920496007965E-17	0.7298222178953	0.36	0.237	6.44154821038592E-13	9	ITGA4
8.46112064899714E-25	0.716478782129461	0.52	0.375	1.38009338905792E-20	9	ARHGEF1
5.49036949769669E-11	0.687656228178751	0.357	0.274	8.95534168769307E-07	9	HCLS1
2.19531804359847E-16	0.67226238433582	0.479	0.374	3.58078326091347E-12	9	NLRC5
8.79981930539019E-13	0.65259895961271	0.542	0.513	1.43533852690219E-08	9	ARGLU1
2.18579869909179E-06	0.649668284519494	0.507	0.544	0.0356525625808862	9	OAZ1
5.53668893607202E-13	0.647436781510205	0.373	0.272	9.03089332362707E-09	9	ARHGAP4
1.46659226554427E-09	0.636664946757268	0.368	0.291	2.39215864432926E-05	9	STK4
9.98818099816899E-15	0.614857264957025	0.39	0.277	1.62917220261134E-10	9	CELF2
7.14569004926976E-54	0.61416098888258	0.396	0.162	1.16553350393639E-49	9	IL7R
3.79683361785472E-23	0.555180587368285	0.533	0.372	6.19301531408283E-19	9	WIPF1
2.59808310036764E-28	0.552238482523431	0.777	0.765	4.23773334500965E-24	9	B2M
2.88983280905206E-09	0.497469067702749	0.49	0.43	4.71360629484481E-05	9	EVL
2.36546834087063E-06	0.49184527344365	0.355	0.297	0.0385831541079409	9	LCP1
2.28089833766946E-12	0.473317713096455	0.666	0.655	3.72037327857265E-08	9	MCL1
8.32976439263997E-08	0.461728266947705	0.592	0.624	0.0013586678700835	9	ACTB
2.55725344365385E-16	0.412309937526881	0.329	0.195	4.1711360919438E-12	9	ACAP1
1.93067222952854E-11	0.334479003199608	0.126	0.26	3.14911947358401E-07	9	FNDC3B
2.35265742563505E-06	0.321253299669004	0.161	0.266	0.0383741952695332	9	LIMD1
2.95272247783257E-07	0.298456470461594	0.173	0.293	0.0048161856335927	9	SUMF2
4.53487417047553E-08	0.263966703060571	0.146	0.259	0.000739683325946264	9	SELENOH
1.79610733990044E-06	0.261908858644434	0.197	0.319	0.0292963068211161	9	EIF3A
0	10.071398690031	0.39	0.002	0	10	IGHD
0	9.6102479028318	0.39	0.002	0	10	FCRL1

0	9.50472700382794	0.328	0.001	0	10	FCRL2
0	9.19755597168739	0.409	0.002	0	10	NIBAN3
0	9.12620740578645	0.339	0.001	0	10	BANK1
0	8.06720784176937	0.252	0.002	0	10	FCRL5
0	7.68176678481883	0.755	0.009	0	10	MS4A1
0	7.6219204109509	0.476	0.004	0	10	CD79A
0	7.32066836888114	0.372	0.012	0	10	CD22
0	6.85724261407377	0.333	0.005	0	10	TNFRSF13C
0	5.35579025708022	0.672	0.017	0	10	IGHM
1.41871751070419E-301	3.88323817040269	0.665	0.117	2.3140701317096E-297	10	CD37
1.56140971597251E-173	3.75886688543363	0.395	0.064	2.54681538772276E-169	10	WDFY4
5.31384109816144E-104	3.73708812610096	0.275	0.049	8.66740621521112E-100	10	SELL
1.98905671167412E-148	3.55380383052926	0.277	0.035	3.24435040241166E-144	10	P2RX5
5.96185088836046E-101	3.19160007162895	0.282	0.051	9.72437498400474E-97	10	PARP15
7.33491078186256E-124	2.90853891223446	0.589	0.201	1.1963972976296E-119	10	CIITA
1.86754300675336E-112	2.86893517499591	0.404	0.095	3.0461493983154E-108	10	RASGRP2
3.01918307540496E-30	2.84199837627378	0.349	0.186	4.92458951429303E-26	10	STX7
0	2.55330896954077	0.277	0.007	0	10	DEPRECATE
4.67390891234612E-50	2.54147224842585	0.416	0.19	7.62361282692775E-46	10	IRF8
3.52577475833826E-64	2.52394668587615	0.286	0.074	5.75089120832553E-60	10	FCMR
0	2.43821766033931	0.27	0.007	0	10	IGKC
9.50494551914879E-54	2.31117404008964	0.256	0.068	1.55035166362836E-49	10	SP140
3.68862080831252E-90	2.29519387089633	0.402	0.105	6.01650940043855E-86	10	ATP2A3
1.07799533681088E-18	2.26822489231651	0.344	0.225	1.75831819387222E-14	10	SYNGR2
9.38508313001032E-35	2.18027894946384	0.291	0.116	1.53080090933598E-30	10	PRKCB
3.50186816396847E-21	2.17199435219216	0.328	0.189	5.71189716224896E-17	10	ELOVL5
1.11252799859439E-12	1.93382725916566	0.314	0.22	1.8146444185073E-08	10	GGA2
2.77729509250578E-94	1.93368314722943	0.464	0.128	4.53004602538618E-90	10	LTB
1.10499216435477E-22	1.89026960533202	0.307	0.16	1.80235271927906E-18	10	SP100
2.27243547571521E-22	1.83816691991897	0.286	0.141	3.70656950443908E-18	10	CD83
6.80651636343803E-27	1.775271825754	0.326	0.16	1.11021088404038E-22	10	MPEG1
1.41995466946635E-15	1.70667806604926	0.254	0.14	2.31608806136657E-11	10	CYBB
1.14848148815712E-12	1.6480196144718	0.314	0.213	1.87328815533309E-08	10	EZR
9.39491273112214E-15	1.61577612659147	0.441	0.371	1.53240421557333E-10	10	MDM4
6.92861878139081E-15	1.52492734809581	0.266	0.15	1.13012700943265E-10	10	IL16
4.6442422412917E-16	1.45805944883036	0.27	0.145	7.5752235197709E-12	10	STK17A
2.40372768994335E-20	1.41046135507482	0.434	0.29	3.9207202350666E-16	10	LAPTM5
6.08061949781926E-21	1.38384579558309	0.376	0.219	9.918098462893E-17	10	LIMD2
1.8062359060589E-12	1.36605823002614	0.298	0.191	2.94615138637268E-08	10	CD53
1.98539411957575E-14	1.34795221102642	0.296	0.173	3.23837634844E-10	10	ARHGAP15
1.33521797965809E-16	1.34702747432849	0.298	0.165	2.17787404662032E-12	10	CCDC88B
1.77288905243391E-09	1.23462729839544	0.367	0.29	2.89175933342496E-05	10	TRIM22
1.11339641648337E-07	1.23249455402969	0.4	0.359	0.00181606089492602	10	OGA
9.01349348232913E-12	1.22689338842239	0.252	0.146	1.4701909219027E-07	10	SASH3
1.29661623739696E-08	1.06565412092403	0.289	0.202	0.000211491074481818	10	DOCK2
2.74173164037468E-18	1.05910600106762	0.413	0.254	4.47203847861513E-14	10	TMC8
2.93065353989583E-90	1.00911673760677	0.947	0.801	4.78018898892409E-86	10	CD74
1.25523108509086E-22	1.00074350396504	0.49	0.296	2.04740742289171E-18	10	CORO1A
4.85383487148237E-32	0.930545122606112	0.658	0.448	7.9170900588749E-28	10	CXCR4
5.31287543676132E-08	0.928939707205228	0.538	0.554	0.00086658311249013 8	10	MT-ND6

**3.16003251353767E-14**	0.928146683316871	0.337	0.197	5.15432903283129E-10	10	ACAP1
**4.63182523840214E-10**	0.816473912898222	0.589	0.594	7.55497014635774E-06	10	LENG8
**9.48749360226843E-07**	0.433691623423141	0.132	0.265	0.01547505081466	10	NPLOC4
**1.07654856238524E-06**	0.432727916295531	0.122	0.25	0.0175595836010657	10	POGZ
**1.75757675577796E-06**	0.324847078409899	0.15	0.288	0.0286678344634943	10	MTCH1
**2.64204197011423E-08**	0.310585810828346	0.127	0.275	0.000430943465745332	10	SELENOF
**3.57250785122861E-07**	0.300095528343175	0.164	0.317	0.00582711755613898	10	EIF3A
**2.93824285072821E-08**	0.289221145935626	0.118	0.261	0.000479256791382278	10	ARL6IP4
**5.93780190410565E-07**	0.288193108724872	0.152	0.298	0.00968514868578673	10	PSME1
**2.24705676757407E-08**	0.282010936378273	0.118	0.264	0.000366517429359007	10	CNDP2
**1.66966637966563E-08**	0.26658564439742	0.164	0.338	0.00027233928318726	10	TBC1D9B
**6.90608815329807E-08**	0.253924140932541	0.125	0.267	0.00112645203868445	10	HNRNPF
**0**	7.64740339803948	0.893	0.03	0	11	HIST1H1B
**0**	6.97575999696809	0.338	0.004	0	11	GTSE1
**0**	6.94718216744057	0.268	0.003	0	11	DLGAP5
**0**	6.91643742355666	0.344	0.003	0	11	HJURP
**0**	6.85069359937201	0.457	0.005	0	11	AURKB
**0**	6.70714840246488	0.347	0.004	0	11	KIF2C
**0**	6.64264362190681	0.322	0.003	0	11	KIF15
**0**	6.59194192563205	0.577	0.009	0	11	RRM2
**0**	6.58548395138855	0.281	0.002	0	11	SPC25
**0**	6.54639465782633	0.562	0.011	0	11	HIST1H2AH
**0**	6.50013861786349	0.599	0.01	0	11	KIFC1
**0**	6.47115221745323	0.256	0.003	0	11	CDCA5
**0**	6.41490883106232	0.356	0.005	0	11	ASPM
**0**	6.33046713224044	0.315	0.003	0	11	KIF18B
**0**	6.26758776872186	0.312	0.005	0	11	HIST1H2BH
**0**	6.23348172617106	0.297	0.006	0	11	FOXM1
**0**	6.19008401586608	0.334	0.005	0	11	ANLN
**0**	6.12851876529441	0.609	0.012	0	11	MKI67
**0**	6.08849307082941	0.53	0.017	0	11	DEPRECATE
**0**	6.07461547341662	0.473	0.006	0	11	NCAPG
**0**	6.05611921921968	0.423	0.007	0	11	TOP2A
**0**	6.02713788024291	0.271	0.005	0	11	CDCA8
**0**	6.01767472333664	0.303	0.006	0	11	MELK
**0**	6.01308281072312	0.347	0.005	0	11	KIF11
**0**	5.89878236508151	0.297	0.005	0	11	BUB1
**0**	5.85761232383811	0.268	0.006	0	11	SHCBP1
**0**	5.85658032512582	0.429	0.009	0	11	SPC24
**0**	5.84750166285564	0.461	0.009	0	11	TPX2
**0**	5.84060483784693	0.42	0.012	0	11	ESPL1
**0**	5.82962635337597	0.303	0.005	0	11	UBE2C
**0**	5.78411681098784	0.271	0.007	0	11	PLK1
**0**	5.78335082153531	0.265	0.004	0	11	KIF23
**0**	5.7741888319057	0.265	0.004	0	11	RAD54L
**0**	5.76397025986571	0.498	0.01	0	11	CENPF
**0**	5.62899941606155	0.432	0.01	0	11	UHRF1
**0**	5.61147161179432	0.274	0.007	0	11	HMMR
**0**	5.55345763969424	0.309	0.007	0	11	ASF1B
**0**	5.51802611711495	0.47	0.019	0	11	TYMS

0	5.46619577824743	0.293	0.006	0	11	NUF2
0	5.43116606773066	0.315	0.006	0	11	DEPDC1B
0	5.4164605978435	0.429	0.01	0	11	CDK1
0	5.4012721192725	0.271	0.009	0	11	PCLAF
0	5.31550143056856	0.394	0.01	0	11	MYBL2
0	5.24889821993137	0.259	0.006	0	11	CIT
0	5.24183125319912	0.293	0.007	0	11	CENPI
0	5.17448439849678	0.372	0.011	0	11	PRC1
0	5.15834340238659	0.574	0.018	0	11	CENPM
0	5.15707469120476	0.363	0.015	0	11	ZWINT
0	5.13741559810977	0.599	0.022	0	11	NUSAP1
0	5.06806192626439	0.347	0.014	0	11	CDT1
0	5.02180413751381	0.274	0.011	0	11	STIL
0	4.93207206528306	0.287	0.011	0	11	CENPU
0	4.87057014285694	0.511	0.031	0	11	H2AFX
0	4.86748110621642	0.293	0.013	0	11	TK1
2.9971222273348E-271	4.81858953988026	0.451	0.041	4.8886060650058E-267	11	HIST1H4C
0	4.81092571412904	0.344	0.01	0	11	CENPE
2.39507439103733E-283	4.80981477649561	0.256	0.011	3.90660583922099E-279	11	CCNF
1.56745357821848E-271	4.75609522834969	0.287	0.015	2.55667353143217E-267	11	SPAG5
4.55643225193234E-275	4.74458445393159	0.297	0.016	7.43199664612683E-271	11	ECT2
0	4.70935088371276	0.584	0.034	0	11	HIST1H1D
0	4.61824898283472	0.82	0.118	0	11	HIST1H1C
0	4.53699680795504	0.448	0.023	0	11	NCAPG2
0	4.50529248141167	0.278	0.01	0	11	UBE2T
0	4.43720386924675	0.7	0.086	0	11	HIST1H1E
0	4.41542736052879	0.454	0.023	0	11	FANCI
0	4.38817015457006	0.382	0.024	0	11	TCF19
0	4.27210542103506	0.407	0.013	0	11	NDC80
5.57768453420469E-246	4.15278072424045	0.404	0.035	9.09776124374127E-242	11	MXD3
3.00598982374236E-232	4.06267046018204	0.407	0.038	4.90307000150617E-228	11	FANCA
0	4.01809317963364	0.653	0.058	0	11	HMGB2
3.94958340481903E-142	4.01605336084945	0.271	0.027	6.44216549160031E-138	11	ARHGAP33
5.92264104315775E-272	4.01031549132864	0.448	0.039	9.6604198054946E-268	11	INCENP
1.57853341612773E-152	3.80941297935178	0.252	0.022	2.57474585504594E-148	11	MCM2
0	3.77941481707848	0.539	0.033	0	11	TACC3
9.17264443381267E-288	3.67085590948373	0.344	0.021	1.49615003359918E-283	11	PTTG1
1.85674563926368E-110	3.61578972319847	0.341	0.054	3.02853781220298E-106	11	TUBB
0	3.61504246564445	0.549	0.042	0	11	LMNB1
1.21635446602262E-307	3.61165493185198	0.521	0.047	1.9839957695295E-303	11	EZH2
4.5032504209834E-169	3.58435858860948	0.256	0.02	7.34525176166603E-165	11	SMC2
2.44277025888607E-241	3.43746623901771	0.498	0.054	3.98440256926908E-237	11	HELLS
3.34302154566966E-293	3.3769118174662	0.644	0.077	5.45280244314179E-289	11	KIF22
3.8597109330731E-214	3.24898184648351	0.521	0.067	6.29557450293553E-210	11	SMC4
2.36899945332684E-195	3.23470931650895	0.577	0.092	3.8640750083214E-191	11	LMNB2
1.99866367801687E-186	3.11661757522182	0.416	0.048	3.26002032521332E-182	11	NCAPD2
2.00917549122677E-101	3.11028010710043	0.3	0.045	3.27716614373999E-97	11	CENPN
5.25915943953349E-192	3.05520552387079	0.647	0.118	8.57821496182308E-188	11	TMPO
1.38903359331557E-132	3.05473321619674	0.287	0.032	2.26565269405703E-128	11	KNTC1
9.76036692955853E-103	3.05090340142384	0.278	0.038	1.59201344988029E-98	11	CHTF18

**2.8845117790397E-126**	2.96634709675659	0.42	0.071	4.70492716279166E-122	11	NCAPD3
**4.90512097979049E-103**	2.87551836249385	0.278	0.038	8.00074283013626E-99	11	TTF2
**3.82193245563425E-122**	2.86023424804871	0.407	0.068	6.23395402838502E-118	11	NEMP1
**1.69554975413421E-105**	2.83932190231328	0.303	0.044	2.76561120396831E-101	11	CHAF1A
**2.93614661060201E-163**	2.83832731312346	0.293	0.027	4.78914873655294E-159	11	KIF20B
**1.23809485637507E-100**	2.82164796632116	0.268	0.036	2.01945652023338E-96	11	TEDC1
**1.07257737472842E-84**	2.78701622168782	0.325	0.061	1.74948095591953E-80	11	MCM3
**4.55626110113471E-126**	2.70991250110296	0.416	0.069	7.43171748206082E-122	11	LIG1
**7.38073158661968E-73**	2.63696874885092	0.328	0.069	1.20387112909354E-68	11	CKAP5
**1.89365644452845E-79**	2.6220176547409	0.287	0.05	3.08874302667036E-75	11	POLD1
**2.24973173542549E-116**	2.60134185310405	0.457	0.089	3.66953743365252E-112	11	ANP32E
**9.44667843421544E-103**	2.59181652213269	0.328	0.052	1.54084771940488E-98	11	NSD2
**2.80847842999461E-63**	2.50263838410948	0.259	0.05	4.58090916716421E-59	11	RRM1
**1.37871504619532E-132**	2.45034398404163	0.372	0.05	2.24882211184919E-128	11	CRIP1
**1.43175566626281E-60**	2.42205112115885	0.356	0.093	2.33533666724127E-56	11	POLE
**2.2312631565035E-50**	2.42043947002476	0.363	0.112	3.63941333457286E-46	11	TMEM106C
**4.43598842629584E-76**	2.29344752353919	0.429	0.109	7.23554072213114E-72	11	MCM7
**9.56492451512731E-187**	2.28800170922683	0.486	0.061	1.56013483766242E-182	11	PHF19
**8.02141215974702E-56**	2.2218977790751	0.312	0.077	1.30837253737634E-51	11	SAE1
**7.60096789877743E-62**	2.18033856192616	0.281	0.058	1.23979387396959E-57	11	CBX5
**1.90573639175309E-114**	2.15387537129777	0.688	0.197	3.10844662858846E-110	11	RBM15
**2.36628155705582E-63**	2.12094104853498	0.404	0.111	3.85964184771375E-59	11	NCAPH2
**2.54406459893177E-94**	2.11555116832276	0.293	0.043	4.14962376731761E-90	11	CEP128
**1.66474371044045E-80**	2.0661286888863	0.479	0.126	2.71536346609943E-76	11	DNMT1
**1.38486033405365E-77**	2.05835440752803	0.416	0.099	2.25884569087491E-73	11	CDK5RAP2
**1.39662744588329E-68**	2.04503719584614	0.385	0.095	2.27803902698024E-64	11	SMC1A
**2.0455068719751E-63**	1.99660412832579	0.401	0.108	3.33642625887858E-59	11	CDC25B
**7.31520769832446E-32**	1.95103690715615	0.262	0.083	1.1931835276737E-27	11	MAD2L2
**6.87741865148877E-31**	1.94536601017976	0.284	0.097	1.12177575624433E-26	11	AHI1
**2.35357984520883E-109**	1.93427680084279	0.413	0.07	3.83892408552012E-105	11	PDCD1
**2.72762596080156E-58**	1.91860592841463	0.331	0.08	4.44903070466343E-54	11	MCM5
**9.301804881443E-43**	1.91383305166379	0.297	0.084	1.51721739421217E-38	11	MPHOSPH9
**6.60422079418212E-57**	1.9126230016108	0.35	0.091	1.07721445373905E-52	11	PCNT
**1.17419020335355E-60**	1.91244963832197	0.413	0.118	1.91522164068998E-56	11	DDX39A
**1.28866456705163E-57**	1.89344645648578	0.309	0.072	2.10194077531792E-53	11	DEK
**5.24745206823648E-70**	1.88659521885666	0.334	0.071	8.55911906850052E-66	11	MCM6
**9.614610333513E-52**	1.86589200771669	0.312	0.079	1.56823909149931E-47	11	SMC3
**3.59373956682863E-99**	1.80805245035233	0.448	0.087	5.86174860745418E-95	11	ITM2A
**5.84705833822521E-57**	1.79659982008016	0.426	0.126	9.53713685547914E-53	11	COTL1
**2.30071632836923E-46**	1.77760993788731	0.252	0.058	3.75269840320306E-42	11	SLC7A5
**7.83132592052081E-60**	1.76832259455468	0.568	0.217	1.27736757089615E-55	11	CALM3
**2.55261776724416E-81**	1.75596088432522	0.741	0.293	4.16357484015195E-77	11	CORO1A
**5.09920882694995E-38**	1.75162315879142	0.293	0.088	8.31731951763806E-34	11	NUDT21
**1.96595199445335E-29**	1.73337537369534	0.271	0.091	3.20666429815286E-25	11	SSRP1
**3.73329211198521E-59**	1.72521999946524	0.612	0.248	6.08937276385907E-55	11	H1FX
**7.07189998042135E-56**	1.72296733644559	0.369	0.099	1.15349760580653E-51	11	FAM111A
**1.00700845394109E-79**	1.71935410425678	0.341	0.064	1.64253148922332E-75	11	DUSP4
**1.16608215175002E-38**	1.70754606172559	0.281	0.081	1.90199659771946E-34	11	RANGAP1
**5.23472664654199E-25**	1.70548848260363	0.287	0.111	8.53836263317464E-21	11	DCTN3
**5.59034978075025E-25**	1.70385376920131	0.274	0.104	9.11841952738174E-21	11	EBP

1.06362447334147E-31	1.69590808023689	0.366	0.148	1.73487787846728E-27	11	SIVA1
2.62721198787845E-33	1.69237876959758	0.252	0.074	4.28524547342854E-29	11	HP1BP3
1.5196888758098E-22	1.69026399813683	0.281	0.113	2.47876452533337E-18	11	PARK7
9.45398633323356E-35	1.69008295421754	0.3	0.097	1.54203971081373E-30	11	MAZ
1.36701066812182E-34	1.68283630728511	0.259	0.075	2.2297311007735E-30	11	CEP192
2.34351114440258E-23	1.61370028422214	0.297	0.122	3.82250102763504E-19	11	ANAPC11
1.0952371252017E-82	1.60563110362839	0.375	0.072	1.78644127491649E-78	11	KLRC4
1.11689814324699E-49	1.57054045949737	0.391	0.12	1.82177256145017E-45	11	WHRN
4.7612016885565E-54	1.5672485206059	0.795	0.495	7.76599607420451E-50	11	PFN1
1.43340170278153E-30	1.56447387682267	0.356	0.14	2.33802151740695E-26	11	NUP50
7.42073624147284E-29	1.5641040601354	0.252	0.081	1.21039628834664E-24	11	CEP57
7.31871923297619E-16	1.56159904288211	0.29	0.144	1.19375629409075E-11	11	COX8A
3.18723875079718E-39	1.55249550392709	0.312	0.093	5.19870512642528E-35	11	CD38
1.47071477120775E-58	1.55121161180713	0.473	0.145	2.39888286331696E-54	11	CNTRL
1.16409122642477E-48	1.54001330116531	0.287	0.07	1.89874919942144E-44	11	PSIP1
6.9786097328756E-77	1.53109483036099	0.634	0.202	1.13828103352934E-72	11	CD8A
2.50599850968181E-20	1.53019462312512	0.278	0.117	4.08753416914201E-16	11	HNRNPAB
1.55605247440285E-39	1.51739058826042	0.297	0.086	2.53807719099849E-35	11	UBALD2
6.97570740991737E-29	1.50378363728347	0.41	0.187	1.13780763563162E-24	11	NUCKS1
2.97180180119455E-46	1.49148233131662	0.461	0.169	4.84730591792842E-42	11	LBR
5.03735873830529E-32	1.4734781952754	0.344	0.125	8.21643583804976E-28	11	PKM
1.80092203879443E-28	1.45708314576355	0.438	0.21	2.93748393747759E-24	11	HDGF
7.23725818610381E-22	1.45451393117009	0.366	0.176	1.18046918273539E-17	11	ENO1
9.71921462907497E-44	1.45299257314072	0.382	0.122	1.58530109814842E-39	11	NUP210
3.01430139758399E-25	1.45028097380318	0.297	0.114	4.91662700959925E-21	11	SLBP
3.57726959721471E-26	1.40711420832691	0.303	0.116	5.83488444001691E-22	11	KHDRBS1
4.40311916223171E-69	1.40431323266405	0.461	0.118	7.18192766551614E-65	11	LAG3
4.86539087137896E-15	1.39096361004246	0.278	0.137	7.93593905030623E-11	11	MTHFD1
1.8848810943095E-31	1.38957923121478	0.293	0.097	3.07442955292823E-27	11	BUB3
1.98325313681891E-35	1.38149088923625	0.59	0.306	3.23488419146532E-31	11	ACTG1
4.83800756440204E-37	1.36712193073741	0.271	0.075	7.89127413829617E-33	11	ADCY3
3.68861374340129E-20	1.35395379932616	0.256	0.102	6.01649787686184E-16	11	SNRPB
4.59468580833952E-23	1.33380683579238	0.271	0.103	7.49439202198259E-19	11	DOT1L
6.26977239653361E-20	1.33202288545505	0.29	0.126	1.0226625755986E-15	11	TUBGCP3
1.33839786250318E-25	1.33179833444208	0.388	0.173	2.18306075352894E-21	11	FZR1
3.58185279122427E-27	1.32966070085621	0.262	0.086	5.8423600877659E-23	11	SCLT1
2.83752337404843E-55	1.32936637830621	0.47	0.142	4.62828437541039E-51	11	RAC2
8.1252888182819E-67	1.3143617107938	0.543	0.159	1.32531585914996E-62	11	NKG7
9.81382742147045E-26	1.30715767634406	0.312	0.123	1.60073339071604E-21	11	SRRT
1.08011971299267E-31	1.30670730456856	0.615	0.355	1.76178326386235E-27	11	CFL1
1.8522881387669E-11	1.30510903437126	0.268	0.147	3.0212671831427E-07	11	CARHSP1
1.65464086549033E-29	1.30264800100119	0.382	0.158	2.69888471570127E-25	11	HNRNPUL2
8.0926687199769E-23	1.30226214780747	0.281	0.112	1.31999519491543E-18	11	SFXN1
1.28161750264324E-33	1.29860872151809	0.448	0.193	2.09044630856138E-29	11	HNRNPUL1
3.05110264303802E-27	1.29413945344189	0.325	0.125	4.97665352105931E-23	11	HNRNPD
6.60903697052719E-21	1.27887097855981	0.331	0.151	1.07800002026269E-16	11	NONO
4.42418753563727E-48	1.2727536859043	0.656	0.304	7.21629228937795E-44	11	HNRNPA0
1.55007663939722E-21	1.2705762932805	0.338	0.153	2.5283300065208E-17	11	UBE2I
8.17178180018423E-17	1.27007810603767	0.265	0.119	1.33289932942805E-12	11	NOP56
1.74518056355812E-29	1.26448964111168	0.284	0.094	2.84656401721965E-25	11	SYNE3

1.16807797471956E-20	1.25309306551601	0.281	0.116	1.90525198456507E-16	11	ZC3H4
6.78483360406758E-29	1.253003080984	0.274	0.091	1.10667420915946E-24	11	LIME1
1.07444930827715E-26	1.25261278555792	0.353	0.144	1.75253426673086E-22	11	CBFB
1.05481152344763E-21	1.24187173841557	0.328	0.144	1.72050307589542E-17	11	NAB1
3.34320093787102E-65	1.24149719952744	0.435	0.106	5.45309504976142E-61	11	ATP2A3
2.4534215239822E-27	1.23998378391803	0.287	0.101	4.00177584776736E-23	11	RNF168
9.3055337664694E-28	1.23573972375419	0.454	0.216	1.51782561264882E-23	11	SUGP2
6.18782901450292E-47	1.22976835967279	0.334	0.09	1.00929679055557E-42	11	NDE1
2.22919837283443E-14	1.22777664820227	0.262	0.126	3.63604546593024E-10	11	MZT2B
8.40170077993513E-19	1.22399104817403	0.274	0.118	1.37040141421522E-14	11	IPO9
2.91417911053216E-57	1.22146461545035	0.341	0.078	4.753317547189E-53	11	PTPN7
2.18536722118064E-18	1.21662754933344	0.29	0.129	3.56455247446774E-14	11	PRRC2A
1.48917485200067E-45	1.21391304748913	0.319	0.082	2.4289931010983E-41	11	SPN
1.03118772195479E-12	1.21268084055958	0.265	0.136	1.68197029328045E-08	11	PI4K2B
8.30360409784182E-43	1.2115587586985	0.423	0.146	1.35440086439898E-38	11	NAP1L4
1.30258690861324E-34	1.20653688773934	0.833	0.62	2.12464950663905E-30	11	ACTB
1.01671216259658E-18	1.19764871187703	0.293	0.131	1.65835920841129E-14	11	HCFC1
7.20177947028822E-26	1.19632445564447	0.3	0.112	1.17468224939871E-21	11	AIP
4.10714017324575E-31	1.19565853145723	0.435	0.188	6.69915633658114E-27	11	NFATC2IP
1.63493975360145E-53	1.1912383413872	0.435	0.122	2.66675023209933E-49	11	GZMA
4.8668015297279E-42	1.19106805950898	0.653	0.308	7.93823997513918E-38	11	LSP1
6.59731648874408E-18	1.18985636950304	0.328	0.158	1.07608829247905E-13	11	TMEM109
3.16587216476949E-20	1.18633322967673	0.278	0.115	5.16385408795551E-16	11	BAZ1B
3.75255168450393E-23	1.17594970543021	0.259	0.092	6.12078705259437E-19	11	ENTPD1
1.06081552171177E-27	1.17288317529666	0.42	0.19	1.73029619746407E-23	11	SRSF2
2.14366421360174E-33	1.17040931851971	0.3	0.092	3.4965306988058E-29	11	S100A4
2.13325760508391E-16	1.16750885344723	0.297	0.143	3.47955647965237E-12	11	TENT4A
2.0027735745082E-18	1.16363994431461	0.363	0.187	3.26672397738033E-14	11	PA2G4
1.0672086733655E-17	1.16066891233624	0.293	0.134	1.74072406712646E-13	11	PPP1CA
2.88900869336768E-20	1.15204411248111	0.259	0.102	4.71226207975202E-16	11	ZCCHC3
7.47559647069955E-38	1.15084115559749	0.508	0.21	1.2193445403358E-33	11	EZR
2.66581169041396E-48	1.14934215272237	0.397	0.118	4.34820544823421E-44	11	RASSF1
5.80474733646944E-44	1.14743498209792	0.681	0.333	9.4681233805153E-40	11	SUN2
6.92004733714186E-14	1.14279219296399	0.306	0.163	1.12872892116121E-09	11	PSMC3
2.56894865780526E-23	1.13876043990073	0.363	0.16	4.19021215574616E-19	11	STAG1
3.58490002408179E-14	1.13747273690553	0.331	0.18	5.8473304292798E-10	11	NACC1
2.81568804830239E-50	1.13462890217154	0.451	0.142	4.59266877558603E-46	11	RAB27A
5.78262763097243E-35	1.12755561022847	0.53	0.233	9.43204392887912E-31	11	IFI16
7.55268461949973E-56	1.12422457360426	0.628	0.223	1.2319183882866E-51	11	TRBC2
2.49040935938232E-26	1.12009401317223	0.429	0.199	4.06210670608851E-22	11	EID1
4.4875634317833E-23	1.11386042977498	0.366	0.164	7.31966471358174E-19	11	PIM1
3.62458345712976E-31	1.11364003448837	0.508	0.239	5.91205807692435E-27	11	ILF3
2.9286028908145E-41	1.11233611609327	0.262	0.064	4.77684417520754E-37	11	GNG2
6.89866340810661E-70	1.08835589737205	0.47	0.115	1.12524098849627E-65	11	TIGIT
1.33654454746509E-12	1.08102426262152	0.297	0.159	2.18003781137031E-08	11	ATP5MG
7.08609802098277E-28	1.07771915412823	0.508	0.261	1.1558134482025E-23	11	HNRNPF
1.03430162079098E-62	1.07703140574772	0.483	0.128	1.68704937367216E-58	11	KLRK1
6.92887902802646E-12	1.07603417154087	0.265	0.137	1.1301694582614E-07	11	ZFP91
1.10277128877559E-08	1.06875084607756	0.265	0.159	0.000179873024912187	11	ATP5MC3
2.63012130895697E-28	1.06621529282811	0.366	0.142	4.28999086703972E-24	11	NOTCH1

8.70644745131757E-35	1.06143354495361	0.546	0.252	1.42010864378441E-30	11	PSMB9
2.68833151589729E-45	1.05297491600047	0.603	0.236	4.38493753558006E-41	11	ITGA4
5.5621630422652E-24	1.05269754463071	0.536	0.285	9.07244413823877E-20	11	METRNL
4.12341311720057E-17	1.0484479120884	0.271	0.119	6.72569913546585E-13	11	HDAC1
2.92981605613679E-14	1.04762632396927	0.271	0.131	4.77882296916471E-10	11	SZRD1
1.59392459094055E-13	1.04753316431057	0.252	0.122	2.59985040028313E-09	11	TRMT112
3.34112153203998E-14	1.03973298814934	0.303	0.158	5.44970333091041E-10	11	ATP5IF1
2.27858483240418E-21	1.03828368816026	0.404	0.197	3.71659972013446E-17	11	HNRNPA3
3.77958640456876E-34	1.03322204761435	0.328	0.104	6.1648833844921E-30	11	CHD3
1.60717875967842E-10	1.02402259856146	0.303	0.177	2.62146927491147E-06	11	TRIOBP
2.39935219286458E-16	1.01876138875144	0.271	0.121	3.91358336178141E-12	11	ZC3H18
1.7754819000728E-16	1.01827413956518	0.303	0.145	2.89598852720874E-12	11	SART1
2.25304955395004E-54	1.01167413848806	0.372	0.092	3.67494912744792E-50	11	SH2D2A
7.70480254547002E-47	1.00961674263114	0.379	0.103	1.25673034319161E-42	11	CD3E
3.00324893674255E-47	1.00918859709647	0.379	0.104	4.89859934072077E-43	11	MYO1G
2.51460286376304E-13	1.00763639201364	0.303	0.159	4.10156873108389E-09	11	SUMO3
8.06292391645961E-29	0.999669363012068	0.334	0.122	1.31514352001373E-24	11	ANKRD36C
1.58352343393542E-43	0.995790191109499	0.309	0.078	2.58288507309207E-39	11	LCK
2.15830730601984E-23	0.993840046732541	0.334	0.135	3.52041504684897E-19	11	CHST12
6.46627917400249E-14	0.989680837823251	0.281	0.141	1.05471479607155E-09	11	SUMO2
8.59034590124052E-21	0.989656603728201	0.505	0.291	1.40117131995134E-16	11	IDH2
6.74863650325749E-23	0.988192946872492	0.366	0.165	1.10077010004633E-18	11	DEPRECATE
8.75664247160001E-41	0.982342245361267	0.35	0.1	1.42829595354268E-36	11	PTPN22
1.68554972459076E-16	0.980417105217213	0.252	0.107	2.74930015578E-12	11	DEPRECATE
1.34153204242339E-28	0.979658263134516	0.587	0.294	2.18817291439679E-24	11	LCP1
1.58248027813251E-31	0.978923962830656	0.593	0.301	2.58118358166193E-27	11	MBD2
1.46197444361917E-10	0.978786293796166	0.256	0.137	2.38462651498723E-06	11	FARSA
7.5131242211256E-17	0.972908457757201	0.3	0.14	1.2254656917078E-12	11	CYB5B
4.50699438703332E-13	0.970916852330179	0.265	0.132	7.35135854469005E-09	11	KANSL3
1.10314824769408E-13	0.96684592373697	0.293	0.152	1.79934510681381E-09	11	HMGN1
1.04990396199995E-31	0.95268954078583	0.571	0.283	1.71249835241812E-27	11	PSMB8
6.42008414358085E-11	0.950820830425942	0.278	0.155	1.04717992465947E-06	11	AP1M1
4.68442983676241E-16	0.945388327454213	0.388	0.215	7.64077350674316E-12	11	TRIM26
1.05559233567181E-12	0.940553658488296	0.347	0.198	1.72177665871429E-08	11	DHX15
3.60351521770434E-16	0.938499840057208	0.256	0.111	5.87769367159755E-12	11	CKLF
3.02512980087131E-17	0.934987637837619	0.252	0.103	4.93428921820119E-13	11	OAS3
1.29050036078517E-12	0.934229582634622	0.278	0.144	2.1049351384767E-08	11	SURF4
2.3190596864199E-09	0.930381265621095	0.297	0.177	3.7826182545195E-05	11	BLOC1S1
1.33751937972783E-13	0.929387793669056	0.353	0.201	2.18162786027407E-09	11	AKT1
1.44373276552385E-18	0.928557261248516	0.495	0.289	2.35487251384596E-14	11	H3F3A
1.63968339614875E-11	0.926313309768152	0.3	0.165	2.67448758745822E-07	11	SEC11A
5.37873869227754E-19	0.925809581945242	0.36	0.174	8.7732606809739E-15	11	YWHAZ
3.15790762868395E-11	0.925283340911617	0.3	0.167	5.1508631331464E-07	11	STK11
2.10297013315461E-28	0.918213032569599	0.3	0.1	3.43015458418848E-24	11	BICDL1
4.54162864888889E-14	0.917370690879134	0.297	0.148	7.40785048920268E-10	11	ANKLE2
1.85217625552308E-08	0.912059746947634	0.256	0.15	0.000302108469038369	11	NDUFA2
1.47540703708427E-19	0.911969617190179	0.334	0.15	2.40653641818815E-15	11	PSPC1
1.56387865769506E-18	0.911819659315537	0.344	0.16	2.55084247856641E-14	11	PPP1R18
9.01056084687247E-08	0.905331532174868	0.284	0.18	0.00146971257973337	11	CALM2
1.11771322645956E-12	0.90420928294861	0.259	0.128	1.8231020436782E-08	11	NUP85

1.22154076376038E-26	0.904173777700298	0.372	0.144	1.99245513976956E-22	11	PRDM1
7.19360179610321E-09	0.903178233992693	0.265	0.154	0.000117334838896239	11	KIF5B
1.99496508469992E-16	0.901663103354152	0.353	0.179	3.25398754965404E-12	11	CREBZF
1.92839349366868E-13	0.900688369269845	0.259	0.124	3.14540262752299E-09	11	FBXO46
8.26223560475352E-26	0.896630498774829	0.467	0.212	1.34765324949135E-21	11	ARHGDIB
1.32947888732014E-41	0.894470004797159	0.369	0.106	2.16851301310788E-37	11	PSTPIP1
4.17339906930452E-12	0.892651594223367	0.287	0.153	6.8072312219426E-08	11	ARHGDIA
9.06652812707902E-12	0.891422705210287	0.309	0.172	1.47884140280786E-07	11	ATP5F1D
1.66465200849389E-24	0.888714616793014	0.379	0.158	2.71521389105439E-20	11	LYST
7.52135565094496E-15	0.888051955265891	0.334	0.174	1.22680832022563E-10	11	PPP6R1
1.9144073075077E-25	0.887234903179428	0.596	0.338	3.12258975927581E-21	11	HNRNPA2B1
3.65196727827116E-10	0.883002099240904	0.268	0.147	5.95672382758809E-06	11	RTF2
1.52281589736044E-09	0.879844518308857	0.271	0.156	2.48386501018461E-05	11	SF3B5
1.03815963877274E-14	0.876816366639123	0.29	0.143	1.69334218680222E-10	11	HDGFL2
8.27521423424058E-10	0.876312305733209	0.312	0.187	1.34977019374698E-05	11	PMVK
7.04500643502724E-10	0.87380794013569	0.259	0.144	1.14911099961729E-05	11	SEM1
2.66586023638401E-12	0.873524068432316	0.265	0.134	4.34828463156596E-08	11	TRA2B
2.11111145679325E-13	0.869565061595262	0.259	0.125	3.44343389717547E-09	11	PRKDC
2.26369770776336E-32	0.86681621696495	0.256	0.07	3.69231733113282E-28	11	CD3D
7.34591906257982E-20	0.863275518342685	0.319	0.139	1.1981928582974E-15	11	RNF4
1.24235389180869E-48	0.86105173699324	0.577	0.192	2.02640343292915E-44	11	TRAC
1.49141592956496E-16	0.851655718184118	0.385	0.204	2.4326485227134E-12	11	CD99
1.48968391716617E-12	0.850762201685054	0.341	0.192	2.42982343728973E-08	11	RHOA
1.06855986248794E-26	0.849875991407306	0.426	0.172	1.74292799170408E-22	11	ARHGAP15
1.01148139970091E-21	0.845895033519009	0.438	0.215	1.64982731105216E-17	11	CSK
1.89153267105227E-27	0.8449523969171	0.379	0.145	3.08527893975335E-23	11	SASH3
5.88848121373685E-11	0.844822761009643	0.287	0.158	9.60470170772618E-07	11	DCAF7
1.94563923693665E-17	0.844115556593636	0.353	0.174	3.17353215936737E-13	11	RBM14
1.33991124752441E-11	0.83901478344518	0.297	0.164	2.18552923583706E-07	11	ENSA
1.97230133430575E-14	0.836691685856654	0.391	0.222	3.21702070638611E-10	11	HBS1L
1.01086374376546E-09	0.835047604873886	0.256	0.139	1.64881985245584E-05	11	PHLDA1
1.27202874837086E-11	0.82973708195027	0.262	0.134	2.07480609146771E-07	11	PURB
1.16917113117736E-19	0.829599262242086	0.401	0.198	1.9070350320634E-15	11	ASXL1
6.27087627370067E-08	0.826497596399312	0.256	0.154	0.00102284262900332	11	PSMA7
1.34297520127022E-06	0.825866475627091	0.29	0.189	0.0219052685079185	11	ANXA5
2.17393581730231E-10	0.825703729458924	0.278	0.154	3.5459067116018E-06	11	TELO2
1.02413068809721E-31	0.824662565911748	0.309	0.096	1.67045956535535E-27	11	PRKCH
3.7132312743542E-23	0.821199991978686	0.328	0.13	6.05665153159913E-19	11	TRAF5
3.5423547143511E-38	0.820990405095971	0.356	0.105	5.77793477457808E-34	11	BCL11B
2.6194976718987E-10	0.820014821995751	0.315	0.184	4.27266265263397E-06	11	GGNBP2
1.50599176348505E-10	0.814434627896306	0.268	0.146	2.45642316542047E-06	11	NELFCD
7.97834029792917E-07	0.812234933671056	0.259	0.165	0.0130134708599523	11	SOD1
2.94114421290256E-30	0.811959288468007	0.278	0.085	4.79730032566537E-26	11	ATP8A1
2.48988881892886E-14	0.809040843309213	0.303	0.153	4.06125765255486E-10	11	PPP1R35
4.964047674096E-19	0.805305887431403	0.483	0.271	8.09685816121799E-15	11	SRSF4
9.63707202063514E-23	0.80387068048769	0.505	0.268	1.5719028172858E-18	11	TRIR
2.90099029314371E-14	0.802933783570474	0.375	0.206	4.7318052671467E-10	11	SLC20A1
3.00676812159887E-18	0.79727814685449	0.353	0.169	4.90433948313992E-14	11	ANXA6
3.9271379193978E-28	0.796206582695817	0.454	0.194	6.40555466032976E-24	11	HAVCR2
1.0088189517571E-17	0.793125284372389	0.457	0.253	1.64548459221101E-13	11	SPTAN1

7.05350795686587E-15	0.792573130250624	0.259	0.118	1.15049768284439E-10	11	HMGN4
2.69123382292546E-12	0.790742535677201	0.29	0.151	4.38967148857371E-08	11	CENPT
5.8689929707302E-33	0.78745797679552	0.309	0.094	9.57291443455803E-29	11	SEMA4D
4.82631276750398E-11	0.786730983469901	0.511	0.357	7.87219875507575E-07	11	PTMS
1.55051081700047E-09	0.781612390614404	0.338	0.21	2.52903819360947E-05	11	YBX1
7.76867561028861E-32	0.777108981439094	0.312	0.098	1.26714867879417E-27	11	LAT
1.15979885859775E-16	0.773485056077687	0.394	0.209	1.89174791825879E-12	11	FUS
5.85528969465414E-12	0.770021320163646	0.309	0.17	9.55056302095036E-08	11	LMF2
7.04066670744896E-12	0.76824090111593	0.303	0.164	1.148403146652E-07	11	ANAPC5
3.95701996726734E-08	0.765915601936709	0.281	0.174	0.000645429526860975	11	GPAA1
2.982590393405E-24	0.762871868647679	0.416	0.188	4.8649031906829E-20	11	ELOVL5
3.67433221863394E-29	0.761490785258499	0.256	0.075	5.99320328181382E-25	11	MAP4K1
3.96607073399179E-17	0.758107714125073	0.268	0.11	6.46905797421402E-13	11	RCSD1
1.24817301342904E-15	0.746610225294012	0.502	0.305	2.0358950022041E-11	11	DRAP1
1.19581766511317E-23	0.745577477145468	0.536	0.285	1.9504981935661E-19	11	SLC2A4RG
2.26860727810942E-20	0.743695261677033	0.473	0.25	3.70032533132427E-16	11	POLR2A
7.725822533718E-14	0.742812203217599	0.259	0.12	1.26015891347474E-09	11	SIN3A
8.06874266751514E-12	0.741933691058513	0.3	0.16	1.31609261649839E-07	11	PITPNM1
1.22675429922642E-18	0.741360300785516	0.416	0.216	2.00095893746821E-14	11	SAFB
2.07417491255692E-09	0.738911576028804	0.331	0.204	3.3831866998716E-05	11	CRELD2
4.67170382359216E-16	0.737356291009969	0.315	0.152	7.62001610666117E-12	11	ADGRG1
1.13860342581724E-21	0.734469974895918	0.615	0.364	1.8571760478505E-17	11	WNK1
3.96553618748393E-38	0.733563336846097	0.382	0.117	6.46818607540503E-34	11	IL2RB
5.54616368970452E-12	0.731802165467626	0.338	0.192	9.04634759427704E-08	11	PNN
2.63279593334924E-13	0.730408641915456	0.338	0.181	4.29435344688595E-09	11	PAXX
2.14292614827602E-15	0.729857301585035	0.271	0.122	3.49532684045302E-11	11	STAG2
2.55819470506451E-12	0.723763715259093	0.334	0.186	4.17267138343073E-08	11	ARF6
1.11438479067318E-18	0.718975078762738	0.426	0.222	1.81767303206703E-14	11	NUMA1
6.96684340539437E-10	0.716617677439094	0.353	0.22	1.13636182785388E-05	11	UQCRC1
3.00794961792859E-06	0.714902749000303	0.274	0.181	0.0490626662180332	11	PRDX2
9.74591440993909E-09	0.707970122600984	0.265	0.153	0.000158965609940517	11	GNB1
2.09079289415524E-29	0.706746729177719	0.584	0.264	3.41029228965661E-25	11	IL2RG
3.40518203316374E-12	0.706504242486672	0.293	0.151	5.55419241429338E-08	11	MSI2
8.92423342199808E-13	0.704097974302509	0.278	0.139	1.45563171346211E-08	11	EXOSC6
1.8545365938352E-19	0.701593202482436	0.603	0.344	3.02493463820459E-15	11	VIM
1.86634800343049E-23	0.697694702966568	0.549	0.288	3.04420022839548E-19	11	SLC9A3R1
1.09012003358698E-12	0.696571813335508	0.278	0.137	1.77809478678373E-08	11	TRAPPC10
6.93852321946788E-24	0.695078483650346	0.423	0.182	1.13174252232741E-19	11	CHST11
5.16976136990745E-13	0.688704324770268	0.429	0.256	8.43239777045605E-09	11	ETFB
1.42035512503509E-08	0.687200674502566	0.274	0.163	0.000231674124444473	11	SCRIB
3.48828565987564E-07	0.682372680979142	0.259	0.159	0.00568974273982315	11	NMT1
5.6959685066286E-08	0.681994018283115	0.262	0.157	0.000929069423116191	11	PSMC5
1.21702155469243E-06	0.680419165280982	0.265	0.171	0.0198508385785883	11	ITPK1
4.8020881788751E-07	0.679746482164737	0.265	0.166	0.00783268602856317	11	KPNA4
4.74999404907342E-10	0.67903945914511	0.268	0.146	7.74771529344366E-06	11	OGDH
1.01659502059374E-17	0.6789140957777	0.502	0.292	1.65816813809045E-13	11	ACTN4
5.03887969153294E-13	0.677863668087195	0.363	0.204	8.21891666485938E-09	11	PRPF3
9.97301732824295E-34	0.676172361327735	0.546	0.216	1.62669885640971E-29	11	ETS1
4.22037748675751E-34	0.671345233868307	0.599	0.267	6.88385771865017E-30	11	RGS1
3.6035951655336E-36	0.668042685458814	0.388	0.122	5.87782407450185E-32	11	IKZF3

1.09051315658064E-11	0.664633664790556	0.315	0.173	1.77873600969869E-07	11	BAX
1.27207563851866E-12	0.664235627353648	0.278	0.138	2.07488257398778E-08	11	S100PBP
1.15923967879087E-14	0.654904456308334	0.489	0.296	1.89083584007579E-10	11	LY6E
3.2835413836315E-25	0.652256548429262	0.688	0.385	5.35578435084134E-21	11	TAP1
1.7108986907164E-22	0.650793035504743	0.445	0.202	2.79064685442752E-18	11	SLA
1.0881886201113E-06	0.6490844203523	0.293	0.197	0.0177494445826354	11	POLR2L
9.04798914426263E-10	0.648405087912431	0.319	0.186	1.47581750932068E-05	11	HNRNPU
2.17155619400608E-06	0.645878580003601	0.369	0.26	0.0354202530804332	11	CYB5R3
7.7714371571336E-28	0.642358746720249	0.442	0.173	1.26759911470006E-23	11	ARHGAP9
6.59714286757018E-17	0.638873144783652	0.656	0.408	1.07605997312937E-12	11	TMSB10
6.78776726539557E-19	0.636230784700833	0.539	0.309	1.10715271865867E-14	11	TCIRG1
2.08358138215822E-25	0.634808558966998	0.338	0.124	3.39852959243827E-21	11	GPRIN3
1.9723960473141E-07	0.630833218081557	0.3	0.193	0.00321717519277402	11	PSMD3
5.92700859779019E-15	0.630470795320053	0.448	0.263	9.66754372385558E-11	11	LUC7L2
1.50595392417244E-12	0.630076545069611	0.429	0.258	2.45636144571767E-08	11	SLC3A2
1.13817562277691E-06	0.629723887967139	0.303	0.201	0.0185647825831142	11	DEPRECATE
3.84128260134783E-39	0.628608170159655	0.407	0.123	6.26551605105845E-35	11	CD2
1.09042373340883E-13	0.626503351540659	0.382	0.216	1.77859015156315E-09	11	ESYT1
1.95709915519829E-22	0.624567760267447	0.685	0.404	3.19222443204392E-18	11	CDKN1B
1.62160504979107E-13	0.62273868052414	0.397	0.225	2.64499999671422E-09	11	SMARCA2
3.40007469110255E-18	0.617238509493695	0.416	0.2	5.54586182865737E-14	11	DOCK2
1.79071616788419E-10	0.61285277162825	0.344	0.201	2.9208371414359E-06	11	SH3BGRL3
1.73275729341378E-14	0.608872548079039	0.382	0.206	2.82630042128722E-10	11	CAPZB
2.19898273944772E-08	0.607632184782775	0.287	0.17	0.000358676074631317	11	SP3
9.66831473578617E-24	0.606575651255656	0.423	0.177	1.57699881655408E-19	11	FMNL1
8.50212415927858E-08	0.60081659175193	0.281	0.172	0.00138678147161993	11	THRAP3
1.49907633785346E-08	0.599903002001582	0.274	0.161	0.000244514341467278	11	TCERG1
2.6031411212879E-10	0.598986295263239	0.281	0.154	4.24598348293269E-06	11	SRSF3
2.4744169286383E-09	0.596345649168613	0.29	0.168	4.03602145230193E-05	11	CASC3
1.06477958963427E-25	0.593130755774119	0.413	0.173	1.73676198865246E-21	11	HNRNPLL
7.01300198608127E-11	0.591462224344238	0.303	0.167	1.14389075394972E-06	11	DDX46
5.81203646159179E-09	0.589641862538093	0.388	0.251	9.48001267250237E-05	11	MSN
1.37334966690884E-10	0.585391070995754	0.401	0.252	2.240070641695E-06	11	PSMB10
8.55029636935976E-24	0.584729209463982	0.413	0.171	1.39463884080627E-19	11	JAK3
5.19593551416379E-35	0.575375769641832	0.457	0.156	8.47509041715255E-31	11	ZAP70
5.79991674993724E-07	0.575086664985006	0.281	0.18	0.00946024421082264	11	RBM3
1.01492881703597E-08	0.572596511324167	0.256	0.143	0.000165545039346737	11	USP48
5.25960460633776E-13	0.571036885667054	0.473	0.292	8.57894107339752E-09	11	PSME1
4.71911453868358E-30	0.564536816574912	0.391	0.137	7.69734772404679E-26	11	RASAL3
1.91406431465741E-07	0.562734447689145	0.325	0.211	0.00312203030363771	11	PPP1R15B
2.2013418975051E-25	0.562255679563479	0.252	0.078	3.59060876902056E-21	11	PPP1R16B
2.39797513357884E-12	0.559910674616296	0.366	0.206	3.91133724038044E-08	11	RSRC2
4.81645527809794E-09	0.556391977080012	0.347	0.216	7.85612020410556E-05	11	EIF4EBP2
1.31476647097908E-16	0.555540167898799	0.558	0.33	2.14451559081398E-12	11	GRK2
4.80118114557541E-11	0.55405385383852	0.325	0.179	7.83120656654806E-07	11	HDAC7
6.14760558223677E-07	0.55033611893131	0.284	0.181	0.0100273594651864	11	IKBKG
8.08636353550105E-29	0.550059449584967	0.344	0.116	1.31896675627558E-24	11	PRF1
5.34689209894597E-12	0.548055046434555	0.438	0.267	8.72131570259077E-08	11	TAP2
2.42121244910472E-06	0.547811438193474	0.29	0.193	0.0394923962573471	11	NDUFB7
1.19705225957202E-12	0.53556035797791	0.319	0.164	1.95251194058792E-08	11	ARPC2

**4.52417270681987E-11**	0.534099786724545	0.454	0.294	7.37937810209388E-07	11	BRD2
**2.81731940124484E-19**	0.533692060898902	0.382	0.168	4.59532967537046E-15	11	PIK3CD
**1.34937126265339E-07**	0.532956237531938	0.3	0.186	0.00220095946651394	11	CCNI
**3.70224198512689E-08**	0.532220712965762	0.262	0.153	0.000603872690194047	11	EDC4
**4.06054784206388E-09**	0.531938510348662	0.306	0.182	6.6231595851904E-05	11	CIC
**1.10755541555174E-10**	0.531486438128219	0.271	0.143	1.80653363830644E-06	11	PIEZO1
**3.20736971400135E-19**	0.531456822808786	0.375	0.165	5.23154074050761E-15	11	CCL5
**2.91599538103366E-12**	0.529654705934686	0.344	0.183	4.75628006600401E-08	11	ARID5B
**2.99511484963158E-19**	0.529177271111298	0.385	0.164	4.88533183123407E-15	11	SPOCK2
**2.62478383703602E-27**	0.525868976329442	0.539	0.229	4.28128491658945E-23	11	CLEC2D
**1.77064633944235E-07**	0.524574855922756	0.297	0.19	0.00288810124426441	11	PMF1
**1.17400827130848E-13**	0.523377165510831	0.369	0.198	1.91492489133127E-09	11	DIDO1
**5.08681188758103E-10**	0.521593849393704	0.353	0.209	8.29709886983342E-06	11	SAFB2
**5.73341062634223E-11**	0.521334307402589	0.315	0.174	9.35176607262681E-07	11	CPSF6
**1.61032599584114E-14**	0.518157226009071	0.871	0.733	2.62660273181648E-10	11	TMSB4X
**2.75424455089751E-14**	0.514709389647898	0.303	0.146	4.49244828696893E-10	11	NIN
**1.1261555283188E-07**	0.507813610191046	0.268	0.162	0.00183687228224079	11	SMARCA4
**1.87802479274287E-07**	0.507711463378027	0.284	0.171	0.0030632462394429	11	CEMIP2
**1.71845642207264E-11**	0.506816254793079	0.382	0.225	2.80297427004268E-07	11	DIAPH1
**2.8107656343236E-06**	0.506715897713263	0.344	0.241	0.0458463982614522	11	EIF4H
**7.2431015712139E-08**	0.50604108011688	0.319	0.199	0.0011814222972807	11	PPP1R12C
**1.51843889658602E-27**	0.505819009040056	0.259	0.077	2.47672568422146E-23	11	CD7
**3.62291725853918E-15**	0.505588801627518	0.47	0.271	5.90934034040326E-11	11	SEPTIN9
**5.79498452719865E-14**	0.503856659734603	0.486	0.283	9.45219926231373E-10	11	FAM118A
**1.65233746147097E-06**	0.497621500610316	0.268	0.17	0.0269512763340531	11	ADD1
**2.80386383462854E-09**	0.495818336022863	0.293	0.169	4.57338230066261E-05	11	OTULIN
**1.61078904005012E-08**	0.494377577589219	0.35	0.22	0.000262735800322575	11	GGA2
**1.51243607578701E-07**	0.493367006088765	0.3	0.187	0.0024669344832162	11	CYTH2
**8.69557413290123E-08**	0.492182771164238	0.356	0.234	0.00141833509681752	11	INTS11
**3.66180076649399E-07**	0.486387846058728	0.315	0.204	0.00597276323022835	11	KMT2B
**1.12451092379694E-06**	0.485739126434326	0.271	0.171	0.0183418976780519	11	HUWE1
**1.62961728307405E-11**	0.483394383371733	0.278	0.14	2.65806875042208E-07	11	RAPGEF1
**3.65372048417422E-27**	0.47748633532646	0.385	0.138	5.95958348173657E-23	11	SEPTIN1
**3.6780895090942E-07**	0.475861855775544	0.265	0.162	0.00599933179828355	11	CDC73
**1.3559042569183E-08**	0.4750733391222	0.451	0.312	0.000221161543345944	11	EIF3A
**2.30754488672193E-07**	0.474724113407509	0.328	0.213	0.00376383646473214	11	PABPC1L
**7.43156238720806E-08**	0.474295710617589	0.322	0.199	0.00121216214097751	11	TACC1
**7.23586243370528E-09**	0.46537378879008	0.262	0.146	0.000118024152156167	11	SART3
**2.56212168325332E-07**	0.464364578408406	0.353	0.237	0.00417907667755449	11	RNF187
**3.06360129880583E-10**	0.463332176222914	0.486	0.314	4.99704007848219E-06	11	ECH1
**5.41374916083202E-11**	0.461737306467394	0.356	0.202	8.83036625623311E-07	11	MAP7D1
**1.3931202712847E-09**	0.460574157436191	0.36	0.22	2.27231847449248E-05	11	ANKRD11
**4.71220529837749E-10**	0.460339679622199	0.328	0.188	7.68607806218352E-06	11	EHMT1
**6.88508796499622E-12**	0.459319466520416	0.319	0.164	1.12302669797053E-07	11	NR4A2
**2.09376381729316E-08**	0.458816314610936	0.41	0.276	0.000341513816238687	11	CYBC1
**4.51745178118028E-26**	0.457771526938662	0.621	0.31	7.36841560028316E-22	11	SYNE2
**3.42642396472248E-08**	0.455406682553946	0.36	0.23	0.000558884012885884	11	ARPC1B
**1.88951648452965E-11**	0.453531832277493	0.445	0.28	3.08199033791631E-07	11	GSDMD
**3.54953197271263E-12**	0.453127292394951	0.372	0.209	5.78964160069158E-08	11	HNRNPDL
**5.54644131491359E-28**	0.449615427109026	0.344	0.115	9.04680042875555E-24	11	CD6

1.64116554612307E-27	0.449324308955524	0.363	0.127	2.67690512228134E-23	11	FYN
2.44175578571734E-07	0.447519364506043	0.259	0.154	0.00398274786208355	11	QRICH1
5.9108700698062E-09	0.443940223181772	0.391	0.256	9.64122017086089E-05	11	ARL6IP4
2.63577028450325E-07	0.44135789795441	0.3	0.188	0.00429920491105325	11	TIAL1
9.29397210089047E-08	0.440609327661327	0.297	0.181	0.00151593978937625	11	PCM1
2.87533551646289E-06	0.434734648763808	0.375	0.264	0.0468995976090262	11	TLN1
3.35347815473613E-21	0.43352727147589	0.382	0.157	5.4698582181901E-17	11	TBC1D10C
7.22665698178322E-07	0.429424467174445	0.328	0.215	0.0117874002029866	11	SBNO2
5.40118849468942E-19	0.423032580299599	0.379	0.165	8.80987855368792E-15	11	CCDC88B
1.59512051193569E-12	0.419736878916762	0.391	0.215	2.6018010670183E-08	11	KCNAB2
2.49988732114965E-06	0.419468054612008	0.356	0.244	0.040775662095272	11	NCOR2
1.70450966188902E-19	0.414850560344507	0.476	0.222	2.78022570950718E-15	11	IKZF1
1.13289837352178E-06	0.413770791398329	0.363	0.252	0.0184787053705137	11	C11orf58
2.45599338590217E-16	0.405099087322492	0.631	0.374	4.00597081174504E-12	11	NLRC5
1.76236886525579E-15	0.404627604671617	0.438	0.225	2.87459985611872E-11	11	ADGRE5
5.53424936579221E-07	0.402491139133414	0.334	0.218	0.00902691414054367	11	CNN2
3.06422122446192E-08	0.399333517798847	0.278	0.158	0.000499805123921983	11	ABCC1
5.58407772923599E-08	0.398821501956469	0.274	0.161	0.000910818918415682	11	BPTF
8.83252067362296E-07	0.397768459749008	0.268	0.166	0.0144067244707464	11	STT3B
3.06682414750714E-13	0.397634178527787	0.792	0.556	5.0022968669989E-09	11	IL32
2.20050122718021E-21	0.397023443996708	0.521	0.249	3.58923755165365E-17	11	ITGAL
7.76682101041739E-12	0.391593169725669	0.385	0.218	1.26684617500918E-07	11	FUBP1
4.00305814120257E-07	0.385944309016442	0.42	0.291	0.00652938813411552	11	RBM6
2.84687433750947E-09	0.385814004609267	0.546	0.372	4.64353673191169E-05	11	EHBP1L1
2.38238677538029E-06	0.384787985109162	0.366	0.256	0.038859110693228	11	NDUFV2
2.08044381777423E-13	0.382700494469981	0.262	0.12	3.39341191117154E-09	11	CCND3
7.27604817547604E-10	0.376496512345065	0.423	0.265	1.1867962179019E-05	11	FAM193B
1.10360827300168E-06	0.375101734239577	0.35	0.239	0.0180009545409304	11	SETD5
4.75274094540835E-12	0.370045454644563	0.438	0.261	7.75219575605556E-08	11	PKN1
3.24468072167781E-24	0.367677003278009	0.372	0.138	5.29239872512868E-20	11	ITK
6.53929131653189E-10	0.361397494308881	0.379	0.226	1.06662380663952E-05	11	DDIT4
3.16665466100763E-07	0.36036021111177	0.319	0.205	0.00516513041756954	11	KANSL1
1.83868801784074E-15	0.35738794258056	0.265	0.109	2.99908402590004E-11	11	FGD3
3.14638747621954E-08	0.353187832250532	0.372	0.238	0.000513207261246169	11	MED15
6.70051739116281E-09	0.352350330530443	0.524	0.369	0.000109292139167257	11	NDUFV1
7.11835522903189E-09	0.346135587388375	0.319	0.189	0.000116107492140739	11	ABCA2
1.96268819405455E-07	0.344091464499766	0.262	0.154	0.00320134071332237	11	CTDSP1
2.64060046738291E-07	0.342761243639036	0.382	0.257	0.00430708342234827	11	PILRB
1.92724691956473E-10	0.342144416394615	0.401	0.24	3.14353245050204E-06	11	CBLB
1.94189231843203E-10	0.341693530659644	0.476	0.303	3.16742056059449E-06	11	RABGAP1L
8.47532810588256E-16	0.339688342588739	0.328	0.148	1.3824107673505E-11	11	FXYD5
3.64943034218218E-09	0.337285282816685	0.375	0.234	5.95258583113336E-05	11	SNRNP200
2.24184612399472E-06	0.332143734862156	0.413	0.297	0.0365667521284779	11	DNM2
4.71757541583622E-07	0.329842694806855	0.347	0.229	0.00769483726077046	11	MAP4
1.71946955000611E-11	0.328871654591239	0.325	0.165	2.80462678301497E-07	11	ARHGAP45
2.13326688626862E-11	0.325509147991121	0.297	0.148	3.47957161819274E-07	11	FLNA
2.63523405238059E-10	0.321962684907644	0.271	0.137	4.29833026283797E-06	11	PTK2B
2.10685052499538E-08	0.321078022220086	0.379	0.244	0.000343648389131996	11	CAPN2
2.31319733779066E-11	0.320617440735317	0.265	0.131	3.77305617767035E-07	11	NFATC2
1.51236163716838E-16	0.319232083232897	0.779	0.51	2.46681306638534E-12	11	ARGLU1

4.86106358526353E-18	0.316997628295811	0.3	0.12	7.92888081392335E-14	11	TRBC1
1.79202044423493E-07	0.316926489149754	0.64	0.491	0.0029229645465916	11	JUND
4.30690952860899E-12	0.313474572498289	0.334	0.167	7.02500013211412E-08	11	ARHGAP30
3.04072437790152E-06	0.299421714610545	0.284	0.181	0.0495972553279518	11	BRD1
3.83228907033372E-14	0.292861483696832	0.347	0.17	6.25084670262133E-10	11	SLC38A1
7.28639877802286E-07	0.290464542301548	0.416	0.289	0.0118848450468331	11	LASP1
3.00900870283E-06	0.277563887610816	0.319	0.213	0.0490799409518602	11	MTDH
3.3062104772294E-07	0.274714330556296	0.41	0.277	0.00539275990940888	11	JAK1
8.57936776748019E-12	0.270408748520079	0.489	0.278	1.39938067655369E-07	11	CELF2
5.99846048478267E-09	0.270265145860465	0.454	0.274	9.78408889672901E-05	11	HCLS1
5.29140672515775E-12	0.267688560925594	0.397	0.22	8.6308135094048E-08	11	MBNL1
4.51782684807351E-07	0.264900226818984	0.328	0.201	0.00736902737189271	11	STK10
2.31181901877908E-06	0.257162396240866	0.338	0.226	0.0377080800153055	11	SLTM
2.45568587129643E-07	0.25459833350462	0.306	0.189	0.0040054692246716	11	DDX6
2.43286384210567E-15	0.254462671228687	0.268	0.11	3.96824421285855E-11	11	DENND1C
6.72281691464119E-14	0.25424103216341	0.366	0.176	1.09655866694712E-09	11	PTPRC
1.89977328878411E-10	0.250962569656336	0.426	0.26	3.09872021133577E-06	11	RNF19A
0	11.1214538488242	0.503	0.001	0	12	XCR1
0	9.70858347192841	0.372	0.001	0	12	CLEC9A
0	8.21070651832142	0.317	0.002	0	12	SLCO5A1
0	8.05184682182558	0.393	0.002	0	12	SLC24A4
0	7.7231839321785	0.814	0.008	0	12	IDO1
0	7.40646234835671	0.276	0.004	0	12	LAMP3
0	7.28056969464909	0.483	0.004	0	12	FLT3
0	6.31788703587222	0.821	0.066	0	12	WDFY4
0	6.18820693106052	0.386	0.008	0	12	ZNF366
3.43323227917535E-81	6.14838943687057	0.317	0.033	5.59994517056291E-77	12	GPR157
6.48909249192069E-140	5.31775341789298	0.876	0.19	1.05843587635718E-135	12	IRF8
1.14840925008124E-244	5.14563178327441	0.331	0.011	1.87317032780752E-240	12	LGALS2
5.64586167898512E-242	5.09851299652369	0.345	0.013	9.20896498459263E-238	12	CCSER1
5.36388082439478E-264	5.06343255059261	0.407	0.016	8.74902601267032E-260	12	BATF3
0	4.86676704292717	0.524	0.015	0	12	CLNK
1.36570297720375E-63	4.65683743123381	0.434	0.079	2.22759812611703E-59	12	ENPP1
5.12520660946225E-133	4.60992084452962	0.379	0.029	8.35972450069387E-129	12	C1orf54
6.38798233061062E-192	4.52389589364206	0.345	0.016	1.0419437979459E-187	12	RUBCNL
2.80204750130871E-221	4.52356530278266	0.538	0.035	4.57041967938464E-217	12	DAPP1
4.49784915958843E-155	4.52181525654932	0.703	0.089	7.33644176420468E-151	12	CSF2RA
1.41563811960057E-149	4.50811878993147	0.572	0.061	2.30904733688049E-145	12	RAB7B
2.12427604736421E-59	4.49994361212102	0.303	0.04	3.46490666085577E-55	12	FSCN1
5.0219802027695E-119	4.33653235577073	0.345	0.026	8.19135190873733E-115	12	SULF2
5.6663619366448E-199	4.31667480540807	0.303	0.012	9.24240295486134E-195	12	CBFA2T3
3.34828562638467E-85	4.28849023400878	0.283	0.025	5.46138868519604E-81	12	LDLRAD3
2.20975230668668E-118	4.28585218427811	0.531	0.065	3.60432698743664E-114	12	LRRK2
1.35588053198932E-87	4.06796843683587	0.359	0.039	2.21157673572777E-83	12	DIPK2A
1.84986875322427E-94	3.98043609355486	0.607	0.112	3.01732092338411E-90	12	CPVL
2.40382741742562E-66	3.94085849937735	0.297	0.034	3.92088290056294E-62	12	LACC1
5.20942276807927E-101	3.92701288296681	0.379	0.037	8.49708947701409E-97	12	LY75
9.5020391807734E-90	3.87255844343361	0.345	0.035	1.54987761077595E-85	12	PLEKHM3
1.08285764026773E-36	3.869945759735	0.255	0.044	1.7662490970407E-32	12	NET1
7.05613864299602E-137	3.86196579646461	0.421	0.034	1.15092677405908E-132	12	HDAC9

1.20469965721794E-71	3.82743817294346	0.49	0.089	1.96498561088817E-67	12	TSPAN33
1.05906791147305E-89	3.70650869035961	0.545	0.088	1.7274456704037E-85	12	NAV1
1.46226408177886E-133	3.67666992891156	0.814	0.139	2.38509894378949E-129	12	CD83
5.1748895814636E-75	3.67530328629198	0.303	0.032	8.44076239632528E-71	12	P2RY6
1.09835324402249E-83	3.6518625664235	0.572	0.105	1.79152397632509E-79	12	BCL6
1.92427543340717E-74	3.64890548403643	0.4	0.055	3.13868565943043E-70	12	LRRK1
1.35717272871123E-61	3.61753749842869	0.428	0.073	2.21368443780089E-57	12	MARCKSL1
8.3323813510721E-83	3.60613095539592	0.407	0.051	1.35909472217337E-78	12	GPR132
2.91025143594676E-27	3.53954018239703	0.31	0.081	4.74691111717275E-23	12	CCND1
2.49754308313198E-77	3.51104386879297	0.662	0.16	4.07374252289657E-73	12	MPEG1
5.31127043877047E-62	3.4817248160211	0.49	0.1	8.66321321267851E-58	12	VAC14
4.18966246939145E-27	3.47406064361723	0.331	0.092	6.8337584538244E-23	12	CADM1
1.41508219510885E-17	3.41114589583936	0.297	0.101	2.30814056844205E-13	12	BIRC3
1.28371786210749E-39	3.39941193029176	0.29	0.051	2.09387220488352E-35	12	TTYH2
3.35932360464175E-111	3.38700161088702	0.572	0.078	5.47939273153116E-107	12	DSE
2.81436720691013E-80	3.380513157527	0.345	0.038	4.59051435119112E-76	12	DEPRECATE
1.09447678546683E-68	3.36726820581454	0.331	0.041	1.78520108477494E-64	12	KCNK6
5.47649890261943E-46	3.36408578384773	0.331	0.058	8.93271736006256E-42	12	IL4I1
1.32094735528854E-36	3.36175921012143	0.414	0.112	2.15459723121114E-32	12	SHTN1
3.60677561994456E-53	3.34848548096242	0.497	0.117	5.88301171369157E-49	12	ZNF516
1.19463223984117E-54	3.34462820240602	0.814	0.389	1.94856464640494E-50	12	CST3
5.11030266936829E-69	3.33777458372051	0.476	0.084	8.33541468400662E-65	12	CCDC88A
2.52174860673101E-99	3.33109568210248	0.469	0.057	4.11322415243895E-95	12	NAAA
1.08377082442554E-86	3.22324029095416	0.579	0.101	1.76773859172049E-82	12	GSTP1
2.44026680065981E-68	3.20482226947147	0.462	0.078	3.98031917855622E-64	12	BASP1
6.31228048955684E-46	3.18498055537639	0.393	0.081	1.02959607065162E-41	12	CERS6
3.61252236100183E-89	3.14983900757765	0.634	0.119	5.89238522303009E-85	12	APOBR
3.43152262942527E-49	3.12874302044441	0.579	0.161	5.59715656085557E-45	12	KDM2B
1.08123178596117E-39	3.09366223304745	0.331	0.066	1.76359716608127E-35	12	CPNE3
1.41871972511775E-40	3.07605757329246	0.6	0.223	2.31407374363956E-36	12	ANPEP
1.99951434397973E-69	3.06447167908343	0.462	0.076	3.26140784646533E-65	12	ST3GAL5
5.6929144946153E-96	3.05627643831548	0.683	0.128	9.28571283216701E-92	12	ETV6
2.91015089035234E-36	3.0466653220495	0.317	0.064	4.7467471172537E-32	12	UVRAG
3.53141648517791E-09	3.02711239502569	0.276	0.134	5.76009342897368E-05	12	AKT1S1
6.93899903753153E-63	2.98865846687835	0.303	0.037	1.13182013301177E-58	12	SPINT2
3.41845737539305E-22	2.96146402803426	0.331	0.1	5.5758458250036E-18	12	ERICH1
3.59735385125755E-43	2.95425330698631	0.255	0.037	5.86764386678619E-39	12	BCL2A1
2.07182518230166E-43	2.88772358106364	0.297	0.049	3.37935405485224E-39	12	SLAMF8
2.17283634804815E-52	2.87489140922156	0.393	0.07	3.54411336730134E-48	12	GRK3
2.4041405757687E-21	2.87379603177087	0.338	0.109	3.92139369313632E-17	12	RASSF4
7.61413249166713E-112	2.86750770577955	0.703	0.114	1.24194115071583E-107	12	ST8SIA4
5.61259051922893E-20	2.86089061687494	0.31	0.099	9.1546963959143E-16	12	CD40
5.72747161792757E-33	2.85789818262025	0.338	0.08	9.34207895600165E-29	12	REPIN1
5.70092273516856E-22	2.85765869472591	0.276	0.075	9.29877507333344E-18	12	SNX3
3.95561840177679E-21	2.85538392517502	0.303	0.091	6.45200917513812E-17	12	PALM2-AKA
2.9554425494017E-97	2.85354066173496	0.634	0.103	4.82062234232912E-93	12	FGD2
2.8998784926107E-87	2.80745749950856	0.924	0.309	4.72999180929731E-83	12	LSP1
3.44681135352045E-17	2.79236188588651	0.366	0.149	5.6220939987272E-13	12	TBC1D8
1.84266019775271E-89	2.77580076617281	0.821	0.204	3.00556304855445E-85	12	CIITA
1.52324069149797E-59	2.7609266466425	0.359	0.052	2.48455789190234E-55	12	THBD

1.25172151460237E-25	2.75661781783327	0.386	0.12	2.04168296246793E-21	12	NUB1
1.50968354246912E-40	2.72480429389224	0.276	0.045	2.46244482612139E-36	12	PLXNC1
2.15368746026567E-41	2.69261928911425	0.331	0.062	3.51287961643933E-37	12	CCNG2
1.49783707827359E-84	2.6902857398193	0.51	0.075	2.44312205837205E-80	12	SLAMF7
6.78065376631977E-34	2.67472947400772	0.352	0.081	1.10599243582442E-29	12	PPT1
5.44834177138029E-37	2.67163288595862	0.379	0.089	8.88679026329839E-33	12	HPS5
5.69405799759723E-26	2.66331257132206	0.262	0.059	9.28757799988085E-22	12	C1orf21
6.02713705975514E-33	2.63006164311877	0.283	0.056	9.8308632581666E-29	12	FARP2
1.20479192708197E-42	2.62743439281003	0.469	0.117	1.9651361122634E-38	12	PTGES2
5.90226537932401E-13	2.62532541933647	0.359	0.175	9.62718506021539E-09	12	MGLL
5.01311818821669E-38	2.62529182667103	0.29	0.051	8.17689707680024E-34	12	ZBTB46
1.49933539731642E-33	2.61252695530831	0.352	0.083	2.4455659665628E-29	12	SLC1A5
4.95083023072235E-89	2.60592505217542	0.786	0.18	8.07529918933123E-85	12	SPI1
1.1267472850661E-29	2.60080826138981	0.29	0.064	1.83783749667131E-25	12	SPATS2
4.38943136274665E-36	2.57406618441934	0.538	0.184	7.15960149577606E-32	12	RUFY3
1.44922562030115E-26	2.57378714746367	0.303	0.075	2.36383190927321E-22	12	MAP3K14
8.46489110978915E-46	2.56666077164067	0.428	0.092	1.38070838891771E-41	12	FCHSD2
6.10102800902802E-28	2.56334957853841	0.276	0.06	9.9513867855256E-24	12	CXCL9
2.50191465107759E-26	2.54884296409826	0.372	0.111	4.08087298737265E-22	12	SCARF1
3.43847215995598E-19	2.54569588710208	0.297	0.093	5.60849194010421E-15	12	STX3
4.56922015260936E-52	2.54563586357497	0.469	0.098	7.45285499092113E-48	12	PTPRE
5.67936465179606E-27	2.54306713042255	0.366	0.105	9.26361168354456E-23	12	BID
3.82005069989605E-35	2.52932385696141	0.366	0.084	6.23088469660044E-31	12	VASH1
1.33629372348611E-40	2.50281164344242	0.345	0.067	2.17962869237819E-36	12	GAS7
1.7451366646581E-49	2.48917681026776	0.414	0.08	2.84649241372383E-45	12	ADAM19
1.1990721349705E-40	2.4878536462937	0.393	0.086	1.95580655935039E-36	12	VOPP1
2.27675206567601E-35	2.46887399305959	0.455	0.126	3.71361029432414E-31	12	BCL2L11
4.67785376002408E-26	2.4227775842719	0.29	0.07	7.63004726797528E-22	12	CAMK2D
8.05533680632276E-43	2.38975019425639	0.338	0.061	1.31390598647931E-38	12	NIBAN1
3.72403063030163E-36	2.36655890764188	0.331	0.068	6.07426636108498E-32	12	HCK
2.47516608002521E-55	2.35342471908271	0.331	0.047	4.03724339312911E-51	12	TRERF1
1.45728342755178E-20	2.34531267292467	0.331	0.107	2.37697499867971E-16	12	FNIP2
1.4315100104925E-18	2.33624492933802	0.269	0.078	2.33493597811431E-14	12	MYO9A
1.20959209497731E-35	2.33288294962799	0.407	0.097	1.97296566611749E-31	12	RFTN1
1.15469880924287E-24	2.31690843076116	0.476	0.182	1.88342922775604E-20	12	ID2
3.34572961902892E-31	2.30745532301668	0.483	0.159	5.45721958159807E-27	12	SFT2D2
2.38377495044874E-82	2.28093577631953	0.669	0.129	3.88817532167694E-78	12	PLEK
3.99741441175381E-58	2.27411962993309	0.979	0.802	6.52018264701164E-54	12	CD74
4.03951780728294E-28	2.27014676729314	0.31	0.072	6.58885749545921E-24	12	KIF2A
1.66943069808417E-84	2.26678244551107	0.441	0.054	2.72300841164509E-80	12	MCOLN2
6.71309180835073E-19	2.24741268691246	0.269	0.078	1.09497240486009E-14	12	DENND1B
1.52784089819718E-23	2.2378651249296	0.255	0.06	2.49206128904943E-19	12	BTN2A2
2.23233999352892E-27	2.21598307077003	0.366	0.102	3.64116976344502E-23	12	ASAP1
2.3412759624324E-58	2.21591239791853	0.331	0.044	3.81885522232349E-54	12	HSH2D
1.68093915434658E-35	2.20498293826375	0.586	0.206	2.74177985465471E-31	12	EDEM1
1.03356962550647E-17	2.16635721158636	0.283	0.089	1.68585541616361E-13	12	AUTS2
1.12559953872434E-32	2.156296620252	0.317	0.067	1.83596540761327E-28	12	FGR
2.82430512000231E-14	2.15452325122406	0.29	0.107	4.60672408123577E-10	12	IFNGR2
1.36556474303712E-23	2.15199193929302	0.269	0.065	2.22737265236785E-19	12	NSMAF
3.04037254221669E-20	2.14912602606152	0.366	0.127	4.95915165360965E-16	12	MED13L

1.13109985977772E-40	2.14084522204613	0.579	0.163	1.84493698128345E-36	12	TRAF1
2.33668507902585E-32	2.1340424332994	0.49	0.152	3.81136703239907E-28	12	GSN
1.43915862738856E-57	2.1248038352442	0.731	0.217	2.34741163713348E-53	12	PLEKHO1
3.72224855092132E-17	2.12163092835018	0.269	0.081	6.07135961140777E-13	12	FAM49A
1.7246275521868E-18	2.08465954443122	0.303	0.092	2.81304000037188E-14	12	NDE1
7.03884974598645E-25	2.08016703545332	0.4	0.128	1.14810678206785E-20	12	GM2A
2.21888493786451E-34	2.05686386710653	0.338	0.073	3.6192232221508E-30	12	INPP5F
7.94004625730541E-42	2.05541822315308	0.338	0.061	1.29510094502909E-37	12	RASSF2
2.02266291447233E-25	2.0305472059321	0.49	0.181	3.29916547979582E-21	12	LGALS1
1.35558761129509E-52	2.01940696272551	0.641	0.173	2.21109895278342E-48	12	FGL2
1.17744305988268E-22	2.01348904555015	0.338	0.102	1.92052737497464E-18	12	ST3GAL2
5.43707284488785E-17	2.00550697504172	0.441	0.201	8.86840951729657E-13	12	DEPRECATE
4.56098591612236E-40	2.00390012222228	0.772	0.359	7.43942412778718E-36	12	LYZ
1.65632050082992E-37	1.99682028709793	0.4	0.09	2.70162436890368E-33	12	CD93
1.61304859889146E-11	1.99639852264496	0.372	0.189	2.63104356965187E-07	12	CTSH
9.75546907042419E-18	1.99041274938816	0.31	0.102	1.59121456007689E-13	12	S100A10
1.61684565640296E-33	1.98834481493266	0.559	0.19	2.63723695015887E-29	12	SERPINB9
1.72569244259363E-20	1.9792730473508	0.421	0.158	2.81477694311446E-16	12	WARS
2.74847066236705E-18	1.96931114770005	0.345	0.123	4.4830304973869E-14	12	DLGAP4
6.29461623347234E-13	1.96923965059688	0.317	0.133	1.02671485384167E-08	12	SRC
2.94651227085028E-29	1.95353253932589	0.414	0.116	4.80605616498389E-25	12	NFKBID
1.15337159626716E-21	1.94596502150674	0.586	0.269	1.88126441067137E-17	12	TNFAIP2
2.38954073118261E-14	1.94344204649612	0.428	0.211	3.89757988663195E-10	12	RAB7A
1.60431618644365E-37	1.94053982669772	0.869	0.622	2.61680013170824E-33	12	ACTB
5.58250238351605E-20	1.93379604997258	0.483	0.198	9.10561963775303E-16	12	NABP1
1.96130600223164E-30	1.92202099614481	0.407	0.109	3.19908622024003E-26	12	RAB8B
2.73324478358959E-54	1.91661751574955	0.69	0.188	4.45819556651298E-50	12	FNBP1
3.65326286735782E-17	1.88937985420682	0.31	0.105	5.95883706294734E-13	12	MTMR10
1.34600714054477E-44	1.88124167137751	0.834	0.371	2.19547224694258E-40	12	EHBP1L1
6.99320551083899E-45	1.87346430014144	0.641	0.191	1.14066175087295E-40	12	MICAL1
3.67498202796654E-11	1.87139431300441	0.386	0.193	5.99426318581622E-07	12	PTPN1
6.73153217027671E-20	1.87121482625388	0.331	0.102	1.09798021229383E-15	12	SLC8A1
1.27791700718196E-25	1.85983442173355	0.49	0.177	2.0844104304145E-21	12	MRTFA
1.10513695413214E-15	1.85791000777662	0.379	0.159	1.80258888588493E-11	12	DOCK5
2.47587074067547E-23	1.85681001177916	0.524	0.222	4.03839276511577E-19	12	LPP
4.76755275342222E-21	1.8541701571989	0.345	0.106	7.77635529610698E-17	12	DPYSL2
6.51892576890542E-22	1.84295891331517	0.359	0.112	1.06330198216616E-17	12	LGALS9
8.83253321990145E-20	1.79399044138919	0.29	0.082	1.44067449349813E-15	12	VSIR
2.82807307827917E-25	1.79389964598284	0.317	0.083	4.61286999798115E-21	12	RAB11FIP4
5.27953610733845E-12	1.78587691440505	0.393	0.197	8.61145134467974E-08	12	ODF3B
5.65602579882357E-26	1.78519677032037	0.262	0.055	9.22554368046112E-22	12	FCHO1
2.97523447389902E-15	1.77448800864043	0.262	0.083	4.85290495037669E-11	12	MCM5
1.49754096431694E-40	1.77330495812757	0.752	0.296	2.44263906689735E-36	12	LCP1
8.16793661936888E-10	1.76550962903944	0.255	0.107	1.33227214198526E-05	12	HMGN3
2.80621016995836E-16	1.76422990809641	0.269	0.081	4.57720940821908E-12	12	MRC2
5.22670152924456E-09	1.76356416456395	0.276	0.13	8.52527286435081E-05	12	FOXN2
1.03610147771072E-10	1.75308720727731	0.379	0.202	1.68998512029396E-06	12	SCARB1
2.07639417921713E-11	1.75246627088885	0.324	0.146	3.38680654572106E-07	12	ZMIZ1
4.82578584308894E-12	1.74992167542645	0.276	0.104	7.87133928866236E-08	12	FMNL2
1.81073655225834E-18	1.74452910095486	0.379	0.135	2.95349239038857E-14	12	SLC6A6

1.64383005056871E-14	1.73813247133499	0.407	0.186	2.68125119548262E-10	12	RAB10
3.56094718907917E-21	1.73800580983832	0.352	0.109	5.80826096010704E-17	12	DYSF
1.24437778070652E-29	1.70554246459746	0.331	0.076	2.0297045981104E-25	12	OSBPL3
6.31292344227426E-17	1.70095459580644	0.386	0.152	1.02970094266935E-12	12	MYD88
1.53940800965062E-18	1.69807013298756	0.483	0.219	2.51092840454113E-14	12	BACH1
1.68547603908719E-14	1.69070088397909	0.407	0.178	2.74917996735512E-10	12	REL
1.70357490765022E-10	1.68028471086159	0.255	0.101	2.77870103186828E-06	12	SNAP23
6.16213931515622E-15	1.67660596738659	0.317	0.117	1.00510654369513E-10	12	SLC12A9
8.10902922255422E-10	1.67321849849773	0.255	0.106	1.32266375649082E-05	12	RAB11FIP1
5.08684891543072E-21	1.65694967324258	0.517	0.225	8.29715926595904E-17	12	SYNGR2
4.13675414907742E-29	1.65526913295512	0.517	0.167	6.74745969256018E-25	12	NBEAL2
2.61195614042065E-14	1.6296856292864	0.483	0.26	4.26036166064011E-10	12	CYB5R3
6.46615539250793E-23	1.62580699461617	0.366	0.109	1.05469460607197E-18	12	FAM49B
2.13129381098329E-14	1.6242384722586	0.372	0.159	3.47635333509485E-10	12	RFX5
3.85093201736948E-16	1.61685516898928	0.359	0.13	6.28125521353136E-12	12	KLHL6
7.35431326473956E-10	1.60164505226515	0.31	0.145	1.19956203661167E-05	12	PITPNA
1.9040109336556E-36	1.58802163562974	0.952	0.734	3.10563223388565E-32	12	TMSB4X
1.94918703539835E-15	1.58297504606172	0.428	0.186	3.17931897343824E-11	12	TYK2
9.78646058658002E-25	1.56399548161915	0.717	0.41	1.59626958627707E-20	12	TMSB10
3.03589328513464E-07	1.55994055580796	0.262	0.125	0.00495184553738311	12	EPSTI1
5.95422846387883E-24	1.55334811654808	0.628	0.307	9.71194204743276E-20	12	RNH1
2.00444535988464E-37	1.54681060615315	0.759	0.32	3.26945082650784E-33	12	CTSZ
2.14441463232424E-27	1.54073270179072	0.676	0.314	3.49775470678407E-23	12	DBNL
1.6348298735983E-14	1.53529154675893	0.276	0.093	2.66657100682619E-10	12	ACTR3
5.17182520414886E-21	1.53147257751936	0.345	0.103	8.43576409048721E-17	12	FAR1
1.13580455031024E-21	1.52321208878703	0.434	0.149	1.85261080201104E-17	12	PARVG
3.51138863790233E-18	1.51131148293674	0.441	0.182	5.7274260072825E-14	12	CIC
1.23920376995179E-29	1.49932486188576	0.366	0.088	2.02126526916837E-25	12	SELPLG
1.3129656471727E-27	1.49325177628015	0.524	0.172	2.14157826710338E-23	12	UCP2
5.87921246238733E-11	1.48926380404993	0.345	0.164	9.58958344739998E-07	12	ENSA
1.95384152392955E-32	1.48578181807369	0.379	0.088	3.18691090968149E-28	12	DOCK10
1.37333689550124E-12	1.48212374794772	0.317	0.129	2.24004981025207E-08	12	LCOR
2.43709135565852E-29	1.47777750644787	0.745	0.353	3.9751397102146E-25	12	TYMP
9.44885001715919E-19	1.47649848535281	0.497	0.219	1.54120192629884E-14	12	SYMPK
6.29443181375186E-28	1.47513074567354	0.786	0.422	1.02668477314107E-23	12	CFLAR
5.92554833302178E-16	1.4506655213704	0.428	0.186	9.66516188599182E-12	12	ARF6
1.15522400110658E-12	1.45021263307092	0.352	0.154	1.88428586820494E-08	12	MED25
9.55858623128931E-33	1.45002153845992	0.303	0.059	1.5591010001856E-28	12	RGCC
1.97308942114858E-14	1.44325504570974	0.545	0.308	3.21830615483545E-10	12	ACTG1
1.00325624775951E-29	1.44173603624773	0.724	0.325	1.63641126572054E-25	12	HSPA1B
1.35851962125426E-49	1.43862189409557	0.669	0.174	2.21588135422783E-45	12	ITGAX
1.33309975296422E-13	1.43677093262975	0.51	0.266	2.17441900705994E-09	12	IRF1
3.45462591579961E-09	1.43338621230291	0.269	0.114	5.63484033126074E-05	12	IL18BP
8.78070006344192E-08	1.42531877423002	0.324	0.178	0.00143221998734801	12	USP12
7.07794120154594E-14	1.41335219127446	0.317	0.12	1.15448298938416E-09	12	ATAD2B
3.00035244008879E-24	1.3966130437214	0.421	0.128	4.89387486502883E-20	12	COTL1
8.41061770680949E-38	1.39616701490561	0.655	0.2	1.3718558541577E-33	12	DOCK2
3.73364531089427E-22	1.39558662823287	0.593	0.255	6.08994886659964E-18	12	MYO9B
1.28569931225913E-34	1.39384350571289	0.497	0.133	2.09710414822586E-30	12	ADCY7
6.63406332721953E-24	1.3872052970743	0.434	0.136	1.08208206930278E-19	12	KCTD12

7.58777698315838E-11	1.38485451214126	0.379	0.188	1.23764230372296E-06	12	STX7
6.67975313951888E-11	1.38232788994894	0.4	0.206	1.08953453458692E-06	12	LAP3
1.29360705712256E-25	1.38225465086387	0.655	0.284	2.11000247087261E-21	12	FAM118A
5.1043946099777E-22	1.37297844432199	0.366	0.107	8.32577804833463E-18	12	CLEC7A
3.63054499977028E-09	1.36910280184694	0.469	0.267	5.92178194912531E-05	12	MARCKS
7.83717142604969E-21	1.3653049963057	0.386	0.126	1.27832103130296E-16	12	PKM
1.25500084015965E-13	1.35255865271051	0.428	0.2	2.04703187038441E-09	12	ZMIZ2
4.81283600068979E-22	1.34970369675376	0.593	0.268	7.85021680072512E-18	12	TAP2
6.87064118122055E-11	1.33593636034216	0.276	0.111	1.12067028306888E-06	12	AFTPH
9.40179208809005E-15	1.32750318884725	0.4	0.165	1.53352630748837E-10	12	PLEKHO2
9.92253250350201E-07	1.32325965500057	0.297	0.163	0.0161846427664621	12	SERPINB6
5.76791751836464E-19	1.3229664391491	0.566	0.27	9.40805026420457E-15	12	PPP1R9B
5.00348353285779E-24	1.31938899880134	0.731	0.387	8.16118199044435E-20	12	TAP1
7.42537332846991E-19	1.3114890471791	0.566	0.264	1.21115264360673E-14	12	SAMHD1
2.43564204817659E-14	1.30245351250902	0.531	0.28	3.97277574478084E-10	12	EFHD2
1.60268365152556E-16	1.2951474069546	0.297	0.093	2.61413730400334E-12	12	TRPS1
3.15619479162085E-33	1.29498041404252	0.317	0.063	5.14806932461276E-29	12	DNASE1L3
1.59178055578159E-07	1.27926966657208	0.29	0.148	0.00259635326453535	12	CEP350
1.24915864537541E-10	1.27531643708174	0.407	0.196	2.03750266647183E-06	12	GNA13
7.32825110775828E-15	1.26132164234982	0.607	0.358	1.19531103818645E-10	12	CFL1
1.93384783049586E-12	1.25133810906292	0.317	0.126	3.1542991963218E-08	12	PRMT2
3.75003083122057E-12	1.24955201813843	0.331	0.136	6.11667528880387E-08	12	PACS1
2.40654630775793E-10	1.24741778861926	0.338	0.155	3.92531768258396E-06	12	PAN3
1.840805443686E-19	1.24649125865426	0.441	0.157	3.00253775919623E-15	12	ADA2
2.1454197209883E-12	1.24516033275095	0.414	0.197	3.49939410690401E-08	12	TRABD
5.95063156976062E-07	1.24409124889502	0.469	0.318	0.00970607515343655	12	PNRC1
6.51871319246808E-16	1.24351020073947	0.372	0.142	1.06326730882347E-11	12	ARPC4
1.14597837954468E-13	1.23460830013086	0.552	0.309	1.86920533487532E-09	12	ZYX
2.09418666677636E-10	1.23164870585593	0.283	0.118	3.41582787217893E-06	12	ARHGAP17
6.3204553393598E-08	1.22895060681476	0.255	0.116	0.00103092947040298	12	HPS1
1.10976712341939E-08	1.22407203325241	0.352	0.184	0.000181014115500937	12	GNAQ
8.70067805346719E-14	1.21227329632051	0.421	0.191	1.41916759730103E-09	12	ITSN2
1.22909392396265E-10	1.21193055439524	0.269	0.104	2.00477509937548E-06	12	TMEM106A
5.68645812156824E-14	1.2112204380245	0.269	0.087	9.27518184208996E-10	12	SEMA4A
5.93992721187369E-13	1.20768011453295	0.455	0.22	9.68861527528717E-09	12	NAGK
2.17221409745247E-11	1.20746660197091	0.331	0.145	3.54309841435472E-07	12	AP1G2
5.00135193679505E-08	1.20288219049385	0.276	0.131	0.000815770514410641	12	RGL2
8.82505353973355E-24	1.19470721077187	0.814	0.497	1.43945448286594E-19	12	PFN1
7.90732440432214E-11	1.19097150473819	0.338	0.153	1.28976368358898E-06	12	HTT
6.70475494961462E-11	1.18979739811961	0.297	0.121	1.09361257983164E-06	12	JAK2
1.34369645621337E-06	1.18192046245851	0.262	0.135	0.0219170328972963	12	KIAA0930
2.49787039526394E-09	1.18079875847522	0.324	0.159	4.07427640171501E-05	12	NMT1
1.09386837213714E-13	1.17459004742738	0.352	0.139	1.78420870179289E-09	12	APOL3
2.10757099430674E-06	1.17103607651279	0.303	0.166	0.0343765904881372	12	RAP2B
2.38771075670408E-09	1.15694640826604	0.372	0.192	3.89459501526002E-05	12	RTN4
2.26010562296411E-08	1.15208268631644	0.359	0.192	0.000368645828161676	12	PLEKHB2
1.65026804884974E-07	1.14964984167703	0.283	0.14	0.00269175221447881	12	PPP3R1
2.4129243628777E-10	1.14120913603556	0.31	0.134	3.93572092828981E-06	12	PURB
2.07677755080089E-10	1.1329204471091	0.49	0.282	3.38743186311133E-06	12	HSPA1A
3.98332093742476E-13	1.12784567781415	0.414	0.19	6.49719478103352E-09	12	NFATC2IP

2.43635914618681E-14	1.11074310991084	0.579	0.304	3.97394540334531E-10	12	RABGAP1L
1.86445387679505E-08	1.11054291114908	0.255	0.11	0.000304111071844041	12	PRDM2
4.300892143544E-13	1.10584968567122	0.352	0.139	7.01518517533461E-09	12	ARHGEF2
2.23023786237776E-22	1.10448755947993	0.69	0.342	3.63774097732437E-18	12	GRN
3.14128495494907E-24	1.10157801650915	0.483	0.154	5.12374989001743E-20	12	NCKAP1L
2.69783601581971E-21	1.0922854471737	0.738	0.345	4.40044032540353E-17	12	VIM
2.25519207485584E-09	1.08995433678884	0.29	0.122	3.67844379329737E-05	12	VCAM1
1.10389288293528E-11	1.08880090575387	0.441	0.224	1.80055968135574E-07	12	AKIRIN2
2.10261499233395E-07	1.08691740534854	0.262	0.122	0.0034295753139959	12	RELT
3.951942341539E-08	1.07402354263044	0.29	0.135	0.000644601315328426	12	MOB3A
6.75743553166465E-07	1.07337797552795	0.31	0.169	0.0110220530956982	12	NRDC
2.25798961023454E-06	1.06450467072523	0.338	0.203	0.0368300685325355	12	LPGAT1
9.65123752746463E-14	1.06257497372423	0.303	0.107	1.57421335310476E-09	12	PLEKHA2
1.05012631244415E-07	1.05793245096393	0.31	0.159	0.00171286102822766	12	WDR33
3.55593835973185E-12	1.05760024829735	0.386	0.173	5.80009105855862E-08	12	KIAA0100
1.06079819695179E-11	1.0521744642542	0.476	0.256	1.73026793904806E-07	12	NDUFV2
8.41215794783863E-13	1.04575561507557	0.407	0.183	1.37210708287196E-08	12	KMT2C
1.84775020449774E-11	1.04214661914613	0.269	0.097	3.01386535855626E-07	12	HAPLN3
1.62988637303282E-09	1.0352031548224	0.421	0.229	2.65850766305383E-05	12	MAP4
4.13742664539818E-27	1.03462148784525	0.841	0.412	6.74855660130898E-23	12	SAT1
1.07055739340806E-06	1.00655230753763	0.303	0.164	0.0174618616438789	12	PCMTD1
1.13633205658973E-08	0.973857747389596	0.393	0.207	0.000185347121750352	12	SLC20A1
2.44058667585574E-09	0.96029183412177	0.407	0.213	3.98084092698829E-05	12	M6PR
2.81030948529707E-06	0.956840122577249	0.4	0.255	0.0458389580146805	12	CLCN7
5.71808829749805E-07	0.954745176961507	0.269	0.132	0.00932677382204906	12	GPBP1
3.40859997503285E-07	0.947351788102436	0.31	0.162	0.00555976741927608	12	TTLL3
1.26919706253008E-11	0.947057727230503	0.441	0.218	2.07018732869281E-07	12	CNN2
4.34203325794357E-09	0.940698741583835	0.366	0.187	7.08229044703176E-05	12	JMJD1C
1.14621787219454E-18	0.921078417180774	0.869	0.654	1.86959597133651E-14	12	MCL1
1.35040247743299E-07	0.920061412787106	0.297	0.145	0.00220264148094094	12	GTPBP1
2.81368876615801E-07	0.916906785408296	0.262	0.123	0.00458940774648032	12	PPP1R12A
1.66720743820547E-08	0.913555924589104	0.297	0.14	0.000271938205245694	12	FLOT2
1.36643797168988E-09	0.883100373276089	0.255	0.1	2.22879697562336E-05	12	STIM2
2.23325657987691E-06	0.870789109863349	0.303	0.165	0.0364266480743723	12	RNASET2
8.52574071542946E-07	0.870501572745965	0.255	0.117	0.013906335680937	12	AOAH
1.92444430706541E-08	0.862521342143187	0.255	0.108	0.000313896110925439	12	DEPRECATE
1.92136847300932E-06	0.854693441853143	0.303	0.166	0.031339441163255	12	KPNA4
2.4186731184566E-10	0.843037980361896	0.607	0.385	3.94509772351456E-06	12	VMP1
2.37572524740444E-13	0.834568653183586	0.331	0.124	3.87504545104139E-09	12	NUP210
1.39995904441941E-08	0.80976811195	0.352	0.177	0.00022834731973525	12	G3BP2
4.57757541826565E-21	0.807998882724728	0.621	0.239	7.46648326473311E-17	12	ITGA4
1.49269436753235E-11	0.792922151614444	0.4	0.173	2.43473378288201E-07	12	PLCB2
1.91650244578443E-07	0.773408710639871	0.483	0.304	0.00312600713931898	12	MBD2
2.94667060790222E-13	0.766836902336237	0.4	0.165	4.80631442854932E-09	12	ARPC2
2.56981303435668E-08	0.765130342319867	0.428	0.234	0.000419162204033918	12	DEPRECATE
3.80218586689052E-08	0.764942655756175	0.331	0.157	0.000620174536748513	12	ANKRD44
2.73305418450452E-08	0.76435498698993	0.352	0.178	0.000445788468034532	12	SUSD6
1.03467369168759E-14	0.760615371708758	0.607	0.279	1.68765625851162E-10	12	CELF2
1.26466803593813E-06	0.759648574139098	0.462	0.301	0.0206280003341868	12	NOTCH2NLC
1.342072749191E-06	0.755672360289023	0.469	0.308	0.0218905486120544	12	HNRNPA0

9.56665553678107E-19	0.749519651591149	0.476	0.166	1.56041718460436E-14	12	CCDC88B
1.16821831198066E-12	0.739499665342564	0.586	0.292	1.90548088867165E-08	12	STK4
1.67298867472909E-07	0.727226209532835	0.538	0.369	0.00272881182735063	12	KDM2A
1.66316130278671E-09	0.726736093605665	0.269	0.106	2.71278240097541E-05	12	EMILIN2
3.11621133957344E-07	0.72598706813769	0.503	0.313	0.00508285231597824	12	MGAT1
3.49863123090896E-07	0.718494225215619	0.4	0.23	0.0057066174007356	12	VPS13C
9.99064433507263E-12	0.70422334609137	0.572	0.287	1.6295739974937E-07	12	ITGB2
2.68820221166239E-07	0.702548762612207	0.352	0.187	0.00438472662744253	12	ZFC3H1
5.8547368369988E-09	0.675575114601935	0.276	0.115	9.54966125482873E-05	12	IGFLR1
4.9744100695855E-07	0.655127808669452	0.572	0.399	0.00811376026450091	12	AHNAK
2.30561040539289E-08	0.652025569733362	0.634	0.429	0.000376068113223634	12	TPP1
2.79815788221885E-07	0.640823627639834	0.566	0.358	0.00456407532168716	12	PTMS
3.6591903390708E-08	0.631902865448357	0.655	0.44	0.000596850536205838	12	OS9
8.74248974515286E-07	0.631101777635132	0.379	0.215	0.0142598750233188	12	SBNO2
3.36432813039632E-07	0.622935522183194	0.331	0.161	0.00548755561348943	12	THEMIS2
6.61395114984104E-12	0.59255020271412	0.676	0.376	1.07880157205057E-07	12	NLRC5
5.37280972673436E-07	0.589305364792624	0.338	0.181	0.00876358994527642	12	BTAF1
1.78898753087557E-07	0.578573959511002	0.469	0.255	0.00291801756161115	12	CD44
9.50588534413363E-09	0.552388565870015	0.8	0.447	0.000155050495848164	12	SRGN
1.50957003715906E-07	0.545453843701818	0.4	0.217	0.00246225968761014	12	CSK
7.92083970442404E-09	0.53662627048878	0.476	0.239	0.000129196816418861	12	IL10RA
2.73004668251951E-12	0.533023352704966	0.434	0.179	4.45297914385757E-08	12	FMNL1
2.35127098831478E-13	0.532440391285478	0.428	0.168	3.83515810904023E-09	12	ARHGAP30
5.64486898735992E-08	0.479196009100089	0.897	0.765	0.000920734580528277	12	B2M
9.20494119319552E-07	0.445336733783227	0.4	0.221	0.0150141795802212	12	MBNL1
6.53586206822923E-07	0.402473208677459	0.276	0.126	0.0106606446194887	12	INPP5D
6.14174936718809E-07	0.396151668332502	0.255	0.114	0.0100178073928205	12	DOCK11
1.96527901607955E-06	0.351203425417206	0.821	0.635	0.0320556660312735	12	TAPBP
1.45653143320835E-06	0.349963736710838	0.338	0.166	0.0237574842070613	12	ARHGAP45
9.3364788766208E-16	0.331953803371448	0.731	0.414	1.52287306956562E-11	12	PSAP
1.03340227496906E-14	0.286295376960498	0.49	0.196	1.68558245070203E-10	12	HAVCR2
0	12.8518120381712	0.396	0.001	0	13	IGHG2
0	12.6687229903637	0.472	0.002	0	13	DEPRECATE
0	12.0066200978035	0.472	0.001	0	13	IGLC1
0	11.6263488051628	0.547	0.003	0	13	IGLC3
0	11.4894276293523	0.717	0.002	0	13	JCHAIN
0	11.2432384808138	0.642	0.007	0	13	IGHG1
0	11.1506801982509	0.453	0.005	0	13	IGHA1
0	11.0368124617212	0.604	0.011	0	13	IGKC
1.39802803139568E-307	10.8700156582981	0.585	0.011	2.28032352200949E-303	13	DEPRECATE
0	10.7965174129567	0.679	0.008	0	13	IGLC2
0	8.65766970190883	0.698	0.002	0	13	DERL3
2.50157310580194E-46	8.36184829371064	0.358	0.029	4.08031589287355E-42	13	IGHM
0	7.61262757217413	0.868	0.008	0	13	MZB1
0	7.36234531681671	0.321	0.002	0	13	JSRP1
0	5.6090103323143	0.679	0.014	0	13	POU2AF1
0	5.50714133104266	0.604	0.006	0	13	FCRL5
1.0237146397777E-184	5.40453570535231	0.264	0.004	1.6697809489414E-180	13	ZNF215
8.68414104351044E-279	5.1477380218718	0.585	0.012	1.41647024560699E-274	13	CD79A
2.35591489520215E-42	5.03429863186397	0.83	0.214	3.84273278556423E-38	13	TXNDC5

4.8537288343202E-63	4.85230537706368	0.283	0.013	7.91691710165968E-59	13	BHLHE41
1.16867542914941E-64	4.72864885116628	0.377	0.023	1.90622649248561E-60	13	RAB30
7.7391705001306E-126	4.36597903797882	0.868	0.065	1.2623361002763E-121	13	ITM2C
2.05459825361237E-62	4.13362654861638	0.623	0.066	3.35125521146714E-58	13	PDK1
1.72065364760756E-23	4.03407605953193	0.264	0.03	2.80655816461269E-19	13	MANEA
1.39096604294107E-44	3.85634541641484	0.358	0.03	2.26880471264118E-40	13	C11orf80
2.13171446230927E-74	3.85535151457388	0.792	0.088	3.47703945947266E-70	13	PIM2
1.59532149811546E-27	3.79878765231702	0.755	0.212	2.60212889557612E-23	13	TP53INP1
8.84619633158705E-22	3.68584961030257	0.396	0.072	1.44290308364516E-17	13	TRAM2
2.5812063893996E-31	3.56084335306603	0.774	0.219	4.21020574174968E-27	13	HERPUD1
1.24944502044664E-43	3.5469018413784	0.283	0.019	2.03796977285052E-39	13	EAF2
3.3887941767224E-15	3.53347754254737	0.264	0.045	5.52746218165191E-11	13	MYO1D
1.06970839625384E-36	3.45412089918059	0.868	0.25	1.74480136512963E-32	13	XBP1
1.55799976172021E-44	3.38814879235048	0.302	0.021	2.54125341134183E-40	13	ST6GALNAC
7.70820621021325E-26	3.35812845971782	0.528	0.109	1.25728551494788E-21	13	SSR4
5.0596721949653E-61	3.26221205451167	0.528	0.046	8.2528313172079E-57	13	CEP128
1.14462834203801E-30	3.17199137923032	0.792	0.216	1.86700328869819E-26	13	BTG2
1.09195157267169E-06	3.03532606108558	0.264	0.086	0.0178108221018479	13	ITGA6
2.97562690715792E-56	2.998556946121	0.415	0.031	4.85354504826528E-52	13	IRF4
3.19771246173278E-17	2.96518075241848	0.396	0.087	5.21578879633234E-13	13	TPD52
1.13149176479887E-65	2.89790126206679	0.547	0.045	1.84557621756344E-61	13	HSH2D
8.59927896040559E-58	2.74750181746042	0.755	0.094	1.40262839123176E-53	13	TENT5C
9.72624972063297E-19	2.7132214587094	0.623	0.195	1.58644859193244E-14	13	SEL1L
1.44950207516686E-09	2.65788987144581	0.377	0.129	2.36428283480466E-05	13	PRDX4
6.08720745113464E-19	2.59526251011821	0.34	0.056	9.9288440735457E-15	13	CHST15
5.09327170052314E-08	2.59518738332566	0.264	0.076	0.000830763547072329	13	MCEE
3.25288955799374E-15	2.57879150650407	0.283	0.05	5.30578815804359E-11	13	EIF2AK3
2.19823191884349E-36	2.54278241966572	0.434	0.048	3.58553608282561E-32	13	ZBP1
3.25828028187714E-14	2.53483051903891	0.283	0.053	5.3145809677698E-10	13	SLC44A1
7.36425642992932E-17	2.52724328525564	0.585	0.191	1.20118386628577E-12	13	HSP90B1
2.08665728020808E-34	2.51245365295702	0.623	0.108	3.40354668974739E-30	13	FKBP11
6.37011944001935E-16	2.48135955567596	0.396	0.085	1.03903018186156E-11	13	LY9
1.00217520836899E-08	2.47256557426457	0.283	0.08	0.000163464798237067	13	BLNK
1.22803748289651E-45	2.43666799055265	0.679	0.095	2.00305193835249E-41	13	CD38
1.43859426041326E-14	2.3989499684751	0.528	0.168	2.34649109816006E-10	13	SEC11C
6.99925911560541E-11	2.398329851291	0.283	0.066	1.1416491543464E-06	13	UBE2J1
3.60874089259775E-17	2.39394548784756	0.528	0.147	5.88621726991619E-13	13	ELL2
8.70483503468479E-26	2.30364884989868	0.623	0.136	1.41984564250744E-21	13	SEL1L3
2.57391543847208E-08	2.28617845174044	0.34	0.114	0.00041983134716918	13	DNAJB9
1.46920741022262E-11	2.26515031117313	0.566	0.221	2.39642420681412E-07	13	TXNDC11
6.6428765125262E-11	2.23714019783206	0.283	0.065	1.08351958795815E-06	13	SPATS2
4.19822736383915E-09	2.21618010729566	0.377	0.116	6.84772865315804E-05	13	DUSP5
5.69820879560201E-15	2.19924681764559	0.396	0.094	9.29434836650643E-11	13	PLPP5
4.2411382010644E-09	2.19321283813871	0.302	0.086	6.91772051975614E-05	13	CHPF
2.58266908459442E-07	2.13878805101501	0.377	0.15	0.00421259154388195	13	SYVN1
3.03375196434477E-19	2.09485573728131	0.642	0.182	4.94835282904276E-15	13	FBXW7
2.52725050290745E-15	2.06419190679081	0.623	0.208	4.12219829529234E-11	13	EDEM1
3.3896567348557E-12	2.04129485078714	0.264	0.051	5.52886910022312E-08	13	SYK
3.04581996697458E-09	2.01032865018288	0.264	0.063	4.96803694813223E-05	13	CSF2RB
2.87589573851848E-11	1.99310487846418	0.472	0.16	4.69087353909749E-07	13	CLPTM1L

**1.29145961079646E-27**	1.98573168580073	0.491	0.077	2.1064997711701E-23	13	SLAMF7
**1.1449141605452E-08**	1.94324939698154	0.377	0.128	0.000186746948726528	13	BCL2L11
**1.3261124756741E-07**	1.93558700804583	0.302	0.097	0.00216302205907202	13	SEC24A
**3.30803190542757E-12**	1.89654452913919	0.396	0.107	5.39573084094292E-08	13	CREB3L2
**1.22720731856268E-11**	1.80497011660111	0.547	0.205	2.0016978573076E-07	13	CRELD2
**1.6172345149449E-10**	1.79936638158352	0.415	0.132	2.63787121732663E-06	13	SELENOS
**1.2356178495708E-10**	1.78926087889282	0.604	0.254	2.01541627443492E-06	13	FNDC3B
**2.29238008497799E-06**	1.67236723830216	0.34	0.135	0.0373910115660759	13	GMPPB
**1.69022696484727E-18**	1.65999895951156	0.679	0.199	2.75692920236238E-14	13	ISG20
**2.30792365601134E-08**	1.65704836012546	0.528	0.229	0.00037644542753201	13	MYDGF
**8.36004958836137E-16**	1.63574688123347	0.566	0.161	1.36360768835762E-11	13	ERN 1.00
**5.7881558057795E-09**	1.57667938293092	0.509	0.21	9.44106093480694E-05	13	HM13
**1.88211845727272E-09**	1.57136172246648	0.302	0.08	3.06992341565754E-05	13	ERLEC1
**3.17040981585564E-10**	1.53534570719229	0.528	0.207	5.17125545064213E-06	13	LMAN1
**6.15757821962969E-08**	1.47792319759918	0.396	0.144	0.0010043625834038	13	SELENOK
**2.13261257084639E-09**	1.46877388687982	0.472	0.175	3.47850436430754E-05	13	SEC61A1
**2.41038755392772E-07**	1.4559654120388	0.547	0.255	0.00393158313921151	13	ST6GAL1
**4.24003338885708E-07**	1.4264976674856	0.283	0.088	0.00691591846056478	13	SPCS3
**1.6905860382889E-08**	1.37901463644526	0.358	0.11	0.000275751488705302	13	SEC14L1
**1.02876522212351E-15**	1.35981538323021	0.604	0.161	1.67801895380565E-11	13	THEMIS2
**8.57498867710338E-13**	1.30858039161264	0.415	0.104	1.39866640312233E-08	13	GSTP1
**3.24340705924528E-07**	1.1568317706584	0.321	0.104	0.00529032125433498	13	NCOA3
**1.56736082427552E-12**	1.08834613477247	0.396	0.092	2.55652224047581E-08	13	SYTL1
**1.08779305743913E-17**	1.08745541767562	0.321	0.05	1.77429925598897E-13	13	CD27
**2.85690403635215E-06**	1.08479352287909	0.321	0.118	0.0465989617369399	13	RABAC1
**2.86701353394187E-09**	1.04112831206926	0.736	0.344	4.67638577521258E-05	13	OGT
**1.02059356402419E-07**	1.01639953568414	0.396	0.131	0.00166469016227985	13	KLHL6
**1.44715336453755E-09**	0.944694776575567	0.472	0.147	2.36045185289719E-05	13	PRDM1
**1.14884320415501E-08**	0.942525414527694	0.566	0.229	0.000187387815029724	13	NFKB2
**7.73543527690438E-08**	0.926591335360011	0.264	0.07	0.00126172684801587	13	ZNF580
**1.68145912379888E-06**	0.840013971480211	0.34	0.115	0.0274262797682835	13	CCDC88C
**2.94286327167795E-07**	0.442356622051136	0.264	0.072	0.0048001042824339	13	CCDC69

SupTable5J_C2_Total_MajorCelltype

p_val	avg_log2FC	pct.1	pct.2	p_val_adj	cluster	gene
0	5.98357534517184	0.975	0.037	0	Hepatocytes	CES1
0	5.8232255820728	0.983	0.056	0	Hepatocytes	RBP4
0	6.01446699990247	0.972	0.056	0	Hepatocytes	APOA1
0	5.9287685066337	0.986	0.071	0	Hepatocytes	TF
0	5.8192530549247	0.979	0.069	0	Hepatocytes	CFB
0	5.82836844138197	0.96	0.053	0	Hepatocytes	TTR
0	5.71077882127586	0.994	0.088	0	Hepatocytes	FGG
0	5.77367611378107	0.947	0.043	0	Hepatocytes	PLA2G2A
0	5.77980559383619	0.949	0.046	0	Hepatocytes	HP
0	5.93362581234073	0.934	0.032	0	Hepatocytes	AGT
0	5.94077012775409	0.929	0.032	0	Hepatocytes	HPX
0	5.61540845184016	0.947	0.051	0	Hepatocytes	CLU
0	5.5477054399513	0.988	0.102	0	Hepatocytes	VTN
0	5.7122269778109	0.992	0.107	0	Hepatocytes	FGA
0	5.50066675786836	0.954	0.074	0	Hepatocytes	APOC2
0	5.27415070273361	0.988	0.114	0	Hepatocytes	C3
0	5.47407976254901	0.99	0.117	0	Hepatocytes	FGB
0	5.36786674662596	0.97	0.105	0	Hepatocytes	APOC3
0	5.36172643057032	0.932	0.068	0	Hepatocytes	GC
0	6.05787512247816	0.89	0.026	0	Hepatocytes	KNG1
0	5.23024265484832	0.911	0.048	0	Hepatocytes	CFH
0	5.83336726388227	0.888	0.028	0	Hepatocytes	APOB
0	6.22831970532495	0.875	0.017	0	Hepatocytes	AMBP
0	5.75748780077697	0.884	0.029	0	Hepatocytes	ORM1
0	5.8852716861153	0.873	0.023	0	Hepatocytes	ITIH2
0	4.05869682155532	0.939	0.092	0	Hepatocytes	FN1
0	5.01309886987725	0.955	0.11	0	Hepatocytes	EPHX1
0	5.90067298547619	0.863	0.023	0	Hepatocytes	AHSG
0	4.77935648003716	0.965	0.129	0	Hepatocytes	MT1B
0	5.965572805322	0.859	0.027	0	Hepatocytes	IGFBP1
0	5.42996824316494	0.868	0.039	0	Hepatocytes	FGL1
0	4.26067817115642	0.94	0.126	0	Hepatocytes	MT1G
0	5.42152640622452	0.859	0.046	0	Hepatocytes	FABP1
0	5.97670548114707	0.821	0.013	0	Hepatocytes	ASGR2
0	6.21003966132079	0.817	0.012	0	Hepatocytes	F2
0	5.60180927487249	0.831	0.026	0	Hepatocytes	APOH
0	4.29752609793232	0.944	0.141	0	Hepatocytes	MT1F
0	6.51278986680383	0.812	0.011	0	Hepatocytes	AGXT
0	6.32443525429296	0.809	0.011	0	Hepatocytes	SERPINF2
0	6.09356049835797	0.815	0.017	0	Hepatocytes	CP
0	3.75083633911074	0.848	0.053	0	Hepatocytes	C1S
0	4.92687478943838	0.83	0.047	0	Hepatocytes	ALDOB
0	6.17319981408213	0.794	0.013	0	Hepatocytes	SERPINC1
0	5.95091594496532	0.787	0.011	0	Hepatocytes	ASGR1
0	3.83015193772755	0.854	0.079	0	Hepatocytes	COL18A1
0	6.0239744804471	0.786	0.014	0	Hepatocytes	ITIH3
0	5.95304079644143	0.783	0.014	0	Hepatocytes	LRG1
0	5.74826672413529	0.786	0.018	0	Hepatocytes	HMGCS2
0	5.3770034120498	0.796	0.028	0	Hepatocytes	C4BPA
0	4.72445861888053	0.99	0.224	0	Hepatocytes	ALB

0	3.73350499945052	0.913	0.149	0	Hepatocytes	MT1X
0	4.90982051117773	0.992	0.241	0	Hepatocytes	SERPINA1
0	6.19420294334117	0.755	0.011	0	Hepatocytes	ORM2
0	5.01958930031206	0.996	0.254	0	Hepatocytes	APOA2
0	6.05675081611499	0.755	0.013	0	Hepatocytes	MAT1A
0	5.95988983289416	0.752	0.013	0	Hepatocytes	SERPINA3
0	5.65100737711262	0.75	0.019	0	Hepatocytes	GSTA1
0	6.43518958770586	0.739	0.009	0	Hepatocytes	TM4SF5
0	6.42887638658634	0.737	0.008	0	Hepatocytes	ITIH1
0	4.448128005684	0.784	0.059	0	Hepatocytes	NUPR1
0	3.49811305699537	0.989	0.267	0	Hepatocytes	MT2A
0	5.51921172243796	0.774	0.053	0	Hepatocytes	CYP 20.00
0	3.74484188315447	0.842	0.123	0	Hepatocytes	MT1E
0	6.09831474563874	0.728	0.011	0	Hepatocytes	APCS
0	5.80269964203703	0.728	0.016	0	Hepatocytes	DHCR24
0	6.36468745952389	0.72	0.008	0	Hepatocytes	HPN
0	5.26300997207342	0.75	0.039	0	Hepatocytes	EFNA1
0	5.84215649526874	0.716	0.012	0	Hepatocytes	TDO2
0	2.74279805792738	0.757	0.054	0	Hepatocytes	C1R
0	3.39543262604303	0.796	0.094	0	Hepatocytes	MT1H
0	3.58070684621064	0.824	0.122	0	Hepatocytes	APOC1
0	5.76907986939731	0.711	0.01	0	Hepatocytes	TFR2
0	6.13558706037904	0.702	0.011	0	Hepatocytes	CYP3A5
0	4.05479764134254	0.757	0.068	0	Hepatocytes	CERS2
0	5.07825647108152	0.715	0.028	0	Hepatocytes	KRT8
0	5.47570972302488	0.706	0.02	0	Hepatocytes	SPINK1
0	6.44908389798405	0.691	0.007	0	Hepatocytes	TM4SF4
0	5.95984946447575	0.692	0.011	0	Hepatocytes	FMO5
0	6.18708679596317	0.687	0.009	0	Hepatocytes	PLG
0	4.36287875692702	0.718	0.04	0	Hepatocytes	OAF
0	4.65785551694261	0.711	0.033	0	Hepatocytes	SDC4
0	2.97701709827307	0.993	0.316	0	Hepatocytes	APOE
0	5.22414443052375	0.698	0.024	0	Hepatocytes	BAAT
0	3.29084374754216	0.953	0.28	0	Hepatocytes	SQSTM1
0	5.28642681157519	0.693	0.022	0	Hepatocytes	CES2
0	6.16264193646591	0.681	0.011	0	Hepatocytes	PAH
0	3.91044121369632	0.738	0.071	0	Hepatocytes	MT1M
0	4.57749865603537	0.687	0.022	0	Hepatocytes	SERPINF1
0	5.41618289396135	0.678	0.014	0	Hepatocytes	AZGP1
0	6.21703132226065	0.669	0.008	0	Hepatocytes	C4BPB
0	5.92259590202075	0.671	0.01	0	Hepatocytes	HPR
0	3.25430807341049	0.781	0.126	0	Hepatocytes	ALDH2
0	6.3363398139813	0.661	0.007	0	Hepatocytes	DEPRECATI
0	5.5117043174913	0.669	0.016	0	Hepatocytes	ADH1C
0	2.71297633227152	0.811	0.161	0	Hepatocytes	C2
0	6.39050966277043	0.657	0.007	0	Hepatocytes	SERPIND1
0	4.45152838047266	0.689	0.04	0	Hepatocytes	CEBPA
0	4.70614455420556	0.671	0.025	0	Hepatocytes	KRT18
0	4.3515275680371	0.689	0.044	0	Hepatocytes	CYB5A
0	5.81040604654194	0.654	0.01	0	Hepatocytes	ELF3
0	5.5944714105336	0.661	0.018	0	Hepatocytes	PRAP1
0	6.4829034636393	0.649	0.007	0	Hepatocytes	BDH1

0	5.90659688519323	0.647	0.01	0	Hepatocytes	AOX1
0	5.66336989341116	0.646	0.012	0	Hepatocytes	UPB1
0	3.80070124951631	0.66	0.029	0	Hepatocytes	SPON2
0	5.66601191646709	0.643	0.012	0	Hepatocytes	LBP
0	5.86097068114016	0.637	0.008	0	Hepatocytes	SERINC2
0	6.14372421754524	0.637	0.008	0	Hepatocytes	TAT
0	2.55415294697546	0.79	0.162	0	Hepatocytes	LMNA
0	3.68903235830275	0.694	0.068	0	Hepatocytes	SHMT1
0	6.05647307390083	0.633	0.011	0	Hepatocytes	HRG
0	3.85247149181768	0.676	0.055	0	Hepatocytes	GDF15
0	2.77666297392566	0.826	0.21	0	Hepatocytes	A2M
0	3.78760937291163	0.673	0.057	0	Hepatocytes	MT1A
0	4.38645257155946	0.643	0.029	0	Hepatocytes	IGFBP2
0	6.39774630801595	0.618	0.006	0	Hepatocytes	SLC38A3
0	3.53668056198018	0.691	0.079	0	Hepatocytes	GATM
0	6.20824344049412	0.618	0.009	0	Hepatocytes	CFHR2
0	5.20100509058936	0.626	0.017	0	Hepatocytes	UGDH
0	5.88171871545093	0.614	0.008	0	Hepatocytes	MLXIPL
0	4.28062805089795	0.639	0.034	0	Hepatocytes	ERRFI1
0	1.10473435485411	0.913	0.308	0	Hepatocytes	FTL
0	2.97934195187064	0.722	0.12	0	Hepatocytes	SCP2
0	6.12855303222763	0.609	0.007	0	Hepatocytes	HABP2
0	6.03364380547524	0.608	0.007	0	Hepatocytes	EPS8L2
0	5.90606544696332	0.608	0.008	0	Hepatocytes	SDC1
0	2.64398576322344	0.763	0.163	0	Hepatocytes	SERPING1
0	6.52573955117992	0.605	0.006	0	Hepatocytes	HGD
0	3.57544892115734	0.661	0.066	0	Hepatocytes	ECHS1
0	6.0716528404288	0.596	0.006	0	Hepatocytes	MGST1
0	6.0633184150029	0.594	0.006	0	Hepatocytes	PROC
0	5.74981423180382	0.596	0.008	0	Hepatocytes	ITIH4
0	3.9836844428021	0.616	0.032	0	Hepatocytes	MTSS2
0	5.25188462920011	0.595	0.012	0	Hepatocytes	CLDN1
0	2.99329584149082	0.669	0.087	0	Hepatocytes	XBP1
0	3.82246351732052	0.628	0.046	0	Hepatocytes	F11R
0	5.62647364703479	0.589	0.007	0	Hepatocytes	MYH14
0	6.11959130884246	0.587	0.006	0	Hepatocytes	HNF4A
0	5.34412062567618	0.593	0.013	0	Hepatocytes	GAMT
0	4.58868255090621	0.598	0.022	0	Hepatocytes	FAH
0	1.3128064538591	0.719	0.143	0	Hepatocytes	IGFBP4
0	2.74556363308992	0.717	0.141	0	Hepatocytes	RRBP1
0	2.58319285719591	0.777	0.203	0	Hepatocytes	ACADVL
0	5.49093083465215	0.581	0.01	0	Hepatocytes	STARD10
0	2.67638311664894	0.642	0.072	0	Hepatocytes	ECHDC2
0	3.62896857410052	0.614	0.044	0	Hepatocytes	ASL
0	2.80857224150785	0.666	0.097	0	Hepatocytes	ETFB
0	2.80050823929144	0.619	0.053	0	Hepatocytes	GPX3
0	4.5192858149149	0.596	0.03	0	Hepatocytes	DEPRECATI
0	6.04383844086343	0.571	0.006	0	Hepatocytes	GPT
0	3.13893384210102	0.631	0.067	0	Hepatocytes	MGAT4B
0	2.33813610082342	0.712	0.149	0	Hepatocytes	IFITM3
0	3.08456909856987	0.646	0.084	0	Hepatocytes	SAT2
0	4.43212485128795	0.585	0.023	0	Hepatocytes	MPST

0	1.61704617000663	0.773	0.212	0	Hepatocytes	SELENOP
0	6.03575649921747	0.567	0.006	0	Hepatocytes	SERPINA7
0	2.62713087656898	0.713	0.153	0	Hepatocytes	DEPRECATI
0	5.7514784803247	0.568	0.009	0	Hepatocytes	AADAC
0	5.33304398865654	0.571	0.014	0	Hepatocytes	OSGIN1
0	4.81266696108755	0.575	0.019	0	Hepatocytes	TCEA3
0	5.88936313741667	0.564	0.008	0	Hepatocytes	MBL2
0	6.32667307813911	0.558	0.005	0	Hepatocytes	PON1
0	2.92430704627636	0.625	0.073	0	Hepatocytes	AGRN
0	6.04762996570623	0.559	0.007	0	Hepatocytes	AKR1B10
0	1.57665675999546	0.751	0.202	0	Hepatocytes	CTSD
0	3.54202068947689	0.584	0.037	0	Hepatocytes	PDIA5
0	4.38900111400135	0.569	0.022	0	Hepatocytes	DEPRECATI
0	6.63342557635382	0.546	0.004	0	Hepatocytes	HSD17B6
0	3.16960561617409	0.645	0.103	0	Hepatocytes	DEPRECATI
0	4.13269340497921	0.567	0.025	0	Hepatocytes	PCSK6
0	6.073473084329	0.547	0.006	0	Hepatocytes	F7
0	3.28862396196013	0.6	0.059	0	Hepatocytes	ADI1
0	3.88086274147864	0.577	0.04	0	Hepatocytes	ACAT1
0	3.6760988780768	0.586	0.049	0	Hepatocytes	PGRMC1
0	4.1532678821133	0.56	0.027	0	Hepatocytes	GGCX
0	2.84240642590303	0.626	0.093	0	Hepatocytes	GLYCTK
0	4.42328112913486	0.551	0.018	0	Hepatocytes	ADH1B
0	3.93127385193736	0.566	0.033	0	Hepatocytes	QPRT
0	6.70946888157699	0.536	0.005	0	Hepatocytes	SLC22A7
0	4.29616953061259	0.553	0.022	0	Hepatocytes	PON2
0	3.38292033582621	0.571	0.04	0	Hepatocytes	TFPI
0	5.69262631321448	0.536	0.006	0	Hepatocytes	CGN
0	4.43907580981222	0.549	0.019	0	Hepatocytes	SEC16B
0	2.47181336099273	0.604	0.076	0	Hepatocytes	ANPEP
0	4.85718702475949	0.542	0.016	0	Hepatocytes	C5
0	2.44020768056769	0.634	0.108	0	Hepatocytes	HYOU1
0	2.10005384639045	0.693	0.168	0	Hepatocytes	ECH1
0	2.36269959885301	0.628	0.104	0	Hepatocytes	VEGFA
0	2.86078957785838	0.585	0.061	0	Hepatocytes	FURIN
0	6.16997473795663	0.528	0.005	0	Hepatocytes	MASP2
0	3.13360000915062	0.574	0.051	0	Hepatocytes	ACOX1
0	2.56154948416297	0.579	0.058	0	Hepatocytes	SLC9A3R2
0	1.78207729604348	0.598	0.078	0	Hepatocytes	THBS1
0	2.98598368830535	0.565	0.045	0	Hepatocytes	S100A16
0	3.61914530880509	0.548	0.029	0	Hepatocytes	CFI
0	5.86505691643434	0.524	0.006	0	Hepatocytes	ONECUT1
0	2.72515107434259	0.595	0.079	0	Hepatocytes	FUOM
0	3.06742511298184	0.573	0.058	0	Hepatocytes	PRDX6
0	4.92130299022028	0.526	0.013	0	Hepatocytes	CHI3L1
0	2.66266385191392	0.598	0.085	0	Hepatocytes	SLC30A1
0	3.137255435473	0.56	0.047	0	Hepatocytes	CLUH
0	1.63109110979725	0.725	0.214	0	Hepatocytes	PTMS
0	4.03858211346632	0.533	0.023	0	Hepatocytes	SELENBP1
0	5.79140580857081	0.516	0.006	0	Hepatocytes	DSP
0	6.26057291244533	0.516	0.006	0	Hepatocytes	SLC38A4
0	3.27968909633336	0.557	0.047	0	Hepatocytes	SEPHS2

0	4.76579455707972	0.523	0.014	0	Hepatocytes	F5
0	2.637864867295	0.596	0.087	0	Hepatocytes	HSD17B4
0	4.46948993522208	0.522	0.013	0	Hepatocytes	LSR
0	1.60204522507699	0.688	0.18	0	Hepatocytes	HSPB1
0	1.9020445562919	0.639	0.132	0	Hepatocytes	ACAA1
0	1.7849603968865	0.562	0.055	0	Hepatocytes	DEPP1
0	6.17182301520852	0.512	0.005	0	Hepatocytes	ANG
0	4.31223787005828	0.527	0.021	0	Hepatocytes	NADK2
0	5.24621004368705	0.513	0.008	0	Hepatocytes	HAO1
0	5.77423443389794	0.51	0.007	0	Hepatocytes	ARG1
0	6.52541431332966	0.507	0.004	0	Hepatocytes	ESRP2
0	5.23248797841085	0.511	0.009	0	Hepatocytes	PTPRF
0	6.18960775099743	0.506	0.004	0	Hepatocytes	C8G
0	2.57908719947097	0.568	0.067	0	Hepatocytes	DDT
0	2.83412563498198	0.551	0.051	0	Hepatocytes	ALDH3A2
0	2.22793510508609	0.551	0.051	0	Hepatocytes	INSR
0	6.05377638045668	0.505	0.005	0	Hepatocytes	GJB1
0	2.54501235763858	0.568	0.069	0	Hepatocytes	NIT1
0	6.17624507743621	0.503	0.005	0	Hepatocytes	SERPINA10
0	5.75911601869691	0.5	0.005	0	Hepatocytes	OGDHL
0	5.35132005967741	0.504	0.009	0	Hepatocytes	GPX2
0	3.93647222029838	0.515	0.02	0	Hepatocytes	PCYT2
0	3.65475647055103	0.528	0.033	0	Hepatocytes	LRP3
0	5.86082712664834	0.501	0.007	0	Hepatocytes	ADH1A
0	5.84928059218191	0.499	0.005	0	Hepatocytes	TMEM45B
0	5.17271645167112	0.502	0.008	0	Hepatocytes	RGN
0	5.83461731153731	0.498	0.005	0	Hepatocytes	HJV
0	1.74653451969431	0.629	0.136	0	Hepatocytes	NDUFS2
0	3.06560764746865	0.555	0.063	0	Hepatocytes	ALDH1A1
0	5.45998339186606	0.498	0.007	0	Hepatocytes	KIAA1522
0	6.35590697990207	0.495	0.004	0	Hepatocytes	C8A
0	4.15017558491829	0.512	0.021	0	Hepatocytes	ENTPD5
0	2.00541666380417	0.574	0.083	0	Hepatocytes	ZBTB7B
0	3.08578930477169	0.542	0.052	0	Hepatocytes	DCXR
0	2.14036388913547	0.585	0.095	0	Hepatocytes	DHRS3
0	6.28336962391063	0.493	0.004	0	Hepatocytes	ACSM2A
0	3.22025551213311	0.537	0.049	0	Hepatocytes	PCK2
0	5.71737287748401	0.494	0.006	0	Hepatocytes	OCLN
0	5.76474382636581	0.495	0.007	0	Hepatocytes	LEAP2
0	1.97228609877921	0.622	0.136	0	Hepatocytes	TMEM176B
0	5.80541277271302	0.489	0.006	0	Hepatocytes	SERPINA5
0	4.8796421517605	0.497	0.014	0	Hepatocytes	SULT2A1
0	6.29137871252225	0.49	0.007	0	Hepatocytes	PCK1
0	4.63663111234035	0.492	0.01	0	Hepatocytes	SLC7A2
0	3.059517699843	0.526	0.048	0	Hepatocytes	SLC37A4
0	6.03539448926822	0.482	0.004	0	Hepatocytes	ABCC2
0	6.41689924669953	0.48	0.004	0	Hepatocytes	DPYS
0	5.10739747640826	0.485	0.009	0	Hepatocytes	HSD17B2
0	5.18567271303141	0.484	0.009	0	Hepatocytes	PON3
0	6.11374830704089	0.479	0.004	0	Hepatocytes	CREB3L3
0	6.35006773190477	0.479	0.005	0	Hepatocytes	UGT2B4
0	3.96886063154627	0.503	0.03	0	Hepatocytes	PTGR1

0	2.24686565619926	0.576	0.103	0	Hepatocytes	P4HB
0	4.64352464543184	0.485	0.012	0	Hepatocytes	PLIN5
0	2.37428963928024	0.551	0.079	0	Hepatocytes	HDGF
0	6.13648675222638	0.476	0.004	0	Hepatocytes	ERBB3
0	1.81605170309567	0.593	0.122	0	Hepatocytes	ST6GAL1
0	3.79180059103595	0.49	0.021	0	Hepatocytes	MYO1B
0	1.97521803259169	0.551	0.083	0	Hepatocytes	TXNDC5
0	1.37143815615204	0.533	0.065	0	Hepatocytes	CALD1
0	3.02046589531709	0.516	0.048	0	Hepatocytes	SPTSSA
0	1.31042296648637	0.757	0.29	0	Hepatocytes	LAMP1
0	3.3301979147897	0.496	0.03	0	Hepatocytes	PGM1
0	2.60166971722766	0.524	0.059	0	Hepatocytes	SLC27A3
0	2.79514959075207	0.523	0.058	0	Hepatocytes	ABCC3
0	5.50685492029412	0.472	0.008	0	Hepatocytes	LEPR
0	2.23199510434352	0.494	0.03	0	Hepatocytes	NR1H4
0	0.419054605385662	0.812	0.349	0	Hepatocytes	FTH1
0	5.28882778614214	0.47	0.007	0	Hepatocytes	LDHD
0	2.6599437960004	0.509	0.047	0	Hepatocytes	AFDN
0	6.4098973238787	0.465	0.003	0	Hepatocytes	TMPRSS6
0	2.25483803230892	0.536	0.075	0	Hepatocytes	MPC1
0	3.25938124619332	0.497	0.036	0	Hepatocytes	SOD1
0	4.91407980691423	0.472	0.011	0	Hepatocytes	MST1
0	2.17109692808201	0.561	0.101	0	Hepatocytes	METTL7A
0	4.98657957708712	0.468	0.008	0	Hepatocytes	CGNL1
0	1.72653802710794	0.595	0.135	0	Hepatocytes	CSRP1
0	2.66136525042998	0.501	0.044	0	Hepatocytes	SPTBN1
0	6.02734913543828	0.461	0.004	0	Hepatocytes	CPB2
0	5.50881391944869	0.463	0.007	0	Hepatocytes	OTC
0	5.54007958268111	0.46	0.005	0	Hepatocytes	ALDH1L1
0	5.28416912107114	0.463	0.008	0	Hepatocytes	KLF15
0	4.36400390036329	0.473	0.018	0	Hepatocytes	FMO3
0	3.44433392482823	0.471	0.018	0	Hepatocytes	PECR
0	0.807769033666611	0.613	0.16	0	Hepatocytes	SLC2A4RG
0	2.76217600621101	0.514	0.062	0	Hepatocytes	GCLC
0	4.65971558500974	0.465	0.014	0	Hepatocytes	HPD
0	2.12823719454894	0.529	0.078	0	Hepatocytes	IRS2
0	4.41651811125607	0.465	0.015	0	Hepatocytes	TSKU
0	2.23954553803351	0.504	0.055	0	Hepatocytes	PRDX2
0	2.43375856046841	0.51	0.062	0	Hepatocytes	WDR13
0	1.93542734909669	0.547	0.1	0	Hepatocytes	CD151
0	6.17732942001442	0.451	0.004	0	Hepatocytes	AKR1C2
0	5.02711659124122	0.456	0.01	0	Hepatocytes	ANGPTL3
0	1.12112765173439	0.673	0.229	0	Hepatocytes	SOD2
0	6.15308441763221	0.448	0.004	0	Hepatocytes	C8B
0	4.24302047919252	0.458	0.014	0	Hepatocytes	NR2F6
0	1.3729311412399	0.679	0.236	0	Hepatocytes	MT-ND4
0	5.55480486960396	0.447	0.006	0	Hepatocytes	A1CF
0	3.89268227008053	0.462	0.022	0	Hepatocytes	MPC2
0	5.0578025734908	0.448	0.008	0	Hepatocytes	KHK
0	1.61400273940163	0.547	0.108	0	Hepatocytes	RHOC
0	6.08210664444161	0.443	0.004	0	Hepatocytes	SLC2A2
0	2.31618400671739	0.516	0.077	0	Hepatocytes	NDUFC2

0	2.22953121583028	0.522	0.083	0	Hepatocytes	ACSL4
0	1.58179855182173	0.573	0.134	0	Hepatocytes	GPX4
0	2.21621134241954	0.515	0.077	0	Hepatocytes	POR
0	6.03424055894122	0.443	0.005	0	Hepatocytes	HAL
0	2.45374802591313	0.487	0.05	0	Hepatocytes	H6PD
0	2.52462792330231	0.486	0.049	0	Hepatocytes	ARHGEF10
0	1.78371069436945	0.536	0.1	0	Hepatocytes	NFIC
0	5.86770215819044	0.441	0.005	0	Hepatocytes	PDZK1IP1
0	5.91356103115465	0.44	0.004	0	Hepatocytes	SLC6A1
0	5.08023898688373	0.442	0.007	0	Hepatocytes	AKR1C3
0	0.98710614357312	0.575	0.14	0	Hepatocytes	PNPLA2
0	1.26277863702894	0.615	0.181	0	Hepatocytes	BRI3
0	4.90383938914905	0.442	0.008	0	Hepatocytes	FBXO17
0	1.03418444149885	0.654	0.221	0	Hepatocytes	LMAN2
0	5.39868762065587	0.44	0.007	0	Hepatocytes	C6
0	6.19124961480414	0.434	0.003	0	Hepatocytes	APOA5
0	1.37710475694503	0.541	0.111	0	Hepatocytes	SHC1
0	2.6444496992772	0.482	0.052	0	Hepatocytes	IL6R
0	1.74411883048228	0.512	0.082	0	Hepatocytes	LPGAT1
0	1.59953136606639	0.466	0.037	0	Hepatocytes	MASP1
0	1.47647065915354	0.519	0.09	0	Hepatocytes	ZKSCAN1
0	0.971711026576404	0.652	0.223	0	Hepatocytes	PLXNB2
0	5.95662550251463	0.439	0.01	0	Hepatocytes	CRP
0	5.95238699031655	0.433	0.004	0	Hepatocytes	MYOM1
0	5.50788702445396	0.433	0.005	0	Hepatocytes	CYP21A2
0	1.00774928058113	0.67	0.243	0	Hepatocytes	SDF4
0	2.01202315619187	0.498	0.071	0	Hepatocytes	HSP90B1
0	1.79962614038269	0.527	0.101	0	Hepatocytes	UQCRC1
0	1.2457513516784	0.561	0.135	0	Hepatocytes	BLVRB
0	5.88838928858731	0.431	0.005	0	Hepatocytes	RNASE4
0	1.1366321723787	0.566	0.14	0	Hepatocytes	MYO18A
0	5.08084787612314	0.432	0.007	0	Hepatocytes	BHMT2
0	2.91850253517598	0.464	0.04	0	Hepatocytes	IDH1
0	1.7661203233203	0.531	0.107	0	Hepatocytes	NECTIN2
0	2.37488968495088	0.482	0.058	0	Hepatocytes	CXXC5
0	4.67361559302246	0.432	0.009	0	Hepatocytes	KLKB1
0	2.22763820618344	0.488	0.065	0	Hepatocytes	ZNHIT1
0	5.49343410015916	0.429	0.006	0	Hepatocytes	APOC4
0	4.29539314394497	0.435	0.013	0	Hepatocytes	CEP72
0	1.16553319263524	0.564	0.142	0	Hepatocytes	CYB5R3
0	1.78414870398999	0.495	0.074	0	Hepatocytes	HAGH
0	5.12067225667001	0.427	0.007	0	Hepatocytes	PLEKHA6
0	1.08365062663442	0.594	0.174	0	Hepatocytes	GRINA
0	1.60896260308728	0.497	0.077	0	Hepatocytes	ETFA
0	1.69820033245197	0.506	0.087	0	Hepatocytes	ADAM15
0	3.78949129614441	0.442	0.023	0	Hepatocytes	PCCB
0	2.26295109069682	0.487	0.068	0	Hepatocytes	CAT
0	1.08611248978035	0.725	0.306	0	Hepatocytes	MT-CO2
0	6.65121462042554	0.421	0.003	0	Hepatocytes	AKR1C8P
0	6.10715594630496	0.422	0.004	0	Hepatocytes	CDHR5
0	0.929725293805319	0.741	0.323	0	Hepatocytes	OS9
0	1.1842001452739	0.547	0.13	0	Hepatocytes	SLC25A39

	0	1.6416843618288	0.508	0.091	0	Hepatocytes	DDRGK1
	0	4.10701864000387	0.431	0.014	0	Hepatocytes	CLDN7
	0	1.57889117534037	0.515	0.099	0	Hepatocytes	CYC1
	0	2.79798082239605	0.457	0.041	0	Hepatocytes	ACAA2
	0	1.0874336867203	0.675	0.26	0	Hepatocytes	MT-ATP6
	0	3.58250089649738	0.442	0.027	0	Hepatocytes	METTL7B
	0	0.901339622051171	0.591	0.176	0	Hepatocytes	IDH2
	0	6.36500639287612	0.417	0.004	0	Hepatocytes	G6PC
	0	4.64660819907635	0.423	0.01	0	Hepatocytes	ANGPTL4
	0	1.43336405925488	0.584	0.171	0	Hepatocytes	SLC12A7
	0	1.37862145628366	0.494	0.082	0	Hepatocytes	SNTB1
	0	1.9990521413798	0.496	0.084	0	Hepatocytes	TXNRD1
	0	3.827473853868	0.432	0.02	0	Hepatocytes	MMP15
	0	2.53265199674585	0.459	0.048	0	Hepatocytes	CHCHD10
	0	4.97019564685037	0.42	0.009	0	Hepatocytes	CAMK2N1
	0	5.81001031259257	0.415	0.004	0	Hepatocytes	DEPRECATI
	0	6.25687905612761	0.413	0.003	0	Hepatocytes	NR1I2
	0	5.10373580408717	0.418	0.008	0	Hepatocytes	EHHADH
	0	2.07119662805607	0.47	0.06	0	Hepatocytes	HTATIP2
	0	1.9265520227397	0.489	0.08	0	Hepatocytes	IFI27
	0	3.18209590631755	0.435	0.026	0	Hepatocytes	DEPRECATI
	0	3.1514472059362	0.439	0.031	0	Hepatocytes	NEDD4L
	0	0.574184994896089	0.556	0.148	0	Hepatocytes	APMAP
	0	5.69617243836175	0.412	0.004	0	Hepatocytes	F13B
	0	5.89413412376032	0.412	0.004	0	Hepatocytes	CHDH
	0	3.30082550867038	0.43	0.023	0	Hepatocytes	ABAT
	0	3.65812464265232	0.44	0.034	0	Hepatocytes	NNMT
	0	0.829778050383146	0.595	0.189	0	Hepatocytes	HSP90AB1
	0	1.79223856031681	0.472	0.066	0	Hepatocytes	DHRS7
	0	3.78142272178306	0.422	0.016	0	Hepatocytes	NIPSNAP1
	0	2.38716018133458	0.451	0.046	0	Hepatocytes	BEX3
	0	1.82687587677616	0.472	0.067	0	Hepatocytes	SPATS2L
	0	0.835631375979647	0.56	0.156	0	Hepatocytes	GOLPH3
	0	3.09717334466519	0.423	0.019	0	Hepatocytes	NR5A2
	0	5.51964025441905	0.409	0.005	0	Hepatocytes	GPT2
	0	0.916508824238012	0.49	0.088	0	Hepatocytes	OBSCN
	0	4.90836174175385	0.408	0.007	0	Hepatocytes	SHROOM1
	0	4.95105123606544	0.411	0.01	0	Hepatocytes	CBS
	0	5.75081545801412	0.405	0.005	0	Hepatocytes	SERPINA4
	0	2.44250349353831	0.443	0.043	0	Hepatocytes	KIF1C
	0	6.23636990353913	0.403	0.003	0	Hepatocytes	SHBG
	0	2.80104950308066	0.426	0.026	0	Hepatocytes	PLIN4
	0	3.02608880460129	0.432	0.033	0	Hepatocytes	DUSP23
	0	1.98286013320186	0.458	0.059	0	Hepatocytes	CYP27A1
	0	1.24183413760978	0.555	0.156	0	Hepatocytes	APLP2
	0	2.96370020799831	0.455	0.057	0	Hepatocytes	UBD
	0	3.84623648761839	0.414	0.016	0	Hepatocytes	TNFRSF12A
	0	2.31961452763341	0.44	0.043	0	Hepatocytes	TOR1AIP2
	0	5.09199485033751	0.406	0.009	0	Hepatocytes	HSD11B1
	0	0.65859688915459	0.548	0.151	0	Hepatocytes	SLC40A1
	0	1.59784539678092	0.489	0.092	0	Hepatocytes	MTSS1
	0	0.485066150170017	0.613	0.217	0	Hepatocytes	LRP1

0	4.99101705796831	0.401	0.006	0	Hepatocytes	PLA1A
0	6.57316028134673	0.398	0.003	0	Hepatocytes	CYP2C9
0	1.27204900102698	0.52	0.125	0	Hepatocytes	PPIB
0	2.77866639725263	0.429	0.034	0	Hepatocytes	RILP
0	1.29304555814144	0.511	0.116	0	Hepatocytes	ETS2
0	3.36521255220694	0.415	0.02	0	Hepatocytes	TSPAN13
0	2.16857104668043	0.46	0.066	0	Hepatocytes	ANXA4
0	4.89811836338872	0.401	0.007	0	Hepatocytes	ACOX2
0	5.62053940203601	0.399	0.005	0	Hepatocytes	ACSM1
0	0.772798137133313	0.693	0.299	0	Hepatocytes	NUCB1
0	2.86697391226735	0.427	0.034	0	Hepatocytes	SCCPDH
0	1.2442228709557	0.507	0.114	0	Hepatocytes	ACSL1
0	5.78103664818517	0.398	0.005	0	Hepatocytes	SERPINA6
0	0.956232463589326	0.566	0.173	0	Hepatocytes	ISG15
0	3.62124798588546	0.407	0.015	0	Hepatocytes	SAMD5
0	3.37801165448325	0.414	0.023	0	Hepatocytes	ACY1
0	5.5427963203459	0.394	0.004	0	Hepatocytes	GCKR
0	5.82720588961729	0.394	0.004	0	Hepatocytes	XDH
0	4.12053831192426	0.399	0.009	0	Hepatocytes	FGFR3
0	1.13425083980266	0.488	0.098	0	Hepatocytes	MLF 2.00
0	5.63973151674281	0.393	0.005	0	Hepatocytes	FNDC4
0	6.20483762086711	0.391	0.003	0	Hepatocytes	AMN
0	6.00884294789697	0.392	0.004	0	Hepatocytes	FGF21
0	1.02845324770673	0.489	0.103	0	Hepatocytes	TMCO1
0	3.64081075363995	0.401	0.015	0	Hepatocytes	MARC2
0	2.18668522468695	0.438	0.052	0	Hepatocytes	SLC29A1
0	1.86871850121176	0.442	0.056	0	Hepatocytes	SLC25A13
0	4.62114312687549	0.396	0.01	0	Hepatocytes	AMDHD1
0	3.54005580794586	0.409	0.023	0	Hepatocytes	MDK
0	5.65606221065085	0.389	0.004	0	Hepatocytes	REEP6
0	1.3718330819883	0.506	0.121	0	Hepatocytes	MYDGF
0	5.63725763388519	0.388	0.003	0	Hepatocytes	FOXA3
0	2.40358372600444	0.418	0.034	0	Hepatocytes	PEX19
0	1.68622065081472	0.462	0.078	0	Hepatocytes	ITGB5
0	0.860945777304531	0.495	0.111	0	Hepatocytes	COPB2
0	1.09467671575286	0.469	0.085	0	Hepatocytes	POLD4
0	0.906915415754949	0.589	0.206	0	Hepatocytes	MT-ND4L
0	5.30521977886582	0.388	0.006	0	Hepatocytes	TUBB2A
0	5.01986132978132	0.387	0.005	0	Hepatocytes	BAIAP2L1
0	2.20295056709401	0.433	0.051	0	Hepatocytes	PAPSS2
0	1.64545158545298	0.471	0.089	0	Hepatocytes	CALR
0	0.407625833129785	0.53	0.149	0	Hepatocytes	NDUFV2
0	1.99871748228323	0.438	0.058	0	Hepatocytes	POLDIP2
0	0.400346791339071	0.616	0.237	0	Hepatocytes	CD14
0	1.3156067222406	0.49	0.111	0	Hepatocytes	PDIA3
0	1.52891371224352	0.479	0.101	0	Hepatocytes	PDIA6
0	6.09454049335636	0.381	0.003	0	Hepatocytes	CYP2C8
0	0.823369421365678	0.458	0.08	0	Hepatocytes	MIA2
0	3.59920896371655	0.392	0.014	0	Hepatocytes	CDH2
0	0.56216354518957	0.588	0.212	0	Hepatocytes	DCAF8
0	6.87700423083361	0.378	0.002	0	Hepatocytes	SLC30A10
0	2.24638058792653	0.407	0.031	0	Hepatocytes	FOXP4

0	0.836719692303052	0.523	0.147	0	Hepatocytes	GNPTG
0	5.01750534240292	0.382	0.006	0	Hepatocytes	PTPRH
0	4.38187289719708	0.384	0.009	0	Hepatocytes	SLC39A14
0	2.79282607210528	0.402	0.027	0	Hepatocytes	DTX4
0	1.9882017200709	0.44	0.066	0	Hepatocytes	MGST3
0	6.39338122253177	0.377	0.003	0	Hepatocytes	AFM
0	0.544673008183304	0.526	0.152	0	Hepatocytes	BAG6
0	0.321150933190144	0.62	0.246	0	Hepatocytes	CEBPD
0	3.33971217505179	0.391	0.017	0	Hepatocytes	KANK1
0	5.92219154683703	0.376	0.003	0	Hepatocytes	F11
0	1.47757115321988	0.433	0.06	0	Hepatocytes	TOB1
0	2.59604168416267	0.395	0.023	0	Hepatocytes	OTUD7B
0	4.61836021286411	0.381	0.009	0	Hepatocytes	BPHL
0	2.21109263159722	0.427	0.055	0	Hepatocytes	PRXL2A
0	0.987420587768959	0.499	0.127	0	Hepatocytes	CTNND1
0	1.50869202794479	0.439	0.067	0	Hepatocytes	DEPRECATI
0	2.23250558411788	0.416	0.045	0	Hepatocytes	SHB
0	4.10671257790708	0.382	0.012	0	Hepatocytes	SPSB1
0	4.33803009675973	0.38	0.01	0	Hepatocytes	EGFR
0	3.07764885314413	0.395	0.025	0	Hepatocytes	OPLAH
0	4.27653513011258	0.38	0.01	0	Hepatocytes	DEPRECATI
0	0.603294999756457	0.495	0.126	0	Hepatocytes	CMPK1
0	1.24736230376116	0.468	0.099	0	Hepatocytes	SCARB1
0	1.51152957439795	0.433	0.064	0	Hepatocytes	SEC11C
0	0.312420875158524	0.617	0.249	0	Hepatocytes	JUN
0	0.670780031239778	0.49	0.122	0	Hepatocytes	TUFM
0	0.770291490420617	0.45	0.083	0	Hepatocytes	GALT
0	2.57459780164701	0.393	0.026	0	Hepatocytes	LRP5
0	6.52306238121546	0.369	0.002	0	Hepatocytes	PALM3
0	1.06927170800327	0.498	0.132	0	Hepatocytes	CREG1
0	1.64703619001001	0.431	0.065	0	Hepatocytes	SIL1
0	6.07638779310544	0.369	0.003	0	Hepatocytes	CYP2C18
0	2.77880423840555	0.389	0.023	0	Hepatocytes	CRLS1
0	2.45459060397921	0.406	0.041	0	Hepatocytes	PINK1
0	4.9932593421237	0.371	0.006	0	Hepatocytes	AJUBA
0	4.17882472588161	0.374	0.009	0	Hepatocytes	HLF
0	6.09672331987196	0.368	0.003	0	Hepatocytes	FTCD
0	1.90262513446676	0.413	0.048	0	Hepatocytes	GUCD1
0	1.22697451537616	0.477	0.112	0	Hepatocytes	LAMP2
0	1.85179006577984	0.396	0.032	0	Hepatocytes	C1RL
0	4.23566198898317	0.372	0.009	0	Hepatocytes	RTKN
0	3.4548693896345	0.383	0.02	0	Hepatocytes	MACROD1
0	4.33090290688679	0.371	0.009	0	Hepatocytes	FGGY
0	3.5217434003162	0.378	0.016	0	Hepatocytes	DTNA
0	5.89721393700153	0.364	0.003	0	Hepatocytes	CFHR4
0	6.1514142368416	0.363	0.002	0	Hepatocytes	GLTPD2
0	0.505799489603877	0.473	0.112	0	Hepatocytes	PABPC1L
0	0.47175666100926	0.549	0.189	0	Hepatocytes	LASP1
0	1.25418012014258	0.433	0.073	0	Hepatocytes	NDST1
0	3.37927079539376	0.379	0.019	0	Hepatocytes	SLC35C1
0	2.87429415842897	0.378	0.019	0	Hepatocytes	PRKAB2
0	4.81681803139837	0.365	0.006	0	Hepatocytes	DNAJC22

	0	0.689348390524667	0.449	0.09	0	Hepatocytes	SELENOO
	0	4.14696540229761	0.37	0.012	0	Hepatocytes	CITED4
	0	0.885725356368168	0.495	0.137	0	Hepatocytes	RNF187
	0	5.99444294997668	0.361	0.003	0	Hepatocytes	PERP
	0	0.52787661524404	0.47	0.112	0	Hepatocytes	TP53INP1
	0	2.70448673864575	0.387	0.029	0	Hepatocytes	SIGMAR1
	0	0.590620715855029	0.463	0.106	0	Hepatocytes	DAP3
	0	1.3382791064822	0.45	0.093	0	Hepatocytes	QSOX1
	0	2.13748838287448	0.398	0.042	0	Hepatocytes	RETSAT
	0	1.70644442334403	0.401	0.045	0	Hepatocytes	C16orf70
	0	2.03509108750652	0.408	0.052	0	Hepatocytes	RAB13
	0	1.68177329935906	0.412	0.056	0	Hepatocytes	NDUFS6
	0	1.42901117496009	0.438	0.082	0	Hepatocytes	CPEB4
	0	1.57891803603531	0.416	0.06	0	Hepatocytes	SLC22A23
	0	1.91230026376626	0.411	0.055	0	Hepatocytes	GRHPR
	0	1.56330888417327	0.426	0.07	0	Hepatocytes	FKBP2
	0	1.71148634013292	0.409	0.053	0	Hepatocytes	PEBP1
	0	0.721994461100946	0.425	0.07	0	Hepatocytes	ECI2
	0	0.57927601122238	0.454	0.099	0	Hepatocytes	ILRUN
	0	0.437643818397507	0.503	0.148	0	Hepatocytes	EXT1
	0	1.22562429794465	0.477	0.122	0	Hepatocytes	MT-CYB
	0	0.771558981184191	0.459	0.105	0	Hepatocytes	CRELD2
	0	1.15494851355387	0.442	0.088	0	Hepatocytes	PMVK
	0	2.3471932231336	0.395	0.042	0	Hepatocytes	QDPR
	0	6.65395551249286	0.355	0.002	0	Hepatocytes	SLC17A4
	0	5.61643919658734	0.357	0.004	0	Hepatocytes	APOM
	0	1.82828300595498	0.403	0.051	0	Hepatocytes	RNF5
	0	1.38900503285418	0.403	0.051	0	Hepatocytes	SYVN1
	0	1.68361163730385	0.412	0.06	0	Hepatocytes	GALNT2
	0	0.856586022077912	0.504	0.152	0	Hepatocytes	CDKN1A
	0	1.08661096426286	0.412	0.06	0	Hepatocytes	HEBP2
	0	0.569416054097653	0.459	0.108	0	Hepatocytes	EPAS1
	0	1.50399175698622	0.4	0.049	0	Hepatocytes	CARHSP1
	0	0.92972047731378	0.425	0.074	0	Hepatocytes	SAR1B
	0	2.79461907556485	0.374	0.023	0	Hepatocytes	SLC41A2
	0	2.17848083052307	0.387	0.037	0	Hepatocytes	COQ8A
	0	1.08013835910376	0.397	0.047	0	Hepatocytes	PLEKHG3
	0	0.910476427410016	0.439	0.089	0	Hepatocytes	EGLN1
	0	1.62164073880984	0.427	0.077	0	Hepatocytes	ASPH
	0	4.78011803391896	0.357	0.007	0	Hepatocytes	SLPI
	0	6.70108926247441	0.351	0.002	0	Hepatocytes	ATP2B2
	0	0.335206569717973	0.5	0.151	0	Hepatocytes	ZFAND3
	0	1.34574269902922	0.43	0.081	0	Hepatocytes	YIPF3
	0	0.89668153856085	0.483	0.134	0	Hepatocytes	BSG
	0	2.08023626250303	0.389	0.041	0	Hepatocytes	ECI1
	0	0.342506125819949	0.463	0.115	0	Hepatocytes	LONP2
	0	4.96775989465797	0.356	0.008	0	Hepatocytes	ALDH4A1
	0	4.34605699905468	0.36	0.012	0	Hepatocytes	ALDH1B1
	0	5.65782173345277	0.351	0.003	0	Hepatocytes	CYP4F3
	0	2.94955128632404	0.371	0.023	0	Hepatocytes	TTC38
	0	2.1561755653657	0.394	0.047	0	Hepatocytes	G0S2
	0	0.728382913172078	0.453	0.106	0	Hepatocytes	FIS1

0	2.36288501469047	0.387	0.04	0	Hepatocytes	NME4
0	1.15090453859109	0.404	0.057	0	Hepatocytes	AKAP1
0	4.67624842403635	0.354	0.007	0	Hepatocytes	PROX1
0	5.82148405936972	0.35	0.003	0	Hepatocytes	CMBL
0	5.09917754316547	0.353	0.006	0	Hepatocytes	PIPOX
0	5.6328708553168	0.351	0.004	0	Hepatocytes	DEPRECATI
0	5.5229669817994	0.349	0.003	0	Hepatocytes	C2orf72
0	0.760502945176843	0.441	0.095	0	Hepatocytes	DGAT1
0	0.6296102630086	0.43	0.084	0	Hepatocytes	LPIN2
0	1.20284295060435	0.426	0.08	0	Hepatocytes	SLC66A2
0	2.60269582622045	0.357	0.012	0	Hepatocytes	RORC
0	4.99327697783131	0.351	0.006	0	Hepatocytes	UGT1A1
0	0.36411983110242	0.476	0.131	0	Hepatocytes	COPG1
0	2.5427506097136	0.369	0.024	0	Hepatocytes	GOT2
0	2.35401259270681	0.384	0.04	0	Hepatocytes	PXDC1
0	1.68130641740502	0.391	0.047	0	Hepatocytes	BCAR1
0	2.2137166800069	0.375	0.031	0	Hepatocytes	UAP1
0	2.67554896949168	0.366	0.022	0	Hepatocytes	PTPRK
0	1.35287929481242	0.428	0.084	0	Hepatocytes	PLOD3
0	4.71175982822681	0.351	0.007	0	Hepatocytes	DECR2
0	3.62735783096202	0.357	0.013	0	Hepatocytes	GPRC5C
0	5.6124680687733	0.346	0.003	0	Hepatocytes	ACOT12
0	4.27018838391541	0.353	0.01	0	Hepatocytes	GOT1
0	4.55220922682655	0.351	0.008	0	Hepatocytes	TM7SF2
0	0.999442252126191	0.422	0.079	0	Hepatocytes	EIF6
0	1.73060595588007	0.391	0.049	0	Hepatocytes	IFRD2
0	0.510733514001137	0.452	0.11	0	Hepatocytes	LMAN1
0	0.94134278708671	0.428	0.086	0	Hepatocytes	FASN
0	5.73494110185074	0.345	0.003	0	Hepatocytes	CAMSAP3
0	0.515143027811648	0.448	0.107	0	Hepatocytes	DTX3L
0	0.804068011771352	0.445	0.104	0	Hepatocytes	MAN1A1
0	1.69960912606685	0.397	0.056	0	Hepatocytes	CIAO2A
0	0.934390636941569	0.433	0.092	0	Hepatocytes	PFDN2
0	1.3360940717976	0.417	0.076	0	Hepatocytes	DNPH1
0	2.48938283092796	0.373	0.032	0	Hepatocytes	AIG1
0	0.861536270148775	0.421	0.082	0	Hepatocytes	ACBD5
0	0.799935428132847	0.434	0.095	0	Hepatocytes	LONP1
0	0.834115252159148	0.45	0.112	0	Hepatocytes	NFE2L1
0	1.86061148096049	0.397	0.059	0	Hepatocytes	H1F0
0	3.94885801704183	0.35	0.012	0	Hepatocytes	AMACR
0	0.628229720457719	0.495	0.157	0	Hepatocytes	INF2
0	1.99877947376951	0.378	0.041	0	Hepatocytes	HMGCL
0	0.726156720891087	0.439	0.102	0	Hepatocytes	HADHA
0	2.04596159201447	0.373	0.036	0	Hepatocytes	CEBPG
0	2.08808224264256	0.361	0.024	0	Hepatocytes	TMEM14A
0	0.660252849725334	0.414	0.077	0	Hepatocytes	TRAP1
0	1.38044046365947	0.391	0.055	0	Hepatocytes	ALDH9A1
0	5.91249874886473	0.339	0.003	0	Hepatocytes	SLC22A1
0	2.43376110808012	0.363	0.027	0	Hepatocytes	LAMC1
0	3.47997142535359	0.352	0.016	0	Hepatocytes	PROS1
0	0.347495049716246	0.458	0.123	0	Hepatocytes	EIF4EBP2
0	5.75174964417382	0.337	0.002	0	Hepatocytes	SEMA4G

0	1.16042372592011	0.407	0.072	0	Hepatocytes	HSPD1
0	2.06124701223377	0.37	0.035	0	Hepatocytes	SHC2
0	1.29226813121316	0.415	0.081	0	Hepatocytes	MGLL
0	5.51335038607949	0.336	0.003	0	Hepatocytes	SGK2
0	3.12719192346423	0.347	0.014	0	Hepatocytes	PCDH1
0	0.339412235641208	0.427	0.094	0	Hepatocytes	IMMP2L
0	1.0182985190745	0.401	0.069	0	Hepatocytes	EIF4G1
0	5.61000890570276	0.338	0.006	0	Hepatocytes	GNMT
0	0.429754412499527	0.384	0.052	0	Hepatocytes	LLGL2
0	0.716827212859187	0.468	0.136	0	Hepatocytes	PRKACA
0	3.47530095951684	0.349	0.017	0	Hepatocytes	ALDH6A1
0	0.311545343883995	0.455	0.124	0	Hepatocytes	TRIM26
0	2.51453519970944	0.36	0.029	0	Hepatocytes	SDHC
0	3.40242680781501	0.35	0.019	0	Hepatocytes	CD24
0	2.15144523214208	0.356	0.025	0	Hepatocytes	ACADSB
0	1.50531939286272	0.372	0.042	0	Hepatocytes	DCAF6
0	5.12282003234464	0.334	0.004	0	Hepatocytes	EVA1A
0	0.739219179236898	0.412	0.082	0	Hepatocytes	GPAA1
0	4.94741910876415	0.334	0.005	0	Hepatocytes	C1orf115
0	1.47596165377766	0.396	0.067	0	Hepatocytes	ATP5MC3
0	1.58511776970277	0.386	0.057	0	Hepatocytes	NAPRT
0	5.68615351929362	0.333	0.004	0	Hepatocytes	GPAM
0	1.49969104842511	0.376	0.047	0	Hepatocytes	ATRN
0	1.14656994782139	0.421	0.092	0	Hepatocytes	RBM47
0	2.25915849359812	0.357	0.028	0	Hepatocytes	MMUT
0	6.09744708798756	0.332	0.004	0	Hepatocytes	CDO1
0	0.415815918299562	0.431	0.103	0	Hepatocytes	REXO2
0	6.21852590197988	0.331	0.003	0	Hepatocytes	CYP2B6
0	3.42410328393669	0.34	0.013	0	Hepatocytes	COBLL1
0	5.44786411550775	0.33	0.003	0	Hepatocytes	PPARGC1A
0	0.93155437374029	0.439	0.112	0	Hepatocytes	TMED9
0	0.711967776204816	0.454	0.127	0	Hepatocytes	GADD45B
0	1.45812594349688	0.4	0.074	0	Hepatocytes	DST
0	0.562973814259709	0.424	0.098	0	Hepatocytes	NUCKS1
0	2.38219132517983	0.351	0.025	0	Hepatocytes	CEACAM1
0	3.13951458255206	0.342	0.017	0	Hepatocytes	FAM50B
0	1.45713462511079	0.397	0.072	0	Hepatocytes	LTBR
0	2.20080717318878	0.346	0.021	0	Hepatocytes	SLC43A1
0	0.662397271678524	0.39	0.066	0	Hepatocytes	NNT
0	5.09975483292501	0.328	0.004	0	Hepatocytes	WWC1
0	3.78586571417816	0.336	0.012	0	Hepatocytes	FAM83H
0	3.05655752862039	0.341	0.017	0	Hepatocytes	PXMP2
0	3.04198685346106	0.344	0.02	0	Hepatocytes	GALK1
0	1.5212997338624	0.379	0.056	0	Hepatocytes	PSMB4
0	0.834167214976662	0.43	0.107	0	Hepatocytes	POLR2L
0	0.510217098191423	0.427	0.104	0	Hepatocytes	SEL1L
0	0.618481346463212	0.444	0.122	0	Hepatocytes	NADK
0	3.37240671968133	0.341	0.019	0	Hepatocytes	STEAP3
0	1.35926685048378	0.383	0.061	0	Hepatocytes	MSRA
0	1.18834586907034	0.386	0.064	0	Hepatocytes	MGMT
0	5.99163841731837	0.325	0.003	0	Hepatocytes	CDH1
0	0.856815853512358	0.425	0.103	0	Hepatocytes	NDUFB7

0	4.70096154869741	0.329	0.007	0	Hepatocytes	DEPRECATI
0	2.20580237804193	0.351	0.029	0	Hepatocytes	TNKS1BP1
0	2.94545462662324	0.333	0.012	0	Hepatocytes	ALDH5A1
0	4.27209176882344	0.329	0.008	0	Hepatocytes	RARRES2
0	4.92994394959611	0.326	0.005	0	Hepatocytes	LGALS4
0	0.559423770776103	0.412	0.091	0	Hepatocytes	CUTA
0	2.12858664452643	0.351	0.03	0	Hepatocytes	SLC17A9
0	3.34444606756955	0.337	0.017	0	Hepatocytes	GALE
0	5.82283747544282	0.322	0.002	0	Hepatocytes	RAB17
0	0.817115608145386	0.442	0.122	0	Hepatocytes	RAB7A
0	1.39394357068488	0.369	0.049	0	Hepatocytes	TSTA3
0	3.72107244781447	0.334	0.014	0	Hepatocytes	PHYH
0	1.88411193836966	0.365	0.045	0	Hepatocytes	CHP1
0	5.85463599283776	0.322	0.002	0	Hepatocytes	CYP2D6
0	1.70187862974631	0.355	0.035	0	Hepatocytes	CRYZ
0	0.697211090534092	0.412	0.092	0	Hepatocytes	STT3A
0	1.35452985858483	0.355	0.036	0	Hepatocytes	NIT2
0	1.27036165232413	0.373	0.054	0	Hepatocytes	SRPRB
0	1.39858856025474	0.389	0.07	0	Hepatocytes	ATOX1
0	1.27065031714192	0.379	0.06	0	Hepatocytes	VARS
0	1.49161973542176	0.386	0.067	0	Hepatocytes	CD59
0	2.80241219697475	0.342	0.023	0	Hepatocytes	HACD3
0	3.60438210618015	0.33	0.011	0	Hepatocytes	COL5A3
0	6.38540312112767	0.321	0.003	0	Hepatocytes	CYP4A11
0	2.6467238972595	0.342	0.024	0	Hepatocytes	ALS2CL
0	0.377407535802649	0.381	0.063	0	Hepatocytes	RGS14
0	1.67319399997451	0.356	0.038	0	Hepatocytes	LHPP
0	0.498560000144889	0.412	0.094	0	Hepatocytes	KLHL21
0	1.24853411991784	0.357	0.04	0	Hepatocytes	IGSF8
0	2.64598060244309	0.343	0.026	0	Hepatocytes	MCFD2
0	1.45994434177296	0.386	0.069	0	Hepatocytes	TRIP6
0	4.08378133959057	0.329	0.012	0	Hepatocytes	PPP1R3B
0	0.558836882212102	0.39	0.073	0	Hepatocytes	PPP2R1B
0	0.553627719618031	0.456	0.139	0	Hepatocytes	EPN1
0	0.477512597669481	0.438	0.121	0	Hepatocytes	ERGIC3
0	0.692621670661454	0.432	0.116	0	Hepatocytes	ADIPOR1
0	3.39435185790526	0.331	0.015	0	Hepatocytes	SNCG
0	5.16155419929629	0.319	0.003	0	Hepatocytes	HNF1A
0	1.7244111721742	0.355	0.039	0	Hepatocytes	AIFM1
0	1.3707407873455	0.345	0.029	0	Hepatocytes	VWA1
0	1.74680456038324	0.344	0.028	0	Hepatocytes	KIAA1671
0	1.12937480431579	0.407	0.091	0	Hepatocytes	MT-ND5
0	1.31395391326506	0.37	0.055	0	Hepatocytes	IMP4
0	1.64709700796664	0.348	0.033	0	Hepatocytes	UGP2
0	5.41529782890571	0.317	0.003	0	Hepatocytes	PDZK1
0	0.655377016676526	0.385	0.071	0	Hepatocytes	EDEM3
0	0.631739574263625	0.388	0.074	0	Hepatocytes	CDC34
0	1.51151592733872	0.363	0.049	0	Hepatocytes	HSD17B10
0	2.29556760810204	0.383	0.07	0	Hepatocytes	P4HA1
0	5.68557046776397	0.314	0.002	0	Hepatocytes	GOLT1A
0	0.350076961821203	0.435	0.123	0	Hepatocytes	REEP5
0	1.80319799875953	0.352	0.04	0	Hepatocytes	TOLLIP

0	6.4151318279535	0.314	0.002	0	Hepatocytes	ABCC6
0	5.89330736620346	0.314	0.002	0	Hepatocytes	NAGS
0	6.0823374049115	0.314	0.003	0	Hepatocytes	CPN2
0	2.51294880044844	0.336	0.025	0	Hepatocytes	ACO1
0	6.48127018192445	0.313	0.002	0	Hepatocytes	SLC22A25
0	4.76679824791982	0.315	0.005	0	Hepatocytes	AGMAT
0	1.51349445512146	0.371	0.061	0	Hepatocytes	GSTO1
0	2.27969072888578	0.328	0.018	0	Hepatocytes	FGFR2
0	3.55683187581358	0.323	0.013	0	Hepatocytes	FN3K
0	1.83485731446152	0.337	0.027	0	Hepatocytes	PTPRS
0	0.454913950800937	0.401	0.092	0	Hepatocytes	TMEM8A
0	0.253657780733927	0.424	0.115	0	Hepatocytes	SSR2
0	2.64710317563816	0.334	0.025	0	Hepatocytes	TXNDC17
0	6.11937714444579	0.311	0.002	0	Hepatocytes	CYP4F11
0	0.329719299853602	0.376	0.067	0	Hepatocytes	PLA2G6
0	2.68493305207781	0.331	0.023	0	Hepatocytes	ABLIM3
0	2.03458288371425	0.347	0.039	0	Hepatocytes	PNPO
0	0.995202784587011	0.401	0.094	0	Hepatocytes	ATP1B1
0	1.24368435747703	0.373	0.066	0	Hepatocytes	CYB5R1
0	0.726140040949075	0.383	0.076	0	Hepatocytes	SCYL1
0	0.988649165137603	0.377	0.07	0	Hepatocytes	SIK2
0	0.352003624244584	0.39	0.083	0	Hepatocytes	TMEM263
0	0.655446307253909	0.387	0.08	0	Hepatocytes	NSFL1C
0	4.53548013879075	0.314	0.007	0	Hepatocytes	TST
0	1.01723676555608	0.355	0.048	0	Hepatocytes	PMM1
0	4.35193988627816	0.313	0.006	0	Hepatocytes	MAOB
0	0.644293260376338	0.394	0.088	0	Hepatocytes	TMED5
0	0.634161661250511	0.467	0.161	0	Hepatocytes	MT-ND3
0	2.67982871313963	0.333	0.027	0	Hepatocytes	FAM162A
0	0.530398954325632	0.379	0.073	0	Hepatocytes	POLR2H
0	4.88097999122593	0.311	0.005	0	Hepatocytes	FST
0	0.916919324981995	0.384	0.078	0	Hepatocytes	PSMC3
0	5.38340664401981	0.31	0.004	0	Hepatocytes	DGAT2
0	3.60788859027912	0.316	0.01	0	Hepatocytes	CEP170B
0	2.1986737832644	0.335	0.03	0	Hepatocytes	FH
0	0.533493464834865	0.446	0.141	0	Hepatocytes	RXRA
0	2.43863498126418	0.33	0.025	0	Hepatocytes	NAXE
0	1.76483830079271	0.342	0.038	0	Hepatocytes	ZCCHC24
0	5.77621729162592	0.307	0.003	0	Hepatocytes	PRG4
0	2.60107572944684	0.328	0.024	0	Hepatocytes	HAAO
0	0.736587901945486	0.355	0.051	0	Hepatocytes	TFG
0	2.12422035981803	0.332	0.028	0	Hepatocytes	DNAJB9
0	1.24964170653841	0.367	0.063	0	Hepatocytes	ROMO1
0	0.285025607414876	0.469	0.165	0	Hepatocytes	CTSA
0	0.422691348064857	0.447	0.144	0	Hepatocytes	AKR1A1
0	1.92344212537185	0.337	0.034	0	Hepatocytes	ACADM
0	0.608848478515854	0.396	0.093	0	Hepatocytes	DNAJB11
0	1.85313758445724	0.333	0.031	0	Hepatocytes	FLNB
0	1.56602182696303	0.346	0.044	0	Hepatocytes	EBNA1BP2
0	3.25179832880214	0.318	0.016	0	Hepatocytes	ALDH7A1
0	5.69377636753034	0.305	0.003	0	Hepatocytes	ABCB4
0	0.701086539397248	0.354	0.052	0	Hepatocytes	NSUN6

0	1.88599194966912	0.349	0.047	0	Hepatocytes	DDIT3
0	0.639912090534925	0.406	0.105	0	Hepatocytes	UBXN6
0	0.40149127981674	0.364	0.063	0	Hepatocytes	AGFG2
0	4.93088137696868	0.306	0.005	0	Hepatocytes	F10
0	5.41247931332128	0.304	0.003	0	Hepatocytes	GRTP1
0	0.417276336504572	0.38	0.08	0	Hepatocytes	APEH
0	1.32867128090946	0.356	0.056	0	Hepatocytes	NDUFB9
0	1.99979448987716	0.345	0.045	0	Hepatocytes	PRDX4
0	1.42726036008648	0.342	0.043	0	Hepatocytes	NRBP2
0	1.08310343369626	0.353	0.054	0	Hepatocytes	KRTCAP2
0	3.72939465412362	0.309	0.01	0	Hepatocytes	RAMP1
0	1.00769891912421	0.36	0.061	0	Hepatocytes	SNRPC
0	4.23636796975933	0.306	0.007	0	Hepatocytes	RND1
0	0.289832063500859	0.409	0.11	0	Hepatocytes	PSMD3
0	0.58648046391696	0.392	0.094	0	Hepatocytes	TMED7
0	0.350460548021419	0.385	0.087	0	Hepatocytes	MAGED2
0	0.72893343039534	0.356	0.058	0	Hepatocytes	MEAF6
0	1.85879912422059	0.325	0.027	0	Hepatocytes	ABCD3
0	2.8156804972966	0.319	0.021	0	Hepatocytes	SMIM14
0	0.985236932578784	0.365	0.067	0	Hepatocytes	SLC39A7
0	2.08683211277993	0.345	0.047	0	Hepatocytes	TMEM176A
0	1.23773400839859	0.356	0.058	0	Hepatocytes	TSR3
0	0.762265884889907	0.369	0.071	0	Hepatocytes	SPATA20
0	4.5928112773955	0.302	0.004	0	Hepatocytes	SLC25A18
0	2.07882082854785	0.33	0.033	0	Hepatocytes	PSMD4
0	1.08272702355592	0.345	0.048	0	Hepatocytes	ZNF444
0	2.49807899969626	0.317	0.02	0	Hepatocytes	PPARA
0	5.27558694292662	0.303	0.007	0	Hepatocytes	BHMT
0	2.39745309208513	0.325	0.029	0	Hepatocytes	ADGRG6
0	0.526953543025517	0.377	0.081	0	Hepatocytes	RMDN3
0	4.37805784102538	0.303	0.007	0	Hepatocytes	SLC25A10
0	2.02937150668645	0.326	0.031	0	Hepatocytes	RIOX2
0	0.375436034268591	0.384	0.089	0	Hepatocytes	ITPK1
0	2.2728101742117	0.317	0.022	0	Hepatocytes	GCLM
0	2.65595480823159	0.318	0.023	0	Hepatocytes	DAG1
0	0.409065206486078	0.436	0.142	0	Hepatocytes	NUDC
0	0.594223198398464	0.384	0.09	0	Hepatocytes	NDUFB5
0	1.22348603473157	0.343	0.049	0	Hepatocytes	JUP
0	0.367786330518737	0.393	0.099	0	Hepatocytes	MAGT1
0	1.29984347856386	0.345	0.051	0	Hepatocytes	SDHB
0	1.19310223002957	0.351	0.057	0	Hepatocytes	NDUFA4
0	3.57786969026075	0.304	0.01	0	Hepatocytes	SOX5
0	1.46787143076194	0.343	0.05	0	Hepatocytes	AURKAIP1
0	1.26224697246419	0.339	0.046	0	Hepatocytes	SLC50A1
0	0.617459294692704	0.392	0.099	0	Hepatocytes	CALM2
0	1.74959871998291	0.333	0.04	0	Hepatocytes	KIFC3
0	4.5112158659734	0.301	0.008	0	Hepatocytes	SLC30A2
0	2.65065573119334	0.311	0.018	0	Hepatocytes	MTHFS
0	1.93678756079796	0.324	0.032	0	Hepatocytes	SLC25A1
0	0.912102550908462	0.321	0.029	0	Hepatocytes	ADHFE1
0	6.05834986969823	0.294	0.002	0	Hepatocytes	KNDC1
0	4.50884211769524	0.298	0.006	0	Hepatocytes	CCDC85C

0	2.53909512425021	0.314	0.022	0	Hepatocytes	NR1D1
0	0.290756847919746	0.388	0.097	0	Hepatocytes	DLD
0	1.70946455947182	0.339	0.048	0	Hepatocytes	MID1IP1
0	1.10360803031755	0.342	0.052	0	Hepatocytes	MANF
0	1.17908495995618	0.339	0.049	0	Hepatocytes	SPCS1
0	0.596034494841224	0.346	0.056	0	Hepatocytes	ZBTB20
0	3.72100392279283	0.303	0.013	0	Hepatocytes	ECHDC3
0	6.26667151965647	0.292	0.002	0	Hepatocytes	ANGPTL8
0	2.02061159039695	0.324	0.035	0	Hepatocytes	NHSL1
0	1.35407232841431	0.351	0.062	0	Hepatocytes	GBA
0	2.33931632762416	0.306	0.017	0	Hepatocytes	HSD17B8
0	0.603295400085192	0.364	0.075	0	Hepatocytes	GHITM
0	6.07555440969472	0.291	0.002	0	Hepatocytes	SLCO1B1
0	2.65846008492829	0.305	0.016	0	Hepatocytes	EBPL
0	3.57597830809807	0.3	0.011	0	Hepatocytes	GPC3
0	0.312445534337925	0.382	0.094	0	Hepatocytes	SEC61A1
0	1.79193307685533	0.322	0.034	0	Hepatocytes	SYNPO
0	0.360382910544863	0.369	0.081	0	Hepatocytes	UFC1
0	0.749112171933403	0.351	0.063	0	Hepatocytes	HES1
0	0.723098006362854	0.349	0.061	0	Hepatocytes	PGRMC2
0	2.84144958171955	0.31	0.022	0	Hepatocytes	SDC2
0	1.06260488776507	0.323	0.036	0	Hepatocytes	HDHD3
0	0.797997298131851	0.336	0.049	0	Hepatocytes	ZNF512B
0	5.8624544234663	0.29	0.003	0	Hepatocytes	HOOK1
0	5.50729660235176	0.292	0.005	0	Hepatocytes	PIGR
0	0.382423829766817	0.363	0.076	0	Hepatocytes	NMD3
0	3.24080028230932	0.298	0.011	0	Hepatocytes	SORBS2
0	2.67258227598762	0.3	0.013	0	Hepatocytes	CADM1
0	0.573365577464615	0.35	0.063	0	Hepatocytes	PCCA
0	4.26911021649751	0.292	0.005	0	Hepatocytes	DHRS2
0	3.54423858907155	0.298	0.011	0	Hepatocytes	DHODH
0	1.43633930292212	0.343	0.057	0	Hepatocytes	PNKD
0	0.758834865411867	0.359	0.073	0	Hepatocytes	KEAP1
0	0.896695058671632	0.335	0.05	0	Hepatocytes	GADD45A
0	2.82704229097922	0.305	0.02	0	Hepatocytes	UQCRQ
0	5.37280018426641	0.287	0.002	0	Hepatocytes	TMEM184A
0	2.13553531815014	0.315	0.031	0	Hepatocytes	IL17RC
0	1.49060490453972	0.32	0.036	0	Hepatocytes	MYO6
0	2.88628459523485	0.299	0.015	0	Hepatocytes	ENAH
0	0.425505405213727	0.362	0.078	0	Hepatocytes	ESRRA
0	2.40580530207037	0.311	0.027	0	Hepatocytes	ACADS
0	5.53832482183733	0.287	0.003	0	Hepatocytes	RHPN2
0	0.377069260599857	0.385	0.102	0	Hepatocytes	NACC1
0	1.50134336516519	0.331	0.048	0	Hepatocytes	CDC42EP1
0	5.11541938391241	0.287	0.004	0	Hepatocytes	TMEM45A
0	3.26757184761097	0.297	0.014	0	Hepatocytes	ACSM5
0	0.968416579904555	0.336	0.054	0	Hepatocytes	ZNF622
0	0.372404585112624	0.374	0.092	0	Hepatocytes	MLEC
0	6.63664022590501	0.284	0.002	0	Hepatocytes	ABCG5
0	0.528885952181436	0.351	0.069	0	Hepatocytes	IFITM2
0	2.71875110229642	0.303	0.021	0	Hepatocytes	CTSF
0	5.73858300836651	0.285	0.003	0	Hepatocytes	SHANK2

	0	1.35107544288141	0.33	0.049	0	Hepatocytes	GOLM1
	0	1.27658571894161	0.329	0.048	0	Hepatocytes	MIF
	0	1.10106552876743	0.304	0.023	0	Hepatocytes	EPHX2
	0	0.258087998477034	0.36	0.079	0	Hepatocytes	QTRT1
	0	0.75690697849485	0.347	0.066	0	Hepatocytes	RAB5IF
	0	0.25903493082358	0.385	0.105	0	Hepatocytes	NDFIP1
	0	1.72276701156418	0.328	0.048	0	Hepatocytes	MSRB2
	0	1.13211828393468	0.334	0.054	0	Hepatocytes	NDUFA1
	0	1.43091296126139	0.302	0.022	0	Hepatocytes	TSTD1
	0	0.434333513610415	0.361	0.081	0	Hepatocytes	GOLIM4
	0	0.727991510806739	0.345	0.065	0	Hepatocytes	AHR
	0	2.9010256338738	0.292	0.012	0	Hepatocytes	DLG5
	0	1.73809672947538	0.3	0.02	0	Hepatocytes	RASSF7
	0	5.0441255459888	0.284	0.004	0	Hepatocytes	GCAT
	0	0.876109450378853	0.319	0.04	0	Hepatocytes	C11orf54
	0	0.320370438956319	0.356	0.077	0	Hepatocytes	SUB1
	0	4.14743711378147	0.287	0.008	0	Hepatocytes	CAPN5
	0	0.631482973838418	0.332	0.054	0	Hepatocytes	HMGCS1
	0	1.38752176823588	0.32	0.042	0	Hepatocytes	NDUFB6
	0	0.810243900474866	0.338	0.06	0	Hepatocytes	MTHFD1
	0	0.769944486983924	0.355	0.077	0	Hepatocytes	TMED10
	0	2.01737195768037	0.308	0.031	0	Hepatocytes	KCTD3
	0	0.79381416542771	0.34	0.063	0	Hepatocytes	SLC4A2
	0	5.69445798658531	0.279	0.002	0	Hepatocytes	GPD1
	0	5.15406778816776	0.281	0.004	0	Hepatocytes	SLC27A5
	0	1.77903303772061	0.311	0.034	0	Hepatocytes	SELENOM
	0	0.933368753542528	0.352	0.075	0	Hepatocytes	FAM20C
	0	5.00954795400681	0.279	0.003	0	Hepatocytes	TUFT1
	0	0.930387032321752	0.329	0.053	0	Hepatocytes	HDHD5
	0	0.757202006970435	0.363	0.087	0	Hepatocytes	LIMK2
	0	1.95803190904785	0.302	0.026	0	Hepatocytes	COMTD1
	0	4.45480533519211	0.283	0.007	0	Hepatocytes	LGR4
	0	2.94763612152691	0.293	0.017	0	Hepatocytes	AQP9
	0	0.412094698233152	0.348	0.072	0	Hepatocytes	STUB1
	0	1.74278766994652	0.294	0.018	0	Hepatocytes	ATP9A
	0	0.256161509846377	0.35	0.074	0	Hepatocytes	DEPRECATI
	0	4.88421077606499	0.278	0.003	0	Hepatocytes	LAD1
	0	1.26287633440848	0.316	0.041	0	Hepatocytes	KIAA1217
	0	0.890265379575442	0.326	0.051	0	Hepatocytes	EI24
	0	0.471354163886438	0.334	0.059	0	Hepatocytes	DCAF11
	0	1.22773516100851	0.311	0.036	0	Hepatocytes	PRPSAP1
	0	6.22059234159376	0.277	0.002	0	Hepatocytes	PFKFB1
	0	4.04411259138182	0.282	0.007	0	Hepatocytes	HOMER2
	0	1.8815040997187	0.299	0.024	0	Hepatocytes	HSF4
	0	6.02472060490753	0.276	0.002	0	Hepatocytes	DIO1
	0	0.377880236079302	0.365	0.091	0	Hepatocytes	POLR2E
	0	3.29148103236393	0.287	0.013	0	Hepatocytes	P4HA2
	0	1.91559265288489	0.295	0.021	0	Hepatocytes	COL27A1
	0	0.529669513883576	0.38	0.107	0	Hepatocytes	NCOA4
	0	1.31121783036415	0.336	0.063	0	Hepatocytes	MT-ND1
	0	2.4953041236851	0.295	0.022	0	Hepatocytes	CPT2
	0	2.32725724494592	0.293	0.02	0	Hepatocytes	MFSD3

0	1.65243674028214	0.301	0.028	0	Hepatocytes	TBC1D24
0	2.26464354527052	0.286	0.013	0	Hepatocytes	SEC14L2
0	5.33102562250924	0.274	0.002	0	Hepatocytes	SLC12A8
0	0.452899928039545	0.326	0.054	0	Hepatocytes	UBA5
0	1.28424930542288	0.315	0.043	0	Hepatocytes	NDUFAB1
0	2.12563490342921	0.298	0.026	0	Hepatocytes	MAP3K13
0	1.65185392983023	0.309	0.038	0	Hepatocytes	ALAS1
0	1.83898480497861	0.302	0.031	0	Hepatocytes	CHPT1
0	6.39352509001502	0.272	0.001	0	Hepatocytes	PGLYRP2
0	0.7330662656662	0.346	0.075	0	Hepatocytes	NDUFA2
0	0.411090155805118	0.348	0.077	0	Hepatocytes	RING1
0	0.865033453350595	0.351	0.08	0	Hepatocytes	MT-ND2
0	5.77850388113516	0.273	0.003	0	Hepatocytes	ADH4
0	0.527747085339844	0.339	0.069	0	Hepatocytes	SEM1
0	1.27645285311501	0.311	0.041	0	Hepatocytes	NFIB
0	3.40504571473117	0.28	0.01	0	Hepatocytes	EXOC3L4
0	0.390744182993729	0.325	0.055	0	Hepatocytes	TRAF4
0	2.73732527519963	0.289	0.019	0	Hepatocytes	CHRD
0	1.28129444890463	0.327	0.058	0	Hepatocytes	PLOD1
0	0.659492326674097	0.331	0.062	0	Hepatocytes	WSB2
0	0.999120222771187	0.329	0.06	0	Hepatocytes	MYO1C
0	2.08650851297228	0.293	0.024	0	Hepatocytes	CPTP
0	0.854393542233648	0.348	0.079	0	Hepatocytes	FUCA1
0	3.50270019281873	0.277	0.009	0	Hepatocytes	XYLB
0	6.09166748066126	0.27	0.002	0	Hepatocytes	CLDN4
0	6.33871676870569	0.269	0.001	0	Hepatocytes	GLS2
0	0.759098728047217	0.345	0.077	0	Hepatocytes	FAM20A
0	5.20348432414088	0.27	0.003	0	Hepatocytes	ALDH8A1
0	1.57958255388047	0.307	0.04	0	Hepatocytes	CRYL1
0	4.84641543507279	0.271	0.004	0	Hepatocytes	TOM1L1
0	3.01711982055137	0.28	0.014	0	Hepatocytes	HYI
0	1.22282547403284	0.304	0.038	0	Hepatocytes	PFDN6
0	1.62145541889128	0.298	0.032	0	Hepatocytes	TMEM205
0	6.5742215682496	0.268	0.002	0	Hepatocytes	DEPRECATI
0	5.87161394239306	0.267	0.002	0	Hepatocytes	UGT2B15
0	0.510464485292711	0.333	0.068	0	Hepatocytes	COMMD4
0	3.67493608737041	0.274	0.009	0	Hepatocytes	ONECUT2
0	0.56272757827922	0.345	0.08	0	Hepatocytes	ARL8A
0	1.83699824094203	0.294	0.029	0	Hepatocytes	HTRA1
0	1.33894539861954	0.31	0.046	0	Hepatocytes	S100A13
0	1.44555661500023	0.302	0.038	0	Hepatocytes	TIMM17A
0	0.56899987195129	0.35	0.086	0	Hepatocytes	RNPEP
0	3.646699750972	0.272	0.008	0	Hepatocytes	ASS1
0	5.83689617805435	0.266	0.002	0	Hepatocytes	CYP2C19
0	0.683455087570948	0.356	0.092	0	Hepatocytes	APOL1
0	1.1168836263893	0.301	0.039	0	Hepatocytes	HAX1
0	3.29312179475937	0.274	0.012	0	Hepatocytes	FZD5
0	5.0618905186985	0.266	0.004	0	Hepatocytes	PACSIN3
0	1.3455712320759	0.303	0.041	0	Hepatocytes	ESD
0	2.05171903611031	0.29	0.028	0	Hepatocytes	FAM114A1
0	1.85648743740331	0.297	0.036	0	Hepatocytes	MGST2
0	2.81559306372754	0.277	0.016	0	Hepatocytes	ACOT13

0	0.489627842474118	0.333	0.072	0	Hepatocytes	SPCS2
0	2.18210238021463	0.294	0.033	0	Hepatocytes	SDSL
0	4.71230337274779	0.267	0.006	0	Hepatocytes	SORD
0	1.42663700521995	0.303	0.042	0	Hepatocytes	PSMC4
0	0.266268253554965	0.345	0.084	0	Hepatocytes	SHARPIN
0	2.42256380216576	0.285	0.024	0	Hepatocytes	DEPRECATI
0	0.481484848881747	0.334	0.074	0	Hepatocytes	APH1A
0	3.15798312198861	0.268	0.008	0	Hepatocytes	ENPP1
0	0.79932875394293	0.332	0.072	0	Hepatocytes	COX8A
0	3.8421909552679	0.269	0.009	0	Hepatocytes	PC
0	4.80773982908354	0.264	0.004	0	Hepatocytes	RHOD
0	2.15890646007912	0.281	0.022	0	Hepatocytes	AP1S1
0	5.88006305884952	0.261	0.002	0	Hepatocytes	HKDC1
0	1.95533538896484	0.284	0.025	0	Hepatocytes	TOMM40L
0	5.69492801513166	0.26	0.002	0	Hepatocytes	GBP7
0	5.53397761248622	0.261	0.003	0	Hepatocytes	FAM110C
0	4.82236385661064	0.261	0.003	0	Hepatocytes	GHR
0	0.878498116785154	0.338	0.08	0	Hepatocytes	HEXB
0	2.98253688506967	0.276	0.018	0	Hepatocytes	PLIN2
0	1.2753160310729	0.308	0.05	0	Hepatocytes	SLC22A18
0	1.12002892025394	0.296	0.038	0	Hepatocytes	NUCB2
0	0.930561023881137	0.3	0.042	0	Hepatocytes	TMEM106C
0	1.88594672849971	0.275	0.017	0	Hepatocytes	TMEM238
0	3.5749033043291	0.266	0.009	0	Hepatocytes	DDAH1
0	1.2189254823858	0.303	0.047	0	Hepatocytes	UROD
0	0.365066874611542	0.32	0.064	0	Hepatocytes	IARS2
0	5.65344394777084	0.258	0.002	0	Hepatocytes	SLC51A
0	5.94170588781142	0.258	0.002	0	Hepatocytes	COBL
0	5.87131344981383	0.258	0.002	0	Hepatocytes	TRIM55
0	0.667294802252178	0.292	0.036	0	Hepatocytes	SLC25A16
0	2.43912240301295	0.279	0.023	0	Hepatocytes	PCBD1
0	0.699942268742988	0.304	0.048	0	Hepatocytes	DEPRECATI
0	1.44805021968924	0.285	0.029	0	Hepatocytes	DCUN1D4
0	1.51928618902159	0.286	0.031	0	Hepatocytes	DVL1
0	3.7388542123948	0.261	0.006	0	Hepatocytes	PLXNB1
0	0.640104860890804	0.341	0.086	0	Hepatocytes	EGR1
0	1.59521611331139	0.275	0.02	0	Hepatocytes	KLC4
0	0.320742605760596	0.312	0.057	0	Hepatocytes	FBXL6
0	1.7934486203945	0.276	0.022	0	Hepatocytes	CUL7
0	0.511763813935508	0.34	0.086	0	Hepatocytes	C7orf50
0	1.47127020247114	0.286	0.032	0	Hepatocytes	ZCCHC14
0	0.348927334702231	0.324	0.07	0	Hepatocytes	PRKCSH
0	0.601730265175139	0.307	0.053	0	Hepatocytes	GIPC1
0	1.18300092672557	0.316	0.063	0	Hepatocytes	HS6ST1
0	1.35901062028686	0.291	0.038	0	Hepatocytes	LRRC59
0	0.469598195423775	0.336	0.084	0	Hepatocytes	PHC2
0	0.413433504856284	0.353	0.101	0	Hepatocytes	CLTA
0	5.71731073809135	0.254	0.002	0	Hepatocytes	MOCOS
0	3.34266772046293	0.262	0.01	0	Hepatocytes	TSPAN6
0	2.60839556642881	0.266	0.015	0	Hepatocytes	CHPF
0	0.579924411721867	0.306	0.055	0	Hepatocytes	COX17
0	1.01338411576696	0.303	0.052	0	Hepatocytes	NUDT16

	0	0.43849307353665	0.312	0.061	0	Hepatocytes	NDUFS4
	0	1.26532517976847	0.296	0.045	0	Hepatocytes	KIF13A
	0	1.51616098790241	0.284	0.033	0	Hepatocytes	AGPAT2
	0	1.19586433639658	0.295	0.044	0	Hepatocytes	CTTN
	0	5.76488789486505	0.254	0.003	0	Hepatocytes	RNF128
	0	1.1691749908194	0.277	0.026	0	Hepatocytes	DEPRECATI
	0	4.76487645188231	0.253	0.003	0	Hepatocytes	RAP1GAP
	0	0.5160261655379	0.293	0.043	0	Hepatocytes	EIF2D
	0	1.88352258593605	0.279	0.029	0	Hepatocytes	PCYOX1
	0	0.506867025468824	0.322	0.072	0	Hepatocytes	LAMB2
	0	0.801767863912141	0.326	0.076	0	Hepatocytes	RTN3
	0	3.85972870278661	0.257	0.007	0	Hepatocytes	STAP2
	0	1.87782294165873	0.275	0.026	0	Hepatocytes	PDGFA
	0	1.91659201808496	0.281	0.032	0	Hepatocytes	CKAP4
	0	0.501677693505257	0.281	0.032	0	Hepatocytes	SLC25A42
	0	0.409560334896965	0.316	0.067	0	Hepatocytes	HADHB
	0	0.936956645105044	0.315	0.066	0	Hepatocytes	HSD3B7
	0	0.336128928756782	0.339	0.091	0	Hepatocytes	ABHD2
	0	0.699509153539329	0.296	0.048	0	Hepatocytes	SMPD1
	0	6.31847291398277	0.25	0.002	0	Hepatocytes	ASPDH
	0	1.17235463310045	0.282	0.034	0	Hepatocytes	EHBP1
	0	2.18500448725442	0.273	0.026	0	Hepatocytes	HHEX
	0	1.19509915603525	0.276	0.029	0	Hepatocytes	LAMA5
	0	2.30271928687104	0.276	0.029	0	Hepatocytes	DEPRECATI
	0	0.385418391768042	0.323	0.076	0	Hepatocytes	FLOT1
	0	5.38039303377234	0.25	0.003	0	Hepatocytes	UNC5CL
	0	4.13423559315801	0.255	0.008	0	Hepatocytes	LOXL4
	0	0.549343746946893	0.301	0.054	0	Hepatocytes	NUDT14
	0	1.24456789067611	0.282	0.035	0	Hepatocytes	YIF1A
	0	1.4680047151598	0.274	0.028	0	Hepatocytes	NQO2
	0	2.0484063635645	0.27	0.024	0	Hepatocytes	EEPD1
	0	1.2406691745121	0.295	0.049	0	Hepatocytes	LAMTOR2
	0	0.47226580831897	0.307	0.061	0	Hepatocytes	FOXN2
	0	0.307618384931225	0.302	0.056	0	Hepatocytes	RNF103
	0	5.20141142154105	0.251	0.005	0	Hepatocytes	SAA1
	0	1.34170400295497	0.359	0.113	0	Hepatocytes	SDS
	0	6.03786530928733	0.248	0.002	0	Hepatocytes	ACSM2B
	0	0.808397185878014	0.295	0.049	0	Hepatocytes	TFIP11
	0	2.07183561878697	0.262	0.017	0	Hepatocytes	ELMO3
	0	2.34595045703832	0.265	0.021	0	Hepatocytes	CAV2
	0	2.17200651310047	0.265	0.021	0	Hepatocytes	FKBP4
	0	0.733677152839706	0.291	0.047	0	Hepatocytes	RNF11
	0	5.39782430323757	0.247	0.003	0	Hepatocytes	PLGLB1
	0	4.6361480092099	0.249	0.005	0	Hepatocytes	STBD1
	0	5.60450001736147	0.246	0.002	0	Hepatocytes	GPLD1
	0	4.66831805650845	0.248	0.004	0	Hepatocytes	PPP1R1A
	0	1.84567969603314	0.269	0.026	0	Hepatocytes	WFS1
	0	2.30716511677827	0.268	0.025	0	Hepatocytes	SRXN1
	0	0.480700278440558	0.302	0.059	0	Hepatocytes	MFN2
	0	3.7594541191517	0.25	0.007	0	Hepatocytes	EPB41L5
	0	1.81760834790254	0.258	0.015	0	Hepatocytes	CEP70
	0	4.46926029040396	0.248	0.005	0	Hepatocytes	RIDA

0	5.82895500548963	0.245	0.002	0	Hepatocytes	GDA
0	2.79111066392031	0.26	0.017	0	Hepatocytes	ZNF219
0	0.754825552088398	0.292	0.049	0	Hepatocytes	ITPKC
0	1.08027363899038	0.282	0.039	0	Hepatocytes	TMEM208
0	1.29086218237539	0.282	0.039	0	Hepatocytes	NAA20
0	5.6385623299216	0.244	0.002	0	Hepatocytes	ZNF648
0	0.927934841203269	0.287	0.045	0	Hepatocytes	TNS2
0	3.88169470466743	0.247	0.005	0	Hepatocytes	CACNA1H
0	0.608607810600528	0.282	0.04	0	Hepatocytes	KLHL20
0	1.29285597443047	0.282	0.04	0	Hepatocytes	CTIF
0	2.05823517870719	0.263	0.022	0	Hepatocytes	TMEM9
0	1.3759690378655	0.281	0.04	0	Hepatocytes	ARHGEF40
0	1.18584509824535	0.278	0.037	0	Hepatocytes	ACSF2
0	2.49742420195986	0.248	0.007	0	Hepatocytes	GLDC
0	0.315572702606523	0.29	0.049	0	Hepatocytes	DENND10
0	0.516679814892376	0.293	0.052	0	Hepatocytes	SLC35B1
0	4.59840510377241	0.246	0.005	0	Hepatocytes	GAGE1
0	1.46002699182632	0.28	0.04	0	Hepatocytes	RHOU
0	1.75985015891063	0.274	0.034	0	Hepatocytes	RHOBTB3
0	1.27017622755391	0.28	0.04	0	Hepatocytes	PVR
0	1.95673827421546	0.268	0.028	0	Hepatocytes	ISOC2
0	1.08869862999379	0.271	0.031	0	Hepatocytes	NECAB3
0	0.935518564159535	0.291	0.051	0	Hepatocytes	SLC25A29
0	1.55084064752597	0.261	0.022	0	Hepatocytes	UMPS
0	1.40385900949167	0.27	0.031	0	Hepatocytes	NDRG2
0	1.13921739812535	0.261	0.022	0	Hepatocytes	CERS4
0	0.35879696655624	0.291	0.052	0	Hepatocytes	JTB
0	2.44366773811233	0.254	0.015	0	Hepatocytes	MAPRE3
0	0.288718111124452	0.287	0.048	0	Hepatocytes	BRPF3
0	5.4087259078508	0.242	0.003	0	Hepatocytes	MYORG
0	3.30087519639248	0.249	0.01	0	Hepatocytes	LRRC75B
0	0.458220287203564	0.281	0.043	0	Hepatocytes	PHKA2
0	4.79475653064554	0.241	0.003	0	Hepatocytes	IL17RB
0	1.6573440137456	0.253	0.015	0	Hepatocytes	FGFRL1
0	5.07907753687093	0.24	0.002	0	Hepatocytes	TJP3
0	1.96219470939148	0.259	0.022	0	Hepatocytes	ZNF595
0	1.53163548215359	0.268	0.031	0	Hepatocytes	HIBADH
0	0.577926568197647	0.291	0.054	0	Hepatocytes	COX7C
0	2.65426454197215	0.252	0.015	0	Hepatocytes	SIAE
0	4.82432446608871	0.24	0.003	0	Hepatocytes	ASPG
0	0.595014131257517	0.303	0.066	0	Hepatocytes	ILVBL
0	1.32579408076926	0.281	0.045	0	Hepatocytes	DUSP3
0	1.01048555382507	0.267	0.031	0	Hepatocytes	SLC25A20
0	1.24505150432469	0.267	0.031	0	Hepatocytes	UQCC2
0	1.90260017015579	0.258	0.022	0	Hepatocytes	MPDZ
0	1.62540673118369	0.269	0.033	0	Hepatocytes	NME1
0	6.76458209561332	0.237	0.001	0	Hepatocytes	CYP2J2
0	5.82349249508389	0.238	0.002	0	Hepatocytes	FETUB
0	0.408963627521575	0.309	0.073	0	Hepatocytes	ATP5ME
0	0.495090491332012	0.3	0.064	0	Hepatocytes	ATP5F1A
0	0.511445186874181	0.3	0.064	0	Hepatocytes	TIMM13
0	0.36137390250654	0.304	0.068	0	Hepatocytes	AKT1S1

0	0.363800772448792	0.325	0.09	0	Hepatocytes	CTNNA1
0	1.28229925807397	0.263	0.028	0	Hepatocytes	SPRYD4
0	3.03419279881053	0.245	0.01	0	Hepatocytes	FAAH
0	0.952205845871149	0.288	0.053	0	Hepatocytes	HSPA5
0	0.629590065224255	0.294	0.059	0	Hepatocytes	SLC25A25
0	2.69457140208957	0.243	0.008	0	Hepatocytes	DEPRECATI
0	0.587709123693087	0.28	0.046	0	Hepatocytes	SNX4
0	0.719273964082052	0.273	0.039	0	Hepatocytes	MUC20
0	1.61369134225359	0.266	0.032	0	Hepatocytes	ALAD
0	2.03674822204692	0.258	0.024	0	Hepatocytes	BCAT2
0	1.62393189695014	0.261	0.027	0	Hepatocytes	ARVCF
0	3.71327337069335	0.243	0.009	0	Hepatocytes	PPP1R3C
0	0.319239213254147	0.307	0.073	0	Hepatocytes	SNF8
0	0.961230471706433	0.264	0.031	0	Hepatocytes	TARS2
0	1.81958297115186	0.257	0.024	0	Hepatocytes	COA3
0	2.2289434243589	0.252	0.019	0	Hepatocytes	DEPRECATI
0	5.94805299578446	0.235	0.002	0	Hepatocytes	IRF6
0	0.253472484607116	0.285	0.052	0	Hepatocytes	SCAP
0	0.421719616392135	0.301	0.068	0	Hepatocytes	NDUFA13
0	1.32600150061142	0.271	0.039	0	Hepatocytes	MYC
0	1.66589595044872	0.255	0.023	0	Hepatocytes	PLS3
0	0.392767346993751	0.286	0.054	0	Hepatocytes	LYPLAL1
0	4.07965371742167	0.237	0.005	0	Hepatocytes	MLPH
0	4.38862893726167	0.236	0.004	0	Hepatocytes	MARVELD2
0	0.288882642766981	0.294	0.062	0	Hepatocytes	DEXI
0	0.483080915041755	0.289	0.057	0	Hepatocytes	PSMD7
0	2.13199285052426	0.249	0.017	0	Hepatocytes	ABCA6
0	1.43654914839789	0.269	0.038	0	Hepatocytes	GLMP
0	1.00220869540625	0.268	0.037	0	Hepatocytes	RNF181
0	0.937809498460288	0.27	0.039	0	Hepatocytes	GLUD1
0	0.805978535911189	0.26	0.029	0	Hepatocytes	FBXO31
0	0.469582764637615	0.325	0.094	0	Hepatocytes	GABARAP
0	5.54708751134269	0.234	0.003	0	Hepatocytes	EEF1A2
0	0.818945719957244	0.289	0.058	0	Hepatocytes	DAP
0	4.33797178196459	0.236	0.005	0	Hepatocytes	OLFM2
0	4.58602228630373	0.241	0.01	0	Hepatocytes	HAMP
0	3.73811085972368	0.239	0.008	0	Hepatocytes	TRIB3
0	0.992385092033006	0.273	0.043	0	Hepatocytes	ST5
0	4.78331697353502	0.233	0.003	0	Hepatocytes	MET
0	1.75219660252983	0.254	0.024	0	Hepatocytes	CISD3
0	0.27147570162699	0.293	0.063	0	Hepatocytes	MZT2B
0	0.541202353001259	0.295	0.065	0	Hepatocytes	TRIO
0	2.79909306245395	0.241	0.011	0	Hepatocytes	PAWR
0	2.26734646403486	0.247	0.017	0	Hepatocytes	FAM174B
0	0.818906966545517	0.274	0.045	0	Hepatocytes	PSMB5
0	1.20814830073333	0.262	0.033	0	Hepatocytes	COX6C
0	0.709972802312592	0.262	0.033	0	Hepatocytes	DAPK2
0	1.55912444162372	0.253	0.024	0	Hepatocytes	TACO1
0	3.968675174363	0.233	0.005	0	Hepatocytes	BTNL8
0	1.98532348798128	0.253	0.025	0	Hepatocytes	AAMDC
0	2.17280889739769	0.251	0.023	0	Hepatocytes	SLC47A1
0	0.76900929739189	0.279	0.052	0	Hepatocytes	GRAMD4

0	1.46911142308582	0.253	0.026		0	Hepatocytes	PLSCR4
0	1.69662610155844	0.247	0.02		0	Hepatocytes	SH3BGRL2
0	1.55189147460188	0.251	0.024		0	Hepatocytes	LACTB2
0	4.14599426097818	0.231	0.004		0	Hepatocytes	CENPV
0	0.920050713253323	0.265	0.038		0	Hepatocytes	RFNG
0	0.606429883969425	0.269	0.042		0	Hepatocytes	TMEM161A
0	0.958253330869199	0.263	0.036		0	Hepatocytes	MAGED1
0	2.59361639446027	0.237	0.01		0	Hepatocytes	LIPG
0	0.495767106908911	0.282	0.055		0	Hepatocytes	PLCXD1
0	0.46024737262804	0.271	0.045		0	Hepatocytes	COQ9
0	5.84557468338133	0.228	0.002		0	Hepatocytes	ADGRV1
0	1.19861944165892	0.261	0.035		0	Hepatocytes	LPCAT3
0	2.31921723682135	0.238	0.012		0	Hepatocytes	TLE1
0	3.14018739647544	0.237	0.011		0	Hepatocytes	MCRIP2
0	1.06358055704188	0.271	0.046		0	Hepatocytes	CANX
0	0.366751605165093	0.272	0.047		0	Hepatocytes	COA4
0	1.43814180017245	0.25	0.025		0	Hepatocytes	SFXN5
0	0.454006877500323	0.312	0.087		0	Hepatocytes	TMEM37
0	1.4328147994311	0.251	0.026		0	Hepatocytes	KDELR2
0	3.68149921497987	0.23	0.005		0	Hepatocytes	FAM83G
0	0.479637125481196	0.261	0.036		0	Hepatocytes	ACBD4
0	3.00188496539052	0.236	0.011		0	Hepatocytes	BOK
0	2.86369838525433	0.235	0.01		0	Hepatocytes	LMO7
0	1.42668424965749	0.25	0.026		0	Hepatocytes	C1orf198
0	2.04275276563156	0.248	0.024		0	Hepatocytes	AIMP2
0	3.46850363469326	0.233	0.009		0	Hepatocytes	SLC6A12
0	0.566837468989508	0.297	0.073		0	Hepatocytes	BAG1
0	0.295858928682244	0.266	0.043		0	Hepatocytes	DNAJA3
0	0.701105193400165	0.258	0.035		0	Hepatocytes	TOMM70
0	0.958682867886604	0.26	0.037		0	Hepatocytes	DCPS
0	5.39995270227014	0.225	0.002		0	Hepatocytes	PLEKHG6
0	3.2622289687479	0.232	0.009		0	Hepatocytes	NOL3
0	1.90566174680671	0.25	0.027		0	Hepatocytes	MPND
0	5.66943420702031	0.225	0.002		0	Hepatocytes	DEPRECATI
0	0.534463836723029	0.303	0.08		0	Hepatocytes	TGM2
0	0.796527868734088	0.27	0.048		0	Hepatocytes	TMBIM6
0	0.986608032832219	0.261	0.039		0	Hepatocytes	SRM
0	5.35350835109211	0.224	0.002		0	Hepatocytes	CFHR5
0	2.1477565832131	0.245	0.023		0	Hepatocytes	DDR1
0	3.62235437784976	0.23	0.008		0	Hepatocytes	MAP7
0	1.13165694119097	0.263	0.041		0	Hepatocytes	ACSS2
0	1.66567532560697	0.246	0.024		0	Hepatocytes	SDF2L1
0	0.857315932320307	0.257	0.035		0	Hepatocytes	POLR2F
0	0.367374827146596	0.289	0.067		0	Hepatocytes	YWHAE
0	0.668159110433602	0.267	0.046		0	Hepatocytes	BCKDHA
0	1.689335097658	0.246	0.025		0	Hepatocytes	GATA4
0	2.42020874062887	0.235	0.014		0	Hepatocytes	SLC25A4
0	1.45573025538687	0.237	0.016		0	Hepatocytes	DMTN
0	3.02537004636208	0.228	0.008		0	Hepatocytes	FAT1
0	1.93851688963747	0.245	0.025		0	Hepatocytes	NLN
0	1.94109589989448	0.243	0.023		0	Hepatocytes	ZFAND2A
0	4.32902074039302	0.225	0.005		0	Hepatocytes	CXADR

	0	3.316692867058	0.228	0.009	0	Hepatocytes	FRRS1
	0	2.56610020774662	0.233	0.014	0	Hepatocytes	THNSL2
	0	3.74319371100509	0.224	0.005	0	Hepatocytes	CAP2
	0	3.37384073944995	0.226	0.007	0	Hepatocytes	RBP5
	0	1.91507769776061	0.245	0.026	0	Hepatocytes	PLEKHH3
	0	1.68355447777617	0.236	0.017	0	Hepatocytes	LNX2
	0	1.71953093397572	0.242	0.024	0	Hepatocytes	POLD2
	0	1.83522184201558	0.236	0.018	0	Hepatocytes	BLNK
	0	1.15420579479435	0.253	0.035	0	Hepatocytes	TSPAN9
	0	2.40189826848686	0.234	0.017	0	Hepatocytes	GNG12
	0	0.28196522399754	0.265	0.048	0	Hepatocytes	PPP1R11
	0	6.09618882578066	0.219	0.002	0	Hepatocytes	PLA2G12B
	0	0.61057537912655	0.258	0.041	0	Hepatocytes	DHCR7
	0	3.06240811140624	0.228	0.011	0	Hepatocytes	SHMT2
	0	4.94170080058277	0.221	0.004	0	Hepatocytes	TSPAN8
	0	3.52063074247106	0.225	0.008	0	Hepatocytes	ERBB2
	0	1.68687084024161	0.242	0.025	0	Hepatocytes	YIPF2
	0	2.10676030961253	0.232	0.016	0	Hepatocytes	GPC1
	0	0.74774680781773	0.271	0.055	0	Hepatocytes	FAM234A
	0	1.03575534175504	0.248	0.032	0	Hepatocytes	GPATCH4
	0	3.95653196395624	0.221	0.005	0	Hepatocytes	SPR
	0	1.27210554239704	0.243	0.027	0	Hepatocytes	GRPEL1
	0	0.857276577081629	0.247	0.031	0	Hepatocytes	MPLKIP
	0	0.808538056376981	0.253	0.037	0	Hepatocytes	ARL 1.00
	0	2.88474708111438	0.226	0.01	0	Hepatocytes	CLMN
	0	4.50763686317385	0.22	0.004	0	Hepatocytes	CCL16
	0	2.03547198093608	0.233	0.018	0	Hepatocytes	MTUS1
	0	0.714865611220987	0.245	0.03	0	Hepatocytes	SLC25A22
	0	0.696652868782442	0.25	0.035	0	Hepatocytes	ZNRD2
	0	0.480726181466801	0.267	0.053	0	Hepatocytes	SAP18
	0	0.3680643111389	0.258	0.044	0	Hepatocytes	RANBP10
	0	5.9998522220107	0.216	0.002	0	Hepatocytes	PKLR
	0	5.57682873830717	0.216	0.002	0	Hepatocytes	GLYATL1
	0	1.70302349732086	0.228	0.014	0	Hepatocytes	SLC25A23
	0	2.3414483418956	0.228	0.015	0	Hepatocytes	AK4
	0	0.837403525603422	0.262	0.049	0	Hepatocytes	IL13RA1
	0	4.12170751750203	0.218	0.005	0	Hepatocytes	VNN 3.00
	0	0.392953208624794	0.255	0.042	0	Hepatocytes	TKFC
	0	2.48621245974326	0.225	0.012	0	Hepatocytes	DOK4
	0	2.94149617620577	0.223	0.01	0	Hepatocytes	RAPGEFL1
	0	0.584110870077122	0.256	0.044	0	Hepatocytes	EPHB4
	0	1.99520773419709	0.258	0.046	0	Hepatocytes	SCD
	0	0.923060275463403	0.266	0.054	0	Hepatocytes	SHTN1
	0	0.499357754909981	0.26	0.048	0	Hepatocytes	ARHGEF12
	0	0.400037606879542	0.277	0.065	0	Hepatocytes	ANAPC11
	0	0.908578992687733	0.27	0.058	0	Hepatocytes	DEPRECATI
	0	1.12603847910635	0.244	0.032	0	Hepatocytes	SF3B4
	0	1.22250754221741	0.25	0.038	0	Hepatocytes	BAIAP2
	0	5.48595587687562	0.214	0.002	0	Hepatocytes	FUT6
	0	0.468679114211878	0.262	0.051	0	Hepatocytes	SSR3
	0	0.603936787748127	0.259	0.048	0	Hepatocytes	SNAP29
	0	0.272467689687138	0.273	0.062	0	Hepatocytes	PES 1.00

0	0.965196231209667	0.236	0.025		0	Hepatocytes	DEPRECATI
0	1.62247503579136	0.232	0.022		0	Hepatocytes	CCT3
0	0.465407716398206	0.263	0.053		0	Hepatocytes	GADD45GIP
0	3.07663412768914	0.22	0.01		0	Hepatocytes	PTGFRN
0	4.88744857922306	0.212	0.002		0	Hepatocytes	ABCB11
0	5.84622226594745	0.212	0.002		0	Hepatocytes	C4orf19
0	6.3978803303347	0.211	0.001		0	Hepatocytes	UGT2B10
0	0.52263589283486	0.252	0.042		0	Hepatocytes	WDR46
0	0.975633388139359	0.238	0.028		0	Hepatocytes	TPD52
0	1.21467484186045	0.236	0.026		0	Hepatocytes	TOP1MT
0	6.10350795108399	0.211	0.001		0	Hepatocytes	TMPRSS2
0	0.648907342947149	0.239	0.029		0	Hepatocytes	MBNL3
0	1.20819660246316	0.224	0.015		0	Hepatocytes	RALGPS2
0	0.423253399575427	0.26	0.051		0	Hepatocytes	SSR4
0	0.524804958937797	0.244	0.035		0	Hepatocytes	FAM20B
0	1.34753675516658	0.237	0.028		0	Hepatocytes	ALG3
0	5.74170599693756	0.211	0.002		0	Hepatocytes	SERPINA11
0	3.47397454058527	0.216	0.008		0	Hepatocytes	SOWAHC
0	1.06872954647251	0.248	0.04		0	Hepatocytes	RPN1
0	1.86703570238621	0.219	0.011		0	Hepatocytes	ENPEP
0	0.860707087010287	0.241	0.033		0	Hepatocytes	GPANK1
0	0.750891001815467	0.228	0.021		0	Hepatocytes	OCIAD2
0	0.622757854049969	0.263	0.056		0	Hepatocytes	CDC42BPB
0	1.1827787262216	0.245	0.038		0	Hepatocytes	FAM174A
0	5.44766402774765	0.21	0.003		0	Hepatocytes	TTPA
0	1.87967021634761	0.22	0.013		0	Hepatocytes	RNF152
0	1.36304662571133	0.229	0.023		0	Hepatocytes	HIGD1A
0	0.35283518575199	0.252	0.046		0	Hepatocytes	GFOD2
0	0.742424160732513	0.264	0.058		0	Hepatocytes	NAGLU
0	0.666926644485641	0.242	0.036		0	Hepatocytes	DPY30
0	0.813165012766969	0.239	0.033		0	Hepatocytes	PCBP4
0	2.68221487843355	0.215	0.009		0	Hepatocytes	ZDHHC11
0	4.83796772258144	0.208	0.002		0	Hepatocytes	CBLC
0	0.395707127036573	0.251	0.046		0	Hepatocytes	POMGNT1
0	2.14435197380575	0.222	0.017		0	Hepatocytes	NFIX
0	1.03028828926551	0.239	0.034		0	Hepatocytes	HSDL2
0	0.526848665775916	0.254	0.05		0	Hepatocytes	FLCN
0	0.275283532003535	0.257	0.053		0	Hepatocytes	OGFOD3
0	0.329143187360866	0.253	0.049		0	Hepatocytes	EBP
0	3.63305755299191	0.21	0.006		0	Hepatocytes	MARC1
0	5.6496368174442	0.207	0.003		0	Hepatocytes	DEFB1
0	4.30121852388251	0.208	0.004		0	Hepatocytes	PTPRD
0	0.735300392543583	0.238	0.034		0	Hepatocytes	DHTKD1
0	0.463366247231186	0.242	0.038		0	Hepatocytes	PTS
0	2.05497106142203	0.218	0.015		0	Hepatocytes	CYP7B1
0	0.858539705592675	0.252	0.049		0	Hepatocytes	SLC46A1
0	2.9040647572226	0.214	0.011		0	Hepatocytes	IGF2BP3
0	0.901553189658316	0.237	0.034		0	Hepatocytes	IFT43
0	1.0666931457406	0.237	0.034		0	Hepatocytes	GLRX5
0	0.903680473315661	0.238	0.035		0	Hepatocytes	TJP1
0	0.367826441284465	0.261	0.059		0	Hepatocytes	TXNL1
0	5.11750945657309	0.204	0.002		0	Hepatocytes	INHBE

0	3.64203215593005	0.209	0.007	0	Hepatocytes	NUDT7
0	0.979693724840102	0.251	0.05	0	Hepatocytes	PRDX1
0	5.23339581408554	0.203	0.002	0	Hepatocytes	SLC17A2
0	0.924484005502362	0.255	0.054	0	Hepatocytes	BLVRA
0	1.0773130745843	0.247	0.047	0	Hepatocytes	ARHGEF11
0	2.12938776774045	0.216	0.016	0	Hepatocytes	TEAD1
0	0.669718425861392	0.234	0.034	0	Hepatocytes	USP35
0	1.60566090855913	0.223	0.023	0	Hepatocytes	DEPRECATI
0	2.56969999864643	0.212	0.012	0	Hepatocytes	PLOD2
0	0.856556542303016	0.236	0.036	0	Hepatocytes	NFKBIL1
0	0.399331290515852	0.241	0.041	0	Hepatocytes	TMEM120A
0	0.324932207926668	0.244	0.045	0	Hepatocytes	KLHDC3
0	0.423131393016582	0.245	0.046	0	Hepatocytes	COX7A2
0	1.1489197111615	0.245	0.046	0	Hepatocytes	TALDO1
0	0.553914561182337	0.23	0.031	0	Hepatocytes	NPIPB15
0	0.361370959955874	0.247	0.048	0	Hepatocytes	UBB
0	5.35402651191812	0.201	0.002	0	Hepatocytes	CCL15
0	3.07292313290117	0.21	0.011	0	Hepatocytes	SLC25A33
0	2.67995475357692	0.21	0.011	0	Hepatocytes	MSRB1
0	0.48124393409497	0.237	0.038	0	Hepatocytes	CDIP1
0	0.409177361735004	0.226	0.028	0	Hepatocytes	SLC35D1
0	5.9229351505271	0.199	0.001	0	Hepatocytes	SLC6A11
0	0.646748866334367	0.233	0.035	0	Hepatocytes	ZSCAN26
0	0.607846140868266	0.257	0.059	0	Hepatocytes	PPFIBP2
0	0.802765538065266	0.234	0.036	0	Hepatocytes	COX6A1
0	0.869811539315313	0.232	0.034	0	Hepatocytes	TEX2
0	1.29671678320443	0.238	0.04	0	Hepatocytes	NEU1
0	2.74963631062649	0.208	0.01	0	Hepatocytes	YAP1
0	0.642936217303261	0.236	0.038	0	Hepatocytes	ATP5PD
0	6.31388445841829	0.201	0.004	0	Hepatocytes	CPS1
0	3.20874591122326	0.207	0.01	0	Hepatocytes	PBLD
0	0.676283689761187	0.235	0.038	0	Hepatocytes	ELOB
0	1.05221034124863	0.229	0.033	0	Hepatocytes	HGH1
0	1.06576202175667	0.223	0.027	0	Hepatocytes	TMED2
0	0.611900830513382	0.242	0.046	0	Hepatocytes	YPEL2
0	1.19417940068076	0.221	0.025	0	Hepatocytes	ADAMTS10
0	2.056014808345	0.21	0.015	0	Hepatocytes	HYAL1
0	1.73051796328656	0.211	0.016	0	Hepatocytes	MAGI1
0	0.474629790490784	0.228	0.033	0	Hepatocytes	MCCC2
0	0.764713315326034	0.228	0.033	0	Hepatocytes	ACAT2
0	1.14940564424572	0.215	0.02	0	Hepatocytes	SERPINE1
0	1.75850635441008	0.213	0.018	0	Hepatocytes	AASS
0	1.34077226562002	0.229	0.034	0	Hepatocytes	TRIB1
0	1.83002607882639	0.21	0.015	0	Hepatocytes	ADAMTSL3
0	1.48998671523817	0.213	0.018	0	Hepatocytes	CX3CL1
0	0.477489523732764	0.25	0.055	0	Hepatocytes	TMEM147
0	0.401133633621066	0.228	0.033	0	Hepatocytes	GABRE
0	0.908267811396232	0.237	0.043	0	Hepatocytes	CTSO
0	3.78035044198172	0.199	0.005	0	Hepatocytes	FGFR4
0	2.3034910933126	0.209	0.015	0	Hepatocytes	HINT2
0	5.46633018749734	0.196	0.002	0	Hepatocytes	DEPRECATI
0	0.286079857976494	0.235	0.041	0	Hepatocytes	GOLGA5

0	1.69755093484054	0.215	0.022		0	Hepatocytes	WLS
0	1.93124294507653	0.213	0.02		0	Hepatocytes	MAP3K20
0	2.59299527878386	0.203	0.01		0	Hepatocytes	TACC2
0	1.89803028945336	0.212	0.019		0	Hepatocytes	ADCY6
0	0.624730506502707	0.225	0.032		0	Hepatocytes	TSFM
0	5.46188770567891	0.195	0.002		0	Hepatocytes	SMOC1
0	2.83385104291212	0.203	0.01		0	Hepatocytes	TMEM220
0	1.414296846721	0.224	0.031		0	Hepatocytes	RGL3
0	2.87689719647628	0.197	0.005		0	Hepatocytes	RETREG1
0	0.53716030500823	0.219	0.027		0	Hepatocytes	BCKDHB
0	5.96283021245488	0.193	0.001		0	Hepatocytes	CLDN3
0	1.87972082329056	0.218	0.026		0	Hepatocytes	PDK4
0	0.972279249824425	0.222	0.03		0	Hepatocytes	PPA1
0	1.12108533825591	0.217	0.025		0	Hepatocytes	C11orf24
0	3.9769776679694	0.196	0.005		0	Hepatocytes	ASAP3
0	2.52111533136917	0.202	0.011		0	Hepatocytes	SMO
0	3.69633141423806	0.196	0.005		0	Hepatocytes	AS3MT
0	2.04913931839109	0.207	0.016		0	Hepatocytes	NAV2
0	0.277346493877723	0.242	0.051		0	Hepatocytes	NDUFS3
0	0.748924745193197	0.217	0.026		0	Hepatocytes	TTC9
0	1.439482289383	0.216	0.026		0	Hepatocytes	SLC16A1
0	4.80203102322025	0.193	0.003		0	Hepatocytes	C1orf56
0	0.484397848923386	0.229	0.039		0	Hepatocytes	ABHD14B
0	5.68936837706825	0.192	0.002		0	Hepatocytes	MUC13
0	3.89187044220289	0.196	0.006		0	Hepatocytes	GGH
0	1.21522979572678	0.212	0.022		0	Hepatocytes	PPP1R16A
0	5.0529035737687	0.192	0.002		0	Hepatocytes	AQP7
0	0.891733896068245	0.224	0.034		0	Hepatocytes	TCEAL8
0	2.31096012910708	0.201	0.012		0	Hepatocytes	KCNT2
0	3.76303767554427	0.194	0.005		0	Hepatocytes	MYRF
0	2.50567156743998	0.201	0.012		0	Hepatocytes	NTHL1
0	0.741082546774265	0.219	0.03		0	Hepatocytes	PDCD5
0	0.386052519372006	0.239	0.05		0	Hepatocytes	NDUFB11
0	2.72003699079248	0.2	0.012		0	Hepatocytes	B4GALT2
0	1.32436582404841	0.21	0.022		0	Hepatocytes	NCKAP1
0	5.92943277913837	0.19	0.002		0	Hepatocytes	F12
0	3.62701235703635	0.193	0.005		0	Hepatocytes	FRK
0	1.06555275148651	0.216	0.029		0	Hepatocytes	C1orf43
0	4.6265739240592	0.19	0.003		0	Hepatocytes	DNAH14
0	0.465152653519375	0.217	0.03		0	Hepatocytes	TMEM63B
0	3.89654574148298	0.192	0.005		0	Hepatocytes	MUC3A
0	1.05065723593078	0.21	0.023		0	Hepatocytes	SLC20A2
0	1.09145410222732	0.206	0.019		0	Hepatocytes	AIFM2
0	1.23950508122409	0.208	0.021		0	Hepatocytes	CPT1A
0	0.359686378480592	0.236	0.049		0	Hepatocytes	NUTF2
0	0.848072459407466	0.218	0.031		0	Hepatocytes	SHPK
0	1.68828339567345	0.21	0.023		0	Hepatocytes	AHCY
0	1.51179582900811	0.216	0.03		0	Hepatocytes	KLF11
0	0.260542116987783	0.231	0.045		0	Hepatocytes	ACAD9
0	3.50053728264256	0.192	0.006		0	Hepatocytes	ADGRA3
0	0.285900712439363	0.225	0.039		0	Hepatocytes	MOCS2
0	3.15026312445487	0.194	0.008		0	Hepatocytes	RDH16

0	6.62467875164498	0.187	0.001	0	Hepatocytes	PRODH2
0	0.333098647702436	0.227	0.041	0	Hepatocytes	IRF2BP1
0	1.70911295285464	0.206	0.02	0	Hepatocytes	SMARCA1
0	1.67429795465946	0.205	0.02	0	Hepatocytes	CKS1B
0	0.871972014131421	0.224	0.039	0	Hepatocytes	PPARG
0	1.1252587918534	0.219	0.034	0	Hepatocytes	MCUR1
0	2.44330787210673	0.201	0.016	0	Hepatocytes	ASB13
0	6.2047552881365	0.187	0.002	0	Hepatocytes	APOF
0	0.484388721772726	0.221	0.036	0	Hepatocytes	NAXD
0	1.07298002281363	0.217	0.032	0	Hepatocytes	DEPRECATI
0	0.48356002380774	0.232	0.048	0	Hepatocytes	SETD7
0	0.301644348098207	0.231	0.047	0	Hepatocytes	AGPAT1
0	2.20398475844481	0.197	0.013	0	Hepatocytes	NT5E
0	0.266394775788389	0.225	0.041	0	Hepatocytes	TCAF1
0	1.13979883224679	0.208	0.024	0	Hepatocytes	MTCH2
0	0.279163803876636	0.236	0.052	0	Hepatocytes	EDNRB
0	0.942126859305351	0.215	0.031	0	Hepatocytes	ALKBH7
0	5.89193267820995	0.186	0.002	0	Hepatocytes	AR
0	5.54407828019554	0.186	0.002	0	Hepatocytes	DEPRECATI
0	0.507332028234579	0.218	0.035	0	Hepatocytes	ZNF281
0	1.34745095140916	0.209	0.026	0	Hepatocytes	ALCAM
0	2.17390228466562	0.197	0.014	0	Hepatocytes	PPIC
0	0.616060415762136	0.217	0.034	0	Hepatocytes	MEA1
0	0.813195273334622	0.197	0.014	0	Hepatocytes	EPHA1
0	1.99167544928222	0.2	0.017	0	Hepatocytes	MTFR1
0	0.660276093671914	0.217	0.034	0	Hepatocytes	WDR18
0	0.390669105602526	0.221	0.039	0	Hepatocytes	HSPA9
0	0.433514702881931	0.214	0.032	0	Hepatocytes	GCDH
0	1.78852936040372	0.2	0.019	0	Hepatocytes	ABTB2
0	5.39527932876662	0.183	0.002	0	Hepatocytes	AGMO
0	6.55889022594796	0.182	0.001	0	Hepatocytes	MOGAT3
0	0.658922974091717	0.224	0.043	0	Hepatocytes	LRMDA
0	2.03320057759344	0.19	0.009	0	Hepatocytes	SLC25A15
0	2.58669519641984	0.194	0.013	0	Hepatocytes	PIR
0	0.628522684142563	0.212	0.032	0	Hepatocytes	MDH2
0	0.267831314982464	0.215	0.035	0	Hepatocytes	STOML2
0	2.50973940601525	0.19	0.01	0	Hepatocytes	PARD3
0	0.618515574572993	0.215	0.035	0	Hepatocytes	RRP12
0	2.04668487207396	0.197	0.017	0	Hepatocytes	ZNF768
0	6.77701581542905	0.181	0.001	0	Hepatocytes	CYP4F22
0	1.33113121954471	0.207	0.027	0	Hepatocytes	TCEAL4
0	0.576934991585313	0.216	0.036	0	Hepatocytes	MORF4L2
0	1.20512681398625	0.197	0.018	0	Hepatocytes	GCHFR
0	0.589038376010421	0.216	0.037	0	Hepatocytes	MED8
0	5.3438736101172	0.181	0.002	0	Hepatocytes	CTH
0	1.21807558820081	0.211	0.032	0	Hepatocytes	HNMT
0	0.637532749529197	0.225	0.046	0	Hepatocytes	RAPH1
0	0.614537348860192	0.218	0.039	0	Hepatocytes	EXOSC4
0	5.24628084253718	0.181	0.002	0	Hepatocytes	GAS2
0	4.87569174694302	0.181	0.002	0	Hepatocytes	RTN4RL2
0	0.723687832781133	0.211	0.032	0	Hepatocytes	DAD1
0	1.82355164611027	0.196	0.017	0	Hepatocytes	ADCY9

0	5.34309207358151	0.179	0.001	0	Hepatocytes	FYB2
0	2.8435504993107	0.185	0.007	0	Hepatocytes	KLHL23
0	1.90369130199311	0.194	0.016	0	Hepatocytes	ABHD11
0	1.47923952228172	0.196	0.019	0	Hepatocytes	CFL2
0	6.78672429188731	0.178	0.001	0	Hepatocytes	NR0B2
0	5.2070592035919	0.179	0.002	0	Hepatocytes	RAB3B
0	1.29324495383561	0.206	0.029	0	Hepatocytes	SLC19A2
0	3.38667365364855	0.184	0.007	0	Hepatocytes	CLDN12
0	2.73206245416705	0.183	0.006	0	Hepatocytes	GLT1D1
0	0.468840223609997	0.197	0.021	0	Hepatocytes	AQP3
0	1.57221171524792	0.195	0.019	0	Hepatocytes	EID3
0	0.320546784775328	0.213	0.037	0	Hepatocytes	INSIG2
0	5.97872294160244	0.177	0.001	0	Hepatocytes	VIL1
0	1.38745844317924	0.189	0.013	0	Hepatocytes	CMTM8
0	5.54924354858732	0.178	0.002	0	Hepatocytes	F9
0	0.378033107794372	0.203	0.028	0	Hepatocytes	CRADD
0	5.86017447272969	0.176	0.001	0	Hepatocytes	MAL2
0	3.50693243270026	0.18	0.005	0	Hepatocytes	ATP7B
0	2.05129240322463	0.188	0.013	0	Hepatocytes	VASN
0	0.682268189149728	0.197	0.023	0	Hepatocytes	ETFDH
0	0.755774313406789	0.203	0.029	0	Hepatocytes	VPS25
0	3.02333116887509	0.179	0.005	0	Hepatocytes	PLGLB2
0	5.23037670922644	0.175	0.001	0	Hepatocytes	SLC26A1
0	2.00251816154881	0.186	0.012	0	Hepatocytes	SH3D19
0	0.364330600673106	0.213	0.039	0	Hepatocytes	SORBS1
0	1.92772564477972	0.191	0.018	0	Hepatocytes	FAXDC2
0	0.842544215547666	0.196	0.023	0	Hepatocytes	MOSPD3
0	2.04056877330536	0.193	0.02	0	Hepatocytes	CBR1
0	3.11249960020784	0.179	0.006	0	Hepatocytes	RASD1
0	2.10948605923439	0.182	0.01	0	Hepatocytes	ZKSCAN3
0	2.16855714389371	0.187	0.015	0	Hepatocytes	CLDN23
0	0.274543166032081	0.204	0.032	0	Hepatocytes	PGAP2
0	1.43940545871951	0.188	0.016	0	Hepatocytes	PDK2
0	0.256170142186	0.205	0.033	0	Hepatocytes	DGCR6L
0	1.22212785149626	0.206	0.034	0	Hepatocytes	MAFF
0	1.38318401398513	0.201	0.03	0	Hepatocytes	KLF10
0	2.6909594529446	0.179	0.008	0	Hepatocytes	TLCD4
0	0.533192857153941	0.209	0.038	0	Hepatocytes	MEGF9
0	5.71450725251146	0.172	0.001	0	Hepatocytes	TM6SF2
0	0.53258147625408	0.204	0.033	0	Hepatocytes	COX6B1
0	3.02494744666364	0.178	0.007	0	Hepatocytes	KDELR3
0	5.37765702304129	0.173	0.002	0	Hepatocytes	ARSE
0	0.603586022130146	0.199	0.029	0	Hepatocytes	GFPT1
0	0.450787467197804	0.196	0.026	0	Hepatocytes	CISH
0	5.76840597679352	0.171	0.001	0	Hepatocytes	DBNDD1
0	1.41047295487048	0.194	0.024	0	Hepatocytes	SOCS6
0	0.992129297904877	0.187	0.017	0	Hepatocytes	AFF3
0	3.26425892585481	0.174	0.004	0	Hepatocytes	NPAS2
0	0.578429399951364	0.212	0.042	0	Hepatocytes	CREB3
0	3.40954017970172	0.177	0.007	0	Hepatocytes	COPZ2
0	0.872141640684794	0.199	0.03	0	Hepatocytes	ACOT7
0	0.988653543979844	0.202	0.033	0	Hepatocytes	SESTD1

	0	0.676207251044844	0.203	0.034	0	Hepatocytes	GPR137
	0	0.417282210292369	0.203	0.034	0	Hepatocytes	DEPRECATI
	0	2.53090842170605	0.178	0.009	0	Hepatocytes	ARHGEF26
	0	3.79262443133021	0.173	0.004	0	Hepatocytes	PROSER2
	0	1.21948465925098	0.195	0.027	0	Hepatocytes	NFIA
	0	1.58317611705856	0.193	0.025	0	Hepatocytes	SIPA1L2
	0	4.36906460088198	0.171	0.003	0	Hepatocytes	AGXT2
	0	1.44430563556904	0.192	0.024	0	Hepatocytes	COPRS
	0	3.36111589230838	0.171	0.003	0	Hepatocytes	RNF165
	0	6.02584112049051	0.17	0.002	0	Hepatocytes	DEPRECATI
	0	0.317057805929548	0.204	0.036	0	Hepatocytes	TPRG1L
	0	1.63324570553144	0.185	0.017	0	Hepatocytes	CCDC9B
	0	0.497465801109602	0.196	0.029	0	Hepatocytes	WASL
	0	4.53823444878893	0.17	0.003	0	Hepatocytes	FOXA2
	0	2.01415591941974	0.183	0.016	0	Hepatocytes	OXER1
	0	2.47521746337835	0.176	0.009	0	Hepatocytes	TEAD3
	0	1.72319094516705	0.183	0.016	0	Hepatocytes	ZNF787
	0	1.36363940087335	0.197	0.031	0	Hepatocytes	TNFRSF21
	0	0.496926716213743	0.196	0.03	0	Hepatocytes	POLR2K
	0	0.44748427093135	0.197	0.031	0	Hepatocytes	PICK1
	0	1.1110208637896	0.189	0.024	0	Hepatocytes	ERGIC1
	0	0.697925255619632	0.194	0.029	0	Hepatocytes	VARS2
	0	0.307065773162355	0.199	0.034	0	Hepatocytes	KLHDC10
	0	0.278598487164285	0.202	0.037	0	Hepatocytes	ZNF664
	0	3.3405778030847	0.17	0.005	0	Hepatocytes	PLEKHA4
	0	2.9680020881576	0.173	0.008	0	Hepatocytes	SPIRE2
	0	0.392262342554743	0.202	0.038	0	Hepatocytes	CAMK1
	0	0.79277289214553	0.186	0.022	0	Hepatocytes	HACD2
	0	4.85319671996928	0.166	0.002	0	Hepatocytes	SMLR1
	0	1.27373463862491	0.186	0.022	0	Hepatocytes	FERMT2
	0	0.607152228868983	0.195	0.031	0	Hepatocytes	GFER
	0	1.22134755631886	0.176	0.012	0	Hepatocytes	NECTIN3
	0	2.17212284879849	0.176	0.012	0	Hepatocytes	CARD10
	0	0.629050161905843	0.199	0.036	0	Hepatocytes	EPHA2
	0	1.29176369413836	0.182	0.019	0	Hepatocytes	GMNN
	0	5.8467943836968	0.164	0.001	0	Hepatocytes	ERICH5
	0	4.97319314511031	0.164	0.002	0	Hepatocytes	ANXA9
	0	0.865785706090787	0.182	0.02	0	Hepatocytes	LARP1B
	0	2.53765606496795	0.171	0.009	0	Hepatocytes	BCO2
	0	2.38630022084217	0.171	0.009	0	Hepatocytes	VSIG10
	0	5.19223217813263	0.164	0.002	0	Hepatocytes	ITPKA
	0	0.930342071269551	0.196	0.034	0	Hepatocytes	BCAM
	0	2.95645300142329	0.167	0.006	0	Hepatocytes	SEMA6C
	0	1.75353391885601	0.171	0.01	0	Hepatocytes	INHBB
	0	1.22762029421845	0.18	0.019	0	Hepatocytes	IL1RAP
	0	1.7180898419532	0.18	0.019	0	Hepatocytes	WWC2
	0	0.991576318325172	0.192	0.031	0	Hepatocytes	CYSTM1
	0	0.686873389854473	0.185	0.024	0	Hepatocytes	SEMA4B
	0	0.86969097506777	0.187	0.026	0	Hepatocytes	NOMO1
	0	0.728782401137517	0.188	0.027	0	Hepatocytes	ATP5MC1
	0	6.65296397992772	0.162	0.001	0	Hepatocytes	GREB1L
	0	1.1780371434467	0.18	0.02	0	Hepatocytes	MAIP1

0	0.788595385846582	0.183	0.023	0	Hepatocytes	FAM8A1
0	0.412104762003119	0.189	0.029	0	Hepatocytes	PGM3
0	5.10464248934503	0.161	0.001	0	Hepatocytes	EPB41L4B
0	1.18738502917591	0.174	0.014	0	Hepatocytes	ZFYVE9
0	2.08226624879387	0.172	0.012	0	Hepatocytes	CCDC125
0	5.87079863829849	0.16	0.001	0	Hepatocytes	NR1I3
0	1.37292712421702	0.184	0.025	0	Hepatocytes	GADD45G
0	1.40759252082176	0.193	0.034	0	Hepatocytes	IFIT2
0	0.417683043112407	0.187	0.028	0	Hepatocytes	SFXN4
0	4.49519174054575	0.161	0.002	0	Hepatocytes	TMEM92
0	1.10791850340472	0.181	0.022	0	Hepatocytes	RBFA
0	2.26320137168665	0.17	0.011	0	Hepatocytes	LPIN3
0	0.932494148332629	0.183	0.025	0	Hepatocytes	MYLK
0	1.959350822957	0.169	0.011	0	Hepatocytes	CRACR2B
0	0.970689794093581	0.187	0.029	0	Hepatocytes	TEAD2
0	1.20133350215155	0.171	0.013	0	Hepatocytes	TSHZ2
0	1.15435583109319	0.186	0.028	0	Hepatocytes	TCEAL9
0	0.984036773475379	0.174	0.016	0	Hepatocytes	ZSCAN2
0	2.02169892021687	0.17	0.013	0	Hepatocytes	RPH3AL
0	0.926689432405324	0.179	0.022	0	Hepatocytes	TICAM1
0	3.10181282193521	0.164	0.007	0	Hepatocytes	ZDHHC9
0	0.607853661363276	0.178	0.022	0	Hepatocytes	GORAB
0	0.439504305852881	0.184	0.028	0	Hepatocytes	ZBTB41
0	1.66063621938275	0.181	0.025	0	Hepatocytes	ROBO1
0	0.311168163148426	0.188	0.032	0	Hepatocytes	TMEM141
0	2.26068760933253	0.166	0.01	0	Hepatocytes	LIN7A
0	0.434456696527587	0.188	0.032	0	Hepatocytes	HDDC3
0	0.819890992114684	0.188	0.032	0	Hepatocytes	NME2
0	5.49495582534882	0.157	0.001	0	Hepatocytes	RIPK4
0	5.829838093882	0.158	0.002	0	Hepatocytes	SEC14L4
0	3.18016527778521	0.161	0.005	0	Hepatocytes	SLC16A2
0	1.97428724907865	0.171	0.016	0	Hepatocytes	SULT1A1
0	0.280582367841253	0.185	0.03	0	Hepatocytes	MZT2A
0	6.01416384168988	0.156	0.001	0	Hepatocytes	SLC28A1
0	0.613457922394821	0.185	0.03	0	Hepatocytes	L3MBTL4
0	0.976750648458643	0.183	0.028	0	Hepatocytes	TP53INP2
0	0.522974035847432	0.177	0.022	0	Hepatocytes	ZNF275
0	0.282518363870209	0.185	0.031	0	Hepatocytes	INPP1
0	2.01918196118812	0.167	0.014	0	Hepatocytes	GBE1
0	0.627930729349555	0.179	0.026	0	Hepatocytes	PRRC1
0	1.90793369326974	0.166	0.013	0	Hepatocytes	PDIA4
0	0.340696352751926	0.176	0.023	0	Hepatocytes	DIP2C
0	0.700626100072865	0.183	0.03	0	Hepatocytes	RCN1
0	4.55045624278641	0.155	0.002	0	Hepatocytes	DEPDC7
0	0.619354412757041	0.179	0.026	0	Hepatocytes	PHB2
0	0.975912547351775	0.183	0.03	0	Hepatocytes	PIK3R2
0	2.54534680302379	0.162	0.009	0	Hepatocytes	SLC2A10
0	1.65189496379824	0.167	0.015	0	Hepatocytes	RBFOX2
0	4.99438164704007	0.154	0.002	0	Hepatocytes	PPP1R9A
0	1.03470945643515	0.18	0.028	0	Hepatocytes	PRDX5
0	2.36579068392316	0.164	0.012	0	Hepatocytes	PNPLA3
0	0.785333972114011	0.183	0.031	0	Hepatocytes	DEPRECATI

	0	3.56264082499314	0.155	0.004	0	Hepatocytes	THRB
	0	0.344584417388323	0.179	0.028	0	Hepatocytes	SSH3
	0	3.68405560760388	0.155	0.004	0	Hepatocytes	PKP2
	0	2.13562572043165	0.163	0.012	0	Hepatocytes	DEPRECATI
	0	1.03226321301011	0.169	0.019	0	Hepatocytes	SNAPIN
	0	1.65384315344905	0.171	0.021	0	Hepatocytes	CDK18
	0	3.28367700008071	0.157	0.007	0	Hepatocytes	MPV17L
	0	1.30241363683204	0.168	0.019	0	Hepatocytes	PCTP
	0	0.90030033983542	0.174	0.025	0	Hepatocytes	PSMB2
	0	0.285236012732024	0.177	0.028	0	Hepatocytes	PUS7
	0	3.09390518074757	0.153	0.004	0	Hepatocytes	ARHGEF5
	0	4.54573326721206	0.151	0.002	0	Hepatocytes	SH2D4A
	0	3.20872842155842	0.153	0.004	0	Hepatocytes	LRRC20
	0	0.481151240796569	0.178	0.029	0	Hepatocytes	RUVBL2
	0	0.64670177909722	0.177	0.028	0	Hepatocytes	TTLL12
	0	2.50250484510768	0.158	0.009	0	Hepatocytes	DEPRECATI
	0	2.4419643716203	0.156	0.008	0	Hepatocytes	CACFD1
	0	0.656323240923952	0.173	0.025	0	Hepatocytes	ZCRB1
	0	1.48236920874114	0.164	0.017	0	Hepatocytes	OBSL1
	0	0.960892363448667	0.169	0.022	0	Hepatocytes	HADH
	0	0.530317830543056	0.171	0.024	0	Hepatocytes	COPZ1
	0	0.272615737029167	0.168	0.021	0	Hepatocytes	RAB3IP
	0	0.89279708832455	0.164	0.017	0	Hepatocytes	NT5C3B
	0	1.15659673009393	0.167	0.02	0	Hepatocytes	IMPA2
	0	0.720187040879318	0.163	0.017	0	Hepatocytes	GRAMD2B
	0	0.927616217028057	0.168	0.022	0	Hepatocytes	SUOX
	0	5.70265653396323	0.147	0.001	0	Hepatocytes	ETNK2
	0	4.87133869432761	0.148	0.002	0	Hepatocytes	GSTA2
	0	3.18775298050459	0.152	0.006	0	Hepatocytes	ACSS3
	0	6.34986597769447	0.147	0.001	0	Hepatocytes	DEPRECATI
	0	1.23697640371483	0.169	0.024	0	Hepatocytes	MPZL1
	0	0.66169049583866	0.171	0.026	0	Hepatocytes	MAD2L1BP
	0	1.17545835033092	0.161	0.016	0	Hepatocytes	MICU3
	0	0.999464488937484	0.161	0.016	0	Hepatocytes	TRPV1
	0	1.62746911440276	0.153	0.008	0	Hepatocytes	C1QTNF3
	0	5.38529553777605	0.146	0.001	0	Hepatocytes	LECT2
	0	1.56447587251174	0.159	0.014	0	Hepatocytes	FDX1
	0	5.17675482226784	0.147	0.002	0	Hepatocytes	SCN8A
	0	4.44327394613376	0.148	0.003	0	Hepatocytes	ETV4
	0	4.68749041304739	0.146	0.001	0	Hepatocytes	CYP4F12
	0	0.579286124648295	0.167	0.023	0	Hepatocytes	TMEM14B
	0	0.351336891684393	0.17	0.026	0	Hepatocytes	PPP1R13B
	0	1.66898655565643	0.157	0.013	0	Hepatocytes	CDC42BPA
	0	5.09950759906956	0.146	0.002	0	Hepatocytes	PSAT1
	0	1.95653288540049	0.151	0.007	0	Hepatocytes	SLC27A2
	0	5.28520927671479	0.145	0.001	0	Hepatocytes	NTN1
	0	4.8989283074563	0.145	0.001	0	Hepatocytes	CYP4F2
	0	3.82799272017349	0.147	0.003	0	Hepatocytes	MAOA
	0	1.50003701027589	0.158	0.015	0	Hepatocytes	RAB11FIP5
	0	0.805381722840201	0.162	0.019	0	Hepatocytes	ALS2
	0	0.449597377661552	0.166	0.023	0	Hepatocytes	ISOC1
	0	1.74040462882139	0.159	0.016	0	Hepatocytes	IGF1

0	1.01192814022486	0.166	0.023	0	Hepatocytes	PEMT
0	0.5700009459605	0.167	0.024	0	Hepatocytes	TUBG2
0	8.97570334255734	0.143	0	0	Hepatocytes	CCDC187
0	6.32808064374079	0.144	0.001	0	Hepatocytes	SPTBN2
0	2.03207145240325	0.155	0.012	0	Hepatocytes	NEDD4
0	4.1898593625457	0.146	0.003	0	Hepatocytes	TLCD2
0	1.56276040414252	0.162	0.02	0	Hepatocytes	TMEM14C
0	1.04696010168746	0.161	0.019	0	Hepatocytes	DEPRECATI
0	3.58026248923457	0.147	0.005	0	Hepatocytes	EPDR1
0	1.85043261013358	0.155	0.013	0	Hepatocytes	FAHD1
0	5.4807854398016	0.143	0.001	0	Hepatocytes	CES3
0	5.03093243929417	0.143	0.001	0	Hepatocytes	FBXO27
0	0.844910785228158	0.162	0.021	0	Hepatocytes	RITA1
0	1.88083122340408	0.154	0.013	0	Hepatocytes	ZNF593
0	3.46781161136764	0.146	0.005	0	Hepatocytes	FMO4
0	2.45621233235444	0.149	0.008	0	Hepatocytes	C8orf82
0	1.91490646199903	0.151	0.01	0	Hepatocytes	JADE3
0	0.754549380206788	0.162	0.022	0	Hepatocytes	PEX5
0	0.888636759515222	0.155	0.015	0	Hepatocytes	RAB15
0	1.26111957474469	0.161	0.021	0	Hepatocytes	MAPK12
0	2.64179627863179	0.148	0.008	0	Hepatocytes	MANEAL
0	2.56901493909046	0.149	0.009	0	Hepatocytes	NUDT12
0	1.50802163751443	0.152	0.012	0	Hepatocytes	GNE
0	4.74541310677442	0.142	0.002	0	Hepatocytes	PTPN3
0	5.5684925143011	0.141	0.001	0	Hepatocytes	SLC6A15
0	1.25073746544924	0.158	0.019	0	Hepatocytes	ERMAP
0	1.33544511589865	0.161	0.022	0	Hepatocytes	SH3RF1
0	5.8259975908878	0.14	0.001	0	Hepatocytes	CSMD1
0	1.03888811913165	0.16	0.021	0	Hepatocytes	UQCC3
0	1.24406018682403	0.159	0.02	0	Hepatocytes	OCRL
0	5.27033080103094	0.141	0.002	0	Hepatocytes	ELOVL2
0	1.62418002174339	0.151	0.012	0	Hepatocytes	SPRYD7
0	1.53597442717484	0.155	0.017	0	Hepatocytes	OPN3
0	1.37362896242608	0.154	0.016	0	Hepatocytes	CNKSR3
0	3.07629237409153	0.143	0.005	0	Hepatocytes	PTPRU
0	2.14876326851968	0.151	0.013	0	Hepatocytes	CTDSPL
0	4.29481863910568	0.14	0.003	0	Hepatocytes	THPO
0	5.64133663620695	0.138	0.001	0	Hepatocytes	NIPAL1
0	1.39496036466276	0.157	0.02	0	Hepatocytes	ZNF704
0	5.49281225035496	0.138	0.001	0	Hepatocytes	ENPP7
0	4.9521060575447	0.139	0.002	0	Hepatocytes	CECR2
0	1.54601826168872	0.149	0.012	0	Hepatocytes	ROBO3
0	1.74813328851591	0.151	0.014	0	Hepatocytes	ITGA7
0	4.88876934418446	0.137	0.001	0	Hepatocytes	IGSF9
0	0.998122903658221	0.156	0.02	0	Hepatocytes	POLR2I
0	2.36125374922686	0.146	0.01	0	Hepatocytes	SIX5
0	1.09190798254314	0.154	0.019	0	Hepatocytes	AMOTL2
0	6.03084756489025	0.136	0.001	0	Hepatocytes	HGFAC
0	0.333604384223992	0.155	0.02	0	Hepatocytes	SIRT5
0	4.04613784441411	0.138	0.003	0	Hepatocytes	TPD52L1
0	4.80363335375446	0.137	0.002	0	Hepatocytes	SAA4
0	4.99904888996256	0.137	0.002	0	Hepatocytes	SALL1

0	2.84430943134811	0.142	0.007	0	Hepatocytes	IGF2BP2
0	1.30760582025728	0.149	0.014	0	Hepatocytes	CBX8
0	1.50933319010638	0.15	0.016	0	Hepatocytes	TMEM150A
0	1.44949425607026	0.151	0.017	0	Hepatocytes	PSD3
0	3.52217849235213	0.138	0.004	0	Hepatocytes	PANK1
0	0.900994610126845	0.154	0.02	0	Hepatocytes	HSPBP1
0	1.94812353352552	0.148	0.014	0	Hepatocytes	FAM89A
0	2.91391046075192	0.142	0.008	0	Hepatocytes	SOX12
0	4.92453493267201	0.135	0.002	0	Hepatocytes	ACADL
0	1.08929251061783	0.149	0.016	0	Hepatocytes	ENTPD7
0	4.12094678576825	0.136	0.003	0	Hepatocytes	CADM4
0	2.40179727394088	0.14	0.007	0	Hepatocytes	LARGE1
0	2.48373313670637	0.138	0.006	0	Hepatocytes	TADA1
0	4.20493118688284	0.134	0.002	0	Hepatocytes	C3orf52
0	1.80150488164289	0.138	0.006	0	Hepatocytes	MPP6
0	0.388399185343435	0.147	0.015	0	Hepatocytes	TMEM8B
0	5.48376186644573	0.133	0.001	0	Hepatocytes	STXBP6
0	4.10523006724849	0.135	0.003	0	Hepatocytes	SLC7A9
0	1.45258217825565	0.142	0.01	0	Hepatocytes	NPR 2.00
0	1.92272772050172	0.142	0.01	0	Hepatocytes	PDXP
0	1.44754295345439	0.147	0.016	0	Hepatocytes	SPRED2
0	5.02986233342036	0.132	0.001	0	Hepatocytes	GRB14
0	4.05755763299148	0.134	0.003	0	Hepatocytes	TBX3
0	1.10798906517686	0.147	0.017	0	Hepatocytes	AKR7A2
0	1.20804849227419	0.147	0.017	0	Hepatocytes	NKIRAS1
0	3.65301707714975	0.133	0.003	0	Hepatocytes	WIPF3
0	1.67266257665614	0.138	0.008	0	Hepatocytes	NT5DC3
0	6.63175994902398	0.13	0.001	0	Hepatocytes	UGT1A9
0	4.80810960712048	0.13	0.001	0	Hepatocytes	MYO5B
0	1.75484930146614	0.141	0.012	0	Hepatocytes	PTGR2
0	1.93728666221479	0.134	0.006	0	Hepatocytes	PAIP2B
0	5.17706364568187	0.129	0.001	0	Hepatocytes	ANKS4B
0	1.96444746440314	0.139	0.011	0	Hepatocytes	CDC42EP4
0	0.821753880306987	0.14	0.012	0	Hepatocytes	SNTA1
0	1.75888596384396	0.141	0.013	0	Hepatocytes	PHETA1
0	5.09202604752046	0.128	0.001	0	Hepatocytes	TMEM82
0	1.03273538774259	0.142	0.015	0	Hepatocytes	LRRC42
0	2.85365592140695	0.133	0.006	0	Hepatocytes	RAPGEF4
0	2.71409281418636	0.134	0.007	0	Hepatocytes	CHST13
0	4.76905436709639	0.129	0.002	0	Hepatocytes	FOXA1
0	2.03109589529918	0.134	0.007	0	Hepatocytes	GNPNAT1
0	2.75899749267026	0.133	0.007	0	Hepatocytes	CCDC24
0	3.99506386056368	0.129	0.003	0	Hepatocytes	C1orf53
0	5.80028195638365	0.127	0.001	0	Hepatocytes	ACMSD
0	1.98185903616959	0.136	0.01	0	Hepatocytes	TXNRD3
0	1.35114574036235	0.14	0.014	0	Hepatocytes	CADPS2
0	4.44446879199044	0.128	0.002	0	Hepatocytes	GJB2
0	2.60280217720262	0.132	0.006	0	Hepatocytes	ABCA10
0	1.31036666598827	0.14	0.014	0	Hepatocytes	ATP5PF
0	1.1246483321089	0.14	0.014	0	Hepatocytes	DEPRECATI
0	1.54993590751499	0.139	0.014	0	Hepatocytes	FBLIM1
0	3.59004575462309	0.129	0.004	0	Hepatocytes	GRHL1

0	5.05224379650617	0.127	0.002	0	Hepatocytes	KLB
0	4.48309153213953	0.127	0.002	0	Hepatocytes	LCN2
0	1.71378896330805	0.138	0.013	0	Hepatocytes	HYPK
0	2.69843167560469	0.131	0.007	0	Hepatocytes	SOX13
0	4.54508493236609	0.126	0.002	0	Hepatocytes	AL135905.2
0	4.78608764497879	0.125	0.001	0	Hepatocytes	SUGCT
0	1.83042672875913	0.134	0.011	0	Hepatocytes	VDAC1
0	1.67103687159618	0.136	0.013	0	Hepatocytes	CRAT
0	0.941462183615414	0.135	0.012	0	Hepatocytes	DUSP8
0	1.85540247765148	0.133	0.01	0	Hepatocytes	GAS8
0	1.29077805945229	0.137	0.014	0	Hepatocytes	HOOK2
0	4.88935767644586	0.124	0.002	0	Hepatocytes	AKR1C4
0	4.11796709643422	0.124	0.002	0	Hepatocytes	MYO5C
0	5.66218432101538	0.122	0.001	0	Hepatocytes	KIF12
0	6.019536231639	0.122	0.001	0	Hepatocytes	PROZ
0	2.7611513840472	0.125	0.005	0	Hepatocytes	MMACHC
0	5.79132226062803	0.121	0.001	0	Hepatocytes	SLC15A1
0	5.14078701530221	0.12	0.001	0	Hepatocytes	PIK3C2G
0	4.79621849843677	0.121	0.002	0	Hepatocytes	PANX2
0	4.65461226200448	0.119	0.002	0	Hepatocytes	SOWAHA
0	1.92316351436487	0.125	0.008	0	Hepatocytes	MROH6
0	4.73663006486187	0.119	0.002	0	Hepatocytes	PLEK2
0	3.98649166560709	0.119	0.003	0	Hepatocytes	ARHGEF16
0	2.13389112785405	0.124	0.008	0	Hepatocytes	BOLA1
0	1.65815898633934	0.123	0.008	0	Hepatocytes	ZDHHC23
0	1.29602526100776	0.123	0.008	0	Hepatocytes	RDH5
0	2.05322642016319	0.122	0.007	0	Hepatocytes	CMTM4
0	5.86339325076187	0.115	0.001	0	Hepatocytes	NAT8
0	1.75669968180337	0.123	0.009	0	Hepatocytes	MSMO1
0	4.81374183386635	0.115	0.001	0	Hepatocytes	FOXQ1
0	6.21847762915928	0.115	0.001	0	Hepatocytes	CYP3A4
0	2.6644290478729	0.121	0.007	0	Hepatocytes	IRS1
0	2.80846630049997	0.121	0.007	0	Hepatocytes	NAV3
0	2.94887799920568	0.119	0.005	0	Hepatocytes	ARL4D
0	4.32216980949852	0.114	0.001	0	Hepatocytes	SEZ6L2
0	2.99652269594726	0.119	0.006	0	Hepatocytes	SHISA4
0	5.19890696958375	0.113	0.001	0	Hepatocytes	CFHR3
0	5.64988541720362	0.113	0.001	0	Hepatocytes	ABCG8
0	2.81582817542628	0.117	0.005	0	Hepatocytes	DIXDC1
0	1.75607524371517	0.121	0.009	0	Hepatocytes	ZNF205
0	1.64517195779093	0.119	0.007	0	Hepatocytes	RYR1
0	3.71003867232702	0.114	0.003	0	Hepatocytes	CLTCL1
0	2.42821959070092	0.116	0.006	0	Hepatocytes	ACKR2
0	5.5647439737143	0.111	0.001	0	Hepatocytes	RTP3
0	5.64601326078088	0.112	0.002	0	Hepatocytes	C9
0	3.85983986864467	0.112	0.002	0	Hepatocytes	SH3RF2
0	2.97298848356421	0.114	0.004	0	Hepatocytes	VNN 1.00
0	2.46085745336933	0.115	0.005	0	Hepatocytes	PEX11A
0	3.12013735441345	0.112	0.004	0	Hepatocytes	TMEM98
0	5.37738960447631	0.108	0.001	0	Hepatocytes	CHAD
0	5.32735315161074	0.108	0.001	0	Hepatocytes	DEPRECATI
0	2.65514374668899	0.111	0.005	0	Hepatocytes	SATB2

0	4.21622388212508	0.108	0.002	0	Hepatocytes	NGEF
0	5.39599591012076	0.107	0.001	0	Hepatocytes	UGT1A4
0	4.78155076503345	0.108	0.002	0	Hepatocytes	DUOX2
0	5.84743926032472	0.107	0.001	0	Hepatocytes	ADM2
0	3.29259549194317	0.108	0.003	0	Hepatocytes	PYCR1
0	4.83330324588916	0.106	0.001	0	Hepatocytes	DEPRECATI
0	5.60544349762297	0.105	0.001	0	Hepatocytes	LONRF2
0	3.94366018461904	0.105	0.002	0	Hepatocytes	C1orf226
0	5.09843978492078	0.104	0.001	0	Hepatocytes	ADH6
0	2.63957601110727	0.108	0.005	0	Hepatocytes	SLC25A27
0	4.00854802640734	0.105	0.002	0	Hepatocytes	HNF4G
0	4.45000548988826	0.104	0.001	0	Hepatocytes	VSTM2L
0	3.82426593132624	0.104	0.002	0	Hepatocytes	SHROOM3
0	3.21627656672576	0.104	0.002	0	Hepatocytes	LCN12
0	5.39265346000793	0.103	0.001	0	Hepatocytes	SAA2
0	2.49557179676523	0.105	0.003	0	Hepatocytes	PARD6B
0	5.29432751065765	0.102	0.001	0	Hepatocytes	IL22RA1
0	5.61912152797251	0.102	0.001	0	Hepatocytes	MTTP
0	5.34582213397737	0.102	0.001	0	Hepatocytes	SRCIN1
0	1.74759761639892	0.105	0.004	0	Hepatocytes	SYNE4
0	4.75656043319931	0.101	0.001	0	Hepatocytes	MAP3K21
0	8.19075190876477	0.1	0	0	Hepatocytes	UGT3A1
0	5.78379892182876	0.101	0.001	0	Hepatocytes	CYP1A2
0	4.44044079589119	0.102	0.002	0	Hepatocytes	HSD17B13
0	4.14301674793803	0.102	0.002	0	Hepatocytes	CCDC198
0	3.62445279409564	0.102	0.002	0	Hepatocytes	LIPC
0	6.61402160748805	0.1	0.001	0	Hepatocytes	KISS1
0	4.99544282277749	0.1	0.001	0	Hepatocytes	ACY3
0	2.37246452403814	0.102	0.003	0	Hepatocytes	RAB27B
0	3.48310120340266	0.101	0.003	0	Hepatocytes	WNT3
0	2.98513126098935	0.1	0.003	0	Hepatocytes	ZNRF3
5.52554067231799E-306	0.382929639492549	0.278	0.073	9.01270939061787E-302	Hepatocytes	SLC38A7
9.73251893484721E-306	0.720494630740442	0.169	0.026	1.58747116346293E-301	Hepatocytes	BANF1
1.72667520868723E-305	1.93359708780004	0.121	0.009	2.81637993288974E-301	Hepatocytes	B9D1
8.20071085105928E-305	0.422276654644893	0.15	0.019	1.33761794691628E-300	Hepatocytes	DXO
1.39326738124098E-304	0.557620097386729	0.166	0.025	2.27255842554217E-300	Hepatocytes	C8orf33
1.84522950869203E-304	1.81177823402998	0.116	0.008	3.00975385162757E-300	Hepatocytes	PALMD
4.52939102689789E-304	1.13231010030424	0.142	0.016	7.38788970397316E-300	Hepatocytes	DSTN
5.24500828026718E-304	0.465571788219568	0.177	0.029	8.5551330059438E-300	Hepatocytes	NFYB
1.22509165160146E-303	1.25933075744601	0.131	0.013	1.99824699292714E-299	Hepatocytes	AARS
3.44779823952943E-302	2.38751221089137	0.104	0.005	5.62370370849646E-298	Hepatocytes	EHF
1.00693380895707E-301	0.688873497610057	0.206	0.042	1.64240973578988E-297	Hepatocytes	NENF
1.12057328763349E-301	0.276614572923302	0.218	0.047	1.82776708945899E-297	Hepatocytes	ELOA
4.25921413839014E-301	1.21143269383151	0.132	0.013	6.94720418112815E-297	Hepatocytes	RAB43
7.84765654386298E-301	0.783779577077513	0.191	0.035	1.28003125886949E-296	Hepatocytes	LAPTM4A
1.10175421450402E-300	1.42595707391187	0.115	0.008	1.79707129927751E-296	Hepatocytes	CCDC58
1.82819341825755E-300	0.779439512735209	0.177	0.03	2.98196628451989E-296	Hepatocytes	SCML1
8.03838382664666E-300	1.21616695204647	0.13	0.013	1.31114078596434E-295	Hepatocytes	SNRPE
8.7488307407796E-300	1.37561601511326	0.13	0.013	1.42702178212856E-295	Hepatocytes	ATP8B1
1.79699823372244E-299	0.290715506034996	0.278	0.074	2.93108381902467E-295	Hepatocytes	SLC43A3
2.53922114195202E-299	0.597660708047369	0.146	0.018	4.14172360463794E-295	Hepatocytes	SSBP3
3.26789509863865E-299	1.7942788572521	0.117	0.009	5.3302636953895E-295	Hepatocytes	GPC6

**5.94222482793163E-299**	0.401302788072095	0.135	0.014	9.69236291683928E-295	Hepatocytes	PDP2
**1.08365383318711E-298**	1.05960389233175	0.19	0.036	1.7675477673115E-294	Hepatocytes	ZNF358
**2.43891474117611E-298**	1.40843906199752	0.109	0.006	3.97811383433236E-294	Hepatocytes	FAM78B
**2.47967227241154E-298**	1.01834231918012	0.138	0.015	4.04459344353046E-294	Hepatocytes	PHLPP1
**2.48292807224737E-298**	0.337292130631352	0.171	0.028	4.04990397864269E-294	Hepatocytes	VPS72
**3.71060974932997E-298**	0.522359207809975	0.601	0.263	6.05237556213211E-294	Hepatocytes	KLF6
**3.73294274073241E-298**	1.85966082561579	0.117	0.009	6.08880290440864E-294	Hepatocytes	RTN4R
**1.81117372471055E-297**	0.390525946521864	0.175	0.029	2.95420546237539E-293	Hepatocytes	PLAAT3
**2.18663513721482E-297**	1.03214459241528	0.125	0.011	3.5666205723111E-293	Hepatocytes	PUS3
**4.01734114398268E-296**	0.467246194910386	0.168	0.027	6.55268513995016E-292	Hepatocytes	RRS1
**7.11602803291495E-296**	1.18195222404177	0.164	0.026	1.16069533244876E-291	Hepatocytes	GNA11
**1.64373846143689E-295**	2.49956633618261	0.108	0.006	2.68110180444971E-291	Hepatocytes	LZTS3
**2.81337916154034E-295**	0.993560149022599	0.164	0.025	4.58890275038845E-291	Hepatocytes	CAMSAP2
**2.94772615449282E-295**	2.16343534064345	0.109	0.006	4.80803613059324E-291	Hepatocytes	BAMBI
**8.07477934169767E-295**	0.894877606151829	0.195	0.038	1.31707725842431E-290	Hepatocytes	CCND1
**2.56393924483575E-294**	2.50457379307879	0.101	0.004	4.1820413022516E-290	Hepatocytes	PRLR
**4.87923418085192E-294**	1.40625706100399	0.127	0.012	7.95851887238757E-290	Hepatocytes	MTX1
**1.17235975682735E-293**	0.318267641447077	0.157	0.023	1.91223599936109E-289	Hepatocytes	UBIAD1
**1.28456773071421E-293**	0.443728594493301	0.185	0.034	2.09525842556794E-289	Hepatocytes	ARF4
**1.83056506250829E-293**	1.66253851220005	0.12	0.01	2.98583467345728E-289	Hepatocytes	FNBP1L
**9.09712980608505E-293**	0.797224442852287	0.183	0.033	1.48383284267053E-288	Hepatocytes	EFNB1
**1.16918235657321E-292**	0.543479393971571	0.15	0.02	1.90705334180656E-288	Hepatocytes	NDUFAF1
**2.02191583268328E-292**	0.845257908593946	0.146	0.019	3.2979469146897E-288	Hepatocytes	GLRX2
**2.72610847716907E-292**	0.265828451608164	0.173	0.029	4.44655553711048E-288	Hepatocytes	RRNAD1
**3.95195752778298E-292**	0.665362182324548	0.145	0.019	6.44603792356682E-288	Hepatocytes	USE1
**6.09777826910719E-292**	1.47218064663237	0.126	0.012	9.94608613474074E-288	Hepatocytes	PARD3B
**9.87100149905966E-292**	0.250875554682698	0.149	0.02	1.61005905451162E-287	Hepatocytes	FAM136A
**1.56516985537345E-291**	0.617399479833829	0.16	0.024	2.55294855109964E-287	Hepatocytes	MLST8
**2.13489186496745E-291**	0.896439440200814	0.133	0.014	3.48222212094841E-287	Hepatocytes	WDR19
**8.20678679946533E-291**	0.894076748712801	0.143	0.018	1.33860899486079E-286	Hepatocytes	POP7
**6.76209982997727E-290**	0.593745274989375	0.212	0.046	1.10296610326759E-285	Hepatocytes	CPPED1
**5.22807887268446E-289**	0.377396905935612	0.172	0.029	8.52751944923562E-285	Hepatocytes	ILF2
**8.66808138591056E-289**	0.602722371286999	0.183	0.034	1.41385075485587E-284	Hepatocytes	EDA
**9.3466458715867E-289**	0.764165208943415	0.153	0.022	1.52453140811451E-284	Hepatocytes	NDUFC1
**4.2900013780184E-288**	0.510237449292948	0.176	0.031	6.99742124768581E-284	Hepatocytes	DEPRECATI
**9.17095350880167E-288**	0.447021812398801	0.157	0.024	1.49587422682064E-283	Hepatocytes	LANCL2
**1.19194867322429E-287**	1.5139589424269	0.124	0.012	1.94418748089615E-283	Hepatocytes	TUSC3
**2.91881946902889E-287**	0.326374781197992	0.179	0.032	4.76088643593302E-283	Hepatocytes	USP5
**3.8627497346303E-287**	0.419113492174596	0.164	0.026	6.30053109215548E-283	Hepatocytes	TCAIM
**2.78020528063757E-286**	0.435993710182883	0.141	0.018	4.53479283324794E-282	Hepatocytes	RPP25L
**4.29132276846454E-286**	1.88104614959172	0.111	0.008	6.99957656764251E-282	Hepatocytes	SPTLC3
**5.28265431258529E-286**	1.11488831410107	0.132	0.015	8.61653744925787E-282	Hepatocytes	SEC61G
**1.74436082219157E-285**	0.846012929285612	0.166	0.028	2.84522693707666E-281	Hepatocytes	FUCA2
**2.02133492628459E-285**	0.442924150590187	0.248	0.063	3.29699939826279E-281	Hepatocytes	CD276
**1.26854950255997E-284**	0.764200577249757	0.114	0.009	2.06913109362557E-280	Hepatocytes	EPPK1
**3.42403350578507E-284**	0.758653106777361	0.141	0.018	5.58494105128602E-280	Hepatocytes	BAD
**4.46282945189379E-284**	1.33402876925581	0.127	0.013	7.27932111898396E-280	Hepatocytes	GATA6
**4.90579388708332E-283**	1.94493992543155	0.123	0.012	8.00184040922161E-279	Hepatocytes	ACOT2
**5.41517477541741E-282**	1.54795123998945	0.126	0.013	8.83269157618334E-278	Hepatocytes	EMP2
**1.13086000330741E-281**	1.24270377491796	0.132	0.015	1.84454575139472E-277	Hepatocytes	TTC8
**1.7780937482511E-281**	0.408118702707683	0.212	0.047	2.90024871277237E-277	Hepatocytes	STX3
**1.01107114018533E-280**	0.478806976193217	0.153	0.023	1.6491581367563E-276	Hepatocytes	NELFE

**1.34330177299263E-280**	1.42177731989525	0.121	0.012	2.19105952192827E-276	Hepatocytes	C1QBP
**1.73042004262014E-280**	2.15287230176706	0.101	0.005	2.82248813151772E-276	Hepatocytes	MORC4
**3.15425750420303E-280**	0.466860709835527	0.14	0.018	5.14490941510556E-276	Hepatocytes	FAHD2A
**7.08412199680048E-280**	1.05483188058474	0.127	0.014	1.15549113889813E-275	Hepatocytes	GTF2H5
**1.47139350556137E-279**	0.308485140863389	0.137	0.017	2.39998994692115E-275	Hepatocytes	SETD6
**1.8274542239826E-279**	0.523325625714139	0.138	0.017	2.98076058473801E-275	Hepatocytes	LRRC28
**1.86311059997284E-279**	1.89658751559555	0.108	0.008	3.0389196996157E-275	Hepatocytes	CKB
**2.73425838668962E-279**	0.387799866347771	0.14	0.018	4.45984885452944E-275	Hepatocytes	AVEN
**5.99572653652551E-279**	0.310055332102599	0.662	0.299	9.77962955372676E-275	Hepatocytes	APOL6
**2.55480651063341E-278**	0.54891792202803	0.702	0.356	4.16714489949415E-274	Hepatocytes	MT-CO3
**2.76902937990005E-278**	1.26248893543319	0.127	0.014	4.51656382155496E-274	Hepatocytes	ZNF697
**1.96980266297037E-277**	1.69356334243041	0.111	0.009	3.21294512357097E-273	Hepatocytes	ZNF408
**3.91643029081377E-277**	1.29579925416537	0.126	0.014	6.38808944734634E-273	Hepatocytes	COX5B
**4.83972617668856E-277**	0.916432018191728	0.153	0.024	7.89407736679671E-273	Hepatocytes	SNX21
**7.41133249671115E-276**	0.991287557832657	0.134	0.017	1.20886244353856E-271	Hepatocytes	PKDCC
**1.01940635518543E-275**	1.04417671096203	0.142	0.02	1.66275370594296E-271	Hepatocytes	PEX10
**1.53298817631572E-275**	1.07570703787986	0.136	0.017	2.50045701438857E-271	Hepatocytes	MCC
**2.61703100032737E-275**	1.31545066900821	0.149	0.022	4.26863926463398E-271	Hepatocytes	TBC1D16
**1.25944591602556E-274**	0.769266983831089	0.132	0.016	2.05428223362929E-270	Hepatocytes	PHB
**2.58704464492534E-274**	1.19902439488523	0.146	0.021	4.21972852033772E-270	Hepatocytes	TUBA1C
**6.13363353594372E-274**	1.28591505853471	0.13	0.016	1.00045696604778E-269	Hepatocytes	SLC66A1
**2.29642723904148E-273**	0.382774577482158	0.212	0.048	3.74570246960056E-269	Hepatocytes	ATP6V0A1
**2.89463181803518E-273**	0.45762309051052	0.152	0.023	4.72143395839719E-269	Hepatocytes	MED18
**9.7744999733393E-273**	0.575172451610388	0.148	0.022	1.59431869065137E-268	Hepatocytes	MMAB
**1.51695285579567E-272**	1.86434622596326	0.102	0.006	2.47430180308832E-268	Hepatocytes	TMEM53
**1.63579306162961E-272**	0.572942651168853	0.232	0.058	2.66814206282405E-268	Hepatocytes	TCF7L2
**1.14048996109364E-271**	0.437092431840031	0.2	0.043	1.86025317553984E-267	Hepatocytes	CLCN5
**3.84178444624306E-271**	0.521982505826446	0.166	0.029	6.26633461026706E-267	Hepatocytes	LONRF3
**1.74339022324601E-270**	1.4653736949386	0.125	0.014	2.84364379313657E-266	Hepatocytes	GTF2IRD1
**1.6033695475059E-269**	0.557252395622273	0.139	0.019	2.61525606893687E-265	Hepatocytes	C6orf136
**4.37365053859773E-269**	0.925652591849944	0.149	0.023	7.13386139350675E-265	Hepatocytes	DCBLD1
**1.02815286883675E-268**	0.499418750152229	0.13	0.016	1.67702014435962E-264	Hepatocytes	CCNB1IP1
**1.57485806245423E-268**	0.297725536258774	0.158	0.027	2.56875098566909E-264	Hepatocytes	MIEF1
**2.13988872342568E-268**	0.553320944537326	0.821	0.489	3.49037249677963E-264	Hepatocytes	MT-CO1
**2.39588062674744E-268**	1.07365215645415	0.124	0.014	3.90792089028775E-264	Hepatocytes	DEPRECATI
**6.97239084259983E-268**	1.01830101474462	0.109	0.009	1.13726667033646E-263	Hepatocytes	ZNF563
**1.29731728758008E-267**	1.75986256800407	0.13	0.016	2.11605422777187E-263	Hepatocytes	IFIT1
**1.69087478520112E-267**	0.254779864309313	0.165	0.029	2.75798586214155E-263	Hepatocytes	SAMM50
**5.91327604098586E-267**	1.90926145284308	0.109	0.009	9.64514455045204E-263	Hepatocytes	LGALSL
**8.06283443074038E-267**	0.465233552114448	0.194	0.042	1.31512892399806E-262	Hepatocytes	STK3
**6.00730992614287E-266**	0.382714431028222	0.147	0.023	9.79852322053163E-262	Hepatocytes	COQ5
**1.02947788111977E-265**	1.53540664388065	0.112	0.01	1.67918137189445E-261	Hepatocytes	PRPF40B
**2.16203804671653E-265**	1.34304102051912	0.126	0.015	3.52650025799933E-261	Hepatocytes	ALPK3
**2.2851205545892E-265**	1.23402382744866	0.114	0.011	3.72726013659045E-261	Hepatocytes	ACACB
**4.11326160390818E-265**	0.619555657610608	0.13	0.016	6.70914100213463E-261	Hepatocytes	ABCB6
**2.39681325899281E-264**	0.792595872550632	0.114	0.011	3.90944210674318E-260	Hepatocytes	TMEM254
**2.51312145226149E-264**	0.427459106011328	0.231	0.059	4.09915240078372E-260	Hepatocytes	ITGAV
**5.60356567733514E-264**	0.250288347598186	0.16	0.028	9.13997597630134E-260	Hepatocytes	BZW1
**7.97222234855293E-264**	1.81932175361109	0.114	0.011	1.30034918727247E-259	Hepatocytes	PHLDA3
**1.17698464473316E-262**	0.931500082010684	0.135	0.019	1.91977965402425E-258	Hepatocytes	CYP51A1
**3.4875276816124E-262**	1.18626373923394	0.139	0.02	5.68850640147798E-258	Hepatocytes	CD302
**1.40089206630204E-261**	0.658329014269393	0.135	0.018	2.28499504934526E-257	Hepatocytes	TEN1

1.61292276781678E-261	2.22726217378986	0.104	0.008	2.63083832658595E-257	Hepatocytes	B4GAT1
2.06310990462542E-261	1.44752392883569	0.112	0.01	3.36513856543452E-257	Hepatocytes	MOCS1
2.54651513040725E-261	0.377710211051163	0.156	0.027	4.15362082920727E-257	Hepatocytes	FOXRED1
9.34295948429621E-261	0.340361279204705	0.132	0.018	1.52393012148355E-256	Hepatocytes	MMAA
8.65733045411448E-260	0.47295974067602	0.146	0.023	1.41209717037061E-255	Hepatocytes	BSCL2
8.51322813942851E-259	0.252743017705065	0.315	0.101	1.38859264182218E-254	Hepatocytes	SLC35F6
9.75319385798552E-259	1.23708404327024	0.109	0.01	1.59084345017602E-254	Hepatocytes	TARS
1.73412541843542E-258	0.74403930421994	0.145	0.023	2.82853197001002E-254	Hepatocytes	CPNE8
1.04628703703408E-256	1.28286479057858	0.126	0.016	1.70659878610629E-252	Hepatocytes	SLC45A3
1.42368499315424E-256	0.449244874303056	0.115	0.012	2.32217259233388E-252	Hepatocytes	BACH2
1.50676315946757E-256	0.847835530155816	0.142	0.022	2.45768138940755E-252	Hepatocytes	TDRD10
2.17485156821537E-256	1.77886556202027	0.101	0.008	3.54740039291609E-252	Hepatocytes	INKA2
5.1427689148506E-256	0.850836204770435	0.13	0.017	8.38837037701281E-252	Hepatocytes	LRP6
7.16440279944886E-256	1.54808290419182	0.112	0.011	1.1685857406181E-251	Hepatocytes	DEPRECATI
2.23675956908332E-255	1.03290516356924	0.154	0.027	3.6483785331318E-251	Hepatocytes	BCKDK
2.66062173954197E-255	0.34132493865496	0.203	0.048	4.3397401193669E-251	Hepatocytes	TAX1BP3
5.65413245891822E-255	0.326051854740479	0.124	0.015	9.2224554537415E-251	Hepatocytes	GPR89A
7.05066508309745E-255	1.22142070202346	0.11	0.01	1.15003398170402E-250	Hepatocytes	WEE1
8.40759515332927E-255	1.72726081418532	0.111	0.011	1.37136284545954E-250	Hepatocytes	SH3BP4
1.89116709827992E-254	0.46651776657553	0.142	0.022	3.08468265400437E-250	Hepatocytes	ST7
2.39733375566546E-254	1.82368167899155	0.103	0.008	3.91029108886593E-250	Hepatocytes	KATNAL2
5.07147194427678E-254	0.466294891364552	0.162	0.03	8.27207788830986E-250	Hepatocytes	RNF26
1.49360422421993E-253	0.52627998276163	0.132	0.018	2.43621785012512E-249	Hepatocytes	TMEM123
2.50132704571763E-253	0.556549232164681	0.124	0.016	4.07991454427002E-249	Hepatocytes	OXSM
2.97253213013914E-253	1.31494140012641	0.109	0.01	4.84849715746996E-249	Hepatocytes	ZNHIT2
3.46361385302876E-253	0.371008799872284	0.156	0.028	5.64950055567521E-249	Hepatocytes	ATP5MF
3.97261316997499E-253	0.475312065853806	0.23	0.061	6.4797293415462E-249	Hepatocytes	FNIP2
4.35207889507823E-253	1.18059839016174	0.121	0.015	7.09867588576209E-249	Hepatocytes	GLCE
6.99763508242514E-253	1.31301881267589	0.116	0.013	1.14138425829436E-248	Hepatocytes	HID1
2.06833757177E-252	0.498081626268989	0.143	0.022	3.37366541331405E-248	Hepatocytes	RPF2
6.02457052939299E-252	0.760673827718832	0.133	0.019	9.8266769904929E-248	Hepatocytes	PSMB6
7.32934594052632E-252	0.277068122253425	0.195	0.044	1.19548961635925E-247	Hepatocytes	TM9SF1
3.163015021624E-251	0.44356307882453	0.183	0.04	5.15919380177091E-247	Hepatocytes	KANK2
4.76729810029574E-251	0.692085103539363	0.116	0.013	7.77593993139238E-247	Hepatocytes	C15orf41
1.00908417592477E-250	1.1795014672894	0.125	0.016	1.64591719935089E-246	Hepatocytes	SDCBP2
3.22214108686533E-250	1.23821143524198	0.134	0.019	5.25563432678605E-246	Hepatocytes	RRAGD
8.31799871652405E-250	1.16226371230906	0.117	0.014	1.35674877065224E-245	Hepatocytes	ATAD3A
1.82140864497128E-249	1.12145188920059	0.123	0.016	2.97089964081265E-245	Hepatocytes	FDX2
1.30208364922517E-248	0.738290799392878	0.113	0.012	2.12382864025118E-244	Hepatocytes	TRMT10C
1.54871740553575E-248	0.320589596279387	0.169	0.034	2.52611296016937E-244	Hepatocytes	UQCR10
1.85892144349259E-248	0.86006756747499	0.119	0.014	3.03208676648076E-244	Hepatocytes	TRMT61A
2.13970718074594E-248	1.35125154022633	0.122	0.016	3.49007638251471E-244	Hepatocytes	HFE
2.25293359344736E-247	1.4398815706472	0.112	0.012	3.67475998427199E-243	Hepatocytes	SYTL4
4.81011596621258E-247	1.19055104559948	0.11	0.011	7.84578015248934E-243	Hepatocytes	DEPRECATI
7.68263203533488E-247	0.432285726077009	0.201	0.048	1.25311411128347E-242	Hepatocytes	DEPRECATI
8.34380486397114E-247	0.381954263781982	0.222	0.058	1.36095801136233E-242	Hepatocytes	PYGL
9.55271992857856E-246	0.251661404565517	0.151	0.027	1.55814414755045E-241	Hepatocytes	C19orf48
3.24903739744857E-245	0.379790983412665	0.146	0.025	5.29950489897837E-241	Hepatocytes	CCT5
4.92876882368259E-245	0.503924728901616	0.152	0.027	8.03931482830867E-241	Hepatocytes	NDUFB2
2.37672531366665E-243	0.92358857175003	0.125	0.017	3.87667665912167E-239	Hepatocytes	PKP4
2.67899069407535E-243	0.856125788749267	0.121	0.015	4.36970172110631E-239	Hepatocytes	HMBS
4.41230770560848E-243	1.91613921837591	0.1	0.009	7.19691509861799E-239	Hepatocytes	SEPTIN5

**4.99096533004606E-243**	0.459999154793455	0.163	0.032	8.14076354983813E-239	Hepatocytes	CTNNBIP1
**1.61770328237111E-242**	0.505717054958441	0.138	0.022	2.63863582387552E-238	Hepatocytes	TOMM6
**1.83246271770963E-242**	0.362747227521811	0.176	0.038	2.98892993885617E-238	Hepatocytes	ST3GAL6
**2.62860796081478E-242**	1.18945587520355	0.115	0.013	4.28752244488498E-238	Hepatocytes	UCK2
**2.94731113273515E-242**	0.262103726238415	0.171	0.036	4.8073591886043E-238	Hepatocytes	MAP1LC3A
**4.42695067483601E-242**	1.10565591809103	0.112	0.013	7.22079924572501E-238	Hepatocytes	DEPRECATI
**4.98351724627252E-242**	0.880239386951388	0.12	0.015	8.12861498039511E-238	Hepatocytes	NET1
**1.97163002451247E-240**	0.251618001810805	0.209	0.053	3.21592573298229E-236	Hepatocytes	MARCH2
**1.33150432745678E-239**	0.484282990858593	0.806	0.461	2.17181670851475E-235	Hepatocytes	IL32
**4.11063663367101E-239**	0.293368312076175	0.109	0.012	6.70485941318079E-235	Hepatocytes	COQ10A
**4.31285494704653E-239**	0.982430899197957	0.112	0.013	7.03469770412759E-235	Hepatocytes	PGBD1
**2.37588178066236E-238**	0.728070254179761	0.116	0.014	3.87530077243838E-234	Hepatocytes	COX7B
**4.88884909461167E-238**	0.590578677599292	0.111	0.013	7.9742017582211E-234	Hepatocytes	NEIL2
**1.03732362535489E-237**	0.455684296326481	0.122	0.016	1.69197856531636E-233	Hepatocytes	CNIH1
**2.44193230093903E-237**	1.60286954776601	0.1	0.009	3.98303577606165E-233	Hepatocytes	PIGZ
**2.59030141367405E-237**	0.908225638080764	0.139	0.023	4.22504063584374E-233	Hepatocytes	TMEM70
**2.66133687952192E-237**	0.370075284582445	0.104	0.01	4.34090658418821E-233	Hepatocytes	ACTR3C
**3.09403092167334E-237**	0.437953031076636	0.121	0.016	5.04667383634139E-233	Hepatocytes	APOOL
**3.99494257756084E-237**	1.77420745524487	0.106	0.011	6.51615083825948E-233	Hepatocytes	HK2
**1.94286506934979E-236**	0.367537286958461	0.132	0.021	3.16900721461644E-232	Hepatocytes	MED9
**2.72555055025164E-236**	0.432091698762851	0.126	0.018	4.44564550251546E-232	Hepatocytes	NEK4
**3.21273150007894E-236**	0.303592608719062	0.135	0.022	5.24028634977875E-232	Hepatocytes	OSTC
**6.69633357608775E-235**	1.26478138193997	0.115	0.014	1.09223896959567E-230	Hepatocytes	SEMA5A
**1.16923163240789E-234**	0.357664470575563	0.112	0.013	1.90713371562051E-230	Hepatocytes	ABHD4
**1.24874824573029E-234**	0.758213480958656	0.154	0.029	2.03683326361068E-230	Hepatocytes	FBP1
**3.91117981389234E-234**	1.42245112508347	0.108	0.012	6.37952539443979E-230	Hepatocytes	TEF
**2.64820417572546E-233**	0.814885713638134	0.147	0.027	4.31948583102579E-229	Hepatocytes	SMCO4
**8.19426745116078E-233**	0.314862874430852	0.185	0.044	1.33656696395883E-228	Hepatocytes	TSPAN15
**1.31349026193745E-232**	0.389192429378369	0.141	0.025	2.14243396624618E-228	Hepatocytes	TIMELESS
**3.7336888550559E-232**	0.260710502000202	0.119	0.016	6.09001989148168E-228	Hepatocytes	LRATD2
**2.46865180097781E-231**	1.35450357512609	0.115	0.015	4.02661795257491E-227	Hepatocytes	ZBED3
**3.47586816064614E-231**	0.459113147107501	0.151	0.029	5.66948855682992E-227	Hepatocytes	TOMM40
**5.36026820536638E-231**	0.9350640061015	0.109	0.013	8.74313346977311E-227	Hepatocytes	ANAPC13
**8.45858184272629E-231**	0.513970853321862	0.149	0.028	1.37967928436709E-226	Hepatocytes	IER3IP1
**1.30038995840742E-230**	1.07273962196058	0.101	0.01	2.12106606115835E-226	Hepatocytes	PAIP1
**1.31828170711284E-230**	1.28698057109681	0.1	0.01	2.15024929247176E-226	Hepatocytes	IQGAP3
**3.56347446675714E-230**	0.383240819876539	0.158	0.032	5.81238320272757E-226	Hepatocytes	TAGLN2
**5.35397183393425E-230**	0.717446861308196	0.122	0.017	8.73286345833015E-226	Hepatocytes	EXOSC5
**7.90411527499834E-230**	0.744912986976981	0.112	0.014	1.28924024250498E-225	Hepatocytes	MECR
**1.94282167754058E-229**	0.686880321096284	0.1	0.01	3.16893643823643E-225	Hepatocytes	HPGD
**2.92487581437925E-229**	1.19357454028624	0.106	0.012	4.770764940834E-225	Hepatocytes	KIF7
**6.77037599370678E-229**	0.29633222408704	0.149	0.029	1.10431602833351E-224	Hepatocytes	PSMC1
**3.08594235400039E-228**	0.345433356793776	0.164	0.035	5.03348057361004E-224	Hepatocytes	RDX
**2.008373728782E-227**	0.998140456627363	0.116	0.016	3.27585838901631E-223	Hepatocytes	SORT1
**2.17883317330646E-227**	0.381252703920066	0.1	0.01	3.55389478898017E-223	Hepatocytes	SLC22A5
**6.40588494898264E-227**	0.439682628627409	0.156	0.031	1.04486389402856E-222	Hepatocytes	USP6NL
**6.66478023127529E-227**	0.733992137163238	0.177	0.041	1.08709230352331E-222	Hepatocytes	TRIM47
**1.88113795866241E-226**	0.455581911765784	0.123	0.018	3.06832412437426E-222	Hepatocytes	RHBDD3
**2.70208481138279E-225**	0.26977966630265	0.129	0.021	4.40737053584648E-221	Hepatocytes	S1PR2
**5.01207067040209E-225**	0.324567910729272	0.116	0.016	8.17518847049285E-221	Hepatocytes	FDXR
**3.23547502985813E-223**	0.774127481001388	0.108	0.013	5.27738332120159E-219	Hepatocytes	ICK
**1.163521480765E-222**	0.408463212414154	0.196	0.051	1.8978198872758E-218	Hepatocytes	CXCL2

2.32840045663398E-222	0.6619202171602	0.111	0.014	3.79785398481568E-218	Hepatocytes	RABEPK
7.6114536753435E-222	0.83254003784113	0.112	0.015	1.24150420898528E-217	Hepatocytes	SMIM13
4.91194157393159E-221	0.281555773293822	0.129	0.021	8.01186790123982E-217	Hepatocytes	HIBCH
7.32459114344494E-221	0.955474752174483	0.109	0.014	1.1947140614073E-216	Hepatocytes	UQCRFS1
1.49136314011294E-218	0.54319533881118	0.112	0.015	2.43256241783821E-214	Hepatocytes	ZBTB42
3.05466990523669E-216	0.395107282096062	0.132	0.023	4.98247208243157E-212	Hepatocytes	GOLPH3L
3.26610453386235E-216	0.579570429060434	0.111	0.015	5.32734310518288E-212	Hepatocytes	ALG8
3.92232720510985E-216	0.872734660229324	0.102	0.012	6.39770790425467E-212	Hepatocytes	CPE
7.21297899382708E-216	0.405115022965227	0.113	0.016	1.17650900368314E-211	Hepatocytes	TMEM256
1.20752326676081E-215	0.588373099713564	0.107	0.014	1.96959120041355E-211	Hepatocytes	NDUFAF4
2.5342004614392E-213	0.592353139514043	0.121	0.019	4.13353437265348E-209	Hepatocytes	BAG3
3.20903997342259E-213	1.53969782079524	0.104	0.013	5.23426510064958E-209	Hepatocytes	SLC48A1
3.87184951597597E-213	0.583937411837665	0.106	0.014	6.3153737455084E-209	Hepatocytes	MCAT
7.58715923221416E-213	0.5362531617124	0.128	0.022	1.23754154236645E-208	Hepatocytes	DEPTOR
5.47511035608476E-212	0.648497859472534	0.1	0.012	8.93045250180985E-208	Hepatocytes	DNAJC30
6.24093481496693E-212	0.845821465614407	0.115	0.017	1.01795887766926E-207	Hepatocytes	PPM1H
6.73003417820149E-211	0.994677195461751	0.133	0.024	1.09773587480644E-206	Hepatocytes	TENT5A
1.49567254687584E-210	0.490534671943811	0.115	0.017	2.43959149120918E-206	Hepatocytes	DEPRECATI
1.77864559099526E-209	0.556822032904416	0.144	0.029	2.90114882347237E-205	Hepatocytes	PPARGC1B
3.16171332043189E-209	0.630368329595747	0.103	0.013	5.15707059695646E-205	Hepatocytes	C12orf45
5.62627700132429E-209	0.637488486192458	0.109	0.015	9.17702041686004E-205	Hepatocytes	MAGEF1
3.67266451328614E-208	0.533397061560174	0.12	0.019	5.99048308762103E-204	Hepatocytes	PAK1IP1
4.75152935187399E-208	0.537818607542823	0.115	0.018	7.75021952584166E-204	Hepatocytes	PSMB3
1.53981254546045E-207	1.19105358207002	0.112	0.016	2.51158824290053E-203	Hepatocytes	MEX3D
5.50620739983496E-207	1.170393792047	0.1	0.012	8.9811748898708E-203	Hepatocytes	RAI14
3.60673244900374E-206	1.01680834579549	0.103	0.013	5.88294129757001E-202	Hepatocytes	CETN2
3.86783181187909E-206	0.803813760298768	0.103	0.013	6.30882046835598E-202	Hepatocytes	MDH1
4.36777069172052E-206	0.42627853801321	0.148	0.032	7.12427077526534E-202	Hepatocytes	NREP
1.5960309180202E-205	0.885124022296099	0.107	0.015	2.60328603038274E-201	Hepatocytes	RNF180
1.96954994735024E-205	0.266734814757054	0.116	0.018	3.21253291912298E-201	Hepatocytes	NDUFA7
2.44266003438518E-205	0.990584103495196	0.11	0.016	3.98422278208567E-201	Hepatocytes	SLIRP
3.92959391018499E-205	0.362462482577295	0.106	0.014	6.40956062690274E-201	Hepatocytes	GPR89B
3.57055553146293E-203	0.573116965953105	0.155	0.035	5.82393312736918E-199	Hepatocytes	ANKRD9
6.8840792299764E-203	0.586798762363643	0.139	0.028	1.12286216320145E-198	Hepatocytes	CLIP2
1.03405256536651E-202	0.319142073684644	0.147	0.032	1.68664313936931E-198	Hepatocytes	ABHD6
1.39341789572595E-202	0.442129392910181	0.123	0.021	2.2728039297186E-198	Hepatocytes	MUL1
4.23942015502939E-201	0.922150730920363	0.107	0.016	6.91491821486844E-197	Hepatocytes	ABHD8
7.20304053412268E-201	0.257296730685428	0.187	0.05	1.17488794152075E-196	Hepatocytes	RGS12
3.99288235751867E-200	1.03618226961755	0.112	0.018	6.5127904133487E-196	Hepatocytes	TPMT
4.43985307777995E-200	0.776997087960402	0.105	0.015	7.24184435516688E-196	Hepatocytes	MEIS2
4.27333601006806E-199	0.356443907845429	0.115	0.019	6.970238366022E-195	Hepatocytes	DEPRECATI
5.39744470313284E-199	0.426360469452657	0.184	0.049	8.80377205527997E-195	Hepatocytes	SAMD4A
5.94074319462463E-199	1.209444545644	0.125	0.023	9.68994622475223E-195	Hepatocytes	IFIT3
7.85819917003711E-199	0.561799450801091	0.154	0.036	1.28175086662475E-194	Hepatocytes	DEPRECATI
3.33427893094415E-196	0.262845019308681	0.122	0.022	5.438542364263E-192	Hepatocytes	SSR1
4.18347796710929E-196	0.574610261740024	0.115	0.019	6.82367091215197E-192	Hepatocytes	LAMTOR5
4.70219368392017E-195	0.465494677708334	0.14	0.03	7.66974811784219E-191	Hepatocytes	PSEN2
5.82279776352252E-195	0.292748136365886	0.118	0.02	9.49756543208159E-191	Hepatocytes	HSD17B12
5.5529683528493E-194	0.332595257291973	0.177	0.047	9.05744668033249E-190	Hepatocytes	PLA2G4C
5.62669951420913E-194	0.55786512587175	0.102	0.014	9.17770957762652E-190	Hepatocytes	SLC25A43
2.80501606640925E-193	0.325962448013934	0.115	0.019	4.57526170592012E-189	Hepatocytes	PGAM5
7.84829638658974E-192	0.259663679598562	0.117	0.021	1.28013562361665E-187	Hepatocytes	UNG

3.96752310445547E-190	0.558917833565007	0.125	0.024	6.47142693567732E-186	Hepatocytes	RUSC2
6.99793248697827E-190	0.288214021099378	0.145	0.033	1.14143276795102E-185	Hepatocytes	TIMM8B
2.69288164126264E-189	0.357859475741428	0.108	0.017	4.3923592450635E-185	Hepatocytes	PTRH2
1.06517303468003E-188	0.723301954787162	0.101	0.015	1.7374037368666E-184	Hepatocytes	FADS3
2.15550310026251E-187	0.675199854294705	0.109	0.018	3.51584110683818E-183	Hepatocytes	PRKN
1.59949666764408E-185	0.531386267936341	0.1	0.015	2.60893901459426E-181	Hepatocytes	PUSL1
2.70056811611228E-184	0.312112619971158	0.142	0.033	4.40489665419074E-180	Hepatocytes	PNPLA7
1.65967792700357E-182	0.582366407928724	0.155	0.039	2.70710066673553E-178	Hepatocytes	CDK2AP1
6.24425546314683E-182	0.617597283037728	0.104	0.017	1.01850050859388E-177	Hepatocytes	EXD2
2.35218683394784E-180	0.418137007116582	0.125	0.026	3.83665194485233E-176	Hepatocytes	CPLX1
2.66533505458053E-178	0.696218887481444	0.109	0.019	4.3474280075263E-174	Hepatocytes	TTC23
6.02083681491354E-176	0.883476285203709	0.101	0.016	9.82058692880548E-172	Hepatocytes	ARSD
4.21129335321548E-174	0.502221858186394	0.122	0.025	6.86904058842977E-170	Hepatocytes	PPP1R14B
3.38943326042945E-173	0.267578276465833	0.101	0.017	5.52850459108648E-169	Hepatocytes	PCGF1
8.33636597645728E-165	0.298332946055661	0.111	0.022	1.35974465441995E-160	Hepatocytes	VAV2
1.14863243737605E-163	0.254165406606238	0.129	0.03	1.87353436860407E-159	Hepatocytes	TUBB
4.40331825846544E-162	0.257190144440694	0.322	0.138	7.18225241138298E-158	Hepatocytes	ATF3
5.43187766858644E-160	0.711467049686836	0.108	0.021	8.85993566523133E-156	Hepatocytes	TP53I3
5.35838494790426E-159	0.695122247593891	0.115	0.025	8.74006168852664E-155	Hepatocytes	ZNF503
4.23203087320722E-158	0.485593999929826	0.119	0.027	6.9028655572883E-154	Hepatocytes	RND3
4.54933666631283E-154	0.588203734692601	0.141	0.038	7.42042303642286E-150	Hepatocytes	SOX4
3.55451109271182E-150	0.321557801734014	0.146	0.041	5.79776304332226E-146	Hepatocytes	CUEDC1
9.17239188997313E-149	0.30387457980193	0.118	0.028	1.49610884117352E-144	Hepatocytes	ROGDI
5.65585849224344E-146	0.276689098274273	0.1	0.02	9.22527078669828E-142	Hepatocytes	ZNF784
1.7272715999507E-142	0.252998181381264	0.11	0.025	2.81735270667959E-138	Hepatocytes	ZNF618
2.18367230487215E-138	0.292297116228128	0.108	0.025	3.56178789647697E-134	Hepatocytes	PALLD
1.51131092548547E-116	0.447092037880648	0.121	0.036	2.46509925055936E-112	Hepatocytes	CXCL10
2.8677859871361E-115	0.258383906895495	0.133	0.043	4.67764572361769E-111	Hepatocytes	HSD17B14
0	6.13333519429503	0.517	0.031	0	T/NK cell	TRBC2
0	6.62724986846311	0.458	0.02	0	T/NK cell	TRAC
0	3.36994783841656	0.549	0.13	0	T/NK cell	CORO1A
0	6.36779452261403	0.453	0.042	0	T/NK cell	CD8A
0	3.26230245959283	0.606	0.201	0	T/NK cell	AKNA
0	3.0825058062942	0.616	0.214	0	T/NK cell	WIPF1
0	4.51266499188621	0.437	0.037	0	T/NK cell	ACAP1
0	4.09038366978125	0.467	0.074	0	T/NK cell	CLEC2D
0	6.23779937248229	0.392	0.014	0	T/NK cell	SPOCK2
0	6.26588175717693	0.389	0.02	0	T/NK cell	IL7R
0	6.19140927330279	0.371	0.016	0	T/NK cell	ZAP70
0	3.328033405316	0.482	0.132	0	T/NK cell	CYTIP
0	3.37490924385676	0.464	0.134	0	T/NK cell	IL2RG
0	3.8192350352105	0.448	0.126	0	T/NK cell	DUSP2
0	5.75081006442281	0.338	0.02	0	T/NK cell	CD69
0	3.44720156760164	0.409	0.092	0	T/NK cell	ETS1
0	6.10217070636226	0.324	0.016	0	T/NK cell	ITK
0	6.9057500436512	0.315	0.008	0	T/NK cell	KLRK1
0	3.04294980696409	0.438	0.133	0	T/NK cell	TMC8
0	5.557897239194	0.347	0.046	0	T/NK cell	CCL5
0	5.76582980193859	0.342	0.042	0	T/NK cell	NKG7
0	6.86747653250425	0.304	0.007	0	T/NK cell	CD2
0	2.70536364943052	0.626	0.333	0	T/NK cell	CXCR4
0	2.92188977647409	0.552	0.263	0	T/NK cell	ARHGEF1

0	4.71631850868577	0.31	0.027	0	T/NK cell	SEPTIN1
0	3.36079700285472	0.439	0.157	0	T/NK cell	RGS1
0	2.8173890193664	0.597	0.32	0	T/NK cell	EVL
0	5.17967743940326	0.298	0.024	0	T/NK cell	PYHIN1
0	6.15595117531938	0.284	0.012	0	T/NK cell	TRBC1
0	6.51120395564108	0.276	0.011	0	T/NK cell	PRF1
0	6.18632086652992	0.284	0.019	0	T/NK cell	GZMA
0	6.50371475957529	0.277	0.013	0	T/NK cell	TIGIT
0	3.72577129197518	0.334	0.07	0	T/NK cell	ARHGAP9
0	2.9021776247821	0.398	0.135	0	T/NK cell	ITGA4
0	6.58222453165541	0.277	0.015	0	T/NK cell	IL2RB
0	4.16186458607319	0.323	0.063	0	T/NK cell	STAT4
0	3.04512448602414	0.38	0.12	0	T/NK cell	IKZF1
0	6.25899646800806	0.272	0.012	0	T/NK cell	CD6
0	3.3497219142083	0.404	0.149	0	T/NK cell	ITGAL
0	4.93083866998624	0.277	0.023	0	T/NK cell	IKZF3
0	6.88949792323528	0.257	0.007	0	T/NK cell	BCL11B
0	5.90894695573476	0.258	0.011	0	T/NK cell	CST7
0	3.79421931003483	0.278	0.036	0	T/NK cell	LTB
0	4.11376877362416	0.281	0.044	0	T/NK cell	RASAL3
0	6.51207774888007	0.243	0.007	0	T/NK cell	CD96
0	3.96569799299665	0.299	0.066	0	T/NK cell	TBC1D10C
0	6.19794775480514	0.245	0.012	0	T/NK cell	CD3E
0	1.84753580587451	0.451	0.219	0	T/NK cell	LSP1
0	3.99700352177521	0.314	0.088	0	T/NK cell	CNOT6L
0	4.2052614609091	0.264	0.038	0	T/NK cell	FYN
0	3.10949752734487	0.369	0.144	0	T/NK cell	TNFAIP3
0	3.48451770052103	0.299	0.075	0	T/NK cell	TRAF1
0	3.8957142695426	0.266	0.052	0	T/NK cell	DGKA
0	4.91313943364843	0.247	0.037	0	T/NK cell	LAG3
0	2.28649791667365	0.404	0.196	0	T/NK cell	CELF2
0	3.05578530686478	0.294	0.086	0	T/NK cell	STK17B
0	5.94309628075313	0.218	0.012	0	T/NK cell	SH2D2A
0	3.09480238555342	0.299	0.096	0	T/NK cell	PTPRC
0	4.71584826276209	0.224	0.022	0	T/NK cell	PTPN22
0	3.14911763804302	0.434	0.232	0	T/NK cell	SYNE2
0	4.14864073258675	0.229	0.028	0	T/NK cell	PSTPIP1
0	2.47323086565376	0.497	0.296	0	T/NK cell	NLRC5
0	3.37713480044979	0.228	0.031	0	T/NK cell	ATP2A3
0	3.11127425789391	0.29	0.094	0	T/NK cell	JAK3
0	3.93379967545762	0.219	0.026	0	T/NK cell	TRAF3IP3
0	6.04238496874296	0.198	0.008	0	T/NK cell	TC2N
0	6.04409870614705	0.197	0.009	0	T/NK cell	CD5
0	6.12524556499216	0.191	0.006	0	T/NK cell	LCK
0	3.7118562914548	0.217	0.032	0	T/NK cell	MYO1G
0	2.91781454233045	0.315	0.131	0	T/NK cell	SLA
0	2.75938993171122	0.274	0.095	0	T/NK cell	CCDC88B
0	6.25153563063165	0.19	0.011	0	T/NK cell	GZMK
0	2.60002033355533	0.4	0.222	0	T/NK cell	STK4
0	3.11134958007516	0.253	0.075	0	T/NK cell	DEF6
0	5.85761273322327	0.191	0.013	0	T/NK cell	KLRG1
0	6.30008525128745	0.186	0.008	0	T/NK cell	CD7

0	6.77473601385896	0.18	0.005	0	T/NK cell	KLRC4
0	4.59857578686439	0.194	0.022	0	T/NK cell	ITM2A
0	6.36936304018898	0.177	0.006	0	T/NK cell	THEMIS
0	5.47895558034785	0.178	0.008	0	T/NK cell	RASGRP1
0	6.19700034311132	0.178	0.009	0	T/NK cell	CD28
0	3.96101597183715	0.192	0.026	0	T/NK cell	SYTL1
0	3.99957866471528	0.208	0.046	0	T/NK cell	CCND2
0	6.40622067793257	0.164	0.005	0	T/NK cell	CD247
0	2.3275201829065	0.501	0.345	0	T/NK cell	CDKN1B
0	5.64510361343255	0.167	0.012	0	T/NK cell	PDCD1
0	6.45586334358205	0.166	0.011	0	T/NK cell	CXCR6
0	5.78964295747431	0.163	0.011	0	T/NK cell	CD3D
0	4.12726880602266	0.171	0.02	0	T/NK cell	PTPN7
0	4.35736825634479	0.164	0.015	0	T/NK cell	NLRC3
0	4.76018022060276	0.179	0.03	0	T/NK cell	SKAP1
0	4.06807392793794	0.187	0.039	0	T/NK cell	PRKCH
0	4.60635596646604	0.167	0.025	0	T/NK cell	SYTL3
0	5.80899919619858	0.152	0.01	0	T/NK cell	SH2D1A
0	4.10290068544629	0.163	0.025	0	T/NK cell	SLAMF6
0	3.25290463090134	0.16	0.022	0	T/NK cell	FCMR
0	4.10138797785424	0.147	0.011	0	T/NK cell	CARMIL2
0	5.77337530816942	0.141	0.006	0	T/NK cell	GRAP2
0	5.27295699172006	0.14	0.006	0	T/NK cell	GPR174
0	5.80606168323623	0.139	0.006	0	T/NK cell	SLFN12L
0	6.29492445287114	0.136	0.004	0	T/NK cell	ICOS
0	4.28411403069627	0.139	0.011	0	T/NK cell	ANO9
0	5.83808438373676	0.129	0.005	0	T/NK cell	ST8SIA1
0	2.37017369548845	0.499	0.382	0	T/NK cell	ZFP36L2
0	7.47367985694225	0.117	0.003	0	T/NK cell	CD3G
0	6.70894501946105	0.115	0.004	0	T/NK cell	PRKCQ
0	0.868111908181914	0.767	0.714	0	T/NK cell	TMSB4X
0	0.876724770627574	0.751	0.776	0	T/NK cell	B2M
3.01283125848256E-307	2.45954137458417	0.328	0.15	4.9142290657109E-303	T/NK cell	LIMD2
1.03655701259157E-303	4.36723827571135	0.136	0.013	1.69072814323811E-299	T/NK cell	RHOH
1.26388803962079E-302	3.06346895286615	0.245	0.079	2.06152778142547E-298	T/NK cell	RAC2
4.00616211126015E-302	6.77628079373888	0.12	0.007	6.53445101967643E-298	T/NK cell	KLRD1
1.35927784192558E-301	3.27697375418373	0.203	0.05	2.21711808796482E-297	T/NK cell	DENND1C
2.64470782511005E-291	5.49039898585282	0.17	0.033	4.31378293353701E-287	T/NK cell	NELL2
3.97909639184411E-291	4.26731998971972	0.187	0.043	6.49030412473692E-287	T/NK cell	LAT
2.57266971337103E-289	3.49483442639911	0.243	0.081	4.19628156947948E-285	T/NK cell	STK17A
1.13113811817634E-285	6.11447015143559	0.108	0.004	1.84499938455743E-281	T/NK cell	SAMD3
5.04924191065029E-285	3.46690109818323	0.159	0.027	8.23581848046169E-281	T/NK cell	PPP1R16B
1.53021269141395E-282	3.51300665325304	0.172	0.035	2.4959299209653E-278	T/NK cell	LBH
2.72800815450595E-282	4.77160849168792	0.187	0.045	4.44965410081465E-278	T/NK cell	BICDL1
1.26398015814626E-280	5.69903044679892	0.121	0.01	2.06167803595236E-276	T/NK cell	TNFRSF9
2.79795067642232E-280	6.40800575769826	0.109	0.005	4.56373734831245E-276	T/NK cell	SLA2
1.55313762315124E-278	5.45149460060249	0.114	0.007	2.53332277712199E-274	T/NK cell	CD27
1.76664847719451E-278	2.52817980456959	0.274	0.109	2.88158033115196E-274	T/NK cell	ARHGAP15
7.15589941132663E-278	4.19772224580747	0.156	0.027	1.16719875298149E-273	T/NK cell	ITGB7
4.41350755020821E-276	4.27618979640254	0.132	0.015	7.19887216514461E-272	T/NK cell	EMB
2.15451371123475E-274	2.70570480168283	0.267	0.105	3.514227314395E-270	T/NK cell	PIK3CD
5.81803650111986E-272	3.78146340692697	0.153	0.026	9.48979933697661E-268	T/NK cell	MAP4K1

1.24030245968507E-269	3.82196954880274	0.266	0.109	2.02305734199231E-265	T/NK cell	SLC38A1
5.60003699994078E-269	3.01039597240289	0.239	0.085	9.13422035060341E-265	T/NK cell	PRDM1
4.45402072789488E-267	4.46868764516215	0.11	0.007	7.26495320926934E-263	T/NK cell	SLAMF1
1.42134078059254E-264	3.70679306988265	0.162	0.033	2.31834894722449E-260	T/NK cell	SPN
4.71057555482888E-260	2.66908378554716	0.409	0.28	7.68341978748139E-256	T/NK cell	BTG1
1.49251794442045E-252	4.64601055949714	0.116	0.012	2.4344460191442E-248	T/NK cell	C16orf54
3.19821484381424E-252	3.89239118061259	0.165	0.037	5.21660823174541E-248	T/NK cell	TNFRSF25
9.83506850002389E-242	4.03257051892474	0.113	0.012	1.6041980230389E-237	T/NK cell	CARD11
5.89201935455652E-239	4.63352673005073	0.107	0.01	9.61047276921714E-235	T/NK cell	ALOX5AP
2.23197657081087E-238	2.43148600073833	0.285	0.133	3.64057698464961E-234	T/NK cell	GBP5
1.48184954721645E-237	6.30455324100769	0.1	0.007	2.41704479646476E-233	T/NK cell	GZMM
5.56478044205689E-236	3.4179179889536	0.195	0.061	9.07671337903899E-232	T/NK cell	CCDC88C
1.01980201881437E-235	2.07099186612682	0.347	0.195	1.66339907288811E-231	T/NK cell	CD44
2.14205427214156E-234	4.13438443494806	0.106	0.01	3.4939047232901E-230	T/NK cell	ZBP1
1.90491094751288E-233	2.43381140167745	0.273	0.123	3.10710024648827E-229	T/NK cell	JAML
1.41569298298486E-232	3.10333414148098	0.19	0.058	2.3091368245466E-228	T/NK cell	FGD3
2.94439901306163E-231	0.280968561086199	0.097	0.311	4.80260923020482E-227	T/NK cell	LPP
3.24690212179095E-230	3.10588562255221	0.139	0.026	5.29602205085322E-226	T/NK cell	SP140
1.54219413058744E-229	3.89552200105707	0.145	0.03	2.51547284640117E-225	T/NK cell	CCR5
8.07993316291398E-228	1.34762512009462	0.59	0.597	1.3179178982029E-223	T/NK cell	LENG8
1.23398277808959E-227	4.03725479813841	0.145	0.031	2.01274930934192E-223	T/NK cell	PCED1B
1.66502694002471E-227	4.09794611784242	0.17	0.046	2.71582544187431E-223	T/NK cell	TENT5C
9.2126855124263E-225	4.12381307521538	0.132	0.024	1.50268113393185E-220	T/NK cell	DUSP4
6.51580484604444E-222	4.17001380968966	0.123	0.02	1.06279292843831E-217	T/NK cell	RUNX3
6.46400940281002E-220	1.79685940750917	0.362	0.217	1.05434457369234E-215	T/NK cell	ARHGAP4
3.35420752161963E-217	3.12980437877112	0.161	0.042	5.47104788851377E-213	T/NK cell	PIM2
3.09412347334997E-215	0.97229842042502	0.609	0.525	5.04682479738114E-211	T/NK cell	IL32
4.8734204124695E-215	2.80632858185245	0.311	0.175	7.94903603477901E-211	T/NK cell	MLLT6
2.77550218434373E-213	0.327594060255228	0.095	0.297	4.52712161288305E-209	T/NK cell	C16orf58
1.9637536048505E-212	2.52271139748797	0.25	0.111	3.20307850487165E-208	T/NK cell	ARHGAP45
1.63205789148718E-210	2.58744316734794	0.251	0.114	2.66204962680473E-206	T/NK cell	FYB1
1.07777982021202E-209	0.256581014152229	0.07	0.247	1.75796666474783E-205	T/NK cell	SPPL2A
3.30869855893163E-209	3.36673625362261	0.168	0.049	5.39681821947338E-205	T/NK cell	SEMA4D
1.07823551499511E-208	2.6496789649534	0.228	0.093	1.75870994850852E-204	T/NK cell	SASH3
2.1638123227261E-208	3.08943574646848	0.204	0.075	3.52939427959854E-204	T/NK cell	GPRIN3
2.80800550368338E-208	0.258746548091808	0.114	0.329	4.58013777705795E-204	T/NK cell	CALCOCO2
4.4766007210491E-208	0.312407147753407	0.056	0.22	7.30178343610318E-204	T/NK cell	KPNA1
3.23485217170994E-205	0.277759990389034	0.115	0.329	5.27636737727608E-201	T/NK cell	EIF4H
1.26975779934517E-204	0.316871782145346	0.099	0.298	2.07110194651191E-200	T/NK cell	SND1
5.22458388379227E-204	2.40014776913414	0.269	0.132	8.52181877285358E-200	T/NK cell	NLRP1
6.95622788830264E-204	0.276734064092523	0.129	0.353	1.13463033086104E-199	T/NK cell	NPLOC4
2.2382443308461E-203	1.81626926965621	0.5	0.468	3.65080032804307E-199	T/NK cell	RNF213
4.83177757583695E-203	0.264833548442974	0.11	0.318	7.88111240394764E-199	T/NK cell	LGALS8
8.44894136427977E-201	0.263729593463558	0.159	0.407	1.37810682592767E-196	T/NK cell	SNX1
1.32750847640334E-200	0.301290999872506	0.078	0.257	2.16529907586149E-196	T/NK cell	SMG5
2.67058596305207E-198	0.259131555382474	0.062	0.226	4.35599276433423E-194	T/NK cell	PARP9
2.73263551259616E-198	0.292053446697736	0.085	0.27	4.4572017845956E-194	T/NK cell	MMP24OS
8.48758262844459E-197	4.9331581951386	0.129	0.028	1.3844096025256E-192	T/NK cell	TCF7
3.34580524514817E-195	2.3787434798109	0.248	0.116	5.45734293536117E-191	T/NK cell	ARHGAP30
7.52237007708897E-195	2.33928782216921	0.401	0.307	1.22697378327398E-190	T/NK cell	OGT
1.32970400137952E-194	0.311921074937504	0.122	0.335	2.16888019665013E-190	T/NK cell	BSDC1
1.32269853802936E-193	0.31017544584226	0.147	0.38	2.15745358537969E-189	T/NK cell	MTCH1

1.87434717638037E-192	0.300506756108457	0.085	0.266	3.05724767939402E-188	T/NK cell	RNF114
1.05457402718406E-191	0.400503777624245	0.079	0.255	1.72011569573992E-187	T/NK cell	VPS45
1.4295702408506E-191	0.310718230409651	0.089	0.273	2.33177201985142E-187	T/NK cell	PSMB1
2.54388121115891E-190	0.264174015290781	0.154	0.391	4.1493246435213E-186	T/NK cell	TAF10
2.49046493178748E-189	0.30038099719828	0.085	0.264	4.06219735023855E-185	T/NK cell	PMF1
5.56580768911174E-189	0.278175230023658	0.068	0.232	9.07838892171016E-185	T/NK cell	APEH
6.96650817761708E-187	0.264047582260774	0.051	0.199	1.13630714885112E-182	T/NK cell	NDUFS1
1.39948704635725E-186	0.408526600865613	0.068	0.232	2.2827033213133E-182	T/NK cell	HSPE1
3.44960812784255E-186	0.348329004859731	0.08	0.253	5.62665581732399E-182	T/NK cell	USP9X
5.13314421566535E-186	2.15218882616465	0.304	0.176	8.37267153017175E-182	T/NK cell	ADGRE5
6.44133266420568E-186	0.355076157629619	0.089	0.269	1.05064577085859E-181	T/NK cell	SNX17
1.01784147082614E-185	0.462223334200309	0.078	0.25	1.66020122306452E-181	T/NK cell	FAM177A1
1.10254624350207E-185	2.16857435513045	0.259	0.128	1.79836317777623E-181	T/NK cell	FMNL1
2.52929223411383E-185	0.315536319054943	0.068	0.23	4.12552856306307E-181	T/NK cell	GNL3
6.00148718924706E-185	0.296355515398802	0.131	0.345	9.78902575438088E-181	T/NK cell	ARL6IP4
2.47996114121612E-184	0.253353893778158	0.08	0.251	4.04506461743762E-180	T/NK cell	CUTA
7.47843745717298E-183	0.43441495402845	0.081	0.253	1.21980793363949E-178	T/NK cell	MIA3
7.68847477255038E-183	0.306905675962877	0.131	0.344	1.25406712015069E-178	T/NK cell	NDUFV2
3.32210203418097E-182	0.285291287274272	0.063	0.22	5.41868062795257E-178	T/NK cell	SUB1
7.10803378964274E-182	0.253930529896485	0.056	0.206	1.15939139142863E-177	T/NK cell	UBE2Q1
1.7752394608711E-181	3.06216903232298	0.16	0.05	2.89559308462685E-177	T/NK cell	S100A4
1.98271305727291E-181	2.99522858425562	0.178	0.064	3.23400326771785E-177	T/NK cell	GIMAP7
2.61700318734329E-181	0.287686068641092	0.049	0.193	4.26859389887565E-177	T/NK cell	HDAC5
3.42594782374593E-181	0.493436484816766	0.035	0.165	5.58806349531199E-177	T/NK cell	ADHFE1
5.61752259781389E-181	4.24811103170888	0.11	0.02	9.16274110929423E-177	T/NK cell	IPCEF1
1.32870151106439E-180	3.08765267352485	0.191	0.073	2.16724503469713E-176	T/NK cell	PDE7A
5.67111451640832E-179	0.347377891019367	0.058	0.209	9.25015488771361E-175	T/NK cell	MPPE1
6.431663812143E-179	0.418864334078811	0.152	0.382	1.04906868439864E-174	T/NK cell	SUMF2
1.5263881140416E-178	0.400904429009925	0.167	0.41	2.48969165281325E-174	T/NK cell	SLC38A10
4.97257086295533E-178	0.269215371311124	0.041	0.175	8.11076033456643E-174	T/NK cell	JTB
1.69274638675176E-177	0.355438966033236	0.039	0.171	2.76103863143079E-173	T/NK cell	DENND10
7.63745515867153E-177	0.334706169021411	0.045	0.182	1.24574531093091E-172	T/NK cell	RNF103
1.0710507231192E-176	0.362781217875448	0.067	0.224	1.74699083447972E-172	T/NK cell	CAST
3.66154699597952E-175	0.262213188045616	0.07	0.228	5.97234930514219E-171	T/NK cell	PPP2R1B
4.24362833322486E-175	0.313755012195915	0.105	0.29	6.92178217432306E-171	T/NK cell	MTDH
9.20154770738372E-175	0.338482899939773	0.08	0.246	1.50086444655136E-170	T/NK cell	USP12
1.49780719537079E-174	0.314754891646337	0.065	0.218	2.4430733163693E-170	T/NK cell	TXLNA
8.81120647225124E-174	0.280964937671286	0.172	0.412	1.4371958876889E-169	T/NK cell	EIF3A
1.52043413126288E-173	0.360140504552066	0.031	0.152	2.47998011150288E-169	T/NK cell	EPHX2
2.09210718656903E-173	0.414322839441722	0.087	0.259	3.41243603201274E-169	T/NK cell	PA2G4
6.74531922339452E-173	0.324478879257223	0.054	0.197	1.10022901852788E-168	T/NK cell	ANXA7
9.1537951005182E-173	0.512178097808126	0.084	0.253	1.49307551884552E-168	T/NK cell	CHTOP
1.60728585749491E-172	0.295267421992397	0.065	0.217	2.62164396215995E-168	T/NK cell	PSMA7
3.05625406549944E-172	0.459089878628449	0.103	0.288	4.98505600623614E-168	T/NK cell	ZSWIM8
8.93491333396016E-172	0.316869285498139	0.057	0.202	1.45737371390224E-167	T/NK cell	TBC1D17
1.16737792203737E-171	0.395345445689002	0.119	0.315	1.90411012863516E-167	T/NK cell	STXBP2
2.2117938422194E-171	0.370094203574187	0.064	0.214	3.60765693604406E-167	T/NK cell	CDIPT
3.89280016317687E-171	0.410338866107917	0.104	0.287	6.34954634615779E-167	T/NK cell	ERO1A
1.4377706792503E-170	0.428112811843221	0.066	0.218	2.34514775492516E-166	T/NK cell	AP1M1
1.50960792508425E-170	2.15548448015488	0.288	0.167	2.46232148660492E-166	T/NK cell	ARHGDIB
2.97828054842364E-170	0.416988259916645	0.072	0.228	4.85787340253379E-166	T/NK cell	PYGB
3.18746540798628E-170	0.265278783979211	0.05	0.188	5.19907482696642E-166	T/NK cell	PWWP3A

3.6040498505929E-170	0.377603050506097	0.074	0.232	5.87856571130208E-166	T/NK cell	PPP4C
7.23948703686185E-170	3.03705816366714	0.232	0.113	1.18083273058254E-165	T/NK cell	LYST
1.03546123076553E-169	0.437502068947836	0.101	0.281	1.68894081350166E-165	T/NK cell	SCAF1
1.19634698601326E-169	0.42662723573082	0.05	0.189	1.95136156888624E-165	T/NK cell	CCS
1.98709795975983E-169	0.333875424366135	0.077	0.237	3.24115548216426E-165	T/NK cell	TNRC18
2.40882407869094E-169	0.333335252409751	0.042	0.173	3.92903295475279E-165	T/NK cell	ACBD6
3.91598240952168E-169	0.437956347717952	0.054	0.194	6.38735890817082E-165	T/NK cell	SCAMP3
6.20546781580176E-169	0.424009953105449	0.093	0.266	1.01217385543543E-164	T/NK cell	MTG1
2.95966019686653E-168	0.396502817810835	0.08	0.242	4.827501747109E-164	T/NK cell	DEPRECATI
3.36989003198283E-168	0.448720538290864	0.088	0.257	5.4966276311672E-164	T/NK cell	AP3D1
3.7212071352621E-168	0.272132172316694	0.087	0.255	6.069660958326E-164	T/NK cell	DNAJC5
4.20974234207372E-168	0.457206282807752	0.131	0.336	6.86651073415645E-164	T/NK cell	SRPRA
4.45507930638206E-168	0.564932244648387	0.077	0.238	7.26667985663977E-164	T/NK cell	TMEM41B
9.92195549243411E-168	0.355456179891288	0.063	0.21	1.61837016037093E-163	T/NK cell	DNAJC4
1.82554243894797E-167	0.426767150893731	0.081	0.244	2.97764227216804E-163	T/NK cell	ATF4
3.82404452873236E-167	0.363943536882701	0.062	0.209	6.23739903081535E-163	T/NK cell	WIZ
1.13766578660027E-166	0.267253403569825	0.043	0.172	1.85564666452369E-162	T/NK cell	TIPRL
1.73807571657242E-166	0.315387091591729	0.059	0.203	2.83497530130127E-162	T/NK cell	TRAPPC3
2.07201110916341E-166	3.51174376263636	0.121	0.029	3.37965732015643E-162	T/NK cell	GNG2
3.05873652649763E-165	0.466003607773413	0.067	0.217	4.98910514837029E-161	T/NK cell	VPS28
1.38174429701521E-164	0.558858901389073	0.09	0.258	2.25376312286151E-160	T/NK cell	UBE2B
1.55270619886937E-164	0.512961215518595	0.103	0.282	2.53261908097582E-160	T/NK cell	GUK1
1.6718449546019E-164	0.433567604751471	0.066	0.214	2.72694630545115E-160	T/NK cell	AUP1
1.56061420989681E-163	0.446990410240378	0.079	0.238	2.54551783776268E-159	T/NK cell	NDUFA3
2.30847243754305E-163	0.453032344353661	0.055	0.194	3.76534939287647E-159	T/NK cell	HECTD1
2.53169470538297E-163	0.31168939715494	0.053	0.19	4.12944723395016E-159	T/NK cell	ULK1
2.54257328440344E-163	0.405818450006759	0.055	0.194	4.14719128419045E-159	T/NK cell	ARHGAP1
2.60864971549642E-163	0.453665325854432	0.073	0.226	4.25496855094621E-159	T/NK cell	PUF60
6.33760421659864E-163	0.494171926443399	0.1	0.277	1.0337266237694E-158	T/NK cell	DYNC1LI2
7.63843753855811E-163	0.351883723116437	0.054	0.192	1.24590554691421E-158	T/NK cell	KDM5B
1.04944818654834E-162	0.462787636877372	0.099	0.274	1.711754937079E-158	T/NK cell	AKT1
1.06175115378414E-162	2.44689644540913	0.383	0.307	1.73182230693732E-158	T/NK cell	SUN2
2.11216653463149E-162	0.529427498035834	0.1	0.275	3.44515483463742E-158	T/NK cell	MROH1
2.29305269691825E-162	0.2797523354299	0.044	0.172	3.74019825394336E-158	T/NK cell	NDUFV3
3.04610877152234E-162	0.456968336576003	0.071	0.223	4.9685080172301E-158	T/NK cell	CLPTM1L
3.62696552466438E-162	0.315688535254408	0.053	0.189	5.91594346728008E-158	T/NK cell	DCTN4
5.32078720268679E-162	0.464232287785488	0.072	0.225	8.67873600630242E-158	T/NK cell	OSBP
6.2250747272484E-162	0.308787993078703	0.076	0.232	1.01537193876149E-157	T/NK cell	FBXL5
6.3308606934128E-162	0.448946100200513	0.082	0.242	1.03262668770256E-157	T/NK cell	FAM50A
7.71975354296823E-162	0.454733885975325	0.041	0.167	1.25916900039355E-157	T/NK cell	BRPF3
9.39390512579405E-162	0.471907574298539	0.092	0.26	1.53223986506827E-157	T/NK cell	IL6ST
2.7599318708679E-161	0.397057584624845	0.111	0.295	4.50172487457264E-157	T/NK cell	APOL2
3.81429387376613E-161	0.511329551058961	0.075	0.228	6.22149473749993E-157	T/NK cell	LRRC47
4.8896385961131E-161	0.482156442690881	0.087	0.251	7.97548951412008E-157	T/NK cell	QARS
5.3966588120033E-161	0.338418947654513	0.079	0.235	8.80249018825858E-157	T/NK cell	TMUB2
5.43985962102089E-161	0.488172767871061	0.076	0.232	8.87295502784717E-157	T/NK cell	TRAM1
9.52069065282911E-161	0.411969422585057	0.044	0.172	1.55291985238296E-156	T/NK cell	CLCC1
1.19097169260737E-160	0.342297025638832	0.121	0.312	1.94259392781188E-156	T/NK cell	RERE
1.32080708895908E-160	2.54700770027847	0.222	0.108	2.15436844280116E-156	T/NK cell	GIMAP4
1.66643956306346E-160	0.532669995945492	0.07	0.219	2.71812957131281E-156	T/NK cell	RSAD1
1.66761295474078E-160	3.60622541273518	0.109	0.024	2.72004349047769E-156	T/NK cell	MATK
2.18965831914575E-160	0.255496271649354	0.044	0.171	3.57155168435863E-156	T/NK cell	SMIM12

4.19724236656183E-160	0.491225377498094	0.056	0.194	6.846122024099E-156	T/NK cell	PI4K2B
4.351049618724E-160	0.265022878757348	0.045	0.174	7.09699703310072E-156	T/NK cell	VDAC3
4.50336164196106E-160	0.491404373805336	0.066	0.212	7.34543317420268E-156	T/NK cell	DEPRECATI
4.63210387485222E-160	0.435249755764282	0.076	0.229	7.55542463027145E-156	T/NK cell	NCLN
7.11718047828013E-160	0.254485215149291	0.051	0.183	1.16088330781227E-155	T/NK cell	RAB2A
7.40736904809727E-160	0.546234354630642	0.103	0.278	1.20821596543515E-155	T/NK cell	INTS1
1.36219347707069E-159	0.500232556775131	0.089	0.253	2.22187378045001E-155	T/NK cell	STAU1
1.44683232597181E-159	0.44196030573078	0.104	0.281	2.35992820689261E-155	T/NK cell	MOV10
1.80825770291603E-159	0.440324318933177	0.073	0.225	2.94944913922634E-155	T/NK cell	MKLN1
1.88003212675952E-159	0.434833200672128	0.141	0.348	3.06652040195745E-155	T/NK cell	APMAP
4.3582664300831E-159	0.51172670599071	0.118	0.307	7.10876837410854E-155	T/NK cell	UBR4
4.99223105836391E-159	0.299578751534978	0.039	0.16	8.14282807929738E-155	T/NK cell	PITRM1
5.15753781489959E-159	0.54691564070827	0.065	0.209	8.41245992988272E-155	T/NK cell	GSE1
5.69826761271661E-159	0.529501919100115	0.102	0.276	9.29444430310206E-155	T/NK cell	HGS
1.09798019522336E-158	0.485927465884798	0.061	0.203	1.79091549642882E-154	T/NK cell	PDHB
1.96731909844341E-158	0.373829628276128	0.043	0.169	3.20889418147104E-154	T/NK cell	SCAP
3.35573156274366E-158	0.4484795864366	0.069	0.216	5.47353375199119E-154	T/NK cell	PITPNB
3.94472092908615E-158	0.411319406930087	0.097	0.266	6.43423430743242E-154	T/NK cell	DEPRECATI
5.05510081676738E-158	0.356034976014882	0.057	0.195	8.24537494222927E-154	T/NK cell	MESD
5.2969719012772E-158	0.390528697225413	0.155	0.37	8.63989086817324E-154	T/NK cell	GSDMD
1.36526207888797E-157	0.319629902994465	0.054	0.189	2.22687897687417E-153	T/NK cell	CAPN1
3.05860046828801E-157	3.06189905681326	0.104	0.022	4.98888322382457E-153	T/NK cell	CRIP1
3.19669399999851E-157	0.304062638789539	0.036	0.155	5.21412758339757E-153	T/NK cell	DESI1
4.8997118304019E-157	0.250655050103472	0.064	0.206	7.99191996656855E-153	T/NK cell	MAVS
5.96090304286687E-157	0.279132622155609	0.072	0.22	9.72282895322016E-153	T/NK cell	KCTD20
8.1809463200682E-157	0.554890792575924	0.104	0.28	1.33439415426632E-152	T/NK cell	NBR1
4.03252011537034E-156	0.450460847729118	0.083	0.241	6.57744356018056E-152	T/NK cell	DENND11
5.3282276601158E-156	0.434781526625447	0.056	0.191	8.69087213641489E-152	T/NK cell	CARS2
1.16973247557945E-155	0.276586616824667	0.078	0.23	1.90795064091764E-151	T/NK cell	TSPO
1.25975818317962E-155	0.27893221169293	0.081	0.235	2.05479157258428E-151	T/NK cell	MAP2K3
1.97258827833624E-155	0.572584055480636	0.108	0.286	3.21748874079425E-151	T/NK cell	SHFL
2.42915519362373E-155	0.624474337334017	0.083	0.24	3.96219503631967E-151	T/NK cell	KLHDC2
2.61339241030553E-155	0.538475822123938	0.062	0.203	4.26270436044936E-151	T/NK cell	RB1CC1
2.6967856819043E-155	0.508805979788557	0.071	0.218	4.39872712575411E-151	T/NK cell	COG3
3.45564902089748E-155	0.491303677813258	0.065	0.207	5.63650911798588E-151	T/NK cell	PET100
4.12039899400567E-155	0.296912896698842	0.113	0.292	6.72078279912264E-151	T/NK cell	BCL3
4.59492844464376E-155	0.404987113613044	0.056	0.19	7.49478778605843E-151	T/NK cell	GMPPB
4.63991184862353E-155	0.485929044343675	0.128	0.321	7.56816021628984E-151	T/NK cell	FLII
9.01853105777434E-155	0.307782319371362	0.048	0.175	1.47101260083357E-150	T/NK cell	AGGF1
1.26970201140877E-154	0.307438176120826	0.051	0.18	2.07101095080884E-150	T/NK cell	CBWD2
1.33720215190421E-154	0.52988104743723	0.123	0.312	2.18111042997095E-150	T/NK cell	CNBP
1.56603545757096E-154	0.55758220755272	0.071	0.218	2.55436043484399E-150	T/NK cell	NPEPPS
2.4073527605309E-154	3.19739374791247	0.242	0.131	3.92663308770195E-150	T/NK cell	HNRNPLL
3.27113298358885E-154	3.23139869097459	0.145	0.048	5.33554500953177E-150	T/NK cell	ATP8A1
3.27447980675493E-154	0.517763891616425	0.108	0.284	5.34100401279796E-150	T/NK cell	PPP1R15B
1.2254651512212E-153	0.537388918818167	0.097	0.264	1.99885620815689E-149	T/NK cell	PSMF1
2.32321070362808E-153	0.578541683170058	0.095	0.262	3.78938897868775E-149	T/NK cell	NSUN2
7.68818796173244E-153	0.424057177805707	0.07	0.214	1.25402033843818E-148	T/NK cell	MED25
8.52639943732633E-153	0.499767236763854	0.037	0.154	1.3907410122223E-148	T/NK cell	DHRS1
1.17626567975222E-152	0.475669143526353	0.104	0.276	1.91860695024384E-148	T/NK cell	GNB2
1.3251644642883E-152	0.65014596672539	0.089	0.249	2.16147575770064E-148	T/NK cell	MAN2A2
3.52820703533965E-152	0.641851036592675	0.081	0.234	5.7548584953425E-148	T/NK cell	RIOK3

**2.63130642692087E-151**	0.350272078127344	0.057	0.189	4.29192391295064E-147	T/NK cell	NCBP2
**5.92989022891966E-151**	0.44204286620124	0.077	0.225	9.67224395239086E-147	T/NK cell	MED10
**8.30283214163383E-151**	0.300192666572491	0.048	0.174	1.35427495062189E-146	T/NK cell	PTPN23
**1.04086738280345E-150**	0.362094792689061	0.046	0.17	1.69775878809071E-146	T/NK cell	UBR3
**2.88487797644996E-150**	0.335967106674079	0.047	0.171	4.70552446738754E-146	T/NK cell	NEDD8
**3.0128817979949E-150**	0.399677930313584	0.05	0.176	4.91431150070947E-146	T/NK cell	MCCC1
**3.63400260941874E-150**	0.480424823674856	0.052	0.181	5.9274216562229E-146	T/NK cell	IVD
**8.99212862578916E-150**	0.527962935930467	0.041	0.161	1.46670610015247E-145	T/NK cell	SLC35D2
**1.49853579509244E-149**	0.414014699488761	0.064	0.203	2.44426173537528E-145	T/NK cell	DECR1
**1.5867638455226E-149**	0.29161165474894	0.053	0.182	2.58817050843191E-145	T/NK cell	UBE2R2
**2.36526033336588E-149**	0.305474662439897	0.043	0.164	3.85797612975309E-145	T/NK cell	CD164
**2.5983911601792E-149**	0.599803695345915	0.099	0.266	4.23823582136829E-145	T/NK cell	DPP9
**4.59084527221593E-149**	0.327975878014553	0.066	0.205	7.4881277235114E-145	T/NK cell	SELENOW
**8.84517096618424E-149**	0.306176439594887	0.057	0.189	1.44273583629431E-144	T/NK cell	HIPK3
**1.18089293085518E-148**	0.490222372564591	0.117	0.296	1.92615445951788E-144	T/NK cell	OGFR
**1.37312778971617E-148**	0.633163651784328	0.107	0.279	2.23970873780604E-144	T/NK cell	DNAJA2
**1.7259536727851E-148**	0.54357010867695	0.089	0.246	2.81520303567977E-144	T/NK cell	LPIN2
**2.48896566723242E-148**	0.425999048817283	0.049	0.174	4.0597518998228E-144	T/NK cell	SEC63
**2.57355349736824E-148**	0.259935604801793	0.04	0.158	4.19772310955733E-144	T/NK cell	PPP1R11
**5.88537099747387E-148**	0.275682633885484	0.048	0.171	9.59962863397964E-144	T/NK cell	ACAP3
**5.0356412778303E-147**	0.55310361509082	0.078	0.226	8.21363448826901E-143	T/NK cell	EIF5
**5.52971693925547E-147**	0.495981690858545	0.064	0.201	9.0195212996196E-143	T/NK cell	DDX54
**6.82204650566121E-147**	0.565244525761237	0.079	0.227	1.1127440055384E-142	T/NK cell	RAB18
**1.40841651871028E-146**	4.80930041382205	0.109	0.028	2.29726818366834E-142	T/NK cell	RNF157
**1.43044593731345E-146**	0.483841181624896	0.044	0.164	2.33320036835196E-142	T/NK cell	MALSU1
**2.38550449897025E-146**	0.283083062113964	0.114	0.288	3.89099638827038E-142	T/NK cell	BTG2
**2.54960634709105E-146**	0.342585880121759	0.04	0.157	4.15866291274022E-142	T/NK cell	FBXO9
**3.37398100432406E-146**	0.399397521220892	0.055	0.183	5.50330041615298E-142	T/NK cell	MAP4K4
**4.43749738236016E-146**	0.561101553215471	0.07	0.211	7.23800198036766E-142	T/NK cell	PPP4R2
**4.68904907683456E-146**	0.609572633578515	0.089	0.246	7.64830794922485E-142	T/NK cell	ZC3H15
**4.89801824276754E-146**	0.53445811580998	0.075	0.219	7.98915755577814E-142	T/NK cell	DDB1
**5.84819534740776E-146**	0.337097303847887	0.053	0.18	9.5389914311568E-142	T/NK cell	SGSM3
**1.95592284132998E-145**	0.592779036956412	0.056	0.186	3.19030574649334E-141	T/NK cell	SMAD4
**2.80292700494038E-145**	0.446496937973144	0.06	0.193	4.57185423775826E-141	T/NK cell	TXNL4A
**4.12098256406802E-145**	0.636259967432725	0.073	0.217	6.72173466025134E-141	T/NK cell	ITFG1
**5.55570427116147E-145**	0.611612482036502	0.132	0.321	9.06190923669148E-141	T/NK cell	PPP6R2
**8.85919539683968E-145**	0.420427314431415	0.091	0.246	1.44502336117852E-140	T/NK cell	FKBP8
**1.61555686399984E-144**	0.541049213591093	0.083	0.233	2.63513480087014E-140	T/NK cell	GTPBP6
**2.45029843759623E-144**	0.294450171168678	0.043	0.161	3.9966817815632E-140	T/NK cell	CCDC107
**2.63704066309977E-144**	0.570979471265563	0.045	0.165	4.30127702558203E-140	T/NK cell	AMT
**5.47149139663368E-144**	0.31424674078401	0.082	0.231	8.92454961704919E-140	T/NK cell	MKNK2
**7.64091211362051E-144**	3.2052547047308	0.176	0.075	1.24630917485264E-139	T/NK cell	VPS37B
**1.48857508940191E-143**	0.372864069672483	0.036	0.147	2.42801482832345E-139	T/NK cell	ACAD11
**1.58082051076253E-143**	0.575258150285623	0.129	0.315	2.57847633510476E-139	T/NK cell	SERINC1
**1.95116351402363E-143**	0.39631155342913	0.049	0.171	3.18254280772395E-139	T/NK cell	CCDC159
**2.35527111988199E-143**	0.606680288627396	0.07	0.21	3.84168272363951E-139	T/NK cell	TPST2
**2.74859513106414E-143**	0.597430037539642	0.082	0.231	4.48323351827872E-139	T/NK cell	TNRC6A
**2.76490224587499E-143**	0.680878787380623	0.081	0.23	4.5098320532467E-139	T/NK cell	TXNRD2
**4.54447313990708E-143**	0.519865702778558	0.151	0.355	7.41249013850243E-139	T/NK cell	SELENOF
**7.48349380309326E-143**	2.90765415926997	0.262	0.159	1.22063267422254E-138	T/NK cell	PPP2R5C
**7.86295696535116E-143**	0.331126494168659	0.058	0.187	1.28252691061843E-138	T/NK cell	TANGO2
**8.80587850017592E-143**	0.569349178566364	0.063	0.196	1.43632684216369E-138	T/NK cell	UHMK1

9.1966593927451E-143	0.3205250277241	0.043	0.16	1.50006711355065E-138	T/NK cell	ATP5F1B
1.11481051717031E-142	0.454832858812506	0.188	0.421	1.8183674345565E-138	T/NK cell	IQSEC1
1.16351635144445E-142	0.262485485825703	0.048	0.169	1.89781152084104E-138	T/NK cell	ACCS
1.35006778680708E-142	0.636040148667598	0.099	0.26	2.20209556706102E-138	T/NK cell	RER1
2.44972748512503E-142	0.40157893169157	0.044	0.161	3.99575050098743E-138	T/NK cell	GPS1
2.72608040879939E-142	0.295723900096644	0.062	0.194	4.44650975479269E-138	T/NK cell	ATN1
6.75965964914635E-142	0.583926062435834	0.079	0.225	1.10256808537226E-137	T/NK cell	ENSA
1.58524869420415E-141	0.448791258351152	0.115	0.287	2.58569914511638E-137	T/NK cell	DENND4B
1.82659089173659E-141	0.304061205831824	0.041	0.156	2.97935240351156E-137	T/NK cell	FAM174C
2.22787327235998E-141	2.73094358740589	0.24	0.135	3.63388409454636E-137	T/NK cell	CDC42SE2
3.59925914451694E-141	0.48172213627368	0.056	0.182	5.87075159062159E-137	T/NK cell	PIGT
5.44552672583177E-141	3.11153552547598	0.195	0.093	8.8821986425042E-137	T/NK cell	PARP8
6.47698058870792E-141	0.462845726973429	0.05	0.171	1.05646030382415E-136	T/NK cell	ZXDC
9.3655272161876E-141	0.479107913328317	0.154	0.357	1.52761114423236E-136	T/NK cell	ANXA11
1.23393423164625E-140	0.325822973119001	0.197	0.418	2.0126701252382E-136	T/NK cell	HSPA1B
1.70183873684824E-140	0.426679266263167	0.053	0.176	2.77586916367316E-136	T/NK cell	ELAC2
1.73353537134693E-140	0.413615294055297	0.179	0.401	2.82756954420398E-136	T/NK cell	ANKRD10
2.17257728736891E-140	1.52820288083793	0.359	0.257	3.54369081342744E-136	T/NK cell	LCP1
2.35761141307803E-140	0.583163114073096	0.076	0.217	3.84549997587157E-136	T/NK cell	ATP5IF1
2.55278356994867E-140	0.634728482012018	0.116	0.289	4.16384528094328E-136	T/NK cell	UBXN4
2.64043030750278E-140	0.448825861328482	0.04	0.152	4.30680587456779E-136	T/NK cell	KIAA0232
5.41560368896972E-140	0.722918398424216	0.074	0.214	8.83339117707851E-136	T/NK cell	NMRAL1
7.84150711791074E-140	3.04692548949607	0.283	0.185	1.27902822600242E-135	T/NK cell	RBL2
9.0295135291349E-140	0.255916038295208	0.029	0.132	1.47280395173719E-135	T/NK cell	ZFAND1
1.63399184537886E-139	0.654727125310297	0.083	0.23	2.66520409899746E-135	T/NK cell	VPS52
1.81681962173597E-139	0.408485179574039	0.063	0.194	2.96341448501354E-135	T/NK cell	DELE1
1.86856077149726E-139	0.529492794063282	0.049	0.169	3.04780947438917E-135	T/NK cell	SCRN2
3.26197083813686E-139	0.323975351936201	0.036	0.145	5.32060063408503E-135	T/NK cell	ZBTB48
4.41572029989827E-139	0.51414490194485	0.055	0.181	7.20248138116407E-135	T/NK cell	RIPK1
8.96878122591747E-139	0.600691770598414	0.123	0.301	1.4628979057594E-134	T/NK cell	IGF2R
1.30174809583527E-138	0.508741213646079	0.08	0.224	2.12328131911691E-134	T/NK cell	EGLN2
1.33360275740793E-138	0.593077527444729	0.061	0.191	2.17523945760808E-134	T/NK cell	KLHL24
1.58670219282685E-138	3.13750184645352	0.102	0.024	2.58806994671988E-134	T/NK cell	ITPR3
4.29715632733278E-138	0.463664681659717	0.062	0.192	7.0090916855125E-134	T/NK cell	SAMD4B
4.96076855707347E-138	0.671409040087406	0.083	0.23	8.09150959344253E-134	T/NK cell	DNAJC21
5.18554090779302E-138	0.394654062852927	0.066	0.198	8.4581357747012E-134	T/NK cell	SELENOK
7.03303933218666E-138	0.720881060190573	0.084	0.232	1.14715904547297E-133	T/NK cell	TNKS
8.37540577549209E-138	0.474779355467021	0.063	0.193	1.36611243604052E-133	T/NK cell	DYNC1I2
1.18845324876243E-137	0.598820284902857	0.122	0.298	1.93848609405641E-133	T/NK cell	B4GALT1
1.55040599094985E-137	0.293017489601597	0.029	0.131	2.5288672118383E-133	T/NK cell	ARFGEF2
1.6475336161105E-137	0.52624677169536	0.057	0.182	2.68729208123784E-133	T/NK cell	LMBRD1
2.66731048217488E-137	0.363901754030663	0.066	0.198	4.35065012747545E-133	T/NK cell	HDGFL2
2.75550037104342E-137	0.596653228648741	0.126	0.305	4.49449665520893E-133	T/NK cell	USF2
2.96341904433834E-137	0.366258913077201	0.053	0.175	4.83363280322027E-133	T/NK cell	XPNPEP1
4.46215947877879E-137	0.6341732805223	0.08	0.222	7.27822832583608E-133	T/NK cell	SCRIB
4.57996625767977E-137	0.43665573539298	0.06	0.186	7.47038296290147E-133	T/NK cell	KXD1
5.4898959330635E-137	0.646506932804681	0.119	0.292	8.95456925641987E-133	T/NK cell	RAB11B
5.91112180755605E-137	0.339558631759073	0.041	0.152	9.64163078030467E-133	T/NK cell	ZNF680
6.11234848429898E-137	0.508700207871837	0.04	0.15	9.96985161274006E-133	T/NK cell	GSS
6.19558343221125E-137	0.372391109314228	0.092	0.245	1.01056161362798E-132	T/NK cell	TWF2
7.10780222572309E-137	0.591130547712423	0.075	0.214	1.15935362103769E-132	T/NK cell	CLIP1
9.07662921002017E-137	0.263539326062488	0.035	0.14	1.48048899044639E-132	T/NK cell	SLC35B2

1.06391694500303E-136	0.595704898388453	0.103	0.263	1.73535492899444E-132	T/NK cell	HSF1
1.32051139386906E-136	1.22256753822327	0.363	0.249	2.15388613453982E-132	T/NK cell	ITPKB
2.10911751124842E-136	3.91583304817227	0.161	0.066	3.4401815725973E-132	T/NK cell	TNIK
2.34631216627311E-136	0.408710708483627	0.057	0.181	3.82706977440807E-132	T/NK cell	TUBGCP2
2.74494528785604E-136	0.52539820690917	0.026	0.124	4.47728025902199E-132	T/NK cell	SLC35D1
4.05963090621797E-136	0.722440651305067	0.115	0.286	6.62166397113213E-132	T/NK cell	ARSA
5.01879547980218E-136	0.376843956889083	0.063	0.191	8.18615730710533E-132	T/NK cell	FARSA
6.80730557267842E-136	0.529598192125411	0.061	0.189	1.11033961195958E-131	T/NK cell	COP 1
7.51752691084461E-136	0.556950892126061	0.045	0.16	1.22618381442786E-131	T/NK cell	PMM2
1.48170271293096E-135	0.416506187029728	0.046	0.161	2.41680529506169E-131	T/NK cell	ZMYM3
1.48263398432133E-135	0.360987809914124	0.056	0.178	2.41832429182653E-131	T/NK cell	GATD1
1.65705188933958E-135	0.498800356781104	0.082	0.226	2.70281733670179E-131	T/NK cell	KPNA4
3.87746100007114E-135	0.402284817020236	0.042	0.154	6.32452663721604E-131	T/NK cell	EIF2S1
4.32456151335304E-135	0.38115585405836	0.035	0.141	7.05379228443014E-131	T/NK cell	DAPK2
4.85123473968375E-135	0.408439701971144	0.064	0.193	7.91284898389817E-131	T/NK cell	MICAL3
5.81917471193683E-135	0.694346561492987	0.07	0.205	9.49165587264016E-131	T/NK cell	PRKAA1
7.36947245522321E-135	0.70490370449802	0.059	0.185	1.20203465217146E-130	T/NK cell	DHX16
1.13397799925728E-134	0.643222011836447	0.093	0.244	1.84963151458854E-130	T/NK cell	IKBKG
1.50116844720431E-134	3.70325039250159	0.102	0.026	2.44855585423495E-130	T/NK cell	MCTP2
1.63119263661552E-134	0.554633528117295	0.048	0.165	2.66063830958357E-130	T/NK cell	EMC2
1.67699566025037E-134	0.566232415288	0.092	0.243	2.73534762143437E-130	T/NK cell	RBM3
1.86873043004769E-134	0.50055640007444	0.058	0.182	3.04808620445079E-130	T/NK cell	UVSSA
1.88224385929432E-134	0.505415898484475	0.072	0.208	3.07012795889496E-130	T/NK cell	IP6K2
2.19340306705458E-134	0.315886452064557	0.047	0.162	3.57765974267273E-130	T/NK cell	ELOVL1
3.26309702668951E-134	0.365869904666783	0.038	0.145	5.32243756023325E-130	T/NK cell	CLPX
1.25688082478695E-133	0.387481796636084	0.115	0.281	2.05009831330999E-129	T/NK cell	TP53INP1
1.7845682108844E-133	0.47271118680677	0.068	0.199	2.91080920877355E-129	T/NK cell	EIF1
2.89994952872345E-133	0.370202289273785	0.047	0.161	4.73010767630082E-129	T/NK cell	TEX264
4.38437033617546E-133	0.353565370729166	0.032	0.133	7.15134645533579E-129	T/NK cell	MOCS2
4.48025134520649E-133	0.291747715283149	0.062	0.187	7.30773796916631E-129	T/NK cell	HIPK2
9.36800370490281E-133	0.365145367849722	0.05	0.167	1.5280150843067E-128	T/NK cell	HNRNPAB
1.16485699109697E-132	0.620512866788588	0.062	0.189	1.89999823817826E-128	T/NK cell	PDCD6
1.25043823253852E-132	0.543608632611344	0.041	0.151	2.03958980109358E-128	T/NK cell	DNAJA3
1.63029862494876E-132	0.398643668199947	0.042	0.152	2.65918008715391E-128	T/NK cell	MIEN1
2.18185299857539E-132	0.397898227126165	0.056	0.177	3.55882042597632E-128	T/NK cell	TUBGCP3
2.45210460777566E-132	0.666095333212288	0.11	0.274	3.99962782574288E-128	T/NK cell	NME3
2.63479517747727E-132	0.680266048077461	0.043	0.154	4.29761441398317E-128	T/NK cell	NFRKB
4.98299354994917E-132	0.272419532739875	0.04	0.148	8.12776077932208E-128	T/NK cell	PIAS2
8.09569160832562E-132	0.519674974736748	0.127	0.301	1.32048825823399E-127	T/NK cell	MAP4
1.07395805716898E-131	0.455358061133992	0.031	0.131	1.75173298704832E-127	T/NK cell	NFS1
1.20376054242604E-131	0.419611408092872	0.046	0.159	1.96345382075112E-127	T/NK cell	WWP1
2.006264053991E-131	0.347117324730701	0.06	0.183	3.27241729846471E-127	T/NK cell	DICER1
2.85388481696037E-131	0.293778297239931	0.033	0.134	4.65497152494406E-127	T/NK cell	PWP1
3.56356143335879E-131	0.299257956510267	0.033	0.135	5.81252505395152E-127	T/NK cell	ZBTB22
5.7632140466146E-131	0.491122175161658	0.054	0.173	9.40037843143308E-127	T/NK cell	PPP2R5D
6.15721665309876E-131	0.430611598586398	0.083	0.224	1.00430360828694E-126	T/NK cell	ARMH3
7.30994696184815E-131	0.432803924778871	0.056	0.177	1.19232544894705E-126	T/NK cell	MZT2B
7.70797152643911E-131	0.605053192788754	0.073	0.207	1.25724723567748E-126	T/NK cell	TRAPPC12
8.60730772434051E-131	0.437179499246931	0.056	0.177	1.40393796291718E-126	T/NK cell	FBXW5
1.99081035967596E-130	0.655640484313764	0.05	0.166	3.24721077766746E-126	T/NK cell	RAF1
3.24798776310247E-130	0.282668524976436	0.035	0.137	5.29779284039644E-126	T/NK cell	CSNK2B
7.27102143828145E-130	0.325860727047878	0.054	0.172	1.18597630679809E-125	T/NK cell	TRMT112

7.55817006009287E-130	0.390198779065932	0.028	0.123	1.23281311850175E-125	T/NK cell	CPOX
9.71019258764044E-130	0.345252775038063	0.039	0.144	1.58382951297003E-125	T/NK cell	PLRG1
9.84275895262747E-130	0.402809464536329	0.052	0.168	1.60545241276307E-125	T/NK cell	SPPL2B
2.29623162316422E-129	0.489953072360001	0.06	0.182	3.74538340054316E-125	T/NK cell	ATP5F1E
4.84721784981495E-129	0.462180730924435	0.038	0.143	7.90629703483317E-125	T/NK cell	GPI
5.86665659390593E-129	0.486028829780075	0.056	0.176	9.56910357031997E-125	T/NK cell	IDH3G
9.57617253845522E-129	0.474238425323557	0.075	0.209	1.56196950274743E-124	T/NK cell	TOP 1.00
1.07019838320479E-128	0.430431753434135	0.046	0.157	1.74560058284534E-124	T/NK cell	COX7A2L
1.18098923376769E-128	0.521333471509619	0.057	0.176	1.92631153919848E-124	T/NK cell	USP14
1.71335512777591E-128	0.44505019458437	0.039	0.143	2.79465354891528E-124	T/NK cell	NUBP2
1.75754894284468E-128	0.658057860254821	0.077	0.212	2.86673808067395E-124	T/NK cell	ABCF1
2.18761173758425E-128	0.368112017279797	0.037	0.141	3.56821350517367E-124	T/NK cell	TSC2
2.91758267516385E-128	0.452607598341759	0.039	0.143	4.75886910145976E-124	T/NK cell	CTPS1
4.45443801461347E-128	0.558344444280941	0.065	0.191	7.26563384563604E-124	T/NK cell	ARFGAP1
6.36622314283501E-128	0.357531763653179	0.13	0.301	1.03839465682782E-123	T/NK cell	ARPC1B
6.6486882493672E-128	0.551361295331121	0.077	0.212	1.08446754035428E-123	T/NK cell	SF3B5
7.59202113554424E-128	0.382623916327225	0.033	0.133	1.23833456741862E-123	T/NK cell	NUDT19
7.94757100384423E-128	0.699881891575321	0.076	0.21	1.29632830643703E-123	T/NK cell	SDHA
8.53553288961914E-128	2.68404681319579	0.204	0.106	1.39223076962578E-123	T/NK cell	PIK3IP1
9.05794105250891E-128	0.707101977784201	0.086	0.228	1.47744076507473E-123	T/NK cell	APRT
1.65088212873939E-127	0.507074064518282	0.051	0.166	2.69275384018681E-123	T/NK cell	UBE2J2
1.72936318019988E-127	0.501479676676246	0.054	0.17	2.82076428322403E-123	T/NK cell	PGP
4.43270488530659E-127	0.394420029880836	0.188	0.404	7.23018493842358E-123	T/NK cell	TNFRSF14
2.28314805682234E-126	0.349003550108233	0.223	0.472	3.72404279548292E-122	T/NK cell	NDUFV1
3.34783822851892E-126	0.670303969262975	0.094	0.241	5.46065893453721E-122	T/NK cell	LEMD2
4.67702115600952E-126	0.301425089258882	0.031	0.129	7.62868920756713E-122	T/NK cell	CC2D1A
5.62299962750798E-126	0.611012471003629	0.16	0.357	9.17167469242827E-122	T/NK cell	DUS1L
6.88569784929094E-126	0.557513288402453	0.074	0.205	1.12312617619785E-121	T/NK cell	CTDSP2
9.3966967202381E-126	0.428705611260828	0.037	0.139	1.53269520203804E-121	T/NK cell	PREB
1.10882562053328E-125	2.42383671167077	0.232	0.133	1.80860546965183E-121	T/NK cell	TSC22D3
1.13176264300854E-125	0.672785697039961	0.09	0.233	1.84601804701123E-121	T/NK cell	PHRF1
1.24062752614059E-125	0.361447587588876	0.027	0.12	2.02358755788792E-121	T/NK cell	SEC23A
2.07894157618298E-125	0.28862128752174	0.056	0.174	3.39096160491205E-121	T/NK cell	PHKB
2.70425929819978E-125	0.392714750692326	0.028	0.121	4.41091734129367E-121	T/NK cell	GCDH
3.93190398325348E-125	0.469195841394749	0.037	0.139	6.41332858708475E-121	T/NK cell	SEC24A
4.86471556187152E-125	0.555791268230972	0.07	0.197	7.93483755296864E-121	T/NK cell	TENT4A
5.53438100371985E-125	0.500805188898093	0.05	0.161	9.02712885516744E-121	T/NK cell	PSMD12
6.86888509361257E-125	0.441859417243529	0.081	0.216	1.12038384761915E-120	T/NK cell	RFX5
6.86968787250539E-125	0.712621595396782	0.127	0.299	1.12051478888435E-120	T/NK cell	CELF1
9.18792414846245E-125	0.623229783107561	0.057	0.176	1.49864230785571E-120	T/NK cell	KTN1
1.18061702767953E-124	0.609939005801676	0.058	0.176	1.92570443384808E-120	T/NK cell	ASCC2
1.33877909087268E-124	0.41394621816648	0.061	0.181	2.18368257512242E-120	T/NK cell	VPS35L
1.54169315621527E-124	0.298196014437297	0.033	0.132	2.51465570710272E-120	T/NK cell	MTFR1L
2.19256693539413E-124	0.293057649094079	0.041	0.145	3.57629592832136E-120	T/NK cell	AGTRAP
2.39693503468591E-124	0.602574985018455	0.087	0.228	3.9096407350762E-120	T/NK cell	PDCD6IP
3.0004508722583E-124	0.332617339951703	0.04	0.143	4.89403541774051E-120	T/NK cell	TIMM10
3.190379532647E-124	0.665895009194601	0.132	0.306	5.20382805570051E-120	T/NK cell	INTS11
5.2813810551007E-124	0.743725131343497	0.075	0.206	8.61446063897475E-120	T/NK cell	DERL1
7.16149569948464E-124	0.304800229344993	0.153	0.338	1.16811156354294E-119	T/NK cell	LPCAT1
7.30162882138079E-124	0.579501033452391	0.051	0.163	1.19096867705542E-119	T/NK cell	PUS1
1.44290859255094E-123	0.680443678803117	0.072	0.201	2.35352820530984E-119	T/NK cell	GNL1
3.23646640306244E-123	0.524221655790164	0.045	0.152	5.27900035003515E-119	T/NK cell	ECHDC1

4.11055909574445E-123	0.504057836338809	0.037	0.138	6.70473294106877E-119	T/NK cell	BBC3
5.03639626442622E-123	0.533282195724708	0.061	0.18	8.21486594690561E-119	T/NK cell	YY1
5.76109501523389E-123	0.612351089069092	0.063	0.184	9.396922079348E-119	T/NK cell	FNDC3A
7.72651984194793E-123	0.631599945894107	0.06	0.179	1.26027265142013E-118	T/NK cell	GSK3B
9.02423771662746E-123	0.407753224306522	0.038	0.139	1.4719434139591E-118	T/NK cell	TBL3
9.04164291053594E-123	0.359502354259851	0.065	0.186	1.47478237513752E-118	T/NK cell	ECE1
1.10365863786896E-122	0.347843147051088	0.038	0.139	1.80017760422806E-118	T/NK cell	EIF1AY
1.180174183714E-122	0.6110115790921	0.043	0.148	1.92498211105591E-118	T/NK cell	EEF1AKNM
1.35429153846538E-122	0.6785439178278	0.062	0.182	2.20898492839088E-118	T/NK cell	IDH3B
1.41583376885856E-122	0.613572131763429	0.158	0.351	2.3093664603852E-118	T/NK cell	MEF2D
1.45040050222122E-122	0.500601611567727	0.078	0.209	2.36574825917303E-118	T/NK cell	TELO2
1.57842792884966E-122	0.553216050718535	0.074	0.203	2.57457379474667E-118	T/NK cell	SIVA1
1.5820734867045E-122	0.704370609472222	0.145	0.328	2.58052006416371E-118	T/NK cell	C11orf58
2.2649192626515E-122	0.351646082699852	0.044	0.151	3.69430980931086E-118	T/NK cell	EIF4E2
2.27871030688759E-122	0.299611502409963	0.052	0.164	3.71680438156434E-118	T/NK cell	SLC12A9
2.56603863307514E-122	0.928337555356498	0.076	0.206	4.18546561440887E-118	T/NK cell	ZBTB16
2.972892548166E-122	0.422468284022377	0.041	0.143	4.84908503531357E-118	T/NK cell	UBAP1
3.31972397896178E-122	0.487795119580374	0.03	0.123	5.41480178208456E-118	T/NK cell	CCNC
3.73281484323401E-122	0.786355603087288	0.09	0.231	6.088594290799E-118	T/NK cell	BET1L
5.60165773171623E-122	0.752838073093143	0.054	0.169	9.13686392620234E-118	T/NK cell	TMEM106E
5.82178103399436E-122	0.588816299591503	0.081	0.215	9.4959070445482E-118	T/NK cell	CCDC9
6.20557303150309E-122	0.526845871283598	0.04	0.142	1.01219101716847E-117	T/NK cell	CDC5L
8.50908776582553E-122	0.559517624146233	0.069	0.193	1.3879173054838E-117	T/NK cell	ZNF592
9.48867554703862E-122	0.354290577640841	0.034	0.131	1.54769786847747E-117	T/NK cell	STRN3
1.10856738716739E-121	0.337959165687708	0.045	0.151	1.80818426520873E-117	T/NK cell	TADA3
1.39089987260848E-121	0.771346701080427	0.041	0.144	2.2686967822117E-117	T/NK cell	SLC25A42
1.59764238523122E-121	0.662864084530713	0.048	0.156	2.60591449455064E-117	T/NK cell	PIGO
2.46356826214365E-121	0.44328233204866	0.025	0.113	4.0183261923825E-117	T/NK cell	TMEM39A
3.77094722706192E-121	0.520428170353413	0.054	0.167	6.15079202206069E-117	T/NK cell	TPGS1
7.30144945703576E-121	0.749254635974763	0.066	0.189	1.1909394209371E-116	T/NK cell	DLG1
7.51607521885748E-121	0.479720195581312	0.189	0.404	1.22594702894784E-116	T/NK cell	DBNL
8.42675635536537E-121	0.36705399919869	0.047	0.154	1.37448822912364E-116	T/NK cell	ZDHHC8
8.58165768963785E-121	0.66244658137041	0.119	0.28	1.39975418575683E-116	T/NK cell	DCTN1
1.07305975931035E-120	0.276017430500706	0.031	0.125	1.75026777341112E-116	T/NK cell	SNUPN
1.16405296095182E-120	0.554920515818305	0.048	0.156	1.89868678460851E-116	T/NK cell	LARS
1.88997042012568E-120	0.370588725195032	0.026	0.116	3.082730752267E-116	T/NK cell	PPOX
2.28870208927864E-120	0.373228916360496	0.025	0.113	3.7331019778224E-116	T/NK cell	NT5DC1
2.62989433869229E-120	0.841972818928101	0.071	0.197	4.28962065584099E-116	T/NK cell	PPP2R5A
2.87232551767001E-120	0.596334498093653	0.093	0.234	4.68505015187155E-116	T/NK cell	EFR3A
2.97351001318568E-120	0.598232234889476	0.053	0.165	4.85009218250717E-116	T/NK cell	FAAP20
3.01804530804909E-120	0.450417880194099	0.044	0.149	4.92273370195887E-116	T/NK cell	YKT6
3.21558940303377E-120	0.612542296478163	0.173	0.378	5.24494787528838E-116	T/NK cell	ADAR
3.63637493245533E-120	0.538492071857496	0.082	0.216	5.93129115232789E-116	T/NK cell	MGRN1
3.88373603751925E-120	0.400190858772068	0.16	0.349	6.33476185079764E-116	T/NK cell	PPP1R9B
3.92366618956628E-120	0.494586935881289	0.046	0.152	6.39989192180155E-116	T/NK cell	DNPEP
5.71752153648226E-120	0.468975556495811	0.05	0.159	9.32584937815622E-116	T/NK cell	TXNDC12
7.20509459828802E-120	0.514010986636168	0.056	0.17	1.17522297992676E-115	T/NK cell	UNC50
7.59502586653454E-120	0.686911153542984	0.051	0.162	1.23882466909045E-115	T/NK cell	EIF2AK1
8.52559807253614E-120	0.634941945149398	0.061	0.179	1.39061030161137E-115	T/NK cell	MYBBP1A
8.95228834087252E-120	0.763825220936595	0.105	0.255	1.46020775127972E-115	T/NK cell	EXOC3
1.02622577291173E-119	0.688929585343069	0.105	0.255	1.67387685819632E-115	T/NK cell	RNF10
1.55333073204115E-119	0.621243718552697	0.05	0.158	2.53363775703232E-115	T/NK cell	TMEM129

1.60423592623213E-119	0.478008711743987	0.132	0.301	2.61666921927723E-115	T/NK cell	PICALM
2.21828375068274E-119	0.387964344776745	0.028	0.118	3.61824262573861E-115	T/NK cell	DHDDS
2.70177417819812E-119	0.62665323840519	0.061	0.177	4.40686386205895E-115	T/NK cell	ELAVL1
4.05152264439918E-119	0.413023581874658	0.029	0.121	6.60843858527951E-115	T/NK cell	DPH2
5.86902762497536E-119	0.504116619030562	0.034	0.129	9.57297095909731E-115	T/NK cell	MACO1
7.19654513338926E-119	0.316232983744689	0.033	0.128	1.17382847670712E-114	T/NK cell	TDP2
8.94259630516776E-119	0.758562978797755	0.111	0.265	1.45862688333591E-114	T/NK cell	DEPRECATI
9.49054437455727E-119	0.526347627481162	0.046	0.151	1.54800269293404E-114	T/NK cell	CWC15
1.01590460587869E-118	0.339244599404694	0.038	0.137	1.65704200264874E-114	T/NK cell	LARP1
1.11306079337239E-118	0.833229042716619	0.093	0.234	1.81551346006971E-114	T/NK cell	RNF115
1.31068034817234E-118	0.740236910866945	0.1	0.245	2.1378507159039E-114	T/NK cell	PSMD13
2.25234524151918E-118	0.6537468536233	0.092	0.231	3.67380032344193E-114	T/NK cell	AHSA1
2.28887526143222E-118	0.480376175440664	0.053	0.163	3.73338443892209E-114	T/NK cell	EDF1
2.3781000270864E-118	0.539432563448781	0.043	0.146	3.87891895418062E-114	T/NK cell	PITPNM2
2.49212744297682E-118	0.68203827189943	0.07	0.194	4.06490907223949E-114	T/NK cell	RPAIN
2.66066372196484E-118	0.396870768244721	0.036	0.132	4.33980859689685E-114	T/NK cell	PKD1
7.15579666102763E-118	0.69527561709606	0.053	0.164	1.16718199338022E-113	T/NK cell	OGFOD2
9.31185657150519E-118	0.629275830292353	0.054	0.165	1.51885692537821E-113	T/NK cell	POLR1D
9.89737325929895E-118	0.557985078129808	0.06	0.175	1.61436055232425E-113	T/NK cell	ZMAT2
1.09426930942666E-117	0.416156676257622	0.034	0.128	1.78486267060582E-113	T/NK cell	ECSIT
1.26504582108001E-117	0.29402907324679	0.027	0.115	2.0634162387636E-113	T/NK cell	ZFYVE1
1.36537834250469E-117	0.480035188242096	0.035	0.13	2.22706861445939E-113	T/NK cell	SLC25A22
1.51776306596913E-117	0.770928383295804	0.082	0.214	2.47562333690225E-113	T/NK cell	NMT1
1.56191078506925E-117	0.740050802744191	0.093	0.233	2.54763268152646E-113	T/NK cell	NDEL1
1.96848300061961E-117	0.439552946912052	0.038	0.135	3.21079262231064E-113	T/NK cell	PQBP1
2.11610503985331E-117	0.446291179595097	0.055	0.166	3.45157893050474E-113	T/NK cell	CUL1
3.03996376609926E-117	0.449329506161706	0.032	0.125	4.95848489888451E-113	T/NK cell	SUCO
3.07388423906636E-117	2.77598606117251	0.16	0.071	5.01381258234113E-113	T/NK cell	CHD3
4.39531138674637E-117	0.366353537933048	0.029	0.118	7.169192402922E-113	T/NK cell	GARS
6.23029258532748E-117	0.688204630027256	0.078	0.207	1.01622302359277E-112	T/NK cell	TM9SF3
6.40053518131031E-117	0.382686446674622	0.05	0.156	1.04399129342352E-112	T/NK cell	ATP6V0E1
7.96347041553015E-117	0.321861934787615	0.029	0.119	1.29892165947712E-112	T/NK cell	ARPC1A
8.89752377854149E-117	3.08452719285636	0.113	0.037	1.4512751035179E-112	T/NK cell	PHF19
1.563093182297E-116	0.700511180264154	0.125	0.288	2.54956128964464E-112	T/NK cell	PLEC
2.28628900000899E-116	0.45382700818186	0.039	0.137	3.72916598791466E-112	T/NK cell	GATD3B
2.6450537973671E-116	0.695606926154016	0.138	0.31	4.31434724888548E-112	T/NK cell	SETD5
3.12025248984257E-116	0.706072623087706	0.057	0.169	5.08944383618221E-112	T/NK cell	SLC35A3
3.73190686843399E-116	0.332805702611771	0.026	0.112	6.08711329310268E-112	T/NK cell	POLR2K
5.20351915152516E-116	0.538783961355546	0.052	0.16	8.48746008805268E-112	T/NK cell	PARK7
6.57322967367732E-116	0.675188968635659	0.077	0.203	1.07215949207351E-111	T/NK cell	CPSF7
7.14120192478825E-116	0.574901469195155	0.055	0.167	1.16480144595221E-111	T/NK cell	XRN2
9.76645301518852E-116	0.666036382089914	0.066	0.184	1.5930061513074E-111	T/NK cell	RRP36
1.13318942294386E-115	0.607059563738185	0.046	0.15	1.84834526776373E-111	T/NK cell	MSTO1
1.56055129296553E-115	0.532503938683693	0.035	0.129	2.54541521395608E-111	T/NK cell	ELMOD2
2.67676571229017E-115	0.726324641708212	0.063	0.179	4.36607255331649E-111	T/NK cell	RBCK1
2.79061750336964E-115	0.403254331734439	0.03	0.119	4.55177620974622E-111	T/NK cell	SHLD2
3.44579964930844E-115	0.590556486021072	0.039	0.137	5.62044380798699E-111	T/NK cell	TMEM183A
3.71459281065566E-115	0.522995169802477	0.029	0.118	6.05887233346044E-111	T/NK cell	DGCR6L
3.96120997606753E-115	0.545691985535314	0.067	0.187	6.46112959196375E-111	T/NK cell	ITGB1
5.5573622912842E-115	0.292183541376279	0.04	0.138	9.06461363331365E-111	T/NK cell	FDPS
6.5199180037922E-115	0.381172663555681	0.026	0.113	1.06346382559855E-110	T/NK cell	ARFIP2
7.38423951556843E-115	0.276029709989432	0.045	0.147	1.20444330738437E-110	T/NK cell	LRCH1

**7.4882813801141E-115**	0.707087293268921	0.074	0.198	1.22141357591041E-110	T/NK cell	COPB1
**1.78743344674853E-114**	0.28942702710166	0.044	0.146	2.91548269499153E-110	T/NK cell	SRF
**1.93630593146455E-114**	0.532212968666399	0.033	0.125	3.15830860481182E-110	T/NK cell	ATIC
**1.9967273744441E-114**	0.680516496253137	0.051	0.158	3.25686202045578E-110	T/NK cell	KCMF1
**2.1151090725692E-114**	0.64451949912498	0.033	0.126	3.44995440826762E-110	T/NK cell	IMPA1
**2.26917819229711E-114**	0.654101887236947	0.061	0.176	3.70125654945582E-110	T/NK cell	NUDT22
**2.29220143128921E-114**	0.564356987061016	0.102	0.247	3.73880975457583E-110	T/NK cell	JMJD1C
**2.43041939286137E-114**	0.28130048989894	0.025	0.111	3.96425707169618E-110	T/NK cell	CYHR1
**4.33244062180595E-114**	0.771415535991187	0.065	0.183	7.06664389822768E-110	T/NK cell	AKT2
**5.09825155484903E-114**	0.533347375161872	0.039	0.135	8.31575811111425E-110	T/NK cell	NOC2L
**7.31709354904075E-114**	0.323623450420318	0.042	0.141	1.19349112878404E-109	T/NK cell	MIIP
**7.70109211532399E-114**	0.742448851701454	0.061	0.175	1.2561251349305E-109	T/NK cell	LRPPRC
**9.83761491220022E-114**	0.319034828172877	0.084	0.214	1.60461336832898E-109	T/NK cell	PML
**1.28835165044474E-113**	0.521973700134219	0.057	0.167	2.10143037704041E-109	T/NK cell	BRAT1
**1.36624336992086E-113**	0.790548588380564	0.086	0.219	2.22847956067792E-109	T/NK cell	TTC3
**1.54522045707822E-113**	0.628669871909809	0.096	0.235	2.52040908754028E-109	T/NK cell	ADAM10
**1.99647429913092E-113**	0.636107101015758	0.062	0.176	3.25644922931244E-109	T/NK cell	SLC25A11
**3.15717005063256E-113**	0.520076276568797	0.051	0.157	5.14966006958677E-109	T/NK cell	DCTN3
**3.51523603887421E-113**	0.529819428632135	0.048	0.152	5.73370150300773E-109	T/NK cell	ARCN1
**5.18535216632096E-113**	0.576445017039222	0.049	0.152	8.45782791848612E-109	T/NK cell	API5
**5.28336902256826E-113**	0.777244156163441	0.058	0.17	8.61770321271109E-109	T/NK cell	SUCLG2
**5.4614813418878E-113**	0.624460340603448	0.067	0.185	8.90822221675319E-109	T/NK cell	MAPK14
**5.62209494344177E-113**	0.725136424358891	0.084	0.214	9.17019906224787E-109	T/NK cell	ATP5MG
**7.57319877094576E-113**	0.349416611601882	0.035	0.127	1.23526445152896E-108	T/NK cell	FBXL15
**8.38970380519195E-113**	0.38772844830573	0.085	0.215	1.36844458766486E-108	T/NK cell	TTLL3
**8.58901009719465E-113**	0.715818013170035	0.055	0.164	1.40095343695342E-108	T/NK cell	EPRS
**8.86039281023089E-113**	0.572628465178393	0.108	0.254	1.44521867127676E-108	T/NK cell	UBE2L6
**1.37167251553268E-112**	0.535362189127372	0.055	0.162	2.23733504008535E-108	T/NK cell	ZC3H7B
**1.45589927297888E-112**	0.668632568061888	0.109	0.257	2.37471730415585E-108	T/NK cell	UBXN1
**1.97740273741293E-112**	1.44285652894647	0.185	0.089	3.22534160499422E-108	T/NK cell	CD37
**2.6880665883884E-112**	0.547478892990943	0.08	0.206	4.38450541232032E-108	T/NK cell	GNB1
**2.73305626010737E-112**	0.650342509885207	0.049	0.152	4.45788806586114E-108	T/NK cell	TBRG4
**2.98355196345801E-112**	0.557021142615143	0.05	0.155	4.86647160759637E-108	T/NK cell	PRPF19
**2.99252617478195E-112**	0.726839682760352	0.126	0.287	4.88110944368684E-108	T/NK cell	ZBTB7A
**3.4476239195146E-112**	0.273362258402821	0.024	0.106	5.62341937512026E-108	T/NK cell	UBL3
**4.23306598876667E-112**	0.534484394118067	0.035	0.127	6.90455393427731E-108	T/NK cell	WDR45
**4.34325546240352E-112**	0.329799819937313	0.077	0.202	7.08428398472638E-108	T/NK cell	SNX6
**4.8932819616838E-112**	0.509151378140439	0.036	0.129	7.98143220770244E-108	T/NK cell	ZNF44
**5.14037567700783E-112**	0.285917948998486	0.029	0.117	8.38446676676748E-108	T/NK cell	DENR
**5.19837408015811E-112**	0.559404001707028	0.077	0.201	8.4790679621459E-108	T/NK cell	CLASRP
**6.85974306728929E-112**	0.325967239104865	0.035	0.127	1.11889269170556E-107	T/NK cell	USP22
**1.02374470360258E-111**	0.46577179026107	0.053	0.16	1.66982998604617E-107	T/NK cell	TARBP1
**1.20113110656832E-111**	0.295360080953767	0.029	0.117	1.95916494792359E-107	T/NK cell	ATG14
**1.69699421937604E-111**	0.599883065465032	0.057	0.166	2.76796727122426E-107	T/NK cell	NPHP3
**1.83927853966853E-111**	0.330627846120109	0.03	0.118	3.00004722605333E-107	T/NK cell	ZNF148
**2.36416104524711E-111**	0.726643144805894	0.113	0.264	3.85618308090256E-107	T/NK cell	MXD4
**2.59646054282097E-111**	3.13074447720316	0.2	0.11	4.23508679139529E-107	T/NK cell	RAB27A
**2.75635970871632E-111**	0.665643604831084	0.071	0.191	4.49589832088719E-107	T/NK cell	SUMO2
**3.14277848762024E-111**	0.784298550674323	0.082	0.211	5.12618599115737E-107	T/NK cell	PSMC5
**4.25068031636956E-111**	0.649765974337573	0.047	0.149	6.93328466403039E-107	T/NK cell	ZNF146
**4.95958998746509E-111**	0.485788095497795	0.03	0.118	8.0895872285543E-107	T/NK cell	GANAB
**5.02776003692575E-111**	0.804134543182961	0.066	0.182	8.20077939622959E-107	T/NK cell	IDUA

5.04091848169578E-111	0.499985809105528	0.048	0.15	8.22224213549398E-107	T/NK cell	HDAC6
6.78850181171566E-111	0.498457070979094	0.046	0.146	1.10727253050894E-106	T/NK cell	TRMT1
7.0931781560312E-111	0.430553264500623	0.047	0.148	1.15696828903025E-106	T/NK cell	SLC35A4
7.23237044613993E-111	0.822379599108799	0.078	0.203	1.17967194346988E-106	T/NK cell	FKBP5
7.23437666260812E-111	0.289092073907651	0.057	0.166	1.17999917743801E-106	T/NK cell	IFFO1
7.98572586907008E-111	0.373533423555776	0.036	0.129	1.30255174650402E-106	T/NK cell	EXOC6
8.731137068487E-111	0.521442792886758	0.051	0.156	1.42413576724091E-106	T/NK cell	ZC3H3
9.30711143162001E-111	0.771835781209493	0.134	0.299	1.51808294561154E-106	T/NK cell	CSNK1D
9.70847357448622E-111	0.638800757997919	0.039	0.134	1.58354912473445E-106	T/NK cell	ACAD8
1.0430070374322E-110	0.707221668134541	0.052	0.157	1.70124877875567E-106	T/NK cell	ATP2C1
1.23490626426135E-110	0.857217736710557	0.063	0.177	2.01425560763669E-106	T/NK cell	SERINC5
1.45257760503784E-110	0.620947451995371	0.058	0.167	2.36929933157723E-106	T/NK cell	YME1L1
1.58114728871212E-110	0.466771571827111	0.047	0.148	2.57900934261834E-106	T/NK cell	EBP
1.94673229859215E-110	0.595206844607235	0.028	0.114	3.17531505223365E-106	T/NK cell	MLYCD
2.04416619180691E-110	0.824589793670097	0.094	0.23	3.33423947545624E-106	T/NK cell	TMEM175
2.11050894433816E-110	0.28885000298087	0.042	0.139	3.44245113910998E-106	T/NK cell	UBE2A
2.35706017093909E-110	0.786370985837127	0.064	0.178	3.84460084481875E-106	T/NK cell	CUL4A
2.75634431940699E-110	0.317181737072402	0.042	0.138	4.49587321938473E-106	T/NK cell	SGPP1
2.80651448076295E-110	0.392060386284912	0.036	0.128	4.57770576957244E-106	T/NK cell	FBH1
5.54191716939755E-110	0.457653993917236	0.033	0.123	9.03942109500434E-106	T/NK cell	SLC15A4
5.93554242597701E-110	0.467104184589244	0.036	0.128	9.6814632510111E-106	T/NK cell	VCP
6.19107586123976E-110	0.350252439693856	0.034	0.124	1.00982638372682E-105	T/NK cell	ZKSCAN8
6.34411302651431E-110	0.357370220460754	0.041	0.137	1.03478827575475E-105	T/NK cell	MAP3K2
6.64140239275622E-110	0.488856908714214	0.047	0.147	1.08327914428247E-105	T/NK cell	HDHD2
7.45090255578929E-110	0.52947041672442	0.131	0.291	1.21531671587479E-105	T/NK cell	AKIRIN2
7.67499139813296E-110	0.656937875609324	0.061	0.172	1.25186784694947E-105	T/NK cell	GTF3C3
8.06274857357412E-110	0.46072806179568	0.04	0.134	1.31511491983567E-105	T/NK cell	RDH11
2.52037543700711E-109	0.393751937936229	0.037	0.129	4.11098437530229E-105	T/NK cell	SERTAD2
2.72028173826146E-109	0.462825997004324	0.039	0.133	4.43705154327826E-105	T/NK cell	VPS9D1
2.78748806304017E-109	0.508177465452962	0.041	0.137	4.54667177962482E-105	T/NK cell	SRP19
2.97657772224755E-109	0.529728571656081	0.066	0.18	4.85509592275797E-105	T/NK cell	WWOX
3.06089824770346E-109	0.504164588687086	0.095	0.23	4.99263113182912E-105	T/NK cell	ELMSAN1
4.44338549369269E-109	0.831159981245908	0.086	0.216	7.24760607876214E-105	T/NK cell	PARP12
5.52932620723919E-109	0.404826709850794	0.032	0.119	9.01888397662784E-105	T/NK cell	UQCRC2
5.65643004654658E-109	0.732958811526875	0.073	0.192	9.22620304892213E-105	T/NK cell	NUP50
5.66772315105653E-109	0.809944762578481	0.054	0.16	9.24462323168831E-105	T/NK cell	PPM1A
5.74128861162282E-109	0.510867385468078	0.042	0.138	9.36461585441798E-105	T/NK cell	USO1
5.87759071707037E-109	0.506851070412553	0.03	0.116	9.58693821861348E-105	T/NK cell	PIK3R4
8.45626062952626E-109	0.72499226205475	0.08	0.205	1.37930067128203E-104	T/NK cell	RABGGTB
8.46090197899943E-109	0.657958490561233	0.044	0.141	1.3800577217946E-104	T/NK cell	CLN3
8.60881655863924E-109	0.80569519279328	0.042	0.138	1.40418406887965E-104	T/NK cell	SMYD2
1.04943197733978E-108	0.815396387562273	0.08	0.204	1.71172849823891E-104	T/NK cell	HMGN1
1.0554990567792E-108	0.485664810342115	0.028	0.112	1.72162451151255E-104	T/NK cell	FASTKD2
1.25580581123932E-108	0.691755062161777	0.078	0.2	2.04834485871245E-104	T/NK cell	MDM2
1.52081837826117E-108	0.406434268913492	0.037	0.128	2.48060685678179E-104	T/NK cell	FUBP3
1.58675802803629E-108	0.544759921359847	0.05	0.151	2.58816101952999E-104	T/NK cell	HNRNPH2
1.60782068198631E-108	0.585902734159925	0.047	0.147	2.62251631438787E-104	T/NK cell	GSR
1.6520697067331E-108	0.652410239456804	0.113	0.261	2.69469089865235E-104	T/NK cell	GPATCH2L
1.7059540790235E-108	0.661882864062813	0.041	0.136	2.78258169829523E-104	T/NK cell	RNASEH1
1.79983127791804E-108	0.542565547701281	0.045	0.144	2.93570479741212E-104	T/NK cell	DNAJC13
2.53555824928577E-108	0.76058156973385	0.058	0.166	4.13574906041003E-104	T/NK cell	GPAT4
3.40496374812805E-108	0.317975680279875	0.034	0.123	5.55383636957166E-104	T/NK cell	SIRT2

4.97141996583845E-108	0.579879648999315	0.05	0.152	8.1088831062791E-104	T/NK cell	DPM1
6.90633264646082E-108	3.26049543554369	0.218	0.131	1.12649191796422E-103	T/NK cell	PGGHG
7.28170129301118E-108	0.850819726632273	0.085	0.214	1.18771829790305E-103	T/NK cell	NUDCD3
7.51846998258343E-108	0.559762499045737	0.076	0.197	1.22633763885918E-103	T/NK cell	CMIP
8.64537014707711E-108	0.616465701278606	0.073	0.191	1.41014632468975E-103	T/NK cell	ZC3H12A
9.40039368535604E-108	0.325037437750316	0.024	0.104	1.53329821401842E-103	T/NK cell	MTIF2
9.49513619507346E-108	0.41926695154107	0.027	0.11	1.54875166477843E-103	T/NK cell	MPHOSPH1
1.1477289743656E-107	0.595288982691213	0.114	0.261	1.87206073008773E-103	T/NK cell	ZMIZ2
1.16010906720328E-107	0.699852130873196	0.043	0.139	1.89225389951528E-103	T/NK cell	XPO5
1.36097176454448E-107	0.264650125635387	0.034	0.123	2.21988104514851E-103	T/NK cell	TM9SF4
1.55191768598768E-107	0.446249408505547	0.044	0.14	2.5313329376145E-103	T/NK cell	PSMD1
1.62899320856054E-107	0.571365111888856	0.041	0.137	2.6570508224831E-103	T/NK cell	TTC37
1.64687073991956E-107	0.378605079458805	0.052	0.154	2.68621086388279E-103	T/NK cell	VPS26A
1.86449840837042E-107	0.300748069498483	0.047	0.147	3.041183353893E-103	T/NK cell	IRGQ
2.65698236671462E-107	0.679782857521326	0.061	0.17	4.33380393834822E-103	T/NK cell	BRMS1
2.95512048779915E-107	0.541631557951427	0.052	0.155	4.82009702764919E-103	T/NK cell	RYBP
3.19020949758999E-107	0.390663801011914	0.025	0.106	5.20355071151903E-103	T/NK cell	COQ8B
3.6511867153772E-107	0.81282071968469	0.1	0.239	5.95545065145175E-103	T/NK cell	SERINC3
4.62450661213623E-107	0.621998440146719	0.055	0.159	7.54303273505541E-103	T/NK cell	MBD6
4.80292622907739E-107	0.580547099343347	0.066	0.179	7.83405297224813E-103	T/NK cell	PPP1R10
5.00554982378142E-107	0.905376804763474	0.078	0.201	8.16455231756988E-103	T/NK cell	STIMATE
5.19638365673491E-107	0.695988061729783	0.057	0.164	8.47582138250032E-103	T/NK cell	C6orf120
5.2192425366296E-107	0.514637099597472	0.064	0.175	8.51310650149654E-103	T/NK cell	RAB8A
5.26746682105059E-107	0.333049971841823	0.032	0.119	8.59176513181561E-103	T/NK cell	TMEM11
5.47915527702846E-107	0.570152116212838	0.035	0.124	8.93705017236112E-103	T/NK cell	ESF1
5.93247987572899E-107	0.280289373331184	0.022	0.1	9.67646792530155E-103	T/NK cell	CPNE3
9.3478321759998E-107	0.571899126606943	0.051	0.154	1.52472490622733E-102	T/NK cell	CBWD1
1.34443170971845E-106	0.676774310607714	0.157	0.334	2.19290256172176E-102	T/NK cell	HSP90AA1
1.4758822142988E-106	0.609743551639898	0.051	0.153	2.40731147974277E-102	T/NK cell	CHERP
1.53442238994926E-106	0.371684442473691	0.027	0.11	2.50279636024624E-102	T/NK cell	ADCK5
1.73641275844507E-106	0.751201742241743	0.101	0.239	2.83226285029976E-102	T/NK cell	RAB21
1.77043094353452E-106	0.482691655449155	0.053	0.157	2.88774991199915E-102	T/NK cell	PBRM1
2.21457211409519E-106	0.751486446956088	0.087	0.215	3.61218857530067E-102	T/NK cell	U2SURP
2.64461928462695E-106	0.77805802955816	0.046	0.143	4.31363851515501E-102	T/NK cell	WDR59
3.18224991296554E-106	0.640224528335847	0.087	0.216	5.19056783303809E-102	T/NK cell	TENT2
3.37515439374959E-106	0.453991511846538	0.028	0.111	5.50521433164495E-102	T/NK cell	MMADHC
6.42039396490697E-106	0.688226393330713	0.067	0.181	1.04723045961598E-101	T/NK cell	MCRS1
6.72599722482887E-106	0.429770889316633	0.031	0.116	1.09707740734184E-101	T/NK cell	SEC23IP
7.02041750279966E-106	0.644834908531136	0.046	0.144	1.14510029888165E-101	T/NK cell	MVP
7.6151056570946E-106	0.799814944045557	0.08	0.202	1.2420998837287E-101	T/NK cell	VPS53
8.47919189363517E-106	0.418733806896723	0.035	0.124	1.38304098977083E-101	T/NK cell	UNC45A
8.95574663627095E-106	0.560851400612517	0.055	0.158	1.46077183384216E-101	T/NK cell	ALKBH5
1.03474220394055E-105	0.410786581598071	0.066	0.178	1.68776800884743E-101	T/NK cell	NXF1
1.10259636742815E-105	0.610734680428973	0.044	0.14	1.79844493491206E-101	T/NK cell	SRP72
1.15802375474122E-105	0.645589901454711	0.059	0.166	1.8888525463584E-101	T/NK cell	SNX14
1.26059145324716E-105	0.73272346975869	0.05	0.151	2.05615071939144E-101	T/NK cell	EIF3G
1.58731719595924E-105	0.648950935350929	0.081	0.204	2.58907307832911E-101	T/NK cell	HTT
1.63572972857363E-105	0.567170803364582	0.051	0.153	2.66803876027644E-101	T/NK cell	MARCH5
1.67745389152374E-105	0.647981088814785	0.057	0.163	2.73609504246438E-101	T/NK cell	COPE
1.77305240316425E-105	0.904690673015431	0.128	0.286	2.89202577480121E-101	T/NK cell	TXNDC11
2.24876368254013E-105	0.40889237526459	0.044	0.14	3.66795844259121E-101	T/NK cell	DNASE1L1
2.28914837294347E-105	0.419009859951904	0.043	0.137	3.73382991110809E-101	T/NK cell	SGK3

**2.52962177317644E-105**	0.653726783930588	0.045	0.141	4.12606607422809E-101	T/NK cell	PSMD6
**2.61833254045595E-105**	0.816789738518278	0.112	0.258	4.27076220673769E-101	T/NK cell	ZNF644
**2.772734977511E-105**	0.552366326351392	0.044	0.139	4.52260802181819E-101	T/NK cell	IFT27
**4.06692698505554E-105**	0.504764800953545	0.04	0.133	6.63356460532408E-101	T/NK cell	CSDE1
**4.65520952957679E-105**	0.585842114322438	0.033	0.12	7.5931122636927E-101	T/NK cell	CDC37L1
**5.32924179273282E-105**	0.69750723783924	0.116	0.264	8.69252628812649E-101	T/NK cell	SHISA5
**6.61760227527159E-105**	0.590252281579994	0.051	0.152	1.07939710711955E-100	T/NK cell	ARAF
**6.93714998533178E-105**	0.372184185766058	0.028	0.11	1.13151853410747E-100	T/NK cell	ATP1A1
**8.90588410273586E-105**	0.790625397678756	0.139	0.304	1.45263875599725E-100	T/NK cell	STAT3
**1.18201858456141E-104**	0.475313860799195	0.039	0.13	1.92799051327811E-100	T/NK cell	TMEM167E
**1.31600890199084E-104**	0.665150860003649	0.089	0.217	2.14654212003725E-100	T/NK cell	PCMTD1
**1.75668044073867E-104**	0.613133601752514	0.042	0.136	2.86532146688884E-100	T/NK cell	CRELD1
**1.92674562167754E-104**	0.855553545664443	0.065	0.177	3.14271478351823E-100	T/NK cell	RCC1L
**2.26566796085347E-104**	0.442626072011626	0.176	0.365	3.6955310109481E-100	T/NK cell	SLC2A4RG
**2.72689216393022E-104**	0.612350235164554	0.052	0.153	4.44783380858658E-100	T/NK cell	DEPRECATI
**3.14190876709525E-104**	0.542206294809901	0.053	0.155	5.12476739000906E-100	T/NK cell	RAB6A
**3.42367152293646E-104**	0.559551150471327	0.052	0.153	5.58435062106166E-100	T/NK cell	SPECC1L
**3.55802821366584E-104**	0.606963334687161	0.044	0.139	5.80349981931035E-100	T/NK cell	POFUT2
**3.72417113523987E-104**	0.560044671554044	0.049	0.148	6.07449553868976E-100	T/NK cell	KLHL22
**3.8617572944155E-104**	0.731106367749018	0.058	0.163	6.29891232292112E-100	T/NK cell	ABCF3
**4.135773927822E-104**	0.646652042680992	0.068	0.181	6.74586085367046E-100	T/NK cell	GLRX3
**4.46137726405451E-104**	0.373796117969306	0.03	0.114	7.27695245539931E-100	T/NK cell	CYP4V2
**5.01709794726815E-104**	2.48727056643859	0.212	0.125	8.18338846178908E-100	T/NK cell	PITPNC1
**5.32973957454468E-104**	0.840060518923466	0.054	0.156	8.69333822003983E-100	T/NK cell	ZBTB37
**6.2785154228826E-104**	0.700044043750955	0.051	0.152	1.02408865062638E-99	T/NK cell	SEC24B
**7.12826526726405E-104**	0.838341446438739	0.092	0.222	1.16269134774344E-99	T/NK cell	ZFR
**8.4801008310856E-104**	0.694662861905577	0.052	0.152	1.38318924655837E-99	T/NK cell	KLHL36
**9.66970605947858E-104**	0.609957883004858	0.03	0.114	1.57722575536155E-99	T/NK cell	FXR2
**1.26859437241566E-103**	0.806734845828088	0.067	0.179	2.06920428084719E-99	T/NK cell	EIF4A2
**1.35982415915122E-103**	0.404119102074697	0.025	0.103	2.21800918599155E-99	T/NK cell	PDHX
**1.64582237902961E-103**	0.781590208886592	0.056	0.159	2.6845008824352E-99	T/NK cell	NCK1
**1.67550347182092E-103**	0.639924753272955	0.029	0.112	2.73291371288711E-99	T/NK cell	MXI1
**1.79297042300761E-103**	0.386053997267643	0.024	0.102	2.9245140569677E-99	T/NK cell	GMPPA
**2.71369787912197E-103**	0.649572210002113	0.046	0.142	4.42631261063585E-99	T/NK cell	SPRYD3
**2.78265058161829E-103**	0.467168170587439	0.045	0.14	4.53878136367759E-99	T/NK cell	STX10
**2.92667614050155E-103**	0.384695754628595	0.026	0.106	4.77370145277208E-99	T/NK cell	ANKRD40
**3.48013852576687E-103**	0.723762692287616	0.058	0.163	5.67645394937835E-99	T/NK cell	MAPKAPK5
**3.59373350053898E-103**	2.95302869739636	0.101	0.033	5.86173871272914E-99	T/NK cell	UNC13D
**3.69877420760309E-103**	0.296013860302535	0.047	0.143	6.03307061002141E-99	T/NK cell	SKIL
**4.24598490561305E-103**	0.525892834895097	0.041	0.132	6.92562597954545E-99	T/NK cell	HPS4
**5.21583214763729E-103**	0.643357825371783	0.059	0.164	8.50754381601119E-99	T/NK cell	STX6
**5.34687632500966E-103**	0.5355325064187	0.029	0.112	8.72128997372326E-99	T/NK cell	PEX26
**5.48565280006073E-103**	0.689368825683585	0.073	0.189	8.94764828217906E-99	T/NK cell	UBE2Z
**6.96529671045607E-103**	0.799446073762272	0.083	0.206	1.13610954644249E-98	T/NK cell	SNU13
**7.51065126535449E-103**	0.307272499268765	0.052	0.153	1.22506232789197E-98	T/NK cell	TBC1D22A
**7.61149795394217E-103**	0.555672731351122	0.036	0.125	1.24151143126751E-98	T/NK cell	SCFD1
**9.2413206582412E-103**	0.811745310352864	0.03	0.113	1.50735181256572E-98	T/NK cell	OCIAD2
**1.11265840234376E-102**	0.427382565407278	0.06	0.165	1.8148571200629E-98	T/NK cell	MLLT1
**1.12465364763601E-102**	0.59083766122519	0.053	0.154	1.83442256465909E-98	T/NK cell	GTPBP4
**1.23243603318906E-102**	0.565676561499207	0.043	0.137	2.01022641373467E-98	T/NK cell	UFM1
**1.27356693320029E-102**	0.553239836295244	0.05	0.148	2.07731502474299E-98	T/NK cell	SRSF9
**1.32691328729408E-102**	0.572695779560198	0.04	0.131	2.16432826290537E-98	T/NK cell	ZNF428

1.52609649926819E-102	0.591417814807419	0.055	0.157	2.48921599995635E-98	T/NK cell	NUP42
1.69792851102914E-102	0.354787355400017	0.03	0.113	2.76949119433963E-98	T/NK cell	UBL7
1.82842095923622E-102	0.537572041098786	0.032	0.116	2.98233742661021E-98	T/NK cell	ATL2
2.15158235301377E-102	0.463712271878186	0.041	0.133	3.50944597600076E-98	T/NK cell	BTBD7
2.33842238534544E-102	0.795654670449164	0.088	0.213	3.81420075273694E-98	T/NK cell	HNRNPC
2.85962345268255E-102	0.400848575024602	0.046	0.142	4.66433181367051E-98	T/NK cell	ST3GAL2
2.99920510926349E-102	0.81472731851796	0.096	0.227	4.89200345371968E-98	T/NK cell	THRAP3
3.16836168987398E-102	0.699455540210388	0.128	0.282	5.16791475235345E-98	T/NK cell	SRRM2
4.40378516946998E-102	0.856269434629552	0.06	0.166	7.18301398992248E-98	T/NK cell	AARS2
5.30987135778849E-102	0.832149810718575	0.068	0.179	8.66093117168881E-98	T/NK cell	TRAPPC4
6.10983617018677E-102	0.721007294313518	0.066	0.175	9.96575377719164E-98	T/NK cell	KDM4B
6.19312201387575E-102	0.749780763220739	0.048	0.145	1.01016013168327E-97	T/NK cell	MED16
6.68609755174664E-102	0.392593700276015	0.231	0.46	1.09056937166539E-97	T/NK cell	CDK5RAP3
1.32852204398449E-101	0.893093701078757	0.07	0.183	2.16695230594309E-97	T/NK cell	ZNF33A
1.32947655053325E-101	0.840456190499711	0.123	0.274	2.16850920157479E-97	T/NK cell	ATF6B
1.38220009237719E-101	0.556338584159332	0.059	0.163	2.25450657067644E-97	T/NK cell	NOP56
1.40743303814882E-101	0.328212739080983	0.026	0.104	2.29566402852454E-97	T/NK cell	BOP 1.00
1.5031898065878E-101	0.487508291931818	0.043	0.136	2.45185289352536E-97	T/NK cell	SMARCB1
1.50995263549626E-101	0.579543117454748	0.05	0.147	2.46288374375795E-97	T/NK cell	WDR83OS
1.9376005590644E-101	0.489638974889374	0.027	0.106	3.16042027188995E-97	T/NK cell	CPD
2.89498199925452E-101	0.807006160630385	0.059	0.164	4.72200513898405E-97	T/NK cell	HDAC10
3.47452004721255E-101	0.377517259371277	0.056	0.158	5.66728964900839E-97	T/NK cell	DUSP22
3.556399869087E-101	0.526575877720008	0.042	0.134	5.80084382646781E-97	T/NK cell	EIF4G3
3.74759624621771E-101	0.470479907003616	0.039	0.128	6.1127042372057E-97	T/NK cell	SPAG7
3.88278373286102E-101	0.567192215194398	0.04	0.129	6.3332085466696E-97	T/NK cell	PRCC
3.95345175908668E-101	0.794404155156964	0.059	0.163	6.44847516424629E-97	T/NK cell	BUD23
4.45536630146222E-101	0.53376911192041	0.03	0.113	7.26714797431503E-97	T/NK cell	DPH6
4.91750051315037E-101	0.924201259484852	0.085	0.209	8.02093508699956E-97	T/NK cell	GPBP1L1
4.98339854619145E-101	0.876376661417096	0.131	0.288	8.12842136869288E-97	T/NK cell	SUPT5H
4.985894273447E-101	0.86896170592341	0.056	0.159	8.13249214941941E-97	T/NK cell	RMND5A
5.99403413268602E-101	0.76102621227203	0.076	0.192	9.77686907382416E-97	T/NK cell	AP5Z1
6.28865873400636E-101	0.766433849924283	0.115	0.258	1.02574312610378E-96	T/NK cell	DHX15
7.53210346042993E-101	1.88059283446068	0.331	0.265	1.22856139543073E-96	T/NK cell	TRIM22
7.63907030779727E-101	0.580943566828391	0.034	0.12	1.24600875790481E-96	T/NK cell	NUS1
8.24896671252264E-101	0.56790354061113	0.045	0.139	1.34548896047957E-96	T/NK cell	MBD4
8.25213446752136E-101	0.425748099212737	0.045	0.138	1.34600565299741E-96	T/NK cell	SPIDR
8.64423945068077E-101	0.36255721475151	0.025	0.102	1.40996189680054E-96	T/NK cell	ABHD10
1.81268662802315E-100	0.256944718822894	0.025	0.103	2.95667315896856E-96	T/NK cell	HMGXB3
1.88276995170475E-100	0.274974429000188	0.112	0.25	3.07098606822561E-96	T/NK cell	PTPN1
3.47474341856629E-100	3.47777597399469	0.106	0.037	5.66765399002347E-96	T/NK cell	CYFIP2
3.63605350775444E-100	0.572712893823723	0.061	0.166	5.93076687649827E-96	T/NK cell	HUS1
4.03412166202706E-100	0.665198076926914	0.044	0.137	6.58005584293234E-96	T/NK cell	EIF3L
4.9817753184608E-100	0.719482490337505	0.051	0.148	8.12577372194142E-96	T/NK cell	CYB561D2
4.9825600658809E-100	0.271812922023463	0.024	0.1	8.12705372345833E-96	T/NK cell	SUPT4H1
5.18739558189832E-100	0.580722635905412	0.041	0.131	8.46116093363435E-96	T/NK cell	EDEM2
5.19351650685603E-100	0.312889254320207	0.05	0.147	8.47114477433288E-96	T/NK cell	HMGN3
5.95851536635532E-100	0.837667712534274	0.103	0.238	9.71893441406216E-96	T/NK cell	ID2
7.43480446541532E-100	0.631289121453105	0.046	0.141	1.21269095635389E-95	T/NK cell	TAPT1
1.34817260220477E-99	0.895322954432284	0.068	0.178	2.1990043314562E-95	T/NK cell	TAX1BP1
1.41515607421952E-99	0.552358076989587	0.039	0.127	2.30826107265947E-95	T/NK cell	LRRC40
1.51644509377546E-99	0.650466813156323	0.052	0.15	2.47347359245715E-95	T/NK cell	WRNIP1
2.19836817123656E-99	0.576432200007075	0.039	0.128	3.58575832410396E-95	T/NK cell	GPR108

**2.26087443476669E-99**	1.10611990565543	0.054	0.153	3.68771229054795E-95	T/NK cell	VWA8
**2.63255333506379E-99**	0.513674446626026	0.041	0.131	4.29395774482254E-95	T/NK cell	PLPP5
**3.13364134227686E-99**	0.507794409564315	0.031	0.112	5.11128239338779E-95	T/NK cell	ANTKMT
**4.76565056567401E-99**	0.830621448844242	0.094	0.222	7.77325263767087E-95	T/NK cell	NRDC
**4.77046093946016E-99**	0.825351224250467	0.053	0.152	7.78109883835347E-95	T/NK cell	ALG12
**5.40533704176047E-99**	0.564944337678221	0.148	0.312	8.8166452488155E-95	T/NK cell	NCOR2
**6.48466859929338E-99**	0.452102360115169	0.039	0.126	1.05771429523074E-94	T/NK cell	GTF2I
**7.34583549674562E-99**	0.642610660583906	0.045	0.138	1.19817922787418E-94	T/NK cell	ABCB8
**7.68488524412355E-99**	0.697331356045309	0.179	0.369	1.25348163216899E-94	T/NK cell	SPG7
**7.97657780746957E-99**	0.459039337564856	0.031	0.113	1.30105960617636E-94	T/NK cell	GRSF1
**8.03124174754266E-99**	0.49691333539518	0.043	0.133	1.30997584144168E-94	T/NK cell	USB1
**1.04195701458226E-98**	0.834139470703196	0.092	0.219	1.69953608648513E-94	T/NK cell	GATAD2A
**1.16782031745431E-98**	0.317333235099223	0.073	0.184	1.90483171979972E-94	T/NK cell	PNPLA6
**1.19486013060557E-98**	0.465379946233484	0.027	0.105	1.94893635903075E-94	T/NK cell	GFM1
**1.3311526008528E-98**	0.555700180927809	0.049	0.145	2.171243007251E-94	T/NK cell	EFTUD2
**1.33990753089207E-98**	0.622172773228915	0.083	0.203	2.18552317363806E-94	T/NK cell	DNAJC7
**1.34722174234988E-98**	0.491670349747311	0.027	0.105	2.19745338394689E-94	T/NK cell	TRMT1L
**1.4212001850673E-98**	0.308379954396206	0.029	0.108	2.31811962186327E-94	T/NK cell	SUGT1
**1.42175415482507E-98**	0.585574889385426	0.027	0.105	2.31902320193518E-94	T/NK cell	RAD50
**1.67797279039746E-98**	0.544906582926634	0.041	0.13	2.7369414184173E-94	T/NK cell	SLC39A9
**2.54970468071536E-98**	0.493036856336241	0.03	0.111	4.15882330471483E-94	T/NK cell	UFL1
**2.75778045775295E-98**	0.545200286687483	0.031	0.112	4.49821570464084E-94	T/NK cell	XIAP
**2.92256909131475E-98**	0.684571653359569	0.047	0.14	4.76700244484349E-94	T/NK cell	USP19
**3.47097447212216E-98**	0.292154700141703	0.027	0.104	5.66150646147845E-94	T/NK cell	SNX12
**4.04742345720365E-98**	0.612743458759812	0.039	0.126	6.60175240104488E-94	T/NK cell	SH3BP5L
**4.35800919291723E-98**	0.87716874538517	0.07	0.181	7.10834879456729E-94	T/NK cell	REPS1
**4.60156120447176E-98**	0.592944649279965	0.096	0.224	7.50560648061388E-94	T/NK cell	SP3
**4.60209605680648E-98**	0.614661723134292	0.041	0.13	7.50647887825705E-94	T/NK cell	COPS6
**5.26658531713466E-98**	0.730071243998243	0.055	0.154	8.59032731077835E-94	T/NK cell	TMEM179E
**5.44304838520875E-98**	0.849425472587646	0.135	0.292	8.878156221114E-94	T/NK cell	TRPC4AP
**7.68429834182898E-98**	0.777881198104681	0.088	0.21	1.25338590253572E-93	T/NK cell	SCAMP2
**8.94709829001708E-98**	0.619601121063025	0.052	0.15	1.45936120208469E-93	T/NK cell	MLLT10
**9.20797800892164E-98**	0.561211490532396	0.035	0.119	1.50191329303521E-93	T/NK cell	DYNLRB1
**1.0698788344618E-97**	0.749427994552811	0.07	0.179	1.74507936689064E-93	T/NK cell	MAF 1.00
**1.14658910361555E-97**	0.561924646843266	0.205	0.413	1.87020148690732E-93	T/NK cell	CCNL1
**1.54239558621401E-97**	0.678666281895695	0.06	0.162	2.51580144067367E-93	T/NK cell	PCBP1
**1.74834042619062E-97**	0.835895742031993	0.121	0.266	2.85171806915951E-93	T/NK cell	C16orf72
**1.94559798822788E-97**	0.710758985206184	0.041	0.13	3.17346487859849E-93	T/NK cell	NR1D2
**2.12128693467367E-97**	0.762317462191273	0.093	0.218	3.46003111914623E-93	T/NK cell	SEC11A
**2.2161710600554E-97**	0.661700892428705	0.126	0.274	3.61479661605636E-93	T/NK cell	TRIM38
**2.28660682582309E-97**	2.5471450578778	0.322	0.266	3.72968439360004E-93	T/NK cell	PDCD4
**2.47112031988186E-97**	0.396513465139552	0.104	0.235	4.03064435375931E-93	T/NK cell	CCDC85B
**2.96404514758672E-97**	0.502587901034615	0.038	0.124	4.83465404022869E-93	T/NK cell	NF1
**3.21907001298139E-97**	0.524351067487773	0.029	0.108	5.25062509817394E-93	T/NK cell	C5orf22
**4.00721531143856E-97**	0.712155764382125	0.136	0.292	6.53616889448743E-93	T/NK cell	CDC37
**5.25166522314664E-97**	0.877419241144961	0.08	0.197	8.56599114547449E-93	T/NK cell	NDUFAF8
**5.38563574511381E-97**	0.792011586642714	0.057	0.158	8.78451046385514E-93	T/NK cell	TBC1D14
**5.44711983457266E-97**	2.78882253063904	0.199	0.116	8.88479716217147E-93	T/NK cell	RESF1
**6.39433676932295E-97**	0.775306442274626	0.072	0.183	1.04298027044427E-92	T/NK cell	EXOC7
**1.13730560188617E-96**	0.882429843752908	0.092	0.217	1.85505916723653E-92	T/NK cell	ANAPC5
**1.14835519934997E-96**	0.713670456203313	0.046	0.139	1.87308216565973E-92	T/NK cell	MTOR
**1.19949143336365E-96**	0.769454446322097	0.053	0.15	1.95649047695945E-92	T/NK cell	RXRB

1.29015099575134E-96	0.556750278545892	0.044	0.135	2.10436528917001E-92	T/NK cell	RUFY2
1.45981678675666E-96	0.815690412312249	0.064	0.17	2.38110716087878E-92	T/NK cell	CCNT2
1.75234037379629E-96	0.349784805022993	0.025	0.101	2.85824238369913E-92	T/NK cell	POFUT1
1.95691698093011E-96	0.290084576482551	0.028	0.106	3.19192728759511E-92	T/NK cell	BAP1
2.04905254792437E-96	0.918079795848828	0.062	0.165	3.34220961091944E-92	T/NK cell	XAB2
2.61534619560737E-96	0.899333238908388	0.071	0.182	4.26589117965519E-92	T/NK cell	RC3H1
2.65626613234647E-96	0.676833546272561	0.058	0.159	4.33263568847033E-92	T/NK cell	RABAC1
3.90093394116678E-96	0.624758587323607	0.047	0.14	6.36281335143713E-92	T/NK cell	ERCC1
4.67630525102104E-96	0.513893620139675	0.053	0.149	7.62752149494041E-92	T/NK cell	RNF138
5.07984918265647E-96	0.824859079852573	0.059	0.16	8.28574200183096E-92	T/NK cell	TTC1
5.26942246634362E-96	0.629988152251375	0.047	0.139	8.59495498485308E-92	T/NK cell	NAA10
6.12549109766022E-96	0.352730482531222	0.043	0.133	9.99128852939358E-92	T/NK cell	ZNF106
7.1106315692452E-96	0.784255281436933	0.072	0.183	1.15981511525958E-91	T/NK cell	GOSR2
7.5013600772805E-96	0.485951309519375	0.032	0.113	1.22354684220522E-91	T/NK cell	ZNF513
9.42957460212911E-96	0.808118739276025	0.055	0.154	1.53805791335328E-91	T/NK cell	MAP2K7
9.57651707492056E-96	1.1149641045637	0.078	0.193	1.56202570009029E-91	T/NK cell	LLGL2
9.93714126529919E-96	0.554180034212122	0.039	0.125	1.62084711178295E-91	T/NK cell	PMPCA
1.13913724946616E-95	0.363518152340754	0.027	0.104	1.85804676760426E-91	T/NK cell	CBR4
1.14945238224409E-95	1.70447619271008	0.254	0.169	1.87487178067834E-91	T/NK cell	DOCK2
1.29701587351059E-95	0.631319775320109	0.068	0.175	2.11556259128312E-91	T/NK cell	SPEN
1.44852746262382E-95	0.867760562101663	0.067	0.174	2.36269314428572E-91	T/NK cell	HMCES
2.0295959509297E-95	0.920507566536631	0.065	0.171	3.31047395556144E-91	T/NK cell	LIN7C
2.1247515967837E-95	0.623228705930492	0.041	0.129	3.4656823295139E-91	T/NK cell	TTC19
2.30446868998555E-95	0.311143134038602	0.037	0.121	3.75881888023543E-91	T/NK cell	ETHE1
2.57335010266312E-95	0.474680225537164	0.033	0.114	4.19739135245382E-91	T/NK cell	HMG20A
3.00242115164431E-95	0.348102608128336	0.027	0.103	4.89724914044703E-91	T/NK cell	TSNAX
3.07773300219939E-95	0.601427362787531	0.043	0.131	5.02009029988742E-91	T/NK cell	FAF1
3.22051284664451E-95	0.944890047761037	0.129	0.28	5.25297850416187E-91	T/NK cell	PJA2
3.31709840144933E-95	0.472970587409049	0.03	0.109	5.410519202604E-91	T/NK cell	YTHDF2
3.86352985788747E-95	0.995938838203195	0.097	0.225	6.30180355120025E-91	T/NK cell	IQGAP2
3.95348604086514E-95	0.546235232208268	0.029	0.107	6.44853108125512E-91	T/NK cell	B4GALT7
5.39126017853844E-95	0.823068296758416	0.081	0.196	8.79368447721405E-91	T/NK cell	CEP350
5.81257026019789E-95	0.479496540486054	0.036	0.12	9.48088335140877E-91	T/NK cell	UFD1
6.436313181618E-95	0.653799511893047	0.055	0.152	1.04982704305371E-90	T/NK cell	SMIM7
6.44746564235507E-95	0.597140684114478	0.043	0.132	1.05164612092454E-90	T/NK cell	YJU2
6.95219806605896E-95	0.539948740299124	0.047	0.139	1.13397302655488E-90	T/NK cell	CCNY
7.52459825908715E-95	0.642844560776963	0.028	0.106	1.2273372220397E-90	T/NK cell	FLAD1
7.7358098955689E-95	0.610610876259521	0.031	0.11	1.26178795206624E-90	T/NK cell	UROS
1.00904808579288E-94	0.429424072482022	0.075	0.185	1.64585833273676E-90	T/NK cell	CERT1
1.03787428044063E-94	0.839650240741311	0.087	0.207	1.69287673882672E-90	T/NK cell	DYNLL2
1.68785696333477E-94	0.537291437787526	0.035	0.117	2.75306349289535E-90	T/NK cell	FBXO22
1.84615433835198E-94	0.797616502699246	0.051	0.145	3.01126234128591E-90	T/NK cell	VPS26B
2.44973102676159E-94	2.96538854811288	0.172	0.092	3.99575627775083E-90	T/NK cell	CASP8
2.57344079095976E-94	0.682891984357185	0.033	0.113	4.19753927413446E-90	T/NK cell	LMBRD2
2.83815109295796E-94	0.431036338446028	0.041	0.128	4.62930824772373E-90	T/NK cell	VPS41
3.0034039425861E-94	0.673092035774723	0.057	0.156	4.8988521707522E-90	T/NK cell	SYF2
3.7090847141072E-94	0.425965407312192	0.026	0.1	6.04988807718026E-90	T/NK cell	MARS
4.64223080627607E-94	0.533057643347413	0.034	0.115	7.5719426681169E-90	T/NK cell	PARL
4.71991062707455E-94	0.851708116104546	0.066	0.171	7.6986462238213E-90	T/NK cell	FASTK
5.02167235472166E-94	1.03349963370873	0.095	0.22	8.1908497777865E-90	T/NK cell	ELP2
7.0864965875194E-94	0.73150030906033	0.162	0.335	1.15587845839029E-89	T/NK cell	HNRNPF
9.03643331861681E-94	0.883028075312055	0.075	0.187	1.47393263859959E-89	T/NK cell	KMT5B

1.07200870628403E-93	0.850409338180095	0.078	0.19	1.74855340081988E-89	T/NK cell	PMPCB
1.25562703623497E-93	0.538018726779534	0.203	0.404	2.04805325880285E-89	T/NK cell	HSPA8
1.53655890098945E-93	0.659333612996182	0.056	0.153	2.50628122340389E-89	T/NK cell	PTBP1
1.85487796746871E-93	0.63762808206988	0.036	0.118	3.02549145273822E-89	T/NK cell	DLST
2.2705771681625E-93	0.779018574855266	0.061	0.161	3.70353841898985E-89	T/NK cell	IPO9
2.67159331384644E-93	0.344247395135007	0.034	0.114	4.35763585421493E-89	T/NK cell	DTNBP1
3.02713926401652E-93	0.797363309789336	0.044	0.132	4.93756685353734E-89	T/NK cell	NUDT5
3.39023329756376E-93	0.873448570227825	0.075	0.185	5.52980953165625E-89	T/NK cell	STX5
3.63847244782479E-93	0.692096728680849	0.076	0.187	5.93471240964702E-89	T/NK cell	UBR5
3.83656350917282E-93	0.702792981491678	0.091	0.212	6.25781873981178E-89	T/NK cell	TCERG1
5.00470842313868E-93	2.76742651944779	0.102	0.037	8.16317990898151E-89	T/NK cell	IL21R
5.14195939351898E-93	0.553030419431357	0.04	0.126	8.38704996676881E-89	T/NK cell	WWP2
5.380953268634E-93	0.982220082921622	0.093	0.216	8.77687287646892E-89	T/NK cell	SRP54
5.78596766067829E-93	0.615582284225485	0.045	0.134	9.43749185133236E-89	T/NK cell	BTBD2
6.91674048284265E-93	0.453190192241696	0.026	0.1	1.12818954015647E-88	T/NK cell	CHAMP1
8.69422533721007E-93	0.605941907648438	0.034	0.115	1.41811509475233E-88	T/NK cell	MARCH8
1.34086820566785E-92	0.408424053368815	0.027	0.102	2.18709013026484E-88	T/NK cell	TIMM23
1.46760482122573E-92	0.250066787332615	0.03	0.108	2.39381022390129E-88	T/NK cell	YIF1B
1.92058764660573E-92	0.535004686148273	0.12	0.258	3.13267051037861E-88	T/NK cell	OBSCN
2.45467874005551E-92	0.64944796777411	0.028	0.103	4.00382649290454E-88	T/NK cell	NDUFA5
2.50864090874199E-92	0.64196845264642	0.044	0.132	4.09184418624906E-88	T/NK cell	RAB5B
2.73205771211319E-92	0.720145202445742	0.17	0.346	4.45625933422782E-88	T/NK cell	SRSF4
2.79928257329885E-92	0.307383089290192	0.03	0.106	4.56590980530776E-88	T/NK cell	SPPL3
2.81445119545642E-92	0.662443273613928	0.042	0.128	4.59065134490897E-88	T/NK cell	PSKH1
2.84162976546344E-92	0.597180930419692	0.044	0.132	4.63498231044741E-88	T/NK cell	PIGQ
3.01020156395171E-92	0.396086545921648	0.031	0.108	4.90993977096164E-88	T/NK cell	MIF4GD
3.39383170246029E-92	0.89594423247439	0.062	0.163	5.53567888988297E-88	T/NK cell	TXNDC15
3.71901891209508E-92	0.756246290887285	0.039	0.122	6.06609174751828E-88	T/NK cell	OARD1
4.28960701536178E-92	2.12336480748103	0.199	0.117	6.9967780027566E-88	T/NK cell	FXYD5
4.95873676285023E-92	0.50539074802671	0.027	0.101	8.08819553388501E-88	T/NK cell	CBWD3
5.46890135331319E-92	0.606701666637608	0.037	0.118	8.92032499738915E-88	T/NK cell	TSR2
7.06118139713914E-92	0.517567361025558	0.072	0.178	1.15174929768736E-87	T/NK cell	STRN4
7.67431400774143E-92	0.702065687281981	0.038	0.121	1.2517573578027E-87	T/NK cell	DNTTIP2
8.28890993800391E-92	0.316755761007297	0.026	0.1	1.35200409998782E-87	T/NK cell	EIF2B3
9.13561082020827E-92	0.391131300416425	0.028	0.102	1.49010948088417E-87	T/NK cell	NBPF12
1.17893786423438E-91	0.533544981031672	0.03	0.108	1.9229655503527E-87	T/NK cell	OSTM1
1.35341934385475E-91	0.749718701420848	0.051	0.144	2.20756229176148E-87	T/NK cell	KDM4A
1.36105196785494E-91	0.311826121874026	0.028	0.103	2.22001186476819E-87	T/NK cell	SEH1L
1.56308814988133E-91	0.943512541545248	0.099	0.225	2.54955308127143E-87	T/NK cell	NRBP1
2.08434519416191E-91	0.532335733626489	0.066	0.168	3.39977544619749E-87	T/NK cell	RUFY1
2.64089132297221E-91	0.565563223817695	0.037	0.119	4.30755783689997E-87	T/NK cell	MAD2L2
3.33277928549688E-91	0.87967725780546	0.097	0.221	5.43609629257395E-87	T/NK cell	SEC16A
3.34311293386141E-91	0.62915406025938	0.039	0.121	5.45295150642135E-87	T/NK cell	RNF123
3.51438505272367E-91	0.759264131954293	0.091	0.21	5.73231345949757E-87	T/NK cell	RAB14
3.68412702706389E-91	0.861054099046583	0.083	0.198	6.00917959384391E-87	T/NK cell	UBE4B
3.6892588007966E-91	0.672705401029941	0.119	0.257	6.01755002997933E-87	T/NK cell	PPP1R12C
3.89286179997737E-91	0.826048055430545	0.069	0.173	6.34964688194309E-87	T/NK cell	HSD17B11
3.9020324382204E-91	0.957687320998176	0.066	0.17	6.3646051099813E-87	T/NK cell	FAM3C
4.56439166428979E-91	0.900052847968034	0.072	0.178	7.44497924362307E-87	T/NK cell	LRP10
4.81532962681513E-91	0.622544387182292	0.057	0.152	7.85428415429815E-87	T/NK cell	RABEP1
5.218173891143E-91	0.791918358573236	0.079	0.191	8.51136343384336E-87	T/NK cell	PARP4
5.45643190043179E-91	0.91202022962342	0.129	0.275	8.89998607279429E-87	T/NK cell	PARP10

6.56066554280093E-91	0.666393977030523	0.048	0.137	1.07011015668626E-86	T/NK cell	TRMT2A
6.6687485354666E-91	0.535733428570841	0.03	0.106	1.08773957361996E-86	T/NK cell	TMEM50B
7.0707232818024E-91	0.594021502238267	0.037	0.118	1.15330567449479E-86	T/NK cell	PSMD9
8.03803193306892E-91	0.630049177928278	0.068	0.171	1.31108338860287E-86	T/NK cell	RBM17
8.59459555762288E-91	0.385546365562957	0.036	0.117	1.40186448140387E-86	T/NK cell	CHMP3
9.00913552135446E-91	0.850994791282639	0.061	0.159	1.46948009488813E-86	T/NK cell	COPS5
1.09491481770174E-90	0.616264003641848	0.033	0.111	1.78591555915331E-86	T/NK cell	DEPRECATI
1.22775276687921E-90	0.659650925034085	0.032	0.11	2.00258753805668E-86	T/NK cell	POLRMT
1.36373201055762E-90	0.558686399595278	0.044	0.131	2.22438328242054E-86	T/NK cell	ANKRD13A
1.56644591103556E-90	0.891174347959791	0.085	0.201	2.5550299254901E-86	T/NK cell	VPS4A
1.66512632407593E-90	0.714154412933236	0.071	0.177	2.71598754720025E-86	T/NK cell	PFDN5
1.76187782371014E-90	0.603000551593403	0.029	0.104	2.87379891825361E-86	T/NK cell	NSMCE1
1.97949288582041E-90	0.497377896541047	0.043	0.128	3.22875084606167E-86	T/NK cell	SNX13
2.24655613321813E-90	0.909012077764008	0.056	0.151	3.66435770889209E-86	T/NK cell	GBF1
2.29745724926184E-90	0.507514216354147	0.028	0.102	3.74738251927098E-86	T/NK cell	ACTR10
2.36948693779716E-90	0.816735887079617	0.058	0.155	3.86487014424095E-86	T/NK cell	UGGT1
3.03685270148896E-90	0.888030919248374	0.048	0.138	4.95341044139865E-86	T/NK cell	COX11
3.61049695371611E-90	0.649065143709208	0.07	0.173	5.88908158120635E-86	T/NK cell	PRRC2A
3.76989838371786E-90	0.749757910807081	0.053	0.146	6.1490812536822E-86	T/NK cell	GID8
3.94573403392216E-90	2.22837665774925	0.245	0.166	6.43588678273044E-86	T/NK cell	HAVCR2
4.63294676462728E-90	0.257929616479613	0.036	0.115	7.55679946778355E-86	T/NK cell	RSU1
5.36390138708179E-90	0.856358930562836	0.054	0.147	8.7490595524691E-86	T/NK cell	WBP1
5.65350781654987E-90	0.71625313618091	0.074	0.182	9.22143659957449E-86	T/NK cell	MAPK8IP3
5.81925753883984E-90	0.583172120344107	0.036	0.116	9.49179097160166E-86	T/NK cell	EMC8
7.80161708413633E-90	0.55452355754682	0.052	0.144	1.27252176259348E-85	T/NK cell	DDX3X
8.78230234653642E-90	0.349585991337794	0.062	0.16	1.43248133574356E-85	T/NK cell	CREBL2
1.07072716621851E-89	0.801919631743455	0.165	0.336	1.74646308081901E-85	T/NK cell	SNRNP70
1.55364200253298E-89	0.785694652125116	0.087	0.202	2.53414547033155E-85	T/NK cell	UBE2I
1.60737959138023E-89	0.805299065323662	0.157	0.322	2.62179685150029E-85	T/NK cell	FBXO7
1.97940726426765E-89	0.853081092596593	0.069	0.172	3.22861118874696E-85	T/NK cell	ERO1B
2.23591035293085E-89	0.825946033860788	0.089	0.205	3.64699337666551E-85	T/NK cell	RBM23
2.41638863322964E-89	0.373992607033887	0.041	0.124	3.94137149966087E-85	T/NK cell	H3F3B
2.92623602083308E-89	0.921351730433987	0.071	0.176	4.77298357358083E-85	T/NK cell	BRF1
2.96460183715417E-89	0.729706739766309	0.041	0.125	4.83556205658217E-85	T/NK cell	NOSIP
3.01446634822324E-89	0.668408162841558	0.082	0.194	4.91689606058692E-85	T/NK cell	RTF2
3.82042628162957E-89	0.927672477720972	0.075	0.182	6.231497307966E-85	T/NK cell	ZFYVE27
3.93487245342546E-89	0.477793424990619	0.028	0.101	6.41817045878227E-85	T/NK cell	ELOF1
4.02534479247912E-89	0.671434983579868	0.034	0.113	6.56573989101269E-85	T/NK cell	C6orf89
4.77982318067609E-89	0.617734336417071	0.036	0.115	7.79636959000077E-85	T/NK cell	FBXO38
6.07711730354297E-89	0.674362791204962	0.03	0.105	9.91238603380895E-85	T/NK cell	FBXL4
6.62480627880727E-89	0.566134946365782	0.039	0.12	1.08057215213625E-84	T/NK cell	FAM199X
6.97303929462165E-89	0.487156275671008	0.036	0.115	1.13737243934574E-84	T/NK cell	WDR55
7.17425572427407E-89	0.696009639828155	0.054	0.147	1.17019285118634E-84	T/NK cell	RPRD1A
8.21229028368035E-89	0.939968877630175	0.117	0.253	1.3395066681711E-84	T/NK cell	STAU2
8.69783282075276E-89	0.488894181544176	0.044	0.129	1.41870351139298E-84	T/NK cell	COMMD10
9.31201022512963E-89	0.379223300047971	0.046	0.133	1.5188819878209E-84	T/NK cell	NAA38
1.11504170808261E-88	0.830242874400187	0.054	0.147	1.81874453005354E-84	T/NK cell	RNF126
1.17875056470256E-88	0.800288109341862	0.048	0.136	1.92266004608635E-84	T/NK cell	GTPBP10
1.28740955999772E-88	0.636386759144401	0.073	0.179	2.09989373331228E-84	T/NK cell	TRA2B
1.89108648150578E-88	0.38841361022028	0.08	0.189	3.08455115998408E-84	T/NK cell	GNAI2
2.07028757997578E-88	1.29849677273791	0.087	0.203	3.37684607169849E-84	T/NK cell	PATJ
2.23997244589104E-88	0.403734927947812	0.036	0.115	3.65361905649287E-84	T/NK cell	NHLRC3

2.81561945552062E-88	0.756232502514963	0.044	0.129	4.59255689389968E-84	T/NK cell	DDX1
2.86138074860999E-88	0.760459639012515	0.197	0.392	4.66719813905776E-84	T/NK cell	TMEM259
2.87551618929801E-88	0.683099570656069	0.069	0.171	4.69025445636398E-84	T/NK cell	NUP85
2.9506719880878E-88	0.951236614633088	0.083	0.195	4.81284107977002E-84	T/NK cell	TAF11
3.06644993163157E-88	0.656960345474257	0.13	0.273	5.00168648348426E-84	T/NK cell	PABPC1L
3.0683699569488E-88	0.56995426072004	0.033	0.11	5.00481823677918E-84	T/NK cell	TBC1D23
3.2081644174857E-88	0.863777730496054	0.072	0.178	5.23283698136092E-84	T/NK cell	MON2
3.692679975552E-88	0.744144173943305	0.05	0.14	6.02313030812286E-84	T/NK cell	DCTD
3.97151513534699E-88	0.940732912968397	0.091	0.209	6.47793833726448E-84	T/NK cell	ARIH2
3.99932767247637E-88	0.671667692037229	0.033	0.11	6.5233033665762E-84	T/NK cell	POLL
4.27522931954314E-88	0.600710731268267	0.03	0.105	6.97332654310681E-84	T/NK cell	HIRA
4.77213958365008E-88	0.502744774672842	0.041	0.124	7.78383687489165E-84	T/NK cell	ABCD4
5.0627832646399E-88	0.594151707916102	0.035	0.113	8.25790578295415E-84	T/NK cell	ERLEC1
6.31660189381268E-88	0.301328121264189	0.041	0.123	1.03030093489979E-83	T/NK cell	RABEP2
6.86701134126374E-88	0.713100555244489	0.057	0.151	1.12007821987353E-83	T/NK cell	PSEN1
7.53205849384671E-88	0.997595009219173	0.086	0.199	1.22855406093134E-83	T/NK cell	RGS14
8.741907618384E-88	0.795534421299733	0.033	0.11	1.42589255163461E-83	T/NK cell	ALG9
8.94003385380053E-88	0.572848074992205	0.055	0.148	1.4582089218934E-83	T/NK cell	DDX21
1.01233011139128E-87	0.654336014263755	0.042	0.125	1.65121164469032E-83	T/NK cell	CHMP6
1.093184450191E-87	0.573278618373278	0.037	0.116	1.78309315670654E-83	T/NK cell	FNTA
1.16917082310585E-87	0.707032922000305	0.043	0.127	1.90703452956795E-83	T/NK cell	OCIAD1
1.21124000784634E-87	0.743719007305274	0.071	0.175	1.97565357679816E-83	T/NK cell	REV1
1.21907179853531E-87	0.60982603706323	0.071	0.174	1.98842801059095E-83	T/NK cell	SYAP1
1.37184736353531E-87	0.777816141763318	0.039	0.121	2.23762023466244E-83	T/NK cell	RO60
1.37403444491876E-87	0.786749185106788	0.066	0.167	2.24118758310699E-83	T/NK cell	C4orf3
1.46008777674991E-87	0.834871894124348	0.079	0.188	2.38154917265678E-83	T/NK cell	SCAF11
2.18285631390632E-87	0.796951080860837	0.101	0.225	3.56045693361259E-83	T/NK cell	ISCU
2.18357529030254E-87	0.575551522140394	0.037	0.116	3.56162965601247E-83	T/NK cell	PRKAB1
2.67389688914335E-87	0.447793269777263	0.034	0.112	4.36139321588171E-83	T/NK cell	COMMD5
2.91082197334771E-87	0.988775094353437	0.089	0.204	4.74784172072746E-83	T/NK cell	LIX1L
3.14643785456398E-87	0.730669504592926	0.039	0.12	5.13215478457931E-83	T/NK cell	GTF2H2C
3.36388965886884E-87	0.617997924100964	0.031	0.106	5.48684042258096E-83	T/NK cell	BCCIP
5.11773153525704E-87	0.69499233155353	0.05	0.139	8.34753190715776E-83	T/NK cell	PHF23
5.55698560458958E-87	1.03015988143956	0.066	0.165	9.06399921964606E-83	T/NK cell	POLR3GL
5.59363636237968E-87	0.805206135188752	0.068	0.169	9.1237802706775E-83	T/NK cell	VPS39
6.77323325427668E-87	0.704320297052271	0.046	0.131	1.10478207610507E-82	T/NK cell	ZNF706
7.56030760565421E-87	0.717670488064949	0.128	0.268	1.23316177355826E-82	T/NK cell	FUS
7.57316037817602E-87	0.786960200796916	0.056	0.148	1.23525818928429E-82	T/NK cell	RNF31
8.61061294181449E-87	0.426389118847672	0.031	0.105	1.40447707693936E-82	T/NK cell	PSMA4
9.22155638667758E-87	0.734797684206023	0.038	0.118	1.50412806223098E-82	T/NK cell	CCDC14
9.78450211574051E-87	0.795831930043078	0.108	0.235	1.59595014009843E-82	T/NK cell	CIC
1.31253812046686E-86	0.791336994155736	0.071	0.174	2.14088092829349E-82	T/NK cell	ECPAS
1.44876108419362E-86	0.776744457783531	0.054	0.144	2.36307420442822E-82	T/NK cell	RBM15B
1.47689846582457E-86	0.46665000847029	0.031	0.105	2.40896908760646E-82	T/NK cell	CCDC6
1.69027095654963E-86	0.749777836914426	0.043	0.126	2.7570009572281E-82	T/NK cell	SAP30L
1.72288663014214E-86	3.10561869410625	0.134	0.063	2.81020038242485E-82	T/NK cell	HELB
1.99160568896588E-86	0.780321256209181	0.051	0.14	3.24850803927225E-82	T/NK cell	RAB5A
2.35985049229122E-86	0.727379132905683	0.034	0.11	3.8491521379762E-82	T/NK cell	TAF4
2.44514596432741E-86	0.786610619959476	0.135	0.28	3.98827758241443E-82	T/NK cell	SYMPK
2.52939175917133E-86	0.503410694128986	0.029	0.102	4.12569089838435E-82	T/NK cell	UBE2E3
2.77144191468857E-86	0.80775179344911	0.048	0.135	4.52049890704852E-82	T/NK cell	E4F1
2.86991356772828E-86	0.735386199737992	0.034	0.111	4.68111602032159E-82	T/NK cell	DENND1B

2.9267993492579E-86	0.932358773166032	0.115	0.248	4.77390241857456E-82	T/NK cell	DEPRECATI
3.78634654498722E-86	0.398246181967335	0.028	0.101	6.17590984952866E-82	T/NK cell	TSNARE1
4.70467563761605E-86	1.01439047648925	0.13	0.274	7.67379643251554E-82	T/NK cell	MARCH6
5.67402901325697E-86	0.807499269078056	0.087	0.201	9.25490872352345E-82	T/NK cell	PRPF40A
6.19616708314778E-86	0.711474864383458	0.053	0.143	1.01065681293223E-81	T/NK cell	TAOK3
6.2956361064639E-86	0.814776460645415	0.053	0.144	1.02688120532533E-81	T/NK cell	KIDINS220
6.48252209961563E-86	0.785062782110464	0.049	0.137	1.05736417966831E-81	T/NK cell	HEXD
7.38790536377428E-86	0.577591410481581	0.033	0.109	1.20504124388522E-81	T/NK cell	NBPF15
7.98634264011313E-86	0.945414574328153	0.07	0.172	1.30265234802885E-81	T/NK cell	TRIM41
7.98849540893428E-86	0.641964248321596	0.029	0.101	1.30300348615127E-81	T/NK cell	LNPK
8.16010500057041E-86	0.677724365070256	0.041	0.123	1.33099472664304E-81	T/NK cell	IMMT
9.15788671516436E-86	0.748223715840333	0.048	0.135	1.49374290211046E-81	T/NK cell	MMS19
9.21139705254621E-86	0.487881055541467	0.043	0.125	1.50247097324081E-81	T/NK cell	LARP4B
1.07571636558207E-85	0.75173315364817	0.087	0.199	1.75460096390092E-81	T/NK cell	YTHDC2
1.21118047914085E-85	0.70352572513934	0.031	0.105	1.97555647952663E-81	T/NK cell	ARL 16.00
1.22664030616243E-85	0.793752561461171	0.034	0.11	2.00077300338155E-81	T/NK cell	NGRN
1.42858265974381E-85	0.539758134029658	0.038	0.117	2.33016117630813E-81	T/NK cell	CHMP1A
1.5147577980373E-85	0.538563323960589	0.037	0.115	2.47072144437863E-81	T/NK cell	HIGD2A
1.84229273109913E-85	0.812026873667243	0.072	0.174	3.00496367369579E-81	T/NK cell	MGAT2
2.26121546302869E-85	0.289113433632619	0.035	0.112	3.6882685417461E-81	T/NK cell	TMEM94
2.50411502449028E-85	0.990065005035894	0.069	0.17	4.0844620164461E-81	T/NK cell	INO80B
4.02625142248095E-85	0.506379660700376	0.085	0.195	6.56721869520867E-81	T/NK cell	ACOT9
4.39833767881553E-85	0.987550670819193	0.028	0.1	7.17412858791602E-81	T/NK cell	AQP3
4.78450879869206E-85	0.759352416851618	0.05	0.138	7.80401230154662E-81	T/NK cell	ARFRP1
6.4780528607963E-85	0.910692931320075	0.071	0.173	1.05663520212448E-80	T/NK cell	CDC16
7.842828645345E-85	0.903898839076782	0.101	0.223	1.27924378034222E-80	T/NK cell	NDUFA10
9.31277411501414E-85	0.44132089016416	0.031	0.104	1.51900658589996E-80	T/NK cell	MKRN1
9.73346259938251E-85	0.69505190846151	0.052	0.141	1.58762508458528E-80	T/NK cell	DEPRECATI
9.96020479227361E-85	0.826699351784497	0.058	0.15	1.62460900366775E-80	T/NK cell	NARF
1.02270158670197E-84	0.707594114615917	0.052	0.14	1.66812855806959E-80	T/NK cell	BCL7B
1.15494930013233E-84	0.76945045902439	0.188	0.371	1.88383780344584E-80	T/NK cell	BRD2
1.7031465344015E-84	0.645130281960307	0.057	0.15	2.77800231226229E-80	T/NK cell	ELL
1.87326472405341E-84	0.425897944574365	0.054	0.143	3.05548209140352E-80	T/NK cell	NLK
1.90401076412965E-84	0.572628285500076	0.03	0.103	3.10563195737188E-80	T/NK cell	SUDS3
2.00901536287992E-84	0.505212391621851	0.042	0.123	3.27690495839343E-80	T/NK cell	HDAC8
2.37701132171535E-84	0.272009901692518	0.039	0.117	3.87714316684991E-80	T/NK cell	ATF7
2.3900758742784E-84	0.572009885387069	0.047	0.132	3.8984527585355E-80	T/NK cell	PIP4P1
2.58661453203284E-84	0.55947799105994	0.031	0.103	4.21902696319877E-80	T/NK cell	DNAJC2
3.01786619346815E-84	0.637047637191377	0.052	0.141	4.9224415481659E-80	T/NK cell	L3HYPDH
3.59448442501774E-84	0.64079058265446	0.05	0.137	5.86296354564643E-80	T/NK cell	MCM3AP
4.14762332236283E-84	0.963540904166411	0.087	0.199	6.76518840110602E-80	T/NK cell	NONO
5.05288341443616E-84	0.392148115281215	0.029	0.1	8.24175813728682E-80	T/NK cell	COMMD1
5.35618996580804E-84	1.16364139962661	0.05	0.137	8.7364814532295E-80	T/NK cell	RDH10
7.52028184854586E-84	0.721308471374299	0.041	0.121	1.22663317231632E-79	T/NK cell	NFYC
7.79001080105603E-84	0.425446581421251	0.044	0.126	1.27062866176025E-79	T/NK cell	VPS8
7.90159256080327E-84	0.621378944643483	0.039	0.118	1.28882876259262E-79	T/NK cell	EHMT2
1.05046705560816E-83	0.628771984949513	0.031	0.103	1.71341681440247E-79	T/NK cell	MZT2A
1.31598180813439E-83	0.689760060899207	0.061	0.154	2.146497927248E-79	T/NK cell	AKAP8
1.47447140192116E-83	3.39958982488696	0.116	0.051	2.4050103036736E-79	T/NK cell	KLF12
1.66980986481853E-83	0.8695715636502	0.047	0.131	2.72362687050551E-79	T/NK cell	NUDT16L1
1.7262872348795E-83	0.77198998063038	0.065	0.162	2.81574710881196E-79	T/NK cell	PHF14
1.83251655925234E-83	0.670554633405426	0.058	0.15	2.9890177597965E-79	T/NK cell	HNRNPM

2.29927705572086E-83	0.822416368064861	0.115	0.246	3.75035080558629E-79	T/NK cell	ITSN2
2.34099923587995E-83	0.479883331926165	0.033	0.108	3.81840385364379E-79	T/NK cell	C14orf119
3.34205453554567E-83	0.809592593637727	0.089	0.201	5.45122515292855E-79	T/NK cell	KIF5B
4.71069262928933E-83	0.717352127482668	0.055	0.145	7.68361074763382E-79	T/NK cell	CHD4
4.7360195896407E-83	0.35596431891849	0.263	0.497	7.72492155266294E-79	T/NK cell	APOL6
6.70750744256953E-83	0.65561734845808	0.042	0.122	1.09406153895752E-78	T/NK cell	CCDC93
7.24963636548921E-83	0.919221073245058	0.06	0.152	1.18248818757495E-78	T/NK cell	TOR1AIP1
8.26831337049133E-83	0.776891488766528	0.057	0.148	1.34864459386084E-78	T/NK cell	DHX30
8.43200763030132E-83	0.559212372862969	0.04	0.119	1.37534476457845E-78	T/NK cell	TMEM185E
9.61776947625181E-83	0.661363800543464	0.045	0.128	1.56875437927143E-78	T/NK cell	AIMP1
1.24283448139143E-82	1.95512470363463	0.198	0.121	2.02718732259756E-78	T/NK cell	IL16
1.2601670592E-82	0.773154504163877	0.071	0.172	2.05545849026113E-78	T/NK cell	ATG4B
1.36625991943774E-82	0.730842350946819	0.053	0.141	2.2285065545949E-78	T/NK cell	MON1B
1.40862057187825E-82	0.777835570016858	0.077	0.181	2.29760101479061E-78	T/NK cell	ZFP91
1.82408480753862E-82	0.969232085864943	0.092	0.206	2.97526472957624E-78	T/NK cell	IK
1.8895151525088E-82	0.854944174141842	0.065	0.16	3.08198816525711E-78	T/NK cell	TMX2
2.41578759612002E-82	0.838003333107409	0.062	0.156	3.94039114803137E-78	T/NK cell	OPA1
2.66059155372862E-82	0.655765356254003	0.073	0.174	4.33969088328676E-78	T/NK cell	E2F4
2.67603253778458E-82	0.997715283075733	0.096	0.213	4.36487667238044E-78	T/NK cell	ITCH
2.8044636631774E-82	0.740116124698701	0.114	0.242	4.57436068100866E-78	T/NK cell	ST3GAL1
3.26520960187983E-82	0.797235449921789	0.073	0.174	5.3258833816262E-78	T/NK cell	HCFC1
3.9972189170347E-82	0.727813826605322	0.031	0.104	6.5198637755753E-78	T/NK cell	SNIP1
4.04235819170202E-82	0.575889613249234	0.047	0.131	6.59349044648517E-78	T/NK cell	MCRIP1
4.27319746968683E-82	2.62519443156492	0.119	0.053	6.9700123928062E-78	T/NK cell	PDE4D
4.74610003747037E-82	0.450017889963614	0.036	0.112	7.74136377111792E-78	T/NK cell	AGO4
5.75658600418921E-82	0.758987429595289	0.055	0.143	9.38956743143301E-78	T/NK cell	GOPC
5.80086451489338E-82	0.429026114893251	0.032	0.104	9.46179011024259E-78	T/NK cell	MLXIP
6.8498116569953E-82	1.03157654638243	0.071	0.171	1.1172727793725E-77	T/NK cell	PAM16
6.98135062939801E-82	0.889709998667465	0.064	0.158	1.13872810116111E-77	T/NK cell	SLC35C2
7.88522671203679E-82	0.647655974019528	0.039	0.116	1.28615932900032E-77	T/NK cell	HDAC3
9.35158278182366E-82	0.543451242777647	0.034	0.108	1.52533666754326E-77	T/NK cell	PITHD1
1.69000538971978E-81	0.731401068548848	0.039	0.116	2.75656779117193E-77	T/NK cell	TRIM28
1.70271120238615E-81	0.817628928046836	0.043	0.123	2.77729224221204E-77	T/NK cell	ADSL
1.88126507364426E-81	0.762433046219575	0.042	0.122	3.06853146162116E-77	T/NK cell	PAPOLA
1.93681981275276E-81	1.00711231823048	0.124	0.26	3.15914679658102E-77	T/NK cell	MOGS
2.48717302906763E-81	0.635555772348626	0.035	0.109	4.0568279277122E-77	T/NK cell	TRIP4
3.34187249854116E-81	0.865695141495971	0.102	0.22	5.45092823237049E-77	T/NK cell	TNIP1
3.3902384359909E-81	0.909226629177074	0.065	0.16	5.52981791294476E-77	T/NK cell	SEC23B
3.71959922914724E-81	0.610975618835915	0.037	0.112	6.06703830266206E-77	T/NK cell	VPS11
4.04670431878206E-81	0.818307359574665	0.071	0.17	6.60057941436543E-77	T/NK cell	MTMR3
4.0749524214785E-81	0.806174886911533	0.055	0.144	6.64665489467359E-77	T/NK cell	ZDHHC14
4.33957723719402E-81	1.01461282683267	0.074	0.175	7.07828443158716E-77	T/NK cell	CLSTN3
4.90055516145451E-81	2.43792726846109	0.188	0.113	7.99329552384845E-77	T/NK cell	LPXN
5.02011126220617E-81	1.2336270684583	0.041	0.119	8.18830347978448E-77	T/NK cell	ABCA5
6.16657819072301E-81	0.556657863283093	0.057	0.147	1.00583056868883E-76	T/NK cell	IFIH1
6.40399461651852E-81	0.862667052608853	0.053	0.141	1.04455556190034E-76	T/NK cell	PCYT1A
7.79501321837365E-81	0.715605713843958	0.053	0.14	1.27144460604893E-76	T/NK cell	ACTR1A
1.0654537183175E-80	0.83839002830369	0.075	0.176	1.73786155994767E-76	T/NK cell	SDR39U1
1.52080003827639E-80	0.72447231880452	0.032	0.103	2.48057694243262E-76	T/NK cell	ZNF839
1.65900962030187E-80	0.622484542781874	0.031	0.103	2.70601059167437E-76	T/NK cell	FAM168B
1.8246637423274E-80	3.71939624155663	0.124	0.058	2.97620903011022E-76	T/NK cell	PTPN4
1.94896359032557E-80	2.93563422014097	0.189	0.117	3.17895451218004E-76	T/NK cell	LEPROTL1

2.13255625237204E-80	0.426241753930628	0.032	0.103	3.47841250324404E-76	T/NK cell	WASHC5
2.65147921141005E-80	0.672509703773702	0.036	0.11	4.32482774173093E-76	T/NK cell	ADNP2
5.07600685049004E-80	0.864964901165665	0.055	0.142	8.2794747738343E-76	T/NK cell	ARFGAP2
5.13053630997526E-80	0.830907320274364	0.051	0.135	8.36841777520064E-76	T/NK cell	YY1AP1
6.2823451016903E-80	0.769574549875072	0.043	0.122	1.0247133095367E-75	T/NK cell	COX14
9.56439162142068E-80	0.970417555259386	0.069	0.165	1.56004791736993E-75	T/NK cell	UXT
9.61329449584868E-80	0.997997786315421	0.105	0.226	1.56802446521788E-75	T/NK cell	DCAF5
1.12307870611099E-79	0.762283462617017	0.053	0.138	1.83185367753764E-75	T/NK cell	SMDT1
1.17428549620457E-79	0.65056755547217	0.042	0.12	1.91537707285928E-75	T/NK cell	C5orf24
1.3632306184245E-79	0.636406344077514	0.169	0.332	2.2235654617122E-75	T/NK cell	TLN1
1.49815807547336E-79	0.720722666678154	0.048	0.13	2.4436456369046E-75	T/NK cell	DEPRECATI
2.15716820625468E-79	0.820693815337399	0.041	0.119	3.518557061222E-75	T/NK cell	MTIF3
2.16665671255577E-79	0.960850944219706	0.051	0.135	3.53403376384971E-75	T/NK cell	CPSF1
3.59681516270392E-79	0.912637978613636	0.032	0.103	5.86676521188637E-75	T/NK cell	CISH
3.74698529991313E-79	0.823384664393671	0.136	0.276	6.11170772268831E-75	T/NK cell	SUGP2
3.76430026544143E-79	0.58630747657556	0.035	0.108	6.13995016296151E-75	T/NK cell	MX1
3.81793762939246E-79	0.843258807230633	0.095	0.207	6.22743806730204E-75	T/NK cell	HNRNPUL2
5.00227097299164E-79	0.638658037862688	0.036	0.109	8.15920418404666E-75	T/NK cell	SLC37A1
6.5970256410169E-79	2.59224431148163	0.176	0.103	1.07604085230627E-74	T/NK cell	TRAF5
6.71692670079529E-79	0.670134608172868	0.044	0.123	1.09559791416672E-74	T/NK cell	NMI
7.63904984038769E-79	0.793070011150863	0.039	0.115	1.24600541946564E-74	T/NK cell	MED24
8.5783589547571E-79	0.50914387538103	0.056	0.144	1.39921612911043E-74	T/NK cell	MAPKAP1
8.60019361819768E-79	1.10290424359536	0.086	0.193	1.40277758106422E-74	T/NK cell	SEC24C
1.22888280393006E-78	0.917097586979027	0.045	0.124	2.00443074149032E-74	T/NK cell	MDC 1.00
1.87224416611189E-78	0.47588469152985	0.033	0.104	3.0538174593451E-74	T/NK cell	TMEM131
2.04966317993443E-78	0.826929433039794	0.038	0.113	3.34320561279105E-74	T/NK cell	ANKRD27
2.4495843548561E-78	0.597989265720028	0.042	0.12	3.99551704120579E-74	T/NK cell	CLEC16A
2.47499407613488E-78	0.905929097130703	0.064	0.156	4.03696283758361E-74	T/NK cell	RNPC3
2.8535848866816E-78	0.824006428030361	0.054	0.14	4.65448230866637E-74	T/NK cell	RNF7
2.86874753608098E-78	0.823562490353551	0.066	0.159	4.67921410610169E-74	T/NK cell	TRPM7
3.20344573962125E-78	0.968006017478917	0.085	0.191	5.22514034589623E-74	T/NK cell	PPIG
3.67681289725749E-78	0.446438314372313	0.032	0.101	5.99724951671668E-74	T/NK cell	HMGXB4
3.75749547407777E-78	0.828171399243321	0.048	0.129	6.12885086776824E-74	T/NK cell	VPS13D
4.46167736735656E-78	0.452408117147279	0.036	0.109	7.27744195389529E-74	T/NK cell	KHSRP
5.7372088096241E-78	1.01813763795282	0.102	0.219	9.35796128937787E-74	T/NK cell	LMF2
7.11913744416005E-78	2.20321079456989	0.202	0.128	1.16120250851695E-73	T/NK cell	ANKRD44
7.93492809315674E-78	0.293930960082896	0.055	0.14	1.2942661212748E-73	T/NK cell	LIMK1
8.21591600480312E-78	2.14664841388477	0.186	0.113	1.34009805954344E-73	T/NK cell	CLEC2B
8.28860959550792E-78	1.08283647257659	0.075	0.174	1.3519551111233E-73	T/NK cell	SORL1
1.13869743318616E-77	0.465734894190817	0.041	0.116	1.85732938326995E-73	T/NK cell	TCF12
1.35460899464801E-77	0.753288019934878	0.037	0.11	2.20950273117037E-73	T/NK cell	TBC1D22B
1.38454166306663E-77	0.641850399248714	0.063	0.154	2.25832590662798E-73	T/NK cell	HPS1
1.98333145440351E-77	2.51906546838096	0.276	0.219	3.23501193527757E-73	T/NK cell	CBLB
2.56605813439517E-77	0.740127507637535	0.075	0.173	4.18549742301196E-73	T/NK cell	LMBR1L
3.06855380719647E-77	0.932252289193279	0.064	0.155	5.00511811491817E-73	T/NK cell	N4BP2L1
3.29169259867794E-77	2.22877538837674	0.205	0.132	5.36907979770358E-73	T/NK cell	BCL2
5.3887785057942E-77	0.872352299025478	0.075	0.173	8.78963662080091E-73	T/NK cell	AKAP8L
6.53403328666716E-77	0.884325752300423	0.075	0.174	1.06576616938828E-72	T/NK cell	WASHC1
6.64430268477047E-77	0.606167000899362	0.108	0.227	1.08375221091291E-72	T/NK cell	MRTFA
6.70209489911438E-77	0.729546793283773	0.038	0.112	1.09317869899455E-72	T/NK cell	PAK2
7.6784822380797E-77	0.722496742955508	0.045	0.124	1.25243723785318E-72	T/NK cell	WDR70
8.65979679185355E-77	1.06429804497853	0.061	0.15	1.41249945471923E-72	T/NK cell	HINT1

1.0662458254984E-76	0.806087630982792	0.036	0.109	1.73915356597044E-72	T/NK cell	CCDC92
1.09407207516418E-76	0.788271231680813	0.047	0.126	1.7845409618003E-72	T/NK cell	DNAJC8
1.20804965513378E-76	0.803628036416239	0.075	0.172	1.97044979248871E-72	T/NK cell	SETD3
1.51336793001371E-76	0.928738043758036	0.138	0.279	2.46845443064536E-72	T/NK cell	PCED1A
1.5464139650129E-76	0.693569134459939	0.054	0.139	2.52235581833254E-72	T/NK cell	PDPR
1.59845796111834E-76	0.755424750842593	0.061	0.15	2.60724478038012E-72	T/NK cell	CTDNEP1
1.67214661345527E-76	0.812421635629191	0.055	0.139	2.72743834120689E-72	T/NK cell	GALNT1
2.52691460241631E-76	0.845046868070855	0.084	0.187	4.12165040800124E-72	T/NK cell	ARIH1
2.71444077683681E-76	0.995842457863224	0.085	0.189	4.42752435109852E-72	T/NK cell	TBC1D15
2.80239280944549E-76	1.00065929159641	0.079	0.18	4.57098291148653E-72	T/NK cell	CCM2
2.92688742331972E-76	0.900024295710276	0.103	0.219	4.77404607617679E-72	T/NK cell	PBX2
3.17356453972849E-76	0.993303567880678	0.073	0.17	5.17640112075114E-72	T/NK cell	TTC17
3.83134939386694E-76	0.806666503081902	0.055	0.14	6.24931399633636E-72	T/NK cell	FBRSL1
4.43335206691269E-76	0.639687651040192	0.048	0.128	7.23124055634129E-72	T/NK cell	PKN2
4.58428748159197E-76	0.756911827434956	0.037	0.109	7.47743131122467E-72	T/NK cell	VPS51
6.10045215270667E-76	0.77348603181484	0.046	0.124	9.95044750627985E-72	T/NK cell	SMPD4
7.33607120161591E-76	1.03306027443637	0.054	0.138	1.19658657369557E-71	T/NK cell	NCOA2
8.02109595981488E-76	0.73786583317977	0.223	0.426	1.3083209620054E-71	T/NK cell	EMC10
8.96180830751543E-76	0.871380120381862	0.051	0.133	1.46176055303884E-71	T/NK cell	ZNF24
8.97872668312044E-76	0.814035954751998	0.042	0.117	1.46452010928378E-71	T/NK cell	DEAF1
1.1121148810319E-75	0.417811979657854	0.044	0.121	1.81397058245114E-71	T/NK cell	ENGASE
1.11814443544545E-75	0.862769116052787	0.053	0.136	1.82380538865508E-71	T/NK cell	EIF3F
1.29740053510229E-75	0.806335519379399	0.051	0.133	2.11619001280535E-71	T/NK cell	TAF8
1.83708365485531E-75	0.404303369358973	0.037	0.109	2.9964671494345E-71	T/NK cell	TAF1C
1.87332085359825E-75	0.984758311127669	0.038	0.111	3.0555736443041E-71	T/NK cell	COG7
1.91481484316128E-75	0.905274966088539	0.039	0.113	3.12325449068036E-71	T/NK cell	EAPP
1.97182202288369E-75	0.366590248969874	0.037	0.108	3.21623890152559E-71	T/NK cell	MYO9A
2.0503826022089E-75	0.85044821176157	0.052	0.134	3.34437906246294E-71	T/NK cell	UBE2H
2.17326261304305E-75	0.833669441183493	0.043	0.118	3.54480864813451E-71	T/NK cell	TADA2B
2.55358007168139E-75	0.567066139080118	0.051	0.132	4.16514445491952E-71	T/NK cell	ACAP2
3.04830464716133E-75	0.973866820003325	0.091	0.198	4.97208970998485E-71	T/NK cell	PUM2
3.2124713570436E-75	1.01061914305814	0.141	0.281	5.23986203047381E-71	T/NK cell	HBS1L
4.18775271232209E-75	0.723201118467985	0.042	0.117	6.83064344906856E-71	T/NK cell	HERC2
4.74019801213706E-75	0.861468561731342	0.044	0.12	7.73173697759675E-71	T/NK cell	DEPRECATI
5.28337938423677E-75	0.472159942045416	0.041	0.115	8.61772011362859E-71	T/NK cell	SEMA4C
5.3437860260901E-75	0.837014006821628	0.035	0.106	8.71624938715556E-71	T/NK cell	UBE2G1
5.3697552193311E-75	0.761534144731799	0.059	0.145	8.75860773825096E-71	T/NK cell	FOXN3
5.56058757994136E-75	0.929926474670223	0.058	0.143	9.06987440164235E-71	T/NK cell	PATZ1
5.74265002762344E-75	0.860796464530113	0.053	0.135	9.3668364600566E-71	T/NK cell	ZRANB1
7.3712507538403E-75	0.546699960845729	0.039	0.112	1.20232471045889E-70	T/NK cell	AZI2
7.76677475115884E-75	1.09172047200681	0.122	0.25	1.26683862966152E-70	T/NK cell	HBP1
8.62473725212291E-75	0.619459935696382	0.038	0.109	1.40678089319377E-70	T/NK cell	DVL2
9.52689423344281E-75	0.845314634270143	0.14	0.279	1.55393171841686E-70	T/NK cell	CALM3
1.02119998421212E-74	0.851358561000424	0.065	0.156	1.66567929424839E-70	T/NK cell	CDS2
1.25872656799257E-74	0.984650859569965	0.074	0.17	2.05310890505267E-70	T/NK cell	EIF2B5
1.94136400820333E-74	0.937751478433836	0.066	0.157	3.16655883378046E-70	T/NK cell	CDK16
2.41771343290921E-74	0.931501775303714	0.069	0.162	3.94353238041821E-70	T/NK cell	EWSR1
2.41869238838563E-74	0.867574148977055	0.092	0.2	3.94512915469581E-70	T/NK cell	PAN3
2.6224033617254E-74	0.987910249854357	0.124	0.252	4.2774021233103E-70	T/NK cell	ZGPAT
2.69830011797625E-74	0.625202784058358	0.038	0.11	4.40119732243105E-70	T/NK cell	ZNF121
2.77117292083351E-74	0.629674024654207	0.041	0.114	4.52006015117154E-70	T/NK cell	EIF1B
3.2976324306893E-74	1.0394686634213	0.072	0.167	5.37876825769732E-70	T/NK cell	MBTPS1

3.8628374568078E-74	0.454008940405234	0.033	0.101	6.3006741757992E-70	T/NK cell	TBC1D5
4.15489110890348E-74	0.736619863005821	0.049	0.127	6.77704288773247E-70	T/NK cell	RETREG2
4.60464779030897E-74	1.90466367710997	0.367	0.354	7.51064101077296E-70	T/NK cell	OGA
4.69250199446956E-74	0.457214092938444	0.055	0.138	7.6539400031793E-70	T/NK cell	ADPGK
4.72145733802483E-74	0.842815505171059	0.056	0.141	7.70116906405231E-70	T/NK cell	AVL9
5.43348914119682E-74	1.13932287580741	0.062	0.15	8.86256413820613E-70	T/NK cell	LANCL1
6.78242215748568E-74	1.05597707071247	0.098	0.208	1.10628087810749E-69	T/NK cell	CDC73
7.64773150127524E-74	0.816047145882504	0.041	0.115	1.247421485173E-69	T/NK cell	SLC9A8
8.6255571308239E-74	0.893325572193041	0.06	0.146	1.40691462360869E-69	T/NK cell	TBC1D20
9.7206393205746E-74	0.811748522405312	0.043	0.119	1.58553347957892E-69	T/NK cell	MFN1
1.15013515721387E-73	0.453209690598912	0.035	0.104	1.87598545493154E-69	T/NK cell	DUS3L
1.28671695971783E-73	0.961516491876916	0.088	0.192	2.09876403299575E-69	T/NK cell	CIAO1
1.98546903615647E-73	1.01548278478562	0.123	0.25	3.23849854487482E-69	T/NK cell	PUM1
2.15427453216816E-73	0.768846885286703	0.096	0.204	3.51383718941949E-69	T/NK cell	DCAF7
2.61155849212447E-73	0.931584697048666	0.051	0.132	4.25971305650423E-69	T/NK cell	EXOSC7
2.85323674186728E-73	0.958313382955036	0.086	0.188	4.65391444965971E-69	T/NK cell	ACSL5
3.11182577309398E-73	0.725676893535666	0.047	0.124	5.07569901849359E-69	T/NK cell	SS18
3.87358871969713E-73	0.403149745214997	0.515	0.404	6.31821056069799E-69	T/NK cell	SRGN
4.03456393284247E-73	0.60089041321157	0.039	0.111	6.58077723085936E-69	T/NK cell	UTP6
4.57650819744708E-73	0.757929293774524	0.076	0.172	7.46474252085594E-69	T/NK cell	USP4
5.33501956647394E-73	0.949454756862731	0.121	0.246	8.70195041487564E-69	T/NK cell	SENP6
8.92535449940657E-73	0.878535152378827	0.1	0.21	1.45581457239821E-68	T/NK cell	TMEM50A
8.92690273813989E-73	0.660400617751165	0.057	0.139	1.456067105618E-68	T/NK cell	DOT1L
1.22817758101655E-72	0.592673226763086	0.033	0.1	2.00328045239609E-68	T/NK cell	SLF2
1.24668793097731E-72	0.744130219834301	0.039	0.11	2.0334726842171E-68	T/NK cell	CUL5
1.28312823805878E-72	0.888382756961656	0.134	0.267	2.09291046909767E-68	T/NK cell	EEF1G
1.30115783530404E-72	0.668942749049359	0.037	0.108	2.12231854516442E-68	T/NK cell	EIF2B1
1.3071671199998E-72	0.590452262584193	0.034	0.103	2.13212028943167E-68	T/NK cell	CDYL
1.32769871512767E-72	0.945264275609205	0.043	0.117	2.16560937424475E-68	T/NK cell	ATG13
2.35637107240545E-72	0.82599342190514	0.075	0.169	3.84347685620053E-68	T/NK cell	LCOR
2.42359496717549E-72	0.758411351570765	0.039	0.111	3.95312575095995E-68	T/NK cell	TMEM68
3.15070653977121E-72	0.909799036890915	0.051	0.13	5.13911743702082E-68	T/NK cell	SSU72
3.60890390371436E-72	0.462544021651352	0.033	0.1	5.8864831573485E-68	T/NK cell	TMEM160
4.11421043728068E-72	0.730494320479699	0.04	0.112	6.71068864424852E-68	T/NK cell	WDR45B
4.27667544889519E-72	0.940963496772763	0.036	0.105	6.97568532469294E-68	T/NK cell	RSPRY1
4.73818644676933E-72	2.65996984700872	0.127	0.064	7.72845591332546E-68	T/NK cell	SELPLG
4.85496821497484E-72	0.845010122335051	0.036	0.105	7.91893865544546E-68	T/NK cell	MBD1
6.92108776245204E-72	0.650934651753611	0.136	0.268	1.12889862493355E-67	T/NK cell	M6PR
1.08099281226746E-71	0.893971859574366	0.06	0.145	1.76320737608946E-67	T/NK cell	SFPQ
1.28670162829879E-71	1.03563199517988	0.09	0.193	2.09873902591815E-67	T/NK cell	TLE5
1.53550689926561E-71	0.680653950106396	0.037	0.106	2.50456530339214E-67	T/NK cell	TRIM21
1.8376926496224E-71	1.0465656658123	0.103	0.215	2.9974604807991E-67	T/NK cell	SMAD2
2.60959981967174E-71	1.05193030098681	0.052	0.132	4.25651826586658E-67	T/NK cell	KIFC2
3.99530808837955E-71	0.973547343934631	0.056	0.138	6.51674702295589E-67	T/NK cell	TTC7A
4.3519975791062E-71	0.927946662011621	0.103	0.214	7.09854325128013E-67	T/NK cell	STK11
4.76136704990453E-71	1.00558563796145	0.138	0.273	7.76626579509928E-67	T/NK cell	ZNRF2
4.83445885160429E-71	0.583243545621212	0.048	0.125	7.88548583285175E-67	T/NK cell	WASF2
5.58972535790089E-71	1.03432065615908	0.092	0.197	9.11740103127214E-67	T/NK cell	PLA2G6
1.017752181372E-70	2.71219572961721	0.146	0.08	1.66005558303586E-66	T/NK cell	RNF166
1.04539825074834E-70	1.06933387202545	0.05	0.127	1.70514908679561E-66	T/NK cell	DMAP1
1.54722726234945E-70	0.893192290257143	0.073	0.166	2.52368238761819E-66	T/NK cell	GALNS
2.32015625060438E-70	0.767569103056705	0.066	0.153	3.7844068603608E-66	T/NK cell	ANKFY1

2.510140823644E-70	0.815594832884765	0.047	0.122	4.09429069744573E-66	T/NK cell	MBTD1
2.66468014111849E-70	2.47657989297248	0.1	0.043	4.34635977817836E-66	T/NK cell	SYNE1
2.98074135455754E-70	0.931672925195378	0.054	0.134	4.86188722341881E-66	T/NK cell	HIP1R
3.06644928356078E-70	0.435220862120392	0.06	0.144	5.00168542641598E-66	T/NK cell	SH3BGRL
3.49044094166483E-70	1.01016806733358	0.056	0.137	5.6932582199495E-66	T/NK cell	BRD9
4.05621618347212E-70	0.574379420532304	0.109	0.222	6.61609421686137E-66	T/NK cell	IFNAR2
4.92463566015817E-70	0.86904722070516	0.035	0.102	8.032573225284E-66	T/NK cell	ERCC6
5.22072964285138E-70	0.881799585678011	0.059	0.142	8.51553212045489E-66	T/NK cell	C17orf49
5.4174837707556E-70	1.0612472012495	0.106	0.218	8.83645777847945E-66	T/NK cell	MAP2K2
5.52234118721348E-70	0.95245530735073	0.052	0.131	9.00749071046391E-66	T/NK cell	HNRNPL
6.65883874703034E-70	0.929784883963462	0.044	0.117	1.08612318802812E-65	T/NK cell	WDR74
6.69942082762571E-70	0.868209670276213	0.053	0.132	1.09274253119403E-65	T/NK cell	POLM
7.05285833350488E-70	0.892604940617928	0.051	0.129	1.15039172277798E-65	T/NK cell	POLE
7.1274475487636E-70	0.874152585323109	0.059	0.142	1.16255796967883E-65	T/NK cell	CREB3L2
9.26046781486777E-70	0.892342513551006	0.051	0.129	1.51047490528308E-65	T/NK cell	AP1G1
1.22085968498446E-69	0.946433480136702	0.062	0.147	1.99134423217815E-65	T/NK cell	FAM160B2
1.23072722016432E-69	1.13579212175372	0.066	0.153	2.00743916881002E-65	T/NK cell	DNAJC1
1.25051217974301E-69	0.831699038595508	0.041	0.112	2.03971041637883E-65	T/NK cell	VAPB
1.6232424533022E-69	1.28905263026587	0.077	0.171	2.64767076558122E-65	T/NK cell	GALM
1.66861699638957E-69	0.878220283555251	0.055	0.136	2.72168118281103E-65	T/NK cell	ISY1
1.78534205932762E-69	0.634962920960874	0.04	0.11	2.91207143296928E-65	T/NK cell	NUDT4
1.9134379050436E-69	0.911746001749744	0.062	0.147	3.12100856691662E-65	T/NK cell	ARMC8
1.97751118108884E-69	0.845960935304174	0.061	0.145	3.225518487474E-65	T/NK cell	MBP
2.03835195483263E-69	0.997856480579381	0.08	0.175	3.32475587352751E-65	T/NK cell	SMARCA5
2.22752132510627E-69	0.80935174609902	0.035	0.102	3.63331003338084E-65	T/NK cell	EEFSEC
2.55106714222277E-69	1.18869349069894	0.304	0.236	4.16104561567957E-65	T/NK cell	ARL4C
2.59798380193825E-69	0.919231733141099	0.048	0.124	4.23757137934148E-65	T/NK cell	SPOP
3.58794700404319E-69	1.26976264415637	0.076	0.17	5.85230035829485E-65	T/NK cell	HS3ST3B1
3.74061709850865E-69	0.380530264060631	0.046	0.12	6.10132054937746E-65	T/NK cell	TAOK2
4.04779555202601E-69	0.906268730232534	0.084	0.181	6.60235932490963E-65	T/NK cell	MTA1
4.30803917518078E-69	0.841035724398488	0.088	0.189	7.02684269863737E-65	T/NK cell	SGSH
5.20727468272858E-69	0.69171240401006	0.038	0.106	8.49358573499859E-65	T/NK cell	BCAS3
5.38426893382259E-69	0.827820443178435	0.059	0.141	8.78228105795802E-65	T/NK cell	PIGG
5.54547971920852E-69	0.808440278926303	0.036	0.103	9.04523197000101E-65	T/NK cell	CUL9
8.44576015411151E-69	0.737722505727956	0.056	0.136	1.37758793873713E-64	T/NK cell	GNL3L
9.89612058962229E-69	0.817312575740642	0.034	0.1	1.61415622937329E-64	T/NK cell	MYNN
1.01564772557593E-68	0.543725475337584	0.128	0.25	1.65662300518691E-64	T/NK cell	TACC1
1.15650748970445E-68	1.1479003377255	0.064	0.149	1.88637936645692E-64	T/NK cell	SPSB2
1.26743537274039E-68	0.991241392890666	0.095	0.2	2.06731383647685E-64	T/NK cell	RNF40
1.34924541067819E-68	0.473212490676953	0.169	0.317	2.2007541893572E-64	T/NK cell	MYO9B
1.5440372407683E-68	0.91025805010474	0.126	0.25	2.51847914341717E-64	T/NK cell	GDI1
1.91156080602547E-68	1.22000317186634	0.05	0.126	3.11794683070814E-64	T/NK cell	MAN1A2
2.29794218253326E-68	1.05225647157805	0.095	0.2	3.74817349393E-64	T/NK cell	ACTR1B
2.53568486972999E-68	1.06709113953409	0.068	0.156	4.13595559101659E-64	T/NK cell	FBXL17
2.74479082169614E-68	1.00379204811203	0.111	0.226	4.47702830926857E-64	T/NK cell	GIGYF1
2.87188017386948E-68	1.05203014221437	0.075	0.167	4.68432375159851E-64	T/NK cell	C19orf12
3.21534121739406E-68	0.979476451788798	0.109	0.222	5.24454305969145E-64	T/NK cell	HECTD4
4.67326489950929E-68	1.15944965568021	0.103	0.213	7.62256237758961E-64	T/NK cell	GCN1
5.8432864847528E-68	0.700444360132909	0.049	0.124	9.5309845852803E-64	T/NK cell	NUP58
5.85685070751753E-68	0.823109423946008	0.048	0.122	9.55310918903185E-64	T/NK cell	MED14
6.45485494301339E-68	1.00331660680426	0.062	0.145	1.05285138975491E-63	T/NK cell	PHKG2
8.02390804745046E-68	0.918322756542836	0.074	0.165	1.30877964161964E-63	T/NK cell	DHX9

**8.5196872828847E-68**	1.04391365589932	0.123	0.245	1.38964619271132E-63	T/NK cell	NDUFB8
**9.0693664582655E-68**	0.954820841042746	0.104	0.214	1.47930436300768E-63	T/NK cell	TBCD
**9.2821360171226E-68**	0.773720701185209	0.041	0.111	1.51400920575287E-63	T/NK cell	SMC6
**1.08086246714002E-67**	0.897928365035739	0.062	0.145	1.76299477015209E-63	T/NK cell	CLK4
**1.15294813816436E-67**	0.861552917965673	0.04	0.11	1.88057370815989E-63	T/NK cell	OXLD1
**1.53987458979723E-67**	0.954703671234226	0.115	0.231	2.51168944341827E-63	T/NK cell	BAZ2A
**1.77764819289428E-67**	0.456136811463381	0.038	0.106	2.89952196742986E-63	T/NK cell	RAI1
**1.98899994739641E-67**	0.65712852954928	0.04	0.109	3.24425781419829E-63	T/NK cell	GTF2F1
**2.26664632369945E-67**	0.964395514939168	0.095	0.198	3.69712681858617E-63	T/NK cell	CHKB
**2.71898148328317E-67**	0.989751515689569	0.077	0.169	4.43493069738318E-63	T/NK cell	CBX4
**2.73098186480824E-67**	0.801736398964342	0.143	0.276	4.45450451968872E-63	T/NK cell	GGA2
**2.80086109289026E-67**	0.99900625408584	0.489	0.531	4.5684845286133E-63	T/NK cell	ARGLU1
**3.01918737094326E-67**	1.06136942280479	0.098	0.203	4.92459652074556E-63	T/NK cell	GON4L
**3.28833622658903E-67**	0.847691735687575	0.037	0.104	5.36360521918937E-63	T/NK cell	ISG20L2
**6.7581444599012E-67**	0.582295515855854	0.037	0.104	1.10232094285448E-62	T/NK cell	PIK3C2A
**7.0907705482057E-67**	0.494268408945206	0.036	0.102	1.15657558411783E-62	T/NK cell	SP1
**8.3100719991098E-67**	0.793405261329177	0.069	0.156	1.3554558437748E-62	T/NK cell	IL17RA
**8.52897744637795E-67**	0.993262384758079	0.059	0.139	1.39116151127871E-62	T/NK cell	OSBPL2
**8.83103709172525E-67**	0.863778008768413	0.039	0.107	1.44043046003131E-62	T/NK cell	SRP9
**9.18515773836429E-67**	0.565784123532642	0.189	0.345	1.4981910787046E-62	T/NK cell	EFHD2
**9.89119394464761E-67**	0.928836142732098	0.043	0.113	1.61335264431147E-62	T/NK cell	ZMYND11
**1.38583567386023E-66**	0.894467152261603	0.055	0.133	2.26043656763342E-62	T/NK cell	SLC30A7
**1.75941754849713E-66**	0.926797124449903	0.043	0.114	2.86978596335366E-62	T/NK cell	EIF3J
**1.86166158125088E-66**	0.84930357394831	0.174	0.328	3.03655620517831E-62	T/NK cell	LAMTOR4
**2.34556569261098E-66**	0.907952058071292	0.094	0.196	3.82585220121776E-62	T/NK cell	AKAP9
**2.46552268052894E-66**	0.939210099719716	0.073	0.162	4.02151404421075E-62	T/NK cell	KDM3B
**2.63579856334533E-66**	0.673176517766991	0.057	0.136	4.29925103667256E-62	T/NK cell	NGLY1
**2.75639409017597E-66**	2.48330585322254	0.235	0.176	4.49595440048602E-62	T/NK cell	ISG20
**2.75655964761148E-66**	0.722063929636504	0.036	0.101	4.49622444121908E-62	T/NK cell	BCL7C
**2.76150441914885E-66**	2.62951593095269	0.106	0.049	4.50428985807369E-62	T/NK cell	IL12RB1
**2.92217444656331E-66**	0.964749148399039	0.104	0.211	4.76635873978941E-62	T/NK cell	MAPK1
**2.9511098777428E-66**	1.07331266353651	0.086	0.183	4.81355532158627E-62	T/NK cell	PCMTD2
**3.11436536810373E-66**	0.875476341827327	0.067	0.151	5.07984135191399E-62	T/NK cell	TNIP2
**3.68880781071363E-66**	1.02700843987837	0.045	0.116	6.016814420055E-62	T/NK cell	COG4
**3.91592235504431E-66**	0.799874896500166	0.046	0.118	6.38726095331277E-62	T/NK cell	UQCRB
**4.01270048794376E-66**	3.85662410243146	0.104	0.048	6.54511576588506E-62	T/NK cell	TSPOAP1
**4.21254503993262E-66**	0.959399225270518	0.072	0.161	6.8710822146341E-62	T/NK cell	UBE2G2
**4.3926860439534E-66**	0.877752039287343	0.042	0.112	7.16491020629239E-62	T/NK cell	CRKL
**5.84514983364773E-66**	1.10565093778079	0.073	0.163	9.53402389366282E-62	T/NK cell	MAN2B2
**5.93806952802712E-66**	0.557684154345053	0.045	0.116	9.68558520716503E-62	T/NK cell	FAM133B
**7.65305358435862E-66**	0.834343463759589	0.042	0.111	1.24828957014473E-61	T/NK cell	PRPF31
**8.41553918212033E-66**	0.598299253101444	0.046	0.118	1.37265859599565E-61	T/NK cell	NUP62
**9.56987630889934E-66**	0.800948056446419	0.112	0.225	1.56094252474457E-61	T/NK cell	SUSD6
**1.01827559801732E-65**	1.05133753311165	0.073	0.162	1.66090932792604E-61	T/NK cell	R3HDM2
**1.55988290892796E-65**	0.766884198879639	0.043	0.112	2.5443250127524E-61	T/NK cell	PPP1R7
**1.62174574997377E-65**	0.570569899620234	0.093	0.192	2.64522949278222E-61	T/NK cell	PXN
**2.23073154564868E-65**	1.11179162548466	0.094	0.196	3.63854622410757E-61	T/NK cell	HERC4
**2.30591255830589E-65**	1.07400220327999	0.037	0.102	3.76117397385273E-61	T/NK cell	TRAPPC6A
**2.78824895093443E-65**	1.12882648983842	0.054	0.13	4.54791286386915E-61	T/NK cell	ATG2B
**2.84112694963251E-65**	0.732756864195918	0.055	0.131	4.63416216754559E-61	T/NK cell	TNPO2
**2.95471481771478E-65**	0.73342858637322	0.205	0.379	4.81943533917458E-61	T/NK cell	DRAP1
**3.18167084003572E-65**	1.03259640146791	0.109	0.22	5.18962330718226E-61	T/NK cell	PI4KA

3.18847524546999E-65	2.09397394845991	0.248	0.19	5.2007219728861E-61	T/NK cell	SIPA1
3.44686102318594E-65	1.3276658543544	0.076	0.166	5.62217501491858E-61	T/NK cell	ARHGAP35
3.8700262175581E-65	0.877761081856281	0.06	0.141	6.31239976345902E-61	T/NK cell	FOXK1
4.77546060023537E-65	0.720577428644563	0.051	0.125	7.78925378504392E-61	T/NK cell	RAB4B
5.50640827646584E-65	1.07605959554915	0.066	0.15	8.98150253974344E-61	T/NK cell	C2orf68
5.84417145241553E-65	1.12191114935705	0.066	0.15	9.53242805603497E-61	T/NK cell	GMDS
6.66452897814232E-65	0.925820074502642	0.041	0.11	1.08705132162479E-60	T/NK cell	NUP88
7.01352733112645E-65	0.979430644040479	0.086	0.181	1.14397644298004E-60	T/NK cell	DAZAP2
8.69694539890598E-65	1.15927397040883	0.084	0.178	1.41855876401556E-60	T/NK cell	BRWD1
9.3221419284695E-65	0.998527508498908	0.047	0.118	1.52053456995266E-60	T/NK cell	MVD
1.05884089356989E-64	1.04326756551121	0.056	0.134	1.72707538150185E-60	T/NK cell	NSMCE4A
1.11365855741822E-64	0.804422709105834	0.061	0.142	1.81648847300487E-60	T/NK cell	DDX42
1.15673542917965E-64	1.22757863614559	0.048	0.12	1.88675115853493E-60	T/NK cell	AUTS2
1.15768314010052E-64	1.07975277962629	0.078	0.168	1.88829696981795E-60	T/NK cell	UBTF
1.57569391113615E-64	0.84764333845939	0.049	0.121	2.57011433845418E-60	T/NK cell	SECISBP2L
1.60056552674368E-64	1.05279814589223	0.089	0.187	2.61068243067162E-60	T/NK cell	SART1
1.70338603234113E-64	0.997481357455123	0.079	0.17	2.77839295735162E-60	T/NK cell	SZRD1
1.73292021583995E-64	0.827026648880404	0.095	0.195	2.82656616405654E-60	T/NK cell	ARHGDIA
1.87549873268799E-64	0.953820812785418	0.053	0.128	3.05912598288739E-60	T/NK cell	TTC31
2.6790128760257E-64	0.880311867169363	0.048	0.12	4.36973790208552E-60	T/NK cell	TMBIM4
3.13546866307384E-64	0.9101150032695	0.058	0.136	5.11426293633974E-60	T/NK cell	PIP4K2B
3.14322472025623E-64	0.642425528086203	0.041	0.109	5.12691384120994E-60	T/NK cell	ZDHHC3
3.94934047440216E-64	0.763313021519247	0.041	0.109	6.44176924779737E-60	T/NK cell	ATXN1L
4.27900118252351E-64	0.538462598988864	0.042	0.111	6.9794788288141E-60	T/NK cell	SLC25A6
4.35870195264086E-64	0.942890550404442	0.068	0.153	7.10947875495251E-60	T/NK cell	HNRNPK
5.82734702020337E-64	1.10004109396156	0.129	0.251	9.50498572465373E-60	T/NK cell	ORMDL3
6.44931280234133E-64	0.730663888938574	0.049	0.122	1.0519474111899E-59	T/NK cell	GPR107
7.19055573249225E-64	0.771260413831131	0.216	0.397	1.17285154552681E-59	T/NK cell	DPP7
8.30757176433105E-64	0.884456469700607	0.048	0.12	1.35504803048004E-59	T/NK cell	FBXO42
9.11787602185195E-64	2.4520522706986	0.153	0.09	1.48721675792427E-59	T/NK cell	PLCG1
9.24143738776266E-64	1.04223277426807	0.087	0.183	1.50737085231797E-59	T/NK cell	SPG11
1.07829290952598E-63	0.751963588057161	0.207	0.382	1.75880356472783E-59	T/NK cell	LTBP3
1.09969005707982E-63	0.560257308589264	0.04	0.106	1.7937044521029E-59	T/NK cell	PCGF3
1.17349236020841E-63	0.938054066227501	0.065	0.147	1.91408338873594E-59	T/NK cell	CHPF2
1.25876226334736E-63	0.859450598620864	0.22	0.406	2.05316712774588E-59	T/NK cell	TCF25
1.42958150691514E-63	0.71217447250345	0.048	0.12	2.33179039592928E-59	T/NK cell	OXSR1
1.78701314863699E-63	0.988295545484925	0.052	0.126	2.91479714674179E-59	T/NK cell	YIPF4
2.16239283416866E-63	1.04108828879684	0.075	0.162	3.5270789518125E-59	T/NK cell	RETREG3
2.221405575518E-63	0.939067023839229	0.038	0.103	3.62333463422742E-59	T/NK cell	FYTTD1
2.28578432573271E-63	1.76475123742019	0.122	0.061	3.72834281370262E-59	T/NK cell	LY9
3.63312780872162E-63	1.13710731852381	0.092	0.191	5.92599476880584E-59	T/NK cell	CALCOCO1
4.07293868469371E-63	0.779128698526515	0.05	0.121	6.64337028860391E-59	T/NK cell	GTF2H1
4.22570092687817E-63	1.0457350958934	0.082	0.175	6.89254078183098E-59	T/NK cell	DENND4A
4.24349444075193E-63	0.925125525635403	0.067	0.149	6.92156378231047E-59	T/NK cell	RAB1B
5.71785822745341E-63	0.8516622392784	0.043	0.111	9.32639855479926E-59	T/NK cell	BORCS8
6.11865598781918E-63	1.09694818251271	0.058	0.136	9.98013978173186E-59	T/NK cell	NBAS
6.12624455989462E-63	1.0503345868496	0.053	0.128	9.99251750164411E-59	T/NK cell	HMOX2
6.13620558768056E-63	0.69768828083759	0.043	0.111	1.00087649340658E-58	T/NK cell	EPS15L1
8.16513912717444E-63	0.953462187341033	0.056	0.132	1.33181584303342E-58	T/NK cell	BRD7
8.27751026029778E-63	1.04779161110237	0.049	0.12	1.35014469855717E-58	T/NK cell	RHBDD2
9.9045301861021E-63	0.936811826744469	0.055	0.13	1.61552791865511E-58	T/NK cell	ATF2
1.03796849213218E-62	0.981137819300085	0.109	0.217	1.6930304075168E-58	T/NK cell	ZNF638

1.11368497990992E-62	0.859152256500578	0.051	0.124	1.81653157073106E-58	T/NK cell	GABARAPL
1.26748813082046E-62	0.786566218964587	0.046	0.116	2.06739989018125E-58	T/NK cell	FAM193A
1.41468764387179E-62	2.11322851407066	0.134	0.073	2.30749701591928E-58	T/NK cell	HAPLN3
2.02458447744891E-62	1.09702003759355	0.08	0.17	3.30229974116692E-58	T/NK cell	CHURC1
2.10189066723639E-62	1.29318140705966	0.058	0.135	3.42839386732927E-58	T/NK cell	LDLRAP1
2.30004916542539E-62	0.841805501818197	0.1	0.202	3.75161019372535E-58	T/NK cell	TMEM109
2.7255438424762E-62	0.853688842048884	0.038	0.102	4.44563456146293E-58	T/NK cell	THOP1
3.15850273981373E-62	0.835386289144647	0.037	0.1	5.15183381891017E-58	T/NK cell	TOP3B
3.25623524566517E-62	2.79971984963933	0.238	0.185	5.31124530920446E-58	T/NK cell	PIK3R1
3.2698293585468E-62	0.943991190027223	0.051	0.124	5.33341866672568E-58	T/NK cell	KDM3A
3.73041137163E-62	0.938791767495486	0.077	0.164	6.08467398826569E-58	T/NK cell	POLR2J3
4.02180279697187E-62	0.868005379106337	0.116	0.226	6.55996254214082E-58	T/NK cell	FAM120A
4.63438332942925E-62	0.885702387426946	0.168	0.313	7.55914264863206E-58	T/NK cell	PSMB10
5.08261040634963E-62	0.858859070583411	0.052	0.125	8.29024583379689E-58	T/NK cell	CANT1
6.008678340364E-62	0.940571669884068	0.04	0.105	9.80075524096771E-58	T/NK cell	RELA
7.02928040259768E-62	1.17240002729277	0.062	0.141	1.14654592646771E-57	T/NK cell	NUP153
7.1546257807582E-62	1.1240454281701	0.14	0.269	1.16699101109947E-57	T/NK cell	PHF1
7.4289714129648E-62	1.04400137196211	0.079	0.169	1.21173952716869E-57	T/NK cell	UBP1
7.44121664556893E-62	1.09804259751086	0.064	0.144	1.21373684705875E-57	T/NK cell	PELP1
7.9008689883056E-62	0.99059143144111	0.071	0.156	1.28871074068253E-57	T/NK cell	PYCR2
7.93083441702488E-62	0.969014338668481	0.049	0.12	1.29359840176093E-57	T/NK cell	COPS2
9.1522508707986E-62	0.676726971395788	0.041	0.107	1.49282363953596E-57	T/NK cell	SAE1
9.64373321880149E-62	1.01451640194266	0.074	0.16	1.57298932531871E-57	T/NK cell	TMEM248
1.09909967469651E-61	0.907081033964036	0.063	0.142	1.79274147939748E-57	T/NK cell	PLEKHJ1
1.12510360274657E-61	0.852175615237489	0.059	0.135	1.83515648643992E-57	T/NK cell	SNRPB
1.13593269411032E-61	0.995662258174302	0.125	0.241	1.85281981736334E-57	T/NK cell	PNN
1.136003947969E-61	0.835979037653583	0.045	0.113	1.85293603953224E-57	T/NK cell	NTMT1
1.2364442902222E-61	1.11532479264215	0.067	0.149	2.01676428178143E-57	T/NK cell	SERBP1
1.36316483480868E-61	1.13763977629978	0.051	0.123	2.22345816205644E-57	T/NK cell	METTL26
1.39922278980593E-61	0.945362224948508	0.081	0.171	2.28227229245245E-57	T/NK cell	FBXW2
1.49348234020736E-61	1.04407242389173	0.099	0.2	2.43601904511222E-57	T/NK cell	IWS1
1.49787762618836E-61	1.53168457883579	0.387	0.391	2.44318819607584E-57	T/NK cell	TAP1
1.91639274649345E-61	0.9299833100739	0.117	0.229	3.12582820880546E-57	T/NK cell	NEMF
1.96242553947057E-61	1.14385761486122	0.048	0.119	3.20091229743045E-57	T/NK cell	ZNF76
2.13839914096352E-61	1.69549030458303	0.23	0.168	3.4879428388256E-57	T/NK cell	CD53
2.15098412911242E-61	1.00581004635708	0.04	0.106	3.50847021299527E-57	T/NK cell	LSG1
2.2244848453773E-61	0.983568732249624	0.092	0.189	3.62835723129491E-57	T/NK cell	SUPT6H
2.29374790028682E-61	1.19983286999252	0.116	0.227	3.74133220015783E-57	T/NK cell	CIB1
2.79942547487323E-61	0.885162626182108	0.045	0.114	4.56614289206572E-57	T/NK cell	SCAF4
2.82833390446941E-61	1.11029429476945	0.122	0.237	4.61329543158006E-57	T/NK cell	FOXP1
2.93855551017866E-61	1.10745187529539	0.08	0.169	4.79307789265242E-57	T/NK cell	TPR
3.27852083675951E-61	1.00622533416817	0.167	0.312	5.34759533683843E-57	T/NK cell	MAPKAPK2
3.36270866810999E-61	1.02693971295771	0.048	0.117	5.48491410855421E-57	T/NK cell	PIH1D1
3.37071181823792E-61	1.42630741205525	0.274	0.217	5.49796804672788E-57	T/NK cell	IL10RA
3.63206970239853E-61	0.907196639628141	0.075	0.161	5.92426889158225E-57	T/NK cell	ACIN1
3.8735787336353E-61	1.06438303696151	0.066	0.146	6.31819427243253E-57	T/NK cell	NOP14
4.43326923666002E-61	0.822069496579081	0.136	0.258	7.23110545191616E-57	T/NK cell	EDEM1
4.46871746083008E-61	0.980690325361893	0.066	0.146	7.28892505035994E-57	T/NK cell	MTR
4.50891140831214E-61	0.965657701941792	0.037	0.1	7.35448539809793E-57	T/NK cell	FBXW8
5.14809113022447E-61	0.999543820423684	0.067	0.148	8.39705144250913E-57	T/NK cell	MYSM1
5.26566167178295E-61	0.864182016890452	0.045	0.113	8.58882075284517E-57	T/NK cell	SENP5
5.46692719638017E-61	0.971868230519252	0.053	0.126	8.9171049500157E-57	T/NK cell	PDCD10

5.60291430895055E-61	0.627122070155416	0.067	0.147	9.13891352932924E-57	T/NK cell	NECAP2
5.80297212460311E-61	0.894573129296304	0.041	0.107	9.46522783244013E-57	T/NK cell	NSF
6.74474295309544E-61	1.02464318468929	0.038	0.101	1.1001350230794E-56	T/NK cell	HEMK1
6.79302495980585E-61	0.92754534983721	0.121	0.233	1.10801030119393E-56	T/NK cell	HNRNPU
6.82117927996737E-61	0.821455200430016	0.054	0.128	1.11260255235548E-56	T/NK cell	IREB2
9.94983910036691E-61	0.919440826937328	0.05	0.121	1.62291825566085E-56	T/NK cell	ANP32A
1.00319814077872E-60	1.06025304297218	0.046	0.114	1.63631648742418E-56	T/NK cell	NAE1
1.0317955869546E-60	0.743028525155527	0.052	0.124	1.68296178188165E-56	T/NK cell	MAPKAPK3
1.1002842255614E-60	0.956028285205853	0.052	0.123	1.7946736003132E-56	T/NK cell	GTF3C1
1.12208258067713E-60	0.987197967474773	0.113	0.221	1.83022889734247E-56	T/NK cell	NUFIP2
1.1890307999633E-60	1.14937116937584	0.077	0.164	1.93942813782014E-56	T/NK cell	TBRG1
1.19333104278436E-60	0.904614313184942	0.05	0.121	1.94644226388556E-56	T/NK cell	SRI
1.25211239586675E-60	0.363178662837789	0.04	0.103	2.04232052889825E-56	T/NK cell	APAF1
1.90807861258267E-60	0.882011593428293	0.057	0.131	3.1122670249836E-56	T/NK cell	TMEM138
2.02284046071355E-60	0.61103007910142	0.039	0.103	3.29945507546988E-56	T/NK cell	PDCL3
2.26652008370633E-60	1.09623743804083	0.091	0.186	3.69692090853339E-56	T/NK cell	MAST3
3.53944142862917E-60	1.03859170808252	0.093	0.188	5.77318291423704E-56	T/NK cell	ZNF410
4.7165752849331E-60	3.44144466483815	0.175	0.115	7.69320594725439E-56	T/NK cell	RORA
4.93793000010253E-60	1.25535906844082	0.07	0.152	8.05425762316724E-56	T/NK cell	RNF167
5.24602035829121E-60	0.835833009622581	0.049	0.118	8.55678380640879E-56	T/NK cell	STXBP3
6.12213316611766E-60	1.08284150179027	0.127	0.244	9.98581140725451E-56	T/NK cell	HNRNPUL1
6.49460206454724E-60	3.14028656225203	0.162	0.103	1.0593345427483E-55	T/NK cell	DENND2D
6.5273671442693E-60	0.842355429675877	0.044	0.11	1.06467885490177E-55	T/NK cell	TRIM5
6.64795792056371E-60	1.17654074853924	0.112	0.22	1.08434841642315E-55	T/NK cell	PHYKPL
7.88662864193818E-60	1.12952811919873	0.106	0.21	1.28638799778654E-55	T/NK cell	KDM4C
9.12896538545071E-60	0.800908736150236	0.052	0.122	1.48902554402087E-55	T/NK cell	SLC25A28
1.01003660029337E-59	0.897071025522274	0.05	0.121	1.64747069873851E-55	T/NK cell	TRAPPC6B
1.1716251556551E-59	1.00715378085758	0.043	0.109	1.91103779138903E-55	T/NK cell	DARS
1.27470805263787E-59	1.05649981856256	0.039	0.102	2.07917630465762E-55	T/NK cell	ABHD15
1.50572841728755E-59	0.832446996651328	0.074	0.158	2.45599362143772E-55	T/NK cell	NCL
1.92921306462725E-59	1.0706459210708	0.097	0.195	3.14673942971351E-55	T/NK cell	SRSF3
2.67959596486394E-59	1.00981333105891	0.083	0.172	4.37068897828958E-55	T/NK cell	WDR6
2.92015874688462E-59	0.840940710036657	0.041	0.106	4.7630709320435E-55	T/NK cell	RAP2C
3.8919622658372E-59	1.0095520854404	0.109	0.214	6.34817965180706E-55	T/NK cell	ADD1
3.91792542965509E-59	1.04709833560254	0.11	0.215	6.39052816831042E-55	T/NK cell	NSD1
5.54906798919417E-59	0.866063702367718	0.329	0.261	9.05108479717461E-55	T/NK cell	ITGB2
6.27343339726392E-59	1.03213976039564	0.08	0.168	1.02325972142772E-54	T/NK cell	SNX9
6.36817718855682E-59	0.910401596284881	0.046	0.113	1.0387133812255E-54	T/NK cell	TM2D3
6.4731410668589E-59	0.840094665604113	0.04	0.104	1.05583403941536E-54	T/NK cell	NEK9
6.52910411122471E-59	1.07791978868903	0.103	0.204	1.06496217158186E-54	T/NK cell	ERBIN
6.64322635109632E-59	0.948572079498465	0.176	0.324	1.08357665012732E-54	T/NK cell	RBM39
6.85994926226494E-59	1.01476388210234	0.084	0.173	1.11892632416803E-54	T/NK cell	PPP1CA
7.14367803773442E-59	0.906477355265062	0.173	0.319	1.16520532473486E-54	T/NK cell	OPTN
7.84985466650353E-59	0.806599019947374	0.112	0.218	1.28038979465339E-54	T/NK cell	KIAA0100
8.95728926878531E-59	1.06736098582035	0.045	0.111	1.46102345263157E-54	T/NK cell	TPGS2
9.73410269208544E-59	1.15144719603931	0.057	0.13	1.58772949010606E-54	T/NK cell	PLXNA3
1.14708864246415E-58	0.867837084967502	0.074	0.157	1.87101628472327E-54	T/NK cell	VHL
1.68191508429739E-58	1.18119220581082	0.073	0.156	2.74337169399747E-54	T/NK cell	ASCC3
1.77176966984655E-58	0.964187271122715	0.048	0.116	2.8899335084867E-54	T/NK cell	MED4
1.7792026411189E-58	0.942810885314459	0.041	0.104	2.90205742792904E-54	T/NK cell	PAF1
1.85351506107961E-58	0.898778613353485	0.039	0.101	3.02326841612696E-54	T/NK cell	PIN1
1.89904137764018E-58	0.927269768416338	0.045	0.11	3.0975263910689E-54	T/NK cell	MLH1

1.99341167674185E-58	0.973300650102433	0.069	0.15	3.25145378593363E-54	T/NK cell	SLBP
2.25075355729084E-58	0.782041109971601	0.041	0.104	3.67120412729708E-54	T/NK cell	FBXO11
2.31045464308603E-58	1.06482028740863	0.107	0.21	3.76858256833763E-54	T/NK cell	DEPRECATI
2.33421701760212E-58	1.30869623585312	0.091	0.185	3.80734137741081E-54	T/NK cell	KIAA0319L
2.36146465289035E-58	0.931760240076117	0.116	0.223	3.85178499532945E-54	T/NK cell	PPP1CB
2.48282311971767E-58	1.8987440611818	0.195	0.134	4.0497327905715E-54	T/NK cell	GPSM3
2.65241132650974E-58	0.975109364929015	0.051	0.12	4.32634811467003E-54	T/NK cell	SMARCD2
3.15905337694651E-58	0.941212522710007	0.079	0.164	5.15273196313745E-54	T/NK cell	STARD3
3.64943309133975E-58	0.860373722977829	0.052	0.122	5.95259031528427E-54	T/NK cell	SSRP1
3.6737132373569E-58	1.05218835995682	0.077	0.161	5.99219366145283E-54	T/NK cell	PRKDC
3.83154168004893E-58	1.76025952655241	0.365	0.36	6.2496276343278E-54	T/NK cell	MAF
4.36917817782631E-58	1.10106288311667	0.09	0.182	7.1265665258525E-54	T/NK cell	KHNYN
4.8306114750362E-58	0.86836773987272	0.044	0.108	7.87921037693154E-54	T/NK cell	LY6G5B
4.8666064091999E-58	1.16711884139863	0.092	0.186	7.93792171404596E-54	T/NK cell	NSMF
6.10118949739021E-58	0.715701925905966	0.041	0.105	9.95165018919317E-54	T/NK cell	RNF169
6.2029060610014E-58	2.06010612981044	0.221	0.164	1.01175600760994E-53	T/NK cell	ZC3HAV1
7.38677375854135E-58	0.564653199870121	0.099	0.195	1.20485666775568E-53	T/NK cell	PGS1
8.2425909156758E-58	0.914080936344725	0.057	0.13	1.34444900425588E-53	T/NK cell	PPP1R21
9.20077465944781E-58	1.01949510612736	0.053	0.123	1.50073835470253E-53	T/NK cell	TRMU
1.04022484350751E-57	1.06090737921352	0.059	0.133	1.69671074224509E-53	T/NK cell	SFSWAP
1.04565335051414E-57	0.794555105238434	0.074	0.155	1.70556518002361E-53	T/NK cell	DDX39A
1.26263617698692E-57	1.14124635239978	0.075	0.159	2.05948586828336E-53	T/NK cell	RPTOR
1.71227736164624E-57	1.09639388467338	0.053	0.123	2.79289560458118E-53	T/NK cell	TOX4
1.87537874329786E-57	1.05438123804911	0.075	0.158	3.05893026819314E-53	T/NK cell	C7orf26
2.02838518864445E-57	1.03922142048742	0.077	0.161	3.30849908119796E-53	T/NK cell	MAEA
2.05719563692489E-57	0.883086943113774	0.072	0.153	3.35549180338818E-53	T/NK cell	AP3B1
2.58147886833419E-57	0.820597854379138	0.049	0.116	4.21065018213989E-53	T/NK cell	TMOD3
2.88537861902958E-57	2.79481661426864	0.117	0.062	4.70634106549915E-53	T/NK cell	SYTL2
3.02835490204858E-57	0.973646221173282	0.145	0.27	4.93954968073144E-53	T/NK cell	SAFB
3.21022239454411E-57	1.05317219295999	0.066	0.144	5.2361937477409E-53	T/NK cell	SMARCC1
3.58500061505145E-57	1.00418464107684	0.134	0.252	5.84749450321043E-53	T/NK cell	GAK
3.71745230439599E-57	1.03619104954967	0.042	0.105	6.0635364537003E-53	T/NK cell	VPS54
4.0270256998518E-57	1.27092538331975	0.079	0.164	6.56848161902827E-53	T/NK cell	PRPF6
5.06722404058406E-57	0.817237913521091	0.055	0.125	8.26514913259666E-53	T/NK cell	STK25
5.10975651396114E-57	0.922274028789226	0.089	0.18	8.33452384992201E-53	T/NK cell	EXOC4
5.20565646670586E-57	1.0777419287068	0.049	0.116	8.49094626284392E-53	T/NK cell	SLMAP
6.18701123954623E-57	0.955588614719616	0.044	0.108	1.00916340328239E-52	T/NK cell	WAPL
7.085294245259E-57	1.25272634805053	0.128	0.243	1.1556823443442E-52	T/NK cell	FBXW4
7.38990182049427E-57	0.751775507953271	0.045	0.109	1.20536688594082E-52	T/NK cell	CCDC82
8.18503716863456E-57	0.986941885882243	0.054	0.125	1.33506141257598E-52	T/NK cell	UBE2O
8.41738017307051E-57	0.507625470535865	0.081	0.166	1.37295888002953E-52	T/NK cell	HMBOX1
8.89244726240099E-57	1.0070631188695	0.091	0.182	1.45044707297023E-52	T/NK cell	PIKFYVE
9.74787488127471E-57	0.569190435980053	0.041	0.103	1.58997587188472E-52	T/NK cell	AGO1
9.79789335009522E-57	0.91881251982025	0.039	0.1	1.59813438433403E-52	T/NK cell	GTF2B
1.01107510967731E-56	0.357541108735333	0.051	0.119	1.64916461139466E-52	T/NK cell	ARHGAP27
1.06579865691888E-56	1.05176632261377	0.072	0.152	1.73842418930039E-52	T/NK cell	UPF1
1.25492188568978E-56	1.11647029755267	0.04	0.102	2.0469030877486E-52	T/NK cell	ZNF282
1.3016750186977E-56	0.998767802702336	0.111	0.214	2.12316212299782E-52	T/NK cell	GRIPAP1
1.5749384586128E-56	1.09706236886616	0.055	0.126	2.56888211984335E-52	T/NK cell	BCR
2.07626910040178E-56	1.00129534194985	0.07	0.149	3.38660252966534E-52	T/NK cell	TAZ
2.18623770757164E-56	1.0955535821354	0.066	0.143	3.56597232482011E-52	T/NK cell	ZNHIT6
2.2341139063047E-56	0.830771801807351	0.039	0.1	3.64406319257359E-52	T/NK cell	USP10

4.50059544835279E-56	1.14348477465962	0.096	0.19	7.34092123580824E-52	T/NK cell	POLR2J2
4.55517512093239E-56	0.678119824784069	0.039	0.1	7.42994613975282E-52	T/NK cell	STRAP
4.64370993256908E-56	1.08787141807182	0.118	0.226	7.57435527101342E-52	T/NK cell	UPK3BL1
4.75541831169783E-56	1.07029838330925	0.044	0.108	7.75656280821033E-52	T/NK cell	BABAM1
5.40666662039272E-56	0.822127254796536	0.041	0.103	8.81881392452257E-52	T/NK cell	BMS1
5.53001467852438E-56	1.18268214835635	0.049	0.117	9.02000694214111E-52	T/NK cell	ZNF91
8.15716035845512E-56	1.08227804977165	0.121	0.23	1.33051442606761E-51	T/NK cell	GGNBP2
8.96943706617252E-56	0.796947308242779	0.232	0.417	1.4630048798634E-51	T/NK cell	HNRNPA2B
9.60340210014191E-56	1.1404834872914	0.052	0.12	1.56641091655415E-51	T/NK cell	VPS4B
1.10222174761243E-55	0.591023840129329	0.051	0.119	1.79783389253063E-51	T/NK cell	SETDB2
1.16247991408036E-55	0.909618822317201	0.042	0.105	1.89612098785647E-51	T/NK cell	RNMT
1.32195752590185E-55	1.26380833658012	0.085	0.173	2.1562449204985E-51	T/NK cell	FMR1
2.64571806032749E-55	0.757474004220163	0.049	0.115	4.31543072820018E-51	T/NK cell	AGO3
3.76311547895044E-55	0.87739683162198	0.058	0.129	6.13801765771607E-51	T/NK cell	TNPO3
4.49557182852105E-55	1.20358199066668	0.069	0.148	7.33272720950068E-51	T/NK cell	MAN2A1
5.34801296527441E-55	1.02574803220602	0.172	0.315	8.72314394765909E-51	T/NK cell	ASH1L
7.64760170025979E-55	1.14247176310161	0.1	0.195	1.24740031332937E-50	T/NK cell	CRTC2
8.02413613428306E-55	0.885097692247706	0.041	0.101	1.30881684486291E-50	T/NK cell	RALBP1
8.76589106793369E-55	1.03512012051785	0.06	0.133	1.42980449209066E-50	T/NK cell	CLK3
9.05002463219206E-55	1.15756128843998	0.065	0.139	1.47614951775685E-50	T/NK cell	SRPK2
9.17581019265456E-55	1.07476385172204	0.049	0.115	1.49666640052389E-50	T/NK cell	ZNF652
9.9358988354369E-55	1.09687449381173	0.075	0.156	1.62064445904811E-50	T/NK cell	MSL1
1.15737036153589E-54	0.810986350809499	0.145	0.265	1.88778679670119E-50	T/NK cell	SBNO2
1.19659518979669E-54	1.16230974199436	0.058	0.128	1.95176641407738E-50	T/NK cell	CMC2
1.32313238087E-54	0.341548894147227	0.046	0.109	2.15816122643705E-50	T/NK cell	VSIR
1.60351472869629E-54	1.0448939590493	0.096	0.189	2.61549287397651E-50	T/NK cell	TAOK1
1.6715176633094E-54	0.941579723811114	0.184	0.331	2.72641246062397E-50	T/NK cell	TRIR
1.77992154544952E-54	1.05012652777764	0.165	0.301	2.90323003278271E-50	T/NK cell	CAPN2
2.01777627736853E-54	0.83718482681669	0.044	0.106	3.2911948860158E-50	T/NK cell	HARS
2.04169169594118E-54	0.994950983085184	0.045	0.108	3.33020332524965E-50	T/NK cell	ZNF26
2.05885387932182E-54	1.13055185927177	0.1	0.195	3.35819656256182E-50	T/NK cell	NIPBL
2.47000411058579E-54	1.08647711119452	0.067	0.143	4.02882370477648E-50	T/NK cell	ATXN2L
3.38631739123631E-54	2.35921593401528	0.124	0.07	5.52342229684554E-50	T/NK cell	ARID5A
3.47442607871191E-54	0.988040884240415	0.059	0.131	5.667136376987E-50	T/NK cell	NOP9
3.61342854310902E-54	1.09697964100955	0.127	0.238	5.89386329666512E-50	T/NK cell	SRSF2
3.68254307383997E-54	1.02134732401596	0.089	0.177	6.00659600774038E-50	T/NK cell	PPP3R1
3.75066044921004E-54	1.06229177330784	0.105	0.203	6.1177022587065E-50	T/NK cell	ZZEF1
4.29810881465511E-54	2.83610507367396	0.114	0.061	7.01064528758395E-50	T/NK cell	RBM38
4.86785158592136E-54	1.13077036223218	0.099	0.193	7.93995272179633E-50	T/NK cell	DNMT3A
5.0322475284269E-54	1.2217762351477	0.067	0.143	8.20809894361711E-50	T/NK cell	ZNRF1
5.79987033287842E-54	1.0230776555695	0.058	0.129	9.460168499958E-50	T/NK cell	CCDC84
6.72975064472192E-54	0.97309161158958	0.073	0.151	1.09768962766059E-49	T/NK cell	CCDC12
7.2559179261403E-54	0.830619987551272	0.042	0.102	1.18351277293274E-49	T/NK cell	SF1
1.05135627722711E-53	0.993306409800106	0.18	0.325	1.71486722378514E-49	T/NK cell	LUC7L2
1.10736576212672E-53	0.715691213245458	0.045	0.108	1.80622429460489E-49	T/NK cell	U2AF1L4
1.16329651693221E-53	0.921645883648199	0.052	0.119	1.89745294876812E-49	T/NK cell	OTUD4
1.52983006918196E-53	1.11874781522495	0.058	0.128	2.49530582584269E-49	T/NK cell	DEPRECATI
1.83292249354122E-53	0.57050631811845	0.048	0.112	2.98967987921509E-49	T/NK cell	PLK3
2.48412698962655E-53	1.07366040966096	0.09	0.178	4.05185953277987E-49	T/NK cell	CYB5B
2.66492236288961E-53	1.03213948913359	0.08	0.163	4.34675486610925E-49	T/NK cell	IRF9
2.85264941261716E-53	1.02666723954188	0.047	0.11	4.65295645691985E-49	T/NK cell	PARP1
2.97667980667345E-53	2.06195324910224	0.202	0.147	4.85526243266506E-49	T/NK cell	NBEAL2

3.25720045497091E-53	1.16080231712295	0.112	0.213	5.31281966210305E-49	T/NK cell	ZMYM2
3.68182830574567E-53	0.976026053006549	0.164	0.297	6.00543014950177E-49	T/NK cell	MPRIP
3.79243777513481E-53	1.23812143231188	0.106	0.203	6.18584525502239E-49	T/NK cell	TSPYL1
3.84259854411137E-53	0.795070242320573	0.061	0.132	6.26766248530006E-49	T/NK cell	S100A10
4.49720132044197E-53	1.24732001158939	0.087	0.174	7.3353850737729E-49	T/NK cell	SLC25A45
5.64520190755622E-53	1.12787028404553	0.089	0.176	9.20788883141496E-49	T/NK cell	EXOSC6
6.08373721067515E-53	1.18109548782144	0.073	0.151	9.92318376433224E-49	T/NK cell	DGCR2
6.19699953893434E-53	0.863886777066923	0.048	0.112	1.01079259479558E-48	T/NK cell	UBA2
6.7824899690902E-53	0.289977802949737	0.073	0.149	1.1062919388583E-48	T/NK cell	AOAH
7.46608090430235E-53	0.986220992696772	0.071	0.148	1.21779245630076E-48	T/NK cell	PHPT1
9.94183431246278E-53	1.02251550595259	0.055	0.123	1.6216125947058E-48	T/NK cell	DNAJB2
1.05343843839723E-52	0.996955985543867	0.073	0.151	1.71826343686973E-48	T/NK cell	CREBBP
1.06276915176363E-52	1.0464662006943	0.062	0.133	1.73348276344165E-48	T/NK cell	CNOT2
1.0727701786079E-52	1.19617188325214	0.055	0.123	1.74979543832735E-48	T/NK cell	IAH1
1.10890547444978E-52	1.01556578487934	0.061	0.132	1.80873571937504E-48	T/NK cell	RANBP3
1.11059756612084E-52	1.21837406218698	0.076	0.155	1.8114956900997E-48	T/NK cell	ABTB1
1.32017979496844E-52	0.960517322251899	0.056	0.125	2.15334526357303E-48	T/NK cell	MAP3K7
2.5234428362799E-52	1.22706770748385	0.064	0.136	4.11598761025615E-48	T/NK cell	C19orf25
2.7266601631099E-52	1.10112990617617	0.078	0.158	4.44745539204855E-48	T/NK cell	SRRT
2.96578983769174E-52	0.891902329289399	0.05	0.115	4.837499804259E-48	T/NK cell	UQCC1
3.24238889040334E-52	0.913009708135188	0.065	0.138	5.28866051913688E-48	T/NK cell	PCF11
4.71208626616971E-52	1.08873563566444	0.106	0.202	7.68588390874942E-48	T/NK cell	SMARCA4
4.80928933305048E-52	1.15479770952563	0.1	0.192	7.84443183113863E-48	T/NK cell	EDC4
5.08271342510851E-52	0.956104378722259	0.048	0.111	8.2904138676945E-48	T/NK cell	CXXC1
5.12338828990884E-52	3.33575804840079	0.114	0.063	8.35675863967031E-48	T/NK cell	ZFYVE28
5.66747593222014E-52	1.08762200792704	0.05	0.114	9.24421999304427E-48	T/NK cell	CNOT11
6.29623788433401E-52	0.877475811355176	0.045	0.106	1.02697936131372E-47	T/NK cell	ANAPC7
6.90813229481581E-52	1.02709302758749	0.142	0.26	1.12678545860741E-47	T/NK cell	ALG13
8.55920227164106E-52	1.0594221003816	0.054	0.121	1.39609148252737E-47	T/NK cell	C1orf52
1.24662031749231E-51	1.02361735030525	0.042	0.101	2.0333623998617E-47	T/NK cell	BUD31
1.5596166823825E-51	1.03690263041164	0.046	0.108	2.5438907706341E-47	T/NK cell	GTF3C2
1.58826582173533E-51	1.04354703649489	0.149	0.271	2.5906203818325E-47	T/NK cell	RBM25
1.64818160140677E-51	0.920043885171921	0.047	0.109	2.68834901005458E-47	T/NK cell	DGUOK
1.66248851011523E-51	1.01167559651355	0.075	0.153	2.71168500884895E-47	T/NK cell	RNPS1
2.21879991390868E-51	1.18451853241317	0.078	0.157	3.61908453957645E-47	T/NK cell	PIGL
2.65228397081898E-51	0.893681868712249	0.044	0.104	4.32614038480284E-47	T/NK cell	RAB11FIP3
2.88455400729552E-51	1.09805408539516	0.056	0.124	4.70499604129972E-47	T/NK cell	EIF5B
3.14497619205698E-51	2.05013243016258	0.148	0.093	5.12977066686413E-47	T/NK cell	DOCK8
3.71977958447557E-51	0.872596908015835	0.042	0.101	6.0673324802381E-47	T/NK cell	STX2
4.15179507105283E-51	1.09938004764415	0.059	0.127	6.77199294039427E-47	T/NK cell	RTCA
5.51412612743537E-51	2.93262113391645	0.136	0.083	8.99409112645983E-47	T/NK cell	RAPGEF6
5.59836234412165E-51	1.85611925206178	0.16	0.104	9.13148881949682E-47	T/NK cell	INPP5D
7.45802534435876E-51	1.15973091022144	0.093	0.181	1.21647851391836E-46	T/NK cell	ZFAND2B
8.82659953911546E-51	1.20866966824513	0.048	0.111	1.43970665082512E-46	T/NK cell	INTS6
9.21085424008313E-51	1.13018080524181	0.07	0.144	1.50238243509996E-46	T/NK cell	PURA
1.00038219343022E-50	1.87834011375564	0.232	0.183	1.63172339570403E-46	T/NK cell	STK10
1.03451140183947E-50	1.17321921544186	0.068	0.142	1.68739154754035E-46	T/NK cell	PSME4
1.09059691668045E-50	1.02718915339333	0.054	0.12	1.77887263079748E-46	T/NK cell	CAPN7
1.11530349609929E-50	0.671336884483087	0.052	0.116	1.81917153248755E-46	T/NK cell	DNM1L
1.12475500893345E-50	0.745902310490533	0.094	0.181	1.83458789507135E-46	T/NK cell	GPCPD1
1.13110569504064E-50	1.07028311653462	0.126	0.232	1.84494649918079E-46	T/NK cell	SREBF2
1.13872292467578E-50	0.839138115651587	0.087	0.171	1.85737096243866E-46	T/NK cell	CASP4

**1.27535933217213E-50**	1.01145361959469	0.085	0.168	2.08023860670595E-46	T/NK cell	RBM5
**1.85198359975963E-50**	1.15694078135354	0.068	0.142	3.02077044956793E-46	T/NK cell	CCDC130
**1.95986696979103E-50**	1.0622004749858	0.173	0.311	3.19673901442614E-46	T/NK cell	SRSF5
**2.01211087371383E-50**	1.09110804336198	0.055	0.121	3.28195404611463E-46	T/NK cell	EEF1B2
**2.13772545753612E-50**	0.995576810259585	0.058	0.125	3.48684399378717E-46	T/NK cell	TFCP2
**3.43767091280496E-50**	1.09852084101391	0.081	0.161	5.60718502587618E-46	T/NK cell	CREBRF
**4.92247316567113E-50**	1.0887858832821	0.084	0.166	8.02904598052618E-46	T/NK cell	ATP13A1
**7.00512514142624E-50**	1.08121353018521	0.069	0.142	1.14260596181803E-45	T/NK cell	AFTPH
**1.3360695609224E-49**	1.07889048136976	0.063	0.132	2.17926306082053E-45	T/NK cell	TMUB1
**1.69591944394802E-49**	0.984678643550699	0.15	0.27	2.76621420502361E-45	T/NK cell	MARCH7
**1.89116588877426E-49**	2.39103065583032	0.279	0.251	3.08468068117969E-45	T/NK cell	RNF19A
**2.08999676372835E-49**	1.27030875696857	0.06	0.128	3.40899372131732E-45	T/NK cell	SENP2
**3.47833812788507E-49**	1.30934319174241	0.08	0.159	5.67351732039333E-45	T/NK cell	PCID2
**6.07791658150828E-49**	0.896339824001285	0.044	0.102	9.91368973609816E-45	T/NK cell	DEPRECATI
**6.52725479928629E-49**	0.922809088080742	0.046	0.105	1.06466053031159E-44	T/NK cell	TBK1
**7.09445144526755E-49**	1.09853374932928	0.091	0.175	1.15717597523759E-44	T/NK cell	TMEM131L
**8.39283728103019E-49**	0.509359598783882	0.051	0.113	1.36895568890883E-44	T/NK cell	VOPP1
**8.60327486550498E-49**	0.853665748840603	0.075	0.15	1.40328016331252E-44	T/NK cell	PTGES2
**9.36894626114491E-49**	0.82866493368532	0.044	0.102	1.52816882465535E-44	T/NK cell	MALT1
**1.01720365667309E-48**	1.14819179658053	0.085	0.166	1.65916088439947E-44	T/NK cell	DDX56
**1.19720313018617E-48**	1.08728992074674	0.051	0.113	1.95275802564665E-44	T/NK cell	RCOR3
**1.22262824939319E-48**	1.17194415578496	0.055	0.12	1.99422893758523E-44	T/NK cell	CC2D1B
**1.29297742856989E-48**	1.10733845478192	0.051	0.113	2.10897548374035E-44	T/NK cell	LRRC14
**1.34863171654995E-48**	0.954440528429066	0.046	0.106	2.19975319286462E-44	T/NK cell	NF2
**1.37169185382585E-48**	1.17428333710952	0.053	0.116	2.23736658277535E-44	T/NK cell	B4GALT3
**1.51108516723877E-48**	1.04848925638727	0.05	0.111	2.46473101628316E-44	T/NK cell	INO80D
**2.13085220561747E-48**	0.631694962636813	0.072	0.145	3.47563303258265E-44	T/NK cell	MAP3K3
**2.59437962136202E-48**	1.43146786584288	0.078	0.155	4.23169260040358E-44	T/NK cell	ATG2A
**3.51735709455852E-48**	1.05697073092085	0.045	0.103	5.7371611569344E-44	T/NK cell	EPG5
**3.79222129859436E-48**	1.87020559925412	0.147	0.093	6.18549216013726E-44	T/NK cell	TAGAP
**3.94997869212588E-48**	1.00462979861672	0.077	0.153	6.44281024472653E-44	T/NK cell	NUB1
**4.01723209818712E-48**	1.14975605625548	0.058	0.123	6.55250727535301E-44	T/NK cell	ELK4
**4.13847425475265E-48**	1.66334036415528	0.216	0.164	6.75026535692705E-44	T/NK cell	CHST11
**4.13956012269568E-48**	1.17861910874035	0.073	0.148	6.75203651612893E-44	T/NK cell	VPS36
**4.23829880083295E-48**	1.03184656295767	0.076	0.151	6.91308917403863E-44	T/NK cell	SF3B1
**4.5082030241708E-48**	0.946916347453416	0.048	0.108	7.353329952725E-44	T/NK cell	USP8
**4.80481778374752E-48**	1.18190688482824	0.048	0.108	7.83713828707058E-44	T/NK cell	CRIPT
**5.99928576170035E-48**	1.21160067576529	0.045	0.103	9.78543500590943E-44	T/NK cell	RUVBL1
**6.7994186592672E-48**	1.00054279970406	0.048	0.108	1.10905317751307E-43	T/NK cell	TRNT1
**7.51425344132612E-48**	0.883748491818493	0.056	0.12	1.2256498788147E-43	T/NK cell	ZNF264
**8.19930903632756E-48**	0.956876715408765	0.065	0.135	1.33738929691539E-43	T/NK cell	NCBP3
**8.22020405589227E-48**	1.0632469050101	0.126	0.23	1.34079748355659E-43	T/NK cell	TYK2
**8.37097738766524E-48**	1.00978128459069	0.073	0.147	1.36539012170208E-43	T/NK cell	RAB35
**8.75227415640517E-48**	0.974398877121318	0.194	0.342	1.42758343765125E-43	T/NK cell	TBC1D2B
**1.05797045240674E-47**	1.10626513952597	0.052	0.113	1.72565560492063E-43	T/NK cell	DAZAP1
**1.93454437962238E-47**	1.05490853616407	0.066	0.135	3.15543533760207E-43	T/NK cell	WIPF2
**2.41528340496083E-47**	0.901422074324436	0.204	0.358	3.9395687618316E-43	T/NK cell	ACTN4
**3.00458156566667E-47**	1.14646920655576	0.06	0.125	4.90077299175891E-43	T/NK cell	RBM10
**3.23287819518225E-47**	0.881989794532533	0.087	0.167	5.27314762416176E-43	T/NK cell	IRF2
**3.74063740529853E-47**	0.978893982508797	0.051	0.111	6.10135367178243E-43	T/NK cell	ZNF384
**5.4213705147552E-47**	0.971000744262511	0.099	0.185	8.8427974466172E-43	T/NK cell	ANKLE2
**5.42253852267714E-47**	0.898528898944492	0.193	0.337	8.84470258433868E-43	T/NK cell	CYBC1

**7.52440084308117E-47**	0.809059020597607	0.06	0.126	1.22730502151497E-42	T/NK cell	SETBP1
**8.40690306165924E-47**	1.19169568723675	0.069	0.14	1.37124995838724E-42	T/NK cell	CBFA2T2
**1.07002395870332E-46**	1.11341340953231	0.115	0.212	1.74531607904098E-42	T/NK cell	ZNF609
**1.12301697593363E-46**	1.29680064188552	0.055	0.118	1.83175298944535E-42	T/NK cell	TASOR2
**1.15101002119639E-46**	1.04904528692677	0.056	0.12	1.87741244557344E-42	T/NK cell	EEF1AKMT
**1.2727540554631E-46**	0.975941301188984	0.057	0.12	2.07598913986587E-42	T/NK cell	KDM7A
**1.49730069759317E-46**	1.15207205651724	0.066	0.135	2.44224716784422E-42	T/NK cell	MED23
**1.50362243990386E-46**	1.0023254981874	0.054	0.116	2.45255856172718E-42	T/NK cell	TAF2
**2.00390328990334E-46**	1.12985183822128	0.138	0.248	3.26856665616134E-42	T/NK cell	SKI
**2.20862164638151E-46**	1.14111227865957	0.093	0.175	3.60248276741288E-42	T/NK cell	NR3C1
**2.26599595114452E-46**	1.05251529743208	0.051	0.111	3.69606599591182E-42	T/NK cell	UBLCP1
**2.51581235898523E-46**	1.39203367181325	0.06	0.126	4.10354153874081E-42	T/NK cell	AEN
**4.07355575058044E-46**	1.23935887119942	0.061	0.127	6.64437678477175E-42	T/NK cell	EIF4B
**4.22533275450138E-46**	1.12398421019739	0.064	0.132	6.89194025586721E-42	T/NK cell	EFCAB14
**4.40256181747976E-46**	0.95690268565374	0.052	0.113	7.18101858049124E-42	T/NK cell	NELFB
**4.86345218668253E-46**	1.27374853858516	0.048	0.106	7.93277686169788E-42	T/NK cell	GOLGA7
**4.93565208334565E-46**	0.95661752236047	0.057	0.121	8.05054211314509E-42	T/NK cell	KBTBD2
**6.57619676313602E-46**	0.965349983716	0.049	0.107	1.07264345403512E-41	T/NK cell	EMG1
**6.9404852465614E-46**	0.879501526358667	0.049	0.107	1.13206254856663E-41	T/NK cell	NHLRC2
**7.11625788998223E-46**	1.02295092208129	0.067	0.136	1.160732824435E-41	T/NK cell	DEPRECATI
**8.288162759575E-46**	1.15907519888104	0.113	0.207	1.35188222771428E-41	T/NK cell	SRCAP
**8.42344706645543E-46**	1.30905669697273	0.077	0.151	1.37394845100954E-41	T/NK cell	UBE4A
**8.856559144282E-46**	1.22006113797	0.133	0.239	1.44459336202384E-41	T/NK cell	TMED4
**8.90753997596033E-46**	1.10349769895345	0.053	0.114	1.45290884547889E-41	T/NK cell	UBA6
**9.86002726060383E-46**	1.16754561308637	0.078	0.153	1.60826904647709E-41	T/NK cell	SLK
**1.00414466643386E-45**	1.28797236811889	0.091	0.173	1.63786036542027E-41	T/NK cell	CMTR1
**1.55052892455338E-45**	0.979073709636359	0.125	0.225	2.52906772883903E-41	T/NK cell	KMT2C
**1.70357856844857E-45**	0.810240148135326	0.052	0.112	2.77870700299646E-41	T/NK cell	ABL1
**1.84843520553047E-45**	1.09364635267623	0.079	0.154	3.01498266374075E-41	T/NK cell	TECPR1
**2.10923099328012E-45**	0.762110779736873	0.077	0.15	3.4403666731392E-41	T/NK cell	RHOG
**2.44926315849478E-45**	1.34174867605747	0.053	0.114	3.99499313782083E-41	T/NK cell	YAF2
**2.47973943324079E-45**	1.21661693813348	0.124	0.224	4.04470298955905E-41	T/NK cell	ANKHD1
**2.70542472503251E-45**	1.21005683358897	0.12	0.217	4.41281826900053E-41	T/NK cell	TRAPPC9
**2.95482749915949E-45**	0.275786674360442	0.14	0.24	4.81961913387905E-41	T/NK cell	NABP1
**3.09454964818776E-45**	1.19191746079354	0.114	0.208	5.04751993115905E-41	T/NK cell	CASC3
**3.82170541404431E-45**	1.18489723687439	0.077	0.15	6.23358370084768E-41	T/NK cell	KIAA2026
**5.54614245307804E-45**	1.0684344250695	0.045	0.1	9.04631295521559E-41	T/NK cell	IP6K1
**6.03546348461213E-45**	0.508652673916705	0.066	0.132	9.84444448975084E-41	T/NK cell	OAS3
**6.63056167156348E-45**	2.8490680158538	0.161	0.112	1.08151091424872E-40	T/NK cell	DUSP16
**7.98412077781353E-45**	1.35506051907033	0.116	0.211	1.30228994006916E-40	T/NK cell	FAM214A
**1.3242191534835E-44**	1.06620918075259	0.054	0.115	2.15993386124694E-40	T/NK cell	OTUB1
**2.33688744863951E-44**	1.19336306417463	0.132	0.236	3.8116971174759E-40	T/NK cell	WDR26
**2.43710542291389E-44**	1.31339623265661	0.086	0.164	3.97516265531485E-40	T/NK cell	CCNDBP1
**2.63745423649477E-44**	1.65583459883485	0.195	0.145	4.30195160514662E-40	T/NK cell	LCP2
**3.13329900706916E-44**	1.23218463561247	0.052	0.112	5.1107240104305E-40	T/NK cell	PPP2R5E
**3.26214842982427E-44**	1.15226590307253	0.049	0.107	5.32089030388636E-40	T/NK cell	ZADH2
**3.31558171325789E-44**	1.1474622067952	0.069	0.138	5.40804533249494E-40	T/NK cell	GRAMD1A
**3.62693974858368E-44**	0.837598045443086	0.256	0.446	5.91590142391485E-40	T/NK cell	GSTK1
**4.13375978876E-44**	1.15686548316146	0.076	0.148	6.74257559144643E-40	T/NK cell	JMJD6
**4.28981397802145E-44**	1.38227590237881	0.067	0.135	6.99711557955079E-40	T/NK cell	PYGO2
**4.30048157956974E-44**	1.30579500641163	0.083	0.159	7.0145155044362E-40	T/NK cell	KAT7
**4.39404407586755E-44**	1.20588860802721	0.108	0.197	7.16712529214756E-40	T/NK cell	TPP2

4.79986455476971E-44	1.11084202995568	0.122	0.22	7.82905907528487E-40	T/NK cell	MARK3
4.95414806813096E-44	1.16009408966522	0.148	0.261	8.08071091392841E-40	T/NK cell	BRD4
7.04486573204521E-44	1.14280202412856	0.056	0.117	1.14908804955389E-39	T/NK cell	SGSM2
8.59975284540783E-44	1.36424323961655	0.101	0.186	1.40270568661447E-39	T/NK cell	GATAD2B
9.06515431429593E-44	1.2710490878976	0.054	0.115	1.47861732020481E-39	T/NK cell	LMF1
1.05603846082086E-43	0.77255301725063	0.21	0.359	1.72250433344491E-39	T/NK cell	LY6E
1.08742415189131E-43	1.15457390306131	0.098	0.182	1.77369753414992E-39	T/NK cell	RELCH
1.10125799824791E-43	1.0939848580252	0.053	0.113	1.79626192094217E-39	T/NK cell	TRIM4
1.17063626144807E-43	1.18142663966573	0.127	0.228	1.90942480604794E-39	T/NK cell	VPS13B
1.47460060085091E-43	1.21380532370588	0.076	0.148	2.40522104004792E-39	T/NK cell	DYM
1.77040291191047E-43	1.14491875728307	0.076	0.147	2.88770418961716E-39	T/NK cell	ZC3H4
1.96188727948855E-43	1.09163976116542	0.103	0.188	3.20003434157377E-39	T/NK cell	CENPT
2.55675123814484E-43	1.36216781223156	0.075	0.146	4.17031694453804E-39	T/NK cell	RANBP9
2.56599348029139E-43	1.00830743726689	0.073	0.142	4.18539196570329E-39	T/NK cell	MUS81
3.13564761514573E-43	1.14535681949856	0.114	0.206	5.11455482506421E-39	T/NK cell	DDX46
3.53168720540716E-43	2.43798391160824	0.194	0.149	5.76053500073961E-39	T/NK cell	BTN3A2
3.68924665083989E-43	1.33164677938175	0.092	0.172	6.01753021218495E-39	T/NK cell	CSTF3
3.72483163531385E-43	1.10441857144279	0.078	0.151	6.07557288036042E-39	T/NK cell	INO80E
4.78850473597271E-43	1.18458824110003	0.048	0.103	7.81053007484509E-39	T/NK cell	RMC1
6.53467309117966E-43	0.868325744074926	0.058	0.119	1.06587052790231E-38	T/NK cell	ACTR3
6.71165046209568E-43	1.16447588220354	0.076	0.146	1.09473730687243E-38	T/NK cell	PHF3
8.58226877809749E-43	1.17525104554358	0.102	0.186	1.39985386039548E-38	T/NK cell	EP300
9.90157825208706E-43	0.976064646859231	0.177	0.306	1.61504642869792E-38	T/NK cell	POLR2A
1.5656426476405E-42	1.44489267292187	0.099	0.182	2.55371972256641E-38	T/NK cell	NELFCD
1.67645584905424E-42	0.750906804382794	0.261	0.449	2.73446713539238E-38	T/NK cell	MDM4
1.86704078537606E-42	1.10266180088157	0.091	0.169	3.04533022502689E-38	T/NK cell	POLDIP3
2.31135711473366E-42	1.09455787834474	0.079	0.15	3.77005458984207E-38	T/NK cell	METTL17
2.86966882948483E-42	1.21376278032534	0.083	0.156	4.6807168277727E-38	T/NK cell	FBXO46
3.82077702248053E-42	1.21474011960937	0.108	0.195	6.23206940136799E-38	T/NK cell	TOMM7
4.99584256428297E-42	1.11526993896838	0.052	0.109	8.14871880660195E-38	T/NK cell	THUMPD1
5.21210953547421E-42	1.13875610276942	0.077	0.147	8.50147186331198E-38	T/NK cell	KHDRBS1
5.47478187888257E-42	1.25628803399267	0.12	0.213	8.92991672264536E-38	T/NK cell	BAX
5.88914734910585E-42	0.851550029686163	0.051	0.107	9.60578824112656E-38	T/NK cell	WDR37
6.15941987648995E-42	1.19858639930552	0.048	0.103	1.00466297605428E-37	T/NK cell	TAF1
6.34861375526733E-42	0.425058866777674	0.271	0.444	1.03552238962165E-37	T/NK cell	EHBP1L1
6.37972264125282E-42	0.934151129587607	0.049	0.104	1.04059656001475E-37	T/NK cell	PPTC7
7.94242403568052E-42	0.983066361257689	0.179	0.308	1.29548878445985E-37	T/NK cell	UBA52
9.05419615314892E-42	1.18490752800002	0.064	0.127	1.47682993454012E-37	T/NK cell	NELFA
1.07985743491548E-41	0.865232656376856	0.046	0.1	1.76135546209064E-37	T/NK cell	RNF111
1.14846457046923E-41	2.18037241698729	0.202	0.157	1.87326056089235E-37	T/NK cell	SMCHD1
1.17993724624192E-41	0.928729780774569	0.065	0.128	1.9245956423452E-37	T/NK cell	SHKBP1
1.37394730301154E-41	1.20015265800767	0.056	0.115	2.24104544594212E-37	T/NK cell	GPS2
1.43782028903754E-41	1.02341593266586	0.11	0.197	2.34522867344913E-37	T/NK cell	TRIP12
1.53928505581056E-41	1.22096993361002	0.093	0.171	2.51072785453261E-37	T/NK cell	PHF20L1
1.79869886614006E-41	1.09647531432247	0.049	0.104	2.93385772056105E-37	T/NK cell	SUPT20H
2.08801987576777E-41	0.668492525798754	0.093	0.169	3.40576921936481E-37	T/NK cell	MAP3K8
2.23454893490005E-41	1.19487303211966	0.07	0.136	3.64477276771547E-37	T/NK cell	PRPF39
2.44005026017263E-41	1.97322397420759	0.309	0.304	3.97996597936757E-37	T/NK cell	RABGAP1L
2.45986199522229E-41	1.22304404461521	0.149	0.26	4.01228090040708E-37	T/NK cell	BIRC6
3.12251688333118E-41	1.56880218167561	0.225	0.181	5.09313728840148E-37	T/NK cell	MYO1F
4.86940356608264E-41	1.04284612159157	0.046	0.1	7.9424841566374E-37	T/NK cell	ERCC3
6.1284460123629E-41	1.17089707303425	0.06	0.121	9.99610829076513E-37	T/NK cell	UBE2V1

6.44299694029143E-41	1.26617709724983	0.058	0.117	1.05091723093094E-36	T/NK cell	SIRT7
7.11388051159192E-41	1.12935978426828	0.1	0.181	1.16034505024576E-36	T/NK cell	OGDH
1.30818253408819E-40	1.05399789101565	0.071	0.138	2.13377653135125E-36	T/NK cell	ZMYND8
1.47811617248299E-40	0.922315798673473	0.162	0.276	2.41095528893701E-36	T/NK cell	NFKB2
2.38226529382611E-40	1.17362979191842	0.069	0.133	3.88571292075976E-36	T/NK cell	RAB11FIP1
2.45470901425332E-40	1.21538035489954	0.081	0.153	4.00387587314859E-36	T/NK cell	PRPF4B
2.68658311714013E-40	1.20902814835785	0.059	0.118	4.38208572236726E-36	T/NK cell	KPNA6
2.84565341555708E-40	1.30279867401269	0.141	0.245	4.64154528611515E-36	T/NK cell	STIM1
4.74776367584884E-40	2.04739010907051	0.242	0.209	7.74407733167704E-36	T/NK cell	MBNL1
4.84414223014695E-40	1.15986472911474	0.078	0.147	7.90128039159269E-36	T/NK cell	SRRM1
6.97739500824248E-40	0.516976374581319	0.177	0.292	1.13808289979443E-35	T/NK cell	FOSL2
7.05344792944491E-40	1.1299048513082	0.059	0.118	1.15048789177176E-35	T/NK cell	UBA3
7.93364817676436E-40	1.12616751315504	0.054	0.111	1.29405735411204E-35	T/NK cell	TMEM35B
7.9738071538546E-40	1.27100945766894	0.052	0.107	1.30060768486522E-35	T/NK cell	RNF220
9.01292240459335E-40	1.03585558675085	0.132	0.229	1.47009777341322E-35	T/NK cell	AKAP17A
9.19355888025604E-40	1.23636323492448	0.068	0.132	1.49956138895856E-35	T/NK cell	FPGS
9.60487919603289E-40	0.934782194322932	0.208	0.354	1.56665184566492E-35	T/NK cell	PSME1
1.11450481605178E-39	1.31582883728617	0.056	0.114	1.81786880546205E-35	T/NK cell	TNKS2
1.31601359739952E-39	0.386555435280949	0.161	0.264	2.14654977871835E-35	T/NK cell	CTSC
1.32005091124867E-39	1.07287360077181	0.146	0.251	2.15313504133771E-35	T/NK cell	MAX
1.6223992885829E-39	0.984562265030946	0.058	0.116	2.64629547960756E-35	T/NK cell	SH3GLB2
2.10287563061799E-39	1.20238029562641	0.13	0.227	3.43000044110101E-35	T/NK cell	ARF6
2.32020432720314E-39	1.53080394901442	0.13	0.228	3.78448527810104E-35	T/NK cell	NEK7
2.57206132457183E-39	3.4176252035585	0.15	0.106	4.1952892265091E-35	T/NK cell	WHRN
2.61030882632491E-39	0.620319067672392	0.048	0.1	4.25767472661855E-35	T/NK cell	DGKE
3.09440672601551E-39	0.907091425778205	0.208	0.351	5.0472868108039E-35	T/NK cell	RBM6
3.88633800720681E-39	1.46152645265073	0.082	0.153	6.33900592355504E-35	T/NK cell	EXOC2
4.3525727879692E-39	0.972807962194196	0.098	0.176	7.09948147445656E-35	T/NK cell	ARPC4
4.39573966850387E-39	1.21702337702485	0.067	0.129	7.16989097329667E-35	T/NK cell	PPP6R3
5.53769031796846E-39	1.10404213404387	0.078	0.146	9.03252667763836E-35	T/NK cell	TBCB
5.5756516452778E-39	0.542330798079824	0.065	0.126	9.09444539861263E-35	T/NK cell	ERICH1
8.06479114484502E-39	0.93025200973012	0.058	0.116	1.31544808363567E-34	T/NK cell	MVB12B
8.09495376660717E-39	1.21299839074842	0.083	0.154	1.3203679088713E-34	T/NK cell	KHDC4
8.1957407328359E-39	0.950113729677592	0.069	0.131	1.33680727093286E-34	T/NK cell	AGFG1
8.65282517773956E-39	2.14431767639436	0.114	0.07	1.4113623147411E-34	T/NK cell	GIMAP6
1.12134661972314E-38	1.40331157632865	0.081	0.15	1.82902847143042E-34	T/NK cell	TP53I13
1.35686272097078E-38	1.07381949304956	0.085	0.156	2.21317878417544E-34	T/NK cell	ANKRD17
1.57089550910531E-38	1.28350870865095	0.145	0.249	2.56228766490167E-34	T/NK cell	PRPF3
1.62452568324633E-38	1.10536000198536	0.061	0.12	2.64976384194308E-34	T/NK cell	ARID1A
1.74886024858259E-38	0.979183242130766	0.058	0.116	2.85256595146306E-34	T/NK cell	CNPPD1
1.81756377575057E-38	1.06803816293386	0.057	0.115	2.96462827462675E-34	T/NK cell	MCMBP
2.48477908468965E-38	1.21288416867202	0.053	0.108	4.05292316503728E-34	T/NK cell	LRIG2
2.73671892928087E-38	1.20115539962295	0.063	0.122	4.46386224555003E-34	T/NK cell	CD2BP2
2.80780452462182E-38	0.531550063328741	0.096	0.171	4.57980996011065E-34	T/NK cell	ARHGEF2
3.00811312716855E-38	0.282394856160737	0.359	0.607	4.90653332172462E-34	T/NK cell	EEF2
3.09963244307594E-38	1.77137118937327	0.128	0.082	5.05581047790117E-34	T/NK cell	RASGRP2
3.86072195958198E-38	0.923068912692915	0.217	0.366	6.29722358827417E-34	T/NK cell	MBD2
5.36768378396618E-38	1.17668908901715	0.056	0.112	8.75522902002723E-34	T/NK cell	DPF2
6.66350957685543E-38	1.20366594538656	0.051	0.104	1.08688504708089E-33	T/NK cell	MAPK9
6.95768045805058E-38	1.17716916998302	0.05	0.103	1.13486725951263E-33	T/NK cell	ZBTB44
7.03117268414489E-38	1.0742836928624	0.049	0.101	1.14685457651087E-33	T/NK cell	ZNF783
9.2721032577723E-38	1.20357001776864	0.074	0.139	1.51237276237524E-33	T/NK cell	AMBRA1

9.90604717774403E-38	2.72636159027115	0.152	0.108	1.61577535516183E-33	T/NK cell	NUP210
1.44079872024448E-37	0.410485863179703	0.166	0.271	2.35008679259077E-33	T/NK cell	DDIT4
1.64495237182781E-37	1.21725812293026	0.065	0.124	2.68308181368834E-33	T/NK cell	MAZ
1.7826460632186E-37	1.72724511734337	0.075	0.141	2.90767399371587E-33	T/NK cell	SFXN1
1.86136527621746E-37	1.2699450163616	0.049	0.101	3.0360729020383E-33	T/NK cell	TMEM87A
1.88594710586211E-37	0.890480010467731	0.096	0.171	3.07616832437168E-33	T/NK cell	PHLDA1
1.97778011657423E-37	1.21914443129945	0.095	0.17	3.22595714814422E-33	T/NK cell	TBL1XR1
2.83280831668644E-37	1.14801170491744	0.065	0.124	4.62059364534725E-33	T/NK cell	TP53
3.29601891187057E-37	1.37576239887702	0.093	0.168	5.37613644715208E-33	T/NK cell	MRFAP1L1
3.43401506564167E-37	1.16678087609388	0.14	0.24	5.60122197356813E-33	T/NK cell	HNRNPA3
3.54860313897119E-37	0.706408720655279	0.256	0.43	5.78812657997591E-33	T/NK cell	CFL1
3.97328345951411E-37	1.29570040401874	0.115	0.201	6.48082265081347E-33	T/NK cell	ACSF3
4.08290233131233E-37	1.1746602466465	0.059	0.116	6.65962199260354E-33	T/NK cell	RALGAPB
4.16023682145134E-37	0.978451376934901	0.054	0.108	6.78576227946927E-33	T/NK cell	NICN1
4.64769838704499E-37	2.7809645406577	0.158	0.115	7.58086083910909E-33	T/NK cell	SIGIRR
4.6996357862207E-37	0.961259320825392	0.069	0.131	7.66557593090459E-33	T/NK cell	ARRDC1
5.20658727768268E-37	1.36258893271815	0.061	0.119	8.49246450862822E-33	T/NK cell	METTL23
6.25889018957603E-37	1.24473317603863	0.146	0.248	1.02088757882175E-32	T/NK cell	CD99
9.87941399539117E-37	1.32586138406172	0.091	0.163	1.61143121678825E-32	T/NK cell	KANSL3
1.06196961305896E-36	1.10432808032938	0.101	0.178	1.73217863586048E-32	T/NK cell	CBFB
1.13524336909865E-36	1.1555929656447	0.059	0.116	1.8516954593368E-32	T/NK cell	FBXL3
1.28471489659649E-36	1.38387430288022	0.117	0.204	2.09549846783853E-32	T/NK cell	FAM107B
1.42302403137942E-36	1.55649820399804	0.055	0.11	2.32109449758297E-32	T/NK cell	SLC25A38
1.49370432903183E-36	0.994010376360734	0.051	0.104	2.43638113108382E-32	T/NK cell	TMEM185A
1.55470885840203E-36	1.29543689980832	0.062	0.119	2.53588561893954E-32	T/NK cell	NOP2
1.58979293820492E-36	1.34221957069454	0.107	0.188	2.59311126150604E-32	T/NK cell	PPP1R35
1.62050880052772E-36	1.19674983167602	0.089	0.16	2.64321190454077E-32	T/NK cell	SON
1.66322162562093E-36	2.09502576726816	0.243	0.215	2.7128807935503E-32	T/NK cell	ATM
1.80163082780958E-36	1.34386605998849	0.139	0.238	2.93864004324021E-32	T/NK cell	SPSB3
2.35764791185247E-36	1.16746912834298	0.068	0.128	3.84555950902256E-32	T/NK cell	SMNDC1
2.45280290257872E-36	0.649576391893994	0.056	0.11	4.00076681439615E-32	T/NK cell	RASSF3
2.5993842255964E-36	1.19091427298077	0.056	0.11	4.23985561037029E-32	T/NK cell	ANAPC4
2.94282426355286E-36	1.1992656456483	0.152	0.257	4.80004065628107E-32	T/NK cell	PAFAH1B1
3.74015471984887E-36	2.42606252922222	0.231	0.201	6.1005663635455E-32	T/NK cell	KLHDC4
3.97812969595142E-36	1.43312331144714	0.1	0.177	6.48872734706636E-32	T/NK cell	SURF4
4.02359720337521E-36	1.30726012259042	0.061	0.118	6.56288939842531E-32	T/NK cell	PCNX3
4.03260044779632E-36	1.16655420403157	0.056	0.11	6.57757459040059E-32	T/NK cell	HEATR5B
4.27670705595723E-36	2.73965240175894	0.158	0.116	6.97573687897184E-32	T/NK cell	NFATC2
4.59337676978569E-36	1.09370818881252	0.056	0.11	7.49225684919744E-32	T/NK cell	SPCS3
4.71421431085922E-36	1.87567753878939	0.115	0.072	7.68935496244248E-32	T/NK cell	S100A6
6.35646236268365E-36	1.27335102555963	0.078	0.143	1.03680257597733E-31	T/NK cell	BAZ1B
7.5844671357091E-36	1.28795958188517	0.058	0.114	1.23710243450551E-31	T/NK cell	RRP1B
1.11862954282618E-35	1.26127176742547	0.076	0.14	1.82459664730378E-31	T/NK cell	RBM19
1.20990381041729E-35	1.21012444462724	0.054	0.107	1.97347410517165E-31	T/NK cell	ZBTB11
1.37631952124189E-35	0.872194046412852	0.062	0.119	2.24491477109765E-31	T/NK cell	CYB5R4
1.38476092138957E-35	1.02488914062515	0.087	0.155	2.25868353887853E-31	T/NK cell	OSBPL8
2.08101002128584E-35	1.15885128723509	0.081	0.146	3.39433544571933E-31	T/NK cell	MAPRE2
2.12019001386028E-35	1.29064718778492	0.074	0.137	3.4582419316075E-31	T/NK cell	TCF20
2.25384845904144E-35	2.2901040628676	0.122	0.08	3.67625222154249E-31	T/NK cell	PHTF2
2.79887360477564E-35	1.19648665731237	0.05	0.101	4.56524273674955E-31	T/NK cell	ARFGEF1
3.05594235332501E-35	1.19771493865785	0.144	0.243	4.98454757250843E-31	T/NK cell	EID1
4.62101849773014E-35	0.592390650586172	0.381	0.339	7.53734327164763E-31	T/NK cell	CYBA

4.75502136350466E-35	1.31412327771616	0.069	0.129	7.75591534601245E-31	T/NK cell	ANAPC16
5.39629067822206E-35	0.654983913182845	0.052	0.103	8.801889725248E-31	T/NK cell	SNX5
6.81451541784543E-35	1.32847837920311	0.091	0.162	1.11151560980477E-30	T/NK cell	CDKAL1
8.26160625769284E-35	1.28032923197355	0.068	0.127	1.34755059669228E-30	T/NK cell	DHPS
8.46987911145943E-35	1.75082896150485	0.15	0.106	1.38152198187015E-30	T/NK cell	SFMBT2
9.17000636273647E-35	1.21988037612467	0.079	0.143	1.49571973782595E-30	T/NK cell	CDK12
1.32922926733734E-34	0.905044403095886	0.064	0.12	2.16810585795394E-30	T/NK cell	ZNF141
1.37208156375273E-34	1.1241378058092	0.071	0.132	2.23800223863708E-30	T/NK cell	HERC1
1.8231183370748E-34	1.32353496780648	0.066	0.125	2.9736883196027E-30	T/NK cell	BDP1
1.90786557708227E-34	0.868836117755155	0.05	0.1	3.11191954277888E-30	T/NK cell	PHACTR2
2.29436594889013E-34	1.26546157482031	0.059	0.114	3.74234029923469E-30	T/NK cell	FIP1L1
2.95726220117642E-34	1.16770437690948	0.137	0.231	4.82359037633885E-30	T/NK cell	RACK1
3.02355278365544E-34	1.29573306794103	0.108	0.187	4.93171694542039E-30	T/NK cell	RAP1A
3.14826196425447E-34	0.916064654983699	0.053	0.103	5.13513008989546E-30	T/NK cell	RELB
3.32437967239336E-34	1.29356779809908	0.062	0.118	5.42239568364081E-30	T/NK cell	BTBD9
3.42560720291107E-34	1.26888694478476	0.068	0.126	5.58750790866825E-30	T/NK cell	DHX38
4.02530462583928E-34	1.64009506802694	0.294	0.281	6.56567437520646E-30	T/NK cell	FAM118A
4.19633390939313E-34	1.22103567147707	0.065	0.122	6.84464023961114E-30	T/NK cell	AKAP11
4.52351648473801E-34	1.4213100728807	0.062	0.117	7.37830773825617E-30	T/NK cell	RICTOR
5.97118310882523E-34	1.44867920591539	0.074	0.135	9.73959676880484E-30	T/NK cell	RPRD2
6.28798925085019E-34	0.80848746657472	0.157	0.258	1.02563392670617E-29	T/NK cell	TGFBR2
6.36232719906441E-34	1.2015163923987	0.136	0.229	1.0377591894394E-29	T/NK cell	NFATC2IP
6.73410228955754E-34	1.03154709238614	0.189	0.313	1.09839942444973E-29	T/NK cell	LUC7L
7.47341523877846E-34	2.05590260652992	0.113	0.072	1.21898875959716E-29	T/NK cell	PIK3R5
7.81810864341577E-34	1.33393874516533	0.066	0.123	1.27521170082755E-29	T/NK cell	MED12
9.48937202674048E-34	1.22395303174772	0.069	0.127	1.54781147128164E-29	T/NK cell	UBE3A
1.08960849587546E-33	0.76781715684622	0.074	0.134	1.77726041762246E-29	T/NK cell	DEPRECATI
1.50957765664135E-33	0.97673603532856	0.068	0.126	2.46227211574771E-29	T/NK cell	GIT2
1.51539525354973E-33	1.38281539662692	0.058	0.112	2.47176119806496E-29	T/NK cell	GOLT1B
1.60353194892316E-33	1.07089395174648	0.1	0.173	2.61552096188857E-29	T/NK cell	CD82
2.18556742437231E-33	0.865162150872152	0.057	0.11	3.56487902589367E-29	T/NK cell	KIF13B
3.06659164227736E-33	1.17874273917202	0.164	0.272	5.0019176277186E-29	T/NK cell	SLTM
3.4827419093922E-33	1.11702454093324	0.087	0.154	5.68070032840962E-29	T/NK cell	STK24
3.4851735654661E-33	1.74607546418713	0.053	0.104	5.68466660263175E-29	T/NK cell	AKTIP
3.63769386385424E-33	0.854135448889246	0.146	0.242	5.93344246133266E-29	T/NK cell	SH3BGRL3
3.66282956943723E-33	0.960422154999115	0.294	0.265	5.97444131070907E-29	T/NK cell	HCLS1
3.92219314264228E-33	1.0356432263903	0.062	0.116	6.39748923496383E-29	T/NK cell	UBE2D3
4.16984509416699E-33	1.085048958164	0.166	0.276	6.80143433309577E-29	T/NK cell	VPS13C
5.78198737079316E-33	1.73670052827282	0.156	0.113	9.43099960050073E-29	T/NK cell	SLC44A2
6.20322493690085E-33	2.49895525992329	0.11	0.07	1.0118080194579E-28	T/NK cell	FAM53B
6.38488150740154E-33	1.09229326237855	0.067	0.124	1.04143802267227E-28	T/NK cell	ARID4A
6.42895201642395E-33	1.24281253223551	0.054	0.105	1.04862636339891E-28	T/NK cell	PCIF1
8.79348454138105E-33	0.851277988231394	0.151	0.248	1.43430526354466E-28	T/NK cell	SLC20A1
8.92329893140663E-33	1.43301311050284	0.051	0.1	1.45547928870174E-28	T/NK cell	TSPAN17
1.15961489720307E-32	2.18110208802869	0.214	0.183	1.89144785882794E-28	T/NK cell	USP36
1.21131043468352E-32	1.36899963689747	0.144	0.241	1.97576845001229E-28	T/NK cell	KDM5D
1.61649008011006E-32	1.23702530405106	0.051	0.1	2.63665696966752E-28	T/NK cell	CBX6
1.71749118782734E-32	1.29234680657817	0.135	0.227	2.80139987646517E-28	T/NK cell	XRN1
1.93034446071584E-32	1.68826876621057	0.34	0.364	3.1485848498736E-28	T/NK cell	CYTH1
2.03515353239002E-32	1.24754227126022	0.12	0.203	3.31953892668136E-28	T/NK cell	NPEPL1
2.09842753288991E-32	1.23191447016892	0.098	0.169	3.42274514889673E-28	T/NK cell	FLOT2
2.56409969880352E-32	1.89909907925007	0.172	0.133	4.18230301871842E-28	T/NK cell	TMC6

**2.93861560231779E-32**	2.74829645885012	0.175	0.138	4.79317590894055E-28	T/NK cell	MSI2
**3.63786870998884E-32**	1.3677032835469	0.103	0.177	5.9337276528628E-28	T/NK cell	INTS4
**3.77032098901864E-32**	2.02254017594543	0.231	0.203	6.14977056518831E-28	T/NK cell	EZR
**3.94114870803254E-32**	2.3150744975483	0.18	0.143	6.42840765767188E-28	T/NK cell	SYNRG
**4.5801946594031E-32**	1.36682340847827	0.065	0.119	7.47075550895239E-28	T/NK cell	DAXX
**5.25131935329202E-32**	1.00535182383556	0.208	0.342	8.56542699715461E-28	T/NK cell	PSMB8
**5.32786480855659E-32**	1.43653389456548	0.052	0.1	8.69028028923665E-28	T/NK cell	GFOD1
**5.94659512979084E-32**	1.230490194663	0.091	0.158	9.69949131620183E-28	T/NK cell	PHF10
**6.03243063110776E-32**	2.20571522737238	0.127	0.087	9.83949760239987E-28	T/NK cell	RASA3
**7.58606551657258E-32**	1.29081448955809	0.083	0.146	1.23736314640815E-27	T/NK cell	NFAT5
**8.97542305167332E-32**	1.61518531280721	0.161	0.119	1.46398125395844E-27	T/NK cell	SAMSN1
**1.11437471582455E-31**	1.35629319321606	0.073	0.132	1.81765659898142E-27	T/NK cell	WDTC1
**1.66828840792076E-31**	1.32423718058132	0.063	0.117	2.72114522215956E-27	T/NK cell	SUPT16H
**2.21813499729842E-31**	1.11801670870744	0.092	0.159	3.61799999409345E-27	T/NK cell	TANK
**2.34267108262135E-31**	1.68524960581624	0.163	0.122	3.82113080286369E-27	T/NK cell	PTK2B
**2.36944151282123E-31**	1.34993828415735	0.126	0.211	3.8647960515627E-27	T/NK cell	KDM5C
**2.44562358907006E-31**	1.15716608739355	0.086	0.15	3.98905663613217E-27	T/NK cell	BOD1L1
**2.62215118991304E-31**	1.72574431157011	0.251	0.228	4.27699080586715E-27	T/NK cell	IFI16
**2.84608691097107E-31**	1.29934242260237	0.077	0.137	4.6422523604849E-27	T/NK cell	USP3
**3.6081185404999E-31**	1.24945345730767	0.097	0.167	5.88520215140939E-27	T/NK cell	ATP9B
**4.31855626279858E-31**	0.915835753907888	0.063	0.115	7.04399712025076E-27	T/NK cell	SSH1
**4.86134305239175E-31**	1.19770790641575	0.077	0.136	7.92933665275618E-27	T/NK cell	TIMM10B
**5.12576285413133E-31**	1.27857631111441	0.102	0.174	8.36063179137362E-27	T/NK cell	USP48
**6.09230570462224E-31**	0.735395464959026	0.307	0.519	9.93715983480934E-27	T/NK cell	TGOLN2
**6.94187099916198E-31**	1.21161757753031	0.131	0.217	1.13228857867331E-26	T/NK cell	BTAF1
**9.14196355050809E-31**	2.4343514253152	0.106	0.068	1.49114567472337E-26	T/NK cell	GRK6
**1.01081885247645E-30**	1.44660729016707	0.078	0.137	1.64874663027434E-26	T/NK cell	EML2
**1.45060020981679E-30**	1.66161312844986	0.067	0.122	2.36607400223217E-26	T/NK cell	AHI1
**1.46994536174473E-30**	1.36175341742873	0.114	0.191	2.39762787954183E-26	T/NK cell	WDR33
**1.64483395531494E-30**	1.34967229005469	0.062	0.115	2.68288866451419E-26	T/NK cell	DUSP11
**1.64774917183376E-30**	1.2750934656223	0.103	0.174	2.68764367417804E-26	T/NK cell	PIEZO1
**1.70631257515908E-30**	1.54237820205888	0.062	0.114	2.78316644134198E-26	T/NK cell	SRPK1
**1.76497147264356E-30**	1.21387803442587	0.053	0.102	2.8788449690289E-26	T/NK cell	CCNK
**1.89324843523993E-30**	1.1296005744282	0.132	0.217	3.08807752271984E-26	T/NK cell	PCM1
**2.02125858208231E-30**	1.41792389290458	0.09	0.155	3.29687487323446E-26	T/NK cell	ATXN7L3B
**2.05474892676377E-30**	1.27858203674666	0.064	0.116	3.35150097444439E-26	T/NK cell	FBL
**2.20677170405198E-30**	1.12735635905637	0.058	0.109	3.59946532647918E-26	T/NK cell	PLCL2
**2.26718599724374E-30**	1.36769331626154	0.158	0.26	3.69800708010427E-26	T/NK cell	ESYT1
**2.44737525417391E-30**	0.839081061690226	0.071	0.127	3.99191377708307E-26	T/NK cell	NCOA3
**3.39329340298411E-30**	1.18792449977476	0.085	0.147	5.53480086960738E-26	T/NK cell	NISCH
**3.83218259142613E-30**	1.17786963327189	0.122	0.202	6.25067302487516E-26	T/NK cell	MED28
**4.92707643907473E-30**	1.36403790851059	0.057	0.107	8.03655437977479E-26	T/NK cell	DNAH1
**5.44599047011771E-30**	1.40605985730808	0.08	0.14	8.882955055809E-26	T/NK cell	NCAPH2
**5.65984830735366E-30**	1.10948533631201	0.074	0.13	9.23177857412455E-26	T/NK cell	SARDH
**6.15894396684954E-30**	1.14916985570491	0.064	0.116	1.00458535043283E-25	T/NK cell	UNK
**6.71447819280509E-30**	1.22393896230263	0.195	0.32	1.09519853802844E-25	T/NK cell	N4BP2L2
**1.0705606017257E-29**	1.47040769746271	0.087	0.151	1.74619139747479E-25	T/NK cell	C12orf57
**1.81903891285107E-29**	1.37347499603346	0.104	0.175	2.96703437075139E-25	T/NK cell	AP1G2
**2.05154326732897E-29**	1.25969277643862	0.055	0.104	3.34627222334028E-25	T/NK cell	CSF1
**2.46373900540396E-29**	1.05747156429737	0.058	0.107	4.01860469171441E-25	T/NK cell	CHFR
**3.32791107153696E-29**	1.32578607419799	0.061	0.112	5.42815574878393E-25	T/NK cell	NUDT21
**3.48552220576751E-29**	1.31867100620749	0.059	0.109	5.68523526982738E-25	T/NK cell	KRIT1

**5.69459396823582E-29**	1.21199060989347	0.151	0.245	9.28845222158945E-25	T/NK cell	FNBP4
**6.05648471354575E-29**	2.91357195629765	0.105	0.07	9.87873221626447E-25	T/NK cell	USP20
**6.25646399722637E-29**	1.18571484572841	0.201	0.327	1.02049184258759E-24	T/NK cell	HNRNPH3
**6.76414054191482E-29**	1.2358062049456	0.085	0.146	1.10329896379173E-24	T/NK cell	DGLUCY
**7.22769840528827E-29**	1.39528076145744	0.121	0.2	1.17890988688657E-24	T/NK cell	STT3B
**8.35430257258513E-29**	1.21915477694359	0.154	0.251	1.36267029261436E-24	T/NK cell	ATP2B4
**8.81968207875387E-29**	1.37234939519556	0.062	0.112	1.43857834386554E-24	T/NK cell	TRIM33
**1.56453673274892E-28**	1.17758020173739	0.078	0.135	2.55191586478677E-24	T/NK cell	ARID1B
**1.64517485632281E-28**	1.2593937276844	0.065	0.116	2.68344470814814E-24	T/NK cell	INTS8
**1.92033698693687E-28**	1.55615678993276	0.189	0.151	3.13226165939273E-24	T/NK cell	NR4A2
**2.02812881499676E-28**	0.685083838951538	0.54	0.702	3.30808091014121E-24	T/NK cell	TAPBP
**2.67280849507926E-28**	1.45308095601842	0.112	0.186	4.35961793632378E-24	T/NK cell	OTUD5
**2.95780714640861E-28**	1.97378321773504	0.159	0.123	4.82447923650709E-24	T/NK cell	CHST12
**3.65386271835292E-28**	1.09562424246831	0.195	0.316	5.95981547990544E-24	T/NK cell	CHD2
**6.03684867756199E-28**	1.31606870424137	0.128	0.209	9.84670387797136E-24	T/NK cell	YWHAZ
**8.005175116765E-28**	1.33033383009368	0.135	0.219	1.30572411329554E-23	T/NK cell	RHOT2
**1.10178959325996E-27**	1.26005126442648	0.103	0.171	1.79712900556632E-23	T/NK cell	EMD
**1.25165543874296E-27**	1.01905589049814	0.082	0.139	2.04157518613364E-23	T/NK cell	CNST
**1.29250592952846E-27**	1.59731790922385	0.096	0.161	2.10820642165386E-23	T/NK cell	PHF8
**1.44468123761646E-27**	1.83197565540116	0.231	0.208	2.3564195666762E-23	T/NK cell	KCNAB2
**2.07257172312182E-27**	1.19578145091253	0.086	0.145	3.380571737584E-23	T/NK cell	HDAC1
**2.44917228506206E-27**	1.31696213626426	0.083	0.141	3.99484491416472E-23	T/NK cell	PHF11
**2.91751913981565E-27**	1.1381599459046	0.069	0.121	4.7587654689533E-23	T/NK cell	ZNFX1
**4.44126581382622E-27**	1.12804805705685	0.153	0.246	7.24414866893195E-23	T/NK cell	CAPZB
**4.6006022217356E-27**	2.1031039197461	0.105	0.07	7.50404228387293E-23	T/NK cell	DEPRECATI
**5.57705565263882E-27**	1.38527162919097	0.093	0.155	9.09673547501918E-23	T/NK cell	PPP2R2D
**5.85476406088484E-27**	1.40132790019583	0.067	0.118	9.54970565970927E-23	T/NK cell	POLR2B
**5.92477187157396E-27**	1.26172791691756	0.083	0.14	9.66389539972428E-23	T/NK cell	TRIM56
**6.00138205999505E-27**	1.09541017441811	0.07	0.122	9.78885427805792E-23	T/NK cell	VTI1A
**7.05419113291129E-27**	1.30767549511096	0.059	0.106	1.15060911568916E-22	T/NK cell	UBAC2
**7.14746759681526E-27**	1.25768739036968	0.169	0.271	1.16582343971654E-22	T/NK cell	SMAD3
**9.26716338188809E-27**	1.34658808836022	0.08	0.137	1.51156701921977E-22	T/NK cell	CDK13
**1.04872714916431E-26**	1.18658077021492	0.086	0.145	1.71057885300191E-22	T/NK cell	ATAD2B
**1.13853159805569E-26**	1.36750491930084	0.102	0.169	1.85705888958864E-22	T/NK cell	USP24
**1.47487857510808E-26**	1.04497619197639	0.229	0.369	2.40567444385879E-22	T/NK cell	TCIRG1
**1.97042441088608E-26**	1.33018833674687	0.151	0.242	3.21395925659629E-22	T/NK cell	KMT2B
**3.82292709629809E-26**	2.17404237683686	0.261	0.257	6.23557638677182E-22	T/NK cell	PCSK7
**4.01691151912333E-26**	1.19572776701005	0.063	0.111	6.55198437884206E-22	T/NK cell	GGA3
**5.16534740453452E-26**	0.608240867455012	0.269	0.419	8.42519815153626E-22	T/NK cell	RNF149
**5.69926676764136E-26**	1.35929384973532	0.108	0.176	9.29607402469982E-22	T/NK cell	MYH9
**6.69481582748712E-26**	1.07130457398726	0.057	0.102	1.09199140962142E-21	T/NK cell	PPP6C
**6.8664350948436E-26**	1.28933593343074	0.145	0.231	1.11998422831994E-21	T/NK cell	TAF1D
**7.27120179677492E-26**	1.27230748903664	0.2	0.321	1.18600572507196E-21	T/NK cell	UBR2
**7.9161243413211E-26**	1.45309569111731	0.079	0.133	1.29119904131288E-21	T/NK cell	PANK4
**9.37841782634964E-26**	1.38381986431582	0.057	0.102	1.52971373165589E-21	T/NK cell	CEP57
**1.22064241098686E-25**	1.44568513275813	0.082	0.139	1.99098983656067E-21	T/NK cell	ERAP1
**1.26271187216705E-25**	0.904272069737091	0.097	0.159	2.05960933469167E-21	T/NK cell	TSPAN14
**1.62540946397746E-25**	0.578280423585244	0.134	0.21	2.65120537669364E-21	T/NK cell	REL
**1.8093089381887E-25**	1.42936374400019	0.067	0.116	2.9511638090796E-21	T/NK cell	TMEM245
**1.82387215044718E-25**	1.22578490169089	0.056	0.1	2.97491786459439E-21	T/NK cell	DPH7
**1.92348148173036E-25**	1.22435198384838	0.07	0.12	3.13739064485039E-21	T/NK cell	ILKAP
**2.40694940108773E-25**	1.22633542845695	0.066	0.115	3.9259751681142E-21	T/NK cell	PCNT

**2.57940598313429E-25**	1.43263182095505	0.076	0.129	4.20726909909034E-21	T/NK cell	PPP3CB
**3.16645545413233E-25**	2.49626813005764	0.146	0.114	5.16480549123524E-21	T/NK cell	ABHD17A
**3.48765613851586E-25**	1.31103123336179	0.191	0.158	5.68871592753322E-21	T/NK cell	DOK2
**3.60002805667904E-25**	1.48271429776816	0.071	0.121	5.87200576324919E-21	T/NK cell	XPC
**4.84272612498163E-25**	1.2250038044437	0.193	0.308	7.89897058245753E-21	T/NK cell	CLK1
**5.0505064431741E-25**	1.45169021183648	0.07	0.12	8.23788105946127E-21	T/NK cell	PPHLN1
**5.46160806261605E-25**	1.3043199242709	0.068	0.117	8.90842891093304E-21	T/NK cell	USP33
**6.32196529724356E-25**	1.3453134974823	0.104	0.168	1.0311757596334E-20	T/NK cell	TRRAP
**6.61294036341231E-25**	1.28348094625214	0.096	0.157	1.07863670267618E-20	T/NK cell	GPBP1
**8.91590608826829E-25**	1.21088350188949	0.07	0.119	1.45427344205744E-20	T/NK cell	SMC1A
**1.05826492028237E-24**	2.53769283973468	0.184	0.157	1.72613591147257E-20	T/NK cell	SARAF
**1.35967798321954E-24**	1.59843847881004	0.056	0.1	2.21777075842939E-20	T/NK cell	GNB5
**1.36264107027455E-24**	1.31915411462544	0.136	0.216	2.22260384972481E-20	T/NK cell	DDX3Y
**1.41705615148557E-24**	1.65012468052333	0.077	0.13	2.31136028868811E-20	T/NK cell	AKIRIN1
**1.42582744068486E-24**	1.32962708598019	0.077	0.129	2.32566713850108E-20	T/NK cell	MARK2
**1.60686863751976E-24**	1.43768005341335	0.146	0.232	2.62096343465848E-20	T/NK cell	TUBGCP6
**1.74286033895662E-24**	1.31715808021621	0.107	0.172	2.84277949887215E-20	T/NK cell	DDX27
**1.97533179023778E-24**	1.3961406762688	0.07	0.119	3.22196368305685E-20	T/NK cell	POLG
**1.98184688949181E-24**	0.822028986906407	0.059	0.103	3.23259046145009E-20	T/NK cell	CSGALNAC
**2.80189275269938E-24**	1.49792464293919	0.127	0.204	4.57016726892796E-20	T/NK cell	HUWE1
**3.20917362182023E-24**	1.22646326895595	0.099	0.16	5.23448309455098E-20	T/NK cell	PURB
**3.30844627952888E-24**	1.12914119092139	0.092	0.151	5.39640672653956E-20	T/NK cell	PRMT2
**4.30203022316748E-24**	1.03048302547309	0.07	0.118	7.01704149700847E-20	T/NK cell	LMNB2
**4.3307938798567E-24**	1.25287718522738	0.155	0.245	7.06395789743427E-20	T/NK cell	RSRC2
**4.91084435528293E-24**	1.42043231246586	0.067	0.115	8.01007822790199E-20	T/NK cell	ZNF800
**4.97365586085742E-24**	1.96675627609895	0.11	0.077	8.11253007464454E-20	T/NK cell	DEPRECATI
**5.25259777095983E-24**	2.64713157204127	0.115	0.083	8.56751222421257E-20	T/NK cell	NSMCE3
**5.4941730930224E-24**	2.73112956218073	0.129	0.097	8.96154573202884E-20	T/NK cell	SFI1
**1.09895300518728E-23**	1.49332170012182	0.068	0.116	1.79250224676097E-19	T/NK cell	N4BP1
**1.22692803918023E-23**	1.30658607384602	0.141	0.223	2.00124232470687E-19	T/NK cell	TIAL1
**1.62132983613838E-23**	1.10354959386667	0.205	0.323	2.64455109572531E-19	T/NK cell	GBP2
**1.94260192896034E-23**	1.64909033596461	0.061	0.105	3.16857800632721E-19	T/NK cell	FBXO44
**1.95586796951152E-23**	1.16449544456546	0.158	0.247	3.19021624507024E-19	T/NK cell	KLC1
**3.26344375936486E-23**	0.979735190585016	0.167	0.259	5.32300311590002E-19	T/NK cell	RIN3
**3.33562129695137E-23**	1.24164470414229	0.064	0.109	5.44073189745737E-19	T/NK cell	RGS3
**3.44404338715747E-23**	1.38699807621989	0.058	0.101	5.61757916879255E-19	T/NK cell	RANGAP1
**3.98004998578734E-23**	0.542280661589742	0.075	0.123	6.49185953181772E-19	T/NK cell	CRIM1
**4.88001169136745E-23**	1.44631298921696	0.086	0.141	7.95978706978945E-19	T/NK cell	UBE3C
**5.38740054930593E-23**	1.25595323773954	0.158	0.247	8.7873890359729E-19	T/NK cell	SAFB2
**8.17409087735596E-23**	1.39888732474808	0.115	0.183	1.33327596300553E-18	T/NK cell	QRICH1
**8.38201993451462E-23**	1.04774970179657	0.21	0.328	1.36719127151868E-18	T/NK cell	SMAP2
**9.45257135709629E-23**	1.73704347428418	0.084	0.138	1.54180891405598E-18	T/NK cell	CNNM3
**9.75413482177202E-23**	1.36220183317606	0.075	0.125	1.59099693077923E-18	T/NK cell	ZNF407
**1.10732008917751E-22**	0.655863106232732	0.063	0.106	1.80614979745744E-18	T/NK cell	SCLT1
**1.75183267091785E-22**	1.28698416614564	0.127	0.2	2.85741426953411E-18	T/NK cell	IST1
**1.77075876333959E-22**	1.0046403787541	0.071	0.119	2.88828461888321E-18	T/NK cell	MADD
**1.83985731020538E-22**	1.1916067113791	0.071	0.119	3.000991258676E-18	T/NK cell	CNOT8
**2.55207265902317E-22**	0.981824129757404	0.154	0.239	4.16268571413269E-18	T/NK cell	MAP7D1
**2.87930830966623E-22**	1.09053752741011	0.087	0.141	4.69643978389659E-18	T/NK cell	RUBCN
**2.91403819457876E-22**	1.52273296895649	0.075	0.124	4.75308769917741E-18	T/NK cell	SIK3
**2.94182941988057E-22**	1.24199882121434	0.062	0.105	4.7984179667672E-18	T/NK cell	DENND6A
**3.10189232553685E-22**	1.44064347093898	0.083	0.135	5.05949657218316E-18	T/NK cell	JOSD1

3.23879637574077E-22	1.40461294943074	0.132	0.207	5.28280076847077E-18	T/NK cell	RBM14
3.74791562548251E-22	1.35213024835533	0.096	0.154	6.11322517672452E-18	T/NK cell	BTN3A3
5.45396157864499E-22	1.378742655053	0.11	0.174	8.89595673092784E-18	T/NK cell	SART3
5.47378562286515E-22	1.53161226555278	0.091	0.146	8.92829172945535E-18	T/NK cell	ZNF217
5.83969305904702E-22	0.889264030185191	0.085	0.136	9.52512334861159E-18	T/NK cell	WDFY1
6.50607250859157E-22	1.13998375413743	0.061	0.103	1.06120548687637E-17	T/NK cell	CNOT9
6.59379055757777E-22	1.20385112347037	0.179	0.279	1.07551317784651E-17	T/NK cell	KMT2D
9.61348264645117E-22	1.85872184722622	0.108	0.077	1.56805515446265E-17	T/NK cell	DOCK10
1.28775308618193E-21	1.12037777139288	0.065	0.108	2.10045405887135E-17	T/NK cell	DCP2
2.06594272244612E-21	1.42239539712639	0.09	0.145	3.36975917458186E-17	T/NK cell	MORC3
2.51522014131301E-21	1.51711517987719	0.131	0.204	4.10257557249565E-17	T/NK cell	DDX24
3.49170526580054E-21	1.0530476535739	0.231	0.362	5.69532045904727E-17	T/NK cell	HNRNPA0
4.33755838571988E-21	0.933592458330106	0.233	0.362	7.0749914829477E-17	T/NK cell	ACTG1
4.66338316365417E-21	1.33399381251168	0.076	0.123	7.60644427823632E-17	T/NK cell	ZCCHC3
4.73072975021036E-21	1.50554307479958	0.168	0.139	7.71629329556812E-17	T/NK cell	FLNA
4.92870376592593E-21	1.34479786151841	0.167	0.259	8.03920871260179E-17	T/NK cell	ANKRD11
5.25621343605589E-21	1.24894248161372	0.291	0.287	8.57340973555076E-17	T/NK cell	METRNL
6.6025146018423E-21	1.2747147504984	0.064	0.107	1.0769361567065E-16	T/NK cell	MTF2
7.14654777441404E-21	1.44140226758818	0.08	0.13	1.16567340748467E-16	T/NK cell	EPC2
7.25402040144046E-21	0.790839440401646	0.065	0.108	1.18320326767895E-16	T/NK cell	NFKB1
7.5964795445136E-21	1.00259730484013	0.072	0.117	1.23906177850561E-16	T/NK cell	IQGAP1
8.32982901528891E-21	1.40059086234642	0.084	0.135	1.35867841068377E-16	T/NK cell	MCM7
9.11809680888647E-21	1.43042476775464	0.076	0.124	1.48725277049747E-16	T/NK cell	TERF2
1.5940407910598E-20	1.34099850076546	0.127	0.197	2.60003993429763E-16	T/NK cell	ORMDL1
1.68025257041767E-20	0.684936542494398	0.085	0.136	2.74065996760826E-16	T/NK cell	CD300A
1.68427217273149E-20	1.20948624679775	0.16	0.246	2.74721634094233E-16	T/NK cell	UBA7
1.81667595916573E-20	0.812883645783949	0.201	0.167	2.96318015699522E-16	T/NK cell	DEPRECATI
1.90026862237692E-20	1.21726754804887	0.06	0.1	3.09952814995899E-16	T/NK cell	TBC1D10B
2.01700939329766E-20	1.26647336134056	0.101	0.159	3.28994402140781E-16	T/NK cell	LRCH3
2.40791729832446E-20	0.931696478369036	0.103	0.16	3.92755390529703E-16	T/NK cell	SLC6A6
2.66486956635923E-20	1.62221983625331	0.062	0.104	4.34666874968855E-16	T/NK cell	TRANK1
2.93832852966241E-20	1.17973697685682	0.224	0.35	4.79270766473236E-16	T/NK cell	DNM2
6.36984181833489E-20	1.42618911421435	0.097	0.153	1.0389848989886E-15	T/NK cell	HELZ
6.45357094511439E-20	1.45809418343361	0.088	0.14	1.05264195685761E-15	T/NK cell	SMG1
1.01787560629868E-19	2.87373629574143	0.126	0.098	1.66025690143378E-15	T/NK cell	STK39
1.16708576747943E-19	1.70453453751264	0.133	0.104	1.90363359533569E-15	T/NK cell	CD84
1.32003835916651E-19	0.817686778897802	0.074	0.118	2.15311456763649E-15	T/NK cell	SMAD7
1.33101574763785E-19	2.63780068181714	0.102	0.073	2.17101978597209E-15	T/NK cell	KIF22
1.48522861636833E-19	1.37103190365234	0.069	0.112	2.42255639615838E-15	T/NK cell	ANP32E
1.58343343948177E-19	1.30242526411059	0.124	0.19	2.58273828313871E-15	T/NK cell	STAG1
1.59160369912326E-19	1.17010886815103	0.069	0.111	2.59606479363995E-15	T/NK cell	ELMO1
1.5981409827682E-19	1.51444912931868	0.13	0.1	2.60672775699321E-15	T/NK cell	RCSD1
1.69515495850465E-19	1.49740614250294	0.063	0.103	2.76496725281693E-15	T/NK cell	SF3A2
1.84018756023226E-19	1.28964994246992	0.186	0.287	3.00152992949484E-15	T/NK cell	DDX39B
2.13346993258658E-19	0.849680364913668	0.061	0.1	3.47990280704196E-15	T/NK cell	MCM5
2.19308651034757E-19	1.61568401598335	0.099	0.155	3.57714340702792E-15	T/NK cell	CERK
2.22693115577224E-19	0.913394031473734	0.187	0.155	3.63234740818011E-15	T/NK cell	SLC2A3
2.25671586653356E-19	0.323872399834031	0.107	0.16	3.68092924990289E-15	T/NK cell	ZNF331
2.591028956956E-19	1.29405580548814	0.149	0.228	4.22622733169093E-15	T/NK cell	TIA1
2.85254355292226E-19	1.21454167766626	0.073	0.118	4.6527837891715E-15	T/NK cell	GNPTAB
2.85816612239114E-19	1.51751231010054	0.077	0.123	4.66195476223219E-15	T/NK cell	PPAN
3.91912717365604E-19	1.41863663732681	0.096	0.15	6.39248833295037E-15	T/NK cell	BCL2L11

5.07044943650166E-19	1.88190245207844	0.103	0.075	8.27041007587786E-15	T/NK cell	GIMAP5
5.26011999971403E-19	1.3915288944214	0.134	0.205	8.57978173153356E-15	T/NK cell	FBRS
5.5415182409932E-19	1.84301651662998	0.131	0.103	9.03877040288401E-15	T/NK cell	DOCK11
7.97275719578595E-19	1.13492232960021	0.146	0.221	1.30043642620465E-14	T/NK cell	EHMT1
1.01669626995602E-18	1.30009989897182	0.087	0.136	1.65833328592526E-14	T/NK cell	KIAA1109
1.26860457170828E-18	1.37754065140975	0.171	0.261	2.06922091691337E-14	T/NK cell	NUMA1
1.41285759397788E-18	1.11890391345156	0.162	0.245	2.30451202153732E-14	T/NK cell	HNRNPDL
1.44902994710353E-18	1.80240580633907	0.1	0.072	2.36351274672056E-14	T/NK cell	SH3BP1
1.4701514987461E-18	1.62420039884596	0.108	0.167	2.39796410960476E-14	T/NK cell	TRADD
1.59258410707626E-18	1.52150074782499	0.102	0.157	2.59766393705208E-14	T/NK cell	AATF
2.37450912847489E-18	1.4981776271765	0.132	0.201	3.8730618394554E-14	T/NK cell	LBR
2.90203800689464E-18	1.66664925418467	0.095	0.147	4.73351419304585E-14	T/NK cell	JMY
3.98434192424779E-18	1.56284338967824	0.094	0.145	6.49886011264057E-14	T/NK cell	MIER1
4.65306491223784E-18	1.58477438855329	0.072	0.115	7.58961417835114E-14	T/NK cell	LETMD1
4.81784538797752E-18	0.94185499741261	0.072	0.114	7.85838761233013E-14	T/NK cell	OSTF1
5.05468199187532E-18	1.40934740850886	0.164	0.249	8.24469179694784E-14	T/NK cell	NCOR1
5.56965250270771E-18	1.50835926474357	0.13	0.197	9.08466019716655E-14	T/NK cell	TNRC6B
6.04959573728133E-18	1.66452579633908	0.103	0.158	9.86749560707958E-14	T/NK cell	HERPUD2
6.16810821907118E-18	1.34542903307054	0.119	0.181	1.0060801316127E-13	T/NK cell	CTDSP1
6.63561962956634E-18	1.56765876251894	0.102	0.157	1.08233591777857E-13	T/NK cell	YPEL3
7.99613634846309E-18	1.69147868011612	0.063	0.102	1.30424979979781E-13	T/NK cell	PLA2G4B
8.47291793967648E-18	1.43751301182815	0.102	0.156	1.38201764514063E-13	T/NK cell	PPM1B
1.1586646073339E-17	1.67326669508328	0.065	0.105	1.88989784102233E-13	T/NK cell	COMMD7
1.17963841678165E-17	1.28718944747982	0.393	0.484	1.92410822161256E-13	T/NK cell	CIRBP
1.27391230848819E-17	2.59916109359565	0.198	0.185	2.07787836637508E-13	T/NK cell	ABCA2
1.49057411740354E-17	1.23714523820909	0.072	0.113	2.43127544289691E-13	T/NK cell	PTBP3
1.63500769826084E-17	1.54218708178029	0.07	0.111	2.66686105663326E-13	T/NK cell	MTO1
1.69385195402737E-17	1.48027086195588	0.09	0.14	2.76284192221404E-13	T/NK cell	PRRC2C
2.15683993372528E-17	1.37937284575193	0.085	0.132	3.5180216158993E-13	T/NK cell	ABI1
2.25761554527697E-17	0.974958839118848	0.457	0.601	3.68239671590126E-13	T/NK cell	OAZ1
2.76773204966918E-17	1.60213728669716	0.084	0.13	4.51444774621539E-13	T/NK cell	URI1
2.79715916690681E-17	1.48990648904371	0.085	0.132	4.56244631714169E-13	T/NK cell	CTDP1
3.10448370928305E-17	1.78613139177827	0.073	0.115	5.06372337821158E-13	T/NK cell	RFFL
3.47226932913153E-17	1.55749917455917	0.15	0.226	5.66361850274643E-13	T/NK cell	SETD1B
3.66064290986413E-17	1.92216012495099	0.11	0.083	5.97087465027939E-13	T/NK cell	NDE1
4.31755752418726E-17	1.7322342996013	0.1	0.153	7.04236807770184E-13	T/NK cell	TUT4
4.59909382129324E-17	0.560701881135879	0.536	0.683	7.5015819319114E-13	T/NK cell	ACTB
4.75427245745678E-17	1.73063153369715	0.1	0.074	7.75469380535775E-13	T/NK cell	ARHGAP25
5.75657567423912E-17	1.64048599271689	0.071	0.112	9.38955058225143E-13	T/NK cell	HIST1H1E
8.60131955171676E-17	1.17019160084859	0.075	0.117	1.40296123208052E-12	T/NK cell	DMWD
1.02725078635276E-16	1.86754564166426	0.096	0.147	1.67554875761999E-12	T/NK cell	EPB41
1.1424148184253E-16	1.20190708182452	0.108	0.162	1.8633928103335E-12	T/NK cell	APOL3
1.15316676832863E-16	0.820625911530742	0.093	0.141	1.88093031582082E-12	T/NK cell	GBP4
1.25149423657164E-16	1.30921479324557	0.076	0.118	2.041312249272E-12	T/NK cell	METTL3
1.61175614322642E-16	1.58129583297242	0.136	0.204	2.62893544521661E-12	T/NK cell	FZR1
3.35317839990392E-16	1.51962673919239	0.111	0.167	5.46936928808328E-12	T/NK cell	ZBTB4
3.37215832869129E-16	1.55339328427665	0.089	0.135	5.50032744992836E-12	T/NK cell	PHC3
3.40044135661645E-16	2.4737523308769	0.129	0.105	5.5464598967771E-12	T/NK cell	BTN3A1
3.95711214527409E-16	1.37456498774265	0.171	0.257	6.45444562015657E-12	T/NK cell	ZRANB2
5.21308196802981E-16	1.88161967305664	0.132	0.198	8.50305799805342E-12	T/NK cell	TTC39C
5.32830289648552E-16	1.6413391736989	0.092	0.139	8.69099485445753E-12	T/NK cell	HMGN4
5.58457700985023E-16	1.52048338657404	0.086	0.132	9.10900356076671E-12	T/NK cell	BAK1

6.12760443186964E-16	1.42823571307971	0.076	0.117	9.99473558882256E-12	T/NK cell	TASOR
7.25609500721806E-16	1.53017648772798	0.159	0.238	1.18354165662734E-11	T/NK cell	KANSL1
9.73401628830084E-16	2.12216032174494	0.129	0.106	1.58771539678475E-11	T/NK cell	APBB1IP
1.22866890839351E-15	1.48633810533095	0.16	0.238	2.00408185648065E-11	T/NK cell	CPNE1
1.26139193107675E-15	2.5396810011688	0.171	0.155	2.05745637877928E-11	T/NK cell	ERN 1.00
1.29389345833928E-15	1.3168022334306	0.11	0.164	2.1104696198972E-11	T/NK cell	RNF4
1.40707173507742E-15	1.4780605334979	0.104	0.157	2.29507470708477E-11	T/NK cell	PDLIM2
1.52645045859161E-15	1.43952413982361	0.091	0.137	2.48979334300877E-11	T/NK cell	ITFG2
1.8022245562097E-15	2.74905088657635	0.103	0.08	2.93960847363364E-11	T/NK cell	OXNAD1
2.67084967558078E-15	0.629977444510906	0.414	0.613	4.35642290583981E-11	T/NK cell	JUNB
3.31117116267639E-15	1.19007942760505	0.076	0.116	5.40085128344145E-11	T/NK cell	CHD1
3.5345705257401E-15	2.3719785749292	0.194	0.184	5.76523798453468E-11	T/NK cell	BCL9L
3.83314361901208E-15	1.32282840454886	0.093	0.139	6.2522405569706E-11	T/NK cell	MTA2
5.34044006936767E-15	1.68990314585543	0.194	0.18	8.71079179714561E-11	T/NK cell	ARID5B
5.58520850758049E-15	1.5317207556782	0.072	0.111	9.11003359671453E-11	T/NK cell	ADSS
5.73594740028719E-15	1.64317876243733	0.074	0.113	9.35590380460844E-11	T/NK cell	DDX23
7.1575286641076E-15	1.42200473050757	0.128	0.189	1.16746450040259E-10	T/NK cell	DMTF1
8.68949229713466E-15	1.45364361835502	0.073	0.112	1.41734308858563E-10	T/NK cell	BCLAF1
1.02235850364857E-14	1.44059880504686	0.132	0.195	1.66756895530119E-10	T/NK cell	USP34
1.16458476198362E-14	1.06227011971952	0.424	0.551	1.89955420527148E-10	T/NK cell	PFN1
1.56469280137238E-14	1.34162542513705	0.08	0.12	2.55217042831849E-10	T/NK cell	RNF168
1.82031645946805E-14	1.33088941123218	0.082	0.124	2.96911817703834E-10	T/NK cell	DDHD1
2.00167394114082E-14	1.36800113456776	0.147	0.216	3.2649303653948E-10	T/NK cell	CCNI
2.58097472495949E-14	1.36504563822993	0.072	0.109	4.20982787388142E-10	T/NK cell	SNRPA
3.69371825154913E-14	0.403019212317198	0.328	0.469	6.02482384010179E-10	T/NK cell	TMSB10
3.80992930283359E-14	1.65411086390034	0.066	0.102	6.21437568585187E-10	T/NK cell	MAN1B1
4.6777233486727E-14	1.40456361277581	0.312	0.348	7.62983455402004E-10	T/NK cell	GRK2
4.94406086708254E-14	1.39076684977651	0.09	0.133	8.06425768029834E-10	T/NK cell	ATRX
5.98170468795593E-14	1.49849237943209	0.078	0.118	9.75675851652491E-10	T/NK cell	PPP4R3A
6.45370241495286E-14	1.52442921956352	0.096	0.142	1.05266340090296E-09	T/NK cell	ZC3H18
7.01984367455436E-14	1.45613942299946	0.097	0.143	1.14500670175656E-09	T/NK cell	STAG2
7.56792853940126E-14	0.972793392245937	0.079	0.118	1.23440482406174E-09	T/NK cell	BIRC3
9.40948964764632E-14	1.48311989308398	0.138	0.202	1.53478185642759E-09	T/NK cell	RNPEPL1
1.24344929258913E-13	2.45450249666672	0.109	0.087	2.02819014114212E-09	T/NK cell	ARAP2
1.31857545208397E-13	1.3096189334263	0.101	0.148	2.15072841989416E-09	T/NK cell	ZBTB1
1.38575850538686E-13	1.37894213428	0.07	0.105	2.26031069813651E-09	T/NK cell	PNRC2
1.5369361710981E-13	1.68316033789332	0.075	0.112	2.50689658867812E-09	T/NK cell	THADA
1.65340912958104E-13	1.45123958470253	0.089	0.132	2.69687563125963E-09	T/NK cell	NUP98
2.05639203055307E-13	1.33935201693149	0.19	0.279	3.35418104103511E-09	T/NK cell	ILF3
2.14848944281202E-13	2.04513075246886	0.228	0.231	3.50440113017069E-09	T/NK cell	HECA
3.08291870072645E-13	1.53610918683067	0.102	0.149	5.02854869275491E-09	T/NK cell	CAMTA2
3.17407211531816E-13	1.79719333014686	0.16	0.142	5.17722902729546E-09	T/NK cell	CNTRL
3.22193203830683E-13	0.732959693661899	0.116	0.165	5.25529334768227E-09	T/NK cell	NOTCH1
3.31909008982408E-13	1.54103305388111	0.099	0.145	5.41376784551205E-09	T/NK cell	USP25
3.56477952757065E-13	0.970673093445712	0.101	0.146	5.81451188742049E-09	T/NK cell	PKM
3.56882747968264E-13	1.10682859847317	0.082	0.12	5.82111450211035E-09	T/NK cell	SNRK
4.02316058931783E-13	1.62086456162931	0.076	0.113	6.56217723723631E-09	T/NK cell	ATP6V0A2
5.01584568925699E-13	1.15148457544044	0.099	0.144	8.18134590374707E-09	T/NK cell	SETX
7.7415307835891E-13	1.3482031419754	0.211	0.313	1.26272108611122E-08	T/NK cell	IRF3
9.74368657657443E-13	1.88155976986381	0.091	0.133	1.58929271750505E-08	T/NK cell	SPATA13
9.93166938612042E-13	1.64391811971879	0.078	0.116	1.6199545935701E-08	T/NK cell	PPP4R3B
1.04111039096052E-12	1.51466085591502	0.081	0.12	1.6981551586957E-08	T/NK cell	HELZ2

1.16326985697335E-12	2.41236623960254	0.158	0.144	1.89740946370924E-08	T/NK cell	NAP1L4
1.28165453636944E-12	1.74302957847377	0.106	0.154	2.0905067142722E-08	T/NK cell	POLR3E
1.33797872223784E-12	1.37908389059422	0.092	0.134	2.18237709384213E-08	T/NK cell	ROCK1
1.43555225732765E-12	1.55354107961628	0.067	0.1	2.34152928692713E-08	T/NK cell	SF3B3
2.17350140359687E-12	0.468460017601322	0.078	0.113	3.54519813940685E-08	T/NK cell	LFNG
2.41394256535554E-12	1.69920668874956	0.095	0.138	3.93738171835142E-08	T/NK cell	TMX4
2.69671521240579E-12	1.33186661383845	0.08	0.117	4.39861218295509E-08	T/NK cell	MAML1
2.73484554110195E-12	1.49278449920987	0.082	0.12	4.4608065620914E-08	T/NK cell	TMF1
2.79406573037108E-12	1.2988209665052	0.153	0.219	4.55740061280826E-08	T/NK cell	TNK2
3.49487404646275E-12	2.00449521550679	0.099	0.144	5.70048905718539E-08	T/NK cell	GSDMB
3.53309495087379E-12	1.39381305611696	0.075	0.11	5.76283117437023E-08	T/NK cell	EZH1
3.71232019949696E-12	0.46012161128711	0.424	0.643	6.05516547739949E-08	T/NK cell	MT-ND6
4.46307612485146E-12	1.13318415557344	0.13	0.185	7.27972346724522E-08	T/NK cell	PPP1R18
4.89073103001878E-12	1.67309585982607	0.136	0.195	7.97727138306364E-08	T/NK cell	OTULIN
5.02407623638614E-12	1.45817964884974	0.158	0.226	8.19477074916944E-08	T/NK cell	TRABD
5.54185574427638E-12	1.48912642210066	0.097	0.14	9.0393209044892E-08	T/NK cell	SIN3A
5.7257652623313E-12	1.76154506236739	0.17	0.157	9.33929571938858E-08	T/NK cell	SP100
6.424994317435E-12	1.53365464708285	0.109	0.156	1.04798082311682E-07	T/NK cell	UTY
6.81631178187133E-12	0.526084286336828	0.095	0.134	1.11180861474103E-07	T/NK cell	ST8SIA4
7.40670383340686E-12	2.25967532984429	0.201	0.199	1.20810746226699E-07	T/NK cell	SATB1
9.19290736722332E-12	1.48767298649063	0.074	0.109	1.4994551206678E-07	T/NK cell	PTAR1
9.75192838767839E-12	1.48673459002816	0.073	0.107	1.59063703931422E-07	T/NK cell	ARPC5L
1.01283935407566E-11	1.01474383429141	0.11	0.156	1.65204227043281E-07	T/NK cell	SEL1L3
1.08787909085593E-11	2.37217259886721	0.112	0.093	1.7744395850951E-07	T/NK cell	SLC7A6
2.02750622462557E-11	1.64844624540162	0.108	0.154	3.30706540298676E-07	T/NK cell	BIN1
2.05138804063794E-11	1.58292449269605	0.123	0.175	3.34601903308455E-07	T/NK cell	R3HDM4
2.34550999382443E-11	1.36184104802679	0.352	0.427	3.82576135092702E-07	T/NK cell	LUC7L3
2.51480473244439E-11	0.755719147812266	0.074	0.107	4.10189799909004E-07	T/NK cell	TRPS1
5.59211871181098E-11	1.60019180434131	0.108	0.153	9.12130483083489E-07	T/NK cell	EPC1
6.1824088814723E-11	1.667226631007	0.09	0.129	1.00841271265695E-06	T/NK cell	MAT2B
6.38434077956691E-11	1.58544172123435	0.086	0.123	1.04134982455516E-06	T/NK cell	FAM172A
7.9659056766452E-11	1.69849533618795	0.123	0.106	1.2993188749176E-06	T/NK cell	RASSF5
7.96659929110436E-11	1.39700488502355	0.099	0.141	1.29943201037203E-06	T/NK cell	PPP1R12A
8.41664871236262E-11	1.51725137761488	0.105	0.149	1.37283957147347E-06	T/NK cell	CSNK1G2
9.24705280326278E-11	1.4968043429774	0.07	0.101	1.50828678274019E-06	T/NK cell	EPM2AIP1
9.54821673157997E-11	1.72495593046854	0.093	0.133	1.55740963108801E-06	T/NK cell	ERCC5
1.12047670985274E-10	1.752414148703	0.116	0.164	1.82760956144081E-06	T/NK cell	DEPRECATI
1.33418441794336E-10	1.66388812358616	0.1	0.142	2.17618820410742E-06	T/NK cell	ATXN7
1.38323184346093E-10	1.14943909374944	0.223	0.323	2.25618945986913E-06	T/NK cell	LRCH4
1.38582180513233E-10	1.79864452277455	0.118	0.167	2.26041394635135E-06	T/NK cell	NAB1
1.42595031664455E-10	1.34440796871895	0.151	0.213	2.32586756147892E-06	T/NK cell	ZFC3H1
1.65244843230351E-10	1.51395023131472	0.113	0.16	2.69530863793026E-06	T/NK cell	AGO2
1.7297722695716E-10	1.58832771505476	0.078	0.112	2.82143154889824E-06	T/NK cell	ZNF292
2.04616803300376E-10	0.764300966836406	0.088	0.124	3.33750467863244E-06	T/NK cell	ABI3
2.58698980821105E-10	1.92517178654646	0.131	0.115	4.21963907617304E-06	T/NK cell	RASSF1
2.59084579197111E-10	1.6729315936568	0.097	0.138	4.22592857128408E-06	T/NK cell	CSTF2T
3.22315853830347E-10	1.35937822672382	0.13	0.112	5.25729389182679E-06	T/NK cell	PRKCB
3.39096029438128E-10	1.5647101174197	0.081	0.115	5.53099533616531E-06	T/NK cell	LSM 8.00
3.50553319856178E-10	2.3611642400098	0.112	0.096	5.71787520017411E-06	T/NK cell	ZBTB25
3.56882795525455E-10	1.41637273387629	0.14	0.198	5.8211152778157E-06	T/NK cell	LDB1
3.8821015157854E-10	1.47226534502536	0.101	0.142	6.33209578239757E-06	T/NK cell	EPS15
3.96101903105572E-10	1.32808443909835	0.191	0.273	6.46081814155499E-06	T/NK cell	MED15

4.13030199258305E-10	1.45287290145779	0.076	0.108	6.73693558010222E-06	T/NK cell	DYRK1A
5.20396707920495E-10	1.53080270040263	0.102	0.142	8.48819070289119E-06	T/NK cell	TMPO
6.45860049385391E-10	1.6095244881634	0.096	0.135	1.05346232655251E-05	T/NK cell	SLC35E2B
6.89433851608064E-10	1.26899833876175	0.209	0.3	1.12453555535791E-05	T/NK cell	PKN1
8.64971712398747E-10	1.94367494639965	0.072	0.103	1.4108553600936E-05	T/NK cell	AK1
9.82328789014265E-10	1.57439061120931	0.326	0.396	1.60227648776117E-05	T/NK cell	WNK1
1.42189135187856E-09	2.03854650093536	0.105	0.089	2.31924698404912E-05	T/NK cell	CRLF3
1.85034699947721E-09	1.38109115709252	0.194	0.276	3.01810099084728E-05	T/NK cell	TRA2A
2.61383498866335E-09	1.79100391087212	0.112	0.097	4.26342625000878E-05	T/NK cell	PREX1
2.85395863217197E-09	1.42513472105309	0.081	0.113	4.6550919249357E-05	T/NK cell	RBM26
3.09586408615623E-09	1.79211264752819	0.086	0.12	5.04966391092943E-05	T/NK cell	LPIN1
3.24150788383776E-09	1.77099282816965	0.09	0.126	5.28722350932778E-05	T/NK cell	KAT6B
3.32300246369594E-09	1.52678422462605	0.085	0.118	5.42014931853445E-05	T/NK cell	KDM5A
3.67261312074748E-09	1.66195732056587	0.103	0.143	5.99039926125121E-05	T/NK cell	DHX36
4.20472502840966E-09	1.4146233184243	0.206	0.295	6.858326993839E-05	T/NK cell	PILRB
4.51702593859611E-09	1.74703594416088	0.077	0.108	7.36772100844411E-05	T/NK cell	ZDHHC18
4.70181393543443E-09	1.50106777947518	0.071	0.1	7.66912871008709E-05	T/NK cell	RAVER1
5.89211525119611E-09	1.10709863722521	0.251	0.365	9.61062918622597E-05	T/NK cell	PNRC1
6.52258380656197E-09	1.98765105880093	0.156	0.148	0.000106389864468832	T/NK cell	TTC14
6.5642105916995E-09	1.76962763808847	0.206	0.21	0.00010706883896121	T/NK cell	CREM
6.60371648495918E-09	1.63288193527303	0.087	0.121	0.000107713219586169	T/NK cell	SLC5A3
8.50941285147949E-09	1.30336785116964	0.084	0.116	0.000138797033020482	T/NK cell	FAM111A
8.53008522861096E-09	1.47362967009259	0.074	0.103	0.000139134220163873	T/NK cell	NR2C2
8.94472891929602E-09	1.39217319344175	0.245	0.259	0.000145897473402637	T/NK cell	TNFRSF1B
1.02880596809808E-08	1.4982167472092	0.152	0.211	0.000167808541456478	T/NK cell	TSC22D4
1.06124512536934E-08	1.62531839857603	0.092	0.127	0.000173099692398992	T/NK cell	CKLF
1.16835268630168E-08	1.65530962065947	0.11	0.151	0.000190570006662667	T/NK cell	TMEM63A
1.86270451636438E-08	1.6977491924313	0.103	0.142	0.000303825733664194	T/NK cell	CGGBP1
1.89713744192887E-08	1.46312213284833	0.163	0.226	0.000309442088153018	T/NK cell	ASXL1
2.26372636110364E-08	1.44309293315001	0.1	0.137	0.000369236406759614	T/NK cell	TATDN2
2.47000402314089E-08	1.03326425330109	0.18	0.171	0.000402882356214511	T/NK cell	UCP2
2.55465267045924E-08	1.78215896379359	0.087	0.121	0.000416689397078606	T/NK cell	C9orf78
3.46337565029176E-08	1.51945138851586	0.151	0.209	0.000564911202319089	T/NK cell	CDK9
3.97174843246488E-08	2.03539087238864	0.171	0.167	0.000647831886819347	T/NK cell	RNF44
4.18674518092468E-08	1.90117891747556	0.105	0.091	0.000682900006460624	T/NK cell	SYNE3
4.67248976154309E-08	1.37969256507432	0.077	0.106	0.000762129805005294	T/NK cell	TRAF3
4.70581087326834E-08	0.613445814154137	0.092	0.124	0.000767564811538799	T/NK cell	CYTH4
4.9351336984532E-08	1.79091372214016	0.23	0.247	0.000804969657554701	T/NK cell	KMT2E
5.012782237554E-08	1.68973407248233	0.075	0.103	0.000817634910767432	T/NK cell	NFATC3
5.36494719232158E-08	1.30474031699407	0.091	0.124	0.000875076536539573	T/NK cell	FAM49B
5.93421986021497E-08	1.6570995230603	0.102	0.139	0.000967930601399663	T/NK cell	CHIC2
6.89552535477386E-08	1.03169461074078	0.265	0.38	0.00112472914061716	T/NK cell	TUT7
7.17167964680359E-08	1.58695422575114	0.077	0.106	0.00116977266719013	T/NK cell	D2HGDH
9.57232438767385E-08	1.35359730109066	0.106	0.144	0.00156134183087348	T/NK cell	ETNK1
1.16341074076431E-07	1.5652430182797	0.142	0.194	0.00189763925926066	T/NK cell	YTHDC1
1.32979205802461E-07	1.27064281530228	0.161	0.153	0.00216902382584394	T/NK cell	NCKAP1L
2.01466981056308E-07	1.49856767984749	0.097	0.132	0.00328612792800944	T/NK cell	ARL6IP5
2.23543600374993E-07	1.67492666220987	0.124	0.112	0.0036462196657165	T/NK cell	KLF2
2.33996958813175E-07	1.76507059308252	0.115	0.156	0.0038167243952017	T/NK cell	IDS
2.95300621306638E-07	1.48948855363294	0.106	0.142	0.00481664843413257	T/NK cell	HNRNPD
3.08267748948663E-07	1.69785098256661	0.147	0.2	0.00502815525310164	T/NK cell	G3BP2
4.48283716452377E-07	1.93806636556032	0.257	0.292	0.00731195569905472	T/NK cell	EML4

5.01577005324402E-07	1.11886869363776	0.16	0.214	0.00818122253384632	T/NK cell	SERPINB9
5.36194004226227E-07	1.57592068962494	0.186	0.257	0.00874586040293399	T/NK cell	SMARCA2
5.93720462001568E-07	1.42201933274471	0.109	0.146	0.00968417445570758	T/NK cell	DNMT1
6.73723542878413E-07	1.55157832562551	0.091	0.122	0.0109891047078898	T/NK cell	SECISBP2
7.35637700949926E-07	0.556901138961225	0.369	0.536	0.0119989865401942	T/NK cell	CCNL2
8.50364045160508E-07	1.44967837401155	0.09	0.12	0.013870287940613	T/NK cell	SH2B1
1.02804242207184E-06	1.34109986831623	0.093	0.124	0.0167683999464138	T/NK cell	PRDM2
1.05516488902087E-06	1.17550312480858	0.119	0.158	0.0172107945048194	T/NK cell	RAPGEF1
1.23402475185588E-06	1.2500645482997	0.235	0.331	0.0201281777275212	T/NK cell	SLC9A3R1
1.39717318906539E-06	1.27793310486955	0.219	0.305	0.0227892918868456	T/NK cell	TAP2
1.41959167585702E-06	2.31984423768427	0.112	0.102	0.0231549598249038	T/NK cell	CCSER2
1.53385524609296E-06	1.77766649353641	0.118	0.158	0.0250187129190223	T/NK cell	DYNLT1
1.70411018288563E-06	1.53920426132744	0.087	0.116	0.0277957411930476	T/NK cell	PTPRJ
1.84454438992224E-06	1.30119760585068	0.155	0.207	0.0300863635440217	T/NK cell	SRSF7
2.063400944762E-06	1.68307463964251	0.08	0.107	0.0336561328100131	T/NK cell	MPHOSPH8
2.337220930695E-06	1.56969323745046	0.119	0.158	0.0381224106005661	T/NK cell	PRPF38B
2.38074846448911E-06	2.10274814718425	0.122	0.113	0.0388323882042819	T/NK cell	STK38
2.4940121043832E-06	1.86869273925297	0.112	0.15	0.0406798314345944	T/NK cell	NOP58
2.88933153422027E-06	1.70512523111337	0.263	0.301	0.0471278866546668	T/NK cell	PLAAT4
0	9.1587305911463	0.925	0.019	0	Fibroblast	COL3A1
0	5.89127585990455	0.946	0.063	0	Fibroblast	IGFBP7
0	9.45116208885606	0.903	0.022	0	Fibroblast	COL1A1
0	9.17007332462631	0.858	0.012	0	Fibroblast	COL1A2
0	6.95258485245285	0.881	0.036	0	Fibroblast	COL6A2
0	4.33375107430521	0.894	0.079	0	Fibroblast	SPARC
0	4.69360879326922	0.872	0.064	0	Fibroblast	COL4A2
0	8.24398221721909	0.816	0.017	0	Fibroblast	BGN
0	4.25424163272668	0.864	0.072	0	Fibroblast	COL4A1
0	7.22588365150943	0.874	0.089	0	Fibroblast	COL6A1
0	8.95756455529533	0.787	0.009	0	Fibroblast	DCN
0	9.35416981137559	0.736	0.006	0	Fibroblast	THBS2
0	7.50907494867531	0.725	0.012	0	Fibroblast	COL5A1
0	6.19914966846667	0.761	0.057	0	Fibroblast	TAGLN
0	7.50962964814776	0.713	0.011	0	Fibroblast	AEBP1
0	7.22006416598186	0.712	0.018	0	Fibroblast	EMILIN1
0	4.92789403156035	0.792	0.103	0	Fibroblast	LTBP4
0	6.71137100050908	0.748	0.07	0	Fibroblast	COLEC11
0	6.72646759729528	0.664	0.015	0	Fibroblast	TNXB
0	6.78970054242759	0.663	0.014	0	Fibroblast	FBN1
0	5.66183706009326	0.652	0.032	0	Fibroblast	ADAMTS2
0	7.34027136259667	0.633	0.014	0	Fibroblast	C7
0	8.06667492633014	0.62	0.006	0	Fibroblast	GGT5
0	9.57562125647862	0.616	0.003	0	Fibroblast	EFEMP1
0	5.83677875649643	0.645	0.039	0	Fibroblast	C11orf96
0	7.33458175996992	0.648	0.044	0	Fibroblast	CCN1
0	7.5902843842791	0.594	0.007	0	Fibroblast	CRISPLD2
0	6.06395539117787	0.596	0.014	0	Fibroblast	COL6A3
0	7.03748290776865	0.669	0.088	0	Fibroblast	IGFBP3
0	4.37906533820534	0.669	0.089	0	Fibroblast	CXCL12
0	7.98642423410805	0.581	0.005	0	Fibroblast	MYL9
0	6.78813529458359	0.595	0.02	0	Fibroblast	FSTL1
0	4.78133383252056	0.701	0.136	0	Fibroblast	TIMP1

0	3.13179860126873	0.796	0.233	0	Fibroblast	C1R
0	4.04914924537969	0.74	0.178	0	Fibroblast	CALD1
0	6.58134539608171	0.599	0.037	0	Fibroblast	ADAMTSL2
0	6.8044929097702	0.538	0.006	0	Fibroblast	CCL21
0	5.15431715030169	0.638	0.109	0	Fibroblast	TPM1
0	4.70628598649024	0.603	0.081	0	Fibroblast	MICALL2
0	8.16580584852335	0.518	0.003	0	Fibroblast	PODN
0	5.03295096871119	0.532	0.022	0	Fibroblast	GEM
0	3.96624064106066	0.53	0.038	0	Fibroblast	LRRC32
0	7.39313574051327	0.512	0.024	0	Fibroblast	PDGFRB
0	2.3657704157201	0.587	0.111	0	Fibroblast	ENG
0	7.24156905118331	0.478	0.004	0	Fibroblast	FBLN5
0	7.44243202428609	0.504	0.031	0	Fibroblast	CCN2
0	4.96924503350576	0.493	0.026	0	Fibroblast	LAMB1
0	9.17049628655996	0.469	0.002	0	Fibroblast	LUM
0	6.50517605883328	0.469	0.006	0	Fibroblast	FBLN2
0	3.10039038322492	0.508	0.051	0	Fibroblast	ITM2C
0	6.61454507246805	0.473	0.016	0	Fibroblast	ACTA2
0	3.93585306356804	0.504	0.05	0	Fibroblast	MEGF6
0	3.36188302089733	0.527	0.079	0	Fibroblast	ITGA9
0	5.78888999246031	0.459	0.012	0	Fibroblast	INMT
0	8.05625960485932	0.452	0.005	0	Fibroblast	COLEC10
0	6.88725630381574	0.453	0.01	0	Fibroblast	COL5A2
0	3.34343669026665	0.519	0.088	0	Fibroblast	CRIM1
0	5.36041907814952	0.438	0.018	0	Fibroblast	ADAMTS1
0	6.69442467691912	0.423	0.009	0	Fibroblast	HGF
0	8.76407044391341	0.406	0.002	0	Fibroblast	SVEP1
0	7.1533044381004	0.419	0.015	0	Fibroblast	ADAMTS13
0	7.73388715701681	0.406	0.003	0	Fibroblast	FBLN1
0	4.58874732815335	0.449	0.047	0	Fibroblast	RBPMS
0	8.83276641127174	0.404	0.003	0	Fibroblast	COL16A1
0	5.51696635907745	0.42	0.02	0	Fibroblast	LOXL2
0	9.22200247367718	0.399	0.002	0	Fibroblast	LAMC3
0	3.64396096374851	0.46	0.065	0	Fibroblast	ITGA1
0	4.80842385739806	0.419	0.026	0	Fibroblast	JAG1
0	6.3673388790301	0.397	0.007	0	Fibroblast	ANGPTL2
0	8.62327881395	0.391	0.001	0	Fibroblast	MFAP4
0	5.64186854174461	0.399	0.012	0	Fibroblast	C1QTNF1
0	3.23790129877404	0.426	0.042	0	Fibroblast	TCIM
0	4.16393605566292	0.425	0.041	0	Fibroblast	PHLDB1
0	5.47696904662515	0.396	0.012	0	Fibroblast	PTGIR
0	4.62805416273089	0.44	0.058	0	Fibroblast	TANC1
0	7.1652668404283	0.385	0.003	0	Fibroblast	ELN
0	7.1962670262085	0.39	0.009	0	Fibroblast	NFASC
0	4.23649668056486	0.424	0.047	0	Fibroblast	LIFR
0	5.88449954335063	0.385	0.011	0	Fibroblast	RIPOR3
0	9.17075724997958	0.372	0.001	0	Fibroblast	PTH1R
0	9.13517515934498	0.369	0.001	0	Fibroblast	ID4
0	8.66985351945702	0.369	0.001	0	Fibroblast	COL14A1
0	6.11621669428883	0.37	0.012	0	Fibroblast	ECM1
0	8.04783270120623	0.358	0.002	0	Fibroblast	NGFR
0	6.47545826154834	0.364	0.01	0	Fibroblast	CCL2

0	4.37030161035343	0.369	0.02	0	Fibroblast	PXDN
0	5.05058161840631	0.389	0.041	0	Fibroblast	MYH10
0	4.96412610806401	0.361	0.014	0	Fibroblast	OSMR
0	5.11033988238604	0.352	0.012	0	Fibroblast	CLEC11A
0	6.39897282580128	0.346	0.009	0	Fibroblast	CCDC102B
0	5.52300038551764	0.348	0.012	0	Fibroblast	NEURL1B
0	5.30228349841991	0.346	0.01	0	Fibroblast	ADGRA2
0	5.2483370540245	0.353	0.017	0	Fibroblast	ARHGEF17
0	8.71363539422	0.335	0.001	0	Fibroblast	MXRA8
0	5.6776719878428	0.348	0.021	0	Fibroblast	RBP1
0	5.63868345454589	0.351	0.027	0	Fibroblast	PCOLCE
0	9.26925170049953	0.325	0.002	0	Fibroblast	TNC
0	3.8620141268574	0.34	0.018	0	Fibroblast	ANK3
0	7.38326803195448	0.323	0.004	0	Fibroblast	OLFML3
0	7.8156375091194	0.319	0.002	0	Fibroblast	PRKG1
0	6.58255819652422	0.318	0.003	0	Fibroblast	COL4A4
0	4.5848624781218	0.331	0.016	0	Fibroblast	RBMS3
0	7.50163386074706	0.317	0.003	0	Fibroblast	CCDC3
0	5.13061368608978	0.335	0.022	0	Fibroblast	LMCD1
0	5.53754132459975	0.335	0.024	0	Fibroblast	RGS16
0	9.15523267521946	0.311	0.002	0	Fibroblast	ALDH1A3
0	6.38627627442516	0.321	0.013	0	Fibroblast	HMCN1
0	7.38369501760132	0.304	0.002	0	Fibroblast	LAMA2
0	3.7765537839697	0.338	0.038	0	Fibroblast	CAVIN1
0	5.62048737179585	0.321	0.023	0	Fibroblast	DMD
0	4.19724988528257	0.337	0.041	0	Fibroblast	TPM2
0	8.54779806739749	0.296	0.001	0	Fibroblast	PDGFRA
0	7.51517708923477	0.297	0.003	0	Fibroblast	FHL2
0	5.17659620955921	0.302	0.01	0	Fibroblast	MMP2
0	7.77459847575682	0.289	0.002	0	Fibroblast	PLA2G5
0	6.43970283670863	0.296	0.009	0	Fibroblast	FSTL3
0	7.72123232674577	0.283	0.002	0	Fibroblast	STEAP4
0	6.25212139541361	0.286	0.005	0	Fibroblast	DENND2A
0	5.08015572022188	0.291	0.011	0	Fibroblast	TBXA2R
0	5.77734357703288	0.311	0.032	0	Fibroblast	LTBP2
0	6.34979687088107	0.277	0.006	0	Fibroblast	SEPTIN4
0	5.42469205199996	0.278	0.009	0	Fibroblast	KAZN
0	5.22811420495878	0.277	0.009	0	Fibroblast	THY1
0	5.1518355205895	0.277	0.009	0	Fibroblast	FKBP10
0	8.10582063293103	0.268	0.002	0	Fibroblast	ITGBL1
0	5.58901671481053	0.284	0.018	0	Fibroblast	VIPR1
0	3.12256069472455	0.283	0.018	0	Fibroblast	IGFBP5
0	8.6346776556345	0.264	0.001	0	Fibroblast	FAM163B
0	5.29086023586347	0.283	0.02	0	Fibroblast	SCARF2
0	8.34078853025633	0.265	0.005	0	Fibroblast	HEYL
0	9.65859791971707	0.259	0.002	0	Fibroblast	PAPPA
0	3.84896846487254	0.279	0.022	0	Fibroblast	PRSS23
0	8.3473361101944	0.258	0.002	0	Fibroblast	HHIP
0	8.69974346967723	0.259	0.003	0	Fibroblast	ADRA1B
0	5.21690039967841	0.273	0.018	0	Fibroblast	MATN2
0	7.23212711420624	0.258	0.006	0	Fibroblast	NOTCH3
0	8.35738179434354	0.252	0.001	0	Fibroblast	FAM180A

	0	6.52582710065431	0.252	0.002	0	Fibroblast	SYNPO2
	0	5.36382892233738	0.269	0.02	0	Fibroblast	PTGDS
	0	8.45608914910752	0.248	0.001	0	Fibroblast	ASPN
	0	5.8729427455315	0.249	0.006	0	Fibroblast	KIRREL1
	0	5.30169616983194	0.252	0.01	0	Fibroblast	EHD2
	0	6.78025339843112	0.244	0.003	0	Fibroblast	CCDC80
	0	6.58883778710502	0.247	0.01	0	Fibroblast	NKD2
	0	6.7965451725224	0.239	0.002	0	Fibroblast	ADAMTS12
	0	7.35540029383531	0.242	0.007	0	Fibroblast	ADCY5
	0	7.48700497131125	0.235	0.001	0	Fibroblast	LOXL1
	0	6.56676599476579	0.239	0.005	0	Fibroblast	DEPRECATI
	0	7.98885320168029	0.234	0.001	0	Fibroblast	PRELP
	0	5.85657310188682	0.238	0.005	0	Fibroblast	PTGIS
	0	4.25085808064168	0.24	0.008	0	Fibroblast	DKK 3.00
	0	5.39542628920094	0.248	0.018	0	Fibroblast	PDLIM3
	0	5.04641673659782	0.236	0.007	0	Fibroblast	SERPINE2
	0	4.52401710956443	0.239	0.011	0	Fibroblast	EFEMP2
	0	8.10538798575043	0.223	0.002	0	Fibroblast	IGF2
	0	4.51063167786143	0.233	0.012	0	Fibroblast	FHL1
	0	6.65957446283391	0.223	0.003	0	Fibroblast	TBX2
	0	3.07523653993785	0.235	0.016	0	Fibroblast	VCAN
	0	4.81617052211593	0.236	0.017	0	Fibroblast	PAPLN
	0	8.91344872996206	0.218	0.001	0	Fibroblast	ISLR
	0	5.40676410439999	0.22	0.006	0	Fibroblast	NID2
	0	7.19809153916578	0.215	0.005	0	Fibroblast	SRPX2
	0	5.60727507888378	0.211	0.005	0	Fibroblast	TGFB1I1
	0	4.08324014418632	0.221	0.016	0	Fibroblast	PMP22
	0	8.34042937075819	0.205	0.001	0	Fibroblast	ITGA11
	0	3.72222146214336	0.218	0.014	0	Fibroblast	ISYNA1
	0	9.97414390940144	0.201	0.001	0	Fibroblast	LRRC7
	0	4.98267078368925	0.208	0.008	0	Fibroblast	WTIP
	0	4.49348374563727	0.204	0.005	0	Fibroblast	MMP23B
	0	7.76090860310215	0.197	0.001	0	Fibroblast	FMO2
	0	6.7245338150905	0.197	0.002	0	Fibroblast	GAS1
	0	7.98908427721038	0.195	0.001	0	Fibroblast	HAND2
	0	5.81040961349077	0.199	0.005	0	Fibroblast	MGP
	0	4.30685423485972	0.206	0.012	0	Fibroblast	LHFPL6
	0	6.59792235441126	0.194	0.003	0	Fibroblast	DEPRECATI
	0	7.65187474730918	0.192	0.002	0	Fibroblast	DAAM2
	0	6.35187311202081	0.191	0.003	0	Fibroblast	DPYSL3
	0	6.43307321591684	0.187	0.002	0	Fibroblast	S1PR3
	0	8.96772438062744	0.181	0.001	0	Fibroblast	PLEKHH2
	0	7.06520653507747	0.186	0.006	0	Fibroblast	CYGB
	0	5.67687002096143	0.184	0.004	0	Fibroblast	PPP1R14A
	0	9.75536921500541	0.179	0	0	Fibroblast	TCF21
	0	7.41655601079573	0.179	0.001	0	Fibroblast	GALNT16
	0	6.31163589086048	0.18	0.003	0	Fibroblast	CERCAM
	0	9.22209269447412	0.176	0	0	Fibroblast	LMOD1
	0	6.59898760928231	0.177	0.001	0	Fibroblast	SSC5D
	0	7.49023833480646	0.174	0.001	0	Fibroblast	MAPK10
	0	6.89306678705704	0.174	0.004	0	Fibroblast	F3
	0	5.99628025449075	0.172	0.003	0	Fibroblast	ADAMTSL1

0	6.14286155279828	0.171	0.003	0	Fibroblast	ANTXR1
0	6.50094751107989	0.169	0.001	0	Fibroblast	PTN
0	8.07527124531827	0.167	0.001	0	Fibroblast	DACT3
0	7.20545518603583	0.164	0.003	0	Fibroblast	SAMD11
0	5.5109386775298	0.166	0.005	0	Fibroblast	GUCY1B1
0	5.25896398565796	0.164	0.003	0	Fibroblast	VEGFC
0	9.62485173353299	0.162	0.001	0	Fibroblast	DLK1
0	7.03623516070733	0.164	0.004	0	Fibroblast	CCL19
0	7.67048021000344	0.158	0.001	0	Fibroblast	BMP10
0	8.44118167180143	0.158	0.001	0	Fibroblast	COL25A1
0	8.9979631415288	0.158	0.001	0	Fibroblast	CCBE1
0	6.77970816193933	0.152	0.002	0	Fibroblast	PLA2R1
0	6.0204438899397	0.153	0.003	0	Fibroblast	CLDN11
0	8.01360117535583	0.148	0	0	Fibroblast	SMOC2
0	8.35767196474768	0.148	0.001	0	Fibroblast	GDF2
0	9.27626146343854	0.146	0	0	Fibroblast	ST6GAL2
0	8.49884957725451	0.147	0.001	0	Fibroblast	PLCXD3
0	6.56705793880257	0.145	0.001	0	Fibroblast	GLI3
0	6.86190989071994	0.145	0.001	0	Fibroblast	SUSD2
0	6.41268611675839	0.145	0.002	0	Fibroblast	PGF
0	6.86637165645521	0.145	0.003	0	Fibroblast	OLFML1
0	8.46922836437768	0.141	0.001	0	Fibroblast	CCN4
0	7.33254112665053	0.141	0.001	0	Fibroblast	MRVI1
0	9.196486046125	0.136	0	0	Fibroblast	ALLC
0	6.49368684397761	0.137	0.002	0	Fibroblast	ARHGEF25
0	8.64175787326641	0.133	0	0	Fibroblast	FMOD
0	5.11301727489529	0.137	0.004	0	Fibroblast	CACNA1C
0	5.01752911396711	0.135	0.004	0	Fibroblast	CHST3
0	8.52660791598219	0.13	0	0	Fibroblast	COL12A1
0	8.50511780378972	0.131	0.001	0	Fibroblast	HSPB6
0	9.20330563691289	0.127	0	0	Fibroblast	PDE3A
0	8.37823055465689	0.127	0.001	0	Fibroblast	CXCL14
0	8.55997700128303	0.125	0	0	Fibroblast	EPHA3
0	6.55919792546731	0.126	0.003	0	Fibroblast	IL34
0	6.93028251059187	0.123	0.002	0	Fibroblast	MXRA5
0	9.633350782315	0.121	0.001	0	Fibroblast	FIBIN
0	9.93119523162844	0.118	0	0	Fibroblast	PAMR1
0	8.66362665760142	0.114	0	0	Fibroblast	CDH19
0	8.6225079785224	0.111	0	0	Fibroblast	DPT
0	8.4034292670018	0.111	0	0	Fibroblast	PDE1A
0	8.50242900889775	0.111	0	0	Fibroblast	SLIT3
0	9.7463002709095	0.111	0	0	Fibroblast	FGF7
0	4.68748542661343	0.112	0.002	0	Fibroblast	CD248
0	6.08699531983277	0.112	0.002	0	Fibroblast	FOXF1
0	8.4672408102075	0.109	0.001	0	Fibroblast	VGLL3
0	6.46543928722394	0.106	0.001	0	Fibroblast	PDZRN3
0	4.77888803894169	0.107	0.002	0	Fibroblast	SPON1
0	7.43528612589878	0.104	0.001	0	Fibroblast	KIAA1211
0	8.42820850565448	0.102	0	0	Fibroblast	KCNE4
0	8.85945286025407	0.102	0.001	0	Fibroblast	GLI2
0	7.79863662476345	0.101	0.001	0	Fibroblast	WFDC1
1.42081737428337E-306	3.63770080874247	0.462	0.082	2.3174952191936E-302	Fibroblast	TIMP3

**1.16237483432657E-304**	3.44080013430427	0.517	0.107	1.89594959227007E-300	Fibroblast	NID1
**2.17191337231311E-303**	4.29027791262468	0.196	0.012	3.54260790157991E-299	Fibroblast	FILIP1L
**3.11012244459766E-303**	3.49131388966264	0.682	0.208	5.07292071938324E-299	Fibroblast	THBS1
**5.24967837283259E-300**	5.28493156757245	0.104	0.002	8.56275039392723E-296	Fibroblast	ZFPM2
**4.36217513133008E-297**	5.43645807720597	0.113	0.003	7.11514385671249E-293	Fibroblast	MAP1B
**6.27131862848338E-296**	2.59801145760156	0.782	0.289	1.02291478149192E-291	Fibroblast	IGFBP4
**4.6429308088186E-295**	4.78479782137799	0.147	0.006	7.57308444226401E-291	Fibroblast	DEPRECATI
**2.1025336515655E-293**	4.04392575547208	0.374	0.056	3.42944263906848E-289	Fibroblast	NR2F1
**9.23521321212896E-292**	3.13613096121035	0.406	0.064	1.50635562703035E-287	Fibroblast	GRK5
**8.37108008392886E-290**	3.59995147645673	0.431	0.075	1.36540687248964E-285	Fibroblast	SERPINH1
**1.7822462439847E-289**	4.8933290065546	0.13	0.005	2.90702184856344E-285	Fibroblast	P3H3
**2.01136561046596E-287**	4.1808821953456	0.201	0.014	3.28073844723102E-283	Fibroblast	CYBRD1
**1.2249558834635E-279**	4.41693624044936	0.254	0.025	1.99802554151731E-275	Fibroblast	PBX1
**1.82226930744567E-279**	4.03489668208465	0.172	0.01	2.97230346737464E-275	Fibroblast	DCLK2
**2.69209686184907E-279**	4.20166291934544	0.551	0.144	4.39107919136202E-275	Fibroblast	MASP1
**3.91129109546365E-277**	3.11073172074022	0.472	0.094	6.37970690581076E-273	Fibroblast	SARDH
**3.38249569079093E-273**	4.74264857513219	0.101	0.002	5.51718872124908E-269	Fibroblast	COL4A3
**4.60216537795938E-270**	5.49840281802377	0.179	0.012	7.50659194798954E-266	Fibroblast	NFATC4
**2.35732639915859E-269**	5.75263523518272	0.22	0.019	3.84503508966758E-265	Fibroblast	TNFRSF11E
**9.0432412082353E-264**	4.41536037774095	0.106	0.003	1.47504307347526E-259	Fibroblast	TRO
**6.55546811550664E-263**	4.91119736608254	0.303	0.04	1.06926240432029E-258	Fibroblast	PKDCC
**1.0027526392818E-260**	5.8181205280996	0.255	0.028	1.63558982993255E-256	Fibroblast	SOD3
**1.27195257649304E-259**	4.47149375476777	0.358	0.059	2.07468184751779E-255	Fibroblast	MYLK
**1.96112062970188E-256**	3.98242452081672	0.255	0.028	3.19878385910673E-252	Fibroblast	VCL
**6.2669460414063E-253**	6.08137127033499	0.107	0.003	1.02220156881378E-248	Fibroblast	FRZB
**8.82936864916035E-251**	4.49457433362906	0.156	0.009	1.44015832036454E-246	Fibroblast	GJA4
**2.84051663812459E-248**	2.03112391336252	0.775	0.26	4.63316668844501E-244	Fibroblast	C1S
**1.42089861811198E-247**	3.16473603700518	0.387	0.068	2.31762773600245E-243	Fibroblast	SMTN
**5.41539381186036E-247**	4.20901426885061	0.189	0.015	8.83304884652544E-243	Fibroblast	TGFB3
**1.50677281207134E-246**	5.23694248361195	0.172	0.012	2.45769713376956E-242	Fibroblast	GLIS3
**1.6481392812686E-241**	4.62548669450887	0.289	0.04	2.68827998167722E-237	Fibroblast	PALLD
**3.3111759278718E-240**	5.95307814214423	0.187	0.016	5.40085905595169E-236	Fibroblast	KIAA1755
**1.14737373627819E-238**	3.86187092026934	0.448	0.103	1.87148130124336E-234	Fibroblast	PTPRS
**7.04482318052487E-235**	3.91779581514607	0.284	0.039	1.14908110897541E-230	Fibroblast	NIBAN2
**1.5039392113221E-232**	3.77816839905566	0.223	0.023	2.45307524758747E-228	Fibroblast	MFGE8
**4.03380504933669E-229**	2.80742723019604	0.609	0.199	6.57953941597307E-225	Fibroblast	GPX3
**2.99779787998205E-227**	3.71905560201365	0.185	0.016	4.88970812203872E-223	Fibroblast	NES
**1.97858387351563E-226**	3.98539005532652	0.384	0.076	3.22726815609135E-222	Fibroblast	IFITM10
**6.17774540570197E-226**	5.18607881427472	0.111	0.005	1.00765205312405E-221	Fibroblast	DACT1
**2.32497999566909E-225**	5.20051543388461	0.141	0.009	3.79227487093585E-221	Fibroblast	GUCY1A1
**8.28733297369828E-224**	3.69501025653142	0.518	0.149	1.35174688133993E-219	Fibroblast	NR1H4
**3.60894698910936E-223**	4.1600144728921	0.123	0.006	5.88655343393629E-219	Fibroblast	EPHB3
**6.12840350434085E-223**	4.56052850736291	0.116	0.005	9.99603895593036E-219	Fibroblast	FILIP1
**5.32300252356062E-219**	4.61706692833707	0.259	0.035	8.68234941617973E-215	Fibroblast	SEMA5A
**5.53669514722541E-217**	3.54786192597365	0.415	0.093	9.03090345463937E-213	Fibroblast	EDNRB
**1.56702165440877E-212**	3.44250270559701	0.314	0.052	2.55596902050614E-208	Fibroblast	DAAM1
**4.83692167878362E-212**	6.33359941780384	0.106	0.005	7.88950295026396E-208	Fibroblast	FIGN
**1.63703347014323E-211**	3.16800611338096	0.205	0.021	2.67016529315062E-207	Fibroblast	NLGN2
**3.47029653320394E-209**	2.74680315140268	0.17	0.014	5.66040067530895E-205	Fibroblast	EVC
**4.19319286646579E-209**	4.05811250425749	0.312	0.053	6.83951688449234E-205	Fibroblast	NR2F2
**6.354600773124E-209**	2.69385646206668	0.42	0.091	1.03649893210425E-204	Fibroblast	RCN3
**1.5957780838312E-208**	6.35149840094714	0.127	0.008	2.60287363253707E-204	Fibroblast	ANGPT1

1.81497238533254E-207	2.72551965466731	0.346	0.062	2.96040145771591E-203	Fibroblast	ID3
1.44521779983617E-205	2.36261095720387	0.506	0.13	2.35729475331278E-201	Fibroblast	TIMP2
1.24131254050364E-203	3.0096932233207	0.4	0.088	2.02470488481549E-199	Fibroblast	PELO
1.76705691485146E-200	2.77613146652907	0.24	0.031	2.88224653381422E-196	Fibroblast	ENC1
2.23513944883777E-200	3.10449013864451	0.345	0.066	3.64573595499928E-196	Fibroblast	LIMA1
6.00914614695997E-199	4.08705647897265	0.366	0.079	9.8015182803064E-195	Fibroblast	C1orf198
3.11482242212409E-198	2.54783551732631	0.367	0.072	5.0805868527266E-194	Fibroblast	MRC2
4.15670536822213E-198	4.54325854323318	0.153	0.012	6.78000212610712E-194	Fibroblast	PCDHGC3
6.61955556342376E-198	3.91062520018327	0.208	0.024	1.07971570795005E-193	Fibroblast	RELN
9.94448809027129E-198	3.17295832480702	0.272	0.04	1.62204545240415E-193	Fibroblast	TINAGL1
3.68003848060419E-196	4.22517997322013	0.201	0.022	6.00251076571349E-192	Fibroblast	PTGS2
4.38512557363877E-196	3.08603612918092	0.433	0.109	7.15257832316219E-192	Fibroblast	CNN3
7.78108985235942E-194	2.50470745263057	0.541	0.169	1.26917356581835E-189	Fibroblast	APP
2.1261227215588E-193	4.2712681817452	0.135	0.009	3.46791877113456E-189	Fibroblast	UNC5B
1.8708873313031E-191	4.9750921063444	0.138	0.01	3.05160432608849E-187	Fibroblast	CACNA2D1
1.6727356952124E-189	2.80707095660394	0.21	0.024	2.72839919246094E-185	Fibroblast	TMEM204
2.11896252686931E-189	3.56136694312908	0.314	0.058	3.45623977757654E-185	Fibroblast	UACA
1.10681706221077E-188	4.29995825487003	0.284	0.049	1.80532931017198E-184	Fibroblast	AMOTL2
4.95666598104237E-188	3.06698305876805	0.506	0.159	8.08481788167821E-184	Fibroblast	ACTN1
1.3612523358671E-187	2.42461381003233	0.63	0.248	2.22033868503282E-183	Fibroblast	LGALS3BP
1.6691763287513E-183	4.14500589767006	0.111	0.006	2.72259350982625E-179	Fibroblast	LPAR1
2.49821319795644E-179	2.68169036895897	0.187	0.021	4.07483554718675E-175	Fibroblast	LAMA4
8.26701559203717E-177	3.82886731102358	0.153	0.014	1.34843291321718E-172	Fibroblast	HSPB8
1.92494626794454E-176	5.8150234325884	0.108	0.006	3.13977985764434E-172	Fibroblast	EFS
2.72838061785863E-176	4.2851409939666	0.102	0.005	4.45026162578921E-172	Fibroblast	DEPRECATI
3.05886802468077E-175	3.30711358453002	0.175	0.018	4.98931963505681E-171	Fibroblast	MYO10
1.87870719580313E-174	3.87834769811739	0.191	0.023	3.06435930707449E-170	Fibroblast	MEIS1
1.15123655823736E-173	1.87887511945276	0.361	0.074	1.87778195014096E-169	Fibroblast	SYTL2
2.71112997239233E-173	2.73929193071841	0.481	0.145	4.42212409796914E-169	Fibroblast	PPP1R12B
6.61857891117462E-173	1.83746033666176	0.503	0.137	1.07955640620169E-168	Fibroblast	FLNA
1.04774743674237E-172	2.72526479201349	0.342	0.072	1.70898084407049E-168	Fibroblast	MXRA7
4.76543986246939E-171	3.01570428974202	0.449	0.131	7.77290895967381E-167	Fibroblast	LAMB2
1.42220775985768E-170	3.05455953947856	0.516	0.182	2.31976307710386E-166	Fibroblast	QSOX1
3.84186656779823E-170	1.6180601972291	0.342	0.068	6.2664685587357E-166	Fibroblast	HSPG2
6.90577802121525E-168	3.14478819856754	0.338	0.074	1.12640145304042E-163	Fibroblast	PDLIM7
4.77218391817067E-167	2.85354719809058	0.283	0.051	7.78390918892818E-163	Fibroblast	SLCO3A1
1.50034579089169E-166	3.36113159159938	0.282	0.052	2.44721401952343E-162	Fibroblast	CPED1
1.85917595599935E-166	4.96101366518994	0.103	0.006	3.03250190183054E-162	Fibroblast	HSPA2
4.85970164019702E-165	5.55185599650325	0.138	0.012	7.92665934532536E-161	Fibroblast	ECM2
4.92649794047827E-165	3.52415351985651	0.326	0.071	8.03561079071411E-161	Fibroblast	KANK2
2.46570617495041E-164	2.47631884653148	0.254	0.041	4.02181334196161E-160	Fibroblast	HIC1
1.2907955541011E-161	2.53816800950927	0.391	0.097	2.1054166282943E-157	Fibroblast	DPYSL2
6.0712761317542E-161	3.29252386385852	0.342	0.079	9.90285849850428E-157	Fibroblast	DLC1
8.38001144126343E-161	3.17329390320641	0.143	0.013	1.36686366618448E-156	Fibroblast	GNA14
1.22415006349019E-158	2.42833327114068	0.283	0.052	1.99671116855885E-154	Fibroblast	FXYD6
3.9991981804903E-154	3.88163004296056	0.218	0.034	6.52309215219772E-150	Fibroblast	EPS8
1.56889033295126E-153	3.25645581836346	0.208	0.031	2.55901702207679E-149	Fibroblast	USP53
1.93099522849434E-153	3.37135499935736	0.37	0.098	3.14964631719713E-149	Fibroblast	ST5
6.05058057680292E-153	2.73406552999997	0.372	0.096	9.86910197882324E-149	Fibroblast	SEPTIN11
1.08686432765046E-143	4.90418092325008	0.165	0.021	1.77278440483067E-139	Fibroblast	KLHL29
4.58110904695056E-143	3.10230097580699	0.13	0.012	7.47224696648105E-139	Fibroblast	BMPER
3.42892342183534E-142	3.03697288299966	0.331	0.082	5.59291699335562E-138	Fibroblast	TNS1

9.4179949607121E-138	2.26640356991246	0.579	0.254	1.53616915804175E-133	Fibroblast	CSRP1
8.19929766693847E-136	4.23939271773676	0.219	0.039	1.33738744245433E-131	Fibroblast	GATA6
9.48644158896929E-136	1.13776430800401	0.795	0.315	1.54733348757678E-131	Fibroblast	FN1
1.26577657906093E-135	3.75495047469176	0.109	0.009	2.06460817810629E-131	Fibroblast	LXN
1.672282565378E-135	2.54045788299903	0.302	0.069	2.72766009238806E-131	Fibroblast	KALRN
4.00137002271423E-134	1.78190583900196	0.208	0.032	6.52663464404917E-130	Fibroblast	F2R
5.51704469514697E-132	2.45600855952048	0.501	0.197	8.99885160225422E-128	Fibroblast	EPAS1
2.79590409145773E-131	1.38409684068812	0.735	0.333	4.5603991635767E-127	Fibroblast	VIM
8.61810024095339E-131	3.20279241468897	0.264	0.057	1.40569833030191E-126	Fibroblast	BMP1
2.53599678103244E-129	3.33468361976275	0.312	0.08	4.13646434954201E-125	Fibroblast	SORBS1
5.8290754356563E-129	4.08961123275452	0.13	0.014	9.50780494309899E-125	Fibroblast	MINDY4
8.28464368449461E-129	3.72291448206419	0.327	0.09	1.35130823137792E-124	Fibroblast	COL27A1
5.77810589852079E-125	2.07257867473573	0.249	0.049	9.42466853107726E-121	Fibroblast	SMAD6
4.69551221948892E-124	2.55262929183195	0.181	0.028	7.65884998120837E-120	Fibroblast	HOXB3
2.68890943374276E-123	2.6741937657629	0.14	0.017	4.38588017737781E-119	Fibroblast	RNF122
8.89365530474725E-122	5.53170243596646	0.104	0.009	1.45064411675732E-117	Fibroblast	BICC1
1.07248222910022E-121	4.19379801892988	0.123	0.013	1.74932576388538E-117	Fibroblast	MEX3B
1.96786942932414E-119	2.04194388236215	0.406	0.128	3.2097918261706E-115	Fibroblast	NRP1
4.43322761752595E-119	2.28669083114928	0.218	0.041	7.23103756694658E-115	Fibroblast	SPRY4
2.21492430263301E-116	4.29904302996457	0.143	0.019	3.61276303002471E-112	Fibroblast	DCBLD2
1.04281416375937E-115	1.52479523487484	0.221	0.042	1.70093418250791E-111	Fibroblast	SPRY1
5.47431848058146E-115	2.46165710507955	0.126	0.014	8.92916087367641E-111	Fibroblast	CCDC146
1.19483210123965E-114	4.55330136374185	0.272	0.068	1.948890640332E-110	Fibroblast	SERPINE1
5.34385965848505E-114	3.13298792789455	0.463	0.189	8.71636948895497E-110	Fibroblast	DEPP1
7.28852717134284E-114	2.47160044131484	0.194	0.034	1.18883166691773E-109	Fibroblast	PKD2
1.75990274537631E-113	2.81224627359703	0.239	0.052	2.8705773679833E-109	Fibroblast	SEPTIN8
1.88886858960745E-113	2.66160765247307	0.195	0.034	3.0809335565087E-109	Fibroblast	ADAMTS4
1.71712946672898E-111	2.80197455024318	0.302	0.082	2.80080987318164E-107	Fibroblast	IL1R1
1.63279969128048E-110	4.04658921186896	0.152	0.023	2.66325957644759E-106	Fibroblast	ART4
3.2372210629068E-110	4.14597560398353	0.108	0.011	5.28023127570729E-106	Fibroblast	TMEM178A
4.63404351890559E-109	1.4587012903437	0.253	0.054	7.5585883836869E-105	Fibroblast	TRIB2
2.8008959788386E-108	3.17459791999007	0.18	0.032	4.56854143108364E-104	Fibroblast	PKIG
1.2685942091997E-106	2.20303470014077	0.38	0.127	2.06920401462563E-102	Fibroblast	SLC12A4
1.53351190301837E-106	2.88983678618011	0.106	0.011	2.50131126501327E-102	Fibroblast	ARHGAP24
3.24148579623321E-106	2.33642686423955	0.15	0.022	5.28718748223599E-102	Fibroblast	EHD3
4.68470441363878E-106	3.17559366618119	0.101	0.01	7.64122136908622E-102	Fibroblast	P3H2
5.40872617124427E-106	3.51398306710289	0.297	0.086	8.82217325791654E-102	Fibroblast	ARVCF
7.47917666953126E-106	3.61042201818399	0.184	0.033	1.21992850656724E-101	Fibroblast	ADAMTS9
2.47681430199827E-104	3.02905184501722	0.323	0.101	4.03993180798937E-100	Fibroblast	CTIF
5.43224765783281E-104	1.9758684360206	0.113	0.013	8.8605391546911E-100	Fibroblast	STMN3
1.00905499672822E-103	2.28228591339024	0.258	0.063	1.64586960516341E-99	Fibroblast	MYADM
3.18680058770128E-103	2.27605724711639	0.19	0.035	5.19799043859956E-99	Fibroblast	DCHS1
4.85900314577351E-103	2.53577469581184	0.201	0.04	7.92552003107117E-99	Fibroblast	MYOF
4.41172438784752E-102	4.45363685011051	0.175	0.032	7.19596364901808E-98	Fibroblast	CPE
1.00086084313093E-101	2.91438901780663	0.142	0.021	1.63250412123087E-97	Fibroblast	ITGA3
5.57094170564054E-101	2.10380176440708	0.146	0.022	9.08676301607029E-97	Fibroblast	APLNR
5.03146730748543E-100	2.11088775981236	0.162	0.027	8.20682632523949E-96	Fibroblast	ARHGAP23
9.23421850587699E-100	3.01906715967351	0.143	0.022	1.5061933804936E-95	Fibroblast	MARVELD1
6.06817433043888E-99	1.68282800171817	0.404	0.14	9.89779915037885E-95	Fibroblast	NNMT
3.38233392080526E-98	3.12381056996311	0.172	0.032	5.51692485822547E-94	Fibroblast	ZNF703
3.79641373409388E-98	2.1293399447036	0.45	0.185	6.19233044168052E-94	Fibroblast	SLC38A2
6.21339258059607E-98	4.25423176521818	0.135	0.02	1.01346646382102E-93	Fibroblast	ABCA9

1.97538109903649E-97	3.05547435945125	0.102	0.011	3.22204411063842E-93	Fibroblast	DEPRECATI
6.66268318507895E-97	4.03409879926755	0.206	0.046	1.08675025431823E-92	Fibroblast	DEPTOR
1.21180979879745E-95	1.28358741889376	0.608	0.3	1.97658296281851E-91	Fibroblast	LTBP3
2.59281652328536E-95	2.83546708944255	0.127	0.018	4.22914303113075E-91	Fibroblast	RAB34
8.93391080016247E-95	2.9834003065874	0.181	0.036	1.4572101906145E-90	Fibroblast	AFAP1
1.21650027034964E-93	2.0068007036322	0.275	0.074	1.9842335909673E-89	Fibroblast	DSE
3.17764784427427E-93	3.90670133119349	0.24	0.064	5.18306139879577E-89	Fibroblast	ADAMTSL3
5.56094447490277E-93	3.76587950532073	0.125	0.018	9.07045653301391E-89	Fibroblast	INHBA
1.19195818181968E-92	3.11165228344407	0.135	0.021	1.94420299036608E-88	Fibroblast	NUMBL
2.34153136280302E-92	1.80125471686381	0.162	0.029	3.819271805868E-88	Fibroblast	DMPK
2.34575917463795E-92	3.75588364426703	0.147	0.025	3.82616778975196E-88	Fibroblast	ANGPTL6
1.15265468793584E-91	4.00992626941498	0.184	0.039	1.88009506149216E-87	Fibroblast	TUSC3
3.95901555225919E-91	1.8810162205324	0.347	0.118	6.45755026728996E-87	Fibroblast	RAPGEF5
7.03560969898471E-91	2.27753837982495	0.35	0.121	1.1475782980014E-86	Fibroblast	CD81
8.11986076526527E-91	1.91313174206813	0.433	0.174	1.32443048942242E-86	Fibroblast	LGALS1
4.00938854043692E-90	2.58650666168029	0.104	0.013	6.53971364830666E-86	Fibroblast	PELI2
1.34894347403172E-88	2.89022487274524	0.208	0.049	2.20026170049314E-84	Fibroblast	PEAR1
1.53817897895625E-88	1.29885359117319	0.273	0.072	2.50892373257554E-84	Fibroblast	TMEM173
2.8051636887853E-88	1.96384404692913	0.369	0.132	4.5755024927777E-84	Fibroblast	PHLDA1
3.86972234113426E-88	2.07903313258365	0.167	0.032	6.31190411062409E-84	Fibroblast	EMP1
8.61105483219021E-87	1.35577993421623	0.55	0.258	1.40454915367855E-82	Fibroblast	MARCKS
3.49734121735041E-86	2.09195105437284	0.254	0.069	5.70451325962025E-82	Fibroblast	MARCKSL1
1.75842426547152E-85	3.78505833652447	0.218	0.056	2.8681658194106E-81	Fibroblast	VASN
5.30741834192597E-85	2.51000820053922	0.175	0.036	8.65693005751545E-81	Fibroblast	SYNJ2
8.92558420377555E-85	3.83232541170038	0.108	0.015	1.45585203947783E-80	Fibroblast	MAP2
9.98124751011112E-85	3.28146202533252	0.199	0.047	1.62804128137422E-80	Fibroblast	RND3
6.68022522244563E-83	2.68879175978035	0.374	0.15	1.08961153603311E-78	Fibroblast	EGR1
1.03746580183919E-80	1.22697402981765	0.304	0.092	1.6922104693799E-76	Fibroblast	RFTN1
5.31407887013777E-80	2.16503717967479	0.14	0.025	8.66779404508172E-76	Fibroblast	HOXB4
1.52714986840952E-77	3.47287255054397	0.239	0.072	2.49093415036277E-73	Fibroblast	GPC1
1.77213102408374E-77	2.64101132401773	0.208	0.053	2.89052291338299E-73	Fibroblast	MTHFD1L
2.50811641868896E-77	1.41487883056016	0.493	0.234	4.09098869052356E-73	Fibroblast	MPRIP
9.65041075812702E-76	2.49550173745304	0.125	0.021	1.5740784987581E-71	Fibroblast	CRIP2
4.56414823091463E-75	4.0013598437256	0.116	0.019	7.44458217944485E-71	Fibroblast	MSRB3
9.11246590799719E-75	2.42821775091947	0.255	0.078	1.48633431425342E-70	Fibroblast	IER5L
4.98844411110342E-74	2.1396481616747	0.309	0.108	8.13665118962079E-70	Fibroblast	NR4A1
1.83534010818502E-73	2.00496176736516	0.415	0.188	2.99362325046059E-69	Fibroblast	TNS3
8.59672445681223E-73	3.96075438837737	0.102	0.015	1.40221172615064E-68	Fibroblast	PHYHD1
1.29020851753211E-72	2.90225658277951	0.162	0.036	2.10445911294662E-68	Fibroblast	ADGRL2
1.90637680167457E-72	3.53412448960464	0.161	0.036	3.1094912012114E-68	Fibroblast	RNF180
2.18359624526053E-72	3.06030930848023	0.233	0.071	3.56166383564444E-68	Fibroblast	WLS
8.29577752211574E-72	3.44879546442249	0.15	0.032	1.3531242716323E-67	Fibroblast	RRAS
1.98329333591001E-71	3.04244451787763	0.254	0.084	3.23494976020282E-67	Fibroblast	PLSCR4
1.61333861485747E-70	2.92278596146203	0.247	0.079	2.63151661469402E-66	Fibroblast	HES4
4.27223794296521E-70	3.4953090580489	0.275	0.1	6.96844730877055E-66	Fibroblast	FGFR2
5.12842770415763E-69	2.06964912576187	0.197	0.052	8.3649784282515E-65	Fibroblast	CHST15
1.22975563913736E-68	2.81028498799635	0.161	0.037	2.00585442299695E-64	Fibroblast	NUAK2
1.23341865087965E-68	1.44484412887749	0.477	0.24	2.0118291614498E-64	Fibroblast	EXT1
1.52768716219233E-67	1.41273327587801	0.233	0.067	2.4918105302519E-63	Fibroblast	PLTP
3.20146209190513E-67	2.26382243638346	0.321	0.127	5.22190481810646E-63	Fibroblast	MMP14
2.18379107772872E-66	2.34016069690454	0.262	0.09	3.56198162688332E-62	Fibroblast	PHLDB2
6.22132811531207E-66	3.11239758367106	0.21	0.063	1.01476082888855E-61	Fibroblast	FERMT2

**3.270910132176E-65**	2.02256683881198	0.272	0.095	5.33518151659228E-61	Fibroblast	FER
**3.32528195652068E-65**	2.0051140086034	0.335	0.137	5.42386739928087E-61	Fibroblast	LZTS2
**6.64075459746835E-65**	1.66878585164767	0.238	0.071	1.08317348239306E-60	Fibroblast	ADCY3
**7.56934416531867E-65**	2.95908828010776	0.259	0.093	1.23463572680513E-60	Fibroblast	NDRG2
**1.55827701296493E-64**	1.40909816321094	0.579	0.331	2.5417056358471E-60	Fibroblast	RHOB
**2.64503495496097E-64**	2.31641037117903	0.171	0.043	4.31431651503684E-60	Fibroblast	ST3GAL4
**3.12589837109025E-64**	2.989058691181	0.137	0.029	5.09865283308531E-60	Fibroblast	APBB2
**3.19660048456441E-64**	1.15531810395584	0.322	0.115	5.21397505037301E-60	Fibroblast	VCAM1
**9.08157142505295E-64**	3.57237103560378	0.201	0.06	1.48129511514039E-59	Fibroblast	KCNT2
**3.18257359032035E-63**	2.88086605272859	0.242	0.082	5.19109578317152E-59	Fibroblast	DEPRECATI
**8.35399727877697E-63**	2.39750648184364	0.181	0.048	1.36262049614131E-58	Fibroblast	TRIP10
**1.47015459245325E-62**	1.78191794417118	0.414	0.2	2.39796915575049E-58	Fibroblast	SPON2
**7.53074724933647E-62**	2.79629478591683	0.117	0.023	1.22834018383927E-57	Fibroblast	NOCT
**7.97135238212968E-61**	3.92214425326639	0.114	0.022	1.30020728704917E-56	Fibroblast	PARVA
**2.66147296697545E-60**	1.20868177244469	0.418	0.195	4.34112855643366E-56	Fibroblast	SKI
**2.712551729637E-60**	0.27648052589094	0.662	0.279	4.4244431262109E-56	Fibroblast	CFH
**7.25331473043829E-60**	2.02114066068568	0.104	0.018	1.18308816568179E-55	Fibroblast	CALCRL
**1.30366163401068E-59**	1.18555276653034	0.552	0.321	2.12640249123482E-55	Fibroblast	LRP1
**2.26046023925775E-59**	2.117766050019	0.15	0.036	3.68703669625331E-55	Fibroblast	BDH2
**2.86267247892471E-59**	2.36867752289619	0.231	0.078	4.6693050803741E-55	Fibroblast	EVI5
**1.1390316167101E-58**	4.0712152054776	0.145	0.035	1.85787447001584E-54	Fibroblast	GPC6
**2.50351124401346E-58**	2.29679414013018	0.268	0.102	4.08347719011036E-54	Fibroblast	ITGAV
**5.81777234410534E-57**	1.61053541203446	0.234	0.078	9.48936847047022E-53	Fibroblast	CSF1
**1.04081950336713E-56**	1.46697227003666	0.386	0.184	1.69768069194212E-52	Fibroblast	IL6ST
**2.29019499765623E-56**	2.3875763186698	0.273	0.108	3.73553706067708E-52	Fibroblast	TNS2
**9.87715025859644E-56**	2.27675145818784	0.116	0.024	1.61106197867967E-51	Fibroblast	GNAO1
**1.26268034641296E-55**	1.49939808079385	0.208	0.063	2.05955791303418E-51	Fibroblast	TCF4
**1.58544756011553E-55**	1.25414752542608	0.151	0.037	2.58602351530443E-51	Fibroblast	ZSCAN18
**1.66085980415579E-55**	1.42745652768994	0.348	0.147	2.70902842655851E-51	Fibroblast	GSN
**2.57588178294447E-55**	2.64924948465497	0.112	0.023	4.20152077616072E-51	Fibroblast	SPHK1
**3.05173300749309E-55**	2.13265156459011	0.116	0.024	4.97768170852197E-51	Fibroblast	PFKM
**8.22788823166458E-55**	3.86946688243681	0.141	0.035	1.34205084946681E-50	Fibroblast	MOCS1
**2.10878442777232E-54**	1.72925923974616	0.191	0.057	3.43963828013944E-50	Fibroblast	PDGFB
**5.56561206030819E-52**	1.59887692169709	0.167	0.047	9.07806983156869E-48	Fibroblast	FAM126A
**1.14580823913778E-51**	2.53135393561742	0.201	0.068	1.86892781885763E-47	Fibroblast	TRAM2
**2.02834214188329E-51**	3.35513300184341	0.199	0.068	3.30842886762584E-47	Fibroblast	CYP7B1
**2.46906910595554E-51**	2.99342133584126	0.165	0.048	4.02729861872409E-47	Fibroblast	RUSC2
**3.71931114661334E-51**	1.56015862123204	0.118	0.026	6.06656841124101E-47	Fibroblast	DTX3
**1.45508235566561E-50**	2.20253688929041	0.122	0.028	2.37338483032618E-46	Fibroblast	DLG4
**3.41609034067521E-50**	1.22571153580323	0.176	0.051	5.57198495467534E-46	Fibroblast	PLEKHG2
**7.09649689255042E-50**	2.39150040174933	0.27	0.115	1.1575096081439E-45	Fibroblast	TNKS1BP1
**2.44969347224162E-49**	2.32048586286347	0.281	0.122	3.99569502257331E-45	Fibroblast	CDC42EP1
**1.01794298725907E-48**	2.05222490726878	0.352	0.181	1.66036680651826E-44	Fibroblast	ITGB5
**3.06678907825881E-48**	2.20624638184856	0.108	0.024	5.00223966554795E-44	Fibroblast	CTSK
**5.17227293569637E-48**	2.22454682139298	0.239	0.094	8.43649438541435E-44	Fibroblast	FARP1
**6.14287320628853E-48**	1.6827691704282	0.221	0.08	1.00196404867772E-43	Fibroblast	SEMA4C
**1.73054617985032E-47**	1.0012978392383	0.57	0.332	2.82269387395385E-43	Fibroblast	LMNA
**2.52773398063771E-47**	2.36398762988007	0.182	0.059	4.12298689581817E-43	Fibroblast	EVA1B
**3.14269109522507E-47**	2.55714271317855	0.184	0.061	5.12604344542162E-43	Fibroblast	RHOBTB1
**8.22822682190442E-47**	1.69155843113645	0.197	0.066	1.34210607692083E-42	Fibroblast	DRAM1
**9.4858423564505E-47**	2.10740249971544	0.23	0.087	1.54723574676064E-42	Fibroblast	PALM2-AKA
**1.28445345808873E-46**	2.23440883193671	0.147	0.042	2.09507203548853E-42	Fibroblast	FHL3

1.5612050901028E-46	2.50612018339681	0.254	0.107	2.54648162246668E-42	Fibroblast	SELENOM
5.05476117704576E-46	4.09011969142878	0.107	0.025	8.24482095587934E-42	Fibroblast	AGTR1
9.08351784413993E-46	3.21494645977207	0.152	0.046	1.48161259555766E-41	Fibroblast	FBLIM1
9.78912635839888E-46	1.67679843094829	0.164	0.049	1.59670440031844E-41	Fibroblast	ZBTB46
1.16088496698801E-45	1.81836657928737	0.172	0.055	1.89351946965415E-41	Fibroblast	AGAP1
1.18527359316175E-45	1.51273650432443	0.25	0.098	1.93329975780613E-41	Fibroblast	ASAP1
9.71628510588676E-45	3.20323246220317	0.126	0.033	1.58482326362119E-40	Fibroblast	TPST1
3.20550658804223E-44	1.07742379919435	0.175	0.054	5.22850179575568E-40	Fibroblast	ZNF83
5.40234613491693E-44	2.95821692355082	0.205	0.078	8.81176678066301E-40	Fibroblast	ABCA6
1.11265201144513E-43	2.16744539338274	0.158	0.049	1.81484669586816E-39	Fibroblast	GAS6
1.64613385798073E-43	2.42410831542621	0.145	0.042	2.68500893575236E-39	Fibroblast	AMOTL1
2.89665797755581E-43	0.875740724236564	0.319	0.139	4.72473882719128E-39	Fibroblast	NOTCH1
3.08296611252845E-43	2.69098291601567	0.118	0.03	5.02862602614516E-39	Fibroblast	ANKRD50
3.37083285947163E-43	1.60059171623821	0.119	0.03	5.49816547708418E-39	Fibroblast	ADARB1
4.50127968008983E-43	2.10357101286193	0.157	0.049	7.34203728619451E-39	Fibroblast	CCDC71L
4.58755840795056E-43	2.08660696492551	0.184	0.063	7.48276651920815E-39	Fibroblast	SOX4
5.18400156463219E-43	1.53178005797368	0.223	0.084	8.45562495207156E-39	Fibroblast	CHSY1
6.05269235867913E-43	1.82903676124654	0.322	0.161	9.87254650624152E-39	Fibroblast	APOL1
1.11817503618186E-42	1.12887922601098	0.54	0.345	1.82385530151622E-38	Fibroblast	CEBPD
1.16104424594919E-42	2.38198580867566	0.172	0.058	1.89377926956772E-38	Fibroblast	DEPRECATI
1.2361873131004E-42	2.67821528990125	0.126	0.034	2.01634512639806E-38	Fibroblast	MCAM
2.72604510810851E-42	1.60608298878002	0.257	0.109	4.44645217583579E-38	Fibroblast	PLPP1
2.98254543306539E-42	1.25318127106647	0.338	0.162	4.86482985587296E-38	Fibroblast	UGCG
3.58064853634775E-42	1.08790007850039	0.242	0.095	5.84039582763682E-38	Fibroblast	DMWD
3.90451371421511E-42	3.18173230342508	0.176	0.062	6.36865231925626E-38	Fibroblast	PPIC
6.26299221704516E-42	1.98186612641955	0.107	0.025	1.02155666052224E-37	Fibroblast	GNG11
1.05840138156772E-41	3.35775841052624	0.158	0.053	1.7263584934751E-37	Fibroblast	TEAD3
1.52252403181578E-41	0.829953150750874	0.59	0.381	2.48338894829471E-37	Fibroblast	CD63
3.51292234715726E-41	1.53451940424236	0.288	0.133	5.7299276404482E-37	Fibroblast	ITGB1
4.84730946369931E-41	0.697581621370045	0.371	0.17	7.90644646623994E-37	Fibroblast	ZEB2
9.38709036578864E-41	2.0945977871777	0.126	0.035	1.53112830956379E-36	Fibroblast	OSBPL10
9.54404095830862E-41	1.6771830927567	0.175	0.059	1.55672852070972E-36	Fibroblast	KCTD10
1.62594943843327E-40	3.70085071458054	0.182	0.068	2.65208612902851E-36	Fibroblast	RNF152
2.59353086092305E-40	0.395127238884993	0.625	0.383	4.23030818725159E-36	Fibroblast	CST3
8.08265047396097E-40	2.03056460489181	0.147	0.046	1.31836111880777E-35	Fibroblast	PTPRG
2.45015850408145E-39	2.77524492712536	0.219	0.092	3.99645353600726E-35	Fibroblast	PDGFA
2.51363401335691E-39	1.6398192657164	0.161	0.052	4.09998843918646E-35	Fibroblast	PEAK1
5.9336287875746E-39	1.43983632507222	0.287	0.135	9.67834191541293E-35	Fibroblast	ATN1
1.00154813591166E-38	2.11765310984836	0.158	0.053	1.6336251644855E-34	Fibroblast	MID1
1.33592825750832E-38	2.19920895013758	0.132	0.039	2.17903258082182E-34	Fibroblast	RASAL2
2.00345272913731E-38	3.99122964333198	0.176	0.066	3.26783174649586E-34	Fibroblast	ENPEP
3.16100905591082E-38	1.75484600969212	0.121	0.033	5.15592187109614E-34	Fibroblast	FGFR1
7.45573724728501E-38	1.46830732383582	0.293	0.14	1.21610530240466E-33	Fibroblast	G0S2
7.45786489035949E-38	1.62361404959623	0.242	0.105	1.21645234226654E-33	Fibroblast	MBNL2
1.40398404658446E-37	3.20853378887303	0.157	0.055	2.29003837838391E-33	Fibroblast	OBSL1
2.30712116108765E-37	2.15119822336206	0.286	0.139	3.76314532585007E-33	Fibroblast	IER3
5.14204777153345E-37	1.92512231394672	0.174	0.063	8.38719412014821E-33	Fibroblast	DNAJB4
5.68828891238136E-37	1.59040816915033	0.265	0.123	9.27816804498524E-33	Fibroblast	ANKS1A
9.19423906789389E-37	1.71889677458619	0.27	0.129	1.49967233436417E-32	Fibroblast	CLSTN3
1.4474405483325E-36	2.66384031871539	0.127	0.038	2.36092027838514E-32	Fibroblast	PDLIM5
2.12710549453247E-36	1.39770199946371	0.155	0.052	3.46952177213191E-32	Fibroblast	ABI2
3.1958324008816E-36	0.98849737816376	0.517	0.338	5.21272222907797E-32	Fibroblast	PLXNB2

3.208367977437E-36	1.59197915456855	0.235	0.101	5.23316900799749E-32	Fibroblast	EPB41L2
3.83297068756002E-36	2.36759652515938	0.101	0.026	6.25195848847915E-32	Fibroblast	SMARCD3
4.7889097596269E-36	2.26043668712306	0.182	0.07	7.81119070892744E-32	Fibroblast	MAP1LC3A
5.517408690412E-36	1.00514296893514	0.233	0.095	8.999445314931E-32	Fibroblast	SMAD7
7.60657836365137E-36	1.69871107791241	0.155	0.052	1.24070899689517E-31	Fibroblast	TPM4
1.17426497677273E-35	2.5235157438916	0.19	0.077	1.91534360361401E-31	Fibroblast	ADAMTS10
1.23503779870966E-35	1.05878267804947	0.196	0.074	2.01447015347533E-31	Fibroblast	KSR1
2.19985295800631E-35	1.48074413436446	0.259	0.12	3.58818015980408E-31	Fibroblast	PTPA
2.28090753301921E-35	1.30412054784256	0.122	0.035	3.72038827710763E-31	Fibroblast	PLAU
3.06037446217154E-35	0.620742647648725	0.361	0.179	4.991776785248E-31	Fibroblast	ARID5B
4.94143437183946E-35	1.86921485712935	0.277	0.14	8.05997360390734E-31	Fibroblast	BCAR1
5.42193612499167E-35	1.08209189672464	0.143	0.046	8.84372001347392E-31	Fibroblast	SYNGAP1
8.91172301739658E-35	2.56676193097935	0.136	0.045	1.45359114136756E-30	Fibroblast	ALPL
1.52846784992831E-33	0.922815326835746	0.462	0.289	2.49308391001806E-29	Fibroblast	ACTN4
5.24863035631118E-33	1.60377412721219	0.113	0.033	8.56104097417916E-29	Fibroblast	KLF7
5.36926411780967E-33	2.03414847149939	0.19	0.078	8.75780670255935E-29	Fibroblast	EPHA2
6.22779385690338E-33	1.96060304756614	0.17	0.065	1.01581545599951E-28	Fibroblast	ROBO1
6.3507906391014E-33	2.25943178808803	0.216	0.098	1.03587746114383E-28	Fibroblast	CKAP4
1.03843237140053E-32	2.43364915866707	0.186	0.077	1.6937870409914E-28	Fibroblast	DOCK1
1.83138918677511E-32	1.86608759805508	0.102	0.028	2.98717890254888E-28	Fibroblast	PLAGL1
4.13563466456373E-32	1.4283924441864	0.143	0.049	6.74563370136989E-28	Fibroblast	ZNF532
4.86050594523137E-32	3.21912138699526	0.142	0.051	7.92797124726688E-28	Fibroblast	LIN7A
5.09833964589901E-32	2.08147364186747	0.103	0.029	8.31590179642588E-28	Fibroblast	INAFM1
5.45957087661307E-32	1.6110835286835	0.303	0.165	8.90510605684359E-28	Fibroblast	CALU
8.99210421760926E-32	1.79586268895592	0.257	0.127	1.46670211893425E-27	Fibroblast	KLF9
1.18489868817219E-31	2.32897117047767	0.169	0.067	1.93268825027766E-27	Fibroblast	NBEAL1
1.60410411919417E-31	2.33993315243579	0.231	0.113	2.6164542288176E-27	Fibroblast	FLNB
1.69723740862787E-31	1.47156829448676	0.171	0.066	2.76836393721292E-27	Fibroblast	CSNK1E
2.04015563713283E-31	1.69868586019059	0.155	0.057	3.32769785972735E-27	Fibroblast	C1orf21
1.13232490421857E-30	2.13936896025959	0.128	0.043	1.8469351512709E-26	Fibroblast	LCAT
1.27260166131956E-30	2.55297583707665	0.117	0.037	2.07574056977834E-26	Fibroblast	FKBP9
1.32129376439652E-30	2.26858903983479	0.236	0.119	2.15516225910716E-26	Fibroblast	KANK1
5.9561478439753E-30	1.18473594437826	0.364	0.216	9.71507274830811E-26	Fibroblast	TRIM8
6.07966294883227E-30	1.18189418139095	0.101	0.028	9.91653823584031E-26	Fibroblast	HECW2
1.28801649849848E-29	1.98033778843318	0.23	0.113	2.10088371070087E-25	Fibroblast	NFIB
3.23033944361835E-29	1.16654295080666	0.479	0.318	5.26900666648589E-25	Fibroblast	HSPB1
3.2890592115499E-29	2.69059642598966	0.123	0.042	5.36478447995905E-25	Fibroblast	PDLIM1
4.70160166226219E-29	1.53612779136877	0.113	0.036	7.66878247131586E-25	Fibroblast	ENDOD1
1.62945889037891E-28	2.25870841360478	0.122	0.042	2.65781039609703E-24	Fibroblast	CLIC4
1.7706491914217E-28	0.884008120494889	0.508	0.348	2.88810589612794E-24	Fibroblast	JUN
4.50735575463535E-28	0.945016132127568	0.323	0.177	7.35194797138572E-24	Fibroblast	CCDC85B
4.86080181966537E-28	0.284634880546631	0.211	0.086	7.92845384805619E-24	Fibroblast	LBH
5.51719028052379E-28	0.65180242846726	0.509	0.291	8.99908906656236E-24	Fibroblast	COL18A1
6.19972479037807E-28	1.56047107174269	0.177	0.074	1.01123711055857E-23	Fibroblast	RHOQ
1.07705207733417E-27	1.80673368672557	0.148	0.057	1.75677964333976E-23	Fibroblast	DOCK6
1.1240806548492E-27	1.48051325681021	0.153	0.059	1.83348795612453E-23	Fibroblast	STK38L
1.46940541617948E-27	2.25768868091361	0.133	0.049	2.39674717433036E-23	Fibroblast	TWSG1
2.13555220332595E-27	2.16297396122007	0.125	0.044	3.48329919884496E-23	Fibroblast	SVIL
4.6565531357937E-27	1.39026539217142	0.14	0.051	7.5953038197931E-23	Fibroblast	THBD
4.68550024353963E-27	2.02408649418686	0.15	0.06	7.64251944723749E-23	Fibroblast	SNX33
4.87523699999648E-27	1.62725538854699	0.132	0.048	7.95199907069426E-23	Fibroblast	BMPR2
6.60185075812424E-27	2.73116311344255	0.167	0.073	1.07682787715764E-22	Fibroblast	PAWR

8.3233231830409E-27	1.21957240437829	0.206	0.095	1.3576172443858E-22	Fibroblast	SETBP1
1.24735723970843E-26	1.18195143264365	0.15	0.058	2.03456439368842E-22	Fibroblast	MAN1C1
1.3029573462965E-26	1.27062342496139	0.118	0.04	2.12525372754421E-22	Fibroblast	KCNK6
1.59231657864984E-26	0.831655994453303	0.467	0.303	2.59722757143576E-22	Fibroblast	IFITM3
2.18109480079721E-26	3.02224675045986	0.137	0.054	3.55758372958034E-22	Fibroblast	TLCD4
2.7310846389897E-26	1.62336725722483	0.108	0.035	4.45467215465609E-22	Fibroblast	CTBP2
3.88873469723122E-26	0.870590798000834	0.369	0.225	6.34291516465384E-22	Fibroblast	MAP4
7.13473213560264E-26	2.18472771778674	0.239	0.127	1.16374615863815E-21	Fibroblast	GADD45A
1.35801587793145E-25	2.03586565758458	0.127	0.047	2.21505969849399E-21	Fibroblast	C2CD2
1.3729178652594E-25	1.48293814441581	0.192	0.089	2.23936633002461E-21	Fibroblast	PKD1
1.9520199088202E-25	1.34702118896578	0.252	0.133	3.18393967327662E-21	Fibroblast	ECE1
2.54755045694107E-25	2.27829599695351	0.122	0.045	4.15530955031658E-21	Fibroblast	TCEAL1
2.76493751859006E-25	1.22489442422993	0.15	0.059	4.50988958657225E-21	Fibroblast	FHOD1
3.13295518424303E-25	2.72746711660116	0.106	0.036	5.1101632010188E-21	Fibroblast	KIF7
3.42262917149402E-25	1.70031367541048	0.27	0.155	5.5826504416239E-21	Fibroblast	TRIP6
5.37113378043151E-25	0.896262111859455	0.119	0.042	8.76085630926183E-21	Fibroblast	ZNF548
5.90682693465785E-25	1.46343711129773	0.239	0.125	9.63462541312043E-21	Fibroblast	CITED2
9.30718602481453E-25	1.80685405906427	0.243	0.132	1.5180951125075E-20	Fibroblast	MYO1C
1.40425732383753E-24	1.43731480054722	0.289	0.168	2.29048412091139E-20	Fibroblast	NEK6
2.75245365314806E-24	2.99387940843245	0.107	0.038	4.48952715364981E-20	Fibroblast	SYTL4
4.03610913628248E-24	1.19426027736355	0.191	0.089	6.58329761219035E-20	Fibroblast	LIMS1
6.36834964504048E-24	2.30208113643514	0.179	0.085	1.03874151060255E-19	Fibroblast	PLEKHH3
9.54814572104204E-24	2.02456510529834	0.236	0.131	1.55739804855917E-19	Fibroblast	PLOD1
1.2786334835163E-23	2.08504110693446	0.138	0.057	2.08557907496343E-19	Fibroblast	CLIP2
1.33546076112741E-23	3.21213170235514	0.132	0.054	2.17827004747492E-19	Fibroblast	ARHGEF26
2.69377962328388E-23	2.06302001371235	0.19	0.093	4.39382394353833E-19	Fibroblast	RAPH1
6.28744153283471E-23	0.772338187508587	0.119	0.043	1.02554458842067E-18	Fibroblast	SLC25A36
3.78513721022666E-22	2.53709127591561	0.136	0.058	6.1739373036007E-18	Fibroblast	SNX21
4.63852527283303E-22	2.50111204613317	0.103	0.037	7.56589857251796E-18	Fibroblast	NAV3
5.13639098920035E-22	1.23306756709006	0.194	0.095	8.3779673424847E-18	Fibroblast	PISD
6.07734325502037E-22	0.839208936374247	0.174	0.079	9.91275458326373E-18	Fibroblast	TMEM185A
9.50014685862675E-22	1.34483797113898	0.113	0.042	1.54956895411061E-17	Fibroblast	FUT4
9.75552179643765E-22	1.20978410608036	0.153	0.067	1.59122316021695E-17	Fibroblast	STXBP5
1.75768090243807E-21	0.973130127494904	0.502	0.382	2.86695331996673E-17	Fibroblast	VMP1
3.494673981901E-21	1.32107501946833	0.108	0.04	5.70016273187872E-17	Fibroblast	SH3YL1
3.67315829567752E-21	2.29724021396684	0.155	0.072	5.9912884960796E-17	Fibroblast	NFIA
4.44159462191712E-21	0.574604165263225	0.14	0.056	7.24468498780901E-17	Fibroblast	ANXA1
7.11759354198588E-21	0.669477511803215	0.445	0.299	1.16095068263332E-16	Fibroblast	RRBP1
9.08379970615867E-21	1.29377134453082	0.24	0.132	1.48165857007154E-16	Fibroblast	JDP2
9.21936029358746E-21	1.94269008292375	0.219	0.123	1.50376985748705E-16	Fibroblast	SAMD5
2.39944976160525E-20	1.12358165192071	0.365	0.249	3.91374250615432E-16	Fibroblast	INF2
2.50807111980346E-20	1.13349522583074	0.142	0.061	4.09091480351143E-16	Fibroblast	FRMD4A
2.6739528589779E-20	1.1854306058091	0.203	0.105	4.36148450827885E-16	Fibroblast	BCL6
3.26480458457229E-20	2.61493429450228	0.143	0.066	5.32522275789586E-16	Fibroblast	PLOD2
3.45913050363271E-20	1.45156626118466	0.327	0.212	5.64218776447531E-16	Fibroblast	ICAM1
6.00447683970603E-20	0.521960992729862	0.258	0.133	9.7939021732445E-16	Fibroblast	KCTD12
7.18861048189195E-20	2.18323527646176	0.151	0.071	1.1725342557014E-15	Fibroblast	TEAD2
1.09374266781369E-19	1.07163592714737	0.147	0.065	1.7840036654709E-15	Fibroblast	MICAL2
1.11545699680188E-19	2.10082864109639	0.19	0.101	1.81942190748355E-15	Fibroblast	HTRA1
1.50481684834141E-19	1.74719877074728	0.166	0.081	2.45450676132967E-15	Fibroblast	PLA2G4C
2.62946733098406E-19	1.47723804552054	0.15	0.069	4.2889241635681E-15	Fibroblast	MAP4K3
2.97809325056094E-19	2.06861947246855	0.164	0.081	4.85756790098995E-15	Fibroblast	DEPRECATI

3.72986180108548E-19	1.65891578206685	0.179	0.091	6.08377758375053E-15	Fibroblast	SOGA1
4.06409686383423E-19	0.421253364886728	0.623	0.463	6.62894839460002E-15	Fibroblast	CCNL2
4.89380326039965E-19	0.568076527134071	0.275	0.158	7.98228249803787E-15	Fibroblast	TTLL3
6.50267123812181E-19	1.70670541441912	0.172	0.086	1.06065070565005E-14	Fibroblast	TSPAN3
8.78654925018095E-19	1.14779583549709	0.211	0.115	1.43317404819701E-14	Fibroblast	H2AFJ
9.72739652196128E-19	0.527417408745803	0.244	0.133	1.5866356466971E-14	Fibroblast	PACS1
1.08243621407147E-18	1.73900345768446	0.174	0.089	1.76556170877197E-14	Fibroblast	TAX1BP3
1.11587584781586E-18	0.736852481145952	0.351	0.23	1.82010509537245E-14	Fibroblast	RERE
1.36177441019992E-18	1.12479703631356	0.247	0.144	2.22119024047709E-14	Fibroblast	FMO3
1.97194394177575E-18	0.877676012589487	0.172	0.084	3.21643776343042E-14	Fibroblast	ABL1
2.05284341912891E-18	1.0422881726774	0.18	0.089	3.34839290094117E-14	Fibroblast	PLXDC2
2.06330712099227E-18	1.52116663731632	0.167	0.084	3.36546024505048E-14	Fibroblast	GABBR1
2.21762739642608E-18	0.64498651955277	0.43	0.306	3.61717204631059E-14	Fibroblast	ANKRD10
3.64107877402611E-18	0.845177569821681	0.104	0.04	5.93896358831398E-14	Fibroblast	LDB2
3.86611413482549E-18	1.06345730406316	0.208	0.111	6.30601876531385E-14	Fibroblast	TMEM165
4.51593644617197E-18	1.2217593191815	0.116	0.047	7.3659439373511E-14	Fibroblast	IRAK3
4.83514447909233E-18	2.59862260693979	0.121	0.053	7.8866041598475E-14	Fibroblast	CNKSR3
5.68827812190094E-18	1.21308493222157	0.153	0.073	9.27815044463263E-14	Fibroblast	SPIN1
5.89293469797939E-18	0.993140846183556	0.282	0.173	9.61196578587419E-14	Fibroblast	DEPRECATI
6.03326139236833E-18	0.748537107790985	0.114	0.046	9.84085265709199E-14	Fibroblast	GYPC
9.5268188369257E-18	0.517200589029174	0.152	0.07	1.55391942049095E-13	Fibroblast	FAS
9.55352657434181E-18	1.50294386664183	0.258	0.163	1.55827571954089E-13	Fibroblast	DST
1.11978421387894E-17	2.04800647904138	0.145	0.07	1.82648003125794E-13	Fibroblast	AASS
1.21197757849793E-17	1.76597533767148	0.106	0.043	1.97685662828797E-13	Fibroblast	ME3
1.41008226489958E-17	2.73349826463019	0.103	0.042	2.29998518227771E-13	Fibroblast	PARD3B
1.48378630612209E-17	1.05368000323727	0.319	0.204	2.42020384391573E-13	Fibroblast	SOCS3
1.70495819178507E-17	1.75758190333426	0.199	0.112	2.78095730662063E-13	Fibroblast	CTTN
1.91805597763214E-17	1.6522353845068	0.169	0.088	3.12854110511578E-13	Fibroblast	AHDC1
2.70966789179264E-17	1.00017845103539	0.107	0.043	4.41973929830298E-13	Fibroblast	TP53I11
2.81911479285446E-17	2.15543098072161	0.143	0.07	4.59825813862491E-13	Fibroblast	TEAD1
3.55883945971819E-17	1.49771459272049	0.281	0.179	5.80482304274634E-13	Fibroblast	FOS
4.17346481089686E-17	2.55457234684087	0.145	0.072	6.80733845305387E-13	Fibroblast	NFIX
4.47467810169825E-17	0.94845900982606	0.199	0.109	7.29864745168001E-13	Fibroblast	SPECC1L
7.65843385405758E-17	1.11129697407687	0.22	0.129	1.24916714593533E-12	Fibroblast	PWWP3A
9.00818996733636E-17	2.91281492804999	0.125	0.059	1.46932586557223E-12	Fibroblast	PARD3
9.03046049454069E-17	0.715439225413675	0.189	0.095	1.47295841126453E-12	Fibroblast	LFNG
1.29587296300792E-16	1.20825694059485	0.264	0.166	2.11369838996223E-12	Fibroblast	ADH1B
1.33239214823367E-16	2.28693366640415	0.131	0.062	2.17326483298393E-12	Fibroblast	MPZL1
1.51573195817023E-16	1.99186214360553	0.205	0.121	2.47231039697147E-12	Fibroblast	ZCCHC24
1.59816039411675E-16	1.94712620795694	0.102	0.042	2.60675941884383E-12	Fibroblast	MRTFB
1.87217337894187E-16	2.50056566299938	0.112	0.05	3.05370199839208E-12	Fibroblast	CTDSPL
1.98183410986898E-16	0.853839887349931	0.313	0.199	3.23256961660729E-12	Fibroblast	IGFBP2
2.80727914989479E-16	0.523154941310102	0.478	0.328	4.57895302139338E-12	Fibroblast	SERPING1
3.08433456340628E-16	0.88263416884285	0.204	0.113	5.03085810637199E-12	Fibroblast	PHPT1
3.49446075942106E-16	0.866604770024144	0.112	0.048	5.69981494469169E-12	Fibroblast	TTC39B
4.9822515614205E-16	0.910435927200257	0.357	0.258	8.12655052183298E-12	Fibroblast	CYB5R3
6.83367397545725E-16	0.630732650896707	0.138	0.064	1.11464056213683E-11	Fibroblast	BICD1
6.86897880141372E-16	1.94447797110143	0.166	0.09	1.12039913229859E-11	Fibroblast	TJP1
1.30794627915815E-15	0.721635143183507	0.345	0.238	2.13339117593486E-11	Fibroblast	CALCOCO2
1.80005729997433E-15	1.91269268094342	0.197	0.119	2.93607346198814E-11	Fibroblast	CDH2
1.81123649423006E-15	1.58579817076163	0.304	0.216	2.95430784573865E-11	Fibroblast	GADD45B
1.85566546851784E-15	1.21784279006043	0.23	0.14	3.02677594569945E-11	Fibroblast	IRF2BPL

2.91798646529328E-15	1.00051801608404	0.112	0.049	4.75952772353987E-11	Fibroblast	DEPRECATI
4.42234940109654E-15	2.27471030250396	0.137	0.07	7.21329410812856E-11	Fibroblast	SMARCA1
4.94969166274728E-15	0.69657814711982	0.109	0.047	8.07344207110709E-11	Fibroblast	NOD1
5.26623426773662E-15	0.887612100256715	0.15	0.075	8.5897547141052E-11	Fibroblast	INPP5B
5.77151454587518E-15	1.15858140415772	0.23	0.141	9.413917375777E-11	Fibroblast	SELENON
7.12476984772653E-15	0.381572753503872	0.431	0.305	1.16212120986267E-10	Fibroblast	MACF1
7.24474255103077E-15	1.20735784102054	0.176	0.098	1.18168995749863E-10	Fibroblast	LIMS2
8.90776764429779E-15	1.69960465604095	0.119	0.057	1.45294598046141E-10	Fibroblast	DAB2IP
9.14808764956765E-15	1.84417724888724	0.197	0.12	1.49214457652098E-10	Fibroblast	LAMC1
1.52058517112877E-14	1.07589112104875	0.184	0.104	2.48022647262814E-10	Fibroblast	DAPK3
1.54860018587322E-14	1.66455836281638	0.167	0.093	2.52592176317781E-10	Fibroblast	RTL8C
1.61364735833695E-14	0.765648967368401	0.24	0.15	2.63202020618339E-10	Fibroblast	ZBTB16
1.7212733688603E-14	0.902093248373993	0.33	0.234	2.80756899194804E-10	Fibroblast	PPIB
1.94507876601655E-14	0.700468792514268	0.14	0.068	3.1726179752496E-10	Fibroblast	PHF2
2.2625192265943E-14	1.65149819051285	0.15	0.079	3.69039511049796E-10	Fibroblast	UBE2E2
2.92665767856089E-14	2.12672478658505	0.142	0.076	4.77367133950067E-10	Fibroblast	TCEAL4
4.25621098621628E-14	1.08714352606789	0.313	0.224	6.94230573961737E-10	Fibroblast	RXRA
4.31891874388777E-14	0.336795176432369	0.132	0.062	7.04458836315534E-10	Fibroblast	HK1
5.8095212110237E-14	0.878423205932	0.162	0.086	9.47591004730076E-10	Fibroblast	BNIP3L
6.5973860765834E-14	1.36055713621275	0.15	0.079	1.07609964295152E-09	Fibroblast	DENND5A
6.96910978433344E-14	1.12518855106348	0.309	0.223	1.13673149692263E-09	Fibroblast	CD151
9.02803406978711E-14	0.593508567488993	0.164	0.087	1.47256263712298E-09	Fibroblast	ST6GALNA
1.17443635719311E-13	2.61392988739748	0.125	0.064	1.91562314221768E-09	Fibroblast	YAP1
1.23339294958738E-13	1.93958261768137	0.125	0.063	2.01178724007197E-09	Fibroblast	GNA11
1.78351489633887E-13	0.815588560857322	0.118	0.056	2.90909114741833E-09	Fibroblast	PHC1
1.90133947310685E-13	0.366034024530634	0.267	0.168	3.10127481458459E-09	Fibroblast	ANXA6
2.96901686647523E-13	1.20922079478071	0.117	0.057	4.84276341090775E-09	Fibroblast	PLPPR2
4.21054080988977E-13	1.74764718476899	0.164	0.094	6.86781311501121E-09	Fibroblast	TSPAN9
5.01582016510125E-13	1.54183704992413	0.107	0.051	8.18130427129665E-09	Fibroblast	KCTD15
5.08272337987045E-13	1.74886629306595	0.136	0.072	8.29043010490668E-09	Fibroblast	PGM3
5.61650882613822E-13	0.415142058385412	0.311	0.207	9.16108754631405E-09	Fibroblast	KLC1
5.76388098377778E-13	0.884246588816535	0.238	0.153	9.40146627263994E-09	Fibroblast	NPTN
6.94102155580255E-13	1.21849435053104	0.166	0.095	1.13215002596695E-08	Fibroblast	KAT2A
8.59809991770661E-13	1.01363837872895	0.162	0.091	1.40243607757712E-08	Fibroblast	TMEM184E
1.04626247134361E-12	0.59318723673188	0.103	0.047	1.70655871700856E-08	Fibroblast	ADM
1.08770949480064E-12	0.698498778308016	0.127	0.063	1.77416295696933E-08	Fibroblast	RNF145
1.35927360336127E-12	1.10247676693184	0.175	0.102	2.21711117444257E-08	Fibroblast	SRF
1.37844231831544E-12	0.62293349952204	0.119	0.058	2.24837726540431E-08	Fibroblast	PLSCR3
1.40820289982933E-12	2.35511016310044	0.133	0.073	2.29691974991161E-08	Fibroblast	BOK
1.87536726599204E-12	0.859873267383591	0.125	0.062	3.05891154755962E-08	Fibroblast	RBMS1
1.89192749016932E-12	0.272678667698423	0.401	0.282	3.08592292921518E-08	Fibroblast	FAM118A
3.12395418373216E-12	1.29642789169269	0.234	0.158	5.09548166908553E-08	Fibroblast	BEX3
3.19686063450658E-12	0.387251092713761	0.107	0.05	5.21439938094369E-08	Fibroblast	NEDD9
3.26574866487915E-12	0.873899844793925	0.118	0.058	5.32676264728439E-08	Fibroblast	YBX3
3.29669982516653E-12	0.967561710971871	0.284	0.199	5.37724708482913E-08	Fibroblast	ALDH1A1
3.35104161184434E-12	2.2651297760587	0.111	0.056	5.4658839730793E-08	Fibroblast	RBFOX2
3.56788501319236E-12	0.808291479690789	0.175	0.102	5.81957724501806E-08	Fibroblast	L3HYPDH
4.00967994256744E-12	0.55142985878365	0.283	0.187	6.54018895432175E-08	Fibroblast	PLXND1
4.47336326721193E-12	2.09590167334978	0.157	0.094	7.29650282514938E-08	Fibroblast	CHRD
5.22002031172639E-12	2.09268052020712	0.138	0.077	8.51437513045692E-08	Fibroblast	ZNF358
5.71120090242305E-12	1.34017131679002	0.177	0.107	9.31553979194223E-08	Fibroblast	NCKAP5L
5.982677988845E-12	0.688368579819144	0.34	0.252	9.75834606760509E-08	Fibroblast	FNDC3B

6.21401510028483E-12	2.56078412385071	0.122	0.066	1.01356800300746E-07	Fibroblast	ZDHHC11
8.46286136323237E-12	0.32161972162869	0.166	0.091	1.38037731695683E-07	Fibroblast	CLSTN1
1.04587375424029E-11	1.71149547560174	0.172	0.106	1.70592468054133E-07	Fibroblast	ARHGEF40
1.14768693989208E-11	2.23772295076972	0.13	0.072	1.87199216765797E-07	Fibroblast	ADCY6
1.15109845355513E-11	2.28660455861767	0.106	0.053	1.87755668759376E-07	Fibroblast	RCAN1
1.79161028309093E-11	0.968694761401987	0.299	0.221	2.92229553274961E-07	Fibroblast	NFIC
1.93737630596075E-11	0.610879850328596	0.253	0.168	3.16005449265258E-07	Fibroblast	ADD1
2.28473712110201E-11	1.31637089718991	0.109	0.055	3.72663471822949E-07	Fibroblast	RIN2
2.29261848338737E-11	0.842029966601578	0.273	0.193	3.73949000825314E-07	Fibroblast	REXO2
2.48000172087888E-11	2.05162304703026	0.14	0.081	4.04513080692554E-07	Fibroblast	MAPRE3
3.21119754391613E-11	0.69876639400024	0.118	0.061	5.2377843138816E-07	Fibroblast	ANTXR2
3.73605121956987E-11	0.618700668289718	0.15	0.084	6.09387314424041E-07	Fibroblast	NICN1
4.41334139269729E-11	0.777379500752939	0.112	0.056	7.19860114562855E-07	Fibroblast	LRRK1
5.41089521619748E-11	0.647348811644325	0.121	0.063	8.8257111871397E-07	Fibroblast	RELL1
6.02897359616777E-11	2.07242806030228	0.146	0.087	9.83385883270925E-07	Fibroblast	PLS3
6.09304127790691E-11	1.33201242304177	0.15	0.088	9.93835962839397E-07	Fibroblast	BTF3L4
6.94478664656332E-11	0.597324384197786	0.185	0.11	1.13276414992094E-06	Fibroblast	SCARF1
7.38323183650191E-11	1.99018188701136	0.152	0.093	1.20427894485183E-06	Fibroblast	RAMP1
8.94512112588457E-11	0.813599893448076	0.116	0.06	1.45903870684303E-06	Fibroblast	SEPTIN2
9.22665620876025E-11	1.6398155069834	0.165	0.102	1.50495989421088E-06	Fibroblast	EHBP1
1.20854335494743E-10	0.420619920149122	0.216	0.135	1.97125506625476E-06	Fibroblast	MAP3K8
1.28244920803284E-10	0.883767576698117	0.123	0.066	2.09180290322236E-06	Fibroblast	SLC37A3
1.51281612294953E-10	2.10345165252466	0.108	0.057	2.46755437814298E-06	Fibroblast	ZNF704
1.5314966718897E-10	1.57382447965775	0.14	0.081	2.49802422151929E-06	Fibroblast	BCL2L2
1.62282550108554E-10	0.796248009550206	0.223	0.149	2.64699067482062E-06	Fibroblast	CTDSP2
1.70149847884141E-10	0.692195583084506	0.376	0.285	2.77531416883823E-06	Fibroblast	BST2
1.81029186116756E-10	1.10601485027257	0.123	0.068	2.9527670547504E-06	Fibroblast	CDK14
2.01329376013878E-10	0.459582315753635	0.119	0.062	3.28388345216237E-06	Fibroblast	PAM
2.47535070859841E-10	1.95077785751089	0.146	0.089	4.03754454079487E-06	Fibroblast	P4HA2
2.50966906875776E-10	1.06779630156086	0.17	0.105	4.09352121805078E-06	Fibroblast	LRRFIP2
2.61837827690881E-10	1.40456959488493	0.146	0.087	4.27083680746596E-06	Fibroblast	PTK2
2.68611400474036E-10	0.936792490925154	0.302	0.23	4.38132055313201E-06	Fibroblast	CTNND1
4.83005928958072E-10	1.3962804211525	0.175	0.112	7.87830970723512E-06	Fibroblast	CRTAP
5.92582835339663E-10	0.307272802697964	0.255	0.171	9.66561862722524E-06	Fibroblast	LDB1
6.1266871437596E-10	0.990762103554827	0.226	0.157	9.99323940018628E-06	Fibroblast	MAFK
6.85048683619805E-10	0.452587563598213	0.131	0.071	1.11738290785226E-05	Fibroblast	ITPR1
7.27416282148053E-10	1.2272920488466	0.151	0.09	1.18648869781169E-05	Fibroblast	ADAM9
7.28788470634601E-10	0.316549856081533	0.585	0.45	1.1887268744521E-05	Fibroblast	MT-CO3
8.66335546136105E-10	0.298169383054922	0.106	0.053	1.4130799093026E-05	Fibroblast	SLFN11
1.03958381224597E-09	1.52093437975405	0.2	0.14	1.69566515615441E-05	Fibroblast	DEPRECATI
1.17381873245361E-09	1.24350525249981	0.147	0.09	1.91461573450508E-05	Fibroblast	PCBP4
1.33830400416523E-09	1.22519225381178	0.167	0.106	2.18290766119391E-05	Fibroblast	CORO1C
1.99588212684202E-09	1.02564428898754	0.156	0.097	3.25548333709202E-05	Fibroblast	GOLGA2
2.08560521324521E-09	2.65231320325175	0.118	0.069	3.40183066332426E-05	Fibroblast	FAT1
2.14810801920377E-09	0.428848982578601	0.301	0.217	3.50377899012327E-05	Fibroblast	CNN2
2.6281801821451E-09	0.626621451686768	0.293	0.221	4.28682469509687E-05	Fibroblast	LPP
3.18572863810553E-09	0.315598637129835	0.289	0.201	5.19624198161393E-05	Fibroblast	MAP7D1
3.37955598106669E-09	1.13548335066271	0.138	0.083	5.51239376071788E-05	Fibroblast	SMURF1
4.57849868202708E-09	0.766617853041639	0.127	0.072	7.46798920025437E-05	Fibroblast	SOCS1
5.85628162744497E-09	0.960122492992315	0.102	0.055	9.5521809625255E-05	Fibroblast	ACOX3
6.3229354305239E-09	0.437571073847214	0.169	0.102	0.000103133399807275	Fibroblast	GSTP1
6.36061839694308E-09	0.334377943635508	0.142	0.083	0.000103748046672539	Fibroblast	NCK2

8.10830314356288E-09	0.329971067542143	0.117	0.065	0.000132254532574654	Fibroblast	ERCC6L2
8.75291703660906E-09	0.88625258611865	0.197	0.135	0.00014276882978413	Fibroblast	ARHGAP1
1.03258313220268E-08	0.911593239098677	0.121	0.069	0.00016842463469358	Fibroblast	MINDY2
1.35263452170432E-08	0.497053261174081	0.141	0.085	0.000220628216835192	Fibroblast	TRANK1
1.35686497274033E-08	0.751919346097403	0.118	0.067	0.000221318245703675	Fibroblast	RABGAP1
1.4025245873098E-08	1.25150110932487	0.137	0.084	0.000228765785436102	Fibroblast	PLXNA1
1.81785591106784E-08	1.02146311747071	0.181	0.122	0.000296510477654275	Fibroblast	DLGAP4
1.99924021495111E-08	1.46096306657139	0.169	0.114	0.000326096071460676	Fibroblast	SYNPO
2.29907977641101E-08	0.651352883223527	0.123	0.071	0.000375002902330399	Fibroblast	TNFSF13B
2.39993803565781E-08	0.393658397561561	0.519	0.419	0.000391453892996145	Fibroblast	LAMP1
2.4233420858489E-08	0.867124163489818	0.257	0.188	0.000395271327622814	Fibroblast	ANXA5
2.77389182993792E-08	2.01501955387938	0.141	0.091	0.000452449496381174	Fibroblast	GPC3
3.53299674304654E-08	0.983819377277737	0.14	0.087	0.000576267098758321	Fibroblast	NCKIPSD
3.75415161673032E-08	1.39546221080099	0.184	0.128	0.000612339670204883	Fibroblast	TRIO
4.01399760495309E-08	0.296209730528608	0.303	0.227	0.000654723149343898	Fibroblast	SMAD3
4.01599505823336E-08	0.971014887917099	0.112	0.064	0.000655048953948443	Fibroblast	GINM1
4.24325774178745E-08	0.75111357811175	0.167	0.108	0.000692117770262951	Fibroblast	SH3BGRL
4.2479579497416E-08	1.13756225900993	0.185	0.127	0.000692884421182353	Fibroblast	SORBS3
5.10295924224561E-08	0.311654377351987	0.102	0.056	0.000832343682002682	Fibroblast	TMEM43
5.21593563004217E-08	1.45188485852728	0.118	0.071	0.000850771260616179	Fibroblast	PER3
5.28872986288562E-08	0.758725662902838	0.147	0.093	0.000862644727935273	Fibroblast	ILK
5.33088116957726E-08	0.741224952271657	0.18	0.12	0.000869520027569747	Fibroblast	MLLT1
6.28601268160622E-08	0.323394462692164	0.146	0.087	0.00102531152849679	Fibroblast	AXL
7.43603094659895E-08	0.764133818972038	0.23	0.17	0.00121289100769976	Fibroblast	MAGED2
8.35170325854864E-08	0.759803642008604	0.122	0.073	0.00136224631850187	Fibroblast	MLXIP
8.66181227788339E-08	0.988388621929223	0.197	0.141	0.00141282820064556	Fibroblast	PGRMC2
8.86108734262467E-08	0.390917980302597	0.291	0.219	0.00144533195645551	Fibroblast	PCED1A
9.6992570098362E-08	0.45215626805895	0.377	0.308	0.00158204581087438	Fibroblast	MYL6
9.75943158559807E-08	0.733188925173103	0.257	0.191	0.0015918608859269	Fibroblast	TSPAN4
1.08936527637618E-07	0.646666143951135	0.296	0.23	0.00177686370229719	Fibroblast	SHC1
1.19020472157189E-07	0.926379096213569	0.142	0.09	0.0019413429213559	Fibroblast	RARA
1.4451199383145E-07	1.4264333033736	0.171	0.119	0.00235713513138477	Fibroblast	S100A13
1.51666276525882E-07	0.442729036716783	0.272	0.204	0.00247382863641367	Fibroblast	CD99
2.03405028675225E-07	0.317460104579737	0.121	0.072	0.0033177394227216	Fibroblast	WWC3
2.06550225755239E-07	0.825786130229377	0.109	0.065	0.00336904073229371	Fibroblast	ACSL3
2.06644656969134E-07	0.730264733430985	0.158	0.103	0.00337058099982354	Fibroblast	PPP1R15A
2.14174628040358E-07	1.0967126707285	0.123	0.076	0.00349340235796628	Fibroblast	MAST4
2.43424147749113E-07	1.08809347249317	0.127	0.08	0.00397049127393578	Fibroblast	LRRC8A
2.55810881714456E-07	0.698958750026101	0.224	0.165	0.00417253129164448	Fibroblast	SEC31A
2.92017316877109E-07	1.4875713864791	0.145	0.097	0.00476309445558252	Fibroblast	CTSO
3.44484063692014E-07	0.436950859695945	0.113	0.067	0.00561887956288044	Fibroblast	RABL6
3.63818620868738E-07	0.790992982508245	0.273	0.214	0.00593424552498999	Fibroblast	TXNDC5
3.99661259420246E-07	0.814499006829214	0.107	0.062	0.00651887480240363	Fibroblast	GNB4
4.12778396673009E-07	1.08774397904858	0.102	0.06	0.00673282842813345	Fibroblast	EXT2
4.62257004881385E-07	0.422209958663651	0.145	0.092	0.00753987400662027	Fibroblast	SSH1
5.74215079004461E-07	1.85176000287476	0.119	0.077	0.00936602215364176	Fibroblast	GNG12
5.86176417351913E-07	2.01094743669068	0.136	0.092	0.00956112354342705	Fibroblast	SOX5
5.86616782795012E-07	1.1347805064224	0.244	0.188	0.00956830634416945	Fibroblast	ATF3
6.17024238139478E-07	1.34397329899544	0.108	0.066	0.010064282348293	Fibroblast	LEPROT
7.38837855761562E-07	0.83532034143	0.131	0.083	0.0120511842653268	Fibroblast	SLC1A5
7.62910654345297E-07	0.747603311558268	0.236	0.18	0.0124438356830261	Fibroblast	DVL3
8.66289230874843E-07	1.28687637400681	0.122	0.078	0.0141300436447996	Fibroblast	SEC24D

9.93198752802783E-07	1.8030878611582	0.131	0.087	0.0162000648569662	Fibroblast	MPDZ
1.00806521051242E-06	0.796697697412738	0.135	0.088	0.0164425516486681	Fibroblast	NF1
1.17435663925416E-06	0.901434867344684	0.127	0.081	0.0191549311428745	Fibroblast	RSU1
1.22941843375571E-06	1.62219365212649	0.13	0.086	0.0200530440729893	Fibroblast	GATA4
1.39317335779319E-06	0.804678554390886	0.19	0.138	0.0227240506389648	Fibroblast	CPQ
1.50775819174693E-06	1.88338702023001	0.132	0.09	0.0245930438655842	Fibroblast	DLG5
1.72312428183561E-06	0.538241506274465	0.24	0.181	0.0281058801610207	Fibroblast	ABCA1
1.84524341961216E-06	0.279458555512411	0.265	0.2	0.0300977654172939	Fibroblast	OBSCN
1.92662623848979E-06	0.961380571670032	0.224	0.172	0.0314252005760069	Fibroblast	SPTBN1
1.99306416790266E-06	1.32738747666486	0.143	0.099	0.0325088696426602	Fibroblast	MAGED1
2.33649680120114E-06	1.35880729736596	0.131	0.088	0.0381105993243919	Fibroblast	SH3PXD2A
2.36341415000336E-06	0.309333451680192	0.047	0.1	0.0385496482007048	Fibroblast	EBPL
2.44010095448033E-06	0.675340310605986	0.104	0.063	0.0398004866685286	Fibroblast	MAPK3
2.55468620052083E-06	1.09479241411489	0.16	0.112	0.0416694866166953	Fibroblast	NCOA7
2.69316045517292E-06	1.77416157326198	0.141	0.099	0.0439281401843256	Fibroblast	PCYOX1
2.76755323790686E-06	1.04787562263096	0.117	0.075	0.0451415608634989	Fibroblast	PTPN12
0	6.79873838069462	0.822	0.025	0	Myeloid	ITGAX
0	6.29481368129336	0.801	0.028	0	Myeloid	FGL2
0	6.73538073772043	0.79	0.044	0	Myeloid	C1QC
0	5.38469213314205	0.784	0.042	0	Myeloid	SPI1
0	6.65691629423137	0.772	0.037	0	Myeloid	CD163
0	5.16881429374554	0.747	0.025	0	Myeloid	MPEG1
0	6.17055411498133	0.769	0.057	0	Myeloid	C1QA
0	6.73998983412855	0.722	0.015	0	Myeloid	CSF1R
0	6.16739798245207	0.763	0.069	0	Myeloid	C1QB
0	7.05323299519636	0.687	0.012	0	Myeloid	MS4A7
0	4.53567508356421	0.894	0.229	0	Myeloid	IFI30
0	4.55157994377358	0.795	0.147	0	Myeloid	TNFAIP2
0	3.96982787036014	0.93	0.293	0	Myeloid	SAT1
0	4.31686520298723	0.93	0.294	0	Myeloid	PSAP
0	3.8218254387674	0.672	0.042	0	Myeloid	THEMIS2
0	4.15441588643549	0.831	0.203	0	Myeloid	CTSZ
0	5.23754954459584	0.717	0.095	0	Myeloid	SDC3
0	5.11172341216442	0.643	0.024	0	Myeloid	CYBB
0	2.88027549884253	0.746	0.128	0	Myeloid	PFKFB3
0	5.34788894770908	0.73	0.112	0	Myeloid	LIPA
0	5.25859601347603	0.697	0.085	0	Myeloid	CD68
0	4.92427759812113	0.781	0.17	0	Myeloid	LGMN
0	7.03713802957555	0.614	0.007	0	Myeloid	MSR1
0	5.83181986248596	0.628	0.022	0	Myeloid	TYROBP
0	5.36273756297177	0.639	0.043	0	Myeloid	FCER1G
0	3.28715984333056	0.686	0.095	0	Myeloid	CIITA
0	3.40359381663699	0.644	0.055	0	Myeloid	MS4A6A
0	4.65800497902448	0.617	0.031	0	Myeloid	CD83
0	7.19569892522563	0.595	0.009	0	Myeloid	LILRB2
0	3.66954049659151	0.818	0.233	0	Myeloid	GRN
0	3.13408995249705	0.834	0.25	0	Myeloid	LYZ
0	5.70030327689512	0.605	0.021	0	Myeloid	PLAUR
0	4.70665201069094	0.605	0.027	0	Myeloid	KCTD12
0	3.76054622157819	0.654	0.077	0	Myeloid	SGK1
0	3.23412482479022	0.817	0.246	0	Myeloid	TYMP
0	3.1110222810165	0.912	0.343	0	Myeloid	CTSB

0	1.92549448627558	0.752	0.185		0	Myeloid	LAPTM5
0	1.9228584772171	0.907	0.341		0	Myeloid	SRGN
0	3.32229068056093	0.848	0.284		0	Myeloid	CST3
0	6.77476696968449	0.565	0.01		0	Myeloid	C5AR1
0	6.60844961504475	0.564	0.012		0	Myeloid	GPNMB
0	2.92279381205477	0.597	0.055		0	Myeloid	ADA2
0	3.44610538598428	0.721	0.18		0	Myeloid	CTSS
0	2.34373152884001	0.637	0.098		0	Myeloid	CD4
0	3.28176208669097	0.735	0.196		0	Myeloid	FCGRT
0	6.59131425434256	0.542	0.007		0	Myeloid	CLEC7A
0	3.45492174712758	0.779	0.254		0	Myeloid	GLUL
0	1.53175419435962	0.781	0.256		0	Myeloid	CYBA
0	6.49371811468263	0.537	0.015		0	Myeloid	CPVL
0	4.85443601868123	0.557	0.039		0	Myeloid	FCGR3A
0	2.29822130213655	0.796	0.278		0	Myeloid	ARAP1
0	2.97775162939937	0.687	0.17		0	Myeloid	MARCKS
0	1.60052419125084	0.706	0.19		0	Myeloid	ITGB2
0	4.79596017141047	0.553	0.041		0	Myeloid	SLC15A3
0	4.40615401998893	0.633	0.121		0	Myeloid	HMOX1
0	6.20101211472713	0.516	0.01		0	Myeloid	EMILIN2
0	1.98823627471212	0.887	0.383		0	Myeloid	FTL
0	3.98118087090424	0.688	0.194		0	Myeloid	PLD3
0	3.57164180389191	0.531	0.038		0	Myeloid	PLEK
0	2.25977468615027	0.619	0.126		0	Myeloid	PLEKHO1
0	0.955547219727265	0.687	0.195		0	Myeloid	METRNL
0	4.81869463404439	0.561	0.07		0	Myeloid	DMXL2
0	2.24390482034753	0.877	0.386		0	Myeloid	FTH1
0	4.64575870106486	0.502	0.013		0	Myeloid	FGD2
0	2.85471092767796	0.615	0.128		0	Myeloid	NAGK
0	1.24742668416868	0.669	0.184		0	Myeloid	HCLS1
0	3.69211041574689	0.617	0.136		0	Myeloid	MFSD1
0	6.52397338507441	0.512	0.037		0	Myeloid	CD5L
0	3.40827563929796	0.514	0.04		0	Myeloid	RGS2
0	5.41439287093978	0.486	0.013		0	Myeloid	SLC8A1
0	2.97797025154614	0.605	0.135		0	Myeloid	TTYH3
0	3.1722721299055	0.633	0.163		0	Myeloid	CTSL
0	2.2352993294598	0.557	0.088		0	Myeloid	ZEB2
0	1.24162568372907	0.642	0.174		0	Myeloid	ARL4C
0	1.82495942719591	0.642	0.177		0	Myeloid	SAMHD1
0	7.51410676301934	0.469	0.004		0	Myeloid	SIGLEC1
0	7.14885351797468	0.469	0.004		0	Myeloid	CSF2RA
0	1.95402480063977	0.793	0.331		0	Myeloid	DUSP1
0	4.22595773956262	0.547	0.085		0	Myeloid	MAFB
0	1.40073916628006	0.719	0.26		0	Myeloid	VIM
0	2.04974388873461	0.568	0.112		0	Myeloid	NABP1
0	2.30817211297491	0.566	0.11		0	Myeloid	GNA13
0	6.83799889654617	0.459	0.005		0	Myeloid	HK3
0	2.26267184510065	0.677	0.229		0	Myeloid	MGAT1
0	4.33838541825783	0.529	0.082		0	Myeloid	MPP1
0	5.21908645818387	0.466	0.023		0	Myeloid	AIF1
0	2.23706001398787	0.703	0.26		0	Myeloid	CD14
0	2.07258425146593	0.788	0.346		0	Myeloid	TPP1

0	2.03786082574334	0.592	0.151	0	Myeloid	DEPRECATI
0	2.36422779662653	0.684	0.246	0	Myeloid	LRP1
0	3.65287860107501	0.582	0.148	0	Myeloid	TGFBI
0	1.47497618182911	0.724	0.291	0	Myeloid	EHBP1L1
0	6.49121400983332	0.44	0.007	0	Myeloid	FOLR2
0	2.26752925149676	0.702	0.269	0	Myeloid	CEBPD
0	2.83114145506899	0.493	0.061	0	Myeloid	TIMP2
0	2.23222604829867	0.734	0.306	0	Myeloid	CD63
0	0.969606148941273	0.585	0.159	0	Myeloid	IL10RA
0	6.66438455624357	0.435	0.01	0	Myeloid	KCNMA1
0	3.27398850002778	0.537	0.113	0	Myeloid	IRF8
0	4.03902374175123	0.438	0.016	0	Myeloid	CD72
0	3.74275165402611	0.431	0.017	0	Myeloid	LAIR1
0	2.60142232314429	0.488	0.076	0	Myeloid	GSN
0	3.0484087494039	0.584	0.173	0	Myeloid	GAA
0	3.48470534413979	0.528	0.118	0	Myeloid	NPC2
0	5.19619912347626	0.441	0.031	0	Myeloid	ADAP2
0	5.83999498952494	0.425	0.017	0	Myeloid	CFD
0	5.63308405353313	0.418	0.012	0	Myeloid	AXL
0	3.01473975679802	0.557	0.152	0	Myeloid	NAMPT
0	1.78101810479303	0.548	0.145	0	Myeloid	CTSC
0	2.21560009083043	0.492	0.089	0	Myeloid	PLEKHO2
0	2.35055792621518	0.456	0.055	0	Myeloid	KLHL6
0	7.12287736106133	0.404	0.003	0	Myeloid	PLA2G7
0	2.79699119100421	0.506	0.106	0	Myeloid	FOS
0	1.60734611726859	0.71	0.31	0	Myeloid	VMP1
0	1.40062104593709	0.681	0.282	0	Myeloid	RNF149
0	2.51785391265484	0.629	0.23	0	Myeloid	MAT2A
0	2.24353384122441	0.441	0.042	0	Myeloid	AOAH
0	1.77056294357128	0.676	0.279	0	Myeloid	SOD2
0	0.504785605069847	0.596	0.2	0	Myeloid	ARHGAP4
0	1.89524976902239	0.725	0.333	0	Myeloid	WSB1
0	3.80570413919942	0.464	0.073	0	Myeloid	ATP6V1B2
0	2.90005335174508	0.433	0.044	0	Myeloid	NFKBID
0	4.62523970825	0.398	0.012	0	Myeloid	LILRB1
0	4.58055207610026	0.416	0.032	0	Myeloid	FMNL2
0	1.83194107856898	0.567	0.184	0	Myeloid	ARRB2
0	6.47133040733715	0.383	0.003	0	Myeloid	CSF3R
0	5.27825748039221	0.399	0.02	0	Myeloid	HSPA6
0	1.24124889941075	0.48	0.102	0	Myeloid	PLCB2
0	3.84363938453211	0.487	0.111	0	Myeloid	ABCA1
0	2.05869157913271	0.442	0.066	0	Myeloid	ZNF331
0	5.02140058033977	0.383	0.011	0	Myeloid	FAM49A
0	0.918864637837912	0.499	0.128	0	Myeloid	MYO1F
0	1.41070459230872	0.449	0.08	0	Myeloid	PARVG
0	6.20921285847922	0.371	0.006	0	Myeloid	ADGRE2
0	0.749190995250032	0.593	0.229	0	Myeloid	LCP1
0	0.84972514131111	0.547	0.184	0	Myeloid	TNFRSF1B
0	1.198982345958	0.467	0.105	0	Myeloid	UCP2
0	1.36730548940889	0.55	0.188	0	Myeloid	MYO9B
0	2.42255310734016	0.517	0.156	0	Myeloid	QKI
0	1.20491150225932	0.462	0.102	0	Myeloid	DOK2

0	1.8418762584361	0.404	0.047	0	Myeloid	CD300A
0	5.2651587479371	0.39	0.033	0	Myeloid	TLR2
0	4.18031962354223	0.41	0.054	0	Myeloid	ASAH1
0	3.05795912666667	0.476	0.126	0	Myeloid	TSPAN4
0	2.3217444886739	0.454	0.105	0	Myeloid	SH3TC1
0	1.3081280849972	0.694	0.345	0	Myeloid	TMSB10
0	1.45552025182962	0.46	0.113	0	Myeloid	REL
0	0.291965255659137	0.477	0.132	0	Myeloid	HAVCR2
0	2.02726195198702	0.4	0.057	0	Myeloid	RELT
0	4.19415598399943	0.407	0.064	0	Myeloid	GM2A
0	4.21280644544894	0.356	0.015	0	Myeloid	BASP1
0	4.16572651187944	0.368	0.027	0	Myeloid	PLXDC2
0	2.46391515793056	0.396	0.058	0	Myeloid	VCAM1
0	2.78225673816372	0.493	0.155	0	Myeloid	CXCL16
0	4.26616831035866	0.38	0.043	0	Myeloid	SLC43A2
0	1.09383620582979	0.856	0.519	0	Myeloid	MT-CO1
0	5.78411679852675	0.341	0.005	0	Myeloid	HCK
0	1.22311364735678	0.726	0.39	0	Myeloid	MT-CO3
0	4.00554620053761	0.353	0.019	0	Myeloid	NR4A3
0	2.37588156632868	0.474	0.14	0	Myeloid	MAN2B1
0	4.17042969074244	0.392	0.059	0	Myeloid	PDE4DIP
0	4.72434371653874	0.353	0.02	0	Myeloid	CFP
0	0.55823978902535	0.472	0.14	0	Myeloid	DOCK2
0	0.967174260785504	0.424	0.093	0	Myeloid	NCKAP1L
0	5.34389323346875	0.335	0.005	0	Myeloid	TFEC
0	1.28570973998149	0.547	0.219	0	Myeloid	EFHD2
0	2.05015372405872	0.527	0.2	0	Myeloid	LGALS3BP
0	3.5676343746897	0.352	0.026	0	Myeloid	SEMA4A
0	6.64751908756312	0.351	0.025	0	Myeloid	MARCO
0	1.99313069908047	0.428	0.104	0	Myeloid	RNASET2
0	2.74204243006268	0.463	0.142	0	Myeloid	GNS
0	2.57647580496136	0.358	0.037	0	Myeloid	SLC16A3
0	1.64740932795034	0.487	0.166	0	Myeloid	SYNGR2
0	2.15171662842657	0.546	0.226	0	Myeloid	CEBPB
0	3.09930066626937	0.374	0.054	0	Myeloid	IL18BP
0	3.66689421416355	0.337	0.017	0	Myeloid	GPR183
0	0.850126267368619	0.79	0.471	0	Myeloid	JUNB
0	2.1754452057383	0.447	0.128	0	Myeloid	STX7
0	6.18840523030085	0.323	0.004	0	Myeloid	EPB41L3
0	3.94247451213993	0.346	0.028	0	Myeloid	LGALS3
0	1.47453541476564	0.558	0.241	0	Myeloid	NOTCH2NL
0	2.12162901329933	0.375	0.058	0	Myeloid	DENND3
0	3.00859494548455	0.424	0.108	0	Myeloid	NINJ1
0	3.75733344583362	0.393	0.077	0	Myeloid	SCO2
0	1.52543013944229	0.598	0.285	0	Myeloid	PLXNB2
0	3.98612531204191	0.398	0.086	0	Myeloid	MCOLN1
0	3.73154192461688	0.378	0.066	0	Myeloid	FCHO2
0	2.11841636508385	0.592	0.28	0	Myeloid	RHOB
0	3.0800063223929	0.428	0.117	0	Myeloid	INSIG1
0	6.23780682340932	0.317	0.006	0	Myeloid	LRRC25
0	3.31458805922532	0.438	0.128	0	Myeloid	SLCO2B1
0	3.37995914251665	0.397	0.088	0	Myeloid	RAB20

	0	3.59442549556384	0.332	0.023	0	Myeloid	MRC2
	0	3.09574751259451	0.422	0.114	0	Myeloid	APBB3
	0	2.54744541867226	0.408	0.101	0	Myeloid	WARS
	0	0.916624272786544	0.696	0.389	0	Myeloid	ZFP36
	0	2.86424461620914	0.438	0.132	0	Myeloid	CTSH
	0	1.65259428951001	0.44	0.134	0	Myeloid	GRB2
	0	5.72548102917084	0.338	0.033	0	Myeloid	IL1RN
	0	1.33999930877612	0.509	0.205	0	Myeloid	LPCAT1
	0	4.8670122626747	0.311	0.008	0	Myeloid	SCIMP
	0	6.27715326309835	0.308	0.005	0	Myeloid	LILRB5
	0	1.97973168067599	0.411	0.11	0	Myeloid	RAP2B
	0	2.40846365850897	0.348	0.047	0	Myeloid	TMEM106A
	0	1.67290094021348	0.354	0.053	0	Myeloid	CYTH4
	0	1.91957904480475	0.353	0.053	0	Myeloid	CXCL12
	0	1.47895725972554	0.6	0.301	0	Myeloid	CTSD
	0	2.01800111396243	0.418	0.119	0	Myeloid	COLGALT1
	0	3.11634600078582	0.371	0.073	0	Myeloid	RASGEF1B
	0	5.5915785573384	0.305	0.007	0	Myeloid	MS4A4A
	0	3.31455677078369	0.4	0.103	0	Myeloid	DOCK5
	0	1.62544811055753	0.358	0.061	0	Myeloid	ST8SIA4
	0	3.60294295169976	0.311	0.014	0	Myeloid	WDFY4
	0	2.68561316118738	0.353	0.057	0	Myeloid	UBXN11
	0	3.30545525619763	0.339	0.044	0	Myeloid	LYN
	0	1.34795700491474	0.359	0.066	0	Myeloid	FERMT3
	0	6.11297047475174	0.296	0.004	0	Myeloid	CALHM6
	0	1.18465496407622	0.481	0.19	0	Myeloid	FOSL2
	0	2.08503612532581	0.383	0.093	0	Myeloid	IFNGR1
	0	2.38881963305403	0.359	0.069	0	Myeloid	HPS3
	0	6.84130269545531	0.292	0.003	0	Myeloid	FPR3
	0	1.09240043727726	0.673	0.385	0	Myeloid	LITAF
	0	2.53280441469938	0.391	0.104	0	Myeloid	PLSCR1
	0	1.57256767180658	0.539	0.254	0	Myeloid	RNH1
	0	1.70943772643329	0.383	0.098	0	Myeloid	PTPN6
	0	4.31874442241463	0.308	0.023	0	Myeloid	HBEGF
	0	1.98055745280432	0.442	0.158	0	Myeloid	STX4
	0	3.61765564406979	0.345	0.062	0	Myeloid	DAPK1
	0	5.17291314999257	0.305	0.022	0	Myeloid	SLC37A2
	0	1.98277307165297	0.343	0.063	0	Myeloid	RILPL2
	0	5.37537858055548	0.291	0.011	0	Myeloid	NCF2
	0	3.58789548638387	0.321	0.042	0	Myeloid	RGL1
	0	1.74950980913521	0.403	0.124	0	Myeloid	SH3BP5
	0	2.09903210822014	0.364	0.085	0	Myeloid	SH2B3
	0	3.05169479937434	0.317	0.039	0	Myeloid	MRC1
	0	6.06864516853645	0.289	0.011	0	Myeloid	MMP19
	0	1.63111709886522	0.357	0.081	0	Myeloid	ATG16L2
	0	2.20578276740422	0.413	0.138	0	Myeloid	ANXA5
	0	3.89487673532733	0.333	0.059	0	Myeloid	RASSF4
	0	2.75490487266484	0.358	0.084	0	Myeloid	ZFYVE16
	0	1.26153103386354	0.511	0.238	0	Myeloid	DPYD
	0	1.52252672313987	0.343	0.07	0	Myeloid	APOBR
	0	3.34594903784024	0.368	0.096	0	Myeloid	KYNU
	0	2.31266391976756	0.334	0.062	0	Myeloid	LGALS9

0	2.6726275414605	0.418	0.146		0	Myeloid	ODF3B
0	4.33884719495971	0.3	0.028		0	Myeloid	LHFPL2
0	2.26319816996377	0.427	0.156		0	Myeloid	LAP3
0	2.78394084775942	0.365	0.095		0	Myeloid	NPL
0	3.09951390115906	0.328	0.058		0	Myeloid	C9orf72
0	1.94488183566053	0.401	0.131		0	Myeloid	SDS
0	6.97350614736852	0.272	0.002		0	Myeloid	CD300LF
0	2.51925072819358	0.344	0.075		0	Myeloid	GSAP
0	0.948809367881877	0.353	0.084		0	Myeloid	ADCY7
0	0.575658276240921	0.343	0.076		0	Myeloid	STAB1
0	1.10887113816523	0.43	0.163		0	Myeloid	M6PR
0	2.07941179935024	0.344	0.077		0	Myeloid	ATG7
0	2.75649733640047	0.351	0.085		0	Myeloid	CLN8
0	3.47744769189691	0.349	0.083		0	Myeloid	SRC
0	0.810703167162366	0.35	0.085		0	Myeloid	SAMSN1
0	1.46917926074113	0.517	0.254		0	Myeloid	BRI3
0	4.17022622244926	0.293	0.03		0	Myeloid	ZNF385A
0	1.65049931682929	0.504	0.243		0	Myeloid	GRINA
0	4.48118488373274	0.277	0.016		0	Myeloid	SLC7A7
0	3.17232663324127	0.347	0.086		0	Myeloid	JDP2
0	1.91641612133796	0.393	0.132		0	Myeloid	ATP6AP1
0	1.67192264496399	0.304	0.044		0	Myeloid	TRPS1
0	5.19546746008655	0.283	0.024		0	Myeloid	MERTK
0	6.83945120585646	0.26	0.002		0	Myeloid	IL18
0	3.64362383748128	0.315	0.057		0	Myeloid	SIRPA
0	2.05324300880404	0.32	0.063		0	Myeloid	STX11
0	1.95102423163132	0.433	0.176		0	Myeloid	CHMP1B
0	2.16294770655277	0.317	0.062		0	Myeloid	DYSF
0	1.93889078661873	0.422	0.167		0	Myeloid	ICAM1
0	2.73738906718887	0.274	0.02		0	Myeloid	GAS7
0	3.90283865936211	0.271	0.018		0	Myeloid	CAPG
0	2.88891652877497	0.286	0.033		0	Myeloid	DSE
0	6.16408210131896	0.26	0.007		0	Myeloid	IGSF6
0	2.06884065269759	0.296	0.043		0	Myeloid	ABR
0	1.9102187832248	0.461	0.208		0	Myeloid	BLVRB
0	2.7999410492324	0.272	0.02		0	Myeloid	FGR
0	2.49923329667929	0.304	0.052		0	Myeloid	PTPRE
0	2.52509941876266	0.289	0.038		0	Myeloid	VASH1
0	4.31699368520612	0.265	0.016		0	Myeloid	FCGR2A
0	4.93476726373749	0.258	0.009		0	Myeloid	SIGLEC9
0	3.16342975025086	0.33	0.082		0	Myeloid	RNF130
0	2.59096485795219	0.36	0.112		0	Myeloid	ACP5
0	3.42689162975954	0.264	0.016		0	Myeloid	CSF2RB
0	3.07690286118157	0.272	0.025		0	Myeloid	GRK3
0	2.64168727589275	0.273	0.027		0	Myeloid	NRROS
0	4.1284787689189	0.298	0.052		0	Myeloid	RNF144B
0	4.21981747442209	0.261	0.015		0	Myeloid	RNF135
0	3.62950627514649	0.259	0.013		0	Myeloid	IL4I1
0	2.05995553576393	0.434	0.189		0	Myeloid	CREG1
0	4.76470694824247	0.25	0.005		0	Myeloid	C3AR1
0	1.83320019167381	0.418	0.173		0	Myeloid	HERPUD1
0	2.72450103423198	0.346	0.102		0	Myeloid	SCPEP1

0	2.9891710291489	0.308	0.064	0	Myeloid	RENBP
0	2.18664720473358	0.391	0.151	0	Myeloid	TNS3
0	1.02515551273561	0.341	0.102	0	Myeloid	RUNX1
0	5.94813126266681	0.256	0.019	0	Myeloid	RAB7B
0	5.61155293594467	0.242	0.005	0	Myeloid	SLAMF8
0	1.78763655326247	0.314	0.077	0	Myeloid	JAK2
0	1.4027112910611	0.296	0.059	0	Myeloid	FAR1
0	1.24181851695363	0.301	0.064	0	Myeloid	ABI3
0	1.00484921616274	0.926	0.69	0	Myeloid	TMSB4X
0	3.32925941340567	0.263	0.028	0	Myeloid	TNFSF13B
0	2.02934397484413	0.297	0.063	0	Myeloid	DPYSL2
0	3.28991183726788	0.353	0.119	0	Myeloid	NR1H3
0	2.82429790002418	0.341	0.108	0	Myeloid	CYFIP1
0	3.83240921391812	0.269	0.037	0	Myeloid	GLA
0	0.723773765972895	0.303	0.071	0	Myeloid	CD84
0	5.86197066497729	0.239	0.009	0	Myeloid	VSIG4
0	2.863918454722	0.291	0.062	0	Myeloid	EPB41L2
0	6.14173437274433	0.231	0.003	0	Myeloid	NFAM1
0	2.96770323542837	0.988	0.76	0	Myeloid	CD74
0	2.44453731503543	0.363	0.136	0	Myeloid	CORO1B
0	2.61452829788503	0.287	0.062	0	Myeloid	HEATR3
0	5.16948538643995	0.233	0.009	0	Myeloid	KMO
0	6.31260461944968	0.23	0.006	0	Myeloid	FPR1
0	2.6830291585559	0.307	0.083	0	Myeloid	DDAH2
0	3.33575289518564	0.266	0.044	0	Myeloid	CCDC88A
0	3.6394294790959	0.236	0.015	0	Myeloid	LRRK1
0	3.49842630916801	0.259	0.039	0	Myeloid	DEPRECATI
0	2.22526057527176	0.28	0.061	0	Myeloid	RCN3
0	1.57459749613484	0.309	0.09	0	Myeloid	ETV6
0	4.04207659081586	0.229	0.011	0	Myeloid	LST1
0	2.98367858249506	0.249	0.031	0	Myeloid	PLTP
0	0.969734559511984	0.29	0.073	0	Myeloid	NR4A1
0	3.00712292457876	0.284	0.067	0	Myeloid	IFNGR2
0	3.15284124141802	0.238	0.023	0	Myeloid	GNB4
0	3.26018794914047	0.225	0.01	0	Myeloid	SYK
0	4.73124840736222	0.226	0.011	0	Myeloid	SLC31A2
0	5.37021495528211	0.217	0.003	0	Myeloid	CD86
0	2.82960792746029	0.264	0.051	0	Myeloid	ADAM9
0	3.5206378606032	0.282	0.069	0	Myeloid	AVPI1
0	2.54761361553118	0.279	0.067	0	Myeloid	DEPRECATI
0	3.14459958798888	0.248	0.036	0	Myeloid	UNC93B1
0	6.376778599087	0.215	0.004	0	Myeloid	SLC1A3
0	3.30430920578457	0.251	0.041	0	Myeloid	MXD1
0	3.9888095522583	0.237	0.027	0	Myeloid	TNFSF13
0	3.99365977617975	0.235	0.028	0	Myeloid	LRRK2
0	1.84427996074303	0.269	0.063	0	Myeloid	MKNK1
0	0.967887318263254	0.258	0.054	0	Myeloid	PTGER4
0	2.59125292269962	0.278	0.075	0	Myeloid	DAB2
0	2.56913406140169	0.235	0.032	0	Myeloid	MANBA
0	3.50560735394097	0.247	0.045	0	Myeloid	KLF4
0	4.41195493733197	0.215	0.015	0	Myeloid	TTYH2
0	3.62226514999529	0.21	0.01	0	Myeloid	DOK3

0	3.1416825147747	0.292	0.093		0	Myeloid	FGD4
0	2.54048229403235	0.242	0.045		0	Myeloid	PPT1
0	1.60558303901352	0.24	0.044		0	Myeloid	NRP2
0	1.81898827873816	0.241	0.046		0	Myeloid	VSIR
0	4.32973543507926	0.217	0.022		0	Myeloid	CPM
0	3.34241044794244	0.206	0.012		0	Myeloid	ALOX5
0	4.03042911256212	0.202	0.009		0	Myeloid	PHACTR1
0	3.12851744889312	0.202	0.01		0	Myeloid	TRPM2
0	5.04002577617509	0.194	0.004		0	Myeloid	CCR1
0	6.11493424819816	0.2	0.01		0	Myeloid	CLEC10A
0	1.97566641924311	0.244	0.055		0	Myeloid	ARRB1
0	1.40940816590399	0.227	0.042		0	Myeloid	SLAMF7
0	1.88216850021556	0.231	0.047		0	Myeloid	FKBP15
0	5.59322877653011	0.184	0.003		0	Myeloid	LOXL3
0	6.73734772592259	0.184	0.003		0	Myeloid	PILRA
0	2.72095544140563	0.21	0.029		0	Myeloid	FRMD4A
0	2.95331123837202	0.207	0.027		0	Myeloid	OTUD1
0	4.22227017308917	0.205	0.025		0	Myeloid	SECTM1
0	4.49122937825581	0.18	0.004		0	Myeloid	BCL2A1
0	2.6803795441663	0.212	0.037		0	Myeloid	DRAM1
0	4.48518433793419	0.176	0.004		0	Myeloid	MARCH1
0	5.80090674949183	0.175	0.003		0	Myeloid	MS4A4E
0	3.42803188207292	0.188	0.017		0	Myeloid	IRAK3
0	3.07252566507633	0.181	0.011		0	Myeloid	TBXAS1
0	7.39751314158417	0.169	0.001		0	Myeloid	HS3ST2
0	3.96390715117163	0.191	0.025		0	Myeloid	NCEH1
0	3.9368464972508	0.174	0.008		0	Myeloid	DEPRECATE
0	2.32069757880136	0.198	0.034		0	Myeloid	ABCC5
0	5.47658824026278	0.17	0.006		0	Myeloid	BEST1
0	2.07289649588113	0.187	0.024		0	Myeloid	CCL4L2
0	4.4805947293387	0.177	0.015		0	Myeloid	LPCAT2
0	5.85169090860423	0.165	0.004		0	Myeloid	PTAFR
0	1.88741418361116	0.19	0.029		0	Myeloid	NAAA
0	4.25382714243443	0.167	0.007		0	Myeloid	RAB31
0	4.54528085294921	0.167	0.008		0	Myeloid	VMO1
0	7.13748130640086	0.16	0.002		0	Myeloid	NAIP
0	5.10608746580827	0.161	0.003		0	Myeloid	CMKLR1
0	2.60289887590066	0.172	0.014		0	Myeloid	DPEP2
0	3.02138563233562	0.178	0.021		0	Myeloid	BMP2K
0	4.93337741597603	0.164	0.008		0	Myeloid	PLAU
0	3.55716220783009	0.161	0.007		0	Myeloid	CD80
0	3.87594562124398	0.178	0.024		0	Myeloid	TMEM86A
0	3.11432803342655	0.169	0.016		0	Myeloid	MYOF
0	3.87362807637019	0.185	0.032		0	Myeloid	CXCL9
0	5.793322577301	0.155	0.003		0	Myeloid	SIGLEC7
0	4.85125498047696	0.172	0.022		0	Myeloid	S100A9
0	2.6160359958237	0.178	0.028		0	Myeloid	RIPK2
0	3.03408455611957	0.163	0.013		0	Myeloid	ITGAM
0	5.03593332889366	0.154	0.005		0	Myeloid	P2RY6
0	2.71047926615663	0.167	0.019		0	Myeloid	SLC49A3
0	3.67595795015892	0.159	0.011		0	Myeloid	TLR4
0	6.29646509791155	0.15	0.002		0	Myeloid	FCAR

0	2.59460950259587	0.169	0.023	0	Myeloid	GASK1B
0	1.76286401123026	0.165	0.019	0	Myeloid	NCF4
0	3.81245232724167	0.153	0.007	0	Myeloid	CD1D
0	4.15444764430888	0.153	0.007	0	Myeloid	MILR1
0	2.7298885742782	0.167	0.023	0	Myeloid	S100A11
0	2.62733320192598	0.158	0.016	0	Myeloid	PYCARD
0	4.61321636789812	0.147	0.005	0	Myeloid	OSM
0	5.30942427817241	0.144	0.003	0	Myeloid	DEPRECATI
0	4.81696887840998	0.152	0.012	0	Myeloid	ALDH3B1
0	4.12159125551481	0.142	0.003	0	Myeloid	LY86
0	4.76797173380501	0.143	0.004	0	Myeloid	BCAT1
0	3.83324341039441	0.145	0.006	0	Myeloid	EBI3
0	3.15700871177265	0.149	0.01	0	Myeloid	DEPRECATI
0	6.50530151896921	0.138	0.002	0	Myeloid	LILRA5
0	7.08154316544412	0.136	0.001	0	Myeloid	CD300E
0	3.27348533635013	0.145	0.01	0	Myeloid	CEP295NL
0	3.75517502078953	0.151	0.018	0	Myeloid	TBC1D2
0	4.49808644145261	0.136	0.004	0	Myeloid	CCRL2
0	5.02213578569879	0.136	0.004	0	Myeloid	SIGLEC11
0	5.86507619749505	0.136	0.004	0	Myeloid	DEPRECATI
0	5.87268787460664	0.13	0.002	0	Myeloid	TM6SF1
0	6.28372860042193	0.128	0.002	0	Myeloid	LILRB4
0	5.48956902506762	0.13	0.005	0	Myeloid	TREM2
0	2.49053270895664	0.139	0.015	0	Myeloid	SPINT2
0	3.44639689220615	0.134	0.011	0	Myeloid	SPIRE1
0	3.06125315814707	0.132	0.009	0	Myeloid	FOSB
0	5.89816742501812	0.126	0.003	0	Myeloid	TRPV4
0	6.37091711164742	0.125	0.002	0	Myeloid	MMP9
0	2.35568262272127	0.134	0.012	0	Myeloid	FMN1
0	5.46130590108082	0.125	0.004	0	Myeloid	PLBD1
0	2.56174741823905	0.129	0.008	0	Myeloid	HVCN1
0	3.91404274676081	0.126	0.008	0	Myeloid	C1orf162
0	3.783459379001	0.124	0.01	0	Myeloid	SLC7A8
0	3.81831578559612	0.116	0.004	0	Myeloid	CR1
0	6.61461517312694	0.111	0.001	0	Myeloid	MEFV
0	6.9485888600576	0.102	0.001	0	Myeloid	CDCP1
0	5.83107494344213	0.102	0.001	0	Myeloid	F13A1
0	3.31110063108673	0.105	0.005	0	Myeloid	BTK
0	4.06844573084248	0.1	0.003	0	Myeloid	SIGLEC10
6.72240710330703E-308	2.0240700134012	0.196	0.036	1.09649182262041E-303	Myeloid	AKR1B1
4.46511901433217E-306	1.6878987977471	0.374	0.14	7.2830556242772E-302	Myeloid	GNAQ
6.13091464323832E-306	1.39341394900815	0.248	0.056	1.0000134874586E-301	Myeloid	CD93
3.64711985907516E-305	1.11265664274533	0.326	0.096	5.94881720213749E-301	Myeloid	ARHGEF2
9.86987536144812E-305	1.2133764476877	0.53	0.259	1.6098753702058E-300	Myeloid	ZYX
6.20849383753372E-304	2.74908673188065	0.147	0.017	1.01266742984012E-299	Myeloid	C5AR2
6.66134049763231E-301	1.64647573061327	0.263	0.067	1.08653124856881E-296	Myeloid	GSTP1
4.72443501214267E-297	1.93412179402289	0.214	0.045	7.70602594830592E-293	Myeloid	ATP1B3
6.03014073806182E-297	1.0182943297428	0.543	0.263	9.83576255785264E-293	Myeloid	TNFRSF14
5.12104035873393E-295	2.21509665279065	0.15	0.019	8.35292892913092E-291	Myeloid	FUT4
8.65867142031381E-295	0.881613731583587	0.409	0.143	1.41231589536738E-290	Myeloid	MICAL1
2.80580510933285E-294	2.23953580867684	0.137	0.015	4.5765487138328E-290	Myeloid	DAPP1
1.31433299585063E-293	3.11976562558129	0.196	0.04	2.14380854953196E-289	Myeloid	STARD13

**5.28728163251507E-293**	0.655547584217517	0.377	0.115	8.62408507079533E-289	Myeloid	LCP2
**1.29865489271027E-292**	1.17717712638569	0.532	0.265	2.11823599549972E-288	Myeloid	NFKBIA
**3.61518795448648E-292**	0.691532661841849	0.409	0.14	5.8967330725629E-288	Myeloid	FNBP1
**3.12208769014739E-291**	2.61930873604863	0.169	0.027	5.0924372313994E-287	Myeloid	THBD
**1.55526265437475E-290**	2.20940020286637	0.312	0.105	2.53678891555066E-286	Myeloid	IER3
**8.54691284968787E-290**	1.518779151383	0.185	0.032	1.39408695491259E-285	Myeloid	UAP1L1
**1.53001533495221E-289**	1.89586475081635	0.181	0.031	2.49560801284055E-285	Myeloid	YBX3
**2.5035994992875E-288**	0.791730876175228	0.253	0.059	4.08362114328785E-284	Myeloid	CD38
**3.88628911079576E-288**	2.84114512249764	0.128	0.013	6.33892616861897E-284	Myeloid	LY96
**7.70942738474163E-288**	2.25592415225322	0.442	0.221	1.25748470072521E-283	Myeloid	SLC40A1
**1.62781053092783E-286**	3.35166397117321	0.224	0.057	2.65512175699639E-282	Myeloid	PLA2G15
**5.69473595535842E-286**	2.26340486149001	0.221	0.052	9.28868381678511E-282	Myeloid	PLD2
**7.59653481584645E-285**	1.75353234081931	0.27	0.075	1.23907079381271E-280	Myeloid	SCARF1
**3.56045031898199E-282**	1.26916990890971	0.454	0.209	5.80745051529153E-278	Myeloid	CLCN7
**6.30538794761401E-282**	1.99264438470355	0.278	0.083	1.02847182813532E-277	Myeloid	SLC26A11
**2.20420231614001E-281**	1.58396184097801	0.259	0.071	3.59527439785597E-277	Myeloid	RHBDF2
**9.11105272423206E-281**	2.57720900144228	0.189	0.039	1.48610380984949E-276	Myeloid	BMF
**1.79526663456335E-280**	2.27141395832835	0.159	0.024	2.92825940763629E-276	Myeloid	LMO2
**1.18463805557093E-279**	2.44533468832862	0.184	0.036	1.93226313244174E-275	Myeloid	ACE
**1.23873622971806E-279**	1.69401488839557	0.237	0.058	2.02050266429313E-275	Myeloid	GRASP
**1.55175663173066E-279**	3.09336811911738	0.149	0.022	2.53107024201588E-275	Myeloid	GAB2
**1.43934337092625E-278**	1.23235134238881	0.245	0.059	2.34771297231781E-274	Myeloid	LPAR6
**1.82911665734727E-278**	0.617161124434017	0.369	0.118	2.98347217979914E-274	Myeloid	NR4A2
**3.82141529754183E-278**	3.4143226547948	0.161	0.027	6.23311049182048E-274	Myeloid	MCTP1
**1.65791918633054E-277**	2.57068269544539	0.104	0.007	2.70423198482375E-273	Myeloid	CABP4
**1.66270201473108E-276**	1.10170146205668	0.609	0.323	2.71203325622786E-272	Myeloid	MT-ATP6
**5.48700700119716E-276**	2.79911027718619	0.234	0.063	8.94985711965269E-272	Myeloid	TCN2
**1.78927940440321E-275**	2.28789860620983	0.215	0.05	2.91849363652208E-271	Myeloid	CD9
**8.4121823543024E-275**	1.38218894223535	0.428	0.194	1.37211106381026E-270	Myeloid	VAMP8
**5.44709471706946E-274**	2.34129261995609	0.207	0.047	8.884756193012E-270	Myeloid	RHOQ
**1.22184575026112E-273**	1.62209318526657	0.155	0.023	1.99295260325091E-269	Myeloid	CCL3
**3.45772621340329E-273**	0.923626729793903	0.253	0.062	5.6398972266821E-269	Myeloid	PECAM1
**1.41309700380842E-272**	0.420375758637843	0.417	0.14	2.30490252291192E-268	Myeloid	CD53
**1.79008013091694E-272**	1.94874310550799	0.357	0.146	2.91979970153861E-268	Myeloid	RUFY3
**3.21270953146133E-272**	3.14775253379667	0.151	0.023	5.24025051676658E-268	Myeloid	INAFM2
**6.24194211118356E-272**	1.24537009544271	0.21	0.045	1.01812317775515E-267	Myeloid	CMTM7
**7.0743120246699E-270**	0.899598698168842	0.673	0.367	1.15389103434391E-265	Myeloid	MT-CO2
**5.87182549428686E-269**	1.34300480610811	0.155	0.023	9.57753456373129E-265	Myeloid	OTULINL
**1.35273178904451E-268**	2.81465209217555	0.134	0.017	2.2064408211105E-264	Myeloid	OGFRL1
**4.42412975737851E-268**	3.43688938121318	0.142	0.021	7.21619804726008E-264	Myeloid	WDFY3
**6.50248731770915E-266**	1.53658153365211	0.179	0.033	1.06062070639154E-261	Myeloid	GLIPR2
**5.81899185081206E-265**	0.87946908059473	0.426	0.173	9.49135760785956E-261	Myeloid	RIN3
**2.64018501401253E-264**	2.34765382530545	0.272	0.087	4.30640577635584E-260	Myeloid	DOCK4
**1.87325825999188E-263**	3.13822919671539	0.104	0.008	3.05547154787276E-259	Myeloid	PTGS1
**4.05721838510697E-263**	0.53715027005055	0.289	0.079	6.61772890794797E-259	Myeloid	PRKCB
**2.31444993364298E-262**	2.00327374445351	0.143	0.02	3.77509928676506E-258	Myeloid	SPART
**7.15837666486897E-262**	3.43401699963082	0.161	0.029	1.16760281780678E-257	Myeloid	SLC29A3
**1.93620062154512E-261**	1.13183359453502	0.358	0.134	3.15813683380225E-257	Myeloid	IFNAR2
**4.92565099572515E-261**	2.53920136755226	0.11	0.01	8.03422933912729E-257	Myeloid	ATP8B4
**3.65378223277175E-260**	1.77837421780172	0.402	0.189	5.959684199874E-256	Myeloid	AKR1A1
**8.57553035860674E-260**	0.719520924431759	0.855	0.608	1.39875475679235E-255	Myeloid	MCL1
**3.26539275731765E-258**	1.68914076036334	0.364	0.149	5.32618212646082E-254	Myeloid	ATF3

6.66540156285979E-258	1.51785142908373	0.451	0.226	1.08719364891806E-253	Myeloid	APLP2
3.06594976766681E-257	1.78952675890355	0.135	0.018	5.00087066604133E-253	Myeloid	LAT2
3.89096509519585E-257	1.62701132718178	0.316	0.115	6.34655316677395E-253	Myeloid	MYD88
1.07790296992225E-256	1.92192360780784	0.287	0.098	1.75816753424019E-252	Myeloid	YTHDF3
8.97744751170843E-256	1.0850786846375	0.459	0.214	1.46431146363476E-251	Myeloid	SLC3A2
5.02390915507437E-255	1.15097743263255	0.303	0.099	8.19449822284181E-251	Myeloid	MAP3K8
5.37881846977037E-255	2.22381061758932	0.29	0.103	8.77339080604245E-251	Myeloid	AMPD2
3.45785161170303E-254	2.04869203693184	0.249	0.074	5.64010176384881E-250	Myeloid	SMS
4.42347562961384E-253	3.48659336249404	0.203	0.052	7.21513109946313E-249	Myeloid	SMPDL3A
6.29878530153835E-252	2.3806533021643	0.196	0.047	1.02739487053392E-247	Myeloid	PRKCD
2.64460457677868E-251	0.894372705408248	0.222	0.052	4.31361452518371E-247	Myeloid	WAS
9.19205094131782E-251	2.18564578909869	0.415	0.209	1.49931542903835E-246	Myeloid	EXT1
3.33386576303806E-250	1.96085451076582	0.394	0.185	5.43786844609138E-246	Myeloid	ACSL1
1.81559839589487E-247	1.67453833471224	0.281	0.094	2.96142254354413E-243	Myeloid	PRNP
2.64775720054143E-247	1.86084961414611	0.149	0.025	4.31875676980312E-243	Myeloid	ARHGAP18
1.4245665651976E-245	2.67610528160046	0.172	0.036	2.32361052449381E-241	Myeloid	P2RX7
1.30565693787163E-244	2.46377370734429	0.119	0.014	2.12965703136242E-240	Myeloid	AP1S2
1.40615886454461E-244	2.15881542336299	0.312	0.123	2.29358572395871E-240	Myeloid	OAZ2
2.16349182358991E-244	1.36471730106211	0.296	0.101	3.52887151345751E-240	Myeloid	PNPLA6
2.10818547261682E-242	2.67458002263084	0.293	0.113	3.4386613243853E-238	Myeloid	ELL2
3.51044036162876E-242	0.908674194178699	0.297	0.091	5.72587927385266E-238	Myeloid	COTL1
5.2113463270407E-242	2.84419154163542	0.218	0.061	8.50022699403608E-238	Myeloid	SCARB2
1.92395956894581E-241	2.28578535726128	0.124	0.016	3.13817045290751E-237	Myeloid	TMEM268
1.30548263945018E-240	2.09826125682506	0.208	0.054	2.12937273320719E-236	Myeloid	CEP170
1.86341176919379E-238	1.50775946461834	0.181	0.038	3.03941093673199E-234	Myeloid	DISC1
7.95315097990707E-238	1.2496344291933	0.311	0.112	1.29723845633264E-233	Myeloid	ACOT9
7.42548769163128E-237	1.53864780004912	0.299	0.109	1.21117129738198E-232	Myeloid	DENND1A
3.98914708050278E-236	1.31693413708266	0.469	0.24	6.50669780300809E-232	Myeloid	HSPA1A
4.64234960126444E-236	1.28645154312177	0.575	0.324	7.57213643462243E-232	Myeloid	SELENOP
8.30556520618465E-234	1.16691635236179	0.44	0.217	1.35472074078078E-229	Myeloid	NFE2L2
9.28442565236794E-233	0.340851807933071	0.322	0.118	1.51438266815773E-228	Myeloid	TIMP1
1.74773386040829E-232	1.52241722846951	0.339	0.143	2.85072869971196E-228	Myeloid	PIK3AP1
1.06671095165866E-231	1.15319751251174	0.448	0.225	1.73991223325044E-227	Myeloid	STAT2
3.54343681925665E-228	1.06171681256584	0.411	0.189	5.77969979588952E-224	Myeloid	ARPC1B
2.15207033570763E-227	1.97315743453947	0.332	0.145	3.51024192457272E-223	Myeloid	PLBD2
6.08722682734645E-227	1.70508244428431	0.261	0.088	9.9288756780848E-223	Myeloid	H2AFY
1.44542909606863E-226	1.28781473607376	0.216	0.058	2.35763939859755E-222	Myeloid	RASSF3
1.56213482756112E-223	3.41693446665803	0.107	0.013	2.54799811723494E-219	Myeloid	FABP5
1.00614036245106E-221	0.947028764977761	0.459	0.228	1.64111554519392E-217	Myeloid	PER1
1.08853269463104E-221	2.49817845376187	0.19	0.05	1.7755056782127E-217	Myeloid	PSTPIP2
1.43878416589423E-221	1.11764528060771	0.235	0.066	2.34680085299009E-217	Myeloid	LFNG
1.85970350172744E-221	1.61005677599234	0.132	0.021	3.03336238166763E-217	Myeloid	SLC9A9
2.62659580820304E-219	1.4618438482211	0.353	0.155	4.28424042275998E-215	Myeloid	RTN4
8.34897090364274E-219	2.44998128801453	0.169	0.039	1.36180064409317E-214	Myeloid	GZF1
1.58887144154511E-218	2.21852223251274	0.105	0.012	2.59160820830424E-214	Myeloid	TIMD4
6.95869377510838E-217	2.74398455864919	0.202	0.058	1.13503254165793E-212	Myeloid	PLXNA1
1.25792270613518E-216	1.31753572986101	0.376	0.172	2.05179772597709E-212	Myeloid	MYO15B
4.05806059043269E-216	2.17795590282726	0.268	0.1	6.61910262905476E-212	Myeloid	ACP2
2.31367177947933E-214	1.4871164033199	0.312	0.127	3.77383003950874E-210	Myeloid	TPD52L2
1.11018425445318E-213	2.04305843564475	0.133	0.023	1.81082153743858E-209	Myeloid	FAM102B
2.31754101796061E-213	1.01698592089655	0.227	0.064	3.78014115439555E-209	Myeloid	ITGA9
4.2859271983688E-212	2.09679249649343	0.237	0.078	6.99077585325935E-208	Myeloid	C15orf39

4.36943984139883E-212	1.51623595672944	0.29	0.112	7.12699332530562E-208	Myeloid	AP1B1
5.99890464000032E-212	2.00282616537856	0.181	0.046	9.78481335830451E-208	Myeloid	MFSD12
1.95943342166106E-211	1.19191121692303	0.182	0.043	3.19603185407135E-207	Myeloid	ALDH16A1
2.71473469545676E-209	0.72659420077622	0.526	0.281	4.42800376175953E-205	Myeloid	HSPA1B
3.65621757607063E-209	1.36463678792649	0.285	0.104	5.9636564883288E-205	Myeloid	NRP1
6.71029103620845E-209	2.43762691513628	0.154	0.034	1.09451557091596E-204	Myeloid	SLC38A6
1.52440327825236E-208	1.0704206441366	0.36	0.155	2.48645418715743E-204	Myeloid	PTPN1
2.43631090795562E-208	1.97455903509015	0.179	0.045	3.97386672196642E-204	Myeloid	DEPRECATI
4.78530882469999E-208	1.45695256881524	0.307	0.127	7.80531722396816E-204	Myeloid	SFT2D2
5.93660536283408E-208	0.774872658628592	0.459	0.228	9.68319700731866E-204	Myeloid	PPP1R9B
2.10096143350237E-207	0.660720133444977	0.288	0.096	3.42687819418572E-203	Myeloid	CMTM3
1.01931753073446E-206	4.07147691160703	0.124	0.022	1.66260882438097E-202	Myeloid	C15orf48
1.26475748081596E-206	0.588183571778018	0.346	0.135	2.06294592695891E-202	Myeloid	CORO7
4.31881547506883E-205	1.70490647124728	0.16	0.036	7.04441992138476E-201	Myeloid	ATP2B1
7.18550072433593E-205	0.909709596311111	0.473	0.25	1.17202702314643E-200	Myeloid	MINK1
4.02591691309863E-204	1.36351940847607	0.409	0.21	6.56667307695518E-200	Myeloid	MT-ND3
1.65211580303273E-203	1.30394145715659	0.255	0.088	2.69476608632668E-199	Myeloid	CREBL2
1.05830891247716E-202	3.37150133588467	0.104	0.014	1.72620766714149E-198	Myeloid	C1orf54
2.88159137684266E-202	1.84368683743896	0.231	0.076	4.70016369476806E-198	Myeloid	SWAP70
5.74858964258404E-202	2.46190608091854	0.184	0.051	9.37652456601883E-198	Myeloid	ME1
2.6400272050844E-200	2.33595011494701	0.284	0.119	4.30614837421316E-196	Myeloid	TBC1D8
3.85310220744745E-200	1.88801277695296	0.206	0.063	6.28479501056754E-196	Myeloid	NOTCH2
1.28266381328758E-199	1.16483095586064	0.322	0.132	2.09215294585338E-195	Myeloid	UGCG
6.05394415954295E-199	1.17019019310989	0.209	0.06	9.8745883186305E-195	Myeloid	SCLT1
1.36119060111792E-198	2.90032071482059	0.142	0.03	2.22023798948343E-194	Myeloid	KLHDC8B
1.49110678716539E-197	1.40145318489928	0.231	0.075	2.43214428054547E-193	Myeloid	LIMK1
3.313069293728E-197	0.701993203627541	0.388	0.166	5.40394732499974E-193	Myeloid	SOCS3
4.01576810971154E-197	1.61340266506938	0.306	0.13	6.55011936375049E-193	Myeloid	SERPINB6
5.21082990074266E-197	1.26461241021719	0.212	0.063	8.49938465110135E-193	Myeloid	ARHGAP27
1.08337095198219E-196	0.774036048296654	0.781	0.586	1.76708635977815E-192	Myeloid	ACTB
1.68385441467099E-196	0.378132824997094	0.371	0.142	2.74653493576986E-192	Myeloid	CHST11
5.02988384326064E-196	1.9913312930929	0.266	0.105	8.20424353674243E-192	Myeloid	KIAA0930
1.11952956363192E-195	2.48468370928039	0.119	0.02	1.82606467124003E-191	Myeloid	SLC25A19
2.82188177968347E-195	2.02795543168712	0.323	0.153	4.6027713708417E-191	Myeloid	RBM47
1.8396419884407E-194	3.58618020964165	0.104	0.015	3.00064004734563E-190	Myeloid	FZD1
2.42384770436517E-194	1.13734635163288	0.296	0.117	3.95353799059003E-190	Myeloid	SNX6
6.46999425459923E-194	1.12838056881525	0.445	0.234	1.05532076286768E-189	Myeloid	TMEM176E
1.74616904047877E-193	2.48914993266723	0.139	0.03	2.84817632192492E-189	Myeloid	SIDT2
9.82804496170309E-193	1.04971154904715	0.386	0.187	1.60305241370339E-188	Myeloid	PARP14
3.40886536289891E-192	1.08413442311177	0.346	0.157	5.56020029342441E-188	Myeloid	PLEKHB2
1.58536324932143E-190	0.834419757404869	0.56	0.315	2.58588599596818E-186	Myeloid	MT-ND4
1.60464906885016E-190	1.66771338889928	0.37	0.193	2.6173430962015E-186	Myeloid	RXRA
2.35559076962256E-190	1.88341915322396	0.109	0.016	3.84220410433136E-186	Myeloid	AGPAT4
3.25784930763282E-189	3.85695950033857	0.118	0.021	5.3138780056799E-185	Myeloid	TMEM150E
4.22391161229681E-189	0.428468364363242	0.214	0.06	6.88962223081734E-185	Myeloid	DOCK10
1.44949822949781E-188	0.953408555421474	0.29	0.112	2.36427656213388E-184	Myeloid	GPCPD1
1.56147965525073E-188	3.16545350822312	0.111	0.018	2.54692946567946E-184	Myeloid	LACC1
3.7804012269939E-188	1.90208399412597	0.164	0.042	6.16621244134975E-184	Myeloid	FRMD4B
6.44458667749796E-188	2.23857081779713	0.319	0.149	1.05117653296669E-183	Myeloid	TFRC
4.11296147067578E-186	2.12332400671785	0.133	0.027	6.70865145481926E-182	Myeloid	CTTNBP2N
1.57625785490334E-184	1.23888434833494	0.226	0.076	2.57103418713284E-180	Myeloid	ADPGK
2.3263876239278E-183	0.549863591238745	0.632	0.375	3.79457085338863E-179	Myeloid	CFLAR

**2.39589573783389E-182**	1.49074819425425	0.327	0.149	3.90794553798086E-178	Myeloid	LGALS1
**3.07121001008841E-182**	1.59972797056801	0.234	0.084	5.00945064745521E-178	Myeloid	SDCBP
**3.69613600532582E-181**	1.244918985418	0.394	0.203	6.02876743828694E-177	Myeloid	DEPRECATI
**3.86094906349546E-181**	2.38954666210952	0.1	0.015	6.29759401746744E-177	Myeloid	CKS2
**6.52360406717381E-181**	2.93402040609075	0.213	0.076	1.06406505939672E-176	Myeloid	ADAMTSL4
**3.22966917995471E-179**	2.24721280461817	0.148	0.035	5.26791339942413E-175	Myeloid	CHST15
**3.4916280248084E-179**	0.906330436603382	0.372	0.18	5.69519447126498E-175	Myeloid	DENND4B
**1.21436596546678E-177**	0.784092461736573	0.329	0.144	1.98075232627286E-173	Myeloid	SUSD6
**1.33590732031322E-177**	1.41455233923495	0.263	0.104	2.1789984301629E-173	Myeloid	TGIF1
**3.80811526156326E-176**	0.335578306527362	0.248	0.08	6.21141680313584E-172	Myeloid	RCSD1
**5.32162418426312E-175**	1.84407223550875	0.11	0.019	8.68010120695158E-171	Myeloid	HDAC9
**8.60414276984624E-173**	1.63467119790987	0.219	0.078	1.40342172718962E-168	Myeloid	SOAT1
**3.76574323238157E-172**	1.16389059643635	0.184	0.054	6.14230378633758E-168	Myeloid	ST3GAL5
**1.46465094013558E-171**	1.02820014726115	0.246	0.091	2.38899214845515E-167	Myeloid	RHOG
**3.08716371445012E-171**	1.62032903852737	0.212	0.073	5.03547273463959E-167	Myeloid	DEPRECATI
**2.01392979598917E-170**	1.55910575126203	0.186	0.057	3.28492089023794E-166	Myeloid	HIF1A
**5.77711956948141E-170**	1.56998386679685	0.203	0.068	9.42305972978112E-166	Myeloid	GPR137B
**3.10885144339416E-169**	0.791363901332668	0.26	0.097	5.07084758932022E-165	Myeloid	PKM
**3.46999626767312E-169**	0.527482899714894	0.877	0.74	5.65991091220163E-165	Myeloid	B2M
**5.54152156187429E-168**	2.05799778670747	0.124	0.027	9.03877581957316E-164	Myeloid	ATP13A2
**7.49654405003138E-168**	0.84596806864065	0.356	0.167	1.22276130000062E-163	Myeloid	SH3BGRL3
**9.36439793664932E-168**	1.65170253048763	0.203	0.069	1.52742694744687E-163	Myeloid	PI4K2A
**1.35345491116666E-167**	2.0866629316398	0.251	0.105	2.20762030560394E-163	Myeloid	PGD
**1.63799729939968E-167**	2.04382209908613	0.167	0.049	2.67173739505082E-163	Myeloid	SLC8B1
**7.8522746171481E-167**	0.307733808462789	0.205	0.061	1.28078451280303E-162	Myeloid	PIK3R5
**1.10848469963903E-166**	0.29672872207178	0.393	0.176	1.80804939358122E-162	Myeloid	KCNAB2
**5.82842606083283E-166**	0.299575177691606	0.247	0.084	9.50674574782443E-162	Myeloid	DOCK11
**1.62218313845594E-165**	0.781491563909739	0.215	0.071	2.64594291713548E-161	Myeloid	IQGAP1
**2.59325839037771E-164**	1.44298978949669	0.278	0.121	4.22986376054508E-160	Myeloid	COMT
**5.01752906293831E-164**	2.10135956373746	0.181	0.058	8.18409165455867E-160	Myeloid	SNX10
**1.8603033410852E-163**	0.845950465822532	0.281	0.116	3.03434077964407E-159	Myeloid	SGSH
**4.46805010172639E-163**	2.30039562449505	0.208	0.076	7.28783652092592E-159	Myeloid	FES
**1.37586024979551E-162**	2.55944194061781	0.141	0.036	2.24416565344146E-158	Myeloid	RAB3IL1
**2.75856202091939E-162**	0.533222106330436	0.211	0.067	4.49949051232162E-158	Myeloid	ENTPD1
**4.95098240139727E-162**	1.44681201765362	0.245	0.099	8.07554739491909E-158	Myeloid	ATP6AP2
**6.30686114876267E-162**	1.23926745655027	0.419	0.229	1.02871212197468E-157	Myeloid	GBP1
**7.38071353195864E-161**	0.609085216092388	0.146	0.035	1.20386818419777E-156	Myeloid	VAV1
**1.71950570844222E-160**	0.93753991986266	0.141	0.034	2.80468576104011E-156	Myeloid	ADAMTS2
**2.92739128385909E-160**	0.806319449136	0.269	0.106	4.77486792310257E-156	Myeloid	SLC6A6
**4.17890895924763E-160**	1.90380773996948	0.283	0.134	6.81621840342881E-156	Myeloid	SLC35F6
**1.91707395714201E-159**	0.365662918420639	0.529	0.287	3.12693933149433E-155	Myeloid	GRK2
**1.68522196232774E-158**	1.27908838961022	0.387	0.22	2.74876554275277E-154	Myeloid	CTSA
**1.68708508636811E-158**	1.42064069082186	0.188	0.061	2.75180448437503E-154	Myeloid	IL10RB
**2.78251447161468E-158**	0.573578280371461	0.491	0.284	4.53855935465071E-154	Myeloid	DPP7
**1.81062573374823E-157**	1.95242270369072	0.301	0.153	2.95331163431673E-153	Myeloid	ATP1B1
**9.03251986792081E-157**	2.3524678992942	0.124	0.028	1.47329431565656E-152	Myeloid	SNAPC1
**1.67325892656375E-156**	1.06759998113951	0.289	0.129	2.72925263511814E-152	Myeloid	PLEKHM2
**5.74891337554422E-156**	0.759624474243877	0.448	0.255	9.37705260685019E-152	Myeloid	H3F3A
**1.61965512677515E-155**	0.688516047798319	0.636	0.407	2.64181947728295E-151	Myeloid	ZFP36L1
**2.30121790228244E-155**	0.446412586524119	0.237	0.083	3.75351652041289E-151	Myeloid	RASSF5
**8.3599078914475E-155**	0.598837210671517	0.358	0.168	1.363584576174E-150	Myeloid	MAP7D1
**1.00541838823389E-153**	1.15117037211266	0.197	0.067	1.6399379330483E-149	Myeloid	SETDB2

3.43051141667583E-153	0.763700670982489	0.483	0.288	5.59550717173995E-149	Myeloid	ITM2B
1.24188083417386E-152	0.91243983775426	0.364	0.19	2.02563182862098E-148	Myeloid	CNPY3
1.88424698286567E-152	1.14088381183566	0.238	0.094	3.0733952537522E-148	Myeloid	IFFO1
3.79278949147452E-151	1.9201911393632	0.146	0.04	6.18641893954409E-147	Myeloid	SNX30
1.18560025610028E-150	1.60418793286447	0.243	0.103	1.93383257772517E-146	Myeloid	CACUL1
1.24131909846991E-150	1.12083786771063	0.191	0.064	2.02471558151427E-146	Myeloid	VOPP1
3.61045523588481E-150	0.607730402341665	0.477	0.279	5.88901353525172E-146	Myeloid	DBNL
9.17989430723613E-150	1.57579034549334	0.122	0.028	1.49733256045329E-145	Myeloid	PLXNC1
1.61683945049975E-149	2.37980368633228	0.264	0.126	2.63722682771014E-145	Myeloid	FAM20A
2.75480638668279E-149	1.07355153562751	0.15	0.041	4.4933646973183E-145	Myeloid	ZNF267
4.76803390039116E-149	0.812321220496475	0.134	0.032	7.77714009492802E-145	Myeloid	GAB3
5.80433620498512E-149	0.848599794583084	0.227	0.083	9.46745278395123E-145	Myeloid	SEC14L1
6.86244269225914E-149	0.707342488071522	0.488	0.298	1.11933302753439E-144	Myeloid	TBC1D9B
4.07598786172479E-148	1.8378724839094	0.267	0.123	6.6483438012593E-144	Myeloid	SRA1
8.07708109789758E-148	1.09055361316813	0.136	0.034	1.31745269787807E-143	Myeloid	GIMAP8
1.51193707370379E-147	1.77412150248437	0.259	0.118	2.46612056091824E-143	Myeloid	ITPRIPL2
2.73582748108336E-147	1.33023414797456	0.145	0.039	4.46240820439508E-143	Myeloid	SNX29
5.62625613666185E-147	1.01065560669887	0.399	0.222	9.17698638450915E-143	Myeloid	IFI6
1.21186558108145E-145	0.576242757856528	0.638	0.395	1.97667394930196E-141	Myeloid	OS9
1.4323892611235E-145	0.924237204433636	0.435	0.253	2.33637012381855E-141	Myeloid	SLC12A7
1.72345492700387E-145	1.70166789049452	0.121	0.028	2.81112733143601E-141	Myeloid	RASA4
2.77642510746733E-145	0.805656364914839	0.368	0.196	4.52862699278996E-141	Myeloid	IGF2R
5.12335976578896E-145	1.02236566481393	0.185	0.061	8.35671211397837E-141	Myeloid	CSGALNAC
7.01588857896387E-145	0.499047869474604	0.188	0.059	1.1443615861148E-140	Myeloid	SH3BP1
3.66113397850038E-144	0.448869071102948	0.224	0.081	5.97167563233197E-140	Myeloid	PLEKHA2
3.71330365295429E-144	0.810643181263339	0.4	0.225	6.05676958833374E-140	Myeloid	EIF4A1
5.32181199498695E-144	3.30871493573632	0.132	0.036	8.68040754502322E-140	Myeloid	TMEM170E
5.9930015149663E-144	0.524241482825901	0.335	0.155	9.77518477106152E-140	Myeloid	ANKRD13D
4.4678643001851E-143	1.70833354954652	0.159	0.049	7.28753346003192E-139	Myeloid	CNEP1R1
6.29770059261577E-143	1.20359254863589	0.286	0.137	1.02721794366156E-138	Myeloid	NOTCH2NL
6.65861376064867E-143	0.4947070257302	0.235	0.088	1.0860864904994E-138	Myeloid	IGFLR1
1.17490576360872E-142	2.79239450552897	0.119	0.029	1.91638879102218E-138	Myeloid	GCA
3.63994772049656E-142	1.0860451888912	0.242	0.1	5.93711872690194E-138	Myeloid	AP2A2
4.40094441509879E-142	0.599244075359056	0.262	0.108	7.17838043546764E-138	Myeloid	SEL1L3
3.67066539193589E-141	0.70897503183473	0.206	0.075	5.98722232078663E-137	Myeloid	GNPTAB
4.2594138806452E-141	1.45116849169593	0.187	0.066	6.94752998072038E-137	Myeloid	BNIP3L
6.48172771162864E-141	0.619044711256008	0.332	0.158	1.05723460704375E-136	Myeloid	PLXND1
6.50781213109801E-141	0.937859183572201	0.194	0.069	1.0614892367034E-136	Myeloid	MVB12B
8.18149639257737E-141	1.79630134870052	0.212	0.084	1.33448387659329E-136	Myeloid	BCL6
1.05326753167751E-140	1.35243756406615	0.119	0.028	1.71798467091918E-136	Myeloid	PACC1
1.13534608780202E-140	0.714292787134235	0.223	0.084	1.85186300381387E-136	Myeloid	FAM49B
2.25867613370685E-140	0.37805376135635	0.238	0.086	3.68412664168924E-136	Myeloid	TAGAP
6.70575607627489E-140	2.57846564465255	0.208	0.086	1.0937758736012E-135	Myeloid	FNIP2
7.31612888538492E-140	1.94690764714591	0.171	0.058	1.19333378249513E-135	Myeloid	CASP9
1.66588565503228E-138	1.334865042339	0.153	0.046	2.71722609192315E-134	Myeloid	KIAA0513
2.24540875178246E-138	1.49913630013531	0.34	0.191	3.66248621503237E-134	Myeloid	GADD45B
6.62286671705969E-138	0.90942626249843	0.259	0.112	1.08025579021961E-133	Myeloid	CERT1
1.00713368354669E-137	1.61095726844643	0.208	0.081	1.64273575123301E-133	Myeloid	PPP1R15A
1.06485762606092E-137	1.71665654834678	0.21	0.085	1.73688927386797E-133	Myeloid	ATP6V0C
6.9139253717044E-137	0.626573089158873	0.414	0.23	1.1277303673787E-132	Myeloid	TLN1
7.27629345929873E-136	1.7348647618471	0.175	0.061	1.18683622614622E-131	Myeloid	CERS6
9.96066715968025E-136	0.697610847753905	0.202	0.072	1.62468442041544E-131	Myeloid	OSTF1

1.98933213170007E-135	0.827995197014054	0.395	0.229	3.24479964001599E-131	Myeloid	CNDP2
2.77715092343715E-135	0.852008908424044	0.217	0.084	4.52981087121834E-131	Myeloid	DEPRECATI
1.02163899386005E-134	0.634584100262398	0.605	0.379	1.66639536288513E-130	Myeloid	LAMP1
5.574317710614E-134	1.06480757877336	0.248	0.107	9.0922696177825E-130	Myeloid	AFF1
2.12178658280597E-133	1.20610293773663	0.329	0.18	3.46084609521481E-129	Myeloid	HEXA
5.3941085408269E-133	1.97002097642676	0.122	0.032	8.79833044094276E-129	Myeloid	SLC18B1
4.37922706622866E-132	1.49251310854317	0.197	0.076	7.14295726772556E-128	Myeloid	GGA1
2.670350211998E-131	0.772919838746373	0.359	0.194	4.35560823078994E-127	Myeloid	AKIRIN2
3.44695216649947E-131	3.07810019949843	0.144	0.046	5.62232367877728E-127	Myeloid	ZMYND15
3.87455486588426E-131	1.53666904254065	0.254	0.119	6.31978644174381E-127	Myeloid	VAMP5
1.40591038479188E-130	2.22684729315306	0.151	0.049	2.29318042863404E-126	Myeloid	PLD1
3.80190955961776E-130	1.4646635328951	0.261	0.127	6.20129468269254E-126	Myeloid	WDR81
1.152309523362E-129	1.88450709518024	0.226	0.101	1.87953206355576E-125	Myeloid	DNASE2
2.44421145484444E-129	2.13035347729616	0.174	0.063	3.98675330399676E-125	Myeloid	STARD8
6.13770172430876E-129	0.783181961538896	0.131	0.035	1.001120528252E-124	Myeloid	GPR132
7.15399705446323E-129	0.445073882137918	0.465	0.276	1.1668884595535E-124	Myeloid	TCIRG1
8.0529102853566E-129	2.10570765454871	0.155	0.052	1.31351019664451E-124	Myeloid	TBC1D9
1.21757673257233E-128	1.1929271594911	0.378	0.222	1.98598940849872E-124	Myeloid	CDKN1A
1.78041749152488E-128	0.461406857105872	0.582	0.378	2.90403897042624E-124	Myeloid	STAT1
5.68202092545555E-128	1.18865004972411	0.276	0.136	9.26794433151055E-124	Myeloid	VEGFB
1.16930332933905E-127	0.861120803305606	0.214	0.085	1.90725066048492E-123	Myeloid	SH3BGRL
1.33327790038503E-127	1.77076026547365	0.173	0.063	2.17470958331802E-123	Myeloid	TPCN1
8.31654014105484E-127	1.62130257832691	0.294	0.159	1.35651086240746E-122	Myeloid	NCOA4
1.66099912111313E-126	1.64911694509798	0.202	0.083	2.70925566644763E-122	Myeloid	CMTM6
4.43124947032582E-126	1.21794713902484	0.236	0.104	7.22781101104844E-122	Myeloid	CD81
1.32889417789515E-125	1.8283310540086	0.104	0.024	2.16755929356477E-121	Myeloid	COQ2
1.70576444496034E-125	2.63797687058092	0.196	0.083	2.78227238617481E-121	Myeloid	PYGL
7.03867863081872E-125	0.800510861852639	0.154	0.048	1.14807887147284E-120	Myeloid	IFI44L
6.12240726406046E-124	1.02886664171743	0.291	0.145	9.98625848840902E-120	Myeloid	KDM6B
6.14400622710605E-124	1.81573204769314	0.196	0.08	1.00214885570327E-119	Myeloid	SNX8
6.70090196291442E-124	0.874716506761209	0.15	0.047	1.09298411917097E-119	Myeloid	RGS19
8.4127183807504E-124	0.642709152919227	0.43	0.255	1.3721984950842E-119	Myeloid	BST2
2.12183344085916E-123	0.538575646796206	0.335	0.169	3.46092252538537E-119	Myeloid	TACC1
3.19050404396904E-123	0.841073238347241	0.303	0.154	5.20403114611791E-119	Myeloid	TWF2
3.66489410018211E-123	1.33729528089452	0.327	0.187	5.97780876680705E-119	Myeloid	NADK
5.61193384529853E-123	0.459407152602069	0.241	0.099	9.15362529506643E-119	Myeloid	EPSTI1
8.39270499806756E-123	1.16781484556166	0.11	0.027	1.3689341122348E-118	Myeloid	PDE4A
1.1582769788171E-122	1.20577622305077	0.316	0.171	1.88926558014857E-118	Myeloid	IER5
3.81875165142101E-121	1.12823181363147	0.155	0.052	6.2287658186328E-117	Myeloid	GPR155
7.18318516783639E-121	0.516849367662636	0.189	0.067	1.17164933272579E-116	Myeloid	EMP3
1.75352686852258E-120	1.16112561483037	0.253	0.121	2.86017767524718E-116	Myeloid	SH3BP2
2.46736423698373E-120	2.25827921842568	0.1	0.024	4.02451780694416E-116	Myeloid	SASH1
8.76189192921884E-120	1.31193193983663	0.162	0.057	1.42915219257489E-115	Myeloid	APAF1
3.24045128952445E-119	1.7874876168997	0.136	0.043	5.28550009834333E-115	Myeloid	SLC36A1
6.6242453458972E-119	1.64803228618767	0.172	0.064	1.08048065836929E-114	Myeloid	IER5L
7.59854129072157E-119	1.35178237294228	0.2	0.083	1.23939806992959E-114	Myeloid	NFKBIE
8.26756225815562E-119	2.97615106203597	0.119	0.035	1.34852207992776E-114	Myeloid	ETV5
1.24582092951314E-118	0.9572540741015	0.157	0.053	2.03205851812888E-114	Myeloid	SFXN3
7.5720854635142E-118	0.358729586450842	0.33	0.165	1.2350828599538E-113	Myeloid	INPP4A
1.08549260933049E-117	1.02152842180318	0.129	0.037	1.77054699507897E-113	Myeloid	STARD4
1.22275456224855E-117	0.561761958074092	0.184	0.067	1.99443496648362E-113	Myeloid	CASP1
1.44525475682105E-116	0.280387906344111	0.164	0.054	2.35735503385081E-112	Myeloid	GIMAP1

2.21029001569595E-116	0.883114044120016	0.279	0.14	3.60520404460167E-112	Myeloid	ARMH3
3.4966802991899E-116	0.448703408621262	0.352	0.191	5.70343523600865E-112	Myeloid	GGA2
3.9401383817298E-116	2.09007891408009	0.253	0.132	6.42675971443948E-112	Myeloid	FUCA1
6.96956685141052E-116	2.30296666685001	0.141	0.047	1.13680604913357E-111	Myeloid	LACTB
1.04854225478817E-115	0.437990856078473	0.485	0.297	1.71027727178498E-111	Myeloid	TUT7
1.1808747248833E-115	0.493496002023751	0.453	0.277	1.92612476375715E-111	Myeloid	ANKRD10
1.33174955113662E-115	0.929908617215466	0.183	0.069	2.17221669285895E-111	Myeloid	NFKB1
1.42445127610177E-115	0.942050228039556	0.123	0.035	2.3234224764496E-111	Myeloid	G6PD
1.48714801792749E-115	1.61716504590111	0.307	0.179	2.42568713204152E-111	Myeloid	SCARB1
2.95649112020398E-115	1.99783395774515	0.175	0.07	4.82233266616472E-111	Myeloid	RNF13
3.12165120217818E-115	0.752340042580938	0.357	0.207	5.09172527587282E-111	Myeloid	STXBP2
4.14081479184624E-115	1.38857614965113	0.17	0.064	6.7540830069804E-111	Myeloid	SLC1A5
6.05276587310786E-115	1.49065642887638	0.146	0.049	9.87266641562624E-111	Myeloid	MIOS
7.26377281523721E-115	2.09323091629114	0.18	0.073	1.18479398389334E-110	Myeloid	VAT1
1.27180757619806E-114	1.31231757829368	0.157	0.055	2.07444533753665E-110	Myeloid	C2CD2L
2.04395515700875E-114	0.886010790087128	0.301	0.16	3.33389525659697E-110	Myeloid	DNAJC5
1.93229649986478E-113	0.843468271569647	0.346	0.199	3.15176882092944E-109	Myeloid	ITPR2
5.7771400486483E-113	0.297594489711585	0.198	0.076	9.42309313335024E-109	Myeloid	GMIP
9.48376604342609E-113	1.0071252240596	0.186	0.073	1.54689707934323E-108	Myeloid	FCHSD2
1.0897229961077E-112	1.99629230555427	0.225	0.109	1.77744717895127E-108	Myeloid	CHKA
8.40872219951483E-112	0.732763726879006	0.216	0.092	1.37154667796286E-107	Myeloid	MAP3K3
1.24989682535167E-111	1.54431442567207	0.142	0.048	2.03870671183111E-107	Myeloid	UVRAG
1.61521860490843E-111	0.617703499278967	0.413	0.255	2.63458306646614E-107	Myeloid	MTCH1
7.72240826483224E-111	0.342898591170641	0.162	0.055	1.25960201207679E-106	Myeloid	HCST
1.73772738322238E-110	1.59942419777448	0.128	0.04	2.83440713477402E-106	Myeloid	FARP2
3.12339118500516E-110	1.79342997981182	0.107	0.029	5.09456336186192E-106	Myeloid	RP2
1.13761352582195E-109	0.939729802255773	0.234	0.109	1.85556142196818E-105	Myeloid	NXF1
3.20620800216234E-109	1.3103189816523	0.188	0.077	5.229645872327E-105	Myeloid	PRCP
4.31316188498574E-109	2.55329151619871	0.104	0.028	7.03519835060025E-105	Myeloid	NANS
6.38468720964647E-109	1.11015082677623	0.177	0.07	1.04140633076544E-104	Myeloid	ME2
1.05408864781468E-108	2.96216999528838	0.126	0.041	1.71932399345053E-104	Myeloid	TBC1D12
1.08551086562467E-108	1.30875469135882	0.218	0.1	1.7705767729204E-104	Myeloid	RIPOR1
1.10821731401468E-108	2.26790952324472	0.227	0.115	1.80761326088935E-104	Myeloid	HSD3B7
1.94248763944457E-108	0.274962126368081	0.482	0.251	3.16839158869804E-104	Myeloid	ITPKB
1.97519544299554E-108	1.42742246738809	0.105	0.028	3.22174128707003E-104	Myeloid	KCNK6
2.62753316352412E-108	1.4840423813525	0.163	0.063	4.2857693430242E-104	Myeloid	FAM53C
2.7957430482908E-108	2.13953508464278	0.135	0.046	4.56013648606712E-104	Myeloid	SLC49A4
4.61471535916786E-108	0.432881119949557	0.164	0.057	7.5270622223387E-104	Myeloid	ADCY3
6.13244161104099E-108	0.436127612977586	0.238	0.104	1.0002625511769E-103	Myeloid	UTRN
8.01599106523708E-108	1.00272678158335	0.259	0.132	1.30748830265082E-103	Myeloid	TMEM127
2.25317006939019E-107	0.36271818048862	0.424	0.249	3.67514570018234E-103	Myeloid	LRCH4
3.40885562802239E-107	2.03475413348059	0.13	0.043	5.56018441486732E-103	Myeloid	SGPL1
5.43761867864103E-104	0.284753045278909	0.221	0.094	8.86929982673138E-100	Myeloid	HERC3
1.42205856716076E-102	0.723178614834388	0.333	0.19	2.31951972889592E-98	Myeloid	BTG2
1.65791842091384E-102	0.923883443516764	0.193	0.08	2.70423073635257E-98	Myeloid	ERICH1
4.20697335709442E-102	2.87266683730743	0.124	0.042	6.86199424275671E-98	Myeloid	PDGFC
4.40947111349507E-102	1.75794689408494	0.104	0.03	7.19228833322181E-98	Myeloid	FIG4
6.22130821065113E-102	0.361399458360826	0.251	0.116	1.0147575822393E-97	Myeloid	RAPGEF1
2.14311486455086E-101	1.18177241763362	0.173	0.071	3.49563465556891E-97	Myeloid	HPS5
4.07894803801228E-101	0.729519880710545	0.137	0.045	6.65317214480183E-97	Myeloid	RASSF2
7.75731177736412E-101	1.50020518349085	0.218	0.106	1.26529512400586E-96	Myeloid	MEF2A
1.1045591797411E-100	0.684500456137271	0.109	0.031	1.80164647807571E-96	Myeloid	MX2

4.26293792849583E-100	1.48709864395031	0.203	0.095	6.95327805516954E-96	Myeloid	SKAP2
1.34993094688892E-99	2.61211518020811	0.127	0.044	2.20187236747051E-95	Myeloid	ATF5
1.47321094639322E-99	1.20720292506536	0.143	0.052	2.40295437466198E-95	Myeloid	CARD19
1.49115838874155E-99	0.55632245672785	0.555	0.377	2.43222844787634E-95	Myeloid	NUCB1
1.77053856146871E-99	2.28362701488565	0.161	0.066	2.88792544761162E-95	Myeloid	TSEN34
1.98685041941994E-99	2.04749152937163	0.242	0.133	3.24075171911587E-95	Myeloid	FAM20C
6.04114919315458E-99	0.751165322881582	0.44	0.285	9.85371844895444E-95	Myeloid	MT-ND4L
1.06781553716169E-98	0.746940474456602	0.324	0.187	1.74171392266443E-94	Myeloid	ARRDC2
1.09832714483089E-98	1.22683877270797	0.211	0.1	1.79148140593367E-94	Myeloid	CEPT1
1.82220469561291E-98	0.955662254263256	0.227	0.109	2.97219807901422E-94	Myeloid	TLNRD1
5.49297304361654E-98	1.35381189976096	0.153	0.059	8.95958833144293E-94	Myeloid	ZSWIM6
7.16032299054898E-98	1.79221778803498	0.241	0.13	1.16792028298844E-93	Myeloid	TMEM37
8.95689849181536E-98	1.47401215559418	0.195	0.089	1.460959713E-93	Myeloid	EIF4A3
1.19355224844823E-97	0.757668474174416	0.276	0.147	1.9468030724439E-93	Myeloid	SP3
1.57537955700877E-97	0.492710298580644	0.158	0.058	2.56960159543701E-93	Myeloid	OSBPL3
8.90796331872183E-97	1.36710761147114	0.159	0.064	1.45297789691672E-92	Myeloid	CYB561A3
1.98788921509974E-96	0.708004503560529	0.181	0.076	3.24244609874919E-92	Myeloid	CYB5R4
8.92216839217483E-96	0.931282087712856	0.315	0.188	1.45529488644764E-91	Myeloid	YBX1
4.21626329517055E-95	0.724119409719012	0.245	0.121	6.87714706075268E-91	Myeloid	GNAI2
4.89710856945241E-95	0.588341736853655	0.282	0.149	7.98767378763383E-91	Myeloid	KIAA0100
1.03821346143696E-94	0.50555431969366	0.417	0.269	1.69342997694983E-90	Myeloid	STAT6
3.2900709986164E-94	1.00044802650647	0.226	0.113	5.3664348058432E-90	Myeloid	TANGO2
5.43930032363544E-94	2.52004420932836	0.122	0.043	8.87204275788177E-90	Myeloid	ITSN1
9.28318310662104E-94	0.549417337269934	0.363	0.218	1.51417999652096E-89	Myeloid	NCOR2
9.60938880095156E-94	0.850328297978818	0.274	0.148	1.56738740732321E-89	Myeloid	VASP
1.0579855209178E-93	2.56804011452273	0.136	0.052	1.72568018316903E-89	Myeloid	ARPIN
3.42769195009864E-93	2.0145760607337	0.168	0.074	5.59090833980589E-89	Myeloid	TSPAN33
6.53898203549623E-93	0.647021901888502	0.411	0.27	1.06657335980979E-88	Myeloid	LRPAP1
1.28153049368221E-92	1.52797707192725	0.236	0.126	2.09030438824505E-88	Myeloid	ZMIZ1
4.06398669296833E-92	2.57539509592361	0.122	0.043	6.62876869490065E-88	Myeloid	SLC31A1
2.85080744570784E-91	0.562270824317466	0.154	0.06	4.64995202469406E-87	Myeloid	AGTPBP1
7.13793428410456E-91	1.27495890947339	0.195	0.093	1.16426846108029E-86	Myeloid	ABCD1
1.54403854749663E-90	0.621542148971704	0.204	0.094	2.51848127482175E-86	Myeloid	NECAP2
2.06917159310478E-90	0.818129669436864	0.155	0.06	3.3750257855132E-86	Myeloid	UPP1
2.4480052414522E-90	1.20104094579528	0.238	0.127	3.99294134933268E-86	Myeloid	AMDHD2
2.45734039190571E-90	1.92308039250144	0.167	0.074	4.0081679132374E-86	Myeloid	NAGA
9.98601499947155E-90	1.15749808109161	0.148	0.059	1.6288189065638E-85	Myeloid	SPIN1
3.50436423912149E-89	0.950690263251381	0.321	0.197	5.71596851043106E-85	Myeloid	BACH1
4.0928137063237E-89	1.00897902810292	0.173	0.076	6.67578843638459E-85	Myeloid	DRAM2
5.04753908242978E-89	0.42666742292113	0.136	0.048	8.23304099735122E-85	Myeloid	HK1
8.54847294221507E-89	1.45192890269012	0.213	0.109	1.3943414216047E-84	Myeloid	ATP6V0D1
1.37382910953695E-88	2.22870248902581	0.119	0.042	2.24085266056572E-84	Myeloid	RIN2
1.88974528311451E-88	2.02103604049824	0.2	0.101	3.08236353128807E-84	Myeloid	ZNF516
3.78302640883737E-88	1.36512369430265	0.214	0.109	6.17049437545463E-84	Myeloid	SORBS3
9.34198727785636E-88	0.534717799376313	0.344	0.205	1.52377154489115E-83	Myeloid	PICALM
1.29065001478974E-87	0.327756178283392	0.402	0.251	2.10517923912354E-83	Myeloid	SMAP2
1.51826811413205E-87	1.51085684749299	0.222	0.118	2.47644712096079E-83	Myeloid	ATP6V0B
5.66271044108658E-87	1.36519308804438	0.111	0.037	9.23644700045631E-83	Myeloid	DEPRECATI
2.1508998959661E-86	1.54970145199502	0.148	0.061	3.50833282031031E-82	Myeloid	CLCN6
2.72687219992706E-86	0.475529508777377	0.293	0.16	4.44780124530103E-82	Myeloid	SRSF7
4.57456687295675E-86	0.38276629472562	0.183	0.077	7.46157602647975E-82	Myeloid	ABCG1
6.26179988370628E-86	2.45633261930026	0.136	0.055	1.02136217903133E-81	Myeloid	CNTLN

1.21787595261447E-85	1.39078941384728	0.184	0.087	1.98647746630946E-81	Myeloid	DHX34
9.9196898621826E-85	0.733834955821213	0.302	0.178	1.6180006134206E-80	Myeloid	SREBF1
1.0830704381206E-84	1.10437410385672	0.201	0.099	1.76659619161851E-80	Myeloid	CSRNP1
1.39164075467141E-84	1.65883035311249	0.114	0.04	2.26990523494453E-80	Myeloid	MOB1B
1.98979459915642E-84	1.01035789143667	0.323	0.207	3.24555397068403E-80	Myeloid	EPN1
4.77949239792563E-84	0.308269060240804	0.482	0.273	7.79583005025649E-80	Myeloid	LSP1
8.22463215242936E-84	1.18399639842973	0.167	0.074	1.34151975038275E-79	Myeloid	GHDC
5.09128054841024E-83	2.21132012021458	0.15	0.066	8.30438770251195E-79	Myeloid	UBE2E2
1.24866098301507E-82	0.387302860771461	0.443	0.298	2.03669092939589E-78	Myeloid	IQSEC1
2.08385046357821E-82	1.94368342099767	0.182	0.089	3.39896849114242E-78	Myeloid	SLC2A9
7.06736114399799E-82	0.522032378235859	0.118	0.04	1.15275727619751E-77	Myeloid	SLFN11
1.04087285050887E-81	1.07667474976348	0.316	0.201	1.69776770646502E-77	Myeloid	MT-CYB
1.16280719510465E-81	1.64744383402179	0.102	0.034	1.8966548159352E-77	Myeloid	C17orf107
1.19634535900406E-81	1.16421077338854	0.135	0.053	1.95135891507152E-77	Myeloid	RABGEF1
5.52310921699957E-81	1.48878023572992	0.147	0.063	9.008743443848E-77	Myeloid	ARL8B
5.88657882561497E-81	0.877242441754586	0.204	0.101	9.60159872246058E-77	Myeloid	IL17RA
7.98957706220673E-81	2.54882322514927	0.132	0.054	1.30317991461654E-76	Myeloid	CSTB
1.2697086573899E-80	0.610062090355159	0.258	0.14	2.07102179106866E-76	Myeloid	TTLL3
2.50337412668121E-80	1.04566523022539	0.107	0.036	4.08325353802972E-76	Myeloid	SLC25A24
4.18352011476543E-80	2.17424382411187	0.112	0.041	6.82373965919389E-76	Myeloid	SLC19A1
4.77268566841101E-80	0.931915213751944	0.277	0.165	7.7847275937452E-76	Myeloid	GUSB
6.18545972276933E-80	2.76707821824526	0.13	0.053	1.00891033538091E-75	Myeloid	CHML
1.56459393525633E-79	0.798049910831006	0.103	0.033	2.5520091677966E-75	Myeloid	DLL1
1.65119007653673E-79	0.255935860762938	0.145	0.056	2.69325613383907E-75	Myeloid	FMNL3
1.91093456595053E-79	1.18505238823846	0.187	0.092	3.1169253705219E-75	Myeloid	C6orf62
6.71780649418451E-79	2.19460112936933	0.187	0.097	1.09574141726643E-74	Myeloid	SHTN1
1.26929568827087E-78	0.908503022891827	0.158	0.069	2.07034819713862E-74	Myeloid	PLK3
2.07808016170267E-78	1.61184921733744	0.184	0.092	3.38955655175322E-74	Myeloid	NCKAP5L
2.27448391192304E-78	1.40746222302803	0.184	0.091	3.70991070873767E-74	Myeloid	MBOAT7
3.36555946719288E-78	0.810859468874953	0.261	0.151	5.48956404693831E-74	Myeloid	TMUB2
4.2013397413151E-78	0.265881756463126	0.392	0.251	6.85280525205906E-74	Myeloid	CYBC1
4.68196135545573E-78	0.332800262970022	0.146	0.058	7.63674716688383E-74	Myeloid	PPM1M
5.19460630393532E-78	1.34463527706197	0.186	0.093	8.47292234234891E-74	Myeloid	PPARD
7.59314886447344E-78	0.993520970552098	0.148	0.063	1.23851851128426E-73	Myeloid	MYO9A
8.31196928184673E-78	0.554450333062554	0.322	0.2	1.35576530956202E-73	Myeloid	OGFR
1.23059188781787E-77	1.51616816758408	0.2	0.106	2.00721842821973E-73	Myeloid	ABHD12
1.2413336010078E-77	1.03695982146068	0.256	0.15	2.02473923660382E-73	Myeloid	ATP2A2
1.3676860351749E-77	0.361181609439536	0.482	0.33	2.23083269197379E-73	Myeloid	CFL1
1.4832245670608E-77	0.654543960939436	0.272	0.157	2.41928759133287E-73	Myeloid	ADAM10
1.89197646496768E-77	0.986268305588739	0.201	0.103	3.08600281200878E-73	Myeloid	FAM91A1
2.48007446541002E-77	1.6823018863754	0.182	0.091	4.04524946053029E-73	Myeloid	CORO1C
3.84386081688664E-77	0.703618647668004	0.288	0.171	6.26972137842379E-73	Myeloid	RHOA
6.22541665972802E-77	0.65207264131701	0.306	0.186	1.01542771136824E-72	Myeloid	EDEM1
1.81374664212129E-76	2.42587427586478	0.176	0.09	2.95840214796404E-72	Myeloid	SLC46A1
7.41009138904926E-76	1.33621005998412	0.185	0.093	1.20866000646782E-71	Myeloid	MEPCE
1.31244159687957E-75	0.736348061620477	0.28	0.169	2.14072348867027E-71	Myeloid	PTTG1IP
2.16401290255463E-75	0.435480405830983	0.233	0.123	3.52972144535686E-71	Myeloid	ARPC4
2.09142370198515E-74	0.545667760434725	0.184	0.088	3.41132120030798E-70	Myeloid	ARRDC1
2.42305750842129E-74	1.10969748734944	0.138	0.058	3.95224910198596E-70	Myeloid	DEGS1
3.19563909028322E-74	0.663286280954053	0.149	0.064	5.21240692016096E-70	Myeloid	LRRFIP1
4.19368458218495E-74	1.23454635094248	0.129	0.052	6.84031892200188E-70	Myeloid	RHOT1
4.49313206670457E-74	1.23101133067878	0.175	0.085	7.32874771400183E-70	Myeloid	SNAP23

1.01578462584794E-73	0.785699791839463	0.256	0.148	1.65684630322058E-69	Myeloid	FBXL5
1.49060580655492E-73	0.886616663584745	0.174	0.084	2.43132713107173E-69	Myeloid	DNASE1L1
4.92908188738761E-73	0.63726881149865	0.251	0.143	8.03982546651793E-69	Myeloid	TENT2
9.00032595638982E-73	1.28611872495551	0.123	0.049	1.46804316674674E-68	Myeloid	STK38L
1.44995772667532E-72	1.21058319565918	0.201	0.107	2.36502604798012E-68	Myeloid	TESK1
1.74926505168626E-72	1.54849787857978	0.105	0.038	2.85322622580546E-68	Myeloid	AGAP3
1.75460357642944E-72	1.00379519052265	0.119	0.047	2.86193389351405E-68	Myeloid	GRAMD1B
1.80207496352813E-72	0.422888226619574	0.181	0.086	2.93936447301073E-68	Myeloid	PTPRJ
1.97880632340776E-72	1.40370068029265	0.209	0.116	3.2276309941104E-68	Myeloid	ATP6V1A
2.50450119508934E-72	0.728881333446225	0.258	0.151	4.08509189931022E-68	Myeloid	TNRC18
2.5152257270434E-72	0.702387304903265	0.108	0.039	4.10258468338048E-68	Myeloid	CAMK1D
2.79703655186895E-72	0.730410567669414	0.275	0.167	4.56224631975345E-68	Myeloid	RAB10
4.22947017674157E-72	1.0135779133774	0.311	0.205	6.89868880528318E-68	Myeloid	NUDC
1.8109740049653E-71	1.03534668651085	0.146	0.064	2.95387969949889E-67	Myeloid	SRGAP2B
1.99710248403193E-71	0.951467964076979	0.12	0.047	3.25747386170448E-67	Myeloid	GALC
3.0655711685913E-71	2.52000894840947	0.102	0.038	5.00025313308927E-67	Myeloid	PFKFB2
6.6243960206987E-71	0.958013770952054	0.124	0.05	1.08050523493617E-66	Myeloid	PLCG2
8.26736582853914E-71	1.14195310233392	0.119	0.047	1.34849004029302E-66	Myeloid	BTN2A2
2.14045591423227E-70	0.263309523616221	0.225	0.115	3.49129764170426E-66	Myeloid	MOB3A
4.61117280007506E-70	0.285162796744796	0.37	0.241	7.52128395420243E-66	Myeloid	LAMTOR4
8.0316253664681E-70	1.27432563981914	0.207	0.115	1.31003841352461E-65	Myeloid	SPNS1
8.32063431313766E-70	0.732213923675698	0.299	0.19	1.35717866281588E-65	Myeloid	INPPL1
4.25770373644255E-69	0.954464447938815	0.219	0.123	6.94474056451145E-65	Myeloid	GNAI3
9.36972333599664E-69	0.79037156660939	0.186	0.094	1.52829557333441E-64	Myeloid	TBC1D22A
1.13217481374483E-68	1.57724820629275	0.251	0.159	1.84669033869919E-64	Myeloid	MGLL
1.36568354479983E-68	1.03278284415757	0.294	0.193	2.22756642992301E-64	Myeloid	RAB7A
3.13992247391539E-68	2.21657043731351	0.15	0.073	5.12152754720339E-64	Myeloid	SAMD4A
3.32228136789356E-68	0.770601571788847	0.142	0.062	5.41897313917118E-64	Myeloid	INPP5B
3.68140339035675E-68	1.27892535306404	0.176	0.09	6.00473707001089E-64	Myeloid	BID
3.76915633598389E-68	0.897215186474203	0.122	0.049	6.14787089962332E-64	Myeloid	RAP2A
4.91272245746521E-68	0.444817789172791	0.131	0.054	8.01314160037151E-64	Myeloid	BEX4
5.57236287852801E-68	1.75604954385017	0.201	0.115	9.08908109116704E-64	Myeloid	SLC38A7
1.04240919975714E-67	0.480896606059473	0.173	0.082	1.70027364572387E-63	Myeloid	MADD
1.27073749160916E-67	0.959912062921398	0.168	0.083	2.0726999225637E-63	Myeloid	SGK3
2.1661254860142E-67	1.712835534703	0.159	0.079	3.53316728023775E-63	Myeloid	SOGA1
2.82952378651547E-67	1.27057641754513	0.134	0.059	4.61523624818538E-63	Myeloid	SNX18
7.46456854499516E-67	0.337397362014069	0.198	0.1	1.21754577537416E-62	Myeloid	RUBCN
1.09466478978636E-66	2.10375655782753	0.131	0.059	1.78550773862053E-62	Myeloid	PTPN18
1.28056259708817E-66	0.553836090189579	0.268	0.158	2.08872565211052E-62	Myeloid	MRTFA
1.95630114879474E-66	0.938008921649799	0.179	0.091	3.1909228037991E-62	Myeloid	RIC1
1.18408108003467E-65	1.41939084615011	0.127	0.055	1.93135464964455E-61	Myeloid	HSPBAP1
4.34167547273229E-65	0.48696042684031	0.266	0.159	7.08170686357364E-61	Myeloid	PPP1CB
6.38747380505844E-65	1.50356249477646	0.108	0.043	1.04186085234308E-60	Myeloid	ZDHHC7
8.27062418658123E-65	0.399824736783513	0.306	0.192	1.34902151107326E-60	Myeloid	TRIM38
8.44908583545738E-65	1.14972902388629	0.234	0.142	1.37813039062145E-60	Myeloid	GABARAP
1.17260709236814E-64	1.14074704721872	0.11	0.044	1.91263942836167E-60	Myeloid	GNG10
3.2426652634889E-64	0.777376087205143	0.338	0.234	5.28911131127674E-60	Myeloid	GNPTG
6.2753266999332E-64	0.962367779147928	0.202	0.111	1.0235685380261E-59	Myeloid	MED13L
8.94433424014484E-64	0.491303204007249	0.242	0.14	1.45891035791002E-59	Myeloid	DCAF7
1.12788877816255E-63	0.276995754212614	0.154	0.07	1.83969938606093E-59	Myeloid	BORCS5
3.00630697273161E-63	1.3794367121004	0.141	0.067	4.90358730322253E-59	Myeloid	RNF19B
6.75832670147751E-63	1.95341476787367	0.18	0.1	1.102350668278E-58	Myeloid	PPFIBP2

**8.39597305057328E-63**	0.401801052622215	0.473	0.332	1.36946716427901E-58	Myeloid	KLF6
**1.68203570203331E-62**	1.12377333553507	0.174	0.091	2.74356843358654E-58	Myeloid	MTMR10
**2.19149340262863E-62**	0.681233708431311	0.189	0.1	3.57454488902756E-58	Myeloid	HPS1
**2.64736917686805E-62**	0.252179331353683	0.216	0.114	4.31812386438947E-58	Myeloid	TSPAN14
**4.53066495815296E-62**	0.546960461187848	0.146	0.067	7.38996761324329E-58	Myeloid	SNX5
**6.73183832070154E-62**	0.615409831754921	0.2	0.109	1.09803014848963E-57	Myeloid	RUFY1
**1.02760942497341E-61**	0.727073331294678	0.109	0.043	1.67613373307413E-57	Myeloid	BICD2
**1.03499249874252E-61**	1.06586176984274	0.104	0.041	1.68817626469892E-57	Myeloid	SLC66A3
**1.11257952568059E-61**	0.435115454637691	0.276	0.169	1.8147284643376E-57	Myeloid	ST3GAL1
**7.19107264290633E-61**	0.747515307900243	0.181	0.096	1.17293585878445E-56	Myeloid	ELL
**7.40423905471083E-61**	0.497060976816992	0.287	0.181	1.20770543221388E-56	Myeloid	ZMIZ2
**8.25199470054528E-61**	0.6846583274637	0.109	0.044	1.34598285560594E-56	Myeloid	GPD2
**1.5966774879058E-60**	0.690062580201868	0.177	0.092	2.60434065052315E-56	Myeloid	MAPKAP1
**2.24524632287713E-60**	1.02394667307314	0.266	0.175	3.66222127724488E-56	Myeloid	RREB1
**7.47525774075885E-60**	0.89337964373041	0.159	0.08	1.21928929009518E-55	Myeloid	MAPKAPK3
**8.9801340515728E-60**	0.415086149078025	0.313	0.206	1.46474966515204E-55	Myeloid	B4GALT1
**1.09620889066214E-59**	1.80461258278675	0.123	0.056	1.78802632155902E-55	Myeloid	SNX24
**1.12337988723054E-59**	0.774390487710522	0.258	0.163	1.83234493406173E-55	Myeloid	SLC23A2
**1.96521225317921E-59**	0.871521637120814	0.18	0.097	3.20545770616061E-55	Myeloid	VPS26A
**6.16036965401684E-59**	0.347703823941063	0.483	0.313	1.00481789426669E-54	Myeloid	C2
**9.96436695593549E-59**	0.260320839770117	0.261	0.157	1.62528789418264E-54	Myeloid	NUFIP2
**1.16223050942621E-58**	0.559837729915436	0.122	0.053	1.89571418392509E-54	Myeloid	C2CD5
**1.44123626096976E-58**	1.98132049944583	0.126	0.059	2.35080046526777E-54	Myeloid	HOMER3
**1.58115765405806E-58**	0.889818287131166	0.124	0.055	2.5790262495341E-54	Myeloid	ZMYM6
**2.18695480026846E-58**	1.5598229101429	0.219	0.137	3.56714197471788E-54	Myeloid	HEXB
**2.7352539266911E-58**	1.53395797529707	0.183	0.103	4.46147267982586E-54	Myeloid	NFIL3
**2.92968703003745E-58**	0.945556724749923	0.164	0.085	4.77861251469409E-54	Myeloid	SNX2
**3.38593942132544E-58**	1.68316305798419	0.116	0.051	5.52280579012392E-54	Myeloid	DEPRECATI
**4.51765669357807E-58**	0.271055008378295	0.13	0.057	7.36874983289519E-54	Myeloid	CD55
**5.99246842334606E-58**	0.549364905480919	0.163	0.082	9.77431524531975E-54	Myeloid	ZNF141
**7.98879828184117E-58**	0.739762886765735	0.341	0.239	1.30305288775111E-53	Myeloid	GPX4
**1.11623186843254E-57**	1.01010592069522	0.12	0.053	1.82068580060032E-53	Myeloid	C20orf194
**1.61240918253433E-57**	0.415134272933374	0.175	0.089	2.63000061763174E-53	Myeloid	OAS3
**2.00800876547272E-57**	0.965447256157427	0.127	0.058	3.27526309736256E-53	Myeloid	TET3
**3.85532128807751E-57**	0.916217972839387	0.136	0.065	6.28841455298322E-53	Myeloid	MALT1
**5.38111781483713E-57**	0.309678030042088	0.204	0.111	8.77714126778084E-53	Myeloid	ZBTB1
**1.48871303690139E-56**	0.773363928802449	0.169	0.088	2.42823983448985E-52	Myeloid	ASAP1
**1.8655991054155E-56**	1.15115036090238	0.135	0.065	3.04297870084323E-52	Myeloid	LTA4H
**2.5484951925812E-56**	0.885242489239442	0.194	0.11	4.1568505086192E-52	Myeloid	OAS1
**2.96984662804856E-56**	0.605349405141498	0.205	0.116	4.84411683501E-52	Myeloid	USP4
**3.51559998243347E-56**	0.845562319500128	0.161	0.084	5.73429513134723E-52	Myeloid	SGPP1
**3.56362567945408E-56**	1.37445112289684	0.116	0.051	5.81262984575755E-52	Myeloid	EVA1B
**1.02963541549142E-55**	1.47444946763393	0.109	0.048	1.67943832620806E-51	Myeloid	BLOC1S6
**1.06774869785076E-55**	0.31464928059165	0.275	0.173	1.74160490106438E-51	Myeloid	ITSN2
**1.37178343715691E-55**	1.2248830267964	0.165	0.089	2.23751596434663E-51	Myeloid	SCAMP4
**1.96355375536135E-55**	1.44524106713712	0.102	0.043	3.20275253036989E-51	Myeloid	ATP6V1F
**2.16428693121083E-55**	0.987362236689857	0.149	0.076	3.53016841349798E-51	Myeloid	WASHC4
**1.28317111738488E-54**	0.579810990920865	0.182	0.099	2.09298040956648E-50	Myeloid	TMEM165
**1.49067613734701E-54**	0.37872137855045	0.359	0.251	2.43144184762672E-50	Myeloid	PRKAR1A
**2.19524916269548E-54**	0.796948125866454	0.15	0.076	3.5806709092726E-50	Myeloid	ENGASE
**3.25355313767687E-54**	1.27147007121111	0.133	0.065	5.30687052286474E-50	Myeloid	RCOR1
**5.34878027139316E-54**	1.37757357505295	0.116	0.053	8.72439550066939E-50	Myeloid	ZFAND5

**1.04599117770945E-53**	0.918822009258463	0.153	0.079	1.70611620996188E-49	Myeloid	VPS8
**8.12100334275737E-53**	0.873991781895266	0.165	0.089	1.32461685523715E-48	Myeloid	RNASEK
**9.79236447961895E-53**	0.734971222856733	0.295	0.204	1.59723257027065E-48	Myeloid	PDXK
**1.89693992938872E-52**	0.600641255402245	0.128	0.06	3.09409871882595E-48	Myeloid	MBOAT1
**2.01251422831307E-52**	1.0070030489936	0.188	0.109	3.28261195780144E-48	Myeloid	P2RX4
**2.11271790349865E-52**	1.02492628427344	0.124	0.059	3.44605417239664E-48	Myeloid	MTMR14
**3.00383141655137E-52**	0.785029226478328	0.176	0.098	4.89954942353694E-48	Myeloid	ATP6V0E1
**3.27856077462158E-52**	1.029254794152	0.117	0.054	5.34766047948526E-48	Myeloid	UBAC1
**6.45057109118053E-52**	0.287204835411118	0.283	0.182	1.05215265068246E-47	Myeloid	PPP1R12C
**8.1656892873666E-52**	0.723064652293817	0.298	0.21	1.33190557966237E-47	Myeloid	NAPA
**9.65661795875511E-52**	2.00336483684233	0.121	0.059	1.57509095525255E-47	Myeloid	CUEDC1
**1.46919538790878E-51**	1.26061911163343	0.151	0.08	2.39640459721801E-47	Myeloid	ANGEL1
**2.3185025871057E-51**	0.347640195124024	0.216	0.127	3.78170956982811E-47	Myeloid	CD82
**6.24196095696115E-51**	1.53472162328437	0.132	0.067	1.01812625168993E-46	Myeloid	ATP6V1C1
**8.63112355165508E-51**	0.331900242773118	0.184	0.099	1.40782256251046E-46	Myeloid	WDFY1
**1.80864451617458E-50**	0.969642641092869	0.124	0.06	2.95008007033236E-46	Myeloid	MAPK7
**3.62957476646035E-50**	0.565382228481795	0.237	0.148	5.92019940157348E-46	Myeloid	SIN3B
**6.69187627321327E-50**	1.0290263077122	0.184	0.108	1.09151193892382E-45	Myeloid	WDFY2
**1.25926651759394E-49**	0.326653403861891	0.124	0.059	2.05398961684748E-45	Myeloid	ARHGEF18
**1.35010704128149E-49**	1.13709928497501	0.101	0.045	2.20215959503424E-45	Myeloid	MOSPD2
**1.35741840978221E-49**	1.39994070923722	0.1	0.044	2.21408516819577E-45	Myeloid	SNN
**2.6656013627588E-49**	0.467170729066555	0.194	0.113	4.34786238279588E-45	Myeloid	GALNS
**7.49849038371366E-49**	0.556318754318259	0.112	0.051	1.22307876648754E-44	Myeloid	TNFSF12
**8.80770368783295E-49**	2.14817004780557	0.148	0.082	1.43662454852243E-44	Myeloid	ATP6V0A1
**1.67621966947897E-48**	0.784755563525718	0.18	0.105	2.73408190288715E-44	Myeloid	CHMP4B
**2.0338604226112E-48**	0.49165029422434	0.393	0.288	3.31742973532112E-44	Myeloid	MIDN
**2.87281488584782E-48**	1.24520727716794	0.195	0.12	4.68584836030638E-44	Myeloid	MMP14
**3.54870255198365E-48**	0.490072110810059	0.289	0.19	5.78828873254054E-44	Myeloid	SLC20A1
**4.08268192826785E-48**	0.586003802195767	0.282	0.194	6.65926249319769E-44	Myeloid	IRAK1
**5.17957561500909E-48**	0.871902028926792	0.272	0.19	8.44840578564133E-44	Myeloid	ADIPOR1
**6.60909765082163E-48**	1.7533471439585	0.166	0.098	1.07800991782552E-43	Myeloid	BLVRA
**7.40225844011902E-48**	0.306662021116007	0.261	0.167	1.20738237416781E-43	Myeloid	KMT2C
**1.00930443758232E-47**	0.889183660941941	0.282	0.2	1.64627646814052E-43	Myeloid	LAMP2
**5.5327821847003E-47**	0.298433690455632	0.163	0.088	9.02452102146465E-43	Myeloid	GIT2
**6.19949536594699E-47**	0.834404468652529	0.239	0.16	1.01119968913961E-42	Myeloid	SPG21
**6.55566800127891E-47**	0.807480716194186	0.148	0.079	1.0692950076886E-42	Myeloid	LIMS1
**1.12703191298468E-46**	1.38977113794941	0.106	0.049	1.83830175326931E-42	Myeloid	PLCB3
**1.30650378030055E-46**	0.348278922322038	0.276	0.183	2.13103831604823E-42	Myeloid	GPATCH2L
**4.49715683906004E-46**	0.784559262331334	0.178	0.105	7.33531252019083E-42	Myeloid	SLC12A9
**1.1394046685875E-45**	0.548961325741557	0.148	0.079	1.85848295493307E-41	Myeloid	GPR107
**1.16002656273518E-45**	0.352309253131829	0.16	0.086	1.89211932647735E-41	Myeloid	CHD1
**1.66102394926106E-45**	0.607151657652194	0.111	0.052	2.70929616363972E-41	Myeloid	CCNG2
**2.03884262429486E-45**	0.477013089272941	0.197	0.119	3.32555620448734E-41	Myeloid	PFDN5
**2.15631426170744E-45**	0.375307772366817	0.27	0.173	3.517164192271E-41	Myeloid	SERPINB9
**3.64743757607311E-45**	0.642089116249807	0.279	0.196	5.94933543033285E-41	Myeloid	RAB5C
**5.77987009228301E-45**	0.858966283194618	0.188	0.114	9.42754610752282E-41	Myeloid	ANKS1A
**7.5679894704041E-45**	0.598113984920741	0.164	0.093	1.23441476251761E-40	Myeloid	DDX3X
**7.61955268858567E-45**	0.750669726442483	0.206	0.13	1.24282523903521E-40	Myeloid	NT5C2
**1.41681068142687E-44**	0.480485334575796	0.106	0.048	2.31095990247536E-40	Myeloid	TGFBR1
**1.7420959995255E-44**	0.538402384419631	0.184	0.109	2.84153278482604E-40	Myeloid	HUS1
**1.92473903121661E-44**	1.93150191975546	0.105	0.05	3.13944183381742E-40	Myeloid	CXCL10
**2.37772458938346E-44**	0.534424999248073	0.179	0.104	3.87830657774336E-40	Myeloid	ARHGAP17

**2.91148374741663E-44**	0.966221740866921	0.188	0.115	4.74892114041126E-40	Myeloid	CITED2
**4.78044026175087E-44**	0.604163580600605	0.108	0.051	7.79737611094185E-40	Myeloid	SESN1
**6.6389790386156E-44**	0.826928176618815	0.223	0.146	1.08288387098859E-39	Myeloid	NAB2
**8.73184858199263E-44**	1.61091840921244	0.12	0.062	1.42425182220882E-39	Myeloid	TTC7B
**1.22672955287544E-43**	0.301806716891556	0.191	0.112	2.00091857369512E-39	Myeloid	PRMT2
**1.64741930508094E-43**	0.793733803708584	0.193	0.12	2.68710562851752E-39	Myeloid	TMBIM1
**2.93003081884936E-43**	0.798490845849591	0.145	0.08	4.7791732686252E-39	Myeloid	PLIN3
**3.34722648363438E-43**	0.457990176825381	0.243	0.161	5.45966111745604E-39	Myeloid	EFR3A
**3.35358061188211E-43**	0.31012347019749	0.124	0.06	5.47002533604091E-39	Myeloid	ID3
**6.59928971689794E-43**	0.832913323531973	0.115	0.057	1.07641014572322E-38	Myeloid	AKAP10
**1.5820162643509E-42**	1.35515562129902	0.124	0.065	2.58042672878275E-38	Myeloid	PTPN12
**2.70963041298152E-42**	1.71803865919906	0.167	0.103	4.41967816661415E-38	Myeloid	CD276
**3.90362194108457E-42**	0.668016776840006	0.123	0.063	6.36719774810304E-38	Myeloid	PIP5K1C
**7.28720056336949E-42**	0.908411028148558	0.164	0.097	1.1886152838912E-37	Myeloid	GLB1
**1.3059538617298E-41**	0.712521015758459	0.152	0.086	2.13014134386747E-37	Myeloid	ACAP2
**2.11068324886921E-41**	0.274058969338754	0.349	0.257	3.44273544723057E-37	Myeloid	ANXA11
**2.37022238183287E-41**	1.28614230757209	0.116	0.06	3.86606972700759E-37	Myeloid	YWHAH
**3.21669617840408E-41**	0.991000934543374	0.109	0.054	5.24675313659489E-37	Myeloid	RIT1
**3.43025099571112E-41**	0.437951726282749	0.345	0.255	5.59508239910442E-37	Myeloid	PIM3
**3.5908320218393E-41**	1.00808584954666	0.214	0.144	5.85700611082209E-37	Myeloid	ABHD16A
**4.31089753278242E-41**	0.394732398412409	0.158	0.089	7.0315049657214E-37	Myeloid	SHKBP1
**4.9122691618975E-41**	1.26397855383842	0.139	0.078	8.01240222997101E-37	Myeloid	TSPAN3
**8.38370664651875E-41**	1.07017273639763	0.229	0.159	1.36746639111367E-36	Myeloid	NEK6
**1.0020592273643E-40**	1.72331852106541	0.112	0.058	1.63445880575391E-36	Myeloid	HSD17B14
**1.63487994046328E-40**	0.58726833011943	0.191	0.119	2.66665267088965E-36	Myeloid	DICER1
**2.45685773115403E-40**	1.6518547211672	0.115	0.06	4.00738064528534E-36	Myeloid	SCN1B
**2.88747627756552E-40**	0.638757717952919	0.318	0.238	4.70976255633712E-36	Myeloid	INF2
**5.1805417748677E-40**	0.388117568989626	0.223	0.146	8.44998168898671E-36	Myeloid	SCAMP2
**6.73812325836669E-40**	1.67869683346767	0.157	0.096	1.09905528467219E-35	Myeloid	TCF7L2
**6.78725979727149E-40**	0.726106983852288	0.291	0.216	1.10706994553295E-35	Myeloid	PRKACA
**9.01017289987597E-40**	1.05636841233281	0.204	0.135	1.46964930169877E-35	Myeloid	ANXA2
**1.1055395606056E-39**	0.444704917366273	0.27	0.19	1.8032455773038E-35	Myeloid	AAMP
**1.25297727285933E-39**	1.15767144858663	0.157	0.094	2.04373122976085E-35	Myeloid	SCD
**1.2759688235952E-39**	1.15488472274842	0.134	0.075	2.08123274816613E-35	Myeloid	VAMP3
**4.31281693429012E-39**	0.454379214275385	0.134	0.072	7.03463570152062E-35	Myeloid	MCM5
**7.41924905663874E-39**	1.64665416185524	0.106	0.054	1.21015371362834E-34	Myeloid	SIK1B
**1.17136721658984E-38**	1.1034396257689	0.214	0.148	1.91061706697968E-34	Myeloid	RNPEP
**1.71323338368485E-38**	0.556374735963717	0.244	0.168	2.79445497212836E-34	Myeloid	BNIP2
**2.83587812563736E-38**	0.35409931332299	0.18	0.11	4.62560081072709E-34	Myeloid	VHL
**5.68684106644909E-38**	0.519663507761705	0.123	0.065	9.27580646348511E-34	Myeloid	ACTR2
**6.24726344199503E-38**	1.21426304453768	0.199	0.135	1.01899114002381E-33	Myeloid	RTN3
**6.69184749148811E-38**	1.03261488394857	0.148	0.088	1.09150724433663E-33	Myeloid	SLC11A2
**1.05395457380037E-37**	0.517587836000515	0.31	0.231	1.71910530532578E-33	Myeloid	TNFRSF1A
**1.356537703167E-37**	0.891633156949963	0.19	0.124	2.21264864763569E-33	Myeloid	USP32
**3.32592892797648E-37**	0.80551888666996	0.13	0.072	5.42492267442244E-33	Myeloid	NHLRC3
**4.73657601587576E-37**	0.32692305244251	0.141	0.079	7.72582913949496E-33	Myeloid	DCP2
**5.61447950430843E-37**	1.06594642065089	0.125	0.069	9.15777751947748E-33	Myeloid	FEZ2
**8.40247395983784E-37**	1.93597260106761	0.134	0.079	1.37052752758915E-32	Myeloid	ZFHX3
**1.58121239195243E-36**	0.826578817464453	0.202	0.134	2.57911553251362E-32	Myeloid	IFRD1
**2.39750308143245E-36**	1.22391186731102	0.217	0.153	3.91056727612446E-32	Myeloid	TNFSF10
**3.02758539556306E-36**	0.643455590138576	0.185	0.117	4.9382945387029E-32	Myeloid	TNFRSF10E
**3.75595481328032E-36**	0.905492627452148	0.106	0.055	6.12633789594153E-32	Myeloid	APPL2

**3.85775939443395E-36**	1.06720646006483	0.131	0.075	6.29239134826122E-32	Myeloid	NAGPA
**4.31842743317548E-36**	0.698400408856361	0.123	0.067	7.04378698625252E-32	Myeloid	AGO1
**2.44853772174724E-35**	0.361958625926359	0.13	0.071	3.99380987794192E-31	Myeloid	JARID2
**3.17014913320188E-35**	0.467319670972171	0.108	0.055	5.17083025116558E-31	Myeloid	SLC12A6
**4.08016920417454E-35**	1.60702161218455	0.119	0.068	6.65516398892909E-31	Myeloid	EIF2AK4
**5.53112216196085E-35**	0.80774670378938	0.193	0.129	9.02181335837434E-31	Myeloid	BCAP31
**7.91592072044906E-35**	0.578498536972654	0.229	0.159	1.29116582871245E-30	Myeloid	MAP2K3
**8.65450758932322E-35**	0.702208817565915	0.209	0.142	1.41163673289451E-30	Myeloid	LAMTOR1
**1.13303722737292E-34**	0.629812465156604	0.241	0.173	1.84809702156796E-30	Myeloid	RAB1A
**1.55906257688825E-34**	0.458390387358938	0.147	0.086	2.54298696916242E-30	Myeloid	IREB2
**2.00113925070721E-34**	0.959102739430099	0.187	0.125	3.26405823182854E-30	Myeloid	MTHFR
**2.37138209550208E-34**	0.326274634191501	0.17	0.104	3.86796133597344E-30	Myeloid	RAB35
**5.70662214485332E-34**	0.839251837950271	0.14	0.083	9.30807138047025E-30	Myeloid	HSPH1
**9.6800683556295E-34**	1.06858461187029	0.146	0.089	1.57891594948673E-29	Myeloid	PABPC4
**1.31137698541121E-33**	0.369891846385671	0.143	0.083	2.13898700090423E-29	Myeloid	ACTR3
**6.31672358161387E-33**	1.69349645212768	0.125	0.074	1.03032078339704E-28	Myeloid	EVI5
**1.90604889091326E-32**	0.541731483535432	0.214	0.147	3.10895634596862E-28	Myeloid	KCTD20
**3.3368242812722E-32**	1.29435665532063	0.144	0.09	5.44269408518308E-28	Myeloid	CD40
**5.25357212993325E-32**	0.778542696664094	0.222	0.159	8.56910150113412E-28	Myeloid	ACTN1
**6.4074289492282E-32**	0.279924729107165	0.139	0.081	1.04511573590861E-27	Myeloid	CNPPD1
**8.10745374441453E-32**	1.16999202875803	0.176	0.119	1.32240678025145E-27	Myeloid	PEA15
**1.49587143928538E-31**	0.95665171238479	0.256	0.194	2.43991590461839E-27	Myeloid	IRS2
**2.37769865048406E-31**	0.43261414582249	0.177	0.114	3.87826426880455E-27	Myeloid	PHKB
**3.00840606708501E-31**	0.638869028189509	0.192	0.129	4.90701113602236E-27	Myeloid	SPAG9
**3.46519364141055E-31**	0.745986542108318	0.148	0.092	5.65207734850474E-27	Myeloid	AGTRAP
**3.82132245672762E-31**	0.92097834823674	0.21	0.15	6.23295905916843E-27	Myeloid	ABHD2
**4.0823229182771E-31**	0.51855053109813	0.16	0.101	6.65867691200177E-27	Myeloid	PSEN1
**6.1783513448661E-31**	0.938935030512151	0.192	0.133	1.00775088786111E-26	Myeloid	CAPZA2
**6.61545931207383E-31**	0.842193535926546	0.138	0.084	1.07904756839236E-26	Myeloid	IFNAR1
**9.09722278933019E-31**	0.553781378275843	0.142	0.087	1.48384800916765E-26	Myeloid	PIP4P1
**1.16794126661295E-30**	1.15365115678821	0.114	0.065	1.90502899997239E-26	Myeloid	SUMF1
**1.52073110435701E-30**	0.53033416824435	0.179	0.118	2.48046450431673E-26	Myeloid	RAB8A
**2.28267250207957E-30**	0.6621485110275	0.167	0.109	3.72326711814199E-26	Myeloid	ACCS
**2.64129796929832E-30**	0.999307293592324	0.139	0.086	4.3082211177225E-26	Myeloid	RAPGEF2
**3.3549468591087E-30**	0.818105292577293	0.221	0.162	5.4722538218922E-26	Myeloid	CLTA
**4.25296764652087E-30**	0.667825732431418	0.195	0.135	6.93701552824018E-26	Myeloid	PITPNA
**7.24416883676842E-30**	1.18504733016685	0.13	0.079	1.1815963789653E-25	Myeloid	ATG3
**1.45323441822357E-29**	1.75992521133471	0.177	0.125	2.37037065956447E-25	Myeloid	AKT1S1
**1.81073054546775E-29**	1.57185251946081	0.124	0.075	2.95348259271245E-25	Myeloid	HES4
**2.95545698533375E-29**	1.42373690073934	0.114	0.068	4.82064588877788E-25	Myeloid	TOR3A
**3.07500501046551E-29**	0.261786882322154	0.208	0.142	5.01564067257029E-25	Myeloid	AKAP9
**3.12039785126202E-29**	1.12765939686298	0.133	0.081	5.08968093519349E-25	Myeloid	NAV1
**3.25087056317717E-29**	0.267301063132682	0.231	0.162	5.30249497559828E-25	Myeloid	ELMSAN1
**5.88251512308823E-29**	0.77855931237883	0.115	0.066	9.59497041726921E-25	Myeloid	PARVB
**1.27832344374094E-28**	1.35310487268836	0.121	0.073	2.08507336908585E-24	Myeloid	DENND5A
**1.67262111801763E-28**	1.56860236043358	0.145	0.095	2.72821230559856E-24	Myeloid	PPIF
**1.73052038646442E-28**	0.395146170059048	0.157	0.1	2.82265180236212E-24	Myeloid	IFIH1
**1.81156269105982E-28**	0.400458645881181	0.115	0.065	2.95483990538768E-24	Myeloid	SPTLC2
**2.36653952370149E-28**	0.434539354616089	0.239	0.175	3.8600626171095E-24	Myeloid	TSC22D2
**2.52221409919156E-28**	1.57367229578886	0.1	0.057	4.11398341719136E-24	Myeloid	RHOBTB1
**5.20142756318524E-28**	0.523844072492697	0.229	0.166	8.48404849831145E-24	Myeloid	TRIOBP
**9.6821254039076E-28**	1.22781740493082	0.104	0.06	1.57925147463137E-23	Myeloid	RALB

1.59489596289434E-27	0.409833366031628	0.136	0.084	2.60143480507696E-23	Myeloid	WASF2
2.04794601524067E-27	0.797749005427643	0.113	0.066	3.34040474545906E-23	Myeloid	SPPL3
2.24064281716827E-27	1.42083234856208	0.159	0.109	3.65471249908317E-23	Myeloid	DEPRECATI
2.61293797842791E-27	0.758991518206279	0.151	0.098	4.26196313661377E-23	Myeloid	CCNYL1
2.99064895682307E-27	0.843390657576797	0.143	0.092	4.87804751347411E-23	Myeloid	CAMKK2
3.53330990026336E-27	0.571658130547222	0.1	0.056	5.76318177831957E-23	Myeloid	TENT4B
4.23061512072186E-27	0.348069794587609	0.243	0.181	6.90055632340943E-23	Myeloid	RNF114
5.2074691064766E-27	0.296619555553757	0.131	0.078	8.49390285957398E-23	Myeloid	KIF13B
5.28390229841362E-27	0.459616354397594	0.11	0.063	8.61857303894245E-23	Myeloid	EXOC1
1.18152125321662E-26	0.335812703359761	0.118	0.069	1.92717931612163E-22	Myeloid	CLIC1
1.46623055685395E-26	0.477842634700745	0.209	0.15	2.39156866128448E-22	Myeloid	SUMO3
2.50560322365808E-26	1.84910564836244	0.124	0.079	4.08688941810869E-22	Myeloid	CLCN5
2.82243907891424E-26	0.426057021426164	0.147	0.094	4.60368038161701E-22	Myeloid	SKIL
3.23857952984635E-26	1.01307658331197	0.101	0.058	5.28244707113238E-22	Myeloid	EAF1
3.33553123535514E-26	0.499950888923629	0.182	0.126	5.44058499798776E-22	Myeloid	PWWP2B
3.47611403258774E-26	0.575076867342844	0.14	0.088	5.66988959855386E-22	Myeloid	CTNNB1
4.27189381186896E-26	0.279358294184251	0.257	0.194	6.96788599653945E-22	Myeloid	GNB2
1.01041575561405E-25	0.705318015146293	0.162	0.111	1.64808913898207E-21	Myeloid	SQOR
1.23169451403923E-25	0.518336207807653	0.162	0.108	2.00901692184939E-21	Myeloid	H2AFJ
2.0139280861186E-25	0.38712351137936	0.208	0.15	3.28491810126805E-21	Myeloid	RFX5
2.20792846938813E-25	1.02094862637979	0.108	0.064	3.60135212641898E-21	Myeloid	RAB24
2.67808482270498E-25	1.50886829124162	0.144	0.098	4.36822415431409E-21	Myeloid	PRDX1
2.92304279471457E-25	1.29166553569897	0.13	0.083	4.76777510245894E-21	Myeloid	CXCL2
4.09950610480325E-25	0.632787370706996	0.119	0.073	6.68670440754459E-21	Myeloid	AGO4
4.11242380488719E-25	0.891591550995432	0.129	0.082	6.7077744681515E-21	Myeloid	B4GALT5
4.62320527415019E-25	0.304189506777449	0.233	0.173	7.54091012266638E-21	Myeloid	QARS
8.47962588863378E-25	0.370248434299152	0.129	0.081	1.38311177869506E-20	Myeloid	STXBP3
1.31657784296611E-24	0.672645996362991	0.335	0.272	2.14747011966202E-20	Myeloid	ISG15
1.70072541206462E-24	0.525988369090878	0.13	0.083	2.7740532196186E-20	Myeloid	LARP4B
1.75449021644359E-24	0.591680602498452	0.173	0.121	2.86174899204114E-20	Myeloid	TUBGCP2
2.15790037330187E-24	0.375015271848152	0.13	0.081	3.51975129889268E-20	Myeloid	DNM1L
2.31799259533987E-24	1.41534366897091	0.127	0.083	3.78087772225886E-20	Myeloid	DEPRECATI
2.38378528063602E-24	0.41976849127794	0.169	0.115	3.88819217124541E-20	Myeloid	PTPA
3.42879170324822E-24	0.828654240621376	0.156	0.107	5.59270214716817E-20	Myeloid	MFHAS1
3.82250415866217E-24	0.602957411759708	0.176	0.122	6.23488653319387E-20	Myeloid	SYAP1
4.9474539108857E-24	0.36613031504143	0.18	0.126	8.06979207404566E-20	Myeloid	TRA2B
4.99301454511239E-24	1.1799486323174	0.171	0.123	8.14410602453282E-20	Myeloid	SLC43A3
5.88575901518229E-24	0.418911047059442	0.241	0.185	9.60026152966384E-20	Myeloid	OXA1L
7.46711829862846E-24	1.0168494068072	0.139	0.093	1.21796166568929E-19	Myeloid	FOXO3
7.83252652321042E-24	0.449393399461256	0.125	0.078	1.27756340120085E-19	Myeloid	FAM133B
1.05461467658855E-23	0.758324951045812	0.105	0.063	1.72018199898358E-19	Myeloid	METTL22
1.83640779746079E-23	0.719001396416793	0.152	0.104	2.99536475843829E-19	Myeloid	CCDC107
2.43978532073057E-23	0.397678993903655	0.133	0.085	3.97953383664363E-19	Myeloid	GABARAPL
4.45134943171736E-23	0.925387000158037	0.158	0.111	7.26059605807419E-19	Myeloid	WBP1L
4.46272363722609E-23	0.639949608190288	0.208	0.157	7.27914852467948E-19	Myeloid	STK40
5.2603961486841E-23	1.18436384530009	0.108	0.067	8.58023215811864E-19	Myeloid	IRAK2
5.27880075534723E-23	0.486941513972434	0.279	0.22	8.61025191204686E-19	Myeloid	METTL7A
8.32787829885377E-23	0.326130424406141	0.199	0.144	1.35836022932604E-18	Myeloid	HTT
8.82326102388179E-23	0.332954574710289	0.162	0.109	1.43916210560536E-18	Myeloid	PTGES2
1.05554753052456E-22	1.37103221543301	0.152	0.108	1.72170357703861E-18	Myeloid	NAGLU
1.63553716472408E-22	0.469915372498807	0.211	0.158	2.66772466938144E-18	Myeloid	TSPO
2.43282912570742E-22	0.488666931587482	0.153	0.104	3.96818758694138E-18	Myeloid	ZC3H3

**4.01747157952232E-22**	0.512893289730875	0.103	0.062	6.55289789335885E-18	Myeloid	PLEKHF2
**4.61102611519049E-22**	0.897855816055456	0.214	0.167	7.5210446964872E-18	Myeloid	ATP13A3
**8.49828660962038E-22**	0.838919744219017	0.142	0.097	1.38615552889518E-17	Myeloid	KLF16
**1.23804750361587E-21**	0.602152824673833	0.137	0.092	2.01937928314785E-17	Myeloid	MIIP
**2.75486566771365E-21**	0.4566610503826	0.133	0.088	4.49346139060774E-17	Myeloid	ZNF106
**2.76680495867136E-21**	0.306636856735391	0.215	0.163	4.51293556808885E-17	Myeloid	IQGAP2
**3.61789431905129E-21**	0.661595745678706	0.138	0.093	5.90114742380456E-17	Myeloid	ZNF330
**3.90959622895396E-21**	0.456019883871101	0.155	0.108	6.37694240904681E-17	Myeloid	TBC1D14
**5.22151864224397E-21**	0.796081563869228	0.2	0.153	8.51681905736414E-17	Myeloid	NUMB
**1.5006185331191E-20**	0.396689663230064	0.122	0.078	2.44765888937057E-16	Myeloid	RB1
**3.85634520635859E-20**	1.34433737301187	0.125	0.086	6.29008466609149E-16	Myeloid	CPPED1
**7.29836905006594E-20**	0.377129369976711	0.137	0.093	1.19043697575626E-15	Myeloid	TMEM138
**1.59073547836335E-19**	0.258731564753505	0.208	0.157	2.59464863875846E-15	Myeloid	SEC11A
**1.68935669863077E-19**	1.00388227539095	0.139	0.097	2.75550971113665E-15	Myeloid	PACSIN2
**1.83776907417439E-19**	0.469794921962241	0.119	0.078	2.99758513688585E-15	Myeloid	TCF12
**2.11533622741639E-19**	0.250790549261204	0.212	0.161	3.45032492053888E-15	Myeloid	PDCD6IP
**2.36182305262037E-19**	0.307342809299232	0.158	0.111	3.85236958112908E-15	Myeloid	AP3B1
**2.5757736759695E-19**	0.423022886635614	0.295	0.245	4.20134444287385E-15	Myeloid	FNDC3B
**2.74712276284058E-19**	0.818475194737919	0.127	0.087	4.48083193846927E-15	Myeloid	UBXN2B
**2.79891647072341E-19**	0.503696581580634	0.122	0.081	4.56531265539695E-15	Myeloid	NUP62
**4.56956381796498E-19**	0.460189305674094	0.173	0.127	7.45341554348268E-15	Myeloid	HIPK3
**5.18301791686594E-19**	0.262794515939025	0.113	0.071	8.45402052420004E-15	Myeloid	PHACTR2
**6.08005394053256E-19**	0.396686413877986	0.249	0.202	9.91717598240267E-15	Myeloid	HM13
**6.27688768037853E-19**	0.942605165714535	0.163	0.121	1.02382314954654E-14	Myeloid	TOM1
**6.44965269758434E-19**	0.446040373051739	0.182	0.135	1.05200285150298E-14	Myeloid	SELENOK
**7.29919060458592E-19**	0.408409527112078	0.116	0.075	1.19057097951401E-14	Myeloid	GANC
**7.30534507760411E-19**	0.421894817323911	0.211	0.165	1.19157483560801E-14	Myeloid	CD46
**8.5407355219697E-19**	0.809738789239918	0.122	0.083	1.39307937098848E-14	Myeloid	PEPD
**1.06700380939133E-18**	0.497080947437107	0.132	0.09	1.7403899134982E-14	Myeloid	PISD
**1.137751304892E-18**	0.368662625029708	0.124	0.083	1.85578615340934E-14	Myeloid	OXSR1
**1.23652217214119E-18**	0.746510612471569	0.216	0.174	2.0168913149795E-14	Myeloid	CALM2
**1.29536684506456E-18**	1.24915509734878	0.17	0.131	2.11287286098481E-14	Myeloid	PNKD
**1.53125751218266E-18**	0.317275874862052	0.17	0.124	2.49763412812114E-14	Myeloid	VPS35L
**2.77567336048706E-18**	0.321770104165137	0.262	0.212	4.52740081829044E-14	Myeloid	TRIM8
**3.46374901637352E-18**	0.683697216794704	0.267	0.224	5.64972102060685E-14	Myeloid	CTNND1
**3.50437620621706E-18**	0.550948991894009	0.125	0.085	5.71598802996065E-14	Myeloid	ERP44
**3.65539053757277E-18**	0.969074725714534	0.137	0.097	5.96230750583495E-14	Myeloid	SLC39A1
**4.13713716521605E-18**	0.819907730530576	0.141	0.101	6.7480844301839E-14	Myeloid	NUDT3
**4.37578937533922E-18**	0.267661021947818	0.131	0.089	7.1373500501158E-14	Myeloid	MCRIP1
**6.54512546332102E-18**	0.307063084996644	0.127	0.086	1.06757541432229E-13	Myeloid	SLC25A28
**1.6039585365658E-17**	1.21981320399108	0.113	0.077	2.61621676899248E-13	Myeloid	TK2
**1.64817959486697E-17**	0.312987201028576	0.1	0.157	2.68834573718752E-13	Myeloid	G0S2
**1.80725338391694E-17**	0.293273029927457	0.209	0.161	2.94781099450692E-13	Myeloid	MKNK2
**2.50138774063636E-17**	0.837932696815143	0.106	0.07	4.08001354375197E-13	Myeloid	KIF16B
**4.6520078140928E-17**	0.458514119633633	0.108	0.071	7.58788994556677E-13	Myeloid	MX1
**7.25420861092681E-17**	0.272627874892397	0.127	0.088	1.18323396652827E-12	Myeloid	RAB4B
**9.35497066537103E-17**	0.772129675710308	0.129	0.091	1.52588926522867E-12	Myeloid	RNF141
**1.44412095555476E-16**	1.51126625784647	0.107	0.073	2.35550569060536E-12	Myeloid	TRIM47
**1.69367450975321E-16**	0.398792967836302	0.124	0.086	2.76255249285846E-12	Myeloid	VPS41
**2.02342209200308E-16**	0.830385950429132	0.235	0.198	3.30040377426623E-12	Myeloid	MTSS1
**2.53268287990407E-16**	0.441287069963829	0.15	0.109	4.13105904541153E-12	Myeloid	DUSP22
**2.54471440302062E-16**	0.29235450842874	0.323	0.265	4.15068366276693E-12	Myeloid	ACAA1

2.85663221789695E-16	0.880107768340359	0.102	0.068	4.65945281061171E-12	Myeloid	DHRSX
3.18031929089248E-16	0.45860578986964	0.228	0.186	5.18741879537472E-12	Myeloid	TMEM219
3.39716333480218E-16	0.351833589421387	0.191	0.148	5.54111311539583E-12	Myeloid	NPTN
3.7614223854908E-16	1.10236299846893	0.173	0.138	6.13525605297404E-12	Myeloid	GBA
5.8621902391202E-16	0.308812166505817	0.226	0.184	9.56181849902896E-12	Myeloid	ARF1
7.41454685686776E-16	1.5216063664759	0.116	0.083	1.2093867378237E-11	Myeloid	RGS12
7.67836306791667E-16	0.266117663697754	0.253	0.208	1.25241780000789E-11	Myeloid	SERPINB1
8.38223950036692E-16	0.32752947086005	0.112	0.075	1.36722708490485E-11	Myeloid	U2AF1L4
1.15131094607867E-15	0.798405437467627	0.188	0.15	1.87790328414892E-11	Myeloid	MT-ND2
1.16726259235171E-15	0.537625917310097	0.15	0.111	1.90392201438488E-11	Myeloid	WDR11
1.30987894586744E-15	0.256659264138003	0.184	0.14	2.13654354860439E-11	Myeloid	CMIP
1.538554460605E-15	0.810675918031307	0.211	0.174	2.50953618069282E-11	Myeloid	MT-ND5
2.03859107584234E-15	0.537278681980237	0.145	0.108	3.32514590380645E-11	Myeloid	WIPI2
3.41041281282037E-15	1.33975587010243	0.105	0.073	5.5627243389913E-11	Myeloid	IFIT2
4.67630420834789E-15	0.437608912381194	0.178	0.137	7.62751979423624E-11	Myeloid	USF1
6.33765900794358E-15	0.403696302879492	0.123	0.087	1.03373556078568E-10	Myeloid	NUP58
7.19621332927865E-15	0.811365025700304	0.153	0.118	1.17377435613864E-10	Myeloid	ZBED1
8.05997387350646E-15	0.549682001298741	0.119	0.084	1.31466233850764E-10	Myeloid	SDE2
1.43267366032673E-14	0.455344046104753	0.164	0.127	2.33683400735892E-10	Myeloid	DCTN4
1.62368028899015E-14	0.322159485747492	0.072	0.119	2.64838491937184E-10	Myeloid	HAAO
1.7078949993635E-14	1.15807374917269	0.149	0.117	2.7857475334618E-10	Myeloid	SLC22A18
1.8208294708002E-14	0.377152281506235	0.111	0.076	2.9699549498222E-10	Myeloid	SLC25A6
2.57160512088373E-14	0.426076517332243	0.138	0.101	4.19454511267345E-10	Myeloid	HMGN3
2.92908629186991E-14	0.359031937028878	0.134	0.097	4.77763265066901E-10	Myeloid	NGLY1
3.225155820861E-14	0.293496291937424	0.225	0.185	5.26055165940637E-10	Myeloid	ATP5MC2
3.4978479959273E-14	0.361325359810188	0.261	0.222	5.70533986615701E-10	Myeloid	IRF7
3.73015836506813E-14	0.393485353264574	0.109	0.075	6.08426130926263E-10	Myeloid	VPS11
4.03098046144473E-14	1.21441690504543	0.196	0.166	6.57493223066249E-10	Myeloid	CYP27A1
4.14931418068514E-14	0.543212979783228	0.129	0.094	6.76794636011553E-10	Myeloid	RAB12
4.97944304061576E-14	0.663909984299254	0.107	0.074	8.12196954354837E-10	Myeloid	DMXL1
6.05914068745333E-14	0.268993826013628	0.111	0.078	9.88306437530512E-10	Myeloid	CHMP3
6.33602453523997E-14	0.281558446379237	0.087	0.138	1.03346896194299E-09	Myeloid	AIG1
6.66123511484059E-14	0.792519055081371	0.104	0.073	1.08651405958165E-09	Myeloid	FEM1B
6.79334977690169E-14	0.28767191816678	0.209	0.17	1.10806328211043E-09	Myeloid	FAM50A
1.41795619938952E-13	0.261259110526297	0.214	0.174	2.31282835682425E-09	Myeloid	IFI35
1.64735048875469E-13	0.483506303667376	0.124	0.089	2.68699338220778E-09	Myeloid	MAP1S
1.8012513705492E-13	0.794099724618428	0.129	0.096	2.9380211105028E-09	Myeloid	PGLS
1.87720608658193E-13	0.383461006129498	0.186	0.149	3.06191084782379E-09	Myeloid	BCL2L1
2.57517797522835E-13	0.258174561947018	0.105	0.072	4.20037279539496E-09	Myeloid	RNF111
2.60402652947423E-13	0.849188471375532	0.207	0.176	4.24742767222542E-09	Myeloid	PLOD3
2.80119981690014E-13	0.312428644823236	0.114	0.081	4.56903702134581E-09	Myeloid	IFITM10
2.82850692538749E-13	0.32144341422024	0.106	0.074	4.61357764599953E-09	Myeloid	COMMD5
3.22793680992069E-13	1.20397925595091	0.127	0.096	5.26508773066164E-09	Myeloid	TALDO1
3.25915081422135E-13	1.09421623443686	0.166	0.134	5.31600089307645E-09	Myeloid	MT-ND1
3.71941667091542E-13	1.34594832440451	0.128	0.098	6.06674053193014E-09	Myeloid	ARHGEF11
3.74803167211227E-13	0.421785263112516	0.1	0.069	6.11341446038233E-09	Myeloid	TMEM131
4.64799148769956E-13	0.352891349861112	0.13	0.094	7.58133891558675E-09	Myeloid	PPM1F
6.60487920411133E-13	0.36042979484721	0.143	0.109	1.0773218469826E-08	Myeloid	ELOVL1
7.50990710532761E-13	0.757584655315475	0.156	0.123	1.22494094794999E-08	Myeloid	KIF1B
8.92117263519386E-13	0.436239678950692	0.134	0.1	1.45513246852647E-08	Myeloid	IRGQ
9.7599391150872E-13	0.526770300481239	0.127	0.094	1.59194366906187E-08	Myeloid	SPIDR
1.04676770226685E-12	0.380502875436088	0.26	0.223	1.70738279916746E-08	Myeloid	MYDGF

**1.36652351311126E-12**	0.404661143796544	0.216	0.183	2.22893650223578E-08	Myeloid	NCSTN
**1.43098027213497E-12**	0.257139072866629	0.109	0.077	2.33407192187934E-08	Myeloid	CCDC82
**1.51412840295912E-12**	0.385683867531448	0.126	0.093	2.46969483806662E-08	Myeloid	UBE2A
**2.23864020934471E-12**	0.633007359803612	0.113	0.082	3.65144604546216E-08	Myeloid	DIAPH2
**2.26228180045162E-12**	0.304007624095201	0.155	0.12	3.69000784471664E-08	Myeloid	IDH3G
**2.27550328759959E-12**	0.535649943238352	0.142	0.108	3.71157341240368E-08	Myeloid	TFE3
**3.58026327278794E-12**	0.772251055747707	0.119	0.089	5.83976742424441E-08	Myeloid	TRAK1
**5.68011203901539E-12**	0.486619210880784	0.133	0.101	9.264830746838E-08	Myeloid	EIF4E2
**5.76445027643538E-12**	0.473546212452931	0.155	0.122	9.40239484589375E-08	Myeloid	GLRX
**6.26261035608753E-12**	0.91901133899954	0.187	0.159	1.02149437518144E-07	Myeloid	LTBR
**7.83314425124985E-12**	0.356263533781625	0.119	0.088	1.27766415882136E-07	Myeloid	SNX13
**9.35897624744721E-12**	0.361212395967015	0.206	0.172	1.52654261572111E-07	Myeloid	ENO1
**1.17092659714667E-11**	0.686006168265509	0.181	0.148	1.90989837260594E-07	Myeloid	TRIM25
**1.4854198786455E-11**	0.589827613711801	0.158	0.127	2.42286836405868E-07	Myeloid	SELENOS
**1.49247069221734E-11**	0.302985929853295	0.136	0.104	2.4343689460757E-07	Myeloid	FOXN3
**2.26801164077496E-11**	0.360733928498208	0.257	0.218	3.69935378726804E-07	Myeloid	FUOM
**2.30746387660141E-11**	0.334785636956835	0.164	0.131	3.76370432912456E-07	Myeloid	COP 1
**2.36856747907088E-11**	0.308470462599485	0.132	0.1	3.86337041511252E-07	Myeloid	TAOK3
**2.39905294909205E-11**	0.531971463762365	0.114	0.085	3.91309526526405E-07	Myeloid	H3F3B
**2.95624691383672E-11**	0.496182012878886	0.121	0.091	4.82193434115908E-07	Myeloid	SGTA
**3.77585292599275E-11**	0.496739222606398	0.137	0.107	6.15879370758677E-07	Myeloid	WWP1
**4.14107687812309E-11**	1.79894021383522	0.103	0.078	6.75451049590657E-07	Myeloid	HNMT
**4.63161956765388E-11**	0.672981673218291	0.126	0.096	7.55463467680025E-07	Myeloid	FER
**5.65992094358087E-11**	0.80416030732882	0.107	0.08	9.23189705107476E-07	Myeloid	AP3S2
**5.70855772841357E-11**	0.364682407394037	0.109	0.08	9.31122851081538E-07	Myeloid	ATF7
**6.15003831750521E-11**	0.291436403734872	0.131	0.1	1.00313274996828E-06	Myeloid	LRCH1
**7.15677930228491E-11**	0.335168768303699	0.116	0.086	1.16734227199569E-06	Myeloid	WDR70
**7.41676937600983E-11**	1.23389661779521	0.121	0.094	1.20974925292096E-06	Myeloid	FARP1
**8.72267084137838E-11**	0.74948969303713	0.132	0.104	1.42275484093723E-06	Myeloid	DBP
**9.18846109112482E-11**	0.796746909299575	0.138	0.109	1.49872988857337E-06	Myeloid	TPRA1
**1.18669620311878E-10**	0.59209648409075	0.123	0.095	1.93562017690705E-06	Myeloid	ADCK2
**1.5248006699762E-10**	0.34068705732098	0.157	0.126	2.48710237279818E-06	Myeloid	DENND4C
**1.62146058408135E-10**	0.640117606353188	0.1	0.073	2.6447643586951E-06	Myeloid	RNF146
**2.87845062227835E-10**	0.482935633785441	0.139	0.11	4.69504080999821E-06	Myeloid	DNAJB12
**3.00858505829057E-10**	0.325029695197649	0.119	0.09	4.90730308857774E-06	Myeloid	PKN2
**4.65588090825643E-10**	0.856850899632832	0.121	0.095	7.59420734945707E-06	Myeloid	RAC1
**6.39166808689179E-10**	0.563043651359539	0.113	0.086	1.04254498165292E-05	Myeloid	APPL1
**7.12825732278304E-10**	0.87330417165608	0.113	0.087	1.16269005191914E-05	Myeloid	ARPC5
**7.93157399849296E-10**	0.566407062407032	0.172	0.145	1.29371903489419E-05	Myeloid	HGSNAT
**8.06873930355141E-10**	0.436496936634236	0.12	0.092	1.31609206780227E-05	Myeloid	MAP3K2
**8.21816661523254E-10**	1.23781947365107	0.116	0.091	1.34046515661058E-05	Myeloid	NEU1
**9.68391971836103E-10**	0.64829349567602	0.162	0.135	1.57954414526187E-05	Myeloid	CPQ
**9.88078407341206E-10**	0.743531516348053	0.116	0.09	1.61165469021424E-05	Myeloid	EHD4
**9.9302535692905E-10**	0.953289253492991	0.125	0.099	1.61972365968697E-05	Myeloid	MAFG
**1.15819678269714E-09**	0.807231841518131	0.103	0.078	1.88913477225731E-05	Myeloid	CARM1
**1.52125643290171E-09**	0.846521393171326	0.109	0.084	2.48132136770597E-05	Myeloid	NBPF26
**1.83022942724916E-09**	0.674122940546019	0.178	0.152	2.9852872187861E-05	Myeloid	CTNNA1
**2.10177831601153E-09**	0.260088608020857	0.109	0.155	3.4282106112464E-05	Myeloid	C16orf70
**2.4013690297857E-09**	0.378256585645583	0.154	0.125	3.91687302448345E-05	Myeloid	HPCAL1
**2.42465987507733E-09**	0.375506755985652	0.137	0.109	3.95486272223863E-05	Myeloid	ZMYM3
**2.78718820378569E-09**	0.761612051147576	0.121	0.096	4.54618267919484E-05	Myeloid	CUX1
**3.64316205736855E-09**	0.55701158314258	0.103	0.077	5.94236163177384E-05	Myeloid	AZI2

3.74495543084083E-09	0.615672160829605	0.11	0.084	6.10839680324448E-05	Myeloid	MYO1E
4.2603007675779E-09	0.316099439438678	0.259	0.224	6.9489765819963E-05	Myeloid	SLC30A1
4.31509009885268E-09	0.570774940843177	0.133	0.108	7.03834346023861E-05	Myeloid	TM7SF3
5.8226168366562E-09	0.545437565376482	0.158	0.132	9.49727032226992E-05	Myeloid	BAG1
5.98294611836492E-09	0.404103240009073	0.228	0.203	9.75878341366501E-05	Myeloid	NFE2L1
6.29644257172279E-09	0.904492205349369	0.171	0.149	0.00010270127478737	Myeloid	CYB5R1
6.94555218870942E-09	0.349097306317006	0.189	0.162	0.000113288901750039	Myeloid	MAP3K11
7.49476139173301E-09	0.804366779934537	0.178	0.156	0.000122247053060557	Myeloid	ATOX1
1.51621514958721E-08	0.33177265168766	0.128	0.102	0.000247309853049169	Myeloid	SLC35A4
2.26582320418669E-08	0.524163870723759	0.112	0.088	0.00036957842283489	Myeloid	RARA
2.33220676247587E-08	0.854949739150628	0.184	0.162	0.00038040624502744	Myeloid	DST
2.57589582735643E-08	0.913493690751473	0.101	0.079	0.000420154368400108	Myeloid	DBI
4.58399265584364E-08	0.25532408472034	0.126	0.101	0.000747695042094657	Myeloid	MON1B
5.44897897756852E-08	0.278793048825021	0.068	0.1	0.000888782961031201	Myeloid	SRXN1
6.05841175949085E-08	0.423938886563092	0.087	0.123	0.000988187542090552	Myeloid	KIF13A
6.77749726491029E-08	0.40122390554127	0.105	0.082	0.00110547757887952	Myeloid	ETHE1
8.03450219582395E-08	0.715532308674755	0.194	0.175	0.00131050765316084	Myeloid	SLC66A2
8.68073494786021E-08	0.714103767382654	0.134	0.113	0.00141591467734548	Myeloid	SLC39A11
9.46716395276282E-08	0.502798132804838	0.173	0.151	0.00154418911233514	Myeloid	ARL8A
1.17962352509706E-07	0.353757791396426	0.223	0.2	0.00192408393178582	Myeloid	TMED9
1.27644282034103E-07	0.962542736068581	0.1	0.079	0.00208200588425826	Myeloid	BCL2L2
1.36255397564536E-07	0.318556497052469	0.101	0.078	0.00222246178967514	Myeloid	RSU1
1.60830970074141E-07	0.749016570347107	0.113	0.092	0.00262331395287931	Myeloid	CTBS
2.35908399955073E-07	0.351306731249942	0.177	0.154	0.0038479019116672	Myeloid	C7orf50
3.19014475603022E-07	0.729920771982182	0.197	0.179	0.00520344511156089	Myeloid	CPEB4
3.53314012327907E-07	0.356987768567986	0.149	0.127	0.0057629048550805	Myeloid	VAPA
3.59912150331608E-07	0.434784839526302	0.145	0.123	0.00587052708405886	Myeloid	TRAPPC5
5.1080615089437E-07	0.362920052006245	0.108	0.086	0.00833175912723807	Myeloid	NMI
5.90303222524119E-07	0.85178607811282	0.126	0.107	0.0096284358625909	Myeloid	TMEM147
7.4400618997242E-07	0.515433204374521	0.244	0.224	0.0121354849646401	Myeloid	ETS2
8.69812015808292E-07	0.267954130972296	0.128	0.169	0.014187503789849	Myeloid	SLC22A23
9.5003594614785E-07	0.361811581520604	0.128	0.106	0.0154960363176176	Myeloid	BAZ2B
9.99045369780901E-07	0.368494410490977	0.122	0.1	0.0162954290264963	Myeloid	PIAS2
1.32066219660449E-06	0.577899511304167	0.072	0.102	0.0215413210888159	Myeloid	PCBD1
1.41317528648991E-06	1.2765411105223	0.133	0.116	0.023050302097937	Myeloid	SLC25A29
2.46083677277981E-06	0.453989271962118	0.111	0.091	0.0401387086008114	Myeloid	WASHC2A
2.46254808987001E-06	0.31862090785313	0.166	0.144	0.0401666218938697	Myeloid	MAVS
2.81255405250968E-06	0.418828100018482	0.1	0.08	0.0458755691504853	Myeloid	PACS2
3.04344715626796E-06	0.293125008822663	0.089	0.12	0.0496416665658867	Myeloid	CDC42BPB
0	7.51754189121164	0.675	0.009	0	B/Plasma cell	MS4A1
0	8.55001537410473	0.638	0.016	0	B/Plasma cell	IGHM
0	8.30289162131934	0.488	0.003	0	B/Plasma cell	CD79A
0	9.02741283205738	0.364	0.002	0	B/Plasma cell	NIBAN3
0	9.91044352372132	0.35	0.002	0	B/Plasma cell	IGHD
0	9.44010476320179	0.348	0.002	0	B/Plasma cell	FCRL1
0	7.15052522925113	0.331	0.012	0	B/Plasma cell	CD22
0	6.75953479040097	0.311	0.005	0	B/Plasma cell	TNFRSF13C
0	9.08077742060994	0.311	0.006	0	B/Plasma cell	DEPRECATI
0	9.28301003494509	0.307	0.005	0	B/Plasma cell	IGKC
0	9.50046524142046	0.302	0.001	0	B/Plasma cell	FCRL2
0	8.95606426615644	0.302	0.001	0	B/Plasma cell	BANK1
0	9.49810736217686	0.29	0.001	0	B/Plasma cell	FCRL5

0	6.11120804188769	0.294	0.009	0	B/Plasma cell	POU2AF1
0	8.75942860343314	0.22	0.004	0	B/Plasma cell	IGHG1
0	5.74610856467059	0.214	0.005	0	B/Plasma cell	TNFRSF13E
0	9.20955616385974	0.198	0.001	0	B/Plasma cell	PAX5
0	7.32286523997235	0.198	0.002	0	B/Plasma cell	BLK
0	9.65716931956088	0.189	0.001	0	B/Plasma cell	FAM30A
0	8.35854893083551	0.189	0.005	0	B/Plasma cell	IGLC2
0	7.7477653445287	0.152	0.002	0	B/Plasma cell	CLEC17A
0	10.4500484289768	0.144	0	0	B/Plasma cell	CD19
0	8.03111281464583	0.121	0.001	0	B/Plasma cell	DERL3
0	9.98426460968465	0.109	0.001	0	B/Plasma cell	COL19A1
6.62031581028337E-286	6.8178094295675	0.121	0.003	1.07983971181532E-281	B/Plasma cell	SPIB
3.25266279013253E-285	5.74529091504253	0.14	0.004	5.30541827698516E-281	B/Plasma cell	CXCR5
3.43742481588811E-280	8.87213701979791	0.101	0.001	5.6067836171951E-276	B/Plasma cell	JCHAIN
9.6490043243755E-265	3.71563400507765	0.603	0.118	1.57384909534889E-260	B/Plasma cell	CD37
6.57480953983955E-254	6.30404686054332	0.115	0.003	1.07241718404323E-249	B/Plasma cell	STAP1
2.58078182343016E-236	5.07455746349733	0.237	0.017	4.20951323219693E-232	B/Plasma cell	ADAM28
1.04885471554613E-234	4.77800247429867	0.224	0.015	1.71078692652729E-230	B/Plasma cell	CD79B
8.48165099718748E-203	5.40462543493766	0.204	0.015	1.38344209415125E-198	B/Plasma cell	BCL11A
1.60317700565936E-200	6.6594862809113	0.109	0.004	2.61494201393097E-196	B/Plasma cell	FCER2
6.43010008519997E-191	8.37667017359505	0.105	0.003	1.04881362489697E-186	B/Plasma cell	IGHA1
1.67262987811105E-169	4.64600601611908	0.185	0.015	2.72822659418693E-165	B/Plasma cell	AIM2
1.5753198506242E-155	4.81973906035642	0.126	0.007	2.56950420835314E-151	B/Plasma cell	MZB1
1.3290887090974E-152	3.42806997382485	0.267	0.035	2.16787659340877E-148	B/Plasma cell	P2RX5
1.44572193429074E-147	3.58872374580362	0.352	0.064	2.35811704702163E-143	B/Plasma cell	WDFY4
6.51250435207018E-101	2.73956765458742	0.529	0.201	1.06225458486617E-96	B/Plasma cell	CIITA
5.74643628004568E-94	2.16871959330603	0.395	0.105	9.37301221638251E-90	B/Plasma cell	ATP2A3
2.93302458323159E-93	2.6987920353659	0.36	0.095	4.78405639770905E-89	B/Plasma cell	RASGRP2
1.5760327486518E-91	3.5723208927694	0.249	0.049	2.57066701632595E-87	B/Plasma cell	SELL
1.69924486333436E-87	4.8822205261909	0.136	0.016	2.77163829658468E-83	B/Plasma cell	RUBCNL
6.29397226106586E-87	3.02432932440907	0.253	0.051	1.02660981550245E-82	B/Plasma cell	PARP15
9.03800510440846E-86	2.93491633125825	0.228	0.042	1.47418901258006E-81	B/Plasma cell	HSH2D
1.52788385473708E-79	0.882139550209365	0.942	0.801	2.49213135546165E-75	B/Plasma cell	CD74
1.61700730831929E-77	1.76696331368781	0.416	0.128	2.6375006205996E-73	B/Plasma cell	LTB
1.0407869848093E-56	3.16644110815756	0.103	0.013	1.69762765092245E-52	B/Plasma cell	FCRL3
1.30068814739573E-56	3.38742559509917	0.165	0.033	2.12155243721718E-52	B/Plasma cell	ORAI2
1.07659292857382E-52	2.35380354624614	0.255	0.074	1.75603072579675E-48	B/Plasma cell	FCMR
1.4975916636485E-52	2.17544733983136	0.245	0.068	2.44272176257707E-48	B/Plasma cell	SP140
5.80843570657297E-50	3.76655052851664	0.183	0.044	9.47413948099117E-46	B/Plasma cell	STRBP
8.16017336952342E-50	3.64397122204538	0.185	0.046	1.33100587830297E-45	B/Plasma cell	ALOX5
2.74828498728199E-48	3.51590581993737	0.189	0.048	4.48272764275565E-44	B/Plasma cell	SYK
3.60786268774375E-46	4.26717986115333	0.107	0.018	5.88478482997884E-42	B/Plasma cell	EAF2
2.34142392498025E-43	2.27035406869947	0.218	0.064	3.81909656403529E-39	B/Plasma cell	ITM2C
1.34050577366342E-42	3.20315056223594	0.117	0.022	2.1864989674224E-38	B/Plasma cell	POU2F2
7.72550811717303E-42	2.50292114218859	0.247	0.082	1.26010762899209E-37	B/Plasma cell	LY9
1.15350253294014E-40	4.15905534159457	0.132	0.029	1.88147798147867E-36	B/Plasma cell	HVCN1
1.5792106730809E-38	2.37223746677397	0.372	0.19	2.57585052886226E-34	B/Plasma cell	IRF8
1.27282842744236E-35	2.71013444387339	0.113	0.024	2.07611044800124E-31	B/Plasma cell	TMEM156
6.7946920711623E-35	2.6949726945961	0.224	0.079	1.10828222372728E-30	B/Plasma cell	ADAM19
4.76402255284984E-34	3.21574012967837	0.189	0.061	7.77059718595337E-30	B/Plasma cell	PLCG2
2.24674549971085E-33	3.41517192635032	0.105	0.022	3.66466658457836E-29	B/Plasma cell	RAB30
3.29815242348632E-32	2.04869007520103	0.276	0.116	5.37961641794853E-28	B/Plasma cell	PRKCB

1.32931306787264E-31	1.52335592360005	0.245	0.09	2.16824254500706E-27	B/Plasma cell	SYTL1
6.26551711267896E-31	2.58092037308116	0.191	0.065	1.02196849624906E-26	B/Plasma cell	TCF4
6.09559629369337E-30	0.824977326282734	0.65	0.448	9.94252711464326E-26	B/Plasma cell	CXCR4
5.50341809051588E-28	2.01497072937542	0.224	0.087	8.97662524744046E-24	B/Plasma cell	PIM2
8.14823472112494E-28	2.11880551746497	0.165	0.053	1.32905856536269E-23	B/Plasma cell	RIPOR2
1.46735061810103E-27	4.43849314880528	0.189	0.071	2.39339559318459E-23	B/Plasma cell	RALGPS2
4.92484401714112E-26	2.69128228562347	0.327	0.186	8.03291307635889E-22	B/Plasma cell	STX7
6.61365693201249E-26	3.02327073333721	0.109	0.028	1.07875358218056E-21	B/Plasma cell	LRMP
2.16364023691977E-24	2.59270690993114	0.179	0.067	3.52911359043983E-20	B/Plasma cell	CHMP7
3.69483652124669E-24	3.86549446509107	0.121	0.036	6.02664784980548E-20	B/Plasma cell	OSBPL10
2.77780108630536E-23	2.64723937437612	0.121	0.036	4.53087135187267E-19	B/Plasma cell	DAPP1
5.57959154031627E-23	2.96934904541656	0.126	0.039	9.10087176140987E-19	B/Plasma cell	MEF2C
1.91469216382913E-21	2.24544548042901	0.21	0.093	3.1230543884217E-17	B/Plasma cell	SP110
3.57239848425374E-21	2.67410064267495	0.206	0.092	5.82693916766628E-17	B/Plasma cell	FCHSD2
1.3564898226413E-20	1.61087698194484	0.294	0.16	2.21257054971023E-16	B/Plasma cell	MPEG1
1.96470880106313E-20	3.18275090420138	0.208	0.098	3.20463652541407E-16	B/Plasma cell	CD40
7.72442798490477E-20	1.67033132569075	0.103	0.03	1.25993144861782E-15	B/Plasma cell	IRF4
1.67373365779367E-19	1.25488748645775	0.366	0.219	2.73002696922726E-15	B/Plasma cell	LIMD2
1.79731359897288E-19	2.80125312846075	0.117	0.039	2.93159821128467E-15	B/Plasma cell	LAT2
2.61447126496229E-19	1.90646258447096	0.257	0.134	4.26446408028E-15	B/Plasma cell	SEL1L3
6.45355717743422E-19	2.40141763023959	0.167	0.069	1.0526397112113E-14	B/Plasma cell	DGKD
1.55350849429952E-18	1.73409981820983	0.284	0.16	2.53392770505195E-14	B/Plasma cell	SP100
1.03649161229241E-17	0.85808431958408	0.459	0.296	1.69062146881015E-13	B/Plasma cell	CORO1A
1.5032773361102E-17	2.37667645150516	0.119	0.042	2.45199566292934E-13	B/Plasma cell	TMEM243
1.77618576439956E-17	2.59828405832121	0.2	0.097	2.89713660031212E-13	B/Plasma cell	SETBP1
3.41784394291963E-17	2.85410754087192	0.185	0.087	5.5748452552962E-13	B/Plasma cell	RABEP2
8.61806883957646E-17	1.67053031046089	0.257	0.141	1.40569320842332E-12	B/Plasma cell	CD83
1.23216821638119E-16	1.22914993073832	0.294	0.165	2.00978957773935E-12	B/Plasma cell	CCDC88B
1.42656168015302E-16	2.11789006784008	0.331	0.225	2.32686475649759E-12	B/Plasma cell	SYNGR2
1.93095713384138E-16	2.62698959691835	0.195	0.098	3.14958418100867E-12	B/Plasma cell	LYN
5.78886518259863E-16	2.0706934103924	0.212	0.109	9.44221799933662E-12	B/Plasma cell	PRDM2
9.29015578325E-16	2.65333951526796	0.136	0.055	1.51531730980591E-11	B/Plasma cell	SESN3
1.54076540287539E-15	2.00515769067579	0.296	0.189	2.51314244863006E-11	B/Plasma cell	ELOVL5
2.88264463594246E-15	1.25413462015027	0.405	0.291	4.70188166568575E-11	B/Plasma cell	LAPTM5
3.77334456456753E-15	1.4075137841518	0.263	0.15	6.1547023192661E-11	B/Plasma cell	IL16
3.83778692336595E-15	2.42962092600924	0.101	0.035	6.2598142507022E-11	B/Plasma cell	HDAC9
7.72231301285342E-15	1.18236780392881	0.126	0.047	1.25958647552652E-10	B/Plasma cell	ZBP1
1.25302279820319E-14	2.82515787427285	0.195	0.104	2.04380548614922E-10	B/Plasma cell	SWAP70
1.27081195618271E-14	1.22498009573921	0.294	0.173	2.07282138172962E-10	B/Plasma cell	ARHGAP15
3.67005330191498E-14	1.9330577462961	0.134	0.056	5.98622394075352E-10	B/Plasma cell	CD52
4.98575279428033E-14	1.51283731761663	0.436	0.371	8.13226138275065E-10	B/Plasma cell	MDM4
5.00459499015755E-14	0.910092558367268	0.385	0.254	8.16299488844599E-10	B/Plasma cell	TMC8
1.02865720770521E-13	1.37142301614654	0.148	0.065	1.67784277148797E-09	B/Plasma cell	CARMIL2
1.71453747922711E-13	1.5458705307962	0.132	0.055	2.79658208236734E-09	B/Plasma cell	MCTP2
1.75980332966689E-13	1.53177617273271	0.317	0.212	2.87041521101967E-09	B/Plasma cell	EZR
2.04585108115547E-13	2.84198110853933	0.146	0.068	3.33698769847269E-09	B/Plasma cell	CDK14
2.08918019714998E-13	1.59310039241074	0.239	0.137	3.40766181957134E-09	B/Plasma cell	PTK2B
2.32366044680968E-13	1.67033048932401	0.187	0.095	3.79012255479127E-09	B/Plasma cell	PDE4B
3.78687149174065E-13	1.30150141278768	0.251	0.145	6.17676609017817E-09	B/Plasma cell	STK17A
4.21402080107893E-13	2.03793428281573	0.144	0.065	6.87348932863985E-09	B/Plasma cell	BICD1
4.60800310915118E-13	1.92243781581207	0.193	0.102	7.51611387133649E-09	B/Plasma cell	NCOA3
5.78611018473002E-12	2.6539372267398	0.156	0.08	9.43772432231313E-08	B/Plasma cell	CYB561A3

7.34020698333515E-12	1.1708749195772	0.247	0.144	1.1972611610518E-07	B/Plasma cell	RAC2
7.67787442946127E-12	0.788957849978436	0.319	0.197	1.25233809818943E-07	B/Plasma cell	ACAP1
9.27873728884886E-12	1.5491552719504	0.23	0.14	1.51345483918414E-07	B/Plasma cell	CYBB
2.23679364626537E-11	1.22389167356932	0.29	0.191	3.64843411642344E-07	B/Plasma cell	CD53
2.60224092520306E-11	1.79589778030305	0.206	0.121	4.24451517309871E-07	B/Plasma cell	RELT
3.93241596321738E-11	1.79448626213131	0.302	0.22	6.41416367760387E-07	B/Plasma cell	GGA2
5.48417337299088E-11	1.79570463297343	0.105	0.044	8.94523518868543E-07	B/Plasma cell	CCDC141
1.41919528333323E-10	1.60509547372927	0.193	0.11	2.31484942664483E-06	B/Plasma cell	RCSD1
1.80113662362543E-10	1.09089218594971	0.241	0.146	2.93783394679544E-06	B/Plasma cell	SASH3
3.37956608080198E-10	0.747434427903183	0.605	0.594	5.51241023439611E-06	B/Plasma cell	LENG8
3.52324450269966E-10	0.283389636840095	0.047	0.158	5.74676410835342E-06	B/Plasma cell	AKAP1
6.08202451895904E-10	1.91429683065397	0.179	0.105	9.92039019287409E-06	B/Plasma cell	FGD2
7.91623674041557E-10	0.333941478415367	0.095	0.232	1.29121737472918E-05	B/Plasma cell	EPN1
9.4153953651717E-10	3.73418020083041	0.126	0.064	1.53574513801316E-05	B/Plasma cell	AFF3
1.82531057095571E-09	2.61501490924458	0.144	0.078	2.97726407228586E-05	B/Plasma cell	CHD7
1.952851791381E-09	2.74346769565928	0.132	0.069	3.18529655692154E-05	B/Plasma cell	PLEKHF2
2.2792542654075E-09	0.256864262678261	0.126	0.271	3.71769163230616E-05	B/Plasma cell	STAT2
3.4587816777594E-09	2.64391260071942	0.101	0.047	5.64161879459335E-05	B/Plasma cell	DOK3
4.01646469863631E-09	2.66955432231105	0.121	0.061	6.55125556994568E-05	B/Plasma cell	PXK
6.41750768200962E-09	0.432229644482344	0.117	0.255	0.000104675967801259	B/Plasma cell	XBP1
6.50819751437035E-09	0.382312589864651	0.091	0.217	0.000106155209656895	B/Plasma cell	NADK
6.65838666771307E-09	1.14999106521231	0.111	0.052	0.000108604944937068	B/Plasma cell	CARD11
7.9610045522723E-09	1.39196739822997	0.226	0.148	0.000129851945252114	B/Plasma cell	CNTRL
9.14487710173983E-09	0.29136810264929	0.101	0.23	0.000149162090406478	B/Plasma cell	TUFM
1.05532030928243E-08	2.82554622720185	0.148	0.085	0.000172133295647056	B/Plasma cell	SIPA1L3
1.17786694854598E-08	2.43210855201534	0.128	0.068	0.000192121877977334	B/Plasma cell	MGAT5
1.94066743778992E-08	0.396777216881784	0.078	0.192	0.000316542265777914	B/Plasma cell	NCSTN
2.12666507019093E-08	1.66803934485286	0.187	0.117	0.000346880339598842	B/Plasma cell	DENND3
2.1461572420137E-08	0.29726598918801	0.066	0.171	0.000350059707744855	B/Plasma cell	POLR2E
2.38099572496997E-08	0.314015020693002	0.037	0.126	0.000388364212699851	B/Plasma cell	NUDT14
2.48175952007229E-08	0.321754790887412	0.091	0.21	0.000404799795318991	B/Plasma cell	INTS1
2.57917209910183E-08	1.50562476245905	0.204	0.131	0.0004206887610845	B/Plasma cell	TRAF5
3.42898688502585E-08	0.255379210711131	0.091	0.207	0.000559302050816566	B/Plasma cell	MPV17
4.07593661406372E-08	0.951794380597239	0.284	0.201	0.000664826021119933	B/Plasma cell	DOCK2
4.60948356478012E-08	0.356868157643487	0.074	0.182	0.000751852864251286	B/Plasma cell	PI4KB
5.07408696626827E-08	0.26280374214003	0.113	0.238	0.000827634325068018	B/Plasma cell	STXBP2
5.10333358152876E-08	0.330462424865993	0.062	0.162	0.000832404740483156	B/Plasma cell	KCTD20
5.1921726920105E-08	1.84558750935432	0.167	0.1	0.000846895287793832	B/Plasma cell	BIRC3
5.21135572775052E-08	2.75393252738765	0.128	0.07	0.000850024232753388	B/Plasma cell	TBC1D9
6.71590205435889E-08	0.337606012427407	0.08	0.189	0.00109543078408648	B/Plasma cell	RAB1A
8.43845215496962E-08	0.340135572062444	0.074	0.179	0.00137639593099709	B/Plasma cell	EFR3A
8.58740989923957E-08	0.304262885532776	0.076	0.181	0.00140069242866497	B/Plasma cell	USP12
1.02020219378182E-07	0.360748727404433	0.041	0.126	0.00166405179827753	B/Plasma cell	ZBED1
1.13836998371704E-07	1.12852326549653	0.403	0.359	0.00185679528044086	B/Plasma cell	OGA
1.15590122316103E-07	3.2407256885731	0.136	0.079	0.00188539048509796	B/Plasma cell	BLNK
1.50868314170508E-07	0.299229659842102	0.045	0.131	0.00246081307243516	B/Plasma cell	EI24
1.52954104748484E-07	0.310088511929498	0.037	0.118	0.00249483440255253	B/Plasma cell	FAM234A
1.54825391803579E-07	0.393898092422161	0.07	0.17	0.00252535696570818	B/Plasma cell	KPNA4
1.90770261294298E-07	1.37470009170424	0.344	0.292	0.0031116537319713	B/Plasma cell	RBM6
2.06197733465086E-07	0.361662080785028	0.099	0.211	0.00336329123054901	B/Plasma cell	HEXA
2.24839927757509E-07	1.12890451348985	0.235	0.16	0.00366736406165273	B/Plasma cell	BCL2
2.35320649770589E-07	0.530013799550619	0.031	0.107	0.00383831511840808	B/Plasma cell	ACSF2

2.37408441484901E-07	0.407170563114354	0.101	0.216	0.00387236908906022	B/Plasma cell	ARRDC2
2.44320900184741E-07	0.591038511093022	0.438	0.354	0.00398511820291331	B/Plasma cell	CYBA
2.4464320289769E-07	0.350783035523821	0.043	0.126	0.00399037528246423	B/Plasma cell	ZBED6
2.54310728849233E-07	0.295772585066888	0.105	0.217	0.00414806229825985	B/Plasma cell	SEC22B
3.1874225781269E-07	0.341248108830336	0.043	0.125	0.00519900496718278	B/Plasma cell	PANK3
3.2202409217952E-07	2.24088827735255	0.107	0.056	0.00525253496754015	B/Plasma cell	LRRK1
3.25862572960058E-07	0.870382143641477	0.239	0.159	0.00531514442755151	B/Plasma cell	TBC1D10C
3.29043875862348E-07	0.250348439946092	0.072	0.168	0.00536703465919076	B/Plasma cell	LARP4
4.04452909175493E-07	1.56453278218889	0.142	0.083	0.00659703140156147	B/Plasma cell	ARHGAP25
4.10817515432661E-07	0.326052176784741	0.128	0.25	0.00670084449422214	B/Plasma cell	POGZ
4.64079123396469E-07	0.390891409281526	0.136	0.265	0.00756959458171981	B/Plasma cell	NPLOC4
4.78199158125363E-07	0.497694653866857	0.084	0.186	0.0077999064681828	B/Plasma cell	PDHA1
4.88711173339507E-07	0.30890696710514	0.101	0.21	0.00797136794834069	B/Plasma cell	SCAF1
5.62419973676681E-07	0.532808224347248	0.035	0.11	0.00917363219064035	B/Plasma cell	CHPT1
5.91370582578168E-07	0.432995954072773	0.064	0.154	0.00964584557243249	B/Plasma cell	WDR81
6.11805694867978E-07	0.286326474934568	0.064	0.153	0.00997916268899159	B/Plasma cell	TLE5
6.22258569375393E-07	0.310429838973417	0.039	0.115	0.010149659525082	B/Plasma cell	EIF2D
6.37454139043105E-07	0.344574798931328	0.126	0.245	0.0103975144619321	B/Plasma cell	EIF4H
7.2562108458285E-07	0.295689057208741	0.041	0.118	0.0118356055106309	B/Plasma cell	PDE8A
8.05516845574289E-07	0.322532406761688	0.107	0.218	0.0131387852681622	B/Plasma cell	PARP10
8.0591289000297E-07	0.827991977899011	0.545	0.554	0.0131452451488384	B/Plasma cell	MT-ND6
8.47407635953403E-07	0.373154716500684	0.123	0.24	0.013822065950036	B/Plasma cell	UBAP2L
8.88808358261267E-07	0.321854528256765	0.113	0.224	0.0144973531315995	B/Plasma cell	PLEC
8.98109432508262E-07	0.51744095123449	0.056	0.139	0.0146490629536423	B/Plasma cell	HECTD1
9.257301940793E-07	0.252889761619099	0.056	0.139	0.0150995851956275	B/Plasma cell	COP 1
9.54104694761725E-07	0.367801744873658	0.049	0.13	0.0155624016762585	B/Plasma cell	MFN2
9.6449154044034E-07	2.4069641758871	0.173	0.116	0.0157318215161224	B/Plasma cell	PIK3C2B
9.6865643837402E-07	0.363492331374053	0.076	0.169	0.0157997551663186	B/Plasma cell	NCLN
9.94899033351922E-07	0.343987989399153	0.051	0.132	0.0162277981330032	B/Plasma cell	RTCB
1.06219930037622E-06	0.358100572735432	0.031	0.1	0.0173255327884364	B/Plasma cell	TOMM70
1.08201414571603E-06	0.290980907544226	0.049	0.129	0.0176487327307742	B/Plasma cell	TRAPPC5
1.101765243828E-06	1.25815227606908	0.193	0.129	0.0179708928920785	B/Plasma cell	SLC44A2
1.20379289082022E-06	0.28107777505249	0.144	0.269	0.0196350658421686	B/Plasma cell	LUC7L2
1.2289666405911E-06	0.951947650108467	0.115	0.062	0.0200456748746815	B/Plasma cell	RHOH
1.25030922027695E-06	0.48180203087253	0.06	0.144	0.0203937936919374	B/Plasma cell	ARNT
1.25688762043439E-06	0.372463415052384	0.064	0.15	0.0205010939769053	B/Plasma cell	AMDHD2
1.33124143493478E-06	0.468124917639151	0.049	0.128	0.0217138790452212	B/Plasma cell	GTF3C3
1.40217876079553E-06	0.274033578425712	0.056	0.137	0.0228709377673358	B/Plasma cell	NCBP2
1.4485002858921E-06	1.97152411276814	0.121	0.07	0.023626488163186	B/Plasma cell	CD55
1.53378010215928E-06	0.267490013964346	0.119	0.231	0.0250174872463201	B/Plasma cell	TRPC4AP
1.65596942464385E-06	0.607632464632411	0.091	0.19	0.0270105172853658	B/Plasma cell	TSC22D2
1.65901117487578E-06	0.304867611180845	0.07	0.157	0.0270601312733989	B/Plasma cell	KIF5B
1.72335993825317E-06	2.43829746633656	0.103	0.056	0.0281097239528475	B/Plasma cell	FARP2
1.73839720583328E-06	0.296475533939818	0.053	0.133	0.0283549968243467	B/Plasma cell	CUL4A
1.78165276532029E-06	0.269219813230247	0.039	0.111	0.0290605382551393	B/Plasma cell	GLB1
1.97116105233442E-06	0.484088964750964	0.074	0.164	0.0321516079246267	B/Plasma cell	RAB14
2.10213585046102E-06	0.418841657619559	0.047	0.123	0.0342879378568697	B/Plasma cell	COX7C
2.10499354647296E-06	0.276315172703298	0.062	0.144	0.0343345497365204	B/Plasma cell	DAZAP2
2.13785046003718E-06	0.377095448813786	0.039	0.11	0.0348704788536665	B/Plasma cell	KLHL20
2.15406518715369E-06	0.279065630858527	0.084	0.178	0.0351349572676639	B/Plasma cell	COLGALT1
2.16436724904774E-06	0.505396699040987	0.058	0.139	0.0353029941992177	B/Plasma cell	IARS2
2.17059193416821E-06	0.364052483181111	0.047	0.123	0.0354045250382176	B/Plasma cell	HDAC10

**2.21952438803001E-06**	0.882119271358167	0.241	0.169	0.0362026622931574	B/Plasma cell	STK17B
**2.31146593939203E-06**	0.458189971421074	0.043	0.117	0.0377023209374234	B/Plasma cell	PPFIBP2
**2.35541601758331E-06**	2.15710520588056	0.146	0.093	0.0384191906628014	B/Plasma cell	DRAM2
**2.35842778782071E-06**	0.259499360921022	0.039	0.109	0.0384683156471436	B/Plasma cell	CORO1C
**2.36567631410773E-06**	0.294773353214209	0.105	0.208	0.0385865463594111	B/Plasma cell	HGS
**2.37564868313966E-06**	0.471246762061345	0.045	0.119	0.038749205670691	B/Plasma cell	PUS1
**2.55689250810534E-06**	0.600932490495147	0.035	0.104	0.0417054736997062	B/Plasma cell	WDR46
**2.57423607033052E-06**	0.392330810132921	0.08	0.172	0.0419883645431612	B/Plasma cell	TXNRD2
**2.58464720053441E-06**	0.305128514825437	0.043	0.116	0.0421581804879167	B/Plasma cell	TIMMDC1
**2.5953822911041E-06**	0.30018848598131	0.091	0.185	0.042333280550199	B/Plasma cell	SERINC3
**2.6657047304238E-06**	0.330920748222894	0.165	0.295	0.0434803098579426	B/Plasma cell	H3F3A
**2.78283960501652E-06**	0.33859034375418	0.041	0.112	0.0453908967974245	B/Plasma cell	PHKA2
**2.84895455482602E-06**	0.255694817517365	0.183	0.317	0.0464692977437672	B/Plasma cell	EIF3A
**2.9451047604519E-06**	0.416372026611369	0.051	0.128	0.048037603747731	B/Plasma cell	ELAC2
**2.95627234673653E-06**	0.276902001241164	0.062	0.143	0.0482197582476195	B/Plasma cell	EXOSC6
**2.97884264281735E-06**	0.54242834545979	0.037	0.106	0.0485879023469938	B/Plasma cell	RANBP10
**2.99472298264905E-06**	0.261217039364295	0.074	0.161	0.0488469265699887	B/Plasma cell	GPBP1L1
**0**	7.19800369189142	0.929	0.092	0	Endothelial	EGFL7
**0**	4.67221399139903	0.867	0.053	0	Endothelial	NPDC1
**0**	3.80626013715626	0.844	0.045	0	Endothelial	COL4A2
**0**	4.19370500733859	0.831	0.054	0	Endothelial	COL4A1
**0**	5.27887306714403	0.851	0.081	0	Endothelial	STAB1
**0**	3.90989456889712	0.831	0.062	0	Endothelial	SPARC
**0**	7.27487477469971	0.781	0.019	0	Endothelial	DNASE1L3
**0**	5.91394848095899	0.784	0.032	0	Endothelial	CDH5
**0**	4.8637243528921	0.832	0.083	0	Endothelial	ENG
**0**	6.40329038415898	0.775	0.033	0	Endothelial	HSPG2
**0**	8.303523285716	0.695	0.006	0	Endothelial	FLT1
**0**	5.00270330647736	0.884	0.196	0	Endothelial	PLPP3
**0**	7.42645364444587	0.698	0.02	0	Endothelial	FCN3
**0**	2.37838842667036	0.713	0.056	0	Endothelial	IGFBP7
**0**	9.06854693055544	0.654	0.004	0	Endothelial	EFNB2
**0**	6.2717658639674	0.654	0.009	0	Endothelial	FLT4
**0**	5.50231085986802	0.64	0.041	0	Endothelial	KALRN
**0**	4.35763887689884	0.654	0.056	0	Endothelial	CD93
**0**	5.17054388658915	0.686	0.09	0	Endothelial	RAPGEF5
**0**	6.78524992238964	0.585	0.007	0	Endothelial	LDB2
**0**	9.10245597737304	0.575	0.003	0	Endothelial	PTPRB
**0**	7.16184692563454	0.581	0.01	0	Endothelial	AFAP1L1
**0**	8.85640352372396	0.572	0.003	0	Endothelial	PCDH17
**0**	7.85536466302178	0.567	0.005	0	Endothelial	MMRN2
**0**	5.87074265383864	0.581	0.023	0	Endothelial	SMAD6
**0**	5.23134204175607	0.577	0.022	0	Endothelial	TCIM
**0**	4.0265937019528	0.704	0.149	0	Endothelial	APP
**0**	5.4726280169739	0.598	0.068	0	Endothelial	HYAL2
**0**	7.61976725915754	0.534	0.004	0	Endothelial	RAMP3
**0**	7.37785551490906	0.52	0.007	0	Endothelial	PDE2A
**0**	7.97792591995352	0.516	0.004	0	Endothelial	ROBO4
**0**	8.36018607186848	0.514	0.006	0	Endothelial	OIT3
**0**	7.21058717138763	0.52	0.015	0	Endothelial	SEMA3F
**0**	7.32654104881496	0.51	0.009	0	Endothelial	CRHBP
**0**	8.05434106999171	0.502	0.008	0	Endothelial	PLVAP

0	8.6512297702329	0.494	0.004	0	Endothelial	VWF
0	7.50631786935024	0.504	0.014	0	Endothelial	SHANK3
0	5.83463131325892	0.5	0.011	0	Endothelial	ADAMTS4
0	8.51788559920429	0.494	0.005	0	Endothelial	FCN2
0	2.69899699610161	0.64	0.162	0	Endothelial	PLXND1
0	2.40398112026354	0.756	0.278	0	Endothelial	IGFBP4
0	4.56695815597299	0.573	0.096	0	Endothelial	ID1
0	4.62364707407527	0.507	0.032	0	Endothelial	FXYD6
0	4.21186331872725	0.524	0.053	0	Endothelial	NRP2
0	2.34747075105895	0.622	0.152	0	Endothelial	DEPRECATI
0	6.02066003420531	0.506	0.038	0	Endothelial	FZD4
0	2.20951591398434	0.859	0.391	0	Endothelial	DUSP1
0	3.59470090878982	0.522	0.064	0	Endothelial	GRASP
0	7.50098940335234	0.463	0.005	0	Endothelial	EXOC3L2
0	6.99007937962436	0.463	0.006	0	Endothelial	ESAM
0	5.68609191874467	0.495	0.039	0	Endothelial	PPFIBP1
0	3.36677290104205	0.564	0.111	0	Endothelial	NRP1
0	3.53678536134903	0.54	0.088	0	Endothelial	NR4A1
0	5.36264359850204	0.462	0.01	0	Endothelial	MCAM
0	3.52198603420989	0.537	0.085	0	Endothelial	SCARF1
0	2.27469833764477	0.593	0.141	0	Endothelial	SLC2A3
0	4.05148494680693	0.492	0.041	0	Endothelial	TCF4
0	8.20261510754433	0.455	0.004	0	Endothelial	RASIP1
0	3.5118487279348	0.532	0.084	0	Endothelial	DYSF
0	3.65179306945516	0.541	0.095	0	Endothelial	NID1
0	4.9660255576683	0.51	0.074	0	Endothelial	LIMS2
0	2.61954765010233	0.686	0.252	0	Endothelial	BHLHE40
0	6.69100066701582	0.442	0.008	0	Endothelial	TMEM255E
0	2.82398513255068	0.619	0.185	0	Endothelial	MYO15B
0	9.39797504129931	0.436	0.003	0	Endothelial	STAB2
0	3.50610845024956	0.5	0.07	0	Endothelial	TIMP3
0	5.06559903347756	0.452	0.023	0	Endothelial	TINAGL1
0	8.49838809818728	0.431	0.002	0	Endothelial	NOSTRIN
0	3.34050427243536	0.467	0.05	0	Endothelial	ITPRIP
0	2.58216185974831	0.505	0.092	0	Endothelial	DUSP5
0	4.21060230040869	0.468	0.055	0	Endothelial	SMTN
0	6.92300220036324	0.417	0.006	0	Endothelial	HECW2
0	3.24084673949468	0.48	0.07	0	Endothelial	ITGA9
0	6.32408754881065	0.431	0.021	0	Endothelial	CCL14
0	3.68175723253506	0.437	0.032	0	Endothelial	LRRC32
0	2.46126908017801	0.589	0.184	0	Endothelial	SOCS3
0	5.88266918057601	0.414	0.011	0	Endothelial	RNASE1
0	5.59827888203182	0.449	0.047	0	Endothelial	ARHGAP29
0	2.19841644002258	0.52	0.121	0	Endothelial	NOTCH1
0	4.67333725745954	0.474	0.077	0	Endothelial	PIEZO2
0	8.59751482628861	0.398	0.002	0	Endothelial	KDR
0	3.16353442595151	0.47	0.075	0	Endothelial	PECAM1
0	4.77004873905073	0.451	0.057	0	Endothelial	SHE
0	3.21756277544164	0.694	0.301	0	Endothelial	IVNS1ABP
0	6.72046548108535	0.397	0.005	0	Endothelial	KANK3
0	6.49694809934007	0.413	0.022	0	Endothelial	PLK2
0	7.27868084640609	0.391	0.004	0	Endothelial	CLDN5

0	3.084826068856	0.57	0.184		0	Endothelial	EPAS1
0	2.75752830524313	0.458	0.074		0	Endothelial	ABCG1
0	2.2677446973095	0.544	0.162		0	Endothelial	STOM
0	4.79487352730396	0.463	0.083		0	Endothelial	MCF2L
0	2.96902378766785	0.466	0.087		0	Endothelial	SEC14L1
0	4.72616753956207	0.393	0.018		0	Endothelial	DCHS1
0	4.59163545672536	0.397	0.023		0	Endothelial	TP53I11
0	6.06687732624267	0.4	0.027		0	Endothelial	ADM
0	5.86443760066236	0.381	0.009		0	Endothelial	ARHGAP23
0	2.3674791847008	0.499	0.129		0	Endothelial	PXN
0	7.87488353394872	0.373	0.005		0	Endothelial	CLEC4G
0	6.36182471307301	0.378	0.011		0	Endothelial	AKAP12
0	9.61951392079353	0.367	0.002		0	Endothelial	CLEC4M
0	5.583244737832	0.377	0.015		0	Endothelial	EMP1
0	5.14630028740239	0.4	0.039		0	Endothelial	DOCK6
0	7.98140476975005	0.363	0.003		0	Endothelial	PCDH12
0	3.50716266384462	0.426	0.067		0	Endothelial	SERPINH1
0	3.66931476075005	0.434	0.076		0	Endothelial	PRCP
0	7.54352354782696	0.359	0.002		0	Endothelial	ADGRL4
0	5.153281885077	0.416	0.061		0	Endothelial	GRB10
0	4.29448655705649	0.434	0.083		0	Endothelial	EPHB4
0	3.56109451461036	0.472	0.122		0	Endothelial	TGM2
0	7.84043317304123	0.35	0.002		0	Endothelial	TIE1
0	3.56723267706978	0.396	0.051		0	Endothelial	ID3
0	3.68376085671109	0.441	0.1		0	Endothelial	CNN3
0	3.57870210667266	0.409	0.071		0	Endothelial	NAV1
0	3.44956654040174	0.39	0.056		0	Endothelial	MARCKSL1
0	7.31535198117163	0.336	0.003		0	Endothelial	CLEC14A
0	5.42749810664024	0.357	0.027		0	Endothelial	SPRY4
0	7.34798092995119	0.329	0.003		0	Endothelial	NPR 1.00
0	4.56530728153202	0.357	0.032		0	Endothelial	TMEM44
0	1.04740403281539	0.37	0.048		0	Endothelial	COL6A2
0	6.37409463396232	0.325	0.004		0	Endothelial	ADGRF5
0	3.81350500642227	0.413	0.096		0	Endothelial	PLPP1
0	2.88143862289243	0.363	0.049		0	Endothelial	IFI44L
0	4.5718854466838	0.342	0.028		0	Endothelial	DLL1
0	8.7346640319069	0.314	0.001		0	Endothelial	NOVA2
0	4.98927948640287	0.324	0.014		0	Endothelial	CD36
0	8.53759341379706	0.309	0.001		0	Endothelial	ARHGEF15
0	5.34547119858874	0.337	0.03		0	Endothelial	SPRY1
0	6.52827047639902	0.311	0.004		0	Endothelial	LIMCH1
0	5.70994741676957	0.317	0.01		0	Endothelial	GNG11
0	7.34729553214517	0.317	0.011		0	Endothelial	STC2
0	6.63778379293753	0.311	0.007		0	Endothelial	ITGB3
0	5.90513840283956	0.311	0.008		0	Endothelial	APLNR
0	3.92917948313353	0.324	0.023		0	Endothelial	FSCN1
0	8.05577047058289	0.303	0.002		0	Endothelial	TEK
0	4.37799161907145	0.329	0.029		0	Endothelial	RASGRP3
0	3.18402147054639	0.362	0.064		0	Endothelial	CD9
0	3.7016921375215	0.332	0.038		0	Endothelial	PHLDB1
0	4.73696821366625	0.365	0.071		0	Endothelial	GABRE
0	4.92970807546716	0.305	0.014		0	Endothelial	PALD1

0	8.12426238989049	0.292	0.003	0	Endothelial	DLL4
0	3.06228440674012	0.349	0.061	0	Endothelial	UPP1
0	5.25534773558046	0.325	0.039	0	Endothelial	TM4SF1
0	5.28418522977808	0.312	0.031	0	Endothelial	WWTR1
0	9.01050330314084	0.279	0.001	0	Endothelial	DEPRECATI
0	4.26764280462591	0.321	0.046	0	Endothelial	PDGFB
0	2.85189764467609	0.308	0.035	0	Endothelial	NEDD9
0	4.61520244959288	0.295	0.022	0	Endothelial	ADAMTS9
0	5.69484032019954	0.283	0.01	0	Endothelial	LAMA4
0	7.61978502303256	0.273	0.001	0	Endothelial	ECSCR
0	4.17029634743963	0.288	0.016	0	Endothelial	PXDN
0	4.72253739526713	0.311	0.04	0	Endothelial	MID1
0	6.15371844877324	0.275	0.006	0	Endothelial	CAVIN2
0	3.51932239425624	0.305	0.037	0	Endothelial	ZBTB46
0	4.93690191231988	0.273	0.006	0	Endothelial	ST6GALNA
0	3.35287534360433	0.304	0.037	0	Endothelial	GDPD5
0	6.1680141611557	0.275	0.008	0	Endothelial	APOLD1
0	7.74552518897372	0.268	0.001	0	Endothelial	SOX18
0	5.8680926234907	0.267	0.005	0	Endothelial	ARAP3
0	6.67597460673075	0.265	0.004	0	Endothelial	LYVE1
0	3.46567884477206	0.306	0.045	0	Endothelial	RGCC
0	6.32407151400087	0.273	0.014	0	Endothelial	SEMA6A
0	5.35247527892338	0.265	0.006	0	Endothelial	ADCY4
0	5.20172138245199	0.29	0.032	0	Endothelial	PLXNA2
0	5.99528069180278	0.261	0.006	0	Endothelial	CALCRL
0	8.72447345947902	0.256	0.002	0	Endothelial	STC1
0	4.78258227050724	0.265	0.012	0	Endothelial	PLEKHG1
0	2.91836859015917	0.279	0.03	0	Endothelial	S1PR1
0	5.04291493931439	0.268	0.019	0	Endothelial	EPB41L4A
0	3.67020012364997	0.265	0.016	0	Endothelial	TMEM204
0	3.42355533726626	0.283	0.035	0	Endothelial	LMO2
0	3.3418921234915	0.289	0.041	0	Endothelial	PEAK1
0	5.20573325972477	0.268	0.022	0	Endothelial	KCNE3
0	2.31569883270635	0.275	0.033	0	Endothelial	SH2D3C
0	3.75174277881825	0.274	0.035	0	Endothelial	CAVIN1
0	3.36798250795445	0.265	0.029	0	Endothelial	LAMB1
0	6.57399161208643	0.24	0.005	0	Endothelial	CDH13
0	8.8965914754816	0.235	0.001	0	Endothelial	EXOC3L1
0	8.48969878957285	0.234	0.001	0	Endothelial	TAL1
0	3.68551171400516	0.255	0.022	0	Endothelial	FGFR1
0	3.33209415041981	0.258	0.027	0	Endothelial	MEF2C
0	3.13630385090528	0.265	0.035	0	Endothelial	HIC1
0	5.72736325697225	0.251	0.024	0	Endothelial	PODXL
0	6.43339699232687	0.234	0.007	0	Endothelial	SPNS2
0	8.09800770045446	0.226	0.001	0	Endothelial	PREX2
0	5.80396092502994	0.228	0.005	0	Endothelial	FAM167B
0	3.76441361422366	0.256	0.033	0	Endothelial	PIK3R3
0	3.57280764976297	0.252	0.03	0	Endothelial	FAM43A
0	6.78435088465935	0.225	0.003	0	Endothelial	SEMA6B
0	4.78084408822857	0.244	0.025	0	Endothelial	MAP3K6
0	8.48169158449411	0.218	0.001	0	Endothelial	MYCT1
0	6.24285538496259	0.222	0.005	0	Endothelial	RAMP2

0	7.0343597444511	0.219	0.003	0	Endothelial	TSPAN7
0	4.30416592814312	0.23	0.015	0	Endothelial	RBMS2
0	5.02045471785643	0.242	0.027	0	Endothelial	JCAD
0	2.57291854116919	0.244	0.029	0	Endothelial	ZNF160
0	5.13268750739343	0.216	0.003	0	Endothelial	TSPAN18
0	4.75407087093973	0.221	0.011	0	Endothelial	IL3RA
0	6.71394340831946	0.219	0.011	0	Endothelial	LRATD1
0	4.21950838060951	0.222	0.014	0	Endothelial	EHD3
0	9.78133405249719	0.208	0	0	Endothelial	SLC5A4
0	3.77557487768589	0.221	0.017	0	Endothelial	KCNC4
0	3.76123429978828	0.22	0.017	0	Endothelial	PLEKHG5
0	7.00989752686699	0.205	0.002	0	Endothelial	RHOJ
0	5.15741353905675	0.216	0.016	0	Endothelial	IGFBP5
0	6.81999975710898	0.203	0.003	0	Endothelial	IL33
0	5.72780082551201	0.219	0.019	0	Endothelial	SOCS2
0	7.16008682757678	0.202	0.002	0	Endothelial	ERG
0	6.18989882482272	0.21	0.012	0	Endothelial	FUT1
0	3.1251373056343	0.218	0.021	0	Endothelial	ADAMTS1
0	7.89414288594572	0.197	0.003	0	Endothelial	SLCO2A1
0	3.66467655954717	0.216	0.023	0	Endothelial	CDC42EP2
0	4.33984487651812	0.205	0.012	0	Endothelial	MYO10
0	4.00563053757886	0.207	0.016	0	Endothelial	CETP
0	8.1077650398994	0.19	0.001	0	Endothelial	CD34
0	6.88295097526976	0.192	0.003	0	Endothelial	FGD5
0	8.58043974311976	0.188	0.001	0	Endothelial	NOS3
0	5.26794318072439	0.193	0.007	0	Endothelial	GNA14
0	4.18292451980802	0.202	0.019	0	Endothelial	RELN
0	7.47564143256772	0.182	0.001	0	Endothelial	EMCN
0	3.50833733836691	0.195	0.016	0	Endothelial	OSMR
0	5.18758110824258	0.184	0.01	0	Endothelial	STXBP1
0	4.10970030284788	0.183	0.011	0	Endothelial	NES
0	4.12591330179673	0.186	0.015	0	Endothelial	CRIP2
0	6.74209922970972	0.171	0.002	0	Endothelial	BCL6B
0	6.87796675709737	0.175	0.006	0	Endothelial	MRO
0	5.27568276480215	0.173	0.006	0	Endothelial	BMPER
0	8.21661693134183	0.166	0.001	0	Endothelial	DIPK2B
0	6.37907339078859	0.167	0.003	0	Endothelial	COL15A1
0	6.05892377603737	0.165	0.002	0	Endothelial	GPR4
0	5.93827927111488	0.164	0.002	0	Endothelial	OLFML2A
0	7.15749805409126	0.162	0.003	0	Endothelial	GJA1
0	7.72361152891659	0.16	0.001	0	Endothelial	CCM2L
0	5.56756380401673	0.162	0.004	0	Endothelial	CA13
0	7.45493379710178	0.158	0.001	0	Endothelial	TM4SF18
0	8.98504551554336	0.155	0	0	Endothelial	DEPRECATI
0	7.2782603591544	0.156	0.002	0	Endothelial	ZNF521
0	6.76793767916896	0.156	0.002	0	Endothelial	CYYR1
0	6.00526986115243	0.156	0.005	0	Endothelial	RAPGEF3
0	5.63831872459295	0.152	0.002	0	Endothelial	KIF19
0	6.03702772897168	0.147	0.002	0	Endothelial	CLEC3B
0	5.19134086014527	0.148	0.003	0	Endothelial	TFPI2
0	7.75919213419522	0.146	0.001	0	Endothelial	C2CD4B
0	5.60414748623534	0.151	0.008	0	Endothelial	TEAD4

0	5.55251986698411	0.146	0.004	0	Endothelial	PDE10A
0	4.83336845240428	0.149	0.007	0	Endothelial	SAMD14
0	5.24439529437962	0.147	0.006	0	Endothelial	PRICKLE2
0	7.17365283132881	0.142	0.001	0	Endothelial	CLEC1B
0	4.64769425801017	0.14	0.005	0	Endothelial	JAM3
0	7.79726545608711	0.133	0.002	0	Endothelial	SOX7
0	6.40576052138259	0.134	0.004	0	Endothelial	FABP4
0	4.02450493097044	0.136	0.007	0	Endothelial	GJA4
0	4.64529008056298	0.133	0.004	0	Endothelial	AKR1E2
0	7.40332632446582	0.129	0.001	0	Endothelial	SOX17
0	5.61505300102404	0.132	0.004	0	Endothelial	ATRNL1
0	4.62865402362089	0.131	0.003	0	Endothelial	C2CD4C
0	5.51460924928607	0.132	0.005	0	Endothelial	PLAT
0	7.20129990247097	0.13	0.003	0	Endothelial	VSIG2
0	5.29097957213897	0.13	0.004	0	Endothelial	A4GALT
0	4.70866367320184	0.128	0.004	0	Endothelial	GJC1
0	5.28300615815151	0.127	0.004	0	Endothelial	FJX1
0	4.62305861483616	0.127	0.005	0	Endothelial	DNM3
0	5.25954485369278	0.124	0.003	0	Endothelial	ZNF462
0	4.79840045111972	0.122	0.005	0	Endothelial	CLEC1A
0	7.72427776627142	0.114	0.001	0	Endothelial	USHBP1
0	6.99140567665364	0.114	0.001	0	Endothelial	HSPA12B
0	8.14483057744934	0.112	0.001	0	Endothelial	GPR182
0	8.88632048115509	0.109	0.001	0	Endothelial	SHROOM4
0	6.71165644794039	0.108	0.001	0	Endothelial	SPAAR
0	7.72106975151542	0.104	0.001	0	Endothelial	CD300LG
7.49984011528781E-308	2.88187576071314	0.389	0.083	1.22329892120459E-303	Endothelial	PPM1F
9.69681327942991E-307	3.95692870257759	0.133	0.007	1.58164721400781E-302	Endothelial	SH3RF3
5.9081214560684E-306	5.33300876187287	0.107	0.003	9.63673690699317E-302	Endothelial	ADAMTS7
6.23081915934902E-304	2.43757091816875	0.477	0.125	1.01630891308142E-299	Endothelial	IFI44
1.42477360773706E-302	5.38239135797777	0.256	0.036	2.32394823157991E-298	Endothelial	CA2
1.8820012670735E-302	2.31869356793619	0.402	0.085	3.06973226672359E-298	Endothelial	SNRK
3.11437804775041E-300	3.4097573203941	0.124	0.006	5.0798620336857E-296	Endothelial	NXPE3
7.71177547813974E-300	3.61863142617279	0.133	0.007	1.25786769823937E-295	Endothelial	KLHL3
7.63372322679357E-295	4.30902692823746	0.237	0.031	1.2451365955223E-290	Endothelial	HLX
2.69363958886103E-294	1.78580394474243	0.858	0.492	4.39359553339123E-290	Endothelial	ARGLU1
7.07789479423779E-291	3.15257998585368	0.359	0.075	1.15447541988813E-286	Endothelial	PALM2-AK/
5.80886577361193E-289	4.7831856633286	0.253	0.037	9.47484096333841E-285	Endothelial	PTPRG
6.47760565653753E-284	3.70180449255781	0.345	0.073	1.05656225863784E-279	Endothelial	DLC1
1.36673266854359E-282	3.03073797806972	0.259	0.038	2.22927765566145E-278	Endothelial	DEPRECATI
1.40745891028439E-281	3.79076066387984	0.123	0.006	2.29570622856487E-277	Endothelial	HDGFL3
2.47252385042457E-277	4.39779210347491	0.2	0.022	4.03293365242752E-273	Endothelial	APBB2
3.66665264191853E-276	4.18832912702707	0.195	0.021	5.98067712423331E-272	Endothelial	EMID1
1.92058019668563E-274	2.44957751032331	0.501	0.157	3.13265835881394E-270	Endothelial	ITGA5
3.10902846062497E-271	3.88169041387068	0.193	0.021	5.07113632212538E-267	Endothelial	PRSS23
7.23013380466085E-270	3.4149748941283	0.155	0.012	1.17930712487823E-265	Endothelial	TBXA2R
3.1971728796154E-269	4.55066905738862	0.12	0.006	5.21490868394068E-265	Endothelial	P3H2
9.02804232346465E-269	4.10782111697402	0.219	0.028	1.47256398338032E-264	Endothelial	ASAP2
3.39585061624284E-266	3.62825547048406	0.275	0.047	5.53897194015369E-262	Endothelial	LIFR
3.28874207415E-265	4.15095848425	0.257	0.041	5.36426719714607E-261	Endothelial	PEAR1
7.86590855779131E-265	4.15761136140987	0.247	0.038	1.28300834486134E-260	Endothelial	DUSP6
2.26316936047118E-264	2.36049108509122	0.324	0.062	3.69145554386454E-260	Endothelial	KSR1

**3.21377278473078E-264**	1.68416905169718	0.304	0.051	5.24198478917437E-260	Endothelial	SYNE1
**4.04859582167087E-260**	3.80050233647902	0.147	0.012	6.60366464472736E-256	Endothelial	FCGR2B
**1.24193405562577E-259**	5.35922467101746	0.193	0.023	2.02571863813119E-255	Endothelial	AQP1
**1.84249827128963E-259**	2.69070326656224	0.318	0.063	3.00529893030052E-255	Endothelial	SLC25A37
**9.10542805941257E-258**	2.73426833191645	0.525	0.186	1.48518637077078E-253	Endothelial	ARRDC3
**2.54179458446842E-257**	2.60877214265189	0.35	0.076	4.14592114672644E-253	Endothelial	MRC1
**1.5470549618239E-253**	3.70860551564375	0.179	0.019	2.52340134823097E-249	Endothelial	GRPEL2
**1.64211900971696E-251**	2.81476335551914	0.315	0.064	2.67846031674934E-247	Endothelial	DGKE
**5.4010721359149E-251**	3.28450813001946	0.239	0.037	8.80968876089079E-247	Endothelial	FRMD8
**9.09025321219641E-251**	3.87014765068581	0.129	0.009	1.48271120144136E-246	Endothelial	DCLK2
**8.62535715210074E-248**	5.93328646898407	0.106	0.005	1.40688200507915E-243	Endothelial	SMAD9
**4.00703645142578E-247**	3.53006032504473	0.113	0.006	6.53587715592059E-243	Endothelial	EVA1C
**2.4974839177174E-245**	4.42520462641898	0.101	0.005	4.07364601818885E-241	Endothelial	PROCR
**1.9675668259425E-244**	3.59223326137024	0.317	0.07	3.20929824979482E-240	Endothelial	TRPM4
**1.24034784251311E-240**	4.834156434302	0.141	0.012	2.02313136592314E-236	Endothelial	CTTNBP2
**2.18556573750485E-239**	2.37506482433221	0.464	0.147	3.56487627444416E-235	Endothelial	ARHGEF7
**2.19942998941232E-239**	2.09164334755518	0.351	0.078	3.58749025573043E-235	Endothelial	LPAR6
**4.23806969103163E-239**	3.07820741229094	0.242	0.039	6.91271547304169E-235	Endothelial	GASK1B
**2.08371906075776E-234**	4.90582878042681	0.187	0.024	3.39875416000198E-230	Endothelial	CASKIN2
**1.89906076989765E-233**	2.53620978441451	0.38	0.101	3.09755802178006E-229	Endothelial	PIK3C2B
**4.61549951405995E-233**	2.49478881453817	0.348	0.084	7.52834125738318E-229	Endothelial	MICALL2
**2.09724813506182E-229**	2.04995648522583	0.265	0.048	3.42082143309934E-225	Endothelial	C11orf96
**9.1451370206499E-229**	4.21336434525376	0.207	0.03	1.4916632994382E-224	Endothelial	ADGRL2
**2.76222271520251E-228**	1.85516521569732	0.545	0.196	4.50546147076681E-224	Endothelial	TGFBR2
**3.74064114913176E-228**	4.9486562290087	0.116	0.008	6.10135977834882E-224	Endothelial	ACKR3
**6.31907856311705E-227**	1.80788912667115	0.385	0.095	1.03070490443002E-222	Endothelial	ARHGAP26
**1.22158308021941E-226**	3.13768584623256	0.109	0.007	1.99252416214588E-222	Endothelial	GRAP
**1.71319339705764E-223**	2.77504120002168	0.477	0.175	2.79438974994072E-219	Endothelial	INSR
**2.28424948832278E-222**	3.06795055238282	0.26	0.049	3.72583934040328E-218	Endothelial	RBPMS
**1.78408008618585E-221**	3.13237975957341	0.21	0.031	2.91001302857773E-217	Endothelial	C5AR2
**7.48130616833604E-220**	4.13347085810875	0.133	0.012	1.22027584911729E-215	Endothelial	HSPB8
**4.18328711625797E-219**	1.91676719960162	0.505	0.167	6.82335961532837E-215	Endothelial	SGK1
**2.0218920595708E-218**	1.86408804686974	0.442	0.139	3.29790813836593E-214	Endothelial	TIMP1
**6.28075733772084E-217**	2.83609337051991	0.403	0.126	1.02445432935565E-212	Endothelial	LAMB2
**7.62554803146834E-216**	3.35478591699744	0.13	0.011	1.2438031394128E-211	Endothelial	FKBP10
**3.9072744519922E-215**	2.04629478298528	0.474	0.159	6.37315535864448E-211	Endothelial	SH3BP5
**4.04864211810375E-213**	4.41026113314924	0.218	0.037	6.60374015883902E-209	Endothelial	TDRP
**1.99399190931706E-209**	2.67213618571909	0.171	0.021	3.25240020328706E-205	Endothelial	NRARP
**3.68747363890551E-208**	1.86908166305292	0.462	0.147	6.01463825241878E-204	Endothelial	PLEKHO2
**6.92157448409772E-208**	1.86601978311736	0.443	0.138	1.12897801410118E-203	Endothelial	PGS1
**8.71299582528951E-207**	3.78402689050909	0.25	0.05	1.42117674906297E-202	Endothelial	DEPRECATI
**1.31475545549094E-205**	2.24172614244227	0.266	0.052	2.14449762345126E-201	Endothelial	DISC1
**1.9407688165267E-205**	2.71786268142105	0.129	0.011	3.1655880166367E-201	Endothelial	RFLNB
**9.27661683638934E-204**	2.59798143310518	0.157	0.018	1.51310897218347E-199	Endothelial	C1QTNF1
**1.15970116227021E-203**	4.05167885629245	0.223	0.04	1.89158856577895E-199	Endothelial	ICA1
**3.94692900498439E-202**	1.92885094159965	0.29	0.063	6.43783590003004E-198	Endothelial	CDK17
**4.55053873663601E-202**	1.971525336895	0.395	0.114	7.42238373332699E-198	Endothelial	HMBOX1
**9.49360806126747E-202**	3.34774867690494	0.229	0.042	1.54850241087334E-197	Endothelial	TRIP10
**2.38209187407499E-201**	4.54932710925757	0.106	0.007	3.88543005580372E-197	Endothelial	ACVRL1
**1.33711743455139E-200**	3.88007657334299	0.229	0.043	2.18097224749677E-196	Endothelial	CPLX1
**2.16685973100626E-199**	3.53561199434059	0.392	0.129	3.53436490724431E-195	Endothelial	HES1
**3.32844955427575E-199**	3.33924500438209	0.118	0.01	5.42903406797917E-195	Endothelial	IGF1R

7.50131862414721E-199	2.80658467942975	0.255	0.052	1.22354008078465E-194	Endothelial	FKBP1A
1.33449584482288E-196	3.34698764572142	0.209	0.035	2.17669617249059E-192	Endothelial	RASA4
3.6508725762751E-195	3.23848991459427	0.127	0.012	5.95493825916232E-191	Endothelial	THY1
8.92408695771201E-195	2.60977597482223	0.248	0.05	1.45560782367241E-190	Endothelial	NOL4L
2.48278085728096E-194	2.25272121800795	0.232	0.042	4.04966385631097E-190	Endothelial	GIMAP8
5.01133874156621E-194	2.19530150441069	0.336	0.088	8.17399462136865E-190	Endothelial	RCN3
4.3922381021109E-191	3.62347594353319	0.284	0.069	7.1641795683531E-187	Endothelial	EPHA2
4.25539491566361E-190	1.44346063343453	0.624	0.279	6.94097464693891E-186	Endothelial	FCGRT
1.00270789299319E-189	3.67822883999054	0.103	0.008	1.63551684426119E-185	Endothelial	DEPRECATI
7.90214824028503E-189	3.13513374586744	0.103	0.008	1.28891939947289E-184	Endothelial	PMEPA1
1.37770526189034E-188	3.20149560921221	0.339	0.1	2.24717505266934E-184	Endothelial	TNS2
3.60391678886412E-188	2.66568041158973	0.229	0.043	5.87834867431627E-184	Endothelial	THBD
2.44533535023801E-187	1.15437489301506	0.192	0.028	3.98858648977322E-183	Endothelial	LTK
2.54975607438092E-187	3.9563661961087	0.157	0.021	4.15890713292271E-183	Endothelial	MEIS1
5.86199112212316E-187	3.07877030666615	0.153	0.019	9.56149371929508E-183	Endothelial	RBMS3
1.395557147535E-186	4.27572423493324	0.164	0.023	2.27629326334435E-182	Endothelial	DEPRECATI
4.20804142095061E-186	3.02111884229648	0.136	0.015	6.86373636171253E-182	Endothelial	ADGRA2
2.28260194602627E-185	1.73306032330584	0.256	0.052	3.72315203416344E-181	Endothelial	ICAM2
1.0603147507416E-184	1.23208401375805	0.664	0.272	1.72947938993463E-180	Endothelial	ITPKB
1.56597027436913E-184	2.29019562726193	0.304	0.076	2.55425411452349E-180	Endothelial	TAOK2
1.66047194852114E-184	1.68368361392069	0.647	0.335	2.70839579523283E-180	Endothelial	JUN
2.57104124936977E-183	3.40164400878228	0.341	0.105	4.19362538184703E-179	Endothelial	KIAA1217
1.09198372561109E-182	2.45420002488509	0.412	0.138	1.78113465484425E-178	Endothelial	GSN
9.62801152526823E-180	3.51220022900442	0.296	0.08	1.5704249598865E-175	Endothelial	TJP1
1.39176329915687E-179	2.75038174380818	0.421	0.159	2.27010511725478E-175	Endothelial	NDST1
1.87239099524387E-179	4.38048516655857	0.192	0.033	3.05405695234228E-175	Endothelial	DIPK1B
2.8130070720536E-178	3.81253473365979	0.216	0.042	4.58829583522663E-174	Endothelial	KCTD15
3.44802880767976E-178	1.81838275487393	0.363	0.103	5.62407978820645E-174	Endothelial	DENND3
3.46067422673672E-175	2.20989511664118	0.271	0.063	5.64470573123027E-171	Endothelial	DOCK9
4.93266640911742E-175	2.17907480873875	0.535	0.235	8.04567217991143E-171	Endothelial	CTSL
2.35281892614821E-173	2.15602851192366	0.24	0.05	3.83768295044035E-169	Endothelial	MLKL
7.11178133968083E-172	1.60973797039628	0.56	0.253	1.16000265431534E-167	Endothelial	XAF1
1.26947705172574E-168	2.54128694391234	0.195	0.035	2.07064401906985E-164	Endothelial	RIMKLB
3.52122636556844E-168	4.00740419204787	0.202	0.039	5.74347232487868E-164	Endothelial	PCGF2
3.12141277043723E-167	2.02286651389618	0.296	0.077	5.09133636986016E-163	Endothelial	RGS3
6.78274113420983E-167	4.71341528914284	0.112	0.011	1.10633290640097E-162	Endothelial	ZNF423
9.52076605739934E-167	1.68080902830303	0.4	0.127	1.55293215162241E-162	Endothelial	TIMP2
2.3522319590117E-166	2.22372068283515	0.322	0.093	3.83672554834399E-162	Endothelial	NLK
1.95263868896576E-165	2.90116459593693	0.176	0.029	3.18494896557205E-161	Endothelial	CTBP2
2.05454312738627E-165	2.4227150554833	0.139	0.017	3.35116529507974E-161	Endothelial	RIPOR3
3.04658201065265E-165	3.06147251974363	0.287	0.078	4.96927991757554E-161	Endothelial	TNS1
1.40266681048095E-164	2.50511799404611	0.155	0.022	2.28788983457547E-160	Endothelial	DEPRECATI
1.89355252184709E-164	2.96964429408369	0.147	0.02	3.08857351838479E-160	Endothelial	ARHGAP31
3.46469969558443E-164	3.01305480454072	0.164	0.026	5.65127167346777E-160	Endothelial	TMOD2
1.57967876581571E-163	2.01052964025706	0.314	0.086	2.576614034922E-159	Endothelial	SMAD7
2.88596830427699E-160	2.19558634154317	0.364	0.122	4.7073029011062E-156	Endothelial	HIPK2
7.5169187859359E-160	3.2530596683512	0.281	0.078	1.226084623174E-155	Endothelial	IL1R1
1.05219548870405E-159	2.06744836969679	0.119	0.013	1.71623606162517E-155	Endothelial	RASGRF2
3.27650067251635E-158	2.71195628983653	0.15	0.022	5.34430024694141E-154	Endothelial	ELK3
1.76418065074786E-157	1.29838189541484	0.453	0.163	2.87755505943483E-153	Endothelial	HDAC7
2.53424520630344E-157	2.16790703774368	0.439	0.181	4.13360735600154E-153	Endothelial	IFI27
1.228599520495E-156	2.02403332858281	0.402	0.144	2.00396867787939E-152	Endothelial	WARS

**2.6345662124815E-156**	1.44125472997976	0.434	0.156	4.29724094917857E-152	Endothelial	CEMIP2
**4.47153642996475E-156**	2.80532967898359	0.363	0.131	7.2935230709155E-152	Endothelial	BCAR1
**5.65770281005377E-156**	2.68711955378929	0.259	0.066	9.22827905347871E-152	Endothelial	MAST4
**6.41706990059863E-156**	3.15195476025103	0.237	0.056	1.04668827148664E-151	Endothelial	NR2F1
**1.92594688102712E-155**	2.55835365936534	0.112	0.012	3.14141195764334E-151	Endothelial	MARCH3
**1.384584131281E-154**	2.44931421708252	0.213	0.045	2.25839517653244E-150	Endothelial	ZEB1
**2.70547994971017E-154**	3.34415962227849	0.287	0.085	4.41290834597225E-150	Endothelial	TSPAN9
**1.25123847123862E-152**	3.1976856660348	0.225	0.052	2.04089507043732E-148	Endothelial	NR2F2
**1.44706560910467E-151**	2.62976115384976	0.333	0.109	2.36030871501063E-147	Endothelial	DOCK4
**4.36460314877027E-148**	4.49227872765251	0.182	0.036	7.11910419595919E-144	Endothelial	MPZL2
**3.03930115985169E-146**	3.07466526557127	0.311	0.103	4.9574041218341E-142	Endothelial	CTTN
**5.06837222883981E-146**	2.83987200687929	0.265	0.073	8.26702194246061E-142	Endothelial	STARD8
**1.3630762531981E-144**	2.89902509193238	0.282	0.084	2.22331367659142E-140	Endothelial	PHLDB2
**4.27850077271543E-144**	2.93722764767481	0.111	0.013	6.97866261037614E-140	Endothelial	KAZN
**1.57857104305399E-142**	3.1397327043153	0.311	0.105	2.57480722832537E-138	Endothelial	NFIB
**2.26226669310579E-142**	1.82475424056449	0.275	0.074	3.68998320312486E-138	Endothelial	VASH1
**3.89656747678293E-142**	1.05284017874941	0.758	0.45	6.35569121138063E-138	Endothelial	CCNL2
**2.21806021845072E-141**	1.38732592991524	0.548	0.251	3.61787802231497E-137	Endothelial	MARCKS
**1.97314011331456E-140**	3.00601222003677	0.169	0.032	3.21838883882737E-136	Endothelial	KIAA0895L
**2.1376860824751E-140**	3.18657663649065	0.308	0.104	3.48677976912514E-136	Endothelial	MYO6
**3.11786443322475E-140**	2.31094793875535	0.265	0.073	5.08554867703289E-136	Endothelial	SEMA4C
**7.91083866367379E-140**	2.47924160342915	0.153	0.026	1.29033689443183E-135	Endothelial	HOXB3
**6.09743583846748E-139**	2.717112839079	0.158	0.028	9.9455275961243E-135	Endothelial	KLF7
**9.03185320672606E-139**	3.54233293908836	0.182	0.037	1.47318557654909E-134	Endothelial	AMOTL1
**3.71319265890451E-138**	3.09988395946509	0.163	0.03	6.05658854593915E-134	Endothelial	OSBPL10
**1.91735027249322E-137**	2.85174449015645	0.216	0.052	3.12739002946368E-133	Endothelial	DAAM1
**5.18498859315476E-137**	2.97593342852375	0.169	0.032	8.45723489429473E-133	Endothelial	JAG1
**6.52699349294056E-137**	3.09151848669589	0.112	0.014	1.06461790863354E-132	Endothelial	FHL1
**7.46507877281039E-137**	2.76225620912627	0.111	0.014	1.2176289986331E-132	Endothelial	LHFPL6
**1.64260722560318E-136**	2.35593771817978	0.217	0.052	2.67925664568135E-132	Endothelial	KIAA0355
**6.92239698775513E-136**	2.93990704252215	0.17	0.033	1.12911217267274E-131	Endothelial	ZNF117
**3.69669994108838E-135**	1.71671367711814	0.294	0.088	6.02968727390925E-131	Endothelial	DMWD
**2.09007818828211E-134**	2.81450149257246	0.289	0.092	3.40912653290695E-130	Endothelial	EDNRB
**1.01898463294466E-133**	2.78378084504385	0.324	0.118	1.66206583479604E-129	Endothelial	SHC2
**1.11970438554055E-133**	2.9877065193977	0.262	0.078	1.82634982325519E-129	Endothelial	PTK2
**2.84000218914026E-131**	3.42050522609883	0.17	0.034	4.63232757070668E-127	Endothelial	RASAL2
**1.03605102205218E-130**	1.05080936623889	0.36	0.116	1.68990282206932E-126	Endothelial	SLC44A2
**2.23344250021224E-130**	2.18685894903201	0.153	0.027	3.64296806209618E-126	Endothelial	LOXL2
**3.74049386710448E-130**	4.55074079491673	0.107	0.014	6.10111954663412E-126	Endothelial	FBXL7
**4.30689266903782E-130**	2.54961795997305	0.137	0.022	7.0249726324676E-126	Endothelial	INMT
**4.42118764180326E-130**	2.28455025430254	0.272	0.082	7.2113991625453E-126	Endothelial	TMEM184E
**1.25772303807335E-129**	2.0148821393969	0.216	0.052	2.05147204740144E-125	Endothelial	FHOD1
**1.75398098434091E-129**	2.6296977811102	0.223	0.058	2.86091838355845E-125	Endothelial	UACA
**7.62215800359968E-129**	1.51689307692739	0.533	0.273	1.24325019196714E-124	Endothelial	BST2
**1.065387587092E-128**	2.92075619590531	0.264	0.081	1.73775369330577E-124	Endothelial	RILPL1
**3.22472384037642E-128**	2.08234386172987	0.389	0.153	5.25984705603798E-124	Endothelial	MS4A6A
**7.29959451520318E-128**	3.51575692498945	0.117	0.017	1.19063686137479E-123	Endothelial	TNFRSF10D
**1.54282147542162E-127**	1.67826309787488	0.41	0.168	2.5164961085602E-123	Endothelial	CCDC85B
**3.27973931183998E-127**	2.49671129533291	0.164	0.032	5.34958279154219E-123	Endothelial	RCBTB2
**1.2326752449494E-126**	1.39763522931197	0.219	0.052	2.01061659203697E-122	Endothelial	TRIB2
**2.61322942259578E-126**	2.06683073246693	0.136	0.022	4.26243851119598E-122	Endothelial	HOXB4
**4.06016674204391E-126**	2.19173351058004	0.17	0.034	6.62253797294783E-122	Endothelial	KCNK6

3.20652090821415E-125	2.11782669771074	0.423	0.192	5.2301562533881E-121	Endothelial	SLC9A3R2
3.79388950629906E-125	4.53575359100343	0.109	0.015	6.1882131737244E-121	Endothelial	PGM5
4.05528680974267E-124	3.72467373328325	0.127	0.02	6.61457831537127E-120	Endothelial	CABLES1
6.32178964817957E-124	3.14594672326893	0.253	0.078	1.03114710951457E-119	Endothelial	SORBS1
1.26952330005386E-123	2.35933141957417	0.3	0.102	2.07071945471785E-119	Endothelial	DAB2
4.98530415405979E-123	1.86277479215694	0.385	0.155	8.13152960568693E-119	Endothelial	UGCG
2.53796947767505E-122	3.59380531285398	0.17	0.037	4.13968201503577E-118	Endothelial	PDLIM1
3.48312197806829E-122	2.74163124071695	0.13	0.021	5.68132025842719E-118	Endothelial	ERV3-1
4.0103252276306E-122	1.15242935487459	0.322	0.102	6.54124147878827E-118	Endothelial	PLCG1
4.93298186225161E-122	3.23341944633377	0.24	0.071	8.0461867155186E-118	Endothelial	DOCK1
5.93557409431508E-122	3.52778385039501	0.125	0.02	9.68151490523733E-118	Endothelial	LDLRAD3
2.33830274752044E-121	2.81574487873672	0.109	0.015	3.81400561148058E-117	Endothelial	MMP2
2.62167316789362E-121	4.40202353624488	0.112	0.016	4.27621110415128E-117	Endothelial	MTCL1
1.31769253405217E-119	3.32246060676149	0.225	0.064	2.14928829229249E-115	Endothelial	TEAD2
1.63029147421124E-119	3.00648815379358	0.314	0.121	2.65916842358596E-115	Endothelial	JUP
2.81477473900333E-119	1.57749835327661	0.321	0.109	4.59117907678832E-115	Endothelial	GBP4
3.24512438297147E-119	3.28553815112078	0.185	0.044	5.29312238106476E-115	Endothelial	NTN4
5.4446089545297E-119	2.3843826757733	0.323	0.122	8.8807016657334E-115	Endothelial	MMP14
3.29936462108738E-118	2.8348585462321	0.29	0.103	5.38159363345562E-114	Endothelial	CDC42BPB
7.74449916059234E-118	1.92215676760935	0.456	0.214	1.26320525808422E-113	Endothelial	DDIT4
1.07812629755344E-117	2.02527682890368	0.441	0.219	1.75853180393941E-113	Endothelial	SHC1
4.71898463318038E-116	1.37198415813261	0.554	0.298	7.69713583518052E-112	Endothelial	IER2
4.70476199389428E-115	3.71622317591831	0.29	0.108	7.67393728824096E-111	Endothelial	VWA1
1.40341412705423E-114	2.72929759316692	0.173	0.04	2.28910878263816E-110	Endothelial	ST3GAL4
3.10015806910086E-114	2.2406202004847	0.226	0.063	5.05666782651042E-110	Endothelial	PLTP
3.63874873885045E-113	1.4418620525802	0.198	0.047	5.93516306793897E-109	Endothelial	FCHO1
1.44883448668431E-112	1.30969786482858	0.345	0.124	2.36319393123078E-108	Endothelial	CHST12
2.14693362370507E-111	3.29559922950574	0.204	0.056	3.50186343362534E-107	Endothelial	NREP
3.72853147195404E-111	2.74470287468299	0.105	0.015	6.08160768390423E-107	Endothelial	PKN3
3.76878633446516E-111	1.33476932499461	0.154	0.03	6.14726739014612E-107	Endothelial	FBN1
3.38656064850023E-110	0.879567589880788	0.557	0.269	5.52381907376872E-106	Endothelial	FAM118A
4.96120867513435E-110	1.70804535696476	0.383	0.167	8.09222747001164E-106	Endothelial	KLHL5
5.52618033502654E-110	2.8682746732618	0.113	0.018	9.0137527444618E-106	Endothelial	DBN1
3.61401879235748E-108	1.62199096381353	0.394	0.176	5.89482605221428E-104	Endothelial	CYTH2
6.94559180566794E-108	2.78674279148594	0.133	0.025	1.1328954794225E-103	Endothelial	DLG4
1.55171604253485E-107	4.03465200598052	0.14	0.028	2.53100403697859E-103	Endothelial	PTPN14
4.96922843989407E-107	1.93533171416364	0.356	0.151	8.10530850831122E-103	Endothelial	SERPINB6
7.10327319112322E-107	3.53719013790774	0.129	0.024	1.15861489020411E-102	Endothelial	SNED1
7.11015792315666E-107	2.54138676451057	0.284	0.103	1.15973785884608E-102	Endothelial	NCOA7
3.02094520590013E-106	1.89999662435552	0.198	0.051	4.92746372534371E-102	Endothelial	YBX3
1.46925550907792E-104	1.90007808639392	0.267	0.09	2.39650266085699E-100	Endothelial	TTLL5
9.42715053015916E-104	2.5854724994409	0.167	0.04	1.53766252297426E-99	Endothelial	CTTNBP2N
1.74622717733353E-103	2.14442029825639	0.227	0.069	2.84827114894871E-99	Endothelial	RAI1
4.34569011961277E-103	1.50620211816105	0.295	0.105	7.08825515410038E-99	Endothelial	CNST
4.6788859101832E-103	2.59843494384052	0.112	0.019	7.63173080809981E-99	Endothelial	NEURL1B
1.21398509250638E-102	2.25859957421058	0.285	0.106	1.98013108438715E-98	Endothelial	PDE8A
1.0236269339933E-101	3.93756512757029	0.167	0.042	1.66963789203648E-97	Endothelial	LRP6
3.18053654161711E-101	3.14876267983933	0.253	0.089	5.18777315303167E-97	Endothelial	LAMA5
7.07579237026931E-101	1.70978481463975	0.308	0.118	1.15413249351463E-96	Endothelial	TNFRSF10E
4.892382282758E-100	2.10931247332129	0.241	0.078	7.97996474140658E-96	Endothelial	SH3GL1
3.37515111203166E-99	0.988686518421474	0.551	0.295	5.50520897883483E-95	Endothelial	MACF1
6.16339753846949E-99	1.07624274462522	0.257	0.08	1.00531177249976E-94	Endothelial	DEPRECATI

**3.985191580963E-98**	1.67961804422109	0.192	0.051	6.50024598770875E-94	Endothelial	CROCC
**1.30984899059335E-97**	1.77769461174332	0.382	0.17	2.13649468855682E-93	Endothelial	FOS
**8.02986153542824E-97**	2.30368826479713	0.235	0.078	1.3097507150437E-92	Endothelial	GABBR1
**1.12648515534068E-96**	1.79908042117807	0.218	0.066	1.83740993687618E-92	Endothelial	SPTLC2
**3.96100393291422E-96**	1.83074825021401	0.415	0.216	6.46079351497638E-92	Endothelial	ETS2
**5.52063096855575E-96**	2.35145082406275	0.282	0.109	9.00470117281128E-92	Endothelial	NDRG1
**5.855098355951E-96**	2.58876006181912	0.207	0.063	9.55025092839168E-92	Endothelial	TANC1
**1.097338377993E-95**	2.63749146002745	0.2	0.059	1.78986862834438E-91	Endothelial	YES1
**1.72767525589216E-95**	1.72614005073344	0.106	0.018	2.8180111098857E-91	Endothelial	TGFBR3
**4.55806089899092E-95**	2.92466360765444	0.185	0.052	7.43465313234409E-91	Endothelial	PTPRM
**6.01215634842435E-95**	2.73387910515588	0.2	0.059	9.80642821991495E-91	Endothelial	SOX4
**1.35508551355898E-94**	3.0716577832058	0.225	0.075	2.21027998116606E-90	Endothelial	DEPRECATI
**6.73192548362782E-94**	0.316130028151586	0.505	0.202	1.09804436563453E-89	Endothelial	ETS1
**7.31254965385285E-94**	1.56285672634798	0.15	0.034	1.19274997403994E-89	Endothelial	ZSCAN18
**1.14187696190567E-93**	2.02598138863284	0.356	0.167	1.86251551256433E-89	Endothelial	AFDN
**1.55924471687625E-93**	1.81515874654846	0.416	0.221	2.54328405769686E-89	Endothelial	CTNND1
**6.82084002196872E-93**	1.32686585077619	0.188	0.05	1.11254721598332E-88	Endothelial	ZNF83
**8.78216968026135E-93**	1.99762050743995	0.315	0.134	1.43245969654743E-88	Endothelial	SELENON
**2.03636334327263E-92**	1.46117089482715	0.534	0.311	3.32151224921199E-88	Endothelial	HSPB1
**2.22511856073291E-90**	2.19446343178032	0.225	0.075	3.62939088441145E-86	Endothelial	PDLIM7
**2.63219548698322E-90**	4.12429072492559	0.1	0.017	4.29337405881833E-86	Endothelial	NUAK1
**3.15230657585095E-90**	1.69373213835947	0.238	0.079	5.14172725587048E-86	Endothelial	CHSY1
**5.095117855857E-90**	1.94825545114364	0.215	0.068	8.31064673468835E-86	Endothelial	PDCL3
**8.23479166921079E-90**	2.83687875449797	0.117	0.023	1.34317686916497E-85	Endothelial	SULF2
**8.29898145406119E-90**	1.34522982609734	0.311	0.122	1.35364686497192E-85	Endothelial	TSPAN14
**9.02055293327279E-90**	2.08505341527673	0.115	0.022	1.47134238894612E-85	Endothelial	NLGN2
**6.11582263768173E-89**	3.81336971751956	0.108	0.02	9.97551830432267E-85	Endothelial	SMOX
**7.20686800937372E-88**	2.37970803852124	0.229	0.08	1.17551224100895E-83	Endothelial	NCKIPSD
**2.24900474424796E-87**	1.69702457728611	0.209	0.065	3.66835163834284E-83	Endothelial	SNTB2
**4.95942476901782E-87**	1.20705232860958	0.141	0.032	8.08931774074496E-83	Endothelial	TNXB
**7.03264887318217E-87**	1.17865180873108	0.264	0.093	1.14709535770474E-82	Endothelial	CRIM1
**7.17418835538142E-87**	1.86668397626805	0.17	0.046	1.17018186264626E-82	Endothelial	IPPK
**1.21644719094097E-86**	1.22997185669159	0.425	0.216	1.98414701314381E-82	Endothelial	CDC37
**1.36207313370665E-86**	1.31492187241829	0.204	0.061	2.22167748838892E-82	Endothelial	TBKBP1
**2.47911385451296E-86**	1.6241240963075	0.297	0.121	4.04368260809609E-82	Endothelial	RGL2
**3.3904532133106E-86**	1.58611512625619	0.128	0.027	5.53016823623092E-82	Endothelial	DMPK
**7.1307034077106E-86**	1.70183422732497	0.256	0.094	1.16308903283168E-81	Endothelial	ASAP1
**7.44315157953936E-86**	1.51136819123993	0.339	0.15	1.21405245413867E-81	Endothelial	PML
**1.03994126809019E-85**	2.43483331515706	0.122	0.026	1.6962482023819E-81	Endothelial	MAPK11
**1.77606743597672E-85**	1.72611026040883	0.162	0.042	2.89694359482162E-81	Endothelial	NOD1
**1.09633640462569E-83**	2.95379412877691	0.149	0.039	1.78823430958496E-79	Endothelial	ME3
**3.11803031592901E-83**	1.93031713286603	0.197	0.061	5.0858192483118E-79	Endothelial	CSNK1E
**9.98740863535482E-83**	1.98725862037163	0.186	0.056	1.62904622251273E-78	Endothelial	FRMD4A
**1.1954797033062E-82**	2.00846654691087	0.246	0.092	1.94994694406274E-78	Endothelial	FER
**1.25063214450718E-82**	1.6722017258705	0.299	0.127	2.03990609090565E-78	Endothelial	ECE1
**7.46495480968551E-82**	1.95030463142413	0.207	0.068	1.2176087790078E-77	Endothelial	ZSWIM6
**6.61912373871862E-81**	2.62194637021658	0.214	0.075	1.07964527302239E-76	Endothelial	TSPAN15
**1.0469024423824E-80**	0.762685076918036	0.335	0.143	1.70760257376993E-76	Endothelial	ADGRG1
**1.46259949032832E-80**	2.36099740238943	0.32	0.148	2.38564602867453E-76	Endothelial	EGR1
**8.2489275781621E-79**	1.52432432927786	0.195	0.061	1.34548257727402E-74	Endothelial	ARL 15.00
**2.23079304997668E-78**	1.13242419901542	0.505	0.296	3.63864654381697E-74	Endothelial	IFITM3
**1.33468345723173E-77**	1.83369633619451	0.239	0.09	2.17700218709068E-73	Endothelial	PELO

4.04240657792482E-77	1.46615720070712	0.253	0.096	6.59356936925318E-73	Endothelial	OAS3
5.77878529469177E-77	0.811432143816656	0.13	0.03	9.42577669417175E-73	Endothelial	C7
8.23469491339792E-77	2.05701786863152	0.112	0.024	1.34316108732433E-72	Endothelial	RNF144A
1.0611310268673E-75	2.307687118455	0.164	0.049	1.73081081792326E-71	Endothelial	FAM241A
1.49029095246354E-75	1.2068350472957	0.135	0.033	2.43081357256328E-71	Endothelial	F2R
1.83288510996837E-75	2.40757230283094	0.24	0.096	2.98961890286942E-71	Endothelial	GFOD2
1.68339567522049E-74	1.5328744369305	0.362	0.184	2.74578668585214E-70	Endothelial	SLC38A2
1.70060553522064E-74	1.52548972149133	0.232	0.085	2.77385768849839E-70	Endothelial	SSH1
2.60303713821281E-74	2.50738450842202	0.127	0.031	4.24581387613892E-70	Endothelial	PKIG
2.88874160081339E-74	2.48078019225386	0.106	0.022	4.71182642508672E-70	Endothelial	PFKM
1.95667555489505E-73	1.90166669054463	0.245	0.096	3.19153349758932E-69	Endothelial	PPP1R15A
8.35112911084413E-73	2.02936393859715	0.101	0.021	1.36215266926979E-68	Endothelial	ANKDD1A
1.06921451134298E-72	2.46761350303	0.146	0.041	1.74399578945154E-68	Endothelial	SVIL
1.56055179589504E-72	2.19076102250605	0.168	0.052	2.54541603428441E-68	Endothelial	MTHFD1L
1.83511572999275E-72	1.76676324979679	0.285	0.127	2.99325726719118E-68	Endothelial	SLC12A4
4.95552940330585E-71	3.366484244319	0.161	0.051	8.08296400973218E-67	Endothelial	TTC28
5.21508858891298E-71	0.643676885526978	0.29	0.113	8.50633099737597E-67	Endothelial	SFMBT2
2.73076365160425E-70	2.20579950931731	0.195	0.069	4.45414859213169E-66	Endothelial	PTPN12
6.1201983771437E-70	3.66430185019228	0.115	0.028	9.98265557295909E-66	Endothelial	CEACAM19
6.16671812767796E-70	2.65186882767458	0.155	0.046	1.00585339380555E-65	Endothelial	GAS6
6.70518994194737E-70	1.72226163822829	0.113	0.026	1.09368353143104E-65	Endothelial	FAM122C
7.63204417190005E-69	1.59762163060202	0.333	0.168	1.24486272487862E-64	Endothelial	TRIOBP
1.34007722547718E-68	2.81330407710644	0.196	0.072	2.18579996247583E-64	Endothelial	BCAM
2.4737512412174E-68	3.40524295663067	0.142	0.041	4.03493564954971E-64	Endothelial	SDCBP2
6.26689797923978E-68	2.10045341257894	0.161	0.05	1.0221937293938E-63	Endothelial	CHST15
2.26366315330758E-67	1.65558356713523	0.227	0.087	3.69226096936E-63	Endothelial	RGL1
2.31798219481302E-67	1.63498575817991	0.172	0.056	3.78086075795952E-63	Endothelial	PLCB1
2.78699068760056E-67	1.8546762556696	0.235	0.096	4.54586051054528E-63	Endothelial	PITPNM2
4.95549371704563E-67	1.64023049471529	0.345	0.183	8.08290580187312E-63	Endothelial	S100A16
5.23811247230356E-67	0.826397196683997	0.22	0.077	8.54388525357434E-63	Endothelial	GIMAP5
9.59580136138767E-67	1.32809320250732	0.265	0.112	1.56517116005594E-62	Endothelial	HDAC1
7.51755281158174E-66	1.20997151523821	0.148	0.042	1.2261880390971E-61	Endothelial	GYPC
8.46155444255352E-66	0.591935614821973	0.606	0.384	1.3801641451249E-61	Endothelial	LUC7L3
1.47548694860224E-65	1.05135824922296	0.607	0.438	2.40666676186511E-61	Endothelial	ZFP36
1.8299923745929E-65	2.61708233486217	0.17	0.058	2.98490056219848E-61	Endothelial	TNFAIP8L1
2.39112262775945E-65	0.913507096575185	0.196	0.066	3.90016011813843E-61	Endothelial	CARD8
3.55375715759491E-65	3.03245491196198	0.135	0.039	5.79653329975307E-61	Endothelial	CLIC4
4.39210741822211E-65	3.56437338843257	0.129	0.036	7.16396640986209E-61	Endothelial	HID1
1.37756538427971E-64	1.61329889960544	0.405	0.242	2.24694689829864E-60	Endothelial	CDKN1A
8.98979403235609E-64	1.46412434284709	0.189	0.066	1.4663253046176E-59	Endothelial	SMURF2
1.22679828437711E-63	1.70695269895528	0.164	0.053	2.0010306816475E-59	Endothelial	WDR27
6.28190014448913E-63	1.30032797733187	0.321	0.159	1.02464073256762E-58	Endothelial	TBCD
6.9252233082996E-63	2.34638154413468	0.27	0.131	1.12957317381675E-58	Endothelial	PLIN4
1.46904820587492E-62	1.42741137518227	0.242	0.099	2.39616452860258E-58	Endothelial	DPYSL2
4.69014250932582E-62	1.10866417072615	0.129	0.035	7.65009144696135E-58	Endothelial	FSTL1
4.81594577919872E-62	1.05036268004041	0.241	0.095	7.85528916045104E-58	Endothelial	FAR1
4.97818434177907E-62	3.54868344685218	0.124	0.035	8.11991647987585E-58	Endothelial	MEIS2
1.95318227350544E-61	1.99351924770018	0.113	0.029	3.18583560631472E-57	Endothelial	CASP10
2.171877833977E-61	1.11802781044403	0.189	0.066	3.54254993499989E-57	Endothelial	FMNL3
2.82338123061022E-61	3.8376953822696	0.137	0.042	4.60521712524832E-57	Endothelial	ALPL
3.00200876121332E-61	3.03815061207962	0.183	0.069	4.89657649041504E-57	Endothelial	EFNB1
3.11916304197246E-61	1.97455403838807	0.205	0.08	5.08766683776127E-57	Endothelial	LDLR

**5.71097083143097E-61**	2.61763315570143	0.121	0.033	9.31516452314705E-57	Endothelial	SASH1
**7.23702476363902E-61**	2.16740378211038	0.18	0.066	1.18043110919716E-56	Endothelial	FAM219A
**1.92042319892909E-60**	1.04374982540899	0.499	0.317	3.13240227977324E-56	Endothelial	HSPA1B
**2.97145769983424E-60**	2.24943619137119	0.166	0.058	4.84674465419963E-56	Endothelial	BMP1
**4.21223607180541E-60**	0.442162652927495	0.133	0.037	6.87057825672181E-56	Endothelial	EMILIN1
**4.53665399378614E-60**	3.71708369808721	0.109	0.029	7.39973632926457E-56	Endothelial	MYRIP
**4.8116390671399E-60**	1.12293262260993	0.105	0.025	7.8482644824119E-56	Endothelial	NBL1
**6.19111181668694E-60**	1.82621058057477	0.283	0.14	1.00983224841981E-55	Endothelial	AGFG2
**7.64049807842789E-60**	2.21741990486873	0.287	0.149	1.24624164157237E-55	Endothelial	CD59
**9.55459830012896E-60**	2.21171744076662	0.111	0.029	1.55845052873404E-55	Endothelial	RPGR
**1.24767354994241E-59**	1.69997522597425	0.192	0.072	2.03508032731106E-55	Endothelial	TAF1C
**9.04215969442264E-59**	1.23863935021791	0.4	0.239	1.47486666775728E-54	Endothelial	BSDC1
**1.60170112561066E-58**	0.474019849975949	0.55	0.283	2.61253470598355E-54	Endothelial	COL18A1
**1.86584697364849E-58**	2.34403130934475	0.158	0.054	3.04338299871806E-54	Endothelial	CPED1
**3.16397748008629E-58**	1.50791302939898	0.121	0.033	5.16076366776875E-54	Endothelial	ENC1
**3.90179163130922E-58**	1.48063466719715	0.11	0.028	6.36421232982847E-54	Endothelial	ADARB1
**7.98734559059612E-58**	1.74893461574067	0.137	0.042	1.30281593928213E-53	Endothelial	SLC49A3
**1.58127794161676E-57**	3.19647744731259	0.112	0.031	2.5792224505711E-53	Endothelial	TCF7L1
**1.70957971014302E-57**	0.972542275457774	0.453	0.282	2.78849546521428E-53	Endothelial	SPG7
**1.92824079830936E-57**	1.74964387290925	0.145	0.046	3.14515356612239E-53	Endothelial	PRKD3
**3.6342586706571E-57**	1.29095617363021	0.32	0.162	5.92783931770879E-53	Endothelial	SH3TC1
**3.76235641485191E-57**	2.12603116615146	0.142	0.045	6.13677954826495E-53	Endothelial	BMPR2
**4.23125763543731E-57**	1.87439028101509	0.205	0.083	6.9016043291618E-53	Endothelial	ME2
**4.6243875517297E-57**	1.62959199239436	0.19	0.073	7.54283853562632E-53	Endothelial	RAB11FIP3
**7.30042653825156E-57**	2.05567992631693	0.226	0.1	1.19077257265421E-52	Endothelial	LRRFIP2
**1.2770768072136E-56**	0.272851646708667	0.379	0.176	2.0830399802461E-52	Endothelial	NLRP1
**1.33961019861025E-56**	0.747533428198826	0.426	0.234	2.18503819495318E-52	Endothelial	FOSL2
**1.38140071847672E-56**	1.54585160593797	0.189	0.072	2.25320271190738E-52	Endothelial	PCGF3
**1.3989585745899E-56**	1.37827974911675	0.374	0.224	2.28184133101359E-52	Endothelial	RHOC
**1.6057393760479E-56**	1.69890454615393	0.124	0.036	2.61912149627173E-52	Endothelial	HEG1
**5.43494598247693E-56**	2.00086037211863	0.121	0.035	8.86494039201812E-52	Endothelial	BDH2
**6.1909237043419E-56**	0.984019807605046	0.255	0.112	1.00980156541521E-51	Endothelial	PRKD2
**8.95288880824518E-56**	1.5028217172779	0.207	0.083	1.46030569351287E-51	Endothelial	ARRB1
**9.85543356875773E-56**	2.51947667248836	0.196	0.081	1.60751976940007E-51	Endothelial	DEPRECATI
**1.4573302851299E-55**	1.28238440700709	0.214	0.087	2.37705142807538E-51	Endothelial	FCHSD2
**1.95536399514584E-55**	2.34557128605636	0.15	0.051	3.18939421248238E-51	Endothelial	RIN2
**2.20981952377008E-55**	2.53193378496913	0.222	0.101	3.60443662522137E-51	Endothelial	PVR
**5.44670413208622E-55**	2.36314457268879	0.204	0.087	8.88411910984583E-51	Endothelial	STX3
**7.85052944855452E-55**	1.533623933121	0.216	0.091	1.28049985835373E-50	Endothelial	CTNNB1
**1.14323723321411E-54**	2.59381384677339	0.103	0.027	1.86473425109554E-50	Endothelial	TNFAIP1
**1.42161490668593E-54**	2.22961144514201	0.206	0.088	2.31879607429542E-50	Endothelial	EHD4
**1.46739732160236E-54**	2.95729418613487	0.154	0.055	2.39347177126561E-50	Endothelial	MAPK12
**1.98110069948559E-54**	1.71418414104446	0.229	0.1	3.23137335093095E-50	Endothelial	C15orf39
**2.23067142182102E-54**	1.79578588841458	0.133	0.042	3.63844815613227E-50	Endothelial	WDR60
**2.65982885777991E-54**	0.851564840906373	0.121	0.034	4.33844684992481E-50	Endothelial	ADORA2A
**2.75690570060622E-54**	1.01522769497014	0.171	0.06	4.49678888825881E-50	Endothelial	BICD1
**2.85233458469946E-54**	0.564098348639981	0.198	0.072	4.65244294110329E-50	Endothelial	PDE4D
**9.63501790194523E-54**	1.86362302259557	0.155	0.055	1.57156776998629E-49	Endothelial	C1orf21
**1.04073835830815E-53**	2.35795354116458	0.121	0.036	1.69754833623642E-49	Endothelial	AFAP1
**1.48412786634456E-53**	1.85612998228862	0.293	0.157	2.42076096279462E-49	Endothelial	LIMK2
**3.21660180608179E-53**	1.4261528458682	0.157	0.054	5.2465992059E-49	Endothelial	PLSCR3
**6.41189202636734E-53**	0.555505368148254	0.32	0.15	1.04584370842078E-48	Endothelial	PITPNC1

6.82062270137994E-53	1.22211118568442	0.127	0.038	1.11251176882208E-48	Endothelial	FLI1
3.55461118127067E-52	1.79646742289515	0.145	0.049	5.7979262977706E-48	Endothelial	EXD3
4.82273400626709E-52	1.9140406316074	0.279	0.148	7.86636143762225E-48	Endothelial	PHC2
9.89067773989345E-52	0.414086599582075	0.772	0.583	1.61326844615402E-47	Endothelial	LENG8
1.16857866561979E-51	1.6848433619472	0.301	0.166	1.90606866149243E-47	Endothelial	SPTBN1
2.65943957056257E-51	1.70519327876824	0.161	0.059	4.33781188354462E-47	Endothelial	MAPK3
5.71490980181083E-51	1.77822643823773	0.225	0.103	9.32158937773364E-47	Endothelial	RNF138
7.47166392640998E-51	1.9999945175089	0.189	0.078	1.21870310303673E-46	Endothelial	IER5L
1.43904827532354E-50	1.22341543806544	0.16	0.056	2.34723164188023E-46	Endothelial	RASSF2
1.64924127128836E-50	1.48290624578481	0.226	0.102	2.69007743759845E-46	Endothelial	BAZ2B
2.11194755113737E-50	0.964421980232224	0.185	0.071	3.44479765066017E-46	Endothelial	DGKH
8.29799863937405E-50	3.37766511448611	0.131	0.045	1.3534865580683E-45	Endothelial	CADPS2
1.62849859359695E-49	2.15228427881827	0.189	0.08	2.65624405601599E-45	Endothelial	ITGA6
1.89650887178022E-49	1.52454038970335	0.252	0.124	3.09339562076072E-45	Endothelial	KLF9
4.08306608596492E-49	1.33394159836237	0.248	0.118	6.65988909281738E-45	Endothelial	FCHO2
4.40055359478519E-49	2.50317100274444	0.179	0.074	7.17774296845412E-45	Endothelial	KANK2
5.76042934951048E-49	2.18332275634593	0.131	0.044	9.39583631198655E-45	Endothelial	TANC2
9.27241474218808E-49	0.961633521788948	0.137	0.044	1.5124235685983E-44	Endothelial	P2RY8
1.25653650327285E-48	2.2384293833366	0.241	0.122	2.04953669048835E-44	Endothelial	GOLM1
1.57771517637841E-48	1.15632665417713	0.425	0.275	2.57341122419082E-44	Endothelial	HSPA1A
1.57850187069693E-48	1.35790161185428	0.182	0.073	2.57469440129376E-44	Endothelial	ITGA1
2.53223185911898E-48	2.52264998114357	0.161	0.063	4.13032338540897E-44	Endothelial	ROBO1
8.26916959774656E-48	1.64806667922826	0.267	0.14	1.34878425308844E-43	Endothelial	CCDC50
2.61005176392498E-47	0.597412459313986	0.428	0.257	4.25725543213804E-43	Endothelial	FAM193B
2.90696303725798E-47	1.5714109501869	0.143	0.05	4.74154741007148E-43	Endothelial	TPM4
5.95551477573514E-47	1.68352428248299	0.127	0.042	9.7140401507016E-43	Endothelial	ZNF606
6.39706778535294E-47	0.881681096044135	0.459	0.299	1.04342572646892E-42	Endothelial	MIDN
8.36218739662951E-47	0.83164314383016	0.421	0.265	1.36395638626424E-42	Endothelial	SRSF4
1.06205657124157E-46	1.34058682666108	0.256	0.127	1.73232047335212E-42	Endothelial	STRN4
1.11202521533591E-46	1.3641380193716	0.289	0.157	1.8138243287344E-42	Endothelial	MED10
1.18051230377833E-46	3.13318184958707	0.105	0.032	1.92553361869284E-42	Endothelial	ZHX3
1.20197666539028E-46	1.51591879525398	0.183	0.076	1.96054413891809E-42	Endothelial	MXRA7
1.38264971679056E-46	1.92723068204317	0.272	0.149	2.25523995305708E-42	Endothelial	CTNNA1
1.4487419021671E-46	1.6387584481462	0.132	0.045	2.36304291662476E-42	Endothelial	IRAK3
1.80333772779782E-46	1.09108591673665	0.241	0.114	2.94142416781102E-42	Endothelial	NISCH
2.37176834722131E-46	2.0950630111066	0.274	0.154	3.86859135115267E-42	Endothelial	SLC22A23
2.66692836774141E-46	2.66791901979602	0.17	0.071	4.35002686062302E-42	Endothelial	ST3GAL6
3.17187772387652E-46	0.416041263549307	0.287	0.133	5.17364975541499E-42	Endothelial	CLEC2B
8.38062713408863E-46	1.04951645332438	0.321	0.18	1.3669640918412E-41	Endothelial	JMJD1C
2.03203267459443E-45	2.19860820783663	0.138	0.05	3.31444849553097E-41	Endothelial	THEM6
4.53571000685471E-45	2.10422176132031	0.115	0.037	7.39819659218072E-41	Endothelial	TCEA2
7.67456381814146E-45	2.14343006778001	0.164	0.067	1.25179810437705E-40	Endothelial	TTC7B
1.15999065040018E-44	1.50565255124764	0.214	0.099	1.89206074986773E-40	Endothelial	EPB41L2
1.43835945079733E-44	1.24383747551092	0.244	0.12	2.34610810019552E-40	Endothelial	AP2A2
4.00458170693155E-44	1.59267253769409	0.175	0.073	6.53187322217606E-40	Endothelial	CHD7
4.56461938177091E-44	1.47809381607987	0.234	0.115	7.44535067360653E-40	Endothelial	RIPOR1
5.61320207810283E-44	3.2454113632033	0.161	0.068	9.15569390959352E-40	Endothelial	CX3CL1
6.12541876273663E-44	1.7245967588675	0.321	0.199	9.99117054389971E-40	Endothelial	ADAM15
6.55945989848087E-44	1.05253010285868	0.36	0.217	1.06991350404121E-39	Endothelial	QKI
1.11272594146507E-43	1.25868932443146	0.281	0.151	1.81496728312368E-39	Endothelial	PLSCR1
1.55137778516509E-43	1.07054665891811	0.269	0.14	2.53045230538277E-39	Endothelial	CMIP
2.03801031087816E-43	2.1351480192334	0.201	0.094	3.32419861807337E-39	Endothelial	FES

**3.17241151577538E-43**	1.76234154867762	0.176	0.075	5.17452042338122E-39	Endothelial	LRRC8A
**3.23120282231533E-43**	0.67239266070733	0.41	0.253	5.27041492347853E-39	Endothelial	LUC7L
**3.94732411919913E-43**	1.03751213512163	0.163	0.064	6.4384803708257E-39	Endothelial	ADD3
**1.0976415709583E-42**	1.58552850990576	0.17	0.071	1.79036316639009E-38	Endothelial	PIK3C2A
**1.14164654178492E-42**	1.98449139187501	0.242	0.129	1.86213967430538E-38	Endothelial	MYO1C
**1.4471836636332E-42**	1.14059144792125	0.354	0.217	2.36050127375211E-38	Endothelial	PARP14
**1.63753767380652E-42**	0.447013228532284	0.144	0.05	2.67098769974581E-38	Endothelial	ITPR3
**1.7097238384613E-42**	2.38149392228729	0.127	0.046	2.78873055291423E-38	Endothelial	JAG2
**2.74678014364871E-42**	1.48767140385565	0.151	0.058	4.48027309230541E-38	Endothelial	GNB4
**3.68151226287732E-42**	1.22202182341157	0.255	0.133	6.0049146519792E-38	Endothelial	ATN1
**4.85963770359762E-42**	1.12267593602427	0.309	0.177	7.92655505833808E-38	Endothelial	GNAQ
**5.27729342657133E-42**	1.75031294068619	0.127	0.045	8.6077933080805E-38	Endothelial	PPP2R5B
**5.32670908908576E-42**	1.25520945379514	0.152	0.059	8.68839519520778E-38	Endothelial	RELL1
**1.23074876904459E-41**	0.85031135074239	0.368	0.223	2.00747431718864E-37	Endothelial	PICALM
**1.66451453620112E-41**	2.69681117539537	0.103	0.033	2.71498965999765E-37	Endothelial	DNMBP
**2.05307731899708E-41**	2.71188418098955	0.113	0.038	3.34877441501613E-37	Endothelial	TSC22D1
**2.97780373619621E-41**	0.467116815342083	0.551	0.382	4.85709567410964E-37	Endothelial	DDX5
**3.46480919584225E-41**	0.928708613557292	0.403	0.263	5.65145027933829E-37	Endothelial	PRKAR1A
**5.81590608766446E-41**	1.28187612746731	0.37	0.246	9.4863244195895E-37	Endothelial	INF2
**1.53961682583845E-40**	1.53257279934357	0.194	0.09	2.5112690046251E-36	Endothelial	MAP1S
**1.60133382485557E-40**	1.51912732361301	0.11	0.036	2.61193560172192E-36	Endothelial	LUZP1
**1.9002504198463E-40**	1.55757506401403	0.112	0.037	3.09949845981129E-36	Endothelial	PNP
**2.54666445767123E-40**	1.49783859107413	0.196	0.091	4.15386439690755E-36	Endothelial	NAA38
**2.62767724516268E-40**	1.94252246084632	0.121	0.043	4.28600435458485E-36	Endothelial	NIBAN2
**2.72697582658632E-40**	2.00119546487087	0.141	0.055	4.44797027074494E-36	Endothelial	PLCB3
**4.09721512442472E-40**	1.30894805164285	0.339	0.22	6.68296758944917E-36	Endothelial	NECTIN2
**6.22710540511762E-40**	3.46779065097389	0.116	0.042	1.01570316262873E-35	Endothelial	ATP8B1
**6.7617230654915E-40**	1.95914902788772	0.252	0.14	1.10290464921232E-35	Endothelial	NPL
**9.14437866296403E-40**	0.510821400828632	0.359	0.191	1.49153960371606E-35	Endothelial	CD4
**1.35636261670511E-39**	0.8909074095768	0.435	0.302	2.21236306410771E-35	Endothelial	MYL6
**1.43793336616463E-39**	0.350154186306691	0.537	0.351	2.34541311355113E-35	Endothelial	MAF
**3.21730487323771E-39**	1.28417788561048	0.17	0.071	5.24774597873803E-35	Endothelial	HBEGF
**3.35259687109092E-39**	1.50021081511914	0.143	0.056	5.4684207564364E-35	Endothelial	MAN1C1
**1.26424808939484E-38**	1.65363241145034	0.278	0.164	2.06211505861192E-34	Endothelial	ACTN1
**6.30919837827382E-38**	3.06657189416135	0.152	0.066	1.02909334748024E-33	Endothelial	MAGI1
**3.620488788114E-37**	1.44312760044056	0.23	0.121	5.90537926229275E-33	Endothelial	ANKS1A
**6.85344934830296E-37**	2.10371112566754	0.153	0.066	1.1178661232017E-32	Endothelial	HOMER3
**6.99371443905536E-37**	0.856709123531126	0.467	0.332	1.14074476215432E-32	Endothelial	RHOB
**7.4689720046855E-37**	0.794364261056063	0.133	0.05	1.21826402368425E-32	Endothelial	RFX3
**1.28693922091163E-36**	0.812170239654546	0.311	0.182	2.09912656322896E-32	Endothelial	AKAP17A
**1.72955889752454E-36**	0.980045065740619	0.256	0.14	2.82108351775227E-32	Endothelial	TBC1D15
**2.31233016750462E-36**	1.51245973908511	0.247	0.138	3.77164173621679E-32	Endothelial	LZTS2
**2.69248376091759E-36**	1.17355432086516	0.172	0.076	4.39171026243267E-32	Endothelial	SNX5
**2.97244419129967E-36**	0.652975735759662	0.339	0.203	4.8483537204289E-32	Endothelial	UBA7
**4.49710328693039E-36**	1.62599417011733	0.163	0.073	7.33522517131216E-32	Endothelial	NBPF15
**1.42643986573201E-35**	1.85362970522976	0.185	0.09	2.32666606499548E-31	Endothelial	TCN2
**1.96072337946059E-35**	0.878835324306934	0.324	0.193	3.19813590423817E-31	Endothelial	TACC1
**1.97958257205787E-35**	2.35135345795169	0.207	0.112	3.22889713328359E-31	Endothelial	CEACAM1
**2.04178980975198E-35**	1.42181196904541	0.16	0.07	3.33036335868645E-31	Endothelial	LIMA1
**2.34577806367487E-35**	1.48436670476797	0.115	0.042	3.82619859966008E-31	Endothelial	DEPRECATI
**2.87050269682804E-35**	1.32437552823451	0.173	0.08	4.68207694879622E-31	Endothelial	ATF7
**6.58587255047892E-35**	1.4643411131975	0.299	0.183	1.07422167170862E-30	Endothelial	ATF3

7.67585844354594E-35	2.16525889648988	0.143	0.062	1.25200927072678E-30	Endothelial	PPP1R13B
8.3848135385588E-35	1.98615630517046	0.198	0.103	1.36764693627433E-30	Endothelial	ARHGEF12
9.43460455171376E-35	1.78497051661279	0.143	0.06	1.53887834843003E-30	Endothelial	SPATS2
1.31669503465091E-34	0.713834346654078	0.201	0.095	2.1476612710191E-30	Endothelial	STIM2
1.61788081931076E-34	0.949078090936379	0.302	0.184	2.63892540437779E-30	Endothelial	IL6ST
2.60142229601546E-34	1.27338862460796	0.179	0.085	4.24317990703082E-30	Endothelial	ABCD4
4.77320376268289E-34	2.37480952590761	0.19	0.099	7.78557265731207E-30	Endothelial	HTRA1
4.78488130578047E-34	1.14891714184099	0.239	0.132	7.80461989785852E-30	Endothelial	GOSR2
5.14722631253479E-34	3.0230214996521	0.111	0.042	8.39564083837549E-30	Endothelial	ALPK3
8.59187221702758E-34	1.45243884512413	0.164	0.075	1.40142027731937E-29	Endothelial	TMEM94
9.33520839933809E-34	0.683653761340789	0.366	0.235	1.52266584201604E-29	Endothelial	MPRIP
1.1286582663188E-33	1.29024914720983	0.123	0.048	1.8409544981926E-29	Endothelial	PELI1
1.30521367610176E-33	2.12623771681607	0.21	0.115	2.12893402708958E-29	Endothelial	S100A13
2.2682465196736E-33	1.04365268195139	0.271	0.158	3.69973689823961E-29	Endothelial	SIN3B
2.80876123804246E-33	2.88291087888223	0.112	0.044	4.58137045537106E-29	Endothelial	PKP4
3.00528607502117E-33	1.40589901132791	0.238	0.135	4.90192211696703E-29	Endothelial	SUN1
3.13738849214649E-33	1.84433333442665	0.164	0.077	5.11739436954013E-29	Endothelial	DENND5A
3.24983276288745E-33	0.619196401726003	0.175	0.078	5.30080221954572E-29	Endothelial	DGKZ
6.31354895281429E-33	1.95417913057108	0.122	0.049	1.02980296969354E-28	Endothelial	MYH10
7.65789481893725E-33	1.52542586289649	0.109	0.041	1.24907922391686E-28	Endothelial	SRGAP2
1.00606389179865E-32	1.54767012504329	0.264	0.161	1.64099081391278E-28	Endothelial	APOL1
1.88250395465919E-32	0.884125928183441	0.143	0.06	3.0705522004446E-28	Endothelial	RNF145
1.9396870300057E-32	2.2723579903133	0.162	0.078	3.16382351464229E-28	Endothelial	MAFF
2.59708499910242E-32	2.26772706376222	0.125	0.052	4.23610534203596E-28	Endothelial	NEIL1
2.84267505620253E-32	0.773119514070217	0.124	0.048	4.63668728417195E-28	Endothelial	DEPRECATI
4.48576525020075E-32	0.86733757492588	0.27	0.156	7.31673169960244E-28	Endothelial	TTLL3
5.63939489626676E-32	1.14135837100451	0.158	0.071	9.19841701530072E-28	Endothelial	GRK5
1.98480394330381E-31	1.08659095302638	0.199	0.101	3.23741371192284E-27	Endothelial	HERC1
2.38338258860575E-31	1.67576510029709	0.111	0.043	3.88753534027483E-27	Endothelial	SNAPC1
2.75356820807347E-31	0.519230049254986	0.42	0.285	4.49134510418864E-27	Endothelial	RBM6
4.20437770443759E-31	2.19583383246348	0.144	0.066	6.85776047370815E-27	Endothelial	SCN1B
6.96955386316098E-31	0.664643108609241	0.491	0.331	1.13680393062019E-26	Endothelial	LMNA
1.34310097517239E-30	0.492989143671826	0.275	0.153	2.19073200060369E-26	Endothelial	ABCC1
1.38753280563276E-30	0.619190715727221	0.315	0.193	2.26320475926759E-26	Endothelial	ASXL1
1.65149444022736E-30	2.91463036928503	0.125	0.054	2.69375258145485E-26	Endothelial	CARD10
1.97205485798306E-30	2.51285169794717	0.15	0.072	3.21661867885616E-26	Endothelial	EDA
2.20888394413585E-30	1.22753609080401	0.224	0.126	3.60291060127999E-26	Endothelial	DENND4C
3.9934151655833E-30	1.21527137009776	0.18	0.091	6.51365947658293E-26	Endothelial	EIF4G3
4.1829463233663E-30	2.86986182885546	0.141	0.066	6.82280374804277E-26	Endothelial	HYAL1
4.84304784434427E-30	1.27425123393649	0.103	0.038	7.89949533890994E-26	Endothelial	SLC9A9
6.56789356940152E-30	1.08408971001856	0.206	0.109	1.07128912010508E-25	Endothelial	TMEM165
1.09866392350969E-29	0.897345767672853	0.428	0.309	1.79203072563666E-25	Endothelial	NFKBIA
1.22053896920844E-29	0.986750115035722	0.153	0.07	1.99082111267589E-25	Endothelial	CLASP1
1.57171880084461E-29	1.9287517049442	0.167	0.084	2.56363053605764E-25	Endothelial	RGS12
1.6394095408954E-29	2.2303264787326	0.107	0.043	2.67404090215448E-25	Endothelial	ANO10
2.04490539179365E-29	2.06697302445359	0.186	0.1	3.33544518455462E-25	Endothelial	MYC
2.14672927695339E-29	1.45617327649047	0.185	0.097	3.50153012363868E-25	Endothelial	MTMR12
2.23350360473837E-29	0.42228432724786	0.238	0.123	3.64306772968875E-25	Endothelial	COTL1
6.4202798171669E-29	2.49062118584087	0.162	0.083	1.04721184097809E-24	Endothelial	GATA4
6.57962708117085E-29	2.68131529233914	0.112	0.047	1.07320297320978E-24	Endothelial	MCC
8.41196284554731E-29	1.03717906416424	0.265	0.164	1.37207525973722E-24	Endothelial	INTS3
9.44725765573317E-29	1.31405068210342	0.121	0.051	1.54094219622664E-24	Endothelial	ABI2

2.02540948453261E-28	2.62334786494034	0.119	0.052	3.30364541022114E-24	Endothelial	TDRD10
2.10553562379248E-28	1.15843511869541	0.166	0.082	3.43433915596791E-24	Endothelial	RB1
2.17219197910614E-28	1.34142617230247	0.156	0.075	3.54306233712003E-24	Endothelial	SRGAP2B
2.47686658232772E-28	2.24835898506875	0.17	0.089	4.04001708243474E-24	Endothelial	LRMDA
3.20585521332402E-28	2.39318430795419	0.124	0.055	5.2290704384528E-24	Endothelial	HEBP1
3.6399677592802E-28	2.46150996908117	0.131	0.06	5.93715141216194E-24	Endothelial	CAMSAP2
7.66725439623135E-28	1.89586321807119	0.164	0.084	1.2506058645693E-23	Endothelial	SH3PXD2A
1.12170776074579E-27	0.87927238724094	0.25	0.147	1.82961752855247E-23	Endothelial	CENPT
1.6418043514066E-27	0.769180410623735	0.149	0.069	2.67794707757931E-23	Endothelial	ITPR1
1.7091765347479E-27	0.785314933451924	0.331	0.224	2.7878378458273E-23	Endothelial	UBR4
1.73616593281351E-27	1.16010417281173	0.112	0.046	2.83186025301211E-23	Endothelial	BBS9
2.67982678659494E-27	0.828580070709504	0.321	0.209	4.37106547161501E-23	Endothelial	SBNO2
2.72254782957059E-27	0.839249637976282	0.107	0.042	4.44074776481259E-23	Endothelial	SLC25A36
2.96854187294684E-27	0.459849420256967	0.398	0.271	4.8419886489636E-23	Endothelial	JAK1
3.44061778443512E-27	1.2046477681594	0.156	0.076	5.61199166819212E-23	Endothelial	ZDHHC3
3.48918661997422E-27	2.51872035206793	0.155	0.08	5.69121229583994E-23	Endothelial	SH3BGRL2
3.8668722438193E-27	2.00202557696589	0.184	0.102	6.30725531689365E-23	Endothelial	SLC2A9
4.43908962692034E-27	0.745076363751836	0.191	0.099	7.24059909046976E-23	Endothelial	NCOA3
4.85268817997289E-27	1.08691558450992	0.158	0.076	7.91521969035379E-23	Endothelial	DSE
5.97629633033809E-27	0.57641534088504	0.321	0.204	9.74793694441446E-23	Endothelial	KLC1
6.54888506647405E-27	0.429716687167254	0.16	0.074	1.06818864319258E-22	Endothelial	TMEM173
6.90867142984237E-27	1.1352400586911	0.121	0.052	1.12687339692159E-22	Endothelial	FUT11
6.98367279649609E-27	1.25177889245633	0.19	0.102	1.13910686983648E-22	Endothelial	DEPRECATI
1.14475842421676E-26	1.38776828642577	0.256	0.164	1.86721546573996E-22	Endothelial	CALU
1.75551120369193E-26	1.61182970207233	0.172	0.091	2.8634143243419E-22	Endothelial	RAPGEF2
2.98794059624308E-26	2.11491412660155	0.207	0.125	4.87362990653208E-22	Endothelial	LRP5
3.1060301805123E-26	1.65226943709113	0.173	0.093	5.06624582743361E-22	Endothelial	KAT2A
4.68275445461888E-26	0.756844740459552	0.235	0.137	7.63804079092885E-22	Endothelial	EXOC4
5.18992141922942E-26	2.49155770345999	0.142	0.071	8.46528082690511E-22	Endothelial	NCKAP1
5.39035152300843E-26	2.33841701856395	0.129	0.061	8.79220236917905E-22	Endothelial	GNA11
5.41959273453959E-26	1.05311490896706	0.228	0.135	8.83989770930753E-22	Endothelial	DELE1
1.34426381329922E-25	0.698940186979417	0.134	0.06	2.19262870587236E-21	Endothelial	HK1
2.07265531561349E-25	1.39315439599054	0.186	0.103	3.38070808529716E-21	Endothelial	CCNYL1
2.11045221183968E-25	1.29753735323637	0.133	0.063	3.4423586027317E-21	Endothelial	TRAF6
2.43055460946088E-25	0.460399807181304	0.23	0.126	3.96447762349164E-21	Endothelial	CMTM3
2.72678691512502E-25	1.42981222279846	0.176	0.095	4.44766213726042E-21	Endothelial	CD40
5.4389837380532E-25	1.54960822182896	0.229	0.143	8.87152637513857E-21	Endothelial	ANXA2
6.82852076571612E-25	0.693932155637577	0.236	0.139	1.11380002209596E-20	Endothelial	PIEZO1
7.22104667729529E-25	0.631547617614199	0.109	0.045	1.17782492353364E-20	Endothelial	SYNGAP1
7.37122938917004E-25	2.09390970629903	0.193	0.113	1.20232122566753E-20	Endothelial	ITPKC
8.41648382298531E-25	1.86015674763397	0.12	0.055	1.37281267636713E-20	Endothelial	DAB2IP
1.28709964565695E-24	2.23241470888263	0.168	0.093	2.09938823203106E-20	Endothelial	BAIAP2
1.50920932596982E-24	1.53103485783838	0.138	0.067	2.46167133158937E-20	Endothelial	TBC1D9
1.89208580380618E-24	1.19744412876153	0.153	0.077	3.08618115458826E-20	Endothelial	HIF1A
1.91246157774523E-24	1.46076152862081	0.112	0.049	3.11941607946025E-20	Endothelial	SDCCAG8
2.16932414713083E-24	0.910008617706144	0.151	0.075	3.5383846163851E-20	Endothelial	PHACTR2
3.20710730926903E-24	2.41109237408447	0.156	0.085	5.23111273214872E-20	Endothelial	MPDZ
3.62656130102839E-24	0.794694077350063	0.224	0.133	5.9152841381074E-20	Endothelial	MAPK8IP3
3.95358414506376E-24	1.19097197047697	0.136	0.065	6.4486910990135E-20	Endothelial	YEATS2
4.18092774069031E-24	0.802193967954604	0.101	0.041	6.81951123783997E-20	Endothelial	REC8
4.47098358911216E-24	0.659925388241105	0.141	0.067	7.29262133220084E-20	Endothelial	DGKD
5.3042052583901E-24	1.18831308559817	0.211	0.125	8.65168919696009E-20	Endothelial	HPCAL1

1.01260750726134E-23	0.316218157682544	0.548	0.416	1.65166410509397E-19	Endothelial	CFLAR
1.19779313913312E-23	1.4062394801563	0.119	0.055	1.95372038924004E-19	Endothelial	AGAP1
3.62598244878429E-23	1.8857432728302	0.168	0.094	5.91433997221206E-19	Endothelial	FARP1
4.69956339404367E-23	1.8587496148777	0.16	0.087	7.66545785202463E-19	Endothelial	TAX1BP3
4.99427939915549E-23	2.29258635201871	0.147	0.078	8.14616912796251E-19	Endothelial	CCND1
6.01756687819954E-23	1.34045140339612	0.131	0.064	9.81525333503126E-19	Endothelial	SLC35A1
6.39550617075962E-23	1.70995674000233	0.124	0.06	1.0431710115126E-18	Endothelial	EVA1B
6.74865219362494E-23	0.691581872547286	0.203	0.115	1.10077265930216E-18	Endothelial	MTA2
9.44087024008794E-23	0.714504175787144	0.197	0.11	1.53990034486074E-18	Endothelial	MAP3K3
1.54058133469159E-22	0.959054097686096	0.167	0.09	2.51284221501545E-18	Endothelial	KBTBD2
2.02587214936586E-22	0.758695724132934	0.115	0.052	3.30440006283065E-18	Endothelial	PLEKHG2
2.27049621001092E-22	2.18757665781862	0.157	0.087	3.70340636814881E-18	Endothelial	PPARG
2.36662684965341E-22	0.354465182473766	0.212	0.119	3.86020505446968E-18	Endothelial	ANKRD36C
2.81615802388897E-22	0.442137993683767	0.173	0.089	4.59343535276529E-18	Endothelial	ENTPD1
5.05409243245776E-22	1.21393129570362	0.159	0.086	8.24373016658185E-18	Endothelial	GTF2I
5.93828903862521E-22	2.78536422504354	0.112	0.054	9.68594325090158E-18	Endothelial	RPH3AL
6.92296979723716E-22	1.03633062912343	0.241	0.156	1.12920560362735E-17	Endothelial	ERF
9.32742236731194E-22	1.36518767445574	0.127	0.062	1.52139586233225E-17	Endothelial	ZNF496
1.05455368492943E-21	1.17029838066698	0.145	0.075	1.72008251548839E-17	Endothelial	IL4R
1.09251757569909E-21	1.01296647030592	0.114	0.052	1.78200541772278E-17	Endothelial	BICD2
1.12090492655477E-21	2.01223632108367	0.127	0.064	1.82830802570349E-17	Endothelial	HSD17B14
2.04924850494399E-21	1.74142057306064	0.198	0.124	3.34252923641414E-17	Endothelial	CDC42EP1
2.22897135140337E-21	2.71606127160519	0.121	0.061	3.63567517127404E-17	Endothelial	NT5E
2.26706803513546E-21	0.614172160710577	0.177	0.097	3.69781467210945E-17	Endothelial	ERICH1
2.59244039511131E-21	1.09478673777107	0.143	0.074	4.22852952846606E-17	Endothelial	DEPRECATI
4.02838361196898E-21	1.19632314420149	0.238	0.157	6.5706965094826E-17	Endothelial	NUMB
4.80428094580471E-21	1.99716441714185	0.101	0.046	7.83626265070207E-17	Endothelial	CTPS2
5.96334394767865E-21	1.69905702091989	0.159	0.089	9.72681031305865E-17	Endothelial	VAT1
1.05824641045402E-20	1.56832991004681	0.228	0.152	1.72610572009155E-16	Endothelial	SIK2
1.21042998984494E-20	1.25537878371862	0.203	0.118	1.97433235643608E-16	Endothelial	VCAM1
1.28900188606148E-20	0.903732455411044	0.142	0.073	2.10249097635487E-16	Endothelial	INPP5B
1.33492263846833E-20	2.25901342967044	0.155	0.088	2.1773923156057E-16	Endothelial	CXCL2
1.41478851962531E-20	1.3491864653903	0.127	0.064	2.30766155436084E-16	Endothelial	PDK1
2.10344673345263E-20	0.402374706937006	0.408	0.305	3.43093196693458E-16	Endothelial	ANKRD10
2.22910932583302E-20	1.33227903688203	0.175	0.102	3.63590022136623E-16	Endothelial	SWAP70
2.308421769139E-20	2.03119651173983	0.131	0.069	3.76526674764263E-16	Endothelial	PER3
2.3430010418064E-20	0.334529622602454	0.19	0.104	3.82166899929042E-16	Endothelial	ABI3
3.21435179003032E-20	0.440590708222815	0.213	0.124	5.24292920471845E-16	Endothelial	UTRN
3.50197984691464E-20	1.99401921639183	0.106	0.05	5.71207932830246E-16	Endothelial	WIPI1
5.03716541898626E-20	1.64624674435645	0.101	0.047	8.21612051490849E-16	Endothelial	C2CD2
6.01004191354666E-20	1.69227402986931	0.219	0.147	9.80297936518596E-16	Endothelial	TMEM37
6.11626256160278E-20	1.53567390909564	0.198	0.125	9.97623586423029E-16	Endothelial	PEA15
6.56136239787868E-20	0.807182483694255	0.21	0.128	1.07022382071799E-15	Endothelial	LMBR1L
8.19244293721568E-20	0.785421231285242	0.354	0.267	1.33626936748925E-15	Endothelial	PIM3
9.33260421421112E-20	0.819733231578252	0.211	0.129	1.52224107337997E-15	Endothelial	AFF1
1.00837254944224E-19	0.489786695803402	0.384	0.287	1.64475646539523E-15	Endothelial	MINK1
1.00951859941976E-19	1.07391058471405	0.106	0.049	1.64662578751357E-15	Endothelial	ZNF532
1.15603604630679E-19	0.734816166965229	0.179	0.104	1.885610395131E-15	Endothelial	PCF11
1.95489990697137E-19	1.03677895428026	0.153	0.085	3.188637238261E-15	Endothelial	NF1
1.95680207696233E-19	1.64488036032633	0.157	0.091	3.19173986773326E-15	Endothelial	RALGAPA2
2.00278152377131E-19	0.768383685865682	0.284	0.2	3.26673694342339E-15	Endothelial	DYNC1LI2
2.17474756979972E-19	0.928284460600325	0.108	0.051	3.54723076110032E-15	Endothelial	GIT1

**2.37382852956933E-19**	1.13841014949826	0.204	0.127	3.87195171458053E-15	Endothelial	TLNRD1
**2.79120037471782E-19**	1.03342004021012	0.216	0.138	4.55272693120224E-15	Endothelial	IRF2BPL
**2.80178691004331E-19**	0.566753183469398	0.244	0.157	4.56999462897165E-15	Endothelial	TRIP12
**4.6161415632101E-19**	0.476318044424375	0.14	0.072	7.529388503752E-15	Endothelial	MFNG
**5.20099875048921E-19**	1.19545310081293	0.133	0.071	8.48334906192295E-15	Endothelial	PIP5K1C
**6.56277312730284E-19**	0.853206457658997	0.241	0.161	1.07045392479437E-14	Endothelial	AFF4
**6.6461465918052E-19**	1.37282842833714	0.153	0.087	1.08405297058935E-14	Endothelial	EXOC6
**6.80350010458777E-19**	1.18783813278325	0.253	0.177	1.10971890205931E-14	Endothelial	SLC23A2
**6.97088351233411E-19**	0.660165803679193	0.101	0.046	1.13702080969682E-14	Endothelial	ZNF544
**7.026016536898E-19**	0.576208817513225	0.19	0.111	1.14601355733343E-14	Endothelial	WDFY1
**7.95608621877402E-19**	0.832193249481109	0.145	0.078	1.29771722314423E-14	Endothelial	TMEM185A
**9.41158965001475E-19**	0.31515116504214	0.164	0.09	1.53512438781391E-14	Endothelial	CLSTN1
**9.53502150205541E-19**	0.260286844739312	0.201	0.117	1.55525735720026E-14	Endothelial	RASSF1
**1.09597460531101E-18**	1.09778384274609	0.152	0.086	1.7876441787228E-14	Endothelial	POLK
**1.12012107033782E-18**	1.06279114020668	0.284	0.211	1.82702947782801E-14	Endothelial	TXNDC5
**1.13291030869538E-18**	0.607882759001499	0.342	0.257	1.84789000451303E-14	Endothelial	NPLOC4
**1.57622969155756E-18**	0.918678905137767	0.151	0.083	2.57098824989954E-14	Endothelial	ABL1
**1.83534610678724E-18**	1.13125384959908	0.199	0.126	2.99363303478067E-14	Endothelial	MAP4K4
**2.44212385842656E-18**	0.92991715268783	0.421	0.339	3.98334822547957E-14	Endothelial	CD14
**2.48821281552024E-18**	1.45080213396048	0.271	0.194	4.05852392339506E-14	Endothelial	DEPP1
**2.73246308482993E-18**	1.31401131691914	0.204	0.133	4.4569205376661E-14	Endothelial	ATP11A
**3.50944872429391E-18**	0.942374694924081	0.212	0.136	5.72426181419579E-14	Endothelial	SPAG9
**4.61468241915605E-18**	0.994914744892982	0.133	0.071	7.52700849388543E-14	Endothelial	C2CD2L
**4.81445984589879E-18**	1.88317113844978	0.177	0.113	7.85286545464552E-14	Endothelial	NHSL1
**5.04570517427417E-18**	0.615094724007245	0.321	0.237	8.23004970975859E-14	Endothelial	CALCOCO2
**5.7183992117222E-18**	0.6793222511522	0.225	0.149	9.32728095424008E-14	Endothelial	ZBTB16
**6.76847057270481E-18**	3.3480433817591	0.104	0.053	1.10400523511388E-13	Endothelial	INHBB
**7.16379240257933E-18**	2.16982321465522	0.176	0.112	1.16848617878471E-13	Endothelial	SYNPO
**1.13315197621676E-17**	0.78069815357359	0.266	0.189	1.84828418840715E-13	Endothelial	CRK
**1.1886096042496E-17**	0.663897291787765	0.345	0.266	1.93874112549153E-13	Endothelial	GOLPH3
**1.2198985870296E-17**	0.66576405773476	0.248	0.167	1.98977658530397E-13	Endothelial	PBX2
**1.37462947025473E-17**	0.694031435439884	0.12	0.061	2.24215812893248E-13	Endothelial	RBMS1
**1.87997170228311E-17**	0.632249554580059	0.165	0.095	3.06642184359399E-13	Endothelial	CNOT8
**2.13886401583165E-17**	1.02763638008962	0.117	0.06	3.488701096223E-13	Endothelial	CEP68
**2.45090468100836E-17**	1.4429613569496	0.12	0.063	3.99767062519273E-13	Endothelial	DNAJB4
**3.03454584680614E-17**	0.835029021802629	0.296	0.221	4.94964773072549E-13	Endothelial	ADRM1
**3.32337661645395E-17**	0.785708151865776	0.203	0.129	5.42075959909804E-13	Endothelial	E2F4
**3.77370831345215E-17**	1.30598450258713	0.213	0.144	6.1552956300718E-13	Endothelial	DEDD
**4.47079232683535E-17**	0.531214728453017	0.319	0.235	7.29230936430114E-13	Endothelial	SETD5
**6.83648874171678E-17**	0.983158893239151	0.26	0.186	1.11509967866142E-12	Endothelial	TFPI
**8.2240528496638E-17**	1.08327595334432	0.1	0.049	1.34142526030866E-12	Endothelial	SLC41A1
**9.12021823520681E-17**	0.872990868063479	0.191	0.12	1.48759879634458E-12	Endothelial	TECPR1
**1.16019224817127E-16**	2.38127679400734	0.102	0.052	1.89238957599216E-12	Endothelial	RAB11FIP5
**1.33085469799604E-16**	2.16385765044397	0.15	0.092	2.17075709790135E-12	Endothelial	WFS1
**1.5457929735333E-16**	0.504129864565954	0.139	0.076	2.52134291913017E-12	Endothelial	MTERF4
**1.67038157539445E-16**	0.788501107024035	0.149	0.084	2.72455938762588E-12	Endothelial	KIF13B
**2.07264170388755E-16**	0.887167018766209	0.116	0.061	3.38068588321098E-12	Endothelial	CSNK1A1
**3.32877812526109E-16**	2.31959291305357	0.118	0.065	5.42957000011337E-12	Endothelial	FERMT2
**3.39735388426687E-16**	1.48168507883699	0.115	0.062	5.5414239206277E-12	Endothelial	PGM2
**3.4688108550191E-16**	0.773556356594223	0.208	0.134	5.65797738562166E-12	Endothelial	SH2B3
**4.02584492377734E-16**	0.834711282323969	0.179	0.109	6.56655565517321E-12	Endothelial	LGALS9
**4.98862252951218E-16**	1.84136252336253	0.146	0.088	8.13694220788732E-12	Endothelial	IFT43

**5.55319996144726E-16**	0.782165445511785	0.128	0.07	9.05782445711663E-12	Endothelial	WWC3
**6.02209370723122E-16**	0.855820461160792	0.122	0.065	9.82263704586484E-12	Endothelial	RABGAP1
**6.09408706402193E-16**	1.14360625057672	0.259	0.195	9.94006541012617E-12	Endothelial	SNTB1
**6.32738736261981E-16**	1.12986869746242	0.166	0.102	1.03206015271692E-11	Endothelial	LRCH1
**9.24108770535105E-16**	1.29383458533504	0.155	0.095	1.50731381561981E-11	Endothelial	GPHN
**9.63077207282843E-16**	1.12937011594423	0.104	0.053	1.57087523279904E-11	Endothelial	GNG10
**1.1156760197284E-15**	0.447527304146523	0.17	0.102	1.81977915577899E-11	Endothelial	DIS3L2
**1.14483968485265E-15**	0.905360377010399	0.179	0.113	1.86734800996316E-11	Endothelial	TNIP2
**1.77640239735112E-15**	1.87544949291623	0.189	0.13	2.8974899503194E-11	Endothelial	NR5A2
**2.21907039285692E-15**	1.88011021791779	0.121	0.068	3.61952571778892E-11	Endothelial	RDX
**2.88606278020117E-15**	0.582913670128756	0.21	0.137	4.70745700078613E-11	Endothelial	RNF4
**3.26788450089629E-15**	1.10320835719223	0.125	0.07	5.33024640941193E-11	Endothelial	CRAMP1
**3.37086484227123E-15**	0.678779205530321	0.357	0.284	5.4982176442286E-11	Endothelial	SLC12A7
**3.75883891107676E-15**	1.1381051028995	0.263	0.201	6.1310421478573E-11	Endothelial	MTSS1
**4.62522969720178E-15**	0.805295773023389	0.232	0.161	7.54421215910582E-11	Endothelial	EGLN2
**4.67822648529333E-15**	1.32858220256231	0.146	0.089	7.63065522016196E-11	Endothelial	TSN
**4.86555587756101E-15**	0.997095740507401	0.217	0.151	7.93620819188977E-11	Endothelial	TRIM25
**5.35584467446686E-15**	0.80805346519735	0.297	0.226	8.73591824852289E-11	Endothelial	AGRN
**8.00884443483795E-15**	1.14826446331589	0.115	0.063	1.30632261576642E-10	Endothelial	C20orf194
**8.41503388123963E-15**	0.85675380566448	0.163	0.101	1.372576176369E-10	Endothelial	L3HYPDH
**9.6261830040301E-15**	0.337780673957982	0.125	0.068	1.57012670978735E-10	Endothelial	CD55
**1.06832301140498E-14**	1.65053620931977	0.185	0.127	1.74254166390267E-10	Endothelial	TRIO
**1.09502664197687E-14**	0.340872210697775	0.173	0.105	1.78609795572847E-10	Endothelial	FRYL
**1.33442789776016E-14**	1.09984687442843	0.103	0.054	2.1765853440366E-10	Endothelial	MAP4K5
**1.388225680522E-14**	0.49931219803613	0.133	0.075	2.26433490749944E-10	Endothelial	ANKRD36B
**1.69343142605297E-14**	0.362615919200616	0.266	0.185	2.76215599903499E-10	Endothelial	EHMT1
**1.88924677256912E-14**	1.04398555601836	0.114	0.062	3.08155041073749E-10	Endothelial	FRMD4B
**2.666707826422E-14**	0.463915647817898	0.2	0.128	4.34966713567693E-10	Endothelial	ETV6
**3.61003952613584E-14**	0.521483165627085	0.383	0.306	5.88833547108016E-10	Endothelial	ZYX
**3.85149172868112E-14**	1.71741523276849	0.169	0.113	6.28216815865177E-10	Endothelial	FLNB
**4.43031187299828E-14**	0.669758470654627	0.206	0.139	7.2262816960475E-10	Endothelial	PCMTD2
**4.66680294991345E-14**	0.577261059955498	0.299	0.226	7.61202229160383E-10	Endothelial	MAP4
**5.72291717735044E-14**	0.271129010495733	0.2	0.128	9.3346502079763E-10	Endothelial	DIP2A
**6.0462401903573E-14**	1.16755053715674	0.107	0.058	9.8620223744918E-10	Endothelial	MRRF
**7.11172162049539E-14**	0.541603547935912	0.106	0.056	1.159992913519E-09	Endothelial	NUP188
**7.33794238966332E-14**	0.375009546443062	0.203	0.129	1.19689178317798E-09	Endothelial	ATG16L2
**9.05802481554238E-14**	1.03448085579108	0.135	0.08	1.47745442766312E-09	Endothelial	CEP170
**9.76786095090341E-14**	0.317609177413481	0.333	0.251	1.59323579970186E-09	Endothelial	SPTAN1
**9.83487573896921E-14**	1.773805626181	0.147	0.094	1.60416658178327E-09	Endothelial	RAPH1
**1.12240381858987E-13**	1.53794769943498	0.121	0.071	1.83075286850194E-09	Endothelial	VPS37A
**1.17827716900449E-13**	0.627234855617493	0.2	0.133	1.92188789036322E-09	Endothelial	CASP4
**1.20398675733902E-13**	1.36955819779896	0.1	0.054	1.96382279989567E-09	Endothelial	PRKCE
**1.2166788334182E-13**	0.824965686903736	0.169	0.109	1.98452484518843E-09	Endothelial	NARF
**1.52829742531382E-13**	0.734270846006926	0.167	0.106	2.49280593042938E-09	Endothelial	GRAMD1A
**1.63688363202305E-13**	0.69702570616988	0.216	0.15	2.6699208921928E-09	Endothelial	TOP 1.00
**1.70630770615695E-13**	1.18991131297469	0.161	0.104	2.78315849951261E-09	Endothelial	DAPK3
**1.79270794990966E-13**	1.15032607082973	0.247	0.187	2.92408593709764E-09	Endothelial	SLC27A3
**1.8373818087899E-13**	1.22096070829255	0.131	0.079	2.9969534683172E-09	Endothelial	ROCK2
**1.85362078801562E-13**	1.23220767879599	0.112	0.063	3.02344086733229E-09	Endothelial	UBE2J1
**2.04592485794788E-13**	0.373947839676217	0.16	0.095	3.33710803579878E-09	Endothelial	LFNG
**3.09945162802944E-13**	0.491587652088308	0.302	0.23	5.05551555047882E-09	Endothelial	RERE
**4.3061471743033E-13**	0.792521072680171	0.301	0.236	7.02375665600611E-09	Endothelial	DEPRECATI

**4.38904417385022E-13**	0.720435027000067	0.133	0.079	7.1589699519671E-09	Endothelial	DPH7
**4.78557743885209E-13**	0.753671646875039	0.206	0.143	7.80575536051164E-09	Endothelial	IFRD1
**5.04785688730538E-13**	0.701935883249237	0.207	0.141	8.2335593688838E-09	Endothelial	GNAI2
**5.20931011190458E-13**	0.269533023037139	0.321	0.241	8.49690572352755E-09	Endothelial	DDX39B
**5.6432496351041E-13**	0.594102782445376	0.244	0.178	9.2047044798183E-09	Endothelial	BNIP2
**6.93500531468601E-13**	0.727215974468844	0.281	0.218	1.13116871687844E-08	Endothelial	TRIM8
**7.7675486141006E-13**	0.590095606426124	0.186	0.123	1.26696485444595E-08	Endothelial	ANKRD17
**8.933806109419E-13**	0.871700947282687	0.158	0.101	1.45719311450733E-08	Endothelial	SKIL
**1.31965348108791E-12**	1.14356906934479	0.14	0.087	2.1524867930025E-08	Endothelial	B4GALT5
**1.57239468937054E-12**	0.734240859636986	0.13	0.077	2.56473297783229E-08	Endothelial	LHFPL2
**1.83865415420535E-12**	0.692140158107219	0.195	0.135	2.99902879092435E-08	Endothelial	ITGB1
**1.86640895698919E-12**	0.978205631412279	0.1	0.055	3.04429964974507E-08	Endothelial	FARP2
**2.13321247550416E-12**	0.561552363996618	0.342	0.278	3.47948286879483E-08	Endothelial	TMEM140
**2.22705723524122E-12**	0.541125791380941	0.118	0.068	3.63255305640195E-08	Endothelial	PHF2
**2.32934433932919E-12**	0.260230721307423	0.157	0.096	3.79939355187984E-08	Endothelial	RC3H2
**2.73799818496721E-12**	1.659683277583	0.128	0.08	4.46594883950002E-08	Endothelial	BCL2L2
**3.031529990096E-12**	0.807705454323768	0.197	0.139	4.94472856684558E-08	Endothelial	CSAD
**3.8157461287635E-12**	1.07185013764882	0.179	0.124	6.22386351062614E-08	Endothelial	MEF2A
**3.856432205177E-12**	1.06172481482906	0.201	0.144	6.2902265698642E-08	Endothelial	ZMIZ1
**4.39066430561973E-12**	0.432541676114639	0.275	0.207	7.16161254889633E-08	Endothelial	FUS
**4.65160024109582E-12**	0.340147493941159	0.263	0.192	7.58722515325139E-08	Endothelial	TIA1
**4.80219839361264E-12**	0.319097927170974	0.175	0.111	7.83286579982157E-08	Endothelial	IL18BP
**5.04290338240075E-12**	0.590868185356276	0.221	0.159	8.22547970703387E-08	Endothelial	TCERG1
**5.77268694453892E-12**	1.72611743640219	0.17	0.12	9.41582967523743E-08	Endothelial	KIFC3
**5.97126593473478E-12**	0.899667887018823	0.114	0.067	9.7397318661459E-08	Endothelial	FAM120B
**7.99810350206321E-12**	0.417947350397471	0.186	0.124	1.30457066222153E-07	Endothelial	HNRNPD
**1.10661084329262E-11**	1.11620349043579	0.14	0.089	1.80499294649459E-07	Endothelial	ADAM9
**1.14385553025648E-11**	0.825646490547259	0.137	0.085	1.86574275540135E-07	Endothelial	TMOD3
**1.20888071310781E-11**	1.63795623622091	0.117	0.072	1.97180533115015E-07	Endothelial	MAP1LC3A
**1.43918576472961E-11**	0.283592792377499	0.183	0.122	2.34745590085046E-07	Endothelial	EPS15
**1.4712086363004E-11**	0.694348978424195	0.183	0.126	2.39968840666959E-07	Endothelial	RBM17
**1.49139321936023E-11**	1.69991414096292	0.106	0.062	2.43261148009847E-07	Endothelial	RHOBTB1
**1.53220606320671E-11**	0.44744653652818	0.262	0.197	2.49918130969646E-07	Endothelial	GPATCH2L
**1.6217690814752E-11**	1.29279113790662	0.157	0.106	2.6452675487942E-07	Endothelial	NCKAP5L
**1.99213318829841E-11**	1.20131197051872	0.1	0.058	3.24936844343353E-07	Endothelial	TTC21B
**2.10850945365341E-11**	0.568960798364524	0.13	0.08	3.43918976985407E-07	Endothelial	FOXO1
**2.12671333022436E-11**	0.314207541867212	0.394	0.307	3.46888211292896E-07	Endothelial	DEPRECATI
**2.18757826995772E-11**	1.67828182605162	0.153	0.105	3.56815891612803E-07	Endothelial	ST5
**2.88514792746631E-11**	0.710016745940185	0.131	0.081	4.7059647844903E-07	Endothelial	WDR37
**3.58443101240156E-11**	0.3705715396788	0.241	0.176	5.84656542432818E-07	Endothelial	MARK3
**3.71706372268681E-11**	0.286740124123903	0.273	0.204	6.06290263807446E-07	Endothelial	MAX
**3.74293442211432E-11**	0.467848599223558	0.219	0.157	6.10510033591067E-07	Endothelial	TMEM109
**4.78125626328985E-11**	0.681810042824383	0.145	0.093	7.79870709105208E-07	Endothelial	STK25
**5.53318512814045E-11**	1.4532734945297	0.151	0.103	9.02517826250989E-07	Endothelial	ACVR1B
**5.58470508264658E-11**	2.24305423063198	0.119	0.076	9.10921246030483E-07	Endothelial	MTUS1
**5.96673087333059E-11**	0.588252894486772	0.232	0.171	9.73233472748953E-07	Endothelial	ELMSAN1
**6.27772250781312E-11**	0.41745829724507	0.34	0.279	1.0239593182494E-06	Endothelial	GSDMD
**8.05853993246285E-11**	0.682068022253432	0.229	0.173	1.31442844838402E-06	Endothelial	DENND11
**8.41482569448677E-11**	0.42855672153999	0.262	0.197	1.37254221902774E-06	Endothelial	PPP1R12C
**9.5973773056059E-11**	0.908189690776463	0.144	0.095	1.56542821231738E-06	Endothelial	RUFY2
**9.66036296676352E-11**	0.777496160166533	0.112	0.067	1.5757018035088E-06	Endothelial	GALK2
**1.00804683150347E-10**	0.791429870928758	0.247	0.19	1.64422518686531E-06	Endothelial	RTN4

1.03387675625362E-10	0.451076047398308	0.206	0.146	1.68635637712528E-06	Endothelial	ANKLE2
1.04272821429607E-10	0.593262495044565	0.169	0.115	1.70079399033831E-06	Endothelial	ANKFY1
1.18785096490437E-10	0.693823002804485	0.273	0.217	1.93750370885552E-06	Endothelial	BACH1
1.58815677169381E-10	0.890838587035566	0.158	0.107	2.59044251030977E-06	Endothelial	MBNL2
1.62002187000601E-10	0.722848928524996	0.218	0.164	2.6424176721668E-06	Endothelial	SEC31A
1.75439656138392E-10	0.681494182123679	0.122	0.076	2.86159623127332E-06	Endothelial	ZNF337
1.99001824176653E-10	0.641529935624297	0.164	0.112	3.24591875414538E-06	Endothelial	MYSM1
2.13643751781987E-10	1.2742478113211	0.133	0.087	3.48474323531599E-06	Endothelial	TSPAN3
2.17412329524548E-10	1.00426433307516	0.223	0.173	3.5462125068749E-06	Endothelial	CFI
2.24401708561445E-10	1.04641889787214	0.14	0.092	3.66021626834572E-06	Endothelial	ILK
2.28224218505998E-10	1.60321800659185	0.143	0.097	3.72256522805133E-06	Endothelial	SETD7
2.37291941283719E-10	0.374450374603475	0.153	0.1	3.87046885427875E-06	Endothelial	FAM111A
2.46287810871699E-10	0.906704621868966	0.235	0.183	4.01720048312828E-06	Endothelial	RUFY3
2.64299677864118E-10	0.360099729624985	0.152	0.099	4.31099204564163E-06	Endothelial	MAML1
2.71157054701567E-10	1.0865023437405	0.147	0.099	4.42284271923727E-06	Endothelial	SNX8
2.93298084244848E-10	0.27019560295367	0.055	0.117	4.78398505211771E-06	Endothelial	ACO1
3.74434330425577E-10	0.707848139384855	0.171	0.12	6.10739836357158E-06	Endothelial	PHF14
3.96345412546705E-10	0.583036749789199	0.168	0.115	6.4647900240493E-06	Endothelial	ARHGAP17
4.1596681657228E-10	1.08584828551437	0.129	0.084	6.78483474511045E-06	Endothelial	INPP5A
5.14212115080815E-10	0.381012822763603	0.25	0.19	8.38731380908317E-06	Endothelial	PNN
5.31553280201494E-10	0.558940158463536	0.25	0.194	8.67016555336656E-06	Endothelial	HSF1
5.64873637462074E-10	0.900559254555472	0.157	0.108	9.21365390064388E-06	Endothelial	MARCH5
6.34160840164662E-10	0.587900222984782	0.268	0.216	1.03437974639258E-05	Endothelial	TRIM27
8.38637003952848E-10	1.90203232717616	0.129	0.088	1.36790081714749E-05	Endothelial	CAV2
8.51287048732126E-10	1.14734622029708	0.219	0.174	1.38853430518697E-05	Endothelial	ASPH
8.5348928588986E-10	1.65131527541983	0.165	0.122	1.39212637421495E-05	Endothelial	ZCCHC24
8.58057859940433E-10	0.437362901809158	0.25	0.195	1.39957817534884E-05	Endothelial	STAU2
8.75634266083651E-10	1.23664042621629	0.125	0.081	1.42824705140904E-05	Endothelial	KLF4
8.79326077214523E-10	0.307117804593088	0.27	0.208	1.43426876454461E-05	Endothelial	ALG13
9.03346467951529E-10	0.853485702258016	0.204	0.153	1.47344842387574E-05	Endothelial	NPTN
9.61578383808899E-10	0.31885343939853	0.331	0.268	1.5684305018307E-05	Endothelial	PER1
9.71784454357178E-10	0.735170181022091	0.158	0.109	1.58507762350199E-05	Endothelial	SPECC1L
1.08479733320075E-09	1.86630010014032	0.111	0.071	1.76941293018375E-05	Endothelial	RCN1
1.11795898530861E-09	1.06764088906245	0.113	0.071	1.82350290093687E-05	Endothelial	WASHC5
1.15455231126237E-09	0.875729039440482	0.176	0.126	1.88319027490006E-05	Endothelial	CD81
1.22793612856378E-09	0.292790483200065	0.281	0.218	2.00288661930038E-05	Endothelial	MARCH7
1.2747878259596E-09	0.307399043304366	0.048	0.104	2.07930642292271E-05	Endothelial	PCYOX1
1.73223534209647E-09	0.778397672329426	0.161	0.113	2.82544906649355E-05	Endothelial	TARBP1
1.83332403043452E-09	0.538741484744264	0.181	0.13	2.99033482604174E-05	Endothelial	REV1
1.89605564736594E-09	1.46349727719571	0.112	0.072	3.09265636641858E-05	Endothelial	INPP1
2.06869513125913E-09	0.257963885480353	0.229	0.172	3.37424862859676E-05	Endothelial	BAX
2.07581941313456E-09	0.54547365654523	0.183	0.131	3.38586904476378E-05	Endothelial	RBM5
2.1567825267148E-09	0.856351027125958	0.266	0.224	3.51792797932452E-05	Endothelial	CD151
2.77891807852992E-09	0.61984685420919	0.175	0.125	4.53269327789016E-05	Endothelial	DHX9
3.1812480285554E-09	1.13452124776349	0.188	0.143	5.18893365937671E-05	Endothelial	FLOT1
3.27465741966264E-09	1.27808045280404	0.124	0.083	5.34129371721173E-05	Endothelial	ARL5B
3.29712429882327E-09	0.51959986213357	0.19	0.139	5.37793944381063E-05	Endothelial	MTA1
3.36182546006878E-09	0.771305786560199	0.186	0.137	5.48347350791819E-05	Endothelial	DYNC1I2
3.3861427110391E-09	0.366815191543197	0.125	0.08	5.52313737597587E-05	Endothelial	CCAR2
3.58143393530333E-09	1.08119943297796	0.114	0.073	5.84167689187326E-05	Endothelial	PARVB
3.73233900152629E-09	0.380832040888144	0.186	0.132	6.08781814538952E-05	Endothelial	LRCH3
4.54187907018487E-09	0.491464116418439	0.204	0.151	7.40825895137854E-05	Endothelial	DNAJC7

**4.98698055801121E-09**	0.397611488925732	0.315	0.257	8.13426398817209E-05	Endothelial	SLC3A2
**5.14934027304779E-09**	1.46636946050325	0.108	0.069	8.39908891936825E-05	Endothelial	PRAG1
**6.19095227065941E-09**	1.07363496855337	0.171	0.126	0.000100980622486726	Endothelial	SORBS3
**6.37144303942098E-09**	0.274635814140889	0.176	0.122	0.000103924607415996	Endothelial	SETX
**7.14006365370106E-09**	1.36795767369512	0.191	0.15	0.000116461578255518	Endothelial	RAB13
**7.25362819131764E-09**	0.287621671711668	0.066	0.126	0.000118313929428582	Endothelial	MACROD1
**7.31826902433865E-09**	0.636270572724316	0.209	0.161	0.000119368286055988	Endothelial	PPP2R1B
**8.09474925163076E-09**	1.13061708189178	0.111	0.072	0.000132033455043349	Endothelial	BCAP29
**9.93165382722038E-09**	0.674738100999315	0.265	0.216	0.000161995205575792	Endothelial	BCL3
**1.00618355622267E-08**	0.466368901249593	0.307	0.252	0.00016411859985548	Endothelial	ST6GAL1
**1.07907385651461E-08**	0.743407703391842	0.125	0.083	0.000176007736736099	Endothelial	TCF12
**1.10881445188577E-08**	0.284836784962603	0.14	0.093	0.000180858725247089	Endothelial	EZH1
**1.11694700376808E-08**	0.536220251382721	0.256	0.206	0.000182185225784611	Endothelial	MOV10
**1.22817206036864E-08**	0.86160990137032	0.234	0.19	0.000200327144766728	Endothelial	PDIA5
**1.36957406370221E-08**	1.14883523773309	0.105	0.067	0.000223391225530467	Endothelial	ATXN3
**1.39735243497696E-08**	0.609110375500413	0.177	0.13	0.000227922155669092	Endothelial	PPP1R10
**1.49534995233471E-08**	0.619320289116033	0.134	0.091	0.000243906530725315	Endothelial	SNX13
**1.61901562109013E-08**	0.904230809956086	0.144	0.102	0.000264077637956012	Endothelial	ATXN2
**1.77286983183839E-08**	0.3560148621603	0.284	0.233	0.000289172798271161	Endothelial	INTS11
**1.85712680904596E-08**	1.13261618703686	0.132	0.091	0.000302915953823486	Endothelial	ANGEL1
**1.85788264239803E-08**	0.812228001175058	0.1	0.063	0.000303039237801543	Endothelial	ADAM17
**1.95953818408422E-08**	0.308530956130505	0.148	0.101	0.000319620273205977	Endothelial	HELZ2
**1.96254134995433E-08**	0.491504358379558	0.16	0.112	0.000320110119591051	Endothelial	NECAP2
**2.03155918605614E-08**	0.726236651209619	0.135	0.093	0.000331367618837616	Endothelial	CANT1
**2.27138968431021E-08**	1.41764150773243	0.107	0.071	0.000370486371407838	Endothelial	CPLANE1
**2.31586613983499E-08**	0.344260115240004	0.304	0.252	0.000377740926068485	Endothelial	FBXO7
**2.5075587117925E-08**	0.818910393694599	0.167	0.123	0.000409007901480475	Endothelial	XPNPEP1
**2.53472508365291E-08**	1.02999059032721	0.105	0.068	0.000413439008394626	Endothelial	GPATCH2
**2.66070644276707E-08**	0.431563031810052	0.22	0.17	0.000433987827879738	Endothelial	ZNF638
**2.70394309524081E-08**	0.323226911647099	0.154	0.106	0.000441040158264728	Endothelial	SECISBP2
**2.81720848994237E-08**	0.323737267136952	0.415	0.355	0.0004595148767945	Endothelial	KLF6
**3.59087690441678E-08**	0.783020554929243	0.109	0.071	0.000585707931879421	Endothelial	DEGS1
**3.60864479194332E-08**	0.759258089461298	0.235	0.194	0.000588606052013874	Endothelial	REXO2
**3.91369382156636E-08**	0.319648625768133	0.1	0.063	0.000638362599235689	Endothelial	CCN1
**3.94554160257134E-08**	0.878381953275642	0.186	0.142	0.000643557290795411	Endothelial	VAMP5
**4.05332006699862E-08**	0.842516544508632	0.195	0.15	0.000661137036128145	Endothelial	IP6K2
**4.4074048844661E-08**	1.02358159205971	0.126	0.087	0.000718891810705265	Endothelial	MYO1E
**4.78261749140604E-08**	1.71967627484597	0.133	0.097	0.000780092739023239	Endothelial	NDRG2
**4.85731553044068E-08**	1.88142464144637	0.106	0.071	0.000792276736170179	Endothelial	TCEAL9
**5.07489132939641E-08**	0.708408832903078	0.162	0.119	0.000827765524737849	Endothelial	NPHP3
**5.3689748778319E-08**	0.587082640090036	0.107	0.069	0.000875733492323161	Endothelial	IPO8
**5.63492289974824E-08**	0.732314885313981	0.154	0.111	0.000919112274177936	Endothelial	RAB6A
**7.07653132565078E-08**	0.721354327278234	0.106	0.068	0.0011542530245269	Endothelial	SOS 2
**7.1626557448334E-08**	0.934188499174465	0.213	0.173	0.00116830077853978	Endothelial	UBC
**7.4905284952055E-08**	0.653709690028486	0.124	0.084	0.00122178010285297	Endothelial	FAM133B
**7.57819493168575E-08**	0.629758488063503	0.298	0.259	0.00123607937530726	Endothelial	CYB5R3
**8.20010754268264E-08**	0.468055691192509	0.168	0.123	0.00133751954128696	Endothelial	KHDC4
**8.28086895810902E-08**	0.497961684271394	0.149	0.103	0.00135069253575716	Endothelial	FMNL2
**8.68176139208712E-08**	0.800766745007189	0.12	0.081	0.00141608210066333	Endothelial	EPS15L1
**8.95699578818451E-08**	0.561683899091859	0.293	0.253	0.00146097558301078	Endothelial	FNDC3B
**9.15439608231979E-08**	0.265681544045595	0.201	0.15	0.00149317354498718	Endothelial	PSPC1
**1.05362987597965E-07**	0.752455903394732	0.205	0.162	0.00171857569071041	Endothelial	TNFSF10

1.25306984069792E-07	0.55093996631013	0.147	0.105	0.00204388221716238	Endothelial	PIGG
1.28294430984587E-07	0.411374442346371	0.132	0.091	0.0020926104637896	Endothelial	DUSP11
1.4122452718874E-07	0.346821389501041	0.159	0.114	0.00230351326297555	Endothelial	TRIM56
1.42884843594743E-07	0.945359870334367	0.1	0.066	0.00233059468387385	Endothelial	EXOC5
1.58083160047549E-07	1.34240419560552	0.183	0.147	0.00257849442353557	Endothelial	CARHSP1
1.73113853161751E-07	0.50559411015707	0.147	0.106	0.00282366005892132	Endothelial	ZDHHC14
2.13341666550866E-07	0.391504638217841	0.244	0.197	0.00347981592311118	Endothelial	DHX15
2.18258995539913E-07	0.978311459769418	0.13	0.092	0.00356002247625152	Endothelial	ZNF621
2.41409372056226E-07	0.692708149225508	0.164	0.123	0.0039376282676091	Endothelial	PTPA
2.46239257219897E-07	0.520128916351196	0.112	0.074	0.00401640852451373	Endothelial	MIGA2
2.50210066429262E-07	0.350301571773109	0.173	0.128	0.0040811763935277	Endothelial	PHF10
2.59285770716664E-07	0.541900624646477	0.163	0.12	0.00422921020615951	Endothelial	HUS1
2.72131986202367E-07	0.305577987006152	0.216	0.168	0.00443874482694681	Endothelial	ADD1
3.08081492659511E-07	1.67146630624092	0.121	0.087	0.00502511722676929	Endothelial	PLS3
3.17515266368564E-07	0.402695542334141	0.344	0.301	0.00517899150973764	Endothelial	HSP90AB1
3.24877200887599E-07	0.691192668911487	0.157	0.116	0.00529907202367763	Endothelial	CSRNP1
3.32426016200922E-07	0.684741394045608	0.118	0.081	0.00542220075025324	Endothelial	ZADH2
3.70236002929267E-07	0.59957298796838	0.229	0.188	0.00603891944377928	Endothelial	PTTG1IP
3.78085649252622E-07	0.307019989430849	0.37	0.322	0.00616695502495951	Endothelial	ITM2B
3.85445797738042E-07	0.448832725212794	0.132	0.092	0.00628700640690521	Endothelial	UNK
3.99666873487196E-07	1.96494391175203	0.121	0.088	0.00651896637344965	Endothelial	PLSCR4
4.20505176014028E-07	1.25203501727068	0.109	0.076	0.00685885992596482	Endothelial	REEP3
4.82593697766463E-07	0.301385501928362	0.065	0.115	0.00787158580426878	Endothelial	DUSP3
4.94462403833789E-07	0.452677810091791	0.308	0.263	0.00806517626893294	Endothelial	CSRP1
5.02300901738058E-07	0.409417939838954	0.14	0.099	0.00819303000824946	Endothelial	METTL3
5.22295925074894E-07	0.90939418161558	0.127	0.09	0.00851916883389659	Endothelial	RARA
5.96939153617188E-07	1.83971752322809	0.103	0.072	0.00973667453464995	Endothelial	ADCY6
6.65802066273294E-07	0.570767785565654	0.107	0.072	0.0108598975029837	Endothelial	TCF3
6.6726582280748E-07	0.719531871760067	0.174	0.134	0.0108837728358128	Endothelial	CLN8
7.0482457990033E-07	1.5189750227684	0.152	0.119	0.0114963937227543	Endothelial	TNKS1BP1
7.32712002532597E-07	0.815049409761245	0.103	0.068	0.0119512654733092	Endothelial	DRAM1
8.03183363021974E-07	0.840938459606891	0.141	0.105	0.0131007238342514	Endothelial	ZNF680
8.22633431263493E-07	1.0398387895709	0.193	0.159	0.0134179738973388	Endothelial	BEX3
8.33707573534328E-07	0.906493067207537	0.152	0.114	0.0135986042319184	Endothelial	DAPK1
8.67098423552486E-07	0.338263073128315	0.225	0.18	0.0141432423865646	Endothelial	UPK3BL1
8.86001995395038E-07	1.69475801849606	0.133	0.1	0.0144515785468885	Endothelial	FAM114A1
9.60337689645734E-07	0.466711009830322	0.299	0.259	0.0156640680558116	Endothelial	MYO18A
1.05415077174994E-06	0.554293779234197	0.133	0.096	0.0171942532380133	Endothelial	PISD
1.11840305785037E-06	0.388279168644271	0.285	0.245	0.0182422722765974	Endothelial	POGZ
1.1643075284931E-06	0.51134002141046	0.217	0.174	0.018991020097251	Endothelial	COLGALT1
1.17443362171688E-06	0.626872962304337	0.163	0.124	0.0191561868038241	Endothelial	PHKB
1.19475553895051E-06	0.763865233534597	0.182	0.144	0.0194876575958218	Endothelial	PITPNA
1.22199257301585E-06	0.560130312266679	0.199	0.157	0.019931920858461 5	Endothelial	ACP5
1.41029841999346E-06	0.559527237357345	0.17	0.131	0.0230033775285134	Endothelial	WWOX
1.42138534578006E-06	1.7807142098945	0.103	0.073	0.0231842163750185	Endothelial	NFIA
1.57998633930529E-06	0.864913032650891	0.103	0.07	0.0257711571804086	Endothelial	MAPK7
1.65002746047085E-06	2.0947585328161	0.1	0.071	0.02691359790774	Endothelial	SMARCA1
1.89198773407048E-06	0.528611430350917	0.142	0.104	0.0308602119304236	Endothelial	NCBP3
1.92493541170886E-06	0.349900831580869	0.417	0.343	0.0313976215003832	Endothelial	VIM
1.93164088859526E-06	0.884382609591011	0.149	0.114	0.0315069945338773	Endothelial	ZMAT3
1.93223374698546E-06	0.430174651736848	0.066	0.114	0.0315166646470798	Endothelial	GPRC5C
2.0083778239266E-06	0.502272384063137	0.105	0.071	0.0327586506860668	Endothelial	SFXN3

**2.17630745461161E-06**	0.463097186455649	0.076	0.128	0.03549775089217	Endothelial	MARC2
**2.29036683324349E-06**	0.521849146369609	0.101	0.069	0.0373581734170345	Endothelial	EXOSC10
**2.48387350444249E-06**	1.37777710449287	0.114	0.082	0.0405144607309614	Endothelial	PLA2G4C
**2.67691619290008E-06**	0.518604517247432	0.127	0.092	0.0436631800223933	Endothelial	ZNF264
**2.89777537165473E-06**	0.562109561964039	0.122	0.087	0.0472656140870602	Endothelial	BNIP3L

SupTable5K_C2_Hep_SeuratCluster

p_val	avg_log2FC	pct.1	pct.2	p_val_adj	cluster	gene
5.87743825199394E-256	1.0490693736867	0.995	0.958	9.58668953282731E-252	0	APOA1
9.07567347187226E-217	0.87854588770807	0.987	0.951	1.48033309999708E-212	0	TTR
1.16578956496286E-13	0.856846384627793	0.607	0.66	1.90151935941092E-09	0	CD14
0	0.82650834842651	1	0.989	0	0	APOE
1.32244128164174E-213	0.785434455661838	0.956	0.935	2.15703397448585E-209	0	GC
7.01122649986784E-155	0.783732870478369	0.973	0.944	1.14360115439344E-150	0	HP
2.344140016397E-16	0.772642315078249	0.624	0.691	3.82352678074515E-12	0	HYOU1
1.12149761047574E-06	0.772476158673343	0.219	0.286	0.0182927475244698	0	YIPF2
4.53186461962575E-56	0.75940031541966	0.755	0.796	7.39192438107157E-52	0	CTSD
4.06427201601772E-79	0.737589735978127	0.812	0.826	6.62923408532651E-75	0	SERPINC1
1.25394653093084E-06	0.72152873047262	0.26	0.337	0.0204531218660129	0	YWHAG
1.19474099192247E-225	0.720863805019408	0.995	0.982	1.94874203192474E-221	0	RBP4
1.41057103899156E-232	0.718992282115897	1	0.997	2.30078242169913E-228	0	APOA2
8.91137795552108E-163	0.712415369587982	0.982	0.98	1.45353485832504E-158	0	CES1
1.03873612672637E-135	0.71019996997538	0.905	0.885	1.69428249630339E-131	0	AMBP
8.01799266705516E-65	0.709514466565738	0.765	0.794	1.30781478392337E-60	0	LAMP1
1.09697374337093E-22	0.702599143425784	0.653	0.712	1.78927387281232E-18	0	CST3
4.07103294357724E-19	0.687142310800376	0.672	0.719	6.64026183426883E-15	0	HPR
2.69556773915921E-181	0.68233396895396	0.966	0.946	4.39674053934259E-177	0	CLU
4.14072300862125E-190	0.675439120420691	0.999	0.985	6.75393329936211E-186	0	ALB
1.57288585714389E-11	0.668127808380243	0.616	0.698	2.5655341215874E-07	0	CD63
2.92612179239347E-27	0.667061775830538	0.684	0.744	4.77279725557299E-23	0	NUCB1
4.57777615855825E-09	0.665880441393195	0.657	0.76	7.46681069222437E-05	0	CEBPA
2.08127221442483E-162	0.659290057735445	0.949	0.949	3.39476310894834E-158	0	AGT
4.79411861333995E-10	0.64735016450845	0.214	0.294	7.81968687021879E-06	0	NDUFS3
7.91582365265487E-27	0.642940909887671	0.702	0.757	1.29114999598454E-22	0	SPINK1
4.76434778700462E-177	0.640389832692164	0.973	0.953	7.77112767538324E-173	0	EPHX1
3.32388703217931E-07	0.639385683473655	0.269	0.349	0.00542159213818768	0	SMPD1
4.95626246073447E-25	0.636683314383589	0.675	0.749	8.08415969970399E-21	0	ECH1
3.83891379851013E-08	0.629608152117489	0.29	0.384	0.000626165229674988	0	PET100
2.45944232485178E-08	0.627445028181001	0.188	0.257	0.000401159637606573	0	KLF16
5.04267138493567E-143	0.625945787673296	0.953	0.953	8.22510129596858E-139	0	MT1G
5.30506804863028E-92	0.62045582967549	0.88	0.902	8.65309649412085E-88	0	ITIH2
5.8040907423968E-147	0.617328138577185	0.995	0.985	9.46705240992342E-143	0	TF
1.01159264853172E-06	0.614172559001748	0.213	0.278	0.0165000876902009	0	EDNRB
3.09577103219423E-18	0.612965312865664	0.639	0.719	5.04951213061201E-14	0	SERPIND1
1.8873283827748E-06	0.610206310213083	0.255	0.328	0.0307842132514398	0	VEGFB
3.27795885751912E-07	0.610168018457591	0.255	0.332	0.00534667869249944	0	CKAP4
6.00858704963803E-08	0.609356210138655	0.273	0.358	0.000980060633666458	0	DNAJC4
2.58480649381065E-13	0.606486268558312	0.643	0.735	4.21607787205454E-09	0	SDF4
4.43069654299414E-07	0.603617526064668	0.21	0.276	0.00722690913127774	0	CCT3
1.42328057515949E-06	0.601599346082615	0.234	0.301	0.0232151294614264	0	RNF128
5.6439003917712E-07	0.600046974110711	0.319	0.411	0.009205765929018	0	FKBP8
3.26093109338374E-07	0.599076414382816	0.232	0.303	0.00531890470641822	0	POLR2F
5.94113863107865E-07	0.595391466244955	0.309	0.403	0.00969059122115239	0	FAM50B
7.42679339326135E-08	0.595137224437977	0.36	0.481	0.00121138427037486	0	UBXN6
4.99696481644303E-165	0.58031379319382	1	0.991	8.15054931210023E-161	0	SERPINA1

3.71055426385686E-40	0.579833963993931	0.761	0.842	6.05228505977692E-36	0	HMGCS2
7.59480799888623E-38	0.57964551465841	0.743	0.797	1.23878913269833E-33	0	GSTA1
4.41742151059702E-66	0.569850194743939	0.814	0.864	7.2052562259348E-62	0	F2
2.90353394675884E-06	0.569539363310042	0.238	0.305	0.0473595422055834	0	CHMP2A
7.48544692323763E-17	0.569523717081423	0.651	0.724	1.22095124764929E-12	0	ETFB
3.05417130315909E-06	0.560214313496356	0.315	0.4	0.0498165881258278	0	EEF1G
3.98099961852912E-148	0.560014298145883	0.997	0.985	6.49340847778285E-144	0	VTN
1.64819727881321E-129	0.559479173870025	0.988	0.965	2.68837458147223E-125	0	APOC3
4.31157084203177E-13	0.557393825792694	0.298	0.42	7.03260320043802E-09	0	STUB1
3.21301232328944E-09	0.55518250772797	0.205	0.281	5.2407444005174E-05	0	SYF2
1.18739433289004E-09	0.554435068999642	0.261	0.35	1.93675889637694E-05	0	HTRA1
1.1182746745748E-07	0.554215418467499	0.292	0.381	0.00182401782169896	0	PKN1
4.36777526091731E-19	0.553059474465654	0.678	0.789	7.12427822808222E-15	0	RRBP1
4.75501619431125E-09	0.552079645733589	0.233	0.315	7.75590691454108E-05	0	ATP5F1E
6.50774849890572E-31	0.550464127183858	0.777	0.731	1.06147885765651E-26	0	CD74
6.8333241697668E-10	0.54550787720403	0.36	0.492	1.11458350533066E-05	0	SERINC1
2.44883471296096E-10	0.545132754192447	0.205	0.289	3.99429430031063E-06	0	NEU1
5.67710608202365E-22	0.542068828509695	0.704	0.79	9.25992773038878E-18	0	EEF2
4.20509573853603E-14	0.54164771853566	0.266	0.384	6.85893165912612E-10	0	HS6ST1
3.31957551632122E-08	0.535285496419443	0.335	0.447	0.000541455962467155	0	CXCL16
3.03973730904581E-06	0.533163072380295	0.357	0.445	0.0495811552478462	0	LY6E
1.17022346203027E-06	0.530514293739772	0.281	0.362	0.0190875148891758	0	PRG4
2.05865892803002E-10	0.528130156558984	0.336	0.459	3.35787857750977E-06	0	NACC1
4.88154396429331E-08	0.527300675781379	0.262	0.346	0.000796228636015882	0	TSPO
3.49794686977999E-10	0.527114804066772	0.211	0.294	5.70550113929814E-06	0	COX7A2
1.54800697211857E-07	0.522446561429266	0.373	0.497	0.0025249541722226	0	CAMK2N1
3.6352612842402E-11	0.522044601355936	0.248	0.342	5.92947468072419E-07	0	RHOA
1.65680103850027E-44	0.521308680485145	0.825	0.87	2.70240817389779E-40	0	ALDOB
1.33456549503887E-07	0.520818764826362	0.202	0.269	0.0021768097789579	0	SCAND1
9.5999411906896E-09	0.519844290623521	0.373	0.503	0.000156584640761338	0	FKBP2
8.60238355471349E-11	0.519123927288944	0.223	0.31	1.40313478160932E-06	0	MGAT2
5.26064902850446E-08	0.518994372370073	0.188	0.254	0.000858064463039363	0	NUDT3
2.44236420853204E-07	0.514414328258976	0.213	0.282	0.00398374026053662	0	DMTN
1.64524112808995E-09	0.514005715185833	0.305	0.399	2.68355280402752E-05	0	C7orf50
9.27007082865942E-11	0.513350650416282	0.182	0.258	1.51204125286264E-06	0	WDR83OS
1.07385033613233E-12	0.512481518565559	0.66	0.767	1.75155728326544E-08	0	CES2
1.2628544669587E-08	0.505583863038209	0.243	0.323	0.000205984192105633	0	PES 1.00
2.12560236056861E-45	0.50557067162636	0.82	0.872	3.46707001032346E-41	0	APOC1
2.01481157361655E-07	0.503915386918412	0.322	0.419	0.00328635915772595	0	NDUFB9
7.11371229177615E-46	0.50347677136782	0.872	0.872	1.16031761191161E-41	0	FABP1
1.7802611531198E-09	0.503082657013143	0.295	0.395	2.90378396685371E-05	0	KIAA2013
4.73413448260111E-09	0.50283736797467	0.237	0.321	7.72184675457067E-05	0	FAM3C
2.37417850965292E-12	0.501523751965981	0.335	0.461	3.87252256709488E-08	0	IRAK1
9.77765864013041E-08	0.500680489248038	0.632	0.742	0.00159483390079167	0	ADH1C
9.66015778428479E-10	0.498663859456836	0.296	0.398	1.57566833619469E-05	0	MXD4
4.84861773024745E-58	0.497908441043158	0.909	0.933	7.90858037980661E-54	0	FTL
1.17646559749475E-07	0.494905873678007	0.289	0.379	0.00191893303607369	0	NDUFB6
2.70685696151821E-08	0.493364104545772	0.31	0.408	0.000441515438993235	0	NDUFA2
1.24084027994972E-18	0.492531633332434	0.7	0.767	2.02393458062598E-14	0	KRT8
1.72621042120837E-11	0.489712087353762	0.232	0.327	2.81562181803298E-07	0	SUMO2

2.44903435157502E-08	0.487684358733193	0.184	0.252	0.000399461993085401	0	TMX4
3.22549087735636E-08	0.486110403431609	0.327	0.43	0.000526109817005595	0	NDUFAF3
4.59054339486657E-09	0.485952539161329	0.225	0.306	7.48763533136686E-05	0	SELENOK
3.08413828688589E-07	0.485622359184666	0.216	0.283	0.00503053795973957	0	TRIB3
2.27536477285901E-07	0.484863412843058	0.28	0.366	0.00371134748101033	0	DGAT2
4.33454636120409E-137	0.483918922858284	1	0.994	7.07007856976E-133	0	FGG
4.30600229404066E-15	0.482772740461754	0.691	0.824	7.02352034180972E-11	0	OS9
1.78971465426468E-33	0.482451023881646	0.889	0.903	2.91920357257112E-29	0	ORM1
7.69114038138176E-15	0.48224612331148	0.755	0.793	1.25450190760718E-10	0	ORM2
4.33351774240716E-11	0.4804238837299	0.346	0.467	7.06840078964032E-07	0	USF2
2.39950335631124E-07	0.480372293484879	0.302	0.391	0.00391382992447927	0	COX8A
1.39008109535088E-06	0.479437828800015	0.305	0.394	0.0226736127462682	0	TXNDC17
8.01973881330432E-09	0.479350361027397	0.23	0.31	0.000130809959783807	0	LIPA
1.21089576979389E-12	0.478067050580209	0.259	0.364	1.97509209011082E-08	0	SLC25A10
1.85581104011935E-07	0.477660246931238	0.281	0.362	0.00302701338753868	0	ATP5ME
4.370841301826E-07	0.476592102853575	0.3	0.384	0.00712927924740839	0	MSRB2
1.78013849107399E-26	0.475756242540021	0.755	0.841	2.90358389279079E-22	0	ALDH2
2.22410891062267E-09	0.4745084656663	0.353	0.477	3.62774404411664E-05	0	RNF5
3.71289842593497E-08	0.473174607052805	0.269	0.356	0.000605610862254252	0	ATP5F1A
7.71573288987017E-08	0.471994051253832	0.362	0.481	0.00125851319166672	0	SERPINA4
3.2982048102806E-07	0.469562802652202	0.309	0.403	0.00537970186604869	0	MCFD2
1.05334363025278E-47	0.462265132375555	0.829	0.887	1.71810879530531E-43	0	MT1E
4.09665719596606E-07	0.460457649241676	0.303	0.389	0.00668205755234025	0	CNPY3
9.56346511756951E-10	0.460288028113145	0.227	0.307	1.55989679532676E-05	0	BRMS1
3.29700893523866E-09	0.459224242859057	0.311	0.422	5.37775127426777E-05	0	GBA
7.41629039602901E-07	0.459103716696262	0.236	0.304	0.0120967112649629	0	MARCKS
9.29787376400998E-13	0.458665243831196	0.743	0.85	1.51657618964767E-08	0	NUPR1
1.57064409158502E-06	0.457384697172472	0.23	0.296	0.0256187757778433	0	TMEM147
4.13028987965072E-07	0.45710119228982	0.245	0.318	0.00673691582269829	0	EMC7
7.51840604677095E-40	0.457038367259856	0.865	0.896	1.22632721028881E-35	0	AHSG
5.6752584628086E-10	0.456829712853618	0.185	0.259	9.25691407868711E-06	0	RPN2
6.70523779765883E-28	0.454440372634275	0.761	0.849	1.09369133717613E-23	0	LMNA
2.74658563608215E-07	0.452535413904173	0.238	0.309	0.0044799558310136	0	COX6C
1.40454749168805E-08	0.451276503773159	0.247	0.326	0.000229095741369238	0	TMEM238
5.98422157046388E-13	0.449326160268562	0.309	0.429	9.76086380358363E-09	0	NCLN
8.93158475082681E-09	0.44676189015431	0.238	0.316	0.000145683078870736	0	PPP1R11
7.42116891682268E-09	0.445945639364049	0.19	0.26	0.000121046686202295	0	GDI2
1.97410059046341E-18	0.444429833079145	0.738	0.842	3.21995547310486E-14	0	SELENOP
2.25634133228624E-13	0.444277293043893	0.208	0.304	3.68031834709209E-09	0	GPATCH4
7.99622207171849E-07	0.442445855361609	0.241	0.312	0.01304263782118	0	SAP18
3.54033665419657E-07	0.442039393416028	0.229	0.3	0.00577464311666002	0	AAMDC
3.20640861725984E-09	0.44168802999095	0.183	0.255	5.22997309561252E-05	0	CREB3
1.83239456009199E-10	0.441610656421194	0.381	0.517	2.98881876696604E-06	0	PFDN2
3.04275755026165E-19	0.441392274218368	0.765	0.831	4.96304184023177E-15	0	LRG1
5.98070861507522E-07	0.440664773148151	0.245	0.317	0.00975513382204919	0	NME1
4.26761912635544E-11	0.440576354861751	0.319	0.435	6.96091355699835E-07	0	GHITM
2.93647695559936E-08	0.440195152219834	0.409	0.562	0.000478968756227811	0	HTATIP2
2.3054575674934E-09	0.439983731464891	0.351	0.475	3.76043183833848E-05	0	PRKAR1A
2.9155330768153E-09	0.437657290350039	0.262	0.349	4.75552600159344E-05	0	COX7C
3.87937851284274E-10	0.437537856250928	0.237	0.323	6.32765429229779E-06	0	CANX

**1.34443768470268E-06**	0.436544899824947	0.628	0.747	0.0219291230751854	0	KRT18
**4.38167473481994E-12**	0.436362920290842	0.274	0.376	7.1469496599648E-08	0	TNIP1
**8.0725111975584E-07**	0.43628708225113	0.403	0.538	0.0131670730143375	0	SELENOH
**4.88416831055888E-09**	0.43376732140662	0.284	0.375	7.96656693135258E-05	0	NDUFAB1
**3.80722496195779E-08**	0.433704789328638	0.188	0.254	0.000620996463544935	0	MDH2
**1.86019590312523E-10**	0.433065474630936	0.399	0.538	3.03416553758756E-06	0	EPN1
**1.9661528500861E-10**	0.432359843387432	0.281	0.381	3.20699191377543E-06	0	CTSH
**7.06190148010642E-11**	0.431918810049705	0.687	0.798	1.15186675042016E-06	0	DHCR24
**2.94382741241942E-07**	0.431635817503579	0.225	0.295	0.00480167689239731	0	MPND
**7.90410598388402E-08**	0.431113360885245	0.231	0.306	0.00128923872703132	0	PARK7
**1.61840940265888E-10**	0.430765819780695	0.701	0.785	2.6397875766769E-06	0	PTMS
**7.33460378190993E-12**	0.430449412555363	0.219	0.31	1.19634722286733E-07	0	ZMAT2
**2.44596934886614E-13**	0.4301744004961	0.228	0.324	3.98962060493557E-09	0	YY1
**3.78957816319799E-07**	0.429769551919726	0.661	0.765	0.00618118094199224	0	TM4SF4
**1.58974188615831E-10**	0.429557354796888	0.198	0.278	2.59302799051281E-06	0	HGH1
**6.09284559134084E-10**	0.428864629568254	0.389	0.525	9.93804044403605E-06	0	PMVK
**3.17506230872609E-14**	0.428163945670998	0.186	0.283	5.17884413176312E-10	0	CDKN1B
**9.6286461948078E-12**	0.427121538281072	0.312	0.431	1.5705284808351E-07	0	DEPRECAT
**7.87089370789955E-09**	0.426960853343811	0.219	0.294	0.00012838214726955	0	PPP1CA
**5.40455581760206E-13**	0.4252660608676	0.31	0.431	8.81537099409072E-09	0	SHISA5
**6.9424117586679E-77**	0.424910638793874	0.946	0.961	1.13237678195632E-72	0	MT1F
**1.31014223805309E-08**	0.423708213276726	0.353	0.471	0.00021369730044884	0	TMED5
**1.3322384367019E-16**	0.423634089335861	0.265	0.382	2.17301411410448E-12	0	UBE2Q1
**5.47507643240118E-10**	0.423617341362119	0.381	0.513	8.93039716888957E-06	0	ADIPOR1
**5.29882112053343E-07**	0.42298137870272	0.393	0.521	0.00864290712970207	0	MGST3
**1.36451205415611E-10**	0.422153872856059	0.285	0.389	2.22565561153403E-06	0	RBM42
**5.50693599777262E-07**	0.421734270694591	0.213	0.279	0.00898236330596692	0	ELOB
**4.48876769058116E-11**	0.421451816365392	0.359	0.493	7.32162898010693E-07	0	GALNT2
**1.29907179399599E-06**	0.421263484837506	0.223	0.288	0.0211891600318685	0	LGALS1
**1.13795938244313E-09**	0.420276203335355	0.248	0.333	1.85612554870298E-05	0	SUMO3
**1.22087289057851E-06**	0.419364830045991	0.399	0.533	0.019913657718226	0	BEX3
**1.83740477017501E-12**	0.418494850798016	0.255	0.36	2.99699092063246E-08	0	CCDC85C
**5.22926291940446E-09**	0.415948113926923	0.287	0.385	8.52945074784061E-05	0	PRKCSH
**5.66199217293908E-11**	0.41578778028034	0.342	0.464	9.23527543328093E-07	0	HNRNPA0
**3.92052987181519E-08**	0.415707175165556	0.207	0.277	0.000639477627391776	0	VAMP5
**4.08236989893268E-07**	0.413717888247306	0.22	0.286	0.0066587535421491	0	C12orf57
**6.40284546445409E-09**	0.413622627874791	0.406	0.541	0.000104436812370711	0	CHCHD10
**4.10835166518378E-07**	0.412622272406941	0.207	0.27	0.00670113240108127	0	RETREG3
**7.27903820853172E-13**	0.412360881899062	0.329	0.456	1.18728392219361E-08	0	TMEM219
**4.0748230250754E-07**	0.411844057629405	0.228	0.295	0.00664644383620048	0	PGD
**1.30308139644559E-12**	0.411765988191359	0.314	0.436	2.12545606574241E-08	0	LGMN
**8.47620138632092E-09**	0.408947096198205	0.224	0.302	0.00013825532081228	0	IRF2BPL
**1.41381706701905E-11**	0.406630901885675	0.296	0.409	2.30607701801477E-07	0	PNKD
**8.0145992487754E-09**	0.405536189481645	0.327	0.431	0.000130726128346776	0	HSD17B10
**2.61573536398752E-13**	0.404426570834795	0.364	0.511	4.26652595220004E-09	0	SSR2
**8.78706110979682E-08**	0.404424089944092	0.418	0.57	0.00143325753761896	0	LAMP2
**3.78740785683588E-14**	0.403682733108266	0.319	0.446	6.177640955285E-10	0	PSMB10
**5.30676377570795E-10**	0.403553190656917	0.298	0.401	8.65586239455723E-06	0	ACADM
**8.03829125884544E-12**	0.403351371166675	0.328	0.449	1.31112568723028E-07	0	SRPRB
**1.76537893812038E-11**	0.403074834649319	0.284	0.39	2.87950958596816E-07	0	GABARAP

1.65765676709877E-15	0.40303771445308	0.372	0.526	2.70380395281481E-11	0	EIF4H
1.31297709219168E-06	0.401367852306807	0.662	0.799	0.0214159693507385	0	TFR2
7.87259066428781E-14	0.400939379586432	0.222	0.321	1.28409826325199E-09	0	LRP10
4.6144854270388E-12	0.400589572992027	0.174	0.258	7.52668718004298E-08	0	MARCH2
6.03603902715745E-17	0.400558746780025	0.272	0.393	9.84538325719652E-13	0	BCL2L13
3.40350405062014E-11	0.400463705747014	0.367	0.497	5.55145545696651E-07	0	GAA
7.11097636630601E-11	0.400015016975541	0.212	0.296	1.15987135510817E-06	0	BIN1
2.45061798262617E-08	0.398477033098704	0.193	0.263	0.000399720299146155	0	TADA3
4.34309820386651E-14	0.398443528274994	0.276	0.386	7.08402748032667E-10	0	UBE2L6
1.64139937788595E-10	0.398397708497954	0.421	0.575	2.67728652526977E-06	0	CXXC5
1.53701095825971E-12	0.398168942765371	0.215	0.304	2.50701857401742E-08	0	MRFAP1L1
1.32128816922597E-06	0.39714832146064	0.516	0.685	0.0215515313282448	0	MYH14
6.00735868238201E-08	0.396520014812921	0.673	0.792	0.00097986027468333	0	IFITM3
1.92525718166285E-06	0.395633343689186	0.471	0.625	0.0314028698901027	0	TAF10
1.562734555388E-15	0.395336673465485	0.266	0.381	2.54897633329336E-11	0	CHID1
2.1106074815153E-09	0.394766154625601	0.229	0.313	3.4426118630996E-05	0	BCAP31
4.12802590801477E-86	0.392592482681989	0.999	0.989	6.7332230585629E-82	0	FGB
6.3746970943113E-14	0.391542281681482	0.24	0.341	1.03977684305312E-09	0	HOMER2
3.42160166808817E-07	0.389913774168605	0.242	0.317	0.00558097448081861	0	NPC2
1.48595186366579E-14	0.388555764159313	0.44	0.621	2.42373608482527E-10	0	DUS1L
8.31497022118529E-67	0.388418298916827	0.942	0.955	1.35625479277753E-62	0	FN1
3.1559988653054E-11	0.388382278344712	0.396	0.543	5.14774974919964E-07	0	LMAN1
2.96171407471305E-13	0.38766686486938	0.243	0.343	4.83085182726445E-09	0	DUSP3
2.64694570683904E-14	0.38753740071108	0.215	0.313	4.31743314242516E-10	0	RAB14
1.25052337732861E-14	0.387274465145801	0.312	0.44	2.0397286807607E-10	0	POLR2E
5.81690342855908E-08	0.386624047245788	0.201	0.269	0.000948795118232272	0	TCEAL8
9.06998833994973E-14	0.386535575362447	0.386	0.525	1.4794057981292E-09	0	POLDIP2
7.29409640393065E-14	0.386516655419242	0.32	0.449	1.18974006444513E-09	0	GUK1
2.57157227761443E-07	0.386423365437112	0.311	0.409	0.00419449154201689	0	FUCA1
1.25946476257877E-09	0.386100163782071	0.181	0.256	2.05431297424223E-05	0	PGLS
2.77410395893679E-11	0.384620059799582	0.295	0.41	4.5248409674218E-07	0	SPCS1
3.5323649820678E-11	0.3823163387841	0.366	0.494	5.76164052225079E-07	0	DNPH1
7.4911161080402E-15	0.381745046193571	0.182	0.276	1.22187594838244E-10	0	SZRD1
6.27755806504921E-17	0.381008893029781	0.745	0.821	1.02393249599018E-12	0	SERPING1
5.97660439162411E-12	0.380860669861116	0.21	0.297	9.74843942317808E-08	0	TALDO1
2.13176940776327E-14	0.380698340577315	0.216	0.317	3.47712908100267E-10	0	SLC25A11
2.45160416723928E-10	0.379693181100877	0.232	0.315	3.99881155718399E-06	0	TSPAN6
2.93153232877498E-78	0.379027439015546	0.993	0.992	4.78162238146487E-74	0	C3
3.60853770902241E-09	0.377473292442922	0.235	0.318	5.88588585718646E-05	0	PIGT
1.64268075988244E-08	0.377221097305957	0.229	0.306	0.000267937658744425	0	NEDD8
3.89665239490759E-62	0.375956619195268	0.97	0.973	6.35582972133377E-58	0	MT1B
5.27851898253035E-12	0.374245213116308	0.274	0.376	8.60979231240525E-08	0	CPN2
4.5888548296637E-13	0.37407441623997	0.332	0.465	7.48488111266446E-09	0	NDFIP1
3.34269658094729E-16	0.37311137110679	0.3	0.427	5.45227239318312E-12	0	LAMTOR4
2.58272188798741E-07	0.372664874385706	0.306	0.394	0.00421267767149627	0	C1orf115
3.38981333506216E-22	0.372047418655792	0.793	0.866	5.5291245308199E-18	0	SERPINF2
9.85544007842351E-10	0.370464250626419	0.299	0.403	1.60752083119166E-05	0	HEXB
3.50533210982498E-11	0.370463731863191	0.187	0.267	5.71754720433552E-07	0	SORBS3
5.99730339483749E-10	0.369867127397098	0.216	0.299	9.78220156731943E-06	0	PPP1R1A
2.92746942214087E-14	0.369421498346346	0.202	0.299	4.77499537445397E-10	0	SLC25A22

1.85253794217168E-07	0.368811131020445	0.418	0.548	0.00302167463747623	0	PRKACA
2.12522132351708E-10	0.368770220573527	0.304	0.411	3.46644850078871E-06	0	GALK1
1.79266187441234E-14	0.367440549459834	0.197	0.291	2.92401078335396E-10	0	ALG3
1.13296619897512E-14	0.367167373224754	0.25	0.357	1.84798116714833E-10	0	CAPZA2
1.41392480030376E-06	0.366928218794484	0.455	0.607	0.0230625274177546	0	HAO1
6.53283054819146E-12	0.365701451264311	0.325	0.443	1.06556999071551E-07	0	YBX1
3.77222595864619E-09	0.365202696230432	0.215	0.29	6.15287776114779E-05	0	G3BP2
5.69237503032187E-08	0.364992606634939	0.185	0.25	0.0009284832911958	0	FRAT2
1.54684360008955E-19	0.363704688726214	0.238	0.364	2.52305659610606E-15	0	TXNL4A
2.35617871640891E-09	0.363689450482643	0.327	0.435	3.84316310433458E-05	0	CITED4
6.57136578094391E-27	0.363531767827437	0.952	0.952	1.07185547252976E-22	0	PLA2G2A
2.5088914730896E-08	0.362860588532063	0.198	0.268	0.000409225288175645	0	TMED2
4.110100548861E-10	0.362575160523448	0.268	0.364	6.70398500524718E-06	0	CTSF
6.48130943099189E-11	0.361379486910954	0.199	0.281	1.05716638128909E-06	0	FLOT2
5.45084570785848E-10	0.36074945407211	0.403	0.539	8.89087443408796E-06	0	CYP27A1
2.49044627735686E-15	0.360739627699395	0.278	0.398	4.06216692299677E-11	0	EI24
3.9636701637811E-33	0.359541359136698	0.847	0.917	6.46514240414335E-29	0	CTSB
4.79838655418003E-12	0.359279561368323	0.202	0.289	7.82664830852304E-08	0	ATP5PD
7.63510196067875E-99	0.359034307590952	0.993	0.992	1.24536148080631E-94	0	MT2A
4.62638428100949E-14	0.358905229324152	0.213	0.309	7.54609540075459E-10	0	FAAP20
1.74933710860381E-13	0.358405222745977	0.259	0.364	2.85334375784367E-09	0	ARF6
1.92513658900602E-14	0.358085194324367	0.306	0.429	3.14009029032772E-10	0	AIFM1
5.93278889185959E-09	0.357478780553098	0.248	0.334	9.67697196151218E-05	0	PCYOX1
9.67785626663525E-16	0.356428637579153	0.195	0.294	1.57855513565088E-11	0	SLC25A4
5.82528163468788E-09	0.356298216143494	0.211	0.285	9.5016168743394E-05	0	ATP6V0D1
5.07282235338117E-15	0.355166548691373	0.246	0.356	8.27428054060003E-11	0	MAN2B1
4.81420968287258E-09	0.355120603495102	0.201	0.274	7.85245741373347E-05	0	PCBP1
1.3806657412686E-10	0.354518896463285	0.35	0.468	2.25200389058322E-06	0	IFI6
1.92112848189122E-07	0.354379569684179	0.211	0.283	0.00313355266681277	0	PHLDA1
1.72077758698313E-10	0.353963519727177	0.226	0.311	2.80676032212818E-06	0	JMJD8
1.03729689534783E-22	0.35217135587805	0.805	0.892	1.69193496600184E-18	0	APOH
3.99045277106855E-15	0.352038572755606	0.26	0.372	6.50882751488992E-11	0	SNF8
6.35089794014542E-10	0.351868895285774	0.224	0.307	1.03589496301712E-05	0	CISD3
3.98280361784104E-25	0.351675766189728	0.791	0.888	6.49635098106053E-21	0	ASGR2
4.16868629730001E-14	0.351395965095748	0.224	0.324	6.79954421952605E-10	0	RHOD
1.1202789596001E-09	0.350872481506804	0.302	0.403	1.82728701100373E-05	0	SLC22A1
8.73499131171926E-15	0.350798576778364	0.336	0.473	1.42476443285453E-10	0	ALDH9A1
3.51926509480452E-17	0.350781679580834	0.252	0.373	5.74027329613566E-13	0	COMTD1
7.64352710925543E-13	0.350049475702316	0.323	0.446	1.24673570679065E-08	0	SPTAN1
2.18137344171344E-14	0.34872522566861	0.207	0.303	3.55803822077879E-10	0	TMEM127
3.33914807893324E-13	0.348524060321091	0.712	0.84	5.44648443154801E-09	0	CERS2
4.45277024829582E-55	0.34686574830163	0.996	0.99	7.26291355199532E-51	0	FGA
2.10288370608878E-15	0.346396385555389	0.303	0.43	3.43001361300141E-11	0	KRTCAP2
5.79055074798095E-10	0.346110488552394	0.409	0.548	9.44496732503172E-06	0	MPC2
2.06212943734472E-10	0.345614756666221	0.315	0.422	3.36353932525297E-06	0	DEPRECAT
3.19396534767214E-10	0.345459980806562	0.189	0.267	5.20967687858803E-06	0	COLEC11
3.41583250787339E-09	0.344339869568212	0.245	0.333	5.57156440359228E-05	0	TOMM7
1.63907354814825E-14	0.342969754851326	0.168	0.256	2.67349286438461E-10	0	CHMP5
9.94375232180276E-12	0.342700387140479	0.256	0.355	1.62192544120925E-07	0	DEXI
1.75254308496254E-13	0.341939329595286	0.196	0.288	2.8585730258824E-09	0	KIAA0930

2.36189467248948E-11	0.341562758973788	0.241	0.333	3.85248640029758E-07	0	ANAPC11
1.42926545539107E-13	0.341526838776279	0.249	0.36	2.33127488428837E-09	0	CPTP
8.10707069765737E-09	0.341359379735412	0.389	0.526	0.000132234430149489	0	METTL7B
1.62984918953805E-13	0.341261521274025	0.216	0.313	2.65844701305551E-09	0	LSM 4.00
1.14208057152571E-09	0.340823338533222	0.253	0.342	1.86284762021559E-05	0	HSPA5
1.63826326226938E-26	0.3401379525147	0.845	0.913	2.67217120708759E-22	0	FGL1
1.80935680212028E-13	0.338221373082404	0.267	0.377	2.95124187993839E-09	0	ATP5MG
3.66414870735163E-08	0.337797383177258	0.221	0.299	0.000597659295656124	0	SELENOW
2.42138653136164E-18	0.337258170527424	0.815	0.869	3.94952357130396E-14	0	A2M
4.00680936075266E-09	0.336187626236586	0.195	0.266	6.53550674832367E-05	0	PYGL
1.4860469877669E-11	0.336067987337674	0.181	0.262	2.42389124174659E-07	0	TXNDC12
4.31006745903529E-11	0.335520561244435	0.335	0.449	7.03015103243247E-07	0	SERP1
5.66297149179322E-12	0.334175583615908	0.344	0.468	9.23687280026392E-08	0	AMN
4.50291979628748E-09	0.333504113759161	0.248	0.329	7.3447124797245E-05	0	NDUFB10
4.23443703156593E-08	0.33329581438219	0.309	0.403	0.000690679024218719	0	HACD3
8.76055646101201E-15	0.332830707477674	0.254	0.365	1.42893436435567E-10	0	MGST2
1.67538847934042E-13	0.332762668073584	0.369	0.506	2.73272614865215E-09	0	NIPSNAP1
4.82846006595326E-12	0.332272938049143	0.349	0.481	7.87570121357637E-08	0	RETSAT
1.6877824395943E-08	0.332148893894612	0.234	0.315	0.000275294193722226	0	GADD45GIF
1.03666901539537E-11	0.332139591317776	0.179	0.26	1.69091083101139E-07	0	RAB1B
3.66848595328539E-15	0.331068778424012	0.175	0.268	5.98366743840379E-11	0	GCDH
1.0071292898809E-10	0.330957969144942	0.189	0.269	1.64272858472474E-06	0	HSPA9
4.08850775444116E-16	0.330809967656324	0.251	0.367	6.66876499826897E-12	0	GATAD2A
7.45006668478068E-14	0.33026941464745	0.18	0.267	1.21518037695458E-09	0	MCOLN1
9.49569209069074E-12	0.329736020396991	0.264	0.365	1.54884233691257E-07	0	PSMC4
2.98845024736918E-09	0.329326568383847	0.189	0.26	4.87446119848388E-05	0	SPRYD3
4.07571629621707E-15	0.329029625308787	0.279	0.398	6.64790085075966E-11	0	PPP4C
3.03845654788919E-15	0.328621190879682	0.299	0.424	4.95602647526205E-11	0	DEPRECAT
1.15759731198247E-17	0.327221978176014	0.288	0.417	1.88815697557461E-13	0	CDIPT
4.82807910434266E-11	0.326909909773242	0.305	0.413	7.87507982709332E-07	0	ATP5MC2
4.024129266671E-12	0.326505951964406	0.23	0.322	6.56375724686707E-08	0	DDAH1
1.68092167320388E-11	0.325721469981306	0.266	0.365	2.74175134116285E-07	0	PFDN6
1.21891618886262E-14	0.325386588547776	0.181	0.273	1.98817419565381E-10	0	ELOA
2.58819853704834E-19	0.323920986028889	0.357	0.523	4.22161063377954E-15	0	SEL1L
3.96588867287196E-21	0.32275568651389	0.319	0.469	6.46876101432145E-17	0	SCYL1
2.78120710141085E-09	0.3226783541236	0.308	0.41	4.53642690311124E-05	0	SDHB
1.71833002256634E-25	0.321544633308728	0.87	0.932	2.80276809980796E-21	0	APOB
1.52069243846444E-12	0.32008609339053	0.231	0.325	2.48040143637935E-08	0	BCKDHA
1.89151014817103E-13	0.319835020033164	0.307	0.426	3.08524220268177E-09	0	CLPTM1
1.84065969600355E-14	0.319062891149762	0.373	0.523	3.0023000301514E-10	0	LONP1
5.93651844688691E-19	0.318504730669592	0.281	0.413	9.68305523871724E-15	0	FAM83H
4.50300188788153E-13	0.318442872394337	0.273	0.381	7.34484637932357E-09	0	APP
3.08058191152901E-10	0.316648677543801	0.307	0.416	5.02473715589497E-06	0	CMBL
7.07549056614023E-16	0.316209180405818	0.306	0.434	1.15408326624313E-11	0	GPRC5C
3.91899991960121E-11	0.315332151046347	0.247	0.34	6.39228076886154E-07	0	AGPAT2
3.13894193218002E-12	0.315168340360631	0.362	0.496	5.11992818557883E-08	0	TSPAN13
2.60473071677467E-16	0.314893745818353	0.335	0.477	4.24857627213117E-12	0	MAGT1
1.03296754243324E-16	0.314546449629627	0.379	0.542	1.68487335846286E-12	0	MAN1A1
1.33036420834266E-09	0.312765566349285	0.214	0.293	2.16995706022771E-05	0	HNRNPAB
9.58095376642676E-10	0.312721148550364	0.379	0.515	1.56274936884187E-05	0	NUDC

4.64326481866107E-15	0.312474000053228	0.302	0.427	7.57362924571808E-11	0	CLTA
4.538534859764E-41	0.312325285603492	0.931	0.948	7.40280420976106E-37	0	HPX
1.35874164338828E-12	0.311028529747988	0.36	0.493	2.21624349453062E-08	0	HEBP2
3.15872214298848E-13	0.310424341802345	0.265	0.371	5.15219168742851E-09	0	PDHB
8.6710885175868E-11	0.309676908044989	0.309	0.416	1.41434124810358E-06	0	NDUFA4
1.32724971226432E-12	0.309112789101794	0.232	0.327	2.16487700567434E-08	0	TOMM20
1.68452310371382E-11	0.308712670106401	0.331	0.454	2.74762563446762E-07	0	DEPRECAT
2.28918476259738E-17	0.307439934732993	0.255	0.376	3.73388926627259E-13	0	EBPL
4.85795334439186E-12	0.307067052824184	0.2	0.285	7.92380770003757E-08	0	NDUFA11
9.43329763834873E-23	0.306957801178607	0.286	0.43	1.53866517779106E-18	0	CLPTM1L
9.26721225411528E-08	0.306644171599993	0.455	0.621	0.00151157499076874	0	LSR
8.29646018357538E-15	0.303078100811751	0.221	0.326	1.35323562054298E-10	0	CAV2
5.58751798479624E-15	0.302865592965073	0.389	0.543	9.11380058500114E-11	0	SUN2
1.09551332350706E-06	0.301660457753892	0.488	0.642	0.0178689178197236	0	LASP1
2.77407991942113E-20	0.301233302192861	0.326	0.482	4.5248017565678E-16	0	CALM2
2.61997466862816E-08	0.301182749837105	0.194	0.259	0.000427344068199939	0	SOWAHC
6.7417772491231E-15	0.30087494133474	0.227	0.33	1.09965128710447E-10	0	DEPRECAT
1.14369807125692E-13	0.300590278219457	0.289	0.406	1.86548592402716E-09	0	ZNF622
2.46173000705075E-11	0.299540343765055	0.35	0.456	4.01532781450047E-07	0	REEP6
1.21741409589462E-12	0.299398618066181	0.39	0.544	1.98572413181372E-08	0	TM9SF2
2.91074234445806E-11	0.299130850500929	0.228	0.316	4.74771183804554E-07	0	SRM
1.21591056215041E-11	0.298577520504372	0.199	0.285	1.98327171792353E-07	0	FAM174C
9.96829419832806E-14	0.296705383221752	0.259	0.365	1.62592846668929E-09	0	NUDT14
1.14608743670416E-21	0.296697620040108	0.2	0.32	1.86938321800816E-17	0	EPHB4
3.81679162758855E-10	0.296622781449024	0.287	0.388	6.22556882375968E-06	0	RTN3
4.69542942720336E-14	0.296085682070639	0.467	0.655	7.6587149387114E-10	0	SLC25A39
3.79871857587577E-14	0.295878210195056	0.326	0.457	6.19608986911096E-10	0	MGAT1
1.34144252510518E-13	0.295541838407896	0.369	0.522	2.18802690269905E-09	0	TRIR
5.02939557157863E-09	0.294763643736894	0.19	0.262	8.2034471168019E-05	0	SLC16A1
4.48880550406514E-14	0.293923713728855	0.211	0.307	7.32169065768066E-10	0	DERL2
7.94393374287762E-21	0.292979602587875	0.314	0.465	1.29573503280077E-16	0	SLC35C1
5.27887491105074E-15	0.292799097256671	0.307	0.436	8.61037286741486E-11	0	SDHC
4.56950527926728E-11	0.292237580539834	0.269	0.363	7.45332006101285E-07	0	CLTB
6.91381839377693E-10	0.291822521949472	0.285	0.381	1.12771291820895E-05	0	DAG1
1.07651141951727E-10	0.291577206956393	0.207	0.287	1.75589777637462E-06	0	VASP
8.32801474257601E-08	0.291071655074294	0.416	0.546	0.00135838248466157	0	OTC
1.19763324128833E-14	0.290762794998674	0.355	0.498	1.9534595798654E-10	0	GUCD1
9.67163678265912E-12	0.290761622627092	0.459	0.641	1.57754067561953E-07	0	EIF3A
4.78881406585093E-10	0.290342200429687	0.379	0.531	7.81103462280945E-06	0	HADHA
3.33097455333119E-10	0.290227571852599	0.179	0.251	5.4331525939385E-06	0	ADCK2
4.37465482448108E-14	0.290139782743925	0.21	0.304	7.13549948421109E-10	0	EXOSC6
1.39870444042725E-12	0.289608764906767	0.212	0.302	2.28142681278088E-08	0	DCTN3
1.57673976209769E-13	0.2893666777737	0.202	0.293	2.57182022595754E-09	0	CRTAP
6.11205162883801E-16	0.289156929362635	0.177	0.275	9.96936741179768E-12	0	OGDH
3.03230210219166E-15	0.28872619589842	0.211	0.313	4.94598795888481E-11	0	SURF4
8.4392001989097E-14	0.287324064171454	0.19	0.279	1.37651794444416E-09	0	SLC25A23
9.41797890668027E-14	0.285468217298117	0.163	0.253	1.53616653946862E-09	0	INHBE
1.48740208832672E-13	0.285033643013039	0.373	0.527	2.42610154626972E-09	0	DEPRECAT
2.6780199101569E-13	0.284305859593516	0.292	0.412	4.36811827545692E-09	0	ATP5IF1
6.72910023350887E-13	0.283992437297855	0.189	0.278	1.09758353908763E-08	0	MMP14

2.46281251743578E-11	0.28391880218222	0.188	0.27	4.01709349718949E-07	0	PTGFRN
1.4251262049229E-18	0.282828045737525	0.386	0.556	2.32452335284974E-14	0	CRELD2
7.17272045835204E-16	0.282246170157788	0.167	0.262	1.1699424339618E-11	0	SYAP1
9.5930597435172E-14	0.281359147447177	0.207	0.301	1.56472397476509E-09	0	UBTF
2.24590900022715E-17	0.280875270951754	0.326	0.47	3.66330217027051E-13	0	ACTG1
1.67670920757781E-08	0.280264364354401	0.237	0.315	0.000273488038848017	0	TMEM9
3.53404211439166E-18	0.279995350697752	0.254	0.373	5.76437609278424E-14	0	NUDT16
2.07506174278498E-08	0.278238827065301	0.23	0.304	0.000338463320865658	0	MESD
3.63118863150345E-13	0.278135999992357	0.258	0.358	5.92283177684527E-09	0	LAMTOR2
2.09034264948408E-16	0.277870416903987	0.282	0.407	3.40955789557348E-12	0	SDHA
1.67929208123526E-06	0.277665903009808	0.497	0.654	0.0273909331370284	0	BLVRB
7.38011690168255E-13	0.277218000799245	0.292	0.404	1.20377086783344E-08	0	PLBD2
1.61779970843664E-18	0.27717946989475	0.275	0.406	2.63879310443101E-14	0	WSB2
2.97857375376353E-13	0.277020319229008	0.2	0.291	4.85835164976369E-09	0	NUBP2
9.1990392207307E-15	0.274135510459995	0.388	0.555	1.50045528729339E-10	0	SERF2
1.98707486182354E-18	0.273753610652077	0.235	0.352	3.24111780712037E-14	0	NAA20
2.39247910418283E-09	0.273432557460838	0.491	0.682	3.90237266683262E-05	0	TFPI
3.52990960490344E-17	0.272544118448046	0.338	0.48	5.757635556558E-13	0	CIAO2A
5.71230408990396E-12	0.272540014772896	0.452	0.633	9.31733920104235E-08	0	NECTIN2
1.17508064460248E-21	0.272318263580661	0.879	0.933	1.91667403941111E-17	0	KNG1
9.28978519267923E-15	0.272069865926346	0.356	0.499	1.51525686277791E-10	0	DPP7
7.7630076607589E-15	0.271989818313744	0.27	0.375	1.26622417954638E-10	0	SDC2
2.29267251664536E-14	0.271880153442303	0.248	0.352	3.73957814190025E-10	0	RNF11
4.67248635287155E-14	0.271605977618958	0.26	0.368	7.62129249016878E-10	0	AKT1S1
8.27702955446188E-12	0.271277237560212	0.218	0.307	1.35006629062828E-07	0	ITPRIPL2
1.7166181824703E-15	0.270388652202647	0.288	0.416	2.79997591742731E-11	0	PXMP2
1.90602385330632E-10	0.269987081534929	0.407	0.549	3.10891550712794E-06	0	CPB2
7.29175171341305E-09	0.26960953106402	0.323	0.423	0.00011893576219748	0	APOM
1.00150890356019E-16	0.269584377498253	0.422	0.596	1.63356117259703E-12	0	CMPK1
7.54427291946064E-17	0.268561851547556	0.316	0.449	1.23054635589322E-12	0	SEC13
2.41154761082072E-13	0.267675848880488	0.32	0.447	3.93347530800968E-09	0	GSTO1
4.24400235814321E-12	0.266439060432698	0.181	0.263	6.92239224636739E-08	0	TIMM50
1.09934303368837E-13	0.266117408320017	0.414	0.589	1.7931384222491E-09	0	CAT
4.21370016807919E-17	0.266044876636771	0.33	0.463	6.87296634415396E-13	0	PSMC3
3.29910028977927E-15	0.265283362790495	0.292	0.416	5.38116248265897E-11	0	CD99
3.89417744630083E-14	0.265007069431812	0.189	0.279	6.35179283266128E-10	0	MOCS2
2.39191528231712E-14	0.263379360841736	0.227	0.326	3.90145301698746E-10	0	ALAD
3.50561428242371E-10	0.263289594704427	0.772	0.869	5.71800745606132E-06	0	FTH1
2.05988521893046E-18	0.263112134801011	0.363	0.524	3.35987878059747E-14	0	PLD3
4.87867613222167E-18	0.262869600186348	0.257	0.378	7.95760863926677E-14	0	GIPC1
1.07863271034984E-14	0.262616151781737	0.233	0.335	1.75935781385162E-10	0	FZD5
1.72855688176845E-16	0.261893377372914	0.24	0.353	2.81944912985252E-12	0	TMEM134
4.79895886845988E-22	0.261819182832476	0.277	0.418	7.82758181034492E-18	0	CDC37
6.43803081680437E-11	0.261676357817671	0.428	0.592	1.05010720652896E-06	0	HAGH
5.00689679159364E-15	0.260884690504538	0.189	0.284	8.16674935676839E-11	0	EZR
2.07849459897811E-14	0.259684184530272	0.436	0.618	3.39023254039319E-10	0	EMC10
9.92851096558747E-23	0.259272558127717	0.287	0.437	1.61943942359697E-18	0	TOLLIP
2.22517045153875E-11	0.259265314642539	0.25	0.346	3.62947552350486E-07	0	P4HA2
4.80315511213811E-10	0.258928137317456	0.443	0.589	7.83442630340847E-06	0	CDKN1A
7.09771761632453E-14	0.25880324932317	0.191	0.282	1.15770872039869E-09	0	SHMT2

2.92715656000354E-14	0.25854113326477	0.366	0.515	4.77448506502177E-10	0	PRXL2A
6.08333216860199E-18	0.258245260673947	0.278	0.406	9.92252310020671E-14	0	DYNLL2
5.355519483916E-16	0.257409091335715	0.322	0.459	8.73538783021538E-12	0	ERO1A
2.96101374490425E-14	0.25724154266093	0.306	0.424	4.82970951931332E-10	0	IFITM2
1.38015197169792E-12	0.25712982363724	0.242	0.339	2.25116588103647E-08	0	ACOT13
2.30368334671935E-20	0.256938660423745	0.244	0.374	3.75753790683394E-16	0	PBXIP1
6.04962464386561E-15	0.256789404097594	0.21	0.319	9.8675427566092E-11	0	SCD
1.05236818060292E-06	0.256751953953459	0.55	0.735	0.0171651773938142	0	MGAT4B
2.89331901772548E-13	0.256707252137902	0.205	0.298	4.71929264981203E-09	0	POLD2
5.59966578760925E-16	0.255242472924798	0.433	0.621	9.13361486616945E-12	0	RNH1
8.07150240594472E-14	0.255197017862216	0.304	0.425	1.31654275743364E-09	0	APOL1
1.1794953172901E-17	0.254736484221277	0.366	0.529	1.92387481203188E-13	0	SIL1
7.84189222648792E-16	0.254474249654074	0.297	0.419	1.27909104106245E-11	0	PNPO
1.3584572640927E-15	0.253990935017903	0.294	0.416	2.21577964346161E-11	0	JUP
2.80471136061454E-13	0.253348639717402	0.362	0.492	4.57476470029837E-09	0	GRHPR
5.43550680180625E-11	0.252912394134159	0.208	0.289	8.86585514442618E-07	0	GNAI3
9.54488462449459E-20	0.252624778827934	0.371	0.536	1.55686613110131E-15	0	KIF1C
1.96745770269796E-15	0.2520214761035	0.188	0.285	3.20912025887064E-11	0	ABHD14B
2.72655420322934E-15	0.251750271858692	0.259	0.368	4.44728256088738E-11	0	ARPC1B
7.42335671901513E-18	0.2514894946665	0.373	0.536	1.21082371443856E-13	0	PSMB8
9.93382635896555E-14	0.251311589248425	0.188	0.279	1.62030641741087E-09	0	CCDC124
1.49814401376218E-15	0.250968362625178	0.215	0.314	2.4436227008475E-11	0	COA3
7.03945107653681E-15	0.250491305586649	0.334	0.465	1.14820486509392E-10	0	RAB5C
5.65275558322538E-18	0.250279429179696	0.327	0.469	9.22020963179892E-14	0	CD59
2.54504791041962E-14	0.250218774793786	0.218	0.315	4.15122764668545E-10	0	ZNF219
6.77251149612485E-105	2.79827583868725	0.273	0.069	1.10466435013292E-100	1	NAV3
6.35466005430821E-105	2.70358096774754	0.294	0.082	1.03650860145821E-100	1	C3orf52
6.86962115133788E-99	2.50809930448061	0.349	0.129	1.12050390599472E-94	1	GABBR1
1.67878267595618E-166	2.45993659626121	0.509	0.194	2.73826242275213E-162	1	CYP2C19
8.39178180912953E-34	2.38943694107435	0.255	0.138	1.36878353088712E-29	1	HSPH1
4.91033513712508E-69	2.33510794794823	0.337	0.155	8.00924764216471E-65	1	EID3
3.73463404842472E-123	2.30244536528114	0.536	0.259	6.09156159638557E-119	1	ATF3
4.35813857250168E-99	2.22637150490373	0.4	0.17	7.1085598256075E-95	1	RNPC3
1.99656335977263E-86	2.22192792806259	0.315	0.115	3.25659449612514E-82	1	MICU3
2.3324155132565E-60	2.19484083572843	0.265	0.107	3.80440294367267E-56	1	DNAH1
2.47239990307104E-81	2.1623416399694	0.278	0.092	4.03273148189917E-77	1	IGSF9
1.95689707166394E-153	2.14898059128884	0.536	0.233	3.19189481359104E-149	1	HSF4
1.32116869464974E-48	2.13205503354925	0.256	0.115	2.15495825784319E-44	1	TTLL5
4.79869732873961E-124	2.11847529545424	0.462	0.19	7.82715521290718E-120	1	FAM118A
1.25467385607158E-52	2.1140139756702	0.255	0.11	2.04649852663836E-48	1	ADAT2
7.12741723800343E-44	2.10943426216622	0.251	0.119	1.16255302569074E-39	1	NEPRO
4.99448538981337E-133	2.08978783072559	0.489	0.209	8.14650511932459E-129	1	GTPBP2
9.57882928200638E-48	2.06532420998457	0.252	0.114	1.56240284418806E-43	1	CHML
1.08823897419416E-253	2.05334939156541	0.779	0.391	1.7750265908081E-249	1	PABPC1L
8.99407042787537E-85	2.043627556738	0.271	0.083	1.46702282749075E-80	1	ADAMTS17
1.06695403682696E-115	2.0316494277353	0.485	0.225	1.74030872946846E-111	1	CSAD
2.62710644032355E-121	2.02761931375061	0.477	0.21	4.28507331481175E-117	1	NFRKB
5.45197401542369E-77	2.02144955874116	0.424	0.218	8.89271481655759E-73	1	HKDC1
1.75463789196634E-87	2.01149216992103	0.386	0.171	2.8619898655863E-83	1	DMTF1
8.28801788182905E-116	2.01009309529089	0.476	0.216	1.35185859670514E-111	1	GSDMB

1.01021914125115E-93	2.00801248446477	0.392	0.164	1.64776844129475E-89	1	APBB3
1.62344879323435E-63	2.00315485775479	0.301	0.13	2.64800732664455E-59	1	TBC1D22B
6.17601139752112E-51	1.9969953385162	0.274	0.127	1.00736921904967E-46	1	FER
1.65285235603162E-270	1.99234213454408	0.795	0.395	2.69596747792318E-266	1	ARGLU1
1.87292263552012E-59	1.96886411697377	0.28	0.119	3.05492411079687E-55	1	PPP1R9A
3.75291455722333E-198	1.96803429166304	0.766	0.423	6.12137893428697E-194	1	TXNIP
1.14768843577649E-42	1.96243224942158	0.253	0.122	1.87199460759503E-38	1	USP36
1.40726380435506E-133	1.95939585140558	0.521	0.236	2.29538799128354E-129	1	DENND4B
1.46009988394971E-47	1.95923555446704	0.257	0.118	2.38156892071037E-43	1	TAF1
1.99364338770351E-128	1.95092950216373	0.54	0.265	3.2518317296832E-124	1	OGT
2.45335950657196E-98	1.95047509353876	0.462	0.229	4.00167469116952E-94	1	FNBP4
2.60868615270285E-54	1.95027486928618	0.283	0.13	4.25502798367363E-50	1	GREB1L
8.47293404479045E-53	1.93806737361521	0.302	0.146	1.38202027204577E-48	1	PLA2G4C
4.59636934494225E-95	1.9379339411998	0.425	0.196	7.4971380385353E-91	1	IFRD1
2.45696165128295E-183	1.92376059985473	0.647	0.308	4.00755014940762E-179	1	PILRB
2.90405138515722E-251	1.91327489409201	0.778	0.403	4.73679821432994E-247	1	WSB1
3.53535869635161E-93	1.90499620106135	0.375	0.152	5.76652356961912E-89	1	SLC17A2
5.16696371025246E-126	1.89665762670091	0.526	0.253	8.42783450779279E-122	1	IP6K2
1.89106730006365E-70	1.8943932032756	0.358	0.169	3.08451987313382E-66	1	CDKAL1
1.06433107513899E-49	1.89043433967532	0.303	0.153	1.73603041665921E-45	1	PRPF39
1.09108769447393E-102	1.88650985857666	0.461	0.22	1.77967313845642E-98	1	PAN2
3.72362859426755E-264	1.88022198542078	0.799	0.403	6.0736106001098E-260	1	LENG8
9.69336697341169E-100	1.87943735204074	0.425	0.187	1.58108508703318E-95	1	USP34
2.05030487864832E-127	1.87780123264164	0.532	0.256	3.34425228756328E-123	1	RBM25
6.87261611485855E-68	1.8767949519354	0.337	0.155	1.12099241449458E-63	1	MTR
8.755111703321E-118	1.87668703287232	0.544	0.276	1.42804626992869E-113	1	PPARGC1A
3.62547836835402E-117	1.87618267640706	0.486	0.223	5.91351776662224E-113	1	PPP1R12B
2.4532137613727E-43	1.8667107476232	0.254	0.122	4.00143696617502E-39	1	CBX8
2.66957239519803E-110	1.85984756687322	0.488	0.238	4.35433953380751E-106	1	PHYKPL
1.26457113770742E-85	1.85188541541365	0.395	0.181	2.06264198271457E-81	1	MAPK8IP3
2.94668666667245E-46	1.84995422368462	0.281	0.139	4.80634062200944E-42	1	TUBG2
4.42128684060062E-102	1.83579587106523	0.483	0.237	7.21156096570367E-98	1	CBLB
4.33912086716503E-54	1.8314631963016	0.312	0.153	7.07754004643289E-50	1	ZKSCAN8
1.81740484116778E-172	1.82634369953495	0.686	0.353	2.96436903642877E-168	1	AKR1C8P
1.69795888742187E-54	1.82363602233702	0.299	0.141	2.76954074127381E-50	1	DHX34
5.74291060641578E-82	1.82267985781518	0.399	0.19	9.36726149012479E-78	1	ZSCAN26
0	1.82135647147819	0.883	0.464	0	1	CCNL2
3.59838955246547E-41	1.82020831150182	0.257	0.127	5.86933319902644E-37	1	C1QTNF3
3.4391965116353E-64	1.82002763903423	0.322	0.146	5.60967343012834E-60	1	METTL17
3.62940218561624E-101	1.81929379316901	0.485	0.249	5.91991790495864E-97	1	TRIM25
3.88619224880614E-64	1.81757234559469	0.333	0.155	6.3387681770277E-60	1	AHI1
1.25651193418892E-59	1.81727065208803	0.317	0.149	2.04949661585555E-55	1	CBFA2T2
1.10215132295358E-135	1.81551912184533	0.583	0.296	1.79771902286958E-131	1	NSUN6
2.13535179888257E-40	1.812469801591	0.278	0.146	3.48297231915737E-36	1	RNF165
1.01203839294448E-43	1.81128905936373	0.272	0.136	1.65073582273173E-39	1	ANKRD27
1.90838111667787E-57	1.81023170593286	0.312	0.148	3.11276043941327E-53	1	ZNF621
3.21197276364539E-93	1.80749170413763	0.44	0.206	5.239048774782E-89	1	CHKB
3.00705854286082E-34	1.80732505673296	0.259	0.142	4.90481318926028E-30	1	BDP1
5.32421372761267E-51	1.80640977997782	0.285	0.135	8.68432501110903E-47	1	LONRF3
9.29675356295086E-58	1.80337211240509	0.324	0.157	1.51639347365292E-53	1	UTY

1.74916330474181E-74	1.80072312492621	0.356	0.161	2.85306026636436E-70	1	FAM160B2
8.35305500151844E-62	1.79487985608436	0.349	0.172	1.36246680129767E-57	1	POLE
1.27292967881121E-45	1.78932383161104	0.29	0.146	2.07627559910896E-41	1	TTLL3
1.09437642122457E-46	1.7879850494309	0.284	0.137	1.7850373806594E-42	1	FOS
1.43012589007125E-74	1.78694779248618	0.378	0.181	2.33267833929522E-70	1	PAN3
6.48645782551567E-86	1.78598353487716	0.452	0.232	1.05800613591986E-81	1	PFKFB1
6.41477585887374E-57	1.7855995852844	0.334	0.167	1.0463140903409E-52	1	KMT2A
5.2784661054152E-53	1.78444997028182	0.292	0.137	8.60970606454273E-49	1	CCDC84
7.52302398316426E-64	1.78298678988316	0.341	0.165	1.22708044189392E-59	1	ORMDL1
6.2525865138282E-41	1.78264854974346	0.287	0.151	1.01985938627052E-36	1	SERPINB9
2.25589432617486E-159	1.78107647827791	0.62	0.302	3.67958923542381E-155	1	RBM6
3.68276741945437E-80	1.78044801625018	0.383	0.175	6.00696193787203E-76	1	ADAMTS10
2.75998748709714E-127	1.77921752908916	0.548	0.273	4.50181559020414E-123	1	PCSK7
1.29007986275722E-59	1.777015587574	0.324	0.154	2.1042492641433E-55	1	DNAH14
1.72333630414428E-58	1.77559211687403	0.344	0.176	2.81093384568973E-54	1	WDFY2
1.56272290508917E-40	1.77261580521746	0.258	0.128	2.54895733049095E-36	1	RCOR3
1.15361471259646E-37	1.7711185657453	0.251	0.128	1.88166095771609E-33	1	EXOC6B
1.51449891524067E-70	1.77050666058155	0.365	0.175	2.47029918064906E-66	1	CBWD1
7.48197438477149E-48	1.76864274219343	0.292	0.143	1.22038484190008E-43	1	TRMU
6.07692752260681E-118	1.75940777430474	0.525	0.261	9.91207648212396E-114	1	KDM5D
5.38746213273133E-54	1.75850708057176	0.333	0.17	8.78748948469807E-50	1	RNF138
1.57112411390212E-61	1.75564156085573	0.372	0.193	2.56266054218575E-57	1	GABRE
7.7774060835707E-94	1.75385899513163	0.463	0.231	1.26857270629122E-89	1	TAF1D
2.59570089629005E-91	1.75331455001085	0.443	0.214	4.2338477319387E-87	1	DAPK2
1.78598004527814E-192	1.75022369010305	0.724	0.376	2.91311205185318E-188	1	CCNL1
4.19677350238E-51	1.74691640008198	0.299	0.144	6.84535725973202E-47	1	KAT6B
1.05241253805161E-113	1.74387095389497	0.557	0.301	1.71659009081599E-109	1	CLK1
8.98600401522332E-151	1.74284991469961	0.63	0.326	1.46570711492308E-146	1	MOV10
6.73388971153287E-92	1.73992502967878	0.436	0.209	1.09836475084813E-87	1	DOCK5
1.78180247862331E-76	1.73867612976849	0.397	0.195	2.90629802288247E-72	1	BTAF1
2.02748645412166E-41	1.73348902784773	0.26	0.13	3.30703315531785E-37	1	ANGEL1
5.83565350874599E-84	1.72850770953964	0.427	0.209	9.51853443811559E-80	1	AKAP17A
5.92900183069705E-183	1.72383950325817	0.689	0.356	9.67079488604996E-179	1	ASH1L
2.69185353978836E-141	1.72359536088711	0.615	0.316	4.3906823087488E-137	1	DTNA
5.46365391258645E-50	1.72006162927836	0.294	0.144	8.91176589681975E-46	1	SIN3A
1.55333999419948E-86	1.71907863229713	0.451	0.232	2.53365286453877E-82	1	HECTD4
1.77400777836075E-61	1.71754530121133	0.364	0.188	2.89358408728422E-57	1	SIN3B
2.96158269406809E-57	1.71752602318463	0.32	0.154	4.83063753229446E-53	1	PIAS2
2.40212713605442E-132	1.71718285007781	0.568	0.285	3.91810957161836E-128	1	ALS2CL
5.10582026420459E-40	1.7164684616294	0.251	0.123	8.32810343294411E-36	1	FAM193A
3.92174011023711E-135	1.71536781861085	0.603	0.316	6.39675029380775E-131	1	ANKRD10
4.16793474267748E-49	1.71230280635313	0.33	0.175	6.79831835878124E-45	1	MBD4
4.17046980562708E-155	1.71151713807119	0.645	0.34	6.80245329995833E-151	1	DST
5.63898970641184E-279	1.71072891323704	0.905	0.555	9.19775611012834E-275	1	VEGFA
2.14102425105346E-65	1.71009146858018	0.39	0.206	3.4922246558933E-61	1	INTS4
7.58593610701646E-73	1.70925833278681	0.42	0.222	1.23734203841545E-68	1	TIA1
2.40196875787005E-65	1.70887419662471	0.371	0.19	3.91785124096184E-61	1	CCNT2
9.75187673039567E-45	1.70854146687654	0.297	0.155	1.59062861349484E-40	1	CCDC130
2.87629954787983E-78	1.70589701560582	0.449	0.241	4.69153219254679E-74	1	MYO15B
1.20702988729221E-111	1.70565841117172	0.541	0.286	1.96878644916233E-107	1	INTS3

**5.56290315740189E-93**	1.70198869460978	0.549	0.317	9.07365134003823E-89	1	HSPA1B
**3.65808837121461E-44**	1.7008352262821	0.29	0.151	5.96670794228816E-40	1	GORAB
**2.32431623966459E-48**	1.70077668566262	0.288	0.142	3.79119221851691E-44	1	LPIN3
**4.06845237801315E-35**	1.69616031180022	0.259	0.139	6.63605267377724E-31	1	BTBD9
**5.18663357658284E-88**	1.69608631405614	0.458	0.232	8.45991802676426E-84	1	TUFT1
**3.89734273914468E-58**	1.6956472382282	0.36	0.182	6.35695574181889E-54	1	VNN 3.00
**7.42438754746974E-35**	1.69179573945052	0.259	0.14	1.21099185286779E-30	1	TRPV1
**2.09132798705148E-163**	1.69046799569585	0.68	0.362	3.41116507967967E-159	1	ATP2B4
**6.50379455341616E-108**	1.68938316966569	0.52	0.254	1.06083392960771E-103	1	ZC3H12A
**2.04073766965567E-77**	1.68620773071606	0.361	0.161	3.32864721297536E-73	1	MEF2A
**1.19519397486543E-63**	1.68539829406155	0.395	0.214	1.949480892403E-59	1	HERC4
**5.92442236671832E-38**	1.6794817028766	0.279	0.153	9.66332532235425E-34	1	PUS7
**9.32026800193848E-141**	1.67840651472156	0.63	0.339	1.52022891379619E-136	1	GCKR
**2.58746946200108E-73**	1.67488857033391	0.405	0.209	4.22042143946997E-69	1	AP1G2
**3.94655366409987E-126**	1.6719659544066	0.574	0.293	6.4372236815133E-122	1	VPS45
**6.75524802956306E-38**	1.66922361402674	0.263	0.137	1.10184850610203E-33	1	SMC6
**1.01483606262644E-44**	1.66856420537801	0.262	0.124	1.65529910174999E-40	1	ATAD2B
**7.51779225400308E-57**	1.66362770035937	0.4	0.23	1.22622709455044E-52	1	KIF1B
**6.67219589342009E-54**	1.66247404206952	0.331	0.169	1.08830187217575E-49	1	SESTD1
**1.1492799317566E-64**	1.65576321842593	0.392	0.209	1.87459049668818E-60	1	TUT4
**1.32978500488708E-63**	1.6547683177478	0.367	0.186	2.16901232147131E-59	1	PIGL
**4.38311252969219E-57**	1.65361880999459	0.437	0.264	7.14929484718093E-53	1	DNAJB1
**2.47284982870597E-220**	1.65347070392871	0.779	0.412	4.0334653556023E-216	1	MDM4
**4.60343462603498E-49**	1.65307644423702	0.259	0.116	7.50866221852565E-45	1	INO80D
**1.01119166002569E-30**	1.65087201228229	0.258	0.146	1.6493547166679E-26	1	NBPF15
**2.43638632771616E-81**	1.65081404879924	0.416	0.205	3.97398973913782E-77	1	UVSSA
**2.47673195550126E-88**	1.64987416686625	0.444	0.217	4.03979749261811E-84	1	DENND4A
**2.91545636713609E-179**	1.64689711812217	0.767	0.442	4.75540088043567E-175	1	EXT1
**1.55193893782402E-114**	1.6464704874494	0.567	0.309	2.53136760148476E-110	1	CHD2
**2.70411095862905E-140**	1.64389260626524	0.586	0.29	4.41067538461985E-136	1	TNKS
**2.24907811580335E-61**	1.64123024275837	0.358	0.182	3.66847131468685E-57	1	AKAP9
**6.75025539858027E-70**	1.64056257689098	0.377	0.183	1.10103415806243E-65	1	RGL3
**5.86566286307444E-61**	1.64050822206758	0.368	0.192	9.56748269596072E-57	1	PIEZO2
**1.59648163459791E-103**	1.63998837962602	0.496	0.249	2.60402119419266E-99	1	TUBGCP6
**3.7607775568282E-72**	1.6396000517587	0.428	0.23	6.13420427294247E-68	1	MTHFR
**4.39250114068834E-90**	1.637943902448	0.448	0.219	7.16460861057675E-86	1	IDUA
**6.53311012639176E-62**	1.63525185363845	0.359	0.181	1.06561559271576E-57	1	TRPM7
**8.92616750284496E-70**	1.6351275104149	0.474	0.267	1.45594718138904E-65	1	GBP1
**3.49666118845873E-45**	1.63507966421691	0.328	0.182	5.70340406449503E-41	1	N4BP2L1
**7.21625318695371E-94**	1.63484155465693	0.426	0.193	1.17704305732402E-89	1	PLEKHH3
**3.58773330491287E-103**	1.6325938229705	0.53	0.28	5.85195179364339E-99	1	SRRM2
**2.47110763854247E-35**	1.6319747939608	0.263	0.142	4.03062366922663E-31	1	NGLY1
**9.00428295414362E-69**	1.63027312694576	0.4	0.21	1.46868859265037E-64	1	GLDC
**2.04095418672042E-61**	1.62958884354336	0.37	0.193	3.32900037395968E-57	1	GTF2H4
**5.30968191540591E-89**	1.62946730351203	0.462	0.232	8.66062217221858E-85	1	SAFB2
**2.58365716083799E-33**	1.6293243994587	0.258	0.141	4.21420319504285E-29	1	DMXL2
**5.96094894721601E-82**	1.62865350127197	0.438	0.223	9.72290382780403E-78	1	ZNF638
**9.39374590554285E-37**	1.62843194580052	0.284	0.157	1.5322138946531E-32	1	ARL5B
**4.11304551235543E-80**	1.62692114926786	0.451	0.236	6.70878853520294E-76	1	KDM4C
**4.14787914714464E-105**	1.6260664885419	0.568	0.318	6.76560567690763E-101	1	MAT2A

9.50805897716507E-112	1.62113974905281	0.529	0.27	1.55085949976539E-107	1	LUC7L
1.31504365446408E-73	1.61967373060329	0.48	0.278	2.14496770479636E-69	1	SLC22A25
4.57557402545716E-70	1.6123073250754	0.341	0.154	7.46321879292318E-66	1	PLXNA3
3.72114232097259E-67	1.61048036806004	0.383	0.198	6.06955523973839E-63	1	NPHP3
1.34126520442682E-147	1.6085923870945	0.717	0.414	2.18773767494059E-143	1	NFKBIZ
4.15458134384424E-119	1.60620892843895	0.58	0.312	6.77653762994434E-115	1	IKBKB
1.12927901070525E-119	1.60393358680951	0.595	0.324	1.84196699436133E-115	1	PLA2G6
4.80222088729963E-78	1.60371941432411	0.442	0.235	7.83290248927442E-74	1	GON4L
3.82659921401844E-89	1.60350803987963	0.483	0.252	6.24156597798547E-85	1	RUFY3
2.7256089415585E-66	1.60328149798616	0.401	0.215	4.44574074457607E-62	1	EPB41
4.1838285772551E-42	1.60297017919328	0.25	0.118	6.82424279236079E-38	1	PPP1R12A
1.00946755294111E-149	1.60239671232196	0.69	0.379	1.64654252560224E-145	1	XAF1
1.84844293766247E-59	1.60225461622895	0.348	0.176	3.01499527562126E-55	1	USP24
1.31173859638401E-47	1.60148307274657	0.31	0.159	2.13957682456196E-43	1	DENND1A
3.69481056884623E-42	1.60107437091435	0.298	0.161	6.02660551884509E-38	1	ATM
2.18705182812016E-47	1.60051055278519	0.254	0.115	3.56730023684679E-43	1	PLA2G4B
1.19385114067539E-49	1.59951317117366	0.323	0.168	1.94729059555563E-45	1	PRPF4B
1.55768269862783E-102	1.59821433293699	0.516	0.266	2.54073624973185E-98	1	DDX39B
7.3525258583693E-69	1.59618476021519	0.414	0.222	1.19927049275862E-64	1	USF1
5.00965281258001E-75	1.59220632884406	0.429	0.225	8.17124470259926E-71	1	NSD1
4.35830760858407E-117	1.59140170639436	0.573	0.311	7.10883554036147E-113	1	PI4KB
1.49084494105112E-87	1.59066427400978	0.467	0.24	2.43171718334848E-83	1	TENT4A
9.63863096141896E-59	1.59048266662588	0.347	0.176	1.57215709611705E-54	1	ADCY6
3.95782026345227E-46	1.59044474231689	0.313	0.166	6.455600631717E-42	1	KAT2A
4.68286654234126E-93	1.58405538257697	0.501	0.272	7.63822361721282E-89	1	MICAL3
1.34327618707437E-40	1.58269172154379	0.287	0.154	2.191017788737E-36	1	RUFY2
3.17249988510437E-46	1.58195036560858	0.295	0.15	5.17466456259373E-42	1	ARFIP2
3.95934034852645E-156	1.58167045594194	0.691	0.386	6.45808004248149E-152	1	DPYD
1.22587573812145E-37	1.57987406690961	0.292	0.163	1.9995259164499E-33	1	CLK4
3.806978479089E-123	1.57798880072687	0.613	0.348	6.20956259724207E-119	1	MPV17
5.54185251276243E-73	1.57743015412393	0.425	0.229	9.0393156335668E-69	1	TCERG1
8.77739910637838E-63	1.5771430426054	0.368	0.188	1.43168156824138E-58	1	CAP2
5.56383181566728E-50	1.57702810038495	0.333	0.177	9.0751660745349E-46	1	KAT6A
2.52135232131049E-34	1.5769846152668	0.276	0.156	4.11257777128954E-30	1	SCML1
1.13664927606045E-38	1.57678194475644	0.278	0.149	1.85398863418219E-34	1	BAZ2B
1.23617029564848E-41	1.57673897747687	0.303	0.163	2.01631736923224E-37	1	MLLT10
2.42251309247675E-126	1.57635716790776	0.602	0.32	3.95136110513882E-122	1	NKTR
1.19634482283026E-63	1.57442306078355	0.348	0.168	1.95135804051844E-59	1	KHDC4
2.1549200739321E-41	1.5731209031685	0.257	0.126	3.51489013259064E-37	1	TOP3B
2.71473886924808E-50	1.57299829746562	0.361	0.204	4.42801056963055E-46	1	NPIPB15
1.16879181571355E-112	1.5727496196862	0.541	0.281	1.90641633061036E-108	1	PRPF3
9.6272226324681E-83	1.57231446914513	0.446	0.228	1.57029628358187E-78	1	KLHDC4
1.55966482049892E-44	1.57198547292744	0.301	0.158	2.54396928871578E-40	1	PSPC1
7.38683461973077E-55	1.57083064657396	0.397	0.23	1.20486659482429E-50	1	MCF2L
4.86914439141641E-50	1.57000253663146	0.305	0.153	7.9420614168393E-46	1	MYSM1
8.67161536781187E-227	1.56948630556147	0.836	0.468	1.41442718264379E-222	1	CDK5RAP3
1.94874621541783E-48	1.56684864538059	0.281	0.135	3.17859995196802E-44	1	SMG1
2.77309707042881E-47	1.56624156612632	0.327	0.176	4.52319863157644E-43	1	ATXN2
6.86182998844354E-45	1.56519905413021	0.352	0.205	1.11923308941503E-40	1	THBS3
8.88399098659522E-199	1.56519104439391	0.787	0.457	1.44906776982355E-194	1	ABCC3

**4.35267641136871E-89**	1.56418264881508	0.452	0.227	7.0996504945835E-85	1	SETDB1
**6.70955764447885E-52**	1.56396123887148	0.28	0.128	1.09439594739095E-47	1	MAPK12
**2.16784688269252E-32**	1.5624130163332	0.272	0.156	3.53597505035977E-28	1	TEAD3
**9.31312244625576E-85**	1.56148451905984	0.459	0.239	1.51906340220878E-80	1	EHBP1
**1.656905827407E-87**	1.56129201580464	0.487	0.266	2.70257909508356E-83	1	SUGP2
**2.24976003412128E-74**	1.56064326099405	0.446	0.244	3.66958359165521E-70	1	CTIF
**1.49482695692608E-70**	1.5599941562484	0.396	0.201	2.43821224944212E-66	1	MARK3
**3.6601703283156E-76**	1.55049396097305	0.487	0.28	5.97010382251557E-72	1	FAM193B
**5.50498254525591E-57**	1.55025907848479	0.347	0.18	8.97917702956691E-53	1	REV1
**1.55693444978953E-53**	1.54837936682263	0.31	0.151	2.5395157810517E-49	1	EDA
**1.17943209616097E-67**	1.54616073929562	0.366	0.178	1.92377169204815E-63	1	NLRC5
**3.59684279689166E-37**	1.54556896924912	0.281	0.153	5.86681028600999E-33	1	PIGG
**3.00490052715201E-49**	1.54311454662075	0.318	0.164	4.90129324983764E-45	1	SFPQ
**1.70383287662362E-65**	1.54233721872633	0.452	0.261	2.77912180506079E-61	1	SLC25A16
**1.85440160820808E-84**	1.54231929618368	0.444	0.226	3.0247144631482E-80	1	RBM15
**1.11272885481745E-29**	1.54217380738646	0.253	0.143	1.81497203509274E-25	1	GRAMD2B
**3.36778971429544E-37**	1.54214142842236	0.31	0.18	5.49320180298729E-33	1	RELCH
**3.49716095201627E-44**	1.53864224248997	0.318	0.173	5.70421922883373E-40	1	KIAA2026
**7.03061735963545E-64**	1.53816024399898	0.392	0.209	1.14676399753014E-59	1	GTF3C3
**6.54662771279899E-38**	1.53745744180823	0.272	0.147	1.06782044623464E-33	1	NUP88
**2.67172415019491E-59**	1.53653194651783	0.358	0.183	4.35784926138292E-55	1	WWOX
**3.84482283565199E-40**	1.53328348042626	0.287	0.152	6.27129052723196E-36	1	KDM3A
**4.39974001886397E-33**	1.53322308052308	0.281	0.161	7.17641594476903E-29	1	CCDC14
**3.20891769440172E-45**	1.53224976231478	0.28	0.14	5.23406565133865E-41	1	LPIN1
**1.34349409555101E-41**	1.53208527664518	0.324	0.183	2.19137321925325E-37	1	ATG4B
**9.03590799471891E-117**	1.5304373945634	0.575	0.306	1.4738469530186E-112	1	RSRP1
**7.31133631787485E-58**	1.52757947842396	0.399	0.227	1.19255206680857E-53	1	MPDZ
**1.89663706899581E-124**	1.52728063440306	0.625	0.352	3.09360472323907E-120	1	DTX4
**4.67118311718793E-57**	1.526940661594	0.386	0.216	7.61916678244523E-53	1	STX7
**1.22449272512758E-78**	1.52599775186276	0.456	0.244	1.99727008395559E-74	1	GBA2
**7.46086013034293E-31**	1.5247462418316	0.266	0.151	1.21694089586024E-26	1	BCO2
**1.23051514766522E-33**	1.52416579940744	0.26	0.143	2.00709325735673E-29	1	MED14
**1.94220828502473E-193**	1.52325696861472	0.774	0.444	3.16793593370384E-189	1	AFDN
**8.39648053497458E-44**	1.5230132889894	0.304	0.162	1.3695499400597E-39	1	HOOK3
**7.54973115557199E-73**	1.52266397495733	0.427	0.227	1.23143664878535E-68	1	IWS1
**5.89794558521866E-65**	1.52229459638184	0.393	0.206	9.62013904405016E-61	1	ELMSAN1
**2.9226442593227E-37**	1.52131520982138	0.307	0.177	4.76712505138125E-33	1	ATG2B
**4.10809539611959E-40**	1.5204285755002	0.281	0.149	6.70071440061066E-36	1	INPP4A
**4.47635227895563E-51**	1.52038120183448	0.356	0.199	7.30137820220452E-47	1	CPSF6
**1.39543191142806E-36**	1.51982826657545	0.263	0.137	2.2760889907303E-32	1	TRMT11
**7.66603699956867E-46**	1.51947147992526	0.34	0.193	1.25040729499965E-41	1	BPTF
**9.59749800221179E-54**	1.51732477513537	0.349	0.182	1.56544789914077E-49	1	KIFC2
**2.39856133953964E-43**	1.51726050352349	0.257	0.123	3.9122934009231E-39	1	PSD3
**1.23874657154669E-86**	1.51690415657299	0.496	0.272	2.0205195328498E-82	1	ZFR
**5.58176626039657E-76**	1.51632092010761	0.282	0.098	9.10441894733285E-72	1	SLC6A15
**9.48983424546229E-78**	1.5156655087083	0.481	0.276	1.54788686377735E-73	1	NCOR1
**6.00841630298534E-139**	1.51535716704819	0.675	0.373	9.80032783179939E-135	1	CEP72
**2.35774891162036E-36**	1.5139242886097	0.279	0.155	3.84572424974396E-32	1	S100PBP
**2.80266103039482E-41**	1.51346876889988	0.266	0.134	4.57142040667699E-37	1	OCRL
**8.98789769827555E-48**	1.51227734756894	0.364	0.211	1.46601599356572E-43	1	RALGAPA2

3.02549169286369E-84	1.51150728871769	0.535	0.316	4.93487950022996E-80	1	SLC17A4
8.26376301475087E-57	1.51008703107927	0.377	0.203	1.34790238533601E-52	1	ARHGAP12
2.32092239223083E-103	1.50753450495816	0.506	0.253	3.78565651396771E-99	1	GIGYF1
5.12797498108377E-50	1.50744431944493	0.367	0.209	8.36423999164574E-46	1	DEPRECAT
1.92674390919171E-43	1.50647213818621	0.3	0.161	3.1427119902826E-39	1	SFXN4
1.42546781189472E-51	1.5052990827399	0.33	0.168	2.32508054798148E-47	1	KLF10
1.75719595591036E-56	1.50520412441713	0.378	0.209	2.86616232368538E-52	1	KMT2E
1.67517754678149E-122	1.50478202426472	0.627	0.354	2.73238209655528E-118	1	LGALS8
1.62613941032286E-31	1.50357605816349	0.263	0.149	2.65239599217762E-27	1	ATG14
8.46333359054877E-50	1.50355597116933	0.327	0.171	1.38045434195441E-45	1	TARBP1
1.23847721664259E-46	1.50050739191529	0.34	0.18	2.02008018806572E-42	1	SERPINE1
9.70280735331285E-64	1.49993456589261	0.381	0.196	1.58262490739886E-59	1	PCM1
1.5868891083483E-64	1.49939375496748	0.351	0.168	2.58837482462691E-60	1	PKD1
1.50977552763273E-42	1.49866448106368	0.273	0.137	2.46259486312175E-38	1	CCDC93
2.00928194662376E-56	1.49849665168347	0.341	0.174	3.27733978313802E-52	1	FEM1C
2.01806579108054E-143	1.49835783239141	0.65	0.354	3.29166711183147E-139	1	SRSF4
2.51655049422485E-42	1.49734503292853	0.296	0.156	4.10474551113016E-38	1	WHRN
3.557497247006E-63	1.49668799478445	0.389	0.206	5.80263375959148E-59	1	YPEL2
8.68417428065888E-42	1.49614944348926	0.295	0.157	1.41647566691827E-37	1	DUSP22
7.21595752747103E-59	1.4953535166333	0.358	0.185	1.1769948323058E-54	1	YTHDC1
9.28964909205455E-53	1.49486350502639	0.346	0.184	1.51523466340502E-48	1	KANSL3
4.68646365670934E-54	1.49454197244885	0.365	0.198	7.6440908704586E-50	1	ARMH3
1.88929529001088E-115	1.49451566900912	0.579	0.315	3.08162954753674E-111	1	MAP4
7.38179796542499E-37	1.49446222108758	0.274	0.15	1.20404506614047E-32	1	SFSWAP
1.89961067995713E-37	1.49413736028406	0.29	0.163	3.09845498007807E-33	1	ANKS1A
3.64973131789648E-40	1.49411015731277	0.322	0.185	5.95307675262096E-36	1	PDE8A
4.10541793391459E-130	1.49137411365834	0.635	0.349	6.69634719200809E-126	1	UBR2
4.08858401702537E-135	1.49125338359548	0.629	0.34	6.66888939017007E-131	1	CHTOP
8.8805760773943E-86	1.49044385380134	0.496	0.275	1.44851076398379E-81	1	ZRANB2
5.01392249857759E-38	1.48986309214868	0.268	0.141	8.1782089874299E-34	1	MED24
6.28538619694469E-42	1.48969054985286	0.297	0.16	1.02520934258365E-37	1	CCDC9B
1.249732202713E-31	1.48957998707451	0.263	0.148	2.03843819584517E-27	1	AP3B1
3.51468210267321E-258	1.48886756105086	0.958	0.69	5.73279797767027E-254	1	EFNA1
1.09639797967625E-34	1.48783074676645	0.297	0.173	1.78833474464993E-30	1	SEPSECS
4.3050329187604E-48	1.48765083736265	0.348	0.194	7.02193919379009E-44	1	XPO5
2.52523109089944E-77	1.48749441159752	0.428	0.221	4.11890443236607E-73	1	ARVCF
3.5898560604831E-35	1.48745821342662	0.261	0.14	5.85541422025398E-31	1	ALS2
1.73104631625432E-32	1.4874472690034	0.254	0.138	2.82350964644242E-28	1	MICALL2
9.1848127212714E-26	1.48606222387963	0.267	0.164	1.49813480296658E-21	1	ATP9B
8.53257752760011E-139	1.48564155174337	0.721	0.428	1.39174872052685E-134	1	VMP1
2.13940290134724E-47	1.48516747735559	0.341	0.189	3.48958007238749E-43	1	ZZEF1
1.16231544269079E-33	1.48439759288301	0.284	0.162	1.89585271857295E-29	1	L3MBTL4
1.1236307025012E-73	1.48417544031825	0.461	0.252	1.83275403884971E-69	1	SLC25A29
1.98565978811375E-130	1.47784407033084	0.623	0.342	3.23880968039233E-126	1	MPRIP
2.70528358273083E-54	1.47653537728642	0.357	0.192	4.41258805179226E-50	1	CENPT
5.52763008999917E-47	1.47378110010113	0.321	0.172	9.01611743979764E-43	1	ZNF44
3.71826233700093E-64	1.47256384872998	0.393	0.21	6.06485769788222E-60	1	HIP1R
2.04918215856278E-46	1.47223992197056	0.315	0.167	3.34242101883175E-42	1	ASAP3
3.62167558402402E-36	1.47119449124549	0.268	0.144	5.90731504510158E-32	1	NXF1
4.7187165458368E-32	1.47066934322354	0.299	0.182	7.6966985579144E-28	1	UBE2G2

1.08699509944419E-30	1.47061392499001	0.253	0.141	1.77299770670342E-26	1	TRANK1
2.08337106833408E-37	1.46965621399804	0.268	0.141	3.39818654955972E-33	1	VPS37A
2.28159125351303E-49	1.46756850651819	0.336	0.182	3.7215034936051E-45	1	RPTOR
2.97857977867467E-37	1.46670972165989	0.272	0.146	4.85836147699625E-33	1	LRCH1
2.51730828512906E-73	1.4664581005313	0.455	0.254	4.105981543874E-69	1	MARCH7
2.00970332374277E-60	1.46563983988677	0.439	0.256	3.27802709135683E-56	1	PARP14
1.03244853033647E-55	1.46366436409315	0.377	0.21	1.68402679783182E-51	1	BRD1
5.42195503030961E-145	1.46299380931313	0.677	0.378	8.843750849938E-141	1	UBAP2L
2.00455294372733E-84	1.46067929355777	0.499	0.277	3.26962630651365E-80	1	BIRC6
1.3520504064811E-32	1.46040452283165	0.279	0.161	2.20532941801133E-28	1	ZKSCAN3
1.44013678468411E-68	1.46040422089324	0.455	0.258	2.34900710949826E-64	1	TFIP11
2.0832889313251E-88	1.45984495806008	0.531	0.302	3.39805257588437E-84	1	RABGAP1L
2.90745370868627E-53	1.4590124744375	0.381	0.217	4.74234774423817E-49	1	ZMYM2
1.96299736513637E-125	1.45803765208497	0.632	0.357	3.20184500227394E-121	1	SETD5
3.3936972890919E-51	1.45801095838418	0.371	0.207	5.5354596482378E-47	1	MLPH
1.00855514593595E-72	1.45658708802799	0.436	0.235	1.64505429853612E-68	1	ARHGEF7
2.13626038616187E-129	1.45619319164627	0.651	0.373	3.48445431586863E-125	1	NR5A2
3.11542303568396E-96	1.45588293419626	0.563	0.326	5.08156651350411E-92	1	MTG1
1.33810627315527E-76	1.4554476708517	0.464	0.253	2.18258514214357E-72	1	DLG5
5.46500456668426E-30	1.45450774616348	0.253	0.143	8.9139689487187E-26	1	CLN8
1.19596407042562E-58	1.45416149933258	0.406	0.23	1.95073699527123E-54	1	ITSN2
3.40748315247907E-72	1.45351637226422	0.431	0.23	5.5579457700086E-68	1	NFATC2IP
2.40289642160895E-50	1.45239635132084	0.362	0.205	3.91936435328636E-46	1	DNAJC10
2.60228970160163E-40	1.45234894337716	0.322	0.184	4.24459473228242E-36	1	PTPRD
7.38285481662264E-134	1.45013968328533	0.67	0.381	1.20421744913932E-129	1	NOTCH2NL
5.53195819249672E-44	1.44986949416755	0.352	0.204	9.0231770077814E-40	1	TCF7L2
5.05616828637518E-44	1.44969030052909	0.283	0.142	8.24711609190655E-40	1	SETBP1
2.78898413894722E-42	1.44925827209855	0.297	0.158	4.54911202903681E-38	1	MED13L
1.2815020378528E-47	1.44714880676124	0.346	0.192	2.0902579739417E-43	1	TBRG1
2.4176743421915E-30	1.4449657063336	0.25	0.14	3.94346861954856E-26	1	NBEAL1
1.05352312882515E-38	1.44236818414674	0.269	0.14	1.7184015754267E-34	1	PGGHG
2.85033913832459E-68	1.43989709668923	0.415	0.223	4.64918816852124E-64	1	ZNF595
2.36522834812614E-57	1.43892929294778	0.358	0.185	3.85792395862855E-53	1	STAG1
1.41331456962356E-52	1.43761138517814	0.333	0.174	2.305257394513E-48	1	ACIN1
3.47303276918174E-54	1.43674733912455	0.383	0.216	5.66486374981233E-50	1	PCID2
1.44243057097604E-41	1.43579466563324	0.287	0.15	2.35274850431902E-37	1	GTF2H2C
2.47684343178354E-71	1.43571596164838	0.417	0.218	4.03997932158214E-67	1	CLASRP
1.99463787749354E-67	1.43568400846208	0.445	0.254	3.25345384197971E-63	1	STX16
2.62953208431336E-100	1.43484379535758	0.549	0.305	4.28902978272352E-96	1	DYNC1LI2
6.24848351915018E-57	1.43435439846559	0.374	0.201	1.01919014680859E-52	1	ACAP3
7.94381980878896E-57	1.43298700059701	0.406	0.234	1.29571644901157E-52	1	PNISR
6.12882599066512E-47	1.43243986102686	0.308	0.159	9.99672807337388E-43	1	HPS4
1.15786617584419E-65	1.43165190106159	0.426	0.236	1.88859551941945E-61	1	GPATCH2L
6.13877056895106E-36	1.43153139239171	0.294	0.168	1.00129486750161E-31	1	GATAD1
1.20857011702533E-66	1.43111826978623	0.417	0.226	1.97129871788001E-62	1	WDR33
5.2486791832484E-51	1.43029903566775	0.341	0.183	8.56112061579646E-47	1	MAP3K20
1.05788965127992E-64	1.42859348146003	0.464	0.277	1.72552381020268E-60	1	FAM214A
6.33832856879207E-32	1.42856660721585	0.3	0.182	1.03384477285567E-27	1	CLCN5
5.07202545240734E-64	1.42842916579941	0.48	0.289	8.27298071542162E-60	1	NAMPT
1.32768284167482E-37	1.42774430700916	0.369	0.232	2.16558348305579E-33	1	SLC20A1

6.86797480186201E-43	1.4270624115921	0.275	0.138	1.12023536993171E-38	1	CAMSAP2
1.26541248779062E-139	1.42653671761138	0.654	0.365	2.06401430883527E-135	1	CDC42SE1
2.5343431482585E-90	1.42606248117281	0.53	0.296	4.13376710912443E-86	1	NRBP2
3.89109724210805E-27	1.42595362646265	0.265	0.159	6.34676871160244E-23	1	ANKRD13A
3.13955117663673E-32	1.42496329784881	0.307	0.187	5.12092192421217E-28	1	SMAD5
1.01497688010385E-59	1.42476593361156	0.418	0.243	1.65552878913738E-55	1	ASXL1
2.20416595945179E-69	1.42278908997025	0.428	0.231	3.59521509646181E-65	1	RBM14
7.56081658220749E-41	1.4222895771788	0.326	0.189	1.23324479272386E-36	1	POLR2J3
1.91355615859923E-50	1.42059434684305	0.333	0.173	3.1212014502912E-46	1	LDB1
2.39555221956781E-147	1.41960953208223	0.697	0.404	3.90738522533705E-143	1	NPLOC4
1.20463614424649E-68	1.41956485045498	0.432	0.237	1.96488201488044E-64	1	ZFYVE27
2.49254256655038E-47	1.41920110498178	0.358	0.206	4.06558618030032E-43	1	CLDND1
5.37211072151994E-20	1.4181886824492	0.257	0.169	8.76244979787117E-16	1	GAS2
1.30468208332162E-73	1.41702014964751	0.452	0.247	2.1280669461059E-69	1	DCUN1D4
1.15287414314573E-80	1.4149933369975	0.51	0.296	1.880453014885E-76	1	RNF149
7.6999091424376E-58	1.41474164879699	0.384	0.21	1.255932180223E-53	1	FGD4
4.96446246903656E-46	1.41421660123885	0.331	0.182	8.09753473324554E-42	1	NUP85
5.30987638001766E-63	1.41216271747339	0.426	0.239	8.6609393634468E-59	1	NQO2
1.05702586443123E-29	1.41197865890061	0.267	0.157	1.72411488747379E-25	1	DENND1B
9.79723398874527E-113	1.41182382858041	0.611	0.356	1.59802683590424E-108	1	C16orf70
2.48046429640598E-51	1.41176805736068	0.353	0.192	4.04588531386779E-47	1	NOP58
2.79656235786032E-41	1.41146081587729	0.317	0.179	4.56147286190597E-37	1	USP48
1.06210289080145E-79	1.40961365512985	0.496	0.285	1.73239602518625E-75	1	PCED1A
2.63263388518216E-133	1.40924541461859	0.654	0.37	4.29408913012062E-129	1	LUC7L3
3.54788978544912E-41	1.40845984025456	0.3	0.161	5.78696302904607E-37	1	TAZ
3.93215065845432E-101	1.40840590152437	0.578	0.332	6.41373093900483E-97	1	PRKAB2
9.53455744664386E-50	1.40750999363311	0.368	0.21	1.55518166512208E-45	1	CSTF3
1.51006852459524E-64	1.40725559033868	0.392	0.205	2.46307277046729E-60	1	WASHC1
4.95982330136608E-21	1.40693491748689	0.257	0.169	8.08996778685821E-17	1	AKIRIN1
9.81911216709738E-41	1.4068276244343	0.286	0.153	1.60159538557525E-36	1	POLM
4.15743964364774E-59	1.40650435395053	0.425	0.247	6.78119980275382E-55	1	UPK3BL1
1.44942229518569E-37	1.40604713887786	0.28	0.153	2.36415270567738E-33	1	INTS6
7.9168677704149E-156	1.4059321218057	0.728	0.429	1.29132030203237E-151	1	ARHGEF10
2.24581517081296E-33	1.40477962915693	0.275	0.154	3.66314912511302E-29	1	TLK1
1.92450900431949E-70	1.40406052079782	0.467	0.267	3.13906663694551E-66	1	NPEPPS
3.90798700926002E-57	1.40397333605582	0.343	0.17	6.37431761080402E-53	1	MAFF
6.46705822837787E-97	1.40204458678961	0.606	0.366	1.05484186763071E-92	1	SHROOM1
1.50235804280953E-49	1.40193032248939	0.366	0.207	2.45049620362662E-45	1	ZNF146
1.86583544765214E-36	1.40055375711914	0.313	0.184	3.04336419866541E-32	1	ECPAS
1.16055701626016E-59	1.39996074146657	0.347	0.173	1.89298454922194E-55	1	MTMR3
1.14113992926277E-36	1.3987330707975	0.29	0.162	1.8613133386205E-32	1	ARMC8
9.61757340137075E-66	1.39803247766328	0.463	0.271	1.56872239749758E-61	1	SOX5
1.14483509672646E-166	1.39720839442249	0.757	0.423	1.86734052627052E-162	1	OBSCN
1.09387846227317E-145	1.39699708498563	0.725	0.426	1.78422515981377E-141	1	IL6R
2.33236333425944E-28	1.39651309544103	0.251	0.145	3.80431783451057E-24	1	DIAPH2
2.78739613591316E-42	1.39614553422081	0.341	0.2	4.54652183728795E-38	1	FNIP1
2.53180338224475E-32	1.39067286931997	0.268	0.152	4.12962449677941E-28	1	COG7
1.42373304519611E-33	1.39020830392561	0.256	0.137	2.32225097001937E-29	1	ZNF507
9.60295169392275E-57	1.39011948312893	0.394	0.223	1.56633745079574E-52	1	NEMF
3.38616113283711E-47	1.38964722549082	0.359	0.206	5.52316742377061E-43	1	IPO9

1.04927580028906E-57	1.38962591506893	0.348	0.178	1.71147375785148E-53	1	STX3
4.853762790746E-31	1.38906037370766	0.274	0.159	7.91697248798581E-27	1	ZNF91
1.93252825553914E-37	1.3885489614153	0.344	0.211	3.1521468376099E-33	1	ACAD11
3.77263515706441E-81	1.38709867044779	0.503	0.285	6.15354520468776E-77	1	ADGRG6
1.16797294491858E-53	1.38638251180337	0.386	0.219	1.9050806704567E-49	1	USP32
7.94553686927928E-46	1.38400829805224	0.413	0.254	1.29599651874814E-41	1	IRF1
3.57524001627122E-46	1.38361543022018	0.337	0.187	5.83157399053999E-42	1	LARS
8.36720425424164E-38	1.38327043869323	0.327	0.194	1.36477468590935E-33	1	ZDHHC11
5.13914895610641E-33	1.3783640125758	0.257	0.14	8.38246586230516E-29	1	TNS1
4.27856575432992E-49	1.37802966861614	0.335	0.181	6.97876860188753E-45	1	ZFC3H1
2.49672212050145E-55	1.37788183643867	0.392	0.22	4.07240345074991E-51	1	POLR2J2
1.04867281290912E-31	1.37752553740611	0.252	0.137	1.71049022513606E-27	1	GSAP
9.5155268355615E-30	1.37566177014574	0.293	0.179	1.55207758214844E-25	1	NUP42
3.89644515205754E-42	1.37516809078048	0.277	0.139	6.35549168752105E-38	1	SBF1
2.82538468298804E-145	1.3744602147762	0.695	0.395	4.6084849564218E-141	1	POGZ
1.46028508772289E-49	1.37389570139284	0.4	0.241	2.3818710065848E-45	1	ABCA1
2.81488881809592E-26	1.37274162606603	0.26	0.157	4.59136515119625E-22	1	ZFYVE9
6.31141435002875E-86	1.37250744468013	0.533	0.305	1.02945479463319E-81	1	ACOT12
1.44081512815597E-40	1.37225233321488	0.366	0.223	2.35011355553521E-36	1	CACNA1H
7.39068137516343E-100	1.3715358336888	0.582	0.337	1.20549403910291E-95	1	POLR2H
1.20574660620249E-55	1.37028602089536	0.396	0.224	1.96669328937688E-51	1	MAPRE3
1.07184394889273E-36	1.37002974751566	0.268	0.143	1.74828466503893E-32	1	BCL2L2
4.72436161154791E-46	1.36969272342244	0.315	0.165	7.70590622459579E-42	1	ABHD11
2.47984353339627E-183	1.36960517660011	0.917	0.685	4.04487278732265E-179	1	MCL1
2.41788293305286E-51	1.36814119585155	0.378	0.216	3.94380885210251E-47	1	CDC16
2.68727789289024E-57	1.36789279806628	0.407	0.235	4.38321897109326E-53	1	SEC24C
5.05116330437469E-31	1.36763179582064	0.269	0.154	8.23895246576555E-27	1	MED23
9.32834990150494E-41	1.36755098014822	0.314	0.177	1.52154715243447E-36	1	TRRAP
8.04428833691922E-72	1.36667513211953	0.469	0.269	1.31210387063489E-67	1	NDEL1
2.22502538315963E-55	1.36663021774814	0.343	0.175	3.62923890247167E-51	1	KIF21B
1.14693842180917E-46	1.36615109578751	0.386	0.233	1.87077125981294E-42	1	CREBZF
3.90275261193683E-93	1.36603284785691	0.559	0.327	6.36577978533017E-89	1	RBM39
3.9844051908583E-37	1.36554554617905	0.291	0.164	6.49896330680898E-33	1	TMEM39A
7.47033202624414E-41	1.36509025759202	0.315	0.175	1.21848585680068E-36	1	ATXN2L
4.00511102811906E-44	1.3645699248768	0.344	0.197	6.532736597965E-40	1	DENND4C
3.15769516116215E-34	1.36154121315333	0.282	0.16	5.15051657737159E-30	1	NFAT5
1.80315575162434E-42	1.36069077829016	0.298	0.159	2.94112734647445E-38	1	FBXO44
1.45536659163161E-73	1.35967909967109	0.461	0.259	2.37384844761033E-69	1	KANSL1
1.43589710030935E-26	1.35794565728999	0.29	0.185	2.34209176031458E-22	1	OARD1
1.02800663599455E-32	1.3569930675906	0.284	0.163	1.6767816239707E-28	1	ZBTB41
8.48202508198184E-42	1.35493332035984	0.269	0.133	1.38350311112206E-37	1	EPC2
7.47899084188603E-84	1.35373965213544	0.476	0.253	1.21989819622003E-79	1	BAZ2A
2.8742825815038E-67	1.35145438131996	0.48	0.286	4.68824231869085E-63	1	FOXP1
7.49233015381194E-100	1.35085194090612	0.562	0.314	1.22207397138827E-95	1	MAN2A2
4.20895389764715E-28	1.35078798345545	0.284	0.176	6.86522470245227E-24	1	ACCS
5.35478267019648E-37	1.35068045095448	0.323	0.192	8.73418601335747E-33	1	ZNF680
2.73908692637131E-38	1.35032045757782	0.285	0.154	4.46772468560424E-34	1	DOT1L
6.67732740377516E-34	1.34939212701937	0.272	0.152	1.08913887282977E-29	1	USP3
4.97516726099434E-68	1.34881871981443	0.48	0.285	8.11499531940787E-64	1	KIAA1217
3.2218246420397E-56	1.34833531637837	0.411	0.24	5.25511817363095E-52	1	ODR4

9.41563184541955E-99	1.34825766425233	0.567	0.315	1.53578371030638E-94	1	LIMK2
1.36878195334744E-59	1.34796416835138	0.435	0.259	2.23262024410502E-55	1	FAM160A2
9.1235962699582E-40	1.34748245720525	0.261	0.13	1.48814978759288E-35	1	SLC28A1
1.6613715694447E-46	1.34729977771582	0.372	0.218	2.70986316692126E-42	1	PCMTD2
2.09588495442385E-43	1.34696417563464	0.328	0.184	3.41859794916074E-39	1	RYBP
1.54261557196986E-91	1.34642859298152	0.538	0.299	2.51616025944004E-87	1	ZNF644
2.20034372749203E-37	1.34514781533353	0.34	0.205	3.58898065391225E-33	1	CLMN
2.4908903626447E-100	1.3444657525485	0.562	0.314	4.06289127050976E-96	1	N4BP2L2
2.73650933906857E-45	1.34378344987013	0.352	0.201	4.46352038295474E-41	1	FRRS1
5.84374165514736E-45	1.3425955290826	0.38	0.232	9.53172701371087E-41	1	RC3H1
7.35860103070377E-72	1.34247786808623	0.457	0.253	1.20026141411809E-67	1	TRIM38
2.62377609967551E-42	1.34206352341259	0.329	0.186	4.27964119618073E-38	1	SORBS1
3.8492253792268E-58	1.34151022800548	0.418	0.244	6.27847151605684E-54	1	RHOBTB3
1.23035168419289E-87	1.3410176577773	0.535	0.309	2.00682663208702E-83	1	SLC17A9
1.73689295589247E-165	1.34100753733318	0.772	0.457	2.83304610035621E-161	1	ZKSCAN1
5.68896061026571E-31	1.34027201285314	0.281	0.165	9.2792636514044E-27	1	KLHL23
5.17708607180507E-73	1.34012553717948	0.461	0.259	8.44434509172125E-69	1	SENP6
1.09924661871988E-44	1.33993438904523	0.359	0.208	1.79298115979399E-40	1	ZBTB37
1.19455628408134E-126	1.33899881229759	0.664	0.382	1.94844075496508E-122	1	ARAP1
1.10436533171829E-46	1.33874510600796	0.372	0.22	1.8013302925657E-42	1	GSK3B
3.46436317082862E-78	1.33838821338801	0.466	0.254	5.65072276793856E-74	1	KIF13A
6.62178955599063E-80	1.33833351630812	0.485	0.268	1.08008009447763E-75	1	COG3
5.39178980028735E-30	1.33646202429812	0.311	0.196	8.7945483432487E-26	1	DEPRECAT
5.30797194047877E-39	1.33636930388471	0.316	0.181	8.65783303211492E-35	1	FBXO46
1.74321246622674E-33	1.33608989559414	0.263	0.143	2.84335385366243E-29	1	HIRA
2.58513719007517E-36	1.33550421732927	0.293	0.165	4.2166172707316E-32	1	MMS19
9.55545203279161E-39	1.33531748044846	0.301	0.168	1.55858978106864E-34	1	NAT9
4.6150562718483E-72	1.33502314795719	0.485	0.282	7.52761828501176E-68	1	SLTM
8.78027653700961E-36	1.33477260166848	0.355	0.225	1.43215090595164E-31	1	FBXO9
3.29684173478284E-32	1.33452482234325	0.293	0.173	5.3774785536043E-28	1	RBM5
4.4044323611263E-22	1.33450405139098	0.262	0.168	7.18406962423311E-18	1	MCEE
6.35003773320039E-61	1.33327091175184	0.389	0.21	1.03575465466232E-56	1	KLC1
4.08457263021007E-57	1.33284856665929	0.372	0.2	6.66234641713565E-53	1	GPHN
1.31579930370843E-72	1.33023073792667	0.47	0.266	2.14620024427882E-68	1	NUMB
8.37557894078471E-41	1.33000157953435	0.34	0.2	1.36614068103139E-36	1	PHLDB2
3.8921221278078E-45	1.32960224562062	0.336	0.187	6.3484404026673E-41	1	VPS39
5.78179664594294E-30	1.32916907116926	0.281	0.169	9.43068850919753E-26	1	NR1D2
4.25736921737273E-41	1.32881393590537	0.328	0.189	6.94419493045666E-37	1	EWSR1
1.11952222594728E-63	1.32828425713236	0.436	0.247	1.82605270274261E-59	1	AAK1
2.57487376065469E-46	1.32580215287595	0.394	0.242	4.19987659100387E-42	1	VPS13B
2.11613046056932E-59	1.32555696150073	0.491	0.305	3.45162039423463E-55	1	SREBF1
1.06341688667286E-30	1.32535756699571	0.256	0.143	1.7345392838521E-26	1	PDPR
3.55669316614456E-40	1.32534559611693	0.331	0.192	5.80132222329839E-36	1	CUL1
1.85976670837988E-34	1.3239612514539	0.263	0.143	3.03346547803843E-30	1	SNIP1
1.4333316586312E-31	1.32333938664635	0.271	0.154	2.33790726839336E-27	1	CNOT2
1.48876861329329E-52	1.32220597931583	0.429	0.264	2.42833048514268E-48	1	XRN1
1.04007389261773E-150	1.32184326542621	0.744	0.438	1.69646452624877E-146	1	TMEM140
1.63177964819006E-84	1.32137248324329	0.518	0.294	2.6615957841628E-80	1	SLC25A44
2.33017033570827E-37	1.32036274158384	0.282	0.152	3.80074083457375E-33	1	CLK3
1.05728823023349E-25	1.31950242636452	0.255	0.154	1.72454283233384E-21	1	HUS1

7.12794235513315E-61	1.31803187915311	0.464	0.282	1.16263867754577E-56	1	TSC22D2
7.23981488974096E-97	1.31716042238894	0.57	0.33	1.18088620666565E-92	1	SLC41A2
9.6153474866335E-62	1.31708776033728	0.418	0.236	1.56835932854479E-57	1	RAF1
5.98311298082474E-27	1.31574682433532	0.274	0.167	9.75905558302323E-23	1	DEDD2
6.89311769579841E-126	1.31506251895944	0.682	0.408	1.12433642736168E-121	1	LIMD1
8.6487920628531E-32	1.31454179304375	0.251	0.137	1.41070447337197E-27	1	C1orf52
2.126178850317E-36	1.31251558039676	0.302	0.171	3.46801032275206E-32	1	STRN3
1.61648841880662E-51	1.31166502548465	0.392	0.227	2.63665425991548E-47	1	CHKA
8.52190291602701E-30	1.31130690565257	0.282	0.169	1.39000758463317E-25	1	SRSF7
6.19954460714458E-31	1.31127532142944	0.286	0.17	1.01120772087135E-26	1	SUSD6
3.31055398250327E-32	1.31017833572807	0.351	0.227	5.39984460086109E-28	1	GDA
8.06795304794191E-51	1.30927557732526	0.376	0.216	1.3159638216498E-46	1	DEPRECAT
3.29706850551386E-66	1.30846738764774	0.455	0.26	5.37784843934365E-62	1	COL27A1
6.45079187742285E-24	1.30840953958546	0.251	0.153	1.05218866312644E-19	1	DOCK4
1.91516995215023E-53	1.30840793505512	0.361	0.191	3.12383370895224E-49	1	PIEZO1
6.48370983448071E-37	1.30793208273045	0.332	0.201	1.05755791110215E-32	1	SPG11
1.1339532679628E-102	1.30735636846079	0.537	0.286	1.84959117537412E-98	1	TIMM44
1.2952835905272E-26	1.30672367556914	0.259	0.154	2.11273706450891E-22	1	MORC3
9.08886795622056E-49	1.30662223911017	0.383	0.224	1.48248525233914E-44	1	BRWD1
3.00048286735495E-52	1.30604923343224	0.415	0.248	4.89408760494267E-48	1	ZNF33A
3.22127898109851E-35	1.30569311664276	0.323	0.194	5.25422814606978E-31	1	SHPK
1.10975710286599E-114	1.30490003716848	0.678	0.412	1.81012481048472E-110	1	RNF213
5.12554957925059E-89	1.30434613230503	0.52	0.291	8.36028391871564E-85	1	USP12
1.44427686439527E-34	1.30407631532324	0.254	0.133	2.35575999351512E-30	1	CBWD3
2.71312803812368E-48	1.30390214454614	0.378	0.221	4.42538314298354E-44	1	TMEM131L
5.62092558088223E-49	1.3038869680623	0.402	0.24	9.16829171497701E-45	1	JMJD1C
9.09610202456619E-65	1.30228289119389	0.451	0.262	1.48366520122699E-60	1	LRCH4
9.40862381111281E-104	1.30211666786906	0.597	0.342	1.53464062983061E-99	1	TBC1D2B
2.46385190336655E-98	1.3019992948131	0.569	0.327	4.01878883958118E-94	1	SMAD3
9.59538324943466E-29	1.30099697737587	0.262	0.151	1.56510296181529E-24	1	NBPF12
1.19724176316239E-42	1.30089678770251	0.333	0.189	1.95282103989417E-38	1	IST1
1.51476618762084E-55	1.30035305602216	0.385	0.214	2.47073512862836E-51	1	ENOSF1
2.53685568273966E-72	1.30034695302541	0.521	0.318	4.13786530411666E-68	1	PCCA
4.45603943269678E-57	1.29886951546301	0.415	0.24	7.26824591867171E-53	1	PLEKHB2
7.87246319369061E-49	1.29866672543782	0.361	0.204	1.28407747152288E-44	1	TENT2
6.15095477946061E-77	1.29848642064515	0.497	0.284	1.00328223407782E-72	1	MACF1
1.19142955611447E-61	1.29798495101496	0.454	0.268	1.94334074897831E-57	1	MKLN1
2.69400720791363E-40	1.29752618210799	0.326	0.186	4.39419515682792E-36	1	MAGI1
2.16432565845348E-90	1.29660142165543	0.548	0.312	3.53023158150347E-86	1	ZSWIM8
1.83957524752715E-131	1.29543283448022	0.726	0.444	3.00053118624153E-127	1	NR1H4
3.563514871834E-181	1.29459600136177	0.874	0.598	5.81244910744844E-177	1	UPB1
2.97040182009887E-39	1.29392525979661	0.318	0.182	4.84502240876328E-35	1	SLC19A2
5.05304549994414E-93	1.29352122815585	0.551	0.312	8.24202251495889E-89	1	MEAF6
3.19891254179638E-29	1.29311698177935	0.262	0.153	5.21774624692407E-25	1	ERCC5
7.33663683522538E-68	1.2928365576121	0.451	0.258	1.19667883419361E-63	1	TRA2A
4.31557039216347E-70	1.29237433248263	0.51	0.312	7.03912686665784E-66	1	TNRC6A
1.61406272643292E-92	1.29143109530878	0.555	0.313	2.63269771308473E-88	1	SRSF5
2.45168283828189E-46	1.28920976527628	0.368	0.213	3.99893987752158E-42	1	TUBGCP3
1.95960819695703E-40	1.28842985267733	0.359	0.217	3.19631693005661E-36	1	PHF8
7.03539411369768E-97	1.28796322210779	0.577	0.337	1.14754313388523E-92	1	CALCOCO2

**5.54505341024187E-45**	1.28787863833897	0.346	0.194	9.04453661744551E-41	1	NPEPL1
**2.87567420124768E-37**	1.28705981912488	0.303	0.172	4.69051218965509E-33	1	LMBR1L
**1.41105026157195E-29**	1.28563608432251	0.257	0.146	2.30156408165001E-25	1	PRPF38B
**6.10169576658465E-43**	1.28541078145985	0.348	0.203	9.95247596487622E-39	1	NCK1
**6.8767015697926E-29**	1.28520372130725	0.257	0.146	1.12165879304887E-24	1	NOP2
**4.67207511976473E-63**	1.28471753687866	0.424	0.235	7.62062172784825E-59	1	EEPD1
**7.49730627792179E-38**	1.28466440278531	0.315	0.182	1.22288562699182E-33	1	EXOC4
**1.32660491674992E-70**	1.28440295161926	0.42	0.217	2.1638252797108E-66	1	MSTO1
**1.85981027297522E-32**	1.28389499389306	0.255	0.14	3.03353653624989E-28	1	PPP1R21
**8.42271130264294E-49**	1.283655597611	0.377	0.219	1.37382844057409E-44	1	COP 1
**6.3698834594676E-134**	1.28361945620146	0.865	0.611	1.03899169107376E-129	1	ELF3
**2.36071255291043E-184**	1.28346161067533	0.81	0.488	3.85055824505221E-180	1	SEC16B
**2.82504611491534E-125**	1.28301734231226	0.704	0.429	4.60793271803842E-121	1	ERBB3
**8.30459706550668E-39**	1.28161674905687	0.313	0.177	1.35456282735479E-34	1	TFE3
**2.79023447593639E-62**	1.2809138563225	0.432	0.248	4.55115145369985E-58	1	TBC1D8
**1.08264744319843E-87**	1.28033584494235	0.597	0.364	1.76590624460095E-83	1	BHLHE40
**2.72055364696738E-60**	1.27878327462452	0.408	0.227	4.4374950535685E-56	1	RPAIN
**1.19212870260155E-43**	1.27874228396107	0.334	0.189	1.94448112681339E-39	1	RRP12
**1.11364326047187E-29**	1.27828550526071	0.293	0.179	1.81646352215567E-25	1	PPOX
**3.96211420533339E-53**	1.27786178035978	0.4	0.23	6.46260448031929E-49	1	CALCOCO1
**8.29851288632551E-72**	1.27703851455604	0.478	0.272	1.35357043688855E-67	1	KDM5B
**2.41067325725116E-35**	1.27663338592841	0.25	0.128	3.93204914990236E-31	1	IRAK2
**2.06016573949484E-33**	1.27583173043097	0.327	0.202	3.36033633769004E-29	1	PPARG
**9.65044270635376E-91**	1.27571151135984	0.546	0.315	1.57408370983336E-86	1	RNF115
**1.53442318671505E-51**	1.2754806038924	0.405	0.236	2.50279765985091E-47	1	UBE4B
**1.56858431386242E-26**	1.27499968706296	0.268	0.163	2.55851787434099E-22	1	NBPF26
**1.70138719187945E-83**	1.27455177589871	0.546	0.32	2.77513264867457E-79	1	QTRT1
**6.57471737504243E-57**	1.27347456844489	0.427	0.251	1.07240215104317E-52	1	KLHL20
**6.60641657332056E-82**	1.27343822056462	0.594	0.379	1.07757260727432E-77	1	HSPA1A
**2.3320639003819E-32**	1.27183666078358	0.327	0.205	3.80382942791291E-28	1	NOP56
**1.13868755852039E-46**	1.27144254872774	0.355	0.2	1.85731327670262E-42	1	SATB1
**5.00668817325158E-104**	1.2713950000792	0.592	0.349	8.16640907939066E-100	1	MARCH6
**6.25533805252442E-43**	1.27019724125367	0.34	0.195	1.02030818974726E-38	1	ZFAND1
**4.48244171008112E-50**	1.26906785059903	0.389	0.227	7.31131067331331E-46	1	DELE1
**2.1317713843592E-30**	1.26886457431365	0.289	0.174	3.4771323050283E-26	1	ADGRA3
**7.37830536051086E-35**	1.26844035075658	0.3	0.174	1.20347538735293E-30	1	RECQL5
**8.04971776262759E-40**	1.2679297628001	0.337	0.2	1.31298946426219E-35	1	MAFG
**1.95053726503094E-50**	1.26778399662852	0.423	0.265	3.18152133299196E-46	1	ZBED6
**1.68965922709583E-20**	1.26745299801252	0.253	0.164	2.75600316531601E-16	1	LRCH3
**2.72355526601797E-35**	1.26644316969463	0.288	0.162	4.44239099440192E-31	1	SEMA4B
**3.49408906969264E-50**	1.26298154327806	0.384	0.221	5.69920868157567E-46	1	TPR
**1.9883019121856E-36**	1.26252905540274	0.282	0.154	3.24311924896593E-32	1	TECPR1
**8.21442995056937E-29**	1.26249423522451	0.263	0.153	1.33985566923737E-24	1	PHF11
**5.03753288373687E-70**	1.26235133093591	0.507	0.304	8.21671988666321E-66	1	GNL3
**1.50672971050365E-24**	1.2616806154473	0.251	0.153	2.4576268308025E-20	1	DPH6
**1.0858286612303E-39**	1.26158434814148	0.294	0.159	1.77109512933274E-35	1	ERC1
**1.01063966349555E-49**	1.26152772436138	0.409	0.251	1.6484543551276E-45	1	ACBD6
**6.32326727575807E-55**	1.26141677111646	0.408	0.234	1.0313881253489E-50	1	DLGAP4
**6.27828859235483E-28**	1.2612420154304	0.282	0.172	1.024051652299E-23	1	HEXD
**5.44370895361272E-61**	1.25994802968541	0.421	0.237	8.87923367423772E-57	1	KDM5C

3.99128104645258E-33	1.25945681709434	0.267	0.147	6.5101785148688E-29	1	ARL 16.00
3.69807737092286E-66	1.2590280878299	0.481	0.287	6.03193399971228E-62	1	SYNE2
2.01171887258547E-32	1.25858503433349	0.281	0.161	3.28131465307416E-28	1	DOCK1
1.10631202422124E-49	1.25855600382997	0.381	0.22	1.80450554270726E-45	1	PPIG
6.6085304599759E-72	1.25842297514486	0.508	0.303	1.07791740332667E-67	1	EHBP1L1
7.59843228078994E-26	1.25830801152747	0.265	0.163	1.23938028931965E-21	1	KIDINS220
3.08843427995014E-31	1.25675720336846	0.266	0.152	5.03754515402667E-27	1	TASOR2
9.86775171683602E-35	1.2557429803855	0.319	0.191	1.60952898253312E-30	1	C4orf19
4.0862180342612E-83	1.25547713435868	0.513	0.295	6.66503023568343E-79	1	INPPL1
1.33919724747381E-33	1.25466525466555	0.277	0.154	2.18436463035453E-29	1	DIP2C
3.2726689487955E-32	1.25446502014877	0.314	0.193	5.33805032238034E-28	1	ABCB11
1.02263478455692E-25	1.25430208743267	0.263	0.16	1.66801959709079E-21	1	GPBP1
3.52398505213458E-57	1.25345780379501	0.421	0.245	5.74797201853671E-53	1	RSRC2
7.15506492731925E-46	1.25250479123141	0.388	0.237	1.16706264029504E-41	1	VWA8
6.52340726027359E-48	1.25229682096944	0.305	0.154	1.06403295822322E-43	1	PSME4
1.02223240459287E-33	1.25131485009851	0.271	0.15	1.66736327513143E-29	1	JMJD6
8.8012285861072E-49	1.25055177173765	0.367	0.209	1.43556839467995E-44	1	RXRB
8.16762117214139E-38	1.25054941856105	0.34	0.204	1.33222068938798E-33	1	WDR6
3.86836707913774E-47	1.24891812235359	0.427	0.273	6.30969354278156E-43	1	U2SURP
3.64873355193989E-96	1.24846516840693	0.596	0.358	5.95144929656916E-92	1	PATJ
8.70446993544573E-54	1.24808444192247	0.39	0.221	1.41978609117055E-49	1	BRF1
3.33479391551071E-24	1.24632297611249	0.257	0.159	5.43938235558951E-20	1	RICTOR
5.07941176854607E-64	1.2462625029314	0.448	0.256	8.28502853567549E-60	1	KMT2D
8.97019240834008E-197	1.24594506943027	0.849	0.532	1.46312808372435E-192	1	DCAF8
3.25551483515949E-26	1.24591328747265	0.257	0.152	5.31007024762864E-22	1	YAF2
1.02414199597168E-19	1.24550151480344	0.252	0.166	1.67047800962941E-15	1	EPC1
1.83499899791435E-47	1.24507309222431	0.484	0.324	2.9930668654981E-43	1	TAP1
9.36668732187296E-23	1.24483733262597	0.282	0.186	1.5278003690707E-18	1	PLCH2
3.08839566983897E-62	1.24481283229079	0.47	0.28	5.03748217707434E-58	1	NFIB
8.26666368416219E-34	1.24479337582558	0.278	0.157	1.34837551352369E-29	1	ATXN7
3.90152793653478E-97	1.24442797731454	0.601	0.368	6.36378221728189E-93	1	SAMD5
9.06072385259073E-22	1.24412519242891	0.26	0.168	1.47789466759607E-17	1	FBXO22
4.13253170016192E-32	1.24356889460049	0.326	0.205	6.74057245613411E-28	1	SEC24B
2.1790149666995E-25	1.24101716880375	0.254	0.153	3.55419131218355E-21	1	PCNX1
1.1408612645502E-58	1.24069975552005	0.414	0.235	1.86085880860783E-54	1	CBWD2
9.25101472183455E-49	1.24063657129087	0.384	0.226	1.50893301127843E-44	1	REPS1
8.10042661218388E-51	1.24033630734509	0.38	0.217	1.32126058471331E-46	1	ARHGEF11
1.00616983523414E-54	1.24019756862857	0.406	0.233	1.6411636182504E-50	1	RABGGTB
6.31779864882246E-55	1.23953351998206	0.382	0.21	1.03049613760943E-50	1	PITPNM2
4.16259441447435E-32	1.23690181333409	0.327	0.206	6.78960774944912E-28	1	SPATA13
9.895668675111E-59	1.23665854548625	0.447	0.269	1.61408251759736E-54	1	DDX17
9.99855576118741E-35	1.23563659720029	0.323	0.195	1.63086443020728E-30	1	FBXL17
1.10326958291947E-47	1.23528272459835	0.366	0.209	1.79954301669995E-43	1	GATD1
3.56646918995893E-36	1.23523945406132	0.288	0.159	5.81726789574202E-32	1	TANK
3.24607800407362E-47	1.23485300252562	0.385	0.228	5.29467783244448E-43	1	RBL2
1.78161681849019E-82	1.23419442455715	0.578	0.36	2.90599519263935E-78	1	LPP
1.4614618928581E-35	1.23388262582616	0.315	0.185	2.38379049344085E-31	1	BRD9
6.4224133644882E-31	1.23306490467756	0.293	0.177	1.04755984388167E-26	1	PHKG2
1.48797866700429E-29	1.23253041321938	0.26	0.147	2.4270420037507E-25	1	NUP210
1.43494270136962E-31	1.23151322905202	0.26	0.146	2.34053504020399E-27	1	SGSH

2.67405849524285E-51	1.23122566409075	0.411	0.243	4.36165681159061E-47	1	CPSF7
3.51711494741496E-36	1.23080254000058	0.315	0.183	5.73676619072854E-32	1	POFUT2
1.68137872329345E-37	1.22962736977256	0.309	0.178	2.74249683556394E-33	1	RBM17
1.96878068168739E-28	1.22894490116948	0.288	0.175	3.21127816990029E-24	1	STK3
1.83847117206836E-98	1.22872338968265	0.596	0.355	2.9987303287607E-94	1	FGFR3
6.25189242737795E-35	1.2286619630301	0.29	0.166	1.01974617382962E-30	1	MDC 1.00
6.46462831177621E-95	1.22762786102957	0.608	0.368	1.05444552393382E-90	1	SNRNP70
1.98176535275831E-48	1.22670574372474	0.387	0.225	3.23245746688408E-44	1	CCDC159
5.54500324134791E-92	1.22546569121808	0.592	0.356	9.04445478696257E-88	1	SMG5
5.8259968232741E-21	1.22289538691904	0.251	0.161	9.50278341844239E-17	1	SS18
1.0327217658941E-39	1.22189758751927	0.335	0.196	1.68447247234987E-35	1	KDM6B
1.16408552506005E-48	1.22175768511797	0.414	0.253	1.89873989992545E-44	1	AKAP13
3.99750623746832E-32	1.22164116353137	0.31	0.19	6.52033242393458E-28	1	NCKAP1
2.41854047195302E-29	1.22071911332331	0.279	0.167	3.94488136380257E-25	1	AFF3
3.60814645821006E-26	1.22066362689324	0.294	0.191	5.88524768798643E-22	1	ARFGAP2
4.43242944405316E-41	1.22064644875377	0.371	0.226	7.22973566619511E-37	1	SFXN5
1.08480922809259E-28	1.21985942477094	0.256	0.147	1.76943233194182E-24	1	TRIM56
1.92051549517486E-22	1.21920635571596	0.26	0.167	3.13255282417971E-18	1	MIER1
1.06492029107465E-63	1.21859225971222	0.42	0.23	1.73699148677187E-59	1	NSMF
2.16148254596675E-115	1.21839171495017	0.716	0.445	3.52559418072637E-111	1	SLC7A2
1.29379941552109E-23	1.2167028138653	0.283	0.184	2.11031622665644E-19	1	PHF3
6.41078086025367E-90	1.21657421046907	0.57	0.341	1.04566246611598E-85	1	CELF1
3.34135211318339E-118	1.2162081752512	0.659	0.391	5.45007943181342E-114	1	FBXO17
9.3323989967919E-67	1.21490645208643	0.508	0.312	1.52220760036673E-62	1	STAU2
2.08724685643321E-50	1.21364133095997	0.371	0.203	3.4045083475282E-46	1	USP35
2.47436795409012E-34	1.21353808259631	0.277	0.155	4.03594156991639E-30	1	AMBRA1
8.39916610631686E-100	1.21290371886944	0.573	0.321	1.36998798360134E-95	1	BACH1
1.27651140023993E-36	1.21278052764367	0.349	0.218	2.08211774493135E-32	1	EIF2B5
3.97147297400879E-35	1.21239355206975	0.308	0.181	6.47786956790574E-31	1	TACC2
3.11095644755174E-75	1.21234605008858	0.527	0.308	5.07428106160164E-71	1	DDIT3
2.83853381636603E-29	1.21198373273165	0.307	0.193	4.62993250787463E-25	1	UBE4A
6.47060718742439E-47	1.21167937970519	0.421	0.266	1.05542073834079E-42	1	ANKHD1
5.46158629316836E-49	1.21154442310709	0.385	0.227	8.90839340278691E-45	1	GLRX3
7.59878284741782E-51	1.21111780364037	0.337	0.178	1.23943747024232E-46	1	P2RX4
2.54346938363931E-85	1.21058791937236	0.519	0.292	4.14865291165408E-81	1	PIP5K1A
6.98025083567759E-18	1.20855075860318	0.251	0.168	1.13854871380737E-13	1	COG4
2.35637169222601E-28	1.20786270176232	0.408	0.285	3.84347786718984E-24	1	HMOX1
2.12104388232485E-72	1.20663210789882	0.519	0.311	3.45963467646006E-68	1	GSE1
1.24170429994671E-67	1.20623825419332	0.499	0.299	2.02534388364308E-63	1	RB1CC1
2.42602756067914E-25	1.2061254689698	0.274	0.171	3.95709355422375E-21	1	ZNF76
3.57484919720543E-51	1.2056196105798	0.426	0.262	5.83093652556178E-47	1	ALG13
4.09357004398729E-31	1.20543310083136	0.284	0.166	6.67702209874767E-27	1	SMPD4
4.10445965202503E-101	1.20503944693926	0.726	0.499	6.69478413841803E-97	1	SLC22A7
6.15189467340386E-32	1.20378341422035	0.302	0.181	1.0034355401789E-27	1	TRMT1
6.9407668928422E-82	1.20349553134013	0.538	0.315	1.13210848789149E-77	1	MROH1
3.7562937991205E-23	1.20297396485974	0.259	0.162	6.12689081574544E-19	1	SF3B1
7.22376878564058E-78	1.20215354839278	0.527	0.312	1.17826892662584E-73	1	OXA1L
3.19523090460289E-94	1.19910145860138	0.591	0.348	5.21174112849777E-90	1	OGA
7.29940784832787E-25	1.19903811290376	0.254	0.15	1.19060641414076E-20	1	LDLR
2.40822354510852E-33	1.19873048822795	0.316	0.191	3.9280534244265E-29	1	ZNF326

5.50214371123332E-25	1.19742526564807	0.277	0.173	8.97454660739268E-21	1	ACP6
4.38524095009608E-38	1.19643993920541	0.339	0.204	7.15276651370172E-34	1	EXOC2
1.83238048233255E-32	1.19597454373381	0.307	0.185	2.98879580473263E-28	1	SRPK2
6.50769047274906E-52	1.19447136529821	0.42	0.253	1.0614693930101E-47	1	PHKA2
1.05453507080904E-27	1.19421303054699	0.341	0.227	1.72005215399662E-23	1	TP53BP2
5.26815864183342E-47	1.19408167719549	0.402	0.245	8.59289356069449E-43	1	SLC5A6
6.19251269129477E-51	1.19403341979803	0.454	0.289	1.01006074507709E-46	1	ABCC6
3.90294311749452E-74	1.19339091158939	0.539	0.339	6.36609051894532E-70	1	CYP2C18
6.1053856580783E-40	1.1931523428764	0.352	0.211	9.95849454689151E-36	1	BLNK
2.30035173699365E-47	1.19200644823649	0.421	0.261	3.75210371821034E-43	1	CRY2
2.48619348444875E-44	1.19127919398299	0.353	0.202	4.05523019248435E-40	1	FNIP2
1.18940524353487E-46	1.1909313729704	0.322	0.172	1.94003889272973E-42	1	PBRM1
1.41159806149607E-34	1.1906549968062	0.302	0.177	2.30245759810624E-30	1	YY1AP1
5.68660758807954E-35	1.19064767275818	0.306	0.179	9.27542563691653E-31	1	RORA
9.40810676196443E-47	1.19024610324778	0.393	0.236	1.53455629394402E-42	1	SLC25A45
3.78046400556234E-30	1.18956444073371	0.263	0.15	6.16631483947273E-26	1	FOSL2
4.74776442115096E-50	1.18937761495108	0.411	0.248	7.74407854733933E-46	1	LAMA5
2.22068478682189E-59	1.18923976814049	0.454	0.274	3.62215895578518E-55	1	SUN1
4.37630669211851E-74	1.1890092536923	0.508	0.3	7.1381938455145E-70	1	KPNA1
6.01873416383253E-85	1.18872120367238	0.573	0.35	9.81715729462724E-81	1	CFLAR
1.3137811401294E-37	1.188112632397	0.333	0.199	2.14290841766506E-33	1	RAPGEFL1
1.35811177569506E-49	1.18785552599528	0.411	0.247	2.21521611733621E-45	1	GATAD2B
1.05400264367314E-24	1.1876796385303	0.279	0.177	1.71918371209526E-20	1	MTFR1L
4.12163065523947E-30	1.1855058178548	0.274	0.158	6.7227917617611E-26	1	PRRC2C
5.99316716019274E-37	1.18536957223065	0.306	0.174	9.77545495499038E-33	1	KIAA0100
1.07908358497948E-41	1.18394052380682	0.344	0.202	1.76009323546003E-37	1	UBP1
3.96354321692535E-78	1.18359090994771	0.572	0.352	6.46493534112694E-74	1	GBP2
2.24884986332148E-41	1.18343566390663	0.347	0.205	3.66809901206366E-37	1	SPSB2
1.5915679526798E-51	1.18188845428847	0.408	0.242	2.59600648761603E-47	1	KMT5B
2.23216594578326E-35	1.18159934655781	0.318	0.187	3.64088587416708E-31	1	VPS35L
9.82725734192892E-65	1.18074187182488	0.495	0.301	1.60292394504203E-60	1	PER1
1.5322771494687E-34	1.18052926952628	0.338	0.21	2.49929725849839E-30	1	MON2
2.42358975647409E-59	1.18030337882244	0.533	0.35	3.95311725178489E-55	1	SLC30A10
7.48126254452792E-40	1.18016305723791	0.304	0.167	1.22026873363795E-35	1	ERN 1.00
1.09256930316337E-44	1.17967442246893	0.348	0.195	1.78208979038977E-40	1	TNK2
1.19000925467766E-30	1.17949144397008	0.313	0.196	1.94102409530474E-26	1	IGF2BP3
1.02075593436703E-32	1.17934717971655	0.292	0.169	1.66495500454606E-28	1	RBM19
3.83877036038707E-53	1.17868971530735	0.34	0.176	6.26141833482735E-49	1	ZDHHC8
4.80425904355196E-60	1.17839301390866	0.436	0.255	7.8362269259376E-56	1	TAF11
6.60633486406739E-47	1.17550431398008	0.402	0.247	1.07755927967803E-42	1	RHOT2
7.96538375645593E-28	1.17345064722149	0.262	0.152	1.29923374451553E-23	1	IFNAR2
2.08920825094561E-39	1.17322756549572	0.322	0.182	3.40770757811739E-35	1	KDM2B
2.96858111353351E-56	1.17183651883173	0.392	0.219	4.84205265428451E-52	1	ISG20
1.09131853830947E-104	1.17167431704314	0.698	0.435	1.78004966783658E-100	1	ABCC2
9.2814474891533E-47	1.17161315464091	0.414	0.255	1.5138968999558E-42	1	PVR
6.78332402332404E-53	1.17086723221985	0.412	0.241	1.10642798144438E-48	1	NECAB3
3.33850787285076E-181	1.16936862497872	0.883	0.55	5.44544019140687E-177	1	MLXIPL
2.21272152398062E-30	1.16804913631504	0.289	0.174	3.6091700777648E-26	1	PPP2R2D
2.9945832166524E-237	1.16781836585458	0.948	0.74	4.88446468468173E-233	1	ACADVL
1.18480541437088E-40	1.1676616865326	0.363	0.221	1.93253611138034E-36	1	FBXL5

5.53526672956854E-44	1.16749392576224	0.368	0.218	9.02857356259924E-40	1	RAPGEF5
1.94679685761627E-41	1.16531548978437	0.414	0.271	3.1754203544579E-37	1	HGSNAT
1.24669220655931E-30	1.16505235741212	0.269	0.154	2.03347965811889E-26	1	SLMAP
1.03130306922605E-51	1.16453418251132	0.446	0.279	1.6821584362146E-47	1	MORC2
1.75584616175768E-21	1.16358743865338	0.265	0.172	2.86396067444295E-17	1	CREB3L2
3.30310199467955E-36	1.16341243788239	0.402	0.268	5.38768966352181E-32	1	ABCG5
1.44930056652768E-75	1.16293884585934	0.533	0.326	2.36395415406329E-71	1	HNRNPH3
8.01627797775395E-25	1.16274254493225	0.266	0.165	1.30753510095145E-20	1	RTCA
2.83908315982496E-69	1.1623880992108	0.49	0.29	4.6308285419905E-65	1	RERE
1.85054060611404E-32	1.16061213228167	0.278	0.158	3.01841678263261E-28	1	CAMTA2
3.07722718886996E-48	1.16058256903647	0.405	0.243	5.01926526776579E-44	1	TOM1L1
4.20829767577629E-29	1.16034597942617	0.269	0.156	6.8641543389587E-25	1	SLC15A4
2.86574116712064E-48	1.16028467585082	0.388	0.229	4.67431041769048E-44	1	MAST3
6.14372115374563E-48	1.1601842898291	0.407	0.244	1.00210235738745E-43	1	RBCK1
7.82349371239288E-27	1.16007221048625	0.273	0.167	1.2760900594284E-22	1	PANK4
3.41594066105654E-86	1.15963432983113	0.583	0.359	5.57174081224932E-82	1	PLEKHG3
8.16815228667891E-45	1.15946264264875	0.393	0.242	1.3323073194802E-40	1	FNDC3A
1.37349816208557E-98	1.15890350453654	0.646	0.398	2.24031285217777E-94	1	SPG7
2.72262310657429E-32	1.15888419317611	0.341	0.219	4.44087054913332E-28	1	HTT
5.02688298507531E-41	1.15864358483895	0.377	0.231	8.19934883695634E-37	1	MAX
2.15023274904528E-41	1.15847160634105	0.335	0.192	3.50724463696775E-37	1	SPAG9
5.77824883636526E-101	1.15681458115078	0.572	0.318	9.42490167699537E-97	1	PHF1
1.61385743201933E-21	1.15592182080481	0.312	0.217	2.63236285736673E-17	1	ABCA5
5.43429465013243E-42	1.15528764583337	0.414	0.262	8.863878003831E-38	1	LSS
2.29035316696955E-36	1.15446838370992	0.352	0.218	3.73579505064403E-32	1	NT5C2
1.2580316147825E-29	1.15377426979806	0.287	0.172	2.05197536687173E-25	1	GRB10
9.3539265004058E-30	1.15277159146417	0.267	0.154	1.52571895148119E-25	1	MUS81
2.08030693606152E-32	1.15224971593192	0.325	0.203	3.39318864340994E-28	1	KAT7
1.05456025200336E-39	1.15210163542302	0.339	0.198	1.72009322704268E-35	1	GBF1
5.72662404137946E-25	1.15190877680529	0.299	0.196	9.34069647389404E-21	1	RNASEH1
5.38898841529154E-60	1.14978504805576	0.502	0.313	8.78997900418204E-56	1	RNF19A
2.57589929122099E-189	1.14956108851476	0.927	0.645	4.20154933391056E-185	1	CYP3A5
6.44549665377767E-31	1.14954770225561	0.271	0.156	1.05132495919768E-26	1	DEPRECAT
5.3798061477955E-51	1.14939523535595	0.44	0.274	8.77500180766923E-47	1	DLG1
1.26563918051034E-63	1.14888101266814	0.542	0.347	2.06438406733041E-59	1	F11
4.09267069511334E-28	1.14878745214953	0.308	0.195	6.67555517079936E-24	1	LRRFIP2
1.74381775093788E-92	1.14858699375276	0.616	0.371	2.84434113355478E-88	1	NR1I2
2.37421720295066E-21	1.14854669041037	0.267	0.175	3.87258567973282E-17	1	DYM
2.33477427801582E-30	1.14789692655988	0.32	0.203	3.8082503248716E-26	1	CDK16
1.82932438420612E-25	1.14690634660153	0.291	0.187	2.9838110030786E-21	1	RPRD2
2.91168359989698E-69	1.14654645483919	0.536	0.337	4.74924711979197E-65	1	UBR4
2.49187315442763E-66	1.14635732609319	0.508	0.314	4.06449430218691E-62	1	ZBTB20
2.81747064567415E-37	1.14569966365173	0.354	0.219	4.59557637015911E-33	1	ZXDC
1.66711924316883E-38	1.14559437030706	0.372	0.233	2.71923819753267E-34	1	KIAA0232
3.34614931960219E-27	1.1455004182507	0.31	0.195	5.45790415520314E-23	1	NID1
4.47723049997419E-31	1.14388376834673	0.289	0.172	7.3028106685079E-27	1	TTC31
4.5192693467937E-150	1.14178212649664	0.878	0.625	7.37138023155521E-146	1	MT1A
4.04825343893483E-34	1.1410860865635	0.317	0.188	6.60310618424661E-30	1	IRF8
1.19652153427648E-55	1.14105132719331	0.407	0.231	1.95164627455836E-51	1	MED15
2.1454040032552E-70	1.14078427411361	0.579	0.37	3.49936846970955E-66	1	BCL3

1.68615984131838E-55	1.1397966753381	0.419	0.246	2.75029531717441E-51	1	SMAD4
1.33002482924176E-19	1.13900363593592	0.26	0.172	2.16940349897624E-15	1	AKTIP
4.7466171860144E-25	1.13862584472711	0.286	0.182	7.7422072921081E-21	1	PHF12
4.68770209671612E-84	1.13714812433486	0.604	0.378	7.64611088995367E-80	1	SLC22A23
9.36450379191191E-43	1.13671056949811	0.308	0.165	1.52744421349875E-38	1	SLC38A1
1.9674092571196E-25	1.13607161512452	0.256	0.152	3.20904123928778E-21	1	CRACR2B
1.05526653686533E-53	1.13545362958605	0.429	0.255	1.72124524828104E-49	1	ZMIZ2
6.40478460495506E-21	1.1348566450657	0.269	0.178	1.04468441691422E-16	1	NT5DC1
4.72363118194158E-86	1.1341655485174	0.632	0.399	7.70471482086491E-82	1	SLC29A1
1.41936866653303E-42	1.1340177926294	0.406	0.254	2.31513223198203E-38	1	CUL7
3.90534383576231E-37	1.13388736214468	0.327	0.192	6.3700063305119E-33	1	TANGO2
1.63047757259489E-22	1.13304662980259	0.274	0.177	2.65947196865952E-18	1	PARD3
2.10165773813248E-98	1.13240937380159	0.652	0.396	3.42801393666789E-94	1	SLC6A1
7.63491255783437E-46	1.13231128204833	0.435	0.281	1.24533058730836E-41	1	GRTP1
7.49124309413099E-59	1.13169142126816	0.458	0.275	1.22189666108371E-54	1	ULK1
9.78733819978208E-50	1.13026893339308	0.437	0.274	1.59641273376646E-45	1	DHX16
1.62028100529412E-34	1.12999886831745	0.281	0.155	2.64284034773524E-30	1	LTBP4
1.31089599452154E-26	1.12964250932312	0.256	0.15	2.13820245666409E-22	1	NCBP3
9.45799786955021E-28	1.12634850664882	0.28	0.17	1.54269403250234E-23	1	SMURF1
1.43292034478193E-41	1.12576380318885	0.369	0.224	2.33723637437381E-37	1	DIDO1
1.97378483120787E-19	1.12561123339755	0.256	0.17	3.21944043818316E-15	1	SPOP
2.95937961978517E-19	1.12546963350038	0.272	0.181	4.82704409783159E-15	1	SIPA1L2
6.98221444895823E-25	1.12473834350196	0.296	0.192	1.13886899876958E-20	1	PHF20L1
1.23123621693862E-53	1.12457228258247	0.414	0.242	2.00826939344859E-49	1	VPS13C
5.42229816971557E-39	1.12454101860043	0.35	0.214	8.84431054462306E-35	1	UBR3
2.51433945067675E-33	1.12442013966547	0.337	0.204	4.10113907799885E-29	1	NCOA7
1.2624858865677E-40	1.12226718029663	0.389	0.243	2.05924072958058E-36	1	TRAPPC9
1.59067848272438E-36	1.12146243616586	0.36	0.223	2.59455567317174E-32	1	CTPS1
5.088098400374E-32	1.12077890870996	0.313	0.189	8.29919730085003E-28	1	SLC25A33
2.2253942098021E-46	1.12047209348189	0.388	0.231	3.62984049560821E-42	1	AARS2
1.51957971336307E-29	1.12044223218814	0.308	0.192	2.47858647046651E-25	1	ADAMTSL3
5.28317420281842E-73	1.12000524484478	0.502	0.285	8.61738544221713E-69	1	FGFR2
2.42516797196352E-51	1.11837115364935	0.495	0.323	3.9556914790697E-47	1	ARRDC3
4.48213652226221E-58	1.11796106778412	0.511	0.329	7.3108128814619E-54	1	RORC
1.31088856907181E-48	1.11771494299597	0.367	0.207	2.13819034501302E-44	1	SPEN
1.69819588779498E-44	1.11741390299078	0.405	0.255	2.7699273125824E-40	1	VPS53
2.86531070718638E-26	1.11696110010105	0.27	0.164	4.6736082944917E-22	1	NISCH
1.02930970681127E-43	1.11601734192431	0.392	0.241	1.67890706277987E-39	1	SHTN1
6.78816068168057E-41	1.11577969899654	0.408	0.264	1.10721688878892E-36	1	ITCH
5.51238645199667E-23	1.11467645526297	0.261	0.165	8.99125354185177E-19	1	HDAC1
1.47576058101503E-56	1.1126987729662	0.427	0.249	2.40711308369361E-52	1	ARHGEF40
5.75271967266125E-35	1.11181068587164	0.291	0.166	9.38326105807776E-31	1	WASHC2A
1.70459075647039E-29	1.11138018804858	0.282	0.167	2.78035798287885E-25	1	CREBRF
2.4226578518959E-49	1.11124077506803	0.407	0.244	3.95159722222741E-45	1	ATP11A
5.06275577950013E-78	1.11093119419211	0.573	0.356	8.25786095194266E-74	1	EML4
8.89090239966736E-44	1.10981776791192	0.378	0.227	1.45019509040974E-39	1	FUBP1
1.34028546235824E-74	1.10890212650558	0.549	0.336	2.18613961765253E-70	1	RTKN
2.6745682421512E-27	1.10869784159767	0.275	0.166	4.36248825977282E-23	1	INPP1
2.83104677672112E-27	1.10707916190985	0.293	0.184	4.61772039750983E-23	1	SPECC1L
1.17439330186639E-24	1.10658589105549	0.261	0.161	1.91555291467427E-20	1	L3HYPDH

4.73779859624481E-64	1.1060985968393	0.473	0.277	7.72782329033491E-60	1	NR1D1
4.10974806209025E-54	1.10573621322024	0.37	0.196	6.70341006407541E-50	1	BBC3
1.80474870224804E-41	1.10503708772591	0.356	0.209	2.94372560823678E-37	1	SLC12A4
3.83230062932422E-79	1.10242976206239	0.589	0.373	6.25086555649074E-75	1	IRF3
3.26396828800488E-31	1.10213503014453	0.267	0.15	5.32385867456476E-27	1	VSIG10
1.05586623513853E-30	1.10077886658105	0.373	0.252	1.72222341613445E-26	1	DUSP10
1.88929672005676E-104	1.10055056117637	0.653	0.395	3.08163188008458E-100	1	NEDD4L
2.53411269102285E-71	1.10003088540193	0.481	0.274	4.13339121032737E-67	1	KMT2B
8.21185830338791E-27	1.09989783337292	0.315	0.207	1.3394362078656E-22	1	YTHDC2
9.16771932613768E-29	1.09795924576432	0.326	0.209	1.49534669928632E-24	1	MSI2
1.16928268946368E-40	1.09791190992336	0.375	0.231	1.90721699478421E-36	1	FLCN
3.95398964067614E-28	1.09783735108426	0.265	0.156	6.44935250290686E-24	1	NPAS2
3.24342596271421E-60	1.09736161035384	0.451	0.268	5.29035208778314E-56	1	GCN1
7.35131723351822E-24	1.09704197274829	0.286	0.187	1.19907335395916E-19	1	TTC37
6.38897362976975E-20	1.09692172304231	0.252	0.164	1.04210548875174E-15	1	HELZ
2.08947455449222E-30	1.096730220402	0.3	0.181	3.40814194583226E-26	1	PML
1.75341422033884E-35	1.09671045843434	0.327	0.195	2.85999393479469E-31	1	MTMR12
1.82317952952733E-73	1.09488978698263	0.549	0.339	2.97378813061203E-69	1	TNFRSF14
2.13446910434092E-41	1.09401086748902	0.407	0.262	3.48153255609047E-37	1	ATP11B
6.44897419816808E-49	1.09244438483898	0.435	0.272	1.0518921814632E-44	1	BNIP2
1.62593413999222E-21	1.09238884555635	0.287	0.191	2.65206117574131E-17	1	ZNF217
1.89657516543759E-23	1.09125519848543	0.293	0.193	3.09350375234526E-19	1	CDC37L1
6.94101404508044E-37	1.09102111615887	0.323	0.188	1.13214880089307E-32	1	MRTFA
3.27771547130695E-25	1.09086004685933	0.355	0.248	5.34628170524877E-21	1	KIAA0319L
9.77829463663253E-59	1.09051615458811	0.47	0.283	1.59493763818113E-54	1	BTG2
4.22356956802502E-93	1.09041565460131	0.627	0.389	6.88906432240561E-89	1	IMMP2L
2.99011674653557E-50	1.09035870306941	0.411	0.244	4.87717942527417E-46	1	DEPRECAT
7.59131554935935E-101	1.09030291794852	0.694	0.417	1.238219479256E-96	1	TP53INP1
1.78627781369236E-49	1.08995939501373	0.37	0.208	2.91359774191361E-45	1	PIK3C2B
2.19897135676258E-43	1.08994092690635	0.376	0.227	3.58674218001544E-39	1	ACSF3
1.41983958743553E-34	1.08936645237179	0.341	0.212	2.31590035106609E-30	1	ZNF410
2.43084717589734E-24	1.08614811158164	0.258	0.159	3.96495482860615E-20	1	ATF2
1.33198702206861E-28	1.08458747960494	0.254	0.145	2.1726040316961E-24	1	TNPO3
1.53298653592438E-104	1.08427725670754	0.685	0.434	2.50045433874626E-100	1	BRD2
7.23480964233942E-47	1.08413954483401	0.421	0.262	1.18006980076198E-42	1	WDR26
1.36287355341107E-53	1.08314022926838	0.482	0.306	2.2229830529688E-49	1	ZNF512B
1.27121564604389E-16	1.08290696159275	0.252	0.173	2.0734798402622E-12	1	C6orf62
2.69088900894158E-49	1.08253638376522	0.433	0.271	4.38910906248462E-45	1	GNL1
8.93331236464662E-25	1.08219607660317	0.291	0.188	1.45711257979751E-20	1	FAF1
2.21561796173654E-26	1.08177269150023	0.296	0.188	3.61389445738847E-22	1	TAPT1
1.03625140351744E-44	1.08097678306916	0.4	0.245	1.6902296642773E-40	1	HUWE1
1.16967424195439E-21	1.08040587965626	0.264	0.169	1.90785565605181E-17	1	CCNYL1
2.62226295171976E-18	1.08024789653996	0.257	0.171	4.27717310055009E-14	1	SLC11A2
1.05766117818128E-32	1.08022046483004	0.327	0.202	1.72515114773149E-28	1	EP300
6.24247070929823E-41	1.07974867730808	0.334	0.19	1.01820939739363E-36	1	CMIP
6.81630079193275E-17	1.07960372197744	0.271	0.191	1.11180682217215E-12	1	MBNL2
1.69556105177823E-36	1.07877776213042	0.347	0.212	2.76562963155547E-32	1	SP3
1.07556861395361E-71	1.07791734478234	0.592	0.378	1.75435996621973E-67	1	CHDH
8.24036342571394E-33	1.07785476833124	0.343	0.217	1.3440856783682E-28	1	UBR5
7.1558222211844E-30	1.07666462081274	0.287	0.171	1.16718616249739E-25	1	PFAS

4.45559292803277E-48	1.07592778365094	0.404	0.24	7.26751762491425E-44	1	TYK2
1.17685091294946E-34	1.07586281239138	0.356	0.226	1.91956152411186E-30	1	TNRC6B
3.89666521862503E-74	1.07488357025649	0.452	0.232	6.35585063809929E-70	1	PLIN2
8.15816068560172E-150	1.07444174429725	0.813	0.511	1.3306775894285E-145	1	MYO18A
3.94238951011586E-37	1.07391737498203	0.355	0.215	6.43043152994998E-33	1	PCBP4
1.52022040939511E-97	1.07333626235791	0.74	0.497	2.47963150976436E-93	1	DUSP1
7.88363045185368E-61	1.07266020318528	0.486	0.301	1.28589896300185E-56	1	ABLIM3
5.79304801819915E-85	1.0723047395447	0.633	0.403	9.44904062248463E-81	1	PLPP3
2.4450268772635E-111	1.07154683230124	0.628	0.326	3.98808333950449E-107	1	FGF21
1.81920161460295E-119	1.0714448370963	0.805	0.555	2.96729975357886E-115	1	KLF6
6.53422473642932E-40	1.07089722786604	0.415	0.272	1.06579739675899E-35	1	NFKB2
9.4768461196845E-30	1.07074462031005	0.344	0.228	1.54576837058174E-25	1	AKAP8L
2.00646230795097E-33	1.06925410988883	0.351	0.223	3.27274067049883E-29	1	CMTR1
1.89342068482998E-23	1.06923124082376	0.272	0.173	3.08835847902619E-19	1	ZYG11B
4.62482907194321E-65	1.06851749391225	0.52	0.321	7.54355869924657E-61	1	TUT7
5.40476291763678E-44	1.06828552923989	0.359	0.208	8.81570879495735E-40	1	WDR59
1.75480175905404E-39	1.06790427505718	0.361	0.22	2.86225714919304E-35	1	UBE2J2
1.16544868882339E-50	1.06699544658108	0.473	0.301	1.90096335633983E-46	1	ODF3B
1.41271444939688E-29	1.06682124380451	0.344	0.223	2.30427853841126E-25	1	PPP1R3C
1.3483429749383E-16	1.06599226939631	0.263	0.185	2.19928222642186E-12	1	CEPT1
1.58624744499261E-34	1.06580633624049	0.371	0.241	2.58732820752745E-30	1	RANBP10
1.51257642998164E-42	1.06546481456114	0.393	0.244	2.46716341494305E-38	1	TRAPPC4
6.27130139870334E-48	1.0644663044717	0.442	0.276	1.0229119711425E-43	1	NAB2
1.41998175631617E-18	1.06433815216554	0.257	0.173	2.31613224272731E-14	1	FBH1
1.60704391157764E-36	1.0626401195731	0.327	0.195	2.62124932417429E-32	1	NUP153
4.54889030616074E-47	1.06261439056629	0.467	0.308	7.41969497837879E-43	1	ELP2
6.69036275016199E-112	1.06241493419617	0.676	0.396	1.09126506817892E-107	1	GALT
1.94306097412276E-19	1.06224210535283	0.305	0.217	3.16932675489163E-15	1	KMT2C
2.15049830667665E-29	1.0622271996897	0.365	0.246	3.50767778802029E-25	1	PLXNB1
5.25348719970544E-63	1.06186902878033	0.483	0.29	8.56896297143954E-59	1	DHX15
1.06264971533966E-28	1.06163608033784	0.323	0.208	1.73328795069052E-24	1	LRMDA
1.99079644914536E-79	1.06144350312642	0.564	0.333	3.247188088201E-75	1	HLF
1.47940791394828E-59	1.06027140837598	0.49	0.31	2.41306224844104E-55	1	RIOK3
1.17333253070681E-21	1.0601656909529	0.256	0.162	1.91382269083588E-17	1	WDR82
1.67624522049861E-59	1.05989397841721	0.49	0.299	2.73412357915529E-55	1	GPAM
3.68414980139176E-29	1.05984619245845	0.298	0.185	6.0092167410501E-25	1	DMAP1
4.60463521388762E-24	1.0592889375861	0.272	0.171	7.5106204973721E-20	1	RGS12
9.70394448356285E-32	1.05866596857474	0.342	0.219	1.58281038471394E-27	1	TPP2
4.86192406264842E-19	1.05864869188786	0.275	0.189	7.93028433858584E-15	1	LRBA
2.83635643191035E-46	1.05829542928285	0.413	0.257	4.62638097608898E-42	1	PLCXD1
2.08601645280461E-30	1.0576025159179	0.302	0.183	3.4025014361696E-26	1	DICER1
7.92583915361123E-24	1.05702122059416	0.293	0.191	1.29278362434553E-19	1	RGL2
1.70133809353684E-29	1.05684646187078	0.309	0.194	2.77505256436794E-25	1	MYO9B
2.63306727151402E-41	1.05674764349528	0.33	0.185	4.29479602656651E-37	1	PACSIN2
1.89174766007632E-32	1.05670521311635	0.279	0.157	3.08562960835049E-28	1	BOD1L1
9.3400418150268E-18	1.05600242171918	0.277	0.191	1.52345422044902E-13	1	KCNT2
1.21688646855892E-60	1.05600176172607	0.476	0.289	1.98486351886646E-56	1	C16orf72
5.49337975489661E-50	1.0550949021688	0.433	0.264	8.96025171821186E-46	1	ITPKC
6.44906688331452E-66	1.05480043093652	0.524	0.324	1.05190729933743E-61	1	NIT2
5.04617983987346E-40	1.05316292110932	0.37	0.23	8.2308239368176E-36	1	MAPK14

1.21505605789164E-18	1.05169591915303	0.303	0.217	1.98187793602705E-14	1	DEPRECAT
2.89350835781928E-57	1.05164004131413	0.456	0.278	4.71960148243903E-53	1	THRAP3
1.49508017769494E-30	1.05150802890961	0.318	0.199	2.43862527783822E-26	1	PHF14
2.3778859773055E-29	1.04998925147154	0.275	0.162	3.878569817583E-25	1	TRAK1
8.92075179741177E-33	1.04903373088471	0.356	0.225	1.45506382567583E-28	1	ZNF648
2.37307170968999E-23	1.04835560972669	0.306	0.203	3.87071726567534E-19	1	IMPA1
1.97932744677053E-65	1.0480030945639	0.538	0.337	3.22848099842742E-61	1	NME3
2.13833028291849E-29	1.04616259446309	0.277	0.162	3.48783052446834E-25	1	TNFRSF10E
1.5298464430696E-34	1.045453359843	0.35	0.219	2.49533253329082E-30	1	PIGO
8.02506615260221E-21	1.0447253296455	0.263	0.171	1.30896854015095E-16	1	TSPAN15
2.11385791007972E-52	1.04213580824354	0.498	0.322	3.44791363713104E-48	1	SLC38A2
2.18929003215525E-29	1.04163937925936	0.3	0.184	3.57095097144844E-25	1	SRC
7.25904965825447E-34	1.04103624214353	0.313	0.188	1.18402358975789E-29	1	PIKFYVE
1.02610750660397E-105	1.04067310555468	0.695	0.437	1.67368395402174E-101	1	PPP6R2
2.28407789237155E-30	1.04012673067899	0.299	0.18	3.72555945024724E-26	1	TRMT2A
1.43421596726311E-27	1.03974909773043	0.284	0.171	2.33934966420285E-23	1	PAG1
3.06598717467327E-43	1.03872120191326	0.405	0.255	5.00093168060956E-39	1	TMUB2
5.15117808133119E-20	1.03870662108225	0.331	0.243	8.4020865684593E-16	1	ABCA6
2.61116811734293E-70	1.03827655580312	0.552	0.343	4.25907631619806E-66	1	UAP1
2.01620043908049E-66	1.03799276686629	0.575	0.364	3.28862453618419E-62	1	IVNS1ABP
5.74517860406323E-34	1.03708063771822	0.372	0.242	9.37096082108754E-30	1	GHR
6.64128190270134E-38	1.03625282191768	0.307	0.171	1.08325949114962E-33	1	ZBTB4
2.36088969681676E-37	1.03569289040694	0.4	0.264	3.85084718447782E-33	1	NRDC
3.48769267800822E-78	1.03540004545043	0.646	0.405	5.6887755270992E-74	1	SAT1
8.34498691689627E-131	1.03530919232839	0.796	0.521	1.36115081601495E-126	1	PCSK6
5.08956811995075E-17	1.03395313095789	0.259	0.177	8.30159456045166E-13	1	SH3D19
1.30942120504702E-46	1.03366053109846	0.439	0.28	2.1357969275522E-42	1	CD46
2.35330374423154E-25	1.03235454272085	0.356	0.244	3.83847373721606E-21	1	KYNU
3.01445468420156E-29	1.03073110256738	0.302	0.187	4.91687703540116E-25	1	RPRD1A
1.2047634102929E-87	1.03070450393715	0.718	0.471	1.96508959852875E-83	1	GCLC
1.00542996387588E-45	1.02942297928271	0.597	0.428	1.63995681407795E-41	1	AKR1C2
3.91364378856123E-32	1.02706843284053	0.319	0.194	6.38354438352222E-28	1	SART3
1.05590981272777E-40	1.02498006870741	0.389	0.242	1.72229449554026E-36	1	MAVS
4.04065768839165E-43	1.02496566664685	0.402	0.252	6.59071675553562E-39	1	ARIH2
1.25923159716895E-32	1.02418726933076	0.344	0.22	2.05393265814228E-28	1	GOSR2
6.26663383269806E-24	1.02382095965559	0.304	0.203	1.02215064445138E-19	1	TEAD1
7.36233656529043E-89	1.02355913996175	0.577	0.347	1.20087071716452E-84	1	BAIAP2L1
2.69825943441291E-42	1.02305824916608	0.427	0.28	4.40113096347089E-38	1	APEX1
8.52074869841657E-27	1.0224130217276	0.253	0.147	1.38981932019873E-22	1	RUFY1
5.36217672302183E-63	1.02222897476509	0.483	0.292	8.74624645292091E-59	1	ARNT
1.85781605031292E-60	1.02125475235462	0.518	0.334	3.03028375966541E-56	1	TRPC4AP
1.08536044947868E-108	1.02015323349213	0.837	0.602	1.77033142914467E-104	1	ERRFI1
1.13159987166871E-66	1.01961040737345	0.556	0.358	1.84575255067883E-62	1	NSFL1C
1.99141075950143E-43	1.01907666123754	0.418	0.27	3.24819008982278E-39	1	HECTD1
4.21183888046358E-42	1.01871970960109	0.402	0.252	6.86993039792414E-38	1	SLC35D2
2.72565886720074E-59	1.01843018973173	0.493	0.313	4.44582217829113E-55	1	RAB1A
5.52267288163107E-37	1.01812740927531	0.381	0.245	9.00803173722843E-33	1	CEP350
1.24315908326889E-46	1.01744689287286	0.467	0.303	2.02771678071989E-42	1	RBM33
4.87645090539661E-25	1.01676371976948	0.292	0.188	7.95397907179241E-21	1	CPOX
1.12303475622797E-95	1.0161556499711	0.703	0.455	1.83178199088344E-91	1	OCLN

8.89830011670529E-65	1.01581599383755	0.478	0.282	1.4514017320358E-60	1	PNN
9.140732679613E-26	1.01477016956843	0.318	0.211	1.49094490737168E-21	1	KDM4B
1.69256498879362E-33	1.01380660472691	0.364	0.237	2.76074275322128E-29	1	TELO2
2.66017308211499E-19	1.01350393919513	0.274	0.184	4.33900831423776E-15	1	NFIA
5.52901583976753E-28	1.01340347837755	0.271	0.16	9.01837773624482E-24	1	CASP4
3.13746609358869E-108	1.01309973783434	0.725	0.463	5.11752094525251E-104	1	SNX1
7.24545728924678E-101	1.01307651616637	0.721	0.466	1.18180653844904E-96	1	WDR13
2.27901036466322E-16	1.01199948670829	0.282	0.203	3.71729380580217E-12	1	SLC20A2
1.94462397454073E-81	1.01110275300638	0.586	0.365	3.17187616487339E-77	1	NFX1
3.55949219885157E-50	1.01044552271231	0.548	0.373	5.80588772554679E-46	1	NFKBIA
1.97561424104648E-38	1.00877974164173	0.377	0.234	3.22242438857091E-34	1	DDX46
7.87043705778472E-26	1.00873064574313	0.29	0.182	1.28374698849527E-21	1	ZRANB1
5.2560473600675E-47	1.0066222112462	0.456	0.298	8.57313884900609E-43	1	SPG21
8.05194320887476E-26	1.00631547479499	0.276	0.168	1.31335245679956E-21	1	AGO2
1.56768172081168E-30	1.00583201157218	0.368	0.248	2.55704565481593E-26	1	ACSS2
1.77392198841647E-21	1.00332599662067	0.295	0.201	2.8934441553061E-17	1	ATP6V0A1
7.8064371171771E-28	1.00233192652141	0.343	0.228	1.27330795818276E-23	1	SPPL2B
7.95184363937599E-91	1.00171882880383	0.721	0.483	1.29702521601862E-86	1	ZFP36
6.58796746570287E-18	1.00008208570627	0.274	0.19	1.0745633733308E-13	1	ZNF277
4.326716195909E-25	1.00003086222062	0.309	0.204	7.05730678714716E-21	1	HERPUD2
4.72695823561927E-24	0.998874865699795	0.297	0.194	7.71014157811859E-20	1	ALCAM
6.11134695695205E-28	0.998403838604893	0.358	0.242	9.9682180214845E-24	1	RIPK1
6.53759902453115E-25	0.997813137600786	0.354	0.248	1.06634777689128E-20	1	CEP70
2.01524437328404E-34	0.997363192213445	0.356	0.227	3.2870650972636E-30	1	TBC1D15
1.0546806568219E-21	0.996887760065614	0.265	0.171	1.72028961934221E-17	1	VPS9D1
1.79633370756377E-24	0.996854757575447	0.281	0.179	2.92999991040727E-20	1	OSBPL2
6.68709414967968E-42	0.995569859890639	0.423	0.273	1.09073192675425E-37	1	TRIO
7.51455106256476E-38	0.994436373794029	0.342	0.209	1.22569842381494E-33	1	ASCC3
2.07958798540683E-23	0.993974487714225	0.29	0.186	3.39201596299708E-19	1	ZCCHC9
3.75297044387255E-26	0.992441157222885	0.493	0.368	6.12147009100051E-22	1	P4HA1
4.85641006370579E-35	0.992337752784523	0.392	0.264	7.92129045491051E-31	1	SLC25A42
1.57804018329055E-21	0.992283155840698	0.273	0.181	2.57394134296521E-17	1	NSMCE4A
1.29473660519741E-49	0.99195901247387	0.494	0.325	2.11184487673749E-45	1	ATP2B2
4.41400551774195E-36	0.991888999831478	0.382	0.247	7.19968439998889E-32	1	FMR1
9.71794017117339E-79	0.990771993166857	0.601	0.376	1.58509322132009E-74	1	INTS11
2.86640223218336E-32	0.990554793521911	0.351	0.225	4.67538868091427E-28	1	MAPK1
3.08673769670429E-17	0.990496087564361	0.289	0.208	5.03477785709437E-13	1	NFS1
6.04815043930777E-32	0.989031649569394	0.3	0.176	9.86513818155491E-28	1	RETREG1
7.52284244916203E-28	0.988805980106514	0.318	0.202	1.22705083188282E-23	1	SH3BP2
3.32102384407868E-48	0.988571279980996	0.5	0.335	5.41692199207673E-44	1	IKBKG
1.12818066086494E-39	0.988179313365617	0.421	0.277	1.8401754759368E-35	1	KLHL5
1.10143258169416E-23	0.987580679855139	0.291	0.187	1.79654668400135E-19	1	LCOR
1.77095785173774E-31	0.987109528154124	0.367	0.243	2.88860935196942E-27	1	FAM110C
5.68743202947754E-158	0.986009863346755	0.92	0.698	9.27677038328081E-154	1	MT1M
1.51200783360072E-58	0.985212253982906	0.451	0.265	2.46623597738614E-54	1	DCAF5
4.15687111614867E-55	0.984686537754853	0.496	0.321	6.7802724775501E-51	1	IBTK
2.41261832627786E-31	0.98456363503296	0.299	0.177	3.93522175199181E-27	1	E2F4
3.87118145546715E-28	0.984201441005872	0.261	0.15	6.31428407201247E-24	1	IRGQ
2.14328122900166E-25	0.983506424695525	0.263	0.158	3.49590601262462E-21	1	VHL
2.3461190018664E-70	0.982145286242398	0.563	0.36	3.82675470394429E-66	1	XDH

2.61802876912229E-30	0.981888656468048	0.306	0.189	4.27026672531537E-26	1	ARFGEF2
5.79291384992716E-35	0.981518469617546	0.316	0.185	9.44882178061619E-31	1	NAV2
1.26405474139509E-30	0.98067669578119	0.316	0.194	2.06179968868953E-26	1	TBC1D20
1.07997650723306E-21	0.978185698939201	0.29	0.195	1.76154968094784E-17	1	LARP1
2.271560405965E-23	0.977974537567605	0.292	0.194	3.70514217816952E-19	1	C7orf26
2.86423677430346E-42	0.977152134318732	0.425	0.275	4.67185660256638E-38	1	ENAH
1.39587426090449E-73	0.975778047730735	0.589	0.368	2.27681050696132E-69	1	LUC7L2
4.65933079769251E-76	0.975557796798416	0.611	0.391	7.59983446411625E-72	1	PLEKHA6
6.03484259681348E-26	0.973018317054878	0.288	0.181	9.84343175966247E-22	1	HMGN4
3.54921102806672E-20	0.972764157688012	0.277	0.185	5.78911810787962E-16	1	PICK1
4.62580460513708E-28	0.972599916399712	0.281	0.17	7.54514989143908E-24	1	PLPP5
2.52587544421409E-27	0.971928129675129	0.327	0.212	4.1199554370576E-23	1	TRIB1
8.59871908179632E-44	0.971832896644894	0.43	0.277	1.4025370694318E-39	1	AFF4
3.95685704723336E-36	0.970907804244983	0.359	0.221	6.45402952974234E-32	1	ERO1B
9.63070996824325E-56	0.970091515304855	0.521	0.338	1.57086510292016E-51	1	PTPRK
5.01099866716129E-31	0.970050661433959	0.362	0.24	8.17343992600678E-27	1	EIF1
4.48041776409983E-23	0.967537376221727	0.251	0.154	7.30800941502323E-19	1	RPH3AL
9.72277604075897E-25	0.967419153542997	0.298	0.193	1.5858820000082E-20	1	NPL
5.37945388976904E-28	0.967088148966435	0.316	0.203	8.77442723960228E-24	1	ZNF281
2.77251731787431E-15	0.965084426335534	0.277	0.201	4.52225299718479E-11	1	TRPT1
2.50999235020394E-46	0.964654355995544	0.392	0.235	4.09404852241764E-42	1	ZNF592
1.85143603825693E-26	0.96448988160154	0.269	0.162	3.01987732200088E-22	1	ZDHHC14
1.74250643257115E-48	0.964476100429468	0.445	0.285	2.84220224216681E-44	1	PITPNB
4.25360927350551E-37	0.963930807175158	0.408	0.27	6.93806208601484E-33	1	HOOK1
7.71679744144054E-63	0.963677032352502	0.563	0.359	1.25868683067337E-58	1	PPP2R1B
8.35305118283031E-36	0.963646344095551	0.266	0.139	1.36246617843145E-31	1	SOGA1
1.6549816650678E-31	0.963578675734362	0.338	0.213	2.69944059389209E-27	1	MTUS1
6.25415995239532E-31	0.963459153542696	0.334	0.212	1.0201160298352E-26	1	CENPV
1.46675888334007E-24	0.96299085798453	0.271	0.168	2.39243041461599E-20	1	USP4
1.67508295096125E-39	0.961284414672292	0.418	0.276	2.73222780131289E-35	1	ATF6
5.84968969521787E-73	0.960620955795278	0.679	0.462	9.54142886186987E-69	1	TXNRD1
1.9589176112256E-39	0.960061812652639	0.398	0.254	3.19519051567008E-35	1	PEX13
1.58885015449769E-65	0.958961815974717	0.539	0.341	2.59157348700118E-61	1	MINK1
2.6224206326285E-25	0.957654971070883	0.299	0.193	4.27743029388035E-21	1	TBC1D14
7.51003033888401E-34	0.955841468677076	0.39	0.259	1.22496104857537E-29	1	DEPRECAT
4.77216924686262E-29	0.955305657647258	0.384	0.266	7.78388525855762E-25	1	EXOC3L4
1.6029751285684E-22	0.95514311093812	0.284	0.185	2.61461273220792E-18	1	AUTS2
8.56200904192798E-26	0.95481501952431	0.322	0.213	1.39654929482887E-21	1	SDR39U1
4.77505805391642E-33	0.954737724189678	0.408	0.278	7.78859719174306E-29	1	KNDC1
2.46305511117384E-42	0.954664765943095	0.453	0.302	4.0174891927565E-38	1	ADHFE1
1.00778099996948E-37	0.95309098559767	0.427	0.287	1.64379158905022E-33	1	CPNE1
3.31400175488626E-54	0.953000568692353	0.52	0.34	5.40546826239498E-50	1	DNAJC22
4.44424065936259E-34	0.95229223276306	0.4	0.268	7.24900093948631E-30	1	LGR4
8.00938319178756E-47	0.952108606951493	0.408	0.25	1.30641049241247E-42	1	EGLN2
3.74739139994009E-25	0.950584759029207	0.256	0.153	6.11237011244228E-21	1	PPP1R13B
9.13847566592763E-24	0.950299808227244	0.27	0.169	1.49057676586946E-19	1	CDK12
2.56965419416718E-27	0.949325513285074	0.37	0.259	4.19136295610608E-23	1	ELOVL5
4.55600434421884E-32	0.948210731469478	0.338	0.214	7.43129868585535E-28	1	PPP1R10
1.39099897941346E-27	0.947965838224429	0.341	0.23	2.26885843532129E-23	1	DDX3Y
1.19284309041008E-40	0.947426471531082	0.403	0.256	1.94564636476788E-36	1	SLC38A7

8.94463163017485E-35	0.945205380909552	0.387	0.253	1.45895886519782E-30	1	OTULIN
3.22969164256795E-31	0.944857429425213	0.43	0.306	5.26795003819259E-27	1	PDZK1
4.72888199418329E-62	0.944702175367628	0.473	0.284	7.71327942071236E-58	1	SETD1B
2.35341553616055E-24	0.941061862688123	0.283	0.18	3.83865608103147E-20	1	MYRF
2.02807381010133E-43	0.940304769916886	0.462	0.303	3.30799119165627E-39	1	SLC23A2
2.42906179201891E-52	0.94013996880361	0.441	0.261	3.96204268896205E-48	1	DHRS2
3.18908987320777E-22	0.93950075511206	0.257	0.162	5.20172449218919E-18	1	ST3GAL6
1.66826932207088E-27	0.939430233493448	0.358	0.242	2.72111409122981E-23	1	PPFIBP2
1.86652319085544E-33	0.939378067702429	0.492	0.356	3.0444859766043E-29	1	FTCD
2.45769835979238E-34	0.939110816496177	0.327	0.197	4.00875179465736E-30	1	STX6
3.04100315536347E-30	0.938959690154414	0.351	0.229	4.96018024671335E-26	1	DNMT3A
7.058959928977E-24	0.938758260911804	0.3	0.202	1.15138695401544E-19	1	DHX9
1.32831253914806E-21	0.938003643889282	0.254	0.16	2.1666105826044E-17	1	WIPF2
3.48396088470713E-13	0.93797837861046	0.262	0.195	5.6826885990458E-09	1	UBXN2B
3.0120184339821E-83	0.937630005012456	0.725	0.486	4.91290326766821E-79	1	ONECUT1
3.9487725207288E-30	0.937363645574497	0.386	0.265	6.44084285856075E-26	1	SMAD2
3.63487555117579E-40	0.93735902509889	0.379	0.235	5.92884551152283E-36	1	EDC4
1.52270038615588E-25	0.937064458608287	0.274	0.167	2.48367659985886E-21	1	PHKB
1.71960953557548E-43	0.935227524598163	0.45	0.3	2.80485511347717E-39	1	PRKAA1
4.95295123961604E-17	0.935112000447477	0.269	0.186	8.07875876693772E-13	1	CISH
1.56234786939864E-27	0.935032169543661	0.279	0.169	2.54834560977612E-23	1	TEAD2
1.68451454780168E-117	0.934747585285722	0.794	0.522	2.74761167891931E-113	1	NIT1
3.56102122978821E-33	0.933122828751772	0.339	0.213	5.80838172790755E-29	1	DNAJC7
1.55306478788296E-36	0.933051237249158	0.403	0.27	2.53320397551589E-32	1	EDEM1
4.24945965033729E-39	0.933034141705679	0.443	0.301	6.93129363566515E-35	1	MYO6
2.12906388566264E-45	0.931673466045119	0.442	0.282	3.47271610390434E-41	1	FOXN2
9.89419018614179E-31	0.931572593410145	0.377	0.253	1.61384136126159E-26	1	PI4KA
4.78259789057964E-34	0.931170029974294	0.441	0.307	7.80089541932445E-30	1	RAB17
2.66367393353828E-83	0.931110890095089	0.626	0.401	4.34471855299429E-79	1	CPEB4
5.48370616421577E-20	0.930943822304879	0.256	0.166	8.94447312445234E-16	1	FASTKD2
4.50432031543493E-33	0.930284639094208	0.359	0.23	7.34699686650592E-29	1	ARFGAP1
3.91827546828729E-34	0.929501284071204	0.352	0.221	6.3910991163234E-30	1	PAWR
2.37722636576112E-47	0.928547787191338	0.504	0.339	3.87749392519296E-43	1	ATP13A3
1.76961031275624E-26	0.926388913849036	0.29	0.183	2.88641138113671E-22	1	AKAP8
2.15502701676158E-66	0.925775073599792	0.535	0.337	3.51506456703981E-62	1	SPATA20
1.17306759476741E-69	0.925264683131012	0.532	0.325	1.91339055382511E-65	1	ESRRA
1.44179028193955E-15	0.925223041284202	0.315	0.237	2.3517041288716E-11	1	IL17RB
5.80284846145982E-38	0.925127179139607	0.327	0.189	9.46502612548712E-34	1	ANKLE2
2.46582183880072E-66	0.924028853322697	0.561	0.357	4.02200200126785E-62	1	NSUN2
4.76823743335472E-63	0.923786585611807	0.551	0.353	7.77747207754488E-59	1	NFE2L2
4.75515605132884E-17	0.922767454703466	0.256	0.176	7.75613503532247E-13	1	EXOC6
1.37462822510791E-27	0.921574990717393	0.328	0.215	2.24215609797352E-23	1	ZNF516
1.26617080879859E-26	0.921131392024409	0.303	0.192	2.06525120623138E-22	1	HDAC6
1.93845081433472E-16	0.92032758443377	0.266	0.187	3.16180712326136E-12	1	INO80E
1.81912853147069E-29	0.919490756977534	0.365	0.247	2.96718054768184E-25	1	UMPS
3.11850995889016E-42	0.918088269360206	0.453	0.302	5.08660159394574E-38	1	NHSL1
4.30293572081841E-19	0.917891346228875	0.255	0.168	7.01851845422691E-15	1	GORASP1
1.59957937726389E-38	0.917482913453451	0.435	0.291	2.60907392225513E-34	1	EIF5
1.03697446722976E-24	0.917053104766547	0.297	0.193	1.69140905349846E-20	1	YAP1
4.53775782964169E-44	0.916556768979089	0.499	0.333	7.40153679592856E-40	1	ARRDC2

8.20407310338684E-51	0.916360079405066	0.481	0.308	1.33816636389343E-46	1	KIFC3
1.02732329462518E-28	0.913580189392275	0.356	0.238	1.67566702586313E-24	1	OSBPL9
3.25661183656938E-16	0.913101575990379	0.269	0.189	5.31185956662831E-12	1	CPSF1
3.06615476475233E-15	0.912205423539984	0.25	0.175	5.00120503678753E-11	1	SAMD4A
5.91129153135003E-21	0.911191748179551	0.253	0.162	9.64190761678503E-17	1	MCM3AP
1.92566199651699E-44	0.910983160670047	0.471	0.305	3.14094728251886E-40	1	TNFAIP2
4.52206771069962E-18	0.910392904477673	0.257	0.174	7.37594464292216E-14	1	VPS13D
5.62021050883901E-40	0.910224526873721	0.479	0.327	9.16712536096731E-36	1	FAM20A
2.14604104889593E-19	0.909877954578041	0.297	0.208	3.50040755485415E-15	1	C11orf24
6.82882245507519E-31	0.909256723294438	0.345	0.225	1.11384923064731E-26	1	SRSF3
1.77238676511925E-29	0.908620628855774	0.356	0.237	2.89094005258601E-25	1	RBM23
2.04348108653383E-46	0.908494040170633	0.442	0.282	3.33312200024534E-42	1	SRP54
1.76404103343928E-44	0.907971547882894	0.501	0.338	2.87732732964281E-40	1	ITPR2
4.3487864699592E-43	0.907424955935472	0.482	0.305	7.09330561115045E-39	1	EGR1
2.96320196948624E-29	0.906673806542251	0.361	0.243	4.833278732429E-25	1	SLC35A3
3.64021376725062E-35	0.90543982650157	0.649	0.506	5.93755267576248E-31	1	CHI3L1
4.74147741843914E-52	0.904860305107189	0.456	0.283	7.73382381721608E-48	1	MAFK
1.50303331482578E-78	0.904854002593218	0.56	0.335	2.45159763981233E-74	1	CEBPG
8.43379528655685E-24	0.903999420556487	0.343	0.24	1.37563634919029E-19	1	ADAM10
3.60869419785082E-46	0.903704845351961	0.42	0.259	5.88614110611447E-42	1	SHANK2
2.32399941297778E-27	0.903698831163836	0.352	0.238	3.79067544250806E-23	1	DEPRECAT
2.58655880379619E-40	0.903624096894135	0.399	0.251	4.21893606487197E-36	1	PANK3
5.07515344569186E-14	0.902969663050673	0.254	0.184	8.278082785268E-10	1	NBAS
1.95127487063677E-23	0.902898474054458	0.262	0.161	3.18272444149564E-19	1	SERTAD2
5.00651296751449E-40	0.90123167411998	0.435	0.291	8.16612330131288E-36	1	SEC31A
7.71191068046874E-51	0.901137429969543	0.534	0.359	1.25788975109126E-46	1	FBXO7
2.10596469968602E-38	0.900938677762309	0.415	0.278	3.43503902165786E-34	1	ANAPC5
2.83003526338457E-28	0.900823007503627	0.384	0.262	4.61607051810658E-24	1	MDM2
2.40460059100282E-32	0.900141013776541	0.383	0.256	3.9221440239847E-28	1	PUM2
6.1400730833504E-103	0.899915534023876	0.853	0.61	1.00150732062528E-98	1	SAT2
6.4550193247845E-25	0.899892115341347	0.29	0.185	1.0528782020656E-20	1	E4F1
3.93640984720757E-34	0.899805035436233	0.316	0.186	6.42067810178026E-30	1	CERK
6.11470011731367E-86	0.89979594568373	0.644	0.41	9.97368736135034E-82	1	A1CF
2.79256026415316E-36	0.898610821651629	0.368	0.233	4.55494504686021E-32	1	MOCOS
1.25278561751697E-33	0.897617328908106	0.355	0.227	2.04341862073192E-29	1	PARP4
1.1631478153549E-26	0.895033248371863	0.343	0.233	1.89721040162538E-22	1	ELAC2
2.3380900775657E-24	0.89495170522486	0.268	0.165	3.81365872551742E-20	1	CGGBP1
6.43353773917788E-53	0.893030054956783	0.492	0.319	1.0493743406373E-48	1	HBP1
1.67487356619984E-29	0.890923555109398	0.338	0.22	2.73188627382856E-25	1	UBE2Z
2.00429646971533E-67	0.890294593128461	0.56	0.36	3.26920797175267E-63	1	BCAR1
7.20228425256186E-68	0.88988211953855	0.619	0.412	1.17476458443536E-63	1	SLC25A13
3.63875308832597E-18	0.889735617339065	0.276	0.191	5.93517016236849E-14	1	MAN2A1
6.04043015716257E-21	0.889283293697371	0.353	0.26	9.85254562934787E-17	1	KLHL24
1.77129713612527E-28	0.888405562952249	0.408	0.285	2.88916275873393E-24	1	MAP3K13
3.56245447652969E-70	0.887983312388872	0.597	0.39	5.81071949666757E-66	1	TRIM27
4.85632690305385E-36	0.885717525027123	0.43	0.298	7.92115481157114E-32	1	PUM1
3.50567644495698E-36	0.885556053222888	0.39	0.252	5.71810884936932E-32	1	ST5
4.90338716176196E-130	0.885367976780226	0.868	0.599	7.99791479954994E-126	1	ECHDC2
5.84449775471476E-29	0.88445801400368	0.331	0.214	9.53296028771525E-25	1	R3HDM2
2.64034249808262E-26	0.884369651140001	0.299	0.189	4.30666264862256E-22	1	RNF31

**5.56267349736977E-46**	0.883831346774265	0.524	0.356	9.07327674155983E-42	1	AFM
**9.49699810283288E-61**	0.883443910613078	0.554	0.364	1.54905536055307E-56	1	KANK1
**3.51113585914037E-71**	0.883223266039476	0.659	0.437	5.72701369984386E-67	1	FNDC3B
**1.26376614587009E-13**	0.882636022147513	0.252	0.181	2.0613289605287E-09	1	FAM91A1
**3.09185813973539E-24**	0.882576509165872	0.343	0.239	5.0431298117224E-20	1	LRRC75B
**1.60460833357761E-31**	0.882309386824555	0.343	0.218	2.61727665289843E-27	1	IFT43
**7.43397742891074E-34**	0.881960888126269	0.423	0.292	1.21255605842963E-29	1	CLSTN3
**3.70234584687198E-47**	0.881137566476024	0.515	0.349	6.03889631083289E-43	1	PALM3
**3.0819621625005E-46**	0.881030739505679	0.468	0.304	5.02698848325457E-42	1	WWC1
**1.68027083711282E-19**	0.880508989615256	0.29	0.198	2.74068976241471E-15	1	MARC1
**3.17391865310226E-21**	0.880118434332244	0.265	0.172	5.17697871507509E-17	1	MSL2
**3.67308656150506E-82**	0.879605798079658	0.685	0.452	5.9911714904709E-78	1	MTSS1
**3.47104402923504E-123**	0.879457914891625	0.951	0.749	5.66161991608527E-119	1	ITIH3
**3.66017576911851E-35**	0.87893899431376	0.455	0.32	5.9701126970092E-31	1	SGK2
**3.15175324236215E-17**	0.87830163210453	0.275	0.191	5.14082471361691E-13	1	WDR45
**7.23313986502772E-67**	0.878296923813141	0.614	0.4	1.17979744338467E-62	1	CYP21A2
**6.3702420033749E-15**	0.8782871098392	0.25	0.175	1.03905017317048E-10	1	SRPK1
**4.7627886821661E-20**	0.878040521097548	0.296	0.203	7.76858461948112E-16	1	ZMYM3
**2.55211700049456E-20**	0.877702705884766	0.315	0.219	4.16275803950668E-16	1	HIGD1A
**3.6802679565788E-23**	0.876639818605086	0.277	0.177	6.00288506397568E-19	1	DEPRECAT
**5.38778269917695E-20**	0.876071414684209	0.263	0.173	8.78801236062752E-16	1	DEPRECAT
**6.53980033922951E-67**	0.875937003908021	0.626	0.396	1.06670683333173E-62	1	MYOM1
**1.43306336724085E-25**	0.875874133116442	0.374	0.269	2.33746965830655E-21	1	TTC3
**8.65628016348772E-20**	0.875635768533108	0.268	0.177	1.41192585746648E-15	1	PGM3
**5.05887458793562E-20**	0.874827740751203	0.28	0.19	8.2515303403818E-16	1	KLHL22
**3.73739515419566E-19**	0.874116815156651	0.251	0.163	6.09606523600854E-15	1	CARD10
**6.98383448135269E-41**	0.874041764667721	0.437	0.29	1.13913324225344E-36	1	NUMA1
**3.40080573856444E-21**	0.873601658346067	0.312	0.218	5.54705424017246E-17	1	EVL
**8.81405736826242E-19**	0.873112455119319	0.314	0.228	1.43766089733728E-14	1	SMARCA5
**8.93110891994567E-44**	0.872281763802368	0.409	0.255	1.45675317593234E-39	1	PARP12
**6.37855055700935E-36**	0.870711841632387	0.327	0.192	1.04040538135379E-31	1	SRRT
**2.07006549243564E-27**	0.869655237160209	0.323	0.208	3.37648382471177E-23	1	MIB2
**8.39211634770071E-18**	0.86956364511249	0.257	0.172	1.36883809747346E-13	1	LARP1B
**3.74432975398524E-21**	0.869216799199535	0.269	0.175	6.10737626172532E-17	1	ESF1
**6.15022041193128E-43**	0.867890278821058	0.453	0.298	1.00316245139011E-38	1	NR1H3
**5.11234682534369E-30**	0.865538583222423	0.288	0.17	8.33874890681809E-26	1	MCM7
**3.81159861592165E-28**	0.86465283673608	0.364	0.246	6.2170985024298E-24	1	DDX24
**4.22493495767977E-36**	0.864468173091762	0.437	0.295	6.89129140947147E-32	1	TNS3
**1.8155669693972E-39**	0.864352214664305	0.414	0.271	2.96137128378377E-35	1	EIF2D
**1.24798909740821E-21**	0.863908493918659	0.299	0.201	2.03559501678253E-17	1	SUCO
**7.84302900969829E-49**	0.863855742597771	0.471	0.303	1.27927646177189E-44	1	ILF3
**5.19816911122244E-25**	0.863192431478192	0.335	0.23	8.47873363731492E-21	1	PTS
**1.24687106943457E-109**	0.861388975457702	0.83	0.565	2.03377140135473E-105	1	EPS8L2
**3.39513931595766E-79**	0.86137541299169	0.695	0.444	5.53781173825854E-75	1	PLIN5
**4.04257103743939E-35**	0.860904938920065	0.446	0.312	6.59383761916738E-31	1	PI4K2B
**1.18907280657394E-46**	0.860515793684006	0.511	0.351	1.93949665480276E-42	1	HEXA
**2.75724722386197E-50**	0.860314753143013	0.556	0.38	4.49734594684126E-46	1	SND1
**2.04357075713708E-51**	0.860056928337647	0.424	0.252	3.33326826196628E-47	1	CRTC2
**3.82910646829151E-23**	0.85963830071963	0.365	0.262	6.24565556043029E-19	1	TRIM44
**1.7725646862953E-56**	0.859282474324757	0.623	0.437	2.89123025981626E-52	1	TNFRSF1A

2.04137845116116E-33	0.858572264564538	0.423	0.288	3.32969239168898E-29	1	CCDC50
1.27716937699317E-24	0.856429347884089	0.364	0.257	2.08319097081356E-20	1	ENPP1
6.34223240514249E-23	0.856048471398833	0.291	0.19	1.03448152760279E-18	1	TAOK1
5.23093375483824E-72	0.855890905479328	0.617	0.399	8.53217604751666E-68	1	NDST1
7.70066596716491E-35	0.855066171263817	0.421	0.285	1.25605562590427E-30	1	ARRB2
9.33234268669003E-87	0.855009828522295	0.62	0.386	1.52219841562601E-82	1	DDX5
5.80416675598489E-26	0.854931097029584	0.371	0.248	9.46717639568695E-22	1	IGFBP3
1.96017779113859E-26	0.854536833975702	0.297	0.186	3.19724599512616E-22	1	AEN
7.05766416214559E-22	0.854519773242924	0.342	0.245	1.15117560148757E-17	1	EXOC7
5.04303484533685E-91	0.854409618937934	0.701	0.463	8.22569413622893E-87	1	ZFAND3
1.33655081227402E-31	0.854218894971917	0.383	0.258	2.18004802990016E-27	1	CUL4A
1.04979344727926E-27	0.853796466643523	0.308	0.196	1.7123180918572E-23	1	WDR11
9.30019328453968E-27	0.853698342342917	0.347	0.237	1.51695452664127E-22	1	NIPBL
3.86078423739901E-21	0.853399361914027	0.259	0.166	6.29732516962153E-17	1	SSH3
4.80486108776702E-33	0.85307696488189	0.333	0.206	7.83720892025679E-29	1	TBRG4
8.2676954854384E-31	0.852610073015465	0.368	0.243	1.34854381062986E-26	1	DDI2
2.1130617595759E-33	0.852277231148146	0.513	0.38	3.44661503604425E-29	1	HSP90AA1
4.89160786566666E-20	0.85182085168635	0.336	0.241	7.97870158968889E-16	1	AP5Z1
3.46047764867865E-129	0.851633653107518	0.904	0.618	5.64438509275975E-125	1	C4BPB
3.04429949381452E-48	0.850878969006529	0.489	0.333	4.96555690436086E-44	1	DCTN2
4.76400312936896E-80	0.849150693141437	0.664	0.433	7.7705655043137E-76	1	WNK1
4.04241307801829E-15	0.848576363779069	0.254	0.179	6.59357997155563E-11	1	ATL2
1.98328164611482E-23	0.84680017482369	0.271	0.17	3.23493069297789E-19	1	SNUPN
1.94403959478119E-19	0.845783497136834	0.262	0.175	3.17092298304759E-15	1	PABPC4
1.01702108259264E-16	0.843890941126183	0.255	0.176	1.65886308781685E-12	1	DNAJC13
8.49499811889369E-24	0.843684098639817	0.293	0.19	1.38561914317275E-19	1	CUX1
1.34753432778982E-21	0.843117252064532	0.31	0.212	2.19796324205798E-17	1	DAPK3
4.02989417781069E-22	0.84297876971465	0.28	0.182	6.57316039342702E-18	1	ARFRP1
2.90091823040457E-22	0.842813673713414	0.278	0.18	4.73168772561289E-18	1	TAF8
5.6012359659044E-42	0.842642404582426	0.445	0.297	9.13617598398666E-38	1	DEDD
1.19779616928272E-44	0.841996089586371	0.479	0.316	1.95372533171705E-40	1	RSAD1
2.41870910211199E-16	0.841817655901345	0.263	0.183	3.94515641645486E-12	1	VPS36
8.59945901153272E-32	0.841063450681726	0.423	0.29	1.4026577593711E-27	1	TMEM175
4.78757701390592E-29	0.840774028939704	0.352	0.232	7.80901686738195E-25	1	PMM2
5.31148384510583E-32	0.840136184667279	0.34	0.214	8.66356129975212E-28	1	FBXW7
3.5033851479932E-27	0.837455481852301	0.315	0.203	5.71437151489171E-23	1	HNRNPDL
3.75681230674078E-20	0.836017699727069	0.363	0.265	6.12773655352488E-16	1	MUC20
3.36352390132547E-47	0.835972646944577	0.461	0.301	5.48624383545197E-43	1	LEMD2
7.41867000391068E-23	0.835914355655497	0.348	0.248	1.21005926433787E-18	1	VDAC3
3.52662829146022E-15	0.835682246012752	0.278	0.2	5.75228340620076E-11	1	SMARCA1
1.31648778734652E-96	0.835487170521639	0.869	0.653	2.1473232299409E-92	1	ZFP36L1
1.98839687235825E-27	0.834025842484187	0.315	0.203	3.24327413850355E-23	1	SEC24A
1.46475534222018E-25	0.834020060560085	0.265	0.159	2.38916243869534E-21	1	TRIM47
9.47770227040585E-16	0.832707412271071	0.259	0.18	1.5459080173259E-11	1	ZFHX3
4.48581378913485E-59	0.831558751443034	0.535	0.347	7.31681087145785E-55	1	STXBP2
1.89571479333525E-21	0.830883622036665	0.261	0.166	3.09210039940913E-17	1	RAB3B
8.85112525937819E-20	0.830139455237011	0.352	0.261	1.44370704105718E-15	1	NDRG2
4.47088421501543E-16	0.829729259694087	0.269	0.189	7.29245924311166E-12	1	MLYCD
4.15766004537578E-24	0.829256305449661	0.332	0.227	6.78155930001244E-20	1	TJP3
8.32593147338475E-56	0.82816788208482	0.552	0.369	1.35804268262379E-51	1	OTUD7B

9.94964981412209E-55	0.826777126447396	0.561	0.385	1.62288738118145E-50	1	TXNDC11
6.90086290518544E-18	0.826452047240619	0.338	0.254	1.1255997484648E-13	1	ELAVL1
5.59651551638272E-19	0.826396201958277	0.381	0.284	9.12847645877186E-15	1	PWWP3A
5.54189997691016E-25	0.825955235891644	0.295	0.187	9.03939305233816E-21	1	JMY
1.96812782257059E-29	0.825501565686585	0.349	0.229	3.21021329139488E-25	1	GPAT4
7.3327483364593E-98	0.824975516413119	0.796	0.567	1.19604458115988E-93	1	SLC30A1
3.1904839968298E-38	0.824814361214976	0.382	0.241	5.20399844722908E-34	1	CASC3
7.89477625768794E-77	0.824380129981551	0.675	0.454	1.28771695539148E-72	1	TCF25
8.278834754633E-18	0.822774847290984	0.297	0.213	1.35036073682819E-13	1	TBL1XR1
8.16627249153607E-11	0.822504023781734	0.255	0.194	1.33200070609445E-06	1	GOPC
5.98400294388311E-32	0.822334532398143	0.391	0.262	9.76050720176774E-28	1	LBR
2.64556788292745E-87	0.821547166348682	0.716	0.471	4.31518577384296E-83	1	PTPRF
1.60861863197179E-31	0.821287840040323	0.49	0.362	2.62381785060919E-27	1	DEPRECAT
2.49122507201E-17	0.820251537276855	0.318	0.232	4.06343721495551E-13	1	NFIL3
1.12061565799684E-21	0.819847032026411	0.306	0.211	1.82783619975865E-17	1	GNL2
1.55978707053294E-18	0.819122653527527	0.272	0.185	2.54416869074628E-14	1	TMEM189
2.05924186104221E-16	0.818735374423131	0.269	0.181	3.35882939954595E-12	1	IFIT2
2.29378857718729E-56	0.818391074910791	0.555	0.368	3.74139854825019E-52	1	OPLAH
1.19529296007583E-75	0.817646896068061	0.644	0.422	1.94964234717969E-71	1	SHFL
1.1635941607419E-11	0.817575118259523	0.262	0.2	1.89793843558612E-07	1	CAMK1
7.64597672507206E-19	0.817315604544588	0.302	0.214	1.2471352636265E-14	1	SNX14
8.75091472865141E-31	0.816934675552041	0.38	0.257	1.42736170139033E-26	1	PPP1R35
3.54995078684515E-30	0.81670198847942	0.396	0.274	5.79032472842312E-26	1	ARHGAP1
8.6443592889E-19	0.81493339789706	0.285	0.197	1.40998144361248E-14	1	CRELD1
2.07976929252409E-29	0.814681560159988	0.357	0.239	3.39231169303605E-25	1	MCRS1
2.46093984540159E-23	0.814574036670576	0.269	0.17	4.01403898183453E-19	1	FERMT2
3.12743658880663E-44	0.814458664358986	0.507	0.344	5.1011618200025E-40	1	MOGS
1.02289436095649E-15	0.813029469693792	0.276	0.198	1.66844299215613E-11	1	CHD4
1.03015427346667E-32	0.812847658901803	0.423	0.294	1.68028463545149E-28	1	KCTD3
2.13818561323421E-23	0.811943078629936	0.285	0.182	3.48759455374632E-19	1	EPHA2
3.12455852510417E-18	0.811197451896106	0.331	0.244	5.09646741029742E-14	1	EPB41L5
1.44533773924153E-14	0.80978332199299	0.308	0.235	2.35749038647686E-10	1	AAAS
2.00372440171294E-16	0.809481139646657	0.284	0.203	3.26827487163398E-12	1	SMARCC1
1.64332218616511E-48	0.809323173798164	0.485	0.316	2.68042281785391E-44	1	EXOC3
1.84971997333061E-41	0.808433913415668	0.464	0.314	3.01707824849955E-37	1	CYTH1
2.99938267388897E-20	0.808317152282235	0.358	0.265	4.8922930793803E-16	1	TRAPPC12
1.14322634094635E-21	0.808229420632254	0.29	0.195	1.8647164847176E-17	1	UBAP1
8.21423817654969E-37	0.807517200964733	0.402	0.26	1.33982438897702E-32	1	GDI1
4.72644693598429E-31	0.806831196954883	0.365	0.238	7.70930759728397E-27	1	YPEL5
3.41179100485495E-59	0.805274097140201	0.536	0.351	5.56497230801891E-55	1	HGS
7.18140419204343E-100	0.804393692703989	0.715	0.456	1.1713588377642E-95	1	SPTBN1
1.63686896562861E-18	0.803847919225566	0.256	0.169	2.66989696983682E-14	1	PELO
2.12281826867536E-48	0.803764524829107	0.493	0.326	3.46252887803638E-44	1	TNKS1BP1
6.81065252082011E-31	0.803441124930841	0.493	0.364	1.11088553267097E-26	1	DEPRECAT
2.37006943547057E-50	0.803327063552511	0.456	0.285	3.86582025619605E-46	1	CLIP1
2.38117250847767E-73	0.802852953835236	0.672	0.457	3.88393047857793E-69	1	H6PD
2.10411470345489E-22	0.802795524792125	0.382	0.279	3.43202149280528E-18	1	LYPLAL1
4.57907669739531E-84	0.801761977724522	0.704	0.468	7.46893200112149E-80	1	ADAM15
6.73709740625732E-28	0.80114308830848	0.282	0.17	1.09888795793463E-23	1	DAXX
7.97523910096169E-21	0.800948972465293	0.342	0.247	1.30084124975786E-16	1	MTMR4

1.64987043203827E-37	0.800941961354707	0.455	0.317	2.69110366169762E-33	1	FLNB
2.11037264649826E-31	0.800834413598986	0.405	0.28	3.44222882370331E-27	1	CDC73
3.36423682352656E-42	0.800569960624566	0.434	0.279	5.48740668285418E-38	1	SRSF2
6.41510767125682E-36	0.800366100227317	0.436	0.298	1.0463682122587E-31	1	CEP170B
1.68342660379503E-22	0.800334195438289	0.299	0.2	2.74583713345007E-18	1	EHMT1
2.02653379379619E-31	0.799558808427231	0.413	0.289	3.30547927106097E-27	1	PAFAH1B1
1.32331666966266E-24	0.799246319388441	0.37	0.264	2.15846181988677E-20	1	XYLB
5.13215038608434E-49	0.798325358945658	0.551	0.378	8.37105049474217E-45	1	RAB11B
4.76505811234998E-15	0.797138827588408	0.335	0.256	7.77228628705406E-11	1	GLRX
1.53087929979398E-12	0.796857001555398	0.25	0.184	2.49701722589396E-08	1	UBE2H
5.05647580690941E-45	0.796590806150736	0.449	0.291	8.24761768864993E-41	1	ANKRD11
1.01570770619917E-26	0.796242876128228	0.359	0.249	1.65672083958147E-22	1	HNRNPU
4.18435332270826E-10	0.795096416964139	0.369	0.306	6.82509870466944E-06	1	RND1
2.11510732950824E-69	0.794887614722252	0.657	0.446	3.44995156516089E-65	1	KDM2A
6.79784468635434E-16	0.794719801027593	0.254	0.178	1.10879644679126E-11	1	AFTPH
3.19489737760932E-26	0.794246070311987	0.385	0.273	5.21119711261856E-22	1	TNS2
3.2204851772435E-67	0.794025431573232	0.652	0.438	5.25293337260187E-63	1	STAT6
3.24446924347498E-14	0.793727372402196	0.26	0.187	5.29205378303204E-10	1	RNF4
2.68756420287746E-30	0.791125877690027	0.354	0.232	4.38368597131342E-26	1	SRCAP
5.69440876690132E-19	0.790150987623185	0.281	0.193	9.28815013969274E-15	1	HNRNPM
2.28287337184614E-54	0.789472749801503	0.65	0.443	3.72359475681824E-50	1	LEPR
1.85652596429619E-35	0.78906160972104	0.389	0.252	3.02817950036352E-31	1	GRK2
4.06429724914805E-17	0.788909971117558	0.316	0.231	6.62927524308539E-13	1	ZBTB18
5.17970877885635E-41	0.788421864794494	0.543	0.384	8.44862298919259E-37	1	MARC2
5.01689297694672E-102	0.787361763334848	0.812	0.537	8.1830541346978E-98	1	SLC12A7
1.31425879639118E-57	0.785603437698705	0.548	0.361	2.14368752279365E-53	1	TMEM263
9.55911359894625E-20	0.785341670663638	0.297	0.206	1.55918701912412E-15	1	DEPRECAT
2.23156219565567E-50	0.784686174627974	0.551	0.377	3.63990109733397E-46	1	UBXN4
6.00553694536011E-26	0.784132232465756	0.288	0.179	9.79563131157687E-22	1	GOLGA2
1.20576093369912E-38	0.784077308177249	0.441	0.3	1.96671665895663E-34	1	GOLGA3
3.46705658591303E-13	0.783682530275369	0.281	0.209	5.65511599728275E-09	1	INSIG2
5.8003477544481E-32	0.783105228264244	0.36	0.236	9.4609472222803E-28	1	SAMD4B
9.22291832484509E-34	0.783031522422039	0.324	0.194	1.50435020796548E-29	1	KLF11
1.50056122809782E-88	0.783000844462	0.676	0.436	2.44756541915036E-84	1	MEF2D
1.04050284182483E-27	0.782801700966642	0.375	0.262	1.69716418530048E-23	1	SLC12A8
5.817753335669E-14	0.78264984376872	0.25	0.179	9.4893374658097E-10	1	ZNF148
1.90763589707812E-15	0.782638309359816	0.297	0.221	3.11154491172412E-11	1	HIPK3
3.60390900079399E-16	0.782492767275887	0.291	0.212	5.87833597119508E-12	1	NUFIP2
1.45282064717907E-30	0.782184655280369	0.346	0.224	2.36969575761377E-26	1	DNAJC11
3.06947276528649E-61	0.781437937607914	0.555	0.361	5.00661702745879E-57	1	TCIRG1
9.72027698698079E-46	0.779862557235413	0.489	0.328	1.58547437934644E-41	1	LARP4
1.58961995470661E-70	0.779792585573323	0.646	0.428	2.59282910812195E-66	1	MIA2
3.8809461814022E-61	0.77967796635988	0.681	0.484	6.33021131648513E-57	1	ETS2
7.80489665003156E-16	0.779402197860266	0.266	0.187	1.27305669258665E-11	1	TRIP12
8.1040032016133E-21	0.777888501392889	0.27	0.176	1.32184396221515E-16	1	PDZD8
1.39931672127966E-28	0.777195342034931	0.341	0.223	2.28242550407925E-24	1	HCFC1
6.85753185861558E-24	0.776659215948041	0.353	0.247	1.11853202145879E-19	1	ADD1
9.84368719352105E-55	0.775827703847262	0.555	0.371	1.60560381813522E-50	1	KLHDC2
7.00599408784461E-19	0.775691611545681	0.258	0.17	1.14274769566833E-14	1	RILPL1
4.82397560136227E-30	0.77453943322988	0.328	0.208	7.868386603382E-26	1	ARIH1

1.41228227643556E-25	0.773690842217487	0.35	0.24	2.30357362109405E-21	1	TRIM41
2.8576407328804E-29	0.773580317154802	0.44	0.312	4.66109779940123E-25	1	RIOX2
8.00751201893457E-40	0.772893824651526	0.518	0.366	1.30610528540842E-35	1	SLC39A14
1.93464423620555E-20	0.772356679196669	0.282	0.187	3.15559821367488E-16	1	ABTB2
1.3567083322795E-31	0.771424791901629	0.432	0.304	2.21292696078109E-27	1	HECTD3
2.14531101966837E-16	0.769936996004327	0.304	0.222	3.49921680418108E-12	1	FAT1
4.33136858816335E-24	0.76921948297414	0.343	0.239	7.06489530415324E-20	1	ACBD3
2.35359756115985E-21	0.769140300964816	0.305	0.207	3.83895298200784E-17	1	AHDC1
1.00842849620292E-40	0.768862301530624	0.454	0.307	1.64484772015659E-36	1	TRAF4
1.51108603279714E-21	0.768562047688347	0.302	0.205	2.46473242809542E-17	1	DHX30
2.92004335059911E-18	0.768092958219652	0.26	0.173	4.76288270916221E-14	1	INO80
5.54874692463213E-12	0.765870884139198	0.28	0.214	9.05056110876747E-08	1	CYB5B
7.75890131570422E-19	0.764860206778715	0.276	0.188	1.26555439360452E-14	1	RAB5A
1.18983959880513E-16	0.763840923290823	0.301	0.218	1.94074736961104E-12	1	TCAF1
6.62183832932706E-30	0.762983682388958	0.411	0.287	1.08008804989654E-25	1	GPBP1L1
3.36983737670242E-21	0.762420570716578	0.26	0.161	5.49654174513931E-17	1	RASD1
6.28415692083156E-19	0.761539075100693	0.309	0.22	1.02500883535684E-14	1	NAB1
4.56145565560622E-20	0.761493971667063	0.299	0.206	7.44019031985931E-16	1	NAGK
1.20236280649141E-30	0.761436460300388	0.417	0.292	1.96117397366813E-26	1	ATP2A2
9.08959465418528E-33	0.761323111614533	0.439	0.303	1.48260378404416E-28	1	HNF1A
5.87169130382167E-42	0.758266608185823	0.575	0.409	9.57731568566353E-38	1	PLIN4
3.43778337454835E-19	0.757536663670732	0.316	0.226	5.60736846222582E-15	1	MET
1.63235315347858E-38	0.757421692295351	0.41	0.264	2.6625312286389E-34	1	CD2AP
2.9317313819212E-21	0.756495144778448	0.368	0.268	4.78194705705167E-17	1	SERINC5
1.54190073548597E-21	0.756286708912547	0.257	0.162	2.51499428965117E-17	1	CEMIP2
8.11268858347552E-24	0.756226310699344	0.419	0.311	1.32326063485069E-19	1	TAP2
1.35215293658508E-16	0.756178894211083	0.285	0.202	2.20549665486393E-12	1	TMPRSS2
1.74619242446903E-45	0.755172897451765	0.488	0.33	2.84821446355143E-41	1	TMEM41B
1.92745805353288E-28	0.755102750291991	0.409	0.284	3.14387683111749E-24	1	SLC30A2
4.07381527069906E-16	0.754960310618646	0.291	0.21	6.64480008803723E-12	1	ZBTB48
1.71074801368077E-21	0.754716370339029	0.257	0.162	2.7904010851147E-17	1	TAF4
2.89089886447533E-39	0.75392170912366	0.463	0.314	4.71534513784572E-35	1	ITGA5
4.77474894525504E-25	0.753842856059918	0.325	0.218	7.78809300460549E-21	1	NOL3
1.9725825598545E-41	0.753684007083459	0.548	0.39	3.21747941337867E-37	1	STAT2
1.28700815677623E-37	0.753520484930015	0.475	0.331	2.09923900451771E-33	1	NEK7
3.85695264094413E-39	0.751378752078344	0.345	0.2	6.29107545264398E-35	1	TGFBR2
3.44236667866609E-18	0.751245259403642	0.31	0.224	5.61484428957226E-14	1	AMPD2
1.11515196800847E-30	0.751017374563441	0.442	0.311	1.81892437501862E-26	1	ID2
3.78784539360505E-25	0.750169521558346	0.357	0.245	6.17835462150919E-21	1	FBXO31
7.71145572043389E-76	0.748349869989255	0.669	0.445	1.25781554255997E-71	1	MAPKAPK2
3.4318548137506E-28	0.748062774669818	0.363	0.245	5.5976983867086E-24	1	PBX2
2.89737647684852E-19	0.747174977162501	0.288	0.198	4.72591077138762E-15	1	KDM4A
1.28382712051049E-29	0.746916730708303	0.381	0.257	2.09405041626466E-25	1	GGA2
1.51335007600447E-22	0.745893496355159	0.31	0.211	2.46842530897089E-18	1	RANBP9
3.94493860733365E-65	0.745602088007108	0.714	0.505	6.43458936242191E-61	1	CGN
3.16215404661179E-23	0.745405999212372	0.357	0.253	5.15778946542849E-19	1	DDX6
8.26655893203131E-13	0.744144328778214	0.306	0.236	1.34835842740363E-08	1	TJP1
1.50134163243156E-41	0.742724369707218	0.507	0.352	2.44883833665912E-37	1	TTC38
2.4812190347594E-07	0.741773115389315	0.287	0.242	0.00404711636759605	1	LIPG
7.75922268562375E-19	0.741600137534858	0.344	0.251	1.26560681225209E-14	1	DCPS

**2.35581679636303E-38**	0.741222799793893	0.451	0.307	3.84257277654773E-34	1	BRD4
**2.27454025517456E-33**	0.741173750143276	0.476	0.337	3.71000261021522E-29	1	IL6ST
**1.20139670728526E-26**	0.738939912410841	0.362	0.251	1.95959816925298E-22	1	USP14
**5.85559435278293E-31**	0.738580172260483	0.341	0.22	9.55105994882424E-27	1	MAPKAPK5
**2.50792410285285E-22**	0.73848469563939	0.309	0.208	4.09067500416328E-18	1	PYGO2
**5.65706608563517E-81**	0.738235546356138	0.757	0.514	9.22724049227953E-77	1	PON2
**3.1141902348741E-41**	0.737528231308135	0.486	0.324	5.07955569210314E-37	1	ZBTB16
**1.36276697762067E-31**	0.736323653431096	0.337	0.21	2.22280921719708E-27	1	FAM83G
**4.68576869548756E-13**	0.735896195194736	0.268	0.198	7.64295731920976E-09	1	ZHX1
**3.62985035483068E-45**	0.735378235783519	0.475	0.299	5.92064891376432E-41	1	PPP1R3B
**2.71671064928817E-21**	0.734278886000997	0.311	0.214	4.43122674005394E-17	1	POLR3E
**1.35653091328408E-19**	0.734240438999159	0.325	0.231	2.21263757265767E-15	1	DCTN4
**3.51847194463748E-23**	0.733808619140866	0.352	0.249	5.73897958889819E-19	1	ASCC2
**1.82701453170624E-22**	0.733689579970591	0.374	0.271	2.98004340266605E-18	1	NUP50
**8.47916352085529E-28**	0.733039957707086	0.395	0.278	1.38303636188671E-23	1	CARS2
**4.3374244031037E-57**	0.732285138178734	0.57	0.383	7.07477294390244E-53	1	KLHL21
**1.99090378966118E-21**	0.731816720109104	0.261	0.166	3.24736317131635E-17	1	PPP6R3
**1.22105269608185E-24**	0.728786529625268	0.325	0.216	1.99165905257911E-20	1	TTYH3
**2.6978576997771E-23**	0.728394257417891	0.274	0.169	4.40047569410643E-19	1	FAM102A
**1.78191795263564E-26**	0.727955113781477	0.388	0.276	2.90648637254399E-22	1	BRPF3
**3.11834112565734E-16**	0.727469951731943	0.34	0.26	5.08632621005969E-12	1	UBA7
**1.03118138428729E-19**	0.726361687454823	0.349	0.256	1.68195995591099E-15	1	SFXN1
**7.09919906201934E-38**	0.726313658310615	0.477	0.324	1.15795035900597E-33	1	HES1
**7.2135263034341E-61**	0.725792240972971	0.631	0.435	1.17659827535314E-56	1	BSDC1
**9.1393856525998E-26**	0.725095123598376	0.301	0.19	1.49072519379555E-21	1	SRF
**1.2880151614081E-65**	0.724387170657067	0.815	0.613	2.10088152977276E-61	1	DEPRECAT
**1.86498262307334E-10**	0.724072432172696	0.26	0.201	3.04197315649493E-06	1	NCOA2
**5.63402804984134E-23**	0.723777306248453	0.324	0.223	9.18966315209621E-19	1	TRIM24
**2.095274961288E-15**	0.723003006020835	0.254	0.178	3.41760298935685E-11	1	CORO1C
**5.07738569262126E-27**	0.722180146733529	0.306	0.194	8.28172380323453E-23	1	YKT6
**3.51117529283319E-19**	0.722026993354069	0.333	0.241	5.72707802014021E-15	1	ERBIN
**2.47838645972248E-57**	0.720494694962171	0.539	0.353	4.04249615445334E-53	1	FBXW4
**2.4180583583257E-11**	0.71897840138746	0.291	0.227	3.94409498826505E-07	1	ITGAV
**2.2220644035928E-11**	0.718970743111471	0.269	0.208	3.62440924870021E-07	1	MARCH5
**1.47150069358239E-12**	0.718792030713534	0.261	0.193	2.40016478130224E-08	1	TNFRSF21
**7.26270493063401E-20**	0.718742244813153	0.338	0.246	1.18461980123571E-15	1	CLCC1
**4.42988532041677E-23**	0.718732337912536	0.328	0.225	7.22558594613179E-19	1	FBRS
**3.64268058771238E-20**	0.71788001768495	0.374	0.279	5.94157630661767E-16	1	SORL1
**7.28095210605217E-12**	0.717670409434256	0.254	0.189	1.18759609801817E-07	1	MAEA
**2.02305419296233E-22**	0.717343680078894	0.306	0.205	3.29980369414086E-18	1	MTOR
**4.43116277279533E-25**	0.717216597556451	0.355	0.245	7.22766959870646E-21	1	RFX5
**2.21474491142443E-18**	0.716881614776866	0.256	0.17	3.61247042502439E-14	1	CTDNEP1
**2.66564893189336E-13**	0.716538144388865	0.268	0.198	4.34793997281127E-09	1	NR3C1
**1.93444467271171E-18**	0.714702928275903	0.275	0.188	3.15527270566008E-14	1	CREBBP
**4.50721391277553E-12**	0.714644035766353	0.258	0.193	7.35171661312816E-08	1	NAP1L4
**7.82253414787276E-19**	0.713715045811671	0.269	0.18	1.27593354485953E-14	1	PHF10
**4.94913955325139E-83**	0.712366243708396	0.8	0.562	8.07254152530835E-79	1	HSD17B4
**5.90720717729869E-31**	0.712261228574006	0.47	0.34	9.6352456268919E-27	1	CYP4F3
**9.25668902776149E-24**	0.711090164306291	0.323	0.218	1.50985854731818E-19	1	ABTB1
**3.58979298797178E-35**	0.709468458347525	0.38	0.243	5.85531134268077E-31	1	PUS1

8.71676762668183E-20	0.709275950570311	0.364	0.272	1.42179196758807E-15	1	PDCD6
8.52617557502291E-29	0.709065167623292	0.422	0.298	1.39070449804199E-24	1	PRPSAP1
6.60397574035673E-70	0.707298099005652	0.818	0.566	1.07717448300959E-65	1	HGD
3.41802742155884E-17	0.707017300158045	0.265	0.181	5.57514452730463E-13	1	STRN4
6.24376484474299E-23	0.70689922381297	0.325	0.222	1.01842048382603E-18	1	KLHL36
4.24055914922061E-72	0.706412843053662	0.776	0.538	6.91677602829373E-68	1	PNPLA2
2.18024730648041E-45	0.705720209479714	0.527	0.358	3.5562013816002E-41	1	RGS14
4.00537878896543E-30	0.70482198103639	0.426	0.299	6.53317334268151E-26	1	NAGS
3.93351041047336E-11	0.704695184044657	0.283	0.222	6.41594883052309E-07	1	RALGPS2
1.26183811607877E-32	0.704679651763405	0.408	0.275	2.05818415113609E-28	1	ATP9A
1.02201784116976E-10	0.704424977131971	0.254	0.195	1.66701330073199E-06	1	ETFDH
1.30017319000659E-23	0.703674373498599	0.328	0.221	2.12071249021976E-19	1	GNAQ
6.56919422699349E-20	0.702922412019901	0.291	0.2	1.07150127036491E-15	1	ATIC
2.32928135904632E-90	0.701674245682624	0.9	0.633	3.79929082474046E-86	1	PAH
3.19310595406017E-69	0.7014197181604	0.669	0.463	5.20827512166755E-65	1	INF2
6.44642385139767E-18	0.70029369613977	0.336	0.248	1.05147619440147E-13	1	KCTD20
1.56453707867314E-17	0.700227905305333	0.278	0.195	2.55191642902376E-13	1	RNF44
1.72310331680186E-35	0.700156205776477	0.633	0.48	2.81055382003552E-31	1	LEAP2
3.07305348859247E-21	0.699499753555085	0.377	0.28	5.01245754524318E-17	1	MAP2K3
4.45987511338754E-26	0.699157042899618	0.287	0.179	7.27450229744642E-22	1	BAZ1B
2.5693462337018E-15	0.699001908286529	0.268	0.192	4.19086064179101E-11	1	USP19
2.42476866673447E-17	0.698983125501896	0.334	0.247	3.9550401723106E-13	1	PRPF40A
7.3192015281898E-53	0.698612078974938	0.613	0.425	1.19383496126304E-48	1	SELENOO
3.42105550068572E-20	0.69738241519277	0.309	0.214	5.58008362716847E-16	1	QRICH1
4.30383124841344E-64	0.697246667302147	0.689	0.478	7.01997914928716E-60	1	ESRP2
2.9454716349616E-23	0.696781610381945	0.358	0.254	4.80435878378586E-19	1	KHNYN
2.08115068492471E-78	0.696259071774439	0.735	0.511	3.39456488218069E-74	1	SHC1
4.03528903377113E-17	0.695428807738232	0.331	0.244	6.58195994298409E-13	1	RAP1GAP
1.02707505319952E-26	0.693183481566493	0.353	0.239	1.67526211927373E-22	1	OTUD5
3.74051205570011E-29	0.692745917939241	0.341	0.222	6.10114921405246E-25	1	PAXX
1.3943264793463E-51	0.692733268565137	0.58	0.403	2.27428592046175E-47	1	APOL2
2.43186340191184E-21	0.692687087248616	0.389	0.279	3.9666123948584E-17	1	CHRD
5.13944025095456E-22	0.692594938822586	0.353	0.25	8.38294099333197E-18	1	CIAO1
5.28417797606272E-25	0.691791239576221	0.353	0.246	8.6190226967559E-21	1	TIAL1
6.01044900898076E-36	0.691672737149706	0.455	0.314	9.80364337854851E-32	1	IGF2R
5.30656950406509E-34	0.691630312951923	0.451	0.317	8.65554551808057E-30	1	ATF4
1.40417963973356E-39	0.691371817735798	0.451	0.304	2.29035741036941E-35	1	SAFB
1.16324394525396E-18	0.691364477539846	0.34	0.251	1.89736719910374E-14	1	COBL
2.80418039800356E-35	0.689925772613301	0.457	0.317	4.5738986471836E-31	1	PHC2
1.7366830888846E-14	0.689394464942642	0.263	0.189	2.83270378627966E-10	1	RABEP1
2.76982767547173E-37	0.688803453816229	0.366	0.223	4.51786592146195E-33	1	PWWP2B
7.53129481697557E-24	0.688750885790338	0.26	0.156	1.22842949759688E-19	1	SPIRE2
9.88711883386556E-47	0.687317248135207	0.58	0.408	1.61268795299181E-42	1	LPIN2
6.32571442683332E-22	0.686292236986395	0.314	0.212	1.03178728016078E-17	1	PLEKHG6
3.35330737954372E-20	0.686059938478825	0.327	0.221	5.46957966677375E-16	1	IER3
4.66137622868334E-44	0.685455637533973	0.608	0.412	7.6031707666054E-40	1	HAL
2.64896889842626E-16	0.685270864169909	0.32	0.237	4.32073317022307E-12	1	TTC17
1.04782766316161E-31	0.68452714852536	0.467	0.333	1.70911170138291E-27	1	SLC43A1
4.12018813546345E-26	0.68445128391032	0.343	0.23	6.72043886775443E-22	1	FAAH
4.75620299718658E-27	0.684272575989304	0.485	0.366	7.75784270871103E-23	1	AIG1

8.43438613387597E-10	0.683601230328013	0.361	0.304	1.37573272229651E-05	1	ACSM5
4.09011144143907E-28	0.68329829090121	0.436	0.315	6.67138077213127E-24	1	UBA5
1.58091643828861E-16	0.682981145547383	0.25	0.168	2.57863280249256E-12	1	TICAM1
9.2020152070568E-18	0.68274637166395	0.324	0.236	1.50094070042303E-13	1	MAPK6
1.62096516916671E-16	0.682720862737724	0.42	0.331	2.64395628742782E-12	1	GADD45A
4.75966040140521E-11	0.682618529781685	0.26	0.202	7.76348208073203E-07	1	TMEM220
2.29323262222517E-20	0.682600391777091	0.274	0.181	3.74049173011147E-16	1	ZNHIT6
1.66886435474083E-36	0.682529752364331	0.465	0.328	2.72208464901776E-32	1	USP9X
3.59709191165504E-38	0.681867219819346	0.466	0.321	5.86721661710054E-34	1	SPSB3
1.53414160672624E-32	0.681232909910027	0.43	0.297	2.50233837473117E-28	1	ASPSCR1
1.35444826319839E-19	0.68036779015317	0.31	0.216	2.20924056210289E-15	1	TPD52L2
3.28077363870045E-13	0.679894792232708	0.3	0.23	5.3512698820843E-09	1	BTNL8
4.47195783746132E-17	0.67933192975701	0.287	0.204	7.29421042868317E-13	1	SETD3
2.30126862165445E-22	0.678973040064757	0.355	0.251	3.75359924878057E-18	1	CDC42BPB
1.75624983436102E-18	0.678399351055675	0.257	0.171	2.86461910482626E-14	1	CD40
9.87277854374463E-53	0.67825806334657	0.492	0.311	1.61034890827019E-48	1	POGK
7.67133576735314E-27	0.678195172172973	0.448	0.329	1.25127157701297E-22	1	SEMA4G
3.63183397795746E-23	0.677230955002648	0.368	0.262	5.92388440144641E-19	1	ONECUT2
6.09958677618256E-21	0.67670808896297	0.306	0.211	9.94903599063138E-17	1	SMYD2
1.62116049219655E-53	0.676006338558829	0.592	0.407	2.64427487882179E-49	1	MIDN
2.92818952074989E-33	0.675791660787787	0.492	0.356	4.77616992729515E-29	1	PNRC1
3.37179956011265E-19	0.6748643925874	0.332	0.239	5.49974226249975E-15	1	GRIPAP1
8.15107538497163E-44	0.674753340437191	0.579	0.413	1.32952190604272E-39	1	CLDN7
5.44185402118194E-22	0.674199613441426	0.369	0.265	8.87620809394986E-18	1	KLC4
2.52382579700464E-21	0.67401655101511	0.449	0.34	4.11661225749427E-17	1	AHR
1.84407112096719E-24	0.673608320921613	0.316	0.207	3.00786440540958E-20	1	TESK1
1.84794808435157E-18	0.673504620030112	0.334	0.243	3.01418812038585E-14	1	STX4
9.9218256265656E-17	0.673375520309932	0.275	0.192	1.61834897794912E-12	1	DPH2
9.210610256977E-14	0.673327293458835	0.251	0.18	1.50234263901552E-09	1	SHLD2
9.77837840897082E-18	0.67263914634414	0.31	0.225	1.59495130228723E-13	1	THNSL2
3.8478531372643E-24	0.672427634289614	0.341	0.232	6.27623325219181E-20	1	MBTPS1
6.42130159574069E-20	0.670005406303469	0.384	0.287	1.04737850328126E-15	1	SREBF2
1.18172787033694E-15	0.667007467170394	0.313	0.234	1.92751632930658E-11	1	EMC2
4.79203692724344E-40	0.666298766486266	0.511	0.362	7.81629143202677E-36	1	PJA2
1.65937275198071E-12	0.66615089133458	0.253	0.187	2.70660289575574E-08	1	EXOSC7
2.42663266874291E-19	0.666064445812415	0.355	0.258	3.95808054598656E-15	1	DNAJC5
5.51802181500798E-19	0.666024687543728	0.302	0.213	9.00044538245951E-15	1	GTPBP4
4.54493571770102E-18	0.664171211528118	0.417	0.328	7.41324464914214E-14	1	TNFSF10
1.28907512350777E-17	0.66367901047297	0.324	0.24	2.10261043395352E-13	1	STX5
6.23343199506006E-12	0.662342882883625	0.281	0.214	1.01673509271425E-07	1	NARF
1.10017247870517E-14	0.661536755891355	0.28	0.204	1.794491330016E-10	1	MLX
1.48038194896379E-31	0.660695603214522	0.436	0.303	2.41465099695483E-27	1	SYNPO
1.92843284411853E-19	0.660284129138739	0.354	0.259	3.14546681204174E-15	1	TNRC18
1.17837720074922E-38	0.659366186434549	0.538	0.382	1.92205105214205E-34	1	ACOX2
7.01036969812406E-10	0.658809415729108	0.252	0.195	1.14346140146101E-05	1	SRP19
1.05253366742064E-14	0.6583517404956	0.278	0.203	1.7167876649298E-10	1	CC2D1A
4.61848797836117E-44	0.657891442833556	0.527	0.366	7.5332157415049E-40	1	CLINT1
2.5492561077237E-19	0.655452450861552	0.33	0.235	4.15809163730813E-15	1	ZMAT3
1.08504434028665E-29	0.653690271932416	0.364	0.238	1.76981582344155E-25	1	LZTS2
1.02728027176784E-69	0.653168229973025	0.671	0.458	1.67559685128052E-65	1	ADAR

3.39387617809128E-12	0.653126878793495	0.318	0.249	5.53575143408469E-08	1	UNC5CL
5.78981609204313E-33	0.652222042075154	0.456	0.322	9.44376902773154E-29	1	PTPRS
2.69025486917114E-29	0.65214128624537	0.39	0.254	4.38807471710504E-25	1	ID1
1.66992810622834E-14	0.651633358309192	0.273	0.199	2.72381973406904E-10	1	PGAP2
4.45093705260041E-13	0.651586730561565	0.285	0.216	7.25992342649652E-09	1	TSC2
4.87300844541894E-13	0.649959504859235	0.258	0.19	7.94836407532283E-09	1	MSL1
3.6713843553101E-10	0.648898527550991	0.251	0.193	5.9883950219463E-06	1	MXI1
1.1972201245811E-31	0.647644449985748	0.383	0.256	1.95278574520423E-27	1	ZNF316
2.90068840995248E-30	0.645899286129331	0.451	0.323	4.73131286547349E-26	1	OGFR
1.92613057043409E-21	0.644098077096132	0.329	0.229	3.14171157343505E-17	1	DYNC1I2
1.14783116740896E-21	0.643782767908539	0.383	0.278	1.87222741716075E-17	1	MAP4K4
1.66203371731274E-26	0.64369513840238	0.39	0.273	2.7109431963088E-22	1	ACTR1B
1.45143659426027E-14	0.643351497381138	0.302	0.23	2.36743822889792E-10	1	ITPRID2
1.01680921381038E-27	0.643082656504019	0.457	0.333	1.65851750864611E-23	1	PDCD4
4.71370443428757E-29	0.642799967587086	0.489	0.361	7.68852330276645E-25	1	DCAF6
6.91707364085316E-16	0.641025697447651	0.3	0.219	1.12824388155956E-11	1	ERBB2
7.38660589779834E-29	0.640463672287765	0.455	0.33	1.20482928798989E-24	1	CIB1
4.79703062516352E-45	0.63938622323493	0.614	0.443	7.82443665270422E-41	1	LONP2
1.26281271663619E-30	0.639082747131659	0.4	0.271	2.05977382210529E-26	1	RHPN2
1.99129463967898E-56	0.638824115920252	0.828	0.587	3.24800068678038E-52	1	GLYCTK
8.07588973948984E-18	0.638623676433827	0.273	0.186	1.31725837540819E-13	1	CBFB
1.52717692137966E-22	0.637664676954473	0.313	0.209	2.49097827646236E-18	1	MBD6
1.27850435639438E-65	0.637459977090754	0.681	0.469	2.08536845571487E-61	1	CTNND1
3.40386479384008E-39	0.635932056361762	0.44	0.29	5.55204386523255E-35	1	RBM3
1.04978844187129E-13	0.635775390142654	0.331	0.256	1.71230992753626E-09	1	DHRS1
2.09001404306425E-55	0.635561729184979	0.536	0.338	3.40902190564209E-51	1	AJUBA
3.55176794875348E-18	0.635551076983981	0.342	0.254	5.7932887012118E-14	1	IDH3B
4.50201734716719E-33	0.634781286280451	0.47	0.331	7.3432404949644E-29	1	SCRIB
6.2777362248266E-42	0.634548386891621	0.538	0.383	1.02396155563147E-37	1	SUPT5H
2.89144817254637E-18	0.633590084656491	0.405	0.313	4.71624111424038E-14	1	PPARA
1.96285969781342E-18	0.632685076985668	0.288	0.199	3.20162045310347E-14	1	GTPBP10
5.71533766776989E-20	0.6323203156162	0.372	0.276	9.32228726989947E-16	1	GAK
4.26824139072267E-19	0.63211044480768	0.352	0.262	6.96192853240774E-15	1	ARHGAP35
5.89383825570445E-25	0.631661281778539	0.455	0.342	9.61343957887953E-21	1	PGPEP1
1.48447961885225E-17	0.63057223660208	0.302	0.216	2.42133470630991E-13	1	MED28
1.20621093607843E-26	0.629778447748797	0.44	0.324	1.96745065783753E-22	1	VPS52
1.55019617037285E-17	0.627788721967839	0.3	0.214	2.52852497349515E-13	1	TP53I13
1.69198090464641E-26	0.627754143566338	0.389	0.273	2.75979005356877E-22	1	TBC1D17
1.25798310021797E-30	0.627693784449591	0.459	0.331	2.05189623476553E-26	1	HERPUD1
1.08187718355832E-33	0.62754450643902	0.477	0.344	1.76464987410197E-29	1	TFG
3.10023539160946E-60	0.626873224838417	0.666	0.473	5.05679394725419E-56	1	KIAA1522
2.60671450191954E-33	0.626247883694184	0.396	0.259	4.25181202408097E-29	1	QKI
4.2344338427076E-17	0.626000337566662	0.269	0.184	6.90678504084037E-13	1	SEPTIN11
4.78673453644454E-27	0.625956226258179	0.433	0.317	7.80764270239469E-23	1	PPP2R5A
2.3103051075847E-14	0.625863723541064	0.282	0.206	3.76833866098141E-10	1	NUDT19
1.39012047332601E-15	0.62563572453741	0.311	0.231	2.26742550404205E-11	1	ACVR1B
1.49253811065861E-30	0.625416194323901	0.641	0.488	2.43447891229526E-26	1	PTGR1
7.19502819087268E-16	0.623096779582405	0.303	0.222	1.17358104821324E-11	1	COPS5
3.03600817214733E-15	0.62308737894464	0.264	0.186	4.9520329295895E-11	1	RAB6A
6.28371784009378E-16	0.62277644193193	0.256	0.177	1.0249372168977E-11	1	FOXK1

4.51287851571282E-42	0.622544910410608	0.437	0.279	7.36095614697918E-38	1	CIC
6.65904285809977E-20	0.621160550599354	0.321	0.227	1.08615648058465E-15	1	NFKBIL1
1.88535086191328E-19	0.619707269205262	0.313	0.222	3.07519579086676E-15	1	GMDS
7.40328091166129E-17	0.619137703920208	0.348	0.264	1.20754914950107E-12	1	PDCD6IP
2.59318398831139E-48	0.618463564593651	0.623	0.445	4.2297424033347E-44	1	CIRBP
2.78274837426906E-12	0.618134642046095	0.301	0.233	4.53894087327027E-08	1	CDC5L
1.07683856740042E-16	0.617607930451829	0.336	0.252	1.75643138728683E-12	1	SEC63
8.85650928166107E-21	0.617082943029714	0.289	0.193	1.44458522893174E-16	1	PLEKHM2
9.56808478273776E-14	0.616975339820063	0.259	0.185	1.56065030891236E-09	1	USO1
1.68503156533515E-15	0.616938011192069	0.285	0.207	2.74845498621817E-11	1	CACUL1
3.08058980081908E-53	0.616596203081147	0.7	0.494	5.02475002411601E-49	1	SLC27A3
6.24890162209739E-20	0.615872632457676	0.35	0.254	1.0192583435803E-15	1	GOLGB1
1.3937759028737E-14	0.612323437737621	0.376	0.297	2.27338787517729E-10	1	SORBS2
1.11132571383405E-12	0.611597060970122	0.301	0.232	1.81268337183471E-08	1	GOLGA5
2.58706956638935E-19	0.611389395468644	0.386	0.293	4.21976916973766E-15	1	ITFG1
1.44890712101092E-49	0.61089573591118	0.675	0.465	2.36331240508092E-45	1	ACSM2A
1.85769914261305E-17	0.609908496053684	0.344	0.256	3.03009307151615E-13	1	CERS4
2.84632603929184E-22	0.606175980540063	0.439	0.332	4.64264240268892E-18	1	SYMPK
4.5538334050117E-39	0.603198186088183	0.624	0.452	7.42775766691458E-35	1	MST1
1.41712000303109E-17	0.602616110776501	0.254	0.169	2.31146443694402E-13	1	MLLT6
3.65340345976753E-29	0.601965308773749	0.415	0.291	5.95906638322682E-25	1	PTTG1IP
4.68183957090598E-16	0.600447069778604	0.29	0.209	7.63654852410475E-12	1	TERF2IP
1.12970489478681E-14	0.599231205945603	0.273	0.197	1.84266165388676E-10	1	FBXW2
6.82237928343096E-25	0.599084253746139	0.386	0.276	1.11279828492042E-20	1	TBCD
1.06896831122649E-11	0.599036241570005	0.282	0.217	1.74359421244153E-07	1	BCKDHB
2.22839775340057E-39	0.598535293376047	0.545	0.391	3.63473957557167E-35	1	PPP1R15B
2.99457877151327E-55	0.59617083228736	0.795	0.562	4.8844574342153E-51	1	ITIH4
3.6112421114519E-12	0.596033631004005	0.282	0.218	5.8902970079892E-08	1	SLC35C2
1.40601688181238E-18	0.595909532419577	0.377	0.286	2.29335413592417E-14	1	DENND10
5.41574783579301E-13	0.595703787537311	0.257	0.187	8.83362629496197E-09	1	SNX27
3.41699192315855E-31	0.595138458223198	0.405	0.277	5.57345552586391E-27	1	HNRNPC
2.31983624784511E-16	0.594986419719555	0.287	0.204	3.78388490386016E-12	1	TRA2B
7.26424563230591E-19	0.594764000092426	0.358	0.266	1.18487110508542E-14	1	PICALM
2.23402483050849E-20	0.592931598033085	0.306	0.211	3.64391790104239E-16	1	XPNPEP1
7.32631438598243E-32	0.592588283304636	0.43	0.3	1.19499513949759E-27	1	IL17RC
1.6323836371411E-20	0.591610145217523	0.361	0.266	2.66258095054085E-16	1	NOTCH2NL
2.86206248994829E-23	0.590862422540939	0.4	0.288	4.66831012735466E-19	1	NDUFAF8
1.53098168086088E-32	0.590470499301362	0.451	0.317	2.49718421965218E-28	1	DENND11
1.12468930727273E-18	0.590331301493522	0.319	0.228	1.83448072909255E-14	1	ABCF3
2.19751492486257E-30	0.58975185219808	0.436	0.305	3.58436659394334E-26	1	LAMB2
1.30072309032149E-36	0.589324827697886	0.528	0.377	2.12160943262338E-32	1	HM13
5.99472167895262E-27	0.588820677957476	0.45	0.334	9.77799053053962E-23	1	DVL3
4.94756081733131E-24	0.587527675633538	0.383	0.274	8.0699664491491E-20	1	NAA60
2.5720017721647E-16	0.584459987994381	0.259	0.18	4.19519209057784E-12	1	FXR2
4.7468748543991E-12	0.584393949249926	0.278	0.21	7.74262757501037E-08	1	C2orf68
2.71648233275187E-15	0.583478612604662	0.258	0.181	4.43085433295158E-11	1	RNF123
1.53573208314443E-09	0.583318003915679	0.319	0.266	2.50493260081688E-05	1	ELMO3
4.25454891371207E-16	0.582673857765858	0.275	0.194	6.93959473315576E-12	1	ADAMTSL4
2.7747875108007E-39	0.581493594121065	0.511	0.36	4.52595590886703E-35	1	ATRN
4.83881332789155E-30	0.581391486478661	0.428	0.302	7.8925884191239E-26	1	MED10

8.35845704588651E-19	0.581078199407849	0.333	0.243	1.36334792875455E-14	1	MTA1
2.09866511915162E-54	0.580929324827666	0.666	0.471	3.42313267584821E-50	1	COPB2
5.55108136685769E-19	0.580597130601274	0.425	0.329	9.05436881748157E-15	1	ALDH5A1
8.35940522767154E-12	0.579670830579079	0.304	0.237	1.3635025866855E-07	1	KCMF1
6.12304734691599E-43	0.579101267482748	0.572	0.408	9.98730252755467E-39	1	PIM3
1.6916396947758E-16	0.578880422735955	0.297	0.214	2.7592335061488E-12	1	PITPNM1
1.26920646334221E-39	0.578352353949611	0.518	0.364	2.07020266235748E-35	1	RREB1
2.69021595379991E-10	0.577725442638201	0.282	0.221	4.38801124224303E-06	1	NOP14
7.71454036177116E-12	0.577623784104229	0.304	0.237	1.25831867840849E-07	1	TLE1
2.30044584225457E-11	0.577274656371167	0.304	0.241	3.75225721330142E-07	1	TOP 1.00
2.88544239709034E-15	0.576852485667404	0.337	0.26	4.70644509389405E-11	1	RARS
1.27041215684329E-39	0.575827025731675	0.62	0.452	2.07216926902709E-35	1	STAT1
3.42417714101657E-38	0.575716187935639	0.495	0.349	5.58517533471213E-34	1	CSNK1D
3.03205617105909E-21	0.575572734028596	0.349	0.251	4.94558682061448E-17	1	TMCO3
1.44021652970277E-17	0.574565626234349	0.26	0.175	2.34913718159819E-13	1	TAOK3
3.19305039556025E-10	0.573853184424498	0.26	0.201	5.20818450019832E-06	1	CRADD
1.48484615965304E-27	0.573851106646242	0.449	0.328	2.42193257101008E-23	1	UBC
4.12617696983459E-35	0.573569715554438	0.51	0.367	6.7302072554972E-31	1	LLGL2
5.86873336543077E-30	0.57279509842538	0.449	0.32	9.57249099235414E-26	1	PARP9
2.65822093122575E-13	0.572342536454508	0.268	0.196	4.33582416092232E-09	1	FOXO3
1.02980117312203E-32	0.571431934022587	0.465	0.332	1.67970869347935E-28	1	CAMSAP3
3.1902628322165E-21	0.571319991222537	0.33	0.229	5.20363770562833E-17	1	ZNRF1
1.06929124820202E-12	0.571249595085961	0.309	0.241	1.74412095494232E-08	1	NDUFV3
2.65377833537716E-37	0.571121023917636	0.607	0.451	4.32857784283369E-33	1	MASP1
6.9104025755278E-13	0.571072197187835	0.256	0.187	1.12715576409434E-08	1	SOCS6
3.52044291995873E-19	0.570806724299751	0.367	0.276	5.74219444674469E-15	1	NELFCD
3.72011095191748E-15	0.570411773865126	0.324	0.245	6.06787297367261E-11	1	AGGF1
2.13140317332313E-17	0.568613857746968	0.369	0.282	3.47653171600736E-13	1	NONO
1.05765876817716E-58	0.568157620277387	0.645	0.443	1.72514721677377E-54	1	SPATS2L
1.88020116116983E-29	0.568069752308311	0.504	0.37	3.06679611398412E-25	1	PTPRH
8.48384571268051E-32	0.567943376837372	0.47	0.338	1.38380007419532E-27	1	DECR2
4.17332517749253E-15	0.567149308281901	0.386	0.299	6.80711069700807E-11	1	SLC25A18
9.36190248549391E-36	0.56584168116224	0.568	0.419	1.52701991440891E-31	1	KLKB1
4.53524629902657E-14	0.565339885928999	0.286	0.211	7.39744023834223E-10	1	NUDT5
8.4158043758501E-59	0.565063904787043	0.727	0.523	1.37270185174491E-54	1	INSR
3.58349588084235E-16	0.564691670201936	0.322	0.239	5.84504013124195E-12	1	ATN1
1.95878996057552E-17	0.564362342147671	0.275	0.188	3.19498230469473E-13	1	SLK
3.85065103751111E-25	0.56259240754717	0.4	0.291	6.28079690728437E-21	1	HMGN1
5.6404444374565E-12	0.561924823348554	0.274	0.208	9.20012892193529E-08	1	HNMT
7.71838557849273E-24	0.561279400625299	0.421	0.314	1.25894587170795E-19	1	FLOT1
3.9456376746924E-38	0.561242231383432	0.541	0.381	6.43572961119077E-34	1	IER2
2.4121101294263E-22	0.560902621008114	0.384	0.281	3.93439283210724E-18	1	ABCA2
2.3530396753763E-25	0.559117880425246	0.523	0.399	3.83804301450629E-21	1	HSPD1
3.06497677489685E-13	0.557179437742371	0.281	0.21	4.99928361753425E-09	1	CDK9
3.38939342070425E-28	0.55620101033354	0.422	0.305	5.52843960851071E-24	1	MPPE1
4.70099346972611E-10	0.556023769975556	0.304	0.24	7.66779044847026E-06	1	ASPG
6.2406108807093E-23	0.555984652652117	0.38	0.274	1.01790604075249E-18	1	ZCCHC14
1.00050152488306E-29	0.554810315429463	0.375	0.25	1.63191803723676E-25	1	SBNO2
1.16207275648899E-10	0.553738415826121	0.265	0.205	1.89545687310918E-06	1	SRP72
1.25886438040135E-19	0.552863387542233	0.418	0.319	2.05333369087264E-15	1	ABCD3

3.27338997662061E-24	0.551948378247341	0.349	0.237	5.33922639086588E-20	1	ZMIZ1
5.29930319685156E-64	0.551634290778076	0.681	0.47	8.64369344438457E-60	1	TMEM259
2.45995824470121E-15	0.549957504953487	0.255	0.175	4.01243789293214E-11	1	WDR74
2.44111543112075E-64	0.548621495349115	0.799	0.595	3.98170337970106E-60	1	AGRN
7.74358748142688E-24	0.548438954066133	0.487	0.371	1.26305655409554E-19	1	TGFBI
1.1796952818388E-30	0.548283844571879	0.442	0.306	1.92420097420727E-26	1	IER5
4.47525915279273E-21	0.546289460215325	0.371	0.269	7.29959520412022E-17	1	ENTPD6
4.08918519291062E-16	0.542660071950947	0.303	0.218	6.66986996815652E-12	1	EML2
2.99153758300466E-39	0.54211639841445	0.799	0.658	4.8794969516389E-35	1	GDF15
5.46713877862029E-10	0.541625835467568	0.251	0.191	8.91745006180755E-06	1	KDM3B
1.37089204772489E-14	0.541113857572635	0.265	0.19	2.23606201904408E-10	1	SNX9
2.77824099428785E-36	0.541096664998973	0.467	0.322	4.53158888578291E-32	1	PHRF1
1.18704683336563E-25	0.540871866069299	0.387	0.27	1.93619208990268E-21	1	MED25
1.00613514024714E-09	0.539237716405308	0.32	0.263	1.64110702725711E-05	1	PITRM1
1.32873751005293E-30	0.538093332210377	0.508	0.376	2.16730375264733E-26	1	HBS1L
6.47228158113367E-13	0.537779112023725	0.311	0.239	1.05569384869871E-08	1	ZFAND2A
1.6420195677029E-25	0.537491022634702	0.43	0.316	2.6782981168802E-21	1	PYGB
5.39703897532141E-41	0.536752164308492	0.556	0.395	8.80311027264676E-37	1	TNFRSF12A
1.53235871040931E-27	0.534891098875496	0.484	0.363	2.49943029254863E-23	1	HSPE1
1.74064191922883E-27	0.533083313790389	0.421	0.301	2.83916103445414E-23	1	FBXL6
7.57997364307992E-25	0.532415159254861	0.338	0.223	1.23636950092277E-20	1	PLPP1
6.07315875711359E-34	0.531716624936895	0.498	0.359	9.90592924872797E-30	1	UBE2B
8.25392511423856E-08	0.531242336280902	0.297	0.25	0.00134629772538345	1	PCMTD1
7.82187618644032E-25	0.530142016567372	0.44	0.325	1.27582622477028E-20	1	HMGCS1
1.65646680585838E-13	0.53007312596695	0.269	0.197	2.7018630070356E-09	1	POLDIP3
1.56560198507756E-61	0.528668430062323	0.748	0.55	2.55365339786001E-57	1	ZBTB7B
4.38076026991077E-13	0.528362808805562	0.34	0.265	7.14545807625145E-09	1	MGRN1
2.67281183231796E-09	0.528298594457928	0.294	0.237	4.35962337969382E-05	1	MARVELD2
2.29406881652974E-09	0.528275615282345	0.293	0.237	3.74185564664166E-05	1	FAM120A
2.97201770662529E-09	0.526243946602717	0.322	0.259	4.84765808127651E-05	1	TRIM55
1.50310218690859E-14	0.525633932002151	0.253	0.179	2.4517099770666E-10	1	BTF3L4
9.67183839061768E-27	0.525351164450928	0.408	0.29	1.57757355989365E-22	1	RNF103
7.16307829859022E-11	0.524843417535934	0.263	0.202	1.16836970128305E-06	1	MAN1A2
1.13383959567059E-12	0.524180477995702	0.374	0.298	1.84940576449829E-08	1	DHODH
1.52052353273054E-25	0.524011301304079	0.41	0.296	2.48012593423679E-21	1	CAST
6.78876260247656E-20	0.523586168473778	0.328	0.231	1.10731506808995E-15	1	BAX
3.21530233848814E-27	0.522599142524264	0.517	0.386	5.24447964430801E-23	1	C1RL
1.74072408163719E-23	0.52124466499027	0.392	0.283	2.83929504955842E-19	1	HDAC5
9.27890203312991E-21	0.521041544484918	0.298	0.2	1.51348171062382E-16	1	RAB11FIP1
5.93401120242463E-28	0.519317047836121	0.497	0.367	9.67896567227482E-24	1	SIK2
7.19187513647652E-17	0.518503321747159	0.403	0.313	1.17306675351068E-12	1	GCLM
6.6441110214087E-32	0.517586966418655	0.508	0.369	1.08372094870197E-27	1	EDEM3
1.30976728770738E-09	0.516900872158487	0.263	0.206	2.13636142297951E-05	1	CBLC
3.215049973801E-13	0.516428140684065	0.314	0.242	5.2440680122668E-09	1	SPNS1
8.4575336875069E-17	0.514816562589701	0.79	0.68	1.37950831976925E-12	1	HSPB1
1.41739556471129E-17	0.514714443948726	0.325	0.235	2.31191390560059E-13	1	MAP2K7
4.54587517326458E-39	0.513678813032191	0.553	0.399	7.41477699511186E-35	1	DBNL
1.37645494368771E-07	0.513463809496532	0.25	0.203	0.00224513565864902	1	C19orf25
3.03579984055971E-29	0.513203733012843	0.456	0.325	4.95169311993694E-25	1	GALE
3.19846410406905E-29	0.51183863719578	0.392	0.266	5.21701480014703E-25	1	FUS

2.11485609024148E-19	0.510823103016857	0.436	0.337	3.44954176879288E-15	1	COBLL1
1.2245894864024E-20	0.510750405807845	0.352	0.247	1.99742791127095E-16	1	CBX4
2.33424253689835E-11	0.51073070824032	0.265	0.198	3.8073830019349E-07	1	PLEKHJ1
1.48438531979483E-17	0.510052022826362	0.291	0.191	2.42118089511734E-13	1	DEFB1
4.05463122369109E-18	0.509234701055578	0.309	0.221	6.61350898896253E-14	1	SERPINB6
5.86144390963737E-12	0.508172193812311	0.299	0.232	9.56060116100952E-08	1	GNG12
7.06803949860406E-08	0.5080117937989	0.285	0.236	0.00115286792261731	1	SLC6A12
4.58363566496891E-21	0.50778280400156	0.449	0.348	7.47636813313079E-17	1	AP1M1
1.78651174200835E-13	0.50695710652738	0.392	0.308	2.91397930238982E-09	1	FST
1.84135305114454E-15	0.50386613483633	0.302	0.221	3.00343096172186E-11	1	TUBGCP2
4.56720599901654E-20	0.503207123064112	0.303	0.205	7.44956970499588E-16	1	MFHAS1
2.78353494414337E-10	0.503113910159861	0.277	0.217	4.54022384739224E-06	1	IDH3G
1.54777332691121E-45	0.502712953060924	0.627	0.45	2.52457307352487E-41	1	HNRNPA2E
4.44177749179611E-13	0.501488303985152	0.369	0.291	7.24498326686863E-09	1	SLC25A25
4.26377276709026E-41	0.497046175260388	0.546	0.354	6.95463976040093E-37	1	G0S2
1.25527983603929E-06	0.495808708142282	0.258	0.216	0.0204748694056369	1	PSD4
8.94744764135699E-12	0.494727213367613	0.285	0.221	1.45941818478174E-07	1	ATP2C1
1.34693565257381E-09	0.494519054532558	0.377	0.317	2.19698674291314E-05	1	CYP4F11
4.33283118121825E-19	0.494369901147392	0.289	0.194	7.06728093968509E-15	1	MAFB
2.25476044878644E-15	0.491272832110038	0.32	0.239	3.67773976801556E-11	1	ZFAND2B
2.06086344867036E-21	0.49119865607111	0.406	0.303	3.36147437112622E-17	1	AUP1
3.50351516901885E-13	0.490553514301072	0.316	0.241	5.71458359218665E-09	1	RRP1
1.15235944526817E-06	0.48943405262972	0.269	0.224	0.0187961349117691	1	NAXD
5.69179122699342E-26	0.487953982507409	0.409	0.29	9.28388067034897E-22	1	CCDC9
1.28329266842467E-07	0.487669756375826	0.259	0.211	0.00209317867146747	1	PQBP1
4.71004331264826E-22	0.487130874826027	0.394	0.292	7.68255164726058E-18	1	RASSF7
8.97130748926602E-19	0.484696175171729	0.375	0.278	1.46330996457418E-14	1	CAPN5
7.19409941087731E-20	0.484559698164814	0.337	0.24	1.1734295549082E-15	1	ARFGAP3
5.88816902873261E-12	0.483532419074784	0.316	0.248	9.60419250276575E-08	1	LIN7C
3.43327821290697E-12	0.482858929561005	0.297	0.228	5.60002009307255E-08	1	LDLRAP1
5.21963647531554E-09	0.482128100947495	0.257	0.206	8.51374905488718E-05	1	TMEM248
3.65497111979452E-40	0.481728008408735	0.548	0.388	5.96162339349684E-36	1	NARS
1.75850884717355E-16	0.481574805440349	0.451	0.362	2.86830378062478E-12	1	NMD3
2.59614902828553E-17	0.481392535851	0.378	0.289	4.23457868003653E-13	1	LIX1L
2.4628726734218E-23	0.480975286637302	0.5	0.386	4.01719161761829E-19	1	DPP9
8.70843299874545E-21	0.480112307972819	0.4	0.304	1.42043250642537E-16	1	UHMK1
2.06438442697858E-40	0.479495087353157	0.847	0.68	3.36721743884476E-36	1	BAAT
2.34058752315945E-35	0.479059024701852	0.568	0.404	3.81773230902537E-31	1	ANGPTL4
8.73976955360387E-22	0.478986388010168	0.451	0.338	1.42554381188833E-17	1	CD24
3.02784075721073E-24	0.478914382167386	0.502	0.384	4.93871105908642E-20	1	NNT
2.48116890769985E-14	0.478728520523354	0.366	0.288	4.04703460534922E-10	1	GGNBP2
3.62691011065918E-30	0.478453627168361	0.524	0.393	5.91585308149619E-26	1	ARSA
3.28709388663214E-69	0.477262755158876	0.973	0.907	5.36157883848569E-65	1	CFH
3.01217955649708E-23	0.4761673560533	0.416	0.309	4.91316607460238E-19	1	TLN1
3.69714885649969E-18	0.475786730718837	0.34	0.248	6.03041949983664E-14	1	LRPPRC
5.98812913019681E-30	0.474072363293707	0.424	0.296	9.76723742426402E-26	1	PPP1R9B
2.11227527698653E-14	0.472526133111551	0.313	0.231	3.44533220429272E-10	1	HDAC10
1.44987054047685E-10	0.472381607457995	0.763	0.664	2.36488383857178E-06	1	SOD2
1.32325324734722E-13	0.47170536141085	0.31	0.237	2.15835837174805E-09	1	HIPK2
1.29119152768072E-06	0.470281238832391	0.301	0.261	0.0210606250080003	1	UBE2R2

**1.34781368921156E-18**	0.468759458082894	0.358	0.264	2.19841890847297E-14	1	PAM16
**2.41181702087143E-31**	0.468137479730593	0.569	0.425	3.93391474274339E-27	1	IRF7
**4.31369217221564E-07**	0.467816162812592	0.282	0.237	0.00703606330210092	1	CLPX
**6.19870914384517E-25**	0.467225825290799	0.368	0.254	1.01107144845259E-20	1	ZNF687
**4.85277168344275E-30**	0.466996587338768	0.496	0.366	7.91535589286347E-26	1	CLCN7
**6.82434145474174E-09**	0.466460595552467	0.318	0.258	0.000111311833468292	1	BCAT2
**6.61962786445645E-12**	0.465870944208383	0.281	0.215	1.07972750097149E-07	1	MORF4L2
**2.40940742332706E-32**	0.46544334505264	0.505	0.368	3.92998444818877E-28	1	NCSTN
**6.46210032576572E-12**	0.465322389532705	0.266	0.2	1.05403318413565E-07	1	IDS
**2.86111507565425E-16**	0.465223170030172	0.364	0.28	4.66676479989965E-12	1	COPB1
**4.06238472422992E-36**	0.463944086841496	0.566	0.412	6.62615572369142E-32	1	PLOD3
**1.22339604167684E-26**	0.46350706985299	0.508	0.385	1.99548128357909E-22	1	PDXK
**5.76341183773075E-21**	0.463083555247072	0.479	0.376	9.40070104852262E-17	1	SPSB1
**3.86390333336476E-10**	0.461965979345509	0.271	0.212	6.30241272705127E-06	1	YPEL3
**2.62411076446539E-22**	0.461017589732178	0.421	0.313	4.2801870679195E-18	1	ZGPAT
**1.25566171196457E-10**	0.460919845919737	0.283	0.221	2.04810981838541E-06	1	API5
**6.79455666824287E-13**	0.46035062679425	0.315	0.245	1.10826013815709E-08	1	EEF1AKNM
**1.40314806623449E-07**	0.459578615875137	0.268	0.221	0.00228867481083508	1	TMEM63B
**1.01263512193658E-18**	0.459355622548777	0.39	0.299	1.65170914739075E-14	1	NUDCD3
**1.52927104831463E-20**	0.45890517691492	0.425	0.321	2.49439400690599E-16	1	MYO1C
**2.21400361564566E-13**	0.458752114046899	0.251	0.18	3.61126129747964E-09	1	ELK4
**2.84430230794375E-13**	0.458443133177414	0.34	0.267	4.63934149448705E-09	1	NDUFA10
**6.13070812437656E-11**	0.457249798417715	0.332	0.268	9.9997980216706E-07	1	C19orf12
**1.74103869631093E-18**	0.457136081318515	0.265	0.175	2.83980821755276E-14	1	FBRSL1
**8.09046614724186E-17**	0.456686012333576	0.259	0.173	1.31963593327662E-12	1	JDP2
**1.72193509623966E-20**	0.456489997923665	0.376	0.274	2.80864833547651E-16	1	TOMM40L
**8.07471618065233E-16**	0.454635018549096	0.382	0.3	1.3170669562262E-11	1	QARS
**6.21714593280462E-14**	0.453831983929711	0.324	0.247	1.01407867309976E-09	1	XAB2
**3.00266646298626E-16**	0.453364957968765	0.384	0.299	4.89764926777689E-12	1	DEPRECAT
**4.71089527177595E-19**	0.45228828770168	0.347	0.252	7.68394127779375E-15	1	SMARCA4
**1.58839113929123E-52**	0.451291532597989	0.927	0.779	2.59082478729793E-48	1	MT1H
**1.75080542231179E-25**	0.449820045976912	0.515	0.392	2.85573872433276E-21	1	PLA1A
**1.77037811017303E-12**	0.449278451471714	0.273	0.203	2.88766373550324E-08	1	MEGF9
**8.01707530866602E-14**	0.449053206140537	0.374	0.294	1.30766515359651E-09	1	TFRC
**1.19000280304277E-35**	0.447334691970508	0.601	0.445	1.94101357204306E-31	1	ORMDL3
**2.01771172830065E-11**	0.446266028039096	0.301	0.233	3.29108960003119E-07	1	IRF6
**2.74311203313676E-17**	0.446086314138683	0.338	0.249	4.47429003724937E-13	1	PPP1R12C
**5.40060325693125E-27**	0.444750612481985	0.451	0.332	8.80892397238056E-23	1	ZC3H15
**5.82478769632831E-08**	0.44472784208833	0.266	0.215	0.000950081121148111	1	LIMS2
**6.82838243743137E-29**	0.444388892151349	0.566	0.421	1.11377745936943E-24	1	BHMT2
**3.07428921143615E-10**	0.443290256047374	0.275	0.214	5.01447313277351E-06	1	GSR
**3.17366373301216E-14**	0.442062468138352	0.323	0.247	5.17656291491614E-10	1	SFT2D2
**1.94038060669219E-07**	0.441118512668022	0.252	0.204	0.00316495480757563	1	DEPRECAT
**1.85901896788225E-08**	0.441023463240735	0.316	0.264	0.000303224583851274	1	ARHGEF12
**1.09134939898822E-11**	0.440971894392365	0.29	0.22	1.78010000468968E-07	1	CXADR
**5.36051248998224E-12**	0.440879629316972	0.25	0.182	8.74353192241003E-08	1	UBQLN4
**4.07539899739945E-20**	0.440438818157177	0.387	0.287	6.64738330465824E-16	1	CTTN
**2.6856188011006E-18**	0.440092730981447	0.284	0.194	4.3805128264752E-14	1	PURA
**4.87838336191183E-22**	0.438880371286485	0.379	0.275	7.95713110161438E-18	1	TRIOBP
**2.54865791569926E-29**	0.438357290238023	0.422	0.29	4.15711592629707E-25	1	ERF

1.98073585672958E-13	0.437307822812675	0.25	0.178	3.23077825591162E-09	1	CDK4
5.89641291828484E-10	0.435708968597675	0.257	0.199	9.6176391110144E-06	1	PTPN1
2.78965803785476E-14	0.435592123372116	0.398	0.319	4.5502112255449E-10	1	C11orf54
9.99762717956825E-11	0.434957614418285	0.259	0.195	1.63071296925938E-06	1	MPP1
1.52917534704136E-26	0.432937976532831	0.522	0.395	2.49423790855916E-22	1	FOXP4
3.56084778943254E-15	0.431581713553479	0.402	0.322	5.80809882934341E-11	1	SERINC3
7.28062877238436E-29	0.430209371344175	0.729	0.582	1.18754335906361E-24	1	THBS1
2.12031253458352E-33	0.430135999760191	0.59	0.435	3.45844177515918E-29	1	GADD45B
7.39617989778355E-13	0.430023648827706	0.28	0.207	1.20639090312748E-08	1	PPP1R16A
4.41951097038594E-55	0.428211077825355	0.867	0.697	7.20866434379651E-51	1	OAF
1.20087724635457E-16	0.427738696153562	0.36	0.272	1.95875087652895E-12	1	CTDSP2
1.11974579939019E-11	0.425473042479975	0.263	0.199	1.82641737338535E-07	1	PFDN5
8.08050041748132E-14	0.424741785578339	0.331	0.256	1.31801042309538E-09	1	EPRS
2.06828261038663E-07	0.424402240444387	0.289	0.243	0.00337357576580163	1	ADGRG1
1.17986971180059E-14	0.424090546278971	0.39	0.312	1.92448548691794E-10	1	SNRNP200
8.60421885038857E-12	0.422848169714343	0.325	0.258	1.40343413668688E-07	1	SELENON
7.85468806703837E-12	0.422817009499289	0.356	0.288	1.28117817061463E-07	1	TMEM184A
3.91980044067754E-10	0.422108577863126	0.325	0.263	6.39358649878913E-06	1	AMT
4.80881551440684E-26	0.420637694389498	0.452	0.331	7.84365898554899E-22	1	ZCCHC24
2.98308987361995E-08	0.419971596711862	0.273	0.221	0.00048657178928615	1	DCAF7
1.57142175043367E-27	0.419400612748332	0.476	0.35	2.56314601713236E-23	1	ATF6B
1.86518648115897E-28	0.418868230631028	0.606	0.462	3.04230566941839E-24	1	PECR
3.30652329171662E-12	0.418032841768365	0.25	0.183	5.39327014111898E-08	1	LRRC40
1.36820658530067E-10	0.416734704759802	0.309	0.244	2.23168176128392E-06	1	DESI1
3.30691580081225E-11	0.416426119259666	0.28	0.213	5.39391036270485E-07	1	TTC7A
3.07939885778164E-17	0.416341418451006	0.335	0.247	5.02280747692764E-13	1	VPS4A
5.05151204118154E-34	0.416009924079965	0.521	0.377	8.23952129037121E-30	1	IFRD2
9.23909143445833E-16	0.415991108438806	0.35	0.268	1.5069882038745E-11	1	MRS2
7.29390631201234E-17	0.415725437786911	0.412	0.324	1.18970905855233E-12	1	CDH1
2.50314724474832E-09	0.414985588291783	0.338	0.284	4.08288347090898E-05	1	SNX4
4.94630979493581E-18	0.414480788054474	0.367	0.273	8.0679259065198E-14	1	HYI
1.11908965376653E-21	0.413355641068124	0.433	0.328	1.82534713425858E-17	1	HSF1
1.2577322602472E-10	0.411391878446462	0.318	0.252	2.0514870896892E-06	1	SLC47A1
4.10124210487455E-40	0.410351639837219	0.779	0.592	6.68953599726089E-36	1	MTSS2
7.880828099561E-36	0.410205173129046	0.781	0.607	1.2854418713194E-31	1	UGDH
9.24601119910956E-14	0.407785618266516	0.303	0.228	1.50811688668676E-09	1	TEX2
6.23548541117318E-18	0.407049752835048	0.395	0.303	1.01707002541646E-13	1	AKIRIN2
9.84386309315897E-09	0.406084062730433	0.347	0.292	0.000160563250912516	1	ELL2
4.50836315495403E-26	0.40603245108992	0.513	0.393	7.35359114204551E-22	1	DNM2
1.36317462121515E-27	0.405856861477639	0.436	0.31	2.22347412466404E-23	1	HDHD3
4.70158515267212E-20	0.404169235609197	0.416	0.317	7.6687555425235E-16	1	SLC35F5
8.42870380907205E-11	0.403078035417297	0.302	0.236	1.37480587829774E-06	1	PGP
9.3407896884966E-13	0.401664958578066	0.336	0.264	1.52357620609068E-08	1	RCC1L
2.21198434810964E-46	0.401501096631749	0.887	0.702	3.60796767020164E-42	1	HPN
4.92108543520138E-26	0.40145419134429	0.416	0.299	8.02678245335698E-22	1	LMF2
7.6975175612237E-08	0.399633123556248	0.271	0.221	0.0012555420894112	1	WRNIP1
1.45491633638635E-27	0.399415014284865	0.91	0.806	2.37311403627978E-23	1	AGXT
1.68568849319804E-18	0.398666481235487	0.349	0.259	2.74952650125532E-14	1	UBE2I
1.67711640721228E-17	0.396821764460212	0.334	0.244	2.73554457180396E-13	1	GFOD2
4.29625301545337E-08	0.393269466819454	0.314	0.262	0.0007007618293506	1	STAP2

**2.12737463938047E-15**	0.392734277387048	0.373	0.29	3.46996077429348E-11	1	SLC35B1
**1.48179313218476E-07**	0.39244830640485	0.289	0.239	0.00241695277790657	1	TOP1MT
**3.11451820332822E-08**	0.392415353380382	0.266	0.216	0.000508009064144866	1	PREB
**4.13970261147054E-12**	0.392178345428615	0.251	0.184	6.7522689295696E-08	1	TCF20
**1.3510951481413E-10**	0.390634973040968	0.356	0.295	2.20377129613328E-06	1	STIMATE
**1.88799354667225E-12**	0.389377983359991	0.303	0.232	3.0795062739771E-08	1	ZFP91
**3.75058431755606E-14**	0.38935741007552	0.454	0.364	6.11757808036569E-10	1	ICAM1
**6.3317759402745E-23**	0.389164905298395	0.393	0.282	1.03277597361817E-18	1	MKNK2
**4.13063359524385E-39**	0.388101309774626	0.735	0.563	6.73747645720225E-35	1	LITAF
**2.01305716428759E-16**	0.388030500719682	0.32	0.232	3.2834975406695E-12	1	FAM3A
**5.06678945187789E-12**	0.387141010069334	0.378	0.309	8.26444027495803E-08	1	TPM1
**2.21534091655658E-10**	0.386861727420119	0.293	0.232	3.61344256899543E-06	1	CCNI
**5.90538376906289E-27**	0.386414896620051	0.505	0.372	9.63227146571848E-23	1	TRIP6
**1.87793868738344E-25**	0.386127232912507	0.43	0.31	3.06310579299113E-21	1	SLC3A2
**1.63948069710535E-27**	0.385941234858362	0.582	0.443	2.67415696504854E-23	1	ILRUN
**2.13309484290802E-33**	0.385707167194551	0.685	0.509	3.47929099826727E-29	1	SLC37A4
**1.99043800793884E-30**	0.385292363532864	0.657	0.494	3.24660343474905E-26	1	LPGAT1
**1.46528902859998E-29**	0.384522635200124	0.538	0.408	2.39003293454943E-25	1	SEC22B
**5.55582874531099E-24**	0.384087934601487	0.54	0.418	9.06211226647675E-20	1	SCCPDH
**4.81489442977929E-11**	0.384032656623679	0.281	0.216	7.853574304413E-07	1	M6PR
**3.12089520311169E-19**	0.383760418838134	0.387	0.287	5.09049216579548E-15	1	TRABD
**1.4194921968868E-14**	0.383613635092878	0.349	0.26	2.31533372234205E-10	1	MYC
**3.71037317055692E-14**	0.3835605030035	0.355	0.276	6.05198967849539E-10	1	RHOU
**2.39964383914988E-13**	0.383481510276072	0.376	0.295	3.91405906603738E-09	1	LPCAT1
**5.833865917026E-22**	0.380110669709124	0.532	0.416	9.51561869726111E-18	1	RBM47
**6.88943694731552E-09**	0.378390942502374	0.263	0.208	0.000112373606047663	1	SEC23B
**2.48539207771379E-09**	0.377960158520982	0.279	0.222	4.05392301795896E-05	1	PPP3R1
**5.46526506795667E-36**	0.376538023690372	0.607	0.449	8.91439385234413E-32	1	IQSEC1
**1.06540572217057E-09**	0.376461001708786	0.348	0.285	1.73778327343242E-05	1	FZR1
**3.87853493982982E-34**	0.376106302459807	0.58	0.429	6.32627834035642E-30	1	NADK
**1.45536114063077E-34**	0.37483476133121	0.561	0.415	2.37383955648284E-30	1	RILP
**1.01705577762605E-15**	0.374391997578125	0.399	0.313	1.65891967888585E-11	1	ABCF1
**7.12513398027322E-09**	0.374128892587684	0.251	0.195	0.000116218060352236	1	SH3PXD2A
**6.27322920458278E-08**	0.373922085594751	0.292	0.241	0.0010232264155595	1	CYP2J2
**3.83046640303442E-12**	0.37377718857694	0.262	0.194	6.24787374998944E-08	1	NCAPH2
**6.71451198643703E-10**	0.373407474367553	0.318	0.261	1.09520405010774E-05	1	TOMM70
**2.22779721862637E-13**	0.370767823797434	0.322	0.247	3.63376004330146E-09	1	SLC33A1
**1.32945940142065E-10**	0.367456722499855	0.282	0.221	2.16848122965722E-06	1	DPM1
**1.65325278895624E-06**	0.366979672341628	0.258	0.217	0.0269662062406652	1	ACAD8
**3.13622658653664E-15**	0.366370579451521	0.38	0.299	5.11549918529992E-11	1	TXLNA
**7.8575530825255E-21**	0.364495176338077	0.35	0.245	1.28164548329073E-16	1	ZNF219
**1.92995640492211E-08**	0.363846006812992	0.263	0.209	0.000314795189206845	1	PYCR2
**2.68288837076379E-12**	0.363350574956753	0.328	0.26	4.37605922155281E-08	1	RMND5A
**3.13992031053679E-29**	0.363273933220823	0.441	0.313	5.12152401851655E-25	1	CYTH2
**1.83118479515179E-09**	0.363071964216192	0.287	0.226	2.98684551937208E-05	1	ADGRV1
**6.45163855288079E-11**	0.363053042630924	0.273	0.208	1.05232676436039E-06	1	CPPED1
**1.749797763273E-11**	0.36263662292057	0.277	0.211	2.85409513167459E-07	1	DDX56
**6.37654502499924E-12**	0.362487854862731	0.369	0.303	1.04007825902763E-07	1	TBC1D24
**1.99458566827712E-33**	0.361891901670387	0.548	0.398	3.2533686835268E-29	1	MGLL
**7.49480259296086E-24**	0.361874199295207	0.485	0.367	1.22247725093785E-19	1	IRF2BP2

3.7470545315114E-17	0.361505739635686	0.395	0.308	6.11182064634825E-13	1	RAB18
6.84953964182267E-11	0.3609002539521	0.261	0.197	1.1172284109777E-06	1	MLLT1
1.00651585556895E-14	0.360082137967478	0.345	0.265	1.64172801201852E-10	1	BCL9L
2.93378487001083E-28	0.35971591542551	0.591	0.451	4.78529650147466E-24	1	DAP3
8.01173870545383E-07	0.35801099755923	0.297	0.252	0.0130679470024657	1	CYFIP1
1.38438258708464E-09	0.357998435805232	0.281	0.221	2.25806643779376E-05	1	PTP4A3
8.86863973234854E-25	0.357668135429999	0.707	0.533	1.44656382674337E-20	1	ALDH3A2
3.35154001312676E-10	0.356892101038921	0.313	0.249	5.46669691541106E-06	1	C1orf198
4.7459555093398E-28	0.35660544429007	0.648	0.498	7.74112803128415E-24	1	JUNB
7.27366162254811E-20	0.356296230440186	0.465	0.362	1.18640694725382E-15	1	CYBC1
2.97807103363373E-24	0.355888463993794	0.439	0.321	4.85753166295997E-20	1	SKI
2.61206760470132E-06	0.355525499664757	0.299	0.258	0.0426054347002831	1	AHCYL1
2.52744551609272E-09	0.354145035226707	0.265	0.208	4.12251638129883E-05	1	DNPEP
1.24284942639628E-09	0.353550528072659	0.277	0.221	2.02721169939497E-05	1	PSMD6
8.16347689765627E-17	0.353297369573119	0.423	0.335	1.33154471677671E-12	1	ITPA
2.92008050312239E-08	0.351834817778834	0.301	0.248	0.000476294330864293	1	PRPF6
1.29178727388504E-11	0.350796816491608	0.376	0.308	2.1070342224339E-07	1	EPHX2
3.69380735197914E-14	0.350033565771348	0.44	0.357	6.02496917181317E-10	1	CRYZ
7.25779835335469E-21	0.349727810695684	0.446	0.339	1.18381948941568E-16	1	RING1
1.65783066919434E-16	0.349437764279655	0.43	0.336	2.70408760452289E-12	1	SLC50A1
5.06377029278704E-22	0.349292865942633	0.569	0.452	8.25951572456495E-18	1	EPAS1
4.14016578020924E-08	0.349179611676246	0.275	0.216	0.000675302440409929	1	PDK4
1.06145873224636E-09	0.345992248152257	0.25	0.193	1.73134533816703E-05	1	RNPS1
4.24337465509503E-15	0.345819117093888	0.356	0.276	6.9213683999255E-11	1	NCBP2
8.73013529170094E-17	0.34533041965312	0.452	0.365	1.42397236742934E-12	1	DNAJC21
1.49630137288881E-15	0.34433030771079	0.341	0.258	2.44061716931894E-11	1	KLF3
9.57436079048591E-22	0.344167280141578	0.501	0.381	1.56167398853616E-17	1	FOXA3
2.95491134238304E-07	0.343513710443265	0.276	0.23	0.00481975589056098	1	CHERP
9.10078440359391E-13	0.341843154099105	0.314	0.243	1.4844289440702E-08	1	ATG2A
1.18264461160729E-24	0.341489440223376	0.623	0.49	1.92901162599265E-20	1	OPTN
1.06279715913742E-13	0.341456143201916	0.379	0.301	1.73352844626904E-09	1	TMED4
5.39591890280723E-24	0.340088213395275	0.484	0.366	8.80128332236887E-20	1	PARP10
1.09685877383867E-10	0.339209749100423	0.296	0.231	1.78908634600826E-06	1	UNC50
5.33807005800639E-08	0.339096355164589	0.253	0.203	0.000870692607161422	1	TAX1BP3
4.4961325902416E-27	0.338899729141167	0.604	0.465	7.33364186794308E-23	1	COPA
4.3309671785319E-19	0.338852896920217	0.454	0.347	7.06424056490338E-15	1	SMAP2
2.19230434681696E-12	0.337790951490153	0.354	0.284	3.57586762009315E-08	1	SCAP
5.17098423314009E-19	0.337376858670844	0.401	0.305	8.4343923826748E-15	1	GMPPB
1.24878855926665E-30	0.337002555946146	0.609	0.453	2.03689901901984E-26	1	CTSA
4.88484272680834E-11	0.336929774651818	0.386	0.306	7.96766697169709E-07	1	DDIT4
1.36940097614412E-10	0.336107349645488	0.305	0.242	2.23362993218867E-06	1	ABHD12
2.05777306437246E-14	0.335714449713558	0.327	0.248	3.35643364529791E-10	1	KXD1
7.80509482314542E-09	0.334804660127965	0.28	0.224	0.000127308901660325	1	ZBTB22
2.36025088374652E-30	0.334800532576244	0.782	0.589	3.84980521647894E-26	1	LRP1
1.52048218838152E-26	0.331568649008979	0.508	0.388	2.4800584974691E-22	1	ZYX
5.09948090292307E-10	0.330920657373501	0.265	0.203	8.31776330075782E-06	1	SLBP
9.17980686889729E-09	0.329803284954562	0.281	0.229	0.000149731829838584	1	OPA1
1.80493715831993E-09	0.328687311423248	0.318	0.259	2.94403299893564E-05	1	RNF40
8.84028165366925E-32	0.328326449658233	0.591	0.447	1.44193834052999E-27	1	CNBP
3.70102151383808E-27	0.328118618216497	0.597	0.451	6.0367361912213E-23	1	CAPN2

4.33558253929415E-11	0.325581738361786	0.3	0.235	7.07176867984268E-07	1	TSC22D4
2.03423230873518E-32	0.325405529365866	0.842	0.674	3.31803631877796E-28	1	SHMT1
8.65607173327065E-22	0.325146582831849	0.597	0.46	1.41189186041378E-17	1	SCARB1
5.35060091868779E-14	0.324884224982516	0.31	0.234	8.72736515847165E-10	1	HECA
7.1996194846558E-15	0.323548412736624	0.458	0.367	1.17432993414221E-10	1	SEPTIN9
1.16570211350402E-14	0.323351177774001	0.471	0.382	1.90137671733641E-10	1	BPHL
5.87263267663788E-22	0.32308057905693	0.746	0.575	9.57885115886404E-18	1	CSRP1
2.43020100974984E-07	0.320593035959185	0.333	0.282	0.00396390086700296	1	DIO1
4.37342392852852E-09	0.320463191974233	0.277	0.221	7.13349176982287E-05	1	MED16
1.80865906552872E-08	0.320194641151735	0.336	0.283	0.00029501038017839	1	ACSF2
3.35581829409679E-19	0.319128121002871	0.44	0.339	5.47367521950127E-15	1	PSMD13
1.97239035510966E-18	0.318005540043758	0.347	0.249	3.21716590821936E-14	1	SELENOS
2.79383883581177E-09	0.317487301488405	0.268	0.212	4.55703052509257E-05	1	UPF1
1.35697483599071E-07	0.31545632577114	0.296	0.247	0.00221336165498445	1	PPP6R1
1.24262014367885E-52	0.315130526003548	0.97	0.908	2.02683771635456E-48	1	MT1X
9.79022760771418E-15	0.314910900950989	0.48	0.393	1.59688402509426E-10	1	CRLS1
5.07107357323845E-10	0.314531809604881	0.305	0.242	8.27142810530924E-06	1	FAM20B
2.26700755052618E-11	0.313944406048869	0.338	0.272	3.69771601566326E-07	1	STT3B
4.45319648693627E-30	0.313092293171645	0.728	0.554	7.26360878984176E-26	1	DDT
1.45320925698493E-10	0.312761779404036	0.299	0.236	2.37032961906811E-06	1	LNX2
3.69483189960668E-07	0.312500106053866	0.263	0.219	0.00602664031144845	1	MACO1
1.44203288219292E-14	0.312086187923365	0.315	0.234	2.35209983414488E-10	1	ZNF609
7.93196626750112E-08	0.312083262815406	0.328	0.275	0.00129378301789211	1	COQ9
1.56906843935172E-10	0.311981283749656	0.37	0.303	2.55930753142658E-06	1	TIMM17A
1.26333924875743E-11	0.31160520149029	0.325	0.259	2.06063264864825E-07	1	KIF5B
4.42245224299264E-12	0.310603363659959	0.316	0.247	7.21346185354529E-08	1	PPM1A
1.19511144439238E-07	0.309965962521453	0.302	0.249	0.00194934627694842	1	GPLD1
1.3894538221662E-09	0.309932232186733	0.493	0.422	2.26633812933529E-05	1	DEPRECAT
1.38690013170219E-10	0.30965335425529	0.269	0.209	2.26217280481944E-06	1	SMIM7
3.71789355511979E-26	0.306816637205381	0.61	0.465	6.06425617775589E-22	1	COPG1
1.79726514551911E-23	0.306435146343174	0.46	0.345	2.93151917885622E-19	1	CRK
6.35439638290249E-08	0.306363164358001	0.254	0.202	0.00103646559401523	1	SMO
5.22585413679659E-07	0.305461583062133	0.272	0.226	0.00852389068252892	1	PRKDC
1.47380374864882E-15	0.303119749034916	0.445	0.36	2.40392129442109E-11	1	OSBP
2.44497177652255E-07	0.301161732903443	0.25	0.204	0.00398799346468592	1	HIST1H1C
2.21966212590781E-06	0.30101045062432	0.251	0.208	0.0362049089356823	1	DDX27
1.21877440107652E-08	0.299519997183123	0.303	0.249	0.000198794292559591	1	CHURC1
8.0142825549838E-27	0.298336917260109	0.701	0.545	1.30720962754341E-22	1	GOLPH3
7.66891484678632E-19	0.298143569232043	0.439	0.342	1.25087670065932E-14	1	SPPL2A
6.50148894363805E-23	0.297987860406201	0.511	0.396	1.0604578615968E-18	1	SYVN1
1.05335637347965E-07	0.29789350279011	0.34	0.28	0.00171812958078265	1	GRAMD4
1.78524283820731E-26	0.297277225798578	0.789	0.612	2.91190959339994E-22	1	F11R
2.61959944271905E-08	0.296817957676876	0.296	0.244	0.000427282865101904	1	PTPN23
6.63751516134415E-12	0.296619516866039	0.302	0.23	1.08264509796684E-07	1	CNNM3
1.74073270057883E-13	0.296595933827998	0.306	0.232	2.83930910791413E-09	1	BRAT1
7.52081776937738E-14	0.293929084925154	0.7	0.562	1.22672058636314E-09	1	OSGIN1
7.82192237159839E-17	0.292283613580827	0.456	0.363	1.27583375803141E-12	1	AGFG2
1.98777931101845E-12	0.292158183302604	0.375	0.299	3.2422668342022E-08	1	FKBP5
2.00912555179036E-14	0.29096827379023	0.41	0.328	3.27708468752526E-10	1	CDC42EP1
7.91989704123239E-18	0.29003435118641	0.553	0.437	1.29181440639542E-13	1	TOR1AIP2

7.57830651778927E-31	0.288745644728499	0.348	0.207	1.23609757611661E-26	1	EEF1A2
1.80309409555868E-12	0.288342438033245	0.375	0.303	2.94102677926577E-08	1	POLR2A
1.23135058115015E-23	0.287902874063251	0.983	0.951	2.008455932914E-19	1	SQSTM1
3.50611658075153E-07	0.287748374479843	0.308	0.259	0.00571882675486382	1	TKFC
4.53792082662908E-18	0.287398501166458	0.421	0.327	7.40180266031469E-14	1	HDHD5
6.80178001914276E-12	0.286277475710632	0.338	0.268	1.10943833892238E-07	1	TMEM161A
1.24116399089588E-12	0.286135736089558	0.668	0.561	2.02446258555028E-08	1	DEPP1
1.45334671278389E-28	0.285390796151828	0.585	0.446	2.37055382322181E-24	1	TRIM26
3.24634687022291E-28	0.285198304127631	0.759	0.597	5.29511638002059E-24	1	JUN
7.11668645876945E-08	0.284374450685705	0.289	0.237	0.00116080272828989	1	DBP
1.50270123377114E-17	0.282927159167156	0.257	0.167	2.45105598240411E-13	1	CYP4F22
1.22248524204971E-20	0.280882080296138	0.517	0.41	1.99399567830728E-16	1	SHB
1.9509692619694E-13	0.280451459633748	0.394	0.311	3.18222596319828E-09	1	HADHB
3.71735224243494E-09	0.279880686408098	0.278	0.219	6.06337324263562E-05	1	PELP1
1.01986190020592E-07	0.278409573370311	0.325	0.272	0.00166349674542588	1	SRXN1
9.81597165367449E-07	0.277911109316543	0.26	0.217	0.0160108313643085	1	IFT27
6.78200477008293E-13	0.274383014759554	0.321	0.241	1.10621279804823E-08	1	FAM174B
9.76211989486419E-19	0.271830565522475	0.533	0.42	1.5922993760513E-14	1	ANXA11
1.52994205805707E-13	0.271061195219395	0.336	0.26	2.49548849089689E-09	1	PAFAH1B2
1.67060611651944E-13	0.270638017972639	0.352	0.275	2.72492563665487E-09	1	TRAPPC3
1.93765645695444E-08	0.270253476413875	0.3	0.246	0.000316051144693839	1	GATA4
1.45031983274776E-14	0.266510961223675	0.358	0.267	2.36561667919487E-10	1	PDGFA
1.83871696039657E-23	0.264846194486726	0.638	0.49	2.99913123410285E-19	1	OGDHL
1.49348646385691E-11	0.263747150613506	0.346	0.278	2.43602577119701E-07	1	EFR3A
2.83007616302028E-10	0.263391436358851	0.275	0.213	4.61613722950238E-06	1	DNAJB12
1.23231100748823E-07	0.2632614879227	0.372	0.317	0.00201002248431405	1	S100A13
2.41973810777116E-10	0.262521607330814	0.294	0.23	3.94683482758554E-06	1	GID8
1.15370102165784E-07	0.26199063961928	0.3	0.251	0.0018818017364261	1	PEA15
1.46820834610083E-08	0.261824658038643	0.331	0.275	0.000239479463332507	1	RRP36
2.37665362940857E-28	0.261371463027932	0.663	0.502	3.87655973492832E-24	1	PCYT2
2.28536825558511E-09	0.261169824728621	0.353	0.293	3.72766416168488E-05	1	PGAP3
4.79777503653139E-14	0.261091933728625	0.37	0.282	7.82565086208634E-10	1	PIM1
4.04954205296746E-10	0.260732274680263	0.314	0.253	6.60520804259523E-06	1	ACO2
1.59369624627659E-22	0.260731240604145	0.493	0.378	2.59947794730174E-18	1	CDC34
1.50592195380098E-19	0.257422613766881	0.375	0.275	2.45630929884478E-15	1	DVL1
2.62086361730149E-08	0.255343906331555	0.274	0.222	0.000427489064618046	1	UBA1
2.12995833341204E-20	0.253650900831366	0.501	0.396	3.47417503878379E-16	1	MTCH1
2.64587331975425E-17	0.25313772688363	0.545	0.429	4.31568397185115E-13	1	PAPSS2
8.49822475581446E-11	0.252353384431459	0.301	0.233	1.3861454399209E-06	1	RNF126
1.15793881179172E-08	0.251808967637743	0.272	0.217	0.000188871399591347	1	ZFYVE21
6.56314180432553E-21	0.251424325254648	0.462	0.35	1.07051405970354E-16	1	IGSF8
6.07878888393252E-18	0.251238419419047	0.471	0.373	9.91511254858234E-14	1	RMDN3
1.43934742314304E-16	0.250621057645971	0.391	0.302	2.34771958188862E-12	1	KPNA4
4.93398276005235E-90	3.34638288180672	0.279	0.071	8.04781927992138E-86	2	CYP1A2
0	3.05309348873917	0.984	0.751	0	2	CYP 20.00
3.56252142430333E-54	2.22415038915071	0.262	0.091	5.81082869518115E-50	2	CYP3A4
3.58502210594627E-147	2.20323322515904	0.85	0.623	5.84752955700896E-143	2	LYZ
3.02810256525866E-74	2.17931360240553	0.338	0.119	4.93913809419341E-70	2	LECT2
4.94299769801032E-61	2.09091442176631	0.35	0.144	8.06252354522464E-57	2	DEPRECAT
9.45540810083762E-110	1.72220011975222	0.702	0.458	1.54227161532762E-105	2	FMO3

2.70971551840156E-61	1.68794894693405	0.305	0.109	4.41981698206478E-57	2	TBX3
6.21701295200064E-69	1.4940240728113	0.494	0.249	1.01405698260082E-64	2	ADH4
6.20085290419218E-35	1.2894512469757	0.276	0.126	1.01142111720279E-30	2	ELOVL2
1.00801443090172E-37	1.20552370247544	0.444	0.264	1.6441723382438E-33	2	ACSL5
2.07323875181796E-50	1.20055284240512	0.49	0.276	3.38165972809027E-46	2	AQP9
1.98332365626454E-30	1.12200008821293	0.416	0.259	3.23499921573309E-26	2	SORD
8.58623263321095E-77	1.10956314172794	0.827	0.657	1.40050040480304E-72	2	PRAP1
6.7663368629694E-29	1.055528959015	0.491	0.334	1.10365720571894E-24	2	MTHFD1
6.29486685975781E-22	1.0329161072042	0.441	0.302	1.0267557334951E-17	2	ABCB4
3.60488538295267E-27	0.979855320825067	0.459	0.304	5.8799285481341E-23	2	ALAS1
1.72486942250673E-51	0.95982070670509	0.729	0.566	2.81343451505073E-47	2	MBL2
3.73160278871369E-31	0.948804002092729	0.438	0.269	6.0866173086709E-27	2	GPD1
1.20473751750205E-25	0.948278029991234	0.364	0.216	1.96504736479759E-21	2	CFHR5
1.99571521696769E-15	0.946804240741059	0.316	0.207	3.255211090396E-11	2	UGT2B10
1.15565834399874E-18	0.93682040036412	0.305	0.186	1.88499432489635E-14	2	CBR1
6.67701536900369E-17	0.933537297326488	0.299	0.19	1.08908797683819E-12	2	CCND1
8.92515757842126E-25	0.927508019637454	0.496	0.35	1.45578245261629E-20	2	UGT1A1
4.44377574012368E-22	0.90738550873136	0.264	0.142	7.24824260971573E-18	2	ACSS3
1.22306788566785E-14	0.90109635083015	0.263	0.161	1.99494602831284E-10	2	LIN7A
5.89951292909366E-15	0.87928259626928	0.282	0.177	9.62269553864468E-11	2	ROBO1
4.67441550887989E-18	0.875262123649683	0.396	0.272	7.624439136534E-14	2	DEPRECAT
4.42986962014899E-21	0.865855278385362	0.484	0.353	7.22556033742502E-17	2	DEPRECAT
2.77085856774905E-17	0.844172372726743	0.387	0.267	4.51954740985547E-13	2	DEPRECAT
8.35534901649443E-16	0.84308645903959	0.325	0.212	1.36284097808041E-11	2	OAS1
4.54344657662565E-33	0.838726584787249	0.563	0.408	7.4108157111341E-29	2	MDK
2.71018064739972E-24	0.820564372751093	0.567	0.424	4.42057565397368E-20	2	CDHR5
2.01009843493755E-71	0.819742896577938	0.94	0.858	3.27867155722664E-67	2	FABP1
2.16767344431956E-09	0.813206520893019	0.405	0.314	3.53569215502963E-05	2	PSMA7
2.66572334958564E-15	0.812780786642371	0.403	0.286	4.34806135550913E-11	2	SEC14L2
1.81866438072044E-15	0.809851144387403	0.326	0.216	2.9664234713931E-11	2	ENPEP
5.84030363728176E-33	0.79503624744236	0.645	0.51	9.52611926277028E-29	2	GPX2
6.68140538026909E-18	0.780784406111813	0.393	0.269	1.08980403157569E-13	2	GLUD1
2.89811851759406E-18	0.779723641474926	0.406	0.279	4.72712111404767E-14	2	SLC27A5
8.75180173542182E-09	0.77242579443736	0.314	0.229	0.000142750638106465	2	PSMC2
2.16066489941114E-13	0.752444363032901	0.416	0.311	3.52426051742952E-09	2	SLC22A18
8.87301445697336E-22	0.750885422108395	0.582	0.451	1.44727738807692E-17	2	SLC2A2
4.16806196599497E-22	0.750016853126678	0.615	0.508	6.79852587273439E-18	2	SULT2A1
4.96658153783649E-09	0.73059867177513	0.342	0.26	8.1009911463651E-05	2	SCD
6.67687059534461E-10	0.729056555364004	0.332	0.241	1.08906436280666E-05	2	PLPP1
1.97366374444693E-25	0.721326287888541	0.645	0.54	3.21924293356739E-21	2	NADK2
2.80292510639856E-19	0.720874408724003	0.733	0.659	4.57185114104669E-15	2	GLUL
4.27929951555573E-16	0.720219651925627	0.517	0.406	6.97996543982295E-12	2	CYP2C9
7.85316504260786E-26	0.718063728939366	0.645	0.505	1.28092975009977E-21	2	ADH1A
1.88373249172897E-15	0.717592963146012	0.348	0.229	3.07255606725912E-11	2	SLC6A12
2.83581646946207E-21	0.709308695777273	0.556	0.418	4.62550024333959E-17	2	LGALS3BP
2.80264631464308E-11	0.704962322844923	0.306	0.211	4.57139640381432E-07	2	SERPINA11
4.40192487786594E-09	0.703834457340938	0.295	0.211	7.17997966828714E-05	2	MSRB1
2.1257917618108E-07	0.699089346960035	0.295	0.22	0.0034673789426896	2	MEA1
1.82850504852442E-12	0.683033788278952	0.447	0.338	2.98247458464818E-08	2	TXNRD2
8.26909800997401E-09	0.663006942245804	0.28	0.197	0.000134877257640686	2	GCHFR

**1.98830743881572E-07**	0.660177175282124	0.306	0.232	0.00324312826345233	2	TEX264
**1.03137507303949E-34**	0.658182669477101	0.765	0.658	1.68227588163471E-30	2	AOX1
**1.08476729801293E-13**	0.650610676661876	0.447	0.332	1.76936393978889E-09	2	PSMD4
**1.83118920512435E-17**	0.630601182004234	0.465	0.335	2.98685271247833E-13	2	EVA1A
**6.20902764343224E-13**	0.629086724207417	0.421	0.309	1.01275449892023E-08	2	F10
**2.94800079743702E-07**	0.628135371857049	0.36	0.281	0.00480848410069952	2	TOM1
**6.33615235947101E-14**	0.62594165144297	0.452	0.338	1.03348981135332E-09	2	C1orf115
**3.82732391676474E-18**	0.623370605806358	0.495	0.358	6.24274804063497E-14	2	APOM
**3.88745165564179E-10**	0.621193986807332	0.407	0.31	6.34082239551732E-06	2	UQCRQ
**2.25317830561137E-22**	0.619987399051173	0.632	0.502	3.6751591342827E-18	2	SOD1
**1.63253090505059E-23**	0.618958435721559	0.682	0.557	2.66282115922801E-19	2	HSD17B6
**1.11408818519561E-16**	0.616910925403304	0.367	0.246	1.81718923887256E-12	2	ASPDH
**2.7366372278873E-08**	0.613616492749788	0.369	0.286	0.000446372898240698	2	PCBD1
**2.45932831921791E-13**	0.611039971352033	0.261	0.164	4.01141042147633E-09	2	DDAH2
**2.23447201116506E-09**	0.604172108696175	0.348	0.256	3.64464729741133E-05	2	TACO1
**4.55613379198627E-11**	0.597777796007872	0.44	0.337	7.4315098281088E-07	2	GPAM
**1.06291468879111E-09**	0.597024815763897	0.316	0.22	1.73372014888718E-05	2	CCL16
**4.37148276699157E-08**	0.592168578289224	0.331	0.248	0.000713032554123995	2	GAGE1
**5.33695536466472E-17**	0.579701411506745	0.663	0.554	8.70510789530462E-13	2	DCXR
**6.75348762936991E-07**	0.5780259305889	0.266	0.195	0.0110156136722653	2	RDH16
**3.34798565279213E-09**	0.575565479779305	0.271	0.188	5.46089939826924E-05	2	H2AFJ
**2.84679615462714E-12**	0.567438510375115	0.508	0.404	4.64340920781232E-08	2	QDPR
**3.29886454688293E-09**	0.56483694482153	0.409	0.315	5.38077796242075E-05	2	ACADS
**4.57753088436454E-07**	0.564737731919303	0.321	0.244	0.00746641062548701	2	HSDL2
**3.61278129458475E-07**	0.560210514419222	0.25	0.181	0.00589280756959718	2	DBI
**6.87551824554417E-10**	0.558943160705262	0.309	0.219	1.12146578103071E-05	2	COLEC11
**3.28231580953074E-09**	0.556837100223421	0.301	0.216	5.3537853169256E-05	2	PKLR
**6.4552200464336E-11**	0.552462685018551	0.317	0.223	1.05291094177379E-06	2	COMT
**8.17160448119464E-19**	0.550578658678844	0.658	0.567	1.33287040692766E-14	2	F7
**6.81745000363563E-09**	0.546283707303279	0.377	0.28	0.000111199427009301	2	TOMM7
**1.79211311170337E-10**	0.544254664821833	0.4	0.299	2.92311569649936E-06	2	LAMTOR2
**1.14630348450413E-08**	0.543060203834466	0.465	0.387	0.000186973561357469	2	HLF
**7.5943591502749E-17**	0.534526252128112	0.594	0.472	1.23871592100134E-12	2	OTC
**2.872131431663E-09**	0.532735014886242	0.398	0.308	4.68473357818552E-05	2	PSMC4
**1.07001112901089E-24**	0.531420729604151	0.724	0.638	1.74529515252966E-20	2	CFHR2
**6.21999732938832E-25**	0.52919795749382	0.819	0.738	1.01454376439653E-20	2	SCP2
**7.66566922538234E-07**	0.528876094483328	0.258	0.189	0.0125034730735211	2	PDLIM2
**2.43527574894241E-09**	0.528632279162912	0.4	0.308	3.97217827409996E-05	2	ECHDC3
**7.66535495457981E-10**	0.521161899996237	0.429	0.335	1.25029604664151E-05	2	MIF
**8.008606262089E-28**	0.52033367485267	0.797	0.704	1.30628376740934E-23	2	PLG
**7.33801052843844E-09**	0.518263499773882	0.465	0.372	0.000119690289729359	2	FCGRT
**7.49793791689811E-07**	0.516684529122142	0.34	0.262	0.0122298865362525	2	SART1
**4.35670991909527E-24**	0.511816567109391	0.798	0.702	7.10622954903629E-20	2	ECH1
**2.81350744441732E-22**	0.511586456721628	0.767	0.672	4.58911199258909E-18	2	ECHS1
**5.01960907898613E-17**	0.508834364692743	0.623	0.494	8.18748436873428E-13	2	IFI27
**6.34573394906263E-07**	0.504345213506328	0.437	0.357	0.0103505266443161	2	PCDH1
**2.13368782924128E-08**	0.503874837009948	0.294	0.213	0.000348025821827545	2	MDH2
**2.11952812134622E-09**	0.500460569271007	0.627	0.557	3.45716231872783E-05	2	PCK2
**1.71435742968045E-10**	0.497109563192785	0.465	0.372	2.79628840355178E-06	2	HSD17B10
**2.27480545353143E-10**	0.493130937574839	0.58	0.49	3.71043517525511E-06	2	UGT2B4

4.0057651814676E-24	0.491481409829698	0.796	0.704	6.5338035874918E-20	2	GATM
2.27233581149963E-08	0.491368504949886	0.406	0.323	0.000370640694213705	2	AGMAT
8.00382483823393E-14	0.491064957526126	0.599	0.501	1.30550386936434E-09	2	CAT
4.22752190802684E-08	0.488667330353158	0.394	0.312	0.000689551098418258	2	HSD17B8
8.94817025435607E-15	0.487564596801292	0.612	0.516	1.45953605018802E-10	2	RGN
1.0112915780467E-11	0.484267612413334	0.551	0.45	1.64951769295197E-07	2	ADRM1
1.35641398303326E-16	0.48407288777573	0.707	0.609	2.21244684772556E-12	2	FUOM
5.10576188441637E-11	0.48319896859249	0.5	0.398	8.32800820967153E-07	2	ECI1
1.69732623144798E-10	0.482949812569209	0.531	0.444	2.7685088161148E-06	2	DUSP23
2.87920231427884E-12	0.480289788139366	0.682	0.606	4.69626689482022E-08	2	HNF4A
2.91472513173122E-11	0.477634104320347	0.623	0.528	4.7542081623668E-07	2	ANG
1.26471255804014E-27	0.475007913268355	0.856	0.795	2.06287265341928E-23	2	ALDH2
4.17145240424999E-08	0.469392674837819	0.493	0.405	0.000680405601657216	2	UBA52
2.85790595752582E-11	0.460874988597372	0.606	0.533	4.66153040732037E-07	2	POR
6.95852942245486E-16	0.45937577754855	0.595	0.467	1.13500573409661E-11	2	HPD
5.53417091908939E-14	0.458215374207508	0.679	0.595	9.02678618612671E-10	2	ACAT1
2.08318650132185E-08	0.457694540676135	0.364	0.271	0.000339788550230608	2	HIBADH
4.89908654496608E-08	0.456715801067378	0.398	0.317	0.000799090006349418	2	RAMP1
6.50181589659353E-09	0.454993760972828	0.467	0.378	0.000106051119089337	2	TSTA3
6.26400938985642E-08	0.452950143059552	0.411	0.32	0.00102172257157948	2	MAOB
3.66885143309488E-09	0.452099269544399	0.595	0.51	5.98426357252106E-05	2	HAGH
2.13655218911947E-06	0.451213590871169	0.411	0.332	0.0348493027567276	2	PPP4R2
2.08053572745415E-08	0.447476181086884	0.359	0.265	0.000339356182505046	2	GADD45GIF
4.41634821194283E-10	0.446814095801412	0.534	0.438	7.20350556849995E-06	2	APOC4
1.17006777781061E-08	0.446218030223934	0.49	0.408	0.000190849755238689	2	H1F0
1.5712134296088E-09	0.443540675600886	0.441	0.34	2.56280622503491E-05	2	TXNDC17
7.11777960289966E-08	0.44222518969212	0.33	0.245	0.00116098103102896	2	EDF1
7.32294250354977E-09	0.437998721519068	0.488	0.398	0.0001194445151754	2	ATOX1
6.99721540498278E-12	0.427259670244739	0.493	0.384	1.14131580470674E-07	2	BST2
1.0875579385237E-11	0.426889546853436	0.523	0.425	1.773915753526E-07	2	DNPH1
9.17816303737506E-14	0.424687778235409	0.692	0.616	1.49705017302625E-09	2	ADI1
1.21536017983859E-07	0.424618305051208	0.451	0.364	0.00198237398933472	2	ALDH4A1
5.88031293265078E-08	0.423533924780793	0.555	0.476	0.000959137842444669	2	MT-ND3
2.39510874183028E-08	0.418863444706093	0.472	0.39	0.000390666186879938	2	PSMB4
1.28920954254699E-10	0.418697347237672	0.374	0.269	2.1028296848484E-06	2	CLDN4
2.67161551756732E-06	0.417236851182295	0.391	0.312	0.0435767207070406	2	COX17
1.96163207325144E-12	0.41702341564058	0.45	0.349	3.19961807468042E-08	2	VWA1
7.97276564166336E-16	0.409717821154013	0.693	0.604	1.30043780381171E-11	2	PGRMC1
4.40054220220223E-08	0.409234927665961	0.263	0.186	0.000717772438601206	2	FOXN3
1.33857516705022E-09	0.408202284760362	0.365	0.271	2.18334995497562E-05	2	CNN3
3.69789516086069E-21	0.408081997593418	0.845	0.778	6.03163679687987E-17	2	SERPING1
4.52010718949367E-10	0.407682569215525	0.651	0.577	7.37274683678312E-06	2	BLVRB
5.66615672212543E-14	0.404832627835063	0.659	0.559	9.24206822945879E-10	2	HDGF
1.30292191912148E-07	0.404051722957677	0.296	0.215	0.00212519594227905	2	INSIG2
1.6543651505463E-06	0.403854341709263	0.469	0.389	0.0269843499705608	2	SERP1
7.84604111360341E-08	0.40251761772536	0.462	0.373	0.00127976776603985	2	ROMO1
3.11036502174054E-09	0.401962115724709	0.597	0.513	5.07331638696099E-05	2	PGM1
2.20285160887766E-06	0.400991371242767	0.436	0.362	0.0359307125924035	2	AMACR
1.89383114801781E-06	0.400876391259738	0.383	0.307	0.0308902798553185	2	ATP5F1A
2.06971414547342E-06	0.397838231150366	0.354	0.278	0.033759107426817	2	FARSA

**1.93769098597425E-07**	0.394118030893569	0.447	0.357	0.0031605677672226	2	ALDH6A1
**1.03286748083447E-09**	0.393577501823898	0.608	0.538	1.6847101479891E-05	2	ACSL4
**2.38366469879614E-10**	0.39254132266942	0.419	0.317	3.88799549020639E-06	2	TST
**8.21658912374769E-09**	0.390600219636059	0.507	0.413	0.000134020785197449	2	SHBG
**1.47458831596322E-06**	0.390436836448541	0.363	0.289	0.0240520100216761	2	SCRN2
**1.21405370341478E-07**	0.39000837061975	0.437	0.353	0.00198024299563984	2	GALK1
**1.13221677832494E-06**	0.387282307708325	0.436	0.353	0.0184675878712582	2	NDUFA2
**5.00296580330874E-11**	0.386373907059986	0.574	0.468	8.16033752177688E-07	2	ALDH1L1
**2.402548921944E-22**	0.385569276549678	0.848	0.765	3.91879754658286E-18	2	CTSD
**4.73714767216504E-08**	0.384363010427943	0.307	0.222	0.00077267615680684	2	NFIX
**2.70084388594343E-11**	0.383894801895599	0.339	0.235	4.40534646236232E-07	2	PHLDA1
**4.51316041568141E-07**	0.382566018112849	0.41	0.326	0.00736141595401795	2	EID1
**5.39484684818618E-09**	0.382314824837488	0.275	0.193	8.79953469407648E-05	2	PIR
**5.16374247093787E-07**	0.382215573859265	0.418	0.335	0.00842258034434676	2	MSRB2
**4.01546859308108E-12**	0.382090011830368	0.568	0.466	6.54963082217456E-08	2	ITM2B
**5.09828394304075E-07**	0.381120557390678	0.708	0.664	0.00831581093949377	2	ACAA1
**1.39255648074844E-06**	0.379180867835647	0.325	0.248	0.0227139887574878	2	TALDO1
**6.83467723329954E-08**	0.377804933427832	0.509	0.428	0.00111480420352349	2	ACY1
**1.55051180329295E-11**	0.377547193375096	0.67	0.588	2.52903980235113E-07	2	OSGIN1
**1.66314809606408E-12**	0.377190841353926	0.745	0.68	2.71276085949012E-08	2	APOL6
**6.60896398724813E-08**	0.376828899765613	0.375	0.29	0.00107798811596004	2	DEPRECAT
**4.0968571000028E-08**	0.376177065976447	0.578	0.507	0.000668238361581457	2	ZNHIT1
**8.49198325384216E-08**	0.37603881984251	0.464	0.381	0.0013851273885342	2	GSTO1
**1.47596004935591E-07**	0.374799313959369	0.52	0.441	0.00240743843650442	2	PRXL2A
**2.6445496674288E-08**	0.374780223757212	0.641	0.57	0.000431352496254311	2	ADH1B
**1.02559536678921E-11**	0.371993967775641	0.507	0.393	1.67284860276987E-07	2	REEP6
**4.36107482292406E-12**	0.369624765591872	0.66	0.585	7.11334914367143E-08	2	SERPINA7
**1.82691691288066E-11**	0.3693545478092	0.763	0.717	2.97988417659965E-07	2	CYB5A
**3.12837273246568E-08**	0.368148705215403	0.407	0.321	0.000510268876392477	2	NAGS
**9.7823207154511E-10**	0.364683771198476	0.541	0.45	1.59559433189723E-05	2	PSME1
**2.34966288720561E-06**	0.360177850407319	0.429	0.349	0.0383253513532107	2	SPCS1
**1.1226020984396E-07**	0.357332966291759	0.781	0.713	0.00183107628276483	2	MT-CO3
**3.91353311838596E-13**	0.357326896270952	0.677	0.574	6.38336386939933E-09	2	ISG15
**3.90970444817978E-11**	0.355887361695263	0.678	0.606	6.37711892542604E-07	2	PFN1
**4.42382124325306E-08**	0.355568253466869	0.257	0.18	0.000721569482987006	2	TLCD4
**1.58242427185772E-09**	0.353350958312135	0.319	0.23	2.58109222982714E-05	2	ABHD14B
**3.60411878805206E-09**	0.351811213116515	0.342	0.249	5.87867815519172E-05	2	MPLKIP
**1.26556090046411E-27**	0.350979354565705	0.863	0.819	2.06425638474701E-23	2	C4BPA
**2.3741760503675E-08**	0.35012844580204	0.356	0.267	0.000387251855575443	2	SAP18
**8.46718918128325E-10**	0.346872629873373	0.603	0.521	1.38108322735911E-05	2	PRDX2
**2.61916287974563E-07**	0.343997703193725	0.444	0.362	0.00427211657315309	2	IFITM2
**1.9429641194758E-07**	0.343719370613405	0.474	0.395	0.00316916877527697	2	PSMC3
**7.11427503494461E-07**	0.343231046716804	0.451	0.367	0.0116040940094982	2	LHPP
**3.72901199245398E-07**	0.343212972677288	0.458	0.377	0.00608239146089169	2	CITED4
**2.03504420415483E-11**	0.341313529500412	0.667	0.591	3.31936060139694E-07	2	TFPI
**1.26418661596564E-16**	0.340776160899854	0.801	0.728	2.06201478930155E-12	2	B2M
**1.62052213785729E-06**	0.33464122606471	0.428	0.346	0.0264323365905902	2	NMRAL1
**4.53863004922468E-07**	0.333154164518954	0.4	0.318	0.00740295947329037	2	PSMC5
**1.5632918609066E-08**	0.329826365967882	0.549	0.464	0.000254988535432475	2	BEX3
**4.82925432802031E-08**	0.329585257909833	0.614	0.531	0.000787699673443392	2	HAO1

5.2453201015776E-07	0.325915068446119	0.535	0.453	0.00855564161768322	2	PCCB
2.0040638113231E-06	0.324493514431982	0.381	0.307	0.0326882848264911	2	MGST2
8.43754166764559E-09	0.323487963947189	0.509	0.421	0.000137624742140967	2	PEBP1
4.56472547507437E-13	0.323056889185	0.751	0.683	7.44552372239381E-09	2	ETFB
4.50474720871453E-08	0.322595446033001	0.517	0.432	0.000734769317213427	2	CAMK2N1
8.34342620349392E-08	0.321459049907213	0.663	0.642	0.00136089624805189	2	LRP1
7.93595003476407E-08	0.316471699216807	0.534	0.449	0.00129443281017037	2	ERGIC3
2.41375763674176E-09	0.316228125934213	0.654	0.58	3.93708008128949E-05	2	DEPP1
2.76855524831864E-08	0.315922850665767	0.635	0.573	0.000451579046553254	2	ALDH3A2
4.07899835346497E-13	0.314509539735171	0.816	0.739	6.65325421433671E-09	2	DHCR24
1.78941484475435E-06	0.314490161855599	0.405	0.321	0.0291871455327883	2	HSD3B7
1.59720867667363E-07	0.311197307506571	0.544	0.471	0.00260520707252235	2	CYP27A1
2.57051587726386E-08	0.307829121237962	0.542	0.454	0.000419276844740508	2	PSMB8
2.70061896860262E-12	0.306381370405942	0.816	0.742	4.40497959968774E-08	2	APCS
2.82732317153169E-17	0.305340965702506	0.773	0.711	4.61164682508535E-13	2	FMO5
6.4823696489979E-19	0.304486708593426	0.86	0.803	1.05733931344805E-14	2	ASGR1
2.48386149127015E-08	0.30269239350567	0.659	0.587	0.000405142647841074	2	QPRT
1.68454942078224E-08	0.301522979471157	0.662	0.577	0.000274766856023791	2	METTL7A
1.09155484469053E-06	0.300390188173438	0.346	0.268	0.0178043510717473	2	ATP5F1E
7.93446342733407E-34	0.299873497896138	0.994	0.992	1.29419032963246E-29	2	C3
1.05097732435596E-14	0.299163807385235	0.852	0.794	1.71424911375701E-10	2	LRG1
8.90042653237196E-08	0.298622428463191	0.496	0.412	0.00145174857169519	2	RNF5
1.06393537872918E-06	0.29554209018922	0.45	0.372	0.0173538499624517	2	ITPR2
9.53176688148219E-07	0.294087375463998	0.501	0.424	0.0155472649603856	2	CUTA
7.69804201263697E-07	0.293699989574395	0.515	0.432	0.0125562763268122	2	H3F3A
2.33380701754829E-06	0.293096446489246	0.37	0.294	0.0380667262632302	2	PIM1
1.12154024348384E-06	0.287313699504919	0.399	0.318	0.018293442911465	2	IQGAP2
6.01506839120522E-08	0.28700741336194	0.284	0.205	0.000981117805289483	2	SARDH
2.7220198681096E-06	0.2865851104756	0.505	0.437	0.0443988660687356	2	ACBD5
1.24683506212921E-06	0.284517526114943	0.513	0.443	0.0203371266983896	2	ECI2
1.20366741213267E-20	0.283761485081613	0.984	0.956	1.9633019159296E-16	2	EPHX1
4.87463001979885E-08	0.281641551788057	0.564	0.476	0.00079510090252939	2	CFL1
2.06688893068887E-06	0.280814402280609	0.427	0.352	0.0337130253484661	2	EEF1G
3.7714738205121E-07	0.279686568575549	0.685	0.621	0.00615165094863728	2	RHOB
7.05217996514106E-08	0.279194966523548	0.705	0.664	0.00115028107411416	2	SERINC2
4.11986502971207E-12	0.278778440653215	0.801	0.741	6.71991184996336E-08	2	PTMS
7.76713057875869E-10	0.276366042896739	0.777	0.737	1.26689666870133E-05	2	IFITM3
2.64067897687588E-06	0.276288446330179	0.474	0.399	0.0430721147918224	2	MAGED2
3.49159220617496E-19	0.272513869648356	0.938	0.883	5.69513604749199E-15	2	AMBP
5.32588535086619E-08	0.267149593692097	0.741	0.716	0.000868705159579784	2	SERPINF1
2.92590372152834E-06	0.259004397644121	0.584	0.516	0.0477244156018487	2	C8A
1.26152656211784E-06	0.25767210933177	0.283	0.213	0.0205767597547042	2	TTPA
1.8477462351836E-17	0.251201191202267	0.998	0.994	3.01385888420797E-13	2	SERPINA1
0	4.12283918099406	0.786	0.057	0	3	HGFAC
0	4.02800670687062	0.842	0.126	0	3	CPS1
1.87908779054543E-263	3.8218891457841	0.375	0.024	3.06498009515865E-259	3	AQP1
3.42458932241029E-279	3.73862436491755	0.611	0.097	5.58584764378342E-275	3	DEPRECAT
6.68573898896994E-259	3.35519332584054	0.846	0.283	1.09051088649089E-254	3	CYP2B6
1.41623981208012E-135	3.09959343204266	0.256	0.027	2.31002875748388E-131	3	NLRP1
1.60878681135059E-190	2.91107787827821	0.394	0.049	2.62409216799394E-186	3	CES4A

1.37146160701792E-219	2.86456995054059	0.638	0.146	2.23699102720693E-215	3	MUC3A
5.0130575740015E-271	2.77741013003292	0.876	0.305	8.17679820895384E-267	3	SDS
6.9964894762696E-187	2.70538291081055	0.316	0.028	1.14119739847433E-182	3	UROC1
6.31558636898042E-209	2.61899683018601	0.576	0.116	1.0301352926444E-204	3	NR1I3
6.39592840294889E-304	2.59881541133863	0.851	0.231	1.04323988180499E-299	3	INSIG1
1.36936818043004E-128	2.54306921306731	0.402	0.083	2.23357643909943E-124	3	CFHR3
2.6130180423629E-164	2.49468027904545	0.856	0.398	4.26209372889813E-160	3	CRP
4.58756085496427E-249	2.42188556217149	0.825	0.253	7.48277051053222E-245	3	BHMT
6.61872814566426E-155	2.39283999326092	0.608	0.177	1.0795807478393E-150	3	AASS
4.13089035042862E-109	2.36222292529886	0.348	0.073	6.73789525058412E-105	3	HPGD
1.40586044844508E-281	2.33622391143276	0.54	0.061	2.29309897745876E-277	3	C9
4.41257029475724E-133	2.2571475246128	0.316	0.046	7.19734340777854E-129	3	DUOXA2
7.49031819545908E-105	2.14317270784759	0.266	0.042	1.22174580086133E-100	3	CYP7A1
3.2720083545289E-204	2.12407618554534	0.754	0.224	5.33697282707209E-200	3	GLS2
1.59142672218496E-176	2.10078407140595	0.438	0.07	2.59577612655588E-172	3	DUOX2
2.41323545457673E-79	2.04766477377798	0.455	0.16	3.9362283499601E-75	3	CTH
4.25027936367887E-126	2.01040808866149	0.355	0.064	6.9326306700966E-122	3	AMOTL1
7.06889883823457E-116	1.97360040184371	0.47	0.126	1.15300808950444E-111	3	DEPDC7
1.40203508329334E-137	1.93374884398747	0.705	0.256	2.28685942435977E-133	3	CHRD
8.86851032065888E-130	1.90319730685955	0.603	0.193	1.44654271840267E-125	3	RAPH1
1.81555372233283E-126	1.80252768288112	0.282	0.037	2.96134967649708E-122	3	LAPTM4B
5.93640578932789E-68	1.74547824674814	0.263	0.062	9.68287148297272E-64	3	NINL
1.00094777144986E-47	1.71650676928787	0.259	0.08	1.63264591001187E-43	3	ADCY10
1.13129360087419E-128	1.6957278680913	0.539	0.151	1.8452529923859E-124	3	OXER1
7.47168867502406E-147	1.66709997005188	0.644	0.201	1.21870713978318E-142	3	FETUB
6.1810636017028E-121	1.66106557500506	0.664	0.247	1.00819328407374E-116	3	GRAMD4
5.39107505139142E-148	1.65322298515071	0.886	0.464	8.79338251632454E-144	3	PCK1
3.22849344232169E-82	1.64196656970545	0.5	0.176	5.26599565377091E-78	3	KCNT2
1.91785289647025E-127	1.64060495457228	0.695	0.259	3.12820985943263E-123	3	PWWP3A
3.61949715804983E-71	1.63201162064545	0.353	0.105	5.90376181449507E-67	3	SDCBP2
1.109899250502E-93	1.59915682587466	0.382	0.097	1.81035666749381E-89	3	SLC15A1
2.22274963252632E-50	1.59385113808943	0.312	0.107	3.62552692561368E-46	3	PROZ
6.87074844301063E-169	1.58801151868485	0.67	0.209	1.12068777853946E-164	3	RIDA
7.15452436958014E-111	1.58488118309586	0.527	0.163	1.16697446992222E-106	3	RDH16
3.70702087812134E-44	1.57880662271967	0.287	0.1	6.04652175430372E-40	3	CLTCL1
2.20982373546413E-176	1.56930007074877	0.886	0.4	3.60444349491554E-172	3	NNMT
8.59640566024875E-107	1.56028360554977	0.359	0.077	1.40215972724317E-102	3	MTTP
3.58672531432622E-127	1.54645596104065	0.848	0.417	5.85030766019749E-123	3	HAL
6.33492629838868E-117	1.54639235238057	0.784	0.376	1.03328982853018E-112	3	CYP2C9
1.51431268312987E-69	1.53992713697493	0.443	0.158	2.46999541745314E-65	3	FYB2
5.79492151703461E-163	1.51710416080272	0.772	0.311	9.45209648643515E-159	3	TMEM176A
2.48557755263344E-188	1.51126883503071	0.951	0.552	4.0542255461004E-184	3	GPT
5.3665125134721E-96	1.50602774357083	0.486	0.15	8.75331856072433E-92	3	ARHGEF26
1.04737488538606E-158	1.46735856952156	0.838	0.365	1.7083731755532E-154	3	AMDHD1
1.51948916767994E-75	1.46561297770942	0.277	0.062	2.47843878140275E-71	3	AGTR1
7.60718206111356E-124	1.46152913918435	0.739	0.304	1.24080746598823E-119	3	CDO1
9.11062861143044E-113	1.46109789197616	0.437	0.108	1.48603463281042E-108	3	SAA4
2.0028086036537E-90	1.45872892683185	0.67	0.286	3.26678111341955E-86	3	FST
3.35003610650042E-65	1.45734822612721	0.394	0.135	5.46424389331284E-61	3	MAST4
3.1523340315407E-120	1.4569135140051	0.816	0.384	5.14177203884604E-116	3	GPT2

1.29971279675789E-53	1.45591718805429	0.28	0.083	2.11996154279179E-49	3	PLEKHF2
3.48634892690265E-121	1.44056311103689	0.884	0.499	5.68658373467091E-117	3	SLC38A4
6.16445011294328E-55	1.4353593784511	0.328	0.108	1.00548345792218E-50	3	DAPK1
6.84209702175185E-152	1.42128458671184	0.818	0.371	1.11601444521794E-147	3	SERPINA6
5.10157140255998E-92	1.42127062375115	0.49	0.158	8.32117311471558E-88	3	AGMO
6.62455072380494E-74	1.41265105418066	0.438	0.149	1.08053046855982E-69	3	MTMR10
2.46276609690888E-114	1.40118291608746	0.892	0.63	4.01701778066808E-110	3	HRG
2.21166386705011E-71	1.39782031464432	0.434	0.149	3.60744493354543E-67	3	RCL1
2.6661665548618E-154	1.39384891952365	0.691	0.23	4.34878426763508E-150	3	ST3GAL1
2.98309083415389E-65	1.36748756255668	0.348	0.107	4.86571945958842E-61	3	LCN2
1.06665051554296E-144	1.35871465076366	0.865	0.437	1.73981365590213E-140	3	HPD
3.92247682512959E-114	1.35615768983835	0.576	0.19	6.39795194946887E-110	3	CCL16
6.77717526510615E-95	1.35524683374083	0.477	0.143	1.10542505749146E-90	3	CDK18
5.23376798817732E-115	1.3533086606253	0.894	0.466	8.53679896551603E-111	3	PLIN5
1.92384653118778E-36	1.35060280402623	0.257	0.093	3.13798607702038E-32	3	LCN12
2.8902518521999E-119	1.34251551729513	0.813	0.39	4.71428979612326E-115	3	G6PC
9.17387270070947E-53	1.33004092465375	0.424	0.175	1.49635037621272E-48	3	MUC13
1.14444740930162E-80	1.31865994111833	0.326	0.081	1.86670816931187E-76	3	SAA2
3.04447822715876E-102	1.31604181838498	0.506	0.161	4.96584843631865E-98	3	APOF
2.72263925837558E-93	1.31245440112724	0.514	0.167	4.44089689433642E-89	3	CXCL2
3.6832996361539E-142	1.30687693873699	0.914	0.486	6.00783003653063E-138	3	ARG1
1.97269720404247E-78	1.30393614885984	0.514	0.188	3.21766640951368E-74	3	PLOD2
5.80531392230903E-107	1.29754889400439	0.874	0.447	9.46904753867826E-103	3	TP53INP1
8.8615053029907E-72	1.29641279598885	0.399	0.13	1.44540012997081E-67	3	MYO1E
4.098713524926E-42	1.28614388495145	0.253	0.082	6.6854116305068E-38	3	TPST1
3.28540650239287E-46	1.28290794883194	0.341	0.126	5.35882654605301E-42	3	GPCPD1
2.13766352652552E-71	1.27687168040276	0.659	0.307	3.48674297811577E-67	3	SLC23A2
9.85982100648653E-45	1.27680800335815	0.32	0.117	1.60823540436802E-40	3	GRB14
4.88783916665474E-83	1.27388288458201	0.615	0.249	7.97255446473055E-79	3	MUC20
1.58186898035232E-44	1.26390716761323	0.486	0.231	2.58018649385266E-40	3	RAPGEF5
9.93278665828131E-58	1.25512352469135	0.389	0.142	1.62013683183226E-53	3	FES
8.66026323113168E-108	1.24174369960258	0.625	0.23	1.41257553562989E-103	3	SLC51A
4.31809967746019E-97	1.24168147238943	0.761	0.356	7.04325238390532E-93	3	P4HA1
7.9836477199552E-40	1.2397755619712	0.295	0.11	1.30221277960189E-35	3	TPCN1
9.89725365219901E-75	1.23526911585766	0.457	0.157	1.61434104321018E-70	3	IL1RAP
2.98535509574151E-92	1.23172831928921	0.826	0.413	4.86941269666398E-88	3	MYOM1
3.54437610484341E-57	1.22188456672636	0.276	0.076	5.78123186461008E-53	3	SNX19
8.84680476495921E-75	1.21586739365985	0.368	0.107	1.4430023252125E-70	3	NDUFAF6
1.22480352982325E-94	1.20534568542728	0.76	0.345	1.9977770374947E-90	3	FTCD
3.03473204871256E-121	1.20484304006224	0.743	0.311	4.94995144465505E-117	3	GNMT
2.55347450460789E-115	1.20136133980224	0.749	0.296	4.16497226446594E-111	3	COL5A3
2.57411786964797E-113	1.20101714210096	0.58	0.196	4.1986436571828E-109	3	RBP5
7.16878766034585E-144	1.19817632109294	0.918	0.618	1.16930095527901E-139	3	TMEM176E
3.06519287570759E-113	1.18888974023316	0.675	0.25	4.99963609956665E-109	3	SLC27A5
4.02329380592519E-101	1.18795358064938	0.682	0.274	6.56239452684458E-97	3	SLC25A18
4.22898383623302E-129	1.18020306612729	0.875	0.433	6.89789553527968E-125	3	ALDH1L1
5.2462497336909E-81	1.17149745615486	0.448	0.148	8.55715794062324E-77	3	AGXT2
1.14532146774767E-57	1.17118935853038	0.254	0.066	1.86813384604322E-53	3	RMDN1
6.36492949891358E-71	1.16495583164112	0.612	0.266	1.03818365056779E-66	3	TMEM184A
5.86620844696725E-81	1.16283973564298	0.432	0.137	9.56837259784828E-77	3	CPNE3

6.81797984963273E-51	1.15046761733013	0.411	0.167	1.1120806932736E-46	3	PPARD
6.16145212287983E-91	1.1495885082338	0.543	0.194	1.00499445576293E-86	3	RNF152
9.33760868981112E-97	1.14856742980252	0.53	0.18	1.52305735339509E-92	3	PBLD
1.01924069156406E-122	1.14602893718069	0.576	0.177	1.66248349201014E-118	3	TTPA
4.23928802193793E-72	1.14527691598359	0.361	0.106	6.91470269258296E-68	3	ACMSD
2.11775503146392E-57	1.14272856272909	0.496	0.21	3.4542702318208E-53	3	ABCA5
2.8176926956401E-51	1.14192567331573	0.412	0.164	4.59593855585857E-47	3	PLGLB2
4.63938602750525E-27	1.13667808468648	0.267	0.115	7.56730254946381E-23	3	DAGLB
7.79635273536509E-74	1.13460013951698	0.552	0.226	1.2716630946654E-69	3	SAA1
1.96606356611616E-36	1.12788883314808	0.292	0.114	3.20684628269208E-32	3	FOXO1
1.67629332734296E-101	1.12524209969652	0.654	0.267	2.7342020462291E-97	3	SDSL
1.7021068836508E-66	1.1232491928696	0.394	0.132	2.77630653792283E-62	3	FBP1
1.45794169002313E-85	1.12308841530581	0.657	0.278	2.37804869059673E-81	3	RND1
6.81596980183205E-49	1.11446916220412	0.461	0.201	1.11175283437683E-44	3	ZDHHC11
5.81696771858595E-41	1.11442968872906	0.355	0.143	9.48805604578554E-37	3	LSG1
3.52045661886498E-102	1.11255479491061	0.534	0.172	5.74221679103067E-98	3	CCL15
1.26009140176839E-67	1.10323665317904	0.516	0.205	2.05533508542442E-63	3	KMT2C
6.11483031794599E-38	1.1022935877332	0.409	0.187	9.9738997316017E-34	3	PLCH2
1.12137464071376E-61	1.09658534849709	0.636	0.306	1.82907417646821E-57	3	ADHFE1
8.94435850914422E-59	1.0964856278985	0.438	0.168	1.45891431642651E-54	3	DEPRECAT
3.02801828660911E-101	1.09622761645881	0.955	0.617	4.93900062728812E-97	3	GLYCTK
1.38680799604334E-76	1.08746119610348	0.563	0.226	2.26202252234629E-72	3	ACSM2B
9.02226633424944E-103	1.08342424146515	0.978	0.674	1.47162186177943E-98	3	PAH
1.51900887203608E-43	1.07917323960716	0.27	0.089	2.47765537117805E-39	3	NIPAL2
2.59511036652328E-57	1.07360145674809	0.254	0.067	4.23288451883612E-53	3	PCOLCE
8.03736352654638E-83	1.07222602134891	0.601	0.244	1.31097436481498E-78	3	PC
8.98689147155856E-73	1.0717557635762	0.707	0.333	1.46585186792592E-68	3	CYP4F3
1.30527507000014E-91	1.06666119135947	0.691	0.305	2.12903416667723E-87	3	HAAO
1.1600656450092E-59	1.06348517893085	0.401	0.145	1.8921830735745E-55	3	CYP4V2
7.05466975914431E-76	1.06204488831869	0.578	0.234	1.15068718441403E-71	3	BCAT2
2.04154575798562E-43	1.05298700532854	0.381	0.156	3.32996528585034E-39	3	NBPF12
1.50876796495226E-51	1.05029452784371	0.671	0.369	2.46095142763364E-47	3	CYP2C8
7.0413798735069E-32	1.04208049454803	0.32	0.14	1.14851947116771E-27	3	VPS54
2.56165002016861E-70	1.03991926783151	0.54	0.22	4.17830734789702E-66	3	ASPG
6.5384625211349E-31	1.03183253881934	0.279	0.115	1.06648862182231E-26	3	SUGCT
3.13469500765566E-58	1.02966169617596	0.3	0.088	5.11300102698715E-54	3	PDE3B
9.31773748593967E-65	1.02492300851413	0.782	0.419	1.51981616133162E-60	3	CYP21A2
6.10732210435868E-62	1.02448729104512	0.602	0.281	9.96165308441944E-58	3	SORBS2
3.65919210232733E-98	1.02424601424553	0.991	0.946	5.9685082381061E-94	3	PLA2G2A
5.76104901985777E-28	1.02392807358177	0.273	0.117	9.39684705629001E-24	3	SLC45A3
1.4072592879035E-79	1.01704795108877	0.909	0.632	2.2953806244994E-75	3	TAT
1.86645563416293E-94	1.0146724050698	0.625	0.242	3.04437578488316E-90	3	ALDH8A1
4.7750018740529E-62	1.01007668793991	0.46	0.178	7.78850555676769E-58	3	FARP1
8.5748946039589E-55	1.00835995886102	0.516	0.224	1.39865105885174E-50	3	IL17RB
4.01133604686841E-93	1.00753037557193	0.876	0.494	6.54289022604707E-89	3	ACSL1
1.10101337942844E-100	0.998284558811858	0.856	0.465	1.79586292318572E-96	3	DPYS
7.11574360978607E-69	0.997195639338154	0.563	0.238	1.16064894019221E-64	3	TRIM55
1.84737133641008E-77	0.991388403699092	0.615	0.266	3.01324738681848E-73	3	TMEM45A
3.12885573138886E-88	0.987195441690761	0.932	0.6	5.10347658346836E-84	3	HGD
7.6113196100261E-67	0.98245326480993	0.682	0.328	1.24148234159136E-62	3	FAM20A

1.71952144129232E-62	0.982183552082306	0.579	0.253	2.8047114228919E-58	3	ADH4
6.18029539504801E-38	0.975389920134864	0.266	0.095	1.00806798188628E-33	3	UBE2D3
4.36582976775701E-32	0.974660547558516	0.267	0.105	7.12110493418846E-28	3	RBM26
2.72458430047589E-73	0.973097671745472	0.835	0.484	4.44406945250622E-69	3	ACSM2A
4.91623548770629E-59	0.972912514399646	0.473	0.191	8.01887170399773E-55	3	NUDT7
7.50287230585891E-31	0.970082358253891	0.272	0.109	1.22379350180865E-26	3	NOTCH2
4.67321985340219E-33	0.969603066507666	0.267	0.102	7.62248890288432E-29	3	ABCG8
1.61536036698933E-56	0.967296424169847	0.315	0.098	2.6348142945963E-52	3	LIMA1
2.54090811689765E-32	0.966185603539983	0.323	0.141	4.14447522947175E-28	3	ITGA7
8.86642700106797E-21	0.965133996242142	0.272	0.132	1.4462029081442E-16	3	SAMD12
1.03264463151122E-75	0.954655525147012	0.698	0.348	1.68434665845795E-71	3	CFHR4
7.58423109476697E-62	0.951897335019053	0.648	0.31	1.23706393386744E-57	3	RBM33
1.08938197435663E-37	0.951367754807598	0.408	0.186	1.77689093837311E-33	3	NUP42
2.49208954189598E-37	0.94911719707431	0.326	0.134	4.06484725178654E-33	3	CYP4F12
1.19254039342362E-42	0.949038919369907	0.3	0.109	1.94515263571326E-38	3	NUDT9
4.38935245888685E-38	0.947841903382162	0.322	0.129	7.15947279569033E-34	3	PLA2G15
1.70321714251566E-34	0.943772447814739	0.293	0.116	2.7781174811573E-30	3	DAB2
2.51180278252239E-87	0.94353220093201	0.776	0.418	4.09700151857226E-83	3	TOB1
7.5393033718952E-31	0.942844025823709	0.267	0.108	1.22973577298983E-26	3	ZC3H13
4.17864580192985E-25	0.942499896520789	0.287	0.133	6.81578916752777E-21	3	ARHGAP29
4.01532013337302E-38	0.942140982770522	0.362	0.155	6.54938866954473E-34	3	SEMA6C
4.56223645741372E-35	0.941825013324128	0.353	0.154	7.44146388568752E-31	3	PNPLA3
1.30009853949597E-38	0.939211690821574	0.493	0.248	2.12059072777187E-34	3	DCPS
6.5183149171295E-44	0.938940455109597	0.392	0.162	1.06320234613299E-39	3	SLC26A1
4.93019356721231E-55	0.937342795082063	0.582	0.269	8.04163872748E-51	3	ABCG5
1.7066321459471E-34	0.936591434170854	0.447	0.226	2.78368769325432E-30	3	USP35
3.69829903688281E-63	0.931704835017279	0.305	0.085	6.03229555905955E-59	3	ADH6
1.2818598227894E-41	0.925836509540397	0.313	0.118	2.0908415569518E-37	3	HSD17B14
8.67451984449906E-45	0.925510675590387	0.58	0.297	1.41490093183624E-40	3	ALAS1
1.79927438701655E-29	0.925267750588253	0.359	0.173	2.9347964526627E-25	3	GAS2
2.14298539577573E-62	0.92458990761405	0.78	0.418	3.4954234790498E-58	3	CEP72
1.33194560677936E-77	0.923019572727338	0.721	0.363	2.17253647921782E-73	3	EGFR
1.20301356107184E-20	0.922684069181844	0.293	0.148	1.96223541946428E-16	3	ZNF280D
6.52815857211272E-69	0.922118886442546	0.684	0.322	1.06480794469731E-64	3	COBLL1
6.3013194041947E-41	0.92193217298647	0.361	0.148	1.0278082080182E-36	3	EPB41L4B
4.55510564860201E-22	0.918701178204983	0.277	0.134	7.42983282343474E-18	3	HMGXB3
1.39926085774196E-18	0.918456125808463	0.273	0.139	2.28233438506292E-14	3	TTC14
6.27002488799916E-52	0.915096291767137	0.468	0.198	1.02270375948154E-47	3	WWP1
4.98566992571454E-78	0.912949842645712	0.931	0.589	8.13212621583299E-74	3	ITIH4
3.16414832783883E-59	0.905044189484106	0.545	0.24	5.16104233753791E-55	3	KYNU
1.11373307221366E-71	0.903741647720996	0.845	0.455	1.8166100140877E-67	3	MST1
1.23751573523542E-61	0.900789996309106	0.375	0.126	2.0185119157425E-57	3	MCRIP1
3.73441982538096E-64	0.900270354712006	0.499	0.199	6.09121217717888E-60	3	CYP7B1
1.55077792758923E-21	0.896219061194353	0.267	0.126	2.52947387769079E-17	3	WDR19
5.13593085382321E-38	0.893656229396982	0.329	0.133	8.37721681567104E-34	3	SNX13
4.36012945070413E-79	0.891681386144368	0.813	0.429	7.1118071470435E-75	3	SAT1
7.14777028447057E-71	0.889268617032008	0.497	0.187	1.1658728111E-66	3	HYAL1
1.17415432931116E-43	0.888925613630169	0.499	0.237	1.91516312653943E-39	3	SLC33A1
5.424596900923E-68	0.88777198458459	0.348	0.103	8.8480600050955E-64	3	AKR1C4
1.17811609086794E-24	0.8860670623147	0.297	0.14	1.9216251558147E-20	3	SGK3

**1.07453108840434E-63**	0.881504534266291	0.361	0.115	1.75266765829633E-59	3	CYP51A1
**7.73616995070564E-94**	0.880552519553423	0.733	0.369	1.2618466806596E-89	3	REEP6
**9.11583760857816E-35**	0.879957860524979	0.28	0.11	1.48688427233518E-30	3	TM4SF1
**7.98642435433858E-64**	0.878737881305754	0.966	0.666	1.30266567643617E-59	3	C4BPB
**2.52230373930735E-42**	0.877058694877233	0.375	0.153	4.11412962918422E-38	3	UBE3C
**2.77374280683923E-35**	0.874887794300344	0.295	0.117	4.52425189223546E-31	3	POLB
**7.81001471527722E-59**	0.871501907480453	0.851	0.475	1.27389150020887E-54	3	OBSCN
**2.8485192686098E-93**	0.867086089999364	0.688	0.306	4.64621977902945E-89	3	RARRES2
**2.7950103303413E-77**	0.865066383694539	0.976	0.757	4.55894134981969E-73	3	MAT1A
**2.91502270200611E-47**	0.864438164393257	0.44	0.186	4.75469352924216E-43	3	C19orf25
**8.12768331802935E-27**	0.863247690856542	0.273	0.118	1.32570642600377E-22	3	NDUFAF7
**1.26271724099504E-50**	0.862316978229791	0.349	0.125	2.059618091787E-46	3	INTS8
**7.80234962589476E-63**	0.861954844885518	0.483	0.187	1.27264124747969E-58	3	SMARCA1
**4.02563145105628E-56**	0.859111253772588	0.647	0.313	6.56620745981789E-52	3	SRRM2
**1.84548265972464E-64**	0.858281422706798	0.53	0.214	3.01016676627687E-60	3	LMO7
**7.27391170952515E-48**	0.857442489795002	0.493	0.224	1.18644773894065E-43	3	TJP1
**2.33916804519646E-71**	0.857190039683425	0.355	0.104	3.81541699851994E-67	3	TIMP3
**4.81420983414416E-92**	0.856419458084399	0.634	0.268	7.85245766047254E-88	3	DECR1
**2.53960368595579E-88**	0.85600382996772	0.691	0.316	4.14234757216249E-84	3	SLC22A1
**7.00526834755814E-40**	0.853119846436606	0.418	0.188	1.14262932017021E-35	3	SLC2A9
**2.25064810237409E-28**	0.849409305621216	0.338	0.157	3.67103211978238E-24	3	PHC3
**1.4373324095993E-39**	0.849129400706077	0.267	0.092	2.34443289329742E-35	3	MYH10
**1.82023156651402E-39**	0.846645651945459	0.389	0.166	2.96897970814102E-35	3	NBPF26
**2.76220666409141E-29**	0.846124808561693	0.263	0.107	4.50543528979949E-25	3	KIAA1191
**6.22766297487914E-20**	0.844522386336896	0.261	0.127	1.01579410783254E-15	3	TBK1
**9.39499184911562E-52**	0.844209478601539	0.625	0.303	1.53241712050925E-47	3	PDZK1
**7.88501819976183E-69**	0.843769239270652	0.603	0.267	1.28612531856315E-64	3	ELL2
**8.16448474208729E-54**	0.84361335869023	0.434	0.175	1.33170910628186E-49	3	FAXDC2
**3.46495058227064E-44**	0.841695813110664	0.274	0.091	5.65168089474164E-40	3	ACTR3C
**4.20123820914359E-29**	0.837691612544565	0.303	0.133	6.8526396429341E-25	3	DGLUCY
**7.40002065291933E-59**	0.837204235091822	0.572	0.261	1.20701736869767E-54	3	SLC43A3
**1.07719021607341E-35**	0.834292834621527	0.392	0.18	1.75700496143735E-31	3	SLC25A15
**3.48081965115234E-26**	0.834109283408986	0.323	0.153	5.67756493299458E-22	3	MFN1
**1.07689663878343E-59**	0.831025111643184	0.736	0.395	1.75652610751965E-55	3	STAT2
**4.93986699100564E-74**	0.829807945719157	0.605	0.26	8.0574170490293E-70	3	CPQ
**3.57474508376777E-40**	0.829018244194816	0.898	0.579	5.83076670613361E-36	3	SLC12A7
**6.49151104534454E-72**	0.828149158736643	0.641	0.287	1.05883036660615E-67	3	SLC22A18
**1.46557674521679E-22**	0.827966218194413	0.283	0.136	2.3905022291231E-18	3	NEIL1
**3.35177793498452E-95**	0.825121319848715	0.678	0.288	5.46708498975326E-91	3	MAOB
**3.97081284754447E-33**	0.824783947275852	0.453	0.23	6.47679283562979E-29	3	NT5C2
**5.07188560039009E-49**	0.821002756761607	0.497	0.222	8.27275260279627E-45	3	LIPG
**4.28932913180331E-46**	0.820775384948839	0.645	0.338	6.99632474688438E-42	3	SLC17A9
**5.09549198601013E-24**	0.813969020213507	0.261	0.116	8.31125697838112E-20	3	BRD8
**1.33478325568679E-70**	0.813052038386355	0.756	0.395	2.17716496835073E-66	3	F13B
**4.19553553805791E-48**	0.810410268567603	0.519	0.241	6.84333801612626E-44	3	MTMR4
**2.33168365839871E-35**	0.809022384735345	0.391	0.178	3.80320921521414E-31	3	ZC3H3
**1.63197293079526E-82**	0.808930252125671	0.575	0.225	2.66191104742014E-78	3	SIAH2
**2.49032758079815E-65**	0.808131054690232	0.665	0.326	4.06197331703986E-61	3	STEAP3
**2.97340081432566E-37**	0.80556698101687	0.405	0.185	4.84991406824658E-33	3	SH3PXD2A
**1.41888989585155E-42**	0.804093648020364	0.603	0.32	2.31435130912346E-38	3	TNFAIP2

**1.77116097387939E-37**	0.797978563696021	0.477	0.234	2.88894066449467E-33	3	GDA
**1.28977444180954E-42**	0.797954140561872	0.516	0.249	2.10375109203554E-38	3	PLXNB1
**1.99936957382381E-21**	0.797510211029963	0.293	0.145	3.26117171186402E-17	3	BCL6
**8.87660418022706E-32**	0.796542262875934	0.283	0.114	1.44786290783684E-27	3	PUS3
**1.30783749745005E-28**	0.796363906304841	0.391	0.194	2.13321374209077E-24	3	NCOA2
**3.44145822209078E-43**	0.790456516495367	0.346	0.133	5.61336250605226E-39	3	UBE3A
**2.54760916640771E-22**	0.788724118584749	0.349	0.181	4.15540531132761E-18	3	ZNF76
**6.19816289996926E-83**	0.788592883725505	0.861	0.568	1.01098235061399E-78	3	DEPRECAT
**6.76475996112491E-26**	0.787414106930319	0.328	0.156	1.10339999725908E-21	3	SS18L1
**9.96521349183683E-50**	0.787383387329314	0.345	0.123	1.62542597265351E-45	3	THPO
**1.08974854963243E-19**	0.78709951949384	0.33	0.178	1.77748885930546E-15	3	CCDC14
**7.6239047928353E-53**	0.785500288472085	0.516	0.231	1.24353511075937E-48	3	PLGLB1
**2.40270962139853E-19**	0.78525050045265	0.25	0.12	3.91905966346315E-15	3	PRKAR2A
**2.52617173573382E-46**	0.783993321155393	0.414	0.173	4.12043871815543E-42	3	PRODH2
**9.36336909559129E-26**	0.781410510705989	0.325	0.154	1.52725913318189E-21	3	ZNF655
**5.42985966342253E-48**	0.774869276656596	0.608	0.3	8.85664409700848E-44	3	SLC22A25
**2.5300432173967E-49**	0.774656767746779	0.539	0.253	4.12675349189576E-45	3	KLHL24
**7.14761734479781E-39**	0.774337710133474	0.292	0.109	1.16584786510997E-34	3	GLCE
**8.7560039050304E-31**	0.774022363169805	0.401	0.198	1.42819179694951E-26	3	PHF20L1
**4.85875573532895E-45**	0.773228280968114	0.345	0.129	7.92511647989505E-41	3	AFF1
**2.72957971273986E-76**	0.770422820124949	0.836	0.537	4.45221746944998E-72	3	DCXR
**7.11390799123137E-62**	0.768558521941693	0.507	0.209	1.16034953244975E-57	3	ACAT2
**2.39888671818559E-51**	0.767048718086322	0.282	0.086	3.91282412603251E-47	3	TMEM64
**1.2416928199216E-52**	0.765994093657117	0.382	0.139	2.02532515857413E-48	3	RNF13
**1.46858397457621E-45**	0.761401081196896	0.562	0.278	2.39540732093125E-41	3	XRN1
**1.47003549271948E-23**	0.759736329974189	0.364	0.188	2.39777489217475E-19	3	KAT2A
**2.82147504743055E-54**	0.759391732909871	0.444	0.178	4.60210794986398E-50	3	AS3MT
**2.25824008245271E-41**	0.758263731605804	0.585	0.307	3.68341539848862E-37	3	FAM193B
**1.28723502697645E-56**	0.755150317793009	0.707	0.363	2.09960905250128E-52	3	F11
**1.91440280609603E-52**	0.754160710957644	0.328	0.108	3.12258241702323E-48	3	ALPK3
**3.53092092968741E-39**	0.751708326488333	0.32	0.126	5.75928512841314E-35	3	ESYT2
**8.325158793936E-85**	0.750792449751002	0.974	0.821	1.3579166508789E-80	3	CP
**1.36908997480301E-56**	0.749142376655198	0.816	0.458	2.2331226579012E-52	3	FNDC3B
**3.52044778549961E-36**	0.746981214978241	0.529	0.277	5.74220238292841E-32	3	CRY2
**7.5942609679673E-34**	0.745595490304733	0.336	0.143	1.23869990648515E-29	3	SYNRG
**3.88180113848553E-59**	0.745453325980892	0.881	0.643	6.33160583698375E-55	3	IGFBP2
**4.58277215158453E-54**	0.744806630743151	0.805	0.462	7.47495965644952E-50	3	LEPR
**1.61918292597244E-57**	0.744761311633909	0.92	0.76	2.64104927055364E-53	3	SERPINA3
**8.25061409300404E-35**	0.743989465122861	0.361	0.16	1.34575766470989E-30	3	MAP1LC3A
**1.36078078121667E-24**	0.743173675460956	0.303	0.143	2.21956953224251E-20	3	DXO
**7.17894351725864E-20**	0.741446938866278	0.378	0.211	1.17095747710006E-15	3	TANGO2
**3.25631879351917E-66**	0.740949150775128	0.636	0.308	5.31138158410912E-62	3	LGALS4
**8.04174371477471E-56**	0.740788263460915	0.55	0.251	1.3116888173169E-51	3	MCCC1
**1.12734008000809E-23**	0.740398841033823	0.33	0.164	1.83880440450119E-19	3	MORC3
**2.69045399528023E-24**	0.740049674665724	0.368	0.191	4.38839951170159E-20	3	CPSF1
**6.60316485895838E-29**	0.73789769195507	0.359	0.169	1.0770422201447E-24	3	MBNL1
**9.31103433122526E-44**	0.737221140542038	0.539	0.265	1.51872280976615E-39	3	ACSF2
**1.67532881235282E-43**	0.73636419435487	0.402	0.17	2.73262882582869E-39	3	GADD45G
**8.26185741951069E-59**	0.735134290733773	0.56	0.249	1.34759156369639E-54	3	CNN3
**1.29758257503262E-24**	0.733976264817854	0.355	0.18	2.11648693813571E-20	3	ATL2

**3.33902228552804E-43**	0.732399771592948	0.343	0.131	5.44627924992478E-39	3	CYP4F2
**4.58695909062036E-33**	0.731955003625648	0.43	0.209	7.48178897271087E-29	3	BCKDHB
**6.34628693868598E-21**	0.730583250159883	0.332	0.173	1.03514286256907E-16	3	ZNF91
**1.12711532064498E-45**	0.72834871783142	0.739	0.419	1.83843779950402E-41	3	PLIN4
**1.79611508424311E-20**	0.727562211074388	0.267	0.129	2.92964331390893E-16	3	SIPA1L1
**2.21901256750408E-54**	0.725322285453614	0.649	0.325	3.6194313988559E-50	3	SLCO2B1
**1.45114089927252E-71**	0.724927138382781	0.874	0.541	2.36695592080341E-67	3	CFI
**5.46826928792289E-54**	0.724601461864536	0.739	0.389	8.91929403553102E-50	3	BCL3
**4.0075317997111E-49**	0.724286968016131	0.57	0.278	6.53668511850877E-45	3	SLC25A25
**7.24435946703389E-57**	0.723759584872558	0.645	0.343	1.1816274726679E-52	3	SLPI
**1.49157374541767E-71**	0.722940323343024	0.553	0.225	2.43290593615076E-67	3	PPP1R1A
**1.260809989277E-16**	0.719190991263752	0.257	0.135	2.05650717350971E-12	3	FBXL8
**6.72273181113128E-80**	0.719186785917493	0.839	0.496	1.09654478571362E-75	3	C8G
**6.56720620778663E-61**	0.717798482440557	0.655	0.321	1.07117700455208E-56	3	ACO1
**1.46453645907679E-38**	0.717748112258078	0.493	0.238	2.38880541840015E-34	3	ABCA6
**3.50106872247085E-64**	0.716625331697181	0.509	0.199	5.7105931932222E-60	3	TSPAN8
**3.17185496595587E-79**	0.716598013720951	0.441	0.144	5.17361263497061E-75	3	LIME1
**2.64572047392399E-34**	0.712343509409724	0.307	0.125	4.31543466501742E-30	3	GLI4
**3.49250308136394E-43**	0.71096629986232	0.484	0.231	5.69662177601272E-39	3	NDRG1
**5.73244509913839E-45**	0.710354387064556	0.299	0.105	9.35019120120463E-41	3	LRATD2
**1.13412221071833E-16**	0.710168523327594	0.273	0.145	1.84986673790266E-12	3	MPHOSPH9
**6.34692395622198E-23**	0.709132875019487	0.259	0.116	1.03524676649937E-18	3	SELENOI
**1.83226062183966E-27**	0.709059537573452	0.427	0.223	2.98860030028267E-23	3	THBS3
**1.37476290672808E-34**	0.708681269616971	0.293	0.116	2.24237577716416E-30	3	KLB
**4.75606523972107E-29**	0.708620975970938	0.366	0.176	7.75761801250903E-25	3	SLC25A38
**2.47346350603848E-51**	0.705472070427557	0.644	0.335	4.03446632469936E-47	3	AHR
**3.9247650699271E-48**	0.705164268128765	0.711	0.378	6.4016843055581E-44	3	PPP2R1B
**4.55581027882457E-25**	0.704137172513324	0.29	0.132	7.43098214579075E-21	3	AVEN
**2.02962282723583E-19**	0.702583179051804	0.351	0.188	3.31051779350436E-15	3	RBM5
**2.36914840613748E-45**	0.702394519688692	0.573	0.283	3.86431796525085E-41	3	U2SURP
**1.0915505217043E-17**	0.701571611099628	0.353	0.199	1.78042805595188E-13	3	DMAP1
**3.69335158297439E-72**	0.698872888515015	0.68	0.335	6.02422576698953E-68	3	PIPOX
**2.9395445962035E-23**	0.696732874778085	0.499	0.291	4.79469119086753E-19	3	KNDC1
**8.57142579103453E-60**	0.696409302464478	0.379	0.127	1.39808526077564E-55	3	LECT2
**6.64586896604771E-71**	0.695808846310817	0.428	0.143	1.08400768705204E-66	3	UQCRB
**1.34512859723809E-69**	0.689588256836292	0.958	0.727	2.19403925495505E-65	3	APCS
**3.70725326502308E-26**	0.68950086604994	0.261	0.112	6.04690080057915E-22	3	SIK1B
**2.4031747206338E-57**	0.68837572190953	0.614	0.296	3.91981828682579E-53	3	CYP4F11
**1.13824033622904E-59**	0.687147856701637	0.894	0.645	1.85658381242318E-55	3	LBP
**7.76413357834519E-36**	0.68266725944449	0.522	0.27	1.26640782796388E-31	3	EXOC3L4
**5.4858809971652E-40**	0.682637948827105	0.601	0.324	8.94802049447616E-36	3	RB1CC1
**1.07897976072639E-35**	0.680516114989185	0.506	0.258	1.75992388772082E-31	3	MTHFR
**7.10109216857479E-57**	0.680450626405521	0.856	0.465	1.15825914361623E-52	3	ABCC2
**1.03165291569549E-29**	0.679470970631073	0.325	0.144	1.68272907079092E-25	3	ZNF7
**3.26259348216344E-27**	0.67905257453103	0.295	0.13	5.32161622875678E-23	3	MLXIP
**1.48254216472023E-42**	0.679036060573216	0.69	0.372	2.41817452487517E-38	3	FBXO7
**5.36322442533728E-19**	0.678542385625824	0.292	0.154	8.74795536016764E-15	3	TIMM10B
**8.22503098411019E-25**	0.678394263510093	0.272	0.122	1.34158480381821E-20	3	LRP6
**1.57444925672427E-45**	0.67830072525777	0.576	0.286	2.56808418264296E-41	3	MAP3K13
**6.9989993908724E-31**	0.677847818698899	0.397	0.189	1.1416067906452E-26	3	MBNL2

8.63290873892146E-58	0.677125949133489	0.29	0.083	1.40811374440548E-53	3	LCAT
3.89013163164053E-72	0.676814761241243	0.632	0.291	6.34519370436887E-68	3	SDC2
2.35716331729745E-33	0.676317976599649	0.364	0.165	3.84476908684387E-29	3	DHX36
1.26305870471126E-33	0.673915805901436	0.384	0.177	2.06017505325454E-29	3	FAM91A1
6.64580119984263E-34	0.67341041550689	0.425	0.207	1.08399663370633E-29	3	IMPA1
1.14667282777319E-23	0.673278085209974	0.322	0.159	1.87033804938085E-19	3	NBPF15
1.27569819443955E-14	0.67114904012279	0.259	0.139	2.08079132495035E-10	3	TDRD10
1.51554078114781E-46	0.671047311270993	0.593	0.294	2.4719985681302E-42	3	CLSTN3
6.67791459409508E-21	0.669774171856844	0.284	0.141	1.08923464944285E-16	3	EPDR1
1.87929106698034E-20	0.669229850272428	0.296	0.147	3.06531165935164E-16	3	ATF7IP2
2.81406975692303E-40	0.667502985511329	0.299	0.11	4.59002918051716E-36	3	MSMO1
1.24434664470729E-45	0.666862391961852	0.369	0.146	2.02965381218206E-41	3	TRIM22
1.16854027701214E-49	0.666574323100498	0.641	0.331	1.9060060458345E-45	3	RNF19A
7.55595244654167E-50	0.666026471408346	0.405	0.162	1.23245140355541E-45	3	F9
5.48015715042727E-33	0.665363471855522	0.369	0.166	8.93868432806192E-29	3	LRCH3
2.72573848847053E-32	0.664927348765075	0.437	0.216	4.44595204854428E-28	3	ERBB2
1.03929566088161E-27	0.664803798214852	0.415	0.211	1.695195152464E-23	3	NUFIP2
7.9212965309458E-46	0.663515779338642	0.614	0.309	1.29204267716257E-41	3	RAB17
6.10495772232836E-24	0.661540271642375	0.284	0.134	9.95779654088979E-20	3	SLC25A30
9.13723419625018E-33	0.660644052245046	0.402	0.188	1.49037426975037E-28	3	UBXN2B
2.3386852466849E-35	0.66054277967514	0.648	0.36	3.81462950586773E-31	3	RBM39
1.27213379009649E-21	0.659853750464634	0.398	0.22	2.07497742502638E-17	3	SPATA13
3.87797509139135E-76	0.659698355781876	0.999	0.992	6.32536517156843E-72	3	FGA
1.02935629514991E-48	0.658532946681945	0.608	0.303	1.67898305301902E-44	3	GCLM
1.21933679748835E-17	0.658330498903015	0.25	0.126	1.98886025038325E-13	3	SOCS3
1.55258037721517E-18	0.655475327014774	0.31	0.164	2.53241385327567E-14	3	CPLANE1
1.54029598603978E-23	0.65412895167042	0.345	0.172	2.51237678282949E-19	3	KIDINS220
2.93254086398806E-56	0.651734578128072	0.659	0.338	4.78326740325092E-52	3	GOT1
6.84299789912937E-34	0.651357942950494	0.552	0.297	1.11616138732699E-29	3	SOX5
1.35389163849655E-17	0.650756305184983	0.381	0.221	2.20833265155173E-13	3	TNK2
5.95243587615143E-19	0.650535873487349	0.341	0.187	9.7090181575906E-15	3	GATAD1
4.30450181910564E-20	0.650035202327986	0.267	0.129	7.02107291714321E-16	3	TMEM87A
1.46022311999878E-29	0.649829360886516	0.32	0.141	2.38176993103001E-25	3	VPS41
7.79481920625447E-61	0.648600824011654	0.777	0.44	1.27141296073217E-56	3	C8B
2.66063750692305E-24	0.6482458922713	0.487	0.276	4.33976583754218E-20	3	TBC1D8
1.24850006096866E-42	0.646646329958697	0.642	0.34	2.03642844944598E-38	3	CLK1
1.90242852660385E-35	0.646197592921083	0.625	0.343	3.10305116974354E-31	3	PCCA
7.669274150317E-25	0.646087832857456	0.45	0.243	1.25093530665821E-20	3	SFXN5
9.18089037542063E-36	0.644361854897023	0.348	0.15	1.49749502913486E-31	3	PARL
2.59921678458845E-40	0.644177365228327	0.684	0.383	4.23958249734222E-36	3	OGA
5.2665191258364E-64	0.642182068513317	0.767	0.421	8.59021934615175E-60	3	PAPSS2
9.61001082292501E-23	0.641861752308232	0.269	0.124	1.5674888653273E-18	3	CCNB1IP1
4.90063286465815E-25	0.640570230143454	0.353	0.176	7.99342226554391E-21	3	AKTIP
6.53260492391396E-12	0.640136185729831	0.26	0.151	1.06553318913961E-07	3	TOP3B
4.869494346942E-28	0.639366199280459	0.497	0.27	7.9426322292971E-24	3	DEPRECAT
2.1985841886327E-22	0.637198162108841	0.376	0.2	3.5861106700788E-18	3	SLC19A2
1.02468548213636E-35	0.636074095548966	0.963	0.695	1.67136448991261E-31	3	CYP3A5
1.96992351153385E-46	0.63560478265925	0.631	0.327	3.21314223966286E-42	3	SEMA4G
6.99969889169892E-30	0.634031941495698	0.428	0.216	1.14172088622501E-25	3	DEPRECAT
1.45502244945563E-40	0.632478636462618	0.648	0.349	2.37328711730707E-36	3	RSRP1

3.72072275160115E-52	0.631626515068476	0.789	0.453	6.06887088013664E-48	3	KLF15
4.54811283148582E-25	0.631581566722748	0.333	0.161	7.41842683943651E-21	3	ZBTB10
2.07659055301635E-14	0.629342210828123	0.315	0.181	3.38712685102498E-10	3	HNRNPL
4.96978806584769E-54	0.627547206794606	0.638	0.319	8.10622131420417E-50	3	ZNRF2
5.2221477594361E-47	0.627130307836427	0.647	0.34	8.51784521041623E-43	3	IL6ST
1.40726147641653E-33	0.62655213404529	0.381	0.173	2.295384194183E-29	3	AVL9
1.9282311749929E-33	0.62396914229345	0.526	0.28	3.14513786953092E-29	3	PARP14
4.32752711166398E-59	0.623626861878953	0.545	0.23	7.05862947183512E-55	3	FASTK
5.02525529498301E-32	0.623041921126096	0.494	0.258	8.19669391164679E-28	3	VPS13B
1.61701263181212E-33	0.622222040095884	0.391	0.182	2.63750930374875E-29	3	SENP2
7.17639662463898E-44	0.621759489353063	0.582	0.294	1.17054205344486E-39	3	TMEM175
3.38274811810026E-23	0.620346506637922	0.51	0.295	5.51760045543333E-19	3	DDX17
5.94923138724148E-44	0.619880349746088	0.559	0.284	9.70379131572957E-40	3	TFRC
1.25990035401186E-29	0.6194724999106	0.376	0.179	2.05502346742874E-25	3	PTK2
5.18072139236744E-36	0.618796129554786	0.494	0.25	8.45027466309052E-32	3	KIAA0319L
1.01362708727019E-24	0.617800409072546	0.323	0.157	1.65332714204641E-20	3	TBC1D23
2.41244308950735E-14	0.615456122343649	0.274	0.153	3.93493592329544E-10	3	SIRT5
1.20059174236908E-54	0.615240060829295	0.754	0.43	1.9582851909782E-50	3	C6
9.5069001096569E-45	0.614725768709194	0.599	0.305	1.55067047688614E-40	3	PPARA
2.07262775690656E-74	0.614619709620135	0.994	0.993	3.38066313429029E-70	3	FGB
2.8248222625761E-21	0.613690677599826	0.339	0.177	4.60756759248788E-17	3	SON
7.20187866605645E-50	0.611915624039648	0.747	0.419	1.17469842922047E-45	3	LPIN2
5.06002419478948E-36	0.61180612375293	0.649	0.36	8.25340546412113E-32	3	MTG1
1.35429918119643E-42	0.611358576991861	0.841	0.475	2.2089973944495E-38	3	MDM4
2.35552089278553E-23	0.609967499377015	0.257	0.114	3.84209012822248E-19	3	B9D1
1.98861044538496E-35	0.609406211994391	0.411	0.187	3.2436224974674E-31	3	LRBA
1.17392065045656E-47	0.608350416536242	0.664	0.35	1.9147819729597E-43	3	NMD3
1.84372066773468E-23	0.608246086566293	0.326	0.159	3.00729278114204E-19	3	LRRC14
1.91236436539657E-37	0.606653546969219	0.895	0.761	3.11925751639835E-33	3	ORM2
1.74530136910405E-24	0.606134051744554	0.404	0.213	2.84676106314562E-20	3	SEC24A
1.00370421321876E-13	0.605795098294507	0.25	0.137	1.63714194218112E-09	3	HERC1
1.23959565504716E-35	0.605487615345426	0.608	0.331	2.02190447294742E-31	3	NRBP2
2.92761293663446E-50	0.605339301579887	0.591	0.283	4.77522946094447E-46	3	ACSM5
3.15536404168942E-22	0.605141437628989	0.369	0.196	5.14671428839962E-18	3	RPRD2
4.61925681657733E-19	0.604032520195689	0.272	0.136	7.53446979351929E-15	3	CASP3
1.11379336946814E-21	0.602760931405536	0.489	0.289	1.81670836493948E-17	3	SLC25A29
3.36201099121165E-52	0.600149284247917	0.622	0.323	5.48377612776532E-48	3	DCAF11
1.17045879934088E-28	0.598633794769608	0.483	0.261	1.90913534760491E-24	3	JMJD1C
1.28588120689523E-36	0.597906528461104	0.487	0.245	2.09740083656681E-32	3	EIF2AK1
2.90608782790688E-49	0.597018673375634	0.385	0.148	4.74011985609892E-45	3	SIGIRR
1.325708765262E-22	0.595874404301119	0.417	0.228	2.16236356701885E-18	3	FBXW7
1.71873071812141E-48	0.595114446108134	0.797	0.455	2.80342167432784E-44	3	MASP1
6.67345342962284E-51	0.594978600638423	0.751	0.424	1.08850698890578E-46	3	BHMT2
1.41420714608658E-80	0.594451502885654	0.49	0.171	2.30671327598182E-76	3	GGH
7.99483119581192E-19	0.594355783406965	0.279	0.141	1.30403691634888E-14	3	ZNF26
2.92014076180232E-35	0.591933742638655	0.273	0.102	4.76304159657576E-31	3	PRKAG2
6.14344920820191E-25	0.590035458877925	0.342	0.169	1.00205800034981E-20	3	ANKRD13A
1.50685175713435E-16	0.588979015078806	0.375	0.222	2.45782590106183E-12	3	CENPT
4.21528829264977E-19	0.586353916961205	0.399	0.225	6.87555673414104E-15	3	GABRE
9.13495846804944E-39	0.580904116015137	0.533	0.27	1.49000307572354E-34	3	GALM

5.5608013746236E-39	0.58087698576036	0.7	0.386	9.07022312214855E-35	3	LPP
1.93525392198043E-44	0.580812741714966	0.619	0.332	3.15659267214229E-40	3	TTC39C
2.34576744641375E-28	0.580046242167976	0.312	0.141	3.82618128184547E-24	3	NF1
8.59685283053356E-27	0.578828741032593	0.516	0.287	1.40223266518833E-22	3	TRA2A
1.93614163912893E-16	0.575657175360109	0.339	0.194	3.1580406275832E-12	3	PICK1
9.27592498574488E-19	0.574814000642553	0.417	0.243	1.51299612442485E-14	3	ZNF648
1.71825721409636E-31	0.573651327343615	0.579	0.324	2.80264934191258E-27	3	PPARGC1A
7.40011083231617E-21	0.573215173490636	0.414	0.233	1.20703207785909E-16	3	DEPRECAT
4.14943317166327E-34	0.572979219282611	0.473	0.236	6.76814044629996E-30	3	CACNA1H
1.23251655647117E-39	0.572844205598785	0.333	0.131	2.01035775526013E-35	3	PDE4DIP
2.1397846978224E-54	0.571261642862031	0.43	0.171	3.49020282061811E-50	3	CIDEB
5.55837960045438E-21	0.570180068562102	0.368	0.195	9.06627296630114E-17	3	TTC37
4.26173105366926E-27	0.567952839394347	0.374	0.182	6.95130952163993E-23	3	F12
1.02641914495802E-23	0.567848018728384	0.44	0.242	1.67419226734102E-19	3	GLDC
4.73242105037078E-44	0.566588564874977	0.626	0.327	7.71905197525978E-40	3	SYMPK
2.40624809028288E-20	0.566587385049898	0.42	0.236	3.92483126006041E-16	3	PHF8
1.40018217687339E-36	0.564717149556202	0.427	0.198	2.28383714869819E-32	3	WIPI2
2.14691363997077E-45	0.564693597051322	0.737	0.404	3.50183083815633E-41	3	SLC22A23
4.86123100885896E-20	0.564335771437036	0.438	0.251	7.92915389854985E-16	3	RBL2
8.19063746108698E-30	0.564330503810782	0.29	0.121	1.3359748762779E-25	3	FBXL3
1.21026874161594E-22	0.563889455487788	0.251	0.112	1.97406934444975E-18	3	C5orf51
1.3642647317793E-56	0.563027091547507	0.651	0.335	2.22525220400522E-52	3	ALDH6A1
1.79301114877979E-39	0.56262541166233	0.389	0.169	2.92458048477471E-35	3	TP53INP2
1.04652716910376E-22	0.56243613409191	0.293	0.139	1.70699046552515E-18	3	SSBP3
1.99366504549317E-33	0.562027544537234	0.417	0.202	3.25186705570392E-29	3	FUT6
3.87587827041632E-47	0.561824262078747	0.445	0.189	6.32194504687607E-43	3	AATF
9.01366214196929E-39	0.561079894941991	0.624	0.335	1.47021843197661E-34	3	TNRC6A
1.28766491510945E-29	0.560648703820433	0.389	0.19	2.10031024303503E-25	3	SLC6A11
1.43522065036858E-21	0.560327491980309	0.418	0.234	2.34098840281619E-17	3	TLE1
4.72874150455278E-45	0.559746708766374	0.58	0.299	7.71305026807604E-41	3	S100A13
2.63988732966753E-19	0.55972703138013	0.392	0.223	4.30592022342071E-15	3	WDR6
2.89614137411129E-20	0.559436249972973	0.289	0.144	4.72389619531293E-16	3	MYNN
5.02970945624956E-37	0.558382528419379	0.963	0.888	8.20395909408865E-33	3	ORM1
2.95753764258525E-25	0.557790630697546	0.289	0.132	4.8240396488208E-21	3	DCUN1D1
2.56820995304117E-35	0.556242340909208	0.941	0.607	4.18900725440546E-31	3	MLXIPL
4.6294148899949E-29	0.554515259652201	0.405	0.2	7.55103862707068E-25	3	ATG4B
6.67347763947589E-41	0.554151922495193	0.272	0.093	1.08851093777491E-36	3	MAD1L1
4.3044248896654E-23	0.553828092939581	0.276	0.13	7.02094743753324E-19	3	KBTBD2
3.66003702063654E-25	0.553444170959724	0.514	0.304	5.96988638436025E-21	3	DDIT4
1.73044736039353E-35	0.552817688622332	0.338	0.143	2.82253268953789E-31	3	CHCHD7
7.16990826731014E-43	0.552703141325398	0.829	0.553	1.16948373748096E-38	3	ALDH1A1
7.81661973933938E-50	0.551436034437918	0.826	0.493	1.27496884568365E-45	3	OGDHL
2.8626822346831E-40	0.549439892174641	0.54	0.266	4.6693209929916E-36	3	TTC3
1.31800231187135E-42	0.546845175175464	0.769	0.43	2.14979357089336E-38	3	SPG7
4.28603785720634E-42	0.546581280070325	0.454	0.207	6.99095634888926E-38	3	PLA2G12B
3.07271229331755E-21	0.5461005187479	0.297	0.146	5.01190102163025E-17	3	DDX3X
1.71753906752572E-21	0.54605683618248	0.428	0.237	2.8014779730412E-17	3	PTS
1.61080487383782E-33	0.545746669157426	0.43	0.208	2.62738382971686E-29	3	HERPUD2
4.84510175776379E-34	0.544806805380038	0.705	0.401	7.90284547708852E-30	3	SNRNP70
4.84318247736226E-20	0.544709177996444	0.32	0.165	7.89971493882558E-16	3	SIK3

7.88340786425431E-37	0.544189127971475	0.534	0.275	1.28586265673852E-32	3	LGR4
4.45480819005435E-15	0.543604987095009	0.29	0.161	7.26623763879765E-11	3	SKIV2L
3.24185130311444E-31	0.542988021654304	0.53	0.283	5.28778366050996E-27	3	HGSNAT
2.11824602820379E-41	0.54294284971701	0.522	0.255	3.45507109660321E-37	3	EIF4A2
8.95321583400487E-25	0.542627978562703	0.309	0.146	1.46035903468453E-20	3	CCT8
1.8735033326116E-44	0.542476926079128	0.346	0.131	3.05587128582278E-40	3	TLCD2
5.38712619671882E-60	0.540636278458132	0.625	0.31	8.78694153946806E-56	3	BTG1
4.89126017711909E-33	0.540452957182856	0.408	0.193	7.97813447489894E-29	3	SARDH
3.51690018852438E-14	0.539524856089466	0.335	0.199	5.73641589750211E-10	3	ZNF44
2.83928236749047E-19	0.538862950659653	0.527	0.321	4.63115346961371E-15	3	LUC7L
1.70432271109955E-42	0.538764506395145	0.636	0.342	2.77992077407447E-38	3	PROX1
3.79820231294957E-20	0.53822498597171	0.438	0.253	6.19524779265205E-16	3	CHKA
1.20095945178354E-22	0.537588402095615	0.345	0.176	1.95888496180413E-18	3	GALNS
5.30338128463451E-31	0.535401386056245	0.632	0.368	8.65034521336735E-27	3	SLC41A2
9.14608761970121E-33	0.535102145029016	0.486	0.251	1.49181835164946E-28	3	MCF2L
1.97116821729469E-71	0.534474049308108	0.655	0.32	3.21517247922937E-67	3	C7orf50
9.66144826352369E-23	0.53324350502554	0.323	0.162	1.57587882626335E-18	3	EIF4G3
9.66383180123467E-52	0.532486413544331	0.532	0.243	1.57626760509939E-47	3	DHCR7
8.80053332063466E-27	0.531916031049171	0.428	0.223	1.43545498992872E-22	3	MON2
3.11566648781031E-26	0.531020717307867	0.533	0.304	5.0819636082674E-22	3	FAM214A
2.35155038418227E-42	0.528318003360755	0.362	0.147	3.83561383163969E-38	3	SLC16A2
2.24038300911242E-49	0.528315360727223	0.911	0.616	3.65428872616326E-45	3	SLC38A3
3.11810243003291E-37	0.527523860669951	0.622	0.342	5.08593687362669E-33	3	LARP4
1.59038245202372E-11	0.5271449029414	0.343	0.219	2.59407281749588E-07	3	VNN 3.00
5.25029775878349E-25	0.526831008561701	0.407	0.209	8.56376067435175E-21	3	GLYATL1
9.31378334630615E-35	0.526150089682803	0.75	0.432	1.519171201616E-30	3	XAF1
4.64472059322814E-29	0.525672607617332	0.496	0.264	7.57600375961442E-25	3	OTULIN
2.86561807750962E-47	0.525593213372875	0.698	0.381	4.67410964622594E-43	3	DPP9
2.96172859211763E-55	0.525046283224912	0.451	0.179	4.83087550660306E-51	3	GCHFR
6.66254764832242E-30	0.524905005754766	0.553	0.308	1.08672814691787E-25	3	ABCC6
1.84511124372608E-20	0.523222429948994	0.296	0.15	3.00956094964161E-16	3	TMEM245
4.68145521260296E-14	0.52322201469169	0.292	0.165	7.63592159727668E-10	3	RBFOX2
1.32484609824926E-16	0.521809308567087	0.261	0.135	2.16095647085436E-12	3	RHEB
1.66448722644613E-08	0.521362405718717	0.254	0.159	0.000271494511505628	3	C1orf52
4.89968576464824E-20	0.521135721113478	0.27	0.132	7.99187745071775E-16	3	TADA1
5.17582064209681E-31	0.520863094299778	0.463	0.237	8.44228104932411E-27	3	GPLD1
1.38851423748441E-18	0.520661966681161	0.296	0.153	2.26480557276081E-14	3	DAZAP1
4.81412672785075E-32	0.520517099509161	0.486	0.25	7.85232210579736E-28	3	RANBP10
1.12081214149205E-19	0.520307484827924	0.303	0.155	1.82815668398769E-15	3	SENP5
7.98918030407633E-72	0.519935605685646	0.687	0.337	1.30311519939789E-67	3	ALDH4A1
6.08727723637199E-16	0.519109560690903	0.27	0.144	9.92895790024635E-12	3	TNKS2
4.17587096270382E-17	0.518549501834438	0.382	0.223	6.8112631272662E-13	3	KDM4B
1.66881383520008E-53	0.518171902161564	0.754	0.419	2.72200224659485E-49	3	ABAT
4.50343210611706E-58	0.51800788835668	0.503	0.209	7.34554810828753E-54	3	AK4
1.19980172860275E-23	0.516272653888538	0.341	0.17	1.95699659952394E-19	3	MAPRE2
2.61228755690694E-22	0.515136379635698	0.339	0.172	4.26090223407092E-18	3	PIP4K2B
3.32945227716588E-26	0.514709452522116	0.292	0.131	5.43066960928527E-22	3	FXR1
8.2215464416334E-33	0.513563049602911	0.424	0.204	1.34101644009482E-28	3	CDK9
6.32877139380135E-35	0.512866659838839	0.486	0.244	1.03228590204294E-30	3	KCTD20
3.21621423396328E-49	0.512826942372915	0.776	0.423	5.2459670370175E-45	3	NOTCH2NL

5.25076418330944E-38	0.512644588396164	0.371	0.159	8.56452145939603E-34	3	SULT1A1
1.7342941145473E-35	0.512498639164562	0.529	0.276	2.8288071302381E-31	3	LSS
6.75483928991728E-33	0.510510767738479	0.654	0.368	1.10178183657841E-28	3	NKTR
2.95413387981192E-26	0.510346711877738	0.295	0.133	4.81848777136123E-22	3	DHRSX
8.45864736861616E-22	0.510249670587006	0.408	0.224	1.37968997229498E-17	3	PLRG1
2.06093565771846E-32	0.508015114180395	0.657	0.374	3.36159215130458E-28	3	POLR2H
3.8570270922313E-16	0.50749964808395	0.299	0.164	6.29119689013847E-12	3	NXF1
5.08025991889886E-26	0.505994648039657	0.511	0.29	8.28641195371592E-22	3	SLC25A16
5.7270571598956E-45	0.505611816851991	0.638	0.338	9.34140293350572E-41	3	TM7SF2
8.78673757137278E-34	0.504695196451189	0.504	0.255	1.43320476526661E-29	3	TRAPPC9
5.63512188448206E-11	0.503853499669897	0.28	0.167	9.19144730577868E-07	3	LPIN1
1.406004478456E-18	0.500707955937584	0.356	0.197	2.29333390480958E-14	3	RORA
1.02504235246606E-21	0.500617175944097	0.348	0.177	1.67194658110739E-17	3	FBH1
2.47065074599233E-22	0.500174315875105	0.401	0.219	4.02987843178809E-18	3	PHLDB2
6.47789889061739E-27	0.499960084703098	0.467	0.253	1.0566100880486E-22	3	CEP70
1.95397182200955E-34	0.498498108097271	0.264	0.098	3.18712343887977E-30	3	KLF13
3.9397444310981E-34	0.498164985578144	0.473	0.235	6.42611714156411E-30	3	PCMTD1
2.10404772497555E-12	0.497528331033741	0.293	0.174	3.43191224420762E-08	3	CNOT2
3.91774285300092E-27	0.497513888392387	0.444	0.237	6.39023036752979E-23	3	AKAP8L
2.15824266343615E-25	0.497400866263537	0.448	0.245	3.52030960833071E-21	3	EPB41L5
2.30119777789638E-15	0.49730472512657	0.48	0.3	3.75348369552679E-11	3	TUBGCP6
1.439866779906E-31	0.496065923862996	0.698	0.402	2.34856670470468E-27	3	SETD5
6.40694432115417E-43	0.495899553903837	0.677	0.362	1.04503668822346E-38	3	AIG1
4.70775155324007E-47	0.495620583514229	0.43	0.176	7.67881355848988E-43	3	AQP7
3.70914589173421E-37	0.494746662976813	0.766	0.438	6.04998786400768E-33	3	CCNL1
3.6488909442278E-18	0.49369919821643	0.32	0.174	5.95170601912996E-14	3	CDK13
4.99823445242374E-44	0.493329699063421	0.507	0.236	8.15262021534835E-40	3	HSD17B11
2.87238293727511E-29	0.493229661771449	0.491	0.264	4.68514380898943E-25	3	ELOVL5
4.28231470343489E-14	0.49193744909565	0.307	0.175	6.98488351277265E-10	3	PSEN1
1.91518067207215E-56	0.491800126250168	0.714	0.401	3.12385119421688E-52	3	GRHPR
5.82192467512455E-38	0.490114729121155	0.894	0.533	9.49614133759565E-34	3	CDK5RAP3
2.24460011055279E-35	0.488774477176969	0.592	0.322	3.66116724032266E-31	3	ELP2
1.02248023198071E-55	0.488667037230265	0.691	0.355	1.66776750638374E-51	3	FGGY
6.61275445260923E-17	0.488634013742554	0.372	0.213	1.07860637876509E-12	3	TEAD1
5.77617243654084E-34	0.488510107901359	0.792	0.497	9.42151486124176E-30	3	PTGR1
6.68254372518419E-36	0.487626767800198	0.478	0.24	1.08998970701479E-31	3	KDELR2
2.50211295553185E-43	0.487144472598186	0.813	0.472	4.08119644176799E-39	3	IL6R
1.40744426815762E-24	0.486830854599809	0.44	0.239	2.29568234579189E-20	3	FBXO9
5.18695794581921E-17	0.486456779809268	0.263	0.135	8.46044710542572E-13	3	IFNGR2
2.06342564586605E-31	0.485853822222818	0.717	0.461	3.36565357097211E-27	3	TSKU
3.58615968626879E-45	0.4857175639529	0.468	0.211	5.84938506427302E-41	3	CFHR5
3.47835775227876E-19	0.48557343765353	0.44	0.258	5.67354932974189E-15	3	CALCOCO1
9.59888677125077E-26	0.484395780178064	0.451	0.247	1.56567442125871E-21	3	DEPRECAT
3.00061203352583E-23	0.48405233822927	0.371	0.191	4.89429828788398E-19	3	KIAA0100
8.03779387759556E-22	0.482845170303184	0.313	0.155	1.31104455937461E-17	3	PLA2G12A
4.88960294544333E-55	0.482727800622365	0.79	0.479	7.97543136431261E-51	3	IFI27
3.50576544529572E-18	0.482595524950753	0.358	0.199	5.71825401782184E-14	3	RELCH
1.52907492839516E-43	0.482510828364154	0.83	0.482	2.49407411570534E-39	3	SLC7A2
2.25753759675892E-10	0.481179682544341	0.257	0.154	3.68226957407347E-06	3	DIP2B
1.05349841448866E-21	0.480752747284788	0.478	0.271	1.71836126387245E-17	3	PFKFB1

9.93126628272559E-20	0.48059714330492	0.326	0.172	1.61988884337537E-15	3	EPC1
3.64888028110315E-27	0.479578089531591	0.378	0.184	5.95168862650734E-23	3	SKAP2
1.06729368366369E-20	0.477990055101278	0.306	0.153	1.74086272742384E-16	3	TMEM63A
3.41422362515744E-22	0.477086571378092	0.481	0.274	5.5689401549943E-18	3	KDM4C
1.73673357521397E-43	0.476779445982626	0.644	0.346	2.83278613453151E-39	3	ACADSB
7.73667263500417E-26	0.47579370714857	0.499	0.275	1.26192867349553E-21	3	PHKA2
4.06103724242545E-13	0.475762730852974	0.254	0.142	6.62395784612016E-09	3	CES3
1.42176814163528E-32	0.475483878915307	0.67	0.392	2.3190460158213E-28	3	IVNS1ABP
1.06057927896837E-20	0.474853816661776	0.3	0.152	1.7299108619253E-16	3	CBR4
3.21576251153695E-29	0.474827980984213	0.579	0.327	5.24523023256791E-25	3	CYTH1
3.90085494599167E-20	0.474721362912534	0.365	0.195	6.36268450240702E-16	3	MAN2A1
1.76634212256511E-22	0.474556760696571	0.361	0.187	2.88108063611595E-18	3	STK3
2.10402975590046E-41	0.473880827625054	0.556	0.28	3.43188293484925E-37	3	SLCO1B1
3.78171559273978E-37	0.473785424503382	0.911	0.543	6.16835630331785E-33	3	CCNL2
4.3904371364643E-26	0.473165467606131	0.264	0.113	7.16124201328691E-22	3	HFE
5.91347135717858E-17	0.473077604072973	0.27	0.139	9.64546313069398E-13	3	SPTBN2
4.08150856200305E-34	0.472642301757374	0.549	0.291	6.65734861548317E-30	3	DLG1
4.44417329078557E-11	0.471910501825696	0.251	0.147	7.24889105460034E-07	3	DDX59
2.07518391740612E-31	0.4717967732804	0.358	0.162	3.38483248768112E-27	3	CMTM6
6.57138282050862E-34	0.470636455663607	0.708	0.407	1.07185825185316E-29	3	NR1I2
2.61741915781559E-23	0.470628293588966	0.391	0.207	4.26927238831301E-19	3	ARAF
2.52074515616344E-32	0.470143942760978	0.292	0.119	4.11158742421818E-28	3	UGT1A9
9.85674895516123E-31	0.469672245630039	0.575	0.321	1.60773432207635E-26	3	PI4K2B
1.46899625274896E-24	0.469401011702273	0.537	0.309	2.39607978785882E-20	3	TNS3
5.51218376180601E-18	0.468510890394566	0.306	0.162	8.99092293388178E-14	3	IL1R1
3.49211231905178E-31	0.4682148660639	0.592	0.328	5.69598440360535E-27	3	SGK2
3.26460739835092E-32	0.467637043973099	0.645	0.367	5.32490112745019E-28	3	TNFRSF14
1.56565199274234E-11	0.467081272711013	0.333	0.207	2.55373496536203E-07	3	SMAD5
8.57960843714577E-25	0.466728342106848	0.5	0.277	1.39941993218285E-20	3	ATP11B
5.56332850795913E-17	0.466418551902788	0.269	0.14	9.07434512933214E-13	3	NPRL3
1.97938100183297E-35	0.465936971532323	0.658	0.37	3.22856835208976E-31	3	SIK2
7.08045982760443E-39	0.465588843711682	0.849	0.516	1.15489380248056E-34	3	ONECUT1
1.37302311668625E-44	0.465367299054796	0.825	0.503	2.23953800562693E-40	3	ETS2
1.21694121513364E-40	0.465356952697841	0.911	0.652	1.98495281600448E-36	3	BDH1
6.8439012120563E-52	0.464803735318158	0.569	0.279	1.1163087266985E-47	3	HTRA1
1.1151580237874E-14	0.46477376020623	0.269	0.145	1.81893425259963E-10	3	MTRR
4.00690337977664E-09	0.463992206535524	0.27	0.17	6.53566010275368E-05	3	ARL 16.00
1.34936653960772E-23	0.462906278468597	0.349	0.176	2.20095176275415E-19	3	MOGAT3
1.98104598578359E-14	0.462201305883228	0.279	0.156	3.23128410741162E-10	3	MTX2
6.49370219948771E-59	0.461289276039769	0.688	0.371	1.05918776575844E-54	3	LY6E
1.06295497816707E-53	0.459869986818016	0.658	0.336	1.73378586488831E-49	3	FAM20C
1.00196043560519E-20	0.459652671671561	0.325	0.166	1.63429766651562E-16	3	CAMKK2
7.34561371853346E-28	0.459479127044323	0.473	0.25	1.19814305362999E-23	3	VDAC3
9.58825841448493E-55	0.458933230418961	0.513	0.221	1.56394082998664E-50	3	DHTKD1
7.76139812207268E-26	0.458488051763663	0.553	0.313	1.26596164769127E-21	3	KIAA1217
6.77044987769705E-34	0.458463034886039	0.483	0.24	1.10432807955117E-29	3	LRRC75B
5.70256722835107E-44	0.457943459880421	0.947	0.833	9.30145740616343E-40	3	A2M
1.60184915839269E-45	0.456175342290087	0.951	0.82	2.61277616225432E-41	3	SERPINF2
2.43335223120336E-33	0.455258379774736	0.632	0.355	3.96904082431579E-29	3	TRPC4AP
1.78441984773777E-10	0.455242315042002	0.299	0.187	2.91056721364507E-06	3	CLK4

1.48279254021882E-14	0.455229434198806	0.397	0.24	2.41858291235091E-10	3	PAWR
2.45319610294327E-18	0.454194380190732	0.414	0.241	4.00140816351076E-14	3	SPPL2B
1.5424849940176E-24	0.452600738224236	0.51	0.29	2.51594727374211E-20	3	TRIO
7.8848810627731E-10	0.451284668329391	0.254	0.155	1.28610295014892E-05	3	CLDN15
6.66253469460038E-33	0.450857212337736	0.295	0.12	1.08672603403627E-28	3	TMEM123
1.12153750830195E-24	0.450853417107842	0.468	0.257	1.82933982979131E-20	3	ACSS2
1.66054060301084E-51	0.45067419930568	0.575	0.277	2.70850777757098E-47	3	MFSD3
6.81375305142854E-50	0.450418181847563	0.77	0.453	1.11139126021851E-45	3	CPB2
4.95171495632974E-20	0.449717819709925	0.401	0.226	8.07674226526943E-16	3	HIGD1A
7.58216181114493E-35	0.449442999090303	0.526	0.271	1.23672641301585E-30	3	SCRN2
2.05544717949026E-34	0.449441230738879	0.399	0.184	3.35263989446656E-30	3	GATD3B
1.53349289131902E-30	0.44815714402473	0.506	0.27	2.50128025503046E-26	3	CUL7
4.30497147085235E-33	0.446884111309692	0.458	0.227	7.02183896610726E-29	3	ABCF3
4.25232711394555E-21	0.445892782641247	0.425	0.24	6.93597075555659E-17	3	TUT4
4.00130467583929E-35	0.444416206646734	0.717	0.402	6.52652805676147E-31	3	CHDH
9.48226794420408E-14	0.443119415306678	0.266	0.147	1.54665272437913E-09	3	ERCC6
1.08222529976431E-24	0.443106995937847	0.325	0.157	1.76521768644556E-20	3	PAK2
3.34736531811153E-21	0.442989733924978	0.277	0.132	5.45988757037172E-17	3	ZNF384
4.22105371544966E-13	0.442889106363616	0.276	0.158	6.88496071526994E-09	3	ZNF507
1.16616233390934E-24	0.441268487410381	0.578	0.346	1.90212738283953E-20	3	SLC38A2
7.55426846052867E-20	0.440815579611761	0.286	0.143	1.23217672859683E-15	3	MMAB
2.42881264305629E-21	0.440795514395673	0.263	0.123	3.96163630208911E-17	3	SNX10
1.37486647686082E-19	0.440763169617559	0.326	0.171	2.24254471040769E-15	3	HELZ
5.25248950721109E-18	0.440757850897585	0.437	0.256	8.56733563521201E-14	3	SLC35A3
5.46094756789807E-17	0.439941880494532	0.302	0.164	8.90735157799854E-13	3	SUOX
4.62769660729875E-16	0.439594280810855	0.323	0.181	7.54823593616499E-12	3	L3MBTL4
5.3052098024405E-27	0.439119144845119	0.328	0.153	8.6533277087607E-23	3	HERC2
1.58162806201854E-07	0.4386412738657	0.273	0.178	0.00257979353195844	3	ST3GAL6
1.19245029732624E-46	0.43792822847199	0.756	0.468	1.94500567996883E-42	3	MT-CYB
1.17280499369235E-30	0.437593589749141	0.474	0.244	1.91296222521159E-26	3	SLC46A1
4.90244908956305E-23	0.43742264543155	0.302	0.144	7.9963847099863E-19	3	PALM2-AK/
6.06923790804285E-30	0.436942502549454	0.264	0.107	9.8995339518087E-26	3	GPC6
1.8001116104179E-43	0.435997260844962	0.642	0.338	2.93616204775263E-39	3	PGPEP1
3.04695546721245E-43	0.43580604325539	0.57	0.289	4.96988906257023E-39	3	TGM2
4.63222397886563E-21	0.435513494437816	0.427	0.233	7.55562053192773E-17	3	EIF2B5
1.49905789258289E-23	0.434407759190486	0.323	0.16	2.44511332859195E-19	3	PPP3CB
7.79178056606232E-18	0.433436350470673	0.272	0.138	1.27091732813042E-13	3	KIAA1109
1.28369021246697E-26	0.433274080589348	0.463	0.246	2.09382710555487E-22	3	OSBPL9
1.32629184812065E-22	0.432486511563304	0.338	0.17	2.16331463346958E-18	3	SF3B1
8.6652305271709E-18	0.432029921964131	0.276	0.142	1.41338575128685E-13	3	PPP4R3A
4.47702807259805E-32	0.431763084292577	0.803	0.473	7.30248048921468E-28	3	WSB1
1.75713958225683E-41	0.431461271303595	0.846	0.595	2.86607037261912E-37	3	GAMT
1.53864332875761E-50	0.431249979480717	0.851	0.57	2.50968113353654E-46	3	SLC9A3R2
1.90261534848024E-26	0.4287696552722	0.978	0.785	3.10335589490612E-22	3	ITIH3
2.51316506407439E-25	0.428003381671927	0.349	0.172	4.09922353601173E-21	3	TLCD4
2.94224096667144E-20	0.427666399299013	0.486	0.286	4.79908924073778E-16	3	STX16
7.56801178741826E-34	0.427318834555388	0.547	0.291	1.23441840264579E-29	3	TMEM106C
3.23341534656274E-11	0.427273501420565	0.272	0.161	5.27402377177849E-07	3	TRPV1
3.74473798662157E-25	0.427214504415938	0.447	0.238	6.10804212997844E-21	3	PCMTD2
8.2631143028818E-19	0.42696358224935	0.468	0.276	1.34779657394305E-14	3	TAF1D

2.21348998641206E-14	0.426751241901826	0.289	0.164	3.61042351683672E-10	3	SMLR1
2.56343753005334E-23	0.423926020404713	0.451	0.254	4.18122295527E-19	3	CREBZF
1.52539677646931E-31	0.423841054936786	0.329	0.141	2.48807468209908E-27	3	IFI16
4.1255874139745E-56	0.423722201664355	0.678	0.355	6.72924563093381E-52	3	MTDH
2.56869114429078E-28	0.423451062329074	0.441	0.228	4.18979212545269E-24	3	UBR5
3.05037958467653E-23	0.423077103723712	0.364	0.185	4.97547414056589E-19	3	MTFR1L
1.03596364437262E-15	0.422682614269469	0.542	0.344	1.68976030033618E-11	3	ALS2CL
3.38036204883399E-10	0.422504965374324	0.284	0.176	5.51370853785311E-06	3	SLC15A4
4.1700500790446E-18	0.422090876064904	0.336	0.186	6.80176868392965E-14	3	HEXD
7.07872046685381E-12	0.421760796066508	0.307	0.186	1.15461009534852E-07	3	TMEM39A
3.37916605900236E-33	0.421205838048235	0.478	0.242	5.51175775883875E-29	3	PLSCR4
2.45037795428716E-32	0.420692812829044	0.487	0.25	3.99681148123779E-28	3	ARHGEF12
1.50433452775094E-28	0.42052306136141	0.635	0.373	2.45372004821456E-24	3	UAP1
2.58069387079133E-23	0.419870662067834	0.322	0.158	4.20936977264775E-19	3	CPEB2
1.15042677791149E-36	0.419387791850394	0.787	0.483	1.87646111745144E-32	3	MTSS1
1.63756919929557E-12	0.419243027464358	0.293	0.173	2.671039120971E-08	3	MED23
6.24514444575289E-31	0.418890691862463	0.338	0.152	1.01864551054675E-26	3	THOP1
8.28179387773436E-52	0.416483835998375	0.603	0.291	1.35084339939725E-47	3	F10
2.72992051342006E-22	0.416435960824064	0.336	0.174	4.45277334943946E-18	3	FPGS
1.35425127653037E-19	0.416212165731333	0.355	0.19	2.20891925714868E-15	3	TRIP12
6.53221296358665E-25	0.415759337454976	0.375	0.193	1.06546925649062E-20	3	KLHDC10
2.69782108750581E-38	0.415371676781626	0.823	0.598	4.40041597583073E-34	3	ST6GAL1
2.63422379845819E-24	0.414567362291885	0.362	0.181	4.29668243766515E-20	3	RNF123
9.75085380014493E-34	0.414438438142938	0.889	0.718	1.59046176334164E-29	3	DEPRECAT
1.03404283934925E-17	0.414205410332659	0.384	0.217	1.68662727526256E-13	3	ZFAND1
5.01853861590278E-22	0.41345140421536	0.473	0.271	8.18573833639903E-18	3	RHOBTB3
7.35160321818924E-32	0.412297863974238	0.631	0.363	1.19912000091885E-27	3	PNRC1
1.22208581485398E-14	0.41188347829393	0.299	0.171	1.99334417260833E-10	3	DEAF1
3.71854914033282E-47	0.41187847686658	0.78	0.468	6.06532550279687E-43	3	UGT2B4
3.99905502517179E-12	0.411548279291459	0.289	0.168	6.52285865155771E-08	3	PCNX1
7.68061298108387E-19	0.410805077998253	0.316	0.167	1.25278478334459E-14	3	BCL2L11
1.2552210305868E-35	0.409904511117573	0.703	0.4	2.04739102299013E-31	3	SHROOM1
3.98694691457005E-15	0.409426857831684	0.32	0.182	6.50310911235521E-11	3	CDK12
2.67923974295432E-20	0.408869770351671	0.453	0.261	4.3701079447328E-16	3	PNISR
2.96617949809761E-36	0.408237866579605	0.542	0.279	4.838135379347E-32	3	GGNBP2
4.70174934183712E-49	0.407987259580187	0.493	0.22	7.66902335147052E-45	3	ALG3
9.50732039963735E-19	0.407295883426893	0.46	0.274	1.55073903038485E-14	3	ARIH2
6.19516114096324E-22	0.405920240732277	0.399	0.218	1.01049273370251E-17	3	RANBP9
3.61402831312464E-44	0.405858594905381	0.687	0.38	5.89484158153759E-40	3	AMN
3.13257880990644E-10	0.405556420540888	0.254	0.152	5.1095492968384E-06	3	CDK14
2.99452401147315E-07	0.405227976049113	0.261	0.172	0.00488436811511386	3	CCDC84
4.74635353762591E-57	0.404027641386794	0.533	0.231	7.74177725522163E-53	3	ASPDH
2.02630406431529E-25	0.404001216300068	0.486	0.269	3.30510455930466E-21	3	KLC4
2.36167174346408E-28	0.403911813878657	0.629	0.366	3.85212278076427E-24	3	TTC38
1.9361035249343E-22	0.403529684667939	0.477	0.27	3.15797845952034E-18	3	GATAD2B
6.9129892845969E-30	0.403411044677523	0.976	0.74	1.1275776822106E-25	3	EFNA1
2.66453486058069E-42	0.402260295422986	0.72	0.411	4.34612281109317E-38	3	INTS1
3.17332898457078E-25	0.401848906516552	0.348	0.169	5.1760169067334E-21	3	CDC25B
2.25933664941634E-39	0.401414655747115	0.829	0.533	3.68520400886299E-35	3	SELENBP1
1.82852863380902E-25	0.401349746337867	0.711	0.43	2.98251305460589E-21	3	ARAP1

2.04238066578036E-27	0.401318445949599	0.48	0.256	3.33132710395434E-23	3	UBA7
2.40844222795332E-32	0.399121681128143	0.751	0.443	3.92841011801466E-28	3	SELENOO
2.07940424973706E-29	0.398390121989493	0.626	0.359	3.39171627174612E-25	3	MAT2A
4.955386951747E-47	0.39835037779374	0.997	0.996	8.08273165699453E-43	3	FGG
9.37487206726098E-26	0.3978591995183	0.517	0.292	1.52913538289094E-21	3	BNIP2
2.79080358229114E-24	0.397427971140301	0.289	0.134	4.55207972307508E-20	3	ZNF106
2.91940039066433E-12	0.397224840014683	0.282	0.165	4.76183397721259E-08	3	USP25
6.04546101608298E-51	0.396868267076134	0.695	0.365	9.86075146333295E-47	3	CTSS
1.22170016490571E-18	0.39675731337071	0.289	0.145	1.9927151389777E-14	3	TRAPPC10
3.22450284574029E-35	0.396630088611114	0.49	0.245	5.25948659168698E-31	3	TAX1BP1
3.0431605404486E-21	0.396211521174472	0.384	0.207	4.96369915752572E-17	3	LRRFIP2
1.61539832698258E-19	0.396043556358435	0.378	0.209	2.63487621114128E-15	3	YPEL3
1.65655612592469E-16	0.395922860314945	0.284	0.151	2.70200869699577E-12	3	FBXW8
4.15995028103801E-36	0.394989785120392	0.665	0.377	6.7852949034011E-32	3	NAPRT
3.3955505009028E-35	0.394848918344642	0.898	0.687	5.53848242202256E-31	3	AZGP1
1.01582913549018E-21	0.393619983511121	0.369	0.195	1.65691890289804E-17	3	KLHL22
2.94283047655368E-17	0.393415507965457	0.266	0.138	4.8000507903067E-13	3	UBA3
4.90327145116212E-35	0.392344821192789	0.797	0.492	7.99772606399054E-31	3	HJV
1.41181304191776E-10	0.39160725906699	0.274	0.167	2.30280825267206E-06	3	NUP210
2.25498171213637E-15	0.391603140464037	0.261	0.137	3.67810067066563E-11	3	SKIL
3.20925306301438E-27	0.391356347033367	0.366	0.178	5.23461267108276E-23	3	PCYT1A
1.34421056917837E-13	0.390489070007379	0.315	0.183	2.19254185938683E-09	3	PEX6
4.78333169660162E-40	0.39012908776132	0.351	0.142	7.8020923303269E-36	3	ERG28
1.26679047067212E-19	0.389561267186155	0.299	0.151	2.06626193671329E-15	3	TM9SF4
9.83996248638096E-23	0.38908091792202	0.596	0.357	1.6049962811536E-18	3	ITPR2
2.49736003664611E-44	0.388240551709317	0.448	0.197	4.07344395577347E-40	3	CD99L2
1.12896522241915E-14	0.388112208388729	0.29	0.162	1.84145517428788E-10	3	NSMCE2
1.57882143294027E-30	0.387518239528658	0.767	0.466	2.57521563926887E-26	3	NFKBIZ
7.41548723895601E-40	0.386723657761094	0.533	0.261	1.20954012354611E-35	3	NOTCH2NL
4.07002373502718E-23	0.386390679182049	0.652	0.4	6.63861571420284E-19	3	MARC2
6.49133895017108E-20	0.386225176924783	0.339	0.179	1.0588022961624E-15	3	PIK3R4
2.88980768490433E-15	0.3857985761326	0.364	0.213	4.71356531484746E-11	3	VPS39
6.67504599105862E-11	0.385338057996873	0.259	0.153	1.08876675160157E-06	3	TRIM4
6.77031534991408E-53	0.38460498670266	0.734	0.407	1.10430613672449E-48	3	EHHADH
1.62199705921622E-08	0.38450054734572	0.273	0.173	0.000264563940328758	3	ERCC5
5.26861952156756E-17	0.384258081253898	0.563	0.362	8.59364530162885E-13	3	QTRT1
2.06639586944192E-19	0.384187659480278	0.431	0.247	3.37049830264672E-15	3	UNC5CL
4.6625957555278E-13	0.384160748515449	0.315	0.184	7.6051599368414E-09	3	ARMC8
1.08281273800092E-35	0.383877324859897	0.404	0.184	1.76617585695331E-31	3	MOSPD3
5.29442714991363E-25	0.383279637467042	0.743	0.455	8.63574012422412E-21	3	RNF213
6.49460022521775E-16	0.382997165313009	0.336	0.19	1.05933424273527E-11	3	MAEA
5.60770135037116E-17	0.38242693877442	0.408	0.238	9.1467216725904E-13	3	ZXDC
3.35498712290631E-34	0.381817770133786	0.733	0.431	5.47231949617248E-30	3	SLC29A1
5.65650479765389E-15	0.381731515821744	0.27	0.145	9.22632497545325E-11	3	FAM136A
3.14170277025171E-37	0.381066204437907	0.655	0.366	5.12443138855756E-33	3	STAT3
2.55842570852631E-09	0.380939212048047	0.391	0.26	4.17304817317727E-05	3	DOCK5
3.84570170836709E-31	0.380548961891917	0.806	0.507	6.27272405651756E-27	3	GCLC
2.04974134150227E-30	0.380469783731436	0.351	0.159	3.34333310212435E-26	3	HADH
2.20168735327406E-12	0.380433717704313	0.286	0.166	3.59117224192532E-08	3	KLHL12
4.18559359820007E-30	0.380285184724281	0.364	0.168	6.82712171802414E-26	3	REPIN1

6.40748857725461E-32	0.379366026186499	0.944	0.801	1.045125461836E-27	3	IL32
1.81488293004766E-25	0.378696155149696	0.599	0.351	2.96025554720073E-21	3	ZSWIM8
1.50332086519515E-30	0.378513277817907	0.52	0.284	2.45206666321981E-26	3	DENND10
3.78858002288797E-43	0.378507318556499	0.516	0.251	6.17955287533258E-39	3	SPRYD4
2.12104988485055E-12	0.378431204651758	0.29	0.17	3.45964446717972E-08	3	FBXO38
8.40011601097798E-16	0.378283504868729	0.295	0.159	1.37014292255062E-11	3	TOX4
5.41067159980811E-11	0.378243122697702	0.395	0.256	8.82534644644702E-07	3	AKAP17A
1.54665932581492E-14	0.37762106469082	0.306	0.174	2.52275602633672E-10	3	TTC19
1.36613831129393E-40	0.376803507901283	0.766	0.465	2.22830819955153E-36	3	LDHD
2.53382250857864E-18	0.37669649130194	0.29	0.15	4.13291789374262E-14	3	FOXRED1
2.32815651471881E-09	0.375992714184693	0.328	0.213	3.79745609115785E-05	3	RPTOR
6.64717597340858E-32	0.375649862883878	0.397	0.189	1.08422087302267E-27	3	MTFR1
9.39554527613076E-20	0.375395183098948	0.335	0.176	1.53250738998969E-15	3	AKIRIN1
2.99420077211118E-21	0.375088090112616	0.392	0.214	4.88384087939055E-17	3	SH3BP2
5.26296878817845E-29	0.374564898787879	0.343	0.159	8.58442839039787E-25	3	STK24
7.93404011870137E-20	0.373480339693262	0.355	0.19	1.29412128376138E-15	3	MSL1
1.1081709872513E-09	0.372930826359894	0.26	0.158	1.80753769730559E-05	3	ZFYVE16
5.94042737690747E-26	0.372712520008506	0.878	0.542	9.68943109447377E-22	3	SEC16B
3.72515686871577E-08	0.371867321095707	0.267	0.173	0.000607610336856229	3	BCO2
4.30754672321355E-27	0.371667433050614	0.348	0.168	7.02603946023362E-23	3	MAL2
2.10703071021856E-28	0.371376398098038	0.51	0.281	3.43677779143749E-24	3	RHPN2
2.62212173623515E-26	0.371078950525195	0.254	0.105	4.27694276397315E-22	3	ELMO1
2.00716597646863E-14	0.37077324509851	0.323	0.186	3.27388842421799E-10	3	LRRC40
3.37213388094501E-20	0.370460895246097	0.513	0.306	5.5002875732094E-16	3	COG3
3.31623988261578E-18	0.370380382577329	0.343	0.189	5.4091188725346E-14	3	ACCS
4.26922842078915E-32	0.369966191509034	0.716	0.414	6.96353847714919E-28	3	AKR1C8P
3.42640736286373E-09	0.369311382564602	0.329	0.216	5.58881304956703E-05	3	EWSR1
6.24989757863521E-28	0.368911028124107	0.353	0.166	1.01942079405119E-23	3	CPD
7.99402993760312E-34	0.368791941545097	0.437	0.215	1.30390622312245E-29	3	DOK4
1.44503889864756E-26	0.368774967958191	0.993	0.988	2.35700294758404E-22	3	TF
5.41950693289589E-12	0.368692340460649	0.259	0.149	8.83975775824648E-08	3	WDR70
3.74682186695536E-39	0.368314922347017	0.736	0.441	6.11144114719089E-35	3	KHK
1.29542303846314E-21	0.367708193705143	0.473	0.273	2.11296451803724E-17	3	ZNF33A
9.31331033882491E-27	0.367370097408536	0.674	0.394	1.51909404936573E-22	3	PATJ
6.65036114194304E-15	0.367001675727895	0.372	0.221	1.08474040586233E-10	3	PIGL
3.45402093977813E-08	0.366954790458371	0.254	0.163	0.000563385355487211	3	TRMT11
2.29569638498152E-12	0.366308280992225	0.32	0.192	3.74451037354336E-08	3	ADGRA3
2.35669564899571E-43	0.366290849899316	0.713	0.4	3.8440062730769E-39	3	CBS
2.23414079537482E-34	0.36594298873279	0.489	0.243	3.64410705133587E-30	3	ENY2
4.65802457831331E-37	0.365799787606416	0.434	0.202	7.59770388968684E-33	3	FDPS
2.78945360128547E-14	0.365162168096993	0.389	0.236	4.54987776905673E-10	3	HTT
3.35831795767948E-24	0.364452898105799	0.655	0.387	5.477752420771E-20	3	KANK1
5.29765572280306E-26	0.363652726289795	0.361	0.176	8.64100624946407E-22	3	YTHDF3
1.79110127914635E-40	0.363435625546558	0.28	0.099	2.92146529641561E-36	3	DEPRECAT
1.5738980476337E-26	0.363346199070655	0.526	0.294	2.56718510549534E-22	3	AFF4
1.34773879723561E-34	0.363342672362932	0.756	0.439	2.198296752171E-30	3	AKR1C2
1.7426722505922E-16	0.362072768938634	0.253	0.128	2.84247270794094E-12	3	GNPNAT1
3.17194807128673E-42	0.361494016984079	0.522	0.25	5.17376449907579E-38	3	ECE1
3.45501659392538E-19	0.361400132810578	0.392	0.218	5.63547756635169E-15	3	SNX14
4.5479355293433E-61	0.361222254759667	0.408	0.147	7.41813764191186E-57	3	UQCR11

**6.08879722285536E-19**	0.360514373335046	0.355	0.194	9.93143715019938E-15	3	PHF3
**5.56384947768162E-14**	0.360132796443823	0.302	0.173	9.07519488304649E-10	3	ADCK5
**5.1982339865431E-32**	0.359453209914985	0.536	0.285	8.47883945545045E-28	3	SREBF2
**4.43042248280378E-20**	0.359428670295385	0.478	0.282	7.22646211170124E-16	3	TNS2
**1.96088368395838E-35**	0.359084630699512	0.351	0.153	3.19839737690451E-31	3	PEMT
**2.34969809686221E-11**	0.35864706208044	0.376	0.242	3.83259256579195E-07	3	TUBGCP3
**1.45992556927146E-40**	0.358602613898343	0.661	0.362	2.38128459603868E-36	3	DCAF6
**1.39351294495705E-18**	0.358553027563035	0.305	0.157	2.27295896451945E-14	3	BABAM1
**4.44477069330003E-37**	0.358543882615347	0.797	0.498	7.24986547784168E-33	3	TMEM45B
**2.70920024542626E-32**	0.358157253097694	0.532	0.276	4.41897652031478E-28	3	SORL1
**3.04948612052527E-20**	0.35806355926699	0.391	0.212	4.97401681118877E-16	3	C11orf24
**1.74659229090453E-30**	0.357502478297031	0.841	0.549	2.84886668569437E-26	3	ALDH3A2
**2.79516708029138E-30**	0.356883676053045	0.477	0.247	4.55919702466327E-26	3	PLS3
**5.87934556937243E-14**	0.356854486319359	0.274	0.151	9.58980055820337E-10	3	CNKSR3
**4.3978785001952E-18**	0.356098872795826	0.381	0.212	7.1733796216684E-14	3	DEPRECAT
**2.30968604726011E-15**	0.35599059520189	0.297	0.164	3.76732891168596E-11	3	TUBG2
**7.99553432607633E-09**	0.3557777736094	0.348	0.229	0.000130415160392631	3	MAPK8IP3
**2.69624533585022E-42**	0.355704606062492	0.625	0.324	4.39784576730529E-38	3	NMRAL1
**2.10261745872031E-19**	0.355520603989267	0.431	0.25	3.4295793369187E-15	3	SLC20A1
**9.1826302613684E-41**	0.354905209639357	0.514	0.256	1.4977788219318E-36	3	HIBADH
**2.01088390827358E-11**	0.354207747952619	0.29	0.175	3.27995274278504E-07	3	KAT6B
**7.43456228343198E-11**	0.353378878657064	0.364	0.235	1.21265145405059E-06	3	NPIPB15
**8.14774627174892E-30**	0.352602924855943	0.714	0.419	1.32897889438497E-25	3	NR5A2
**2.50821005353119E-14**	0.352345746724831	0.261	0.141	4.09114141831472E-10	3	NLK
**4.53195066591984E-27**	0.352095882539529	0.411	0.207	7.39206473118186E-23	3	MACO1
**3.80398857351783E-36**	0.351852076753739	0.514	0.26	6.20468576226494E-32	3	MAF
**3.13877598252113E-20**	0.351155549163036	0.329	0.172	5.11965750509022E-16	3	NFYC
**5.27526643631216E-36**	0.351034980248424	0.638	0.349	8.60448708426876E-32	3	IKBKG
**1.05970232263237E-13**	0.350941212971437	0.253	0.139	1.72848045844566E-09	3	ST7
**1.19466513060415E-50**	0.350881984752041	0.678	0.365	1.94861829452842E-46	3	BST2
**8.5636642326382E-21**	0.350699010113439	0.858	0.678	1.39681927298562E-16	3	MT-ATP6
**9.56282178394842E-15**	0.350215263109506	0.312	0.178	1.55979186117983E-10	3	GMNN
**3.63961937883466E-25**	0.349594010376925	0.568	0.326	5.93658316881721E-21	3	ABLIM3
**1.30427843673202E-21**	0.349342135868064	0.717	0.455	2.12740855815336E-17	3	SHFL
**1.0292730267596E-20**	0.349127617853625	0.547	0.333	1.67884723394759E-16	3	HMGCS1
**7.00725105830647E-25**	0.348993038603235	0.374	0.188	1.14295272012037E-20	3	CISH
**1.18488567967647E-34**	0.348820668690788	0.444	0.209	1.93266703212029E-30	3	TBL1XR1
**1.21251428627105E-14**	0.348323545394444	0.341	0.198	1.97773205233671E-10	3	KIAA2026
**1.59699022596922E-30**	0.348026580640684	0.282	0.115	2.60485075757839E-26	3	MICOS10
**2.98526210237647E-26**	0.348012434543044	0.253	0.106	4.86926101518626E-22	3	SEMA5A
**2.71236751996554E-36**	0.346544485337377	0.667	0.393	4.4241426618158E-32	3	ACSM1
**1.10156924145557E-23**	0.345853667564614	0.576	0.341	1.79676958973817E-19	3	STAU2
**1.14133816265071E-14**	0.345319812043074	0.261	0.14	1.86163667709957E-10	3	DMXL1
**3.17890156694259E-49**	0.34508472094197	0.463	0.199	5.18510634584005E-45	3	GCDH
**1.63176710718954E-44**	0.34465079335352	0.388	0.159	2.66157532853685E-40	3	PLAAT3
**9.41295197676708E-56**	0.344417515866421	0.734	0.403	1.53534659693048E-51	3	LGALS3BP
**8.22999609646654E-20**	0.344410647177129	0.457	0.265	1.34239466329466E-15	3	ENPP1
**4.77405446586503E-21**	0.344285584702973	0.385	0.209	7.78696023927244E-17	3	WLS
**2.61056125682911E-29**	0.343621519288143	0.362	0.17	4.25808646601395E-25	3	RTN4RL2
**8.75087682841674E-34**	0.343450683242015	0.658	0.379	1.42735551948305E-29	3	MAGED2

5.36047124986053E-15	0.343008254520162	0.316	0.179	8.7434646556475E-11	3	SPOP
2.88686253617429E-13	0.342437170384188	0.342	0.203	4.70876148275389E-09	3	TAPT1
6.52566848750808E-18	0.342060048518104	0.346	0.191	1.06440178699744E-13	3	STRN3
1.05167672045966E-22	0.341990855939097	0.631	0.381	1.71538989874175E-18	3	NFE2L2
1.86677958596415E-35	0.341243230628622	0.478	0.236	3.04490418266612E-31	3	RDH10
1.12303625975034E-16	0.34068062997034	0.31	0.169	1.83178444327878E-12	3	TSHZ2
8.18843409122719E-20	0.340283857978247	0.318	0.163	1.33561548462007E-15	3	DOCK4
1.288863402262E-24	0.340102437326954	0.346	0.172	2.10226509542954E-20	3	WDTC1
1.268171089859E-35	0.33992479815209	0.591	0.316	2.06851386466902E-31	3	B4GALT1
3.62505961702848E-10	0.339553726008367	0.375	0.244	5.91283474133515E-06	3	SRSF3
4.17834806509265E-16	0.338980845143394	0.292	0.157	6.81530352897262E-12	3	LMF1
2.14572915818057E-32	0.338970374053268	0.805	0.464	3.49989882990833E-28	3	PABPC1L
4.00401286212446E-12	0.338469254303031	0.307	0.182	6.5309453794112E-08	3	MYSM1
1.71853037564058E-32	0.337997591033208	0.539	0.28	2.80309489570735E-28	3	ABCA2
9.58828297963904E-29	0.337596639585642	0.428	0.22	1.56394483680892E-24	3	OCIAD2
3.90059173952986E-23	0.337566723759185	0.639	0.387	6.36225518634715E-19	3	TCIRG1
1.27553906900503E-15	0.337326260428215	0.319	0.179	2.08053177545411E-11	3	C6orf62
1.16232413447402E-18	0.337206057259527	0.309	0.161	1.89586689574057E-14	3	TRMT1L
7.78567698940172E-43	0.336740084223005	0.636	0.347	1.26992177374132E-38	3	APOM
1.33763451522865E-25	0.336605231322772	0.362	0.177	2.18181565778945E-21	3	AR
4.08465740330919E-19	0.33596205459517	0.549	0.336	6.66248469053762E-15	3	KPNA1
1.27948906054384E-54	0.335535386837333	0.614	0.303	2.08697460665306E-50	3	TRAM1
1.59927688894214E-16	0.335290464703041	0.272	0.141	2.60858053355353E-12	3	OMA1
5.7167704826642E-18	0.33494196459821	0.503	0.309	9.32462433427357E-14	3	KDM5B
1.70047401049743E-20	0.334874722920457	0.256	0.121	2.77364315852236E-16	3	ASB8
4.2771708224973E-26	0.334630571082582	0.437	0.229	6.97649332857535E-22	3	ACVR1B
8.7419185785868E-45	0.33455471785188	0.388	0.156	1.42589433935329E-40	3	S100A10
1.01547726844214E-13	0.334545074319832	0.254	0.139	1.65634497255597E-09	3	XPR1
1.53617839218736E-18	0.334090088309256	0.583	0.364	2.50566057549681E-14	3	IKBKB
4.03407604656857E-24	0.333022190811624	0.595	0.351	6.57998143955799E-20	3	MROH1
3.31555162833287E-22	0.33300041518627	0.886	0.56	5.40799626097374E-18	3	MYO18A
1.04784665592567E-11	0.33288443670872	0.253	0.143	1.70914268048035E-07	3	GGA1
6.87414120183929E-26	0.332813957740712	0.454	0.243	1.12124117143201E-21	3	AP5Z1
3.55184623139862E-29	0.332512376316888	0.359	0.169	5.79341638803429E-25	3	CHPF2
1.05549297034376E-13	0.332422271588629	0.312	0.181	1.72161458392771E-09	3	LARP1B
1.95717913888476E-41	0.331618250997919	0.693	0.401	3.19235489343493E-37	3	PEBP1
1.17108838721036E-27	0.331414872196658	0.499	0.27	1.91016226837882E-23	3	EFR3A
5.67628375964043E-31	0.330636999208988	0.506	0.269	9.2585864403495E-27	3	CTDSP2
2.29194020050955E-15	0.330538839479177	0.305	0.172	3.73838366105112E-11	3	CYHR1
3.84295304718901E-41	0.329880802197814	0.582	0.301	6.26824071527E-37	3	TST
5.21407233848044E-26	0.329831034524871	0.776	0.483	8.50467339129545E-22	3	TCF25
4.23096613395148E-18	0.329763258072544	0.306	0.163	6.90112886108826E-14	3	SCO2
4.44646411060866E-11	0.329119415002435	0.273	0.161	7.25262761081379E-07	3	BTBD9
9.48829233407541E-37	0.328817716518359	0.483	0.241	1.54763536261104E-32	3	LACTB2
4.34168875534279E-17	0.32856181502574	0.497	0.308	7.08172852883962E-13	3	SUGP2
3.33442262630096E-30	0.328490639625177	0.606	0.34	5.4387767457595E-26	3	SLC43A1
2.08185888672327E-26	0.327941058458319	0.319	0.149	3.39572003013432E-22	3	PLEKHF1
6.33491078544717E-19	0.32784661802278	0.316	0.169	1.03328729821429E-14	3	GNB5
1.72906476924959E-11	0.327348660315895	0.345	0.212	2.82027754512301E-07	3	ZNF326
1.58905021182001E-34	0.326836783137378	0.665	0.374	2.59189980049961E-30	3	PTPRH

2.977520891816E-43	0.326638391587315	0.83	0.58	4.85663432664108E-39	3	STARD10
9.44292043476682E-15	0.325759599569786	0.365	0.216	1.54023475211482E-10	3	ZZEF1
1.235106903047E-12	0.325596488474772	0.432	0.278	2.01458286955996E-08	3	SMAD4
2.30237316141161E-23	0.324832486293554	0.305	0.144	3.75540086357848E-19	3	SDCBP
7.92154705043301E-12	0.324485768329072	0.415	0.268	1.29208353939613E-07	3	ZNF638
2.56750772059534E-16	0.324244652428645	0.474	0.293	4.18786184306306E-12	3	DLG5
3.61960750949875E-11	0.323991006840794	0.282	0.169	5.90394180874342E-07	3	ATG14
3.93011012687171E-23	0.323758212949624	0.487	0.273	6.41040262794044E-19	3	UPK3BL1
2.8508609007535E-24	0.323623516067346	0.575	0.343	4.65003921521903E-20	3	EBNA1BP2
1.65440756637064E-29	0.323467332572571	0.417	0.205	2.69850418150715E-25	3	STOM
1.78815177321788E-29	0.322625857577802	0.864	0.592	2.91665435729568E-25	3	CSRP1
2.00692299766111E-17	0.322490899502154	0.3	0.158	3.27349210148504E-13	3	GRAMD2B
5.20651343492493E-26	0.321317765082426	0.641	0.371	8.49234406370606E-22	3	PLA2G6
3.17930847953021E-16	0.32107922759287	0.277	0.149	5.18577006096172E-12	3	SLC2A13
3.75503082327783E-37	0.320925328754611	0.424	0.194	6.12483077584847E-33	3	CD68
2.06913824985267E-10	0.320598657221053	0.257	0.154	3.37497139933469E-06	3	CLEC16A
2.02956274839128E-33	0.320413611637391	0.589	0.322	3.31041979890101E-29	3	TNFSF10
7.97314303454498E-22	0.319917554199482	0.504	0.292	1.30049936036463E-17	3	ANAPC5
4.39377419509891E-15	0.319011357688709	0.323	0.185	7.16668508962583E-11	3	PIGQ
9.94524069702002E-21	0.31824366923836	0.295	0.144	1.62216821009094E-16	3	TMEM50B
4.33834568160917E-26	0.317962901542892	0.306	0.138	7.07627564127272E-22	3	DEPRECAT
2.92991881755948E-14	0.317843114087018	0.415	0.253	4.77899058332127E-10	3	NEMF
3.75489467989081E-18	0.316850831969393	0.335	0.182	6.1246087123699E-14	3	NCKAP5L
8.42122231401882E-32	0.316743579477782	0.693	0.421	1.37358557163961E-27	3	ASPH
1.42256897036911E-10	0.316217750473964	0.33	0.206	2.32035224756906E-06	3	KHDC4
7.0836952353944E-30	0.316127482732093	0.807	0.488	1.15542152984518E-25	3	NR1H4
1.32632178541295E-35	0.315626648374441	0.501	0.248	2.16336346418706E-31	3	GBP7
4.85891058617837E-24	0.314889282428003	0.642	0.393	7.92536905711554E-20	3	OPLAH
2.22766353453096E-13	0.314875118765217	0.32	0.187	3.63354199117346E-09	3	PIAS2
1.64480120351847E-09	0.314691554338589	0.283	0.178	2.68283524305898E-05	3	SCML1
1.6337778585832E-08	0.314189401015486	0.323	0.215	0.000266485506513505	3	SORBS1
2.32401330346791E-30	0.31404245322928	0.67	0.391	3.79069809928651E-26	3	C1RL
1.64261122658998E-09	0.314022363086955	0.27	0.166	2.67926317169091E-05	3	MED24
1.72430022588635E-15	0.313079979089046	0.316	0.177	2.81250609844323E-11	3	USP22
9.99714396379663E-22	0.312870330108916	0.256	0.115	1.63063415193487E-17	3	ZBTB21
4.07173386871669E-14	0.312308048768472	0.292	0.162	6.6414051132638E-10	3	FBXL18
9.76074564561215E-48	0.31218151122055	0.588	0.293	1.5920752222558E-43	3	PRG4
4.51244838526157E-34	0.31113568733165	0.576	0.316	7.36025456120014E-30	3	SLC25A1
3.61335441239643E-30	0.311098039553863	0.336	0.151	5.89374238205982E-26	3	RITA1
7.60183249003826E-19	0.310908533105986	0.316	0.167	1.23993489745014E-14	3	TRAM2
3.24808959837696E-26	0.310707668753375	0.493	0.271	5.29795894391266E-22	3	AKT2
1.97630248623154E-07	0.310125283852729	0.287	0.189	0.00322354698529226	3	CREBRF
4.87804924854464E-17	0.309539756222106	0.325	0.181	7.95658612930116E-13	3	VPS13D
2.22082365324455E-14	0.309297750811118	0.54	0.346	3.62238546080719E-10	3	RABGAP1L
6.66260915266127E-45	0.30921311508168	0.522	0.251	1.08673817889058E-40	3	NDUFS7
8.50222998643084E-12	0.30915918843133	0.356	0.219	1.38679873308673E-07	3	BPTF
5.45865689654106E-28	0.308200594664153	0.364	0.172	8.90361526394813E-24	3	C6orf89
6.34143338766865E-15	0.30793893430808	0.371	0.221	1.03435119986263E-10	3	SIN3B
9.95714374016382E-11	0.307926214727677	0.32	0.2	1.62410971545812E-06	3	ABTB2
2.8635499438565E-12	0.306440595257736	0.376	0.234	4.67073631342433E-08	3	DNAJC10

2.94491675543335E-39	0.306404634412989	0.624	0.335	4.80345371978734E-35	3	MT-ND2
8.72586600241905E-09	0.306279134132809	0.279	0.178	0.000142327600365457	3	L3HYPDH
1.98614473959083E-32	0.306217436779913	0.345	0.152	3.2396006847466E-28	3	OBSL1
8.65111698369817E-18	0.30560373915287	0.444	0.264	1.41108369121101E-13	3	UBE4B
8.00350161367858E-28	0.305253160241405	0.634	0.365	1.30545114820711E-23	3	CYP2C18
7.84185673225483E-28	0.305108760509345	0.32	0.146	1.27908525159808E-23	3	OPN3
2.0342470052298E-21	0.304711398622156	0.557	0.33	3.31806029023033E-17	3	FLNB
6.28980112313194E-09	0.304414584942993	0.33	0.215	0.000102592946119405	3	WDR11
2.17461853386096E-22	0.304153812266236	0.431	0.237	3.54702029058061E-18	3	TP53BP2
1.05832545984836E-13	0.303768650436547	0.31	0.18	1.72623465755866E-09	3	NFAT5
1.38237626929548E-26	0.303487978517353	0.414	0.212	2.25479393284786E-22	3	TSC2
6.95542473081207E-31	0.302600636081382	0.822	0.498	1.13449932784276E-26	3	EXT1
7.65906176584463E-11	0.30251947099464	0.253	0.148	1.24926956462692E-06	3	WAPL
2.17019116039383E-12	0.302391871507576	0.341	0.206	3.53979880171837E-08	3	KAT6A
4.64872964844014E-40	0.302263822960718	0.636	0.346	7.58254292957071E-36	3	MMUT
2.63322917125835E-09	0.30223704891859	0.261	0.162	4.2950601012395E-05	3	TNS1
9.38984206423275E-21	0.301861660932912	0.511	0.303	1.531577139097E-16	3	SRP54
1.91773955360322E-39	0.301539276819711	0.463	0.215	3.12802498588222E-35	3	ABHD14B
3.79201471122447E-21	0.301056806560566	0.399	0.218	6.18515519547823E-17	3	RALGPS2
2.62179116393874E-16	0.300888554178719	0.431	0.261	4.27640356750047E-12	3	VWA8
6.28204239670547E-20	0.300878231534084	0.401	0.224	1.02466393532663E-15	3	FAT1
6.79807885476405E-17	0.300850139045744	0.25	0.127	1.10883464200056E-12	3	PPIA
2.0864253366311E-24	0.300477231470075	0.329	0.158	3.40316836657899E-20	3	RAPGEF2
2.41149621414387E-36	0.300435960111197	0.569	0.304	3.93339147489006E-32	3	RAB10
1.52976519876029E-12	0.300368835849249	0.348	0.215	2.49520001569791E-08	3	KIFC2
3.14182449676116E-14	0.298968083798475	0.385	0.229	5.12462993666713E-10	3	R3HDM2
7.32492093273368E-35	0.298577510699818	0.483	0.245	1.19476785333819E-30	3	COA3
1.26442458134094E-09	0.298433024601273	0.322	0.211	2.0624029346252E-05	3	PIKFYVE
1.10520877995525E-13	0.298210469771236	0.316	0.185	1.80270604098501E-09	3	SH3D19
1.82085013563944E-13	0.29771682145387	0.359	0.216	2.96998865624149E-09	3	MTMR12
2.89141022847393E-36	0.297682375734497	0.807	0.575	4.71617922366383E-32	3	P4HB
3.5869577200715E-28	0.297672075051598	0.444	0.232	5.85068673720862E-24	3	NFIL3
5.36968437948388E-11	0.2970967701666	0.266	0.157	8.75849219137616E-07	3	MBTD1
3.85255887026519E-16	0.296870285583168	0.282	0.15	6.28390877328954E-12	3	BTN3A2
6.85092687841825E-22	0.296505752648684	0.509	0.291	1.1174546831388E-17	3	COL27A1
8.97714645452606E-24	0.296094370581663	0.371	0.192	1.46426235819775E-19	3	ANKH
2.67808625980927E-18	0.295996979699716	0.441	0.259	4.3682264983749E-14	3	CDC42BPB
8.34879484480896E-08	0.295271302573047	0.293	0.197	0.00136177192713679	3	SEPTIN11
2.58641215420163E-10	0.295251102409643	0.293	0.181	4.21869686471827E-06	3	RTCA
4.1596768632071E-33	0.295196496622197	0.443	0.214	6.7848489315771E-29	3	SUPT6H
1.22242356168385E-37	0.29313098150526	0.792	0.497	1.99389507146253E-33	3	RGN
4.56232552946867E-20	0.293013532465978	0.611	0.376	7.44160917111634E-16	3	DTNA
5.61339099300022E-31	0.291977219427591	0.72	0.44	9.15600204868266E-27	3	SLC2A2
2.44599942711816E-19	0.29085960435175	0.333	0.176	3.98966966557243E-15	3	RAB11FIP4
5.42108019115893E-19	0.290689682646644	0.261	0.127	8.84232389979934E-15	3	VDAC1
2.16293598779007E-09	0.290572000493881	0.299	0.19	3.52796488968439E-05	3	ZFHX3
1.75748532015403E-16	0.290462683399671	0.309	0.169	2.86663430570324E-12	3	CCDC125
1.41483687146816E-19	0.290448410831647	0.319	0.166	2.30774042105171E-15	3	CRKL
1.87249175113598E-30	0.290413310807283	0.825	0.592	3.0542212952779E-26	3	IDH2
5.3010092935075E-27	0.290203073255804	0.428	0.218	8.64647625864008E-23	3	VAC14

7.47866124426841E-17	0.290032665932915	0.315	0.171	1.21984443555262E-12	3	PIK3CB
1.1790706502714E-15	0.290019961642436	0.326	0.182	1.92318213765768E-11	3	DNAJC13
1.0817668590684E-22	0.289701884929924	0.3	0.145	1.76446992382647E-18	3	GATD3A
7.26159150239783E-15	0.289098249882399	0.289	0.156	1.18443818995611E-10	3	FCSK
8.95693920995639E-29	0.28824533810759	0.816	0.52	1.46096635453599E-24	3	F5
3.05151123575521E-21	0.288092732065227	0.477	0.275	4.97731997664032E-17	3	NCBP2
1.27110657393211E-18	0.287987318201057	0.496	0.299	2.07330193274067E-14	3	MKLN1
9.72966488293476E-09	0.28679530057284	0.261	0.164	0.000158700563905549	3	HDAC8
7.00270695064218E-12	0.285829194987936	0.338	0.205	1.14221153071925E-07	3	ARFGAP2
8.03036872002202E-19	0.285733882231767	0.44	0.256	1.30983344192279E-14	3	ADD1
5.19617523951923E-29	0.285638155387505	0.7	0.44	8.47548143317981E-25	3	DGAT1
5.03342876403228E-28	0.285175234355664	0.305	0.134	8.21002565701306E-24	3	C16orf87
3.69997647299568E-25	0.28380699404076	0.529	0.3	6.03503162510325E-21	3	CCDC50
2.9126733507183E-38	0.283191588814706	0.514	0.256	4.75086150235662E-34	3	TWF2
7.42949237750679E-19	0.283148595113605	0.279	0.138	1.21182450169513E-14	3	RSRC1
1.88553016103519E-11	0.282019651822531	0.276	0.162	3.07548824566451E-07	3	ADNP2
8.00739007791241E-31	0.282017619754933	0.483	0.252	1.30608539560829E-26	3	RAB2A
3.06468622760592E-15	0.281435243030003	0.445	0.27	4.99880970584802E-11	3	SLC5A6
2.81272410169664E-11	0.28115355676836	0.375	0.238	4.58783428227739E-07	3	UBR3
2.09533351718786E-19	0.280759015943875	0.514	0.301	3.41769849988512E-15	3	CD46
1.14306359127609E-22	0.280392762004278	0.555	0.32	1.86445102373043E-18	3	ABCD3
3.07324941707396E-10	0.280238557894481	0.26	0.156	5.01277712418934E-06	3	OSBPL8
1.6545473961291E-14	0.280222134959706	0.391	0.238	2.69873225782617E-10	3	CSTF3
2.91382729677983E-29	0.27993668780488	0.434	0.22	4.75274370377758E-25	3	COPS5
2.51739072055464E-21	0.279899937294062	0.28	0.134	4.10611600429667E-17	3	GABARAPL
1.91548079325952E-27	0.279867627729771	0.591	0.341	3.12434072188561E-23	3	ZBTB16
7.17902238850427E-21	0.279327388861834	0.529	0.318	1.17097034178893E-16	3	FOXP1
3.32140358224262E-23	0.278335552729444	0.369	0.191	5.41754138299594E-19	3	ELMOD2
2.0034180674303E-24	0.277586542525538	0.774	0.477	3.26777520978557E-20	3	PPP6R2
9.54961753777396E-33	0.277518422552371	0.5	0.264	1.55763811658631E-28	3	PGLYRP2
7.18420476887265E-15	0.277182690019604	0.29	0.159	1.17181563985082E-10	3	MED4
3.91915680144583E-29	0.276387620690578	0.793	0.482	6.3925366588383E-25	3	H6PD
2.84418292462592E-14	0.27579120052915	0.269	0.145	4.63914676835734E-10	3	KCNAB2
7.57085840865205E-41	0.275213469481421	0.718	0.413	1.23488271503524E-36	3	NIPSNAP1
2.5856882105622E-21	0.275127759856775	0.427	0.235	4.21751604024801E-17	3	HIPK2
1.56140649514803E-10	0.274673065138274	0.29	0.178	2.54681013423594E-06	3	PLA2G4C
1.23386933206548E-16	0.274566519555932	0.437	0.258	2.01256426753201E-12	3	TIA1
5.52390900429013E-12	0.273780864511154	0.379	0.238	9.01004797689763E-08	3	MLPH
1.23522619610878E-26	0.27367246600057	0.332	0.157	2.01477744847303E-22	3	C8orf33
7.20061539981495E-11	0.273397730896199	0.303	0.186	1.17449237786382E-06	3	ZNF148
4.21479581478983E-15	0.273299164044124	0.328	0.186	6.8747534535037E-11	3	PDZD8
3.84520885167054E-25	0.27317270487096	0.58	0.338	6.27192015795982E-21	3	DVL3
8.39435297012078E-11	0.272619223609194	0.338	0.211	1.3692029129564E-06	3	USP24
2.9524323957324E-07	0.272553128597537	0.277	0.186	0.00481571248067911	3	WHRN
3.56592026353673E-11	0.272124698127491	0.353	0.223	5.81637254185476E-07	3	TP53I13
3.44536291533525E-14	0.271809805157464	0.28	0.153	5.61973145120332E-10	3	OTUB1
1.34582349129808E-17	0.271524105236335	0.513	0.316	2.1951726966563E-13	3	ZRANB2
2.91583977159096E-14	0.271507940204057	0.361	0.211	4.75602625144201E-10	3	POLR2J3
2.25448915039727E-20	0.27130200584641	0.307	0.156	3.67729725321299E-16	3	DEPRECAT
3.20950798088681E-23	0.271178419114557	0.79	0.474	5.23502846762447E-19	3	ARGLU1

**9.45896500340086E-24**	0.270934793276855	0.506	0.284	1.54285178170471E-19	3	FAM160A2
**5.21041635638234E-24**	0.270620462114569	0.296	0.139	8.49871011889524E-20	3	MAOA
**6.35941439230867E-13**	0.270467059038478	0.263	0.147	1.03728408152947E-08	3	BLZF1
**2.33804767458609E-16**	0.270291559740538	0.339	0.19	3.81358956201737E-12	3	FRK
**6.25111268257681E-41**	0.270153872435644	0.674	0.388	1.0196189896551E-36	3	QDPR
**9.9311008976465E-17**	0.269491087770874	0.441	0.266	1.61986186741512E-12	3	TNRC18
**1.09435209649242E-17**	0.269253332807334	0.527	0.321	1.78499770458878E-13	3	OGT
**4.43209374047386E-20**	0.268870372442138	0.307	0.155	7.22918810008692E-16	3	NCKIPSD
**5.82275342963989E-14**	0.268196358928308	0.447	0.282	9.49749311908562E-10	3	MYO15B
**2.73453224916419E-11**	0.267990019246656	0.297	0.181	4.46029555161172E-07	3	NISCH
**8.91623344003986E-15**	0.267864550585401	0.447	0.279	1.4543268364049E-10	3	VPS53
**5.53729952222263E-20**	0.267836936333284	0.371	0.202	9.03188925069733E-16	3	CPT1A
**2.4781632339878E-14**	0.267678662423826	0.29	0.161	4.0421320509575E-10	3	FBXL4
**2.40200082978233E-15**	0.266714242211344	0.447	0.273	3.91790355345796E-11	3	ZFYVE27
**1.27096665326533E-13**	0.26669533710412	0.359	0.214	2.07307370814109E-09	3	NFS1
**1.73956448834828E-17**	0.266479022801792	0.284	0.147	2.83740363694488E-13	3	GNE
**6.95766115653004E-21**	0.266378909899285	0.47	0.268	1.13486411124161E-16	3	PICALM
**1.00065326116681E-26**	0.266327800494796	0.26	0.108	1.63216553428919E-22	3	ACKR2
**3.68450097981516E-35**	0.266166092835656	0.364	0.157	6.0097895481765E-31	3	TRAPPC6A
**1.22809234745469E-20**	0.265905818470587	0.94	0.744	2.00314142793335E-16	3	ITIH1
**2.4494903303384E-35**	0.265556500925642	0.779	0.482	3.99536367781496E-31	3	PON3
**5.42029942803163E-36**	0.265538894783065	0.533	0.272	8.8410503970624E-32	3	GCAT
**1.56600717870247E-24**	0.2651894729828	0.286	0.129	2.55431430918159E-20	3	MPP6
**7.54871911076531E-19**	0.265187152843146	0.295	0.151	1.23127157415693E-14	3	RIPK4
**2.1631051321486E-09**	0.264923478540912	0.372	0.248	3.52824078104757E-05	3	CTPS1
**2.34101881519437E-23**	0.263761612372447	0.391	0.204	3.81843578946354E-19	3	RTL8C
**3.91016671418553E-15**	0.263286996438326	0.315	0.177	6.37787292750801E-11	3	HDAC1
**1.13630310429705E-24**	0.263282058882829	0.44	0.237	1.85342399341891E-20	3	MAPK6
**2.13408282321858E-21**	0.263162828796458	0.364	0.192	3.48090249295182E-17	3	SIPA1L3
**6.13512007900947E-24**	0.263149080252672	0.527	0.306	1.00069943608723E-19	3	EIF5
**7.19154831579418E-11**	0.262928349592998	0.338	0.211	1.17301344578919E-06	3	NUP85
**3.32038731586054E-15**	0.262444438769203	0.336	0.192	5.41588375090013E-11	3	OSBPL2
**3.2872796675875E-11**	0.262388486336214	0.267	0.157	5.36188186580197E-07	3	RSPRY1
**1.41181153328752E-20**	0.262273742099733	0.43	0.24	2.30280579194528E-16	3	DEPRECAT
**2.45965003556194E-19**	0.261685151895431	0.385	0.213	4.01193517300508E-15	3	ZBTB48
**1.52158177805869E-26**	0.261309237838128	0.33	0.155	2.48185203819153E-22	3	GFM1
**2.20327827260752E-11**	0.261297123499119	0.302	0.18	3.59376719045013E-07	3	FBXO22
**3.12694342947852E-17**	0.261242231274916	0.315	0.17	5.10035742782241E-13	3	RICTOR
**3.88381606281828E-39**	0.259532928797267	0.45	0.211	6.33489238006289E-35	3	DNAJC3
**4.83531930578631E-20**	0.259502224492468	0.481	0.278	7.88688931966806E-16	3	AAK1
**1.35695631926473E-17**	0.259447162995168	0.369	0.207	2.2133314523527E-13	3	YKT6
**2.30838764337904E-21**	0.259202731513342	0.519	0.303	3.76521108511556E-17	3	FOXN2
**3.42524538103135E-33**	0.258857839660568	0.675	0.409	5.58691774100023E-29	3	GPAA1
**5.97946397185328E-21**	0.258568492334278	0.253	0.115	9.75310368448988E-17	3	USP38
**4.53130458282844E-20**	0.258447926860337	0.381	0.207	7.39101090505147E-16	3	ATP6V0A1
**9.97215351558279E-27**	0.257107792406967	0.356	0.173	1.62655795992671E-22	3	ZNF24
**5.9672713225162E-22**	0.256685937103089	0.322	0.16	9.73321625415618E-18	3	CD47
**1.03710783300906E-11**	0.256005765240138	0.394	0.249	1.69162658642108E-07	3	UVSSA
**3.53424472310917E-18**	0.255016650439093	0.272	0.136	5.76470656786337E-14	3	PTGR2
**7.13773196756289E-28**	0.254885499386118	0.782	0.477	1.16423546122918E-23	3	VMP1

**8.60748842417633E-20**	0.254801354387256	0.519	0.312	1.4039674368674E-15	3	PUM1
**3.75842964375843E-16**	0.254719427305836	0.251	0.129	6.13037459193438E-12	3	ARFGEF1
**2.99414638389679E-13**	0.254704726775226	0.303	0.175	4.88375216677405E-09	3	GPBP1
**1.45219009689208E-08**	0.254521249754306	0.343	0.229	0.000236866726704067	3	CCNT2
**8.95700272902315E-33**	0.254345019180978	0.455	0.228	1.46097671513097E-28	3	CTSO
**1.32138713733659E-22**	0.253508505554878	0.889	0.613	2.15531455970971E-18	3	ASL
**8.99779158646988E-11**	0.253263670376002	0.251	0.147	1.4676297856691E-06	3	MLH1
**1.94777564626225E-18**	0.253161945427721	0.96	0.904	3.17701685661835E-14	3	KNG1
**7.47479255430407E-30**	0.252954898447871	0.721	0.436	1.21921341353254E-25	3	TOR1AIP2
**5.20118183585502E-20**	0.252909546805627	0.629	0.386	8.48364769246312E-16	3	MOV10
**2.61295573514617E-20**	0.2528510671255	0.434	0.244	4.26199209959692E-16	3	PMM2
**3.09001467688668E-15**	0.252413224719453	0.411	0.251	5.04012293946986E-11	3	POLR2J2
**1.92464617609901E-08**	0.252103164788278	0.25	0.156	0.00031392903778351	3	DNASE1L1
**5.59269360981267E-20**	0.251771074796004	0.471	0.277	9.12224254696545E-16	3	AKAP13
**1.25247064128343E-35**	0.251121646849598	0.727	0.44	2.04290486299741E-31	3	UBD
**1.69131954586351E-23**	0.251067318267503	0.566	0.329	2.75871131125797E-19	3	PER1
**7.1015725747198E-35**	0.250953191368083	0.565	0.31	1.15833750266255E-30	3	SMIM14
**6.15700205888036E-27**	0.250880419580777	0.391	0.196	1.00426860582398E-22	3	RAC1
**1.67636329977841E-21**	0.250378600870172	0.46	0.264	2.73431617826856E-17	3	DUSP10
**3.26155056998519E-16**	0.250261813357361	0.376	0.218	5.31991513470284E-12	3	TBRG1
**3.98718228950645E-29**	0.250228484212213	0.841	0.6	6.50349303241398E-25	3	ADI1

SupTable5L_C2_Hep_HepatoSubtype

p_val	avg_log2FC	pct.1	pct.2	p_val_adj	cluster	gene
0	0.82650834842651	1	0.989	0	Mature metabolic hepatocytes	APOE
5.87743825199394E-256	1.0490693736867	0.995	0.958	9.58668953282731E-252	Mature metabolic hepatocytes	APOA1
1.41057103899156E-232	0.718992282115897	1	0.997	2.30078242169913E-228	Mature metabolic hepatocytes	APOA2
1.19474099192247E-225	0.720863805019408	0.995	0.982	1.94874203192474E-221	Mature metabolic hepatocytes	RBP4
9.07567347187226E-217	0.87854588770807	0.987	0.951	1.48033309999708E-212	Mature metabolic hepatocytes	TTR
1.32244128164174E-213	0.785434455661838	0.956	0.935	2.15703397448585E-209	Mature metabolic hepatocytes	GC
4.14072300862125E-190	0.675439120420691	0.999	0.985	6.75393329936211E-186	Mature metabolic hepatocytes	ALB
2.69556773915921E-181	0.68233396895396	0.966	0.946	4.39674053934259E-177	Mature metabolic hepatocytes	CLU
4.76434778700462E-177	0.640389832692164	0.973	0.953	7.77112767538324E-173	Mature metabolic hepatocytes	EPHX1
4.99696481644303E-165	0.58031379319382	1	0.991	8.15054931210023E-161	Mature metabolic hepatocytes	SERPINA1
8.91137795552108E-163	0.712415369587982	0.982	0.98	1.45353485832504E-158	Mature metabolic hepatocytes	CES1
2.08127221442483E-162	0.659290057735445	0.949	0.949	3.39476310894834E-158	Mature metabolic hepatocytes	AGT
7.01122649986784E-155	0.783732870478369	0.973	0.944	1.14360115439344E-150	Mature metabolic hepatocytes	HP
3.98099961852912E-148	0.560014298145883	0.997	0.985	6.49340847778285E-144	Mature metabolic hepatocytes	VTN
5.8040907423968E-147	0.617328138577185	0.995	0.985	9.46705240992342E-143	Mature metabolic hepatocytes	TF
5.04267138493567E-143	0.625945787673296	0.953	0.953	8.22510129596858E-139	Mature metabolic hepatocytes	MT1G
4.33454636120409E-137	0.483918922858284	1	0.994	7.07007856976E-133	Mature metabolic hepatocytes	FGG
1.03873612672637E-135	0.71019996997538	0.905	0.885	1.69428249630339E-131	Mature metabolic hepatocytes	AMBP
1.64819727881321E-129	0.559479173870025	0.988	0.965	2.68837458147223E-125	Mature metabolic hepatocytes	APOC3
7.63510196067875E-99	0.359034307590952	0.993	0.992	1.24536148080631E-94	Mature metabolic hepatocytes	MT2A
5.30506804863028E-92	0.62045582967549	0.88	0.902	8.65309649412085E-88	Mature metabolic hepatocytes	ITIH2
4.12802590801477E-86	0.392592482681989	0.999	0.989	6.7332230585629E-82	Mature metabolic hepatocytes	FGB
4.06427201601772E-79	0.737589735978127	0.812	0.826	6.62923408532651E-75	Mature metabolic hepatocytes	SERPINC1
2.93153232877498E-78	0.379027439015546	0.993	0.992	4.78162238146487E-74	Mature metabolic hepatocytes	C3
6.9424117586679E-77	0.424910638793874	0.946	0.961	1.13237678195632E-72	Mature metabolic hepatocytes	MT1F
8.31497022118529E-67	0.388418298916827	0.942	0.955	1.35625479277753E-62	Mature metabolic hepatocytes	FN1
4.41742151059702E-66	0.569850194743939	0.814	0.864	7.2052562259348E-62	Mature metabolic hepatocytes	F2
8.01799266705516E-65	0.709514466565738	0.765	0.794	1.30781478392337E-60	Mature metabolic hepatocytes	LAMP1
3.89665239490759E-62	0.375956619195268	0.97	0.973	6.35582972133377E-58	Mature metabolic hepatocytes	MT1B
4.84861773024745E-58	0.497908441043158	0.909	0.933	7.90858037980661E-54	Mature metabolic hepatocytes	FTL
4.53186461962575E-56	0.75940031541966	0.755	0.796	7.39192438107157E-52	Mature metabolic hepatocytes	CTSD
4.45277024829582E-55	0.34686574830163	0.996	0.99	7.26291355199532E-51	Mature metabolic hepatocytes	FGA
1.05334363025278E-47	0.462265132375555	0.829	0.887	1.71810879530531E-43	Mature metabolic hepatocytes	MT1E
7.11371229177615E-46	0.50347677136782	0.872	0.872	1.16031761191161E-41	Mature metabolic hepatocytes	FABP1
2.12560236056861E-45	0.50557067162636	0.82	0.872	3.46707001032346E-41	Mature metabolic hepatocytes	APOC1
1.65680103850027E-44	0.521308680485145	0.825	0.87	2.70240817389779E-40	Mature metabolic hepatocytes	ALDOB
4.538534859764E-41	0.312325285603492	0.931	0.948	7.40280420976106E-37	Mature metabolic hepatocytes	HPX
3.71055426385686E-40	0.579833963993931	0.761	0.842	6.05228505977692E-36	Mature metabolic hepatocytes	HMGCS2
7.51840604677095E-40	0.457038367259856	0.865	0.896	1.22632721028881E-35	Mature metabolic hepatocytes	AHSG
7.59480799888623E-38	0.57964551465841	0.743	0.797	1.23878913269833E-33	Mature metabolic hepatocytes	GSTA1
1.78971465426468E-33	0.482451023881646	0.889	0.903	2.91920357257112E-29	Mature metabolic hepatocytes	ORM1
3.9636701637811E-33	0.359541359136698	0.847	0.917	6.46514240414335E-29	Mature metabolic hepatocytes	CTSB
6.50774849890572E-31	0.550464127183858	0.777	0.731	1.06147885765651E-26	Mature metabolic hepatocytes	CD74
6.70523779765883E-28	0.454440372634275	0.761	0.849	1.09369133717613E-23	Mature metabolic hepatocytes	LMNA
2.92612179239347E-27	0.667061775830538	0.684	0.744	4.77279725557299E-23	Mature metabolic hepatocytes	NUCB1
6.57136578094391E-27	0.363531767827437	0.952	0.952	1.07185547252976E-22	Mature metabolic hepatocytes	PLA2G2A
7.91582365265487E-27	0.642940909887671	0.702	0.757	1.29114999598454E-22	Mature metabolic hepatocytes	SPINK1
1.63826326226938E-26	0.3401379525147	0.845	0.913	2.67217120708759E-22	Mature metabolic hepatocytes	FGL1
1.78013849107399E-26	0.475756242540021	0.755	0.841	2.90358389279079E-22	Mature metabolic hepatocytes	ALDH2
1.71833002256634E-25	0.321544633308728	0.87	0.932	2.80276809980796E-21	Mature metabolic hepatocytes	APOB
3.98280361784104E-25	0.351675766189728	0.791	0.888	6.49635098106053E-21	Mature metabolic hepatocytes	ASGR2
4.95626246073447E-25	0.636683314383589	0.675	0.749	8.08415969970399E-21	Mature metabolic hepatocytes	ECH1
9.43329763834873E-23	0.306957801178607	0.286	0.43	1.53866517779106E-18	Mature metabolic hepatocytes	CLPTM1L
9.92851096558747E-23	0.259272558127717	0.287	0.437	1.61943942359697E-18	Mature metabolic hepatocytes	TOLLIP
1.03729689534783E-22	0.35217135587805	0.805	0.892	1.69193496600184E-18	Mature metabolic hepatocytes	APOH
1.09697374337093E-22	0.702599143425784	0.653	0.712	1.78927387281232E-18	Mature metabolic hepatocytes	CST3
3.38981333506216E-22	0.372047418655792	0.793	0.866	5.5291245308199E-18	Mature metabolic hepatocytes	SERPINF2
4.79895886845988E-22	0.261819182832476	0.277	0.418	7.82758181034492E-18	Mature metabolic hepatocytes	CDC37

5.67710608202365E-22	0.542068828509695	0.704	0.79	9.25992773038878E-18	Mature metabolic hepatocytes	EEF2
1.14608743670416E-21	0.296697620040108	0.2	0.32	1.86938321800816E-17	Mature metabolic hepatocytes	EPHB4
1.17508064460248E-21	0.272318263580661	0.879	0.933	1.91667403941111E-17	Mature metabolic hepatocytes	KNG1
3.96588867287196E-21	0.32275568651389	0.319	0.469	6.46876101432145E-17	Mature metabolic hepatocytes	SCYL1
7.94393374287762E-21	0.292979602587875	0.314	0.465	1.29573503280077E-16	Mature metabolic hepatocytes	SLC35C1
2.30368334671935E-20	0.256938660423745	0.244	0.374	3.75753790683394E-16	Mature metabolic hepatocytes	PBXIP1
2.77407991942113E-20	0.301233302192861	0.326	0.482	4.5248017565678E-16	Mature metabolic hepatocytes	CALM2
9.54488462449459E-20	0.252624778827934	0.371	0.536	1.55686613110131E-15	Mature metabolic hepatocytes	KIF1C
1.54684360008955E-19	0.363704688726214	0.238	0.364	2.52305659610606E-15	Mature metabolic hepatocytes	TXNL4A
2.58819853704834E-19	0.323920986028889	0.357	0.523	4.22161063377954E-15	Mature metabolic hepatocytes	SEL1L
3.04275755026165E-19	0.441392274218368	0.765	0.831	4.96304184023177E-15	Mature metabolic hepatocytes	LRG1
4.07103294357724E-19	0.687142310800376	0.672	0.719	6.64026183426883E-15	Mature metabolic hepatocytes	HPR
4.36777526091731E-19	0.553059474465654	0.678	0.789	7.12427822808222E-15	Mature metabolic hepatocytes	RRBP1
5.93651844688691E-19	0.318504730669592	0.281	0.413	9.68305523871724E-15	Mature metabolic hepatocytes	FAM83H
1.24084027994972E-18	0.492531633332434	0.7	0.767	2.02393458062598E-14	Mature metabolic hepatocytes	KRT8
1.4251262049229E-18	0.282828045737525	0.386	0.556	2.32452335284974E-14	Mature metabolic hepatocytes	CRELD2
1.61779970843664E-18	0.27717946989475	0.275	0.406	2.63879310443101E-14	Mature metabolic hepatocytes	WSB2
1.97410059046341E-18	0.444429833079145	0.738	0.842	3.21995547310486E-14	Mature metabolic hepatocytes	SELENOP
1.98707486182354E-18	0.273753610652077	0.235	0.352	3.24111780712037E-14	Mature metabolic hepatocytes	NAA20
2.03912292645395E-18	0.336845485283285	0.084	0.166	3.32601340533904E-14	Mature metabolic hepatocytes	AKR1C4
2.05988521893046E-18	0.263112134801011	0.363	0.524	3.35987878059747E-14	Mature metabolic hepatocytes	PLD3
2.42138653136164E-18	0.337258170527424	0.815	0.869	3.94952357130396E-14	Mature metabolic hepatocytes	A2M
3.09577103219423E-18	0.612965312865664	0.639	0.719	5.04951213061201E-14	Mature metabolic hepatocytes	SERPIND1
3.53404211439166E-18	0.279995350697752	0.254	0.373	5.76437609278424E-14	Mature metabolic hepatocytes	NUDT16
4.87867613222167E-18	0.262869600186348	0.257	0.378	7.95760863926677E-14	Mature metabolic hepatocytes	GIPC1
5.65275558322538E-18	0.250279429179696	0.327	0.469	9.22020963179892E-14	Mature metabolic hepatocytes	CD59
6.08333216860199E-18	0.258245260673947	0.278	0.406	9.92252310020671E-14	Mature metabolic hepatocytes	DYNLL2
6.20213298841939E-18	0.286651841884265	0.15	0.246	1.01162991174109E-13	Mature metabolic hepatocytes	ELP5
7.42335671901513E-18	0.2514894946665	0.373	0.536	1.21082371443856E-13	Mature metabolic hepatocytes	PSMB8
1.15759731198247E-17	0.327221978176014	0.288	0.417	1.88815697557461E-13	Mature metabolic hepatocytes	CDIPT
1.1794953172901E-17	0.254736484221277	0.366	0.529	1.92387481203188E-13	Mature metabolic hepatocytes	SIL1
1.76852542525033E-17	0.251762254908943	0.122	0.21	2.88464182112581E-13	Mature metabolic hepatocytes	SNAP23
2.24590900022715E-17	0.280875270951754	0.326	0.47	3.66330217027051E-13	Mature metabolic hepatocytes	ACTG1
2.28918476259738E-17	0.307439934732993	0.255	0.376	3.73388926627259E-13	Mature metabolic hepatocytes	EBPL
3.51926509480452E-17	0.350781679580834	0.252	0.373	5.74027329613566E-13	Mature metabolic hepatocytes	COMTD1
3.52990960490344E-17	0.272544118448046	0.338	0.48	5.757635556558E-13	Mature metabolic hepatocytes	CIAO2A
4.21370016807919E-17	0.266044876636771	0.33	0.463	6.87296634415396E-13	Mature metabolic hepatocytes	PSMC3
4.7842614995871E-17	0.251414958403715	0.138	0.229	7.80360893197652E-13	Mature metabolic hepatocytes	TMUB1
6.03603902715745E-17	0.400558746780025	0.272	0.393	9.84538325719652E-13	Mature metabolic hepatocytes	BCL2L13
6.27755806504921E-17	0.381008893029781	0.745	0.821	1.02393249599018E-12	Mature metabolic hepatocytes	SERPING1
7.48544692323763E-17	0.569523717081423	0.651	0.724	1.22095124764929E-12	Mature metabolic hepatocytes	ETFB
7.54427291946064E-17	0.268561851547556	0.316	0.449	1.23054635589322E-12	Mature metabolic hepatocytes	SEC13
1.00150890356019E-16	0.269584377498253	0.422	0.596	1.63356117259703E-12	Mature metabolic hepatocytes	CMPK1
1.03296754243324E-16	0.314546449629627	0.379	0.542	1.68487335846286E-12	Mature metabolic hepatocytes	MAN1A1
1.3322384367019E-16	0.423634089335861	0.265	0.382	2.17301411410448E-12	Mature metabolic hepatocytes	UBE2Q1
1.72855688176845E-16	0.261893377372914	0.24	0.353	2.81944912985252E-12	Mature metabolic hepatocytes	TMEM134
2.03248805828302E-16	0.250204726251821	0.154	0.245	3.31519127186544E-12	Mature metabolic hepatocytes	PIR
2.09034264948408E-16	0.277870416903987	0.282	0.407	3.40955789557348E-12	Mature metabolic hepatocytes	SDHA
2.344140016397E-16	0.772642315078249	0.624	0.691	3.82352678074515E-12	Mature metabolic hepatocytes	HYOU1
2.60473071677467E-16	0.314893745818353	0.335	0.477	4.24857627213117E-12	Mature metabolic hepatocytes	MAGT1
3.34269658094729E-16	0.37311137110679	0.3	0.427	5.45227239318312E-12	Mature metabolic hepatocytes	LAMTOR4
4.08850775444116E-16	0.330809967656324	0.251	0.367	6.66876499826897E-12	Mature metabolic hepatocytes	GATAD2A
5.355519483916E-16	0.257409091335715	0.322	0.459	8.73538783021538E-12	Mature metabolic hepatocytes	ERO1A
5.59966578760925E-16	0.255242472924798	0.433	0.621	9.13361486616945E-12	Mature metabolic hepatocytes	RNH1
6.11205162883801E-16	0.289156929362635	0.177	0.275	9.96936741179768E-12	Mature metabolic hepatocytes	OGDH
7.07549056614023E-16	0.316209180405818	0.306	0.434	1.15408326624313E-11	Mature metabolic hepatocytes	GPRC5C
7.17272045835204E-16	0.282246170157788	0.167	0.262	1.1699424339618E-11	Mature metabolic hepatocytes	SYAP1
7.84189222648792E-16	0.254474249654074	0.297	0.419	1.27909104106245E-11	Mature metabolic hepatocytes	PNPO
9.67785626663525E-16	0.356428637579153	0.195	0.294	1.57855513565088E-11	Mature metabolic hepatocytes	SLC25A4
1.3584572640927E-15	0.253990935017903	0.294	0.416	2.21577964346161E-11	Mature metabolic hepatocytes	JUP

**1.49814401376218E-15**	0.250968362625178	0.215	0.314	2.4436227008475E-11	Mature metabolic hepatocytes	COA3
**1.562734555388E-15**	0.395336673465485	0.266	0.381	2.54897633329336E-11	Mature metabolic hepatocytes	CHID1
**1.64192333577133E-15**	0.276215174356463	0.131	0.214	2.67814115297662E-11	Mature metabolic hepatocytes	LIME1
**1.65765676709877E-15**	0.40303771445308	0.372	0.526	2.70380395281481E-11	Mature metabolic hepatocytes	EIF4H
**1.7166181824703E-15**	0.270388652202647	0.288	0.416	2.79997591742731E-11	Mature metabolic hepatocytes	PXMP2
**1.96745770269796E-15**	0.2520214761035	0.188	0.285	3.20912025887064E-11	Mature metabolic hepatocytes	ABHD14B
**2.10288370608878E-15**	0.346396385555389	0.303	0.43	3.43001361300141E-11	Mature metabolic hepatocytes	KRTCAP2
**2.49044627735686E-15**	0.360739627699395	0.278	0.398	4.06216692299677E-11	Mature metabolic hepatocytes	EI24
**2.72655420322934E-15**	0.251750271858692	0.259	0.368	4.44728256088738E-11	Mature metabolic hepatocytes	ARPC1B
**3.03230210219166E-15**	0.28872619589842	0.211	0.313	4.94598795888481E-11	Mature metabolic hepatocytes	SURF4
**3.03845654788919E-15**	0.328621190879682	0.299	0.424	4.95602647526205E-11	Mature metabolic hepatocytes	DEPRECAT
**3.29910028977927E-15**	0.265283362790495	0.292	0.416	5.38116248265897E-11	Mature metabolic hepatocytes	CD99
**3.66848595328539E-15**	0.331068778424012	0.175	0.268	5.98366743840379E-11	Mature metabolic hepatocytes	GCDH
**3.99045277106855E-15**	0.352038572755606	0.26	0.372	6.50882751488992E-11	Mature metabolic hepatocytes	SNF8
**4.07571629621707E-15**	0.329029625308787	0.279	0.398	6.64790085075966E-11	Mature metabolic hepatocytes	PPP4C
**4.30600229404066E-15**	0.482772740461754	0.691	0.824	7.02352034180972E-11	Mature metabolic hepatocytes	OS9
**4.64326481866107E-15**	0.312474000053228	0.302	0.427	7.57362924571808E-11	Mature metabolic hepatocytes	CLTA
**5.00689679159364E-15**	0.260884690504538	0.189	0.284	8.16674935676839E-11	Mature metabolic hepatocytes	EZR
**5.07282235338117E-15**	0.355166548691373	0.246	0.356	8.27428054060003E-11	Mature metabolic hepatocytes	MAN2B1
**5.27887491105074E-15**	0.292799097256671	0.307	0.436	8.61037286741486E-11	Mature metabolic hepatocytes	SDHC
**5.58751798479624E-15**	0.302865592965073	0.389	0.543	9.11380058500114E-11	Mature metabolic hepatocytes	SUN2
**5.67461376816018E-15**	0.275560149455825	0.152	0.238	9.25586251724606E-11	Mature metabolic hepatocytes	SLC12A9
**6.04962464386561E-15**	0.256789404097594	0.21	0.319	9.8675427566092E-11	Mature metabolic hepatocytes	SCD
**6.7417772491231E-15**	0.30087494133474	0.227	0.33	1.09965128710447E-10	Mature metabolic hepatocytes	DEPRECAT
**7.03945107653681E-15**	0.250491305586649	0.334	0.465	1.14820486509392E-10	Mature metabolic hepatocytes	RAB5C
**7.4911161080402E-15**	0.381745046193571	0.182	0.276	1.22187594838244E-10	Mature metabolic hepatocytes	SZRD1
**7.69114038138176E-15**	0.48224612331148	0.755	0.793	1.25450190760718E-10	Mature metabolic hepatocytes	ORM2
**7.7630076607589E-15**	0.271989818313744	0.27	0.375	1.26622417954638E-10	Mature metabolic hepatocytes	SDC2
**8.29646018357538E-15**	0.303078100811751	0.221	0.326	1.35323562054298E-10	Mature metabolic hepatocytes	CAV2
**8.73499131171926E-15**	0.350798576778364	0.336	0.473	1.42476443285453E-10	Mature metabolic hepatocytes	ALDH9A1
**8.76055646101201E-15**	0.332830707477674	0.254	0.365	1.42893436435567E-10	Mature metabolic hepatocytes	MGST2
**9.1990392207307E-15**	0.274135510459995	0.388	0.555	1.50045528729339E-10	Mature metabolic hepatocytes	SERF2
**9.28978519267923E-15**	0.272069865926346	0.356	0.499	1.51525686277791E-10	Mature metabolic hepatocytes	DPP7
**1.07863271034984E-14**	0.262616151781737	0.233	0.335	1.75935781385162E-10	Mature metabolic hepatocytes	FZD5
**1.13296619897512E-14**	0.367167373224754	0.25	0.357	1.84798116714833E-10	Mature metabolic hepatocytes	CAPZA2
**1.19763324128833E-14**	0.290762794998674	0.355	0.498	1.9534595798654E-10	Mature metabolic hepatocytes	GUCD1
**1.21891618886262E-14**	0.325386588547776	0.181	0.273	1.98817419565381E-10	Mature metabolic hepatocytes	ELOA
**1.25052337732861E-14**	0.387274465145801	0.312	0.44	2.0397286807607E-10	Mature metabolic hepatocytes	POLR2E
**1.43031791994364E-14**	0.284707209136986	0.14	0.222	2.33299155922008E-10	Mature metabolic hepatocytes	RAB12
**1.48595186366579E-14**	0.388555764159313	0.44	0.621	2.42373608482527E-10	Mature metabolic hepatocytes	DUS1L
**1.52082868761454E-14**	0.255687819285421	0.06	0.122	2.48062367236808E-10	Mature metabolic hepatocytes	MYRIP
**1.63907354814825E-14**	0.342969754851326	0.168	0.256	2.67349286438461E-10	Mature metabolic hepatocytes	CHMP5
**1.79266187441234E-14**	0.367440549459834	0.197	0.291	2.92401078335396E-10	Mature metabolic hepatocytes	ALG3
**1.84065969600355E-14**	0.319062891149762	0.373	0.523	3.0023000301514E-10	Mature metabolic hepatocytes	LONP1
**1.92513658900602E-14**	0.358085194324367	0.306	0.429	3.14009029032772E-10	Mature metabolic hepatocytes	AIFM1
**2.07849459897811E-14**	0.259684184530272	0.436	0.618	3.39023254039319E-10	Mature metabolic hepatocytes	EMC10
**2.13176940776327E-14**	0.380698340577315	0.216	0.317	3.47712908100267E-10	Mature metabolic hepatocytes	SLC25A11
**2.18137344171344E-14**	0.34872522566861	0.207	0.303	3.55803822077879E-10	Mature metabolic hepatocytes	TMEM127
**2.29267251664536E-14**	0.271880153442303	0.248	0.352	3.73957814190025E-10	Mature metabolic hepatocytes	RNF11
**2.29544267049783E-14**	0.277660977228859	0.109	0.184	3.74409653984901E-10	Mature metabolic hepatocytes	PLIN3
**2.39191528231712E-14**	0.263379360841736	0.227	0.326	3.90145301698746E-10	Mature metabolic hepatocytes	ALAD
**2.4869506418411E-14**	0.250288564298604	0.148	0.231	4.05646519190702E-10	Mature metabolic hepatocytes	FLAD1
**2.54504791041962E-14**	0.250218774793786	0.218	0.315	4.15122764668545E-10	Mature metabolic hepatocytes	ZNF219
**2.64694570683904E-14**	0.38753740071108	0.215	0.313	4.31743314242516E-10	Mature metabolic hepatocytes	RAB14
**2.92715656000354E-14**	0.25854113326477	0.366	0.515	4.77448506502177E-10	Mature metabolic hepatocytes	PRXL2A
**2.92746942214087E-14**	0.369421498346346	0.202	0.299	4.77499537445397E-10	Mature metabolic hepatocytes	SLC25A22
**2.96101374490425E-14**	0.25724154266093	0.306	0.424	4.82970951931332E-10	Mature metabolic hepatocytes	IFITM2
**3.17506230872609E-14**	0.428163945670998	0.186	0.283	5.17884413176312E-10	Mature metabolic hepatocytes	CDKN1B
**3.31409234899217E-14**	0.250106384967096	0.15	0.236	5.40561603044114E-10	Mature metabolic hepatocytes	SLC39A9
**3.78740785683588E-14**	0.403682733108266	0.319	0.446	6.177640955285E-10	Mature metabolic hepatocytes	PSMB10

3.79871857587577E-14	0.295878210195056	0.326	0.457	6.19608986911096E-10	Mature metabolic hepatocytes	MGAT1
3.89417744630083E-14	0.265007069431812	0.189	0.279	6.35179283266128E-10	Mature metabolic hepatocytes	MOCS2
4.16868629730001E-14	0.351395965095748	0.224	0.324	6.79954421952605E-10	Mature metabolic hepatocytes	RHOD
4.20509573853603E-14	0.54164771853566	0.266	0.384	6.85893165912612E-10	Mature metabolic hepatocytes	HS6ST1
4.34309820386651E-14	0.398443528274994	0.276	0.386	7.08402748032667E-10	Mature metabolic hepatocytes	UBE2L6
4.37465482448108E-14	0.290139782743925	0.21	0.304	7.13549948421109E-10	Mature metabolic hepatocytes	EXOSC6
4.48880550406514E-14	0.293923713728855	0.211	0.307	7.32169065768066E-10	Mature metabolic hepatocytes	DERL2
4.62638428100949E-14	0.358905229324152	0.213	0.309	7.54609540075459E-10	Mature metabolic hepatocytes	FAAP20
4.67248635287155E-14	0.271605977618958	0.26	0.368	7.62129249016878E-10	Mature metabolic hepatocytes	AKT1S1
4.69542942720336E-14	0.296085682070639	0.467	0.655	7.6587149387114E-10	Mature metabolic hepatocytes	SLC25A39
6.3746970943113E-14	0.391542281681482	0.24	0.341	1.03977684305312E-09	Mature metabolic hepatocytes	HOMER2
7.09771761632453E-14	0.25880324932317	0.191	0.282	1.15770872039869E-09	Mature metabolic hepatocytes	SHMT2
7.18282193535872E-14	0.368615472273797	0.113	0.189	1.17159008587636E-09	Mature metabolic hepatocytes	NFKBIE
7.29409640393065E-14	0.386516655419242	0.32	0.449	1.18974006444513E-09	Mature metabolic hepatocytes	GUK1
7.45006668478068E-14	0.33026941464745	0.18	0.267	1.21518037695458E-09	Mature metabolic hepatocytes	MCOLN1
7.87259066428781E-14	0.400939379586432	0.222	0.321	1.28409826325199E-09	Mature metabolic hepatocytes	LRP10
8.07150240594472E-14	0.255197017862216	0.304	0.425	1.31654275743364E-09	Mature metabolic hepatocytes	APOL1
8.4392001989097E-14	0.287324064171454	0.19	0.279	1.37651794444416E-09	Mature metabolic hepatocytes	SLC25A23
9.06998833994973E-14	0.386535575362447	0.386	0.525	1.4794057981292E-09	Mature metabolic hepatocytes	POLDIP2
9.18993440373832E-14	0.325044952361072	0.149	0.231	1.49897020059376E-09	Mature metabolic hepatocytes	MAZ
9.41797890668027E-14	0.285468217298117	0.163	0.253	1.53616653946862E-09	Mature metabolic hepatocytes	INHBE
9.5930597435172E-14	0.281359147447177	0.207	0.301	1.56472397476509E-09	Mature metabolic hepatocytes	UBTF
9.93382635896555E-14	0.251311589248425	0.188	0.279	1.62030641741087E-09	Mature metabolic hepatocytes	CCDC124
9.96829419832806E-14	0.296705383221752	0.259	0.365	1.62592846668929E-09	Mature metabolic hepatocytes	NUDT14
1.09934303368837E-13	0.266117408320017	0.414	0.589	1.7931384222491E-09	Mature metabolic hepatocytes	CAT
1.14369807125692E-13	0.300590278219457	0.289	0.406	1.86548592402716E-09	Mature metabolic hepatocytes	ZNF622
1.16578956496286E-13	0.856846384627793	0.607	0.66	1.90151935941092E-09	Mature metabolic hepatocytes	CD14
1.34144252510518E-13	0.295541838407896	0.369	0.522	2.18802690269905E-09	Mature metabolic hepatocytes	TRIR
1.42926545539107E-13	0.341526838776279	0.249	0.36	2.33127488428837E-09	Mature metabolic hepatocytes	CPTP
1.48740208832672E-13	0.285033643013039	0.373	0.527	2.42610154626972E-09	Mature metabolic hepatocytes	DEPRECAT
1.57673976209769E-13	0.2893666777737	0.202	0.293	2.57182022595754E-09	Mature metabolic hepatocytes	CRTAP
1.58348766233995E-13	0.282441786662852	0.121	0.195	2.58282672604269E-09	Mature metabolic hepatocytes	DTNBP1
1.62984918953805E-13	0.341261521274025	0.216	0.313	2.65844701305551E-09	Mature metabolic hepatocytes	LSM 4.00
1.67538847934042E-13	0.332762668073584	0.369	0.506	2.73272614865215E-09	Mature metabolic hepatocytes	NIPSNAP1
1.74933710860381E-13	0.358405222745977	0.259	0.364	2.85334375784367E-09	Mature metabolic hepatocytes	ARF6
1.75254308496254E-13	0.341939329595286	0.196	0.288	2.8585730258824E-09	Mature metabolic hepatocytes	KIAA0930
1.80935680212028E-13	0.338221373082404	0.267	0.377	2.95124187993839E-09	Mature metabolic hepatocytes	ATP5MG
1.89151014817103E-13	0.319835020033164	0.307	0.426	3.08524220268177E-09	Mature metabolic hepatocytes	CLPTM1
2.20714879877471E-13	0.266943436741431	0.14	0.219	3.60008040568142E-09	Mature metabolic hepatocytes	NCBP2AS2
2.25634133228624E-13	0.444277293043893	0.208	0.304	3.68031834709209E-09	Mature metabolic hepatocytes	GPATCH4
2.41154761082072E-13	0.267675848880488	0.32	0.447	3.93347530800968E-09	Mature metabolic hepatocytes	GSTO1
2.44596934886614E-13	0.4301744004961	0.228	0.324	3.98962060493557E-09	Mature metabolic hepatocytes	YY1
2.58480649381065E-13	0.606486268558312	0.643	0.735	4.21607787205454E-09	Mature metabolic hepatocytes	SDF4
2.61573536398752E-13	0.404426570834795	0.364	0.511	4.26652595220004E-09	Mature metabolic hepatocytes	SSR2
2.6780199101569E-13	0.284305859593516	0.292	0.412	4.36811827545692E-09	Mature metabolic hepatocytes	ATP5IF1
2.80471136061454E-13	0.253348639717402	0.362	0.492	4.57476470029837E-09	Mature metabolic hepatocytes	GRHPR
2.86603295259365E-13	0.287229445070305	0.144	0.224	4.6747863489755E-09	Mature metabolic hepatocytes	PEX26
2.89331901772548E-13	0.256707252137902	0.205	0.298	4.71929264981203E-09	Mature metabolic hepatocytes	POLD2
2.96171407471305E-13	0.38766686486938	0.243	0.343	4.83085182726445E-09	Mature metabolic hepatocytes	DUSP3
2.97857375376353E-13	0.277020319229008	0.2	0.291	4.85835164976369E-09	Mature metabolic hepatocytes	NUBP2
3.15872214298848E-13	0.310424341802345	0.265	0.371	5.15219168742851E-09	Mature metabolic hepatocytes	PDHB
3.33914807893324E-13	0.348524060321091	0.712	0.84	5.44648443154801E-09	Mature metabolic hepatocytes	CERS2
3.63118863150345E-13	0.278135999992357	0.258	0.358	5.92283177684527E-09	Mature metabolic hepatocytes	LAMTOR2
4.31157084203177E-13	0.557393825792694	0.298	0.42	7.03260320043802E-09	Mature metabolic hepatocytes	STUB1
4.50300188788153E-13	0.318442872394337	0.273	0.381	7.34484637932357E-09	Mature metabolic hepatocytes	APP
4.5888548296637E-13	0.37407441623997	0.332	0.465	7.48488111266446E-09	Mature metabolic hepatocytes	NDFIP1
5.40455581760206E-13	0.4252660608676	0.31	0.431	8.81537099409072E-09	Mature metabolic hepatocytes	SHISA5
5.98422157046388E-13	0.449326160268562	0.309	0.429	9.76086380358363E-09	Mature metabolic hepatocytes	NCLN
6.72910023350887E-13	0.283992437297855	0.189	0.278	1.09758353908763E-08	Mature metabolic hepatocytes	MMP14
7.27903820853172E-13	0.412360881899062	0.329	0.456	1.18728392219361E-08	Mature metabolic hepatocytes	TMEM219

7.38011690168255E-13	0.277218000799245	0.292	0.404	1.20377086783344E-08	Mature metabolic hepatocytes	PLBD2
7.64352710925543E-13	0.350049475702316	0.323	0.446	1.24673570679065E-08	Mature metabolic hepatocytes	SPTAN1
9.09304375935609E-13	0.271985968776767	0.142	0.222	1.48316636758857E-08	Mature metabolic hepatocytes	COX14
9.29787376400998E-13	0.458665243831196	0.743	0.85	1.51657618964767E-08	Mature metabolic hepatocytes	NUPR1
1.07385033613233E-12	0.512481518565559	0.66	0.767	1.75155728326544E-08	Mature metabolic hepatocytes	CES2
1.21089576979389E-12	0.478067050580209	0.259	0.364	1.97509209011082E-08	Mature metabolic hepatocytes	SLC25A10
1.21741409589462E-12	0.299398618066181	0.39	0.544	1.98572413181372E-08	Mature metabolic hepatocytes	TM9SF2
1.30308139644559E-12	0.411765988191359	0.314	0.436	2.12545606574241E-08	Mature metabolic hepatocytes	LGMN
1.32724971226432E-12	0.309112789101794	0.232	0.327	2.16487700567434E-08	Mature metabolic hepatocytes	TOMM20
1.35874164338828E-12	0.311028529747988	0.36	0.493	2.21624349453062E-08	Mature metabolic hepatocytes	HEBP2
1.38015197169792E-12	0.25712982363724	0.242	0.339	2.25116588103647E-08	Mature metabolic hepatocytes	ACOT13
1.39870444042725E-12	0.289608764906767	0.212	0.302	2.28142681278088E-08	Mature metabolic hepatocytes	DCTN3
1.52069243846444E-12	0.32008609339053	0.231	0.325	2.48040143637935E-08	Mature metabolic hepatocytes	BCKDHA
1.53701095825971E-12	0.398168942765371	0.215	0.304	2.50701857401742E-08	Mature metabolic hepatocytes	MRFAP1L1
1.76168350885844E-12	0.334530860184792	0.104	0.173	2.873481971299E-08	Mature metabolic hepatocytes	CYP51A1
1.83740477017501E-12	0.418494850798016	0.255	0.36	2.99699092063246E-08	Mature metabolic hepatocytes	CCDC85C
1.94144100802617E-12	0.33090631969617	0.113	0.182	3.16668442819149E-08	Mature metabolic hepatocytes	MTG2
2.21033990753097E-12	0.32697324154373	0.085	0.146	3.60528542317377E-08	Mature metabolic hepatocytes	ESS2
2.37417850965292E-12	0.501523751965981	0.335	0.461	3.87252256709488E-08	Mature metabolic hepatocytes	IRAK1
2.63141409483074E-12	0.279830597654797	0.158	0.233	4.29209953007843E-08	Mature metabolic hepatocytes	CUEDC2
3.13894193218002E-12	0.315168340360631	0.362	0.496	5.11992818557883E-08	Mature metabolic hepatocytes	TSPAN13
3.16695786197088E-12	0.349260747965037	0.141	0.216	5.1656249686607E-08	Mature metabolic hepatocytes	CD2BP2
4.024129266671E-12	0.326505951964406	0.23	0.322	6.56375724686707E-08	Mature metabolic hepatocytes	DDAH1
4.24400235814321E-12	0.266439060432698	0.181	0.263	6.92239224636739E-08	Mature metabolic hepatocytes	TIMM50
4.38167473481994E-12	0.436362920290842	0.274	0.376	7.1469496599648E-08	Mature metabolic hepatocytes	TNIP1
4.41357406538518E-12	0.611910607393837	0.136	0.208	7.19898065804977E-08	Mature metabolic hepatocytes	UQCR11
4.6144854270388E-12	0.400589572992027	0.174	0.258	7.52668718004298E-08	Mature metabolic hepatocytes	MARCH2
4.79838655418003E-12	0.359279561368323	0.202	0.289	7.82664830852304E-08	Mature metabolic hepatocytes	ATP5PD
4.82846006595326E-12	0.332272938049143	0.349	0.481	7.87570121357637E-08	Mature metabolic hepatocytes	RETSAT
4.85795334439186E-12	0.307067052824184	0.2	0.285	7.92380770003757E-08	Mature metabolic hepatocytes	NDUFA11
5.27851898253035E-12	0.374245213116308	0.274	0.376	8.60979231240525E-08	Mature metabolic hepatocytes	CPN2
5.66297149179322E-12	0.334175583615908	0.344	0.468	9.23687280026392E-08	Mature metabolic hepatocytes	AMN
5.71230408990396E-12	0.272540014772896	0.452	0.633	9.31733920104235E-08	Mature metabolic hepatocytes	NECTIN2
5.97660439162411E-12	0.380860669861116	0.21	0.297	9.74843942317808E-08	Mature metabolic hepatocytes	TALDO1
6.53283054819146E-12	0.365701451264311	0.325	0.443	1.06556999071551E-07	Mature metabolic hepatocytes	YBX1
6.5930400651407E-12	0.526621370535216	0.141	0.216	1.0753907650251E-07	Mature metabolic hepatocytes	VPS51
6.7979827159301E-12	0.425115510443001	0.134	0.206	1.10881896079536E-07	Mature metabolic hepatocytes	SDC3
7.33460378190993E-12	0.430449412555363	0.219	0.31	1.19634722286733E-07	Mature metabolic hepatocytes	ZMAT2
8.03829125884544E-12	0.403351371166675	0.328	0.449	1.31112568723028E-07	Mature metabolic hepatocytes	SRPRB
8.0596756507664E-12	0.282201433147457	0.151	0.225	1.31461369539651E-07	Mature metabolic hepatocytes	MMADHC
8.27702955446188E-12	0.271277237560212	0.218	0.307	1.35006629062828E-07	Mature metabolic hepatocytes	ITPRIPL2
8.96756861032671E-12	0.256221179838677	0.162	0.238	1.46270011603039E-07	Mature metabolic hepatocytes	BCL7B
9.49569209069074E-12	0.329736020396991	0.264	0.365	1.54884233691257E-07	Mature metabolic hepatocytes	PSMC4
9.6286461948078E-12	0.427121538281072	0.312	0.431	1.5705284808351E-07	Mature metabolic hepatocytes	DEPRECAT
9.67163678265912E-12	0.290761622627092	0.459	0.641	1.57754067561953E-07	Mature metabolic hepatocytes	EIF3A
9.94375232180276E-12	0.342700387140479	0.256	0.355	1.62192544120925E-07	Mature metabolic hepatocytes	DEXI
1.03666901539537E-11	0.332139591317776	0.179	0.26	1.69091083101139E-07	Mature metabolic hepatocytes	RAB1B
1.07923116359518E-11	0.292697503857813	0.163	0.242	1.76033395094009E-07	Mature metabolic hepatocytes	GGH
1.21591056215041E-11	0.298577520504372	0.199	0.285	1.98327171792353E-07	Mature metabolic hepatocytes	FAM174C
1.41381706701905E-11	0.406630901885675	0.296	0.409	2.30607701801477E-07	Mature metabolic hepatocytes	PNKD
1.4860469877669E-11	0.336067987337674	0.181	0.262	2.42389124174659E-07	Mature metabolic hepatocytes	TXNDC12
1.57288585714389E-11	0.668127808380243	0.616	0.698	2.5655341215874E-07	Mature metabolic hepatocytes	CD63
1.68092167320388E-11	0.325721469981306	0.266	0.365	2.74175134116285E-07	Mature metabolic hepatocytes	PFDN6
1.68452310371382E-11	0.308712670106401	0.331	0.454	2.74762563446762E-07	Mature metabolic hepatocytes	DEPRECAT
1.72621042120837E-11	0.489712087353762	0.232	0.327	2.81562181803298E-07	Mature metabolic hepatocytes	SUMO2
1.76537893812038E-11	0.403074834649319	0.284	0.39	2.87950958596816E-07	Mature metabolic hepatocytes	GABARAP
1.88605251013922E-11	0.314556203260186	0.16	0.234	3.07634024928807E-07	Mature metabolic hepatocytes	TACC1
2.22517045153875E-11	0.259265314642539	0.25	0.346	3.62947552350486E-07	Mature metabolic hepatocytes	P4HA2
2.36189467248948E-11	0.341562758973788	0.241	0.333	3.85248640029758E-07	Mature metabolic hepatocytes	ANAPC11
2.46173000705075E-11	0.299540343765055	0.35	0.456	4.01532781450047E-07	Mature metabolic hepatocytes	REEP6

2.46281251743578E-11	0.28391880218222	0.188	0.27	4.01709349718949E-07	Mature metabolic hepatocytes	PTGFRN
2.65589437698621E-11	0.432037890038352	0.119	0.185	4.33202931830221E-07	Mature metabolic hepatocytes	PAF1
2.77410395893679E-11	0.384620059799582	0.295	0.41	4.5248409674218E-07	Mature metabolic hepatocytes	SPCS1
2.91074234445806E-11	0.299130850500929	0.228	0.316	4.74771183804554E-07	Mature metabolic hepatocytes	SRM
3.1559988653054E-11	0.388382278344712	0.396	0.543	5.14774974919964E-07	Mature metabolic hepatocytes	LMAN1
3.34507139772726E-11	0.499499302961222	0.122	0.19	5.45614595683293E-07	Mature metabolic hepatocytes	TSPAN3
3.40350405062014E-11	0.400463705747014	0.367	0.497	5.55145545696651E-07	Mature metabolic hepatocytes	GAA
3.50533210982498E-11	0.370463731863191	0.187	0.267	5.71754720433552E-07	Mature metabolic hepatocytes	SORBS3
3.5323649820678E-11	0.3823163387841	0.366	0.494	5.76164052225079E-07	Mature metabolic hepatocytes	DNPH1
3.6352612842402E-11	0.522044601355936	0.248	0.342	5.92947468072419E-07	Mature metabolic hepatocytes	RHOA
3.82683872309782E-11	0.276586594185726	0.145	0.214	6.24195664124485E-07	Mature metabolic hepatocytes	PROSER2
3.91899991960121E-11	0.315332151046347	0.247	0.34	6.39228076886154E-07	Mature metabolic hepatocytes	AGPAT2
4.26761912635544E-11	0.440576354861751	0.319	0.435	6.96091355699835E-07	Mature metabolic hepatocytes	GHITM
4.31006745903529E-11	0.335520561244435	0.335	0.449	7.03015103243247E-07	Mature metabolic hepatocytes	SERP1
4.33351774240716E-11	0.4804238837299	0.346	0.467	7.06840078964032E-07	Mature metabolic hepatocytes	USF2
4.48876769058116E-11	0.421451816365392	0.359	0.493	7.32162898010693E-07	Mature metabolic hepatocytes	GALNT2
4.54132880432266E-11	0.392644720689056	0.16	0.235	7.40736141273069E-07	Mature metabolic hepatocytes	TXN2
4.56950527926728E-11	0.292237580539834	0.269	0.363	7.45332006101285E-07	Mature metabolic hepatocytes	CLTB
4.82807910434266E-11	0.326909909773242	0.305	0.413	7.87507982709332E-07	Mature metabolic hepatocytes	ATP5MC2
5.13612790974626E-11	0.291371424148794	0.144	0.213	8.37753823358713E-07	Mature metabolic hepatocytes	RETREG2
5.43550680180625E-11	0.252912394134159	0.208	0.289	8.86585514442618E-07	Mature metabolic hepatocytes	GNAI3
5.66199217293908E-11	0.41578778028034	0.342	0.464	9.23527543328093E-07	Mature metabolic hepatocytes	HNRNPA0
5.80349943785423E-11	0.259911994603005	0.171	0.244	9.46608793308404E-07	Mature metabolic hepatocytes	SMARCB1
6.19632639249497E-11	0.255605789942671	0.098	0.158	1.01068279787985E-06	Mature metabolic hepatocytes	GLYR1
6.43803081680437E-11	0.261676357817671	0.428	0.592	1.05010720652896E-06	Mature metabolic hepatocytes	HAGH
6.48130943099189E-11	0.361379486910954	0.199	0.281	1.05716638128909E-06	Mature metabolic hepatocytes	FLOT2
7.06190148010642E-11	0.431918810049705	0.687	0.798	1.15186675042016E-06	Mature metabolic hepatocytes	DHCR24
7.11097636630601E-11	0.400015016975541	0.212	0.296	1.15987135510817E-06	Mature metabolic hepatocytes	BIN1
7.47868796391369E-11	0.34601121173658	0.113	0.177	1.21984879379396E-06	Mature metabolic hepatocytes	CSTB
8.60238355471349E-11	0.519123927288944	0.223	0.31	1.40313478160932E-06	Mature metabolic hepatocytes	MGAT2
8.6710885175868E-11	0.309676908044989	0.309	0.416	1.41434124810358E-06	Mature metabolic hepatocytes	NDUFA4
9.27007082865942E-11	0.513350650416282	0.182	0.258	1.51204125286264E-06	Mature metabolic hepatocytes	WDR83OS
9.82920342050368E-11	0.311538393866713	0.158	0.231	1.60324136991835E-06	Mature metabolic hepatocytes	PDLIM2
1.0071292898809E-10	0.330957969144942	0.189	0.269	1.64272858472474E-06	Mature metabolic hepatocytes	HSPA9
1.07349157493793E-10	0.375141126179473	0.084	0.141	1.75097210788126E-06	Mature metabolic hepatocytes	SLC31A1
1.07651141951727E-10	0.291577206956393	0.207	0.287	1.75589777637462E-06	Mature metabolic hepatocytes	VASP
1.08391247985624E-10	0.574346657389782	0.133	0.2	1.76796964589352E-06	Mature metabolic hepatocytes	DEPRECAT
1.25564357963304E-10	0.444703996705294	0.133	0.2	2.04808024273946E-06	Mature metabolic hepatocytes	RITA1
1.36451205415611E-10	0.422153872856059	0.285	0.389	2.22565561153403E-06	Mature metabolic hepatocytes	RBM42
1.3806657412686E-10	0.354518896463285	0.35	0.468	2.25200389058322E-06	Mature metabolic hepatocytes	IFI6
1.44032393513973E-10	0.314542121959152	0.111	0.173	2.34931237060641E-06	Mature metabolic hepatocytes	LMNB2
1.45390307225205E-10	0.334140693200764	0.171	0.247	2.37146130115032E-06	Mature metabolic hepatocytes	DEPRECAT
1.58974188615831E-10	0.429557354796888	0.198	0.278	2.59302799051281E-06	Mature metabolic hepatocytes	HGH1
1.61840940265888E-10	0.430765819780695	0.701	0.785	2.6397875766769E-06	Mature metabolic hepatocytes	PTMS
1.62520281124613E-10	0.390560975876764	0.148	0.217	2.65086830542356E-06	Mature metabolic hepatocytes	ZNF275
1.64139937788595E-10	0.398397708497954	0.421	0.575	2.67728652526977E-06	Mature metabolic hepatocytes	CXXC5
1.72077758698313E-10	0.353963519727177	0.226	0.311	2.80676032212818E-06	Mature metabolic hepatocytes	JMJD8
1.8147636700707E-10	0.268383353402974	0.114	0.177	2.96006102225232E-06	Mature metabolic hepatocytes	DEPRECAT
1.83239456009199E-10	0.441610656421194	0.381	0.517	2.98881876696604E-06	Mature metabolic hepatocytes	PFDN2
1.86019590312523E-10	0.433065474630936	0.399	0.538	3.03416553758756E-06	Mature metabolic hepatocytes	EPN1
1.90602385330632E-10	0.269987081534929	0.407	0.549	3.10891550712794E-06	Mature metabolic hepatocytes	CPB2
1.9661528500861E-10	0.432359843387432	0.281	0.381	3.20699191377543E-06	Mature metabolic hepatocytes	CTSH
2.05865892803002E-10	0.528130156558984	0.336	0.459	3.35787857750977E-06	Mature metabolic hepatocytes	NACC1
2.06212943734472E-10	0.345614756666221	0.315	0.422	3.36353932525297E-06	Mature metabolic hepatocytes	DEPRECAT
2.12522132351708E-10	0.368770220573527	0.304	0.411	3.46644850078871E-06	Mature metabolic hepatocytes	GALK1
2.3156208769347E-10	0.341022253436349	0.171	0.24	3.77700921236819E-06	Mature metabolic hepatocytes	PPIC
2.44883471296096E-10	0.545132754192447	0.205	0.289	3.99429430031063E-06	Mature metabolic hepatocytes	NEU1
2.45160416723928E-10	0.379693181100877	0.232	0.315	3.99881155718399E-06	Mature metabolic hepatocytes	TSPAN6
2.48814514505492E-10	0.359735260833634	0.057	0.106	4.05841354609907E-06	Mature metabolic hepatocytes	ACSM3
3.08058191152901E-10	0.316648677543801	0.307	0.416	5.02473715589497E-06	Mature metabolic hepatocytes	CMBL

3.19396534767214E-10	0.345459980806562	0.189	0.267	5.20967687858803E-06	Mature metabolic hepatocytes	COLEC11
3.31689447547049E-10	0.323518846475553	0.09	0.146	5.41018657893991E-06	Mature metabolic hepatocytes	LIMK1
3.33097455333119E-10	0.290227571852599	0.179	0.251	5.4331525939385E-06	Mature metabolic hepatocytes	ADCK2
3.49794686977999E-10	0.527114804066772	0.211	0.294	5.70550113929814E-06	Mature metabolic hepatocytes	COX7A2
3.50561428242371E-10	0.263289594704427	0.772	0.869	5.71800745606132E-06	Mature metabolic hepatocytes	FTH1
3.69806173327851E-10	0.315458962676716	0.145	0.212	6.03190849315057E-06	Mature metabolic hepatocytes	NAGA
3.70955438604845E-10	0.323109106348201	0.122	0.184	6.05065415908363E-06	Mature metabolic hepatocytes	C8orf82
3.81679162758855E-10	0.296622781449024	0.287	0.388	6.22556882375968E-06	Mature metabolic hepatocytes	RTN3
3.87937851284274E-10	0.437537856250928	0.237	0.323	6.32765429229779E-06	Mature metabolic hepatocytes	CANX
4.110100548861E-10	0.362575160523448	0.268	0.364	6.70398500524718E-06	Mature metabolic hepatocytes	CTSF
4.78881406585093E-10	0.290342200429687	0.379	0.531	7.81103462280945E-06	Mature metabolic hepatocytes	HADHA
4.79411861333995E-10	0.64735016450845	0.214	0.294	7.81968687021879E-06	Mature metabolic hepatocytes	NDUFS3
4.80315511213811E-10	0.258928137317456	0.443	0.589	7.83442630340847E-06	Mature metabolic hepatocytes	CDKN1A
5.30676377570795E-10	0.403553190656917	0.298	0.401	8.65586239455723E-06	Mature metabolic hepatocytes	ACADM
5.45084570785848E-10	0.36074945407211	0.403	0.539	8.89087443408796E-06	Mature metabolic hepatocytes	CYP27A1
5.47507643240118E-10	0.423617341362119	0.381	0.513	8.93039716888957E-06	Mature metabolic hepatocytes	ADIPOR1
5.51108132613676E-10	0.443800412534231	0.086	0.139	8.98912475106167E-06	Mature metabolic hepatocytes	MAP2K1
5.6752584628086E-10	0.456829712853618	0.185	0.259	9.25691407868711E-06	Mature metabolic hepatocytes	RPN2
5.79055074798095E-10	0.346110488552394	0.409	0.548	9.44496732503172E-06	Mature metabolic hepatocytes	MPC2
5.99730339483749E-10	0.369867127397098	0.216	0.299	9.78220156731943E-06	Mature metabolic hepatocytes	PPP1R1A
6.09284559134084E-10	0.428864629568254	0.389	0.525	9.93804044403605E-06	Mature metabolic hepatocytes	PMVK
6.14812507765652E-10	0.30973299666964	0.114	0.174	1.00282068141655E-05	Mature metabolic hepatocytes	TMEM70
6.35089794014542E-10	0.351868895285774	0.224	0.307	1.03589496301712E-05	Mature metabolic hepatocytes	CISD3
6.8333241697668E-10	0.54550787720403	0.36	0.492	1.11458350533066E-05	Mature metabolic hepatocytes	SERINC1
6.91381839377693E-10	0.291822521949472	0.285	0.381	1.12771291820895E-05	Mature metabolic hepatocytes	DAG1
7.7205344134408E-10	0.383586557882809	0.156	0.223	1.25929636817633E-05	Mature metabolic hepatocytes	GPR108
8.55909466556457E-10	0.357565707032603	0.159	0.227	1.39607393090024E-05	Mature metabolic hepatocytes	GALNT1
8.62811213503564E-10	0.571414273710508	0.134	0.2	1.40733137034566E-05	Mature metabolic hepatocytes	CIAO2B
9.23266138529321E-10	0.261457089312428	0.162	0.231	1.50593939855518E-05	Mature metabolic hepatocytes	H2AFJ
9.56346511756951E-10	0.460288028113145	0.227	0.307	1.55989679532676E-05	Mature metabolic hepatocytes	BRMS1
9.58095376642676E-10	0.312721148550364	0.379	0.515	1.56274936884187E-05	Mature metabolic hepatocytes	NUDC
9.66015778428479E-10	0.498663859456836	0.296	0.398	1.57566833619469E-05	Mature metabolic hepatocytes	MXD4
9.85544007842351E-10	0.370464250626419	0.299	0.403	1.60752083119166E-05	Mature metabolic hepatocytes	HEXB
1.06507356819486E-09	0.534658349863503	0.136	0.201	1.73724149708264E-05	Mature metabolic hepatocytes	CTNNBIP1
1.1202789596001E-09	0.350872481506804	0.302	0.403	1.82728701100373E-05	Mature metabolic hepatocytes	SLC22A1
1.13431176788701E-09	0.253100307555804	0.095	0.15	1.85017592460049E-05	Mature metabolic hepatocytes	CEP131
1.13795938244313E-09	0.420276203335355	0.248	0.333	1.85612554870298E-05	Mature metabolic hepatocytes	SUMO3
1.14208057152571E-09	0.340823338533222	0.253	0.342	1.86284762021559E-05	Mature metabolic hepatocytes	HSPA5
1.14644119977204E-09	0.473051635550798	0.144	0.213	1.86996024094817E-05	Mature metabolic hepatocytes	RAP2B
1.16606081845619E-09	0.455334130657307	0.126	0.186	1.90196180098388E-05	Mature metabolic hepatocytes	TOMM40
1.18739433289004E-09	0.554435068999642	0.261	0.35	1.93675889637694E-05	Mature metabolic hepatocytes	HTRA1
1.22958731882231E-09	0.327857402504413	0.17	0.239	2.00557987573108E-05	Mature metabolic hepatocytes	ARPC5
1.23051530972212E-09	0.324117703774186	0.122	0.183	2.00709352168776E-05	Mature metabolic hepatocytes	PET117
1.25946476257877E-09	0.386100163782071	0.181	0.256	2.05431297424223E-05	Mature metabolic hepatocytes	PGLS
1.27163061412303E-09	0.315576896394356	0.154	0.22	2.07415669469608E-05	Mature metabolic hepatocytes	DBI
1.33036420834266E-09	0.312765566349285	0.214	0.293	2.16995706022771E-05	Mature metabolic hepatocytes	HNRNPAB
1.35483832019731E-09	0.29679377279922	0.149	0.212	2.20987678407384E-05	Mature metabolic hepatocytes	PLAAT3
1.45261572015652E-09	0.407998738066247	0.172	0.242	2.3693615011473E-05	Mature metabolic hepatocytes	ACOT7
1.63422015829931E-09	0.299678853968855	0.166	0.236	2.665576500202E-05	Mature metabolic hepatocytes	GFER
1.64524112808995E-09	0.514005715185833	0.305	0.399	2.68355280402752E-05	Mature metabolic hepatocytes	C7orf50
1.73362718874292E-09	0.26670020851812	0.118	0.177	2.82771930755857E-05	Mature metabolic hepatocytes	BAHD1
1.7802611531198E-09	0.503082657013143	0.295	0.395	2.90378396685371E-05	Mature metabolic hepatocytes	KIAA2013
2.1106074815153E-09	0.394766154625601	0.229	0.313	3.4426118630996E-05	Mature metabolic hepatocytes	BCAP31
2.12294488260734E-09	0.40178453638687	0.137	0.201	3.46273539802083E-05	Mature metabolic hepatocytes	GNA11
2.20745657076342E-09	0.296292835460821	0.128	0.191	3.60058241257222E-05	Mature metabolic hepatocytes	BCKDK
2.22410891062267E-09	0.4745084656663	0.353	0.477	3.62774404411664E-05	Mature metabolic hepatocytes	RNF5
2.29495427344154E-09	0.35945410970728	0.136	0.197	3.7432999154105E-05	Mature metabolic hepatocytes	SGPP1
2.3054575674934E-09	0.439983731464891	0.351	0.475	3.76043183833848E-05	Mature metabolic hepatocytes	PRKAR1A
2.34239550237164E-09	0.373700843720544	0.145	0.21	3.82068130391839E-05	Mature metabolic hepatocytes	ANP32E
2.35617871640891E-09	0.363689450482643	0.327	0.435	3.84316310433458E-05	Mature metabolic hepatocytes	CITED4

**2.3899285001993E-09**	0.488490800248682	0.149	0.215	3.89821237667508E-05	Mature metabolic hepatocytes	MAP7D1
**2.39247910418283E-09**	0.273432557460838	0.491	0.682	3.90237266683262E-05	Mature metabolic hepatocytes	TFPI
**2.47671857594816E-09**	0.528954437127583	0.16	0.227	4.03977566922904E-05	Mature metabolic hepatocytes	MBP
**2.6246393074882E-09**	0.746312088709065	0.092	0.148	4.281049174444E-05	Mature metabolic hepatocytes	C1QA
**2.78120710141085E-09**	0.3226783541236	0.308	0.41	4.53642690311124E-05	Mature metabolic hepatocytes	SDHB
**2.9155330768153E-09**	0.437657290350039	0.262	0.349	4.75552600159344E-05	Mature metabolic hepatocytes	COX7C
**2.98845024736918E-09**	0.329326568383847	0.189	0.26	4.87446119848388E-05	Mature metabolic hepatocytes	SPRYD3
**3.08026822101937E-09**	0.306006168036339	0.104	0.16	5.0242254953047E-05	Mature metabolic hepatocytes	YIF1B
**3.15533918268131E-09**	0.28086377625351	0.159	0.225	5.14667374087148E-05	Mature metabolic hepatocytes	YJU2
**3.20640861725984E-09**	0.44168802999095	0.183	0.255	5.22997309561252E-05	Mature metabolic hepatocytes	CREB3
**3.21301232328944E-09**	0.55518250772797	0.205	0.281	5.2407444005174E-05	Mature metabolic hepatocytes	SYF2
**3.29700893523866E-09**	0.459224242859057	0.311	0.422	5.37775127426777E-05	Mature metabolic hepatocytes	GBA
**3.41583250787339E-09**	0.344339869568212	0.245	0.333	5.57156440359228E-05	Mature metabolic hepatocytes	TOMM7
**3.60853770902241E-09**	0.377473292442922	0.235	0.318	5.88588585718646E-05	Mature metabolic hepatocytes	PIGT
**3.77222595864619E-09**	0.365202696230432	0.215	0.29	6.15287776114779E-05	Mature metabolic hepatocytes	G3BP2
**4.00680936075266E-09**	0.336187626236586	0.195	0.266	6.53550674832367E-05	Mature metabolic hepatocytes	PYGL
**4.50291979628748E-09**	0.333504113759161	0.248	0.329	7.3447124797245E-05	Mature metabolic hepatocytes	NDUFB10
**4.57777615855825E-09**	0.665880441393195	0.657	0.76	7.46681069222437E-05	Mature metabolic hepatocytes	CEBPA
**4.59054339486657E-09**	0.485952539161329	0.225	0.306	7.48763533136686E-05	Mature metabolic hepatocytes	SELENOK
**4.73413448260111E-09**	0.50283736797467	0.237	0.321	7.72184675457067E-05	Mature metabolic hepatocytes	FAM3C
**4.75501619431125E-09**	0.552079645733589	0.233	0.315	7.75590691454108E-05	Mature metabolic hepatocytes	ATP5F1E
**4.81420968287258E-09**	0.355120603495102	0.201	0.274	7.85245741373347E-05	Mature metabolic hepatocytes	PCBP1
**4.83536557032927E-09**	0.480192839183242	0.148	0.21	7.88696478176407E-05	Mature metabolic hepatocytes	RNASET2
**4.88416831055888E-09**	0.43376732140662	0.284	0.375	7.96656693135258E-05	Mature metabolic hepatocytes	NDUFAB1
**5.02939557157863E-09**	0.294763643736894	0.19	0.262	8.2034471168019E-05	Mature metabolic hepatocytes	SLC16A1
**5.0976666368361E-09**	0.25618559010152	0.129	0.187	8.31480405134336E-05	Mature metabolic hepatocytes	NDUFC1
**5.22375354427966E-09**	0.410755395551639	0.094	0.148	8.52046440607455E-05	Mature metabolic hepatocytes	SELENOT
**5.22926291940446E-09**	0.415948113926923	0.287	0.385	8.52945074784061E-05	Mature metabolic hepatocytes	PRKCSH
**5.82528163468788E-09**	0.356298216143494	0.211	0.285	9.5016168743394E-05	Mature metabolic hepatocytes	ATP6V0D1
**5.93278889185959E-09**	0.357478780553098	0.248	0.334	9.67697196151218E-05	Mature metabolic hepatocytes	PCYOX1
**6.40284546445409E-09**	0.413622627874791	0.406	0.541	0.000104436812370711	Mature metabolic hepatocytes	CHCHD10
**6.96135155765615E-09**	0.471312238457634	0.131	0.192	0.000113546605256929	Mature metabolic hepatocytes	ATP5MF
**7.29175171341305E-09**	0.26960953106402	0.323	0.423	0.00011893576219748	Mature metabolic hepatocytes	APOM
**7.42116891682268E-09**	0.445945639364049	0.19	0.26	0.000121046686202295	Mature metabolic hepatocytes	GDI2
**7.44626354449122E-09**	0.293944411867494	0.164	0.229	0.000121456004674196	Mature metabolic hepatocytes	SNRPB
**7.87089370789955E-09**	0.426960853343811	0.219	0.294	0.00012838214726955	Mature metabolic hepatocytes	PPP1CA
**8.0145992487754E-09**	0.405536189481645	0.327	0.431	0.000130726128346776	Mature metabolic hepatocytes	HSD17B10
**8.01973881330432E-09**	0.479350361027397	0.23	0.31	0.000130809959783807	Mature metabolic hepatocytes	LIPA
**8.10707069765737E-09**	0.341359379735412	0.389	0.526	0.000132234430149489	Mature metabolic hepatocytes	METTL7B
**8.3629317544915E-09**	0.371429050659856	0.158	0.222	0.000136407779847511	Mature metabolic hepatocytes	PIK3R2
**8.47620138632092E-09**	0.408947096198205	0.224	0.302	0.00013825532081228	Mature metabolic hepatocytes	IRF2BPL
**8.60408373317888E-09**	0.2618922731155	0.169	0.236	0.000140341209771881	Mature metabolic hepatocytes	HMOX2
**8.93158475082681E-09**	0.44676189015431	0.238	0.316	0.000145683078870736	Mature metabolic hepatocytes	PPP1R11
**9.09574478536207E-09**	0.307751473093495	0.172	0.24	0.000148360693194041	Mature metabolic hepatocytes	NOSIP
**9.5999411906896E-09**	0.519844290623521	0.373	0.503	0.000156584640761338	Mature metabolic hepatocytes	FKBP2
**9.70045919358252E-09**	0.284698774999107	0.149	0.211	0.000158224189906524	Mature metabolic hepatocytes	PSMB2
**1.03480951622559E-08**	0.410020422729634	0.13	0.188	0.000168787780191556	Mature metabolic hepatocytes	HYAL2
**1.22226430533522E-08**	0.378376869173057	0.143	0.204	0.000199363530843228	Mature metabolic hepatocytes	IMPA2
**1.2628544669587E-08**	0.505583863038209	0.243	0.323	0.000205984192105633	Mature metabolic hepatocytes	PES 1.00
**1.31014223805309E-08**	0.423708213276726	0.353	0.471	0.00021369730044884	Mature metabolic hepatocytes	TMED5
**1.36125856208988E-08**	0.339447798897384	0.163	0.227	0.000222034884062481	Mature metabolic hepatocytes	NME2
**1.40454749168805E-08**	0.451276503773159	0.247	0.326	0.000229095741369238	Mature metabolic hepatocytes	TMEM238
**1.49125656695732E-08**	0.302388427935054	0.14	0.199	0.000243238858636409	Mature metabolic hepatocytes	ANXA9
**1.49392586641162E-08**	0.275578857477145	0.136	0.193	0.0002436742480704	Mature metabolic hepatocytes	ILK
**1.51420934756325E-08**	0.533130934641813	0.166	0.233	0.000246982686681041	Mature metabolic hepatocytes	LAPTM4A
**1.55190854906262E-08**	0.408294797275397	0.098	0.149	0.000253131803437605	Mature metabolic hepatocytes	LRATD2
**1.63969612766901E-08**	0.427628721144878	0.116	0.171	0.000267450835384092	Mature metabolic hepatocytes	CHMP3
**1.64268075988244E-08**	0.377221097305957	0.229	0.306	0.000267937658744425	Mature metabolic hepatocytes	NEDD8
**1.65839730980682E-08**	0.317968614784075	0.131	0.189	0.00027050118520259	Mature metabolic hepatocytes	SUPT4H1
**1.67670920757781E-08**	0.280264364354401	0.237	0.315	0.000273488038848017	Mature metabolic hepatocytes	TMEM9

1.6877824395943E-08	0.332148893894612	0.234	0.315	0.000275294193722226	Mature metabolic hepatocytes	GADD45GII
1.87880462804989E-08	0.330823599381449	0.152	0.216	0.000306451822881217	Mature metabolic hepatocytes	SPAG7
1.93523059111351E-08	0.342913737296939	0.123	0.177	0.000315655461716524	Mature metabolic hepatocytes	USP10
2.03796668507259E-08	0.530276223085462	0.091	0.141	0.00033241274600219	Mature metabolic hepatocytes	PHLDA3
2.07506174278498E-08	0.278238827065301	0.23	0.304	0.000338463320865658	Mature metabolic hepatocytes	MESD
2.07848272568842E-08	0.340001103233101	0.124	0.18	0.000339021317387038	Mature metabolic hepatocytes	TLCD2
2.15281379004224E-08	0.41840925501922	0.152	0.215	0.00035114545729379	Mature metabolic hepatocytes	ALG5
2.16808815637933E-08	0.419963024240796	0.146	0.204	0.000353636859187033	Mature metabolic hepatocytes	SRP9
2.19459646053669E-08	0.292777043039663	0.107	0.16	0.00035796062867814	Mature metabolic hepatocytes	ELOC
2.33175878437259E-08	0.298220781800538	0.073	0.118	0.000380333175319013	Mature metabolic hepatocytes	LCP1
2.44903435157502E-08	0.487684358733193	0.184	0.252	0.000399461993085401	Mature metabolic hepatocytes	TMX4
2.45061798262617E-08	0.398477033098704	0.193	0.263	0.000399720299146155	Mature metabolic hepatocytes	TADA3
2.45944232485178E-08	0.627445028181001	0.188	0.257	0.000401159637606573	Mature metabolic hepatocytes	KLF16
2.5088914730896E-08	0.362860588532063	0.198	0.268	0.000409225288175645	Mature metabolic hepatocytes	TMED2
2.61997466862816E-08	0.301182749837105	0.194	0.259	0.000427344068199939	Mature metabolic hepatocytes	SOWAHC
2.69708602717747E-08	0.321163982035046	0.141	0.2	0.000439921701892917	Mature metabolic hepatocytes	PRNP
2.70685696151821E-08	0.493364104545772	0.31	0.408	0.000441515438993235	Mature metabolic hepatocytes	NDUFA2
2.93647695559936E-08	0.440195152219834	0.409	0.562	0.000478968756227811	Mature metabolic hepatocytes	HTATIP2
3.01118944531963E-08	0.45213683438866	0.128	0.183	0.000491155110426085	Mature metabolic hepatocytes	PEX16
3.01865569141558E-08	0.367965860755545	0.078	0.124	0.000492372929826795	Mature metabolic hepatocytes	BLOC1S3
3.08636959964603E-08	0.540329658391382	0.177	0.24	0.000503417745398263	Mature metabolic hepatocytes	ASB13
3.22549087735636E-08	0.486110403431609	0.327	0.43	0.000526109817005595	Mature metabolic hepatocytes	NDUFAF3
3.31957551632122E-08	0.535285496419443	0.335	0.447	0.000541455962467155	Mature metabolic hepatocytes	CXCL16
3.35336969467501E-08	0.399209961337802	0.132	0.188	0.000546968130898441	Mature metabolic hepatocytes	DEPRECAT
3.47727329812447E-08	0.437777762781659	0.141	0.199	0.000567178047657082	Mature metabolic hepatocytes	GNAI2
3.5127198113545E-08	0.456395993500368	0.068	0.113	0.000572959728430032	Mature metabolic hepatocytes	DEPRECAT
3.66414870735163E-08	0.337797383177258	0.221	0.299	0.000597659295656124	Mature metabolic hepatocytes	SELENOW
3.67542868199177E-08	0.422280182415763	0.139	0.197	0.000599499172319677	Mature metabolic hepatocytes	RNF26
3.71289842593497E-08	0.473174607052805	0.269	0.356	0.000605610862254252	Mature metabolic hepatocytes	ATP5F1A
3.80722496195779E-08	0.433704789328638	0.188	0.254	0.000620996463544935	Mature metabolic hepatocytes	MDH2
3.83891379851013E-08	0.629608152117489	0.29	0.384	0.000626165229674988	Mature metabolic hepatocytes	PET100
3.92052987181519E-08	0.415707175165556	0.207	0.277	0.000639477627391776	Mature metabolic hepatocytes	VAMP5
4.14839343581899E-08	0.25464816367546	0.146	0.203	0.000676644453316435	Mature metabolic hepatocytes	RRS1
4.22547739996157E-08	0.262758932216711	0.097	0.148	0.000689217618707732	Mature metabolic hepatocytes	ARL4D
4.23443703156593E-08	0.33329581438219	0.309	0.403	0.000690679024218719	Mature metabolic hepatocytes	HACD3
4.88112377641971E-08	0.510382813185848	0.075	0.121	0.000796160099171818	Mature metabolic hepatocytes	MAPK3
4.88154396429331E-08	0.527300675781379	0.262	0.346	0.000796228636015882	Mature metabolic hepatocytes	TSPO
4.88187665686921E-08	0.426681956875862	0.164	0.225	0.000796282901501937	Mature metabolic hepatocytes	HDDC3
5.03488102987777E-08	0.251201723786859	0.129	0.184	0.000821239444783363	Mature metabolic hepatocytes	KHSRP
5.26064902850446E-08	0.518994372370073	0.188	0.254	0.000858064463039363	Mature metabolic hepatocytes	NUDT3
5.35506040911657E-08	0.321504370058353	0.13	0.184	0.000873463903331004	Mature metabolic hepatocytes	NACC2
5.46156427399062E-08	0.432412088674737	0.106	0.157	0.000890835748730611	Mature metabolic hepatocytes	EXOSC3
5.53620705399912E-08	0.51542801831729	0.121	0.174	0.000903010732577796	Mature metabolic hepatocytes	SCARB2
5.69237503032187E-08	0.364992606634939	0.185	0.25	0.0009284832911958	Mature metabolic hepatocytes	FRAT2
5.81690342855908E-08	0.386624047245788	0.201	0.269	0.000948795118232272	Mature metabolic hepatocytes	TCEAL8
6.00735868238201E-08	0.396520014812921	0.673	0.792	0.00097986027468333	Mature metabolic hepatocytes	IFITM3
6.00858704963803E-08	0.609356210138655	0.273	0.358	0.000980060633666458	Mature metabolic hepatocytes	DNAJC4
6.19285664841503E-08	0.390388957756258	0.17	0.234	0.00101011684792298	Mature metabolic hepatocytes	DEPRECAT
6.74061598414914E-08	0.503774759006707	0.131	0.188	0.00109946187317457	Mature metabolic hepatocytes	VAT1
7.08954234116421E-08	0.313855514315345	0.148	0.205	0.00115637525126729	Mature metabolic hepatocytes	TRAPPC6A
7.42679339326135E-08	0.595137224437977	0.36	0.481	0.00121138427037486	Mature metabolic hepatocytes	UBXN6
7.61831695269743E-08	0.588830185238152	0.177	0.242	0.00124262367815448	Mature metabolic hepatocytes	PSENEN
7.71573288987017E-08	0.471994051253832	0.362	0.481	0.00125851319166672	Mature metabolic hepatocytes	SERPINA4
7.90410598388402E-08	0.431113360885245	0.231	0.306	0.00128923872703132	Mature metabolic hepatocytes	PARK7
8.17387220558687E-08	0.33908579118166	0.128	0.182	0.00133324029545327	Mature metabolic hepatocytes	IER3IP1
8.32801474257601E-08	0.291071655074294	0.416	0.546	0.00135838248466157	Mature metabolic hepatocytes	OTC
8.78706110979682E-08	0.404424089944092	0.418	0.57	0.00143325753761896	Mature metabolic hepatocytes	LAMP2
8.91423553835305E-08	0.380033125484552	0.1	0.149	0.00145400095866077	Mature metabolic hepatocytes	PPP1R14B
9.26721225411528E-08	0.306644171599993	0.455	0.621	0.00151157499076874	Mature metabolic hepatocytes	LSR
9.46323426498864E-08	0.336490069395917	0.118	0.171	0.0015435481409623	Mature metabolic hepatocytes	DEPRECAT

9.77765864013041E-08	0.500680489248038	0.632	0.742	0.00159483390079167	Mature metabolic hepatocytes	ADH1C
9.94122159449686E-08	0.382335921951571	0.059	0.1	0.00162151265427838	Mature metabolic hepatocytes	UBE2D4
1.01677928482248E-07	0.448761467051838	0.156	0.217	0.00165846869147395	Mature metabolic hepatocytes	PHB2
1.05921984329757E-07	0.303879934193446	0.113	0.165	0.00172769348640266	Mature metabolic hepatocytes	TIMP1
1.1182746745748E-07	0.554215418467499	0.292	0.381	0.00182401782169896	Mature metabolic hepatocytes	PKN1
1.17646559749475E-07	0.494905873678007	0.289	0.379	0.00191893303607369	Mature metabolic hepatocytes	NDUFB6
1.18619775465312E-07	0.419403525876338	0.131	0.184	0.00193480715761471	Mature metabolic hepatocytes	SNX12
1.24708466755145E-07	0.466810364705431	0.117	0.17	0.00203411980124318	Mature metabolic hepatocytes	BORCS6
1.25165961625742E-07	0.294706054430354	0.094	0.14	0.00204158200007748	Mature metabolic hepatocytes	POLR3H
1.25189188198311E-07	0.258803087199309	0.104	0.151	0.00204196084870265	Mature metabolic hepatocytes	GDE1
1.28213119534177E-07	0.279773882435622	0.073	0.115	0.00209128419272197	Mature metabolic hepatocytes	UBAC1
1.28877771587539E-07	0.445854204707541	0.144	0.199	0.00210212533236434	Mature metabolic hepatocytes	ZCCHC3
1.29585192534565E-07	0.25180362896358	0.141	0.197	0.00211366407543129	Mature metabolic hepatocytes	FIBP
1.32465200438259E-07	0.328941367653936	0.065	0.107	0.00216063988434844	Mature metabolic hepatocytes	DEPRECAT
1.33456549503887E-07	0.520818764826362	0.202	0.269	0.0021768097789579	Mature metabolic hepatocytes	SCAND1
1.42302585179673E-07	0.354290769323252	0.119	0.169	0.00232109746686565	Mature metabolic hepatocytes	NOB1
1.45141607099895E-07	0.266301914640186	0.143	0.195	0.00236740475340638	Mature metabolic hepatocytes	RDX
1.51040372396944E-07	0.478062372078311	0.092	0.139	0.00246361951416656	Mature metabolic hepatocytes	SH3BP4
1.54800697211857E-07	0.522446561429266	0.373	0.497	0.0025249541722226	Mature metabolic hepatocytes	CAMK2N1
1.57110380289402E-07	0.380082612917654	0.148	0.205	0.00256262741290044	Mature metabolic hepatocytes	AIP
1.80440318286508E-07	0.338247759020421	0.148	0.204	0.00294316203157122	Mature metabolic hepatocytes	BANF1
1.85253794217168E-07	0.368811131020445	0.418	0.548	0.00302167463747623	Mature metabolic hepatocytes	PRKACA
1.85581104011935E-07	0.477660246931238	0.281	0.362	0.00302701338753868	Mature metabolic hepatocytes	ATP5ME
1.92112848189122E-07	0.354379569684179	0.211	0.283	0.00313355266681277	Mature metabolic hepatocytes	PHLDA1
2.01481157361655E-07	0.503915386918412	0.322	0.419	0.00328635915772595	Mature metabolic hepatocytes	NDUFB9
2.18994911845585E-07	0.499770455355139	0.104	0.153	0.00357202600711333	Mature metabolic hepatocytes	RHOG
2.27536477285901E-07	0.484863412843058	0.28	0.366	0.00371134748101033	Mature metabolic hepatocytes	DGAT2
2.2988041870247E-07	0.260212637932868	0.14	0.193	0.003749579509456	Mature metabolic hepatocytes	H3F3B
2.39950335631124E-07	0.480372293484879	0.302	0.391	0.00391382992447927	Mature metabolic hepatocytes	COX8A
2.44236420853204E-07	0.514414328258976	0.213	0.282	0.00398374026053662	Mature metabolic hepatocytes	DMTN
2.47712142529532E-07	0.297870590834168	0.103	0.149	0.0040404327567992	Mature metabolic hepatocytes	EXOSC5
2.51730645428968E-07	0.544281479479287	0.099	0.146	0.00410597855759189	Mature metabolic hepatocytes	TIMM29
2.57075288632873E-07	0.293671294597858	0.1	0.147	0.0041931550328908	Mature metabolic hepatocytes	MFAP1
2.57157227761443E-07	0.386423365437112	0.311	0.409	0.00419449154201689	Mature metabolic hepatocytes	FUCA1
2.58272188798741E-07	0.372664874385706	0.306	0.394	0.00421267767149627	Mature metabolic hepatocytes	C1orf115
2.61588603191017E-07	0.288074317238888	0.12	0.17	0.00426677170664868	Mature metabolic hepatocytes	CDC42EP4
2.65455106895409E-07	0.304619950506341	0.172	0.23	0.00432983824857101	Mature metabolic hepatocytes	COPRS
2.74658563608215E-07	0.452535413904173	0.238	0.309	0.0044799558310136	Mature metabolic hepatocytes	COX6C
2.9129849828575E-07	0.319344596084427	0.094	0.139	0.00475136980553886	Mature metabolic hepatocytes	DEPRECAT
2.94382741241942E-07	0.431635817503579	0.225	0.295	0.00480167689239731	Mature metabolic hepatocytes	MPND
3.02778668812072E-07	0.302073555060404	0.097	0.144	0.00493862286699371	Mature metabolic hepatocytes	NDUFA7
3.08413828688589E-07	0.485622359184666	0.216	0.283	0.00503053795973957	Mature metabolic hepatocytes	TRIB3
3.11035073822618E-07	0.281217084534566	0.15	0.205	0.00507329308912072	Mature metabolic hepatocytes	UQCR10
3.26093109338374E-07	0.599076414382816	0.232	0.303	0.00531890470641822	Mature metabolic hepatocytes	POLR2F
3.27795885751912E-07	0.610168018457591	0.255	0.332	0.00534667869249944	Mature metabolic hepatocytes	CKAP4
3.2982048102806E-07	0.469562802652202	0.309	0.403	0.00537970186604869	Mature metabolic hepatocytes	MCFD2
3.32388703217931E-07	0.639385683473655	0.269	0.349	0.00542159213818768	Mature metabolic hepatocytes	SMPD1
3.36422255493156E-07	0.319662015110472	0.091	0.135	0.00548738340934887	Mature metabolic hepatocytes	DMAC2
3.42160166808817E-07	0.389913774168605	0.242	0.317	0.00558097448081861	Mature metabolic hepatocytes	NPC2
3.54033665419657E-07	0.442039393416028	0.229	0.3	0.00577464311666002	Mature metabolic hepatocytes	AAMDC
3.74652487242718E-07	0.499476060462849	0.164	0.224	0.00611095671941598	Mature metabolic hepatocytes	CD82
3.78957816319799E-07	0.429769551919726	0.661	0.765	0.00618118094199224	Mature metabolic hepatocytes	TM4SF4
4.01092419514005E-07	0.250555714927528	0.124	0.173	0.00654221845469294	Mature metabolic hepatocytes	HARS
4.0748230250754E-07	0.411844057629405	0.228	0.295	0.00664644383620048	Mature metabolic hepatocytes	PGD
4.08236989893268E-07	0.413717888247306	0.22	0.286	0.0066587535421491	Mature metabolic hepatocytes	C12orf57
4.09665719596606E-07	0.460457649241676	0.303	0.389	0.00668205755234025	Mature metabolic hepatocytes	CNPY3
4.10835166518378E-07	0.412622272406941	0.207	0.27	0.00670113240108127	Mature metabolic hepatocytes	RETREG3
4.1139477333491E-07	0.315986552106173	0.114	0.163	0.00671026014786571	Mature metabolic hepatocytes	ALG1
4.13028987965072E-07	0.45710119228982	0.245	0.318	0.00673691582269829	Mature metabolic hepatocytes	EMC7
4.14901079772685E-07	0.257675070726599	0.168	0.222	0.00676745151217227	Mature metabolic hepatocytes	CLDN23

4.370841301826E-07	0.476592102853575	0.3	0.384	0.00712927924740839	Mature metabolic hepatocytes	MSRB2
4.37859033787188E-07	0.356084622834171	0.095	0.14	0.00714191870010283	Mature metabolic hepatocytes	MICOS13
4.43069654299414E-07	0.603617526064668	0.21	0.276	0.00722690913127774	Mature metabolic hepatocytes	CCT3
4.4733167400653E-07	0.504623807946623	0.14	0.193	0.00729642693472052	Mature metabolic hepatocytes	SNX8
4.82987035278062E-07	0.257716468413013	0.087	0.13	0.00787800153242047	Mature metabolic hepatocytes	VSTM2L
4.91148360757424E-07	0.363790505697176	0.131	0.181	0.00801112091231435	Mature metabolic hepatocytes	NUDCD2
5.15757190640496E-07	0.412151757205255	0.093	0.138	0.00841251553653713	Mature metabolic hepatocytes	PSMA2
5.29882112053343E-07	0.42298137870272	0.393	0.521	0.00864290712970207	Mature metabolic hepatocytes	MGST3
5.47142353267903E-07	0.336175523944729	0.151	0.205	0.00892443892415277	Mature metabolic hepatocytes	COPZ1
5.50693599777262E-07	0.421734270694591	0.213	0.279	0.00898236330596692	Mature metabolic hepatocytes	ELOB
5.6439003917712E-07	0.600046974110711	0.319	0.411	0.009205765929018	Mature metabolic hepatocytes	FKBP8
5.94113863107865E-07	0.595391466244955	0.309	0.403	0.00969059122115239	Mature metabolic hepatocytes	FAM50B
5.98070861507522E-07	0.440664773148151	0.245	0.317	0.00975513382204919	Mature metabolic hepatocytes	NME1
6.6971931889533E-07	0.367149250281429	0.107	0.152	0.0109237918105017	Mature metabolic hepatocytes	SLC27A4
6.9841118269571E-07	0.340520886093795	0.12	0.168	0.0113917848009497	Mature metabolic hepatocytes	CLIP2
7.41629039602901E-07	0.459103716696262	0.236	0.304	0.0120967112649629	Mature metabolic hepatocytes	MARCKS
7.69766318120811E-07	0.355010909675876	0.137	0.187	0.0125556584148685	Mature metabolic hepatocytes	POLR2I
7.99622207171849E-07	0.442445855361609	0.241	0.312	0.01304263782118	Mature metabolic hepatocytes	SAP18
8.0725111975584E-07	0.43628708225113	0.403	0.538	0.0131670730143375	Mature metabolic hepatocytes	SELENOH
8.60606309540522E-07	0.403016745278449	0.126	0.176	0.0140373495149155	Mature metabolic hepatocytes	USE1
8.79940507578955E-07	0.581923676823571	0.158	0.212	0.0143527096191203	Mature metabolic hepatocytes	TSPAN33
9.06014142736555E-07	0.478837395463394	0.098	0.142	0.014777996682176	Mature metabolic hepatocytes	UXS1
9.35969681081183E-07	0.567331650563507	0.184	0.243	0.0152666014681152	Mature metabolic hepatocytes	EIF3F
9.91085205877509E-07	0.499009645823548	0.103	0.148	0.0161655907930681	Mature metabolic hepatocytes	TUSC2
1.01159264853172E-06	0.614172559001748	0.213	0.278	0.0165000876902009	Mature metabolic hepatocytes	EDNRB
1.02266075693123E-06	0.595031523423698	0.124	0.173	0.0166806196063054	Mature metabolic hepatocytes	ARPC5L
1.05236818060292E-06	0.256751953953459	0.55	0.735	0.0171651773938142	Mature metabolic hepatocytes	MGAT4B
1.08453780795778E-06	0.262540527778498	0.094	0.136	0.0176898961855993	Mature metabolic hepatocytes	TCTN3
1.09551332350706E-06	0.301660457753892	0.488	0.642	0.0178689178197236	Mature metabolic hepatocytes	LASP1
1.10106617918359E-06	0.466486191769133	0.147	0.198	0.0179594904486636	Mature metabolic hepatocytes	ITPKA
1.12149761047574E-06	0.772476158673343	0.219	0.286	0.0182927475244698	Mature metabolic hepatocytes	YIPF2
1.16168470164801E-06	0.387832472769345	0.141	0.191	0.0189482391685807	Mature metabolic hepatocytes	RPP21
1.17022346203027E-06	0.530514293739772	0.281	0.362	0.0190875148891758	Mature metabolic hepatocytes	PRG4
1.22087289057851E-06	0.419364830045991	0.399	0.533	0.019913657718226	Mature metabolic hepatocytes	BEX3
1.25394653093084E-06	0.72152873047262	0.26	0.337	0.0204531218660129	Mature metabolic hepatocytes	YWHAG
1.25744179886542E-06	0.363219198838808	0.185	0.243	0.0205101331812939	Mature metabolic hepatocytes	NENF
1.29907179399599E-06	0.421263484837506	0.223	0.288	0.0211891600318685	Mature metabolic hepatocytes	LGALS1
1.31297709219168E-06	0.401367852306807	0.662	0.799	0.0214159693507385	Mature metabolic hepatocytes	TFR2
1.32128816922597E-06	0.39714832146064	0.516	0.685	0.0215515313282448	Mature metabolic hepatocytes	MYH14
1.34443768470268E-06	0.436544899824947	0.628	0.747	0.0219291230751854	Mature metabolic hepatocytes	KRT18
1.37650125752688E-06	0.468872234203264	0.174	0.232	0.0224521120115209	Mature metabolic hepatocytes	TM9SF1
1.39008109535088E-06	0.479437828800015	0.305	0.394	0.0226736127462682	Mature metabolic hepatocytes	TXNDC17
1.41392480030376E-06	0.366928218794484	0.455	0.607	0.0230625274177546	Mature metabolic hepatocytes	HAO1
1.42328057515949E-06	0.601599346082615	0.234	0.301	0.0232151294614264	Mature metabolic hepatocytes	RNF128
1.45399127637205E-06	0.511586029086	0.167	0.221	0.0237160517089046	Mature metabolic hepatocytes	TADA2B
1.46918902207034E-06	0.487110700671174	0.091	0.132	0.0239639421389893	Mature metabolic hepatocytes	TMEM19
1.57064409158502E-06	0.457384697172472	0.23	0.296	0.0256187757778433	Mature metabolic hepatocytes	TMEM147
1.64332681832944E-06	0.558748567693282	0.1	0.143	0.0268043037337715	Mature metabolic hepatocytes	PTRH1
1.67169474706884E-06	0.552633224799177	0.16	0.215	0.0272670130194398	Mature metabolic hepatocytes	ATP6V0C
1.67929208123526E-06	0.277665903009808	0.497	0.654	0.0273909331370284	Mature metabolic hepatocytes	BLVRB
1.82780400092495E-06	0.277144475041993	0.064	0.1	0.0298133110590868	Mature metabolic hepatocytes	FAM210B
1.8873283827748E-06	0.610206310213083	0.255	0.328	0.0307842132514398	Mature metabolic hepatocytes	VEGFB
1.88830893874558E-06	0.418244880826945	0.133	0.18	0.0308002070998791	Mature metabolic hepatocytes	TMEM185E
1.91689732735434E-06	0.28064035996284	0.159	0.211	0.0312665123064767	Mature metabolic hepatocytes	DEPRECAT
1.92210916682804E-06	0.673246550115849	0.181	0.239	0.0313515226201321	Mature metabolic hepatocytes	CSDE1
1.92525718166285E-06	0.395633343689186	0.471	0.625	0.0314028698901027	Mature metabolic hepatocytes	TAF10
1.94632826603715E-06	0.606508791386911	0.126	0.176	0.031746560347332	Mature metabolic hepatocytes	NDUFS5
2.47330414136179E-06	0.754863256204315	0.182	0.238	0.0403420638497521	Mature metabolic hepatocytes	B4GALT2
2.53562006116579E-06	0.340265491904423	0.121	0.167	0.0413584988176752	Mature metabolic hepatocytes	CD302
2.66629010497598E-06	0.34940170794303	0.186	0.243	0.0434898579022632	Mature metabolic hepatocytes	DGCR6L

2.81316808400143E-06	0.656406888309336	0.094	0.136	0.0458855846181474	Mature metabolic hepatocytes	THEM6
2.90353394675884E-06	0.569539363310042	0.238	0.305	0.0473595422055834	Mature metabolic hepatocytes	CHMP2A
3.03973730904581E-06	0.533163072380295	0.357	0.445	0.0495811552478462	Mature metabolic hepatocytes	LY6E
3.05417130315909E-06	0.560214313496356	0.315	0.4	0.0498165881258278	Mature metabolic hepatocytes	EEF1G
0	1.82135647147819	0.883	0.464	0	Mid-zonal hepatocytes	CCNL2
5.63898970641184E-279	1.71072891323704	0.905	0.555	9.19775611012834E-275	Mid-zonal hepatocytes	VEGFA
1.65285235603162E-270	1.99234213454408	0.795	0.395	2.69596747792318E-266	Mid-zonal hepatocytes	ARGLU1
3.72362859426755E-264	1.88022198542078	0.799	0.403	6.0736106001098E-260	Mid-zonal hepatocytes	LENG8
3.51468210267321E-258	1.48886756105086	0.958	0.69	5.73279797767027E-254	Mid-zonal hepatocytes	EFNA1
1.08823897419416E-253	2.05334939156541	0.779	0.391	1.7750265908081E-249	Mid-zonal hepatocytes	PABPC1L
2.90405138515722E-251	1.91327489409201	0.778	0.403	4.73679821432994E-247	Mid-zonal hepatocytes	WSB1
2.9945832166524E-237	1.16781836585458	0.948	0.74	4.88446468468173E-233	Mid-zonal hepatocytes	ACADVL
8.67161536781187E-227	1.56948630556147	0.836	0.468	1.41442718264379E-222	Mid-zonal hepatocytes	CDK5RAP3
2.47284982870597E-220	1.65347070392871	0.779	0.412	4.0334653556023E-216	Mid-zonal hepatocytes	MDM4
8.88399098659522E-199	1.56519104439391	0.787	0.457	1.44906776982355E-194	Mid-zonal hepatocytes	ABCC3
3.75291455722333E-198	1.96803429166304	0.766	0.423	6.12137893428697E-194	Mid-zonal hepatocytes	TXNIP
8.97019240834008E-197	1.24594506943027	0.849	0.532	1.46312808372435E-192	Mid-zonal hepatocytes	DCAF8
1.94220828502473E-193	1.52325696861472	0.774	0.444	3.16793593370384E-189	Mid-zonal hepatocytes	AFDN
1.78598004527814E-192	1.75022369010305	0.724	0.376	2.91311205185318E-188	Mid-zonal hepatocytes	CCNL1
2.57589929122099E-189	1.14956108851476	0.927	0.645	4.20154933391056E-185	Mid-zonal hepatocytes	CYP3A5
2.36071255291043E-184	1.28346161067533	0.81	0.488	3.85055824505221E-180	Mid-zonal hepatocytes	SEC16B
2.45696165128295E-183	1.92376059985473	0.647	0.308	4.00755014940762E-179	Mid-zonal hepatocytes	PILRB
2.47984353339627E-183	1.36960517660011	0.917	0.685	4.04487278732265E-179	Mid-zonal hepatocytes	MCL1
5.92900183069705E-183	1.72383950325817	0.689	0.356	9.67079488604996E-179	Mid-zonal hepatocytes	ASH1L
3.33850787285076E-181	1.16936862497872	0.883	0.55	5.44544019140687E-177	Mid-zonal hepatocytes	MLXIPL
3.563514871834E-181	1.29459600136177	0.874	0.598	5.81244910744844E-177	Mid-zonal hepatocytes	UPB1
2.91545636713609E-179	1.64689711812217	0.767	0.442	4.75540088043567E-175	Mid-zonal hepatocytes	EXT1
1.81740484116778E-172	1.82634369953495	0.686	0.353	2.96436903642877E-168	Mid-zonal hepatocytes	AKR1C8P
1.14483509672646E-166	1.39720839442249	0.757	0.423	1.86734052627052E-162	Mid-zonal hepatocytes	OBSCN
1.67878267595618E-166	2.45993659626121	0.509	0.194	2.73826242275213E-162	Mid-zonal hepatocytes	CYP2C19
1.73689295589247E-165	1.34100753733318	0.772	0.457	2.83304610035621E-161	Mid-zonal hepatocytes	ZKSCAN1
2.09132798705148E-163	1.69046799569585	0.68	0.362	3.41116507967967E-159	Mid-zonal hepatocytes	ATP2B4
2.25589432617486E-159	1.78107647827791	0.62	0.302	3.67958923542381E-155	Mid-zonal hepatocytes	RBM6
5.68743202947754E-158	0.986009863346755	0.92	0.698	9.27677038328081E-154	Mid-zonal hepatocytes	MT1M
3.95934034852645E-156	1.58167045594194	0.691	0.386	6.45808004248149E-152	Mid-zonal hepatocytes	DPYD
7.9168677704149E-156	1.4059321218057	0.728	0.429	1.29132030203237E-151	Mid-zonal hepatocytes	ARHGEF10
4.17046980562708E-155	1.71151713807119	0.645	0.34	6.80245329995833E-151	Mid-zonal hepatocytes	DST
1.95689707166394E-153	2.14898059128884	0.536	0.233	3.19189481359104E-149	Mid-zonal hepatocytes	HSF4
8.98600401522332E-151	1.74284991469961	0.63	0.326	1.46570711492308E-146	Mid-zonal hepatocytes	MOV10
1.04007389261773E-150	1.32184326542621	0.744	0.438	1.69646452624877E-146	Mid-zonal hepatocytes	TMEM140
4.5192693467937E-150	1.14178212649664	0.878	0.625	7.37138023155521E-146	Mid-zonal hepatocytes	MT1A
8.15816068560172E-150	1.07444174429725	0.813	0.511	1.3306775894285E-145	Mid-zonal hepatocytes	MYO18A
1.00946755294111E-149	1.60239671232196	0.69	0.379	1.64654252560224E-145	Mid-zonal hepatocytes	XAF1
1.34126520442682E-147	1.6085923870945	0.717	0.414	2.18773767494059E-143	Mid-zonal hepatocytes	NFKBIZ
2.39555221956781E-147	1.41960953208223	0.697	0.404	3.90738522533705E-143	Mid-zonal hepatocytes	NPLOC4
1.09387846227317E-145	1.39699708498563	0.725	0.426	1.78422515981377E-141	Mid-zonal hepatocytes	IL6R
2.82538468298804E-145	1.3744602147762	0.695	0.395	4.6084849564218E-141	Mid-zonal hepatocytes	POGZ
5.42195503030961E-145	1.46299380931313	0.677	0.378	8.843750849938E-141	Mid-zonal hepatocytes	UBAP2L
2.01806579108054E-143	1.49835783239141	0.65	0.354	3.29166711183147E-139	Mid-zonal hepatocytes	SRSF4
2.69185353978836E-141	1.72359536088711	0.615	0.316	4.3906823087488E-137	Mid-zonal hepatocytes	DTNA
9.32026800193848E-141	1.67840651472156	0.63	0.339	1.52022891379619E-136	Mid-zonal hepatocytes	GCKR
2.70411095862905E-140	1.64389260626524	0.586	0.29	4.41067538461985E-136	Mid-zonal hepatocytes	TNKS
1.26541248779062E-139	1.42653671761138	0.654	0.365	2.06401430883527E-135	Mid-zonal hepatocytes	CDC42SE1
6.00841630298534E-139	1.51535716704819	0.675	0.373	9.80032783179939E-135	Mid-zonal hepatocytes	CEP72
8.53257752760011E-139	1.48564155174337	0.721	0.428	1.39174872052685E-134	Mid-zonal hepatocytes	VMP1
1.10215132295358E-135	1.81551912184533	0.583	0.296	1.79771902286958E-131	Mid-zonal hepatocytes	NSUN6
3.92174011023711E-135	1.71536781861085	0.603	0.316	6.39675029380775E-131	Mid-zonal hepatocytes	ANKRD10
4.08858401702537E-135	1.49125338359548	0.629	0.34	6.66888939017007E-131	Mid-zonal hepatocytes	CHTOP
6.3698834594676E-134	1.28361945620146	0.865	0.611	1.03899169107376E-129	Mid-zonal hepatocytes	ELF3
7.38285481662264E-134	1.45013968328533	0.67	0.381	1.20421744913932E-129	Mid-zonal hepatocytes	NOTCH2NL

1.40726380435506E-133	1.95939585140558	0.521	0.236	2.29538799128354E-129	Mid-zonal hepatocytes	DENND4B
2.63263388518216E-133	1.40924541461859	0.654	0.37	4.29408913012062E-129	Mid-zonal hepatocytes	LUC7L3
4.99448538981337E-133	2.08978783072559	0.489	0.209	8.14650511932459E-129	Mid-zonal hepatocytes	GTPBP2
2.40212713605442E-132	1.71718285007781	0.568	0.285	3.91810957161836E-128	Mid-zonal hepatocytes	ALS2CL
1.83957524752715E-131	1.29543283448022	0.726	0.444	3.00053118624153E-127	Mid-zonal hepatocytes	NR1H4
8.34498691689627E-131	1.03530919232839	0.796	0.521	1.36115081601495E-126	Mid-zonal hepatocytes	PCSK6
1.98565978811375E-130	1.47784407033084	0.623	0.342	3.23880968039233E-126	Mid-zonal hepatocytes	MPRIP
4.10541793391459E-130	1.49137411365834	0.635	0.349	6.69634719200809E-126	Mid-zonal hepatocytes	UBR2
4.90338716176196E-130	0.885367976780226	0.868	0.599	7.99791479954994E-126	Mid-zonal hepatocytes	ECHDC2
2.13626038616187E-129	1.45619319164627	0.651	0.373	3.48445431586863E-125	Mid-zonal hepatocytes	NR5A2
3.46047764867865E-129	0.851633653107518	0.904	0.618	5.64438509275975E-125	Mid-zonal hepatocytes	C4BPB
1.99364338770351E-128	1.95092950216373	0.54	0.265	3.2518317296832E-124	Mid-zonal hepatocytes	OGT
2.05030487864832E-127	1.87780123264164	0.532	0.256	3.34425228756328E-123	Mid-zonal hepatocytes	RBM25
2.75998748709714E-127	1.77921752908916	0.548	0.273	4.50181559020414E-123	Mid-zonal hepatocytes	PCSK7
1.19455628408134E-126	1.33899881229759	0.664	0.382	1.94844075496508E-122	Mid-zonal hepatocytes	ARAP1
2.42251309247675E-126	1.57635716790776	0.602	0.32	3.95136110513882E-122	Mid-zonal hepatocytes	NKTR
3.94655366409987E-126	1.6719659544066	0.574	0.293	6.4372236815133E-122	Mid-zonal hepatocytes	VPS45
5.16696371025246E-126	1.89665762670091	0.526	0.253	8.42783450779279E-122	Mid-zonal hepatocytes	IP6K2
6.89311769579841E-126	1.31506251895944	0.682	0.408	1.12433642736168E-121	Mid-zonal hepatocytes	LIMD1
1.96299736513637E-125	1.45803765208497	0.632	0.357	3.20184500227394E-121	Mid-zonal hepatocytes	SETD5
2.82504611491534E-125	1.28301734231226	0.704	0.429	4.60793271803842E-121	Mid-zonal hepatocytes	ERBB3
1.89663706899581E-124	1.52728063440306	0.625	0.352	3.09360472323907E-120	Mid-zonal hepatocytes	DTX4
4.79869732873961E-124	2.11847529545424	0.462	0.19	7.82715521290718E-120	Mid-zonal hepatocytes	FAM118A
3.47104402923504E-123	0.879457914891625	0.951	0.749	5.66161991608527E-119	Mid-zonal hepatocytes	ITIH3
3.73463404842472E-123	2.30244536528114	0.536	0.259	6.09156159638557E-119	Mid-zonal hepatocytes	ATF3
3.806978479089E-123	1.57798880072687	0.613	0.348	6.20956259724207E-119	Mid-zonal hepatocytes	MPV17
1.67517754678149E-122	1.50478202426472	0.627	0.354	2.73238209655528E-118	Mid-zonal hepatocytes	LGALS8
2.62710644032355E-121	2.02761931375061	0.477	0.21	4.28507331481175E-117	Mid-zonal hepatocytes	NFRKB
1.12927901070525E-119	1.60393358680951	0.595	0.324	1.84196699436133E-115	Mid-zonal hepatocytes	PLA2G6
1.81920161460295E-119	1.0714448370963	0.805	0.555	2.96729975357886E-115	Mid-zonal hepatocytes	KLF6
4.15458134384424E-119	1.60620892843895	0.58	0.312	6.77653762994434E-115	Mid-zonal hepatocytes	IKBKB
3.34135211318339E-118	1.2162081752512	0.659	0.391	5.45007943181342E-114	Mid-zonal hepatocytes	FBXO17
6.07692752260681E-118	1.75940777430474	0.525	0.261	9.91207648212396E-114	Mid-zonal hepatocytes	KDM5D
8.755111703321E-118	1.87668703287232	0.544	0.276	1.42804626992869E-113	Mid-zonal hepatocytes	PPARGC1A
1.68451454780168E-117	0.934747585285722	0.794	0.522	2.74761167891931E-113	Mid-zonal hepatocytes	NIT1
3.62547836835402E-117	1.87618267640706	0.486	0.223	5.91351776662224E-113	Mid-zonal hepatocytes	PPP1R12B
4.35830760858407E-117	1.59140170639436	0.573	0.311	7.10883554036147E-113	Mid-zonal hepatocytes	PI4KB
9.03590799471891E-117	1.5304373945634	0.575	0.306	1.4738469530186E-112	Mid-zonal hepatocytes	RSRP1
8.28801788182905E-116	2.01009309529089	0.476	0.216	1.35185859670514E-111	Mid-zonal hepatocytes	GSDMB
1.06695403682696E-115	2.0316494277353	0.485	0.225	1.74030872946846E-111	Mid-zonal hepatocytes	CSAD
1.88929529001088E-115	1.49451566900912	0.579	0.315	3.08162954753674E-111	Mid-zonal hepatocytes	MAP4
2.16148254596675E-115	1.21839171495017	0.716	0.445	3.52559418072637E-111	Mid-zonal hepatocytes	SLC7A2
1.10975710286599E-114	1.30490003716848	0.678	0.412	1.81012481048472E-110	Mid-zonal hepatocytes	RNF213
1.55193893782402E-114	1.6464704874494	0.567	0.309	2.53136760148476E-110	Mid-zonal hepatocytes	CHD2
1.05241253805161E-113	1.74387095389497	0.557	0.301	1.71659009081599E-109	Mid-zonal hepatocytes	CLK1
9.79723398874527E-113	1.41182382858041	0.611	0.356	1.59802683590424E-108	Mid-zonal hepatocytes	C16orf70
1.16879181571355E-112	1.5727496196862	0.541	0.281	1.90641633061036E-108	Mid-zonal hepatocytes	PRPF3
6.69036275016199E-112	1.06241493419617	0.676	0.396	1.09126506817892E-107	Mid-zonal hepatocytes	GALT
9.50805897716507E-112	1.62113974905281	0.529	0.27	1.55085949976539E-107	Mid-zonal hepatocytes	LUC7L
1.20702988729221E-111	1.70565841117172	0.541	0.286	1.96878644916233E-107	Mid-zonal hepatocytes	INTS3
2.4450268772635E-111	1.07154683230124	0.628	0.326	3.98808333950449E-107	Mid-zonal hepatocytes	FGF21
2.66957239519803E-110	1.85984756687322	0.488	0.238	4.35433953380751E-106	Mid-zonal hepatocytes	PHYKPL
1.24687106943457E-109	0.861388975457702	0.83	0.565	2.03377140135473E-105	Mid-zonal hepatocytes	EPS8L2
1.08536044947868E-108	1.02015323349213	0.837	0.602	1.77033142914467E-104	Mid-zonal hepatocytes	ERRFI1
3.13746609358869E-108	1.01309973783434	0.725	0.463	5.11752094525251E-104	Mid-zonal hepatocytes	SNX1
6.50379455341616E-108	1.68938316966569	0.52	0.254	1.06083392960771E-103	Mid-zonal hepatocytes	ZC3H12A
1.02610750660397E-105	1.04067310555468	0.695	0.437	1.67368395402174E-101	Mid-zonal hepatocytes	PPP6R2
4.14787914714464E-105	1.6260664885419	0.568	0.318	6.76560567690763E-101	Mid-zonal hepatocytes	MAT2A
6.35466005430821E-105	2.70358096774754	0.294	0.082	1.03650860145821E-100	Mid-zonal hepatocytes	C3orf52
6.77251149612485E-105	2.79827583868725	0.273	0.069	1.10466435013292E-100	Mid-zonal hepatocytes	NAV3

1.09131853830947E-104	1.17167431704314	0.698	0.435	1.78004966783658E-100	Mid-zonal hepatocytes	ABCC2
1.53298653592438E-104	1.08427725670754	0.685	0.434	2.50045433874626E-100	Mid-zonal hepatocytes	BRD2
1.88929672005676E-104	1.10055056117637	0.653	0.395	3.08163188008458E-100	Mid-zonal hepatocytes	NEDD4L
5.00668817325158E-104	1.2713950000792	0.592	0.349	8.16640907939066E-100	Mid-zonal hepatocytes	MARCH6
9.40862381111281E-104	1.30211666786906	0.597	0.342	1.53464062983061E-99	Mid-zonal hepatocytes	TBC1D2B
1.59648163459791E-103	1.63998837962602	0.496	0.249	2.60402119419266E-99	Mid-zonal hepatocytes	TUBGCP6
2.32092239223083E-103	1.50753450495816	0.506	0.253	3.78565651396771E-99	Mid-zonal hepatocytes	GIGYF1
3.58773330491287E-103	1.6325938229705	0.53	0.28	5.85195179364339E-99	Mid-zonal hepatocytes	SRRM2
6.1400730833504E-103	0.899915534023876	0.853	0.61	1.00150732062528E-98	Mid-zonal hepatocytes	SAT2
1.09108769447393E-102	1.88650985857666	0.461	0.22	1.77967313845642E-98	Mid-zonal hepatocytes	PAN2
1.1339532679628E-102	1.30735636846079	0.537	0.286	1.84959117537412E-98	Mid-zonal hepatocytes	TIMM44
1.55768269862783E-102	1.59821433293699	0.516	0.266	2.54073624973185E-98	Mid-zonal hepatocytes	DDX39B
4.42128684060062E-102	1.83579587106523	0.483	0.237	7.21156096570367E-98	Mid-zonal hepatocytes	CBLB
5.01689297694672E-102	0.787361763334848	0.812	0.537	8.1830541346978E-98	Mid-zonal hepatocytes	SLC12A7
3.62940218561624E-101	1.81929379316901	0.485	0.249	5.91991790495864E-97	Mid-zonal hepatocytes	TRIM25
3.93215065845432E-101	1.40840590152437	0.578	0.332	6.41373093900483E-97	Mid-zonal hepatocytes	PRKAB2
4.10445965202503E-101	1.20503944693926	0.726	0.499	6.69478413841803E-97	Mid-zonal hepatocytes	SLC22A7
5.77824883636526E-101	1.15681458115078	0.572	0.318	9.42490167699537E-97	Mid-zonal hepatocytes	PHF1
7.24545728924678E-101	1.01307651616637	0.721	0.466	1.18180653844904E-96	Mid-zonal hepatocytes	WDR13
7.59131554935935E-101	1.09030291794852	0.694	0.417	1.238219479256E-96	Mid-zonal hepatocytes	TP53INP1
2.4908903626447E-100	1.3444657525485	0.562	0.314	4.06289127050976E-96	Mid-zonal hepatocytes	N4BP2L2
2.62953208431336E-100	1.43484379535758	0.549	0.305	4.28902978272352E-96	Mid-zonal hepatocytes	DYNC1LI2
7.18140419204343E-100	0.804393692703989	0.715	0.456	1.1713588377642E-95	Mid-zonal hepatocytes	SPTBN1
7.39068137516343E-100	1.3715358336888	0.582	0.337	1.20549403910291E-95	Mid-zonal hepatocytes	POLR2H
7.49233015381194E-100	1.35085194090612	0.562	0.314	1.22207397138827E-95	Mid-zonal hepatocytes	MAN2A2
8.39916610631686E-100	1.21290371886944	0.573	0.321	1.36998798360134E-95	Mid-zonal hepatocytes	BACH1
9.69336697341169E-100	1.87943735204074	0.425	0.187	1.58108508703318E-95	Mid-zonal hepatocytes	USP34
4.35813857250168E-99	2.22637150490373	0.4	0.17	7.1085598256075E-95	Mid-zonal hepatocytes	RNPC3
6.86962115133788E-99	2.50809930448061	0.349	0.129	1.12050390599472E-94	Mid-zonal hepatocytes	GABBR1
9.41563184541955E-99	1.34825766425233	0.567	0.315	1.53578371030638E-94	Mid-zonal hepatocytes	LIMK2
1.37349816208557E-98	1.15890350453654	0.646	0.398	2.24031285217777E-94	Mid-zonal hepatocytes	SPG7
1.83847117206836E-98	1.22872338968265	0.596	0.355	2.9987303287607E-94	Mid-zonal hepatocytes	FGFR3
2.10165773813248E-98	1.13240937380159	0.652	0.396	3.42801393666789E-94	Mid-zonal hepatocytes	SLC6A1
2.45335950657196E-98	1.95047509353876	0.462	0.229	4.00167469116952E-94	Mid-zonal hepatocytes	FNBP4
2.46385190336655E-98	1.3019992948131	0.569	0.327	4.01878883958118E-94	Mid-zonal hepatocytes	SMAD3
7.3327483364593E-98	0.824975516413119	0.796	0.567	1.19604458115988E-93	Mid-zonal hepatocytes	SLC30A1
1.52022040939511E-97	1.07333626235791	0.74	0.497	2.47963150976436E-93	Mid-zonal hepatocytes	DUSP1
3.90152793653478E-97	1.24442797731454	0.601	0.368	6.36378221728189E-93	Mid-zonal hepatocytes	SAMD5
6.46705822837787E-97	1.40204458678961	0.606	0.366	1.05484186763071E-92	Mid-zonal hepatocytes	SHROOM1
7.03539411369768E-97	1.28796322210779	0.577	0.337	1.14754313388523E-92	Mid-zonal hepatocytes	CALCOCO2
7.23981488974096E-97	1.31716042238894	0.57	0.33	1.18088620666565E-92	Mid-zonal hepatocytes	SLC41A2
1.31648778734652E-96	0.835487170521639	0.869	0.653	2.1473232299409E-92	Mid-zonal hepatocytes	ZFP36L1
3.11542303568396E-96	1.45588293419626	0.563	0.326	5.08156651350411E-92	Mid-zonal hepatocytes	MTG1
3.64873355193989E-96	1.24846516840693	0.596	0.358	5.95144929656916E-92	Mid-zonal hepatocytes	PATJ
1.12303475622797E-95	1.0161556499711	0.703	0.455	1.83178199088344E-91	Mid-zonal hepatocytes	OCLN
4.59636934494225E-95	1.9379339411998	0.425	0.196	7.4971380385353E-91	Mid-zonal hepatocytes	IFRD1
6.46462831177621E-95	1.22762786102957	0.608	0.368	1.05444552393382E-90	Mid-zonal hepatocytes	SNRNP70
3.19523090460289E-94	1.19910145860138	0.591	0.348	5.21174112849777E-90	Mid-zonal hepatocytes	OGA
7.21625318695371E-94	1.63484155465693	0.426	0.193	1.17704305732402E-89	Mid-zonal hepatocytes	PLEKHH3
7.7774060835707E-94	1.75385899513163	0.463	0.231	1.26857270629122E-89	Mid-zonal hepatocytes	TAF1D
1.01021914125115E-93	2.00801248446477	0.392	0.164	1.64776844129475E-89	Mid-zonal hepatocytes	APBB3
3.21197276364539E-93	1.80749170413763	0.44	0.206	5.239048774782E-89	Mid-zonal hepatocytes	CHKB
3.53535869635161E-93	1.90499620106135	0.375	0.152	5.76652356961912E-89	Mid-zonal hepatocytes	SLC17A2
3.90275261193683E-93	1.36603284785691	0.559	0.327	6.36577978533017E-89	Mid-zonal hepatocytes	RBM39
4.22356956802502E-93	1.09041565460131	0.627	0.389	6.88906432240561E-89	Mid-zonal hepatocytes	IMMP2L
4.68286654234126E-93	1.58405538257697	0.501	0.272	7.63822361721282E-89	Mid-zonal hepatocytes	MICAL3
5.05304549994414E-93	1.29352122815585	0.551	0.312	8.24202251495889E-89	Mid-zonal hepatocytes	MEAF6
5.56290315740189E-93	1.70198869460978	0.549	0.317	9.07365134003823E-89	Mid-zonal hepatocytes	HSPA1B
1.61406272643292E-92	1.29143109530878	0.555	0.313	2.63269771308473E-88	Mid-zonal hepatocytes	SRSF5
1.74381775093788E-92	1.14858699375276	0.616	0.371	2.84434113355478E-88	Mid-zonal hepatocytes	NR1I2

5.54500324134791E-92	1.22546569121808	0.592	0.356	9.04445478696257E-88	Mid-zonal hepatocytes	SMG5
6.73388971153287E-92	1.73992502967878	0.436	0.209	1.09836475084813E-87	Mid-zonal hepatocytes	DOCK5
1.54261557196986E-91	1.34642859298152	0.538	0.299	2.51616025944004E-87	Mid-zonal hepatocytes	ZNF644
2.59570089629005E-91	1.75331455001085	0.443	0.214	4.2338477319387E-87	Mid-zonal hepatocytes	DAPK2
5.04303484533685E-91	0.854409618937934	0.701	0.463	8.22569413622893E-87	Mid-zonal hepatocytes	ZFAND3
7.95184363937599E-91	1.00171882880383	0.721	0.483	1.29702521601862E-86	Mid-zonal hepatocytes	ZFP36
9.65044270635376E-91	1.27571151135984	0.546	0.315	1.57408370983336E-86	Mid-zonal hepatocytes	RNF115
2.16432565845348E-90	1.29660142165543	0.548	0.312	3.53023158150347E-86	Mid-zonal hepatocytes	ZSWIM8
2.32928135904632E-90	0.701674245682624	0.9	0.633	3.79929082474046E-86	Mid-zonal hepatocytes	PAH
2.5343431482585E-90	1.42606248117281	0.53	0.296	4.13376710912443E-86	Mid-zonal hepatocytes	NRBP2
4.39250114068834E-90	1.637943902448	0.448	0.219	7.16460861057675E-86	Mid-zonal hepatocytes	IDUA
6.41078086025367E-90	1.21657421046907	0.57	0.341	1.04566246611598E-85	Mid-zonal hepatocytes	CELF1
3.82659921401844E-89	1.60350803987963	0.483	0.252	6.24156597798547E-85	Mid-zonal hepatocytes	RUFY3
4.35267641136871E-89	1.56418264881508	0.452	0.227	7.0996504945835E-85	Mid-zonal hepatocytes	SETDB1
5.12554957925059E-89	1.30434613230503	0.52	0.291	8.36028391871564E-85	Mid-zonal hepatocytes	USP12
5.30968191540591E-89	1.62946730351203	0.462	0.232	8.66062217221858E-85	Mid-zonal hepatocytes	SAFB2
7.36233656529043E-89	1.02355913996175	0.577	0.347	1.20087071716452E-84	Mid-zonal hepatocytes	BAIAP2L1
1.50056122809782E-88	0.783000844462	0.676	0.436	2.44756541915036E-84	Mid-zonal hepatocytes	MEF2D
2.0832889313251E-88	1.45984495806008	0.531	0.302	3.39805257588437E-84	Mid-zonal hepatocytes	RABGAP1L
2.47673195550126E-88	1.64987416686625	0.444	0.217	4.03979749261811E-84	Mid-zonal hepatocytes	DENND4A
5.18663357658284E-88	1.69608631405614	0.458	0.232	8.45991802676426E-84	Mid-zonal hepatocytes	TUFT1
1.08264744319843E-87	1.28033584494235	0.597	0.364	1.76590624460095E-83	Mid-zonal hepatocytes	BHLHE40
1.2047634102929E-87	1.03070450393715	0.718	0.471	1.96508959852875E-83	Mid-zonal hepatocytes	GCLC
1.23035168419289E-87	1.3410176577773	0.535	0.309	2.00682663208702E-83	Mid-zonal hepatocytes	SLC17A9
1.49084494105112E-87	1.59066427400978	0.467	0.24	2.43171718334848E-83	Mid-zonal hepatocytes	TENT4A
1.656905827407E-87	1.56129201580464	0.487	0.266	2.70257909508356E-83	Mid-zonal hepatocytes	SUGP2
1.75463789196634E-87	2.01149216992103	0.386	0.171	2.8619898655863E-83	Mid-zonal hepatocytes	DMTF1
2.64556788292745E-87	0.821547166348682	0.716	0.471	4.31518577384296E-83	Mid-zonal hepatocytes	PTPRF
9.33234268669003E-87	0.855009828522295	0.62	0.386	1.52219841562601E-82	Mid-zonal hepatocytes	DDX5
1.23874657154669E-86	1.51690415657299	0.496	0.272	2.0205195328498E-82	Mid-zonal hepatocytes	ZFR
1.55333999419948E-86	1.71907863229713	0.451	0.232	2.53365286453877E-82	Mid-zonal hepatocytes	HECTD4
1.99656335977263E-86	2.22192792806259	0.315	0.115	3.25659449612514E-82	Mid-zonal hepatocytes	MICU3
3.41594066105654E-86	1.15963432983113	0.583	0.359	5.57174081224932E-82	Mid-zonal hepatocytes	PLEKHG3
4.72363118194158E-86	1.1341655485174	0.632	0.399	7.70471482086491E-82	Mid-zonal hepatocytes	SLC29A1
6.11470011731367E-86	0.89979594568373	0.644	0.41	9.97368736135034E-82	Mid-zonal hepatocytes	A1CF
6.31141435002875E-86	1.37250744468013	0.533	0.305	1.02945479463319E-81	Mid-zonal hepatocytes	ACOT12
6.48645782551567E-86	1.78598353487716	0.452	0.232	1.05800613591986E-81	Mid-zonal hepatocytes	PFKFB1
8.8805760773943E-86	1.49044385380134	0.496	0.275	1.44851076398379E-81	Mid-zonal hepatocytes	ZRANB2
1.26457113770742E-85	1.85188541541365	0.395	0.181	2.06264198271457E-81	Mid-zonal hepatocytes	MAPK8IP3
2.54346938363931E-85	1.21058791937236	0.519	0.292	4.14865291165408E-81	Mid-zonal hepatocytes	PIP5K1A
5.79304801819915E-85	1.0723047395447	0.633	0.403	9.44904062248463E-81	Mid-zonal hepatocytes	PLPP3
6.01873416383253E-85	1.18872120367238	0.573	0.35	9.81715729462724E-81	Mid-zonal hepatocytes	CFLAR
8.99407042787537E-85	2.043627556738	0.271	0.083	1.46702282749075E-80	Mid-zonal hepatocytes	ADAMTS17
9.31312244625576E-85	1.56148451905984	0.459	0.239	1.51906340220878E-80	Mid-zonal hepatocytes	EHBP1
1.63177964819006E-84	1.32137248324329	0.518	0.294	2.6615957841628E-80	Mid-zonal hepatocytes	SLC25A44
1.85440160820808E-84	1.54231929618368	0.444	0.226	3.0247144631482E-80	Mid-zonal hepatocytes	RBM15
2.00455294372733E-84	1.46067929355777	0.499	0.277	3.26962630651365E-80	Mid-zonal hepatocytes	BIRC6
3.02549169286369E-84	1.51150728871769	0.535	0.316	4.93487950022996E-80	Mid-zonal hepatocytes	SLC17A4
4.57907669739531E-84	0.801761977724522	0.704	0.468	7.46893200112149E-80	Mid-zonal hepatocytes	ADAM15
4.68770209671612E-84	1.13714812433486	0.604	0.378	7.64611088995367E-80	Mid-zonal hepatocytes	SLC22A23
5.83565350874599E-84	1.72850770953964	0.427	0.209	9.51853443811559E-80	Mid-zonal hepatocytes	AKAP17A
7.47899084188603E-84	1.35373965213544	0.476	0.253	1.21989819622003E-79	Mid-zonal hepatocytes	BAZ2A
1.70138719187945E-83	1.27455177589871	0.546	0.32	2.77513264867457E-79	Mid-zonal hepatocytes	QTRT1
2.66367393353828E-83	0.931110890095089	0.626	0.401	4.34471855299429E-79	Mid-zonal hepatocytes	CPEB4
3.0120184339821E-83	0.937630005012456	0.725	0.486	4.91290326766821E-79	Mid-zonal hepatocytes	ONECUT1
4.0862180342612E-83	1.25547713435868	0.513	0.295	6.66503023568343E-79	Mid-zonal hepatocytes	INPPL1
4.94913955325139E-83	0.712366243708396	0.8	0.562	8.07254152530835E-79	Mid-zonal hepatocytes	HSD17B4
9.6272226324681E-83	1.57231446914513	0.446	0.228	1.57029628358187E-78	Mid-zonal hepatocytes	KLHDC4
1.78161681849019E-82	1.23419442455715	0.578	0.36	2.90599519263935E-78	Mid-zonal hepatocytes	LPP
3.67308656150506E-82	0.879605798079658	0.685	0.452	5.9911714904709E-78	Mid-zonal hepatocytes	MTSS1

5.74291060641578E-82	1.82267985781518	0.399	0.19	9.36726149012479E-78	Mid-zonal hepatocytes	ZSCAN26
5.96094894721601E-82	1.62865350127197	0.438	0.223	9.72290382780403E-78	Mid-zonal hepatocytes	ZNF638
6.60641657332056E-82	1.27343822056462	0.594	0.379	1.07757260727432E-77	Mid-zonal hepatocytes	HSPA1A
6.9407668928422E-82	1.20349553134013	0.538	0.315	1.13210848789149E-77	Mid-zonal hepatocytes	MROH1
1.94462397454073E-81	1.01110275300638	0.586	0.365	3.17187616487339E-77	Mid-zonal hepatocytes	NFX1
2.43638632771616E-81	1.65081404879924	0.416	0.205	3.97398973913782E-77	Mid-zonal hepatocytes	UVSSA
2.47239990307104E-81	2.1623416399694	0.278	0.092	4.03273148189917E-77	Mid-zonal hepatocytes	IGSF9
3.77263515706441E-81	1.38709867044779	0.503	0.285	6.15354520468776E-77	Mid-zonal hepatocytes	ADGRG6
5.65706608563517E-81	0.738235546356138	0.757	0.514	9.22724049227953E-77	Mid-zonal hepatocytes	PON2
1.15287414314573E-80	1.4149933369975	0.51	0.296	1.880453014885E-76	Mid-zonal hepatocytes	RNF149
3.68276741945437E-80	1.78044801625018	0.383	0.175	6.00696193787203E-76	Mid-zonal hepatocytes	ADAMTS10
4.11304551235543E-80	1.62692114926786	0.451	0.236	6.70878853520294E-76	Mid-zonal hepatocytes	KDM4C
4.76400312936896E-80	0.849150693141437	0.664	0.433	7.7705655043137E-76	Mid-zonal hepatocytes	WNK1
6.62178955599063E-80	1.33833351630812	0.485	0.268	1.08008009447763E-75	Mid-zonal hepatocytes	COG3
1.06210289080145E-79	1.40961365512985	0.496	0.285	1.73239602518625E-75	Mid-zonal hepatocytes	PCED1A
1.99079644914536E-79	1.06144350312642	0.564	0.333	3.247188088201E-75	Mid-zonal hepatocytes	HLF
3.39513931595766E-79	0.86137541299169	0.695	0.444	5.53781173825854E-75	Mid-zonal hepatocytes	PLIN5
3.83230062932422E-79	1.10242976206239	0.589	0.373	6.25086555649074E-75	Mid-zonal hepatocytes	IRF3
9.71794017117339E-79	0.990771993166857	0.601	0.376	1.58509322132009E-74	Mid-zonal hepatocytes	INTS11
1.22449272512758E-78	1.52599775186276	0.456	0.244	1.99727008395559E-74	Mid-zonal hepatocytes	GBA2
1.50303331482578E-78	0.904854002593218	0.56	0.335	2.45159763981233E-74	Mid-zonal hepatocytes	CEBPG
2.08115068492471E-78	0.696259071774439	0.735	0.511	3.39456488218069E-74	Mid-zonal hepatocytes	SHC1
2.87629954787983E-78	1.70589701560582	0.449	0.241	4.69153219254679E-74	Mid-zonal hepatocytes	MYO15B
3.46436317082862E-78	1.33838821338801	0.466	0.254	5.65072276793856E-74	Mid-zonal hepatocytes	KIF13A
3.48769267800822E-78	1.03540004545043	0.646	0.405	5.6887755270992E-74	Mid-zonal hepatocytes	SAT1
3.96354321692535E-78	1.18359090994771	0.572	0.352	6.46493534112694E-74	Mid-zonal hepatocytes	GBP2
4.80222088729963E-78	1.60371941432411	0.442	0.235	7.83290248927442E-74	Mid-zonal hepatocytes	GON4L
5.06275577950013E-78	1.11093119419211	0.573	0.356	8.25786095194266E-74	Mid-zonal hepatocytes	EML4
7.22376878564058E-78	1.20215354839278	0.527	0.312	1.17826892662584E-73	Mid-zonal hepatocytes	OXA1L
9.48983424546229E-78	1.5156655087083	0.481	0.276	1.54788686377735E-73	Mid-zonal hepatocytes	NCOR1
2.04073766965567E-77	1.68620773071606	0.361	0.161	3.32864721297536E-73	Mid-zonal hepatocytes	MEF2A
2.52523109089944E-77	1.48749441159752	0.428	0.221	4.11890443236607E-73	Mid-zonal hepatocytes	ARVCF
5.45197401542369E-77	2.02144955874116	0.424	0.218	8.89271481655759E-73	Mid-zonal hepatocytes	HKDC1
6.15095477946061E-77	1.29848642064515	0.497	0.284	1.00328223407782E-72	Mid-zonal hepatocytes	MACF1
7.89477625768794E-77	0.824380129981551	0.675	0.454	1.28771695539148E-72	Mid-zonal hepatocytes	TCF25
1.33810627315527E-76	1.4554476708517	0.464	0.253	2.18258514214357E-72	Mid-zonal hepatocytes	DLG5
1.78180247862331E-76	1.73867612976849	0.397	0.195	2.90629802288247E-72	Mid-zonal hepatocytes	BTAF1
3.6601703283156E-76	1.55049396097305	0.487	0.28	5.97010382251557E-72	Mid-zonal hepatocytes	FAM193B
4.65933079769251E-76	0.975557796798416	0.611	0.391	7.59983446411625E-72	Mid-zonal hepatocytes	PLEKHA6
5.58176626039657E-76	1.51632092010761	0.282	0.098	9.10441894733285E-72	Mid-zonal hepatocytes	SLC6A15
7.71145572043389E-76	0.748349869989255	0.669	0.445	1.25781554255997E-71	Mid-zonal hepatocytes	MAPKAPK2
1.19529296007583E-75	0.817646896068061	0.644	0.422	1.94964234717969E-71	Mid-zonal hepatocytes	SHFL
1.44930056652768E-75	1.16293884585934	0.533	0.326	2.36395415406329E-71	Mid-zonal hepatocytes	HNRNPH3
3.11095644755174E-75	1.21234605008858	0.527	0.308	5.07428106160164E-71	Mid-zonal hepatocytes	DDIT3
5.00965281258001E-75	1.59220632884406	0.429	0.225	8.17124470259926E-71	Mid-zonal hepatocytes	NSD1
1.34028546235824E-74	1.10890212650558	0.549	0.336	2.18613961765253E-70	Mid-zonal hepatocytes	RTKN
1.43012589007125E-74	1.78694779248618	0.378	0.181	2.33267833929522E-70	Mid-zonal hepatocytes	PAN3
1.74916330474181E-74	1.80072312492621	0.356	0.161	2.85306026636436E-70	Mid-zonal hepatocytes	FAM160B2
2.24976003412128E-74	1.56064326099405	0.446	0.244	3.66958359165521E-70	Mid-zonal hepatocytes	CTIF
3.89666521862503E-74	1.07488357025649	0.452	0.232	6.35585063809929E-70	Mid-zonal hepatocytes	PLIN2
3.90294311749452E-74	1.19339091158939	0.539	0.339	6.36609051894532E-70	Mid-zonal hepatocytes	CYP2C18
4.37630669211851E-74	1.1890092536923	0.508	0.3	7.1381938455145E-70	Mid-zonal hepatocytes	KPNA1
1.1236307025012E-73	1.48417544031825	0.461	0.252	1.83275403884971E-69	Mid-zonal hepatocytes	SLC25A29
1.30468208332162E-73	1.41702014964751	0.452	0.247	2.1280669461059E-69	Mid-zonal hepatocytes	DCUN1D4
1.31504365446408E-73	1.61967373060329	0.48	0.278	2.14496770479636E-69	Mid-zonal hepatocytes	SLC22A25
1.39587426090449E-73	0.975778047730735	0.589	0.368	2.27681050696132E-69	Mid-zonal hepatocytes	LUC7L2
1.45536659163161E-73	1.35967909967109	0.461	0.259	2.37384844761033E-69	Mid-zonal hepatocytes	KANSL1
1.82317952952733E-73	1.09488978698263	0.549	0.339	2.97378813061203E-69	Mid-zonal hepatocytes	TNFRSF14
2.38117250847767E-73	0.802852953835236	0.672	0.457	3.88393047857793E-69	Mid-zonal hepatocytes	H6PD
2.51730828512906E-73	1.4664581005313	0.455	0.254	4.105981543874E-69	Mid-zonal hepatocytes	MARCH7

2.58746946200108E-73	1.67488857033391	0.405	0.209	4.22042143946997E-69	Mid-zonal hepatocytes	AP1G2
5.17708607180507E-73	1.34012553717948	0.461	0.259	8.44434509172125E-69	Mid-zonal hepatocytes	SENP6
5.28317420281842E-73	1.12000524484478	0.502	0.285	8.61738544221713E-69	Mid-zonal hepatocytes	FGFR2
5.54185251276243E-73	1.57743015412393	0.425	0.229	9.0393156335668E-69	Mid-zonal hepatocytes	TCERG1
5.84968969521787E-73	0.960620955795278	0.679	0.462	9.54142886186987E-69	Mid-zonal hepatocytes	TXNRD1
7.54973115557199E-73	1.52266397495733	0.427	0.227	1.23143664878535E-68	Mid-zonal hepatocytes	IWS1
7.58593610701646E-73	1.70925833278681	0.42	0.222	1.23734203841545E-68	Mid-zonal hepatocytes	TIA1
1.00855514593595E-72	1.45658708802799	0.436	0.235	1.64505429853612E-68	Mid-zonal hepatocytes	ARHGEF7
1.31579930370843E-72	1.33023073792667	0.47	0.266	2.14620024427882E-68	Mid-zonal hepatocytes	NUMB
2.12104388232485E-72	1.20663210789882	0.519	0.311	3.45963467646006E-68	Mid-zonal hepatocytes	GSE1
2.53685568273966E-72	1.30034695302541	0.521	0.318	4.13786530411666E-68	Mid-zonal hepatocytes	PCCA
3.40748315247907E-72	1.45351637226422	0.431	0.23	5.5579457700086E-68	Mid-zonal hepatocytes	NFATC2IP
3.7607775568282E-72	1.6396000517587	0.428	0.23	6.13420427294247E-68	Mid-zonal hepatocytes	MTHFR
4.24055914922061E-72	0.706412843053662	0.776	0.538	6.91677602829373E-68	Mid-zonal hepatocytes	PNPLA2
4.6150562718483E-72	1.33502314795719	0.485	0.282	7.52761828501176E-68	Mid-zonal hepatocytes	SLTM
5.23093375483824E-72	0.855890905479328	0.617	0.399	8.53217604751666E-68	Mid-zonal hepatocytes	NDST1
6.6085304599759E-72	1.25842297514486	0.508	0.303	1.07791740332667E-67	Mid-zonal hepatocytes	EHBP1L1
7.35860103070377E-72	1.34247786808623	0.457	0.253	1.20026141411809E-67	Mid-zonal hepatocytes	TRIM38
8.04428833691922E-72	1.36667513211953	0.469	0.269	1.31210387063489E-67	Mid-zonal hepatocytes	NDEL1
8.29851288632551E-72	1.27703851455604	0.478	0.272	1.35357043688855E-67	Mid-zonal hepatocytes	KDM5B
1.07556861395361E-71	1.07791734478234	0.592	0.378	1.75435996621973E-67	Mid-zonal hepatocytes	CHDH
2.47684343178354E-71	1.43571596164838	0.417	0.218	4.03997932158214E-67	Mid-zonal hepatocytes	CLASRP
2.53411269102285E-71	1.10003088540193	0.481	0.274	4.13339121032737E-67	Mid-zonal hepatocytes	KMT2B
3.51113585914037E-71	0.883223266039476	0.659	0.437	5.72701369984386E-67	Mid-zonal hepatocytes	FNDC3B
1.32660491674992E-70	1.28440295161926	0.42	0.217	2.1638252797108E-66	Mid-zonal hepatocytes	MSTO1
1.49482695692608E-70	1.5599941562484	0.396	0.201	2.43821224944212E-66	Mid-zonal hepatocytes	MARK3
1.51449891524067E-70	1.77050666058155	0.365	0.175	2.47029918064906E-66	Mid-zonal hepatocytes	CBWD1
1.58961995470661E-70	0.779792585573323	0.646	0.428	2.59282910812195E-66	Mid-zonal hepatocytes	MIA2
1.7754475330712E-70	2.6248036191307	0.233	0.074	2.89593247119244E-66	Mid-zonal hepatocytes	SLC22A15
1.89106730006365E-70	1.8943932032756	0.358	0.169	3.08451987313382E-66	Mid-zonal hepatocytes	CDKAL1
1.92450900431949E-70	1.40406052079782	0.467	0.267	3.13906663694551E-66	Mid-zonal hepatocytes	NPEPPS
2.1454040032552E-70	1.14078427411361	0.579	0.37	3.49936846970955E-66	Mid-zonal hepatocytes	BCL3
2.3461190018664E-70	0.982145286242398	0.563	0.36	3.82675470394429E-66	Mid-zonal hepatocytes	XDH
2.61116811734293E-70	1.03827655580312	0.552	0.343	4.25907631619806E-66	Mid-zonal hepatocytes	UAP1
3.56245447652969E-70	0.887983312388872	0.597	0.39	5.81071949666757E-66	Mid-zonal hepatocytes	TRIM27
4.31557039216347E-70	1.29237433248263	0.51	0.312	7.03912686665784E-66	Mid-zonal hepatocytes	TNRC6A
4.57557402545716E-70	1.6123073250754	0.341	0.154	7.46321879292318E-66	Mid-zonal hepatocytes	PLXNA3
5.03753288373687E-70	1.26235133093591	0.507	0.304	8.21671988666321E-66	Mid-zonal hepatocytes	GNL3
6.60397574035673E-70	0.707298099005652	0.818	0.566	1.07717448300959E-65	Mid-zonal hepatocytes	HGD
6.75025539858027E-70	1.64056257689098	0.377	0.183	1.10103415806243E-65	Mid-zonal hepatocytes	RGL3
8.92616750284496E-70	1.6351275104149	0.474	0.267	1.45594718138904E-65	Mid-zonal hepatocytes	GBP1
1.02728027176784E-69	0.653168229973025	0.671	0.458	1.67559685128052E-65	Mid-zonal hepatocytes	ADAR
1.17306759476741E-69	0.925264683131012	0.532	0.325	1.91339055382511E-65	Mid-zonal hepatocytes	ESRRA
2.11510732950824E-69	0.794887614722252	0.657	0.446	3.44995156516089E-65	Mid-zonal hepatocytes	KDM2A
2.20416595945179E-69	1.42278908997025	0.428	0.231	3.59521509646181E-65	Mid-zonal hepatocytes	RBM14
2.83908315982496E-69	1.1623880992108	0.49	0.29	4.6308285419905E-65	Mid-zonal hepatocytes	RERE
2.91168359989698E-69	1.14654645483919	0.536	0.337	4.74924711979197E-65	Mid-zonal hepatocytes	UBR4
3.19310595406017E-69	0.7014197181604	0.669	0.463	5.20827512166755E-65	Mid-zonal hepatocytes	INF2
3.28709388663214E-69	0.477262755158876	0.973	0.907	5.36157883848569E-65	Mid-zonal hepatocytes	CFH
4.91033513712508E-69	2.33510794794823	0.337	0.155	8.00924764216471E-65	Mid-zonal hepatocytes	EID3
7.3525258583693E-69	1.59618476021519	0.414	0.222	1.19927049275862E-64	Mid-zonal hepatocytes	USF1
9.00428295414362E-69	1.63027312694576	0.4	0.21	1.46868859265037E-64	Mid-zonal hepatocytes	GLDC
1.20463614424649E-68	1.41956485045498	0.432	0.237	1.96488201488044E-64	Mid-zonal hepatocytes	ZFYVE27
1.44013678468411E-68	1.46040422089324	0.455	0.258	2.34900710949826E-64	Mid-zonal hepatocytes	TFIP11
2.85033913832459E-68	1.43989709668923	0.415	0.223	4.64918816852124E-64	Mid-zonal hepatocytes	ZNF595
4.97516726099434E-68	1.34881871981443	0.48	0.285	8.11499531940787E-64	Mid-zonal hepatocytes	KIAA1217
6.87261611485855E-68	1.8767949519354	0.337	0.155	1.12099241449458E-63	Mid-zonal hepatocytes	MTR
7.20228425256186E-68	0.88988211953855	0.619	0.412	1.17476458443536E-63	Mid-zonal hepatocytes	SLC25A13
7.33663683522538E-68	1.2928365576121	0.451	0.258	1.19667883419361E-63	Mid-zonal hepatocytes	TRA2A
1.17943209616097E-67	1.54616073929562	0.366	0.178	1.92377169204815E-63	Mid-zonal hepatocytes	NLRC5

1.24170429994671E-67	1.20623825419332	0.499	0.299	2.02534388364308E-63	Mid-zonal hepatocytes	RB1CC1
1.99463787749354E-67	1.43568400846208	0.445	0.254	3.25345384197971E-63	Mid-zonal hepatocytes	STX16
2.00429646971533E-67	0.890294593128461	0.56	0.36	3.26920797175267E-63	Mid-zonal hepatocytes	BCAR1
2.8742825815038E-67	1.35145438131996	0.48	0.286	4.68824231869085E-63	Mid-zonal hepatocytes	FOXP1
3.2204851772435E-67	0.794025431573232	0.652	0.438	5.25293337260187E-63	Mid-zonal hepatocytes	STAT6
3.72114232097259E-67	1.61048036806004	0.383	0.198	6.06955523973839E-63	Mid-zonal hepatocytes	NPHP3
6.53980033922951E-67	0.875937003908021	0.626	0.396	1.06670683333173E-62	Mid-zonal hepatocytes	MYOM1
7.23313986502772E-67	0.878296923813141	0.614	0.4	1.17979744338467E-62	Mid-zonal hepatocytes	CYP21A2
9.3323989967919E-67	1.21490645208643	0.508	0.312	1.52220760036673E-62	Mid-zonal hepatocytes	STAU2
1.13159987166871E-66	1.01961040737345	0.556	0.358	1.84575255067883E-62	Mid-zonal hepatocytes	NSFL1C
1.20857011702533E-66	1.43111826978623	0.417	0.226	1.97129871788001E-62	Mid-zonal hepatocytes	WDR33
2.01620043908049E-66	1.03799276686629	0.575	0.364	3.28862453618419E-62	Mid-zonal hepatocytes	IVNS1ABP
2.15502701676158E-66	0.925775073599792	0.535	0.337	3.51506456703981E-62	Mid-zonal hepatocytes	SPATA20
2.46582183880072E-66	0.924028853322697	0.561	0.357	4.02200200126785E-62	Mid-zonal hepatocytes	NSUN2
2.49187315442763E-66	1.14635732609319	0.508	0.314	4.06449430218691E-62	Mid-zonal hepatocytes	ZBTB20
2.7256089415585E-66	1.60328149798616	0.401	0.215	4.44574074457607E-62	Mid-zonal hepatocytes	EPB41
3.29706850551386E-66	1.30846738764774	0.455	0.26	5.37784843934365E-62	Mid-zonal hepatocytes	COL27A1
3.69807737092286E-66	1.2590280878299	0.481	0.287	6.03193399971228E-62	Mid-zonal hepatocytes	SYNE2
6.44906688331452E-66	1.05480043093652	0.524	0.324	1.05190729933743E-61	Mid-zonal hepatocytes	NIT2
9.61757340137075E-66	1.39803247766328	0.463	0.271	1.56872239749758E-61	Mid-zonal hepatocytes	SOX5
1.15786617584419E-65	1.43165190106159	0.426	0.236	1.88859551941945E-61	Mid-zonal hepatocytes	GPATCH2L
1.27850435639438E-65	0.637459977090754	0.681	0.469	2.08536845571487E-61	Mid-zonal hepatocytes	CTNND1
1.2880151614081E-65	0.724387170657067	0.815	0.613	2.10088152977276E-61	Mid-zonal hepatocytes	DEPRECAT
1.58885015449769E-65	0.958961815974717	0.539	0.341	2.59157348700118E-61	Mid-zonal hepatocytes	MINK1
1.70383287662362E-65	1.54233721872633	0.452	0.261	2.77912180506079E-61	Mid-zonal hepatocytes	SLC25A16
1.97932744677053E-65	1.0480030945639	0.538	0.337	3.22848099842742E-61	Mid-zonal hepatocytes	NME3
2.14102425105346E-65	1.71009146858018	0.39	0.206	3.4922246558933E-61	Mid-zonal hepatocytes	INTS4
2.40196875787005E-65	1.70887419662471	0.371	0.19	3.91785124096184E-61	Mid-zonal hepatocytes	CCNT2
3.94493860733365E-65	0.745602088007108	0.714	0.505	6.43458936242191E-61	Mid-zonal hepatocytes	CGN
4.62482907194321E-65	1.06851749391225	0.52	0.321	7.54355869924657E-61	Mid-zonal hepatocytes	TUT7
5.89794558521866E-65	1.52229459638184	0.393	0.206	9.62013904405016E-61	Mid-zonal hepatocytes	ELMSAN1
8.89830011670529E-65	1.01581599383755	0.478	0.282	1.4514017320358E-60	Mid-zonal hepatocytes	PNN
9.09610202456619E-65	1.30228289119389	0.451	0.262	1.48366520122699E-60	Mid-zonal hepatocytes	LRCH4
9.82725734192892E-65	1.18074187182488	0.495	0.301	1.60292394504203E-60	Mid-zonal hepatocytes	PER1
1.05788965127992E-64	1.42859348146003	0.464	0.277	1.72552381020268E-60	Mid-zonal hepatocytes	FAM214A
1.1492799317566E-64	1.65576321842593	0.392	0.209	1.87459049668818E-60	Mid-zonal hepatocytes	TUT4
1.51006852459524E-64	1.40725559033868	0.392	0.205	2.46307277046729E-60	Mid-zonal hepatocytes	WASHC1
1.5868891083483E-64	1.49939375496748	0.351	0.168	2.58837482462691E-60	Mid-zonal hepatocytes	PKD1
2.44111543112075E-64	0.548621495349115	0.799	0.595	3.98170337970106E-60	Mid-zonal hepatocytes	AGRN
3.4391965116353E-64	1.82002763903423	0.322	0.146	5.60967343012834E-60	Mid-zonal hepatocytes	METTL17
3.71826233700093E-64	1.47256384872998	0.393	0.21	6.06485769788222E-60	Mid-zonal hepatocytes	HIP1R
3.88619224880614E-64	1.81757234559469	0.333	0.155	6.3387681770277E-60	Mid-zonal hepatocytes	AHI1
4.30383124841344E-64	0.697246667302147	0.689	0.478	7.01997914928716E-60	Mid-zonal hepatocytes	ESRP2
4.73779859624481E-64	1.1060985968393	0.473	0.277	7.72782329033491E-60	Mid-zonal hepatocytes	NR1D1
5.07202545240734E-64	1.42842916579941	0.48	0.289	8.27298071542162E-60	Mid-zonal hepatocytes	NAMPT
5.07941176854607E-64	1.2462625029314	0.448	0.256	8.28502853567549E-60	Mid-zonal hepatocytes	KMT2D
5.29930319685156E-64	0.551634290778076	0.681	0.47	8.64369344438457E-60	Mid-zonal hepatocytes	TMEM259
7.03061735963545E-64	1.53816024399898	0.392	0.209	1.14676399753014E-59	Mid-zonal hepatocytes	GTF3C3
7.52302398316426E-64	1.78298678988316	0.341	0.165	1.22708044189392E-59	Mid-zonal hepatocytes	ORMDL1
9.70280735331285E-64	1.49993456589261	0.381	0.196	1.58262490739886E-59	Mid-zonal hepatocytes	PCM1
1.06492029107465E-63	1.21859225971222	0.42	0.23	1.73699148677187E-59	Mid-zonal hepatocytes	NSMF
1.11952222594728E-63	1.32828425713236	0.436	0.247	1.82605270274261E-59	Mid-zonal hepatocytes	AAK1
1.19519397486543E-63	1.68539829406155	0.395	0.214	1.949480892403E-59	Mid-zonal hepatocytes	HERC4
1.19634482283026E-63	1.57442306078355	0.348	0.168	1.95135804051844E-59	Mid-zonal hepatocytes	KHDC4
1.26563918051034E-63	1.14888101266814	0.542	0.347	2.06438406733041E-59	Mid-zonal hepatocytes	F11
1.32978500488708E-63	1.6547683177478	0.367	0.186	2.16901232147131E-59	Mid-zonal hepatocytes	PIGL
1.62344879323435E-63	2.00315485775479	0.301	0.13	2.64800732664455E-59	Mid-zonal hepatocytes	TBC1D22B
3.557497247006E-63	1.49668799478445	0.389	0.206	5.80263375959148E-59	Mid-zonal hepatocytes	YPEL2
4.67207511976473E-63	1.28471753687866	0.424	0.235	7.62062172784825E-59	Mid-zonal hepatocytes	EEPD1
4.76823743335472E-63	0.923786585611807	0.551	0.353	7.77747207754488E-59	Mid-zonal hepatocytes	NFE2L2

5.25348719970544E-63	1.06186902878033	0.483	0.29	8.56896297143954E-59	Mid-zonal hepatocytes	DHX15
5.30987638001766E-63	1.41216271747339	0.426	0.239	8.6609393634468E-59	Mid-zonal hepatocytes	NQO2
5.36217672302183E-63	1.02222897476509	0.483	0.292	8.74624645292091E-59	Mid-zonal hepatocytes	ARNT
7.71679744144054E-63	0.963677032352502	0.563	0.359	1.25868683067337E-58	Mid-zonal hepatocytes	PPP2R1B
8.77739910637838E-63	1.5771430426054	0.368	0.188	1.43168156824138E-58	Mid-zonal hepatocytes	CAP2
2.79023447593639E-62	1.2809138563225	0.432	0.248	4.55115145369985E-58	Mid-zonal hepatocytes	TBC1D8
3.08839566983897E-62	1.24481283229079	0.47	0.28	5.03748217707434E-58	Mid-zonal hepatocytes	NFIB
4.72888199418329E-62	0.944702175367628	0.473	0.284	7.71327942071236E-58	Mid-zonal hepatocytes	SETD1B
6.53311012639176E-62	1.63525185363845	0.359	0.181	1.06561559271576E-57	Mid-zonal hepatocytes	TRPM7
8.35305500151844E-62	1.79487985608436	0.349	0.172	1.36246680129767E-57	Mid-zonal hepatocytes	POLE
9.6153474866335E-62	1.31708776033728	0.418	0.236	1.56835932854479E-57	Mid-zonal hepatocytes	RAF1
1.19142955611447E-61	1.29798495101496	0.454	0.268	1.94334074897831E-57	Mid-zonal hepatocytes	MKLN1
1.56560198507756E-61	0.528668430062323	0.748	0.55	2.55365339786001E-57	Mid-zonal hepatocytes	ZBTB7B
1.57112411390212E-61	1.75564156085573	0.372	0.193	2.56266054218575E-57	Mid-zonal hepatocytes	GABRE
1.77400777836075E-61	1.71754530121133	0.364	0.188	2.89358408728422E-57	Mid-zonal hepatocytes	SIN3B
2.04095418672042E-61	1.62958884354336	0.37	0.193	3.32900037395968E-57	Mid-zonal hepatocytes	GTF2H4
2.24907811580335E-61	1.64123024275837	0.358	0.182	3.66847131468685E-57	Mid-zonal hepatocytes	AKAP9
3.06947276528649E-61	0.781437937607914	0.555	0.361	5.00661702745879E-57	Mid-zonal hepatocytes	TCIRG1
3.8809461814022E-61	0.779677966359881	0.681	0.484	6.33021131648513E-57	Mid-zonal hepatocytes	ETS2
5.44370895361272E-61	1.25994802968541	0.421	0.237	8.87923367423772E-57	Mid-zonal hepatocytes	KDM5C
5.86566286307444E-61	1.64050822206758	0.368	0.192	9.56748269596072E-57	Mid-zonal hepatocytes	PIEZO2
6.35003773320039E-61	1.33327091175184	0.389	0.21	1.03575465466232E-56	Mid-zonal hepatocytes	KLC1
7.12794235513315E-61	1.31803187915311	0.464	0.282	1.16263867754577E-56	Mid-zonal hepatocytes	TSC22D2
7.2135263034341E-61	0.725792240972971	0.631	0.435	1.17659827535314E-56	Mid-zonal hepatocytes	BSDC1
7.88363045185368E-61	1.07266020318528	0.486	0.301	1.28589896300185E-56	Mid-zonal hepatocytes	ABLIM3
9.49699810283288E-61	0.883443910613078	0.554	0.364	1.54905536055307E-56	Mid-zonal hepatocytes	KANK1
1.21688646855892E-60	1.05600176172607	0.476	0.289	1.98486351886646E-56	Mid-zonal hepatocytes	C16orf72
1.85781605031292E-60	1.02125475235462	0.518	0.334	3.03028375966541E-56	Mid-zonal hepatocytes	TRPC4AP
2.00970332374277E-60	1.46563983988677	0.439	0.256	3.27802709135683E-56	Mid-zonal hepatocytes	PARP14
2.3324155132565E-60	2.19484083572843	0.265	0.107	3.80440294367267E-56	Mid-zonal hepatocytes	DNAH1
2.72055364696738E-60	1.27878327462452	0.408	0.227	4.4374950535685E-56	Mid-zonal hepatocytes	RPAIN
3.10023539160946E-60	0.626873224838417	0.666	0.473	5.05679394725419E-56	Mid-zonal hepatocytes	KIAA1522
3.24342596271421E-60	1.09736161035384	0.451	0.268	5.29035208778314E-56	Mid-zonal hepatocytes	GCN1
4.80425904355196E-60	1.17839301390866	0.436	0.255	7.8362269259376E-56	Mid-zonal hepatocytes	TAF11
5.38898841529154E-60	1.14978504805576	0.502	0.313	8.78997900418204E-56	Mid-zonal hepatocytes	RNF19A
1.01497688010385E-59	1.42476593361156	0.418	0.243	1.65552878913738E-55	Mid-zonal hepatocytes	ASXL1
1.16055701626016E-59	1.39996074146657	0.347	0.173	1.89298454922194E-55	Mid-zonal hepatocytes	MTMR3
1.25651193418892E-59	1.81727065208803	0.317	0.149	2.04949661585555E-55	Mid-zonal hepatocytes	CBFA2T2
1.29007986275722E-59	1.777015587574	0.324	0.154	2.1042492641433E-55	Mid-zonal hepatocytes	DNAH14
1.36878195334744E-59	1.34796416835138	0.435	0.259	2.23262024410502E-55	Mid-zonal hepatocytes	FAM160A2
1.47940791394828E-59	1.06027140837598	0.49	0.31	2.41306224844104E-55	Mid-zonal hepatocytes	RIOK3
1.67624522049861E-59	1.05989397841721	0.49	0.299	2.73412357915529E-55	Mid-zonal hepatocytes	GPAM
1.84844293766247E-59	1.60225461622895	0.348	0.176	3.01499527562126E-55	Mid-zonal hepatocytes	USP24
1.87292263552012E-59	1.96886411697377	0.28	0.119	3.05492411079687E-55	Mid-zonal hepatocytes	PPP1R9A
2.11613046056932E-59	1.32555696150073	0.491	0.305	3.45162039423463E-55	Mid-zonal hepatocytes	SREBF1
2.22068478682189E-59	1.18923976814049	0.454	0.274	3.62215895578518E-55	Mid-zonal hepatocytes	SUN1
2.42358975647409E-59	1.18030337882244	0.533	0.35	3.95311725178489E-55	Mid-zonal hepatocytes	SLC30A10
2.67172415019491E-59	1.53653194651783	0.358	0.183	4.35784926138292E-55	Mid-zonal hepatocytes	WWOX
2.72565886720074E-59	1.01843018973173	0.493	0.313	4.44582217829113E-55	Mid-zonal hepatocytes	RAB1A
3.41179100485495E-59	0.805274097140201	0.536	0.351	5.56497230801891E-55	Mid-zonal hepatocytes	HGS
4.15743964364774E-59	1.40650435395053	0.425	0.247	6.78119980275382E-55	Mid-zonal hepatocytes	UPK3BL1
4.48581378913485E-59	0.831558751443034	0.535	0.347	7.31681087145785E-55	Mid-zonal hepatocytes	STXBP2
7.21595752747103E-59	1.4953535166333	0.358	0.185	1.1769948323058E-54	Mid-zonal hepatocytes	YTHDC1
7.49124309413099E-59	1.13169142126816	0.458	0.275	1.22189666108371E-54	Mid-zonal hepatocytes	ULK1
8.4158043758501E-59	0.565063904787043	0.727	0.523	1.37270185174491E-54	Mid-zonal hepatocytes	INSR
9.63863096141896E-59	1.59048266662588	0.347	0.176	1.57215709611705E-54	Mid-zonal hepatocytes	ADCY6
9.77829463663253E-59	1.09051615458811	0.47	0.283	1.59493763818113E-54	Mid-zonal hepatocytes	BTG2
9.895668675111E-59	1.23665854548625	0.447	0.269	1.61408251759736E-54	Mid-zonal hepatocytes	DDX17
1.05765876817716E-58	0.568157620277387	0.645	0.443	1.72514721677377E-54	Mid-zonal hepatocytes	SPATS2L
1.1408612645502E-58	1.24069975552005	0.414	0.235	1.86085880860783E-54	Mid-zonal hepatocytes	CBWD2

1.19596407042562E-58	1.45416149933258	0.406	0.23	1.95073699527123E-54	Mid-zonal hepatocytes	ITSN2
1.51200783360072E-58	0.985212253982906	0.451	0.265	2.46623597738614E-54	Mid-zonal hepatocytes	DCAF5
1.72333630414428E-58	1.77559211687403	0.344	0.176	2.81093384568973E-54	Mid-zonal hepatocytes	WDFY2
3.8492253792268E-58	1.34151022800548	0.418	0.244	6.27847151605684E-54	Mid-zonal hepatocytes	RHOBTB3
3.89734273914468E-58	1.6956472382282	0.36	0.182	6.35695574181889E-54	Mid-zonal hepatocytes	VNN 3.00
4.48213652226221E-58	1.11796106778412	0.511	0.329	7.3108128814619E-54	Mid-zonal hepatocytes	RORC
7.31133631787485E-58	1.52757947842396	0.399	0.227	1.19255206680857E-53	Mid-zonal hepatocytes	MPDZ
7.6999091424376E-58	1.41474164879699	0.384	0.21	1.255932180223E-53	Mid-zonal hepatocytes	FGD4
9.29675356295086E-58	1.80337211240509	0.324	0.157	1.51639347365292E-53	Mid-zonal hepatocytes	UTY
1.04927580028906E-57	1.38962591506893	0.348	0.178	1.71147375785148E-53	Mid-zonal hepatocytes	STX3
1.31425879639118E-57	0.785603437698705	0.548	0.361	2.14368752279365E-53	Mid-zonal hepatocytes	TMEM263
1.90838111667787E-57	1.81023170593286	0.312	0.148	3.11276043941327E-53	Mid-zonal hepatocytes	ZNF621
2.36522834812614E-57	1.43892929294778	0.358	0.185	3.85792395862855E-53	Mid-zonal hepatocytes	STAG1
2.47838645972248E-57	0.720494694962171	0.539	0.353	4.04249615445334E-53	Mid-zonal hepatocytes	FBXW4
2.68727789289024E-57	1.36789279806628	0.407	0.235	4.38321897109326E-53	Mid-zonal hepatocytes	SEC24C
2.89350835781928E-57	1.05164004131413	0.456	0.278	4.71960148243903E-53	Mid-zonal hepatocytes	THRAP3
2.96158269406809E-57	1.71752602318463	0.32	0.154	4.83063753229446E-53	Mid-zonal hepatocytes	PIAS2
3.52398505213458E-57	1.25345780379501	0.421	0.245	5.74797201853671E-53	Mid-zonal hepatocytes	RSRC2
3.90798700926002E-57	1.40397333605582	0.343	0.17	6.37431761080402E-53	Mid-zonal hepatocytes	MAFF
4.08457263021007E-57	1.33284856665929	0.372	0.2	6.66234641713565E-53	Mid-zonal hepatocytes	GPHN
4.3374244031037E-57	0.732285138178734	0.57	0.383	7.07477294390244E-53	Mid-zonal hepatocytes	KLHL21
4.38311252969219E-57	1.65361880999459	0.437	0.264	7.14929484718093E-53	Mid-zonal hepatocytes	DNAJB1
4.45603943269678E-57	1.29886951546301	0.415	0.24	7.26824591867171E-53	Mid-zonal hepatocytes	PLEKHB2
4.67118311718793E-57	1.526940661594	0.386	0.216	7.61916678244523E-53	Mid-zonal hepatocytes	STX7
5.50498254525591E-57	1.55025907848479	0.347	0.18	8.97917702956691E-53	Mid-zonal hepatocytes	REV1
6.24848351915018E-57	1.43435439846559	0.374	0.201	1.01919014680859E-52	Mid-zonal hepatocytes	ACAP3
6.41477585887374E-57	1.7855995852844	0.334	0.167	1.0463140903409E-52	Mid-zonal hepatocytes	KMT2A
6.57471737504243E-57	1.27347456844489	0.427	0.251	1.07240215104317E-52	Mid-zonal hepatocytes	KLHL20
7.51779225400308E-57	1.66362770035937	0.4	0.23	1.22622709455044E-52	Mid-zonal hepatocytes	KIF1B
7.94381980878896E-57	1.43298700059701	0.406	0.234	1.29571644901157E-52	Mid-zonal hepatocytes	PNISR
8.26376301475087E-57	1.51008703107927	0.377	0.203	1.34790238533601E-52	Mid-zonal hepatocytes	ARHGAP12
9.60295169392275E-57	1.39011948312893	0.394	0.223	1.56633745079574E-52	Mid-zonal hepatocytes	NEMF
1.47576058101503E-56	1.1126987729662	0.427	0.249	2.40711308369361E-52	Mid-zonal hepatocytes	ARHGEF40
1.75719595591036E-56	1.50520412441713	0.378	0.209	2.86616232368538E-52	Mid-zonal hepatocytes	KMT2E
1.7725646862953E-56	0.859282474324757	0.623	0.437	2.89123025981626E-52	Mid-zonal hepatocytes	TNFRSF1A
1.99129463967898E-56	0.638824115920252	0.828	0.587	3.24800068678038E-52	Mid-zonal hepatocytes	GLYCTK
2.00928194662376E-56	1.49849665168347	0.341	0.174	3.27733978313802E-52	Mid-zonal hepatocytes	FEM1C
2.29378857718729E-56	0.818391074910791	0.555	0.368	3.74139854825019E-52	Mid-zonal hepatocytes	OPLAH
2.96858111353351E-56	1.17183651883173	0.392	0.219	4.84205265428451E-52	Mid-zonal hepatocytes	ISG20
3.2218246420397E-56	1.34833531637837	0.411	0.24	5.25511817363095E-52	Mid-zonal hepatocytes	ODR4
8.32593147338475E-56	0.82816788208482	0.552	0.369	1.35804268262379E-51	Mid-zonal hepatocytes	OTUD7B
9.00913451559525E-56	2.6631609489847	0.154	0.039	1.46947993083874E-51	Mid-zonal hepatocytes	SCUBE3
9.63070996824325E-56	0.970091515304855	0.521	0.338	1.57086510292016E-51	Mid-zonal hepatocytes	PTPRK
1.03244853033647E-55	1.46366436409315	0.377	0.21	1.68402679783182E-51	Mid-zonal hepatocytes	BRD1
1.19652153427648E-55	1.14105132719331	0.407	0.231	1.95164627455836E-51	Mid-zonal hepatocytes	MED15
1.20574660620249E-55	1.37028602089536	0.396	0.224	1.96669328937688E-51	Mid-zonal hepatocytes	MAPRE3
1.51476618762084E-55	1.30035305602216	0.385	0.214	2.47073512862836E-51	Mid-zonal hepatocytes	ENOSF1
1.68615984131838E-55	1.1397966753381	0.419	0.246	2.75029531717441E-51	Mid-zonal hepatocytes	SMAD4
2.09001404306425E-55	0.635561729184979	0.536	0.338	3.40902190564209E-51	Mid-zonal hepatocytes	AJUBA
2.22502538315963E-55	1.36663021774814	0.343	0.175	3.62923890247167E-51	Mid-zonal hepatocytes	KIF21B
2.49672212050145E-55	1.37788183643867	0.392	0.22	4.07240345074991E-51	Mid-zonal hepatocytes	POLR2J2
2.99457877151327E-55	0.59617083228736	0.795	0.562	4.8844574342153E-51	Mid-zonal hepatocytes	ITIH4
4.15687111614867E-55	0.984686537754853	0.496	0.321	6.7802724775501E-51	Mid-zonal hepatocytes	IBTK
4.41951097038594E-55	0.428211077825355	0.867	0.697	7.20866434379651E-51	Mid-zonal hepatocytes	OAF
6.31779864882246E-55	1.23953351998206	0.382	0.21	1.03049613760943E-50	Mid-zonal hepatocytes	PITPNM2
6.32326727575807E-55	1.26141677111646	0.408	0.234	1.0313881253489E-50	Mid-zonal hepatocytes	DLGAP4
7.38683461973077E-55	1.57083064657396	0.397	0.23	1.20486659482429E-50	Mid-zonal hepatocytes	MCF2L
9.84368719352105E-55	0.775827703847262	0.555	0.371	1.60560381813522E-50	Mid-zonal hepatocytes	KLHDC2
9.94964981412209E-55	0.826777126447396	0.561	0.385	1.62288738118145E-50	Mid-zonal hepatocytes	TXNDC11
1.00616983523414E-54	1.24019756862857	0.406	0.233	1.6411636182504E-50	Mid-zonal hepatocytes	RABGGTB

**1.69795888742187E-54**	1.82363602233702	0.299	0.141	2.76954074127381E-50	Mid-zonal hepatocytes	DHX34
**2.09866511915162E-54**	0.580929324827666	0.666	0.471	3.42313267584821E-50	Mid-zonal hepatocytes	COPB2
**2.28287337184614E-54**	0.789472749801503	0.65	0.443	3.72359475681824E-50	Mid-zonal hepatocytes	LEPR
**2.60868615270285E-54**	1.95027486928618	0.283	0.13	4.25502798367363E-50	Mid-zonal hepatocytes	GREB1L
**2.70528358273083E-54**	1.47653537728642	0.357	0.192	4.41258805179226E-50	Mid-zonal hepatocytes	CENPT
**3.31400175488626E-54**	0.953000568692353	0.52	0.34	5.40546826239498E-50	Mid-zonal hepatocytes	DNAJC22
**3.47303276918174E-54**	1.43674733912455	0.383	0.216	5.66486374981233E-50	Mid-zonal hepatocytes	PCID2
**4.10974806209025E-54**	1.10573621322024	0.37	0.196	6.70341006407541E-50	Mid-zonal hepatocytes	BBC3
**4.33912086716503E-54**	1.8314631963016	0.312	0.153	7.07754004643289E-50	Mid-zonal hepatocytes	ZKSCAN8
**4.68646365670934E-54**	1.49454197244885	0.365	0.198	7.6440908704586E-50	Mid-zonal hepatocytes	ARMH3
**5.38746213273133E-54**	1.75850708057176	0.333	0.17	8.78748948469807E-50	Mid-zonal hepatocytes	RNF138
**6.67219589342009E-54**	1.66247404206952	0.331	0.169	1.08830187217575E-49	Mid-zonal hepatocytes	SESTD1
**8.70446993544573E-54**	1.24808444192247	0.39	0.221	1.41978609117055E-49	Mid-zonal hepatocytes	BRF1
**9.59749800221179E-54**	1.51732477513537	0.349	0.182	1.56544789914077E-49	Mid-zonal hepatocytes	KIFC2
**1.05526653686533E-53**	1.13545362958605	0.429	0.255	1.72124524828104E-49	Mid-zonal hepatocytes	ZMIZ2
**1.16797294491858E-53**	1.38638251180337	0.386	0.219	1.9050806704567E-49	Mid-zonal hepatocytes	USP32
**1.23123621693862E-53**	1.12457228258247	0.414	0.242	2.00826939344859E-49	Mid-zonal hepatocytes	VPS13C
**1.36287355341107E-53**	1.08314022926838	0.482	0.306	2.2229830529688E-49	Mid-zonal hepatocytes	ZNF512B
**1.55693444978953E-53**	1.54837936682263	0.31	0.151	2.5395157810517E-49	Mid-zonal hepatocytes	EDA
**1.62116049219655E-53**	0.676006338558829	0.592	0.407	2.64427487882179E-49	Mid-zonal hepatocytes	MIDN
**1.91516995215023E-53**	1.30840793505512	0.361	0.191	3.12383370895224E-49	Mid-zonal hepatocytes	PIEZO1
**2.90745370868627E-53**	1.4590124744375	0.381	0.217	4.74234774423817E-49	Mid-zonal hepatocytes	ZMYM2
**3.08058980081908E-53**	0.616596203081147	0.7	0.494	5.02475002411601E-49	Mid-zonal hepatocytes	SLC27A3
**3.83877036038707E-53**	1.17868971530735	0.34	0.176	6.26141833482735E-49	Mid-zonal hepatocytes	ZDHHC8
**3.96211420533339E-53**	1.27786178035978	0.4	0.23	6.46260448031929E-49	Mid-zonal hepatocytes	CALCOCO1
**5.2784661054152E-53**	1.78444997028182	0.292	0.137	8.60970606454273E-49	Mid-zonal hepatocytes	CCDC84
**6.43353773917788E-53**	0.893030054956783	0.492	0.319	1.0493743406373E-48	Mid-zonal hepatocytes	HBP1
**6.78332402332404E-53**	1.17086723221985	0.412	0.241	1.10642798144438E-48	Mid-zonal hepatocytes	NECAB3
**7.3192015281898E-53**	0.698612078974938	0.613	0.425	1.19383496126304E-48	Mid-zonal hepatocytes	SELENOO
**8.47293404479045E-53**	1.93806737361521	0.302	0.146	1.38202027204577E-48	Mid-zonal hepatocytes	PLA2G4C
**9.28964909205455E-53**	1.49486350502639	0.346	0.184	1.51523466340502E-48	Mid-zonal hepatocytes	KANSL3
**9.87277854374463E-53**	0.67825806334657	0.492	0.311	1.61034890827019E-48	Mid-zonal hepatocytes	POGK
**1.24262014367885E-52**	0.315130526003548	0.97	0.908	2.02683771635456E-48	Mid-zonal hepatocytes	MT1X
**1.25467385607158E-52**	2.1140139756702	0.255	0.11	2.04649852663836E-48	Mid-zonal hepatocytes	ADAT2
**1.41331456962356E-52**	1.43761138517814	0.333	0.174	2.305257394513E-48	Mid-zonal hepatocytes	ACIN1
**1.48876861329329E-52**	1.32220597931583	0.429	0.264	2.42833048514268E-48	Mid-zonal hepatocytes	XRN1
**1.58839113929123E-52**	0.451291532597989	0.927	0.779	2.59082478729793E-48	Mid-zonal hepatocytes	MT1H
**2.11385791007972E-52**	1.04213580824354	0.498	0.322	3.44791363713104E-48	Mid-zonal hepatocytes	SLC38A2
**2.42906179201891E-52**	0.94013996880361	0.441	0.261	3.96204268896205E-48	Mid-zonal hepatocytes	DHRS2
**3.00048286735495E-52**	1.30604923343224	0.415	0.248	4.89408760494267E-48	Mid-zonal hepatocytes	ZNF33A
**4.74147741843914E-52**	0.904860305107189	0.456	0.283	7.73382381721608E-48	Mid-zonal hepatocytes	MAFK
**6.50769047274906E-52**	1.19447136529821	0.42	0.253	1.0614693930101E-47	Mid-zonal hepatocytes	PHKA2
**6.70955764447885E-52**	1.56396123887148	0.28	0.128	1.09439594739095E-47	Mid-zonal hepatocytes	MAPK12
**1.03130306922605E-51**	1.16453418251132	0.446	0.279	1.68215843621 46E-47	Mid-zonal hepatocytes	MORC2
**1.3943264793463E-51**	0.692733268565137	0.58	0.403	2.27428592046175E-47	Mid-zonal hepatocytes	APOL2
**1.42546781189472E-51**	1.5052990827399	0.33	0.168	2.32508054798148E-47	Mid-zonal hepatocytes	KLF10
**1.53442318671505E-51**	1.2754806038924	0.405	0.236	2.50279765985091E-47	Mid-zonal hepatocytes	UBE4B
**1.5915679526798E-51**	1.18188845428847	0.408	0.242	2.59600648761603E-47	Mid-zonal hepatocytes	KMT5B
**1.61648841880662E-51**	1.31166502548465	0.392	0.227	2.63665425991548E-47	Mid-zonal hepatocytes	CHKA
**2.04357075713708E-51**	0.860056928337647	0.424	0.252	3.33326826196628E-47	Mid-zonal hepatocytes	CRTC2
**2.41788293305286E-51**	1.36814119585155	0.378	0.216	3.94380885210251E-47	Mid-zonal hepatocytes	CDC16
**2.42516797196352E-51**	1.11837115364935	0.495	0.323	3.9556914790697E-47	Mid-zonal hepatocytes	ARRDC3
**2.48046429640598E-51**	1.41176805736068	0.353	0.192	4.04588531386779E-47	Mid-zonal hepatocytes	NOP58
**2.67405849524285E-51**	1.23122566409075	0.411	0.243	4.36165681159061E-47	Mid-zonal hepatocytes	CPSF7
**3.32600658567653E-51**	1.78306998832478	0.229	0.09	5.42504934189699E-47	Mid-zonal hepatocytes	LYSMD4
**3.3936972890919E-51**	1.45801095838418	0.371	0.207	5.5354596482378E-47	Mid-zonal hepatocytes	MLPH
**3.57484919720543E-51**	1.2056196105798	0.426	0.262	5.83093652556178E-47	Mid-zonal hepatocytes	ALG13
**4.19677350238E-51**	1.74691640008198	0.299	0.144	6.84535725973202E-47	Mid-zonal hepatocytes	KAT6B
**4.47635227895563E-51**	1.52038120183448	0.356	0.199	7.30137820220452E-47	Mid-zonal hepatocytes	CPSF6
**5.2486791832484E-51**	1.43029903566775	0.341	0.183	8.56112061579646E-47	Mid-zonal hepatocytes	MAP3K20

**5.32421372761267E-51**	1.80640977997782	0.285	0.135	8.68432501110903E-47	Mid-zonal hepatocytes	LONRF3
**5.3798061477955E-51**	1.14939523535595	0.44	0.274	8.77500180766923E-47	Mid-zonal hepatocytes	DLG1
**6.17601139752112E-51**	1.9969953385162	0.274	0.127	1.00736921904967E-46	Mid-zonal hepatocytes	FER
**6.19251269129477E-51**	1.19403341979803	0.454	0.289	1.01006074507709E-46	Mid-zonal hepatocytes	ABCC6
**7.59878284741782E-51**	1.21111780364037	0.337	0.178	1.23943747024232E-46	Mid-zonal hepatocytes	P2RX4
**7.71191068046874E-51**	0.901137429969543	0.534	0.359	1.25788975109126E-46	Mid-zonal hepatocytes	FBXO7
**8.06795304794191E-51**	1.30927557732526	0.376	0.216	1.3159638216498E-46	Mid-zonal hepatocytes	DEPRECAT
**8.10042661218388E-51**	1.24033630734509	0.38	0.217	1.32126058471331E-46	Mid-zonal hepatocytes	ARHGEF11
**8.20407310338684E-51**	0.916360079405066	0.481	0.308	1.33816636389343E-46	Mid-zonal hepatocytes	KIFC3
**1.16544868882339E-50**	1.06699544658108	0.473	0.301	1.90096335633983E-46	Mid-zonal hepatocytes	ODF3B
**1.91355615859923E-50**	1.42059434684305	0.333	0.173	3.1212014502912E-46	Mid-zonal hepatocytes	LDB1
**1.95053726503094E-50**	1.26778399662852	0.423	0.265	3.18152133299196E-46	Mid-zonal hepatocytes	ZBED6
**2.08724685643321E-50**	1.21364133095997	0.371	0.203	3.4045083475282E-46	Mid-zonal hepatocytes	USP35
**2.23156219565567E-50**	0.784686174627974	0.551	0.377	3.63990109733397E-46	Mid-zonal hepatocytes	UBXN4
**2.37006943547057E-50**	0.803327063552511	0.456	0.285	3.86582025619605E-46	Mid-zonal hepatocytes	CLIP1
**2.40289642160895E-50**	1.45239635132084	0.362	0.205	3.91936435328636E-46	Mid-zonal hepatocytes	DNAJC10
**2.71473886924808E-50**	1.57299829746562	0.361	0.204	4.42801056963055E-46	Mid-zonal hepatocytes	NPIPB15
**2.75724722386197E-50**	0.860314753143013	0.556	0.38	4.49734594684126E-46	Mid-zonal hepatocytes	SND1
**2.99011674653557E-50**	1.09035870306941	0.411	0.244	4.87717942527417E-46	Mid-zonal hepatocytes	DEPRECAT
**3.49408906969264E-50**	1.26298154327806	0.384	0.221	5.69920868157567E-46	Mid-zonal hepatocytes	TPR
**3.55949219885157E-50**	1.01044552271231	0.548	0.373	5.80588772554679E-46	Mid-zonal hepatocytes	NFKBIA
**4.48244171008112E-50**	1.26906785059903	0.389	0.227	7.31131067331331E-46	Mid-zonal hepatocytes	DELE1
**4.74776442115096E-50**	1.18937761495108	0.411	0.248	7.74407854733933E-46	Mid-zonal hepatocytes	LAMA5
**4.86914439141641E-50**	1.57000253663146	0.305	0.153	7.9420614168393E-46	Mid-zonal hepatocytes	MYSM1
**5.12797498108377E-50**	1.50744431944493	0.367	0.209	8.36423999164574E-46	Mid-zonal hepatocytes	DEPRECAT
**5.46365391258645E-50**	1.72006162927836	0.294	0.144	8.91176589681975E-46	Mid-zonal hepatocytes	SIN3A
**5.49337975489661E-50**	1.0550949021688	0.433	0.264	8.96025171821186E-46	Mid-zonal hepatocytes	ITPKC
**5.56383181566728E-50**	1.57702810038495	0.333	0.177	9.0751660745349E-46	Mid-zonal hepatocytes	KAT6A
**8.46333359054877E-50**	1.50355597116933	0.327	0.171	1.38045434195441E-45	Mid-zonal hepatocytes	TARBP1
**8.95954924275631E-50**	2.5400578886375	0.162	0.048	1.46139207698598E-45	Mid-zonal hepatocytes	SLC4A3
**9.53455744664386E-50**	1.40750999363311	0.368	0.21	1.55518166512208E-45	Mid-zonal hepatocytes	CSTF3
**9.78733819978208E-50**	1.13026893339308	0.437	0.274	1.59641273376646E-45	Mid-zonal hepatocytes	DHX16
**1.01063966349555E-49**	1.26152772436138	0.409	0.251	1.6484543551276E-45	Mid-zonal hepatocytes	ACBD6
**1.06433107513899E-49**	1.89043433967532	0.303	0.153	1.73603041665921E-45	Mid-zonal hepatocytes	PRPF39
**1.10631202422124E-49**	1.25855600382997	0.381	0.22	1.80450554270726E-45	Mid-zonal hepatocytes	PPIG
**1.19385114067539E-49**	1.59951317117366	0.323	0.168	1.94729059555563E-45	Mid-zonal hepatocytes	PRPF4B
**1.29473660519741E-49**	0.99195901247387	0.494	0.325	2.11184487673749E-45	Mid-zonal hepatocytes	ATP2B2
**1.35811177569506E-49**	1.18785552599528	0.411	0.247	2.21521611733621E-45	Mid-zonal hepatocytes	GATAD2B
**1.44890712101092E-49**	0.61089573591118	0.675	0.465	2.36331240508092E-45	Mid-zonal hepatocytes	ACSM2A
**1.46028508772289E-49**	1.37389570139284	0.4	0.241	2.3818710065848E-45	Mid-zonal hepatocytes	ABCA1
**1.50235804280953E-49**	1.40193032248939	0.366	0.207	2.45049620362662E-45	Mid-zonal hepatocytes	ZNF146
**1.78627781369236E-49**	1.08995939501373	0.37	0.208	2.91359774191361E-45	Mid-zonal hepatocytes	PIK3C2B
**2.28159125351303E-49**	1.46756850651819	0.336	0.182	3.7215034936051E-45	Mid-zonal hepatocytes	RPTOR
**2.4226578518959E-49**	1.11124077506803	0.407	0.244	3.95159722222741E-45	Mid-zonal hepatocytes	ATP11A
**2.69088900894158E-49**	1.08253638376522	0.433	0.271	4.38910906248462E-45	Mid-zonal hepatocytes	GNL1
**3.00490052715201E-49**	1.54311454662075	0.318	0.164	4.90129324983764E-45	Mid-zonal hepatocytes	SFPQ
**4.16793474267748E-49**	1.71230280635313	0.33	0.175	6.79831835878124E-45	Mid-zonal hepatocytes	MBD4
**4.27856575432992E-49**	1.37802966861614	0.335	0.181	6.97876860188753E-45	Mid-zonal hepatocytes	ZFC3H1
**4.60343462603498E-49**	1.65307644423702	0.259	0.116	7.50866221852565E-45	Mid-zonal hepatocytes	INO80D
**5.13215038608434E-49**	0.798325358945658	0.551	0.378	8.37105049474217E-45	Mid-zonal hepatocytes	RAB11B
**5.46158629316836E-49**	1.21154442310709	0.385	0.227	8.90839340278691E-45	Mid-zonal hepatocytes	GLRX3
**5.62092558088223E-49**	1.3038869680623	0.402	0.24	9.16829171497701E-45	Mid-zonal hepatocytes	JMJD1C
**6.44897419816808E-49**	1.09244438483898	0.435	0.272	1.0518921814632E-44	Mid-zonal hepatocytes	BNIP2
**7.84302900969829E-49**	0.863855742597771	0.471	0.303	1.27927646177189E-44	Mid-zonal hepatocytes	ILF3
**7.87246319369061E-49**	1.29866672543782	0.361	0.204	1.28407747152288E-44	Mid-zonal hepatocytes	TENT2
**8.42271130264294E-49**	1.283655597611	0.377	0.219	1.37382844057409E-44	Mid-zonal hepatocytes	COP 1
**8.8012285861072E-49**	1.25055177173765	0.367	0.209	1.43556839467995E-44	Mid-zonal hepatocytes	RXRB
**9.08886795622056E-49**	1.30662223911017	0.383	0.224	1.48248525233914E-44	Mid-zonal hepatocytes	BRWD1
**9.25101472183455E-49**	1.24063657129087	0.384	0.226	1.50893301127843E-44	Mid-zonal hepatocytes	REPS1
**1.16408552506005E-48**	1.22175768511797	0.414	0.253	1.89873989992545E-44	Mid-zonal hepatocytes	AKAP13

1.31088856907181E-48	1.11771494299597	0.367	0.207	2.13819034501302E-44	Mid-zonal hepatocytes	SPEN
1.32116869464974E-48	2.13205503354925	0.256	0.115	2.15495825784319E-44	Mid-zonal hepatocytes	TTLL5
1.64332218616511E-48	0.809323173798164	0.485	0.316	2.68042281785391E-44	Mid-zonal hepatocytes	EXOC3
1.74250643257115E-48	0.964476100429468	0.445	0.285	2.84220224216681E-44	Mid-zonal hepatocytes	PITPNB
1.94874621541783E-48	1.56684864538059	0.281	0.135	3.17859995196802E-44	Mid-zonal hepatocytes	SMG1
1.98176535275831E-48	1.22670574372474	0.387	0.225	3.23245746688408E-44	Mid-zonal hepatocytes	CCDC159
2.12281826867536E-48	0.803764524829107	0.493	0.326	3.46252887803638E-44	Mid-zonal hepatocytes	TNKS1BP1
2.32431623966459E-48	1.70077668566262	0.288	0.142	3.79119221851691E-44	Mid-zonal hepatocytes	LPIN3
2.59318398831139E-48	0.618463564593651	0.623	0.445	4.2297424033347E-44	Mid-zonal hepatocytes	CIRBP
2.71312803812368E-48	1.30390214454614	0.378	0.221	4.42538314298354E-44	Mid-zonal hepatocytes	TMEM131L
2.86574116712064E-48	1.16028467585082	0.388	0.229	4.67431041769048E-44	Mid-zonal hepatocytes	MAST3
3.04429949381452E-48	0.850878969006529	0.489	0.333	4.96555690436086E-44	Mid-zonal hepatocytes	DCTN2
3.07722718886996E-48	1.16058256903647	0.405	0.243	5.01926526776579E-44	Mid-zonal hepatocytes	TOM1L1
3.32102384407868E-48	0.988571279980996	0.5	0.335	5.41692199207673E-44	Mid-zonal hepatocytes	IKBKG
4.3050329187604E-48	1.48765083736265	0.348	0.194	7.02193919379009E-44	Mid-zonal hepatocytes	XPO5
4.45559292803277E-48	1.07592778365094	0.404	0.24	7.26751762491425E-44	Mid-zonal hepatocytes	TYK2
6.14372115374563E-48	1.1601842898291	0.407	0.244	1.00210235738745E-43	Mid-zonal hepatocytes	RBCK1
6.27130139870334E-48	1.0644663044717	0.442	0.276	1.0229119711425E-43	Mid-zonal hepatocytes	NAB2
6.52340726027359E-48	1.25229682096944	0.305	0.154	1.06403295822322E-43	Mid-zonal hepatocytes	PSME4
7.48197438477149E-48	1.76864274219343	0.292	0.143	1.22038484190008E-43	Mid-zonal hepatocytes	TRMU
8.98789769827555E-48	1.51227734756894	0.364	0.211	1.46601599356572E-43	Mid-zonal hepatocytes	RALGAPA2
9.57882928200638E-48	2.06532420998457	0.252	0.114	1.56240284418806E-43	Mid-zonal hepatocytes	CHML
1.10326958291947E-47	1.23528272459835	0.366	0.209	1.79954301669995E-43	Mid-zonal hepatocytes	GATD1
1.2815020378528E-47	1.44714880676124	0.346	0.192	2.0902579739417E-43	Mid-zonal hepatocytes	TBRG1
1.31173859638401E-47	1.60148307274657	0.31	0.159	2.13957682456196E-43	Mid-zonal hepatocytes	DENND1A
1.46009988394971E-47	1.95923555446704	0.257	0.118	2.38156892071037E-43	Mid-zonal hepatocytes	TAF1
1.78897618097191E-47	1.74890203885704	0.239	0.103	2.91799904878328E-43	Mid-zonal hepatocytes	NEURL4
1.83499899791435E-47	1.24507309222431	0.484	0.324	2.9930668654981E-43	Mid-zonal hepatocytes	TAP1
2.13940290134724E-47	1.48516747735559	0.341	0.189	3.48958007238749E-43	Mid-zonal hepatocytes	ZZEF1
2.18705182812016E-47	1.60051055278519	0.254	0.115	3.56730023684679E-43	Mid-zonal hepatocytes	PLA2G4B
2.30035173699365E-47	1.19200644823649	0.421	0.261	3.75210371821034E-43	Mid-zonal hepatocytes	CRY2
2.37722636576112E-47	0.928547787191338	0.504	0.339	3.87749392519296E-43	Mid-zonal hepatocytes	ATP13A3
2.49254256655038E-47	1.41920110498178	0.358	0.206	4.06558618030032E-43	Mid-zonal hepatocytes	CLDND1
2.77309707042881E-47	1.56624156612632	0.327	0.176	4.52319863157644E-43	Mid-zonal hepatocytes	ATXN2
3.24607800407362E-47	1.23485300252562	0.385	0.228	5.29467783244448E-43	Mid-zonal hepatocytes	RBL2
3.36352390132547E-47	0.835972646944577	0.461	0.301	5.48624383545197E-43	Mid-zonal hepatocytes	LEMD2
3.38616113283711E-47	1.38964722549082	0.359	0.206	5.52316742377061E-43	Mid-zonal hepatocytes	IPO9
3.70234584687198E-47	0.881137566476024	0.515	0.349	6.03889631083289E-43	Mid-zonal hepatocytes	PALM3
3.86836707913774E-47	1.24891812235359	0.427	0.273	6.30969354278156E-43	Mid-zonal hepatocytes	U2SURP
4.54889030616074E-47	1.06261439056629	0.467	0.308	7.41969497837879E-43	Mid-zonal hepatocytes	ELP2
5.2560473600675E-47	1.0066222112462	0.456	0.298	8.57313884900609E-43	Mid-zonal hepatocytes	SPG21
5.26815864183342E-47	1.19408167719549	0.402	0.245	8.59289356069449E-43	Mid-zonal hepatocytes	SLC5A6
5.52763008999917E-47	1.47378110010113	0.321	0.172	9.01611743979764E-43	Mid-zonal hepatocytes	ZNF44
6.12882599066512E-47	1.43243986102686	0.308	0.159	9.99672807337388E-43	Mid-zonal hepatocytes	HPS4
6.47060718742439E-47	1.21167937970519	0.421	0.266	1.05542073834079E-42	Mid-zonal hepatocytes	ANKHD1
6.60633486406739E-47	1.17550431398008	0.402	0.247	1.07755927967803E-42	Mid-zonal hepatocytes	RHOT2
7.23480964233942E-47	1.08413954483401	0.421	0.262	1.18006980076198E-42	Mid-zonal hepatocytes	WDR26
8.00938319178756E-47	0.952108606951493	0.408	0.25	1.30641049241247E-42	Mid-zonal hepatocytes	EGLN2
9.2814474891533E-47	1.17161315464091	0.414	0.255	1.5138968999558E-42	Mid-zonal hepatocytes	PVR
9.40810676196443E-47	1.19024610324778	0.393	0.236	1.53455629394402E-42	Mid-zonal hepatocytes	SLC25A45
9.88711883386556E-47	0.687317248135207	0.58	0.408	1.61268795299181E-42	Mid-zonal hepatocytes	LPIN2
1.09437642122457E-46	1.7879850494309	0.284	0.137	1.7850373806594E-42	Mid-zonal hepatocytes	FOS
1.10436533171829E-46	1.33874510600796	0.372	0.22	1.8013302925657E-42	Mid-zonal hepatocytes	GSK3B
1.13868755852039E-46	1.27144254872774	0.355	0.2	1.85731327670262E-42	Mid-zonal hepatocytes	SATB1
1.14693842180917E-46	1.36615109578751	0.386	0.233	1.87077125981294E-42	Mid-zonal hepatocytes	CREBZF
1.18907280657394E-46	0.860515793684006	0.511	0.351	1.93949665480276E-42	Mid-zonal hepatocytes	HEXA
1.18940524353487E-46	1.1909313729704	0.322	0.172	1.94003889272973E-42	Mid-zonal hepatocytes	PBRM1
1.23847721664259E-46	1.50050739191529	0.34	0.18	2.02008018806572E-42	Mid-zonal hepatocytes	SERPINE1
1.24315908326889E-46	1.01744689287286	0.467	0.303	2.02771678071989E-42	Mid-zonal hepatocytes	RBM33
1.30942120504702E-46	1.03366053109846	0.439	0.28	2.1357969275522E-42	Mid-zonal hepatocytes	CD46

1.48916602207193E-46	1.75752688336533	0.232	0.098	2.42897869860152E-42	Mid-zonal hepatocytes	PPP1R15A
1.6613715694447E-46	1.34729977771582	0.372	0.218	2.70986316692126E-42	Mid-zonal hepatocytes	PCMTD2
2.04348108653383E-46	0.908494040170633	0.442	0.282	3.33312200024534E-42	Mid-zonal hepatocytes	SRP54
2.04918215856278E-46	1.47223992197056	0.315	0.167	3.34242101883175E-42	Mid-zonal hepatocytes	ASAP3
2.21198434810964E-46	0.401501096631749	0.887	0.702	3.60796767020164E-42	Mid-zonal hepatocytes	HPN
2.2253942098021E-46	1.12047209348189	0.388	0.231	3.62984049560821E-42	Mid-zonal hepatocytes	AARS2
2.45168283828189E-46	1.28920976527628	0.368	0.213	3.99893987752158E-42	Mid-zonal hepatocytes	TUBGCP3
2.50999235020394E-46	0.964654355995544	0.392	0.235	4.09404852241764E-42	Mid-zonal hepatocytes	ZNF592
2.57487376065469E-46	1.32580215287595	0.394	0.242	4.19987659100387E-42	Mid-zonal hepatocytes	VPS13B
2.83635643191035E-46	1.05829542928285	0.413	0.257	4.62638097608898E-42	Mid-zonal hepatocytes	PLCXD1
2.94668666667245E-46	1.84995422368462	0.281	0.139	4.80634062200944E-42	Mid-zonal hepatocytes	TUBG2
3.0819621625005E-46	0.881030739505679	0.468	0.304	5.02698848325457E-42	Mid-zonal hepatocytes	WWC1
3.17249988510437E-46	1.58195036560858	0.295	0.15	5.17466456259373E-42	Mid-zonal hepatocytes	ARFIP2
3.57524001627122E-46	1.38361543022018	0.337	0.187	5.83157399053999E-42	Mid-zonal hepatocytes	LARS
3.60869419785082E-46	0.903704845351961	0.42	0.259	5.88614110611447E-42	Mid-zonal hepatocytes	SHANK2
3.95782026345227E-46	1.59044474231689	0.313	0.166	6.455600631717E-42	Mid-zonal hepatocytes	KAT2A
4.72436161154791E-46	1.36969272342244	0.315	0.165	7.70590622459579E-42	Mid-zonal hepatocytes	ABHD11
4.96446246903656E-46	1.41421660123885	0.331	0.182	8.09753473324554E-42	Mid-zonal hepatocytes	NUP85
5.56267349736977E-46	0.883831346774265	0.524	0.356	9.07327674155983E-42	Mid-zonal hepatocytes	AFM
7.15506492731925E-46	1.25250479123141	0.388	0.237	1.16706264029504E-41	Mid-zonal hepatocytes	VWA8
7.63491255783437E-46	1.13231128204833	0.435	0.281	1.24533058730836E-41	Mid-zonal hepatocytes	GRTP1
7.66603699956867E-46	1.51947147992526	0.34	0.193	1.25040729499965E-41	Mid-zonal hepatocytes	BPTF
7.94553686927928E-46	1.38400829805224	0.413	0.254	1.29599651874814E-41	Mid-zonal hepatocytes	IRF1
9.72027698698079E-46	0.779862557235413	0.489	0.328	1.58547437934644E-41	Mid-zonal hepatocytes	LARP4
1.00542996387588E-45	1.02942297928271	0.597	0.428	1.63995681407795E-41	Mid-zonal hepatocytes	AKR1C2
1.27292967881121E-45	1.78932383161104	0.29	0.146	2.07627559910896E-41	Mid-zonal hepatocytes	TTLL3
1.54777332691121E-45	0.502712953060924	0.627	0.45	2.52457307352487E-41	Mid-zonal hepatocytes	HNRNPA2E
1.74619242446903E-45	0.755172897451765	0.488	0.33	2.84821446355143E-41	Mid-zonal hepatocytes	TMEM41B
2.12906388566264E-45	0.931673466045119	0.442	0.282	3.47271610390434E-41	Mid-zonal hepatocytes	FOXN2
2.18024730648041E-45	0.705720209479714	0.527	0.358	3.5562013816002E-41	Mid-zonal hepatocytes	RGS14
2.73650933906857E-45	1.34378344987013	0.352	0.201	4.46352038295474E-41	Mid-zonal hepatocytes	FRRS1
3.20891769440172E-45	1.53224976231478	0.28	0.14	5.23406565133865E-41	Mid-zonal hepatocytes	LPIN1
3.49666118845873E-45	1.63507966421691	0.328	0.182	5.70340406449503E-41	Mid-zonal hepatocytes	N4BP2L1
3.62985035483068E-45	0.735378235783519	0.475	0.299	5.92064891376432E-41	Mid-zonal hepatocytes	PPP1R3B
3.8921221278078E-45	1.32960224562062	0.336	0.187	6.3484404026673E-41	Mid-zonal hepatocytes	VPS39
4.15345371512195E-45	2.15955286740794	0.2	0.078	6.77469835473541E-41	Mid-zonal hepatocytes	LONRF2
4.79703062516352E-45	0.63938622323493	0.614	0.443	7.82443665270422E-41	Mid-zonal hepatocytes	LONP2
5.05647580690941E-45	0.796590806150736	0.449	0.291	8.24761768864993E-41	Mid-zonal hepatocytes	ANKRD11
5.54505341024187E-45	1.28787863833897	0.346	0.194	9.04453661744551E-41	Mid-zonal hepatocytes	NPEPL1
5.84374165514736E-45	1.3425955290826	0.38	0.232	9.53172701371087E-41	Mid-zonal hepatocytes	RC3H1
6.86182998844354E-45	1.56519905413021	0.352	0.205	1.11923308941503E-40	Mid-zonal hepatocytes	THBS3
8.16815228667891E-45	1.15946264264875	0.393	0.242	1.3323073194802E-40	Mid-zonal hepatocytes	FNDC3A
9.75187673039567E-45	1.70854146687654	0.297	0.155	1.59062861349484E-40	Mid-zonal hepatocytes	CCDC130
1.01483606262644E-44	1.66856420537801	0.262	0.124	1.65529910174999E-40	Mid-zonal hepatocytes	ATAD2B
1.03625140351744E-44	1.08097678306916	0.4	0.245	1.6902296642773E-40	Mid-zonal hepatocytes	HUWE1
1.07535792803767E-44	2.47991100988137	0.179	0.064	1.75401631642225E-40	Mid-zonal hepatocytes	PPP4R4
1.09256930316337E-44	1.17967442246893	0.348	0.195	1.78208979038977E-40	Mid-zonal hepatocytes	TNK2
1.09924661871988E-44	1.33993438904523	0.359	0.208	1.79298115979399E-40	Mid-zonal hepatocytes	ZBTB37
1.19779616928272E-44	0.841996089586371	0.479	0.316	1.95372533171705E-40	Mid-zonal hepatocytes	RSAD1
1.55966482049892E-44	1.57198547292744	0.301	0.158	2.54396928871578E-40	Mid-zonal hepatocytes	PSPC1
1.69819588779498E-44	1.11741390299078	0.405	0.255	2.7699273125824E-40	Mid-zonal hepatocytes	VPS53
1.76404103343928E-44	0.907971547882894	0.501	0.338	2.87732732964281E-40	Mid-zonal hepatocytes	ITPR2
1.7912327022598E-44	2.10551649445797	0.216	0.089	2.92167966065595E-40	Mid-zonal hepatocytes	CTC1
1.92566199651699E-44	0.910983160670047	0.471	0.305	3.14094728251886E-40	Mid-zonal hepatocytes	TNFAIP2
2.48619348444875E-44	1.19127919398299	0.353	0.202	4.05523019248435E-40	Mid-zonal hepatocytes	FNIP2
3.12743658880663E-44	0.814458664358986	0.507	0.344	5.1011618200025E-40	Mid-zonal hepatocytes	MOGS
3.49716095201627E-44	1.53864224248997	0.318	0.173	5.70421922883373E-40	Mid-zonal hepatocytes	KIAA2026
3.65808837121461E-44	1.7008352262821	0.29	0.151	5.96670794228816E-40	Mid-zonal hepatocytes	GORAB
4.00511102811906E-44	1.3645699248768	0.344	0.197	6.532736597965E-40	Mid-zonal hepatocytes	DENND4C
4.53775782964169E-44	0.916556768979089	0.499	0.333	7.40153679592856E-40	Mid-zonal hepatocytes	ARRDC2

4.61848797836117E-44	0.657891442833556	0.527	0.366	7.5332157415049E-40	Mid-zonal hepatocytes	CLINT1
4.66137622868334E-44	0.685455637533973	0.608	0.412	7.6031707666054E-40	Mid-zonal hepatocytes	HAL
5.05616828637518E-44	1.44969030052909	0.283	0.142	8.24711609190655E-40	Mid-zonal hepatocytes	SETBP1
5.40476291763678E-44	1.06828552923989	0.359	0.208	8.81570879495735E-40	Mid-zonal hepatocytes	WDR59
5.53195819249672E-44	1.44986949416755	0.352	0.204	9.0231770077814E-40	Mid-zonal hepatocytes	TCF7L2
5.53526672956854E-44	1.16749392576224	0.368	0.218	9.02857356259924E-40	Mid-zonal hepatocytes	RAPGEF5
7.12741723800343E-44	2.10943426216622	0.251	0.119	1.16255302569074E-39	Mid-zonal hepatocytes	NEPRO
7.55112998658049E-44	1.97093342508977	0.22	0.093	1.23166481211114E-39	Mid-zonal hepatocytes	ZNF783
8.15107538497163E-44	0.674753340437191	0.579	0.413	1.32952190604272E-39	Mid-zonal hepatocytes	CLDN7
8.39648053497458E-44	1.5230132889894	0.304	0.162	1.3695499400597E-39	Mid-zonal hepatocytes	HOOK3
8.59871908179632E-44	0.971832896644894	0.43	0.277	1.4025370694318E-39	Mid-zonal hepatocytes	AFF4
8.89090239966736E-44	1.10981776791192	0.378	0.227	1.45019509040974E-39	Mid-zonal hepatocytes	FUBP1
8.93110891994567E-44	0.872281763802368	0.409	0.255	1.45675317593234E-39	Mid-zonal hepatocytes	PARP12
1.01203839294448E-43	1.81128905936373	0.272	0.136	1.65073582273173E-39	Mid-zonal hepatocytes	ANKRD27
1.02930970681127E-43	1.11601734192431	0.392	0.241	1.67890706277987E-39	Mid-zonal hepatocytes	SHTN1
1.19212870260155E-43	1.27874228396107	0.334	0.189	1.94448112681339E-39	Mid-zonal hepatocytes	RRP12
1.71960953557548E-43	0.935227524598163	0.45	0.3	2.80485511347717E-39	Mid-zonal hepatocytes	PRKAA1
1.92674390919171E-43	1.50647213818621	0.3	0.161	3.1427119902826E-39	Mid-zonal hepatocytes	SFXN4
1.99141075950143E-43	1.01907666123754	0.418	0.27	3.24819008982278E-39	Mid-zonal hepatocytes	HECTD1
2.02807381010133E-43	0.940304769916886	0.462	0.303	3.30799119165627E-39	Mid-zonal hepatocytes	SLC23A2
2.09588495442385E-43	1.34696417563464	0.328	0.184	3.41859794916074E-39	Mid-zonal hepatocytes	RYBP
2.19897135676258E-43	1.08994092690635	0.376	0.227	3.58674218001544E-39	Mid-zonal hepatocytes	ACSF3
2.39856133953964E-43	1.51726050352349	0.257	0.123	3.9122934009231E-39	Mid-zonal hepatocytes	PSD3
2.4532137613727E-43	1.8667107476232	0.254	0.122	4.00143696617502E-39	Mid-zonal hepatocytes	CBX8
3.06598717467327E-43	1.03872120191326	0.405	0.255	5.00093168060956E-39	Mid-zonal hepatocytes	TMUB2
4.04065768839165E-43	1.02496566664685	0.402	0.252	6.59071675553562E-39	Mid-zonal hepatocytes	ARIH2
4.3487864699592E-43	0.907424955935472	0.482	0.305	7.09330561115045E-39	Mid-zonal hepatocytes	EGR1
6.10169576658465E-43	1.28541078145985	0.348	0.203	9.95247596487622E-39	Mid-zonal hepatocytes	NCK1
6.12304734691599E-43	0.579101267482748	0.572	0.408	9.98730252755467E-39	Mid-zonal hepatocytes	PIM3
6.15022041193128E-43	0.867890278821058	0.453	0.298	1.00316245139011E-38	Mid-zonal hepatocytes	NR1H3
6.25533805252442E-43	1.27019724125367	0.34	0.195	1.02030818974726E-38	Mid-zonal hepatocytes	ZFAND1
6.86797480186201E-43	1.4270624115921	0.275	0.138	1.12023536993171E-38	Mid-zonal hepatocytes	CAMSAP2
9.36450379191191E-43	1.13671056949811	0.308	0.165	1.52744421349875E-38	Mid-zonal hepatocytes	SLC38A1
1.14768843577649E-42	1.96243224942158	0.253	0.122	1.87199460759503E-38	Mid-zonal hepatocytes	USP36
1.19724176316239E-42	1.30089678770251	0.333	0.189	1.95282103989417E-38	Mid-zonal hepatocytes	IST1
1.41936866653303E-42	1.1340177926294	0.406	0.254	2.31513223198203E-38	Mid-zonal hepatocytes	CUL7
1.50977552763273E-42	1.49866448106368	0.273	0.137	2.46259486312175E-38	Mid-zonal hepatocytes	CCDC93
1.51257642998164E-42	1.06546481456114	0.393	0.244	2.46716341494305E-38	Mid-zonal hepatocytes	TRAPPC4
1.57883923855512E-42	1.82931838999086	0.245	0.114	2.57524468200726E-38	Mid-zonal hepatocytes	LY6G5B
1.80315575162434E-42	1.36069077829016	0.298	0.159	2.94112734647445E-38	Mid-zonal hepatocytes	FBXO44
1.86412719762693E-42	2.33994672335842	0.206	0.085	3.04057787204928E-38	Mid-zonal hepatocytes	PGBD1
2.4630551117384E-42	0.954664765943095	0.453	0.302	4.0174891927565E-38	Mid-zonal hepatocytes	ADHFE1
2.51655049422485E-42	1.49734503292853	0.296	0.156	4.10474551113016E-38	Mid-zonal hepatocytes	WHRN
2.62377609967551E-42	1.34206352341259	0.329	0.186	4.27964119618073E-38	Mid-zonal hepatocytes	SORBS1
2.69825943441291E-42	1.02305824916608	0.427	0.28	4.40113096347089E-38	Mid-zonal hepatocytes	APEX1
2.70217049085332E-42	1.63876987609572	0.225	0.098	4.40751028763085E-38	Mid-zonal hepatocytes	MTMR9
2.78739613591316E-42	1.39614553422081	0.341	0.2	4.54652183728795E-38	Mid-zonal hepatocytes	FNIP1
2.78898413894722E-42	1.44925827209855	0.297	0.158	4.54911202903681E-38	Mid-zonal hepatocytes	MED13L
2.86423677430346E-42	0.977152134318732	0.425	0.275	4.67185660256638E-38	Mid-zonal hepatocytes	ENAH
3.11850995889016E-42	0.918088269360206	0.453	0.302	5.08660159394574E-38	Mid-zonal hepatocytes	NHSL1
3.36423682352656E-42	0.800569960624566	0.434	0.279	5.48740668285418E-38	Mid-zonal hepatocytes	SRSF2
3.69481056884623E-42	1.60107437091435	0.298	0.161	6.02660551884509E-38	Mid-zonal hepatocytes	ATM
3.89644515205754E-42	1.37516809078048	0.277	0.139	6.35549168752105E-38	Mid-zonal hepatocytes	SBF1
4.1838285772551E-42	1.60297017919328	0.25	0.118	6.82424279236079E-38	Mid-zonal hepatocytes	PPP1R12A
4.21183888046358E-42	1.01871970960109	0.402	0.252	6.86993039792414E-38	Mid-zonal hepatocytes	SLC35D2
4.51287851571282E-42	0.622544910410608	0.437	0.279	7.36095614697918E-38	Mid-zonal hepatocytes	CIC
5.43429465013243E-42	1.15528764583337	0.414	0.262	8.863878003831E-38	Mid-zonal hepatocytes	LSS
5.6012359659044E-42	0.842642404582426	0.445	0.297	9.13617598398666E-38	Mid-zonal hepatocytes	DEDD
5.87169130382167E-42	0.758266608185823	0.575	0.409	9.57731568566353E-38	Mid-zonal hepatocytes	PLIN4
6.2777362248266E-42	0.634548386891621	0.538	0.383	1.02396155563147E-37	Mid-zonal hepatocytes	SUPT5H

6.28538619694469E-42	1.48969054985286	0.297	0.16	1.02520934258365E-37	Mid-zonal hepatocytes	CCDC9B
6.68709414967968E-42	0.995569859890639	0.423	0.273	1.09073192675425E-37	Mid-zonal hepatocytes	TRIO
8.48202508198184E-42	1.35493332035984	0.269	0.133	1.38350311112206E-37	Mid-zonal hepatocytes	EPC2
8.68417428065888E-42	1.49614944348926	0.295	0.157	1.41647566691827E-37	Mid-zonal hepatocytes	DUSP22
1.07908358497948E-41	1.18394052380682	0.344	0.202	1.76009323546003E-37	Mid-zonal hepatocytes	UBP1
1.23617029564848E-41	1.57673897747687	0.303	0.163	2.01631736923224E-37	Mid-zonal hepatocytes	MLLT10
1.34349409555101E-41	1.53208527664518	0.324	0.183	2.19137321925325E-37	Mid-zonal hepatocytes	ATG4B
1.43292034478193E-41	1.12576380318885	0.369	0.224	2.33723637437381E-37	Mid-zonal hepatocytes	DIDO1
1.44243057097604E-41	1.43579466563324	0.287	0.15	2.35274850431902E-37	Mid-zonal hepatocytes	GTF2H2C
1.50134163243156E-41	0.742724369707218	0.507	0.352	2.44883833665912E-37	Mid-zonal hepatocytes	TTC38
1.80474870224804E-41	1.10503708772591	0.356	0.209	2.94372560823678E-37	Mid-zonal hepatocytes	SLC12A4
1.84971997333061E-41	0.808433913415668	0.464	0.314	3.01707824849955E-37	Mid-zonal hepatocytes	CYTH1
1.94679685761627E-41	1.16531548978437	0.414	0.271	3.1754203544579E-37	Mid-zonal hepatocytes	HGSNAT
1.9725825598545E-41	0.753684007083459	0.548	0.39	3.21747941337867E-37	Mid-zonal hepatocytes	STAT2
2.02748645412166E-41	1.73348902784773	0.26	0.13	3.30703315531785E-37	Mid-zonal hepatocytes	ANGEL1
2.13446910434092E-41	1.09401086748902	0.407	0.262	3.48153255609047E-37	Mid-zonal hepatocytes	ATP11B
2.15023274904528E-41	1.15847160634105	0.335	0.192	3.50724463696775E-37	Mid-zonal hepatocytes	SPAG9
2.1549200739321E-41	1.5731209031685	0.257	0.126	3.51489013259064E-37	Mid-zonal hepatocytes	TOP3B
2.24884986332148E-41	1.18343566390663	0.347	0.205	3.66809901206366E-37	Mid-zonal hepatocytes	SPSB2
2.63306727151402E-41	1.05674764349528	0.33	0.185	4.29479602656651E-37	Mid-zonal hepatocytes	PACSIN2
2.79656235786032E-41	1.41146081587729	0.317	0.179	4.56147286190597E-37	Mid-zonal hepatocytes	USP48
2.80266103039482E-41	1.51346876889988	0.266	0.134	4.57142040667699E-37	Mid-zonal hepatocytes	OCRL
3.1141902348741E-41	0.737528231308135	0.486	0.324	5.07955569210314E-37	Mid-zonal hepatocytes	ZBTB16
3.54788978544912E-41	1.40845984025456	0.3	0.161	5.78696302904607E-37	Mid-zonal hepatocytes	TAZ
3.59838955246547E-41	1.82020831150182	0.257	0.127	5.86933319902644E-37	Mid-zonal hepatocytes	C1QTNF3
3.70530587934257E-41	1.85771625294715	0.241	0.114	6.04372441979567E-37	Mid-zonal hepatocytes	CSMD1
4.25736921737273E-41	1.32881393590537	0.328	0.189	6.94419493045666E-37	Mid-zonal hepatocytes	EWSR1
4.26377276709026E-41	0.497046175260388	0.546	0.354	6.95463976040093E-37	Mid-zonal hepatocytes	G0S2
4.43242944405316E-41	1.22064644875377	0.371	0.226	7.22973566619511E-37	Mid-zonal hepatocytes	SFXN5
5.02688298507531E-41	1.15864358483895	0.377	0.231	8.19934883695634E-37	Mid-zonal hepatocytes	MAX
5.17970877885635E-41	0.788421864794494	0.543	0.384	8.44862298919259E-37	Mid-zonal hepatocytes	MARC2
5.39703897532141E-41	0.536752164308492	0.556	0.395	8.80311027264676E-37	Mid-zonal hepatocytes	TNFRSF12/
6.24247070929823E-41	1.07974867730808	0.334	0.19	1.01820939739363E-36	Mid-zonal hepatocytes	CMIP
6.2525865138282E-41	1.78264854974346	0.287	0.151	1.01985938627052E-36	Mid-zonal hepatocytes	SERPINB9
6.78816068168057E-41	1.11577969899654	0.408	0.264	1.10721688878892E-36	Mid-zonal hepatocytes	ITCH
6.98383448135269E-41	0.874041764667721	0.437	0.29	1.13913324225344E-36	Mid-zonal hepatocytes	NUMA1
7.47033202624414E-41	1.36509025759202	0.315	0.175	1.21848585680068E-36	Mid-zonal hepatocytes	ATXN2L
7.56081658220749E-41	1.4222895771788	0.326	0.189	1.23324479272386E-36	Mid-zonal hepatocytes	POLR2J3
7.69148348967206E-41	1.88925852458809	0.246	0.119	1.25455787200041E-36	Mid-zonal hepatocytes	IL11RA
8.37557894078471E-41	1.33000157953435	0.34	0.2	1.36614068103139E-36	Mid-zonal hepatocytes	PHLDB2
9.32834990150494E-41	1.36755098014822	0.314	0.177	1.52154715243447E-36	Mid-zonal hepatocytes	TRRAP
9.81911216709738E-41	1.4068276244343	0.286	0.153	1.60159538557525E-36	Mid-zonal hepatocytes	POLM
1.00842849620292E-40	0.768862301530624	0.454	0.307	1.64484772015659E-36	Mid-zonal hepatocytes	TRAF4
1.05590981272777E-40	1.02498006870741	0.389	0.242	1.72229449554026E-36	Mid-zonal hepatocytes	MAVS
1.16928268946368E-40	1.09791190992336	0.375	0.231	1.90721699478421E-36	Mid-zonal hepatocytes	FLCN
1.18480541437088E-40	1.1676616865326	0.363	0.221	1.93253611138034E-36	Mid-zonal hepatocytes	FBXL5
1.19284309041008E-40	0.947426471531082	0.403	0.256	1.94564636476788E-36	Mid-zonal hepatocytes	SLC38A7
1.2624858865677E-40	1.12226718029663	0.389	0.243	2.05924072958058E-36	Mid-zonal hepatocytes	TRAPPC9
1.34327618707437E-40	1.58269172154379	0.287	0.154	2.191017788737E-36	Mid-zonal hepatocytes	RUFY2
1.44081512815597E-40	1.37225233321488	0.366	0.223	2.35011355553521E-36	Mid-zonal hepatocytes	CACNA1H
1.56272290508917E-40	1.77261580521746	0.258	0.128	2.54895733049095E-36	Mid-zonal hepatocytes	RCOR3
1.95960819695703E-40	1.28842985267733	0.359	0.217	3.19631693005661E-36	Mid-zonal hepatocytes	PHF8
2.06438442697858E-40	0.479495087353157	0.847	0.68	3.36721743884476E-36	Mid-zonal hepatocytes	BAAT
2.13535179888257E-40	1.812469801591	0.278	0.146	3.48297231915737E-36	Mid-zonal hepatocytes	RNF165
2.23653680102455E-40	1.92111064770755	0.209	0.089	3.64801517615114E-36	Mid-zonal hepatocytes	DCUN1D2
2.58655880379619E-40	0.903624096894135	0.399	0.251	4.21893606487197E-36	Mid-zonal hepatocytes	PANK3
2.60228970160163E-40	1.45234894337716	0.322	0.184	4.24459473228242E-36	Mid-zonal hepatocytes	PTPRD
2.69400720791363E-40	1.29752618210799	0.326	0.186	4.39419515682792E-36	Mid-zonal hepatocytes	MAGI1
3.23113713836335E-40	1.98562520492134	0.209	0.089	5.27030778638447E-36	Mid-zonal hepatocytes	CLK2
3.55669316614456E-40	1.32534559611693	0.331	0.192	5.80132222329839E-36	Mid-zonal hepatocytes	CUL1

3.63487555117579E-40	0.93735902509889	0.379	0.235	5.92884551152283E-36	Mid-zonal hepatocytes	EDC4
3.64973131789648E-40	1.49411015731277	0.322	0.185	5.95307675262096E-36	Mid-zonal hepatocytes	PDE8A
3.65497111979452E-40	0.481728008408735	0.548	0.388	5.96162339349684E-36	Mid-zonal hepatocytes	NARS
3.84482283565199E-40	1.53328348042626	0.287	0.152	6.27129052723196E-36	Mid-zonal hepatocytes	KDM3A
4.10124210487455E-40	0.410351639837219	0.779	0.592	6.68953599726089E-36	Mid-zonal hepatocytes	MTSS2
4.10809539611959E-40	1.5204285755002	0.281	0.149	6.70071440061066E-36	Mid-zonal hepatocytes	INPP4A
4.79203692724344E-40	0.666298766486266	0.511	0.362	7.81629143202677E-36	Mid-zonal hepatocytes	PJA2
5.00651296751449E-40	0.90123167411998	0.435	0.291	8.16612330131288E-36	Mid-zonal hepatocytes	SEC31A
5.04617983987346E-40	1.05316292110932	0.37	0.23	8.2308239368176E-36	Mid-zonal hepatocytes	MAPK14
5.10582026420459E-40	1.7164684616294	0.251	0.123	8.32810343294411E-36	Mid-zonal hepatocytes	FAM193A
5.62021050883901E-40	0.910224526873721	0.479	0.327	9.16712536096731E-36	Mid-zonal hepatocytes	FAM20A
6.1053856580783E-40	1.1931523428764	0.352	0.211	9.95849454689151E-36	Mid-zonal hepatocytes	BLNK
6.53422473642932E-40	1.07089722786604	0.415	0.272	1.06579739675899E-35	Mid-zonal hepatocytes	NFKB2
7.48126254452792E-40	1.18016305723791	0.304	0.167	1.22026873363795E-35	Mid-zonal hepatocytes	ERN 1.00
8.00751201893457E-40	0.772893824651526	0.518	0.366	1.30610528540842E-35	Mid-zonal hepatocytes	SLC39A14
8.04971776262759E-40	1.2679297628001	0.337	0.2	1.31298946426219E-35	Mid-zonal hepatocytes	MAFG
8.64715854060574E-40	1.89394058886963	0.197	0.081	1.4104380295582E-35	Mid-zonal hepatocytes	MICAL1
9.1235962699582E-40	1.34748245720525	0.261	0.13	1.48814978759288E-35	Mid-zonal hepatocytes	SLC28A1
1.0327217658941E-39	1.22189758751927	0.335	0.196	1.68447247234987E-35	Mid-zonal hepatocytes	KDM6B
1.05456025200336E-39	1.15210163542302	0.339	0.198	1.72009322704268E-35	Mid-zonal hepatocytes	GBF1
1.0858286612303E-39	1.26158434814148	0.294	0.159	1.77109512933274E-35	Mid-zonal hepatocytes	ERC1
1.12818066086494E-39	0.988179313365617	0.421	0.277	1.8401754759368E-35	Mid-zonal hepatocytes	KLHL5
1.17973873622385E-39	1.79203498777414	0.222	0.101	1.92427185265472E-35	Mid-zonal hepatocytes	THOC1
1.26920646334221E-39	0.578352353949611	0.518	0.364	2.07020266235748E-35	Mid-zonal hepatocytes	RREB1
1.27041215684329E-39	0.575827025731675	0.62	0.452	2.07216926902709E-35	Mid-zonal hepatocytes	STAT1
1.40417963973356E-39	0.691371817735798	0.451	0.304	2.29035741036941E-35	Mid-zonal hepatocytes	SAFB
1.67508295096125E-39	0.961284414672292	0.418	0.276	2.73222780131289E-35	Mid-zonal hepatocytes	ATF6
1.75480175905404E-39	1.06790427505718	0.361	0.22	2.86225714919304E-35	Mid-zonal hepatocytes	UBE2J2
1.8155669693972E-39	0.864352214664305	0.414	0.271	2.96137128378377E-35	Mid-zonal hepatocytes	EIF2D
1.9589176112256E-39	0.960061812652639	0.398	0.254	3.19519051567008E-35	Mid-zonal hepatocytes	PEX13
2.08920825094561E-39	1.17322756549572	0.322	0.182	3.40770757811739E-35	Mid-zonal hepatocytes	KDM2B
2.22839775340057E-39	0.598535293376047	0.545	0.391	3.63473957557167E-35	Mid-zonal hepatocytes	PPP1R15B
2.28485960984688E-39	1.89173110198268	0.15	0.05	3.72683450962125E-35	Mid-zonal hepatocytes	CRYBG2
2.68921187239453E-39	1.51014787275632	0.237	0.112	4.38637348506272E-35	Mid-zonal hepatocytes	CEP83
2.7747875108007E-39	0.581493594121065	0.511	0.36	4.52595590886703E-35	Mid-zonal hepatocytes	ATRN
2.89089886447533E-39	0.75392170912366	0.463	0.314	4.71534513784572E-35	Mid-zonal hepatocytes	ITGA5
2.97040182009887E-39	1.29392525979661	0.318	0.182	4.84502240876328E-35	Mid-zonal hepatocytes	SLC19A2
2.99153758300466E-39	0.54211639841445	0.799	0.658	4.8794969516389E-35	Mid-zonal hepatocytes	GDF15
3.40386479384008E-39	0.635932056361762	0.44	0.29	5.55204386523255E-35	Mid-zonal hepatocytes	RBM3
3.57756250383932E-39	1.58286636371377	0.247	0.121	5.83536220001232E-35	Mid-zonal hepatocytes	SMCHD1
3.85695264094413E-39	0.751378752078344	0.345	0.2	6.29107545264398E-35	Mid-zonal hepatocytes	TGFBR2
4.13063359524385E-39	0.388101309774626	0.735	0.563	6.73747645720225E-35	Mid-zonal hepatocytes	LITAF
4.24945965033729E-39	0.933034141705679	0.443	0.301	6.93129363566515E-35	Mid-zonal hepatocytes	MYO6
4.54587517326458E-39	0.513678813032191	0.553	0.399	7.41477699511186E-35	Mid-zonal hepatocytes	DBNL
4.5538334050117E-39	0.603198186088183	0.624	0.452	7.42775766691458E-35	Mid-zonal hepatocytes	MST1
5.30797194047877E-39	1.33636930388471	0.316	0.181	8.65783303211492E-35	Mid-zonal hepatocytes	FBXO46
5.42229816971557E-39	1.12454101860043	0.35	0.214	8.84431054462306E-35	Mid-zonal hepatocytes	UBR3
6.99134655279843E-39	1.67902740014751	0.248	0.123	1.14035853622695E-34	Mid-zonal hepatocytes	NFATC2
7.14771886726574E-39	2.39937421001618	0.163	0.06	1.16586442443971E-34	Mid-zonal hepatocytes	DEPRECAT
8.30459706550668E-39	1.28161674905687	0.313	0.177	1.35456282735479E-34	Mid-zonal hepatocytes	TFE3
9.55545203279161E-39	1.33531748044846	0.301	0.168	1.55858978106864E-34	Mid-zonal hepatocytes	NAT9
1.05352312882515E-38	1.44236818414674	0.269	0.14	1.7184015754267E-34	Mid-zonal hepatocytes	PGGHG
1.13664927606045E-38	1.57678194475644	0.278	0.149	1.85398863418219E-34	Mid-zonal hepatocytes	BAZ2B
1.17837720074922E-38	0.659366186434549	0.538	0.382	1.92205105214205E-34	Mid-zonal hepatocytes	ACOX2
1.20576093369912E-38	0.784077308177249	0.441	0.3	1.96671665895663E-34	Mid-zonal hepatocytes	GOLGA3
1.36646391782681E-38	2.17341857256504	0.213	0.095	2.22883929636731E-34	Mid-zonal hepatocytes	DDX31
1.59957937726389E-38	0.917482913453451	0.435	0.291	2.60907392225513E-34	Mid-zonal hepatocytes	EIF5
1.63235315347858E-38	0.757421692295351	0.41	0.264	2.6625312286389E-34	Mid-zonal hepatocytes	CD2AP
1.66711924316883E-38	1.14559437030706	0.372	0.233	2.71923819753267E-34	Mid-zonal hepatocytes	KIAA0232
1.97561424104648E-38	1.00877974164173	0.377	0.234	3.22242438857091E-34	Mid-zonal hepatocytes	DDX46

2.10596469968602E-38	0.900938677762309	0.415	0.278	3.43503902165786E-34	Mid-zonal hepatocytes	ANAPC5
2.35581679636303E-38	0.741222799793893	0.451	0.307	3.84257277654773E-34	Mid-zonal hepatocytes	BRD4
2.73908692637131E-38	1.35032045757782	0.285	0.154	4.46772468560424E-34	Mid-zonal hepatocytes	DOT1L
3.1904839968298E-38	0.824814361214976	0.382	0.241	5.20399844722908E-34	Mid-zonal hepatocytes	CASC3
3.42417714101657E-38	0.575716187935639	0.495	0.349	5.58517533471213E-34	Mid-zonal hepatocytes	CSNK1D
3.59709191165504E-38	0.681867219819346	0.466	0.321	5.86721661710054E-34	Mid-zonal hepatocytes	SPSB3
3.9456376746924E-38	0.561242231383432	0.541	0.381	6.43572961119077E-34	Mid-zonal hepatocytes	IER2
3.96556892764153E-38	1.58644294797124	0.231	0.11	6.4682394778761E-34	Mid-zonal hepatocytes	ZNF441
4.14899212481443E-38	1.71392892948399	0.214	0.096	6.76742105478482E-34	Mid-zonal hepatocytes	MICA
4.38524095009608E-38	1.19643993920541	0.339	0.204	7.15276651370172E-34	Mid-zonal hepatocytes	EXOC2
4.96263906489301E-38	1.92744235777836	0.204	0.089	8.09456057874699E-34	Mid-zonal hepatocytes	SLC7A6
5.01392249857759E-38	1.48986309214868	0.268	0.141	8.1782089874299E-34	Mid-zonal hepatocytes	MED24
5.80284846145982E-38	0.925127179139607	0.327	0.189	9.46502612548712E-34	Mid-zonal hepatocytes	ANKLE2
5.92442236671832E-38	1.6794817028766	0.279	0.153	9.66332532235425E-34	Mid-zonal hepatocytes	PUS7
6.54662771279899E-38	1.53745744180823	0.272	0.147	1.06782044623464E-33	Mid-zonal hepatocytes	NUP88
6.64128190270134E-38	1.03625282191768	0.307	0.171	1.08325949114962E-33	Mid-zonal hepatocytes	ZBTB4
6.75524802956306E-38	1.66922361402674	0.263	0.137	1.10184850610203E-33	Mid-zonal hepatocytes	SMC6
7.09919906201934E-38	0.726313658310615	0.477	0.324	1.15795035900597E-33	Mid-zonal hepatocytes	HES1
7.49730627792179E-38	1.28466440278531	0.315	0.182	1.22288562699182E-33	Mid-zonal hepatocytes	EXOC4
7.51455106256476E-38	0.994436373794029	0.342	0.209	1.22569842381494E-33	Mid-zonal hepatocytes	ASCC3
7.82735224778657E-38	1.75591016534574	0.249	0.126	1.27671942513647E-33	Mid-zonal hepatocytes	SGSM2
8.16762117214139E-38	1.25054941856105	0.34	0.204	1.33222068938798E-33	Mid-zonal hepatocytes	WDR6
8.36720425424164E-38	1.38327043869323	0.327	0.194	1.36477468590935E-33	Mid-zonal hepatocytes	ZDHHC11
1.00778099996948E-37	0.95309098559767	0.427	0.287	1.64379158905022E-33	Mid-zonal hepatocytes	CPNE1
1.15361471259646E-37	1.7711185657453	0.251	0.128	1.88166095771609E-33	Mid-zonal hepatocytes	EXOC6B
1.21218123447808E-37	2.2538878678565	0.14	0.046	1.9771888115572E-33	Mid-zonal hepatocytes	MCOLN3
1.22587573812145E-37	1.57987406690961	0.292	0.163	1.9995259164499E-33	Mid-zonal hepatocytes	CLK4
1.23467533387098E-37	1.48781842256815	0.24	0.117	2.01387893707696E-33	Mid-zonal hepatocytes	EPS15L1
1.28700815677623E-37	0.753520484930015	0.475	0.331	2.09923900451771E-33	Mid-zonal hepatocytes	NEK7
1.30406634991775E-37	1.83455847624804	0.231	0.112	2.12706262335084E-33	Mid-zonal hepatocytes	TRMT44
1.3137811401294E-37	1.188112632397	0.333	0.199	2.14290841766506E-33	Mid-zonal hepatocytes	RAPGEFL1
1.32768284167482E-37	1.42774430700916	0.369	0.232	2.16558348305579E-33	Mid-zonal hepatocytes	SLC20A1
1.44942229518569E-37	1.40604713887786	0.28	0.153	2.36415270567738E-33	Mid-zonal hepatocytes	INTS6
1.64987043203827E-37	0.800941961354707	0.455	0.317	2.69110366169762E-33	Mid-zonal hepatocytes	FLNB
1.68137872329345E-37	1.22962736977256	0.309	0.178	2.74249683556394E-33	Mid-zonal hepatocytes	RBM17
1.76204413604617E-37	1.69550798493406	0.221	0.102	2.87407019030491E-33	Mid-zonal hepatocytes	SCART1
1.89961067995713E-37	1.49413736028406	0.29	0.163	3.09845498007807E-33	Mid-zonal hepatocytes	ANKS1A
1.93252825553914E-37	1.3885489614153	0.344	0.211	3.1521468376099E-33	Mid-zonal hepatocytes	ACAD11
2.08337106833408E-37	1.46965621399804	0.268	0.141	3.39818654955972E-33	Mid-zonal hepatocytes	VPS37A
2.20034372749203E-37	1.34514781533353	0.34	0.205	3.58898065391225E-33	Mid-zonal hepatocytes	CLMN
2.33017033570827E-37	1.32036274158384	0.282	0.152	3.80074083457375E-33	Mid-zonal hepatocytes	CLK3
2.36088969681676E-37	1.03569289040694	0.4	0.264	3.85084718447782E-33	Mid-zonal hepatocytes	NRDC
2.65377833537716E-37	0.571121023917636	0.607	0.451	4.32857784283369E-33	Mid-zonal hepatocytes	MASP1
2.74268013542944E-37	1.52787060175194	0.247	0.123	4.47358556889896E-33	Mid-zonal hepatocytes	ROBO3
2.76982767547173E-37	0.688803453816229	0.366	0.223	4.51786592146195E-33	Mid-zonal hepatocytes	PWWP2B
2.81747064567415E-37	1.14569966365173	0.354	0.219	4.59557637015911E-33	Mid-zonal hepatocytes	ZXDC
2.87567420124768E-37	1.28705981912488	0.303	0.172	4.69051218965509E-33	Mid-zonal hepatocytes	LMBR1L
2.9226442593227E-37	1.52131520982138	0.307	0.177	4.76712505138125E-33	Mid-zonal hepatocytes	ATG2B
2.97857977867467E-37	1.46670972165989	0.272	0.146	4.85836147699625E-33	Mid-zonal hepatocytes	LRCH1
3.36778971429544E-37	1.54214142842236	0.31	0.18	5.49320180298729E-33	Mid-zonal hepatocytes	RELCH
3.59684279689166E-37	1.54556896924912	0.281	0.153	5.86681028600999E-33	Mid-zonal hepatocytes	PIGG
3.90534383576231E-37	1.13388736214468	0.327	0.192	6.3700063305119E-33	Mid-zonal hepatocytes	TANGO2
3.94238951011586E-37	1.07391737498203	0.355	0.215	6.43043152994998E-33	Mid-zonal hepatocytes	PCBP4
3.9844051908583E-37	1.36554554617905	0.291	0.164	6.49896330680898E-33	Mid-zonal hepatocytes	TMEM39A
4.25360927350551E-37	0.963930807175158	0.408	0.27	6.93806208601484E-33	Mid-zonal hepatocytes	HOOK1
5.35478267019648E-37	1.35068045095448	0.323	0.192	8.73418601335747E-33	Mid-zonal hepatocytes	ZNF680
5.52267288163107E-37	1.01812740927531	0.381	0.245	9.00803173722843E-33	Mid-zonal hepatocytes	CEP350
5.99316716019274E-37	1.18536957223065	0.306	0.174	9.77545495499038E-33	Mid-zonal hepatocytes	KIAA0100
6.48370983448071E-37	1.30793208273045	0.332	0.201	1.05755791110215E-32	Mid-zonal hepatocytes	SPG11
6.94101404508044E-37	1.09102111615887	0.323	0.188	1.13214880089307E-32	Mid-zonal hepatocytes	MRTFA

7.38179796542499E-37	1.49446222108758	0.274	0.15	1.20404506614047E-32	Mid-zonal hepatocytes	SFSWAP
8.21423817654969E-37	0.807517200964733	0.402	0.26	1.33982438897702E-32	Mid-zonal hepatocytes	GDI1
9.39374590554285E-37	1.62843194580052	0.284	0.157	1.5322138946531E-32	Mid-zonal hepatocytes	ARL5B
1.07184394889273E-36	1.37002974751566	0.268	0.143	1.74828466503893E-32	Mid-zonal hepatocytes	BCL2L2
1.14113992926277E-36	1.3987330707975	0.29	0.162	1.8613133386205E-32	Mid-zonal hepatocytes	ARMC8
1.221469525689E-36	1.89179423386662	0.195	0.084	1.99233894335133E-32	Mid-zonal hepatocytes	ZSCAN9
1.27651140023993E-36	1.21278052764367	0.349	0.218	2.08211774493135E-32	Mid-zonal hepatocytes	EIF2B5
1.30072309032149E-36	0.589324827697886	0.528	0.377	2.12160943262338E-32	Mid-zonal hepatocytes	HM13
1.33359949547818E-36	1.63806804536844	0.232	0.112	2.17523413707446E-32	Mid-zonal hepatocytes	ABL2
1.39543191142806E-36	1.51982826657545	0.263	0.137	2.2760889907303E-32	Mid-zonal hepatocytes	TRMT11
1.55306478788296E-36	0.933051237249158	0.403	0.27	2.53320397551589E-32	Mid-zonal hepatocytes	EDEM1
1.59067848272438E-36	1.12146243616586	0.36	0.223	2.59455567317174E-32	Mid-zonal hepatocytes	CTPS1
1.60704391157764E-36	1.0626401195731	0.327	0.195	2.62124932417429E-32	Mid-zonal hepatocytes	NUP153
1.66886435474083E-36	0.682529752364331	0.465	0.328	2.72208464901776E-32	Mid-zonal hepatocytes	USP9X
1.6795177502185E-36	2.12350826889313	0.195	0.085	2.7394614023814E-32	Mid-zonal hepatocytes	TTC23
1.69556105177823E-36	1.07877776213042	0.347	0.212	2.76562963155547E-32	Mid-zonal hepatocytes	SP3
1.86583544765214E-36	1.40055375711914	0.313	0.184	3.04336419866541E-32	Mid-zonal hepatocytes	ECPAS
1.9883019121856E-36	1.26252905540274	0.282	0.154	3.24311924896593E-32	Mid-zonal hepatocytes	TECPR1
2.126178850317E-36	1.31251558039676	0.302	0.171	3.46801032275206E-32	Mid-zonal hepatocytes	STRN3
2.29035316696955E-36	1.15446838370992	0.352	0.218	3.73579505064403E-32	Mid-zonal hepatocytes	NT5C2
2.35774891162036E-36	1.5139242886097	0.279	0.155	3.84572424974396E-32	Mid-zonal hepatocytes	S100PBP
2.58513719007517E-36	1.33550421732927	0.293	0.165	4.2166172707316E-32	Mid-zonal hepatocytes	MMS19
2.61843721816222E-36	2.11115937379717	0.168	0.066	4.2709329465444E-32	Mid-zonal hepatocytes	DEPRECAT
2.77824099428785E-36	0.541096664998973	0.467	0.322	4.53158888578291E-32	Mid-zonal hepatocytes	PHRF1
2.79256026415316E-36	0.898610821651629	0.368	0.233	4.55494504686021E-32	Mid-zonal hepatocytes	MOCOS
3.09449292055637E-36	1.89670686474731	0.178	0.072	5.04742740271949E-32	Mid-zonal hepatocytes	PLK3
3.30310199467955E-36	1.16341243788239	0.402	0.268	5.38768966352181E-32	Mid-zonal hepatocytes	ABCG5
3.50523210054347E-36	1.69094817914851	0.238	0.12	5.71738407919645E-32	Mid-zonal hepatocytes	AGFG1
3.50567644495698E-36	0.885556053222888	0.39	0.252	5.71810884936932E-32	Mid-zonal hepatocytes	ST5
3.51711494741496E-36	1.23080254000058	0.315	0.183	5.73676619072854E-32	Mid-zonal hepatocytes	POFUT2
3.56646918995893E-36	1.23523945406132	0.288	0.159	5.81726789574202E-32	Mid-zonal hepatocytes	TANK
3.62167558402402E-36	1.47119449124549	0.268	0.144	5.90731504510158E-32	Mid-zonal hepatocytes	NXF1
3.95685704723336E-36	0.970907804244983	0.359	0.221	6.45402952974234E-32	Mid-zonal hepatocytes	ERO1B
4.06238472422992E-36	0.463944086841496	0.566	0.412	6.62615572369142E-32	Mid-zonal hepatocytes	PLOD3
4.22493495767977E-36	0.864468173091762	0.437	0.295	6.89129140947147E-32	Mid-zonal hepatocytes	TNS3
4.41400551774195E-36	0.991888999831478	0.382	0.247	7.19968439998889E-32	Mid-zonal hepatocytes	FMR1
4.85632690305385E-36	0.885717525027123	0.43	0.298	7.92115481157114E-32	Mid-zonal hepatocytes	PUM1
5.46526506795667E-36	0.376538023690372	0.607	0.449	8.91439385234413E-32	Mid-zonal hepatocytes	IQSEC1
6.01044900898076E-36	0.691672737149706	0.455	0.314	9.80364337854851E-32	Mid-zonal hepatocytes	IGF2R
6.13877056895106E-36	1.43153139239171	0.294	0.168	1.00129486750161E-31	Mid-zonal hepatocytes	GATAD1
6.37855055700935E-36	0.870711841632387	0.327	0.192	1.04040538135379E-31	Mid-zonal hepatocytes	SRRT
6.41510767125682E-36	0.800366100227317	0.436	0.298	1.0463682122587E-31	Mid-zonal hepatocytes	CEP170B
6.82437828924291E-36	2.07187308749457	0.197	0.087	1.11312434275841E-31	Mid-zonal hepatocytes	ANKRD36C
7.880828099561E-36	0.410205173129046	0.781	0.607	1.2854418713194E-31	Mid-zonal hepatocytes	UGDH
8.35305118283031E-36	0.963646344095551	0.266	0.139	1.36246617843145E-31	Mid-zonal hepatocytes	SOGA1
8.78027653700961E-36	1.33477260166848	0.355	0.225	1.43215090595164E-31	Mid-zonal hepatocytes	FBXO9
9.36190248549391E-36	0.56584168116224	0.568	0.419	1.52701991440891E-31	Mid-zonal hepatocytes	KLKB1
1.19000280304277E-35	0.447334691970508	0.601	0.445	1.94101357204306E-31	Mid-zonal hepatocytes	ORMDL3
1.4614618928581E-35	1.23388262582616	0.315	0.185	2.38379049344085E-31	Mid-zonal hepatocytes	BRD9
1.72310331680186E-35	0.700156205776477	0.633	0.48	2.81055382003552E-31	Mid-zonal hepatocytes	LEAP2
1.75341422033884E-35	1.09671045843434	0.327	0.195	2.85999393479469E-31	Mid-zonal hepatocytes	MTMR12
1.81648012522097E-35	1.6619575609464	0.204	0.093	2.96286073224793E-31	Mid-zonal hepatocytes	XPOT
1.85652596429619E-35	0.78906160972104	0.389	0.252	3.02817950036352E-31	Mid-zonal hepatocytes	GRK2
2.12651950110548E-35	1.69729857678301	0.242	0.124	3.46856595825315E-31	Mid-zonal hepatocytes	ZBTB11
2.23216594578326E-35	1.18159934655781	0.318	0.187	3.64088587416708E-31	Mid-zonal hepatocytes	VPS35L
2.34058752315945E-35	0.479059024701852	0.568	0.404	3.81773230902537E-31	Mid-zonal hepatocytes	ANGPTL4
2.354115795886E-35	1.91487955209688	0.219	0.105	3.83979827466965E-31	Mid-zonal hepatocytes	GRHL1
2.41067325725116E-35	1.27663338592841	0.25	0.128	3.93204914990236E-31	Mid-zonal hepatocytes	IRAK2
2.47110763854247E-35	1.6319747939608	0.263	0.142	4.03062366922663E-31	Mid-zonal hepatocytes	NGLY1
2.59862034414087E-35	1.96381151170277	0.17	0.068	4.23860964332817E-31	Mid-zonal hepatocytes	TEP1

**2.72355526601797E-35**	1.26644316969463	0.288	0.162	4.44239099440192E-31	Mid-zonal hepatocytes	SEMA4B
**2.7434017103033E-35**	1.59469411647955	0.24	0.121	4.47476252967571E-31	Mid-zonal hepatocytes	ANKZF1
**2.80418039800356E-35**	0.689925772613301	0.457	0.317	4.5738986471836E-31	Mid-zonal hepatocytes	PHC2
**3.22127898109851E-35**	1.30569311664276	0.323	0.194	5.25422814606978E-31	Mid-zonal hepatocytes	SHPK
**3.58979298797178E-35**	0.709468458347525	0.38	0.243	5.85531134268077E-31	Mid-zonal hepatocytes	PUS1
**3.5898560604831E-35**	1.48745821342662	0.261	0.14	5.85541422025398E-31	Mid-zonal hepatocytes	ALS2
**3.64021376725062E-35**	0.90543982650157	0.649	0.506	5.93755267576248E-31	Mid-zonal hepatocytes	CHI3L1
**3.66017576911851E-35**	0.87893899431376	0.455	0.32	5.9701126970092E-31	Mid-zonal hepatocytes	SGK2
**3.97147297400879E-35**	1.21239355206975	0.308	0.181	6.47786956790574E-31	Mid-zonal hepatocytes	TACC2
**4.04257103743939E-35**	0.860904938920065	0.446	0.312	6.59383761916738E-31	Mid-zonal hepatocytes	PI4K2B
**4.06845237801315E-35**	1.69616031180022	0.259	0.139	6.63605267377724E-31	Mid-zonal hepatocytes	BTBD9
**4.12617696983459E-35**	0.573569715554438	0.51	0.367	6.7302072554972E-31	Mid-zonal hepatocytes	LLGL2
**4.16407961266964E-35**	1.58352231448192	0.202	0.091	6.79203025622545E-31	Mid-zonal hepatocytes	KANSL2
**4.3291794718046E-35**	1.58106515267454	0.229	0.114	7.06132463646048E-31	Mid-zonal hepatocytes	USP21
**4.85641006370579E-35**	0.992337752784523	0.392	0.264	7.92129045491051E-31	Mid-zonal hepatocytes	SLC25A42
**5.68660758807954E-35**	1.19064767275818	0.306	0.179	9.27542563691653E-31	Mid-zonal hepatocytes	RORA
**5.75271967266125E-35**	1.11181068587164	0.291	0.166	9.38326105807776E-31	Mid-zonal hepatocytes	WASHC2A
**5.79291384992716E-35**	0.981518469617546	0.316	0.185	9.44882178061619E-31	Mid-zonal hepatocytes	NAV2
**6.25189242737795E-35**	1.2286619630301	0.29	0.166	1.01974617382962E-30	Mid-zonal hepatocytes	MDC 1.00
**7.37830536051086E-35**	1.26844035075658	0.3	0.174	1.20347538735293E-30	Mid-zonal hepatocytes	RECQL5
**7.42438754746974E-35**	1.69179573945052	0.259	0.14	1.21099185286779E-30	Mid-zonal hepatocytes	TRPV1
**7.70066596716491E-35**	0.855066171263817	0.421	0.285	1.25605562590427E-30	Mid-zonal hepatocytes	ARRB2
**8.2681296278743E-35**	1.50349303863502	0.241	0.123	1.34861462360258E-30	Mid-zonal hepatocytes	CUL9
**8.94463163017485E-35**	0.945205380909552	0.387	0.253	1.45895886519782E-30	Mid-zonal hepatocytes	OTULIN
**9.86775171683602E-35**	1.2557429803855	0.319	0.191	1.60952898253312E-30	Mid-zonal hepatocytes	C4orf19
**9.99855576118741E-35**	1.23563659720029	0.323	0.195	1.63086443020728E-30	Mid-zonal hepatocytes	FBXL17
**1.09639797967625E-34**	1.48783074676645	0.297	0.173	1.78833474464993E-30	Mid-zonal hepatocytes	SEPSECS
**1.17685091294946E-34**	1.07586281239138	0.356	0.226	1.91956152411186E-30	Mid-zonal hepatocytes	TNRC6B
**1.41159806149607E-34**	1.1906549968062	0.302	0.177	2.30245759810624E-30	Mid-zonal hepatocytes	YY1AP1
**1.41983958743553E-34**	1.08936645237179	0.341	0.212	2.31590035106609E-30	Mid-zonal hepatocytes	ZNF410
**1.44427686439527E-34**	1.30407631532324	0.254	0.133	2.35575999351512E-30	Mid-zonal hepatocytes	CBWD3
**1.45536114063077E-34**	0.37483476133121	0.561	0.415	2.37383955648284E-30	Mid-zonal hepatocytes	RILP
**1.5298464430696E-34**	1.045453359843	0.35	0.219	2.49533253329082E-30	Mid-zonal hepatocytes	PIGO
**1.5322771494687E-34**	1.18052926952628	0.338	0.21	2.49929725849839E-30	Mid-zonal hepatocytes	MON2
**1.58624744499261E-34**	1.06580633624049	0.371	0.241	2.58732820752745E-30	Mid-zonal hepatocytes	RANBP10
**1.62028100529412E-34**	1.12999886831745	0.281	0.155	2.64284034773524E-30	Mid-zonal hepatocytes	LTBP4
**1.77017712993432E-34**	1.20795217208618	0.237	0.119	2.88733591663586E-30	Mid-zonal hepatocytes	PGS1
**1.85976670837988E-34**	1.3239612514539	0.263	0.143	3.03346547803843E-30	Mid-zonal hepatocytes	SNIP1
**1.90529284950418E-34**	2.03790149335985	0.199	0.09	3.10772316682626E-30	Mid-zonal hepatocytes	DUSP5
**2.01524437328404E-34**	0.997363192213445	0.356	0.227	3.2870650972636E-30	Mid-zonal hepatocytes	TBC1D15
**2.45769835979238E-34**	0.939110816496177	0.327	0.197	4.00875179465736E-30	Mid-zonal hepatocytes	STX6
**2.47436795409012E-34**	1.21353808259631	0.277	0.155	4.03594156991639E-30	Mid-zonal hepatocytes	AMBRA1
**2.52135232131049E-34**	1.5769846152668	0.276	0.156	4.11257777128954E-30	Mid-zonal hepatocytes	SCML1
**2.88288765740394E-34**	1.77384762893333	0.232	0.117	4.70227805799157E-30	Mid-zonal hepatocytes	LRIG2
**3.00705854286082E-34**	1.80732505673296	0.259	0.142	4.90481318926028E-30	Mid-zonal hepatocytes	BDP1
**3.15769516116215E-34**	1.36154121315333	0.282	0.16	5.15051657737159E-30	Mid-zonal hepatocytes	NFAT5
**3.87853493982982E-34**	0.376106302459807	0.58	0.429	6.32627834035642E-30	Mid-zonal hepatocytes	NADK
**3.91827546828729E-34**	0.929501284071204	0.352	0.221	6.3910991163234E-30	Mid-zonal hepatocytes	PAWR
**3.93640984720757E-34**	0.899805035436233	0.316	0.186	6.42067810178026E-30	Mid-zonal hepatocytes	CERK
**4.04825343893483E-34**	1.1410860865635	0.317	0.188	6.60310618424661E-30	Mid-zonal hepatocytes	IRF8
**4.44424065936259E-34**	0.95229223276306	0.4	0.268	7.24900093948631E-30	Mid-zonal hepatocytes	LGR4
**4.78259789057964E-34**	0.931170029974294	0.441	0.307	7.80089541932445E-30	Mid-zonal hepatocytes	RAB17
**4.90943969658941E-34**	1.88373482724423	0.235	0.121	8.00778708910698E-30	Mid-zonal hepatocytes	PTPN3
**5.05151204118154E-34**	0.416009924079965	0.521	0.377	8.23952129037121E-30	Mid-zonal hepatocytes	IFRD2
**5.30656950406509E-34**	0.691630312951923	0.451	0.317	8.65554551808057E-30	Mid-zonal hepatocytes	ATF4
**5.74517860406323E-34**	1.03708063771822	0.372	0.242	9.37096082108754E-30	Mid-zonal hepatocytes	GHR
**5.80484337146678E-34**	1.77965498012162	0.192	0.086	9.46828002319947E-30	Mid-zonal hepatocytes	PLCB1
**6.07315875711359E-34**	0.531716624936895	0.498	0.359	9.90592924872797E-30	Mid-zonal hepatocytes	UBE2B
**6.67732740377516E-34**	1.34939212701937	0.272	0.152	1.08913887282977E-29	Mid-zonal hepatocytes	USP3
**7.25904965825447E-34**	1.04103624214353	0.313	0.188	1.18402358975789E-29	Mid-zonal hepatocytes	PIKFYVE

7.43397742891074E-34	0.881960888126269	0.423	0.292	1.21255605842963E-29	Mid-zonal hepatocytes	CLSTN3
7.51003033888401E-34	0.955841468677076	0.39	0.259	1.22496104857537E-29	Mid-zonal hepatocytes	DEPRECAT
8.26666368416219E-34	1.24479337582558	0.278	0.157	1.34837551352369E-29	Mid-zonal hepatocytes	ATXN7
8.39178180912953E-34	2.38943694107435	0.255	0.138	1.36878353088712E-29	Mid-zonal hepatocytes	HSPH1
9.22291832484509E-34	0.783031522422039	0.324	0.194	1.50435020796548E-29	Mid-zonal hepatocytes	KLF11
1.02223240459287E-33	1.25131485009851	0.271	0.15	1.66736327513143E-29	Mid-zonal hepatocytes	JMJD6
1.08187718355832E-33	0.62754450643902	0.477	0.344	1.76464987410197E-29	Mid-zonal hepatocytes	TFG
1.16231544269079E-33	1.48439759288301	0.284	0.162	1.89585271857295E-29	Mid-zonal hepatocytes	L3MBTL4
1.23051514766522E-33	1.52416579940744	0.26	0.143	2.00709325735673E-29	Mid-zonal hepatocytes	MED14
1.25278561751697E-33	0.897617328908106	0.355	0.227	2.04341862073192E-29	Mid-zonal hepatocytes	PARP4
1.31707544315322E-33	1.67941437685929	0.235	0.121	2.14828175532722E-29	Mid-zonal hepatocytes	CCDC187
1.33919724747381E-33	1.25466525466555	0.277	0.154	2.18436463035453E-29	Mid-zonal hepatocytes	DIP2C
1.42373304519611E-33	1.39020830392561	0.256	0.137	2.32225097001937E-29	Mid-zonal hepatocytes	ZNF507
1.69256498879362E-33	1.01380660472691	0.364	0.237	2.76074275322128E-29	Mid-zonal hepatocytes	TELO2
1.74321246622674E-33	1.33608989559414	0.263	0.143	2.84335385366243E-29	Mid-zonal hepatocytes	HIRA
1.86652319085544E-33	0.939378067702429	0.492	0.356	3.0444859766043E-29	Mid-zonal hepatocytes	FTCD
1.94662302612734E-33	1.87891710223382	0.213	0.104	3.1751368179163E-29	Mid-zonal hepatocytes	U2AF1L4
1.99458566827712E-33	0.361891901670387	0.548	0.398	3.2533686835268E-29	Mid-zonal hepatocytes	MGLL
2.00646230795097E-33	1.06925410988883	0.351	0.223	3.27274067049883E-29	Mid-zonal hepatocytes	CMTR1
2.04137845116116E-33	0.858572264564538	0.423	0.288	3.32969239168898E-29	Mid-zonal hepatocytes	CCDC50
2.06016573949484E-33	1.27583173043097	0.327	0.202	3.36033633769004E-29	Mid-zonal hepatocytes	PPARG
2.1130617595759E-33	0.852277231148146	0.513	0.38	3.44661503604425E-29	Mid-zonal hepatocytes	HSP90AA1
2.12031253458352E-33	0.430135999760191	0.59	0.435	3.45844177515918E-29	Mid-zonal hepatocytes	GADD45B
2.13309484290802E-33	0.385707167194551	0.685	0.509	3.47929099826727E-29	Mid-zonal hepatocytes	SLC37A4
2.24114067240264E-33	1.54883491263165	0.24	0.125	3.65552455075594E-29	Mid-zonal hepatocytes	FAM219A
2.24581517081296E-33	1.40477962915693	0.275	0.154	3.66314912511302E-29	Mid-zonal hepatocytes	TLK1
2.27454025517456E-33	0.741173750143276	0.476	0.337	3.71000261021522E-29	Mid-zonal hepatocytes	IL6ST
2.40822354510852E-33	1.19873048822795	0.316	0.191	3.9280534244265E-29	Mid-zonal hepatocytes	ZNF326
2.51433945067675E-33	1.12442013966547	0.337	0.204	4.10113907799885E-29	Mid-zonal hepatocytes	NCOA7
2.58365716083799E-33	1.6293243994587	0.258	0.141	4.21420319504285E-29	Mid-zonal hepatocytes	DMXL2
2.60671450191954E-33	0.626247883694184	0.396	0.259	4.25181202408097E-29	Mid-zonal hepatocytes	QKI
2.92818952074989E-33	0.675791660787787	0.492	0.356	4.77616992729515E-29	Mid-zonal hepatocytes	PNRC1
3.56102122978821E-33	0.933122828751772	0.339	0.213	5.80838172790755E-29	Mid-zonal hepatocytes	DNAJC7
3.99128104645258E-33	1.25945681709434	0.267	0.147	6.5101785148688E-29	Mid-zonal hepatocytes	ARL 16.00
4.39974001886397E-33	1.53322308052308	0.281	0.161	7.17641594476903E-29	Mid-zonal hepatocytes	CCDC14
4.50201734716719E-33	0.634781286280451	0.47	0.331	7.3432404949644E-29	Mid-zonal hepatocytes	SCRIB
4.50432031543493E-33	0.930284639094208	0.359	0.23	7.34699686650592E-29	Mid-zonal hepatocytes	ARFGAP1
4.77505805391642E-33	0.954737724189678	0.408	0.278	7.78859719174306E-29	Mid-zonal hepatocytes	KNDC1
4.80486108776702E-33	0.85307696488189	0.333	0.206	7.83720892025679E-29	Mid-zonal hepatocytes	TBRG4
4.91884878105096E-33	1.63102333207935	0.226	0.114	8.02313424677223E-29	Mid-zonal hepatocytes	DENND5A
5.13914895610641E-33	1.3783640125758	0.257	0.14	8.38246586230516E-29	Mid-zonal hepatocytes	TNS1
5.78981609204313E-33	0.652222042075154	0.456	0.322	9.44376902773154E-29	Mid-zonal hepatocytes	PTPRS
6.93635606706797E-33	1.69196506637447	0.243	0.129	1.13138903809946E-28	Mid-zonal hepatocytes	WDR70
8.24036342571394E-33	1.07785476833124	0.343	0.217	1.3440856783682E-28	Mid-zonal hepatocytes	UBR5
8.92075179741177E-33	1.04903373088471	0.356	0.225	1.45506382567583E-28	Mid-zonal hepatocytes	ZNF648
9.08959465418528E-33	0.761323111614533	0.439	0.303	1.48260378404416E-28	Mid-zonal hepatocytes	HNF1A
1.02075593436703E-32	1.17934717971655	0.292	0.169	1.66495500454606E-28	Mid-zonal hepatocytes	RBM19
1.02800663599455E-32	1.3569930675906	0.284	0.163	1.6767816239707E-28	Mid-zonal hepatocytes	ZBTB41
1.02980117312203E-32	0.571431934022587	0.465	0.332	1.67970869347935E-28	Mid-zonal hepatocytes	CAMSAP3
1.03015427346667E-32	0.812847658901803	0.423	0.294	1.68028463545149E-28	Mid-zonal hepatocytes	KCTD3
1.05766117818128E-32	1.08022046483004	0.327	0.202	1.72515114773149E-28	Mid-zonal hepatocytes	EP300
1.08165333658574E-32	1.47893214116119	0.208	0.1	1.76428475730499E-28	Mid-zonal hepatocytes	PTPRM
1.25923159716895E-32	1.02418726933076	0.344	0.22	2.05393265814228E-28	Mid-zonal hepatocytes	GOSR2
1.26183811607877E-32	0.704679651763405	0.408	0.275	2.05818415113609E-28	Mid-zonal hepatocytes	ATP9A
1.3520504064811E-32	1.46040452283165	0.279	0.161	2.20532941801133E-28	Mid-zonal hepatocytes	ZKSCAN3
1.53098168086088E-32	0.590470499301362	0.451	0.317	2.49718421965218E-28	Mid-zonal hepatocytes	DENND11
1.53414160672624E-32	0.681232909910027	0.43	0.297	2.50233837473117E-28	Mid-zonal hepatocytes	ASPSCR1
1.73104631625432E-32	1.4874472690034	0.254	0.138	2.82350964644242E-28	Mid-zonal hepatocytes	MICALL2
1.74304317926529E-32	1.82260828084726	0.228	0.118	2.84307772969961E-28	Mid-zonal hepatocytes	MRNIP
1.83238048233255E-32	1.19597454373381	0.307	0.185	2.98879580473263E-28	Mid-zonal hepatocytes	SRPK2

1.85054060611404E-32	1.16061213228167	0.278	0.158	3.01841678263261E-28	Mid-zonal hepatocytes	CAMTA2
1.85981027297522E-32	1.28389499389306	0.255	0.14	3.03353653624989E-28	Mid-zonal hepatocytes	PPP1R21
1.89174766007632E-32	1.05670521311635	0.279	0.157	3.08562960835049E-28	Mid-zonal hepatocytes	BOD1L1
1.90089556247367E-32	1.75979379633306	0.217	0.108	3.10055075195081E-28	Mid-zonal hepatocytes	ORC2
1.92523290386002E-32	1.48744419969436	0.216	0.107	3.14024738948609E-28	Mid-zonal hepatocytes	SIAH1
2.01171887258547E-32	1.25858503433349	0.281	0.161	3.28131465307416E-28	Mid-zonal hepatocytes	DOCK1
2.03423230873518E-32	0.325405529365866	0.842	0.674	3.31803631877796E-28	Mid-zonal hepatocytes	SHMT1
2.08030693606152E-32	1.15224971593192	0.325	0.203	3.39318864340994E-28	Mid-zonal hepatocytes	KAT7
2.16784688269252E-32	1.5624130163332	0.272	0.156	3.53597505035977E-28	Mid-zonal hepatocytes	TEAD3
2.3320639003819E-32	1.27183666078358	0.327	0.205	3.80382942791291E-28	Mid-zonal hepatocytes	NOP56
2.3747981959302E-32	1.37917568862452	0.184	0.082	3.87353333738176E-28	Mid-zonal hepatocytes	ANKS3
2.40460059100282E-32	0.900141013776541	0.383	0.256	3.9221440239847E-28	Mid-zonal hepatocytes	PUM2
2.40940742332706E-32	0.46544334505264	0.505	0.368	3.92998444818877E-28	Mid-zonal hepatocytes	NCSTN
2.4147942800931E-32	1.45343810295576	0.247	0.132	3.93877095025986E-28	Mid-zonal hepatocytes	CERT1
2.53180338224475E-32	1.39067286931997	0.268	0.152	4.12962449677941E-28	Mid-zonal hepatocytes	COG7
2.56384231579663E-32	1.58577190600236	0.198	0.093	4.18188320129588E-28	Mid-zonal hepatocytes	AGAP1
2.56775335317589E-32	1.76978935084865	0.182	0.081	4.18826249436519E-28	Mid-zonal hepatocytes	BCLAF3
2.72262310657429E-32	1.15888419317611	0.341	0.219	4.44087054913332E-28	Mid-zonal hepatocytes	HTT
2.86640223218336E-32	0.990554793521911	0.351	0.225	4.67538868091427E-28	Mid-zonal hepatocytes	MAPK1
2.88953088609527E-32	1.63560893192301	0.245	0.132	4.71311382831E-28	Mid-zonal hepatocytes	ATF7IP2
3.13955117663673E-32	1.42496329784881	0.307	0.187	5.12092192421217E-28	Mid-zonal hepatocytes	SMAD5
3.2726689487955E-32	1.25446502014877	0.314	0.193	5.33805032238034E-28	Mid-zonal hepatocytes	ABCB11
3.29238615171974E-32	1.72306213041824	0.214	0.105	5.37021105207007E-28	Mid-zonal hepatocytes	ADAM19
3.29684173478284E-32	1.33452482234325	0.293	0.173	5.3774785536043E-28	Mid-zonal hepatocytes	RBM5
3.31055398250327E-32	1.31017833572807	0.351	0.227	5.39984460086109E-28	Mid-zonal hepatocytes	GDA
3.91364378856123E-32	1.02706843284053	0.319	0.194	6.38354438352222E-28	Mid-zonal hepatocytes	SART3
3.99750623746832E-32	1.22164116353137	0.31	0.19	6.52033242393458E-28	Mid-zonal hepatocytes	NCKAP1
4.13253170016192E-32	1.24356889460049	0.326	0.205	6.74057245613411E-28	Mid-zonal hepatocytes	SEC24B
4.16259441447435E-32	1.23690181333409	0.327	0.206	6.78960774944912E-28	Mid-zonal hepatocytes	SPATA13
4.55600434421884E-32	0.948210731469478	0.338	0.214	7.43129868585535E-28	Mid-zonal hepatocytes	PPP1R10
4.7187165458368E-32	1.47066934322354	0.299	0.182	7.6966985579144E-28	Mid-zonal hepatocytes	UBE2G2
5.088098400374E-32	1.12077890870996	0.313	0.189	8.29919730085003E-28	Mid-zonal hepatocytes	SLC25A33
5.31148384510583E-32	0.840136184667279	0.34	0.214	8.66356129975212E-28	Mid-zonal hepatocytes	FBXW7
5.8003477544481E-32	0.783105228264244	0.36	0.236	9.4609472222803E-28	Mid-zonal hepatocytes	SAMD4B
5.98400294388311E-32	0.822334532398143	0.391	0.262	9.76050720176774E-28	Mid-zonal hepatocytes	LBR
6.04815043930777E-32	0.989031649569394	0.3	0.176	9.86513818155491E-28	Mid-zonal hepatocytes	RETREG1
6.15189467340386E-32	1.20378341422035	0.302	0.181	1.0034355401789E-27	Mid-zonal hepatocytes	TRMT1
6.33832856879207E-32	1.42856660721585	0.3	0.182	1.03384477285567E-27	Mid-zonal hepatocytes	CLCN5
6.61465900104932E-32	1.32511151512119	0.231	0.12	1.07891702966115E-27	Mid-zonal hepatocytes	RBM28
6.6441110214087E-32	0.517586966418655	0.508	0.369	1.08372094870197E-27	Mid-zonal hepatocytes	EDEM3
7.17844392275895E-32	1.67955426481141	0.219	0.111	1.17087598824121E-27	Mid-zonal hepatocytes	TTC8
7.32631438598243E-32	0.592588283304636	0.43	0.3	1.19499513949759E-27	Mid-zonal hepatocytes	IL17RC
8.48384571268051E-32	0.567943376837372	0.47	0.338	1.38380007419532E-27	Mid-zonal hepatocytes	DECR2
8.59945901153272E-32	0.841063450681726	0.423	0.29	1.4026577593711E-27	Mid-zonal hepatocytes	TMEM175
8.6487920628531E-32	1.31454179304375	0.251	0.137	1.41070447337197E-27	Mid-zonal hepatocytes	C1orf52
8.84028165366925E-32	0.328326449658233	0.591	0.447	1.44193834052999E-27	Mid-zonal hepatocytes	CNBP
9.70394448356285E-32	1.05866596857474	0.342	0.219	1.58281038471394E-27	Mid-zonal hepatocytes	TPP2
1.02559399537117E-31	1.4076050483975	0.228	0.117	1.67284636584992E-27	Mid-zonal hepatocytes	ZBTB5
1.03655326729907E-31	1.38443196971821	0.221	0.111	1.69072203429152E-27	Mid-zonal hepatocytes	PGM2
1.04782766316161E-31	0.68452714852536	0.467	0.333	1.70911170138291E-27	Mid-zonal hepatocytes	SLC43A1
1.04867281290912E-31	1.37752553740611	0.252	0.137	1.71049022513606E-27	Mid-zonal hepatocytes	GSAP
1.1972201245811E-31	0.647644449985748	0.383	0.256	1.95278574520423E-27	Mid-zonal hepatocytes	ZNF316
1.249732202713E-31	1.48957998707451	0.263	0.148	2.03843819584517E-27	Mid-zonal hepatocytes	AP3B1
1.33655081227402E-31	0.854218894971917	0.383	0.258	2.18004802990016E-27	Mid-zonal hepatocytes	CUL4A
1.3567083322795E-31	0.771424791901629	0.432	0.304	2.21292696078109E-27	Mid-zonal hepatocytes	HECTD3
1.36276697762067E-31	0.736323653431096	0.337	0.21	2.22280921719708E-27	Mid-zonal hepatocytes	FAM83G
1.40447339698634E-31	1.92715553718229	0.188	0.087	2.29083655782441E-27	Mid-zonal hepatocytes	SLC25A27
1.4333316586312E-31	1.32333938664635	0.271	0.154	2.33790726839336E-27	Mid-zonal hepatocytes	CNOT2
1.43494270136962E-31	1.23151322905202	0.26	0.146	2.34053504020399E-27	Mid-zonal hepatocytes	SGSH
1.44774376032734E-31	1.72393327666548	0.207	0.101	2.36141484746992E-27	Mid-zonal hepatocytes	ZDHHC23

1.46635302605368E-31	1.34363476025333	0.237	0.124	2.39176842079615E-27	Mid-zonal hepatocytes	RDH13
1.48038194896379E-31	0.660695603214522	0.436	0.303	2.41465099695483E-27	Mid-zonal hepatocytes	SYNPO
1.60460833357761E-31	0.882309386824555	0.343	0.218	2.61727665289843E-27	Mid-zonal hepatocytes	IFT43
1.60861863197179E-31	0.821287840040323	0.49	0.362	2.62381785060919E-27	Mid-zonal hepatocytes	DEPRECAT
1.62613941032286E-31	1.50357605816349	0.263	0.149	2.65239599217762E-27	Mid-zonal hepatocytes	ATG14
1.64704646751767E-31	1.12947936789292	0.248	0.135	2.68649749316807E-27	Mid-zonal hepatocytes	LONRF1
1.6549816650678E-31	0.963578675734362	0.338	0.213	2.69944059389209E-27	Mid-zonal hepatocytes	MTUS1
1.73922824387005E-31	2.20049084877076	0.129	0.046	2.83685518857643E-27	Mid-zonal hepatocytes	MAPKBP1
1.77095785173774E-31	0.987109528154124	0.367	0.243	2.88860935196942E-27	Mid-zonal hepatocytes	FAM110C
1.776770868746E-31	1.34985446440118	0.231	0.121	2.8980909640116E-27	Mid-zonal hepatocytes	SFI1
1.8485830433091E-31	1.4410545614219	0.246	0.133	3.01522380194147E-27	Mid-zonal hepatocytes	ATPSCKMT
2.02653379379619E-31	0.799558808427231	0.413	0.289	3.30547927106097E-27	Mid-zonal hepatocytes	PAFAH1B1
2.10847414379259E-31	1.56893208953133	0.225	0.116	3.4391321759401E-27	Mid-zonal hepatocytes	BCAS3
2.11037264649826E-31	0.800834413598986	0.405	0.28	3.44222882370331E-27	Mid-zonal hepatocytes	CDC73
2.39723403883752E-31	1.95493565372594	0.195	0.093	3.91012844074788E-27	Mid-zonal hepatocytes	FAM133B
2.41181702087143E-31	0.468137479730593	0.569	0.425	3.93391474274339E-27	Mid-zonal hepatocytes	IRF7
2.41261832627786E-31	0.98456363503296	0.299	0.177	3.93522175199181E-27	Mid-zonal hepatocytes	E2F4
2.4792137314552E-31	1.93766392564876	0.188	0.087	4.04384551737658E-27	Mid-zonal hepatocytes	FAM219B
2.63492559517901E-31	1.62334415543123	0.238	0.128	4.29782713829648E-27	Mid-zonal hepatocytes	FAM172A
2.7705569336336E-31	1.52777273561962	0.19	0.087	4.51905541444976E-27	Mid-zonal hepatocytes	WEE1
2.85917749861599E-31	1.52480323048644	0.232	0.121	4.66360441799254E-27	Mid-zonal hepatocytes	ANO8
2.95324141478136E-31	1.98862445343405	0.198	0.095	4.81703207164987E-27	Mid-zonal hepatocytes	ZMAT1
3.08843427995014E-31	1.25675720336846	0.266	0.152	5.03754515402667E-27	Mid-zonal hepatocytes	TASOR2
3.22969164256795E-31	0.944857429425213	0.43	0.306	5.26795003819259E-27	Mid-zonal hepatocytes	PDZK1
3.26396828800488E-31	1.10213503014453	0.267	0.15	5.32385867456476E-27	Mid-zonal hepatocytes	VSIG10
3.41699192315855E-31	0.595138458223198	0.405	0.277	5.57345552586391E-27	Mid-zonal hepatocytes	HNRNPC
3.70755047431638E-31	1.46225070671875	0.244	0.132	6.04738557865744E-27	Mid-zonal hepatocytes	CDK14
4.09357004398729E-31	1.20543310083136	0.284	0.166	6.67702209874767E-27	Mid-zonal hepatocytes	SMPD4
4.47723049997419E-31	1.14388376834673	0.289	0.172	7.3028106685079E-27	Mid-zonal hepatocytes	TTC31
4.72644693598429E-31	0.806831196954883	0.365	0.238	7.70930759728397E-27	Mid-zonal hepatocytes	YPEL5
4.853762790746E-31	1.38906037370766	0.274	0.159	7.91697248798581E-27	Mid-zonal hepatocytes	ZNF91
5.01099866716129E-31	0.970050661433959	0.362	0.24	8.17343992600678E-27	Mid-zonal hepatocytes	EIF1
5.05116330437469E-31	1.36763179582064	0.269	0.154	8.23895246576555E-27	Mid-zonal hepatocytes	MED23
5.68896061026571E-31	1.34027201285314	0.281	0.165	9.2792636514044E-27	Mid-zonal hepatocytes	KLHL23
5.85559435278293E-31	0.738580172260483	0.341	0.22	9.55105994882424E-27	Mid-zonal hepatocytes	MAPKAPK5
5.90720717729869E-31	0.712261228574006	0.47	0.34	9.6352456268919E-27	Mid-zonal hepatocytes	CYP4F3
6.19954460714458E-31	1.31127532142944	0.286	0.17	1.01120772087135E-26	Mid-zonal hepatocytes	SUSD6
6.25415995239532E-31	0.963459153542696	0.334	0.212	1.0201160298352E-26	Mid-zonal hepatocytes	CENPV
6.4224133644882E-31	1.23306490467756	0.293	0.177	1.04755984388167E-26	Mid-zonal hepatocytes	PHKG2
6.44549665377767E-31	1.14954770225561	0.271	0.156	1.05132495919768E-26	Mid-zonal hepatocytes	DEPRECAT
6.63696932783669E-31	1.70151404889436	0.219	0.114	1.08255606706344E-26	Mid-zonal hepatocytes	SCFD2
6.81065252082011E-31	0.803441124930841	0.493	0.364	1.11088553267097E-26	Mid-zonal hepatocytes	DEPRECAT
6.82882245507519E-31	0.909256723294438	0.345	0.225	1.11384923064731E-26	Mid-zonal hepatocytes	SRSF3
7.46086013034293E-31	1.5247462418316	0.266	0.151	1.21694089586024E-26	Mid-zonal hepatocytes	BCO2
7.57830651778927E-31	0.288745644728499	0.348	0.207	1.23609757611661E-26	Mid-zonal hepatocytes	EEF1A2
8.10658518200571E-31	1.48328406062049	0.227	0.119	1.32226510903695E-26	Mid-zonal hepatocytes	HERC1
8.2676954854384E-31	0.852610073015465	0.368	0.243	1.34854381062986E-26	Mid-zonal hepatocytes	DDI2
8.75091472865141E-31	0.816934675552041	0.38	0.257	1.42736170139033E-26	Mid-zonal hepatocytes	PPP1R35
9.89419018614179E-31	0.931572593410145	0.377	0.253	1.61384136126159E-26	Mid-zonal hepatocytes	PI4KA
1.01119166002569E-30	1.65087201228229	0.258	0.146	1.6493547166679E-26	Mid-zonal hepatocytes	NBPF15
1.05586623513853E-30	1.10077886658105	0.373	0.252	1.72222341613445E-26	Mid-zonal hepatocytes	DUSP10
1.06341688667286E-30	1.32535756699571	0.256	0.143	1.7345392838521E-26	Mid-zonal hepatocytes	PDPR
1.08699509944419E-30	1.47061392499001	0.253	0.141	1.77299770670342E-26	Mid-zonal hepatocytes	TRANK1
1.11515196800847E-30	0.751017374563441	0.442	0.311	1.81892437501862E-26	Mid-zonal hepatocytes	ID2
1.1796952818388E-30	0.548283844571879	0.442	0.306	1.92420097420727E-26	Mid-zonal hepatocytes	IER5
1.19000925467766E-30	1.17949144397008	0.313	0.196	1.94102409530474E-26	Mid-zonal hepatocytes	IGF2BP3
1.20236280649141E-30	0.761436460300388	0.417	0.292	1.96117397366813E-26	Mid-zonal hepatocytes	ATP2A2
1.24669220655931E-30	1.16505235741212	0.269	0.154	2.03347965811889E-26	Mid-zonal hepatocytes	SLMAP
1.24878855926665E-30	0.337002555946146	0.609	0.453	2.03689901901984E-26	Mid-zonal hepatocytes	CTSA
1.25798310021797E-30	0.627693784449591	0.459	0.331	2.05189623476553E-26	Mid-zonal hepatocytes	HERPUD1

1.26281271663619E-30	0.639082747131659	0.4	0.271	2.05977382210529E-26	Mid-zonal hepatocytes	RHPN2
1.26405474139509E-30	0.98067669578119	0.316	0.194	2.06179968868953E-26	Mid-zonal hepatocytes	TBC1D20
1.32873751005293E-30	0.538093332210377	0.508	0.376	2.16730375264733E-26	Mid-zonal hepatocytes	HBS1L
1.34385981744635E-30	1.29825304724479	0.247	0.134	2.19196974823674E-26	Mid-zonal hepatocytes	SH3TC1
1.45282064717907E-30	0.782184655280369	0.346	0.224	2.36969575761377E-26	Mid-zonal hepatocytes	DNAJC11
1.49253811065861E-30	0.625416194323901	0.641	0.488	2.43447891229526E-26	Mid-zonal hepatocytes	PTGR1
1.49508017769494E-30	1.05150802890961	0.318	0.199	2.43862527783822E-26	Mid-zonal hepatocytes	PHF14
1.56768172081168E-30	1.00583201157218	0.368	0.248	2.55704565481593E-26	Mid-zonal hepatocytes	ACSS2
1.90662178369751E-30	1.50584695191455	0.228	0.121	3.10989079138901E-26	Mid-zonal hepatocytes	IGF2BP2
1.95248550781798E-30	1.43005146970582	0.216	0.11	3.18469911180191E-26	Mid-zonal hepatocytes	MAP4K3
1.9635737591645E-30	2.13057705528086	0.124	0.043	3.20278515857322E-26	Mid-zonal hepatocytes	CRIP3
1.98504235569195E-30	1.65858902171003	0.215	0.11	3.23780258636913E-26	Mid-zonal hepatocytes	SNHG32
1.99043800793884E-30	0.385292363532864	0.657	0.494	3.24660343474905E-26	Mid-zonal hepatocytes	LPGAT1
2.0152369248821E-30	2.45199227418574	0.107	0.033	3.2870529481752E-26	Mid-zonal hepatocytes	FMNL2
2.08601645280461E-30	1.0576025159179	0.302	0.183	3.4025014361696E-26	Mid-zonal hepatocytes	DICER1
2.08947455449222E-30	1.096730220402	0.3	0.181	3.40814194583226E-26	Mid-zonal hepatocytes	PML
2.1317713843592E-30	1.26886457431365	0.289	0.174	3.4771323050283E-26	Mid-zonal hepatocytes	ADGRA3
2.19751492486257E-30	0.58975185219808	0.436	0.305	3.58436659394334E-26	Mid-zonal hepatocytes	LAMB2
2.21272152398062E-30	1.16804913631504	0.289	0.174	3.6091700777648E-26	Mid-zonal hepatocytes	PPP2R2D
2.28407789237155E-30	1.04012673067899	0.299	0.18	3.72555945024724E-26	Mid-zonal hepatocytes	TRMT2A
2.33477427801582E-30	1.14789692655988	0.32	0.203	3.8082503248716E-26	Mid-zonal hepatocytes	CDK16
2.36025088374652E-30	0.334800532576244	0.782	0.589	3.84980521647894E-26	Mid-zonal hepatocytes	LRP1
2.40516626632372E-30	1.51862730175041	0.216	0.111	3.92306669700063E-26	Mid-zonal hepatocytes	ATAD3B
2.4176743421915E-30	1.4449657063336	0.25	0.14	3.94346861954856E-26	Mid-zonal hepatocytes	NBEAL1
2.49587016206954E-30	1.59934521315873	0.229	0.122	4.07101382135163E-26	Mid-zonal hepatocytes	KDM7A
2.61802876912229E-30	0.981888656468048	0.306	0.189	4.27026672531537E-26	Mid-zonal hepatocytes	ARFGEF2
2.68756420287746E-30	0.791125877690027	0.354	0.232	4.38368597131342E-26	Mid-zonal hepatocytes	SRCAP
2.82470061167873E-30	1.63694372349927	0.203	0.1	4.60736916770918E-26	Mid-zonal hepatocytes	SCMH1
2.90068840995248E-30	0.645899286129331	0.451	0.323	4.73131286547349E-26	Mid-zonal hepatocytes	OGFR
2.91624466244501E-30	1.41365737755425	0.244	0.134	4.75668666891406E-26	Mid-zonal hepatocytes	DOCK7
3.04100315536347E-30	0.938959690154414	0.351	0.229	4.96018024671335E-26	Mid-zonal hepatocytes	DNMT3A
3.54995078684515E-30	0.81670198847942	0.396	0.274	5.79032472842312E-26	Mid-zonal hepatocytes	ARHGAP1
3.62691011065918E-30	0.478453627168361	0.524	0.393	5.91585308149619E-26	Mid-zonal hepatocytes	ARSA
3.78046400556234E-30	1.18956444073371	0.263	0.15	6.16631483947273E-26	Mid-zonal hepatocytes	FOSL2
3.9487725207288E-30	0.937363645574497	0.386	0.265	6.44084285856075E-26	Mid-zonal hepatocytes	SMAD2
3.95789668093857E-30	1.62044992539678	0.207	0.105	6.4557252762789E-26	Mid-zonal hepatocytes	DDHD1
4.00537878896543E-30	0.70482198103639	0.426	0.299	6.53317334268151E-26	Mid-zonal hepatocytes	NAGS
4.02107091950727E-30	1.73077466853073	0.225	0.119	6.55876877680831E-26	Mid-zonal hepatocytes	CECR2
4.12163065523947E-30	1.1855058178548	0.274	0.158	6.7227917617611E-26	Mid-zonal hepatocytes	PRRC2C
4.45319648693627E-30	0.313092293171645	0.728	0.554	7.26360878984176E-26	Mid-zonal hepatocytes	DDT
4.6355783839591E-30	1.56818510927944	0.185	0.086	7.56109190207568E-26	Mid-zonal hepatocytes	CNTRL
4.76735280358588E-30	1.43928201965022	0.215	0.109	7.77602915792893E-26	Mid-zonal hepatocytes	HMBOX1
4.82397560136227E-30	0.77453943322988	0.328	0.208	7.868386603382E-26	Mid-zonal hepatocytes	ARIH1
4.83881332789155E-30	0.581391486478661	0.428	0.302	7.8925884191239E-26	Mid-zonal hepatocytes	MED10
4.85277168344275E-30	0.466996587338768	0.496	0.366	7.91535589286347E-26	Mid-zonal hepatocytes	CLCN7
5.11234682534369E-30	0.865538583222423	0.288	0.17	8.33874890681809E-26	Mid-zonal hepatocytes	MCM7
5.39178980028735E-30	1.33646202429812	0.311	0.196	8.7945483432487E-26	Mid-zonal hepatocytes	DEPRECAT
5.44323372396975E-30	1.52734979515011	0.205	0.102	8.87845852716706E-26	Mid-zonal hepatocytes	FAM76B
5.46500456668426E-30	1.45450774616348	0.253	0.143	8.9139689487187E-26	Mid-zonal hepatocytes	CLN8
5.47096552802588E-30	2.29131372526684	0.132	0.049	8.92369187276301E-26	Mid-zonal hepatocytes	MPP3
5.78179664594294E-30	1.32916907116926	0.281	0.169	9.43068850919753E-26	Mid-zonal hepatocytes	NR1D2
5.86873336543077E-30	0.57279509842538	0.449	0.32	9.57249099235414E-26	Mid-zonal hepatocytes	PARP9
5.98812913019681E-30	0.474072363293707	0.424	0.296	9.76723742426402E-26	Mid-zonal hepatocytes	PPP1R9B
6.21562959004886E-30	1.42896232177792	0.228	0.121	1.01383134243287E-25	Mid-zonal hepatocytes	CHD7
6.54747206684477E-30	1.52188047776467	0.209	0.104	1.06795816882305E-25	Mid-zonal hepatocytes	MDN1
6.62183832932706E-30	0.762983682388958	0.411	0.287	1.08008804989654E-25	Mid-zonal hepatocytes	GPBP1L1
7.1558222211844E-30	1.07666462081274	0.287	0.171	1.16718616249739E-25	Mid-zonal hepatocytes	PFAS
7.3189348216321E-30	1.57657294687879	0.2	0.097	1.19379145875641E-25	Mid-zonal hepatocytes	SLC6A6
8.52190291602701E-30	1.31130690565257	0.282	0.169	1.39000758463317E-25	Mid-zonal hepatocytes	SRSF7
9.3539265004058E-30	1.15277159146417	0.267	0.154	1.52571895148119E-25	Mid-zonal hepatocytes	MUS81

9.4768461196845E-30	1.07074462031005	0.344	0.228	1.54576837058174E-25	Mid-zonal hepatocytes	AKAP8L
9.5155268355615E-30	1.37566177014574	0.293	0.179	1.55207758214844E-25	Mid-zonal hepatocytes	NUP42
9.59263784893156E-30	1.79634822958811	0.168	0.074	1.56465515953923E-25	Mid-zonal hepatocytes	NEMP1
1.00050152488306E-29	0.554810315429463	0.375	0.25	1.63191803723676E-25	Mid-zonal hepatocytes	SBNO2
1.05702586443123E-29	1.41197865890061	0.267	0.157	1.72411488747379E-25	Mid-zonal hepatocytes	DENND1B
1.08504434028665E-29	0.653690271932416	0.364	0.238	1.76981582344155E-25	Mid-zonal hepatocytes	LZTS2
1.11272885481745E-29	1.54217380738646	0.253	0.143	1.81497203509274E-25	Mid-zonal hepatocytes	GRAMD2B
1.11364326047187E-29	1.27828550526071	0.293	0.179	1.81646352215567E-25	Mid-zonal hepatocytes	PPOX
1.14357703584679E-29	1.60095387595497	0.229	0.122	1.8652885031697E-25	Mid-zonal hepatocytes	PER3
1.24322309838404E-29	2.20970010238813	0.203	0.103	2.02782119577421E-25	Mid-zonal hepatocytes	TMX3
1.2580316147825E-29	1.15377426979806	0.287	0.172	2.05197536687173E-25	Mid-zonal hepatocytes	GRB10
1.28263799559463E-29	1.49677825094098	0.208	0.105	2.0921108346144E-25	Mid-zonal hepatocytes	PARD3B
1.28382712051049E-29	0.746916730708303	0.381	0.257	2.09405041626466E-25	Mid-zonal hepatocytes	GGA2
1.41105026157195E-29	1.28563608432251	0.257	0.146	2.30156408165001E-25	Mid-zonal hepatocytes	PRPF38B
1.41271444939688E-29	1.06682124380451	0.344	0.223	2.30427853841126E-25	Mid-zonal hepatocytes	PPP1R3C
1.46528902859998E-29	0.384522635200124	0.538	0.408	2.39003293454943E-25	Mid-zonal hepatocytes	SEC22B
1.48797866700429E-29	1.23253041321938	0.26	0.147	2.4270420037507E-25	Mid-zonal hepatocytes	NUP210
1.51957971336307E-29	1.12044223218814	0.308	0.192	2.47858647046651E-25	Mid-zonal hepatocytes	ADAMTSL3
1.67487356619984E-29	0.890923555109398	0.338	0.22	2.73188627382856E-25	Mid-zonal hepatocytes	UBE2Z
1.70133809353684E-29	1.05684646187078	0.309	0.194	2.77505256436794E-25	Mid-zonal hepatocytes	MYO9B
1.70459075647039E-29	1.11138018804858	0.282	0.167	2.78035798287885E-25	Mid-zonal hepatocytes	CREBRF
1.77238676511925E-29	0.908620628855774	0.356	0.237	2.89094005258601E-25	Mid-zonal hepatocytes	RBM23
1.81912853147069E-29	0.919490756977534	0.365	0.247	2.96718054768184E-25	Mid-zonal hepatocytes	UMPS
1.88020116116983E-29	0.568069752308311	0.504	0.37	3.06679611398412E-25	Mid-zonal hepatocytes	PTPRH
1.92916609661343E-29	1.50124061615574	0.241	0.133	3.14666282018617E-25	Mid-zonal hepatocytes	TRIM33
1.96812782257059E-29	0.825501565686585	0.349	0.229	3.21021329139488E-25	Mid-zonal hepatocytes	GPAT4
2.07976929252409E-29	0.814681560159988	0.357	0.239	3.39231169303605E-25	Mid-zonal hepatocytes	MCRS1
2.13833028291849E-29	1.04616259446309	0.277	0.162	3.48783052446834E-25	Mid-zonal hepatocytes	TNFRSF10B
2.15049830667665E-29	1.0622271996897	0.365	0.246	3.50767778802029E-25	Mid-zonal hepatocytes	PLXNB1
2.18929003215525E-29	1.04163937925936	0.3	0.184	3.57095097144844E-25	Mid-zonal hepatocytes	SRC
2.28350668969277E-29	1.80885238232022	0.124	0.044	3.72462776155787E-25	Mid-zonal hepatocytes	MCTP1
2.3778859773055E-29	1.04998925147154	0.275	0.162	3.878569817583E-25	Mid-zonal hepatocytes	TRAK1
2.41854047195302E-29	1.22071911332331	0.279	0.167	3.94488136380257E-25	Mid-zonal hepatocytes	AFF3
2.54865791569926E-29	0.438357290238023	0.422	0.29	4.15711592629707E-25	Mid-zonal hepatocytes	ERF
2.69025486917114E-29	0.65214128624537	0.39	0.254	4.38807471710504E-25	Mid-zonal hepatocytes	ID1
2.83853381636603E-29	1.21198373273165	0.307	0.193	4.62993250787463E-25	Mid-zonal hepatocytes	UBE4A
2.8576407328804E-29	0.773580317154802	0.44	0.312	4.66109779940123E-25	Mid-zonal hepatocytes	RIOX2
2.96320196948624E-29	0.906673806542251	0.361	0.243	4.833278732429E-25	Mid-zonal hepatocytes	SLC35A3
3.01445468420156E-29	1.03073110256738	0.302	0.187	4.91687703540116E-25	Mid-zonal hepatocytes	RPRD1A
3.03579984055971E-29	0.513203733012843	0.456	0.325	4.95169311993694E-25	Mid-zonal hepatocytes	GALE
3.13992031053679E-29	0.363273933220823	0.441	0.313	5.12152401851655E-25	Mid-zonal hepatocytes	CYTH2
3.19846410406905E-29	0.51183863719578	0.392	0.266	5.21701480014703E-25	Mid-zonal hepatocytes	FUS
3.19891254179638E-29	1.29311698177935	0.262	0.153	5.21774624692407E-25	Mid-zonal hepatocytes	ERCC5
3.65340345976753E-29	0.601965308773749	0.415	0.291	5.95906638322682E-25	Mid-zonal hepatocytes	PTTG1IP
3.68414980139176E-29	1.05984619245845	0.298	0.185	6.0092167410501E-25	Mid-zonal hepatocytes	DMAP1
3.74051205570011E-29	0.692745917939241	0.341	0.222	6.10114921405246E-25	Mid-zonal hepatocytes	PAXX
3.91570089822886E-29	1.82898458711761	0.181	0.085	6.3868997351011E-25	Mid-zonal hepatocytes	SRSF11
3.92820105400705E-29	1.81399923049488	0.172	0.079	6.4072887391909E-25	Mid-zonal hepatocytes	RRAGB
4.13186798811462E-29	1.57943387489094	0.184	0.086	6.73948987541375E-25	Mid-zonal hepatocytes	CEP85
4.20829767577629E-29	1.16034597942617	0.269	0.156	6.8641543389587E-25	Mid-zonal hepatocytes	SLC15A4
4.4438050521451E-29	2.33621433676436	0.125	0.045	7.24829042055388E-25	Mid-zonal hepatocytes	LAMA3
4.71370443428757E-29	0.642799967587086	0.489	0.361	7.68852330276645E-25	Mid-zonal hepatocytes	DCAF6
4.77216924686262E-29	0.955305657647258	0.384	0.266	7.78388525855762E-25	Mid-zonal hepatocytes	EXOC3L4
4.78757701390592E-29	0.840774028939704	0.352	0.232	7.80901686738195E-25	Mid-zonal hepatocytes	PMM2
4.8132251755645E-29	2.04697256569321	0.164	0.072	7.85085158386326E-25	Mid-zonal hepatocytes	TPM2
4.82847560832699E-29	1.68294214616922	0.219	0.115	7.87572656474216E-25	Mid-zonal hepatocytes	DEPRECAT
5.30138296552695E-29	1.43061571653323	0.238	0.132	8.64708575507101E-25	Mid-zonal hepatocytes	ARHGAP17
5.84449775471476E-29	0.88445801400368	0.331	0.214	9.53296028771525E-25	Mid-zonal hepatocytes	R3HDM2
6.15498361514872E-29	1.44511914557761	0.222	0.117	1.00393937746691E-24	Mid-zonal hepatocytes	UCKL1
6.41244029042341E-29	1.58431496071597	0.205	0.104	1.04593313577096E-24	Mid-zonal hepatocytes	POLR3A

**6.82838243743137E-29**	0.444388892151349	0.566	0.421	1.11377745936943E-24	Mid-zonal hepatocytes	BHMT2
**6.84172399450343E-29**	1.61620706927131	0.234	0.127	1.11595360074346E-24	Mid-zonal hepatocytes	SCN8A
**6.8767015697926E-29**	1.28520372130725	0.257	0.146	1.12165879304887E-24	Mid-zonal hepatocytes	NOP2
**6.9221538646823E-29**	1.39844679354248	0.224	0.119	1.12907251686833E-24	Mid-zonal hepatocytes	APTX
**7.28062877238436E-29**	0.430209371344175	0.729	0.582	1.18754335906361E-24	Mid-zonal hepatocytes	THBS1
**7.38660589779834E-29**	0.640463672287765	0.455	0.33	1.20482928798989E-24	Mid-zonal hepatocytes	CIB1
**7.79581116734589E-29**	1.16826863394134	0.243	0.135	1.27157475950579E-24	Mid-zonal hepatocytes	DSTYK
**8.21442995056937E-29**	1.26249423522451	0.263	0.153	1.33985566923737E-24	Mid-zonal hepatocytes	PHF11
**8.52617557502291E-29**	0.709065167623292	0.422	0.298	1.39070449804199E-24	Mid-zonal hepatocytes	PRPSAP1
**8.54818372261031E-29**	1.25972065872455	0.195	0.096	1.39429424699497E-24	Mid-zonal hepatocytes	FRS2
**9.16771932613768E-29**	1.09795924576432	0.326	0.209	1.49534669928632E-24	Mid-zonal hepatocytes	MSI2
**9.33078774802868E-29**	1.76809853618574	0.159	0.069	1.52194478958096E-24	Mid-zonal hepatocytes	VRK2
**9.59538324943466E-29**	1.30099697737587	0.262	0.151	1.56510296181529E-24	Mid-zonal hepatocytes	NBPF12
**1.02732329462518E-28**	0.913580189392275	0.356	0.238	1.67566702586313E-24	Mid-zonal hepatocytes	OSBPL9
**1.034513417767E-28**	1.20751529038801	0.237	0.131	1.68739483571976E-24	Mid-zonal hepatocytes	PISD
**1.06264971533966E-28**	1.06163608033784	0.323	0.208	1.73328795069052E-24	Mid-zonal hepatocytes	LRMDA
**1.08480922809259E-28**	1.21985942477094	0.256	0.147	1.76943233194182E-24	Mid-zonal hepatocytes	TRIM56
**1.28355251184728E-28**	1.44467384022768	0.246	0.14	2.0936025020741E-24	Mid-zonal hepatocytes	CHIC2
**1.33198702206861E-28**	1.08458747960494	0.254	0.145	2.1726040316961E-24	Mid-zonal hepatocytes	TNPO3
**1.39931672127966E-28**	0.777195342034931	0.341	0.223	2.28242550407925E-24	Mid-zonal hepatocytes	HCFC1
**1.45334671278389E-28**	0.285390796151828	0.585	0.446	2.37055382322181E-24	Mid-zonal hepatocytes	TRIM26
**1.66497600090522E-28**	1.75538638818216	0.181	0.086	2.7157423550765E-24	Mid-zonal hepatocytes	DEPRECAT
**1.77129713612527E-28**	0.888405562952249	0.408	0.285	2.88916275873393E-24	Mid-zonal hepatocytes	MAP3K13
**1.86518648115897E-28**	0.418868230631028	0.606	0.462	3.04230566941839E-24	Mid-zonal hepatocytes	PECR
**1.92745805353288E-28**	0.755102750291991	0.409	0.284	3.14387683111749E-24	Mid-zonal hepatocytes	SLC30A2
**1.96878068168739E-28**	1.22894490116948	0.288	0.175	3.21127816990029E-24	Mid-zonal hepatocytes	STK3
**1.9735913908345E-28**	1.44592872388731	0.194	0.095	3.21912491759016E-24	Mid-zonal hepatocytes	CORO7
**1.99362390721755E-28**	1.68409354005754	0.223	0.121	3.25179995506255E-24	Mid-zonal hepatocytes	NR2C1
**2.30623985344862E-28**	1.97969780113115	0.139	0.056	3.76170782496004E-24	Mid-zonal hepatocytes	PLEKHH1
**2.33236333425944E-28**	1.39651309544103	0.251	0.145	3.80431783451057E-24	Mid-zonal hepatocytes	DIAPH2
**2.35637169222601E-28**	1.20786270176232	0.408	0.285	3.84347786718984E-24	Mid-zonal hepatocytes	HMOX1
**2.37665362940857E-28**	0.261371463027932	0.663	0.502	3.87655973492832E-24	Mid-zonal hepatocytes	PCYT2
**2.55154634292556E-28**	1.69477414153073	0.195	0.097	4.16182723994588E-24	Mid-zonal hepatocytes	ZNF791
**2.83003526338457E-28**	0.900823007503627	0.384	0.262	4.61607051810658E-24	Mid-zonal hepatocytes	MDM2
**2.93378487001083E-28**	0.35971591542551	0.591	0.451	4.78529650147466E-24	Mid-zonal hepatocytes	DAP3
**3.10786357904192E-28**	1.59668149312528	0.226	0.124	5.06923628377527E-24	Mid-zonal hepatocytes	DMXL1
**3.24634687022291E-28**	0.285198304127631	0.759	0.597	5.29511638002059E-24	Mid-zonal hepatocytes	JUN
**3.38939342070425E-28**	0.55620101033354	0.422	0.305	5.52843960851071E-24	Mid-zonal hepatocytes	MPPE1
**3.4318548137506E-28**	0.748062774669818	0.363	0.245	5.5976983867086E-24	Mid-zonal hepatocytes	PBX2
**3.81159861592165E-28**	0.86465283673608	0.364	0.246	6.2170985024298E-24	Mid-zonal hepatocytes	DDX24
**3.87118145546715E-28**	0.984201441005872	0.261	0.15	6.31428407201247E-24	Mid-zonal hepatocytes	IRGQ
**3.95398964067614E-28**	1.09783735108426	0.265	0.156	6.44935250290686E-24	Mid-zonal hepatocytes	NPAS2
**4.09011144143907E-28**	0.68329829090121	0.436	0.315	6.67138077213127E-24	Mid-zonal hepatocytes	UBA5
**4.09267069511334E-28**	1.14878745214953	0.308	0.195	6.67555517079936E-24	Mid-zonal hepatocytes	LRRFIP2
**4.20895389764715E-28**	1.35078798345545	0.284	0.176	6.86522470245227E-24	Mid-zonal hepatocytes	ACCS
**4.3650475038836E-28**	1.87830768584957	0.153	0.065	7.11982898358454E-24	Mid-zonal hepatocytes	USP45
**4.62580460513708E-28**	0.972599916399712	0.281	0.17	7.54514989143908E-24	Mid-zonal hepatocytes	PLPP5
**4.68920903996109E-28**	1.17679507812647	0.216	0.113	7.64856886508053E-24	Mid-zonal hepatocytes	MED9
**4.7459555093398E-28**	0.35660544429007	0.648	0.498	7.74112803128415E-24	Mid-zonal hepatocytes	JUNB
**5.37945388976904E-28**	0.967088148966435	0.316	0.203	8.77442723960228E-24	Mid-zonal hepatocytes	ZNF281
**5.52901583976753E-28**	1.01340347837755	0.271	0.16	9.01837773624482E-24	Mid-zonal hepatocytes	CASP4
**5.58866556065547E-28**	1.61391524185062	0.182	0.088	9.11567239598514E-24	Mid-zonal hepatocytes	FBF1
**5.68135396258808E-28**	1.4103418778251	0.203	0.104	9.26685644837742E-24	Mid-zonal hepatocytes	MYO5C
**5.93401120242463E-28**	0.519317047836121	0.497	0.367	9.67896567227482E-24	Mid-zonal hepatocytes	SIK2
**6.11134695695205E-28**	0.998403838604893	0.358	0.242	9.9682180214845E-24	Mid-zonal hepatocytes	RIPK1
**6.27828859235483E-28**	1.2612420154304	0.282	0.172	1.024051652299E-23	Mid-zonal hepatocytes	HEXD
**6.71582344354412E-28**	1.53607962770355	0.18	0.085	1.09541796187648E-23	Mid-zonal hepatocytes	RAD9A
**6.73709740625732E-28**	0.80114308830848	0.282	0.17	1.09888795793463E-23	Mid-zonal hepatocytes	DAXX
**6.8515618171933E-28**	1.63680758634832	0.157	0.069	1.1175582480024E-23	Mid-zonal hepatocytes	GKAP1
**7.52284244916203E-28**	0.988805980106514	0.318	0.202	1.22705083188282E-23	Mid-zonal hepatocytes	SH3BP2

7.7010918027688E-28	1.16180366827441	0.241	0.137	1.25612508394962E-23	Mid-zonal hepatocytes	LETMD1
7.8064371171771E-28	1.00233192652141	0.343	0.228	1.27330795818276E-23	Mid-zonal hepatocytes	SPPL2B
7.96538375645593E-28	1.17345064722149	0.262	0.152	1.29923374451553E-23	Mid-zonal hepatocytes	IFNAR2
8.1780404578114E-28	1.57508134615694	0.19	0.094	1.33392017907362E-23	Mid-zonal hepatocytes	PARP6
8.39650392854228E-28	1.80597869284651	0.221	0.12	1.36955375578453E-23	Mid-zonal hepatocytes	NT5DC3
8.47916352085529E-28	0.733039957707086	0.395	0.278	1.38303636188671E-23	Mid-zonal hepatocytes	CARS2
9.45799786955021E-28	1.12634850664882	0.28	0.17	1.54269403250234E-23	Mid-zonal hepatocytes	SMURF1
1.01680921381038E-27	0.643082656504019	0.457	0.333	1.65851750864611E-23	Mid-zonal hepatocytes	PDCD4
1.04050284182483E-27	0.782801700966642	0.375	0.262	1.69716418530048E-23	Mid-zonal hepatocytes	SLC12A8
1.04979344727926E-27	0.853796466643523	0.308	0.196	1.7123180918572E-23	Mid-zonal hepatocytes	WDR11
1.05453507080904E-27	1.19421303054699	0.341	0.227	1.72005215399662E-23	Mid-zonal hepatocytes	TP53BP2
1.22410090238303E-27	1.27412193946013	0.246	0.141	1.99663098187696E-23	Mid-zonal hepatocytes	PCF11
1.24442978634259E-27	1.59685733503945	0.17	0.078	2.0297894245034E-23	Mid-zonal hepatocytes	FIGNL2
1.36317462121515E-27	0.405856861477639	0.436	0.31	2.22347412466404E-23	Mid-zonal hepatocytes	HDHD3
1.37462822510791E-27	0.921574990717393	0.328	0.215	2.24215609797352E-23	Mid-zonal hepatocytes	ZNF516
1.39099897941346E-27	0.947965838224429	0.341	0.23	2.26885843532129E-23	Mid-zonal hepatocytes	DDX3Y
1.43421596726311E-27	1.03974909773043	0.284	0.171	2.33934966420285E-23	Mid-zonal hepatocytes	PAG1
1.45491633638635E-27	0.399415014284865	0.91	0.806	2.37311403627978E-23	Mid-zonal hepatocytes	AGXT
1.47209262737776E-27	1.55992636346353	0.184	0.09	2.40113028451586E-23	Mid-zonal hepatocytes	MAP3K4
1.48484615965304E-27	0.573851106646242	0.449	0.328	2.42193257101008E-23	Mid-zonal hepatocytes	UBC
1.53235871040931E-27	0.534891098875496	0.484	0.363	2.49943029254863E-23	Mid-zonal hepatocytes	HSPE1
1.56234786939864E-27	0.935032169543661	0.279	0.169	2.54834560977612E-23	Mid-zonal hepatocytes	TEAD2
1.57142175043367E-27	0.419400612748332	0.476	0.35	2.56314601713236E-23	Mid-zonal hepatocytes	ATF6B
1.62165104035165E-27	2.0522475628095	0.147	0.062	2.64507501191757E-23	Mid-zonal hepatocytes	MXD3
1.63948069710535E-27	0.385941234858362	0.582	0.443	2.67415696504854E-23	Mid-zonal hepatocytes	ILRUN
1.66826932207088E-27	0.939430233493448	0.358	0.242	2.72111409122981E-23	Mid-zonal hepatocytes	PPFIBP2
1.74064191922883E-27	0.533083313790389	0.421	0.301	2.83916103445414E-23	Mid-zonal hepatocytes	FBXL6
1.94518885955371E-27	1.51255482048711	0.217	0.117	3.17279754881806E-23	Mid-zonal hepatocytes	SIPA1L1
1.98839687235825E-27	0.834025842484187	0.315	0.203	3.24327413850355E-23	Mid-zonal hepatocytes	SEC24A
2.07006549243564E-27	0.869655237160209	0.323	0.208	3.37648382471177E-23	Mid-zonal hepatocytes	MIB2
2.16794689142336E-27	1.59207605750724	0.185	0.091	3.53613817460064E-23	Mid-zonal hepatocytes	POLI
2.20799242253458E-27	1.3722395277484	0.167	0.077	3.60145644039615E-23	Mid-zonal hepatocytes	MTHFD1L
2.32399941297778E-27	0.903698831163836	0.352	0.238	3.79067544250806E-23	Mid-zonal hepatocytes	DEPRECAT
2.52587544421409E-27	0.971928129675129	0.327	0.212	4.1199554370576E-23	Mid-zonal hepatocytes	TRIB1
2.56965419416718E-27	0.949325513285074	0.37	0.259	4.19136295610608E-23	Mid-zonal hepatocytes	ELOVL5
2.6745682421512E-27	1.10869784159767	0.275	0.166	4.36248825977282E-23	Mid-zonal hepatocytes	INPP1
2.83104677672112E-27	1.10707916190985	0.293	0.184	4.61772039750983E-23	Mid-zonal hepatocytes	SPECC1L
3.21530233848814E-27	0.522599142524264	0.517	0.386	5.24447964430801E-23	Mid-zonal hepatocytes	C1RL
3.34614931960219E-27	1.1455004182507	0.31	0.195	5.45790415520314E-23	Mid-zonal hepatocytes	NID1
3.4587212053738E-27	1.30100082414431	0.234	0.131	5.64152015808521E-23	Mid-zonal hepatocytes	CDK5RAP2
3.48331618235789E-27	1.94872133185883	0.159	0.071	5.68163702504396E-23	Mid-zonal hepatocytes	ME3
3.5033851479932E-27	0.837455481852301	0.315	0.203	5.71437151489171E-23	Mid-zonal hepatocytes	HNRNPDL
3.5372562134634E-27	1.39525489647658	0.235	0.133	5.76961860978015E-23	Mid-zonal hepatocytes	PKN2
3.58408524380133E-27	1.84615303178291	0.156	0.07	5.84600144116435E-23	Mid-zonal hepatocytes	CCDC7
3.70102151383808E-27	0.328118618216497	0.597	0.451	6.0367361912213E-23	Mid-zonal hepatocytes	CAPN2
3.79236099608005E-27	1.42889313892821	0.196	0.098	6.18572002070617E-23	Mid-zonal hepatocytes	DIXDC1
3.89109724210805E-27	1.42595362646265	0.265	0.159	6.34676871160244E-23	Mid-zonal hepatocytes	ANKRD13A
4.01473530978309E-27	1.4031213458936	0.201	0.105	6.54843476378719E-23	Mid-zonal hepatocytes	CAD
4.11286355609332E-27	1.57807132118228	0.223	0.123	6.70849174634381E-23	Mid-zonal hepatocytes	ABI1
4.25604013518767E-27	1.25600784726699	0.245	0.14	6.9420270645046E-23	Mid-zonal hepatocytes	ELL
4.4961325902416E-27	0.338899729141167	0.604	0.465	7.33364186794308E-23	Mid-zonal hepatocytes	COPA
4.75620299718658E-27	0.684272575989304	0.485	0.366	7.75784270871103E-23	Mid-zonal hepatocytes	AIG1
4.78673453644454E-27	0.625956226258179	0.433	0.317	7.80764270239469E-23	Mid-zonal hepatocytes	PPP2R5A
5.07738569262126E-27	0.722180146733529	0.306	0.194	8.28172380323453E-23	Mid-zonal hepatocytes	YKT6
5.40060325693125E-27	0.444750612481985	0.451	0.332	8.80892397238056E-23	Mid-zonal hepatocytes	ZC3H15
5.90538376906289E-27	0.386414896620051	0.505	0.372	9.63227146571848E-23	Mid-zonal hepatocytes	TRIP6
5.98311298082474E-27	1.31574682433532	0.274	0.167	9.75905558302323E-23	Mid-zonal hepatocytes	DEDD2
5.99472167895262E-27	0.588820677957476	0.45	0.334	9.77799053053962E-23	Mid-zonal hepatocytes	DVL3
7.41052970142588E-27	1.17627173027353	0.173	0.081	1.20873149959958E-22	Mid-zonal hepatocytes	MAP3K3
7.67133576735314E-27	0.678195172172973	0.448	0.329	1.25127157701297E-22	Mid-zonal hepatocytes	SEMA4G

**7.82349371239288E-27**	1.16007221048625	0.273	0.167	1.2760900594284E-22	Mid-zonal hepatocytes	PANK4
**8.00735039647428E-27**	1.56497903188858	0.219	0.119	1.30607892316892E-22	Mid-zonal hepatocytes	SAMD12
**8.0142825549838E-27**	0.298336917260109	0.701	0.545	1.30720962754341E-22	Mid-zonal hepatocytes	GOLPH3
**8.21185830338791E-27**	1.09989783337292	0.315	0.207	1.3394362078656E-22	Mid-zonal hepatocytes	YTHDC2
**8.52074869841657E-27**	1.0224130217276	0.253	0.147	1.38981932019873E-22	Mid-zonal hepatocytes	RUFY1
**8.6237968787522E-27**	1.92716144815666	0.132	0.052	1.40662750889327E-22	Mid-zonal hepatocytes	SCARF1
**9.30019328453968E-27**	0.853698342342917	0.347	0.237	1.51695452664127E-22	Mid-zonal hepatocytes	NIPBL
**9.52243579614689E-27**	1.41292930490118	0.243	0.14	1.55320450270952E-22	Mid-zonal hepatocytes	ZNF839
**9.67183839061768E-27**	0.525351164450928	0.408	0.29	1.57757355989365E-22	Mid-zonal hepatocytes	RNF103
**1.01570770619917E-26**	0.796242876128228	0.359	0.249	1.65672083958147E-22	Mid-zonal hepatocytes	HNRNPU
**1.02707505319952E-26**	0.693183481566493	0.353	0.239	1.67526211927373E-22	Mid-zonal hepatocytes	OTUD5
**1.06553745395645E-26**	1.29023046203198	0.226	0.125	1.73799814114836E-22	Mid-zonal hepatocytes	USP33
**1.07710843596169E-26**	1.33409447100942	0.232	0.131	1.75687156989712E-22	Mid-zonal hepatocytes	FBXO42
**1.11177341008797E-26**	1.22559317559581	0.228	0.127	1.81341360919449E-22	Mid-zonal hepatocytes	ZNF292
**1.13515156581529E-26**	1.59422332675087	0.223	0.124	1.85154571900132E-22	Mid-zonal hepatocytes	TDRD10
**1.1631478153549E-26**	0.895033248371863	0.343	0.233	1.89721040162538E-22	Mid-zonal hepatocytes	ELAC2
**1.20139670728526E-26**	0.738939912410841	0.362	0.251	1.95959816925298E-22	Mid-zonal hepatocytes	USP14
**1.20621093607843E-26**	0.629778447748797	0.44	0.324	1.96745065783753E-22	Mid-zonal hepatocytes	VPS52
**1.22339604167684E-26**	0.46350706985299	0.508	0.385	1.99548128357909E-22	Mid-zonal hepatocytes	PDXK
**1.26617080879859E-26**	0.921131392024409	0.303	0.192	2.06525120623138E-22	Mid-zonal hepatocytes	HDAC6
**1.2952835905272E-26**	1.30672367556914	0.259	0.154	2.11273706450891E-22	Mid-zonal hepatocytes	MORC3
**1.31089599452154E-26**	1.12964250932312	0.256	0.15	2.13820245666409E-22	Mid-zonal hepatocytes	NCBP3
**1.4133096792692E-26**	0.946109988856538	0.191	0.094	2.30524941785599E-22	Mid-zonal hepatocytes	KCTD15
**1.43589710030935E-26**	1.35794565728999	0.29	0.185	2.34209176031458E-22	Mid-zonal hepatocytes	OARD1
**1.4554022130136E-26**	1.86874045211849	0.117	0.043	2.37390654964648E-22	Mid-zonal hepatocytes	NALCN
**1.49818506663026E-26**	1.15416417141946	0.229	0.127	2.44368966218061E-22	Mid-zonal hepatocytes	CDYL
**1.52048218838152E-26**	0.331568649008979	0.508	0.388	2.4800584974691E-22	Mid-zonal hepatocytes	ZYX
**1.52917534704136E-26**	0.432937976532831	0.522	0.395	2.49423790855916E-22	Mid-zonal hepatocytes	FOXP4
**1.56858431386242E-26**	1.27499968706296	0.268	0.163	2.55851787434099E-22	Mid-zonal hepatocytes	NBPF26
**1.66203371731274E-26**	0.64369513840238	0.39	0.273	2.7109431963088E-22	Mid-zonal hepatocytes	ACTR1B
**1.69198090464641E-26**	0.627754143566338	0.389	0.273	2.75979005356877E-22	Mid-zonal hepatocytes	TBC1D17
**1.76961031275624E-26**	0.926388913849036	0.29	0.183	2.88641138113671E-22	Mid-zonal hepatocytes	AKAP8
**1.773918885993E-26**	2.35283709446695	0.127	0.05	2.89343909494318E-22	Mid-zonal hepatocytes	SPATA6L
**1.78191795263564E-26**	0.727955113781477	0.388	0.276	2.90648637254399E-22	Mid-zonal hepatocytes	BRPF3
**1.78524283820731E-26**	0.297277225798578	0.789	0.612	2.91190959339994E-22	Mid-zonal hepatocytes	F11R
**1.85143603825693E-26**	0.96448988160154	0.269	0.162	3.01987732200088E-22	Mid-zonal hepatocytes	ZDHHC14
**1.94409222397083E-26**	1.84342155399419	0.154	0.069	3.17100882651881E-22	Mid-zonal hepatocytes	REL
**1.96017779113859E-26**	0.854536833975702	0.297	0.186	3.19724599512616E-22	Mid-zonal hepatocytes	AEN
**1.96497216197512E-26**	1.66663027320819	0.181	0.089	3.20506609339761E-22	Mid-zonal hepatocytes	POU2F1
**2.04029099865465E-26**	1.62795033986881	0.172	0.082	3.32791864790559E-22	Mid-zonal hepatocytes	UPF2
**2.14043347037244E-26**	2.09269989370743	0.146	0.063	3.49126103352448E-22	Mid-zonal hepatocytes	SEC31B
**2.21561796173654E-26**	1.08177269150023	0.296	0.188	3.61389445738847E-22	Mid-zonal hepatocytes	TAPT1
**2.3754459049392E-26**	2.05867828717666	0.184	0.092	3.87458981554632E-22	Mid-zonal hepatocytes	ZNF618
**2.64034249808262E-26**	0.884369651140001	0.299	0.189	4.30666264862256E-22	Mid-zonal hepatocytes	RNF31
**2.81488881809592E-26**	1.37274162606603	0.26	0.157	4.59136515119625E-22	Mid-zonal hepatocytes	ZFYVE9
**2.85923653589009E-26**	1.30491196898878	0.237	0.136	4.66370071369033E-22	Mid-zonal hepatocytes	MAP3K7
**2.86531070718638E-26**	1.11696110010105	0.27	0.164	4.6736082944917E-22	Mid-zonal hepatocytes	NISCH
**2.91672344216404E-26**	1.74224549008058	0.161	0.074	4.75746760651377E-22	Mid-zonal hepatocytes	FOXP2
**3.19489737760932E-26**	0.794246070311987	0.385	0.273	5.21119711261856E-22	Mid-zonal hepatocytes	TNS2
**3.25551483515949E-26**	1.24591328747265	0.257	0.152	5.31007024762864E-22	Mid-zonal hepatocytes	YAF2
**3.34371436229848E-26**	1.58202181456371	0.218	0.12	5.45393249634505E-22	Mid-zonal hepatocytes	IFT88
**3.4334007789349E-26**	1.49386079884427	0.225	0.127	5.60022001052071E-22	Mid-zonal hepatocytes	KRIT1
**3.60814645821006E-26**	1.22066362689324	0.294	0.191	5.88524768798643E-22	Mid-zonal hepatocytes	ARFGAP2
**3.71789355511979E-26**	0.306816637205381	0.61	0.465	6.06425617775589E-22	Mid-zonal hepatocytes	COPG1
**3.75297044387255E-26**	0.992441157222885	0.493	0.368	6.12147009100051E-22	Mid-zonal hepatocytes	P4HA1
**4.12018813546345E-26**	0.68445128391032	0.343	0.23	6.72043886775443E-22	Mid-zonal hepatocytes	FAAH
**4.15454165428267E-26**	1.35761887808638	0.199	0.104	6.77647289230047E-22	Mid-zonal hepatocytes	CMTM4
**4.36978182545097E-26**	0.944841301698091	0.206	0.107	7.12755113549307E-22	Mid-zonal hepatocytes	TTC7B
**4.45987511338754E-26**	0.699157042899618	0.287	0.179	7.27450229744642E-22	Mid-zonal hepatocytes	BAZ1B
**4.50836315495403E-26**	0.40603245108992	0.513	0.393	7.35359114204551E-22	Mid-zonal hepatocytes	DNM2

4.80881551440684E-26	0.420637694389498	0.452	0.331	7.84365898554899E-22	Mid-zonal hepatocytes	ZCCHC24
4.83212777949504E-26	1.48512069317255	0.231	0.131	7.88168362113436E-22	Mid-zonal hepatocytes	SMNDC1
4.92108543520138E-26	0.40145419134429	0.416	0.299	8.02678245335698E-22	Mid-zonal hepatocytes	LMF2
5.2918582141913E-26	1.68319012887639	0.189	0.097	8.63154993316742E-22	Mid-zonal hepatocytes	SZT2
5.69179122699342E-26	0.487953982507409	0.409	0.29	9.28388067034897E-22	Mid-zonal hepatocytes	CCDC9
5.8011354804349E-26	1.62664127555417	0.2	0.106	9.46223208213737E-22	Mid-zonal hepatocytes	THOC2
5.80416675598489E-26	0.854931097029584	0.371	0.248	9.46717639568695E-22	Mid-zonal hepatocytes	IGFBP3
6.00553694536011E-26	0.784132232465756	0.288	0.179	9.79563131157687E-22	Mid-zonal hepatocytes	GOLGA2
6.03484259681348E-26	0.973018317054878	0.288	0.181	9.84343175966247E-22	Mid-zonal hepatocytes	HMGN4
6.1649460557005E-26	0.638543408404682	0.222	0.12	1.00556435114531E-21	Mid-zonal hepatocytes	FBLIM1
6.35800422015606E-26	1.95105196775797	0.165	0.078	1.03705406834966E-21	Mid-zonal hepatocytes	NSD2
6.47033678448015E-26	1.88797496459592	0.119	0.045	1.05537663291656E-21	Mid-zonal hepatocytes	NUAK2
6.51439200712877E-26	1.38388086277465	0.173	0.084	1.06256248028277E-21	Mid-zonal hepatocytes	ADAMTSL2
6.90087086130427E-26	1.18831940432673	0.234	0.134	1.12560104618734E-21	Mid-zonal hepatocytes	POLK
6.96806033878878E-26	1.7651732250518	0.155	0.07	1.13656032185984E-21	Mid-zonal hepatocytes	PHF21A
7.58830257164139E-26	2.12300538646501	0.129	0.052	1.23772803246043E-21	Mid-zonal hepatocytes	AP002495.
7.59843228078994E-26	1.25830801152747	0.265	0.163	1.23938028931965E-21	Mid-zonal hepatocytes	KIDINS220
7.87043705778472E-26	1.00873064574313	0.29	0.182	1.28374698849527E-21	Mid-zonal hepatocytes	ZRANB1
8.05194320887476E-26	1.00631547479499	0.276	0.168	1.31335245679956E-21	Mid-zonal hepatocytes	AGO2
8.5162765551498E-26	1.22448204153851	0.244	0.143	1.38908986891048E-21	Mid-zonal hepatocytes	FBXL12
8.56200904192798E-26	0.95481501952431	0.322	0.213	1.39654929482887E-21	Mid-zonal hepatocytes	SDR39U1
8.70667405240222E-26	1.52220408645527	0.197	0.104	1.42014560468733E-21	Mid-zonal hepatocytes	COX10
8.8021276210471E-26	1.53720990909502	0.229	0.131	1.43571503626899E-21	Mid-zonal hepatocytes	TNKS2
8.8027226352114E-26	1.3974404524956	0.218	0.121	1.43581208902933E-21	Mid-zonal hepatocytes	RBM4
9.1393856525998E-26	0.725095123598376	0.301	0.19	1.49072519379555E-21	Mid-zonal hepatocytes	SRF
9.140732679613E-26	1.01477016956843	0.318	0.211	1.49094490737168E-21	Mid-zonal hepatocytes	KDM4B
9.1848127212714E-26	1.48606222387963	0.267	0.164	1.49813480296658E-21	Mid-zonal hepatocytes	ATP9B
1.01781886809453E-25	1.35001321774968	0.246	0.145	1.66016435574899E-21	Mid-zonal hepatocytes	ZMYND8
1.02263478455692E-25	1.25430208743267	0.263	0.16	1.66801959709079E-21	Mid-zonal hepatocytes	GPBP1
1.05728823023349E-25	1.31950242636452	0.255	0.154	1.72454283233384E-21	Mid-zonal hepatocytes	HUS1
1.06590740725611E-25	1.53330869199142	0.192	0.099	1.73860157197544E-21	Mid-zonal hepatocytes	NR2C2
1.07826720573166E-25	1.30514010674682	0.242	0.141	1.75876163926891E-21	Mid-zonal hepatocytes	ZFYVE16
1.07846328570238E-25	1.50203757801745	0.18	0.09	1.75908146530915E-21	Mid-zonal hepatocytes	TRAF5
1.16711808632393E-25	1.08591234749351	0.225	0.124	1.90368631060297E-21	Mid-zonal hepatocytes	ANKRD13D
1.18704683336563E-25	0.540871866069299	0.387	0.27	1.93619208990268E-21	Mid-zonal hepatocytes	MED25
1.2228700532445E-25	1.69295262907114	0.173	0.084	1.9946233438471E-21	Mid-zonal hepatocytes	CCDC198
1.28064886929184E-25	1.95612626948515	0.138	0.059	2.08886637070193E-21	Mid-zonal hepatocytes	ANKS1B
1.41228227643556E-25	0.773690842217487	0.35	0.24	2.30357362109405E-21	Mid-zonal hepatocytes	TRIM41
1.43306336724085E-25	0.875874133116442	0.374	0.269	2.33746965830655E-21	Mid-zonal hepatocytes	TTC3
1.4372944592489E-25	1.39146203288221	0.215	0.118	2.34437099248087E-21	Mid-zonal hepatocytes	ZCCHC7
1.46475534222018E-25	0.834020060560085	0.265	0.159	2.38916243869534E-21	Mid-zonal hepatocytes	TRIM47
1.47218465458198E-25	1.57843300250691	0.182	0.092	2.40128039008866E-21	Mid-zonal hepatocytes	PIAS3
1.47574513701383E-25	1.79621768159879	0.169	0.082	2.40708789298325E-21	Mid-zonal hepatocytes	B3GALNT2
1.50931264692314E-25	1.46353737249506	0.163	0.077	2.46183985839633E-21	Mid-zonal hepatocytes	GRB7
1.52052353273054E-25	0.524011301304079	0.41	0.296	2.48012593423679E-21	Mid-zonal hepatocytes	CAST
1.52270038615588E-25	0.937064458608287	0.274	0.167	2.48367659985886E-21	Mid-zonal hepatocytes	PHKB
1.62244685190325E-25	1.55119089452964	0.184	0.093	2.6463730601394E-21	Mid-zonal hepatocytes	SYTL4
1.6420195677029E-25	0.537491022634702	0.43	0.316	2.6782981168802E-21	Mid-zonal hepatocytes	PYGB
1.75080542231179E-25	0.449820045976912	0.515	0.392	2.85573872433276E-21	Mid-zonal hepatocytes	PLA1A
1.82932438420612E-25	1.14690634660153	0.291	0.187	2.9838110030786E-21	Mid-zonal hepatocytes	RPRD2
1.87793868738344E-25	0.386127232912507	0.43	0.31	3.06310579299113E-21	Mid-zonal hepatocytes	SLC3A2
1.9674092571196E-25	1.13607161512452	0.256	0.152	3.20904123928778E-21	Mid-zonal hepatocytes	CRACR2B
1.98099810903884E-25	1.2042125286049	0.232	0.133	3.23120601565325E-21	Mid-zonal hepatocytes	DCBLD1
2.01098517847661E-25	1.45839833661022	0.194	0.102	3.2801179246132E-21	Mid-zonal hepatocytes	C9orf72
2.09922434857455E-25	1.51209323218421	0.226	0.13	3.42404483495994E-21	Mid-zonal hepatocytes	ZNF26
2.14328122900166E-25	0.983506424695525	0.263	0.158	3.49590601262462E-21	Mid-zonal hepatocytes	VHL
2.1790149666995E-25	1.24101716880375	0.254	0.153	3.55419131218355E-21	Mid-zonal hepatocytes	PCNX1
2.3530396753763E-25	0.559117880425246	0.523	0.399	3.83804301450629E-21	Mid-zonal hepatocytes	HSPD1
2.35330374423154E-25	1.03235454272085	0.356	0.244	3.83847373721606E-21	Mid-zonal hepatocytes	KYNU
2.42602756067914E-25	1.2061254689698	0.274	0.171	3.95709355422375E-21	Mid-zonal hepatocytes	ZNF76

**2.6224206326285E-25**	0.957654971070883	0.299	0.193	4.27743029388035E-21	Mid-zonal hepatocytes	TBC1D14
**2.7346012690741E-25**	1.43557296234677	0.188	0.096	4.46040812998677E-21	Mid-zonal hepatocytes	ADPGK
**2.80360276785557E-25**	1.72039069924047	0.17	0.083	4.57295647464922E-21	Mid-zonal hepatocytes	MAST2
**3.05250837780934E-25**	1.48999039995504	0.222	0.125	4.97894641504481E-21	Mid-zonal hepatocytes	PNPLA7
**3.1160126135455E-25**	1.31213125042809	0.197	0.104	5.08252817395407E-21	Mid-zonal hepatocytes	PRDM2
**3.16749174021887E-25**	2.34050754147229	0.11	0.041	5.16649577747099E-21	Mid-zonal hepatocytes	SLAMF9
**3.27771547130695E-25**	1.09086004685933	0.355	0.248	5.34628170524877E-21	Mid-zonal hepatocytes	KIAA0319L
**3.2923779436947E-25**	1.60597691442123	0.204	0.109	5.37019766396043E-21	Mid-zonal hepatocytes	EPG5
**3.72973169249672E-25**	1.47964685455625	0.181	0.091	6.0835653636314E-21	Mid-zonal hepatocytes	FRYL
**3.74739139994009E-25**	0.950584759029207	0.256	0.153	6.11237011244228E-21	Mid-zonal hepatocytes	PPP1R13B
**3.75947764777452E-25**	1.45409383954852	0.153	0.07	6.13208399128503E-21	Mid-zonal hepatocytes	CLCF1
**3.78630759429455E-25**	1.91539531779385	0.144	0.063	6.17584631705384E-21	Mid-zonal hepatocytes	LRSAM1
**3.78784539360505E-25**	0.750169521558346	0.357	0.245	6.17835462150919E-21	Mid-zonal hepatocytes	FBXO31
**3.85065103751111E-25**	0.56259240754717	0.4	0.291	6.28079690728437E-21	Mid-zonal hepatocytes	HMGN1
**3.90411131176456E-25**	1.90940912849603	0.138	0.059	6.36799596061917E-21	Mid-zonal hepatocytes	HOXC6
**4.326716195909E-25**	1.00003086222062	0.309	0.204	7.05730678714716E-21	Mid-zonal hepatocytes	HERPUD2
**4.43116277279533E-25**	0.717216597556451	0.355	0.245	7.22766959870646E-21	Mid-zonal hepatocytes	RFX5
**4.7466171860144E-25**	1.13862584472711	0.286	0.182	7.7422072921081E-21	Mid-zonal hepatocytes	PHF12
**4.77474894525504E-25**	0.753842856059918	0.325	0.218	7.78809300460549E-21	Mid-zonal hepatocytes	NOL3
**4.87645090539661E-25**	1.01676371976948	0.292	0.188	7.95397907179241E-21	Mid-zonal hepatocytes	CPOX
**5.11083270158637E-25**	1.10250586276917	0.247	0.145	8.33627921955752E-21	Mid-zonal hepatocytes	HES4
**5.19816911122244E-25**	0.863192431478192	0.335	0.23	8.47873363731492E-21	Mid-zonal hepatocytes	PTS
**5.28417797606272E-25**	0.691791239576221	0.353	0.246	8.6190226967559E-21	Mid-zonal hepatocytes	TIAL1
**5.50214371123332E-25**	1.19742526564807	0.277	0.173	8.97454660739268E-21	Mid-zonal hepatocytes	ACP6
**5.54189997691016E-25**	0.825955235891644	0.295	0.187	9.03939305233816E-21	Mid-zonal hepatocytes	JMY
**5.72662404137946E-25**	1.15190877680529	0.299	0.196	9.34069647389404E-21	Mid-zonal hepatocytes	RNASEH1
**5.7698026010305E-25**	1.25641471549422	0.196	0.103	9.41112502254085E-21	Mid-zonal hepatocytes	ENTPD4
**5.78365076471277E-25**	1.83317429275579	0.163	0.077	9.433712762323E-21	Mid-zonal hepatocytes	KCTD9
**5.89383825570445E-25**	0.631661281778539	0.455	0.342	9.61343957887953E-21	Mid-zonal hepatocytes	PGPEP1
**6.19870914384517E-25**	0.467225825290799	0.368	0.254	1.01107144845259E-20	Mid-zonal hepatocytes	ZNF687
**6.4550193247845E-25**	0.899892115341347	0.29	0.185	1.0528782020656E-20	Mid-zonal hepatocytes	E4F1
**6.53759902453115E-25**	0.997813137600786	0.354	0.248	1.06634777689128E-20	Mid-zonal hepatocytes	CEP70
**6.82237928343096E-25**	0.599084253746139	0.386	0.276	1.11279828492042E-20	Mid-zonal hepatocytes	TBCD
**6.98221444895823E-25**	1.12473834350196	0.296	0.192	1.13886899876958E-20	Mid-zonal hepatocytes	PHF20L1
**7.24700093425337E-25**	2.05419168223299	0.139	0.06	1.18205832238607E-20	Mid-zonal hepatocytes	DCAF4
**7.29940784832787E-25**	1.19903811290376	0.254	0.15	1.19060641414076E-20	Mid-zonal hepatocytes	LDLR
**7.57997364307992E-25**	0.532415159254861	0.338	0.223	1.23636950092277E-20	Mid-zonal hepatocytes	PLPP1
**7.82187618644032E-25**	0.530142016567372	0.44	0.325	1.27582622477028E-20	Mid-zonal hepatocytes	HMGCS1
**8.01627797775395E-25**	1.16274254493225	0.266	0.165	1.30753510095145E-20	Mid-zonal hepatocytes	RTCA
**8.03218851084642E-25**	1.66425007909995	0.169	0.083	1.31013026800416E-20	Mid-zonal hepatocytes	SLC22A5
**8.86863973234854E-25**	0.357668135429999	0.707	0.533	1.44656382674337E-20	Mid-zonal hepatocytes	ALDH3A2
**8.93331236464662E-25**	1.08219607660317	0.291	0.188	1.45711257979751E-20	Mid-zonal hepatocytes	FAF1
**9.29460067608754E-25**	1.09755426615828	0.226	0.128	1.51604231627664E-20	Mid-zonal hepatocytes	STK4
**9.3593894449909E-25**	1.63119418060901	0.153	0.07	1.52661001237247E-20	Mid-zonal hepatocytes	ZNF512
**9.50343520091998E-25**	1.87411440535614	0.184	0.095	1.55010531562206E-20	Mid-zonal hepatocytes	LTN1
**9.63724569255566E-25**	1.37929179004122	0.23	0.133	1.57193114491275E-20	Mid-zonal hepatocytes	TANC1
**9.72277604075897E-25**	0.967419153542997	0.298	0.193	1.5858820000082E-20	Mid-zonal hepatocytes	NPL
**9.85133963104392E-25**	1.21633386473836	0.206	0.11	1.60685200721957E-20	Mid-zonal hepatocytes	KCTD13
**9.91612067731776E-25**	1.80999215583466	0.197	0.106	1.6174184436773E-20	Mid-zonal hepatocytes	GPATCH2
**1.03697446722976E-24**	0.917053104766547	0.297	0.193	1.69140905349846E-20	Mid-zonal hepatocytes	YAP1
**1.05400264367314E-24**	1.1876796385303	0.279	0.177	1.71918371209526E-20	Mid-zonal hepatocytes	MTFR1L
**1.06668486320425E-24**	1.47577490137563	0.222	0.126	1.73986968037245E-20	Mid-zonal hepatocytes	NEIL1
**1.11318318086519E-24**	1.2211510938593	0.199	0.107	1.81571308630922E-20	Mid-zonal hepatocytes	RUSC2
**1.14790432506322E-24**	1.30118410910727	0.217	0.122	1.87234674461062E-20	Mid-zonal hepatocytes	PDCD11
**1.15178654473243E-24**	1.07087775583307	0.211	0.115	1.87867903311307E-20	Mid-zonal hepatocytes	PKDCC
**1.15918969240781E-24**	1.36951322891876	0.185	0.094	1.89075430728637E-20	Mid-zonal hepatocytes	PMS1
**1.17439330186639E-24**	1.10658589105549	0.261	0.161	1.91555291467427E-20	Mid-zonal hepatocytes	L3HYPDH
**1.18264461160729E-24**	0.341489440223376	0.623	0.49	1.92901162599265E-20	Mid-zonal hepatocytes	OPTN
**1.22105269608185E-24**	0.728786529625268	0.325	0.216	1.99165905257911E-20	Mid-zonal hepatocytes	TTYH3
**1.23291719939425E-24**	1.31092586482329	0.192	0.101	2.01101124393197E-20	Mid-zonal hepatocytes	RCCD1

1.25496883644663E-24	1.54265609825037	0.17	0.083	2.0469796691281E-20	Mid-zonal hepatocytes	MAP3K21
1.27716937699317E-24	0.856429347884089	0.364	0.257	2.08319097081356E-20	Mid-zonal hepatocytes	ENPP1
1.28251936926352E-24	1.53783215564152	0.201	0.108	2.09191734320572E-20	Mid-zonal hepatocytes	TAF1C
1.32331666966266E-24	0.799246319388441	0.37	0.264	2.15846181988677E-20	Mid-zonal hepatocytes	XYLB
1.33483944423066E-24	1.52946895619912	0.195	0.105	2.17725661748463E-20	Mid-zonal hepatocytes	MARF1
1.37460562802707E-24	1.19342258124955	0.219	0.123	2.24211923987496E-20	Mid-zonal hepatocytes	CADPS2
1.46675888334007E-24	0.96299085798453	0.271	0.168	2.39243041461599E-20	Mid-zonal hepatocytes	USP4
1.50672971050365E-24	1.2616806154473	0.251	0.153	2.4576268308025E-20	Mid-zonal hepatocytes	DPH6
1.68389520619888E-24	1.32414753759088	0.222	0.126	2.746601470831E-20	Mid-zonal hepatocytes	HMGXB4
1.74382416168288E-24	1.14992649373007	0.182	0.092	2.84435159012094E-20	Mid-zonal hepatocytes	WHAMM
1.79310036909355E-24	1.03837654569786	0.241	0.141	2.9247260120285E-20	Mid-zonal hepatocytes	EVI5
1.79633370756377E-24	0.996854757575447	0.281	0.179	2.92999991040727E-20	Mid-zonal hepatocytes	OSBPL2
1.84407112096719E-24	0.673608320921613	0.316	0.207	3.00786440540958E-20	Mid-zonal hepatocytes	TESK1
1.87126251283875E-24	1.14653130377994	0.249	0.15	3.05221628469129E-20	Mid-zonal hepatocytes	CHD9
2.05738579310043E-24	1.63541827164474	0.201	0.109	3.35580196712611E-20	Mid-zonal hepatocytes	POLG2
2.27001846521463E-24	1.2879744455316	0.198	0.106	3.70262711861158E-20	Mid-zonal hepatocytes	AXIN1
2.27445127140539E-24	1.28816752545222	0.208	0.114	3.70985746878933E-20	Mid-zonal hepatocytes	TENT5A
2.3380900775657E-24	0.89495170522486	0.268	0.165	3.81365872551742E-20	Mid-zonal hepatocytes	CGGBP1
2.35341553616055E-24	0.941061862688123	0.283	0.18	3.83865608103147E-20	Mid-zonal hepatocytes	MYRF
2.43084717589734E-24	1.08614811158164	0.258	0.159	3.96495482860615E-20	Mid-zonal hepatocytes	ATF2
2.66215595266234E-24	1.59666245259938	0.153	0.071	4.34224257438754E-20	Mid-zonal hepatocytes	PLCG1
2.78219415870576E-24	1.62181801238026	0.175	0.088	4.53803689226497E-20	Mid-zonal hepatocytes	SETD4
2.97807103363373E-24	0.355888463993794	0.439	0.321	4.85753166295997E-20	Mid-zonal hepatocytes	SKI
3.01961772460633E-24	1.50312763611521	0.188	0.098	4.92529847060539E-20	Mid-zonal hepatocytes	EXOSC8
3.02784075721073E-24	0.478914382167386	0.502	0.384	4.93871105908642E-20	Mid-zonal hepatocytes	NNT
3.09185813973539E-24	0.882576509165872	0.343	0.239	5.0431298117224E-20	Mid-zonal hepatocytes	LRRC75B
3.09696243685763E-24	1.52443904672791	0.162	0.078	5.05145543075848E-20	Mid-zonal hepatocytes	DUSP12
3.27338997662061E-24	0.551948378247341	0.349	0.237	5.33922639086588E-20	Mid-zonal hepatocytes	ZMIZ1
3.27759015795972E-24	1.65798914937015	0.197	0.105	5.3460773066481E-20	Mid-zonal hepatocytes	TMCO6
3.33479391551071E-24	1.24632297611249	0.257	0.159	5.43938235558951E-20	Mid-zonal hepatocytes	RICTOR
3.60619310154114E-24	1.33730971727437	0.203	0.112	5.88206156792375E-20	Mid-zonal hepatocytes	ZNF263
3.71432532935172E-24	1.40109030412012	0.211	0.118	6.05843604470559E-20	Mid-zonal hepatocytes	IFITM10
3.73125626627112E-24	1.46845735566422	0.191	0.101	6.08605209591482E-20	Mid-zonal hepatocytes	MTF2
3.8478531372643E-24	0.672427634289614	0.341	0.232	6.27623325219181E-20	Mid-zonal hepatocytes	MBTPS1
3.92312259272139E-24	1.03303685623343	0.247	0.147	6.39900526098786E-20	Mid-zonal hepatocytes	CSRNP1
4.15766004537578E-24	0.829256305449661	0.332	0.227	6.78155930001244E-20	Mid-zonal hepatocytes	TJP3
4.33136858816335E-24	0.76921948297414	0.343	0.239	7.06489530415324E-20	Mid-zonal hepatocytes	ACBD3
4.60463521388762E-24	1.0592889375861	0.272	0.171	7.5106204973721E-20	Mid-zonal hepatocytes	RGS12
4.72695823561927E-24	0.998874865699795	0.297	0.194	7.71014157811859E-20	Mid-zonal hepatocytes	ALCAM
4.94756081733131E-24	0.587527675633538	0.383	0.274	8.0699664491491E-20	Mid-zonal hepatocytes	NAA60
5.39591890280723E-24	0.340088213395275	0.484	0.366	8.80128332236887E-20	Mid-zonal hepatocytes	PARP10
5.45692488730581E-24	1.24302986606232	0.216	0.121	8.90079018368451E-20	Mid-zonal hepatocytes	C6orf136
5.55582874531099E-24	0.384087934601487	0.54	0.418	9.06211226647675E-20	Mid-zonal hepatocytes	SCCPDH
5.63114043706075E-24	1.20607836754119	0.225	0.129	9.18495316688979E-20	Mid-zonal hepatocytes	ZNF121
5.68256363805143E-24	1.43887797973507	0.173	0.087	9.26882955002569E-20	Mid-zonal hepatocytes	CWF19L2
5.87204105162295E-24	1.22466098281135	0.178	0.09	9.57788615930219E-20	Mid-zonal hepatocytes	PXK
5.97624223577615E-24	1.22074527543039	0.246	0.148	9.74784871077448E-20	Mid-zonal hepatocytes	SS18L1
6.26663383269806E-24	1.02382095965559	0.304	0.203	1.02215064445138E-19	Mid-zonal hepatocytes	TEAD1
6.42197591883349E-24	1.38637094576199	0.235	0.14	1.04748849212093E-19	Mid-zonal hepatocytes	MED12
6.45079187742285E-24	1.30840953958546	0.251	0.153	1.05218866312644E-19	Mid-zonal hepatocytes	DOCK4
6.82300042874072E-24	1.50968905547315	0.195	0.105	1.1128995999319E-19	Mid-zonal hepatocytes	ATG5
6.85753185861558E-24	0.776659215948041	0.353	0.247	1.11853202145879E-19	Mid-zonal hepatocytes	ADD1
7.02057057744621E-24	1.24533971021515	0.229	0.133	1.14512526688725E-19	Mid-zonal hepatocytes	ARID4A
7.058959928977E-24	0.938758260911804	0.3	0.202	1.15138695401544E-19	Mid-zonal hepatocytes	DHX9
7.09882258648542E-24	1.13755981468955	0.228	0.132	1.15788895208164E-19	Mid-zonal hepatocytes	GTF2H1
7.35131723351822E-24	1.09704197274829	0.286	0.187	1.19907335395916E-19	Mid-zonal hepatocytes	TTC37
7.49480259296086E-24	0.361874199295207	0.485	0.367	1.22247725093785E-19	Mid-zonal hepatocytes	IRF2BP2
7.53129481697557E-24	0.688750885790338	0.26	0.156	1.22842949759688E-19	Mid-zonal hepatocytes	SPIRE2
7.71838557849273E-24	0.561279400625299	0.421	0.314	1.25894587170795E-19	Mid-zonal hepatocytes	FLOT1
7.74358748142688E-24	0.548438954066133	0.487	0.371	1.26305655409554E-19	Mid-zonal hepatocytes	TGFBI

**7.92583915361123E-24**	1.05702122059416	0.293	0.191	1.29278362434553E-19	Mid-zonal hepatocytes	RGL2
**8.11268858347552E-24**	0.756226310699344	0.419	0.311	1.32326063485069E-19	Mid-zonal hepatocytes	TAP2
**8.43379528655685E-24**	0.903999420556487	0.343	0.24	1.37563634919029E-19	Mid-zonal hepatocytes	ADAM10
**8.49499811889369E-24**	0.843684098639817	0.293	0.19	1.38561914317275E-19	Mid-zonal hepatocytes	CUX1
**8.71688514943108E-24**	1.79669759013446	0.184	0.096	1.4218111367237E-19	Mid-zonal hepatocytes	SH3RF2
**8.97383934969716E-24**	1.35879432683657	0.197	0.107	1.4637229363291E-19	Mid-zonal hepatocytes	CRTC3
**9.13847566592763E-24**	0.950299808227244	0.27	0.169	1.49057676586946E-19	Mid-zonal hepatocytes	CDK12
**9.25668902776149E-24**	0.711090164306291	0.323	0.218	1.50985854731818E-19	Mid-zonal hepatocytes	ABTB1
**1.10143258169416E-23**	0.987580679855139	0.291	0.187	1.79654668400135E-19	Mid-zonal hepatocytes	LCOR
**1.15877946516397E-23**	1.10332307665884	0.198	0.106	1.89008518562896E-19	Mid-zonal hepatocytes	PPRC1
**1.22013323575252E-23**	1.67616020362249	0.187	0.099	1.99015932083593E-19	Mid-zonal hepatocytes	METTL22
**1.2211199203459E-23**	1.44513605201014	0.178	0.091	1.99176870207619E-19	Mid-zonal hepatocytes	ICA1
**1.23135058115015E-23**	0.287902874063251	0.983	0.951	2.008455932914E-19	Mid-zonal hepatocytes	SQSTM1
**1.24973864193423E-23**	1.30663762701331	0.234	0.139	2.03844869885893E-19	Mid-zonal hepatocytes	CLEC16A
**1.264646119637E-23**	1.77519545365127	0.148	0.068	2.06276428573992E-19	Mid-zonal hepatocytes	ARRDC4
**1.27445586930605E-23**	1.29551395353997	0.223	0.13	2.07876496842509E-19	Mid-zonal hepatocytes	CPNE8
**1.29379941552109E-23**	1.2167028138653	0.283	0.184	2.11031622665644E-19	Mid-zonal hepatocytes	PHF3
**1.30017319000659E-23**	0.703674373498599	0.328	0.221	2.12071249021976E-19	Mid-zonal hepatocytes	GNAQ
**1.58972958479641E-23**	1.31669210785104	0.216	0.124	2.59300792576142E-19	Mid-zonal hepatocytes	DIP2A
**1.65470793825774E-23**	1.36780712528022	0.213	0.121	2.6989941180922E-19	Mid-zonal hepatocytes	SLC26A6
**1.74072408163719E-23**	0.52124466499027	0.392	0.283	2.83929504955842E-19	Mid-zonal hepatocytes	HDAC5
**1.79726514551911E-23**	0.306435146343174	0.46	0.345	2.93151917885622E-19	Mid-zonal hepatocytes	CRK
**1.83871696039657E-23**	0.264846194486726	0.638	0.49	2.99913123410285E-19	Mid-zonal hepatocytes	OGDHL
**1.89342068482998E-23**	1.06923124082376	0.272	0.173	3.08835847902619E-19	Mid-zonal hepatocytes	ZYG11B
**1.89657516543759E-23**	1.09125519848543	0.293	0.193	3.09350375234526E-19	Mid-zonal hepatocytes	CDC37L1
**1.95127487063677E-23**	0.902898474054458	0.262	0.161	3.18272444149564E-19	Mid-zonal hepatocytes	SERTAD2
**1.98328164611482E-23**	0.84680017482369	0.271	0.17	3.23493069297789E-19	Mid-zonal hepatocytes	SNUPN
**2.01808464832796E-23**	1.48769020272348	0.17	0.085	3.29169786988774E-19	Mid-zonal hepatocytes	NBEAL2
**2.07958798540683E-23**	0.993974487714225	0.29	0.186	3.39201596299708E-19	Mid-zonal hepatocytes	ZCCHC9
**2.13818561323421E-23**	0.811943078629936	0.285	0.182	3.48759455374632E-19	Mid-zonal hepatocytes	EPHA2
**2.23067192794391E-23**	1.31346623565506	0.198	0.109	3.63844898166932E-19	Mid-zonal hepatocytes	GTF2IRD1
**2.271560405965E-23**	0.977974537567605	0.292	0.194	3.70514217816952E-19	Mid-zonal hepatocytes	C7orf26
**2.37307170968999E-23**	1.04835560972669	0.306	0.203	3.87071726567534E-19	Mid-zonal hepatocytes	IMPA1
**2.42055070231614E-23**	1.55223758299521	0.192	0.104	3.94816025054785E-19	Mid-zonal hepatocytes	AGO3
**2.46093984540159E-23**	0.814574036670576	0.269	0.17	4.01403898183453E-19	Mid-zonal hepatocytes	FERMT2
**2.46203350386123E-23**	1.73399422437772	0.187	0.1	4.01582284814805E-19	Mid-zonal hepatocytes	DPH7
**2.4628726734218E-23**	0.480975286637302	0.5	0.386	4.01719161761829E-19	Mid-zonal hepatocytes	DPP9
**2.52650849486761E-23**	1.18974069272825	0.249	0.154	4.12098800597856E-19	Mid-zonal hepatocytes	CPLANE1
**2.57894731408546E-23**	1.46811307100671	0.171	0.086	4.20652096400479E-19	Mid-zonal hepatocytes	KATNAL2
**2.6978576997771E-23**	0.728394257417891	0.274	0.169	4.40047569410643E-19	Mid-zonal hepatocytes	FAM102A
**2.82965019500623E-23**	1.75778720091299	0.129	0.055	4.61544243307466E-19	Mid-zonal hepatocytes	RALGPS1
**2.86206248994829E-23**	0.590862422540939	0.4	0.288	4.66831012735466E-19	Mid-zonal hepatocytes	NDUFAF8
**2.9454716349616E-23**	0.696781610381945	0.358	0.254	4.80435878378586E-19	Mid-zonal hepatocytes	KHNYN
**2.98284124655485E-23**	1.35885015452891	0.191	0.103	4.86531235725562E-19	Mid-zonal hepatocytes	SRBD1
**3.01217955649708E-23**	0.4761673560533	0.416	0.309	4.91316607460238E-19	Mid-zonal hepatocytes	TLN1
**3.16215404661179E-23**	0.745405999212372	0.357	0.253	5.15778946542849E-19	Mid-zonal hepatocytes	DDX6
**3.24589918654332E-23**	1.44806776969307	0.232	0.139	5.29438616317081E-19	Mid-zonal hepatocytes	CNKSR3
**3.35003733316365E-23**	1.73946177443723	0.182	0.096	5.46424589412323E-19	Mid-zonal hepatocytes	DCP1A
**3.36428234479841E-23**	1.24983029136972	0.24	0.145	5.48748093260069E-19	Mid-zonal hepatocytes	SH3RF1
**3.51847194463748E-23**	0.733808619140866	0.352	0.249	5.73897958889819E-19	Mid-zonal hepatocytes	ASCC2
**3.63183397795746E-23**	0.677230955002648	0.368	0.262	5.92388440144641E-19	Mid-zonal hepatocytes	ONECUT2
**3.6802679565788E-23**	0.876639818605086	0.277	0.177	6.00288506397568E-19	Mid-zonal hepatocytes	DEPRECAT
**3.7562937991205E-23**	1.20297396485974	0.259	0.162	6.12689081574544E-19	Mid-zonal hepatocytes	SF3B1
**3.82910646829151E-23**	0.85963830071963	0.365	0.262	6.24565556043029E-19	Mid-zonal hepatocytes	TRIM44
**3.85135119799901E-23**	1.37561071478878	0.174	0.089	6.28193893905619E-19	Mid-zonal hepatocytes	DTNB
**4.42988532041677E-23**	0.718732337912536	0.328	0.225	7.22558594613179E-19	Mid-zonal hepatocytes	FBRS
**4.48041776409983E-23**	0.967537376221727	0.251	0.154	7.30800941502323E-19	Mid-zonal hepatocytes	RPH3AL
**4.76744715942004E-23**	1.37029945472982	0.191	0.103	7.77618306173002E-19	Mid-zonal hepatocytes	IKZF5
**4.93329109551654E-23**	1.73792412289798	0.16	0.079	8.04669110589704E-19	Mid-zonal hepatocytes	DOP1A
**5.09915026338922E-23**	1.36727714235471	0.175	0.089	8.31722399461416E-19	Mid-zonal hepatocytes	HK2

5.3361955699733E-23	1.83108544070007	0.189	0.103	8.70386859418345E-19	Mid-zonal hepatocytes	TTC21B
5.3608483850715E-23	1.33026387963124	0.224	0.131	8.74407980089012E-19	Mid-zonal hepatocytes	NUP58
5.51238645199667E-23	1.11467645526297	0.261	0.165	8.99125354185177E-19	Mid-zonal hepatocytes	HDAC1
5.60418358351165E-23	1.3254352527291	0.202	0.113	9.14098384306585E-19	Mid-zonal hepatocytes	PHLPP2
5.63402804984134E-23	0.723777306248453	0.324	0.223	9.18966315209621E-19	Mid-zonal hepatocytes	TRIM24
5.66376805225223E-23	1.37728508260743	0.21	0.121	9.23817207002861E-19	Mid-zonal hepatocytes	AHCYL2
5.79133866318836E-23	1.18577462866711	0.216	0.124	9.44625249352654E-19	Mid-zonal hepatocytes	RMC1
6.12475939300464E-23	1.85568760841468	0.157	0.077	9.99009504592987E-19	Mid-zonal hepatocytes	WDR27
6.2406108807093E-23	0.555984652652117	0.38	0.274	1.01790604075249E-18	Mid-zonal hepatocytes	ZCCHC14
6.24376484474299E-23	0.70689922381297	0.325	0.222	1.01842048382603E-18	Mid-zonal hepatocytes	KLHL36
6.3317759402745E-23	0.389164905298395	0.393	0.282	1.03277597361817E-18	Mid-zonal hepatocytes	MKNK2
6.34223240514249E-23	0.856048471398833	0.291	0.19	1.03448152760279E-18	Mid-zonal hepatocytes	TAOK1
6.50148894363805E-23	0.297987860406201	0.511	0.396	1.0604578615968E-18	Mid-zonal hepatocytes	SYVN1
6.97742164385111E-23	1.6736767768224	0.173	0.089	1.13808724432856E-18	Mid-zonal hepatocytes	MAP3K8
7.41867000391068E-23	0.835914355655497	0.348	0.248	1.21005926433787E-18	Mid-zonal hepatocytes	VDAC3
7.76219035690468E-23	1.35053852505332	0.182	0.096	1.26609086911472E-18	Mid-zonal hepatocytes	PRELID3B
8.34948328841205E-23	0.850045249260988	0.215	0.122	1.36188421917289E-18	Mid-zonal hepatocytes	AACS
8.35739877929583E-23	2.08129967218926	0.137	0.062	1.36317531489094E-18	Mid-zonal hepatocytes	ZSWIM6
9.3501184427829E-23	2.01765414495593	0.146	0.069	1.52509781920232E-18	Mid-zonal hepatocytes	ANKRD36E
9.36668732187296E-23	1.24483733262597	0.282	0.186	1.5278003690707E-18	Mid-zonal hepatocytes	PLCH2
9.42451962269646E-23	1.83973488686661	0.147	0.07	1.53723339565802E-18	Mid-zonal hepatocytes	AKAP10
9.86672442322906E-23	1.16531705238861	0.221	0.129	1.60936142067289E-18	Mid-zonal hepatocytes	PPARGC1B
1.02432380403647E-22	1.4703906716307	0.216	0.128	1.67077455676388E-18	Mid-zonal hepatocytes	ATG12
1.0536409008629E-22	1.5257944742764	0.204	0.115	1.71859367339748E-18	Mid-zonal hepatocytes	VPS8
1.0582392762936E-22	1.5866632789351	0.188	0.102	1.72609408356249E-18	Mid-zonal hepatocytes	FAM122B
1.07947375068788E-22	1.11555372669756	0.209	0.118	1.760729634747E-18	Mid-zonal hepatocytes	GGA3
1.1517109662613E-22	1.89847604664192	0.192	0.106	1.8785557570688E-18	Mid-zonal hepatocytes	KIF12
1.28578497341444E-22	1.32979805156439	0.225	0.133	2.09724387013629E-18	Mid-zonal hepatocytes	MPHOSPH
1.31357949016902E-22	1.31175394407454	0.2	0.111	2.14257950641468E-18	Mid-zonal hepatocytes	WDR90
1.3974688308162E-22	1.57916699798616	0.182	0.097	2.27941140994431E-18	Mid-zonal hepatocytes	PDS5A
1.43875545150304E-22	1.47797210917012	0.206	0.117	2.3467540169466E-18	Mid-zonal hepatocytes	VTI1A
1.51335007600447E-22	0.745893496355159	0.31	0.211	2.46842530897089E-18	Mid-zonal hepatocytes	RANBP9
1.52717692137966E-22	0.637664676954473	0.313	0.209	2.49097827646236E-18	Mid-zonal hepatocytes	MBD6
1.59369624627659E-22	0.260731240604145	0.493	0.378	2.59947794730174E-18	Mid-zonal hepatocytes	CDC34
1.6029751285684E-22	0.95514311093812	0.284	0.185	2.61461273220792E-18	Mid-zonal hepatocytes	AUTS2
1.63047757259489E-22	1.13304662980259	0.274	0.177	2.65947196865952E-18	Mid-zonal hepatocytes	PARD3
1.65092075425392E-22	1.36595683056633	0.197	0.109	2.69281684226358E-18	Mid-zonal hepatocytes	GATA6
1.68342660379503E-22	0.800334195438289	0.299	0.2	2.74583713345007E-18	Mid-zonal hepatocytes	EHMT1
1.70634033198533E-22	1.283356986033	0.24	0.146	2.78321171550127E-18	Mid-zonal hepatocytes	MFN1
1.74824256218281E-22	1.66846031336233	0.151	0.073	2.85155844317638E-18	Mid-zonal hepatocytes	UIMC1
1.76935013096679E-22	1.50875737573154	0.213	0.123	2.88598699861993E-18	Mid-zonal hepatocytes	LRRC28
1.78351476339891E-22	1.26579662086462	0.21	0.12	2.90909093057996E-18	Mid-zonal hepatocytes	SETD6
1.82701453170624E-22	0.733689579970591	0.374	0.271	2.98004340266605E-18	Mid-zonal hepatocytes	NUP50
1.8644919281677E-22	1.0591808668616	0.234	0.139	3.04117278403433E-18	Mid-zonal hepatocytes	PIP4P1
1.91420243420756E-22	1.68710705842293	0.23	0.137	3.12225559043594E-18	Mid-zonal hepatocytes	BIRC3
1.92051549517486E-22	1.21920635571596	0.26	0.167	3.13255282417971E-18	Mid-zonal hepatocytes	MIER1
1.96726474853894E-22	1.77092617405187	0.16	0.08	3.20880553134186E-18	Mid-zonal hepatocytes	NAA25
2.02305419296233E-22	0.717343680078894	0.306	0.205	3.29980369414086E-18	Mid-zonal hepatocytes	MTOR
2.09204371769008E-22	1.29309381911477	0.212	0.121	3.41233250792428E-18	Mid-zonal hepatocytes	ZBTB1
2.10411470345489E-22	0.802795524792125	0.382	0.279	3.43202149280528E-18	Mid-zonal hepatocytes	LYPLAL1
2.13955073650657E-22	2.09010507015572	0.141	0.066	3.48982120631587E-18	Mid-zonal hepatocytes	CEP170
2.20528749767864E-22	1.5373836575006	0.162	0.08	3.59704443746363E-18	Mid-zonal hepatocytes	SLC25A37
2.24132861356875E-22	1.90080937426493	0.108	0.043	3.65583110159198E-18	Mid-zonal hepatocytes	AMY2B
2.30126862165445E-22	0.678973040064757	0.355	0.251	3.75359924878057E-18	Mid-zonal hepatocytes	CDC42BPB
2.37596092007021E-22	1.70634799440738	0.182	0.098	3.87542985672652E-18	Mid-zonal hepatocytes	SCYL3
2.38527642522498E-22	1.06719380693009	0.246	0.152	3.89062437718446E-18	Mid-zonal hepatocytes	ACVR1
2.3981799369016E-22	1.77472299868041	0.154	0.076	3.9116712950802E-18	Mid-zonal hepatocytes	PTBP2
2.4121101294263E-22	0.560902621008114	0.384	0.281	3.93439283210724E-18	Mid-zonal hepatocytes	ABCA2
2.4651447663378E-22	1.1740477291522	0.173	0.088	4.02089762837359E-18	Mid-zonal hepatocytes	KIF7
2.49283484133847E-22	1.59844698511698	0.179	0.095	4.06606290970718E-18	Mid-zonal hepatocytes	CCDC186

2.50792410285285E-22	0.73848469563939	0.309	0.208	4.09067500416328E-18	Mid-zonal hepatocytes	PYGO2
2.53076086755326E-22	0.977382975856201	0.246	0.152	4.12792405106613E-18	Mid-zonal hepatocytes	PLXND1
2.53159708121783E-22	1.21379711602116	0.222	0.131	4.1292879991744E-18	Mid-zonal hepatocytes	NUP98
2.62411076446539E-22	0.461017589732178	0.421	0.313	4.2801870679195E-18	Mid-zonal hepatocytes	ZGPAT
2.72644201484891E-22	1.41277920815182	0.113	0.045	4.44709957042006E-18	Mid-zonal hepatocytes	PLEKHO1
2.84632603929184E-22	0.606175980540063	0.439	0.332	4.64264240268892E-18	Mid-zonal hepatocytes	SYMPK
2.90091823040457E-22	0.842813673713414	0.278	0.18	4.73168772561289E-18	Mid-zonal hepatocytes	TAF8
2.98799686289848E-22	1.63539216672884	0.159	0.079	4.87372168307371E-18	Mid-zonal hepatocytes	TRMT10B
3.14237852407726E-22	1.33089411419605	0.246	0.154	5.12553361062242E-18	Mid-zonal hepatocytes	SPIDR
3.18908987320777E-22	0.93950075511206	0.257	0.162	5.20172449218919E-18	Mid-zonal hepatocytes	ST3GAL6
3.32064514722547E-22	1.35579965381381	0.195	0.108	5.41630429963946E-18	Mid-zonal hepatocytes	GDAP2
3.36187307415896E-22	1.43374931528672	0.233	0.142	5.48355117126069E-18	Mid-zonal hepatocytes	MLH3
3.41030783679653E-22	1.76230526348075	0.176	0.094	5.56255311259882E-18	Mid-zonal hepatocytes	DPP8
3.76003755992736E-22	1.50860383303284	0.15	0.072	6.13299726399751E-18	Mid-zonal hepatocytes	VCPKMT
3.8870496556215E-22	1.52234013888481	0.164	0.084	6.34016669328422E-18	Mid-zonal hepatocytes	CEP44
4.02989417781069E-22	0.84297876971465	0.28	0.182	6.57316039342702E-18	Mid-zonal hepatocytes	ARFRP1
4.05803393661715E-22	1.34797435755907	0.177	0.092	6.61905915401623E-18	Mid-zonal hepatocytes	MAML1
4.21982025271367E-22	1.26821911457649	0.228	0.138	6.88294881420127E-18	Mid-zonal hepatocytes	RIC1
4.39489207217732E-22	1.56363742235495	0.167	0.085	7.16850845892843E-18	Mid-zonal hepatocytes	CHFR
4.40423174683769E-22	1.1630953082959	0.242	0.15	7.18374240226695E-18	Mid-zonal hepatocytes	ITFG2
4.4044323611263E-22	1.33450405139098	0.262	0.168	7.18406962423311E-18	Mid-zonal hepatocytes	MCEE
4.71004331264826E-22	0.487130874826027	0.394	0.292	7.68255164726058E-18	Mid-zonal hepatocytes	RASSF7
4.87838336191183E-22	0.438880371286485	0.379	0.275	7.95713110161438E-18	Mid-zonal hepatocytes	TRIOBP
5.06377029278704E-22	0.349292865942633	0.569	0.452	8.25951572456495E-18	Mid-zonal hepatocytes	EPAS1
5.07238267785057E-22	0.870558840179639	0.241	0.146	8.27356338584207E-18	Mid-zonal hepatocytes	STAG2
5.13944025095456E-22	0.692594938822586	0.353	0.25	8.38294099333197E-18	Mid-zonal hepatocytes	CIAO1
5.44185402118194E-22	0.674199613441426	0.369	0.265	8.87620809394986E-18	Mid-zonal hepatocytes	KLC4
5.71355169713887E-22	1.1896525635182	0.212	0.122	9.31937417320322E-18	Mid-zonal hepatocytes	DEPRECAT
5.833865917026E-22	0.380110669709124	0.532	0.416	9.51561869726111E-18	Mid-zonal hepatocytes	RBM47
5.86766972031752E-22	1.41990479081864	0.218	0.128	9.5707560808099E-18	Mid-zonal hepatocytes	ZNF407
5.87263267663788E-22	0.32308057905693	0.746	0.575	9.57885115886404E-18	Mid-zonal hepatocytes	CSRP1
5.89418332594291E-22	1.36837384114862	0.226	0.136	9.61400242294548E-18	Mid-zonal hepatocytes	DXO
6.00235436550392E-22	1.08647538711658	0.195	0.108	9.79044020557345E-18	Mid-zonal hepatocytes	SOCS7
6.03697127620936E-22	1.13808338312479	0.2	0.112	9.84690384862509E-18	Mid-zonal hepatocytes	ILKAP
6.32571442683332E-22	0.686292236986395	0.314	0.212	1.03178728016078E-17	Mid-zonal hepatocytes	PLEKHG6
6.62547531916477E-22	1.04695160824109	0.232	0.14	1.08068127930897E-17	Mid-zonal hepatocytes	DVL2
7.01818301588678E-22	1.64812187167816	0.105	0.041	1.14473583172129E-17	Mid-zonal hepatocytes	NIBAN1
7.05766416214559E-22	0.854519773242924	0.342	0.245	1.15117560148757E-17	Mid-zonal hepatocytes	EXOC7
7.57428283481601E-22	1.20020634055435	0.21	0.121	1.23544127318684E-17	Mid-zonal hepatocytes	GABPB2
7.95772767684862E-22	1.49779174433032	0.133	0.06	1.29798496137078E-17	Mid-zonal hepatocytes	TMEM74B
8.1018096260062E-22	1.14867194245529	0.223	0.134	1.32148616809787E-17	Mid-zonal hepatocytes	KCNAB2
8.64090758981998E-22	1.38526944095419	0.19	0.105	1.40941843697554E-17	Mid-zonal hepatocytes	HEATR1
8.65607173327065E-22	0.325146582831849	0.597	0.46	1.41189186041378E-17	Mid-zonal hepatocytes	SCARB1
8.68490878287995E-22	1.59410409215531	0.195	0.109	1.41659547157555E-17	Mid-zonal hepatocytes	PARN
8.73976955360387E-22	0.478986388010168	0.451	0.338	1.42554381188833E-17	Mid-zonal hepatocytes	CD24
8.75305287813672E-22	1.27787511482177	0.215	0.126	1.42771045495288E-17	Mid-zonal hepatocytes	GTF3C2
8.79417929910318E-22	1.43997145579046	0.187	0.101	1.43441858547672E-17	Mid-zonal hepatocytes	ZNF276
8.89191152416536E-22	1.26377094526986	0.224	0.135	1.45035968870661E-17	Mid-zonal hepatocytes	ERCC6
8.90104150293255E-22	1.28985080527736	0.123	0.053	1.45184887954333E-17	Mid-zonal hepatocytes	PTPN21
9.06072385259073E-22	1.24412519242891	0.26	0.168	1.47789466759607E-17	Mid-zonal hepatocytes	FBXO22
9.08149526847355E-22	1.36221130902474	0.235	0.143	1.48128269324072E-17	Mid-zonal hepatocytes	MBTD1
9.20130016042786E-22	1.28043768180003	0.235	0.144	1.50082406916739E-17	Mid-zonal hepatocytes	COMMD10
9.47548318543575E-22	1.71484566186655	0.163	0.083	1.54554606237642E-17	Mid-zonal hepatocytes	ZDHHC17
9.56151027772993E-22	1.10791329072522	0.185	0.099	1.55957794140053E-17	Mid-zonal hepatocytes	JAG2
9.57436079048591E-22	0.344167280141578	0.501	0.381	1.56167398853616E-17	Mid-zonal hepatocytes	FOXA3
9.89340715426073E-22	1.20406003238732	0.246	0.153	1.61371364093147E-17	Mid-zonal hepatocytes	RBFOX2
1.01031869353922E-21	1.50201232732335	0.146	0.07	1.64793082103182E-17	Mid-zonal hepatocytes	RNF207
1.0546806568219E-21	0.996887760065614	0.265	0.171	1.72028961934221E-17	Mid-zonal hepatocytes	VPS9D1
1.07997650723306E-21	0.978185698939201	0.29	0.195	1.76154968094784E-17	Mid-zonal hepatocytes	LARP1
1.09328646235812E-21	1.43912108195062	0.17	0.089	1.78325954875232E-17	Mid-zonal hepatocytes	AC093323.

1.0979432261175E-21	1.02228039089825	0.219	0.128	1.79085519612025E-17	Mid-zonal hepatocytes	WARS
1.11908965376653E-21	0.413355641068124	0.433	0.328	1.82534713425858E-17	Mid-zonal hepatocytes	HSF1
1.12061565799684E-21	0.819847032026411	0.306	0.211	1.82783619975865E-17	Mid-zonal hepatocytes	GNL2
1.14322634094635E-21	0.808229420632254	0.29	0.195	1.8647164847176E-17	Mid-zonal hepatocytes	UBAP1
1.14783116740896E-21	0.643782767908539	0.383	0.278	1.87222741716075E-17	Mid-zonal hepatocytes	MAP4K4
1.16967424195439E-21	1.08040587965626	0.264	0.169	1.90785565605181E-17	Mid-zonal hepatocytes	CCNYL1
1.17333253070681E-21	1.0601656909529	0.256	0.162	1.91382269083588E-17	Mid-zonal hepatocytes	WDR82
1.24798909740821E-21	0.863908493918659	0.299	0.201	2.03559501678253E-17	Mid-zonal hepatocytes	SUCO
1.32831253914806E-21	0.938003643889282	0.254	0.16	2.1666105826044E-17	Mid-zonal hepatocytes	WIPF2
1.34753432778982E-21	0.843117252064532	0.31	0.212	2.19796324205798E-17	Mid-zonal hepatocytes	DAPK3
1.35932465310368E-21	1.65457258898444	0.192	0.107	2.21719444167741E-17	Mid-zonal hepatocytes	DNAJB4
1.36870187453116E-21	1.31771349817781	0.133	0.06	2.23248962754778E-17	Mid-zonal hepatocytes	KIAA1328
1.49409941884526E-21	1.17315715884145	0.195	0.109	2.4370255620785E-17	Mid-zonal hepatocytes	NOL6
1.51108603279714E-21	0.768562047688347	0.302	0.205	2.46473242809542E-17	Mid-zonal hepatocytes	DHX30
1.52933705712451E-21	0.914436979332576	0.229	0.136	2.49450167387579E-17	Mid-zonal hepatocytes	ARID1A
1.54190073548597E-21	0.756286708912547	0.257	0.162	2.51499428965117E-17	Mid-zonal hepatocytes	CEMIP2
1.57804018329055E-21	0.992283155840698	0.273	0.181	2.57394134296521E-17	Mid-zonal hepatocytes	NSMCE4A
1.61385743201933E-21	1.15592182080481	0.312	0.217	2.63236285736673E-17	Mid-zonal hepatocytes	ABCA5
1.61438594193682E-21	1.80342721447063	0.166	0.087	2.63322490989315E-17	Mid-zonal hepatocytes	TBP
1.62593413999222E-21	1.09238884555635	0.287	0.191	2.65206117574131E-17	Mid-zonal hepatocytes	ZNF217
1.67006765314355E-21	1.11709520526255	0.19	0.105	2.72404734904244E-17	Mid-zonal hepatocytes	CEP192
1.67013635079716E-21	1.38952542958757	0.199	0.114	2.72415940178525E-17	Mid-zonal hepatocytes	HARS2
1.68143168152913E-21	1.31171473440391	0.222	0.133	2.74258321574216E-17	Mid-zonal hepatocytes	TMEM138
1.71074801368077E-21	0.754716370339029	0.257	0.162	2.7904010851147E-17	Mid-zonal hepatocytes	TAF4
1.75584616175768E-21	1.16358743865338	0.265	0.172	2.86396067444295E-17	Mid-zonal hepatocytes	CREB3L2
1.77392198841647E-21	1.00332599662067	0.295	0.201	2.8934441553061E-17	Mid-zonal hepatocytes	ATP6V0A1
1.78727019392488E-21	1.33374228514219	0.178	0.095	2.91521641331087E-17	Mid-zonal hepatocytes	PHIP
1.89571479333525E-21	0.830883622036665	0.261	0.166	3.09210039940913E-17	Mid-zonal hepatocytes	RAB3B
1.92613057043409E-21	0.644098077096132	0.329	0.229	3.14171157343505E-17	Mid-zonal hepatocytes	DYNC1I2
1.99090378966118E-21	0.731816720109104	0.261	0.166	3.24736317131635E-17	Mid-zonal hepatocytes	PPP6R3
2.06086344867036E-21	0.49119865607111	0.406	0.303	3.36147437112622E-17	Mid-zonal hepatocytes	AUP1
2.35359756115985E-21	0.769140300964816	0.305	0.207	3.83895298200784E-17	Mid-zonal hepatocytes	AHDC1
2.37421720295066E-21	1.14854669041037	0.267	0.175	3.87258567973282E-17	Mid-zonal hepatocytes	DYM
2.43186340191184E-21	0.692687087248616	0.389	0.279	3.9666123948584E-17	Mid-zonal hepatocytes	CHRD
2.52382579700464E-21	0.67401655101511	0.449	0.34	4.11661225749427E-17	Mid-zonal hepatocytes	AHR
2.57070410058013E-21	1.27193942495274	0.24	0.151	4.193075458456 26E-17	Mid-zonal hepatocytes	BTBD7
2.70893816591226E-21	1.53259448922125	0.172	0.091	4.41854904241949E-17	Mid-zonal hepatocytes	ANKRD12
2.71671064928817E-21	0.734278886000997	0.311	0.214	4.43122674005394E-17	Mid-zonal hepatocytes	POLR3E
2.78108166334372E-21	1.26683174609565	0.21	0.123	4.53622230107994E-17	Mid-zonal hepatocytes	TMCO4
2.9317313819212E-21	0.756495144778448	0.368	0.268	4.78194705705167E-17	Mid-zonal hepatocytes	SERINC5
3.02547726725718E-21	1.08712758687649	0.173	0.091	4.93485597062318E-17	Mid-zonal hepatocytes	DCUN1D3
3.03085793211112E-21	1.44287127976155	0.121	0.052	4.94363237306644E-17	Mid-zonal hepatocytes	AASDH
3.03205617105909E-21	0.575572734028596	0.349	0.251	4.94558682061448E-17	Mid-zonal hepatocytes	TMCO3
3.05344599265799E-21	1.77617366931057	0.143	0.069	4.980475758624 45E-17	Mid-zonal hepatocytes	TMEM128
3.07305348859247E-21	0.699499753555085	0.377	0.28	5.01245754524318E-17	Mid-zonal hepatocytes	MAP2K3
3.17391865310226E-21	0.880118434332244	0.265	0.172	5.17697871507509E-17	Mid-zonal hepatocytes	MSL2
3.1902628322165E-21	0.571319991222537	0.33	0.229	5.20363770562833E-17	Mid-zonal hepatocytes	ZNRF1
3.20042753771405E-21	1.41514692845435	0.209	0.122	5.22021735676539E-17	Mid-zonal hepatocytes	BIVM
3.36983737670242E-21	0.762420570716578	0.26	0.161	5.49654174513931E-17	Mid-zonal hepatocytes	RASD1
3.39834829161745E-21	1.20590394743109	0.202	0.116	5.54304589845723E-17	Mid-zonal hepatocytes	NHLRC2
3.40080573856444E-21	0.873601658346067	0.312	0.218	5.54705424017246E-17	Mid-zonal hepatocytes	EVL
3.48116537910835E-21	0.909319463611235	0.235	0.144	5.67812884986363E-17	Mid-zonal hepatocytes	FIP1L1
3.53478426247092E-21	1.51317898821224	0.182	0.099	5.76558661051632E-17	Mid-zonal hepatocytes	NRF1
3.71533257294484E-21	0.98592549577788	0.213	0.125	6.06007895973032E-17	Mid-zonal hepatocytes	NUP62
3.74432975398524E-21	0.869216799199535	0.269	0.175	6.10737626172532E-17	Mid-zonal hepatocytes	ESF1
3.86078423739901E-21	0.853399361914027	0.259	0.166	6.29732516962153E-17	Mid-zonal hepatocytes	SSH3
4.1937480112208E-21	1.20182452282112	0.189	0.105	6.84042238110225E-17	Mid-zonal hepatocytes	EXOSC10
4.41366220477419E-21	1.27783704409786	0.19	0.106	7.19912442220718E-17	Mid-zonal hepatocytes	DIS3L2
4.44530408387481E-21	1.68935857535156	0.155	0.078	7.2507354912082E-17	Mid-zonal hepatocytes	KALRN
4.47525915279273E-21	0.546289460215325	0.371	0.269	7.29959520412022E-17	Mid-zonal hepatocytes	ENTPD6

4.58363566496891E-21	0.50778280400156	0.449	0.348	7.47636813313079E-17	Mid-zonal hepatocytes	AP1M1
4.61385477680413E-21	1.460349934332	0.154	0.077	7.52565852644521E-17	Mid-zonal hepatocytes	QSER1
4.72402290417017E-21	1.5612501958751	0.174	0.093	7.70535375899197E-17	Mid-zonal hepatocytes	CHAD
4.95982330136608E-21	1.40693491748689	0.257	0.169	8.08996778685821E-17	Mid-zonal hepatocytes	AKIRIN1
5.04111486953946E-21	0.958804949456626	0.225	0.136	8.22256246370582E-17	Mid-zonal hepatocytes	RPAP1
5.52657474582344E-21	1.74037259201086	0.205	0.121	9.01439606791262E-17	Mid-zonal hepatocytes	TMEM87A
5.55357118976652E-21	1.08665241504399	0.244	0.153	9.05842996762818E-17	Mid-zonal hepatocytes	CYLD
5.61397184592293E-21	1.64627210977599	0.139	0.066	9.15694947788489E-17	Mid-zonal hepatocytes	EFCAB2
5.72681759033001E-21	1.44405779578213	0.227	0.14	9.34101217158727E-17	Mid-zonal hepatocytes	ZNF280D
5.72910694425069E-21	0.943428528626428	0.241	0.15	9.3447463367673E-17	Mid-zonal hepatocytes	UGCG
5.76341183773075E-21	0.463083555247072	0.479	0.376	9.40070104852262E-17	Mid-zonal hepatocytes	SPSB1
5.8259968232741E-21	1.22289538691904	0.251	0.161	9.50278341844239E-17	Mid-zonal hepatocytes	SS18
5.91129153135003E-21	0.911191748179551	0.253	0.162	9.64190761678503E-17	Mid-zonal hepatocytes	MCM3AP
5.94589287220404E-21	1.45659811364534	0.174	0.094	9.69834586385201E-17	Mid-zonal hepatocytes	PPWD1
6.04043015716257E-21	0.889283293697371	0.353	0.26	9.85254562934787E-17	Mid-zonal hepatocytes	KLHL24
6.09958677618256E-21	0.67670808896297	0.306	0.211	9.94903599063138E-17	Mid-zonal hepatocytes	SMYD2
6.10398136874915E-21	1.38856694676629	0.19	0.106	9.95620401056673E-17	Mid-zonal hepatocytes	GANC
6.40478460495506E-21	1.1348566450657	0.269	0.178	1.04468441691422E-16	Mid-zonal hepatocytes	NT5DC1
6.56314180432553E-21	0.251424325254648	0.462	0.35	1.07051405970354E-16	Mid-zonal hepatocytes	IGSF8
6.62944265939795E-21	1.58942706745092	0.184	0.102	1.0813283921744E-16	Mid-zonal hepatocytes	NICN1
6.85648468307007E-21	1.57120284170184	0.153	0.077	1.11836121665556E-16	Mid-zonal hepatocytes	IRAK4
7.20278699553806E-21	1.24661835080626	0.246	0.156	1.17484658684221E-16	Mid-zonal hepatocytes	FNTA
7.25779835335469E-21	0.349727810695684	0.446	0.339	1.18381948941568E-16	Mid-zonal hepatocytes	RING1
7.26923712566082E-21	1.20167364512501	0.216	0.129	1.18568526756654E-16	Mid-zonal hepatocytes	ZC3H14
7.31748198016447E-21	1.08064317838149	0.218	0.13	1.19355448578463E-16	Mid-zonal hepatocytes	UBA6
7.50227118435732E-21	1.41021290212893	0.175	0.094	1.22369545288052E-16	Mid-zonal hepatocytes	DCAF1
7.61295243458271E-21	1.28810788877141	0.187	0.103	1.24174867160479E-16	Mid-zonal hepatocytes	XPO1
7.6866550159027E-21	1.17446558688801	0.197	0.113	1.25377029964389E-16	Mid-zonal hepatocytes	PREPL
7.72591567357462E-21	1.53974603062446	0.174	0.093	1.26017410551676E-16	Mid-zonal hepatocytes	HSPBAP1
7.8575530825255E-21	0.364495176338077	0.35	0.245	1.28164548329073E-16	Mid-zonal hepatocytes	ZNF219
7.97523910096169E-21	0.800948972465293	0.342	0.247	1.30084124975786E-16	Mid-zonal hepatocytes	MTMR4
8.02506615260221E-21	1.0447253296455	0.263	0.171	1.30896854015095E-16	Mid-zonal hepatocytes	TSPAN15
8.1040032016133E-21	0.777888501392889	0.27	0.176	1.32184396221515E-16	Mid-zonal hepatocytes	PDZD8
8.70843299874545E-21	0.480112307972819	0.4	0.304	1.42043250642537E-16	Mid-zonal hepatocytes	UHMK1
8.7805165838373E-21	1.52813455908907	0.191	0.109	1.4321900599897E-16	Mid-zonal hepatocytes	USP40
8.85650928166107E-21	0.617082943029714	0.289	0.193	1.44458522893174E-16	Mid-zonal hepatocytes	PLEKHM2
9.15888383674176E-21	1.32634932892203	0.207	0.122	1.49390554261095E-16	Mid-zonal hepatocytes	TMF1
9.27890203312991E-21	0.521041544484918	0.298	0.2	1.51348171062382E-16	Mid-zonal hepatocytes	RAB11FIP1
9.44387643934752E-21	1.12384307171507	0.221	0.134	1.54039068602197E-16	Mid-zonal hepatocytes	PCIF1
9.59472649212848E-21	1.14208921037887	0.21	0.123	1.56499583813108E-16	Mid-zonal hepatocytes	POLR1A
1.01081319653768E-20	1.65837955647752	0.188	0.106	1.64873740487261E-16	Mid-zonal hepatocytes	PIK3C2G
1.06436451440018E-20	1.56175808431822	0.14	0.067	1.73608495943813E-16	Mid-zonal hepatocytes	MAP3K9
1.07056335507666E-20	1.01207679580093	0.227	0.139	1.74619588846554E-16	Mid-zonal hepatocytes	N4BP1
1.08883019399894E-20	1.30824544310777	0.195	0.112	1.77599092943167E-16	Mid-zonal hepatocytes	DDX55
1.09365098141845E-20	1.43993987429759	0.162	0.084	1.78385411579163E-16	Mid-zonal hepatocytes	PAXBP1
1.12029880812166E-20	1.39428636630283	0.193	0.11	1.82731938592724E-16	Mid-zonal hepatocytes	GYS1
1.12462857637329E-20	1.06224469234172	0.229	0.14	1.83438167092247E-16	Mid-zonal hepatocytes	DIP2B
1.13978379264776E-20	1.42871762418305	0.147	0.072	1.85910134418775E-16	Mid-zonal hepatocytes	SLC19A3
1.19264007409763E-20	1.58281367835156	0.185	0.102	1.94531522486064E-16	Mid-zonal hepatocytes	CLCN6
1.22248524204971E-20	0.280882080296138	0.517	0.41	1.99399567830728E-16	Mid-zonal hepatocytes	SHB
1.2245894864024E-20	0.510750405807845	0.352	0.247	1.99742791127095E-16	Mid-zonal hepatocytes	CBX4
1.25057170968221E-20	1.11603789135603	0.245	0.154	2.03980751566266E-16	Mid-zonal hepatocytes	XPC
1.30621588281671E-20	1.70489308136531	0.125	0.056	2.13056872646233E-16	Mid-zonal hepatocytes	POLA1
1.36862092473796E-20	1.90730199593521	0.124	0.056	2.23235759034008E-16	Mid-zonal hepatocytes	IARS
1.38331882997545E-20	1.39472391672976	0.191	0.109	2.25633134357295E-16	Mid-zonal hepatocytes	AGO4
1.39430947043562E-20	1.51320763974431	0.195	0.113	2.27425817722753E-16	Mid-zonal hepatocytes	TYW3
1.52927104831463E-20	0.45890517691492	0.425	0.321	2.49439400690599E-16	Mid-zonal hepatocytes	MYO1C
1.6323836371411E-20	0.591610145217523	0.361	0.266	2.66258095054085E-16	Mid-zonal hepatocytes	NOTCH2NL
1.68965922709583E-20	1.26745299801252	0.253	0.164	2.75600316531601E-16	Mid-zonal hepatocytes	LRCH3
1.72193509623966E-20	0.456489997923665	0.376	0.274	2.80864833547651E-16	Mid-zonal hepatocytes	TOMM40L

1.73985154934254E-20	0.881483785762823	0.202	0.116	2.83787186213261E-16	Mid-zonal hepatocytes	CEP250
1.93464423620555E-20	0.772356679196669	0.282	0.187	3.15559821367488E-16	Mid-zonal hepatocytes	ABTB2
1.95196639540729E-20	1.80166599909808	0.144	0.071	3.18385238754883E-16	Mid-zonal hepatocytes	GIGYF2
2.01821693572339E-20	0.979917315231361	0.237	0.148	3.29191364385843E-16	Mid-zonal hepatocytes	GTPBP1
2.09413588763948E-20	1.36211421397164	0.2	0.117	3.41574504632875E-16	Mid-zonal hepatocytes	OFD1
2.1299583341204E-20	0.253650900831366	0.501	0.396	3.47417503878379E-16	Mid-zonal hepatocytes	MTCH1
2.13891889077404E-20	1.13994213234589	0.219	0.132	3.48879060274154E-16	Mid-zonal hepatocytes	ATRX
2.19576772082578E-20	1.34140226437863	0.213	0.128	3.58151672943893E-16	Mid-zonal hepatocytes	THADA
2.23402483050849E-20	0.592931598033085	0.306	0.211	3.64391790104239E-16	Mid-zonal hepatocytes	XPNPEP1
2.29323262222517E-20	0.682600391777091	0.274	0.181	3.74049173011147E-16	Mid-zonal hepatocytes	ZNHIT6
2.33550400726631E-20	1.44616071907871	0.146	0.072	3.80944058625208E-16	Mid-zonal hepatocytes	CCDC88A
2.43938737446037E-20	1.28940360123378	0.181	0.1	3.97888474648231E-16	Mid-zonal hepatocytes	BACH2
2.45730200946635E-20	1.78754773007559	0.142	0.07	4.00810530764057E-16	Mid-zonal hepatocytes	SENP7
2.55211700049456E-20	0.877702705884766	0.315	0.219	4.16275803950668E-16	Mid-zonal hepatocytes	HIGD1A
2.58639113343686E-20	1.31037620830941	0.178	0.098	4.21866257774887E-16	Mid-zonal hepatocytes	TTC27
2.5917348102839E-20	1.16344449539024	0.145	0.072	4.22737864905406E-16	Mid-zonal hepatocytes	CDK8
2.64629483126775E-20	1.0317999985458	0.243	0.154	4.31637149928082E-16	Mid-zonal hepatocytes	ROCK2
2.90884060867895E-20	1.42314301499493	0.218	0.133	4.74460991681624E-16	Mid-zonal hepatocytes	PPP4R3A
2.99938267388897E-20	0.808317152282235	0.358	0.265	4.8922930793803E-16	Mid-zonal hepatocytes	TRAPPC12
3.1401975733672E-20	1.04843049854191	0.187	0.105	5.12197626191924E-16	Mid-zonal hepatocytes	FNBP1L
3.17879479333557E-20	1.6127355954175	0.163	0.085	5.18493218740964E-16	Mid-zonal hepatocytes	CAMKMT
3.21132242108221E-20	0.81795472395633	0.241	0.151	5.2379880010272E-16	Mid-zonal hepatocytes	ANKRD40
3.35330737954372E-20	0.686059938478825	0.327	0.221	5.46957966677375E-16	Mid-zonal hepatocytes	IER3
3.42105550068572E-20	0.69738241519277	0.309	0.214	5.58008362716847E-16	Mid-zonal hepatocytes	QRICH1
3.43233847368839E-20	1.84298669482325	0.154	0.079	5.59848728443313E-16	Mid-zonal hepatocytes	UBN2
3.49335439319879E-20	1.37943692368007	0.119	0.052	5.69801035074655E-16	Mid-zonal hepatocytes	MELTF
3.54921102806672E-20	0.972764157688012	0.277	0.185	5.78911810787962E-16	Mid-zonal hepatocytes	PICK1
3.64268058771238E-20	0.71788001768495	0.374	0.279	5.94157630661767E-16	Mid-zonal hepatocytes	SORL1
3.75681230674078E-20	0.836017699727069	0.363	0.265	6.12773655352488E-16	Mid-zonal hepatocytes	MUC20
3.7866061829062E-20	1.80110608615311	0.153	0.078	6.1763333449383E-16	Mid-zonal hepatocytes	JAK2
3.94640976903666E-20	1.20388870010523	0.179	0.099	6.4369889742757E-16	Mid-zonal hepatocytes	GIT2
4.07539899739945E-20	0.440438818157177	0.387	0.287	6.64738330465824E-16	Mid-zonal hepatocytes	CTTN
4.32800276791309E-20	1.0318468493027	0.227	0.142	7.05940531474304E-16	Mid-zonal hepatocytes	AKAP11
4.36418536382099E-20	1.30455423411198	0.189	0.108	7.11842274692842E-16	Mid-zonal hepatocytes	TRMT13
4.56145565560622E-20	0.761493971667063	0.299	0.206	7.44019031985931E-16	Mid-zonal hepatocytes	NAGK
4.56720599901654E-20	0.503207123064112	0.303	0.205	7.44956970499588E-16	Mid-zonal hepatocytes	MFHAS1
4.65615250146634E-20	1.46225031613114	0.141	0.069	7.59465034514175E-16	Mid-zonal hepatocytes	C20orf194
4.70158515267212E-20	0.404169235609197	0.416	0.317	7.6687555425235E-16	Mid-zonal hepatocytes	SLC35F5
4.7627886821661E-20	0.878040521097548	0.296	0.203	7.76858461948112E-16	Mid-zonal hepatocytes	ZMYM3
4.89160786566666E-20	0.85182085168635	0.336	0.241	7.97870158968889E-16	Mid-zonal hepatocytes	AP5Z1
5.05887458793562E-20	0.874827740751203	0.28	0.19	8.2515303403818E-16	Mid-zonal hepatocytes	KLHL22
5.13506100556718E-20	1.50764167782858	0.187	0.106	8.37579800618062E-16	Mid-zonal hepatocytes	UHRF1BP1
5.15117808133119E-20	1.03870662108225	0.331	0.243	8.4020865684593E-16	Mid-zonal hepatocytes	ABCA6
5.37211072151994E-20	1.4181886824492	0.257	0.169	8.76244979787117E-16	Mid-zonal hepatocytes	GAS2
5.38778269917695E-20	0.876071414684209	0.263	0.173	8.78801236062752E-16	Mid-zonal hepatocytes	DEPRECAT
5.45189170911681E-20	1.13489417448609	0.247	0.159	8.89258056674044E-16	Mid-zonal hepatocytes	FBXO38
5.48370616421577E-20	0.930943822304879	0.256	0.166	8.94447312445234E-16	Mid-zonal hepatocytes	FASTKD2
5.621681767574E-20	0.874924265384738	0.219	0.132	9.16952513108995E-16	Mid-zonal hepatocytes	GTPBP3
5.71533766776989E-20	0.6323203156162	0.372	0.276	9.32228726989947E-16	Mid-zonal hepatocytes	GAK
5.73696632376107E-20	1.47400152039292	0.148	0.074	9.35756577068669E-16	Mid-zonal hepatocytes	PHC1
6.24890162209739E-20	0.615872632457676	0.35	0.254	1.0192583435803E-15	Mid-zonal hepatocytes	GOLGB1
6.38897362976975E-20	1.09692172304231	0.252	0.164	1.04210548875174E-15	Mid-zonal hepatocytes	HELZ
6.42130159574069E-20	0.670005406303469	0.384	0.287	1.04737850328126E-15	Mid-zonal hepatocytes	SREBF2
6.47054540922416E-20	0.989578365122269	0.201	0.118	1.05541066169855E-15	Mid-zonal hepatocytes	RGP1
6.56919422699349E-20	0.702922412019901	0.291	0.2	1.07150127036491E-15	Mid-zonal hepatocytes	ATIC
6.65904285809977E-20	0.621160550599354	0.321	0.227	1.08615648058465E-15	Mid-zonal hepatocytes	NFKBIL1
6.78876260247656E-20	0.523586168473778	0.328	0.231	1.10731506808995E-15	Mid-zonal hepatocytes	BAX
7.02174946241377E-20	0.975975606280887	0.188	0.106	1.14531755481431E-15	Mid-zonal hepatocytes	IL4R
7.03974421427099E-20	1.15210557265921	0.195	0.112	1.14825267878974E-15	Mid-zonal hepatocytes	CCDC91
7.04262070786681E-20	1.07546396683922	0.216	0.131	1.14872186366016E-15	Mid-zonal hepatocytes	RCBTB1

7.10340087596517E-20	1.29102468100345	0.215	0.131	1.15863571687868E-15	Mid-zonal hepatocytes	METTL3
7.19409941087731E-20	0.484559698164814	0.337	0.24	1.1734295549082E-15	Mid-zonal hepatocytes	ARFGAP3
7.26270493063401E-20	0.718742244813153	0.338	0.246	1.18461980123571E-15	Mid-zonal hepatocytes	CLCC1
7.27366162254811E-20	0.356296230440186	0.465	0.362	1.18640694725382E-15	Mid-zonal hepatocytes	CYBC1
7.28473606096476E-20	0.830694592733288	0.247	0.158	1.18821329890396E-15	Mid-zonal hepatocytes	IFI44
7.54777069450039E-20	0.992365823180984	0.225	0.139	1.23111687797996E-15	Mid-zonal hepatocytes	FEM1B
7.6968063000594E-20	1.06370124679788	0.235	0.149	1.25542607560269E-15	Mid-zonal hepatocytes	POLR3C
8.06676872635587E-20	1.3782479183054	0.202	0.12	1.31577064695591E-15	Mid-zonal hepatocytes	SHPRH
8.09649238530752E-20	1.78448080529337	0.136	0.066	1.32061887296751E-15	Mid-zonal hepatocytes	CAPN3
8.1219623724968E-20	1.29989540143258	0.185	0.105	1.32477328257795E-15	Mid-zonal hepatocytes	IFT122
8.19116037771802E-20	1.12101051562043	0.185	0.105	1.33606016920959E-15	Mid-zonal hepatocytes	TTL
8.45906872829846E-20	1.08857646808739	0.221	0.135	1.37975870027276E-15	Mid-zonal hepatocytes	MTO1
8.51437885907428E-20	1.11940164091277	0.237	0.152	1.38878033570361E-15	Mid-zonal hepatocytes	FBXL4
8.65628016348772E-20	0.875635768533108	0.268	0.177	1.41192585746648E-15	Mid-zonal hepatocytes	PGM3
8.71676762668183E-20	0.709275950570311	0.364	0.272	1.42179196758807E-15	Mid-zonal hepatocytes	PDCD6
8.85112525937819E-20	0.830139455237011	0.352	0.261	1.44370704105718E-15	Mid-zonal hepatocytes	NDRG2
9.05913976534416E-20	1.86037424924508	0.138	0.067	1.47763628712529E-15	Mid-zonal hepatocytes	TSC1
9.55911359894625E-20	0.785341670663638	0.297	0.206	1.55918701912412E-15	Mid-zonal hepatocytes	DEPRECAT
9.9631886796401E-20	1.41018113289072	0.108	0.046	1.6250957055361E-15	Mid-zonal hepatocytes	ZNF449
1.02414199597168E-19	1.24550151480344	0.252	0.166	1.67047800962941E-15	Mid-zonal hepatocytes	EPC1
1.03118138428729E-19	0.726361687454823	0.349	0.256	1.68195995591099E-15	Mid-zonal hepatocytes	SFXN1
1.0907461369918E-19	1.63888583380157	0.107	0.045	1.77911602404733E-15	Mid-zonal hepatocytes	NOD2
1.12357989752481E-19	0.869594701582461	0.231	0.143	1.83267117085272E-15	Mid-zonal hepatocytes	OSBPL8
1.1496554836637E-19	1.60851550832849	0.167	0.09	1.87520305940386E-15	Mid-zonal hepatocytes	CCNT1
1.25886438040135E-19	0.552863387542233	0.418	0.319	2.05333369087264E-15	Mid-zonal hepatocytes	ABCD3
1.31328072191651E-19	1.2414580468765	0.173	0.096	2.14209218551802E-15	Mid-zonal hepatocytes	DEPRECAT
1.33002482924176E-19	1.13900363593592	0.26	0.172	2.16940349897624E-15	Mid-zonal hepatocytes	AKTIP
1.35444826319839E-19	0.68036779015317	0.31	0.216	2.20924056210289E-15	Mid-zonal hepatocytes	TPD52L2
1.35653091328408E-19	0.734240438999159	0.325	0.231	2.21263757265767E-15	Mid-zonal hepatocytes	DCTN4
1.40961544723317E-19	0.844523729087781	0.204	0.121	2.29922375598203E-15	Mid-zonal hepatocytes	DUSP8
1.42952202295807E-19	1.40090751406229	0.216	0.134	2.3316933716469E-15	Mid-zonal hepatocytes	OTUD4
1.47091650524987E-19	1.340104165907	0.176	0.097	2.39921191171306E-15	Mid-zonal hepatocytes	RAB28
1.50592195380098E-19	0.257422613766881	0.375	0.275	2.45630929884478E-15	Mid-zonal hepatocytes	DVL1
1.5818322963992E-19	1.06024454515873	0.229	0.144	2.58012665865673E-15	Mid-zonal hepatocytes	HDAC7
1.62799105321711E-19	1.60008245103387	0.128	0.06	2.65541620690242E-15	Mid-zonal hepatocytes	PLEKHN1
1.62910903533687E-19	1.22799996341162	0.194	0.113	2.65723974753798E-15	Mid-zonal hepatocytes	BCLAF1
1.68027083711282E-19	0.880508989615256	0.29	0.198	2.74068976241471E-15	Mid-zonal hepatocytes	MARC1
1.72131618263439E-19	1.54694765724694	0.111	0.048	2.80763882549496E-15	Mid-zonal hepatocytes	RWDD2A
1.73294310302071E-19	1.00344978327224	0.249	0.162	2.82660349533708E-15	Mid-zonal hepatocytes	KAT2B
1.77428254882105E-19	1.43494596258293	0.163	0.087	2.89403226538202E-15	Mid-zonal hepatocytes	WBP4
1.83915556662934E-19	1.18951536927439	0.181	0.102	2.99984664472912E-15	Mid-zonal hepatocytes	STARD8
1.84612554884966E-19	1.45132706099846	0.136	0.066	3.01121538272868E-15	Mid-zonal hepatocytes	ZDHHC7
1.87982120269879E-19	1.1667567304011	0.212	0.129	3.066176363722E-15	Mid-zonal hepatocytes	THUMPD1
1.88535086191328E-19	0.619707269205262	0.313	0.222	3.07519579086676E-15	Mid-zonal hepatocytes	GMDS
1.92843284411853E-19	0.660284129138739	0.354	0.259	3.14546681204174E-15	Mid-zonal hepatocytes	TNRC18
1.94306097412276E-19	1.06224210535283	0.305	0.217	3.16932675489163E-15	Mid-zonal hepatocytes	KMT2C
1.94403959478119E-19	0.845783497136834	0.262	0.175	3.17092298304759E-15	Mid-zonal hepatocytes	PABPC4
1.96473257657135E-19	1.40984519716508	0.187	0.107	3.20467530564553E-15	Mid-zonal hepatocytes	ZNF337
1.97378483120787E-19	1.12561123339755	0.256	0.17	3.21944043818316E-15	Mid-zonal hepatocytes	SPOP
2.00131613211446E-19	1.32393986528486	0.178	0.1	3.2643467430919E-15	Mid-zonal hepatocytes	SCAPER
2.11485609024148E-19	0.510823103016857	0.436	0.337	3.44954176879288E-15	Mid-zonal hepatocytes	COBLL1
2.12875191850234E-19	1.5975753415925	0.147	0.075	3.47220725426917E-15	Mid-zonal hepatocytes	CEP120
2.14604104889593E-19	0.909877954578041	0.297	0.208	3.50040755485415E-15	Mid-zonal hepatocytes	C11orf24
2.15637818919906E-19	0.926124000828941	0.235	0.148	3.51726846440259E-15	Mid-zonal hepatocytes	TNPO2
2.19416536862791E-19	1.36217579371414	0.162	0.086	3.57890313276898E-15	Mid-zonal hepatocytes	PRDM4
2.29259530692188E-19	1.71778697645422	0.125	0.058	3.73945220512028E-15	Mid-zonal hepatocytes	MCPH1
2.37746352026385E-19	1.04166439160298	0.238	0.153	3.87788074790237E-15	Mid-zonal hepatocytes	NSMCE2
2.39670387521796E-19	1.53639657390181	0.162	0.086	3.90926369086802E-15	Mid-zonal hepatocytes	TMEM168
2.42663266874291E-19	0.666064445812415	0.355	0.258	3.95808054598656E-15	Mid-zonal hepatocytes	DNAJC5
2.5492561077237E-19	0.655452450861552	0.33	0.235	4.15809163730813E-15	Mid-zonal hepatocytes	ZMAT3

2.58706956638935E-19	0.611389395468644	0.386	0.293	4.21976916973766E-15	Mid-zonal hepatocytes	ITFG1
2.6040443372623E-19	1.19070004189961	0.226	0.14	4.24745671850854E-15	Mid-zonal hepatocytes	USP15
2.66017308211499E-19	1.01350393919513	0.274	0.184	4.33900831423776E-15	Mid-zonal hepatocytes	NFIA
2.67747642107849E-19	0.993465245904071	0.237	0.15	4.36723179042113E-15	Mid-zonal hepatocytes	ADNP2
2.67806388967782E-19	1.01957914453768	0.245	0.16	4.36819001045349E-15	Mid-zonal hepatocytes	GTF2I
2.68831436595128E-19	1.32153772389228	0.22	0.137	4.38490956230313E-15	Mid-zonal hepatocytes	MYNN
2.81635718896262E-19	1.41921522071979	0.112	0.048	4.59376021091693E-15	Mid-zonal hepatocytes	MTMR11
2.83510690341192E-19	1.12842738337927	0.212	0.128	4.62434287015519E-15	Mid-zonal hepatocytes	ERMP1
2.89737647684852E-19	0.747174977162501	0.288	0.198	4.72591077138762E-15	Mid-zonal hepatocytes	KDM4A
2.90408243321779E-19	0.862476695720968	0.23	0.143	4.73684885682153E-15	Mid-zonal hepatocytes	B4GALT5
2.95937961978517E-19	1.12546963350038	0.272	0.181	4.82704409783159E-15	Mid-zonal hepatocytes	SIPA1L2
3.03090768960006E-19	1.32778914744295	0.175	0.096	4.94371353250665E-15	Mid-zonal hepatocytes	CIITA
3.06968804870241E-19	1.65570497906163	0.166	0.091	5.0069681762385E-15	Mid-zonal hepatocytes	TUBE1
3.12089520311169E-19	0.383760418838134	0.387	0.287	5.09049216579548E-15	Mid-zonal hepatocytes	TRABD
3.2134316443089E-19	0.423629599101574	0.218	0.13	5.24142835503224E-15	Mid-zonal hepatocytes	RENBP
3.21467142657748E-19	0.901538269313718	0.228	0.141	5.24345056389053E-15	Mid-zonal hepatocytes	EARS2
3.35581829409679E-19	0.319128121002871	0.44	0.339	5.47367521950127E-15	Mid-zonal hepatocytes	PSMD13
3.37179956011265E-19	0.6748643925874	0.332	0.239	5.49974226249975E-15	Mid-zonal hepatocytes	GRIPAP1
3.39183316625183E-19	1.40782995119765	0.153	0.079	5.53241907747337E-15	Mid-zonal hepatocytes	FARSB
3.43778337454835E-19	0.757536663670732	0.316	0.226	5.60736846222582E-15	Mid-zonal hepatocytes	MET
3.48792495377343E-19	1.76899155220237	0.15	0.078	5.68915439209984E-15	Mid-zonal hepatocytes	TRIM3
3.49576319092874E-19	1.1775018276675	0.213	0.132	5.70193934072387E-15	Mid-zonal hepatocytes	RALGAPB
3.51117529283319E-19	0.722026993354069	0.333	0.241	5.72707802014021E-15	Mid-zonal hepatocytes	ERBIN
3.52044291995873E-19	0.570806724299751	0.367	0.276	5.74219444674469E-15	Mid-zonal hepatocytes	NELFCD
3.55460328054836E-19	1.4574559301273	0.147	0.075	5.79791341090243E-15	Mid-zonal hepatocytes	TRPV3
3.58149288486161E-19	1.58822440810175	0.158	0.084	5.84177304449778E-15	Mid-zonal hepatocytes	NPHP4
3.67250929018158E-19	1.66489576063007	0.157	0.084	5.99022990321518E-15	Mid-zonal hepatocytes	RBBP6
3.73739515419566E-19	0.874116815156651	0.251	0.163	6.09606523600854E-15	Mid-zonal hepatocytes	CARD10
3.80023926363217E-19	1.47583578965991	0.151	0.078	6.19857026291044E-15	Mid-zonal hepatocytes	PRMT3
3.81107211408485E-19	1.28368607557673	0.163	0.087	6.21623972528381E-15	Mid-zonal hepatocytes	TBC1D12
3.86652030714132E-19	1.1710568968907	0.184	0.104	6.30668127297821E-15	Mid-zonal hepatocytes	CCDC82
3.959127530833E-19	1.30741784991748	0.2	0.12	6.45773291554172E-15	Mid-zonal hepatocytes	ANAPC4
4.26824139072267E-19	0.63211044480768	0.352	0.262	6.96192853240774E-15	Mid-zonal hepatocytes	ARHGAP35
4.30293572081841E-19	0.917891346228875	0.255	0.168	7.01851845422691E-15	Mid-zonal hepatocytes	GORASP1
4.3309671785319E-19	0.338852896920217	0.454	0.347	7.06424056490338E-15	Mid-zonal hepatocytes	SMAP2
4.33283118121825E-19	0.494369901147392	0.289	0.194	7.06728093968509E-15	Mid-zonal hepatocytes	MAFB
4.63939869462226E-19	1.88415574298867	0.133	0.065	7.56732321079837E-15	Mid-zonal hepatocytes	MPPED1
4.71089527177595E-19	0.45228828770168	0.347	0.252	7.68394127779375E-15	Mid-zonal hepatocytes	SMARCA4
4.72495127680013E-19	1.11486014615154	0.249	0.163	7.70686802758869E-15	Mid-zonal hepatocytes	ADCK5
4.86192406264842E-19	1.05864869188786	0.275	0.189	7.93028433858584E-15	Mid-zonal hepatocytes	LRBA
4.99756803944347E-19	0.857544103707457	0.195	0.113	8.15153322913624E-15	Mid-zonal hepatocytes	SCN1B
5.17098423314009E-19	0.337376858670844	0.401	0.305	8.4343923826748E-15	Mid-zonal hepatocytes	GMPPB
5.51802181500798E-19	0.666024687543728	0.302	0.213	9.00044538245951E-15	Mid-zonal hepatocytes	GTPBP4
5.55108136685769E-19	0.580597130601274	0.425	0.329	9.05436881748157E-15	Mid-zonal hepatocytes	ALDH5A1
5.58699868025733E-19	0.869222012782895	0.225	0.141	9.11295354736773E-15	Mid-zonal hepatocytes	NEDD4
5.59651551638272E-19	0.826396201958277	0.381	0.284	9.12847645877186E-15	Mid-zonal hepatocytes	PWWP3A
5.66210734889213E-19	1.03807084145278	0.191	0.111	9.23546329677796E-15	Mid-zonal hepatocytes	PKP4
5.69440876690132E-19	0.790150987623185	0.281	0.193	9.28815013969274E-15	Mid-zonal hepatocytes	HNRNPM
5.72467965984262E-19	1.6075040604896	0.166	0.091	9.3375249931693E-15	Mid-zonal hepatocytes	RWDD3
5.87054106470765E-19	1.54082895363525	0.201	0.122	9.57543953064464E-15	Mid-zonal hepatocytes	TTC28
5.88946440979928E-19	1.89647106654127	0.107	0.046	9.60630539882361E-15	Mid-zonal hepatocytes	SGSM1
5.90379444671622E-19	0.813538233139605	0.175	0.096	9.62967912203883E-15	Mid-zonal hepatocytes	FAM53C
6.16993580757523E-19	1.25234572071361	0.219	0.137	1.0063782295736E-14	Mid-zonal hepatocytes	TRIP4
6.28415692083156E-19	0.761539075100693	0.309	0.22	1.02500883535684E-14	Mid-zonal hepatocytes	NAB1
6.32960964510475E-19	1.2472348549115	0.213	0.131	1.03242262921304E-14	Mid-zonal hepatocytes	ARID4B
6.47529011236498E-19	1.07482120515281	0.192	0.112	1.05618457022785E-14	Mid-zonal hepatocytes	PATL1
6.63506941894488E-19	1.1066572909717	0.226	0.145	1.0822461729241E-14	Mid-zonal hepatocytes	USP6NL
6.64621056024396E-19	1.45136631835178	0.15	0.078	1.08406340448139E-14	Mid-zonal hepatocytes	BCS1L
6.94968479378453E-19	1.25442882779863	0.199	0.119	1.13356308671419E-14	Mid-zonal hepatocytes	RAPGEF4
7.00599408784461E-19	0.775691611545681	0.258	0.17	1.14274769566833E-14	Mid-zonal hepatocytes	RILPL1

7.26424563230591E-19	0.594764000092426	0.358	0.266	1.18487110508542E-14	Mid-zonal hepatocytes	PICALM
7.28432212283806E-19	1.62864092244551	0.133	0.065	1.18814578145612E-14	Mid-zonal hepatocytes	LPXN
7.64597672507206E-19	0.817315604544588	0.302	0.214	1.2471352636265E-14	Mid-zonal hepatocytes	SNX14
7.66891484678632E-19	0.298143569232043	0.439	0.342	1.25087670065932E-14	Mid-zonal hepatocytes	SPPL2A
7.75890131570422E-19	0.764860206778715	0.276	0.188	1.26555439360452E-14	Mid-zonal hepatocytes	RAB5A
7.75922268562375E-19	0.741600137534858	0.344	0.251	1.26560681225209E-14	Mid-zonal hepatocytes	DCPS
7.82253414787276E-19	0.713715045811671	0.269	0.18	1.27593354485953E-14	Mid-zonal hepatocytes	PHF10
8.00906248132164E-19	1.13385558381753	0.178	0.1	1.30635818132837E-14	Mid-zonal hepatocytes	BICRAL
8.06967314157748E-19	1.4894100928368	0.139	0.07	1.3162443861227E-14	Mid-zonal hepatocytes	SLC7A1
8.35845704588651E-19	0.581078199407849	0.333	0.243	1.36334792875455E-14	Mid-zonal hepatocytes	MTA1
8.58041629516454E-19	1.08527275503725	0.175	0.097	1.39955170190429E-14	Mid-zonal hepatocytes	TP53BP1
8.62222343264899E-19	1.82841399395631	0.152	0.081	1.40637086409938E-14	Mid-zonal hepatocytes	MYO9A
8.6443592889E-19	0.81493339789706	0.285	0.197	1.40998144361248E-14	Mid-zonal hepatocytes	CRELD1
8.81405736826242E-19	0.873112455119319	0.314	0.228	1.43766089733728E-14	Mid-zonal hepatocytes	SMARCA5
8.94245805397488E-19	1.0607437199171	0.244	0.159	1.45860433318384E-14	Mid-zonal hepatocytes	MAP3K2
8.97130748926602E-19	0.484696175171729	0.375	0.278	1.46330996457418E-14	Mid-zonal hepatocytes	CAPN5
9.06144467291684E-19	1.07821806627314	0.207	0.125	1.47801224059947E-14	Mid-zonal hepatocytes	OXSR1
9.46160390587995E-19	1.33192335233974	0.181	0.104	1.54328221308808E-14	Mid-zonal hepatocytes	RCOR1
9.76211989486419E-19	0.271830565522475	0.533	0.42	1.5922993760513E-14	Mid-zonal hepatocytes	ANXA11
1.01263512193658E-18	0.459355622548777	0.39	0.299	1.65170914739075E-14	Mid-zonal hepatocytes	NUDCD3
1.01913443472475E-18	1.06065114399168	0.2	0.119	1.66231017647953E-14	Mid-zonal hepatocytes	UNK
1.03619664429853E-18	1.22230885572414	0.24	0.157	1.69014034651534E-14	Mid-zonal hepatocytes	GNL3L
1.04203792978732E-18	1.6430167369763	0.159	0.085	1.6996680672761E-14	Mid-zonal hepatocytes	ZNF236
1.12468930727273E-18	0.590331301493522	0.319	0.228	1.83448072909255E-14	Mid-zonal hepatocytes	ABCF3
1.16324394525396E-18	0.691364477539846	0.34	0.251	1.89736719910374E-14	Mid-zonal hepatocytes	COBL
1.18454806318497E-18	1.05961412749756	0.194	0.113	1.932116345861E-14	Mid-zonal hepatocytes	NTN4
1.21505605789164E-18	1.05169591915303	0.303	0.217	1.98187793602705E-14	Mid-zonal hepatocytes	DEPRECAT
1.29313546633233E-18	1.5279692564477	0.176	0.1	2.10923325913467E-14	Mid-zonal hepatocytes	NAA40
1.31624062560086E-18	0.717611010727145	0.172	0.093	2.14692008441756E-14	Mid-zonal hepatocytes	CKB
1.31790437689109E-18	1.09181434516201	0.198	0.117	2.14963382914706E-14	Mid-zonal hepatocytes	URGCP
1.34781368921156E-18	0.468759458082894	0.358	0.264	2.19841890847297E-14	Mid-zonal hepatocytes	PAM16
1.36613559521915E-18	1.0379423895558	0.2	0.119	2.22830376936196E-14	Mid-zonal hepatocytes	ZSCAN29
1.40601688181238E-18	0.595909532419577	0.377	0.286	2.29335413592417E-14	Mid-zonal hepatocytes	DENND10
1.41998175631617E-18	1.06433815216554	0.257	0.173	2.31613224272731E-14	Mid-zonal hepatocytes	FBH1
1.55978707053294E-18	0.819122653527527	0.272	0.185	2.54416869074628E-14	Mid-zonal hepatocytes	TMEM189
1.56681911778625E-18	1.25071899182858	0.18	0.102	2.55563866302115E-14	Mid-zonal hepatocytes	PEX11A
1.58564042891593E-18	1.09496184404924	0.231	0.148	2.58633810360478E-14	Mid-zonal hepatocytes	ZC3H11A
1.63686896562861E-18	0.803847919225566	0.256	0.169	2.66989696983682E-14	Mid-zonal hepatocytes	PELO
1.65530834397984E-18	1.05687182528213	0.185	0.108	2.69997343986552E-14	Mid-zonal hepatocytes	PIK3C2A
1.68568849319804E-18	0.398666481235487	0.349	0.259	2.74952650125532E-14	Mid-zonal hepatocytes	UBE2I
1.74103869631093E-18	0.457136081318515	0.265	0.175	2.83980821755276E-14	Mid-zonal hepatocytes	FBRSL1
1.75624983436102E-18	0.678399351055675	0.257	0.171	2.86461910482626E-14	Mid-zonal hepatocytes	CD40
1.7773512728488E-18	0.82246254677136	0.226	0.143	2.89903766114368E-14	Mid-zonal hepatocytes	DNASE1L1
1.84794808435157E-18	0.673504620030112	0.334	0.243	3.01418812038585E-14	Mid-zonal hepatocytes	STX4
1.9095788627153E-18	1.16740187332668	0.22	0.137	3.11471408297492E-14	Mid-zonal hepatocytes	NF1
1.93444467271171E-18	0.714702928275903	0.275	0.188	3.15527270566008E-14	Mid-zonal hepatocytes	CREBBP
1.96240512151287E-18	1.56256973434645	0.2	0.121	3.20087899369964E-14	Mid-zonal hepatocytes	ARFGEF1
1.96285969781342E-18	0.632685076985668	0.288	0.199	3.20162045310347E-14	Mid-zonal hepatocytes	GTPBP10
1.97239035510966E-18	0.318005540043758	0.347	0.249	3.21716590821936E-14	Mid-zonal hepatocytes	SELENOS
2.05764732983461E-18	0.816740411920081	0.197	0.116	3.35622855969324E-14	Mid-zonal hepatocytes	SMYD5
2.08757983236237E-18	1.3093020720503	0.241	0.157	3.40505146456626E-14	Mid-zonal hepatocytes	RAB3IP
2.13701862297723E-18	1.28381720023675	0.192	0.114	3.48569107593815E-14	Mid-zonal hepatocytes	RNGTT
2.14572607974118E-18	1.37519481606027	0.161	0.087	3.49989380866584E-14	Mid-zonal hepatocytes	RIC8B
2.21474491142443E-18	0.716881614776866	0.256	0.17	3.61247042502439E-14	Mid-zonal hepatocytes	CTDNEP1
2.35909675479332E-18	0.810939188607446	0.218	0.134	3.84792271674338E-14	Mid-zonal hepatocytes	ZDHHC18
2.37820978115789E-18	1.6188966617072	0.124	0.059	3.87909797404663E-14	Mid-zonal hepatocytes	SLX4
2.39324940295006E-18	1.09523717618794	0.212	0.13	3.90362910115184E-14	Mid-zonal hepatocytes	PTPN18
2.40364867690775E-18	1.24494297717661	0.225	0.143	3.92059135690423E-14	Mid-zonal hepatocytes	CAPN7
2.42832110041467E-18	1.02896373284371	0.221	0.139	3.96083454688637E-14	Mid-zonal hepatocytes	ARID1B
2.48720117174846E-18	0.98312318152544	0.232	0.147	4.05687383123892E-14	Mid-zonal hepatocytes	SCAF4

2.48891141080032E-18	1.19663986458933	0.172	0.096	4.05966340215641E-14	Mid-zonal hepatocytes	RIOK1
2.60807742367069E-18	1.320093124954	0.14	0.071	4.25403508574926E-14	Mid-zonal hepatocytes	DAGLA
2.62226295171976E-18	1.08024789653996	0.257	0.171	4.27717310055009E-14	Mid-zonal hepatocytes	SLC11A2
2.66485575056834E-18	1.43129333201374	0.188	0.111	4.34664621475201E-14	Mid-zonal hepatocytes	MTPAP
2.6856188011006E-18	0.440092730981447	0.284	0.194	4.3805128264752E-14	Mid-zonal hepatocytes	PURA
2.6915168319374E-18	0.934210348578559	0.241	0.157	4.39013310457309E-14	Mid-zonal hepatocytes	EIF4G3
2.89144817254637E-18	0.633590084656491	0.405	0.313	4.71624111424038E-14	Mid-zonal hepatocytes	PPARA
2.92004335059911E-18	0.768092958219652	0.26	0.173	4.76288270916221E-14	Mid-zonal hepatocytes	INO80
2.92873472651565E-18	1.86754081814481	0.114	0.052	4.77705921241968E-14	Mid-zonal hepatocytes	RIC3
2.99301931314153E-18	1.33700533465667	0.139	0.07	4.88191380166516E-14	Mid-zonal hepatocytes	SYNE3
3.12455852510417E-18	0.811197451896106	0.331	0.244	5.09646741029742E-14	Mid-zonal hepatocytes	EPB41L5
3.17968523078402E-18	1.16467318307074	0.151	0.08	5.18638457993182E-14	Mid-zonal hepatocytes	ZC3H8
3.3866975384928E-18	1.43813103923353	0.121	0.057	5.52404235503561E-14	Mid-zonal hepatocytes	USH1C
3.44236667866609E-18	0.751245259403642	0.31	0.224	5.61484428957226E-14	Mid-zonal hepatocytes	AMPD2
3.55176794875348E-18	0.635551076983981	0.342	0.254	5.7932887012118E-14	Mid-zonal hepatocytes	IDH3B
3.63875308832597E-18	0.889735617339065	0.276	0.191	5.93517016236849E-14	Mid-zonal hepatocytes	MAN2A1
3.69714885649969E-18	0.475786730718837	0.34	0.248	6.03041949983664E-14	Mid-zonal hepatocytes	LRPPRC
3.74505701076743E-18	0.94492588663305	0.163	0.089	6.10856249026276E-14	Mid-zonal hepatocytes	TDRD3
3.75273853524442E-18	1.45091232723182	0.173	0.099	6.12109182483718E-14	Mid-zonal hepatocytes	PTAR1
3.76735005117838E-18	0.913782701428882	0.249	0.167	6.14492466847705E-14	Mid-zonal hepatocytes	PSEN1
3.8551946157617E-18	1.3821030066521	0.147	0.077	6.28820793776891E-14	Mid-zonal hepatocytes	ADAM17
3.89360475514975E-18	1.21206704974397	0.211	0.132	6.35085871612476E-14	Mid-zonal hepatocytes	NFATC3
4.05463122369109E-18	0.509234701055578	0.309	0.221	6.61350898896253E-14	Mid-zonal hepatocytes	SERPINB6
4.2235781898602E-18	1.14216062876599	0.159	0.086	6.88907838548097E-14	Mid-zonal hepatocytes	CHD1
4.27506779430318E-18	1.76661745680494	0.123	0.059	6.97306307928791E-14	Mid-zonal hepatocytes	CDRT4
4.28745857988391E-18	1.35463063521176	0.223	0.143	6.99327368964864E-14	Mid-zonal hepatocytes	HMG20A
4.33008972120879E-18	1.45528831115546	0.169	0.096	7.06280934426366E-14	Mid-zonal hepatocytes	SLC35A1
4.52206771069962E-18	0.910392904477673	0.257	0.174	7.37594464292216E-14	Mid-zonal hepatocytes	VPS13D
4.53792082662908E-18	0.287398501166458	0.421	0.327	7.40180266031469E-14	Mid-zonal hepatocytes	HDHD5
4.54493571770102E-18	0.664171211528118	0.417	0.328	7.41324464914214E-14	Mid-zonal hepatocytes	TNFSF10
4.58811641258742E-18	1.14636876486526	0.126	0.06	7.48367668057135E-14	Mid-zonal hepatocytes	BCL2
4.61148293783903E-18	1.52361865264873	0.154	0.083	7.52178981990924E-14	Mid-zonal hepatocytes	MRRF
4.70461725120032E-18	1.39732875353226	0.149	0.079	7.67370119843285E-14	Mid-zonal hepatocytes	LRRC31
4.72084116118756E-18	1.25336577843666	0.184	0.107	7.70016401801303E-14	Mid-zonal hepatocytes	BMP1
4.94630979493581E-18	0.414480788054474	0.367	0.273	8.0679259065198E-14	Mid-zonal hepatocytes	HYI
5.00390930326179E-18	1.68514905070361	0.172	0.098	8.16187646455031E-14	Mid-zonal hepatocytes	PPFIBP1
5.00511314026387E-18	1.04589413312287	0.162	0.088	8.1638400430844E-14	Mid-zonal hepatocytes	DOCK6
5.19109599946844E-18	2.03397769372257	0.126	0.062	8.46719668473297E-14	Mid-zonal hepatocytes	CACNA1D
5.26318667482346E-18	1.29101477886062	0.152	0.081	8.58478378530454E-14	Mid-zonal hepatocytes	CEP95
5.33293558910994E-18	0.942276729587793	0.228	0.145	8.69855123939722E-14	Mid-zonal hepatocytes	WDR55
5.74245834084118E-18	1.00340425099876	0.214	0.134	9.36652379974605E-14	Mid-zonal hepatocytes	TFCP2
5.85009683374092E-18	0.98681964270194	0.225	0.143	9.54209294551482E-14	Mid-zonal hepatocytes	SLC9A8
6.07878888393252E-18	0.251238419419047	0.471	0.373	9.91511254858234E-14	Mid-zonal hepatocytes	RMDN3
6.1633863065234E-18	1.00619390438206	0.239	0.158	1.00530994045703E-13	Mid-zonal hepatocytes	RAD50
6.23548541117318E-18	0.407049752835048	0.395	0.303	1.01707002541646E-13	Mid-zonal hepatocytes	AKIRIN2
6.44642385139767E-18	0.70029369613977	0.336	0.248	1.05147619440147E-13	Mid-zonal hepatocytes	KCTD20
6.52398898232875E-18	1.42560389677138	0.117	0.054	1.06412784290764E-13	Mid-zonal hepatocytes	GPSM1
6.58796746570287E-18	1.00008208570627	0.274	0.19	1.0745633733308E-13	Mid-zonal hepatocytes	ZNF277
6.77339892767235E-18	1.46320961204482	0.19	0.114	1.10480909909264E-13	Mid-zonal hepatocytes	CNTLN
6.85360178927857E-18	1.06714096915584	0.219	0.138	1.11789098784923E-13	Mid-zonal hepatocytes	TRAPPC6B
6.90086290518544E-18	0.826452047240619	0.338	0.254	1.1255997484648E-13	Mid-zonal hepatocytes	ELAVL1
6.96602396045579E-18	0.938153850711473	0.218	0.138	1.13622816818994E-13	Mid-zonal hepatocytes	NUDT12
6.98025083567759E-18	1.20855075860318	0.251	0.168	1.13854871380737E-13	Mid-zonal hepatocytes	COG4
7.10792966603765E-18	1.4582645559033	0.163	0.09	1.1593744078274E-13	Mid-zonal hepatocytes	RIT1
7.13350383241508E-18	1.33352042136294	0.159	0.087	1.16354581010522E-13	Mid-zonal hepatocytes	PINX1
7.40287130791053E-18	1.34654950584621	0.184	0.108	1.20748233903329E-13	Mid-zonal hepatocytes	TMEM260
7.41464069133524E-18	1.11296192187321	0.238	0.157	1.20940204316369E-13	Mid-zonal hepatocytes	PPHLN1
7.52767312438353E-18	1.17782956351702	0.201	0.123	1.2278387633182E-13	Mid-zonal hepatocytes	ANKRD52
7.54726344827246E-18	1.2594431909201	0.169	0.094	1.23103414104772E-13	Mid-zonal hepatocytes	KLHL25
7.63608836199698E-18	1.03645725715789	0.231	0.149	1.24552237272533E-13	Mid-zonal hepatocytes	NPC1

7.91989704123239E-18	0.29003435118641	0.553	0.437	1.29181440639542E-13	Mid-zonal hepatocytes	TOR1AIP2
8.06817769133965E-18	1.72750747387533	0.108	0.048	1.31600046323441E-13	Mid-zonal hepatocytes	B3GAT1
8.07588973948984E-18	0.638623676433827	0.273	0.186	1.31725837540819E-13	Mid-zonal hepatocytes	CBFB
8.25995363503514E-18	1.12861594925598	0.206	0.127	1.34728103741058E-13	Mid-zonal hepatocytes	MED20
8.278834754633E-18	0.822774847290984	0.297	0.213	1.35036073682819E-13	Mid-zonal hepatocytes	TBL1XR1
8.35923232993935E-18	1.28195572448046	0.17	0.096	1.36347438533641E-13	Mid-zonal hepatocytes	PEAR1
8.39211634770071E-18	0.86956364511249	0.257	0.172	1.36883809747346E-13	Mid-zonal hepatocytes	LARP1B
8.56254905489702E-18	1.34437947944813	0.198	0.12	1.39663737634425E-13	Mid-zonal hepatocytes	SUPT20H
9.03117706140595E-18	1.77533486195561	0.151	0.082	1.47307529048592E-13	Mid-zonal hepatocytes	ARL17A
9.2020152070568E-18	0.68274637166395	0.324	0.236	1.50094070042303E-13	Mid-zonal hepatocytes	MAPK6
9.3400418150268E-18	1.05600242171918	0.277	0.191	1.52345422044902E-13	Mid-zonal hepatocytes	KCNT2
9.77837840897082E-18	0.67263914634414	0.31	0.225	1.59495130228723E-13	Mid-zonal hepatocytes	THNSL2
9.85014719084327E-18	1.28816442796771	0.222	0.142	1.60665750829845E-13	Mid-zonal hepatocytes	TRIM4
1.01495140394697E-17	1.10233704516778	0.237	0.155	1.6554872349779E-13	Mid-zonal hepatocytes	USP25
1.02042545672633E-17	1.54747602927288	0.116	0.054	1.66441596246632E-13	Mid-zonal hepatocytes	PARP8
1.02327614462193E-17	1.5886474549041	0.156	0.085	1.66906571949283E-13	Mid-zonal hepatocytes	TNFAIP3
1.04741893505506E-17	1.39047286142655	0.146	0.077	1.70844502496831E-13	Mid-zonal hepatocytes	WDR73
1.05269693433235E-17	2.1102239337304	0.105	0.047	1.71705396958949E-13	Mid-zonal hepatocytes	KLHL17
1.06497392705362E-17	1.02390869729499	0.202	0.124	1.73707897241717E-13	Mid-zonal hepatocytes	RNF144B
1.12749225761631E-17	1.38769438522957	0.133	0.068	1.83905262139796E-13	Mid-zonal hepatocytes	NBPF11
1.14546441100081E-17	0.969602643902441	0.238	0.155	1.86836700078343E-13	Mid-zonal hepatocytes	RAPGEF2
1.18765221196887E-17	1.48656973227057	0.152	0.082	1.93717952294242E-13	Mid-zonal hepatocytes	SYT17
1.21738179814553E-17	0.902798169646677	0.179	0.104	1.98567145095517E-13	Mid-zonal hepatocytes	SHOC2
1.22035755958452E-17	1.38354641898256	0.15	0.081	1.99052521543831E-13	Mid-zonal hepatocytes	DEPRECAT
1.24188492314717E-17	1.10261268225783	0.198	0.119	2.02563849814535E-13	Mid-zonal hepatocytes	GAS8
1.2643182830184E-17	1.77327360516409	0.136	0.07	2.06222955143132E-13	Mid-zonal hepatocytes	ZEB1
1.28907512350777E-17	0.66367901047297	0.324	0.24	2.10261043395352E-13	Mid-zonal hepatocytes	STX5
1.32262877191896E-17	0.955740840862588	0.178	0.102	2.15733978987701E-13	Mid-zonal hepatocytes	HID1
1.32512987615302E-17	0.991684587995065	0.221	0.14	2.16141934099319E-13	Mid-zonal hepatocytes	PCNT
1.32666453100314E-17	0.941625780358329	0.218	0.138	2.16392251651922E-13	Mid-zonal hepatocytes	ATP6V1B2
1.33213944253761E-17	1.25078416084619	0.173	0.1	2.1728526447231E-13	Mid-zonal hepatocytes	DNM1L
1.34023367193575E-17	1.06383335371224	0.191	0.115	2.18605514229441E-13	Mid-zonal hepatocytes	GGT7
1.41712000303109E-17	0.602616110776501	0.254	0.169	2.31146443694402E-13	Mid-zonal hepatocytes	MLLT6
1.41739556471129E-17	0.514714443948726	0.325	0.235	2.31191390560059E-13	Mid-zonal hepatocytes	MAP2K7
1.41868259494343E-17	1.59754268063487	0.125	0.062	2.31401318061222E-13	Mid-zonal hepatocytes	C11orf80
1.44021652970277E-17	0.574565626234349	0.26	0.175	2.34913718159819E-13	Mid-zonal hepatocytes	TAOK3
1.47988101420941E-17	1.63872342944525	0.136	0.07	2.41383392227697E-13	Mid-zonal hepatocytes	RAB2B
1.48438531979483E-17	0.510052022826362	0.291	0.191	2.42118089511734E-13	Mid-zonal hepatocytes	DEFB1
1.48447961885225E-17	0.63057223660208	0.302	0.216	2.42133470630991E-13	Mid-zonal hepatocytes	MED28
1.49354304055184E-17	1.13401080995695	0.208	0.129	2.4361180534441E-13	Mid-zonal hepatocytes	PRPSAP2
1.50270123377114E-17	0.282927159167156	0.257	0.167	2.45105598240411E-13	Mid-zonal hepatocytes	CYP4F22
1.53730428376452E-17	1.27397972336679	0.156	0.085	2.50749701724831E-13	Mid-zonal hepatocytes	UTP25
1.55019617037285E-17	0.627788721967839	0.3	0.214	2.52852497349515E-13	Mid-zonal hepatocytes	TP53I13
1.55751318599086E-17	1.20069846091904	0.225	0.146	2.54045975766969E-13	Mid-zonal hepatocytes	CDC42BPA
1.56453707867314E-17	0.700227905305333	0.278	0.195	2.55191642902376E-13	Mid-zonal hepatocytes	RNF44
1.66194173248866E-17	1.78877754121552	0.13	0.066	2.71079315986226E-13	Mid-zonal hepatocytes	SLX4IP
1.67711640721228E-17	0.396821764460212	0.334	0.244	2.73554457180396E-13	Mid-zonal hepatocytes	GFOD2
1.71566748280451E-17	1.53147969652937	0.128	0.063	2.79842523120244E-13	Mid-zonal hepatocytes	SP140L
1.73635486841909E-17	1.08658973067424	0.181	0.106	2.83216842587837E-13	Mid-zonal hepatocytes	NT5C
1.76333116520961E-17	1.41531749723344	0.166	0.093	2.87616946357339E-13	Mid-zonal hepatocytes	TCP11L2
1.85769914261305E-17	0.609908496053684	0.344	0.256	3.03009307151615E-13	Mid-zonal hepatocytes	CERS4
1.90881779152376E-17	1.11201732529699	0.181	0.105	3.11347269975441E-13	Mid-zonal hepatocytes	MGME1
1.91573455226209E-17	1.25467977489485	0.213	0.135	3.12475462819469E-13	Mid-zonal hepatocytes	SOAT1
1.95878996057552E-17	0.564362342147671	0.275	0.188	3.19498230469473E-13	Mid-zonal hepatocytes	SLK
1.97312023064639E-17	1.13776861893705	0.209	0.132	3.21835640820733E-13	Mid-zonal hepatocytes	ZNF800
2.01844046588055E-17	1.10815010446018	0.239	0.159	3.29227824389776E-13	Mid-zonal hepatocytes	UBE2O
2.06321854257813E-17	1.15947412413765	0.24	0.158	3.36531576479918E-13	Mid-zonal hepatocytes	ZBTB10
2.07746485910344E-17	1.36426437452861	0.144	0.076	3.38855293168362E-13	Mid-zonal hepatocytes	POGLUT1
2.12256458608192E-17	1.2854761204574	0.186	0.111	3.46211509635822E-13	Mid-zonal hepatocytes	FCHO2
2.13140317332313E-17	0.568613857746968	0.369	0.282	3.47653171600736E-13	Mid-zonal hepatocytes	NONO

**2.16177722630649E-17**	1.03181703805114	0.204	0.127	3.52607483382852E-13	Mid-zonal hepatocytes	TRNT1
**2.2098576408877E-17**	1.36951084591847	0.184	0.11	3.60449879805192E-13	Mid-zonal hepatocytes	HPS5
**2.21447357596131E-17**	1.50861430514085	0.198	0.122	3.61202784975049E-13	Mid-zonal hepatocytes	RASGEF1B
**2.21888004095589E-17**	0.864699772242731	0.231	0.15	3.61921523480316E-13	Mid-zonal hepatocytes	NCKIPSD
**2.31507290223535E-17**	1.36294476947904	0.148	0.079	3.77611541083607E-13	Mid-zonal hepatocytes	SVIL
**2.33563067254919E-17**	1.40075095254113	0.164	0.093	3.80964718999498E-13	Mid-zonal hepatocytes	AEBP2
**2.36286233207887E-17**	0.991237248852938	0.243	0.161	3.85406474985384E-13	Mid-zonal hepatocytes	BCL2L11
**2.37496958052898E-17**	0.84158068666273	0.234	0.151	3.87381288280083E-13	Mid-zonal hepatocytes	FAM199X
**2.39341759154895E-17**	1.23679553439008	0.174	0.101	3.90390343357549E-13	Mid-zonal hepatocytes	GNA13
**2.42476866673447E-17**	0.698983125501896	0.334	0.247	3.9550401723106E-13	Mid-zonal hepatocytes	PRPF40A
**2.43512298839683E-17**	1.27893641776668	0.162	0.091	3.97192910637408E-13	Mid-zonal hepatocytes	KMT5C
**2.46074328839083E-17**	0.951924320997952	0.207	0.129	4.01371837769428E-13	Mid-zonal hepatocytes	SKIL
**2.4794863423115E-17**	1.94580547245824	0.119	0.057	4.0442901729443E-13	Mid-zonal hepatocytes	CPT1B
**2.49122507201E-17**	0.820251537276855	0.318	0.232	4.06343721495551E-13	Mid-zonal hepatocytes	NFIL3
**2.50477185521895E-17**	1.05343745808667	0.215	0.138	4.08553337304764E-13	Mid-zonal hepatocytes	SUPV3L1
**2.59614902828553E-17**	0.481392535851	0.378	0.289	4.23457868003653E-13	Mid-zonal hepatocytes	LIX1L
**2.62824899699876E-17**	1.22994084388769	0.199	0.123	4.28693693900468E-13	Mid-zonal hepatocytes	MCC
**2.64587331975425E-17**	0.25313772688363	0.545	0.429	4.31568397185115E-13	Mid-zonal hepatocytes	PAPSS2
**2.74311203313676E-17**	0.446086314138683	0.338	0.249	4.47429003724937E-13	Mid-zonal hepatocytes	PPP1R12C
**2.75866736141638E-17**	1.04278514087937	0.229	0.151	4.49966233320625E-13	Mid-zonal hepatocytes	ANKRD17
**2.8576879456277E-17**	0.743505759190558	0.187	0.11	4.66117480811333E-13	Mid-zonal hepatocytes	TUSC3
**2.93107949559342E-17**	1.29003402652013	0.195	0.119	4.78088376526242E-13	Mid-zonal hepatocytes	ZNF692
**3.04359769884662E-17**	1.52325911910902	0.163	0.093	4.96441220658872E-13	Mid-zonal hepatocytes	LYST
**3.07939885778164E-17**	0.416341418451006	0.335	0.247	5.02280747692764E-13	Mid-zonal hepatocytes	VPS4A
**3.08673769670429E-17**	0.990496087564361	0.289	0.208	5.03477785709437E-13	Mid-zonal hepatocytes	NFS1
**3.15175324236215E-17**	0.87830163210453	0.275	0.191	5.14082471361691E-13	Mid-zonal hepatocytes	WDR45
**3.243834548712E-17**	0.917111042230076	0.226	0.147	5.29101853240415E-13	Mid-zonal hepatocytes	SUDS3
**3.2696376872348E-17**	1.1176870177265	0.226	0.149	5.33310603164868E-13	Mid-zonal hepatocytes	RNF8
**3.35061390150912E-17**	1.29295811688536	0.181	0.107	5.46518633475153E-13	Mid-zonal hepatocytes	NCOA6
**3.35156670856032E-17**	1.5059348151967	0.195	0.122	5.46674045833273E-13	Mid-zonal hepatocytes	WDR19
**3.37379357900897E-17**	1.33235515542426	0.187	0.113	5.50299470672153E-13	Mid-zonal hepatocytes	SBF2
**3.39600820582726E-17**	1.6849041273916	0.154	0.085	5.53922898452484E-13	Mid-zonal hepatocytes	TRIM15
**3.41802742155884E-17**	0.707017300158045	0.265	0.181	5.57514452730463E-13	Mid-zonal hepatocytes	STRN4
**3.43462142713008E-17**	1.48839766288847	0.146	0.078	5.60221100979187E-13	Mid-zonal hepatocytes	ZNF84
**3.57311342733245E-17**	1.70432665197986	0.126	0.063	5.82810531132195E-13	Mid-zonal hepatocytes	L3MBTL1
**3.59572612917825E-17**	1.85704456016021	0.109	0.05	5.86498888930265E-13	Mid-zonal hepatocytes	TAF1A
**3.59652243561918E-17**	0.784789410027902	0.222	0.142	5.86628774473844E-13	Mid-zonal hepatocytes	CXXC1
**3.7470545315114E-17**	0.361505739635686	0.395	0.308	6.11182064634825E-13	Mid-zonal hepatocytes	RAB18
**3.81907038881579E-17**	0.966953245873433	0.183	0.108	6.22928571119744E-13	Mid-zonal hepatocytes	REXO1
**3.96906307131824E-17**	1.02988363434943	0.243	0.163	6.47393877562717E-13	Mid-zonal hepatocytes	DEAF1
**4.03528903377113E-17**	0.695428807738232	0.331	0.244	6.58195994298409E-13	Mid-zonal hepatocytes	RAP1GAP
**4.05298023282161E-17**	1.03547080588602	0.241	0.162	6.61081605775533E-13	Mid-zonal hepatocytes	GTF3C1
**4.06429724914805E-17**	0.788909971117558	0.316	0.231	6.62927524308539E-13	Mid-zonal hepatocytes	ZBTB18
**4.06868730718848E-17**	0.672669367850236	0.21	0.129	6.63643586675514E-13	Mid-zonal hepatocytes	PHETA1
**4.11135734946195E-17**	1.25041526299221	0.2	0.125	6.70603497270739E-13	Mid-zonal hepatocytes	NEK9
**4.2344338427076E-17**	0.626000337566662	0.269	0.184	6.90678504084037E-13	Mid-zonal hepatocytes	SEPTIN11
**4.3482730205385E-17**	1.4428561937763	0.136	0.071	7.09246812380036E-13	Mid-zonal hepatocytes	EME2
**4.35385662364997E-17**	1.17045902356929	0.184	0.11	7.10157553883546E-13	Mid-zonal hepatocytes	ENTR1
**4.47195783746132E-17**	0.67933192975701	0.287	0.204	7.29421042868317E-13	Mid-zonal hepatocytes	SETD3
**4.75515605132884E-17**	0.922767454703466	0.256	0.176	7.75613503532247E-13	Mid-zonal hepatocytes	EXOC6
**4.80121813316344E-17**	1.28245159639905	0.175	0.103	7.83126689700288E-13	Mid-zonal hepatocytes	EPM2AIP1
**4.82393490353393E-17**	1.4915458403126	0.168	0.097	7.86832022115419E-13	Mid-zonal hepatocytes	PRKN
**4.84622779320658E-17**	1.44796669674698	0.139	0.074	7.90468215349925E-13	Mid-zonal hepatocytes	TAMM41
**4.88961312187138E-17**	1.29539715589427	0.186	0.112	7.9754479630844E-13	Mid-zonal hepatocytes	BRD8
**4.95295123961604E-17**	0.935112000447477	0.269	0.186	8.07875876693772E-13	Mid-zonal hepatocytes	CISH
**4.98650103115007E-17**	0.757890591056322	0.238	0.155	8.13348183190887E-13	Mid-zonal hepatocytes	STK39
**5.02964715778633E-17**	1.03215478514687	0.164	0.093	8.20385747906528E-13	Mid-zonal hepatocytes	RTEL1
**5.08956811995075E-17**	1.03395313095789	0.259	0.177	8.30159456045166E-13	Mid-zonal hepatocytes	SH3D19
**5.43932231670045E-17**	1.44108473525436	0.194	0.119	8.87207863077011E-13	Mid-zonal hepatocytes	SNX24
**5.45374306140172E-17**	1.01212616861541	0.234	0.154	8.89560030745234E-13	Mid-zonal hepatocytes	FBXL18

5.45942922835188E-17	1.43780356077197	0.131	0.067	8.90487501436475E-13	Mid-zonal hepatocytes	OTUD3
5.47211726336268E-17	1.10777377823138	0.17	0.098	8.92557046827088E-13	Mid-zonal hepatocytes	ULK3
5.55791814256786E-17	1.22291636235694	0.236	0.158	9.06552028234244E-13	Mid-zonal hepatocytes	KLHL12
5.76401281891786E-17	1.57400822757706	0.144	0.077	9.40168130893693E-13	Mid-zonal hepatocytes	ULK4
5.88680031044137E-17	1.12424547513372	0.19	0.115	9.60195998636091E-13	Mid-zonal hepatocytes	RNMT
6.54540843840689E-17	1.20356959147385	0.153	0.084	1.06762157038855E-12	Mid-zonal hepatocytes	CDAN1
6.57515498388178E-17	1.02091771566314	0.245	0.166	1.07247352942096E-12	Mid-zonal hepatocytes	ALG9
6.67854208805601E-17	1.17337061695546	0.216	0.139	1.08933699998282E-12	Mid-zonal hepatocytes	VPS54
6.80068734978577E-17	1.663312376928	0.116	0.056	1.10926011362356E-12	Mid-zonal hepatocytes	GLS
6.81630079193275E-17	1.07960372197744	0.271	0.191	1.11180682217215E-12	Mid-zonal hepatocytes	MBNL2
6.8601768044443E-17	1.4537657571205	0.127	0.064	1.11896343857291E-12	Mid-zonal hepatocytes	ZBED5
6.890862569769E-17	1.13086395713632	0.218	0.14	1.12396859375502E-12	Mid-zonal hepatocytes	TRAPPC10
7.01612693241922E-17	1.00290444610714	0.19	0.114	1.1444004639469E-12	Mid-zonal hepatocytes	DUSP16
7.19187513647652E-17	0.518503321747159	0.403	0.313	1.17306675351068E-12	Mid-zonal hepatocytes	GCLM
7.25096893815299E-17	1.57191810941801	0.14	0.075	1.18270554350213E-12	Mid-zonal hepatocytes	LCORL
7.29390631201234E-17	0.415725437786911	0.412	0.324	1.18970905855233E-12	Mid-zonal hepatocytes	CDH1
7.40328091166129E-17	0.619137703920208	0.348	0.264	1.20754914950107E-12	Mid-zonal hepatocytes	PDCD6IP
7.82192237159839E-17	0.292283613580827	0.456	0.363	1.27583375803141E-12	Mid-zonal hepatocytes	AGFG2
8.09046614724186E-17	0.456686012333576	0.259	0.173	1.31963593327662E-12	Mid-zonal hepatocytes	JDP2
8.16347689765627E-17	0.353297369573119	0.423	0.335	1.33154471677671E-12	Mid-zonal hepatocytes	ITPA
8.35362272531079E-17	1.31006492634452	0.17	0.098	1.36255940272544E-12	Mid-zonal hepatocytes	PRPF40B
8.43385336990466E-17	1.49585297551505	0.148	0.082	1.37564582316515E-12	Mid-zonal hepatocytes	SETDB2
8.4575336875069E-17	0.514816562589701	0.79	0.68	1.37950831976925E-12	Mid-zonal hepatocytes	HSPB1
8.54113219429105E-17	0.843589146772828	0.188	0.112	1.39314407221081E-12	Mid-zonal hepatocytes	NAV1
8.73013529170094E-17	0.34533041965312	0.452	0.365	1.42397236742934E-12	Mid-zonal hepatocytes	DNAJC21
8.83564598818652E-17	1.05506338133339	0.228	0.148	1.4411822171331E-12	Mid-zonal hepatocytes	PPP2R5E
9.11263922418006E-17	1.39240965448205	0.164	0.093	1.48636258385601E-12	Mid-zonal hepatocytes	GPR89B
9.17480249944432E-17	1.09423884752779	0.131	0.067	1.49650203568436E-12	Mid-zonal hepatocytes	ZNF500
9.32135378079085E-17	1.28611237169175	0.164	0.093	1.5204060151848E-12	Mid-zonal hepatocytes	ESCO1
9.70497739631847E-17	1.45670782080779	0.162	0.092	1.58297886311351E-12	Mid-zonal hepatocytes	GTF2IRD2E
9.9218256265656E-17	0.673375520309932	0.275	0.192	1.61834897794912E-12	Mid-zonal hepatocytes	DPH2
1.01702108259264E-16	0.843890941126183	0.255	0.176	1.65886308781685E-12	Mid-zonal hepatocytes	DNAJC13
1.02557905574971E-16	0.909929492059401	0.194	0.119	1.67282199783335E-12	Mid-zonal hepatocytes	ATP8B1
1.06481320179981E-16	1.08154852966394	0.176	0.104	1.73681681345566E-12	Mid-zonal hepatocytes	RYK
1.06676343669638E-16	1.04925865134765	0.176	0.103	1.73999784159547E-12	Mid-zonal hepatocytes	SMARCC2
1.07683856740042E-16	0.617607930451829	0.336	0.252	1.75643138728683E-12	Mid-zonal hepatocytes	SEC63
1.08652522316454E-16	0.742142677786245	0.249	0.168	1.77223129150368E-12	Mid-zonal hepatocytes	SH3BP5L
1.16208848059698E-16	0.743066887971002	0.208	0.13	1.89548252070174E-12	Mid-zonal hepatocytes	CABIN1
1.1638645655269E-16	0.874319815194011	0.174	0.1	1.89837949283092E-12	Mid-zonal hepatocytes	RAVER1
1.16926234337457E-16	0.81442309397378	0.23	0.151	1.90718380827826E-12	Mid-zonal hepatocytes	GPS2
1.18983959880513E-16	0.763840923290823	0.301	0.218	1.94074736961104E-12	Mid-zonal hepatocytes	TCAF1
1.20087724635457E-16	0.427738696153562	0.36	0.272	1.95875087652895E-12	Mid-zonal hepatocytes	CTDSP2
1.26427190476803E-16	0.907251817162328	0.239	0.159	2.06215390386714E-12	Mid-zonal hepatocytes	SEC23IP
1.27121564604389E-16	1.08290696159275	0.252	0.173	2.0734798402622E-12	Mid-zonal hepatocytes	C6orf62
1.28529580271049E-16	0.63262002864856	0.241	0.16	2.09644598380108E-12	Mid-zonal hepatocytes	RUSC1
1.33320511068761E-16	1.20113200540141	0.235	0.157	2.17459085604256E-12	Mid-zonal hepatocytes	IL1R1
1.3483429749383E-16	1.06599226939631	0.263	0.185	2.19928222642186E-12	Mid-zonal hepatocytes	CEPT1
1.35215293658508E-16	0.756178894211083	0.285	0.202	2.20549665486393E-12	Mid-zonal hepatocytes	TMPRSS2
1.36621882572029E-16	1.11274925496422	0.217	0.139	2.22843952663237E-12	Mid-zonal hepatocytes	ABLIM1
1.36808906685286E-16	0.956622571847773	0.191	0.117	2.2314900769437E-12	Mid-zonal hepatocytes	TMEM94
1.40559185769379E-16	1.3123115283786	0.162	0.092	2.29266087908433E-12	Mid-zonal hepatocytes	HP1BP3
1.42199050909676E-16	1.42138158644442	0.146	0.079	2.31940871938773E-12	Mid-zonal hepatocytes	ATG10
1.42610767097211E-16	1.22615998075983	0.206	0.131	2.32612422212261E-12	Mid-zonal hepatocytes	UQCC1
1.43934742314304E-16	0.250621057645971	0.391	0.302	2.34771958188862E-12	Mid-zonal hepatocytes	KPNA4
1.48101103487391E-16	1.38714772954254	0.122	0.061	2.41567709898283E-12	Mid-zonal hepatocytes	CEP85L
1.50767282518958E-16	1.24605987245698	0.213	0.136	2.45916514516673E-12	Mid-zonal hepatocytes	FMO4
1.51473629472037E-16	1.6467400291312	0.125	0.063	2.4706863703184E-12	Mid-zonal hepatocytes	IFT81
1.52605812843676E-16	1.0447317543919	0.21	0.133	2.48915341329 32E-12	Mid-zonal hepatocytes	TTC14
1.58091643828861E-16	0.682981145547383	0.25	0.168	2.57863280249256E-12	Mid-zonal hepatocytes	TICAM1
1.62096516916671E-16	0.682720862737724	0.42	0.331	2.64395628742782E-12	Mid-zonal hepatocytes	GADD45A

**1.65783066919434E-16**	0.349437764279655	0.43	0.336	2.70408760452289E-12	Mid-zonal hepatocytes	SLC50A1
**1.67478882002816E-16**	1.49943520118053	0.133	0.07	2.73174804434793E-12	Mid-zonal hepatocytes	CSNK1G1
**1.68225219597487E-16**	0.809209075018374	0.215	0.137	2.74392155685461E-12	Mid-zonal hepatocytes	DUSP11
**1.6916396947758E-16**	0.578880422735955	0.297	0.214	2.7592335061488E-12	Mid-zonal hepatocytes	PITPNM1
**1.74355391081502E-16**	1.13700169532965	0.243	0.165	2.84391078393038E-12	Mid-zonal hepatocytes	PIK3CB
**1.7461964342346E-16**	1.1177425678189	0.212	0.136	2.84822100388006E-12	Mid-zonal hepatocytes	LARP4B
**1.75850884717355E-16**	0.481574805440349	0.451	0.362	2.86830378062478E-12	Mid-zonal hepatocytes	NMD3
**1.81281934690104E-16**	1.22061586526019	0.192	0.118	2.95688963673028E-12	Mid-zonal hepatocytes	LRP6
**1.8130228957809E-16**	0.705323665560089	0.205	0.128	2.95722164530823E-12	Mid-zonal hepatocytes	SLC26A11
**1.93845081433472E-16**	0.92032758443377	0.266	0.187	3.16180712326136E-12	Mid-zonal hepatocytes	INO80E
**1.98574479971507E-16**	1.46743178152661	0.182	0.11	3.23894834281525E-12	Mid-zonal hepatocytes	TCF12
**2.00372440171294E-16**	0.809481139646657	0.284	0.203	3.26827487163398E-12	Mid-zonal hepatocytes	SMARCC1
**2.01305716428759E-16**	0.388030500719682	0.32	0.232	3.2834975406695E-12	Mid-zonal hepatocytes	FAM3A
**2.05924186104221E-16**	0.818735374423131	0.269	0.181	3.35882939954595E-12	Mid-zonal hepatocytes	IFIT2
**2.14531101966837E-16**	0.769936996004327	0.304	0.222	3.49921680418108E-12	Mid-zonal hepatocytes	FAT1
**2.2045480577423E-16**	1.60650803200495	0.167	0.098	3.59583833698346E-12	Mid-zonal hepatocytes	ACTR5
**2.21854097177378E-16**	1.19108196511115	0.156	0.087	3.61866217906021E-12	Mid-zonal hepatocytes	NUP205
**2.26183717758617E-16**	1.21593656053135	0.2	0.126	3.6892826203608E-12	Mid-zonal hepatocytes	ADSS
**2.27901036466322E-16**	1.01199948670829	0.282	0.203	3.71729380580217E-12	Mid-zonal hepatocytes	SLC20A2
**2.28937907761232E-16**	1.23056385449048	0.173	0.102	3.73420621349345E-12	Mid-zonal hepatocytes	SETX
**2.31983624784511E-16**	0.594986419719555	0.287	0.204	3.78388490386016E-12	Mid-zonal hepatocytes	TRA2B
**2.32161431554756E-16**	0.981234916075602	0.216	0.138	3.78678511008962E-12	Mid-zonal hepatocytes	PDLIM7
**2.36992444897277E-16**	0.78578305574977	0.238	0.159	3.86558376871948E-12	Mid-zonal hepatocytes	PPP2R5C
**2.38772045925022E-16**	1.04922346477037	0.159	0.09	3.89461084108304E-12	Mid-zonal hepatocytes	NUP107
**2.39890414672851E-16**	2.10193950145754	0.121	0.061	3.91285255372888E-12	Mid-zonal hepatocytes	KCNMB3
**2.41870910211199E-16**	0.841817655901345	0.263	0.183	3.94515641645486E-12	Mid-zonal hepatocytes	VPS36
**2.53932433080729E-16**	1.73761770114532	0.116	0.057	4.14189191597977E-12	Mid-zonal hepatocytes	GTDC1
**2.56761854894884E-16**	1.93915274445883	0.1	0.045	4.18804261519045E-12	Mid-zonal hepatocytes	RABL2A
**2.5720017721647E-16**	0.584459987994381	0.259	0.18	4.19519209057784E-12	Mid-zonal hepatocytes	FXR2
**2.61993654743573E-16**	1.29308686076922	0.177	0.106	4.27337850252242E-12	Mid-zonal hepatocytes	RUBCN
**2.62416078706504E-16**	0.998200480680659	0.216	0.14	4.28026865978179E-12	Mid-zonal hepatocytes	THOC6
**2.64896889842626E-16**	0.685270864169909	0.32	0.237	4.32073317022307E-12	Mid-zonal hepatocytes	TTC17
**2.665773854307E-16**	1.4621071040837	0.192	0.12	4.34814373376014E-12	Mid-zonal hepatocytes	BOD1
**2.81837879512478E-16**	1.25715422305018	0.178	0.107	4.59705765272802E-12	Mid-zonal hepatocytes	RAB24
**2.86111507565425E-16**	0.465223170030172	0.364	0.28	4.66676479989965E-12	Mid-zonal hepatocytes	COPB1
**2.89297247208013E-16**	0.836758733893007	0.208	0.132	4.7187273992099E-12	Mid-zonal hepatocytes	RAB11FIP3
**2.90048049994647E-16**	0.918406402996467	0.18	0.107	4.73097374346269E-12	Mid-zonal hepatocytes	STX2
**3.00266646298626E-16**	0.453364957968765	0.384	0.299	4.89764926777689E-12	Mid-zonal hepatocytes	DEPRECAT
**3.04182930076361E-16**	1.17755885861806	0.2	0.126	4.96152777247552E-12	Mid-zonal hepatocytes	WDR36
**3.0695602684486E-16**	1.42829208109007	0.156	0.088	5.0067597538665E-12	Mid-zonal hepatocytes	PIGZ
**3.11834112565734E-16**	0.727469951731943	0.34	0.26	5.08632621005969E-12	Mid-zonal hepatocytes	UBA7
**3.17796053372498E-16**	0.649222970736143	0.183	0.109	5.18357142655881E-12	Mid-zonal hepatocytes	MAP3K14
**3.19581730107837E-16**	1.3753509927662	0.166	0.096	5.21269759978893E-12	Mid-zonal hepatocytes	WDR37
**3.23561661434705E-16**	1.16405801111428	0.204	0.13	5.27761425966147E-12	Mid-zonal hepatocytes	SH3GLB2
**3.25661183656938E-16**	0.913101575990379	0.269	0.189	5.31185956662831E-12	Mid-zonal hepatocytes	CPSF1
**3.30264394060575E-16**	1.22472949266483	0.148	0.081	5.38694253152204E-12	Mid-zonal hepatocytes	ZNF496
**3.4131361157997E-16**	0.94764987685462	0.188	0.115	5.56716631848089E-12	Mid-zonal hepatocytes	SMPD2
**3.41328818582113E-16**	1.49201083144301	0.105	0.049	5.56741435989284E-12	Mid-zonal hepatocytes	IPPK
**3.41402388675846E-16**	1.41610096766001	0.184	0.112	5.56861436169172E-12	Mid-zonal hepatocytes	LDAH
**3.44663323054453E-16**	0.679978031525166	0.163	0.093	5.62180346234118E-12	Mid-zonal hepatocytes	PARD6B
**3.55669431966223E-16**	1.85742129402876	0.136	0.073	5.80132410480106E-12	Mid-zonal hepatocytes	MXD1
**3.58349588084235E-16**	0.564691670201936	0.322	0.239	5.84504013124195E-12	Mid-zonal hepatocytes	ATN1
**3.60390900079399E-16**	0.782492767275887	0.291	0.212	5.87833597119508E-12	Mid-zonal hepatocytes	NUFIP2
**3.635494840245E-16**	1.17637779505401	0.23	0.154	5.92985563392362E-12	Mid-zonal hepatocytes	HDAC8
**3.84754272096527E-16**	1.51311060155197	0.157	0.09	6.27572693216645E-12	Mid-zonal hepatocytes	ZNF529
**3.9094909974374E-16**	1.28954415606967	0.185	0.113	6.37677076592014E-12	Mid-zonal hepatocytes	D2HGDH
**3.9674366609194E-16**	1.21495998974783	0.198	0.125	6.47128593762564E-12	Mid-zonal hepatocytes	SLF2
**4.03780540091459E-16**	1.2376237797725	0.161	0.092	6.58606438943179E-12	Mid-zonal hepatocytes	TNRC6C
**4.07381527069906E-16**	0.754960310618646	0.291	0.21	6.64480008803723E-12	Mid-zonal hepatocytes	ZBTB48
**4.08918519291062E-16**	0.542660071950947	0.303	0.218	6.66986996815652E-12	Mid-zonal hepatocytes	EML2

4.09793458109603E-16	0.710198251647374	0.219	0.141	6.68414109522573E-12	Mid-zonal hepatocytes	SNX21
4.25454891371207E-16	0.582673857765858	0.275	0.194	6.93959473315576E-12	Mid-zonal hepatocytes	ADAMTSL4
4.35327473652194E-16	1.55677865698714	0.105	0.049	7.10062642274093E-12	Mid-zonal hepatocytes	TRIM52
4.46875596330814E-16	1.37860115041706	0.144	0.078	7.2889878517519E-12	Mid-zonal hepatocytes	CWC25
4.47088421501543E-16	0.829729259694087	0.269	0.189	7.29245924311166E-12	Mid-zonal hepatocytes	MLYCD
4.62657698735278E-16	0.994358716866769	0.153	0.085	7.54640972407112E-12	Mid-zonal hepatocytes	RASA1
4.68183957090598E-16	0.600447069778604	0.29	0.209	7.63654852410475E-12	Mid-zonal hepatocytes	TERF2IP
4.8385627030716E-16	1.20558362070435	0.188	0.115	7.89217962498009E-12	Mid-zonal hepatocytes	RESF1
4.92605362940632E-16	1.4926478683222	0.126	0.064	8.03488607492465E-12	Mid-zonal hepatocytes	STXBP4
5.04643919395141E-16	0.877367446300092	0.225	0.148	8.23124696925415E-12	Mid-zonal hepatocytes	TIMM10B
5.05599832333728E-16	1.39675087212908	0.166	0.097	8.24683886519544E-12	Mid-zonal hepatocytes	ZNF224
5.40208804289236E-16	1.25098410432615	0.162	0.094	8.81134580676172E-12	Mid-zonal hepatocytes	NIPSNAP2
5.61449155526721E-16	1.52746895087471	0.138	0.074	9.15779717579635E-12	Mid-zonal hepatocytes	PLD2
5.93256820151553E-16	0.929503032541835	0.12	0.06	9.67661199349198E-12	Mid-zonal hepatocytes	VIM
5.99643352162952E-16	0.694327337443341	0.212	0.135	9.7807827171299E-12	Mid-zonal hepatocytes	RANGAP1
6.2353323906348E-16	1.4640421510209	0.167	0.099	1.01704506623644E-11	Mid-zonal hepatocytes	ZNF131
6.28371784009378E-16	0.62277644193193	0.256	0.177	1.0249372168977E-11	Mid-zonal hepatocytes	FOXK1
6.3046324394838E-16	1.77218928554112	0.107	0.051	1.0283485972042E-11	Mid-zonal hepatocytes	KIAA1257
6.62228797531768E-16	1.20741778529126	0.135	0.072	1.08016139165407E-11	Mid-zonal hepatocytes	KIAA0355
6.62504029337692E-16	0.857920572225255	0.219	0.144	1.08061032225271E-11	Mid-zonal hepatocytes	YARS
6.74561191307804E-16	1.11058518409383	0.169	0.099	1.10027675914216E-11	Mid-zonal hepatocytes	SEL1L3
6.79784468635434E-16	0.794719801027593	0.254	0.178	1.10879644679126E-11	Mid-zonal hepatocytes	AFTPH
6.86051293191553E-16	0.839271145982336	0.226	0.149	1.11901826432474E-11	Mid-zonal hepatocytes	GFOD1
6.91707364085316E-16	0.641025697447651	0.3	0.219	1.12824388155956E-11	Mid-zonal hepatocytes	ERBB2
7.16252007234177E-16	0.947970770212771	0.223	0.149	1.16827864899967E-11	Mid-zonal hepatocytes	RSPRY1
7.19502819087268E-16	0.623096779582405	0.303	0.222	1.17358104821324E-11	Mid-zonal hepatocytes	COPS5
7.43197120358316E-16	1.35309286125289	0.136	0.073	1.21222882301645E-11	Mid-zonal hepatocytes	ZMYM6
7.45083692768117E-16	0.937532825396382	0.212	0.137	1.21530601127408E-11	Mid-zonal hepatocytes	TJAP1
7.64721846785235E-16	0.961485292754411	0.232	0.156	1.2473378042914E-11	Mid-zonal hepatocytes	PCNX3
7.69487686474462E-16	1.15946083373525	0.166	0.097	1.2551113654085E-11	Mid-zonal hepatocytes	RAD54L2
7.80489665003156E-16	0.779402197860266	0.266	0.187	1.27305669258665E-11	Mid-zonal hepatocytes	TRIP12
7.82165555935531E-16	0.981054073411123	0.231	0.154	1.27579023828644E-11	Mid-zonal hepatocytes	SKIV2L
7.85816812763265E-16	1.45779620636707	0.144	0.079	1.28174580329816E-11	Mid-zonal hepatocytes	TOGARAM1
8.06766359746482E-16	1.28072436322662	0.204	0.131	1.31591660938249E-11	Mid-zonal hepatocytes	GPCPD1
8.07471618065233E-16	0.454635018549096	0.382	0.3	1.3170669562262E-11	Mid-zonal hepatocytes	QARS
8.20838232755145E-16	1.4457535369079	0.153	0.087	1.33886924144692E-11	Mid-zonal hepatocytes	GABPB1
8.35225867699443E-16	1.44907183770115	0.162	0.094	1.36233691280456E-11	Mid-zonal hepatocytes	MEIS2
8.38436216474734E-16	0.925943514978347	0.178	0.107	1.36757331269194E-11	Mid-zonal hepatocytes	RND3
9.0298845632814E-16	1.52575418639043	0.116	0.057	1.47286447111683E-11	Mid-zonal hepatocytes	ZNF324
9.06714021445996E-16	1.19104392730507	0.161	0.093	1.47894124038056E-11	Mid-zonal hepatocytes	RPUSD4
9.11248886149189E-16	0.917956806252112	0.18	0.109	1.48613580179794E-11	Mid-zonal hepatocytes	AP5M1
9.23909143445833E-16	0.415991108438806	0.35	0.268	1.5069882038745E-11	Mid-zonal hepatocytes	MRS2
9.25399429399478E-16	1.46502519574336	0.103	0.048	1.50941900929349E-11	Mid-zonal hepatocytes	ZNF778
9.46809677259044E-16	1.16576652239624	0.157	0.09	1.54434126457723E-11	Mid-zonal hepatocytes	RNF38
9.47770227040585E-16	0.832707412271071	0.259	0.18	1.5459080173259E-11	Mid-zonal hepatocytes	ZFHX3
9.62302806510447E-16	0.679109132674445	0.222	0.145	1.56961210769919E-11	Mid-zonal hepatocytes	ZNF704
9.92673661285539E-16	1.23867359032379	0.122	0.062	1.61915000892284E-11	Mid-zonal hepatocytes	PPP2R2A
1.00158624160699E-15	0.555901019162707	0.184	0.11	1.63368731868517E-11	Mid-zonal hepatocytes	SH2B3
1.00577815430603E-15	0.944413334424058	0.196	0.123	1.64052474748857E-11	Mid-zonal hepatocytes	LZTR1
1.01705577762605E-15	0.374391997578125	0.399	0.313	1.65891967888585E-11	Mid-zonal hepatocytes	ABCF1
1.02283357044692E-15	1.53378445032071	0.113	0.056	1.66834383675598E-11	Mid-zonal hepatocytes	ZFYVE26
1.02289436095649E-15	0.813029469693792	0.276	0.198	1.66844299215613E-11	Mid-zonal hepatocytes	CHD4
1.03041685550331E-15	1.36542185672278	0.205	0.133	1.68071293301146E-11	Mid-zonal hepatocytes	KIAA1109
1.03725761108679E-15	1.54087876051764	0.117	0.058	1.69187088944367E-11	Mid-zonal hepatocytes	DDTL
1.04604739675974E-15	0.733029081445225	0.238	0.16	1.70620790885481E-11	Mid-zonal hepatocytes	NOP9
1.16183828264577E-15	1.44665332659402	0.16	0.094	1.89507442282352E-11	Mid-zonal hepatocytes	RNF111
1.18172787033694E-15	0.667007467170394	0.313	0.234	1.92751632930658E-11	Mid-zonal hepatocytes	EMC2
1.19739656973112E-15	0.736181529527836	0.223	0.147	1.95307354488844E-11	Mid-zonal hepatocytes	DNAJB2
1.21917187103709E-15	0.961306473856359	0.125	0.064	1.9885912388486E-11	Mid-zonal hepatocytes	BTBD3
1.22412422816781E-15	1.12888659726894	0.169	0.101	1.99666902856452E-11	Mid-zonal hepatocytes	ZCCHC4

1.22566020019055E-15	0.675550369504411	0.244	0.166	1.9991743525308E-11	Mid-zonal hepatocytes	DDX21
1.23652713169453E-15	0.864444012108736	0.203	0.13	2.01689940450694E-11	Mid-zonal hepatocytes	AZI2
1.33088872135869E-15	1.10484074007459	0.129	0.067	2.17081259340816E-11	Mid-zonal hepatocytes	GAS6
1.35112326755492E-15	0.697268756560131	0.181	0.11	2.20381716170883E-11	Mid-zonal hepatocytes	PPTC7
1.35999973439783E-15	0.720198513142741	0.244	0.167	2.21829556677631E-11	Mid-zonal hepatocytes	DDX42
1.38351558582722E-15	1.06937505435626	0.145	0.08	2.25665227204278E-11	Mid-zonal hepatocytes	CSRNP2
1.38887093236033E-15	1.19348622743272	0.217	0.143	2.26538737777293E-11	Mid-zonal hepatocytes	USF3
1.39012047332601E-15	0.62563572453741	0.311	0.231	2.26742550404205E-11	Mid-zonal hepatocytes	ACVR1B
1.40746977877285E-15	1.01045453789157	0.219	0.146	2.29572395615639E-11	Mid-zonal hepatocytes	EEF1AKMT
1.44066214150033E-15	0.845377840520097	0.246	0.169	2.34986401900119E-11	Mid-zonal hepatocytes	CDK13
1.44179028193955E-15	0.925223041284202	0.315	0.237	2.3517041288716E-11	Mid-zonal hepatocytes	IL17RB
1.47380374864882E-15	0.303119749034916	0.445	0.36	2.40392129442109E-11	Mid-zonal hepatocytes	OSBP
1.47537036631679E-15	1.40760160094116	0.14	0.077	2.40647660449932E-11	Mid-zonal hepatocytes	RAB6B
1.49630137288881E-15	0.34433030771079	0.341	0.258	2.44061716931894E-11	Mid-zonal hepatocytes	KLF3
1.51525911867027E-15	0.726695628212858	0.18	0.109	2.47153914846308E-11	Mid-zonal hepatocytes	BCOR
1.52624700629754E-15	1.34961122841182	0.195	0.123	2.48946149197191E-11	Mid-zonal hepatocytes	USP8
1.52772372815928E-15	1.19483033549666	0.166	0.098	2.49187017300061E-11	Mid-zonal hepatocytes	EIF4ENIF1
1.61737336888431E-15	1.19615134697268	0.141	0.078	2.6380977019872E-11	Mid-zonal hepatocytes	MCM9
1.64770226894677E-15	0.642290354542363	0.172	0.103	2.68756717087908E-11	Mid-zonal hepatocytes	KLC2
1.65970225608779E-15	1.41340500669088	0.13	0.069	2.70714034990479E-11	Mid-zonal hepatocytes	ZMYM5
1.68503156533515E-15	0.616938011192069	0.285	0.207	2.74845498621817E-11	Mid-zonal hepatocytes	CACUL1
1.69071741662493E-15	1.11558526338228	0.157	0.09	2.75772917825692E-11	Mid-zonal hepatocytes	R3HCC1L
1.70043005605096E-15	1.49160417944724	0.104	0.049	2.77357146442472E-11	Mid-zonal hepatocytes	KIAA0895L
1.75986413810073E-15	0.635274609626726	0.191	0.118	2.87051439565611E-11	Mid-zonal hepatocytes	TATDN2
1.83099575812381E-15	1.10847437006178	0.207	0.136	2.98653718107574E-11	Mid-zonal hepatocytes	RINT1
1.84135305114454E-15	0.50386613483633	0.302	0.221	3.00343096172186E-11	Mid-zonal hepatocytes	TUBGCP2
1.8941399466777E-15	0.849912582243745	0.216	0.14	3.089531667026E-11	Mid-zonal hepatocytes	SH2D4A
1.90763589707812E-15	0.782638309359816	0.297	0.221	3.11154491172412E-11	Mid-zonal hepatocytes	HIPK3
1.90839259369025E-15	0.986690690870629	0.15	0.084	3.11277915956817E-11	Mid-zonal hepatocytes	MVB12B
1.91532406037987E-15	1.59839419309839	0.148	0.084	3.12408507488561E-11	Mid-zonal hepatocytes	ARID2
2.00062152369922E-15	1.32969906027028	0.152	0.086	3.2632137673058E-11	Mid-zonal hepatocytes	MOB3B
2.095274961288E-15	0.723003006020835	0.254	0.178	3.41760298935685E-11	Mid-zonal hepatocytes	CORO1C
2.10125478909748E-15	0.985045676549442	0.212	0.138	3.4273566864969E-11	Mid-zonal hepatocytes	TMEM8B
2.12737463938047E-15	0.392734277387048	0.373	0.29	3.46996077429348E-11	Mid-zonal hepatocytes	SLC35B1
2.24187160233916E-15	0.976427861755587	0.193	0.121	3.6567167705754E-11	Mid-zonal hepatocytes	RABGGTA
2.25476044878644E-15	0.491272832110038	0.32	0.239	3.67773976801556E-11	Mid-zonal hepatocytes	ZFAND2B
2.29845910804112E-15	1.43484976371432	0.147	0.083	3.74901665112588E-11	Mid-zonal hepatocytes	ELP1
2.30630004489307E-15	1.51734958666556	0.139	0.077	3.76180600322508E-11	Mid-zonal hepatocytes	CYP2R1
2.32149653076113E-15	1.89105099536365	0.101	0.047	3.78659299132448E-11	Mid-zonal hepatocytes	TLCD5
2.36550652655178E-15	2.03768805669247	0.1	0.047	3.85837769545861E-11	Mid-zonal hepatocytes	TRIM66
2.43451725054977E-15	1.8118606854234	0.102	0.048	3.97094108737173E-11	Mid-zonal hepatocytes	PRELID2
2.44412232834635E-15	1.73841732177105	0.122	0.063	3.98660792976573E-11	Mid-zonal hepatocytes	FAM13B
2.45995824470121E-15	0.549957504953487	0.255	0.175	4.01243789293214E-11	Mid-zonal hepatocytes	WDR74
2.49849743801313E-15	1.26238171807969	0.151	0.086	4.07529917114322E-11	Mid-zonal hepatocytes	SOS 1
2.56734821502385E-15	0.898199871613135	0.179	0.109	4.1876016735254E-11	Mid-zonal hepatocytes	DDX51
2.5693462337018E-15	0.699001908286529	0.268	0.192	4.19086064179101E-11	Mid-zonal hepatocytes	USP19
2.66363311886907E-15	1.13467224165964	0.184	0.115	4.34465198018734E-11	Mid-zonal hepatocytes	PPIL2
2.71648233275187E-15	0.583478612604662	0.258	0.181	4.43085433295158E-11	Mid-zonal hepatocytes	RNF123
2.7266823222451E-15	0.806833732596668	0.231	0.156	4.44749153581399E-11	Mid-zonal hepatocytes	PRKAB1
2.77251731787431E-15	0.965084426335534	0.277	0.201	4.52225299718479E-11	Mid-zonal hepatocytes	TRPT1
2.81689847363264E-15	1.69899144225698	0.118	0.06	4.5946431003422E-11	Mid-zonal hepatocytes	ARHGAP32
2.88544239709034E-15	0.576852485667404	0.337	0.26	4.70644509389405E-11	Mid-zonal hepatocytes	RARS
2.88947464845822E-15	0.760393344731331	0.241	0.165	4.71302209910021E-11	Mid-zonal hepatocytes	ZSCAN2
2.93050509382721E-15	1.37390436624932	0.135	0.074	4.77994685854156E-11	Mid-zonal hepatocytes	LEMD3
2.94345208455111E-15	0.8655681367608	0.237	0.161	4.80106469511131E-11	Mid-zonal hepatocytes	EFCAB14
2.99795738029776E-15	1.50252273044724	0.101	0.048	4.88996828300367E-11	Mid-zonal hepatocytes	TTF2
3.03600817214733E-15	0.62308737894464	0.264	0.186	4.9520329295895E-11	Mid-zonal hepatocytes	RAB6A
3.03920444506171E-15	0.590112505982299	0.222	0.147	4.95724637034015E-11	Mid-zonal hepatocytes	RFC1
3.04100998590188E-15	1.17881392469613	0.16	0.094	4.96019138800455E-11	Mid-zonal hepatocytes	DEPRECAT
3.06615476475233E-15	0.912205423539984	0.25	0.175	5.00120503678753E-11	Mid-zonal hepatocytes	SAMD4A

3.12933317745335E-15	0.582839774769609	0.229	0.152	5.10425534574416E-11	Mid-zonal hepatocytes	RAB27A
3.13107069999863E-15	0.621450396312519	0.231	0.154	5.10708941876776E-11	Mid-zonal hepatocytes	JOSD1
3.13622658653664E-15	0.366370579451521	0.38	0.299	5.11549918529992E-11	Mid-zonal hepatocytes	TXLNA
3.13847282650684E-15	1.73239174215884	0.123	0.064	5.1191630273153E-11	Mid-zonal hepatocytes	CEACAM19
3.22447436192217E-15	0.949632327167919	0.192	0.122	5.25944013173125E-11	Mid-zonal hepatocytes	ERI1
3.32291391201124E-15	0.86593013746367	0.191	0.121	5.42000488188153E-11	Mid-zonal hepatocytes	WDR43
3.34284371199799E-15	1.22271232932392	0.128	0.067	5.45251237863992E-11	Mid-zonal hepatocytes	ING5
3.39380802648842E-15	0.655522483601353	0.243	0.168	5.53564027200526E-11	Mid-zonal hepatocytes	INPP5A
3.4330374846275E-15	1.47300164346194	0.154	0.089	5.59962744117591E-11	Mid-zonal hepatocytes	ZNF740
3.43392401572641E-15	1.89640939292957	0.107	0.052	5.60107346205135E-11	Mid-zonal hepatocytes	FRMD8
3.47295829605036E-15	1.10207359245543	0.158	0.091	5.66474227668774E-11	Mid-zonal hepatocytes	SLC1A5
3.5185508447007E-15	1.39146433216996	0.182	0.114	5.7391082827913E-11	Mid-zonal hepatocytes	CHD6
3.52255695828588E-15	1.01644461125642	0.19	0.12	5.7456426546601E-11	Mid-zonal hepatocytes	RNFT1
3.52662829146022E-15	0.835682246012752	0.278	0.2	5.75228340620076E-11	Mid-zonal hepatocytes	SMARCA1
3.55631889184326E-15	1.39330582936919	0.157	0.091	5.80071174448554E-11	Mid-zonal hepatocytes	MTERF2
3.55687111403933E-15	1.36959346538195	0.188	0.119	5.80161247410955E-11	Mid-zonal hepatocytes	SLC10A7
3.56084778943254E-15	0.431581713553479	0.402	0.322	5.80809882934341E-11	Mid-zonal hepatocytes	SERINC3
3.63031210868983E-15	1.05365149089048	0.145	0.081	5.92140208048398E-11	Mid-zonal hepatocytes	BEND7
3.64928874137496E-15	1.72977443341438	0.11	0.054	5.9523548660567E-11	Mid-zonal hepatocytes	MIPOL1
3.65538817873275E-15	0.929099844615803	0.228	0.155	5.96230365833098E-11	Mid-zonal hepatocytes	POLL
3.65850843116698E-15	0.833887059383763	0.221	0.147	5.96739310207646E-11	Mid-zonal hepatocytes	MARK2
3.67982245554908E-15	1.32548817752594	0.151	0.086	6.0021584072461E-11	Mid-zonal hepatocytes	UHRF2
3.72011095191748E-15	0.570411773865126	0.324	0.245	6.06787297367261E-11	Mid-zonal hepatocytes	AGGF1
3.80843859321323E-15	1.02773456500249	0.122	0.062	6.21194418939009E-11	Mid-zonal hepatocytes	RRAD
3.80993957662684E-15	1.04189504663556	0.212	0.14	6.21439244343605E-11	Mid-zonal hepatocytes	ADAT1
3.93774618534627E-15	0.764967500828938	0.231	0.156	6.42285780291831E-11	Mid-zonal hepatocytes	SNX6
3.95748478790089E-15	1.06230738975247	0.157	0.091	6.45505343754514E-11	Mid-zonal hepatocytes	FAM111A
4.00955497207053E-15	1.28436689476535	0.128	0.068	6.53998511494424E-11	Mid-zonal hepatocytes	XPO7
4.04241307801829E-15	0.848576363779069	0.254	0.179	6.59357997155563E-11	Mid-zonal hepatocytes	ATL2
4.10949923523539E-15	0.830306139513044	0.192	0.12	6.70300420259244E-11	Mid-zonal hepatocytes	PHACTR4
4.17332517749253E-15	0.567149308281901	0.386	0.299	6.80711069700807E-11	Mid-zonal hepatocytes	SLC25A18
4.24337465509503E-15	0.345819117093888	0.356	0.276	6.9213683999255E-11	Mid-zonal hepatocytes	NCBP2
4.2904330168348E-15	0.947310289018942	0.154	0.089	6.99812529375924E-11	Mid-zonal hepatocytes	SBNO1
4.35203131774271E-15	0.932798092640956	0.209	0.136	7.09859828237014E-11	Mid-zonal hepatocytes	SPRED2
4.36118583459792E-15	1.20042386632472	0.209	0.138	7.11353021481267E-11	Mid-zonal hepatocytes	CNST
4.53321364796995E-15	1.29994405550463	0.146	0.082	7.39412478120379E-11	Mid-zonal hepatocytes	GOLGA1
4.64384450973638E-15	0.709744796231972	0.248	0.172	7.57457477983101E-11	Mid-zonal hepatocytes	PURB
4.67431111126488E-15	1.32335140595898	0.148	0.084	7.62426885358415E-11	Mid-zonal hepatocytes	DEPRECAT
4.76505811234998E-15	0.797138827588408	0.335	0.256	7.77228628705406E-11	Mid-zonal hepatocytes	GLRX
4.81325279490538E-15	1.45909468687152	0.138	0.077	7.85089663377016E-11	Mid-zonal hepatocytes	SMG7
4.84272885245479E-15	0.92213877173227	0.237	0.163	7.89897503123901E-11	Mid-zonal hepatocytes	TRAM2
4.91686731226745E-15	1.65615670852157	0.102	0.049	8.01990227303943E-11	Mid-zonal hepatocytes	NAA16
5.29569678307669E-15	0.820780590835187	0.21	0.137	8.63781102287638E-11	Mid-zonal hepatocytes	NKIRAS1
5.51000006724953E-15	1.47679719249091	0.128	0.068	8.98736110969071E-11	Mid-zonal hepatocytes	ZCCHC8
5.77358514975978E-15	0.889652742634782	0.229	0.155	9.41729473777318E-11	Mid-zonal hepatocytes	TBC1D23
5.78297111986051E-15	1.2576621506013	0.148	0.084	9.43260419360448E-11	Mid-zonal hepatocytes	AP4B1
5.79804692892409E-15	0.842961651512175	0.229	0.156	9.45719434576808E-11	Mid-zonal hepatocytes	ZNF652
5.8210289287952E-15	1.39190156744048	0.134	0.073	9.49468028575785E-11	Mid-zonal hepatocytes	CDK7
5.84862679501067E-15	1.15694165496167	0.176	0.108	9.53969516534191E-11	Mid-zonal hepatocytes	NFXL1
5.95343305654211E-15	1.20917591208128	0.202	0.131	9.71064465852584E-11	Mid-zonal hepatocytes	ARHGAP29
6.3702420033749E-15	0.8782871098392	0.25	0.175	1.03905017317048E-10	Mid-zonal hepatocytes	SRPK1
6.51063685095473E-15	1.69856760301622	0.126	0.067	1.06194997675923E-10	Mid-zonal hepatocytes	ZNF248
6.69450413496756E-15	1.69837837643476	0.107	0.052	1.09194056945456E-10	Mid-zonal hepatocytes	FANCL
6.74021277954646E-15	1.08333533440026	0.19	0.12	1.09939610647182E-10	Mid-zonal hepatocytes	CRCP
6.87135236114113E-15	1.40239261220318	0.132	0.072	1.12078628362573E-10	Mid-zonal hepatocytes	SMURF2
7.11553910934562E-15	0.832145596098991	0.206	0.134	1.16061558412536E-10	Mid-zonal hepatocytes	AP2A2
7.17542749084151E-15	1.38403129830522	0.147	0.084	1.17038397803116E-10	Mid-zonal hepatocytes	ALMS1
7.19489801808873E-15	1.25486080386851	0.118	0.061	1.17355981573045E-10	Mid-zonal hepatocytes	UNC13B
7.1996194846558E-15	0.323548412736624	0.458	0.367	1.17432993414221E-10	Mid-zonal hepatocytes	SEPTIN9
7.48790654412731E-15	0.900276986301952	0.224	0.15	1.22135243641261E-10	Mid-zonal hepatocytes	TMEM63A

8.00415501552985E-15	1.43125403221806	0.154	0.091	1.30555772458307E-10	Mid-zonal hepatocytes	RAPGEF6
8.39989846647781E-15	1.5355255636849	0.1	0.047	1.3701074388672E-10	Mid-zonal hepatocytes	DUSP2
8.65531711383588E-15	1.38343283657706	0.128	0.069	1.41176877443777E-10	Mid-zonal hepatocytes	TUBGCP4
8.93561452252152E-15	1.41787934638468	0.141	0.079	1.45748808476849E-10	Mid-zonal hepatocytes	FLVCR1
9.0288486809527E-15	1.37468802978958	0.16	0.095	1.47269550835019E-10	Mid-zonal hepatocytes	MINDY2
9.24407825895408E-15	1.55446413001415	0.111	0.055	1.507801604818E-10	Mid-zonal hepatocytes	SAMD9L
9.28530499091439E-15	1.81733213364312	0.101	0.049	1.51452609706805E-10	Mid-zonal hepatocytes	POU5F2
9.35041855557792E-15	0.911024528513457	0.164	0.098	1.52514677060031E-10	Mid-zonal hepatocytes	NUP155
9.40763302389735E-15	1.02866995496443	0.174	0.107	1.5344790225279E-10	Mid-zonal hepatocytes	SERPINH1
9.48409229633378E-15	1.44270361071488	0.128	0.069	1.546950294455E-10	Mid-zonal hepatocytes	FBXO4
9.6424276373569E-15	1.10341654145885	0.202	0.133	1.57277637192928E-10	Mid-zonal hepatocytes	TAF2
9.67831140159102E-15	0.810519057846437	0.212	0.14	1.57862937271351E-10	Mid-zonal hepatocytes	POLR2B
9.79022760771418E-15	0.314910900950989	0.48	0.393	1.59688402509426E-10	Mid-zonal hepatocytes	CRLS1
1.00651585556895E-14	0.360082137967478	0.345	0.265	1.64172801201852E-10	Mid-zonal hepatocytes	BCL9L
1.03759103581388E-14	1.33875662881138	0.159	0.093	1.69241473851603E-10	Mid-zonal hepatocytes	STK38
1.05253366742064E-14	0.6583517404956	0.278	0.203	1.7167876649298E-10	Mid-zonal hepatocytes	CC2D1A
1.07659340053276E-14	1.09259237465986	0.179	0.111	1.75603149560898E-10	Mid-zonal hepatocytes	ZER1
1.08120924125344E-14	1.24461075065307	0.145	0.083	1.76356039340849E-10	Mid-zonal hepatocytes	KDM6A
1.09456099695177E-14	1.07871160777535	0.116	0.059	1.78533844212802E-10	Mid-zonal hepatocytes	PHF2
1.0959255282969E-14	1.16984700133495	0.167	0.102	1.78756412920508E-10	Mid-zonal hepatocytes	ABHD4
1.10017247870517E-14	0.661536755891355	0.28	0.204	1.794491330016E-10	Mid-zonal hepatocytes	MLX
1.11378315595945E-14	0.7173565889889	0.245	0.171	1.81669170568547E-10	Mid-zonal hepatocytes	RBM10
1.12970489478681E-14	0.599231205945603	0.273	0.197	1.84266165388676E-10	Mid-zonal hepatocytes	FBXW2
1.15564025401412E-14	0.670160947976241	0.223	0.149	1.88496481832244E-10	Mid-zonal hepatocytes	POLG
1.15726376364625E-14	1.2083133934909	0.194	0.125	1.8876129248834E-10	Mid-zonal hepatocytes	NFYA
1.16570211350402E-14	0.323351177774001	0.471	0.382	1.90137671733641E-10	Mid-zonal hepatocytes	BPHL
1.17293049105284E-14	1.76125471389464	0.126	0.068	1.91316692395629E-10	Mid-zonal hepatocytes	ZNF823
1.17986971180059E-14	0.424090546278971	0.39	0.312	1.92448548691794E-10	Mid-zonal hepatocytes	SNRNP200
1.19481675585428E-14	0.955652751700924	0.2	0.129	1.94886561047391E-10	Mid-zonal hepatocytes	LARGE1
1.20403214123571E-14	1.96239800206345	0.101	0.048	1.96389682556957E-10	Mid-zonal hepatocytes	TBC1D19
1.25478955836002E-14	1.74008600592115	0.155	0.091	2.04668724864102E-10	Mid-zonal hepatocytes	NEK3
1.29228872527047E-14	1.48854101071305	0.134	0.074	2.10785213978867E-10	Mid-zonal hepatocytes	ZNF18
1.35055290707085E-14	1.02246439955913	0.184	0.116	2.20288684672327E-10	Mid-zonal hepatocytes	HCFC2
1.3673578667738E-14	1.0087836543128	0.204	0.135	2.23029741649474E-10	Mid-zonal hepatocytes	CXorf56
1.37089204772489E-14	0.541113857572635	0.265	0.19	2.23606201904408E-10	Mid-zonal hepatocytes	SNX9
1.39117935708504E-14	0.774855119125087	0.214	0.143	2.26915264934141E-10	Mid-zonal hepatocytes	C19orf48
1.3937759028737E-14	0.612323437737621	0.376	0.297	2.27338787517729E-10	Mid-zonal hepatocytes	SORBS2
1.4194921968868E-14	0.383613635092878	0.349	0.26	2.31533372234205E-10	Mid-zonal hepatocytes	MYC
1.41950371356568E-14	1.09074173564136	0.182	0.114	2.31535250719698E-10	Mid-zonal hepatocytes	DEPRECAT
1.42157840914881E-14	0.743264354075333	0.212	0.139	2.31873654316263E-10	Mid-zonal hepatocytes	C1orf35
1.44203288219292E-14	0.312086187923365	0.315	0.234	2.35209983414488E-10	Mid-zonal hepatocytes	ZNF609
1.44533773924153E-14	0.80978332199299	0.308	0.235	2.35749038647686E-10	Mid-zonal hepatocytes	AAAS
1.45031983274776E-14	0.266510961223675	0.358	0.267	2.36561667919487E-10	Mid-zonal hepatocytes	PDGFA
1.45143659426027E-14	0.643351497381138	0.302	0.23	2.36743822889792E-10	Mid-zonal hepatocytes	ITPRID2
1.45598604705147E-14	1.58758744552674	0.162	0.096	2.37485884134565E-10	Mid-zonal hepatocytes	RNF180
1.45708947062868E-14	1.35709389104213	0.148	0.085	2.37665863554244E-10	Mid-zonal hepatocytes	EXOC1
1.4833214655531E-14	1.13340622688969	0.173	0.107	2.41944564246367E-10	Mid-zonal hepatocytes	STX17
1.48447144295635E-14	1.61259543813899	0.108	0.054	2.42132137060611E-10	Mid-zonal hepatocytes	LATS1
1.50310218690859E-14	0.525633932002151	0.253	0.179	2.4517099770666E-10	Mid-zonal hepatocytes	BTF3L4
1.53438315295402E-14	1.48795058436969	0.103	0.05	2.5027323607833E-10	Mid-zonal hepatocytes	CPEB3
1.54881667504612E-14	1.1359482189692	0.168	0.102	2.52627487866772E-10	Mid-zonal hepatocytes	ZNF445
1.5664936000124E-14	1.25812103186584	0.154	0.091	2.55510771098023E-10	Mid-zonal hepatocytes	SSH1
1.5830218047379E-14	0.335956987766699	0.217	0.143	2.58206686570798E-10	Mid-zonal hepatocytes	DTNBP1
1.60895915292695E-14	1.67794234259323	0.103	0.05	2.62437327433915E-10	Mid-zonal hepatocytes	RASAL2
1.64582467253057E-14	1.40729294153408	0.121	0.063	2.68450462336461E-10	Mid-zonal hepatocytes	UTRN
1.66992810622834E-14	0.651633358309192	0.273	0.199	2.72381973406904E-10	Mid-zonal hepatocytes	PGAP2
1.69360996363343E-14	1.23225888648335	0.138	0.077	2.76244721168249E-10	Mid-zonal hepatocytes	PHACTR2
1.70400448953236E-14	1.23182547180826	0.23	0.16	2.77940172287624E-10	Mid-zonal hepatocytes	SEMA6C
1.72512358684561E-14	0.949013045644131	0.209	0.137	2.81384908250388E-10	Mid-zonal hepatocytes	RNF146
1.7366830888846E-14	0.689394464942642	0.263	0.189	2.83270378627966E-10	Mid-zonal hepatocytes	RABEP1

1.74786604792031E-14	1.60962030040763	0.163	0.099	2.85094431076281E-10	Mid-zonal hepatocytes	FAM78B
1.75370956082996E-14	0.940272825472069	0.146	0.083	2.86047566466976E-10	Mid-zonal hepatocytes	TMEM120E
1.77227894221367E-14	1.34757774762575	0.167	0.102	2.89076418264472E-10	Mid-zonal hepatocytes	NCAPD3
1.79704292138398E-14	0.92239876056032	0.213	0.142	2.93115670906942E-10	Mid-zonal hepatocytes	JADE3
1.82781362063133E-14	1.56654706818307	0.105	0.052	2.98134679661176E-10	Mid-zonal hepatocytes	KIAA0753
1.86699980508064E-14	1.86657868508684	0.144	0.083	3.04526338206704E-10	Mid-zonal hepatocytes	TIAF1
1.90759638836664E-14	1.9200064449767	0.109	0.055	3.11148046906483E-10	Mid-zonal hepatocytes	MAMLD1
1.93954428112717E-14	1.02440848893274	0.187	0.119	3.16359067694653E-10	Mid-zonal hepatocytes	C15orf39
1.94115921335893E-14	0.544141418594645	0.238	0.162	3.16622479290976E-10	Mid-zonal hepatocytes	PRAG1
1.9557903203013E-14	0.626230002926094	0.209	0.136	3.19008959144345E-10	Mid-zonal hepatocytes	TRPM4
1.99224722898569E-14	0.731963974351797	0.209	0.139	3.24955445519857E-10	Mid-zonal hepatocytes	DDX59
1.99361467357173E-14	1.05265775828623	0.194	0.126	3.25178489406284E-10	Mid-zonal hepatocytes	TXNRD3
2.00912555179036E-14	0.29096827379023	0.41	0.328	3.27708468752526E-10	Mid-zonal hepatocytes	CDC42EP1
2.04214692665771E-14	1.52529833306809	0.135	0.075	3.33094585207139E-10	Mid-zonal hepatocytes	DGKH
2.05777306437246E-14	0.335714449713558	0.327	0.248	3.35643364529791E-10	Mid-zonal hepatocytes	KXD1
2.097832554784E-14	1.21959426448112	0.214	0.144	3.42177468010818E-10	Mid-zonal hepatocytes	TBC1D22A
2.11227527698653E-14	0.472526133111551	0.313	0.231	3.44533220429272E-10	Mid-zonal hepatocytes	HDAC10
2.1167537825267E-14	1.38750267741041	0.111	0.056	3.45263709467931E-10	Mid-zonal hepatocytes	KLHL29
2.11903174379853E-14	1.20748537982856	0.191	0.123	3.45635267730979E-10	Mid-zonal hepatocytes	HEATR5B
2.14924292845353E-14	0.781367524609134	0.244	0.172	3.50563014060056E-10	Mid-zonal hepatocytes	C5orf22
2.15168036258596E-14	1.52374572983287	0.175	0.109	3.50960583941395E-10	Mid-zonal hepatocytes	ST3GAL3
2.28608401876193E-14	0.352024542151704	0.238	0.162	3.72883164300259E-10	Mid-zonal hepatocytes	B4GALT3
2.3103051075847E-14	0.625863723541064	0.282	0.206	3.76833866098141E-10	Mid-zonal hepatocytes	NUDT19
2.31700890334375E-14	0.401701473052555	0.219	0.144	3.779273222244E-10	Mid-zonal hepatocytes	ANKRD9
2.35078223141224E-14	1.49522511841829	0.115	0.059	3.83436089765651E-10	Mid-zonal hepatocytes	P2RX7
2.39957398002511E-14	1.30653793742495	0.202	0.133	3.91394511881896E-10	Mid-zonal hepatocytes	DGLUCY
2.40398271300315E-14	1.51450582843291	0.115	0.059	3.92113620317944E-10	Mid-zonal hepatocytes	RABGAP1
2.40931539985705E-14	0.774962110942251	0.16	0.094	3.92983434870683E-10	Mid-zonal hepatocytes	CDK6
2.42708526110567E-14	0.619561261151447	0.213	0.142	3.95881876938946E-10	Mid-zonal hepatocytes	DGKQ
2.46035486473263E-14	1.427042295914	0.113	0.058	4.01308481986539E-10	Mid-zonal hepatocytes	ZMYM1
2.46851930076131E-14	1.52888784855668	0.116	0.06	4.02640183147177E-10	Mid-zonal hepatocytes	PPP1R3E
2.48116890769985E-14	0.478728520523354	0.366	0.288	4.04703460534922E-10	Mid-zonal hepatocytes	GGNBP2
2.50496230157174E-14	1.01076316530874	0.172	0.106	4.08584401009367E-10	Mid-zonal hepatocytes	C15orf41
2.5797808930001E-14	1.05701095838777	0.185	0.118	4.20788061457246E-10	Mid-zonal hepatocytes	SNRPA
2.60771101797206E-14	1.00389277426045	0.136	0.076	4.25343744141423E-10	Mid-zonal hepatocytes	SMG6
2.62722446215177E-14	1.08872958547217	0.175	0.109	4.28526582021576E-10	Mid-zonal hepatocytes	NUP133
2.66726908035932E-14	0.752721362960921	0.138	0.077	4.35058259697408E-10	Mid-zonal hepatocytes	EPHB6
2.77577724527716E-14	0.93110284721547	0.23	0.159	4.52757026477157E-10	Mid-zonal hepatocytes	HMGN3
2.78965803785476E-14	0.435592123372116	0.398	0.319	4.5502112255449E-10	Mid-zonal hepatocytes	C11orf54
2.80131342530209E-14	0.473407507169163	0.224	0.151	4.56922232801024E-10	Mid-zonal hepatocytes	SLC37A1
2.83544696307454E-14	1.28347332245461	0.156	0.093	4.62489754147088E-10	Mid-zonal hepatocytes	TMEM234
2.8494869168646E-14	0.969564005215696	0.229	0.158	4.64779811009785E-10	Mid-zonal hepatocytes	PHC3
2.86914935530406E-14	1.04062889346384	0.179	0.112	4.67986951343645E-10	Mid-zonal hepatocytes	METTL15
2.8839598582342E-14	1.55887147110463	0.131	0.073	4.70402692476581E-10	Mid-zonal hepatocytes	DCP1B
2.94709279355268E-14	1.27197231010446	0.178	0.111	4.80700305556377E-10	Mid-zonal hepatocytes	ELF1
2.95925604010974E-14	1.22562167555678	0.176	0.111	4.82684252702299E-10	Mid-zonal hepatocytes	GTF2H2
2.96709310138544E-14	1.25363505510509	0.163	0.098	4.83962555766979E-10	Mid-zonal hepatocytes	DGCR8
3.07616444397785E-14	0.822287524288803	0.135	0.075	5.01753182457226E-10	Mid-zonal hepatocytes	TOX2
3.10016593718412E-14	1.02318065700394	0.203	0.134	5.05668066014101E-10	Mid-zonal hepatocytes	SEMA4C
3.15060259101791E-14	0.941545372894016	0.212	0.142	5.13894788620932E-10	Mid-zonal hepatocytes	TMEM50B
3.17366373301216E-14	0.442062468138352	0.323	0.247	5.17656291491614E-10	Mid-zonal hepatocytes	SFT2D2
3.18585053524534E-14	1.05686717162182	0.173	0.106	5.19644080803867E-10	Mid-zonal hepatocytes	ERMARD
3.24446924347498E-14	0.793727372402196	0.26	0.187	5.29205378303204E-10	Mid-zonal hepatocytes	RNF4
3.34771319275018E-14	1.09762940910913	0.189	0.122	5.46045498869481E-10	Mid-zonal hepatocytes	MRI1
3.40863281735862E-14	0.731612488964849	0.241	0.169	5.55982098839364E-10	Mid-zonal hepatocytes	ZC3H18
3.59639262398125E-14	0.680596216878629	0.206	0.135	5.86607600897582E-10	Mid-zonal hepatocytes	KIF16B
3.69380735197914E-14	0.350033565771348	0.44	0.357	6.02496917181317E-10	Mid-zonal hepatocytes	CRYZ
3.71037317055692E-14	0.3835605030035	0.355	0.276	6.05198967849539E-10	Mid-zonal hepatocytes	RHOU
3.72121157696948E-14	1.41946737043163	0.142	0.081	6.06966820319492E-10	Mid-zonal hepatocytes	UBAP2
3.74294868072922E-14	0.79154236772032	0.162	0.096	6.10512359313743E-10	Mid-zonal hepatocytes	NIPA1

3.75058431755606E-14	0.38935741007552	0.454	0.364	6.11757808036569E-10	Mid-zonal hepatocytes	ICAM1
4.22693114804865E-14	1.63542460933357	0.108	0.054	6.89454739558216E-10	Mid-zonal hepatocytes	POMT2
4.23317004990723E-14	1.18364213789504	0.139	0.079	6.90472366840369E-10	Mid-zonal hepatocytes	REV3L
4.29956109687642E-14	1.2298960660295	0.121	0.064	7.01301410511513E-10	Mid-zonal hepatocytes	FANCA
4.42465218150846E-14	1.40817343290047	0.106	0.053	7.21705017325845E-10	Mid-zonal hepatocytes	PUS10
4.53524629902657E-14	0.565339885928999	0.286	0.211	7.39744023834223E-10	Mid-zonal hepatocytes	NUDT5
4.66789473278987E-14	1.64884360310595	0.113	0.058	7.61380309865355E-10	Mid-zonal hepatocytes	PFKFB3
4.73851932081025E-14	1.17090948459531	0.138	0.079	7.7289988641736E-10	Mid-zonal hepatocytes	LRRC8B
4.75344977735708E-14	1.00810443334806	0.188	0.12	7.75335193184713E-10	Mid-zonal hepatocytes	INO80C
4.79777503653139E-14	0.261091933728625	0.37	0.282	7.82565086208634E-10	Mid-zonal hepatocytes	PIM1
4.85315981597868E-14	1.37213471109419	0.132	0.074	7.91598897584283E-10	Mid-zonal hepatocytes	LLGL1
4.92323658471723E-14	1.0403196579782	0.194	0.127	8.03029119333228E-10	Mid-zonal hepatocytes	ANAPC7
4.94471219483009E-14	1.28919104571376	0.116	0.061	8.06532006098737E-10	Mid-zonal hepatocytes	CRLF3
4.95276422782095E-14	1.18410203053408	0.168	0.104	8.07845373199875E-10	Mid-zonal hepatocytes	HDDC2
5.07515344569186E-14	0.902969663050673	0.254	0.184	8.278082785268E-10	Mid-zonal hepatocytes	NBAS
5.35060091868779E-14	0.324884224982516	0.31	0.234	8.72736515847165E-10	Mid-zonal hepatocytes	HECA
5.52518376852027E-14	1.11076228582239	0.191	0.124	9.01212724483341E-10	Mid-zonal hepatocytes	RAB3GAP2
5.59238769423441E-14	1.13049519722194	0.129	0.071	9.12174356806575E-10	Mid-zonal hepatocytes	USP31
5.80962403072747E-14	1.0278181653272	0.179	0.113	9.47607775651958E-10	Mid-zonal hepatocytes	ZBTB21
5.817753335669E-14	0.78264984376872	0.25	0.179	9.4893374658097E-10	Mid-zonal hepatocytes	ZNF148
5.82391436324566E-14	0.930402552250088	0.173	0.107	9.49938671789E-10	Mid-zonal hepatocytes	C18orf25
5.87091570064809E-14	1.80214470864216	0.115	0.06	9.5760505993271E-10	Mid-zonal hepatocytes	ZNF566
5.88068764914846E-14	1.03451858466812	0.124	0.067	9.59198962452605E-10	Mid-zonal hepatocytes	ZNF343
5.89070194320372E-14	1.17030924476732	0.16	0.097	9.60832393955958E-10	Mid-zonal hepatocytes	ZFAND5
6.17122500421153E-14	1.08458240245602	0.141	0.081	1.00658851043694E-09	Mid-zonal hepatocytes	TRMT2B
6.21714593280462E-14	0.453831983929711	0.324	0.247	1.01407867309976E-09	Mid-zonal hepatocytes	XAB2
6.37052837988182E-14	1.07832520973991	0.195	0.128	1.03909688404252E-09	Mid-zonal hepatocytes	TSNARE1
6.38908877191815E-14	0.602664587609499	0.247	0.176	1.04212426958757E-09	Mid-zonal hepatocytes	MON1B
6.41868259945882E-14	0.860418168338035	0.202	0.134	1.04695131879773E-09	Mid-zonal hepatocytes	MAPK9
6.4351668745898E-14	0.724005459459673	0.209	0.14	1.04964006891434E-09	Mid-zonal hepatocytes	MTRR
6.61182488332059E-14	0.82190319330271	0.182	0.116	1.07845475671842E-09	Mid-zonal hepatocytes	CCNK
6.6758120707844E-14	0.671682804807896	0.2	0.131	1.08889170686564E-09	Mid-zonal hepatocytes	UBLCP1
6.68984650820832E-14	1.28377169519521	0.11	0.057	1.09118086395386E-09	Mid-zonal hepatocytes	TANC2
6.83523175692134E-14	1.15848854719712	0.181	0.116	1.11489465187144E-09	Mid-zonal hepatocytes	TMEM131
6.84378578520653E-14	1.44050265326344	0.103	0.051	1.11628989942504E-09	Mid-zonal hepatocytes	RBM45
6.88042029428371E-14	1.34469950805185	0.116	0.061	1.12226535420062E-09	Mid-zonal hepatocytes	HELB
6.90074222098758E-14	1.54807588793862	0.167	0.104	1.12558006366528E-09	Mid-zonal hepatocytes	ACACB
6.93013136812866E-14	1.12277833746049	0.154	0.092	1.13037372745547E-09	Mid-zonal hepatocytes	CTPS2
6.95554089872244E-14	0.407974868411757	0.207	0.136	1.13451827599062E-09	Mid-zonal hepatocytes	SARS
6.97088367596125E-14	0.924143934798384	0.182	0.116	1.13702083638604E-09	Mid-zonal hepatocytes	DHX8
7.12755741682928E-14	1.33774185154908	0.139	0.08	1.16257589025902E-09	Mid-zonal hepatocytes	TEPSIN
7.16489691051709E-14	0.998807480344507	0.179	0.113	1.16866633507444E-09	Mid-zonal hepatocytes	SLC37A3
7.28924869979527E-14	0.816145101277035	0.238	0.167	1.18894935542361E-09	Mid-zonal hepatocytes	CYHR1
7.49607772431816E-14	0.787300615978847	0.218	0.148	1.22268523761353E-09	Mid-zonal hepatocytes	YTHDF2
7.52081776937738E-14	0.293929084925154	0.7	0.562	1.22672058636314E-09	Mid-zonal hepatocytes	OSGIN1
7.61488687225017E-14	0.745396669553352	0.156	0.093	1.24206419773273E-09	Mid-zonal hepatocytes	CSNK1E
7.8723999261626E-14	1.25380663356303	0.156	0.094	1.28406715195638E-09	Mid-zonal hepatocytes	STK17A
8.01707530866602E-14	0.449053206140537	0.374	0.294	1.30766515359651E-09	Mid-zonal hepatocytes	TFRC
8.08050041748132E-14	0.424741785578339	0.331	0.256	1.31801042309538E-09	Mid-zonal hepatocytes	EPRS
8.13373903072671E-14	1.0161720099555	0.182	0.116	1.32669417330183E-09	Mid-zonal hepatocytes	NEK4
8.33376242480392E-14	1.40369447197426	0.187	0.123	1.35931998910977E-09	Mid-zonal hepatocytes	CCNB1IP1
8.6054308940776E-14	0.950771053836634	0.175	0.106	1.403631833133E-09	Mid-zonal hepatocytes	CXCL10
9.210610256977E-14	0.673327293458835	0.251	0.18	1.50234263901552E-09	Mid-zonal hepatocytes	SHLD2
9.22723393336295E-14	1.30715719560414	0.141	0.081	1.50505412687083E-09	Mid-zonal hepatocytes	UBXN11
9.24601119910956E-14	0.407785618266516	0.303	0.228	1.50811688668676E-09	Mid-zonal hepatocytes	TEX2
9.25696851659252E-14	1.13802467226317	0.157	0.095	1.50990413474141E-09	Mid-zonal hepatocytes	HIF1AN
9.28625115456515E-14	0.867526278519618	0.237	0.165	1.51468042582112E-09	Mid-zonal hepatocytes	TSHZ2
9.36923231127107E-14	0.961840574540875	0.175	0.11	1.52821548229142E-09	Mid-zonal hepatocytes	DEPRECAT
9.56808478273776E-14	0.616975339820063	0.259	0.185	1.56065030891236E-09	Mid-zonal hepatocytes	USO1
9.6239948691523E-14	0.862840853015948	0.206	0.138	1.56976980310743E-09	Mid-zonal hepatocytes	TMEM184E

9.63637682981805E-14	1.46667820666998	0.139	0.079	1.57178942471162E-09	Mid-zonal hepatocytes	INPP5E
9.71821371203719E-14	0.967648065400005	0.154	0.092	1.58513783857039E-09	Mid-zonal hepatocytes	CCDC174
9.97481801210268E-14	1.22028737216495	0.131	0.073	1.62699256595407E-09	Mid-zonal hepatocytes	HSD11B2
1.0207310547248E-13	0.853578949175704	0.224	0.156	1.66491442336162E-09	Mid-zonal hepatocytes	SECISBP2L
1.0304794355069E-13	0.827040346434518	0.172	0.108	1.68081500725531E-09	Mid-zonal hepatocytes	CBX7
1.04925341731621E-13	1.40995839644426	0.165	0.103	1.71143724898447E-09	Mid-zonal hepatocytes	DGKE
1.04978844187129E-13	0.635775390142654	0.331	0.256	1.71230992753626E-09	Mid-zonal hepatocytes	DHRS1
1.06279715913742E-13	0.341456143201916	0.379	0.301	1.73352844626904E-09	Mid-zonal hepatocytes	TMED4
1.09192583074795E-13	0.987692680091075	0.15	0.088	1.78104022253299E-09	Mid-zonal hepatocytes	ADRA1A
1.11778900614727E-13	0.86375754895755	0.181	0.114	1.82322564792682E-09	Mid-zonal hepatocytes	MROH6
1.17281638399093E-13	0.623683207456831	0.179	0.113	1.91298080392761E-09	Mid-zonal hepatocytes	PNRC2
1.17297495077416E-13	1.56602711421305	0.131	0.074	1.91323944220774E-09	Mid-zonal hepatocytes	FOXJ3
1.17452354859373E-13	0.764912922062146	0.241	0.17	1.91576536011123E-09	Mid-zonal hepatocytes	NAE1
1.22318555873134E-13	1.37726836457669	0.119	0.064	1.99513796484668E-09	Mid-zonal hepatocytes	PTPN2
1.24742457039851E-13	1.09568786955648	0.198	0.132	2.03467421677701E-09	Mid-zonal hepatocytes	TIMELESS
1.2474376215872E-13	1.09252347084024	0.192	0.127	2.03469550457087E-09	Mid-zonal hepatocytes	FAM160B1
1.25320346373715E-13	1.40029427083069	0.143	0.084	2.04410016970166E-09	Mid-zonal hepatocytes	BRAF
1.26376614587009E-13	0.882636022147513	0.252	0.181	2.0613289605287E-09	Mid-zonal hepatocytes	FAM91A1
1.26811894762212E-13	1.03650694917928	0.171	0.106	2.06842881546644E-09	Mid-zonal hepatocytes	GPALPP1
1.26812375286913E-13	0.48125503871905	0.231	0.158	2.06843665330483E-09	Mid-zonal hepatocytes	IL17RA
1.27444551036857E-13	1.41295818484391	0.11	0.057	2.07874807196217E-09	Mid-zonal hepatocytes	PGBD2
1.32325324734722E-13	0.47170536141085	0.31	0.237	2.15835837174805E-09	Mid-zonal hepatocytes	HIPK2
1.32918902627804E-13	0.601771490113412	0.222	0.151	2.16804022076211E-09	Mid-zonal hepatocytes	PPP1R18
1.33402222618518E-13	1.39856901616404	0.128	0.071	2.17592365313065E-09	Mid-zonal hepatocytes	MYLIP
1.35665606469255E-13	0.494585241732395	0.243	0.171	2.21284170712003E-09	Mid-zonal hepatocytes	SEC24D
1.40322629390622E-13	0.954142740719464	0.209	0.141	2.28880240799044E-09	Mid-zonal hepatocytes	IFNGR1
1.42257009536761E-13	1.26875134567595	0.163	0.101	2.3203540825541E-09	Mid-zonal hepatocytes	SNAPC4
1.47206640935255E-13	1.05503884807498	0.231	0.161	2.40108752029494E-09	Mid-zonal hepatocytes	METTL23
1.49608198379994E-13	0.596412046553551	0.163	0.099	2.44025932377608E-09	Mid-zonal hepatocytes	ABL1
1.52994205805707E-13	0.271061195219395	0.336	0.26	2.49548849089689E-09	Mid-zonal hepatocytes	PAFAH1B2
1.53743442958208E-13	0.898508620739376	0.154	0.092	2.50770929809133E-09	Mid-zonal hepatocytes	TET3
1.5425445490314E-13	1.43538548778171	0.122	0.067	2.51604441392512E-09	Mid-zonal hepatocytes	ATXN7L1
1.55409761833445E-13	1.27394226199028	0.111	0.058	2.53488862526531E-09	Mid-zonal hepatocytes	TSTD2
1.55909727282396E-13	1.0656935309088	0.142	0.082	2.54304356170315E-09	Mid-zonal hepatocytes	SMC4
1.57470116511814E-13	0.756487279133461	0.209	0.14	2.56849507042419E-09	Mid-zonal hepatocytes	TULP4
1.59386503529635E-13	1.53072729118028	0.101	0.051	2.59975325907188E-09	Mid-zonal hepatocytes	DEPRECAT
1.62424252887423E-13	0.991314082407775	0.166	0.103	2.64930198884675E-09	Mid-zonal hepatocytes	PTBP3
1.63562079714937E-13	1.0647473869364	0.184	0.117	2.66786108223033E-09	Mid-zonal hepatocytes	ZNF264
1.65646680585838E-13	0.53007312596695	0.269	0.197	2.7018630070356E-09	Mid-zonal hepatocytes	POLDIP3
1.67060611651944E-13	0.270638017972639	0.352	0.275	2.72492563665487E-09	Mid-zonal hepatocytes	TRAPPC3
1.68181806033396E-13	0.56550299479901	0.23	0.16	2.74321343821073E-09	Mid-zonal hepatocytes	CC2D1B
1.74073270057883E-13	0.296595933827998	0.306	0.232	2.83930910791413E-09	Mid-zonal hepatocytes	BRAT1
1.78651174200835E-13	0.50695710652738	0.392	0.308	2.91397930238982E-09	Mid-zonal hepatocytes	FST
1.80456403790309E-13	1.0744469334797	0.178	0.114	2.94342440222373E-09	Mid-zonal hepatocytes	GPR89A
1.81461964333828E-13	0.930930946779834	0.191	0.124	2.95982610024906E-09	Mid-zonal hepatocytes	CCDC24
1.86294285702905E-13	1.00022115004329	0.203	0.136	3.03864609410008E-09	Mid-zonal hepatocytes	SP100
1.93757568460435E-13	0.685330348616858	0.247	0.176	3.16037969915815E-09	Mid-zonal hepatocytes	RAB3GAP1
1.9509692619694E-13	0.280451459633748	0.394	0.311	3.18222596319828E-09	Mid-zonal hepatocytes	HADHB
1.96643574749821E-13	1.10536307886547	0.117	0.063	3.20745334774434E-09	Mid-zonal hepatocytes	FAM126B
1.98073585672958E-13	0.437307822812675	0.25	0.178	3.23077825591162E-09	Mid-zonal hepatocytes	CDK4
2.072283879962E-13	1.29691118949692	0.15	0.09	3.38010223660602E-09	Mid-zonal hepatocytes	SPATS2
2.0772115993624E-13	1.07580441593709	0.222	0.155	3.38813983972001E-09	Mid-zonal hepatocytes	ZNF655
2.09560921623981E-13	1.27009998959464	0.119	0.064	3.41814819260875E-09	Mid-zonal hepatocytes	CSPP1
2.12988106776302E-13	0.57669497167833	0.215	0.147	3.47404900962826E-09	Mid-zonal hepatocytes	BAK1
2.13954354730139E-13	1.34575421236717	0.122	0.067	3.48980948000329E-09	Mid-zonal hepatocytes	METAP1D
2.21400361564566E-13	0.458752114046899	0.251	0.18	3.61126129747964E-09	Mid-zonal hepatocytes	ELK4
2.22779721862637E-13	0.370767823797434	0.322	0.247	3.63376004330146E-09	Mid-zonal hepatocytes	SLC33A1
2.28600047229316E-13	1.10898970684849	0.17	0.107	3.72869537035737E-09	Mid-zonal hepatocytes	FGFR1OP
2.30677769405491E-13	1.10161706776392	0.16	0.098	3.76258509677296E-09	Mid-zonal hepatocytes	MALT1
2.35821918414355E-13	1.22732080192437	0.17	0.107	3.84649131125654E-09	Mid-zonal hepatocytes	GCNT2

**2.37262776927558E-13**	0.466640112943182	0.226	0.155	3.8699931544654E-09	Mid-zonal hepatocytes	UFL1
**2.39964383914988E-13**	0.383481510276072	0.376	0.295	3.91405906603738E-09	Mid-zonal hepatocytes	LPCAT1
**2.48995339331854E-13**	1.24362715926959	0.168	0.105	4.06136297984187E-09	Mid-zonal hepatocytes	B4GALT4
**2.49231054767818E-13**	0.990963758778038	0.11	0.057	4.06520773431788E-09	Mid-zonal hepatocytes	LURAP1L
**2.52522931471504E-13**	1.08231330548305	0.176	0.113	4.1189015352317E-09	Mid-zonal hepatocytes	ABCA7
**2.52901219942458E-13**	0.903445671334686	0.218	0.15	4.12507179848144E-09	Mid-zonal hepatocytes	ARHGEF1
**2.63729808586943E-13**	0.91434644502796	0.133	0.075	4.30169690786162E-09	Mid-zonal hepatocytes	GLIS2
**2.65518287768872E-13**	0.63867689892726	0.245	0.174	4.33086879179808E-09	Mid-zonal hepatocytes	DDX39A
**2.65822093122575E-13**	0.572342536454508	0.268	0.196	4.33582416092232E-09	Mid-zonal hepatocytes	FOXO3
**2.66564893189336E-13**	0.716538144388865	0.268	0.198	4.34793997281127E-09	Mid-zonal hepatocytes	NR3C1
**2.67910762186246E-13**	1.23147950248967	0.14	0.081	4.36989244201986E-09	Mid-zonal hepatocytes	FAM189A2
**2.68657642694887E-13**	1.15459974794839	0.197	0.132	4.3820748099963E-09	Mid-zonal hepatocytes	HMGXB3
**2.70675687073249E-13**	1.20145552676858	0.176	0.112	4.41499113185176E-09	Mid-zonal hepatocytes	CRAMP1
**2.76643853501722E-13**	0.639907506100591	0.226	0.157	4.51233789446659E-09	Mid-zonal hepatocytes	CDR2
**2.84430230794375E-13**	0.458443133177414	0.34	0.267	4.63934149448705E-09	Mid-zonal hepatocytes	NDUFA10
**2.90918331432888E-13**	0.888394475457018	0.215	0.147	4.74516890400184E-09	Mid-zonal hepatocytes	FBXW8
**2.98368911841447E-13**	1.33646276472771	0.119	0.064	4.86669532104584E-09	Mid-zonal hepatocytes	RBPJ
**3.05764866553213E-13**	1.0112053791759	0.197	0.132	4.98733073834946E-09	Mid-zonal hepatocytes	ANKRD28
**3.06497677489685E-13**	0.557179437742371	0.281	0.21	4.99928361753425E-09	Mid-zonal hepatocytes	CDK9
**3.07729417737436E-13**	0.677881713615846	0.249	0.178	5.01937453271531E-09	Mid-zonal hepatocytes	AVL9
**3.09079743581801E-13**	0.927959335598626	0.228	0.159	5.04139969756276E-09	Mid-zonal hepatocytes	XIAP
**3.117723980221E-13**	1.20156036021413	0.111	0.058	5.08531958413847E-09	Mid-zonal hepatocytes	ARHGEF2
**3.215049973801E-13**	0.516428140684065	0.314	0.242	5.2440680122668E-09	Mid-zonal hepatocytes	SPNS1
**3.2583448964978E-13**	1.03534855374402	0.122	0.066	5.31468636067756E-09	Mid-zonal hepatocytes	CHIC1
**3.28077363870045E-13**	0.679894792232708	0.3	0.23	5.3512698820843E-09	Mid-zonal hepatocytes	BTNL8
**3.30808452641955E-13**	0.586430970834444	0.212	0.145	5.39581667104293E-09	Mid-zonal hepatocytes	EIF2AK4
**3.46705658591303E-13**	0.783682530275369	0.281	0.209	5.65511599728275E-09	Mid-zonal hepatocytes	INSIG2
**3.48396088470713E-13**	0.93797837861046	0.262	0.195	5.6826885990458E-09	Mid-zonal hepatocytes	UBXN2B
**3.50351516901885E-13**	0.490553514301072	0.316	0.241	5.71458359218665E-09	Mid-zonal hepatocytes	RRP1
**3.53517079980227E-13**	0.77167197582888	0.191	0.126	5.76621709155749E-09	Mid-zonal hepatocytes	ROCK1
**3.60459181156968E-13**	0.987184648633694	0.173	0.11	5.8794497038513E-09	Mid-zonal hepatocytes	PDCD7
**3.72470191520608E-13**	0.945782705083073	0.227	0.158	6.07536129389263E-09	Mid-zonal hepatocytes	MTIF2
**3.8631163610046E-13**	0.627767181713871	0.173	0.109	6.30112909643461E-09	Mid-zonal hepatocytes	ASAH1
**3.8902577625937E-13**	1.17015920226363	0.142	0.083	6.34539943656658E-09	Mid-zonal hepatocytes	EIF2AK3
**4.2196108369718E-13**	1.29485080605869	0.137	0.08	6.8826072361847E-09	Mid-zonal hepatocytes	CHORDC1
**4.30231200146895E-13**	1.35824086273774	0.166	0.104	7.017501105596E-09	Mid-zonal hepatocytes	ENGASE
**4.38076026991077E-13**	0.528362808805562	0.34	0.265	7.14545807625145E-09	Mid-zonal hepatocytes	MGRN1
**4.44177749179611E-13**	0.501488303985152	0.369	0.291	7.24498326686863E-09	Mid-zonal hepatocytes	SLC25A25
**4.45093705260041E-13**	0.651586730561565	0.285	0.216	7.25992342649652E-09	Mid-zonal hepatocytes	TSC2
**4.55767045887739E-13**	0.911958880967043	0.143	0.083	7.43401628547492E-09	Mid-zonal hepatocytes	PRR14
**4.63144721622547E-13**	1.07687951554278	0.14	0.081	7.55435355438537E-09	Mid-zonal hepatocytes	ZFYVE28
**4.68576869548756E-13**	0.735896195194736	0.268	0.198	7.64295731920976E-09	Mid-zonal hepatocytes	ZHX1
**4.77370972414389E-13**	0.844868847095665	0.163	0.101	7.7863979310511E-09	Mid-zonal hepatocytes	BAZ1A
**4.87300844541894E-13**	0.649959504859235	0.258	0.19	7.94836407532283E-09	Mid-zonal hepatocytes	MSL1
**5.07340372777044E-13**	0.631673344133171	0.164	0.1	8.27522882036636E-09	Mid-zonal hepatocytes	JADE1
**5.16378387760001E-13**	0.725674565592696	0.243	0.175	8.42264788275338E-09	Mid-zonal hepatocytes	UFM1
**5.21142559020357E-13**	1.11831762087672	0.189	0.125	8.50035628018104E-09	Mid-zonal hepatocytes	MED17
**5.30745662064784E-13**	1.28792959646172	0.173	0.111	8.6569924939387E-09	Mid-zonal hepatocytes	ETAA1
**5.32263498814887E-13**	1.20419297267264	0.184	0.12	8.68174992916963E-09	Mid-zonal hepatocytes	FBXO11
**5.41574783579301E-13**	0.595703787537311	0.257	0.187	8.83362629496197E-09	Mid-zonal hepatocytes	SNX27
**5.47028773313216E-13**	1.07877740068955	0.186	0.123	8.92258632151187E-09	Mid-zonal hepatocytes	AC118549.
**5.52840911012459E-13**	1.24852880060095	0.183	0.12	9.01738809952422E-09	Mid-zonal hepatocytes	TBC1D5
**5.53588400335402E-13**	0.573940771188906	0.106	0.054	9.02958039787074E-09	Mid-zonal hepatocytes	CD163
**5.53756849353551E-13**	1.44168337884277	0.103	0.053	9.03232796980577E-09	Mid-zonal hepatocytes	WDR41
**5.61774414340671E-13**	1.17863792179042	0.2	0.137	9.16310247231069E-09	Mid-zonal hepatocytes	NLK
**5.68512736867198E-13**	1.08444780180538	0.136	0.078	9.27301125104087E-09	Mid-zonal hepatocytes	RALGAPA1
**5.77129903558066E-13**	0.789114645044552	0.224	0.157	9.41356585693561E-09	Mid-zonal hepatocytes	APPL1
**5.9459951569617E-13**	0.886363879118368	0.221	0.155	9.69851270052023E-09	Mid-zonal hepatocytes	HDAC3
**6.25628016481219E-13**	1.0090156974559	0.188	0.124	1.02046185768252E-08	Mid-zonal hepatocytes	CEP63
**6.32988542547658E-13**	1.45648248328651	0.104	0.053	1.03246761174948E-08	Mid-zonal hepatocytes	METTL1

6.45615408630276E-13	0.972417164374909	0.2	0.136	1.05306329301684E-08	Mid-zonal hepatocytes	ATP6V0A2
6.47228158113367E-13	0.537779112023725	0.311	0.239	1.05569384869871E-08	Mid-zonal hepatocytes	ZFAND2A
6.51966795444914E-13	0.551722914948519	0.208	0.141	1.0634230400502E-08	Mid-zonal hepatocytes	BLZF1
6.73682315748068E-13	1.22291435487556	0.204	0.14	1.09884322521667E-08	Mid-zonal hepatocytes	SGK3
6.77376035730253E-13	1.5494816679152	0.101	0.052	1.10486805187962E-08	Mid-zonal hepatocytes	DEPRECAT
6.78200477008293E-13	0.274383014759554	0.321	0.241	1.10621279804823E-08	Mid-zonal hepatocytes	FAM174B
6.79455666824287E-13	0.46035062679425	0.315	0.245	1.10826013815709E-08	Mid-zonal hepatocytes	EEF1AKNM
6.9104025755278E-13	0.571072197187835	0.256	0.187	1.12715576409434E-08	Mid-zonal hepatocytes	SOCS6
6.94244538896121E-13	0.966814230918142	0.195	0.13	1.13238226739346E-08	Mid-zonal hepatocytes	CSF1
7.04197956630673E-13	1.56861163071558	0.102	0.052	1.14861728706029E-08	Mid-zonal hepatocytes	DEPRECAT
7.39617989778355E-13	0.430023648827706	0.28	0.207	1.20639090312748E-08	Mid-zonal hepatocytes	PPP1R16A
7.40171548558682E-13	1.71492012534093	0.116	0.064	1.20729381285407E-08	Mid-zonal hepatocytes	SPICE1
7.4636091757809E-13	0.857569739957577	0.157	0.096	1.21738929266162E-08	Mid-zonal hepatocytes	PEX1
7.56958430991541E-13	1.06708407210408	0.15	0.09	1.2346748967903E-08	Mid-zonal hepatocytes	PRKCE
7.5976850713086E-13	0.693439968805809	0.228	0.161	1.23925841198115E-08	Mid-zonal hepatocytes	CHAMP1
7.83734724994987E-13	0.540268872362903	0.238	0.168	1.27834970993932E-08	Mid-zonal hepatocytes	NELFA
8.26655893203131E-13	0.744144328778214	0.306	0.236	1.34835842740363E-08	Mid-zonal hepatocytes	TJP1
8.34549482032372E-13	0.75253586696428	0.249	0.18	1.361233660143E-08	Mid-zonal hepatocytes	NCKAP5L
8.46348186386842E-13	0.823941113997276	0.219	0.154	1.38047852681558E-08	Mid-zonal hepatocytes	SENP5
8.50849690478352E-13	0.685484285810408	0.234	0.166	1.38782093013924E-08	Mid-zonal hepatocytes	SIRT7
8.61210776869708E-13	1.58504621300693	0.147	0.089	1.40472089815218E-08	Mid-zonal hepatocytes	NABP1
8.7282830090401E-13	1.88522784836571	0.105	0.056	1.42367024160453E-08	Mid-zonal hepatocytes	CD58
8.90359555564858E-13	1.03093853542739	0.173	0.112	1.45226547108184E-08	Mid-zonal hepatocytes	ABHD3
9.09999148132217E-13	1.0341421328921	0.19	0.126	1.48429961051846E-08	Mid-zonal hepatocytes	ACADL
9.10078440359391E-13	0.341843154099105	0.314	0.243	1.4844289440702E-08	Mid-zonal hepatocytes	ATG2A
9.3407896884966E-13	0.401664958578066	0.336	0.264	1.52357620609068E-08	Mid-zonal hepatocytes	RCC1L
9.53872112858937E-13	0.796145194709581	0.135	0.078	1.55586080328421E-08	Mid-zonal hepatocytes	MIEF2
9.84841688312638E-13	1.41964511882372	0.111	0.06	1.60637527780674E-08	Mid-zonal hepatocytes	ZDHHC21
1.06929124820202E-12	0.571249595085961	0.309	0.241	1.74412095494232E-08	Mid-zonal hepatocytes	NDUFV3
1.07262822620534E-12	1.62076117116453	0.119	0.066	1.74956389976353E-08	Mid-zonal hepatocytes	RBMXL1
1.11132571383405E-12	0.611597060970122	0.301	0.232	1.81268337183471E-08	Mid-zonal hepatocytes	GOLGA5
1.13383959567059E-12	0.524180477995702	0.374	0.298	1.84940576449829E-08	Mid-zonal hepatocytes	DHODH
1.14155761946312E-12	1.22616004061958	0.145	0.088	1.86199463310629E-08	Mid-zonal hepatocytes	SMTN
1.15603072483162E-12	1.27269301710002	0.118	0.065	1.88560171527286E-08	Mid-zonal hepatocytes	LIN52
1.16111360321109E-12	0.822309087761212	0.244	0.179	1.8938923981976E-08	Mid-zonal hepatocytes	PIK3R4
1.17173323848956E-12	1.39083205561954	0.147	0.09	1.91121408530032E-08	Mid-zonal hepatocytes	IQGAP3
1.186734202398E-12	1.35682625718964	0.138	0.082	1.93568215753138E-08	Mid-zonal hepatocytes	MGA
1.20423915106191E-12	1.25269207593025	0.135	0.079	1.96423447929709E-08	Mid-zonal hepatocytes	ELMOD3
1.21457320899991E-12	0.54048555605789	0.223	0.156	1.98109036119976E-08	Mid-zonal hepatocytes	RMND5B
1.23157538470927E-12	0.723900558581067	0.222	0.155	2.00882260999929E-08	Mid-zonal hepatocytes	ZNF513
1.23621473369986E-12	0.539095294721519	0.232	0.164	2.01638985213784E-08	Mid-zonal hepatocytes	BLCAP
1.24116399089588E-12	0.286135736089558	0.668	0.561	2.02446258555028E-08	Mid-zonal hepatocytes	DEPP1
1.25618750808708E-12	0.840450026928879	0.2	0.135	2.04896744444084E-08	Mid-zonal hepatocytes	DYRK2
1.25710709686382E-12	1.3495794599582	0.147	0.089	2.05046738569457E-08	Mid-zonal hepatocytes	SEPTIN10
1.26583166146506E-12	1.06341608302098	0.168	0.107	2.06469802301566E-08	Mid-zonal hepatocytes	BICRA
1.27994569739212E-12	0.966770034991076	0.182	0.119	2.08771942701629E-08	Mid-zonal hepatocytes	DAB2
1.29817075705057E-12	0.734638765689242	0.241	0.174	2.11744632182518E-08	Mid-zonal hepatocytes	URI1
1.3759272953154E-12	1.53111259547976	0.101	0.052	2.24427501138895E-08	Mid-zonal hepatocytes	PARP2
1.3797879914663E-12	0.771037690881584	0.212	0.146	2.25057219288068E-08	Mid-zonal hepatocytes	MTA2
1.38663216890843E-12	0.788608067101197	0.151	0.092	2.26173573070654E-08	Mid-zonal hepatocytes	WNT3
1.39013982790239E-12	1.11970982657671	0.158	0.098	2.26745707329159E-08	Mid-zonal hepatocytes	PCGF3
1.39226493814384E-12	0.829915881431369	0.173	0.111	2.27092334060642E-08	Mid-zonal hepatocytes	DEPRECAT
1.41200410738346E-12	0.708419582962561	0.236	0.167	2.30311989955316E-08	Mid-zonal hepatocytes	RRNAD1
1.42833976647889E-12	1.50727327380379	0.104	0.055	2.32976499310373E-08	Mid-zonal hepatocytes	WRN
1.43328574135155E-12	1.21786213384342	0.14	0.083	2.33783237271852E-08	Mid-zonal hepatocytes	PARP3
1.45647650674696E-12	1.32308702651175	0.124	0.07	2.37565883015497E-08	Mid-zonal hepatocytes	DHX32
1.47150069358239E-12	0.718792030713534	0.261	0.193	2.40016478130224E-08	Mid-zonal hepatocytes	TNFRSF21
1.50012713085671E-12	1.06439440808513	0.148	0.09	2.44685736314038E-08	Mid-zonal hepatocytes	INPP5F
1.53087929979398E-12	0.796857001555398	0.25	0.184	2.49701722589396E-08	Mid-zonal hepatocytes	UBE2H
1.65438720263648E-12	1.26685066462621	0.156	0.097	2.69847096622037E-08	Mid-zonal hepatocytes	KIZ

**1.65937275198071E-12**	0.66615089133458	0.253	0.187	2.70660289575574E-08	Mid-zonal hepatocytes	EXOSC7
**1.66262011765002E-12**	1.09000342161283	0.185	0.121	2.71189967389894E-08	Mid-zonal hepatocytes	ERCC3
**1.71659960350194E-12**	1.38389727382356	0.101	0.052	2.79994561327202E-08	Mid-zonal hepatocytes	NELL2
**1.76010999019015E-12**	0.705523688377186	0.189	0.125	2.87091540499916E-08	Mid-zonal hepatocytes	SF3B3
**1.76513313973562E-12**	0.68757846400983	0.2	0.136	2.87910866422277E-08	Mid-zonal hepatocytes	STK25
**1.76931541268663E-12**	0.992810746718787	0.162	0.102	2.88593036963316E-08	Mid-zonal hepatocytes	BTBD1
**1.77037811017303E-12**	0.449278451471714	0.273	0.203	2.88766373550324E-08	Mid-zonal hepatocytes	MEGF9
**1.80309409555868E-12**	0.288342438033245	0.375	0.303	2.94102677926577E-08	Mid-zonal hepatocytes	POLR2A
**1.80444696531782E-12**	1.06897009315526	0.181	0.119	2.9432334451299E-08	Mid-zonal hepatocytes	DDX19A
**1.8073201956867E-12**	0.712413014325897	0.214	0.148	2.94791997118458E-08	Mid-zonal hepatocytes	PPP4R3B
**1.88799354667225E-12**	0.389377983359991	0.303	0.232	3.0795062739771E-08	Mid-zonal hepatocytes	ZFP91
**1.89291021312885E-12**	1.04596396206129	0.128	0.074	3.08752584863447E-08	Mid-zonal hepatocytes	IQSEC2
**1.98777931101845E-12**	0.292158183302604	0.375	0.299	3.2422668342022E-08	Mid-zonal hepatocytes	FKBP5
**1.99351864733941E-12**	1.16250479113181	0.128	0.074	3.25162826567532E-08	Mid-zonal hepatocytes	HHAT
**2.01524591241314E-12**	1.2018652770387	0.164	0.104	3.28706760773708E-08	Mid-zonal hepatocytes	ATR
**2.03677848107744E-12**	0.887545937973606	0.225	0.16	3.32218938048541E-08	Mid-zonal hepatocytes	WWP2
**2.04590255496425E-12**	1.24158704631881	0.159	0.1	3.33707165740219E-08	Mid-zonal hepatocytes	SLC2A11
**2.12566734154096E-12**	0.825574102196775	0.167	0.107	3.46717600078746E-08	Mid-zonal hepatocytes	INTS10
**2.14792635091265E-12**	1.54052808652485	0.114	0.062	3.50348267097363E-08	Mid-zonal hepatocytes	SARM1
**2.16295008135106E-12**	1.17769547201414	0.144	0.088	3.52798787769171E-08	Mid-zonal hepatocytes	ZNF641
**2.19230434681696E-12**	0.337790951490153	0.354	0.284	3.57586762009315E-08	Mid-zonal hepatocytes	SCAP
**2.20586500791031E-12**	1.25034258140967	0.135	0.079	3.59798641440251E-08	Mid-zonal hepatocytes	COG2
**2.21147161911686E-12**	0.958069642182483	0.174	0.114	3.60713135794151E-08	Mid-zonal hepatocytes	LIAS
**2.21789208392787E-12**	1.50147777695324	0.154	0.097	3.61760377809475E-08	Mid-zonal hepatocytes	FPGT
**2.27605705219223E-12**	1.02037055875446	0.167	0.107	3.71247665783074E-08	Mid-zonal hepatocytes	ATF7
**2.30751985641202E-12**	1.54991489386083	0.126	0.073	3.76379563779364E-08	Mid-zonal hepatocytes	METTL2B
**2.3408730030192E-12**	1.11111219455024	0.144	0.087	3.81819795522461E-08	Mid-zonal hepatocytes	SLC8B1
**2.34239710418105E-12**	1.62349008428647	0.103	0.054	3.82068391662971E-08	Mid-zonal hepatocytes	DEPRECAT
**2.35356891179354E-12**	0.872757175760897	0.226	0.162	3.83890625202644E-08	Mid-zonal hepatocytes	AP3S2
**2.40040540597273E-12**	0.902076009797851	0.16	0.1	3.91530125768211E-08	Mid-zonal hepatocytes	SAP130
**2.41343766006898E-12**	0.880022300420573	0.167	0.107	3.93655816733852E-08	Mid-zonal hepatocytes	FOXK2
**2.42317416501532E-12**	0.632644701762474	0.243	0.177	3.95243938055649E-08	Mid-zonal hepatocytes	RNF185
**2.46244074688773E-12**	0.881067456241163	0.181	0.119	4.01648710224857E-08	Mid-zonal hepatocytes	MED13
**2.4867769422181E-12**	1.39569289071146	0.167	0.108	4.05618187045195E-08	Mid-zonal hepatocytes	SNRNP48
**2.5187689031204E-12**	1.28959959973053	0.131	0.076	4.10836395787968E-08	Mid-zonal hepatocytes	TTI1
**2.58492310462658E-12**	1.05504828097849	0.134	0.079	4.21626807595641E-08	Mid-zonal hepatocytes	VEZT
**2.58862265344085E-12**	1.1357982455478	0.143	0.087	4.22230241002737E-08	Mid-zonal hepatocytes	PAQR5
**2.59116419394362E-12**	1.04626093080738	0.195	0.133	4.22644791674145E-08	Mid-zonal hepatocytes	SAP30BP
**2.63557403182987E-12**	0.675067507673154	0.241	0.174	4.2988848033177E-08	Mid-zonal hepatocytes	AP1G1
**2.65343217944557E-12**	1.55052525564634	0.144	0.087	4.32801322789367E-08	Mid-zonal hepatocytes	DNAJC25
**2.65480378199145E-12**	1.20358597328224	0.122	0.069	4.33025044880626E-08	Mid-zonal hepatocytes	COL4A5
**2.68288837076379E-12**	0.363350574956753	0.328	0.26	4.37605922155281E-08	Mid-zonal hepatocytes	RMND5A
**2.75647465523866E-12**	1.4447299468088	0.128	0.074	4.49608581015978E-08	Mid-zonal hepatocytes	ATG16L1
**2.78274837426906E-12**	0.618134642046095	0.301	0.233	4.53894087327027E-08	Mid-zonal hepatocytes	CDC5L
**2.83612326537287E-12**	1.45811808073546	0.118	0.067	4.62600065814969E-08	Mid-zonal hepatocytes	DDX11
**2.92122607522503E-12**	0.852312957204732	0.152	0.093	4.76481185129954E-08	Mid-zonal hepatocytes	KIF22
**2.97704074467186E-12**	0.82466391863448	0.181	0.118	4.85585115863427E-08	Mid-zonal hepatocytes	ZNFX1
**3.02692837718508E-12**	1.1541977937117	0.122	0.07	4.93722287602658E-08	Mid-zonal hepatocytes	CWF19L1
**3.08191625147941E-12**	1.13659502266372	0.15	0.092	5.02691359778806E-08	Mid-zonal hepatocytes	CYB5RL
**3.11032338670973E-12**	0.790159081825877	0.152	0.093	5.07324847606224E-08	Mid-zonal hepatocytes	ASB1
**3.1163190571791E-12**	0.602338477974807	0.249	0.181	5.08302801416484E-08	Mid-zonal hepatocytes	TGIF1
**3.17774310761577E-12**	1.00518285961243	0.2	0.139	5.18321678283208E-08	Mid-zonal hepatocytes	STAT5B
**3.19299815948708E-12**	0.869387041644248	0.172	0.111	5.20809929793937E-08	Mid-zonal hepatocytes	CASP9
**3.21956606848032E-12**	1.35631447346532	0.107	0.057	5.25143421429825E-08	Mid-zonal hepatocytes	DCAF17
**3.25316124816593E-12**	1.22674496225734	0.162	0.103	5.30623131188345E-08	Mid-zonal hepatocytes	PSTPIP2
**3.29568005247255E-12**	1.19298074893029	0.129	0.075	5.37558373358798E-08	Mid-zonal hepatocytes	TLR2
**3.30652329171662E-12**	0.418032841768365	0.25	0.183	5.39327014111898E-08	Mid-zonal hepatocytes	LRRC40
**3.32466803439912E-12**	0.744894737596754	0.24	0.175	5.4228660309084E-08	Mid-zonal hepatocytes	WWC2
**3.32838708536699E-12**	1.50020430763713	0.104	0.055	5.4289321749421E-08	Mid-zonal hepatocytes	CSGALNAC
**3.39387617809128E-12**	0.653126878793495	0.318	0.249	5.53575143408469E-08	Mid-zonal hepatocytes	UNC5CL

3.43327821290697E-12	0.482858929561005	0.297	0.228	5.60002009307255E-08	Mid-zonal hepatocytes	LDLRAP1
3.43377581014253E-12	1.06362779011772	0.202	0.138	5.60083172392348E-08	Mid-zonal hepatocytes	SLAIN2
3.49456043941362E-12	1.01121654548571	0.138	0.082	5.69997753272756E-08	Mid-zonal hepatocytes	FBXW11
3.55266315316224E-12	1.02707889992725	0.171	0.111	5.79474886912292E-08	Mid-zonal hepatocytes	ZC3H7A
3.6112421114519E-12	0.596033631004005	0.282	0.218	5.8902970079892E-08	Mid-zonal hepatocytes	SLC35C2
3.77281096797256E-12	1.18319819034195	0.179	0.12	6.15383196986005E-08	Mid-zonal hepatocytes	LYSMD3
3.83046640303442E-12	0.37377718857694	0.262	0.194	6.24787374998944E-08	Mid-zonal hepatocytes	NCAPH2
3.8608531321816E-12	0.784972138937852	0.142	0.085	6.29743754390141E-08	Mid-zonal hepatocytes	LMNTD2
3.8890986387912E-12	1.05632724446189	0.187	0.126	6.34350878973233E-08	Mid-zonal hepatocytes	CRBN
3.92500326889676E-12	1.00180490762816	0.129	0.075	6.40207283189751E-08	Mid-zonal hepatocytes	EOGT
3.92734458845167E-12	1.19217413601013	0.117	0.065	6.40589175822352E-08	Mid-zonal hepatocytes	ZKSCAN4
3.94543071311425E-12	1.14720822017829	0.169	0.111	6.43539203616065E-08	Mid-zonal hepatocytes	SMARCE1
4.06279164615747E-12	1.24013468964399	0.158	0.1	6.62681945404745E-08	Mid-zonal hepatocytes	TMEM126B
4.0745637071298E-12	1.6237729515121	0.105	0.056	6.64602086269941E-08	Mid-zonal hepatocytes	ZNF841
4.08813536678184E-12	0.801086494569808	0.225	0.161	6.66815759675786E-08	Mid-zonal hepatocytes	UBE3C
4.13970261147054E-12	0.392178345428615	0.251	0.184	6.7522689295696E-08	Mid-zonal hepatocytes	TCF20
4.14410106914049E-12	0.677034354742577	0.182	0.12	6.75944325387505E-08	Mid-zonal hepatocytes	GTF2A1
4.16443049250694E-12	1.34422417052491	0.129	0.076	6.79260257632808E-08	Mid-zonal hepatocytes	ZNF335
4.24942298732612E-12	1.01857021867625	0.167	0.107	6.93123383462763E-08	Mid-zonal hepatocytes	EZH1
4.3064214270225E-12	1.25446409854124	0.12	0.068	7.02420398961639E-08	Mid-zonal hepatocytes	SEPTIN2
4.41309077356652E-12	1.28592903064157	0.131	0.077	7.19819236076435E-08	Mid-zonal hepatocytes	AMMECR1L
4.42245224299264E-12	0.310603363659959	0.316	0.247	7.21346185354529E-08	Mid-zonal hepatocytes	PPM1A
4.48287032959782E-12	0.986856195350692	0.14	0.084	7.31200979460701E-08	Mid-zonal hepatocytes	EPN2
4.50721391277553E-12	0.714644035766353	0.258	0.193	7.35171661312816E-08	Mid-zonal hepatocytes	NAP1L4
4.70408673907071E-12	0.828306368560721	0.139	0.083	7.67283588009824E-08	Mid-zonal hepatocytes	SETD1A
4.7468748543991E-12	0.584393949249926	0.278	0.21	7.74262757501037E-08	Mid-zonal hepatocytes	C2orf68
4.78489656983438E-12	1.29167114766679	0.107	0.058	7.80464479505686E-08	Mid-zonal hepatocytes	MEX3C
4.91557977146104E-12	1.40418959972707	0.122	0.069	8.01780216523011E-08	Mid-zonal hepatocytes	MREG
4.92804631228489E-12	1.22379280986258	0.115	0.064	8.03813633996788E-08	Mid-zonal hepatocytes	CDK3
5.06678945187789E-12	0.387141010069334	0.378	0.309	8.26444027495803E-08	Mid-zonal hepatocytes	TPM1
5.14042668337502E-12	1.0393295082726	0.19	0.128	8.38454996325299E-08	Mid-zonal hepatocytes	CLYBL
5.27721904062007E-12	0.830191392131042	0.166	0.107	8.6076719771554E-08	Mid-zonal hepatocytes	WRAP73
5.28882317684797E-12	0.536782765044976	0.208	0.142	8.62659948375673E-08	Mid-zonal hepatocytes	CXCR4
5.36051248998224E-12	0.440879629316972	0.25	0.182	8.74353192241003E-08	Mid-zonal hepatocytes	UBQLN4
5.36522738761138E-12	0.430195229269037	0.247	0.18	8.75122239193292E-08	Mid-zonal hepatocytes	TCEAL9
5.41006251014178E-12	1.16043118303614	0.166	0.107	8.82435296029225E-08	Mid-zonal hepatocytes	UBN1
5.482447313886E-12	0.902159158796145	0.201	0.139	8.94241981367945E-08	Mid-zonal hepatocytes	UBA2
5.54874692463213E-12	0.765870884139198	0.28	0.214	9.05056110876747E-08	Mid-zonal hepatocytes	CYB5B
5.6404444374565E-12	0.561924823348554	0.274	0.208	9.20012892193529E-08	Mid-zonal hepatocytes	HNMT
5.75802930937479E-12	1.10702721404865	0.104	0.056	9.39192160652121E-08	Mid-zonal hepatocytes	TRIM65
5.86144390963737E-12	0.508172193812311	0.299	0.232	9.56060116100952E-08	Mid-zonal hepatocytes	GNG12
5.87771941380954E-12	0.853547282982885	0.204	0.143	9.58714813586474E-08	Mid-zonal hepatocytes	NIN
5.88816902873261E-12	0.483532419074784	0.316	0.248	9.60419250276575E-08	Mid-zonal hepatocytes	LIN7C
5.96759775794645E-12	0.682982274805892	0.162	0.102	9.73374870298646E-08	Mid-zonal hepatocytes	NEIL2
6.06011111207401E-12	0.645189601657528	0.246	0.178	9.88464723490391E-08	Mid-zonal hepatocytes	LMBRD2
6.20989011442286E-12	0.750350982046082	0.235	0.171	1.01289517656351E-07	Mid-zonal hepatocytes	PHPT1
6.21884250044023E-12	0.68592667421829	0.185	0.123	1.01435540024681E-07	Mid-zonal hepatocytes	PLXNA1
6.23343199506006E-12	0.662342882883625	0.281	0.214	1.01673509271425E-07	Mid-zonal hepatocytes	NARF
6.31978191001937E-12	0.715098270918977	0.177	0.117	1.03081962734326E-07	Mid-zonal hepatocytes	CCSER2
6.37654502499924E-12	0.362487854862731	0.369	0.303	1.04007825902763E-07	Mid-zonal hepatocytes	TBC1D24
6.46210032576572E-12	0.465322389532705	0.266	0.2	1.05403318413565E-07	Mid-zonal hepatocytes	IDS
6.48293934560169E-12	0.763282395507641	0.188	0.127	1.05743223666109E-07	Mid-zonal hepatocytes	L3MBTL2
6.57129779054563E-12	0.866761144121947	0.191	0.13	1.0718443826159E-07	Mid-zonal hepatocytes	HOOK2
6.61242797689471E-12	1.48098703298232	0.135	0.081	1.0785531273113E-07	Mid-zonal hepatocytes	ZDHHC6
6.61962786445645E-12	0.465870944208383	0.281	0.215	1.07972750097149E-07	Mid-zonal hepatocytes	MORF4L2
6.63751516134415E-12	0.296619516866039	0.302	0.23	1.08264509796684E-07	Mid-zonal hepatocytes	CNNM3
6.69401598367771E-12	0.692317218890136	0.188	0.125	1.09186094709767E-07	Mid-zonal hepatocytes	ETV3
6.80178001914276E-12	0.286277475710632	0.338	0.268	1.10943833892238E-07	Mid-zonal hepatocytes	TMEM161A
6.8046204034836E-12	1.00957173152013	0.151	0.094	1.10990163401221E-07	Mid-zonal hepatocytes	IL22RA1
6.85063887535049E-12	1.05248782816965	0.155	0.098	1.11740770695842E-07	Mid-zonal hepatocytes	ORC4

6.98633617278596E-12	0.964195739946693	0.204	0.142	1.13954129314312E-07	Mid-zonal hepatocytes	TERF2
7.25057441501446E-12	0.767242941460146	0.221	0.157	1.18264119283301E-07	Mid-zonal hepatocytes	SLC35E2B
7.28095210605217E-12	0.717670409434256	0.254	0.189	1.18759609801817E-07	Mid-zonal hepatocytes	MAEA
7.35069986312516E-12	0.685443542598105	0.221	0.157	1.19897265467434E-07	Mid-zonal hepatocytes	TOX4
7.38552555599368E-12	1.08303463004949	0.177	0.118	1.20465307343813E-07	Mid-zonal hepatocytes	MED6
7.52713218964999E-12	1.22453153235354	0.142	0.086	1.22775053145381E-07	Mid-zonal hepatocytes	NOL4L
7.58603235932544E-12	0.812428095903801	0.164	0.105	1.23735773812957E-07	Mid-zonal hepatocytes	TWSG1
7.61077382584796E-12	0.774397335935384	0.21	0.148	1.24139331873406E-07	Mid-zonal hepatocytes	ZNF7
7.63338267780633E-12	1.09919456781073	0.103	0.055	1.24508104857699E-07	Mid-zonal hepatocytes	GAB1
7.71454036177116E-12	0.577623784104229	0.304	0.237	1.25831867840849E-07	Mid-zonal hepatocytes	TLE1
7.75519515507121E-12	1.14418955938066	0.157	0.101	1.26494988174367E-07	Mid-zonal hepatocytes	KRR1
7.85468806703837E-12	0.422817009499289	0.356	0.288	1.28117817061463E-07	Mid-zonal hepatocytes	TMEM184A
7.86307693090557E-12	0.911970306242885	0.149	0.092	1.28254647820001E-07	Mid-zonal hepatocytes	INKA2
7.92443800393715E-12	0.887166328852693	0.164	0.106	1.29255508282219E-07	Mid-zonal hepatocytes	SNRNP40
8.10874417865177E-12	1.07870101561136	0.191	0.131	1.32261726297989E-07	Mid-zonal hepatocytes	EDC3
8.11068189036262E-12	0.970112792027475	0.133	0.079	1.32293332313705E-07	Mid-zonal hepatocytes	LIG4
8.16992281162447E-12	1.19723270012233	0.181	0.122	1.33259610980407E-07	Mid-zonal hepatocytes	ATP8B2
8.26037504717085E-12	0.4744925549566	0.217	0.153	1.34734977394404E-07	Mid-zonal hepatocytes	SDE2
8.35940522767154E-12	0.579670830579079	0.304	0.237	1.3635025866855E-07	Mid-zonal hepatocytes	KCMF1
8.60421885038857E-12	0.422848169714343	0.325	0.258	1.40343413668688E-07	Mid-zonal hepatocytes	SELENON
8.7356992243669E-12	0.798366800873997	0.178	0.117	1.42487990048649E-07	Mid-zonal hepatocytes	VAMP2
8.76755235159573E-12	0.721848013637153	0.151	0.095	1.43007546406878E-07	Mid-zonal hepatocytes	POM121
8.81774303573186E-12	1.05934067842968	0.119	0.068	1.43826206655822E-07	Mid-zonal hepatocytes	RASSF1
8.90334584158033E-12	0.61576853262354	0.219	0.155	1.45222474022017E-07	Mid-zonal hepatocytes	IRF2
8.94744764135699E-12	0.494727213367613	0.285	0.221	1.45941818478174E-07	Mid-zonal hepatocytes	ATP2C1
8.9679151446393E-12	1.24008707011639	0.117	0.067	1.46275663924212E-07	Mid-zonal hepatocytes	SUPT3H
9.43665136108376E-12	1.17662343099858	0.162	0.104	1.53921220350637E-07	Mid-zonal hepatocytes	SMARCD1
9.7819289463717E-12	0.832553685298669	0.187	0.126	1.59553043044269E-07	Mid-zonal hepatocytes	AAGAB
9.89385393095234E-12	0.635529224920918	0.185	0.124	1.61378651467764E-07	Mid-zonal hepatocytes	SNX33
9.92080763227808E-12	1.22514847092273	0.109	0.06	1.61818293290088E-07	Mid-zonal hepatocytes	RGS7BP
1.02134795894818E-11	0.995262145141295	0.187	0.127	1.66592065584038E-07	Mid-zonal hepatocytes	RBBP5
1.04097295542015E-11	1.01640678168196	0.142	0.087	1.6979309875858E-07	Mid-zonal hepatocytes	DEPDC5
1.04835243698332E-11	1.37121433093038	0.106	0.057	1.7099676599635E-07	Mid-zonal hepatocytes	WDR91
1.04954470351353E-11	1.27814692436898	0.126	0.074	1.71191236590091E-07	Mid-zonal hepatocytes	ZC3H6
1.05995745443307E-11	0.873556122999237	0.21	0.149	1.72889660392578E-07	Mid-zonal hepatocytes	KPNA6
1.06662959361671E-11	1.44969173712795	0.153	0.097	1.73977953014821E-07	Mid-zonal hepatocytes	MTM1
1.06896831122649E-11	0.599036241570005	0.282	0.217	1.74359421244153E-07	Mid-zonal hepatocytes	BCKDHB
1.09134939898822E-11	0.440971894392365	0.29	0.22	1.78010000468968E-07	Mid-zonal hepatocytes	CXADR
1.11067375928786E-11	1.20067370461154	0.162	0.105	1.81161996877443E-07	Mid-zonal hepatocytes	STXBP5
1.11555941402723E-11	1.57782353699736	0.122	0.071	1.81958896021982E-07	Mid-zonal hepatocytes	THEM4
1.11974579939019E-11	0.425473042479975	0.263	0.199	1.82641737338535E-07	Mid-zonal hepatocytes	PFDN5
1.12803487548241E-11	0.534903394631052	0.198	0.134	1.83993768539936E-07	Mid-zonal hepatocytes	FBXO27
1.14561477760364E-11	0.455710121605676	0.247	0.182	1.8686122637493E-07	Mid-zonal hepatocytes	ITGA6
1.14647245102298E-11	1.20880702312899	0.108	0.059	1.87001121486357E-07	Mid-zonal hepatocytes	BAIAP2L2
1.1635941607419E-11	0.817575118259523	0.262	0.2	1.89793843558612E-07	Mid-zonal hepatocytes	CAMK1
1.16768076553525E-11	0.98804684344731	0.159	0.101	1.90460409666455E-07	Mid-zonal hepatocytes	CCDC97
1.18656808649709E-11	0.825003476469266	0.213	0.151	1.9354112058854E-07	Mid-zonal hepatocytes	LSG1
1.22583061106744E-11	1.55128757819611	0.126	0.074	1.99945230971211E-07	Mid-zonal hepatocytes	TTBK2
1.26333924875743E-11	0.31160520149029	0.325	0.259	2.06063264864825E-07	Mid-zonal hepatocytes	KIF5B
1.27461209225982E-11	1.18614130063889	0.15	0.094	2.07901978368499E-07	Mid-zonal hepatocytes	SFMBT1
1.29135959590885E-11	1.48930220379302	0.111	0.062	2.10633663688693E-07	Mid-zonal hepatocytes	PRDM10
1.29178727388504E-11	0.350796816491608	0.376	0.308	2.1070342224339E-07	Mid-zonal hepatocytes	EPHX2
1.30285940570714E-11	1.75886882752626	0.139	0.086	2.12509397664892E-07	Mid-zonal hepatocytes	MSH5
1.32298589624669E-11	0.692344470864414	0.241	0.178	2.15792229536798E-07	Mid-zonal hepatocytes	SON
1.32353917937949E-11	0.914083318803496	0.166	0.108	2.15882475548589E-07	Mid-zonal hepatocytes	PIGN
1.35606960715516E-11	0.809838763853479	0.142	0.087	2.21188513623079E-07	Mid-zonal hepatocytes	PRR14L
1.39692968583046E-11	0.839398838787041	0.14	0.085	2.27853201055806E-07	Mid-zonal hepatocytes	RAB8B
1.40382994080863E-11	0.894684303070409	0.177	0.118	2.28978701645296E-07	Mid-zonal hepatocytes	TAF6L
1.41507092767584E-11	0.495067911558065	0.212	0.148	2.30812219013206E-07	Mid-zonal hepatocytes	TOR3A
1.41587353659151E-11	0.697446538963696	0.221	0.159	2.30943132553441E-07	Mid-zonal hepatocytes	CKLF

1.43131989882761E-11	1.0914728531392	0.173	0.115	2.33462588697771E-07	Mid-zonal hepatocytes	INTS7
1.43429802605563E-11	1.32268959738714	0.106	0.058	2.33948351029934E-07	Mid-zonal hepatocytes	IQGAP1
1.43523337938776E-11	0.893131046667733	0.197	0.138	2.34100916511937E-07	Mid-zonal hepatocytes	FN3KRP
1.4365074467891E-11	1.03316894871945	0.114	0.064	2.3430872964577E-07	Mid-zonal hepatocytes	CEP164
1.46110449773674E-11	1.04563433201537	0.13	0.078	2.38320754625839E-07	Mid-zonal hepatocytes	DHX35
1.49348646385691E-11	0.263747150613506	0.346	0.278	2.43602577119701E-07	Mid-zonal hepatocytes	EFR3A
1.51046044299287E-11	0.889945036768119	0.165	0.107	2.46371202856567E-07	Mid-zonal hepatocytes	RPRD1B
1.51373471868148E-11	1.45660906516682	0.133	0.079	2.46905269964136E-07	Mid-zonal hepatocytes	ZNF561
1.54569537043014E-11	1.42541369948646	0.105	0.058	2.52118371870861E-07	Mid-zonal hepatocytes	PIAS1
1.58936076602978E-11	1.06079936753534	0.171	0.112	2.59240634547117E-07	Mid-zonal hepatocytes	CUL4B
1.59010552988975E-11	1.05455296255889	0.157	0.1	2.59362112980317E-07	Mid-zonal hepatocytes	FAM204A
1.60514515989307E-11	0.912198955254819	0.196	0.136	2.61815227030158E-07	Mid-zonal hepatocytes	MAPKAP1
1.62838825151488E-11	0.512295851405354	0.188	0.127	2.65606407704592E-07	Mid-zonal hepatocytes	LSM 8.00
1.66081681519238E-11	1.54851292281849	0.114	0.065	2.70895830726029E-07	Mid-zonal hepatocytes	TRIT1
1.66585238866475E-11	1.31467373279898	0.116	0.066	2.71717183115107E-07	Mid-zonal hepatocytes	SPIN3
1.70173441678242E-11	0.995120466033498	0.242	0.181	2.77569900721381E-07	Mid-zonal hepatocytes	YTHDF3
1.7355795738941E-11	0.774104089187657	0.244	0.181	2.83090384297867E-07	Mid-zonal hepatocytes	SLC25A38
1.749797763273E-11	0.36263662292057	0.277	0.211	2.85409513167459E-07	Mid-zonal hepatocytes	DDX56
1.7784355149282E-11	1.06917549379802	0.169	0.112	2.90080616839939E-07	Mid-zonal hepatocytes	ZNF3
1.78509964293676E-11	0.803435410690601	0.234	0.172	2.91167602759415E-07	Mid-zonal hepatocytes	MAPRE2
1.84379995205444E-11	1.3616656063624	0.105	0.058	3.007422101796E-07	Mid-zonal hepatocytes	FAM200B
1.87904271684392E-11	0.78513123659619	0.206	0.146	3.06490657544412E-07	Mid-zonal hepatocytes	EEA1
1.87950095816266E-11	1.36909939455406	0.13	0.078	3.06565401285912E-07	Mid-zonal hepatocytes	CREB1
1.88956485618578E-11	0.69057990555899	0.153	0.096	3.08206923692463E-07	Mid-zonal hepatocytes	ARHGAP21
1.94968344434134E-11	1.16078252529478	0.154	0.098	3.18012866606516E-07	Mid-zonal hepatocytes	MICAL2
1.95148620124522E-11	0.807309559714095	0.168	0.11	3.18306914285108E-07	Mid-zonal hepatocytes	DRAM2
1.97372296242586E-11	1.01549746582375	0.136	0.083	3.21933952401281E-07	Mid-zonal hepatocytes	PPCDC
2.00212584122479E-11	0.799182770231926	0.141	0.085	3.26566745962176E-07	Mid-zonal hepatocytes	KLF4
2.01771172830065E-11	0.446266028039096	0.301	0.233	3.29108960003119E-07	Mid-zonal hepatocytes	IRF6
2.1175994010957E-11	0.637167560525961	0.19	0.13	3.4540163831272E-07	Mid-zonal hepatocytes	SLC25A28
2.14562039710343E-11	0.499353951912901	0.222	0.159	3.4997214297154E-07	Mid-zonal hepatocytes	DEPRECAT
2.2220644035928E-11	0.718970743111471	0.269	0.208	3.62440924870021E-07	Mid-zonal hepatocytes	MARCH5
2.23165566073696E-11	0.819646795842724	0.175	0.117	3.64005354822806E-07	Mid-zonal hepatocytes	LSM14B
2.25687971207507E-11	0.902464586124198	0.185	0.125	3.68119649836564E-07	Mid-zonal hepatocytes	SECISBP2
2.26700755052618E-11	0.313944406048869	0.338	0.272	3.69771601566326E-07	Mid-zonal hepatocytes	STT3B
2.27699850589923E-11	0.721425393666406	0.228	0.166	3.71401226297223E-07	Mid-zonal hepatocytes	ANKFY1
2.30044584225457E-11	0.577274656371167	0.304	0.241	3.75225721330142E-07	Mid-zonal hepatocytes	TOP 1.00
2.33424253689835E-11	0.51073070824032	0.265	0.198	3.8073830019349E-07	Mid-zonal hepatocytes	PLEKHJ1
2.36609902633363E-11	1.07257985318754	0.186	0.126	3.85934412185279E-07	Mid-zonal hepatocytes	PDK1
2.38412343826796E-11	0.946040690080397	0.152	0.096	3.88874374015887E-07	Mid-zonal hepatocytes	SMAP1
2.39105656441957E-11	0.778884804313249	0.193	0.133	3.90005236222476E-07	Mid-zonal hepatocytes	GFM2
2.3959914367653E-11	0.396997198691385	0.197	0.133	3.90810163250789E-07	Mid-zonal hepatocytes	ARID3A
2.4180583583257E-11	0.71897840138746	0.291	0.227	3.94409498826505E-07	Mid-zonal hepatocytes	ITGAV
2.52010233099841E-11	0.878537942169924	0.147	0.092	4.1105389120915E-07	Mid-zonal hepatocytes	KIF21A
2.53063578948878E-11	0.889685593162985	0.18	0.121	4.12772003623515E-07	Mid-zonal hepatocytes	CNOT8
2.5452470208896E-11	0.96426530784866	0.172	0.115	4.15155241577303E-07	Mid-zonal hepatocytes	NCOA1
2.56644950924316E-11	1.3641652244955	0.151	0.095	4.18613579452652E-07	Mid-zonal hepatocytes	EHF
2.60811492208222E-11	0.515289355691016	0.235	0.171	4.25409624940831E-07	Mid-zonal hepatocytes	DEPRECAT
2.64402311567839E-11	1.05206929288187	0.113	0.064	4.31266610398303E-07	Mid-zonal hepatocytes	CHTF18
2.67209051081501E-11	1.27433422921664	0.158	0.102	4.35844683219036E-07	Mid-zonal hepatocytes	VAV2
2.7115125741993E-11	1.58834982019933	0.116	0.067	4.42274815977648E-07	Mid-zonal hepatocytes	ZNF557
2.74047429092137E-11	1.47629337767007	0.105	0.058	4.46998761592184E-07	Mid-zonal hepatocytes	E2F6
2.74246630250809E-11	0.837870772760331	0.203	0.143	4.47323678602094E-07	Mid-zonal hepatocytes	HNRNPD
2.79464279491292E-11	1.48076898915389	0.139	0.087	4.55834186278246E-07	Mid-zonal hepatocytes	CNOT6
2.81124403828088E-11	1.19863456411943	0.171	0.115	4.58542015083994E-07	Mid-zonal hepatocytes	DROSHA
2.8980519937599E-11	0.900468034581255	0.128	0.075	4.72701260702178E-07	Mid-zonal hepatocytes	TNPO1
2.95526722734656E-11	0.972743279369523	0.2	0.141	4.82033637452498E-07	Mid-zonal hepatocytes	CAND1
2.95841055279657E-11	1.40384586383669	0.135	0.082	4.82546345266649E-07	Mid-zonal hepatocytes	C11orf95
2.97573767206788E-11	1.06467580442873	0.116	0.067	4.85372571690992E-07	Mid-zonal hepatocytes	ZNF254
2.99972128988171E-11	1.20130826611359	0.148	0.094	4.89284539592606E-07	Mid-zonal hepatocytes	SEC22C

3.10139984441865E-11	1.16313116608977	0.135	0.083	5.05869328623127E-07	Mid-zonal hepatocytes	KIAA0556
3.11768115986099E-11	1.31552372859603	0.125	0.074	5.08524973984927E-07	Mid-zonal hepatocytes	NECAP1
3.17136945326129E-11	0.700595648516967	0.227	0.165	5.17282071521449E-07	Mid-zonal hepatocytes	CASK
3.22039606930796E-11	0.729971466267558	0.183	0.125	5.25278802864822E-07	Mid-zonal hepatocytes	TMEM165
3.2349137770442E-11	1.14024340748926	0.16	0.105	5.2764678617368E-07	Mid-zonal hepatocytes	TGS1
3.27706894363017E-11	0.796111839324305	0.101	0.055	5.34522715395517E-07	Mid-zonal hepatocytes	WDR44
3.30550138973123E-11	1.34672874132038	0.12	0.071	5.39160331679062E-07	Mid-zonal hepatocytes	EYA3
3.30691580081225E-11	0.416426119259666	0.28	0.213	5.39391036270485E-07	Mid-zonal hepatocytes	TTC7A
3.30944741173296E-11	0.926978567091594	0.118	0.068	5.39803967327763E-07	Mid-zonal hepatocytes	SNRPA1
3.47945148106581E-11	1.36188931728524	0.119	0.069	5.67533331076645E-07	Mid-zonal hepatocytes	ZFP30
3.49788890819776E-11	0.640503103551705	0.201	0.14	5.70540659816137E-07	Mid-zonal hepatocytes	RAB4B
3.52442080572448E-11	0.648350124605525	0.183	0.125	5.7486827762172E-07	Mid-zonal hepatocytes	SF1
3.5666585668551E-11	0.996483692906015	0.122	0.071	5.81757678839736E-07	Mid-zonal hepatocytes	PTPN14
3.57029027844372E-11	1.03090348619717	0.154	0.099	5.82350047316955E-07	Mid-zonal hepatocytes	NCOA3
3.70676806568638E-11	1.25703124854726	0.111	0.062	6.04610939194105E-07	Mid-zonal hepatocytes	RELL1
3.93351041047336E-11	0.704695184044657	0.283	0.222	6.41594883052309E-07	Mid-zonal hepatocytes	RALGPS2
3.95704224580173E-11	0.586160302458573	0.206	0.145	6.45433160712721E-07	Mid-zonal hepatocytes	BMS1
4.09458201306285E-11	0.904312550388205	0.139	0.086	6.67867272150681E-07	Mid-zonal hepatocytes	TRAF2
4.23472433450104E-11	1.24387842339063	0.135	0.083	6.90725886200465E-07	Mid-zonal hepatocytes	NKAPD1
4.33558253929415E-11	0.325581738361786	0.3	0.235	7.07176867984268E-07	Mid-zonal hepatocytes	TSC22D4
4.33990560646503E-11	0.875569546533594	0.139	0.086	7.07882003470512E-07	Mid-zonal hepatocytes	U2AF2
4.34750072245624E-11	0.943688616552514	0.203	0.145	7.09120842839837E-07	Mid-zonal hepatocytes	CEP57
4.39304324753953E-11	1.09322016517487	0.148	0.094	7.16549284106173E-07	Mid-zonal hepatocytes	PRKCA
4.55090902636518E-11	1.06722325619909	0.236	0.176	7.42298771290425E-07	Mid-zonal hepatocytes	PLGLB2
4.59785555886447E-11	0.791951402442661	0.196	0.136	7.49956220206384E-07	Mid-zonal hepatocytes	PTPRU
4.59888148967435E-11	1.60815376558607	0.129	0.078	7.50123559780784E-07	Mid-zonal hepatocytes	ZNF700
4.62520110370375E-11	1.46586823522961	0.125	0.075	7.54416552025119E-07	Mid-zonal hepatocytes	APAF1
4.67033552774592E-11	0.865485745149383	0.153	0.098	7.61778427930638E-07	Mid-zonal hepatocytes	MECP2
4.75966040140521E-11	0.682618529781685	0.26	0.202	7.76348208073203E-07	Mid-zonal hepatocytes	TMEM220
4.79624182705721E-11	0.929058326061638	0.159	0.103	7.82315004411301E-07	Mid-zonal hepatocytes	OXNAD1
4.81489442977929E-11	0.384032656623679	0.281	0.216	7.853574304413E-07	Mid-zonal hepatocytes	M6PR
4.83315550338068E-11	0.92217016060178	0.159	0.103	7.88335994156422E-07	Mid-zonal hepatocytes	ZNF408
4.83967593242119E-11	1.06388330622029	0.173	0.117	7.89399541337221E-07	Mid-zonal hepatocytes	DAGLB
4.84260918978089E-11	0.619972163771747	0.216	0.155	7.8987798494516E-07	Mid-zonal hepatocytes	HPS1
4.88484272680834E-11	0.336929774651818	0.386	0.306	7.96766697169709E-07	Mid-zonal hepatocytes	DDIT4
5.17670547128816E-11	0.960247561258165	0.104	0.057	8.44372429421812E-07	Mid-zonal hepatocytes	PRKD3
5.18857347841195E-11	1.10229413120676	0.194	0.136	8.46308220063774E-07	Mid-zonal hepatocytes	XPR1
5.23482564840305E-11	1.13199780542043	0.147	0.094	8.53852411511021E-07	Mid-zonal hepatocytes	DENND6A
5.36411031417107E-11	1.25938008074092	0.114	0.066	8.74940033344443E-07	Mid-zonal hepatocytes	STRADA
5.39553610352796E-11	0.716565660237816	0.175	0.117	8.80065893846445E-07	Mid-zonal hepatocytes	DEPRECAT
5.42753657093201E-11	0.945954923519602	0.166	0.11	8.8528549008472E-07	Mid-zonal hepatocytes	RAB29
5.46314865797727E-11	0.919491330859197	0.14	0.087	8.91094177602672E-07	Mid-zonal hepatocytes	TRNAU1AF
5.55533373884427E-11	1.14858205732523	0.124	0.074	9.06130486142888E-07	Mid-zonal hepatocytes	SOCS4
5.64662728112837E-11	0.786368862550006	0.163	0.107	9.21021375824848E-07	Mid-zonal hepatocytes	DEPRECAT
5.67619828552333E-11	0.958001619416905	0.164	0.107	9.2584470235171E-07	Mid-zonal hepatocytes	PPM1H
5.7029748137442E-11	0.905597488671092	0.16	0.104	9.30212221869817E-07	Mid-zonal hepatocytes	PPIL3
5.71174810134298E-11	1.09700250296445	0.143	0.09	9.31643232810053E-07	Mid-zonal hepatocytes	CLASP1
5.7444398612455E-11	0.976490151808788	0.153	0.098	9.36975585767753E-07	Mid-zonal hepatocytes	IPO13
5.92364381542672E-11	0.48362238348989	0.182	0.123	9.66205542734252E-07	Mid-zonal hepatocytes	PPM1F
6.02382993554723E-11	1.22990708952251	0.133	0.082	9.82546900787108E-07	Mid-zonal hepatocytes	YEATS2
6.0315400284794E-11	1.33755445375381	0.103	0.057	9.83804494045275E-07	Mid-zonal hepatocytes	ARHGAP26
6.13070812437656E-11	0.457249798417715	0.332	0.268	9.9997980216706E-07	Mid-zonal hepatocytes	C19orf12
6.21610762894584E-11	1.30790986378815	0.114	0.066	1.01390931535736E-06	Mid-zonal hepatocytes	TJP2
6.22516747123689E-11	1.35298646003226	0.123	0.074	1.01538706623345E-06	Mid-zonal hepatocytes	GEN1
6.4212543677793E-11	1.19689302274757	0.127	0.076	1.04737079992848E-06	Mid-zonal hepatocytes	ELMO2
6.43889405330229E-11	0.637557984738579	0.181	0.124	1.05024800903414E-06	Mid-zonal hepatocytes	COMMD1
6.45163855288079E-11	0.363053042630924	0.273	0.208	1.05232676436039E-06	Mid-zonal hepatocytes	CPPED1
6.54836199438555E-11	1.65294520864416	0.109	0.062	1.06810332490423E-06	Mid-zonal hepatocytes	SLC12A6
6.56810415261405E-11	0.891245605890517	0.148	0.094	1.07132346833288E-06	Mid-zonal hepatocytes	CYB5R4
6.59266711547632E-11	1.07540596535844	0.1	0.055	1.07532993320534E-06	Mid-zonal hepatocytes	RAB9A

6.84953964182267E-11	0.3609002539521	0.261	0.197	1.1172284109777E-06	Mid-zonal hepatocytes	MLLT1
6.86362231207404E-11	0.740503707672368	0.172	0.116	1.1195254353224E-06	Mid-zonal hepatocytes	SRFBP1
6.9077656090279E-11	0.642916513747718	0.142	0.088	1.12672564848854E-06	Mid-zonal hepatocytes	GTF2F2
7.03366457764435E-11	0.693255829172492	0.234	0.175	1.14726102925957E-06	Mid-zonal hepatocytes	MBNL1
7.13612400930399E-11	0.955057843979651	0.201	0.143	1.16397318715757E-06	Mid-zonal hepatocytes	FAM136A
7.16307829859022E-11	0.524843417535934	0.263	0.202	1.16836970128305E-06	Mid-zonal hepatocytes	MAN1A2
7.40187665609868E-11	1.14270305993979	0.103	0.057	1.20732010137626E-06	Mid-zonal hepatocytes	SECTM1
7.49060885403591E-11	0.80444838295431	0.109	0.061	1.2217932101818E-06	Mid-zonal hepatocytes	TBKBP1
7.58906733204012E-11	0.956831949756455	0.11	0.063	1.23785277252906E-06	Mid-zonal hepatocytes	WDR4
7.75802763598709E-11	0.739755213768505	0.235	0.175	1.26541188770585E-06	Mid-zonal hepatocytes	MTIF3
8.16627249153607E-11	0.822504023781734	0.255	0.194	1.33200070609445E-06	Mid-zonal hepatocytes	GOPC
8.26258646468705E-11	0.92675566458704	0.205	0.147	1.3477104782551E-06	Mid-zonal hepatocytes	DDX3X
8.2995146827951E-11	1.04396440474587	0.109	0.062	1.35373383991071E-06	Mid-zonal hepatocytes	ZNF580
8.37350789300418E-11	0.465908966586219	0.197	0.137	1.36580287242791E-06	Mid-zonal hepatocytes	DENND2D
8.42565881247207E-11	1.01366779088082	0.141	0.088	1.37430920890232E-06	Mid-zonal hepatocytes	TGFBRAP1
8.42870380907205E-11	0.403078035417297	0.302	0.236	1.37480587829774E-06	Mid-zonal hepatocytes	PGP
8.49822475581446E-11	0.252353384431459	0.301	0.233	1.3861454399209E-06	Mid-zonal hepatocytes	RNF126
8.53042131627459E-11	0.670950531945552	0.228	0.168	1.39139702089755E-06	Mid-zonal hepatocytes	AGTRAP
8.56489429032492E-11	1.01571235514792	0.154	0.1	1.3970199076949E-06	Mid-zonal hepatocytes	TDRP
8.78429137424328E-11	0.671838690503266	0.247	0.185	1.43280576605282E-06	Mid-zonal hepatocytes	PTK2
8.94322996081094E-11	1.45428887727323	0.13	0.08	1.45873023890787E-06	Mid-zonal hepatocytes	TLK2
9.03111691826681E-11	0.920952357571623	0.148	0.095	1.4730654805385E-06	Mid-zonal hepatocytes	GLT8D1
9.17505470295942E-11	1.42885739325755	0.128	0.077	1.49654317259971E-06	Mid-zonal hepatocytes	MTX3
9.21170274016991E-11	1.48524833164415	0.121	0.072	1.50252083394911E-06	Mid-zonal hepatocytes	RAD51B
9.29057794405266E-11	1.83639051591767	0.1	0.056	1.51538616845443E-06	Mid-zonal hepatocytes	SCG5
9.2923310274034E-11	1.26668867326358	0.105	0.059	1.51567211387977E-06	Mid-zonal hepatocytes	PRIM2
9.55971363147673E-11	1.32146532066388	0.133	0.083	1.55928489043017E-06	Mid-zonal hepatocytes	ITSN1
9.64626659663026E-11	1.17088565662665	0.111	0.064	1.57340254457636E-06	Mid-zonal hepatocytes	THAP6
9.76924376326841E-11	0.96532364654089	0.119	0.07	1.59346135022671E-06	Mid-zonal hepatocytes	ASB16
9.99762717956825E-11	0.434957614418285	0.259	0.195	1.63071296925938E-06	Mid-zonal hepatocytes	MPP1
1.00637183566978E-10	0.990001045995223	0.145	0.092	1.64149310116098E-06	Mid-zonal hepatocytes	SHROOM2
1.00928798057044E-10	0.922624450276407	0.131	0.08	1.64624962510845E-06	Mid-zonal hepatocytes	INTS14
1.01090662266298E-10	1.18372724695072	0.144	0.09	1.64888979222559E-06	Mid-zonal hepatocytes	DDX58
1.02201784116976E-10	0.704424977131971	0.254	0.195	1.66701330073199E-06	Mid-zonal hepatocytes	ETFDH
1.06078067456894E-10	1.08077182680237	0.204	0.148	1.7302393582894E-06	Mid-zonal hepatocytes	HNRNPLL
1.06280710454278E-10	1.49229081442219	0.181	0.127	1.73354466821973E-06	Mid-zonal hepatocytes	ABCA10
1.07140017421647E-10	0.748889228788591	0.177	0.12	1.74756082416448E-06	Mid-zonal hepatocytes	ZNF697
1.09393701355306E-10	1.08117291272067	0.138	0.086	1.78432066280639E-06	Mid-zonal hepatocytes	SPAG16
1.09685877383867E-10	0.339209749100423	0.296	0.231	1.78908634600826E-06	Mid-zonal hepatocytes	UNC50
1.1302332472543E-10	1.19358437166605	0.137	0.086	1.84352344959649E-06	Mid-zonal hepatocytes	GOLGA8A
1.16207275648899E-10	0.553738415826121	0.265	0.205	1.89545687310918E-06	Mid-zonal hepatocytes	SRP72
1.18198694166213E-10	0.374152979759836	0.207	0.148	1.92793890054511E-06	Mid-zonal hepatocytes	FAM168B
1.20469594748355E-10	1.17771676605442	0.163	0.109	1.96497955994042E-06	Mid-zonal hepatocytes	RBM26
1.21889139824677E-10	0.250894160393206	0.156	0.099	1.98813375968031E-06	Mid-zonal hepatocytes	ADM2
1.22461392805764E-10	1.16540853881344	0.122	0.073	1.99746777805481E-06	Mid-zonal hepatocytes	IQCB1
1.25353576869713E-10	1.91776197225766	0.102	0.057	2.04464219232189E-06	Mid-zonal hepatocytes	ERCC6L2
1.25566171196457E-10	0.460919845919737	0.283	0.221	2.04810981838541E-06	Mid-zonal hepatocytes	API5
1.25589987926419E-10	0.963778017059057	0.197	0.14	2.04849829306783E-06	Mid-zonal hepatocytes	SNX13
1.2577322602472E-10	0.411391878446462	0.318	0.252	2.0514870896892E-06	Mid-zonal hepatocytes	SLC47A1
1.26084640713862E-10	0.615237037538015	0.138	0.085	2.0565665746838E-06	Mid-zonal hepatocytes	CCN2
1.26364238547363E-10	0.436501078330046	0.216	0.154	2.06112709494604E-06	Mid-zonal hepatocytes	B4GALT7
1.26571452271982E-10	0.941490766291401	0.123	0.074	2.0645069580083E-06	Mid-zonal hepatocytes	KPNB1
1.30193877645106E-10	1.02433285207898	0.212	0.156	2.12359233826933E-06	Mid-zonal hepatocytes	FCSK
1.32945940142065E-10	0.367456722499855	0.282	0.221	2.16848122965722E-06	Mid-zonal hepatocytes	DPM1
1.3510951481413E-10	0.390634973040968	0.356	0.295	2.20377129613328E-06	Mid-zonal hepatocytes	STIMATE
1.35310707555725E-10	1.1511883766412	0.13	0.079	2.20705295094143E-06	Mid-zonal hepatocytes	XPO4
1.36820658530067E-10	0.416734704759802	0.309	0.244	2.23168176128392E-06	Mid-zonal hepatocytes	DESI1
1.36940097614412E-10	0.336107349645488	0.305	0.242	2.23362993218867E-06	Mid-zonal hepatocytes	ABHD12
1.38690013170219E-10	0.30965335425529	0.269	0.209	2.26217280481944E-06	Mid-zonal hepatocytes	SMIM7
1.42024586917818E-10	0.9794367098018	0.126	0.077	2.31656303721653E-06	Mid-zonal hepatocytes	SYNJ2

1.4317329839085E-10	1.24170209037129	0.126	0.076	2.33529967005316E-06	Mid-zonal hepatocytes	ZNF564
1.44987054047685E-10	0.472381607457995	0.763	0.664	2.36488383857178E-06	Mid-zonal hepatocytes	SOD2
1.45320925698493E-10	0.312761779404036	0.299	0.236	2.37032961906811E-06	Mid-zonal hepatocytes	LNX2
1.45913239684919E-10	1.38206234826458	0.128	0.078	2.37999085250072E-06	Mid-zonal hepatocytes	ORC3
1.4969709226232E-10	1.05530827177174	0.162	0.107	2.4417092718907E-06	Mid-zonal hepatocytes	ZNF251
1.50510438351024E-10	0.7610975388784	0.172	0.117	2.45497575994354E-06	Mid-zonal hepatocytes	RAI1
1.517065866081E-10	0.867821380167878	0.183	0.127	2.47448613416473E-06	Mid-zonal hepatocytes	MEGF8
1.53206153362632E-10	0.79277278184295	0.197	0.14	2.49894556749789E-06	Mid-zonal hepatocytes	GGA1
1.56906843935172E-10	0.311981283749656	0.37	0.303	2.55930753142658E-06	Mid-zonal hepatocytes	TIMM17A
1.5695316910187E-10	1.16183337232132	0.149	0.096	2.56006314122061E-06	Mid-zonal hepatocytes	DOCK9
1.5777608776462E-10	0.760078296631235	0.134	0.08	2.57348576752871E-06	Mid-zonal hepatocytes	CCL20
1.58251737005812E-10	0.635916566904625	0.247	0.19	2.5812440823018E-06	Mid-zonal hepatocytes	RFFL
1.60096009623927E-10	1.3476504359271	0.102	0.057	2.61132601297587E-06	Mid-zonal hepatocytes	DEPRECAT
1.6115466515285E-10	1.09005830709764	0.108	0.061	2.62859374330814E-06	Mid-zonal hepatocytes	CAPS
1.62680465294633E-10	0.911398068359386	0.248	0.19	2.65348106942075E-06	Mid-zonal hepatocytes	AQP7
1.63155002734802E-10	1.37470738737673	0.121	0.073	2.66122124960735E-06	Mid-zonal hepatocytes	PIP4K2A
1.6672715547257E-10	0.992334049885052	0.111	0.064	2.71948663291309E-06	Mid-zonal hepatocytes	KIF2A
1.67300180602275E-10	0.65994153306851	0.229	0.17	2.72883324580371E-06	Mid-zonal hepatocytes	SRRM1
1.67759220425408E-10	1.76528189458322	0.104	0.059	2.73632064435883E-06	Mid-zonal hepatocytes	INPP5B
1.68672280132292E-10	0.856040101467464	0.154	0.1	2.75121356123781E-06	Mid-zonal hepatocytes	CHD8
1.71860451533687E-10	0.828552764129963	0.203	0.146	2.80321582496598E-06	Mid-zonal hepatocytes	VPS41
1.72975689864003E-10	0.715321227598617	0.2	0.143	2.82140647737175E-06	Mid-zonal hepatocytes	BORCS7
1.73169631196003E-10	1.2855552345964	0.13	0.08	2.824569854438E-06	Mid-zonal hepatocytes	SPATA5
1.7534574523284E-10	0.87878000909493	0.179	0.124	2.86006445049286E-06	Mid-zonal hepatocytes	MCMBP
1.80222851624883E-10	0.883640497505186	0.17	0.115	2.93961493285347E-06	Mid-zonal hepatocytes	COG1
1.84254570023518E-10	0.919231294995031	0.12	0.071	3.0053762916536E-06	Mid-zonal hepatocytes	ZNF646
1.84412339455586E-10	1.24942606901792	0.135	0.085	3.00794966886006E-06	Mid-zonal hepatocytes	CDA
1.84425252403499E-10	1.27184860672469	0.134	0.084	3.00816029195348E-06	Mid-zonal hepatocytes	TTC13
1.8555724691965E-10	0.681013458240478	0.234	0.176	3.02662425450642E-06	Mid-zonal hepatocytes	DHPS
1.86229305353924E-10	1.34650719148914	0.116	0.069	3.03758619962786E-06	Mid-zonal hepatocytes	URB1
1.86498262307334E-10	0.724072432172696	0.26	0.201	3.04197315649493E-06	Mid-zonal hepatocytes	NCOA2
1.89166090128174E-10	0.734637523070619	0.144	0.092	3.08548809608065E-06	Mid-zonal hepatocytes	MFSD14A
1.92773977524756E-10	1.4779776199158	0.125	0.077	3.1443363474063E-06	Mid-zonal hepatocytes	ZNF266
1.95097610024806E-10	1.0405111018614	0.131	0.081	3.18223711711461E-06	Mid-zonal hepatocytes	WDR20
1.95267097234916E-10	0.671994966033462	0.19	0.133	3.18500162299871E-06	Mid-zonal hepatocytes	PLAA
1.9669892866922E-10	1.02538829183888	0.232	0.176	3.20835622552366E-06	Mid-zonal hepatocytes	FYB2
1.9915315837915E-10	0.860218037629342	0.129	0.079	3.24838716632232E-06	Mid-zonal hepatocytes	CERS5
1.99600939778547E-10	1.50352503121988	0.104	0.059	3.25569092872789E-06	Mid-zonal hepatocytes	BBS9
2.02918572773712E-10	0.558877981239251	0.206	0.147	3.30980484051201E-06	Mid-zonal hepatocytes	SLC2A13
2.04856228435642E-10	0.960421378495371	0.156	0.103	3.34140994201376E-06	Mid-zonal hepatocytes	TBC1D10B
2.08162876771438E-10	0.930395748278555	0.167	0.113	3.39534468301892E-06	Mid-zonal hepatocytes	TRAPPC8
2.0896609836785E-10	1.01248990267941	0.117	0.07	3.408446030478E-06	Mid-zonal hepatocytes	TXLNG
2.10213191045051E-10	0.779975290383241	0.123	0.074	3.42878735913583E-06	Mid-zonal hepatocytes	MDGA1
2.12309541528597E-10	0.801669234371595	0.194	0.138	3.46298093187295E-06	Mid-zonal hepatocytes	UTP6
2.12719093282459E-10	1.02980099298815	0.187	0.13	3.46966113053019E-06	Mid-zonal hepatocytes	MEN1
2.13271800187796E-10	0.468088043627471	0.198	0.138	3.47867633286314E-06	Mid-zonal hepatocytes	NTN1
2.13695112132414E-10	0.795723651331056	0.148	0.095	3.48558097399181E-06	Mid-zonal hepatocytes	CPSF2
2.18070666224244E-10	1.15236022443181	0.142	0.091	3.55695063678364E-06	Mid-zonal hepatocytes	RHBDF2
2.18394933429272E-10	1.20779419499987	0.142	0.091	3.56223975916485E-06	Mid-zonal hepatocytes	ANKRD6
2.19343414867085E-10	1.2694308258014	0.104	0.059	3.57771043989703E-06	Mid-zonal hepatocytes	SPATA33
2.21534091655658E-10	0.386861727420119	0.293	0.232	3.61344256899543E-06	Mid-zonal hepatocytes	CCNI
2.23831679300276E-10	1.00486061444846	0.103	0.058	3.65091852106681E-06	Mid-zonal hepatocytes	FGFR1OP2
2.28305459542856E-10	0.863148763436267	0.172	0.117	3.72389035060353E-06	Mid-zonal hepatocytes	OSER1
2.30099007827598E-10	0.938674383095083	0.121	0.072	3.75314491667594E-06	Mid-zonal hepatocytes	PIDD1
2.34136121163118E-10	1.16975008614995	0.142	0.091	3.81899427229161E-06	Mid-zonal hepatocytes	ABHD18
2.40482296125546E-10	0.663068423702045	0.175	0.118	3.92250673210378E-06	Mid-zonal hepatocytes	TMEM170A
2.41973810777116E-10	0.262521607330814	0.294	0.23	3.94683482758554E-06	Mid-zonal hepatocytes	GID8
2.43902032346076E-10	1.32968816062687	0.153	0.101	3.97828604959685E-06	Mid-zonal hepatocytes	EBLN2
2.46293655271613E-10	0.617600616636464	0.158	0.104	4.01729581113529E-06	Mid-zonal hepatocytes	CUL2
2.50345346199952E-10	1.61030494462284	0.107	0.062	4.08338294186742E-06	Mid-zonal hepatocytes	SYTL2

2.56232894730196E-10	0.444305738720721	0.166	0.11	4.17941474594422E-06	Mid-zonal hepatocytes	CAPN15
2.57856562757724E-10	1.19882470312088	0.126	0.077	4.20589839514124E-06	Mid-zonal hepatocytes	WASF3
2.58914252353972E-10	0.950693592547325	0.125	0.076	4.22315037014564E-06	Mid-zonal hepatocytes	ATRIP
2.61395402265556E-10	1.5038867824075	0.113	0.067	4.26362040635349E-06	Mid-zonal hepatocytes	PPP2R3A
2.62285535864498E-10	0.769524022262067	0.188	0.134	4.27813937548582E-06	Mid-zonal hepatocytes	GART
2.64158916878733E-10	1.12851892823295	0.19	0.133	4.30869609320901E-06	Mid-zonal hepatocytes	FBXL8
2.69021595379991E-10	0.577725442638201	0.282	0.221	4.38801124224303E-06	Mid-zonal hepatocytes	NOP14
2.72010380339709E-10	0.641914015975195	0.212	0.154	4.436761313721E-06	Mid-zonal hepatocytes	GMPPA
2.73451308692601E-10	0.743511638472447	0.164	0.109	4.46026429608501E-06	Mid-zonal hepatocytes	SMIM29
2.73762613522782E-10	0.832619838857025	0.201	0.144	4.46534198917009E-06	Mid-zonal hepatocytes	ATP6V1C1
2.77658449109176E-10	0.984370196407785	0.119	0.071	4.52888696341977E-06	Mid-zonal hepatocytes	SLC36A1
2.78353494414337E-10	0.503113910159861	0.277	0.217	4.54022384739224E-06	Mid-zonal hepatocytes	IDH3G
2.78788703272807E-10	1.09832095306952	0.197	0.141	4.54732253908276E-06	Mid-zonal hepatocytes	CUL5
2.79658533421195E-10	0.477127818875642	0.133	0.082	4.56151033863312E-06	Mid-zonal hepatocytes	CHSY1
2.80191323036662E-10	0.794945467029618	0.166	0.112	4.570200670051E-06	Mid-zonal hepatocytes	CAAP1
2.83007616302028E-10	0.263391436358851	0.275	0.213	4.61613722950238E-06	Mid-zonal hepatocytes	DNAJB12
2.85392633626816E-10	1.24714861774384	0.157	0.104	4.655039247087E-06	Mid-zonal hepatocytes	NSUN4
2.85875193815431E-10	1.05051022375308	0.16	0.107	4.6629102863235E-06	Mid-zonal hepatocytes	PLEKHA1
2.91396752515611E-10	1.16308597128231	0.122	0.074	4.75297243028213E-06	Mid-zonal hepatocytes	ABI2
3.03085600543088E-10	0.778318726545584	0.185	0.128	4.9436292304583E-06	Mid-zonal hepatocytes	IREB2
3.06333335814181E-10	0.78419030488427	0.16	0.107	4.99660304046511E-06	Mid-zonal hepatocytes	ATE1
3.07428921143615E-10	0.443290256047374	0.275	0.214	5.01447313277351E-06	Mid-zonal hepatocytes	GSR
3.19305039556025E-10	0.573853184424498	0.26	0.201	5.20818450019832E-06	Mid-zonal hepatocytes	CRADD
3.22390580844349E-10	1.18502023665955	0.149	0.097	5.25851276415217E-06	Mid-zonal hepatocytes	PDPK1
3.35021386094647E-10	0.66825524444481	0.17	0.116	5.46453382858978E-06	Mid-zonal hepatocytes	PLCL2
3.35075692731542E-10	0.722001562994182	0.186	0.13	5.46541962414419E-06	Mid-zonal hepatocytes	BBX
3.35154001312676E-10	0.356892101038921	0.313	0.249	5.46669691541106E-06	Mid-zonal hepatocytes	C1orf198
3.35989847660621E-10	0.98496772777404	0.122	0.073	5.48033040519238E-06	Mid-zonal hepatocytes	ZNF274
3.55492044955486E-10	0.956469231589776	0.144	0.093	5.79843074526893E-06	Mid-zonal hepatocytes	NAT10
3.6128922284171E-10	0.831825073420091	0.181	0.126	5.89298851377112E-06	Mid-zonal hepatocytes	GTF3A
3.6713843553101E-10	0.648898527550991	0.251	0.193	5.9883950219463E-06	Mid-zonal hepatocytes	MXI1
3.681727177189E-10	1.21568783886214	0.122	0.074	6.00526519871298E-06	Mid-zonal hepatocytes	PACRGL
3.83573703331919E-10	0.472477327354902	0.235	0.175	6.25647067504693E-06	Mid-zonal hepatocytes	GRAMD1A
3.86390333336476E-10	0.461965979345509	0.271	0.212	6.30241272705127E-06	Mid-zonal hepatocytes	YPEL3
3.86433500468474E-10	1.07978272134322	0.156	0.105	6.30311682614128E-06	Mid-zonal hepatocytes	CERS6
3.91980044067754E-10	0.422108577863126	0.325	0.263	6.39358649878913E-06	Mid-zonal hepatocytes	AMT
4.02866896325694E-10	0.996293927733867	0.163	0.11	6.57116194596839E-06	Mid-zonal hepatocytes	CEP104
4.03052864009631E-10	0.555857079038625	0.24	0.18	6.57419526486109E-06	Mid-zonal hepatocytes	ISY1
4.04954205296746E-10	0.260732274680263	0.314	0.253	6.60520804259523E-06	Mid-zonal hepatocytes	ACO2
4.09275859137990E-10	1.05996502818132	0.154	0.102	6.67569853839976E-06	Mid-zonal hepatocytes	GK5
4.15645390832288E-10	0.923578180260599	0.136	0.086	6.77959196986545E-06	Mid-zonal hepatocytes	MFSD11
4.16440821351712E-10	0.892400226867485	0.185	0.13	6.79256623706778E-06	Mid-zonal hepatocytes	SLC25A26
4.18435332270826E-10	0.795096416964139	0.369	0.306	6.82509870466944E-06	Mid-zonal hepatocytes	RND1
4.19548359627519E-10	0.638880210420742	0.216	0.159	6.84325329388446E-06	Mid-zonal hepatocytes	NAGPA
4.23020894349335E-10	0.851663478497203	0.18	0.125	6.89989380773201E-06	Mid-zonal hepatocytes	RNF19B
4.30868087596296E-10	0.616030938313194	0.196	0.14	7.02788937678318E-06	Mid-zonal hepatocytes	CIAO3
4.31507962437926E-10	1.16270559758204	0.129	0.08	7.03832637532501E-06	Mid-zonal hepatocytes	FNBP1
4.32004778249549E-10	0.510050260528333	0.154	0.101	7.04642993802839E-06	Mid-zonal hepatocytes	IL15RA
4.41297312861151E-10	1.06680434769919	0.141	0.09	7.19800047007823E-06	Mid-zonal hepatocytes	TRAF6
4.53812897170752E-10	0.693350054296663	0.209	0.152	7.40214216575214E-06	Mid-zonal hepatocytes	H2AFY
4.54894837052274E-10	1.08005549925778	0.186	0.132	7.41978968715964E-06	Mid-zonal hepatocytes	NIPAL1
4.69092058086898E-10	1.10781015507005	0.147	0.095	7.6513605594554E-06	Mid-zonal hepatocytes	ARID5B
4.70099346972611E-10	0.556023769975556	0.304	0.24	7.66779044847026E-06	Mid-zonal hepatocytes	ASPG
4.71661294586794E-10	1.11867652730799	0.122	0.074	7.6932673760052E-06	Mid-zonal hepatocytes	ERI2
4.72644063663156E-10	0.825645909740365	0.133	0.084	7.70929732240973E-06	Mid-zonal hepatocytes	MRTFB
4.7703974732318E-10	1.17435939482815	0.113	0.067	7.78099531858839E-06	Mid-zonal hepatocytes	PLD6
4.77997940206316E-10	0.560801211837812	0.199	0.143	7.79662440270522E-06	Mid-zonal hepatocytes	PACS2
4.97330537873109E-10	1.73147393391207	0.101	0.057	8.11195840324828E-06	Mid-zonal hepatocytes	CAPRIN2
4.97765546299795E-10	1.30474221229166	0.113	0.067	8.11905382569596E-06	Mid-zonal hepatocytes	PPP2R5B
5.01421911124861E-10	0.728739258551322	0.167	0.112	8.17869279235761E-06	Mid-zonal hepatocytes	TRMT61A

5.07107357323845E-10	0.314531809604881	0.305	0.242	8.27142810530924E-06	Mid-zonal hepatocytes	FAM20B
5.09948090292307E-10	0.330920657373501	0.265	0.203	8.31776330075782E-06	Mid-zonal hepatocytes	SLBP
5.28373974586374E-10	0.963042888924102	0.161	0.107	8.61830789947834E-06	Mid-zonal hepatocytes	CLTCL1
5.36324782504396E-10	0.744466417854974	0.108	0.063	8.74799352742921E-06	Mid-zonal hepatocytes	USP39
5.46713877862029E-10	0.541625835467568	0.251	0.191	8.91745006180755E-06	Mid-zonal hepatocytes	KDM3B
5.52523321992979E-10	0.709716491226861	0.229	0.172	9.01220790502748E-06	Mid-zonal hepatocytes	DHX36
5.62051247146349E-10	0.771950576913914	0.159	0.106	9.1676178922041E-06	Mid-zonal hepatocytes	ARPC2
5.77512472902758E-10	1.14247936160815	0.117	0.071	9.41980594551689E-06	Mid-zonal hepatocytes	TMEM143
5.79740898523971E-10	0.443648043195785	0.244	0.186	9.45615379582449E-06	Mid-zonal hepatocytes	IAH1
5.79751096582562E-10	0.919781629304575	0.153	0.101	9.45632013635818E-06	Mid-zonal hepatocytes	TRIM23
5.89408559746159E-10	1.02071237542724	0.171	0.118	9.6138430180196E-06	Mid-zonal hepatocytes	TIMM8A
5.89641291828484E-10	0.435708968597675	0.257	0.199	9.6176391110144E-06	Mid-zonal hepatocytes	PTPN1
5.94584765336621E-10	1.06308693263292	0.122	0.075	9.69827210740562E-06	Mid-zonal hepatocytes	ZNF133
5.94712799582162E-10	0.596007341776598	0.231	0.174	9.70036047398464E-06	Mid-zonal hepatocytes	CMC2
5.97419036510174E-10	1.10747935158622	0.156	0.104	9.74450190451744E-06	Mid-zonal hepatocytes	BBS2
6.11367994900835E-10	0.874849902473897	0.169	0.116	9.97202336482751E-06	Mid-zonal hepatocytes	SH2B1
6.39535097241476E-10	0.775109670448287	0.115	0.069	1.04314569711057E-05	Mid-zonal hepatocytes	RBM38
6.41632818594687E-10	0.777803382104714	0.164	0.111	1.04656729040979E-05	Mid-zonal hepatocytes	ARHGEF16
6.45114427371073E-10	1.10755048432122	0.113	0.068	1.05224614248496E-05	Mid-zonal hepatocytes	TMEM44
6.5754553850854E-10	1.20539175183712	0.113	0.068	1.07252252786128E-05	Mid-zonal hepatocytes	RHOQ
6.5876550170391E-10	0.600611507268224	0.237	0.181	1.07451240982925E-05	Mid-zonal hepatocytes	HNRNPL
6.64905391425145E-10	0.672136466048529	0.191	0.135	1.08452718395355E-05	Mid-zonal hepatocytes	DNMT1
6.65628147782643E-10	1.28511887887698	0.126	0.078	1.08570607184827E-05	Mid-zonal hepatocytes	SLC44A3
6.70569468776536E-10	1.15443742658716	0.182	0.129	1.09376586052141E-05	Mid-zonal hepatocytes	WASHC5
6.71451198643703E-10	0.373407474367553	0.318	0.261	1.09520405010774E-05	Mid-zonal hepatocytes	TOMM70
6.74847278808059E-10	0.971410304387173	0.17	0.117	1.10074339646382E-05	Mid-zonal hepatocytes	DEPRECAT
7.01036969812406E-10	0.658809415729108	0.252	0.195	1.14346140146101E-05	Mid-zonal hepatocytes	SRP19
7.08252369381735E-10	1.30125823649006	0.131	0.084	1.15523043969855E-05	Mid-zonal hepatocytes	BTRC
7.13262269798787E-10	0.947501069198225	0.16	0.108	1.1634020882688E-05	Mid-zonal hepatocytes	PPP4R1
7.23627760184631E-10	0.878047135165069	0.147	0.096	1.18030923963715E-05	Mid-zonal hepatocytes	PRRC2B
7.2491893064153E-10	0.811053326322321	0.195	0.141	1.1824152677694E-05	Mid-zonal hepatocytes	DNAJC2
7.55065243879806E-10	0.641618965630054	0.213	0.158	1.23158691929235E-05	Mid-zonal hepatocytes	HERC2
7.73311227605014E-10	1.01714110422418	0.159	0.107	1.26134794334654E-05	Mid-zonal hepatocytes	ATG7
7.73792992622457E-10	0.282764815620709	0.215	0.154	1.26213375026649E-05	Mid-zonal hepatocytes	ERLEC1
7.95086652893075E-10	0.919341341759415	0.139	0.09	1.29686583953389E-05	Mid-zonal hepatocytes	PFKFB2
8.23520088169177E-10	1.08761951081803	0.108	0.063	1.34324361581275E-05	Mid-zonal hepatocytes	AC073111.
8.24009684200817E-10	0.649883546599004	0.171	0.118	1.34404219589995E-05	Mid-zonal hepatocytes	SP1
8.28986425785925E-10	1.18134902113156	0.103	0.06	1.35215975909942E-05	Mid-zonal hepatocytes	RASA2
8.43438613387597E-10	0.683601230328013	0.361	0.304	1.37573272229651E-05	Mid-zonal hepatocytes	ACSM5
8.47906714547727E-10	0.906543616976397	0.144	0.094	1.3830206420988E-05	Mid-zonal hepatocytes	TIMM9
8.49465416287931E-10	1.04572914412057	0.159	0.107	1.38556304050724E-05	Mid-zonal hepatocytes	KDM1A
8.59499783068683E-10	1.32226367737004	0.128	0.08	1.40193009616333E-05	Mid-zonal hepatocytes	NSMAF
8.62189050416254E-10	0.826503091349963	0.132	0.083	1.40631656013395E-05	Mid-zonal hepatocytes	NOTCH1
9.01925115614534E-10	0.778457158218184	0.206	0.152	1.47113005607887E-05	Mid-zonal hepatocytes	SIRT5
9.23334843056292E-10	1.3569725434903	0.12	0.074	1.50605146250912E-05	Mid-zonal hepatocytes	SNAPC3
9.27678267419479E-10	0.635258551438691	0.166	0.114	1.51313602198791E-05	Mid-zonal hepatocytes	ZBTB17
9.44838908424069E-10	1.32877400256361	0.125	0.078	1.5411267435305E-05	Mid-zonal hepatocytes	NDUFAF5
9.52890498516278E-10	1.07975584842264	0.141	0.091	1.5542596921299E-05	Mid-zonal hepatocytes	ASXL2
9.65750459939626E-10	0.765203165354533	0.224	0.17	1.57523557520752E-05	Mid-zonal hepatocytes	CAMKK2
9.71418931218105E-10	0.582250160948307	0.18	0.125	1.58448141870985E-05	Mid-zonal hepatocytes	ATXN3
1.00613514024714E-09	0.539237716405308	0.32	0.263	1.64110702725711E-05	Mid-zonal hepatocytes	PITRM1
1.01024868340871E-09	1.09009353478823	0.167	0.115	1.64781662750795E-05	Mid-zonal hepatocytes	ZMYND15
1.01051081773758E-09	0.393392667556835	0.163	0.11	1.64824419481177E-05	Mid-zonal hepatocytes	PWWP2A
1.01135100443603E-09	1.01279598575081	0.166	0.114	1.64961462333561E-05	Mid-zonal hepatocytes	TINF2
1.02497116704084E-09	0.747897770359294	0.169	0.116	1.67183047056032E-05	Mid-zonal hepatocytes	CMTR2
1.03798312485221E-09	1.14483192556814	0.17	0.117	1.69305427494643E-05	Mid-zonal hepatocytes	SRSF8
1.05831531834992E-09	0.991974649237303	0.194	0.142	1.72621811576056E-05	Mid-zonal hepatocytes	SLC5A3
1.06145873224636E-09	0.345992248152257	0.25	0.193	1.73134533816703E-05	Mid-zonal hepatocytes	RNPS1
1.06540572217057E-09	0.376461001708786	0.348	0.285	1.73778327343242E-05	Mid-zonal hepatocytes	FZR1
1.06706477992769E-09	0.760064663757641	0.172	0.119	1.74048936254006E-05	Mid-zonal hepatocytes	MTREX

1.06933542854325E-09	0.572898533251001	0.213	0.155	1.7441930174969E-05	Mid-zonal hepatocytes	C5orf24
1.0777698910139E-09	0.951494121394062	0.177	0.123	1.75795046923277E-05	Mid-zonal hepatocytes	MPHOSPH8
1.07942603966901E-09	1.01664584635253	0.119	0.073	1.76065181330413E-05	Mid-zonal hepatocytes	CCDC32
1.09515518233162E-09	1.19864535893302	0.132	0.084	1.78630761790111E-05	Mid-zonal hepatocytes	MADD
1.11500411612968E-09	1.58364802235416	0.103	0.06	1.81868321381911E-05	Mid-zonal hepatocytes	MED22
1.11912085464478E-09	0.917265066039976	0.142	0.093	1.8253980260111E-05	Mid-zonal hepatocytes	TMEM242
1.17854475654267E-09	0.456696652585694	0.238	0.179	1.92232435239676E-05	Mid-zonal hepatocytes	GALNS
1.19191397363353E-09	1.37082493300836	0.137	0.088	1.94413088239365E-05	Mid-zonal hepatocytes	DDX60L
1.19757667945079E-09	0.666711064096673	0.248	0.193	1.95336732185218E-05	Mid-zonal hepatocytes	PPCS
1.20671338433654E-09	1.38400945317326	0.141	0.093	1.96827020119133E-05	Mid-zonal hepatocytes	PLD1
1.21475459144953E-09	0.57708777405602	0.198	0.144	1.98138621411333E-05	Mid-zonal hepatocytes	ZPR1
1.22449478840824E-09	0.760675764617508	0.189	0.135	1.99727344937268E-05	Mid-zonal hepatocytes	MED11
1.23940263658735E-09	0.60914241925585	0.186	0.132	2.02158964053763E-05	Mid-zonal hepatocytes	CUTC
1.24284942639628E-09	0.353550528072659	0.277	0.221	2.02721169939497E-05	Mid-zonal hepatocytes	PSMD6
1.25157770730934E-09	1.19756897583318	0.115	0.07	2.04144839839227E-05	Mid-zonal hepatocytes	CARD19
1.27046901885957E-09	0.60204814316896	0.184	0.131	2.07226201666185E-05	Mid-zonal hepatocytes	BET1
1.28912803905144E-09	1.14628703108354	0.128	0.08	2.10269674449681E-05	Mid-zonal hepatocytes	RRP8
1.29420648745763E-09	1.58701518682431	0.118	0.073	2.11098020169215E-05	Mid-zonal hepatocytes	DDX10
1.30196561082424E-09	1.11874794783565	0.125	0.078	2.12363610781542E-05	Mid-zonal hepatocytes	STRN
1.30976728770738E-09	0.516900872158487	0.263	0.206	2.13636142297951E-05	Mid-zonal hepatocytes	CBLC
1.33535479847198E-09	0.545658937339116	0.18	0.125	2.17809721178764E-05	Mid-zonal hepatocytes	SPATA2L
1.34693565257381E-09	0.494519054532558	0.377	0.317	2.19698674291314E-05	Mid-zonal hepatocytes	CYP4F11
1.3677656935571E-09	0.670403950805932	0.136	0.087	2.23096262276099E-05	Mid-zonal hepatocytes	SLC41A1
1.37013997544373E-09	0.741282322181674	0.178	0.126	2.23483531394627E-05	Mid-zonal hepatocytes	KDM5A
1.38438258708464E-09	0.357998435805232	0.281	0.221	2.25806643779376E-05	Mid-zonal hepatocytes	PTP4A3
1.3894538221662E-09	0.309932232186733	0.493	0.422	2.26633812933529E-05	Mid-zonal hepatocytes	DEPRECAT
1.44521769659017E-09	0.479560943256995	0.186	0.131	2.35729458490823E-05	Mid-zonal hepatocytes	DUS3L
1.47103687444103E-09	0.769040963835211	0.138	0.088	2.39940824590077E-05	Mid-zonal hepatocytes	TRNP1
1.49908150041925E-09	0.7753256344615	0.135	0.086	2.44515183533384E-05	Mid-zonal hepatocytes	GNPDA1
1.53573208314443E-09	0.583318003915679	0.319	0.266	2.50493260081688E-05	Mid-zonal hepatocytes	ELMO3
1.55901534015496E-09	1.02597063509905	0.111	0.067	2.54290992132675E-05	Mid-zonal hepatocytes	RABGEF1
1.56221050403725E-09	1.10711926344933	0.215	0.163	2.54812155313516E-05	Mid-zonal hepatocytes	PNPLA3
1.58534188458934E-09	0.796260166723529	0.117	0.071	2.58585114795368E-05	Mid-zonal hepatocytes	C2CD2L
1.58623037653685E-09	1.31501005445304	0.105	0.062	2.58730036716925E-05	Mid-zonal hepatocytes	COG5
1.60109235343303E-09	0.766875852368663	0.107	0.064	2.61154173768462E-05	Mid-zonal hepatocytes	FAM192A
1.61577664980754E-09	0.827005595913043	0.14	0.091	2.63549329350107E-05	Mid-zonal hepatocytes	PLEKHA7
1.62815159563481E-09	0.588716472904515	0.235	0.18	2.65567806763993E-05	Mid-zonal hepatocytes	GLT1D1
1.65293823822968E-09	0.86951837057903	0.2	0.148	2.69610756037643E-05	Mid-zonal hepatocytes	DGUOK
1.68279312816857E-09	1.05859166242455	0.125	0.078	2.74480387135576E-05	Mid-zonal hepatocytes	NUP93
1.68996360999304E-09	0.874758409900202	0.179	0.126	2.75649964425964E-05	Mid-zonal hepatocytes	TCOF1
1.7023154652759E-09	1.11706379940285	0.147	0.098	2.77664675541153E-05	Mid-zonal hepatocytes	TBCE.1
1.71562874629956E-09	0.795704865984531	0.144	0.095	2.79836204808921E-05	Mid-zonal hepatocytes	PSIP1
1.74462372993536E-09	1.0482896480086	0.107	0.064	2.84565576589757E-05	Mid-zonal hepatocytes	MAGI3
1.76461153589008E-09	0.583499546709523	0.196	0.142	2.87825787619031E-05	Mid-zonal hepatocytes	TMOD3
1.76469518426203E-09	0.89554367602872	0.163	0.112	2.8783943150498E-05	Mid-zonal hepatocytes	RBM27
1.77002307890065E-09	1.28348722712812	0.135	0.088	2.88708464399485E-05	Mid-zonal hepatocytes	DCP2
1.7984270555727E-09	1.22638853998599	0.139	0.092	2.93341437034463E-05	Mid-zonal hepatocytes	TNIK
1.79863846546975E-09	0.95278155803829	0.147	0.098	2.93375920102771E-05	Mid-zonal hepatocytes	ZNF141
1.80493715831993E-09	0.328687311423248	0.318	0.259	2.94403299893564E-05	Mid-zonal hepatocytes	RNF40
1.8224568805712E-09	1.27629843400254	0.12	0.075	2.97260941789969E-05	Mid-zonal hepatocytes	TSPOAP1
1.83118479515179E-09	0.363071964216192	0.287	0.226	2.98684551937208E-05	Mid-zonal hepatocytes	ADGRV1
1.89902955601536E-09	0.662018740883107	0.176	0.124	3.09750710881665E-05	Mid-zonal hepatocytes	UBAC2
2.00410910732966E-09	0.982127797824049	0.111	0.067	3.26890236496541E-05	Mid-zonal hepatocytes	SIRT1
2.01168886309556E-09	0.549981276097302	0.246	0.191	3.28126570459517E-05	Mid-zonal hepatocytes	EFTUD2
2.07636599511917E-09	0.36341846445541	0.233	0.176	3.38676057463888E-05	Mid-zonal hepatocytes	EHD4
2.08066767596518E-09	0.827400394572313	0.234	0.179	3.39377704626681E-05	Mid-zonal hepatocytes	RAB11FIP4
2.16508971343255E-09	0.630349990011343	0.232	0.177	3.53147783157984E-05	Mid-zonal hepatocytes	ATP7B
2.17604329821693E-09	1.00232289900351	0.148	0.099	3.54934422372164E-05	Mid-zonal hepatocytes	NUP43
2.1898646377191E-09	0.959391466711915	0.159	0.109	3.57188821058363E-05	Mid-zonal hepatocytes	PSMA1
2.20641225829242E-09	0.856985092449924	0.133	0.085	3.59887903450077E-05	Mid-zonal hepatocytes	RFX1

**2.20963311814866E-09**	0.712226872946025	0.182	0.13	3.60413257901228E-05	Mid-zonal hepatocytes	PTPN6
**2.2231162234358E-09**	0.677215027766496	0.159	0.109	3.62612487204613E-05	Mid-zonal hepatocytes	ADK
**2.28536825558511E-09**	0.261169824728621	0.353	0.293	3.72766416168488E-05	Mid-zonal hepatocytes	PGAP3
**2.29406881652974E-09**	0.528275615282345	0.293	0.237	3.74185564664166E-05	Mid-zonal hepatocytes	FAM120A
**2.29480504575485E-09**	0.72931450263623	0.176	0.124	3.74305651013073E-05	Mid-zonal hepatocytes	CEBPZ
**2.33119393490519E-09**	0.73388079437993	0.205	0.15	3.80241042722385E-05	Mid-zonal hepatocytes	ACOT9
**2.34126791860568E-09**	1.35354661888032	0.114	0.07	3.81884210203773E-05	Mid-zonal hepatocytes	ZNF398
**2.37624731631098E-09**	0.507012062709675	0.222	0.168	3.87589699763484E-05	Mid-zonal hepatocytes	SIK3
**2.39162003761548E-09**	0.763777106377331	0.174	0.123	3.90097144335461E-05	Mid-zonal hepatocytes	SLC16A7
**2.40653409341495E-09**	0.992809958202843	0.101	0.059	3.92529775976912E-05	Mid-zonal hepatocytes	OSBPL10
**2.45124117729955E-09**	0.98308606013078	0.128	0.082	3.9982194842933E-05	Mid-zonal hepatocytes	MAP4K5
**2.46512918201223E-09**	1.31339973487571	0.142	0.094	4.02087220878015E-05	Mid-zonal hepatocytes	BIRC2
**2.46677189251975E-09**	0.88057990566856	0.114	0.069	4.02355163388896E-05	Mid-zonal hepatocytes	ASB7
**2.47782759909248E-09**	1.19375129991328	0.114	0.069	4.04158459687974E-05	Mid-zonal hepatocytes	PRRG1
**2.48539207771379E-09**	0.377960158520982	0.279	0.222	4.05392301795896E-05	Mid-zonal hepatocytes	PPP3R1
**2.50314724474832E-09**	0.414985588291783	0.338	0.284	4.08288347090898E-05	Mid-zonal hepatocytes	SNX4
**2.5158455308308E-09**	0.453457509424225	0.237	0.181	4.10359564533812E-05	Mid-zonal hepatocytes	CCNY
**2.52732185775055E-09**	1.28820888390606	0.105	0.062	4.12231468217693E-05	Mid-zonal hepatocytes	AC253572.
**2.52744551609272E-09**	0.354145035226707	0.265	0.208	4.12251638129883E-05	Mid-zonal hepatocytes	DNPEP
**2.56428457710476E-09**	0.863938787034588	0.204	0.152	4.18260457371558E-05	Mid-zonal hepatocytes	UCHL5
**2.57449834497511E-09**	0.452386288655551	0.247	0.191	4.19926425048891E-05	Mid-zonal hepatocytes	SENP2
**2.65489934835946E-09**	1.47450689438444	0.106	0.063	4.33040632710911E-05	Mid-zonal hepatocytes	ZFP62
**2.67281183231796E-09**	0.528298594457928	0.294	0.237	4.35962337969382E-05	Mid-zonal hepatocytes	MARVELD2
**2.6772820459762E-09**	0.851644463651046	0.121	0.076	4.36691474519179E-05	Mid-zonal hepatocytes	CLASP2
**2.74721562137795E-09**	0.761935679617456	0.195	0.143	4.48098340002957E-05	Mid-zonal hepatocytes	HLCS
**2.77115232944065E-09**	0.448911132416593	0.244	0.188	4.52002656455064E-05	Mid-zonal hepatocytes	IRF9
**2.79383883581177E-09**	0.317487301488405	0.268	0.212	4.55703052509257E-05	Mid-zonal hepatocytes	UPF1
**2.83550978021671E-09**	0.760612914180444	0.186	0.134	4.62500000251148E-05	Mid-zonal hepatocytes	SLC35E2A
**2.85118036118152E-09**	0.548650755293173	0.229	0.174	4.65056028712317E-05	Mid-zonal hepatocytes	ACTR1A
**2.88456012444733E-09**	1.03546862587424	0.151	0.102	4.70500601898604E-05	Mid-zonal hepatocytes	LIG3
**2.89796880540205E-09**	0.621295660772957	0.162	0.111	4.72687691849129E-05	Mid-zonal hepatocytes	CSNK2A2
**2.9013613778404E-09**	0.607918089791076	0.225	0.171	4.73241054339547E-05	Mid-zonal hepatocytes	MIIP
**2.91681841718668E-09**	0.652090767877943	0.236	0.183	4.75762252027319E-05	Mid-zonal hepatocytes	CLDN12
**2.92528744527979E-09**	1.18216023532064	0.125	0.079	4.77143635199586E-05	Mid-zonal hepatocytes	CARMIL1
**2.9283138800531E-09**	1.19057905830099	0.149	0.1	4.7763727697546E-05	Mid-zonal hepatocytes	MYO19
**2.96911009805662E-09**	1.06846161258731	0.14	0.093	4.84291548094016E-05	Mid-zonal hepatocytes	TRAFD1
**2.97201770662529E-09**	0.526243946602717	0.322	0.259	4.84765808127651E-05	Mid-zonal hepatocytes	TRIM55
**3.01775644268869E-09**	0.339984558613754	0.223	0.169	4.92226253366952E-05	Mid-zonal hepatocytes	COPS2
**3.02646381799333E-09**	0.717259471748407	0.149	0.099	4.93646513352893E-05	Mid-zonal hepatocytes	METTL2A
**3.0446269353838E-09**	0.572905085337821	0.181	0.129	4.96609099430451E-05	Mid-zonal hepatocytes	MOB2
**3.18745496822722E-09**	0.775282568306198	0.214	0.16	5.19905779867541E-05	Mid-zonal hepatocytes	UBE2G1
**3.18883746901763E-09**	1.50493099376367	0.106	0.064	5.20131279571465E-05	Mid-zonal hepatocytes	DRG1
**3.28188511509968E-09**	0.914989763088853	0.164	0.114	5.35308281123909E-05	Mid-zonal hepatocytes	ARL14EP
**3.29901009292123E-09**	0.753689749124202	0.235	0.182	5.38101536256382E-05	Mid-zonal hepatocytes	DHDDS
**3.47281486079634E-09**	0.829831375460432	0.105	0.063	5.6645083194449E-05	Mid-zonal hepatocytes	ZFPL1
**3.5065632413612E-09**	0.637489699467214	0.197	0.144	5.71955530298426E-05	Mid-zonal hepatocytes	MLH1
**3.53693970782572E-09**	0.859516706168894	0.179	0.128	5.76910235743454E-05	Mid-zonal hepatocytes	NHLRC3
**3.54490780104085E-09**	0.74162843154539	0.168	0.117	5.78209911427773E-05	Mid-zonal hepatocytes	RNF169
**3.58020777786734E-09**	0.9477598133495	0.12	0.075	5.83967690647942E-05	Mid-zonal hepatocytes	MID2
**3.61931778866079E-09**	0.620361427915352	0.16	0.109	5.90346924508462E-05	Mid-zonal hepatocytes	SLC44A1
**3.68785569118338E-09**	0.51628552860109	0.19	0.137	6.01526141788921E-05	Mid-zonal hepatocytes	IDH3A
**3.70233572909257E-09**	0.563934330848318	0.226	0.172	6.03887980772289E-05	Mid-zonal hepatocytes	ZMYND11
**3.71735224243494E-09**	0.279880686408098	0.278	0.219	6.06337324263562E-05	Mid-zonal hepatocytes	PELP1
**3.72446735098911E-09**	1.22959758081166	0.119	0.074	6.07497869619834E-05	Mid-zonal hepatocytes	USP28
**3.82371652964291E-09**	1.25511610801863	0.111	0.068	6.23686403150055E-05	Mid-zonal hepatocytes	C1QTNF6
**3.83810638251479E-09**	0.890539746544241	0.188	0.137	6.26033532051988E-05	Mid-zonal hepatocytes	TASOR
**3.89437901795244E-09**	1.05466272579502	0.14	0.092	6.35212161618222E-05	Mid-zonal hepatocytes	STRIP1
**4.0845140652257E-09**	0.526969816166598	0.231	0.178	6.66225089178964E-05	Mid-zonal hepatocytes	RBFA
**4.10119149445304E-09**	1.19982345107263	0.119	0.075	6.68945344660235E-05	Mid-zonal hepatocytes	USP2
**4.21759450918161E-09**	0.372692451517843	0.232	0.177	6.87931840392613E-05	Mid-zonal hepatocytes	ZFYVE1

**4.22829235822601E-09**	0.521213840263832	0.206	0.152	6.89676766550245E-05	Mid-zonal hepatocytes	RRP1B
**4.28088741617644E-09**	0.890704023240542	0.113	0.069	6.98255546452539E-05	Mid-zonal hepatocytes	YOD1
**4.32371048560308E-09**	0.665997506343325	0.179	0.128	7.05240417306719E-05	Mid-zonal hepatocytes	GTF2B
**4.37342392852852E-09**	0.320463191974233	0.277	0.221	7.13349176982287E-05	Mid-zonal hepatocytes	MED16
**4.41774876726798E-09**	1.02583832645975	0.154	0.105	7.2057900142908E-05	Mid-zonal hepatocytes	CAMK2G
**4.48014138412665E-09**	1.1917104503566	0.1	0.058	7.30755861164898E-05	Mid-zonal hepatocytes	STK38L
**4.60050436445565E-09**	0.733890640920247	0.187	0.136	7.50388266886362E-05	Mid-zonal hepatocytes	FAHD2A
**4.6097628459644E-09**	1.48550404238787	0.107	0.065	7.51898417805253E-05	Mid-zonal hepatocytes	CASP2
**4.63183495662175E-09**	1.17539180180578	0.104	0.062	7.55498599774574E-05	Mid-zonal hepatocytes	ANKRD49
**4.64668544183983E-09**	0.859450703112683	0.2	0.148	7.57920862418495E-05	Mid-zonal hepatocytes	BCL6
**4.80764421316389E-09**	0.802137926515375	0.207	0.155	7.84174847609161E-05	Mid-zonal hepatocytes	ERMAP
**4.85628639773476E-09**	1.15857316207396	0.14	0.093	7.92108874334516E-05	Mid-zonal hepatocytes	CDC40
**4.87812149860606E-09**	0.79682841905117	0.119	0.074	7.95670397637635E-05	Mid-zonal hepatocytes	ZNF446
**4.89276836480657E-09**	0.965100297622194	0.123	0.078	7.98059447983599E-05	Mid-zonal hepatocytes	CNEP1R1
**4.90111626836256E-09**	0.912931275695268	0.116	0.072	7.99421074532617E-05	Mid-zonal hepatocytes	RIF1
**5.10286040663791E-09**	0.819217271384003	0.117	0.073	8.32327560926709E-05	Mid-zonal hepatocytes	CNOT3
**5.21963647531554E-09**	0.482128100947495	0.257	0.206	8.51374905488718E-05	Mid-zonal hepatocytes	TMEM248
**5.25958059674913E-09**	0.550268655304024	0.16	0.11	8.57890191135751E-05	Mid-zonal hepatocytes	HRAS
**5.38407942351618E-09**	1.10116610409595	0.137	0.09	8.78197194769724E-05	Mid-zonal hepatocytes	MKNK1
**5.44534488330537E-09**	0.873673275411303	0.159	0.11	8.88190203915939E-05	Mid-zonal hepatocytes	CCDC58
**5.44924048581756E-09**	0.780965598051803	0.241	0.188	8.88825615641701E-05	Mid-zonal hepatocytes	ZC3H3
**5.56532943290551E-09**	1.14146650995747	0.126	0.081	9.07760883801218E-05	Mid-zonal hepatocytes	TBCCD1
**5.65866826641416E-09**	0.907239489721598	0.161	0.112	9.22985380934813E-05	Mid-zonal hepatocytes	TRIM39
**5.80666990299959E-09**	0.765846046820584	0.141	0.093	9.47125927878264E-05	Mid-zonal hepatocytes	ETF1
**5.84463213253288E-09**	0.536173052055102	0.135	0.088	9.53317947137437E-05	Mid-zonal hepatocytes	TMEM18
**5.89076479406684E-09**	0.327947151844564	0.223	0.166	9.60842645560242E-05	Mid-zonal hepatocytes	TLNRD1
**6.14312294286551E-09**	0.527229418142957	0.206	0.154	0.000100200478321079	Mid-zonal hepatocytes	BMI1
**6.21608405745709E-09**	1.04101264735338	0.169	0.121	0.000101390547061183	Mid-zonal hepatocytes	DAPK1
**6.22099116174813E-09**	0.918859151991414	0.175	0.125	0.000101470586839274	Mid-zonal hepatocytes	STK35
**6.31471685393157E-09**	1.04549536434265	0.127	0.081	0.000102999346604478	Mid-zonal hepatocytes	GBP4
**6.39297000518881E-09**	0.711801100902129	0.144	0.096	0.000104275733754635	Mid-zonal hepatocytes	UPF3A
**6.39580810005944E-09**	1.21081381986996	0.132	0.086	0.000104322025920069	Mid-zonal hepatocytes	GPR157
**6.44461074620468E-09**	0.894736974764164	0.107	0.064	0.000105118045881345	Mid-zonal hepatocytes	DEPRECAT
**6.44585156162549E-09**	0.782186369041756	0.181	0.131	0.000105138284821673	Mid-zonal hepatocytes	ACAP2
**6.82434145474174E-09**	0.466460595552467	0.318	0.258	0.000111311833468292	Mid-zonal hepatocytes	BCAT2
**6.88730815391904E-09**	1.10394056153842	0.112	0.069	0.000112338883298573	Mid-zonal hepatocytes	MTMR1
**6.88943694731552E-09**	0.378390942502374	0.263	0.208	0.000112373606047663	Mid-zonal hepatocytes	SEC23B
**6.90571471194649E-09**	0.867956751396144	0.109	0.067	0.000112639112666559	Mid-zonal hepatocytes	ZBTB45
**6.92280462089888E-09**	0.789449078471619	0.148	0.1	0.000112917866171482	Mid-zonal hepatocytes	SPTY2D1
**7.02456588039679E-09**	0.821592699030991	0.196	0.146	0.000114577694075152	Mid-zonal hepatocytes	UBE2E2
**7.12513398027322E-09**	0.374128892587684	0.251	0.195	0.000116218060352236	Mid-zonal hepatocytes	SH3PXD2A
**7.1407589984937E-09**	1.04557357983161	0.137	0.09	0.000116472920024431	Mid-zonal hepatocytes	DHX58
**7.30981040822194E-09**	0.558371818859511	0.181	0.13	0.000119230317568508	Mid-zonal hepatocytes	SPCS3
**7.34651339785501E-09**	0.770055435158871	0.185	0.136	0.000119828980032413	Mid-zonal hepatocytes	PITHD1
**7.56087611686375E-09**	0.667218712017677	0.133	0.087	0.000123325450342165	Mid-zonal hepatocytes	PIP5K1C
**7.69163104424426E-09**	0.888530421636585	0.102	0.061	0.000125458193962668	Mid-zonal hepatocytes	ZBTB47
**7.74092296222419E-09**	0.578951430108425	0.209	0.155	0.000126262194436839	Mid-zonal hepatocytes	TM2D3
**7.76817698550056E-09**	1.17870610861974	0.125	0.081	0.0001267067348105	Mid-zonal hepatocytes	ANKRD44
**7.80509482314542E-09**	0.334804660127965	0.28	0.224	0.000127308901660325	Mid-zonal hepatocytes	ZBTB22
**7.93560024317644E-09**	1.06616390268655	0.101	0.06	0.000129437575566451	Mid-zonal hepatocytes	MOB1B
**7.99814468525173E-09**	0.467570104797164	0.234	0.181	0.000130457737961141	Mid-zonal hepatocytes	MARCH8
**8.02189178891633E-09**	1.18829469760496	0.127	0.083	0.000130845076969014	Mid-zonal hepatocytes	JARID2
**8.12033635373313E-09**	1.07474218848156	0.156	0.108	0.000132450806265741	Mid-zonal hepatocytes	MGAT5
**8.19955132741252E-09**	0.952421624514834	0.15	0.102	0.000133742881701426	Mid-zonal hepatocytes	TRAF3
**8.20502150662201E-09**	0.893639737043611	0.13	0.084	0.000133832105794512	Mid-zonal hepatocytes	RBM7
**8.28356168611488E-09**	0.656165881120491	0.188	0.137	0.00013511317466222	Mid-zonal hepatocytes	IFFO1
**8.32453964397991E-09**	0.793772679923409	0.173	0.124	0.000135781566132956	Mid-zonal hepatocytes	FBXO3
**8.33003809685825E-09**	0.540427024269828	0.14	0.092	0.000135871251397855	Mid-zonal hepatocytes	MAPK7
**8.37299098549501E-09**	0.848932049625778	0.101	0.06	0.000136571855964409	Mid-zonal hepatocytes	TDP1
**8.38865414534096E-09**	1.02214425931441	0.101	0.06	0.000136827337764656	Mid-zonal hepatocytes	NVL

8.46284138087857E-09	0.825760263308797	0.154	0.106	0.00013803740576351	Mid-zonal hepatocytes	AUH
8.88585268297681E-09	0.807630303465574	0.172	0.122	0.000144937143112035	Mid-zonal hepatocytes	LTV1
8.90654119699708E-09	1.24337317273985	0.11	0.068	0.000145274593464219	Mid-zonal hepatocytes	COPS7B
8.9339036889313E-09	0.744113295757177	0.157	0.109	0.000145720903070158	Mid-zonal hepatocytes	ABCB7
9.02367507417104E-09	1.29027191760147	0.125	0.081	0.000147185164134804	Mid-zonal hepatocytes	TRIP11
9.06650315317233E-09	1.05696154357611	0.159	0.112	0.000147883732931394	Mid-zonal hepatocytes	RPP30
9.17980686889729E-09	0.329803284954562	0.281	0.229	0.000149731829838584	Mid-zonal hepatocytes	OPA1
9.62812822746766E-09	0.846125246451468	0.146	0.099	0.000157044399518225	Mid-zonal hepatocytes	CLN6
9.65800329568237E-09	1.14009812779488	0.151	0.105	0.000157531691755875	Mid-zonal hepatocytes	GCC2
9.71466232704792E-09	0.815545581107716	0.101	0.06	0.000158455857216479	Mid-zonal hepatocytes	KCNK1
9.73772078581058E-09	0.813131179872844	0.177	0.127	0.000158831963737356	Mid-zonal hepatocytes	PDSS2
9.74268483658886E-09	0.956290294185976	0.144	0.097	0.000158912932369601	Mid-zonal hepatocytes	SRCIN1
9.79376253389142E-09	0.673953185853808	0.213	0.161	0.000159746060690303	Mid-zonal hepatocytes	VAPB
9.80715281957279E-09	0.664205964267314	0.207	0.155	0.000159964469640052	Mid-zonal hepatocytes	SEC14L4
9.84386309315897E-09	0.406084062730433	0.347	0.292	0.000160563250912516	Mid-zonal hepatocytes	ELL2
1.01419211565371E-08	0.598963000118754	0.214	0.162	0.000165424875984277	Mid-zonal hepatocytes	TMEM68
1.01450387063946E-08	0.807607474156284	0.172	0.122	0.000165475726340003	Mid-zonal hepatocytes	MPP5
1.02223959556892E-08	0.709200065894324	0.181	0.131	0.000166737500433247	Mid-zonal hepatocytes	OGG1
1.02894571965557E-08	1.27344954540041	0.109	0.068	0.000167831336333019	Mid-zonal hepatocytes	WDR48
1.03792436902808E-08	0.569900920087756	0.141	0.094	0.000169295843832171	Mid-zonal hepatocytes	FADS3
1.04612139204488E-08	0.915064413011184	0.187	0.138	0.000170632860256441	Mid-zonal hepatocytes	CASP3
1.05337336436909E-08	0.984138151325567	0.132	0.087	0.000171815729462243	Mid-zonal hepatocytes	WAC
1.07964312114926E-08	0.708041848439536	0.185	0.135	0.000176100589490656	Mid-zonal hepatocytes	ZADH2
1.08034401761355E-08	0.72600274819519	0.156	0.108	0.000176214912712947	Mid-zonal hepatocytes	MFF
1.11121223565064E-08	1.09022643668431	0.16	0.113	0.000181249827756976	Mid-zonal hepatocytes	ZMYM4
1.13936790256675E-08	0.957424558432903	0.141	0.094	0.000185842298587663	Mid-zonal hepatocytes	ZNF451
1.15793881179172E-08	0.251808967637743	0.272	0.217	0.000188871399591347	Mid-zonal hepatocytes	ZFYVE21
1.16400603021834E-08	0.894698186078856	0.153	0.106	0.000189861023588914	Mid-zonal hepatocytes	SPOPL
1.17538500276087E-08	0.806304157217477	0.106	0.064	0.000191717047800325	Mid-zonal hepatocytes	DEPRECAT
1.17624845438158E-08	1.06880546107481	0.114	0.071	0.00019185788539418	Mid-zonal hepatocytes	SNTB2
1.17646876716147E-08	1.07498838541111	0.153	0.107	0.000191893820611708	Mid-zonal hepatocytes	FAM120B
1.17855610370738E-08	0.538371075982575	0.215	0.163	0.00019223428607571	Mid-zonal hepatocytes	ZNF282
1.18909360186908E-08	0.794065658748991	0.196	0.146	0.000193953057400866	Mid-zonal hepatocytes	TMEM150A
1.1905802887478E-08	1.02307714139066	0.141	0.094	0.000194195550897654	Mid-zonal hepatocytes	MTAP
1.20604512653858E-08	1.54668957188695	0.128	0.084	0.000196718020589707	Mid-zonal hepatocytes	ANKRD26
1.21247182887765E-08	1.072444468064	0.13	0.086	0.000197766280008234	Mid-zonal hepatocytes	CCAR2
1.21638457261848E-08	0.660372339906982	0.203	0.152	0.0001984044876398	Mid-zonal hepatocytes	SYNRG
1.21877440107652E-08	0.299519997183123	0.303	0.249	0.000198794292559591	Mid-zonal hepatocytes	CHURC1
1.2273201858663E-08	0.657336800882762	0.179	0.13	0.000200188195516651	Mid-zonal hepatocytes	NAA30
1.24308845866996E-08	1.43732124020829	0.12	0.077	0.000202760158493657	Mid-zonal hepatocytes	WDYHV1
1.27030960324317E-08	0.902237278690027	0.142	0.096	0.000207200199384993	Mid-zonal hepatocytes	SOS 2
1.30976879792008E-08	0.701012643316675	0.142	0.096	0.000213636388628744	Mid-zonal hepatocytes	NUP214
1.31160546009121E-08	0.8467944157349	0.154	0.106	0.000213935966595478	Mid-zonal hepatocytes	CNOT10
1.33495995875205E-08	0.456937029293449	0.125	0.08	0.000217745318872047	Mid-zonal hepatocytes	PELI3
1.38485876179283E-08	0.883685721840821	0.176	0.129	0.000225884312636028	Mid-zonal hepatocytes	WASF2
1.40186091780366E-08	0.797485756388095	0.16	0.112	0.000228657534302956	Mid-zonal hepatocytes	ABHD17A
1.42913132311467E-08	0.804341654244003	0.179	0.131	0.000233105610113235	Mid-zonal hepatocytes	THUMPD3
1.42955677617486E-08	1.25798855438142	0.175	0.127	0.000233175005761882	Mid-zonal hepatocytes	ZC3HAV1
1.43661812474673E-08	0.61165410015686	0.186	0.136	0.000234326782327438	Mid-zonal hepatocytes	SACM1L
1.43954712788083E-08	1.0689546471792	0.129	0.085	0.000234804532028642	Mid-zonal hepatocytes	WDR25
1.4463295902243E-08	0.849642126220631	0.129	0.084	0.000235910819461486	Mid-zonal hepatocytes	BRAP
1.45748500731755E-08	0.598849903662019	0.185	0.136	0.000237730379543566	Mid-zonal hepatocytes	RAP2C
1.46692701702318E-08	0.539279639793199	0.195	0.144	0.000239270465746652	Mid-zonal hepatocytes	TBC1D16
1.46820834610083E-08	0.261824658038643	0.331	0.275	0.000239479463332507	Mid-zonal hepatocytes	RRP36
1.49242174749119E-08	0.631343527672493	0.155	0.107	0.000243428911233288	Mid-zonal hepatocytes	RB1
1.49635367687257E-08	0.967845751694832	0.15	0.104	0.000244070248234685	Mid-zonal hepatocytes	PTPRJ
1.50599531036208E-08	0.931003220501442	0.147	0.1	0.000245642895073159	Mid-zonal hepatocytes	NUP160
1.56577380454913E-08	0.825606315067867	0.153	0.107	0.000255393365260009	Mid-zonal hepatocytes	PCGF2
1.56693235617745E-08	1.04833401165183	0.14	0.095	0.000255582336616103	Mid-zonal hepatocytes	RBM22
1.58098958142148E-08	1.34488742589812	0.142	0.097	0.000257875210625658	Mid-zonal hepatocytes	PRUNE1

1.58474665945445E-08	1.10962051359821	0.117	0.075	0.000258488027623616	Mid-zonal hepatocytes	CSNK1G3
1.60366216580313E-08	1.19380763660087	0.104	0.064	0.000261573335864149	Mid-zonal hepatocytes	GPR155
1.60819412274682E-08	0.606711585149561	0.211	0.161	0.000262312543361234	Mid-zonal hepatocytes	PIP4K2C
1.61079259341412E-08	0.858893558318868	0.148	0.102	0.000262736379911778	Mid-zonal hepatocytes	PLEKHA3
1.63955807486174E-08	0.660109867478876	0.2	0.149	0.000267428317590699	Mid-zonal hepatocytes	ITGA7
1.67184968052033E-08	1.49689109359523	0.108	0.068	0.000272695401389672	Mid-zonal hepatocytes	EXD3
1.69082129298789E-08	0.701067810593118	0.132	0.085	0.000275789861099254	Mid-zonal hepatocytes	SERTM2
1.71064595872947E-08	0.939877876896002	0.178	0.13	0.000279023462328364	Mid-zonal hepatocytes	SIRT3
1.79316816217476E-08	0.403079907233885	0.233	0.18	0.000292483658932326	Mid-zonal hepatocytes	POLRMT
1.79394250252939E-08	0.899639543146555	0.14	0.095	0.000292609961587569	Mid-zonal hepatocytes	GPATCH8
1.80865906552872E-08	0.320194641151735	0.336	0.283	0.00029501038017839	Mid-zonal hepatocytes	ACSF2
1.85901896788225E-08	0.441023463240735	0.316	0.264	0.000303224583851274	Mid-zonal hepatocytes	ARHGEF12
1.90833135143221E-08	0.820972885284972	0.148	0.102	0.000311267926732108	Mid-zonal hepatocytes	TBL1X
1.92995640492211E-08	0.363846006812992	0.263	0.209	0.000314795189206845	Mid-zonal hepatocytes	PYCR2
1.93765645695444E-08	0.270253476413875	0.3	0.246	0.000316051144693839	Mid-zonal hepatocytes	GATA4
1.9495276077479E-08	1.30789928410548	0.117	0.076	0.00031798744809976	Mid-zonal hepatocytes	SCAF8
1.95603336266989E-08	1.14661952272946	0.148	0.102	0.000319048601785086	Mid-zonal hepatocytes	UBE3B
2.02090492782551E-08	0.501857060713658	0.234	0.183	0.000329629802777619	Mid-zonal hepatocytes	EIF4B
2.03569723361846E-08	1.37487574740995	0.108	0.068	0.000332042575775507	Mid-zonal hepatocytes	CCDC68
2.06330069372837E-08	1.07252459221186	0.108	0.067	0.000336544976154035	Mid-zonal hepatocytes	PITPNC1
2.06437001322021E-08	0.571469579794877	0.174	0.126	0.000336719392856349	Mid-zonal hepatocytes	GPR137B
2.0794629786131E-08	1.43994188473585	0.129	0.085	0.000339181206441582	Mid-zonal hepatocytes	TTC39B
2.10686194043006E-08	0.737439947926782	0.231	0.183	0.000343650251103548	Mid-zonal hepatocytes	EAPP
2.11043049009685E-08	1.05745488879446	0.133	0.088	0.000344232317239698	Mid-zonal hepatocytes	TIRAP
2.12034826721028E-08	0.743922944135482	0.142	0.097	0.000345850005864669	Mid-zonal hepatocytes	DHX29
2.18533769987123E-08	1.6076440224404	0.103	0.063	0.000356450432225997	Mid-zonal hepatocytes	ZNF552
2.20524582383451E-08	1.09958768637213	0.148	0.104	0.000359697646325647	Mid-zonal hepatocytes	CNOT9
2.21503880570137E-08	0.729387397309131	0.166	0.119	0.00036129497959795	Mid-zonal hepatocytes	FTO
2.24094285915128E-08	0.91642447777269	0.113	0.071	0.000365520189756165	Mid-zonal hepatocytes	PDGFRB
2.25106377573798E-08	0.571210853349933	0.194	0.144	0.000367171012460622	Mid-zonal hepatocytes	EIF2B1
2.25349341487896E-08	0.774907395535938	0.154	0.107	0.000367567310900907	Mid-zonal hepatocytes	MBIP
2.28123505709519E-08	1.5379252519839	0.104	0.065	0.000372092250162797	Mid-zonal hepatocytes	TMEM267
2.28263731025479E-08	0.696968121453251	0.201	0.152	0.000372320971675658	Mid-zonal hepatocytes	CTDP1
2.29316293070471E-08	0.713157091790252	0.107	0.066	0.000374037805627244	Mid-zonal hepatocytes	NCBP1
2.29796314855355E-08	0.919297323910163	0.113	0.071	0.000374820769160569	Mid-zonal hepatocytes	LGALS9
2.33389700198073E-08	0.721282820071321	0.171	0.123	0.000380681939993077	Mid-zonal hepatocytes	LIG1
2.45882882509508E-08	0.787098893807071	0.136	0.091	0.000401059569661258	Mid-zonal hepatocytes	KIF13B
2.54436944788364E-08	0.686980218314969	0.181	0.133	0.000415012100644301	Mid-zonal hepatocytes	RTF1
2.59638823608742E-08	1.05623403880926	0.153	0.108	0.00042349688518822	Mid-zonal hepatocytes	TRMT61B
2.61959944271905E-08	0.296817957676876	0.296	0.244	0.000427282865101904	Mid-zonal hepatocytes	PTPN23
2.62086361730149E-08	0.255343906331555	0.274	0.222	0.000427489064618046	Mid-zonal hepatocytes	UBA1
2.78565480158723E-08	1.05886468309822	0.117	0.075	0.000454368154686893	Mid-zonal hepatocytes	CENPC
2.79472624371669E-08	0.652717105311062	0.12	0.077	0.00045584779761263	Mid-zonal hepatocytes	BICDL1
2.82067726028563E-08	1.62280748246852	0.123	0.081	0.000460080667925189	Mid-zonal hepatocytes	GOLGA8B
2.82174449741722E-08	0.751754838255841	0.117	0.076	0.000460254744973723	Mid-zonal hepatocytes	HAUS2
2.84702059579976E-08	1.03667240808591	0.121	0.078	0.0004643775293809	Mid-zonal hepatocytes	ABCC4
2.85848022574047E-08	1.43863873785833	0.126	0.083	0.000466246709620528	Mid-zonal hepatocytes	MAPK8
2.92008050312239E-08	0.351834817778834	0.301	0.248	0.000476294330864293	Mid-zonal hepatocytes	PRPF6
2.98308987361995E-08	0.419971596711862	0.273	0.221	0.00048657178928615	Mid-zonal hepatocytes	DCAF7
3.00306481880591E-08	0.912254682257291	0.135	0.09	0.000489829902595432	Mid-zonal hepatocytes	UACA
3.05241744872344E-08	0.558119599511056	0.177	0.129	0.000497879810061281	Mid-zonal hepatocytes	WIPF3
3.11451820332822E-08	0.392415353380382	0.266	0.216	0.000508009064144866	Mid-zonal hepatocytes	PREB
3.12173324233656E-08	0.758091694339394	0.159	0.112	0.000509185909157516	Mid-zonal hepatocytes	RSBN1L
3.15838060391881E-08	0.865304396235056	0.191	0.143	0.000515163460305197	Mid-zonal hepatocytes	EIF1B
3.20144370433677E-08	0.937348144066243	0.129	0.086	0.00052218748261437	Mid-zonal hepatocytes	IPO8
3.20161861116963E-08	0.992307970981629	0.144	0.099	0.000522216011667879	Mid-zonal hepatocytes	TBCEL
3.22950062849493E-08	0.649056887391511	0.159	0.112	0.000526763847513808	Mid-zonal hepatocytes	HAUS5
3.25441585739071E-08	0.919905793304774	0.153	0.108	0.000530827770498999	Mid-zonal hepatocytes	ABHD13
3.26347479528781E-08	0.590299289643604	0.208	0.16	0.000532305373859395	Mid-zonal hepatocytes	SUPT16H
3.31640136011758E-08	0.89269158567769	0.114	0.073	0.000540938225848779	Mid-zonal hepatocytes	DUSP6

3.35536254844993E-08	1.01411519232124	0.133	0.089	0.000547293185277668	Mid-zonal hepatocytes	NAA15
3.37300690259794E-08	0.367716319015757	0.241	0.192	0.00055017115588275	Mid-zonal hepatocytes	TNIP2
3.43230789124981E-08	0.77824674415584	0.132	0.088	0.000559843740141756	Mid-zonal hepatocytes	IFIT5
3.50902786307018E-08	0.719143108380396	0.142	0.097	0.000572357534745376	Mid-zonal hepatocytes	RBM41
3.57351574443352E-08	0.528674321297966	0.159	0.112	0.000582876153074551	Mid-zonal hepatocytes	CDK5RAP1
3.59545779443162E-08	1.20203807272908	0.131	0.088	0.000586455120849742	Mid-zonal hepatocytes	APBB1IP
3.70537747298919E-08	1.19290293536391	0.125	0.082	0.000604384119619267	Mid-zonal hepatocytes	SRGAP2B
3.78191104195103E-08	1.20670587465046	0.117	0.076	0.000616867510052632	Mid-zonal hepatocytes	DEPRECAT
3.80696343566454E-08	0.977377494901187	0.104	0.064	0.000620953805991243	Mid-zonal hepatocytes	UBOX5
3.90920758489624E-08	0.863385507538545	0.156	0.11	0.000637630849172426	Mid-zonal hepatocytes	GPR107
3.93541921172788E-08	1.04074973469389	0.147	0.102	0.000641906227624934	Mid-zonal hepatocytes	RARS2
3.9504340027666E-08	0.847108060175874	0.182	0.136	0.00064435529019126	Mid-zonal hepatocytes	FAM98B
3.96005031251953E-08	0.556742932576571	0.145	0.1	0.000645923806475061	Mid-zonal hepatocytes	CNTROB
3.9849198836899E-08	0.62483165815112	0.155	0.11	0.00064998028222866	Mid-zonal hepatocytes	DEPRECAT
4.02177831137494E-08	0.302192639277074	0.209	0.158	0.000655992260368367	Mid-zonal hepatocytes	PHAX
4.04080189223333E-08	0.656931683190609	0.209	0.159	0.000659095196642179	Mid-zonal hepatocytes	TIMM23
4.04160303146478E-08	0.854663982068198	0.16	0.115	0.00065922587046222	Mid-zonal hepatocytes	TM2D1
4.09710493456198E-08	1.09119783730142	0.163	0.117	0.000668278785876405	Mid-zonal hepatocytes	CPSF3
4.14016578020924E-08	0.349179611676246	0.275	0.216	0.000675302440409929	Mid-zonal hepatocytes	PDK4
4.15849623363274E-08	0.906659008352328	0.103	0.064	0.000678292320667836	Mid-zonal hepatocytes	CWC27
4.26772875567684E-08	0.728257048854819	0.184	0.136	0.00069610923733845	Mid-zonal hepatocytes	IFNGR2
4.26831872935244E-08	0.420150253138291	0.208	0.158	0.000696205467944677	Mid-zonal hepatocytes	EPS15
4.29625301545337E-08	0.393269466819454	0.314	0.262	0.0007007618293506	Mid-zonal hepatocytes	STAP2
4.31694871087025E-08	0.818018894346673	0.178	0.131	0.000704137504230047	Mid-zonal hepatocytes	WASHC4
4.33862192563192E-08	1.02653131282042	0.138	0.094	0.000707672622289822	Mid-zonal hepatocytes	ZW10
4.33962472503486E-08	0.560543991353562	0.236	0.186	0.000707836188900436	Mid-zonal hepatocytes	PSMD1
4.36671185611763E-08	0.932828254596097	0.131	0.087	0.000712254370851347	Mid-zonal hepatocytes	AAR2
4.39819258954274E-08	0.759294405313968	0.187	0.139	0.000717389193280317	Mid-zonal hepatocytes	UBA3
4.52703903126874E-08	0.680062385256691	0.229	0.181	0.000738405336390244	Mid-zonal hepatocytes	EDEM2
4.57891119028885E-08	1.34904179487261	0.107	0.067	0.000746866204248015	Mid-zonal hepatocytes	DCAKD
4.69594249671099E-08	0.743179547955448	0.148	0.103	0.000765955180638529	Mid-zonal hepatocytes	MOB4
4.69622672110033E-08	0.37470146852783	0.142	0.097	0.000766001540478676	Mid-zonal hepatocytes	CDKN2B
4.75010520947153E-08	0.836629050915331	0.145	0.1	0.000774789660716901	Mid-zonal hepatocytes	DAAM1
4.77817524320407E-08	0.633076272753902	0.196	0.148	0.000779368163919015	Mid-zonal hepatocytes	ACTR10
4.79577601613523E-08	1.05300728989596	0.12	0.079	0.000782239025991817	Mid-zonal hepatocytes	ARHGEF28
4.9396444412415E-08	0.461177755610841	0.239	0.191	0.0008057054048109	Mid-zonal hepatocytes	STX10
4.94151734826253E-08	0.686835372799064	0.161	0.114	0.000806010894675102	Mid-zonal hepatocytes	TOM1L2
4.96216111029351E-08	0.406476436256052	0.178	0.13	0.000809378098699974	Mid-zonal hepatocytes	SLC4A1AP
4.96565358020795E-08	0.686991358815393	0.169	0.122	0.000809947755467718	Mid-zonal hepatocytes	FAF2
4.97705511873045E-08	0.459871602395754	0.249	0.195	0.000811807460416123	Mid-zonal hepatocytes	BTBD2
5.0643465304079E-08	0.521324750407791	0.148	0.102	0.000826045562574832	Mid-zonal hepatocytes	LZTS3
5.0971055476881E-08	0.982156605585985	0.126	0.083	0.000831388885883406	Mid-zonal hepatocytes	ZNF608
5.32714876150005E-08	1.36105025836168	0.1	0.062	0.000868911234488273	Mid-zonal hepatocytes	USP42
5.33807005800639E-08	0.339096355164589	0.253	0.203	0.000870692607161422	Mid-zonal hepatocytes	TAX1BP3
5.41372740165803E-08	1.08442956408355	0.116	0.075	0.000883033076484441	Mid-zonal hepatocytes	PNPLA8
5.45077315223575E-08	0.714012153890309	0.221	0.171	0.000889075608861173	Mid-zonal hepatocytes	CCDC125
5.48298946133134E-08	0.474356691113709	0.235	0.185	0.000894330411037756	Mid-zonal hepatocytes	FOXN3
5.57168234783065E-08	0.84214923725948	0.141	0.097	0.000908797107754657	Mid-zonal hepatocytes	USP16
5.68600082292248E-08	0.54592624444454	0.151	0.105	0.000927443594226886	Mid-zonal hepatocytes	SEPTIN8
5.70347898492742E-08	1.00049373223209	0.124	0.081	0.000930294457231511	Mid-zonal hepatocytes	SGK1
5.81134055583953E-08	0.889166509639404	0.13	0.087	0.000947887758062985	Mid-zonal hepatocytes	STX8
5.82478769632831E-08	0.44472784208833	0.266	0.215	0.000950081121148111	Mid-zonal hepatocytes	LIMS2
5.84876928022421E-08	0.898296644061509	0.136	0.092	0.00095399275729737	Mid-zonal hepatocytes	CNNM2
5.95985508450232E-08	0.808144326230521	0.145	0.1	0.000972111962833174	Mid-zonal hepatocytes	MIGA2
6.00945752617619E-08	1.31265277183068	0.137	0.094	0.000980202617094598	Mid-zonal hepatocytes	WDR75
6.05519141775578E-08	0.447132260246228	0.133	0.089	0.000987662272150145	Mid-zonal hepatocytes	WIPI1
6.20006936921997E-08	1.41725830404475	0.105	0.066	0.00101129331481347	Mid-zonal hepatocytes	KANSL1L
6.27322920458278E-08	0.373922085594751	0.292	0.241	0.0010232264155595	Mid-zonal hepatocytes	CYP2J2
6.3109683684528E-08	0.664139656930362	0.226	0.178	0.00102938205057834	Mid-zonal hepatocytes	PIP4K2B
6.31849991703631E-08	0.959974965304212	0.137	0.094	0.00103061052146779	Mid-zonal hepatocytes	TMEM181

6.31965165221642E-08	0.982735891189452	0.111	0.071	0.00103079838099302	Mid-zonal hepatocytes	BTN2A2
6.32291173133268E-08	0.53147097780793	0.156	0.109	0.00103133013249767	Mid-zonal hepatocytes	FBXO25
6.35439638290249E-08	0.306363164358001	0.254	0.202	0.00103646559401523	Mid-zonal hepatocytes	SMO
6.3643771844815E-08	0.745173146138947	0.111	0.071	0.00103809356256078	Mid-zonal hepatocytes	BAHCC1
6.38348008545392E-08	0.620314646142193	0.13	0.086	0.00104120943673839	Mid-zonal hepatocytes	COQ7
6.65895193786694E-08	0.514988531776631	0.192	0.146	0.00108614165058548	Mid-zonal hepatocytes	NAA50
6.68719928746576E-08	0.642439560706235	0.167	0.121	0.00109074907577854	Mid-zonal hepatocytes	PIGH
6.77881958283255E-08	0.86393592529329	0.124	0.081	0.00110569326215582	Mid-zonal hepatocytes	CIZ1
6.90641512869357E-08	1.31292768188898	0.1	0.062	0.00112650537164121	Mid-zonal hepatocytes	RUNDC1
6.91155162578152E-08	1.08449916189088	0.111	0.071	0.00112734318568122	Mid-zonal hepatocytes	SLC35B4
7.03055828334093E-08	1.16275097882202	0.107	0.067	0.00114675436159574	Mid-zonal hepatocytes	RABL6
7.06803949860406E-08	0.5080117937989	0.285	0.236	0.00115286792261731	Mid-zonal hepatocytes	SLC6A12
7.11668645876945E-08	0.284374450685705	0.289	0.237	0.00116080272828989	Mid-zonal hepatocytes	DBP
7.13658489228359E-08	1.03010868586582	0.122	0.08	0.00116404836178038	Mid-zonal hepatocytes	FBXO30
7.18784073604613E-08	0.593215304078164	0.182	0.136	0.00117240870245648	Mid-zonal hepatocytes	SEH1L
7.24217083335725E-08	0.496127598279384	0.183	0.136	0.0011812704846289	Mid-zonal hepatocytes	TAF6
7.40260418082538E-08	0.937373032727973	0.139	0.096	0.00120743876793443	Mid-zonal hepatocytes	ZBTB38
7.45114307232152E-08	0.840994246609765	0.108	0.069	0.00121535594652636	Mid-zonal hepatocytes	WASF1
7.55181439760825E-08	0.594577260642186	0.219	0.17	0.00123177644639388	Mid-zonal hepatocytes	MAP1LC3A
7.56783730498655E-08	1.34151112668179	0.123	0.082	0.00123438994281636	Mid-zonal hepatocytes	SMARCAL1
7.6975175612237E-08	0.399633123556248	0.271	0.221	0.0012555420894112	Mid-zonal hepatocytes	WRNIP1
7.7258948940285E-08	0.539891172383217	0.2	0.151	0.00126017071616499	Mid-zonal hepatocytes	MBD1
7.86869674546059E-08	0.52412382811195	0.173	0.127	0.00128346312615208	Mid-zonal hepatocytes	SH3GLB1
7.87309406737588E-08	0.626815273189802	0.187	0.14	0.00128418037332968	Mid-zonal hepatocytes	SMC1A
7.93196626750112E-08	0.312083262815406	0.328	0.275	0.00129378301789211	Mid-zonal hepatocytes	COQ9
7.93672453931059E-08	1.49637133324731	0.111	0.072	0.00129455913960695	Mid-zonal hepatocytes	STARD13
7.94279385095435E-08	0.564795068369774	0.194	0.147	0.00129554910502916	Mid-zonal hepatocytes	WAPL
7.97228937095707E-08	0.600646276115934	0.225	0.178	0.00130036011929681	Mid-zonal hepatocytes	UQCRC2
8.13145727068853E-08	0.771366033844841	0.172	0.126	0.00132632199542201	Mid-zonal hepatocytes	EIF2B4
8.25392511423856E-08	0.531242336280902	0.297	0.25	0.00134629772538345	Mid-zonal hepatocytes	PCMTD1
8.49643784115979E-08	0.255439639477105	0.231	0.181	0.00138585397627157	Mid-zonal hepatocytes	LRRC58
8.5133323872973E-08	1.09654944237592	0.145	0.102	0.00138860964569206	Mid-zonal hepatocytes	HNF4G
8.51818941977896E-08	0.705321609398836	0.23	0.183	0.00138940187626015	Mid-zonal hepatocytes	MOGAT3
8.628154233478E-08	0.601610545292095	0.184	0.137	0.0014073382370226	Mid-zonal hepatocytes	MKRN1
8.74739348184889E-08	0.85178872325315	0.194	0.148	0.00142678735082437	Mid-zonal hepatocytes	ENTPD7
8.81067036650481E-08	0.394786158410455	0.131	0.087	0.0014371084434806	Mid-zonal hepatocytes	LCP1
8.893448741827E-08	1.25575032820383	0.112	0.073	0.0014506104242794	Mid-zonal hepatocytes	CNOT6L
8.91495780125477E-08	0.820322966996748	0.1	0.062	0.00145411876696267	Mid-zonal hepatocytes	NDE1
8.95128863717988E-08	0.814628197105856	0.181	0.136	0.00146004468961041	Mid-zonal hepatocytes	CTU2
9.19989199422143E-08	0.530783895325005	0.187	0.14	0.00150059438317746	Mid-zonal hepatocytes	ATAD1
9.25328664958375E-08	0.931093791683595	0.132	0.089	0.0015093035854136	Mid-zonal hepatocytes	PLXNA2
9.2924307831039E-08	0.352633910793507	0.104	0.064	0.00151568838503208	Mid-zonal hepatocytes	AVPR1A
9.34615241423712E-08	0.806941374050545	0.139	0.097	0.00152445092028622	Mid-zonal hepatocytes	PCGF1
9.44028902410663E-08	0.861067566298408	0.153	0.109	0.00153980554272203	Mid-zonal hepatocytes	SDHAF2
9.52476515515643E-08	0.864967205070481	0.153	0.109	0.00155358444445757	Mid-zonal hepatocytes	LNPEP
9.69017201520034E-08	0.586655776640956	0.21	0.164	0.00158056395739933	Mid-zonal hepatocytes	CPEB2
9.78128613732223E-08	1.13765472348985	0.112	0.073	0.00159542558185863	Mid-zonal hepatocytes	BTBD8
9.84163954460382E-08	0.94083927478418	0.138	0.095	0.00160526982612033	Mid-zonal hepatocytes	ZNF318
9.89031925667405E-08	0.903813260387745	0.172	0.127	0.0016132099739561	Mid-zonal hepatocytes	SLU7
9.91865977880517E-08	0.944191765960265	0.132	0.09	0.00161783259652091	Mid-zonal hepatocytes	RAB11FIP2
1.00761511109882E-07	0.413629207235462	0.167	0.121	0.00164352100771328	Mid-zonal hepatocytes	RNF168
1.01731983794148E-07	0.629655608035458	0.172	0.126	0.00165935038766635	Mid-zonal hepatocytes	BTN3A1
1.01986190020592E-07	0.278409573370311	0.325	0.272	0.00166349674542588	Mid-zonal hepatocytes	SRXN1
1.05335637347965E-07	0.29789350279011	0.34	0.28	0.00171812958078265	Mid-zonal hepatocytes	GRAMD4
1.06196727067969E-07	0.306096852481957	0.12	0.078	0.00173217481520565	Mid-zonal hepatocytes	FHL3
1.08033768382835E-07	0.733070486425003	0.156	0.112	0.00176213879609242	Mid-zonal hepatocytes	TNFRSF1B
1.0825030330429E-07	0.466555303179981	0.123	0.081	0.00176567069719627	Mid-zonal hepatocytes	POLR2D
1.09056803480109E-07	0.535875259676909	0.199	0.151	0.00177882552156407	Mid-zonal hepatocytes	ARHGEF5
1.10010104635602E-07	1.13153595235818	0.123	0.082	0.00179437481671131	Mid-zonal hepatocytes	STAM2
1.10386046781512E-07	0.587721426934815	0.212	0.166	0.00180050680905324	Mid-zonal hepatocytes	BCCIP

1.10860100865097E-07	1.05193909407406	0.141	0.099	0.0018082391052106	Mid-zonal hepatocytes	MGAT4A
1.11401215475693E-07	0.614308662928049	0.135	0.092	0.00181706522562403	Mid-zonal hepatocytes	RNF214
1.14223198846286E-07	0.382540545032799	0.171	0.125	0.00186309459638177	Mid-zonal hepatocytes	AP5S1
1.15370102165784E-07	0.26199063961928	0.3	0.251	0.0018818017364261	Mid-zonal hepatocytes	PEA15
1.15455198891188E-07	0.453920642683436	0.2	0.151	0.00188318974911417	Mid-zonal hepatocytes	RABEP2
1.16637650678317E-07	0.850435320102329	0.136	0.093	0.00190247672021402	Mid-zonal hepatocytes	TRIM14
1.17619350353012E-07	0.735510355840851	0.147	0.103	0.00191848922360797	Mid-zonal hepatocytes	TSEN54
1.17996815873488E-07	0.914027780460359	0.114	0.075	0.00192464606371246	Mid-zonal hepatocytes	TOPBP1
1.19511144439238E-07	0.309965962521453	0.302	0.249	0.00194934627694842	Mid-zonal hepatocytes	GPLD1
1.1981244448896E-07	0.504635252543009	0.244	0.196	0.00195426078205942	Mid-zonal hepatocytes	SEC23A
1.20612517750551E-07	0.926836226029261	0.165	0.121	0.00196731077702924	Mid-zonal hepatocytes	RTL10
1.21504411980028E-07	0.572169064432877	0.154	0.109	0.00198185846380624	Mid-zonal hepatocytes	SNRK
1.22181889398234E-07	0.491222370113721	0.162	0.117	0.0019929087979746	Mid-zonal hepatocytes	XYLT2
1.22252657430458E-07	0.475359036956709	0.163	0.117	0.0019940630953482	Mid-zonal hepatocytes	PLPPR2
1.22805711102599E-07	0.264845139512554	0.192	0.143	0.0020030839537945	Mid-zonal hepatocytes	CUEDC1
1.22996093632121E-07	0.812107010291042	0.133	0.092	0.00200618928323352	Mid-zonal hepatocytes	LATS2
1.23231100748823E-07	0.2632614879227	0.372	0.317	0.00201002248431405	Mid-zonal hepatocytes	S100A13
1.2396144387291E-07	1.18035879852802	0.11	0.071	0.00202193511101103	Mid-zonal hepatocytes	SUPT7L
1.28329266842467E-07	0.487669756375826	0.259	0.211	0.00209317867146747	Mid-zonal hepatocytes	PQBP1
1.29250694733751E-07	0.968628699555213	0.135	0.094	0.00210820808180222	Mid-zonal hepatocytes	TMEM161E
1.2925658389874E-07	0.538445239311153	0.155	0.11	0.00210830413997235	Mid-zonal hepatocytes	RHOBTB1
1.35697483599071E-07	0.31545632577114	0.296	0.247	0.00221336165498445	Mid-zonal hepatocytes	PPP6R1
1.36914508468577E-07	1.24435146877798	0.109	0.071	0.00223321254763095	Mid-zonal hepatocytes	MFSD8
1.37645494368771E-07	0.513463809496532	0.25	0.203	0.00224513565864902	Mid-zonal hepatocytes	C19orf25
1.39805895683473E-07	0.689170188718024	0.177	0.131	0.00228037396449313	Mid-zonal hepatocytes	COQ4
1.40314806623449E-07	0.459578615875137	0.268	0.221	0.00228867481083508	Mid-zonal hepatocytes	TMEM63B
1.43803484635278E-07	0.666351629211425	0.138	0.095	0.00234557863788601	Mid-zonal hepatocytes	DLAT
1.46133108371515E-07	0.966995522683884	0.116	0.077	0.00238357713064778	Mid-zonal hepatocytes	LCMT1
1.46255630084965E-07	0.453896816042657	0.199	0.152	0.00238557558231587	Mid-zonal hepatocytes	RAB35
1.47967650364509E-07	0.893936412563935	0.11	0.071	0.00241350034509551	Mid-zonal hepatocytes	RAE1
1.4800256149672E-07	0.959797484142926	0.118	0.078	0.002414069780573	Mid-zonal hepatocytes	PLCB3
1.48179313218476E-07	0.39244830640485	0.289	0.239	0.00241695277790657	Mid-zonal hepatocytes	TOP1MT
1.50364280785647E-07	0.673315683399487	0.107	0.069	0.00245259178389469	Mid-zonal hepatocytes	PNPT1
1.5090578158168E-07	0.799327830735117	0.149	0.106	0.00246142420337879	Mid-zonal hepatocytes	DCAF15
1.51016669427168E-07	0.948521097102876	0.104	0.066	0.00246323289502654	Mid-zonal hepatocytes	BCL9
1.51257389716092E-07	0.745146777213882	0.178	0.133	0.00246715928365917	Mid-zonal hepatocytes	GNPAT
1.62821012281236E-07	0.406507668123814	0.159	0.113	0.00265577353131925	Mid-zonal hepatocytes	RELB
1.64347535988854E-07	0.33451646189795	0.226	0.178	0.0026806726595142	Mid-zonal hepatocytes	STARD3
1.65414162061291E-07	0.959625662822327	0.115	0.076	0.00269807039738172	Mid-zonal hepatocytes	RNF41
1.70055986567859E-07	0.928611839431269	0.127	0.085	0.00277378319690835	Mid-zonal hepatocytes	CRYBG1
1.7067023086031E-07	0.537028165191479	0.205	0.157	0.00278380213556251	Mid-zonal hepatocytes	IFIH1
1.72463817030837E-07	0.545497190958372	0.219	0.172	0.00281305731958998	Mid-zonal hepatocytes	TMEM167E
1.74855629369177E-07	0.538404857510119	0.209	0.163	0.00285207017064064	Mid-zonal hepatocytes	BAP1
1.81581357537297E-07	0.553592884404457	0.213	0.165	0.00296177352279086	Mid-zonal hepatocytes	LRRC14
1.83486986878922E-07	1.03346254871235	0.132	0.091	0.00299285624298209	Mid-zonal hepatocytes	UMAD1
1.83576870377378E-07	0.363099271720532	0.247	0.201	0.00299432233272542	Mid-zonal hepatocytes	EIF3L
1.84780596345633E-07	0.807427862037851	0.138	0.095	0.00301395630699362	Mid-zonal hepatocytes	MNAT1
1.85038922605737E-07	0.692023599405913	0.238	0.195	0.00301816986662217	Mid-zonal hepatocytes	SKAP2
1.85074704632515E-07	0.385142047693471	0.24	0.192	0.00301875350726095	Mid-zonal hepatocytes	NUB1
1.87059958131785E-07	0.568252275044615	0.215	0.17	0.00305113497708754	Mid-zonal hepatocytes	VPS26A
1.87679053094306E-07	0.895707116922282	0.147	0.103	0.00306123303502123	Mid-zonal hepatocytes	MEMO1
1.94038060669219E-07	0.441118512668022	0.252	0.204	0.00316495480757563	Mid-zonal hepatocytes	DEPRECAT
1.94527196363867E-07	0.660490759798951	0.158	0.113	0.00317293309989104	Mid-zonal hepatocytes	LMO4
1.95506608355969E-07	0.870811696624613	0.113	0.074	0.00318890828889421	Mid-zonal hepatocytes	SP2
1.95802284116369E-07	0.271969929736928	0.224	0.175	0.00319373105622209	Mid-zonal hepatocytes	UBL3
2.01972241243422E-07	0.642378176701507	0.144	0.102	0.00329436922692146	Mid-zonal hepatocytes	FRA10AC1
2.0369287143402E-07	0.450032028506631	0.2	0.154	0.00332243442596029	Mid-zonal hepatocytes	PRKD2
2.06828261038663E-07	0.424402240444387	0.289	0.243	0.00337357576580163	Mid-zonal hepatocytes	ADGRG1
2.13989056129596E-07	0.734855908464996	0.116	0.077	0.00349037549452983	Mid-zonal hepatocytes	XPA
2.18566542797486E-07	0.7933545724677	0.191	0.145	0.0035650388795698	Mid-zonal hepatocytes	UBE3A

2.21746195380751E-07	1.17669039010073	0.11	0.072	0.00361690219285543	Mid-zonal hepatocytes	KCTD6
2.28725883076351E-07	0.585795970907695	0.137	0.095	0.00373074787885836	Mid-zonal hepatocytes	SARS2
2.30706185158888E-07	0.57453067582755	0.119	0.079	0.00376304858612663	Mid-zonal hepatocytes	C15orf40
2.32781982906927E-07	0.715889142524156	0.167	0.124	0.00379690692319489	Mid-zonal hepatocytes	CAMK2D
2.34025553984972E-07	0.75910663009271	0.188	0.144	0.00381719081104888	Mid-zonal hepatocytes	OMA1
2.36261038848202E-07	0.593794508769891	0.199	0.154	0.00385365380465302	Mid-zonal hepatocytes	BORCS8
2.37502637001943E-07	0.55059631574374	0.181	0.136	0.00387390551213868	Mid-zonal hepatocytes	VPS11
2.3766408504597E-07	0.71895048799811	0.131	0.09	0.00387653889118481	Mid-zonal hepatocytes	TBC1D9
2.38722239142071E-07	0.952375960219017	0.117	0.078	0.00389379844264631	Mid-zonal hepatocytes	STX18
2.40391698140534E-07	1.21271791772858	0.104	0.067	0.00392102898837025	Mid-zonal hepatocytes	METTL9
2.43020100974984E-07	0.320593035959185	0.333	0.282	0.00396390086700296	Mid-zonal hepatocytes	DIO1
2.44497177652255E-07	0.301161732903443	0.25	0.204	0.00398799346468592	Mid-zonal hepatocytes	HIST1H1C
2.45225334099409E-07	0.891873311313356	0.132	0.091	0.00399987042449547	Mid-zonal hepatocytes	DEPRECAT
2.4812190347594E-07	0.741773115389315	0.287	0.242	0.00404711636759605	Mid-zonal hepatocytes	LIPG
2.50804686298141E-07	0.790472158951704	0.112	0.073	0.00409087523820898	Mid-zonal hepatocytes	AFAP1
2.51170493072806E-07	0.930800728832393	0.16	0.116	0.00409684191251054	Mid-zonal hepatocytes	NOTCH2
2.51273179020938E-07	1.12519194304785	0.136	0.095	0.00409851682301052	Mid-zonal hepatocytes	TCP11L1
2.61222509224661E-07	0.504054658386834	0.139	0.097	0.00426080034796345	Mid-zonal hepatocytes	SERGEF
2.63513127292575E-07	0.546083091977303	0.206	0.161	0.0042981626192692	Mid-zonal hepatocytes	EPB41L4B
2.67991956793702E-07	0.45006931239246	0.209	0.164	0.00437121680726207	Mid-zonal hepatocytes	TK2
2.72139137310556E-07	1.12560285541776	0.111	0.073	0.00443886146867247	Mid-zonal hepatocytes	MITD1
2.74671897959736E-07	0.555402903705463	0.157	0.113	0.00448017332762126	Mid-zonal hepatocytes	NOC4L
2.78589299392282E-07	0.562363825897437	0.215	0.17	0.00454407006238751	Mid-zonal hepatocytes	EIF3J
2.79587595846281E-07	1.0190459366104	0.147	0.106	0.00456035327584868	Mid-zonal hepatocytes	C1orf21
2.81743207623953E-07	0.366146531775667	0.162	0.118	0.0045955134595543	Mid-zonal hepatocytes	OSGEP
2.82874379372293E-07	0.937468877307293	0.187	0.143	0.00461396400194147	Mid-zonal hepatocytes	CES3
2.86598300612482E-07	0.767634491935818	0.234	0.188	0.00467470488129019	Mid-zonal hepatocytes	DEPRECAT
2.87305726778747E-07	0.322206198233544	0.2	0.154	0.00468624370948814	Mid-zonal hepatocytes	CACFD1
2.92199640165616E-07	0.772467141079415	0.154	0.112	0.00476606833074136	Mid-zonal hepatocytes	DR1
2.92444601222672E-07	0.375735139371973	0.229	0.181	0.00477006389054301	Mid-zonal hepatocytes	MPHOSPH
2.92669232662705E-07	1.02096128940182	0.128	0.088	0.00477372785396138	Mid-zonal hepatocytes	SLC25A17
2.95491134238304E-07	0.343513710443265	0.276	0.23	0.00481975589056098	Mid-zonal hepatocytes	CHERP
2.96855532120067E-07	1.08294603740781	0.147	0.106	0.00484201058441042	Mid-zonal hepatocytes	COQ10A
3.07339564924046E-07	0.714682499688982	0.12	0.081	0.00501301564347611	Mid-zonal hepatocytes	KCNC3
3.09815041354825E-07	1.22462702381413	0.122	0.083	0.00505339313953855	Mid-zonal hepatocytes	PTPN4
3.34270013330887E-07	1.01669473012773	0.12	0.081	0.0054522781874401	Mid-zonal hepatocytes	MIOS
3.35897277750265E-07	0.398721735485064	0.239	0.194	0.00547882049738456	Mid-zonal hepatocytes	CFDP1
3.42883361813081E-07	0.491798801087619	0.2	0.154	0.00559277051453316	Mid-zonal hepatocytes	LNPK
3.48625250852307E-07	0.721855156996205	0.198	0.155	0.00568642646665198	Mid-zonal hepatocytes	ADIPOR2
3.50611658075153E-07	0.287748374479843	0.308	0.259	0.00571882675486382	Mid-zonal hepatocytes	TKFC
3.5925909349235E-07	1.30693966524643	0.105	0.069	0.00585987507395372	Mid-zonal hepatocytes	MFSD4B
3.67765081765789E-07	0.835913035915999	0.131	0.091	0.00599861624868179	Mid-zonal hepatocytes	BLOC1S6
3.69483189960668E-07	0.312500106053866	0.263	0.219	0.00602664031144845	Mid-zonal hepatocytes	MACO1
3.7456387468801E-07	0.604444959378874	0.136	0.095	0.00610951136003613	Mid-zonal hepatocytes	WDR77
3.86266249458911E-07	1.17624651592637	0.126	0.086	0.0063003887949243	Mid-zonal hepatocytes	ACACA
3.86721875293723E-07	0.832529373754409	0.142	0.101	0.00630782050791591	Mid-zonal hepatocytes	PGGT1B
3.96620527020803E-07	1.34179925004934	0.1	0.064	0.00646927741623632	Mid-zonal hepatocytes	HOMER1
3.96746711889009E-07	0.625329787189664	0.211	0.167	0.00647133561762163	Mid-zonal hepatocytes	PPP3CB
3.97900706736986E-07	0.810144512582904	0.194	0.15	0.00649015842758697	Mid-zonal hepatocytes	PALM2-AK
3.98740878641104E-07	0.372037322734102	0.248	0.201	0.00650386247151504	Mid-zonal hepatocytes	ZC3H4
4.08926281051065E-07	0.710053094978963	0.15	0.107	0.00666999657022392	Mid-zonal hepatocytes	BPNT1
4.12240658413149E-07	0.789479559005872	0.164	0.122	0.00672405737937687	Mid-zonal hepatocytes	EXOSC9
4.12283123431563E-07	0.60421290989798	0.13	0.089	0.00672475002629222	Mid-zonal hepatocytes	PIGF
4.1527883969921E-07	0.554993324835724	0.179	0.135	0.00677361315433382	Mid-zonal hepatocytes	HIPK1
4.20858944329502E-07	0.58808349663022	0.122	0.082	0.00686463024095851	Mid-zonal hepatocytes	FGF13
4.27586277185153E-07	0.574652599051101	0.131	0.09	0.00697435976716702	Mid-zonal hepatocytes	GRK5
4.30324596879932E-07	0.804183578583685	0.104	0.068	0.00701902449970857	Mid-zonal hepatocytes	POLR3B
4.31369217221564E-07	0.467816162812592	0.282	0.237	0.00703606330210092	Mid-zonal hepatocytes	CLPX
4.39267416426665E-07	0.512667186709604	0.227	0.181	0.00716489082933533	Mid-zonal hepatocytes	UNC45A
4.42042229503697E-07	0.297816299876221	0.14	0.098	0.0072101508054348	Mid-zonal hepatocytes	FKRP

4.45311101128918E-07	0.995263280772251	0.107	0.07	0.00726346937051378	Mid-zonal hepatocytes	RHPN1
4.55817793936669E-07	0.589892715768314	0.202	0.157	0.007434844036901	Mid-zonal hepatocytes	SNX2
4.70854192324343E-07	0.827451262617177	0.151	0.11	0.00768010273100236	Mid-zonal hepatocytes	TMEM135
4.73746010530472E-07	0.597137782051467	0.138	0.097	0.00772727117776252	Mid-zonal hepatocytes	NOL9
4.74278297742176E-07	0.306057714746808	0.243	0.197	0.00773595331447264	Mid-zonal hepatocytes	WASL
4.7487499415169E-07	0.604232635690645	0.146	0.105	0.00774568602960821	Mid-zonal hepatocytes	EIF4G2
4.78256828151448E-07	0.609043045910268	0.127	0.087	0.00780084712397827	Mid-zonal hepatocytes	WWC3
5.00970388938704E-07	0.572382758915926	0.163	0.121	0.0081713280139792	Mid-zonal hepatocytes	MANBAL
5.0326336963956E-07	0.752318337099868	0.15	0.108	0.00820872882219086	Mid-zonal hepatocytes	C1orf174
5.03543270028152E-07	0.925967791991041	0.121	0.082	0.00821329427742918	Mid-zonal hepatocytes	SPIN1
5.06114920925214E-07	1.08035849695885	0.134	0.095	0.00825524047521117	Mid-zonal hepatocytes	TAB1
5.08506690255429E-07	0.92956402131514	0.113	0.076	0.0082942526247563	Mid-zonal hepatocytes	CHRNB1
5.21835564662826E-07	0.74359636746025	0.122	0.083	0.00851165989521536	Mid-zonal hepatocytes	DEPRECAT
5.22585413679659E-07	0.305461583062133	0.272	0.226	0.00852389068252892	Mid-zonal hepatocytes	PRKDC
5.31596641576767E-07	0.748019944952806	0.135	0.094	0.00867087282075865	Mid-zonal hepatocytes	CXorf38
5.34280586938489E-07	1.16369082672868	0.119	0.081	0.0087146506535537	Mid-zonal hepatocytes	MTHFSD
5.42043442780513E-07	0.944552742866802	0.142	0.101	0.00884127059519295	Mid-zonal hepatocytes	ANAPC2
5.88256060941442E-07	0.778560631472194	0.126	0.087	0.00959504461001586	Mid-zonal hepatocytes	COQ10B
6.03163953468443E-07	0.644065294562424	0.187	0.143	0.00983820724502378	Mid-zonal hepatocytes	MRGBP
6.24997306806666E-07	0.848498761708161	0.15	0.11	0.0101943310713235	Mid-zonal hepatocytes	TARDBP
6.31440435533112E-07	0.440250530657622	0.235	0.191	0.0102994249439806	Mid-zonal hepatocytes	GORASP2
6.35855081997597E-07	0.788942123820478	0.128	0.089	0.0103714322424628	Mid-zonal hepatocytes	RFT1
6.60057038203774E-07	0.612628593990844	0.159	0.116	0.0107661903501418	Mid-zonal hepatocytes	ZNF205
6.84051452539827E-07	0.575421176039298	0.213	0.169	0.0111575632423771	Mid-zonal hepatocytes	DNAJC8
6.8584689567484E-07	0.50515196145913	0.219	0.175	0.0111868487153523	Mid-zonal hepatocytes	SLC30A7
7.00818708141754E-07	0.819553571827798	0.179	0.138	0.0114310539485002	Mid-zonal hepatocytes	ZNF384
7.0402561542398E-07	0.679192926227629	0.146	0.106	0.0114833618131805	Mid-zonal hepatocytes	RNF215
7.16394491955861E-07	0.703044193700187	0.15	0.109	0.0116851105582921	Mid-zonal hepatocytes	UBE2J1
7.24466929755017E-07	1.43176178069776	0.103	0.067	0.0118167800912341	Mid-zonal hepatocytes	ELF2
7.28594488640389E-07	1.02562180837072	0.123	0.085	0.0118841047042134	Mid-zonal hepatocytes	DHRS12
7.38804112794682E-07	0.59701037644924	0.105	0.069	0.0120506338837941	Mid-zonal hepatocytes	JADE2
7.42711159722753E-07	1.12440759467287	0.138	0.098	0.0121143617262378	Mid-zonal hepatocytes	OTUD6B
7.49389102887772E-07	0.623446246010842	0.176	0.134	0.0122232856572025	Mid-zonal hepatocytes	NELFB
7.62114131237812E-07	0.690152809632004	0.174	0.132	0.0124308435946199	Mid-zonal hepatocytes	MMAA
7.62711533350454E-07	0.559446483952617	0.164	0.123	0.0124405878204793	Mid-zonal hepatocytes	ARL2BP
7.95906934095878E-07	0.324960465983276	0.188	0.144	0.0129820380020379	Mid-zonal hepatocytes	NTMT1
8.01173870545383E-07	0.35801099755923	0.297	0.252	0.0130679470024657	Mid-zonal hepatocytes	CYFIP1
8.14956769793632E-07	1.46942566997834	0.119	0.083	0.0132927598721039	Mid-zonal hepatocytes	USP30
8.27329538386697E-07	0.811462394292019	0.14	0.1	0.0134945721006254	Mid-zonal hepatocytes	UBE2Q2
8.37606180989248E-07	0.385869705444857	0.126	0.087	0.0136621944181156	Mid-zonal hepatocytes	ELFN1
8.50235237270028E-07	0.463853527449424	0.144	0.104	0.0138681869551114	Mid-zonal hepatocytes	PRPF18
8.64865803800988E-07	0.631652277283629	0.116	0.078	0.0141068261257979	Mid-zonal hepatocytes	RPE
8.70831977127117E-07	0.554835498630276	0.116	0.078	0.0142041403789204	Mid-zonal hepatocytes	ABCB10
8.74567750502896E-07	1.33195776103261	0.108	0.073	0.0142650745784527	Mid-zonal hepatocytes	NOC3L
8.80555611653265E-07	0.57934921414214	0.17	0.128	0.0143627425816764	Mid-zonal hepatocytes	MX1
8.97197604470455E-07	0.775572375766731	0.175	0.134	0.0146341901265176	Mid-zonal hepatocytes	PDP2
9.06590208027469E-07	0.752590664810293	0.128	0.089	0.0147873928831361	Mid-zonal hepatocytes	C2orf49
9.27629566668636E-07	0.653075076677805	0.138	0.099	0.0151305658619321	Mid-zonal hepatocytes	SLC25A43
9.29310372418706E-07	1.17561597189795	0.101	0.067	0.0151579814845215	Mid-zonal hepatocytes	C19orf54
9.5495642469766E-07	0.581083303788149	0.239	0.195	0.0155762942432435	Mid-zonal hepatocytes	FRK
9.55415376729615E-07	0.505885087083161	0.173	0.131	0.0155837802098368	Mid-zonal hepatocytes	PPP1R37
9.65334050185495E-07	0.544368956644752	0.119	0.081	0.0157455636925756	Mid-zonal hepatocytes	DSG2
9.72424472009007E-07	0.411573973620852	0.194	0.15	0.0158612155629389	Mid-zonal hepatocytes	CTDSPL
9.79266718069489E-07	0.381096849472768	0.114	0.077	0.0159728194384314	Mid-zonal hepatocytes	ATG4D
9.81597165367449E-07	0.277911109316543	0.26	0.217	0.0160108313643085	Mid-zonal hepatocytes	IFT27
9.83809199529077E-07	0.520067872404737	0.217	0.172	0.0160469118535188	Mid-zonal hepatocytes	NOP16
9.88964956842717E-07	0.626839435605152	0.154	0.113	0.0161310074110615	Mid-zonal hepatocytes	TMA16
1.00292060159448E-06	0.775006033684238	0.125	0.086	0.0163586379326075	Mid-zonal hepatocytes	MED1
1.06282803784418E-06	0.422445306368593	0.232	0.187	0.0173357881252764	Mid-zonal hepatocytes	ADSL
1.0789578990147E-06	0.495072442488387	0.157	0.117	0.0175988822908288	Mid-zonal hepatocytes	PAK1IP1

1.0947883960086E-06	1.01130496125	0.111	0.076	0.0178570935272963	Mid-zonal hepatocytes	PIK3C3
1.09903028322747E-06	0.387640907722996	0.148	0.108	0.0179262829497233	Mid-zonal hepatocytes	FYCO1
1.10340463639741E-06	1.00009175095716	0.132	0.093	0.0179976330242781	Mid-zonal hepatocytes	LIFR
1.10593691652147E-06	0.43285443641765	0.164	0.121	0.0180389370453818	Mid-zonal hepatocytes	IFIT3
1.10959962943824E-06	0.695789203927868	0.163	0.123	0.0180986795557672	Mid-zonal hepatocytes	P3H1
1.11249710215923E-06	0.562983411939244	0.176	0.132	0.0181459402333192	Mid-zonal hepatocytes	TEN1
1.1225468311686E-06	1.0436773912806	0.137	0.099	0.018309861363191	Mid-zonal hepatocytes	POLH
1.1334939303896E-06	0.727096427773544	0.126	0.088	0.0184884194985848	Mid-zonal hepatocytes	SUZ12
1.13454473379559E-06	0.495929740196071	0.157	0.117	0.0185055591529398	Mid-zonal hepatocytes	ECD
1.15165233287778E-06	0.543833500414116	0.132	0.092	0.0187846012015694	Mid-zonal hepatocytes	BFAR
1.15235944526817E-06	0.48943405262972	0.269	0.224	0.0187961349117691	Mid-zonal hepatocytes	NAXD
1.16936254057751E-06	0.537015179832707	0.147	0.107	0.0190734723993598	Mid-zonal hepatocytes	PTPN12
1.17109314274522E-06	0.746204558640917	0.119	0.082	0.0191017002513173	Mid-zonal hepatocytes	COA8
1.17496899105171E-06	0.377729600418883	0.208	0.165	0.0191649192130445	Mid-zonal hepatocytes	NMI
1.17500419092376E-06	0.779659056087527	0.144	0.105	0.0191654933581574	Mid-zonal hepatocytes	ASAP1
1.21882578052015E-06	0.842183136919304	0.181	0.141	0.0198802673060642	Mid-zonal hepatocytes	PTGR2
1.21925557337856E-06	0.804506854224161	0.132	0.093	0.0198872776573777	Mid-zonal hepatocytes	C2CD2
1.22319912716601E-06	0.360401749122587	0.114	0.077	0.0199516009632048	Mid-zonal hepatocytes	BMF
1.25527983603929E-06	0.495808708142282	0.258	0.216	0.0204748694056369	Mid-zonal hepatocytes	PSD4
1.26043804353703E-06	0.819681173622908	0.138	0.098	0.0205590049281325	Mid-zonal hepatocytes	CASP8
1.27515733091701E-06	0.809270061213102	0.129	0.09	0.0207990912245873	Mid-zonal hepatocytes	WDFY1
1.28702356023236E-06	1.01927187494122	0.113	0.077	0.0209926412909501	Mid-zonal hepatocytes	XRRA1
1.29119152768072E-06	0.470281238832391	0.301	0.261	0.0210606250080003	Mid-zonal hepatocytes	UBE2R2
1.29450916355902E-06	0.809225135440613	0.208	0.167	0.0211147389668112	Mid-zonal hepatocytes	TRMT1L
1.30199956326141E-06	0.517721989570022	0.112	0.076	0.0212369148763568	Mid-zonal hepatocytes	LRRC57
1.30235471159298E-06	1.12853518476723	0.125	0.087	0.0212427077007931	Mid-zonal hepatocytes	PMS2
1.3065603980624E-06	0.512878374701416	0.18	0.138	0.0213113066527959	Mid-zonal hepatocytes	SNTA1
1.34343612967355E-06	1.1288130071424	0.1	0.065	0.0219127867111053	Mid-zonal hepatocytes	FAM184A
1.35106254786797E-06	0.34611709495552	0.169	0.129	0.0220371812182745	Mid-zonal hepatocytes	PI4K2A
1.35750439135543E-06	0.476035306874695	0.234	0.192	0.0221422541273984	Mid-zonal hepatocytes	USB1
1.36852473423515E-06	1.17055746226541	0.125	0.088	0.0223220069401095	Mid-zonal hepatocytes	ZNF862
1.40353488581738E-06	0.735104381810497	0.141	0.102	0.0228930575225673	Mid-zonal hepatocytes	CAMTA1
1.40534703562148E-06	1.28175102530918	0.106	0.071	0.022922615498022	Mid-zonal hepatocytes	ZNF587B
1.42847675553059E-06	0.792250793099175	0.195	0.154	0.0232998843594595	Mid-zonal hepatocytes	CCT8
1.45532744434511E-06	0.901967303549581	0.108	0.073	0.0237378459447132	Mid-zonal hepatocytes	CTNS
1.48842634393901E-06	0.670411095616803	0.161	0.122	0.0242777220959893	Mid-zonal hepatocytes	ZDHHC16
1.49865189905056E-06	0.59805518191806	0.166	0.126	0.0244445111254137	Mid-zonal hepatocytes	MIB1
1.52664002825585E-06	0.861508365300476	0.151	0.112	0.0249010255008812	Mid-zonal hepatocytes	ARHGAP5
1.54086136879928E-06	0.299153510007987	0.151	0.11	0.0251329897864851	Mid-zonal hepatocytes	MFSD6
1.55460165600779E-06	0.771146584235865	0.134	0.096	0.0253571076111431	Mid-zonal hepatocytes	ANO10
1.55752795783833E-06	1.03062231842157	0.143	0.105	0.025404838520301	Mid-zonal hepatocytes	ZBTB25
1.57150455448438E-06	1.08881085970792	0.114	0.078	0.0256328107881947	Mid-zonal hepatocytes	ANKRD13C
1.57206843676025E-06	1.00915925029193	0.128	0.091	0.0256420082719965	Mid-zonal hepatocytes	TBC1D7
1.57558403333573E-06	0.941664766327025	0.179	0.136	0.0256993511677391	Mid-zonal hepatocytes	SOX4
1.5769696151407E-06	1.01289296278023	0.142	0.104	0.0257219513925599	Mid-zonal hepatocytes	NSUN3
1.58623431691074E-06	1.00248605905176	0.125	0.088	0.0258730679431311	Mid-zonal hepatocytes	WDR3
1.61329697982581E-06	0.77005170128314	0.144	0.105	0.0263144870379387	Mid-zonal hepatocytes	PBDC1
1.61626191443369E-06	1.04727109013156	0.142	0.104	0.026362848086328	Mid-zonal hepatocytes	GALK2
1.62068551063789E-06	0.707495982887522	0.1	0.065	0.0264350013640146	Mid-zonal hepatocytes	SNX30
1.6514410070538E-06	0.924189870960703	0.113	0.077	0.0269366542660546	Mid-zonal hepatocytes	ERICH1
1.65325278895624E-06	0.366979672341628	0.258	0.217	0.0269662062406652	Mid-zonal hepatocytes	ACAD8
1.72887753423515E-06	0.821759392792307	0.139	0.101	0.0281997214609095	Mid-zonal hepatocytes	C8orf76
1.75758116699159E-06	0.753388699462523	0.129	0.091	0.0286679064147998	Mid-zonal hepatocytes	PPP5C
1.76129545323733E-06	0.72387195659502	0.119	0.082	0.0287284901377541	Mid-zonal hepatocytes	POMT1
1.7901771157479E-06	0.767085307740514	0.15	0.111	0.0291995789349641	Mid-zonal hepatocytes	COIL
1.79916940034817E-06	0.975682957879204	0.119	0.082	0.029346252089079	Mid-zonal hepatocytes	FADS6
1.80506273223714E-06	0.997167596098872	0.113	0.077	0.0294423782255199	Mid-zonal hepatocytes	ALG14
1.83447449240317E-06	0.780814712096178	0.124	0.087	0.0299221134455881	Mid-zonal hepatocytes	ETV6
1.84252328479768E-06	0.807608910279509	0.156	0.116	0.030053397298335	Mid-zonal hepatocytes	HEATR3
1.86080443417453E-06	0.255984259070132	0.188	0.146	0.0303515811258207	Mid-zonal hepatocytes	TSNAX

1.86095649507224E-06	0.638526375107155	0.181	0.141	0.0303540613911234	Mid-zonal hepatocytes	PLA2G15
1.86581368563938E-06	0.857945990191962	0.164	0.124	0.0304332870264639	Mid-zonal hepatocytes	FOXO1
1.86877495055745E-06	0.646440403855595	0.179	0.138	0.0304815882185426	Mid-zonal hepatocytes	RHEB
1.88542531443592E-06	0.30416670180008	0.174	0.132	0.0307531723037644	Mid-zonal hepatocytes	ZNF598
1.91498982645971E-06	0.66163322404306	0.179	0.138	0.0312353990593843	Mid-zonal hepatocytes	TSR1
1.94771169319688E-06	0.571070396421682	0.149	0.11	0.0317691254277344	Mid-zonal hepatocytes	STIP1
1.97068413359505E-06	0.661719835928136	0.102	0.068	0.0321438289030689	Mid-zonal hepatocytes	TUBG1
1.98730946304835E-06	0.61557260920602	0.107	0.072	0.0324150046517817	Mid-zonal hepatocytes	NAIF1
2.02529189961527E-06	1.23437518901843	0.121	0.085	0.0330345361746247	Mid-zonal hepatocytes	ZNF75A
2.03092517545629E-06	0.892316859783162	0.147	0.109	0.0331264205368675	Mid-zonal hepatocytes	NOL12
2.03689424696654E-06	0.804012776623934	0.139	0.101	0.0332237820622712	Mid-zonal hepatocytes	IGBP1
2.03832225925312E-06	1.16359896662458	0.125	0.088	0.0332470743706776	Mid-zonal hepatocytes	PRPF38A
2.05157388710986E-06	0.654388298220957	0.2	0.159	0.0334632216726489	Mid-zonal hepatocytes	MTX2
2.09273996284821E-06	0.656462904867129	0.121	0.084	0.0341346815340171	Mid-zonal hepatocytes	PRR12
2.14796946246708E-06	0.701995161187141	0.179	0.14	0.0350355299023005	Mid-zonal hepatocytes	PRMT2
2.21966212590781E-06	0.30101045062432	0.251	0.208	0.0362049089356823	Mid-zonal hepatocytes	DDX27
2.24042297159706E-06	0.443271404910745	0.207	0.167	0.0365435390897197	Mid-zonal hepatocytes	GBE1
2.2773477661186E-06	0.509632452402541	0.135	0.097	0.0371458194131605	Mid-zonal hepatocytes	ARAP2
2.29370822133787E-06	1.0852388738755	0.108	0.074	0.0374126747982421	Mid-zonal hepatocytes	STRBP
2.32226682095441E-06	0.657977631024569	0.105	0.071	0.0378784941165874	Mid-zonal hepatocytes	COPG2
2.3273003717051E-06	0.442702164487769	0.159	0.12	0.037960596362882	Mid-zonal hepatocytes	DYRK1A
2.4050695712085E-06	0.660219327330799	0.116	0.08	0.0392290897759818	Mid-zonal hepatocytes	ZNF212
2.40711072631404E-06	0.791618837452439	0.176	0.136	0.0392623830569083	Mid-zonal hepatocytes	ABCD4
2.42743729754016E-06	1.11063269991243	0.125	0.089	0.0395939297601775	Mid-zonal hepatocytes	FAM49B
2.49226130069836E-06	1.10666703149399	0.11	0.075	0.0406512740756909	Mid-zonal hepatocytes	TBCK
2.51383580682609E-06	0.692392674793023	0.137	0.098	0.0410031758451404	Mid-zonal hepatocytes	LARS2
2.53455120748653E-06	0.611331896743172	0.176	0.136	0.0413410647453128	Mid-zonal hepatocytes	GCFC2
2.53936983680632E-06	1.00238994630394	0.127	0.091	0.0414196614081478	Mid-zonal hepatocytes	PKNOX1
2.56203398940106E-06	0.42337380055753	0.218	0.177	0.0417893364011207	Mid-zonal hepatocytes	VIL1
2.57839881492999E-06	0.661352394897622	0.144	0.106	0.0420562630703231	Mid-zonal hepatocytes	FAM131A
2.58318238721637E-06	0.349067703236858	0.188	0.149	0.0421342879178862	Mid-zonal hepatocytes	GOLT1B
2.61206760470132E-06	0.355525499664757	0.299	0.258	0.0426054347002831	Mid-zonal hepatocytes	AHCYL1
2.67768864861305E-06	0.403960737371805	0.244	0.203	0.0436757795475275	Mid-zonal hepatocytes	KLHDC10
2.70800564209884E-06	0.986955079724392	0.164	0.126	0.0441702800282741	Mid-zonal hepatocytes	AL135905.
2.71928398788864E-06	0.532128225651561	0.119	0.083	0.0443542411264516	Mid-zonal hepatocytes	B3GNT2
2.72697369829941E-06	0.373928626128242	0.197	0.157	0.0444796679929617	Mid-zonal hepatocytes	TM9SF4
2.73497399245493E-06	0.825258368942305	0.129	0.092	0.0446101607909324	Mid-zonal hepatocytes	DEPRECAT
2.73835498834804E-06	0.684209140747721	0.113	0.077	0.0446653082149449	Mid-zonal hepatocytes	SMAD1
2.82602170415624E-06	0.573052671878587	0.115	0.079	0.0460952400164924	Mid-zonal hepatocytes	DDB2
2.83725141908936E-06	1.10797176145694	0.108	0.074	0.0462784078967665	Mid-zonal hepatocytes	TTC12
2.89221340703444E-06	0.909351847589999	0.105	0.071	0.0471748928821387	Mid-zonal hepatocytes	C3orf38
2.89780019932228E-06	0.927328950970048	0.12	0.084	0.0472660190511456	Mid-zonal hepatocytes	E2F3
2.90598262386364E-06	0.789985848286828	0.17	0.132	0.0473994825778398	Mid-zonal hepatocytes	TBK1
2.92661695035329E-06	0.373570979390518	0.195	0.154	0.0477360490772125	Mid-zonal hepatocytes	DDX23
2.98927471554202E-06	0.84383104354966	0.181	0.141	0.0487580598852059	Mid-zonal hepatocytes	ST7
2.99689851099382E-06	0.466645979899873	0.106	0.072	0.0488824116128202	Mid-zonal hepatocytes	THRA
3.00022127032419E-06	0.585783880584943	0.126	0.089	0.0489366091402578	Mid-zonal hepatocytes	ZSCAN21
3.01528654385482E-06	0.655037251861177	0.123	0.087	0.049182338816816	Mid-zonal hepatocytes	MDFIC
3.03461024292117E-06	0.685398116980586	0.117	0.081	0.0494975276722871	Mid-zonal hepatocytes	DHX33
3.04004264831903E-06	0.499320124616897	0.192	0.152	0.0495861356367317	Mid-zonal hepatocytes	TSPAN17
0	3.05309348873917	0.984	0.751	0	Pericentral hepatocytes	CYP 20.00
3.58502210594627E-147	2.20323322515904	0.85	0.623	5.84752955700896E-143	Pericentral hepatocytes	LYZ
9.45540810083762E-110	1.72220011975222	0.702	0.458	1.54227161532762E-105	Pericentral hepatocytes	FMO3
4.93398276005235E-90	3.34638288180672	0.279	0.071	8.04781927992138E-86	Pericentral hepatocytes	CYP1A2
8.58623263321095E-77	1.10956314172794	0.827	0.657	1.40050040480304E-72	Pericentral hepatocytes	PRAP1
3.02810256525866E-74	2.17931360240553	0.338	0.119	4.93913809419341E-70	Pericentral hepatocytes	LECT2
9.65676369403025E-73	2.14075040643243	0.227	0.054	1.57511472613327E-68	Pericentral hepatocytes	CLDN2
2.01009843493755E-71	0.819742896577938	0.94	0.858	3.27867155722664E-67	Pericentral hepatocytes	FABP1
6.21701295200064E-69	1.4940240728113	0.494	0.249	1.01405698260082E-64	Pericentral hepatocytes	ADH4
2.70971551840156E-61	1.68794894693405	0.305	0.109	4.41981698206478E-57	Pericentral hepatocytes	TBX3

4.94299769801032E-61	2.09091442176631	0.35	0.144	8.06252354522464E-57	Pericentral hepatocytes	DEPRECAT
1.19030164742185E-58	2.47277995910657	0.148	0.028	1.94150101710978E-54	Pericentral hepatocytes	RHBG
3.56252142430333E-54	2.22415038915071	0.262	0.091	5.81082869518115E-50	Pericentral hepatocytes	CYP3A4
1.72486942250673E-51	0.95982070670509	0.729	0.566	2.81343451505073E-47	Pericentral hepatocytes	MBL2
2.07323875181796E-50	1.20055284240512	0.49	0.276	3.38165972809027E-46	Pericentral hepatocytes	AQP9
1.65377586428844E-47	2.70792355130889	0.104	0.016	2.69747381224087E-43	Pericentral hepatocytes	ALDH3A1
1.00801443090172E-37	1.20552370247544	0.444	0.264	1.6441723382438E-33	Pericentral hepatocytes	ACSL5
6.20085290419218E-35	1.2894512469757	0.276	0.126	1.01142111720279E-30	Pericentral hepatocytes	ELOVL2
1.03137507303949E-34	0.658182669477101	0.765	0.658	1.68227588163471E-30	Pericentral hepatocytes	AOX1
7.93446342733407E-34	0.299873497896138	0.994	0.992	1.29419032963246E-29	Pericentral hepatocytes	C3
4.54344657662565E-33	0.838726584787249	0.563	0.408	7.4108157111341E-29	Pericentral hepatocytes	MDK
5.84030363728176E-33	0.79503624744236	0.645	0.51	9.52611926277028E-29	Pericentral hepatocytes	GPX2
3.73160278871369E-31	0.948804002092729	0.438	0.269	6.0866173086709E-27	Pericentral hepatocytes	GPD1
1.98332365626454E-30	1.12200008821293	0.416	0.259	3.23499921573309E-26	Pericentral hepatocytes	SORD
6.7663368629694E-29	1.055528959015	0.491	0.334	1.10365720571894E-24	Pericentral hepatocytes	MTHFD1
8.008606262089E-28	0.52033367485267	0.797	0.704	1.30628376740934E-23	Pericentral hepatocytes	PLG
1.26471255804014E-27	0.475007913268355	0.856	0.795	2.06287265341928E-23	Pericentral hepatocytes	ALDH2
1.26556090046411E-27	0.350979354565705	0.863	0.819	2.06425638474701E-23	Pericentral hepatocytes	C4BPA
3.60488538295267E-27	0.979855320825067	0.459	0.304	5.8799285481341E-23	Pericentral hepatocytes	ALAS1
7.85316504260786E-26	0.718063728939366	0.645	0.505	1.28092975009977E-21	Pericentral hepatocytes	ADH1A
1.20473751750205E-25	0.948278029991234	0.364	0.216	1.96504736479759E-21	Pericentral hepatocytes	CFHR5
1.97366374444693E-25	0.721326287888541	0.645	0.54	3.21924293356739E-21	Pericentral hepatocytes	NADK2
6.21999732938832E-25	0.52919795749382	0.819	0.738	1.01454376439653E-20	Pericentral hepatocytes	SCP2
8.92515757842126E-25	0.927508019637454	0.496	0.35	1.45578245261629E-20	Pericentral hepatocytes	UGT1A1
1.07001112901089E-24	0.531420729604151	0.724	0.638	1.74529515252966E-20	Pericentral hepatocytes	CFHR2
2.71018064739972E-24	0.820564372751093	0.567	0.424	4.42057565397368E-20	Pericentral hepatocytes	CDHR5
4.0057651814676E-24	0.491481409829698	0.796	0.704	6.5338035874918E-20	Pericentral hepatocytes	GATM
4.35670991909527E-24	0.511816567109391	0.798	0.702	7.10622954903629E-20	Pericentral hepatocytes	ECH1
1.63253090505059E-23	0.618958435721559	0.682	0.557	2.66282115922801E-19	Pericentral hepatocytes	HSD17B6
2.25317830561137E-22	0.619987399051173	0.632	0.502	3.6751591342827E-18	Pericentral hepatocytes	SOD1
2.402548921944E-22	0.385569276549678	0.848	0.765	3.91879754658286E-18	Pericentral hepatocytes	CTSD
2.81350744441732E-22	0.511586456721628	0.767	0.672	4.58911199258909E-18	Pericentral hepatocytes	ECHS1
4.16806196599497E-22	0.750016853126678	0.615	0.508	6.79852587273439E-18	Pericentral hepatocytes	SULT2A1
4.44377574012368E-22	0.90738550873136	0.264	0.142	7.24824260971573E-18	Pericentral hepatocytes	ACSS3
6.29486685975781E-22	1.0329161072042	0.441	0.302	1.0267557334951E-17	Pericentral hepatocytes	ABCB4
8.87301445697336E-22	0.750885422108395	0.582	0.451	1.44727738807692E-17	Pericentral hepatocytes	SLC2A2
2.83581646946207E-21	0.709308695777273	0.556	0.418	4.62550024333959E-17	Pericentral hepatocytes	LGALS3BP
3.69789516086069E-21	0.408081997593418	0.845	0.778	6.03163679687987E-17	Pericentral hepatocytes	SERPING1
4.42986962014899E-21	0.865855278385362	0.484	0.353	7.22556033742502E-17	Pericentral hepatocytes	DEPRECAT
1.20366741213267E-20	0.283761485081613	0.984	0.956	1.9633019159296E-16	Pericentral hepatocytes	EPHX1
2.80292510639856E-19	0.720874408724003	0.733	0.659	4.57185114104669E-15	Pericentral hepatocytes	GLUL
2.85535068774295E-19	0.772896884802518	0.187	0.09	4.65736250677753E-15	Pericentral hepatocytes	ZNRF3
3.49159220617496E-19	0.272513869648356	0.938	0.883	5.69513604749199E-15	Pericentral hepatocytes	AMBP
6.4823696489979E-19	0.304486708593426	0.86	0.803	1.05733931344805E-14	Pericentral hepatocytes	ASGR1
8.17160448119464E-19	0.550578658678844	0.658	0.567	1.33287040692766E-14	Pericentral hepatocytes	F7
8.37896717985958E-19	1.22241066824653	0.138	0.058	1.3666933367069E-14	Pericentral hepatocytes	ADIRF
1.15565834399874E-18	0.93682040036412	0.305	0.186	1.88499432489635E-14	Pericentral hepatocytes	CBR1
2.62559112159055E-18	0.582609257113101	0.164	0.076	4.28260167842634E-14	Pericentral hepatocytes	RNF43
2.89811851759406E-18	0.779723641474926	0.406	0.279	4.72712111404767E-14	Pericentral hepatocytes	SLC27A5
3.82732391676474E-18	0.623370605806358	0.495	0.358	6.24274804063497E-14	Pericentral hepatocytes	APOM
4.67441550887989E-18	0.875262123649683	0.396	0.272	7.624439136534E-14	Pericentral hepatocytes	DEPRECAT
4.96133307824463E-18	0.845348292948321	0.104	0.038	8.09243038392481E-14	Pericentral hepatocytes	TPRG1
6.68140538026909E-18	0.780784406111813	0.393	0.269	1.08980403157569E-13	Pericentral hepatocytes	GLUD1
1.83118920512435E-17	0.630601182004234	0.465	0.335	2.98685271247833E-13	Pericentral hepatocytes	EVA1A
1.8477462351836E-17	0.251201191202267	0.998	0.994	3.01385888420797E-13	Pericentral hepatocytes	SERPINA1
2.77085856774905E-17	0.844172372726743	0.387	0.267	4.51954740985547E-13	Pericentral hepatocytes	DEPRECAT
2.82732317153169E-17	0.305340965702506	0.773	0.711	4.61164682508535E-13	Pericentral hepatocytes	FMO5
5.01960907898613E-17	0.508834364692743	0.623	0.494	8.18748436873428E-13	Pericentral hepatocytes	IFI27
5.33695536466472E-17	0.579701411506745	0.663	0.554	8.70510789530462E-13	Pericentral hepatocytes	DCXR
6.67701536900369E-17	0.933537297326488	0.299	0.19	1.08908797683819E-12	Pericentral hepatocytes	CCND1

7.5943591502749E-17	0.534526252128112	0.594	0.472	1.23871592100134E-12	Pericentral hepatocytes	OTC
1.11408818519561E-16	0.616910925403304	0.367	0.246	1.81718923887256E-12	Pericentral hepatocytes	ASPDH
1.26418661596564E-16	0.340776160899854	0.801	0.728	2.06201478930155E-12	Pericentral hepatocytes	B2M
1.35641398303326E-16	0.48407288777573	0.707	0.609	2.21244684772556E-12	Pericentral hepatocytes	FUOM
2.56412768374486E-16	1.171845434135	0.184	0.096	4.18234866495625E-12	Pericentral hepatocytes	ADH6
4.27929951555573E-16	0.720219651925627	0.517	0.406	6.97996543982295E-12	Pericentral hepatocytes	CYP2C9
4.95355405623239E-16	1.0304289624799	0.149	0.071	8.07974202112065E-12	Pericentral hepatocytes	SLC16A13
6.95852942245486E-16	0.45937577754855	0.595	0.467	1.13500573409661E-11	Pericentral hepatocytes	HPD
7.97276564166336E-16	0.409717821154013	0.693	0.604	1.30043780381171E-11	Pericentral hepatocytes	PGRMC1
8.35534901649443E-16	0.84308645903959	0.325	0.212	1.36284097808041E-11	Pericentral hepatocytes	OAS1
1.81866438072044E-15	0.809851144387403	0.326	0.216	2.9664234713931E-11	Pericentral hepatocytes	ENPEP
1.88373249172897E-15	0.717592963146012	0.348	0.229	3.07255606725912E-11	Pericentral hepatocytes	SLC6A12
1.99571521696769E-15	0.946804240741059	0.316	0.207	3.255211090396E-11	Pericentral hepatocytes	UGT2B10
2.66572334958564E-15	0.812780786642371	0.403	0.286	4.34806135550913E-11	Pericentral hepatocytes	SEC14L2
3.80181609032661E-15	0.695506212104151	0.197	0.107	6.20114222493173E-11	Pericentral hepatocytes	RHOBTB1
5.89951292909366E-15	0.87928259626928	0.282	0.177	9.62269553864468E-11	Pericentral hepatocytes	ROBO1
8.94817025435607E-15	0.487564596801292	0.612	0.516	1.45953605018802E-10	Pericentral hepatocytes	RGN
1.05097732435596E-14	0.299163807385235	0.852	0.794	1.71424911375701E-10	Pericentral hepatocytes	LRG1
1.22306788566785E-14	0.90109635083015	0.263	0.161	1.99494602831284E-10	Pericentral hepatocytes	LIN7A
3.68951972848718E-14	1.32739073559864	0.102	0.043	6.01797562913543E-10	Pericentral hepatocytes	SLC4A4
5.53417091908939E-14	0.458215374207508	0.679	0.595	9.02678618612671E-10	Pericentral hepatocytes	ACAT1
5.66615672212543E-14	0.404832627835063	0.659	0.559	9.24206822945879E-10	Pericentral hepatocytes	HDGF
6.33615235947101E-14	0.62594165144297	0.452	0.338	1.03348981135332E-09	Pericentral hepatocytes	C1orf115
8.00382483823393E-14	0.491064957526126	0.599	0.501	1.30550386936434E-09	Pericentral hepatocytes	CAT
9.17816303737506E-14	0.424687778235409	0.692	0.616	1.49705017302625E-09	Pericentral hepatocytes	ADI1
1.08476729801293E-13	0.650610676661876	0.447	0.332	1.76936393978889E-09	Pericentral hepatocytes	PSMD4
1.66317896561759E-13	1.15532668805199	0.205	0.121	2.71281121081885E-09	Pericentral hepatocytes	LCN2
1.77650436223849E-13	0.452285181086954	0.186	0.101	2.89765626524721E-09	Pericentral hepatocytes	MID1
2.01192525365262E-13	1.43381060718032	0.112	0.051	3.28165128123279E-09	Pericentral hepatocytes	AQP11
2.16066489941114E-13	0.752444363032901	0.416	0.311	3.52426051742952E-09	Pericentral hepatocytes	SLC22A18
2.45932831921791E-13	0.611039971352033	0.261	0.164	4.01141042147633E-09	Pericentral hepatocytes	DDAH2
3.91353311838596E-13	0.357326896270952	0.677	0.574	6.38336386939933E-09	Pericentral hepatocytes	ISG15
4.07899835346497E-13	0.314509539735171	0.816	0.739	6.65325421433671E-09	Pericentral hepatocytes	DHCR24
4.56472547507437E-13	0.323056889185	0.751	0.683	7.44552372239381E-09	Pericentral hepatocytes	ETFB
6.20902764343224E-13	0.629086724207417	0.421	0.309	1.01275449892023E-08	Pericentral hepatocytes	F10
8.05183745485754E-13	1.1564559500474	0.115	0.054	1.31333520726181E-08	Pericentral hepatocytes	SHF
1.65641557034374E-12	0.648605617569524	0.161	0.087	2.70177943678767E-08	Pericentral hepatocytes	SOD3
1.66314809606408E-12	0.377190841353926	0.745	0.68	2.71276085949012E-08	Pericentral hepatocytes	APOL6
1.82850504852442E-12	0.683033788278952	0.447	0.338	2.98247458464818E-08	Pericentral hepatocytes	TXNRD2
1.96163207325144E-12	0.41702341564058	0.45	0.349	3.19961807468042E-08	Pericentral hepatocytes	VWA1
2.70061896860262E-12	0.306381370405942	0.816	0.742	4.40497959968774E-08	Pericentral hepatocytes	APCS
2.84679615462714E-12	0.567438510375115	0.508	0.404	4.64340920781232E-08	Pericentral hepatocytes	QDPR
2.87920231427884E-12	0.480289788139366	0.682	0.606	4.69626689482022E-08	Pericentral hepatocytes	HNF4A
3.50155819790999E-12	0.755279319597884	0.233	0.143	5.71139157661098E-08	Pericentral hepatocytes	ETV4
4.01546859308108E-12	0.382090011830368	0.568	0.466	6.54963082217456E-08	Pericentral hepatocytes	ITM2B
4.11986502971207E-12	0.278778440653215	0.801	0.741	6.71991184996336E-08	Pericentral hepatocytes	PTMS
4.36107482292406E-12	0.369624765591872	0.66	0.585	7.11334914367143E-08	Pericentral hepatocytes	SERPINA7
6.99721540498278E-12	0.427259670244739	0.493	0.384	1.14131580470674E-07	Pericentral hepatocytes	BST2
1.0112915780467E-11	0.484267612413334	0.551	0.45	1.64951769295197E-07	Pericentral hepatocytes	ADRM1
1.02559536678921E-11	0.371993967775641	0.507	0.393	1.67284860276987E-07	Pericentral hepatocytes	REEP6
1.08077103752502E-11	0.636544627163011	0.17	0.095	1.76284563930706E-07	Pericentral hepatocytes	MORC4
1.0875579385237E-11	0.426889546853436	0.523	0.425	1.773915753526E-07	Pericentral hepatocytes	DNPH1
1.55051180329295E-11	0.377547193375096	0.67	0.588	2.52903980235113E-07	Pericentral hepatocytes	OSGIN1
1.82691691288066E-11	0.3693545478092	0.763	0.717	2.97988417659965E-07	Pericentral hepatocytes	CYB5A
2.03504420415483E-11	0.341313529500412	0.667	0.591	3.31936060139694E-07	Pericentral hepatocytes	TFPI
2.70084388594343E-11	0.383894801895599	0.339	0.235	4.40534646236232E-07	Pericentral hepatocytes	PHLDA1
2.80264631464308E-11	0.704962322844923	0.306	0.211	4.57139640381432E-07	Pericentral hepatocytes	SERPINA11
2.85790595752582E-11	0.460874988597372	0.606	0.533	4.66153040732037E-07	Pericentral hepatocytes	POR
2.91472513173122E-11	0.477634104320347	0.623	0.528	4.7542081623668E-07	Pericentral hepatocytes	ANG
3.90970444817978E-11	0.355887361695263	0.678	0.606	6.37711892542604E-07	Pericentral hepatocytes	PFN1

**4.55613379198627E-11**	0.597777796007872	0.44	0.337	7.4315098281088E-07	Pericentral hepatocytes	GPAM
**5.00296580330874E-11**	0.386373907059986	0.574	0.468	8.16033752177688E-07	Pericentral hepatocytes	ALDH1L1
**5.10576188441637E-11**	0.48319896859249	0.5	0.398	8.32800820967153E-07	Pericentral hepatocytes	ECI1
**6.4552200464336E-11**	0.552462685018551	0.317	0.223	1.05291094177379E-06	Pericentral hepatocytes	COMT
**7.62033957391496E-11**	0.671307468078956	0.149	0.083	1.24295358790127E-06	Pericentral hepatocytes	DEPRECAT
**1.28920954254699E-10**	0.418697347237672	0.374	0.269	2.1028296848484E-06	Pericentral hepatocytes	CLDN4
**1.69732623144798E-10**	0.482949812569209	0.531	0.444	2.7685088161148E-06	Pericentral hepatocytes	DUSP23
**1.71435742968045E-10**	0.497109563192785	0.465	0.372	2.79628840355178E-06	Pericentral hepatocytes	HSD17B10
**1.79211311170337E-10**	0.544254664821833	0.4	0.299	2.92311569649936E-06	Pericentral hepatocytes	LAMTOR2
**2.27480545353143E-10**	0.493130937574839	0.58	0.49	3.71043517525511E-06	Pericentral hepatocytes	UGT2B4
**2.38366469879614E-10**	0.39254132266942	0.419	0.317	3.88799549020639E-06	Pericentral hepatocytes	TST
**3.88745165564179E-10**	0.621193986807332	0.407	0.31	6.34082239551732E-06	Pericentral hepatocytes	UQCRQ
**4.41634821194283E-10**	0.446814095801412	0.534	0.438	7.20350556849995E-06	Pericentral hepatocytes	APOC4
**4.52010718949367E-10**	0.407682569215525	0.651	0.577	7.37274683678312E-06	Pericentral hepatocytes	BLVRB
**5.04878420780898E-10**	1.27637196424636	0.18	0.109	8.23507192135723E-06	Pericentral hepatocytes	ZNF503
**6.67687059534461E-10**	0.729056555364004	0.332	0.241	1.08906436280666E-05	Pericentral hepatocytes	PLPP1
**6.87551824554417E-10**	0.558943160705262	0.309	0.219	1.12146578103071E-05	Pericentral hepatocytes	COLEC11
**7.66535495457981E-10**	0.521161899996237	0.429	0.335	1.25029604664151E-05	Pericentral hepatocytes	MIF
**7.76713057875869E-10**	0.276366042896739	0.777	0.737	1.26689666870133E-05	Pericentral hepatocytes	IFITM3
**8.46718918128325E-10**	0.346872629873373	0.603	0.521	1.38108322735911E-05	Pericentral hepatocytes	PRDX2
**9.7823207154511E-10**	0.364683771198476	0.541	0.45	1.59559433189723E-05	Pericentral hepatocytes	PSME1
**1.03286748083447E-09**	0.393577501823898	0.608	0.538	1.6847101479891E-05	Pericentral hepatocytes	ACSL4
**1.06291468879111E-09**	0.597024815763897	0.316	0.22	1.73372014888718E-05	Pericentral hepatocytes	CCL16
**1.33857516705022E-09**	0.408202284760362	0.365	0.271	2.18334995497562E-05	Pericentral hepatocytes	CNN3
**1.5712134296088E-09**	0.443540675600886	0.441	0.34	2.56280622503491E-05	Pericentral hepatocytes	TXNDC17
**1.58242427185772E-09**	0.353350958312135	0.319	0.23	2.58109222982714E-05	Pericentral hepatocytes	ABHD14B
**2.05854117052196E-09**	0.573681344952295	0.139	0.079	3.35768650323837E-05	Pericentral hepatocytes	SOX9
**2.11952812134622E-09**	0.500460569271007	0.627	0.557	3.45716231872783E-05	Pericentral hepatocytes	PCK2
**2.16767344431956E-09**	0.813206520893019	0.405	0.314	3.53569215502963E-05	Pericentral hepatocytes	PSMA7
**2.23447201116506E-09**	0.604172108696175	0.348	0.256	3.64464729741133E-05	Pericentral hepatocytes	TACO1
**2.35616566737058E-09**	0.950058644801594	0.117	0.063	3.84314182004815E-05	Pericentral hepatocytes	SLC22A18A
**2.41375763674176E-09**	0.316228125934213	0.654	0.58	3.93708008128949E-05	Pericentral hepatocytes	DEPP1
**2.43527574894241E-09**	0.528632279162912	0.4	0.308	3.97217827409996E-05	Pericentral hepatocytes	ECHDC3
**2.872131431663E-09**	0.532735014886242	0.398	0.308	4.68473357818552E-05	Pericentral hepatocytes	PSMC4
**3.11036502174054E-09**	0.401962115724709	0.597	0.513	5.07331638696099E-05	Pericentral hepatocytes	PGM1
**3.28231580953074E-09**	0.556837100223421	0.301	0.216	5.3537853169256E-05	Pericentral hepatocytes	PKLR
**3.29886454688293E-09**	0.56483694482153	0.409	0.315	5.38077796242075E-05	Pericentral hepatocytes	ACADS
**3.34798565279213E-09**	0.575565479779305	0.271	0.188	5.46089939826924E-05	Pericentral hepatocytes	H2AFJ
**3.60411878805206E-09**	0.351811213116515	0.342	0.249	5.87867815519172E-05	Pericentral hepatocytes	MPLKIP
**3.66885143309488E-09**	0.452099269544399	0.595	0.51	5.98426357252106E-05	Pericentral hepatocytes	HAGH
**4.40192487786594E-09**	0.703834457340938	0.295	0.211	7.17997966828714E-05	Pericentral hepatocytes	MSRB1
**4.43080467768295E-09**	0.43461032932955	0.246	0.164	7.22708550976867E-05	Pericentral hepatocytes	SMLR1
**4.96658153783649E-09**	0.73059867177513	0.342	0.26	8.1009911463651E-05	Pericentral hepatocytes	SCD
**5.39484684818618E-09**	0.382314824837488	0.275	0.193	8.79953469407648E-05	Pericentral hepatocytes	PIR
**6.50181589659353E-09**	0.454993760972828	0.467	0.378	0.000106051119089337	Pericentral hepatocytes	TSTA3
**6.81745000363563E-09**	0.546283707303279	0.377	0.28	0.000111199427009301	Pericentral hepatocytes	TOMM7
**7.32294250354977E-09**	0.437998721519068	0.488	0.398	0.0001194445151754	Pericentral hepatocytes	ATOX1
**7.33801052843844E-09**	0.518263499773882	0.465	0.372	0.000119690289729359	Pericentral hepatocytes	FCGRT
**8.21658912374769E-09**	0.390600219636059	0.507	0.413	0.000134020785197449	Pericentral hepatocytes	SHBG
**8.26909800997401E-09**	0.663006942245804	0.28	0.197	0.000134877257640686	Pericentral hepatocytes	GCHFR
**8.43754166764559E-09**	0.323487963947189	0.509	0.421	0.000137624742140967	Pericentral hepatocytes	PEBP1
**8.75180173542182E-09**	0.77242579443736	0.314	0.229	0.000142750638106465	Pericentral hepatocytes	PSMC2
**1.04421036158069E-08**	0.785254023908477	0.147	0.088	0.000170321152077426	Pericentral hepatocytes	SERTM2
**1.14630348450413E-08**	0.543060203834466	0.465	0.387	0.000186973561357469	Pericentral hepatocytes	HLF
**1.17006777781061E-08**	0.446218030223934	0.49	0.408	0.000190849755238689	Pericentral hepatocytes	H1F0
**1.29224737559473E-08**	0.901625373049963	0.107	0.058	0.000210778469433256	Pericentral hepatocytes	NRXN2
**1.5632918609066E-08**	0.329826365967882	0.549	0.464	0.000254988535432475	Pericentral hepatocytes	BEX3
**1.68454942078224E-08**	0.301522979471157	0.662	0.577	0.000274766856023791	Pericentral hepatocytes	METTL7A
**1.71308161470083E-08**	0.351783032571416	0.237	0.163	0.000279420742173853	Pericentral hepatocytes	SAMM50
**2.08053572745415E-08**	0.447476181086884	0.359	0.265	0.000339356182505046	Pericentral hepatocytes	GADD45GIF

2.08318650132185E-08	0.457694540676135	0.364	0.271	0.000339788550230608	Pericentral hepatocytes	HIBADH
2.13368782924128E-08	0.503874837009948	0.294	0.213	0.000348025821827545	Pericentral hepatocytes	MDH2
2.23794611483053E-08	0.520052563625762	0.204	0.137	0.000365031390790007	Pericentral hepatocytes	ENPP7
2.27233581149963E-08	0.491368504949886	0.406	0.323	0.000370640694213705	Pericentral hepatocytes	AGMAT
2.3741760503675E-08	0.35012844580204	0.356	0.267	0.000387251855575443	Pericentral hepatocytes	SAP18
2.39510874183028E-08	0.418863444706093	0.472	0.39	0.000390666186879938	Pericentral hepatocytes	PSMB4
2.48386149127015E-08	0.30269239350567	0.659	0.587	0.000405142647841074	Pericentral hepatocytes	QPRT
2.57051587726386E-08	0.307829121237962	0.542	0.454	0.000419276844740508	Pericentral hepatocytes	PSMB8
2.58902765202021E-08	0.544104719096477	0.127	0.072	0.000422296300321016	Pericentral hepatocytes	SEMA7A
2.6445496674288E-08	0.374780223757212	0.641	0.57	0.000431352496254311	Pericentral hepatocytes	ADH1B
2.7366372278873E-08	0.613616492749788	0.369	0.286	0.000446372898240698	Pericentral hepatocytes	PCBD1
2.76855524831864E-08	0.315922850665767	0.635	0.573	0.000451579046553254	Pericentral hepatocytes	ALDH3A2
3.12837273246568E-08	0.368148705215403	0.407	0.321	0.000510268876392477	Pericentral hepatocytes	NAGS
3.41342723877095E-08	1.15411184592499	0.102	0.056	0.00055676411691593	Pericentral hepatocytes	CYP8B1
4.0968571000028E-08	0.376177065976447	0.578	0.507	0.000668238361581457	Pericentral hepatocytes	ZNHIT1
4.17145240424999E-08	0.469392674837819	0.493	0.405	0.000680405601657216	Pericentral hepatocytes	UBA52
4.22752190802684E-08	0.488667330353158	0.394	0.312	0.000689551098418258	Pericentral hepatocytes	HSD17B8
4.35826516482413E-08	0.25706597724568	0.167	0.104	0.000710876631034464	Pericentral hepatocytes	NMT2
4.37148276699157E-08	0.592168578289224	0.331	0.248	0.000713032554123995	Pericentral hepatocytes	GAGE1
4.40054220220223E-08	0.409234927665961	0.263	0.186	0.000717772438601206	Pericentral hepatocytes	FOXN3
4.42382124325306E-08	0.355568253466869	0.257	0.18	0.000721569482987006	Pericentral hepatocytes	TLCD4
4.50474720871453E-08	0.322595446033001	0.517	0.432	0.000734769317213427	Pericentral hepatocytes	CAMK2N1
4.73714767216504E-08	0.384363010427943	0.307	0.222	0.00077267615680684	Pericentral hepatocytes	NFIX
4.82925432802031E-08	0.329585257909833	0.614	0.531	0.000787699673443392	Pericentral hepatocytes	HAO1
4.87463001979885E-08	0.281641551788057	0.564	0.476	0.00079510090252939	Pericentral hepatocytes	CFL1
4.89908654496608E-08	0.456715801067378	0.398	0.317	0.000799090006349418	Pericentral hepatocytes	RAMP1
5.32588535086619E-08	0.267149593692097	0.741	0.716	0.000868705159579784	Pericentral hepatocytes	SERPINF1
5.88031293265078E-08	0.423533924780793	0.555	0.476	0.000959137842444669	Pericentral hepatocytes	MT-ND3
6.01506839120522E-08	0.28700741336194	0.284	0.205	0.000981117805289483	Pericentral hepatocytes	SARDH
6.26400938985642E-08	0.452950143059552	0.411	0.32	0.00102172257157948	Pericentral hepatocytes	MAOB
6.60896398724813E-08	0.376828899765613	0.375	0.29	0.00107798811596004	Pericentral hepatocytes	DEPRECAT
6.74541585113782E-08	0.457485334331664	0.238	0.164	0.00110024477947909	Pericentral hepatocytes	PEMT
6.83467723329954E-08	0.377804933427832	0.509	0.428	0.00111480420352349	Pericentral hepatocytes	ACY1
7.05217996514106E-08	0.279194966523548	0.705	0.664	0.00115028107411416	Pericentral hepatocytes	SERINC2
7.11777960289966E-08	0.44222518969212	0.33	0.245	0.00116098103102896	Pericentral hepatocytes	EDF1
7.84604111360341E-08	0.40251761772536	0.462	0.373	0.00127976776603985	Pericentral hepatocytes	ROMO1
7.93595003476407E-08	0.316471699216807	0.534	0.449	0.00129443281017037	Pericentral hepatocytes	ERGIC3
8.34342620349392E-08	0.321459049907213	0.663	0.642	0.00136089624805189	Pericentral hepatocytes	LRP1
8.49198325384216E-08	0.37603881984251	0.464	0.381	0.0013851273885342	Pericentral hepatocytes	GSTO1
8.90042653237196E-08	0.298622428463191	0.496	0.412	0.00145174857169519	Pericentral hepatocytes	RNF5
1.0394421988149E-07	0.398781366956982	0.174	0.112	0.00169543417048698	Pericentral hepatocytes	NRP1
1.1226020984396E-07	0.357332966291759	0.781	0.713	0.00183107628276483	Pericentral hepatocytes	MT-CO3
1.21405370341478E-07	0.39000837061975	0.437	0.353	0.00198024299563984	Pericentral hepatocytes	GALK1
1.21536017983859E-07	0.424618305051208	0.451	0.364	0.00198237398933472	Pericentral hepatocytes	ALDH4A1
1.28627287082811E-07	0.383714259225215	0.171	0.111	0.00209803967960774	Pericentral hepatocytes	SEZ6L2
1.30292191912148E-07	0.404051722957677	0.296	0.215	0.00212519594227905	Pericentral hepatocytes	INSIG2
1.36239626757317E-07	0.773667565657502	0.211	0.145	0.0022222045520386	Pericentral hepatocytes	DPP4
1.47596004935591E-07	0.374799313959369	0.52	0.441	0.00240743843650442	Pericentral hepatocytes	PRXL2A
1.59720867667363E-07	0.311197307506571	0.544	0.471	0.00260520707252235	Pericentral hepatocytes	CYP27A1
1.78206592478346E-07	0.485964503106041	0.229	0.16	0.0029067277299143	Pericentral hepatocytes	TMEM92
1.83580629304304E-07	0.641976149523865	0.14	0.087	0.00299438364458251	Pericentral hepatocytes	CCL20
1.88718150919603E-07	0.36513110406829	0.222	0.154	0.00307818175964965	Pericentral hepatocytes	FDFT1
1.93769098597425E-07	0.394118030893569	0.447	0.357	0.0031605677672226	Pericentral hepatocytes	ALDH6A1
1.9429641194758E-07	0.343719370613405	0.474	0.395	0.00316916877527697	Pericentral hepatocytes	PSMC3
1.98830743881572E-07	0.660177175282124	0.306	0.232	0.00324312826345233	Pericentral hepatocytes	TEX264
1.99369113057477E-07	0.743498674229785	0.158	0.101	0.00325190960308051	Pericentral hepatocytes	MTTP
2.1257917618108E-07	0.699089346960035	0.295	0.22	0.0034673789426896	Pericentral hepatocytes	MEA1
2.37464580799747E-07	0.554668694143045	0.125	0.075	0.00387328477742468	Pericentral hepatocytes	PPL
2.61916287974563E-07	0.343997703193725	0.444	0.362	0.00427211657315309	Pericentral hepatocytes	IFITM2
2.68935197680572E-07	0.396949640376945	0.135	0.082	0.00438660200936781	Pericentral hepatocytes	ARHGEF28

2.94800079743702E-07	0.628135371857049	0.36	0.281	0.00480848410069952	Pericentral hepatocytes	TOM1
3.09540102826206E-07	0.641604716520041	0.226	0.158	0.00504890861719825	Pericentral hepatocytes	DEPRECAT
3.1206759177409E-07	0.517040882886203	0.206	0.14	0.00509013448942719	Pericentral hepatocytes	DUT
3.57645057905675E-07	0.656686666222627	0.172	0.113	0.00583354853949947	Pericentral hepatocytes	NAT8
3.61278129458475E-07	0.560210514419222	0.25	0.181	0.00589280756959718	Pericentral hepatocytes	DBI
3.72901199245398E-07	0.343212972677288	0.458	0.377	0.00608239146089169	Pericentral hepatocytes	CITED4
3.7714738205121E-07	0.279686568575549	0.685	0.621	0.00615165094863728	Pericentral hepatocytes	RHOB
4.51316041568141E-07	0.382566018112849	0.41	0.326	0.00736141595401795	Pericentral hepatocytes	EID1
4.53863004922468E-07	0.333154164518954	0.4	0.318	0.00740295947329037	Pericentral hepatocytes	PSMC5
4.57753088436454E-07	0.564737731919303	0.321	0.244	0.00746641062548701	Pericentral hepatocytes	HSDL2
5.09828394304075E-07	0.381120557390678	0.708	0.664	0.00831581093949377	Pericentral hepatocytes	ACAA1
5.16374247093787E-07	0.382215573859265	0.418	0.335	0.00842258034434676	Pericentral hepatocytes	MSRB2
5.2453201015776E-07	0.325915068446119	0.535	0.453	0.00855564161768322	Pericentral hepatocytes	PCCB
6.34573394906263E-07	0.504345213506328	0.437	0.357	0.0103505266443161	Pericentral hepatocytes	PCDH1
6.75348762936991E-07	0.5780259305889	0.266	0.195	0.0110156136722653	Pericentral hepatocytes	RDH16
7.11427503494461E-07	0.343231046716804	0.451	0.367	0.0116040940094982	Pericentral hepatocytes	LHPP
7.49793791689811E-07	0.516684529122142	0.34	0.262	0.0122298865362525	Pericentral hepatocytes	SART1
7.66566922538234E-07	0.528876094483328	0.258	0.189	0.0125034730735211	Pericentral hepatocytes	PDLIM2
7.69804201263697E-07	0.293699989574395	0.515	0.432	0.0125562763268122	Pericentral hepatocytes	H3F3A
8.68579742959709E-07	0.486074452309124	0.218	0.152	0.0141674041874158	Pericentral hepatocytes	LRRC20
8.71375000969552E-07	0.729374971303495	0.115	0.069	0.0142129976408144	Pericentral hepatocytes	RBKS
9.53176688148219E-07	0.294087375463998	0.501	0.424	0.0155472649603856	Pericentral hepatocytes	CUTA
1.00037839559009E-06	0.553721061969055	0.21	0.146	0.01631717201047	Pericentral hepatocytes	TMEM160
1.06393537872918E-06	0.29554209018922	0.45	0.372	0.0173538499624517	Pericentral hepatocytes	ITPR2
1.09155484469053E-06	0.300390188173438	0.346	0.268	0.0178043510717473	Pericentral hepatocytes	ATP5F1E
1.12154024348384E-06	0.287313699504919	0.399	0.318	0.018293442911465	Pericentral hepatocytes	IQGAP2
1.13221677832494E-06	0.387282307708325	0.436	0.353	0.0184675878712582	Pericentral hepatocytes	NDUFA2
1.24683506212921E-06	0.284517526114943	0.513	0.443	0.0203371266983896	Pericentral hepatocytes	ECI2
1.26152656211784E-06	0.25767210933177	0.283	0.213	0.0205767597547042	Pericentral hepatocytes	TTPA
1.39255648074844E-06	0.379180867835647	0.325	0.248	0.0227139887574878	Pericentral hepatocytes	TALDO1
1.47458831596322E-06	0.390436836448541	0.363	0.289	0.0240520100216761	Pericentral hepatocytes	SCRN2
1.62052213785729E-06	0.33464122606471	0.428	0.346	0.0264323365905902	Pericentral hepatocytes	NMRAL1
1.6543651505463E-06	0.403854341709263	0.469	0.389	0.0269843499705608	Pericentral hepatocytes	SERP1
1.78941484475435E-06	0.314490161855599	0.405	0.321	0.0291871455327883	Pericentral hepatocytes	HSD3B7
1.89383114801781E-06	0.400876391259738	0.383	0.307	0.0308902798553185	Pericentral hepatocytes	ATP5F1A
1.9382355890546E-06	0.348063457533336	0.162	0.107	0.0316145606930696	Pericentral hepatocytes	ACSL3
2.0040638113231E-06	0.324493514431982	0.381	0.307	0.0326882848264911	Pericentral hepatocytes	MGST2
2.06688893068887E-06	0.280814402280609	0.427	0.352	0.0337130253484661	Pericentral hepatocytes	EEF1G
2.06971414547342E-06	0.397838231150366	0.354	0.278	0.033759107426817	Pericentral hepatocytes	FARSA
2.13655218911947E-06	0.451213590871169	0.411	0.332	0.0348493027567276	Pericentral hepatocytes	PPP4R2
2.14442085622055E-06	0.311330741961864	0.246	0.179	0.0349776485858134	Pericentral hepatocytes	USP5
2.20285160887766E-06	0.400991371242767	0.436	0.362	0.0359307125924035	Pericentral hepatocytes	AMACR
2.33380701754829E-06	0.293096446489246	0.37	0.294	0.0380667262632302	Pericentral hepatocytes	PIM1
2.34966288720561E-06	0.360177850407319	0.429	0.349	0.0383253513532107	Pericentral hepatocytes	SPCS1
2.50226080430372E-06	0.596742427239489	0.138	0.09	0.040814375978998	Pericentral hepatocytes	FOXRED2
2.64067897687588E-06	0.276288446330179	0.474	0.399	0.0430721147918224	Pericentral hepatocytes	MAGED2
2.64777959004858E-06	0.414065244672076	0.188	0.132	0.0431879328932823	Pericentral hepatocytes	PHB
2.67161551756732E-06	0.417236851182295	0.391	0.312	0.0435767207070406	Pericentral hepatocytes	COX17
2.7220198681096E-06	0.2865851104756	0.505	0.437	0.0443988660687356	Pericentral hepatocytes	ACBD5
2.92590372152834E-06	0.259004397644121	0.584	0.516	0.0477244156018487	Pericentral hepatocytes	C8A
0	4.12283918099406	0.786	0.057	0	Periportal hepatocytes	HGFAC
0	4.02800670687062	0.842	0.126	0	Periportal hepatocytes	CPS1
6.39592840294889E-304	2.59881541133863	0.851	0.231	1.04323988180499E-299	Periportal hepatocytes	INSIG1
1.40586044844508E-281	2.33622391143276	0.54	0.061	2.29309897745876E-277	Periportal hepatocytes	C9
3.42458932241029E-279	3.73862436491755	0.611	0.097	5.58584764378342E-275	Periportal hepatocytes	DEPRECAT
5.0130575740015E-271	2.77741013003292	0.876	0.305	8.17679820895384E-267	Periportal hepatocytes	SDS
1.87908779054543E-263	3.8218891457841	0.375	0.024	3.06498009515865E-259	Periportal hepatocytes	AQP1
6.68573898896994E-259	3.35519332584054	0.846	0.283	1.09051088649089E-254	Periportal hepatocytes	CYP2B6
4.58756085496427E-249	2.42188556217149	0.825	0.253	7.48277051053222E-245	Periportal hepatocytes	BHMT
1.37146160701792E-219	2.86456995054059	0.638	0.146	2.23699102720693E-215	Periportal hepatocytes	MUC3A

6.31558636898042E-209	2.61899683018601	0.576	0.116	1.0301352926444E-204	Periportal hepatocytes	NR1I3
3.2720083545289E-204	2.12407618554534	0.754	0.224	5.33697282707209E-200	Periportal hepatocytes	GLS2
1.60878681135059E-190	2.91107787827821	0.394	0.049	2.62409216799394E-186	Periportal hepatocytes	CES4A
2.48557755263344E-188	1.51126883503071	0.951	0.552	4.0542255461004E-184	Periportal hepatocytes	GPT
6.9964894762696E-187	2.70538291081055	0.316	0.028	1.14119739847433E-182	Periportal hepatocytes	UROC1
1.59142672218496E-176	2.10078407140595	0.438	0.07	2.59577612655588E-172	Periportal hepatocytes	DUOX2
2.20982373546413E-176	1.56930007074877	0.886	0.4	3.60444349491554E-172	Periportal hepatocytes	NNMT
6.87074844301063E-169	1.58801151868485	0.67	0.209	1.12068777853946E-164	Periportal hepatocytes	RIDA
2.6130180423629E-164	2.49468027904545	0.856	0.398	4.26209372889813E-160	Periportal hepatocytes	CRP
5.79492151703461E-163	1.51710416080272	0.772	0.311	9.45209648643515E-159	Periportal hepatocytes	TMEM176A
7.11602203556625E-163	3.51923605271747	0.247	0.017	1.16069435422121E-158	Periportal hepatocytes	SULT1C2
1.04737488538606E-158	1.46735856952156	0.838	0.365	1.7083731755532E-154	Periportal hepatocytes	AMDHD1
6.61872814566426E-155	2.39283999326092	0.608	0.177	1.0795807478393E-150	Periportal hepatocytes	AASS
2.6661665548618E-154	1.39384891952365	0.691	0.23	4.34878426763508E-150	Periportal hepatocytes	ST3GAL1
6.84209702175185E-152	1.42128458671184	0.818	0.371	1.11601444521794E-147	Periportal hepatocytes	SERPINA6
5.39107505139142E-148	1.65322298515071	0.886	0.464	8.79338251632454E-144	Periportal hepatocytes	PCK1
7.47168867502406E-147	1.66709997005188	0.644	0.201	1.21870713978318E-142	Periportal hepatocytes	FETUB
1.06665051554296E-144	1.35871465076366	0.865	0.437	1.73981365590213E-140	Periportal hepatocytes	HPD
7.16878766034585E-144	1.19817632109294	0.918	0.618	1.16930095527901E-139	Periportal hepatocytes	TMEM176B
3.6832996361539E-142	1.30687693873699	0.914	0.486	6.00783003653063E-138	Periportal hepatocytes	ARG1
1.40203508329334E-137	1.93374884398747	0.705	0.256	2.28685942435977E-133	Periportal hepatocytes	CHRD
1.41623981208012E-135	3.09959343204266	0.256	0.027	2.31002875748388E-131	Periportal hepatocytes	NLRP1
4.41257029475724E-133	2.2571475246128	0.316	0.046	7.19734340777854E-129	Periportal hepatocytes	DUOXA2
8.86851032065888E-130	1.90319730685955	0.603	0.193	1.44654271840267E-125	Periportal hepatocytes	RAPH1
4.22898383623302E-129	1.18020306612729	0.875	0.433	6.89789553527968E-125	Periportal hepatocytes	ALDH1L1
1.13129360087419E-128	1.6957278680913	0.539	0.151	1.8452529923859E-124	Periportal hepatocytes	OXER1
1.36936818043004E-128	2.54306921306731	0.402	0.083	2.23357643909943E-124	Periportal hepatocytes	CFHR3
1.91785289647025E-127	1.64060495457228	0.695	0.259	3.12820985943263E-123	Periportal hepatocytes	PWWP3A
3.58672531432622E-127	1.54645596104065	0.848	0.417	5.85030766019749E-123	Periportal hepatocytes	HAL
6.8620779567132E-127	3.55450876842255	0.187	0.012	1.11927353551949E-122	Periportal hepatocytes	UPP2
1.81555372233283E-126	1.80252768288112	0.282	0.037	2.96134967649708E-122	Periportal hepatocytes	LAPTM4B
4.25027936367887E-126	2.01040808866149	0.355	0.064	6.9326306700966E-122	Periportal hepatocytes	AMOTL1
7.60718206111356E-124	1.46152913918435	0.739	0.304	1.24080746598823E-119	Periportal hepatocytes	CDO1
1.01924069156406E-122	1.14602893718069	0.576	0.177	1.66248349201014E-118	Periportal hepatocytes	TTPA
3.03473204871256E-121	1.20484304006224	0.743	0.311	4.94995144465505E-117	Periportal hepatocytes	GNMT
3.48634892690265E-121	1.44056311103689	0.884	0.499	5.68658373467091E-117	Periportal hepatocytes	SLC38A4
6.1810636017028E-121	1.66106557500506	0.664	0.247	1.00819328407374E-116	Periportal hepatocytes	GRAMD4
3.1523340315407E-120	1.4569135140051	0.816	0.384	5.14177203884604E-116	Periportal hepatocytes	GPT2
2.8902518521999E-119	1.34251551729513	0.813	0.39	4.71428979612326E-115	Periportal hepatocytes	G6PC
6.33492629838868E-117	1.54639235238057	0.784	0.376	1.03328982853018E-112	Periportal hepatocytes	CYP2C9
7.06889883823457E-116	1.97360040184371	0.47	0.126	1.15300808950444E-111	Periportal hepatocytes	DEPDC7
2.55347450460789E-115	1.20136133980224	0.749	0.296	4.16497226446594E-111	Periportal hepatocytes	COL5A3
5.23376798817732E-115	1.3533086606253	0.894	0.466	8.53679896551603E-111	Periportal hepatocytes	PLIN5
2.46276609690888E-114	1.40118291608746	0.892	0.63	4.01701778066808E-110	Periportal hepatocytes	HRG
3.92247682512959E-114	1.35615768983835	0.576	0.19	6.39795194946887E-110	Periportal hepatocytes	CCL16
2.57411786964797E-113	1.20101714210096	0.58	0.196	4.1986436571828E-109	Periportal hepatocytes	RBP5
3.06519287570759E-113	1.18888974023316	0.675	0.25	4.99963609956665E-109	Periportal hepatocytes	SLC27A5
9.11062861143044E-113	1.46109789197616	0.437	0.108	1.48603463281042E-108	Periportal hepatocytes	SAA4
7.15452436958014E-111	1.58488118309586	0.527	0.163	1.16697446992222E-106	Periportal hepatocytes	RDH16
4.13089035042862E-109	2.36222292529886	0.348	0.073	6.73789525058412E-105	Periportal hepatocytes	HPGD
8.66026323113168E-108	1.24174369960258	0.625	0.23	1.41257553562989E-103	Periportal hepatocytes	SLC51A
5.80531392230903E-107	1.29754889400439	0.874	0.447	9.46904753867826E-103	Periportal hepatocytes	TP53INP1
8.59640566024875E-107	1.56028360554977	0.359	0.077	1.40215972724317E-102	Periportal hepatocytes	MTTP
7.49031819545908E-105	2.14317270784759	0.266	0.042	1.22174580086133E-100	Periportal hepatocytes	CYP7A1
9.02226633424944E-103	1.08342424146515	0.978	0.674	1.47162186177943E-98	Periportal hepatocytes	PAH
3.04447822715876E-102	1.31604181838498	0.506	0.161	4.96584843631865E-98	Periportal hepatocytes	APOF
3.52045661886498E-102	1.11255479491061	0.534	0.172	5.74221679103067E-98	Periportal hepatocytes	CCL15
1.67629332734296E-101	1.12524209969652	0.654	0.267	2.7342020462291E-97	Periportal hepatocytes	SDSL
3.02801828660911E-101	1.09622761645881	0.955	0.617	4.93900062728812E-97	Periportal hepatocytes	GLYCTK
4.02329380592519E-101	1.18795358064938	0.682	0.274	6.56239452684458E-97	Periportal hepatocytes	SLC25A18

1.10101337942844E-100	0.998284558811858	0.856	0.465	1.79586292318572E-96	Periportal hepatocytes	DPYS
3.65919210232733E-98	1.02424601424553	0.991	0.946	5.9685082381061E-94	Periportal hepatocytes	PLA2G2A
4.31809967746019E-97	1.24168147238943	0.761	0.356	7.04325238390532E-93	Periportal hepatocytes	P4HA1
9.33760868981112E-97	1.14856742980252	0.53	0.18	1.52305735339509E-92	Periportal hepatocytes	PBLD
5.3665125134721E-96	1.50602774357083	0.486	0.15	8.75331856072433E-92	Periportal hepatocytes	ARHGEF26
3.35177793498452E-95	0.825121319848715	0.678	0.288	5.46708498975326E-91	Periportal hepatocytes	MAOB
6.77717526510615E-95	1.35524683374083	0.477	0.143	1.10542505749146E-90	Periportal hepatocytes	CDK18
1.22480352982325E-94	1.20534568542728	0.76	0.345	1.9977770374947E-90	Periportal hepatocytes	FTCD
1.86645563416293E-94	1.0146724050698	0.625	0.242	3.04437578488316E-90	Periportal hepatocytes	ALDH8A1
7.73616995070564E-94	0.880552519553423	0.733	0.369	1.2618466806596E-89	Periportal hepatocytes	REEP6
1.109899250502E-93	1.59915682587466	0.382	0.097	1.81035666749381E-89	Periportal hepatocytes	SLC15A1
2.72263925837558E-93	1.31245440112724	0.514	0.167	4.44089689433642E-89	Periportal hepatocytes	CXCL2
2.8485192686098E-93	0.867086089999364	0.688	0.306	4.64621977902945E-89	Periportal hepatocytes	RARRES2
4.01133604686841E-93	1.00753037557193	0.876	0.494	6.54289022604707E-89	Periportal hepatocytes	ACSL1
4.9176702580737E-93	2.6736684038988	0.244	0.04	8.02121195794401E-89	Periportal hepatocytes	SPATA21
2.98535509574151E-92	1.23172831928921	0.826	0.413	4.86941269666398E-88	Periportal hepatocytes	MYOM1
4.81420983414416E-92	0.856419458084399	0.634	0.268	7.85245766047254E-88	Periportal hepatocytes	DECR1
5.10157140255998E-92	1.42127062375115	0.49	0.158	8.32117311471558E-88	Periportal hepatocytes	AGMO
1.30527507000014E-91	1.06666119135947	0.691	0.305	2.12903416667723E-87	Periportal hepatocytes	HAAO
1.55473585407699E-91	3.82784109410887	0.22	0.033	2.53592965158497E-87	Periportal hepatocytes	DEPRECAT
6.16145212287983E-91	1.1495885082338	0.543	0.194	1.00499445576293E-86	Periportal hepatocytes	RNF152
1.28236482771036E-90	2.36991364178389	0.241	0.04	2.09166527047837E-86	Periportal hepatocytes	SLC22A31
2.0028086036537E-90	1.45872892683185	0.67	0.286	3.26678111341955E-86	Periportal hepatocytes	FST
1.50732989025631E-88	2.43525272679138	0.226	0.036	2.45860578399707E-84	Periportal hepatocytes	DEPRECAT
2.53960368595579E-88	0.85600382996772	0.691	0.316	4.14234757216249E-84	Periportal hepatocytes	SLC22A1
3.12885573138886E-88	0.987195441690761	0.932	0.6	5.10347658346836E-84	Periportal hepatocytes	HGD
2.51180278252239E-87	0.94353220093201	0.776	0.418	4.09700151857226E-83	Periportal hepatocytes	TOB1
1.45794169002313E-85	1.12308841530581	0.657	0.278	2.37804869059673E-81	Periportal hepatocytes	RND1
8.325158793936E-85	0.750792449751002	0.974	0.821	1.3579166508789E-80	Periportal hepatocytes	CP
4.88783916665474E-83	1.27388288458201	0.615	0.249	7.97255446473055E-79	Periportal hepatocytes	MUC20
6.19816289996926E-83	0.788592883725505	0.861	0.568	1.01098235061399E-78	Periportal hepatocytes	DEPRECAT
8.03736352654638E-83	1.07222602134891	0.601	0.244	1.31097436481498E-78	Periportal hepatocytes	PC
1.63197293079526E-82	0.808930252125671	0.575	0.225	2.66191104742014E-78	Periportal hepatocytes	SIAH2
3.22849344232169E-82	1.64196656970545	0.5	0.176	5.26599565377091E-78	Periportal hepatocytes	KCNT2
5.2462497336909E-81	1.17149745615486	0.448	0.148	8.55715794062324E-77	Periportal hepatocytes	AGXT2
5.86620844696725E-81	1.16283973564298	0.432	0.137	9.56837259784828E-77	Periportal hepatocytes	CPNE3
1.14444740930162E-80	1.31865994111833	0.326	0.081	1.86670816931187E-76	Periportal hepatocytes	SAA2
1.41420714608658E-80	0.594451502885654	0.49	0.171	2.30671327598182E-76	Periportal hepatocytes	GGH
6.72273181113128E-80	0.719186785917493	0.839	0.496	1.09654478571362E-75	Periportal hepatocytes	C8G
1.4072592879035E-79	1.01704795108877	0.909	0.632	2.2953806244994E-75	Periportal hepatocytes	TAT
2.41323545457673E-79	2.04766477377798	0.455	0.16	3.9362283499601E-75	Periportal hepatocytes	CTH
3.17185496595587E-79	0.716598013720951	0.441	0.144	5.17361263497061E-75	Periportal hepatocytes	LIME1
4.36012945070413E-79	0.891681386144368	0.813	0.429	7.1118071470435E-75	Periportal hepatocytes	SAT1
8.80781290122298E-79	3.12479882645854	0.141	0.013	1.43664236231848E-74	Periportal hepatocytes	PCSK9
1.97269720404247E-78	1.30393614885984	0.514	0.188	3.21766640951368E-74	Periportal hepatocytes	PLOD2
4.98566992571454E-78	0.912949842645712	0.931	0.589	8.13212621583299E-74	Periportal hepatocytes	ITIH4
1.33194560677936E-77	0.923019572727338	0.721	0.363	2.17253647921782E-73	Periportal hepatocytes	EGFR
1.84737133641008E-77	0.991388403699092	0.615	0.266	3.01324738681848E-73	Periportal hepatocytes	TMEM45A
2.7950103303413E-77	0.865066383694539	0.976	0.757	4.55894134981969E-73	Periportal hepatocytes	MAT1A
1.38680799604334E-76	1.08746119610348	0.563	0.226	2.26202252234629E-72	Periportal hepatocytes	ACSM2B
2.72957971273986E-76	0.770422820124949	0.836	0.537	4.45221746944998E-72	Periportal hepatocytes	DCXR
3.87797509139135E-76	0.659698355781876	0.999	0.992	6.32536517156843E-72	Periportal hepatocytes	FGA
7.05466975914431E-76	1.06204488831869	0.578	0.234	1.15068718441403E-71	Periportal hepatocytes	BCAT2
1.03264463151122E-75	0.954655525147012	0.698	0.348	1.68434665845795E-71	Periportal hepatocytes	CFHR4
1.51948916767994E-75	1.46561297770942	0.277	0.062	2.47843878140275E-71	Periportal hepatocytes	AGTR1
8.84680476495921E-75	1.21586739365985	0.368	0.107	1.4430023252125E-70	Periportal hepatocytes	NDUFAF6
9.89725365219901E-75	1.23526911585766	0.457	0.157	1.61434104321018E-70	Periportal hepatocytes	IL1RAP
2.07262775690656E-74	0.614619709620135	0.994	0.993	3.38066313429029E-70	Periportal hepatocytes	FGB
4.93986699100564E-74	0.829807945719157	0.605	0.26	8.0574170490293E-70	Periportal hepatocytes	CPQ
5.19840013327989E-74	2.42614625066196	0.157	0.019	8.47911045739283E-70	Periportal hepatocytes	CLRN3

6.62455072380494E-74	1.41265105418066	0.438	0.149	1.08053046855982E-69	Periportal hepatocytes	MTMR10
7.79635273536509E-74	1.13460013951698	0.552	0.226	1.2716630946654E-69	Periportal hepatocytes	SAA1
2.72458430047589E-73	0.973097671745472	0.835	0.484	4.44406945250622E-69	Periportal hepatocytes	ACSM2A
8.98689147155856E-73	1.0717557635762	0.707	0.333	1.46585186792592E-68	Periportal hepatocytes	CYP4F3
3.69335158297439E-72	0.698872888515015	0.68	0.335	6.02422576698953E-68	Periportal hepatocytes	PIPOX
3.89013163164053E-72	0.676814761241243	0.632	0.291	6.34519370436887E-68	Periportal hepatocytes	SDC2
4.23928802193793E-72	1.14527691598359	0.361	0.106	6.91470269258296E-68	Periportal hepatocytes	ACMSD
6.14182539096347E-72	1.4650977756759	0.193	0.031	1.00179313952005E-67	Periportal hepatocytes	PHGDH
6.49151104534454E-72	0.828149158736643	0.641	0.287	1.05883036660615E-67	Periportal hepatocytes	SLC22A18
7.98918030407633E-72	0.519935605685646	0.687	0.337	1.30311519939789E-67	Periportal hepatocytes	ALDH4A1
8.8615053029907E-72	1.29641279598885	0.399	0.13	1.44540012997081E-67	Periportal hepatocytes	MYO1E
1.11373307221366E-71	0.903741647720996	0.845	0.455	1.8166100140877E-67	Periportal hepatocytes	MST1
1.45114089927252E-71	0.724927138382781	0.874	0.541	2.36695592080341E-67	Periportal hepatocytes	CFI
1.49157374541767E-71	0.722940323343024	0.553	0.225	2.43290593615076E-67	Periportal hepatocytes	PPP1R1A
1.97116821729469E-71	0.534474049308108	0.655	0.32	3.21517247922937E-67	Periportal hepatocytes	C7orf50
2.13766352652552E-71	1.27687168040276	0.659	0.307	3.48674297811577E-67	Periportal hepatocytes	SLC23A2
2.21166386705011E-71	1.39782031464432	0.434	0.149	3.60744493354543E-67	Periportal hepatocytes	RCL1
2.33916804519646E-71	0.857190039683425	0.355	0.104	3.81541699851994E-67	Periportal hepatocytes	TIMP3
3.61949715804983E-71	1.63201162064545	0.353	0.105	5.90376181449507E-67	Periportal hepatocytes	SDCBP2
5.89140551098055E-71	2.57109249530557	0.112	0.008	9.60947152896037E-67	Periportal hepatocytes	CREB3L1
6.36492949891358E-71	1.16495583164112	0.612	0.266	1.03818365056779E-66	Periportal hepatocytes	TMEM184A
6.64586896604771E-71	0.695808846310817	0.428	0.143	1.08400768705204E-66	Periportal hepatocytes	UQCRB
7.14777028447057E-71	0.889268617032008	0.497	0.187	1.1658728111E-66	Periportal hepatocytes	HYAL1
1.33478325568679E-70	0.813052038386355	0.756	0.395	2.17716496835073E-66	Periportal hepatocytes	F13B
2.56165002016861E-70	1.03991926783151	0.54	0.22	4.17830734789702E-66	Periportal hepatocytes	ASPG
3.31984372427597E-70	2.08781189729022	0.172	0.025	5.41499709866653E-66	Periportal hepatocytes	ECM2
6.61870128828768E-70	2.29069146849543	0.151	0.019	1.0795763671326E-65	Periportal hepatocytes	AATK
1.34512859723809E-69	0.689588256836292	0.958	0.727	2.19403925495505E-65	Periportal hepatocytes	APCS
1.51431268312987E-69	1.53992713697493	0.443	0.158	2.46999541745314E-65	Periportal hepatocytes	FYB2
6.52815857211272E-69	0.922118886442546	0.684	0.322	1.06480794469731E-64	Periportal hepatocytes	COBLL1
7.11574360978607E-69	0.997195639338154	0.563	0.238	1.16064894019221E-64	Periportal hepatocytes	TRIM55
7.88501819976183E-69	0.843769239270652	0.603	0.267	1.28612531856315E-64	Periportal hepatocytes	ELL2
5.424596900923E-68	0.88777198458459	0.348	0.103	8.8480600050955E-64	Periportal hepatocytes	AKR1C4
5.93640578932789E-68	1.74547824674814	0.263	0.062	9.68287148297272E-64	Periportal hepatocytes	NINL
1.26009140176839E-67	1.10323665317904	0.516	0.205	2.05533508542442E-63	Periportal hepatocytes	KMT2C
7.6113196100261E-67	0.98245326480993	0.682	0.328	1.24148234159136E-62	Periportal hepatocytes	FAM20A
1.7021068836508E-66	1.1232491928696	0.394	0.132	2.77630653792283E-62	Periportal hepatocytes	FBP1
3.25631879351917E-66	0.740949150775128	0.636	0.308	5.31138158410912E-62	Periportal hepatocytes	LGALS4
2.49032758079815E-65	0.808131054690232	0.665	0.326	4.06197331703986E-61	Periportal hepatocytes	STEAP3
2.98309083415389E-65	1.36748756255668	0.348	0.107	4.86571945958842E-61	Periportal hepatocytes	LCN2
3.35003610650042E-65	1.45734822612721	0.394	0.135	5.46424389331284E-61	Periportal hepatocytes	MAST4
6.26763900765308E-65	1.81985850711475	0.19	0.034	1.02231459853829E-60	Periportal hepatocytes	WDR72
9.31773748593967E-65	1.02492300851413	0.782	0.419	1.51981616133162E-60	Periportal hepatocytes	CYP21A2
1.84548265972464E-64	0.858281422706798	0.53	0.214	3.01016676627687E-60	Periportal hepatocytes	LMO7
3.50106872247085E-64	0.716625331697181	0.509	0.199	5.7105931932222E-60	Periportal hepatocytes	TSPAN8
3.73441982538096E-64	0.900270354712006	0.499	0.199	6.09121217717888E-60	Periportal hepatocytes	CYP7B1
5.2665191258364E-64	0.642182068513317	0.767	0.421	8.59021934615175E-60	Periportal hepatocytes	PAPSS2
7.98642435433858E-64	0.878737881305754	0.966	0.666	1.30266567643617E-59	Periportal hepatocytes	C4BPB
1.07453108840434E-63	0.881504534266291	0.361	0.115	1.75266765829633E-59	Periportal hepatocytes	CYP51A1
3.69829903688281E-63	0.931704835017279	0.305	0.085	6.03229555905955E-59	Periportal hepatocytes	ADH6
4.61705045990099E-63	2.35087599171908	0.201	0.04	7.53087100514451E-59	Periportal hepatocytes	ASB9
5.8854621499591E-63	1.03279765027276	0.211	0.043	9.59977731279829E-59	Periportal hepatocytes	METRN
7.80234962589476E-63	0.861954844885518	0.483	0.187	1.27264124747969E-58	Periportal hepatocytes	SMARCA1
1.71952144129232E-62	0.982183552082306	0.579	0.253	2.8047114228919E-58	Periportal hepatocytes	ADH4
2.14298539577573E-62	0.92458990761405	0.78	0.418	3.4954234790498E-58	Periportal hepatocytes	CEP72
3.63408363012741E-62	1.50471423602455	0.184	0.034	5.92755380910082E-58	Periportal hepatocytes	FXYD1
4.7750018740529E-62	1.01007668793991	0.46	0.178	7.78850555676769E-58	Periportal hepatocytes	FARP1
6.10732210435868E-62	1.02448729104512	0.602	0.281	9.96165308441944E-58	Periportal hepatocytes	SORBS2
7.11390799123137E-62	0.768558521941693	0.507	0.209	1.16034953244975E-57	Periportal hepatocytes	ACAT2
7.58423109476697E-62	0.951897335019053	0.648	0.31	1.23706393386744E-57	Periportal hepatocytes	RBM33

**1.12137464071376E-61**	1.09658534849709	0.636	0.306	1.82907417646821E-57	Periportal hepatocytes	ADHFE1
**1.23751573523542E-61**	0.900789996309106	0.375	0.126	2.0185119157425E-57	Periportal hepatocytes	MCRIP1
**4.5479355293433E-61**	0.361222254759667	0.408	0.147	7.41813764191186E-57	Periportal hepatocytes	UQCR11
**6.56720620778663E-61**	0.717798482440557	0.655	0.321	1.07117700455208E-56	Periportal hepatocytes	ACO1
**7.79481920625447E-61**	0.648600824011654	0.777	0.44	1.27141296073217E-56	Periportal hepatocytes	C8B
**2.57208322092972E-60**	1.29046667136203	0.164	0.027	4.19532494165846E-56	Periportal hepatocytes	ALDOC
**5.38712619671882E-60**	0.540636278458132	0.625	0.31	8.78694153946806E-56	Periportal hepatocytes	BTG1
**6.55134135677002E-60**	1.59675521858037	0.187	0.036	1.06858928870276E-55	Periportal hepatocytes	SLC2A1
**8.57142579103453E-60**	0.696409302464478	0.379	0.127	1.39808526077564E-55	Periportal hepatocytes	LECT2
**1.07689663878343E-59**	0.831025111643184	0.736	0.395	1.75652610751965E-55	Periportal hepatocytes	STAT2
**1.13824033622904E-59**	0.687147856701637	0.894	0.645	1.85658381242318E-55	Periportal hepatocytes	LBP
**1.1600656450092E-59**	1.06348517893085	0.401	0.145	1.8921830735745E-55	Periportal hepatocytes	CYP4V2
**1.18129237146883E-59**	2.08096668246845	0.148	0.022	1.92680598710281E-55	Periportal hepatocytes	HEPACAM
**3.16414832783883E-59**	0.905044189484106	0.545	0.24	5.16104233753791E-55	Periportal hepatocytes	KYNU
**3.51825022414638E-59**	1.62150622751353	0.138	0.019	5.73861794060515E-55	Periportal hepatocytes	CHST7
**3.88180113848553E-59**	0.745453325980892	0.881	0.643	6.33160583698375E-55	Periportal hepatocytes	IGFBP2
**4.32752711166398E-59**	0.623626861878953	0.545	0.23	7.05862947183512E-55	Periportal hepatocytes	FASTK
**4.91623548770629E-59**	0.972912514399646	0.473	0.191	8.01887170399773E-55	Periportal hepatocytes	NUDT7
**6.49370219948771E-59**	0.461289276039769	0.688	0.371	1.05918776575844E-54	Periportal hepatocytes	LY6E
**7.40002065291933E-59**	0.837204235091822	0.572	0.261	1.20701736869767E-54	Periportal hepatocytes	SLC43A3
**7.81001471527722E-59**	0.871501907480453	0.851	0.475	1.27389150020887E-54	Periportal hepatocytes	OBSCN
**8.26185741951069E-59**	0.735134290733773	0.56	0.249	1.34759156369639E-54	Periportal hepatocytes	CNN3
**8.94435850914422E-59**	1.0964856278985	0.438	0.168	1.45891431642651E-54	Periportal hepatocytes	DEPRECAT
**3.13469500765566E-58**	1.02966169617596	0.3	0.088	5.11300102698715E-54	Periportal hepatocytes	PDE3B
**3.35448933307975E-58**	1.39810980356867	0.249	0.064	5.47150755118638E-54	Periportal hepatocytes	SYBU
**4.50343210611706E-58**	0.51800788835668	0.503	0.209	7.34554810828753E-54	Periportal hepatocytes	AK4
**8.63290873892146E-58**	0.677125949133489	0.29	0.083	1.40811374440548E-53	Periportal hepatocytes	LCAT
**9.93278665828131E-58**	1.25512352469135	0.389	0.142	1.62013683183226E-53	Periportal hepatocytes	FES
**1.14532146774767E-57**	1.17118935853038	0.254	0.066	1.86813384604322E-53	Periportal hepatocytes	RMDN1
**1.61918292597244E-57**	0.744761311633909	0.92	0.76	2.64104927055364E-53	Periportal hepatocytes	SERPINA3
**2.11775503146392E-57**	1.14272856272909	0.496	0.21	3.4542702318208E-53	Periportal hepatocytes	ABCA5
**2.4031747206338E-57**	0.68837572190953	0.614	0.296	3.91981828682579E-53	Periportal hepatocytes	CYP4F11
**2.59511036652328E-57**	1.07360145674809	0.254	0.067	4.23288451883612E-53	Periportal hepatocytes	PCOLCE
**3.33355135607671E-57**	2.16729537896863	0.132	0.018	5.43735561689671E-53	Periportal hepatocytes	SLC39A5
**3.54437610484341E-57**	1.22188456672636	0.276	0.076	5.78123186461008E-53	Periportal hepatocytes	SNX19
**4.74635353762591E-57**	0.404027641386794	0.533	0.231	7.74177725522163E-53	Periportal hepatocytes	ASPDH
**7.10109216857479E-57**	0.680450626405521	0.856	0.465	1.15825914361623E-52	Periportal hepatocytes	ABCC2
**7.24435946703389E-57**	0.723759584872558	0.645	0.343	1.1816274726679E-52	Periportal hepatocytes	SLPI
**1.28723502697645E-56**	0.755150317793009	0.707	0.363	2.09960905250128E-52	Periportal hepatocytes	F11
**1.3642647317793E-56**	0.563027091547507	0.651	0.335	2.22525220400522E-52	Periportal hepatocytes	ALDH6A1
**1.36908997480301E-56**	0.749142376655198	0.816	0.458	2.2331226579012E-52	Periportal hepatocytes	FNDC3B
**1.61536036698933E-56**	0.967296424169847	0.315	0.098	2.6348142945963E-52	Periportal hepatocytes	LIMA1
**1.91518067207215E-56**	0.491800126250168	0.714	0.401	3.12385119421688E-52	Periportal hepatocytes	GRHPR
**2.93254086398806E-56**	0.651734578128072	0.659	0.338	4.78326740325092E-52	Periportal hepatocytes	GOT1
**4.02563145105628E-56**	0.859111253772588	0.647	0.313	6.56620745981789E-52	Periportal hepatocytes	SRRM2
**4.1255874139745E-56**	0.423722201664355	0.678	0.355	6.72924563093381E-52	Periportal hepatocytes	MTDH
**8.04174371477471E-56**	0.740788263460915	0.55	0.251	1.3116888173169E-51	Periportal hepatocytes	MCCC1
**9.41295197676708E-56**	0.344417515866421	0.734	0.403	1.53534659693048E-51	Periportal hepatocytes	LGALS3BP
**1.02248023198071E-55**	0.488667037230265	0.691	0.355	1.66776750638374E-51	Periportal hepatocytes	FGGY
**1.04365541603273E-55**	1.63278641089287	0.213	0.049	1.70230634909099E-51	Periportal hepatocytes	MAP3K5
**1.37902414402011E-55**	1.36800140820427	0.234	0.059	2.2493262813112E-51	Periportal hepatocytes	LDLRAD1
**2.96172859211763E-55**	0.525046283224912	0.451	0.179	4.83087550660306E-51	Periportal hepatocytes	GCHFR
**4.88960294544333E-55**	0.482727800622365	0.79	0.479	7.97543136431261E-51	Periportal hepatocytes	IFI27
**4.93019356721231E-55**	0.937342795082063	0.582	0.269	8.04163872748E-51	Periportal hepatocytes	ABCG5
**5.58744604096881E-55**	0.805371036199845	0.24	0.061	9.11368323742423E-51	Periportal hepatocytes	VIRMA
**6.16445011294328E-55**	1.4353593784511	0.328	0.108	1.00548345792218E-50	Periportal hepatocytes	DAPK1
**8.5748946039589E-55**	1.00835995886102	0.516	0.224	1.39865105885174E-50	Periportal hepatocytes	IL17RB
**9.58825841448493E-55**	0.458933230418961	0.513	0.221	1.56394082998664E-50	Periportal hepatocytes	DHTKD1
**1.20059174236908E-54**	0.615240060829295	0.754	0.43	1.9582851909782E-50	Periportal hepatocytes	C6
**1.27948906054384E-54**	0.335535386837333	0.614	0.303	2.08697460665306E-50	Periportal hepatocytes	TRAM1

**1.53210857012729E-54**	2.11195125198386	0.151	0.026	2.49902228873461E-50	Periportal hepatocytes	DCDC1
**2.1397846978224E-54**	0.571261642862031	0.43	0.171	3.49020282061811E-50	Periportal hepatocytes	CIDEB
**2.21901256750408E-54**	0.725322285453614	0.649	0.325	3.6194313988559E-50	Periportal hepatocytes	SLCO2B1
**2.82147504743055E-54**	0.759391732909871	0.444	0.178	4.60210794986398E-50	Periportal hepatocytes	AS3MT
**4.58277215158453E-54**	0.744806630743151	0.805	0.462	7.47495965644952E-50	Periportal hepatocytes	LEPR
**4.96978806584769E-54**	0.627547206794606	0.638	0.319	8.10622131420417E-50	Periportal hepatocytes	ZNRF2
**5.46826928792289E-54**	0.724601461864536	0.739	0.389	8.91929403553102E-50	Periportal hepatocytes	BCL3
**5.47900505299236E-54**	2.77556369794936	0.128	0.018	8.93680514193585E-50	Periportal hepatocytes	MRC1
**8.16448474208729E-54**	0.84361335869023	0.434	0.175	1.33170910628186E-49	Periportal hepatocytes	FAXDC2
**1.06295497816707E-53**	0.459869986818016	0.658	0.336	1.73378586488831E-49	Periportal hepatocytes	FAM20C
**1.29971279675789E-53**	1.45591718805429	0.28	0.083	2.11996154279179E-49	Periportal hepatocytes	PLEKHF2
**1.66881383520008E-53**	0.518171902161564	0.754	0.419	2.72200224659485E-49	Periportal hepatocytes	ABAT
**6.77031534991408E-53**	0.38460498670266	0.734	0.407	1.10430613672449E-48	Periportal hepatocytes	EHHADH
**7.6239047928353E-53**	0.785500288472085	0.516	0.231	1.24353511075937E-48	Periportal hepatocytes	PLGLB1
**9.17387270070947E-53**	1.33004092465375	0.424	0.175	1.49635037621272E-48	Periportal hepatocytes	MUC13
**1.2416928199216E-52**	0.765994093657117	0.382	0.139	2.02532515857413E-48	Periportal hepatocytes	RNF13
**1.91440280609603E-52**	0.754160710957644	0.328	0.108	3.12258241702323E-48	Periportal hepatocytes	ALPK3
**3.36201099121165E-52**	0.600149284247917	0.622	0.323	5.48377612776532E-48	Periportal hepatocytes	DCAF11
**3.72072275160115E-52**	0.631626515068476	0.789	0.453	6.06887088013664E-48	Periportal hepatocytes	KLF15
**5.09940922685732E-52**	1.70656644122881	0.134	0.021	8.31764638992697E-48	Periportal hepatocytes	SNRPN
**6.27002488799916E-52**	0.915096291767137	0.468	0.198	1.02270375948154E-47	Periportal hepatocytes	WWP1
**6.8439012120563E-52**	0.464803735318158	0.569	0.279	1.1163087266985E-47	Periportal hepatocytes	HTRA1
**8.28179387773436E-52**	0.416483835998375	0.603	0.291	1.35084339939725E-47	Periportal hepatocytes	F10
**9.39499184911562E-52**	0.844209478601539	0.625	0.303	1.53241712050925E-47	Periportal hepatocytes	PDZK1
**9.66383180123467E-52**	0.532486413544331	0.532	0.243	1.57626760509939E-47	Periportal hepatocytes	DHCR7
**1.50876796495226E-51**	1.05029452784371	0.671	0.369	2.46095142763364E-47	Periportal hepatocytes	CYP2C8
**1.66054060301084E-51**	0.45067419930568	0.575	0.277	2.70850777757098E-47	Periportal hepatocytes	MFSD3
**2.39888671818559E-51**	0.767048718086322	0.282	0.086	3.91282412603251E-47	Periportal hepatocytes	TMEM64
**2.47346350603848E-51**	0.705472070427557	0.644	0.335	4.03446632469936E-47	Periportal hepatocytes	AHR
**2.8176926956401E-51**	1.14192567331573	0.412	0.164	4.59593855585857E-47	Periportal hepatocytes	PLGLB2
**3.77375139986386E-51**	1.13829430650991	0.177	0.037	6.15536590831794E-47	Periportal hepatocytes	CPM
**4.706073040441E-51**	1.69911755485034	0.131	0.02	7.67607573626331E-47	Periportal hepatocytes	MOGAT2
**6.04546101608298E-51**	0.396868267076134	0.695	0.365	9.86075146333295E-47	Periportal hepatocytes	CTSS
**6.67345342962284E-51**	0.594978600638423	0.751	0.424	1.08850698890578E-46	Periportal hepatocytes	BHMT2
**6.81797984963273E-51**	1.15046761733013	0.411	0.167	1.1120806932736E-46	Periportal hepatocytes	PPARD
**1.19466513060415E-50**	0.350881984752041	0.678	0.365	1.94861829452842E-46	Periportal hepatocytes	BST2
**1.26271724099504E-50**	0.862316978229791	0.349	0.125	2.059618091787E-46	Periportal hepatocytes	INTS8
**1.53864332875761E-50**	0.431249979480717	0.851	0.57	2.50968113353654E-46	Periportal hepatocytes	SLC9A3R2
**1.97161283335013E-50**	1.83526206446498	0.121	0.017	3.2158976924774E-46	Periportal hepatocytes	LAMB1
**2.22274963252632E-50**	1.59385113808943	0.312	0.107	3.62552692561368E-46	Periportal hepatocytes	PROZ
**2.92761293663446E-50**	0.605339301579887	0.591	0.283	4.77522946094447E-46	Periportal hepatocytes	ACSM5
**6.81375305142854E-50**	0.450418181847563	0.77	0.453	1.11139126021851E-45	Periportal hepatocytes	CPB2
**7.20187866605645E-50**	0.611915624039648	0.747	0.419	1.17469842922047E-45	Periportal hepatocytes	LPIN2
**7.55595244654167E-50**	0.666026471408346	0.405	0.162	1.23245140355541E-45	Periportal hepatocytes	F9
**7.81661973933938E-50**	0.551436034437918	0.826	0.493	1.27496884568365E-45	Periportal hepatocytes	OGDHL
**9.96521349183683E-50**	0.787383387329314	0.345	0.123	1.62542597265351E-45	Periportal hepatocytes	THPO
**1.16854027701214E-49**	0.666574323100498	0.641	0.331	1.9060060458345E-45	Periportal hepatocytes	RNF19A
**2.24038300911242E-49**	0.528315360727223	0.911	0.616	3.65428872616326E-45	Periportal hepatocytes	SLC38A3
**2.5300432173967E-49**	0.774656767746779	0.539	0.253	4.12675349189576E-45	Periportal hepatocytes	KLHL24
**2.90608782790688E-49**	0.597018673375634	0.385	0.148	4.74011985609892E-45	Periportal hepatocytes	SIGIRR
**3.17890156694259E-49**	0.34508472094197	0.463	0.199	5.18510634584005E-45	Periportal hepatocytes	GCDH
**3.21621423396328E-49**	0.512826942372915	0.776	0.423	5.2459670370175E-45	Periportal hepatocytes	NOTCH2NL
**4.0075317997111E-49**	0.724286968016131	0.57	0.278	6.53668511850877E-45	Periportal hepatocytes	SLC25A25
**4.70174934183712E-49**	0.407987259580187	0.493	0.22	7.66902335147052E-45	Periportal hepatocytes	ALG3
**5.07188560039009E-49**	0.821002756761607	0.497	0.222	8.27275260279627E-45	Periportal hepatocytes	LIPG
**6.81596980183205E-49**	1.11446916220412	0.461	0.201	1.11175283437683E-44	Periportal hepatocytes	ZDHHC11
**1.02935629514991E-48**	0.658532946681945	0.608	0.303	1.67898305301902E-44	Periportal hepatocytes	GCLM
**1.71873071812141E-48**	0.595114446108134	0.797	0.455	2.80342167432784E-44	Periportal hepatocytes	MASP1
**3.9247650699271E-48**	0.705164268128765	0.711	0.378	6.4016843055581E-44	Periportal hepatocytes	PPP2R1B
**4.19553553805791E-48**	0.810410268567603	0.519	0.241	6.84333801612626E-44	Periportal hepatocytes	MTMR4

5.42985966342253E-48	0.774869276656596	0.608	0.3	8.85664409700848E-44	Periportal hepatocytes	SLC22A25
7.27391170952515E-48	0.857442489795002	0.493	0.224	1.18644773894065E-43	Periportal hepatocytes	TJP1
9.12248953879999E-48	1.55170020933478	0.111	0.015	1.48796926867367E-43	Periportal hepatocytes	BBOX1
9.76074564561215E-48	0.31218151122055	0.588	0.293	1.5920752222558E-43	Periportal hepatocytes	PRG4
1.00094777144986E-47	1.71650676928787	0.259	0.08	1.63264591001187E-43	Periportal hepatocytes	ADCY10
1.17392065045656E-47	0.608350416536242	0.664	0.35	1.9147819729597E-43	Periportal hepatocytes	NMD3
2.86561807750962E-47	0.525593213372875	0.698	0.381	4.67410964622594E-43	Periportal hepatocytes	DPP9
2.91502270200611E-47	0.864438164393257	0.44	0.186	4.75469352924216E-43	Periportal hepatocytes	C19orf25
3.71854914033282E-47	0.41187847686658	0.78	0.468	6.06532550279687E-43	Periportal hepatocytes	UGT2B4
3.87587827041632E-47	0.561824262078747	0.445	0.189	6.32194504687607E-43	Periportal hepatocytes	AATF
4.70775155324007E-47	0.495620583514229	0.43	0.176	7.67881355848988E-43	Periportal hepatocytes	AQP7
4.955386951747E-47	0.39835037779374	0.997	0.996	8.08273165699453E-43	Periportal hepatocytes	FGG
5.2221477594361E-47	0.627130307836427	0.647	0.34	8.51784521041623E-43	Periportal hepatocytes	IL6ST
7.49518339993375E-47	1.40328974894047	0.216	0.059	1.22253936436319E-42	Periportal hepatocytes	SRD5A1
1.19245029732624E-46	0.43792822847199	0.756	0.468	1.94500567996883E-42	Periportal hepatocytes	MT-CYB
1.44878303504766E-46	0.841596158616285	0.205	0.053	2.36311000846623E-42	Periportal hepatocytes	LAPTM5
1.51554078114781E-46	0.671047311270993	0.593	0.294	2.4719985681302E-42	Periportal hepatocytes	CLSTN3
1.96992351153385E-46	0.63560478265925	0.631	0.327	3.21314223966286E-42	Periportal hepatocytes	SEMA4G
2.52617173573382E-46	0.783993321155393	0.414	0.173	4.12043871815543E-42	Periportal hepatocytes	PRODH2
3.28540650239287E-46	1.28290794883194	0.341	0.126	5.35882654605301E-42	Periportal hepatocytes	GPCPD1
4.28932913180331E-46	0.820775384948839	0.645	0.338	6.99632474688438E-42	Periportal hepatocytes	SLC17A9
7.9212965309458E-46	0.663515779338642	0.614	0.309	1.29204267716257E-41	Periportal hepatocytes	RAB17
1.06751182115193E-45	2.06247295717463	0.111	0.016	1.74121853148091E-41	Periportal hepatocytes	SULF2
1.12711532064498E-45	0.72834871783142	0.739	0.419	1.83843779950402E-41	Periportal hepatocytes	PLIN4
1.24434664470729E-45	0.666862391961852	0.369	0.146	2.02965381218206E-41	Periportal hepatocytes	TRIM22
1.46858397457621E-45	0.761401081196896	0.562	0.278	2.39540732093125E-41	Periportal hepatocytes	XRN1
1.57444925672427E-45	0.67830072525777	0.576	0.286	2.56808418264296E-41	Periportal hepatocytes	MAP3K13
1.60184915839269E-45	0.456175342290087	0.951	0.82	2.61277616225432E-41	Periportal hepatocytes	SERPINF2
2.14691363997077E-45	0.564693597051322	0.737	0.404	3.50183083815633E-41	Periportal hepatocytes	SLC22A23
2.36914840613748E-45	0.702394519688692	0.573	0.283	3.86431796525085E-41	Periportal hepatocytes	U2SURP
3.58615968626879E-45	0.4857175639529	0.468	0.211	5.84938506427302E-41	Periportal hepatocytes	CFHR5
4.72874150455278E-45	0.559746708766374	0.58	0.299	7.71305026807604E-41	Periportal hepatocytes	S100A13
4.85875573532895E-45	0.773228280968114	0.345	0.129	7.92511647989505E-41	Periportal hepatocytes	AFF1
5.7270571598956E-45	0.505611816851991	0.638	0.338	9.34140293350572E-41	Periportal hepatocytes	TM7SF2
5.73244509913839E-45	0.710354387064556	0.299	0.105	9.35019120120463E-41	Periportal hepatocytes	LRATD2
6.66260915266127E-45	0.30921311508168	0.522	0.251	1.08673817889058E-40	Periportal hepatocytes	NDUFS7
6.85169021838652E-45	2.11084395226539	0.122	0.02	1.11757919152103E-40	Periportal hepatocytes	ANO1
8.67451984449906E-45	0.925510675590387	0.58	0.297	1.41490093183624E-40	Periportal hepatocytes	ALAS1
8.7419185785868E-45	0.33455471785188	0.388	0.156	1.42589433935329E-40	Periportal hepatocytes	S100A10
9.5069001096569E-45	0.614725768709194	0.599	0.305	1.55067047688614E-40	Periportal hepatocytes	PPARA
9.55726290281732E-45	0.460885685272487	0.223	0.062	1.55888515207853E-40	Periportal hepatocytes	CA2
9.85982100648653E-45	1.27680800335815	0.32	0.117	1.60823540436802E-40	Periportal hepatocytes	GRB14
1.10721588286223E-44	2.11143456811506	0.121	0.02	1.80597982653659E-40	Periportal hepatocytes	CLDN14
1.37302311668625E-44	0.465367299054796	0.825	0.503	2.23953800562693E-40	Periportal hepatocytes	ETS2
1.58186898035232E-44	1.26390716761323	0.486	0.231	2.58018649385266E-40	Periportal hepatocytes	RAPGEF5
1.63176710718954E-44	0.34465079335352	0.388	0.159	2.66157532853685E-40	Periportal hepatocytes	PLAAT3
1.8735033326116E-44	0.542476926079128	0.346	0.131	3.05587128582278E-40	Periportal hepatocytes	TLCD2
1.93525392198043E-44	0.580812741714966	0.619	0.332	3.15659267214229E-40	Periportal hepatocytes	TTC39C
2.49736003664611E-44	0.388240551709317	0.448	0.197	4.07344395577347E-40	Periportal hepatocytes	CD99L2
3.46495058227064E-44	0.841695813110664	0.274	0.091	5.65168089474164E-40	Periportal hepatocytes	ACTR3C
3.61402831312464E-44	0.405858594905381	0.687	0.38	5.89484158153759E-40	Periportal hepatocytes	AMN
3.70702087812134E-44	1.57880662271967	0.287	0.1	6.04652175430372E-40	Periportal hepatocytes	CLTCL1
3.77691924767136E-44	1.77784444939486	0.103	0.014	6.16053298487676E-40	Periportal hepatocytes	CRIP2
4.23035266870034E-44	1.5823167099958	0.211	0.059	6.90012823791713E-40	Periportal hepatocytes	SLC22A10
4.73242105037078E-44	0.566588564874977	0.626	0.327	7.71905197525978E-40	Periportal hepatocytes	SYMPK
4.99823445242374E-44	0.493329699063421	0.507	0.236	8.15262021534835E-40	Periportal hepatocytes	HSD17B11
5.70256722835107E-44	0.457943459880421	0.947	0.833	9.30145740616343E-40	Periportal hepatocytes	A2M
5.94923138724148E-44	0.619880349746088	0.559	0.284	9.70379131572957E-40	Periportal hepatocytes	TFRC
6.5183149171295E-44	0.938940455109597	0.392	0.162	1.06320234613299E-39	Periportal hepatocytes	SLC26A1
7.17639662463898E-44	0.621759489353063	0.582	0.294	1.17054205344486E-39	Periportal hepatocytes	TMEM175

9.31103433122526E-44	0.737221140542038	0.539	0.265	1.51872280976615E-39	Periportal hepatocytes	ACSF2
1.08530798586051E-43	1.09445573230371	0.175	0.042	1.77024585573708E-39	Periportal hepatocytes	DYSF
1.17415432931116E-43	0.888925613630169	0.499	0.237	1.91516312653943E-39	Periportal hepatocytes	SLC33A1
1.38726374670524E-43	1.9720459024543	0.132	0.025	2.26276589725091E-39	Periportal hepatocytes	PLXNC1
1.51900887203608E-43	1.07917323960716	0.27	0.089	2.47765537117805E-39	Periportal hepatocytes	NIPAL2
1.52907492839516E-43	0.482510828364154	0.83	0.482	2.49407411570534E-39	Periportal hepatocytes	SLC7A2
1.67532881235282E-43	0.73636419435487	0.402	0.17	2.73262882582869E-39	Periportal hepatocytes	GADD45G
1.73673357521397E-43	0.476779445982626	0.644	0.346	2.83278613453151E-39	Periportal hepatocytes	ACADSB
1.8001116104179E-43	0.435997260844962	0.642	0.338	2.93616204775263E-39	Periportal hepatocytes	PGPEP1
2.04154575798562E-43	1.05298700532854	0.381	0.156	3.32996528585034E-39	Periportal hepatocytes	NBPF12
2.35669564899571E-43	0.366290849899316	0.713	0.4	3.8440062730769E-39	Periportal hepatocytes	CBS
2.50211295553185E-43	0.487144472598186	0.813	0.472	4.08119644176799E-39	Periportal hepatocytes	IL6R
2.52505786218315E-43	1.05622584001619	0.185	0.047	4.11862187900693E-39	Periportal hepatocytes	CYP8B1
2.977520891816E-43	0.326638391587315	0.83	0.58	4.85663432664108E-39	Periportal hepatocytes	STARD10
3.04695546721245E-43	0.43580604325539	0.57	0.289	4.96988906257023E-39	Periportal hepatocytes	TGM2
3.33902228552804E-43	0.732399771592948	0.343	0.131	5.44627924992478E-39	Periportal hepatocytes	CYP4F2
3.44145822209078E-43	0.790456516495367	0.346	0.133	5.61336250605226E-39	Periportal hepatocytes	UBE3A
3.49250308136394E-43	0.71096629986232	0.484	0.231	5.69662177601272E-39	Periportal hepatocytes	NDRG1
3.78858002288797E-43	0.378507318556499	0.516	0.251	6.17955287533258E-39	Periportal hepatocytes	SPRYD4
4.39315081980718E-43	0.79354274701612	0.246	0.076	7.16566830218749E-39	Periportal hepatocytes	ODC1
6.40694432115417E-43	0.495899553903837	0.677	0.362	1.04503668822346E-38	Periportal hepatocytes	AIG1
7.16990826731014E-43	0.552703141325398	0.829	0.553	1.16948373748096E-38	Periportal hepatocytes	ALDH1A1
7.78567698940172E-43	0.336740084223005	0.636	0.347	1.26992177374132E-38	Periportal hepatocytes	APOM
1.19254039342362E-42	0.949038919369907	0.3	0.109	1.94515263571326E-38	Periportal hepatocytes	NUDT9
1.24850006096866E-42	0.646646329958697	0.642	0.34	2.03642844944598E-38	Periportal hepatocytes	CLK1
1.28977444180954E-42	0.797954140561872	0.516	0.249	2.10375109203554E-38	Periportal hepatocytes	PLXNB1
1.31800231187135E-42	0.546845175175464	0.769	0.43	2.14979357089336E-38	Periportal hepatocytes	SPG7
1.35429918119643E-42	0.611358576991861	0.841	0.475	2.2089973944495E-38	Periportal hepatocytes	MDM4
1.41888989585155E-42	0.804093648020364	0.603	0.32	2.31435130912346E-38	Periportal hepatocytes	TNFAIP2
1.48254216472023E-42	0.679036060573216	0.69	0.372	2.41817452487517E-38	Periportal hepatocytes	FBXO7
1.53067412009595E-42	1.79306115472297	0.101	0.014	2.4966825572885E-38	Periportal hepatocytes	IGSF1
1.70432271109955E-42	0.538764506395145	0.636	0.342	2.77992077407447E-38	Periportal hepatocytes	PROX1
2.35155038418227E-42	0.528318003360755	0.362	0.147	3.83561383163969E-38	Periportal hepatocytes	SLC16A2
2.52230373930735E-42	0.877058694877233	0.375	0.153	4.11412962918422E-38	Periportal hepatocytes	UBE3C
2.66453486058069E-42	0.402260295422986	0.72	0.411	4.34612281109317E-38	Periportal hepatocytes	INTS1
2.69624533585022E-42	0.355704606062492	0.625	0.324	4.39784576730529E-38	Periportal hepatocytes	NMRAL1
3.17194807128673E-42	0.361494016984079	0.522	0.25	5.17376449907579E-38	Periportal hepatocytes	ECE1
4.098713524926E-42	1.28614388495145	0.253	0.082	6.6854116305068E-38	Periportal hepatocytes	TPST1
4.28603785720634E-42	0.546581280070325	0.454	0.207	6.99095634888926E-38	Periportal hepatocytes	PLA2G12B
1.2818598227894E-41	0.925836509540397	0.313	0.118	2.0908415569518E-37	Periportal hepatocytes	HSD17B14
1.75713958225683E-41	0.431461271303595	0.846	0.595	2.86607037261912E-37	Periportal hepatocytes	GAMT
1.95717913888476E-41	0.331618250997919	0.693	0.401	3.19235489343493E-37	Periportal hepatocytes	PEBP1
2.10402975590046E-41	0.473880827625054	0.556	0.28	3.43188293484925E-37	Periportal hepatocytes	SLCO1B1
2.11824602820379E-41	0.54294284971701	0.522	0.255	3.45507109660321E-37	Periportal hepatocytes	EIF4A2
2.25824008245271E-41	0.758263731605804	0.585	0.307	3.68341539848862E-37	Periportal hepatocytes	FAM193B
3.84295304718901E-41	0.329880802197814	0.582	0.301	6.26824071527E-37	Periportal hepatocytes	TST
5.81696771858595E-41	1.11442968872906	0.355	0.143	9.48805604578554E-37	Periportal hepatocytes	LSG1
6.25111268257681E-41	0.270153872435644	0.674	0.388	1.0196189896551E-36	Periportal hepatocytes	QDPR
6.3013194041947E-41	0.92193217298647	0.361	0.148	1.0278082080182E-36	Periportal hepatocytes	EPB41L4B
6.67347763947589E-41	0.554151922495193	0.272	0.093	1.08851093777491E-36	Periportal hepatocytes	MAD1L1
7.57085840865205E-41	0.275213469481421	0.718	0.413	1.23488271503524E-36	Periportal hepatocytes	NIPSNAP1
9.1826302613684E-41	0.354905209639357	0.514	0.256	1.4977788219318E-36	Periportal hepatocytes	HIBADH
1.05097639901689E-40	1.82962087237604	0.147	0.032	1.71424760443644E-36	Periportal hepatocytes	SLC16A9
1.21694121513364E-40	0.465356952697841	0.911	0.652	1.98495281600448E-36	Periportal hepatocytes	BDH1
1.36613831129393E-40	0.376803507901283	0.766	0.465	2.22830819955153E-36	Periportal hepatocytes	LDHD
1.45502244945563E-40	0.632478636462618	0.648	0.349	2.37328711730707E-36	Periportal hepatocytes	RSRP1
1.45992556927146E-40	0.358602613898343	0.661	0.362	2.38128459603868E-36	Periportal hepatocytes	DCAF6
1.79110127914635E-40	0.363435625546558	0.28	0.099	2.92146529641561E-36	Periportal hepatocytes	DEPRECAT
2.59921678458845E-40	0.644177365228327	0.684	0.383	4.23958249734222E-36	Periportal hepatocytes	OGA
2.81406975692303E-40	0.667502985511329	0.299	0.11	4.59002918051716E-36	Periportal hepatocytes	MSMO1

2.8626822346831E-40	0.549439892174641	0.54	0.266	4.6693209929916E-36	Periportal hepatocytes	TTC3
3.57474508376777E-40	0.829018244194816	0.898	0.579	5.83076670613361E-36	Periportal hepatocytes	SLC12A7
4.3085971377486E-40	1.99601513634001	0.132	0.027	7.02775279138175E-36	Periportal hepatocytes	SLC25A47
4.64872964844014E-40	0.302263822960718	0.636	0.346	7.58254292957071E-36	Periportal hepatocytes	MMUT
4.78333169660162E-40	0.39012908776132	0.351	0.142	7.8020923303269E-36	Periportal hepatocytes	ERG28
5.37933010271745E-40	1.40004473056421	0.223	0.068	8.77422533054243E-36	Periportal hepatocytes	MYO7B
5.4858809971652E-40	0.682637948827105	0.601	0.324	8.94802049447616E-36	Periportal hepatocytes	RB1CC1
7.00526834755814E-40	0.853119846436606	0.418	0.188	1.14262932017021E-35	Periportal hepatocytes	SLC2A9
7.41548723895601E-40	0.386723657761094	0.533	0.261	1.20954012354611E-35	Periportal hepatocytes	NOTCH2NL
7.9836477199552E-40	1.2397755619712	0.295	0.11	1.30221277960189E-35	Periportal hepatocytes	TPCN1
1.23251655647117E-39	0.572844205598785	0.333	0.131	2.01035775526013E-35	Periportal hepatocytes	PDE4DIP
1.4373324095993E-39	0.849129400706077	0.267	0.092	2.34443289329742E-35	Periportal hepatocytes	MYH10
1.79301114877979E-39	0.56262541166233	0.389	0.169	2.92458048477471E-35	Periportal hepatocytes	TP53INP2
1.82023156651402E-39	0.846645651945459	0.389	0.166	2.96897970814102E-35	Periportal hepatocytes	NBPF26
1.91773955360322E-39	0.301539276819711	0.463	0.215	3.12802498588222E-35	Periportal hepatocytes	ABHD14B
2.25933664941634E-39	0.401414655747115	0.829	0.533	3.68520400886299E-35	Periportal hepatocytes	SELENBP1
2.57449076475688E-39	1.9266053770365	0.112	0.02	4.19925188639495E-35	Periportal hepatocytes	HSH2D
2.94491675543335E-39	0.306404634412989	0.624	0.335	4.80345371978734E-35	Periportal hepatocytes	MT-ND2
3.53092092968741E-39	0.751708326488333	0.32	0.126	5.75928512841314E-35	Periportal hepatocytes	ESYT2
3.74682186695536E-39	0.368314922347017	0.736	0.441	6.11144114719089E-35	Periportal hepatocytes	KHK
3.88381606281828E-39	0.259532928797267	0.45	0.211	6.33489238006289E-35	Periportal hepatocytes	DNAJC3
5.5608013746236E-39	0.58087698576036	0.7	0.386	9.07022312214855E-35	Periportal hepatocytes	LPP
7.08045982760443E-39	0.465588843711682	0.849	0.516	1.15489380248056E-34	Periportal hepatocytes	ONECUT1
7.14761734479781E-39	0.774337710133474	0.292	0.109	1.16584786510997E-34	Periportal hepatocytes	GLCE
9.01366214196929E-39	0.561079894941991	0.624	0.335	1.47021843197661E-34	Periportal hepatocytes	TNRC6A
9.13495846804944E-39	0.580904116015137	0.533	0.27	1.49000307572354E-34	Periportal hepatocytes	GALM
1.30009853949597E-38	0.939211690821574	0.493	0.248	2.12059072777187E-34	Periportal hepatocytes	DCPS
1.46453645907679E-38	0.717748112258078	0.493	0.238	2.38880541840015E-34	Periportal hepatocytes	ABCA6
2.69782108750581E-38	0.415371676781626	0.823	0.598	4.40041597583073E-34	Periportal hepatocytes	ST6GAL1
2.9126733507183E-38	0.283191588814706	0.514	0.256	4.75086150235662E-34	Periportal hepatocytes	TWF2
4.01532013337302E-38	0.942140982770522	0.362	0.155	6.54938866954473E-34	Periportal hepatocytes	SEMA6C
4.38935245888685E-38	0.947841903382162	0.322	0.129	7.15947279569033E-34	Periportal hepatocytes	PLA2G15
5.13593085382321E-38	0.893656229396982	0.329	0.133	8.37721681567104E-34	Periportal hepatocytes	SNX13
5.25076418330944E-38	0.512644588396164	0.371	0.159	8.56452145939603E-34	Periportal hepatocytes	SULT1A1
5.82192467512455E-38	0.490114729121155	0.894	0.533	9.49614133759565E-34	Periportal hepatocytes	CDK5RAP3
6.11483031794599E-38	1.1022935877332	0.409	0.187	9.9738997316017E-34	Periportal hepatocytes	PLCH2
6.18029539504801E-38	0.975389920134864	0.266	0.095	1.00806798188628E-33	Periportal hepatocytes	UBE2D3
1.08938197435663E-37	0.951367754807598	0.408	0.186	1.77689093837311E-33	Periportal hepatocytes	NUP42
1.22242356168385E-37	0.29313098150526	0.792	0.497	1.99389507146253E-33	Periportal hepatocytes	RGN
1.30388445356379E-37	1.02425981762241	0.223	0.072	2.1267659322079E-33	Periportal hepatocytes	ETNPPL
1.77116097387939E-37	0.797978563696021	0.477	0.234	2.88894066449467E-33	Periportal hepatocytes	GDA
1.91236436539657E-37	0.606653546969219	0.895	0.761	3.11925751639835E-33	Periportal hepatocytes	ORM2
2.49208954189598E-37	0.94911719707431	0.326	0.134	4.06484725178654E-33	Periportal hepatocytes	CYP4F12
2.97340081432566E-37	0.80556698101687	0.405	0.185	4.84991406824658E-33	Periportal hepatocytes	SH3PXD2A
3.00817777431405E-37	1.52849900007178	0.168	0.044	4.90663876768365E-33	Periportal hepatocytes	KSR1
3.11810243003291E-37	0.527523860669951	0.622	0.342	5.08593687362669E-33	Periportal hepatocytes	LARP4
3.14170277025171E-37	0.381066204437907	0.655	0.366	5.12443138855756E-33	Periportal hepatocytes	STAT3
3.39230453108031E-37	1.62033267678304	0.118	0.023	5.5331879206451E-33	Periportal hepatocytes	OAT
3.70914589173421E-37	0.494746662976813	0.766	0.438	6.04998786400768E-33	Periportal hepatocytes	CCNL1
3.75503082327783E-37	0.320925328754611	0.424	0.194	6.12483077584847E-33	Periportal hepatocytes	CD68
3.78171559273978E-37	0.473785424503382	0.911	0.543	6.16835630331785E-33	Periportal hepatocytes	CCNL2
4.34796064772182E-37	1.39177523204157	0.185	0.053	7.09195861249906E-33	Periportal hepatocytes	FAM126A
4.44477069330003E-37	0.358543882615347	0.797	0.498	7.24986547784168E-33	Periportal hepatocytes	TMEM45B
4.65802457831331E-37	0.365799787606416	0.434	0.202	7.59770388968684E-33	Periportal hepatocytes	FDPS
4.668171700765E-37	1.13375949385468	0.217	0.069	7.6142548611178E-33	Periportal hepatocytes	ADD3
5.02970945624956E-37	0.558382528419379	0.963	0.888	8.20395909408865E-33	Periportal hepatocytes	ORM1
7.88340786425431E-37	0.544189127971475	0.534	0.275	1.28586265673852E-32	Periportal hepatocytes	LGR4
9.48829233407541E-37	0.328817716518359	0.483	0.241	1.54763536261104E-32	Periportal hepatocytes	LACTB2
1.15042677791149E-36	0.419387791850394	0.787	0.483	1.87646111745144E-32	Periportal hepatocytes	MTSS1
1.28588120689523E-36	0.597906528461104	0.487	0.245	2.09740083656681E-32	Periportal hepatocytes	EIF2AK1

1.40018217687339E-36	0.564717149556202	0.427	0.198	2.28383714869819E-32	Periportal hepatocytes	WIPI2
1.92384653118778E-36	1.35060280402623	0.257	0.093	3.13798607702038E-32	Periportal hepatocytes	LCN12
1.96606356611616E-36	1.12788883314808	0.292	0.114	3.20684628269208E-32	Periportal hepatocytes	FOXO1
2.41149621414387E-36	0.300435960111197	0.569	0.304	3.93339147489006E-32	Periportal hepatocytes	RAB10
2.71236751996554E-36	0.346544485337377	0.667	0.393	4.4241426618158E-32	Periportal hepatocytes	ACSM1
2.89141022847393E-36	0.297682375734497	0.807	0.575	4.71617922366383E-32	Periportal hepatocytes	P4HB
2.96617949809761E-36	0.408237866579605	0.542	0.279	4.838135379347E-32	Periportal hepatocytes	GGNBP2
3.36048467991955E-36	0.454376087878642	0.236	0.079	5.48128656141678E-32	Periportal hepatocytes	NDFIP2
3.52044778549961E-36	0.746981214978241	0.529	0.277	5.74220238292841E-32	Periportal hepatocytes	CRY2
3.58537912438234E-36	1.05338998562531	0.207	0.065	5.84811188978003E-32	Periportal hepatocytes	GCH1
3.80398857351783E-36	0.351852076753739	0.514	0.26	6.20468576226494E-32	Periportal hepatocytes	MAF
4.15995028103801E-36	0.394989785120392	0.665	0.377	6.7852949034011E-32	Periportal hepatocytes	NAPRT
5.06002419478948E-36	0.61180612375293	0.649	0.36	8.25340546412113E-32	Periportal hepatocytes	MTG1
5.18072139236744E-36	0.618796129554786	0.494	0.25	8.45027466309052E-32	Periportal hepatocytes	KIAA0319L
5.27526643631216E-36	0.351034980248424	0.638	0.349	8.60448708426876E-32	Periportal hepatocytes	IKBKG
5.42029942803163E-36	0.265538894783065	0.533	0.272	8.8410503970624E-32	Periportal hepatocytes	GCAT
6.68254372518419E-36	0.487626767800198	0.478	0.24	1.08998970701479E-31	Periportal hepatocytes	KDELR2
7.76413357834519E-36	0.68266725944449	0.522	0.27	1.26640782796388E-31	Periportal hepatocytes	EXOC3L4
9.18089037542063E-36	0.644361854897023	0.348	0.15	1.49749502913486E-31	Periportal hepatocytes	PARL
1.02468548213636E-35	0.636074095548966	0.963	0.695	1.67136448991261E-31	Periportal hepatocytes	CYP3A5
1.07719021607341E-35	0.834292834621527	0.392	0.18	1.75700496143735E-31	Periportal hepatocytes	SLC25A15
1.07897976072639E-35	0.680516114989185	0.506	0.258	1.75992388772082E-31	Periportal hepatocytes	MTHFR
1.08281273800092E-35	0.383877324859897	0.404	0.184	1.76617585695331E-31	Periportal hepatocytes	MOSPD3
1.23959565504716E-35	0.605487615345426	0.608	0.331	2.02190447294742E-31	Periportal hepatocytes	NRBP2
1.25247064128343E-35	0.251121646849598	0.727	0.44	2.04290486299741E-31	Periportal hepatocytes	UBD
1.2552210305868E-35	0.409904511117573	0.703	0.4	2.04739102299013E-31	Periportal hepatocytes	SHROOM1
1.268171089859E-35	0.33992479815209	0.591	0.316	2.06851386466902E-31	Periportal hepatocytes	B4GALT1
1.29855198519451E-35	0.927876332332613	0.177	0.05	2.11806814305076E-31	Periportal hepatocytes	DPY19L4
1.32632178541295E-35	0.315626648374441	0.501	0.248	2.16336346418706E-31	Periportal hepatocytes	GBP7
1.73044736039353E-35	0.552817688622332	0.338	0.143	2.82253268953789E-31	Periportal hepatocytes	CHCHD7
1.7342941145473E-35	0.512498639164562	0.529	0.276	2.8288071302381E-31	Periportal hepatocytes	LSS
1.86677958596415E-35	0.341243230628622	0.478	0.236	3.04490418266612E-31	Periportal hepatocytes	RDH10
1.90242852660385E-35	0.646197592921083	0.625	0.343	3.10305116974354E-31	Periportal hepatocytes	PCCA
1.96088368395838E-35	0.359084630699512	0.351	0.153	3.19839737690451E-31	Periportal hepatocytes	PEMT
1.97938100183297E-35	0.465936971532323	0.658	0.37	3.22856835208976E-31	Periportal hepatocytes	SIK2
1.98861044538496E-35	0.609406211994391	0.411	0.187	3.2436224974674E-31	Periportal hepatocytes	LRBA
2.24460011055279E-35	0.488774477176969	0.592	0.322	3.66116724032266E-31	Periportal hepatocytes	ELP2
2.33168365839871E-35	0.809022384735345	0.391	0.178	3.80320921521414E-31	Periportal hepatocytes	ZC3H3
2.3386852466849E-35	0.66054277967514	0.648	0.36	3.81462950586773E-31	Periportal hepatocytes	RBM39
2.4494903303384E-35	0.265556500925642	0.779	0.482	3.99536367781496E-31	Periportal hepatocytes	PON3
2.56820995304117E-35	0.556242340909208	0.941	0.607	4.18900725440546E-31	Periportal hepatocytes	MLXIPL
2.77374280683923E-35	0.874887794300344	0.295	0.117	4.52425189223546E-31	Periportal hepatocytes	POLB
2.92014076180232E-35	0.591933742638655	0.273	0.102	4.76304159657576E-31	Periportal hepatocytes	PRKAG2
3.22450284574029E-35	0.396630088611114	0.49	0.245	5.25948659168698E-31	Periportal hepatocytes	TAX1BP1
3.3955505009028E-35	0.394848918344642	0.898	0.687	5.53848242202256E-31	Periportal hepatocytes	AZGP1
3.68450097981516E-35	0.266166092835656	0.364	0.157	6.0097895481765E-31	Periportal hepatocytes	TRAPPC6A
4.00130467583929E-35	0.444416206646734	0.717	0.402	6.52652805676147E-31	Periportal hepatocytes	CHDH
4.11202494377422E-35	0.777046064373702	0.188	0.056	6.70712388579013E-31	Periportal hepatocytes	DNALI1
4.56223645741372E-35	0.941825013324128	0.353	0.154	7.44146388568752E-31	Periportal hepatocytes	PNPLA3
4.90327145116212E-35	0.392344821192789	0.797	0.492	7.99772606399054E-31	Periportal hepatocytes	HJV
6.32877139380135E-35	0.512866659838839	0.486	0.244	1.03228590204294E-30	Periportal hepatocytes	KCTD20
7.1015725747198E-35	0.250953191368083	0.565	0.31	1.15833750266255E-30	Periportal hepatocytes	SMIM14
7.32492093273368E-35	0.298577510699818	0.483	0.245	1.19476785333819E-30	Periportal hepatocytes	COA3
7.58216181114493E-35	0.449442999090303	0.526	0.271	1.23672641301585E-30	Periportal hepatocytes	SCRN2
8.25061409300404E-35	0.743989465122861	0.361	0.16	1.34575766470989E-30	Periportal hepatocytes	MAP1LC3A
9.11583760857816E-35	0.879957860524979	0.28	0.11	1.48688427233518E-30	Periportal hepatocytes	TM4SF1
9.31378334630615E-35	0.526150089682803	0.75	0.432	1.519171201616E-30	Periportal hepatocytes	XAF1
1.18488567967647E-34	0.348820668690788	0.444	0.209	1.93266703212029E-30	Periportal hepatocytes	TBL1XR1
1.34773879723561E-34	0.363342672362932	0.756	0.439	2.198296752171E-30	Periportal hepatocytes	AKR1C2
1.37476290672808E-34	0.708681269616971	0.293	0.116	2.24237577716416E-30	Periportal hepatocytes	KLB

1.58905021182001E-34	0.326836783137378	0.665	0.374	2.59189980049961E-30	Periportal hepatocytes	PTPRH
1.59486340949137E-34	1.32112474207442	0.157	0.042	2.60138170722137E-30	Periportal hepatocytes	KIAA1755
1.70321714251566E-34	0.943772447814739	0.293	0.116	2.7781174811573E-30	Periportal hepatocytes	DAB2
1.7066321459471E-34	0.936591434170854	0.447	0.226	2.78368769325432E-30	Periportal hepatocytes	USP35
1.95397182200955E-34	0.498498108097271	0.264	0.098	3.18712343887977E-30	Periportal hepatocytes	KLF13
2.05544717949026E-34	0.449441230738879	0.399	0.184	3.35263989446656E-30	Periportal hepatocytes	GATD3B
2.23414079537482E-34	0.36594298873279	0.489	0.243	3.64410705133587E-30	Periportal hepatocytes	ENY2
2.64572047392399E-34	0.712343509409724	0.307	0.125	4.31543466501742E-30	Periportal hepatocytes	GLI4
2.8900148918587E-34	0.705174770853658	0.24	0.085	4.71390329011072E-30	Periportal hepatocytes	TRIQK
3.35498712290631E-34	0.381817770133786	0.733	0.431	5.47231949617248E-30	Periportal hepatocytes	SLC29A1
3.9397444310981E-34	0.498164985578144	0.473	0.235	6.42611714156411E-30	Periportal hepatocytes	PCMTD1
4.08150856200305E-34	0.472642301757374	0.549	0.291	6.65734861548317E-30	Periportal hepatocytes	DLG1
4.14943317166327E-34	0.572979219282611	0.473	0.236	6.76814044629996E-30	Periportal hepatocytes	CACNA1H
4.51244838526157E-34	0.31113568733165	0.576	0.316	7.36025456120014E-30	Periportal hepatocytes	SLC25A1
4.84510175776379E-34	0.544806805380038	0.705	0.401	7.90284547708852E-30	Periportal hepatocytes	SNRNP70
5.77617243654084E-34	0.488510107901359	0.792	0.497	9.42151486124176E-30	Periportal hepatocytes	PTGR1
6.57138282050862E-34	0.470636455663607	0.708	0.407	1.07185825185316E-29	Periportal hepatocytes	NR1I2
6.64580119984263E-34	0.67341041550689	0.425	0.207	1.08399663370633E-29	Periportal hepatocytes	IMPA1
6.77044987769705E-34	0.458463034886039	0.483	0.24	1.10432807955117E-29	Periportal hepatocytes	LRRC75B
6.84299789912937E-34	0.651357942950494	0.552	0.297	1.11616138732699E-29	Periportal hepatocytes	SOX5
7.48507649088482E-34	1.4286456807352	0.187	0.057	1.22089082642822E-29	Periportal hepatocytes	KLF12
7.56801178741826E-34	0.427318834555388	0.547	0.291	1.23441840264579E-29	Periportal hepatocytes	TMEM106C
7.5942609679673E-34	0.745595490304733	0.336	0.143	1.23869990648515E-29	Periportal hepatocytes	SYNRG
7.99402993760312E-34	0.368791941545097	0.437	0.215	1.30390622312245E-29	Periportal hepatocytes	DOK4
8.75087682841674E-34	0.343450683242015	0.658	0.379	1.42735551948305E-29	Periportal hepatocytes	MAGED2
8.78673757137278E-34	0.504695196451189	0.504	0.255	1.43320476526661E-29	Periportal hepatocytes	TRAPPC9
9.75085380014493E-34	0.414438438142938	0.889	0.718	1.59046176334164E-29	Periportal hepatocytes	DEPRECAT
1.26305870471126E-33	0.673915805901436	0.384	0.177	2.06017505325454E-29	Periportal hepatocytes	FAM91A1
1.40726147641653E-33	0.62655213404529	0.381	0.173	2.295384194183E-29	Periportal hepatocytes	AVL9
1.61080487383782E-33	0.545746669157426	0.43	0.208	2.62738382971686E-29	Periportal hepatocytes	HERPUD2
1.61701263181212E-33	0.622222040095884	0.391	0.182	2.63750930374875E-29	Periportal hepatocytes	SENP2
1.9282311749929E-33	0.62396914229345	0.526	0.28	3.14513786953092E-29	Periportal hepatocytes	PARP14
1.99366504549317E-33	0.562027544537234	0.417	0.202	3.25186705570392E-29	Periportal hepatocytes	FUT6
2.02956274839128E-33	0.320413611637391	0.589	0.322	3.31041979890101E-29	Periportal hepatocytes	TNFSF10
2.35716331729745E-33	0.676317976599649	0.364	0.165	3.84476908684387E-29	Periportal hepatocytes	DHX36
2.43335223120336E-33	0.455258379774736	0.632	0.355	3.96904082431579E-29	Periportal hepatocytes	TRPC4AP
3.37916605900236E-33	0.421205838048235	0.478	0.242	5.51175775883875E-29	Periportal hepatocytes	PLSCR4
3.42524538103135E-33	0.258857839660568	0.675	0.409	5.58691774100023E-29	Periportal hepatocytes	GPAA1
3.97081284754447E-33	0.824783947275852	0.453	0.23	6.47679283562979E-29	Periportal hepatocytes	NT5C2
4.1596768632071E-33	0.295196496622197	0.443	0.214	6.7848489315771E-29	Periportal hepatocytes	SUPT6H
4.30497147085235E-33	0.446884111309692	0.458	0.227	7.02183896610726E-29	Periportal hepatocytes	ABCF3
4.40957487989477E-33	0.727903083551265	0.216	0.073	7.19245758659636E-29	Periportal hepatocytes	DMGDH
4.51813073136905E-33	0.926485500225956	0.216	0.073	7.36952303593605E-29	Periportal hepatocytes	TBC1D10C
4.58695909062036E-33	0.731955003625648	0.43	0.209	7.48178897271087E-29	Periportal hepatocytes	BCKDHB
4.67321985340219E-33	0.969603066507666	0.267	0.102	7.62248890288432E-29	Periportal hepatocytes	ABCG8
4.89126017711909E-33	0.540452957182856	0.408	0.193	7.97813447489894E-29	Periportal hepatocytes	SARDH
5.48015715042727E-33	0.665363471855522	0.369	0.166	8.93868432806192E-29	Periportal hepatocytes	LRCH3
5.54314180125167E-33	0.660551891043295	0.226	0.079	9.0414185920216E-29	Periportal hepatocytes	MYRIP
6.66253469460038E-33	0.450857212337736	0.295	0.12	1.08672603403627E-28	Periportal hepatocytes	TMEM123
6.75483928991728E-33	0.510510767738479	0.654	0.368	1.10178183657841E-28	Periportal hepatocytes	NKTR
7.20368978132183E-33	1.20527085299262	0.224	0.078	1.1749938402314E-28	Periportal hepatocytes	DNHD1
7.33071489814437E-33	0.729199713478509	0.185	0.057	1.19571290703633E-28	Periportal hepatocytes	PTDSS1
8.2215464416334E-33	0.513563049602911	0.424	0.204	1.34101644009482E-28	Periportal hepatocytes	CDK9
8.90814996038737E-33	1.22568143324379	0.22	0.077	1.45300834003878E-28	Periportal hepatocytes	CYP4X1
8.95700272902315E-33	0.254345019180978	0.455	0.228	1.46097671513097E-28	Periportal hepatocytes	CTSO
9.13723419625018E-33	0.660644052245046	0.402	0.188	1.49037426975037E-28	Periportal hepatocytes	UBXN2B
9.14608761970121E-33	0.535102145029016	0.486	0.251	1.49181835164946E-28	Periportal hepatocytes	MCF2L
9.54961753777396E-33	0.277518422552371	0.5	0.264	1.55763811658631E-28	Periportal hepatocytes	PGLYRP2
1.32101493435597E-32	0.548378165657031	0.223	0.077	2.15470745942803E-28	Periportal hepatocytes	ITGAL
1.42176814163528E-32	0.475483878915307	0.67	0.392	2.3190460158213E-28	Periportal hepatocytes	IVNS1ABP

**1.44219392313369E-32**	0.695347357640618	0.243	0.09	2.35236250802335E-28	Periportal hepatocytes	UGT3A1
**1.71853037564058E-32**	0.337997591033208	0.539	0.28	2.80309489570735E-28	Periportal hepatocytes	ABCA2
**1.98614473959083E-32**	0.306217436779913	0.345	0.152	3.2396006847466E-28	Periportal hepatocytes	OBSL1
**2.06093565771846E-32**	0.508015114180395	0.657	0.374	3.36159215130458E-28	Periportal hepatocytes	POLR2H
**2.14572915818057E-32**	0.338970374053268	0.805	0.464	3.49989882990833E-28	Periportal hepatocytes	PABPC1L
**2.40844222795332E-32**	0.399121681128143	0.751	0.443	3.92841011801466E-28	Periportal hepatocytes	SELENOO
**2.45037795428716E-32**	0.420692812829044	0.487	0.25	3.99681148123779E-28	Periportal hepatocytes	ARHGEF12
**2.52074515616344E-32**	0.470143942760978	0.292	0.119	4.11158742421818E-28	Periportal hepatocytes	UGT1A9
**2.54090811689765E-32**	0.966185603539983	0.323	0.141	4.14447522947175E-28	Periportal hepatocytes	ITGA7
**2.70920024542626E-32**	0.358157253097694	0.532	0.276	4.41897652031478E-28	Periportal hepatocytes	SORL1
**2.72573848847053E-32**	0.664927348765075	0.437	0.216	4.44595204854428E-28	Periportal hepatocytes	ERBB2
**2.95950178585563E-32**	1.06363095192149	0.135	0.034	4.82724336290912E-28	Periportal hepatocytes	TGDS
**2.97702995450403E-32**	0.824074527703437	0.182	0.056	4.85583355879153E-28	Periportal hepatocytes	ATP6V0E2
**3.19004760255082E-32**	1.95078727104382	0.115	0.025	5.20328664452064E-28	Periportal hepatocytes	ADTRP
**3.26460739835092E-32**	0.467637043973099	0.645	0.367	5.32490112745019E-28	Periportal hepatocytes	TNFRSF14
**4.26922842078915E-32**	0.369966191509034	0.716	0.414	6.96353847714919E-28	Periportal hepatocytes	AKR1C8P
**4.36582976775701E-32**	0.974660547558516	0.267	0.105	7.12110493418846E-28	Periportal hepatocytes	RBM26
**4.47702807259805E-32**	0.431763084292577	0.803	0.473	7.30248048921468E-28	Periportal hepatocytes	WSB1
**4.81412672785075E-32**	0.520517099509161	0.486	0.25	7.85232210579736E-28	Periportal hepatocytes	RANBP10
**5.02525529498301E-32**	0.623041921126096	0.494	0.258	8.19669391164679E-28	Periportal hepatocytes	VPS13B
**5.1982339865431E-32**	0.359453209914985	0.536	0.285	8.47883945545045E-28	Periportal hepatocytes	SREBF2
**6.40748857725461E-32**	0.379366026186499	0.944	0.801	1.045125461836E-27	Periportal hepatocytes	IL32
**6.64717597340858E-32**	0.375649862883878	0.397	0.189	1.08422087302267E-27	Periportal hepatocytes	MTFR1
**7.0413798735069E-32**	1.04208049454803	0.32	0.14	1.14851947116771E-27	Periportal hepatocytes	VPS54
**7.35160321818924E-32**	0.412297863974238	0.631	0.363	1.19912000091885E-27	Periportal hepatocytes	PNRC1
**8.42122231401882E-32**	0.316743579477782	0.693	0.421	1.37358557163961E-27	Periportal hepatocytes	ASPH
**8.87660418022706E-32**	0.796542262875934	0.283	0.114	1.44786290783684E-27	Periportal hepatocytes	PUS3
**1.06023214779828E-31**	1.28395450544604	0.111	0.024	1.72934465627377E-27	Periportal hepatocytes	CARD6
**1.439866779906E-31**	0.496065923862996	0.698	0.402	2.34856670470468E-27	Periportal hepatocytes	SETD5
**1.52539677646931E-31**	0.423841054936786	0.329	0.141	2.48807468209908E-27	Periportal hepatocytes	IFI16
**1.71825721409636E-31**	0.573651327343615	0.579	0.324	2.80264934191258E-27	Periportal hepatocytes	PPARGC1A
**1.80738527635481E-31**	1.49353787368348	0.122	0.029	2.94802612426232E-27	Periportal hepatocytes	GFRA1
**1.82422001593444E-31**	1.06450572809735	0.241	0.092	2.97548526799066E-27	Periportal hepatocytes	RBPMS
**1.98962406863863E-31**	1.19270330128188	0.102	0.02	3.24527581835646E-27	Periportal hepatocytes	DEPRECAT
**2.06342564586605E-31**	0.485853822222818	0.717	0.461	3.36565357097211E-27	Periportal hepatocytes	TSKU
**2.07518391740612E-31**	0.4717967732804	0.358	0.162	3.38483248768112E-27	Periportal hepatocytes	CMTM6
**3.00758742860514E-31**	1.66877794492245	0.182	0.059	4.90567585479785E-27	Periportal hepatocytes	ZNF333
**3.24185130311444E-31**	0.542988021654304	0.53	0.283	5.28778366050996E-27	Periportal hepatocytes	HGSNAT
**3.49211231905178E-31**	0.4682148660639	0.592	0.328	5.69598440360535E-27	Periportal hepatocytes	SGK2
**3.84570170836709E-31**	0.380548961891917	0.806	0.507	6.27272405651756E-27	Periportal hepatocytes	GCLC
**5.17582064209681E-31**	0.520863094299778	0.463	0.237	8.44228104932411E-27	Periportal hepatocytes	GPLD1
**5.30338128463451E-31**	0.535401386056245	0.632	0.368	8.65034521336735E-27	Periportal hepatocytes	SLC41A2
**5.61339099300022E-31**	0.291977219427591	0.72	0.44	9.15600204868266E-27	Periportal hepatocytes	SLC2A2
**5.67628375964043E-31**	0.330636999208988	0.506	0.269	9.2585864403495E-27	Periportal hepatocytes	CTDSP2
**6.24514444575289E-31**	0.418890691862463	0.338	0.152	1.01864551054675E-26	Periportal hepatocytes	THOP1
**6.5384625211349E-31**	1.03183253881934	0.279	0.115	1.06648862182231E-26	Periportal hepatocytes	SUGCT
**6.60723309326866E-31**	0.93893328361575	0.193	0.064	1.07770578984305E-26	Periportal hepatocytes	MARCKSL1
**6.95542473081207E-31**	0.302600636081382	0.822	0.498	1.13449932784276E-26	Periportal hepatocytes	EXT1
**6.9989993908724E-31**	0.677847818698899	0.397	0.189	1.1416067906452E-26	Periportal hepatocytes	MBNL2
**7.50287230585891E-31**	0.970082358253891	0.272	0.109	1.22379350180865E-26	Periportal hepatocytes	NOTCH2
**7.5393033718952E-31**	0.942844025823709	0.267	0.108	1.22973577298983E-26	Periportal hepatocytes	ZC3H13
**8.00739007791241E-31**	0.282017619754933	0.483	0.252	1.30608539560829E-26	Periportal hepatocytes	RAB2A
**8.7560039050304E-31**	0.774022363169805	0.401	0.198	1.42819179694951E-26	Periportal hepatocytes	PHF20L1
**9.85674895516123E-31**	0.469672245630039	0.575	0.321	1.60773432207635E-26	Periportal hepatocytes	PI4K2B
**1.021509226773E-30**	0.872546022653369	0.17	0.052	1.66618369978943E-26	Periportal hepatocytes	NCALD
**1.17280499369235E-30**	0.437593589749141	0.474	0.244	1.91296222521159E-26	Periportal hepatocytes	SLC46A1
**1.50332086519515E-30**	0.378513277817907	0.52	0.284	2.45206666321981E-26	Periportal hepatocytes	DENND10
**1.53349289131902E-30**	0.44815714402473	0.506	0.27	2.50128025503046E-26	Periportal hepatocytes	CUL7
**1.57882143294027E-30**	0.387518239528658	0.767	0.466	2.57521563926887E-26	Periportal hepatocytes	NFKBIZ
**1.59699022596922E-30**	0.348026580640684	0.282	0.115	2.60485075757839E-26	Periportal hepatocytes	MICOS10

**1.74659229090453E-30**	0.357502478297031	0.841	0.549	2.84886668569437E-26	Periportal hepatocytes	ALDH3A2
**1.87249175113598E-30**	0.290413310807283	0.825	0.592	3.0542212952779E-26	Periportal hepatocytes	IDH2
**2.04974134150227E-30**	0.380469783731436	0.351	0.159	3.34333310212435E-26	Periportal hepatocytes	HADH
**2.32401330346791E-30**	0.31404245322928	0.67	0.391	3.79069809928651E-26	Periportal hepatocytes	C1RL
**2.79516708029138E-30**	0.356883676053045	0.477	0.247	4.55919702466327E-26	Periportal hepatocytes	PLS3
**3.33442262630096E-30**	0.328490639625177	0.606	0.34	5.4387767457595E-26	Periportal hepatocytes	SLC43A1
**3.61335441239643E-30**	0.311098039553863	0.336	0.151	5.89374238205982E-26	Periportal hepatocytes	RITA1
**4.18559359820007E-30**	0.380285184724281	0.364	0.168	6.82712171802414E-26	Periportal hepatocytes	REPIN1
**6.06923790804285E-30**	0.436942502549454	0.264	0.107	9.8995339518087E-26	Periportal hepatocytes	GPC6
**6.66254764832242E-30**	0.524905005754766	0.553	0.308	1.08672814691787E-25	Periportal hepatocytes	ABCC6
**6.9129892845969E-30**	0.403411044677523	0.976	0.74	1.1275776822106E-25	Periportal hepatocytes	EFNA1
**6.99969889169892E-30**	0.634031941495698	0.428	0.216	1.14172088622501E-25	Periportal hepatocytes	DEPRECAT
**7.0836952353944E-30**	0.316127482732093	0.807	0.488	1.15542152984518E-25	Periportal hepatocytes	NR1H4
**7.47479255430407E-30**	0.252954898447871	0.721	0.436	1.21921341353254E-25	Periportal hepatocytes	TOR1AIP2
**7.86000741736202E-30**	0.981953316927863	0.188	0.062	1.28204580984592E-25	Periportal hepatocytes	CNGA1
**8.14774627174892E-30**	0.352602924855943	0.714	0.419	1.32897889438497E-25	Periportal hepatocytes	NR5A2
**8.19063746108698E-30**	0.564330503810782	0.29	0.121	1.3359748762779E-25	Periportal hepatocytes	FBXL3
**1.03165291569549E-29**	0.679470970631073	0.325	0.144	1.68272907079092E-25	Periportal hepatocytes	ZNF7
**1.25990035401186E-29**	0.6194724999106	0.376	0.179	2.05502346742874E-25	Periportal hepatocytes	PTK2
**1.28766491510945E-29**	0.560648703820433	0.389	0.19	2.10031024303503E-25	Periportal hepatocytes	SLC6A11
**1.46022311999878E-29**	0.649829360886516	0.32	0.141	2.38176993103001E-25	Periportal hepatocytes	VPS41
**1.65440756637064E-29**	0.323467332572571	0.417	0.205	2.69850418150715E-25	Periportal hepatocytes	STOM
**1.7099895815928E-29**	1.08192175741101	0.171	0.054	2.78916400653601E-25	Periportal hepatocytes	RBAK
**1.72553169566132E-29**	1.3424383391414	0.175	0.057	2.81451474879317E-25	Periportal hepatocytes	ARSB
**1.78815177321788E-29**	0.322625857577802	0.864	0.592	2.91665435729568E-25	Periportal hepatocytes	CSRP1
**1.79927438701655E-29**	0.925267750588253	0.359	0.173	2.9347964526627E-25	Periportal hepatocytes	GAS2
**2.07940424973706E-29**	0.398390121989493	0.626	0.359	3.39171627174612E-25	Periportal hepatocytes	MAT2A
**2.0976694551644E-29**	1.26170787387577	0.178	0.059	3.42150864831865E-25	Periportal hepatocytes	FAN1
**2.61056125682911E-29**	0.343621519288143	0.362	0.17	4.25808646601395E-25	Periportal hepatocytes	RTN4RL2
**2.76220666409141E-29**	0.846124808561693	0.263	0.107	4.50543528979949E-25	Periportal hepatocytes	KIAA1191
**2.87238293727511E-29**	0.493229661771449	0.491	0.264	4.68514380898943E-25	Periportal hepatocytes	ELOVL5
**2.91382729677983E-29**	0.27993668780488	0.434	0.22	4.75274370377758E-25	Periportal hepatocytes	COPS5
**3.21576251153695E-29**	0.474827980984213	0.579	0.327	5.24523023256791E-25	Periportal hepatocytes	CYTH1
**3.55184623139862E-29**	0.332512376316888	0.359	0.169	5.79341638803429E-25	Periportal hepatocytes	CHPF2
**3.91915680144583E-29**	0.276387620690578	0.793	0.482	6.3925366588383E-25	Periportal hepatocytes	H6PD
**3.98718228950645E-29**	0.250228484212213	0.841	0.6	6.50349303241398E-25	Periportal hepatocytes	ADI1
**4.20123820914359E-29**	0.837691612544565	0.303	0.133	6.8526396429341E-25	Periportal hepatocytes	DGLUCY
**4.6294148899949E-29**	0.554515259652201	0.405	0.2	7.55103862707068E-25	Periportal hepatocytes	ATG4B
**4.64472059322814E-29**	0.525672607617332	0.496	0.264	7.57600375961442E-25	Periportal hepatocytes	OTULIN
**4.75606523972107E-29**	0.708620975970938	0.366	0.176	7.75761801250903E-25	Periportal hepatocytes	SLC25A38
**4.81939938365543E-29**	1.02259259273694	0.161	0.049	7.86092233468038E-25	Periportal hepatocytes	SOCS2
**5.19617523951923E-29**	0.285638155387505	0.7	0.44	8.47548143317981E-25	Periportal hepatocytes	DGAT1
**5.26296878817845E-29**	0.374564898787879	0.343	0.159	8.58442839039787E-25	Periportal hepatocytes	STK24
**6.60316485895838E-29**	0.73789769195507	0.359	0.169	1.0770422201447E-24	Periportal hepatocytes	MBNL1
**8.95693920995639E-29**	0.28824533810759	0.816	0.52	1.46096635453599E-24	Periportal hepatocytes	F5
**9.37270082194355E-29**	1.50394406946475	0.128	0.034	1.52878123106721E-24	Periportal hepatocytes	ABCA8
**9.40454682467762E-29**	1.25417738623323	0.188	0.065	1.53397563257317E-24	Periportal hepatocytes	FAM221A
**9.58828297963904E-29**	0.337596639585642	0.428	0.22	1.56394483680892E-24	Periportal hepatocytes	OCIAD2
**1.17045879934088E-28**	0.598633794769608	0.483	0.261	1.90913534760491E-24	Periportal hepatocytes	JMJD1C
**1.30783749745005E-28**	0.796363906304841	0.391	0.194	2.13321374209077E-24	Periportal hepatocytes	NCOA2
**1.50433452775094E-28**	0.42052306136141	0.635	0.373	2.45372004821456E-24	Periportal hepatocytes	UAP1
**1.80953348811263E-28**	1.10694269351124	0.213	0.079	2.95153007246051E-24	Periportal hepatocytes	ZNF12
**2.05642320344292E-28**	0.498647212667746	0.197	0.069	3.35423188713574E-24	Periportal hepatocytes	GGCT
**2.09984008743211E-28**	1.03967447039756	0.138	0.039	3.42504916661052E-24	Periportal hepatocytes	LRRC3
**2.10703071021856E-28**	0.371376398098038	0.51	0.281	3.43677779143749E-24	Periportal hepatocytes	RHPN2
**2.25064810237409E-28**	0.849409305621216	0.338	0.157	3.67103211978238E-24	Periportal hepatocytes	PHC3
**2.34576744641375E-28**	0.580046242167976	0.312	0.141	3.82618128184547E-24	Periportal hepatocytes	NF1
**2.36167174346408E-28**	0.403911813878657	0.629	0.366	3.85212278076427E-24	Periportal hepatocytes	TTC38
**2.56869114429078E-28**	0.423451062329074	0.441	0.228	4.18979212545269E-24	Periportal hepatocytes	UBR5
**2.91707169457142E-28**	1.12182579522509	0.227	0.087	4.75803564101545E-24	Periportal hepatocytes	DEPRECAT

3.34270999930332E-28	1.09207410733906	0.233	0.091	5.45229427986364E-24	Periportal hepatocytes	C1GALT1
3.5869577200715E-28	0.297672075051598	0.444	0.232	5.85068673720862E-24	Periportal hepatocytes	NFIL3
4.78600671482816E-28	0.795771383630974	0.168	0.054	7.8064555525562E-24	Periportal hepatocytes	KRT7
4.869494346942E-28	0.639366199280459	0.497	0.27	7.9426322292971E-24	Periportal hepatocytes	DEPRECAT
5.03342876403228E-28	0.285175234355664	0.305	0.134	8.21002565701306E-24	Periportal hepatocytes	C16orf87
5.45865689654106E-28	0.308200594664153	0.364	0.172	8.90361526394813E-24	Periportal hepatocytes	C6orf89
5.76104901985777E-28	1.02392807358177	0.273	0.117	9.39684705629001E-24	Periportal hepatocytes	SLC45A3
6.0852333844346E-28	0.317723403065725	0.244	0.097	9.92562417335128E-24	Periportal hepatocytes	PALLD
6.24989757863521E-28	0.368911028124107	0.353	0.166	1.01942079405119E-23	Periportal hepatocytes	CPD
6.32752757337785E-28	1.0162825774715	0.187	0.065	1.03208302249366E-23	Periportal hepatocytes	FAAH2
7.13773196756289E-28	0.254885499386118	0.782	0.477	1.16423546122918E-23	Periportal hepatocytes	VMP1
7.34561371853346E-28	0.459479127044323	0.473	0.25	1.19814305362999E-23	Periportal hepatocytes	VDAC3
7.84185673225483E-28	0.305108760509345	0.32	0.146	1.27908525159808E-23	Periportal hepatocytes	OPN3
8.00350161367858E-28	0.305253160241405	0.634	0.365	1.30545114820711E-23	Periportal hepatocytes	CYP2C18
8.58254521482493E-28	1.19192641962204	0.184	0.063	1.39989894999009E-23	Periportal hepatocytes	DEPRECAT
1.03929566088161E-27	0.664803798214852	0.415	0.211	1.695195152464E-23	Periportal hepatocytes	NUFIP2
1.17108838721036E-27	0.331414872196658	0.499	0.27	1.91016226837882E-23	Periportal hepatocytes	EFR3A
1.20530576908526E-27	0.561874725798647	0.175	0.058	1.96597423995497E-23	Periportal hepatocytes	SLC22A18/
1.22316883217922E-27	1.02854811652555	0.201	0.073	1.99511068216752E-23	Periportal hepatocytes	ZHX3
1.82099848603148E-27	1.19883495110565	0.18	0.061	2.97023063056594E-23	Periportal hepatocytes	ANGPTL6
1.83226062183966E-27	0.709059537573452	0.427	0.223	2.98860030028267E-23	Periportal hepatocytes	THBS3
1.91548079325952E-27	0.279867627729771	0.591	0.341	3.12434072188561E-23	Periportal hepatocytes	ZBTB16
2.04238066578036E-27	0.401318445949599	0.48	0.256	3.33132710395434E-23	Periportal hepatocytes	UBA7
2.10537835646149E-27	1.02460561335102	0.155	0.048	3.43408263722433E-23	Periportal hepatocytes	MTERF3
2.31586530702617E-27	1.36732776985768	0.115	0.029	3.77740790229039E-23	Periportal hepatocytes	DSC2
3.20925306301438E-27	0.391356347033367	0.366	0.178	5.23461267108276E-23	Periportal hepatocytes	PCYT1A
3.26259348216344E-27	0.67905257453103	0.295	0.13	5.32161622875678E-23	Periportal hepatocytes	MLXIP
3.64888028110315E-27	0.479578089531591	0.378	0.184	5.95168862650734E-23	Periportal hepatocytes	SKAP2
3.70402794540678E-27	2.07021411468552	0.109	0.027	6.041639981753E-23	Periportal hepatocytes	HMCN1
3.91774285300092E-27	0.497513888392387	0.444	0.237	6.39023036752979E-23	Periportal hepatocytes	AKAP8L
4.26173105366926E-27	0.567952839394347	0.374	0.182	6.95130952163993E-23	Periportal hepatocytes	F12
4.30754672321355E-27	0.371667433050614	0.348	0.168	7.02603946023362E-23	Periportal hepatocytes	MAL2
4.53195066591984E-27	0.352095882539529	0.411	0.207	7.39206473118186E-23	Periportal hepatocytes	MACO1
4.63938602750525E-27	1.13667808468648	0.267	0.115	7.56730254946381E-23	Periportal hepatocytes	DAGLB
5.3010092935075E-27	0.290203073255804	0.428	0.218	8.64647625864008E-23	Periportal hepatocytes	VAC14
5.3052098024405E-27	0.439119144845119	0.328	0.153	8.6533277087607E-23	Periportal hepatocytes	HERC2
6.14279707464036E-27	0.949808659604271	0.165	0.055	1.00195163084459E-22	Periportal hepatocytes	HOGA1
6.15700205888036E-27	0.250880419580777	0.391	0.196	1.00426860582398E-22	Periportal hepatocytes	RAC1
6.39072900831817E-27	0.942724718770222	0.207	0.078	1.04239180854678E-22	Periportal hepatocytes	MINDY1
6.47789889061739E-27	0.499960084703098	0.467	0.253	1.0566100880486E-22	Periportal hepatocytes	CEP70
8.12768331802935E-27	0.863247690856542	0.273	0.118	1.32570642600377E-22	Periportal hepatocytes	NDUFAF7
8.59685283053356E-27	0.578828741032593	0.516	0.287	1.40223266518833E-22	Periportal hepatocytes	TRA2A
8.80053332063466E-27	0.531916031049171	0.428	0.223	1.43545498992872E-22	Periportal hepatocytes	MON2
9.31331033882491E-27	0.367370097408536	0.674	0.394	1.51909404936573E-22	Periportal hepatocytes	PATJ
9.31572323432838E-27	1.05747761301536	0.118	0.031	1.5194876167513E-22	Periportal hepatocytes	HMGB3
9.97215351558279E-27	0.257107792406967	0.356	0.173	1.62655795992671E-22	Periportal hepatocytes	ZNF24
1.00065326116681E-26	0.266327800494796	0.26	0.108	1.63216553428919E-22	Periportal hepatocytes	ACKR2
1.23182485453272E-26	1.11037195786501	0.226	0.09	2.00922952022832E-22	Periportal hepatocytes	PRG2
1.23522619610878E-26	0.27367246600057	0.332	0.157	2.01477744847303E-22	Periportal hepatocytes	C8orf33
1.28369021246697E-26	0.433274080589348	0.463	0.246	2.09382710555487E-22	Periportal hepatocytes	OSBPL9
1.38237626929548E-26	0.303487978517353	0.414	0.212	2.25479393284786E-22	Periportal hepatocytes	TSC2
1.44503889864756E-26	0.368774967958191	0.993	0.988	2.35700294758404E-22	Periportal hepatocytes	TF
1.52158177805869E-26	0.261309237838128	0.33	0.155	2.48185203819153E-22	Periportal hepatocytes	GFM1
1.56470034021127E-26	0.505181650460663	0.211	0.08	2.5521827249186E-22	Periportal hepatocytes	WWTR1
1.5738980476337E-26	0.363346199070655	0.526	0.294	2.56718510549534E-22	Periportal hepatocytes	AFF4
1.90261534848024E-26	0.4287696552722	0.978	0.785	3.10335589490612E-22	Periportal hepatocytes	ITIH3
2.08185888672327E-26	0.327941058458319	0.319	0.149	3.39572003013432E-22	Periportal hepatocytes	PLEKHF1
2.19226285015746E-26	0.815986594520238	0.177	0.061	3.57579993489184E-22	Periportal hepatocytes	FBXO32
2.62212173623515E-26	0.371078950525195	0.254	0.105	4.27694276397315E-22	Periportal hepatocytes	ELMO1
2.95413387981192E-26	0.510346711877738	0.295	0.133	4.81848777136123E-22	Periportal hepatocytes	DHRSX

2.98526210237647E-26	0.348012434543044	0.253	0.106	4.86926101518626E-22	Periportal hepatocytes	SEMA5A
3.11566648781031E-26	0.531020717307867	0.533	0.304	5.0819636082674E-22	Periportal hepatocytes	FAM214A
3.24808959837696E-26	0.310707668753375	0.493	0.271	5.29795894391266E-22	Periportal hepatocytes	AKT2
3.32945227716588E-26	0.514709452522116	0.292	0.131	5.43066960928527E-22	Periportal hepatocytes	FXR1
3.48081965115234E-26	0.834109283408986	0.323	0.153	5.67756493299458E-22	Periportal hepatocytes	MFN1
3.70725326502308E-26	0.68950086604994	0.261	0.112	6.04690080057915E-22	Periportal hepatocytes	SIK1B
4.2771708224973E-26	0.334630571082582	0.437	0.229	6.97649332857535E-22	Periportal hepatocytes	ACVR1B
4.33834568160917E-26	0.317962901542892	0.306	0.138	7.07627564127272E-22	Periportal hepatocytes	DEPRECAT
4.3904371364643E-26	0.473165467606131	0.264	0.113	7.16124201328691E-22	Periportal hepatocytes	HFE
5.08025991889886E-26	0.505994648039657	0.511	0.29	8.28641195371592E-22	Periportal hepatocytes	SLC25A16
5.20651343492493E-26	0.321317765082426	0.641	0.371	8.49234406370606E-22	Periportal hepatocytes	PLA2G6
5.21407233848044E-26	0.329831034524871	0.776	0.483	8.50467339129545E-22	Periportal hepatocytes	TCF25
5.29765572280306E-26	0.363652726289795	0.361	0.176	8.64100624946407E-22	Periportal hepatocytes	YTHDF3
5.94042737690747E-26	0.372712520008506	0.878	0.542	9.68943109447377E-22	Periportal hepatocytes	SEC16B
6.50896602948869E-26	0.49402606844418	0.223	0.087	1.0616774490699E-21	Periportal hepatocytes	DIS3
6.76475996112491E-26	0.787414106930319	0.328	0.156	1.10339999725908E-21	Periportal hepatocytes	SS18L1
6.87414120183929E-26	0.332813957740712	0.454	0.243	1.12124117143201E-21	Periportal hepatocytes	AP5Z1
6.93033177081022E-26	1.17936757178983	0.165	0.056	1.13040641513685E-21	Periportal hepatocytes	GK
7.73667263500417E-26	0.47579370714857	0.499	0.275	1.26192867349553E-21	Periportal hepatocytes	PHKA2
7.76139812207268E-26	0.458488051763663	0.553	0.313	1.26596164769127E-21	Periportal hepatocytes	KIAA1217
9.36336909559129E-26	0.781410510705989	0.325	0.154	1.52725913318189E-21	Periportal hepatocytes	ZNF655
9.37487206726098E-26	0.3978591995183	0.517	0.292	1.52913538289094E-21	Periportal hepatocytes	BNIP2
9.43934889768269E-26	0.54540233073674	0.223	0.087	1.53965219870102E-21	Periportal hepatocytes	HAO2
9.59888677125077E-26	0.484395780178064	0.451	0.247	1.56567442125871E-21	Periportal hepatocytes	DEPRECAT
1.03677490498274E-25	0.955539923863084	0.136	0.041	1.69108354751735E-21	Periportal hepatocytes	NRN1
1.31507217712674E-25	0.789974212717366	0.244	0.102	2.14501422811143E-21	Periportal hepatocytes	ZSCAN30
1.33763451522865E-25	0.336605231322772	0.362	0.177	2.18181565778945E-21	Periportal hepatocytes	AR
1.40297217047226E-25	0.986347850198523	0.208	0.08	2.2883879072573E-21	Periportal hepatocytes	ZNF75A
1.81488293004766E-25	0.378696155149696	0.599	0.351	2.96025554720073E-21	Periportal hepatocytes	ZSWIM8
1.82852863380902E-25	0.401349746337867	0.711	0.43	2.98251305460589E-21	Periportal hepatocytes	ARAP1
2.02630406431529E-25	0.404001216300068	0.486	0.269	3.30510455930466E-21	Periportal hepatocytes	KLC4
2.15824266343615E-25	0.497400866263537	0.448	0.245	3.52030960833071E-21	Periportal hepatocytes	EPB41L5
2.41229146797365E-25	1.3008729391783	0.164	0.056	3.93468861341182E-21	Periportal hepatocytes	GPR88
2.51316506407439E-25	0.428003381671927	0.349	0.172	4.09922353601173E-21	Periportal hepatocytes	TLCD4
2.95753764258525E-25	0.557790630697546	0.289	0.132	4.8240396488208E-21	Periportal hepatocytes	DCUN1D1
3.17332898457078E-25	0.401848906516552	0.348	0.169	5.1760169067334E-21	Periportal hepatocytes	CDC25B
3.61266651532527E-25	0.29133116255028	0.161	0.054	5.89262035314705E-21	Periportal hepatocytes	NRXN2
3.63961937883466E-25	0.349594010376925	0.568	0.326	5.93658316881721E-21	Periportal hepatocytes	ABLIM3
3.66003702063654E-25	0.553444170959724	0.514	0.304	5.96988638436025E-21	Periportal hepatocytes	DDIT4
3.69997647299568E-25	0.28380699404076	0.529	0.3	6.03503162510325E-21	Periportal hepatocytes	CCDC50
3.74473798662157E-25	0.427214504415938	0.447	0.238	6.10804212997844E-21	Periportal hepatocytes	PCMTD2
3.84520885167054E-25	0.27317270487096	0.58	0.338	6.27192015795982E-21	Periportal hepatocytes	DVL3
4.17864580192985E-25	0.942499896520789	0.287	0.133	6.81578916752777E-21	Periportal hepatocytes	ARHGAP29
4.34851009674371E-25	0.596700515989188	0.157	0.052	7.09285481879866E-21	Periportal hepatocytes	ABCF2
4.54811283148582E-25	0.631581566722748	0.333	0.161	7.41842683943651E-21	Periportal hepatocytes	ZBTB10
4.55581027882457E-25	0.704137172513324	0.29	0.132	7.43098214579075E-21	Periportal hepatocytes	AVEN
4.90063286465815E-25	0.640570230143454	0.353	0.176	7.99342226554391E-21	Periportal hepatocytes	AKTIP
5.25029775878349E-25	0.526831008561701	0.407	0.209	8.56376067435175E-21	Periportal hepatocytes	GLYATL1
5.29442714991363E-25	0.383279637467042	0.743	0.455	8.63574012422412E-21	Periportal hepatocytes	RNF213
6.14344920820191E-25	0.590035458877925	0.342	0.169	1.00205800034981E-20	Periportal hepatocytes	ANKRD13A
6.53221296358665E-25	0.415759337454976	0.375	0.193	1.06546925649062E-20	Periportal hepatocytes	KLHDC10
7.00725105830647E-25	0.348993038603235	0.374	0.188	1.14295272012037E-20	Periportal hepatocytes	CISH
7.669274150317E-25	0.646087832857456	0.45	0.243	1.25093530665821E-20	Periportal hepatocytes	SFXN5
8.0506816771797E-25	0.728279473067956	0.213	0.084	1.31314668836478E-20	Periportal hepatocytes	DDX52
8.22503098411019E-25	0.678394263510093	0.272	0.122	1.34158480381821E-20	Periportal hepatocytes	LRP6
8.54719147882739E-25	0.822299294139877	0.181	0.066	1.39413240211154E-20	Periportal hepatocytes	SLC23A1
8.57960843714577E-25	0.466728342106848	0.5	0.277	1.39941993218285E-20	Periportal hepatocytes	ATP11B
8.95321583400487E-25	0.542627978562703	0.309	0.146	1.46035903468453E-20	Periportal hepatocytes	CCT8
1.01362708727019E-24	0.617800409072546	0.323	0.157	1.65332714204641E-20	Periportal hepatocytes	TBC1D23
1.08222529976431E-24	0.443106995937847	0.325	0.157	1.76521768644556E-20	Periportal hepatocytes	PAK2

1.12153750830195E-24	0.450853417107842	0.468	0.257	1.82933982979131E-20	Periportal hepatocytes	ACSS2
1.13630310429705E-24	0.263282058882829	0.44	0.237	1.85342399341891E-20	Periportal hepatocytes	MAPK6
1.16616233390934E-24	0.441268487410381	0.578	0.346	1.90212738283953E-20	Periportal hepatocytes	SLC38A2
1.17811609086794E-24	0.8860670623147	0.297	0.14	1.9216251558147E-20	Periportal hepatocytes	SGK3
1.1926696889072E-24	0.490382723604826	0.223	0.09	1.94536352957654E-20	Periportal hepatocytes	TMEM192
1.288863402262E-24	0.340102437326954	0.346	0.172	2.10226509542954E-20	Periportal hepatocytes	WDTC1
1.29758257503262E-24	0.733976264817854	0.355	0.18	2.11648693813571E-20	Periportal hepatocytes	ATL2
1.36078078121667E-24	0.743173675460956	0.303	0.143	2.21956953224251E-20	Periportal hepatocytes	DXO
1.40744426815762E-24	0.486830854599809	0.44	0.239	2.29568234579189E-20	Periportal hepatocytes	FBXO9
1.43287535159597E-24	0.343173743248127	0.217	0.087	2.33716298598819E-20	Periportal hepatocytes	TMEM41A
1.46899625274896E-24	0.469401011702273	0.537	0.309	2.39607978785882E-20	Periportal hepatocytes	TNS3
1.5424849940176E-24	0.452600738224236	0.51	0.29	2.51594727374211E-20	Periportal hepatocytes	TRIO
1.56600717870247E-24	0.2651894729828	0.286	0.129	2.55431430918159E-20	Periportal hepatocytes	MPP6
1.58221067349691E-24	0.96433788202901	0.21	0.083	2.58074382954081E-20	Periportal hepatocytes	ZNF862
1.74530136910405E-24	0.606134051744554	0.404	0.213	2.84676106314562E-20	Periportal hepatocytes	SEC24A
2.0034180674303E-24	0.277586542525538	0.774	0.477	3.26777520978557E-20	Periportal hepatocytes	PPP6R2
2.0864253366311E-24	0.300477231470075	0.329	0.158	3.40316836657899E-20	Periportal hepatocytes	RAPGEF2
2.63422379845819E-24	0.414567362291885	0.362	0.181	4.29668243766515E-20	Periportal hepatocytes	RNF123
2.66063750692305E-24	0.6482458922713	0.487	0.276	4.33976583754218E-20	Periportal hepatocytes	TBC1D8
2.69045399528023E-24	0.740049674665724	0.368	0.191	4.38839951170159E-20	Periportal hepatocytes	CPSF1
2.79080358229114E-24	0.397427971140301	0.289	0.134	4.55207972307508E-20	Periportal hepatocytes	ZNF106
2.8508609007535E-24	0.323623516067346	0.575	0.343	4.65003921521903E-20	Periportal hepatocytes	EBNA1BP2
2.89415830321456E-24	0.73235516977265	0.24	0.103	4.72066160837328E-20	Periportal hepatocytes	GOSR1
3.35831795767948E-24	0.364452898105799	0.655	0.387	5.477752420771E-20	Periportal hepatocytes	KANK1
3.43265890386068E-24	1.00258297097029	0.165	0.058	5.59900993808716E-20	Periportal hepatocytes	KRT86
4.03407604656857E-24	0.333022190811624	0.595	0.351	6.57998143955799E-20	Periportal hepatocytes	MROH1
4.10005952186159E-24	0.857279952674198	0.205	0.082	6.68760708610844E-20	Periportal hepatocytes	SLC43A2
4.85891058617837E-24	0.314889282428003	0.642	0.393	7.92536905711554E-20	Periportal hepatocytes	OPLAH
5.09549198601013E-24	0.813969020213507	0.261	0.116	8.31125697838112E-20	Periportal hepatocytes	BRD8
5.21041635638234E-24	0.270620462114569	0.296	0.139	8.49871011889524E-20	Periportal hepatocytes	MAOA
6.10495772232836E-24	0.661540271642375	0.284	0.134	9.95779654088979E-20	Periportal hepatocytes	SLC25A30
6.13512007900947E-24	0.263149080252672	0.527	0.306	1.00069943608723E-19	Periportal hepatocytes	EIF5
6.4022564651475E-24	1.16223925936711	0.181	0.067	1.04427205203021E-19	Periportal hepatocytes	LRIF1
7.12230616256145E-24	0.3488729850845	0.221	0.091	1.1617193581754E-19	Periportal hepatocytes	AGA
8.52427147803267E-24	1.27323617927197	0.201	0.08	1.39039392078191E-19	Periportal hepatocytes	ZNF436
8.7585564685849E-24	1.60154041333514	0.106	0.028	1.42860814559088E-19	Periportal hepatocytes	DEPRECAT
8.97714645452606E-24	0.296094370581663	0.371	0.192	1.46426235819775E-19	Periportal hepatocytes	ANKH
9.45896500340086E-24	0.270934793276855	0.506	0.284	1.54285178170471E-19	Periportal hepatocytes	FAM160A2
1.02641914495802E-23	0.567848018728384	0.44	0.242	1.67419226734102E-19	Periportal hepatocytes	GLDC
1.10156924145557E-23	0.345853667564614	0.576	0.341	1.79676958973817E-19	Periportal hepatocytes	STAU2
1.12734008000809E-23	0.740398841033823	0.33	0.164	1.83880440450119E-19	Periportal hepatocytes	MORC3
1.14667282777319E-23	0.673278085209974	0.322	0.159	1.87033804938085E-19	Periportal hepatocytes	NBPF15
1.19980172860275E-23	0.516272653888538	0.341	0.17	1.95699659952394E-19	Periportal hepatocytes	MAPRE2
1.22089037269429E-23	0.981651466640629	0.162	0.057	1.99139428690166E-19	Periportal hepatocytes	SLC1A1
1.34936653960772E-23	0.462906278468597	0.349	0.176	2.20095176275415E-19	Periportal hepatocytes	MOGAT3
1.47003549271948E-23	0.759736329974189	0.364	0.188	2.39777489217475E-19	Periportal hepatocytes	KAT2A
1.49905789258289E-23	0.434407759190486	0.323	0.16	2.44511332859195E-19	Periportal hepatocytes	PPP3CB
1.54029598603978E-23	0.65412895167042	0.345	0.172	2.51237678282949E-19	Periportal hepatocytes	KIDINS220
1.69131954586351E-23	0.251067318267503	0.566	0.329	2.75871131125797E-19	Periportal hepatocytes	PER1
1.84372066773468E-23	0.608246086566293	0.326	0.159	3.00729278114204E-19	Periportal hepatocytes	LRRC14
2.30237316141161E-23	0.324832486293554	0.305	0.144	3.75540086357848E-19	Periportal hepatocytes	SDCBP
2.34101881519437E-23	0.263761612372447	0.391	0.204	3.81843578946354E-19	Periportal hepatocytes	RTL8C
2.35552089278553E-23	0.609967499377015	0.257	0.114	3.84209012822248E-19	Periportal hepatocytes	B9D1
2.53986133186512E-23	0.80606146573439	0.118	0.034	4.1427678184052E-19	Periportal hepatocytes	IPMK
2.56343753005334E-23	0.423926020404713	0.451	0.254	4.18122295527E-19	Periportal hepatocytes	CREBZF
2.58069387079133E-23	0.419870662067834	0.322	0.158	4.20936977264775E-19	Periportal hepatocytes	CPEB2
2.61741915781559E-23	0.470628293588966	0.391	0.207	4.26927238831301E-19	Periportal hepatocytes	ARAF
2.64683155666341E-23	0.497793446133952	0.227	0.096	4.31724695207369E-19	Periportal hepatocytes	SHROOM3
2.9395445962035E-23	0.696732874778085	0.499	0.291	4.79469119086753E-19	Periportal hepatocytes	KNDC1
3.00061203352583E-23	0.48405233822927	0.371	0.191	4.89429828788398E-19	Periportal hepatocytes	KIAA0100

3.05037958467653E-23	0.423077103723712	0.364	0.185	4.97547414056589E-19	Periportal hepatocytes	MTFR1L
3.20950798088681E-23	0.271178419114557	0.79	0.474	5.23502846762447E-19	Periportal hepatocytes	ARGLU1
3.32140358224262E-23	0.278335552729444	0.369	0.191	5.41754138299594E-19	Periportal hepatocytes	ELMOD2
3.33082037238868E-23	0.848430396278253	0.168	0.061	5.43290110940317E-19	Periportal hepatocytes	DEPRECAT
3.38274811810026E-23	0.620346506637922	0.51	0.295	5.51760045543333E-19	Periportal hepatocytes	DDX17
3.74301658278525E-23	0.770456855554084	0.115	0.033	6.10523434818103E-19	Periportal hepatocytes	RBP1
3.90059173952986E-23	0.337566723759185	0.639	0.387	6.36225518634715E-19	Periportal hepatocytes	TCIRG1
3.93011012687171E-23	0.323758212949624	0.487	0.273	6.41040262794044E-19	Periportal hepatocytes	UPK3BL1
4.03996403406855E-23	1.36249209433636	0.102	0.027	6.58958533596921E-19	Periportal hepatocytes	ACTR3B
4.07002373502718E-23	0.386390679182049	0.652	0.4	6.63861571420284E-19	Periportal hepatocytes	MARC2
4.3044248896654E-23	0.553828092939581	0.276	0.13	7.02094743753324E-19	Periportal hepatocytes	KBTBD2
4.43720225491328E-23	0.925470418424943	0.217	0.09	7.23752059798904E-19	Periportal hepatocytes	CRYM
4.804072014405E-23	1.12485451699148	0.17	0.062	7.835921862696E-19	Periportal hepatocytes	SLC17A1
4.90244908956305E-23	0.43742264543155	0.302	0.144	7.9963847099863E-19	Periportal hepatocytes	PALM2-AKA
5.83630902945028E-23	0.43510417865989	0.207	0.084	9.51960365793636E-19	Periportal hepatocytes	BMP2
6.11913826115811E-23	0.799600790803471	0.205	0.083	9.98092641777499E-19	Periportal hepatocytes	ACACA
6.34692395622198E-23	0.709132875019487	0.259	0.116	1.03524676649937E-18	Periportal hepatocytes	SELENOI
6.69800761752588E-23	0.589398931821328	0.129	0.04	1.09251202249465E-18	Periportal hepatocytes	ADH5
9.61001082292501E-23	0.641861752308232	0.269	0.124	1.5674888653273E-18	Periportal hepatocytes	CCNB1IP1
9.66144826352369E-23	0.53324350502554	0.323	0.162	1.57587882626335E-18	Periportal hepatocytes	EIF4G3
9.83996248638096E-23	0.38908091792202	0.596	0.357	1.6049962811536E-18	Periportal hepatocytes	ITPR2
1.00908003004917E-22	0.355162754136895	0.2	0.08	1.6459104370132E-18	Periportal hepatocytes	EEF1AKMT
1.04652716910376E-22	0.56243613409191	0.293	0.139	1.70699046552515E-18	Periportal hepatocytes	SSBP3
1.05167672045966E-22	0.341990855939097	0.631	0.381	1.71538989874175E-18	Periportal hepatocytes	NFE2L2
1.0817668590684E-22	0.289701884929924	0.3	0.145	1.76446992382647E-18	Periportal hepatocytes	GATD3A
1.14306359127609E-22	0.280392762004278	0.555	0.32	1.86445102373043E-18	Periportal hepatocytes	ABCD3
1.20095945178354E-22	0.537588402095615	0.345	0.176	1.95888496180413E-18	Periportal hepatocytes	GALNS
1.21026874161594E-22	0.563889455487788	0.251	0.112	1.97406934444975E-18	Periportal hepatocytes	C5orf51
1.25160976105337E-22	0.431219617057692	0.228	0.098	2.04150068125415E-18	Periportal hepatocytes	PDGFC
1.32138713733659E-22	0.253508505554878	0.889	0.613	2.15531455970971E-18	Periportal hepatocytes	ASL
1.325708765262E-22	0.595874404301119	0.417	0.228	2.16236356701885E-18	Periportal hepatocytes	FBXW7
1.32629184812065E-22	0.432486511563304	0.338	0.17	2.16331463346958E-18	Periportal hepatocytes	SF3B1
1.43535240009883E-22	0.583748157240265	0.214	0.088	2.3412032998012E-18	Periportal hepatocytes	MYO1D
1.46557674521679E-22	0.827966218194413	0.283	0.136	2.3905022291231E-18	Periportal hepatocytes	NEIL1
1.76634212256511E-22	0.474556760696571	0.361	0.187	2.88108063611595E-18	Periportal hepatocytes	STK3
1.87414187741589E-22	0.948067494556521	0.187	0.073	3.05691281625305E-18	Periportal hepatocytes	SLC7A5
1.91135978825773E-22	0.265949366259919	0.246	0.109	3.11761895062718E-18	Periportal hepatocytes	RTN4R
1.9361035249343E-22	0.403529684667939	0.477	0.27	3.15797845952034E-18	Periportal hepatocytes	GATAD2B
2.17461853386096E-22	0.304153812266236	0.431	0.237	3.54702029058061E-18	Periportal hepatocytes	TP53BP2
2.1985841886327E-22	0.637198162108841	0.376	0.2	3.5861106700788E-18	Periportal hepatocytes	SLC19A2
2.41330932306757E-22	0.738274856425082	0.115	0.034	3.93634883685552E-18	Periportal hepatocytes	COMMD6
2.47065074599233E-22	0.500174315875105	0.401	0.219	4.02987843178809E-18	Periportal hepatocytes	PHLDB2
2.54760916640771E-22	0.788724118584749	0.349	0.181	4.15540531132761E-18	Periportal hepatocytes	ZNF76
2.61228755690694E-22	0.515136379635698	0.339	0.172	4.26090223407092E-18	Periportal hepatocytes	PIP4K2B
2.72992051342006E-22	0.416435960824064	0.336	0.174	4.45277334943946E-18	Periportal hepatocytes	FPGS
3.11028272491224E-22	1.11386956932172	0.151	0.053	5.07318215260436E-18	Periportal hepatocytes	TLE3
3.15536404168942E-22	0.605141437628989	0.369	0.196	5.14671428839962E-18	Periportal hepatocytes	RPRD2
3.31555162833287E-22	0.33300041518627	0.886	0.56	5.40799626097374E-18	Periportal hepatocytes	MYO18A
3.38736901873164E-22	0.982894129979528	0.167	0.062	5.52513760645318E-18	Periportal hepatocytes	LMBR1
3.41422362515744E-22	0.477086571378092	0.481	0.274	5.5689401549943E-18	Periportal hepatocytes	KDM4C
4.207104694584E-22	0.585048192119255	0.241	0.106	6.86220846733596E-18	Periportal hepatocytes	ACACB
4.30585288334425E-22	1.48731522700914	0.165	0.062	7.0232766380228E-18	Periportal hepatocytes	ZNF558
4.51730198429083E-22	2.24825781369453	0.102	0.028	7.36817126657677E-18	Periportal hepatocytes	KCNIP2
4.55510564860201E-22	0.918701178204983	0.277	0.134	7.42983282343474E-18	Periportal hepatocytes	HMGXB3
5.01853861590278E-22	0.41345140421536	0.473	0.271	8.18573833639903E-18	Periportal hepatocytes	RHOBTB3
5.16783162736566E-22	0.470636706259136	0.139	0.046	8.42925016739613E-18	Periportal hepatocytes	ANXA13
5.4925101746174E-22	0.886485486953669	0.164	0.06	8.95883334581844E-18	Periportal hepatocytes	HERC3
5.9672713225162E-22	0.256685937103089	0.322	0.16	9.73321625415618E-18	Periportal hepatocytes	CD47
6.19516114096324E-22	0.405920240732277	0.399	0.218	1.01049273370251E-17	Periportal hepatocytes	RANBP9
6.85092687841825E-22	0.296505752648684	0.509	0.291	1.1174546831388E-17	Periportal hepatocytes	COL27A1

7.97314303454498E-22	0.319917554199482	0.504	0.292	1.30049936036463E-17	Periportal hepatocytes	ANAPC5
8.03779387759556E-22	0.482845170303184	0.313	0.155	1.31104455937461E-17	Periportal hepatocytes	PLA2G12A
8.33854105666455E-22	0.520491164550433	0.22	0.093	1.36009943175255E-17	Periportal hepatocytes	AP2M1
8.45864736861616E-22	0.510249670587006	0.408	0.224	1.37968997229498E-17	Periportal hepatocytes	PLRG1
9.99714396379663E-22	0.312870330108916	0.256	0.115	1.63063415193487E-17	Periportal hepatocytes	ZBTB21
1.01582913549018E-21	0.393619983511121	0.369	0.195	1.65691890289804E-17	Periportal hepatocytes	KLHL22
1.02504235246606E-21	0.500617175944097	0.348	0.177	1.67194658110739E-17	Periportal hepatocytes	FBH1
1.05349841448866E-21	0.480752747284788	0.478	0.271	1.71836126387245E-17	Periportal hepatocytes	PFKFB1
1.05835436986146E-21	1.18777079559201	0.151	0.053	1.72628181268103E-17	Periportal hepatocytes	IFT172
1.06928192128753E-21	0.615864022968221	0.154	0.055	1.74410574181209E-17	Periportal hepatocytes	PLS1
1.11379336946814E-21	0.602760931405536	0.489	0.289	1.81670836493948E-17	Periportal hepatocytes	SLC25A29
1.11829971756713E-21	1.09642964664554	0.105	0.03	1.82405866932374E-17	Periportal hepatocytes	CRPPA
1.21726259265274E-21	1.27952152254614	0.142	0.049	1.98547701487588E-17	Periportal hepatocytes	TRIM50
1.27213379009649E-21	0.659853750464634	0.398	0.22	2.07497742502638E-17	Periportal hepatocytes	SPATA13
1.29542303846314E-21	0.367708193705143	0.473	0.273	2.11296451803724E-17	Periportal hepatocytes	ZNF33A
1.30427843673202E-21	0.349342135868064	0.717	0.455	2.1274085581536E-17	Periportal hepatocytes	SHFL
1.34697505252618E-21	0.491493932308279	0.207	0.086	2.19705100817546E-17	Periportal hepatocytes	ZNF639
1.38184873995543E-21	1.12235118334534	0.116	0.035	2.25393347974131E-17	Periportal hepatocytes	SEMA6A
1.43522065036858E-21	0.560327491980309	0.418	0.234	2.34098840281619E-17	Periportal hepatocytes	TLE1
1.44512301384224E-21	0.817008538168038	0.141	0.048	2.35714014787807E-17	Periportal hepatocytes	CD9
1.55077792758923E-21	0.896219061194353	0.267	0.126	2.52947387769079E-17	Periportal hepatocytes	WDR19
1.60981109869857E-21	0.669790112878164	0.171	0.065	2.62576288308724E-17	Periportal hepatocytes	GALNT11
1.61063772183206E-21	0.740862940529933	0.122	0.038	2.62711118808027E-17	Periportal hepatocytes	ARID3C
1.67636329977841E-21	0.250378600870172	0.46	0.264	2.73431617826856E-17	Periportal hepatocytes	DUSP10
1.71753906752572E-21	0.54605683618248	0.428	0.237	2.8014779730412E-17	Periportal hepatocytes	PTS
1.86653223779505E-21	0.769832124032549	0.102	0.028	3.0445007330675E-17	Periportal hepatocytes	ANKRD37
1.95082073831067E-21	0.530031680334576	0.246	0.111	3.18198370625854E-17	Periportal hepatocytes	RCAN1
1.99936957382381E-21	0.797510211029963	0.293	0.145	3.26117171186402E-17	Periportal hepatocytes	BCL6
2.0342470052298E-21	0.304711398622156	0.557	0.33	3.31806029023033E-17	Periportal hepatocytes	FLNB
2.13408282321858E-21	0.263162828796458	0.364	0.192	3.48090249295182E-17	Periportal hepatocytes	SIPA1L3
2.30838764337904E-21	0.259202731513342	0.519	0.303	3.76521108511556E-17	Periportal hepatocytes	FOXN2
2.42881264305629E-21	0.440795514395673	0.263	0.123	3.96163630208911E-17	Periportal hepatocytes	SNX10
2.51739072055464E-21	0.279899937294062	0.28	0.134	4.10611600429667E-17	Periportal hepatocytes	GABARAPL
2.54434270186402E-21	0.849259017698394	0.135	0.045	4.1500773810104E-17	Periportal hepatocytes	NBN
2.5856882105622E-21	0.275127759856775	0.427	0.235	4.21751604024801E-17	Periportal hepatocytes	HIPK2
2.73523228169033E-21	0.58915290973904	0.181	0.072	4.4614373746651E-17	Periportal hepatocytes	FAM210B
2.8248222625761E-21	0.613690677599826	0.339	0.177	4.60756759248788E-17	Periportal hepatocytes	SON
2.82837651313729E-21	1.03497916046467	0.102	0.028	4.61336493057824E-17	Periportal hepatocytes	SYT7
2.99420077211118E-21	0.375088090112616	0.392	0.214	4.88384087939055E-17	Periportal hepatocytes	SH3BP2
3.0431605404486E-21	0.396211521174472	0.384	0.207	4.96369915752572E-17	Periportal hepatocytes	LRRFIP2
3.05151123575521E-21	0.288092732065227	0.477	0.275	4.97731997664032E-17	Periportal hepatocytes	NCBP2
3.07271229331755E-21	0.5461005187479	0.297	0.146	5.01190102163025E-17	Periportal hepatocytes	DDX3X
3.34736531811153E-21	0.442989733924978	0.277	0.132	5.45988757037172E-17	Periportal hepatocytes	ZNF384
3.36814416568765E-21	0.872197544238868	0.161	0.06	5.49377994865312E-17	Periportal hepatocytes	SPRY2
3.37900463176625E-21	0.604818854882593	0.249	0.115	5.51149445487394E-17	Periportal hepatocytes	SH2B1
3.58529718369369E-21	1.12324745980373	0.115	0.035	5.84797823632277E-17	Periportal hepatocytes	ZNF268
3.78415577589844E-21	0.812631241453771	0.223	0.098	6.17233648606794E-17	Periportal hepatocytes	DAAM1
3.79201471122447E-21	0.301056806560566	0.399	0.218	6.18515519547823E-17	Periportal hepatocytes	RALGPS2
3.88656731582003E-21	0.319295864575813	0.243	0.111	6.33937994883406E-17	Periportal hepatocytes	HSD17B12
4.25232711394555E-21	0.445892782641247	0.425	0.24	6.93597075555659E-17	Periportal hepatocytes	TUT4
4.33759345864943E-21	0.8420725772869	0.214	0.092	7.07504869040308E-17	Periportal hepatocytes	TCP11L1
4.43209462502305E-21	0.457725687307461	0.227	0.101	7.22918954287509E-17	Periportal hepatocytes	PBDC1
4.63222397886563E-21	0.435513494437816	0.427	0.233	7.55562053192773E-17	Periportal hepatocytes	EIF2B5
4.77405446586503E-21	0.344285584702973	0.385	0.209	7.78696023927244E-17	Periportal hepatocytes	WLS
5.55837960045438E-21	0.570180068562102	0.368	0.195	9.06627296630114E-17	Periportal hepatocytes	TTC37
5.97946397185328E-21	0.258568492334278	0.253	0.115	9.75310368448988E-17	Periportal hepatocytes	USP38
6.09720917928379E-21	1.08345160654235	0.149	0.054	9.94515789232978E-17	Periportal hepatocytes	HSPA4L
6.34628693868598E-21	0.730583250159883	0.332	0.173	1.03514286256907E-16	Periportal hepatocytes	ZNF91
6.67791459409508E-21	0.669774171856844	0.284	0.141	1.08923464944285E-16	Periportal hepatocytes	EPDR1
6.95766115653004E-21	0.266378909899285	0.47	0.268	1.13486411124161E-16	Periportal hepatocytes	PICALM

7.10453620644241E-21	0.640301152770172	0.177	0.069	1.15882090063282E-16	Periportal hepatocytes	RBM12B
7.17902238850427E-21	0.279327388861834	0.529	0.318	1.17097034178893E-16	Periportal hepatocytes	FOXP1
7.40011083231617E-21	0.573215173490636	0.414	0.233	1.20703207785909E-16	Periportal hepatocytes	DEPRECAT
8.45712746806693E-21	0.793705914595301	0.217	0.095	1.3794420613164E-16	Periportal hepatocytes	OTUD6B
8.5636642326382E-21	0.350699010113439	0.858	0.678	1.39681927298562E-16	Periportal hepatocytes	MT-ATP6
8.86642700106797E-21	0.965133996242142	0.272	0.132	1.4462029081442E-16	Periportal hepatocytes	SAMD12
9.38984206423275E-21	0.301861660932912	0.511	0.303	1.531577139097E-16	Periportal hepatocytes	SRP54
9.94524069702002E-21	0.31824366923836	0.295	0.144	1.62216821009094E-16	Periportal hepatocytes	TMEM50B
1.00196043560519E-20	0.459652671671561	0.325	0.166	1.63429766651562E-16	Periportal hepatocytes	CAMKK2
1.0292730267596E-20	0.349127617853625	0.547	0.333	1.67884723394759E-16	Periportal hepatocytes	HMGCS1
1.06057927896837E-20	0.474853816661776	0.3	0.152	1.7299108619253E-16	Periportal hepatocytes	CBR4
1.06729368366369E-20	0.477990055101278	0.306	0.153	1.74086272742384E-16	Periportal hepatocytes	TMEM63A
1.11051619060958E-20	0.784263326669869	0.138	0.048	1.81136295850329E-16	Periportal hepatocytes	SIDT2
1.20301356107184E-20	0.922684069181844	0.293	0.148	1.96223541946428E-16	Periportal hepatocytes	ZNF280D
1.22809234745469E-20	0.265905818470587	0.94	0.744	2.00314142793335E-16	Periportal hepatocytes	ITIH1
1.23647661352624E-20	0.284859889674538	0.116	0.036	2.01681700432264E-16	Periportal hepatocytes	ITGB2
1.41181153328752E-20	0.262273742099733	0.43	0.24	2.30280579194528E-16	Periportal hepatocytes	DEPRECAT
1.63599585518178E-20	0.264965379089738	0.228	0.102	2.668472839387E-16	Periportal hepatocytes	TAB3
1.67865190588741E-20	0.675507365862194	0.171	0.067	2.73804912369295E-16	Periportal hepatocytes	SPP2
1.70047401049743E-20	0.334874722920457	0.256	0.121	2.77364315852236E-16	Periportal hepatocytes	ASB8
1.79611508424311E-20	0.727562211074388	0.267	0.129	2.92964331390893E-16	Periportal hepatocytes	SIPA1L1
1.84511124372608E-20	0.523222429948994	0.296	0.15	3.00956094964161E-16	Periportal hepatocytes	TMEM245
1.87929106698034E-20	0.669229850272428	0.296	0.147	3.06531165935164E-16	Periportal hepatocytes	ATF7IP2
1.89717762366036E-20	0.38908003631939	0.19	0.078	3.09448642195242E-16	Periportal hepatocytes	DEPRECAT
2.17856344999191E-20	0.384285404508658	0.241	0.11	3.55345484328181E-16	Periportal hepatocytes	SORT1
2.25448915039727E-20	0.27130200584641	0.307	0.156	3.67729725321299E-16	Periportal hepatocytes	DEPRECAT
2.40624809028288E-20	0.566587385049898	0.42	0.236	3.92483126006041E-16	Periportal hepatocytes	PHF8
2.51981547104517E-20	0.702346955453363	0.168	0.065	4.11007101482178E-16	Periportal hepatocytes	PDLIM5
2.61295573514617E-20	0.2528510671255	0.434	0.244	4.26199209959692E-16	Periportal hepatocytes	PMM2
2.67923974295432E-20	0.408869770351671	0.453	0.261	4.3701079447328E-16	Periportal hepatocytes	PNISR
2.78049846292327E-20	0.812419940716872	0.129	0.044	4.53527104287415E-16	Periportal hepatocytes	ARNTL
2.89614137411129E-20	0.559436249972973	0.289	0.144	4.72389619531293E-16	Periportal hepatocytes	MYNN
2.94224096667144E-20	0.427666399299013	0.486	0.286	4.79908924073778E-16	Periportal hepatocytes	STX16
3.04948612052527E-20	0.35806355926699	0.391	0.212	4.97401681118877E-16	Periportal hepatocytes	C11orf24
3.13877598252113E-20	0.351155549163036	0.329	0.172	5.11965750509022E-16	Periportal hepatocytes	NFYC
3.37213388094501E-20	0.370460895246097	0.513	0.306	5.5002875732094E-16	Periportal hepatocytes	COG3
3.79820231294957E-20	0.53822498597171	0.438	0.253	6.19524779265205E-16	Periportal hepatocytes	CHKA
3.90085494599167E-20	0.474721362912534	0.365	0.195	6.36268450240702E-16	Periportal hepatocytes	MAN2A1
4.01580033100246E-20	0.780802834044577	0.23	0.105	6.55017191989811E-16	Periportal hepatocytes	TUT1
4.30450181910564E-20	0.650035202327986	0.267	0.129	7.02107291714321E-16	Periportal hepatocytes	TMEM87A
4.43042248280378E-20	0.359428670295385	0.478	0.282	7.22646211170124E-16	Periportal hepatocytes	TNS2
4.43209374047386E-20	0.268870372442138	0.307	0.155	7.22918810008692E-16	Periportal hepatocytes	NCKIPSD
4.53130458282844E-20	0.258447926860337	0.381	0.207	7.39101090505147E-16	Periportal hepatocytes	ATP6V0A1
4.56232552946867E-20	0.293013532465978	0.611	0.376	7.44160917111634E-16	Periportal hepatocytes	DTNA
4.83531930578631E-20	0.259502224492468	0.481	0.278	7.88688931966806E-16	Periportal hepatocytes	AAK1
4.84318247736226E-20	0.544709177996444	0.32	0.165	7.89971493882558E-16	Periportal hepatocytes	SIK3
4.86123100885896E-20	0.564335771437036	0.438	0.251	7.92915389854985E-16	Periportal hepatocytes	RBL2
4.89968576464824E-20	0.521135721113478	0.27	0.132	7.99187745071775E-16	Periportal hepatocytes	TADA1
4.90968762748364E-20	0.526163337794586	0.18	0.072	8.00819148918856E-16	Periportal hepatocytes	TBCK
4.935988607739E-20	0.31075551736868	0.174	0.069	8.05109101808309E-16	Periportal hepatocytes	JAZF1
4.95171495632974E-20	0.449717819709925	0.401	0.226	8.07674226526943E-16	Periportal hepatocytes	HIGD1A
5.12178659671227E-20	0.383701501069737	0.152	0.056	8.35414611789738E-16	Periportal hepatocytes	TEDC1
5.20118183585502E-20	0.252909546805627	0.629	0.386	8.48364769246312E-16	Periportal hepatocytes	MOV10
5.53729952222263E-20	0.267836936333284	0.371	0.202	9.03188925069733E-16	Periportal hepatocytes	CPT1A
5.59269360981267E-20	0.251771074796004	0.471	0.277	9.12224254696545E-16	Periportal hepatocytes	AKAP13
6.22766297487914E-20	0.844522386336896	0.261	0.127	1.01579410783254E-15	Periportal hepatocytes	TBK1
6.28204239670547E-20	0.300878231534084	0.401	0.224	1.02466393532663E-15	Periportal hepatocytes	FAT1
6.49133895017108E-20	0.386225176924783	0.339	0.179	1.0588022961624E-15	Periportal hepatocytes	PIK3R4
7.17894351725864E-20	0.741446938866278	0.378	0.211	1.17095747710006E-15	Periportal hepatocytes	TANGO2
7.55426846052867E-20	0.440815579611761	0.286	0.143	1.23217672859683E-15	Periportal hepatocytes	MMAB

**7.93404011870137E-20**	0.373480339693262	0.355	0.19	1.29412128376138E-15	Periportal hepatocytes	MSL1
**8.18843409122719E-20**	0.340283857978247	0.318	0.163	1.33561548462007E-15	Periportal hepatocytes	DOCK4
**8.22999609646654E-20**	0.344410647177129	0.457	0.265	1.34239466329466E-15	Periportal hepatocytes	ENPP1
**8.49974676924721E-20**	0.335726141474364	0.187	0.077	1.38639369553191E-15	Periportal hepatocytes	DEPRECAT
**8.60748842417633E-20**	0.254801354387256	0.519	0.312	1.4039674368674E-15	Periportal hepatocytes	PUM1
**9.14291177332641E-20**	0.681070728542973	0.22	0.1	1.49130033934727E-15	Periportal hepatocytes	MYO19
**9.39554527613076E-20**	0.375395183098948	0.335	0.176	1.53250738998969E-15	Periportal hepatocytes	AKIRIN1
**9.65152215561126E-20**	0.388964280247937	0.239	0.112	1.57425977880175E-15	Periportal hepatocytes	RYR1
**9.93126628272559E-20**	0.48059714330492	0.326	0.172	1.61988884337537E-15	Periportal hepatocytes	EPC1
**1.08941766180478E-19**	0.390620632921071	0.203	0.087	1.77694914816978E-15	Periportal hepatocytes	KLHL2
**1.08974854963243E-19**	0.78709951949384	0.33	0.178	1.77748885930546E-15	Periportal hepatocytes	CCDC14
**1.12081214149205E-19**	0.520307484827924	0.303	0.155	1.82815668398769E-15	Periportal hepatocytes	SENP5
**1.21179880142195E-19**	0.78643608533727	0.234	0.109	1.97656502499935E-15	Periportal hepatocytes	FZD4
**1.26679047067212E-19**	0.389561267186155	0.299	0.151	2.06626193671329E-15	Periportal hepatocytes	TM9SF4
**1.29775773324006E-19**	0.937588441174952	0.138	0.049	2.11677263868786E-15	Periportal hepatocytes	ATF7IP
**1.35425127653037E-19**	0.416212165731333	0.355	0.19	2.20891925714868E-15	Periportal hepatocytes	TRIP12
**1.37486647686082E-19**	0.440763169617559	0.326	0.171	2.24254471040769E-15	Periportal hepatocytes	HELZ
**1.41483687146816E-19**	0.290448410831647	0.319	0.166	2.30774042105171E-15	Periportal hepatocytes	CRKL
**1.58371468068234E-19**	0.586577023240925	0.135	0.048	2.58319701566097E-15	Periportal hepatocytes	NINJ2
**1.61539832698258E-19**	0.396043556358435	0.378	0.209	2.63487621114128E-15	Periportal hepatocytes	YPEL3
**1.85240773612636E-19**	0.979512564617325	0.102	0.03	3.0214622583957E-15	Periportal hepatocytes	RWDD2B
**1.89035898139447E-19**	0.268668974161432	0.171	0.069	3.08336453455252E-15	Periportal hepatocytes	RRAS
**2.02962282723583E-19**	0.702583179051804	0.351	0.188	3.31051779350436E-15	Periportal hepatocytes	RBM5
**2.0614262925602E-19**	0.717701856938639	0.174	0.07	3.36239242579494E-15	Periportal hepatocytes	HIF1A
**2.06639586944192E-19**	0.384187659480278	0.431	0.247	3.37049830264672E-15	Periportal hepatocytes	UNC5CL
**2.09533351718786E-19**	0.280759015943875	0.514	0.301	3.41769849988512E-15	Periportal hepatocytes	CD46
**2.10261745872031E-19**	0.355520603989267	0.431	0.25	3.4295793369187E-15	Periportal hepatocytes	SLC20A1
**2.40270962139853E-19**	0.78525050045265	0.25	0.12	3.91905966346315E-15	Periportal hepatocytes	PRKAR2A
**2.44599942711816E-19**	0.29085960435175	0.333	0.176	3.98966966557243E-15	Periportal hepatocytes	RAB11FIP4
**2.45965003556194E-19**	0.261685151895431	0.385	0.213	4.01193517300508E-15	Periportal hepatocytes	ZBTB48
**2.63988732966753E-19**	0.55972703138013	0.392	0.223	4.30592022342071E-15	Periportal hepatocytes	WDR6
**2.7123698726303E-19**	0.731981193210106	0.223	0.102	4.42414649924728E-15	Periportal hepatocytes	ASAP1
**2.75851368758057E-19**	0.37385370732452	0.18	0.074	4.49941167581267E-15	Periportal hepatocytes	TTPAL
**2.83928236749047E-19**	0.538862950659653	0.527	0.321	4.63115346961371E-15	Periportal hepatocytes	LUC7L
**2.88705228963457E-19**	0.984391850041022	0.122	0.041	4.70907098962295E-15	Periportal hepatocytes	GPER1
**3.12715279584522E-19**	0.326030393451397	0.228	0.105	5.10069892530314E-15	Periportal hepatocytes	ATF5
**3.23541453096114E-19**	1.38200186754506	0.182	0.078	5.27728464145072E-15	Periportal hepatocytes	IL1RN
**3.23634287239112E-19**	0.442345134527255	0.205	0.09	5.27879885915715E-15	Periportal hepatocytes	C2CD2
**3.45501659392538E-19**	0.361400132810578	0.392	0.218	5.63547756635169E-15	Periportal hepatocytes	SNX14
**3.47835775227876E-19**	0.48557343765353	0.44	0.258	5.67354932974189E-15	Periportal hepatocytes	CALCOCO1
**4.08465740330919E-19**	0.33596205459517	0.549	0.336	6.66248469053762E-15	Periportal hepatocytes	KPNA1
**4.21528829264977E-19**	0.586353916961205	0.399	0.225	6.87555673414104E-15	Periportal hepatocytes	GABRE
**4.61925681657733E-19**	0.604032520195689	0.272	0.136	7.53446979351929E-15	Periportal hepatocytes	CASP3
**4.95536576051795E-19**	0.331234474937834	0.197	0.085	8.08269709198083E-15	Periportal hepatocytes	RNF216
**5.046582304795E-19**	0.406822920641243	0.241	0.115	8.23148039735112E-15	Periportal hepatocytes	METTL14
**5.36322442533728E-19**	0.678542385625824	0.292	0.154	8.74795536016764E-15	Periportal hepatocytes	TIMM10B
**5.42108019115893E-19**	0.290689682646644	0.261	0.127	8.84232389979934E-15	Periportal hepatocytes	VDAC1
**5.83267399256461E-19**	0.541135925519746	0.201	0.089	9.51367454927214E-15	Periportal hepatocytes	DDX60L
**5.95243587615143E-19**	0.650535873487349	0.341	0.187	9.7090181575906E-15	Periportal hepatocytes	GATAD1
**6.08879722285536E-19**	0.360514373335046	0.355	0.194	9.93143715019938E-15	Periportal hepatocytes	PHF3
**6.27511880083016E-19**	0.344386649312888	0.144	0.053	1.02353462760341E-14	Periportal hepatocytes	LRRC61
**6.33491078544717E-19**	0.32784661802278	0.316	0.169	1.03328729821429E-14	Periportal hepatocytes	GNB5
**7.42949237750679E-19**	0.283148595113605	0.279	0.138	1.21182450169513E-14	Periportal hepatocytes	RSRC1
**7.53531394544967E-19**	0.875051667467484	0.111	0.035	1.22908505764229E-14	Periportal hepatocytes	FAM110B
**7.54871911076531E-19**	0.265187152843146	0.295	0.151	1.23127157415693E-14	Periportal hepatocytes	RIPK4
**7.60183249003826E-19**	0.310908533105986	0.316	0.167	1.23993489745014E-14	Periportal hepatocytes	TRAM2
**7.68061298108387E-19**	0.410805077998253	0.316	0.167	1.25278478334459E-14	Periportal hepatocytes	BCL2L11
**7.99483119581192E-19**	0.594355783406965	0.279	0.141	1.30403691634888E-14	Periportal hepatocytes	ZNF26
**8.03036872002202E-19**	0.285733882231767	0.44	0.256	1.30983344192279E-14	Periportal hepatocytes	ADD1
**8.05017909481267E-19**	0.87582847501245	0.182	0.077	1.31306471215489E-14	Periportal hepatocytes	UGGT2

8.2631143028818E-19	0.42696358224935	0.468	0.276	1.34779657394305E-14	Periportal hepatocytes	TAF1D
9.27592498574488E-19	0.574814000642553	0.417	0.243	1.51299612442485E-14	Periportal hepatocytes	ZNF648
9.42442072695044E-19	0.522475735696688	0.175	0.073	1.53721726477289E-14	Periportal hepatocytes	SYT12
9.50732039963735E-19	0.407295883426893	0.46	0.274	1.55073903038485E-14	Periportal hepatocytes	ARIH2
1.0640117056727E-18	0.834068657001342	0.101	0.031	1.73550949312274E-14	Periportal hepatocytes	S1PR1
1.12789374135793E-18	1.31748807037525	0.122	0.042	1.83970748152892E-14	Periportal hepatocytes	VIPR1
1.13246847570044E-18	0.60920550112541	0.208	0.094	1.84716933071499E-14	Periportal hepatocytes	EBAG9
1.15566354795273E-18	0.622263282664708	0.231	0.109	1.88500281306569E-14	Periportal hepatocytes	TFDP2
1.16232413447402E-18	0.337206057259527	0.309	0.161	1.89586689574057E-14	Periportal hepatocytes	TRMT1L
1.22170016490571E-18	0.39675731337071	0.289	0.145	1.9927151389777E-14	Periportal hepatocytes	TRAPPC10
1.27110657393211E-18	0.287987318201057	0.496	0.299	2.07330193274067E-14	Periportal hepatocytes	MKLN1
1.2712737152282E-18	0.813070935499493	0.175	0.073	2.07357455690871E-14	Periportal hepatocytes	RUNX1
1.3306416547004E-18	0.865444477199459	0.105	0.033	2.17040960298182E-14	Periportal hepatocytes	DPF3
1.33492407360146E-18	0.84239978179183	0.233	0.112	2.17739465645135E-14	Periportal hepatocytes	CLCN6
1.36002350690526E-18	0.57851119553211	0.205	0.092	2.21833434211317E-14	Periportal hepatocytes	SLC30A9
1.38851423748441E-18	0.520661966681161	0.296	0.153	2.26480557276081E-14	Periportal hepatocytes	DAZAP1
1.39351294495705E-18	0.358553027563035	0.305	0.157	2.27295896451945E-14	Periportal hepatocytes	BABAM1
1.39926085774196E-18	0.918456125808463	0.273	0.139	2.28233438506292E-14	Periportal hepatocytes	TTC14
1.406004478456E-18	0.500707955937584	0.356	0.197	2.29333390480958E-14	Periportal hepatocytes	RORA
1.53617839218736E-18	0.334090088309256	0.583	0.364	2.50566057549681E-14	Periportal hepatocytes	IKBKB
1.55258037721517E-18	0.655475327014774	0.31	0.164	2.53241385327567E-14	Periportal hepatocytes	CPLANE1
1.94777564626225E-18	0.253161945427721	0.96	0.904	3.17701685661835E-14	Periportal hepatocytes	KNG1
2.08379688846256E-18	0.355981027879793	0.194	0.085	3.39888110477129E-14	Periportal hepatocytes	OST4
2.45319610294327E-18	0.454194380190732	0.414	0.241	4.00140816351076E-14	Periportal hepatocytes	SPPL2B
2.49436943191101E-18	0.978308321484389	0.227	0.107	4.06856598039004E-14	Periportal hepatocytes	ATR
2.53382250857864E-18	0.37669649130194	0.29	0.15	4.13291789374262E-14	Periportal hepatocytes	FOXRED1
2.67808625980927E-18	0.295996979699716	0.441	0.259	4.3682264983749E-14	Periportal hepatocytes	CDC42BPB
2.74134965967372E-18	0.707402769715936	0.205	0.093	4.4714154298938E-14	Periportal hepatocytes	SEC22A
2.8498935653544E-18	0.878636802253562	0.24	0.115	4.64846139444957E-14	Periportal hepatocytes	PIK3C2G
2.90183115972438E-18	0.674842899503816	0.218	0.103	4.73317680462644E-14	Periportal hepatocytes	GK5
3.31623988261578E-18	0.370380382577329	0.343	0.189	5.4091188725346E-14	Periportal hepatocytes	ACCS
3.43626659032917E-18	0.654120862780647	0.214	0.099	5.60489443548591E-14	Periportal hepatocytes	SYNE4
3.50220258197151E-18	0.398859750928065	0.231	0.11	5.71244263145373E-14	Periportal hepatocytes	SLC44A1
3.50576544529572E-18	0.482595524950753	0.358	0.199	5.71825401782184E-14	Periportal hepatocytes	RELCH
3.53424472310917E-18	0.255016650439093	0.272	0.136	5.76470656786337E-14	Periportal hepatocytes	PTGR2
3.6488909442278E-18	0.49369919821643	0.32	0.174	5.95170601912996E-14	Periportal hepatocytes	CDK13
3.75489467989081E-18	0.316850831969393	0.335	0.182	6.1246087123699E-14	Periportal hepatocytes	NCKAP5L
4.1700500790446E-18	0.422090876064904	0.336	0.186	6.80176868392965E-14	Periportal hepatocytes	HEXD
4.23096613395148E-18	0.329763258072544	0.306	0.163	6.90112886108826E-14	Periportal hepatocytes	SCO2
4.38105620702016E-18	0.933706772595308	0.19	0.084	7.14594077927058E-14	Periportal hepatocytes	ZNF518A
4.3978785001952E-18	0.356098872795826	0.381	0.212	7.1733796216684E-14	Periportal hepatocytes	DEPRECAT
4.55707629700916E-18	1.12764139848135	0.174	0.074	7.43304714805164E-14	Periportal hepatocytes	FCHSD2
4.72101865856349E-18	0.362280579714914	0.136	0.051	7.70045353398291E-14	Periportal hepatocytes	ORAI3
4.74785831449615E-18	0.69506288147707	0.221	0.104	7.74423169677467E-14	Periportal hepatocytes	COQ10A
5.13125007390813E-18	0.753191167212711	0.247	0.124	8.36958199555155E-14	Periportal hepatocytes	SLC10A7
5.25248950721109E-18	0.440757850897585	0.437	0.256	8.56733563521201E-14	Periportal hepatocytes	SLC35A3
5.51218376180601E-18	0.468510890394566	0.306	0.162	8.99092293388178E-14	Periportal hepatocytes	IL1R1
5.67317824875198E-18	0.617129330468482	0.228	0.108	9.25352104153936E-14	Periportal hepatocytes	VNN 1.00
5.7167704826642E-18	0.33494196459821	0.503	0.309	9.32462433427357E-14	Periportal hepatocytes	KDM5B
6.06245989949056E-18	0.688667984149795	0.201	0.091	9.88847834205905E-14	Periportal hepatocytes	FOCAD
6.52566848750808E-18	0.342060048518104	0.346	0.191	1.06440178699744E-13	Periportal hepatocytes	STRN3
7.57148934306626E-18	0.644677423890605	0.182	0.079	1.23498562674754E-13	Periportal hepatocytes	COMMD2
7.79178056606232E-18	0.433436350470673	0.272	0.138	1.27091732813042E-13	Periportal hepatocytes	KIAA1109
8.65111698369817E-18	0.30560373915287	0.444	0.264	1.41108369121101E-13	Periportal hepatocytes	UBE4B
8.6652305271709E-18	0.432029921964131	0.276	0.142	1.41338575128685E-13	Periportal hepatocytes	PPP4R3A
9.77356072530636E-18	0.475179391328936	0.126	0.046	1.59416548990472E-13	Periportal hepatocytes	SUSD3
1.03404283934925E-17	0.414205410332659	0.384	0.217	1.68662727526256E-13	Periportal hepatocytes	ZFAND1
1.05360284880191E-17	0.37299326129383	0.175	0.075	1.7185316066808E-13	Periportal hepatocytes	ASNSD1
1.0915505217043E-17	0.701571611099628	0.353	0.199	1.78042805595188E-13	Periportal hepatocytes	DMAP1
1.09435209649242E-17	0.269253332807334	0.527	0.321	1.78499770458878E-13	Periportal hepatocytes	OGT

1.17524748724968E-17	0.556740856256492	0.128	0.046	1.91694617645295E-13	Periportal hepatocytes	EMID1
1.21933679748835E-17	0.658330498903015	0.25	0.126	1.98886025038325E-13	Periportal hepatocytes	SOCS3
1.24752362826172E-17	0.566562450044782	0.147	0.057	2.03483579005768E-13	Periportal hepatocytes	SAYSD1
1.34582349129808E-17	0.271524105236335	0.513	0.316	2.1951726966563E-13	Periportal hepatocytes	ZRANB2
1.35389163849655E-17	0.650756305184983	0.381	0.221	2.20833265155173E-13	Periportal hepatocytes	TNK2
1.35695631926473E-17	0.259447162995168	0.369	0.207	2.2133314523527E-13	Periportal hepatocytes	YKT6
1.52549219956892E-17	0.582544592463155	0.203	0.092	2.48823032671686E-13	Periportal hepatocytes	TNIK
1.63786892690111E-17	0.929749942756175	0.149	0.059	2.6715280066684E-13	Periportal hepatocytes	IFT80
1.64429427169139E-17	0.837434301895313	0.112	0.038	2.68200838655583E-13	Periportal hepatocytes	POP1
1.67521774577342E-17	0.809782513675005	0.171	0.073	2.73244766513103E-13	Periportal hepatocytes	TRAPPC11
1.73956448834828E-17	0.266479022801792	0.284	0.147	2.83740363694488E-13	Periportal hepatocytes	GNE
1.76939609760785E-17	0.340176740964926	0.23	0.11	2.88606197480817E-13	Periportal hepatocytes	COX19
2.00692299766111E-17	0.322490899502154	0.3	0.158	3.27349210148504E-13	Periportal hepatocytes	GRAMD2B
2.31769900870755E-17	1.11678246978705	0.122	0.044	3.78039885310288E-13	Periportal hepatocytes	TBC1D4
2.44193029951286E-17	0.725526817658614	0.233	0.113	3.98303251153543E-13	Periportal hepatocytes	SLC6A6
2.72143991928842E-17	0.826917825223316	0.115	0.04	4.43894065235133E-13	Periportal hepatocytes	NUAK1
2.75347974331156E-17	0.322203599129306	0.226	0.108	4.49120080931548E-13	Periportal hepatocytes	VCPIP1
2.7815953648786E-17	0.629048885309417	0.145	0.058	4.53706019965349E-13	Periportal hepatocytes	FNDC5
2.92335026024814E-17	0.263621152963959	0.223	0.106	4.76827660949074E-13	Periportal hepatocytes	RABEPK
2.94283047655368E-17	0.393415507965457	0.266	0.138	4.8000507903067E-13	Periportal hepatocytes	UBA3
2.99093993222404E-17	0.431078709379754	0.19	0.084	4.87852212345064E-13	Periportal hepatocytes	BACE1
3.08884724378204E-17	0.314224444631961	0.135	0.051	5.03821873933288E-13	Periportal hepatocytes	TMEM97
3.12694342947852E-17	0.261242231274916	0.315	0.17	5.10035742782241E-13	Periportal hepatocytes	RICTOR
3.94494909482025E-17	0.485814640385168	0.139	0.054	6.4346064685613E-13	Periportal hepatocytes	ACE
4.17587096270382E-17	0.518549501834438	0.382	0.223	6.8112631272662E-13	Periportal hepatocytes	KDM4B
4.25764540195283E-17	0.680593616286744	0.154	0.064	6.94464541512526E-13	Periportal hepatocytes	TMOD1
4.34168875534279E-17	0.32856181502574	0.497	0.308	7.08172852883962E-13	Periportal hepatocytes	SUGP2
4.44356945537778E-17	0.742343535891573	0.147	0.059	7.2479061386667E-13	Periportal hepatocytes	DGKD
4.570592002135E-17	0.84264832089888	0.236	0.117	7.4550926146824E-13	Periportal hepatocytes	COG1
4.87804924854464E-17	0.309539756222106	0.325	0.181	7.95658612930116E-13	Periportal hepatocytes	VPS13D
5.18695794581921E-17	0.486456779809268	0.263	0.135	8.46044710542572E-13	Periportal hepatocytes	IFNGR2
5.26861952156756E-17	0.384258081253898	0.563	0.362	8.59364530162885E-13	Periportal hepatocytes	QTRT1
5.32829706493621E-17	1.36981810713787	0.159	0.067	8.69098534261745E-13	Periportal hepatocytes	ZNF397
5.35516108618809E-17	0.579138026424813	0.226	0.109	8.73480324768139E-13	Periportal hepatocytes	ZNF251
5.39308793573558E-17	0.636805673869902	0.128	0.048	8.7966657319783E-13	Periportal hepatocytes	ZNF544
5.46094756789807E-17	0.439941880494532	0.302	0.164	8.90735157799854E-13	Periportal hepatocytes	SUOX
5.56332850795913E-17	0.466418551902788	0.269	0.14	9.07434512933214E-13	Periportal hepatocytes	NPRL3
5.60770135037116E-17	0.38242693877442	0.408	0.238	9.1467216725904E-13	Periportal hepatocytes	ZXDC
5.91347135717858E-17	0.473077604072973	0.27	0.139	9.64546313069398E-13	Periportal hepatocytes	SPTBN2
6.22146948956625E-17	0.294097449528951	0.246	0.121	1.01478388844315E-12	Periportal hepatocytes	DCAF10
6.61275445260923E-17	0.488634013742554	0.372	0.213	1.07860637876509E-12	Periportal hepatocytes	TEAD1
6.79807885476405E-17	0.300850139045744	0.25	0.127	1.10883464200056E-12	Periportal hepatocytes	PPIA
7.47866124426841E-17	0.290032665932915	0.315	0.171	1.21984443555262E-12	Periportal hepatocytes	PIK3CB
7.7430615303858E-17	0.506599815278678	0.197	0.09	1.26297076622123E-12	Periportal hepatocytes	IL10RB
7.94541410274629E-17	0.254408383888715	0.205	0.095	1.29597649429895E-12	Periportal hepatocytes	SOCS1
8.30257513118121E-17	0.362799518433708	0.158	0.066	1.35423302964697E-12	Periportal hepatocytes	RBKS
9.9311008976465E-17	0.269491087770874	0.441	0.266	1.61986186741512E-12	Periportal hepatocytes	TNRC18
9.99086326237475E-17	0.70786175427747	0.139	0.055	1.62960970672595E-12	Periportal hepatocytes	NECTIN1
1.00748468427869E-16	1.02430107153709	0.116	0.042	1.64330826852697E-12	Periportal hepatocytes	PIBF1
1.12303625975034E-16	0.34068062997034	0.31	0.169	1.83178444327878E-12	Periportal hepatocytes	TSHZ2
1.13412221071833E-16	0.710168523327594	0.273	0.145	1.84986673790266E-12	Periportal hepatocytes	MPHOSPH
1.18250364880551E-16	0.605712259168451	0.188	0.086	1.92878170156667E-12	Periportal hepatocytes	PMS2
1.23386933206548E-16	0.274566519555932	0.437	0.258	2.01256426753201E-12	Periportal hepatocytes	TIA1
1.260809989277E-16	0.719190991263752	0.257	0.135	2.05650717350971E-12	Periportal hepatocytes	FBXL8
1.32484609824926E-16	0.521809308567087	0.261	0.135	2.16095647085436E-12	Periportal hepatocytes	RHEB
1.34736695348768E-16	0.540296150145549	0.223	0.109	2.19769023783376E-12	Periportal hepatocytes	MGAT5
1.35359676118794E-16	1.00212054376284	0.181	0.081	2.20785167717365E-12	Periportal hepatocytes	MTERF4
1.50685175713435E-16	0.588979015078806	0.375	0.222	2.45782590106183E-12	Periportal hepatocytes	CENPT
1.55802907285283E-16	0.299229569719552	0.161	0.068	2.54130122073026E-12	Periportal hepatocytes	IGFLR1
1.59927688894214E-16	0.335290464703041	0.272	0.141	2.60858053355353E-12	Periportal hepatocytes	OMA1

1.63112509012055E-16	0.453972921104756	0.207	0.098	2.66052813449563E-12	Periportal hepatocytes	ACOT8
1.65655612592469E-16	0.395922860314945	0.284	0.151	2.70200869699577E-12	Periportal hepatocytes	FBXW8
1.67768973228119E-16	0.256464524507468	0.19	0.087	2.73647972232385E-12	Periportal hepatocytes	FAM149A
1.7426722505922E-16	0.362072768938634	0.253	0.128	2.84247270794094E-12	Periportal hepatocytes	GNPNAT1
1.75748532015403E-16	0.290462683399671	0.309	0.169	2.86663430570324E-12	Periportal hepatocytes	CCDC125
1.93614163912893E-16	0.575657175360109	0.339	0.194	3.1580406275832E-12	Periportal hepatocytes	PICK1
1.97660994017063E-16	0.612212861804135	0.128	0.048	3.22404847341232E-12	Periportal hepatocytes	PPA2
2.00528691927281E-16	0.290278216479245	0.129	0.049	3.27082349402588E-12	Periportal hepatocytes	DEPRECAT
2.0547177642194E-16	0.60445980222373	0.207	0.098	3.35145014521827E-12	Periportal hepatocytes	CLCN3
2.05820424050583E-16	0.437385864680005	0.204	0.095	3.35713693668907E-12	Periportal hepatocytes	LMAN2L
2.13336096217294E-16	0.538729573515336	0.247	0.125	3.47972506540028E-12	Periportal hepatocytes	CFAP97
2.33804767458609E-16	0.270291559740538	0.339	0.19	3.81358956201737E-12	Periportal hepatocytes	FRK
2.45463458965826E-16	1.42432094538797	0.115	0.042	4.00375447919159E-12	Periportal hepatocytes	PSORS1C1
2.56750772059534E-16	0.324244652428645	0.474	0.293	4.18786184306306E-12	Periportal hepatocytes	DLG5
2.58527435016617E-16	0.374585575156549	0.141	0.056	4.21684099255604E-12	Periportal hepatocytes	SNRPD2
2.62179116393874E-16	0.300888554178719	0.431	0.261	4.27640356750047E-12	Periportal hepatocytes	VWA8
3.17930847953021E-16	0.32107922759287	0.277	0.149	5.18577006096172E-12	Periportal hepatocytes	SLC2A13
3.20543229034625E-16	0.302948688268629	0.205	0.096	5.22838060878377E-12	Periportal hepatocytes	PRLR
3.26155056998519E-16	0.250261813357361	0.376	0.218	5.31991513470284E-12	Periportal hepatocytes	TBRG1
3.61237340265542E-16	0.85518412611426	0.108	0.037	5.89214225707125E-12	Periportal hepatocytes	DEPRECAT
3.65261812158307E-16	0.700315386912919	0.129	0.05	5.95778541811414E-12	Periportal hepatocytes	FITM2
3.75842964375843E-16	0.254719427305836	0.251	0.129	6.13037459193438E-12	Periportal hepatocytes	ARFGEF1
3.85255887026519E-16	0.296870285583168	0.282	0.15	6.28390877328954E-12	Periportal hepatocytes	BTN3A2
3.8570270922313E-16	0.50749964808395	0.299	0.164	6.29119689013847E-12	Periportal hepatocytes	NXF1
4.17834806509265E-16	0.338980845143394	0.292	0.157	6.81530352897262E-12	Periportal hepatocytes	LMF1
4.1979834949321E-16	0.850572286656202	0.177	0.079	6.84733087858375E-12	Periportal hepatocytes	GYG2
4.19915446743907E-16	0.874452897013437	0.226	0.112	6.84924085183986E-12	Periportal hepatocytes	RSBN1L
4.45550768296431E-16	0.486518706889617	0.194	0.091	7.26737858168309E-12	Periportal hepatocytes	UACA
4.55191629005388E-16	1.04282916323264	0.151	0.063	7.42463066070689E-12	Periportal hepatocytes	SLC22A3
4.62769660729875E-16	0.439594280810855	0.323	0.181	7.54823593616499E-12	Periportal hepatocytes	L3MBTL4
4.91242981043228E-16	0.372273447934013	0.198	0.093	8.01266426379609E-12	Periportal hepatocytes	NABP1
5.26218498811383E-16	0.469406043750129	0.195	0.091	8.58314993411247E-12	Periportal hepatocytes	DNAJC25
5.46563263084104E-16	0.831026279928764	0.246	0.127	8.91499338416482E-12	Periportal hepatocytes	ZNF692
5.51991799641479E-16	0.611075697264344	0.181	0.082	9.00353824395217E-12	Periportal hepatocytes	IFFO2
5.58336410658439E-16	0.700775847678028	0.185	0.084	9.1070251942498E-12	Periportal hepatocytes	YEATS2
6.08727723637199E-16	0.519109560690903	0.27	0.144	9.92895790024635E-12	Periportal hepatocytes	TNKS2
6.28274827881677E-16	0.607565119555982	0.134	0.053	1.0247790717578E-11	Periportal hepatocytes	SEPTIN7
6.49460022521775E-16	0.382997165313009	0.336	0.19	1.05933424273527E-11	Periportal hepatocytes	MAEA
8.40011601097798E-16	0.378283504868729	0.295	0.159	1.37014292255062E-11	Periportal hepatocytes	TOX4
9.16202619772987E-16	0.475705507168113	0.134	0.053	1.49441809311172E-11	Periportal hepatocytes	FBXL20
9.48366461427924E-16	0.739750039905565	0.178	0.081	1.54688053523509E-11	Periportal hepatocytes	EIF2A
9.55076729610664E-16	0.602097145670754	0.24	0.123	1.55782565366795E-11	Periportal hepatocytes	AL135905.
9.96272426283647E-16	0.956168245019687	0.138	0.056	1.62501995451126E-11	Periportal hepatocytes	FRMD4B
1.00808429893723E-15	0.930556211701364	0.211	0.104	1.64428629999652E-11	Periportal hepatocytes	SLC2A11
1.02452814304806E-15	0.697616300544835	0.164	0.072	1.67110785412569E-11	Periportal hepatocytes	KLHL7
1.03596364437262E-15	0.422682614269469	0.542	0.344	1.68976030033618E-11	Periportal hepatocytes	ALS2CL
1.06785826605832E-15	0.557869558094941	0.244	0.125	1.74178361776773E-11	Periportal hepatocytes	ATP8B2
1.08510326546769E-15	0.981037237382406	0.126	0.049	1.76991193630435E-11	Periportal hepatocytes	TESC
1.13719126244027E-15	0.301083918279804	0.116	0.043	1.85487266816633E-11	Periportal hepatocytes	STS
1.1790706502714E-15	0.290019961642436	0.326	0.182	1.92318213765768E-11	Periportal hepatocytes	DNAJC13
1.22054819413747E-15	0.256669878785076	0.165	0.072	1.99083615945763E-11	Periportal hepatocytes	COA5
1.26632533713764E-15	0.515036697246964	0.216	0.106	2.0655032574052E-11	Periportal hepatocytes	ARPP19
1.27553906900503E-15	0.337326260428215	0.319	0.179	2.08053177545411E-11	Periportal hepatocytes	C6orf62
1.3216363755699E-15	0.549499625290491	0.122	0.046	2.15572109219206E-11	Periportal hepatocytes	GLYAT
1.32581626366313E-15	0.575562498222058	0.246	0.127	2.16253890766093E-11	Periportal hepatocytes	VPS8
1.34004801452516E-15	0.507190891650696	0.23	0.115	2.18575231649199E-11	Periportal hepatocytes	DBT
1.57256700845835E-15	0.554012014262497	0.178	0.081	2.56501404749641E-11	Periportal hepatocytes	GSTM3
1.58946198106232E-15	0.403696901292678	0.208	0.101	2.59257143731075E-11	Periportal hepatocytes	SMUG1
1.65137466552967E-15	0.277543064431613	0.22	0.11	2.69355721694544E-11	Periportal hepatocytes	PLEKHA1
1.72430022588635E-15	0.313079979089046	0.316	0.177	2.81250609844323E-11	Periportal hepatocytes	USP22

1.96383895468869E-15	0.958224172792829	0.175	0.08	3.20321771899272E-11	Periportal hepatocytes	MBOAT1
1.99172495376165E-15	0.933806264027875	0.118	0.044	3.24870257208063E-11	Periportal hepatocytes	SSH2
2.25498171213637E-15	0.391603140464037	0.261	0.137	3.67810067066563E-11	Periportal hepatocytes	SKIL
2.29194020050955E-15	0.330538839479177	0.305	0.172	3.73838366105112E-11	Periportal hepatocytes	CYHR1
2.30119777789638E-15	0.49730472512657	0.48	0.3	3.75348369552679E-11	Periportal hepatocytes	TUBGCP6
2.30968604726011E-15	0.35599059520189	0.297	0.164	3.76732891168596E-11	Periportal hepatocytes	TUBG2
2.35756437613224E-15	0.599845165548129	0.184	0.086	3.84542325390929E-11	Periportal hepatocytes	MPZ
2.40200082978233E-15	0.266714242211344	0.447	0.273	3.91790355345796E-11	Periportal hepatocytes	ZFYVE27
2.70858755933718E-15	0.463515634247932	0.164	0.072	4.41797716803488E-11	Periportal hepatocytes	LDLRAD4
2.77354293798452E-15	0.366365389185761	0.154	0.066	4.52392588614656E-11	Periportal hepatocytes	SLC15A3
2.81497131176944E-15	1.60352356699278	0.111	0.041	4.59149970662713E-11	Periportal hepatocytes	TPRG1
2.82251951945246E-15	0.341053330463233	0.172	0.077	4.60381158817891E-11	Periportal hepatocytes	KLHDC9
2.88980768490433E-15	0.3857985761326	0.364	0.213	4.71356531484746E-11	Periportal hepatocytes	VPS39
2.90297475389388E-15	0.40658856584549	0.182	0.085	4.73504212107631E-11	Periportal hepatocytes	ABCB1
3.06468622760592E-15	0.281435243030003	0.445	0.27	4.99880970584802E-11	Periportal hepatocytes	SLC5A6
3.09001467688668E-15	0.252413224719453	0.411	0.251	5.04012293946986E-11	Periportal hepatocytes	POLR2J2
3.32038731586054E-15	0.262444438769203	0.336	0.192	5.41588375090013E-11	Periportal hepatocytes	OSBPL2
3.45948975090869E-15	0.487661302198243	0.114	0.042	5.64277373270717E-11	Periportal hepatocytes	GAREM1
3.58762951050413E-15	0.987240052188344	0.152	0.066	5.85178249458329E-11	Periportal hepatocytes	USP53
3.72776216085132E-15	0.500279771664045	0.19	0.089	6.0803528605646E-11	Periportal hepatocytes	PKNOX1
3.87175950979159E-15	0.50065738048377	0.119	0.045	6.31522693642106E-11	Periportal hepatocytes	MYCBP2
3.91016671418553E-15	0.263286996438326	0.315	0.177	6.37787292750801E-11	Periportal hepatocytes	HDAC1
3.98694691457005E-15	0.409426857831684	0.32	0.182	6.50310911235521E-11	Periportal hepatocytes	CDK12
4.19401110409384E-15	0.310741219593311	0.227	0.114	6.84085151188747E-11	Periportal hepatocytes	TOM1L2
4.21479581478983E-15	0.273299164044124	0.328	0.186	6.8747534535037E-11	Periportal hepatocytes	PDZD8
4.39377419509891E-15	0.319011357688709	0.323	0.185	7.16668508962583E-11	Periportal hepatocytes	PIGQ
4.45480819005435E-15	0.543604987095009	0.29	0.161	7.26623763879765E-11	Periportal hepatocytes	SKIV2L
4.92349108303328E-15	0.772661238032567	0.141	0.059	8.03070630553559E-11	Periportal hepatocytes	PIK3CA
5.24554370329145E-15	0.59652406828119	0.233	0.121	8.55600633443868E-11	Periportal hepatocytes	MTA3
5.36047124986053E-15	0.343008254520162	0.316	0.179	8.7434646556475E-11	Periportal hepatocytes	SPOP
5.65650479765389E-15	0.381731515821744	0.27	0.145	9.22632497545325E-11	Periportal hepatocytes	FAM136A
5.88493199000264E-15	0.739846530018124	0.154	0.067	9.59891256889331E-11	Periportal hepatocytes	USPL1
6.34143338766865E-15	0.30793893430808	0.371	0.221	1.03435119986263E-10	Periportal hepatocytes	SIN3B
6.45588122841275E-15	0.444848710831959	0.17	0.077	1.0530187871664E-10	Periportal hepatocytes	PLCG2
6.52988036375641E-15	1.03171227111032	0.126	0.05	1.06508878613231E-10	Periportal hepatocytes	MFNG
6.65036114194304E-15	0.367001675727895	0.372	0.221	1.08474040586233E-10	Periportal hepatocytes	PIGL
6.78184857236166E-15	0.553384366075855	0.191	0.09	1.10618732063791E-10	Periportal hepatocytes	AP3M1
7.1281565133574E-15	0.60688460922572	0.182	0.086	1.16267360889373E-10	Periportal hepatocytes	HPS3
7.18420476887265E-15	0.277182690019604	0.29	0.159	1.17181563985082E-10	Periportal hepatocytes	MED4
7.26159150239783E-15	0.289098249882399	0.289	0.156	1.18443818995611E-10	Periportal hepatocytes	FCSK
7.64790172968018E-15	0.852625142845165	0.159	0.071	1.24744925112813E-10	Periportal hepatocytes	MFSD8
7.8119682893522E-15	1.24004230976876	0.149	0.066	1.27421014767624E-10	Periportal hepatocytes	TCEA2
8.46266772510089E-15	0.259618487474946	0.193	0.092	1.38034573264121E-10	Periportal hepatocytes	SLC25A32
8.91623344003986E-15	0.267864550585401	0.447	0.279	1.4543268364049E-10	Periportal hepatocytes	VPS53
9.43440341240419E-15	0.459979329894262	0.138	0.057	1.53884554059725E-10	Periportal hepatocytes	CLCN4
9.44292043476682E-15	0.325759599569786	0.365	0.216	1.54023475211482E-10	Periportal hepatocytes	ZZEF1
9.56282178394842E-15	0.350215263109506	0.312	0.178	1.55979186117983E-10	Periportal hepatocytes	GMNN
1.01754745429068E-14	0.359738095098143	0.237	0.124	1.65972165269353E-10	Periportal hepatocytes	AZIN1
1.04563359392524E-14	0.482865744781426	0.24	0.126	1.70553295505146E-10	Periportal hepatocytes	ERCC3
1.1151580237874E-14	0.46477376020623	0.269	0.145	1.81893425259963E-10	Periportal hepatocytes	MTRR
1.12896522241915E-14	0.388112208388729	0.29	0.162	1.84145517428788E-10	Periportal hepatocytes	NSMCE2
1.14133816265071E-14	0.345319812043074	0.261	0.14	1.86163667709957E-10	Periportal hepatocytes	DMXL1
1.21251428627105E-14	0.348323545394444	0.341	0.198	1.97773205233671E-10	Periportal hepatocytes	KIAA2026
1.22208581485398E-14	0.41188347829393	0.299	0.171	1.99334417260833E-10	Periportal hepatocytes	DEAF1
1.27569819443955E-14	0.67114904012279	0.259	0.139	2.08079132495035E-10	Periportal hepatocytes	TDRD10
1.28743092663768E-14	0.275210725078702	0.194	0.094	2.09992858443872E-10	Periportal hepatocytes	ANKRD6
1.2977892160543E-14	0.430618407425944	0.239	0.123	2.11682399030616E-10	Periportal hepatocytes	MED13
1.38394853712326E-14	0.449192407910428	0.22	0.113	2.25735845890176E-10	Periportal hepatocytes	PALMD
1.42092616611922E-14	0.338515875505807	0.149	0.065	2.31767266955706E-10	Periportal hepatocytes	CDK17
1.42276254263057E-14	0.386458913506289	0.159	0.072	2.32066798328472E-10	Periportal hepatocytes	EPS8

1.48279254021882E-14	0.455229434198806	0.397	0.24	2.41858291235091E-10	Periportal hepatocytes	PAWR
1.54665932581492E-14	0.37762106469082	0.306	0.174	2.52275602633672E-10	Periportal hepatocytes	TTC19
1.6168009916583E-14	0.573699088228515	0.216	0.11	2.63716409749385E-10	Periportal hepatocytes	NAA40
1.6545473961291E-14	0.280222134959706	0.391	0.238	2.69873225782617E-10	Periportal hepatocytes	CSTF3
1.80143362029071E-14	0.543228856992217	0.151	0.066	2.93831837805618E-10	Periportal hepatocytes	ARMC1
1.87133478710538E-14	0.33839485344491	0.233	0.121	3.05233417124758E-10	Periportal hepatocytes	SFXN2
1.98104598578359E-14	0.462201305883228	0.279	0.156	3.23128410741162E-10	Periportal hepatocytes	MTX2
2.00716597646863E-14	0.37077324509851	0.323	0.186	3.27388842421799E-10	Periportal hepatocytes	LRRC40
2.02469662567048E-14	0.361765688144452	0.142	0.061	3.30248266613112E-10	Periportal hepatocytes	PFN2
2.07659055301635E-14	0.629342210828123	0.315	0.181	3.38712685102498E-10	Periportal hepatocytes	HNRNPL
2.21348998641206E-14	0.426751241901826	0.289	0.164	3.61042351683672E-10	Periportal hepatocytes	SMLR1
2.22082365324455E-14	0.309297750811118	0.54	0.346	3.62238546080719E-10	Periportal hepatocytes	RABGAP1L
2.41244308950735E-14	0.615456122343649	0.274	0.153	3.93493592329544E-10	Periportal hepatocytes	SIRT5
2.4781632339878E-14	0.267678662423826	0.29	0.161	4.0421320509575E-10	Periportal hepatocytes	FBXL4
2.50821005353119E-14	0.352345746724831	0.261	0.141	4.09114141831472E-10	Periportal hepatocytes	NLK
2.59252362081053E-14	0.361580220475521	0.195	0.096	4.22866527790405E-10	Periportal hepatocytes	SLC49A4
2.78945360128547E-14	0.365162168096993	0.389	0.236	4.54987776905673E-10	Periportal hepatocytes	HTT
2.79591146980444E-14	0.896010772674296	0.101	0.036	4.56041119839803E-10	Periportal hepatocytes	NCDN
2.84418292462592E-14	0.27579120052915	0.269	0.145	4.63914676835734E-10	Periportal hepatocytes	KCNAB2
2.89661112740104E-14	0.560482639268697	0.125	0.051	4.72466240990384E-10	Periportal hepatocytes	DEPRECAT
2.91583977159096E-14	0.271507940204057	0.361	0.211	4.75602625144201E-10	Periportal hepatocytes	POLR2J3
2.92991881755948E-14	0.317843114087018	0.415	0.253	4.77899058332127E-10	Periportal hepatocytes	NEMF
3.14182449676116E-14	0.298968083798475	0.385	0.229	5.12462993666713E-10	Periportal hepatocytes	R3HDM2
3.44536291533525E-14	0.271809805157464	0.28	0.153	5.61973145120332E-10	Periportal hepatocytes	OTUB1
3.51690018852438E-14	0.539524856089466	0.335	0.199	5.73641589750211E-10	Periportal hepatocytes	ZNF44
4.07173386871669E-14	0.312308048768472	0.292	0.162	6.6414051132638E-10	Periportal hepatocytes	FBXL18
4.08473253014432E-14	0.619580501732785	0.201	0.101	6.6626072299184E-10	Periportal hepatocytes	NUP43
4.28231470343489E-14	0.49193744909565	0.307	0.175	6.98488351277265E-10	Periportal hepatocytes	PSEN1
4.54544462673574E-14	0.395570861673952	0.187	0.09	7.41407473066867E-10	Periportal hepatocytes	TBC1D9
4.55119542329474E-14	0.79278618461628	0.154	0.069	7.42345485493605E-10	Periportal hepatocytes	TIPARP
4.68145521260296E-14	0.52322201469169	0.292	0.165	7.63592159727668E-10	Periportal hepatocytes	RBFOX2
4.82577512769739E-14	0.536230433454028	0.223	0.116	7.87132181078722E-10	Periportal hepatocytes	CASP9
5.56384947768162E-14	0.360132796443823	0.302	0.173	9.07519488304649E-10	Periportal hepatocytes	ADCK5
5.82275342963989E-14	0.268196358928308	0.447	0.282	9.49749311908562E-10	Periportal hepatocytes	MYO15B
5.87934556937243E-14	0.356854486319359	0.274	0.151	9.58980055820337E-10	Periportal hepatocytes	CNKSR3
5.91371138208357E-14	0.348010437960341	0.228	0.119	9.64585463531651E-10	Periportal hepatocytes	DEPRECAT
5.9801441341263E-14	0.8445875754597	0.195	0.098	9.75421309717341E-10	Periportal hepatocytes	LARS2
7.11811301875842E-14	0.546981342814838	0.208	0.105	1.16103541448969E-09	Periportal hepatocytes	PEAR1
7.30198015213257E-14	0.350595747354662	0.191	0.094	1.19102598261434E-09	Periportal hepatocytes	DDX58
7.67878597765227E-14	0.34717801388231	0.203	0.103	1.25248678081486E-09	Periportal hepatocytes	AP1AR
8.6004271147811E-14	0.870985359521005	0.139	0.061	1.40281566669194E-09	Periportal hepatocytes	ASCC1
9.48226794420408E-14	0.443119415306678	0.266	0.147	1.54665272437913E-09	Periportal hepatocytes	ERCC6
9.90712508372175E-14	0.873428530704166	0.167	0.078	1.61595117240585E-09	Periportal hepatocytes	WASHC3
9.9132595032525E-14	0.58828067997964	0.228	0.121	1.61695175757551E-09	Periportal hepatocytes	GDAP2
1.00370421321876E-13	0.605795098294507	0.25	0.137	1.63714194218112E-09	Periportal hepatocytes	HERC1
1.01547726844214E-13	0.334545074319832	0.254	0.139	1.65634497255597E-09	Periportal hepatocytes	XPR1
1.01632704385054E-13	0.354010035219624	0.247	0.134	1.65773104122462E-09	Periportal hepatocytes	ABCD4
1.05549297034376E-13	0.332422271588629	0.312	0.181	1.72161458392771E-09	Periportal hepatocytes	LARP1B
1.05832545984836E-13	0.303768650436547	0.31	0.18	1.72623465755866E-09	Periportal hepatocytes	NFAT5
1.05970232263237E-13	0.350941212971437	0.253	0.139	1.72848045844566E-09	Periportal hepatocytes	ST7
1.10520877995525E-13	0.298210469771236	0.316	0.185	1.80270604098501E-09	Periportal hepatocytes	SH3D19
1.20366576749009E-13	0.841320595038387	0.168	0.08	1.96329923335308E-09	Periportal hepatocytes	ABCC4
1.27096665326533E-13	0.26669533710412	0.359	0.214	2.07307370814109E-09	Periportal hepatocytes	NFS1
1.27209588722023E-13	0.474403506459469	0.184	0.09	2.07491560164492E-09	Periportal hepatocytes	DEPRECAT
1.30711924912242E-13	0.828038428600578	0.115	0.045	2.13204220724358E-09	Periportal hepatocytes	DZIP3
1.34421056917837E-13	0.390489070007379	0.315	0.183	2.19254185938683E-09	Periportal hepatocytes	PEX6
1.39491599696163E-13	0.351952372572726	0.191	0.095	2.27524748264411E-09	Periportal hepatocytes	TRAFD1
1.47827505546814E-13	0.758314227807807	0.182	0.09	2.41121444297408E-09	Periportal hepatocytes	ELP4
1.52116950175451E-13	0.480896208735568	0.155	0.07	2.48117957431179E-09	Periportal hepatocytes	UTRN
1.6767725935867E-13	0.534235073983598	0.172	0.082	2.73498377739926E-09	Periportal hepatocytes	GTF2E2

1.82085013563944E-13	0.29771682145387	0.359	0.216	2.96998865624149E-09	Periportal hepatocytes	MTMR12
1.95823142509914E-13	0.800758149996788	0.144	0.065	3.19407127747921E-09	Periportal hepatocytes	MFSD2A
2.06476140744852E-13	0.4275358774723	0.148	0.068	3.36783233168928E-09	Periportal hepatocytes	CPN1
2.14992189870772E-13	0.724825655205126	0.139	0.062	3.50673760898216E-09	Periportal hepatocytes	TSC22D3
2.17265979780696E-13	0.278386044173071	0.111	0.044	3.54382539620293E-09	Periportal hepatocytes	CLN5
2.20279495113775E-13	0.614339649920172	0.249	0.137	3.59297884480079E-09	Periportal hepatocytes	RAP2C
2.22766353453096E-13	0.314875118765217	0.32	0.187	3.63354199117346E-09	Periportal hepatocytes	PIAS2
2.38249839028211E-13	0.629850665406555	0.134	0.057	3.88609312438914E-09	Periportal hepatocytes	AP3S1
2.39699885810683E-13	0.412680500035506	0.241	0.131	3.90974483745805E-09	Periportal hepatocytes	RASGEF1B
2.60154614060234E-13	0.314551700968709	0.236	0.128	4.24338190993648E-09	Periportal hepatocytes	RNFT1
2.61629808317633E-13	1.10438386649556	0.102	0.039	4.26744380346892E-09	Periportal hepatocytes	KYAT3
2.88686253617429E-13	0.342437170384188	0.342	0.203	4.70876148275389E-09	Periportal hepatocytes	TAPT1
2.93933441206408E-13	1.19701835682172	0.122	0.051	4.79434835951772E-09	Periportal hepatocytes	STK36
2.99414638389679E-13	0.254704726775226	0.303	0.175	4.88375216677405E-09	Periportal hepatocytes	GPBP1
3.01117691448103E-13	0.545556890895486	0.194	0.098	4.91153066521001E-09	Periportal hepatocytes	ZNF529
3.06302054315481E-13	0.57414108814885	0.136	0.06	4.99609280793981E-09	Periportal hepatocytes	TNFAIP1
3.08230000178938E-13	0.540005211687438	0.204	0.104	5.02753953291866E-09	Periportal hepatocytes	DEPRECAT
3.1213442598992E-13	0.579072347763242	0.155	0.073	5.09122462232158E-09	Periportal hepatocytes	CD44
3.23216838306828E-13	0.261534821993204	0.155	0.071	5.27198984962267E-09	Periportal hepatocytes	UBE2W
3.39970336982275E-13	0.485082571321297	0.116	0.048	5.54525616651789E-09	Periportal hepatocytes	TMC6
3.54060807234943E-13	0.277633573854959	0.226	0.122	5.77508582680915E-09	Periportal hepatocytes	CPLX1
4.06103724242545E-13	0.475762730852974	0.254	0.142	6.62395784612016E-09	Periportal hepatocytes	CES3
4.14176474841618E-13	0.607735629150365	0.102	0.039	6.75563248114164E-09	Periportal hepatocytes	C12orf4
4.22105371544966E-13	0.442889106363616	0.276	0.158	6.88496071526994E-09	Periportal hepatocytes	ZNF507
4.35348432184361E-13	0.870435460312432	0.124	0.052	7.10096827735911E-09	Periportal hepatocytes	ZNF567
4.4397048625808E-13	0.816827027043687	0.203	0.107	7.24160260135555E-09	Periportal hepatocytes	NRIP1
4.6625957555278E-13	0.384160748515449	0.315	0.184	7.6051599368414E-09	Periportal hepatocytes	ARMC8
4.94390253771896E-13	0.893918891024886	0.126	0.054	8.06399942927339E-09	Periportal hepatocytes	ZBED6CL
5.23184986133416E-13	0.546571893538047	0.249	0.137	8.53367030882214E-09	Periportal hepatocytes	PITHD1
5.38014580290919E-13	0.557642376452704	0.168	0.08	8.77555581912517E-09	Periportal hepatocytes	ZNF266
5.41391627450766E-13	0.263674042115131	0.244	0.133	8.83063883534944E-09	Periportal hepatocytes	FAM98A
5.64396086715588E-13	0.710988904292392	0.124	0.053	9.20586457041796E-09	Periportal hepatocytes	IFNLR1
5.65762383891283E-13	0.790563068556032	0.171	0.083	9.22815024365071E-09	Periportal hepatocytes	NSMAF
6.35941439230867E-13	0.270467059038478	0.263	0.147	1.03728408152947E-08	Periportal hepatocytes	BLZF1
6.49489676602124E-13	0.462620614531978	0.195	0.099	1.05938261150573E-08	Periportal hepatocytes	PRRC2B
6.54136557988694E-13	0.293581268132366	0.227	0.121	1.06696213973536E-08	Periportal hepatocytes	LMTK2
6.655101976104E-13	0.605665221106156	0.155	0.073	1.08551368332232E-08	Periportal hepatocytes	PPID
6.75000518349879E-13	0.56764575132079	0.115	0.047	1.10099334548049E-08	Periportal hepatocytes	TPRN
7.51407035671281E-13	0.322074047679132	0.241	0.132	1.22562001588343E-08	Periportal hepatocytes	NFYA
7.80933291275624E-13	0.362413716786826	0.158	0.075	1.27378029139967E-08	Periportal hepatocytes	NSRP1
8.30948545563081E-13	0.340241098852136	0.152	0.07	1.35536017266794E-08	Periportal hepatocytes	DEPRECAT
8.46435350655984E-13	0.320502532594541	0.148	0.068	1.38062070045498E-08	Periportal hepatocytes	FAM114A2
8.62704345937951E-13	0.912786091358137	0.154	0.073	1.40715705865939E-08	Periportal hepatocytes	KCTD6
8.96642334363521E-13	0.998014644740755	0.105	0.041	1.46251331158034E-08	Periportal hepatocytes	RHBG
1.02326903213443E-12	0.534534259407233	0.208	0.109	1.66905411831446E-08	Periportal hepatocytes	LTN1
1.18055893843068E-12	0.50541489304259	0.177	0.087	1.92560968447428E-08	Periportal hepatocytes	ITSN1
1.22650179699458E-12	0.312804972455609	0.197	0.101	2.00054708107786E-08	Periportal hepatocytes	C8orf76
1.235106903047E-12	0.325596488474772	0.432	0.278	2.01458286955996E-08	Periportal hepatocytes	SMAD4
1.398306473402E-12	0.426504350836271	0.2	0.104	2.280777688766E-08	Periportal hepatocytes	UBE3B
1.41906390276048E-12	0.346720828327299	0.155	0.073	2.31463513179263E-08	Periportal hepatocytes	TERF1
1.46721027287784E-12	0.399712813101335	0.158	0.076	2.39316667609104E-08	Periportal hepatocytes	DEPRECAT
1.52976519876029E-12	0.300368835849249	0.348	0.215	2.49520001569791E-08	Periportal hepatocytes	KIFC2
1.62203155478756E-12	0.343994189843966	0.211	0.111	2.64569566901399E-08	Periportal hepatocytes	ARHGAP5
1.62767007725924E-12	0.531311483338441	0.149	0.07	2.65489266301755E-08	Periportal hepatocytes	YAE1
1.63756919929557E-12	0.419243027464358	0.293	0.173	2.671039120971E-08	Periportal hepatocytes	MED23
1.71154459211581E-12	0.40746986342375	0.161	0.078	2.7917003842001E-08	Periportal hepatocytes	ARFIP1
1.71732572709452E-12	0.292983927618027	0.139	0.063	2.80112999346387E-08	Periportal hepatocytes	NIFK
1.79850192745179E-12	0.320730919779727	0.178	0.088	2.93353649386662E-08	Periportal hepatocytes	FAM49B
1.83238925753201E-12	0.422391502806095	0.204	0.109	2.98881011796047E-08	Periportal hepatocytes	PPIL3
1.87989096403363E-12	0.30091456016099	0.125	0.054	3.06629015143525E-08	Periportal hepatocytes	CYFIP2

1.91694145616971E-12	0.45446411795499	0.228	0.127	3.12672320915842E-08	Periportal hepatocytes	CEBPZ
2.0537423214457E-12	0.579368965382426	0.128	0.056	3.34985910051008E-08	Periportal hepatocytes	POU5F1
2.10404772497555E-12	0.497528331033741	0.293	0.174	3.43191224420762E-08	Periportal hepatocytes	CNOT2
2.12104988485055E-12	0.378431204651758	0.29	0.17	3.45964446717972E-08	Periportal hepatocytes	FBXO38
2.14998187237152E-12	0.840466159242049	0.134	0.06	3.50683543202519E-08	Periportal hepatocytes	DMD
2.15770672899307E-12	0.509580582300239	0.203	0.108	3.5194354456606E-08	Periportal hepatocytes	PPFIBP1
2.17019116039383E-12	0.302391871507576	0.341	0.206	3.53979880171837E-08	Periportal hepatocytes	KAT6A
2.20168735327406E-12	0.380433717704313	0.286	0.166	3.59117224192532E-08	Periportal hepatocytes	KLHL12
2.29569638498152E-12	0.366308280992225	0.32	0.192	3.74451037354336E-08	Periportal hepatocytes	ADGRA3
2.32277527018178E-12	0.926440927874473	0.116	0.049	3.7886787431935E-08	Periportal hepatocytes	FP565260.(
2.32618375608106E-12	0.257580738156902	0.249	0.14	3.79423832454382E-08	Periportal hepatocytes	MAPKAP1
2.45776108560976E-12	0.394710565369338	0.201	0.105	4.00885410673808E-08	Periportal hepatocytes	CCDC28A
2.75149854886129E-12	0.445242999370427	0.116	0.049	4.48796928304764E-08	Periportal hepatocytes	ZNF460
2.78747526231172E-12	0.714967546061426	0.105	0.042	4.54665090035665E-08	Periportal hepatocytes	TRPC1
2.8635499438565E-12	0.306440595257736	0.376	0.234	4.67073631342433E-08	Periportal hepatocytes	DNAJC10
2.91940039066433E-12	0.397224840014683	0.282	0.165	4.76183397721259E-08	Periportal hepatocytes	USP25
3.16863196751549E-12	0.401475719426	0.185	0.095	5.16835560221452E-08	Periportal hepatocytes	APPL2
3.37498248663447E-12	0.705788838872099	0.122	0.053	5.50493393394949E-08	Periportal hepatocytes	FASTKD3
3.62280412352151E-12	0.824864452642368	0.187	0.096	5.90915580587593E-08	Periportal hepatocytes	CEP44
3.68139956294554E-12	0.567935269359523	0.135	0.061	6.00473082712047E-08	Periportal hepatocytes	UBE2K
3.79093011839506E-12	0.852090360938373	0.108	0.044	6.18338611611418E-08	Periportal hepatocytes	FAM43A
3.88983363977447E-12	0.338565941674694	0.141	0.065	6.34470764983613E-08	Periportal hepatocytes	PFKFB3
3.96157440511799E-12	0.568868767070175	0.152	0.073	6.46172401218796E-08	Periportal hepatocytes	SLC35B4
3.99905502517179E-12	0.411548279291459	0.289	0.168	6.52285865155771E-08	Periportal hepatocytes	PCNX1
4.00401286212446E-12	0.338469254303031	0.307	0.182	6.5309453794112E-08	Periportal hepatocytes	MYSM1
4.10404411288314E-12	0.396161972451819	0.178	0.09	6.69410635252369E-08	Periportal hepatocytes	SGPL1
4.4128056184675E-12	0.51204265580399	0.18	0.091	7.19772724428233E-08	Periportal hepatocytes	NAA15
4.63346011233731E-12	0.407743790398249	0.201	0.106	7.5576367892334E-08	Periportal hepatocytes	ACAD10
5.08059729027301E-12	0.378297071385214	0.237	0.132	8.2869622401643E-08	Periportal hepatocytes	TTC28
5.41950693289589E-12	0.368692340460649	0.259	0.149	8.83975775824648E-08	Periportal hepatocytes	WDR70
5.52390900429013E-12	0.273780864511154	0.379	0.238	9.01004797689763E-08	Periportal hepatocytes	MLPH
5.76065956528838E-12	0.348685323728365	0.193	0.101	9.39621181694188E-08	Periportal hepatocytes	PPIP5K2
5.8573019824676E-12	0.692301899291882	0.131	0.059	9.5538452636029E-08	Periportal hepatocytes	WDR60
5.98802630428026E-12	0.473594318451892	0.101	0.04	9.76706970491153E-08	Periportal hepatocytes	RIPK2
6.30425069346703E-12	0.731509950262213	0.171	0.087	1.02828633061141E-07	Periportal hepatocytes	ANKRD26
6.395848925722E-12	0.414926065632912	0.119	0.052	1.04322691827451E-07	Periportal hepatocytes	ST3GAL5
6.53260492391396E-12	0.640136185729831	0.26	0.151	1.06553318913961E-07	Periportal hepatocytes	TOP3B
6.56659986389048E-12	0.463914187750469	0.168	0.084	1.07107810379918E-07	Periportal hepatocytes	ANKRD44
6.94106640523524E-12	0.546889296580235	0.142	0.067	1.13215734135792E-07	Periportal hepatocytes	SLC10A5
6.99928620043016E-12	0.269195642601348	0.19	0.099	1.14165357215216E-07	Periportal hepatocytes	LIPC
7.00270695064218E-12	0.285829194987936	0.338	0.205	1.14221153071925E-07	Periportal hepatocytes	ARFGAP2
7.07872046685381E-12	0.421760796066508	0.307	0.186	1.15461009534852E-07	Periportal hepatocytes	TMEM39A
7.21429000395536E-12	0.420470255071286	0.162	0.08	1.17672284254516E-07	Periportal hepatocytes	DDB2
7.21522611587695E-12	0.982968274785213	0.125	0.056	1.17687553176069E-07	Periportal hepatocytes	RALGDS
7.54532518164994E-12	0.327990855080874	0.17	0.085	1.23071799037892E-07	Periportal hepatocytes	E2F3
7.67107485758081E-12	0.283410480248146	0.243	0.137	1.25122902002001E-07	Periportal hepatocytes	SEH1L
7.82371087647025E-12	0.384450220245053	0.2	0.105	1.27612548106106E-07	Periportal hepatocytes	CNOT9
7.83000945027402E-12	0.429191888662474	0.246	0.14	1.27715284143419E-07	Periportal hepatocytes	PNPLA7
7.8412720512324E-12	0.302956141280246	0.168	0.084	1.27898988427652E-07	Periportal hepatocytes	NMNAT3
7.92154705043301E-12	0.324485768329072	0.415	0.268	1.29208353939613E-07	Periportal hepatocytes	ZNF638
7.96250201110975E-12	0.435206747441161	0.217	0.119	1.29876370303211E-07	Periportal hepatocytes	PIK3C2A
8.21190156237716E-12	0.384052220561457	0.187	0.097	1.33944326383934E-07	Periportal hepatocytes	RBM22
8.25710382675708E-12	0.500922749638642	0.112	0.047	1.34681620518235E-07	Periportal hepatocytes	SNRPG
8.49654380243225E-12	0.482884578044421	0.132	0.06	1.38587125961472E-07	Periportal hepatocytes	CDKN2AIP
8.50222998643084E-12	0.30915918843133	0.356	0.219	1.38679873308673E-07	Periportal hepatocytes	BPTF
8.51548007775541E-12	0.472406013495951	0.208	0.112	1.38895995548268E-07	Periportal hepatocytes	ADK
8.55504678902478E-12	0.433493634557266	0.121	0.053	1.39541368175783E-07	Periportal hepatocytes	CLCN2
8.7304435550368E-12	0.466713281685502	0.185	0.096	1.42402264826205E-07	Periportal hepatocytes	PLD1
8.97674679160106E-12	0.434101245646522	0.21	0.112	1.46419716917805E-07	Periportal hepatocytes	CDC123
1.00234831205102E-11	0.33975017720564	0.131	0.06	1.63493033178642E-07	Periportal hepatocytes	TMEM30B

1.0222404708006E-11	0.388105796294737	0.181	0.093	1.66737643192286E-07	Periportal hepatocytes	ITGB1BP1
1.02556048788141E-11	0.295128849156431	0.136	0.063	1.67279171178337E-07	Periportal hepatocytes	TRUB2
1.03710783300906E-11	0.256005765240138	0.394	0.249	1.69162658642108E-07	Periportal hepatocytes	UVSSA
1.04784665592567E-11	0.33288443670872	0.253	0.143	1.70914268048035E-07	Periportal hepatocytes	GGA1
1.05586538114555E-11	0.331354666292747	0.148	0.071	1.7222220231865E-07	Periportal hepatocytes	AGL
1.07196714061865E-11	0.531432666899158	0.158	0.078	1.74848560306308E-07	Periportal hepatocytes	LRRC8D
1.07321450669661E-11	0.683901292212055	0.136	0.063	1.75052018187285E-07	Periportal hepatocytes	ZNF207
1.09149556040902E-11	0.595789665127845	0.136	0.063	1.78033840858316E-07	Periportal hepatocytes	ZFX
1.12306816276708E-11	0.642078267665496	0.144	0.068	1.83183648028939E-07	Periportal hepatocytes	ELF2
1.13761578635643E-11	0.45537869249473	0.216	0.119	1.85556510912597E-07	Periportal hepatocytes	TMEM260
1.20128797109402E-11	0.399482541098551	0.161	0.079	1.95942080965146E-07	Periportal hepatocytes	CLASP2
1.21552151307349E-11	0.684950840678061	0.158	0.078	1.98263713997417E-07	Periportal hepatocytes	DEPRECAT
1.36951350035902E-11	0.622942347915083	0.237	0.134	2.2338134704356E-07	Periportal hepatocytes	SETD6
1.47209734841803E-11	0.26994705910055	0.236	0.13	2.40113798500465E-07	Periportal hepatocytes	SECISBP2
1.47797399385511E-11	0.472536360569673	0.205	0.111	2.41072338137706E-07	Periportal hepatocytes	ATG7
1.50793740617738E-11	0.493304271356886	0.122	0.054	2.45959670321592E-07	Periportal hepatocytes	EBF4
1.5260208738035E-11	0.399671445480661	0.101	0.041	2.48909264726088E-07	Periportal hepatocytes	NIPAL3
1.55679083955038E-11	0.251674024033383	0.161	0.079	2.53928153839063E-07	Periportal hepatocytes	RAD23B
1.56187014114695E-11	0.345443505530593	0.249	0.141	2.5475663872248E-07	Periportal hepatocytes	ATAD1
1.56565199274234E-11	0.467081272711013	0.333	0.207	2.55373496536203E-07	Periportal hepatocytes	SMAD5
1.59038245202372E-11	0.5271449029414	0.343	0.219	2.59407281749588E-07	Periportal hepatocytes	VNN 3.00
1.60395481364517E-11	0.884057554138358	0.158	0.078	2.61621069653663E-07	Periportal hepatocytes	ZNF554
1.66975915159274E-11	0.45396387820446	0.21	0.114	2.72354415216292E-07	Periportal hepatocytes	NICN1
1.72906476924959E-11	0.327348660315895	0.345	0.212	2.82027754512301E-07	Periportal hepatocytes	ZNF326
1.74562772599271E-11	0.476471205971249	0.125	0.056	2.8472933838667E-07	Periportal hepatocytes	SMARCD3
1.83853317010292E-11	0.570095992837819	0.138	0.065	2.99883145375487E-07	Periportal hepatocytes	RNF217
1.88553016103519E-11	0.282019651822531	0.276	0.162	3.07548824566451E-07	Periportal hepatocytes	ADNP2
1.90638991582418E-11	0.954787123244825	0.182	0.096	3.10951259170081E-07	Periportal hepatocytes	WDR75
2.00957251806832E-11	0.361548063544817	0.101	0.041	3.27781373422123E-07	Periportal hepatocytes	SCLT1
2.01088390827358E-11	0.354207747952619	0.29	0.175	3.27995274278504E-07	Periportal hepatocytes	KAT6B
2.10220464443251E-11	0.521190097759557	0.111	0.047	3.42890599553387E-07	Periportal hepatocytes	ANKMY2
2.20327827260752E-11	0.261297123499119	0.302	0.18	3.59376719045013E-07	Periportal hepatocytes	FBXO22
2.34969809686221E-11	0.35864706208044	0.376	0.242	3.83259256579195E-07	Periportal hepatocytes	TUBGCP3
2.41344799883641E-11	0.723547216437512	0.167	0.085	3.93657503090207E-07	Periportal hepatocytes	TBCCD1
2.43872265990262E-11	0.523317995982709	0.207	0.114	3.97780053056716E-07	Periportal hepatocytes	GCNT2
2.65888359789824E-11	0.359450562645926	0.136	0.064	4.33690503653183E-07	Periportal hepatocytes	CASP6
2.67932361127275E-11	0.615964772837757	0.19	0.101	4.37024474234698E-07	Periportal hepatocytes	EHF
2.71083343350659E-11	0.337015796316153	0.132	0.061	4.42164041339259E-07	Periportal hepatocytes	PIGS
2.73453224916419E-11	0.267990019246656	0.297	0.181	4.46029555161172E-07	Periportal hepatocytes	NISCH
2.81272410169664E-11	0.28115355676836	0.375	0.238	4.58783428227739E-07	Periportal hepatocytes	UBR3
2.91793218370679E-11	0.37967488238943	0.126	0.057	4.75943918484415E-07	Periportal hepatocytes	AKR1C1
3.01617696387374E-11	0.774240708795813	0.122	0.055	4.91968624577446E-07	Periportal hepatocytes	PELI1
3.01909053218684E-11	0.603395554242857	0.216	0.12	4.92443856704995E-07	Periportal hepatocytes	CMTR2
3.19899058099951E-11	0.824851587917988	0.157	0.078	5.2178735366683E-07	Periportal hepatocytes	RAB33B
3.23341534656274E-11	0.427273501420565	0.272	0.161	5.27402377177849E-07	Periportal hepatocytes	TRPV1
3.2872796675875E-11	0.262388486336214	0.267	0.157	5.36188186580197E-07	Periportal hepatocytes	RSPRY1
3.36959837386606E-11	0.661832698843149	0.126	0.058	5.49615190761293E-07	Periportal hepatocytes	ATG9A
3.56592026353673E-11	0.272124698127491	0.353	0.223	5.81637254185476E-07	Periportal hepatocytes	TP53I13
3.61960750949875E-11	0.323991006840794	0.282	0.169	5.90394180874342E-07	Periportal hepatocytes	ATG14
3.64221290793028E-11	0.340471524593212	0.132	0.061	5.94081347412508E-07	Periportal hepatocytes	STYXL1
4.02599726160266E-11	0.340554816201646	0.164	0.083	6.5668041334001E-07	Periportal hepatocytes	STIM2
4.09234279833277E-11	0.509714461958096	0.224	0.125	6.67502033836058E-07	Periportal hepatocytes	LYSMD3
4.39252249174155E-11	0.264546486945204	0.136	0.065	7.16464343627965E-07	Periportal hepatocytes	ALG6
4.44417329078557E-11	0.471910501825696	0.251	0.147	7.24889105460034E-07	Periportal hepatocytes	DDX59
4.44646411060866E-11	0.329119415002435	0.273	0.161	7.25262761081379E-07	Periportal hepatocytes	BTBD9
4.5289813441278E-11	0.427351227017057	0.167	0.086	7.38722147040685E-07	Periportal hepatocytes	MAPK8
4.74987335738611E-11	0.760152746858783	0.138	0.066	7.74751843323248E-07	Periportal hepatocytes	AC253572.
4.94671881953614E-11	0.904438160873276	0.119	0.054	8.0685930665454E-07	Periportal hepatocytes	SLC2A12
5.34667014081927E-11	0.6507878116367	0.216	0.121	8.72095366669032E-07	Periportal hepatocytes	FAM76B
5.36968437948388E-11	0.2970967701666	0.266	0.157	8.75849219137616E-07	Periportal hepatocytes	MBTD1

5.41067159980811E-11	0.378243122697702	0.395	0.256	8.82534644644702E-07	Periportal hepatocytes	AKAP17A
5.4972408448596E-11	1.12405556262372	0.106	0.046	8.9665495420505E-07	Periportal hepatocytes	HSPG2
5.63512188448206E-11	0.503853499669897	0.28	0.167	9.19144730577868E-07	Periportal hepatocytes	LPIN1
5.65170388795197E-11	0.253945460104612	0.194	0.105	9.21849421163846E-07	Periportal hepatocytes	PRPF18
5.75171380642145E-11	0.340437007434681	0.234	0.133	9.38162038965403E-07	Periportal hepatocytes	PTPN6
5.78368322418538E-11	0.53588544827434	0.236	0.135	9.43376570696878E-07	Periportal hepatocytes	TMF1
5.82688176972965E-11	0.588064865546527	0.164	0.083	9.50422685460603E-07	Periportal hepatocytes	GOLGA8B
6.02653969446646E-11	0.48229550593316	0.124	0.056	9.82988889564424E-07	Periportal hepatocytes	FGD6
6.10012523292426E-11	0.368604696150098	0.158	0.08	9.94991426742276E-07	Periportal hepatocytes	CPED1
6.3010025134343E-11	0.284976353246377	0.247	0.144	1.02775651996627E-06	Periportal hepatocytes	EIF1B
6.38455727991391E-11	0.596441026263348	0.155	0.078	1.04138513792676E-06	Periportal hepatocytes	ZNF274
6.41972783393154E-11	0.574140109719054	0.194	0.106	1.04712180699257E-06	Periportal hepatocytes	WDR37
6.67504599105862E-11	0.385338057996873	0.259	0.153	1.08876675160157E-06	Periportal hepatocytes	TRIM4
6.6808848258289E-11	0.752246218552826	0.115	0.051	1.08971912394095E-06	Periportal hepatocytes	ITPR1
6.76373123379854E-11	0.425220775676764	0.105	0.045	1.10323220154488E-06	Periportal hepatocytes	PNMA6A
6.82802565505468E-11	0.904619723336328	0.106	0.046	1.11371926459597E-06	Periportal hepatocytes	ALPK1
6.963318620785E-11	0.57196209908874	0.18	0.096	1.13578690023624E-06	Periportal hepatocytes	TMEM161E
7.19154831579418E-11	0.262928349592998	0.338	0.211	1.17301344578919E-06	Periportal hepatocytes	NUP85
7.20061539981495E-11	0.273397730896199	0.303	0.186	1.17449237786382E-06	Periportal hepatocytes	ZNF148
7.43456228343198E-11	0.353378878657064	0.364	0.235	1.21265145405059E-06	Periportal hepatocytes	NPIPB15
7.46301630942949E-11	0.320460660858678	0.247	0.146	1.21729259023104E-06	Periportal hepatocytes	CAND1
7.65906176584463E-11	0.30251947099464	0.253	0.148	1.24926956462692E-06	Periportal hepatocytes	WAPL
7.68563067194246E-11	0.345233441252085	0.203	0.111	1.25360321890053E-06	Periportal hepatocytes	TARDBP
8.0310369177229E-11	0.37831114196696	0.234	0.134	1.30994243164978E-06	Periportal hepatocytes	FAM160B1
8.39435297012078E-11	0.272619223609194	0.338	0.211	1.3692029129564E-06	Periportal hepatocytes	USP24
8.50837141630118E-11	0.514881599495735	0.191	0.104	1.38780046171289E-06	Periportal hepatocytes	FRA10AC1
8.53062184356529E-11	0.492270061853807	0.167	0.087	1.39142972890393E-06	Periportal hepatocytes	SLC22A9
8.71248062951315E-11	0.429765522449403	0.17	0.087	1.42109271547989E-06	Periportal hepatocytes	ZNF197
8.86949736077749E-11	0.375346548400171	0.145	0.071	1.44670371451642E-06	Periportal hepatocytes	LYN
8.9521804359702E-11	0.342481527005277	0.122	0.057	1.4601901509111E-06	Periportal hepatocytes	EPB41L1
8.97389782984282E-11	0.336134115473055	0.237	0.137	1.46373247502566E-06	Periportal hepatocytes	SLC35E2A
8.99779158646988E-11	0.253263670376002	0.251	0.147	1.4676297856691E-06	Periportal hepatocytes	MLH1
9.2073817282914E-11	0.431558532959427	0.106	0.046	1.50181603370161E-06	Periportal hepatocytes	VSTM4
9.25902534971214E-11	0.695959121146162	0.154	0.078	1.51023962479155E-06	Periportal hepatocytes	NBPF11
9.41756628416724E-11	0.887631469144475	0.109	0.048	1.53609923661052E-06	Periportal hepatocytes	KIAA0586
9.58392786575151E-11	1.35689754268156	0.111	0.049	1.56323447418273E-06	Periportal hepatocytes	MFSD13A
9.81292652415374E-11	0.268308995627891	0.223	0.126	1.60058644535472E-06	Periportal hepatocytes	GGT7
9.82524444696826E-11	0.483843626008083	0.128	0.06	1.60259562174499E-06	Periportal hepatocytes	ANKRD50
9.95714374016382E-11	0.307926214727677	0.32	0.2	1.62410971545812E-06	Periportal hepatocytes	ABTB2
1.01678742173961E-10	0.479831496646135	0.149	0.074	1.65848196359948E-06	Periportal hepatocytes	SEPTIN2
1.02320304899906E-10	0.750434549574224	0.116	0.053	1.66894649322236E-06	Periportal hepatocytes	LRIG3
1.10784491215699E-10	0.462651847644771	0.237	0.138	1.80700583621926E-06	Periportal hepatocytes	CECR2
1.15099890985097E-10	0.393880592512922	0.201	0.112	1.87739432185791E-06	Periportal hepatocytes	FBXO25
1.2927319035019E-10	0.359249135845322	0.243	0.142	2.10857500780194E-06	Periportal hepatocytes	ZNF407
1.38717058738304E-10	0.567864278963504	0.22	0.124	2.26261394508048E-06	Periportal hepatocytes	HCFC2
1.41181304191776E-10	0.39160725906699	0.274	0.167	2.30280825267206E-06	Periportal hepatocytes	NUP210
1.42256897036911E-10	0.316217750473964	0.33	0.206	2.32035224756906E-06	Periportal hepatocytes	KHDC4
1.44388500726853E-10	0.781507491784794	0.112	0.05	2.3551208353557E-06	Periportal hepatocytes	SNX22
1.5351580079066E-10	0.487995147218852	0.21	0.119	2.50399622669646E-06	Periportal hepatocytes	ZNF337
1.56090288749906E-10	0.365533139341264	0.198	0.109	2.54598869979971E-06	Periportal hepatocytes	CCDC186
1.56140649514803E-10	0.274673065138274	0.29	0.178	2.54681013423594E-06	Periportal hepatocytes	PLA2G4C
1.61002373152935E-10	0.330626143267081	0.151	0.075	2.62610970849752E-06	Periportal hepatocytes	FBXO21
1.6110330230465E-10	0.287580055761445	0.182	0.098	2.62775596389114E-06	Periportal hepatocytes	PTPRG
1.63115415989612E-10	0.381832086881973	0.111	0.049	2.66057555020656E-06	Periportal hepatocytes	TMEM86A
1.73398242941653E-10	0.84332635844402	0.121	0.057	2.8282987406213E-06	Periportal hepatocytes	IPO5
1.78441984773777E-10	0.455242315042002	0.299	0.187	2.91056721364507E-06	Periportal hepatocytes	CLK4
1.87099824486151E-10	0.371825403094665	0.216	0.122	3.05178523719361E-06	Periportal hepatocytes	THOC2
1.94161082402093E-10	0.566316939597773	0.138	0.068	3.16696141506054E-06	Periportal hepatocytes	NOL11
1.99983526126886E-10	0.293946172285166	0.17	0.09	3.26193129465565E-06	Periportal hepatocytes	PPP2R3B
2.01909482559249E-10	0.925449187427139	0.126	0.06	3.29334557002391E-06	Periportal hepatocytes	PUM3

**2.0263699552742E-10**	0.617613743686436	0.162	0.085	3.30521203404775E-06	Periportal hepatocytes	PTPN4
**2.06913824985267E-10**	0.320598657221053	0.257	0.154	3.37497139933469E-06	Periportal hepatocytes	CLEC16A
**2.25012157806278E-10**	0.5822940713781	0.118	0.054	3.6701733059782E-06	Periportal hepatocytes	RALA
**2.25753759675892E-10**	0.481179682544341	0.257	0.154	3.68226957407347E-06	Periportal hepatocytes	DIP2B
**2.35188854387899E-10**	0.320027955119901	0.19	0.104	3.83616540392103E-06	Periportal hepatocytes	IPO13
**2.35685764526724E-10**	0.63322909381576	0.132	0.064	3.8442705051954E-06	Periportal hepatocytes	NR3C2
**2.36097234953722E-10**	0.642313454715227	0.181	0.098	3.85098199933016E-06	Periportal hepatocytes	BIRC2
**2.38531358620223E-10**	1.00288242607466	0.114	0.051	3.89068499045446E-06	Periportal hepatocytes	APBB2
**2.39310514199789E-10**	0.613825027662614	0.141	0.07	3.90339379711275E-06	Periportal hepatocytes	ACTR6
**2.41143610780895E-10**	0.59070177597069	0.125	0.06	3.93329343544718E-06	Periportal hepatocytes	NFKB1
**2.58641215420163E-10**	0.295251102409643	0.293	0.181	4.21869686471827E-06	Periportal hepatocytes	RTCA
**2.69628238000935E-10**	0.31899782533485	0.181	0.098	4.39790619003325E-06	Periportal hepatocytes	SBNO1
**2.74324934323883E-10**	0.747438313656929	0.224	0.131	4.47451400375686E-06	Periportal hepatocytes	MRI1
**2.75593867953321E-10**	0.696541758414289	0.154	0.079	4.49521158018662E-06	Periportal hepatocytes	PPME1
**2.76526875985744E-10**	0.628625925061181	0.197	0.112	4.51042987420346E-06	Periportal hepatocytes	CYP3A4
**2.78794111232767E-10**	0.356144049781344	0.234	0.136	4.54741074831767E-06	Periportal hepatocytes	TMEM86B
**2.81137920843121E-10**	0.542604301891821	0.159	0.083	4.58564062687215E-06	Periportal hepatocytes	MIOS
**2.91474869599458E-10**	0.392270277017523	0.193	0.107	4.75424659803676E-06	Periportal hepatocytes	HSPBAP1
**3.03985004985579E-10**	0.297867670269542	0.249	0.149	4.95829941631978E-06	Periportal hepatocytes	TMEM150A
**3.07324941707396E-10**	0.280238557894481	0.26	0.156	5.01277712418934E-06	Periportal hepatocytes	OSBPL8
**3.10939930935873E-10**	0.512929013068847	0.121	0.057	5.07174121349503E-06	Periportal hepatocytes	LRRK2
**3.13257880990644E-10**	0.405556420540888	0.254	0.152	5.1095492968384E-06	Periportal hepatocytes	CDK14
**3.16457361459608E-10**	0.694554198503256	0.106	0.048	5.16173602276767E-06	Periportal hepatocytes	TMEM63C
**3.16835263779393E-10**	0.339958021437704	0.155	0.079	5.16789998750567E-06	Periportal hepatocytes	DCAF13
**3.27184322907446E-10**	0.99227209756067	0.114	0.052	5.33670349094336E-06	Periportal hepatocytes	ZFP41
**3.34486255434267E-10**	0.450032664835579	0.135	0.066	5.45580531238833E-06	Periportal hepatocytes	HOMER1
**3.38036204883399E-10**	0.422504965374324	0.284	0.176	5.51370853785311E-06	Periportal hepatocytes	SLC15A4
**3.605299934031E-10**	0.483658065079185	0.164	0.086	5.88060472239797E-06	Periportal hepatocytes	GNPTAB
**3.62505961702848E-10**	0.339553726008367	0.375	0.244	5.91283474133515E-06	Periportal hepatocytes	SRSF3
**3.65578858664945E-10**	0.376248231586862	0.246	0.147	5.96295676368392E-06	Periportal hepatocytes	TMEM138
**3.669267585284E-10**	0.394336001750828	0.128	0.062	5.98494235835674E-06	Periportal hepatocytes	EPSTI1
**3.68150972993061E-10**	0.273087971636943	0.218	0.124	6.00491052048981E-06	Periportal hepatocytes	NEK4
**3.74471907937872E-10**	0.506721221355128	0.223	0.13	6.10801129037464E-06	Periportal hepatocytes	KDM5A
**3.86249952743807E-10**	0.523985153083815	0.118	0.055	6.30012297920424E-06	Periportal hepatocytes	N4BP2
**4.21057835976255E-10**	0.653708616138728	0.112	0.051	6.8678743626087E-06	Periportal hepatocytes	NTAN1
**4.26312309854857E-10**	0.961918037020022	0.122	0.058	6.95358008604258E-06	Periportal hepatocytes	PLEKHM3
**4.32004569237217E-10**	0.428245976746414	0.214	0.123	7.04642652882825E-06	Periportal hepatocytes	GYS1
**4.42863942651505E-10**	0.326689557250381	0.109	0.049	7.2235537685887E-06	Periportal hepatocytes	RP9
**4.81726496680986E-10**	0.26279803697596	0.118	0.055	7.85744088736357E-06	Periportal hepatocytes	TMEM150B
**4.89039819784559E-10**	0.605589964439476	0.152	0.078	7.97672850050595E-06	Periportal hepatocytes	BABAM2
**4.89319209500863E-10**	0.854808676282967	0.126	0.061	7.98128562616858E-06	Periportal hepatocytes	METTL6
**5.07296456280626E-10**	0.69341158028559	0.128	0.062	8.27451249839329E-06	Periportal hepatocytes	ART4
**5.11374714771404E-10**	0.434446748526873	0.214	0.122	8.34103297263638E-06	Periportal hepatocytes	GPR89A
**5.52000282975205E-10**	0.50118592455718	0.151	0.077	9.00367661560857E-06	Periportal hepatocytes	GLG1
**6.38129051910289E-10**	0.37907980470147	0.247	0.148	1.04085229657087E-05	Periportal hepatocytes	TMEM8B
**7.01623347076563E-10**	1.21050579974241	0.109	0.05	1.14441784141658E-05	Periportal hepatocytes	PRKCI
**7.06686912381483E-10**	0.307235529076788	0.218	0.126	1.15267702278544E-05	Periportal hepatocytes	EPG5
**7.15928555372545E-10**	0.443038982586964	0.171	0.093	1.16775106666816E-05	Periportal hepatocytes	OXR1
**7.34081752689408E-10**	0.27451938319937	0.165	0.088	1.19736074681169E-05	Periportal hepatocytes	DDX60
**7.36789315173117E-10**	0.419058689155301	0.165	0.09	1.20177705197887E-05	Periportal hepatocytes	SUZ12
**7.7075562190638E-10**	0.385089020745933	0.193	0.108	1.2571794948915E-05	Periportal hepatocytes	PTPN12
**7.8848810627731E-10**	0.451284668329391	0.254	0.155	1.28610295014892E-05	Periportal hepatocytes	CLDN15
**8.0353295604264E-10**	0.440236619273008	0.243	0.146	1.31064260460115E-05	Periportal hepatocytes	RASSF4
**8.9504268974676E-10**	0.699977799323239	0.177	0.097	1.45990413124594E-05	Periportal hepatocytes	ABHD18
**9.23183910085518E-10**	0.554995785106904	0.178	0.098	1.50580527574049E-05	Periportal hepatocytes	ARAP2
**9.71808679343024E-10**	0.263687489058735	0.217	0.126	1.58511713687641E-05	Periportal hepatocytes	BCLAF1
**9.72159323290738E-10**	0.263575092645615	0.18	0.098	1.58568907221952E-05	Periportal hepatocytes	ZBTB38
**1.02559627293412E-09**	0.640616251029089	0.207	0.121	1.67285008078285E-05	Periportal hepatocytes	ABCA7
**1.07238314038541E-09**	0.415028693022714	0.175	0.096	1.74916414028264E-05	Periportal hepatocytes	CLASP1
**1.1081709872513E-09**	0.372930826359894	0.26	0.158	1.80753769730559E-05	Periportal hepatocytes	ZFYVE16

1.14686024808938E-09	0.691508092907645	0.154	0.081	1.87064375065858E-05	Periportal hepatocytes	RAB2B
1.20095701235399E-09	0.671088250307086	0.151	0.079	1.95888098285059E-05	Periportal hepatocytes	DEPRECAT
1.25062210557888E-09	0.389223482033057	0.2	0.114	2.03988971640971E-05	Periportal hepatocytes	METTL22
1.26442458134094E-09	0.298433024601273	0.322	0.211	2.0624029346252E-05	Periportal hepatocytes	PIKFYVE
1.28407244943203E-09	0.322845318482719	0.129	0.064	2.09445057226858E-05	Periportal hepatocytes	OPA3
1.30450756175266E-09	0.295979300843934	0.224	0.132	2.12778228397476E-05	Periportal hepatocytes	HEATR5B
1.3088468984265E-09	0.274602849367631	0.155	0.081	2.13486017602347E-05	Periportal hepatocytes	FOXJ3
1.31731212767672E-09	0.335841367941954	0.193	0.109	2.14866781145351E-05	Periportal hepatocytes	CAMK2G
1.32834929389564E-09	0.441559171932486	0.149	0.077	2.16667053327317E-05	Periportal hepatocytes	DDX10
1.45184474682451E-09	0.273264243581524	0.198	0.113	2.36810396654546E-05	Periportal hepatocytes	PSMA1
1.50346591705238E-09	0.379006254575943	0.208	0.121	2.45230325730414E-05	Periportal hepatocytes	RNF169
1.59828584617126E-09	0.289841870051522	0.145	0.074	2.60696404368994E-05	Periportal hepatocytes	TOPORS
1.60714350877845E-09	0.595499652367412	0.106	0.049	2.62141177716853E-05	Periportal hepatocytes	TMEM184C
1.62044398294965E-09	0.520943651485935	0.239	0.144	2.64310618058918E-05	Periportal hepatocytes	KRIT1
1.64261122658998E-09	0.314022363086955	0.27	0.166	2.67926317169091E-05	Periportal hepatocytes	MED24
1.64480120351847E-09	0.314691554338589	0.283	0.178	2.68283524305898E-05	Periportal hepatocytes	SCML1
1.66520991127196E-09	0.675341390855249	0.109	0.051	2.71612388627569E-05	Periportal hepatocytes	PPIP5K1
1.70854973687248E-09	0.315765193858552	0.188	0.106	2.78681547581269E-05	Periportal hepatocytes	PCGF3
1.84631684078414E-09	0.262564003478768	0.131	0.066	3.01152739900302E-05	Periportal hepatocytes	WNT4
1.86741727496762E-09	0.40853519220193	0.139	0.071	3.04594431719968E-05	Periportal hepatocytes	GLA
1.93643299108043E-09	0.533275937248181	0.155	0.083	3.1585158517513E-05	Periportal hepatocytes	TMEM218
1.9449782293052E-09	0.323729094187917	0.184	0.104	3.17245398981971E-05	Periportal hepatocytes	RWDD3
2.01494360139205E-09	0.256705576996662	0.181	0.1	3.28657450823058E-05	Periportal hepatocytes	RBM41
2.04382714841144E-09	0.307101160152581	0.178	0.098	3.3336864617739E-05	Periportal hepatocytes	DPY19L3
2.11715048823874E-09	0.41944975646661	0.125	0.061	3.45328416136621E-05	Periportal hepatocytes	SCG5
2.15410670018708E-09	0.266905994691656	0.23	0.137	3.51356343867514E-05	Periportal hepatocytes	NIPAL1
2.15794175554417E-09	0.48948103460278	0.177	0.098	3.5198187974681E-05	Periportal hepatocytes	ZNF451
2.16293598779007E-09	0.290572000493881	0.299	0.19	3.52796488968439E-05	Periportal hepatocytes	ZFHX3
2.1631051321486E-09	0.264923478540912	0.372	0.248	3.52824078104757E-05	Periportal hepatocytes	CTPS1
2.26358640392584E-09	0.344781912173736	0.134	0.068	3.69213578344344E-05	Periportal hepatocytes	TMEM65
2.32815651471881E-09	0.375992714184693	0.328	0.213	3.79745609115785E-05	Periportal hepatocytes	RPTOR
2.3301493491573E-09	0.340922823375696	0.136	0.069	3.80070660341048E-05	Periportal hepatocytes	RWDD1
2.37041811194133E-09	0.442758833359992	0.126	0.063	3.8663889823875E-05	Periportal hepatocytes	NME7
2.37252202190734E-09	0.316662343713591	0.132	0.066	3.86982066993306E-05	Periportal hepatocytes	PREP
2.38948431817595E-09	0.355457425179808	0.18	0.1	3.89748787137679E-05	Periportal hepatocytes	CASP8
2.50330759380709E-09	0.45294432653249	0.208	0.121	4.08314501625874E-05	Periportal hepatocytes	INTS7
2.54590112240039E-09	0.283609772293438	0.231	0.137	4.15261932074728E-05	Periportal hepatocytes	TMCO4
2.55842570852631E-09	0.380939212048047	0.391	0.26	4.17304817317727E-05	Periportal hepatocytes	DOCK5
2.55931986099165E-09	0.32005847264518	0.2	0.116	4.17450662526347E-05	Periportal hepatocytes	ERMARD
2.62027817601189E-09	0.324954587870568	0.207	0.121	4.27393573289299E-05	Periportal hepatocytes	KIF12
2.62212554177719E-09	0.26967894519209	0.224	0.133	4.27694897119277E-05	Periportal hepatocytes	PDK1
2.63322917125835E-09	0.30223704891859	0.261	0.162	4.2950601012395E-05	Periportal hepatocytes	TNS1
2.6375860104062E-09	0.421714651055174	0.142	0.074	4.30216654157356E-05	Periportal hepatocytes	CMC4
3.01681167069935E-09	0.32808638004551	0.223	0.132	4.92072151607771E-05	Periportal hepatocytes	UNK
3.05450857723425E-09	0.345272730610859	0.21	0.123	4.98220894032678E-05	Periportal hepatocytes	USP40
3.17562368520161E-09	0.513846731182734	0.151	0.081	5.17975979293234E-05	Periportal hepatocytes	TTBK2
3.22573755992309E-09	0.849644681799388	0.139	0.072	5.26150053399056E-05	Periportal hepatocytes	COPS7B
3.31808315600144E-09	0.550466451450713	0.162	0.089	5.41212543575395E-05	Periportal hepatocytes	ETV6
3.34677957016443E-09	0.285101548889988	0.21	0.124	5.4589321568952E-05	Periportal hepatocytes	PKP4
3.34995968113373E-09	0.475092392316266	0.125	0.063	5.46411923589723E-05	Periportal hepatocytes	NOM1
3.40063869571499E-09	0.294641898850838	0.228	0.138	5.54678177658072E-05	Periportal hepatocytes	EDC3
3.42640736286373E-09	0.369311382564602	0.329	0.216	5.58881304956703E-05	Periportal hepatocytes	EWSR1
3.49283697866073E-09	0.623384887222535	0.119	0.058	5.69716639589352E-05	Periportal hepatocytes	CACHD1
3.64600735829112E-09	0.5991592588751	0.22	0.131	5.94700260210864E-05	Periportal hepatocytes	OFD1
3.72651423351809E-09	0.602444839866383	0.124	0.062	6.07831736629136E-05	Periportal hepatocytes	KIAA1958
4.00690337977664E-09	0.463992206535524	0.27	0.17	6.53566010275368E-05	Periportal hepatocytes	ARL 16.00
4.07631883031516E-09	0.355999120654625	0.152	0.081	6.64888364412705E-05	Periportal hepatocytes	RHOT1
4.3065551080934E-09	0.604057899309625	0.195	0.113	7.02442203681114E-05	Periportal hepatocytes	SNRNP40
4.34678130203375E-09	0.368493743993353	0.119	0.058	7.09003498174725E-05	Periportal hepatocytes	PEX3
4.35069433557231E-09	0.345572816625933	0.184	0.105	7.096417530752E-05	Periportal hepatocytes	PYCR1

4.59067099540991E-09	0.279388660696961	0.184	0.106	7.48784346061311E-05	Periportal hepatocytes	NSUN3
4.86739645470735E-09	0.274285345393605	0.24	0.147	7.93921035727315E-05	Periportal hepatocytes	NUP98
4.92932133975975E-09	0.410155923018033	0.149	0.08	8.04021603728212E-05	Periportal hepatocytes	DEPRECAT
4.97542736629879E-09	0.640763804661832	0.131	0.067	8.11541957716995E-05	Periportal hepatocytes	HACL1
5.24931601405568E-09	0.285743656733516	0.124	0.062	8.56215935052621E-05	Periportal hepatocytes	NCAPD2
5.40862493311254E-09	0.442571886913615	0.204	0.12	8.82200812839986E-05	Periportal hepatocytes	ENTPD4
5.58304647895647E-09	0.377674047283631	0.142	0.075	9.1065071118259E-05	Periportal hepatocytes	C11orf52
5.65985576413665E-09	0.590806987299221	0.207	0.121	9.23179073688329E-05	Periportal hepatocytes	LIAS
5.77994912565953E-09	0.476327667002049	0.213	0.126	9.42767501886326E-05	Periportal hepatocytes	RESF1
5.82922425688984E-09	0.323674680885078	0.22	0.133	9.50804768541302E-05	Periportal hepatocytes	MEGF8
5.86884002602504E-09	0.283046620209982	0.102	0.047	9.57266496644944E-05	Periportal hepatocytes	TSPYL4
6.01771723209475E-09	0.295106382407335	0.171	0.096	9.81549857726974E-05	Periportal hepatocytes	CNNM2
6.1649915144258E-09	0.312807783958415	0.201	0.119	0.000100557176591799	Periportal hepatocytes	TRAPPC8
6.28980112313194E-09	0.304414584942993	0.33	0.215	0.000102592946119405	Periportal hepatocytes	WDR11
6.85405992737099E-09	0.411201947700543	0.17	0.095	0.000111796571475348	Periportal hepatocytes	PFKFB2
7.61942598876329E-09	0.57126113157278	0.134	0.07	0.000124280457302718	Periportal hepatocytes	NAA80
7.8546588539559E-09	0.601450882541221	0.114	0.056	0.000128117340566875	Periportal hepatocytes	GNAI1
7.99553432607633E-09	0.3557777736094	0.348	0.229	0.000130415160392631	Periportal hepatocytes	MAPK8IP3
8.06472560368992E-09	0.380207325507777	0.114	0.056	0.000131543739321786	Periportal hepatocytes	AREL1
8.58165023860086E-09	0.642965961027145	0.129	0.067	0.000139975297041819	Periportal hepatocytes	FAM184A
8.72586600241905E-09	0.306279134132809	0.279	0.178	0.000142327600365457	Periportal hepatocytes	L3HYPDH
8.77315681998469E-09	0.571763943524584	0.181	0.104	0.00014309896089077	Periportal hepatocytes	CCNT1
8.81570921457333E-09	0.946763356820757	0.125	0.064	0.000143793032998906	Periportal hepatocytes	WDR91
8.84714146783316E-09	0.434118059760508	0.171	0.096	0.000144305724481827	Periportal hepatocytes	TTC9C
9.18690239360704E-09	0.641323250401606	0.115	0.057	0.000149847564942124	Periportal hepatocytes	TADA2A
9.72966488293476E-09	0.28679530057284	0.261	0.164	0.000158700563905549	Periportal hepatocytes	HDAC8
9.76718479769745E-09	0.347196664196412	0.155	0.084	0.000159312551235243	Periportal hepatocytes	ATAD2
9.84789807449365E-09	0.857369722370938	0.121	0.061	0.000160629065493066	Periportal hepatocytes	MAP2K4
1.00845850617366E-08	0.268590899737637	0.239	0.146	0.000164489666941985	Periportal hepatocytes	ATRX
1.05544314069222E-08	0.28185308772386	0.24	0.146	0.000172153330678307	Periportal hepatocytes	ZNF121
1.0880572387989E-08	0.513432493310774	0.193	0.113	0.000177473016220489	Periportal hepatocytes	ENGASE
1.14247418387389E-08	0.434160366478898	0.185	0.108	0.00018634896413167	Periportal hepatocytes	TMEM126B
1.20867668121527E-08	0.68965975299695	0.122	0.062	0.000197147253473022	Periportal hepatocytes	LRRC1
1.21430069538513E-08	0.547181429231292	0.182	0.108	0.000198064586424269	Periportal hepatocytes	RAD54L2
1.22003039968223E-08	0.504159760843451	0.135	0.071	0.000198999158492168	Periportal hepatocytes	FAM126B
1.22526441885411E-08	0.611998269595635	0.141	0.075	0.000199852879359294	Periportal hepatocytes	PIGC
1.2392670460421E-08	0.607614585300102	0.171	0.098	0.000202136847879927	Periportal hepatocytes	ZNF236
1.25178750141613E-08	0.390774115061191	0.165	0.093	0.000204179059355985	Periportal hepatocytes	MSH5
1.28923646201866E-08	0.800396335481478	0.108	0.053	0.000210287359319863	Periportal hepatocytes	FHIT
1.30017472927806E-08	0.283034383213255	0.162	0.09	0.000212071500092544	Periportal hepatocytes	PTBP2
1.33706910899674E-08	0.459328436875505	0.203	0.121	0.000218089342368458	Periportal hepatocytes	GANC
1.33792757071625E-08	0.374301320580147	0.155	0.085	0.000218229366059527	Periportal hepatocytes	PSMG1
1.35554337418557E-08	0.388506755549432	0.165	0.092	0.000221102679763409	Periportal hepatocytes	WAC
1.36849972223237E-08	0.453362429270497	0.204	0.122	0.000223215989693322	Periportal hepatocytes	TRMT13
1.3931968650646E-08	0.346173294741424	0.144	0.078	0.000227244340660687	Periportal hepatocytes	TMEM104
1.39558678936738E-08	0.361004928103434	0.208	0.124	0.000227634161213713	Periportal hepatocytes	CCSER2
1.45219009689208E-08	0.254521249754306	0.343	0.229	0.000236866726704067	Periportal hepatocytes	CCNT2
1.62199705921622E-08	0.38450054734572	0.273	0.173	0.000264563940328758	Periportal hepatocytes	ERCC5
1.63076281860808E-08	0.337297469944783	0.205	0.124	0.000265993723343163	Periportal hepatocytes	SOCS7
1.6337778585832E-08	0.314189401015486	0.323	0.215	0.000266485506513505	Periportal hepatocytes	SORBS1
1.66448722644613E-08	0.521362405718717	0.254	0.159	0.000271494511505628	Periportal hepatocytes	C1orf52
1.77439405545558E-08	1.16717263040706	0.106	0.052	0.000289421414385359	Periportal hepatocytes	FASTKD1
1.78626988290296E-08	0.862891242649091	0.181	0.107	0.000291358480600301	Periportal hepatocytes	MALT1
1.81265498669942E-08	0.41202311445838	0.112	0.056	0.000295662154880542	Periportal hepatocytes	CSTF1
1.87687817906279E-08	0.638627780715964	0.125	0.064	0.000306137599786932	Periportal hepatocytes	TEFM
1.92464617609901E-08	0.252103164788278	0.25	0.156	0.00031392903778351	Periportal hepatocytes	DNASE1L1
1.93678526404849E-08	0.268581670937879	0.165	0.093	0.000315909044418948	Periportal hepatocytes	PRRG4
1.99971055710977E-08	0.569442871246306	0.171	0.097	0.000326172788970174	Periportal hepatocytes	TMEM181
2.08450200202628E-08	0.547554129296771	0.109	0.054	0.000340003121550506	Periportal hepatocytes	FARP2
2.14452485822906E-08	0.620141488608555	0.152	0.085	0.000349793449625741	Periportal hepatocytes	ZNF700

2.14531504539288E-08	0.85777946929699	0.118	0.06	0.000349922337054033	Periportal hepatocytes	MTURN
2.1470578167391E-08	0.595649318391645	0.181	0.105	0.000350206600488314	Periportal hepatocytes	MICAL2
2.23348972173222E-08	0.602579858927613	0.114	0.057	0.000364304508511743	Periportal hepatocytes	AFG1L
2.25084504270051E-08	0.437854330352558	0.132	0.07	0.00036713533491488	Periportal hepatocytes	KANSL1L
2.26261615386375E-08	0.617677874789645	0.151	0.083	0.000369055320856716	Periportal hepatocytes	CLIC4
2.284491333281E-08	0.410061316603195	0.165	0.094	0.000372623381371465	Periportal hepatocytes	TRMT10B
2.34993239490586E-08	0.586806970354093	0.125	0.065	0.000383297472933094	Periportal hepatocytes	KLHL29
2.5288360240275E-08	0.495255907582513	0.157	0.088	0.000412478443879125	Periportal hepatocytes	INPP5E
2.54120263871009E-08	0.419912621089185	0.23	0.143	0.000414495562400002	Periportal hepatocytes	CABIN1
2.56457272645982E-08	1.00060052945976	0.126	0.067	0.00041830745741286	Periportal hepatocytes	COX18
2.72982355847999E-08	0.36386475255306	0.145	0.079	0.000445261520623671	Periportal hepatocytes	SNAPC3
2.90743954402558E-08	0.73524115442644	0.136	0.073	0.000474232464026012	Periportal hepatocytes	CEACAM19
2.90780116217802E-08	0.413119396854958	0.144	0.079	0.000474291447562857	Periportal hepatocytes	ST3GAL4
3.06135983357455E-08	0.361099245146964	0.147	0.08	0.000499338402454345	Periportal hepatocytes	ANKRD13C
3.16269096762366E-08	0.58942424985776	0.208	0.127	0.000515866523729095	Periportal hepatocytes	WDR90
3.18328367229776E-08	0.490377338367723	0.145	0.079	0.000519225399788488	Periportal hepatocytes	PNPLA8
3.34962597189784E-08	0.6927726587178	0.122	0.063	0.000546357492276257	Periportal hepatocytes	HAUS3
3.38831131640055E-08	0.410089852368031	0.177	0.104	0.000552667458818094	Periportal hepatocytes	STK38
3.40134878485835E-08	0.673113005960837	0.102	0.049	0.000554794000298245	Periportal hepatocytes	MYL5
3.45402093977813E-08	0.366954790458371	0.254	0.163	0.000563385355487211	Periportal hepatocytes	TRMT11
3.53085689556861E-08	0.530143537733508	0.108	0.054	0.000575918068236196	Periportal hepatocytes	STK19
3.72515686871577E-08	0.371867321095707	0.267	0.173	0.000607610336856229	Periportal hepatocytes	BCO2
3.75745197926736E-08	0.8446260599201	0.164	0.096	0.000612877992338299	Periportal hepatocytes	LIFR
4.05699330771518E-08	0.300046625190404	0.131	0.069	0.000661736178421424	Periportal hepatocytes	ACTR8
4.153552334174E-08	0.628337595441457	0.161	0.092	0.00067748592122712	Periportal hepatocytes	GOLGA8A
4.16907675559616E-08	0.279765439297508	0.164	0.093	0.00068001810960529	Periportal hepatocytes	ELP1
4.2746131637173E-08	0.557320396426324	0.125	0.065	0.00069723215313393	Periportal hepatocytes	OSBP2
4.31381499437916E-08	0.417646971904668	0.128	0.067	0.000703626363733184	Periportal hepatocytes	CDC27
4.35743447209592E-08	0.268850122074192	0.128	0.067	0.000710741136743566	Periportal hepatocytes	CBLL1
4.36462843217694E-08	0.347218216905046	0.224	0.139	0.000711914543572381	Periportal hepatocytes	OXSR1
4.6417066364106E-08	0.50336631855852	0.178	0.104	0.000757108769464933	Periportal hepatocytes	RPUSD4
4.73065990922918E-08	0.520442183006073	0.121	0.063	0.000771617937794371	Periportal hepatocytes	UNC119
4.84118998965509E-08	0.483740239679219	0.193	0.116	0.000789646499212642	Periportal hepatocytes	ATF7
4.85266881320751E-08	0.615004224715152	0.124	0.065	0.000791518810122277	Periportal hepatocytes	RASA2
4.85829543457409E-08	0.321027571891513	0.159	0.09	0.00079243656833338	Periportal hepatocytes	DYNLT3
5.0966836234761E-08	0.543337775251138	0.185	0.109	0.000831320065825187	Periportal hepatocytes	GCC2
5.11820666527072E-08	0.349559024691726	0.154	0.087	0.000834830689172307	Periportal hepatocytes	SRGAP2B
5.11959138276939E-08	0.71072596325826	0.122	0.064	0.000835056550443515	Periportal hepatocytes	ZNF841
5.17557296504866E-08	0.395171921419842	0.147	0.081	0.000844187706329087	Periportal hepatocytes	RMND1
5.21404707511127E-08	0.602716642022429	0.125	0.066	0.000850463218421399	Periportal hepatocytes	EXOSC1
5.37188585254111E-08	0.844177883956534	0.118	0.061	0.00087620830140798	Periportal hepatocytes	RAD52
5.58622322364251E-08	0.578608488337484	0.102	0.05	0.00091116887000833	Periportal hepatocytes	DEPRECAT
5.60529959223794E-08	0.45186054315758	0.142	0.079	0.00091428041648993	Periportal hepatocytes	RAB11A
5.80400760285225E-08	0.68796768195087	0.108	0.054	0.000946691680101231	Periportal hepatocytes	SNX25
5.83966599806145E-08	0.279225517186372	0.101	0.049	0.000952507920943804	Periportal hepatocytes	TGFBR1
6.10664520327889E-08	0.329686237859092	0.178	0.105	0.000996054899106819	Periportal hepatocytes	RNF111
6.35077391510022E-08	0.75163792447806	0.139	0.077	0.001035874733292	Periportal hepatocytes	ZNF823
6.44898584736801E-08	0.375850877510968	0.159	0.09	0.0010518940815642	Periportal hepatocytes	DDO
6.55640845086604E-08	0.287904977802409	0.24	0.151	0.00106941578242076	Periportal hepatocytes	TRAPPC6B
6.56831911171114E-08	0.322624847657542	0.214	0.133	0.0010713585303112	Periportal hepatocytes	ABCA10
6.68563406163959E-08	0.408311524474024	0.244	0.158	0.00109049377179403	Periportal hepatocytes	SLC28A1
6.98632128282229E-08	0.289626692795158	0.226	0.139	0.00113953886444114	Periportal hepatocytes	TRNT1
7.12296543883332E-08	0.327698155509479	0.131	0.071	0.0011618268927281	Periportal hepatocytes	SYNJ2BP
7.13238344888401E-08	0.493420832859164	0.171	0.101	0.00116336306434747	Periportal hepatocytes	NUP214
7.21779605853204E-08	0.318226828404103	0.148	0.083	0.00117729471510716	Periportal hepatocytes	SENP7
7.30007995583059E-08	0.255286570544609	0.109	0.055	0.00119071604159553	Periportal hepatocytes	S100A14
7.47838574775898E-08	0.519936755730115	0.128	0.068	0.00121979949931697	Periportal hepatocytes	UFSP2
7.50796883473282E-08	0.338614482802852	0.185	0.11	0.00122462479663327	Periportal hepatocytes	BPNT1
7.65341868625472E-08	0.520592200039361	0.134	0.072	0.00124834912191501	Periportal hepatocytes	EXD3
7.79941116845069E-08	0.2730916956483	0.22	0.137	0.00127216195568599	Periportal hepatocytes	ZCCHC7

**7.93647161763312E-08**	0.344824055467575	0.132	0.072	0.00129451788555214	Periportal hepatocytes	GPN1
**8.13983470284264E-08**	0.365009759937471	0.135	0.074	0.00132768843838066	Periportal hepatocytes	GMEB1
**8.2470582604462E-08**	0.799029357200855	0.106	0.053	0.00134517767286138	Periportal hepatocytes	EDRF1
**8.34879484480896E-08**	0.295271302573047	0.293	0.197	0.00136177192713679	Periportal hepatocytes	SEPTIN11
**8.43776170894736E-08**	0.440763783021646	0.115	0.059	0.0013762833123464	Periportal hepatocytes	ADSSL1
**8.72791912268474E-08**	0.728530771310916	0.125	0.067	0.00142361088810111	Periportal hepatocytes	ACVR2A
**9.2326945814396E-08**	0.836976121918754	0.119	0.063	0.00150594481317861	Periportal hepatocytes	STK38L
**9.67978916369217E-08**	0.252079831264058	0.101	0.049	0.00157887041048983	Periportal hepatocytes	APPBP2
**1.01893960456321E-07**	0.332457193084844	0.152	0.087	0.00166199238900306	Periportal hepatocytes	PHACTR2
**1.08611896484875E-07**	0.258231788684068	0.139	0.078	0.00177156864356479	Periportal hepatocytes	HRCT1
**1.10754701289686E-07**	0.613038165180464	0.103	0.052	0.00180651993273608	Periportal hepatocytes	ZNF142
**1.16004452514672E-07**	0.438125131206729	0.221	0.139	0.00189214862496682	Periportal hepatocytes	CEP83
**1.19949163801816E-07**	0.34647515466606	0.193	0.118	0.00195649081077141	Periportal hepatocytes	TBC1D10A
**1.21158708448415E-07**	0.585362237821129	0.151	0.086	0.0019762196935021	Periportal hepatocytes	STAM2
**1.21625885915963E-07**	0.403367003550841	0.162	0.094	0.00198383982517527	Periportal hepatocytes	SYT17
**1.22120590078675E-07**	0.276406428638498	0.187	0.111	0.00199190894477326	Periportal hepatocytes	MECR
**1.25312779828804E-07**	0.612641088180027	0.102	0.051	0.00204397675178763	Periportal hepatocytes	MED30
**1.26893309590221E-07**	0.609997422928012	0.149	0.085	0.00206975677272609	Periportal hepatocytes	CARMIL1
**1.40835360300829E-07**	1.04972337063633	0.101	0.051	0.00229716556186682	Periportal hepatocytes	DENND2C
**1.43504089506755E-07**	0.332994078608145	0.221	0.138	0.00234069520394467	Periportal hepatocytes	SLC26A6
**1.50854133279137E-07**	0.408027879681893	0.126	0.068	0.00246058176791601	Periportal hepatocytes	P2RX7
**1.56451050674018E-07**	0.315638457816606	0.149	0.085	0.00255187308754391	Periportal hepatocytes	REST
**1.56559222917474E-07**	0.271007830018994	0.178	0.107	0.00255363748500692	Periportal hepatocytes	NCOA3
**1.58162806201854E-07**	0.4386412738657	0.273	0.178	0.00257979353195844	Periportal hepatocytes	ST3GAL6
**1.58820597360312E-07**	0.682555289225753	0.105	0.054	0.00259052276354404	Periportal hepatocytes	EFHC1
**1.59945569632557E-07**	0.35497679624397	0.19	0.116	0.00260887218627663	Periportal hepatocytes	TNFRSF1B
**1.61979818746436E-07**	0.43638650320803	0.193	0.118	0.00264205282357312	Periportal hepatocytes	XPO1
**1.78826262043634E-07**	0.75018297496644	0.118	0.063	0.00291683516019371	Periportal hepatocytes	OLA1
**1.81142288943969E-07**	0.570835289674657	0.149	0.085	0.00295461187496507	Periportal hepatocytes	RAB40C
**1.82089357158214E-07**	0.459522235766483	0.121	0.065	0.00297005950460764	Periportal hepatocytes	DDX19B
**1.90771771232014E-07**	0.488531936663312	0.152	0.088	0.00311167836056538	Periportal hepatocytes	RPAP3
**1.91346812544982E-07**	0.575035703415902	0.103	0.053	0.0031210578594212	Periportal hepatocytes	SFXN3
**1.93004320136244E-07**	0.3208683609882	0.217	0.138	0.00314809346574228	Periportal hepatocytes	UCKL1
**1.9581278238457E-07**	0.619146809972702	0.138	0.077	0.00319390229347472	Periportal hepatocytes	ZFP64
**1.97630248623154E-07**	0.310125283852729	0.287	0.189	0.00322354698529226	Periportal hepatocytes	CREBRF
**1.9929171249407E-07**	0.422423649344252	0.152	0.088	0.00325064712249077	Periportal hepatocytes	DHRS12
**2.0722670918908E-07**	0.662008256574509	0.101	0.051	0.00338007485358308	Periportal hepatocytes	VAMP1
**2.365528407625E-07**	0.511411522582546	0.157	0.092	0.00385841338567713	Periportal hepatocytes	FOXP2
**2.41521284837469E-07**	0.352235304173472	0.158	0.093	0.00393945367698395	Periportal hepatocytes	TIAF1
**2.68867544633208E-07**	0.254199667367716	0.103	0.053	0.00438549852051225	Periportal hepatocytes	TYW5
**2.9524323957324E-07**	0.272553128597537	0.277	0.186	0.00481571248067911	Periportal hepatocytes	WHRN
**2.99452401147315E-07**	0.405227976049113	0.261	0.172	0.00488436811511386	Periportal hepatocytes	CCDC84
**3.0359615515256E-07**	0.317224241269484	0.154	0.091	0.0049519568866934	Periportal hepatocytes	PRPF38A
**3.036883276207E-07**	0.417025982581275	0.151	0.087	0.00495346031182124	Periportal hepatocytes	SPATA5
**3.08022319098137E-07**	0.373403278030451	0.213	0.134	0.00502415204680971	Periportal hepatocytes	NHLRC3
**3.11850812580977E-07**	0.339997121110106	0.188	0.116	0.00508659860400832	Periportal hepatocytes	RPRD1B
**3.12708507074192E-07**	0.406126168717716	0.134	0.075	0.00510058845888714	Periportal hepatocytes	RAE1
**3.1307797541928E-07**	0.463782802004321	0.124	0.067	0.00510661485706388	Periportal hepatocytes	STARD3NL
**3.16226349896361E-07**	0.66703707759017	0.118	0.063	0.00515796799315954	Periportal hepatocytes	ARHGAP26
**3.25397567081916E-07**	0.641816536174431	0.152	0.089	0.00530755971667312	Periportal hepatocytes	ZDHHC6
**3.31247070551681E-07**	0.332335273724166	0.112	0.059	0.00540297096776847	Periportal hepatocytes	POLR1C
**3.48258467311944E-07**	0.515305666298974	0.136	0.079	0.00568044386032511	Periportal hepatocytes	SNW1
**3.49907097880033E-07**	0.555084101644475	0.147	0.085	0.00570733467352121	Periportal hepatocytes	FBXO30
**3.54210149994685E-07**	0.384519255342479	0.152	0.089	0.00577752175656331	Periportal hepatocytes	ME3
**3.67627810273994E-07**	0.515606517013631	0.138	0.078	0.00599637721337911	Periportal hepatocytes	LPXN
**3.69285745661175E-07**	0.547408536990784	0.157	0.092	0.00602341979747943	Periportal hepatocytes	WDR27
**3.76120172720179E-07**	0.39533289067502	0.115	0.062	0.00613489613723883	Periportal hepatocytes	C2CD5
**3.91615643243767E-07**	0.4871428499978	0.141	0.08	0.00638764275694909	Periportal hepatocytes	AGK
**4.18065507121694E-07**	0.311747169196585	0.198	0.126	0.00681906648666195	Periportal hepatocytes	NAV1
**4.33840785156147E-07**	0.515732092794504	0.205	0.131	0.00707637704668192	Periportal hepatocytes	HMBOX1

4.53246571421595E-07	0.260278162830146	0.141	0.08	0.00739290482645764	Periportal hepatocytes	RRAS2
4.74448318841721E-07	0.497896434692626	0.105	0.055	0.00773872652862731	Periportal hepatocytes	MTF1
4.84748850301784E-07	0.398545573629374	0.119	0.065	0.00790673849727239	Periportal hepatocytes	ZNF576
4.89856092899984E-07	0.553870894149278	0.108	0.057	0.00799004273129164	Periportal hepatocytes	SMIM8
5.13756106226427E-07	0.253630824539838	0.193	0.12	0.00837987584865926	Periportal hepatocytes	ZC3H7A
5.29754392433466E-07	0.527250765097719	0.132	0.074	0.00864082389498227	Periportal hepatocytes	ZNF587B
6.5443691585411E-07	0.602554802310198	0.159	0.096	0.0106745205344964	Periportal hepatocytes	EXOC1
7.27629619758872E-07	0.27809162566383	0.167	0.101	0.011868366727887	Periportal hepatocytes	DEPRECAT
7.29911752336359E-07	0.29477441589451	0.152	0.09	0.0119055905923584	Periportal hepatocytes	EVI5L
7.37794784394518E-07	0.403425312895786	0.111	0.059	0.012034170728259	Periportal hepatocytes	CLIP4
7.55958870882542E-07	0.359725650524721	0.19	0.119	0.0123304451429651	Periportal hepatocytes	MTF2
7.63632004610278E-07	0.752074993852937	0.122	0.068	0.0124556016271983	Periportal hepatocytes	CDC14B
7.8066431043405E-07	0.856750464483336	0.134	0.077	0.0127334155674898	Periportal hepatocytes	BTBD8
8.07486538809917E-07	0.425623845493171	0.155	0.092	0.0131709129345286	Periportal hepatocytes	SLC25A17
8.12420561057275E-07	0.490218294500528	0.129	0.073	0.0132513917714052	Periportal hepatocytes	CSPP1
8.13436653832508E-07	0.43242406606066	0.124	0.069	0.013267965260662	Periportal hepatocytes	GPR155
8.21488197469154E-07	0.610546417608321	0.131	0.075	0.0133992939889194	Periportal hepatocytes	TBCE
8.22663998779505E-07	0.369123453098347	0.175	0.109	0.0134184724840925	Periportal hepatocytes	ETNK1
8.27331156995798E-07	0.367462957102854	0.201	0.127	0.0134945985017585	Periportal hepatocytes	POLG2
8.58570577219474E-07	0.818007787610561	0.102	0.054	0.0140041446850268	Periportal hepatocytes	CHM
8.6597350839397E-07	0.629810794496535	0.175	0.108	0.014124893895414	Periportal hepatocytes	KLHL25
9.20045854776532E-07	0.641064000791315	0.158	0.095	0.01500686793726	Periportal hepatocytes	DOP1A
9.30963915057365E-07	0.288854738862578	0.147	0.086	0.0151849524185007	Periportal hepatocytes	PLD2
9.33851663120469E-07	0.269951182508606	0.19	0.12	0.015232054477158	Periportal hepatocytes	TM2D1
9.38030620850541E-07	0.519109353390878	0.115	0.063	0.0153002174566932	Periportal hepatocytes	ICE2
9.59485365265225E-07	0.294207895011979	0.197	0.124	0.0156501657928411	Periportal hepatocytes	TMX3
1.05367089485106E-06	0.349074472889314	0.144	0.084	0.0171864259659157	Periportal hepatocytes	ZNF18
1.05693383749077E-06	0.478912833104824	0.185	0.117	0.0172396478233119	Periportal hepatocytes	ZMAT1
1.11086756767162E-06	0.279599675478289	0.155	0.092	0.0181193608962917	Periportal hepatocytes	AAR2
1.14753494844803E-06	0.283154281388388	0.148	0.087	0.0187174425441358	Periportal hepatocytes	SLC30A6
1.16251840802514E-06	0.361199887243011	0.174	0.109	0.0189618377532981	Periportal hepatocytes	MID1
1.18342487633321E-06	0.282437560875566	0.224	0.147	0.019302843157871	Periportal hepatocytes	PLA2G4B
1.19251766549559E-06	0.397247837201745	0.217	0.142	0.0194511556418986	Periportal hepatocytes	NR2C1
1.23718293748676E-06	0.282863741348957	0.195	0.124	0.0201796908933466	Periportal hepatocytes	FCHO2
1.30097989682405E-06	0.597389297285129	0.158	0.095	0.0212202830970971	Periportal hepatocytes	DEPDC5
1.34933507705735E-06	0.539455179184105	0.114	0.063	0.0220090044418824	Periportal hepatocytes	MPHOSPH6
1.3503173510296E-06	0.699179086939554	0.109	0.06	0.0220250263126438	Periportal hepatocytes	ABCA9
1.35501417411264E-06	0.62913902064687	0.128	0.073	0.0221016361939512	Periportal hepatocytes	WASF1
1.41150445220846E-06	0.553229528019763	0.109	0.059	0.0230230491199722	Periportal hepatocytes	CBY1
1.46287526551302E-06	0.309320830031353	0.118	0.066	0.0238609584557828	Periportal hepatocytes	ZNF707
1.49755155134562E-06	0.498932555166754	0.154	0.093	0.0244265633539984	Periportal hepatocytes	GOLGA1
1.55900791491254E-06	0.283033128638194	0.164	0.1	0.0254289781001384	Periportal hepatocytes	PRKCE
1.638830879422E-06	0.550216982428469	0.207	0.136	0.0267309704742522	Periportal hepatocytes	GGA3
1.68239361732014E-06	0.370310640440416	0.198	0.127	0.0274415222921089	Periportal hepatocytes	TAF6L
1.7529860742332E-06	0.269061768494241	0.116	0.065	0.0285929558568178	Periportal hepatocytes	PTER
1.77663859546145E-06	0.675822509035043	0.115	0.064	0.0289787521305717	Periportal hepatocytes	ORC5
1.88911683624761E-06	0.300810457524556	0.126	0.072	0.0308133847160347	Periportal hepatocytes	DCAKD
1.96758332589866E-06	0.37469192971211	0.154	0.093	0.0320932516287331	Periportal hepatocytes	KALRN
2.04030677594231E-06	0.284256102478784	0.223	0.148	0.033279443822395	Periportal hepatocytes	ZNF292
2.09305437963024E-06	0.486699471327718	0.172	0.109	0.0341398099861488	Periportal hepatocytes	EBLN2
2.23778584463203E-06	0.316970709619561	0.187	0.118	0.036500524911793	Periportal hepatocytes	TRIM39
2.24326382729507E-06	0.612981820486188	0.114	0.064	0.0365898762870099	Periportal hepatocytes	NAPB
2.37070972422617E-06	0.310977280342839	0.112	0.063	0.038668646311853	Periportal hepatocytes	ARL 15.00
2.69235733302719E-06	0.348057735908499	0.216	0.143	0.0439150404590065	Periportal hepatocytes	PRPSAP2
2.73849890806333E-06	0.542940018356421	0.17	0.107	0.044667655689421	Periportal hepatocytes	KIZ
2.78272063239121E-06	0.370571046311973	0.106	0.059	0.045388956234933	Periportal hepatocytes	R3HDM1
2.80780117913224E-06	0.778610348468941	0.152	0.094	0.0457980450328259	Periportal hepatocytes	EIF2AK3
2.8223204357385E-06	0.263812535921842	0.174	0.109	0.0460348686273307	Periportal hepatocytes	FAM204A
2.85411606221939E-06	0.307939958887133	0.246	0.168	0.0465534870908604	Periportal hepatocytes	ZNF652
2.9076551242323E-06	0.296160048162989	0.191	0.123	0.047426762731353	Periportal hepatocytes	HPS5

**3.00169666728671E-06**	0.367795862120821	0.152	0.093	0.0489606743401135	Periportal hepatocytes	SMC4

SupTable5M_C2_TNK_SeuratCluster

p_val	avg_log2FC	pct.1	pct.2	p_val_adj	cluster	gene
0	2.96726315239092	0.834	0.177	0	0	IL7R
2.31451599967439E-262	1.98679652915935	0.51	0.168	3.77520704706889E-258	0	LTB
2.2734475834329E-131	1.70494951294022	0.349	0.131	3.70822035333741E-127	0	SATB1
6.66178670206962E-98	1.67942788341475	0.283	0.109	1.08660402897458E-93	0	TNFRSF25
3.00573042890447E-291	1.43084214791672	0.823	0.534	4.90264690258608E-287	0	CXCR4
2.29200365235458E-63	1.25832025082176	0.267	0.125	3.73848715735555E-59	0	ERN 1.00
2.65506169814869E-64	1.09818301843256	0.304	0.15	4.33067113585033E-60	0	TC2N
2.33930223180189E-88	1.05281939672438	0.447	0.249	3.81563587029207E-84	0	AHNAK
1.44495124152201E-114	1.04355389163152	0.635	0.43	2.35685997004655E-110	0	ZFP36L2
4.28617555687836E-43	0.996985683863366	0.279	0.157	6.99118095082429E-39	0	PARP8
8.67288715878607E-37	0.986346611199645	0.26	0.153	1.4146346244696E-32	0	SLC2A3
1.88848090430483E-49	0.985729893152303	0.499	0.367	3.0803012030116E-45	0	JUNB
8.73924985495908E-70	0.883753022420388	0.521	0.355	1.42545904384238E-65	0	BTG1
6.822437747144E-20	0.80139809514154	0.25	0.176	1.11280782093666E-15	0	FXYD5
9.19455959009043E-41	0.787970621998239	0.408	0.282	1.49972461473965E-36	0	PDCD4
4.10083218000737E-32	0.77821846397316	0.329	0.222	6.68886736881002E-28	0	EML4
3.52343899151966E-32	0.767640128462903	0.441	0.337	5.74708133906772E-28	0	TNFAIP3
5.4428129974923E-40	0.747142630252061	0.278	0.154	8.87777228020969E-36	0	KLRG1
1.22049697171864E-53	0.740176488044478	0.498	0.346	1.99075261057028E-49	0	SPOCK2
2.44620131032252E-26	0.73274008262803	0.304	0.207	3.98999895726706E-22	0	PIK3R1
1.91704597683414E-23	0.719534531012223	0.285	0.199	3.12689369281417E-19	0	HECA
3.86071393748085E-34	0.716271052214982	0.389	0.276	6.29721050342501E-30	0	VIM
1.23067804694217E-27	0.685546034262041	0.343	0.242	2.00735896236737E-23	0	CD6
5.07053422502485E-20	0.684277085754821	0.259	0.179	8.27054837443803E-16	0	PIK3IP1
2.12472313317619E-44	0.653431010243306	0.526	0.393	3.46563590252368E-40	0	SYNE2
7.08778200773992E-19	0.610025012815792	0.256	0.177	1.15608812328246E-14	0	LTBP3
2.26662783757787E-25	0.598878480339959	0.343	0.246	3.69709666587327E-21	0	TXNIP
1.30560711754868E-17	0.595930918087617	0.32	0.245	2.12957576943365E-13	0	DGKA
1.6608171144757E-16	0.590050174497435	0.266	0.194	2.70895879542132E-12	0	NOP53
3.40264213776993E-18	0.55079508024154	0.364	0.286	5.55004959091654E-14	0	CDC42SE1
5.69521045176292E-18	0.541656618697503	0.298	0.217	9.2894577678705E-14	0	ATM
3.96854310178428E-23	0.536636325459429	0.393	0.294	6.47309065332034E-19	0	STAT4
1.66470553527015E-13	0.532522458335777	0.38	0.32	2.71530119857915E-09	0	STAT1
2.62950740917758E-18	0.525588109814921	0.399	0.313	4.28898953510955E-14	0	CD69
4.49663698544751E-11	0.497120142438181	0.272	0.212	7.33446458696344E-07	0	TSC22D3
3.13045811529716E-18	0.484542140325621	0.434	0.359	5.1060902318612E-14	0	IKZF1
2.74310415783105E-09	0.459433285102147	0.256	0.204	4.47427719183823E-05	0	ZC3HAV1
7.16308367813388E-09	0.455186910515375	0.263	0.216	0.000116837057874042	0	STX16
8.4357581363667E-11	0.453397758642868	0.281	0.224	1.37595650962277E-06	0	CDC42SE2
2.03153127790835E-09	0.449238295543441	0.287	0.236	3.31363066739632E-05	0	GSTK1
1.18834181773138E-12	0.444211739219684	0.393	0.333	1.93830433890166E-08	0	EEF2
1.60676253461339E-15	0.432881007917254	0.399	0.324	2.6207903702079E-11	0	CD44
5.97992829393978E-09	0.426601883370285	0.282	0.233	9.75386104024517E-05	0	PNRC1
5.94156656850218E-08	0.399432140191653	0.302	0.257	0.00096912892298839	0	NLRP1
6.16597709559509E-07	0.392855449706138	0.295	0.254	0.0100573252406252	0	RNF149
4.90239967812806E-07	0.370962170082071	0.287	0.245	0.00799630411499468	0	BCL11B
2.79126161711838E-06	0.362028352903993	0.274	0.234	0.0455282682368179	0	CD96
2.31364010837348E-10	0.359548103320109	0.381	0.323	3.77377838076799E-06	0	CYTH1

1.08073918865058E-09	0.276389373924076	0.565	0.523	1.76279369060797E-05	0	TAPBP
7.92307964053373E-07	0.275523691620792	0.467	0.432	0.0129233352016746	0	TMC8
0	3.61133664789886	0.631	0.087	0	1	HAVCR2
5.55116366560857E-270	2.73004646885348	0.384	0.079	9.05450305497414E-266	1	PDCD1
9.11717120251333E-285	2.72651074915583	0.371	0.064	1.48710179484195E-280	1	ADGRG1
8.42044657118331E-174	2.56227149372083	0.277	0.06	1.37345904022571E-169	1	TNFRSF9
0	2.35111711582071	0.516	0.141	0	1	LAG3
0	2.1636342561532	0.566	0.177	0	1	METRNL
4.68291301006045E-200	2.12591971401783	0.375	0.102	7.6382994107096E-196	1	KLRC4
0	2.10558286568072	0.805	0.295	0	1	RGS1
2.08958204842104E-148	2.02550958687504	0.306	0.088	3.40831727917955E-144	1	WHRN
0	1.76926353105869	0.803	0.314	0	1	CD8A
2.8548787490314E-158	1.67762163574434	0.419	0.157	4.65659272754512E-154	1	LYST
1.81818586695861E-110	1.5876513632297	0.335	0.131	2.96564296759618E-106	1	NR4A2
5.68876731504384E-96	1.54613045443526	0.297	0.115	9.27894836756801E-92	1	CXCR6
6.03697351363471E-74	1.41747526278716	0.281	0.122	9.84690749808958E-70	1	CEMIP2
6.04594900094449E-167	1.4057111347828	0.488	0.195	9.86154741544055E-163	1	TIGIT
3.59036572780809E-179	1.37543439334027	0.564	0.255	5.85624553862778E-175	1	NKG7
6.82360856538045E-159	1.31081045972501	0.529	0.233	1.11299879309921E-154	1	KLRK1
1.22523462664456E-120	1.26178947617257	0.522	0.279	1.99848019951995E-116	1	CCL5
9.70643101150195E-73	1.24974518579869	0.327	0.158	1.58321596228608E-68	1	CREM
1.5331473103133E-60	1.22100744174192	0.267	0.124	2.50071657785202E-56	1	RIN3
1.09819709572474E-73	1.21755314679754	0.349	0.175	1.79126928283662E-69	1	PTPN22
4.31436042252188E-62	1.20076391738522	0.294	0.144	7.03715328517544E-58	1	CD7
6.3253231128108E-67	1.1580889421065	0.357	0.191	1.03172345293057E-62	1	PRDM1
1.91839517289142E-54	1.12024508711137	0.298	0.155	3.12909436650319E-50	1	ABCA2
8.18803897176788E-57	1.09869535673215	0.363	0.209	1.33555103668506E-52	1	ZFP36
3.44692588494124E-47	1.09462220835746	0.281	0.15	5.62228081092766E-43	1	BICDL1
1.52512575808102E-76	1.07275134709881	0.41	0.219	2.48763262400596E-72	1	JAML
7.98303618308674E-54	1.07126948624612	0.297	0.154	1.30211303182328E-49	1	ITM2A
5.71479964692175E-68	1.04746097256965	0.401	0.226	9.32140970409407E-64	1	CBLB
6.61537566048571E-79	0.998464025057486	0.478	0.279	1.07903392398182E-74	1	ARHGAP9
1.64270334318131E-37	0.99537119243753	0.257	0.144	2.67941342306303E-33	1	TRAF5
1.19485102439686E-70	0.99298803910725	0.411	0.225	1.94892150589371E-66	1	PRF1
3.97655705249929E-41	0.986654760576963	0.254	0.135	6.48616220833159E-37	1	SYTL3
3.02526171476912E-38	0.980633253494721	0.26	0.145	4.93450438295991E-34	1	VPS37B
2.31482090796338E-54	0.939459184019154	0.391	0.234	3.77570438297907E-50	1	IKZF3
6.76837839522216E-53	0.930810343115083	0.426	0.273	1.10399020004469E-48	1	SLA
4.57537135416915E-82	0.912759594988135	0.55	0.342	7.4628882157853E-78	1	ITGA4
2.36642717477087E-36	0.887431265929259	0.283	0.167	3.85987936476876E-32	1	RAB27A
1.83849666708224E-44	0.863729748156967	0.378	0.236	2.99877191367784E-40	1	RNF19A
2.82005736425322E-103	0.861946956457079	0.67	0.463	4.59979556683342E-99	1	TRBC2
5.64809483714867E-48	0.835673992311127	0.425	0.272	9.21260748887319E-44	1	CNOT6L
1.0418516126944E-32	0.829039586523308	0.29	0.176	1.69936416546584E-28	1	CCND2
7.99970003204836E-14	0.811393898534241	0.263	0.199	1.30483107222741E-09	1	PGGHG
6.97739527049582E-57	0.760687106286262	0.56	0.413	1.13808294257057E-52	1	LSP1
7.77903835471084E-36	0.745644269821769	0.335	0.21	1.26883894603689E-31	1	TNFRSF1B
3.3872629821697E-62	0.7451814340908	0.599	0.419	5.52496465021699E-58	1	CLEC2D
2.10708501499578E-54	0.69972725352201	0.621	0.474	3.43686636795961E-50	1	SRGN
3.32664510456242E-33	0.697022199387121	0.376	0.251	5.42609083005177E-29	1	GZMA

1.97244733025173E-20	0.662971038089489	0.281	0.193	3.2172588403736E-16	1	ZFP36L1
2.6220940820848E-31	0.653202785849079	0.41	0.289	4.27689765728852E-27	1	WNK1
4.58708231219608E-20	0.626862704283669	0.262	0.176	7.48198995942303E-16	1	ACTN4
5.33241289966813E-21	0.619620187278394	0.289	0.197	8.69769868064868E-17	1	SPTAN1
4.80122720852553E-24	0.609512446305965	0.316	0.214	7.83128169982599E-20	1	AKAP13
3.94917843478368E-17	0.559814049997737	0.267	0.186	6.44150494497565E-13	1	MSN
4.86915315557519E-33	0.542154415859203	0.508	0.377	7.94207571205869E-29	1	ETS1
3.18727777149342E-26	0.534595429017023	0.485	0.376	5.19876877308292E-22	1	ITGAL
1.36973404382125E-11	0.463774157944494	0.309	0.244	2.23417319887685E-07	1	PLAAT4
3.64786164014179E-11	0.447619969493104	0.267	0.201	5.95002712123527E-07	1	SH2D2A
2.84637997759849E-14	0.442718064589992	0.335	0.254	4.6427303814609E-10	1	CCDC88B
4.32208374809784E-09	0.433030920022965	0.255	0.2	7.04975080152239E-05	1	MBD2
6.67467355948456E-12	0.41723727801275	0.274	0.207	1.08870600428753E-07	1	TCIRG1
1.326592449477E-09	0.415094715579669	0.312	0.252	2.16380494434194E-05	1	EHBP1L1
4.43020996844481E-11	0.411084753743329	0.35	0.281	7.22611547953033E-07	1	PYHIN1
6.84276554028442E-16	0.407459661158446	0.454	0.365	1.11612348727579E-11	1	JUND
1.42960741959636E-10	0.403787073718948	0.294	0.23	2.33183266210362E-06	1	IFI16
1.66032532515643E-09	0.392171318700612	0.35	0.288	2.70815663786266E-05	1	ADGRE5
3.51993106845274E-20	0.38829621129069	0.441	0.336	5.74135956575327E-16	1	MAF
8.22537535319485E-11	0.385102127549438	0.353	0.283	1.34164097385961E-06	1	PTPRC
5.74271981975065E-07	0.383776446629789	0.254	0.204	0.00936695029799529	1	CHST11
4.11206730513342E-12	0.377529980562439	0.384	0.309	6.70719298140311E-08	1	ITGB2
1.71764049032028E-11	0.365581493542906	0.312	0.241	2.80164340376141E-07	1	CST7
9.4834498243598E-19	0.319543014578967	0.349	0.253	1.54684550085133E-14	1	IL2RB
4.30137674480202E-14	0.312223961799077	0.527	0.438	7.01597560844657E-10	1	TRAC
9.2910838122215E-08	0.309288431872185	0.424	0.37	0.00151546868061145	1	TAP1
1.06647724776436E-11	0.298732688312529	0.504	0.434	1.73953103882844E-07	1	OAZ1
1.19644419780928E-09	0.29804529619473	0.455	0.387	1.95152013104672E-05	1	CELF2
2.20982250167804E-13	0.290845535889671	0.582	0.504	3.60444148248705E-09	1	MCL1
3.58321886194418E-09	0.281079111826832	0.536	0.472	5.84458828571716E-05	1	ARGLU1
3.4901264721248E-09	0.278998874855605	0.423	0.354	5.69274528868276E-05	1	ZAP70
2.65019838059831E-07	0.276291523228432	0.326	0.268	0.0043227385785939	1	RASAL3
1.14132869030643E-12	0.269757190964269	0.653	0.58	1.86162122675881E-08	1	EVL
5.58653952667891E-10	0.265260017395589	0.569	0.517	9.11220462196597E-06	1	ACTB
1.54446548607813E-15	0.262848403507471	0.756	0.702	2.51917765434204E-11	1	CD74
6.43713112661411E-161	1.93385723150072	0.458	0.148	1.04996045806203E-156	2	GZMK
8.87441295367144E-97	1.53827750445143	0.403	0.159	1.44750549687335E-92	2	KLRG1
1.91142169225661E-23	0.968846374146679	0.272	0.164	3.11771992223975E-19	2	THEMIS
3.40865137860661E-33	0.942423964193681	0.384	0.24	5.55985126364525E-29	2	CST7
1.55403423104729E-27	0.899414029223678	0.31	0.182	2.53478523426123E-23	2	TC2N
1.26740732892402E-30	0.833953275866539	0.406	0.267	2.06726809420798E-26	2	GZMA
4.85332360967103E-28	0.764115284952791	0.557	0.433	7.91625613973441E-24	2	DUSP2
2.96150136758443E-15	0.708243565574882	0.316	0.226	4.83050488066696E-11	2	PIK3R1
5.180673900388E-11	0.675025679849449	0.273	0.203	8.45019719892287E-07	2	PITPNC1
8.11552890127097E-11	0.638316314199714	0.26	0.188	1.32372391908631E-06	2	RESF1
1.18871219952707E-12	0.619275189058426	0.36	0.275	1.93890846864861E-08	2	TRBC1
1.1702916523462E-12	0.601100602919574	0.251	0.171	1.90886271414189E-08	2	DNAJB1
9.50402572675151E-13	0.568279586453826	0.359	0.273	1.55020163629044E-08	2	RBL2
2.82141965375823E-10	0.566015532336253	0.306	0.233	4.60201759724504E-06	2	STK17A
2.50072675182491E-09	0.563349776770772	0.28	0.213	4.07893540490161E-05	2	GIMAP4

2.76841186477832E-15	0.559180768501636	0.425	0.327	4.51555659263992E-11	2	CD69
3.55538348489898E-07	0.551510224910113	0.304	0.253	0.00579918600221872	2	LITAF
2.72925856911921E-10	0.522754542927843	0.294	0.22	4.45169365209035E-06	2	KLHDC4
1.24963418252795E-11	0.522522783248995	0.369	0.289	2.03827831512135E-07	2	PYHIN1
1.24419901245131E-44	0.497764712779208	0.782	0.603	2.02941300920933E-40	2	CXCR4
3.54878490191489E-21	0.477762877787911	0.461	0.33	5.78842305351338E-17	2	CCL5
7.80031538522157E-11	0.42333859771675	0.392	0.301	1.27230944248349E-06	2	AHNAK
5.42676488943231E-08	0.41972601735166	0.375	0.314	0.000885159621115304	2	PDCD4
2.68558955050194E-21	0.404366753638596	0.766	0.709	4.38046511582371E-17	2	CD74
2.5246622039344E-06	0.380098559464701	0.388	0.342	0.041179765208374	2	CD44
3.2620041604485E-11	0.360858132831659	0.646	0.605	5.32065498610755E-07	2	AKNA
4.70314668477617E-07	0.327047683646389	0.531	0.495	0.00767130255753842	2	NLRC5
1.27158938507E-06	0.305310357531566	0.375	0.309	0.0207408944598768	2	KLRK1
1.15304427388067E-09	0.277714583911131	0.671	0.615	1.88073051512676E-05	2	WIPF1
0	7.10366857242646	0.5	0.005	0	3	FOXP3
0	5.3940512816	0.281	0.011	0	3	TNFRSF4
0	4.4806895615814	0.579	0.041	0	3	TNFRSF18
1.57200005913069E-217	4.4408105173899	0.259	0.019	2.56408929644806E-213	3	MAGEH1
2.49259821393375E-212	3.9617617899335	0.253	0.018	4.06567694674735E-208	3	GADD45A
2.29794376782541E-154	3.20830062536428	0.266	0.033	3.74817607970003E-150	3	TBC1D4
6.72265410300578E-156	3.1382246729381	0.299	0.042	1.09653211074127E-151	3	NTNG2
1.47492239980749E-118	2.67724058445732	0.271	0.045	2.405745926326E-114	3	STAM
2.5369355884401E-185	2.62482697328786	0.442	0.081	4.13799563830464E-181	3	CD4
2.4653766043645E-114	2.62121229194318	0.254	0.041	4.02127577937894E-110	3	IKZF2
2.01471697521141E-97	2.28774540600386	0.314	0.073	3.28620485826734E-93	3	CTLA4
3.02173096517341E-91	2.28356728396866	0.274	0.059	4.92874537729434E-87	3	ZC3H12D
7.52207647319528E-74	2.00066618184864	0.305	0.085	1.22692589354288E-69	3	ADAT2
1.10001644674602E-58	1.99951616697201	0.251	0.072	1.79423682628743E-54	3	CASP1
3.30299555229946E-67	1.97248568371911	0.298	0.087	5.38751604535565E-63	3	IL21R
4.363988644676E-82	1.97014740257291	0.295	0.073	7.11810187833101E-78	3	ENTPD1
2.78554622411515E-114	1.86689081594775	0.58	0.218	4.54350444615422E-110	3	TNFRSF1B
1.04204110493701E-74	1.86268715932899	0.4	0.14	1.69967324626276E-70	3	CTSC
1.63888946000064E-82	1.83784975053344	0.381	0.116	2.67319259820704E-78	3	ICOS
2.61931925876844E-71	1.79444422323155	0.399	0.142	4.27237164297721E-67	3	PIM2
7.08143419109902E-51	1.756799261998	0.315	0.117	1.15505273091016E-46	3	DUSP4
3.4282485762922E-131	1.7178161301995	0.665	0.247	5.5918162527902E-127	3	TIGIT
1.75096227611597E-63	1.63151879595207	0.371	0.132	2.85599456857276E-59	3	WHRN
1.3586215851542E-58	1.54847345252739	0.32	0.106	2.21604766754502E-54	3	TNFRSF9
2.61804479140856E-39	1.4909345173598	0.28	0.109	4.2702928592665E-35	3	PHTF2
2.02657630238085E-65	1.48563831407154	0.397	0.143	3.30554860681341E-61	3	DUSP16
1.31498543266987E-38	1.47490487117352	0.263	0.098	2.14487273922783E-34	3	FAM53B
3.59037673574363E-40	1.44806574925384	0.344	0.152	5.85626349367144E-36	3	TPP1
1.64665174668013E-96	1.43048496229466	0.702	0.338	2.68585366400995E-92	3	MAF
5.9602077062958E-36	1.42839329675122	0.327	0.147	9.72169478973908E-32	3	SERPINB9
3.29555523662381E-34	1.37724757555438	0.259	0.102	5.37538014645709E-30	3	CD27
3.36093250645049E-47	1.36909702808924	0.362	0.148	5.4820170112714E-43	3	SAT1
3.73417815485241E-33	1.36365502752824	0.295	0.128	6.09081798837976E-29	3	NABP1
4.63723477913836E-34	1.3464613839281	0.293	0.126	7.56379364825258E-30	3	KDM2B
3.50714754417545E-44	1.33973947728764	0.42	0.201	5.72050835930457E-40	3	ZFP36L1
1.47925678848519E-55	1.31491481880067	0.438	0.183	2.41281574769819E-51	3	LTBP3

1.59421160461009E-82	1.20156528729368	0.817	0.589	2.60031854827952E-78	3	IL32
8.31995994417816E-28	1.15849515595108	0.274	0.123	1.3570686664949E-23	3	KAT2B
5.44712516573483E-44	1.1098198800734	0.534	0.285	8.88480585783009E-40	3	TRAF1
1.71664131961919E-19	1.06706668295191	0.302	0.168	2.80001365643086E-15	3	ANKRD10
2.65962338841024E-24	1.06157342143427	0.265	0.124	4.33811170883594E-20	3	ZNRF2
8.75319216744829E-34	1.05036839276806	0.384	0.188	1.42773317443249E-29	3	GBP2
4.52391520598399E-27	1.03024323695117	0.314	0.149	7.37895809248049E-23	3	TNIK
6.66507747192781E-18	1.01440642854874	0.299	0.168	1.08714078644615E-13	3	HDAC7
5.08290602614857E-22	0.991523783885211	0.29	0.149	8.29072801925094E-18	3	S100A4
1.0476359833253E-21	0.976753088844298	0.304	0.158	1.7087990524019E-17	3	VMP1
1.50881452230374E-30	0.962673859494911	0.369	0.181	2.46102736732964E-26	3	ARID5B
7.74768933742752E-46	0.951243472572205	0.533	0.26	1.2637256078278E-41	3	LTB
7.02750898275004E-27	0.949084021311394	0.372	0.193	1.14625699017636E-22	3	BCL2
5.4709520296415E-44	0.941902486429885	0.632	0.376	8.92366985554826E-40	3	SPOCK2
1.2540839894616E-15	0.901676337070285	0.251	0.137	2.04553639521081E-11	3	OS9
2.19451391885151E-24	0.854679670638387	0.408	0.228	3.5794716530387E-20	3	HNRNPLL
3.56745952321094E-19	0.844609200797088	0.345	0.198	5.81888322830936E-15	3	CCND2
7.03685372887506E-19	0.837670800233111	0.298	0.159	1.14778121171681E-14	3	PBXIP1
1.0717490000469E-18	0.770864167953239	0.491	0.324	1.74812979397649E-14	3	CFLAR
8.6596036196756E-13	0.763581566339679	0.277	0.166	1.41246794640529E-08	3	MSI2
4.49522360239801E-10	0.74546958068417	0.269	0.174	7.33215921787139E-06	3	UCP2
3.87351049223237E-13	0.744612369153541	0.293	0.177	6.31808296388022E-09	3	WSB1
1.04568023070157E-15	0.724156344360054	0.333	0.199	1.70560902429733E-11	3	SMAP2
1.27764733756869E-12	0.721274981875367	0.281	0.168	2.08397057230829E-08	3	SAMHD1
1.74239811477062E-24	0.716394653840051	0.455	0.266	2.84202556500235E-20	3	IL2RB
2.9682776587545E-15	0.712243099433353	0.305	0.177	4.84155768919447E-11	3	CD7
2.12716533384959E-14	0.711774174991334	0.35	0.222	3.46961937604206E-10	3	TSC22D3
9.31086534670922E-13	0.664849292479043	0.314	0.195	1.51869524670174E-08	3	NBEAL2
5.3263719605253E-20	0.641710431107267	0.613	0.451	8.68784530481281E-16	3	TRAC
1.58933400889741E-22	0.63442992674179	0.631	0.455	2.59236270191256E-18	3	IL2RG
1.74056608012573E-09	0.633150411605713	0.269	0.175	2.83903733329308E-05	3	SKAP1
2.12192708659977E-13	0.619572535084991	0.333	0.209	3.46107527095289E-09	3	CHST11
6.99162377130843E-10	0.585433411810554	0.384	0.267	1.14040375333812E-05	3	IL10RA
1.62383147801882E-14	0.581265673936488	0.432	0.288	2.64863152379649E-10	3	MT-CO1
4.66522443790329E-09	0.576617034362412	0.251	0.16	7.60944758066405E-05	3	CD247
6.10596337910834E-11	0.565251243097028	0.31	0.196	9.95943686766361E-07	3	PIK3IP1
6.66207424589927E-10	0.546066288768811	0.412	0.298	1.08665093024863E-05	3	CD2
4.94771572268162E-11	0.54040623843193	0.262	0.158	8.070219115266E-07	3	TNFRSF25
2.40513416001828E-17	0.509015046870218	0.683	0.514	3.92301432840582E-13	3	MCL1
1.36949396112917E-07	0.480916572658484	0.379	0.279	0.00223378159999778	3	GBP5
2.97888302980855E-12	0.476343548264276	0.442	0.307	4.85885610992072E-08	3	SLA
2.14530269659707E-07	0.428768293759064	0.265	0.18	0.00349920322841948	3	EFHD2
1.98982476800143E-08	0.401572387283082	0.362	0.248	0.000324560317908713	3	DOCK2
2.82480497192063E-06	0.392755504084042	0.254	0.175	0.0460753938969973	3	CD28
9.25582697840655E-09	0.379122652802037	0.612	0.493	0.000150971793844789	3	RNF213
9.39092087775496E-10	0.334994655181626	0.701	0.583	1.53175310437061E-05	3	LENG8
1.61946832805374E-06	0.310793458729228	0.583	0.481	0.0264151478988846	3	CYTIP
3.97978447494426E-07	0.29310198105046	0.496	0.384	0.00649142645708159	3	CIRBP
0	4.13792024054591	0.982	0.158	0	4	HSPA1B
2.06881542615668E-167	3.44083886337679	0.469	0.072	3.37444484160416E-163	4	HSPA1A

9.03589584832948E-172	2.85374215472849	0.684	0.159	1.47384497182102E-167	4	DNAJB1
2.01108579682516E-48	2.01916283111767	0.399	0.142	3.28028204320152E-44	4	HSP90AA1
8.36003321344428E-16	1.1777111718449	0.352	0.193	1.3636050174449E-11	4	HSPA8
1.14572701475215E-13	0.925862637175483	0.339	0.188	1.86879533376224E-09	4	KLRG1
6.14978821476984E-10	0.76027653371156	0.319	0.188	1.00309195571111E-05	4	GZMK
2.46422465593204E-16	0.739040373028053	0.505	0.306	4.01939683629076E-12	4	AHNAK
1.55110493950285E-10	0.622879051468666	0.425	0.274	2.53000726682311E-06	4	LTB
5.87248113732913E-07	0.586001809726832	0.303	0.196	0.00957860398309754	4	TC2N
1.75691903892812E-16	0.572605757651607	0.609	0.384	2.86571064439566E-12	4	IL7R
2.88917259697767E-06	0.566414400359437	0.293	0.193	0.0471252942293028	4	PARP8
0	8.07750807416909	0.413	0.003	0	5	FGFBP2
0	7.90833726206531	0.558	0.005	0	5	GNLY
3.33829249414183E-109	3.15639291552719	0.324	0.042	5.44508888719474E-105	5	ADRB2
2.14516906414795E-92	3.07702037558587	0.301	0.043	3.49898526053173E-88	5	FCRL6
9.21852965783455E-150	3.0212995316088	0.529	0.086	1.50363437248939E-145	5	SYNE1
1.95510500658162E-75	2.80066753452594	0.343	0.067	3.18897177623528E-71	5	GZMH
5.49154714815891E-97	2.61538167272	0.59	0.171	8.95726255336199E-93	5	EFHD2
7.2509426959354E-56	2.60419708581798	0.263	0.052	1.18270126313402E-51	5	PLEK
5.14152145302731E-72	2.2024921236141	0.494	0.14	8.38633564203284E-68	5	ADGRG1
7.33902632181438E-40	2.16565997322869	0.272	0.072	1.19706858335114E-35	5	TBX21
5.11191938876651E-55	2.0756859642529	0.397	0.111	8.33805171501705E-51	5	KLRD1
4.00093433586301E-41	2.01076030307886	0.324	0.095	6.52592399522616E-37	5	ZEB2
1.69557761389933E-37	1.93713913907555	0.285	0.079	2.7656566460312E-33	5	CD300A
2.5300265274204E-98	1.92276178230529	0.814	0.327	4.12672626887542E-94	5	NKG7
4.38341761576014E-27	1.90253130020497	0.26	0.086	7.14979247306636E-23	5	PXN
2.95609893549796E-40	1.84984157818628	0.397	0.141	4.82169297369072E-36	5	LPCAT1
1.77015459558713E-50	1.71302559478219	0.401	0.114	2.88729916086217E-46	5	KLF2
3.5422491721856E-24	1.62810432039244	0.282	0.102	5.77776262475193E-20	5	SAMD3
7.15101912135286E-30	1.50660528907092	0.462	0.217	1.16640272888387E-25	5	MYO1F
7.41537030939879E-38	1.47811372124783	0.561	0.268	1.20952105116604E-33	5	PRF1
1.47178183761006E-16	1.38693610291924	0.279	0.123	2.40062335532578E-12	5	RASGRP2
6.8707378830505E-24	1.38629760267069	0.439	0.221	1.12068605610437E-19	5	SLC9A3R1
4.70297312253769E-14	1.36360894978799	0.26	0.123	7.67101946017123E-10	5	RASA3
5.99810395495961E-16	1.29997098550205	0.317	0.158	9.78350736093462E-12	5	SPN
9.01294842858202E-17	1.2779077831261	0.359	0.189	1.47010201818601E-12	5	BCL9L
2.48674058144359E-15	1.27018637016341	0.263	0.117	4.05612256239265E-11	5	STK38
1.03059017722337E-15	1.23473874938737	0.359	0.187	1.68099563806904E-11	5	SYTL1
3.21477188224694E-35	1.21875040554646	0.612	0.303	5.24361441713299E-31	5	AHNAK
8.77514941813259E-23	1.16620147605559	0.481	0.253	1.43131462159161E-18	5	LITAF
4.99426165436095E-20	1.13507479806588	0.394	0.187	8.14614018442815E-16	5	KLRG1
4.65911007957077E-10	1.07411042914131	0.285	0.164	7.59947445078788E-06	5	FLNA
5.39422368741598E-13	1.02394966397945	0.375	0.214	8.7985182565442E-09	5	MYO1G
4.29349872849418E-15	0.994839542420202	0.455	0.271	7.00312577604686E-11	5	TXNIP
4.45603710795827E-09	0.966451836701547	0.276	0.155	7.26824212679074E-05	5	SIGIRR
3.72403026544961E-15	0.957493663750478	0.484	0.294	6.07426576597486E-11	5	PYHIN1
5.8110020302786E-08	0.946157456045613	0.304	0.193	0.000947832541158743	5	CCDC88C
4.14360261741657E-10	0.794632306718154	0.452	0.306	6.75863022926816E-06	5	CDC42SE1
3.54997599819592E-09	0.785979746596456	0.455	0.326	5.79036585065737E-05	5	ITGB2
1.12667734713278E-13	0.755140388501439	0.558	0.376	1.83772342090828E-09	5	SUN2
3.22601243448953E-11	0.657298968909549	0.59	0.431	5.26194888189586E-07	5	SYNE2

1.73696318652017E-09	0.641170201764548	0.538	0.403	2.83316065353304E-05	5	ITGAL
1.1827864345724E-06	0.585038812804026	0.436	0.314	0.0192924295343104	5	KLRK1
3.30275242718103E-74	4.80088014358873	0.266	0.036	5.38711948397498E-70	6	CCL4L2
6.93225598785621E-143	4.25953848500914	0.262	0.016	1.13072027417923E-138	6	TRDC
6.16235530817474E-133	4.09763596091097	0.329	0.03	1.00514177431638E-128	6	CCL3
2.07800128258625E-128	4.04200559110613	0.278	0.021	3.38942789202643E-124	6	FGR
1.12148012827708E-92	3.4975593870746	0.25	0.024	1.82924623723275E-88	6	TXK
4.56617754945506E-55	3.46032034062671	0.317	0.067	7.44789220091615E-51	6	CCL4
9.91879005353378E-110	3.33644772259546	0.377	0.049	1.6178538456319E-105	6	NCR1
1.56487367676894E-109	3.00361232396228	0.429	0.063	2.55246545417782E-105	6	ZBTB16
2.03764572100061E-65	2.69419395238626	0.345	0.065	3.3236039355241E-61	6	AOAH
3.71553058542168E-91	2.69394680161398	0.516	0.109	6.0604019378813E-87	6	KLRD1
8.03442007916478E-70	2.57559413299036	0.667	0.269	1.31049425911257E-65	6	IL2RB
2.0562006215349E-53	2.47812999627438	0.385	0.095	3.35386883378557E-49	6	MCTP2
3.18049118976081E-41	2.43053095838171	0.274	0.062	5.18769917961886E-37	6	FASLG
9.5286147803443E-46	2.34397429660899	0.377	0.104	1.55421235682196E-41	6	MATK
3.31225032137954E-42	2.27688576778919	0.317	0.079	5.40261149920216E-38	6	EOMES
9.4242732793194E-20	1.89171332395753	0.25	0.088	1.53719321458979E-15	6	MAP3K8
5.46093334625251E-22	1.73045788486041	0.405	0.183	8.90732838107246E-18	6	NR4A2
1.15477565980222E-17	1.67609518552001	0.25	0.093	1.8835545787034E-13	6	CD38
1.62562738600137E-20	1.60271792989871	0.313	0.122	2.65156082930684E-16	6	APOBR
8.56307815340605E-25	1.57126920142346	0.421	0.18	1.39672367760206E-20	6	CD7
1.59773682872357E-11	1.49035201139347	0.282	0.14	2.60606854133101E-07	6	NFKBIA
1.32571602497474E-18	1.40585240002132	0.357	0.16	2.16237540833629E-14	6	RIN3
1.10979814813427E-15	1.39540383967306	0.413	0.221	1.81019175942181E-11	6	TCIRG1
3.40500229307332E-14	1.37969532474047	0.278	0.122	5.55389924023189E-10	6	CLDND1
1.85988789880123E-15	1.36696977201985	0.333	0.162	3.03366315173469E-11	6	CD247
2.63971785996479E-13	1.3154016973659	0.425	0.249	4.30564380138856E-09	6	ZFP36
3.5267891434551E-13	1.1965836612658	0.369	0.198	5.75254577188961E-09	6	LTBP3
3.55341153090143E-09	1.16252857954094	0.274	0.145	5.79596954805333E-05	6	TAGAP
8.2134255372591E-09	1.12310195089825	0.254	0.134	0.000133969183938233	6	APMAP
6.94047211767424E-25	1.11912868594055	0.599	0.31	1.13206040711385E-20	6	KLRK1
7.01660142764444E-11	1.08882655664605	0.294	0.148	1.14447785886308E-06	6	SFMBT2
1.42065664454911E-08	1.07951876986963	0.27	0.147	0.000231723305292406	6	LPCAT1
5.48740289490672E-11	1.07787481314855	0.504	0.337	8.95050286188235E-07	6	CD69
4.70170630701974E-11	1.07389537084457	0.329	0.174	7.6689531573799E-07	6	VPS37B
6.43501001883478E-10	0.994851841784901	0.337	0.192	1.04961448417214E-05	6	ABCA2
1.77953209798435E-24	0.984557297691678	0.631	0.336	2.90259480502227E-20	6	NKG7
2.69065578218729E-10	0.957019768841194	0.397	0.239	4.38872864632569E-06	6	STK17A
2.94884089624541E-09	0.893426739913232	0.357	0.209	4.80985438586589E-05	6	PITPNC1
1.84145873947778E-07	0.886758654493619	0.329	0.201	0.00300360334996221	6	PILRB
1.47591381100614E-06	0.876798635300245	0.29	0.178	0.0240736301713212	6	KLRC4
1.42345534920303E-07	0.871440935472551	0.282	0.16	0.00232179802008506	6	DENND2D
4.39005794490642E-08	0.86851652884319	0.44	0.302	0.000716062351393687	6	ADGRE5
7.84278744407311E-09	0.849386875334547	0.381	0.235	0.000127923706000277	6	PIK3R1
3.00403988339513E-11	0.808701438189298	0.504	0.332	4.89988945380579E-07	6	ARHGAP9
1.05573589922103E-13	0.790477570151411	0.683	0.511	1.72201082521943E-09	6	SRGN
7.0504235676318E-11	0.789604893717363	0.46	0.282	1.14999458811642E-06	6	GZMA
2.08196486742452E-08	0.778955070870599	0.417	0.274	0.000339589289525614	6	PRF1
2.14384208461756E-06	0.543227711625574	0.429	0.286	0.034968208242197	6	METRNL

0	8.2696826629026	0.925	0.015	0	7	HIST1H1B
0	7.41255847649297	0.289	0.001	0	7	CDCA3
0	7.06329382958059	0.281	0.001	0	7	DLGAP5
0	7.06326417041273	0.469	0.003	0	7	AURKB
0	7.02164978488717	0.605	0.005	0	7	RRM2
0	6.92834384033718	0.259	0.001	0	7	SPC25
0	6.85696115935177	0.325	0.003	0	7	KIF15
0	6.77562286680208	0.329	0.002	0	7	GTSE1
0	6.63502818042101	0.25	0.002	0	7	CDCA5
0	6.52193433294803	0.36	0.002	0	7	HJURP
0	6.43336900713337	0.482	0.004	0	7	ESPL1
0	6.42324418524127	0.338	0.003	0	7	KIF2C
0	6.37763323848133	0.311	0.003	0	7	ANLN
0	6.37696915335856	0.57	0.007	0	7	KIFC1
0	6.3351885289101	0.623	0.008	0	7	HIST1H2AH
0	6.23930683344175	0.364	0.003	0	7	ASPM
0	6.22994679532287	0.32	0.003	0	7	KIF18B
0	6.15585623152593	0.425	0.005	0	7	HIST1H2BH
0	6.13997333893848	0.263	0.002	0	7	SHCBP1
0	6.09717501429979	0.491	0.005	0	7	NCAPG
0	6.08955933911941	0.276	0.002	0	7	FOXM1
0	5.9977696506294	0.298	0.003	0	7	MELK
0	5.89196073129744	0.272	0.003	0	7	PLK1
0	5.88654046359692	0.412	0.004	0	7	SPC24
0	5.86542574935309	0.268	0.003	0	7	CDCA8
0	5.86250572174348	0.417	0.005	0	7	TOP2A
0	5.76729919388788	0.504	0.006	0	7	TYMS
0	5.71098934276044	0.338	0.004	0	7	ZWINT
0	5.65503747383229	0.333	0.005	0	7	ASF1B
0	5.635269645188	0.25	0.002	0	7	CDCA2
0	5.63500567340627	0.268	0.004	0	7	RAD54L
0	5.53049643040723	0.294	0.004	0	7	UBE2C
0	5.47306352740785	0.36	0.005	0	7	KIF11
0	5.47199625717727	0.311	0.004	0	7	CENPI
0	5.47167117432157	0.618	0.011	0	7	MKI67
0	5.4074632540893	0.425	0.007	0	7	TPX2
0	5.39697302352154	0.364	0.006	0	7	MYBL2
7.47649814043284E-303	5.21094919152704	0.268	0.004	1.219491611686E-298	7	STIL
0	5.16250538758271	0.452	0.01	0	7	HIST1H4C
0	5.11907797747278	0.412	0.008	0	7	CDK1
3.39277314381336E-275	5.04149845183006	0.268	0.005	5.53395227487396E-271	7	BUB1
1.70126141816661E-290	5.03227092627987	0.272	0.005	2.77492749917156E-286	7	SPAG5
0	5.01425360355465	0.417	0.009	0	7	UHRF1
4.22968529724393E-253	5.00609666591001	0.263	0.006	6.89903968833457E-249	7	CIT
0	5.00064075340099	0.469	0.01	0	7	CENPF
4.32405915031914E-264	4.96930841093496	0.254	0.005	7.05297288008555E-260	7	NUF2
0	4.96333972279205	0.32	0.006	0	7	PRC1
0	4.95895904870246	0.5	0.013	0	7	H2AFX
2.34164545834867E-294	4.83693779046848	0.281	0.005	3.81945790711252E-290	7	ECT2
1.19728227590536E-271	4.80346057685613	0.276	0.006	1.95288712022923E-267	7	CENPU

1.02646341144439E-267	4.7656577870328	0.272	0.006	1.67426447040694E-263	7	DEPDC1B
0	4.6686347792488	0.636	0.02	0	7	CENPM
1.53445762095708E-274	4.64998542315418	0.307	0.008	2.50285382554309E-270	7	CDT1
0	4.34478623464715	0.382	0.012	0	7	CENPE
9.75454984664593E-243	4.30319902241051	0.311	0.01	1.59106462548642E-238	7	TUBB
0	4.27387218161158	0.601	0.023	0	7	NUSAP1
0	4.25635086166874	0.868	0.087	0	7	HIST1H1C
0	4.21397670498172	0.482	0.016	0	7	NCAPG2
9.40303523345084E-286	4.16216380821114	0.36	0.011	1.53372907692817E-281	7	MXD3
3.7749975511191E-290	4.14208516099234	0.417	0.017	6.15739850563036E-286	7	FANCA
0	4.12230837972818	0.526	0.021	0	7	FANCI
0	4.09045928279481	0.728	0.049	0	7	HIST1H1E
2.20687897329912E-269	4.03160087729706	0.364	0.013	3.59964029334819E-265	7	TCF19
0	3.99008061771393	0.737	0.06	0	7	HIST1H1D
1.05261349349801E-282	3.70063165899338	0.461	0.022	1.71691786924461E-278	7	INCENP
1.15772296033871E-133	3.67645185372036	0.259	0.015	1.88836192060847E-129	7	ARHGAP33
9.66293942998017E-175	3.49059938568689	0.355	0.022	1.57612205042407E-170	7	HDGF
1.66942545856222E-185	3.44328067769728	0.285	0.012	2.72299986546083E-181	7	SMC2
2.09535981862937E-212	3.32328405889662	0.373	0.019	3.41774140016637E-208	7	NDC80
2.18460463616285E-248	3.28959966833801	0.667	0.06	3.56330862204523E-244	7	HMGB2
2.55791442326976E-135	3.25549549966269	0.25	0.014	4.1722142157953E-131	7	SCP2
5.68804831798258E-151	3.21038931386556	0.272	0.015	9.27777561146139E-147	7	CHTF18
2.19886837762968E-272	3.13910244569336	0.592	0.04	3.58657421075177E-268	7	TACC3
2.13658328529826E-133	3.12945016872708	0.254	0.015	3.48498099664999E-129	7	CENPN
7.57186427619198E-225	3.10014754909831	0.544	0.042	1.23504678208967E-220	7	EZH2
1.84969197782633E-187	2.98521820059559	0.553	0.053	3.01703258503253E-183	7	LMNB2
1.3700784818086E-199	2.97060062517273	0.513	0.042	2.23473501167801E-195	7	LMNB1
4.67804775798858E-141	2.91550680534112	0.32	0.023	7.63036369805517E-137	7	CHAF1A
6.76024545817005E-125	2.88900730494783	0.294	0.022	1.10266363668212E-120	7	KNTC1
4.74931287939596E-139	2.87998515122843	0.329	0.024	7.74660423758275E-135	7	PTTG1
6.37269340401872E-150	2.86098505589237	0.522	0.06	1.03945002112949E-145	7	HELLS
1.11812856279757E-113	2.82864809060508	0.325	0.03	1.82377949877911E-109	7	APOA2
2.73140231656384E-117	2.74766522011111	0.316	0.027	4.45519031854727E-113	7	TMEM106C
3.80461068986595E-175	2.69772393314623	0.557	0.057	6.20570049624036E-171	7	SMC4
6.87950873280695E-185	2.69341867818314	0.684	0.083	1.12211666940814E-180	7	KIF22
1.45118674905885E-85	2.67998554190113	0.276	0.029	2.36703070638989E-81	7	ANXA5
5.99805908581126E-143	2.67350306185417	0.434	0.042	9.78343417486674E-139	7	NCAPD3
1.17766806782106E-174	2.65976654624625	0.667	0.083	1.92089438542294E-170	7	TMPO
1.43654174498352E-137	2.64800326413131	0.421	0.041	2.34314324024262E-133	7	NEMP1
1.9519523618831E-102	2.59020225285502	0.338	0.036	3.18382949746753E-98	7	ETFB
1.88639312116307E-94	2.49173514595264	0.272	0.025	3.07689581992908E-90	7	UQCRC1
3.03819505465993E-104	2.4445469842523	0.285	0.025	4.95559995365581E-100	7	TTF2
2.58729449208157E-79	2.43339875206838	0.259	0.027	4.22013604603425E-75	7	COX8A
2.74667023293748E-113	2.410949849035	0.377	0.04	4.48009381694432E-109	7	POLE
2.01161024346542E-119	2.40072326102412	0.346	0.032	3.28113746811644E-115	7	NSD2
2.13418897859303E-116	2.38060058722998	0.386	0.041	3.48107564298309E-112	7	NCAPD2
1.31229288934357E-75	2.32782192997825	0.25	0.027	2.1404809318083E-71	7	POLD1
5.04623630908797E-96	2.31070982234632	0.303	0.03	8.23091604375339E-92	7	CKAP5
1.78822554093202E-91	2.1952169818785	0.377	0.049	2.91677467981421E-87	7	LIG1
2.31777997713503E-71	2.15030545887667	0.281	0.035	3.78053092070494E-67	7	MCM3

3.84544615500508E-79	2.14178087445807	0.272	0.03	6.27230722342879E-75	7	ANAPC11
2.92802889614425E-71	2.11395013054008	0.276	0.034	4.77590793250089E-67	7	KIF20B
7.7017749688026E-77	2.02313972598704	0.276	0.031	1.25623651516139E-72	7	SAE1
6.98292367474477E-55	2.00776444970678	0.25	0.036	1.13898468058762E-50	7	HYOU1
3.26508362918134E-68	1.927509153623	0.285	0.037	5.32567790755769E-64	7	CBX5
6.19414002678497E-83	1.90443418201811	0.39	0.056	1.0103261797689E-78	7	ANP32E
6.03814700514457E-52	1.86131178012921	0.276	0.045	9.84882158009131E-48	7	MYEF2
7.41395901262997E-52	1.83532410462824	0.294	0.05	1.20929085455007E-47	7	CYB5R3
7.10661758539804E-73	1.79681582521366	0.509	0.101	1.15916039435428E-68	7	MT-CO2
5.44758143367232E-55	1.7664678987659	0.276	0.042	8.88555007646293E-51	7	DCTN3
1.10264324903762E-60	1.75297656439645	0.386	0.072	1.79852140350526E-56	7	MCM7
4.74920327768805E-55	1.74521360737946	0.281	0.044	7.74642546623698E-51	7	PARK7
3.5646677840719E-96	1.74431326624482	0.741	0.158	5.81432962259968E-92	7	RBM15
3.00436223205623E-57	1.73568049912939	0.268	0.039	4.90041523670691E-53	7	EBP
9.89633113705696E-55	1.70461679989123	0.338	0.061	1.61419057176536E-50	7	MT-ATP6
1.3062365179362E-77	1.70441616069401	0.399	0.061	2.13060238440573E-73	7	DDX39A
3.01296200019992E-48	1.68424105495877	0.281	0.049	4.91444231852609E-44	7	MT-ND4
1.14535504199756E-63	1.66557728002624	0.386	0.068	1.86818860900222E-59	7	NCAPH2
5.87298928355659E-57	1.64761688358145	0.443	0.095	9.57943282040916E-53	7	CRIP1
1.24531094825849E-68	1.64375654383722	0.439	0.08	2.03122668770443E-64	7	CDK5RAP2
2.09816848974752E-55	1.55205336035163	0.351	0.064	3.42232262362718E-51	7	SIVA1
9.88573764918143E-67	1.55068956313806	0.487	0.098	1.61246266795798E-62	7	DNMT1
1.18956862822524E-56	1.54844705749459	0.496	0.115	1.94030538949819E-52	7	COTL1
8.06581763247859E-65	1.5458527741467	0.364	0.06	1.31561551403358E-60	7	SMC1A
1.30507866680473E-79	1.53544132233541	0.531	0.098	2.12871381342519E-75	7	PHF19
3.81228808017489E-54	1.51447354576778	0.325	0.057	6.21822308757326E-50	7	PCNT
2.80816090490337E-56	1.50741407274985	0.289	0.045	4.58039125198788E-52	7	ACADVL
2.27716509407111E-42	1.50561673422631	0.298	0.06	3.71428398493939E-38	7	AHI1
2.5306864125697E-45	1.50388201703444	0.285	0.052	4.12780260754243E-41	7	MCM5
1.76255865321143E-59	1.50027998102401	0.377	0.068	2.87490941925317E-55	7	NUCKS1
6.62524570782853E-44	1.48013361508818	0.412	0.102	1.08064382740391E-39	7	PTMS
7.03955278588873E-39	1.44875665950589	0.289	0.06	1.14822145490631E-34	7	ENO1
2.02240935344961E-43	1.42093345349828	0.294	0.057	3.29875189641166E-39	7	MAZ
5.96485716548007E-45	1.41470606439776	0.25	0.042	9.72927852261454E-41	7	HNRNPAB
2.50649326254513E-38	1.40709900916468	0.259	0.05	4.08834116053736E-34	7	EEF1AKMT2
1.07142744313649E-57	1.40274650509299	0.535	0.125	1.74760530249992E-53	7	CALM3
9.80257171129272E-48	1.39602541727305	0.276	0.047	1.59889747182896E-43	7	TUBGCP3
7.31194269188118E-50	1.39317909493643	0.316	0.057	1.19265097247274E-45	7	SMC3
1.80414945680385E-51	1.3889540744847	0.39	0.08	2.94274817899275E-47	7	ECH1
6.88886517786065E-36	1.38549029016486	0.25	0.05	1.12364279916085E-31	7	TRIOBP
4.28501086935695E-54	1.37606447062988	0.325	0.056	6.98928122900813E-50	7	CEP128
1.3671059705018E-45	1.37309253776839	0.272	0.047	2.22988654848549E-41	7	POLR2L
4.61272622194766E-50	1.36894692636043	0.399	0.085	7.52381774061884E-46	7	LAMP1
6.5580247474037E-50	1.36100279165751	0.364	0.073	1.06967941654902E-45	7	FAM111A
1.17014179406629E-39	1.35783521475087	0.259	0.049	1.90861828030153E-35	7	DOT1L
2.00119474512656E-42	1.34211336344752	0.259	0.046	3.26414874877594E-38	7	DEK
1.17020362016687E-37	1.31480807622673	0.25	0.048	1.90871912485418E-33	7	PI4K2B
1.10588912424556E-32	1.29687078559604	0.25	0.053	1.80381575055693E-28	7	GSTP1
6.97520713044457E-40	1.28684337628426	0.259	0.048	1.13772603504681E-35	7	NDUFB7
7.57909502705222E-40	1.26618902053786	0.303	0.063	1.23622618986249E-35	7	MT-ND4L

**1.20284220229304E-36**	1.25889709272556	0.254	0.05	1.96195591616018E-32	7	HP1BP3
**2.78521320304433E-33**	1.25216963258511	0.303	0.072	4.54296125548561E-29	7	ADCY3
**1.5516690130372E-37**	1.22786467861485	0.482	0.142	2.53092732716497E-33	7	MT-CO3
**3.12617191481932E-31**	1.20512825166865	0.281	0.066	5.09909901026179E-27	7	MPHOSPH9
**2.17949215360505E-33**	1.18855939168527	0.285	0.065	3.55496965174519E-29	7	YBX1
**6.71574652942848E-38**	1.18670166087342	0.456	0.127	1.09540541641508E-33	7	IDH2
**1.70298080489929E-35**	1.18503582551176	0.263	0.054	2.77773199087123E-31	7	NUDT21
**4.1249279426831E-30**	1.18364050913992	0.25	0.056	6.7281699673104E-26	7	SCLT1
**2.12766860883688E-33**	1.18344290884225	0.785	0.412	3.47044026787384E-29	7	PFN1
**6.48128698688941E-34**	1.17332448483489	0.583	0.208	1.05716272043153E-29	7	H1FX
**8.47296061648434E-33**	1.14109123477512	0.276	0.062	1.38202460615476E-28	7	SUMO3
**1.00861866039853E-38**	1.12066392952647	0.364	0.086	1.64515789697605E-34	7	CDC25B
**1.58396007947314E-34**	1.11667842770291	0.364	0.093	2.58359728562863E-30	7	PKM
**4.18052555579532E-26**	1.11619932799956	0.25	0.062	6.81885523405774E-22	7	SLBP
**6.4334362583909E-37**	1.1140993317854	0.338	0.079	1.04935778810614E-32	7	UBE2I
**2.08512960249071E-45**	1.10595931308331	0.487	0.123	3.4010548946226E-41	7	SUGP2
**1.07819726743667E-34**	1.09820018622954	0.285	0.063	1.75864756291595E-30	7	NUP50
**1.33351816180852E-31**	1.08785627434725	0.39	0.111	2.17510147372588E-27	7	MAT2A
**7.6017676806979E-28**	1.06759238057397	0.311	0.084	1.23992432639863E-23	7	ENTPD1
**7.36258935985648E-24**	1.04421839101689	0.807	0.525	1.20091195048619E-19	7	ACTB
**5.93061618002654E-33**	1.04190552483279	0.25	0.052	9.67342805124129E-29	7	SNRPB
**1.66716422353191E-35**	1.04135885871682	0.36	0.09	2.7193115650029E-31	7	CD38
**4.65766842140191E-34**	1.02983054090505	0.259	0.054	7.59712296214866E-30	7	TBC1D10B
**4.72013988895351E-29**	1.02622410422521	0.263	0.062	7.69902017287208E-25	7	TENT4A
**5.71465185989555E-32**	1.02246989650824	0.316	0.078	9.32116864867562E-28	7	PA2G4
**8.60950423018351E-26**	1.0102227069174	0.289	0.078	1.40429623498523E-21	7	NONO
**3.77485768464672E-34**	1.00733926304339	0.377	0.099	6.15717036942726E-30	7	HNRNPD
**3.56302204952963E-25**	0.996473284792656	0.588	0.245	5.81164526498779E-21	7	CFL1
**9.2726643652781E-27**	0.989100264883775	0.263	0.066	1.51246428462051E-22	7	NACC1
**9.08469220795363E-37**	0.987711170131361	0.311	0.069	1.48180414603932E-32	7	SRRT
**6.77407192998195E-43**	0.986667345464062	0.382	0.086	1.10491887249936E-38	7	HNRNPUL2
**2.85743392105341E-34**	0.972982892689168	0.338	0.083	4.66076046863022E-30	7	TRIM26
**7.30102846063963E-30**	0.970599958839037	0.289	0.071	1.19087075221493E-25	7	SLC7A5
**4.70754333940307E-29**	0.968273148858255	0.417	0.126	7.67847394090034E-25	7	FZR1
**2.02892369890295E-28**	0.966609778455727	0.268	0.064	3.30937744528061E-24	7	ATP5F1D
**1.77186853021915E-32**	0.960414069113479	0.325	0.081	2.89009475964046E-28	7	MCM6
**1.62204196527946E-39**	0.960147072158635	0.447	0.116	2.64571264956732E-35	7	HNRNPUL1
**2.0518788253926E-30**	0.948100871828188	0.289	0.07	3.34681955209788E-26	7	KHDRBS1
**1.35004866360497E-35**	0.944376405548013	0.504	0.15	2.20206437520607E-31	7	CNTRL
**9.8679577434496E-24**	0.944178430133277	0.553	0.222	1.60956258753406E-19	7	ACTG1
**1.01077851702628E-31**	0.937895166725275	0.268	0.06	1.64868083912157E-27	7	FTH1
**2.33646388400272E-25**	0.931031231755101	0.32	0.092	3.81100624119684E-21	7	TMEM109
**3.4704441700766E-38**	0.928110757552126	0.421	0.109	5.66064148581193E-34	7	NOTCH1
**9.75857112036236E-28**	0.926654483691286	0.254	0.061	1.5917205354423E-23	7	PPP6R3
**8.72667689140172E-34**	0.921180906032403	0.456	0.131	1.42340826775654E-29	7	NUCB1
**3.82114946618306E-31**	0.914310804140244	0.285	0.067	6.2326768942912E-27	7	PFKP
**2.60387779632437E-30**	0.913723568914944	0.281	0.067	4.24718507358467E-26	7	ATP5IF1
**1.10730406955221E-29**	0.906374587377243	0.346	0.093	1.80612366784661E-25	7	CBFB
**7.24705996542581E-25**	0.898520445299598	0.263	0.068	1.1820679509606E-20	7	RNH1
**4.49813937891733E-27**	0.894520842585538	0.281	0.072	7.33691514095206E-23	7	UBALD2

2.34935504117964E-28	0.892181643194992	0.25	0.058	3.83203300766811E-24	7	AP1M1
4.209606043203E-33	0.878076884963392	0.636	0.217	6.86628841706842E-29	7	HNRNPA0
5.74495100611217E-29	0.873818340266939	0.478	0.159	9.37058958606957E-25	7	PDCD1
2.06297795366395E-28	0.872222267392381	0.329	0.088	3.36492334022127E-24	7	RNF187
8.41958325176919E-26	0.872106985745074	0.43	0.141	1.37331822419607E-21	7	H3F3A
2.8995979699998E-23	0.870906431402291	0.25	0.066	4.72953424886668E-19	7	RAVER1
8.08103453859973E-28	0.862197628162167	0.272	0.067	1.318097543591E-23	7	HCFC1
6.67723263857188E-27	0.861237877418394	0.281	0.072	1.08912341567746E-22	7	BAZ1B
1.04406092340545E-31	0.852504297801019	0.303	0.073	1.70296777216663E-27	7	RNF168
4.07047266728572E-24	0.849586616204885	0.64	0.28	6.63934796760974E-20	7	METRNL
1.00410216771576E-29	0.847179037382637	0.478	0.15	1.63779104576118E-25	7	HNRNPF
3.78615684074257E-32	0.845359615890746	0.434	0.126	6.1756004229352E-28	7	NFATC2IP
2.16099733254385E-23	0.844580169062673	0.263	0.071	3.52480274911228E-19	7	SF3B5
1.8538699351413E-25	0.837208091335637	0.272	0.071	3.02384725120898E-21	7	CRELD2
3.99080327859963E-20	0.825967477840688	0.259	0.076	6.50939922772386E-16	7	ATP5MG
6.02938641490859E-24	0.822654118962549	0.675	0.303	9.8345321813574E-20	7	VIM
6.11946069268028E-29	0.807064970307855	0.544	0.186	9.9814523358308E-25	7	ITM2A
5.81501469031419E-26	0.805295156143197	0.259	0.065	9.48487046137148E-22	7	PSIP1
9.20271386081558E-23	0.798611771083802	0.873	0.548	1.50105465783763E-18	7	CORO1A
4.6599415891354E-33	0.795526826288959	0.43	0.12	7.60083072603875E-29	7	SLC3A2
2.6158485558103E-33	0.79308406993397	0.434	0.122	4.26671057938219E-29	7	LBR
3.11859686105247E-26	0.792012855059064	0.325	0.091	5.08674334006269E-22	7	AKT1
1.45430619439331E-23	0.785360578145331	0.25	0.065	2.37211883367493E-19	7	TAF15
2.00972591038605E-26	0.778552607155524	0.417	0.131	3.27806393243069E-22	7	HNRNPA3
2.17852032260243E-19	0.77705733945606	0.254	0.076	3.55338449819683E-15	7	DGKH
7.03197277774027E-25	0.770106427145394	0.36	0.11	1.14698507977721E-20	7	NAB1
3.44342629520486E-24	0.768879452784134	0.281	0.077	5.61657263010864E-20	7	PPP1CA
1.5754738359631E-21	0.754007373321232	0.355	0.117	2.56975537383941E-17	7	STAG1
1.30267247463509E-22	0.752355323061201	0.254	0.069	2.12478907337729E-18	7	ZC3H4
2.73102435416326E-21	0.750125172293214	0.285	0.085	4.4545738240757E-17	7	SEC11A
1.64057743133768E-31	0.742149595940251	0.465	0.142	2.67594584825489E-27	7	WHRN
7.06251451944719E-25	0.738341377136699	0.268	0.07	1.15196674326703E-20	7	PSMD3
4.31833666723502E-23	0.729968519302464	0.456	0.161	7.04363893792704E-19	7	VMP1
1.13454630099812E-24	0.72064979620714	0.434	0.144	1.85055847155803E-20	7	NUP210
1.59058505215593E-25	0.712113956282466	0.259	0.065	2.59440327857154E-21	7	SUMO2
3.86561993479888E-24	0.709838334962325	0.504	0.179	6.30521267565046E-20	7	ILF3
4.61425199640898E-22	0.707727490960173	0.338	0.106	7.52630643134268E-18	7	DHX15
2.92371280734575E-23	0.707516092418013	0.263	0.071	4.76886796006165E-19	7	ZCCHC3
5.40578565368713E-29	0.702476414303198	0.281	0.068	8.81737697972908E-25	7	SFXN1
7.51096820164541E-27	0.692778730645616	0.39	0.117	1.22511402337038E-22	7	SRSF2
4.14998095365558E-21	0.682563625404685	0.316	0.099	6.76903393350762E-17	7	SYNE3
2.72607801119522E-16	0.681953461905801	0.268	0.09	4.44650584406052E-12	7	PHLDA1
3.33738031742987E-18	0.67893614427561	0.325	0.113	5.44360103575985E-14	7	PIM3
5.4570252716234E-23	0.677396518928073	0.303	0.088	8.90095392054492E-19	7	ARHGDIA
9.1076712510204E-20	0.676179571597325	0.25	0.073	1.48555225775394E-15	7	SCRIB
1.69479180747788E-21	0.67148867373266	0.285	0.084	2.76437491717717E-17	7	KANSL3
1.52500487586501E-25	0.669169025675218	0.474	0.16	2.48743545302341E-21	7	SRSF4
9.73518604744588E-21	0.666048155383785	0.294	0.09	1.5879061961989E-16	7	GOLPH3
6.2843344688188E-24	0.662323368849075	0.298	0.084	1.02503779520903E-19	7	RHOA
4.23436452675473E-24	0.661700121096182	0.482	0.173	6.90667197958964E-20	7	KLRC4

1.58242820187963E-20	0.649908186818214	0.583	0.24	2.58109864008586E-16	7	IFI16
3.42488280566009E-26	0.646134515574784	0.399	0.125	5.58632634431217E-22	7	DUSP4
1.11529194029439E-23	0.643879403797883	0.408	0.134	1.81915268381418E-19	7	EID1
1.48852484453468E-19	0.630828147255546	0.268	0.081	2.42793287392052E-15	7	VAMP1
5.65621984336952E-27	0.621917495678797	0.513	0.171	9.22586018652002E-23	7	TRIR
6.37780773045894E-20	0.621194315577846	0.404	0.144	1.04028421891516E-15	7	SLC20A1
3.95155575060741E-21	0.621043118462296	0.386	0.132	6.44538258481574E-17	7	PIM1
6.73359412463539E-33	0.614246777061426	0.434	0.122	1.09831653766928E-28	7	RASSF1
2.2805507059513E-19	0.599346281581232	0.36	0.124	3.71980625647716E-15	7	RBM14
1.44227227132339E-17	0.595995890841864	0.281	0.092	2.35249030175559E-13	7	ANKLE2
5.88822716854353E-25	0.589814020616531	0.561	0.201	9.60428733461136E-21	7	PSMB9
1.26797799646271E-17	0.580602378106859	0.329	0.115	2.06819891003033E-13	7	GGNBP2
1.48401038167246E-19	0.573232446427219	0.373	0.129	2.42056933354594E-15	7	LMAN2
1.27379190527794E-25	0.572115913660753	0.579	0.206	2.07768197669886E-21	7	MBD2
2.96478801688573E-18	0.571575992503002	0.364	0.13	4.83586573434232E-14	7	SDF4
2.8881380689833E-23	0.568671339926779	0.373	0.118	4.71084200431866E-19	7	FUS
1.59241548416412E-21	0.568526797583406	0.329	0.102	2.59738889622009E-17	7	RNF4
1.065547906972E-24	0.566384689069384	0.342	0.101	1.73801519106203E-20	7	LRPAP1
1.90520163367234E-21	0.566034901501365	0.316	0.097	3.10757438468296E-17	7	AIP
2.46578908787974E-21	0.564082268102704	0.579	0.221	4.02194858124065E-17	7	HNRNPA2B1
3.16186398002008E-20	0.560141582074374	0.276	0.083	5.15731633781075E-16	7	SART1
2.22127153519202E-19	0.555423531834893	0.268	0.081	3.6231160010517E-15	7	HDAC1
2.90361169323625E-13	0.553500188601348	0.276	0.104	4.73608103283764E-09	7	HSP90AB1
2.57020079424545E-20	0.549831363357341	0.32	0.102	4.19225451549376E-16	7	NDE1
1.05308315386661E-21	0.544085916524295	0.382	0.126	1.71768393227182E-17	7	PSPC1
5.88158113852025E-16	0.541200380326576	0.518	0.223	9.59344699504038E-12	7	EZR
3.92841852837698E-20	0.53980952011965	0.36	0.121	6.40764346163568E-16	7	YWHAZ
3.60158820077003E-20	0.535360185596356	0.382	0.132	5.874550514276E-16	7	HBS1L
1.86056781456442E-25	0.535204109607697	0.456	0.149	3.03477216233603E-21	7	NAP1L4
8.96655489601414E-20	0.526351733926316	0.355	0.119	1.46253476908887E-15	7	TACC1
2.11736003632957E-13	0.508442537681266	0.259	0.094	3.45362595525717E-09	7	SIRPG
3.76656527706216E-14	0.508359022450564	0.654	0.314	6.14364462341609E-10	7	TMSB10
9.25880280685934E-17	0.502998272996646	0.386	0.145	1.51020332582683E-12	7	PPP6R1
6.32622961427859E-13	0.495729287804334	0.254	0.094	1.03187131238498E-08	7	PURB
2.98332610667582E-20	0.495185696870266	0.263	0.077	4.86610321259893E-16	7	FBXO46
9.29922193673816E-17	0.487298742322425	0.259	0.083	1.51679609010136E-12	7	CYB5B
5.83188353739429E-16	0.485978400131559	0.443	0.179	9.51238523784382E-12	7	WSB1
6.75970216390124E-18	0.483842696281158	0.303	0.1	1.10257501995393E-13	7	PPP1R15B
1.42100953824084E-12	0.483623882859547	0.259	0.098	2.31780865782463E-08	7	ITPR3
3.41167445801251E-15	0.479732230466695	0.614	0.291	5.5647822084642E-11	7	MT-CO1
1.42670030649243E-21	0.479228802705784	0.404	0.135	2.3270908699198E-17	7	SAFB
4.59000697691893E-14	0.478614787629029	0.768	0.447	7.48676038005247E-10	7	CD8A
1.21045757128274E-15	0.469272564418822	0.377	0.147	1.97437734451927E-11	7	CREBZF
4.57649640584006E-09	0.466007628403557	0.939	0.712	7.46472328756572E-05	7	CD74
2.56704293585582E-17	0.463910705945579	0.268	0.086	4.18710373267443E-13	7	EIF3B
4.36395910382675E-18	0.463383320303408	0.329	0.112	7.11805369425181E-14	7	PPP1R12C
3.74493994369929E-17	0.463196382339242	0.289	0.097	6.10837154216792E-13	7	CENPT
1.86321879652505E-20	0.458573684923701	0.289	0.087	3.03909617901201E-16	7	LIME1
1.89398778286272E-14	0.452871105756318	0.294	0.107	3.08928347262739E-10	7	EIF4H
2.41073628427026E-18	0.452676808425402	0.342	0.118	3.93215195327322E-14	7	PNN

7.30075576746643E-18	0.450672404451026	0.272	0.086	1.19082627323145E-13	7	WAS
1.02365917760829E-22	0.449246671271097	0.377	0.12	1.66969048459688E-18	7	ARL6IP4
1.23075223365696E-16	0.446299794753922	0.281	0.094	2.00747996831786E-12	7	SCAF1
1.02767703749943E-10	0.443575857545544	0.754	0.448	1.67624401586532E-06	7	LSP1
3.97686060288618E-21	0.44299020825351	0.526	0.195	6.48665732936765E-17	7	PSMB8
6.16957529855403E-15	0.440743632096618	0.259	0.089	1.00631942694715E-10	7	DCAF7
4.42253001180755E-23	0.44007574890091	0.303	0.087	7.2135887022593E-19	7	IFI6
1.01224966897848E-14	0.433319134940485	0.272	0.096	1.65108043507081E-10	7	STK11
8.01188095197184E-18	0.431831946208452	0.289	0.094	1.30681790207613E-13	7	SURF4
3.06359196233338E-16	0.429029193932415	0.285	0.097	4.99702484976198E-12	7	MOV10
1.77452335442098E-21	0.428454853090516	0.469	0.166	2.89442504339607E-17	7	POLR2A
2.06181676609223E-15	0.42738278943431	0.368	0.141	3.36302932717303E-11	7	MYL6
2.66507666227338E-16	0.426193884859516	0.272	0.091	4.3470065438341E-12	7	ZC3H18
9.05231713558961E-15	0.420850003753935	0.254	0.088	1.47652344798602E-10	7	CTLA4
3.56536109957455E-21	0.418174161075547	0.316	0.098	5.81546048951604E-17	7	ZEB2
5.63254584933973E-16	0.412283448259738	0.364	0.137	9.18724553485804E-12	7	CD99
6.82983431584661E-19	0.411669865888533	0.285	0.089	1.11401427525774E-14	7	EIF4EBP2
1.68517529442193E-17	0.404637979256433	0.342	0.121	2.7486894227316E-13	7	PABPC1L
4.65511814159018E-19	0.403268132590785	0.281	0.087	7.59296320074774E-15	7	RBM3
7.43619879746328E-16	0.402709359673863	0.566	0.24	1.21291838585423E-11	7	RAC2
2.21202445269878E-14	0.39797790479492	0.36	0.14	3.60803308479697E-10	7	SH3BGRL3
2.96419805846012E-10	0.397147559065418	0.658	0.352	4.83490345315431E-06	7	LCP1
4.25171094763924E-18	0.392798479843152	0.351	0.122	6.93496572669437E-14	7	ANKRD36C
5.18942491043643E-12	0.391317862294888	0.715	0.373	8.46447097141286E-08	7	SUN2
1.95038976093676E-13	0.383942653889811	0.452	0.195	3.18128073906394E-09	7	DRAP1
1.59185287272924E-15	0.380701423099628	0.254	0.084	2.59647122070866E-11	7	ABI3
1.96674881858574E-16	0.380150884849788	0.259	0.084	3.2079639979952E-12	7	TCERG1
1.32437615056659E-14	0.378402049705766	0.325	0.121	2.16018993918917E-10	7	PRKAR1A
1.91858941770479E-13	0.37755559642177	0.351	0.14	3.12941119921828E-09	7	NCOR2
4.73059722167153E-15	0.376263086929844	0.281	0.099	7.71607712826844E-11	7	CCDC85B
2.67984071749232E-14	0.375975987505622	0.443	0.186	4.37108819430173E-10	7	CYBC1
1.03714395475464E-16	0.374502888847787	0.561	0.241	1.69168550460029E-12	7	LAG3
4.98802267992142E-11	0.367149964283723	0.25	0.1	8.13596379321983E-07	7	IL21R
7.44946404637486E-21	0.364596107645647	0.461	0.164	1.2150820806042E-16	7	SLC2A4RG
7.61261860167624E-15	0.36226900664983	0.333	0.125	1.24169422011941E-10	7	PPP1R18
2.30015189073208E-16	0.35892637183961	0.342	0.124	3.7517777489731E-12	7	INTS11
1.74641674970431E-15	0.358689411116707	0.254	0.085	2.8485803604427E-11	7	ANAPC5
8.24154844662755E-13	0.351997995502018	0.294	0.113	1.34427896712942E-08	7	HNRNPU
5.16344849735272E-13	0.351647666589127	0.25	0.09	8.42210084403203E-09	7	SP3
1.00871724396499E-17	0.349519189521761	0.289	0.094	1.6453186966313E-13	7	LMF2
1.16210852282579E-14	0.347176812911341	0.263	0.091	1.89551521158115E-10	7	SRSF3
2.55468890739253E-10	0.339452473322547	0.728	0.393	4.16695307684796E-06	7	ITGA4
6.37621079511237E-12	0.338667176013864	0.553	0.261	1.04002374279078E-07	7	EHBP1L1
3.43975595626149E-15	0.338109190885193	0.268	0.091	5.61058594025811E-11	7	STAG2
5.60834694020113E-16	0.332608197679384	0.417	0.163	9.14777469416206E-12	7	NUMA1
3.76391321911793E-16	0.33138474560125	0.364	0.135	6.13931885170326E-12	7	GGA2
3.60039273543919E-09	0.329670496180174	0.294	0.131	5.87260059077486E-05	7	EDEM1
1.4668300544388E-14	0.329337217666874	0.303	0.111	2.39254650179512E-10	7	BST2
4.45348725805724E-13	0.325211763058297	0.254	0.092	7.26408306661717E-09	7	ARPC4
5.29597182634118E-13	0.3226383886998	0.382	0.158	8.6382596459451E-09	7	SPN

4.08485043237208E-17	0.321589150416606	0.539	0.218	6.6627995402421E-13	7	TCIRG1
3.07137420595174E-17	0.318328667966413	0.386	0.141	5.00971846732789E-13	7	LASP1
2.35866302346845E-13	0.315612718348375	0.504	0.223	3.8472152575794E-09	7	ATP2A3
8.18493064719932E-15	0.301986301909822	0.272	0.094	1.33504403786468E-10	7	OGDH
9.86927082594166E-12	0.300194150035957	0.36	0.154	1.60977676441934E-07	7	CHST12
1.13047918156503E-14	0.296592584207432	0.465	0.191	1.84392459305072E-10	7	CSK
1.24909318590395E-12	0.295337718996103	0.25	0.091	2.03739589552794E-08	7	SIN3A
1.23562463910192E-13	0.29022959859109	0.386	0.158	2.01542734883914E-09	7	DDIT4
1.14438075376403E-13	0.288537003113604	0.276	0.101	1.86659944746452E-09	7	SMARCA4
1.16502538119101E-11	0.287052133927154	0.531	0.25	1.90027289926065E-07	7	DOCK2
1.81919952206108E-12	0.283126278378919	0.298	0.116	2.96729634043383E-08	7	EIF4A1
3.78922563582008E-17	0.282374962298534	0.395	0.145	6.18060593458612E-13	7	GSDMD
2.97590762421986E-14	0.277644562334529	0.338	0.13	4.85400292586501E-10	7	SETD5
1.70840392982499E-10	0.274713582670077	0.263	0.107	2.78657764993754E-06	7	GRIPAP1
9.22841148459764E-13	0.27215782176898	0.316	0.125	1.50524619725272E-08	7	ARRB2
4.67454064175698E-15	0.271206559125602	0.36	0.137	7.62464324076982E-11	7	PRPF3
1.67735517397129E-13	0.269791255928677	0.39	0.159	2.73593402426458E-09	7	EIF3A
4.68176852264452E-10	0.268166709634496	0.627	0.337	7.63643263728547E-06	7	NKG7
3.97953293585966E-16	0.2663321963083	0.294	0.1	6.4910161716807E-12	7	PPP1R35
4.41741585185466E-13	0.265487034845002	0.259	0.094	7.20524699596014E-09	7	EDC4
7.94757629545029E-12	0.263493640030553	0.25	0.094	1.2963291695509E-07	7	TMEM50A
8.53395303268512E-11	0.260147536260929	0.281	0.119	1.39197307916127E-06	7	TNFRSF9
7.37481211669205E-14	0.259968440273324	0.329	0.127	1.20290560435364E-09	7	RCSD1
7.84470983683609E-12	0.259332402272911	0.254	0.097	1.27955062148634E-07	7	SH3BP1
1.5886929611656E-09	0.258391513950599	0.596	0.305	2.59131708895722E-05	7	GRK2
6.6756807818761E-09	0.25615847272422	0.25	0.108	0.000108887029233181	7	BCL3
6.12381528585854E-18	0.254692485889156	0.272	0.085	9.98855511276387E-14	7	HMGN4
4.17373693659249E-12	0.253998357929243	0.368	0.156	6.807782317276E-08	7	ASXL1
9.83770221983524E-16	0.252596130015092	0.478	0.193	1.60462760907733E-11	7	RAB27A
7.08824760389938E-12	0.251081042195351	0.439	0.194	1.15616406667203E-07	7	ACTN4
3.83085883616875E-139	4.89043998065372	0.424	0.031	6.24851384767485E-135	8	CXCL13
6.57107968681798E-90	3.7159447290718	0.361	0.034	1.07180880771688E-85	8	SESN3
2.17878517422181E-60	2.82518669264923	0.472	0.082	3.5538164976732E-56	8	IL6ST
1.5634065473755E-55	2.60602750612047	0.549	0.123	2.55007241942417E-51	8	ST8SIA1
2.22324490028603E-34	2.5187292947859	0.34	0.07	3.62633475685654E-30	8	THADA
9.8955305504546E-36	2.4545404942656	0.347	0.071	1.61405998808465E-31	8	FKBP5
2.04268676762111E-45	2.21050576944089	0.521	0.121	3.33182638666679E-41	8	TCF7
4.53467688770535E-34	2.12491947775044	0.5	0.146	7.3965114715362E-30	8	SH2D1A
4.37074907036099E-24	2.0442799647072	0.306	0.076	7.12912880866581E-20	8	NPDC1
7.06977678275937E-20	2.02330325604992	0.264	0.068	1.15315129103588E-15	8	NFATC1
1.30706990761735E-21	2.00566962989339	0.319	0.088	2.13196172631466E-17	8	NR3C1
1.67735791777269E-28	1.97862984785584	0.403	0.107	2.73593849967904E-24	8	TP53INP1
3.4266541722019E-26	1.96967371478391	0.431	0.132	5.58921562027851E-22	8	ICOS
3.35346111975582E-20	1.9036964535945	0.278	0.072	5.46983043243371E-16	8	ZC3H12D
6.92299616419434E-22	1.86191291496687	0.34	0.096	1.12920990434174E-17	8	TRAT1
4.79164643958223E-19	1.84380519927413	0.306	0.087	7.81565450760258E-15	8	KIAA0319L
5.8552753706874E-15	1.83679036150177	0.278	0.089	9.55053965712822E-11	8	CTLA4
3.40351456726343E-20	1.76380659556442	0.479	0.19	5.55147261066337E-16	8	ITM2A
1.95413997161463E-11	1.63654415473112	0.278	0.107	3.18739770770063E-07	8	IFNAR2
1.03770255663925E-17	1.63269184990207	0.507	0.213	1.69259664013428E-13	8	PGGHG

**3.58390818900047E-27**	1.60159878456884	0.736	0.362	5.84571264707866E-23	8	ITPKB
**2.70329708957394E-12**	1.59546448398833	0.271	0.097	4.40934788280405E-08	8	CD82
**1.64728196468231E-19**	1.54479029624624	0.576	0.264	2.68688161259332E-15	8	DGKA
**2.00700720069565E-16**	1.54199093257306	0.417	0.162	3.27362944505468E-12	8	TNFRSF25
**1.87107327921896E-10**	1.44312678596998	0.306	0.13	3.05190762573404E-06	8	CD84
**2.66594733868954E-12**	1.43074708206794	0.396	0.178	4.34842670413651E-08	8	UCP2
**1.26116379099273E-11**	1.39345223061407	0.319	0.129	2.05708425948825E-07	8	DUSP4
**1.0222043898202E-09**	1.35530868788239	0.278	0.113	1.66731758023572E-05	8	STAT5B
**2.80241142111721E-11**	1.32705325466629	0.285	0.107	4.57101326898428E-07	8	CD4
**2.5068086919172E-15**	1.31960880134525	0.444	0.183	4.08885565738614E-11	8	BICDL1
**1.61573205222519E-13**	1.31458945915154	0.389	0.158	2.6354205503845E-09	8	TNIK
**1.34255335872378E-11**	1.30162414637259	0.312	0.12	2.18983878341436E-07	8	SREBF2
**1.01498893370733E-10**	1.27162990238947	0.312	0.127	1.65554844977003E-06	8	SFI1
**4.12748451379691E-19**	1.26312262081453	0.701	0.361	6.73233999045413E-15	8	MAF
**1.98525072679456E-07**	1.25929399469856	0.306	0.15	0.00323814246047461	8	PFKFB3
**4.96119890466949E-09**	1.248468209623	0.375	0.193	8.0922115334064E-05	8	ARID5B
**1.14075137719772E-09**	1.23000530781218	0.382	0.185	1.8606795713472E-05	8	LAT
**4.4972949086394E-14**	1.17833531795222	0.569	0.272	7.33553772548172E-10	8	RNF19A
**4.60376841271334E-13**	1.17084976002084	0.458	0.205	7.50920665797673E-09	8	SMAP2
**1.89644996820428E-06**	1.11021408438797	0.299	0.154	0.0309329954313801	8	NAP1L4
**1.56594672522879E-08**	1.09218166204386	0.431	0.25	0.000255421570352069	8	FYB1
**6.7787740550397E-11**	1.08767749012742	0.424	0.2	1.10568583611753E-06	8	LTBP3
**3.2493747247257E-11**	1.06893176098997	0.507	0.276	5.30005511350009E-07	8	TIGIT
**6.5498039161308E-07**	1.04588231362412	0.25	0.114	0.0106833851676009	8	NAB1
**1.53715266640082E-07**	0.976606412040605	0.382	0.202	0.00250724971416637	8	PIK3IP1
**1.24470841662101E-08**	0.973845624894264	0.528	0.323	0.000203024389835053	8	ITK
**7.0131331431436E-11**	0.950930163757086	0.736	0.513	1.14391214697815E-06	8	SRGN
**5.9195940643879E-07**	0.924089673692423	0.5	0.314	0.00965544987842311	8	SLA
**1.63930814216882E-06**	0.850021820850484	0.417	0.239	0.0267387551069156	8	HNRNPLL
**1.38283723131765E-06**	0.816164547534966	0.472	0.292	0.0225554580800222	8	FAM118A
**4.87781403938939E-07**	0.782017611358943	0.326	0.16	0.00795620247964803	8	SNRNP70

SupTable5N_C2_TNK_TNKSubtype

p_val	avg_log2FC	pct.1	pct.2	p_val_adj	cluster	gene
0	2.59415074059274	0.646	0.163	0	Naive/Memory T cell	IL7R
0	1.85094852032207	0.81	0.464	0	Naive/Memory T cell	CXCR4
2.834133006571E-164	2.02399158960243	0.317	0.083	4.62275434701796E-160	Naive/Memory T cell	KLRG1
9.80635413442769E-154	1.60328866591224	0.411	0.162	1.5995144228665E-149	Naive/Memory T cell	LTB
5.73340553328008E-124	1.85141966447783	0.307	0.107	9.35175776533313E-120	Naive/Memory T cell	SATB1
6.51494356461451E-124	1.776199296228	0.306	0.104	1.06265244482427E-119	Naive/Memory T cell	TC2N
5.73032566602699E-121	1.3116425309503	0.43	0.209	9.34673419385663E-117	Naive/Memory T cell	AHNAK
9.00367612999445E-106	2.0842743975993	0.21	0.053	1.46858961356339E-101	Naive/Memory T cell	TCF7
2.01257903600055E-97	1.58479953505854	0.285	0.111	3.2827176656205E-93	Naive/Memory T cell	GZMK
1.11422930933217E-95	4.18887491821825	0.117	0.01	1.8174194264517E-91	Naive/Memory T cell	LTK
1.30763848578305E-91	0.963949731749392	0.584	0.419	2.13288913416074E-87	Naive/Memory T cell	ZFP36L2
1.17947526464553E-79	1.59473733438691	0.25	0.1	1.92384210416332E-75	Naive/Memory T cell	ERN 1.00
3.77620365138346E-75	2.10744915306232	0.158	0.041	6.15936577577156E-71	Naive/Memory T cell	MGAT4A
1.6926309097493E-73	1.93537572080017	0.191	0.064	2.76085027689208E-69	Naive/Memory T cell	KLF2
1.7901935682277E-68	0.911410909343015	0.488	0.339	2.9199847291362E-64	Naive/Memory T cell	BTG1
7.91217029831361E-61	1.00905012502242	0.398	0.255	1.29055409735793E-56	Naive/Memory T cell	PDCD4
1.4442662761317E-60	1.52786804078883	0.19	0.072	2.35574272299842E-56	Naive/Memory T cell	RASGRP2
6.03752216770198E-55	1.15747629451007	0.308	0.176	9.8478024077387E-51	Naive/Memory T cell	PIK3R1
8.71220724276446E-55	1.83896396359552	0.152	0.053	1.42104812336731E-50	Naive/Memory T cell	TRAT1
1.72390117922266E-54	1.82308452219521	0.134	0.04	2.81185521343008E-50	Naive/Memory T cell	PATJ
1.17885751486299E-49	1.54712037960098	0.184	0.081	1.92283449249302E-45	Naive/Memory T cell	TTC39C
1.20233854967896E-48	1.65539164040997	0.159	0.063	1.96113440838135E-44	Naive/Memory T cell	TMEM63A
1.73373886210644E-48	0.674733275150184	0.503	0.376	2.82790145798181E-44	Naive/Memory T cell	SYNE2
5.44947842099936E-47	2.13953647737483	0.103	0.027	8.88864425249206E-43	Naive/Memory T cell	ITGA5
1.48554277349728E-43	1.54702950554202	0.145	0.057	2.42306881785141E-39	Naive/Memory T cell	RIPOR2
1.5280020507123E-43	0.825864184504853	0.407	0.282	2.49232414491682E-39	Naive/Memory T cell	CD69
1.43487894886192E-39	1.01095241510644	0.254	0.146	2.34043105348868E-35	Naive/Memory T cell	PARP8
2.25219450228817E-38	1.64369667775431	0.111	0.039	3.67355445268224E-34	Naive/Memory T cell	SORL1
3.33851190759691E-34	0.758105382505508	0.332	0.228	5.44544677248132E-30	Naive/Memory T cell	TXNIP
6.44911733137219E-34	1.10530857050379	0.215	0.122	1.05191552792012E-29	Naive/Memory T cell	TNFRSF25
7.40518115194935E-34	1.43749328855799	0.149	0.071	1.20785909769446E-29	Naive/Memory T cell	APOL3
2.33768678731258E-33	1.38678689360309	0.148	0.071	3.81300091878555E-29	Naive/Memory T cell	TRADD
4.50728615394271E-31	0.648538699716158	0.382	0.276	7.35183444569596E-27	Naive/Memory T cell	STAT4
1.0961675099335E-29	1.69118352603977	0.104	0.042	1.78795882545253E-25	Naive/Memory T cell	LRIG1
1.62420030193747E-29	1.38040785735045	0.147	0.074	2.64923311249021E-25	Naive/Memory T cell	FAM102A
1.24473913463545E-28	0.744659798345964	0.294	0.198	2.03029400250388E-24	Naive/Memory T cell	ATM
1.99514358587737E-27	0.615363556831659	0.396	0.305	3.25427870292457E-23	Naive/Memory T cell	CD44
3.80435516021712E-27	1.23362085772188	0.14	0.071	6.20528370183014E-23	Naive/Memory T cell	OXNAD1
3.73623520712886E-26	0.895674221309545	0.223	0.14	6.09417324634789E-22	Naive/Memory T cell	THEMIS
5.0499362687636E-26	1.38745460361218	0.115	0.053	8.2369510479803E-22	Naive/Memory T cell	DENND11
9.02512177712611E-26	0.914880531105522	0.21	0.129	1.47208761306704E-21	Naive/Memory T cell	SMAD3
9.42444401013081E-26	0.747193420178375	0.453	0.372	1.53722106249244E-21	Naive/Memory T cell	JUNB
8.38308875647783E-25	0.864292788667739	0.244	0.162	1.3673656070691E-20	Naive/Memory T cell	DEPRECATED-ENSG00000059804
1.15666978919123E-24	0.744834432371707	0.301	0.217	1.88664409314982E-20	Naive/Memory T cell	EML4
1.51796778120918E-24	1.39014262922971	0.102	0.045	2.47595724793029E-20	Naive/Memory T cell	PLXND1
5.3856475059726E-24	0.564265565463443	0.423	0.347	8.78452964699191E-20	Naive/Memory T cell	IKZF1
1.47021377220426E-23	1.08881490789605	0.158	0.09	2.39806568384236E-19	Naive/Memory T cell	STK38
1.98013661100538E-23	1.25809141462239	0.108	0.051	3.22980082621088E-19	Naive/Memory T cell	CDC14A
5.17343721660877E-23	0.821815739081221	0.228	0.152	8.43839344401056E-19	Naive/Memory T cell	SLC2A3
6.15244991079045E-23	0.812665089855098	0.266	0.191	1.00352610494903E-18	Naive/Memory T cell	HECA
1.55595716558823E-22	0.678828051990884	0.316	0.238	2.53792173279097E-18	Naive/Memory T cell	CD6
1.58398938344905E-22	0.797628277720932	0.219	0.144	2.58364508334374E-18	Naive/Memory T cell	GIMAP7
1.729053135159E-22	1.13654082146821	0.107	0.051	2.82025856875785E-18	Naive/Memory T cell	MYBL1
3.22180528096616E-21	0.972303718869117	0.141	0.079	5.25508659378391E-17	Naive/Memory T cell	SAMD3
4.01093935998526E-21	1.0196838181391	0.144	0.082	6.54224319007195E-17	Naive/Memory T cell	DEPRECATED-ENSG00000196329
7.18460196816887E-21	0.968208260201954	0.17	0.105	1.17188042702802E-16	Naive/Memory T cell	PER1
7.45010703548146E-21	0.87462368941801	0.218	0.148	1.21518695855738E-16	Naive/Memory T cell	CD28
9.16513016381929E-21	0.598917014605878	0.328	0.248	1.49492438102056E-16	Naive/Memory T cell	RBL2
2.41454769544265E-20	0.569394223282319	0.353	0.276	3.93836874603651E-16	Naive/Memory T cell	VIM
2.54318419825409E-20	0.81836029830333	0.237	0.166	4.14818774577224E-16	Naive/Memory T cell	CD5
1.32977410192613E-18	0.594879037250715	0.478	0.426	2.1689945376517E-14	Naive/Memory T cell	DUSP2
2.63878448232864E-18	0.712700502928016	0.231	0.162	4.30412136912625E-14	Naive/Memory T cell	BCL9L
3.42793976392659E-18	0.859917732595117	0.176	0.113	5.59131254894067E-14	Naive/Memory T cell	GRAP2
3.52540060524235E-18	0.977560286663515	0.133	0.077	5.75028092721079E-14	Naive/Memory T cell	GIMAP5

3.86204897144514E-18	0.818515508940347	0.182	0.119	6.29938807732416E-14	Naive/Memory T cell	TAGAP
6.20403014285032E-18	0.8520907533434	0.165	0.105	1.01193935660032E-13	Naive/Memory T cell	EMB
1.95206656049207E-17	0.926473923523917	0.166	0.107	3.18401576681862E-13	Naive/Memory T cell	SKI
4.99107879672406E-17	1.16451696554589	0.101	0.054	8.14094862533662E-13	Naive/Memory T cell	ADAM19
5.78141986263606E-17	0.73236446338738	0.203	0.139	9.43007393794567E-13	Naive/Memory T cell	PBXIP1
9.63832569097311E-17	0.548417249026766	0.346	0.28	1.57210730345462E-12	Naive/Memory T cell	CDC42SE1
3.69230770016827E-16	0.39188290250706	0.218	0.15	6.02252308974447E-12	Naive/Memory T cell	DNAJB1
4.40223984522681E-16	0.756531350351486	0.13	0.077	7.18049341154945E-12	Naive/Memory T cell	SYNE1
1.95915029066887E-15	0.861235348282972	0.177	0.121	3.19557003911E-11	Naive/Memory T cell	JUN
2.49109136819392E-15	0.79850804071819	0.157	0.102	4.0632191306611E-11	Naive/Memory T cell	RASA3
5.40421130341577E-15	0.841776914187932	0.141	0.089	8.81480905700146E-11	Naive/Memory T cell	ZFYVE28
5.73844945744847E-15	0.344636659371002	0.559	0.516	9.3599849100442E-11	Naive/Memory T cell	TAPBP
1.31506553846387E-14	0.893695725963117	0.149	0.098	2.14500339978843E-10	Naive/Memory T cell	R3HDM4
2.94128673145296E-14	0.868126614083091	0.142	0.092	4.79753278767292E-10	Naive/Memory T cell	SOCS3
5.21531688420164E-14	1.0375703187949	0.103	0.061	8.5067033698213E-10	Naive/Memory T cell	RCAN3
6.08661373426301E-14	1.0106087562646	0.109	0.065	9.9278756619564E-10	Naive/Memory T cell	ITM2C
1.22480676239209E-13	0.752163700534258	0.164	0.112	1.99778231013774E-09	Naive/Memory T cell	DEDD2
1.41452461912011E-13	0.884068298588227	0.152	0.102	2.30723110624681E-09	Naive/Memory T cell	CLDND1
1.81988073616858E-13	0.462686374855577	0.38	0.328	2.96840746876456E-09	Naive/Memory T cell	EEF2
2.24728494436572E-13	0.570569901205936	0.277	0.22	3.66554647275492E-09	Naive/Memory T cell	CD96
3.3648966473114E-13	0.948224294154166	0.106	0.064	5.48848292142963E-09	Naive/Memory T cell	GIMAP1
5.4772071500733E-13	0.850914703271457	0.121	0.077	8.93387258248456E-09	Naive/Memory T cell	NCK2
8.16775568395029E-13	0.568277689624263	0.247	0.191	1.33224262960913E-08	Naive/Memory T cell	NOP53
8.37337139108845E-13	0.638939694327413	0.228	0.175	1.36578060760044E-08	Naive/Memory T cell	IL16
1.03122547278644E-12	0.378400638140654	0.426	0.369	1.68203186866197E-08	Naive/Memory T cell	SPOCK2
1.03256098691734E-12	0.566951887984137	0.281	0.227	1.68421022576087E-08	Naive/Memory T cell	GSTK1
3.16609639685479E-12	0.591867295372164	0.21	0.157	5.16421983290985E-08	Naive/Memory T cell	AAK1
4.07894977971203E-12	0.877787111964094	0.108	0.067	6.6531749856883E-08	Naive/Memory T cell	ZNF276
1.11557796332607E-11	0.492145061801793	0.271	0.217	1.81961921598115E-07	Naive/Memory T cell	CDC42SE2
1.52333039513194E-11	0.625440577944113	0.209	0.16	2.4847042074997E-07	Naive/Memory T cell	CNN2
2.50704997611843E-11	0.780150915386034	0.107	0.068	4.08924921604678E-07	Naive/Memory T cell	DYRK2
2.94898137457159E-11	0.805466207010874	0.151	0.107	4.81008352006372E-07	Naive/Memory T cell	NEK7
3.21251728109338E-11	0.522903109405458	0.242	0.189	5.23993693719141E-07	Naive/Memory T cell	USP36
3.67695269086731E-11	0.614436668443822	0.197	0.148	5.99747753407367E-07	Naive/Memory T cell	NELL2
5.23110827430756E-11	0.476559168317696	0.296	0.246	8.53246070622307E-07	Naive/Memory T cell	DGKA
5.46650653079513E-11	0.49635642149313	0.22	0.169	8.91641880237994E-07	Naive/Memory T cell	SYTL1
7.38253684080374E-11	0.83774033035325	0.107	0.069	1.2041655841035E-06	Naive/Memory T cell	LIX1L
9.99794829327564E-11	0.575571236042801	0.165	0.118	1.63076534611619E-06	Naive/Memory T cell	ANO9
1.56103522077594E-10	0.572926916623204	0.179	0.132	2.54620454860764E-06	Naive/Memory T cell	YPEL5
2.84235074837303E-10	0.910719178793728	0.1	0.064	4.63615830567125E-06	Naive/Memory T cell	DEPRECATED-ENSG00000183943
3.76384982286535E-10	0.710886753246353	0.154	0.112	6.13921544607567E-06	Naive/Memory T cell	EEF1G
5.46267115615151E-10	0.588595069661385	0.106	0.068	8.91016292279873E-06	Naive/Memory T cell	CD300A
5.47673273270832E-10	0.71897330783872	0.13	0.09	8.93309876032054E-06	Naive/Memory T cell	RNF157
5.51480687847444E-10	0.461947779297378	0.269	0.22	8.99520149947965E-06	Naive/Memory T cell	STK17A
5.82269447010637E-10	0.544251976809091	0.247	0.201	9.49739695019049E-06	Naive/Memory T cell	GIMAP4
8.25532064699531E-10	0.786090325685072	0.112	0.075	1.34652535073141E-05	Naive/Memory T cell	STOM
1.13993873816114E-09	0.716072474710275	0.142	0.101	1.85935407581464E-05	Naive/Memory T cell	FOXP1
2.5904732050556E-09	0.636473030608048	0.16	0.118	4.22532084476618E-05	Naive/Memory T cell	PHF1
4.6819185834676E-09	0.727545804522131	0.112	0.076	7.636677401494E-05	Naive/Memory T cell	FKBP11
6.43626474238007E-09	0.708945544058342	0.117	0.081	0.000104981914212961	Naive/Memory T cell	CERK
6.82637901120479E-09	0.55954506416489	0.151	0.11	0.000111345068051761	Naive/Memory T cell	LTB4R
8.52688455317908E-09	0.705976025266939	0.129	0.092	0.000139082013946904	Naive/Memory T cell	HERC3
1.06791591275869E-08	0.563715235503669	0.138	0.099	0.00017418776453007	Naive/Memory T cell	OBSCN
1.07479039245437E-08	0.633714615461832	0.137	0.099	0.000175309060913233	Naive/Memory T cell	DPYD
1.19018611914566E-08	0.537261704814045	0.216	0.176	0.000194131257893848	Naive/Memory T cell	GPSM3
1.43282571960429E-08	0.465569979673803	0.271	0.23	0.000233708203124656	Naive/Memory T cell	PNRC1
1.4527885623728E-08	0.442360341085181	0.359	0.322	0.000236964342408628	Naive/Memory T cell	STAT1
1.50848991731557E-08	0.839130703373841	0.111	0.078	0.000246049790413342	Naive/Memory T cell	EMP3
1.66039003455486E-08	0.714815131076135	0.11	0.077	0.000270826218536244	Naive/Memory T cell	ADAT1
5.23494612460278E-08	0.605591423033067	0.14	0.103	0.000853872062383959	Naive/Memory T cell	EIF4E3
5.76448333946464E-08	0.416001148728102	0.308	0.274	0.000940244877500078	Naive/Memory T cell	ARHGDIB
7.21823953536188E-08	0.47942928344766	0.22	0.18	0.00117736705061288	Naive/Memory T cell	RESF1
1.07984367757057E-07	0.303176857996882	0.421	0.387	0.00176133302248535	Naive/Memory T cell	STK4
1.30232373745073E-07	0.432323750407353	0.226	0.186	0.00212422024815588	Naive/Memory T cell	PIK3IP1
1.30475398061005E-07	0.606630194174675	0.193	0.156	0.00212818421777306	Naive/Memory T cell	RORA
1.30711774464034E-07	0.388103810113049	0.285	0.244	0.00213203975328286	Naive/Memory T cell	PPP2R5C

1.36152356430355E-07	0.512277152071189	0.21	0.172	0.00222078108573553	Naive/Memory T cell	LEPROTL1
1.77931446395598E-07	0.442865236057929	0.28	0.243	0.0029022398221586	Naive/Memory T cell	LITAF
3.02682427936106E-07	0.779882975704139	0.103	0.074	0.00493705308206583	Naive/Memory T cell	GIMAP2
3.43142187184134E-07	0.633073484645306	0.117	0.086	0.0055969922151604	Naive/Memory T cell	GZMM
5.77524530165147E-07	0.415714607798484	0.277	0.242	0.00942000261152371	Naive/Memory T cell	BCL11B
6.05700905906484E-07	0.637569641786855	0.103	0.074	0.00987958747624066	Naive/Memory T cell	ZNF101
6.09687703370996E-07	0.461091261305245	0.385	0.359	0.00994461612968431	Naive/Memory T cell	TNFAIP3
6.3130632226776E-07	0.597343696239996	0.173	0.139	0.0102972374225094	Naive/Memory T cell	ITM2B
6.38150953539515E-07	0.626177309158352	0.124	0.093	0.010408880203183	Naive/Memory T cell	PCNX1
7.91535531523319E-07	0.26756598461722	0.455	0.431	0.0129107360546769	Naive/Memory T cell	ACAP1
8.28296411666729E-07	0.589316730066937	0.122	0.091	0.013510342770696	Naive/Memory T cell	KDM4C
1.13451352329785E-06	0.607780810909748	0.125	0.094	0.0185050500785112	Naive/Memory T cell	SP140L
1.13986933986184E-06	0.437290286341411	0.239	0.204	0.0185924088024865	Naive/Memory T cell	ZC3HAV1
1.30017003800712E-06	0.701085121415523	0.101	0.073	0.0212070734899341	Naive/Memory T cell	BIN2
1.8359616400479E-06	0.532951664210076	0.216	0.186	0.0299463703108212	Naive/Memory T cell	FXYD5
1.90027245563295E-06	0.374205712144251	0.289	0.256	0.0309953440238291	Naive/Memory T cell	NLRP1
2.32525481973089E-06	0.709228904585277	0.101	0.074	0.0379272313646305	Naive/Memory T cell	MFNG
0	3.61133664789886	0.631	0.087	0	CD8+ Teff/Tex	HAVCR2
0	2.10558286568072	0.805	0.295	0	CD8+ Teff/Tex	RGS1
0	1.76926353105869	0.803	0.314	0	CD8+ Teff/Tex	CD8A
0	2.1636342561532	0.566	0.177	0	CD8+ Teff/Tex	METRNL
0	2.35111711582071	0.516	0.141	0	CD8+ Teff/Tex	LAG3
9.11717120251333E-285	2.72651074915583	0.371	0.064	1.48710179484195E-280	CD8+ Teff/Tex	ADGRG1
5.55116366560857E-270	2.73004646885348	0.384	0.079	9.05450305497414E-266	CD8+ Teff/Tex	PDCD1
4.68291301006045E-200	2.12591971401783	0.375	0.102	7.6382994107096E-196	CD8+ Teff/Tex	KLRC4
3.59036572780809E-179	1.37543439334027	0.564	0.255	5.85624553862778E-175	CD8+ Teff/Tex	NKG7
8.42044657118331E-174	2.56227149372083	0.277	0.06	1.37345904022571E-169	CD8+ Teff/Tex	TNFRSF9
1.18851607251648E-170	4.28795370581364	0.161	0.011	1.93858856588163E-166	CD8+ Teff/Tex	ITGAD
6.04594900094449E-167	1.4057111347828	0.488	0.195	9.86154741544055E-163	CD8+ Teff/Tex	TIGIT
6.82360856538045E-159	1.31081045972501	0.529	0.233	1.11299879309921E-154	CD8+ Teff/Tex	KLRK1
2.8548787490314E-158	1.67762163574434	0.419	0.157	4.65659272754512E-154	CD8+ Teff/Tex	LYST
9.77653825480265E-158	2.5654329016233	0.249	0.053	1.59465115474086E-153	CD8+ Teff/Tex	PTMS
2.08958204842104E-148	2.02550958687504	0.306	0.088	3.40831727917955E-144	CD8+ Teff/Tex	WHRN
1.65609412202044E-144	3.41312911972286	0.172	0.022	2.70125512242753E-140	CD8+ Teff/Tex	AKAP5
1.22523462664456E-120	1.26178947617257	0.522	0.279	1.99848019951995E-116	CD8+ Teff/Tex	CCL5
1.81818586695861E-110	1.5876513632297	0.335	0.131	2.96564296759618E-106	CD8+ Teff/Tex	NR4A2
1.20878997861466E-107	2.18353888773405	0.207	0.053	1.97165733411837E-103	CD8+ Teff/Tex	CD38
1.23960241173525E-106	2.25870901980454	0.204	0.052	2.02191549378136E-102	CD8+ Teff/Tex	BCL2L11
2.82005736425322E-103	0.861946956457079	0.67	0.463	4.59979556683342E-99	CD8+ Teff/Tex	TRBC2
1.56252953630903E-102	2.704705065591	0.156	0.03	2.54864192667365E-98	CD8+ Teff/Tex	AHI1
3.81311117587377E-99	3.12104325027611	0.129	0.019	6.2195656389677E-95	CD8+ Teff/Tex	ETV1
3.02268669065844E-98	3.2429444416635	0.131	0.021	4.93030426113297E-94	CD8+ Teff/Tex	CEBPB
3.9542410509186E-98	2.40688637879752	0.168	0.037	6.44976257815332E-94	CD8+ Teff/Tex	CRIM1
4.50120164403511E-97	2.45369360860594	0.165	0.036	7.34191000158566E-93	CD8+ Teff/Tex	SARDH
5.68876731504384E-96	1.54613045443526	0.297	0.115	9.27894836756801E-92	CD8+ Teff/Tex	CXCR6
8.7626599896692E-94	2.64209348386728	0.145	0.028	1.42927747091494E-89	CD8+ Teff/Tex	LAYN
4.57537135416915E-82	0.912759594988135	0.55	0.342	7.4628882157853E-78	CD8+ Teff/Tex	ITGA4
6.61537566048571E-79	0.998464025057486	0.478	0.279	1.07903392398182E-74	CD8+ Teff/Tex	ARHGAP9
1.52512575808102E-76	1.07275134709881	0.41	0.219	2.48763262400596E-72	CD8+ Teff/Tex	JAML
5.6222352810024E-76	2.13289013814821	0.165	0.047	9.17042796684301E-72	CD8+ Teff/Tex	DGKH
6.03697351363471E-74	1.41747526278716	0.281	0.122	9.84690749808958E-70	CD8+ Teff/Tex	CEMIP2
1.09819709572474E-73	1.21755314679754	0.349	0.175	1.79126928283662E-69	CD8+ Teff/Tex	PTPN22
3.35978244259202E-73	2.74805003564736	0.113	0.022	5.48014114211184E-69	CD8+ Teff/Tex	RDH10
9.70643101150195E-73	1.24974518579869	0.327	0.158	1.58321596228608E-68	CD8+ Teff/Tex	CREM
1.18874806117678E-70	2.49549282421816	0.117	0.024	1.93896696258545E-66	CD8+ Teff/Tex	GFOD1
1.19485102439686E-70	0.99298803910725	0.411	0.225	1.94892150589371E-66	CD8+ Teff/Tex	PRF1
5.71479964692175E-68	1.04746097256965	0.401	0.226	9.32140970409407E-64	CD8+ Teff/Tex	CBLB
6.3253231128108E-67	1.1580889421065	0.357	0.191	1.03172345293057E-62	CD8+ Teff/Tex	PRDM1
5.00336654034473E-66	1.79083972913244	0.179	0.061	8.1609911639563E-62	CD8+ Teff/Tex	PHLDA1
2.69392471844498E-64	2.3319808598865	0.129	0.034	4.3940606082556E-60	CD8+ Teff/Tex	RGS2
7.66433307528017E-64	1.95017535026233	0.125	0.031	1.25012936790895E-59	CD8+ Teff/Tex	NCR1
5.69683697208817E-63	2.47773912601087	0.103	0.021	9.29211078517302E-59	CD8+ Teff/Tex	VCAM1
3.3872629821697E-62	0.7451814340908	0.599	0.419	5.52496465021699E-58	CD8+ Teff/Tex	CLEC2D
3.59755456544555E-62	1.47784731083977	0.203	0.078	5.86797125169823E-58	CD8+ Teff/Tex	CD27
4.31436042252188E-62	1.20076391738522	0.294	0.144	7.03715328517544E-58	CD8+ Teff/Tex	CD7
6.16011575618141E-61	1.79655365573986	0.169	0.058	1.00477648099075E-56	CD8+ Teff/Tex	ENTPD1

6.23374083795676E-61	2.12289216941285	0.126	0.033	1.01678546807913E-56	CD8+ Teff/Tex	TTYH3
1.5331473103133E-60	1.22100744174192	0.267	0.124	2.50071657785202E-56	CD8+ Teff/Tex	RIN3
2.30445235289769E-60	1.48325947325303	0.204	0.081	3.75879223281142E-56	CD8+ Teff/Tex	NAB1
2.28868870808675E-59	1.55359144876013	0.193	0.074	3.7330801517603E-55	CD8+ Teff/Tex	DEPRECATED-ENSG00000115738
6.97739527049582E-57	0.760687106286262	0.56	0.413	1.13808294257057E-52	CD8+ Teff/Tex	LSP1
8.18803897176788E-57	1.09869535673215	0.363	0.209	1.33555103668506E-52	CD8+ Teff/Tex	ZFP36
6.12215296852384E-55	2.14380246802286	0.115	0.031	9.98584370695924E-51	CD8+ Teff/Tex	TTN
1.29234661557739E-54	1.8760182609956	0.128	0.038	2.10794656466828E-50	CD8+ Teff/Tex	CCDC141
1.91839517289142E-54	1.12024508711137	0.298	0.155	3.12909436650319E-50	CD8+ Teff/Tex	ABCA2
2.10708501499578E-54	0.69972725352201	0.621	0.474	3.43686636795961E-50	CD8+ Teff/Tex	SRGN
2.31482090796338E-54	0.939459184019154	0.391	0.234	3.77570438297907E-50	CD8+ Teff/Tex	IKZF3
7.98303618308674E-54	1.07126948624612	0.297	0.154	1.30211303182328E-49	CD8+ Teff/Tex	ITM2A
6.76837839522216E-53	0.930810343115083	0.426	0.273	1.10399020004469E-48	CD8+ Teff/Tex	SLA
1.84096581408648E-52	1.88083569602033	0.125	0.037	3.00279933935647E-48	CD8+ Teff/Tex	SSH1
3.85306497909734E-52	1.92408576149687	0.127	0.039	6.28473428740566E-48	CD8+ Teff/Tex	FAM3C
5.64809483714867E-48	0.835673992311127	0.425	0.272	9.21260748887319E-44	CD8+ Teff/Tex	CNOT6L
1.06816792166063E-47	2.02350842915528	0.114	0.034	1.74228869702066E-43	CD8+ Teff/Tex	SNAP47
3.44692588494124E-47	1.09462220835746	0.281	0.15	5.62228081092766E-43	CD8+ Teff/Tex	BICDL1
3.50898579931627E-47	1.14166378503919	0.215	0.099	5.72350673726477E-43	CD8+ Teff/Tex	DUSP4
1.66017542595833E-46	1.368119711254	0.17	0.07	2.70791213728064E-42	CD8+ Teff/Tex	SIRPG
3.18585098617711E-46	1.46615226864149	0.174	0.073	5.19644154355349E-42	CD8+ Teff/Tex	ID2
7.85534686165467E-46	1.03205247315719	0.247	0.123	1.28128562660449E-41	CD8+ Teff/Tex	CHST12
1.28489749294493E-45	1.35960329428922	0.176	0.074	2.09579630074247E-41	CD8+ Teff/Tex	ZEB2
6.04301378496278E-45	1.35025128494974	0.16	0.064	9.85675978465279E-41	CD8+ Teff/Tex	CTLA4
1.83849666708224E-44	0.863729748156967	0.378	0.236	2.99877191367784E-40	CD8+ Teff/Tex	RNF19A
4.52310924336834E-44	1.51460415528818	0.143	0.054	7.3776434868581E-40	CD8+ Teff/Tex	SNX9
2.1396671960079E-43	1.28924796354943	0.248	0.131	3.49001116340849E-39	CD8+ Teff/Tex	SAT1
3.97655705249929E-41	0.986654760576963	0.254	0.135	6.48616220833159E-37	CD8+ Teff/Tex	SYTL3
5.51896934759664E-41	1.1772347478619	0.18	0.08	9.00199090286488E-37	CD8+ Teff/Tex	DUSP5
1.04193330974415E-40	1.09779528284722	0.195	0.091	1.69949742152368E-36	CD8+ Teff/Tex	KLRD1
4.76385558547532E-40	1.07421713598009	0.209	0.102	7.77032484546879E-36	CD8+ Teff/Tex	CD84
7.9783766637291E-39	1.75046293908149	0.105	0.035	1.30135301762085E-34	CD8+ Teff/Tex	TOX
3.02526171476912E-38	0.980633253494721	0.26	0.145	4.93450438295991E-34	CD8+ Teff/Tex	VPS37B
1.64270334318131E-37	0.99537119243753	0.257	0.144	2.67941342306303E-33	CD8+ Teff/Tex	TRAF5
2.36642717477087E-36	0.887431265929259	0.283	0.167	3.85987936476876E-32	CD8+ Teff/Tex	RAB27A
3.2453430280918E-36	1.12997873104888	0.185	0.089	5.29347901312054E-32	CD8+ Teff/Tex	NOTCH1
3.59424940034255E-36	1.06063737464618	0.227	0.121	5.86258019689873E-32	CD8+ Teff/Tex	SFMBT2
7.77903835471084E-36	0.745644269821769	0.335	0.21	1.26883894603689E-31	CD8+ Teff/Tex	TNFRSF1B
2.82503855496305E-34	0.96564781126429	0.237	0.131	4.60792038700023E-30	CD8+ Teff/Tex	SAMSN1
3.32664510456242E-33	0.697022199387121	0.376	0.251	5.42609083005177E-29	CD8+ Teff/Tex	GZMA
4.86915315557519E-33	0.542154415859203	0.508	0.377	7.94207571205869E-29	CD8+ Teff/Tex	ETS1
7.22188000468372E-33	1.10224139371965	0.179	0.089	1.17796084756396E-28	CD8+ Teff/Tex	IGFLR1
1.0418516126944E-32	0.829039586523308	0.29	0.176	1.69936416546584E-28	CD8+ Teff/Tex	CCND2
2.6220940820848E-31	0.653202785849079	0.41	0.289	4.27689765728852E-27	CD8+ Teff/Tex	WNK1
2.82542971191853E-30	1.14670654444854	0.172	0.087	4.60855840311031E-26	CD8+ Teff/Tex	SLA2
2.6659557163283E-28	1.48302613725711	0.104	0.042	4.3484403689031E-24	CD8+ Teff/Tex	IRF4
1.28783515368453E-27	1.16599213724002	0.126	0.057	2.10058791917484E-23	CD8+ Teff/Tex	D2HGDH
1.05348416831722E-26	0.945218060095744	0.21	0.122	1.71833802694221E-22	CD8+ Teff/Tex	ATP8A1
3.18727777149342E-26	0.534595429017023	0.485	0.376	5.19876877308292E-22	CD8+ Teff/Tex	ITGAL
3.91819494724964E-25	1.2575567367136	0.113	0.051	6.39096777845 89E-21	CD8+ Teff/Tex	PAM
4.80122720852553E-24	0.609512446305965	0.316	0.214	7.83128169982599E-20	CD8+ Teff/Tex	AKAP13
2.33830261988326E-23	1.10202572527328	0.11	0.051	3.81400540329159E-19	CD8+ Teff/Tex	FASLG
9.48193011963797E-23	0.992759858571072	0.128	0.063	1.54659762181415E-18	CD8+ Teff/Tex	P2RY11
9.6965023013278E-23	1.01092584525942	0.159	0.088	1.58159649036958E-18	CD8+ Teff/Tex	ZNF331
1.23662565133332E-22	1.07084850279197	0.123	0.06	2.01706009988977E-18	CD8+ Teff/Tex	ADCY3
3.92074288963927E-22	1.16637529264408	0.115	0.056	6.39512372729062E-18	CD8+ Teff/Tex	SLF1
4.24383708967979E-21	0.740536625253608	0.203	0.123	6.9221226769767E-17	CD8+ Teff/Tex	CCR5
5.33241289966813E-21	0.619620187278394	0.289	0.197	8.69769868064868E-17	CD8+ Teff/Tex	SPTAN1
1.40453009081035E-20	0.950937155927836	0.119	0.06	2.29092903112077E-16	CD8+ Teff/Tex	GZMH
1.48906744381588E-20	0.798588167353073	0.201	0.124	2.42881790760808E-16	CD8+ Teff/Tex	PCED1B
1.97244733025173E-20	0.662971038089489	0.281	0.193	3.2172588403736E-16	CD8+ Teff/Tex	ZFP36L1
2.674805291781E-20	0.93731091818634	0.169	0.099	4.36287491142398E-16	CD8+ Teff/Tex	ATXN1
3.51993106845274E-20	0.38829621129069	0.441	0.336	5.74135956575327E-16	CD8+ Teff/Tex	MAF
4.10252587716278E-20	0.670129039952964	0.116	0.058	6.69162995824021E-16	CD8+ Teff/Tex	CCL4
4.58708231219608E-20	0.626862704283669	0.262	0.176	7.48198995942303E-16	CD8+ Teff/Tex	ACTN4
5.09345491176629E-20	0.701702658500353	0.22	0.14	8.307934306582E-16	CD8+ Teff/Tex	DUSP16

6.39000275312995E-20	0.808381935627731	0.16	0.091	1.04227334906303E-15	CD8+ Teff/Tex	FAM53B
7.0944646235741E-20	1.06869577633903	0.118	0.06	1.15717812475117E-15	CD8+ Teff/Tex	SLC7A5
1.40275418741932E-19	1.08386076847711	0.112	0.056	2.28803235509966E-15	CD8+ Teff/Tex	ASXL2
4.57954230453679E-19	0.647550513744691	0.249	0.166	7.46969145292995E-15	CD8+ Teff/Tex	PRKCH
5.73216348609341E-19	1.03229622508344	0.125	0.067	9.34973186216697E-15	CD8+ Teff/Tex	ACOT9
9.4834498243598E-19	0.319543014578967	0.349	0.253	1.54684550085133E-14	CD8+ Teff/Tex	IL2RB
1.81116703331251E-18	1.09440178440887	0.101	0.05	2.95419454803603E-14	CD8+ Teff/Tex	GMDS
2.12181824933241E-18	0.718148093885377	0.196	0.123	3.46089774648609E-14	CD8+ Teff/Tex	ABCC1
1.54007517413283E-17	0.846461205839686	0.134	0.075	2.51201661652806E-13	CD8+ Teff/Tex	NSMF
3.94917843478368E-17	0.559814049997737	0.267	0.186	6.44150494497565E-13	CD8+ Teff/Tex	MSN
5.13616000387982E-16	0.758319790221438	0.156	0.095	8.37759058232837E-12	CD8+ Teff/Tex	SUSD6
5.77313075244894E-16	0.901545169322425	0.125	0.072	9.41655357031947E-12	CD8+ Teff/Tex	PTPRJ
6.84276554028442E-16	0.407459661158446	0.454	0.365	1.11612348727579E-11	CD8+ Teff/Tex	JUND
1.54446548607813E-15	0.262848403507471	0.756	0.702	2.51917765434204E-11	CD8+ Teff/Tex	CD74
2.42503735868311E-15	0.697825423224295	0.175	0.112	3.95547843574802E-11	CD8+ Teff/Tex	S100PBP
4.06111980384874E-15	0.737901290446329	0.15	0.091	6.62409251205767E-11	CD8+ Teff/Tex	ALOX5AP
4.27482977290705E-15	0.929091093234193	0.106	0.058	6.97267484258868E-11	CD8+ Teff/Tex	HELLS
5.27703420461478E-15	0.586480091238288	0.201	0.134	8.60737049114717E-11	CD8+ Teff/Tex	PFKFB3
7.1576527867747E-15	0.762282340191483	0.174	0.113	1.16748474605082E-10	CD8+ Teff/Tex	RASSF1
1.88053916954324E-14	0.66302254562932	0.139	0.084	3.06734743944198E-10	CD8+ Teff/Tex	DTHD1
2.84637997759849E-14	0.442718064589992	0.335	0.254	4.6427303814609E-10	CD8+ Teff/Tex	CCDC88B
4.30137674480202E-14	0.312223961799077	0.527	0.438	7.01597560844657E-10	CD8+ Teff/Tex	TRAC
4.91412371591533E-14	0.748356019754622	0.136	0.082	8.0154271930295E-10	CD8+ Teff/Tex	DNMT3A
5.38281604699867E-14	0.595547883318515	0.212	0.146	8.77991125425953E-10	CD8+ Teff/Tex	CD3D
5.64027793671618E-14	0.909563162930397	0.112	0.064	9.19985734257776E-10	CD8+ Teff/Tex	FNDC3B
5.87768843468818E-14	0.63607733912916	0.191	0.129	9.58709760581989E-10	CD8+ Teff/Tex	ABHD17A
6.82006893666051E-14	0.824744539222495	0.111	0.063	1.1124214442587E-09	CD8+ Teff/Tex	OSBPL3
7.99970003204836E-14	0.811393898534241	0.263	0.199	1.30483107222741E-09	CD8+ Teff/Tex	PGGHG
8.09699371570978E-14	0.672526625014934	0.211	0.149	1.32070064496942E-09	CD8+ Teff/Tex	IVNS1ABP
9.26581347111792E-14	0.672378663046539	0.185	0.124	1.51134683527404E-09	CD8+ Teff/Tex	GGA2
1.10509892337694E-13	0.619693740985233	0.185	0.123	1.80252685392013E-09	CD8+ Teff/Tex	EID1
1.23571891888556E-13	0.723670293276924	0.146	0.091	2.01558112859423E-09	CD8+ Teff/Tex	PLEKHA2
2.20982250167804E-13	0.290845535889671	0.582	0.504	3.60444148248705E-09	CD8+ Teff/Tex	MCL1
2.66657979029553E-13	0.6719448309136	0.146	0.093	4.34945829595104E-09	CD8+ Teff/Tex	ZBP1
3.72951019781145E-13	0.668749105626328	0.2	0.139	6.08320408365025E-09	CD8+ Teff/Tex	NAP1L4
4.40093282495463E-13	0.714135694668979	0.188	0.13	7.1783615307835E-09	CD8+ Teff/Tex	LASP1
5.17125403773823E-13	0.487881410532002	0.206	0.142	8.43483246095482E-09	CD8+ Teff/Tex	CTSC
5.19477795305736E-13	0.605782120246199	0.205	0.143	8.47320231923186E-09	CD8+ Teff/Tex	INPP5D
5.84558587234292E-13	0.746091490284601	0.115	0.068	9.53473511637854E-09	CD8+ Teff/Tex	TRIM8
9.7413476345901E-13	0.843640854646874	0.121	0.073	1.58891121267799E-08	CD8+ Teff/Tex	MCM6
1.11463732364322E-12	0.599125540836282	0.17	0.114	1.81808493859446E-08	CD8+ Teff/Tex	ST8SIA1
1.14132869030643E-12	0.269757190964269	0.653	0.58	1.86162122675881E-08	CD8+ Teff/Tex	EVL
1.28236494495466E-12	0.906868129003403	0.112	0.067	2.09166546171555E-08	CD8+ Teff/Tex	MAPRE2
2.01194501077535E-12	0.529085045286772	0.222	0.159	3.28168350707567E-08	CD8+ Teff/Tex	FOSL2
2.74079385909265E-12	0.460487165681334	0.244	0.176	4.47050886356603E-08	CD8+ Teff/Tex	ARID5B
2.77255869396365E-12	0.65605100479218	0.14	0.089	4.52232048572411E-08	CD8+ Teff/Tex	MCTP2
4.11206730513342E-12	0.377529980562439	0.384	0.309	6.70719298140311E-08	CD8+ Teff/Tex	ITGB2
4.26228416606291E-12	0.792667803147963	0.106	0.063	6.95221170326521E-08	CD8+ Teff/Tex	GALM
5.0196456085976E-12	0.787735868340066	0.119	0.073	8.18754395218354E-08	CD8+ Teff/Tex	VAMP1
6.0286990922429E-12	0.540592289342254	0.202	0.141	9.8334110893574E-08	CD8+ Teff/Tex	ITGB7
6.67467355948456E-12	0.41723727801275	0.274	0.207	1.08870600428753E-07	CD8+ Teff/Tex	TCIRG1
9.69867415452923E-12	0.906502019068045	0.104	0.062	1.58195074134526E-07	CD8+ Teff/Tex	HS3ST3B1
1.06647724776436E-11	0.298732688312529	0.504	0.434	1.73953103882844E-07	CD8+ Teff/Tex	OAZ1
1.20972003374454E-11	0.537148935937932	0.21	0.151	1.97317434704071E-07	CD8+ Teff/Tex	MYO9B
1.36973404382125E-11	0.463774157944494	0.309	0.244	2.23417319887685E-07	CD8+ Teff/Tex	PLAAT4
1.66211909449889E-11	0.583095203357213	0.157	0.105	2.71108245503714E-07	CD8+ Teff/Tex	IGF2R
1.71764049032028E-11	0.365581493542906	0.312	0.241	2.80164340376141E-07	CD8+ Teff/Tex	CST7
3.64786164014179E-11	0.447619969493104	0.267	0.201	5.95002712123527E-07	CD8+ Teff/Tex	SH2D2A
3.87913405088827E-11	0.749197029844784	0.118	0.074	6.32725555040386E-07	CD8+ Teff/Tex	TSPYL2
4.43020996844481E-11	0.411084753743329	0.35	0.281	7.22611547953033E-07	CD8+ Teff/Tex	PYHIN1
6.02011601162294E-11	0.647548584259804	0.153	0.104	9.81941122655818E-07	CD8+ Teff/Tex	RAPGEF1
8.22537535319485E-11	0.385102127549438	0.353	0.283	1.34164097385961E-06	CD8+ Teff/Tex	PTPRC
1.08980236297774E-10	0.701267828767227	0.124	0.08	1.77757663425299E-06	CD8+ Teff/Tex	MAP3K8
1.42960741959636E-10	0.403787073718948	0.294	0.23	2.33183266210362E-06	CD8+ Teff/Tex	IFI16
2.06079140486802E-10	0.617152777799679	0.162	0.113	3.36135686048022E-06	CD8+ Teff/Tex	LRBA
2.25764232670258E-10	0.673636413412484	0.129	0.085	3.68244039908458E-06	CD8+ Teff/Tex	NELFCD

2.6178180461061E-10	0.66282107663286	0.128	0.084	4.26992301500366E-06	CD8+ Teff/Tex	RALGDS
2.7808096717422E-10	0.660203425607351	0.119	0.076	4.53577865557871E-06	CD8+ Teff/Tex	ABI3
4.64767457344267E-10	0.489241322677523	0.179	0.126	7.58082199674233E-06	CD8+ Teff/Tex	SLFN12L
5.58653952667891E-10	0.265260017395589	0.569	0.517	9.11220462196597E-06	CD8+ Teff/Tex	ACTB
6.99430957177798E-10	0.56659790280412	0.167	0.119	1.14084183425271E-05	CD8+ Teff/Tex	TMEM140
8.36841202383927E-10	0.588570483367282	0.16	0.111	1.36497168520842E-05	CD8+ Teff/Tex	COTL1
1.19644419780928E-09	0.29804529619473	0.455	0.387	1.95152013104672E-05	CD8+ Teff/Tex	CELF2
1.326592449477E-09	0.415094715579669	0.312	0.252	2.16380494434194E-05	CD8+ Teff/Tex	EHBP1L1
1.40169028630493E-09	0.631239273218319	0.144	0.099	2.28629702599197E-05	CD8+ Teff/Tex	PHYKPL
1.66032532515643E-09	0.392171318700612	0.35	0.288	2.70815663786266E-05	CD8+ Teff/Tex	ADGRE5
1.85895257849318E-09	0.594263210699664	0.144	0.099	3.03213755078023E-05	CD8+ Teff/Tex	PREX1
2.23034307099433E-09	0.545415774849921	0.133	0.09	3.63791258309885E-05	CD8+ Teff/Tex	ADAT2
3.4901264721248E-09	0.278998874855605	0.423	0.354	5.69274528868276E-05	CD8+ Teff/Tex	ZAP70
3.58321886194418E-09	0.281079111826832	0.536	0.472	5.84458828571716E-05	CD8+ Teff/Tex	ARGLU1
3.92330669198825E-09	0.476061074560636	0.181	0.132	6.39930554530203E-05	CD8+ Teff/Tex	RNF166
4.17883066840531E-09	0.507289885165707	0.151	0.105	6.8160907032359E-05	CD8+ Teff/Tex	CD3G
4.32208374809784E-09	0.433030920022965	0.255	0.2	7.04975080152239E-05	CD8+ Teff/Tex	MBD2
4.7266431158665E-09	0.450768429237641	0.245	0.191	7.70962758628985E-05	CD8+ Teff/Tex	BCL2
1.81883000133271E-08	0.590028011315901	0.145	0.103	0.000296669361517379	CD8+ Teff/Tex	RBM38
1.95476081582714E-08	0.658827692301792	0.141	0.1	0.000318841036669565	CD8+ Teff/Tex	TP53INP1
1.96721853584838E-08	0.46750845824925	0.189	0.142	0.000320873015382229	CD8+ Teff/Tex	MEF2D
2.13329968836779E-08	0.451425167961277	0.195	0.147	0.00034796251216967	CD8+ Teff/Tex	PPP1R16B
3.16171792418115E-08	0.593049176408758	0.115	0.078	0.000515707810613187	CD8+ Teff/Tex	IL6ST
3.48719832377406E-08	0.536269220232141	0.146	0.104	0.000568796918590788	CD8+ Teff/Tex	SYTL2
3.6508422521137E-08	0.465474291429879	0.166	0.122	0.000595488879742266	CD8+ Teff/Tex	HAPLN3
5.50722769087955E-08	0.421220237915843	0.24	0.189	0.000898283908659363	CD8+ Teff/Tex	GBP2
6.02457809587179E-08	0.583264461793854	0.156	0.115	0.000982668933217648	CD8+ Teff/Tex	PABPC1L
7.81154952821773E-08	0.437699465828755	0.161	0.118	0.00127414184354759	CD8+ Teff/Tex	NIN
8.4002658948195E-08	0.434639915865967	0.104	0.069	0.00137016737010401	CD8+ Teff/Tex	PPM1M
8.75062283534765E-08	0.385791100265341	0.223	0.172	0.00142731409067356	CD8+ Teff/Tex	CLEC2B
9.2910838122215E-08	0.309288431872185	0.424	0.37	0.00151546868061145	CD8+ Teff/Tex	TAP1
1.62733268925148E-07	0.546973602396112	0.118	0.082	0.0026543423494381	CD8+ Teff/Tex	IFI6
1.80553301409509E-07	0.598514810642519	0.109	0.074	0.0029450048992905	CD8+ Teff/Tex	ACSL5
2.03248782456373E-07	0.565728509370409	0.114	0.078	0.00331519089064589	CD8+ Teff/Tex	ECH1
2.28239954687769E-07	0.471007118226803	0.142	0.103	0.0037228219009122	CD8+ Teff/Tex	EPSTI1
2.65019838059831E-07	0.276291523228432	0.326	0.268	0.0043227385785939	CD8+ Teff/Tex	RASAL3
2.70578044701344E-07	0.509992534298187	0.116	0.08	0.00441339848712362	CD8+ Teff/Tex	HCST
3.34630798250788E-07	0.500380614860207	0.125	0.089	0.00545816295026861	CD8+ Teff/Tex	SURF4
3.40830733012686E-07	0.541352555481417	0.128	0.092	0.00555929008616993	CD8+ Teff/Tex	EMD
3.52779097351422E-07	0.493273173130025	0.143	0.104	0.00575417985689904	CD8+ Teff/Tex	GIMAP6
5.20796650138129E-07	0.34469777954215	0.224	0.177	0.00849471416040303	CD8+ Teff/Tex	MED15
5.74271981975065E-07	0.383776446629789	0.254	0.204	0.00936695029799529	CD8+ Teff/Tex	CHST11
7.20705733141075E-07	0.473689728283723	0.14	0.103	0.0117554312132641	CD8+ Teff/Tex	PIK3R5
8.06592402178267E-07	0.497029315788231	0.133	0.097	0.0131563286719297	CD8+ Teff/Tex	ETNK1
1.09638761962286E-06	0.386865924611516	0.203	0.161	0.0178831784636685	CD8+ Teff/Tex	PTPN7
1.29931633376583E-06	0.485848760548791	0.105	0.073	0.0211931487200545	CD8+ Teff/Tex	NFAT5
1.33113452921789E-06	0.516207356813963	0.116	0.083	0.0217121353060731	CD8+ Teff/Tex	NRDC
1.36833289967428E-06	0.491262856451392	0.109	0.076	0.0223188779265872	CD8+ Teff/Tex	CDR2
1.40593389967416E-06	0.322931208927319	0.242	0.195	0.0229321878375852	CD8+ Teff/Tex	LY6E
1.71528694763821E-06	0.461187789242747	0.12	0.086	0.0279780454029269	CD8+ Teff/Tex	MCOLN2
1.9820150280894E-06	0.474589600582884	0.154	0.117	0.0323286471231662	CD8+ Teff/Tex	ORMDL3
2.21765134483793E-06	0.488523748884081	0.128	0.094	0.0361721110856514	CD8+ Teff/Tex	GNA13
2.29119704428889E-06	0.537173299254277	0.126	0.093	0.037371714989396	CD8+ Teff/Tex	USP24
2.75240155043741E-06	0.545051059847056	0.122	0.089	0.0448944216891847	CD8+ Teff/Tex	ICAM3
7.6561135512229E-143	6.44316099600553	0.132	0.002	1.24878868133997E-138	CD4+ Tpex	ANKRD55
3.83085883616875E-139	4.89043998065372	0.424	0.031	6.24851384767485E-135	CD4+ Tpex	CXCL13
6.57107968681798E-90	3.7159447290718	0.361	0.034	1.07180880771688E-85	CD4+ Tpex	SESN3
7.73057782968487E-78	4.9302448629489	0.132	0.005	1.2609345497999E-73	CD4+ Tpex	PTPN13
2.05885466521558E-64	5.08379680482448	0.118	0.005	3.35819784443314E-60	CD4+ Tpex	NMB
2.17878517422181E-60	2.82518669264923	0.472	0.082	3.5538164976732E-56	CD4+ Tpex	IL6ST
1.5634065473755E-55	2.60602750612047	0.549	0.123	2.55007241942417E-51	CD4+ Tpex	ST8SIA1
6.28845716939029E-47	3.34402221377575	0.222	0.024	1.02571024889925E-42	CD4+ Tpex	TIAM1
2.04268676762111E-45	2.21050576944089	0.521	0.121	3.33182638666679E-41	CD4+ Tpex	TCF7
1.64219377128321E-36	4.01976297673376	0.104	0.007	2.67858226034004E-32	CD4+ Tpex	CXCR5
1.86393614107784E-36	3.57685040615869	0.111	0.008	3.04026623971207E-32	CD4+ Tpex	TSHZ2
9.8955305504546E-36	2.4545404942656	0.347	0.071	1.61405998808465E-31	CD4+ Tpex	FKBP5

2.22324490028603E-34	2.5187292947859	0.34	0.07	3.62633475685654E-30	CD4+ Tpex	THADA
4.53467688770535E-34	2.12491947775044	0.5	0.146	7.3965114715362E-30	CD4+ Tpex	SH2D1A
5.63879454928133E-33	3.68055087108391	0.111	0.009	9.19743778933277E-29	CD4+ Tpex	KIAA1324
1.29096865195601E-28	3.00593131933868	0.153	0.019	2.10569896820545E-24	CD4+ Tpex	EVC
1.67735791777269E-28	1.97862984785584	0.403	0.107	2.73593849967904E-24	CD4+ Tpex	TP53INP1
3.58390818900047E-27	1.60159878456884	0.736	0.362	5.84571264707866E-23	CD4+ Tpex	ITPKB
2.50445501666944E-26	3.33743518481551	0.16	0.022	4.08501657768953E-22	CD4+ Tpex	COL6A3
3.4266541722019E-26	1.96967371478391	0.431	0.132	5.58921562027851E-22	CD4+ Tpex	ICOS
6.806605999037E-25	3.31796093257112	0.104	0.01	1.11022550450292E-20	CD4+ Tpex	CD200
3.0630549173867E-24	2.31585959073687	0.201	0.036	4.99614887574944E-20	CD4+ Tpex	SELENON
4.37074907036099E-24	2.0442799647072	0.306	0.076	7.12912880866581E-20	CD4+ Tpex	NPDC1
6.50836116395487E-22	2.76300820326782	0.153	0.024	1.06157878945268E-17	CD4+ Tpex	KCNK5
6.92299616419434E-22	1.86191291496687	0.34	0.096	1.12920990434174E-17	CD4+ Tpex	TRAT1
1.30706990761735E-21	2.00566962989339	0.319	0.088	2.13196172631466E-17	CD4+ Tpex	NR3C1
3.35346111975582E-20	1.9036964535945	0.278	0.072	5.46983043243371E-16	CD4+ Tpex	ZC3H12D
3.40351456726343E-20	1.76380659556442	0.479	0.19	5.55147261066337E-16	CD4+ Tpex	ITM2A
7.06977678275937E-20	2.02330325604992	0.264	0.068	1.15315129103588E-15	CD4+ Tpex	NFATC1
1.64728196468231E-19	1.54479029624624	0.576	0.264	2.68688161259332E-15	CD4+ Tpex	DGKA
4.12748451379691E-19	1.26312262081453	0.701	0.361	6.73233999045413E-15	CD4+ Tpex	MAF
4.79164643958223E-19	1.84380519927413	0.306	0.087	7.81565450760258E-15	CD4+ Tpex	KIAA0319L
1.03770255663925E-17	1.63269184990207	0.507	0.213	1.69259664013428E-13	CD4+ Tpex	PGGHG
2.48721123767025E-17	2.41767672723984	0.174	0.037	4.05689024976394E-13	CD4+ Tpex	CCDC50
1.93328896125062E-16	2.56211941532563	0.125	0.021	3.15338762469589E-12	CD4+ Tpex	CCR7
2.00700720069565E-16	1.54199093257306	0.417	0.162	3.27362944505468E-12	CD4+ Tpex	TNFRSF25
3.84733164341693E-16	2.49375750290934	0.194	0.048	6.27538264357735E-12	CD4+ Tpex	TOX2
6.64182153550619E-16	1.96137757971204	0.188	0.044	1.08334751065642E-11	CD4+ Tpex	CD226
2.5068086919172E-15	1.31960880134525	0.444	0.183	4.08885565738614E-11	CD4+ Tpex	BICDL1
5.8552753706874E-15	1.83679036150177	0.278	0.089	9.55053965712822E-11	CD4+ Tpex	CTLA4
2.61825362006403E-14	2.48866924237936	0.104	0.017	4.27063347968644E-10	CD4+ Tpex	ZBTB10
2.95326178657968E-14	1.62617897802559	0.236	0.069	4.81706530009011E-10	CD4+ Tpex	HELLS
3.83724474633025E-14	2.7046635692705	0.111	0.02	6.25892990573928E-10	CD4+ Tpex	AHR
4.44780766306364E-14	2.07235382609754	0.188	0.049	7.25481907922311E-10	CD4+ Tpex	SELL
4.4972949086394E-14	1.17833531795222	0.569	0.272	7.33553772548172E-10	CD4+ Tpex	RNF19A
8.93382244390016E-14	1.60517526298601	0.236	0.071	1.45719577882456E-09	CD4+ Tpex	SARDH
1.14089240450073E-13	2.48481807421817	0.111	0.02	1.86090960098114E-09	CD4+ Tpex	FAAH2
1.61573205222519E-13	1.31458945915154	0.389	0.158	2.6354205503845E-09	CD4+ Tpex	TNIK
4.60376841271334E-13	1.17084976002084	0.458	0.205	7.50920665797673E-09	CD4+ Tpex	SMAP2
5.75807670445087E-13	1.94053956493128	0.208	0.061	9.39199891262982E-09	CD4+ Tpex	CEP128
7.98726407892994E-13	1.94920362841517	0.215	0.065	1.30280264391426E-08	CD4+ Tpex	AGFG1
2.01401334519465E-12	1.83999881540117	0.174	0.046	3.28505716734699E-08	CD4+ Tpex	NCALD
2.66594733868954E-12	1.43074708206794	0.396	0.178	4.34842670413651E-08	CD4+ Tpex	UCP2
2.70329708957394E-12	1.59546448398833	0.271	0.097	4.40934788280405E-08	CD4+ Tpex	CD82
4.54247324842864E-12	2.34145585349106	0.118	0.025	7.40922811551196E-08	CD4+ Tpex	QSOX2
1.26116379099273E-11	1.39345223061407	0.319	0.129	2.05708425948825E-07	CD4+ Tpex	DUSP4
1.34255335872378E-11	1.30162414637259	0.312	0.12	2.18983878341436E-07	CD4+ Tpex	SREBF2
1.95413997161463E-11	1.63654415473112	0.278	0.107	3.18739770770063E-07	CD4+ Tpex	IFNAR2
2.09763780777277E-11	1.38835331344574	0.132	0.031	3.42145702825817E-07	CD4+ Tpex	TNFRSF4
2.66849619263987E-11	1.79481661516397	0.188	0.055	4.35258413981488E-07	CD4+ Tpex	IRF2BP2
2.80241142111721E-11	1.32705325466629	0.285	0.107	4.57101326898428E-07	CD4+ Tpex	CD4
3.2493747247257E-11	1.06893176098997	0.507	0.276	5.30005511350009E-07	CD4+ Tpex	TIGIT
6.7787740550397E-11	1.08767749012742	0.424	0.2	1.10568583611753E-06	CD4+ Tpex	LTBP3
7.0131331431436E-11	0.950930163757086	0.736	0.513	1.14391214697815E-06	CD4+ Tpex	SRGN
1.01498893370733E-10	1.27162990238947	0.312	0.127	1.65554844977003E-06	CD4+ Tpex	SFI1
1.45609523940558E-10	1.57748044907914	0.167	0.048	2.37503694499445E-06	CD4+ Tpex	CCR4
1.87107327921896E-10	1.44312678596998	0.306	0.13	3.05190762573404E-06	CD4+ Tpex	CD84
2.44467098238013E-10	2.06809362486263	0.118	0.028	3.98750283936023E-06	CD4+ Tpex	NCOA7
2.75519385625906E-10	1.38865410006411	0.243	0.089	4.49399669894416E-06	CD4+ Tpex	PCNX2
7.22971672380748E-10	1.79145513488465	0.181	0.058	1.17923909482024E-05	CD4+ Tpex	INPP5F
9.06352023330227E-10	1.30685797989739	0.215	0.075	1.47835078525393E-05	CD4+ Tpex	ZNF831
1.0222043898202E-09	1.35530868788239	0.278	0.113	1.66731758023572E-05	CD4+ Tpex	STAT5B
1.14075137719772E-09	1.23000530781218	0.382	0.185	1.8606795713472E-05	CD4+ Tpex	LAT
2.13435822068517E-09	1.54098969929667	0.194	0.067	3.48135169375959E-05	CD4+ Tpex	RNASET2
3.04988665057834E-09	1.55890390144739	0.132	0.036	4.97467011575833E-05	CD4+ Tpex	MAGEH1
4.30892451513846E-09	1.83975342822656	0.139	0.04	7.02828677664234E-05	CD4+ Tpex	SH3TC1
4.96119890466949E-09	1.248468209623	0.375	0.193	8.0922115334064E-05	CD4+ Tpex	ARID5B
6.98970515918702E-09	1.64988117861809	0.111	0.028	0.0001140090808515	CD4+ Tpex	CD40LG

1.24470841662101E-08	0.973845624894264	0.528	0.323	0.000203024389835053	CD4+ Tpex	ITK
1.45114901273773E-08	1.37884919560748	0.188	0.066	0.000236696915467651	CD4+ Tpex	ELMO1
1.56594672522879E-08	1.09218166204386	0.431	0.25	0.000255421570352069	CD4+ Tpex	FYB1
1.60811948221265E-08	1.81631860571335	0.104	0.026	0.000262300368743706	CD4+ Tpex	N4BP2
1.64049054488356E-08	1.70255480636452	0.132	0.039	0.000267580412775958	CD4+ Tpex	NCDN
3.29770645668573E-08	1.32678836669947	0.208	0.079	0.00053788890015001	CD4+ Tpex	TRIM8
3.37473750260916E-08	1.41783498633373	0.153	0.049	0.000550453434050581	CD4+ Tpex	TBC1D4
3.76805304450519E-08	1.70215922390652	0.104	0.027	0.000614607132089241	CD4+ Tpex	FBXO34
4.42546794586635E-08	1.28986568860728	0.236	0.098	0.00072183807665026	CD4+ Tpex	PKM
5.79115875721694E-08	1.01175579502625	0.257	0.107	0.000944595904889655	CD4+ Tpex	DEPRECATED-ENSG00000026297
6.47026268202714E-08	1.32939388693495	0.181	0.065	0.00105536454606545	CD4+ Tpex	INO80B
8.50076319401843E-08	1.15601223516024	0.181	0.065	0.00138655948457635	CD4+ Tpex	CHMP7
8.74292364164348E-08	1.2450272446724	0.194	0.073	0.00142605827518847	CD4+ Tpex	TRPS1
1.53715266640082E-07	0.976606412040605	0.382	0.202	0.00250724971416637	CD4+ Tpex	PIK3IP1
1.98525072679456E-07	1.25929399469856	0.306	0.15	0.00323814246047461	CD4+ Tpex	PFKFB3
2.45876058278608E-07	1.57233402893495	0.139	0.046	0.00401048438658237	CD4+ Tpex	GOLGA7
3.40993231553752E-07	1.44322056195009	0.139	0.047	0.00556194059987325	CD4+ Tpex	RDH10
4.08339704082394E-07	1.82436860500663	0.125	0.041	0.00666042891328792	CD4+ Tpex	ARSG
4.79767838861696E-07	1.43685882055583	0.132	0.044	0.00782549321967313	CD4+ Tpex	TRMT1
4.87781403938939E-07	0.782017611358943	0.326	0.16	0.00795620247964803	CD4+ Tpex	SNRNP70
4.87904928080097E-07	1.32851567001297	0.16	0.058	0.00795821728191446	CD4+ Tpex	RBPJ
5.9195940643879E-07	0.924089673692423	0.5	0.314	0.00965544987842311	CD4+ Tpex	SLA
6.5498039161308E-07	1.04588231362412	0.25	0.114	0.0106833851676009	CD4+ Tpex	NAB1
7.5907645353567E-07	1.51614210369702	0.104	0.031	0.0123812960336203	CD4+ Tpex	ZNF827
1.34457439713186E-06	1.5583751530517	0.139	0.05	0.0219313529916177	CD4+ Tpex	MYEF2
1.38283723131765E-06	0.816164547534966	0.472	0.292	0.0225554580800222	CD4+ Tpex	FAM118A
1.63930814216882E-06	0.850021820850484	0.417	0.239	0.0267387551069156	CD4+ Tpex	HNRNPLL
1.65914079075078E-06	1.57180197709643	0.104	0.032	0.027062245437936	CD4+ Tpex	DAPK2
1.89644996820428E-06	1.11021408438797	0.299	0.154	0.0309329954313801	CD4+ Tpex	NAP1L4
0	4.4806895615814	0.579	0.041	0	Treg	TNFRSF18
0	7.10366857242646	0.5	0.005	0	Treg	FOXP3
0	5.3940512816	0.281	0.011	0	Treg	TNFRSF4
2.891303506361E-290	5.93258228464858	0.208	0.004	4.71600514922543E-286	Treg	IL2RA
1.54186197611997E-246	7.62722990184792	0.152	0.001	2.51493106924929E-242	Treg	CCR8
1.57200005913069E-217	4.4408105173899	0.259	0.019	2.56408929644806E-213	Treg	MAGEH1
2.49259821393375E-212	3.9617617899335	0.253	0.018	4.06567694674735E-208	Treg	GADD45A
2.5369355884401E-185	2.62482697328786	0.442	0.081	4.13799563830464E-181	Treg	CD4
1.74226234831368E-180	4.95667802842165	0.155	0.005	2.84180411633444E-176	Treg	RTKN2
8.66045374657745E-173	6.52051734827289	0.118	0.002	1.41260661060425E-168	Treg	TNFRSF13B
6.72265410300578E-156	3.1382246729381	0.299	0.042	1.09653211074127E-151	Treg	NTNG2
2.29794376782541E-154	3.20830062536428	0.266	0.033	3.74817607970003E-150	Treg	TBC1D4
1.62041979443971E-140	4.47117129054429	0.141	0.007	2.64306672671061E-136	Treg	COL9A2
3.4282485762922E-131	1.7178161301995	0.665	0.247	5.5918162527902E-127	Treg	TIGIT
1.21440407586152E-129	4.79616193105111	0.122	0.005	1.98081448813773E-125	Treg	LRRC32
1.64461247343921E-125	4.22305663597347	0.137	0.008	2.6825274054267E-121	Treg	VDR
2.54606254589023E-119	3.10637944456858	0.228	0.031	4.15288261860156E-115	Treg	BATF
1.47492239980749E-118	2.67724058445732	0.271	0.045	2.4057459263260E-114	Treg	STAM
2.4653766043645E-114	2.62121229194318	0.254	0.041	4.02127577937894E-110	Treg	IKZF2
2.78554622411515E-114	1.86689081594775	0.58	0.218	4.54350444615422E-110	Treg	TNFRSF1B
1.84044892708748E-112	4.81382645534521	0.107	0.005	3.0019562449724E-108	Treg	TBC1D8
2.84450283183264E-103	3.93560745001654	0.124	0.009	4.63966856900222E-99	Treg	FAM184A
2.01471697521141E-97	2.28774540600386	0.314	0.073	3.28620485826734E-93	Treg	CTLA4
1.64665174668013E-96	1.43048496229466	0.702	0.338	2.68585366400995E-92	Treg	MAF
3.02173096517341E-91	2.28356728396866	0.274	0.059	4.92874537729434E-87	Treg	ZC3H12D
1.37377116219168E-82	4.06455499990142	0.101	0.008	2.24075814265086E-78	Treg	TNFRSF8
1.59421160461009E-82	1.20156528729368	0.817	0.589	2.60031854827952E-78	Treg	IL32
1.63888946000064E-82	1.83784975053344	0.381	0.116	2.67319259820704E-78	Treg	ICOS
4.363988644676E-82	1.97014740257291	0.295	0.073	7.11810187833101E-78	Treg	ENTPD1
1.90412622828345E-80	2.39290480420298	0.229	0.047	3.10582029095314E-76	Treg	IL12RB2
5.84332188191276E-76	2.84179869871567	0.179	0.03	9.5310423215879E-72	Treg	TYMP
1.04204110493701E-74	1.86268715932899	0.4	0.14	1.69967324626276E-70	Treg	CTSC
2.47089284195136E-74	2.79765936168081	0.141	0.019	4.03027331450686E-70	Treg	EPHX2
7.52207647319528E-74	2.00066618184864	0.305	0.085	1.22692589354288E-69	Treg	ADAT2
7.80079146722983E-72	2.24806857339036	0.214	0.045	1.27238709621986E-67	Treg	CREB3L2
2.61931925876844E-71	1.79444422323155	0.399	0.142	4.27237164297721E-67	Treg	PIM2
3.30299555229946E-67	1.97248568371911	0.298	0.087	5.38751604535565E-63	Treg	IL21R

2.02657630238085E-65	1.48563831407154	0.397	0.143	3.30554860681341E-61	Treg	DUSP16
1.75096227611597E-63	1.63151879595207	0.371	0.132	2.85599456857276E-59	Treg	WHRN
1.10001644674602E-58	1.99951616697201	0.251	0.072	1.79423682628743E-54	Treg	CASP1
1.3586215851542E-58	1.54847345252739	0.32	0.106	2.21604766754502E-54	Treg	TNFRSF9
1.47925678848519E-55	1.31491481880067	0.438	0.183	2.41281574769819E-51	Treg	LTBP3
2.10015035230471E-55	3.0153358710223	0.104	0.014	3.42555523964421E-51	Treg	LINC02694
3.38326246922856E-55	2.66626190670206	0.134	0.023	5.5184394135587E-51	Treg	CLNK
3.1004467612311E-54	2.03995782734384	0.247	0.073	5.05713871224404E-50	Treg	SYNGR2
4.86387275168237E-54	1.92389932234686	0.225	0.062	7.93346284526911E-50	Treg	HS3ST3B1
7.08143419109902E-51	1.756799261998	0.315	0.117	1.15505273091016E-46	Treg	DUSP4
2.09994914446694E-50	1.65980550046257	0.226	0.064	3.42522704954002E-46	Treg	TRAF3
2.71992823949989E-50	2.34366094856972	0.15	0.032	4.43647495144827E-46	Treg	SGPP1
3.74300725811961E-49	1.82519704510032	0.22	0.063	6.10521913871889E-45	Treg	GLCCI1
2.54990616659105E-48	2.15000503101945	0.188	0.049	4.15915194832666E-44	Treg	INPP5F
3.36093250645049E-47	1.36909702808924	0.362	0.148	5.4820170112714E-43	Treg	SAT1
7.87207176741298E-47	2.49790098382116	0.118	0.021	1.28401362598273E-42	Treg	PELI1
7.74768933742752E-46	0.951243472572205	0.533	0.26	1.2637256078278E-41	Treg	LTB
3.50714754417545E-44	1.33973947728764	0.42	0.201	5.72050835930457E-40	Treg	ZFP36L1
5.44712516573483E-44	1.1098198800734	0.534	0.285	8.88480585783009E-40	Treg	TRAF1
5.4709520296415E-44	0.941902486429885	0.632	0.376	8.92366985554826E-40	Treg	SPOCK2
5.70409909327914E-44	2.46178007497483	0.131	0.028	9.30395603104761E-40	Treg	PMAIP1
7.45474146544253E-43	1.80691682375063	0.185	0.051	1.21594288042833E-38	Treg	LAYN
3.59037673574363E-40	1.44806574925384	0.344	0.152	5.85626349367144E-36	Treg	TPP1
5.25794235272447E-40	1.91420058487011	0.156	0.04	8.57622977152888E-36	Treg	PHACTR2
2.61804479140856E-39	1.4909345173598	0.28	0.109	4.2702928592665E-35	Treg	PHTF2
3.35686606829095E-39	2.42991455077563	0.109	0.021	5.47538424398937E-35	Treg	ERI1
5.12896403757881E-39	2.29556540752915	0.107	0.021	8.3658532416948E-35	Treg	TIAM1
1.31498543266987E-38	1.47490487117352	0.263	0.098	2.14487273922783E-34	Treg	FAM53B
6.16434530446628E-38	1.86231593379226	0.152	0.04	1.00546636261149E-33	Treg	LY75
4.55626168097514E-37	1.68797951647374	0.24	0.089	7.43171842783855E-33	Treg	PKM
8.54603311162817E-37	2.06689405719091	0.138	0.035	1.39394346083767E-32	Treg	MALT1
5.9602077062958E-36	1.42839329675122	0.327	0.147	9.72169478973908E-32	Treg	SERPINB9
6.22221770753178E-36	2.40656107290793	0.112	0.025	1.01490593027551E-31	Treg	DNPH1
2.78217506421272E-35	2.32109941247135	0.122	0.029	4.53800574723736E-31	Treg	ZBTB32
3.29555523662381E-34	1.37724757555438	0.259	0.102	5.37538014645709E-30	Treg	CD27
4.63723477913836E-34	1.3464613839281	0.293	0.126	7.56379364825258E-30	Treg	KDM2B
8.75319216744829E-34	1.05036839276806	0.384	0.188	1.42773317443249E-29	Treg	GBP2
2.13469891310061E-33	2.16819969049766	0.104	0.023	3.48190739715841E-29	Treg	ZBED2
3.73417815485241E-33	1.36365502752824	0.295	0.128	6.09081798837976E-29	Treg	NABP1
8.85286106641542E-33	1.46362420218394	0.213	0.077	1.44399016854302E-28	Treg	PTPRJ
1.50881452230374E-30	0.962673859494911	0.369	0.181	2.46102736732964E-26	Treg	ARID5B
2.42803562709604E-30	1.8106257293112	0.132	0.037	3.96036891135635E-26	Treg	GLRX
1.84591025944448E-29	1.33127707970525	0.243	0.1	3.0108642241799E-25	Treg	OTUD5
5.83314110802775E-29	1.7006767407394	0.141	0.043	9.51443646130406E-25	Treg	MAN1A2
1.34900832377292E-28	1.58538706354623	0.14	0.042	2.20036747690601E-24	Treg	CCR4
8.31995994417816E-28	1.15849515595108	0.274	0.123	1.3570686664949E-23	Treg	KAT2B
1.65634449069589E-27	1.95333391407594	0.118	0.033	2.70166349877406E-23	Treg	OAS1
4.52391520598399E-27	1.03024323695117	0.314	0.149	7.37895809248049E-23	Treg	TNIK
6.08792685497737E-27	1.26468689331445	0.243	0.104	9.9300174931536E-23	Treg	IGFLR1
7.02750898275004E-27	0.949084021311394	0.372	0.193	1.14625699017636E-22	Treg	BCL2
7.08541924482036E-27	1.43105426123936	0.185	0.068	1.15570273302265E-22	Treg	USP12
1.0926220932845E-26	1.51999217706348	0.141	0.045	1.78217589635635E-22	Treg	KSR1
4.97353559078331E-26	1.59755471199361	0.156	0.054	8.11233390212665E-22	Treg	BACH1
9.96685973619776E-25	1.65895488084973	0.14	0.047	1.62569449157122E-20	Treg	PCYOX1L
1.74239811477062E-24	0.716394653840051	0.455	0.266	2.84202556500235E-20	Treg	IL2RB
2.19451391885151E-24	0.854679670638387	0.408	0.228	3.5794716530387E-20	Treg	HNRNPLL
2.65962338841024E-24	1.06157342143427	0.265	0.124	4.33811170883594E-20	Treg	ZNRF2
2.93585298750246E-24	1.53787267956781	0.144	0.049	4.78866980791526E-20	Treg	BCL2L1
2.47265300022335E-23	1.41353706242512	0.177	0.07	4.03314430866431E-19	Treg	ADCY3
4.87786683785289E-23	1.64045306232337	0.113	0.034	7.95628859922185E-19	Treg	UXS1
7.77118007647431E-23	1.68549798071628	0.126	0.042	1.26755718227372E-18	Treg	TMEM154
7.80295265374577E-23	1.31114890021028	0.18	0.072	1.27273960735247E-18	Treg	CEP120
8.38822159513533E-23	1.19249866155367	0.202	0.086	1.36820282438252E-18	Treg	PHLDA1
1.58933400889741E-22	0.63442992674179	0.631	0.455	2.59236270191256E-18	Treg	IL2RG
2.18615765435202E-22	1.61998651676485	0.112	0.034	3.56584175001359E-18	Treg	ZBTB7B
2.70230027445853E-22	1.13333424547692	0.217	0.095	4.4077219776693E-18	Treg	RFTN1

5.02057554526317E-22	1.22340374176278	0.193	0.081	8.18906077187876E-18	Treg	TMEM173
5.08290602614857E-22	0.991523783885211	0.29	0.149	8.29072801925094E-18	Treg	S100A4
1.0476359833253E-21	0.976753088844298	0.304	0.158	1.7087990524019E-17	Treg	VMP1
3.31527251640124E-21	1.58794493084787	0.129	0.045	5.40754100150206E-17	Treg	STAMBPL1
4.97545712609783E-21	1.41846217901717	0.135	0.048	8.11546811837817E-17	Treg	CSF1
2.28201732341977E-20	1.07463106534478	0.214	0.097	3.72219845622999E-16	Treg	CASK
5.21153532235771E-20	1.12921641501734	0.201	0.09	8.50053526429766E-16	Treg	SIRPG
5.3263719605253E-20	0.641710431107267	0.613	0.451	8.68784530481281E-16	Treg	TRAC
7.58755240468194E-20	1.08854502224168	0.21	0.096	1.23760567272767E-15	Treg	SYNE3
9.29347977396194E-20	1.22498965973821	0.168	0.07	1.51585948593093E-15	Treg	ZNF292
1.71058047235725E-19	1.10303953778727	0.213	0.099	2.79012780846191E-15	Treg	MDFIC
1.71664131961919E-19	1.06706668295191	0.302	0.168	2.80001365643086E-15	Treg	ANKRD10
3.56745952321094E-19	0.844609200797088	0.345	0.198	5.81888322830936E-15	Treg	CCND2
4.94226663563518E-19	1.11368659368673	0.199	0.091	8.06133110938454E-15	Treg	CD82
7.03685372887506E-19	0.837670800233111	0.298	0.159	1.14778121171681E-14	Treg	PBXIP1
1.0717490000469E-18	0.770864167953239	0.491	0.324	1.74812979397649E-14	Treg	CFLAR
3.4541922979239E-18	1.24369864325071	0.165	0.071	5.63413305714367E-14	Treg	LFNG
3.70451063846392E-18	1.14435712206829	0.201	0.095	6.0424273023985E-14	Treg	SLC6A6
5.75734135024118E-18	1.55175916989968	0.116	0.042	9.3907994763784E-14	Treg	RHBDD2
5.91077420520953E-18	1.59798159851722	0.109	0.038	9.64106380611727E-14	Treg	CCNG2
6.66507747192781E-18	1.01440642854874	0.299	0.168	1.08714078644615E-13	Treg	HDAC7
8.90178459242713E-18	0.99845645481004	0.188	0.085	1.45197008487079E-13	Treg	PRDM2
9.39781033361315E-18	0.985176256204282	0.244	0.127	1.53287684351564E-13	Treg	SDF4
1.25844771930782E-17	1.16647301927882	0.164	0.071	2.05265407496299E-13	Treg	BIRC3
1.94614822780039E-17	1.44556250066252	0.118	0.044	3.17436237436522E-13	Treg	PMVK
2.40513416001828E-17	0.509015046870218	0.683	0.514	3.92301432840582E-13	Treg	MCL1
5.50452250501054E-17	1.20292058252253	0.164	0.072	8.9784266579227E-13	Treg	MAP2K3
6.44231276557348E-17	1.07941261463254	0.155	0.066	1.05080563519269E-12	Treg	WDTC1
4.32410076836461E-16	1.26574826543347	0.15	0.066	7.05304076327952E-12	Treg	TFRC
5.80720332306805E-16	1.18363014785295	0.146	0.063	9.47212934025629E-12	Treg	FOXO1
1.04568023070157E-15	0.724156344360054	0.333	0.199	1.70560902429733E-11	Treg	SMAP2
1.2540839894616E-15	0.901676337070285	0.251	0.137	2.04553639521081E-11	Treg	OS9
1.79747795264272E-15	1.39416624366197	0.122	0.049	2.93186628855554E-11	Treg	ZDHHC14
2.06352959963451E-15	1.15766517504678	0.153	0.069	3.36582312996385E-11	Treg	TRPS1
2.4126146448274E-15	1.10237943693377	0.159	0.073	3.93521574717796E-11	Treg	SNX9
2.9682776587545E-15	0.712243099433353	0.305	0.177	4.84155768919447E-11	Treg	CD7
3.01393264958634E-15	1.26730228293787	0.119	0.048	4.91602554474029E-11	Treg	MICAL2
4.49113611403683E-15	1.07255298087424	0.132	0.056	7.32549211560547E-11	Treg	MICAL3
4.60045152804807E-15	0.958296350077176	0.116	0.045	7.5037964873992E-11	Treg	SELL
4.89929768127154E-15	1.15703588002503	0.121	0.049	7.99124444792201E-11	Treg	IL18R1
5.03714017660014E-15	1.1410207017169	0.132	0.056	8.21607934205249E-11	Treg	DYNC1I2
5.86732246200178E-15	1.0433982172952	0.165	0.077	9.57018966777111E-11	Treg	ACOT9
9.91459990216264E-15	0.965978589985302	0.153	0.069	1.61717039004175E-10	Treg	ZDHHC18
1.07132167531111E-14	0.914782984504121	0.204	0.104	1.74743278459995E-10	Treg	ZMIZ2
1.62383147801882E-14	0.581265673936488	0.432	0.288	2.64863152379649E-10	Treg	MT-CO1
2.12716533384959E-14	0.711774174991334	0.35	0.222	3.46961937604206E-10	Treg	TSC22D3
3.16595146956012E-14	0.888658830206295	0.22	0.117	5.16398344199951E-10	Treg	TXNDC11
3.81743126949959E-14	0.960162527413901	0.161	0.076	6.22661214368078E-10	Treg	CNST
4.30849529707334E-14	1.30940727133328	0.109	0.043	7.02758667905632E-10	Treg	CXCR3
8.95661036586371E-14	1.15343511335114	0.146	0.067	1.46091271677603E-09	Treg	IER5
1.60447052458767E-13	0.964594165352918	0.177	0.089	2.61705187265495E-09	Treg	TLK1
1.61309740080238E-13	0.880226904077793	0.229	0.127	2.63112317044876E-09	Treg	HAPLN3
1.65061577662131E-13	1.02234112611838	0.134	0.059	2.69231939324702E-09	Treg	CBX7
1.84839070256555E-13	1.196844399927	0.118	0.05	3.01491007495466E-09	Treg	PHKB
2.03763321782765E-13	1.26638030486403	0.116	0.049	3.32358354159868E-09	Treg	TTC7A
2.12192708659977E-13	0.619572535084991	0.333	0.209	3.46107527095289E-09	Treg	CHST11
2.35362662037121E-13	1.32934102045	0.1	0.039	3.83900038048748E-09	Treg	SLC12A6
3.87351049223237E-13	0.744612369153541	0.293	0.177	6.31808296388022E-09	Treg	WSB1
4.3212539352542E-13	1.05507683843519	0.149	0.071	7.04839729379313E-09	Treg	RHOG
5.03878818377403E-13	1.01830863119484	0.137	0.063	8.21876740655383E-09	Treg	PRPF39
6.30293146151856E-13	0.978524205137667	0.132	0.06	1.02807115068829E-08	Treg	PBX4
8.6596036196756E-13	0.763581566339679	0.277	0.166	1.41246794640529E-08	Treg	MSI2
9.31086534670922E-13	0.664849292479043	0.314	0.195	1.51869524670174E-08	Treg	NBEAL2
1.27764733756869E-12	0.721274981875367	0.281	0.168	2.08397057230829E-08	Treg	SAMHD1
2.28002965531516E-12	1.10093024683436	0.153	0.077	3.71895637078456E-08	Treg	NFAT5
2.31862741432887E-12	0.798949133157696	0.213	0.118	3.78191317551182E-08	Treg	MOB3A

2.97888302980855E-12	0.476343548264276	0.442	0.307	4.85885610992072E-08	Treg	SLA
5.13381643764621E-12	0.957913950403318	0.162	0.083	8.37376799144474E-08	Treg	IL6ST
5.60908010132895E-12	1.20655054498246	0.124	0.057	9.14897055327764E-08	Treg	LGALS1
5.91052659624911E-12	1.20751112320787	0.107	0.046	9.64065993114193E-08	Treg	UBASH3B
6.97074919731546E-12	0.982298626591308	0.137	0.065	1.13699890157413E-07	Treg	AK1
1.02645998891674E-11	0.849878402923144	0.192	0.104	1.6742588879221E-07	Treg	PTPN1
1.09931121532975E-11	0.956055709734603	0.199	0.112	1.79308652332436E-07	Treg	PIM3
1.51249476025396E-11	0.699941789803827	0.195	0.105	2.46703020345023E-07	Treg	TP53INP1
1.77147354245988E-11	0.71576382375609	0.232	0.136	2.8894504951063E-07	Treg	NFKBIA
1.85173426017727E-11	1.16919312924139	0.128	0.061	3.02036375177514E-07	Treg	ZNRF1
2.70022357173632E-11	0.961924863299904	0.159	0.083	4.40433466785912E-07	Treg	KAT6B
3.2833483969113E-11	0.976103654385881	0.144	0.072	5.35546957020202E-07	Treg	FNDC3B
4.47706683664822E-11	0.899868851427741	0.152	0.077	7.30254371725692E-07	Treg	FAS
4.94771572268162E-11	0.54040623843193	0.262	0.158	8.070219115266E-07	Treg	TNFRSF25
5.48042419332403E-11	0.956985799289016	0.135	0.067	8.93911990173083E-07	Treg	NCOA3
5.566108989195E-11	0.771298701543224	0.188	0.103	9.07888037227596E-07	Treg	IFNAR2
6.10596337910834E-11	0.565251243097028	0.31	0.196	9.95943686766361E-07	Treg	PIK3IP1
1.13219369922598E-10	0.834347581584716	0.174	0.094	1.8467211428075E-06	Treg	USP48
1.15530876370237E-10	1.14179038560371	0.104	0.047	1.88442412447494E-06	Treg	RYBP
1.31147160428721E-10	0.759908019082627	0.207	0.119	2.13914133375286E-06	Treg	ARPC1B
2.09928829168816E-10	0.790516588772107	0.141	0.071	3.42414913257256E-06	Treg	ST6GALNAC6
4.49522360239801E-10	0.74546958068417	0.269	0.174	7.33215921787139E-06	Treg	UCP2
6.66207424589927E-10	0.546066288768811	0.412	0.298	1.08665093024863E-05	Treg	CD2
6.99162377130843E-10	0.585433411810554	0.384	0.267	1.14040375333812E-05	Treg	IL10RA
9.39092087775496E-10	0.334994655181626	0.701	0.583	1.53175310437061E-05	Treg	LENG8
9.40684232082836E-10	0.770733765039564	0.159	0.086	1.53435005095031E-05	Treg	TANK
1.11818843380451E-09	0.6084726695892	0.229	0.139	1.82387715437854E-05	Treg	PCED1B
1.14710302123741E-09	0.880043086518343	0.131	0.067	1.87103973794034E-05	Treg	NIBAN1
1.42300747850016E-09	0.748583365191066	0.177	0.101	2.32106749818161E-05	Treg	RAP1A
1.58947781016886E-09	1.1009837006936	0.109	0.052	2.59259725616642E-05	Treg	NPTN
1.6776091650712E-09	0.985782713872731	0.112	0.054	2.73634830914764E-05	Treg	NDFIP1
1.74056608012573E-09	0.633150411605713	0.269	0.175	2.83903733329308E-05	Treg	SKAP1
1.7562317743076E-09	0.909052166433983	0.149	0.081	2.86458964707312E-05	Treg	TAB2
1.79146609392459E-09	0.817846694693557	0.147	0.079	2.9220603458004E-05	Treg	WDFY1
1.88524074691105E-09	0.652385104493167	0.185	0.105	3.07501618228662E-05	Treg	SLAMF1
2.1667332081869E-09	0.683080844202632	0.202	0.12	3.53415853587365E-05	Treg	HUWE1
2.26643315351895E-09	0.686223013925267	0.179	0.101	3.69677911670476E-05	Treg	IL12RB1
2.4638850784124E-09	0.879823677941357	0.106	0.05	4.01884295139847E-05	Treg	MT-ND4
2.52162090866875E-09	0.981587269706791	0.14	0.075	4.11301586412959E-05	Treg	ISG15
2.83609835159585E-09	0.935823472399214	0.121	0.061	4.62596002128799E-05	Treg	REXO2
3.16648425445054E-09	1.06837497121579	0.132	0.07	5.16485246743428E-05	Treg	THADA
3.39973936482893E-09	0.616678881004131	0.244	0.153	5.54531487797246E-05	Treg	NFKB2
3.82266822073771E-09	0.950525050789986	0.124	0.064	6.23515413484529E-05	Treg	AFTPH
4.0177244949137E-09	0.909235201494611	0.156	0.087	6.55331042365374E-05	Treg	IFI6
4.66522443790329E-09	0.576617034362412	0.251	0.16	7.60944758066405E-05	Treg	CD247
4.68628084309002E-09	1.06861200719889	0.113	0.057	7.64379268316413E-05	Treg	CD58
6.79681872390043E-09	0.830703227251831	0.147	0.081	0.00011086291020554	Treg	TSPYL2
9.25582697840655E-09	0.379122652802037	0.612	0.493	0.000150971793844789	Treg	RNF213
9.74297499030703E-09	0.642701377455481	0.153	0.084	0.000158917665066898	Treg	MAT2B
9.90259922410202E-09	0.743238587387263	0.112	0.056	0.000161521295944328	Treg	IRF4
1.01905735942878E-08	0.824794326549913	0.137	0.074	0.000166218445896428	Treg	RFX5
1.35715312648798E-08	0.801379329745296	0.147	0.082	0.000221365246461455	Treg	SLC5A3
1.98982476800143E-08	0.401572387283082	0.362	0.248	0.000324560317908713	Treg	DOCK2
2.76364891788612E-08	0.860741548857933	0.109	0.055	0.000450778774996404	Treg	NAGK
3.02578771504948E-08	0.825320841939438	0.132	0.072	0.00049353623420172	Treg	STIMATE
3.05189415427633E-08	0.920812996220941	0.131	0.072	0.000497794455504012	Treg	EML2
3.83218612078084E-08	0.644696404528898	0.164	0.095	0.000625067878160562	Treg	RAB21
4.24276704196111E-08	0.660362038913542	0.235	0.155	0.000692037732214277	Treg	CHD3
4.53246844503639E-08	0.789599375612072	0.161	0.094	0.000739290928069886	Treg	IKBKE
4.99307599799229E-08	0.897270991671709	0.104	0.053	0.000814420626032523	Treg	TFCP2
5.71633045454478E-08	0.717374060319706	0.176	0.106	0.000932390660440799	Treg	MGAT1
5.79477999521618E-08	0.513780751773832	0.222	0.141	0.000945186565019711	Treg	LASP1
6.10552388543183E-08	0.580268731307326	0.156	0.09	0.000995872000952785	Treg	ADAM10
7.04683725586879E-08	0.74418691138707	0.143	0.081	0.00114940962480476	Treg	VAMP1
7.39372826729428E-08	0.972499662453312	0.103	0.053	0.00120599101767837	Treg	PPP1R12B
7.60465193457107E-08	0.825398915837922	0.155	0.092	0.00124039477704789	Treg	SAMD9L

8.56654688381263E-08	0.797923100016943	0.144	0.083	0.00139728946221868	Treg	SGSH
1.17596511211152E-07	0.85271384569101	0.144	0.084	0.0019181166943651	Treg	ECH1
1.1900597491468E-07	0.886469455948918	0.115	0.062	0.00194110645683335	Treg	TNIP2
1.23181072954313E-07	0.64937947216545	0.162	0.096	0.0020092064809578	Treg	TNIP1
1.26940148753571E-07	0.840571413753562	0.14	0.08	0.0020705207663195	Treg	LMAN1
1.36949396112917E-07	0.480916572658484	0.379	0.279	0.00223378159999778	Treg	GBP5
1.4226838593003E-07	0.668496709390793	0.193	0.123	0.00232053964290472	Treg	AKIRIN2
1.84961713407374E-07	0.749980400496219	0.1	0.051	0.00301691050738768	Treg	SLC25A38
1.93281263650851E-07	0.637747340429139	0.217	0.142	0.00315261069140902	Treg	SETD1B
2.14530269659707E-07	0.428768293759064	0.265	0.18	0.00349920322841948	Treg	EFHD2
2.26235727553489E-07	0.717599347356125	0.158	0.095	0.00369013095212495	Treg	PGS1
2.44911380787077E-07	0.795951267214821	0.128	0.072	0.00399474953201801	Treg	CUTA
3.21849797430887E-07	0.56367528453319	0.156	0.093	0.0052496920458952	Treg	ARL6IP5
3.25218028234683E-07	0.687066399449289	0.158	0.095	0.00530463125853591	Treg	ISCU
3.42130267702989E-07	0.515592586075025	0.174	0.107	0.00558048679650345	Treg	MT-CO2
3.84299343858241E-07	0.568763578388869	0.186	0.117	0.00626830659767176	Treg	PLEC
3.97978447494426E-07	0.29310198105046	0.496	0.384	0.00649142645708159	Treg	CIRBP
4.20910912225478E-07	0.710759089740882	0.104	0.055	0.00686547788930977	Treg	CSGALNACT2
5.55994918600363E-07	0.520151677333485	0.18	0.113	0.00906883311729052	Treg	OBSCN
5.74906509498988E-07	0.585055739784125	0.146	0.085	0.00937730007643799	Treg	P2RY10
5.79046258092086E-07	0.569976383256908	0.214	0.142	0.00944482351574002	Treg	MYL6
5.86204243100435E-07	0.802500362130743	0.122	0.069	0.0095615774092112	Treg	RNH1
6.0436667467945E-07	0.557436818540657	0.192	0.123	0.00985782483069651	Treg	SLC3A2
6.08333812919849E-07	0.871341097142173	0.109	0.06	0.00992253282253566	Treg	FMNL3
7.57012455443411E-07	0.537705205685453	0.124	0.069	0.0123476301607375	Treg	GNPTAB
7.76076671186302E-07	0.673323260737233	0.122	0.069	0.0126585865837198	Treg	DGCR2
1.03365436904466E-06	0.610059967065671	0.17	0.106	0.0168599364134874	Treg	MXD4
1.25102381081261E-06	0.877918019976294	0.107	0.059	0.0204054493781645	Treg	BSG
1.3255985287342E-06	0.555966358340042	0.223	0.151	0.0216218376021835	Treg	ESYT1
1.61946832805374E-06	0.310793458729228	0.583	0.481	0.0264151478988846	Treg	CYTIP
1.91296250866178E-06	0.679428018988343	0.115	0.065	0.0312023314787823	Treg	PI4KB
2.00887188876843E-06	0.409926350880564	0.217	0.145	0.0327667093777019	Treg	MT-CO3
2.34706154263978E-06	0.659921388522811	0.164	0.104	0.0382829208219975	Treg	HSP90AB1
2.50212014409542E-06	0.651805212372406	0.101	0.056	0.0408120816703405	Treg	SETBP1
2.78424251025159E-06	0.667508132416859	0.113	0.064	0.0454137795847137	Treg	MT-ATP6
2.82480497192063E-06	0.392755504084042	0.254	0.175	0.0460753938969973	Treg	CD28
0	4.13792024054591	0.982	0.158	0	Stressed T cell	HSPA1B
9.03589584832948E-172	2.85374215472849	0.684	0.159	1.47384497182102E-167	Stressed T cell	DNAJB1
2.06881542615668E-167	3.44083886337679	0.469	0.072	3.37444484160416E-163	Stressed T cell	HSPA1A
5.93449937803323E-134	3.20418227209246	0.381	0.057	9.67976193551E-130	Stressed T cell	DEPRECATED-ENSG00000204389
1.08556997044967E-127	2.94853226669418	0.482	0.098	1.77067317880045E-123	Stressed T cell	DEPRECATED-ENSG00000132002
2.01108579682516E-48	2.01916283111767	0.399	0.142	3.28028204320152E-44	Stressed T cell	HSP90AA1
3.53133591345746E-26	1.90576577542566	0.249	0.09	5.75996200844047E-22	Stressed T cell	DUSP1
1.75691903892812E-16	0.572605757651607	0.609	0.384	2.86571064439566E-12	Stressed T cell	IL7R
2.46422465593204E-16	0.739040373028053	0.505	0.306	4.01939683629076E-12	Stressed T cell	AHNAK
8.36003321344428E-16	1.1777111718449	0.352	0.193	1.3636050174449E-11	Stressed T cell	HSPA8
1.14572701475215E-13	0.925862637175483	0.339	0.188	1.86879533376224E-09	Stressed T cell	KLRG1
1.35566931730736E-13	1.12537207334038	0.225	0.103	2.21123222346004E-09	Stressed T cell	SAMD3
1.04288021103588E-12	1.49234065164332	0.155	0.064	1.70104191222062E-08	Stressed T cell	KCNA3
1.55110493950285E-10	0.622879051468666	0.425	0.274	2.53000726682311E-06	Stressed T cell	LTB
1.90411094022642E-10	1.00789835920708	0.244	0.132	3.10579535460332E-06	Stressed T cell	DEDD2
2.51332412299966E-10	1.06966842241245	0.174	0.081	4.09948297702475E-06	Stressed T cell	PATJ
5.90049295783403E-10	1.28979204938518	0.142	0.063	9.62429406352309E-06	Stressed T cell	HSPE1
6.14978821476984E-10	0.76027653371156	0.319	0.188	1.00309195571111E-05	Stressed T cell	GZMK
2.02303998383929E-08	0.799507364732463	0.223	0.123	0.000329978051764026	Stressed T cell	TCF7
2.65828090269842E-08	0.952772080762852	0.233	0.135	0.000433592198039139	Stressed T cell	PIM1
6.90182357719211E-08	0.717531458420388	0.22	0.124	0.00112575644367581	Stressed T cell	RASA3
1.00429256765613E-07	0.824245621181368	0.238	0.143	0.00163810160710391	Stressed T cell	JUN
3.56833654625977E-07	1.08467679190891	0.145	0.075	0.00582031374060431	Stressed T cell	MYBL1
5.87248113732913E-07	0.586001809726832	0.303	0.196	0.00957860398309754	Stressed T cell	TC2N
1.22302960116968E-06	0.770600739798342	0.176	0.099	0.0199488358246787	Stressed T cell	SYNE1
1.41428870049546E-06	0.604413073511091	0.231	0.139	0.0230684629937815	Stressed T cell	MAX
2.88917259697767E-06	0.566414400359437	0.293	0.193	0.0471252942293028	Stressed T cell	PARP8
0	7.62450608784322	0.326	0.004	0	NK cell	GNLY
0	7.33478044294084	0.232	0.002	0	NK cell	FGFBP2
7.08425471529199E-205	4.44259954629223	0.236	0.014	1.15551278661128E-200	NK cell	FGR

2.89057894167375E-200	5.70023813171448	0.156	0.004	4.71482331176406E-196	NK cell	KLRF1
1.68758418456376E-144	2.61325079982717	0.45	0.098	2.75261856344195E-140	NK cell	KLRD1
1.34762835607513E-115	1.65755097845523	0.732	0.317	2.19811661159415E-111	NK cell	NKG7
3.43620283293462E-106	2.938593272305	0.248	0.038	5.60479044079967E-102	NK cell	FCRL6
3.52208397556607E-80	3.38624120357483	0.14	0.015	5.74487117254582E-76	NK cell	TRDC
4.99461883613808E-74	2.95766370761608	0.17	0.025	8.14672278362483E-70	NK cell	S1PR5
3.71732464028294E-72	2.22858581291497	0.317	0.087	6.0633282207655E-68	NK cell	SYNE1
2.05076342059442E-67	2.81044756711655	0.165	0.026	3.34500021533155E-63	NK cell	PTGDR
5.64057842010755E-63	2.04191355364857	0.422	0.17	9.20034746103742E-59	NK cell	EFHD2
4.28894672918484E-61	3.12402370640314	0.167	0.03	6.99570100997339E-57	NK cell	CCL3
1.56379959491185E-59	2.80727277855257	0.145	0.023	2.55071351926072E-55	NK cell	TXK
7.87744632035483E-59	2.15677091679404	0.243	0.062	1.28489026931308E-54	NK cell	ZBTB16
2.02467501590052E-57	2.4116577723233	0.195	0.042	3.30244741843534E-53	NK cell	ADRB2
7.62514314029107E-57	2.46644496176868	0.207	0.048	1.24373709761288E-52	NK cell	NCR1
2.35173700222266E-51	2.78194546575029	0.115	0.017	3.83591822432538E-47	NK cell	NMUR1
7.33557627154152E-51	1.94588623280045	0.255	0.074	1.19650584565114E-46	NK cell	CD300A
1.48826512456325E-45	1.86214346975841	0.287	0.099	2.42750924467511E-41	NK cell	MATK
3.62554554785946E-44	2.1986252582946	0.191	0.05	5.91362734311356E-40	NK cell	PLEK
6.67098780477375E-44	1.63959324490749	0.34	0.137	1.08810482083665E-39	NK cell	LPCAT1
1.51926266442162E-43	2.662700112903	0.138	0.028	2.4780693319381E-39	NK cell	GZMB
2.02472959173463E-43	1.60426272333807	0.346	0.139	3.30253643707835E-39	NK cell	ADGRG1
9.85048158764048E-42	1.26307771077286	0.496	0.263	1.60671205176004E-37	NK cell	PRF1
5.93913298394963E-41	3.78596362546944	0.151	0.035	9.68731981012024E-37	NK cell	CCL4L2
2.29696748241323E-40	2.09274745035691	0.218	0.067	3.74658366056421E-36	NK cell	GZMH
6.36945260259234E-40	1.81385632544301	0.264	0.093	1.03892141400884E-35	NK cell	MCTP2
1.73578139672496E-36	2.04131364995048	0.174	0.048	2.83123303619809E-32	NK cell	DEPRECATED-ENSG00000100450
1.92060082401992E-35	1.85090148382651	0.204	0.064	3.1326920040589E-31	NK cell	AOAH
2.01135412137835E-35	2.29077776122465	0.142	0.035	3.28071970738023E-31	NK cell	PLEKHG3
5.12994311103837E-32	1.92811969222251	0.149	0.04	8.36745020841468E-28	NK cell	PLAC8
1.73620514758405E-29	2.53269486115994	0.19	0.066	2.83192421622434E-25	NK cell	CCL4
1.91348829250169E-28	2.26615674931015	0.126	0.033	3.1210907538995E-24	NK cell	CLDND2
4.04844617559336E-28	2.23001857643925	0.117	0.03	6.60342055701032E-24	NK cell	CTSW
1.24404580209497E-27	1.9563066190844	0.133	0.037	2.02916310779711E-23	NK cell	DEPRECATED-ENSG00000145287
1.15127359662543E-26	0.888428004102356	0.509	0.305	1.87784236345575E-22	NK cell	KLRK1
1.97853064103654E-26	1.52077613046699	0.23	0.094	3.2271813285947E-22	NK cell	ZEB2
1.70019613767079E-25	1.46674051934272	0.191	0.07	2.77318992015483E-21	NK cell	MYBL1
7.38447537066635E-25	1.59604936151744	0.19	0.071	1.20448177770939E-20	NK cell	TBX21
1.16437749297512E-24	1.81304365053835	0.145	0.047	1.89921612879172E-20	NK cell	THEMIS2
1.6144561049214E-23	1.63311139260145	0.415	0.271	2.63333935273729E-19	NK cell	IL2RB
1.95338702922375E-23	1.55833741342051	0.197	0.078	3.18616958336686E-19	NK cell	EOMES
6.45272326794223E-23	1.34858428897263	0.232	0.1	1.05250369223406E-18	NK cell	SAMD3
3.86383991350245E-21	0.918890164134027	0.417	0.25	6.30230928291385E-17	NK cell	LITAF
8.62518335072431E-21	1.12264023208349	0.367	0.216	1.40685365633664E-16	NK cell	MYO1F
7.00307031244696E-19	1.46938150825842	0.17	0.07	1.14227079866322E-14	NK cell	LLGL2
7.0674890676569E-18	1.55417062626885	0.154	0.062	1.15277814182552E-13	NK cell	FASLG
1.0687202661608E-16	1.16007568200505	0.236	0.12	1.74318962613488E-12	NK cell	APOBR
4.2435125619476E-16	1.36681254687534	0.168	0.074	6.92159333979272E-12	NK cell	ADAM8
1.77731478861127E-15	1.77969486014493	0.112	0.041	2.89897815170385E-11	NK cell	KLRC3
2.48761874394841E-15	1.27132976419227	0.199	0.097	4.05755493325424E-11	NK cell	MAPK1
3.05621649383149E-15	0.755929532328362	0.436	0.291	4.98499472308854E-11	NK cell	PYHIN1
3.04504823844942E-14	1.13048330909465	0.268	0.16	4.96677818173485E-10	NK cell	CD247
1.24871537092896E-13	1.37646659844371	0.131	0.055	2.03677964152223E-09	NK cell	AGTPBP1
1.30806145579981E-13	0.832978410106457	0.225	0.118	2.13357904055508E-09	NK cell	KLF2
2.55677592742559E-13	1.12739636526535	0.193	0.098	4.17035721522388E-09	NK cell	TSPOAP1
4.85160517239611E-13	1.04750489502617	0.225	0.124	7.91345319669529E-09	NK cell	RASSF1
1.64310884937432E-12	1.4694443420897	0.124	0.054	2.68007484421445E-08	NK cell	KLRB1
3.39012764273688E-12	1.43552686726896	0.131	0.059	5.52963719806813E-08	NK cell	DGKD
6.55893499666462E-12	0.800975262086385	0.3	0.189	1.06982788730597E-07	NK cell	ABCA2
1.07486604242248E-11	1.35528471766517	0.115	0.049	1.75321400179531E-07	NK cell	USP28
5.24187029009751E-11	1.0556675126468	0.179	0.095	8.55001463017804E-07	NK cell	GZMM
1.14698543682997E-10	0.837026348191384	0.259	0.159	1.87084794601336E-06	NK cell	RIN3
1.26725363572049E-10	1.17307032312746	0.165	0.087	2.06701740522369E-06	NK cell	PXN
2.72506118594027E-10	1.14612027129129	0.147	0.074	4.44484730038717E-06	NK cell	LTBP4
5.45291101360316E-10	0.903655593360683	0.22	0.131	8.89424315428811E-06	NK cell	APMAP
7.39780521856315E-10	1.30254281480716	0.128	0.063	1.20665600919983E-05	NK cell	PLEKHF1
7.68143220682675E-10	1.38224488016366	0.126	0.061	1.25291840725551E-05	NK cell	HSH2D

9.89031822911502E-10	1.03208072324103	0.137	0.068	1.61320980635095E-05	NK cell	SLAMF7
1.21505225635303E-09	0.839139946031116	0.317	0.223	1.98187173533742E-05	NK cell	SLC9A3R1
1.38177119018141E-09	1.33579181751933	0.113	0.053	2.2538069883049E-05	NK cell	TES
1.77513548208565E-09	1.14052209343763	0.121	0.058	2.89542348482991E-05	NK cell	PRR5L
4.69134873772856E-09	0.841604652441891	0.28	0.187	7.65205892610905E-05	NK cell	SYTL1
4.92560725750051E-09	0.645467798818784	0.326	0.225	8.03415799770909E-05	NK cell	KLHDC4
6.52949493767985E-09	1.12268769613172	0.126	0.063	0.000106502591928496	NK cell	TBKBP1
8.0221039941045E-09	1.10966638729551	0.158	0.088	0.000130848538247839	NK cell	MAP3K8
9.15767433136183E-09	0.519112879067872	0.495	0.402	0.000149370826018843	NK cell	ITGAL
9.72430132095013E-09	1.11050094639744	0.128	0.066	0.000158613078846018	NK cell	RAP2B
1.02271404380248E-08	0.418265753837826	0.459	0.342	0.000166814887684622	NK cell	CCL5
1.40099595193945E-08	1.37627388433781	0.117	0.059	0.000228516449720844	NK cell	RGS3
2.30497601820055E-08	0.872546976728831	0.239	0.159	0.000375964638328692	NK cell	SPN
1.06443030497445E-07	0.483576082696033	0.465	0.376	0.00173619227044382	NK cell	SUN2
1.74604579776483E-07	0.779756310703403	0.23	0.154	0.00284797530073422	NK cell	CHST12
2.63625504965392E-07	0.480089922655111	0.376	0.281	0.00429999561149051	NK cell	GZMA
3.26244887420293E-07	1.02605996568412	0.119	0.064	0.00532138035871241	NK cell	RAB11FIP1
3.64335031667904E-07	1.11349748189406	0.126	0.07	0.00594266870153519	NK cell	GNPTAB
4.13620232154453E-07	0.997165946013645	0.121	0.066	0.00674655960667129	NK cell	TNFRSF1A
5.30699600527735E-07	0.625679118780427	0.296	0.215	0.00865624118420789	NK cell	MYO1G
1.15533689033588E-06	0.69140398847084	0.229	0.154	0.0188447000182686	NK cell	SIGIRR
1.44486921584224E-06	0.580322839020376	0.28	0.201	0.0235672617796028	NK cell	PKN1
2.34799993997413E-06	0.839452703385059	0.121	0.068	0.0382982270209181	NK cell	DEPRECATED-ENSG00000204389
2.49697265564639E-06	0.85419360211001	0.142	0.085	0.0407281209862482	NK cell	ABI3
0	8.2696826629026	0.925	0.015	0	Proliferating T/NK	HIST1H1B
0	4.25635086166874	0.868	0.087	0	Proliferating T/NK	HIST1H1C
0	4.09045928279481	0.728	0.049	0	Proliferating T/NK	HIST1H1E
0	3.99008061771393	0.737	0.06	0	Proliferating T/NK	HIST1H1D
0	4.6686347792488	0.636	0.02	0	Proliferating T/NK	CENPM
0	6.3351885289101	0.623	0.008	0	Proliferating T/NK	HIST1H2AH
0	5.47167117432157	0.618	0.011	0	Proliferating T/NK	MKI67
0	7.02164978488717	0.605	0.005	0	Proliferating T/NK	RRM2
0	4.27387218161158	0.601	0.023	0	Proliferating T/NK	NUSAP1
0	6.37696915335856	0.57	0.007	0	Proliferating T/NK	KIFC1
0	6.21640608443054	0.544	0.007	0	Proliferating T/NK	DEPRECATED-ENSG00000188486
0	4.12230837972818	0.526	0.021	0	Proliferating T/NK	FANCI
0	5.76729919388788	0.504	0.006	0	Proliferating T/NK	TYMS
0	4.95895904870246	0.5	0.013	0	Proliferating T/NK	H2AFX
0	6.09717501429979	0.491	0.005	0	Proliferating T/NK	NCAPG
0	6.43336900713337	0.482	0.004	0	Proliferating T/NK	ESPL1
0	4.21397670498172	0.482	0.016	0	Proliferating T/NK	NCAPG2
0	7.06326417041273	0.469	0.003	0	Proliferating T/NK	AURKB
0	5.00064075340099	0.469	0.01	0	Proliferating T/NK	CENPF
0	5.16250538758271	0.452	0.01	0	Proliferating T/NK	HIST1H4C
0	6.15585623152593	0.425	0.005	0	Proliferating T/NK	HIST1H2BH
0	5.4074632540893	0.425	0.007	0	Proliferating T/NK	TPX2
0	5.86250572174348	0.417	0.005	0	Proliferating T/NK	TOP2A
0	5.01425360355465	0.417	0.009	0	Proliferating T/NK	UHRF1
0	5.88654046359692	0.412	0.004	0	Proliferating T/NK	SPC24
0	5.11907797747278	0.412	0.008	0	Proliferating T/NK	CDK1
0	4.34478623464715	0.382	0.012	0	Proliferating T/NK	CENPE
0	6.23930683344175	0.364	0.003	0	Proliferating T/NK	ASPM
0	6.52193433294803	0.36	0.002	0	Proliferating T/NK	HJURP
0	5.39697302352154	0.364	0.006	0	Proliferating T/NK	MYBL2
0	5.47306352740785	0.36	0.005	0	Proliferating T/NK	KIF11
0	6.42324418524127	0.338	0.003	0	Proliferating T/NK	KIF2C
0	5.71098934276044	0.338	0.004	0	Proliferating T/NK	ZWINT
0	5.65503747383229	0.333	0.005	0	Proliferating T/NK	ASF1B
0	6.77562286680208	0.329	0.002	0	Proliferating T/NK	GTSE1
0	6.85696115935177	0.325	0.003	0	Proliferating T/NK	KIF15
0	6.22994679532287	0.32	0.003	0	Proliferating T/NK	KIF18B
0	4.96333972279205	0.32	0.006	0	Proliferating T/NK	PRC1
0	6.37763323848133	0.311	0.003	0	Proliferating T/NK	ANLN
0	5.47199625717727	0.311	0.004	0	Proliferating T/NK	CENPI
0	5.9977696506294	0.298	0.003	0	Proliferating T/NK	MELK
0	5.53049643040723	0.294	0.004	0	Proliferating T/NK	UBE2C

0	7.41255847649297	0.289	0.001	0	Proliferating T/NK	CDCA3
0	7.06329382958059	0.281	0.001	0	Proliferating T/NK	DLGAP5
0	6.08955933911941	0.276	0.002	0	Proliferating T/NK	FOXM1
0	5.89196073129744	0.272	0.003	0	Proliferating T/NK	PLK1
0	5.86542574935309	0.268	0.003	0	Proliferating T/NK	CDCA8
0	5.63500567340627	0.268	0.004	0	Proliferating T/NK	RAD54L
0	6.13997333893848	0.263	0.002	0	Proliferating T/NK	SHCBP1
0	6.92834384033718	0.259	0.001	0	Proliferating T/NK	SPC25
0	5.635269645188	0.25	0.002	0	Proliferating T/NK	CDCA2
0	6.63502818042101	0.25	0.002	0	Proliferating T/NK	CDCA5
0	6.3376057720543	0.246	0.002	0	Proliferating T/NK	POLQ
0	5.79655491190265	0.241	0.003	0	Proliferating T/NK	TROAP
0	6.09270819351572	0.232	0.002	0	Proliferating T/NK	BIRC5
0	6.47670687701254	0.215	0.001	0	Proliferating T/NK	CENPA
7.47649814043284E-303	5.21094919152704	0.268	0.004	1.219491611686E-298	Proliferating T/NK	STIL
6.0027180551639E-301	5.64073362157533	0.246	0.003	9.79103341977783E-297	Proliferating T/NK	KIF23
2.67377662845327E-295	5.77996601401219	0.232	0.003	4.36119705867012E-291	Proliferating T/NK	HMMR
2.34164545834867E-294	4.83693779046848	0.281	0.005	3.81945790711252E-290	Proliferating T/NK	ECT2
5.80402249617927E-291	5.3695621104489	0.232	0.003	9.46694109351801E-287	Proliferating T/NK	CCNA2
1.70126141816661E-290	5.03227092627987	0.272	0.005	2.77492749917156E-286	Proliferating T/NK	SPAG5
3.7749975511191E-290	4.14208516099234	0.417	0.017	6.15739850563036E-286	Proliferating T/NK	FANCA
9.40303523345084E-286	4.16216380821114	0.36	0.011	1.53372907692817E-281	Proliferating T/NK	MXD3
1.05261349349801E-282	3.70063165899338	0.461	0.022	1.71691786924461E-278	Proliferating T/NK	INCENP
3.39277314381336E-275	5.04149845183006	0.268	0.005	5.53395227487396E-271	Proliferating T/NK	BUB1
1.53445762095708E-274	4.64998542315418	0.307	0.008	2.50285382554309E-270	Proliferating T/NK	CDT1
2.19886837762968E-272	3.13910244569336	0.592	0.04	3.58657421075177E-268	Proliferating T/NK	TACC3
5.36268934378036E-272	5.33280466041168	0.241	0.004	8.74708258864014E-268	Proliferating T/NK	PCLAF
1.19728227590536E-271	4.80346057685613	0.276	0.006	1.95288712022923E-267	Proliferating T/NK	CENPU
2.20687897329912E-269	4.03160087729706	0.364	0.013	3.59964029334819E-265	Proliferating T/NK	TCF19
1.02646341144439E-267	4.7656577870328	0.272	0.006	1.67426447040694E-263	Proliferating T/NK	DEPDC1B
4.32405915031914E-264	4.96930841093496	0.254	0.005	7.05297288008555E-260	Proliferating T/NK	NUF2
4.22968529724393E-253	5.00609666591001	0.263	0.006	6.89903968833457E-249	Proliferating T/NK	CIT
2.18460463616285E-248	3.28959966833801	0.667	0.06	3.56330862204523E-244	Proliferating T/NK	HMGB2
9.34248313778807E-246	6.30671534997881	0.197	0.002	1.52385242460461E-241	Proliferating T/NK	PKMYT1
2.56673365261468E-243	5.77163918649309	0.189	0.002	4.1865992607798E-239	Proliferating T/NK	KIF14
9.75454984664593E-243	4.30319902241051	0.311	0.01	1.59106462548642E-238	Proliferating T/NK	TUBB
2.48364578436271E-242	5.5286218987676	0.228	0.004	4.05107463887402E-238	Proliferating T/NK	TK1
3.53163195908768E-239	6.36778647499857	0.167	0.001	5.76044488846792E-235	Proliferating T/NK	E2F7
5.99146363127543E-239	7.8419515873466	0.154	0.001	9.77267632897335E-235	Proliferating T/NK	ESCO2
7.33535026952079E-239	7.46472834784495	0.154	0.001	1.19646898246154E-234	Proliferating T/NK	CKAP2L
6.66280152018823E-237	6.56835683178037	0.162	0.001	1.0867695559579E-232	Proliferating T/NK	CDC25C
7.57186427619198E-225	3.10014754909831	0.544	0.042	1.23504678208967E-220	Proliferating T/NK	EZH2
2.61211438556579E-224	5.85298296120863	0.167	0.002	4.26061977429636E-220	Proliferating T/NK	TTK
7.65395182804993E-224	4.70448154613524	0.211	0.004	1.24843608267322E-219	Proliferating T/NK	WDR62
1.01846521333193E-223	5.03298360980803	0.206	0.004	1.66121860946572E-219	Proliferating T/NK	C21orf58
1.71383124343216E-212	6.11565360656418	0.162	0.002	2.7954301411622E-208	Proliferating T/NK	TICRR
2.09535981862937E-212	3.32328405889662	0.373	0.019	3.41774140016637E-208	Proliferating T/NK	NDC80
1.4196493339336E-211	4.70254162281378	0.206	0.004	2.31559002857909E-207	Proliferating T/NK	BUB1B
2.69346258422323E-209	3.8876475251319	0.246	0.007	4.39330682112651E-205	Proliferating T/NK	UBE2T
5.57391268477658E-207	4.56490466435143	0.219	0.005	9.09160898013908E-203	Proliferating T/NK	FBXO5
7.77554723628817E-207	5.02817195305713	0.167	0.002	1.26826950971096E-202	Proliferating T/NK	NEIL3
9.78227655131197E-204	4.41005754840675	0.224	0.006	1.59558712828449E-199	Proliferating T/NK	CCNF
1.3700784818086E-199	2.97060062517273	0.513	0.042	2.23473501167801E-195	Proliferating T/NK	LMNB1
2.33867474275537E-199	4.36290037284762	0.211	0.005	3.81461237290828E-195	Proliferating T/NK	ARHGAP11A
9.56961411075992E-199	6.8353249143508	0.14	0.001	1.56089975760605E-194	Proliferating T/NK	CDC20
2.94897109542177E-193	5.59432028025842	0.154	0.002	4.81006675374245E-189	Proliferating T/NK	HIST1H2BB
3.98297935747689E-192	5.51025735228392	0.167	0.003	6.49663762998055E-188	Proliferating T/NK	CCNB2
7.57553832828603E-192	5.0291426873259	0.171	0.003	1.23564605672673E-187	Proliferating T/NK	KNL1
1.22531626670927E-188	6.66114915244922	0.132	0.001	1.99861336262949E-184	Proliferating T/NK	KIF4A
1.84969197782633E-187	2.98521820059559	0.553	0.053	3.01703258503253E-183	Proliferating T/NK	LMNB2
4.1351343627094E-186	6.18971228667666	0.136	0.001	6.74481765901531E-182	Proliferating T/NK	E2F8
1.66942545856222E-185	3.44328067769728	0.285	0.012	2.72299986546083E-181	Proliferating T/NK	SMC2
6.87950873280695E-185	2.69341867818314	0.684	0.083	1.12211666940814E-180	Proliferating T/NK	KIF22
2.81896584165961E-183	5.21979943977489	0.175	0.003	4.59801518433099E-179	Proliferating T/NK	STMN1
1.60313050580359E-180	7.05685447229899	0.114	0	2.61486616801624E-176	Proliferating T/NK	PBK
6.00525060880032E-180	5.70192639208411	0.14	0.002	9.79516426801421E-176	Proliferating T/NK	KIF20A

1.54620334708949E-178	4.72449824683559	0.167	0.003	2.52201227943767E-174	Proliferating T/NK	HIST1H2BN
2.02630959395748E-178	5.71655209163833	0.149	0.002	3.30511357870404E-174	Proliferating T/NK	POC1A
3.80461068986595E-175	2.69772393314623	0.557	0.057	6.20570049624036E-171	Proliferating T/NK	SMC4
9.66293942998017E-175	3.49059938568689	0.355	0.022	1.57612205042407E-170	Proliferating T/NK	HDGF
1.17766806782106E-174	2.65976654624625	0.667	0.083	1.92089438542294E-170	Proliferating T/NK	TMPO
6.79465873758868E-174	5.86001538079321	0.132	0.001	1.10827678668809E-169	Proliferating T/NK	EXO1
1.01857938260321E-172	7.87186200052284	0.11	0	1.6614048309641E-168	Proliferating T/NK	SHMT1
8.19888011011549E-172	4.10250706548364	0.202	0.006	1.33731933476094E-167	Proliferating T/NK	NCAPH
7.13341291137287E-169	3.57573925745913	0.237	0.009	1.16353097997403E-164	Proliferating T/NK	HIST1H2AE
4.4891143390775E-168	4.3619338760489	0.171	0.004	7.3221943984693E-164	Proliferating T/NK	RECQL4
1.41104012123043E-167	4.85729987789629	0.149	0.002	2.30154754173896E-163	Proliferating T/NK	RAD51AP1
1.73194824850989E-164	4.56927768104593	0.167	0.004	2.82498078814449E-160	Proliferating T/NK	CDC45
1.45641653367972E-163	5.03213784817487	0.171	0.004	2.37556100808499E-159	Proliferating T/NK	DTL
6.98426028397511E-161	6.117645726497	0.11	0.001	1.13920269491918E-156	Proliferating T/NK	SKA3
3.8034588647777E-158	5.51018524868364	0.127	0.002	6.20382175433891E-154	Proliferating T/NK	DIAPH3
8.02076523276812E-157	3.70296979143631	0.241	0.011	1.30826701711681E-152	Proliferating T/NK	MCM2
5.59260620657452E-154	5.63443345965581	0.132	0.002	9.1220999835437E-150	Proliferating T/NK	FAM72C
8.7104932286372E-153	3.56016249391327	0.211	0.008	1.42076855052301E-148	Proliferating T/NK	PIF1
2.60656957754442E-152	3.73563152296637	0.206	0.007	4.25157563793271E-148	Proliferating T/NK	CEP55
2.98402032318926E-152	4.43181532401848	0.167	0.004	4.86723554915401E-148	Proliferating T/NK	CEP72
6.6797358425477E-152	5.62646123029536	0.118	0.001	1.08953171327796E-147	Proliferating T/NK	DSCC1
5.68804831798258E-151	3.21038931386556	0.272	0.015	9.27777561146139E-147	Proliferating T/NK	CHTF18
6.37269340401872E-150	2.86098505589237	0.522	0.06	1.03945002112949E-145	Proliferating T/NK	HELLS
4.72966681291017E-148	3.46007306017672	0.228	0.01	7.71455953853777E-144	Proliferating T/NK	C1orf112
1.01849574371455E-144	6.1408150281416	0.114	0.001	1.6612684075728E-140	Proliferating T/NK	MCM10
5.99805908581126E-143	2.67350306185417	0.434	0.042	9.78343417486674E-139	Proliferating T/NK	NCAPD3
4.67804775798858E-141	2.91550680534112	0.32	0.023	7.63036369805517E-137	Proliferating T/NK	CHAF1A
4.47594554266931E-140	5.12600456517554	0.118	0.002	7.3007147746479E-136	Proliferating T/NK	MND1
5.96947533899557E-140	5.36089009191869	0.123	0.002	9.73681122543568E-136	Proliferating T/NK	IQGAP3
4.74931287939596E-139	2.87998515122843	0.329	0.024	7.74660423758275E-135	Proliferating T/NK	PTTG1
9.33316606286029E-139	4.42078827748313	0.154	0.004	1.52233271651314E-134	Proliferating T/NK	ORC6
1.43654174498352E-137	2.64800326413131	0.421	0.041	2.34314324024262E-133	Proliferating T/NK	NEMP1
5.75996551206277E-137	4.56184735804338	0.127	0.002	9.39507974672559E-133	Proliferating T/NK	PSRC1
1.0764847898949E-136	5.35011997243704	0.123	0.002	1.75585434079757E-132	Proliferating T/NK	SMTN
2.55791442326976E-135	3.25549549966269	0.25	0.014	4.1722142157953E-131	Proliferating T/NK	SCP2
2.20923059333421E-134	3.48107456916044	0.202	0.009	3.60347602078743E-130	Proliferating T/NK	DEPRECATED-ENSG00000132646
3.15246618586923E-134	5.46833759163678	0.105	0.001	5.1419875957713E-130	Proliferating T/NK	XRCC2
4.21776433521986E-134	3.62994211477716	0.206	0.009	6.87959540717711E-130	Proliferating T/NK	CDKN2C
1.15772296033871E-133	3.67645185372036	0.259	0.015	1.88836192060847E-129	Proliferating T/NK	ARHGAP33
1.90524587098444E-133	5.63423297206753	0.11	0.001	3.10764654016272E-129	Proliferating T/NK	FAM111B
2.13658328529826E-133	3.12945016872708	0.254	0.015	3.48498099664999E-129	Proliferating T/NK	CENPN
2.65596081769448E-131	6.30103070071053	0.101	0.001	4.33213768974147E-127	Proliferating T/NK	E2F2
9.92187759658929E-131	3.66366832027631	0.232	0.012	1.61835745477968E-126	Proliferating T/NK	MT2A
7.74170819426164E-130	5.13076076015967	0.118	0.002	1.26275002356602E-125	Proliferating T/NK	SAPCD2
1.66359601730786E-127	3.61611367932746	0.211	0.01	2.71349146383086E-123	Proliferating T/NK	MCM4
2.44039080556091E-127	4.44965110287595	0.123	0.002	3.98052144295041E-123	Proliferating T/NK	CENPW
3.88888907176357E-126	5.26453378515288	0.11	0.002	6.34316696495356E-122	Proliferating T/NK	RAD51
6.76024545817005E-125	2.88900730494783	0.294	0.022	1.10266363668212E-120	Proliferating T/NK	KNTC1
9.92609696766654E-125	4.89540168547455	0.123	0.003	1.61904567639609E-120	Proliferating T/NK	CLSPN
1.74046111958357E-124	4.10367130260703	0.145	0.004	2.83886613215276E-120	Proliferating T/NK	SGO2
8.3011331752191E-123	3.967828862733	0.14	0.004	1.35399783220999E-118	Proliferating T/NK	TXNDC5
2.0674382842474E-122	3.43314132305628	0.184	0.008	3.37219858543594E-118	Proliferating T/NK	UBE2S
4.03901757983791E-120	3.27898870285506	0.215	0.011	6.58804157447362E-116	Proliferating T/NK	ATAD5
2.01161024346542E-119	2.40072326102412	0.346	0.032	3.28113746811644E-115	Proliferating T/NK	NSD2
2.19776002220948E-118	3.16934376031186	0.206	0.011	3.58476637222589E-114	Proliferating T/NK	CENPO
2.73140231656384E-117	2.74766522011111	0.316	0.027	4.45519031854727E-113	Proliferating T/NK	TMEM106C
2.13418897859303E-116	2.38060058722998	0.386	0.041	3.48107564298309E-112	Proliferating T/NK	NCAPD2
4.15900330810547E-115	4.53223953758565	0.14	0.004	6.78375029585083E-111	Proliferating T/NK	WDR34
5.61910877165135E-115	4.05828592906125	0.136	0.004	9.16532831744051E-111	Proliferating T/NK	PARPBP
2.78727446724235E-114	4.10136740666918	0.114	0.002	4.54632338351899E-110	Proliferating T/NK	HIST1H2BM
9.23574450415897E-114	3.14741409342341	0.215	0.012	1.50644228607337E-109	Proliferating T/NK	DTYMK
1.11812856279757E-113	2.82864809060508	0.325	0.03	1.82377949877911E-109	Proliferating T/NK	APOA2
2.74667023293748E-113	2.410949849035	0.377	0.04	4.48009381694432E-109	Proliferating T/NK	POLE
3.13352481473605E-112	4.53863889398691	0.123	0.003	5.11109232531597E-108	Proliferating T/NK	GINS1
7.13167053657623E-112	4.76691186159254	0.114	0.003	1.16324678122095E-107	Proliferating T/NK	CCNB1
2.92543546197011E-109	5.12648494259928	0.101	0.002	4.77167778201944E-105	Proliferating T/NK	SAC3D1

5.79168956429703E-108	3.26483858192298	0.189	0.01	9.44682484832489E-104	Proliferating T/NK	DNA2
5.98414304643202E-108	4.03730657388013	0.14	0.005	9.76073572303527E-104	Proliferating T/NK	SLC27A3
3.34037497872119E-106	4.57823020537985	0.118	0.003	5.44848562779213E-102	Proliferating T/NK	BRCA1
1.06585469940437E-105	2.89874076057933	0.241	0.017	1.73851560019846E-101	Proliferating T/NK	CENPP
3.03819505465993E-104	2.4445469842523	0.285	0.025	4.95559995365581E-100	Proliferating T/NK	TTF2
4.77465309087126E-104	3.56255770642986	0.154	0.006	7.78793665652011E-100	Proliferating T/NK	PCNA
1.9519523618831E-102	2.59020225285502	0.338	0.036	3.18382949746753E-98	Proliferating T/NK	ETFB
4.44236940408737E-102	3.33933687805887	0.193	0.011	7.24594873500691E-98	Proliferating T/NK	TIMELESS
3.00661086541706E-100	2.49332921387256	0.246	0.019	4.90408298258176E-96	Proliferating T/NK	TEDC1
2.32890556307127E-99	4.71655980080509	0.105	0.003	3.79867786392555E-95	Proliferating T/NK	FAM83D
8.64961081914767E-99	3.87610243274294	0.132	0.005	1.41083802071118E-94	Proliferating T/NK	POLE2
3.4618980095167E-97	3.60343719311086	0.132	0.005	5.64670184332268E-93	Proliferating T/NK	ORC1
3.75023226943372E-97	3.19648997450219	0.184	0.011	6.11700385467334E-93	Proliferating T/NK	RFC3
3.06254087338079E-96	2.52380923443943	0.241	0.019	4.99531041857141E-92	Proliferating T/NK	H2AFZ
3.5646677840719E-96	1.74431326624482	0.741	0.158	5.81432962259968E-92	Proliferating T/NK	RBM15
5.04623630908797E-96	2.31070982234632	0.303	0.03	8.23091604375339E-92	Proliferating T/NK	CKAP5
1.88639312116307E-94	2.49173514595264	0.272	0.025	3.07689581992908E-90	Proliferating T/NK	UQCRC1
2.46341279476816E-94	4.19227766995938	0.11	0.003	4.01807260954635E-90	Proliferating T/NK	MT1E
1.23459484014119E-93	2.93621793354666	0.228	0.018	2.01374764375429E-89	Proliferating T/NK	CSRP1
2.37111857343032E-93	2.49539056594608	0.215	0.016	3.8675315051222E-89	Proliferating T/NK	RCCD1
1.32758290740625E-92	4.43221817656205	0.105	0.003	2.16542048027033E-88	Proliferating T/NK	CERS2
1.78822554093202E-91	2.1952169818785	0.377	0.049	2.91677467981421E-87	Proliferating T/NK	LIG1
9.20613726701582E-91	2.85415318175737	0.211	0.015	1.50161304962295E-86	Proliferating T/NK	ACOT7
2.38546036738469E-90	3.8996570587674	0.11	0.003	3.89092440524116E-86	Proliferating T/NK	TUBA1C
3.52064089124148E-89	2.44695712013355	0.228	0.019	5.74251735770397E-85	Proliferating T/NK	CDK2
5.39998934774004E-89	2.78938337350573	0.211	0.016	8.80792262509879E-85	Proliferating T/NK	ATAD2
8.78996525374528E-89	3.14927879722948	0.162	0.009	1.43373123253839E-84	Proliferating T/NK	GEN1
2.7114309922545E-87	2.5311613740743	0.228	0.019	4.42261509146632E-83	Proliferating T/NK	DEPRECATED-ENSG00000120802
1.30258506146259E-86	3.72280573148482	0.101	0.003	2.12464649375163E-82	Proliferating T/NK	HIST1H2AK
1.45118674905885E-85	2.67998554190113	0.276	0.029	2.36703070638989E-81	Proliferating T/NK	ANXA5
8.10363000508254E-85	3.95094419496559	0.105	0.003	1.32178309012901E-80	Proliferating T/NK	SLC37A4
9.19361495636839E-85	3.79313038520477	0.105	0.003	1.49957053553325E-80	Proliferating T/NK	CIP2A
4.58519012570244E-84	3.06864885234081	0.145	0.007	7.47890361403326E-80	Proliferating T/NK	DBF4B
6.19414002678497E-83	1.90443418201811	0.39	0.056	1.0103261797689E-78	Proliferating T/NK	ANP32E
6.78931925634709E-83	4.1897898554219	0.101	0.003	1.10740586390277E-78	Proliferating T/NK	CKS1B
1.60775621419661E-82	3.77426308473487	0.114	0.004	2.62241116097608E-78	Proliferating T/NK	FEN1
4.62976413525948E-82	2.72971107892615	0.175	0.012	7.55160828102174E-78	Proliferating T/NK	BARD1
5.29846262848867E-82	2.72101898433551	0.211	0.017	8.64232239332787E-78	Proliferating T/NK	MASTL
8.24561168754609E-80	2.71822895946848	0.193	0.015	1.34494172235564E-75	Proliferating T/NK	TXNRD1
1.30507866680473E-79	1.53544132233541	0.531	0.098	2.12871381342519E-75	Proliferating T/NK	PHF19
2.57275044456387E-79	3.76568742174843	0.101	0.003	4.19641325012813E-75	Proliferating T/NK	KIF18A
2.58729449208157E-79	2.43339875206838	0.259	0.027	4.22013604603425E-75	Proliferating T/NK	COX8A
3.42917375039582E-79	2.5463910568832	0.215	0.019	5.59332530427062E-75	Proliferating T/NK	SLC43A3
3.84544615500508E-79	2.14178087445807	0.272	0.03	6.27230722342879E-75	Proliferating T/NK	ANAPC11
5.1192979859006E-79	3.15321807126933	0.149	0.009	8.35008694480248E-75	Proliferating T/NK	TUBB4B
6.43019168366787E-79	3.14191296175577	0.162	0.01	1.04882856552307E-74	Proliferating T/NK	GMNN
1.2729907481753E-78	3.35238214559157	0.145	0.008	2.07637520934873E-74	Proliferating T/NK	CENPJ
2.64243058063875E-78	3.80996061635209	0.114	0.005	4.31006852007986E-74	Proliferating T/NK	BRIP1
6.22298969042636E-78	3.17954309244303	0.14	0.008	1.01503184840544E-73	Proliferating T/NK	SUV39H2
1.3062365179362E-77	1.70441616069401	0.399	0.061	2.13060238440573E-73	Proliferating T/NK	DDX39A
2.287085512548E-77	3.61374401181945	0.11	0.004	3.73046517951705E-73	Proliferating T/NK	BRCA2
2.90338631260062E-77	2.81621859246542	0.162	0.011	4.73571341448288E-73	Proliferating T/NK	DDX11
7.7017749688026E-77	2.02313972598704	0.276	0.031	1.25623651516139E-72	Proliferating T/NK	SAE1
1.44749489465743E-76	3.62578383496104	0.105	0.004	2.36100892267574E-72	Proliferating T/NK	CDCA7
9.40140535467672E-76	2.94950180131818	0.189	0.015	1.53346322740132E-71	Proliferating T/NK	P4HB
1.31229288934357E-75	2.32782192997825	0.25	0.027	2.1404809318083E-71	Proliferating T/NK	POLD1
6.14952733465543E-74	3.3967276856898	0.136	0.008	1.00304940355565E-69	Proliferating T/NK	E2F1
7.3625363418315E-74	2.58534931109584	0.202	0.018	1.20090330271614E-69	Proliferating T/NK	NDUFC2
8.94832406779285E-74	2.50127654793655	0.171	0.013	1.45956113869769E-69	Proliferating T/NK	FANCG
3.90177275670932E-73	3.24732548298872	0.114	0.005	6.36418154346857E-69	Proliferating T/NK	KNSTRN
5.22956419840635E-73	2.72617845625295	0.158	0.011	8.52994216402059E-69	Proliferating T/NK	RCC1
7.10661758539804E-73	1.79681582521366	0.509	0.101	1.15916039435428E-68	Proliferating T/NK	MT-CO2
3.0690500516243E-72	3.16255972857793	0.132	0.007	5.00592753920439E-68	Proliferating T/NK	CEP152
2.31777997713503E-71	2.15030545887667	0.281	0.035	3.78053092070494E-67	Proliferating T/NK	MCM3
2.92802889614425E-71	2.11395013054008	0.276	0.034	4.77590793250089E-67	Proliferating T/NK	KIF20B
1.07446968632376E-70	2.65727998737657	0.197	0.018	1.75256750536269E-66	Proliferating T/NK	ZGRF1

1.06803075605028E-69	3.55916898583902	0.105	0.005	1.74206496619361E-65	Proliferating T/NK	MGST3
2.52418393674747E-69	3.08652516709904	0.123	0.007	4.11719641922879E-65	Proliferating T/NK	CENPH
3.29300516063028E-69	2.50931584312111	0.14	0.009	5.37122071750406E-65	Proliferating T/NK	CDCA4
5.26504780314211E-69	2.89872719638387	0.149	0.01	8.5878194717051E-65	Proliferating T/NK	SUV39H1
1.17783870702729E-68	2.01221461521251	0.215	0.022	1.92117271503222E-64	Proliferating T/NK	CNTROB
1.24531094825849E-68	1.64375654383722	0.439	0.08	2.03122668770443E-64	Proliferating T/NK	CDK5RAP2
3.26508362918134E-68	1.927509153623	0.285	0.037	5.32567790755769E-64	Proliferating T/NK	CBX5
9.88573764918143E-67	1.55068956313806	0.487	0.098	1.61246266795798E-62	Proliferating T/NK	DNMT1
8.06581763247859E-65	1.5458527741467	0.364	0.06	1.31561551403358E-60	Proliferating T/NK	SMC1A
1.3264582681381E-64	2.80742821749746	0.132	0.008	2.16358608116006E-60	Proliferating T/NK	PGM1
1.48721788812033E-64	3.26197243565545	0.14	0.01	2.42580109731308E-60	Proliferating T/NK	NME1
1.78279058572109E-64	2.00874922069432	0.189	0.018	2.90790972436968E-60	Proliferating T/NK	MCM8
9.78542300305661E-64	2.64859417493225	0.158	0.013	1.59610034602856E-59	Proliferating T/NK	ITGB3BP
1.14535504199756E-63	1.66557728002624	0.386	0.068	1.86818860900222E-59	Proliferating T/NK	NCAPH2
3.3918312144835E-63	2.69074775285653	0.154	0.012	5.53241589394403E-59	Proliferating T/NK	RNF26
3.79958130699727E-63	2.34138222587808	0.162	0.013	6.19749706984325E-59	Proliferating T/NK	NUDT1
1.12733162997279E-62	2.55816448850574	0.145	0.011	1.83879062164862E-58	Proliferating T/NK	CKS2
1.23166432972243E-62	3.39899326195344	0.101	0.005	2.00896768821026E-58	Proliferating T/NK	C18orf54
1.45383182879518E-62	2.30907064557521	0.211	0.023	2.37134509594782E-58	Proliferating T/NK	CARHSP1
1.82377957306374E-62	3.07842045044599	0.127	0.008	2.97476686162426E-58	Proliferating T/NK	ACAA2
2.69490425142897E-62	1.89550663447	0.228	0.027	4.3956583245058E-58	Proliferating T/NK	HAUS5
1.14972954530572E-61	2.10575475242657	0.206	0.022	1.87532386134816E-57	Proliferating T/NK	ALYREF
2.31262845307047E-61	2.69350287740358	0.175	0.016	3.77212826980324E-57	Proliferating T/NK	SOD1
3.83818088738979E-61	2.53327193834643	0.158	0.013	6.26045684542149E-57	Proliferating T/NK	KPNA2
5.59783792681566E-61	3.08847214150839	0.114	0.007	9.13063344242903E-57	Proliferating T/NK	ZNF367
1.10264324903762E-60	1.75297656439645	0.386	0.072	1.79852140350526E-56	Proliferating T/NK	MCM7
1.59678666709354E-60	2.46594259143416	0.18	0.017	2.60451873269628E-56	Proliferating T/NK	WDR76
1.15699823317244E-59	1.95780836858775	0.232	0.029	1.88717981812757E-55	Proliferating T/NK	RRM1
1.72468755171021E-59	3.34451875549403	0.101	0.005	2.81313786559452E-55	Proliferating T/NK	PHGDH
1.76255865321143E-59	1.50027998102401	0.377	0.068	2.87490941925317E-55	Proliferating T/NK	NUCKS1
7.26592284234913E-59	2.69798499546855	0.136	0.01	1.18514467481557E-54	Proliferating T/NK	HIRIP3
4.68407292272188E-58	2.14715001176157	0.171	0.016	7.64019134425165E-54	Proliferating T/NK	CEP131
1.07142744313649E-57	1.40274650509299	0.535	0.125	1.74760530249992E-53	Proliferating T/NK	CALM3
2.31500534314453E-57	3.07941232814192	0.123	0.008	3.77600521520304E-53	Proliferating T/NK	SLC19A1
3.00436223205623E-57	1.73568049912939	0.268	0.039	4.90041523670691E-53	Proliferating T/NK	EBP
3.37681611848598E-57	2.52686687040892	0.158	0.014	5.50792477086249E-53	Proliferating T/NK	SPATS2L
5.87298928355659E-57	1.64761688358145	0.443	0.095	9.57943282040916E-53	Proliferating T/NK	CRIP1
6.00072072923641E-57	2.78435151021449	0.105	0.006	9.78777558145751E-53	Proliferating T/NK	JPT1
1.18956862822524E-56	1.54844705749459	0.496	0.115	1.94030538949819E-52	Proliferating T/NK	COTL1
1.38113585041223E-56	2.78375637374466	0.136	0.011	2.2527706856074E-52	Proliferating T/NK	SPIN4
2.80816090490337E-56	1.50741407274985	0.289	0.045	4.58039125198788E-52	Proliferating T/NK	ACADVL
3.91052796838474E-56	2.67511952648263	0.118	0.008	6.37846216923235E-52	Proliferating T/NK	ARHGAP19
5.63777400528742E-56	1.99774642044013	0.162	0.015	9.19577318002431E-52	Proliferating T/NK	MFGE8
1.628280430323E-55	2.74659833370238	0.118	0.008	2.65588820989984E-51	Proliferating T/NK	CHEK2
2.09816848974752E-55	1.55205336035163	0.351	0.064	3.42232262362718E-51	Proliferating T/NK	SIVA1
4.74920327768805E-55	1.74521360737946	0.281	0.044	7.74642546623698E-51	Proliferating T/NK	PARK7
5.31226026712609E-55	2.05849873345612	0.184	0.02	8.66482772170936E-51	Proliferating T/NK	ILVBL
5.44758143367232E-55	1.7664678987659	0.276	0.042	8.88555007646293E-51	Proliferating T/NK	DCTN3
6.98292367474477E-55	2.00776444970678	0.25	0.036	1.13898468058762E-50	Proliferating T/NK	HYOU1
9.89633113705696E-55	1.70461679989123	0.338	0.061	1.61419057176536E-50	Proliferating T/NK	MT-ATP6
1.60416294600889E-54	2.96946246449801	0.127	0.01	2.6165501812351E-50	Proliferating T/NK	WDR90
2.17933351247142E-54	1.95294008798426	0.228	0.03	3.55471352192133E-50	Proliferating T/NK	CYC1
3.81228808017489E-54	1.51447354576778	0.325	0.057	6.21822308757326E-50	Proliferating T/NK	PCNT
4.28501086935695E-54	1.37606447062988	0.325	0.056	6.98928122900813E-50	Proliferating T/NK	CEP128
7.73515774585245E-54	2.35810261446164	0.123	0.009	1.26168157992599E-49	Proliferating T/NK	GINS4
2.96307081246326E-53	2.1030398150468	0.232	0.032	4.83306480220882E-49	Proliferating T/NK	PDIA6
5.67137658230648E-53	2.96110298157809	0.101	0.006	9.2505823434001E-49	Proliferating T/NK	MGAT4B
1.02976169704217E-52	2.64612131880893	0.154	0.015	1.67964430404549E-48	Proliferating T/NK	DCXR
1.75033444494117E-52	1.79770001838135	0.219	0.029	2.85497051314354E-48	Proliferating T/NK	MAD2L2
3.93633737531143E-52	2.50277187147128	0.118	0.009	6.42055989287048E-48	Proliferating T/NK	BCL2L12
5.15141445999155E-52	1.93031203224462	0.211	0.027	8.40247212569222E-48	Proliferating T/NK	CD63
6.03814700514457E-52	1.86131178012921	0.276	0.045	9.84882158009131E-48	Proliferating T/NK	MYEF2
7.41395901262997E-52	1.83532410462824	0.294	0.05	1.20929085455007E-47	Proliferating T/NK	CYB5R3
8.99071601594895E-52	2.53782702823574	0.136	0.012	1.46647568936143E-47	Proliferating T/NK	BORA
1.31899406116613E-51	2.16125919727498	0.184	0.021	2.15141121316807E-47	Proliferating T/NK	SERPINA1
1.80414945680385E-51	1.3889540744847	0.39	0.08	2.94274817899275E-47	Proliferating T/NK	ECH1

2.44535798392713E-51	2.56248781569194	0.105	0.007	3.98862340758354E-47	Proliferating T/NK	MT1B
2.63663123125964E-51	2.73465132899711	0.101	0.006	4.3006092013076E-47	Proliferating T/NK	WDHD1
4.45457756682888E-51	1.76994692997846	0.202	0.025	7.26586146925459E-47	Proliferating T/NK	SLC7A1
4.68810386611884E-51	2.43192855488455	0.158	0.016	7.64676621602645E-47	Proliferating T/NK	PRPSAP1
1.826289375527E-50	1.81390394592983	0.162	0.017	2.97886060042209E-46	Proliferating T/NK	HIST3H2BB
2.59053703187845E-50	2.54875354761766	0.123	0.01	4.22542495269693E-46	Proliferating T/NK	ECI1
4.61272622194766E-50	1.36894692636043	0.399	0.085	7.52381774061884E-46	Proliferating T/NK	LAMP1
6.5580247474037E-50	1.36100279165751	0.364	0.073	1.06967941654902E-45	Proliferating T/NK	FAM111A
7.31194269188118E-50	1.39317909493643	0.316	0.057	1.19265097247274E-45	Proliferating T/NK	SMC3
1.15019076069224E-49	2.06594010183	0.149	0.015	1.8760761497651E-45	Proliferating T/NK	FTL
2.54554180015582E-49	2.46024718497344	0.127	0.011	4.15203323023416E-45	Proliferating T/NK	SASS6
7.3471896982683E-49	1.82680993229233	0.189	0.023	1.19840011168454E-44	Proliferating T/NK	TOPBP1
1.23141360945167E-48	1.86170553376699	0.197	0.025	2.00855873837661E-44	Proliferating T/NK	ATP5MC3
1.37907035876433E-48	2.42495208824481	0.132	0.012	2.24940166218049E-44	Proliferating T/NK	ZNHIT1
1.51003942468613E-48	2.06269603288873	0.206	0.028	2.46302530560555E-44	Proliferating T/NK	POLA1
3.01296200019992E-48	1.68424105495877	0.281	0.049	4.91444231852609E-44	Proliferating T/NK	MT-ND4
3.02007115613678E-48	2.63101101143226	0.154	0.016	4.92603806277469E-44	Proliferating T/NK	RTN3
4.9938323924803E-48	1.74752560180821	0.171	0.02	8.14544001537461E-44	Proliferating T/NK	TFDP1
9.80257171129272E-48	1.39602541727305	0.276	0.047	1.59889747182896E-43	Proliferating T/NK	TUBGCP3
1.36427883924644E-47	1.58540643484507	0.224	0.033	2.22527521469486E-43	Proliferating T/NK	CIITA
3.43833866529164E-47	1.86676286015767	0.219	0.032	5.60827419695719E-43	Proliferating T/NK	CXCL13
3.0494090779768E-46	1.89811850669543	0.14	0.014	4.97389114708796E-42	Proliferating T/NK	WRAP53
1.25101286291103E-45	1.96940241793489	0.154	0.017	2.04052708069418E-41	Proliferating T/NK	CEP85
1.3671059705018E-45	1.37309253776839	0.272	0.047	2.22988654848549E-41	Proliferating T/NK	POLR2L
2.08512960249071E-45	1.10595931308331	0.487	0.123	3.4010548946226E-41	Proliferating T/NK	SUGP2
2.5306864125697E-45	1.50388201703444	0.285	0.052	4.12780260754243E-41	Proliferating T/NK	MCM5
5.96485716548007E-45	1.41470606439776	0.25	0.042	9.72927852261454E-41	Proliferating T/NK	HNRNPAB
6.97617957735968E-45	2.82625025599187	0.105	0.008	1.13788465086314E-40	Proliferating T/NK	SIGMAR1
2.58264779888459E-44	2.78690977280978	0.105	0.008	4.21255682476066E-40	Proliferating T/NK	TP73
5.03077176723337E-44	2.39987456190755	0.118	0.01	8.20569182953434E-40	Proliferating T/NK	BANF1
6.62524570782853E-44	1.48013361508818	0.412	0.102	1.08064382740391E-39	Proliferating T/NK	PTMS
1.68077012703762E-43	2.67150544045034	0.114	0.01	2.74150415421107E-39	Proliferating T/NK	CCDC18
2.02240935344961E-43	1.42093345349828	0.294	0.057	3.29875189641166E-39	Proliferating T/NK	MAZ
2.32791079057469E-43	1.90742849695897	0.154	0.018	3.79705529050637E-39	Proliferating T/NK	YWHAH
2.33690618828661E-43	1.58071026688669	0.193	0.027	3.8117276837143E-39	Proliferating T/NK	RBL1
4.27137041910267E-43	2.43317684842886	0.101	0.008	6.96703229059836E-39	Proliferating T/NK	FANCD2
5.10926379184845E-43	2.15478855914334	0.14	0.015	8.333720170884E-39	Proliferating T/NK	CIAO2A
6.77407192998195E-43	0.986667345464062	0.382	0.086	1.10491887249936E-38	Proliferating T/NK	HNRNPUL2
2.00119474512656E-42	1.34211336344752	0.259	0.046	3.26414874877594E-38	Proliferating T/NK	DEK
2.27716509407111E-42	1.50561673422631	0.298	0.06	3.71428398493939E-38	Proliferating T/NK	AHI1
1.08523877067792E-41	2.10547496747389	0.145	0.016	1.77013295885276E-37	Proliferating T/NK	DPYSL2
1.18725143238436E-41	1.91043822322272	0.154	0.018	1.93652581136213E-37	Proliferating T/NK	CDC7
2.76961300614781E-41	1.51703267309334	0.232	0.039	4.51751577432768E-37	Proliferating T/NK	PSMC3
2.81532053881851E-41	2.13192837453781	0.145	0.017	4.59206933086687E-37	Proliferating T/NK	DDT
3.34086829096303E-41	2.29956734919614	0.11	0.01	5.4492902693898E-37	Proliferating T/NK	ATP8B3
5.45205981659559E-41	2.36328466561617	0.105	0.009	8.89285476684906E-37	Proliferating T/NK	IFI27L1
1.41724661827849E-40	1.85744138471497	0.158	0.02	2.31167095907405E-36	Proliferating T/NK	SQSTM1
2.18214391587271E-40	1.62688882061513	0.189	0.028	3.55929494117997E-36	Proliferating T/NK	FANCL
3.69925568153337E-40	1.47246583394311	0.202	0.031	6.03385594214908E-36	Proliferating T/NK	THOP1
4.29063751753403E-40	2.55065812643997	0.105	0.009	6.99845885484976E-36	Proliferating T/NK	MPC1
4.84852778820296E-40	1.85693506475993	0.189	0.028	7.90843367533785E-36	Proliferating T/NK	MYDGF
6.97520713044457E-40	1.28684337628426	0.259	0.048	1.13772603504681E-35	Proliferating T/NK	NDUFB7
7.57909502705222E-40	1.26618902053786	0.303	0.063	1.23622618986249E-35	Proliferating T/NK	MT-ND4L
1.11639605379884E-39	1.76197026190205	0.145	0.017	1.8209536033513E-35	Proliferating T/NK	EMC9
1.17014179406629E-39	1.35783521475087	0.259	0.049	1.90861828030153E-35	Proliferating T/NK	DOT1L
1.56998737407949E-39	1.44341296354939	0.224	0.038	2.56080640586105E-35	Proliferating T/NK	EIF4A3
1.62204196527946E-39	0.960147072158635	0.447	0.116	2.64571264956732E-35	Proliferating T/NK	HNRNPUL1
1.64292192040989E-39	1.95458906850649	0.162	0.021	2.67976994438058E-35	Proliferating T/NK	MSH5
1.98562986029899E-39	2.04185897827274	0.145	0.017	3.23876086513368E-35	Proliferating T/NK	RNF5
2.16101598752587E-39	1.86995184488201	0.136	0.015	3.52483317725345E-35	Proliferating T/NK	RAD18
7.03955278588873E-39	1.44875665950589	0.289	0.06	1.14822145490631E-34	Proliferating T/NK	ENO1
1.00861866039853E-38	1.12066392952647	0.364	0.086	1.64515789697605E-34	Proliferating T/NK	CDC25B
1.43656893779613E-38	1.51294483628828	0.241	0.044	2.34318759443926E-34	Proliferating T/NK	PPIB
1.81520465257706E-38	1.49168781704239	0.232	0.042	2.96078030881844E-34	Proliferating T/NK	H2AFY
2.3637650391029E-38	2.43626322556802	0.101	0.009	3.85553715528074E-34	Proliferating T/NK	CHP1
2.50649326254513E-38	1.40709900916468	0.259	0.05	4.08834116053736E-34	Proliferating T/NK	EEF1AKMT2

3.37749179942674E-38	1.76020666417565	0.167	0.023	5.50902687404496E-34	Proliferating T/NK	NUCB2
3.4704441700766E-38	0.928110757552126	0.421	0.109	5.66064148581193E-34	Proliferating T/NK	NOTCH1
3.97177551026316E-38	2.51911862341933	0.11	0.01	6.47836303479023E-34	Proliferating T/NK	INSIG1
3.98278332687044E-38	1.26803378995022	0.241	0.044	6.49631788445838E-34	Proliferating T/NK	PMVK
4.3800938426703E-38	1.73660268386077	0.193	0.03	7.14437106677953E-34	Proliferating T/NK	ROMO1
5.80893800795915E-38	1.65461393098286	0.175	0.026	9.47495878478217E-34	Proliferating T/NK	POLA2
6.71574652942848E-38	1.18670166087342	0.456	0.127	1.09540541641508E-33	Proliferating T/NK	IDH2
7.75490768244318E-38	1.6580042201165	0.18	0.027	1.26490299208331E-33	Proliferating T/NK	ZMIZ1
1.17020362016687E-37	1.31480807622673	0.25	0.048	1.90871912485418E-33	Proliferating T/NK	PI4K2B
1.5516690130372E-37	1.22786467861485	0.482	0.142	2.53092732716497E-33	Proliferating T/NK	MT-CO3
2.03149481222528E-37	1.55932132413096	0.211	0.036	3.31357118822066E-33	Proliferating T/NK	CALM2
4.1957549401615E-37	1.34305926898753	0.241	0.045	6.84369588289742E-33	Proliferating T/NK	CEP192
5.0475213288973E-37	1.75795246470041	0.145	0.018	8.23301203956439E-33	Proliferating T/NK	DHFR
6.4334362583909E-37	1.1140993317854	0.338	0.079	1.04935778810614E-32	Proliferating T/NK	UBE2I
8.63769432261916E-37	1.8965850174352	0.149	0.02	1.40889432096241E-32	Proliferating T/NK	SLX4
9.08469220795363E-37	0.987711170131361	0.311	0.069	1.48180414603932E-32	Proliferating T/NK	SRRT
1.12375563112577E-36	1.45225371840044	0.224	0.04	1.83295780992925E-32	Proliferating T/NK	TMED9
1.20284220229304E-36	1.25889709272556	0.254	0.05	1.96195591616018E-32	Proliferating T/NK	HP1BP3
2.35230428753403E-36	1.97811480430327	0.127	0.015	3.83684352339676E-32	Proliferating T/NK	C19orf48
2.52447918936121E-36	1.31090191003279	0.202	0.034	4.11767800576706E-32	Proliferating T/NK	NDUFA2
3.16067822293487E-36	1.66460950067244	0.167	0.024	5.15538224942906E-32	Proliferating T/NK	HSD17B10
4.89208967139118E-36	2.22370819124996	0.136	0.017	7.97948746300616E-32	Proliferating T/NK	MT-ND5
6.88886517786065E-36	1.38549029016486	0.25	0.05	1.12364279916085E-31	Proliferating T/NK	TRIOBP
7.3142757670553E-36	1.44457139063031	0.175	0.027	1.19303152036439E-31	Proliferating T/NK	NDUFB9
9.50212004474973E-36	1.84406323977496	0.145	0.019	1.54989080049913E-31	Proliferating T/NK	ARPC5
9.73265675909174E-36	1.77243903067779	0.145	0.019	1.58749364397545E-31	Proliferating T/NK	NDUFAB1
1.00301409118666E-35	1.7194240441364	0.162	0.023	1.63601628413456E-31	Proliferating T/NK	PSMB4
1.35004866360497E-35	0.944376405548013	0.504	0.15	2.20206437520607E-31	Proliferating T/NK	CNTRL
1.66716422353191E-35	1.04135885871682	0.36	0.09	2.7193115650029E-31	Proliferating T/NK	CD38
1.70298080489929E-35	1.18503582551176	0.263	0.054	2.77773199087123E-31	Proliferating T/NK	NUDT21
2.57926698851764E-35	1.51172351212226	0.18	0.028	4.20704238497112E-31	Proliferating T/NK	LRP8
4.44383494778794E-35	1.75298644203259	0.127	0.015	7.24833918333691E-31	Proliferating T/NK	KMT5A
1.07819726743667E-34	1.09820018622954	0.285	0.063	1.75864756291595E-30	Proliferating T/NK	NUP50
1.09371981667403E-34	1.67858716337476	0.167	0.025	1.783966392977E-30	Proliferating T/NK	ACSL4
1.58396007947314E-34	1.11667842770291	0.364	0.093	2.58359728562863E-30	Proliferating T/NK	PKM
1.9215381938587E-34	2.03727301455568	0.114	0.012	3.13422094800293E-30	Proliferating T/NK	PGD
2.44927276598372E-34	1.18591180719532	0.224	0.042	3.99500880859604E-30	Proliferating T/NK	PPM1G
2.80407278760186E-34	1.28462666391609	0.202	0.036	4.5737231238574E-30	Proliferating T/NK	GOLIM4
2.85743392105341E-34	0.972982892689168	0.338	0.083	4.66076046863022E-30	Proliferating T/NK	TRIM26
3.77485768464672E-34	1.00733926304339	0.377	0.099	6.15717036942726E-30	Proliferating T/NK	HNRNPD
3.87739973059025E-34	1.45659269814086	0.175	0.028	6.32442670056576E-30	Proliferating T/NK	NDUFB11
4.65766842140191E-34	1.02983054090505	0.259	0.054	7.59712296214866E-30	Proliferating T/NK	TBC1D10B
6.48128698688941E-34	1.17332448483489	0.583	0.208	1.05716272043153E-29	Proliferating T/NK	H1FX
7.02746655098694E-34	1.24055324314855	0.219	0.041	1.14625006913148E-29	Proliferating T/NK	NDUFB5
7.78504016562491E-34	1.28090495629124	0.237	0.047	1.26981790141508E-29	Proliferating T/NK	NUDC
8.72667689140172E-34	0.921180906032403	0.456	0.131	1.42340826775654E-29	Proliferating T/NK	NUCB1
1.99598084817183E-33	1.32390007123821	0.211	0.039	3.25564436145307E-29	Proliferating T/NK	NDUFA13
2.12766860883688E-33	1.18344290884225	0.785	0.412	3.47044026787384E-29	Proliferating T/NK	PFN1
2.17949215360505E-33	1.18855939168527	0.285	0.065	3.55496965174519E-29	Proliferating T/NK	YBX1
2.44814026076453E-33	1.57772652081589	0.145	0.02	3.99316157933303E-29	Proliferating T/NK	PPP2R3B
2.5657998083815E-33	1.77311241403096	0.132	0.017	4.18507606745106E-29	Proliferating T/NK	EBNA1BP2
2.6158485558103E-33	0.79308406993397	0.434	0.122	4.26671057938219E-29	Proliferating T/NK	LBR
2.78521320304433E-33	1.25216963258511	0.303	0.072	4.54296125548561E-29	Proliferating T/NK	ADCY3
3.00301722378405E-33	1.52526797962458	0.175	0.028	4.89822139371417E-29	Proliferating T/NK	GDI2
3.43473681076654E-33	1.89932779469328	0.101	0.01	5.6023992120413E-29	Proliferating T/NK	SNRNP25
3.66236555796338E-33	1.93178928323985	0.114	0.013	5.97368446159407E-29	Proliferating T/NK	COMTD1
4.209606043203E-33	0.878076884963392	0.636	0.217	6.86628841706842E-29	Proliferating T/NK	HNRNPA0
4.6599415891354E-33	0.795526826288959	0.43	0.12	7.60083072603875E-29	Proliferating T/NK	SLC3A2
5.93061618002654E-33	1.04190552483279	0.25	0.052	9.67342805124129E-29	Proliferating T/NK	SNRPB
6.73359412463539E-33	0.614246777061426	0.434	0.122	1.09831653766928E-28	Proliferating T/NK	RASSF1
7.35054360211704E-33	1.70213614701858	0.171	0.028	1.19894716694131E-28	Proliferating T/NK	CDK14
8.47296061648434E-33	1.14109123477512	0.276	0.062	1.38202460615476E-28	Proliferating T/NK	SUMO3
1.07024356428781E-32	1.04812927965111	0.241	0.05	1.74567427770985E-28	Proliferating T/NK	RABL6
1.10588912424556E-32	1.29687078559604	0.25	0.053	1.80381575055693E-28	Proliferating T/NK	GSTP1
1.19030853703818E-32	1.375686912814	0.162	0.025	1.94151225476297E-28	Proliferating T/NK	YWHAQ
1.2925312792112E-32	1.85976130310191	0.127	0.016	2.10824776952138E-28	Proliferating T/NK	GRHPR

1.31767889102459E-32	1.65884718218407	0.154	0.023	2.1492660391502E-28	Proliferating T/NK	RFWD3
1.43052600387894E-32	1.22354740536219	0.202	0.037	2.33333096492694E-28	Proliferating T/NK	PMM1
1.77186853021915E-32	0.960414069113479	0.325	0.081	2.89009475964046E-28	Proliferating T/NK	MCM6
3.13448527422274E-32	1.44211872388231	0.18	0.031	5.11265893078472E-28	Proliferating T/NK	CLTA
3.29041586061831E-32	1.84253646355717	0.14	0.02	5.36699731025453E-28	Proliferating T/NK	GPX4
3.4135792959564E-32	1.67254685623227	0.14	0.02	5.56788918963448E-28	Proliferating T/NK	MPDU1
3.78615684074257E-32	0.845359615890746	0.434	0.126	6.1756004229352E-28	Proliferating T/NK	NFATC2IP
5.71465185989555E-32	1.02246989650824	0.316	0.078	9.32116864867562E-28	Proliferating T/NK	PA2G4
7.63227369032567E-32	1.12895504629293	0.237	0.049	1.24490016162902E-27	Proliferating T/NK	MZT2B
8.31406269282435E-32	1.36618872820617	0.14	0.02	1.35610676582658E-27	Proliferating T/NK	FIBP
1.01077851702628E-31	0.937895166725275	0.268	0.06	1.64868083912157E-27	Proliferating T/NK	FTH1
1.04406092340545E-31	0.852504297801019	0.303	0.073	1.70296777216663E-27	Proliferating T/NK	RNF168
1.08818952503874E-31	1.80994482212861	0.158	0.025	1.77494593429068E-27	Proliferating T/NK	PRIM2
1.15025700699291E-31	1.39717941568297	0.175	0.03	1.87618420410614E-27	Proliferating T/NK	YWHAE
1.31763103589628E-31	1.66202797289875	0.114	0.014	2.14918798265042E-27	Proliferating T/NK	NDC1
1.33351816180852E-31	1.08785627434725	0.39	0.111	2.17510147372588E-27	Proliferating T/NK	MAT2A
1.64057743133768E-31	0.742149595940251	0.465	0.142	2.67594584825489E-27	Proliferating T/NK	WHRN
2.37216724728864E-31	1.38925140296787	0.206	0.04	3.8692419970525E-27	Proliferating T/NK	MTHFD1
2.4407016047178E-31	2.08866275580953	0.11	0.013	3.98102838745521E-27	Proliferating T/NK	TMEM201
2.6869521087242E-31	1.27282433696978	0.206	0.04	4.38268758454004E-27	Proliferating T/NK	CCDC82
3.12617191481932E-31	1.20512825166865	0.281	0.066	5.09909901026179E-27	Proliferating T/NK	MPHOSPH9
3.40096730216382E-31	1.68327572695221	0.167	0.028	5.5473177665594E-27	Proliferating T/NK	DNPH1
3.82114946618306E-31	0.914310804140244	0.285	0.067	6.2326768942912E-27	Proliferating T/NK	PFKP
4.5318842388701E-31	1.16340736213681	0.241	0.052	7.39195638202102E-27	Proliferating T/NK	NOP56
7.5625481461802E-31	1.59873193465456	0.136	0.019	1.23352722812345E-26	Proliferating T/NK	HAUS4
8.80288282915539E-31	1.22276746478038	0.175	0.03	1.43583821826354E-26	Proliferating T/NK	DEPRECATED-ENSG00000163463
9.89999564273182E-31	1.50675330066248	0.162	0.027	1.61478828928599E-26	Proliferating T/NK	NUTF2
1.07395272408248E-30	1.12978968748648	0.158	0.025	1.75172428825094E-26	Proliferating T/NK	USP1
1.5423080992786E-30	1.45748611180736	0.175	0.031	2.51565874073332E-26	Proliferating T/NK	RAB7A
2.03179308234245E-30	1.46893143731073	0.167	0.028	3.31405769660877E-26	Proliferating T/NK	RTEL1
2.0518788253926E-30	0.948100871828188	0.289	0.07	3.34681955209788E-26	Proliferating T/NK	KHDRBS1
2.60387779632437E-30	0.913723568914944	0.281	0.067	4.24718507358467E-26	Proliferating T/NK	ATP5IF1
2.97887095690347E-30	0.986739728516063	0.211	0.042	4.85883641780526E-26	Proliferating T/NK	SEM1
3.37907836440996E-30	1.84760972570198	0.118	0.015	5.51161472018908E-26	Proliferating T/NK	CENPK
4.1249279426831E-30	1.18364050913992	0.25	0.056	6.7281699673104E-26	Proliferating T/NK	SCLT1
6.36890475028101E-30	1.55139272887205	0.14	0.021	1.03883205381834E-25	Proliferating T/NK	HLTF
7.30102846063963E-30	0.970599958839037	0.289	0.071	1.19087075221493E-25	Proliferating T/NK	SLC7A5
8.84879636753714E-30	1.55457416231604	0.193	0.037	1.44332717550898E-25	Proliferating T/NK	PRDX2
9.4328463269033E-30	0.963636837000116	0.219	0.045	1.5385915643812E-25	Proliferating T/NK	TOX2
1.00410216771576E-29	0.847179037382637	0.478	0.15	1.63779104576118E-25	Proliferating T/NK	HNRNPF
1.07807144813043E-29	1.4555751167481	0.14	0.021	1.75844233904555E-25	Proliferating T/NK	POLR3K
1.10730406955221E-29	0.906374587377243	0.346	0.093	1.80612366784661E-25	Proliferating T/NK	CBFB
1.83746590467882E-29	1.93908425621927	0.101	0.011	2.99709063712162E-25	Proliferating T/NK	FADS2
2.14683150489222E-29	1.16447164531062	0.215	0.045	3.5016968676297E-25	Proliferating T/NK	HMGN3
2.15138215190134E-29	1.27454309175061	0.206	0.042	3.50911942796627E-25	Proliferating T/NK	SUZ12
2.60751093714552E-29	1.03952752433214	0.246	0.055	4.25311108957806E-25	Proliferating T/NK	GUSB
2.98902589113699E-29	1.79147940831097	0.118	0.016	4.87540013103354E-25	Proliferating T/NK	MID1IP1
3.08762121603939E-29	1.267764918899	0.167	0.029	5.03621896548185E-25	Proliferating T/NK	APOE
3.13437697974986E-29	1.07757061605067	0.224	0.048	5.11248229167E-25	Proliferating T/NK	TLNRD1
3.60322048448247E-29	1.60047730205124	0.114	0.015	5.87721293223935E-25	Proliferating T/NK	POLD3
4.07016691177647E-29	1.7652466719951	0.132	0.019	6.6388492497986E-25	Proliferating T/NK	LRRC45
4.70754333940307E-29	0.968273148858255	0.417	0.126	7.67847394090034E-25	Proliferating T/NK	FZR1
4.72013988895351E-29	1.02622410422521	0.263	0.062	7.69902017287208E-25	Proliferating T/NK	TENT4A
5.21195121469E-29	1.29480043794893	0.215	0.045	8.50121362628087E-25	Proliferating T/NK	SSRP1
5.40578565368713E-29	0.702476414303198	0.281	0.068	8.81737697972908E-25	Proliferating T/NK	SFXN1
5.74495100611217E-29	0.873818340266939	0.478	0.159	9.37058958606957E-25	Proliferating T/NK	PDCD1
6.11946069268028E-29	0.807064970307855	0.544	0.186	9.9814523358308E-25	Proliferating T/NK	ITM2A
8.48213970701956E-29	1.32305296609859	0.136	0.02	1.38352180761196E-24	Proliferating T/NK	ACAT2
9.64468805958977E-29	1.48667306731452	0.132	0.019	1.57314506939969E-24	Proliferating T/NK	CDK4
1.64364407102596E-28	0.987259966061131	0.228	0.05	2.68094784425044E-24	Proliferating T/NK	PTBP1
1.86647995826304E-28	1.59078025474249	0.154	0.026	3.04441545992284E-24	Proliferating T/NK	ODF2
2.02892369890295E-28	0.966609778455727	0.268	0.064	3.30937744528061E-24	Proliferating T/NK	ATP5F1D
2.06297795366395E-28	0.872222267392381	0.329	0.088	3.36492334022127E-24	Proliferating T/NK	RNF187
2.33355248373126E-28	1.92728994982474	0.132	0.02	3.80625745621406E-24	Proliferating T/NK	GUCD1
2.34935504117964E-28	0.892181643194992	0.25	0.058	3.83203300766811E-24	Proliferating T/NK	AP1M1
2.76616282078968E-28	1.6752051121214	0.118	0.016	4.51188817699005E-24	Proliferating T/NK	BRI3BP

5.71634198189526E-28	1.64779792406877	0.114	0.015	9.32392540666936E-24	Proliferating T/NK	RUVBL2
7.6017676806979E-28	1.06759238057397	0.311	0.084	1.23992432639863E-23	Proliferating T/NK	ENTPD1
8.08103453859973E-28	0.862197628162167	0.272	0.067	1.318097543591E-23	Proliferating T/NK	HCFC1
9.75857112036236E-28	0.926654483691286	0.254	0.061	1.5917205354423E-23	Proliferating T/NK	PPP6R3
9.80768627422805E-28	1.13668928896823	0.241	0.056	1.59973170818934E-23	Proliferating T/NK	NUP205
9.96769291624098E-28	1.43357636482709	0.127	0.019	1.62583039156807E-23	Proliferating T/NK	DEPRECATED-ENSG00000071564
1.36373023690019E-27	1.10603247816727	0.149	0.025	2.2243803894079E-23	Proliferating T/NK	C1orf35
1.57298585453331E-27	1.01102043169537	0.162	0.029	2.56569722732929E-23	Proliferating T/NK	NUP107
1.67078834448675E-27	1.63884465717226	0.118	0.017	2.72522286869234E-23	Proliferating T/NK	SF3B4
2.50809307039264E-27	1.28683899565	0.193	0.039	4.09095060711744E-23	Proliferating T/NK	C2CD5
2.62352731897262E-27	1.24225860901778	0.154	0.026	4.27923540997625E-23	Proliferating T/NK	CKAP2
3.21343560636254E-27	1.38052655276304	0.162	0.029	5.24143481753793E-23	Proliferating T/NK	DDRGK1
4.08699398363747E-27	0.951912774411173	0.228	0.051	6.66629588671107E-23	Proliferating T/NK	POLR2E
4.49813937891733E-27	0.894520842585538	0.281	0.072	7.33691514095206E-23	Proliferating T/NK	UBALD2
5.29492086627123E-27	1.89668201387788	0.118	0.017	8.63654542497499E-23	Proliferating T/NK	SPINDOC
5.65621984336952E-27	0.621917495678797	0.513	0.171	9.22586018652002E-23	Proliferating T/NK	TRIR
6.67723263857188E-27	0.861237877418394	0.281	0.072	1.08912341567746E-22	Proliferating T/NK	BAZ1B
7.26279502661485E-27	1.30403542013287	0.171	0.032	1.18463449679115E-22	Proliferating T/NK	SMS
7.51096820164541E-27	0.692778730645616	0.39	0.117	1.22511402337038E-22	Proliferating T/NK	SRSF2
9.2726643652781E-27	0.989100264883775	0.263	0.066	1.51246428462051E-22	Proliferating T/NK	NACC1
1.00425314932023E-26	1.35576514005347	0.184	0.037	1.63803731185623E-22	Proliferating T/NK	LSM 4.00
1.05004707382633E-26	0.996920481112898	0.193	0.04	1.71273178211813E-22	Proliferating T/NK	CCNG2
1.08826706705005E-26	1.62503309228782	0.145	0.024	1.77507241306534E-22	Proliferating T/NK	CALR
1.43662166353961E-26	1.52147871069523	0.114	0.016	2.34327359539946E-22	Proliferating T/NK	UBR7
1.89889179295041E-26	1.11377922142545	0.145	0.024	3.09728240348141E-22	Proliferating T/NK	GABARAP
2.00972591038605E-26	0.778552607155524	0.417	0.131	3.27806393243069E-22	Proliferating T/NK	HNRNPA3
2.72894845826411E-26	1.21830879626077	0.184	0.037	4.45118783027459E-22	Proliferating T/NK	MIS18BP1
3.11859686105247E-26	0.792012855059064	0.325	0.091	5.08674334006269E-22	Proliferating T/NK	AKT1
3.42488280566009E-26	0.646134515574784	0.399	0.125	5.58632634431217E-22	Proliferating T/NK	DUSP4
4.18052555579532E-26	1.11619932799956	0.25	0.062	6.81885523405774E-22	Proliferating T/NK	SLBP
5.18871504009314E-26	1.49426418332711	0.118	0.017	8.46331310189592E-22	Proliferating T/NK	ANXA2
5.57896600528603E-26	1.0888158324171	0.202	0.044	9.09985145122204E-22	Proliferating T/NK	NDUFS2
5.81501469031419E-26	0.805295156143197	0.259	0.065	9.48487046137148E-22	Proliferating T/NK	PSIP1
7.06349761727184E-26	1.34428339034744	0.184	0.038	1.15212709635321E-21	Proliferating T/NK	PRKACA
8.40261630883905E-26	1.00183466375932	0.232	0.055	1.37055074613474E-21	Proliferating T/NK	IRF4
8.41958325176919E-26	0.872106985745074	0.43	0.141	1.37331822419607E-21	Proliferating T/NK	H3F3A
8.60950423018351E-26	1.0102227069174	0.289	0.078	1.40429623498523E-21	Proliferating T/NK	NONO
9.09454403171396E-26	1.93094378419597	0.14	0.024	1.48341107701286E-21	Proliferating T/NK	ALB
9.1177330461744E-26	0.967234684743832	0.215	0.049	1.48719343716151E-21	Proliferating T/NK	GMPPB
1.27379190527794E-25	0.572115913660753	0.579	0.206	2.07768197669886E-21	Proliferating T/NK	MBD2
1.49424849164315E-25	1.83912596915214	0.118	0.018	2.43726871471915E-21	Proliferating T/NK	GALNT2
1.52500487586501E-25	0.669169025675218	0.474	0.16	2.48743545302341E-21	Proliferating T/NK	SRSF4
1.59058505215593E-25	0.712113956282466	0.259	0.065	2.59440327857154E-21	Proliferating T/NK	SUMO2
1.8538699351413E-25	0.837208091335637	0.272	0.071	3.02384725120898E-21	Proliferating T/NK	CRELD2
1.86056781456442E-25	0.535204109607697	0.456	0.149	3.03477216233603E-21	Proliferating T/NK	NAP1L4
2.05266017659025E-25	0.974341786550233	0.215	0.049	3.34809401403636E-21	Proliferating T/NK	DCK
2.33646388400272E-25	0.931031231755101	0.32	0.092	3.81100624119684E-21	Proliferating T/NK	TMEM109
2.51231324565752E-25	1.07487505615575	0.189	0.04	4.09783413499198E-21	Proliferating T/NK	TTLL5
2.61429240843986E-25	1.17663741222745	0.171	0.034	4.26417234740625E-21	Proliferating T/NK	EPN1
3.07225437062386E-25	1.25623579961366	0.154	0.028	5.01115410392459E-21	Proliferating T/NK	CHCHD2
3.15065106229645E-25	1.41691424468427	0.118	0.018	5.13902694771175E-21	Proliferating T/NK	TESC
3.20822487983187E-25	1.02000418833654	0.219	0.051	5.23293560149376E-21	Proliferating T/NK	RANGAP1
3.29306285140177E-25	1.59989853581315	0.11	0.016	5.37131481692142E-21	Proliferating T/NK	PDCD5
3.56302204952963E-25	0.996473284792656	0.588	0.245	5.81164526498779E-21	Proliferating T/NK	CFL1
5.00185128737324E-25	1.35048916937854	0.127	0.02	8.1585196348345E-21	Proliferating T/NK	SLC38A5
5.31976004797665E-25	0.880584354525385	0.154	0.028	8.67706061425472E-21	Proliferating T/NK	URM1
5.36879842831003E-25	1.57844756933374	0.127	0.02	8.7570471164165E-21	Proliferating T/NK	KMT5C
5.88822716854353E-25	0.589814020616531	0.561	0.201	9.60428733461136E-21	Proliferating T/NK	PSMB9
5.93502777904848E-25	1.37437036288032	0.132	0.022	9.68062381040598E-21	Proliferating T/NK	POLH
6.18514782058846E-25	1.47429540623383	0.123	0.019	1.00885946101618E-20	Proliferating T/NK	DEPRECATED-ENSG00000188529
7.03197277774027E-25	0.770106427145394	0.36	0.11	1.14698507977721E-20	Proliferating T/NK	NAB1
7.06251451944719E-25	0.738341377136699	0.268	0.07	1.15196674326703E-20	Proliferating T/NK	PSMD3
7.24705996542581E-25	0.898520445299598	0.263	0.068	1.1820679509606E-20	Proliferating T/NK	RNH1
7.44515443566633E-25	1.54766651855484	0.118	0.018	1.21437914000154E-20	Proliferating T/NK	MEA1
8.59799799101746E-25	1.05509565402418	0.18	0.037	1.40241945231486E-20	Proliferating T/NK	ALDH16A1
9.00662281133987E-25	0.888292637226219	0.197	0.043	1.46907024675765E-20	Proliferating T/NK	TDP1

9.23839836253836E-25	1.11711644691923	0.215	0.05	1.50687515691363E-20	Proliferating T/NK	CEP57
1.02969955258708E-24	1.58909744088948	0.127	0.021	1.67954294022479E-20	Proliferating T/NK	NDUFS6
1.05484990459011E-24	1.01905362467908	0.162	0.031	1.72056567937693E-20	Proliferating T/NK	EXOSC8
1.065547906972E-24	0.566384689069384	0.342	0.101	1.73801519106203E-20	Proliferating T/NK	LRPAP1
1.13454630099812E-24	0.72064979620714	0.434	0.144	1.85055847155803E-20	Proliferating T/NK	NUP210
1.18495833366809E-24	1.35661945915309	0.158	0.03	1.93278553804602E-20	Proliferating T/NK	MTHFD2
1.24378902851914E-24	1.20386578576121	0.18	0.038	2.02874428441756E-20	Proliferating T/NK	SPATA5
1.47185023656456E-24	0.886394188314174	0.184	0.039	2.40073492086045E-20	Proliferating T/NK	DUT
2.45900182409927E-24	1.23657771118714	0.145	0.026	4.01087787528832E-20	Proliferating T/NK	COX7A2
2.57957598378889E-24	1.51868697346045	0.11	0.016	4.20754638715806E-20	Proliferating T/NK	NDUFS8
3.44342629520486E-24	0.768879452784134	0.281	0.077	5.61657263010864E-20	Proliferating T/NK	PPP1CA
3.86561993479888E-24	0.709838334962325	0.504	0.179	6.30521267565046E-20	Proliferating T/NK	ILF3
4.07047266728572E-24	0.849586616204885	0.64	0.28	6.63934796760974E-20	Proliferating T/NK	METRNL
4.23436452675473E-24	0.661700121096182	0.482	0.173	6.90667197958964E-20	Proliferating T/NK	KLRC4
5.04329912276223E-24	1.06520611332644	0.114	0.017	8.22612519913747E-20	Proliferating T/NK	GTF3C6
5.59046811884955E-24	1.17762022018609	0.162	0.032	9.1186125486555E-20	Proliferating T/NK	ETFA
5.89293935369528E-24	0.793865983761673	0.197	0.044	9.61197337981237E-20	Proliferating T/NK	TCF3
6.02938641490859E-24	0.822654118962549	0.675	0.303	9.8345321813574E-20	Proliferating T/NK	VIM
6.2843344688188E-24	0.662323368849075	0.298	0.084	1.02503779520903E-19	Proliferating T/NK	RHOA
6.61309153417882E-24	1.46723861370037	0.11	0.016	1.07866136013991E-19	Proliferating T/NK	ARL6IP6
6.98807181788611E-24	0.832107247248205	0.202	0.046	1.1398243942154E-19	Proliferating T/NK	DAZAP1
7.36258935985648E-24	1.04421839101689	0.807	0.525	1.20091195048619E-19	Proliferating T/NK	ACTB
7.37917868065365E-24	0.708389336799186	0.259	0.068	1.20361783460142E-19	Proliferating T/NK	DEPRECATED-ENSG00000124831
7.44772574738801E-24	1.14773147187785	0.171	0.035	1.21479854665646E-19	Proliferating T/NK	RAD9A
9.76498814491168E-24	1.08057603831358	0.202	0.047	1.59276721631654E-19	Proliferating T/NK	RTN4
9.8679577434496E-24	0.944178430133277	0.553	0.222	1.60956258753406E-19	Proliferating T/NK	ACTG1
1.01738518527958E-23	1.29287147348491	0.105	0.015	1.65945697570953E-19	Proliferating T/NK	HPRT1
1.11529194029439E-23	0.643879403797883	0.408	0.134	1.81915268381418E-19	Proliferating T/NK	EID1
1.16368315209116E-23	0.89093521841662	0.219	0.053	1.89808358937588E-19	Proliferating T/NK	IPO9
1.4168346544715E-23	1.56264525508298	0.123	0.02	2.31099900490847E-19	Proliferating T/NK	RCC2
1.45430619439331E-23	0.785360578145331	0.25	0.065	2.37211883367493E-19	Proliferating T/NK	TAF15
1.55128683617606E-23	0.804806578760663	0.241	0.062	2.53030395848676E-19	Proliferating T/NK	NUP85
1.80126513054232E-23	1.59040566509398	0.132	0.023	2.93804355442758E-19	Proliferating T/NK	PIDD1
2.16099733254385E-23	0.844580169062673	0.263	0.071	3.52480274911228E-19	Proliferating T/NK	SF3B5
2.49636443925829E-23	1.91926832328536	0.11	0.017	4.0718200368742E-19	Proliferating T/NK	AIFM1
2.8881380689833E-23	0.568671339926779	0.373	0.118	4.71084200431866E-19	Proliferating T/NK	FUS
2.8995979699998E-23	0.870906431402291	0.25	0.066	4.72953424886668E-19	Proliferating T/NK	RAVER1
2.92371280734575E-23	0.707516092418013	0.263	0.071	4.76886796006165E-19	Proliferating T/NK	ZCCHC3
3.71736204970378E-23	1.07707889371009	0.18	0.039	6.06338923927184E-19	Proliferating T/NK	SEC11C
3.75728844554079E-23	0.842671367869617	0.237	0.061	6.12851318352158E-19	Proliferating T/NK	AKAP5
4.31833666723502E-23	0.729968519302464	0.456	0.161	7.04363893792704E-19	Proliferating T/NK	VMP1
4.42253001180755E-23	0.44007574890091	0.303	0.087	7.2135887022593E-19	Proliferating T/NK	IFI6
5.4570252716234E-23	0.677396518928073	0.303	0.088	8.90095392054492E-19	Proliferating T/NK	ARHGDIA
5.51382508334112E-23	1.87643511623281	0.11	0.017	8.99360009343769E-19	Proliferating T/NK	MS4A6A
5.86196734701446E-23	0.725563027540438	0.211	0.051	9.56145493971529E-19	Proliferating T/NK	TOX
6.63621889408548E-23	1.33030441858281	0.132	0.023	1.08243366381428E-18	Proliferating T/NK	PYM1
7.5005258709221E-23	1.36263255822478	0.136	0.025	1.2234107748061E-18	Proliferating T/NK	FGFR1OP
9.20271386081558E-23	0.798611771083802	0.873	0.548	1.50105465783763E-18	Proliferating T/NK	CORO1A
9.34058961950958E-23	0.677846367846418	0.202	0.047	1.52354357283821E-18	Proliferating T/NK	HNRNPR
1.02365917760829E-22	0.449246671271097	0.377	0.12	1.66969048459688E-18	Proliferating T/NK	ARL6IP4
1.03231837595011E-22	0.854472005586139	0.184	0.041	1.68381450301222E-18	Proliferating T/NK	PARP1
1.11025990776148E-22	1.02298542060891	0.171	0.037	1.81094493554976E-18	Proliferating T/NK	PDIA3
1.14262193805244E-22	0.659437922943177	0.202	0.047	1.86373064315733E-18	Proliferating T/NK	TRMT112
1.15759421317739E-22	1.21519617788673	0.136	0.025	1.88815192111364E-18	Proliferating T/NK	NUP93
1.17073065614898E-22	0.695537371377855	0.184	0.041	1.9095787732446E-18	Proliferating T/NK	MBD3
1.30267247463509E-22	0.752355323061201	0.254	0.069	2.12478907337729E-18	Proliferating T/NK	ZC3H4
1.45260223251452E-22	1.19115874617805	0.145	0.028	2.36933950145443E-18	Proliferating T/NK	SLF2
1.63615244582445E-22	1.78550732525397	0.123	0.021	2.66872825438426E-18	Proliferating T/NK	CDKN2D
1.8647956084594E-22	1.72760339003601	0.114	0.019	3.04166811695813E-18	Proliferating T/NK	COX6A1
2.32777100974594E-22	1.25432897018106	0.132	0.024	3.7968272939966E-18	Proliferating T/NK	SNAP29
2.41221068144723E-22	1.02566717702247	0.228	0.059	3.93455684250857E-18	Proliferating T/NK	BSG
2.48769085670503E-22	0.854194229469772	0.162	0.034	4.05767255637158E-18	Proliferating T/NK	SNRPC
2.5206956778073E-22	1.33688951449548	0.114	0.019	4.11150672007149E-18	Proliferating T/NK	HSPA13
3.02973747556871E-22	0.865628077987419	0.202	0.048	4.94180479640012E-18	Proliferating T/NK	DEPDC5
3.10594712552115E-22	1.30826170363547	0.123	0.021	5.06611035643754E-18	Proliferating T/NK	GMEB1
3.14637130458487E-22	1.01786160182911	0.167	0.036	5.13204623490838E-18	Proliferating T/NK	SF3B2

3.37610572948738E-22	1.29210267431337	0.14	0.027	5.50676605536686E-18	Proliferating T/NK	BAG1
3.77646964104147E-22	1.00948047290306	0.228	0.059	6.15979963150275E-18	Proliferating T/NK	SLC25A39
3.93710293471568E-22	1.33586160574706	0.132	0.024	6.42180859681474E-18	Proliferating T/NK	AKR1A1
4.18478253773628E-22	0.851252638807551	0.149	0.03	6.82579879730165E-18	Proliferating T/NK	LDLR
4.61425199640898E-22	0.707727490960173	0.338	0.106	7.52630643134268E-18	Proliferating T/NK	DHX15
5.02338404869737E-22	1.55135156116079	0.118	0.02	8.19364172183028E-18	Proliferating T/NK	USP5
5.69495198552878E-22	1.51906841328131	0.114	0.019	9.28903618359599E-18	Proliferating T/NK	PSMC4
6.28512775040502E-22	1.02681345969434	0.167	0.036	1.02516718736856E-17	Proliferating T/NK	BRD8
6.46152817144494E-22	0.798897136393205	0.197	0.047	1.05393986004438E-17	Proliferating T/NK	GFOD1
6.46731084520605E-22	1.16799597245564	0.14	0.027	1.05488307196156E-17	Proliferating T/NK	HMGXB4
7.33765103395334E-22	0.857020006225435	0.167	0.036	1.19684426014813E-17	Proliferating T/NK	ATP5F1A
8.01853840614883E-22	0.66186993562747	0.228	0.059	1.30790379942694E-17	Proliferating T/NK	HDGFL2
8.67971917949249E-22	0.808708194420515	0.211	0.053	1.41574899536702E-17	Proliferating T/NK	C17orf49
9.02261704595142E-22	1.0605910694247	0.145	0.028	1.47167906636514E-17	Proliferating T/NK	MIF
1.05308315386661E-21	0.544085916524295	0.382	0.126	1.71768393227182E-17	Proliferating T/NK	PSPC1
1.09424271727177E-21	0.406505165296131	0.325	0.099	1.78481929614199E-17	Proliferating T/NK	DEPRECATED-ENSG00000161547
1.24271056518729E-21	1.53377852062551	0.101	0.015	2.02698520287698E-17	Proliferating T/NK	SFXN2
1.32588972282462E-21	0.992090704306829	0.145	0.029	2.16265872689924E-17	Proliferating T/NK	TMEM160
1.38732666418951E-21	0.742728442674046	0.219	0.056	2.26286852195951E-17	Proliferating T/NK	CTDNEP1
1.42670030649243E-21	0.479228802705784	0.404	0.135	2.3270908699198E-17	Proliferating T/NK	SAFB
1.43749588440452E-21	0.934212030305817	0.18	0.041	2.34469953705222E-17	Proliferating T/NK	NAA38
1.43758604809097E-21	0.947437514605586	0.241	0.066	2.34484660304118E-17	Proliferating T/NK	BLOC1S1
1.46328060919414E-21	1.21022527480339	0.105	0.017	2.38675700165657E-17	Proliferating T/NK	G2E3
1.51347739734846E-21	0.683500118422614	0.246	0.067	2.46863298281507E-17	Proliferating T/NK	TRA2B
1.5754738359631E-21	0.754007373321232	0.355	0.117	2.56975537383941E-17	Proliferating T/NK	STAG1
1.59241548416412E-21	0.568526797583406	0.329	0.102	2.59738889622009E-17	Proliferating T/NK	RNF4
1.69479180747788E-21	0.67148867373266	0.285	0.084	2.76437491717717E-17	Proliferating T/NK	KANSL3
1.77452335442098E-21	0.428454853090516	0.469	0.166	2.89442504339607E-17	Proliferating T/NK	POLR2A
1.82066761859354E-21	0.932529630204206	0.158	0.033	2.96969095268792E-17	Proliferating T/NK	ATP1B3
1.90520163367234E-21	0.566034901501365	0.316	0.097	3.10757438468296E-17	Proliferating T/NK	AIP
2.24326883471253E-21	1.31525048779674	0.136	0.026	3.65899579629962E-17	Proliferating T/NK	ZBED2
2.46578908787974E-21	0.564082268102704	0.579	0.221	4.02194858124065E-17	Proliferating T/NK	HNRNPA2B1
2.73102435416326E-21	0.750125172293214	0.285	0.085	4.4545738240757E-17	Proliferating T/NK	SEC11A
2.91612060066809E-21	1.46715587486118	0.101	0.016	4.75648431174973E-17	Proliferating T/NK	CDKN2A
2.94992859102416E-21	0.889410827108974	0.202	0.05	4.81162852481951E-17	Proliferating T/NK	STX11
3.16256712818573E-21	0.934970705564609	0.219	0.057	5.15846324278374E-17	Proliferating T/NK	SUPT16H
3.48593968553319E-21	1.00946538834668	0.211	0.054	5.68591622107319E-17	Proliferating T/NK	FASN
3.56536109957455E-21	0.418174161075547	0.316	0.098	5.81546048951604E-17	Proliferating T/NK	ZEB2
3.95155575060741E-21	0.621043118462296	0.386	0.132	6.44538258481574E-17	Proliferating T/NK	PIM1
3.97686060288618E-21	0.44299020825351	0.526	0.195	6.48665732936765E-17	Proliferating T/NK	PSMB8
4.05360383640703E-21	0.663757959517164	0.215	0.055	6.6118332175635E-17	Proliferating T/NK	HIPK2
4.14998095365558E-21	0.682563625404685	0.316	0.099	6.76903393350762E-17	Proliferating T/NK	SYNE3
4.19644673687394E-21	1.53062860912235	0.118	0.021	6.84482427251509E-17	Proliferating T/NK	TMEM14A
4.55657857254616E-21	1.4692698539132	0.114	0.02	7.43223530968004E-17	Proliferating T/NK	TTL
5.01918668999187E-21	1.26199452783046	0.154	0.033	8.18679541004574E-17	Proliferating T/NK	STRN
5.05918068384014E-21	0.831838214228192	0.224	0.06	8.25202961341165E-17	Proliferating T/NK	WWOX
5.56690520782364E-21	1.17673602586712	0.127	0.024	9.08017908448113E-17	Proliferating T/NK	CYB561A3
5.6627307022033E-21	0.846470974227218	0.193	0.047	9.23648004836381E-17	Proliferating T/NK	PFDN2
6.15659733530752E-21	0.842797472882573	0.193	0.047	1.00420259136201E-16	Proliferating T/NK	PTPA
6.21725873947857E-21	1.04927687305995	0.167	0.037	1.01409707299635E-16	Proliferating T/NK	LLGL1
6.7982787804941E-21	0.975933544990395	0.145	0.029	1.10886725188639E-16	Proliferating T/NK	CEP78
7.1423619398211E-21	0.888179514570972	0.189	0.045	1.16499065600422E-16	Proliferating T/NK	CLIC1
7.27273606302484E-21	1.23219192322034	0.136	0.027	1.18625597923998E-16	Proliferating T/NK	SAP30
7.44946404637486E-21	0.364596107645647	0.461	0.164	1.2150820806042E-16	Proliferating T/NK	SLC2A4RG
8.53236235584064E-21	1.66783500299465	0.105	0.017	1.39171362386117E-16	Proliferating T/NK	SLC2A13
8.53737492238658E-21	0.982159178570665	0.149	0.031	1.39253122359048E-16	Proliferating T/NK	KHSRP
9.50799383493429E-21	0.855012165729121	0.149	0.031	1.55084887441613E-16	Proliferating T/NK	PHACTR4
9.73518604744588E-21	0.666048155383785	0.294	0.09	1.5879061961989E-16	Proliferating T/NK	GOLPH3
1.00042888760363E-20	0.675118862096743	0.215	0.056	1.63179955857029E-16	Proliferating T/NK	LRRFIP1
1.10072187909246E-20	0.941846472481152	0.167	0.037	1.79538745698772E-16	Proliferating T/NK	RPA1
1.44694467643309E-20	0.730359833770142	0.193	0.048	2.36011146173001E-16	Proliferating T/NK	CASP2
1.4568104935115E-20	0.99082738994252	0.158	0.034	2.37620359596661E-16	Proliferating T/NK	FDPS
1.58242820187963E-20	0.649908186818214	0.583	0.24	2.58109864008586E-16	Proliferating T/NK	IFI16
1.61935390431484E-20	1.60013938619476	0.11	0.019	2.64132815332794E-16	Proliferating T/NK	MSH2
1.86321879652505E-20	0.458573684923701	0.289	0.087	3.03909617901201E-16	Proliferating T/NK	LIME1
2.06916320903513E-20	0.68485834277399	0.189	0.046	3.3750121102572E-16	Proliferating T/NK	ZDHHC20

2.10977275372214E-20	0.855448806263883	0.154	0.033	3.44125033859618E-16	Proliferating T/NK	PSMD9
2.57020079424545E-20	0.549831363357341	0.32	0.102	4.19225451549376E-16	Proliferating T/NK	NDE1
2.84742563401375E-20	1.80700327838146	0.101	0.016	4.64443595163982E-16	Proliferating T/NK	PPIF
2.91334263877309E-20	1.04268074412576	0.118	0.021	4.75195317810278E-16	Proliferating T/NK	PDS5B
2.98332610667582E-20	0.495185696870266	0.263	0.077	4.86610321259893E-16	Proliferating T/NK	FBXO46
3.16186398002008E-20	0.560141582074374	0.276	0.083	5.15731633781075E-16	Proliferating T/NK	SART1
3.26450714436545E-20	1.32128576155141	0.105	0.017	5.32473760317448E-16	Proliferating T/NK	ACYP1
3.60158820077003E-20	0.535360185596356	0.382	0.132	5.874550514276E-16	Proliferating T/NK	HBS1L
3.92841852837698E-20	0.53980952011965	0.36	0.121	6.40764346163568E-16	Proliferating T/NK	YWHAZ
3.99080327859963E-20	0.825967477840688	0.259	0.076	6.50939922772386E-16	Proliferating T/NK	ATP5MG
4.05118190211631E-20	0.778067659971124	0.241	0.068	6.60788280054192E-16	Proliferating T/NK	ARPC5L
5.97403425681336E-20	1.20905662643977	0.114	0.02	9.74424727628827E-16	Proliferating T/NK	SKP2
6.0272012037647E-20	1.24974045412708	0.136	0.028	9.8309678834606E-16	Proliferating T/NK	CD70
6.37780773045894E-20	0.621194315577846	0.404	0.144	1.04028421891516E-15	Proliferating T/NK	SLC20A1
7.87665121459554E-20	0.930486151950298	0.171	0.04	1.28476057961268E-15	Proliferating T/NK	TPGS2
8.88642237630351E-20	0.949126733930634	0.118	0.022	1.44946435379887E-15	Proliferating T/NK	SCMH1
8.96655489601414E-20	0.526351733926316	0.355	0.119	1.46253476908887E-15	Proliferating T/NK	TACC1
9.1076712510204E-20	0.676179571597325	0.25	0.073	1.48555225775394E-15	Proliferating T/NK	SCRIB
1.28222717933847E-19	1.75593146029155	0.11	0.019	2.09144075221898E-15	Proliferating T/NK	GPANK1
1.45900375458293E-19	1.36173360386509	0.14	0.03	2.37978102410022E-15	Proliferating T/NK	NADK
1.48401038167246E-19	0.573232446427219	0.373	0.129	2.42056933354594E-15	Proliferating T/NK	LMAN2
1.48852484453468E-19	0.630828147255546	0.268	0.081	2.42793287392052E-15	Proliferating T/NK	VAMP1
1.5101860903216E-19	0.994598428580716	0.127	0.025	2.46326453192357E-15	Proliferating T/NK	DEPRECATED-ENSG00000179051
1.53967078099628E-19	1.60744413262353	0.105	0.018	2.51135701088303E-15	Proliferating T/NK	AURKAIP1
1.59338627717539E-19	0.801762043451433	0.175	0.042	2.59897235670078E-15	Proliferating T/NK	SET
1.7369316521287E-19	0.931485967055354	0.224	0.062	2.83310921778712E-15	Proliferating T/NK	PRRC2A
2.01788719835802E-19	0.61005872363442	0.202	0.053	3.29137580924177E-15	Proliferating T/NK	HADHA
2.10797263644754E-19	0.748505705294947	0.171	0.041	3.43831416730959E-15	Proliferating T/NK	VDAC3
2.17852032260243E-19	0.77705733945606	0.254	0.076	3.55338449819683E-15	Proliferating T/NK	DGKH
2.22127153519202E-19	0.555423531834893	0.268	0.081	3.6231160010517E-15	Proliferating T/NK	HDAC1
2.27110379127799E-19	0.591646352231619	0.246	0.072	3.70439739395352E-15	Proliferating T/NK	TELO2
2.2805507059513E-19	0.599346281581232	0.36	0.124	3.71980625647716E-15	Proliferating T/NK	RBM14
2.60869732745874E-19	0.72680914882554	0.18	0.044	4.25504621081796E-15	Proliferating T/NK	OXSR1
2.61481396262756E-19	0.619482801293198	0.202	0.053	4.26502305444181E-15	Proliferating T/NK	NT5C2
3.36843285184041E-19	1.55821518207738	0.101	0.017	5.49425082463689E-15	Proliferating T/NK	HSP90B1
3.54819602215988E-19	0.643309639724327	0.219	0.061	5.78746253174499E-15	Proliferating T/NK	OAS3
4.65511814159018E-19	0.403268132590785	0.281	0.087	7.59296320074774E-15	Proliferating T/NK	RBM3
4.81470425373156E-19	0.599805535047765	0.232	0.066	7.85326410826156E-15	Proliferating T/NK	PPP4C
5.0456342888911E-19	0.602020749241261	0.184	0.046	8.22993408861027E-15	Proliferating T/NK	DEPRECATED-ENSG00000265241
5.34022190899232E-19	1.05723171593709	0.127	0.026	8.71043595575738E-15	Proliferating T/NK	VRK1
5.39350388511557E-19	0.801926402779735	0.241	0.071	8.79734418701201E-15	Proliferating T/NK	ZFP91
5.45805148172065E-19	1.1384645333469	0.167	0.04	8.90262777183455E-15	Proliferating T/NK	CEP44
5.81026578925365E-19	1.22821471204445	0.101	0.017	9.47712452885163E-15	Proliferating T/NK	DEPRECATED-ENSG00000140365
6.30513102732574E-19	0.896539565077404	0.154	0.035	1.0284299218671E-14	Proliferating T/NK	RAB5IF
6.82983431584661E-19	0.411669865888533	0.285	0.089	1.11401427525774E-14	Proliferating T/NK	EIF4EBP2
7.93924927771386E-19	0.702645917920974	0.175	0.043	1.29497094968791E-14	Proliferating T/NK	B3GAT3
8.4700941768887E-19	1.22733976058028	0.101	0.017	1.38155706119232E-14	Proliferating T/NK	GLRX5
9.28134616553527E-19	1.01422268742021	0.136	0.029	1.51388037306046E-14	Proliferating T/NK	MYO1E
1.20027799337639E-18	0.53322374385583	0.211	0.057	1.95777343499623E-14	Proliferating T/NK	PSMA7
1.23084423632533E-18	0.689302118147631	0.219	0.062	2.00763003387025E-14	Proliferating T/NK	RBM17
1.34242480908692E-18	0.44405422428625	0.202	0.054	2.18962910610167E-14	Proliferating T/NK	ACTR3
1.34709820023197E-18	0.708013233370102	0.18	0.045	2.19725187439837E-14	Proliferating T/NK	NUP58
1.47384002745264E-18	1.16677778068952	0.11	0.02	2.40398046877799E-14	Proliferating T/NK	DCLRE1C
1.64764023258625E-18	1.19184363103053	0.101	0.017	2.68746598337144E-14	Proliferating T/NK	TOMM22
1.83857874736211E-18	1.16379788970379	0.127	0.026	2.99890579482234E-14	Proliferating T/NK	HTRA2
1.88285621981954E-18	0.551804874435253	0.206	0.056	3.07112678014765E-14	Proliferating T/NK	OPA1
1.97117999922126E-18	1.13300869479848	0.118	0.023	3.2151916967298E-14	Proliferating T/NK	CSTF1
2.24048801764604E-18	1.10535466637422	0.175	0.045	3.65446000558246E-14	Proliferating T/NK	RDH10
2.41073628427026E-18	0.452676808425402	0.342	0.118	3.93215195327322E-14	Proliferating T/NK	PNN
2.79300243236286E-18	0.602993575026864	0.246	0.074	4.55566626742706E-14	Proliferating T/NK	ATP5MC2
2.96478801688573E-18	0.571575992503002	0.364	0.13	4.83586573434232E-14	Proliferating T/NK	SDF4
3.0339443757279E-18	1.45171882672024	0.101	0.018	4.94866667124979E-14	Proliferating T/NK	ASH2L
3.33738031742987E-18	0.67893614427561	0.325	0.113	5.44360103575985E-14	Proliferating T/NK	PIM3
3.52772150898704E-18	1.00273797564563	0.154	0.036	5.75406655330876E-14	Proliferating T/NK	CCDC50
3.87224338469283E-18	1.03050343640295	0.215	0.062	6.31601618477248E-14	Proliferating T/NK	FAR1
4.25171094763924E-18	0.392798479843152	0.351	0.122	6.93496572669437E-14	Proliferating T/NK	ANKRD36C

4.32598005979305E-18	0.576208052222098	0.232	0.068	7.05610607552845E-14	Proliferating T/NK	SLC38A2
4.36395910382675E-18	0.463383320303408	0.329	0.112	7.11805369425181E-14	Proliferating T/NK	PPP1R12C
4.47264348394249E-18	0.815822120429851	0.136	0.03	7.29532878665859E-14	Proliferating T/NK	ELP5
4.78839504894658E-18	1.31507459818422	0.105	0.019	7.81035116433676E-14	Proliferating T/NK	TOP3A
5.15308440031309E-18	0.672389964561864	0.237	0.071	8.40519596535068E-14	Proliferating T/NK	TSPO
5.623410997018E-18	0.917513910571314	0.154	0.036	9.17234567723607E-14	Proliferating T/NK	ATP5ME
5.97570024382865E-18	0.899596377891347	0.162	0.04	9.74696466770892E-14	Proliferating T/NK	MLH1
6.12381528585854E-18	0.254692485889156	0.272	0.085	9.98855511276387E-14	Proliferating T/NK	HMGN4
6.75970216390124E-18	0.483842696281158	0.303	0.1	1.10257501995393E-13	Proliferating T/NK	PPP1R15B
7.17102912963816E-18	1.13673734278266	0.105	0.019	1.16966656133528E-13	Proliferating T/NK	RHEB
7.27329787834592E-18	0.680135400745734	0.193	0.052	1.186347616937E-13	Proliferating T/NK	RBM42
7.30075576746643E-18	0.450672404451026	0.272	0.086	1.19082627323145E-13	Proliferating T/NK	WAS
7.57521036600937E-18	0.932548496812515	0.14	0.031	1.23559256279979E-13	Proliferating T/NK	SAP18
7.61300617008961E-18	0.875548622302809	0.215	0.062	1.24175743640332E-13	Proliferating T/NK	ZNRF1
7.89370328955146E-18	1.09659908885755	0.105	0.019	1.28754194355874E-13	Proliferating T/NK	AGPAT5
8.01188095197184E-18	0.431831946208452	0.289	0.094	1.30681790207613E-13	Proliferating T/NK	SURF4
8.48701939992182E-18	0.9923280195242	0.11	0.021	1.38431773432125E-13	Proliferating T/NK	TMEM189
9.92640039627353E-18	0.572789432173956	0.241	0.073	1.61909516863618E-13	Proliferating T/NK	HMGN1
1.00085651862213E-17	1.04303860351108	0.127	0.027	1.63249706752456E-13	Proliferating T/NK	TSR3
1.00871724396499E-17	0.349519189521761	0.289	0.094	1.6453186966313E-13	Proliferating T/NK	LMF2
1.03777538008797E-17	0.773020926471933	0.132	0.028	1.69271542246149E-13	Proliferating T/NK	DCPS
1.08975423010809E-17	1.45349235024919	0.101	0.018	1.77749812472931E-13	Proliferating T/NK	CAMK1
1.17219763091052E-17	0.887604552441381	0.167	0.042	1.91197155577815E-13	Proliferating T/NK	KLRC3
1.1971927664446E-17	0.847774753611341	0.184	0.049	1.95274112134779E-13	Proliferating T/NK	CEBPB
1.26797799646271E-17	0.580602378106859	0.329	0.115	2.06819891003033E-13	Proliferating T/NK	GGNBP2
1.37225397229979E-17	0.875696105728233	0.18	0.047	2.23828345421819E-13	Proliferating T/NK	UBASH3B
1.37598017869712E-17	1.23378958695759	0.118	0.024	2.24436126947286E-13	Proliferating T/NK	NDUFA4
1.44004191318511E-17	0.653935705646302	0.246	0.076	2.34885236459624E-13	Proliferating T/NK	RTF2
1.44227227132339E-17	0.595995890841864	0.281	0.092	2.35249030175559E-13	Proliferating T/NK	ANKLE2
1.63003300551083E-17	0.901193518193508	0.123	0.026	2.65874683528871E-13	Proliferating T/NK	TRAF7
1.68517529442193E-17	0.404637979256433	0.342	0.121	2.7486894227316E-13	Proliferating T/NK	PABPC1L
1.92668939981508E-17	1.10598288979435	0.101	0.018	3.14262308003838E-13	Proliferating T/NK	NCOA4
2.2004363255834E-17	0.974068395579646	0.193	0.053	3.58913169065908E-13	Proliferating T/NK	MCMBP
2.36650122464393E-17	0.766085937911554	0.136	0.03	3.86000014751672E-13	Proliferating T/NK	HAT1
2.56704293585582E-17	0.463910705945579	0.268	0.086	4.18710373267443E-13	Proliferating T/NK	EIF3B
2.58083408034354E-17	0.57117131260136	0.184	0.049	4.20959846844835E-13	Proliferating T/NK	TNPO2
3.07137420595174E-17	0.318328667966413	0.386	0.141	5.00971846732789E-13	Proliferating T/NK	LASP1
3.26309096476318E-17	1.22090539803837	0.105	0.02	5.32242767262522E-13	Proliferating T/NK	JPT2
3.74493994369929E-17	0.463196382339242	0.289	0.097	6.10837154216792E-13	Proliferating T/NK	CENPT
3.78922563582008E-17	0.282374962298534	0.395	0.145	6.18060593458612E-13	Proliferating T/NK	GSDMD
3.83067594188864E-17	0.404710100498292	0.246	0.076	6.24821552881456E-13	Proliferating T/NK	PSMC5
4.08485043237208E-17	0.321589150416606	0.539	0.218	6.6627995402421E-13	Proliferating T/NK	TCIRG1
4.27173634826825E-17	1.02485127365603	0.14	0.032	6.96762915766034E-13	Proliferating T/NK	UBB
4.57975397684676E-17	0.405702359146918	0.237	0.072	7.47003671163474E-13	Proliferating T/NK	TUBA4A
4.8236494336139E-17	0.469474616985459	0.175	0.046	7.86785459116763E-13	Proliferating T/NK	ANP32A
4.94291556311209E-17	0.615903751205235	0.228	0.069	8.06238957499213E-13	Proliferating T/NK	NCL
5.13541187133774E-17	0.6165780149696	0.162	0.041	8.37637030333899E-13	Proliferating T/NK	RUVBL1
5.59944512185956E-17	0.779349815878631	0.154	0.038	9.13325493826513E-13	Proliferating T/NK	EEF1AKNMT
6.1498076747493E-17	0.753148083106131	0.14	0.033	1.00309512982836E-12	Proliferating T/NK	DERL2
6.39037055209935E-17	0.832636886574625	0.18	0.049	1.04233334075292E-12	Proliferating T/NK	PBRM1
6.58409642211832E-17	0.427641576653867	0.171	0.044	1.07393196741172E-12	Proliferating T/NK	SRSF9
6.86947163987127E-17	0.885552174567726	0.127	0.028	1.1204795191794E-12	Proliferating T/NK	VARS
7.0445850241329E-17	0.805456463763843	0.145	0.034	1.14904226328632E-12	Proliferating T/NK	YWHAG
7.41463349833747E-17	0.980140663248829	0.118	0.025	1.20940086991383E-12	Proliferating T/NK	ARL6IP1
7.54430034585254E-17	0.794362519828426	0.197	0.056	1.23055082941201E-12	Proliferating T/NK	FARSA
7.72242923259931E-17	0.737757807587953	0.175	0.047	1.25960543212927E-12	Proliferating T/NK	ZNF384
7.95288480181628E-17	0.468160323283241	0.241	0.075	1.29719504002425E-12	Proliferating T/NK	MAP2K3
8.05145638808099E-17	1.05690171132584	0.118	0.025	1.31327305145989E-12	Proliferating T/NK	ACAD9
8.12026181256814E-17	0.927994572390771	0.175	0.047	1.32449590424799E-12	Proliferating T/NK	CBX6
8.35966611362215E-17	0.546914987639837	0.193	0.054	1.36354513979291E-12	Proliferating T/NK	SFPQ
8.5922596752112E-17	0.313962439435463	0.175	0.046	1.4014834756237E-12	Proliferating T/NK	SMARCD2
8.99928280534206E-17	0.960741098556857	0.136	0.031	1.46787301837934E-12	Proliferating T/NK	ADO
9.25880280685934E-17	0.502998272996646	0.386	0.145	1.51020332582683E-12	Proliferating T/NK	PPP6R1
9.29922193673816E-17	0.487298742322425	0.259	0.083	1.51679609010136E-12	Proliferating T/NK	CYB5B
9.38028116690651E-17	0.795423105058563	0.136	0.031	1.53001766113412E-12	Proliferating T/NK	SEPTIN11
9.66183285052491E-17	0.742462861652361	0.171	0.045	1.57594155624912E-12	Proliferating T/NK	CXCR3

9.82654239553328E-17	0.403744854108934	0.197	0.056	1.60280733013543E-12	Proliferating T/NK	KIAA2013
1.02244289605371E-16	1.31779831814384	0.127	0.028	1.66770660775321E-12	Proliferating T/NK	PHTF1
1.03714395475464E-16	0.374502888847787	0.561	0.241	1.69168550460029E-12	Proliferating T/NK	LAG3
1.06900264593038E-16	0.811869313500175	0.145	0.035	1.74365021577705E-12	Proliferating T/NK	IFI27L2
1.23075223365696E-16	0.446299794753922	0.281	0.094	2.00747996831786E-12	Proliferating T/NK	SCAF1
1.28011399802065E-16	0.797534086234712	0.149	0.036	2.08799394217148E-12	Proliferating T/NK	RABEP2
1.33502435459169E-16	0.868240129009715	0.123	0.027	2.1775582247745E-12	Proliferating T/NK	MZT2A
1.52519050742459E-16	1.26893227584046	0.11	0.022	2.48773823666026E-12	Proliferating T/NK	NFIL3
1.58959316565399E-16	0.692910511712677	0.184	0.051	2.59278541249823E-12	Proliferating T/NK	NNT
1.60111682855618E-16	0.769145985658619	0.149	0.037	2.61158165905799E-12	Proliferating T/NK	TIPRL
1.81285294212731E-16	1.02076875674832	0.114	0.024	2.95694443390385E-12	Proliferating T/NK	KCNK5
1.85521040702154E-16	0.592583200194512	0.18	0.049	3.02603369489284E-12	Proliferating T/NK	GPAA1
1.88352340838879E-16	1.27173052187663	0.101	0.019	3.07221503142296E-12	Proliferating T/NK	XRCC6
1.92603148774036E-16	0.882588996773283	0.136	0.032	3.1415499596533E-12	Proliferating T/NK	NUDCD2
1.94876301905813E-16	0.849981537275328	0.136	0.032	3.17862736038572E-12	Proliferating T/NK	PCMT1
1.96674881858574E-16	0.380150884849788	0.259	0.084	3.2079639979952E-12	Proliferating T/NK	TCERG1
2.049082065225E-16	0.44862950073892	0.211	0.062	3.3422577565885E-12	Proliferating T/NK	RAB1B
2.16757011328554E-16	0.795934380693661	0.105	0.021	3.53552361178004E-12	Proliferating T/NK	ARMC6
2.23932309002431E-16	0.426280203769724	0.241	0.076	3.65255989213866E-12	Proliferating T/NK	LDHB
2.30015189073208E-16	0.35892637183961	0.342	0.124	3.7517777489731E-12	Proliferating T/NK	INTS11
2.38387387658255E-16	0.972319705008173	0.162	0.042	3.8883366800938E-12	Proliferating T/NK	TAOK2
2.66507666227338E-16	0.426193884859516	0.272	0.091	4.3470065438341E-12	Proliferating T/NK	ZC3H18
2.72607801119522E-16	0.681953461905801	0.268	0.09	4.44650584406052E-12	Proliferating T/NK	PHLDA1
2.84430524325977E-16	0.663056251596498	0.18	0.049	4.63934628228101E-12	Proliferating T/NK	DNAJB11
2.99181624583193E-16	0.299309776787545	0.232	0.072	4.87995147857646E-12	Proliferating T/NK	ENSA
3.04556554054234E-16	0.880752526377014	0.101	0.019	4.96762195317861E-12	Proliferating T/NK	NUDT2
3.06359196233338E-16	0.429029193932415	0.285	0.097	4.99702484976198E-12	Proliferating T/NK	MOV10
3.7117751305342E-16	0.565058377351147	0.127	0.029	6.05427641541434E-12	Proliferating T/NK	FADD
3.76391321911793E-16	0.33138474560125	0.364	0.135	6.13931885170326E-12	Proliferating T/NK	GGA2
3.91802893577354E-16	0.421193729924172	0.237	0.074	6.39069699714023E-12	Proliferating T/NK	GNAI2
3.97953293585966E-16	0.2663321963083	0.294	0.1	6.4910161716807E-12	Proliferating T/NK	PPP1R35
4.05464290350257E-16	0.447465925229799	0.228	0.071	6.61352803990304E-12	Proliferating T/NK	PRKDC
4.23495958312745E-16	0.602464418689839	0.232	0.073	6.90764257603918E-12	Proliferating T/NK	INO80E
4.43432681265214E-16	1.05300502580791	0.114	0.024	7.23283046411691E-12	Proliferating T/NK	KATNAL1
4.84168784921314E-16	0.975472656087384	0.127	0.029	7.89727705085156E-12	Proliferating T/NK	RAD21
4.85685341228668E-16	0.841586397075619	0.105	0.021	7.9220136007808E-12	Proliferating T/NK	NEDD1
5.09713900610968E-16	0.498788521160548	0.149	0.037	8.31394343286551E-12	Proliferating T/NK	FAF1
5.12355248665373E-16	0.514469916283249	0.171	0.046	8.3570264609809E-12	Proliferating T/NK	SRI
5.224474226458E-16	0.725754427356929	0.132	0.031	8.52163991077565E-12	Proliferating T/NK	INTS9
5.39796802628476E-16	0.508078235364853	0.193	0.055	8.80462564767308E-12	Proliferating T/NK	ITPK1
5.49794060725735E-16	0.946690653045704	0.118	0.026	8.96769092449747E-12	Proliferating T/NK	INF2
5.60834694020113E-16	0.332608197679384	0.417	0.163	9.14777469416206E-12	Proliferating T/NK	NUMA1
5.63254584933973E-16	0.412283448259738	0.364	0.137	9.18724553485804E-12	Proliferating T/NK	CD99
5.83188353739429E-16	0.485978400131559	0.443	0.179	9.51238523784382E-12	Proliferating T/NK	WSB1
5.88158113852025E-16	0.541200380326576	0.518	0.223	9.59344699504038E-12	Proliferating T/NK	EZR
5.95354596836824E-16	0.738425149739786	0.197	0.058	9.71082882900543E-12	Proliferating T/NK	TXLNA
6.02098369855444E-16	0.4300388130808	0.162	0.043	9.82082651071214E-12	Proliferating T/NK	SLC35A4
6.59456681677344E-16	0.471967346732608	0.197	0.058	1.07563979348392E-11	Proliferating T/NK	TMUB1
7.43619879746328E-16	0.402709359673863	0.566	0.24	1.21291838585423E-11	Proliferating T/NK	RAC2
8.7141149876781E-16	0.820947747238544	0.136	0.033	1.42135929564017E-11	Proliferating T/NK	EIF1AY
8.92783818263662E-16	0.853775208992938	0.158	0.042	1.45621968596986E-11	Proliferating T/NK	ZNF706
9.08099124240408E-16	0.852067792465277	0.114	0.024	1.48120048154853E-11	Proliferating T/NK	TIMM50
9.19564136698857E-16	0.905656694480556	0.136	0.033	1.4999010633695E-11	Proliferating T/NK	OXCT1
9.83770221983524E-16	0.252596130015092	0.478	0.193	1.60462760907733E-11	Proliferating T/NK	RAB27A
9.89912641612319E-16	0.792836569056097	0.118	0.026	1.61464650973385E-11	Proliferating T/NK	NRROS
1.03162286596519E-15	0.878833906298804	0.136	0.033	1.68268005667583E-11	Proliferating T/NK	TOR1A
1.08548926954133E-15	0.797087524201965	0.118	0.026	1.77054154754886E-11	Proliferating T/NK	ELP6
1.10700760706005E-15	0.384859228829548	0.228	0.071	1.80564010787565E-11	Proliferating T/NK	AGPAT3
1.12314391659772E-15	1.00890142245199	0.127	0.029	1.83196004236253E-11	Proliferating T/NK	PLCXD1
1.12880484561793E-15	0.38443596643234	0.232	0.074	1.8411935836874E-11	Proliferating T/NK	SZRD1
1.21045757128274E-15	0.469272564418822	0.377	0.147	1.97437734451927E-11	Proliferating T/NK	CREBZF
1.25569634655636E-15	0.440646650199787	0.237	0.076	2.04816631086808E-11	Proliferating T/NK	IFI35
1.28679456659415E-15	0.344756318557893	0.197	0.058	2.09889061757171E-11	Proliferating T/NK	RAB8A
1.34995850757569E-15	0.724192608121976	0.149	0.038	2.2019173217067E-11	Proliferating T/NK	SMARCB1
1.44953295579455E-15	0.682379603483205	0.132	0.031	2.36433320419649E-11	Proliferating T/NK	EEFSEC
1.59185287272924E-15	0.380701423099628	0.254	0.084	2.59647122070866E-11	Proliferating T/NK	ABI3

1.74641674970431E-15	0.358689411116707	0.254	0.085	2.8485803604427E-11	Proliferating T/NK	ANAPC5
1.95502620279241E-15	0.485675478558183	0.228	0.072	3.1888432393747E-11	Proliferating T/NK	GNB1
1.9793230571939E-15	0.847722845177401	0.145	0.037	3.22847383858897E-11	Proliferating T/NK	CEP135
2.06181676609223E-15	0.42738278943431	0.368	0.141	3.36302932717303E-11	Proliferating T/NK	MYL6
2.37751563470243E-15	0.629049019108109	0.162	0.044	3.87796575176314E-11	Proliferating T/NK	MED4
2.65275300958181E-15	0.558357549008482	0.114	0.025	4.32690543392889E-11	Proliferating T/NK	TMX1
2.78881094183428E-15	1.14161032691867	0.136	0.034	4.5488295272259E-11	Proliferating T/NK	ZBTB32
2.96103323952493E-15	0.508799528886946	0.189	0.055	4.82974131698911E-11	Proliferating T/NK	PCBP1
2.99648856809763E-15	1.03158200116871	0.145	0.037	4.88757250342404E-11	Proliferating T/NK	NEMP2
3.03503802124674E-15	0.749683718820207	0.145	0.037	4.95045051645555E-11	Proliferating T/NK	CD8B
3.13664725597263E-15	1.04277338023123	0.105	0.022	5.11618533921696E-11	Proliferating T/NK	LRMP
3.1498888061149E-15	0.871361081906193	0.123	0.028	5.13778363165401E-11	Proliferating T/NK	IRF8
3.24030397888734E-15	1.33419000678557	0.101	0.021	5.28525981996313E-11	Proliferating T/NK	HTATIP2
3.28066094016383E-15	0.739856112737363	0.145	0.037	5.35108605950122E-11	Proliferating T/NK	PRPF31
3.41167445801251E-15	0.479732230466695	0.614	0.291	5.5647822084642E-11	Proliferating T/NK	MT-CO1
3.43975595626149E-15	0.338109190885193	0.268	0.091	5.61058594025811E-11	Proliferating T/NK	STAG2
3.70332992747193E-15	0.628413420001128	0.132	0.032	6.04050144469946E-11	Proliferating T/NK	WDR1
3.81701998898988E-15	0.504848194803674	0.171	0.048	6.22594130404139E-11	Proliferating T/NK	NELFB
3.94874389539573E-15	0.686328227504157	0.197	0.06	6.44079616777997E-11	Proliferating T/NK	CDK16
4.11750690664597E-15	0.488875028683667	0.193	0.057	6.71606551543025E-11	Proliferating T/NK	SF3A2
4.67454064175698E-15	0.271206559125602	0.36	0.137	7.62464324076982E-11	Proliferating T/NK	PRPF3
4.73059722167153E-15	0.376263086929844	0.281	0.099	7.71607712826844E-11	Proliferating T/NK	CCDC85B
5.11182501792946E-15	1.27280325502736	0.101	0.021	8.33789778674474E-11	Proliferating T/NK	PEBP1
5.17371101102966E-15	0.926289034124288	0.123	0.029	8.43884003009048E-11	Proliferating T/NK	DGCR8
5.39259114267071E-15	0.600375538248866	0.154	0.041	8.7958554128102E-11	Proliferating T/NK	SMPD4
5.57761713355752E-15	0.753322045013011	0.14	0.036	9.09765130654567E-11	Proliferating T/NK	PES 1.00
5.6474990583028E-15	0.513631740011401	0.175	0.05	9.2116357139977E-11	Proliferating T/NK	SLC2A1
6.16957529855403E-15	0.440743632096618	0.259	0.089	1.00631942694715E-10	Proliferating T/NK	DCAF7
6.50465989123744E-15	0.684817028713859	0.105	0.022	1.06097507485974E-10	Proliferating T/NK	RALY
7.61261860167624E-15	0.36226900664983	0.333	0.125	1.24169422011941E-10	Proliferating T/NK	PPP1R18
7.75072708874981E-15	0.620836252306206	0.202	0.062	1.26422109544598E-10	Proliferating T/NK	SF3B3
8.18493064719932E-15	0.301986301909822	0.272	0.094	1.33504403786468E-10	Proliferating T/NK	OGDH
8.51168278153014E-15	0.475304254224935	0.136	0.034	1.38834057849538E-10	Proliferating T/NK	TRIM28
8.75318617422353E-15	0.335290806997476	0.202	0.062	1.4277321968776E-10	Proliferating T/NK	MCRS1
8.91470158123538E-15	0.757077492459237	0.127	0.031	1.4540769749153E-10	Proliferating T/NK	CSNK2B
8.96304731400738E-15	0.749537992575622	0.154	0.041	1.46196264738774E-10	Proliferating T/NK	CGAS
9.05231713558961E-15	0.420850003753935	0.254	0.088	1.47652344798602E-10	Proliferating T/NK	CTLA4
9.20613487910351E-15	0.394973534086054	0.197	0.06	1.50161266013057E-10	Proliferating T/NK	NCBP3
9.719060697237E-15	0.648216709705416	0.202	0.063	1.58527599032633E-10	Proliferating T/NK	ITGB1
9.99484089240606E-15	0.817695289629142	0.101	0.021	1.63025849796035E-10	Proliferating T/NK	RAC1
1.01224966897848E-14	0.433319134940485	0.272	0.096	1.65108043507081E-10	Proliferating T/NK	STK11
1.08255902140578E-14	0.546257685358456	0.118	0.027	1.76576201981497E-10	Proliferating T/NK	YIF1B
1.13047918156503E-14	0.296592584207432	0.465	0.191	1.84392459305072E-10	Proliferating T/NK	CSK
1.16210852282579E-14	0.347176812911341	0.263	0.091	1.89551521158115E-10	Proliferating T/NK	SRSF3
1.16843435399411E-14	0.311760334415115	0.241	0.08	1.9058332747998E-10	Proliferating T/NK	SERF2
1.18442272677981E-14	0.323943499301153	0.197	0.06	1.93191190965056E-10	Proliferating T/NK	TP53
1.29431470344916E-14	0.59858238529114	0.162	0.045	2.11115671279592E-10	Proliferating T/NK	KEAP1
1.29606982782613E-14	1.22643902990545	0.11	0.024	2.1140194961672E-10	Proliferating T/NK	SHC1
1.32437615056659E-14	0.378402049705766	0.325	0.121	2.16018993918917E-10	Proliferating T/NK	PRKAR1A
1.4668300544388E-14	0.329337217666874	0.303	0.111	2.39254650179512E-10	Proliferating T/NK	BST2
1.57842860687064E-14	0.793233861765744	0.101	0.021	2.5745749006667E-10	Proliferating T/NK	CNP
1.62235236814937E-14	0.657834912745881	0.167	0.047	2.64621894768844E-10	Proliferating T/NK	RING1
1.63231013973786E-14	1.02355013725794	0.189	0.058	2.66246106892642E-10	Proliferating T/NK	LGALS1
1.83985547530375E-14	0.703013050444432	0.14	0.036	3.00098826576795E-10	Proliferating T/NK	COMMD4
1.88544543685406E-14	0.585376478200269	0.167	0.048	3.07535005205266E-10	Proliferating T/NK	VOPP1
1.89398778286272E-14	0.452871105756318	0.294	0.107	3.08928347262739E-10	Proliferating T/NK	EIF4H
2.03655873402803E-14	0.773545864296512	0.132	0.033	3.32183095107312E-10	Proliferating T/NK	ACAA1
2.20553238631846E-14	0.59103368818997	0.228	0.076	3.59744387532405E-10	Proliferating T/NK	ISG15
2.21202445269878E-14	0.39797790479492	0.36	0.14	3.60803308479697E-10	Proliferating T/NK	SH3BGRL3
2.48951659664011E-14	0.390697660576465	0.219	0.071	4.06065052077969E-10	Proliferating T/NK	CLASRP
2.49963671434976E-14	0.941482473028053	0.114	0.026	4.07715744477589E-10	Proliferating T/NK	CCT6A
2.67984071749232E-14	0.375975987505622	0.443	0.186	4.37108819430173E-10	Proliferating T/NK	CYBC1
2.70887529082076E-14	0.390367195725436	0.18	0.053	4.41844648685775E-10	Proliferating T/NK	TNPO3
2.73480892818975E-14	0.489266339745348	0.184	0.055	4.4607468427703E-10	Proliferating T/NK	MLLT1
2.78927870508359E-14	0.846402988993313	0.136	0.035	4.54959249586185E-10	Proliferating T/NK	EHMT2
2.86163706474057E-14	0.762060784542195	0.118	0.028	4.66761621629835E-10	Proliferating T/NK	SRSF1

2.90242625965357E-14	0.477345507980194	0.14	0.037	4.73414747212094E-10	Proliferating T/NK	COPS6
2.97590762421986E-14	0.277644562334529	0.338	0.13	4.85400292586501E-10	Proliferating T/NK	SETD5
3.20600787707367E-14	0.768724270005585	0.118	0.028	5.22931944829486E-10	Proliferating T/NK	RAP2A
3.22930267333308E-14	0.285644175249433	0.228	0.075	5.26731559047359E-10	Proliferating T/NK	CYTH2
3.61996093769705E-14	0.286699197814097	0.167	0.048	5.90451828547765E-10	Proliferating T/NK	DCTN2
3.64093698718198E-14	0.632928035481718	0.189	0.058	5.93873231979252E-10	Proliferating T/NK	PELP1
3.76656527706216E-14	0.508359022450564	0.654	0.314	6.14364462341609E-10	Proliferating T/NK	TMSB10
4.20175076212012E-14	0.324551471440447	0.145	0.039	6.85347566809412E-10	Proliferating T/NK	USB1
4.32260999213507E-14	0.479817353387093	0.224	0.074	7.05060915817151E-10	Proliferating T/NK	HEXIM1
4.3707254776372E-14	0.380987724127973	0.202	0.064	7.12909032657403E-10	Proliferating T/NK	PNRC2
4.59000697691893E-14	0.478614787629029	0.768	0.447	7.48676038005247E-10	Proliferating T/NK	CD8A
4.61194994519906E-14	0.841742343483128	0.162	0.047	7.52255155561418E-10	Proliferating T/NK	CANT1
4.74000434620246E-14	0.807577521377292	0.145	0.039	7.73142108909083E-10	Proliferating T/NK	CAND1
5.187200419714E-14	0.597058357132933	0.118	0.028	8.4608426045955E-10	Proliferating T/NK	AZIN1
5.39876420571174E-14	0.608743555741738	0.136	0.035	8.80592429593642E-10	Proliferating T/NK	ABHD2
5.59145860469877E-14	0.752095308764197	0.114	0.027	9.12022813012416E-10	Proliferating T/NK	DBF4
5.73192380200907E-14	0.747496394189221	0.193	0.061	9.349340913457E-10	Proliferating T/NK	FAM3C
5.8158894239175E-14	0.765523437612725	0.11	0.025	9.48629723935183E-10	Proliferating T/NK	RTL10
6.13889009848354E-14	0.916267926381782	0.11	0.025	1.00131436396365E-09	Proliferating T/NK	SUGT1
6.20754740662053E-14	0.57654546860717	0.175	0.052	1.01251305749387E-09	Proliferating T/NK	SPCS3
6.53403323527212E-14	0.799902811422375	0.127	0.032	1.06576616100524E-09	Proliferating T/NK	SLC4A2
6.5525253528456E-14	0.551363369500646	0.162	0.047	1.06878241030265E-09	Proliferating T/NK	EIF2AK1
6.98102946718593E-14	0.725899490144436	0.202	0.065	1.1386757163927E-09	Proliferating T/NK	PAM
7.31958438981387E-14	0.796154984872479	0.114	0.027	1.19389740982254E-09	Proliferating T/NK	NDUFA1
7.37481211669205E-14	0.259968440273324	0.329	0.127	1.20290560435364E-09	Proliferating T/NK	RCSD1
7.54573825659734E-14	0.618099465369855	0.14	0.037	1.23078536703359E-09	Proliferating T/NK	FAM174C
7.76099304983694E-14	1.10989801313241	0.101	0.022	1.26589557763589E-09	Proliferating T/NK	TIMM17B
8.1305016231892E-14	1.13548016854076	0.114	0.027	1.32616611975839E-09	Proliferating T/NK	CNOT6
8.18980747699559E-14	0.628469559498521	0.171	0.051	1.33583949757275E-09	Proliferating T/NK	RECQL
8.36098405862078E-14	0.72393593848576	0.114	0.027	1.36376010980164E-09	Proliferating T/NK	MIF4GD
8.39369027229335E-14	0.366033385748094	0.127	0.032	1.36909482031377E-09	Proliferating T/NK	XRCC1
8.4291323997507E-14	0.489115114379478	0.149	0.041	1.37487578572334E-09	Proliferating T/NK	ABCB8
8.48590426775701E-14	0.576699301593042	0.184	0.057	1.38413584511385E-09	Proliferating T/NK	TTYH3
8.73394303436795E-14	0.664612144201962	0.101	0.022	1.42459344833576E-09	Proliferating T/NK	PSME2
8.92507142994355E-14	0.453569145303132	0.162	0.047	1.45576840093809E-09	Proliferating T/NK	MLEC
9.00071430380597E-14	0.393835921003562	0.184	0.056	1.46810651009379E-09	Proliferating T/NK	CNOT9
9.25568869990195E-14	0.886673623768468	0.118	0.029	1.50969538384101E-09	Proliferating T/NK	ZNF672
9.4309769668643E-14	0.50052434069883	0.114	0.027	1.53828665306524E-09	Proliferating T/NK	NDOR1
9.88080899676662E-14	0.712883908256035	0.171	0.051	1.6116587554626E-09	Proliferating T/NK	TTC7A
1.03062697432004E-13	0.820865406429975	0.145	0.04	1.68105565781342E-09	Proliferating T/NK	IFT27
1.04964760113596E-13	0.734059795811127	0.162	0.047	1.71208020221287E-09	Proliferating T/NK	GLYCTK
1.07656523477693E-13	0.458849984241617	0.224	0.075	1.75598555444465E-09	Proliferating T/NK	CCNT1
1.10424023711301E-13	0.375325484734431	0.171	0.051	1.80112625075503E-09	Proliferating T/NK	MLF 2.00
1.14438075376403E-13	0.288537003113604	0.276	0.101	1.86659944746452E-09	Proliferating T/NK	SMARCA4
1.15853941316439E-13	0.363365766876031	0.202	0.065	1.88969363681243E-09	Proliferating T/NK	RELT
1.23562463910192E-13	0.29022959859109	0.386	0.158	2.01542734883914E-09	Proliferating T/NK	DDIT4
1.25207387078567E-13	0.958322928217963	0.154	0.044	2.0422576906385E-09	Proliferating T/NK	HDAC6
1.26970920639966E-13	0.546984953261467	0.132	0.034	2.07102268655848E-09	Proliferating T/NK	AKAP10
1.28662521864733E-13	1.3324159018599	0.101	0.023	2.09861439413565E-09	Proliferating T/NK	COL6A3
1.30738208160988E-13	0.428136870945214	0.224	0.076	2.13247091331388E-09	Proliferating T/NK	KPNA4
1.3727552978294E-13	0.833912644196293	0.105	0.024	2.23910116628954E-09	Proliferating T/NK	TPM4
1.42056511749081E-13	0.439300336533099	0.215	0.071	2.31708376313926E-09	Proliferating T/NK	TMEM41B
1.48295876238653E-13	0.952760560604152	0.132	0.035	2.41885403732866E-09	Proliferating T/NK	UBXN11
1.50573387079057E-13	0.344577643585737	0.132	0.034	2.45600251664649E-09	Proliferating T/NK	LRRC40
1.51735916564794E-13	0.932885050147377	0.123	0.031	2.47496453508836E-09	Proliferating T/NK	TRAPPC1
1.57443709461824E-13	0.625046686709523	0.14	0.038	2.56806434503181E-09	Proliferating T/NK	DHX8
1.66248257603172E-13	0.877517450232179	0.132	0.035	2.71167532976533E-09	Proliferating T/NK	INTS7
1.67735517397129E-13	0.269791255928677	0.39	0.159	2.73593402426458E-09	Proliferating T/NK	EIF3A
1.78787184634809E-13	0.294116675215416	0.149	0.042	2.91619776857836E-09	Proliferating T/NK	SPCS2
1.79012874162027E-13	1.02647503549921	0.136	0.037	2.91987899045682E-09	Proliferating T/NK	ADIPOR1
1.79781638952557E-13	0.534973418698737	0.206	0.068	2.93241831295516E-09	Proliferating T/NK	AP3B1
1.86541486197154E-13	0.602124319780754	0.175	0.054	3.04267818136177E-09	Proliferating T/NK	SNAP47
1.91858941770479E-13	0.37755559642177	0.351	0.14	3.12941119921828E-09	Proliferating T/NK	NCOR2
1.95038976093676E-13	0.383942653889811	0.452	0.195	3.18128073906394E-09	Proliferating T/NK	DRAP1
2.09039184233676E-13	0.429468649312437	0.171	0.051	3.4096381340355E-09	Proliferating T/NK	JADE1
2.11736003632957E-13	0.508442537681266	0.259	0.094	3.45362595525717E-09	Proliferating T/NK	SIRPG

2.35866302346845E-13	0.315612718348375	0.504	0.223	3.8472152575794E-09	Proliferating T/NK	ATP2A3
2.36035090978303E-13	0.53680398471552	0.246	0.087	3.84996836894711E-09	Proliferating T/NK	PLA2G6
2.40909099857003E-13	1.07314913858956	0.118	0.029	3.92946832776758E-09	Proliferating T/NK	SLC39A7
2.54495937135063E-13	0.715359185562189	0.193	0.062	4.15108323061001E-09	Proliferating T/NK	ATXN2L
2.69101861391563E-13	0.934056268740763	0.105	0.024	4.38932046115778E-09	Proliferating T/NK	CPSF3
2.90361169323625E-13	0.553500188601348	0.276	0.104	4.73608103283764E-09	Proliferating T/NK	HSP90AB1
3.26110358025895E-13	0.470453681124401	0.114	0.028	5.31918604976038E-09	Proliferating T/NK	PSMA4
3.36962953063145E-13	0.74988752653158	0.154	0.044	5.49620272741296E-09	Proliferating T/NK	GTPBP2
3.4274430544155E-13	0.686182473608317	0.127	0.033	5.59050236605712E-09	Proliferating T/NK	PMAIP1
3.56441481692866E-13	0.53470131170573	0.167	0.05	5.81391700789234E-09	Proliferating T/NK	UBE2O
3.57999060898158E-13	0.90672265631384	0.101	0.023	5.83932268230985E-09	Proliferating T/NK	TCP1
3.84935462493345E-13	0.686421033336324	0.127	0.033	6.27868232872894E-09	Proliferating T/NK	GPR137B
3.88956282054727E-13	0.562726827467332	0.171	0.053	6.34426591659465E-09	Proliferating T/NK	NSMCE4A
4.09506312144405E-13	0.539163675563723	0.14	0.039	6.67945745738739E-09	Proliferating T/NK	TADA3
4.11026060684192E-13	0.762558971688067	0.105	0.025	6.70424607581985E-09	Proliferating T/NK	CEP83
4.1224681814915E-13	0.544523961255837	0.14	0.039	6.72415785083078E-09	Proliferating T/NK	APH1A
4.17712571733646E-13	0.612448167043971	0.101	0.023	6.81330975754751E-09	Proliferating T/NK	PFAS
4.33763674262071E-13	1.14199694559666	0.127	0.034	7.07511929088864E-09	Proliferating T/NK	TSTA3
4.41741585185466E-13	0.265487034845002	0.259	0.094	7.20524699596014E-09	Proliferating T/NK	EDC4
4.45348725805724E-13	0.325211763058297	0.254	0.092	7.26408306661717E-09	Proliferating T/NK	ARPC4
4.46246836399777E-13	0.81420454634475	0.114	0.028	7.27873214851676E-09	Proliferating T/NK	VDAC2
4.55002740697617E-13	0.352305219888509	0.202	0.067	7.42154970351883E-09	Proliferating T/NK	PUF60
4.75300786260558E-13	1.00499410470739	0.101	0.023	7.75263112469597E-09	Proliferating T/NK	WARS
5.10748311682612E-13	0.371432203029113	0.158	0.046	8.33081571185508E-09	Proliferating T/NK	RAD23A
5.16054122686552E-13	0.398777579028842	0.149	0.043	8.41735879514036E-09	Proliferating T/NK	NEDD8
5.16344849735272E-13	0.351647666589127	0.25	0.09	8.42210084403203E-09	Proliferating T/NK	SP3
5.29597182634118E-13	0.3226383886998	0.382	0.158	8.6382596459451E-09	Proliferating T/NK	SPN
5.38536562719002E-13	0.616526983345088	0.167	0.051	8.78406987450964E-09	Proliferating T/NK	CHD4
5.52663191710112E-13	0.534589632300069	0.232	0.083	9.01448931998364E-09	Proliferating T/NK	KIF5B
5.68256614043411E-13	0.252932305022437	0.241	0.086	9.26883363166207E-09	Proliferating T/NK	RAB14
5.69170914917669E-13	0.563185778959935	0.145	0.041	9.2837467932221E-09	Proliferating T/NK	SRA1
5.85018842532772E-13	0.909392591942022	0.118	0.03	9.54224234055204E-09	Proliferating T/NK	YIPF3
5.98664304603349E-13	0.481555076260126	0.127	0.033	9.76481347238523E-09	Proliferating T/NK	RIC8A
6.05960821018159E-13	0.75818607746454	0.105	0.025	9.8838269516272E-09	Proliferating T/NK	HTATSF1
6.32622961427859E-13	0.495729287804334	0.254	0.094	1.03187131238498E-08	Proliferating T/NK	PURB
6.55525640712103E-13	0.59099162278494	0.149	0.043	1.06922787256551E-08	Proliferating T/NK	ELOVL1
6.63277248004029E-13	0.39522908275155	0.158	0.047	1.08187151921937E-08	Proliferating T/NK	PGP
6.8084230704117E-13	0.830469272313943	0.127	0.034	1.11052188701485E-08	Proliferating T/NK	QKI
7.28307385210255E-13	0.3131661066346	0.193	0.063	1.18794217601645E-08	Proliferating T/NK	HSD17B11
7.97339476183002E-13	0.29754465420988	0.241	0.087	1.30054041960209E-08	Proliferating T/NK	CDKAL1
8.24154844662755E-13	0.351997995502018	0.294	0.113	1.34427896712942E-08	Proliferating T/NK	HNRNPU
9.22841148459764E-13	0.27215782176898	0.316	0.125	1.50524619725272E-08	Proliferating T/NK	ARRB2
9.29375462525019E-13	0.592561553025601	0.114	0.028	1.51590431692456E-08	Proliferating T/NK	MTLN
9.48375970400138E-13	0.30224343288225	0.215	0.074	1.54689604531967E-08	Proliferating T/NK	PANK4
9.50071348407861E-13	0.678339056783057	0.132	0.036	1.54966137638806E-08	Proliferating T/NK	SRP9
9.50946656857541E-13	0.531096937226814	0.184	0.06	1.55108909200034E-08	Proliferating T/NK	RGS3
1.00457989302868E-12	0.89710895046943	0.101	0.023	1.63857026351908E-08	Proliferating T/NK	ARF5
1.05568231174191E-12	0.463069402007042	0.14	0.039	1.72192341868222E-08	Proliferating T/NK	ENY2
1.05981893494477E-12	0.270077519055219	0.193	0.063	1.72867066478842E-08	Proliferating T/NK	RAB5C
1.06275359505068E-12	0.457946241337562	0.118	0.03	1.73345738888716E-08	Proliferating T/NK	PPP1CC
1.09883506846109E-12	0.429409201170844	0.171	0.053	1.79230988016688E-08	Proliferating T/NK	NARF
1.10589296755864E-12	0.632676808447427	0.105	0.025	1.80382201938489E-08	Proliferating T/NK	CD320
1.11896018929738E-12	0.540690439048473	0.14	0.04	1.82513596476295E-08	Proliferating T/NK	L3MBTL2
1.17311995761386E-12	0.625478245217887	0.149	0.044	1.91347596286397E-08	Proliferating T/NK	C9orf16
1.17532222165292E-12	0.635119228543295	0.132	0.036	1.91706807573807E-08	Proliferating T/NK	USP10
1.21210216365494E-12	0.53717822708977	0.149	0.043	1.97705983913757E-08	Proliferating T/NK	UBA2
1.23730245535828E-12	0.531266361995794	0.132	0.036	2.01816403493489E-08	Proliferating T/NK	ADK
1.2475040653077E-12	0.338099190149553	0.171	0.053	2.03480388092339E-08	Proliferating T/NK	COPE
1.24909318590395E-12	0.295337718996103	0.25	0.091	2.03739589552794E-08	Proliferating T/NK	SIN3A
1.32561765991163E-12	0.471971088585546	0.202	0.068	2.16221496521235E-08	Proliferating T/NK	MUS81
1.33260059171823E-12	0.550831478467891	0.189	0.062	2.17360482515161E-08	Proliferating T/NK	ANKFY1
1.4054754430023E-12	0.315948454214107	0.149	0.043	2.29247099508106E-08	Proliferating T/NK	EIF6
1.40887172379334E-12	0.795240343249367	0.127	0.034	2.29801066867932E-08	Proliferating T/NK	CCDC14
1.42100953824084E-12	0.483623882859547	0.259	0.098	2.31780865782463E-08	Proliferating T/NK	ITPR3
1.45437741215162E-12	0.501557363519962	0.241	0.089	2.37223499696052E-08	Proliferating T/NK	BUB3
1.56337703619198E-12	0.40878061709716	0.158	0.047	2.55002428373273E-08	Proliferating T/NK	BCL7B

1.5818367475391E-12	0.925555037809956	0.123	0.033	2.58013391891102E-08	Proliferating T/NK	PSMD7
1.7564412967646E-12	0.402618445335933	0.158	0.048	2.86493139915273E-08	Proliferating T/NK	PPP2R5D
1.79947199946698E-12	0.309778540249858	0.197	0.066	2.93511877833059E-08	Proliferating T/NK	STRN4
1.81919952206108E-12	0.283126278378919	0.298	0.116	2.96729634043383E-08	Proliferating T/NK	EIF4A1
2.08503600679185E-12	0.31201065444628	0.14	0.04	3.40090223067819E-08	Proliferating T/NK	PPP1R7
2.12013347514114E-12	0.411399093887665	0.232	0.084	3.45814971130271E-08	Proliferating T/NK	STK24
2.19832309486187E-12	0.321697900278252	0.237	0.086	3.5856848000292E-08	Proliferating T/NK	IKBKG
2.41835914125263E-12	0.878760001628264	0.11	0.028	3.94458559529717E-08	Proliferating T/NK	DNAJC15
2.41955326554449E-12	0.408006375467025	0.145	0.042	3.94653333142961E-08	Proliferating T/NK	ZMYM3
2.48012077632792E-12	0.358677683059399	0.158	0.048	4.04532499826847E-08	Proliferating T/NK	FAM13B
2.49868486790026E-12	0.391137799359202	0.237	0.086	4.07560488803211E-08	Proliferating T/NK	P2RY10
2.50702666493995E-12	0.433143523994381	0.18	0.058	4.08921119318355E-08	Proliferating T/NK	SRPK1
2.69630203262115E-12	0.38332645181407	0.136	0.038	4.39793824540836E-08	Proliferating T/NK	XPO5
2.72635004738153E-12	0.386247839134531	0.158	0.048	4.44694956228402E-08	Proliferating T/NK	NAA40
2.80154724609863E-12	0.689374095545372	0.118	0.031	4.56960371311147E-08	Proliferating T/NK	SRGAP2B
2.89547611376523E-12	0.627473113695074	0.127	0.035	4.72281108916247E-08	Proliferating T/NK	CCDC124
3.21198374539906E-12	0.774964175104068	0.105	0.026	5.2390666871204E-08	Proliferating T/NK	THOC6
3.25240362129233E-12	0.417917605276518	0.189	0.063	5.30499554668992E-08	Proliferating T/NK	DCAF15
3.26412809138313E-12	0.442924544427365	0.154	0.046	5.32411932985503E-08	Proliferating T/NK	CPSF1
3.2702423069238E-12	0.437432559589525	0.127	0.035	5.33409222682341E-08	Proliferating T/NK	NUBP2
3.71029616865559E-12	0.48276998271688	0.162	0.051	6.05186408069414E-08	Proliferating T/NK	HSPB11
3.73788550794603E-12	0.470567084093931	0.193	0.065	6.09686505201078E-08	Proliferating T/NK	TMCO1
3.75086041093799E-12	0.651804093320061	0.175	0.057	6.11802841628096E-08	Proliferating T/NK	INPP5F
3.80580631474253E-12	0.34091319683698	0.219	0.077	6.20765067997654E-08	Proliferating T/NK	VASP
3.84003162130824E-12	0.396873177317	0.145	0.043	6.26347557751587E-08	Proliferating T/NK	NF2
3.85401413333254E-12	0.52711185516076	0.145	0.043	6.28628245287871E-08	Proliferating T/NK	NUP62
4.11478868706184E-12	0.935144299268685	0.127	0.035	6.71163182746656E-08	Proliferating T/NK	SLC12A4
4.17234234388029E-12	0.9051422696694	0.101	0.024	6.80550759710314E-08	Proliferating T/NK	ABHD12
4.17373693659249E-12	0.253998357929243	0.368	0.156	6.807782317276E-08	Proliferating T/NK	ASXL1
5.02159251665892E-12	0.70322276639693	0.114	0.03	8.19071955392237E-08	Proliferating T/NK	FN3KRP
5.18942491043643E-12	0.391317862294888	0.715	0.373	8.46447097141286E-08	Proliferating T/NK	SUN2
5.8388879953548E-12	0.451312128328624	0.171	0.055	9.52381020922322E-08	Proliferating T/NK	YY1
6.06743272577007E-12	0.582777394256617	0.105	0.026	9.89658951900356E-08	Proliferating T/NK	DENR
6.19160834306551E-12	0.503974850908444	0.11	0.028	1.00991323683742E-07	Proliferating T/NK	MFF
6.22418478608326E-12	0.295785588339865	0.154	0.047	1.01522678045804E-07	Proliferating T/NK	DEPRECATED-ENSG00000205937
6.37621079511237E-12	0.338667176013864	0.553	0.261	1.04002374279078E-07	Proliferating T/NK	EHBP1L1
6.38409649944232E-12	0.428507701385343	0.118	0.031	1.04130998002404E-07	Proliferating T/NK	ATG9A
6.53757280449686E-12	0.450418776480511	0.158	0.049	1.06634350014148E-07	Proliferating T/NK	TTC31
7.08824760389938E-12	0.251081042195351	0.439	0.194	1.15616406667203E-07	Proliferating T/NK	ACTN4
7.25134033348037E-12	0.340471147327743	0.184	0.061	1.18276612179398E-07	Proliferating T/NK	CDS2
7.26573884804119E-12	0.987704077167358	0.105	0.026	1.185114663504E-07	Proliferating T/NK	NSMCE2
7.34263027531371E-12	0.565598533770875	0.101	0.025	1.19765642420642E-07	Proliferating T/NK	GMPS
7.36985438771184E-12	0.870600700629725	0.127	0.036	1.20209694917968E-07	Proliferating T/NK	HAGH
7.45099342219183E-12	0.393648067426605	0.127	0.035	1.21533153709371E-07	Proliferating T/NK	CBL
7.4903071236672E-12	0.652754040983349	0.132	0.038	1.22174399494136E-07	Proliferating T/NK	GPD2
7.84470983683609E-12	0.259332402272911	0.254	0.097	1.27955062148634E-07	Proliferating T/NK	SH3BP1
7.94757629545029E-12	0.263493640030553	0.25	0.094	1.2963291695509E-07	Proliferating T/NK	TMEM50A
8.04237521350716E-12	0.516223108857718	0.123	0.034	1.31179182107515E-07	Proliferating T/NK	PLRG1
8.28969320148274E-12	0.645501740832968	0.101	0.025	1.35213185809385E-07	Proliferating T/NK	SFMBT1
8.29679636964354E-12	0.404671361713597	0.167	0.054	1.35329045585256E-07	Proliferating T/NK	PPP1R12B
8.33204977548739E-12	0.317673725442037	0.189	0.064	1.35904063887975E-07	Proliferating T/NK	SPEN
9.49942546390265E-12	0.626830208258388	0.154	0.048	1.54945128741716E-07	Proliferating T/NK	ETV1
9.86927082594166E-12	0.300194150035957	0.36	0.154	1.60977676441934E-07	Proliferating T/NK	CHST12
1.04132656482986E-11	0.378036065832497	0.123	0.034	1.69850775989398E-07	Proliferating T/NK	UPF2
1.09014098190135E-11	0.377448465732773	0.154	0.047	1.77812895557929E-07	Proliferating T/NK	MAN1A1
1.10146565747562E-11	0.390539540039819	0.11	0.028	1.79660063390848E-07	Proliferating T/NK	TIMM13
1.15497543860827E-11	0.426008560740915	0.197	0.069	1.88388043791396E-07	Proliferating T/NK	TFRC
1.16502538119101E-11	0.287052133927154	0.531	0.25	1.90027289926065E-07	Proliferating T/NK	DOCK2
1.26763852722423E-11	0.628172648615343	0.101	0.025	2.06764520175544E-07	Proliferating T/NK	RRP7A
1.30748947003358E-11	0.90042551250991	0.101	0.025	2.13264607457178E-07	Proliferating T/NK	MGMT
1.31716935129586E-11	0.675601704988585	0.101	0.025	2.14843492889867E-07	Proliferating T/NK	SEMA4A
1.37753392906112E-11	0.27317102905596	0.145	0.044	2.24689559169159E-07	Proliferating T/NK	MCRIP1
1.38707377438195E-11	0.382822350148741	0.211	0.076	2.2624560333944E-07	Proliferating T/NK	NMT1
1.4120520278881E-11	0.460968350994297	0.127	0.036	2.30319806268828E-07	Proliferating T/NK	G6PD
1.44024524087807E-11	0.278298719622397	0.18	0.06	2.34918401239622E-07	Proliferating T/NK	PET100
1.55131408275597E-11	0.410357596986613	0.241	0.092	2.53034840038326E-07	Proliferating T/NK	LNPEP

1.60989755787304E-11	0.317829530360909	0.154	0.048	2.62590390664671E-07	Proliferating T/NK	GNAI3
1.65209345855455E-11	0.284063372015469	0.167	0.054	2.69472964024833E-07	Proliferating T/NK	PPP6C
1.7875400099503E-11	0.576737317990331	0.132	0.038	2.91565651022993E-07	Proliferating T/NK	HDAC8
1.86369748895036E-11	0.581595084293434	0.11	0.029	3.03987697422693E-07	Proliferating T/NK	LSM 7.00
1.95571417407265E-11	0.878426796209132	0.105	0.027	3.18996538932991E-07	Proliferating T/NK	ATL2
2.01858743172619E-11	0.374833482449495	0.162	0.052	3.29251795988859E-07	Proliferating T/NK	GLMN
2.03048769494414E-11	0.442181115666394	0.145	0.044	3.31192847922338E-07	Proliferating T/NK	APOBEC3C
2.03155959789266E-11	0.262265170131406	0.202	0.071	3.31367686012272E-07	Proliferating T/NK	SMG5
2.06144283608084E-11	0.451304150586827	0.158	0.051	3.36241940993146E-07	Proliferating T/NK	HAUS3
2.06928302243737E-11	0.310365876306157	0.132	0.038	3.37520753789759E-07	Proliferating T/NK	NDUFB10
2.27905882634052E-11	0.626843729048509	0.101	0.025	3.71737285164402E-07	Proliferating T/NK	PSMC6
2.29148599145193E-11	0.712693263873909	0.105	0.027	3.73764280065724E-07	Proliferating T/NK	ITPRID2
2.30333072831826E-11	0.370067247020023	0.171	0.057	3.75696275095991E-07	Proliferating T/NK	NUP153
2.43069743391532E-11	0.318078111066086	0.162	0.052	3.96471058445928E-07	Proliferating T/NK	TRAP1
2.51362221007213E-11	0.379866390494648	0.228	0.086	4.09996918684865E-07	Proliferating T/NK	POLDIP3
2.55050533782436E-11	0.357880228171848	0.175	0.059	4.16012925652532E-07	Proliferating T/NK	BACH1
2.60726361394566E-11	1.09667786689336	0.114	0.031	4.25270768070676E-07	Proliferating T/NK	GLE1
2.78221941469544E-11	0.393978999639139	0.127	0.037	4.53807808730973E-07	Proliferating T/NK	SQOR
2.9215431665446E-11	0.79679873326221	0.127	0.037	4.76532905895089E-07	Proliferating T/NK	SNTB1
3.13842642558644E-11	0.849177721806559	0.14	0.043	5.11908734277404E-07	Proliferating T/NK	NBL1
3.55131264992366E-11	0.535359480733742	0.101	0.026	5.79254606329048E-07	Proliferating T/NK	LNPK
3.76790817304854E-11	0.324437313272858	0.167	0.055	6.14583502105947E-07	Proliferating T/NK	AARS2
3.89012400389941E-11	0.549447771924275	0.145	0.045	6.34518126276033E-07	Proliferating T/NK	API5
3.93952871785369E-11	0.862397278539554	0.114	0.031	6.42576529169116E-07	Proliferating T/NK	ZEB1
4.0609894670906E-11	0.699169512569624	0.105	0.028	6.62387991977147E-07	Proliferating T/NK	SRSF10
4.14636430761595E-11	0.664590021289282	0.149	0.047	6.76313482215238E-07	Proliferating T/NK	SPECC1L
4.22725139813438E-11	0.349417626143837	0.206	0.074	6.89506975549699E-07	Proliferating T/NK	UHRF2
4.37453126077906E-11	0.497932332384223	0.136	0.041	7.13529793945673E-07	Proliferating T/NK	RAB5B
4.44671616774626E-11	0.526845976650387	0.171	0.058	7.25303874121092E-07	Proliferating T/NK	PLEKHJ1
4.6195385333972E-11	0.72335759692609	0.123	0.035	7.53492930182417E-07	Proliferating T/NK	NFE2L1
4.78296867806499E-11	0.625881592718613	0.127	0.037	7.80150021079181E-07	Proliferating T/NK	DNAJB12
4.95396305974695E-11	0.626938628214708	0.123	0.035	8.08040914675324E-07	Proliferating T/NK	SLC35B1
4.96597029168902E-11	0.575529202104486	0.145	0.045	8.09999414277397E-07	Proliferating T/NK	GTDC1
4.98802267992142E-11	0.367149964283723	0.25	0.1	8.13596379321983E-07	Proliferating T/NK	IL21R
5.32166222858313E-11	0.632054444425132	0.123	0.035	8.68016326104195E-07	Proliferating T/NK	MT-ND3
6.12415397745197E-11	0.529282788622662	0.162	0.054	9.98910755262191E-07	Proliferating T/NK	SNRNP40
6.30510516934589E-11	0.300373309260293	0.202	0.073	1.02842570417201E-06	Proliferating T/NK	TM9SF3
6.43518334158382E-11	0.530159971736012	0.232	0.091	1.04964275484574E-06	Proliferating T/NK	CHKB
6.46632337301612E-11	0.434040041599225	0.162	0.054	1.05472200537266E-06	Proliferating T/NK	CNPPD1
6.73357337772072E-11	0.45032478440037	0.158	0.052	1.09831315364003E-06	Proliferating T/NK	RMND5A
6.8782489355937E-11	0.460030465111496	0.132	0.039	1.12191118388469E-06	Proliferating T/NK	WSB2
6.97005725475199E-11	0.886007643099039	0.123	0.036	1.1368860388226E-06	Proliferating T/NK	DDX55
7.0876351858061E-11	0.261517761647967	0.184	0.064	1.15606417515683E-06	Proliferating T/NK	HNRNPK
7.41818061889611E-11	0.687435767408511	0.101	0.026	1.20997944074815E-06	Proliferating T/NK	TRIM24
7.75053928659033E-11	0.427919812549938	0.14	0.043	1.26419046303575E-06	Proliferating T/NK	NAA60
7.76794900296553E-11	0.352564086822254	0.202	0.074	1.26703016187371E-06	Proliferating T/NK	SRRM1
7.99915728088964E-11	0.572153688682346	0.123	0.036	1.30474254408591E-06	Proliferating T/NK	FCHSD2
8.03543895999592E-11	0.449854449318677	0.149	0.048	1.31066044876494E-06	Proliferating T/NK	TAF8
8.53395303268512E-11	0.260147536260929	0.281	0.119	1.39197307916127E-06	Proliferating T/NK	TNFRSF9
8.7901411113005E-11	0.682766413038178	0.132	0.04	1.43375991666422E-06	Proliferating T/NK	NAB2
8.89934897202706E-11	0.656904471591341	0.162	0.055	1.45157281082733E-06	Proliferating T/NK	ADD3
8.97609005296815E-11	0.591740758694413	0.127	0.038	1.46409004853964E-06	Proliferating T/NK	RBM12
9.20135068695425E-11	1.07304092688111	0.101	0.026	1.50083231054911E-06	Proliferating T/NK	RHOC
9.54935606004567E-11	0.302380106008932	0.189	0.067	1.55759546695405E-06	Proliferating T/NK	PYCR2
9.75007349293412E-11	0.286532051358749	0.259	0.104	1.59033448743248E-06	Proliferating T/NK	DEPRECATED-ENSG00000115738
9.89777929170615E-11	0.30826156709176	0.123	0.036	1.61442678027019E-06	Proliferating T/NK	TRIM11
1.01056785426156E-10	0.638278831523883	0.136	0.042	1.64833722708604E-06	Proliferating T/NK	LRCH1
1.02767703749943E-10	0.443575857545544	0.754	0.448	1.67624401586532E-06	Proliferating T/NK	LSP1
1.04389920184117E-10	0.405149927659859	0.193	0.07	1.70270398812314E-06	Proliferating T/NK	NR2C2
1.05348057121418E-10	0.254457017001587	0.219	0.083	1.71833215970744E-06	Proliferating T/NK	UBE3C
1.09874802962933E-10	0.637306037757261	0.101	0.026	1.7921679111284E-06	Proliferating T/NK	ZBED1
1.25787767133112E-10	0.284575258251414	0.118	0.034	2.05172426970818E-06	Proliferating T/NK	CTPS1
1.28991140510722E-10	0.612465576804333	0.105	0.028	2.10397449287038E-06	Proliferating T/NK	TMEM11
1.30904626544627E-10	0.473287642785681	0.136	0.042	2.13518536356941E-06	Proliferating T/NK	VCAM1
1.34750696905579E-10	0.314398171723072	0.175	0.061	2.1979186172269E-06	Proliferating T/NK	AXIN1
1.48305424424355E-10	0.713616964017724	0.105	0.029	2.41900977778565E-06	Proliferating T/NK	SGMS1

1.63823505793691E-10	0.366451704358109	0.145	0.046	2.67212520300089E-06	Proliferating T/NK	TMOD3
1.67645055961588E-10	0.450705637796255	0.11	0.031	2.73445850778946E-06	Proliferating T/NK	PHF5A
1.70840392982499E-10	0.274713582670077	0.263	0.107	2.78657764993754E-06	Proliferating T/NK	GRIPAP1
1.98232029758314E-10	0.716544257198857	0.105	0.029	3.23336263738786E-06	Proliferating T/NK	CALU
2.02079549313203E-10	0.892858390709288	0.114	0.033	3.29611952884765E-06	Proliferating T/NK	RFC1
2.13664101930947E-10	0.423166056779207	0.145	0.047	3.48507516659568E-06	Proliferating T/NK	ARFRP1
2.22423815101099E-10	0.399718978908919	0.127	0.039	3.62795484811403E-06	Proliferating T/NK	TIMMDC1
2.2488117272385E-10	0.284013078709317	0.118	0.034	3.66803680829872E-06	Proliferating T/NK	KRR1
2.28682277194509E-10	0.474714196971316	0.105	0.029	3.73003662331964E-06	Proliferating T/NK	SYT11
2.46751925797701E-10	0.264942166304904	0.175	0.062	4.0247706616863E-06	Proliferating T/NK	APOBEC3G
2.53571517932913E-10	0.398880540844245	0.114	0.033	4.13600502900375E-06	Proliferating T/NK	DLST
2.55468890739253E-10	0.339452473322547	0.728	0.393	4.16695307684796E-06	Proliferating T/NK	ITGA4
2.58252189189075E-10	0.528217993739295	0.11	0.031	4.212351457863E-06	Proliferating T/NK	MAP1S
2.65664657492306E-10	0.749061318518127	0.101	0.027	4.33325622835701E-06	Proliferating T/NK	PPP5C
2.67390230730903E-10	0.659923800945162	0.184	0.068	4.36140205345175E-06	Proliferating T/NK	RILPL2
2.8323262891895E-10	0.478175655006887	0.136	0.043	4.61980741029699E-06	Proliferating T/NK	OASL
2.94042564444835E-10	0.548572483898239	0.127	0.039	4.79612826865971E-06	Proliferating T/NK	LARP4B
2.96419805846012E-10	0.397147559065418	0.658	0.352	4.83490345315431E-06	Proliferating T/NK	LCP1
3.13432752471483E-10	0.749936802339896	0.101	0.027	5.11240162556236E-06	Proliferating T/NK	SPCS1
3.19938106112563E-10	0.632386436983352	0.114	0.033	5.21851044880201E-06	Proliferating T/NK	CAMKK2
3.2816410813349E-10	0.676506637763938	0.132	0.041	5.35268476776536E-06	Proliferating T/NK	DHRS7
3.38875860173448E-10	0.432502869970444	0.132	0.041	5.52740415528911E-06	Proliferating T/NK	ENOSF1
3.46555982469981E-10	0.492757195944589	0.114	0.033	5.65267463006786E-06	Proliferating T/NK	CCDC7
3.81887881479829E-10	0.484693632557482	0.123	0.037	6.2289732348175E-06	Proliferating T/NK	HSPD1
3.96514432155239E-10	0.279487831231681	0.132	0.041	6.46754690288411E-06	Proliferating T/NK	PEX13
4.20921378699891E-10	0.426297217367516	0.189	0.07	6.86564860797393E-06	Proliferating T/NK	IQGAP1
4.31041738179454E-10	0.482049636525338	0.11	0.031	7.03072179144507E-06	Proliferating T/NK	PPP2R3C
4.53790619559894E-10	0.307970781725291	0.127	0.039	7.40177879564143E-06	Proliferating T/NK	PGRMC2
4.60777610242375E-10	0.378054442356147	0.11	0.031	7.51574360066337E-06	Proliferating T/NK	COX7C
4.68176852264452E-10	0.268166709634496	0.627	0.337	7.63643263728547E-06	Proliferating T/NK	NKG7
5.15970405495606E-10	0.524700807090966	0.114	0.034	8.41599328403883E-06	Proliferating T/NK	CERS5
5.18043699448257E-10	0.477488934009105	0.101	0.028	8.44981078170052E-06	Proliferating T/NK	USP37
5.24746990376741E-10	0.75227420190338	0.101	0.028	8.55914816003502E-06	Proliferating T/NK	MPG
5.38662504547848E-10	0.46001871183584	0.136	0.044	8.78612411167995E-06	Proliferating T/NK	ATP13A3
5.85713279561903E-10	0.561564875652624	0.114	0.034	9.5535693029342E-06	Proliferating T/NK	SP1
6.03408545706018E-10	0.260001929804372	0.127	0.04	9.84219678901085E-06	Proliferating T/NK	PSMD1
6.06900837110253E-10	0.726374446403058	0.105	0.03	9.89915955410534E-06	Proliferating T/NK	AHCYL1
6.15419080433855E-10	0.418570165157542	0.145	0.048	1.00381006209566E-05	Proliferating T/NK	DDX50
6.62254989636407E-10	0.460598015100326	0.123	0.038	1.08020411359594E-05	Proliferating T/NK	IMMT
6.64472044982172E-10	0.322562002313863	0.211	0.082	1.08382035257042E-05	Proliferating T/NK	PTPN6
6.74146897303638E-10	0.409990113405662	0.224	0.089	1.09960100419196E-05	Proliferating T/NK	MTA2
6.78485657940285E-10	0.262826647292862	0.18	0.066	1.1066779566664E-05	Proliferating T/NK	PRPF39
7.02622287983885E-10	0.305995405980822	0.132	0.042	1.14604721393051E-05	Proliferating T/NK	PSMD6
7.03984537606895E-10	0.38806988114916	0.127	0.04	1.14826917929061E-05	Proliferating T/NK	HMG20B
7.27685396021158E-10	0.774634048321373	0.101	0.028	1.18692764945011E-05	Proliferating T/NK	AFG3L2
7.43753187111406E-10	0.446830456331955	0.18	0.066	1.21313582349741E-05	Proliferating T/NK	ILKAP
8.30600106394032E-10	0.562239583968681	0.105	0.03	1.35479183353931E-05	Proliferating T/NK	IRF2BP1
8.38095891939698E-10	0.449792555017359	0.158	0.055	1.36701820934284E-05	Proliferating T/NK	KRIT1
8.38786702576106E-10	0.478912026967488	0.145	0.048	1.36814499057189E-05	Proliferating T/NK	SLC12A9
8.75819867901801E-10	0.345341506142517	0.114	0.034	1.42854978653463E-05	Proliferating T/NK	LARP1
9.86102096650528E-10	0.365002010831037	0.158	0.055	1.60843112984668E-05	Proliferating T/NK	DGAT1
9.9284649417804E-10	0.473102595772113	0.101	0.028	1.6194319166538E-05	Proliferating T/NK	PTPN11
1.0045847341049E-09	0.373460476131912	0.14	0.046	1.6385781597985E-05	Proliferating T/NK	VPS26B
1.02273471949392E-09	0.405071792579037	0.158	0.055	1.66818260096654E-05	Proliferating T/NK	AGPS
1.02792629228133E-09	0.37185176955763	0.105	0.03	1.67665057534008E-05	Proliferating T/NK	POLE3
1.03946095786287E-09	0.364447490112538	0.114	0.034	1.69546476837012E-05	Proliferating T/NK	RRP1
1.20952903267081E-09	0.268491779036578	0.167	0.06	1.97286280518936E-05	Proliferating T/NK	TMX2
1.31083370211569E-09	0.307105034848122	0.118	0.036	2.1381008515209E-05	Proliferating T/NK	GTF2H2C
1.42927396982908E-09	0.621404386424892	0.14	0.047	2.33128877218822E-05	Proliferating T/NK	RBM12B
1.44288754318918E-09	0.581505240282519	0.11	0.033	2.35349387169587E-05	Proliferating T/NK	SLC9A1
1.554776994569E-09	0.363945981052671	0.158	0.056	2.5359967558415E-05	Proliferating T/NK	ELAVL1
1.5886929611656E-09	0.258391513950599	0.596	0.305	2.59131708895722E-05	Proliferating T/NK	GRK2
1.59190446431438E-09	0.471508028345026	0.136	0.045	2.59655537174318E-05	Proliferating T/NK	MED14
1.70361905202991E-09	0.400376903791832	0.145	0.049	2.77877303576599E-05	Proliferating T/NK	UBA6
1.72507068616386E-09	0.730864835484585	0.105	0.031	2.81376279620187E-05	Proliferating T/NK	USP42
1.83610840055408E-09	0.620675106003674	0.127	0.041	2.99487641214376E-05	Proliferating T/NK	STX10

1.95840401064104E-09	0.307652314338467	0.184	0.07	3.1943527817566E-05	Proliferating T/NK	SLF1
2.00635462910364E-09	0.296285782807475	0.118	0.037	3.27256503553095E-05	Proliferating T/NK	PSMG3
2.11626564158077E-09	0.272569388388479	0.149	0.052	3.45184088798239E-05	Proliferating T/NK	USP14
2.15302639826368E-09	0.29502512297223	0.184	0.069	3.51180135820788E-05	Proliferating T/NK	E2F4
2.37133273893417E-09	0.364421646607547	0.118	0.037	3.86788083047552E-05	Proliferating T/NK	WAC
2.42885409654812E-09	0.427616986300795	0.101	0.029	3.96170391687964E-05	Proliferating T/NK	TJAP1
2.64782000752132E-09	0.277156691041288	0.193	0.075	4.31885921426803E-05	Proliferating T/NK	NDUFAF8
2.6715502599692E-09	0.49339431013612	0.118	0.037	4.35756562903577E-05	Proliferating T/NK	GRK5
2.76925433434193E-09	0.364407710595412	0.118	0.037	4.51693074474513E-05	Proliferating T/NK	CHMP2A
2.82261533280912E-09	0.606143463579474	0.114	0.035	4.60396786934495E-05	Proliferating T/NK	SPAG7
3.13058218557253E-09	0.279918227427633	0.114	0.035	5.10629260288735E-05	Proliferating T/NK	ZNRD1
3.1546326226962E-09	0.511002227019225	0.132	0.044	5.14552127087977E-05	Proliferating T/NK	MTX3
3.17259502677154E-09	0.280817864576795	0.11	0.033	5.17481974816705E-05	Proliferating T/NK	LSM 6.00
3.39705410284452E-09	0.568091889701306	0.101	0.029	5.54093494714969E-05	Proliferating T/NK	SNRNP48
3.5349884917282E-09	0.271528639295983	0.123	0.039	5.76591972885787E-05	Proliferating T/NK	SLC25A6
3.53911460574724E-09	0.411552108165283	0.136	0.046	5.77264983343432E-05	Proliferating T/NK	STT3A
3.60039273543919E-09	0.329670496180174	0.294	0.131	5.87260059077486E-05	Proliferating T/NK	EDEM1
3.76423146763347E-09	0.312964364773011	0.123	0.04	6.13983794685695E-05	Proliferating T/NK	FAM98B
3.77952502637127E-09	0.33088151901953	0.11	0.033	6.16478327051417E-05	Proliferating T/NK	PAGR1
3.93089160308655E-09	0.55472313122826	0.123	0.04	6.41167729379447E-05	Proliferating T/NK	TESK1
3.99198901973738E-09	0.403669110378852	0.14	0.048	6.51133329009364E-05	Proliferating T/NK	DNM1L
4.25240173650351E-09	0.273691709845075	0.123	0.04	6.93609247241088E-05	Proliferating T/NK	SCAP
4.26956365345371E-09	0.303126164472358	0.14	0.048	6.96408527514835E-05	Proliferating T/NK	ACBD5
4.3244161117978E-09	0.35364116318438	0.145	0.05	7.05355511995339E-05	Proliferating T/NK	CCNK
4.38596632637457E-09	0.30709371080948	0.123	0.04	7.15394967494956E-05	Proliferating T/NK	YKT6
4.47254305623414E-09	0.35319273666424	0.14	0.048	7.2951649790235E-05	Proliferating T/NK	COLGALT1
4.57649640584006E-09	0.466007628403557	0.939	0.712	7.46472328756572E-05	Proliferating T/NK	CD74
4.6283270792784E-09	0.279640716835535	0.167	0.062	7.549264299011E-05	Proliferating T/NK	PPP1R10
4.7904079785919E-09	0.314718514249992	0.171	0.064	7.81363445388126E-05	Proliferating T/NK	ZNF592
5.31240223252638E-09	0.255021289546538	0.118	0.038	8.66505928147378E-05	Proliferating T/NK	MIIP
5.62975401024571E-09	0.421752511253888	0.101	0.03	9.18269176611178E-05	Proliferating T/NK	RPP30
6.19172268528646E-09	0.702137978129164	0.105	0.032	0.000100993188719707	Proliferating T/NK	UNC45A
6.6756807818761E-09	0.25615847272422	0.25	0.108	0.000108887029233181	Proliferating T/NK	BCL3
6.75490199148177E-09	0.300885752121621	0.127	0.042	0.000110179206383059	Proliferating T/NK	SENP5
7.44031144615481E-09	0.351992970676108	0.114	0.036	0.000121358919998231	Proliferating T/NK	AZI2
7.97026093680357E-09	0.278056106245411	0.219	0.091	0.000130002926140203	Proliferating T/NK	TMX4
8.03420174262006E-09	0.272136320960936	0.11	0.034	0.000131045864623876	Proliferating T/NK	TTC9C
8.36098433671411E-09	0.404348478787529	0.132	0.045	0.000136376015516144	Proliferating T/NK	ZNF830
8.5158136123265E-09	0.427243690402136	0.132	0.045	0.000138901435830658	Proliferating T/NK	NHLRC2
8.75245525922745E-09	0.360010282967758	0.114	0.036	0.000142761297733259	Proliferating T/NK	INTS6L
9.29124137410707E-09	0.350839522399833	0.118	0.038	0.00015154943805306	Proliferating T/NK	UXS1
9.44706193734881E-09	0.348593046424577	0.101	0.03	0.000154091027260097	Proliferating T/NK	TET3
9.64063238837387E-09	0.345210455716771	0.149	0.054	0.000157248354886766	Proliferating T/NK	GHITM
1.02751920355086E-08	0.285546528496005	0.154	0.056	0.00016759865729118	Proliferating T/NK	UFC1
1.16006645514383E-08	0.662794754223991	0.101	0.031	0.00018921843949851	Proliferating T/NK	FHOD1
1.17942715447365E-08	0.480590755084525	0.11	0.035	0.000192376363166196	Proliferating T/NK	TAF6
1.1857688711834E-08	0.425284804407249	0.105	0.033	0.000193410760578725	Proliferating T/NK	PSMG2
1.29808273563324E-08	0.539119763052675	0.123	0.041	0.000211730275009139	Proliferating T/NK	CDYL2
1.42199618335537E-08	0.386222594799572	0.127	0.043	0.000231941797467094	Proliferating T/NK	POLD4
1.45234423857129E-08	0.266033028402505	0.149	0.054	0.000236891868753363	Proliferating T/NK	NPTN
1.5423085929132E-08	0.393909197849906	0.171	0.066	0.000251565954590072	Proliferating T/NK	SYAP1
1.75292931353792E-08	0.588457260474667	0.118	0.04	0.000285920300331169	Proliferating T/NK	SCYL1
1.75719092308684E-08	0.424876772296985	0.136	0.048	0.000286615411464694	Proliferating T/NK	GSPT1
1.77228522450796E-08	0.277544416025472	0.11	0.035	0.000289077442969493	Proliferating T/NK	LARP7
1.88983209645228E-08	0.335431242195158	0.154	0.057	0.000308250513252332	Proliferating T/NK	ATN1
2.13547640760327E-08	0.314255503306134	0.189	0.076	0.000348317556844169	Proliferating T/NK	TSC22D2
2.19957779083434E-08	0.316992694817799	0.14	0.051	0.000358773133462989	Proliferating T/NK	PPM1A
2.27149394225997E-08	0.268112243989991	0.132	0.046	0.000370503376922023	Proliferating T/NK	NDUFS7
2.49893833531026E-08	0.569954473054062	0.105	0.033	0.000407601831872456	Proliferating T/NK	DVL2
2.93102127744053E-08	0.301849649029246	0.123	0.042	0.000478078880563325	Proliferating T/NK	NFKBID
2.98600830845825E-08	0.353814128648332	0.14	0.051	0.000487047815192625	Proliferating T/NK	TMEM219
3.0074320300292E-08	0.391212551115656	0.206	0.088	0.000490542238418063	Proliferating T/NK	FRYL
3.16129293222528E-08	0.256280928596015	0.105	0.034	0.000515638490175265	Proliferating T/NK	PREB
3.21098035750527E-08	0.306918044520351	0.127	0.044	0.000523743006112684	Proliferating T/NK	ARNT
3.21607958789183E-08	0.439729823062323	0.136	0.049	0.000524574741581037	Proliferating T/NK	UBE2R2
3.24665252772486E-08	0.361572996349601	0.101	0.031	0.000529561493797201	Proliferating T/NK	WDR55

3.56864567086919E-08	0.323067813150927	0.175	0.07	0.000582081795375473	Proliferating T/NK	P2RX5
3.63908791695888E-08	0.367036344112736	0.105	0.034	0.000593571630135162	Proliferating T/NK	CRAMP1
3.82715389139241E-08	0.273259721536874	0.145	0.054	0.000624247071225016	Proliferating T/NK	IFFO1
3.8661896412884E-08	0.359529723672831	0.145	0.054	0.000630614192390551	Proliferating T/NK	DHX30
4.33244686649431E-08	0.309853384592125	0.11	0.036	0.000706665408393886	Proliferating T/NK	ACO2
4.51792819939807E-08	0.260173612642964	0.175	0.07	0.000736919268603819	Proliferating T/NK	DDX23
4.82747122512438E-08	0.293380978008074	0.118	0.04	0.000787408831530038	Proliferating T/NK	NUDT5
5.06486951690791E-08	0.30303903606999	0.105	0.034	0.00082613086690285	Proliferating T/NK	DNAJC11
5.21805197808236E-08	0.371826532957595	0.123	0.043	0.000851116458145014	Proliferating T/NK	ERCC3
5.33705677408783E-08	0.305146692361194	0.105	0.034	0.000870527330421467	Proliferating T/NK	MANF
5.50642235749624E-08	0.493152945994636	0.105	0.034	0.000898152550731212	Proliferating T/NK	TBC1D22B
5.70668241840301E-08	0.253020212951962	0.101	0.032	0.000930816969265715	Proliferating T/NK	SELENOS
5.75136251910723E-08	0.411241596120277	0.114	0.039	0.00093810474049158	Proliferating T/NK	EIF4G1
6.34628015294816E-08	0.535106567348182	0.105	0.034	0.00103514175574737	Proliferating T/NK	CHMP1A
6.76346996618923E-08	0.570809652665612	0.105	0.035	0.00110318958618512	Proliferating T/NK	TSC2
7.3742282853073E-08	0.400368962142089	0.123	0.043	0.00120281037561647	Proliferating T/NK	AP2A1
7.70401782081065E-08	0.768879746054019	0.123	0.044	0.00125660234675242	Proliferating T/NK	ACAP3
7.96822953373692E-08	0.321872888568903	0.123	0.043	0.00129969791924783	Proliferating T/NK	NUP188
8.13427207908127E-08	0.342465222872348	0.136	0.05	0.00132678111881895	Proliferating T/NK	LRIG2
9.26201794606816E-08	0.400646867306014	0.123	0.044	0.00151072774718318	Proliferating T/NK	GNPTG
9.3864981266042E-08	0.339193478400594	0.114	0.039	0.00153103170943041	Proliferating T/NK	AGK
1.04616140582057E-07	0.39876651990179	0.123	0.044	0.00170639386903394	Proliferating T/NK	DNAJC8
1.05389952447348E-07	0.351540421557782	0.101	0.033	0.0017190155143687	Proliferating T/NK	PRKAB1
1.18412765917226E-07	0.271967672989057	0.162	0.065	0.00193143062487587	Proliferating T/NK	ELMO1
1.18444658285506E-07	0.513047899078312	0.105	0.035	0.00193195082129489	Proliferating T/NK	KCTD5
1.21618265543324E-07	0.457112834247747	0.101	0.033	0.00198371552927716	Proliferating T/NK	MFN2
1.3177106792723E-07	0.426135857431448	0.114	0.04	0.00214931788896105	Proliferating T/NK	SUPT7L
1.33240430438438E-07	0.273049821357109	0.149	0.058	0.00217328466088136	Proliferating T/NK	ARID1A
1.44127075316137E-07	0.261538301777301	0.118	0.042	0.00235085672548151	Proliferating T/NK	COG4
1.45757936215518E-07	0.388789623047177	0.132	0.049	0.00237745769761131	Proliferating T/NK	SLC25A28
1.49484553443794E-07	0.253634712877552	0.118	0.042	0.00243824255122172	Proliferating T/NK	PIK3CG
1.75144647668388E-07	0.281914556118156	0.132	0.049	0.00285678434811907	Proliferating T/NK	VPS26A
1.79084997435295E-07	0.327931085568655	0.114	0.04	0.0029210553931671	Proliferating T/NK	ARSG
1.96956795936051E-07	0.380908225473476	0.101	0.034	0.00321256229851292	Proliferating T/NK	POLR1E
1.98554512466588E-07	0.428662718955961	0.105	0.036	0.00323862265284252	Proliferating T/NK	EED
2.00628208159704E-07	0.302635823337201	0.105	0.036	0.00327244670329294	Proliferating T/NK	GALK2
2.04833528185523E-07	0.25567581575355	0.154	0.061	0.00334103967823406	Proliferating T/NK	DCP2
2.05818934798506E-07	0.268014501850556	0.136	0.052	0.00335711264549844	Proliferating T/NK	CARS2
2.53713540734854E-07	0.326523209063293	0.145	0.057	0.0041383215629262	Proliferating T/NK	GTF3C3
2.74337561835443E-07	0.359416856495449	0.158	0.064	0.0044747199710979	Proliferating T/NK	CPT1B
2.76238525480512E-07	0.284516439543906	0.105	0.036	0.00450572658911264	Proliferating T/NK	SNHG32
2.91416663073425E-07	0.349708019372698	0.167	0.069	0.00475329719139064	Proliferating T/NK	PTBP3
3.01499287148281E-07	0.426520637074075	0.11	0.039	0.00491775487267562	Proliferating T/NK	MAP3K2
3.06588350048547E-07	0.340816315622567	0.132	0.05	0.00500076257764185	Proliferating T/NK	PHF2
3.34780884749899E-07	0.393435886356476	0.114	0.041	0.00546061101115561	Proliferating T/NK	ASB1
3.37510057432226E-07	0.412711143064664	0.127	0.048	0.00550512654677704	Proliferating T/NK	ABL1
3.37537157020482E-07	0.333316236607115	0.127	0.048	0.00550556856816109	Proliferating T/NK	F2R
3.42483146826649E-07	0.261519008160119	0.114	0.041	0.00558624260788948	Proliferating T/NK	DDX1
3.8975357822725E-07	0.312869619812325	0.145	0.058	0.00635727061446467	Proliferating T/NK	NUDT22
4.0046472740555E-07	0.546402931810081	0.127	0.049	0.00653198016871193	Proliferating T/NK	MAP3K14
4.34470062683603E-07	0.466710633788484	0.105	0.037	0.00708664119243225	Proliferating T/NK	RDH11
4.71711824654396E-07	0.270024009046771	0.158	0.065	0.00769409157193785	Proliferating T/NK	RSBN1L
5.80140543174545E-07	0.530620057856768	0.101	0.035	0.00946267239972001	Proliferating T/NK	PIN1
6.3429298403576E-07	0.265310816039027	0.114	0.042	0.0103459528626073	Proliferating T/NK	IP6K1
6.84731527983048E-07	0.345192792361906	0.149	0.061	0.0111686559529315	Proliferating T/NK	CCDC141
6.85778169914112E-07	0.350508353592968	0.136	0.054	0.0111857277294691	Proliferating T/NK	TRAF2
7.51618112608698E-07	0.639181678981221	0.114	0.042	0.0122596430347605	Proliferating T/NK	THOC2
7.6802943218863E-07	0.325684076547347	0.101	0.035	0.0125273280684287	Proliferating T/NK	FBF1
7.95328876216542E-07	0.365038139409877	0.105	0.038	0.012972609299968	Proliferating T/NK	TMCO4
8.05810052902577E-07	0.277478371632263	0.105	0.037	0.0131435677728939	Proliferating T/NK	ZNF680
9.07792777905895E-07	0.260223700177923	0.101	0.036	0.0148070080004231	Proliferating T/NK	NF1
1.01185479748875E-06	0.587860061517751	0.101	0.036	0.0165043636018391	Proliferating T/NK	MXRA7
1.04976088086617E-06	0.501173679539371	0.101	0.036	0.017122649727808	Proliferating T/NK	NUDT4
1.85045073881672E-06	0.327386251654255	0.118	0.046	0.0301827020008395	Proliferating T/NK	PSMD12
1.99688686980809E-06	0.251129352037088	0.101	0.037	0.0325712217334397	Proliferating T/NK	ZNF326

SupTable5O_C2_EC_SeuratCluster

p_val	avg_log2FC	pct.1	pct.2	p_val_adj	cluster	gene
6.53391932446114E-61	8.04584635795252	0.36	0.003	1.06574758101286E-56	0	MRO
4.13024836763781E-46	6.65181821615907	0.286	0.005	6.73684811245403E-42	0	CLEC1B
1.46341820848641E-135	5.44548543516883	0.722	0.04	2.38698143986218E-131	0	CLEC4G
2.24507479730868E-131	5.32329674930016	0.713	0.039	3.66194150189018E-127	0	CLEC4M
2.55447526977863E-138	5.26502481516913	0.746	0.056	4.16660461253593E-134	0	MS4A6A
5.51914029915704E-78	4.77901361617571	0.491	0.031	9.00226974195505E-74	0	NPL
4.87111548854101E-159	4.7772740220175	0.83	0.073	7.94527647335925E-155	0	STAB2
2.96956826698595E-41	4.70954757766097	0.278	0.012	4.84366280028079E-37	0	RIPOR3
2.65174780532828E-55	4.17248925932484	0.385	0.031	4.32526584527095E-51	0	RELN
1.73477846616165E-35	4.0839962150502	0.258	0.019	2.82959715615627E-31	0	ATRNL1
5.50410172131546E-55	3.92383764329626	0.387	0.031	8.97774031763765E-51	0	FCHO1
1.7958935253184E-160	3.90028280485146	0.884	0.165	2.92928192914685E-156	0	OIT3
7.65557590337134E-146	3.883909177629	0.849	0.163	1.2487009855989E-141	0	FCN2
5.32221273898205E-37	3.62620054702792	0.281	0.025	8.68106119855363E-33	0	FCGR2B
3.57840017786896E-47	3.44859581526064	0.377	0.05	5.83672853012206E-43	0	ME2
3.11341577518974E-40	3.44636419588913	0.323	0.039	5.07829247091199E-36	0	BMPER
2.7706609161161E-83	3.26457123293739	0.668	0.173	4.51922502027698E-79	0	CD14
8.22269116769477E-48	3.23372289899862	0.396	0.059	1.34120315636269E-43	0	PLTP
8.22793043906872E-41	3.1538922682927	0.38	0.076	1.3420577339165E-36	0	EHD3
4.83523785502063E-31	3.10830759613091	0.276	0.04	7.88675646532415E-27	0	RAB20
2.01898226297842E-57	3.07620360356151	0.47	0.075	3.2931619691441E-53	0	LYVE1
4.83388283786756E-61	2.99693094324176	0.528	0.11	7.88454629684577E-57	0	ITGB3
6.9997408176233E-94	2.96957669861168	0.78	0.3	1.14172772476254E-89	0	CTSL
3.20368570423364E-31	2.9584275262851	0.286	0.048	5.22553175217549E-27	0	GATA4
1.90616888944849E-31	2.81141768398689	0.348	0.087	3.10915207557943E-27	0	GDF15
8.17831476158266E-32	2.80450085340122	0.319	0.065	1.33396492076175E-27	0	NTN4
1.26479437762977E-27	2.76964638362763	0.26	0.044	2.06300610935191E-23	0	ANPEP
6.57402767080586E-84	2.66357562804199	0.759	0.283	1.07228965338514E-79	0	CRHBP
5.37668335030099E-36	2.63290173948161	0.401	0.107	8.76990821267595E-32	0	SOD2
3.50646349071728E-30	2.62729277312847	0.268	0.037	5.71939259970895E-26	0	TFPI2
1.04585701331244E-24	2.5855716006168	0.287	0.075	1.70589737441392E-20	0	SNX5
1.30007688290371E-25	2.51313629224489	0.278	0.061	2.12055540370425E-21	0	CA13
2.07743493853767E-22	2.37925905646417	0.258	0.061	3.38850412824879E-18	0	KIF19
6.17898915260731E-29	2.34672070981152	0.319	0.073	1.00785492068178E-24	0	ACP5
2.68884185341133E-26	2.31693551277547	0.315	0.084	4.38576994709922E-22	0	LTK
2.10940776316488E-27	2.25430666187812	0.343	0.101	3.44065500249824E-23	0	DAAM1
1.99970061514885E-46	2.19305557350068	0.498	0.138	3.26171167336928E-42	0	SYNE1
2.00987641961267E-28	2.10855494111174	0.355	0.1	3.27830942803022E-24	0	LRATD1
7.97411284469887E-30	2.06486676150447	0.469	0.196	1.30065754609883E-25	0	LGMN
2.48326719992334E-25	2.02496032048366	0.308	0.079	4.05045712979495E-21	0	NR5A2
6.16199989668065E-26	2.02485425700695	0.345	0.106	1.00508380314758E-21	0	TMEM37
3.00705089606385E-106	1.99345586705548	0.928	0.468	4.90480071656974E-102	0	FCN3
9.68199241585106E-107	1.99064815759939	0.944	0.792	1.57922978294947E-102	0	STAB1
7.9429178358043E-42	1.94498429296522	0.53	0.187	1.29556932819804E-37	0	MRC1
2.26488953628293E-19	1.85082196007076	0.284	0.09	3.69426132263109E-15	0	PARP4
1.94082703052545E-34	1.79616705070909	0.437	0.129	3.16568296949007E-30	0	LIFR
6.40574195363412E-15	1.79099713740677	0.319	0.149	1.04484057005726E-10	0	SERPINB1

6.43710599884231E-16	1.78892184751535	0.294	0.114	1.04995635947117E-11	0	APOE
4.00849146864338E-18	1.74616031055656	0.326	0.131	6.53825043450422E-14	0	TRIB2
1.04564443426539E-21	1.73450842318641	0.334	0.114	1.70555063673028E-17	0	CXCL16
1.09343677960617E-33	1.73294873350445	0.485	0.171	1.78350473121562E-29	0	SH3TC1
5.45207122740577E-41	1.717493475565	0.615	0.275	8.89287337902155E-37	0	TIMP1
5.03605093685709E-33	1.71311685495942	0.44	0.135	8.2143026831076E-29	0	IL1R1
1.23616637470787E-32	1.69699767478601	0.543	0.25	2.016310973786E-28	0	UGCG
7.35091960028312E-39	1.68114490794169	0.581	0.246	1.19900849600218E-34	0	BSDC1
2.85492666087815E-16	1.66473283061376	0.268	0.095	4.65667087655835E-12	0	ST3GAL4
2.79120396169162E-11	1.65238222683374	0.265	0.128	4.5527327819152E-07	0	ITPKC
4.3469716009528E-17	1.62778849672936	0.252	0.076	7.09034537831412E-13	0	C1QTNF1
1.113482373753E-18	1.5828143468112	0.327	0.124	1.81620109982852E-14	0	TRIM25
7.74835440865228E-30	1.55293662302314	0.498	0.208	1.26383408759527E-25	0	LITAF
7.05453370065148E-24	1.53238552313218	0.483	0.246	1.15066499191326E-19	0	CD4
1.27765588487739E-19	1.48968733563071	0.34	0.126	2.08398451382351E-15	0	LRRFIP2
5.35609991795924E-46	1.48829711494545	0.685	0.309	8.73633457618331E-42	0	ITGA9
2.63477183088259E-19	1.48144414417497	0.422	0.202	4.2975763333526E-15	0	DAB2
5.45216617342237E-14	1.45301273825521	0.302	0.129	8.89302824546923E-10	0	C5AR2
3.6433441358275E-10	1.43058657212146	0.274	0.138	5.94265861994824E-06	0	MT2A
1.14650054476026E-12	1.42895025911846	0.278	0.123	1.87005703855847E-08	0	GNA14
3.15321970301164E-17	1.4199889635767	0.371	0.166	5.14321665758228E-13	0	BCL3
8.79225883920303E-10	1.41535323722868	0.265	0.137	1.43410533926241E-05	0	CTSB
4.02880251286816E-15	1.40720775548691	0.348	0.163	6.57137977873926E-11	0	SERPINB9
1.30783221350242E-16	1.38755138540918	0.332	0.138	2.1332051234438E-12	0	ANKS1A
5.74150275591015E-14	1.3708171500862	0.294	0.129	9.36496514516505E-10	0	NFKBIZ
3.16382258581083E-32	1.35832562298852	0.657	0.364	5.16051101971604E-28	0	ADAMTS4
3.61269820269862E-67	1.33818839206633	0.915	0.666	5.89267203842173E-63	0	DNASE1L3
4.17576833720474E-17	1.33762832437312	0.327	0.131	6.81109573481465E-13	0	LGALS3BP
1.91520641064261E-17	1.33669641451603	0.358	0.151	3.12389317639916E-13	0	FCHO2
1.30506085544643E-22	1.33439995860584	0.453	0.193	2.12868476131868E-18	0	MID1
2.10636069673064E-13	1.25257398758251	0.294	0.126	3.43568493243735E-09	0	IL33
8.0263827384976E-22	1.25058994450385	0.555	0.306	1.30918328847634E-17	0	SQSTM1
1.28426725431044E-17	1.2419781053631	0.457	0.244	2.09476831850575E-13	0	TMEM140
7.98399682140997E-15	1.2395640305396	0.417	0.226	1.30226972154018E-10	0	PNRC1
2.02168307565713E-13	1.22685936710609	0.31	0.14	3.29756726470435E-09	0	TSPAN7
4.0411729613523E-12	1.20152014286234	0.364	0.198	6.59155721726174E-08	0	SERPINA1
3.31310441452746E-13	1.1729732322008	0.422	0.24	5.40400461053573E-09	0	CD36
1.80121196406103E-09	1.17211995582644	0.286	0.149	2.93795683457994E-05	0	FUT1
6.87971477703043E-14	1.16631969293429	0.422	0.235	1.12215027728143E-09	0	SBNO2
2.55526317055906E-18	1.14430041495616	0.485	0.261	4.16788975749888E-14	0	SLC38A2
1.41397575981113E-11	1.14345421974125	0.326	0.17	2.30633586182793E-07	0	PELO
5.94819462669784E-15	1.13948438911181	0.388	0.194	9.70210025560684E-11	0	AGFG2
5.3641350529681E-11	1.12058560515921	0.284	0.135	8.74944068489628E-07	0	STX3
6.05730541900574E-12	1.11371021304479	0.31	0.149	9.88007086894026E-08	0	DENND4C
2.64325248541916E-34	1.09338492380705	0.738	0.423	4.31140912896718E-30	0	APOA2
2.03379628586445E-16	1.08611463997562	0.464	0.255	3.31732512187351E-12	0	CHST12
4.34425717446011E-07	1.06416530271498	0.318	0.21	0.00708591787726188	0	FGFR1
1.49191489196281E-10	1.04665513534237	0.361	0.212	2.43346238028054E-06	0	PLSCR1
5.25903086355423E-14	1.03658420828183	0.358	0.17	8.57800524154331E-10	0	SORBS1
4.1295682919059E-16	1.02620189611713	0.425	0.207	6.73573884092771E-12	0	CBLB

1.05139015027268E-16	1.02485928179913	0.536	0.328	1.71492247410977E-12	0	NFKBIA
3.97206195219518E-10	1.00904789481458	0.372	0.221	6.47883025022556E-06	0	PXDN
2.44585939303197E-11	1.00132230608085	0.395	0.235	3.98944125597445E-07	0	IL6ST
3.26042955077719E-07	0.996516005193817	0.263	0.149	0.00531808664027267	0	YES1
8.50169010016603E-24	0.988622186039269	0.615	0.336	1.38671067223808E-19	0	MCAM
3.48633161585474E-07	0.977896007680285	0.279	0.165	0.00568655549862067	0	FCHSD2
3.6425135134963E-21	0.977305712843285	0.584	0.337	5.94130379186381E-17	0	TINAGL1
5.98143901493577E-11	0.977257687402886	0.368	0.208	9.75632517726174E-07	0	ACTN1
8.09499810625582E-12	0.967734768345553	0.409	0.236	1.32037514111139E-07	0	AKAP17A
1.584721601304E-09	0.963356501490648	0.315	0.173	2.58483940388696E-05	0	LGALS8
2.32060539856598E-14	0.958596180067965	0.543	0.372	3.78513946560098E-10	0	IL32
1.43415555295836E-13	0.952901645082927	0.403	0.218	2.33925112243037E-09	0	ST6GAL1
2.13815823803285E-07	0.948638371230584	0.318	0.201	0.00348754990205537	0	CALU
2.78667917999775E-13	0.935290885445942	0.421	0.226	4.54535241049433E-09	0	DGKE
2.46891355623237E-08	0.919613302429659	0.313	0.182	0.000402704490157062	0	NAMPT
1.7092852997583E-12	0.917882314739335	0.34	0.168	2.78801525243576E-08	0	ZNF160
5.59483748234708E-08	0.888040256872972	0.313	0.185	0.000912573941745633	0	NISCH
3.95902590492137E-09	0.884146063719326	0.364	0.221	6.45756715351725E-05	0	NCOA7
4.87270301633094E-09	0.877050985103905	0.361	0.222	7.94786588993739E-05	0	PHLDB2
1.82416323724621E-17	0.871563145751931	0.523	0.299	2.9753926562723E-13	0	WARS
7.48374148247773E-10	0.866965774591525	0.335	0.185	1.22067307320694E-05	0	SLC23A2
1.52288779766088E-09	0.85256002080869	0.327	0.179	2.48398228676467E-05	0	AP2A2
2.97676250862202E-17	0.846747515133095	0.615	0.409	4.85539732781337E-13	0	PPFIBP1
5.9921382165608E-14	0.846665690607921	0.502	0.311	9.77377664503232E-10	0	HMBOX1
4.03020637849586E-13	0.835993372301632	0.465	0.274	6.5736696239646E-09	0	TMEM44
1.89414857027851E-06	0.826335227045429	0.287	0.177	0.0308954573298128	0	LRCH4
5.19628820891434E-12	0.814972303014255	0.526	0.358	8.47566569756018E-08	0	RSRP1
1.92022788279706E-06	0.811443047101117	0.308	0.196	0.0313208369963028	0	MAP3K6
7.645602477856E-07	0.803197480941965	0.265	0.152	0.0124707422016309	0	RASSF1
2.93486348622131E-06	0.802723572646441	0.294	0.188	0.0478705583237559	0	CHSY1
1.85303860810491E-23	0.800141702448682	0.73	0.481	3.02249127367992E-19	0	SOCS3
5.64079759564698E-09	0.786970879074698	0.462	0.32	9.20070495825979E-05	0	ARHGAP23
5.99380928977393E-08	0.783048693524551	0.305	0.176	0.000977650233255025	0	RNF149
1.30888437779352E-14	0.779559748733332	0.549	0.348	2.13492130861901E-10	0	CEMIP2
2.58020688769482E-09	0.772267526344893	0.488	0.344	4.20857545451902E-05	0	SELENOP
1.47005262210962E-07	0.754569440271035	0.416	0.294	0.002397802831923	0	DLC1
3.0708780337897E-13	0.744445320218797	0.647	0.467	5.00890916091437E-09	0	WSB1
2.51092673882175E-12	0.740218111988507	0.64	0.462	4.09557260369216E-08	0	NID1
1.68530579386581E-08	0.73659088730795	0.39	0.244	0.000274890228037452	0	GSTK1
4.94218465481497E-07	0.735876242646462	0.347	0.219	0.00806119739046869	0	SEMA6A
6.82764128742614E-11	0.73580798068261	0.486	0.311	1.11365657039208E-06	0	ARHGAP26
2.30264085490492E-15	0.735333692210769	0.693	0.498	3.75583749843541E-11	0	PTPRB
6.10997045615456E-09	0.720690918291222	0.403	0.267	9.9659728110337E-05	0	SHC2
1.09667515100129E-09	0.719698102907591	0.445	0.283	1.7887868387982E-05	0	AFDN
5.66762197699258E-07	0.710418542761084	0.43	0.302	0.0092444582066726	0	PARP14
5.5926512448471E-08	0.694958468497495	0.334	0.194	0.000912217344547011	0	SNTB1
9.46453999683971E-11	0.67402927681233	0.563	0.392	1.54376111888453E-06	0	ARHGEF7
1.6589774372942E-12	0.674028208834678	0.618	0.429	2.70595809797056E-08	0	DUSP5
1.47937533796917E-26	0.661115544213826	0.917	0.848	2.41300911376152E-22	0	ARGLU1
1.89525047135528E-10	0.658928325661388	0.623	0.473	3.0913430438276E-06	0	MAF

5.52978654146645E-08	0.656494136272777	0.421	0.274	0.000901963482778593	0	RCN3
3.01592356176859E-06	0.651572291094796	0.318	0.205	0.0491927292160074	0	TBC1D15
7.78219505984375E-11	0.649603590642968	0.535	0.364	1.26935383621111E-06	0	NOSTRIN
1.22090323759257E-12	0.635740053757589	0.457	0.26	1.99141527083725E-08	0	UPP1
1.04147674091166E-07	0.629163325013923	0.52	0.39	0.001698752712101	0	PGS1
7.47626734993264E-09	0.614007957631208	0.469	0.314	0.000121945396744751	0	AKAP12
1.83407033441063E-07	0.60240385537827	0.348	0.218	0.00299155212245718	0	ABCC1
2.05129500179407E-06	0.589194085071316	0.416	0.3	0.0334586727742631	0	TPP1
8.67489161805025E-08	0.581679152824159	0.472	0.319	0.00141496157182018	0	CCNL1
1.16403225175621E-06	0.579780405673869	0.339	0.213	0.0189865300583956	0	TTLL5
9.66191544878374E-07	0.576522571522493	0.361	0.236	0.0157595502885112	0	MED10
3.00794071523409E-11	0.569201547681266	0.657	0.477	4.90625210061833E-07	0	CFLAR
1.53896652516876E-07	0.565158502878625	0.465	0.322	0.00251020829920277	0	NLRP1
1.31304931215966E-06	0.561174785319943	0.419	0.299	0.0214171473306361	0	OGT
2.84463576762635E-09	0.532037686473456	0.493	0.327	4.63988540057534E-05	0	ADM
2.04591900237244E-07	0.516849089737601	0.401	0.258	0.00333709848476968	0	GBP4
2.60860077074259E-08	0.511537959263132	0.698	0.579	0.000425488871715824	0	FCGRT
6.98958315765635E-08	0.472468987294239	0.384	0.232	0.00114007090884533	0	ATF3
8.939339397682E-08	0.37221956066274	0.477	0.32	0.00145809564915591	0	CLDN5
1.12503026667088E-06	0.301169134992491	0.828	0.768	0.0183503686796687	0	LENG8
4.33529509093368E-95	5.15771634374058	0.552	0.036	7.07129982282192E-91	1	CA2
5.68623538311999E-63	4.97967522972475	0.37	0.011	9.27481853340702E-59	1	COL15A1
7.06143955961762E-123	4.86184741507227	0.66	0.034	1.15179140656923E-118	1	RGCC
1.62712421842407E-89	3.91632983983553	0.58	0.07	2.6540023126715E-85	1	VWA1
9.5752901673517E-158	3.6604041356544	0.896	0.197	1.56182557919674E-153	1	PLVAP
1.62348341071002E-92	3.51539308715018	0.646	0.109	2.64806379120911E-88	1	SPRY1
3.85058415251186E-36	3.31148142950155	0.307	0.049	6.28068781116209E-32	1	CX3CL1
9.31532292468114E-42	3.07395601972214	0.353	0.056	1.51942232224474E-37	1	MPZL2
4.14682429180084E-53	3.05695393756478	0.469	0.099	6.76388510235636E-49	1	STC1
4.89747989855696E-32	3.05379190846898	0.265	0.036	7.98827946253625E-28	1	FABP4
2.31602471751174E-64	2.98510635357072	0.561	0.137	3.7776679167334E-60	1	PDGFB
1.10919693823577E-57	2.98371642601431	0.42	0.047	1.80921112595637E-53	1	IGFBP5
3.14915962766596E-45	2.79946242552108	0.381	0.059	5.13659426868595E-41	1	SLCO2A1
7.21214594330074E-60	2.55360393275672	0.63	0.253	1.17637312481178E-55	1	GSN
1.16442419318739E-26	2.473382741135	0.298	0.076	1.89929230150794E-22	1	JAG1
2.66846362487813E-32	2.40894479608503	0.342	0.079	4.35253101853871E-28	1	AQP1
1.51338608186519E-94	2.38251364648834	0.808	0.265	2.46848403813032E-90	1	VWF
7.26621703628118E-31	2.37928283596145	0.303	0.06	1.18519266078782E-26	1	GJA1
1.53644659117103E-65	2.35184300462996	0.64	0.187	2.50609803485907E-61	1	EMP1
3.1718265532392E-38	2.28177349075212	0.439	0.134	5.17356629098846E-34	1	LAMB1
2.22926213996216E-97	2.22026895440534	0.892	0.572	3.63614947649228E-93	1	IVNS1ABP
5.19444915774372E-31	2.18125594611557	0.337	0.08	8.47266602119578E-27	1	CD34
2.93484492132865E-41	2.1588723287345	0.51	0.18	4.78702555117916E-37	1	STC2
1.68914714037417E-21	2.12679244012806	0.307	0.103	2.75516790066431E-17	1	HTRA1
1.66815364486717E-35	2.11283069541058	0.446	0.152	2.72092541014285E-31	1	APOLD1
2.34930215887382E-23	2.10459158604044	0.316	0.099	3.83194675133908E-19	1	ITGA6
7.29243129660734E-25	2.07467665478257	0.302	0.079	1.18946846878962E-20	1	SYNPO
3.61769879784345E-30	2.0597255061747	0.406	0.137	5.90082850916245E-26	1	PODXL
1.85045114231021E-65	2.02054040798633	0.776	0.382	3.01827085822219E-61	1	NR4A1
2.96125027235203E-22	1.92225330864842	0.319	0.103	4.8300953192334E-18	1	TNK2

**1.12067423121378E-21**	1.87584389863668	0.374	0.156	1.8279317385328E-17	1	DUSP6
**1.65101833610919E-18**	1.84027988495462	0.282	0.096	2.6929760080277E-14	1	ENTPD1
**5.90553811605196E-25**	1.83477135657746	0.287	0.07	9.63252322109234E-21	1	OLFML2A
**1.45869182674668E-27**	1.80498217527668	0.425	0.162	2.37927223860651E-23	1	DOCK9
**1.34969417647614E-42**	1.79049596679526	0.656	0.336	2.20148617125024E-38	1	MCF2L
**1.58619002709555E-25**	1.78670107093086	0.384	0.139	2.58723455319555E-21	1	CDH13
**9.89364213380249E-17**	1.78596272469683	0.266	0.093	1.61375196844452E-12	1	TMOD2
**1.22759071859473E-23**	1.77748547251232	0.437	0.203	2.00232322109986E-19	1	ADAMTS9
**9.69910280506766E-17**	1.71845880664545	0.317	0.136	1.58202065853459E-12	1	RASA4
**2.75639096708573E-23**	1.69938324962513	0.381	0.143	4.49594930641354E-19	1	JCAD
**3.0348458172764E-29**	1.65324002733008	0.511	0.23	4.95013701255954E-25	1	DLL1
**2.78035663149444E-30**	1.65309298925384	0.501	0.219	4.53503970163059E-26	1	CLEC14A
**3.52871058940244E-16**	1.62064016254451	0.284	0.109	5.75567984237431E-12	1	EMCN
**6.77562431221875E-34**	1.58453229511401	0.586	0.293	1.105172081566E-29	1	PLPP1
**2.43018457895215E-21**	1.54743772264344	0.476	0.246	3.96387406672885E-17	1	FN1
**1.16832180068049E-20**	1.50939481249509	0.418	0.189	1.90564968908994E-16	1	LAMA4
**4.91402964366277E-25**	1.48417094828523	0.556	0.325	8.01527375177835E-21	1	GRB10
**1.2652756308753E-23**	1.44471804468699	0.508	0.266	2.0637910815207E-19	1	PCDH12
**2.97452400052186E-13**	1.43840915115684	0.404	0.237	4.8517460972512E-09	1	TNFAIP3
**2.0037601131664E-23**	1.41773749306034	0.478	0.229	3.26833312058571E-19	1	ADGRG1
**1.67076454236959E-10**	1.39192969031245	0.268	0.134	2.72518404505904E-06	1	IER5L
**2.48226366151829E-35**	1.39181156250778	0.64	0.345	4.04882025830249E-31	1	PECAM1
**3.7935611304686E-10**	1.37943254790212	0.302	0.166	6.18767755990734E-06	1	TDRP
**1.21573423216486E-42**	1.37745520023815	0.824	0.619	1.9829841060841E-38	1	FLT1
**6.57397306964736E-20**	1.3610255028094	0.423	0.202	1.07228074739018E-15	1	AGRN
**4.08023229278424E-12**	1.34023807690825	0.305	0.15	6.65526689276038E-08	1	TSPAN15
**5.63479931633319E-58**	1.32896653818646	0.922	0.775	9.19092116487107E-54	1	COL4A1
**7.75224095924771E-13**	1.3278457508414	0.399	0.242	1.26446802286289E-08	1	METRNL
**3.65640572436912E-11**	1.30525852027523	0.326	0.179	5.96396337701847E-07	1	HLX
**9.79735365278524E-56**	1.30177161310955	0.889	0.791	1.5980463543058E-51	1	SPARC
**1.67396201320369E-14**	1.26477274574373	0.39	0.209	2.73039943973654E-10	1	NDRG1
**7.87292386622449E-08**	1.25387314834316	0.422	0.322	0.00128415261181988	1	SPRY4
**3.49254024812052E-30**	1.24116240631686	0.732	0.511	5.69668239870938E-26	1	SLC2A3
**2.98958921367762E-14**	1.23372737720526	0.434	0.247	4.87631896642957E-10	1	PITPNC1
**1.69185827189015E-08**	1.22994958773911	0.265	0.149	0.000275959002728002	1	RIN3
**4.07223107921981E-10**	1.21338618631167	0.298	0.162	6.64221611331542E-06	1	RAMP2
**1.60989626493013E-14**	1.20726788427537	0.422	0.239	2.62590179772753E-10	1	JUP
**1.38528058227146E-47**	1.2018978585756	0.64	0.233	2.25953115774298E-43	1	VIM
**4.01906795191078E-10**	1.19118183311075	0.272	0.136	6.55550173636167E-06	1	FMNL3
**7.62829347325281E-16**	1.17877920513389	0.427	0.232	1.24425094842227E-11	1	APLNR
**9.48216730527522E-08**	1.178520623992	0.333	0.222	0.00154663630916344	1	LIMD1
**1.28392799604082E-18**	1.17671462539918	0.446	0.23	2.09421495434219E-14	1	EGR1
**2.25680377199242E-09**	1.15300542797247	0.277	0.146	3.68107263249684E-05	1	SOX4
**6.91044468869346E-12**	1.13875874983411	0.356	0.199	1.12716263317279E-07	1	CALCRL
**2.10466694794819E-11**	1.13256383939735	0.33	0.175	3.4329222587983E-07	1	LTBP3
**3.29602792420814E-17**	1.12205541286898	0.4	0.195	5.37615114717589E-13	1	AHNAK
**1.42821538523915E-14**	1.11035523505485	0.591	0.448	2.32956211486357E-10	1	HSPA1B
**1.02150689534559E-14**	1.09764031617937	0.504	0.33	1.66617989699819E-10	1	TP53I11
**1.11490367693376E-10**	1.07201821413004	0.372	0.225	1.81851938744666E-06	1	S1PR1
**5.98500435923075E-07**	1.06556378643306	0.268	0.163	0.00976214061034128	1	EHD4

1.12906655998335E-09	1.06029161321663	0.272	0.139	1.84162046598885E-05	1	BCAM
2.55398974874433E-11	1.05616023109065	0.358	0.206	4.16581267917688E-07	1	SOX18
7.4744690858484E-07	1.0433735280196	0.342	0.235	0.0121916065259273	1	TMEM184E
5.57997116760865E-10	1.03709344478817	0.328	0.183	9.10149097148648E-06	1	GASK1B
3.68756403218938E-08	1.03520057833113	0.395	0.276	0.000601478569290409	1	PLCG1
2.23133006595019E-07	1.03462758528492	0.28	0.173	0.00363952247057136	1	ZEB1
9.82950796712003E-47	1.03417822159276	0.919	0.798	1.60329104451695E-42	1	COL4A2
4.2819953218781E-10	1.03217571032165	0.349	0.209	6.98436256951536E-06	1	TMEM204
3.83878807657347E-38	1.02601281211867	0.852	0.691	6.26144723169898E-34	1	IGFBP4
1.40387964205812E-09	1.02177000251907	0.451	0.316	2.289868084161E-05	1	GABRE
5.45576519048528E-09	1.01377428823666	0.354	0.216	8.89889860220054E-05	1	KCNE3
9.60912254283555E-07	1.01079424091576	0.295	0.186	0.0156734397796191	1	C15orf39
2.07919303779011E-09	1.00317556277488	0.321	0.19	3.39137176393944E-05	1	DPYSL2
1.76322039210149E-08	0.992919528753665	0.384	0.265	0.000287598878155674	1	ARHGEF15
1.08192551214931E-20	0.984499331399648	0.661	0.476	1.76472870286675E-16	1	A2M
6.68875890894058E-07	0.980685723793498	0.282	0.175	0.010910034656373	1	ASPH
7.84648087369678E-17	0.965828437597492	0.578	0.383	1.27983949530868E-12	1	EXOC3L2
1.9938142024417E-18	0.950369879696035	0.646	0.481	3.25211034560265E-14	1	MARCKS
7.58690405162243E-17	0.939563247188744	0.633	0.459	1.23749991986014E-12	1	SEMA3F
5.10129004257551E-15	0.937814453447181	0.573	0.375	8.32071418844492E-11	1	DDIT4
3.24196111223481E-08	0.932610800858392	0.485	0.376	0.00052879627701662	1	PLK2
1.98622530593286E-12	0.916730586043633	0.571	0.412	3.23973209650709E-08	1	INSR
2.30708446452077E-06	0.916266714486942	0.448	0.351	0.0376308547007982	1	FOS
2.41561116710146E-09	0.887357485711273	0.34	0.2	3.9401033746592E-05	1	FKBP1A
4.1235341913119E-10	0.88128165266162	0.52	0.398	6.72589661944884E-06	1	SHC1
7.5168293241255E-07	0.87465539003211	0.337	0.222	0.0122607003105811	1	HIC1
1.29308578366185E-07	0.873599328751879	0.393	0.268	0.00210915222173084	1	SMAD7
1.57553409500331E-09	0.86959571337594	0.494	0.348	2.5698536623599E-05	1	KDR
1.35921353415644E-12	0.84917474975457	0.64	0.525	2.21701319556257E-08	1	EPAS1
6.71254429487957E-07	0.847679582196537	0.342	0.23	0.0109488309993781	1	ECSCR
5.52213641990102E-12	0.821976711972362	0.395	0.226	9.00715671450055E-08	1	DLL4
2.00733954034329E-19	0.81963912978272	0.868	0.83	3.27417152425394E-15	1	ENG
1.79512813347789E-08	0.815857946743739	0.517	0.399	0.000292803349851578	1	LRRC32
6.91637477312493E-24	0.810042026053988	0.845	0.748	1.12812988924441E-19	1	HSPG2
1.30957962777653E-11	0.801902641308974	0.497	0.332	2.13605533086629E-07	1	ID3
1.76615272187409E-11	0.801447799261536	0.624	0.501	2.88077170464882E-07	1	TGFBR2
5.66317314328757E-11	0.798174136989116	0.614	0.489	9.23720171401635E-07	1	COL18A1
2.59713389252594E-06	0.796747483322682	0.411	0.305	0.0423618509209906	1	PML
8.54490320468033E-11	0.784841004928881	0.58	0.451	1.39375916171541E-06	1	TCF4
3.18180163890529E-32	0.781476229426408	0.926	0.831	5.18983665321841E-28	1	DUSP1
1.09774152963994E-06	0.7761906073161	0.295	0.185	0.0179052620899571	1	SEMA6B
3.37810164131686E-10	0.765680381238902	0.608	0.498	5.51002158715194E-06	1	DYSF
2.96928474046064E-08	0.73914541139183	0.668	0.597	0.000484320034016535	1	JUNB
1.55403428643631E-13	0.687001446958442	0.771	0.682	2.53478532460627E-09	1	IGFBP7
3.5717482450435E-07	0.685968380724985	0.504	0.385	0.00582587856249045	1	HSPA1A
1.04991877299123E-07	0.685216718065696	0.654	0.544	0.001712522510626	1	TCIM
1.48937697419048E-09	0.683497631312464	0.552	0.428	2.42932278260209E-05	1	LMNA
1.99297497958542E-11	0.681441810984737	0.596	0.454	3.25074148920178E-07	1	ITGA5
4.25784141950582E-08	0.669965884657942	0.598	0.486	0.000694496513935594	1	ARRDC3
2.55072427027862E-06	0.664812063326648	0.437	0.323	0.0416048635725146	1	DENND3

**4.5500343385982E-10**	0.64814359868428	0.631	0.505	7.42156100968753E-06	1	ACTB
**1.86613773052133E-10**	0.623567628344743	0.704	0.624	3.04385725225334E-06	1	PLXND1
**1.36922219622458E-08**	0.610276875337493	0.589	0.451	0.000223333832426192	1	PXN
**8.8835627270284E-09**	0.605225425820581	0.63	0.512	0.00014489979164056	1	IER2
**8.75540724664784E-08**	0.577571621443219	0.372	0.24	0.00142809447600073	1	MT-ATP6
**1.64790110264341E-07**	0.515692931482984	0.728	0.678	0.00268789148852166	1	BHLHE40
**2.7106708303035E-06**	0.4980026464631	0.601	0.519	0.0442137519130803	1	SCARF1
**5.57921123320044E-09**	0.489634767458313	0.644	0.535	9.10025144247324E-05	1	MMRN2
**7.43440568413343E-07**	0.486232941484663	0.519	0.412	0.01212625911139	1	MT-ND6
**2.63440815404689E-09**	0.448521353244708	0.905	0.896	4.29698314006589E-05	1	CD74
**1.66404054118594E-06**	0.405987752692087	0.709	0.632	0.0271421652672838	1	CD93
**5.09266174793106E-87**	8.48726129209971	0.343	0.001	8.30664057705036E-83	2	CADM3
**5.06218305418105E-179**	8.11533980170598	0.829	0.006	8.25692677967471E-175	2	LYPD2
**6.75049417142984E-104**	7.16327715292794	0.6	0.01	1.10107310430192E-99	2	NTS
**2.68003883315683E-130**	6.99134697653118	0.571	0.003	4.37141134076211E-126	2	RSPO3
**2.03640575451881E-65**	6.68989275371765	0.286	0.002	3.32158142619563E-61	2	P2RX1
**1.55257494530459E-128**	6.6405141103311	0.686	0.009	2.53240499328632E-124	2	CPE
**3.57293170152155E-82**	5.97950691308618	0.486	0.008	5.82780889835181E-78	2	ACKR1
**8.50717099250085E-92**	5.84033802548411	0.8	0.032	1.38760466058681E-87	2	PTGDS
**6.70841822163905E-138**	5.69106495657389	0.714	0.008	1.09421009613154E-133	2	SELP
**9.88445164990122E-59**	5.45235951640595	0.343	0.006	1.61225290861539E-54	2	COL13A1
**2.50992009399211E-67**	5.40506741048302	0.743	0.042	4.09393066531054E-63	2	SELE
**3.43905597854501E-98**	5.33789858197955	0.857	0.034	5.60944420660477E-94	2	FBLN2
**2.35320268708107E-63**	5.0720879758378	0.514	0.017	3.83830890289794E-59	2	CDH23
**1.50384285367903E-65**	5.06907436285698	0.4	0.007	2.45291807863586E-61	2	CLDN11
**4.99605056125052E-80**	5.00546981205413	0.514	0.011	8.14905807045573E-76	2	WNT2
**1.01488649688914E-38**	4.89373196651538	0.457	0.028	1.65538136507587E-34	2	EMP3
**1.67025029540567E-53**	4.65930221547914	0.429	0.014	2.72434525683619E-49	2	LSR
**5.07465587291595E-28**	4.58451061339555	0.257	0.011	8.27727119431321E-24	2	SAMD5
**1.39733701929724E-49**	4.485586956228	0.914	0.11	2.27919641217573E-45	2	CLU
**2.03235075496991E-39**	4.39907662873541	0.6	0.05	3.31496731643141E-35	2	TAGLN
**8.24576776797049E-52**	4.3205766116339	0.457	0.017	1.34496718063367E-47	2	LHX6
**7.04860683059391E-54**	4.27758309860881	0.514	0.022	1.14969826013817E-49	2	PTGIS
**7.22974490285896E-32**	4.16985420929523	0.514	0.046	1.17924369110533E-27	2	IER3
**4.74618698811727E-36**	4.00578672880609	0.914	0.179	7.74150559631809E-32	2	PRSS23
**1.47312007427642E-34**	3.99767440637349	0.343	0.015	2.40280615315226E-30	2	ARL4D
**1.11306282544317E-39**	3.85099039747571	0.486	0.029	1.81551677458036E-35	2	LTC4S
**2.07201433376675E-43**	3.65743307058365	0.714	0.063	3.37966257980695E-39	2	PTGS1
**1.44119424297936E-43**	3.65237201252609	0.629	0.047	2.35073192972363E-39	2	LRATD2
**2.87371911552765E-22**	3.58583142213773	0.257	0.015	4.68732324933715E-18	2	SYT15
**3.67668404870431E-43**	3.5630137895526	0.514	0.03	5.9970393518416E-39	2	VCAN
**1.76704750537391E-40**	3.53732764673974	0.371	0.015	2.88223118601538E-36	2	SCARA3
**4.75421598762557E-30**	3.49623007588371	0.343	0.019	7.75460169741607E-26	2	CCDC69
**2.52671699591858E-22**	3.48620071603048	0.257	0.015	4.1213280920428E-18	2	RNF112
**3.91386875551322E-38**	3.45628912583638	0.8	0.1	6.38391132711761E-34	2	ACKR3
**1.73539027994788E-19**	3.44534923956531	0.286	0.022	2.83059508562299E-15	2	LRG1
**1.12182402396355E-36**	3.3569748226749	0.4	0.02	1.82980716548694E-32	2	PLA1A
**3.35505000344527E-35**	3.33265752982663	0.829	0.119	5.47242206061958E-31	2	ALPL
**1.04679834085074E-33**	3.3320800999966	0.4	0.023	1.70743277376164E-29	2	DPYSL3
**1.6672569668076E-18**	3.28004122573032	0.286	0.024	2.71946283855988E-14	2	SLC8A1

3.83050130323718E-28	3.2714982655145	0.429	0.034	6.24793067571016E-24	2	HS3ST1
1.18073509228318E-33	3.24688503261206	0.543	0.046	1.92589700902309E-29	2	RASSF9
5.43380751663838E-28	3.18533427595402	0.514	0.05	8.86308344038887E-24	2	NDUFA8
9.2573072030293E-29	3.17086797892265	0.743	0.111	1.50995937788611E-24	2	C7
1.28711980188116E-19	3.16348247648075	0.4	0.045	2.09942110884836E-15	2	HRCT1
1.66721715000602E-22	3.15232873087522	0.343	0.028	2.71939789337482E-18	2	SRPX
3.53828394342013E-21	3.12459916079596	0.257	0.015	5.77129494011257E-17	2	PALLD
6.74974375628085E-25	3.12103628019867	0.4	0.034	1.10095070408697E-20	2	DUSP23
6.31993779487266E-33	3.10548692286807	0.743	0.094	1.03084505372168E-28	2	PGM5
7.43976118881384E-30	3.07037343319151	0.314	0.015	1.21349944750743E-25	2	SELENBP1
2.50290264477884E-15	3.06793744074361	0.257	0.024	4.08248450389877E-11	2	LINGO1
7.30935441576057E-25	2.95961796699536	1	0.409	1.19222879875471E-20	2	RNASE1
4.5040490867198E-27	2.8543622589538	0.486	0.046	7.34655446534866E-23	2	FGL2
1.75772257702391E-21	2.83127511275627	0.457	0.052	2.8670212953837E-17	2	SLC22A17
2.22747484993536E-20	2.80736488747573	0.514	0.07	3.63323422772957E-16	2	COL5A1
3.63154590000082E-23	2.781819397369	0.429	0.041	5.92341451749133E-19	2	FGFR3
1.06710482890287E-11	2.76124874270373	0.314	0.048	1.74055468642347E-07	2	CCL2
3.12301129573462E-20	2.76099185319898	0.457	0.054	5.09394372447273E-16	2	PLAC8
1.6053577078444E-19	2.74846290301683	0.371	0.037	2.61849895726501E-15	2	TSHZ2
6.47617329670414E-23	2.65834240264919	0.4	0.037	1.05632862642541E-18	2	ADIRF
8.91765387767113E-18	2.64347982637925	0.371	0.041	1.45455852398694E-13	2	PROS1
2.16240688771983E-27	2.62057632916651	0.571	0.062	3.52710187455981E-23	2	JAM2
7.82050660313507E-26	2.6115138673299	0.8	0.145	1.27560283203736E-21	2	C1QTNF1
1.43324672096252E-14	2.60896769053602	0.629	0.147	2.33776872656197E-10	2	CXCL2
4.61331069859058E-24	2.54700602905043	0.971	0.266	7.5247710804711E-20	2	IL1R1
1.13271483430111E-26	2.5053736454655	0.771	0.122	1.84757116622854E-22	2	INMT
1.56982352982413E-18	2.48317940964209	0.714	0.16	2.56053915949614E-14	2	GLUL
8.16298475377558E-16	2.48205207880148	0.486	0.081	1.33146444318833E-11	2	PALM
1.76222807196253E-10	2.47691590446715	0.286	0.044	2.87437020817809E-06	2	ETV5
1.56718940766666E-08	2.44032272411011	0.257	0.044	0.000255624264284509	2	GBA
4.44145250009211E-17	2.43104118128625	0.314	0.032	7.24445317290023E-13	2	COL3A1
2.95212774112635E-18	2.39545645719072	0.257	0.019	4.81521555855118E-14	2	FAM107A
2.79848312951582E-20	2.37894395933859	0.886	0.257	4.56460583255326E-16	2	LY6E
3.47069128393816E-19	2.37624094988316	0.914	0.262	5.66104455323152E-15	2	LIFR
3.57895262509677E-19	2.36340785468543	0.543	0.081	5.83762962679534E-15	2	CCND3
1.48269689032033E-14	2.36254589911214	0.314	0.037	2.41842689780149E-10	2	ALDH2
5.34278296223183E-14	2.34988731209428	0.314	0.038	8.71461328969634E-10	2	TNFRSF6B
1.63109317497275E-11	2.34404860555939	0.286	0.039	2.66047607769806E-07	2	TBL2
1.75992673971083E-14	2.33278875978758	0.714	0.193	2.87061650514233E-10	2	VCAM1
1.69571337523498E-18	2.31187781439784	0.343	0.033	2.76587808634578E-14	2	MARVELD1
6.40027786920589E-13	2.28407922078856	0.314	0.041	1.04394932324617E-08	2	RDH10
1.93781325313308E-21	2.27674450557572	0.714	0.128	3.16076719718536E-17	2	TNXB
5.27121884690366E-23	2.27102327426639	0.857	0.175	8.59788506118456E-19	2	ACP5
6.62361644965525E-12	2.2461440371802	0.371	0.063	1.08037807910327E-07	2	FOXC1
9.47894922878532E-12	2.22762937353709	0.314	0.045	1.54611140870717E-07	2	NMI
1.79683743762212E-13	2.22376487797809	0.4	0.065	2.93082154450544E-09	2	C1R
3.12643699883041E-17	2.21701212615092	0.429	0.056	5.09953138879229E-13	2	ECHDC2
2.28943639982189E-13	2.19112184317316	0.257	0.027	3.73429971174948E-09	2	NHP2
3.89150434783458E-09	2.17965371331207	0.286	0.05	6.34743274175298E-05	2	SESN3
2.16707595682398E-09	2.15965660167521	0.286	0.048	3.53471759317559E-05	2	KRAS

4.31142793893339E-15	2.15677479398302	0.371	0.049	7.03237011119425E-11	2	S100A6
3.34427025165748E-11	2.15578375828552	0.257	0.032	5.45483920747851E-07	2	CENPB
9.08527011633998E-11	2.14871584162653	0.314	0.05	1.48189840867621E-06	2	PELI2
1.02352243336075E-13	2.11668019805562	0.314	0.039	1.66946744105472E-09	2	MT1E
1.7746538035679E-11	2.09791638711724	0.343	0.055	2.8946378189996E-07	2	TSTA3
3.26452126689273E-10	2.09513833230041	0.257	0.036	5.32476063842872E-06	2	ATP5MC3
2.7277109614474E-10	2.07572762787812	0.286	0.043	4.44916934921685E-06	2	METTL7A
3.50555906471127E-12	2.06387846897731	0.286	0.037	5.71791739045055E-08	2	COX7A1
2.12954471518844E-09	2.04553911415793	0.314	0.057	3.47350038494386E-05	2	KCTD17
3.66964661659668E-15	2.04288117643512	0.6	0.125	5.98556059633084E-11	2	SERPING1
2.10800184527699E-12	2.04210258101663	0.286	0.036	3.43836180983129E-08	2	NUPR1
1.12229120593111E-11	2.03397555053399	0.286	0.038	1.83056918599423E-07	2	TTR
1.04016634038755E-10	2.01779644239144	0.314	0.05	1.69661531780612E-06	2	CTBS
3.07684013943855E-11	2.00521046664727	0.743	0.258	5.01863395143822E-07	2	CEBPD
3.7907397727957E-10	2.00375124489828	0.371	0.072	6.18307564340707E-06	2	DGKG
4.69008086196259E-17	1.97328587793398	1	0.535	7.64999089394718E-13	2	RAMP3
3.37548325681461E-07	1.96262528074792	0.286	0.061	0.00550575074019031	2	TFPT
1.98668789489893E-10	1.9599763133333	0.257	0.035	3.24048662536964E-06	2	PLCD1
4.75671007173444E-09	1.94545742694858	0.286	0.049	7.75866979800605E-05	2	MAP3K8
8.23807782846447E-12	1.93759986351242	0.457	0.09	1.34371287460084E-07	2	ANXA4
5.4410070726645E-17	1.93293069056433	0.343	0.037	8.87482663622307E-13	2	ARL4C
1.32996782925952E-14	1.9318149481858	0.543	0.103	2.16931052630521E-10	2	PLAAT4
7.72634555168877E-15	1.90833455408345	0.857	0.253	1.26024422293596E-10	2	CRIM1
3.59529426180582E-10	1.89972170881855	0.657	0.221	5.86428447043147E-06	2	THBD
7.47745838704251E-10	1.87594512443972	0.286	0.045	1.2196482375105E-05	2	CTNNBL1
1.55435511026359E-08	1.86718750645369	0.257	0.042	0.000253530862035095	2	TAGLN2
2.00546800383071E-10	1.85572044999359	0.314	0.051	3.27111886104827E-06	2	SAP18
6.20823753860844E-08	1.83991316406885	0.257	0.045	0.00101262562492242	2	FAM110D
5.75718980097408E-08	1.83559261776798	0.257	0.045	0.000939055228436882	2	C19orf12
2.25733946763089E-12	1.81918398810119	0.571	0.132	3.68194640565275E-08	2	HYAL1
2.5284748284448E-09	1.81501022979649	0.6	0.201	4.12419529267632E-05	2	FXYD5
1.44692731541634E-07	1.80727045805452	0.286	0.058	0.00236008314417559	2	MEX3D
6.0882834218205E-14	1.79829465047026	0.514	0.097	9.93059908933142E-10	2	NBL1
5.59042484578512E-10	1.79204513436053	0.257	0.037	9.11854196596011E-06	2	MMRN1
2.12310462956933E-09	1.78468303679967	0.257	0.038	3.46299596129053E-05	2	C1RL
5.27127142271713E-18	1.7739893099288	0.486	0.067	8.59797081759391E-14	2	MT1B
9.76111867834012E-08	1.77240615836013	0.286	0.056	0.00159213606762406	2	TMX4
8.51440010241613E-17	1.77201666512266	0.543	0.086	1.38878380070509E-12	2	RHOU
4.55042510015996E-08	1.76189595202197	0.286	0.054	0.00074221983808709	2	CBX5
2.4678162230553E-09	1.76058210824331	0.686	0.275	4.0252550414255E-05	2	CD59
4.50720525994721E-12	1.74286294859392	0.457	0.085	7.35170249949989E-08	2	PMP22
2.29356428238781E-08	1.74048783283037	0.343	0.072	0.000374103270100276	2	EXT1
2.31282618042714E-13	1.73666006236364	0.914	0.468	3.77245078289471E-09	2	TGM2
2.24662004106066E-08	1.73539213253461	0.257	0.043	0.000366446194897404	2	DBP
7.62043600788338E-10	1.71832237068279	0.371	0.072	1.24296931724586E-05	2	CHMP4B
1.16097992461668E-10	1.71444395138327	0.457	0.097	1.89367435504227E-06	2	MAPK7
5.14731857203215E-11	1.71437278180784	0.514	0.116	8.39579132284164E-07	2	MEIS2
5.7229592330915E-08	1.71264756733064	0.286	0.054	0.000933471880509555	2	CD320
6.30969224064919E-11	1.71225890233415	0.457	0.095	1.02917390137229E-06	2	PROCR
5.13526560646652E-11	1.70584474522547	0.486	0.107	8.37613173070754E-07	2	HSPA12B

4.71855924321944E-07	1.70218535039478	0.314	0.073	0.00769644198161522	2	HAGH
2.1552261654823E-07	1.70145819189024	0.286	0.059	0.00351538939851818	2	SLC25A11
4.9930219572175E-12	1.69740278488898	0.857	0.427	8.14411811441747E-08	2	IFI27
1.24419308419967E-17	1.69287589582418	0.829	0.18	2.02940333963809E-13	2	LTK
1.28972093279652E-15	1.69254876690426	0.543	0.095	2.10366381348441E-11	2	APOC3
3.67987587326471E-12	1.68521418405485	0.714	0.214	6.00224553688207E-08	2	LGALS3BP
5.72766911268244E-09	1.68214806857886	0.371	0.08	9.34240108969633E-05	2	PCDH1
2.04376474163254E-09	1.6787269009593	0.314	0.055	3.33358467007684E-05	2	INPP5K
1.5638359413714E-07	1.67250113658334	0.486	0.141	0.00255077280397089	2	ANPEP
1.44481137503909E-10	1.67046240788498	0.457	0.097	2.35663183382625E-06	2	HNRNPAB
9.99806205005064E-09	1.66708695271882	0.371	0.08	0.000163078390098376	2	GNAI3
1.65364908741501E-06	1.6592805611689	0.257	0.055	0.0269726702648262	2	CYB5A
6.27163608612728E-12	1.65370921369577	0.857	0.292	1.02296656200822E-07	2	ECE1
4.11138845893227E-10	1.64039542901306	0.571	0.155	6.70608571536443E-06	2	EID1
1.90118724136829E-09	1.636652796858	0.6	0.193	3.10102650939582E-05	2	TLNRD1
3.63406772418119E-07	1.63521493429494	0.657	0.287	0.00592752786491194	2	IFI6
3.30034251918591E-13	1.62828293262713	0.8	0.251	5.38318868304414E-09	2	APOL1
7.58598934316963E-11	1.62309757101131	0.686	0.201	1.2373507217644E-06	2	ICAM1
4.89565239375236E-07	1.622965926916	0.257	0.05	0.00798529861944947	2	NAA20
2.75759518919366E-11	1.62211646318815	0.457	0.09	4.49791351309378E-07	2	TIAM1
1.6310616792424E-07	1.61528769281881	0.457	0.126	0.00266042470501228	2	SIGIRR
9.75144589697634E-13	1.59998095972425	0.771	0.227	1.59055834025581E-08	2	GOLM1
5.18896311213422E-07	1.58613066554044	0.343	0.083	0.00846371773220212	2	NNMT
1.32911191052337E-08	1.58416469349832	0.429	0.104	0.000216791443725467	2	KAZN
4.03147610328813E-08	1.57649387566658	0.343	0.072	0.000657574067207326	2	MCU
1.82011985492836E-08	1.5734123048234	0.457	0.115	0.000296879749537364	2	PLSCR4
2.80664749611671E-13	1.57084574606829	0.971	0.427	4.57792273091597E-09	2	TIMP1
2.36463378463462E-08	1.56665569629528	0.6	0.19	0.000385695416611753	2	OSMR
6.53606088548619E-08	1.56663743873262	0.286	0.054	0.00106609689103165	2	C7orf50
3.09372575331935E-07	1.55639833883136	0.343	0.08	0.00504617607623919	2	CLIC1
2.04067693586702E-09	1.54514320376392	0.8	0.345	3.32854815009269E-05	2	CHST12
2.2707873870343E-06	1.54413491181099	0.371	0.102	0.0370388130699164	2	SELENOS
5.39396357515853E-08	1.53919443200628	0.257	0.045	0.000879809398744108	2	TPRG1L
2.48740957545285E-06	1.53400212340551	0.286	0.067	0.0405721375852114	2	ATXN7L3B
3.12864671604188E-09	1.53119249154968	0.543	0.147	5.10313565853592E-05	2	BSG
2.76008080095367E-12	1.52855024772051	0.971	0.495	4.50196779443553E-08	2	VWF
2.49261115330253E-07	1.52735612099365	0.543	0.178	0.00406569805215176	2	TCN2
8.06906565941365E-14	1.51538094349251	0.6	0.123	1.31614529970696E-09	2	HSPB8
6.44259431230214E-07	1.5105897907123	0.257	0.051	0.010508515582796	2	CHCHD2
1.22603647162432E-08	1.50878877792107	0.429	0.103	0.000199978808886642	2	PA2G4
1.41950612466375E-10	1.5077250856219	0.714	0.22	2.31535643993905E-06	2	SLC40A1
6.20967181256389E-08	1.49878102454747	0.314	0.064	0.0010128595693473	2	CDC123
2.04867933047885E-09	1.49831016981775	0.943	0.596	3.34160085594405E-05	2	HYAL2
2.05876406491303E-06	1.4876642995956	0.257	0.055	0.0335805006627964	2	GPX3
2.49698077321638E-07	1.48639840038929	0.257	0.048	0.00407282533919324	2	DTNBP1
4.31301039799839E-09	1.48466504665861	0.4	0.089	7.03495126017518E-05	2	MT1F
2.88139210548749E-06	1.48084835158098	0.286	0.067	0.0469983866326064	2	RAB5IF
2.62883098273823E-10	1.47629819688179	0.429	0.088	4.28788621594433E-06	2	FLOT2
1.3440040156628E-07	1.46395330930756	0.257	0.047	0.00219220494994759	2	FAM189A2
6.42545639773487E-07	1.46342092421149	0.286	0.061	0.0104805619303453	2	BRMS1

2.61421992801325E-07	1.46261552967876	0.314	0.069	0.00426405412458241	2	NDUFB9
5.99014017857932E-14	1.45914377647971	1	0.897	9.77051764528073E-10	2	CD74
1.79342648478794E-06	1.45511216792585	0.286	0.065	0.0292525793933761	2	GADD45GIF
4.24308455897356E-09	1.44680270564503	0.371	0.076	6.92089522414177E-05	2	LRP10
2.89445259147951E-10	1.4451187621285	0.486	0.106	4.72114162196222E-06	2	SDCBP
1.18692110866428E-10	1.44235514956331	0.771	0.265	1.9359870203423E-06	2	CAVIN1
2.91520936212015E-06	1.43487166128755	0.6	0.227	0.0475499799055417	2	ISG15
1.26292878628919E-07	1.43233605158561	0.286	0.056	0.0020599631433163	2	DNASE1L1
1.01458647851903E-08	1.43110849114017	0.429	0.1	0.00016548920051124	2	FBXL7
9.63654660140537E-07	1.42911978428928	0.4	0.108	0.0157181711615523	2	RAB35
8.03375602413578E-09	1.42704970929196	0.457	0.11	0.000131038594509679	2	IFI35
9.19648084275664E-07	1.42408022077777	0.257	0.052	0.0150003799026204	2	GTF2F1
7.88607984927999E-11	1.41715585886412	0.571	0.147	1.28629848421606E-06	2	CST3
5.95192727400491E-13	1.40648146501991	0.457	0.082	9.70818857662942E-09	2	KCTD12
4.52333666978349E-10	1.4026864773197	0.857	0.421	7.37801444208385E-06	2	TMSB10
7.75070905058169E-08	1.3982090074744	0.4	0.097	0.00126421815324038	2	S100A10
1.01290667370769E-06	1.39537068106658	0.286	0.063	0.0165215207548462	2	EEA1
7.72787080957157E-07	1.39039129182352	0.314	0.073	0.0126049300774922	2	CDC25B
2.53921563519563E-07	1.38391476418106	0.4	0.104	0.00414171462256759	2	MYRIP
1.81354002541987E-06	1.37827195194101	0.486	0.163	0.0295806513546234	2	TMEM50A
5.77452826674018E-07	1.3695405891138	0.257	0.05	0.0094188330558799	2	SPIN4
2.35692345717943E-07	1.3656272681825	0.257	0.048	0.00384437785100537	2	DARS
2.81019570478457E-10	1.36226092826753	0.457	0.098	4.58371021407412E-06	2	SLC6A6
8.54741349188082E-08	1.35611787346034	0.486	0.142	0.00139416861466068	2	REEP5
9.02184696160293E-08	1.35467364024998	0.514	0.151	0.00147155345790705	2	TAX1BP3
2.5243691949587E-09	1.35056655515604	0.743	0.281	4.11749859389714E-05	2	MT-CO2
2.79147301706384E-06	1.3421513222232	0.371	0.101	0.0455317163813283	2	OGDH
1.70831183235445E-08	1.34123909506004	0.486	0.125	0.000278642742975334	2	IFITM2
1.14455370892771E-07	1.33826816393083	0.571	0.179	0.00186688155463198	2	SERINC1
1.75898145694485E-07	1.33356355580903	0.4	0.102	0.00286907465442274	2	CYC1
7.60259415217557E-08	1.32484738352859	0.286	0.054	0.00124005913216136	2	DCUN1D3
6.24779057499751E-08	1.32440966133713	0.343	0.073	0.00101907712068784	2	CEBPG
9.2064227517724E-09	1.32289647231566	0.343	0.067	0.00015016596150416	2	RARB
2.12710018126365E-08	1.31799285219762	0.571	0.165	0.000346951310565914	2	TSC22D2
4.15843902493791E-08	1.31328278949039	0.771	0.361	0.000678282989357622	2	ITM2B
1.32196970987855E-06	1.30563873444112	0.257	0.053	0.0215626479378291	2	DDOST
3.26434928201322E-07	1.30372821351729	0.314	0.07	0.00532448011389176	2	KLF10
2.26554306659032E-07	1.28240638911586	0.743	0.281	0.00369532729591546	2	TSPAN9
3.58390560740109E-07	1.28120777627528	0.743	0.324	0.00584570843623191	2	GRN
6.14587298581002E-09	1.28021128758477	0.743	0.282	0.000100245334271547	2	FTL
4.6496857433203E-08	1.27527774300499	0.514	0.148	0.000758410241592974	2	PLEC
7.71474881910137E-07	1.27356018213962	0.286	0.063	0.0125835267988363	2	RBP4
4.3416922746455E-08	1.25850245217783	0.771	0.301	0.000708173426917428	2	TEK
6.32923538693525E-11	1.25093158645645	1	0.705	1.03236158396301E-06	2	APP
9.02446094590617E-08	1.24852507542957	0.371	0.085	0.00147197982488675	2	ECHS1
4.09811050853261E-08	1.24527805010435	0.886	0.526	0.000668442805046755	2	BST2
2.60232479249694E-08	1.23974026706492	0.829	0.412	0.000424465196904176	2	SLC9A3R2
1.7594405781709E-08	1.23746789598435	0.686	0.227	0.000286982352705456	2	CHSY1
3.45877078882616E-09	1.23233317133016	0.4	0.083	5.64160103365435E-05	2	EHD2
3.7818658461681E-07	1.23176498829623	0.429	0.115	0.00616860138168479	2	AFAP1

8.36499068247079E-07	1.22818771080906	0.343	0.083	0.0136441363021781	2	ALS2CL
1.45526543897248E-08	1.22737487822492	0.429	0.097	0.000237368345750801	2	TMEM115
6.93663654633554E-09	1.22719947814504	0.886	0.357	0.000113143478707279	2	SLC38A2
3.58920168959405E-08	1.21653912221125	0.629	0.197	0.000585434687589686	2	LAMTOR4
1.4204991670635E-06	1.20403800986777	0.371	0.097	0.0231697619139727	2	MYBBP1A
4.78976452058424E-07	1.20358930423408	0.4	0.104	0.00781258490952496	2	ZNHIT1
1.23837409495E-07	1.20163474306724	0.571	0.179	0.00201991198627295	2	STXBP1
7.15657962075829E-07	1.19501462267185	0.6	0.217	0.0116730970194189	2	NR2F2
3.00946089596481E-06	1.19406327761483	0.4	0.116	0.049087316674082	2	OAS1
4.37535771884527E-07	1.19334507472941	0.686	0.299	0.00713664597520852	2	CSRP1
2.09128729078935E-07	1.18928849502329	0.486	0.136	0.00341109870000651	2	BMPR2
1.4438458426465E-07	1.18917532046	0.314	0.067	0.0023550569539407	2	ETFB
8.61315753741777E-08	1.17807414229326	0.743	0.274	0.00140489212592821	2	ACTN1
1.67475230046737E-08	1.17503840194854	0.943	0.513	0.000273168847729233	2	LIMS2
6.94027455360805E-08	1.17334973306188	0.857	0.4	0.00113202818243901	2	MT-CO1
4.99246660174445E-08	1.16995309999737	0.514	0.146	0.000814321227410538	2	ZFP36L2
1.39197653942121E-06	1.16659437742783	0.343	0.086	0.0227045293344993	2	VAMP8
1.57021907784354E-09	1.16199656508382	0.286	0.047	2.5611843378706E-05	2	IGFBP3
1.53146506629686E-06	1.16095641333791	0.4	0.11	0.0249797266963682	2	SH3BGRL3
2.88618395427635E-07	1.15646384126532	0.857	0.494	0.00470765464782015	2	IFITM3
1.98722184190974E-06	1.14969026183542	0.543	0.184	0.0324135754633898	2	PPIB
1.68753939836647E-08	1.14856570404342	0.743	0.284	0.000275254551267555	2	EDNRB
1.8022833118609E-06	1.14820501441718	0.771	0.35	0.0293970430997631	2	PIM3
3.16788044627396E-08	1.1381490512029	0.543	0.146	0.000516712979591746	2	TMED9
2.58423628969032E-07	1.13627646813531	0.543	0.167	0.00421514781211388	2	CTDSP1
5.66540755447235E-07	1.13062345525544	0.371	0.091	0.00924084626209985	2	TRAM2
7.5039832875645E-07	1.11918618846064	0.343	0.081	0.0122397471403465	2	SNRPB
1.72430661076878E-07	1.11590623998917	0.857	0.475	0.00281251651282496	2	SH3BP5
2.1416549412642E-09	1.11492021534241	0.714	0.207	3.49325337469604E-05	2	FCHSD2
3.71338106680929E-07	1.11292872178703	0.686	0.265	0.00605689585807263	2	CAVIN2
7.36472276308916E-08	1.10819163005278	0.514	0.143	0.00120125992988747	2	RIN2
1.04794990777106E-08	1.10470354910263	0.457	0.106	0.000170931109456537	2	NCSTN
2.32631120167286E-06	1.10444175936168	0.686	0.279	0.037944462010486	2	NDRG1
4.19892462447298E-07	1.10423515276348	0.771	0.303	0.00684886595497788	2	HNRNPA0
1.28848384709618E-07	1.10182153890743	0.457	0.12	0.00210164600299858	2	UBXN6
6.48190859537634E-08	1.10125666112487	0.6	0.182	0.00105726411099183	2	APLP2
1.03867326952813E-06	1.0963689612773	0.4	0.11	0.0169417996992734	2	MT-CYB
5.95139398343467E-08	1.0931818463278	0.829	0.308	0.000970731872638029	2	SELENON
8.27523144275201E-07	1.08239837469849	0.429	0.123	0.0134977300062728	2	FJX1
2.5559418071212E-08	1.08055901810198	0.686	0.22	0.000416899668159539	2	SERPINB1
1.6055338861038E-06	1.07171095862926	0.343	0.085	0.026187863216239	2	CDR2L
4.65663166438894E-11	1.06714021735014	0.686	0.192	7.5954319077848E-07	2	MT2A
3.63595001838126E-07	1.0644844247113	0.4	0.102	0.00593059807498167	2	NCOA4
2.17781741865272E-07	1.06400796151967	0.457	0.127	0.00355223799156445	2	FGA
4.13672930714103E-07	1.03938066934908	0.886	0.465	0.00674741917287774	2	PECAM1
1.5529320551504E-07	1.03142545435966	0.514	0.144	0.00253298747515583	2	ITPR1
1.37971740918937E-06	1.03092244052949	0.714	0.313	0.0225045706612879	2	PTMS
1.10183412695056E-06	1.0166568870359	0.6	0.199	0.0179720164446906	2	RHOA
3.80024355205871E-07	1.01097539997259	0.686	0.244	0.00619857725776296	2	BRI3
4.16124976814664E-07	0.99869426442599	0.429	0.113	0.00678741449682398	2	MGST3

2.87807475647965E-07	0.974265846015444	0.943	0.799	0.00469442773529396	2	B2M
2.42738722819401E-06	0.972106840417238	0.514	0.17	0.0395931130790726	2	ENTPD1
1.59151586868307E-07	0.941092538625574	0.686	0.223	0.00259592153340895	2	EGLN2
1.98933426643445E-06	0.937493522823418	0.429	0.123	0.0324480312198124	2	SUN2
1.27070494774398E-06	0.935514615201796	0.429	0.118	0.020726468402652	2	HEG1
1.26596017449608E-06	0.910879757288289	0.457	0.131	0.0206490764062055	2	RABAC1
3.18225105627618E-07	0.84064077881619	0.943	0.716	0.00519056969789207	2	IGFBP7
1.63349152543793E-08	0.835696198883135	0.743	0.266	0.00026643880271418	2	SERPINA1
3.05315850847573E-06	0.826936381379687	0.314	0.076	0.0498000684317476	2	MT-ND2
1.99091834295277E-06	0.820541331854644	0.514	0.155	0.0324738690919026	2	GATA4
1.71531527495053E-08	0.730008625509114	0.914	0.361	0.000279785074497182	2	ALB
2.75876174175906E-183	9.37573143370163	0.957	0.008	4.49981627698321E-179	3	CCL21
1.05125425373209E-228	9.18829781443578	0.87	0.001	1.71470081326242E-224	3	PDPN
5.54606114487303E-74	8.92183675327057	0.304	0.001	9.0461803334024E-70	3	SEMA3D
5.98714926776312E-74	8.64738069816689	0.304	0.001	9.76563917064842E-70	3	ADAMTS15
4.91775540347817E-142	8.24812992698508	0.652	0.003	8.02135083861325E-138	3	ABI3BP
3.3566584376302E-81	8.24068345324758	0.391	0.002	5.47504557761862E-77	3	CDCP2
4.0907480935129E-59	7.06570812577253	0.304	0.002	6.67241921532889E-55	3	EFNA5
5.62085135115602E-122	6.93047302714561	0.652	0.006	9.16817063887058E-118	3	TBX1
8.29525677598475E-60	6.76779470647092	0.435	0.008	1.35303933273087E-55	3	MFAP2
5.35863312247621E-55	6.73894960702902	0.348	0.005	8.74046648607095E-51	3	PARD6G
8.66092799104576E-75	6.71785846200468	0.391	0.003	1.41268396461947E-70	3	MPP7
7.08494976319302E-59	6.64834978972772	0.304	0.002	1.15562615587441E-54	3	GAS7
3.30947159745136E-95	6.38838535158512	0.826	0.022	5.39807912260292E-91	3	SPHK1
2.64962040976931E-81	6.37386921904365	0.739	0.021	4.32179585037472E-77	3	CRTAC1
7.05091746391181E-57	6.3371586730398	0.391	0.006	1.15007514753866E-52	3	TC2N
1.35836103091908E-83	6.17434271854793	0.565	0.009	2.2156226775321E-79	3	PTX3
7.7615122373609E-69	6.07553959530406	0.609	0.016	1.26598026103594E-64	3	TFF3
4.96350337090099E-42	5.9528432939352	0.304	0.006	8.09597034827661E-38	3	IGF1
2.09352662663857E-68	5.87656596937982	0.478	0.008	3.41475128071018E-64	3	PRKG1
1.83647584790738E-94	5.69764357451856	0.783	0.019	2.99547575552173E-90	3	ELN
6.16551313988611E-42	5.69090195463023	0.304	0.006	1.00565684824682E-37	3	PLEKHG3
5.45496171724617E-57	5.53527008218869	0.696	0.031	8.89758805700022E-53	3	MMRN1
1.94839321251161E-30	5.38694291218022	0.348	0.014	3.17802416892769E-26	3	FRMD6
1.34234738826021E-58	5.284354023292	0.739	0.034	2.18950282499123E-54	3	ADIRF
1.87137082187597E-32	5.20860812174479	0.304	0.009	3.0523929475619E-28	3	MYO5C
6.9339031495898E-31	5.13294990808521	0.261	0.006	1.13098894272959E-26	3	SEMA3A
2.01144892223038E-51	5.08680281588938	0.522	0.017	3.28087433704997E-47	3	PROX1
2.99807463896763E-38	4.98694595299864	0.522	0.027	4.89015954362009E-34	3	UNC5B
8.60754635045614E-35	4.9806290027155	0.435	0.019	1.4039768852229E-30	3	DMTN
2.7801906029339E-39	4.93928369853239	0.435	0.016	4.53476889244548E-35	3	CELSR1
1.27469662473681E-42	4.88241833030316	0.609	0.032	2.07915766460821E-38	3	FLRT2
1.24626720677431E-21	4.85997828693809	0.304	0.016	2.03278644096958E-17	3	RNF112
1.51003204730228E-17	4.67134362527977	0.391	0.036	2.46301327235475E-13	3	NUDT4
1.29731412307863E-18	4.6146036959958	0.304	0.019	2.11604906615355E-14	3	PLXNA4
1.14811997532114E-22	4.59686933002735	0.261	0.01	1.87269849174631E-18	3	PTPRS
9.9871398863293E-19	4.58765458829554	0.348	0.026	1.62900238685917E-14	3	GJC2
1.13915812230052E-20	4.50508771877051	0.435	0.038	1.85808081328437E-16	3	ACKR2
4.29201741274561E-38	4.48980698196846	0.435	0.017	7.00070960192937E-34	3	SLC38A1
1.12208166703584E-20	4.42786271665331	0.435	0.038	1.83022740710216E-16	3	HS3ST1

1.33709647625656E-18	4.42514428549812	0.261	0.014	2.18093806242207E-14	3	FAM89A
1.69419588284342E-38	4.42071862068611	0.783	0.068	2.76340290450591E-34	3	DOCK5
1.81068743703064E-16	4.38622651089685	0.261	0.016	2.95341227854067E-12	3	VSTM4
3.158427076856E-37	4.36220202650483	0.565	0.032	5.15171040505982E-33	3	ADAM19
5.59135681040735E-22	4.34270425608615	0.391	0.027	9.12006209345542E-18	3	COL23A1
5.2706124483707E-23	4.27053181493731	0.522	0.049	8.59689596453745E-19	3	S100A6
4.9032145789372E-22	4.2352249293792	0.304	0.015	7.99763329970447E-18	3	TLE1
1.09583047275444E-17	4.14311697088391	0.261	0.014	1.78740908410976E-13	3	CCDC102B
1.25293928164201E-24	4.13349750932306	0.435	0.03	2.04366926228629E-20	3	GLIPR2
4.66219079594312E-17	4.10604119528669	0.261	0.015	7.60449940726283E-13	3	ITGB4
1.54132443688312E-18	4.08453603130332	0.304	0.019	2.51405428900006E-14	3	ROBO3
8.02999570951307E-29	4.04388076993067	0.652	0.061	1.30977260017868E-24	3	LTBP4
3.08810220094903E-21	3.99671644637784	0.391	0.028	5.03700349996797E-17	3	PDGFC
8.36338062168043E-27	3.98500606852454	0.478	0.033	1.3641510132023E-22	3	STON2
5.02465628929963E-14	3.94741253312131	0.261	0.019	8.19571687347662E-10	3	LTBP2
3.42285324940636E-15	3.85798886142287	0.435	0.053	5.58301593510672E-11	3	RASSF9
9.56016924661597E-15	3.772002446519	0.565	0.093	1.55935920581553E-10	3	MEGF6
2.95680315286992E-16	3.7572507811647	0.304	0.023	4.82284162264613E-12	3	KLHL4
1.32519568509603E-17	3.71688631091778	0.261	0.014	2.16152668196014E-13	3	KRT18
8.2138471904126E-17	3.71112437684689	0.304	0.022	1.3397606152282E-12	3	CDKN2C
6.9519448325776E-17	3.68094166363929	0.696	0.134	1.13393172164173E-12	3	TNXB
2.19925053021053E-23	3.60118861554261	0.739	0.101	3.5871975398264E-19	3	PGM5
9.7328601791976E-13	3.55192312709333	0.304	0.03	1.58752682382892E-08	3	ATP9A
3.01916741455881E-11	3.52224787813965	0.261	0.025	4.92456396988687E-07	3	PPP1R2
1.15639773236402E-19	3.50106509990357	0.565	0.068	1.88620034125895E-15	3	LIMS1
1.84788446718859E-13	3.48988593871136	0.304	0.028	3.01408435443131E-09	3	ASNSD1
3.67779740617879E-26	3.41940933024577	0.565	0.047	5.99885534921822E-22	3	FBLN2
4.20155536279711E-17	3.41888385494622	0.652	0.115	6.85315695225837E-13	3	SNCG
2.9580162113936E-12	3.35556361788225	0.348	0.041	4.8248202424041E-08	3	ARL4A
3.28564917483681E-15	3.32586679629695	0.391	0.04	5.35922236907632E-11	3	HP
4.29462742474853E-16	3.28608026408673	0.565	0.083	7.00496679250733E-12	3	EHD2
6.97675811350425E-16	3.28183529642407	0.522	0.07	1.13797901589368E-11	3	MGP
3.01021534420724E-08	3.27248260613225	0.304	0.049	0.000490996224793643	3	SMAD1
1.53944901358393E-16	3.2693359390789	0.565	0.081	2.51099528605676E-12	3	NNMT
1.45095317279276E-11	3.25042180370779	0.304	0.033	2.36664972014226E-07	3	CFAP54
1.60880223526551E-23	3.21949069862063	0.87	0.145	2.62411732594157E-19	3	FBN1
3.23629451404778E-15	3.18621584162345	0.478	0.061	5.27871998186334E-11	3	PAPLN
1.50451255166657E-16	3.11622993820729	0.435	0.045	2.45401042302334E-12	3	TSPAN11
2.35697842086841E-13	3.06781923029896	0.696	0.158	3.84446750227847E-09	3	OLFML2A
3.40571927006914E-14	3.0669077341909	0.304	0.026	5.55506870140977E-10	3	CDH23
9.29583246009991E-10	3.0578577182247	0.261	0.029	1.5162432325669E-05	3	PDGFA
6.07748532278782E-14	3.0512440945439	0.391	0.044	9.91298630999921E-10	3	RTKN
1.82395105168281E-10	3.04345350149594	0.261	0.027	2.97504656039982E-06	3	CFH
8.46808758564144E-10	3.0409964475637	0.261	0.029	1.38122976609398E-05	3	GNB5
2.82312067853247E-09	3.01685490968345	0.261	0.031	4.60479213875431E-05	3	CCDC102A
5.71082238061979E-07	2.98926102835446	0.261	0.041	0.00931492238502893	3	HECTD2
4.61533936316437E-09	2.96145282081852	0.261	0.031	7.5280800352574E-05	3	NFIX
7.0815097151695E-09	2.95811338184334	0.261	0.032	0.00011550650496413	3	PPP1R13L
9.81742519080572E-12	2.94569625708031	0.304	0.032	1.60132022287232E-07	3	BCL2
2.33784171222249E-09	2.94382704955691	0.261	0.031	3.8132536168061E-05	3	CREB5

5.675690546643E-07	2.91055222568991	0.261	0.043	0.00925761885062939	3	ZFP30
1.39928755099848E-13	2.9016106541262	0.522	0.083	2.28237792443363E-09	3	PTPRE
1.68815878205063E-09	2.80607391803555	0.261	0.03	2.75355578940278E-05	3	THAP12
3.47479898094268E-07	2.77779541851562	0.261	0.04	0.0056677446178156	3	ZMAT1
3.52473817194708E-12	2.7748387139288	0.565	0.108	5.74920043226288E-08	3	DST
4.84761442154427E-13	2.7018096954317	0.304	0.028	7.90694388298086E-09	3	KIAA0513
1.05686773349641E-09	2.67701093506597	0.696	0.224	1.723856960106E-05	3	THBD
6.16246971747188E-09	2.65251662124136	0.391	0.07	0.000100516043561684	3	CTSZ
1.98422624165744E-09	2.63013310208962	0.522	0.117	3.23647142276745E-05	3	PLSCR4
5.16023120100964E-10	2.59201825100617	0.304	0.038	8.41685311196683E-06	3	SLCO2B1
6.7429196644577E-08	2.56614262764816	0.435	0.094	0.0010998376264697	3	RAB11FIP5
1.27272125530122E-07	2.55114745095272	0.435	0.101	0.00207593563952183	3	MAPK7
5.97767330312333E-07	2.52597918078536	0.261	0.041	0.00975018292472446	3	KNOP1
3.04638261097445E-07	2.51679146604062	0.304	0.053	0.00496895467676042	3	ZNF358
2.10179181074527E-07	2.51335330336432	0.391	0.084	0.00342823262250661	3	MICAL2
8.11088122056213E-07	2.48241156723975	0.304	0.056	0.0132296583588589	3	DOC2B
4.15489667906356E-09	2.47589240330522	0.391	0.068	6.77705197322057E-05	3	C1R
2.63072651086481E-09	2.46052907545861	0.435	0.082	4.2909780118716E-05	3	ELOVL1
1.32816130445218E-08	2.42619178524203	0.348	0.057	0.000216636390369195	3	DNASE1L1
5.9644085359566E-09	2.39351416495362	0.391	0.069	9.72854676299881E-05	3	GC
1.35975540760807E-06	2.38821862406367	0.348	0.076	0.0221789704534952	3	SIRPA
2.16144669423187E-06	2.37064352473179	0.348	0.073	0.0352553570296161	3	PLCG2
3.85281103957338E-07	2.36410756280226	0.609	0.207	0.00628432008664814	3	IGFBP5
2.59210061327496E-07	2.35936594050278	0.391	0.083	0.00422797531031279	3	MDFIC
1.92116327246117E-19	2.34866546340875	0.478	0.046	3.13360941371141E-15	3	PTGDS
5.98398971286496E-14	2.33977537319917	1	0.405	9.76048562065403E-10	3	VIM
5.65087614129749E-09	2.2842831749012	0.783	0.315	9.21714407407033E-05	3	PTMS
7.22125936679586E-09	2.28014930529623	0.609	0.161	0.000117785961531807	3	GJA1
9.62940239232596E-07	2.26214206143098	0.261	0.042	0.0157065182421229	3	CITED4
5.8283966883298E-08	2.25483832551118	0.522	0.129	0.000950669783833473	3	FGA
2.58388783493337E-06	2.25028993770246	0.261	0.044	0.0421457944755981	3	OBSL1
1.20699111936052E-08	2.22558468122003	0.652	0.204	0.000196872321478894	3	OAF
1.43964678357433E-08	2.17080026046474	0.478	0.103	0.00023482078686881	3	C3
4.19029978204709E-10	2.1565206541465	0.304	0.037	6.83479797449701E-06	3	FABP1
1.16377453888558E-06	2.15151547263357	0.348	0.074	0.0189823265037626	3	FAF1
9.08282806174784E-07	2.14072824234749	0.261	0.043	0.0148150008515169	3	MT1E
2.47305261928377E-07	2.13492176150221	0.565	0.164	0.00403379612731376	3	LAPTM5
1.07822676326642E-07	2.09026746783615	0.696	0.279	0.00175869567356386	3	AHNAK
2.91972021493978E-07	2.01463577593996	0.565	0.175	0.00476235564258827	3	TTYH3
1.53930347644897E-09	2.01181843877219	0.739	0.22	2.51075790043591E-05	3	CALD1
8.4063159989293E-07	2.00392128984037	0.348	0.069	0.0137115420258536	3	ARHGEF3
2.3773788636393E-07	2.00121025975056	0.435	0.1	0.00387774266448206	3	ATXN3
8.20499021397228E-09	2.00096364242396	0.522	0.126	0.000133831595380102	3	CLU
1.88354723696028E-09	1.97697078582472	0.783	0.282	3.07225389820591E-05	3	LAMA4
7.20661676687785E-09	1.96797814038951	0.87	0.476	0.000117547126084545	3	LMNA
1.91138283550527E-06	1.89420069594153	0.435	0.11	0.0311765654299265	3	LHFPL6
1.88173822537075E-08	1.89097771260782	0.478	0.106	0.000306930321940223	3	LGALS1
1.42771748594558E-06	1.88395006206141	0.348	0.074	0.0232874999132584	3	FGG
2.75473716337249E-07	1.84045908982484	0.435	0.099	0.00449325178717687	3	PROCR
1.82569297402938E-07	1.82550244312228	0.478	0.113	0.00297788780993931	3	TRIP6

**1.9491216207974E-06**	1.80900915817278	0.609	0.231	0.0317921227568265	3	FGB
**2.89492014044576E-10**	1.74426968117542	0.87	0.33	4.72190424108108E-06	3	TRIOBP
**2.83368026370642E-06**	1.71011645070868	0.391	0.096	0.0462201587813154	3	VTN
**1.75968245454752E-09**	1.69342891808844	0.87	0.34	2.87021805161246E-05	3	FN1
**1.34061313906197E-06**	1.68749952381591	0.348	0.07	0.0218667409112398	3	SH3PXD2B
**4.2216384419025E-07**	1.67889379621638	0.478	0.116	0.00688591446258717	3	SULF2
**1.36491667282484E-07**	1.66544446232002	0.913	0.487	0.00222631558504459	3	ITGA9
**2.9766381537871E-06**	1.62528792704308	0.609	0.211	0.0485519449264213	3	EPB41L2
**3.55861241804718E-10**	1.57828105628941	0.609	0.13	5.80445271507675E-06	3	FABP4
**7.77024191693693E-07**	1.4357267540562	0.87	0.445	0.0126740415907158	3	SHC1
**6.97940394008331E-08**	1.42117884160801	0.87	0.404	0.00113841057666699	3	MT-CO1
**3.05888583871655E-06**	1.39947530024117	0.826	0.541	0.0498934869153056	3	MAF
**1.34064001613914E-69**	8.47223705919446	0.333	0.002	2.18671793032456E-65	4	GUCY1A1
**1.09846660956831E-152**	8.24040639314299	0.722	0.003	1.79170888686687E-148	4	GJA5
**1.30919281617142E-75**	8.11595842462588	0.389	0.002	2.13542440245721E-71	4	TMEM100
**1.77413208396859E-55**	7.72042695551477	0.278	0.002	2.89378684216116E-51	4	CPAMD8
**2.23194329471407E-108**	7.62825533103533	0.778	0.011	3.64052270800811E-104	4	FBLN5
**3.51852135158326E-43**	6.89992685415407	0.278	0.003	5.73906017656745E-39	4	PDZRN3
**2.89456711881065E-39**	6.54723876866679	0.722	0.044	4.72132842749205E-35	4	IGFBP3
**1.15510411638532E-39**	6.40201831859581	0.333	0.006	1.8840903242361E-35	4	MET
**1.92582361192602E-69**	5.99124589005344	0.556	0.01	3.14121089341253E-65	4	SLC45A4
**2.57592959837238E-32**	5.6747890531701	0.278	0.006	4.20159876790519E-28	4	ELOVL7
**4.00938155737075E-45**	5.63177300875867	0.556	0.019	6.53970225822743E-41	4	RNF144B
**4.09282470537498E-54**	5.51849916662941	0.778	0.034	6.67580637693712E-50	4	SEMA3G
**9.45543625611199E-24**	5.40965214052265	0.722	0.083	1.54227620773443E-19	4	KCTD12
**4.51575702575606E-40**	5.38825172907167	0.611	0.029	7.3656512847107E-36	4	VEGFC
**6.54007226567132E-34**	4.77942867943488	0.778	0.062	1.06675118725365E-29	4	LTBP4
**1.03960976381277E-29**	4.69165142750524	0.5	0.026	1.695707485755E-25	4	ELN
**1.44429039224036E-18**	4.61810912269002	0.5	0.046	2.35578205878325E-14	4	HEY1
**1.87186198375174E-15**	4.52736874469251	0.278	0.016	3.05319408169747E-11	4	ITGB4
**7.41096828103754E-20**	4.49293817530284	0.444	0.033	1.20880303632003E-15	4	PCSK5
**1.82451095625997E-11**	4.37835061876365	0.278	0.023	2.97595982075564E-07	4	COL8A1
**1.68505876136133E-07**	4.25670866822374	0.333	0.054	0.00274849934565646	4	FGL2
**6.64719375480222E-17**	4.22437017311683	0.556	0.065	1.08422377334579E-12	4	RCAN1
**6.09140775296972E-11**	4.12977666063843	0.556	0.102	9.93569518586892E-07	4	BTG2
**1.13427872270685E-11**	3.99668494380143	0.278	0.022	1.85012202460714E-07	4	SETBP1
**2.2918129923777E-11**	3.9924763383611	0.556	0.098	3.73817617186726E-07	4	CCN1
**1.09719290554902E-15**	3.76048267747777	0.5	0.054	1.789631348241E-11	4	FBLIM1
**1.39150654994821E-11**	3.72113409692529	0.389	0.044	2.26968633362052E-07	4	LMCD1
**1.72387319567168E-09**	3.70595413844236	0.389	0.057	2.81180956946007E-05	4	AIF1L
**1.87661033096535E-29**	3.67257401506114	0.667	0.048	3.06093911083757E-25	4	FBLN2
**2.96804257164261E-15**	3.63534253409782	0.556	0.071	4.84117423860625E-11	4	MGP
**4.83893129340382E-14**	3.63071978171166	0.278	0.018	7.89278083267097E-10	4	SEPTIN5
**1.04223414207347E-12**	3.57998676572595	0.444	0.054	1.69998810913604E-08	4	EGR3
**1.95790007570726E-08**	3.57700756002054	0.333	0.047	0.000319353081348612	4	KCNJ2
**2.667846885928E-12**	3.56501843352633	0.889	0.329	4.35152505563715E-08	4	TM4SF1
**5.32997360123461E-07**	3.56299562488131	0.278	0.041	0.00869371994097377	4	INSIG1
**7.33259521280749E-10**	3.50702220840236	0.444	0.071	1.19601960516103E-05	4	ACE
**6.56464726847875E-09**	3.49428718433785	0.444	0.081	0.000107075961596157	4	HIP1R
**5.31597548120954E-10**	3.47588093400909	0.722	0.218	8.67088760740088E-06	4	ADAMTS1

**1.17928402694039E-06**	3.43235821906113	0.278	0.042	0.0192353017634246	4	CITED4
**1.7549978638735E-11**	3.43152148192476	0.333	0.033	2.86257701576407E-07	4	ENPP2
**1.00560273232455E-09**	3.36094675304865	0.778	0.267	1.64023861669457E-05	4	DEPP1
**2.96792033025268E-13**	3.25805540581387	0.722	0.15	4.84097485067514E-09	4	FBN1
**6.04753131977904E-13**	3.21204381980751	0.667	0.129	9.8641283356916E-09	4	GJA4
**1.12047023631524E-09**	3.16917601108122	0.833	0.294	1.82759900245379E-05	4	DLL4
**8.63554364849316E-07**	3.16302138880869	0.278	0.04	0.0140854352450572	4	MECOM
**6.9859973042625E-09**	3.11085075688311	0.833	0.391	0.000113948602029826	4	SAT1
**4.96604552642138E-10**	2.89230870685639	0.722	0.191	8.1001168581459E-06	4	BCAM
**2.43626010681359E-10**	2.8793313215685	0.5	0.085	3.97378386022365E-06	4	BGN
**1.36039619163363E-10**	2.8361187749276	0.611	0.123	2.21894222817361E-06	4	JAG2
**6.52205490419337E-07**	2.8036500896368	0.389	0.075	0.0106381237542298	4	GFOD1
**3.52348806957159E-09**	2.76530830805927	0.833	0.391	5.74716139027822E-05	4	CLDN5
**7.11555710921448E-08**	2.75707118825908	0.5	0.109	0.00116061852008397	4	CXCL12
**2.29938176581358E-06**	2.71686951538052	0.333	0.061	0.0375052159821853	4	ARHGAP4
**7.39974855498576E-09**	2.68964495410854	0.833	0.409	0.000120697298680373	4	VIM
**4.31132247705957E-08**	2.60909370267872	0.667	0.195	0.000703219809233187	4	ARL 15.00
**1.09238080291419E-07**	2.58050770679321	0.833	0.462	0.00178178232763333	4	RHOB
**8.08622449794035E-08**	2.56725436449598	0.278	0.034	0.00131894407785905	4	ZC3H10
**5.75055538812787E-07**	2.16744463464506	0.556	0.147	0.00937973089357537	4	RAPGEF1
**4.83140134522483E-08**	1.96828968673646	1	0.578	0.000788049873419622	4	MMRN2
**1.11827506598004E-07**	1.77677200327507	0.889	0.554	0.00182401846012005	4	A2M
**1.43475857016999E-06**	1.77285792368194	0.778	0.339	0.0234023470380427	4	CLEC14A

SupTable5P_C2_EC_ECSubtype

p_val	avg_log2FC	pct.1	pct.2	p_val_adj	cluster	gene
1.7958935253184E-160	3.90028280485146	0.884	0.165	2.92928192914685E-156	LSEC	OIT3
4.87111548854101E-159	4.7772740220175	0.83	0.073	7.94527647335925E-155	LSEC	STAB2
7.65557590337134E-146	3.883909177629	0.849	0.163	1.2487009855989E-141	LSEC	FCN2
2.55447526977863E-138	5.26502481516913	0.746	0.056	4.16660461253593E-134	LSEC	MS4A6A
1.46341820848641E-135	5.44548543516883	0.722	0.04	2.38698143986218E-131	LSEC	CLEC4G
2.24507479730868E-131	5.32329674930016	0.713	0.039	3.66194150189018E-127	LSEC	CLEC4M
9.68199241585106E-107	1.99064815759939	0.944	0.792	1.57922978294947E-102	LSEC	STAB1
3.00705089606385E-106	1.99345586705548	0.928	0.468	4.90480071656974E-102	LSEC	FCN3
2.31454977162083E-99	5.77554806864804	0.562	0.017	3.77526213249074E-95	LSEC	DEPRECATED-ENSG00000104938
6.9997408176233E-94	2.96957669861168	0.78	0.3	1.14172772476254E-89	LSEC	CTSL
6.57402767080586E-84	2.66357562804199	0.759	0.283	1.07228965338514E-79	LSEC	CRHBP
2.7706609161161E-83	3.26457123293739	0.668	0.173	4.51922502027698E-79	LSEC	CD14
5.51914029915704E-78	4.77901361617571	0.491	0.031	9.00226974195505E-74	LSEC	NPL
3.61269820269862E-67	1.33818839206633	0.915	0.666	5.89267203842173E-63	LSEC	DNASE1L3
4.83388283786756E-61	2.99693094324176	0.528	0.11	7.88454629684577E-57	LSEC	ITGB3
6.53391932446114E-61	8.04584635795252	0.36	0.003	1.06574758101286E-56	LSEC	MRO
2.01898226297842E-57	3.07620360356151	0.47	0.075	3.2931619691441E-53	LSEC	LYVE1
2.65174780532828E-55	4.17248925932484	0.385	0.031	4.32526584527095E-51	LSEC	RELN
5.50410172131546E-55	3.92383764329626	0.387	0.031	8.97774031763765E-51	LSEC	FCHO1
3.02953841778338E-48	5.44121446825318	0.308	0.009	4.94148011324648E-44	LSEC	DEPRECATED-ENSG00000165682
8.22269116769477E-48	3.23372289899862	0.396	0.059	1.34120315636269E-43	LSEC	PLTP
3.57840017786896E-47	3.44859581526064	0.377	0.05	5.83672853012206E-43	LSEC	ME2
1.99970061514885E-46	2.19305557350068	0.498	0.138	3.26171167336928E-42	LSEC	SYNE1
4.13024836763781E-46	6.65181821615907	0.286	0.005	6.73684811245403E-42	LSEC	CLEC1B
5.35609991795924E-46	1.48829711494545	0.685	0.309	8.73633457618331E-42	LSEC	ITGA9
7.9429178358043E-42	1.94498429296522	0.53	0.187	1.29556932819804E-37	LSEC	MRC1
2.96956826698595E-41	4.70954757766097	0.278	0.012	4.84366280028079E-37	LSEC	RIPOR3
5.45207122740577E-41	1.717493475565	0.615	0.275	8.89287337902155E-37	LSEC	TIMP1
8.22793043906872E-41	3.1538922682927	0.38	0.076	1.3420577339165E-36	LSEC	EHD3
3.11341577518974E-40	3.44636419588913	0.323	0.039	5.07829247091199E-36	LSEC	BMPER
7.35091960028312E-39	1.68114490794169	0.581	0.246	1.19900849600218E-34	LSEC	BSDC1
5.32221273898205E-37	3.62620054702792	0.281	0.025	8.68106119855363E-33	LSEC	FCGR2B
5.37668335030099E-36	2.63290173948161	0.401	0.107	8.76990821267595E-32	LSEC	SOD2
1.73477846616165E-35	4.0839962150502	0.258	0.019	2.82959715615627E-31	LSEC	ATRNL1
1.94082703052545E-34	1.79616705070909	0.437	0.129	3.16568296949007E-30	LSEC	LIFR
2.64325248541916E-34	1.09338492380705	0.738	0.423	4.31140912896718E-30	LSEC	APOA2
1.09343677960617E-33	1.73294873350445	0.485	0.171	1.78350473121562E-29	LSEC	SH3TC1
5.03605093685709E-33	1.71311685495942	0.44	0.135	8.2143026831076E-29	LSEC	IL1R1
1.23616637470787E-32	1.69699767478601	0.543	0.25	2.016310973786E-28	LSEC	UGCG
3.16382258581083E-32	1.35832562298852	0.657	0.364	5.16051101971604E-28	LSEC	ADAMTS4
8.17831476158266E-32	2.80450085340122	0.319	0.065	1.33396492076175E-27	LSEC	NTN4
1.90616888944849E-31	2.81141768398689	0.348	0.087	3.10915207557943E-27	LSEC	GDF15
3.20368570423364E-31	2.9584275262851	0.286	0.048	5.22553175217549E-27	LSEC	GATA4
4.83523785502063E-31	3.10830759613091	0.276	0.04	7.88675646532415E-27	LSEC	RAB20
3.50646349071728E-30	2.62729277312847	0.268	0.037	5.71939259970895E-26	LSEC	TFPI2
7.74835440865228E-30	1.55293662302314	0.498	0.208	1.26383408759527E-25	LSEC	LITAF
7.97411284469887E-30	2.06486676150447	0.469	0.196	1.30065754609883E-25	LSEC	LGMN
6.17898915260731E-29	2.34672070981152	0.319	0.073	1.00785492068178E-24	LSEC	ACP5
2.00987641961267E-28	2.10855494111174	0.355	0.1	3.27830942803022E-24	LSEC	LRATD1
1.26479437762977E-27	2.76964638362763	0.26	0.044	2.06300610935191E-23	LSEC	ANPEP
2.10940776316488E-27	2.25430666187812	0.343	0.101	3.44065500249824E-23	LSEC	DAAM1
3.18366469820434E-27	4.34261101685789	0.197	0.012	5.19287548924109E-23	LSEC	DEPRECATED-ENSG00000072694
1.47937533796917E-26	0.661115544213826	0.917	0.848	2.41300911376152E-22	LSEC	ARGLU1
2.68884185341133E-26	2.31693551277547	0.315	0.084	4.38576994709922E-22	LSEC	LTK
6.16199989668065E-26	2.02485425700695	0.345	0.106	1.00508380314758E-21	LSEC	TMEM37
1.30007688290371E-25	2.51313629224489	0.278	0.061	2.12055540370425E-21	LSEC	CA13
2.48326719992334E-25	2.02496032048366	0.308	0.079	4.05045712979495E-21	LSEC	NR5A2
1.04585701331244E-24	2.5855716006168	0.287	0.075	1.70589737441392E-20	LSEC	SNX5

7.05453370065148E-24	1.53238552313218	0.483	0.246	1.15066499191326E-19	LSEC	CD4
8.50169010016603E-24	0.988622186039269	0.615	0.336	1.38671067223808E-19	LSEC	MCAM
1.16839263179194E-23	4.80712895872466	0.172	0.011	1.90576522171583E-19	LSEC	ACSM3
1.85303860810491E-23	0.800141702448682	0.73	0.481	3.02249127367992E-19	LSEC	SOCS3
1.30506085544643E-22	1.33439995860584	0.453	0.193	2.12868476131868E-18	LSEC	MID1
2.07743493853767E-22	2.37925905646417	0.258	0.061	3.38850412824879E-18	LSEC	KIF19
2.55638667832373E-22	3.40887591314211	0.172	0.014	4.16972231101384E-18	LSEC	PITPNM3
2.67386490681876E-22	2.66032891526113	0.23	0.045	4.36134104951207E-18	LSEC	HSPB8
8.0263827384976E-22	1.25058994450385	0.555	0.306	1.30918328847634E-17	LSEC	SQSTM1
1.04564443426539E-21	1.73450842318641	0.334	0.114	1.70555063673028E-17	LSEC	CXCL16
2.00794178836243E-21	2.62252499142631	0.236	0.053	3.27515385099796E-17	LSEC	MAN1C1
3.48554357933953E-21	3.09616044194384	0.196	0.03	5.68527013226071E-17	LSEC	IFITM10
3.6425135134963E-21	0.977305712843285	0.584	0.337	5.94130379186381E-17	LSEC	TINAGL1
5.69562967619374E-21	3.98512963737916	0.162	0.014	9.29014156483962E-17	LSEC	NXF3
3.42948493828279E-20	2.48676777815061	0.239	0.059	5.59383288283306E-16	LSEC	SNX8
3.71058944519831E-20	3.22298517459141	0.177	0.023	6.05234244406297E-16	LSEC	FCGR2A
1.27765588487739E-19	1.48968733563071	0.34	0.126	2.08398451382351E-15	LSEC	LRRFIP2
2.26488953628293E-19	1.85082196007076	0.284	0.09	3.69426132263109E-15	LSEC	PARP4
2.63477183088259E-19	1.48144414417497	0.422	0.202	4.2975763333526E-15	LSEC	DAB2
1.113482373753E-18	1.5828143468112	0.327	0.124	1.81620109982852E-14	LSEC	TRIM25
2.55526317055906E-18	1.14430041495616	0.485	0.261	4.16788975749888E-14	LSEC	SLC38A2
4.00849146864338E-18	1.74616031055656	0.326	0.131	6.53825043450422E-14	LSEC	TRIB2
6.77501332806853E-18	2.13908977868063	0.225	0.058	1.10507242394126E-13	LSEC	INMT
1.28426725431044E-17	1.2419781053631	0.457	0.244	2.09476831850575E-13	LSEC	TMEM140
1.60783382569195E-17	2.40383227926384	0.21	0.051	2.62253775308614E-13	LSEC	HS3ST3B1
1.82416323724621E-17	0.871563145751931	0.523	0.299	2.9753926562723E-13	LSEC	WARS
1.91520641064261E-17	1.33669641451603	0.358	0.151	3.12389317639916E-13	LSEC	FCHO2
2.80694307412559E-17	2.65000206296687	0.164	0.025	4.57840484820626E-13	LSEC	RASGEF1B
2.97676250862202E-17	0.846747515133095	0.615	0.409	4.85539732781337E-13	LSEC	PPFIBP1
3.15321970301164E-17	1.4199889635767	0.371	0.166	5.14321665758228E-13	LSEC	BCL3
4.17576833720474E-17	1.33762832437312	0.327	0.131	6.81109573481465E-13	LSEC	LGALS3BP
4.3469716009528E-17	1.62778849672936	0.252	0.076	7.09034537831412E-13	LSEC	C1QTNF1
1.05139015027268E-16	1.02485928179913	0.536	0.328	1.71492247410977E-12	LSEC	NFKBIA
1.30783221350242E-16	1.38755138540918	0.332	0.138	2.1332051234438E-12	LSEC	ANKS1A
2.03379628586445E-16	1.08611463997562	0.464	0.255	3.31732512187351E-12	LSEC	CHST12
2.85492666087815E-16	1.66473283061376	0.268	0.095	4.65667087655835E-12	LSEC	ST3GAL4
4.1295682919059E-16	1.02620189611713	0.425	0.207	6.73573884092771E-12	LSEC	CBLB
4.44196652596629E-16	1.92308441132108	0.239	0.076	7.24529160050361E-12	LSEC	DAPK1
5.18838771107929E-16	1.84958423366468	0.239	0.075	8.46277919554143E-12	LSEC	EDA
6.43710599884231E-16	1.78892184751535	0.294	0.114	1.04995635947117E-11	LSEC	APOE
1.91499737735432E-15	2.84960117571245	0.128	0.014	3.12355222220263E-11	LSEC	SLC7A8
1.99690949192889E-15	4.10350727364517	0.117	0.009	3.25715907228522E-11	LSEC	ANKRD55
2.30264085490492E-15	0.735333692210769	0.693	0.498	3.75583749843541E-11	LSEC	PTPRB
3.5124327948833E-15	2.40086492992248	0.173	0.039	5.72912913173416E-11	LSEC	C2
3.99094400055264E-15	2.03338589745946	0.212	0.061	6.50962875930141E-11	LSEC	ERV3-1
4.02880251286816E-15	1.40720775548691	0.348	0.163	6.57137977873926E-11	LSEC	SERPINB9
4.28680852467447E-15	2.21437403623132	0.205	0.059	6.99221338459654E-11	LSEC	EMILIN1
5.94819462669784E-15	1.13948438911181	0.388	0.194	9.70210025560684E-11	LSEC	AGFG2
6.40574195363412E-15	1.79099713740677	0.319	0.149	1.04484057005726E-10	LSEC	SERPINB1
7.98399682140997E-15	1.2395640305396	0.417	0.226	1.30226972154018E-10	LSEC	PNRC1
1.30888437779352E-14	0.779559748733332	0.549	0.348	2.13492130861901E-10	LSEC	CEMIP2
2.10349659186232E-14	2.78440101511963	0.119	0.012	3.43101329098663E-10	LSEC	SCN9A
2.32060539856598E-14	0.958596180067965	0.543	0.372	3.78513946560098E-10	LSEC	IL32
2.38905472651501E-14	2.83948698394019	0.14	0.023	3.89678716441863E-10	LSEC	DGKG
3.18588680999752E-14	2.47993134477103	0.161	0.036	5.19649997578696E-10	LSEC	RAB3IL1
5.25903086355423E-14	1.03658420828183	0.358	0.17	8.57800524154331E-10	LSEC	SORBS1
5.45216617342237E-14	1.45301273825521	0.302	0.129	8.89302824546923E-10	LSEC	C5AR2
5.74150275591015E-14	1.3708171500862	0.294	0.129	9.36496514516505E-10	LSEC	NFKBIZ
5.9921382165608E-14	0.846665690607921	0.502	0.311	9.77377664503232E-10	LSEC	HMBOX1
6.87971477703043E-14	1.16631969293429	0.422	0.235	1.12215027728143E-09	LSEC	SBNO2

1.43415555295836E-13	0.952901645082927	0.403	0.218	2.33925112243037E-09	LSEC	ST6GAL1
2.02168307565713E-13	1.22685936710609	0.31	0.14	3.29756726470435E-09	LSEC	TSPAN7
2.10636069673064E-13	1.25257398758251	0.294	0.126	3.43568493243735E-09	LSEC	IL33
2.78667917999775E-13	0.935290885445942	0.421	0.226	4.54535241049433E-09	LSEC	DGKE
3.0708780337897E-13	0.744445320218797	0.647	0.467	5.00890916091437E-09	LSEC	WSB1
3.31310441452746E-13	1.1729732322008	0.422	0.24	5.40400461053573E-09	LSEC	CD36
4.03020637849586E-13	0.835993372301632	0.465	0.274	6.5736696239646E-09	LSEC	TMEM44
4.4287250652708E-13	1.9748577878224	0.169	0.044	7.2236934539632E-09	LSEC	CSF1
9.39704399633939E-13	2.12043701513992	0.161	0.042	1.53275184624292E-08	LSEC	RENBP
1.12634716069865E-12	2.01401672361972	0.178	0.053	1.83718485381556E-08	LSEC	PAPSS2
1.14650054476026E-12	1.42895025911846	0.278	0.123	1.87005703855847E-08	LSEC	GNA14
1.22090323759257E-12	0.635740053757589	0.457	0.26	1.99141527083725E-08	LSEC	UPP1
1.56994947197452E-12	1.98448130930292	0.249	0.106	2.56074458373765E-08	LSEC	ZNF117
1.62751766139739E-12	2.64317841590588	0.119	0.019	2.65464405750528E-08	LSEC	NPY1R
1.6589774372942E-12	0.674028208834678	0.618	0.429	2.70595809797056E-08	LSEC	DUSP5
1.66570561288613E-12	2.51179401656526	0.122	0.02	2.71693242517856E-08	LSEC	ABCA6
1.7092852997583E-12	0.917882314739335	0.34	0.168	2.78801525243576E-08	LSEC	ZNF160
1.91959030467334E-12	2.25757169353039	0.141	0.031	3.13104374595268E-08	LSEC	P2RX4
2.51092673882175E-12	0.740218111988507	0.64	0.462	4.09557260369216E-08	LSEC	NID1
3.28657094374728E-12	2.2447655161119	0.12	0.02	5.36072586634619E-08	LSEC	SLC26A5
3.30708954735161E-12	1.95160035752995	0.14	0.031	5.39419376068521E-08	LSEC	LIPG
3.39851308451708E-12	1.36050677316264	0.196	0.064	5.54331469215581E-08	LSEC	C7
4.0411729613523E-12	1.20152014286234	0.364	0.198	6.59155721726174E-08	LSEC	SERPINA1
4.28323110375748E-12	1.71367300405155	0.204	0.072	6.98637825333883E-08	LSEC	C2CD2L
4.86337482405953E-12	2.86424518144265	0.132	0.028	7.93265067552349E-08	LSEC	ERRFI1
5.19628820891434E-12	0.814972303014255	0.526	0.358	8.47566569756018E-08	LSEC	RSRP1
5.46726225231641E-12	1.6847258699053	0.189	0.062	8.9176514597533E-08	LSEC	SEC16B
6.05730541900574E-12	1.11371021304479	0.31	0.149	9.88007086894026E-08	LSEC	DENND4C
6.35482011999157E-12	2.08053446428146	0.133	0.028	1.03653470977183E-07	LSEC	FEZ1
8.09499810625582E-12	0.967734768345553	0.409	0.236	1.32037514111139E-07	LSEC	AKAP17A
1.14719712172316E-11	1.2880359421653	0.223	0.084	1.87119322524265E-07	LSEC	PRICKLE2
1.41397575981113E-11	1.14345421974125	0.326	0.17	2.30633586182793E-07	LSEC	PELO
2.44585939303197E-11	1.00132230608085	0.395	0.235	3.98944125597445E-07	LSEC	IL6ST
2.79120396169162E-11	1.65238222683374	0.265	0.128	4.5527327819152E-07	LSEC	ITPKC
3.00794071523409E-11	0.569201547681266	0.657	0.477	4.90625210061833E-07	LSEC	CFLAR
4.46734912882207E-11	1.45826435320945	0.212	0.081	7.28669316402167E-07	LSEC	CTTNBP2
5.3641350529681E-11	1.12058560515921	0.284	0.135	8.74944068489628E-07	LSEC	STX3
5.98143901493577E-11	0.977257687402886	0.368	0.208	9.75632517726174E-07	LSEC	ACTN1
6.82764128742614E-11	0.73580798068261	0.486	0.311	1.11365657039208E-06	LSEC	ARHGAP26
6.84473149702066E-11	1.68636331698139	0.161	0.05	1.11644415447904E-06	LSEC	SLC9A9
6.8583461068847E-11	1.90127274417909	0.143	0.039	1.11866483349396E-06	LSEC	ECM1
7.24253955183112E-11	1.67676773872648	0.173	0.058	1.18133062629917E-06	LSEC	ANKRD44
7.78219505984375E-11	0.649603590642968	0.535	0.364	1.26935383621111E-06	LSEC	NOSTRIN
9.46453999683971E-11	0.67402927681233	0.563	0.392	1.54376111888453E-06	LSEC	ARHGEF7
9.8795028333254E-11	1.36939788704195	0.222	0.092	1.61144570714371E-06	LSEC	C1orf21
1.00869546989478E-10	1.21363247071847	0.194	0.072	1.64528318094538E-06	LSEC	HSD17B14
1.42291347986344E-10	1.27754724274577	0.246	0.112	2.32091417700526E-06	LSEC	DHX9
1.49191489196281E-10	1.04665513534237	0.361	0.212	2.43346238028054E-06	LSEC	PLSCR1
1.89525047135528E-10	0.658928325661388	0.623	0.473	3.0913430438276E-06	LSEC	MAF
2.11041869517191E-10	2.50918851220195	0.106	0.02	3.4423039336949E-06	LSEC	ONECUT2
2.14305678040821E-10	1.49311843986244	0.173	0.059	3.49553991452384E-06	LSEC	GPR182
3.48019130140554E-10	1.55679796570029	0.21	0.089	5.67654003172258E-06	LSEC	TMEM185A
3.6433441358275E-10	1.43058657212146	0.274	0.138	5.94265861994824E-06	LSEC	MT2A
3.97206195219518E-10	1.00904789481458	0.372	0.221	6.47883025022556E-06	LSEC	PXDN
4.00521244938245E-10	1.60309600322103	0.169	0.059	6.53290202618771E-06	LSEC	STXBP2
4.22627226169494E-10	1.59548814403933	0.175	0.062	6.89347268605062E-06	LSEC	POLE
4.37200270449489E-10	1.86025893070619	0.119	0.028	7.13117361130162E-06	LSEC	VMO1
6.33542873837994E-10	1.57890145018395	0.17	0.061	1.03337178151715E-05	LSEC	MTCL1
6.59091124500122E-10	1.62385104631796	0.193	0.078	1.07504353317215E-05	LSEC	LHFPL2
6.60800499580958E-10	1.89879005152882	0.159	0.054	1.0778316948665E-05	LSEC	ARRB2

6.97906512050371E-10	2.44012287152088	0.114	0.026	1.13835531180536E-05	LSEC	PPM1H
7.23302547002119E-10	1.3501052360463	0.185	0.07	1.17977878441516E-05	LSEC	SMAP2
7.33711893524721E-10	2.09733756432761	0.117	0.028	1.19675746952817E-05	LSEC	IL18R1
7.48374148247773E-10	0.866965774591525	0.335	0.185	1.22067307320694E-05	LSEC	SLC23A2
8.79225883920303E-10	1.41535323722868	0.265	0.137	1.43410533926241E-05	LSEC	CTSB
1.09667515100129E-09	0.719698102907591	0.445	0.283	1.7887868387982E-05	LSEC	AFDN
1.21068613868308E-09	1.16220787055861	0.262	0.131	1.97475016080596E-05	LSEC	DEPRECATED-ENSG00000188313
1.32817526090726E-09	1.62655487344007	0.185	0.073	2.16638666806583E-05	LSEC	FAM102A
1.52288779766088E-09	0.85256002080869	0.327	0.179	2.48398228676467E-05	LSEC	AP2A2
1.584721601304E-09	0.963356501490648	0.315	0.173	2.58483940388696E-05	LSEC	LGALS8
1.6540186268282E-09	1.49605668867616	0.193	0.079	2.69786978221948E-05	LSEC	C7orf26
1.80121196406103E-09	1.17211995582644	0.286	0.149	2.93795683457994E-05	LSEC	FUT1
1.85056234220739E-09	1.38984761785289	0.149	0.048	3.01845223637448E-05	LSEC	TOX2
2.0571366975023E-09	1.22967743988097	0.212	0.092	3.355395667296E-05	LSEC	HELZ2
2.14560199260197E-09	1.82703033264549	0.151	0.051	3.49969141013307E-05	LSEC	TIAM1
2.1503954603035E-09	1.09572948268511	0.138	0.042	3.50751003530104E-05	LSEC	RGS16
2.58020688769482E-09	0.772267526344893	0.488	0.344	4.20857545451902E-05	LSEC	SELENOP
2.84463576762635E-09	0.532037686473456	0.493	0.327	4.63988540057534E-05	LSEC	ADM
3.72558586272342E-09	1.55351282255616	0.132	0.039	6.07680310068817E-05	LSEC	ATP1B1
3.95902590492137E-09	0.884146063719326	0.364	0.221	6.45756715351725E-05	LSEC	NCOA7
4.04764156042231E-09	1.27529477371485	0.23	0.109	6.60210814920483E-05	LSEC	SREBF1
4.87270301633094E-09	0.877050985103905	0.361	0.222	7.94786588993739E-05	LSEC	PHLDB2
5.50882618494596E-09	1.50937984108998	0.173	0.068	8.98544639026536E-05	LSEC	PCBP4
5.64079759564698E-09	0.786970879074698	0.462	0.32	9.20070495825979E-05	LSEC	ARHGAP23
6.10997045615456E-09	0.720690918291222	0.403	0.267	9.9659728110337E-05	LSEC	SHC2
6.43581646871208E-09	0.996634842347418	0.234	0.112	0.000104974602421163	LSEC	KCNK6
6.7641625414287E-09	2.33010834923812	0.106	0.026	0.000110330255213244	LSEC	PLAC8
7.15016198602715E-09	2.48439780126317	0.106	0.026	0.000116626292154089	LSEC	BMP2
7.47626734993264E-09	0.614007957631208	0.469	0.314	0.000121945396744751	LSEC	AKAP12
1.66171998682687E-08	1.259990687359	0.186	0.079	0.000271043147051331	LSEC	CABLES1
1.67638107717178E-08	1.85931227825634	0.135	0.045	0.000273434517497489	LSEC	TGFB3
1.68530579386581E-08	0.73659088730795	0.39	0.244	0.000274890228037452	LSEC	GSTK1
1.83184659489397E-08	1.50306529510605	0.161	0.064	0.000298792498093155	LSEC	MARCH2
1.86800607067977E-08	1.29017824824518	0.167	0.065	0.000304690470188577	LSEC	WASHC5
1.89030495005443E-08	1.56399654914178	0.138	0.047	0.000308327640403379	LSEC	PLIN5
2.45900688124108E-08	0.975070348698895	0.331	0.204	0.000401088612399233	LSEC	DEPRECATED-ENSG00000196329
2.46891355623237E-08	0.919613302429659	0.313	0.182	0.000402704490157062	LSEC	NAMPT
2.60860077074259E-08	0.511537959263132	0.698	0.579	0.000425488871715824	LSEC	FCGRT
3.06715312977636E-08	2.72122187466329	0.109	0.031	0.000500283346997822	LSEC	RBP4
3.53403522875606E-08	1.3805661993608	0.181	0.079	0.000576436486162401	LSEC	NXPE3
4.56144827298603E-08	1.35887941245909	0.191	0.089	0.000744017827806751	LSEC	TRAF6
4.8368412054925E-08	1.74754713372074	0.114	0.034	0.000788937169027882	LSEC	MGME1
5.52978654146645E-08	0.656494136272777	0.421	0.274	0.000901963482778593	LSEC	RCN3
5.5926512448471E-08	0.694958468497495	0.334	0.194	0.000912217344547011	LSEC	SNTB1
5.59483748234708E-08	0.888040256872972	0.313	0.185	0.000912573941745633	LSEC	NISCH
5.68360592710214E-08	1.44783353757423	0.127	0.042	0.000927052962769631	LSEC	MAP7D3
5.99380928977393E-08	0.783048693524551	0.305	0.176	0.000977650233255025	LSEC	RNF149
6.08208366964301E-08	1.55636594458062	0.119	0.037	0.000992048667355471	LSEC	DEPRECATED-ENSG00000145287
6.98958315765635E-08	0.472468987294239	0.384	0.232	0.00114007090884533	LSEC	ATF3
8.67489161805025E-08	0.581679152824159	0.472	0.319	0.00141496157182018	LSEC	CCNL1
8.939339397682E-08	0.37221956066274	0.477	0.32	0.00145809564915591	LSEC	CLDN5
1.04147674091166E-07	0.629163325013923	0.52	0.39	0.001698752712101	LSEC	PGS1
1.15996601047184E-07	1.01835199098174	0.233	0.12	0.00189202055968062	LSEC	IPPK
1.47005262210962E-07	0.754569440271035	0.416	0.294	0.002397802831923	LSEC	DLC1
1.5003684237305E-07	1.22388667024443	0.177	0.079	0.00244725093594682	LSEC	ZNF462
1.53896652516876E-07	0.565158502878625	0.465	0.322	0.00251020829920277	LSEC	NLRP1
1.55601542330773E-07	1.99413792303473	0.116	0.039	0.00253801675695725	LSEC	PRUNE2
1.65139871166097E-07	1.04932087535468	0.236	0.126	0.00269359643859021	LSEC	GADD45B
1.78693250901336E-07	0.982544053372202	0.236	0.124	0.00291466561545169	LSEC	CLN8
1.83407033441063E-07	0.60240385537827	0.348	0.218	0.00299155212245718	LSEC	ABCC1

1.98853338211314E-07	0.857451146784558	0.217	0.107	0.00324349679956474	LSEC	MEIS1
2.04591900237244E-07	0.516849089737601	0.401	0.258	0.00333709848476968	LSEC	GBP4
2.13815823803285E-07	0.948638371230584	0.318	0.201	0.00348754990205537	LSEC	CALU
2.17166746631166E-07	1.25249505248504	0.204	0.103	0.00354220680430095	LSEC	CTSD
2.33177706107091E-07	1.13683893881217	0.205	0.101	0.00380336156431276	LSEC	RAPH1
2.39398029248745E-07	1.21849128403549	0.144	0.056	0.00390482125507628	LSEC	UAP1L1
2.83425266052667E-07	1.95162828973379	0.109	0.036	0.00462294951458505	LSEC	SLC7A1
2.92489023984055E-07	1.35194225767032	0.136	0.053	0.00477078847020393	LSEC	ARHGAP12
3.11302972730469E-07	1.63047876740367	0.146	0.062	0.00507766278820668	LSEC	TMEM106C
3.26042955077719E-07	0.996516005193817	0.263	0.149	0.00531808664027267	LSEC	YES1
3.37026651946675E-07	1.03379024108052	0.186	0.089	0.00549724171990221	LSEC	GJA4
3.40025442850036E-07	2.00013880926318	0.135	0.053	0.00554615499832693	LSEC	FGF23
3.48633161585474E-07	0.977896007680285	0.279	0.165	0.00568655549862067	LSEC	FCHSD2
3.72352571432583E-07	1.24083003769829	0.183	0.087	0.00607344279263686	LSEC	SLC35F6
4.34425717446011E-07	1.06416530271498	0.318	0.21	0.00708591787726188	LSEC	FGFR1
4.94218465481497E-07	0.735876242646462	0.347	0.219	0.00806119739046869	LSEC	SEMA6A
5.04474179449947E-07	1.20924315774977	0.197	0.101	0.00822847834100808	LSEC	TSN
5.66762197699258E-07	0.710418542761084	0.43	0.302	0.0092444582066726	LSEC	PARP14
6.10500627136953E-07	1.52797212627825	0.124	0.047	0.00995787572923084	LSEC	BMP6
6.48528410428753E-07	1.03425497277196	0.18	0.086	0.0105781469025034	LSEC	NUP42
6.57064045691561E-07	1.25399799672295	0.177	0.084	0.0107173716492751	LSEC	CFP
7.645602477856E-07	0.803197480941965	0.265	0.152	0.0124707422016309	LSEC	RASSF1
9.66191544878374E-07	0.576522571522493	0.361	0.236	0.0157595502885112	LSEC	MED10
9.69811376690165E-07	0.94852671500305	0.209	0.109	0.0158185933651933	LSEC	GPHN
1.03988621242187E-06	1.2729611274475	0.154	0.068	0.0169615840108131	LSEC	TGFBR3
1.12503026667088E-06	0.301169134992491	0.828	0.768	0.0183503686796687	LSEC	LENG8
1.16403225175621E-06	0.579780405673869	0.339	0.213	0.0189865300583956	LSEC	TTLL5
1.29958233614427E-06	1.00082568873948	0.202	0.104	0.0211974874848491	LSEC	GNA13
1.31304931215966E-06	0.561174785319943	0.419	0.299	0.0214171473306361	LSEC	OGT
1.36665422293714E-06	1.57557007768932	0.103	0.034	0.0222914970303277	LSEC	CXorf40A
1.5203053932582E-06	1.06283478378845	0.209	0.112	0.0247977012694345	LSEC	RALGAPA2
1.55122620383839E-06	0.815395424434959	0.234	0.132	0.0253020506108079	LSEC	STK38
1.74795220536359E-06	1.56887714029131	0.128	0.053	0.0285108484216856	LSEC	SLC37A4
1.89414857027851E-06	0.826335227045429	0.287	0.177	0.0308954573298128	LSEC	LRCH4
1.92022788279706E-06	0.811443047101117	0.308	0.196	0.0313208369963028	LSEC	MAP3K6
2.05129500179407E-06	0.589194085071316	0.416	0.3	0.0334586727742631	LSEC	TPP1
2.1411406604928E-06	1.2220428663655	0.146	0.065	0.034924145313298	LSEC	ABLIM3
2.14606547722212E-06	1.53792614153215	0.138	0.059	0.03500447399897	LSEC	MT1F
2.73056953783343E-06	0.724201532188514	0.215	0.117	0.044538319731601	LSEC	CPED1
2.93486348622131E-06	0.802723572646441	0.294	0.188	0.0478705583237559	LSEC	CHSY1
2.95481636047539E-06	0.992384955323854	0.204	0.112	0.048196009655714	LSEC	MAPK12
3.00053522575435E-06	1.3661153455019	0.154	0.073	0.0489417300672792	LSEC	ACAA1
3.01592356176859E-06	0.651572291094796	0.318	0.205	0.0491927292160074	LSEC	TBC1D15
3.0559034657425E-06	0.716201138819235	0.189	0.095	0.0498448414297259	LSEC	WDFY2
9.5752901673517E-158	3.6604041356544	0.896	0.197	1.56182557919674E-153	Tumor E	PLVAP
7.06143955961762E-123	4.86184741507227	0.66	0.034	1.15179140656923E-118	Tumor E	RGCC
2.22926213996216E-97	2.22026895440534	0.892	0.572	3.63614947649228E-93	Tumor E	IVNS1ABP
4.33529509093368E-95	5.15771634374058	0.552	0.036	7.07129982282192E-91	Tumor E	CA2
1.51338608186519E-94	2.38251364648834	0.808	0.265	2.46848403813032E-90	Tumor E	VWF
1.62348341071002E-92	3.51539308715018	0.646	0.109	2.64806379120911E-88	Tumor E	SPRY1
1.62712421842407E-89	3.91632983983553	0.58	0.07	2.6540023126715E-85	Tumor E	VWA1
1.53644659117103E-65	2.35184300462996	0.64	0.187	2.50609803485907E-61	Tumor E	EMP1
1.85045114231021E-65	2.02054040798633	0.776	0.382	3.01827085822219E-61	Tumor E	NR4A1
2.31602471751174E-64	2.98510635357072	0.561	0.137	3.7776679167334E-60	Tumor E	PDGFB
5.68623538311999E-63	4.97967522972475	0.37	0.011	9.27481853340702E-59	Tumor E	COL15A1
7.21214594330074E-60	2.55360393275672	0.63	0.253	1.17637312481178E-55	Tumor E	GSN
5.63479931633319E-58	1.32896653818646	0.922	0.775	9.19092116487107E-54	Tumor E	COL4A1
1.10919693823577E-57	2.98371642601431	0.42	0.047	1.80921112595637E-53	Tumor E	IGFBP5
9.79735365278524E-56	1.30177161310955	0.889	0.791	1.5980463543058E-51	Tumor E	SPARC
4.14682429180084E-53	3.05695393756478	0.469	0.099	6.76388510235636E-49	Tumor E	STC1

1.38528058227146E-47	1.2018978585756	0.64	0.233	2.25953115774298E-43	Tumor E	VIM
9.82950796712003E-47	1.03417822159276	0.919	0.798	1.60329104451695E-42	Tumor E	COL4A2
3.14915962766596E-45	2.79946242552108	0.381	0.059	5.13659426868595E-41	Tumor E	SLCO2A1
1.21573423216486E-42	1.37745520023815	0.824	0.619	1.9829841060841E-38	Tumor E	FLT1
1.34969417647614E-42	1.79049596679526	0.656	0.336	2.20148617125024E-38	Tumor E	MCF2L
9.31532292468114E-42	3.07395601972214	0.353	0.056	1.51942232224474E-37	Tumor E	MPZL2
2.93484492132865E-41	2.1588723287345	0.51	0.18	4.78702555117916E-37	Tumor E	STC2
2.06617021877568E-39	1.33230565113422	0.79	0.524	3.37013024384501E-35	Tumor E	DEPRECATED-ENSG00000059804
3.1718265532392E-38	2.28177349075212	0.439	0.134	5.17356629098846E-34	Tumor E	LAMB1
3.83878807657347E-38	1.02601281211867	0.852	0.691	6.26144723169898E-34	Tumor E	IGFBP4
3.85058415251186E-36	3.31148142950155	0.307	0.049	6.28068781116209E-32	Tumor E	CX3CL1
1.66815364486717E-35	2.11283069541058	0.446	0.152	2.72092541014285E-31	Tumor E	APOLD1
2.48226366151829E-35	1.39181156250778	0.64	0.345	4.04882025830249E-31	Tumor E	PECAM1
6.77562431221875E-34	1.58453229511401	0.586	0.293	1.105172081566E-29	Tumor E	PLPP1
2.66846362487813E-32	2.40894479608503	0.342	0.079	4.35253101853871E-28	Tumor E	AQP1
3.18180163890529E-32	0.781476229426408	0.926	0.831	5.18983665321841E-28	Tumor E	DUSP1
4.89747989855696E-32	3.05379190846898	0.265	0.036	7.98827946253625E-28	Tumor E	FABP4
5.19444915774372E-31	2.18125594611557	0.337	0.08	8.47266602119578E-27	Tumor E	CD34
7.26621703628118E-31	2.37928283596145	0.303	0.06	1.18519266078782E-26	Tumor E	GJA1
2.78035663149444E-30	1.65309298925384	0.501	0.219	4.53503970163059E-26	Tumor E	CLEC14A
3.49254024812052E-30	1.24116240631686	0.732	0.511	5.69668239870938E-26	Tumor E	SLC2A3
3.61769879784345E-30	2.0597255061747	0.406	0.137	5.90082850916245E-26	Tumor E	PODXL
1.45487149543338E-29	8.03172384992963	0.173	0.001	2.37304089620138E-25	Tumor E	KCNK5
3.0348458172764E-29	1.65324002733008	0.511	0.23	4.95013701255954E-25	Tumor E	DLL1
1.45869182674668E-27	1.80498217527668	0.425	0.162	2.37927223860651E-23	Tumor E	DOCK9
4.95329208856472E-27	3.68805217735266	0.201	0.019	8.07931472565792E-23	Tumor E	CPNE5
4.96816321249309E-27	3.74800134690742	0.21	0.023	8.10357101589748E-23	Tumor E	CD300LG
1.16442419318739E-26	2.473382741135	0.298	0.076	1.89929230150794E-22	Tumor E	JAG1
1.51149966940767E-26	3.64643231728299	0.183	0.011	2.46540711077085E-22	Tumor E	RBP7
1.58619002709555E-25	1.78670107093086	0.384	0.139	2.58723455319555E-21	Tumor E	CDH13
4.53199360940421E-25	2.48195995392205	0.247	0.047	7.3921347762992E-21	Tumor E	PLAT
4.91402964366277E-25	1.48417094828523	0.556	0.325	8.01527375177835E-21	Tumor E	GRB10
5.90553811605196E-25	1.83477135657746	0.287	0.07	9.63252322109234E-21	Tumor E	OLFML2A
7.29243129660734E-25	2.07467665478257	0.302	0.079	1.18946846878962E-20	Tumor E	SYNPO
4.95845259689383E-24	2.75144327855366	0.224	0.039	8.08773203079353E-20	Tumor E	RASGRF2
6.91637477312493E-24	0.810042026053988	0.845	0.748	1.12812988924441E-19	Tumor E	HSPG2
1.22759071859473E-23	1.77748547251232	0.437	0.203	2.00232322109986E-19	Tumor E	ADAMTS9
1.2652756308753E-23	1.44471804468699	0.508	0.266	2.0637910815207E-19	Tumor E	PCDH12
2.0037601131664E-23	1.41773749306034	0.478	0.229	3.26833312058571E-19	Tumor E	ADGRG1
2.34930215887382E-23	2.10459158604044	0.316	0.099	3.83194675133908E-19	Tumor E	ITGA6
2.75639096708573E-23	1.69938324962513	0.381	0.143	4.49594930641354E-19	Tumor E	JCAD
1.82376845460781E-22	2.47324694589033	0.233	0.047	2.9747487263108E-18	Tumor E	THY1
2.0056770709014E-22	3.31311446365192	0.178	0.02	3.27145987034728E-18	Tumor E	COL21A1
2.58039680243668E-22	2.91664679378246	0.201	0.031	4.20888522445446E-18	Tumor E	INHBB
2.96125027235203E-22	1.92225330864842	0.319	0.103	4.8300953192334E-18	Tumor E	TNK2
1.09060860436142E-21	3.51295963593234	0.164	0.016	1.77889169457391E-17	Tumor E	SPARCL1
1.12067423121378E-21	1.87584389863668	0.374	0.156	1.8279317385328E-17	Tumor E	DUSP6
1.68914714037417E-21	2.12679244012806	0.307	0.103	2.75516790066431E-17	Tumor E	HTRA1
2.43018457895215E-21	1.54743772264344	0.476	0.246	3.96387406672885E-17	Tumor E	FN1
1.08192551214931E-20	0.984499331399648	0.661	0.476	1.76472870286675E-16	Tumor E	A2M
1.16832180068049E-20	1.50939481249509	0.418	0.189	1.90564968908994E-16	Tumor E	LAMA4
6.57397306964736E-20	1.3610255028094	0.423	0.202	1.07228074739018E-15	Tumor E	AGRN
9.41186628378256E-20	3.2051711402678	0.162	0.02	1.53516950954777E-15	Tumor E	NID2
1.08635390928926E-19	2.65439424884444	0.215	0.05	1.7719518614417E-15	Tumor E	DHRS3
2.00733954034329E-19	0.81963912978272	0.868	0.83	3.27417152425394E-15	Tumor E	ENG
4.03286168845249E-19	2.30200551142323	0.235	0.063	6.578000700003485E-15	Tumor E	P2RY8
1.28392799604082E-18	1.17671462539918	0.446	0.23	2.09421495434219E-14	Tumor E	EGR1
1.65101833610919E-18	1.84027988495462	0.282	0.096	2.6929760080277E-14	Tumor E	ENTPD1
1.75226743734387E-18	2.24172339154051	0.198	0.041	2.85812341705158E-14	Tumor E	MMP2
1.9938142024417E-18	0.950369879696035	0.646	0.481	3.25211034560265E-14	Tumor E	MARCKS

2.36606754577601E-18	1.1298260626781	0.506	0.298	3.85929277391524E-14	Tumor E	DEPRECATED-ENSG00000175899
2.41978277684447E-18	2.00628260128935	0.266	0.087	3.94690768731102E-14	Tumor E	DEPRECATED-ENSG00000139318
2.5816713398737E-18	2.85700835878645	0.196	0.043	4.21096412246799E-14	Tumor E	SMOX
3.06304538376008E-18	2.67470325775577	0.15	0.019	4.99613332545106E-14	Tumor E	MAFB
9.65002963096215E-18	4.37334356020008	0.123	0.009	1.57401633310624E-13	Tumor E	JPH1
1.40330472045871E-17	1.85003489158083	0.22	0.057	2.28893032954021E-13	Tumor E	JAG2
2.21210246308284E-17	2.13766897520025	0.222	0.062	3.60816032753441E-13	Tumor E	DNM3
3.29602792420814E-17	1.12205541286898	0.4	0.195	5.37615114717589E-13	Tumor E	AHNAK
7.58690405162243E-17	0.939563247188744	0.633	0.459	1.23749991986014E-12	Tumor E	SEMA3F
7.84648087369678E-17	0.965828437597492	0.578	0.383	1.27983949530868E-12	Tumor E	EXOC3L2
8.49523402037612E-17	2.17053120383641	0.194	0.046	1.38565762106355E-12	Tumor E	ITM2A
9.69910280506766E-17	1.71845880664545	0.317	0.136	1.58202065853459E-12	Tumor E	RASA4
9.89364213380249E-17	1.78596272469683	0.266	0.093	1.61375196844452E-12	Tumor E	TMOD2
3.14116501178312E-16	4.0329717369648	0.115	0.009	5.12355425071944E-12	Tumor E	CD200
3.52871058940244E-16	1.62064016254451	0.284	0.109	5.75567984237431E-12	Tumor E	EMCN
6.10150537752703E-16	2.44503573263438	0.164	0.033	9.95216542128435E-12	Tumor E	ANGPTL2
7.62829347325281E-16	1.17877920513389	0.427	0.232	1.24425094842227E-11	Tumor E	APLNR
8.78436206008869E-16	2.35072060846609	0.182	0.044	1.43281729562107E-11	Tumor E	LBH
1.34490007014752E-15	3.93877029989027	0.111	0.009	2.19366650441761E-11	Tumor E	KCNN3
1.47334724645575E-15	3.57244908114463	0.108	0.007	2.40317669369398E-11	Tumor E	PDGFD
1.85563504216169E-15	3.46167541058467	0.143	0.024	3.02672631726993E-11	Tumor E	TSPAN12
2.02728046357393E-15	2.11426942885247	0.168	0.036	3.30669716413543E-11	Tumor E	MGLL
4.49611455072387E-15	2.13045803920202	0.21	0.064	7.33361244368571E-11	Tumor E	NR4A2
5.10129004257551E-15	0.937814453447181	0.573	0.375	8.32071418844492E-11	Tumor E	DDIT4
9.19456784062792E-15	2.06087778746017	0.19	0.053	1.49972596048482E-10	Tumor E	NEURL1B
1.02150689534559E-14	1.09764031617937	0.504	0.33	1.66617989699819E-10	Tumor E	TP53I11
1.29733062814744E-14	2.85869034653836	0.131	0.02	2.11607598757129E-10	Tumor E	ABCG2
1.42821538523915E-14	1.11035523505485	0.591	0.448	2.32956211486357E-10	Tumor E	HSPA1B
1.60989626493013E-14	1.20726788427537	0.422	0.239	2.62590179772753E-10	Tumor E	JUP
1.67396201320369E-14	1.26477274574373	0.39	0.209	2.73039943973654E-10	Tumor E	NDRG1
2.30262892262897E-14	2.15532878322342	0.192	0.056	3.75581803570012E-10	Tumor E	TSC22D1
2.98958921367762E-14	1.23372737720526	0.434	0.247	4.87631896642957E-10	Tumor E	PITPNC1
1.27723464776856E-13	3.61924098039982	0.102	0.01	2.0832974339753E-09	Tumor E	CDKN2B
1.55403428643631E-13	0.687001446958442	0.771	0.682	2.53478532460627E-09	Tumor E	IGFBP7
2.08710700266596E-13	1.99258208516629	0.171	0.046	3.40428023204845E-09	Tumor E	SLC16A3
2.09102140864891E-13	3.36459277180362	0.16	0.041	3.41066501964724E-09	Tumor E	ESM1
2.46026234223413E-13	3.4281218103921	0.101	0.01	4.01293390641808E-09	Tumor E	MDFI
2.56648408149533E-13	2.17414335169353	0.123	0.02	4.18619218532703E-09	Tumor E	CCND2
2.97452400052186E-13	1.43840915115684	0.404	0.237	4.8517460972512E-09	Tumor E	TNFAIP3
7.75224095924771E-13	1.3278457508414	0.399	0.242	1.26446802286289E-08	Tumor E	METRNL
8.8468726941677E-13	1.78732347635411	0.178	0.053	1.44301340514569E-08	Tumor E	ACVRL1
9.27079706368415E-13	2.95171031130741	0.101	0.011	1.51215970905752E-08	Tumor E	MRC2
1.35921353415644E-12	0.84917474975457	0.64	0.525	2.21701319556257E-08	Tumor E	EPAS1
1.98622530593286E-12	0.916730586043633	0.571	0.412	3.23973209650709E-08	Tumor E	INSR
3.54525719127275E-12	2.76453030426482	0.125	0.026	5.78266900468499E-08	Tumor E	ITGA10
4.08023229278424E-12	1.34023807690825	0.305	0.15	6.65526689276038E-08	Tumor E	TSPAN15
5.52213641990102E-12	0.821976711972362	0.395	0.226	9.00715671450055E-08	Tumor E	DLL4
5.63907293078949E-12	3.32971380030221	0.118	0.023	9.19789185741074E-08	Tumor E	NR4A3
6.91044468869346E-12	1.13875874983411	0.356	0.199	1.12716263317279E-07	Tumor E	CALCRL
8.16072669346248E-12	1.62774215119151	0.247	0.107	1.33109613097067E-07	Tumor E	DIPK2B
8.3622762717509E-12	1.54224687092269	0.192	0.066	1.36397088268529E-07	Tumor E	SULF2
1.30957962777653E-11	0.801902641308974	0.497	0.332	2.13605533086629E-07	Tumor E	ID3
1.76615272187409E-11	0.801447799261536	0.624	0.501	2.88077170464882E-07	Tumor E	TGFBR2
1.99297497958542E-11	0.681441810984737	0.596	0.454	3.25074148920178E-07	Tumor E	ITGA5
2.10466694794819E-11	1.13256383939735	0.33	0.175	3.4329222587983E-07	Tumor E	LTBP3
2.55398974874433E-11	1.05616023109065	0.358	0.206	4.16581267917688E-07	Tumor E	SOX18
2.82662439485741E-11	1.92132566204204	0.145	0.039	4.61050705045192E-07	Tumor E	GNAI1
3.48114651057075E-11	1.97459271394464	0.148	0.041	5.67809807339195E-07	Tumor E	ZFYVE28
3.65640572436912E-11	1.30525852027523	0.326	0.179	5.96396337701847E-07	Tumor E	HLX
3.94902744579551E-11	1.6477218213439	0.229	0.099	6.44125866683706E-07	Tumor E	GAS6

5.66317314328757E-11	0.798174136989116	0.614	0.489	9.23720171401635E-07	Tumor E	COL18A1
8.54490320468033E-11	0.784841004928881	0.58	0.451	1.39375916171541E-06	Tumor E	TCF4
1.11490367693376E-10	1.07201821413004	0.372	0.225	1.81851938744666E-06	Tumor E	S1PR1
1.67076454236959E-10	1.39192969031245	0.268	0.134	2.72518404505904E-06	Tumor E	IER5L
1.86613773052133E-10	0.623567628344743	0.704	0.624	3.04385725225334E-06	Tumor E	PLXND1
2.98292677660383E-10	1.80283747354802	0.176	0.064	4.8654518653185E-06	Tumor E	CXCL12
3.37810164131686E-10	0.765680381238902	0.608	0.498	5.51002158715194E-06	Tumor E	DYSF
3.7935611304686E-10	1.37943254790212	0.302	0.166	6.18767755990734E-06	Tumor E	TDRP
4.01906795191078E-10	1.19118183311075	0.272	0.136	6.55550173636167E-06	Tumor E	FMNL3
4.07223107921981E-10	1.21338618631167	0.298	0.162	6.64221611331542E-06	Tumor E	RAMP2
4.1235341913119E-10	0.88128165266162	0.52	0.398	6.72589661944884E-06	Tumor E	SHC1
4.2819953218781E-10	1.03217571032165	0.349	0.209	6.98436256951536E-06	Tumor E	TMEM204
4.5500343385982E-10	0.64814359868428	0.631	0.505	7.42156100968753E-06	Tumor E	ACTB
5.57997116760865E-10	1.03709344478817	0.328	0.183	9.10149097148648E-06	Tumor E	GASK1B
6.45518557165654E-10	1.79578424717958	0.145	0.044	1.0529053185929E-05	Tumor E	BDKRB2
9.69722118844643E-10	2.49094550428063	0.104	0.023	1.5817137480475E-05	Tumor E	OSBPL3
1.08794323685875E-09	1.48235996113299	0.229	0.107	1.77454421364031E-05	Tumor E	ZNF521
1.12906655998335E-09	1.06029161321663	0.272	0.139	1.84162046598885E-05	Tumor E	BCAM
1.16794245210148E-09	1.50545154170285	0.224	0.104	1.90503093362273E-05	Tumor E	LOXL2
1.29480030656504E-09	1.53449308309049	0.175	0.066	2.11194878003824E-05	Tumor E	ALPK3
1.40387964205812E-09	1.02177000251907	0.451	0.316	2.289868084161E-05	Tumor E	GABRE
1.47306267944351E-09	1.73073926369295	0.217	0.102	2.4027125364403E-05	Tumor E	SLC29A1
1.48937697419048E-09	0.683497631312464	0.552	0.428	2.42932278260209E-05	Tumor E	LMNA
1.57553409500331E-09	0.86959571337594	0.494	0.348	2.5698536623599E-05	Tumor E	KDR
1.62454294438206E-09	1.64282277718364	0.162	0.059	2.64979199658158E-05	Tumor E	PMEPA1
1.97868553622605E-09	1.5284733685502	0.152	0.052	3.22743397813831E-05	Tumor E	PLD1
2.07919303779011E-09	1.00317556277488	0.321	0.19	3.39137176393944E-05	Tumor E	DPYSL2
2.25680377199242E-09	1.15300542797247	0.277	0.146	3.68107263249684E-05	Tumor E	SOX4
2.41561116710146E-09	0.887357485711273	0.34	0.2	3.9401033746592E-05	Tumor E	FKBP1A
2.60121080432988E-09	2.06490393406243	0.12	0.033	4.24283494294246E-05	Tumor E	DIAPH2
2.63440815404689E-09	0.448521353244708	0.905	0.896	4.29698314006589E-05	Tumor E	CD74
3.32037848828465E-09	1.11343223868957	0.236	0.113	5.41586935224108E-05	Tumor E	MAFF
3.95798882001277E-09	1.53273809717401	0.187	0.079	6.45587556432283E-05	Tumor E	MYO1E
5.45576519048528E-09	1.01377428823666	0.354	0.216	8.89889860220054E-05	Tumor E	KCNE3
5.57921123320044E-09	0.489634767458313	0.644	0.535	9.10025144247324E-05	Tumor E	MMRN2
8.8835627270284E-09	0.605225425820581	0.63	0.512	0.00014489979164056	Tumor E	IER2
1.36922219622458E-08	0.610276875337493	0.589	0.451	0.000223333832426192	Tumor E	PXN
1.53931976650383E-08	1.40046194904082	0.166	0.066	0.000251078447114439	Tumor E	PTPRK
1.69185827189015E-08	1.22994958773911	0.265	0.149	0.000275959002728002	Tumor E	RIN3
1.76322039210149E-08	0.992919528753665	0.384	0.265	0.000287598878155674	Tumor E	ARHGEF15
1.79512813347789E-08	0.815857946743739	0.517	0.399	0.000292803349851578	Tumor E	LRRC32
1.92919683529782E-08	2.17888919436828	0.101	0.026	0.000314671295805427	Tumor E	EGR3
2.96928474046064E-08	0.73914541139183	0.668	0.597	0.000484320034016535	Tumor E	JUNB
3.24196111223481E-08	0.932610800858392	0.485	0.376	0.00052879627701662	Tumor E	PLK2
3.68756403218938E-08	1.03520057833113	0.395	0.276	0.000601478569290409	Tumor E	PLCG1
4.25784141950582E-08	0.669965884657942	0.598	0.486	0.000694496513935594	Tumor E	ARRDC3
4.33539643142022E-08	1.36361482459926	0.229	0.119	0.000707146511928951	Tumor E	ROBO1
6.30052573608014E-08	1.34835145866931	0.132	0.047	0.00102767875281203	Tumor E	PALMD
6.7881436714855E-08	1.65210257728077	0.136	0.05	0.001107214114256	Tumor E	PCDH1
7.39457853795038E-08	1.55298993496403	0.146	0.057	0.00120612970532509	Tumor E	TSPAN13
7.87292386622449E-08	1.25387314834316	0.422	0.322	0.00128415261181988	Tumor E	SPRY4
8.75540724664784E-08	0.577571621443219	0.372	0.24	0.00142809447600073	Tumor E	MT-ATP6
9.48216730527522E-08	1.178520623992	0.333	0.222	0.00154663630916344	Tumor E	LIMD1
1.04991877299123E-07	0.685216718065696	0.654	0.544	0.001712522510626	Tumor E	TCIM
1.19001908541478E-07	1.58413213212685	0.139	0.054	0.00194104013022005	Tumor E	ZNF366
1.29308578366185E-07	0.873599328751879	0.393	0.268	0.00210915222173084	Tumor E	SMAD7
1.3560675180532E-07	1.03998010640527	0.208	0.103	0.00221188172869658	Tumor E	CCND1
1.64790110264341E-07	0.515692931482984	0.728	0.678	0.00268789148852166	Tumor E	BHLHE40
1.68756594737417E-07	1.59840872108282	0.12	0.041	0.002752588816762	Tumor E	ARHGAP18
2.00051307227543E-07	1.24376338423874	0.229	0.124	0.00326303687218846	Tumor E	GPR4

2.14604830839736E-07	1.11370910555137	0.187	0.089	0.00350041939582693	Tumor E	MAST3
2.23133006595019E-07	1.03462758528492	0.28	0.173	0.00363952247057136	Tumor E	ZEB1
2.35740857880893E-07	1.75246577034752	0.132	0.052	0.00384516913289525	Tumor E	CTSH
2.71960530999444E-07	2.23485248710179	0.101	0.031	0.00443594822113194	Tumor E	LRRFIP1
3.18784620550453E-07	1.53227364624362	0.129	0.049	0.00519969594579843	Tumor E	DEPRECATED-ENSG00000124831
3.5717482450435E-07	0.685968380724985	0.504	0.385	0.00582587856249045	Tumor E	HSPA1A
5.54620842893338E-07	1.71377633697909	0.115	0.041	0.00904642056843324	Tumor E	GALNT15
5.98500435923075E-07	1.06556378643306	0.268	0.163	0.00976214061034128	Tumor E	EHD4
6.68875890894058E-07	0.980685723793498	0.282	0.175	0.010910034656373	Tumor E	ASPH
6.71254429487957E-07	0.847679582196537	0.342	0.23	0.0109488309993781	Tumor E	ECSCR
6.90038876991287E-07	1.63376921262371	0.123	0.047	0.0112552241226049	Tumor E	ADAMTSL2
7.43440568413343E-07	0.486232941484663	0.519	0.412	0.01212625911139	Tumor E	MT-ND6
7.4744690858484E-07	1.0433735280196	0.342	0.235	0.0121916065259273	Tumor E	TMEM184B
7.5168293241255E-07	0.87465539003211	0.337	0.222	0.0122607003105811	Tumor E	HIC1
7.8124911257168E-07	0.945490471282758	0.212	0.11	0.0127429542751567	Tumor E	ST5
8.33173089239994E-07	1.33959297735309	0.106	0.036	0.0135898862585935	Tumor E	HES4
8.37381293714897E-07	1.31520911276067	0.189	0.094	0.0136585262817837	Tumor E	TANC2
8.66611076721051E-07	1.62801936635708	0.102	0.034	0.0141352932723971	Tumor E	LYST
9.60912254283555E-07	1.01079424091576	0.295	0.186	0.0156734397796191	Tumor E	C15orf39
1.09774152963994E-06	0.7761906073161	0.295	0.185	0.0179052620899571	Tumor E	SEMA6B
1.1809943607823E-06	1.5968706722522	0.178	0.09	0.0192631990187201	Tumor E	PRDM1
1.66404054118594E-06	0.405987752692087	0.709	0.632	0.0271421652672838	Tumor E	CD93
1.93049092200125E-06	1.19576596442154	0.18	0.09	0.0314882374287624	Tumor E	LAMC1
2.10220214562979E-06	1.63795958088206	0.108	0.04	0.0342890191973676	Tumor E	DOK4
2.17001155757704E-06	1.08951256731446	0.148	0.069	0.035395058515639	Tumor E	CCN1
2.30708446452077E-06	0.916266714486942	0.448	0.351	0.0376308547007982	Tumor E	FOS
2.55072427027862E-06	0.664812063326648	0.437	0.323	0.0416048635725146	Tumor E	DENND3
2.59713389252594E-06	0.796747483322682	0.411	0.305	0.0423618509209906	Tumor E	PML
2.7106708303035E-06	0.4980026464631	0.601	0.519	0.0442137519130803	Tumor E	SCARF1
2.94511310672437E-06	1.25886332628225	0.131	0.056	0.0480377398837813	Tumor E	THSD7A
5.06218305418105E-179	8.11533980170598	0.829	0.006	8.25692677967471E-175	Venous I	LYPD2
6.70841822163905E-138	5.69106495657389	0.714	0.008	1.09421009613154E-133	Venous I	SELP
2.68003883315683E-130	6.99134697653118	0.571	0.003	4.37141134076211E-126	Venous I	RSPO3
1.55257494530459E-128	6.6405141103311	0.686	0.009	2.53240499328632E-124	Venous I	CPE
6.75049417142984E-104	7.16327715292794	0.6	0.01	1.10107310430192E-99	Venous I	NTS
3.43905597854501E-98	5.33789858197955	0.857	0.034	5.60944420660477E-94	Venous I	FBLN2
8.50717099250085E-92	5.84033802548411	0.8	0.032	1.38760466058681E-87	Venous I	PTGDS
5.09266174793106E-87	8.48726129209971	0.343	0.001	8.30664057705036E-83	Venous I	CADM3
3.57293170152155E-82	5.97950691308618	0.486	0.008	5.82780889835181E-78	Venous I	ACKR1
4.99605056125052E-80	5.00546981205413	0.514	0.011	8.14905807045573E-76	Venous I	WNT2
2.50992009399211E-67	5.40506741048302	0.743	0.042	4.09393066531054E-63	Venous I	SELE
1.50384285367903E-65	5.06907436285698	0.4	0.007	2.45291807863586E-61	Venous I	CLDN11
2.03640575451881E-65	6.68989275371765	0.286	0.002	3.32158142619563E-61	Venous I	P2RX1
2.35320268708107E-63	5.0720879758378	0.514	0.017	3.83830890289794E-59	Venous I	CDH23
9.88445164990122E-59	5.45235951640595	0.343	0.006	1.61225290861539E-54	Venous I	COL13A1
7.04860683059391E-54	4.27758309860881	0.514	0.022	1.14969826013817E-49	Venous I	PTGIS
1.67025029540567E-53	4.65930221547914	0.429	0.014	2.72434525683619E-49	Venous I	LSR
8.24576776797049E-52	4.3205766116339	0.457	0.017	1.34496718063367E-47	Venous I	LHX6
1.39733701929724E-49	4.485586956228	0.914	0.11	2.27919641217573E-45	Venous I	CLU
5.54143015316611E-48	10.2372275994016	0.171	0	9.03862672282924E-44	Venous I	FAM43B
1.44119424297936E-43	3.65237201252609	0.629	0.047	2.35073192972363E-39	Venous I	LRATD2
2.07201433376675E-43	3.65743307058365	0.714	0.063	3.37966257980695E-39	Venous I	PTGS1
3.67668404870431E-43	3.5630137895526	0.514	0.03	5.9970393518416E-39	Venous I	VCAN
1.76704750537391E-40	3.53732764673974	0.371	0.015	2.88223118601538E-36	Venous I	SCARA3
3.02641561057612E-40	9.8675494514536	0.143	0	4.93638650241071E-36	Venous I	SMPDL3B
1.11306282544317E-39	3.85099039747571	0.486	0.029	1.81551677458036E-35	Venous I	LTC4S
2.03235075496991E-39	4.39907662873541	0.6	0.05	3.31496731643141E-35	Venous I	TAGLN
1.01488649688914E-38	4.89373196651538	0.457	0.028	1.65538136507587E-34	Venous I	EMP3
3.91386875551322E-38	3.45628912583638	0.8	0.1	6.38391132711761E-34	Venous I	ACKR3
1.12182402396355E-36	3.3569748226749	0.4	0.02	1.82980716548694E-32	Venous I	PLA1A

4.74618698811727E-36	4.00578672880609	0.914	0.179	7.74150559631809E-32	Venous I	PRSS23
8.03024793096288E-36	5.63839692138814	0.171	0.002	1.30981374001935E-31	Venous I	PLAC9
9.95250658764284E-36	5.68646164881556	0.171	0.002	1.62335334951042E-31	Venous I	CELF4
3.35505000344527E-35	3.33265752982663	0.829	0.119	5.47242206061958E-31	Venous I	ALPL
1.47312007427642E-34	3.99767440637349	0.343	0.015	2.40280615315226E-30	Venous I	ARL4D
1.04679834085074E-33	3.3320800999966	0.4	0.023	1.70743277376164E-29	Venous I	DPYSL3
1.18073509228318E-33	3.24688503261206	0.543	0.046	1.92589700902309E-29	Venous I	RASSF9
6.31993779487266E-33	3.10548692286807	0.743	0.094	1.03084505372168E-28	Venous I	PGM5
1.63966956597841E-32	9.22511137391595	0.114	0	2.67446502906739E-28	Venous I	SLURP1
7.22974490285896E-32	4.16985420929523	0.514	0.046	1.17924369110533E-27	Venous I	IER3
1.24270250818801E-31	4.26462777183305	0.171	0.002	2.02697206110546E-27	Venous I	LCNL1
2.67020073095332E-31	3.292497455062	0.6	0.063	4.35536441225797E-27	Venous I	DEPRECATED-ENSG00000145287
4.75421598762557E-30	3.49623007588371	0.343	0.019	7.75460169741607E-26	Venous I	CCDC69
7.43976118881384E-30	3.07037343319151	0.314	0.015	1.21349944750743E-25	Venous I	SELENBP1
7.18457057350126E-29	3.56023702578699	0.229	0.007	1.17187530624379E-24	Venous I	RGN
9.2573072030293E-29	3.17086797892265	0.743	0.111	1.50995937788611E-24	Venous I	C7
1.34509403031128E-28	5.64883554626338	0.143	0.002	2.19398287284073E-24	Venous I	WNT9B
1.45947980597648E-28	4.68823014951163	0.143	0.002	2.38055751152824E-24	Venous I	GDNF
3.83050130323718E-28	3.2714982655145	0.429	0.034	6.24793067571016E-24	Venous I	HS3ST1
5.07465587291595E-28	4.58451061339555	0.257	0.011	8.27727119431321E-24	Venous I	SAMD5
5.43380751663838E-28	3.18533427595402	0.514	0.05	8.86308344038887E-24	Venous I	NDUFA8
2.16240688771983E-27	2.62057632916651	0.571	0.062	3.52710187455981E-23	Venous I	JAM2
4.5040490867198E-27	2.8543622589538	0.486	0.046	7.34655446534866E-23	Venous I	FGL2
1.13271483430111E-26	2.5053736454655	0.771	0.122	1.84757116622854E-22	Venous I	INMT
7.82050660313507E-26	2.6115138673299	0.8	0.145	1.27560283203736E-21	Venous I	C1QTNF1
1.68194547894708E-25	5.27205926977572	0.2	0.006	2.74342127071058E-21	Venous I	TGFB2
6.74974375628085E-25	3.12103628019867	0.4	0.034	1.10095070408697E-20	Venous I	DUSP23
7.30935441576057E-25	2.95961796699536	1	0.409	1.19222879875471E-20	Venous I	RNASE1
4.61331069859058E-24	2.54700602905043	0.971	0.266	7.5247710804711E-20	Venous I	IL1R1
1.2539505942686E-23	4.77483792884861	0.171	0.005	2.04531881431151E-19	Venous I	CPXM2
3.63154590000082E-23	2.781819397369	0.429	0.041	5.92341451749133E-19	Venous I	FGFR3
5.27121884690366E-23	2.27102327426639	0.857	0.175	8.59788506118456E-19	Venous I	ACP5
6.47617329670414E-23	2.65834240264919	0.4	0.037	1.05632862642541E-18	Venous I	ADIRF
1.66721715000602E-22	3.15232873087522	0.343	0.028	2.71939789337482E-18	Venous I	SRPX
2.52671699591858E-22	3.48620071603048	0.257	0.015	4.1213280920428E-18	Venous I	RNF112
2.87371911552765E-22	3.58583142213773	0.257	0.015	4.68732324933715E-18	Venous I	SYT15
4.07983399382945E-22	4.41515439195224	0.143	0.003	6.65461722733522E-18	Venous I	HJV
1.22506140914409E-21	5.41451444711529	0.114	0.002	1.99819766445492E-17	Venous I	CHCHD4
1.75772257702391E-21	2.83127511275627	0.457	0.052	2.8670212953837E-17	Venous I	SLC22A17
1.93781325313308E-21	2.27674450557572	0.714	0.128	3.16076719718536E-17	Venous I	TNXB
3.53828394342013E-21	3.12459916079596	0.257	0.015	5.77129494011257E-17	Venous I	PALLD
7.11781100020892E-21	3.99241741626639	0.2	0.009	1.16098615224408E-16	Venous I	TMOD1
7.11781100020892E-21	3.94060813932665	0.2	0.009	1.16098615224408E-16	Venous I	ST8SIA6
2.22747484993536E-20	2.80736488747573	0.514	0.07	3.63323422772957E-16	Venous I	COL5A1
2.79848312951582E-20	2.37894395933859	0.886	0.257	4.56460583255326E-16	Venous I	LY6E
3.12301129573462E-20	2.76099185319898	0.457	0.054	5.09394372447273E-16	Venous I	PLAC8
7.20314445000626E-20	4.63327390490649	0.143	0.004	1.17490489124052E-15	Venous I	DEPRECATED-ENSG00000159228
1.0746392274995E-19	3.89819404985732	0.2	0.01	1.75284404397444E-15	Venous I	PRICKLE1
1.28711980188116E-19	3.16348247648075	0.4	0.045	2.09942110884836E-15	Venous I	HRCT1
1.6053577078444E-19	2.74846290301683	0.371	0.037	2.61849895726501E-15	Venous I	TSHZ2
1.73539027994788E-19	3.44534923956531	0.286	0.022	2.83059508562299E-15	Venous I	LRG1
3.47069128393816E-19	2.37624094988316	0.914	0.262	5.66104455323152E-15	Venous I	LIFR
3.57895262509677E-19	2.36340785468543	0.543	0.081	5.83762962679534E-15	Venous I	CCND3
1.5406071868359E-18	3.99998458492736	0.114	0.002	2.51288438244803E-14	Venous I	NRXN2
1.56982352982413E-18	2.48317940964209	0.714	0.16	2.56053915949614E-14	Venous I	GLUL
1.6672569668076E-18	3.28004122573032	0.286	0.024	2.71946283855988E-14	Venous I	SLC8A1
1.69571337523498E-18	2.31187781439784	0.343	0.033	2.76587808634578E-14	Venous I	MARVELD1
2.58410155362531E-18	3.0875435065358	0.229	0.015	4.21492804411824E-14	Venous I	CES2
2.95212774112635E-18	2.39545645719072	0.257	0.019	4.81521555855118E-14	Venous I	FAM107A
5.27127142271713E-18	1.7739893099288	0.486	0.067	8.59797081759391E-14	Venous I	MT1B

8.91765387767113E-18	2.64347982637925	0.371	0.041	1.45455852398694E-13	Venous I	PROS1
1.24419308419967E-17	1.69287589582418	0.829	0.18	2.02940333963809E-13	Venous I	LTK
3.12643699883041E-17	2.21701212615092	0.429	0.056	5.09953138879229E-13	Venous I	ECHDC2
4.44145250009211E-17	2.43104118128625	0.314	0.032	7.24445317290023E-13	Venous I	COL3A1
4.69008086196259E-17	1.97328587793398	1	0.535	7.64999089394718E-13	Venous I	RAMP3
5.4410070726645E-17	1.93293069056433	0.343	0.037	8.87482663622307E-13	Venous I	ARL4C
8.51440010241613E-17	1.77201666512266	0.543	0.086	1.38878380070509E-12	Venous I	RHOU
9.21586846194981E-17	2.82740031124069	0.229	0.016	1.50320030482863E-12	Venous I	NOL7
3.02383099316293E-16	3.24492532631133	0.171	0.009	4.93217073294806E-12	Venous I	CMKLR1
3.1861422519312E-16	4.82650293841193	0.114	0.003	5.19691662712497E-12	Venous I	ADGRV1
4.94416368392765E-16	3.3780933737761	0.2	0.013	8.0644253848544E-12	Venous I	SLC26A10
8.16298475377558E-16	2.48205207880148	0.486	0.081	1.33146444318833E-11	Venous I	PALM
1.04253905453828E-15	1.97475428796198	0.514	0.085	1.7004854518574E-11	Venous I	DEPRECATED-ENSG00000184500
1.28972093279652E-15	1.69254876690426	0.543	0.095	2.10366381348441E-11	Venous I	APOC3
2.50290264477884E-15	3.06793744074361	0.257	0.024	4.08248450389877E-11	Venous I	LINGO1
3.66964661659668E-15	2.04288117643512	0.6	0.125	5.98556059633084E-11	Venous I	SERPING1
3.70275202934432E-15	3.72605028304507	0.143	0.006	6.03955883506352E-11	Venous I	STARD10
4.30182841316232E-15	3.65386471704035	0.143	0.006	7.01671232470907E-11	Venous I	MYO7B
4.31142793893339E-15	2.15677479398302	0.371	0.049	7.03237011119425E-11	Venous I	S100A6
7.72634555168877E-15	1.90833455408345	0.857	0.253	1.26024422293596E-10	Venous I	CRIM1
1.32996782925952E-14	1.9318149481858	0.543	0.103	2.16931052630521E-10	Venous I	PLAAT4
1.33775306160936E-14	3.42019422865892	0.2	0.015	2.18200901879103E-10	Venous I	SULT1C2
1.43324672096252E-14	2.60896769053602	0.629	0.147	2.33776872656197E-10	Venous I	CXCL2
1.48269689032033E-14	2.36254589911214	0.314	0.037	2.41842689780149E-10	Venous I	ALDH2
1.75992673971083E-14	2.33278875978758	0.714	0.193	2.87061650514233E-10	Venous I	VCAM1
1.81696327202598E-14	3.07558807466401	0.171	0.011	2.96364879300158E-10	Venous I	GATA6
2.17470026052301E-14	2.67902142591565	0.229	0.019	3.54715359493908E-10	Venous I	LYNX1
2.41454082411694E-14	3.94213783367381	0.114	0.004	3.93835753821713E-10	Venous I	WFDC1
2.60232646473013E-14	3.46341137108925	0.114	0.004	4.24465469662132E-10	Venous I	CDH11
5.28908285524671E-14	3.54715142315058	0.143	0.007	8.62702304519291E-10	Venous I	BMX
5.34278296223183E-14	2.34988731209428	0.314	0.038	8.71461328969634E-10	Venous I	TNFRSF6B
5.99014017857932E-14	1.45914377647971	1	0.897	9.77051764528073E-10	Venous I	CD74
6.0882834218205E-14	1.79829465047026	0.514	0.097	9.93059908933142E-10	Venous I	NBL1
6.31965694727697E-14	2.48033634689177	0.143	0.007	1.03079924467035E-09	Venous I	TNFRSF12A
7.55825441065491E-14	2.2467045693469	0.229	0.02	1.23282687692192E-09	Venous I	PGF
7.7115250322064E-14	4.08358911975547	0.171	0.011	1.25782684800319E-09	Venous I	TMEM223
8.06906565941365E-14	1.51538094349251	0.6	0.123	1.31614529970696E-09	Venous I	HSPB8
1.02352243336075E-13	2.11668019805562	0.314	0.039	1.66946744105472E-09	Venous I	MT1E
1.49595468485488E-13	2.66925966221059	0.229	0.021	2.4400516864668E-09	Venous I	NPR 2.00
1.79683743762212E-13	2.22376487797809	0.4	0.065	2.93082154450544E-09	Venous I	C1R
2.28943639982189E-13	2.19112184317316	0.257	0.027	3.73429971174948E-09	Venous I	NHP2
2.31282618042714E-13	1.73666006236364	0.914	0.468	3.77245078289471E-09	Venous I	TGM2
2.80664749611671E-13	1.57084574606829	0.971	0.427	4.57792273091597E-09	Venous I	TIMP1
3.30034251918591E-13	1.62828293262713	0.8	0.251	5.38318868304414E-09	Venous I	APOL1
5.64360325382248E-13	4.2173757596966	0.114	0.005	9.20528126730985E-09	Venous I	NDUFB3
5.95192727400491E-13	1.40648146501991	0.457	0.082	9.70818857662942E-09	Venous I	KCTD12
6.40027786920589E-13	2.28407922078856	0.314	0.041	1.04394932324617E-08	Venous I	RDH10
6.85009605532074E-13	2.41182240674048	0.171	0.012	1.11731916758337E-08	Venous I	MORN4
6.90484833004728E-13	2.96931847284484	0.114	0.005	1.12624981111401E-08	Venous I	STMN3
6.90484833004728E-13	3.96342825196111	0.114	0.005	1.12624981111401E-08	Venous I	SHISAL1
9.75144589697634E-13	1.59998095972425	0.771	0.227	1.59055834025581E-08	Venous I	GOLM1
1.85376675056619E-12	2.3121614066532	0.229	0.023	3.02367894684851E-08	Venous I	RRAS
2.10800184527699E-12	2.04210258101663	0.286	0.036	3.43836180983129E-08	Venous I	NUPR1
2.25733946763089E-12	1.81918398810119	0.571	0.132	3.68194640565275E-08	Venous I	HYAL1
2.76008080095367E-12	1.52855024772051	0.971	0.495	4.50196779443553E-08	Venous I	VWF
3.50555906471127E-12	2.06387846897731	0.286	0.037	5.71791739045055E-08	Venous I	COX7A1
3.66120371460943E-12	3.31480305415678	0.143	0.009	5.97178937889945E-08	Venous I	ABAT
3.67987587326471E-12	1.68521418405485	0.714	0.214	6.00224553688207E-08	Venous I	LGALS3BP
4.50720525994721E-12	1.74286294859392	0.457	0.085	7.35170249949989E-08	Venous I	PMP22
4.9930219572175E-12	1.69740278488898	0.857	0.427	8.14411811441747E-08	Venous I	IFI27

5.78161745760987E-12	2.90243565471586	0.2	0.019	9.43039623510746E-08	Venous I	UBL5
6.27163608612728E-12	1.65370921369577	0.857	0.292	1.02296656200822E-07	Venous I	ECE1
6.62361644965525E-12	2.2461440371802	0.371	0.063	1.08037807910327E-07	Venous I	FOXC1
7.79745639215516E-12	2.52426027751825	0.2	0.019	1.27184311212443E-07	Venous I	DHH
8.23807782846447E-12	1.93759986351242	0.457	0.09	1.34371287460084E-07	Venous I	ANXA4
8.31551517441752E-12	2.59176932706532	0.171	0.014	1.35634368009924E-07	Venous I	RGS5
9.47894922878532E-12	2.22762937353709	0.314	0.045	1.54611140870717E-07	Venous I	NMI
1.06710482890287E-11	2.76124874270373	0.314	0.048	1.74055468642347E-07	Venous I	CCL2
1.12229120593111E-11	2.03397555053399	0.286	0.038	1.83056918599423E-07	Venous I	TTR
1.34781116972958E-11	3.25781882796039	0.2	0.019	2.19841479894592E-07	Venous I	TMTC1
1.63109317497275E-11	2.34404860555939	0.286	0.039	2.66047607769806E-07	Venous I	TBL2
1.7746538035679E-11	2.09791638711724	0.343	0.055	2.8946378189996E-07	Venous I	TSTA3
2.16337727617231E-11	1.75522744535641	0.229	0.025	3.52868467516466E-07	Venous I	ORMDL2
2.75759518919366E-11	1.62211646318815	0.457	0.09	4.49791351309378E-07	Venous I	TIAM1
3.07684013943855E-11	2.00521046664727	0.743	0.258	5.01863395143822E-07	Venous I	CEBPD
3.34427025165748E-11	2.15578375828552	0.257	0.032	5.45483920747851E-07	Venous I	CENPB
3.39623447332779E-11	2.70655950337194	0.171	0.015	5.53959804944496E-07	Venous I	ARHGEF25
4.65663166438894E-11	1.06714021735014	0.686	0.192	7.5954319077848E-07	Venous I	MT2A
5.13526560646652E-11	1.70584474522547	0.486	0.107	8.37613173070754E-07	Venous I	HSPA12B
5.14731857203215E-11	1.71437278180784	0.514	0.116	8.39579132284164E-07	Venous I	MEIS2
6.30969224064919E-11	1.71225890233415	0.457	0.095	1.02917390137229E-06	Venous I	PROCR
6.32923538693525E-11	1.25093158645645	1	0.705	1.03236158396301E-06	Venous I	APP
7.58598934316963E-11	1.62309757101131	0.686	0.201	1.2373507217644E-06	Venous I	ICAM1
7.83412261845814E-11	3.13936027331292	0.171	0.015	1.27782374029671E-06	Venous I	DEPRECATED-ENSG00000204397
7.88607984927999E-11	1.41715585886412	0.571	0.147	1.28629848421606E-06	Venous I	CST3
9.05334136591274E-11	3.03319591115883	0.143	0.011	1.47669051019403E-06	Venous I	FAH
9.08527011633998E-11	2.14871584162653	0.314	0.05	1.48189840867621E-06	Venous I	PELI2
9.26147902204235E-11	3.43971293913456	0.143	0.011	1.51063984328533E-06	Venous I	ADCY1
1.04016634038755E-10	2.01779644239144	0.314	0.05	1.69661531780612E-06	Venous I	CTBS
1.05393307041757E-10	2.17855147543664	0.171	0.015	1.71907023115811E-06	Venous I	TMEM8B
1.10492879273885E-10	2.5737273653267	0.114	0.006	1.80224935383634E-06	Venous I	DHRS13
1.11880417295221E-10	1.97792574063438	0.143	0.011	1.82488148650235E-06	Venous I	DBNDD2
1.16097992461668E-10	1.71444395138327	0.457	0.097	1.89367435504227E-06	Venous I	MAPK7
1.18692110866428E-10	1.44235514956331	0.771	0.265	1.9359870203423E-06	Venous I	CAVIN1
1.26454339584375E-10	2.03744421542694	0.2	0.021	2.06259673296074E-06	Venous I	DEPRECATED-ENSG00000108691
1.41950612466375E-10	1.5077250856219	0.714	0.22	2.31535643993905E-06	Venous I	SLC40A1
1.44481137503909E-10	1.67046240788498	0.457	0.097	2.35663183382625E-06	Venous I	HNRNPAB
1.76222807196253E-10	2.47691590446715	0.286	0.044	2.87437020817809E-06	Venous I	ETV5
1.98668789489893E-10	1.9599763133333	0.257	0.035	3.24048662536964E-06	Venous I	PLCD1
2.00546800383071E-10	1.85572044999359	0.314	0.051	3.27111886104827E-06	Venous I	SAP18
2.62883098273823E-10	1.47629819688179	0.429	0.088	4.28788621594433E-06	Venous I	FLOT2
2.7277109614474E-10	2.07572762787812	0.286	0.043	4.44916934921685E-06	Venous I	METTL7A
2.81019570478457E-10	1.36226092826753	0.457	0.098	4.58371021407412E-06	Venous I	SLC6A6
2.89445259147951E-10	1.4451187621285	0.486	0.106	4.72114162196222E-06	Venous I	SDCBP
3.26452126689273E-10	2.09513833230041	0.257	0.036	5.32476063842872E-06	Venous I	ATP5MC3
3.59529426180582E-10	1.89972170881855	0.657	0.221	5.86428447043147E-06	Venous I	THBD
3.7907397727957E-10	2.00375124489828	0.371	0.072	6.18307564340707E-06	Venous I	DGKG
4.11138845893227E-10	1.64039542901306	0.571	0.155	6.70608571536443E-06	Venous I	EID1
4.52333666978349E-10	1.4026864773197	0.857	0.421	7.37801444208385E-06	Venous I	TMSB10
5.59042484578512E-10	1.79204513436053	0.257	0.037	9.11854196596011E-06	Venous I	MMRN1
5.93602251528327E-10	2.05078255621286	0.2	0.023	9.68224632467855E-06	Venous I	KLKB1
7.47745838704251E-10	1.87594512443972	0.286	0.045	1.2196482375105E-05	Venous I	CTNNBL1
7.54551193818401E-10	3.2395860209423	0.114	0.007	1.23074845223719E-05	Venous I	CBR1
7.62043600788338E-10	1.71832237068279	0.371	0.072	1.24296931724586E-05	Venous I	CHMP4B
1.28262786690965E-09	2.81661707512239	0.143	0.012	2.09209431371633E-05	Venous I	MRAS
1.35383372717034E-09	2.80292204822204	0.143	0.012	2.20823819238753E-05	Venous I	TLR3
1.35509267524635E-09	2.10582304254194	0.2	0.024	2.21029166259432E-05	Venous I	RRS1
1.39686761971771E-09	1.83054016678837	0.2	0.024	2.27843077452155E-05	Venous I	NHEJ1
1.44827335016296E-09	2.67275128079229	0.143	0.012	2.3622786614508E-05	Venous I	GPD1L
1.57021907784354E-09	1.16199656508382	0.286	0.047	2.5611843378706E-05	Venous I	IGFBP3

1.90118724136829E-09	1.636652796858	0.6	0.193	3.10102650939582E-05	Venous I	TLNRD1
2.04067693586702E-09	1.54514320376392	0.8	0.345	3.32854815009269E-05	Venous I	CHST12
2.04376474163254E-09	1.6787269009593	0.314	0.055	3.33358467007684E-05	Venous I	INPP5K
2.04867933047885E-09	1.49831016981775	0.943	0.596	3.34160085594405E-05	Venous I	HYAL2
2.12310462956933E-09	1.78468303679967	0.257	0.038	3.46299596129053E-05	Venous I	C1RL
2.12954471518844E-09	2.04553911415793	0.314	0.057	3.47350038494386E-05	Venous I	KCTD17
2.1416549412642E-09	1.11492021534241	0.714	0.207	3.49325337469604E-05	Venous I	FCHSD2
2.16707595682398E-09	2.15965660167521	0.286	0.048	3.53471759317559E-05	Venous I	KRAS
2.4678162230553E-09	1.76058210824331	0.686	0.275	4.0252550414255E-05	Venous I	CD59
2.5243691949587E-09	1.35056655515604	0.743	0.281	4.11749859389714E-05	Venous I	MT-CO2
2.5284748284448E-09	1.81501022979649	0.6	0.201	4.12419529267632E-05	Venous I	FXYD5
3.12864671604188E-09	1.53119249154968	0.543	0.147	5.10313565853592E-05	Venous I	BSG
3.45877078882616E-09	1.23233317133016	0.4	0.083	5.64160103365435E-05	Venous I	EHD2
3.57114588120492E-09	3.71588979819794	0.114	0.008	5.82489604683334E-05	Venous I	PCYT2
3.89150434783458E-09	2.17965371331207	0.286	0.05	6.34743274175298E-05	Venous I	SESN3
3.97171294437422E-09	2.75040356700541	0.143	0.013	6.47826098356879E-05	Venous I	ITGA2B
4.1766791281524E-09	2.90602721950701	0.114	0.008	6.81258132592939E-05	Venous I	ERBB2
4.24308455897356E-09	1.44680270564503	0.371	0.076	6.92089522414177E-05	Venous I	LRP10
4.31301039799839E-09	1.48466504665861	0.4	0.089	7.03495126017518E-05	Venous I	MT1F
4.44591412138974E-09	2.40893104166919	0.114	0.008	7.25173052339881E-05	Venous I	LYPD5
4.75671007173444E-09	1.94545742694858	0.286	0.049	7.75866979800605E-05	Venous I	MAP3K8
4.9200680628175E-09	2.11241371572616	0.2	0.025	8.02512301726163E-05	Venous I	ELK1
5.72766911268244E-09	1.68214806857886	0.371	0.08	9.34240108969633E-05	Venous I	PCDH1
6.14587298581002E-09	1.28021128758477	0.743	0.282	0.000100245334271547	Venous I	FTL
6.39737737678337E-09	1.53207964509897	0.229	0.032	0.000104347622392713	Venous I	BLOC1S3
6.93663654633554E-09	1.22719947814504	0.886	0.357	0.000113143478707279	Venous I	SLC38A2
8.03375602413578E-09	1.42704970929196	0.457	0.11	0.000131038594509679	Venous I	IFI35
8.54482495636684E-09	2.76348258784679	0.2	0.027	0.0001393746398633	Venous I	CXCL3
8.91030899333306E-09	2.71225265347741	0.143	0.014	0.000145336049990256	Venous I	IL6
9.2064227517724E-09	1.32289647231566	0.343	0.067	0.00015016596150416	Venous I	RARB
9.99806205005064E-09	1.66708695271882	0.371	0.08	0.000163078390098376	Venous I	GNAI3
1.01458647851903E-08	1.43110849114017	0.429	0.1	0.00016548920051124	Venous I	FBXL7
1.04794990777106E-08	1.10470354910263	0.457	0.106	0.000170931109456537	Venous I	NCSTN
1.08824887274635E-08	2.09465834704188	0.2	0.026	0.000177504273633658	Venous I	B4GALT2
1.22603647162432E-08	1.50878877792107	0.429	0.103	0.000199978808886642	Venous I	PA2G4
1.26894460380129E-08	2.4425360276832	0.143	0.014	0.000206977554326029	Venous I	TBC1D2
1.32911191052337E-08	1.58416469349832	0.429	0.104	0.000216791443725467	Venous I	KAZN
1.36817480511904E-08	1.40638481903893	0.343	0.069	0.000223162992462966	Venous I	DEPRECATED-ENSG00000205937
1.37672487929993E-08	3.17234515956083	0.114	0.009	0.000224557595062611	Venous I	CALCA
1.38414883940289E-08	2.05898694878993	0.2	0.027	0.000225768517195005	Venous I	UBFD1
1.41754856667154E-08	3.84663483466627	0.114	0.009	0.000231216346709795	Venous I	GSTM3
1.45526543897248E-08	1.22737487822492	0.429	0.097	0.000237368345750801	Venous I	TMEM115
1.55435511026359E-08	1.86718750645369	0.257	0.042	0.000253530862035095	Venous I	TAGLN2
1.56718940766666E-08	2.44032272411011	0.257	0.044	0.000255624264284509	Venous I	GBA
1.63349152543793E-08	0.835696198883135	0.743	0.266	0.00026643880271418	Venous I	SERPINA1
1.66397995265993E-08	2.99203578151969	0.114	0.009	0.000271411770078362	Venous I	NRG3
1.67475230046737E-08	1.17503840194854	0.943	0.513	0.000273168847729233	Venous I	LIMS2
1.68753939836647E-08	1.14856570404342	0.743	0.284	0.000275254551267555	Venous I	EDNRB
1.70831183235445E-08	1.34123909506004	0.486	0.125	0.000278642742975334	Venous I	IFITM2
1.71531527495053E-08	0.730008625509114	0.914	0.361	0.000279785074497182	Venous I	ALB
1.7594405781709E-08	1.23746789598435	0.686	0.227	0.000286982352705456	Venous I	CHSY1
1.76352451272727E-08	2.82492020209179	0.114	0.009	0.000287648483270945	Venous I	PLPP6
1.76352451272727E-08	2.39593350717545	0.114	0.009	0.000287648483270945	Venous I	ZNHIT2
1.82011985492836E-08	1.5734123048234	0.457	0.115	0.000296879749537364	Venous I	PLSCR4
2.12710018126365E-08	1.31799285219762	0.571	0.165	0.000346951310565914	Venous I	TSC22D2
2.24662004106066E-08	1.73539213253461	0.257	0.043	0.000366446194897404	Venous I	DBP
2.29356428238781E-08	1.74048783283037	0.343	0.072	0.000374103270100276	Venous I	EXT1
2.36463378463462E-08	1.56665569629528	0.6	0.19	0.000385695416611753	Venous I	OSMR
2.5559418071212E-08	1.08055901810198	0.686	0.22	0.000416899668159539	Venous I	SERPINB1
2.60232479249694E-08	1.23974026706492	0.829	0.412	0.000424465196904176	Venous I	SLC9A3R2

2.80657045823898E-08	1.98535188321111	0.229	0.036	0.000457779707443359	Venous I	PDZD4
3.16788044627396E-08	1.1381490512029	0.543	0.146	0.000516712979591746	Venous I	TMED9
3.18680489705647E-08	2.53503138529797	0.143	0.015	0.000519799746758881	Venous I	RASSF5
3.26128357270033E-08	2.32086099263887	0.143	0.015	0.000531947963543151	Venous I	CYP1B1
3.58920168959405E-08	1.21653912221125	0.629	0.197	0.000585434687589686	Venous I	LAMTOR4
3.65418558251584E-08	2.16673596503675	0.171	0.021	0.000596034210364158	Venous I	TMED1
4.03147610328813E-08	1.57649387566658	0.343	0.072	0.000657574067207326	Venous I	MCU
4.09811050853261E-08	1.24527805010435	0.886	0.526	0.000668442805046755	Venous I	BST2
4.15843902493791E-08	1.31328278949039	0.771	0.361	0.000678282989357622	Venous I	ITM2B
4.3416922746455E-08	1.25850245217783	0.771	0.301	0.000708173426917428	Venous I	TEK
4.55042510015996E-08	1.76189595202197	0.286	0.054	0.00074221983808709	Venous I	CBX5
4.6496857433203E-08	1.27527774300499	0.514	0.148	0.000758410241592974	Venous I	PLEC
4.99246660174445E-08	1.16995309999737	0.514	0.146	0.000814321227410538	Venous I	ZFP36L2
5.39396357515853E-08	1.53919443200628	0.257	0.045	0.000879809398744108	Venous I	TPRG1L
5.7229592330915E-08	1.71264756733064	0.286	0.054	0.000933471880509555	Venous I	CD320
5.75718980097408E-08	1.83559261776798	0.257	0.045	0.000939055228436882	Venous I	C19orf12
5.88486260453905E-08	1.84050815971434	0.229	0.037	0.000959879939426364	Venous I	DDR2
5.95139398343467E-08	1.0931818463278	0.829	0.308	0.000970731872638029	Venous I	SELENON
6.06014774576162E-08	3.01092768098125	0.114	0.01	0.000988470698811179	Venous I	CKB
6.20823753860844E-08	1.83991316406885	0.257	0.045	0.00101262562492242	Venous I	FAM110D
6.20967181256389E-08	1.49878102454747	0.314	0.064	0.0010128595693473	Venous I	CDC123
6.24779057499751E-08	1.32440966133713	0.343	0.073	0.00101907712068784	Venous I	CEBPG
6.48190859537634E-08	1.10125666112487	0.6	0.182	0.00105726411099183	Venous I	APLP2
6.48440504808037E-08	2.39134055247369	0.114	0.01	0.00105767130739239	Venous I	OCIAD2
6.51187318714972E-08	2.20048859622133	0.2	0.029	0.00106215163555599	Venous I	BOK
6.53606088548619E-08	1.56663743873262	0.286	0.054	0.00106609689103165	Venous I	C7orf50
6.57263586211483E-08	2.47802664885222	0.114	0.01	0.00107206263546955	Venous I	PFN2
6.94027455360805E-08	1.17334973306188	0.857	0.4	0.00113202818243901	Venous I	MT-CO1
6.9469016759596E-08	1.05610603985945	0.229	0.037	0.00113310913236577	Venous I	FABP1
7.36472276308916E-08	1.10819163005278	0.514	0.143	0.00120125992988747	Venous I	RIN2
7.4495284366113E-08	2.4130177658181	0.2	0.03	0.00121509258329567	Venous I	CLIC2
7.60259415217557E-08	1.32484738352859	0.286	0.054	0.00124005913216136	Venous I	DCUN1D3
7.75070905058169E-08	1.3982090074744	0.4	0.097	0.00126421815324038	Venous I	S100A10
8.13248736277717E-08	1.38398406299628	0.229	0.037	0.00132649001374258	Venous I	FBXO34
8.15236832940734E-08	2.38011939757634	0.2	0.03	0.00132973279820963	Venous I	IGFBP1
8.54741349188082E-08	1.35611787346034	0.486	0.142	0.00139416861466068	Venous I	REEP5
8.61315753741777E-08	1.17807414229326	0.743	0.274	0.00140489212592821	Venous I	ACTN1
8.99817731650818E-08	1.81662429770305	0.2	0.029	0.00146769270209565	Venous I	PGM3
9.02184696160293E-08	1.35467364024998	0.514	0.151	0.00147155345790705	Venous I	TAX1BP3
9.02446094590617E-08	1.24852507542957	0.371	0.085	0.00147197982488675	Venous I	ECHS1
9.34719508258649E-08	2.34929034646826	0.229	0.038	0.00152462098992068	Venous I	IFIT3
9.76111867834012E-08	1.77240615836013	0.286	0.056	0.00159213606762406	Venous I	TMX4
1.07018648941834E-07	1.60927319721105	0.229	0.037	0.00174558118289026	Venous I	ATP1A1
1.14455370892771E-07	1.33826816393083	0.571	0.179	0.00186688155463198	Venous I	SERINC1
1.17571906191599E-07	1.82209386814827	0.2	0.03	0.00191771536189117	Venous I	LXN
1.23837409495E-07	1.20163474306724	0.571	0.179	0.00201991198627295	Venous I	STXBP1
1.26292878628919E-07	1.43233605158561	0.286	0.056	0.0020599631433163	Venous I	DNASE1L1
1.28848384709618E-07	1.10182153890743	0.457	0.12	0.00210164600299858	Venous I	UBXN6
1.3440040156628E-07	1.46395330930756	0.257	0.047	0.00219220494994759	Venous I	FAM189A2
1.39219992010344E-07	1.75101323367489	0.171	0.023	0.00227081728968072	Venous I	FLT3LG
1.4438458426465E-07	1.18917532046	0.314	0.067	0.0023550569539407	Venous I	ETFB
1.44692731541634E-07	1.80727045805452	0.286	0.058	0.00236008314417559	Venous I	MEX3D
1.552819914562E-07	1.41743047964942	0.171	0.023	0.00253280456264208	Venous I	UQCR10
1.5529320551504E-07	1.03142545435966	0.514	0.144	0.00253298747515583	Venous I	ITPR1
1.5638359413714E-07	1.67250113658334	0.486	0.141	0.00255077280397089	Venous I	ANPEP
1.59151586868307E-07	0.941092538625574	0.686	0.223	0.00259592153340895	Venous I	EGLN2
1.59593847694681E-07	2.17755836279393	0.143	0.016	0.00260313524974794	Venous I	SLC25A1
1.60815651685759E-07	3.05768035627654	0.114	0.011	0.00262306409464642	Venous I	EMC6
1.62994108192978E-07	2.28342193772163	0.143	0.016	0.00265859689873567	Venous I	OSBPL11
1.6310616792424E-07	1.61528769281881	0.457	0.126	0.00266042470501228	Venous I	SIGIRR

1.65464022775069E-07	1.76931528072578	0.171	0.023	0.00269888367548414	Venous I	SIDT2
1.72430661076878E-07	1.11590623998917	0.857	0.475	0.00281251651282496	Venous I	SH3BP5
1.75898145694485E-07	1.33356355580903	0.4	0.102	0.00286907465442274	Venous I	CYC1
1.99561562064182E-07	2.06042173092366	0.114	0.011	0.00325504863882888	Venous I	NR2C2AP
2.09128729078935E-07	1.18928849502329	0.486	0.136	0.00341109870000651	Venous I	BMPR2
2.1552261654823E-07	1.70145819189024	0.286	0.059	0.00351538939851818	Venous I	SLC25A11
2.17781741865272E-07	1.06400796151967	0.457	0.127	0.00355223799156445	Venous I	FGA
2.26554306659032E-07	1.28240638911586	0.743	0.281	0.00369532729591546	Venous I	TSPAN9
2.30490643392294E-07	0.460765844011268	0.2	0.031	0.00375953288437171	Venous I	LYZ
2.35692345717943E-07	1.3656272681825	0.257	0.048	0.00384437785100537	Venous I	DARS
2.49261115330253E-07	1.52735612099365	0.543	0.178	0.00406569805215176	Venous I	TCN2
2.49698077321638E-07	1.48639840038929	0.257	0.048	0.00407282533919324	Venous I	DTNBP1
2.53921563519563E-07	1.38391476418106	0.4	0.104	0.00414171462256759	Venous I	MYRIP
2.58423628969032E-07	1.13627646813531	0.543	0.167	0.00421514781211388	Venous I	CTDSP1
2.61421992801325E-07	1.46261552967876	0.314	0.069	0.00426405412458241	Venous I	NDUFB9
2.87807475647965E-07	0.974265846015444	0.943	0.799	0.00469442773529396	Venous I	B2M
2.88618395427635E-07	1.15646384126532	0.857	0.494	0.00470765464782015	Venous I	IFITM3
2.98770190636576E-07	2.54885469427565	0.143	0.017	0.0048732405794732	Venous I	C19orf53
3.07982196997087E-07	2.41198971499637	0.143	0.017	0.00502349761521949	Venous I	GTF2H5
3.09372575331935E-07	1.55639833883136	0.343	0.08	0.00504617607623919	Venous I	CLIC1
3.17468158412755E-07	2.35200980472447	0.143	0.017	0.00517822313187045	Venous I	SS18L2
3.18225105627618E-07	0.84064077881619	0.943	0.716	0.00519056969789207	Venous I	IGFBP7
3.26434928201322E-07	1.30372821351729	0.314	0.07	0.00532448011389176	Venous I	KLF10
3.37548325681461E-07	1.96262528074792	0.286	0.061	0.00550575074019031	Venous I	TFPT
3.45601434573644E-07	1.8092999999854	0.2	0.032	0.00563710499933071	Venous I	USP5
3.49352864168343E-07	1.57244165254977	0.229	0.041	0.00569829456744984	Venous I	UCK1
3.58390560740109E-07	1.28120777627528	0.743	0.324	0.00584570843623191	Venous I	GRN
3.63406772418119E-07	1.63521493429494	0.657	0.287	0.00592752786491194	Venous I	IFI6
3.63595001838126E-07	1.0644844247113	0.4	0.102	0.00593059807498167	Venous I	NCOA4
3.65594086970255E-07	2.04240330799788	0.143	0.017	0.00596320515257183	Venous I	SFT2D3
3.71338106680929E-07	1.11292872178703	0.686	0.265	0.00605689585807263	Venous I	CAVIN2
3.76578126491765E-07	1.75045287284809	0.229	0.041	0.00614236582120718	Venous I	UNG
3.7818658461681E-07	1.23176498829623	0.429	0.115	0.00616860138168479	Venous I	AFAP1
3.80024355205871E-07	1.01097539997259	0.686	0.244	0.00619857725776296	Venous I	BRI3
3.87989210641305E-07	2.04554982999313	0.171	0.024	0.00632849201477032	Venous I	TRIQK
4.0494885089321E-07	2.22718619434733	0.171	0.024	0.00660512070691916	Venous I	SLX4
4.05895824741387E-07	1.86294333526988	0.229	0.041	0.00662056679735676	Venous I	AMOTL2
4.13672930714103E-07	1.03938066934908	0.886	0.465	0.00674741917287774	Venous I	PECAM1
4.16124976814664E-07	0.99869426442599	0.429	0.113	0.00678741449682398	Venous I	MGST3
4.19892462447298E-07	1.10423515276348	0.771	0.303	0.00684886595497788	Venous I	HNRNPA0
4.37535771884527E-07	1.19334507472941	0.686	0.299	0.00713664597520852	Venous I	CSRP1
4.40246869121895E-07	2.72603762708143	0.114	0.011	0.00718086668224723	Venous I	CRIP1
4.64775410438766E-07	1.49696992991956	0.229	0.041	0.00758095171966671	Venous I	ANP32E
4.68130916049448E-07	1.06185287658519	0.171	0.024	0.00763568337168255	Venous I	ALDOB
4.701718317207E-07	2.50709630757942	0.114	0.011	0.00766897274719633	Venous I	LRRC4B
4.71855924321944E-07	1.70218535039478	0.314	0.073	0.00769644198161522	Venous I	HAGH
4.78976452058424E-07	1.20358930423408	0.4	0.104	0.00781258490952496	Venous I	ZNHIT1
4.89565239375236E-07	1.622965926916	0.257	0.05	0.00798529861944947	Venous I	NAA20
5.08262543648976E-07	2.10896676837822	0.229	0.041	0.00829027034945845	Venous I	SLCO4A1
5.08867310547627E-07	2.60573979666737	0.143	0.018	0.00830013470234234	Venous I	FSTL3
5.18896311213422E-07	1.58613066554044	0.343	0.083	0.00846371773220212	Venous I	NNMT
5.66540755447235E-07	1.13062345525544	0.371	0.091	0.00924084626209985	Venous I	TRAM2
5.77452826674018E-07	1.3695405891138	0.257	0.05	0.0094188330558799	Venous I	SPIN4
5.83168228816535E-07	2.879615193816	0.143	0.018	0.0095120569802265	Venous I	DEPRECATED-ENSG00000102349
6.12157038883616E-07	2.06139290969236	0.143	0.018	0.00998489346123066	Venous I	TYSND1
6.16374710654826E-07	2.05275816109883	0.171	0.025	0.0100536879054909	Venous I	DSTN
6.21518673533483E-07	2.19556708104959	0.171	0.025	0.0101375910840046	Venous I	JADE1
6.36343338917628E-07	2.33350616842132	0.143	0.018	0.0103793962010854	Venous I	PLEKHF1
6.42545639773487E-07	1.46342092421149	0.286	0.061	0.0104805619303453	Venous I	BRMS1
6.44259431230214E-07	1.5105897907123	0.257	0.051	0.010508515582796	Venous I	CHCHD2

6.91852035433148E-07	2.06544357847492	0.2	0.033	0.0112847985499501	Venous I	LRIG3
7.00906272120266E-07	1.92287636306525	0.143	0.018	0.0114324822045537	Venous I	GRK3
7.15657962075829E-07	1.19501462267185	0.6	0.217	0.0116730970194189	Venous I	NR2F2
7.5039832875645E-07	1.11918618846064	0.343	0.081	0.0122397471403465	Venous I	SNRPB
7.56977677551803E-07	1.13803295627429	0.229	0.041	0.0123470628985475	Venous I	GSR
7.71474881910137E-07	1.27356018213962	0.286	0.063	0.0125835267988363	Venous I	RBP4
7.72787080957157E-07	1.39039129182352	0.314	0.073	0.0126049300774922	Venous I	CDC25B
8.27523144275201E-07	1.08239837469849	0.429	0.123	0.0134977300062728	Venous I	FJX1
8.36499068247079E-07	1.22818771080906	0.343	0.083	0.0136441363021781	Venous I	ALS2CL
9.19648084275664E-07	1.42408022077777	0.257	0.052	0.0150003799026204	Venous I	GTF2F1
9.63654660140537E-07	1.42911978428928	0.4	0.108	0.0157181711615523	Venous I	RAB35
9.89843027893265E-07	1.70918801193811	0.2	0.034	0.016145329627967	Venous I	SERTAD2
1.01290667370769E-06	1.39537068106658	0.286	0.063	0.0165215207548462	Venous I	EEA1
1.03867326952813E-06	1.0963689612773	0.4	0.11	0.0169417996992734	Venous I	MT-CYB
1.06530228468649E-06	2.40435255731147	0.143	0.019	0.0173761455655214	Venous I	SLC16A14
1.09747369094424E-06	1.89425841929875	0.2	0.034	0.0179008933729914	Venous I	LTF
1.10183412695056E-06	1.0166568870359	0.6	0.199	0.0179720164446906	Venous I	RHOA
1.13184117766648E-06	1.86292080140302	0.171	0.026	0.0184614614489179	Venous I	PRAF2
1.16052172023348E-06	2.6324559511088	0.114	0.012	0.0189292697787283	Venous I	SRC
1.17813320435514E-06	1.77462540160668	0.171	0.026	0.0192165306962366	Venous I	B3GLCT
1.23837198210001E-06	2.47121116882956	0.171	0.027	0.0201990854000332	Venous I	SEC11C
1.24783468089516E-06	1.70883835423271	0.143	0.019	0.0203534314800809	Venous I	MYO1F
1.24880116264944E-06	1.64052518175066	0.229	0.044	0.0203691957639751	Venous I	UQCR11
1.26596017449608E-06	0.910879757288289	0.457	0.131	0.0206490764062055	Venous I	RABAC1
1.27070494774398E-06	0.935514615201796	0.429	0.118	0.020726468402652	Venous I	HEG1
1.31175376562988E-06	1.3349419406286	0.229	0.043	0.021396015671189	Venous I	IFITM1
1.32196970987855E-06	1.30563873444112	0.257	0.053	0.0215626479378291	Venous I	DDOST
1.37971740918937E-06	1.03092244052949	0.714	0.313	0.0225045706612879	Venous I	PTMS
1.39197653942121E-06	1.16659437742783	0.343	0.086	0.0227045293344993	Venous I	VAMP8
1.4204991670635E-06	1.20403800986777	0.371	0.097	0.0231697619139727	Venous I	MYBBP1A
1.53146506629686E-06	1.16095641333791	0.4	0.11	0.0249797266963682	Venous I	SH3BGRL3
1.6055338861038E-06	1.07171095862926	0.343	0.085	0.026187863216239	Venous I	CDR2L
1.65364908741501E-06	1.6592805611689	0.257	0.055	0.0269726702648262	Venous I	CYB5A
1.77992199813282E-06	1.44278463014064	0.2	0.035	0.0290323077115444	Venous I	CYB561D2
1.79342648478794E-06	1.45511216792585	0.286	0.065	0.0292525793933761	Venous I	GADD45GIP1
1.8022833118609E-06	1.14820501441718	0.771	0.35	0.0293970430997631	Venous I	PIM3
1.81354002541987E-06	1.37827195194101	0.486	0.163	0.0295806513546234	Venous I	TMEM50A
1.98711593857938E-06	2.23528296789987	0.2	0.037	0.0324118480741683	Venous I	TMEM181
1.98722184190974E-06	1.14969026183542	0.543	0.184	0.0324135754633898	Venous I	PPIB
1.98829849556283E-06	1.80989520810091	0.143	0.019	0.0324311367611253	Venous I	ATP2A3
1.98933426643445E-06	0.937493522823418	0.429	0.123	0.0324480312198124	Venous I	SUN2
1.99091834295277E-06	0.820541331854644	0.514	0.155	0.0324738690919026	Venous I	GATA4
1.99210310642267E-06	1.58040804025118	0.229	0.045	0.0324931937688602	Venous I	CAT
2.0424069711649E-06	1.72490802583219	0.143	0.019	0.0333137001066706	Venous I	APOB
2.05876406491303E-06	1.4876642995956	0.257	0.055	0.0335805006627964	Venous I	GPX3
2.09792832018188E-06	2.14201036363277	0.143	0.019	0.0342193088304866	Venous I	SMIM19
2.16983341052213E-06	2.29084743138	0.171	0.028	0.0353921527590265	Venous I	TUSC1
2.19369964089137E-06	1.31822441955971	0.143	0.019	0.0357814348425791	Venous I	MPST
2.2707873870343E-06	1.54413491181099	0.371	0.102	0.0370388130699164	Venous I	SELENOS
2.32631120167286E-06	1.10444175936168	0.686	0.279	0.037944462010486	Venous I	NDRG1
2.37753659264197E-06	2.15350457387983	0.171	0.028	0.0387799993625832	Venous I	RBM7
2.42738722819401E-06	0.972106840417238	0.514	0.17	0.0395931130790726	Venous I	ENTPD1
2.45632122188249E-06	2.68479164740694	0.114	0.013	0.0400650554501253	Venous I	SMIM30
2.48471809412224E-06	1.36473744487553	0.143	0.019	0.0405282368332278	Venous I	BMERB1
2.48740957545285E-06	1.53400212340551	0.286	0.067	0.0405721375852114	Venous I	ATXN7L3B
2.58192235079904E-06	1.86531886130724	0.2	0.037	0.0421137354638831	Venous I	PSMB2
2.59057740524507E-06	2.56100336901915	0.114	0.013	0.0422549080569523	Venous I	SKP1
2.64622842668769E-06	2.39325943823403	0.114	0.013	0.0431626318677028	Venous I	FNDC4
2.79042084228682E-06	2.08852669926006	0.114	0.013	0.0455145543585403	Venous I	HTR2B
2.79147301706384E-06	1.3421513222232	0.371	0.101	0.0455317163813283	Venous I	OGDH

2.82014832911313E-06	2.301555667835	0.114	0.013	0.0459994393961642	Venous I	PRMT6
2.82015037238687E-06	2.20057251218374	0.114	0.013	0.0459994727240022	Venous I	RHNO1
2.88139210548749E-06	1.48084835158098	0.286	0.067	0.0469983866326064	Venous I	RAB5IF
2.91520936212015E-06	1.43487166128755	0.6	0.227	0.0475499799055417	Venous I	ISG15
2.97341466727823E-06	1.63838463873101	0.114	0.013	0.0484993666379752	Venous I	CCDC117
3.00946089596481E-06	1.19406327761483	0.4	0.116	0.049087316674082	Venous I	OAS1
3.05315850847573E-06	0.826936381379687	0.314	0.076	0.0498000684317476	Venous I	MT-ND2
1.09846660956831E-152	8.24040639314299	0.722	0.003	1.79170888686687E-148	Arterial I	GJA5
2.23194329471407E-108	7.62825533103533	0.778	0.011	3.64052270800811E-104	Arterial I	FBLN5
1.30919281617142E-75	8.11595842462588	0.389	0.002	2.13542440245721E-71	Arterial I	TMEM100
1.34064001613914E-69	8.47223705919446	0.333	0.002	2.18671793032456E-65	Arterial I	GUCY1A1
1.92582361192602E-69	5.99124589005344	0.556	0.01	3.14121089341253E-65	Arterial I	SLC45A4
2.17523542648753E-62	11.9614188303597	0.222	0	3.54802650414382E-58	Arterial I	TSPAN2
1.77413208396859E-55	7.72042695551477	0.278	0.002	2.89378684216116E-51	Arterial I	CPAMD8
4.09282470537498E-54	5.51849916662941	0.778	0.034	6.67580637693712E-50	Arterial I	SEMA3G
3.36286051139882E-47	10.1991902352302	0.167	0	5.48516178014262E-43	Arterial I	LRRC3B
3.36286051139882E-47	10.2347641051322	0.167	0	5.48516178014262E-43	Arterial I	DEPRECATED-ENSG00000164116
4.00938155737075E-45	5.63177300875867	0.556	0.019	6.53970225822743E-41	Arterial I	RNF144B
3.51852135158326E-43	6.89992685415407	0.278	0.003	5.73906017656745E-39	Arterial I	PDZRN3
1.13937354299025E-41	7.53001783681739	0.222	0.002	1.8584321859714E-37	Arterial I	SLC4A4
4.51575702575606E-40	5.38825172907167	0.611	0.029	7.3656512847107E-36	Arterial I	VEGFC
1.15510411638532E-39	6.40201831859581	0.333	0.006	1.8840903242361E-35	Arterial I	MET
2.89456711881065E-39	6.54723876866679	0.722	0.044	4.72132842749205E-35	Arterial I	IGFBP3
1.37458641365293E-35	9.77360823911823	0.167	0.001	2.2420878993093E-31	Arterial I	SPEG
6.54007226567132E-34	4.77942867943488	0.778	0.062	1.06675118725365E-29	Arterial I	LTBP4
2.57592959837238E-32	5.6747890531701	0.278	0.006	4.20159876790519E-28	Arterial I	ELOVL7
5.2135489174322E-32	9.62122365267832	0.111	0	8.50381963922366E-28	Arterial I	BEND4
5.2135489174322E-32	9.68603429463789	0.111	0	8.50381963922366E-28	Arterial I	DKK 2.00
5.2135489174322E-32	10.2845581798899	0.111	0	8.50381963922366E-28	Arterial I	SSTR1
5.2135489174322E-32	9.43332507282879	0.111	0	8.50381963922366E-28	Arterial I	AHNAK2
5.2135489174322E-32	9.81136736246546	0.111	0	8.50381963922366E-28	Arterial I	ACTL10
5.2135489174322E-32	10.0451502655231	0.111	0	8.50381963922366E-28	Arterial I	DEPRECATED-ENSG00000102934
1.03960976381277E-29	4.69165142750524	0.5	0.026	1.695707485755E-25	Arterial I	ELN
1.87661033096535E-29	3.67257401506114	0.667	0.048	3.06093911083757E-25	Arterial I	FBLN2
1.2282221519957E-27	4.23891679311637	0.222	0.004	2.00335315212018E-23	Arterial I	KCNT2
9.45543625611199E-24	5.40965214052265	0.722	0.083	1.54227620773443E-19	Arterial I	KCTD12
4.97332197123231E-23	6.6870948566998	0.222	0.006	8.11198546727703E-19	Arterial I	CCDC3
1.21374432740968E-21	7.80582721160311	0.111	0.001	1.97973837243793E-17	Arterial I	HEY2
1.30755188047386E-21	6.71310901819485	0.111	0.001	2.13274787224092E-17	Arterial I	CHAC1
1.62466211180227E-20	6.03607418978013	0.167	0.003	2.64998637056068E-16	Arterial I	SNCAIP
1.70362435878323E-20	6.18536876160474	0.167	0.003	2.77878169161133E-16	Arterial I	LOX
7.41096828103754E-20	4.49293817530284	0.444	0.033	1.20880303632003E-15	Arterial I	PCSK5
1.44429039224036E-18	4.61810912269002	0.5	0.046	2.35578205878325E-14	Arterial I	HEY1
6.64719375480222E-17	4.22437017311683	0.556	0.065	1.08422377334579E-12	Arterial I	RCAN1
2.15795131564764E-16	5.67688597605167	0.111	0.002	3.51983439095286E-12	Arterial I	LCN10
2.15795131564764E-16	6.3261768346032	0.111	0.002	3.51983439095286E-12	Arterial I	SLC25A52
2.41180845688318E-16	4.49371523656266	0.111	0.002	3.93390077402215E-12	Arterial I	JCHAIN
4.88485826137835E-16	5.46019312343851	0.167	0.005	7.96769231013422E-12	Arterial I	RAB40B
1.09719290554902E-15	3.76048267747777	0.5	0.054	1.789631348241E-11	Arterial I	FBLIM1
1.87186198375174E-15	4.52736874469251	0.278	0.016	3.05319408169747E-11	Arterial I	ITGB4
2.96804257164261E-15	3.63534253409782	0.556	0.071	4.84117423860625E-11	Arterial I	MGP
4.83893129340382E-14	3.63071978171166	0.278	0.018	7.89278083267097E-10	Arterial I	SEPTIN5
2.92091436804828E-13	4.65460023375733	0.222	0.012	4.76430342572356E-09	Arterial I	HMCN2
2.96792033025268E-13	3.25805540581387	0.722	0.15	4.84097485067514E-09	Arterial I	FBN1
3.12188838679852E-13	5.17701795129329	0.111	0.002	5.09211214770707E-09	Arterial I	TRABD2A
3.26343148024878E-13	4.7447418703852	0.111	0.002	5.32298308743378E-09	Arterial I	TSNAXIP1
6.04753131977904E-13	3.21204381980751	0.667	0.129	9.8641283356916E-09	Arterial I	GJA4
1.04223414207347E-12	3.57998676572595	0.444	0.054	1.69998810913604E-08	Arterial I	EGR3
2.667846885928E-12	3.56501843352633	0.889	0.329	4.35152505563715E-08	Arterial I	TM4SF1
3.49437293798195E-12	6.38081387389755	0.167	0.007	5.69967169914236E-08	Arterial I	NPR 3.00

4.11886950182596E-12	5.3002950031271	0.167	0.007	6.71828804442833E-08	Arterial I	CPXM2
8.76286406121698E-12	3.59122362152334	0.222	0.014	1.4293107570251E-07	Arterial I	GIPC2
1.13427872270685E-11	3.99668494380143	0.278	0.022	1.85012202460714E-07	Arterial I	SETBP1
1.39150654994821E-11	3.72113409692529	0.389	0.044	2.26968633362052E-07	Arterial I	LMCD1
1.7549978638735E-11	3.43152148192476	0.333	0.033	2.86257701576407E-07	Arterial I	ENPP2
1.82451095625997E-11	4.37835061876365	0.278	0.023	2.97595982075564E-07	Arterial I	COL8A1
2.2918129923777E-11	3.9924763383611	0.556	0.098	3.73817617186726E-07	Arterial I	CCN1
3.25065531788088E-11	6.75271342437301	0.111	0.003	5.30214388899551E-07	Arterial I	ENPP4
3.25065531788088E-11	7.32293583486628	0.111	0.003	5.30214388899551E-07	Arterial I	PHLDA2
3.50015414847565E-11	5.57773334227822	0.111	0.003	5.70910143157864E-07	Arterial I	SULF1
3.76836277996182E-11	5.28338527348331	0.111	0.003	6.14657653039572E-07	Arterial I	SERPINE2
3.90990725197787E-11	4.7169459456457	0.111	0.003	6.3774497187011E-07	Arterial I	SV2C
4.05664989220446E-11	5.08154675741847	0.111	0.003	6.61680163917469E-07	Arterial I	PLLP
6.09140775296972E-11	4.12977666063843	0.556	0.102	9.93569518586892E-07	Arterial I	BTG2
1.36039619163363E-10	2.8361187749276	0.611	0.123	2.21894222817361E-06	Arterial I	JAG2
1.64184451236043E-10	5.07897247129568	0.167	0.009	2.6780125841111E-06	Arterial I	BMX
2.17340518562002E-10	4.37197702399478	0.167	0.009	3.54504119826482E-06	Arterial I	FGF2
2.43626010681359E-10	2.8793313215685	0.5	0.085	3.97378386022365E-06	Arterial I	BGN
4.96604552642138E-10	2.89230870685639	0.722	0.191	8.1001168581459E-06	Arterial I	BCAM
5.31597548120954E-10	3.47588093400909	0.722	0.218	8.67088760740088E-06	Arterial I	ADAMTS1
7.19648000128881E-10	7.07811815625029	0.167	0.01	1.17381785301022E-05	Arterial I	EGR2
7.33259521280749E-10	3.50702220840236	0.444	0.071	1.19601960516103E-05	Arterial I	ACE
1.00560273232455E-09	3.36094675304865	0.778	0.267	1.64023861669457E-05	Arterial I	DEPP1
1.01983608121561E-09	3.67748948469805	0.167	0.01	1.66345463207079E-05	Arterial I	MMP28
1.09811028066228E-09	5.43435002657284	0.111	0.004	1.79112767878824E-05	Arterial I	COL4A4
1.09811028066228E-09	5.54975598181079	0.111	0.004	1.79112767878824E-05	Arterial I	FLNC
1.12047023631524E-09	3.16917601108122	0.833	0.294	1.82759900245379E-05	Arterial I	DLL4
1.20717207869345E-09	5.3063068481177	0.111	0.004	1.96901837755689E-05	Arterial I	EPPK1
1.24581984179889E-09	4.6158842736936	0.111	0.004	2.03205674395817E-05	Arterial I	APLP1
1.26558913415434E-09	4.5091545750717	0.111	0.004	2.06430243671914E-05	Arterial I	ANKRD29
1.32676757490697E-09	3.97784074558573	0.111	0.004	2.16409059143076E-05	Arterial I	LURAP1
1.72387319567168E-09	3.70595413844236	0.389	0.057	2.81180956946007E-05	Arterial I	AIF1L
3.06378625633013E-09	4.82331496327025	0.167	0.01	4.99734176270008E-05	Arterial I	PFN2
3.09524612187785E-09	4.03164942660049	0.167	0.01	5.04865594939496E-05	Arterial I	LRIG1
3.52348806957159E-09	2.76530830805927	0.833	0.391	5.74716139027822E-05	Arterial I	CLDN5
6.56464726847875E-09	3.49428718433785	0.444	0.081	0.000107075961596157	Arterial I	HIP1R
6.9859973042625E-09	3.11085075688311	0.833	0.391	0.000113948602029826	Arterial I	SAT1
7.39974855498576E-09	2.68964495410854	0.833	0.409	0.000120697298680373	Arterial I	VIM
1.16636450940658E-08	4.69824701061029	0.167	0.011	0.000190245715129308	Arterial I	EDN1
1.23516523415717E-08	4.731556210402	0.167	0.011	0.000201467801343376	Arterial I	NR2C2AP
1.59464171819281E-08	5.62423065359669	0.111	0.005	0.000260102010654429	Arterial I	PDLIM4
1.82943463430097E-08	4.11411699638357	0.111	0.005	0.000298399083200832	Arterial I	VAX2
1.95790007570726E-08	3.57700756002054	0.333	0.047	0.000319353081348612	Arterial I	KCNJ2
2.29509707324839E-08	3.93754430827738	0.222	0.021	0.000374353283617545	Arterial I	DHH
4.31132247705957E-08	2.60909370267872	0.667	0.195	0.000703219809233187	Arterial I	ARL 15.00
4.83140134522483E-08	1.96828968673646	1	0.578	0.000788049873419622	Arterial I	MMRN2
5.58122057595203E-08	4.05751679331785	0.222	0.022	0.000910352888143536	Arterial I	FAM107A
7.11555710921448E-08	2.75707118825908	0.5	0.109	0.00116061852008397	Arterial I	CXCL12
8.08622449794035E-08	2.56725436449598	0.278	0.034	0.00131894407785905	Arterial I	ZC3H10
1.05729529010212E-07	6.65088569827834	0.111	0.006	0.00172455434768557	Arterial I	IGKC
1.09238080291419E-07	2.58050770679321	0.833	0.462	0.00178178232763333	Arterial I	RHOB
1.11827506598004E-07	1.77677200327507	0.889	0.554	0.00182401846012005	Arterial I	A2M
1.25460522138239E-07	5.36515902439402	0.111	0.006	0.00204638657659681	Arterial I	DDR1
1.25460522138239E-07	4.5290320700659	0.111	0.006	0.00204638657659681	Arterial I	RTN4R
1.38288424798402E-07	4.12056893583553	0.111	0.006	0.00225562249688673	Arterial I	SAR1A
1.68505876136133E-07	4.25670866822374	0.333	0.054	0.00274849934565646	Arterial I	FGL2
2.25684374747182E-07	2.305835385729	0.222	0.023	0.00368113783650129	Arterial I	PCK2
5.32997360123461E-07	3.56299562488131	0.278	0.041	0.00869371994097377	Arterial I	INSIG1
5.75055538812787E-07	2.16744463464506	0.556	0.147	0.00937973089357537	Arterial I	RAPGEF1
5.75759971720406E-07	5.74573428956907	0.111	0.006	0.00939122089873155	Arterial I	B3GALNT1

5.97912616814603E-07	2.65788665507808	0.222	0.025	0.00975255269286298	Arterial I	SYT11
6.52205490419337E-07	2.8036500896368	0.389	0.075	0.0106381237542298	Arterial I	GFOD1
8.63554364849316E-07	3.16302138880869	0.278	0.04	0.0140854352450572	Arterial I	MECOM
9.31852673069924E-07	4.30183742252891	0.167	0.015	0.0151994489504435	Arterial I	IL6
1.17928402694039E-06	3.43235821906113	0.278	0.042	0.0192353017634246	Arterial I	CITED4
1.43475857016999E-06	1.77285792368194	0.778	0.339	0.0234023470380427	Arterial I	CLEC14A
1.79055197091071E-06	4.04458678984957	0.167	0.016	0.0292056931975246	Arterial I	CYP1B1
2.29938176581358E-06	2.71686951538052	0.333	0.061	0.0375052159821853	Arterial I	ARHGAP4
2.40640214157117E-06	4.05582818472812	0.111	0.007	0.0392508253311673	Arterial I	ABCB9
2.45460691293175E-06	3.95026192871871	0.111	0.007	0.0400370933568297	Arterial I	SARM1
2.5037379374755E-06	3.77671879080896	0.111	0.007	0.0408384694981628	Arterial I	KITLG
2.7098686384334E-06	3.25795269311229	0.111	0.007	0.0442006673614871	Arterial I	KLF12
1.05125425373209E-228	9.18829781443578	0.87	0.001	1.71470081326242E-224	Lymphat	PDPN
2.75876174175906E-183	9.37573143370163	0.957	0.008	4.49981627698321E-179	Lymphat	CCL21
4.91775540347817E-142	8.24812992698508	0.652	0.003	8.02135083861325E-138	Lymphat	ABI3BP
5.62085135115602E-122	6.93047302714561	0.652	0.006	9.16817063887058E-118	Lymphat	TBX1
3.68788861659414E-121	9.63161480426536	0.478	0.001	6.0153151225267E-117	Lymphat	DEPRECATED-ENSG00000184058
3.30947159745136E-95	6.38838535158512	0.826	0.022	5.39807912260292E-91	Lymphat	SPHK1
1.83647584790738E-94	5.69764357451856	0.783	0.019	2.99547575552176E-90	Lymphat	ELN
1.35836103091908E-83	6.17434271854793	0.565	0.009	2.2156226775321E-79	Lymphat	PTX3
2.64962040976931E-81	6.37386921904365	0.739	0.021	4.32179585037472E-77	Lymphat	CRTAC1
3.3566584376302E-81	8.24068345324758	0.391	0.002	5.47504557761862E-77	Lymphat	CDCP2
8.66092799104576E-75	6.71785846200468	0.391	0.003	1.41268396461947E-70	Lymphat	MPP7
5.54606114487303E-74	8.92183675327057	0.304	0.001	9.0461803334024E-70	Lymphat	SEMA3D
5.98714926776312E-74	8.64738069816689	0.304	0.001	9.76563917064842E-70	Lymphat	ADAMTS15
7.7615122373609E-69	6.07553959530406	0.609	0.016	1.26598026103594E-64	Lymphat	TFF3
2.09352662663857E-68	5.87656596937982	0.478	0.008	3.41475128071018E-64	Lymphat	PRKG1
8.29525677598475E-60	6.76779470647092	0.435	0.008	1.35303933273087E-55	Lymphat	MFAP2
4.0907480935129E-59	7.06570812577253	0.304	0.002	6.67241921532889E-55	Lymphat	EFNA5
7.08494976319302E-59	6.64834978972772	0.304	0.002	1.15562615587441E-54	Lymphat	GAS7
1.34234738826021E-58	5.284354023292	0.739	0.034	2.18950282499123E-54	Lymphat	ADIRF
5.45496171724617E-57	5.53527008218869	0.696	0.031	8.89758805700022E-53	Lymphat	MMRN1
7.05091746391181E-57	6.3371586730398	0.391	0.006	1.15007514753866E-52	Lymphat	TC2N
5.35863312247621E-55	6.73894960702902	0.348	0.005	8.74046648607095E-51	Lymphat	PARD6G
2.01144892223038E-51	5.08680281588938	0.522	0.017	3.28087433704997E-47	Lymphat	PROX1
4.98934685546771E-49	10.317668448757	0.174	0	8.13812365595339E-45	Lymphat	LRRN4CL
2.30847360454939E-43	6.32176986060462	0.217	0.002	3.76535129638051E-39	Lymphat	FLNC
1.27469662473681E-42	4.88241833030316	0.609	0.032	2.07915766460821E-38	Lymphat	FLRT2
4.96350337090099E-42	5.9528432939352	0.304	0.006	8.09597034827661E-38	Lymphat	IGF1
6.16551313988611E-42	5.69090195463023	0.304	0.006	1.00565684824682E-37	Lymphat	PLEKHG3
2.7801906029339E-39	4.93928369853239	0.435	0.016	4.53476889244548E-35	Lymphat	CELSR1
1.69419588284342E-38	4.42071862068611	0.783	0.068	2.76340290450591E-34	Lymphat	DOCK5
2.99807463896763E-38	4.98694595299864	0.522	0.027	4.89015954362009E-34	Lymphat	UNC5B
4.29201741274561E-38	4.48980698196846	0.435	0.017	7.00070960192937E-34	Lymphat	SLC38A1
5.33104619597976E-38	7.19264378621379	0.217	0.002	8.69546945026259E-34	Lymphat	TNFRSF11A
3.158427076856E-37	4.36220202650483	0.565	0.032	5.15171040505982E-33	Lymphat	ADAM19
3.55784499496497E-37	9.88016883818189	0.13	0	5.80320097128736E-33	Lymphat	CSMD2
3.55784499496497E-37	10.4106075402482	0.13	0	5.80320097128736E-33	Lymphat	SNTG2
3.55784499496497E-37	10.5069133957128	0.13	0	5.80320097128736E-33	Lymphat	SRGAP3
3.55784499496497E-37	10.4615807190343	0.13	0	5.80320097128736E-33	Lymphat	PSORS1C1
3.55784499496497E-37	10.2331061639221	0.13	0	5.80320097128736E-33	Lymphat	PTPN3
3.55784499496497E-37	10.0404942550053	0.13	0	5.80320097128736E-33	Lymphat	CATSPERB
8.60754635045614E-35	4.9806290027155	0.435	0.019	1.4039768852229E-30	Lymphat	DMTN
1.04930012349254E-32	5.75642108034079	0.174	0.002	1.71151343142868E-28	Lymphat	MEDAG
1.11197703217709E-32	6.6173807672919	0.174	0.002	1.81374573718405E-28	Lymphat	NMNAT2
1.87137082187597E-32	5.20860812174479	0.304	0.009	3.0523929475619E-28	Lymphat	MYO5C
6.9339031495898E-31	5.13294990808521	0.261	0.006	1.13098894272959E-26	Lymphat	SEMA3A
1.94839321251161E-30	5.38694291218022	0.348	0.014	3.17802416892769E-26	Lymphat	FRMD6
8.02999570951307E-29	4.04388076993067	0.652	0.061	1.30977260017868E-24	Lymphat	LTBP4
4.13775116978689E-28	7.29510135871965	0.174	0.002	6.7490859330394E-24	Lymphat	MYCN

4.87336558755782E-28	8.40094055997071	0.13	0.001	7.94894660986556E-24	Lymphat	IGSF3
5.3027436703467E-28	5.67124916568861	0.174	0.002	8.6493052007025E-24	Lymphat	THSD4
5.3027436703467E-28	5.62798985856083	0.174	0.002	8.6493052007025E-24	Lymphat	PPL
5.55548046387199E-28	7.11786819590524	0.13	0.001	9.06154418462161E-24	Lymphat	LRRC15
8.36338062168043E-27	3.98500606852454	0.478	0.033	1.3641510132023E-22	Lymphat	STON2
3.67779740617879E-26	3.41940933024577	0.565	0.047	5.99885534921822E-22	Lymphat	FBLN2
1.25293928164201E-24	4.13349750932306	0.435	0.03	2.04366926228629E-20	Lymphat	GLIPR2
1.60880223526551E-23	3.21949069862063	0.87	0.145	2.62411732594157E-19	Lymphat	FBN1
2.19925053021053E-23	3.60118861554261	0.739	0.101	3.5871975398264E-19	Lymphat	PGM5
5.2706124483707E-23	4.27053181493731	0.522	0.049	8.59689596453745E-19	Lymphat	S100A6
1.14811997532114E-22	4.59686933002735	0.261	0.01	1.87269849174631E-18	Lymphat	PTPRS
1.68197360517138E-22	7.45555568577548	0.13	0.002	2.74346714739504E-18	Lymphat	APOD
1.77215010970002E-22	6.39829227500773	0.13	0.002	2.8905540439317E-18	Lymphat	LRCOL1
1.86710939870528E-22	6.86921362010089	0.13	0.002	3.04544214022818E-18	Lymphat	CADM4
4.9032145789372E-22	4.2352249293792	0.304	0.015	7.99763329970447E-18	Lymphat	TLE1
5.59135681040735E-22	4.34270425608615	0.391	0.027	9.12006209345542E-18	Lymphat	COL23A1
1.16140033741012E-21	5.42155269209305	0.217	0.007	1.89436009034965E-17	Lymphat	EFEMP1
1.23493996138001E-21	4.77883494940783	0.217	0.007	2.01431057100694E-17	Lymphat	SFRP1
1.24626720677431E-21	4.85997828693809	0.304	0.016	2.03278644096958E-17	Lymphat	RNF112
2.07772502655467E-21	3.59050808548266	0.217	0.007	3.38897729081332E-17	Lymphat	BHMT2
3.08810220094903E-21	3.99671644637784	0.391	0.028	5.03700349996797E-17	Lymphat	PDGFC
1.12208166703584E-20	4.42786271665331	0.435	0.038	1.83022740710216E-16	Lymphat	HS3ST1
1.13915812230052E-20	4.50508771877051	0.435	0.038	1.85808081328437E-16	Lymphat	ACKR2
6.45267261936734E-20	4.16138532082826	0.217	0.008	1.05249543094501E-15	Lymphat	SPATA6L
1.15639773236402E-19	3.50106509990357	0.565	0.068	1.88620034125895E-15	Lymphat	LIMS1
1.66068961389244E-19	4.14452994779527	0.174	0.005	2.70875082921995E-15	Lymphat	SLC45A3
1.92116327246117E-19	2.34866546340875	0.478	0.046	3.13360941371141E-15	Lymphat	PTGDS
8.04856299368129E-19	6.0535421495511	0.13	0.002	1.31280110989936E-14	Lymphat	RTN1
9.9871398863293E-19	4.58765458829554	0.348	0.026	1.62900238685917E-14	Lymphat	GJC2
1.29731412307863E-18	4.6146036959958	0.304	0.019	2.11604906615355E-14	Lymphat	PLXNA4
1.33709647625656E-18	4.42514428549812	0.261	0.014	2.18093806242207E-14	Lymphat	FAM89A
1.54132443688312E-18	4.08453603130332	0.304	0.019	2.51405428900006E-14	Lymphat	ROBO3
7.00331411259727E-18	5.15389830716593	0.174	0.006	1.14231056490574E-13	Lymphat	CBFA2T3
8.34103360686111E-18	4.51564376274007	0.217	0.01	1.36050599161512E-13	Lymphat	DHODH
9.27322681543465E-18	4.71837358559065	0.174	0.006	1.51255602586555E-13	Lymphat	NPNT
1.09583047275444E-17	4.14311697088391	0.261	0.014	1.78740908410976E-13	Lymphat	CCDC102B
1.30971463020473E-17	4.14834425974422	0.217	0.01	2.13627553332693E-13	Lymphat	GLIS3
1.32519568509603E-17	3.71688631091778	0.261	0.014	2.16152668196014E-13	Lymphat	KRT18
1.51003204730228E-17	4.67134362527977	0.391	0.036	2.46301327235475E-13	Lymphat	NUDT4
4.20155536279711E-17	3.41888385494622	0.652	0.115	6.85315695225837E-13	Lymphat	SNCG
4.66219079594312E-17	4.10604119528669	0.261	0.015	7.60449940726283E-13	Lymphat	ITGB4
6.9519448325776E-17	3.68094166363929	0.696	0.134	1.13393172164173E-12	Lymphat	TNXB
8.2138471904126E-17	3.71112437684689	0.304	0.022	1.3397606152282E-12	Lymphat	CDKN2C
8.83578988921278E-17	4.65407330172091	0.217	0.01	1.4412056888295E-12	Lymphat	TMOD1
1.50451255166657E-16	3.11622993820729	0.435	0.045	2.45401042302334E-12	Lymphat	TSPAN11
1.53944901358393E-16	3.2693359390789	0.565	0.081	2.51099528605676E-12	Lymphat	NNMT
1.81068743703064E-16	4.38622651089685	0.261	0.016	2.95341227854067E-12	Lymphat	VSTM4
2.95680315286992E-16	3.7572507811647	0.304	0.023	4.82284162264613E-12	Lymphat	KLHL4
3.212402928437E-16	6.72541046499917	0.13	0.003	5.23975041657358E-12	Lymphat	MFAP4
3.33386942162998E-16	5.80639014057074	0.13	0.003	5.43787441362067E-12	Lymphat	LURAP1
3.72608446633385E-16	5.53286933019741	0.13	0.003	6.07761637303714E-12	Lymphat	NALCN
4.29462742474853E-16	3.28608026408673	0.565	0.083	7.00496679250733E-12	Lymphat	EHD2
6.97675811350425E-16	3.28183529642407	0.522	0.07	1.13797901589368E-11	Lymphat	MGP
3.23629451404778E-15	3.18621584162345	0.478	0.061	5.27871998186334E-11	Lymphat	PAPLN
3.28564917483681E-15	3.32586679629695	0.391	0.04	5.35922236907632E-11	Lymphat	HP
3.42285324940636E-15	3.85798886142287	0.435	0.053	5.58301593510672E-11	Lymphat	RASSF9
4.8053780834603E-15	4.04407253875408	0.174	0.007	7.8380521919321E-11	Lymphat	TNIK
8.84101776210158E-15	3.08364973731986	0.217	0.012	1.44205840717639E-10	Lymphat	SERPINA3
9.56016924661597E-15	3.772002446519	0.565	0.093	1.55935920581553E-10	Lymphat	MEGF6
3.40571927006914E-14	3.0669077341909	0.304	0.026	5.55506870140977E-10	Lymphat	CDH23

**3.51340198029944E-14**	4.96655953232533	0.13	0.004	5.73070997006641E-10	Lymphat	BATF
**3.8707537736075E-14**	4.2301061550581	0.13	0.004	6.31358648013118E-10	Lymphat	HPR
**3.8707537736075E-14**	3.87058492190408	0.13	0.004	6.31358648013118E-10	Lymphat	ADAM33
**3.99763446650528E-14**	4.74425910488488	0.13	0.004	6.52054157831676E-10	Lymphat	TMEM176A
**5.02465628929963E-14**	3.94741253312131	0.261	0.019	8.19571687347662E-10	Lymphat	LTBP2
**5.98398971286496E-14**	2.33977537319917	1	0.405	9.76048562065403E-10	Lymphat	VIM
**6.07748532278782E-14**	3.0512440945439	0.391	0.044	9.91298630999921E-10	Lymphat	RTKN
**1.39928755099848E-13**	2.9016106541262	0.522	0.083	2.28237792443363E-09	Lymphat	PTPRE
**1.84788446718859E-13**	3.48988593871136	0.304	0.028	3.01408435443131E-09	Lymphat	ASNSD1
**2.35697842086841E-13**	3.06781923029896	0.696	0.158	3.84446750227847E-09	Lymphat	OLFML2A
**4.84761442154427E-13**	2.7018096954317	0.304	0.028	7.90694388298086E-09	Lymphat	KIAA0513
**9.7328601791976E-13**	3.55192312709333	0.304	0.03	1.58752682382892E-08	Lymphat	ATP9A
**1.48274464220637E-12**	3.76075084433876	0.13	0.005	2.41850478590282E-08	Lymphat	FRY
**2.65064597167174E-12**	4.39428127823894	0.174	0.01	4.32346864439377E-08	Lymphat	OSBP2
**2.9580162113936E-12**	3.35556361788225	0.348	0.041	4.8248202424041E-08	Lymphat	ARL4A
**3.52473817194708E-12**	2.7748387139288	0.565	0.108	5.74920043226288E-08	Lymphat	DST
**9.81742519080572E-12**	2.94569625708031	0.304	0.032	1.60132022287232E-07	Lymphat	BCL2
**1.45095317279276E-11**	3.25042180370779	0.304	0.033	2.36664972014226E-07	Lymphat	CFAP54
**1.8397587626293E-11**	3.84971899959612	0.174	0.01	3.00083051772465E-07	Lymphat	HSD17B8
**2.42073062012601E-11**	3.86340541828355	0.13	0.006	3.94845371448753E-07	Lymphat	SDC4
**3.01916741455881E-11**	3.52224787813965	0.261	0.025	4.92456396988687E-07	Lymphat	PPP1R2
**3.13574035135384E-11**	4.52883981024157	0.217	0.018	5.11470608709325E-07	Lymphat	IL7
**4.21398884381652E-11**	3.89666278572153	0.217	0.018	6.87343720314913E-07	Lymphat	WASF3
**5.34534519799672E-11**	4.51920974932727	0.174	0.011	8.71879255245245E-07	Lymphat	CGNL1
**6.3928203714952E-11**	2.86324607811967	0.217	0.018	1.04273293079458E-06	Lymphat	SPINK1
**6.57214465465638E-11**	3.90841640661006	0.174	0.011	1.071982514621E-06	Lymphat	RDH5
**7.684393397388886E-11**	2.44465946086824	0.217	0.018	1.2534014070481E-06	Lymphat	AHSG
**1.82395105168281E-10**	3.04345350149594	0.261	0.027	2.97504656039982E-06	Lymphat	CFH
**2.02950662553909E-10**	4.66309352639817	0.13	0.006	3.31032825691681E-06	Lymphat	MUC1
**2.27999331040708E-10**	4.38064157709387	0.13	0.006	3.71889708860498E-06	Lymphat	SCGB3A1
**2.54379840924433E-10**	4.00924458204723	0.174	0.012	4.14918958531843E-06	Lymphat	TXNL4B
**2.89492014044576E-10**	1.74426968117542	0.87	0.33	4.72190424108108E-06	Lymphat	TRIOBP
**3.55861241804718E-10**	1.57828105628941	0.609	0.13	5.80445271507675E-06	Lymphat	FABP4
**4.19029978204709E-10**	2.1565206541465	0.304	0.037	6.83479797449701E-06	Lymphat	FABP1
**5.16023120100964E-10**	2.59201825100617	0.304	0.038	8.41685311196683E-06	Lymphat	SLCO2B1
**8.18499362830911E-10**	3.85321855879781	0.174	0.013	1.3350543107135E-05	Lymphat	HSD3B7
**8.46808758564144E-10**	3.0409964475637	0.261	0.029	1.38122976609398E-05	Lymphat	GNB5
**9.29583246009991E-10**	3.0578577182247	0.261	0.029	1.5162432325669E-05	Lymphat	PDGFA
**1.05686773349641E-09**	2.67701093506597	0.696	0.224	1.723856960106E-05	Lymphat	THBD
**1.53930347644897E-09**	2.01181843877219	0.739	0.22	2.51075790043591E-05	Lymphat	CALD1
**1.68815878205063E-09**	2.80607391803555	0.261	0.03	2.75355578940278E-05	Lymphat	THAP12
**1.75968245454752E-09**	1.69342891808844	0.87	0.34	2.87021805161246E-05	Lymphat	FN1
**1.8728114567972E-09**	2.79159803233785	0.13	0.007	3.05474276718191E-05	Lymphat	DEPRECATED-ENSG00000106538
**1.88354723696028E-09**	1.97697078582472	0.783	0.282	3.07225389820591E-05	Lymphat	LAMA4
**1.98422624165744E-09**	2.63013310208962	0.522	0.117	3.23647142276745E-05	Lymphat	PLSCR4
**2.33784171222249E-09**	2.94382704955691	0.261	0.031	3.8132536168061E-05	Lymphat	CREB5
**2.63072651086481E-09**	2.46052907545861	0.435	0.082	4.2909780118716E-05	Lymphat	ELOVL1
**2.82312067853247E-09**	3.01685490968345	0.261	0.031	4.60479213875431E-05	Lymphat	CCDC102A
**3.37076311221796E-09**	3.36392162528163	0.217	0.022	5.49805171233872E-05	Lymphat	LYNX1
**4.15489667906356E-09**	2.47589240330522	0.391	0.068	6.77705197322057E-05	Lymphat	C1R
**4.61533936316437E-09**	2.96145282081852	0.261	0.031	7.5280800352574E-05	Lymphat	NFIX
**5.64013660413968E-09**	3.97152239928302	0.174	0.014	9.19962681501223E-05	Lymphat	COLEC12
**5.65087614129749E-09**	2.2842831749012	0.783	0.315	9.21714407407033E-05	Lymphat	PTMS
**5.9644085359566E-09**	2.39351416495362	0.391	0.069	9.72854676299881E-05	Lymphat	GC
**6.16246971747188E-09**	2.65251662124136	0.391	0.07	0.000100516043561684	Lymphat	CTSZ
**7.0815097151695E-09**	2.95811338184334	0.261	0.032	0.00011550650496413	Lymphat	PPP1R13L
**7.20661676687785E-09**	1.96797814038951	0.87	0.476	0.000117547126084545	Lymphat	LMNA
**7.22125936679586E-09**	2.28014930529623	0.609	0.161	0.000117785961531807	Lymphat	GJA1
**8.20499021397228E-09**	2.00096364242396	0.522	0.126	0.000133831595380102	Lymphat	CLU
**9.17181686017561E-09**	3.68062896481235	0.13	0.008	0.000149601504806324	Lymphat	BACH2

1.0917775475558E-08	3.30266833133081	0.217	0.023	0.000178079835781826	Lymphat	SAMD4A
1.20699111936052E-08	2.22558468122003	0.652	0.204	0.000196872321478894	Lymphat	OAF
1.32816130445218E-08	2.42619178524203	0.348	0.057	0.000216636390369195	Lymphat	DNASE1L1
1.43964678357433E-08	2.17080026046474	0.478	0.103	0.00023482078686881	Lymphat	C3
1.79828475401334E-08	3.43086234439313	0.174	0.015	0.000293318226227116	Lymphat	GSAP
1.88173822537075E-08	1.89097771260782	0.478	0.106	0.000306930321940223	Lymphat	LGALS1
2.62321015897245E-08	3.30431562644815	0.217	0.024	0.000427871809029996	Lymphat	EPB41L1
3.01021534420724E-08	3.27248260613225	0.304	0.049	0.000490996224793643	Lymphat	SMAD1
3.16202544954663E-08	3.91775904466403	0.13	0.009	0.00051575797107555	Lymphat	CDK20
3.39946666768567E-08	3.773756417927	0.13	0.009	0.000554487008166209	Lymphat	HOXD8
3.52458442293827E-08	3.63519901611041	0.13	0.009	0.000574894965225462	Lymphat	TARSL2
4.50103235238114E-08	3.52553707912756	0.217	0.026	0.000734163386996888	Lymphat	DKK 3.00
5.8283966883298E-08	2.25483832551118	0.522	0.129	0.000950669783833473	Lymphat	FGA
6.3191212067413E-08	3.38923690894721	0.174	0.017	0.00103071186003157	Lymphat	SEC14L2
6.7429196644577E-08	2.56614262764816	0.435	0.094	0.0010998376264697	Lymphat	RAB11FIP5
6.97940394008331E-08	1.42117884160801	0.87	0.404	0.00113841057666699	Lymphat	MT-CO1
1.06049259379476E-07	4.20228036959425	0.13	0.01	0.00172976946973863	Lymphat	AVPI1
1.07822676326642E-07	2.09026746783615	0.696	0.279	0.00175869567356386	Lymphat	AHNAK
1.15368612368396E-07	3.60193106986889	0.13	0.01	0.0018817774363409	Lymphat	PTPRF
1.27272125530122E-07	2.55114745095272	0.435	0.101	0.00207593563952183	Lymphat	MAPK7
1.36442493830291E-07	2.91889142424442	0.13	0.01	0.00222551351686588	Lymphat	RTN4RL1
1.36491667282484E-07	1.66544446232002	0.913	0.487	0.00222631558504459	Lymphat	ITGA9
1.82569297402938E-07	1.82550244312228	0.478	0.113	0.00297788780993931	Lymphat	TRIP6
2.04388172165656E-07	3.02422305615585	0.174	0.018	0.00333377547619401	Lymphat	C1orf198
2.10179181074527E-07	2.51335330336432	0.391	0.084	0.00342823262250661	Lymphat	MICAL2
2.3773788636393E-07	2.00121025975056	0.435	0.1	0.00387774266448206	Lymphat	ATXN3
2.47305261928377E-07	2.13492176150221	0.565	0.164	0.00403379612731376	Lymphat	LAPTM5
2.59210061327496E-07	2.35936594050278	0.391	0.083	0.00422797531031279	Lymphat	MDFIC
2.75473716337249E-07	1.84045908982484	0.435	0.099	0.00449325178717687	Lymphat	PROCR
2.91972021493978E-07	2.01463577593996	0.565	0.175	0.00476235564258827	Lymphat	TTYH3
3.01505528579931E-07	3.84733967290345	0.13	0.01	0.00491785667666725	Lymphat	EIF3M
3.04638261097445E-07	2.51679146604062	0.304	0.053	0.00496895467676042	Lymphat	ZNF358
3.16111029618776E-07	3.75145200891805	0.13	0.01	0.00515608700411185	Lymphat	ADAMTSL3
3.47479898094268E-07	2.77779541851562	0.261	0.04	0.0056677446178156	Lymphat	ZMAT1
3.4973182324439E-07	3.07653480064135	0.174	0.019	0.00570447576893925	Lymphat	SYT15
3.64142843410058E-07	3.50736469949219	0.13	0.01	0.00593953391886145	Lymphat	DNAJB5
3.75735672852162E-07	4.05399127956411	0.13	0.01	0.00612862455989162	Lymphat	GPR146
3.85281103957338E-07	2.36410756280226	0.609	0.207	0.00628432008664814	Lymphat	IGFBP5
4.14854086556709E-07	2.53000317353036	0.217	0.028	0.00676668500582648	Lymphat	COL5A2
4.2216384419025E-07	1.67889379621638	0.478	0.116	0.00688591446258717	Lymphat	SULF2
5.49863621385589E-07	3.3087093209324	0.174	0.019	0.00896882552842035	Lymphat	EBAG9
5.675690546643E-07	2.91055222568991	0.261	0.043	0.00925761885062939	Lymphat	ZFP30
5.71082238061979E-07	2.98926102835446	0.261	0.041	0.00931492238502893	Lymphat	HECTD2
5.97767330312333E-07	2.52597918078536	0.261	0.041	0.00975018292472446	Lymphat	KNOP1
7.77024191693693E-07	1.4357267540562	0.87	0.445	0.0126740415907158	Lymphat	SHC1
8.11088122056213E-07	2.48241156723975	0.304	0.056	0.0132296583588589	Lymphat	DOC2B
8.4063159989293E-07	2.00392128984037	0.348	0.069	0.0137115420258536	Lymphat	ARHGEF3
8.49849360011168E-07	2.46472755210218	0.217	0.03	0.0138618929111422	Lymphat	HOXD4
9.08282806174784E-07	2.14072824234749	0.261	0.043	0.0148150008515169	Lymphat	MT1E
9.62940239232596E-07	2.26214206143098	0.261	0.042	0.0157065182421229	Lymphat	CITED4
1.06547363101045E-06	2.66259946582665	0.13	0.011	0.0173789403954114	Lymphat	OCLN
1.16377453888558E-06	2.15151547263357	0.348	0.074	0.0189823265037626	Lymphat	FAF1
1.34061313906197E-06	1.68749952381591	0.348	0.07	0.0218667409112398	Lymphat	SH3PXD2B
1.35740964576332E-06	3.46218684748542	0.174	0.021	0.0221407087320456	Lymphat	DHH
1.35975540760807E-06	2.38821862406367	0.348	0.076	0.0221789704534952	Lymphat	SIRPA
1.42771748594558E-06	1.88395006206141	0.348	0.074	0.0232874999132584	Lymphat	FGG
1.54513754825046E-06	4.33913627651415	0.13	0.012	0.0252027385495132	Lymphat	C6orf141
1.91138283550527E-06	1.89420069594153	0.435	0.11	0.0311765654299265	Lymphat	LHFPL6
1.9491216207974E-06	1.80900915817278	0.609	0.231	0.0317921227568265	Lymphat	FGB
2.12928195566395E-06	3.60228641387183	0.13	0.012	0.0347307179788347	Lymphat	TLE4

**2.16144669423187E-06**	2.37064352473179	0.348	0.073	0.0352553570296161	Lymphat	PLCG2
**2.22529463288656E-06**	2.14402539439002	0.217	0.032	0.0362967807570127	Lymphat	LYZ
**2.28179901304771E-06**	2.48728205312057	0.13	0.012	0.0372184237018212	Lymphat	MT1M
**2.31353638370946E-06**	2.72216123034825	0.13	0.012	0.0377360919546849	Lymphat	TMEM176B
**2.58388783493337E-06**	2.25028993770246	0.261	0.044	0.0421457944755981	Lymphat	OBSL1
**2.83368026370642E-06**	1.71011645070868	0.391	0.096	0.0462201587813154	Lymphat	VTN
**2.95577659367581E-06**	2.8279377196196	0.174	0.022	0.0482116720194461	Lymphat	KIAA1522
**2.95577659367581E-06**	3.08213965964128	0.174	0.022	0.0482116720194461	Lymphat	HPS6
**2.9766381537871E-06**	1.62528792704308	0.609	0.211	0.0485519449264213	Lymphat	EPB41L2
**3.05888583871655E-06**	1.39947530024117	0.826	0.541	0.0498934869153056	Lymphat	MAF
