# Supplementary material for: Spatiotemporal dynamics of the tumor microenvironment in hepatocellular carcinoma during combination immunotherapy
Source: Hepatol Commun. 2026 Aug 7;10(9):e1014. doi: 10.1097/HC9.0000000000001014 (PMC13456720; doi:10.1097/HC9.0000000000001014)
Supplement: Supplementary file 6 [file hc9-10-e1014-s005.docx]

Supplementary Table. 1 Clinicopathological characteristics of three hepato cases subjected to single-cell RNA sequence: one upfront resection case (Pat one recurrent case after ATZ/BEV, and one case with paired tumor edge sampling (Patient 2).

	Patient 1<br>1st surgery	Patient 1<br>2nd surgery
Age (years)	74	75
Sex (male/female)	Female	Female
Etiology (HBV/HCV/alcohol/MASH)	HCV	HCV
Cirrhosis	Yes	Yes
ALBI score	1	2b
AFP (ng/mL)	3.7	3.1
PIVKA_II (mAU/mL)	294	4797
ICG-R15 (%)	14.7	24.6
Child–Pugh score (A/B/C)	A	A
Histological differentiation (well/moderate/poor/others)	Moderate	Moderate
Maximum tumor size (cm)	3.0	5.3
Tumor count	2	1
Microvascular invasion	None	None
BCLC stage (0/A/B/C/D)	A	A
ECOG PS (0/1/2/3/4)	0	0
ATZ/BEV exposure (cycles)	0	5

Supplementary Table. 2 Summary of cell numbers in each major cell typ[illegible] ATZ/BEV therapy.

Cell type	Before treatment	After treatment	To[illegible]
Hepatocytes	7,682	14,448	22[illegible]
T/NK cell	846	1,167	2,[illegible]

Supplementary Table. 3 Summary of cell numbers in each major cell type i and tumor margin.

Cell type	Tumor center (cells)	Tumor margin (cells)
Hepatocytes	2,766	3,475
T/NK cell	3,922	5,004

Supplementary Table. 4 Baseline clinicopathological characteristics of p[illegible] upfront hepatectomy (n = 31) versus post-atezolizumab/bevacizumab hepat[illegible] 8)

	Upfront (n=31)	ATZ[illegible]
Age (years)	71 (21–84)	73.5[illegible]
Sex (male/female)	17/14	6/2
Etiology (HBV/HCV/alcohol/MASH)	14/9/3/5	4/3/[illegible]
Platelet count (10³/μL)	169 (82–327)	172 [illegible]
Albumin (g/dL)	4 (3.3–4.8)	3.8 ([illegible]
Total bilirubin (mg/dL)	0.9 (0.3–1.5)	0.9 ([illegible]
Modified ALBI grade (1/2a/2b/3)	18/9/4/0	2/3/[illegible]
%PT (%)	98 (63–137)	91 ([illegible]
AFP (ng/mL)	37.6 (1.5–5024)	2.9 ([illegible]
PIVKA_II (mAU/mL)	238 (15–35855)	991.[illegible]
ICG-R15 (%)	8 (0.1–38.3)	14.8[illegible]
Child–Pugh score (A/B/C)	29/2/0	8/0/[illegible]
Histological differentiation (well/moderate/poor/others)	0/14/17/0	0/4/[illegible]
Maximum tumor size (cm)	2.5 (1–8.5)	5.3 ([illegible]
Tumor count	1: 27, 2: 3	1: 5,[illegible]
Microvascular invasion	7 (23%)	2 (2[illegible]
BCLC stage (0/A/B/C/D)	10/20/1/0/0	0/5/[illegible]
