# Supplementary material for: Spatiotemporal dynamics of the tumor microenvironment in hepatocellular carcinoma during combination immunotherapy
Source: Hepatol Commun. 2026 Aug 7;10(9):e1014. doi: 10.1097/HC9.0000000000001014 (PMC13456720; doi:10.1097/HC9.0000000000001014)
Supplement: Supplementary file 6 [file hc9-10-e1014-s004.docx]

**1. Single-cell sample collection**

For each case, a 5-mm square tissue specimen was excised from the tumor, immediately snap-frozen in liquid nitrogen, and stored until analysis. For spatial sampling, the tumor margin was defined as the region within 1,000 μm of the boundary between the tumor and the adjacent non-tumorous liver tissue, whereas all other tumor areas were defined as the tumor center. Specimens from both regions were collected under identical conditions.

**2. Preparation of single-cell suspensions**

Stored specimens were processed at a later time point and mechanically dissociated to generate single-cell suspensions. The suspensions were filtered through a sterile 30-µm cell strainer (CellTrics, Sysmex; 04-004-2326). Cell number and viability were assessed both manually using OLYMPUS APX100 with ReadyCount Blue Nuclear Stain (Invitrogen; A49904) and automatically using Countess 3 FL with ReadyCount Green/Red Viability Stain (Invitrogen; A49905).

**3. Library construction and sequencing**

Approximately 10,000 cells were used for library preparation. Gene expression libraries were generated using the Chromium Fixed RNA Kit, Human Transcriptome (10x Genomics; PN-1000475), Chromium Next GEM Chip Q Single Cell Kit (PN-1000418), and Dual Index Kit TS Set A (PN-1000251), according to the manufacturer’s protocol (CG000527, Rev. E). Libraries were sequenced on the NovaSeq X Plus system (Illumina) with paired-end 150-bp reads (R1: 150 bp; R2: 150 bp), yielding a total of 745,962,497 paired-end reads.

**4. Software environment**

Initial processing was performed using Cell Ranger (v8.0.1). Ambient RNA removal was conducted using CellBender (v0.2.1). Downstream analyses were performed using R (v4.4.2), Bioconductor (v3.20), Seurat (v5.4.0), SeuratObject (v5.3.0), and Harmony (v1.2.4). Cell-cell communication analysis was conducted using CellChat (v2.2.0.9001).

**5. Ambient RNA removal**

CellBender was applied to the raw count matrix of each sample generated by Cell Ranger to remove ambient RNA contamination. The CellBender-corrected cellbender_output_filtered.h5 files were used for downstream analyses.

**6. Seurat object generation and initial filtering**

Seurat objects were generated from the CellBender-corrected count matrices with min.cells = 3 and min.features = 200. For each cell, the proportions of mitochondrial genes (percent.mt) and ribosomal genes (percent.rb) were calculated. When duplicated gene names were detected, they were made unique using make.unique().

**7. Sample-specific quality control**

Quality control was performed separately for each sample. Thresholds for nFeature_RNA were defined as the mean ± 3 standard deviations within each sample, with a lower bound of 200 genes. For nCount_RNA, thresholds were determined by applying mean ± 3 standard deviations to log10-transformed values and then back-transforming to the original scale. Cells with percent.mt > 10 were excluded. Distributions before and after quality control were examined by violin plots, and thresholds and excluded cell numbers were recorded for each sample.

**8. Doublet detection and removal**

Doublets were identified using scDblFinder (v1.22.0). After conversion of each Seurat object to a SingleCellExperiment object, scDblFinder was run for each sample, and cells classified as doublets were removed. The distribution of doublet scores was assessed using violin plots (Sup Fig. 4A/B, 14A/B). In addition, before doublet removal, NormalizeData, FindVariableFeatures, ScaleData, RunPCA, and RunUMAP were performed to visualize the distribution of singlets and doublets on UMAP (Sup Fig. 4C/D, 14C/D).

**9. Data merging, normalization, and dimensionality reduction**

After quality control and doublet removal, the filtered samples were merged using merge(), and sample IDs were added to cell barcodes using add.cell.ids. After adding sample and condition metadata, normalization and variance stabilization were performed using SCTransform (v2), and the effect of mitochondrial gene content was regressed out using vars.to.regress = "percent.mt". Principal component analysis was performed using RunPCA. After inspection of the elbow plot up to 50 dimensions, the top 40 principal components were used for downstream analyses (Sup Fig. 4E, 14E).

**10. Batch correction and clustering**

Before batch correction, a PCA-based UMAP was generated to assess the distribution of cells across conditions. Harmony was then applied with group.by.vars = "sample", and UMAP was recalculated based on the Harmony embedding. Clustering was performed in the Harmony-corrected low-dimensional space using FindNeighbors(dims = 1:40, reduction = "harmony") and FindClusters(resolution = 0.3).

**11. Cell type annotation and subclustering**

Initial cluster annotation was performed using a three-step approach. First, reference-based automated annotation was conducted using SingleR (v2.8.0). Second, cluster-specific marker genes were identified using Seurat’s FindAllMarkers function and used to support cell identity assignment. Third, the final annotation was manually confirmed by examining the expression patterns of known canonical marker genes on UMAPs, feature plots, and heatmaps, with reference to public cell marker resources, including CellMarker 2.0. Each major cell population was then extracted separately and subjected to repeated dimensionality reduction, UMAP visualization, and subclustering analysis. Subcluster identities were assigned using the same marker-based strategy, based on cluster-specific marker genes, canonical marker expression, and CellMarker 2.0-supported marker information. During lineage-specific subclustering, residual clusters showing low-quality profiles or mixed lineage marker expression were identified based on cluster-specific marker genes, canonical marker expression patterns, and overall transcriptional profiles. These clusters were excluded from subsequent lineage-specific downstream analyses to minimize their influence on cell-state characterization and biological interpretation. After exclusion of these residual clusters, the remaining lineage-specific cells were reprocessed by SCTransform normalization, principal component analysis, clustering, and UMAP visualization before cell-state annotation, FeaturePlot generation, and downstream analyses.

**12. Differential expression analysis**

Differential expression analysis was performed using Seurat with assay = "RNA", test.use = "wilcox", only.pos = FALSE, min.pct = 0.1, and logfc.threshold = 0.1. Differentially expressed genes (DEGs) were defined as those with p_val_adj < 0.05. Genes satisfying both |avg_log2FC| > 0.25 and p_val_adj < 0.05 were labeled in volcano plots.

**13. GO-BP over-representation analysis**

Gene Ontology biological process (GO-BP) analysis was performed by over-representation analysis using the enrichGO function. Input genes were defined as significantly upregulated DEGs with avg_log2FC > 0.25. The analysis was conducted using org.Hs.eg.db with ontology = "BP", pAdjustMethod = "BH", pvalueCutoff = 0.05, and qvalueCutoff = 0.2.

**14. Module score analysis**

To evaluate functional states within each lineage, gene signature scores were calculated using Seurat’s AddModuleScore. In T/NK cells, exhaustion and effector scores were evaluated, whereas in myeloid cells, M1 and M2 scores were assessed. The gene sets used for each score were as follows:

Exhaustion score (T/NK cells): PDCD1, TIGIT, LAG3, HAVCR2, CTLA4, TOX, ENTPD1, CXCL13, PHLDA1, TNFRSF9

Effector score (T/NK cells): GZMB, GZMA, PRF1, IFNG, TNF, NKG7, GNLY, FGFBP2, GZMH, CX3CR1

M1 score (myeloid cells): TNF, IL1B, IL6, CXCL8, IL12A, IL12B, CXCL9, CXCL10, CXCL11, NOS2, CD86, CD80, HLA-DRA, HLA-DRB1, CCL2, CCL3, CCL4, CCL5, STAT1, IRF5, SOCS1

M2 score (myeloid cells): CD163, MRC1, IL10, TGFB1, ARG1, VEGFA, MMP9, CCL17, CCL22, CCL24, CD209, IL4R, PPARG, MSR1, FOLR2, LYVE1, STAB1, CLEC4F, TIMD4, C1QA, C1QB, C1QC

**15. Cell-cell communication analysis**

Cell-cell communication was inferred using CellChat (v2.2.0.9001) with the human ligand-receptor database, CellChatDB.human, using the final annotated high-quality major cell populations. For each cohort, the major annotated cell populations were analyzed together to reconstruct intercellular communication networks. Overexpressed genes were identified using identifyOverExpressedGenes(do.fast = FALSE). Communication probability was calculated using computeCommunProb(type = "triMean"), and groups with insufficient cell numbers were excluded using filterCommunication(min.cells = 10). Network centrality scores were calculated using netAnalysis_computeCentrality(slot.name = "netP"). Information flow was compared using rankNet(mode = "comparison", stacked = TRUE, do.stat = TRUE). For pathway functional embedding, type = "functional" and umap.method = "uwot" were used consistently. The number of clusters (k) for functional embedding was set to 4 for both the before and after sample in Cohort 1, and to 4 for both the center and margin samples in Cohort 2. Comparisons were performed between before and after in Cohort 1, and between center and margin in Cohort 2.

**16. In silico analysis**

In silico analyses were performed using publicly available datasets. Immune infiltration of CD8⁺ T cells and regulatory T cells (Tregs) was evaluated using TIMER2.0, in which gene expression levels of PVR and NECTIN2 were correlated with estimated immune cell abundances across multiple cancer types, including LIHC. For survival analysis, RNA expression data and clinical information from the LIHC cohort in the Human Protein Atlas were utilized. Patients were stratified into high- and low-expression groups based on median gene expression, and overall survival was compared between groups using Kaplan–Meier analysis with log-rank testing.
